# Comparative phylogeography of two commensal rat species (*Rattus tanezumi* and *Rattus norvegicus*) in China: Insights from mitochondrial DNA, microsatellite, and 2b‐RAD data

**DOI:** 10.1002/ece3.9409

**Published:** 2022-10-13

**Authors:** Meidong Jing, Yingjie Chen, Keying Yao, Youming Wang, Ling Huang

**Affiliations:** ^1^ School of Life Sciences Nantong University Nantong China

**Keywords:** colonization history, demographic history, *Rattus norvegicus*, *Rattus tanezumi*

## Abstract

*Rattus norvegicus* and *Rattus tanezumi* are dominant species of Chinese house rats, but the colonization and demographic history of two species in China have not been thoroughly explored. Phylogenetic analyses with mitochondrial DNA including 486 individuals from 31 localities revealed that *R. norvegicus* is widely distributed in China, *R. tanezumi* is mainly distributed in southern China with currently invading northward; northeast China was the natal region of *R. norvegicus*, while the spread of *R. tanezumi* in China most likely started from the southeast coast. A total of 123 individuals from 18 localities were subjected to 2b‐RAD analyses. In neighbor‐joining tree, individuals of *R. tanezumi* grouped into geographic‐specific branches, and populations from southeast coast were ancestral groups, which confirmed the colonization route from southeast coast to central and western China. However, individuals of *R. norvegicus* were generally grouped into two clusters instead of geographic‐specific branches. One cluster comprised inland populations, and another cluster included both southeast coast and inland populations, which indicated that spread history of *R. norvegicus* in China was complex; in addition to on‐land colonization, shipping transportation also have played great roles. ADMIXTURE and principal component analyses provided further supports for the colonization history. Demographic analyses revealed that climate changes at ~40,000 to 18,000 years ago and ~4000 years ago had led to population declines of both species; the *R. norvegicus* declined rapidly while the population of *R. tanezumi* continuously expanded since ~1500 years ago, indicating the importance of interspecies' competition in their population size changes. Our study provided a valuable framework for further investigation on phylogeography of two species in China.

## Graphical abstract

We investigated the phylogeography of *Rattus tanezumi* and *Rattus norvegicus* in China; 486 individuals collected from 31 localities were initially analyzed with mitochondrial DNA and 10 microsatellite loci.

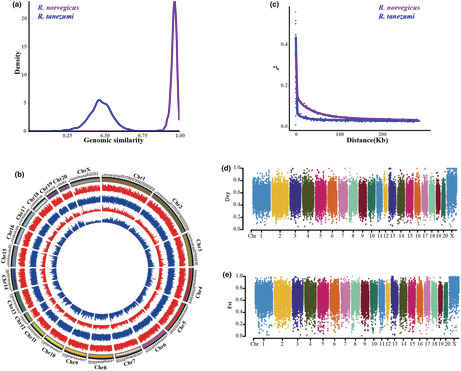

## 1 INTRODUCTION

*Rattus*, consisting of >60 recognized species (Musser & Carleton, 2005), originated in Asia at the center of Pliocene‐Pleistocene boundary (Chaimanee & Jaeger, 2000; Rowe et al., 2011), with various species diverging in disparate areas of Asia (Aplin et al., 2011; Rowe et al., 2011). Four species in this genus (the black rat: *Rattus rattus*; the Asian house rat: *R. tanezumi*; the Pacific rat: *R. exulans*; and the brown rat: *R. norvegicus*) became human commensals and expanded globally with human migration and trade contact (Aplin et al., 2011; Innes, 1990; Lack et al., 2012; Matisoo‐Smith et al., 1998; Matisoo‐Smith & Robins, 2004; Puckett et al., 2016, 2020; Puckett & Munshi‐South, 2019; Rowe et al., 2011).

In the past three decades, investigations on phylogeography of house rats have provided valuable information on their origin, colonization, and demographic histories, as well as relationships with human activities. Studies based on mitochondrial DNA suggested that *R. tanzumi* belongs to the *R. rattus* species complex (RrC), which consists of four lineages: the black rat (RrC I), the Asian house rat (RrC II), an unnamed RrC III, and RrC IV (Aplin et al., 2011), whereas analyses based on nuclear gene markers and morphological characters suggested that RrC II, RrC III, and RrC IV all belong to *R. tanzumi* (Pagès et al., 2013). The species composition and migration history of RrC have been explored in Madagascar (Brouat et al., 2014; Hingston et al., 2005; Tollenaere et al., 2010), Europe and America (Abdelkrim et al., 2005; Lack et al., 2012), south and western Africa (Bastos et al., 2011; Konec̆ný et al., 2013), Southeast Asia (Pagès et al., 2013), Western Mediterranean basin (Colangelo et al., 2015), New Zealand (Robins et al., 2016), and India (Baig et al., 2019), using mitochondrial DNA markers (*Cytb*, *COI* or D‐loop) and/or microsatellite loci. Hybridization between species of RrC has been confirmed in both south‐east Asia and America, causing discordance between mitochondrial and nuclear phylogenetic trees (Lack et al., 2012; Pagès et al., 2013).

**FIGURE 1:**
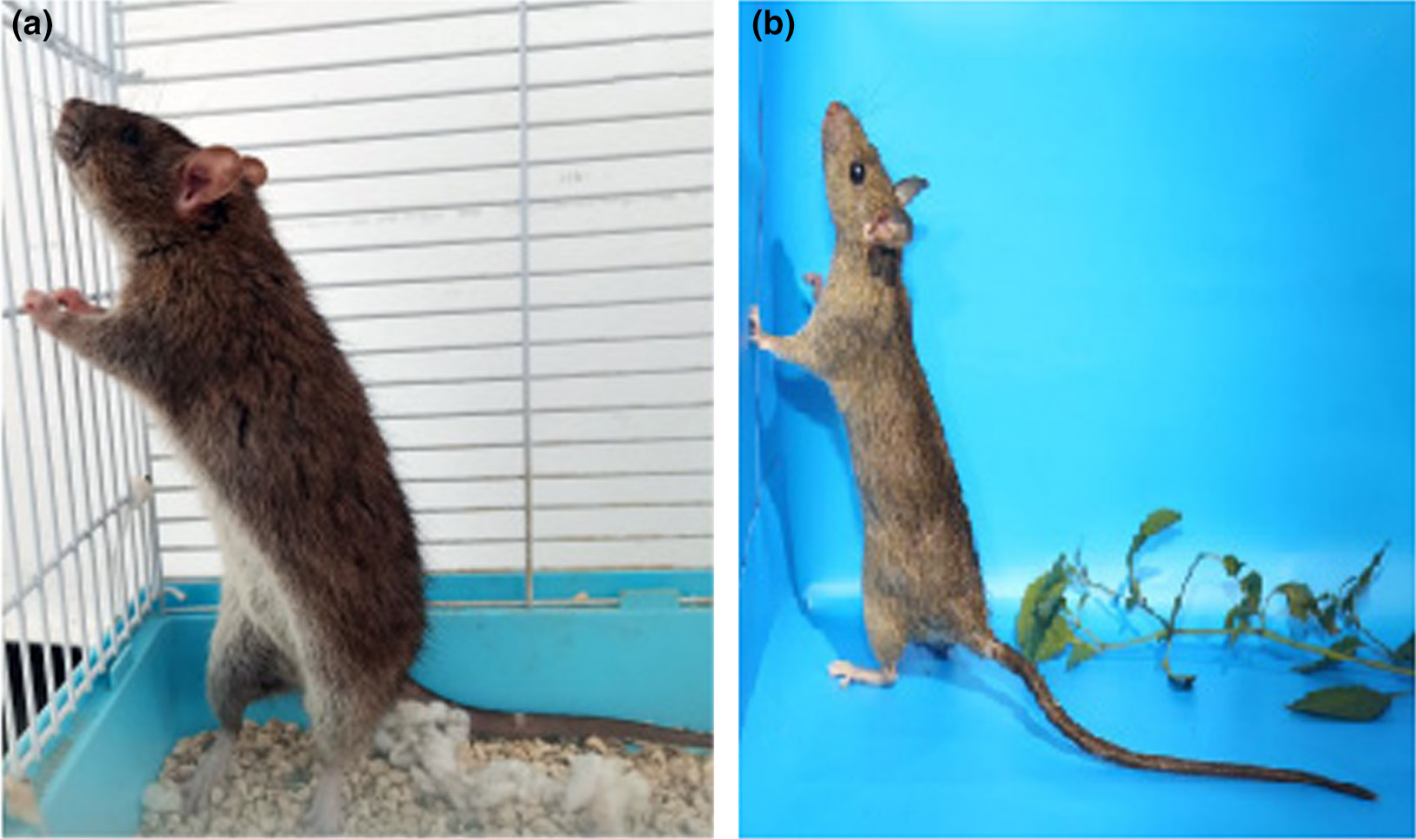
Photograph of (a) *Rattus norvegicus* and (b) *Rattus tanezumi*.

Both *R. rattus* and *R. tanezumi* (Figure 1) are distributed in China (Wei et al., 2021), and *R. tanezumi* is one of the dominant species of Chinese house rats (Wang, 2003). Based on microsatellite loci and mitochondrial DNA, *R. tanezumi* likely immigrated into China by shipping transportation over the sea and then expanded into Central China from coastal areas along the Yangtze River (Guo et al., 2019). Recent investigations with genome sequence data explored the possible genetic basis contributing to the northward invasion of *R. tanezumi* from southern China in recent decades, as well as the possible genetic basis favoring adaptation to high‐altitude hypoxia in Tibet (Chen et al., 2021). However, there are still key issues remaining to be solved, such as when *R. tanezumi* immigrated to China and the demographic history of this species. In addition, *R. norvegicus* is another dominant house rat species in China (Wang, 2003). Some studies proposed that *R. norvegicus* originated in southeast Asia (Zeng et al., 2017), but a recent investigation using genome data (Puckett & Munshi‐South, 2019) supported the long‐time consideration that *R. norvegicus* originated in northern China and Mongolia (Allen, 1940; Silver, 1941; Wilson & Reeder, 2005). From its natal range, this species migrated to southeast Asia and coastal ports and then expanded into the Middle East and Europe, and subsequently invaded western Africa, eastern North America, South America, and New Zealand; furthermore, this species also expanded eastward into Russia and finally to western North America (Puckett et al., 2016, 2020; Puckett & Munshi‐South, 2019; Russell et al., 2019). The genetic structure and demographic history of *R. norvegicus* throughout China have not been explored systematically with molecular markers, although *several samples were involved in previous genomic analyses (*Deinum et al., 2015; Puckett et al., 2016; Puckett & Munshi‐South, 2019 *)*.

Herein, 486 house rat samples were randomly collected from 31 localities in China. Analyses based on mitochondrial sequences (*COI*, *Cyt b* & D‐loop), microsatellite loci, and 2b‐RAD sequences provided insights on population structure, migration route, and demographic history of *R. tanezumi* and *R. norvegicus* in China.

## 2 MATERIALS AND METHODS

### 2.1 Sampling

**FIGURE 2:**
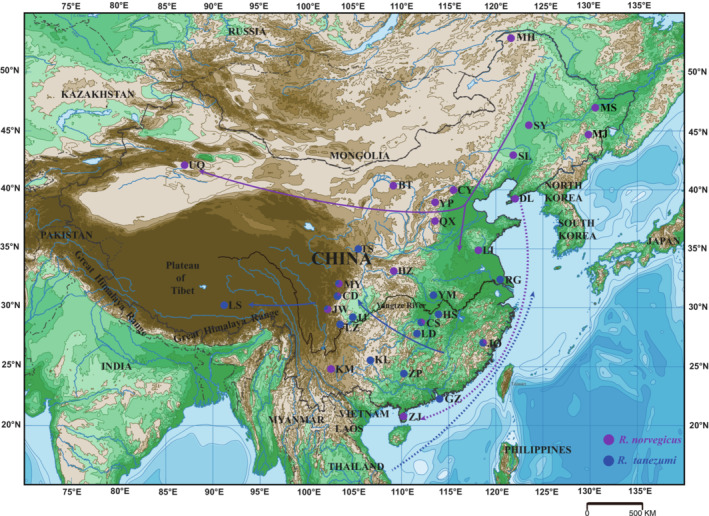
Geographic distribution of *Rattus norvegicus* (purple circles) and *Rattus tanezumi* (blue circles) used in this study. The migration routes for *R. norvegicus* (purple arrow lines) and *R. tanezumi* (blue arrow lines) were roughly indicated. Circles in the map indicated the sampling locations. The circles with both colors also showed the proportion of individuals from the two species sampled at the location. The detailed individual numbers of two species from each location are presented in Table S1. The green regions in the map represent plains; the oyster white and beige regions represent the hilly mountains; the brown regions represent the plateaus; the sapphire blue lines on land represent rivers; the thick black line across the east and west in southern China represents the Yangtze River; the celeste regions represent the coastal sea area; the blue regions represent the deep‐sea areas. BT, Baotou; CD, Chengdu; CS, Changsha; CY, Chaoyang; DL, Dalian; GZ, Guangzhou; HS, Huangshi; HZ, Hanzhong; JJ, Jiangjin; JO, Jian'ou; JW, Jianwei; KL, Kaili; KM, Kunming; LD, Loudi; LI, Linshu; LS, Lhasa; LZ, Luzhou; MH, Mohe; MJ, Mudanjiang; MS, Jiamusi; MY, Mianyang; QX, Qinxian; RG, Rugao; SL, Shuangliao; SY, Songyuan; TS, Tianshui; UQ, Urumqi; YM, Yunmeng; YP, Yuanping; ZJ, Zhanjiang; ZP, Zhaoping.

During 2013 to 2019, a total of 486 house rats were randomly live‐trapped around human settlements (houses, yards, grain storage, etc.) from 31 localities (Figure 2). Sampling sites were at least 100 m apart from each other in each locality. All specimens were first identified based on external characteristics (length of head and body, length of tail, color of venter, Figure 1; Corbet & Hill, 1992; Pan et al., 2007). Then, mitochondrial DNA barcoding analyses were used to furtherly define species. Localities and sample sizes are shown (Table S1).

### 2.2 Mitochonrial DNA analyses

#### 2.2.1 Polymerase chain reaction (PCR) amplification and sequencing of mitochondrial gene markers

Genomic DNA was extracted from muscle or tail tips using a DNeasy Tissue Kit (QIAGEN). Three mtDNA fragments (*COI*: 681 bp, D‐loop: 1048 bp, and *cyt b*: 1143 bp), extensively used in phylogenetic analyses of *Rattus*, were amplified separately with primers listed in Table S2.

The PCR was done in a 50 μl reaction volume, including 5 μl of 10× ^EX^Taq buffer (Mg^2+^ Free; Takara Biotech), 10 mM of each dNTP, 75 mM MgCl_2_, 10 μM of each primer, 1.5 U ^EX^Taq polymerase (Takara Biotech) and ~20–50 ng total genomic DNA. Amplifications were done as described (Pagès et al., 2010) with some modifications of annealing temperatures. Each round of PCR included one negative control to avoid contamination, and the negative control yielded no product. The PCR products were purified with a gel extraction kit (Sangon BioMedical), and directly sequenced (both directions) with an ABI 3730XL automatic sequencer (Perkin‐Elmer) using the ABI PRISM BigDye Terminator Cycle Sequencing Ready Reaction Kit (with AmpliTaq DNA polymerase FS, Applied Biosystems).

#### 2.2.2 Mitochondrial DNA data analyses

All mtDNA sequences (*COI*, D‐loop and *cyt b*) were separately edited, managed, and aligned with BioEdit 7.0.9.0 (Hall, 1999) and CLUSTAL W 1.83 (Thompson et al., 1994). For species identification, a barcoding NeighbourNet network was constructed including 486 *COI* sequences obtained in this study and 507 available *COI* sequences for all *Rattus* species downloaded from GenBank (Table S3). The network was established with a hypothesis‐poor algorithm implemented in SPLITSTREE V4.10 (Huson & Bryant, 2006) with default setting (*P* distance).

All haplotypes defined by 486 combined mitochondrial sequences were used in phylogenetic analyses. Sequences for *R. rattus* (EU273707) were used as an outgroup, with downloaded sequences for *R. norvegicus* (KM114605) and *R. tanezumi* (KF011916) used as references. A Bayesian method was used to construct phylogenetic trees. MRBAYES 3.2.7a (Ronquist & Huelsenbeck, 2003) was applied to perform Bayesian inference (BI), and jMODELTEST 2.1.7 (Darriba et al., 2012) was performed to determine appropriate parameters, based on hierarchical likelihood ratio test (hLRTs). For this, HKY + I + G was chosen as the Best‐fit model. Four Markov chains were run simultaneously; each chain was started from a random tree and run for 2 × 10^8^ cycles, with sampling every 1000 cycles. The first 5000 trees (25%) were discarded as burn‐in values. The entire procedure was repeated twice, starting with different random trees. It was noteworthy that the same tree topologies were obtained. Mean sequence divergences among major lineages were calculated using MEGA X (Kumar et al., 2018) and the pairwise Kimura 2‐parameter (K2P) model.

According to the results of species definition, diversity parameters (nucleotide diversity: *π*, haplotype diversity: *h*, Tajima's D, Fu's Fs) for each species were estimated based on combined mtDNA sequences of *COI*, D‐loop & *cyt b* with Arlequin V 3.5.2.2 (Excoffier & Lischer, 2010). Tajima's D and Fu's Fs were each calculated with 10,000 simulations.

### 2.3 Microsatellite DNA analyses

#### 2.3.1 Microsatellite genotyping

Ten microsatellite loci located on 10 chromosomes (R158, R8, R29, R203, R102, R7, R137, R36, R60, R145) were selected based on previous information (Hilbert et al., 1991; Serikawa et al., 1992). For each primer set, the forward primer was 5′‐fluorescent labeled with one of two dyes (6‐FAM or HEX). Amplifications were carried out in 10‐μl reaction volumes, using reagents, concentrations, and procedures for PCR reactions as described (Jing et al., 2014). Each round included one negative control, and the negative control yielded no product. Product sizes were estimated relative to an internal standard (GeneScan‐500 ROX) in polyacrylamide gels using an ABI PRISM 3730XL automated DNA sequencer, GENESCAN 3.1, and GENOTYPER 2.5 software (all from PerkinElmer Applied Biosystems).

#### 2.3.2 Microsatellite data analyses

Ten microsatellite loci were screened for null alleles and large allele dropouts using MICRO‐CHECKER 2.2.3 (Van Oosterhout et al., 2004). GENALEX 6.41 (Peakall & Smouse, 2006) was used to characterize genetic diversity (N*a*, N*e*, H*o*, and H*e*) for all loci at each site and for each population. GENEPOP 4.7.5 (Rousset, 2008) was used to test for deviations from the Hardy–Weinberg equilibrium (HWE). Each test was run for 10,000 dememorization steps, followed by 100 batches of 5000 steps each.

The relatedness of individuals within populations with *n* ≥ 5 (unrelated, half‐siblings, full‐siblings, and parent‐offspring) were estimated by maximum‐likelihood methods using ML‐Relate (Kalinowski et al., 2006).

The Bayesian inference‐based program STRUCTURE 2.3.4 (Hubisz et al., 2009; Pritchard et al., 2000) was used to detect clusters of similar genotypes in 28 populations with *n* ≥ 5. The population admixture model with default settings was used, and 20 runs were performed with K = 1–28 for each run (*K* was the user‐defined number of clusters). Each run consisted of 1 × 10^6^ Markov Chain‐Monte Carlo repetitions with a 100,000 repetitions burn‐in period. The ln Prob (data) for each run was recorded, and the ln Prob (data) across runs were averaged for each value of *K*. The *ΔK* metric (Evanno et al., 2005) was calculated to evaluate the optimal number of clusters within the data for each analysis. A threshold posterior probability (Q‐value) of .90 was employed to differentiate non‐admixed and admixed (potentially hybrid) rats, and individuals with a Q‐value < .90 were considered as a potential hybrid.

### 2.4 2b‐RAD analyses

#### 2.4.1 2b‐RAD sequencing procedures and bioinformatics analysis

To further explore the genetic structure, colonization and demographic history of two species with nuclear genome data, 142 individuals from 18 localities (Table S1) were selected based on the distribution information and structure results inferred from microsatellite data and were involved in subsequent 2b‐RAD analyses. The 2b‐RAD libraries were prepared as described (Wang et al., 2016). For each sample, 100–200 ng genomic DNA was digested with one unit of BsaXI (New England Biolabs, cat. no. R0609) in a 15 μl reaction at 37°C for 45 min. Then, 4 μl of digested DNA (~50 ng) was separated on a 1% agarose gel to verify the effectiveness of digestion. The ligation master mix (volume, 20 μl) contained 0.2 μM of each specific adaptor (five pairs of adaptors for every five samples), 0.5 mM ATP (New England Biolabs), 200 U T4 DNA ligase (New England Biolabs), 2 μl 10 × T4 ligase buffer, 5.9 μl nuclease‐free water, and 10 μl digestion product. Each reaction tube was incubated at 16°C for 1 h. Ligation products were amplified in 50 μl PCRs, comprised of 0.16 μM of each primer, 0.24 mM dNTP, 10 μl 5 × HF buffer, 0.8 U Phusion high‐fidelity DNA polymerase (New England BioLabs), 18.8 μl nuclease‐free water, and 18 μl ligation products. Then, the PCR was conducted in a MyCycler thermal cycler (Bio‐Rad) with 16 cycles of 98°C for 5 s, 60°C for 20 s, and 72°C for 10 s. Thereafter, 50 μl of each PCR product was placed on an 8% polyacrylamide gel, and the DNA was diffused into nuclease‐free water at 37°C for 30 min. For each tube, 12 μl of supernatant was used as a template, and the PCR steps described above were repeated for 4–6 PCR cycles to improve yield. Thereafter, PCR products from five samples were combined and the mixture was purified using a MinElute PCR Purification Kit. For this, 30 μl of digestion master mix containing 1 mM ATP, 3 μl 10 × CutSmart buffer, 2 U SapI (New England Biolabs), 10 μl purified mixed PCR product, and 13.8 μl nuclease‐free water was prepared and incubated at 37°C for 30 min. Digested product was added to the tube containing pretreated magnetic beads and incubated at room temperature. Then, a magnet was applied, the supernatant transferred to a new tube, 200 U T4 DNA ligase added to the supernatant, and the mixture incubated at 16°C for 45 min, followed by gel purification as described above. Barcodes were introduced by PCR with barcode‐bearing primers, and PCR products were purified using a MinElute PCR Purification Kit and pooled for sequencing using an Illumina PE sequencing platform. Library preparation, sequencing, and processing of raw reads were done by Qingdao OE Biotech Co., Ltd.

Raw reads were processed using a custom Perl script to trim adaptor sequences. The terminal 3‐bp positions were excluded from each read to eliminate artifacts that may have arisen from ligation sites. Reads with ambiguous bases (N), long homopolymer regions (˃10 bp), poor quality (15% nucleotide positions with a Phred quality ˂ 30), or without restriction sites were removed. A total of 1,404,872 tags with BsaXI digestion site were selected from the reference genome RGSC 5.0 and used as reference sequences. Sequencing reads with high quality were mapped to reference sequences with SOAP2 (Li et al., 2009), and unique tags were selected. Then, SNP genotyping was performed with the RAD typing program (Fu et al., 2013). Only tags with SNP number ≤ 3 and with sequence size <500 bp were retained, and only loci with two alleles were retained. The missing rates of loci in each sample were calculated, and individuals with missing rate > 30% were removed. Finally, SNPs of remaining samples were re‐genotyped.

The GENEPOP V4.7.5 (Rousset, 2008) was used to calculate deviations from the Hardy–Weinberg equilibrium (*p* value), the expected heterozygosity (He), the observed heterozygosity (Ho), polymorphism information content (PIC), effective number of alleles (Ne), and the fixation index (*F*st) of each SNP.

#### 2.4.2 Phylogenetic and population structure analysis

The neighbor‐joining tree was constructed with TreeBeST 1.9.2 (Vilella et al., 2009) under the *p*‐distances model, with bootstrapping (1000). The software MEGA X (Kumar et al., 2018) was used to visualize the phylogenetic tree. Population‐genetic clusters were determined using ADMIXTURE 1.3.0 (Alexander et al., 2009) with K = 1–18; analyses were repeated 10 times with 10 different seeds. The optimal K value was selected based on CV (cross‐validation error). Principal component analysis (PCA) of the SNPs was performed with Plink V 1.9066.21 (Purcell et al., 2007).

#### 2.4.3 D‐statistic

We used a D‐statistic approach (“ABBA‐BABA test”) to distinguish gene flow from incomplete lineage sorting and to estimate gene flow in a four‐taxon framework (Durand et al., 2011). The D‐statistic is used to detect gene flow between an inner‐group P1/P2 and a third inner‐group P3. We set various individuals of *R. tanezumi* (or *R. norvegicus*) as P1 and P2, *R. norvegicus* (or *R. tanezumi*) as P3, and *R. nitidus* as the outgroup. All D‐statistics were computed using Dsuite (Malinsky et al., 2021).

#### 2.4.4 Estimation of population genomic parameters

Identical scores (ISs) were calculated as described (Ai et al., 2015) to evaluate similarities of sequenced genomes to the reference genome. The estimation was carried out with 1 M non‐overlapping windows. The ISs of each window in all chromosomes of each sample were generated and transformed to an ISs heatmap. The pi (intra‐species nucleotide diversity), Tajima's D, and the interspecies' fixation index (*F*_*ST*_) and Dxy (inter‐species nucleotide divergence) were estimated using VCFtools V 0.1.16 (Danecek et al., 2011). The pi and Tajima's D were estimated with 100 kb non‐overlapping windows along each chromosome, and distribution maps of pi and Tajima's D were drawn with Circus 0.69.6 (Krzywinski et al., 2009). Interspecies differentiation analyses (*F*_*ST*_ and Dxy) were done with 500‐kb sliding windows with 100‐kb stepping.

#### 2.4.5 Linkage disequilibrium decay analysis

Linkage disequilibrium (LD) decay of two species was estimated using PopLDdecay (https://github.com/BGI‐shenzhen/PopLDdecay). Loci without polymorphism were removed from analyses. The correlation coefficient (*r* ^2^) between any two SNPs was calculated, and the average *r* ^2^ with 300 kb windows was plotted against physical distance with R script.

#### 2.4.6 Demographic inference

Demographic histories of *R. norvegicus* and *R. tanezumi* were inferred with Stairway Plot 2 (Liu & Fu, 2020) based on the composite likelihood of SNP frequency spectrum (SFS). This method uses the expected number of mutation(s) per base pair to measure time and uses θ per base pair to measure population size (θ = 4Neμ, where Ne is the effective population size and μ is the mutation rate per generation). We used μ = 2.96 × 10^−9^ per base pair per generation (Deinum et al., 2015; Ness et al., 2012) and a generation time of 0.5 years. All SNPs with no missing data (by population) were used in the SFS calculations, due to the difficulty of integrating missing data when modeling SFS under coalescent approaches. Folded SFSs for each species were generated by the vcf2sfs R script (Liu et al., 2018).

## 3 RESULTS

### 3.1 Genetic structure based on mitochondrial markers

The *COI* sequences were obtained from all 486 samples. A total of 57 haplotypes were defined (for these haplotypes, GenBank accession numbers were OP164163–OP164173 and OP164266–OP164309). Based on the barcoding unrooted Neighbor‐joining network tree, all *COI* haplotypes from our samples were clustered into two groups and were nested with reference sequences of *R. tanezumi* or *R. norvegicus* (Figure S1).

A total of 139 haplotypes were defined from 486 combined sequences of *COI*, *cyt b*, & D‐loop (Tables S1 and S4). The sequences of *cyt b* and D‐loop also have been deposited in GenBank (accession numbers were OP149390–OP149528 for *cyt b* and OP149620–OP149758 for D‐loop).

In a phylogenetic tree constructed with combined sequences for 139 haplotypes, all haplotypes were apparently clustered into two monophyletic lineages (the mean K2P distance between them was 0.147; Figure S2). The reference sequence of *R. tanezumi* (KF011916) from GenBank and haplotypes T1–T36 (T = *tanezumi*) from 15 localities (TS, RG, YM, MY, CD, HS, JW, CS, LD, KL, JO, ZP, KM, GZ, and LS) clustered into one lineage. In this lineage, haplotype T17 from GZ formed a subclade; the other subclade comprised two branches, and haplotypes from the southeast coast of China (GZ, RG) diverged firstly in each branch. The reference sequence of *R. norvegicus* (KM114605) from GenBank and haplotypes N1 ‐ N103 (N = *norvegicus*) from 24 localities (MH, MS, SY, MJ, SL, CY, DL, UQ, BT, YP, QX, LI, HZ, MY, CD, HS, JJ, JW, LZ, CS, JO, GZ, ZJ, and KM) grouped into the other lineage with two sublineages. Some haplotypes from northeast China (CY, DL, MJ, MS, MH, SL, and SY) clustered into one subclade; in the second subclade, some haplotypes from CD, GZ, JJ, KM, LZ, MY, and ZJ grouped into one branch, and the remaining haplotypes from various localities grouped into several sub‐branches in the other branch.

The phylogenetic analyses with mtDNA markers confirmed the identification results based on external characteristics that 486 rat samples contained 306 individuals of *R. norvegicus* from 24 localities representing most areas of China except Tibet. Furthermore, 180 individuals of *R. tanezumi* were distributed in 15 localities representing most areas of southern China and a part of northern China (Figure 2; Figure S2; Tables S1 and S4). Among 31 localities, eight localities (MY, CD, HS, JW, CS, JO, KM, and GZ) contained individuals of both species (Figure 2; Figure S2; Table S1). For *R. tanezumi*, three mitochondrial haplotypes (T6, T9, and T29) were shared by 12, three and four populations, respectively. For *R. norvegicus*, 14 haplotypes (N1, N15, N25, N29, N42, N45, N50, N61, N65, N67, N72, N75, N98, and N100) were shared by two to four populations (Figure S2, Tables S1 and S4).

Based on combined mtDNA sequences, haplotype diversity (*h*) was 0.9763 ± 0.0027 (*R. norvegicus*) and 0.8091 ± 0.0276 (*R. tanezumi*), nucleotide diversity (π) was 0.0089 ± 0.0041 (*R. norvegicus*) and 0.0052 ± 0.0023 (*R. tanezumi*; Table S5). The values of Tajima's D and Fu's Fs for the two species were all negative; however, the *p* value for Fu's Fs of *R. tanezumi* was >.05.

### 3.2 Variations in microsatellites and population structure

Microsatellite genotyping was successful in 462 individuals (average genotyping success for the 10 microsatellite loci was >95%). Alleles per locus ranged from 6.000 to 10.214 (Table S6). The values of H*o* (0.586–0.813, mean 0.652) were slightly smaller than those of H*e* (0.619–0.981, mean 0.707). The F*st* values ranged from 0.133 to 0.247, indicating a high level of genetic divergence. The excess test for deviations of each locus from Hardy–Weinberg equilibrium (HWE) did not deviate from Hardy–Weinberg proportions for all samples (P ≈ 1).

Ratios of unrelated individuals in 28 populations (with *n* ≥ 5) ranged from 86.10% to 100% (mean 94.92%; Table S7); therefore, all results based on these samples were persuasive.

**FIGURE 3:**
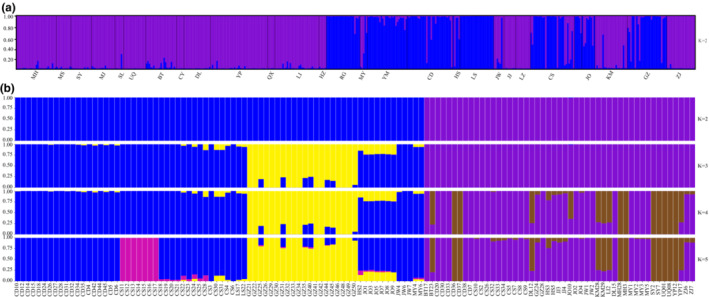
Genetic structure of *Rattus norvegicus* and *Rattus tanezumi*. (a) Genetic structure of 486 individuals inferred from 10 microsatellite loci data (K = 2). Red and blue represent *R. norvegicus* and *R. tanezumi*, respectively. (b) Genetic structure of 123 individuals based on 2b‐RAD sequences (K = 2–5). When K = 2, red and blue represent *R. norvegicus* and *R. tanezumi*, respectively. Locality abbreviations correspond to those in Figure 2 and Table S1.

Based on STRUCTURE analyses, microsatellite data had a maximum log likelihood of posterior probability at K = 2 [lnp(X/K) = 22.21], and the *ΔK* value was maximum (91.57) at K = 2 (Figure S3a), which suggested that two genetic clusters were defined with the data (Figure 3a). In addition, STRUCTURE patterns indicated that some individuals from 16 populations (GZ, KM, JO, LD, CS, JW, HS, CD, YM, MY, RG, LI, YP, BT, SL, and MH) contained microsatellite sequences that originated from both species.

The Q‐values for each individual and each population were estimated based on microsatellite data. In total, 43 individuals from 16 populations had a Q‐value < 0.9. Furthermore, the Q‐values of 8 populations (GZ, JO, CS, JW, HS, MY, BT, and SL) were <0.9 (Table S8).

### 3.3 Genetic structure based on 2b‐RAD

#### 3.3.1 RAD‐seq data output

A total of 123 samples were retained in quality control, and the statistical data for 2b‐RAD sequencing quality are shown in Table S9. These samples generated ~3694.47 × 10^6^ reads with BsaXI digestion site (range, 12.30–43.18 × 10^6^ reads). An average of 720,436 unique tags for each sample were obtained (range, 450,988–995,213). The average level of sequencing depth was 26.76 for each sample (range, 12.55–39.80), and the average mapping rate was 64.12% (range, 55.91%–76.11%). Ultimately, 187,483 SNPs were defined in 123 samples. Genetic parameters of each SNP are shown (Table S10).

#### 3.3.2 Population genetic structure

**FIGURE 4:**
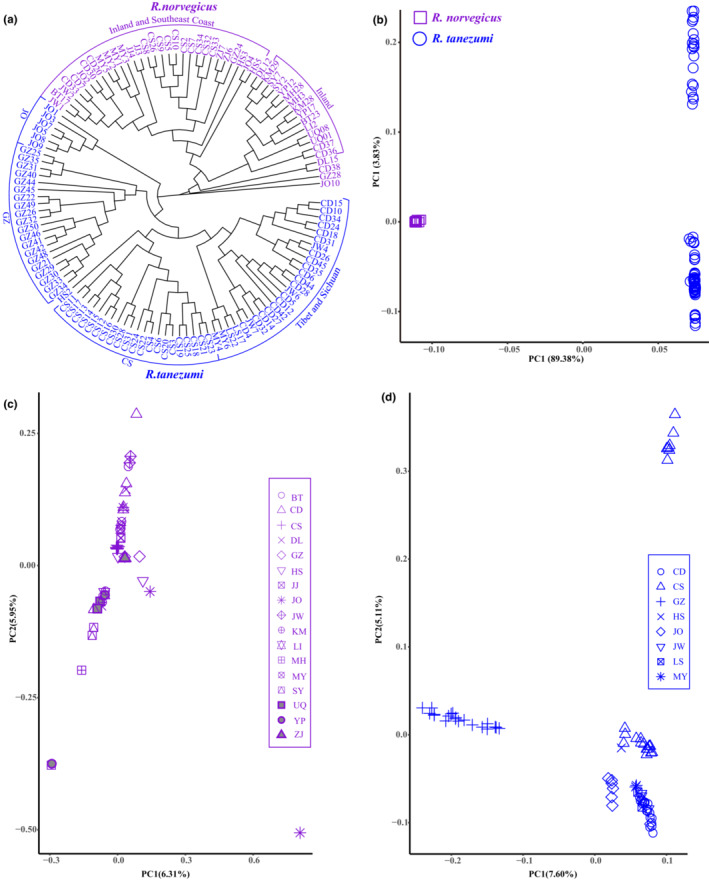
The phylogenetic tree and principal component analyses of *Rattus norvegicus* and *Rattus tanezumi* inferred from 2b‐RAD sequences. (a) Neighbor‐joining tree including 123 samples from 18 localities. (b) Principal component analysis (PCA) of the first two components including 123 samples. (c) Principal component analysis of the first two components including 49 individuals of *R. norvegicus* from 17 localities. (d) Principal component analysis of the first two components including 74 individuals of *R. tanezumi* from 8 localities.

The NJ tree including 123 sequenced individuals featured two lineages (Figure 4a). Combining the distribution of mitochondrial haplotypes (Table S4; Figure S2), the lineage including 49 samples from 17 localities (BT, CS, DL, GZ, HS, JJ, JO, JW, KM, LI, MH, MY, SC, SY, UQ, YP, ZJ) represented to *R. norvegicus*, whereas the other lineage comprising 74 samples from eight localities (CS, GZ, HS, JO, JW, LS, MY, SC) represented to *R. tanezumi*. In the *R. norvegicus* lineage, except for four individuals from DL, CD, GZ, and JO, other 45 individuals were grouped into two clusters; one cluster comprised individuals from DL and eight inland populations (MH, SY, YP, BT, UQ, LI, CD, and KM), whereas the other cluster included individuals from southeast coast populations (JO, GZ, ZJ) and seven inland population (CS, HS, JJ, MY, CD, JW, and BT). In the *R. tanezumi* lineage, 74 individuals from Fujian province (JO), Guangdong province (GZ), Hunan province (CS), Hubei province (HS), and Sichang Province (CD, MY, JW) were grouped into geographic‐specific branches, respectively. Individuals from LS (Tibet) had a close relationship with individuals from Sichang Province (MY).

In ADMIXTURE analyses including all 123 samples, the optimal value of parameter K was K = 2 (Figure S3b), indicating two distinct genetic clusters. However, the population structure inferred from 2b‐RAD data was different from that inferred from microsatellite data (Figure 3a), and no apparent genetic admixture between two clusters was detected at the genome‐wide level (Figure 3b). Species‐specific ADMIXTURE analyses were also carried out, and the optimal value of parameter K was K = 1 for each species. In order to show the genetic structure within species, the results with K = 2 ~ 5 were presented (Figure S4). For *R. norvegicus*, at K = 2, one individuals from DL and 14 individuals from eight inland populations (MH, SY, YP, BT, UQ, LI, CD, and KM) were classified into one cluster (purple); one individuals from DL, four individuals from southeast coast populations (JO and GZ) and 22 individuals from five inland population (CS, MY, CD, JW and BT) were classified into another cluster (gray); seven samples from four populations (GZ, HS, JJ and ZJ) showed genetic mixture of two clusters (Figure S4a). For *R. tanezumi*, at K = 2–5, individuals from GZ were always classified into one distinct cluster (blue; Figure S4b). With the increase of K value, individuals from CS and JO formed specific clusters and some samples from CS, GZ, HS, LS, and MY showed genetic mixture among different clusters.

In the principal components analysis (PCA), all 123 samples were clearly differentiated in two distinct plots, corresponding to *R. norvegicus* and *R. tanezumi*, along with principal component 1 (PC1, 89.38% of variance; Figure 4b). Furthermore, PC2 (3.83% of variance) and PC3 (2.61% of variance) corresponded to variation within *R. tanezumi* (Figure S5a,b). In species‐specific PCA analyses, most individuals of *R. norvegicus* and *R. tanezumi* were differentiated along all three principal components (Figure 4c,d; Figure S5c–f). Based on the differentiation along PC1 (6.31% of variance) and PC2 (5.95% of variance), two plots were generally detected in *R. norvegicus*: samples from DL and eight inland populations (MH, SY, DL, YP, BT, UQ, LI, CD, and KM) were scattered in one plot, some individuals from DL, southeast coast populations (GZ, JO, ZJ) and seven inland populations (HS, CS, JJ, CD, MY, JW, and BT) were scattered in the other plot (Figure 4c). The PCA results were broadly consistent with the clustering results in species‐specific ADMIXTURE analyses with K = 2 (Figure S4a) and the results of NJ tree (Figure 4a). For *R. tanezumi*, based on the differentiation along PC1 (7.60% of variance) and PC2 (5.11% of variance), samples from different localities clustered into geographic‐specific plots, but seven individuals from CS differentiated greatly from other samples from CS along PC2 (Figure 4d). The PCA results were consistent with the clustering results in ADMIXTURE analyses for *R. tanezumi* with K = 5 (Figure S4b) and the results of NJ tree (Figure 4a).

Based on D‐statistical analyses, the Z scores of D values for all individuals were greatly smaller than three (Max: 0.308953), and all *p* values were >.05 (Min: 0.050006; Table S11).

#### 3.3.3 Genetic diversity and genetic differentiation between two species

**FIGURE 5:**
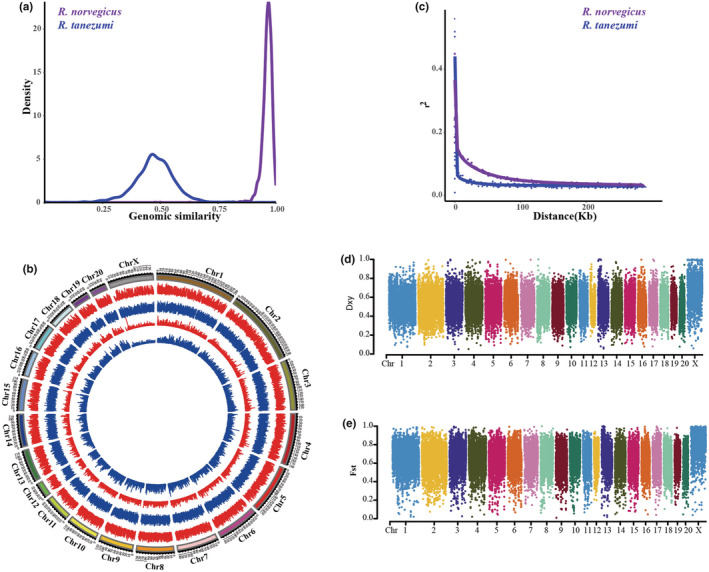
Genomic variation in *Rattus norvegicus* and *Rattus tanezumi*. (a) Genomic similarity of *R. norvegicus* and *R. tanezumi* to the RGSC 5.0 reference genome. (b) Genome‐wide distribution of nucleotide diversity, Tajima's D value in *R. norvegicus* and *R. tanezumi*. The two circles adjacent to the karyotypes show lines representing Tajima's D value in *R. norvegicus* (red) and *R. tanezumi* (blue). The inner two circles show lines representing nucleotide diversity in *R. norvegicus* (red) and *R. tanezumi* (blue). (c) Decay of linkage disequilibrium of *R. norvegicus* and *R. tanezumi* measured by *r* ^2^. (d) Mean pairwise sequence divergence (dxy) between *R. norvegicus* and *R. tanezumi*. (e) Population differentiation (Fst) between *R. norvegicus* and *R. tanezumi*. D and E are shown for SNPs within each 100‐kb windows.

Since the population genetic structure analyses based on 2b‐RAD data did not support apparent gene flow between *R. norvegicus* and *R. tanezumi*, both genetic diversity of the two species and genetic differentiation between them were calculated. Among 187,483 SNPs identified in 123 individuals with RGSC 5.0 as reference genome, 142,809 SNPs were specific for *R. tanezumi*, 19,553 SNPs were specific for *R. norvegicus*, and 25,121 SNPs were shared by two species. Identity score analyses clearly illustrated that the mean genomic similarity between *R. norvegicus* and the reference genome was 0.96190, whereas mean genomic similarity between *R. tanezumi* and reference genome was only 0.47539 (Figure 5a; Figure S6). Nucleotide diversity of *R. tanezumi* exceeded that of *R. norvegicus* in both autosomes (mean: 0.0092/0.0025) and X chromosome (mean: 0.0028/0.0010; Figure 5b). The pairwise linkage disequilibrium (LD) between polymorphic sites for all autosome regions in each species was calculated; the correlation coefficient of LD in *R. tanezumi* decayed more rapidly than those in *R. norvegicus* (Figure 5c). The mean pairwise sequence divergences (Dxy) between *R. tanezumi* and *R. norvegicus* were 0.5181 in autosomes and 0.6588 in the X chromosome (Figure 5d; Table S12). The inter‐species' fixation index (*Fst*) was 0.6447 in autosomes and 0.7892 in the X chromosome (Figure 5e; Table S12).

#### 3.3.4 Demographic history of the two species

Based on the 2b‐RAD sequences, the average Tajimas' D values of *R. norvegicus* were negative in both autosomes (mean: −0.2869) and the X chromosome (mean: −0.4640; Figure 5b; Table S5). However, the average Tajimas' D values of *R. tanezumi* were positive in both autosomes (mean: 0.7205) and the X chromosome (mean: 0.2244), which did not support a standard population expansion event.

**FIGURE 6:**
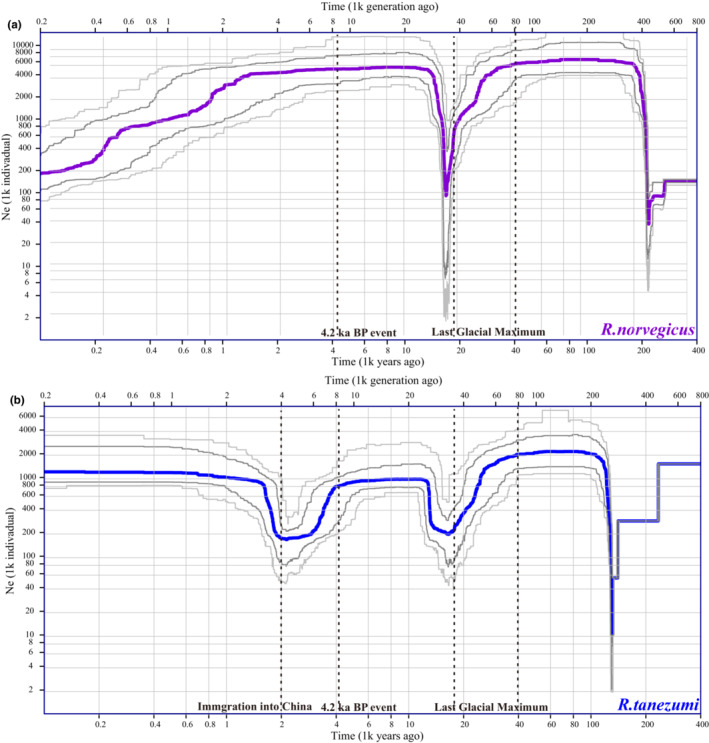
Estimation of the variation of effective population size through time for *Rattus norvegicus* (a) and *Rattus tanezumi* (b) using stairway plot 2. The purple and blue thick lines represent the median effective population sizes, and the dark and gray lines represent the 95% and 75% confidence intervals, respectively. The climate change events and migration event that greatly influenced the population sizes of two species were indicated by gray vertical dotted lines.

The demographic history of each species was inferred through Stairway Plot 2, using 44,674 SNPs (*R. norvegicus*) and 167,930 SNPs (*R. tanezumi*), respectively. The results presented confidently the population size changes within the last 100,000 years (Figure 6). From 100,000 to 60,000 years ago, the *Ne* of *R. norvegicus* was ~6 × 10^6^, and the *Ne* of *R. tanezumi* was ~2 × 10^6^. Both species experienced a severe bottleneck at ~40,000 to 18,000 years ago, followed by a period of rapid population growth from 18,000 to ~13,000 years ago. Thereafter, *R. norvegicus* experienced a gradual decline since ~4000 years ago, and the decline began to accelerate since ~1500 years ago. *R. tanezumi* underwent a second bottleneck from ~4000 to 2200 years ago, followed by a period of rapid population growth from 2200 to ~1500 years ago. At present, the *Ne* of *R. tanezumi* (~1.2 × 10^6^) is significantly greater than that of *R. norvegicus* (~2 × 10^5^) in China.

## 4 DISCUSSION

### 4.1 Species origin and migration history shaped the extant distribution patterns and population structures of *R. norvegicus* and *R. tanezumi* in China

Expansion histories of *R. norvegicus* and *R. tanezumi* in China (this country's two dominant species of house rat) have not been thoroughly explored. In the present study of 486 Chinese rats, there were 306 individuals of *R. norvegicus* from 24 localities representing most of China except Tibet. The other 180 individuals belonging to *R. tanezumi* were distributed in 15 localities representing most areas of southern China and a part of northern China (Figure 2; Figures S1 and S2; Tables S1 and S4). The distribution patterns of the two species were consistent with previous records (Chen et al., 2021; Guo et al., 2019; Wang, 2003).

It was believed that *R. tanezumi* originated in southeast Asian (Aplin et al., 2011; Chen et al., 2021; Guo et al., 2019). Based on mitochondrial DNA markers and several microsatellite loci, *R. tanezumi* immigrated into southeast coast of China by shipping transport and subsequently expanded to central China (Guo et al., 2019). In our phylogenetic tree including 36 mitochondrial haplotypes from 180 individuals of *R. tanezumi*, haplotype T17 from southeast coast (GZ) formed a specific subclade. Furthermore, haplotypes from southeast coast (GZ, RG) diverged firstly in both branches of the other subclade (Figure S2). In the NJ tree based on 2b‐RAD data, all individuals grouped into geographic‐specific branches, and populations from JO and GZ were ancestral branches (Figure 4a). Therefore, we inferred that the southeast coast was the early location of *R. tanezumi* in China. Extensively shared mitochondrial haplotypes (T6, T9 and T29) provided additional evidence for population expansion and colonization (Figure S2, Table S4). In addition, PCA (Figure 4d; Figure S5e,f) and ADMIXTURE analyses (Figure S4b, K = 5) indicated that, during colonization, populations settled in different regions had undergone specific differentiation, and there was gene flow between different geographic populations.

In phylogenetic tree including 103 mitochondrial haplotypes from 306 individuals of *R. norvegicus*, some haplotypes from northeast China (CY, DL, MH, MJ, MS, SL, SY) formed an ancestral subclade, other haplotypes from northeast China scattered in different branches of the other subclade (Figure S2), which supported a previous conclusion that northern Asia was the ancestral range for *R. norvegicus* (Puckett et al., 2016; Puckett & Munshi‐South, 2019). In the NJ tree (Figure 4a) and PCA (Figure 4c) based on 2b‐RAD data, 49 individuals of *R. norvegicus* from 17 localities were generally gathered into two clusters (inland populations; southeast coast and inland populations) instead of geographic‐specific groups. The ADMIXTURE analyses also got similar clustering results (Figure S4a, K = 2). Furthermore, the four individuals (CD 38, DL15, GZ28, and JO10) that diverged firstly in the NJ tree (Figure 4a) belonged to cluster comprising southeast coast and inland populations in PCA (Figure 4c) and ADMIXTURE analyses (Figure S4a, K = 2). These results indicated that the spread history of *R. norvegicus* in China, probably more complex than sole on‐land colonization mode, maybe shipping transportation and also have played great roles. The cluster comprising individuals from MH, SY, DL, YP, BT, UQ, LI, CD, and KM indicated the westward and southward expansion of *R. norvegicus* on land, whereas the cluster including individuals from DL, JO, GZ, ZJ, CS, HS, JJ, MY, CD, JW, and BT indicated a new migration route, comprising shipping transport from important port‐city of northeast China (DL) to the southeast coast (GZ, JO, ZJ) and subsequent on‐land expansion to central China (Figure 2). Populations from two spread routes converged, and gene flow between individuals from the two routes occurred (Figure 3b, k > 3; Figure S4a, K = 2).

Natural immigration of *R. tanezumi* into China through Yunnan province was blocked by high mountains and valleys (Guo et al., 2019). Similarly, the Gobi desert in north China and the mountains in northeast China hindered the natural southward and westward migration of *R. norvegicus* from natal regions. Moreover, given their large body size and substantial demand for food, rats needed relatively large transport vehicles to cross substantial geographic barriers (sea, desert, plateau, etc.) during colonization. It was proposed that the expansion of *R. tanezumi* in West Pacific was facilitated by shipping transportation of Austronesian speakers during their diaspora about 4000 years ago (Aplin et al., 2011). The dispersal of *R. norvegicus* to America, the Caribbean, West Africa, and Australasia from European was also attributed to ocean shipping transportation during the age of European imperialism at 1600s–1800s (Puckett et al., 2016). These supported the possible contribution of shipping transportation in colonization history of *R. tanezumi* and *R. norvegicus* in China. Since the Spring and Autumn periods and the Warring States period (770 BC ‐ 221 BC), shipping transportation along coast of China has emerged (Feng, 1981). Since Nanyue Kingdom period (204 BC–111 BC, with Guangdong as the center), the southeast coastal cities of China have had extensive maritime trade with southeast Asia (Peng, 2004). Ancient DNA investigation also suggested that many farmers from south China immigrated into various countries of southeast Asian during that period (Lipson et al., 2018). The development of Chinese coastal shipping probably facilitated the spread of *R. norvegicus* along the southeast coast from northeast China and the immigration of *R. tanezumi*, but the precise time of the colonization events need further investigation.

Both the Ancient Tea Horse Road (connecting southwest region and Tibet of China to Southeast Asia and South Asia) and the Land Silk Road (connecting central and northwest China to central and West Asia) began in the Western Han Dynasty (202 BC), but the transportation using horses or camels did not promote the spread of house rats in China. There were no house rats in Xinjiang and Tibet plateau before the 1960s (Yu et al., 1980; Zhang et al., 2000). Currently, *R. norvegicus* is the dominant house rat species in Xinjiang, whereas *R. tanezumi* is the dominant house rat species in the Tibet plateau (Figure 2; Table S1). Fifteen individuals of *R. norvegicus* from Xinjiang (UQ) defined only one mitochondrial haplotype (N4), and 23 individuals of *R. tanezumi* from Tibet plateau (LS) defined only two mitochondrial haplotypes (T4 and T6; Tables S1 and S4), which supported their short colonization histories in local regions. Based on phylogenetic analyses, mitochondrial haplotype of *R. norvegicus* in Xinjiang was closely related to haplotype from DL (Figure S2), whereas *R. tanezumi* in Tibet plateau had close relationships with individuals from Sichuan province (CD and MY; Figure 4a; Figure S2). Our results indicated that the invasion of *R. norvegicus* into Xinjiang was concurrent with development of railway transportation connecting north China and northwest China, which were consisted with the record of species survey (Yu et al., 1980). The results also suggested that the expansion of *R. tanezumi* into the Tibet plateau was in accordance with development of highway transportation connecting Chengdu and Lhasa (Sichuan‐Tibet Highway) during the last half‐century.

### 4.2 The demographic histories of the two species were closely correlated to climate change, migration history and interspecific competition

Ancestral populations of *R. tanezumi* were distributed from Southeast Asia to Yunnan of China (Guo et al., 2019), whereas ancestral populations of *R. norvegicus* were distributed from Inner Mongolia to northern China (Allen, 1940; Puckett et al., 2020; Silver, 1941; Wilson & Reeder, 2005). Before large‐scale expansion with human shipping transportations, the demographic histories of the two species were closely related to climate changes. Analyses based on SFS suggested that both species experienced a severe bottleneck event ~40,000 to 18,000 years ago (Figure 6), apparently consistent with the period of Last Glacial Maximum (LGM). During this event, the *Ne* of the two species decreased dramatically (*R. norvegicus*: from 6 × 10^6^ to 9 × 10^4^, *R. tanezumi*: from 2 × 10^6^ to 2 × 10^5^). Then, both species underwent rapid population recovery during the subsequent 5000 years, although neither *Ne* reached the initial level.

Before 4000 years ago, *R. tanezumi* began to undergo a second bottleneck event (Figure 6b), consistent with abrupt climate changes at that period. The “4.2 ka BP abrupt climatic change event” characterized by extreme drought had led to the collapse of civilization in southwest Asia (Ön et al., 2021; Weiss et al., 1993). At that period, the climate in China was also dry and cold, which intensified expansion of deserts and a decline in vegetation (Li et al., 2014). Correspondingly, populations of *R. norvegicus* also began to decline (Figure 6a). Approximately 2200 to 1500 years ago, the population of *R. tanezumi* expanded rapidly, the time was highly congruent with the maritime trade between southeast coast of China and southeast Asia at Nanyue Kingdom period (Peng, 2004). So, *R. tanezumi* likely immigrated into China about 2200 years ago.

With the population expansion of *R. tanezumi*, the population decline of *R. norvegicus* started to accelerate ~1500 years ago, which indicated that interspecies competition had an important role in population changes of two species in the last 1500 years. Although it is uncertain which species entered southern China earlier, according to their distribution patterns, *R. tanezumi* and *R. norvegicus* met in southern China and began interspecies competition during invasion of the new entrants. As a species more adapted to tropical and subtropical climates, *R. tanezumi* had greater advantages during invasion. It was suggested that *R. tanezumi* had evolved to resist commonly used rodenticides (Andru et al., 2013; Huang et al., 2012; Teng et al., 2016) and that nonphysical/chronic interspecific interactions (chemical stimuli) may have contributed to the invasion of *R. tanezumi* into the range of *R. norvegicus* (Guo et al., 2017). In addition, global warming is promoting the continued northward invasion of *R. tanezumi* in China, which contributes to the continuous increase of its *Ne* (Figure 6b). Both current results (Figure 2; Table S1) and previous studies (Chen et al., 2021; Zhang et al., 2000) indicated that *R. tanezumi* has established stable populations in Gansu (TS), Shanxi, Hebei, and Tianjin during recent decades.

Migration and demographic histories have shaped the present genetic diversity of the two species in China. Ancestral populations often have the highest genetic diversity, whereas biological invasions are often associated with a reduction in genetic diversity in colonizing populations (Dlugosch & Parker, 2008). Therefore, it is easy to understand that the genetic diversity of mitochondrial DNA for *R. norvegicus* (h: 0.9763 ± 0.0027, π: 0.0089 ± 0.0041) was greater than that of *R. tanezumi* (h: 0.8091 ± 0.0276, π: 0.0052 ± 0.0023; Table S5). The nucleotide diversity of nuclear genome for *R. norvegicus* was ~0.0025 in autosomes and 0.0010 in X chromosome (Figure 5b), similar to results inferred from whole‐genome sequences (Teng et al., 2017). Nucleotide diversity of the nuclear genome for *R. tanezumi* was 0.0092 in autosomes and 0.0028 in X chromosome (Figure 5b). The rapid population decline of *R. norvegicus* in the past 1500 years must have decreased its genomic polymorphism, causing the comparative low genomic polymorphism in *R. norvegicus* compared to the Himalayan field rat (*R. nitidus*; Teng et al., 2017) and *R. tanezumi*. The population decline also was possible reason for the absence of extensively shared mitochondrial haplotypes among different populations. Conversely, continuous population expansion of *R. tanezumi* during the past 2200 years contributed to the comparatively high genomic polymorphism in this species.

### 4.3 Discordant results on population structure inferred from microsatellite markers and 2b‐RAD data

Based on previous studies, invasion of *R. tanezumi* in America was unsuccessful, the huge population of *R. rattus* was a great impediment to the invasion of *R. tanezumi*, and gene introgression was unidirectional from *R. rattus* into *R. tanezumi*, which ultimately caused complete loss of the nuclear genetic signal of *R. tanezumi* (Lack et al., 2012). However, in China, invasion of *R. tanezumi* was very successful, and it is still invading northward. Population structure analyses and subsequent Q‐value estimation based on 10 microsatellite loci from 462 individuals indicated that 43 individuals from 16 populations (GZ, KM, JO, LD, CS, JW, HS, CD, YM, MY, RG, LI, YP, BT, SL, and MH) contained microsatellite sequences originated from both species (Figure 3a; Table S8). Moreover, the niches of the two species are overlapped, and both species in China have a karyotype of 2n = 42 (Pang et al., 1994). All these raise the question whether it is possible that gene introgression also has an important role in interspecies competition between *R. tanezumi* and *R. norvegicus*? However, all results of further analyses with 2b‐RAD data from 123 individuals (NJ tree, Admixture, PCA, and D‐statistic) did not support gene flow between the two species (Figure 3b; Table S11). *R. norvegicus* and the lineage comprising *R. tanezumi* diverged ~3.5 million years ago (Aplin et al., 2011), and great genomic divergence between *R. tanezumi* and *R. norvegicus* (Figure 5a,d,e; Figure S6, Table S12) may be the key reason hindering interspecies gene flow. So, the successful invasion of *R. tanezumi* in China should be attributed to potential reasons discussed above rather than interspecies' gene introgression. The pattern of population structure inferred from microsatellite data may be a consequence of shared ancestral polymorphism or homoplasy in microsatellites between two species.

Compared to microsatellites, SNP loci are more evenly and densely distributed across the genome (Xing et al., 2005), yielding lower error rates in genotyping (Montgomery et al., 2005). In addition, SNPs have less homoplasy than microsatellites (Morin et al., 2004), making them fine molecular markers for many evolutionary analyses, including screening outlier loci (Hohenlohe et al., 2012), analyzing population structures, or selecting small regions of introgression in the genome (Hohenlohe et al., 2013). Moreover, the number of populations or the number of samples per population had no apparent impact on the robustness of results based on SNPs (Jeffries et al., 2016). In contrast, the high rates of homoplasy in microsatellites accumulating over a long interval (Van Oppen et al., 2000) make microsatellites unreliable and error prone in inferring phylogeography over long time scales (Cornuet et al., 2010). As microsatellite loci have been widely used to detect gene flow between species or subspecies, based on our results, previous investigations on hybridization based solely on microsatellite loci should be verified with more densely distributed SNPs.

## AUTHOR CONTRIBUTIONS

**Ling Huang:** Writing – original draft (equal); writing – review and editing (equal). **Jing Meidong:** Resources (equal); software (equal); writing – original draft (equal); writing – review and editing (equal). **Yingjie Chen:** Resources (equal); software (equal); writing – original draft (equal). **Keying Yao:** Software (supporting). **Youming Wang:** Software (supporting).

## FUNDING INFORMATION

This work was funded by grants from the National Natural Science Foundation of China (No. 31171189) and the Second Tibetan Plateau Scientific Expedition and Research Program (STEP) (2019QZKK05010303).

## CONFLICT OF INTEREST

The authors declare that they have no competing interests.

## Supporting information


Figure S1

Figure S2

Figure S3

Figure S4

Figure S5

Figure S6

Table S1

Table S2

Table S3

Table S4

Table S5

Table S6

Table S7

Table S8

Table S9

Table S10

Table S11

Table S12

## Data Availability

The 2b‐RAD reads data and microsatellite data are available at https://datadryad.org/stash/dataset/doi:10.5061/dryad.8gtht76s2. Mitochondrial DNA sequences are available at NCBI (*COI*: OP164163–OP164173 and OP164266–OP164309; *cyt b*: OP149390–OP149528; D‐loop: OP149620–OP149758).
