# Supplementary figures and images for: Comparative phylogeography of two commensal rat species (Rattus tanezumi and Rattus norvegicus) in China: Insights from mitochondrial DNA, microsatellite, and 2b‐RAD data
Source: Ecol Evol. 2022 Oct 13;12(10):e9409. doi: 10.1002/ece3.9409 (PMC9557235; doi:10.1002/ece3.9409)

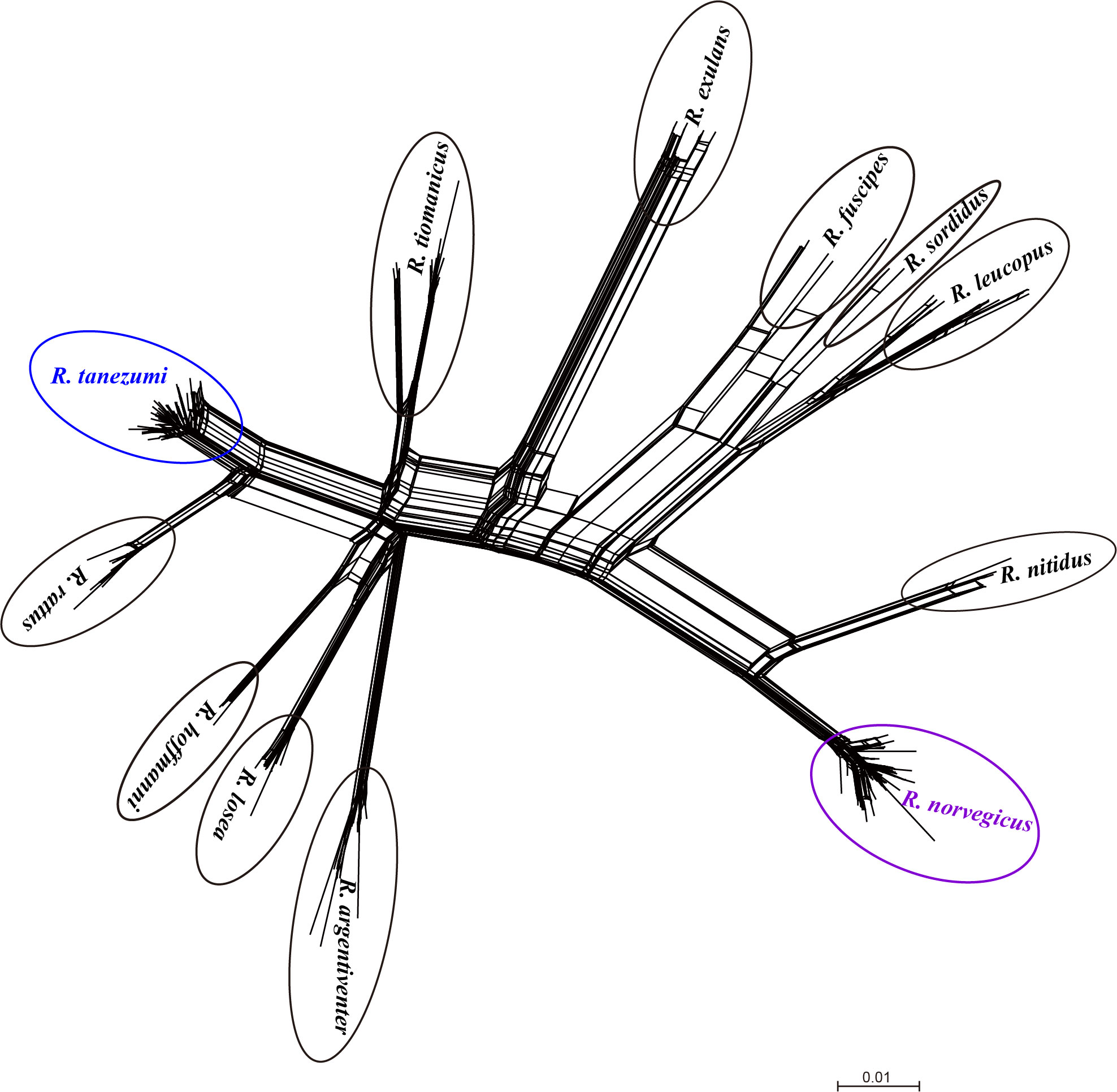

Supplement: Supplementary file 1 — Figure S1 [file ECE3-12-e9409-s015.jpg]

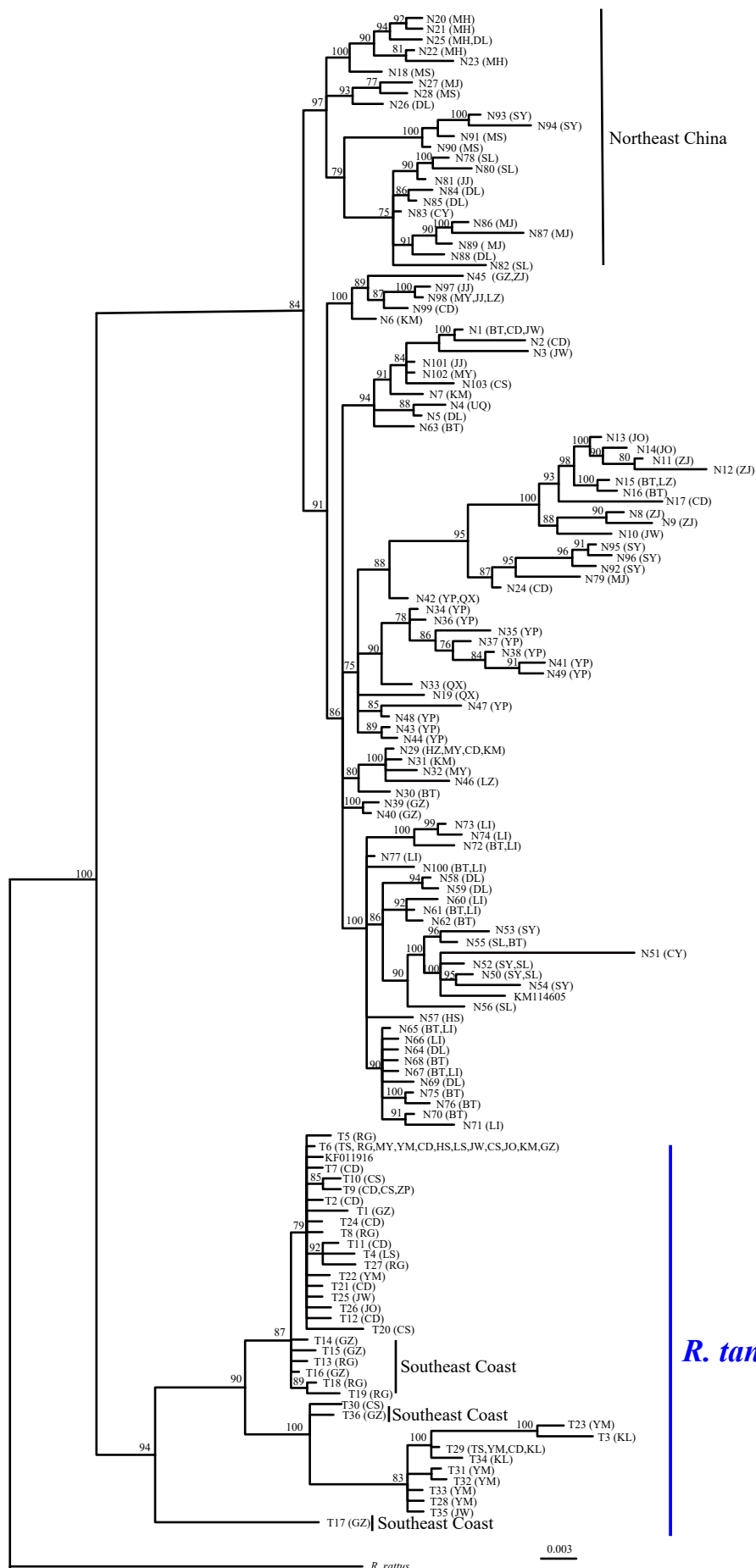

*R. norvegicus*

*R. tanezumi*

Supplement: Supplementary file 2 — Figure S2 [file ECE3-12-e9409-s003.pdf]

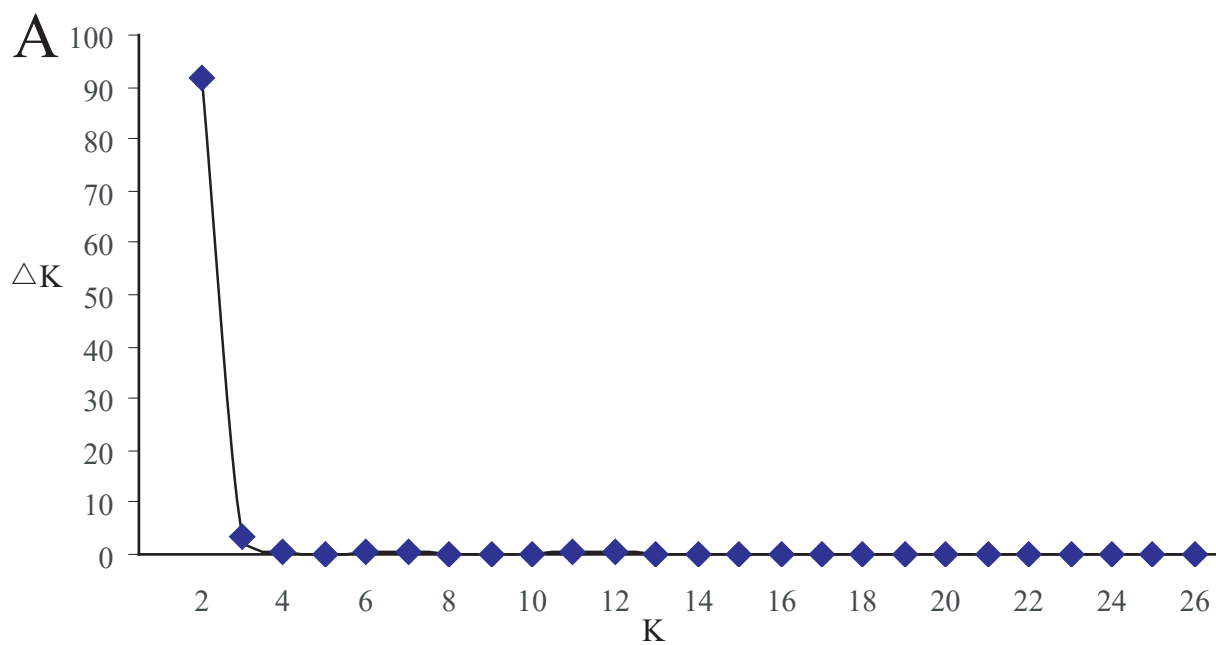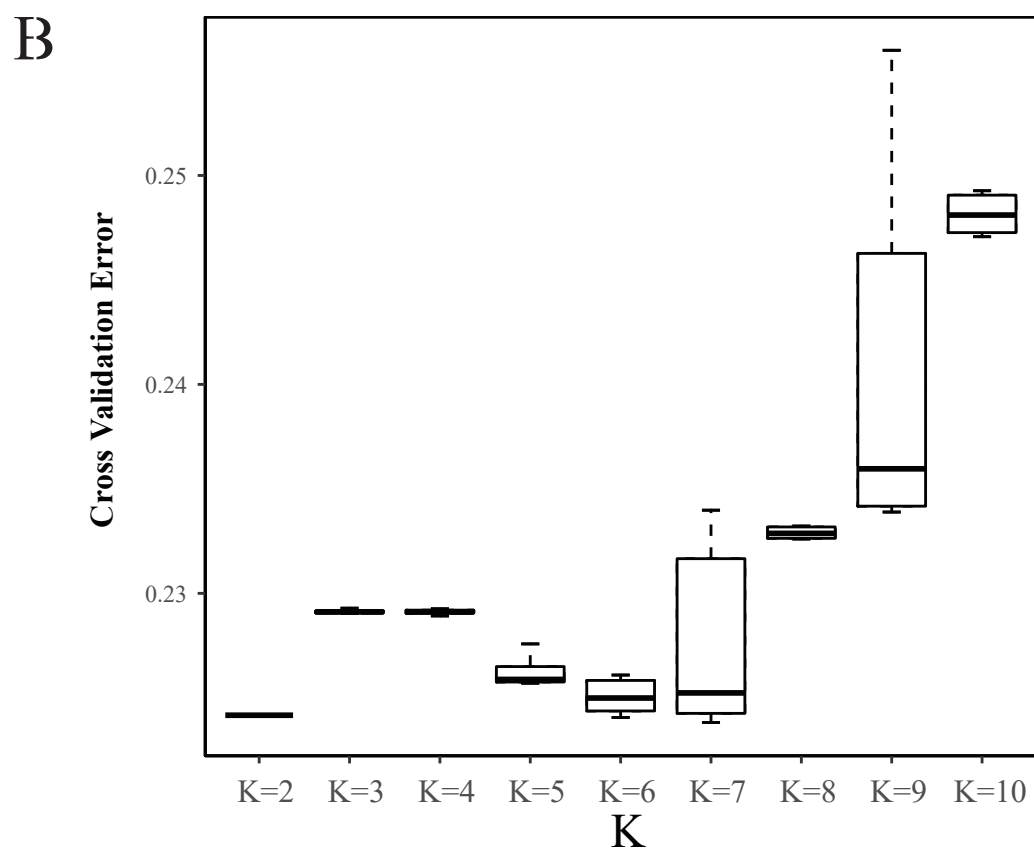

Supplement: Supplementary file 3 — Figure S3 [file ECE3-12-e9409-s002.pdf]

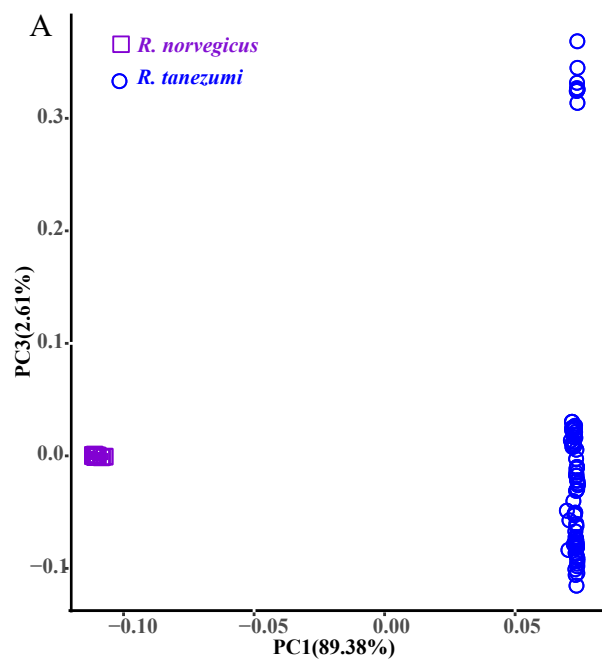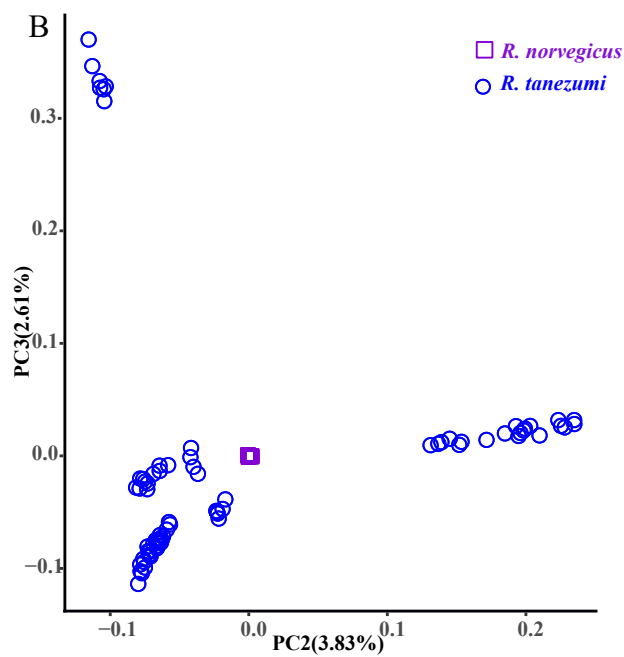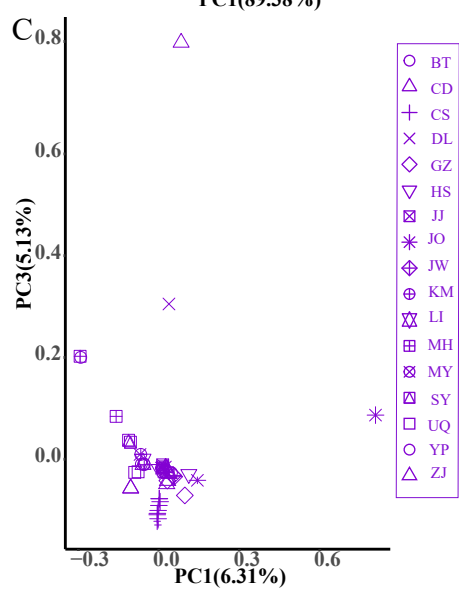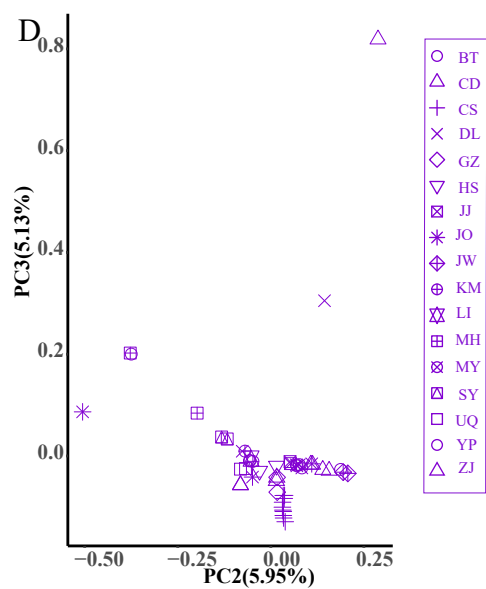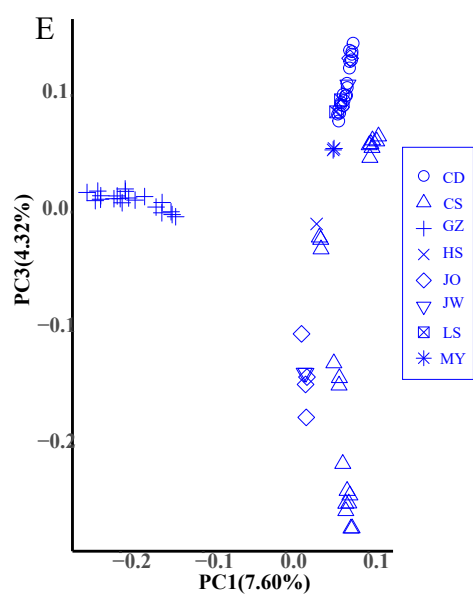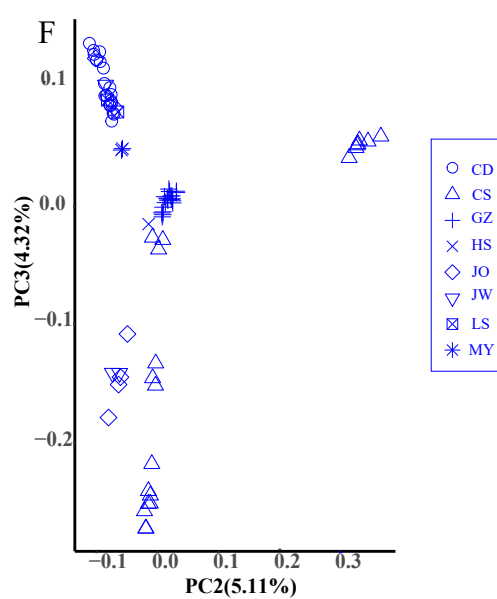

Supplement: Supplementary file 5 — Figure S5 [file ECE3-12-e9409-s008.pdf]

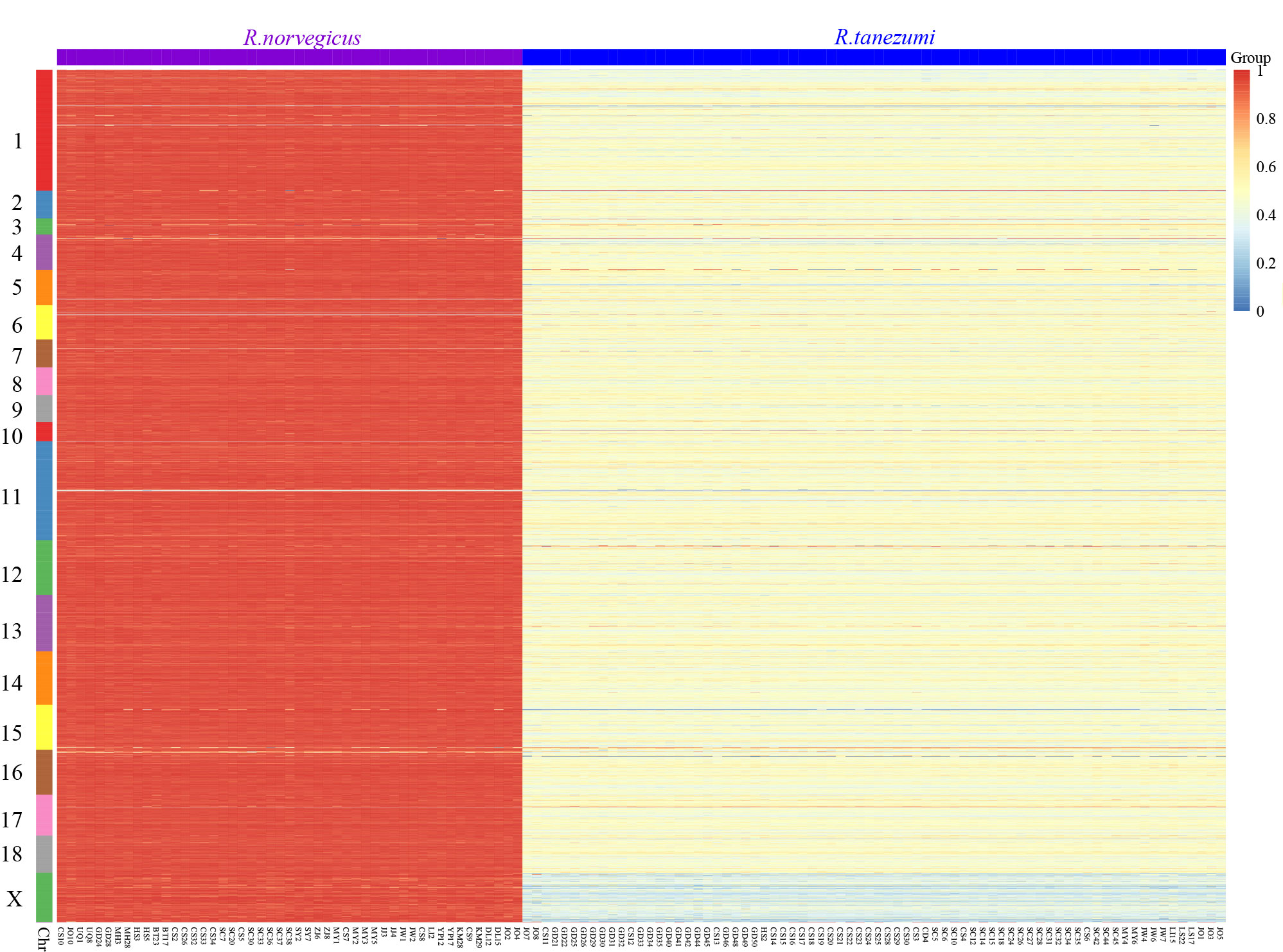

Supplement: Supplementary file 6 — Figure S6 [file ECE3-12-e9409-s018.jpg]
