# Supplementary material for: Comparative phylogeography of two commensal rat species (Rattus tanezumi and Rattus norvegicus) in China: Insights from mitochondrial DNA, microsatellite, and 2b‐RAD data
Source: Ecol Evol. 2022 Oct 13;12(10):e9409. doi: 10.1002/ece3.9409 (PMC9557235; doi:10.1002/ece3.9409)
Supplement: Supplementary file 7 — Table S1 [file ECE3-12-e9409-s013.pdf]

Table S1 Collection details for Chinese house rats specimens use in this study. Note that code for location corresponds to Fig. 1 and that clade corresponds to the lineage (*R. norvegicus*: N and *R. tanezumi*: T)

Code	County, province	Coordinates	No.	Clade	mtDNA Haplotype no.	RADSseq
MH	Mohe, Heilongjiang	52º97`N; 122º53`E	27	N	N20-23, N25	2
MS	Jiamusi, Heilongjiang	46º81`N; 130º28`E	10	N	N18, N28, N90-91	
SY	Songyuan, Jilin	45º11`N; 124º49`E	14	N	N50, N53-54, N92-96	1
MJ	Mudanjiang, Heilongjiang	44º58`N; 129º60`E	16	N	N27, N79, N86-87, N89	
SL	Shuangliao, Jilin	43º76`N; 123º70`E	6	N	N50, N56, N78, N80, N82	
UQ	Urumqi, Xinjiang	43º46`N; 87º36`E	15	N	N4	2
BT	Baotou, Inner Mongolia	40º64`N; 109º84`E	21	N	N1, N15-16, N30, N55, N61-63, N65, N67-68, N70, N72, N75-76, N100	1
CY	Chaoyang, Liaoning	39º95`N; 116º49`E	5	N	N51-52, N83	
DL	Dalian, Liaoning	38º94`N; 121º59`E	20	N	N5, N25-26, N58-59, N64, N69, N84-85, N88	2
YP	Yuanping, Shanxi	38°83`N; 112°68`E	39	N	N34-38, N41-44, N47-49	1
QX	Qinxian, Shanxi	36º70`N; 112º65`E	5	N	N19, N33, N42	
LI	Linshu, Shandong	34º89`N; 118º73`E	30	N	N15, N60-61, N65-67, N71-74, N77, N100	2
TS	Tianshui, Gansu	34º58`N; 105º73`E	2	T	T6	
HZ	Hanzhong, Shaanxi	33º08`N; 107º04`E	5	N	N29	
RG	Rugao, Jiangsu	32º27`N; 120º58`E	23	T	T5-6, T8, T13, T18-19, T27	
MY	Mianyang, Sichuan	31º50`N; 104º70`E	6	N:4; T:2	N29, N32, N98, N102, T6	6
YM	Yunmeng, Hubei	31º00`N; 113º77`E	32	T	T6, T22-23, T28-29, T31-33	
CD	Chengdu, Sichuan	30º67`N; 104º06`E	34	N:6; T:28	N1-2, N17, N24, N29, N99, T2, T6-7, T9, T11-12, T21, T24	30
HS	Huangshi, Hubei	30º21`N; 115º05`E	5	N:2; T:3	N57, T6	3
LS	Lhasa, Tibet	29º66`N; 91º11`E	23	T	T4, T6	2
JW	Jianwei, Sichuan	29º23`N; 103º98`E	7	N:5; T:2	N1, N3, N10, T6, T25	5
JJ	Jiangjin, Chongqing	29º03`N; 106º26`E	8	N	N81, N97-98, N101	2
LZ	Luzhou, Sichuan	28º89`N; 105º44`E	10	N	N46, N98	
CS	Changsha, Hunan	28º21`N; 112º97`E	33	N:12; T:21	N103, T6, T9-10, T20, T30	31
LD	Loudi, Hunan	27º74`N; 111º99`E	5	T	T29, T35	
JO	Jian`ou, Fujian	27º04`N; 118º48`E	9	N:3; T:6	N13-14, N75, T6, T26	8
KL	Kaili, Guizhou	26º63`N; 107º94`E	4	T	T29, T3, T34	
KM	Kunming, Yunnan	25º04`N; 102º72`E	22	N:21, T:1	N6-7, N29, N31, T6	2
ZP	Zhaoping, Guangxi	24º01`N; 110º97`E	3	T	T9	
GZ	Guangzhou, Guangdong	23º13`N; 113º25`E	27	N:3; T:24	N39-40, N45, T1, T6, T14-T17, T36	22
ZJ	Zhanjiang, Guangdong	21º25`N; 110º36`E	20	N	N8-9, N11-12, N45	2
