# Supplementary material for: Comparative phylogeography of two commensal rat species (Rattus tanezumi and Rattus norvegicus) in China: Insights from mitochondrial DNA, microsatellite, and 2b‐RAD data
Source: Ecol Evol. 2022 Oct 13;12(10):e9409. doi: 10.1002/ece3.9409 (PMC9557235; doi:10.1002/ece3.9409)
Supplement: Supplementary file 8 — Table S2 [file ECE3-12-e9409-s004.pdf]

Table S2 Primers list of this study

Gene symbol	Primer sequence	Reference
COI	CCTACTCRGCCATTTTACCTATG	Robins *et al*. 2007
	ACTTCTGGGTGTCCAAAGAATCA	
Cytochrome b	ACCAATGACATGAAAAATCATCGTT	Irwin *et al*. 1991
	TCTCCATTTCTGGTTTACAAGAC	
D-loop	ATAAACATTACTCTGGTCTTGTAAACC	Nachman *et al*. 1994
	ATTAATTATAAGGCAGGACCAAACCT	
R158	AGAACCCTTCACTGCTCACC	Hilbert *et al*.1991
	AGAAAGTCCCAGAAAGTGGC	
R8	GATGAGAGATTCTGGTACGGAG	Hilbert *et al*.1991
	TTTTCAGTTTTCATTCTTGTGC	
R29	CTTATGTTACCTCACAGCCTGG	Hilbert *et al*.1991
	GGGTGGGCCATCTTTATAATC	
R203	GGACTCTCGTCTCCGATTTC	Hilbert *et al*.1991
	GCGATTCGGTTGAGTTCTAC	
R102	GATTTGAAGCGATTGTCCAT	Serikawa et al. 1992
	GTCTAGCTGCCCACAGGAG	
R7	ACCCCACAATCCAACACTATTAC	Hilbert *et al*.1991
	GCAGGATCTAGTCTCAGCCC	
R137	GGGATCTTGCCAAGGTGA	Serikawa et al. 1992
	CGGCTTCTGAATGTATTGGA	
R36	GATTTCTCGAAAGGCTCCAC	Hilbert *et al*. 1991
	GACAGTGAAACGGCTTTGG	
R60	TATGTAACTCAACGCCAGCC	Mori *et al*.1989
	GAAGCCCTAGTGGCAGATG	
R145	AGGAAATGGGTTTCAGTTCC	Serikawa et al. 1992
	CAGGATTCTGTGGCAATCTG	

Reference

Hilbert P, Lindpaintner S, Beckmann T, Serikawa F, Soubrier C *et al* (1991) Chromosomal mapping of two genetic loci associated with blood-pressure regulation in hereditary hypertensive rats. *Nature*, 353, 521-529.

Irwin DM, Kocher TD, Wilson AC (1991) Evolution of the cytochrome *b* gene of mammals. *J Mol Evol*, 32, 128-144.

Mori M, Ishizakit K, Yamada T, Sugiyama T, Serikawa T, Yamada J (1989) Restriction fragment length polymorphisms of the angiotensinogen gene in inbred rats trains and mapping of the gene on chromosome 19q. *Cytogenet Cell Genet*, 50, 42-45.

Nachman MW, Boyer SN, Searle JB, Aquadro CF (1994) Mitochondrial DNA variation and the

evolution of Robertsonian chromosomal races of house mice, *Mus domesticus*. *Genetics*, 136, 1105–1120.

Robins J, Hingston M, Matisoo-Smith E, Ross H (2007) Identifying *Rattus* species using mitochondrial DNA. *Mol Ecol Notes*, 7, 717-729.

Serikawa T, Kuramoto T, Hilbert P, Mori M, Yamada J *et al*. (1992) Rat Gene Mapping Using PCR-Analyzed Microsatellites. Genetics, 131, 701-721.
