# Supplementary material for: Comparative phylogeography of two commensal rat species (Rattus tanezumi and Rattus norvegicus) in China: Insights from mitochondrial DNA, microsatellite, and 2b‐RAD data
Source: Ecol Evol. 2022 Oct 13;12(10):e9409. doi: 10.1002/ece3.9409 (PMC9557235; doi:10.1002/ece3.9409)
Supplement: Supplementary file 9 — Table S3 [file ECE3-12-e9409-s007.pdf]

Table S3. Localities information of genetic sequences downloaded from GenBank.

Taxon Name	Number	Genes	Source of samples	Reference
R. argentiventer	FR775823	COI	Viet Nam: Vinh Long pr. near t. Vinh Long	(Balakirev & Rozhnov, 2012)
R. argentiventer	FR775824	COI	Viet Nam: Vinh Long pr. near t. Vinh Long	(Balakirev & Rozhnov, 2012)
R. argentiventer	FR775825	COI	Viet Nam: Vinh Long pr. near t. Vinh Long	(Balakirev & Rozhnov, 2012)
R. argentiventer	FR775826	COI	Viet Nam: Vinh Long pr. near t. Vinh Long	(Balakirev & Rozhnov, 2012)
R. argentiventer	FR775827	COI	Viet Nam: Vinh Long pr. near t. Vinh Long	(Balakirev & Rozhnov, 2012)
R. argentiventer	FR775829	COI	Viet Nam: Vinh Long pr. near t. Vinh Long	(Balakirev & Rozhnov, 2012)
R. argentiventer	FR775830	COI	Viet Nam: Vinh Long pr. near t. Vinh Long	(Balakirev & Rozhnov, 2012)
R. argentiventer	FR775831	COI	Viet Nam: Vinh Long pr. near t. Vinh Long	(Balakirev & Rozhnov, 2012)
R. argentiventer	FR775832	COI	Viet Nam: Vinh Long pr. near t. Vinh Long	(Balakirev & Rozhnov, 2012)
R. argentiventer	FR775833	COI	Viet Nam: Vinh Long pr. near t. Vinh Long	(Balakirev & Rozhnov, 2012)
R. argentiventer	FR775834	COI	Viet Nam: Vinh Long pr. near t. Vinh Long	(Balakirev & Rozhnov, 2012)
R. argentiventer	FR775835	COI	Viet Nam: Vinh Long pr. near t. Vinh Long	(Balakirev & Rozhnov, 2012)
R. exulans	FR775836	COI	Viet Nam: Vinh Long pr. near t. Vinh Long	(Balakirev & Rozhnov, 2012)
R. exulans	FR775837	COI	Viet Nam: Vinh Long pr. near t. Vinh Long	(Balakirev & Rozhnov, 2012)
R. exulans	FR775838	COI	Viet Nam: Vinh Long pr. near t. Vinh Long	(Balakirev & Rozhnov, 2012)
R. exulans	FR775839	COI	Viet Nam: Vinh Long pr. near t. Vinh Long	(Balakirev & Rozhnov, 2012)
R. exulans	FR775840	COI	Viet Nam: Vinh Long pr. near t. Vinh Long	(Balakirev & Rozhnov, 2012)
R. exulans	FR775841	COI	Viet Nam: Vinh Long pr. near t. Vinh Long	(Balakirev & Rozhnov, 2012)
R. exulans	FR775842	COI	Viet Nam: Vinh Long pr. near t. Vinh Long	(Balakirev & Rozhnov, 2012)
R. exulans	FR775843	COI	Viet Nam: Vinh Long pr. near t. Vinh Long	(Balakirev & Rozhnov, 2012)
R. tiomanicus	FR775808	COI	Viet Nam: Dong Nai pr. Nam Cat Tien Natural	(Balakirev & Rozhnov, 2012)
R. tiomanicus	FR775809	COI	Viet Nam: Dong Nai pr. Nam Cat Tien Natural	(Balakirev & Rozhnov, 2012)
R. tiomanicus	FR775810	COI	Viet Nam: Dong Nai pr. Nam Cat Tien Natural	(Balakirev & Rozhnov, 2012)
R. tiomanicus	FR775812	COI	Viet Nam: Dong Nai pr. Nam Cat Tien Natural	(Balakirev & Rozhnov, 2012)
R. tiomanicus	FR775813	COI	Viet Nam: Dong Nai pr. Nam Cat Tien Natural	(Balakirev & Rozhnov, 2012)
R. tiomanicus	FR775814	COI	Viet Nam: Dong Nai pr. Nam Cat Tien Natural	(Balakirev & Rozhnov, 2012)
R. tiomanicus	FR775815	COI	Viet Nam: Dong Nai pr. Nam Cat Tien Natural	(Balakirev & Rozhnov, 2012)
R. tiomanicus	FR775816	COI	Viet Nam: Dong Nai pr. Nam Cat Tien Natural	(Balakirev & Rozhnov, 2012)
R. tiomanicus	FR775817	COI	Viet Nam: Dong Nai pr. Nam Cat Tien Natural	(Balakirev & Rozhnov, 2012)
R. tiomanicus	FR775818	COI	Viet Nam: Dong Nai pr. Nam Cat Tien Natural	(Balakirev & Rozhnov, 2012)
R. tiomanicus	FR775819	COI	Viet Nam: Dong Nai pr. Nam Cat Tien Natural	(Balakirev & Rozhnov, 2012)
R. tiomanicus	FR775820	COI	Viet Nam: Dong Nai pr. Nam Cat Tien Natural	(Balakirev & Rozhnov, 2012)
R. nitidus	JQ918374	COI	India: Ch. Dhananjoy Singh	Dhananjoy *et al.*, unpubl. data
R. tanezumi	JQ906931	COI	India	Dhananjoy *et al.*, unpubl. data
R. exulans	JF445250	COI	Viet Nam: Dac Lac	Eger *et al.*, unpubl. data
R. exulans	JF445251	COI	Viet Nam: Dac Lac	Eger *et al.*, unpubl. data
R. exulans	JF445252	COI	Viet Nam: Dac Lac	Eger *et al.*, unpubl. data
R. exulans	JF445253	COI	Viet Nam: Dac Lac	Eger *et al.*, unpubl. data
R. exulans	JF445254	COI	Viet Nam: Dac Lac	Eger *et al.*, unpubl. data
R. norvegicus	JF457096	COI	Canada: Ontario	Eger *et al.*, unpubl. data

Taxon Name	Number	Genes	Source of samples	Reference
R. norvegicus	JF457097	COI	Canada: Ontario	Eger *et al.*, unpubl. data
R. norvegicus	JF457098	COI	Canada: Ontario	Eger *et al.*, unpubl. data
R. rattus	JF444222	COI	Cote d'Ivoire: Parc National De Mont Peko	Eger *et al.*, unpubl. data
R. rattus	JF444941	COI	Cote d'Ivoire: Parc National De Mont Peko	Eger *et al.*, unpubl. data
R. rattus	JF444942	COI	Cote d'Ivoire: Parc National De Mont Peko	Eger *et al.*, unpubl. data
R. rattus	JF444943	COI	Cote d'Ivoire: Parc National De Mont Peko	Eger *et al.*, unpubl. data
R. rattus	JF444944	COI	Cote d'Ivoire: Parc National De Mont Peko	Eger *et al.*, unpubl. data
R. rattus	JF445257	COI	Cambodia: Kaoh Kong	Eger *et al.*, unpubl. data
R. rattus	JF445258	COI	Cambodia: Kaoh Kong	Eger *et al.*, unpubl. data
R. tanezumi	JF445285	COI	China: Guangxi, Nian Wei	Eger *et al.*, unpubl. data
R. tanezumi	JF445286	COI	China: Guizhou, Libo	Eger *et al.*, unpubl. data
R. tanezumi	JF445287	COI	China: Guizhou, Libo	Eger *et al.*, unpubl. data
R. exulans	JF459863	COI	Malaysia: Johor	Engstrom *et al.*, unpubl.data
R. rattus	JF459864	COI	Malaysia: Johor	Engstrom *et al.*, unpubl.data
R. rattus	JF459865	COI	Malaysia: Johor	Engstrom *et al.*, unpubl.data
R. exulans	KC617850	COI	USA	(Jones *et al.*, 2013)
R. exulans	KC617851	COI	USA	(Jones *et al.*, 2013)
R. norvegicus	KC617853	COI	USA	(Jones *et al.*, 2013)
R. norvegicus	KC617854	COI	USA	(Jones *et al.*, 2013)
R. norvegicus	KC617855	COI	USA	(Jones *et al.*, 2013)
R. norvegicus	KC617856	COI	USA	(Jones *et al.*, 2013)
R. rattus	KC617857	COI	USA	(Jones *et al.*, 2013)
R. rattus	KC617858	COI	USA	(Jones *et al.*, 2013)
R. rattus	KC617859	COI	USA	(Jones *et al.*, 2013)
R. tanezumi	JN105102	COI	Viet Nam	Balakirev AE. Unpubl.data
R. tanezumi	KC010257	COI	Thailand	(Latinne *et al.*, 2012)
R. tanezumi	KC010258	COI	Thailand	(Latinne *et al.*, 2012)
R. tanezumi	KC010259	COI	Thailand	(Latinne *et al.*, 2012)
R. tanezumi	KC010260	COI	Thailand	(Latinne *et al.*, 2012)
R. tanezumi	KC010261	COI	Thailand	(Latinne *et al.*, 2012)
R. tanezumi	KC010262	COI	Thailand	(Latinne *et al.*, 2012)
R. tanezumi	KC010263	COI	Thailand	(Latinne *et al.*, 2012)
R. tanezumi	KC010264	COI	Thailand	(Latinne *et al.*, 2012)
R. tanezumi	KC010265	COI	Thailand	(Latinne *et al.*, 2012)
R. tanezumi	KC010266	COI	Thailand	(Latinne *et al.*, 2012)
R. tanezumi	KC010267	COI	Thailand	(Latinne *et al.*, 2012)
R. tanezumi	KC010268	COI	Thailand	(Latinne *et al.*, 2012)
R. tanezumi	KC010269	COI	Thailand	(Latinne *et al.*, 2012)
R. tanezumi	KC010270	COI	Thailand	(Latinne *et al.*, 2012)
R. tanezumi	KC010271	COI	Thailand	(Latinne *et al.*, 2012)
R. tanezumi	KC010272	COI	Thailand	(Latinne *et al.*, 2012)

Taxon Name	Number	Genes	Source of samples	Reference
R. tanezumi	KC010273	COI	Thailand	(Latinne *et al.*, 2012)
R. tanezumi	KC010274	COI	Thailand	(Latinne *et al.*, 2012)
R. tanezumi	KC010275	COI	Thailand	(Latinne *et al.*, 2012)
R. tanezumi	KC010276	COI	Thailand	(Latinne *et al.*, 2012)
R. tiomanicus	KC010289	COI	Thailand	(Latinne *et al.*, 2012)
R. tiomanicus	KC010290	COI	Thailand	(Latinne *et al.*, 2012)
R. tiomanicus	KC010291	COI	Thailand	(Latinne *et al.*, 2012)
R. nitidus	KF999100	COI	China: Sichuan	(Li *et al.*, 2014)
R. nitidus	KF999101	COI	China: Sichuan	(Li *et al.*, 2014)
R. nitidus	KF999102	COI	China: Sichuan	(Li *et al.*, 2014)
R. nitidus	KF999103	COI	China: Sichuan	(Li *et al.*, 2014)
R. nitidus	KF999104	COI	China: Sichuan	(Li *et al.*, 2014)
R. nitidus	KF999105	COI	China: Sichuan	(Li *et al.*, 2014)
R. nitidus	KF999106	COI	China: Sichuan	(Li *et al.*, 2014)
R. norvegicus	KF999117	COI	China: Sichuan	(Li *et al.*, 2014)
R. norvegicus	KF999118	COI	China: Sichuan	(Li *et al.*, 2014)
R. norvegicus	KF999119	COI	China: Sichuan	(Li *et al.*, 2014)
R. norvegicus	KF999120	COI	China: Sichuan	(Li *et al.*, 2014)
R. norvegicus	KF999121	COI	China: Sichuan	(Li *et al.*, 2014)
R. tanezumi	KF999133	COI	China: Sichuan,Anxian	(Li *et al.*, 2014)
R. tanezumi	KF999134	COI	China: Sichuan,Pengzhou	(Li *et al.*, 2014)
R. tanezumi	KF999135	COI	China: Sichuan,Mianzhu	(Li *et al.*, 2014)
R. tanezumi	KF999136	COI	China: Sichuan,Pengzhou	(Li *et al.*, 2014)
R. tanezumi	KF999137	COI	China: Sichuan,Mianzhu	(Li *et al.*, 2014)
R. rattus	JF459256	COI	Guyana: Barima-Waini	Lim *et al.*, unpubl.data
R. rattus	JF459257	COI	Guyana: Barima-Waini	Lim *et al.*, unpubl.data
R. rattus	JF459258	COI	Guyana: Barima-Waini	Lim *et al.*, unpubl.data
R. rattus	JF459259	COI	Guyana: Barima-Waini	Lim *et al.*, unpubl.data
R. norvegicus	JF499336	COI	Russia: Chita Region	Lissovsky *et al.*, unpubl.data
R. norvegicus	JF499337	COI	Russia: Amur Region	Lissovsky *et al.*, unpubl.data
R. norvegicus	JF499338	COI	Russia: Chita Region	Lissovsky *et al.*, unpubl.data
R. norvegicus	JF499339	COI	Russia: Chita Region	Lissovsky *et al.*, unpubl.data
R. losea	HM031871	COI	China: Hainan	(Lu *et al.*, 2012)
R. losea	HM031872	COI	China: Hainan	(Lu *et al.*, 2012)
R. losea	HM031873	COI	China: Hainan	(Lu *et al.*, 2012)
R. losea	HM031874	COI	China: Hainan	(Lu *et al.*, 2012)
R. losea	HM031875	COI	China: Hainan	(Lu *et al.*, 2012)
R. losea	HM031876	COI	China: Hainan	(Lu *et al.*, 2012)
R. losea	HM031877	COI	China: Hainan	(Lu *et al.*, 2012)
R. losea	HM031878	COI	China: Hainan	(Lu *et al.*, 2012)
R. losea	HM031879	COI	China: Hainan	(Lu *et al.*, 2012)

Taxon Name	Number	Genes	Source of samples	Reference
R. losea	HM031880	COI	China: Hainan	(Lu *et al*., 2012)
R. losea	HM031881	COI	China: Hainan	(Lu *et al*., 2012)
R. losea	HM031882	COI	China: Hainan	(Lu *et al*., 2012)
R. losea	HM031883	COI	China: Hainan	(Lu *et al*., 2012)
R. losea	HM031884	COI	China: Hainan	(Lu *et al*., 2012)
R. losea	HM031886	COI	China: Hainan	(Lu *et al*., 2012)
R. losea	HM031887	COI	China: Hainan	(Lu *et al*., 2012)
R. losea	HM031888	COI	China: Hainan	(Lu *et al*., 2012)
R. losea	HM031889	COI	China: Hainan	(Lu *et al*., 2012)
R. losea	HM031890	COI	China: Hainan	(Lu *et al*., 2012)
R. losea	HM031891	COI	China: Hainan	(Lu *et al*., 2012)
R. losea	HM031892	COI	China: Hainan	(Lu *et al*., 2012)
R. losea	HM031893	COI	China: Hainan	(Lu *et al*., 2012)
R. losea	HM031894	COI	China: Hainan	(Lu *et al*., 2012)
R. losea	HM031895	COI	China: Hainan	(Lu *et al*., 2012)
R. losea	HM031896	COI	China: Hainan	(Lu *et al*., 2012)
R. norvegicus	HM031897	COI	China: Hainan	(Lu *et al*., 2012)
R. norvegicus	HM031898	COI	China: Hainan	(Lu *et al*., 2012)
R. norvegicus	HM031899	COI	China: Hainan	(Lu *et al*., 2012)
R. norvegicus	HM031900	COI	China: Hainan	(Lu *et al*., 2012)
R. norvegicus	HM031901	COI	China: Hainan	(Lu *et al*., 2012)
R. norvegicus	HM031902	COI	China: Hainan	(Lu *et al*., 2012)
R. norvegicus	HM031903	COI	China: Hainan	(Lu *et al*., 2012)
R. norvegicus	HM031904	COI	China: Hainan	(Lu *et al*., 2012)
R. norvegicus	HM031905	COI	China: Hainan	(Lu *et al*., 2012)
R. norvegicus	HM031906	COI	China: Hainan	(Lu *et al*., 2012)
R. norvegicus	HM031907	COI	China: Hainan	(Lu *et al*., 2012)
R. norvegicus	HM031908	COI	China: Hainan	(Lu *et al*., 2012)
R. norvegicus	HM031909	COI	China: Hainan	(Lu *et al*., 2012)
R. norvegicus	HM031910	COI	China: Hainan	(Lu *et al*., 2012)
R. norvegicus	JX962219	COI	China: Qinghai	Ma & Lu, unpubl.data
R. norvegicus	JQ043459	COI	China: Qinghai	Ma & Lu, unpubl.data
R. norvegicus	JQ043460	COI	China: Qinghai	Ma & Lu, unpubl.data
R. norvegicus	JQ043461	COI	China: Qinghai	Ma & Lu, unpubl.data
R. norvegicus	JQ043462	COI	China: Qinghai	Ma & Lu, unpubl.data
R. norvegicus	AB451019	COI	Japan	Nakamura & Aiko, unpubl.data
R. rattus	AB752785	COI	Zambia: Lusaka	(Nakamura *et al*., 2013)
R. rattus	AB752786	COI	Zambia: Lusaka	(Nakamura *et al*., 2013)
R. rattus	AB752787	COI	Zambia: Lusaka	(Nakamura *et al*., 2013)
R. rattus	AB752788	COI	Zambia: Lusaka	(Nakamura *et al*., 2013)
R. rattus	AB752789	COI	Zambia: Lusaka	(Nakamura *et al*., 2013)

Taxon Name	Number	Genes	Source of samples	Reference
R. rattus	AB752790	COI	Zambia: Lusaka	(Nakamura *et al.*, 2013)
R. rattus	AB752791	COI	Zambia: Lusaka	(Nakamura *et al.*, 2013)
R. rattus	AB752792	COI	Zambia: Lusaka	(Nakamura *et al.*, 2013)
R. rattus	AB752793	COI	Zambia: Lusaka	(Nakamura *et al.*, 2013)
R. rattus	AB752794	COI	Zambia: Lusaka	(Nakamura *et al.*, 2013)
R. rattus	AB752795	COI	Zambia: Namwala, Katoshi	(Nakamura *et al.*, 2013)
R. rattus	AB752796	COI	Zambia: Lusaka	(Nakamura *et al.*, 2013)
R. rattus	AB752797	COI	Zambia: Namwala, Katoshi	(Nakamura *et al.*, 2013)
R. rattus	AB752798	COI	Zambia: Namwala, Katoshi	(Nakamura *et al.*, 2013)
R. rattus	AB752799	COI	Zambia: Namwala, Katoshi	(Nakamura *et al.*, 2013)
R. rattus	AB752800	COI	Zambia: Namwala, Katoshi	(Nakamura *et al.*, 2013)
R. rattus	AB752801	COI	Zambia: Namwala, Katoshi	(Nakamura *et al.*, 2013)
R. rattus	AB752802	COI	Zambia: Namwala, Katoshi	(Nakamura *et al.*, 2013)
R. rattus	AB752803	COI	Zambia: Namwala, Katoshi	(Nakamura *et al.*, 2013)
R. rattus	AB752804	COI	Zambia: Namwala, Katoshi	(Nakamura *et al.*, 2013)
R. rattus	AB752805	COI	Zambia: Namwala, Kantengwa	(Nakamura *et al.*, 2013)
R. rattus	AB752806	COI	Zambia: Namwala, Banamaiya	(Nakamura *et al.*, 2013)
R. rattus	JQ668025	COI	Niger: Niamey	(Nicolas *et al.*, 2012)
R. tanezumi	HM217489	COI	Laos: Luang Prabang	(Pagès *et al.*, 2010)
R. tanezumi	HM217494	COI	Laos: Luang Prabang	(Pagès *et al.*, 2010)
R. tanezumi	HM217502	COI	Thailand: Ratchaburi	(Pagès *et al.*, 2010)
R. tanezumi	HM217529	COI	Thailand: Ratchaburi	(Pagès *et al.*, 2010)
R. tanezumi	HM217540	COI	Thailand: Kanchanaburi	(Pagès *et al.*, 2010)
R. tanezumi	HM217555	COI	Thailand: Phrae	(Pagès *et al.*, 2010)
R. tanezumi	HM217579	COI	Thailand: Nan	(Pagès *et al.*, 2010)
R. tanezumi	HM217582	COI	Thailand: Nan	(Pagès *et al.*, 2010)
R. tanezumi	HM217583	COI	Thailand: Nan	(Pagès *et al.*, 2010)
R. tanezumi	HM217584	COI	Thailand: Nan	(Pagès *et al.*, 2010)
R. tanezumi	HM217593	COI	Thailand: Nan	(Pagès *et al.*, 2010)
R. tanezumi	JX533940	COI	Laos: Luang Prabang	(Pagès *et al.*, 2013)
R. tanezumi	JX533941	COI	Laos: Luang Prabang	(Pagès *et al.*, 2013)
R. tanezumi	JX533942	COI	Laos: Luang Prabang	(Pagès *et al.*, 2013)
R. tanezumi	JX533943	COI	Laos: Luang Prabang	(Pagès *et al.*, 2013)
R. tanezumi	JX533944	COI	Laos: Luang Prabang	(Pagès *et al.*, 2013)
R. tanezumi	JX533945	COI	Laos: Luang Prabang	(Pagès *et al.*, 2013)
R. tanezumi	JX533946	COI	Laos: Luang Prabang	(Pagès *et al.*, 2013)
R. tanezumi	JX533947	COI	Laos: Luang Prabang	(Pagès *et al.*, 2013)
R. tanezumi	JX533948	COI	Laos: Luang Prabang	(Pagès *et al.*, 2013)
R. tanezumi	JX533949	COI	Laos: Luang Prabang	(Pagès *et al.*, 2013)
R. tanezumi	JX533950	COI	Laos: Luang Prabang	(Pagès *et al.*, 2013)
R. tanezumi	JX533951	COI	Laos: Luang Prabang	(Pagès *et al.*, 2013)

Taxon Name	Number	Genes	Source of samples	Reference
R. tanezumi	JX533952	COI	Thailand: Kanchanaburi	(Pagès *et al.*, 2013)
R. tanezumi	JX533953	COI	Thailand: Loei	(Pagès *et al.*, 2013)
R. tanezumi	JX533954	COI	Thailand: Loei	(Pagès *et al.*, 2013)
R. tanezumi	JX533956	COI	Thailand: Loei	(Pagès *et al.*, 2013)
R. tanezumi	JX533959	COI	Thailand: Loei	(Pagès *et al.*, 2013)
R. tanezumi	JX533960	COI	Thailand: Loei	(Pagès *et al.*, 2013)
R. tanezumi	JX533961	COI	Thailand: Loei	(Pagès *et al.*, 2013)
R. tanezumi	JX533962	COI	Thailand: Loei	(Pagès *et al.*, 2013)
R. tanezumi	JX533963	COI	Thailand: Loei	(Pagès *et al.*, 2013)
R. tanezumi	JX533964	COI	Thailand: Phrae	(Pagès *et al.*, 2013)
R. tanezumi	JX533965	COI	Thailand: Phrae	(Pagès *et al.*, 2013)
R. tanezumi	JX533966	COI	Thailand: Phrae	(Pagès *et al.*, 2013)
R. tanezumi	JX533967	COI	Thailand: Phrae	(Pagès *et al.*, 2013)
R. tanezumi	JX533968	COI	Thailand: Phrae	(Pagès *et al.*, 2013)
R. tanezumi	JX533969	COI	Thailand: Phrae	(Pagès *et al.*, 2013)
R. tanezumi	JX533970	COI	Thailand: Phrae	(Pagès *et al.*, 2013)
R. tanezumi	JX533971	COI	Thailand: Phrae	(Pagès *et al.*, 2013)
R. tanezumi	JX533972	COI	Thailand: Phrae	(Pagès *et al.*, 2013)
R. tanezumi	JX533973	COI	Thailand: Phrae	(Pagès *et al.*, 2013)
R. tanezumi	JX533974	COI	Thailand: Phrae	(Pagès *et al.*, 2013)
R. tanezumi	JX533975	COI	Thailand: Phrae	(Pagès *et al.*, 2013)
R. tanezumi	JX533976	COI	Thailand: Phrae	(Pagès *et al.*, 2013)
R. tanezumi	JX533977	COI	Thailand: Phrae	(Pagès *et al.*, 2013)
R. tanezumi	JX533978	COI	Thailand: Phrae	(Pagès *et al.*, 2013)
R. tanezumi	JX533979	COI	Thailand: Phrae	(Pagès *et al.*, 2013)
R. tanezumi	JX533980	COI	Thailand: Phrae	(Pagès *et al.*, 2013)
R. tanezumi	JX533981	COI	Thailand: Phrae	(Pagès *et al.*, 2013)
R. tanezumi	JX533982	COI	Thailand: Loei	(Pagès *et al.*, 2013)
R. tanezumi	JX533983	COI	Thailand: Loei	(Pagès *et al.*, 2013)
R. tanezumi	JX533984	COI	Thailand: Loei	(Pagès *et al.*, 2013)
R. tanezumi	JX533985	COI	Thailand: Loei	(Pagès *et al.*, 2013)
R. tanezumi	JX533986	COI	Thailand: Loei	(Pagès *et al.*, 2013)
R. tanezumi	JX533987	COI	Thailand: Loei	(Pagès *et al.*, 2013)
R. tanezumi	JX533988	COI	Thailand: Loei	(Pagès *et al.*, 2013)
R. tanezumi	JX533989	COI	Thailand: Loei	(Pagès *et al.*, 2013)
R. tanezumi	JX533990	COI	Thailand: Loei	(Pagès *et al.*, 2013)
R. tanezumi	JX533991	COI	Thailand: Loei	(Pagès *et al.*, 2013)
R. tanezumi	JX533992	COI	Thailand: Loei	(Pagès *et al.*, 2013)
R. tanezumi	JX533993	COI	Thailand: Loei	(Pagès *et al.*, 2013)
R. tanezumi	JX533994	COI	Thailand: Loei	(Pagès *et al.*, 2013)
R. tanezumi	JX533995	COI	Thailand: Loei	(Pagès *et al.*, 2013)

Taxon Name	Number	Genes	Source of samples	Reference
R. tanezumi	JX533996	COI	Thailand: Loei	(Pagès *et al.*, 2013)
R. tanezumi	JX533997	COI	Thailand: Loei	(Pagès *et al.*, 2013)
R. tanezumi	JX533998	COI	Thailand: Loei	(Pagès *et al.*, 2013)
R. tanezumi	JX533999	COI	Thailand: Loei	(Pagès *et al.*, 2013)
R. tanezumi	JX534000	COI	Thailand: Loei	(Pagès *et al.*, 2013)
R. tanezumi	JX534001	COI	Thailand: Loei	(Pagès *et al.*, 2013)
R. tanezumi	JX534002	COI	Thailand: Loei	(Pagès *et al.*, 2013)
R. tanezumi	JX534003	COI	Thailand: Loei	(Pagès *et al.*, 2013)
R. tanezumi	JX534004	COI	Thailand: Loei	(Pagès *et al.*, 2013)
R. tanezumi	JX534005	COI	Thailand: Loei	(Pagès *et al.*, 2013)
R. tanezumi	JX534006	COI	Thailand: Loei	(Pagès *et al.*, 2013)
R. tanezumi	JX534007	COI	Thailand: Loei	(Pagès *et al.*, 2013)
R. tanezumi	JX534008	COI	Thailand: Loei	(Pagès *et al.*, 2013)
R. tanezumi	JX534009	COI	Thailand: Loei	(Pagès *et al.*, 2013)
R. tanezumi	JX534010	COI	Thailand: Loei	(Pagès *et al.*, 2013)
R. tanezumi	JX534011	COI	Thailand: Loei	(Pagès *et al.*, 2013)
R. tanezumi	JX534012	COI	Thailand: Loei	(Pagès *et al.*, 2013)
R. tanezumi	JX534013	COI	Thailand: Loei	(Pagès *et al.*, 2013)
R. rattus	JF827644	COI	India	Rengarajan & Archunan, unpubl.data
R. rattus	JF827645	COI	India	Rengarajan & Archunan, unpubl.data
R. rattus	JF827646	COI	India	Rengarajan & Archunan, unpubl.data
R. rattus	JF827647	COI	India	Rengarajan & Archunan, unpubl.data
R. rattus	JF827648	COI	India	Rengarajan & Archunan, unpubl.data
R. rattus	JN547787	COI	India	Rengarajan & Archunan, unpubl.data
R. rattus	JN547788	COI	India	Rengarajan & Archunan, unpubl.data
R. rattus	JN547789	COI	India	Rengarajan & Archunan, unpubl.data
R. rattus	JN547790	COI	India	Rengarajan & Archunan, unpubl.data
R. rattus	JN547791	COI	India	Rengarajan & Archunan, unpubl.data
R. rattus	JN547792	COI	India	Rengarajan & Archunan, unpubl.data
R. rattus	JN547793	COI	India	Rengarajan & Archunan, unpubl.data
R. rattus	JN547794	COI	India	Rengarajan & Archunan, unpubl.data
R. rattus	JN547795	COI	India	Rengarajan & Archunan, unpubl.data
R. rattus	JN547796	COI	India	Rengarajan & Archunan, unpubl.data
R. rattus	JN547797	COI	India	Rengarajan & Archunan, unpubl.data
R. exulans	EF186529	COI	Cook Islands: Aitutaki	(Robins *et al.*, 2007)
R. exulans	EF186530	COI	Cook Islands: Aitutaki	(Robins *et al.*, 2007)
R. exulans	EF186531	COI	Takutea: Cook Islands	(Robins *et al.*, 2007)
R. exulans	EF186532	COI	Fiji	(Robins *et al.*, 2007)
R. exulans	EF186533	COI	Hawaii	(Robins *et al.*, 2007)
R. exulans	EF186534	COI	Hawaii	(Robins *et al.*, 2007)
R. exulans	EF186535	COI	Society Islands	(Robins *et al.*, 2007)

Taxon Name	Number	Genes	Source of samples	Reference
R. exulans	EF186536	COI	Indonesia	(Robins *et al.*, 2007)
R. exulans	EF186537	COI	Marquesas Islands	(Robins *et al.*, 2007)
R. exulans	EF186538	COI	Marquesas Islands	(Robins *et al.*, 2007)
R. exulans	EF186539	COI	New Zealand	(Robins *et al.*, 2007)
R. exulans	EF186540	COI	New Zealand	(Robins *et al.*, 2007)
R. exulans	EF186541	COI	New Zealand	(Robins *et al.*, 2007)
R. exulans	EF186542	COI	Papua New Guinea	(Robins *et al.*, 2007)
R. exulans	EF186543	COI	Papua New Guinea	(Robins *et al.*, 2007)
R. exulans	EF186544	COI	Society Islands	(Robins *et al.*, 2007)
R. exulans	EF186545	COI	Samoa	(Robins *et al.*, 2007)
R. exulans	EF186546	COI	Samoa	(Robins *et al.*, 2007)
R. exulans	EF186547	COI	Thailand	(Robins *et al.*, 2007)
R. exulans	EF186548	COI	Thailand	(Robins *et al.*, 2007)
R. exulans	EF186549	COI	Thailand	(Robins *et al.*, 2007)
R. exulans	EF186599	COI	Thailand	(Robins *et al.*, 2007)
R. exulans	EF186638	COI	Thailand	(Robins *et al.*, 2007)
R. fuscipes	EF186550	COI	Australia	(Robins *et al.*, 2007)
R. fuscipes	EF186551	COI	Australia	(Robins *et al.*, 2007)
R. fuscipes	EF186552	COI	Australia	(Robins *et al.*, 2007)
R. fuscipes	EF186553	COI	Australia	(Robins *et al.*, 2007)
R. fuscipes	EF186554	COI	Australia	(Robins *et al.*, 2007)
R. hoffmanni	EF186556	COI	Indonesia	(Robins *et al.*, 2007)
R. hoffmanni	EF186557	COI	Indonesia	(Robins *et al.*, 2007)
R. hoffmanni	EF186558	COI	Indonesia	(Robins *et al.*, 2007)
R. leucopus	EF186562	COI	Australia	(Robins *et al.*, 2007)
R. leucopus	EF186563	COI	Australia	(Robins *et al.*, 2007)
R. leucopus	EF186566	COI	Australia	(Robins *et al.*, 2007)
R. leucopus	EF186567	COI	Australia	(Robins *et al.*, 2007)
R. norvegicus	EF186576	COI	French Polynesia	(Robins *et al.*, 2007)
R. norvegicus	EF186577	COI	French Polynesia	(Robins *et al.*, 2007)
R. rattus	EF186584	COI	French Polynesia	(Robins *et al.*, 2007)
R. rattus	EF186585	COI	New Zealand	(Robins *et al.*, 2007)
R. rattus	EF186587	COI	Society Islands	(Robins *et al.*, 2007)
R. rattus	EF186589	COI	New Zealand	(Robins *et al.*, 2007)
R. rattus	EF186590	COI	Papua New Guinea	(Robins *et al.*, 2007)
R. sordidus	EF186595	COI	Australia	(Robins *et al.*, 2007)
R. sordidus	EF186596	COI	Australia	(Robins *et al.*, 2007)
R. sordidus	EF186597	COI	Australia	(Robins *et al.*, 2007)
R. sordidus	EF186598	COI	Australia	(Robins *et al.*, 2007)
R. tiomanicus	EF186628	COI	Indonesia	(Robins *et al.*, 2007)
R. tiomanicus	EF186629	COI	Indonesia	(Robins *et al.*, 2007)

Taxon Name	Number	Genes	Source of samples	Reference
R. leucopus	KF510037	COI	Papua New Guinea	(Robins *et al*., 2014)
R. leucopus	KF510038	COI	Papua New Guinea	(Robins *et al*., 2014)
R. leucopus	KF510039	COI	Papua New Guinea	(Robins *et al*., 2014)
R. leucopus	KF510040	COI	Papua New Guinea	(Robins *et al*., 2014)
R. leucopus	KF510041	COI	Papua New Guinea	(Robins *et al*., 2014)
R. leucopus	KF510042	COI	Papua New Guinea	(Robins *et al*., 2014)
R. leucopus	KF510043	COI	Papua New Guinea	(Robins *et al*., 2014)
R. leucopus	KF510044	COI	Papua New Guinea	(Robins *et al*., 2014)
R. leucopus	KF510045	COI	Papua New Guinea	(Robins *et al*., 2014)
R. leucopus	KF510046	COI	Papua New Guinea	(Robins *et al*., 2014)
R. leucopus	KF510047	COI	Papua New Guinea	(Robins *et al*., 2014)
R. tanezumi	KT335544	COI	China	Sun *et al*., unpubl.data
R. tanezumi	KT335545	COI	China	Sun *et al*., Unpubl.data
R. tanezumi	KT335546	COI	China	Sun *et al*., Unpubl.data
R. tanezumi	KT335557	COI	China	Sun *et al*., Unpubl.data
R. tanezumi	KT335558	COI	China	Sun *et al*., Unpubl.data
R. tanezumi	KT335559	COI	China	Sun *et al*., Unpubl.data
R. tanezumi	KT335561	COI	China	Sun *et al*., Unpubl.data
R. tanezumi	KT335562	COI	China	Sun *et al*., Unpubl.data
R. tanezumi	KT335563	COI	China	Sun *et al*., Unpubl.data
R. tanezumi	KT335564	COI	China	Sun *et al*., Unpubl.data
R. tanezumi	KT335580	COI	China	Sun *et al*., Unpubl.data
R. tanezumi	KT335581	COI	China	Sun *et al*., Unpubl.data
R. tanezumi	KT335582	COI	China	Sun *et al*., Unpubl.data
R. tanezumi	KT335583	COI	China	Sun *et al*., Unpubl.data
R. tanezumi	KT335595	COI	China	Sun *et al*., Unpubl.data
R. tanezumi	KT335597	COI	China	Sun *et al*., Unpubl.data
R. tanezumi	KT335598	COI	China	Sun *et al*., Unpubl.data
R. tanezumi	KT335599	COI	China	Sun *et al*., Unpubl.data
R. tanezumi	KT335601	COI	China	Sun *et al*., Unpubl.data
R. tanezumi	KT335602	COI	China	Sun *et al*., Unpubl.data
R. tanezumi	KT335605	COI	China	Sun *et al*., Unpubl.data
R. tanezumi	KT335606	COI	China	Sun *et al*., Unpubl.data
R. norvegicus	KT335532	COI	China	Sun *et al*., Unpubl.data
R. norvegicus	KT335533	COI	China	Sun *et al*., Unpubl.data
R. norvegicus	KT335552	COI	China	Sun *et al*., Unpubl.data
R. norvegicus	KT335553	COI	China	Sun *et al*., Unpubl.data
R. norvegicus	KT335554	COI	China	Sun *et al*., Unpubl.data
R. norvegicus	KT335555	COI	China	Sun *et al*., Unpubl.data
R. norvegicus	KT335587	COI	China	Sun *et al*., Unpubl.data
R. norvegicus	KT335588	COI	China	Sun *et al*., Unpubl.data

Taxon Name	Number	Genes	Source of samples	Reference
R. norvegicus	KT335589	COI	China	Sun *et al.*, Unpubl.data
R. norvegicus	KT335590	COI	China	Sun *et al.*, Unpubl.data
R. norvegicus	KT335591	COI	China	Sun *et al.*, Unpubl.data
R. norvegicus	KT335596	COI	China	Sun *et al.*, Unpubl.data
R. norvegicus	KT335604	COI	China	Sun *et al.*, Unpubl.data
R. norvegicus	KT335608	COI	China	Sun *et al.*, Unpubl.data
R. norvegicus	KT763036	COI	China	Sun *et al.*, Unpubl.data
R. norvegicus	KT763037	COI	China	Sun *et al.*, Unpubl.data
R. norvegicus	KU182943	COI	China	Yin *et al.*, Unpubl.data
R. norvegicus	KU182944	COI	China	Yin *et al.*, Unpubl.data
R. norvegicus	KM497428	COI	China	Yue QY, Unpubl.data
R. norvegicus	KM657952	mtgenome	China	Ji C. Unpubl.data
R. rattus	EU273707	mtgenome	New Zealand	(Robins *et al.*, 2008)
R. tanezumi	NC_011638	mtgenome	New Zealand	(Robins *et al.*, 2008)
