# Supplementary material for: Comparative phylogeography of two commensal rat species (Rattus tanezumi and Rattus norvegicus) in China: Insights from mitochondrial DNA, microsatellite, and 2b‐RAD data
Source: Ecol Evol. 2022 Oct 13;12(10):e9409. doi: 10.1002/ece3.9409 (PMC9557235; doi:10.1002/ece3.9409)
Supplement: Supplementary file 10 — Table S4 [file ECE3-12-e9409-s016.pdf]

Table S4 Haplotype distribution of Chinese house rats inferred from 486 complete mitochondrial D-loop sequences

	MH	MS	SY	MJ	SL	UQ	BT	CY	DL	YP	QX	LI	TS	HZ	RG	MY	YM	CD	HS	LS	JW	JJ	LZ
n	27	10	14	16	6	15	21	5	20	39	5	30	2	5	23	6	32	34	5	23	7	8	10
N1							1											1			3		
N2																		1					
N3																					1		
N4						15																	
N5									1														
N6																							
N7																							
N8																							
N9																							
N10																					1		
N11																							
N12																							
N13																							
N14																							
N15							2					5											
N16							1																
N17																		1					
N18		2																					
N19											1												
N20	2																						
N21	1																						
N22	22																						
N23	1																						
N24																		1					
N25	1								2														
N26									3														
N27				8																			
N28		1																					
N29														5		1		1					
N30							1																
N31																							
N32																1							
N33											3												
N34										14													
N35										1													
N36										2													
N37										1													
N38										1													
N39																							
N40																							
N41										3													
N42										2	1												
N43										2													
N44										5													
N45																							
N46																							1
N47										1													
N48										6													
N49										1													
N50			4		2																		
N51								1															

N52								1															
N53			1																				
N54			1																				
N55							1																
N56					1																		
N57																			2				
N58									2														
N59									1														
N60												2											
N61							1					3											
N62							1																
N63							1																
N64									4														
N65							1					5											
N66												1											
N67							4					1											
N68							2																
N69									1														
N70							1																
N71												1											
N72							1					1											
N73												8											
N74												1											
N75							1																
N76							1																
N77												1											
N78					1																		
N79				1																			
N80					1																		
N81																						1	
N82					1																		
N83								3															
N84									1														
N85									4														
N86				5																			
N87				1																			
N88									1														
N89				1																			
N90		6																					
N91		1																					
N92			1																				
N93			2																				
N94			1																				
N95			3																				
N96			1																				
N97																						3	
N98																1						3	9
N99																		1					
N100							1					1											
N101																						1	
N102																1							
N103																							
T1																							
T2																		1					

T3									
T4								1	
T5			1						
T6	1		11	2	1	19	3	22	1
T7						1			
T8			2						
T9						1			
T10									
T11						1			
T12						3			
T13			2						
T14									
T15									
T16									
T17									
T18			5						
T19			1						
T20									
T21						1			
T22					18				
T23					1				
T24						1			
T25									1
T26									
T27			1						
T28					1				
T29	1				8				
T30									
T31					1				
T32					1				
T33					1				
T34									
T35									
T36									

CS	LD	KL	JO	KM	ZP	GZ	ZJ	
33	5	4	9	22	3	27	20	n
								5
								1
								1
								15
								1
				2				2
				3				3
							1	1
							1	1
								1
							8	8
							1	1
			1					1
			1					1
								7
								1
								1
								2
								1
								2
								1
								22
								1
								1
								3
								3
								8
								1
				15				22
								1
				1				1
								1
								3
								14
								1
								2
								1
								1
						1		1
						1		1
								3
								3
								2
								5
						1	9	10
								1
								1
								6
								1
								6
								1

			1
			1
			1
			1
			1
			2
			2
			1
			2
			4
			1
			1
			4
			6
			1
			5
			2
			1
			1
			1
			2
			8
			1
	1		2
			1
			1
			1
			1
			1
			1
			1
			3
			1
			4
			5
			1
			1
			1
			6
			1
			1
			2
			1
			3
			1
			3
			13
			1
			2
			1
			1
12			12
		1	1
			1

		1					1
							1
							1
6			4	1		2	73
							1
							2
2					3		6
1							1
							1
							3
							2
						1	1
						10	10
						8	8
						1	1
							5
							1
1							1
							1
							18
							1
							1
							1
			2				2
							1
							1
	3	2					14
11							11
							1
							1
							1
		1					1
	2						2
						1	1
