# Supplementary material for: Comparative phylogeography of two commensal rat species (Rattus tanezumi and Rattus norvegicus) in China: Insights from mitochondrial DNA, microsatellite, and 2b‐RAD data
Source: Ecol Evol. 2022 Oct 13;12(10):e9409. doi: 10.1002/ece3.9409 (PMC9557235; doi:10.1002/ece3.9409)
Supplement: Supplementary file 11 — Table S5 [file ECE3-12-e9409-s009.pdf]

Table S5 Genetic diversity and demographic statistics for mtDNA (combining cytochondrial *b* gene and d - loop) and 2b-Rad sequences of Chinese house rats

	mtDNA						2b-Rad sequences		
Grouping	n	N	*h*±SD	*π*±SD	Tajima’s D	Fu's	n	*π*	Tajima’s D
R. norvegicus	307	106	0.9763 ± 0.0027	0.0089 ± 0.0041	-1.7795**	- 24.1172**	49	0.00246	-0.28688
R. tanezumi	179	36	0.8091 ± 0.0276	0.0052 ± 0.0023	-1.4188*	- 2.137ns	74	0.00921	0.72045

n, number of individuals; N, number of haplotypes; *h*, haplotype diversity; *π*, nucleotide diversity; Tajima’s D, Tajima’s D value; SSD, sum of squared deviations (goodness-of-fit to a simulated population expansion); Raggedness, raggedness index; Fu's, F*s* test of selective neutrality (ns: P> 0.05, *: P < 0.05, **: P < 0.01, ***: P < 0.001).
