# Supplementary material for: Comparative phylogeography of two commensal rat species (Rattus tanezumi and Rattus norvegicus) in China: Insights from mitochondrial DNA, microsatellite, and 2b‐RAD data
Source: Ecol Evol. 2022 Oct 13;12(10):e9409. doi: 10.1002/ece3.9409 (PMC9557235; doi:10.1002/ece3.9409)
Supplement: Supplementary file 12 — Table S6 [file ECE3-12-e9409-s012.pdf]

Table 2 Characteristics of 10 microsatellite loci genotyped in 462 Chinese house rats from 28 localities

Locus	Chromosome no.	Allele size (range)	N_A	N_E	H_O	H_E	F_{St}	P
R158	1	85 - 148	8.929	5.606	0.801	0.776	0.133	0.985
R8	2	197 - 384	10.214	7.330	0.813	0.981	0.172	1.000
R29	3	215 - 327	7.857	4.830	0.662	0.708	0.237	1.000
R203	7	80 - 274	7.393	4.958	0.728	0.716	0.212	1.000
R102	8	108 - 142	7.250	4.390	0.726	0.744	0.171	1.000
R7	10	88 - 148	8.607	5.875	0.781	0.774	0.179	1.000
R137	12	108 - 203	6.571	3.885	0.627	0.619	0.247	0.989
R36	18	86 - 131	6.000	3.604	0.621	0.690	0.200	1.000
R60	19	58 - 103	7.964	4.857	0.586	0.716	0.233	1.000
R145	20	90 - 136	6.714	4.547	0.758	0.741	0.158	0.999

N_A, number of alleles; N_E, number of effective alleles; H_O, observed heterozygosity; H_E, expected heterozygosity; F_{St}, A measure of allele frequency differences across populations; P, p value of Hardy-Weinberg test in all populations.
