# Supplementary material for: Comparative phylogeography of two commensal rat species (Rattus tanezumi and Rattus norvegicus) in China: Insights from mitochondrial DNA, microsatellite, and 2b‐RAD data
Source: Ecol Evol. 2022 Oct 13;12(10):e9409. doi: 10.1002/ece3.9409 (PMC9557235; doi:10.1002/ece3.9409)
Supplement: Supplementary file 13 — Table S7 [file ECE3-12-e9409-s017.pdf]

Table S7 Percentage of pairwise comparisons of individuals that fell into each relatedness category (e.g. unrelated, half-sibling, full-sibling) as calculated in ML-Relate (Kalinowski *et al*. 2006). Comparisons were made among all individuals and among individuals within populations.

| | **All** | **MH (27)** | **MS (10)** | **SY (14)** | **MJ (16)** | **SL (6)** | **UQ (15)** | **BT (21)** | **CY (5)** | **DL (18)** | **YP (39)** | **QX (5)** | **LI (30)** | **HZ (5)** | **RG (23)** | **MY (5)** | **YM (27)** | **CD (31)** | **HS (5)** | **LS (23)** | **JW (7)** | **JJ (8)** | **LZ (10)** | **CS (30)** | **LD (5)** | **JO (9)** | **KM (22)** | **GZ (27)** | **ZJ (19)** |
|---|---|---|---|---|---|---|---|---|---|---|---|---|---|---|---|---|---|---|---|---|---|---|---|---|---|---|---|---|---|
| Unrelated | 94.92 | 88.61 | 95.55 | 96.70 | 94.17 | 100 | 88.57 | 96.22 | 100 | 92.81 | 96.63 | 100 | 93.78 | 100 | 93.28 | 100 | 91.17 | 89.24 | 100 | 86.10 | 100 | 96.55 | 93.34 | 95.18 | 100 | 97.22 | 95.67 | 96.02 | 91.81 |
| Half-sibling | 4.70 | 7.12 | 2.22 | 1.10 | 3.33 | 0 | 7.62 | 2.86 | 0 | 7.19 | 2.02 | 0 | 1.61 | 0 | 4.74 | 0 | 5.13 | 4.09 | 0 | 9.95 | 0 | 3.45 | 3.33 | 2.99 | 0 | 0 | 1.30 | 2.56 | 4.68 |
| Full-sibling | 0.36 | 2.85 | 2.22 | 2.20 | 2.50 | 0 | 3.81 | 0.95 | 0 | 0 | 1.35 | 0 | 4.61 | 0 | 1.98 | 0 | 3.70 | 6.67 | 0 | 3.95 | 0 | 0 | 3.33 | 1.83 | 0 | 2.78 | 3.03 | 1.42 | 3.51 |
| Paren-Offspring | 0.02 | 1.42 | 0 | 0 | 0 | 0 | 0 | 0 | 0 | 0 | 0 | 0 | 0 | 0 | 0 | 0 | 0 | 0 | 0 | 0 | 0 | 0 | 0 | 0 | 0 | 0 | 0 | 0 | 0 |
