# Supplementary material for: Comparative phylogeography of two commensal rat species (Rattus tanezumi and Rattus norvegicus) in China: Insights from mitochondrial DNA, microsatellite, and 2b‐RAD data
Source: Ecol Evol. 2022 Oct 13;12(10):e9409. doi: 10.1002/ece3.9409 (PMC9557235; doi:10.1002/ece3.9409)
Supplement: Supplementary file 14 — Table S8 [file ECE3-12-e9409-s001.pdf]

Table S9 Q value and ratio of hybrid for each population of Chinese house rats calculated by STRUCTURE

Population	Inferred clusters		Assignment		
	1	2	Number of pure	Number of hybrid	Percent of hybrid
MH	0.022	0.978	27	2	6.70%
MS	0.018	0.982	10	0	0
SY	0.011	0.989	14	0	0
MJ	0.071	0.929	16	0	0
SL	0.125	0.875	5	1	16.67%
UQ	0.005	0.995	15	0	0
BT	0.128	0.872	15	6	28.57%
CY	0.007	0.993	5	0	0
DL	0.008	0.992	18	0	0
YP	0.015	0.985	38	1	2.56%
QX	0.009	0.991	5	0	0
LI	0.044	0.956	29	2	6.45%
HZ	0.016	0.984	5	0	0
RG	0.962	0.038	22	1	4.34%
MY	0.400	0.600	3	2	40.00%
YM	0.905	0.095	23	5	17.86%
CD	0.907	0.093	26	3	10.34%
HS	0.707	0.293	4	1	20.00%
LS	0.996	0.004	23	0	0
JW	0.199	0.801	5	2	28.57%
JJ	0.008	0.992	8	0	0
LZ	0.006	0.994	10	0	0
CS	0.732	0.268	25	5	16.67%
LD	0.922	0.078	4	1	20%
JO	0.799	0.201	6	3	33.33%
KM	0.067	0.933	20	2	9.09%
GZ	0.830	0.170	21	6	22.22%
ZJ	0.012	0.988	19	0	0
