# Supplementary material for: Comparative phylogeography of two commensal rat species (Rattus tanezumi and Rattus norvegicus) in China: Insights from mitochondrial DNA, microsatellite, and 2b‐RAD data
Source: Ecol Evol. 2022 Oct 13;12(10):e9409. doi: 10.1002/ece3.9409 (PMC9557235; doi:10.1002/ece3.9409)
Supplement: Supplementary file 15 — Table S9 [file ECE3-12-e9409-s011.pdf]

Sample_ID	species	Clean data	Enzyme	Percentage	Tag number	Depth	Mapping rate
LI 2	*R. norvegicus*	29,771,843	20,549,043	69.02%	923,413	15.91	72.20%
YP12	*R. norvegicus*	32,073,738	30,073,535	93.76%	984,106	22.85	74.97%
YP17	*R. norvegicus*	32,073,738	30,357,843	94.65%	725,475	31.55	76.11%
KM28	*R. norvegicus*	32,073,738	30,726,868	95.80%	719,168	31.83	75.26%
KM29	*R. norvegicus*	37,656,651	32,385,388	86.00%	821,883	28.66	73.34%
DL12	*R. norvegicus*	37,656,651	36,512,279	96.96%	993,771	27.67	75.47%
JO2	*R. norvegicus*	32,073,738	21,002,007	65.48%	849,221	17.84	73.02%
JO4	*R. norvegicus*	31,547,315	20,256,215	64.21%	708,993	20.63	73.48%
JO10	*R. norvegicus*	28,162,076	27,309,975	96.97%	547,004	37.09	74.93%
UQ01	*R. norvegicus*	28,162,076	20,611,872	73.19%	925,482	16.35	74.05%
UQ08	*R. norvegicus*	42,492,177	36,508,135	85.92%	832,249	32.21	73.91%
GZ24	*R. norvegicus*	37,843,815	32,804,776	86.68%	731,366	31.43	70.78%
GZ28	*R. norvegicus*	37,843,815	35,142,111	92.86%	626,105	39.76	71.60%
MH3	*R. norvegicus*	30,196,748	25,692,934	85.09%	834,682	22.61	74.16%
MH28	*R. norvegicus*	30,196,748	28,539,173	94.51%	975,729	21.91	75.14%
HS3	*R. norvegicus*	30,196,748	28,654,455	94.89%	839,213	24.66	72.87%
HS5	*R. norvegicus*	24,620,532	12,295,736	49.94%	692,736	12.55	73.16%
BT23	*R. norvegicus*	24,620,532	23,590,877	95.82%	669,206	25.46	73.15%
BT17	*R. norvegicus*	24,620,532	23,717,175	96.33%	821,387	20.84	73.00%
CS2	*R. norvegicus*	38,671,934	30,487,705	78.84%	763,087	28.78	72.76%
CS5	*R. norvegicus*	38,671,934	37,775,951	97.68%	809,741	33.92	73.18%
CS7	*R. norvegicus*	37,346,052	28,930,312	77.47%	772,674	26.17	70.67%
CS8	*R. norvegicus*	37,346,052	36,134,402	96.76%	984,498	27.00	73.74%
CS9	*R. norvegicus*	37,346,052	35,768,298	95.78%	916,910	27.93	71.95%
CS10	*R. norvegicus*	37,346,052	35,968,191	96.31%	890,734	28.55	71.08%
CS26	*R. norvegicus*	44,714,526	31,657,963	70.80%	995,231	23.60	74.38%
CS32	*R. norvegicus*	41,744,899	39,938,728	95.67%	913,801	32.01	73.54%
CS33	*R. norvegicus*	41,744,899	29,040,503	69.57%	984,569	21.48	73.08%
CS34	*R. norvegicus*	33,813,044	25,941,500	76.72%	753,132	25.20	74.08%
CD7	*R. norvegicus*	33,813,044	22,844,485	67.56%	954,514	17.32	72.83%
CD20	*R. norvegicus*	33,240,990	26,079,889	78.46%	756,407	24.80	72.85%
CD30	*R. norvegicus*	36,393,245	28,688,408	78.83%	753,245	26.40	70.12%
CD33	*R. norvegicus*	36,393,245	35,119,525	96.50%	874,540	28.54	71.50%
CD36	*R. norvegicus*	31,547,315	30,391,564	96.34%	959,846	23.60	74.85%
CD37	*R. norvegicus*	31,547,315	30,775,724	97.55%	805,712	27.99	73.93%
CD38	*R. norvegicus*	31,547,315	27,859,528	88.31%	576,031	27.62	58.17%
SY2	*R. norvegicus*	32,613,716	25,459,958	78.07%	734,251	24.94	72.88%
SY7	*R. norvegicus*	32,613,716	31,180,539	95.61%	890,101	26.40	75.84%
ZJ6	*R. norvegicus*	32,613,716	29,817,081	91.42%	892,024	22.56	68.02%
ZJ7	*R. norvegicus*	32,613,716	30,327,054	92.99%	836,020	23.78	66.18%
MY1	*R. norvegicus*	32,613,716	22,243,722	68.20%	953,921	16.95	73.17%
MY2	*R. norvegicus*	35,768,233	27,440,838	76.72%	766,739	26.34	74.44%
MY3	*R. norvegicus*	35,768,233	34,034,731	95.15%	984,970	25.64	74.41%
MY5	*R. norvegicus*	35,768,233	33,099,906	92.54%	907,473	26.38	72.72%
JJ3	*R. norvegicus*	29,771,843	25,372,482	85.22%	760,937	24.36	73.97%
JJ4	*R. norvegicus*	29,771,843	28,562,251	95.94%	971,015	21.89	74.73%
JW1	*R. norvegicus*	29,771,843	28,812,787	96.78%	866,236	22.76	69.03%
JW2	*R. norvegicus*	29,771,843	28,587,763	96.02%	853,174	22.52	67.83%
DL15	*R. norvegicus*	37,656,651	36,877,973	97.93%	658,474	32.97	59.23%
MY4	*R. tanezumi*	35,768,233	34,634,449	96.83%	680,964	29.33	58.02%
GZ48	*R. tanezumi*	33,982,164	31,916,739	93.92%	534,324	34.85	58.90%
LS22	*R. tanezumi*	37,656,651	27,796,358	73.82%	813,027	24.33	71.92%
CS3	*R. tanezumi*	38,671,934	36,759,499	95.05%	688,142	30.23	56.90%

CS4	*R. tanezumi*	38,671,934	37,759,768	97.64%	681,564	32.28	58.60%
CS6	*R. tanezumi*	38,671,934	25,150,712	65.04%	668,648	22.07	59.21%
CS11	*R. tanezumi*	37,346,052	24,397,322	65.33%	715,109	20.03	59.06%
CS12	*R. tanezumi*	37,083,746	28,973,238	78.13%	590,923	28.48	58.73%
CS13	*R. tanezumi*	37,083,746	35,627,060	96.07%	710,498	30.12	60.32%
CS14	*R. tanezumi*	37,083,746	35,858,492	96.70%	676,399	30.91	58.63%
CS15	*R. tanezumi*	37,083,746	35,928,411	96.88%	675,664	30.66	58.03%
CS16	*R. tanezumi*	37,083,746	24,314,853	65.57%	737,588	19.22	58.59%
CS17	*R. tanezumi*	41,826,997	32,861,827	78.57%	568,236	34.34	59.90%
CS18	*R. tanezumi*	41,826,997	40,231,696	96.19%	732,756	32.65	59.65%
CS19	*R. tanezumi*	41,826,997	39,446,736	94.31%	617,881	37.77	59.53%
CS20	*R. tanezumi*	41,826,997	40,644,415	97.17%	697,544	33.64	58.00%
CS21	*R. tanezumi*	41,826,997	29,238,320	69.90%	731,444	23.32	58.68%
CS22	*R. tanezumi*	44,714,526	35,414,240	79.20%	643,890	32.13	58.81%
CS23	*R. tanezumi*	44,714,526	42,908,268	95.96%	752,681	33.44	58.78%
CS24	*R. tanezumi*	44,714,526	43,063,681	96.31%	683,895	37.21	59.34%
CS25	*R. tanezumi*	44,714,526	43,182,832	96.57%	641,214	39.80	59.43%
CS28	*R. tanezumi*	41,744,899	33,270,679	79.70%	564,883	35.22	60.34%
CS30	*R. tanezumi*	41,744,899	39,681,159	95.06%	729,753	32.19	59.39%
CS31	*R. tanezumi*	41,744,899	40,436,815	96.87%	697,636	33.60	58.22%
CD4	*R. tanezumi*	33,813,044	32,599,970	96.41%	733,691	26.06	58.87%
CD5	*R. tanezumi*	33,813,044	32,456,535	95.99%	639,330	29.94	59.39%
CD6	*R. tanezumi*	33,813,044	32,555,246	96.28%	648,816	28.88	58.00%
CD10	*R. tanezumi*	34,588,980	26,735,150	77.29%	571,177	26.34	57.03%
CD12	*R. tanezumi*	34,588,980	33,809,812	97.75%	731,988	27.29	59.31%
CD14	*R. tanezumi*	34,588,980	33,721,190	97.49%	670,850	29.34	58.73%
CD15	*R. tanezumi*	34,588,980	33,673,954	97.35%	663,540	29.17	57.84%
CD18	*R. tanezumi*	34,588,980	24,894,943	71.97%	659,561	22.37	59.87%
CD24	*R. tanezumi*	33,240,990	31,877,150	95.90%	715,606	26.23	59.16%
CD26	*R. tanezumi*	33,240,990	32,259,890	97.05%	676,142	27.88	58.79%
CD27	*R. tanezumi*	33,240,990	31,710,520	95.40%	675,589	26.87	57.62%
CD28	*R. tanezumi*	33,240,990	22,526,002	67.77%	719,586	18.13	58.36%
CD31	*R. tanezumi*	36,393,245	34,992,722	96.15%	746,869	27.41	58.75%
CD32	*R. tanezumi*	36,393,245	35,031,330	96.26%	693,310	29.17	58.07%
CD34	*R. tanezumi*	36,393,245	24,566,286	67.50%	675,005	21.39	59.37%
CD35	*R. tanezumi*	28,284,941	27,145,368	95.97%	723,265	21.97	58.93%
CD42	*R. tanezumi*	28,162,076	24,231,557	86.04%	551,856	25.44	58.95%
CD44	*R. tanezumi*	28,162,076	27,016,393	95.93%	721,359	21.62	58.17%
CD45	*R. tanezumi*	28,162,076	26,864,090	95.39%	659,868	23.30	57.80%
MY6	*R. tanezumi*	35,768,233	25,757,375	72.01%	752,067	19.99	58.64%
JW4	*R. tanezumi*	20,864,677	19,748,556	94.65%	560,060	19.40	56.17%
JW6	*R. tanezumi*	20,864,677	13,311,950	63.80%	551,084	13.64	58.12%
JW7	*R. tanezumi*	24,620,532	15,985,965	64.93%	606,336	15.50	59.90%
LS17	*R. tanezumi*	37,656,651	36,717,766	97.51%	647,623	33.04	58.72%
JO1	*R. tanezumi*	28,284,941	27,097,972	95.80%	621,848	25.31	58.77%
JO3	*R. tanezumi*	28,284,941	18,618,462	65.82%	567,092	19.34	60.01%
JO5	*R. tanezumi*	28,284,941	27,279,450	96.45%	617,352	25.43	58.22%
JO7	*R. tanezumi*	31,547,315	15,755,034	49.94%	529,851	16.84	58.09%
JO8	*R. tanezumi*	28,284,941	14,347,083	50.72%	499,724	16.13	57.86%
GZ21	*R. tanezumi*	42,492,177	41,099,968	96.72%	628,560	37.25	57.41%
GZ22	*R. tanezumi*	42,492,177	31,875,628	75.02%	698,727	26.17	57.83%
GZ25	*R. tanezumi*	37,843,815	34,694,607	91.68%	626,523	32.21	58.69%
GZ26	*R. tanezumi*	37,843,815	35,885,350	94.82%	597,186	33.29	55.91%
GZ29	*R. tanezumi*	37,843,815	27,785,679	73.42%	619,140	25.76	58.14%

GZ30	*R. tanezumi*	33,671,093	29,109,245	86.45%	450,988	35.66	56.08%
GZ31	*R. tanezumi*	33,671,093	30,987,296	92.03%	613,155	28.72	57.41%
GZ32	*R. tanezumi*	33,671,093	32,853,370	97.57%	514,244	38.23	60.45%
GZ33	*R. tanezumi*	33,671,093	32,484,648	96.48%	644,028	29.04	58.03%
GZ34	*R. tanezumi*	33,671,093	25,910,459	76.95%	723,608	20.48	57.57%
GZ35	*R. tanezumi*	33,968,172	29,621,770	87.20%	613,536	27.91	58.39%
GZ40	*R. tanezumi*	33,968,172	32,941,909	96.98%	718,196	26.86	58.88%
GZ41	*R. tanezumi*	33,968,172	33,040,323	97.27%	629,548	29.84	57.32%
GZ42	*R. tanezumi*	33,968,172	32,897,515	96.85%	612,715	30.31	56.98%
GZ44	*R. tanezumi*	33,968,172	25,001,823	73.60%	619,423	22.94	57.54%
GZ45	*R. tanezumi*	33,982,164	28,861,289	84.93%	497,369	33.28	58.14%
GZ46	*R. tanezumi*	33,982,164	32,294,244	95.03%	705,886	26.01	57.20%
GZ49	*R. tanezumi*	33,982,164	29,180,087	85.87%	484,667	34.72	58.44%
GZ50	*R. tanezumi*	33,982,164	24,448,423	71.94%	535,225	26.29	58.43%
HS2	*R. tanezumi*	30,196,748	28,699,159	95.04%	665,752	25.48	59.55%
JO9	*R. tanezumi*	20,864,677	20,064,798	96.17%	588,794	19.52	58.30%
		34,561,844	30,036,366	86.75%	88,613,574	39.80	64.12%
