# Supplementary material for: Comparative phylogeography of two commensal rat species (Rattus tanezumi and Rattus norvegicus) in China: Insights from mitochondrial DNA, microsatellite, and 2b‐RAD data
Source: Ecol Evol. 2022 Oct 13;12(10):e9409. doi: 10.1002/ece3.9409 (PMC9557235; doi:10.1002/ece3.9409)
Supplement: Supplementary file 18 — Table S12 [file ECE3-12-e9409-s006.pdf]

SNP	chr	pos	Dxy	Fst
NC_005100.4_1400001	1	1400001	0.709	0.7911
NC_005100.4_1500001	1	1500001	0.77	0.8116
NC_005100.4_1600001	1	1600001	0.77	0.8116
NC_005100.4_1700001	1	1700001	0.7691	0.8183
NC_005100.4_1800001	1	1800001	0.6761	0.731
NC_005100.4_1900001	1	1900001	0.5956	0.6496
NC_005100.4_2000001	1	2000001	0.4301	0.4923
NC_005100.4_2100001	1	2100001	0.49	0.5501
NC_005100.4_2200001	1	2200001	0.5368	0.5651
NC_005100.4_2300001	1	2300001	0.5607	0.6345
NC_005100.4_2400001	1	2400001	0.5502	0.6279
NC_005100.4_2500001	1	2500001	0.7091	0.756
NC_005100.4_2600001	1	2600001	0.7179	0.7922
NC_005100.4_2700001	1	2700001	0.6683	0.7823
NC_005100.4_2800001	1	2800001	0.7405	0.8101
NC_005100.4_2900001	1	2900001	0.7864	0.8833
NC_005100.4_3000001	1	3000001	0.6922	0.8405
NC_005100.4_3100001	1	3100001	0.6136	0.7675
NC_005100.4_3200001	1	3200001	0.5888	0.7777
NC_005100.4_3300001	1	3300001	0.5678	0.7215
NC_005100.4_3400001	1	3400001	0.5311	0.6543
NC_005100.4_3500001	1	3500001	0.5364	0.634
NC_005100.4_3600001	1	3600001	0.5673	0.6482
NC_005100.4_3700001	1	3700001	0.5504	0.6199
NC_005100.4_3800001	1	3800001	0.5467	0.6479
NC_005100.4_3900001	1	3900001	0.5808	0.6974
NC_005100.4_4000001	1	4000001	0.6003	0.7229
NC_005100.4_4100001	1	4100001	0.591	0.7323
NC_005100.4_4200001	1	4200001	0.6192	0.6928
NC_005100.4_4300001	1	4300001	0.5857	0.6591
NC_005100.4_4400001	1	4400001	0.6229	0.7282
NC_005100.4_4500001	1	4500001	0.5989	0.656
NC_005100.4_4600001	1	4600001	0.6343	0.6787
NC_005100.4_4700001	1	4700001	0.7118	0.7994
NC_005100.4_4800001	1	4800001	0.6714	0.7616
NC_005100.4_4900001	1	4900001	0.6843	0.7019
NC_005100.4_5000001	1	5000001	0.6843	0.7156
NC_005100.4_5100001	1	5100001	0.6341	0.6761
NC_005100.4_5200001	1	5200001	0.6136	0.6554
NC_005100.4_5300001	1	5300001	0.6816	0.6912
NC_005100.4_5400001	1	5400001	0.6342	0.6203
NC_005100.4_5500001	1	5500001	0.6325	0.6662
NC_005100.4_5600001	1	5600001	0.6958	0.7358
NC_005100.4_5700001	1	5700001	0.5654	0.6373
NC_005100.4_5800001	1	5800001	0.4751	0.5894
NC_005100.4_5900001	1	5900001	0.4097	0.568
NC_005100.4_6000001	1	6000001	0.4088	0.5489
NC_005100.4_6100001	1	6100001	0.4292	0.5596
NC_005100.4_6200001	1	6200001	0.5235	0.6495
NC_005100.4_6300001	1	6300001	0.5162	0.617
NC_005100.4_6400001	1	6400001	0.5117	0.5911
NC_005100.4_6500001	1	6500001	0.5032	0.6089
NC_005100.4_6600001	1	6600001	0.4762	0.5913
NC_005100.4_6700001	1	6700001	0.4258	0.55
NC_005100.4_6800001	1	6800001	0.4211	0.5461
NC_005100.4_6900001	1	6900001	0.5878	0.7564
NC_005100.4_7000001	1	7000001	0.6036	0.789

NC_005100.4_7100001	1	7100001	0.6459	0.8002
NC_005100.4_7200001	1	7200001	0.5971	0.7727
NC_005100.4_7300001	1	7300001	0.6189	0.8025
NC_005100.4_7400001	1	7400001	0.5523	0.7362
NC_005100.4_7500001	1	7500001	0.5002	0.6957
NC_005100.4_7600001	1	7600001	0.4706	0.6934
NC_005100.4_7700001	1	7700001	0.5493	0.7483
NC_005100.4_7800001	1	7800001	0.5522	0.7146
NC_005100.4_7900001	1	7900001	0.5139	0.6687
NC_005100.4_8000001	1	8000001	0.6519	0.7408
NC_005100.4_8100001	1	8100001	0.6426	0.7451
NC_005100.4_8200001	1	8200001	0.5411	0.6871
NC_005100.4_8300001	1	8300001	0.4876	0.7436
NC_005100.4_8400001	1	8400001	0.5514	0.825
NC_005100.4_8500001	1	8500001	0.2786	0.7347
NC_005100.4_8600001	1	8600001	0.3957	0.7989
NC_005100.4_8700001	1	8700001	0.4426	0.8114
NC_005100.4_8800001	1	8800001	0.6837	0.8971
NC_005100.4_8900001	1	8900001	0.3783	0.6992
NC_005100.4_9000001	1	9000001	0.4659	0.7643
NC_005100.4_9100001	1	9100001	0.2561	0.4185
NC_005100.4_9200001	1	9200001	0.362	0.577
NC_005100.4_9300001	1	9300001	0.2969	0.4799
NC_005100.4_9400001	1	9400001	0.4713	0.5522
NC_005100.4_9500001	1	9500001	0.4368	0.5417
NC_005100.4_9600001	1	9600001	0.4751	0.5994
NC_005100.4_9700001	1	9700001	0.4178	0.4882
NC_005100.4_9800001	1	9800001	0.414	0.3948
NC_005100.4_9900001	1	9900001	0.5107	0.5044
NC_005100.4_10000001	1	10000001	0.6523	0.5879
NC_005100.4_10100001	1	10100001	0.6539	0.6455
NC_005100.4_10200001	1	10200001	0.6464	0.6161
NC_005100.4_10300001	1	10300001	0.7029	0.681
NC_005100.4_10400001	1	10400001	0.6577	0.6777
NC_005100.4_10500001	1	10500001	0.5893	0.6239
NC_005100.4_10600001	1	10600001	0.5697	0.5666
NC_005100.4_10700001	1	10700001	0.657	0.6939
NC_005100.4_10800001	1	10800001	0.6876	0.7874
NC_005100.4_10900001	1	10900001	0.6361	0.7223
NC_005100.4_11000001	1	11000001	0.7611	0.8661
NC_005100.4_11100001	1	11100001	0.7611	0.8661
NC_005100.4_11200001	1	11200001	0.4996	0.7732
NC_005100.4_11300001	1	11300001	0.5022	0.7593
NC_005100.4_11400001	1	11400001	0.5381	0.8382
NC_005100.4_11500001	1	11500001	0.4247	0.777
NC_005100.4_11600001	1	11600001	0.4247	0.777
NC_005100.4_12100001	1	12100001	0.6582	0.8098
NC_005100.4_12200001	1	12200001	0.6984	0.8152
NC_005100.4_12300001	1	12300001	0.6518	0.8242
NC_005100.4_12400001	1	12400001	0.6261	0.8121
NC_005100.4_12500001	1	12500001	0.651	0.8548
NC_005100.4_12600001	1	12600001	0.7142	0.8649
NC_005100.4_12700001	1	12700001	0.609	0.8169
NC_005100.4_12800001	1	12800001	0.5764	0.8081
NC_005100.4_12900001	1	12900001	0.5717	0.8047
NC_005100.4_13000001	1	13000001	0.581	0.7685
NC_005100.4_13100001	1	13100001	0.567	0.7295
NC_005100.4_13200001	1	13200001	0.6275	0.7622

NC_005100.4_13300001	1	13300001	0.7049	0.757
NC_005100.4_13400001	1	13400001	0.5887	0.708
NC_005100.4_13500001	1	13500001	0.5013	0.6876
NC_005100.4_13600001	1	13600001	0.3915	0.6928
NC_005100.4_13700001	1	13700001	0.3215	0.6574
NC_005100.4_13800001	1	13800001	0.3559	0.6821
NC_005100.4_13900001	1	13900001	0.4768	0.7746
NC_005100.4_14000001	1	14000001	0.5455	0.7716
NC_005100.4_14100001	1	14100001	0.734	0.8122
NC_005100.4_14200001	1	14200001	0.725	0.7916
NC_005100.4_14300001	1	14300001	0.6936	0.7546
NC_005100.4_14400001	1	14400001	0.559	0.7169
NC_005100.4_14500001	1	14500001	0.6528	0.7622
NC_005100.4_14600001	1	14600001	0.6414	0.7254
NC_005100.4_14700001	1	14700001	0.6418	0.7179
NC_005100.4_14800001	1	14800001	0.6384	0.7495
NC_005100.4_14900001	1	14900001	0.6951	0.7574
NC_005100.4_15000001	1	15000001	0.6391	0.7483
NC_005100.4_15100001	1	15100001	0.6186	0.777
NC_005100.4_15200001	1	15200001	0.6573	0.8237
NC_005100.4_15300001	1	15300001	0.637	0.716
NC_005100.4_15400001	1	15400001	0.6654	0.7442
NC_005100.4_15500001	1	15500001	0.5861	0.6813
NC_005100.4_15600001	1	15600001	0.5887	0.6466
NC_005100.4_15700001	1	15700001	0.5219	0.595
NC_005100.4_15800001	1	15800001	0.5488	0.7227
NC_005100.4_15900001	1	15900001	0.5183	0.7342
NC_005100.4_16000001	1	16000001	0.5009	0.7611
NC_005100.4_16100001	1	16100001	0.5355	0.747
NC_005100.4_16200001	1	16200001	0.4971	0.7067
NC_005100.4_16300001	1	16300001	0.4245	0.6668
NC_005100.4_16400001	1	16400001	0.4153	0.6467
NC_005100.4_16500001	1	16500001	0.4567	0.6819
NC_005100.4_16600001	1	16600001	0.4189	0.6719
NC_005100.4_16700001	1	16700001	0.429	0.6448
NC_005100.4_16800001	1	16800001	0.5189	0.6675
NC_005100.4_16900001	1	16900001	0.3926	0.545
NC_005100.4_17000001	1	17000001	0.427	0.6229
NC_005100.4_17100001	1	17100001	0.4949	0.6917
NC_005100.4_17200001	1	17200001	0.4519	0.7076
NC_005100.4_17300001	1	17300001	0.4366	0.72
NC_005100.4_17400001	1	17400001	0.4686	0.7394
NC_005100.4_17500001	1	17500001	0.4584	0.6797
NC_005100.4_17600001	1	17600001	0.3909	0.6519
NC_005100.4_17700001	1	17700001	0.4402	0.7288
NC_005100.4_17800001	1	17800001	0.4994	0.7813
NC_005100.4_17900001	1	17900001	0.4888	0.7577
NC_005100.4_18000001	1	18000001	0.4934	0.8311
NC_005100.4_18100001	1	18100001	0.5314	0.8489
NC_005100.4_18200001	1	18200001	0.5733	0.6961
NC_005100.4_18300001	1	18300001	0.683	0.7171
NC_005100.4_18400001	1	18400001	0.7229	0.7575
NC_005100.4_18500001	1	18500001	0.7922	0.8228
NC_005100.4_18600001	1	18600001	0.7695	0.8033
NC_005100.4_18700001	1	18700001	0.8227	0.8831
NC_005100.4_18800001	1	18800001	0.6475	0.8272
NC_005100.4_18900001	1	18900001	0.5907	0.7185
NC_005100.4_19000001	1	19000001	0.5509	0.6441

NC_005100.4_19100001	1	19100001	0.5375	0.6353
NC_005100.4_19200001	1	19200001	0.5651	0.6537
NC_005100.4_19300001	1	19300001	0.6471	0.66
NC_005100.4_19400001	1	19400001	0.7803	0.8083
NC_005100.4_19500001	1	19500001	0.7516	0.8572
NC_005100.4_19600001	1	19600001	0.7754	0.8746
NC_005100.4_19700001	1	19700001	0.6487	0.8389
NC_005100.4_19800001	1	19800001	0.5407	0.6859
NC_005100.4_19900001	1	19900001	0.4987	0.6396
NC_005100.4_20000001	1	20000001	0.5024	0.5974
NC_005100.4_20100001	1	20100001	0.457	0.545
NC_005100.4_20200001	1	20200001	0.4678	0.4735
NC_005100.4_20300001	1	20300001	0.5319	0.5517
NC_005100.4_20400001	1	20400001	0.613	0.6465
NC_005100.4_20500001	1	20500001	0.6577	0.701
NC_005100.4_20600001	1	20600001	0.7755	0.8077
NC_005100.4_20700001	1	20700001	0.8146	0.8834
NC_005100.4_20800001	1	20800001	0.7176	0.838
NC_005100.4_20900001	1	20900001	0.6818	0.7868
NC_005100.4_21000001	1	21000001	0.6452	0.7712
NC_005100.4_21100001	1	21100001	0.574	0.7232
NC_005100.4_21200001	1	21200001	0.5021	0.6899
NC_005100.4_21300001	1	21300001	0.6462	0.8153
NC_005100.4_21400001	1	21400001	0.6012	0.7849
NC_005100.4_21500001	1	21500001	0.6077	0.7941
NC_005100.4_21600001	1	21600001	0.6196	0.7547
NC_005100.4_21700001	1	21700001	0.6623	0.7576
NC_005100.4_21800001	1	21800001	0.6923	0.7566
NC_005100.4_21900001	1	21900001	0.6997	0.7779
NC_005100.4_22000001	1	22000001	0.6774	0.7639
NC_005100.4_22100001	1	22100001	0.6563	0.7643
NC_005100.4_22200001	1	22200001	0.6643	0.8
NC_005100.4_22300001	1	22300001	0.5285	0.6703
NC_005100.4_22400001	1	22400001	0.5243	0.6498
NC_005100.4_22500001	1	22500001	0.5851	0.6873
NC_005100.4_22600001	1	22600001	0.6348	0.7065
NC_005100.4_22700001	1	22700001	0.6001	0.6667
NC_005100.4_22800001	1	22800001	0.6344	0.6932
NC_005100.4_22900001	1	22900001	0.6974	0.7129
NC_005100.4_23000001	1	23000001	0.5714	0.664
NC_005100.4_23100001	1	23100001	0.4802	0.5408
NC_005100.4_23200001	1	23200001	0.5373	0.5681
NC_005100.4_23300001	1	23300001	0.5135	0.5943
NC_005100.4_23400001	1	23400001	0.4934	0.5873
NC_005100.4_23500001	1	23500001	0.506	0.6306
NC_005100.4_23600001	1	23600001	0.4508	0.6059
NC_005100.4_23700001	1	23700001	0.4499	0.6518
NC_005100.4_23800001	1	23800001	0.4569	0.6692
NC_005100.4_23900001	1	23900001	0.4578	0.6582
NC_005100.4_24000001	1	24000001	0.5239	0.7029
NC_005100.4_24100001	1	24100001	0.5951	0.7622
NC_005100.4_24200001	1	24200001	0.6273	0.7962
NC_005100.4_24300001	1	24300001	0.6499	0.7946
NC_005100.4_24400001	1	24400001	0.7054	0.849
NC_005100.4_24500001	1	24500001	0.6591	0.8436
NC_005100.4_24600001	1	24600001	0.7532	0.8434
NC_005100.4_24800001	1	24800001	0.5099	0.4902
NC_005100.4_24900001	1	24900001	0.5599	0.5763

NC_005100.4_25000001	1	25000001	0.5759	0.6067
NC_005100.4_25100001	1	25100001	0.5874	0.5778
NC_005100.4_25200001	1	25200001	0.5218	0.5306
NC_005100.4_25300001	1	25300001	0.6194	0.6473
NC_005100.4_25400001	1	25400001	0.4732	0.4641
NC_005100.4_27100001	1	27100001	0.6708	0.7846
NC_005100.4_27200001	1	27200001	0.6203	0.7583
NC_005100.4_27300001	1	27300001	0.701	0.8182
NC_005100.4_27400001	1	27400001	0.6183	0.7672
NC_005100.4_27500001	1	27500001	0.5899	0.7594
NC_005100.4_27600001	1	27600001	0.546	0.7531
NC_005100.4_27700001	1	27700001	0.6475	0.8458
NC_005100.4_27800001	1	27800001	0.5896	0.8115
NC_005100.4_27900001	1	27900001	0.7633	0.9094
NC_005100.4_28000001	1	28000001	0.7816	0.9335
NC_005100.4_28100001	1	28100001	0.6595	0.879
NC_005100.4_28200001	1	28200001	0.5993	0.8304
NC_005100.4_28300001	1	28300001	0.4511	0.7035
NC_005100.4_28400001	1	28400001	0.5046	0.7249
NC_005100.4_28500001	1	28500001	0.4587	0.6085
NC_005100.4_28600001	1	28600001	0.4696	0.5808
NC_005100.4_28700001	1	28700001	0.4874	0.5428
NC_005100.4_28800001	1	28800001	0.6295	0.6621
NC_005100.4_28900001	1	28900001	0.5347	0.5667
NC_005100.4_29000001	1	29000001	0.7076	0.7376
NC_005100.4_29100001	1	29100001	0.6738	0.7038
NC_005100.4_29200001	1	29200001	0.7202	0.7869
NC_005100.4_29400001	1	29400001	0.7011	0.8326
NC_005100.4_29500001	1	29500001	0.5313	0.6571
NC_005100.4_29800001	1	29800001	0.7185	0.8114
NC_005100.4_29900001	1	29900001	0.5069	0.6936
NC_005100.4_30000001	1	30000001	0.3969	0.661
NC_005100.4_30100001	1	30100001	0.3974	0.6316
NC_005100.4_30200001	1	30200001	0.4481	0.7027
NC_005100.4_30300001	1	30300001	0.4561	0.736
NC_005100.4_30400001	1	30400001	0.5194	0.7711
NC_005100.4_30500001	1	30500001	0.5846	0.8391
NC_005100.4_30600001	1	30600001	0.5957	0.8652
NC_005100.4_30700001	1	30700001	0.6617	0.9022
NC_005100.4_30800001	1	30800001	0.6575	0.9009
NC_005100.4_31100001	1	31100001	0.5327	0.8568
NC_005100.4_31200001	1	31200001	0.4376	0.6697
NC_005100.4_31300001	1	31300001	0.4401	0.6573
NC_005100.4_31400001	1	31400001	0.5521	0.7651
NC_005100.4_31500001	1	31500001	0.6493	0.7779
NC_005100.4_31600001	1	31600001	0.7146	0.8164
NC_005100.4_31700001	1	31700001	0.8045	0.8709
NC_005100.4_31800001	1	31800001	0.843	0.8902
NC_005100.4_31900001	1	31900001	0.7054	0.7994
NC_005100.4_32000001	1	32000001	0.6028	0.797
NC_005100.4_32100001	1	32100001	0.5453	0.7229
NC_005100.4_32200001	1	32200001	0.4928	0.7021
NC_005100.4_32300001	1	32300001	0.4622	0.6722
NC_005100.4_32400001	1	32400001	0.4635	0.7221
NC_005100.4_32500001	1	32500001	0.5222	0.7445
NC_005100.4_32600001	1	32600001	0.5175	0.6949
NC_005100.4_32700001	1	32700001	0.6328	0.7811
NC_005100.4_32800001	1	32800001	0.6962	0.8066

NC_005100.4_32900001	1	32900001	0.7088	0.8065
NC_005100.4_33000001	1	33000001	0.7014	0.8033
NC_005100.4_33100001	1	33100001	0.6901	0.8143
NC_005100.4_33200001	1	33200001	0.6903	0.7762
NC_005100.4_33300001	1	33300001	0.6116	0.7181
NC_005100.4_33400001	1	33400001	0.6943	0.7274
NC_005100.4_33500001	1	33500001	0.6096	0.6865
NC_005100.4_33600001	1	33600001	0.6723	0.7261
NC_005100.4_33700001	1	33700001	0.5796	0.6287
NC_005100.4_33800001	1	33800001	0.5716	0.5902
NC_005100.4_33900001	1	33900001	0.6627	0.7002
NC_005100.4_34000001	1	34000001	0.6836	0.7267
NC_005100.4_34100001	1	34100001	0.6252	0.7036
NC_005100.4_34200001	1	34200001	0.6674	0.7526
NC_005100.4_34300001	1	34300001	0.6246	0.7445
NC_005100.4_34400001	1	34400001	0.5236	0.6458
NC_005100.4_34500001	1	34500001	0.5459	0.6625
NC_005100.4_34600001	1	34600001	0.5559	0.6612
NC_005100.4_34700001	1	34700001	0.5204	0.587
NC_005100.4_34800001	1	34800001	0.5428	0.6315
NC_005100.4_34900001	1	34900001	0.5189	0.589
NC_005100.4_35000001	1	35000001	0.5139	0.5593
NC_005100.4_35100001	1	35100001	0.5352	0.619
NC_005100.4_35200001	1	35200001	0.5475	0.6535
NC_005100.4_35300001	1	35300001	0.5947	0.7165
NC_005100.4_35400001	1	35400001	0.6583	0.7947
NC_005100.4_35500001	1	35500001	0.6531	0.7833
NC_005100.4_35600001	1	35600001	0.5934	0.74
NC_005100.4_35700001	1	35700001	0.5909	0.7408
NC_005100.4_35800001	1	35800001	0.5761	0.7413
NC_005100.4_35900001	1	35900001	0.6052	0.7735
NC_005100.4_36000001	1	36000001	0.6155	0.731
NC_005100.4_36100001	1	36100001	0.6337	0.7383
NC_005100.4_36200001	1	36200001	0.7138	0.797
NC_005100.4_36300001	1	36300001	0.658	0.7684
NC_005100.4_36400001	1	36400001	0.5281	0.6911
NC_005100.4_36500001	1	36500001	0.5287	0.836
NC_005100.4_36600001	1	36600001	0.5227	0.8519
NC_005100.4_36700001	1	36700001	0.4443	0.8326
NC_005100.4_36800001	1	36800001	0.5151	0.8162
NC_005100.4_36900001	1	36900001	0.4826	0.7741
NC_005100.4_37000001	1	37000001	0.4873	0.7873
NC_005100.4_37100001	1	37100001	0.5327	0.7528
NC_005100.4_37200001	1	37200001	0.6135	0.7094
NC_005100.4_37300001	1	37300001	0.5569	0.7663
NC_005100.4_37400001	1	37400001	0.6479	0.8615
NC_005100.4_37500001	1	37500001	0.6759	0.8762
NC_005100.4_37600001	1	37600001	0.7622	0.9545
NC_005100.4_37700001	1	37700001	0.7622	0.9545
NC_005100.4_37800001	1	37800001	0.8403	0.9615
NC_005100.4_38500001	1	38500001	0.4816	0.6656
NC_005100.4_39500001	1	39500001	0.4425	0.8659
NC_005100.4_39600001	1	39600001	0.522	0.8878
NC_005100.4_39700001	1	39700001	0.636	0.9044
NC_005100.4_39800001	1	39800001	0.6202	0.8319
NC_005100.4_39900001	1	39900001	0.6118	0.7731
NC_005100.4_40000001	1	40000001	0.6391	0.6923
NC_005100.4_40100001	1	40100001	0.5718	0.682

NC_005100.4_40200001	1	40200001	0.5691	0.6881
NC_005100.4_40300001	1	40300001	0.6726	0.7206
NC_005100.4_40400001	1	40400001	0.6321	0.7527
NC_005100.4_40500001	1	40500001	0.539	0.7296
NC_005100.4_40600001	1	40600001	0.6257	0.753
NC_005100.4_40700001	1	40700001	0.6036	0.733
NC_005100.4_40800001	1	40800001	0.6036	0.733
NC_005100.4_40900001	1	40900001	0.6211	0.732
NC_005100.4_41000001	1	41000001	0.8413	0.8877
NC_005100.4_41100001	1	41100001	0.6391	0.8081
NC_005100.4_41200001	1	41200001	0.678	0.8081
NC_005100.4_41300001	1	41300001	0.6166	0.774
NC_005100.4_41400001	1	41400001	0.581	0.7689
NC_005100.4_41500001	1	41500001	0.5256	0.707
NC_005100.4_41600001	1	41600001	0.5004	0.6666
NC_005100.4_41700001	1	41700001	0.4409	0.5781
NC_005100.4_41800001	1	41800001	0.4771	0.5852
NC_005100.4_41900001	1	41900001	0.5255	0.596
NC_005100.4_42000001	1	42000001	0.5575	0.5378
NC_005100.4_42100001	1	42100001	0.7024	0.5895
NC_005100.4_42200001	1	42200001	0.3999	0.3488
NC_005100.4_42300001	1	42300001	0.5897	0.5876
NC_005100.4_42400001	1	42400001	0.5685	0.551
NC_005100.4_42500001	1	42500001	0.5853	0.6108
NC_005100.4_42600001	1	42600001	0.5659	0.622
NC_005100.4_42700001	1	42700001	0.7107	0.7461
NC_005100.4_42800001	1	42800001	0.6205	0.6852
NC_005100.4_42900001	1	42900001	0.6867	0.7993
NC_005100.4_43000001	1	43000001	0.626	0.7169
NC_005100.4_43100001	1	43100001	0.5493	0.6515
NC_005100.4_43200001	1	43200001	0.5157	0.6387
NC_005100.4_43300001	1	43300001	0.5171	0.6588
NC_005100.4_43400001	1	43400001	0.4834	0.66
NC_005100.4_43500001	1	43500001	0.4815	0.6903
NC_005100.4_43600001	1	43600001	0.5251	0.7601
NC_005100.4_43700001	1	43700001	0.5282	0.7532
NC_005100.4_43800001	1	43800001	0.4655	0.6367
NC_005100.4_43900001	1	43900001	0.4693	0.5727
NC_005100.4_44000001	1	44000001	0.5235	0.6032
NC_005100.4_44100001	1	44100001	0.5212	0.5807
NC_005100.4_44200001	1	44200001	0.4793	0.5238
NC_005100.4_44300001	1	44300001	0.5162	0.5561
NC_005100.4_44400001	1	44400001	0.4885	0.5973
NC_005100.4_44500001	1	44500001	0.3992	0.5133
NC_005100.4_44600001	1	44600001	0.3331	0.4778
NC_005100.4_44700001	1	44700001	0.3438	0.5566
NC_005100.4_44800001	1	44800001	0.3417	0.592
NC_005100.4_44900001	1	44900001	0.369	0.6301
NC_005100.4_45000001	1	45000001	0.3496	0.5456
NC_005100.4_45100001	1	45100001	0.3331	0.5137
NC_005100.4_45200001	1	45200001	0.3658	0.4587
NC_005100.4_45300001	1	45300001	0.3871	0.4982
NC_005100.4_45400001	1	45400001	0.3942	0.4952
NC_005100.4_45500001	1	45500001	0.4773	0.5994
NC_005100.4_45600001	1	45600001	0.5421	0.6966
NC_005100.4_45700001	1	45700001	0.5441	0.7878
NC_005100.4_45800001	1	45800001	0.5213	0.7859
NC_005100.4_45900001	1	45900001	0.488	0.7758

NC_005100.4_46000001	1	46000001	0.4552	0.7353
NC_005100.4_46100001	1	46100001	0.3417	0.6046
NC_005100.4_46200001	1	46200001	0.3641	0.5785
NC_005100.4_46300001	1	46300001	0.3608	0.5823
NC_005100.4_46400001	1	46400001	0.426	0.6368
NC_005100.4_46500001	1	46500001	0.4329	0.662
NC_005100.4_46600001	1	46600001	0.5635	0.7526
NC_005100.4_46700001	1	46700001	0.4629	0.7007
NC_005100.4_46800001	1	46800001	0.5599	0.7529
NC_005100.4_46900001	1	46900001	0.5291	0.6912
NC_005100.4_47000001	1	47000001	0.4923	0.6488
NC_005100.4_47100001	1	47100001	0.4807	0.6255
NC_005100.4_47200001	1	47200001	0.5707	0.6835
NC_005100.4_47300001	1	47300001	0.5132	0.5975
NC_005100.4_47400001	1	47400001	0.5348	0.5979
NC_005100.4_47500001	1	47500001	0.574	0.6495
NC_005100.4_47600001	1	47600001	0.5229	0.6695
NC_005100.4_47700001	1	47700001	0.5084	0.6589
NC_005100.4_47800001	1	47800001	0.6825	0.8178
NC_005100.4_47900001	1	47900001	0.5962	0.7808
NC_005100.4_48000001	1	48000001	0.6236	0.8088
NC_005100.4_48100001	1	48100001	0.6524	0.8044
NC_005100.4_48200001	1	48200001	0.6446	0.8166
NC_005100.4_48300001	1	48300001	0.5255	0.7651
NC_005100.4_48400001	1	48400001	0.5991	0.83
NC_005100.4_48500001	1	48500001	0.6323	0.8486
NC_005100.4_48600001	1	48600001	0.6322	0.9
NC_005100.4_48700001	1	48700001	0.4805	0.7764
NC_005100.4_48800001	1	48800001	0.5353	0.8119
NC_005100.4_48900001	1	48900001	0.486	0.7324
NC_005100.4_49000001	1	49000001	0.4474	0.6377
NC_005100.4_49100001	1	49100001	0.4249	0.5936
NC_005100.4_49200001	1	49200001	0.5111	0.6462
NC_005100.4_49300001	1	49300001	0.5358	0.6331
NC_005100.4_49400001	1	49400001	0.5837	0.6791
NC_005100.4_49500001	1	49500001	0.6145	0.7096
NC_005100.4_49600001	1	49600001	0.6751	0.7447
NC_005100.4_49700001	1	49700001	0.6809	0.7458
NC_005100.4_49800001	1	49800001	0.5515	0.7092
NC_005100.4_49900001	1	49900001	0.6318	0.7766
NC_005100.4_50000001	1	50000001	0.5499	0.7233
NC_005100.4_50100001	1	50100001	0.5237	0.705
NC_005100.4_50200001	1	50200001	0.4974	0.6816
NC_005100.4_50300001	1	50300001	0.585	0.6977
NC_005100.4_50400001	1	50400001	0.4508	0.5694
NC_005100.4_50500001	1	50500001	0.5029	0.634
NC_005100.4_50800001	1	50800001	0.5605	0.7223
NC_005100.4_50900001	1	50900001	0.515	0.643
NC_005100.4_51000001	1	51000001	0.3602	0.5228
NC_005100.4_51100001	1	51100001	0.4199	0.5584
NC_005100.4_51200001	1	51200001	0.3711	0.4707
NC_005100.4_51300001	1	51300001	0.3477	0.4494
NC_005100.4_51400001	1	51400001	0.3672	0.535
NC_005100.4_51500001	1	51500001	0.5153	0.6557
NC_005100.4_51600001	1	51600001	0.4155	0.6149
NC_005100.4_51700001	1	51700001	0.4608	0.6691
NC_005100.4_51800001	1	51800001	0.521	0.6837
NC_005100.4_51900001	1	51900001	0.5677	0.6774

NC_005100.4_52000001	1	52000001	0.5769	0.645
NC_005100.4_52100001	1	52100001	0.6678	0.6588
NC_005100.4_52200001	1	52200001	0.6685	0.6868
NC_005100.4_52300001	1	52300001	0.6064	0.6491
NC_005100.4_52400001	1	52400001	0.5386	0.6494
NC_005100.4_52500001	1	52500001	0.5363	0.6779
NC_005100.4_52600001	1	52600001	0.4767	0.6659
NC_005100.4_52700001	1	52700001	0.4424	0.6306
NC_005100.4_52800001	1	52800001	0.4959	0.6684
NC_005100.4_52900001	1	52900001	0.5049	0.6548
NC_005100.4_53000001	1	53000001	0.4447	0.6002
NC_005100.4_53100001	1	53100001	0.4459	0.616
NC_005100.4_53200001	1	53200001	0.5051	0.647
NC_005100.4_53300001	1	53300001	0.4501	0.6294
NC_005100.4_53400001	1	53400001	0.4839	0.6144
NC_005100.4_53500001	1	53500001	0.3848	0.6513
NC_005100.4_53600001	1	53600001	0.3366	0.5091
NC_005100.4_53700001	1	53700001	0.396	0.5389
NC_005100.4_53800001	1	53800001	0.3731	0.5059
NC_005100.4_53900001	1	53900001	0.4971	0.6112
NC_005100.4_54000001	1	54000001	0.5993	0.6208
NC_005100.4_54100001	1	54100001	0.8223	0.7257
NC_005100.4_54200001	1	54200001	0.7728	0.7209
NC_005100.4_54300001	1	54300001	0.7728	0.7209
NC_005100.4_54400001	1	54400001	0.6546	0.5987
NC_005100.4_54500001	1	54500001	0.6024	0.5698
NC_005100.4_54600001	1	54600001	0.6024	0.5698
NC_005100.4_55200001	1	55200001	0.261	0.1657
NC_005100.4_55300001	1	55300001	0.2428	0.1847
NC_005100.4_55400001	1	55400001	0.2489	0.2098
NC_005100.4_55900001	1	55900001	0.6045	0.6565
NC_005100.4_56000001	1	56000001	0.512	0.6454
NC_005100.4_56100001	1	56100001	0.5273	0.67
NC_005100.4_56200001	1	56200001	0.4875	0.6407
NC_005100.4_56300001	1	56300001	0.4838	0.6469
NC_005100.4_56400001	1	56400001	0.5153	0.6916
NC_005100.4_56500001	1	56500001	0.4615	0.6072
NC_005100.4_56600001	1	56600001	0.4495	0.5796
NC_005100.4_56700001	1	56700001	0.5036	0.589
NC_005100.4_56800001	1	56800001	0.486	0.5683
NC_005100.4_56900001	1	56900001	0.506	0.5703
NC_005100.4_57000001	1	57000001	0.5788	0.6103
NC_005100.4_57100001	1	57100001	0.6442	0.6761
NC_005100.4_57200001	1	57200001	0.6701	0.7279
NC_005100.4_57300001	1	57300001	0.7485	0.8094
NC_005100.4_57400001	1	57400001	0.7712	0.9288
NC_005100.4_57500001	1	57500001	0.7169	0.8582
NC_005100.4_57600001	1	57600001	0.5246	0.6685
NC_005100.4_57700001	1	57700001	0.5221	0.6378
NC_005100.4_57800001	1	57800001	0.4866	0.6333
NC_005100.4_57900001	1	57900001	0.5253	0.6698
NC_005100.4_58000001	1	58000001	0.5458	0.6845
NC_005100.4_58100001	1	58100001	0.5931	0.7513
NC_005100.4_58200001	1	58200001	0.5931	0.7513
NC_005100.4_58300001	1	58300001	0.6915	0.8096
NC_005100.4_58400001	1	58400001	0.6477	0.7348
NC_005100.4_58600001	1	58600001	0.4917	0.64
NC_005100.4_58700001	1	58700001	0.6341	0.7203

NC_005100.4_58800001	1	58800001	0.6606	0.684
NC_005100.4_58900001	1	58900001	0.6405	0.6626
NC_005100.4_59000001	1	59000001	0.6178	0.6515
NC_005100.4_59100001	1	59100001	0.7436	0.7336
NC_005100.4_59300001	1	59300001	0.2938	0.4117
NC_005100.4_59400001	1	59400001	0.335	0.5069
NC_005100.4_59500001	1	59500001	0.4117	0.6325
NC_005100.4_59600001	1	59600001	0.4686	0.6944
NC_005100.4_59700001	1	59700001	0.5357	0.7551
NC_005100.4_59800001	1	59800001	0.566	0.6865
NC_005100.4_59900001	1	59900001	0.5744	0.6592
NC_005100.4_60000001	1	60000001	0.3964	0.4547
NC_005100.4_60100001	1	60100001	0.3266	0.3387
NC_005100.4_60200001	1	60200001	0.2574	0.2077
NC_005100.4_61000001	1	61000001	0.6198	0.7367
NC_005100.4_61100001	1	61100001	0.5691	0.6854
NC_005100.4_61200001	1	61200001	0.6496	0.6991
NC_005100.4_61300001	1	61300001	0.6947	0.7444
NC_005100.4_61400001	1	61400001	0.7729	0.8008
NC_005100.4_61900001	1	61900001	0.1952	0.1516
NC_005100.4_62000001	1	62000001	0.2649	0.2279
NC_005100.4_62100001	1	62100001	0.2489	0.2247
NC_005100.4_62200001	1	62200001	0.3325	0.3594
NC_005100.4_62300001	1	62300001	0.3322	0.4115
NC_005100.4_62400001	1	62400001	0.394	0.4609
NC_005100.4_62500001	1	62500001	0.3553	0.4903
NC_005100.4_63700001	1	63700001	0.0962	0.062
NC_005100.4_63800001	1	63800001	0.202	0.339
NC_005100.4_63900001	1	63900001	0.5622	0.6516
NC_005100.4_64000001	1	64000001	0.5569	0.6869
NC_005100.4_64100001	1	64100001	0.5563	0.7019
NC_005100.4_64200001	1	64200001	0.641	0.7461
NC_005100.4_64300001	1	64300001	0.6395	0.7692
NC_005100.4_64400001	1	64400001	0.5042	0.812
NC_005100.4_64500001	1	64500001	0.4721	0.8509
NC_005100.4_64600001	1	64600001	0.4571	0.8328
NC_005100.4_64700001	1	64700001	0.3027	0.736
NC_005100.4_65300001	1	65300001	0.5549	0.5457
NC_005100.4_65400001	1	65400001	0.5715	0.5419
NC_005100.4_65500001	1	65500001	0.474	0.5606
NC_005100.4_65600001	1	65600001	0.4766	0.5348
NC_005100.4_65700001	1	65700001	0.5088	0.5387
NC_005100.4_65800001	1	65800001	0.4447	0.5014
NC_005100.4_65900001	1	65900001	0.3789	0.4522
NC_005100.4_66000001	1	66000001	0.4023	0.3661
NC_005100.4_66100001	1	66100001	0.4014	0.3689
NC_005100.4_66200001	1	66200001	0.5184	0.5408
NC_005100.4_66300001	1	66300001	0.4927	0.5891
NC_005100.4_66400001	1	66400001	0.6033	0.5309
NC_005100.4_66500001	1	66500001	0.7596	0.6515
NC_005100.4_66600001	1	66600001	0.7512	0.6394
NC_005100.4_66700001	1	66700001	0.6785	0.5359
NC_005100.4_66800001	1	66800001	0.5892	0.5689
NC_005100.4_66900001	1	66900001	0.4828	0.6445
NC_005100.4_67000001	1	67000001	0.2929	0.489
NC_005100.4_67100001	1	67100001	0.3883	0.7429
NC_005100.4_67200001	1	67200001	0.3883	0.7429
NC_005100.4_67300001	1	67300001	0.2582	0.7308

NC_005100.4_69100001	1	69100001	0.5724	0.6645
NC_005100.4_69200001	1	69200001	0.6508	0.7473
NC_005100.4_69300001	1	69300001	0.6804	0.728
NC_005100.4_69400001	1	69400001	0.5609	0.7128
NC_005100.4_69500001	1	69500001	0.4446	0.6022
NC_005100.4_69600001	1	69600001	0.4634	0.7274
NC_005100.4_69700001	1	69700001	0.4114	0.7111
NC_005100.4_69800001	1	69800001	0.472	0.7597
NC_005100.4_69900001	1	69900001	0.5804	0.8119
NC_005100.4_70000001	1	70000001	0.6473	0.8506
NC_005100.4_70100001	1	70100001	0.6284	0.8156
NC_005100.4_70200001	1	70200001	0.6902	0.8074
NC_005100.4_70300001	1	70300001	0.6081	0.8009
NC_005100.4_70400001	1	70400001	0.463	0.6708
NC_005100.4_70800001	1	70800001	0.3761	0.4218
NC_005100.4_70900001	1	70900001	0.407	0.5123
NC_005100.4_71000001	1	71000001	0.5417	0.6781
NC_005100.4_71100001	1	71100001	0.549	0.7002
NC_005100.4_71200001	1	71200001	0.5332	0.7254
NC_005100.4_71300001	1	71300001	0.6543	0.8901
NC_005100.4_71400001	1	71400001	0.6739	0.8911
NC_005100.4_71500001	1	71500001	0.5128	0.7601
NC_005100.4_71600001	1	71600001	0.4733	0.7572
NC_005100.4_71700001	1	71700001	0.5788	0.803
NC_005100.4_71900001	1	71900001	0.6875	0.8811
NC_005100.4_72000001	1	72000001	0.6424	0.8544
NC_005100.4_72100001	1	72100001	0.6721	0.8564
NC_005100.4_72200001	1	72200001	0.6803	0.8463
NC_005100.4_72300001	1	72300001	0.653	0.8286
NC_005100.4_72400001	1	72400001	0.5721	0.7909
NC_005100.4_72500001	1	72500001	0.5631	0.7723
NC_005100.4_72600001	1	72600001	0.4433	0.7141
NC_005100.4_72700001	1	72700001	0.4284	0.6311
NC_005100.4_72800001	1	72800001	0.46	0.6633
NC_005100.4_72900001	1	72900001	0.4851	0.5372
NC_005100.4_73000001	1	73000001	0.5441	0.5802
NC_005100.4_73100001	1	73100001	0.7018	0.5993
NC_005100.4_73200001	1	73200001	0.7813	0.7629
NC_005100.4_73300001	1	73300001	0.6485	0.6946
NC_005100.4_73400001	1	73400001	0.7608	0.8031
NC_005100.4_73500001	1	73500001	0.7748	0.8165
NC_005100.4_73600001	1	73600001	0.7743	0.8317
NC_005100.4_73700001	1	73700001	0.7563	0.8161
NC_005100.4_73800001	1	73800001	0.9212	0.9077
NC_005100.4_74600001	1	74600001	0.7179	0.8408
NC_005100.4_75400001	1	75400001	0.5716	0.5013
NC_005100.4_75500001	1	75500001	0.5893	0.5944
NC_005100.4_75600001	1	75600001	0.5893	0.5944
NC_005100.4_75700001	1	75700001	0.621	0.6221
NC_005100.4_75800001	1	75800001	0.645	0.7108
NC_005100.4_75900001	1	75900001	0.5915	0.675
NC_005100.4_76900001	1	76900001	0.4804	0.4473
NC_005100.4_77000001	1	77000001	0.4804	0.4473
NC_005100.4_77100001	1	77100001	0.4501	0.4074
NC_005100.4_77200001	1	77200001	0.4501	0.4074
NC_005100.4_77300001	1	77300001	0.4631	0.4232
NC_005100.4_77400001	1	77400001	0.3599	0.3139
NC_005100.4_77500001	1	77500001	0.4926	0.5236

NC_005100.4_77600001	1	77600001	0.4776	0.5125
NC_005100.4_77700001	1	77700001	0.4776	0.5125
NC_005100.4_77800001	1	77800001	0.5457	0.6476
NC_005100.4_77900001	1	77900001	0.6643	0.7522
NC_005100.4_78000001	1	78000001	0.639	0.7254
NC_005100.4_78100001	1	78100001	0.6413	0.8644
NC_005100.4_78200001	1	78200001	0.5803	0.7521
NC_005100.4_78300001	1	78300001	0.5353	0.7362
NC_005100.4_78400001	1	78400001	0.5128	0.7016
NC_005100.4_78500001	1	78500001	0.5482	0.7216
NC_005100.4_78600001	1	78600001	0.6169	0.7396
NC_005100.4_78700001	1	78700001	0.7101	0.8332
NC_005100.4_78800001	1	78800001	0.7348	0.8339
NC_005100.4_78900001	1	78900001	0.6901	0.7947
NC_005100.4_79000001	1	79000001	0.5099	0.6731
NC_005100.4_79100001	1	79100001	0.4595	0.6532
NC_005100.4_79200001	1	79200001	0.3337	0.4594
NC_005100.4_79300001	1	79300001	0.4056	0.4935
NC_005100.4_79400001	1	79400001	0.4836	0.6718
NC_005100.4_79500001	1	79500001	0.5543	0.7101
NC_005100.4_79600001	1	79600001	0.6067	0.7529
NC_005100.4_79700001	1	79700001	0.6434	0.7607
NC_005100.4_79800001	1	79800001	0.595	0.7743
NC_005100.4_79900001	1	79900001	0.6647	0.8007
NC_005100.4_80000001	1	80000001	0.7233	0.8498
NC_005100.4_80100001	1	80100001	0.7147	0.8229
NC_005100.4_80200001	1	80200001	0.7278	0.8563
NC_005100.4_80300001	1	80300001	0.7135	0.8435
NC_005100.4_80400001	1	80400001	0.6247	0.7503
NC_005100.4_80500001	1	80500001	0.5605	0.7071
NC_005100.4_80600001	1	80600001	0.4928	0.6633
NC_005100.4_80700001	1	80700001	0.4939	0.6757
NC_005100.4_80800001	1	80800001	0.5229	0.5632
NC_005100.4_80900001	1	80900001	0.6087	0.5886
NC_005100.4_81000001	1	81000001	0.6421	0.6301
NC_005100.4_81100001	1	81100001	0.6036	0.5868
NC_005100.4_81200001	1	81200001	0.6156	0.5673
NC_005100.4_81300001	1	81300001	0.6818	0.697
NC_005100.4_81400001	1	81400001	0.7874	0.8477
NC_005100.4_81500001	1	81500001	0.8054	0.8483
NC_005100.4_81600001	1	81600001	0.8435	0.843
NC_005100.4_81700001	1	81700001	0.8401	0.8678
NC_005100.4_81800001	1	81800001	0.7816	0.8547
NC_005100.4_81900001	1	81900001	0.6595	0.8147
NC_005100.4_82000001	1	82000001	0.6601	0.7707
NC_005100.4_82100001	1	82100001	0.5734	0.7418
NC_005100.4_82200001	1	82200001	0.5269	0.6637
NC_005100.4_82300001	1	82300001	0.5438	0.6434
NC_005100.4_82400001	1	82400001	0.6361	0.659
NC_005100.4_82500001	1	82500001	0.5229	0.64
NC_005100.4_82600001	1	82600001	0.6894	0.7791
NC_005100.4_82700001	1	82700001	0.7249	0.8726
NC_005100.4_82800001	1	82800001	0.7233	0.8679
NC_005100.4_82900001	1	82900001	0.7218	0.8552
NC_005100.4_83000001	1	83000001	0.6022	0.7707
NC_005100.4_83100001	1	83100001	0.6022	0.7707
NC_005100.4_83200001	1	83200001	0.5333	0.687
NC_005100.4_83300001	1	83300001	0.5029	0.6786

NC_005100.4_83400001	1	83400001	0.3754	0.4289
NC_005100.4_83500001	1	83500001	0.635	0.6474
NC_005100.4_83600001	1	83600001	0.5257	0.5886
NC_005100.4_83700001	1	83700001	0.6599	0.7054
NC_005100.4_83800001	1	83800001	0.6954	0.743
NC_005100.4_83900001	1	83900001	0.6804	0.832
NC_005100.4_84000001	1	84000001	0.6063	0.819
NC_005100.4_84100001	1	84100001	0.6535	0.8542
NC_005100.4_84200001	1	84200001	0.616	0.8452
NC_005100.4_84300001	1	84300001	0.5945	0.8176
NC_005100.4_84400001	1	84400001	0.6267	0.7534
NC_005100.4_84500001	1	84500001	0.5567	0.7425
NC_005100.4_84600001	1	84600001	0.4584	0.7224
NC_005100.4_84700001	1	84700001	0.4884	0.7098
NC_005100.4_84800001	1	84800001	0.4844	0.7144
NC_005100.4_84900001	1	84900001	0.4404	0.722
NC_005100.4_85000001	1	85000001	0.5723	0.7906
NC_005100.4_85100001	1	85100001	0.6466	0.801
NC_005100.4_85200001	1	85200001	0.6449	0.8134
NC_005100.4_85300001	1	85300001	0.7046	0.8468
NC_005100.4_85400001	1	85400001	0.811	0.8602
NC_005100.4_85600001	1	85600001	0.6448	0.5858
NC_005100.4_85800001	1	85800001	0.7628	0.6654
NC_005100.4_85900001	1	85900001	0.6809	0.5776
NC_005100.4_86000001	1	86000001	0.6414	0.5605
NC_005100.4_86100001	1	86100001	0.6733	0.6155
NC_005100.4_86200001	1	86200001	0.6492	0.6165
NC_005100.4_86300001	1	86300001	0.4861	0.3971
NC_005100.4_86400001	1	86400001	0.5782	0.5803
NC_005100.4_86500001	1	86500001	0.5844	0.5799
NC_005100.4_86600001	1	86600001	0.5738	0.6274
NC_005100.4_86700001	1	86700001	0.5577	0.6676
NC_005100.4_86800001	1	86800001	0.5689	0.6947
NC_005100.4_86900001	1	86900001	0.652	0.8029
NC_005100.4_87000001	1	87000001	0.6807	0.9012
NC_005100.4_87100001	1	87100001	0.713	0.9024
NC_005100.4_87200001	1	87200001	0.8549	0.9303
NC_005100.4_87300001	1	87300001	0.8541	0.9057
NC_005100.4_87400001	1	87400001	0.7236	0.817
NC_005100.4_87500001	1	87500001	0.7349	0.8291
NC_005100.4_87600001	1	87600001	0.6741	0.802
NC_005100.4_87700001	1	87700001	0.6716	0.829
NC_005100.4_87800001	1	87800001	0.6799	0.8343
NC_005100.4_87900001	1	87900001	0.6799	0.8343
NC_005100.4_88000001	1	88000001	0.6612	0.8303
NC_005100.4_88100001	1	88100001	0.8319	0.9683
NC_005100.4_88400001	1	88400001	0.5602	0.7074
NC_005100.4_88500001	1	88500001	0.5581	0.7472
NC_005100.4_88600001	1	88600001	0.5042	0.7379
NC_005100.4_88700001	1	88700001	0.545	0.7566
NC_005100.4_88800001	1	88800001	0.5782	0.7735
NC_005100.4_88900001	1	88900001	0.6367	0.7791
NC_005100.4_89000001	1	89000001	0.6116	0.7298
NC_005100.4_89100001	1	89100001	0.6712	0.7689
NC_005100.4_89200001	1	89200001	0.6481	0.7667
NC_005100.4_89300001	1	89300001	0.6697	0.7669
NC_005100.4_89400001	1	89400001	0.6568	0.7955
NC_005100.4_89500001	1	89500001	0.746	0.8555

NC_005100.4_89600001	1	89600001	0.7686	0.873
NC_005100.4_89700001	1	89700001	0.7888	0.869
NC_005100.4_89800001	1	89800001	0.7968	0.8515
NC_005100.4_89900001	1	89900001	0.8148	0.8652
NC_005100.4_90000001	1	90000001	0.7484	0.8437
NC_005100.4_90100001	1	90100001	0.5698	0.6743
NC_005100.4_90200001	1	90200001	0.5224	0.6333
NC_005100.4_90300001	1	90300001	0.4438	0.5618
NC_005100.4_90400001	1	90400001	0.3822	0.4675
NC_005100.4_90500001	1	90500001	0.4113	0.4923
NC_005100.4_90600001	1	90600001	0.504	0.6157
NC_005100.4_90700001	1	90700001	0.4519	0.4777
NC_005100.4_90800001	1	90800001	0.2795	0.3307
NC_005100.4_90900001	1	90900001	0.4496	0.5922
NC_005100.4_91000001	1	91000001	0.4258	0.5272
NC_005100.4_91100001	1	91100001	0.4668	0.5694
NC_005100.4_91200001	1	91200001	0.4322	0.5727
NC_005100.4_91300001	1	91300001	0.4538	0.5827
NC_005100.4_91400001	1	91400001	0.4414	0.565
NC_005100.4_91500001	1	91500001	0.4816	0.6383
NC_005100.4_91600001	1	91600001	0.458	0.5903
NC_005100.4_91700001	1	91700001	0.4517	0.5613
NC_005100.4_91800001	1	91800001	0.5255	0.5989
NC_005100.4_91900001	1	91900001	0.4705	0.5446
NC_005100.4_92000001	1	92000001	0.4672	0.5404
NC_005100.4_92100001	1	92100001	0.4572	0.5205
NC_005100.4_92200001	1	92200001	0.5027	0.537
NC_005100.4_92300001	1	92300001	0.4384	0.5073
NC_005100.4_92400001	1	92400001	0.4983	0.5221
NC_005100.4_92500001	1	92500001	0.5538	0.5565
NC_005100.4_92600001	1	92600001	0.5867	0.6114
NC_005100.4_92700001	1	92700001	0.5555	0.6297
NC_005100.4_92800001	1	92800001	0.5622	0.6395
NC_005100.4_92900001	1	92900001	0.5434	0.6706
NC_005100.4_93000001	1	93000001	0.4838	0.6372
NC_005100.4_93100001	1	93100001	0.5006	0.6017
NC_005100.4_93200001	1	93200001	0.5485	0.5712
NC_005100.4_93300001	1	93300001	0.5577	0.5484
NC_005100.4_93400001	1	93400001	0.5692	0.5556
NC_005100.4_93500001	1	93500001	0.5919	0.6499
NC_005100.4_93600001	1	93600001	0.5463	0.7218
NC_005100.4_93700001	1	93700001	0.5357	0.7635
NC_005100.4_93800001	1	93800001	0.5374	0.724
NC_005100.4_93900001	1	93900001	0.5801	0.7471
NC_005100.4_94000001	1	94000001	0.6041	0.7196
NC_005100.4_94100001	1	94100001	0.6462	0.7035
NC_005100.4_94200001	1	94200001	0.6738	0.7495
NC_005100.4_94300001	1	94300001	0.7064	0.7706
NC_005100.4_94400001	1	94400001	0.6865	0.7369
NC_005100.4_94500001	1	94500001	0.6437	0.702
NC_005100.4_94600001	1	94600001	0.5908	0.664
NC_005100.4_94700001	1	94700001	0.5007	0.5377
NC_005100.4_94800001	1	94800001	0.5685	0.7052
NC_005100.4_94900001	1	94900001	0.5133	0.6657
NC_005100.4_95000001	1	95000001	0.5208	0.6775
NC_005100.4_95100001	1	95100001	0.5021	0.7032
NC_005100.4_95200001	1	95200001	0.5295	0.7808
NC_005100.4_96300001	1	96300001	0.5527	0.6787

NC_005100.4_96400001	1	96400001	0.4676	0.6174
NC_005100.4_96500001	1	96500001	0.4319	0.5708
NC_005100.4_96600001	1	96600001	0.419	0.5539
NC_005100.4_96700001	1	96700001	0.4157	0.5337
NC_005100.4_96800001	1	96800001	0.4124	0.465
NC_005100.4_96900001	1	96900001	0.4599	0.4645
NC_005100.4_97000001	1	97000001	0.4853	0.4542
NC_005100.4_97100001	1	97100001	0.4857	0.483
NC_005100.4_97200001	1	97200001	0.5521	0.6016
NC_005100.4_97300001	1	97300001	0.554	0.6391
NC_005100.4_97400001	1	97400001	0.5409	0.6318
NC_005100.4_97500001	1	97500001	0.4647	0.5624
NC_005100.4_97600001	1	97600001	0.473	0.5825
NC_005100.4_97700001	1	97700001	0.3371	0.3646
NC_005100.4_97800001	1	97800001	0.3371	0.3646
NC_005100.4_97900001	1	97900001	0.4006	0.4216
NC_005100.4_98000001	1	98000001	0.4131	0.5786
NC_005100.4_98100001	1	98100001	0.375	0.4543
NC_005100.4_98200001	1	98200001	0.3343	0.3836
NC_005100.4_98300001	1	98300001	0.3352	0.4225
NC_005100.4_98400001	1	98400001	0.2978	0.4128
NC_005100.4_98500001	1	98500001	0.3062	0.3187
NC_005100.4_98700001	1	98700001	0.3526	0.5028
NC_005100.4_99100001	1	99100001	0.4142	0.4895
NC_005100.4_99200001	1	99200001	0.5491	0.68
NC_005100.4_99300001	1	99300001	0.5014	0.6755
NC_005100.4_99400001	1	99400001	0.4866	0.6751
NC_005100.4_99500001	1	99500001	0.4973	0.6975
NC_005100.4_99600001	1	99600001	0.5161	0.699
NC_005100.4_99700001	1	99700001	0.4203	0.6277
NC_005100.4_99800001	1	99800001	0.3749	0.561
NC_005100.4_99900001	1	99900001	0.4351	0.6404
NC_005100.4_100000001	1	100000001	0.492	0.6998
NC_005100.4_100100001	1	100100001	0.5457	0.7636
NC_005100.4_100200001	1	100200001	0.5535	0.7505
NC_005100.4_100300001	1	100300001	0.5865	0.7621
NC_005100.4_100400001	1	100400001	0.5746	0.7586
NC_005100.4_100500001	1	100500001	0.59	0.769
NC_005100.4_100600001	1	100600001	0.5441	0.6951
NC_005100.4_100700001	1	100700001	0.5647	0.7496
NC_005100.4_100800001	1	100800001	0.502	0.7208
NC_005100.4_100900001	1	100900001	0.4771	0.7081
NC_005100.4_101000001	1	101000001	0.5142	0.7206
NC_005100.4_101100001	1	101100001	0.4871	0.7771
NC_005100.4_101200001	1	101200001	0.4842	0.7591
NC_005100.4_101300001	1	101300001	0.5243	0.8171
NC_005100.4_101400001	1	101400001	0.5988	0.8595
NC_005100.4_101500001	1	101500001	0.4744	0.7806
NC_005100.4_101600001	1	101600001	0.4862	0.6783
NC_005100.4_101700001	1	101700001	0.5158	0.6336
NC_005100.4_101800001	1	101800001	0.5539	0.6326
NC_005100.4_101900001	1	101900001	0.5122	0.5492
NC_005100.4_102000001	1	102000001	0.5471	0.6072
NC_005100.4_102100001	1	102100001	0.5607	0.6492
NC_005100.4_102200001	1	102200001	0.5551	0.6626
NC_005100.4_102300001	1	102300001	0.5444	0.6732
NC_005100.4_102400001	1	102400001	0.548	0.7039
NC_005100.4_102500001	1	102500001	0.5213	0.6944

NC_005100.4_102600001	1	102600001	0.5204	0.6508
NC_005100.4_102700001	1	102700001	0.4948	0.662
NC_005100.4_102800001	1	102800001	0.449	0.5566
NC_005100.4_102900001	1	102900001	0.5289	0.6147
NC_005100.4_103000001	1	103000001	0.5319	0.4451
NC_005100.4_103100001	1	103100001	0.4228	0.4509
NC_005100.4_103200001	1	103200001	0.4003	0.3799
NC_005100.4_103300001	1	103300001	0.4003	0.3799
NC_005100.4_103400001	1	103400001	0.2887	0.2688
NC_005100.4_103500001	1	103500001	0.4059	0.4766
NC_005100.4_103600001	1	103600001	0.5713	0.7434
NC_005100.4_103700001	1	103700001	0.6162	0.7643
NC_005100.4_103800001	1	103800001	0.5534	0.745
NC_005100.4_103900001	1	103900001	0.5288	0.7053
NC_005100.4_104000001	1	104000001	0.4827	0.6117
NC_005100.4_104100001	1	104100001	0.495	0.5693
NC_005100.4_104200001	1	104200001	0.4908	0.5816
NC_005100.4_104300001	1	104300001	0.4951	0.5606
NC_005100.4_104400001	1	104400001	0.5364	0.5534
NC_005100.4_104500001	1	104500001	0.6052	0.635
NC_005100.4_104600001	1	104600001	0.5664	0.596
NC_005100.4_104700001	1	104700001	0.5626	0.6249
NC_005100.4_104800001	1	104800001	0.5292	0.6287
NC_005100.4_104900001	1	104900001	0.4826	0.6396
NC_005100.4_105000001	1	105000001	0.4099	0.603
NC_005100.4_105100001	1	105100001	0.4804	0.7147
NC_005100.4_105200001	1	105200001	0.5157	0.7099
NC_005100.4_105300001	1	105300001	0.5367	0.7054
NC_005100.4_105400001	1	105400001	0.6427	0.7988
NC_005100.4_105500001	1	105500001	0.6389	0.7612
NC_005100.4_105600001	1	105600001	0.5439	0.6767
NC_005100.4_105700001	1	105700001	0.5199	0.6579
NC_005100.4_105800001	1	105800001	0.5755	0.6971
NC_005100.4_105900001	1	105900001	0.4666	0.5879
NC_005100.4_106000001	1	106000001	0.4641	0.6242
NC_005100.4_106100001	1	106100001	0.4788	0.8048
NC_005100.4_106200001	1	106200001	0.5416	0.8606
NC_005100.4_106300001	1	106300001	0.2893	0.3915
NC_005100.4_106400001	1	106400001	0.2465	0.3697
NC_005100.4_106500001	1	106500001	0.4056	0.6119
NC_005100.4_106600001	1	106600001	0.4062	0.5934
NC_005100.4_106700001	1	106700001	0.3618	0.5431
NC_005100.4_106800001	1	106800001	0.4876	0.7221
NC_005100.4_106900001	1	106900001	0.5512	0.7679
NC_005100.4_107000001	1	107000001	0.4583	0.7219
NC_005100.4_107100001	1	107100001	0.5263	0.7792
NC_005100.4_107200001	1	107200001	0.5607	0.7888
NC_005100.4_107300001	1	107300001	0.5224	0.7688
NC_005100.4_107400001	1	107400001	0.4211	0.5903
NC_005100.4_107500001	1	107500001	0.3809	0.4072
NC_005100.4_107600001	1	107600001	0.2504	0.1915
NC_005100.4_107700001	1	107700001	0.2579	0.2718
NC_005100.4_107800001	1	107800001	0.2029	0.244
NC_005100.4_107900001	1	107900001	0.2311	0.2697
NC_005100.4_108000001	1	108000001	0.2142	0.2909
NC_005100.4_108100001	1	108100001	0.2526	0.3494
NC_005100.4_108200001	1	108200001	0.2597	0.1872
NC_005100.4_108600001	1	108600001	0.3584	0.4463

NC_005100.4_108700001	1	108700001	0.5993	0.7902
NC_005100.4_108800001	1	108800001	0.5993	0.7902
NC_005100.4_108900001	1	108900001	0.5993	0.7902
NC_005100.4_109600001	1	109600001	0.4327	0.3956
NC_005100.4_109700001	1	109700001	0.5182	0.5179
NC_005100.4_109800001	1	109800001	0.4407	0.5172
NC_005100.4_109900001	1	109900001	0.4203	0.5074
NC_005100.4_110000001	1	110000001	0.4164	0.5674
NC_005100.4_110100001	1	110100001	0.4133	0.6168
NC_005100.4_110200001	1	110200001	0.3251	0.4887
NC_005100.4_110600001	1	110600001	0.4137	0.4494
NC_005100.4_110700001	1	110700001	0.3527	0.4127
NC_005100.4_110800001	1	110800001	0.3527	0.4127
NC_005100.4_110900001	1	110900001	0.3752	0.5659
NC_005100.4_111000001	1	111000001	0.3669	0.5336
NC_005100.4_111100001	1	111100001	0.3706	0.5658
NC_005100.4_111200001	1	111200001	0.4555	0.6413
NC_005100.4_111300001	1	111300001	0.4555	0.6413
NC_005100.4_111700001	1	111700001	0.3405	0.4304
NC_005100.4_111800001	1	111800001	0.327	0.4063
NC_005100.4_111900001	1	111900001	0.4407	0.5686
NC_005100.4_112000001	1	112000001	0.5034	0.627
NC_005100.4_112100001	1	112100001	0.5097	0.6089
NC_005100.4_112200001	1	112200001	0.5679	0.6129
NC_005100.4_112300001	1	112300001	0.5632	0.6227
NC_005100.4_112400001	1	112400001	0.6555	0.6999
NC_005100.4_112500001	1	112500001	0.6307	0.6878
NC_005100.4_112600001	1	112600001	0.5416	0.6866
NC_005100.4_112700001	1	112700001	0.501	0.6658
NC_005100.4_112800001	1	112800001	0.4917	0.6784
NC_005100.4_112900001	1	112900001	0.4293	0.6164
NC_005100.4_113000001	1	113000001	0.3904	0.5714
NC_005100.4_113100001	1	113100001	0.4553	0.6151
NC_005100.4_113200001	1	113200001	0.4419	0.651
NC_005100.4_113300001	1	113300001	0.4817	0.6333
NC_005100.4_113400001	1	113400001	0.4294	0.5722
NC_005100.4_113500001	1	113500001	0.4734	0.6532
NC_005100.4_113600001	1	113600001	0.4923	0.6236
NC_005100.4_113700001	1	113700001	0.4482	0.4503
NC_005100.4_113800001	1	113800001	0.4184	0.4357
NC_005100.4_113900001	1	113900001	0.5307	0.5866
NC_005100.4_114000001	1	114000001	0.4464	0.5153
NC_005100.4_114100001	1	114100001	0.4829	0.5902
NC_005100.4_114200001	1	114200001	0.4845	0.7543
NC_005100.4_114300001	1	114300001	0.4649	0.64
NC_005100.4_114400001	1	114400001	0.3782	0.5313
NC_005100.4_114500001	1	114500001	0.4339	0.476
NC_005100.4_114600001	1	114600001	0.3407	0.3179
NC_005100.4_114700001	1	114700001	0.5339	0.5485
NC_005100.4_114800001	1	114800001	0.6279	0.5984
NC_005100.4_114900001	1	114900001	0.6018	0.5965
NC_005100.4_115000001	1	115000001	0.5992	0.6529
NC_005100.4_115100001	1	115100001	0.6634	0.7165
NC_005100.4_115200001	1	115200001	0.5165	0.6126
NC_005100.4_115300001	1	115300001	0.5214	0.6628
NC_005100.4_115400001	1	115400001	0.5205	0.6829
NC_005100.4_115500001	1	115500001	0.4521	0.646
NC_005100.4_115600001	1	115600001	0.4083	0.5492

NC_005100.4_115700001	1	115700001	0.3938	0.5738
NC_005100.4_115800001	1	115800001	0.3547	0.4898
NC_005100.4_115900001	1	115900001	0.3547	0.4898
NC_005100.4_116000001	1	116000001	0.4045	0.5342
NC_005100.4_116100001	1	116100001	0.4807	0.7098
NC_005100.4_119200001	1	119200001	0.6404	0.5913
NC_005100.4_119300001	1	119300001	0.6404	0.5913
NC_005100.4_119400001	1	119400001	0.6404	0.5913
NC_005100.4_119500001	1	119500001	0.4772	0.5264
NC_005100.4_120400001	1	120400001	0.6801	0.928
NC_005100.4_120500001	1	120500001	0.5724	0.8992
NC_005100.4_120600001	1	120600001	0.5724	0.8992
NC_005100.4_120700001	1	120700001	0.6267	0.9178
NC_005100.4_120800001	1	120800001	0.6362	0.9206
NC_005100.4_122200001	1	122200001	0.4642	0.646
NC_005100.4_122300001	1	122300001	0.5542	0.7387
NC_005100.4_122400001	1	122400001	0.5436	0.7246
NC_005100.4_122500001	1	122500001	0.7114	0.8361
NC_005100.4_122600001	1	122600001	0.6426	0.8126
NC_005100.4_122700001	1	122700001	0.6773	0.8567
NC_005100.4_122800001	1	122800001	0.6463	0.8132
NC_005100.4_122900001	1	122900001	0.6347	0.8036
NC_005100.4_123000001	1	123000001	0.4534	0.6599
NC_005100.4_123100001	1	123100001	0.5632	0.7198
NC_005100.4_123200001	1	123200001	0.392	0.5417
NC_005100.4_123300001	1	123300001	0.3971	0.6523
NC_005100.4_123400001	1	123400001	0.5834	0.7554
NC_005100.4_123500001	1	123500001	0.5834	0.7554
NC_005100.4_123600001	1	123600001	0.4359	0.5532
NC_005100.4_123700001	1	123700001	0.455	0.5253
NC_005100.4_123800001	1	123800001	0.4924	0.4981
NC_005100.4_123900001	1	123900001	0.4923	0.6167
NC_005100.4_124000001	1	124000001	0.5366	0.664
NC_005100.4_124100001	1	124100001	0.6069	0.7675
NC_005100.4_124200001	1	124200001	0.6675	0.8132
NC_005100.4_124300001	1	124300001	0.7497	0.8929
NC_005100.4_124400001	1	124400001	0.7693	0.8697
NC_005100.4_124500001	1	124500001	0.7191	0.8528
NC_005100.4_124600001	1	124600001	0.6749	0.7787
NC_005100.4_124700001	1	124700001	0.5874	0.6989
NC_005100.4_124800001	1	124800001	0.4462	0.5126
NC_005100.4_124900001	1	124900001	0.4176	0.4932
NC_005100.4_125000001	1	125000001	0.4228	0.4955
NC_005100.4_125100001	1	125100001	0.4353	0.5739
NC_005100.4_125200001	1	125200001	0.4798	0.6432
NC_005100.4_125300001	1	125300001	0.4929	0.6808
NC_005100.4_125400001	1	125400001	0.4906	0.6679
NC_005100.4_125500001	1	125500001	0.5331	0.7166
NC_005100.4_125600001	1	125600001	0.5575	0.7185
NC_005100.4_125700001	1	125700001	0.632	0.7973
NC_005100.4_125800001	1	125800001	0.652	0.731
NC_005100.4_125900001	1	125900001	0.6934	0.8065
NC_005100.4_126000001	1	126000001	0.6515	0.7411
NC_005100.4_126100001	1	126100001	0.5888	0.6923
NC_005100.4_126200001	1	126200001	0.5032	0.6047
NC_005100.4_126300001	1	126300001	0.4909	0.6775
NC_005100.4_126400001	1	126400001	0.4941	0.628
NC_005100.4_126500001	1	126500001	0.4657	0.6128

NC_005100.4_126600001	1	126600001	0.4364	0.6097
NC_005100.4_126700001	1	126700001	0.5131	0.6588
NC_005100.4_126800001	1	126800001	0.5431	0.6776
NC_005100.4_126900001	1	126900001	0.5901	0.725
NC_005100.4_127000001	1	127000001	0.5726	0.7482
NC_005100.4_127100001	1	127100001	0.6232	0.7464
NC_005100.4_127200001	1	127200001	0.5704	0.7498
NC_005100.4_127300001	1	127300001	0.5243	0.7214
NC_005100.4_127400001	1	127400001	0.5121	0.6014
NC_005100.4_127500001	1	127500001	0.4978	0.5082
NC_005100.4_127600001	1	127600001	0.5712	0.5646
NC_005100.4_127700001	1	127700001	0.5983	0.5738
NC_005100.4_127800001	1	127800001	0.6211	0.5464
NC_005100.4_127900001	1	127900001	0.5915	0.5591
NC_005100.4_128000001	1	128000001	0.6877	0.6826
NC_005100.4_128100001	1	128100001	0.5981	0.6826
NC_005100.4_128200001	1	128200001	0.602	0.6936
NC_005100.4_128300001	1	128300001	0.5939	0.696
NC_005100.4_128400001	1	128400001	0.5574	0.6617
NC_005100.4_128500001	1	128500001	0.5193	0.652
NC_005100.4_128600001	1	128600001	0.516	0.6772
NC_005100.4_128700001	1	128700001	0.4946	0.6851
NC_005100.4_128800001	1	128800001	0.446	0.6624
NC_005100.4_128900001	1	128900001	0.4352	0.6266
NC_005100.4_129000001	1	129000001	0.451	0.6346
NC_005100.4_129100001	1	129100001	0.4624	0.6211
NC_005100.4_129200001	1	129200001	0.439	0.5492
NC_005100.4_129300001	1	129300001	0.4856	0.5238
NC_005100.4_129400001	1	129400001	0.5733	0.6102
NC_005100.4_129500001	1	129500001	0.5674	0.6108
NC_005100.4_129600001	1	129600001	0.5297	0.5636
NC_005100.4_129700001	1	129700001	0.4276	0.4925
NC_005100.4_129800001	1	129800001	0.3101	0.5091
NC_005100.4_129900001	1	129900001	0.3101	0.5091
NC_005100.4_130000001	1	130000001	0.1192	0.0971
NC_005100.4_130100001	1	130100001	0.1233	0.0956
NC_005100.4_130200001	1	130200001	0.2016	0.3202
NC_005100.4_130300001	1	130300001	0.2662	0.4996
NC_005100.4_130400001	1	130400001	0.3618	0.6887
NC_005100.4_130500001	1	130500001	0.422	0.6476
NC_005100.4_130600001	1	130600001	0.4419	0.635
NC_005100.4_130700001	1	130700001	0.4717	0.6561
NC_005100.4_130800001	1	130800001	0.493	0.658
NC_005100.4_130900001	1	130900001	0.5174	0.6334
NC_005100.4_131000001	1	131000001	0.5317	0.6848
NC_005100.4_131100001	1	131100001	0.4769	0.6295
NC_005100.4_131200001	1	131200001	0.4535	0.6135
NC_005100.4_131300001	1	131300001	0.4337	0.5534
NC_005100.4_131400001	1	131400001	0.3861	0.4422
NC_005100.4_131500001	1	131500001	0.3555	0.3908
NC_005100.4_131600001	1	131600001	0.3679	0.431
NC_005100.4_131700001	1	131700001	0.3587	0.4157
NC_005100.4_131800001	1	131800001	0.4544	0.5376
NC_005100.4_131900001	1	131900001	0.4379	0.5774
NC_005100.4_132000001	1	132000001	0.566	0.6516
NC_005100.4_132100001	1	132100001	0.6012	0.6551
NC_005100.4_132200001	1	132200001	0.5523	0.6235
NC_005100.4_132300001	1	132300001	0.5905	0.7254

NC_005100.4_132400001	1	132400001	0.6019	0.7868
NC_005100.4_132500001	1	132500001	0.6058	0.8399
NC_005100.4_132600001	1	132600001	0.6058	0.8399
NC_005100.4_132700001	1	132700001	0.6522	0.8872
NC_005100.4_132800001	1	132800001	0.5356	0.8326
NC_005100.4_132900001	1	132900001	0.5398	0.6781
NC_005100.4_133000001	1	133000001	0.5621	0.6448
NC_005100.4_133100001	1	133100001	0.5479	0.6525
NC_005100.4_133200001	1	133200001	0.508	0.6378
NC_005100.4_133300001	1	133300001	0.5519	0.6409
NC_005100.4_133400001	1	133400001	0.5559	0.7149
NC_005100.4_133500001	1	133500001	0.5868	0.7403
NC_005100.4_133600001	1	133600001	0.6326	0.7323
NC_005100.4_133700001	1	133700001	0.6654	0.7323
NC_005100.4_133800001	1	133800001	0.6851	0.7233
NC_005100.4_133900001	1	133900001	0.6784	0.7089
NC_005100.4_134000001	1	134000001	0.5864	0.6738
NC_005100.4_134100001	1	134100001	0.5327	0.6436
NC_005100.4_134200001	1	134200001	0.5385	0.6622
NC_005100.4_134300001	1	134300001	0.5064	0.6831
NC_005100.4_134400001	1	134400001	0.5552	0.728
NC_005100.4_134500001	1	134500001	0.558	0.7469
NC_005100.4_134600001	1	134600001	0.5967	0.7925
NC_005100.4_134700001	1	134700001	0.6264	0.7976
NC_005100.4_134800001	1	134800001	0.7011	0.8453
NC_005100.4_134900001	1	134900001	0.5575	0.7261
NC_005100.4_135000001	1	135000001	0.5712	0.6905
NC_005100.4_135100001	1	135100001	0.6205	0.7499
NC_005100.4_135200001	1	135200001	0.5685	0.7113
NC_005100.4_135300001	1	135300001	0.6057	0.67
NC_005100.4_135400001	1	135400001	0.6502	0.7413
NC_005100.4_135500001	1	135500001	0.6452	0.7198
NC_005100.4_135600001	1	135600001	0.5253	0.6762
NC_005100.4_135700001	1	135700001	0.473	0.6288
NC_005100.4_135800001	1	135800001	0.3526	0.5371
NC_005100.4_135900001	1	135900001	0.4746	0.6375
NC_005100.4_136000001	1	136000001	0.4649	0.6737
NC_005100.4_136100001	1	136100001	0.6225	0.7296
NC_005100.4_136200001	1	136200001	0.7496	0.8185
NC_005100.4_136300001	1	136300001	0.6921	0.7242
NC_005100.4_136400001	1	136400001	0.4833	0.4635
NC_005100.4_136500001	1	136500001	0.5132	0.4962
NC_005100.4_136600001	1	136600001	0.444	0.4415
NC_005100.4_136700001	1	136700001	0.5826	0.6214
NC_005100.4_136800001	1	136800001	0.5982	0.711
NC_005100.4_136900001	1	136900001	0.5806	0.8189
NC_005100.4_137000001	1	137000001	0.5043	0.8073
NC_005100.4_137100001	1	137100001	0.4946	0.7825
NC_005100.4_137200001	1	137200001	0.4521	0.7208
NC_005100.4_137300001	1	137300001	0.4285	0.6875
NC_005100.4_137400001	1	137400001	0.4727	0.6699
NC_005100.4_137500001	1	137500001	0.5493	0.7004
NC_005100.4_137600001	1	137600001	0.6708	0.7854
NC_005100.4_137700001	1	137700001	0.5923	0.6556
NC_005100.4_137800001	1	137800001	0.5254	0.6554
NC_005100.4_137900001	1	137900001	0.5113	0.655
NC_005100.4_138000001	1	138000001	0.6024	0.6686
NC_005100.4_138100001	1	138100001	0.6001	0.7074

NC_005100.4_138200001	1	138200001	0.5548	0.6844
NC_005100.4_138300001	1	138300001	0.5877	0.7169
NC_005100.4_138400001	1	138400001	0.5796	0.6945
NC_005100.4_138500001	1	138500001	0.5064	0.6289
NC_005100.4_138600001	1	138600001	0.4452	0.5044
NC_005100.4_138700001	1	138700001	0.4671	0.48
NC_005100.4_138800001	1	138800001	0.4342	0.4175
NC_005100.4_138900001	1	138900001	0.4449	0.4387
NC_005100.4_139000001	1	139000001	0.4682	0.5129
NC_005100.4_139100001	1	139100001	0.5658	0.6803
NC_005100.4_139200001	1	139200001	0.5637	0.6738
NC_005100.4_139300001	1	139300001	0.5802	0.6413
NC_005100.4_139400001	1	139400001	0.6136	0.68
NC_005100.4_139500001	1	139500001	0.4919	0.6648
NC_005100.4_139600001	1	139600001	0.4849	0.6019
NC_005100.4_139700001	1	139700001	0.5228	0.6218
NC_005100.4_139800001	1	139800001	0.4816	0.62
NC_005100.4_139900001	1	139900001	0.4153	0.5143
NC_005100.4_140000001	1	140000001	0.4236	0.5232
NC_005100.4_140100001	1	140100001	0.4216	0.5361
NC_005100.4_140200001	1	140200001	0.3995	0.5025
NC_005100.4_140300001	1	140300001	0.4244	0.5072
NC_005100.4_140400001	1	140400001	0.4687	0.6002
NC_005100.4_140500001	1	140500001	0.5063	0.599
NC_005100.4_140600001	1	140600001	0.5138	0.573
NC_005100.4_140700001	1	140700001	0.4995	0.5937
NC_005100.4_140800001	1	140800001	0.4528	0.5831
NC_005100.4_140900001	1	140900001	0.5075	0.6368
NC_005100.4_141000001	1	141000001	0.5589	0.7045
NC_005100.4_141100001	1	141100001	0.4727	0.6624
NC_005100.4_141200001	1	141200001	0.5288	0.6795
NC_005100.4_141300001	1	141300001	0.5651	0.698
NC_005100.4_141400001	1	141400001	0.5452	0.6447
NC_005100.4_141500001	1	141500001	0.493	0.5747
NC_005100.4_141600001	1	141600001	0.6195	0.6517
NC_005100.4_141700001	1	141700001	0.5645	0.6557
NC_005100.4_141800001	1	141800001	0.5425	0.6478
NC_005100.4_141900001	1	141900001	0.5877	0.7218
NC_005100.4_142000001	1	142000001	0.4625	0.7473
NC_005100.4_142100001	1	142100001	0.4646	0.7483
NC_005100.4_142200001	1	142200001	0.4271	0.8086
NC_005100.4_142300001	1	142300001	0.458	0.7653
NC_005100.4_142400001	1	142400001	0.4882	0.6987
NC_005100.4_142500001	1	142500001	0.6321	0.7104
NC_005100.4_142600001	1	142600001	0.6065	0.7299
NC_005100.4_142700001	1	142700001	0.5478	0.6952
NC_005100.4_142800001	1	142800001	0.4962	0.7022
NC_005100.4_142900001	1	142900001	0.4233	0.6734
NC_005100.4_143000001	1	143000001	0.3988	0.662
NC_005100.4_143100001	1	143100001	0.4148	0.6473
NC_005100.4_143200001	1	143200001	0.4631	0.6571
NC_005100.4_143300001	1	143300001	0.5357	0.6891
NC_005100.4_143400001	1	143400001	0.564	0.7099
NC_005100.4_143500001	1	143500001	0.6046	0.7156
NC_005100.4_143600001	1	143600001	0.6118	0.7285
NC_005100.4_143700001	1	143700001	0.4575	0.6897
NC_005100.4_143800001	1	143800001	0.3573	0.595
NC_005100.4_143900001	1	143900001	0.3372	0.4658

NC_005100.4_144000001	1	144000001	0.372	0.5291
NC_005100.4_144100001	1	144100001	0.4111	0.5662
NC_005100.4_144200001	1	144200001	0.4436	0.5737
NC_005100.4_144300001	1	144300001	0.5409	0.641
NC_005100.4_144400001	1	144400001	0.6459	0.7618
NC_005100.4_144500001	1	144500001	0.6271	0.74
NC_005100.4_144600001	1	144600001	0.6023	0.7042
NC_005100.4_144700001	1	144700001	0.6023	0.7042
NC_005100.4_144800001	1	144800001	0.6051	0.7185
NC_005100.4_145000001	1	145000001	0.5379	0.618
NC_005100.4_145100001	1	145100001	0.5544	0.6212
NC_005100.4_145200001	1	145200001	0.5221	0.6051
NC_005100.4_145300001	1	145300001	0.4246	0.5203
NC_005100.4_145400001	1	145400001	0.4609	0.5014
NC_005100.4_145500001	1	145500001	0.4682	0.5309
NC_005100.4_145600001	1	145600001	0.3904	0.464
NC_005100.4_145700001	1	145700001	0.3755	0.4301
NC_005100.4_145800001	1	145800001	0.3577	0.403
NC_005100.4_145900001	1	145900001	0.3513	0.4621
NC_005100.4_146000001	1	146000001	0.2766	0.3267
NC_005100.4_146100001	1	146100001	0.2787	0.3295
NC_005100.4_146200001	1	146200001	0.3613	0.5056
NC_005100.4_146300001	1	146300001	0.4207	0.5973
NC_005100.4_146400001	1	146400001	0.3709	0.4872
NC_005100.4_146500001	1	146500001	0.4784	0.6416
NC_005100.4_146600001	1	146600001	0.5527	0.7176
NC_005100.4_146700001	1	146700001	0.5189	0.6627
NC_005100.4_146800001	1	146800001	0.5632	0.6267
NC_005100.4_147300001	1	147300001	0.5783	0.4578
NC_005100.4_147400001	1	147400001	0.5783	0.4578
NC_005100.4_147500001	1	147500001	0.5783	0.4578
NC_005100.4_147600001	1	147600001	0.5783	0.4578
NC_005100.4_147700001	1	147700001	0.4935	0.4962
NC_005100.4_149900001	1	149900001	0.528	0.7479
NC_005100.4_150000001	1	150000001	0.528	0.7479
NC_005100.4_150100001	1	150100001	0.4831	0.7147
NC_005100.4_150200001	1	150200001	0.4444	0.6843
NC_005100.4_150300001	1	150300001	0.4436	0.7411
NC_005100.4_150400001	1	150400001	0.6161	0.8527
NC_005100.4_150500001	1	150500001	0.637	0.7451
NC_005100.4_150600001	1	150600001	0.6505	0.7329
NC_005100.4_150700001	1	150700001	0.5484	0.6565
NC_005100.4_150800001	1	150800001	0.5798	0.6322
NC_005100.4_150900001	1	150900001	0.5208	0.5521
NC_005100.4_151000001	1	151000001	0.4228	0.5525
NC_005100.4_151100001	1	151100001	0.4651	0.5448
NC_005100.4_151200001	1	151200001	0.5417	0.5835
NC_005100.4_151300001	1	151300001	0.4985	0.5847
NC_005100.4_151400001	1	151400001	0.4181	0.5509
NC_005100.4_151500001	1	151500001	0.3812	0.4921
NC_005100.4_151600001	1	151600001	0.4548	0.6023
NC_005100.4_151700001	1	151700001	0.4991	0.6372
NC_005100.4_151800001	1	151800001	0.489	0.6771
NC_005100.4_151900001	1	151900001	0.6812	0.7703
NC_005100.4_152000001	1	152000001	0.8765	0.8552
NC_005100.4_152100001	1	152100001	0.8635	0.891
NC_005100.4_152200001	1	152200001	0.7311	0.8658
NC_005100.4_152300001	1	152300001	0.5618	0.7262

NC_005100.4_152400001	1	152400001	0.5253	0.6777
NC_005100.4_152500001	1	152500001	0.5099	0.6568
NC_005100.4_152600001	1	152600001	0.513	0.6847
NC_005100.4_152700001	1	152700001	0.477	0.6523
NC_005100.4_152800001	1	152800001	0.5121	0.6742
NC_005100.4_152900001	1	152900001	0.4768	0.6087
NC_005100.4_153000001	1	153000001	0.3622	0.4731
NC_005100.4_153100001	1	153100001	0.3557	0.4258
NC_005100.4_153200001	1	153200001	0.4991	0.5555
NC_005100.4_153300001	1	153300001	0.5488	0.603
NC_005100.4_153400001	1	153400001	0.551	0.6217
NC_005100.4_153500001	1	153500001	0.594	0.6451
NC_005100.4_153600001	1	153600001	0.6385	0.6879
NC_005100.4_153700001	1	153700001	0.617	0.7081
NC_005100.4_153800001	1	153800001	0.5642	0.6471
NC_005100.4_153900001	1	153900001	0.6268	0.6633
NC_005100.4_154000001	1	154000001	0.5796	0.6064
NC_005100.4_154100001	1	154100001	0.52	0.5384
NC_005100.4_154200001	1	154200001	0.4816	0.454
NC_005100.4_154300001	1	154300001	0.4432	0.4978
NC_005100.4_154400001	1	154400001	0.2197	0.2749
NC_005100.4_154500001	1	154500001	0.3841	0.4444
NC_005100.4_154600001	1	154600001	0.3855	0.4903
NC_005100.4_154700001	1	154700001	0.4812	0.615
NC_005100.4_154800001	1	154800001	0.6005	0.6938
NC_005100.4_154900001	1	154900001	0.6005	0.6938
NC_005100.4_155000001	1	155000001	0.5338	0.719
NC_005100.4_155100001	1	155100001	0.5164	0.7377
NC_005100.4_155200001	1	155200001	0.4211	0.6605
NC_005100.4_155300001	1	155300001	0.3784	0.6085
NC_005100.4_155400001	1	155400001	0.3784	0.6085
NC_005100.4_155500001	1	155500001	0.2647	0.6582
NC_005100.4_155600001	1	155600001	0.5316	0.8408
NC_005100.4_155700001	1	155700001	0.5927	0.8807
NC_005100.4_155800001	1	155800001	0.5927	0.8807
NC_005100.4_155900001	1	155900001	0.5927	0.8807
NC_005100.4_156000001	1	156000001	0.5582	0.6198
NC_005100.4_156100001	1	156100001	0.3795	0.3716
NC_005100.4_156200001	1	156200001	0.2984	0.3496
NC_005100.4_156300001	1	156300001	0.2497	0.3341
NC_005100.4_156400001	1	156400001	0.3897	0.5501
NC_005100.4_156500001	1	156500001	0.4333	0.6904
NC_005100.4_156600001	1	156600001	0.4185	0.6872
NC_005100.4_156700001	1	156700001	0.5582	0.7631
NC_005100.4_156800001	1	156800001	0.5826	0.7795
NC_005100.4_156900001	1	156900001	0.5187	0.8067
NC_005100.4_157000001	1	157000001	0.5187	0.8067
NC_005100.4_157100001	1	157100001	0.4872	0.7703
NC_005100.4_157200001	1	157200001	0.3785	0.7066
NC_005100.4_157300001	1	157300001	0.369	0.6345
NC_005100.4_157400001	1	157400001	0.4349	0.5484
NC_005100.4_157500001	1	157500001	0.4523	0.572
NC_005100.4_157600001	1	157600001	0.5997	0.6424
NC_005100.4_157700001	1	157700001	0.5997	0.6424
NC_005100.4_157800001	1	157800001	0.5922	0.5786
NC_005100.4_157900001	1	157900001	0.5123	0.5579
NC_005100.4_158000001	1	158000001	0.4701	0.52
NC_005100.4_158100001	1	158100001	0.2632	0.3959

NC_005100.4_158200001	1	158200001	0.2632	0.3959
NC_005100.4_158300001	1	158300001	0.241	0.6291
NC_005100.4_158400001	1	158400001	0.4183	0.8042
NC_005100.4_158500001	1	158500001	0.4481	0.8106
NC_005100.4_158600001	1	158600001	0.5648	0.8671
NC_005100.4_158700001	1	158700001	0.6171	0.8925
NC_005100.4_158800001	1	158800001	0.7284	0.9628
NC_005100.4_158900001	1	158900001	0.4401	0.9092
NC_005100.4_159000001	1	159000001	0.3708	0.8903
NC_005100.4_159100001	1	159100001	0.342	0.6229
NC_005100.4_159200001	1	159200001	0.2853	0.5378
NC_005100.4_159300001	1	159300001	0.2656	0.4366
NC_005100.4_159400001	1	159400001	0.3579	0.5813
NC_005100.4_159500001	1	159500001	0.362	0.5232
NC_005100.4_159600001	1	159600001	0.3716	0.5553
NC_005100.4_159700001	1	159700001	0.4123	0.4906
NC_005100.4_159800001	1	159800001	0.4123	0.4906
NC_005100.4_159900001	1	159900001	0.3747	0.2525
NC_005100.4_160000001	1	160000001	0.507	0.4428
NC_005100.4_160100001	1	160100001	0.4941	0.4691
NC_005100.4_160200001	1	160200001	0.4743	0.4632
NC_005100.4_160300001	1	160300001	0.4956	0.5081
NC_005100.4_160400001	1	160400001	0.5451	0.6403
NC_005100.4_160500001	1	160500001	0.5014	0.6162
NC_005100.4_160600001	1	160600001	0.668	0.7122
NC_005100.4_160700001	1	160700001	0.7027	0.7583
NC_005100.4_160800001	1	160800001	0.6619	0.7709
NC_005100.4_160900001	1	160900001	0.6734	0.8039
NC_005100.4_161000001	1	161000001	0.5681	0.6917
NC_005100.4_161100001	1	161100001	0.446	0.6189
NC_005100.4_161200001	1	161200001	0.4518	0.6468
NC_005100.4_161300001	1	161300001	0.4327	0.6113
NC_005100.4_161400001	1	161400001	0.4767	0.6427
NC_005100.4_161500001	1	161500001	0.519	0.7007
NC_005100.4_161600001	1	161600001	0.5366	0.7206
NC_005100.4_161700001	1	161700001	0.4525	0.6504
NC_005100.4_161800001	1	161800001	0.5699	0.7138
NC_005100.4_161900001	1	161900001	0.6561	0.7391
NC_005100.4_162000001	1	162000001	0.6385	0.74
NC_005100.4_162100001	1	162100001	0.6752	0.7345
NC_005100.4_162200001	1	162200001	0.7135	0.7391
NC_005100.4_162300001	1	162300001	0.6809	0.7292
NC_005100.4_162400001	1	162400001	0.5912	0.6877
NC_005100.4_162500001	1	162500001	0.5922	0.7147
NC_005100.4_162600001	1	162600001	0.532	0.6615
NC_005100.4_162700001	1	162700001	0.4957	0.6392
NC_005100.4_162800001	1	162800001	0.4578	0.6003
NC_005100.4_162900001	1	162900001	0.4689	0.5972
NC_005100.4_163000001	1	163000001	0.478	0.6047
NC_005100.4_163100001	1	163100001	0.6211	0.6843
NC_005100.4_163200001	1	163200001	0.602	0.7029
NC_005100.4_163300001	1	163300001	0.5119	0.6502
NC_005100.4_163400001	1	163400001	0.4634	0.6404
NC_005100.4_163500001	1	163500001	0.4261	0.6353
NC_005100.4_163600001	1	163600001	0.4202	0.6807
NC_005100.4_163700001	1	163700001	0.5064	0.6979
NC_005100.4_163800001	1	163800001	0.5421	0.7422
NC_005100.4_163900001	1	163900001	0.4938	0.6804

NC_005100.4_164000001	1	164000001	0.5202	0.6471
NC_005100.4_164100001	1	164100001	0.4965	0.5986
NC_005100.4_164200001	1	164200001	0.4641	0.5496
NC_005100.4_164300001	1	164300001	0.4495	0.501
NC_005100.4_164400001	1	164400001	0.5143	0.5676
NC_005100.4_164500001	1	164500001	0.5155	0.5945
NC_005100.4_164600001	1	164600001	0.5111	0.6024
NC_005100.4_164700001	1	164700001	0.4903	0.6096
NC_005100.4_164800001	1	164800001	0.5269	0.6258
NC_005100.4_164900001	1	164900001	0.5441	0.6364
NC_005100.4_165000001	1	165000001	0.5237	0.6319
NC_005100.4_165100001	1	165100001	0.6068	0.7126
NC_005100.4_165200001	1	165200001	0.5742	0.7237
NC_005100.4_165300001	1	165300001	0.6064	0.7659
NC_005100.4_165400001	1	165400001	0.5934	0.7699
NC_005100.4_165500001	1	165500001	0.5932	0.7481
NC_005100.4_165600001	1	165600001	0.4304	0.6625
NC_005100.4_165700001	1	165700001	0.4604	0.7072
NC_005100.4_165800001	1	165800001	0.3834	0.6295
NC_005100.4_165900001	1	165900001	0.4343	0.6701
NC_005100.4_166000001	1	166000001	0.434	0.5998
NC_005100.4_166100001	1	166100001	0.5527	0.6542
NC_005100.4_166200001	1	166200001	0.5524	0.6014
NC_005100.4_166300001	1	166300001	0.5903	0.6612
NC_005100.4_166400001	1	166400001	0.573	0.6495
NC_005100.4_166500001	1	166500001	0.5787	0.7015
NC_005100.4_166600001	1	166600001	0.5871	0.7249
NC_005100.4_166700001	1	166700001	0.5618	0.783
NC_005100.4_166800001	1	166800001	0.4988	0.733
NC_005100.4_166900001	1	166900001	0.4842	0.7363
NC_005100.4_167000001	1	167000001	0.525	0.7542
NC_005100.4_167100001	1	167100001	0.5398	0.6874
NC_005100.4_167200001	1	167200001	0.607	0.6219
NC_005100.4_167300001	1	167300001	0.7454	0.7228
NC_005100.4_167400001	1	167400001	0.6451	0.7086
NC_005100.4_167500001	1	167500001	0.6508	0.6828
NC_005100.4_167600001	1	167600001	0.5507	0.6875
NC_005100.4_167700001	1	167700001	0.5908	0.6998
NC_005100.4_167800001	1	167800001	0.4435	0.5209
NC_005100.4_167900001	1	167900001	0.5879	0.6011
NC_005100.4_168000001	1	168000001	0.4913	0.545
NC_005100.4_168200001	1	168200001	0.4981	0.5863
NC_005100.4_168300001	1	168300001	0.4981	0.5863
NC_005100.4_168400001	1	168400001	0.5799	0.6837
NC_005100.4_168500001	1	168500001	0.466	0.685
NC_005100.4_168600001	1	168600001	0.547	0.6806
NC_005100.4_168700001	1	168700001	0.6906	0.7707
NC_005100.4_168800001	1	168800001	0.6356	0.7646
NC_005100.4_168900001	1	168900001	0.5836	0.6389
NC_005100.4_169000001	1	169000001	0.6028	0.6299
NC_005100.4_169100001	1	169100001	0.5553	0.6151
NC_005100.4_169200001	1	169200001	0.4314	0.5098
NC_005100.4_169300001	1	169300001	0.4373	0.4975
NC_005100.4_169400001	1	169400001	0.3639	0.4741
NC_005100.4_169500001	1	169500001	0.3281	0.4513
NC_005100.4_169600001	1	169600001	0.3669	0.5449
NC_005100.4_169700001	1	169700001	0.5397	0.6165
NC_005100.4_169800001	1	169800001	0.5309	0.6327

NC_005100.4_169900001	1	169900001	0.6732	0.7767
NC_005100.4_170000001	1	170000001	0.78	0.849
NC_005100.4_170100001	1	170100001	0.778	0.8422
NC_005100.4_170200001	1	170200001	0.7454	0.8914
NC_005100.4_170300001	1	170300001	0.7725	0.9059
NC_005100.4_170400001	1	170400001	0.6984	0.8673
NC_005100.4_170500001	1	170500001	0.6837	0.8878
NC_005100.4_170600001	1	170600001	0.5633	0.8865
NC_005100.4_171200001	1	171200001	0.6508	0.8367
NC_005100.4_171300001	1	171300001	0.5438	0.7704
NC_005100.4_171400001	1	171400001	0.5215	0.6332
NC_005100.4_171500001	1	171500001	0.5784	0.7121
NC_005100.4_171600001	1	171600001	0.5055	0.6321
NC_005100.4_171700001	1	171700001	0.4748	0.5396
NC_005100.4_171800001	1	171800001	0.4427	0.5033
NC_005100.4_171900001	1	171900001	0.4178	0.5162
NC_005100.4_172000001	1	172000001	0.3315	0.3329
NC_005100.4_172100001	1	172100001	0.4191	0.4382
NC_005100.4_172200001	1	172200001	0.4171	0.5013
NC_005100.4_172600001	1	172600001	0.4109	0.2585
NC_005100.4_172700001	1	172700001	0.5167	0.4676
NC_005100.4_172800001	1	172800001	0.5128	0.55
NC_005100.4_172900001	1	172900001	0.5593	0.5753
NC_005100.4_173000001	1	173000001	0.6473	0.6803
NC_005100.4_173100001	1	173100001	0.5417	0.7073
NC_005100.4_173200001	1	173200001	0.4847	0.6394
NC_005100.4_173300001	1	173300001	0.4644	0.5943
NC_005100.4_173400001	1	173400001	0.4625	0.6235
NC_005100.4_173500001	1	173500001	0.3963	0.5466
NC_005100.4_173600001	1	173600001	0.4433	0.5352
NC_005100.4_173700001	1	173700001	0.4673	0.5926
NC_005100.4_173800001	1	173800001	0.4969	0.6368
NC_005100.4_173900001	1	173900001	0.4984	0.6434
NC_005100.4_174000001	1	174000001	0.4637	0.6084
NC_005100.4_174100001	1	174100001	0.4624	0.6675
NC_005100.4_174200001	1	174200001	0.4458	0.6345
NC_005100.4_174300001	1	174300001	0.4345	0.6202
NC_005100.4_174400001	1	174400001	0.4443	0.6265
NC_005100.4_174500001	1	174500001	0.5641	0.6937
NC_005100.4_174600001	1	174600001	0.6255	0.6974
NC_005100.4_174700001	1	174700001	0.7546	0.8214
NC_005100.4_174800001	1	174800001	0.7809	0.8177
NC_005100.4_174900001	1	174900001	0.7574	0.7953
NC_005100.4_175000001	1	175000001	0.8392	0.8483
NC_005100.4_175100001	1	175100001	0.6975	0.8283
NC_005100.4_175200001	1	175200001	0.6232	0.7
NC_005100.4_175300001	1	175300001	0.6529	0.7245
NC_005100.4_175400001	1	175400001	0.6084	0.7027
NC_005100.4_175500001	1	175500001	0.5915	0.6748
NC_005100.4_175600001	1	175600001	0.5799	0.6452
NC_005100.4_175700001	1	175700001	0.4599	0.6286
NC_005100.4_175800001	1	175800001	0.3878	0.5655
NC_005100.4_175900001	1	175900001	0.4295	0.6651
NC_005100.4_176000001	1	176000001	0.4738	0.7131
NC_005100.4_176100001	1	176100001	0.506	0.7277
NC_005100.4_176200001	1	176200001	0.4976	0.7391
NC_005100.4_176300001	1	176300001	0.4896	0.7007
NC_005100.4_176400001	1	176400001	0.4623	0.6266

NC_005100.4_176500001	1	176500001	0.4583	0.65
NC_005100.4_176600001	1	176600001	0.425	0.5992
NC_005100.4_176700001	1	176700001	0.5345	0.6025
NC_005100.4_176800001	1	176800001	0.5303	0.5828
NC_005100.4_176900001	1	176900001	0.6024	0.6418
NC_005100.4_177000001	1	177000001	0.5619	0.5979
NC_005100.4_177100001	1	177100001	0.588	0.6204
NC_005100.4_177200001	1	177200001	0.5931	0.6926
NC_005100.4_177300001	1	177300001	0.6695	0.7618
NC_005100.4_177400001	1	177400001	0.6192	0.7652
NC_005100.4_177500001	1	177500001	0.5949	0.7508
NC_005100.4_177600001	1	177600001	0.5524	0.7709
NC_005100.4_177700001	1	177700001	0.5433	0.7396
NC_005100.4_177800001	1	177800001	0.5129	0.7292
NC_005100.4_177900001	1	177900001	0.5524	0.7887
NC_005100.4_178000001	1	178000001	0.5758	0.8355
NC_005100.4_178100001	1	178100001	0.6881	0.8724
NC_005100.4_178200001	1	178200001	0.4835	0.6798
NC_005100.4_178300001	1	178300001	0.4131	0.6078
NC_005100.4_178400001	1	178400001	0.3389	0.5186
NC_005100.4_178500001	1	178500001	0.3638	0.5507
NC_005100.4_178600001	1	178600001	0.3618	0.5467
NC_005100.4_178700001	1	178700001	0.3831	0.5913
NC_005100.4_178800001	1	178800001	0.403	0.622
NC_005100.4_178900001	1	178900001	0.515	0.7207
NC_005100.4_183200001	1	183200001	0.5408	0.7349
NC_005100.4_183300001	1	183300001	0.4934	0.6501
NC_005100.4_183400001	1	183400001	0.4934	0.6501
NC_005100.4_183500001	1	183500001	0.4775	0.5683
NC_005100.4_183600001	1	183600001	0.4907	0.5166
NC_005100.4_183700001	1	183700001	0.2333	0.1955
NC_005100.4_183800001	1	183800001	0.3278	0.4094
NC_005100.4_183900001	1	183900001	0.4555	0.5333
NC_005100.4_184000001	1	184000001	0.498	0.5912
NC_005100.4_184100001	1	184100001	0.4562	0.6307
NC_005100.4_184200001	1	184200001	0.4717	0.6118
NC_005100.4_184300001	1	184300001	0.486	0.5901
NC_005100.4_184400001	1	184400001	0.5177	0.6183
NC_005100.4_184500001	1	184500001	0.5267	0.678
NC_005100.4_184600001	1	184600001	0.5549	0.664
NC_005100.4_184700001	1	184700001	0.6174	0.6633
NC_005100.4_184800001	1	184800001	0.6196	0.674
NC_005100.4_184900001	1	184900001	0.584	0.6653
NC_005100.4_185000001	1	185000001	0.5717	0.593
NC_005100.4_185100001	1	185100001	0.5964	0.6044
NC_005100.4_185200001	1	185200001	0.5424	0.547
NC_005100.4_185300001	1	185300001	0.625	0.6579
NC_005100.4_185400001	1	185400001	0.6547	0.6768
NC_005100.4_185500001	1	185500001	0.6899	0.7242
NC_005100.4_185600001	1	185600001	0.7334	0.7897
NC_005100.4_185700001	1	185700001	0.8703	0.9716
NC_005100.4_185800001	1	185800001	0.6078	0.7373
NC_005100.4_185900001	1	185900001	0.5617	0.6961
NC_005100.4_186000001	1	186000001	0.5614	0.7149
NC_005100.4_186100001	1	186100001	0.4458	0.5946
NC_005100.4_186200001	1	186200001	0.3502	0.4157
NC_005100.4_186300001	1	186300001	0.3907	0.4729
NC_005100.4_186400001	1	186400001	0.4135	0.4726

NC_005100.4_186500001	1	186500001	0.3781	0.4175
NC_005100.4_186600001	1	186600001	0.4268	0.4506
NC_005100.4_186700001	1	186700001	0.4823	0.5195
NC_005100.4_186800001	1	186800001	0.4892	0.5562
NC_005100.4_186900001	1	186900001	0.4705	0.5806
NC_005100.4_187000001	1	187000001	0.472	0.6085
NC_005100.4_187100001	1	187100001	0.4899	0.6504
NC_005100.4_187200001	1	187200001	0.4409	0.6672
NC_005100.4_187300001	1	187300001	0.4494	0.6896
NC_005100.4_187400001	1	187400001	0.4492	0.7052
NC_005100.4_187500001	1	187500001	0.5814	0.7299
NC_005100.4_187600001	1	187600001	0.5712	0.6774
NC_005100.4_187700001	1	187700001	0.5217	0.6106
NC_005100.4_187800001	1	187800001	0.4833	0.538
NC_005100.4_187900001	1	187900001	0.5139	0.4997
NC_005100.4_188000001	1	188000001	0.3878	0.4163
NC_005100.4_188100001	1	188100001	0.475	0.6164
NC_005100.4_188200001	1	188200001	0.619	0.7729
NC_005100.4_188300001	1	188300001	0.6374	0.7907
NC_005100.4_188400001	1	188400001	0.5896	0.7502
NC_005100.4_188500001	1	188500001	0.5981	0.7461
NC_005100.4_188600001	1	188600001	0.5715	0.689
NC_005100.4_188700001	1	188700001	0.5121	0.6814
NC_005100.4_188800001	1	188800001	0.4921	0.7284
NC_005100.4_188900001	1	188900001	0.5358	0.7374
NC_005100.4_189000001	1	189000001	0.5672	0.7471
NC_005100.4_189100001	1	189100001	0.5463	0.7451
NC_005100.4_189200001	1	189200001	0.6065	0.744
NC_005100.4_189300001	1	189300001	0.7399	0.7433
NC_005100.4_189400001	1	189400001	0.7163	0.7531
NC_005100.4_189500001	1	189500001	0.7874	0.864
NC_005100.4_189600001	1	189600001	0.7388	0.7809
NC_005100.4_189700001	1	189700001	0.6987	0.7399
NC_005100.4_189800001	1	189800001	0.6987	0.7399
NC_005100.4_190100001	1	190100001	0.3403	0.3713
NC_005100.4_190200001	1	190200001	0.461	0.6427
NC_005100.4_190300001	1	190300001	0.5162	0.6996
NC_005100.4_190400001	1	190400001	0.5162	0.6996
NC_005100.4_190500001	1	190500001	0.5908	0.7905
NC_005100.4_190600001	1	190600001	0.594	0.8529
NC_005100.4_190700001	1	190700001	0.6034	0.7806
NC_005100.4_190800001	1	190800001	0.5647	0.7376
NC_005100.4_190900001	1	190900001	0.569	0.7352
NC_005100.4_191000001	1	191000001	0.5615	0.7067
NC_005100.4_191100001	1	191100001	0.572	0.6487
NC_005100.4_191200001	1	191200001	0.5189	0.6626
NC_005100.4_191300001	1	191300001	0.5603	0.6939
NC_005100.4_191400001	1	191400001	0.5177	0.6695
NC_005100.4_191500001	1	191500001	0.4823	0.6335
NC_005100.4_191600001	1	191600001	0.4326	0.5806
NC_005100.4_191700001	1	191700001	0.4542	0.5997
NC_005100.4_191800001	1	191800001	0.4276	0.5892
NC_005100.4_191900001	1	191900001	0.449	0.5866
NC_005100.4_192000001	1	192000001	0.3898	0.5277
NC_005100.4_192100001	1	192100001	0.4598	0.5572
NC_005100.4_192200001	1	192200001	0.5914	0.6555
NC_005100.4_192300001	1	192300001	0.6087	0.6566
NC_005100.4_192400001	1	192400001	0.5975	0.6922

NC_005100.4_192500001	1	192500001	0.6262	0.7202
NC_005100.4_192600001	1	192600001	0.6571	0.7823
NC_005100.4_192700001	1	192700001	0.6147	0.7509
NC_005100.4_192800001	1	192800001	0.5301	0.7223
NC_005100.4_192900001	1	192900001	0.5769	0.7647
NC_005100.4_193000001	1	193000001	0.5134	0.7036
NC_005100.4_193100001	1	193100001	0.4926	0.6771
NC_005100.4_193200001	1	193200001	0.4744	0.6702
NC_005100.4_193300001	1	193300001	0.5237	0.6756
NC_005100.4_193400001	1	193400001	0.4456	0.6055
NC_005100.4_193500001	1	193500001	0.4645	0.5956
NC_005100.4_193600001	1	193600001	0.5359	0.6746
NC_005100.4_193700001	1	193700001	0.5098	0.6792
NC_005100.4_193800001	1	193800001	0.4853	0.709
NC_005100.4_193900001	1	193900001	0.4956	0.7091
NC_005100.4_194000001	1	194000001	0.4245	0.7343
NC_005100.4_194100001	1	194100001	0.3478	0.6519
NC_005100.4_194200001	1	194200001	0.1836	0.4394
NC_005100.4_194300001	1	194300001	0.0836	0.0395
NC_005100.4_194400001	1	194400001	0.075	0.0221
NC_005100.4_194600001	1	194600001	0.1298	0.081
NC_005100.4_194700001	1	194700001	0.3521	0.5087
NC_005100.4_194800001	1	194800001	0.3946	0.4711
NC_005100.4_194900001	1	194900001	0.3775	0.48
NC_005100.4_195000001	1	195000001	0.3775	0.48
NC_005100.4_195100001	1	195100001	0.454	0.5571
NC_005100.4_195200001	1	195200001	0.3268	0.3918
NC_005100.4_195300001	1	195300001	0.3468	0.4924
NC_005100.4_195700001	1	195700001	0.5849	0.7329
NC_005100.4_195800001	1	195800001	0.6117	0.7931
NC_005100.4_195900001	1	195900001	0.6194	0.7807
NC_005100.4_196000001	1	196000001	0.5791	0.7278
NC_005100.4_196100001	1	196100001	0.4847	0.6581
NC_005100.4_196200001	1	196200001	0.46	0.6305
NC_005100.4_196300001	1	196300001	0.5183	0.6651
NC_005100.4_196400001	1	196400001	0.4351	0.5954
NC_005100.4_196500001	1	196500001	0.4318	0.6022
NC_005100.4_196600001	1	196600001	0.5508	0.7298
NC_005100.4_196700001	1	196700001	0.5609	0.7371
NC_005100.4_196800001	1	196800001	0.4178	0.6454
NC_005100.4_196900001	1	196900001	0.4982	0.6685
NC_005100.4_197000001	1	197000001	0.5363	0.6986
NC_005100.4_197100001	1	197100001	0.5194	0.6805
NC_005100.4_197200001	1	197200001	0.5397	0.667
NC_005100.4_197300001	1	197300001	0.6645	0.7352
NC_005100.4_197400001	1	197400001	0.6544	0.7813
NC_005100.4_197500001	1	197500001	0.6337	0.7538
NC_005100.4_197600001	1	197600001	0.6006	0.7108
NC_005100.4_197700001	1	197700001	0.4619	0.6711
NC_005100.4_197800001	1	197800001	0.444	0.6334
NC_005100.4_197900001	1	197900001	0.4179	0.5487
NC_005100.4_198000001	1	198000001	0.4301	0.6044
NC_005100.4_198100001	1	198100001	0.4834	0.6252
NC_005100.4_198200001	1	198200001	0.5562	0.7249
NC_005100.4_198300001	1	198300001	0.5639	0.6463
NC_005100.4_198400001	1	198400001	0.6079	0.6887
NC_005100.4_198500001	1	198500001	0.6131	0.6767
NC_005100.4_198600001	1	198600001	0.6472	0.7075

NC_005100.4_198700001	1	198700001	0.71	0.6833
NC_005100.4_198800001	1	198800001	0.7243	0.762
NC_005100.4_198900001	1	198900001	0.687	0.7902
NC_005100.4_199000001	1	199000001	0.7231	0.7894
NC_005100.4_199100001	1	199100001	0.6006	0.7749
NC_005100.4_199200001	1	199200001	0.575	0.781
NC_005100.4_199300001	1	199300001	0.5625	0.7172
NC_005100.4_199400001	1	199400001	0.5546	0.7068
NC_005100.4_199500001	1	199500001	0.527	0.6985
NC_005100.4_199600001	1	199600001	0.5815	0.6833
NC_005100.4_199700001	1	199700001	0.5718	0.6464
NC_005100.4_199800001	1	199800001	0.5904	0.6815
NC_005100.4_199900001	1	199900001	0.6065	0.6556
NC_005100.4_200000001	1	200000001	0.6051	0.6331
NC_005100.4_200100001	1	200100001	0.5144	0.591
NC_005100.4_200200001	1	200200001	0.6924	0.7952
NC_005100.4_200300001	1	200300001	0.7175	0.8098
NC_005100.4_200400001	1	200400001	0.7175	0.8098
NC_005100.4_200500001	1	200500001	0.7367	0.8702
NC_005100.4_200600001	1	200600001	0.7945	0.887
NC_005100.4_200700001	1	200700001	0.6765	0.7815
NC_005100.4_200800001	1	200800001	0.5116	0.5501
NC_005100.4_200900001	1	200900001	0.547	0.61
NC_005100.4_201000001	1	201000001	0.5428	0.5974
NC_005100.4_201100001	1	201100001	0.5356	0.5986
NC_005100.4_201200001	1	201200001	0.5493	0.6047
NC_005100.4_201300001	1	201300001	0.5413	0.6002
NC_005100.4_201400001	1	201400001	0.5123	0.6041
NC_005100.4_201500001	1	201500001	0.479	0.5835
NC_005100.4_201600001	1	201600001	0.5162	0.6018
NC_005100.4_201700001	1	201700001	0.4758	0.6053
NC_005100.4_201800001	1	201800001	0.4698	0.5799
NC_005100.4_201900001	1	201900001	0.4709	0.5264
NC_005100.4_202000001	1	202000001	0.4578	0.5183
NC_005100.4_202100001	1	202100001	0.3959	0.4587
NC_005100.4_202200001	1	202200001	0.4339	0.4272
NC_005100.4_202300001	1	202300001	0.461	0.5328
NC_005100.4_202400001	1	202400001	0.492	0.6458
NC_005100.4_202500001	1	202500001	0.6639	0.7542
NC_005100.4_202600001	1	202600001	0.6712	0.7495
NC_005100.4_202700001	1	202700001	0.6661	0.785
NC_005100.4_202800001	1	202800001	0.6709	0.7743
NC_005100.4_202900001	1	202900001	0.5918	0.675
NC_005100.4_203000001	1	203000001	0.4139	0.5756
NC_005100.4_203100001	1	203100001	0.4496	0.5749
NC_005100.4_203200001	1	203200001	0.4603	0.6144
NC_005100.4_203300001	1	203300001	0.4475	0.6077
NC_005100.4_203400001	1	203400001	0.4751	0.6243
NC_005100.4_203500001	1	203500001	0.487	0.6285
NC_005100.4_203600001	1	203600001	0.5315	0.7026
NC_005100.4_203700001	1	203700001	0.5528	0.6881
NC_005100.4_203800001	1	203800001	0.6723	0.7608
NC_005100.4_203900001	1	203900001	0.6936	0.7706
NC_005100.4_204000001	1	204000001	0.5967	0.7085
NC_005100.4_204100001	1	204100001	0.5785	0.6994
NC_005100.4_204200001	1	204200001	0.5881	0.6998
NC_005100.4_204300001	1	204300001	0.6191	0.7378
NC_005100.4_204400001	1	204400001	0.591	0.7639

NC_005100.4_204500001	1	204500001	0.6797	0.8363
NC_005100.4_204600001	1	204600001	0.6524	0.7759
NC_005100.4_204700001	1	204700001	0.5812	0.7734
NC_005100.4_204800001	1	204800001	0.5442	0.7508
NC_005100.4_204900001	1	204900001	0.5412	0.7238
NC_005100.4_205000001	1	205000001	0.5145	0.6453
NC_005100.4_205100001	1	205100001	0.4654	0.636
NC_005100.4_205200001	1	205200001	0.4919	0.6324
NC_005100.4_205300001	1	205300001	0.4705	0.6129
NC_005100.4_205400001	1	205400001	0.5426	0.6843
NC_005100.4_205500001	1	205500001	0.514	0.7024
NC_005100.4_205600001	1	205600001	0.5811	0.7484
NC_005100.4_205700001	1	205700001	0.5433	0.6803
NC_005100.4_205800001	1	205800001	0.5149	0.6422
NC_005100.4_205900001	1	205900001	0.4731	0.6193
NC_005100.4_206000001	1	206000001	0.525	0.6944
NC_005100.4_206100001	1	206100001	0.4756	0.6487
NC_005100.4_206200001	1	206200001	0.5642	0.8178
NC_005100.4_206300001	1	206300001	0.5356	0.7338
NC_005100.4_206400001	1	206400001	0.5144	0.6893
NC_005100.4_206500001	1	206500001	0.4833	0.5935
NC_005100.4_206600001	1	206600001	0.49	0.6022
NC_005100.4_206700001	1	206700001	0.5418	0.6433
NC_005100.4_206800001	1	206800001	0.5817	0.6538
NC_005100.4_206900001	1	206900001	0.6231	0.7037
NC_005100.4_207000001	1	207000001	0.6325	0.7265
NC_005100.4_207100001	1	207100001	0.6488	0.7105
NC_005100.4_207200001	1	207200001	0.5893	0.6618
NC_005100.4_207300001	1	207300001	0.5951	0.6966
NC_005100.4_207400001	1	207400001	0.5761	0.6429
NC_005100.4_207500001	1	207500001	0.6078	0.6629
NC_005100.4_207600001	1	207600001	0.6046	0.7131
NC_005100.4_207700001	1	207700001	0.6314	0.7658
NC_005100.4_207800001	1	207800001	0.6178	0.7448
NC_005100.4_207900001	1	207900001	0.5359	0.6659
NC_005100.4_208000001	1	208000001	0.5367	0.6496
NC_005100.4_208100001	1	208100001	0.5046	0.6284
NC_005100.4_208200001	1	208200001	0.4853	0.5955
NC_005100.4_208300001	1	208300001	0.4637	0.5827
NC_005100.4_208400001	1	208400001	0.5536	0.6691
NC_005100.4_208500001	1	208500001	0.5683	0.6968
NC_005100.4_208600001	1	208600001	0.5951	0.7183
NC_005100.4_208700001	1	208700001	0.5837	0.7348
NC_005100.4_208800001	1	208800001	0.652	0.7958
NC_005100.4_208900001	1	208900001	0.6217	0.8261
NC_005100.4_209000001	1	209000001	0.6735	0.8641
NC_005100.4_209100001	1	209100001	0.7126	0.8566
NC_005100.4_209200001	1	209200001	0.7215	0.8206
NC_005100.4_209300001	1	209300001	0.6744	0.7698
NC_005100.4_209400001	1	209400001	0.6625	0.7462
NC_005100.4_209500001	1	209500001	0.6078	0.6606
NC_005100.4_209600001	1	209600001	0.5959	0.6279
NC_005100.4_209700001	1	209700001	0.5682	0.6552
NC_005100.4_209800001	1	209800001	0.6063	0.7083
NC_005100.4_209900001	1	209900001	0.5416	0.6886
NC_005100.4_210000001	1	210000001	0.5011	0.6447
NC_005100.4_210100001	1	210100001	0.4422	0.6237
NC_005100.4_210200001	1	210200001	0.5111	0.639

NC_005100.4_210300001	1	210300001	0.5558	0.6844
NC_005100.4_210400001	1	210400001	0.6328	0.6952
NC_005100.4_210500001	1	210500001	0.6495	0.7147
NC_005100.4_210600001	1	210600001	0.6477	0.6808
NC_005100.4_210700001	1	210700001	0.6651	0.673
NC_005100.4_210800001	1	210800001	0.6307	0.63
NC_005100.4_210900001	1	210900001	0.5259	0.5994
NC_005100.4_211000001	1	211000001	0.5312	0.6345
NC_005100.4_211100001	1	211100001	0.4949	0.6549
NC_005100.4_211200001	1	211200001	0.4317	0.611
NC_005100.4_211300001	1	211300001	0.4249	0.5782
NC_005100.4_211400001	1	211400001	0.4691	0.6379
NC_005100.4_211500001	1	211500001	0.448	0.5831
NC_005100.4_211600001	1	211600001	0.4526	0.5707
NC_005100.4_211700001	1	211700001	0.4902	0.6014
NC_005100.4_211800001	1	211800001	0.5454	0.734
NC_005100.4_211900001	1	211900001	0.5409	0.7339
NC_005100.4_212000001	1	212000001	0.553	0.7728
NC_005100.4_212100001	1	212100001	0.5938	0.7527
NC_005100.4_212200001	1	212200001	0.6002	0.7548
NC_005100.4_212300001	1	212300001	0.6029	0.7113
NC_005100.4_212400001	1	212400001	0.5259	0.6662
NC_005100.4_212500001	1	212500001	0.4663	0.6342
NC_005100.4_212600001	1	212600001	0.4074	0.5448
NC_005100.4_212700001	1	212700001	0.3557	0.4786
NC_005100.4_212800001	1	212800001	0.3217	0.4489
NC_005100.4_212900001	1	212900001	0.4267	0.5166
NC_005100.4_213000001	1	213000001	0.57	0.6302
NC_005100.4_213100001	1	213100001	0.5777	0.7221
NC_005100.4_213200001	1	213200001	0.5777	0.7221
NC_005100.4_213300001	1	213300001	0.6389	0.7713
NC_005100.4_213400001	1	213400001	0.6377	0.8129
NC_005100.4_213500001	1	213500001	0.5714	0.7778
NC_005100.4_213600001	1	213600001	0.6272	0.8616
NC_005100.4_213700001	1	213700001	0.5992	0.8002
NC_005100.4_213800001	1	213800001	0.5215	0.7639
NC_005100.4_213900001	1	213900001	0.5498	0.7184
NC_005100.4_214000001	1	214000001	0.6129	0.7456
NC_005100.4_214100001	1	214100001	0.5987	0.7081
NC_005100.4_214200001	1	214200001	0.5785	0.6856
NC_005100.4_214300001	1	214300001	0.5396	0.6798
NC_005100.4_214400001	1	214400001	0.5045	0.6817
NC_005100.4_214500001	1	214500001	0.5948	0.7539
NC_005100.4_214600001	1	214600001	0.5609	0.7317
NC_005100.4_214700001	1	214700001	0.5827	0.7621
NC_005100.4_214800001	1	214800001	0.6279	0.7757
NC_005100.4_214900001	1	214900001	0.6587	0.7576
NC_005100.4_215000001	1	215000001	0.4669	0.5642
NC_005100.4_215100001	1	215100001	0.6492	0.7043
NC_005100.4_215200001	1	215200001	0.7052	0.7205
NC_005100.4_215300001	1	215300001	0.6937	0.7107
NC_005100.4_215400001	1	215400001	0.6599	0.7277
NC_005100.4_215500001	1	215500001	0.6729	0.7386
NC_005100.4_215600001	1	215600001	0.6373	0.7045
NC_005100.4_215700001	1	215700001	0.4296	0.555
NC_005100.4_215800001	1	215800001	0.5282	0.6875
NC_005100.4_215900001	1	215900001	0.5236	0.6428
NC_005100.4_216000001	1	216000001	0.544	0.6646

NC_005100.4_216100001	1	216100001	0.5668	0.7247
NC_005100.4_216200001	1	216200001	0.6552	0.7532
NC_005100.4_216300001	1	216300001	0.5285	0.6232
NC_005100.4_216400001	1	216400001	0.5578	0.6453
NC_005100.4_216500001	1	216500001	0.5447	0.6358
NC_005100.4_216600001	1	216600001	0.556	0.6239
NC_005100.4_216700001	1	216700001	0.5335	0.5955
NC_005100.4_216800001	1	216800001	0.5127	0.596
NC_005100.4_216900001	1	216900001	0.4567	0.5431
NC_005100.4_217000001	1	217000001	0.3925	0.4975
NC_005100.4_217100001	1	217100001	0.3668	0.4721
NC_005100.4_217200001	1	217200001	0.3606	0.5455
NC_005100.4_217300001	1	217300001	0.406	0.5784
NC_005100.4_217400001	1	217400001	0.4426	0.6308
NC_005100.4_217500001	1	217500001	0.4832	0.6633
NC_005100.4_217600001	1	217600001	0.4677	0.6409
NC_005100.4_217700001	1	217700001	0.4589	0.6054
NC_005100.4_217800001	1	217800001	0.5185	0.6463
NC_005100.4_217900001	1	217900001	0.5501	0.6859
NC_005100.4_218000001	1	218000001	0.6013	0.7127
NC_005100.4_218100001	1	218100001	0.6224	0.6895
NC_005100.4_218200001	1	218200001	0.6296	0.6961
NC_005100.4_218300001	1	218300001	0.6145	0.6955
NC_005100.4_218400001	1	218400001	0.6251	0.6941
NC_005100.4_218500001	1	218500001	0.5718	0.616
NC_005100.4_218600001	1	218600001	0.6014	0.6305
NC_005100.4_218700001	1	218700001	0.6269	0.7005
NC_005100.4_218800001	1	218800001	0.6119	0.6816
NC_005100.4_218900001	1	218900001	0.6025	0.6858
NC_005100.4_219000001	1	219000001	0.6388	0.7174
NC_005100.4_219100001	1	219100001	0.6237	0.7681
NC_005100.4_219200001	1	219200001	0.6528	0.7219
NC_005100.4_219300001	1	219300001	0.5995	0.6854
NC_005100.4_219400001	1	219400001	0.61	0.6707
NC_005100.4_219500001	1	219500001	0.6125	0.7052
NC_005100.4_219600001	1	219600001	0.6081	0.684
NC_005100.4_219700001	1	219700001	0.6017	0.6977
NC_005100.4_219800001	1	219800001	0.6934	0.7474
NC_005100.4_219900001	1	219900001	0.67	0.7231
NC_005100.4_220000001	1	220000001	0.6453	0.6977
NC_005100.4_220100001	1	220100001	0.8256	0.8752
NC_005100.4_220200001	1	220200001	0.8168	0.9841
NC_005100.4_220300001	1	220300001	0.6118	0.6559
NC_005100.4_220400001	1	220400001	0.7115	0.7612
NC_005100.4_220500001	1	220500001	0.6838	0.7688
NC_005100.4_220600001	1	220600001	0.6308	0.7522
NC_005100.4_220700001	1	220700001	0.6381	0.7365
NC_005100.4_220800001	1	220800001	0.7304	0.8782
NC_005100.4_220900001	1	220900001	0.6949	0.7995
NC_005100.4_221000001	1	221000001	0.7334	0.7963
NC_005100.4_221100001	1	221100001	0.5291	0.6487
NC_005100.4_221200001	1	221200001	0.5417	0.6982
NC_005100.4_221300001	1	221300001	0.4999	0.6935
NC_005100.4_221400001	1	221400001	0.417	0.6309
NC_005100.4_221500001	1	221500001	0.4567	0.6508
NC_005100.4_221600001	1	221600001	0.5424	0.7059
NC_005100.4_221700001	1	221700001	0.59	0.6755
NC_005100.4_221800001	1	221800001	0.6041	0.6825

NC_005100.4_221900001	1	221900001	0.6342	0.7317
NC_005100.4_222000001	1	222000001	0.6361	0.7509
NC_005100.4_222100001	1	222100001	0.6401	0.7553
NC_005100.4_222200001	1	222200001	0.5974	0.8389
NC_005100.4_222300001	1	222300001	0.5912	0.804
NC_005100.4_222400001	1	222400001	0.5347	0.7514
NC_005100.4_222500001	1	222500001	0.5329	0.6867
NC_005100.4_222600001	1	222600001	0.4782	0.6511
NC_005100.4_222700001	1	222700001	0.4407	0.6099
NC_005100.4_222800001	1	222800001	0.3636	0.4959
NC_005100.4_224200001	1	224200001	0.8124	0.7932
NC_005100.4_224300001	1	224300001	0.6459	0.767
NC_005100.4_224400001	1	224400001	0.5766	0.6916
NC_005100.4_224500001	1	224500001	0.6167	0.7401
NC_005100.4_224600001	1	224600001	0.6684	0.782
NC_005100.4_224700001	1	224700001	0.6571	0.7683
NC_005100.4_224800001	1	224800001	0.6104	0.6883
NC_005100.4_224900001	1	224900001	0.6488	0.7156
NC_005100.4_225000001	1	225000001	0.6097	0.6742
NC_005100.4_225100001	1	225100001	0.4421	0.4726
NC_005100.4_225200001	1	225200001	0.3477	0.3406
NC_005100.4_225300001	1	225300001	0.4096	0.4614
NC_005100.4_225500001	1	225500001	0.4836	0.6218
NC_005100.4_225600001	1	225600001	0.5393	0.6483
NC_005100.4_225700001	1	225700001	0.5487	0.7546
NC_005100.4_225800001	1	225800001	0.612	0.6542
NC_005100.4_225900001	1	225900001	0.5045	0.6328
NC_005100.4_226000001	1	226000001	0.4848	0.5968
NC_005100.4_226100001	1	226100001	0.4666	0.5978
NC_005100.4_226200001	1	226200001	0.4711	0.6021
NC_005100.4_226300001	1	226300001	0.3701	0.579
NC_005100.4_226400001	1	226400001	0.4371	0.5702
NC_005100.4_226500001	1	226500001	0.4664	0.5554
NC_005100.4_226600001	1	226600001	0.4757	0.4975
NC_005100.4_226700001	1	226700001	0.4976	0.5049
NC_005100.4_226800001	1	226800001	0.4976	0.5049
NC_005100.4_226900001	1	226900001	0.4521	0.4786
NC_005100.4_227000001	1	227000001	0.4391	0.5137
NC_005100.4_227100001	1	227100001	0.4665	0.5884
NC_005100.4_227200001	1	227200001	0.3785	0.5441
NC_005100.4_227300001	1	227300001	0.3785	0.5441
NC_005100.4_227400001	1	227400001	0.3865	0.6003
NC_005100.4_227500001	1	227500001	0.3326	0.5968
NC_005100.4_227600001	1	227600001	0.3374	0.6426
NC_005100.4_227700001	1	227700001	0.5959	0.8206
NC_005100.4_227800001	1	227800001	0.5902	0.8202
NC_005100.4_227900001	1	227900001	0.6776	0.8059
NC_005100.4_228000001	1	228000001	0.7076	0.7695
NC_005100.4_228100001	1	228100001	0.6287	0.7328
NC_005100.4_228200001	1	228200001	0.3866	0.5072
NC_005100.4_228300001	1	228300001	0.3027	0.36
NC_005100.4_228400001	1	228400001	0.3954	0.5259
NC_005100.4_228500001	1	228500001	0.3929	0.5579
NC_005100.4_228600001	1	228600001	0.3849	0.5895
NC_005100.4_228700001	1	228700001	0.3669	0.6342
NC_005100.4_228800001	1	228800001	0.6085	0.8977
NC_005100.4_228900001	1	228900001	0.4969	0.8641
NC_005100.4_229000001	1	229000001	0.4258	0.7263

NC_005100.4_229100001	1	229100001	0.4662	0.6533
NC_005100.4_229200001	1	229200001	0.6671	0.7321
NC_005100.4_229300001	1	229300001	0.5563	0.6544
NC_005100.4_229400001	1	229400001	0.5563	0.6544
NC_005100.4_229900001	1	229900001	0.4782	0.4297
NC_005100.4_230000001	1	230000001	0.4265	0.4254
NC_005100.4_230100001	1	230100001	0.4851	0.4783
NC_005100.4_230200001	1	230200001	0.3376	0.305
NC_005100.4_230300001	1	230300001	0.3957	0.4175
NC_005100.4_230400001	1	230400001	0.528	0.6557
NC_005100.4_230500001	1	230500001	0.659	0.7271
NC_005100.4_230600001	1	230600001	0.6356	0.678
NC_005100.4_230700001	1	230700001	0.5803	0.6438
NC_005100.4_230800001	1	230800001	0.5758	0.6478
NC_005100.4_230900001	1	230900001	0.5652	0.6913
NC_005100.4_231000001	1	231000001	0.5189	0.6291
NC_005100.4_231100001	1	231100001	0.4978	0.6472
NC_005100.4_231200001	1	231200001	0.5288	0.6177
NC_005100.4_231300001	1	231300001	0.5106	0.6256
NC_005100.4_231400001	1	231400001	0.377	0.4301
NC_005100.4_231500001	1	231500001	0.4099	0.4433
NC_005100.4_231600001	1	231600001	0.3663	0.3967
NC_005100.4_231700001	1	231700001	0.3903	0.4711
NC_005100.4_231800001	1	231800001	0.338	0.3983
NC_005100.4_231900001	1	231900001	0.4198	0.4915
NC_005100.4_232000001	1	232000001	0.3201	0.5057
NC_005100.4_232100001	1	232100001	0.372	0.495
NC_005100.4_232200001	1	232200001	0.3488	0.5083
NC_005100.4_232300001	1	232300001	0.4168	0.5255
NC_005100.4_232400001	1	232400001	0.3987	0.5047
NC_005100.4_232500001	1	232500001	0.4055	0.4661
NC_005100.4_232600001	1	232600001	0.4276	0.4548
NC_005100.4_232700001	1	232700001	0.4803	0.5307
NC_005100.4_232800001	1	232800001	0.4947	0.5942
NC_005100.4_232900001	1	232900001	0.4834	0.5796
NC_005100.4_233000001	1	233000001	0.4922	0.6294
NC_005100.4_233100001	1	233100001	0.4834	0.7482
NC_005100.4_233200001	1	233200001	0.462	0.7088
NC_005100.4_233300001	1	233300001	0.3627	0.5889
NC_005100.4_233400001	1	233400001	0.3758	0.631
NC_005100.4_233500001	1	233500001	0.349	0.5976
NC_005100.4_233600001	1	233600001	0.3758	0.5913
NC_005100.4_233700001	1	233700001	0.2508	0.1858
NC_005100.4_233800001	1	233800001	0.4645	0.4598
NC_005100.4_233900001	1	233900001	0.4734	0.4819
NC_005100.4_234000001	1	234000001	0.4941	0.5473
NC_005100.4_234100001	1	234100001	0.4294	0.5011
NC_005100.4_234200001	1	234200001	0.4323	0.5504
NC_005100.4_234300001	1	234300001	0.3904	0.4905
NC_005100.4_234400001	1	234400001	0.4454	0.5293
NC_005100.4_234500001	1	234500001	0.4236	0.4984
NC_005100.4_234600001	1	234600001	0.6081	0.699
NC_005100.4_234700001	1	234700001	0.6232	0.7308
NC_005100.4_234800001	1	234800001	0.627	0.7036
NC_005100.4_234900001	1	234900001	0.5989	0.7458
NC_005100.4_235000001	1	235000001	0.5721	0.7209
NC_005100.4_235100001	1	235100001	0.5245	0.6704
NC_005100.4_235200001	1	235200001	0.5282	0.6594

NC_005100.4_235300001	1	235300001	0.5096	0.8252
NC_005100.4_235400001	1	235400001	0.5422	0.8255
NC_005100.4_235500001	1	235500001	0.4941	0.8322
NC_005100.4_235600001	1	235600001	0.3744	0.6337
NC_005100.4_235700001	1	235700001	0.41	0.5677
NC_005100.4_235800001	1	235800001	0.38	0.5225
NC_005100.4_235900001	1	235900001	0.3812	0.5235
NC_005100.4_236000001	1	236000001	0.4375	0.5078
NC_005100.4_236100001	1	236100001	0.5155	0.5691
NC_005100.4_236200001	1	236200001	0.5053	0.6
NC_005100.4_236300001	1	236300001	0.6439	0.7006
NC_005100.4_236400001	1	236400001	0.6021	0.6683
NC_005100.4_236500001	1	236500001	0.6114	0.6988
NC_005100.4_236600001	1	236600001	0.5576	0.6722
NC_005100.4_236700001	1	236700001	0.5646	0.6569
NC_005100.4_236800001	1	236800001	0.4993	0.5415
NC_005100.4_236900001	1	236900001	0.5204	0.5502
NC_005100.4_237000001	1	237000001	0.5262	0.4905
NC_005100.4_237100001	1	237100001	0.4842	0.4666
NC_005100.4_237200001	1	237200001	0.4172	0.428
NC_005100.4_237300001	1	237300001	0.4188	0.5144
NC_005100.4_237400001	1	237400001	0.4188	0.5144
NC_005100.4_237500001	1	237500001	0.4237	0.5869
NC_005100.4_237600001	1	237600001	0.448	0.606
NC_005100.4_237700001	1	237700001	0.4475	0.6234
NC_005100.4_237900001	1	237900001	0.5527	0.7785
NC_005100.4_238000001	1	238000001	0.4865	0.5579
NC_005100.4_238100001	1	238100001	0.5264	0.5994
NC_005100.4_238200001	1	238200001	0.5317	0.5179
NC_005100.4_238300001	1	238300001	0.4628	0.4613
NC_005100.4_238400001	1	238400001	0.4312	0.4564
NC_005100.4_238500001	1	238500001	0.4606	0.5469
NC_005100.4_238600001	1	238600001	0.5038	0.5714
NC_005100.4_238700001	1	238700001	0.519	0.6388
NC_005100.4_238800001	1	238800001	0.6102	0.7335
NC_005100.4_238900001	1	238900001	0.6557	0.7541
NC_005100.4_239000001	1	239000001	0.6209	0.7098
NC_005100.4_239100001	1	239100001	0.5773	0.6637
NC_005100.4_239200001	1	239200001	0.5399	0.6585
NC_005100.4_239300001	1	239300001	0.4775	0.6986
NC_005100.4_239400001	1	239400001	0.4617	0.7178
NC_005100.4_239500001	1	239500001	0.5612	0.7894
NC_005100.4_239600001	1	239600001	0.4878	0.7564
NC_005100.4_239700001	1	239700001	0.5689	0.7832
NC_005100.4_239800001	1	239800001	0.5494	0.7202
NC_005100.4_239900001	1	239900001	0.4761	0.6579
NC_005100.4_240000001	1	240000001	0.4413	0.6076
NC_005100.4_240100001	1	240100001	0.491	0.6175
NC_005100.4_240200001	1	240200001	0.4276	0.5436
NC_005100.4_240300001	1	240300001	0.3739	0.5222
NC_005100.4_240400001	1	240400001	0.5	0.6357
NC_005100.4_240500001	1	240500001	0.4926	0.6893
NC_005100.4_240600001	1	240600001	0.4453	0.7327
NC_005100.4_240700001	1	240700001	0.4376	0.6019
NC_005100.4_240800001	1	240800001	0.4828	0.6196
NC_005100.4_240900001	1	240900001	0.4594	0.5672
NC_005100.4_241000001	1	241000001	0.4607	0.5406
NC_005100.4_241100001	1	241100001	0.4912	0.5285

NC_005100.4_241200001	1	241200001	0.5037	0.5701
NC_005100.4_241300001	1	241300001	0.5032	0.5769
NC_005100.4_241400001	1	241400001	0.5202	0.6327
NC_005100.4_241500001	1	241500001	0.4743	0.5846
NC_005100.4_241600001	1	241600001	0.4304	0.5426
NC_005100.4_241700001	1	241700001	0.4236	0.5476
NC_005100.4_241800001	1	241800001	0.4044	0.5306
NC_005100.4_241900001	1	241900001	0.3681	0.4843
NC_005100.4_242000001	1	242000001	0.3715	0.4806
NC_005100.4_242100001	1	242100001	0.5272	0.6494
NC_005100.4_242200001	1	242200001	0.5627	0.6725
NC_005100.4_242300001	1	242300001	0.569	0.6995
NC_005100.4_242400001	1	242400001	0.6082	0.7145
NC_005100.4_242500001	1	242500001	0.6434	0.7595
NC_005100.4_242600001	1	242600001	0.5773	0.6898
NC_005100.4_242700001	1	242700001	0.6027	0.7254
NC_005100.4_242800001	1	242800001	0.6646	0.7339
NC_005100.4_242900001	1	242900001	0.5307	0.6677
NC_005100.4_243000001	1	243000001	0.4858	0.5832
NC_005100.4_243100001	1	243100001	0.4005	0.4863
NC_005100.4_243200001	1	243200001	0.342	0.3893
NC_005100.4_243300001	1	243300001	0.3591	0.3734
NC_005100.4_243400001	1	243400001	0.3668	0.376
NC_005100.4_243500001	1	243500001	0.3369	0.3453
NC_005100.4_243600001	1	243600001	0.4691	0.537
NC_005100.4_243700001	1	243700001	0.4649	0.5586
NC_005100.4_243800001	1	243800001	0.4877	0.6451
NC_005100.4_243900001	1	243900001	0.5788	0.7434
NC_005100.4_244000001	1	244000001	0.6118	0.7439
NC_005100.4_244100001	1	244100001	0.62	0.7606
NC_005100.4_244200001	1	244200001	0.5973	0.7642
NC_005100.4_244300001	1	244300001	0.6411	0.7629
NC_005100.4_244400001	1	244400001	0.5369	0.6123
NC_005100.4_244500001	1	244500001	0.5035	0.616
NC_005100.4_244600001	1	244600001	0.5035	0.616
NC_005100.4_244700001	1	244700001	0.5499	0.6201
NC_005100.4_244800001	1	244800001	0.4506	0.448
NC_005100.4_244900001	1	244900001	0.5465	0.5772
NC_005100.4_245000001	1	245000001	0.5887	0.6744
NC_005100.4_245100001	1	245100001	0.467	0.5892
NC_005100.4_245200001	1	245200001	0.4422	0.5812
NC_005100.4_245300001	1	245300001	0.4789	0.6734
NC_005100.4_245400001	1	245400001	0.4499	0.6705
NC_005100.4_245500001	1	245500001	0.456	0.6652
NC_005100.4_245600001	1	245600001	0.6088	0.806
NC_005100.4_245700001	1	245700001	0.6852	0.8435
NC_005100.4_245800001	1	245800001	0.7663	0.9093
NC_005100.4_245900001	1	245900001	0.7408	0.7732
NC_005100.4_246000001	1	246000001	0.69	0.6917
NC_005100.4_246100001	1	246100001	0.5578	0.5963
NC_005100.4_246200001	1	246200001	0.4606	0.5507
NC_005100.4_246300001	1	246300001	0.4589	0.5702
NC_005100.4_246400001	1	246400001	0.4397	0.6176
NC_005100.4_246500001	1	246500001	0.3839	0.5629
NC_005100.4_246600001	1	246600001	0.3735	0.5625
NC_005100.4_246700001	1	246700001	0.3984	0.6112
NC_005100.4_246800001	1	246800001	0.3421	0.5249
NC_005100.4_246900001	1	246900001	0.3034	0.4451

NC_005100.4_247000001	1	247000001	0.4485	0.6492
NC_005100.4_247100001	1	247100001	0.4934	0.6469
NC_005100.4_247200001	1	247200001	0.6042	0.7136
NC_005100.4_247300001	1	247300001	0.5988	0.7361
NC_005100.4_247400001	1	247400001	0.6152	0.7592
NC_005100.4_247500001	1	247500001	0.5355	0.7409
NC_005100.4_247600001	1	247600001	0.5857	0.7209
NC_005100.4_247700001	1	247700001	0.5328	0.6418
NC_005100.4_247800001	1	247800001	0.597	0.6866
NC_005100.4_247900001	1	247900001	0.5909	0.6828
NC_005100.4_248000001	1	248000001	0.5493	0.6455
NC_005100.4_248100001	1	248100001	0.5365	0.6698
NC_005100.4_248200001	1	248200001	0.495	0.6584
NC_005100.4_248300001	1	248300001	0.4032	0.5506
NC_005100.4_248400001	1	248400001	0.3423	0.4441
NC_005100.4_248600001	1	248600001	0.6053	0.6524
NC_005100.4_248700001	1	248700001	0.6053	0.6524
NC_005100.4_248800001	1	248800001	0.7166	0.698
NC_005100.4_248900001	1	248900001	0.3854	0.4779
NC_005100.4_249000001	1	249000001	0.3817	0.5231
NC_005100.4_249100001	1	249100001	0.3421	0.5653
NC_005100.4_249200001	1	249200001	0.3639	0.5788
NC_005100.4_249300001	1	249300001	0.3448	0.5651
NC_005100.4_249400001	1	249400001	0.5072	0.7635
NC_005100.4_249500001	1	249500001	0.5614	0.7712
NC_005100.4_249600001	1	249600001	0.5414	0.7342
NC_005100.4_249700001	1	249700001	0.5066	0.7428
NC_005100.4_249800001	1	249800001	0.618	0.8009
NC_005100.4_249900001	1	249900001	0.576	0.72
NC_005100.4_250000001	1	250000001	0.5406	0.701
NC_005100.4_250100001	1	250100001	0.5766	0.6405
NC_005100.4_250200001	1	250200001	0.5606	0.6066
NC_005100.4_250300001	1	250300001	0.4747	0.5303
NC_005100.4_250400001	1	250400001	0.5	0.6165
NC_005100.4_250500001	1	250500001	0.489	0.6074
NC_005100.4_250600001	1	250600001	0.4913	0.6308
NC_005100.4_250700001	1	250700001	0.5008	0.6359
NC_005100.4_250800001	1	250800001	0.4782	0.6142
NC_005100.4_250900001	1	250900001	0.3621	0.4711
NC_005100.4_251000001	1	251000001	0.3502	0.4494
NC_005100.4_251100001	1	251100001	0.3573	0.4699
NC_005100.4_251200001	1	251200001	0.4214	0.5223
NC_005100.4_251300001	1	251300001	0.4382	0.5532
NC_005100.4_251400001	1	251400001	0.571	0.6477
NC_005100.4_251500001	1	251500001	0.5436	0.6664
NC_005100.4_251600001	1	251600001	0.5317	0.6519
NC_005100.4_251700001	1	251700001	0.4804	0.6277
NC_005100.4_251800001	1	251800001	0.4631	0.6697
NC_005100.4_251900001	1	251900001	0.3956	0.539
NC_005100.4_252000001	1	252000001	0.4161	0.5456
NC_005100.4_252100001	1	252100001	0.3348	0.459
NC_005100.4_252200001	1	252200001	0.3062	0.4224
NC_005100.4_252300001	1	252300001	0.3249	0.4582
NC_005100.4_252400001	1	252400001	0.2876	0.5328
NC_005100.4_252500001	1	252500001	0.3256	0.5454
NC_005100.4_252600001	1	252600001	0.3933	0.6392
NC_005100.4_252700001	1	252700001	0.4653	0.63
NC_005100.4_252800001	1	252800001	0.4562	0.661

NC_005100.4_252900001	1	252900001	0.4452	0.6532
NC_005100.4_253000001	1	253000001	0.4227	0.657
NC_005100.4_253100001	1	253100001	0.3927	0.6254
NC_005100.4_253200001	1	253200001	0.3007	0.6636
NC_005100.4_253300001	1	253300001	0.2274	0.509
NC_005100.4_253400001	1	253400001	0.2843	0.5804
NC_005100.4_254500001	1	254500001	0.4143	0.5071
NC_005100.4_254600001	1	254600001	0.4432	0.5086
NC_005100.4_254700001	1	254700001	0.3607	0.4921
NC_005100.4_254800001	1	254800001	0.3096	0.5123
NC_005100.4_254900001	1	254900001	0.366	0.5477
NC_005100.4_255000001	1	255000001	0.4353	0.6475
NC_005100.4_255100001	1	255100001	0.4525	0.7013
NC_005100.4_255200001	1	255200001	0.5618	0.7511
NC_005100.4_255300001	1	255300001	0.7663	0.8582
NC_005100.4_255400001	1	255400001	0.8865	0.9714
NC_005100.4_255500001	1	255500001	0.8734	0.9882
NC_005100.4_255600001	1	255600001	0.8415	0.9634
NC_005100.4_255700001	1	255700001	0.8235	0.9673
NC_005100.4_255800001	1	255800001	0.7669	0.8962
NC_005100.4_255900001	1	255900001	0.757	0.8915
NC_005100.4_256000001	1	256000001	0.7803	0.836
NC_005100.4_256100001	1	256100001	0.6183	0.7116
NC_005100.4_256200001	1	256200001	0.4859	0.5724
NC_005100.4_256300001	1	256300001	0.4394	0.619
NC_005100.4_256400001	1	256400001	0.4604	0.6391
NC_005100.4_256500001	1	256500001	0.4012	0.5794
NC_005100.4_256600001	1	256600001	0.4932	0.6964
NC_005100.4_256700001	1	256700001	0.5773	0.7608
NC_005100.4_256800001	1	256800001	0.5834	0.7254
NC_005100.4_256900001	1	256900001	0.5555	0.6903
NC_005100.4_257000001	1	257000001	0.5813	0.6714
NC_005100.4_257100001	1	257100001	0.5276	0.6323
NC_005100.4_257200001	1	257200001	0.4854	0.5845
NC_005100.4_257300001	1	257300001	0.5607	0.625
NC_005100.4_257400001	1	257400001	0.5764	0.6312
NC_005100.4_258800001	1	258800001	0.4436	0.4511
NC_005100.4_258900001	1	258900001	0.5858	0.6284
NC_005100.4_259000001	1	259000001	0.6872	0.686
NC_005100.4_259100001	1	259100001	0.6872	0.686
NC_005100.4_259200001	1	259200001	0.6872	0.686
NC_005100.4_259300001	1	259300001	0.8947	0.8209
NC_005100.4_259700001	1	259700001	0.8142	0.9138
NC_005100.4_259800001	1	259800001	0.7	0.8447
NC_005100.4_259900001	1	259900001	0.6091	0.8502
NC_005100.4_260000001	1	260000001	0.5761	0.8201
NC_005100.4_260100001	1	260100001	0.5155	0.7259
NC_005100.4_260200001	1	260200001	0.5101	0.7082
NC_005100.4_260300001	1	260300001	0.5217	0.7244
NC_005100.4_260400001	1	260400001	0.5624	0.7228
NC_005100.4_260500001	1	260500001	0.6311	0.7574
NC_005100.4_260600001	1	260600001	0.5839	0.7858
NC_005100.4_260700001	1	260700001	0.5685	0.79
NC_005100.4_260800001	1	260800001	0.5547	0.7812
NC_005100.4_260900001	1	260900001	0.5327	0.7725
NC_005100.4_261000001	1	261000001	0.497	0.7781
NC_005100.4_261100001	1	261100001	0.5755	0.7905
NC_005100.4_261200001	1	261200001	0.5175	0.7276

NC_005100.4_261300001	1	261300001	0.4977	0.6648
NC_005100.4_261400001	1	261400001	0.5554	0.6755
NC_005100.4_261500001	1	261500001	0.4043	0.537
NC_005100.4_261600001	1	261600001	0.541	0.6314
NC_005100.4_261700001	1	261700001	0.5831	0.6828
NC_005100.4_261800001	1	261800001	0.6085	0.7139
NC_005100.4_261900001	1	261900001	0.5878	0.7005
NC_005100.4_262000001	1	262000001	0.709	0.7364
NC_005100.4_262100001	1	262100001	0.7304	0.8506
NC_005100.4_262600001	1	262600001	0.7928	0.9823
NC_005100.4_262700001	1	262700001	0.6453	0.9284
NC_005100.4_262800001	1	262800001	0.4801	0.7606
NC_005100.4_262900001	1	262900001	0.5151	0.725
NC_005100.4_263000001	1	263000001	0.5397	0.7585
NC_005100.4_263100001	1	263100001	0.4505	0.6959
NC_005100.4_263200001	1	263200001	0.4732	0.6526
NC_005100.4_263300001	1	263300001	0.4374	0.632
NC_005100.4_263400001	1	263400001	0.4134	0.598
NC_005100.4_263500001	1	263500001	0.439	0.5426
NC_005100.4_263600001	1	263600001	0.4274	0.5393
NC_005100.4_263700001	1	263700001	0.4562	0.5095
NC_005100.4_263800001	1	263800001	0.4453	0.4874
NC_005100.4_263900001	1	263900001	0.4774	0.5766
NC_005100.4_264000001	1	264000001	0.4859	0.5775
NC_005100.4_264100001	1	264100001	0.5082	0.5689
NC_005100.4_264200001	1	264200001	0.5121	0.5995
NC_005100.4_264300001	1	264300001	0.561	0.6332
NC_005100.4_264400001	1	264400001	0.6157	0.6589
NC_005100.4_264500001	1	264500001	0.6472	0.7242
NC_005100.4_264600001	1	264600001	0.6515	0.7613
NC_005100.4_264700001	1	264700001	0.6508	0.7749
NC_005100.4_264800001	1	264800001	0.6218	0.8027
NC_005100.4_264900001	1	264900001	0.5225	0.7584
NC_005100.4_265000001	1	265000001	0.4736	0.7066
NC_005100.4_265100001	1	265100001	0.557	0.7685
NC_005100.4_265200001	1	265200001	0.6454	0.8292
NC_005100.4_265300001	1	265300001	0.7186	0.7823
NC_005100.4_265400001	1	265400001	0.7357	0.7728
NC_005100.4_265500001	1	265500001	0.6978	0.7271
NC_005100.4_265600001	1	265600001	0.5869	0.7102
NC_005100.4_265700001	1	265700001	0.5406	0.6552
NC_005100.4_265800001	1	265800001	0.568	0.7215
NC_005100.4_265900001	1	265900001	0.5544	0.7061
NC_005100.4_266000001	1	266000001	0.5645	0.7125
NC_005100.4_266100001	1	266100001	0.6532	0.7494
NC_005100.4_266200001	1	266200001	0.7293	0.8097
NC_005100.4_266300001	1	266300001	0.6524	0.8032
NC_005100.4_266400001	1	266400001	0.6647	0.8438
NC_005100.4_266500001	1	266500001	0.6085	0.8248
NC_005100.4_266600001	1	266600001	0.6469	0.7712
NC_005100.4_266700001	1	266700001	0.5153	0.6733
NC_005100.4_266800001	1	266800001	0.4346	0.5817
NC_005100.4_266900001	1	266900001	0.4109	0.5449
NC_005100.4_267000001	1	267000001	0.3961	0.5169
NC_005100.4_267100001	1	267100001	0.3713	0.4732
NC_005100.4_267200001	1	267200001	0.3767	0.4891
NC_005100.4_267300001	1	267300001	0.3874	0.4744
NC_005100.4_267400001	1	267400001	0.3965	0.4923

NC_005100.4_267500001	1	267500001	0.4513	0.5192
NC_005100.4_267800001	1	267800001	0.4606	0.7982
NC_005100.4_267900001	1	267900001	0.4373	0.702
NC_005100.4_268000001	1	268000001	0.3156	0.5154
NC_005100.4_268100001	1	268100001	0.4297	0.5315
NC_005100.4_268200001	1	268200001	0.4207	0.5206
NC_005100.4_268300001	1	268300001	0.3776	0.4496
NC_005100.4_268400001	1	268400001	0.3966	0.4637
NC_005100.4_268500001	1	268500001	0.4302	0.5151
NC_005100.4_268600001	1	268600001	0.3881	0.472
NC_005100.4_268700001	1	268700001	0.4892	0.6421
NC_005100.4_268800001	1	268800001	0.5547	0.7365
NC_005100.4_268900001	1	268900001	0.5491	0.7106
NC_005100.4_269000001	1	269000001	0.6292	0.7831
NC_005100.4_269100001	1	269100001	0.6671	0.8085
NC_005100.4_269200001	1	269200001	0.629	0.7555
NC_005100.4_269300001	1	269300001	0.5787	0.6376
NC_005100.4_269400001	1	269400001	0.6093	0.6883
NC_005100.4_269500001	1	269500001	0.4849	0.5403
NC_005100.4_269600001	1	269600001	0.632	0.6929
NC_005100.4_269700001	1	269700001	0.7576	0.7651
NC_005100.4_269800001	1	269800001	0.8457	0.8509
NC_005100.4_269900001	1	269900001	0.7396	0.7791
NC_005100.4_270000001	1	270000001	0.7372	0.7605
NC_005100.4_270100001	1	270100001	0.5841	0.6651
NC_005100.4_270200001	1	270200001	0.526	0.6008
NC_005100.4_270300001	1	270300001	0.5325	0.6323
NC_005100.4_270400001	1	270400001	0.5763	0.7067
NC_005100.4_270500001	1	270500001	0.5036	0.677
NC_005100.4_270600001	1	270600001	0.472	0.6507
NC_005100.4_270700001	1	270700001	0.4698	0.6884
NC_005100.4_270800001	1	270800001	0.4129	0.6075
NC_005100.4_271600001	1	271600001	0.6491	0.7074
NC_005100.4_271700001	1	271700001	0.6491	0.7074
NC_005100.4_271800001	1	271800001	0.5881	0.7028
NC_005100.4_271900001	1	271900001	0.5895	0.6503
NC_005100.4_272000001	1	272000001	0.5455	0.6469
NC_005100.4_272100001	1	272100001	0.2156	0.1767
NC_005100.4_272200001	1	272200001	0.3327	0.3762
NC_005100.4_272300001	1	272300001	0.3445	0.3579
NC_005100.4_272400001	1	272400001	0.5145	0.6184
NC_005100.4_272500001	1	272500001	0.5595	0.5981
NC_005100.4_272600001	1	272600001	0.5383	0.5759
NC_005100.4_272700001	1	272700001	0.5393	0.6193
NC_005100.4_272800001	1	272800001	0.5727	0.6423
NC_005100.4_272900001	1	272900001	0.422	0.49
NC_005100.4_273000001	1	273000001	0.3591	0.584
NC_005100.4_273100001	1	273100001	0.5006	0.7473
NC_005100.4_273200001	1	273200001	0.5491	0.7616
NC_005100.4_273300001	1	273300001	0.4276	0.6787
NC_005100.4_273400001	1	273400001	0.3863	0.6173
NC_005100.4_273500001	1	273500001	0.4396	0.6042
NC_005100.4_273600001	1	273600001	0.4364	0.5948
NC_005100.4_273700001	1	273700001	0.5175	0.6601
NC_005100.4_273800001	1	273800001	0.5873	0.6923
NC_005100.4_273900001	1	273900001	0.6164	0.6826
NC_005100.4_274000001	1	274000001	0.5831	0.6757
NC_005100.4_274100001	1	274100001	0.5573	0.652

NC_005100.4_274200001	1	274200001	0.5122	0.5972
NC_005100.4_274300001	1	274300001	0.5172	0.6047
NC_005100.4_274400001	1	274400001	0.56	0.6409
NC_005100.4_274500001	1	274500001	0.5989	0.6786
NC_005100.4_274600001	1	274600001	0.6438	0.713
NC_005100.4_274700001	1	274700001	0.5962	0.6801
NC_005100.4_274800001	1	274800001	0.5779	0.6646
NC_005100.4_274900001	1	274900001	0.5396	0.6954
NC_005100.4_275000001	1	275000001	0.5626	0.7143
NC_005100.4_275100001	1	275100001	0.4094	0.6274
NC_005100.4_275200001	1	275200001	0.4563	0.6493
NC_005100.4_275300001	1	275300001	0.5463	0.6836
NC_005100.4_275400001	1	275400001	0.622	0.7532
NC_005100.4_275500001	1	275500001	0.5827	0.7042
NC_005100.4_275600001	1	275600001	0.6059	0.7049
NC_005100.4_275700001	1	275700001	0.5825	0.6679
NC_005100.4_275800001	1	275800001	0.5341	0.6796
NC_005100.4_275900001	1	275900001	0.4903	0.6359
NC_005100.4_276000001	1	276000001	0.5586	0.7337
NC_005100.4_276100001	1	276100001	0.6246	0.7664
NC_005100.4_276200001	1	276200001	0.6756	0.8128
NC_005100.4_276300001	1	276300001	0.7223	0.748
NC_005100.4_276400001	1	276400001	0.6031	0.6952
NC_005100.4_276500001	1	276500001	0.4681	0.5269
NC_005100.4_276600001	1	276600001	0.4568	0.5758
NC_005100.4_276700001	1	276700001	0.4449	0.5682
NC_005100.4_276800001	1	276800001	0.407	0.4931
NC_005100.4_276900001	1	276900001	0.4445	0.4647
NC_005100.4_277000001	1	277000001	0.4496	0.4998
NC_005100.4_277100001	1	277100001	0.4737	0.4802
NC_005100.4_277200001	1	277200001	0.5688	0.6003
NC_005100.4_277300001	1	277300001	0.5648	0.6295
NC_005100.4_277400001	1	277400001	0.5525	0.6634
NC_005100.4_277500001	1	277500001	0.6039	0.6961
NC_005100.4_277600001	1	277600001	0.5561	0.6364
NC_005100.4_277700001	1	277700001	0.5246	0.6077
NC_005100.4_277800001	1	277800001	0.5281	0.6291
NC_005100.4_277900001	1	277900001	0.5625	0.6748
NC_005100.4_278000001	1	278000001	0.5211	0.6475
NC_005100.4_278100001	1	278100001	0.522	0.7464
NC_005100.4_278200001	1	278200001	0.4386	0.6174
NC_005100.4_278300001	1	278300001	0.4394	0.5765
NC_005100.4_278400001	1	278400001	0.4411	0.6046
NC_005100.4_278500001	1	278500001	0.4433	0.6159
NC_005100.4_278600001	1	278600001	0.4342	0.5816
NC_005100.4_278700001	1	278700001	0.4624	0.6896
NC_005100.4_278800001	1	278800001	0.4844	0.7394
NC_005100.4_278900001	1	278900001	0.4879	0.735
NC_005100.4_279000001	1	279000001	0.4683	0.718
NC_005100.4_279100001	1	279100001	0.4933	0.8215
NC_005100.4_279200001	1	279200001	0.3629	0.6497
NC_005100.4_279300001	1	279300001	0.5634	0.7194
NC_005100.4_279400001	1	279400001	0.5932	0.717
NC_005100.4_279500001	1	279500001	0.678	0.6987
NC_005100.4_279600001	1	279600001	0.6018	0.6906
NC_005100.4_279700001	1	279700001	0.6312	0.7175
NC_005100.4_279800001	1	279800001	0.5536	0.6887
NC_005100.4_279900001	1	279900001	0.5344	0.6746

NC_005100.4_280000001	1	280000001	0.4312	0.6417
NC_005100.4_280100001	1	280100001	0.4345	0.6156
NC_005100.4_280200001	1	280200001	0.4403	0.5701
NC_005100.4_280300001	1	280300001	0.4407	0.5708
NC_005100.4_280400001	1	280400001	0.4135	0.5515
NC_005100.4_280500001	1	280500001	0.4265	0.5142
NC_005100.4_280600001	1	280600001	0.4191	0.4959
NC_005100.4_280700001	1	280700001	0.4849	0.5989
NC_005100.4_280800001	1	280800001	0.5069	0.5667
NC_005100.4_280900001	1	280900001	0.5178	0.6087
NC_005100.4_281000001	1	281000001	0.5303	0.6807
NC_005100.4_281100001	1	281100001	0.4767	0.6625
NC_005100.4_281200001	1	281200001	0.3909	0.5629
NC_005100.4_281300001	1	281300001	0.3106	0.6047
NC_005100.4_281400001	1	281400001	0.3471	0.6048
NC_005100.4_281500001	1	281500001	0.359	0.5085
NC_005100.4_281600001	1	281600001	0.3897	0.3873
NC_005100.4_281700001	1	281700001	0.4069	0.4238
NC_005100.4_281800001	1	281800001	0.4048	0.5108
NC_005100.4_281900001	1	281900001	0.3353	0.3802
NC_005100.4_282000001	1	282000001	0.343	0.4216
NC_005100.4_282100001	1	282100001	0.337	0.4649
NC_005100.4_282200001	1	282200001	0.3094	0.4599
NC_005100.4_282300001	1	282300001	0.3494	0.4103
NC_005101.4_1200001	2	1200001	0.7534	0.8296
NC_005101.4_1300001	2	1300001	0.668	0.74
NC_005101.4_1400001	2	1400001	0.7085	0.7786
NC_005101.4_1500001	2	1500001	0.7402	0.7447
NC_005101.4_1600001	2	1600001	0.7073	0.7069
NC_005101.4_1700001	2	1700001	0.7294	0.756
NC_005101.4_2300001	2	2300001	0.7235	0.7514
NC_005101.4_2400001	2	2400001	0.7121	0.6832
NC_005101.4_2500001	2	2500001	0.7121	0.6832
NC_005101.4_2600001	2	2600001	0.7121	0.6832
NC_005101.4_2700001	2	2700001	0.7275	0.7465
NC_005101.4_3200001	2	3200001	0.36	0.5145
NC_005101.4_3300001	2	3300001	0.3978	0.4753
NC_005101.4_3400001	2	3400001	0.6482	0.6893
NC_005101.4_3500001	2	3500001	0.7023	0.7429
NC_005101.4_3600001	2	3600001	0.6813	0.7693
NC_005101.4_3700001	2	3700001	0.7439	0.7919
NC_005101.4_3800001	2	3800001	0.7536	0.8294
NC_005101.4_3900001	2	3900001	0.776	0.8112
NC_005101.4_4000001	2	4000001	0.7341	0.7308
NC_005101.4_4100001	2	4100001	0.6216	0.6292
NC_005101.4_4400001	2	4400001	0.4707	0.4579
NC_005101.4_5000001	2	5000001	0.5017	0.7286
NC_005101.4_5100001	2	5100001	0.6559	0.8732
NC_005101.4_5200001	2	5200001	0.5694	0.8113
NC_005101.4_5300001	2	5300001	0.5379	0.7288
NC_005101.4_5400001	2	5400001	0.5433	0.7669
NC_005101.4_5500001	2	5500001	0.5398	0.782
NC_005101.4_5600001	2	5600001	0.4584	0.6846
NC_005101.4_5700001	2	5700001	0.5186	0.7319
NC_005101.4_5800001	2	5800001	0.7015	0.9758
NC_005101.4_5900001	2	5900001	0.8173	0.9848
NC_005101.4_6000001	2	6000001	0.7464	0.8861
NC_005101.4_6100001	2	6100001	0.7475	0.8869

NC_005101.4_6200001	2	6200001	0.7475	0.8869
NC_005101.4_6300001	2	6300001	0.6154	0.7817
NC_005101.4_6400001	2	6400001	0.5642	0.689
NC_005101.4_6500001	2	6500001	0.5381	0.6942
NC_005101.4_6600001	2	6600001	0.4357	0.5751
NC_005101.4_6700001	2	6700001	0.3677	0.5687
NC_005101.4_6900001	2	6900001	0.4121	0.928
NC_005101.4_7000001	2	7000001	0.3253	0.8208
NC_005101.4_7100001	2	7100001	0.3253	0.8208
NC_005101.4_7200001	2	7200001	0.46	0.8751
NC_005101.4_7300001	2	7300001	0.46	0.8751
NC_005101.4_7600001	2	7600001	0.2394	0.6555
NC_005101.4_7800001	2	7800001	0.215	0.6308
NC_005101.4_7900001	2	7900001	0.2122	0.4487
NC_005101.4_8000001	2	8000001	0.2433	0.4715
NC_005101.4_8100001	2	8100001	0.6093	0.6797
NC_005101.4_8200001	2	8200001	0.6729	0.7396
NC_005101.4_8300001	2	8300001	0.7015	0.7462
NC_005101.4_8400001	2	8400001	0.712	0.741
NC_005101.4_8500001	2	8500001	0.6752	0.7385
NC_005101.4_8600001	2	8600001	0.6117	0.6751
NC_005101.4_8700001	2	8700001	0.5457	0.6234
NC_005101.4_8800001	2	8800001	0.5157	0.5509
NC_005101.4_8900001	2	8900001	0.5808	0.6377
NC_005101.4_9000001	2	9000001	0.7001	0.7108
NC_005101.4_9100001	2	9100001	0.6366	0.7513
NC_005101.4_9200001	2	9200001	0.6592	0.7609
NC_005101.4_9300001	2	9300001	0.6531	0.7308
NC_005101.4_9400001	2	9400001	0.5983	0.6691
NC_005101.4_9500001	2	9500001	0.5368	0.6367
NC_005101.4_9600001	2	9600001	0.5363	0.6046
NC_005101.4_9700001	2	9700001	0.5286	0.5946
NC_005101.4_9800001	2	9800001	0.4481	0.5687
NC_005101.4_9900001	2	9900001	0.4052	0.5248
NC_005101.4_10000001	2	10000001	0.3316	0.377
NC_005101.4_10100001	2	10100001	0.4514	0.3847
NC_005101.4_10200001	2	10200001	0.4165	0.3597
NC_005101.4_10300001	2	10300001	0.4737	0.3822
NC_005101.4_10400001	2	10400001	0.5329	0.5099
NC_005101.4_10500001	2	10500001	0.5649	0.5213
NC_005101.4_10600001	2	10600001	0.6388	0.6269
NC_005101.4_10700001	2	10700001	0.6141	0.6608
NC_005101.4_10800001	2	10800001	0.5303	0.6443
NC_005101.4_10900001	2	10900001	0.4289	0.5911
NC_005101.4_11000001	2	11000001	0.4108	0.5546
NC_005101.4_11100001	2	11100001	0.3799	0.5845
NC_005101.4_11200001	2	11200001	0.3796	0.5416
NC_005101.4_11300001	2	11300001	0.538	0.6791
NC_005101.4_11400001	2	11400001	0.567	0.7123
NC_005101.4_11500001	2	11500001	0.5634	0.7241
NC_005101.4_11600001	2	11600001	0.5086	0.6829
NC_005101.4_11700001	2	11700001	0.5086	0.6829
NC_005101.4_11800001	2	11800001	0.4983	0.6851
NC_005101.4_11900001	2	11900001	0.3967	0.574
NC_005101.4_12000001	2	12000001	0.4399	0.6419
NC_005101.4_12100001	2	12100001	0.4688	0.658
NC_005101.4_12200001	2	12200001	0.4374	0.6089
NC_005101.4_12300001	2	12300001	0.4159	0.5523

NC_005101.4_12400001	2	12400001	0.5838	0.7354
NC_005101.4_12500001	2	12500001	0.6174	0.7028
NC_005101.4_12600001	2	12600001	0.5532	0.7098
NC_005101.4_12700001	2	12700001	0.6951	0.758
NC_005101.4_12800001	2	12800001	0.6864	0.7505
NC_005101.4_12900001	2	12900001	0.5693	0.6718
NC_005101.4_13000001	2	13000001	0.5449	0.6108
NC_005101.4_13100001	2	13100001	0.5503	0.5567
NC_005101.4_13200001	2	13200001	0.4617	0.559
NC_005101.4_13300001	2	13300001	0.4933	0.6374
NC_005101.4_13400001	2	13400001	0.4065	0.5971
NC_005101.4_13500001	2	13500001	0.4414	0.7219
NC_005101.4_13600001	2	13600001	0.4458	0.6272
NC_005101.4_13700001	2	13700001	0.5202	0.6549
NC_005101.4_13900001	2	13900001	0.6961	0.5784
NC_005101.4_14000001	2	14000001	0.5729	0.4942
NC_005101.4_14100001	2	14100001	0.5604	0.5682
NC_005101.4_14200001	2	14200001	0.5907	0.5839
NC_005101.4_14300001	2	14300001	0.4896	0.5538
NC_005101.4_14400001	2	14400001	0.412	0.5658
NC_005101.4_14500001	2	14500001	0.3764	0.5681
NC_005101.4_14800001	2	14800001	0.7941	0.8524
NC_005101.4_14900001	2	14900001	0.8229	0.8752
NC_005101.4_15000001	2	15000001	0.6721	0.7844
NC_005101.4_15100001	2	15100001	0.6812	0.7778
NC_005101.4_15200001	2	15200001	0.6812	0.7778
NC_005101.4_15300001	2	15300001	0.4548	0.5947
NC_005101.4_15400001	2	15400001	0.2298	0.3258
NC_005101.4_15500001	2	15500001	0.4211	0.5884
NC_005101.4_15600001	2	15600001	0.4729	0.677
NC_005101.4_15700001	2	15700001	0.4729	0.677
NC_005101.4_15800001	2	15800001	0.4458	0.6102
NC_005101.4_15900001	2	15900001	0.4537	0.5858
NC_005101.4_16000001	2	16000001	0.4806	0.6617
NC_005101.4_16100001	2	16100001	0.4442	0.6146
NC_005101.4_16200001	2	16200001	0.4155	0.6033
NC_005101.4_16300001	2	16300001	0.5027	0.6949
NC_005101.4_16400001	2	16400001	0.6547	0.794
NC_005101.4_16500001	2	16500001	0.5851	0.7456
NC_005101.4_16600001	2	16600001	0.6351	0.7746
NC_005101.4_16700001	2	16700001	0.601	0.7113
NC_005101.4_16800001	2	16800001	0.4871	0.5376
NC_005101.4_16900001	2	16900001	0.3235	0.3845
NC_005101.4_17000001	2	17000001	0.378	0.533
NC_005101.4_17100001	2	17100001	0.378	0.533
NC_005101.4_17200001	2	17200001	0.5045	0.6978
NC_005101.4_17300001	2	17300001	0.4965	0.7054
NC_005101.4_17400001	2	17400001	0.4622	0.6716
NC_005101.4_17500001	2	17500001	0.5641	0.6603
NC_005101.4_17600001	2	17600001	0.6231	0.7233
NC_005101.4_17700001	2	17700001	0.6199	0.7203
NC_005101.4_17800001	2	17800001	0.7782	0.8898
NC_005101.4_17900001	2	17900001	0.679	0.8098
NC_005101.4_18000001	2	18000001	0.6507	0.7712
NC_005101.4_18100001	2	18100001	0.626	0.7327
NC_005101.4_18200001	2	18200001	0.5929	0.6558
NC_005101.4_18300001	2	18300001	0.5194	0.5837
NC_005101.4_18400001	2	18400001	0.5323	0.5591

NC_005101.4_18500001	2	18500001	0.5366	0.5897
NC_005101.4_18600001	2	18600001	0.4568	0.5329
NC_005101.4_18700001	2	18700001	0.4994	0.6569
NC_005101.4_18800001	2	18800001	0.5919	0.7339
NC_005101.4_18900001	2	18900001	0.556	0.7077
NC_005101.4_19000001	2	19000001	0.5112	0.6663
NC_005101.4_19100001	2	19100001	0.484	0.5457
NC_005101.4_19200001	2	19200001	0.3859	0.4394
NC_005101.4_19300001	2	19300001	0.3595	0.3861
NC_005101.4_19400001	2	19400001	0.3573	0.3547
NC_005101.4_19500001	2	19500001	0.3096	0.2697
NC_005101.4_19600001	2	19600001	0.3375	0.408
NC_005101.4_19700001	2	19700001	0.3357	0.4379
NC_005101.4_19800001	2	19800001	0.3214	0.4575
NC_005101.4_19900001	2	19900001	0.3723	0.5889
NC_005101.4_20000001	2	20000001	0.4032	0.6024
NC_005101.4_20100001	2	20100001	0.3738	0.5676
NC_005101.4_20200001	2	20200001	0.4434	0.5949
NC_005101.4_20300001	2	20300001	0.516	0.6745
NC_005101.4_20400001	2	20400001	0.4921	0.5715
NC_005101.4_20500001	2	20500001	0.4735	0.5132
NC_005101.4_20600001	2	20600001	0.5026	0.5261
NC_005101.4_20700001	2	20700001	0.4739	0.5091
NC_005101.4_20800001	2	20800001	0.4739	0.5091
NC_005101.4_21100001	2	21100001	0.4744	0.6357
NC_005101.4_21200001	2	21200001	0.5117	0.6123
NC_005101.4_21300001	2	21300001	0.5403	0.6448
NC_005101.4_21400001	2	21400001	0.5251	0.5867
NC_005101.4_21500001	2	21500001	0.4456	0.5049
NC_005101.4_21600001	2	21600001	0.5113	0.5214
NC_005101.4_21700001	2	21700001	0.4701	0.5416
NC_005101.4_21800001	2	21800001	0.464	0.5073
NC_005101.4_21900001	2	21900001	0.4652	0.5301
NC_005101.4_22000001	2	22000001	0.5448	0.5935
NC_005101.4_22100001	2	22100001	0.5277	0.5834
NC_005101.4_22200001	2	22200001	0.5289	0.5774
NC_005101.4_22300001	2	22300001	0.5277	0.5951
NC_005101.4_22400001	2	22400001	0.4819	0.5956
NC_005101.4_22500001	2	22500001	0.4454	0.6163
NC_005101.4_22600001	2	22600001	0.39	0.5413
NC_005101.4_22700001	2	22700001	0.4853	0.6619
NC_005101.4_22800001	2	22800001	0.5905	0.7629
NC_005101.4_22900001	2	22900001	0.618	0.7276
NC_005101.4_23000001	2	23000001	0.5987	0.6915
NC_005101.4_23100001	2	23100001	0.5843	0.6807
NC_005101.4_23200001	2	23200001	0.4767	0.5875
NC_005101.4_23300001	2	23300001	0.4112	0.5221
NC_005101.4_23400001	2	23400001	0.3829	0.5332
NC_005101.4_23500001	2	23500001	0.3605	0.4826
NC_005101.4_23600001	2	23600001	0.3669	0.4971
NC_005101.4_23700001	2	23700001	0.3994	0.5419
NC_005101.4_23800001	2	23800001	0.372	0.522
NC_005101.4_23900001	2	23900001	0.4317	0.544
NC_005101.4_24000001	2	24000001	0.4741	0.6375
NC_005101.4_24100001	2	24100001	0.5278	0.6662
NC_005101.4_24200001	2	24200001	0.5068	0.6381
NC_005101.4_24300001	2	24300001	0.5435	0.6484
NC_005101.4_24400001	2	24400001	0.5451	0.6809

NC_005101.4_24500001	2	24500001	0.5995	0.7121
NC_005101.4_24600001	2	24600001	0.5441	0.7352
NC_005101.4_24700001	2	24700001	0.5115	0.7012
NC_005101.4_24800001	2	24800001	0.4603	0.6554
NC_005101.4_24900001	2	24900001	0.4648	0.6583
NC_005101.4_25000001	2	25000001	0.4116	0.5989
NC_005101.4_25100001	2	25100001	0.4088	0.5057
NC_005101.4_25200001	2	25200001	0.3841	0.549
NC_005101.4_25300001	2	25300001	0.4196	0.5707
NC_005101.4_25400001	2	25400001	0.3874	0.5406
NC_005101.4_25500001	2	25500001	0.3897	0.5471
NC_005101.4_25600001	2	25600001	0.3418	0.5533
NC_005101.4_25700001	2	25700001	0.416	0.5294
NC_005101.4_25800001	2	25800001	0.4034	0.5467
NC_005101.4_25900001	2	25900001	0.3851	0.5213
NC_005101.4_26000001	2	26000001	0.3542	0.4995
NC_005101.4_26100001	2	26100001	0.3292	0.4632
NC_005101.4_26200001	2	26200001	0.3243	0.5457
NC_005101.4_26300001	2	26300001	0.3798	0.5763
NC_005101.4_26400001	2	26400001	0.3347	0.5368
NC_005101.4_26500001	2	26500001	0.3391	0.5524
NC_005101.4_26600001	2	26600001	0.4012	0.6202
NC_005101.4_26700001	2	26700001	0.412	0.5869
NC_005101.4_26800001	2	26800001	0.3323	0.4915
NC_005101.4_26900001	2	26900001	0.4135	0.5733
NC_005101.4_27000001	2	27000001	0.4779	0.5808
NC_005101.4_27100001	2	27100001	0.4634	0.4884
NC_005101.4_27200001	2	27200001	0.3824	0.4009
NC_005101.4_27300001	2	27300001	0.4346	0.5014
NC_005101.4_27400001	2	27400001	0.3659	0.3634
NC_005101.4_27500001	2	27500001	0.4984	0.5509
NC_005101.4_27600001	2	27600001	0.4984	0.6082
NC_005101.4_27700001	2	27700001	0.4697	0.6055
NC_005101.4_27800001	2	27800001	0.4948	0.6289
NC_005101.4_27900001	2	27900001	0.4912	0.6618
NC_005101.4_28000001	2	28000001	0.2794	0.3877
NC_005101.4_28100001	2	28100001	0.3737	0.5121
NC_005101.4_28200001	2	28200001	0.4576	0.6209
NC_005101.4_28300001	2	28300001	0.4474	0.6047
NC_005101.4_28400001	2	28400001	0.4665	0.641
NC_005101.4_28500001	2	28500001	0.5555	0.7193
NC_005101.4_28600001	2	28600001	0.6308	0.8207
NC_005101.4_28700001	2	28700001	0.7561	0.8797
NC_005101.4_28800001	2	28800001	0.6375	0.9668
NC_005101.4_28900001	2	28900001	0.4682	0.7412
NC_005101.4_29000001	2	29000001	0.3824	0.6257
NC_005101.4_29100001	2	29100001	0.3591	0.5903
NC_005101.4_29200001	2	29200001	0.2833	0.4666
NC_005101.4_29300001	2	29300001	0.3687	0.4829
NC_005101.4_29400001	2	29400001	0.4348	0.5617
NC_005101.4_29500001	2	29500001	0.4734	0.5231
NC_005101.4_29600001	2	29600001	0.5163	0.5499
NC_005101.4_29700001	2	29700001	0.5298	0.5393
NC_005101.4_29800001	2	29800001	0.5703	0.6011
NC_005101.4_29900001	2	29900001	0.5575	0.5689
NC_005101.4_30000001	2	30000001	0.576	0.6498
NC_005101.4_30100001	2	30100001	0.5207	0.6097
NC_005101.4_30200001	2	30200001	0.4982	0.6178

NC_005101.4_30300001	2	30300001	0.4214	0.5284
NC_005101.4_30400001	2	30400001	0.4317	0.5378
NC_005101.4_30500001	2	30500001	0.4193	0.5063
NC_005101.4_30600001	2	30600001	0.4387	0.5084
NC_005101.4_30700001	2	30700001	0.4504	0.5236
NC_005101.4_30800001	2	30800001	0.5339	0.6549
NC_005101.4_30900001	2	30900001	0.504	0.6533
NC_005101.4_31000001	2	31000001	0.5039	0.6991
NC_005101.4_31100001	2	31100001	0.4963	0.7252
NC_005101.4_31200001	2	31200001	0.5328	0.7997
NC_005101.4_31300001	2	31300001	0.4082	0.7994
NC_005101.4_31400001	2	31400001	0.3893	0.7629
NC_005101.4_31500001	2	31500001	0.4022	0.7132
NC_005101.4_31600001	2	31600001	0.4079	0.7233
NC_005101.4_31700001	2	31700001	0.3114	0.5747
NC_005101.4_31800001	2	31800001	0.4401	0.6497
NC_005101.4_31900001	2	31900001	0.5039	0.7
NC_005101.4_32000001	2	32000001	0.5021	0.7899
NC_005101.4_32100001	2	32100001	0.6458	0.7131
NC_005101.4_32200001	2	32200001	0.6546	0.7007
NC_005101.4_32300001	2	32300001	0.5466	0.64
NC_005101.4_32400001	2	32400001	0.5914	0.6186
NC_005101.4_32500001	2	32500001	0.5459	0.5938
NC_005101.4_32600001	2	32600001	0.4609	0.5648
NC_005101.4_32700001	2	32700001	0.3841	0.4932
NC_005101.4_32800001	2	32800001	0.4969	0.585
NC_005101.4_32900001	2	32900001	0.4901	0.6123
NC_005101.4_33000001	2	33000001	0.4568	0.6082
NC_005101.4_33100001	2	33100001	0.464	0.6105
NC_005101.4_33200001	2	33200001	0.5009	0.6513
NC_005101.4_33300001	2	33300001	0.4602	0.6647
NC_005101.4_33400001	2	33400001	0.4873	0.6672
NC_005101.4_33500001	2	33500001	0.5979	0.7121
NC_005101.4_33600001	2	33600001	0.6091	0.7478
NC_005101.4_33700001	2	33700001	0.5617	0.7346
NC_005101.4_33800001	2	33800001	0.5911	0.7261
NC_005101.4_33900001	2	33900001	0.5257	0.7714
NC_005101.4_34000001	2	34000001	0.6102	0.8567
NC_005101.4_34100001	2	34100001	0.7291	0.9149
NC_005101.4_34200001	2	34200001	0.6457	0.8883
NC_005101.4_34300001	2	34300001	0.5955	0.8806
NC_005101.4_34400001	2	34400001	0.6272	0.8945
NC_005101.4_34500001	2	34500001	0.538	0.7771
NC_005101.4_34600001	2	34600001	0.5714	0.7883
NC_005101.4_34700001	2	34700001	0.6047	0.7493
NC_005101.4_34800001	2	34800001	0.622	0.7261
NC_005101.4_34900001	2	34900001	0.584	0.6868
NC_005101.4_35000001	2	35000001	0.6323	0.7771
NC_005101.4_35300001	2	35300001	0.5434	0.4941
NC_005101.4_35400001	2	35400001	0.4435	0.4533
NC_005101.4_35500001	2	35500001	0.4435	0.4533
NC_005101.4_35600001	2	35600001	0.4435	0.4533
NC_005101.4_35700001	2	35700001	0.4064	0.4708
NC_005101.4_35800001	2	35800001	0.2983	0.3935
NC_005101.4_35900001	2	35900001	0.4018	0.558
NC_005101.4_36000001	2	36000001	0.3773	0.5208
NC_005101.4_36100001	2	36100001	0.3336	0.4387
NC_005101.4_36200001	2	36200001	0.2955	0.3612

NC_005101.4_36300001	2	36300001	0.4563	0.6511
NC_005101.4_36400001	2	36400001	0.4493	0.5505
NC_005101.4_36500001	2	36500001	0.4372	0.525
NC_005101.4_36600001	2	36600001	0.4429	0.4456
NC_005101.4_36700001	2	36700001	0.4759	0.4764
NC_005101.4_36800001	2	36800001	0.3685	0.2788
NC_005101.4_36900001	2	36900001	0.3549	0.2785
NC_005101.4_37000001	2	37000001	0.3855	0.3006
NC_005101.4_37200001	2	37200001	0.7371	0.6741
NC_005101.4_37300001	2	37300001	0.6307	0.6376
NC_005101.4_37400001	2	37400001	0.5732	0.5861
NC_005101.4_37500001	2	37500001	0.4851	0.5573
NC_005101.4_37600001	2	37600001	0.5068	0.5706
NC_005101.4_37700001	2	37700001	0.482	0.6104
NC_005101.4_37800001	2	37800001	0.4719	0.6643
NC_005101.4_37900001	2	37900001	0.6014	0.7416
NC_005101.4_38000001	2	38000001	0.615	0.7466
NC_005101.4_38100001	2	38100001	0.5041	0.7056
NC_005101.4_38200001	2	38200001	0.4951	0.6424
NC_005101.4_38300001	2	38300001	0.4543	0.6229
NC_005101.4_38400001	2	38400001	0.2778	0.5043
NC_005101.4_38500001	2	38500001	0.3447	0.5207
NC_005101.4_38600001	2	38600001	0.3616	0.5661
NC_005101.4_38700001	2	38700001	0.3561	0.6024
NC_005101.4_38800001	2	38800001	0.3985	0.6459
NC_005101.4_38900001	2	38900001	0.5002	0.7455
NC_005101.4_39000001	2	39000001	0.4715	0.7605
NC_005101.4_39100001	2	39100001	0.5689	0.7823
NC_005101.4_39200001	2	39200001	0.5166	0.6313
NC_005101.4_39300001	2	39300001	0.5765	0.6877
NC_005101.4_39400001	2	39400001	0.5633	0.6176
NC_005101.4_39500001	2	39500001	0.5874	0.6193
NC_005101.4_39600001	2	39600001	0.5634	0.6263
NC_005101.4_39700001	2	39700001	0.5846	0.7099
NC_005101.4_39800001	2	39800001	0.4504	0.5767
NC_005101.4_39900001	2	39900001	0.435	0.5359
NC_005101.4_40000001	2	40000001	0.3819	0.5309
NC_005101.4_40100001	2	40100001	0.4014	0.5311
NC_005101.4_40200001	2	40200001	0.5343	0.5801
NC_005101.4_40300001	2	40300001	0.6473	0.7405
NC_005101.4_40400001	2	40400001	0.6973	0.8041
NC_005101.4_40500001	2	40500001	0.6973	0.8041
NC_005101.4_40600001	2	40600001	0.6878	0.8487
NC_005101.4_40700001	2	40700001	0.5829	0.8564
NC_005101.4_40800001	2	40800001	0.5035	0.7123
NC_005101.4_40900001	2	40900001	0.4398	0.6114
NC_005101.4_41000001	2	41000001	0.4592	0.6017
NC_005101.4_41100001	2	41100001	0.4366	0.5771
NC_005101.4_41200001	2	41200001	0.4532	0.5353
NC_005101.4_41300001	2	41300001	0.4366	0.5465
NC_005101.4_41400001	2	41400001	0.3909	0.4929
NC_005101.4_41500001	2	41500001	0.4048	0.5611
NC_005101.4_41600001	2	41600001	0.4524	0.5817
NC_005101.4_41700001	2	41700001	0.439	0.6144
NC_005101.4_41800001	2	41800001	0.4533	0.6394
NC_005101.4_41900001	2	41900001	0.4737	0.6741
NC_005101.4_42000001	2	42000001	0.4588	0.6058
NC_005101.4_42100001	2	42100001	0.4031	0.6332

NC_005101.4_42200001	2	42200001	0.5732	0.822
NC_005101.4_42300001	2	42300001	0.5205	0.7125
NC_005101.4_42400001	2	42400001	0.506	0.688
NC_005101.4_42500001	2	42500001	0.457	0.6958
NC_005101.4_42600001	2	42600001	0.5072	0.7071
NC_005101.4_42700001	2	42700001	0.4138	0.5277
NC_005101.4_42800001	2	42800001	0.4238	0.5626
NC_005101.4_42900001	2	42900001	0.5056	0.6693
NC_005101.4_43000001	2	43000001	0.5648	0.6539
NC_005101.4_43100001	2	43100001	0.5254	0.6261
NC_005101.4_43200001	2	43200001	0.5806	0.7068
NC_005101.4_43300001	2	43300001	0.4723	0.593
NC_005101.4_43400001	2	43400001	0.4068	0.4712
NC_005101.4_43500001	2	43500001	0.3814	0.4374
NC_005101.4_43600001	2	43600001	0.3721	0.4069
NC_005101.4_43700001	2	43700001	0.3171	0.3481
NC_005101.4_43800001	2	43800001	0.4599	0.5032
NC_005101.4_43900001	2	43900001	0.4989	0.5945
NC_005101.4_44000001	2	44000001	0.4694	0.5298
NC_005101.4_44100001	2	44100001	0.5211	0.5999
NC_005101.4_44200001	2	44200001	0.5063	0.5652
NC_005101.4_44300001	2	44300001	0.4601	0.5742
NC_005101.4_44400001	2	44400001	0.423	0.5393
NC_005101.4_44500001	2	44500001	0.4259	0.5906
NC_005101.4_44600001	2	44600001	0.3287	0.4496
NC_005101.4_44700001	2	44700001	0.3624	0.5493
NC_005101.4_44800001	2	44800001	0.4228	0.5857
NC_005101.4_44900001	2	44900001	0.4397	0.5796
NC_005101.4_45000001	2	45000001	0.4966	0.6307
NC_005101.4_45100001	2	45100001	0.5022	0.6541
NC_005101.4_45200001	2	45200001	0.5886	0.7135
NC_005101.4_45300001	2	45300001	0.6178	0.7568
NC_005101.4_45400001	2	45400001	0.6504	0.8115
NC_005101.4_45500001	2	45500001	0.6532	0.8076
NC_005101.4_45600001	2	45600001	0.6139	0.7268
NC_005101.4_45700001	2	45700001	0.5909	0.6947
NC_005101.4_45800001	2	45800001	0.5138	0.6265
NC_005101.4_45900001	2	45900001	0.389	0.4896
NC_005101.4_46000001	2	46000001	0.3696	0.5142
NC_005101.4_46100001	2	46100001	0.4448	0.6757
NC_005101.4_46200001	2	46200001	0.4797	0.6532
NC_005101.4_46300001	2	46300001	0.5349	0.6596
NC_005101.4_46400001	2	46400001	0.6431	0.7118
NC_005101.4_46500001	2	46500001	0.6188	0.6261
NC_005101.4_46600001	2	46600001	0.5821	0.6487
NC_005101.4_46700001	2	46700001	0.5384	0.6432
NC_005101.4_46800001	2	46800001	0.5469	0.6895
NC_005101.4_46900001	2	46900001	0.544	0.6507
NC_005101.4_47000001	2	47000001	0.5299	0.6762
NC_005101.4_47100001	2	47100001	0.4981	0.6111
NC_005101.4_47200001	2	47200001	0.5535	0.6254
NC_005101.4_47300001	2	47300001	0.5481	0.592
NC_005101.4_47400001	2	47400001	0.556	0.6392
NC_005101.4_47500001	2	47500001	0.6841	0.6101
NC_005101.4_47600001	2	47600001	0.5525	0.6506
NC_005101.4_47700001	2	47700001	0.5192	0.6792
NC_005101.4_47800001	2	47800001	0.416	0.6602
NC_005101.4_47900001	2	47900001	0.416	0.6602

NC_005101.4_48000001	2	48000001	0.5108	0.7334
NC_005101.4_48100001	2	48100001	0.5308	0.7629
NC_005101.4_48200001	2	48200001	0.5417	0.8146
NC_005101.4_48300001	2	48300001	0.5908	0.71
NC_005101.4_48400001	2	48400001	0.5584	0.7054
NC_005101.4_48500001	2	48500001	0.5576	0.6716
NC_005101.4_48600001	2	48600001	0.5664	0.7075
NC_005101.4_48700001	2	48700001	0.575	0.7184
NC_005101.4_48800001	2	48800001	0.6181	0.7717
NC_005101.4_48900001	2	48900001	0.648	0.7819
NC_005101.4_49000001	2	49000001	0.655	0.8576
NC_005101.4_49100001	2	49100001	0.6821	0.7638
NC_005101.4_49200001	2	49200001	0.582	0.6486
NC_005101.4_49300001	2	49300001	0.5272	0.5879
NC_005101.4_49400001	2	49400001	0.5007	0.581
NC_005101.4_49500001	2	49500001	0.423	0.4574
NC_005101.4_49600001	2	49600001	0.4715	0.5427
NC_005101.4_49700001	2	49700001	0.4565	0.5328
NC_005101.4_50000001	2	50000001	0.5734	0.7986
NC_005101.4_50100001	2	50100001	0.4814	0.7262
NC_005101.4_50200001	2	50200001	0.4126	0.6977
NC_005101.4_50300001	2	50300001	0.4126	0.6977
NC_005101.4_50400001	2	50400001	0.4367	0.8524
NC_005101.4_50500001	2	50500001	0.4324	0.8394
NC_005101.4_50600001	2	50600001	0.2928	0.7553
NC_005101.4_50700001	2	50700001	0.3054	0.6032
NC_005101.4_50800001	2	50800001	0.28	0.5748
NC_005101.4_50900001	2	50900001	0.3225	0.512
NC_005101.4_51000001	2	51000001	0.2572	0.3631
NC_005101.4_51100001	2	51100001	0.4741	0.5856
NC_005101.4_51200001	2	51200001	0.5139	0.6281
NC_005101.4_51300001	2	51300001	0.5978	0.6858
NC_005101.4_51400001	2	51400001	0.5787	0.6796
NC_005101.4_51500001	2	51500001	0.6738	0.7424
NC_005101.4_51600001	2	51600001	0.7097	0.7672
NC_005101.4_51700001	2	51700001	0.745	0.7444
NC_005101.4_51800001	2	51800001	0.7048	0.6896
NC_005101.4_51900001	2	51900001	0.7181	0.7034
NC_005101.4_52000001	2	52000001	0.6323	0.6877
NC_005101.4_52100001	2	52100001	0.598	0.6456
NC_005101.4_52200001	2	52200001	0.5178	0.6241
NC_005101.4_52300001	2	52300001	0.571	0.6936
NC_005101.4_52400001	2	52400001	0.5196	0.6744
NC_005101.4_52500001	2	52500001	0.6603	0.7477
NC_005101.4_52600001	2	52600001	0.4163	0.6663
NC_005101.4_52700001	2	52700001	0.4551	0.6055
NC_005101.4_52800001	2	52800001	0.4618	0.6384
NC_005101.4_52900001	2	52900001	0.4884	0.6811
NC_005101.4_53000001	2	53000001	0.4911	0.6845
NC_005101.4_53100001	2	53100001	0.53	0.7063
NC_005101.4_53200001	2	53200001	0.5872	0.8154
NC_005101.4_53300001	2	53300001	0.7594	0.8858
NC_005101.4_53400001	2	53400001	0.784	0.8873
NC_005101.4_53500001	2	53500001	0.8548	0.8947
NC_005101.4_53600001	2	53600001	0.808	0.8974
NC_005101.4_53700001	2	53700001	0.7844	0.8952
NC_005101.4_53800001	2	53800001	0.7532	0.9111
NC_005101.4_53900001	2	53900001	0.7732	0.8974

NC_005101.4_54000001	2	54000001	0.2223	0.5164
NC_005101.4_54100001	2	54100001	0.3428	0.5185
NC_005101.4_54200001	2	54200001	0.391	0.5097
NC_005101.4_54300001	2	54300001	0.5413	0.6177
NC_005101.4_54400001	2	54400001	0.5892	0.6501
NC_005101.4_54500001	2	54500001	0.7128	0.703
NC_005101.4_54600001	2	54600001	0.7423	0.7259
NC_005101.4_54700001	2	54700001	0.7363	0.7589
NC_005101.4_54800001	2	54800001	0.7655	0.827
NC_005101.4_54900001	2	54900001	0.7838	0.8917
NC_005101.4_55000001	2	55000001	0.6541	0.8136
NC_005101.4_55100001	2	55100001	0.5527	0.7225
NC_005101.4_55200001	2	55200001	0.5696	0.6733
NC_005101.4_55300001	2	55300001	0.5901	0.7358
NC_005101.4_55400001	2	55400001	0.5377	0.7092
NC_005101.4_55500001	2	55500001	0.6303	0.7974
NC_005101.4_55600001	2	55600001	0.6811	0.8219
NC_005101.4_55700001	2	55700001	0.6102	0.7116
NC_005101.4_55800001	2	55800001	0.574	0.6404
NC_005101.4_55900001	2	55900001	0.6462	0.6912
NC_005101.4_56000001	2	56000001	0.6469	0.6639
NC_005101.4_56100001	2	56100001	0.6428	0.7053
NC_005101.4_56200001	2	56200001	0.6409	0.7748
NC_005101.4_56300001	2	56300001	0.6236	0.7836
NC_005101.4_56400001	2	56400001	0.5998	0.7285
NC_005101.4_56500001	2	56500001	0.546	0.7162
NC_005101.4_56600001	2	56600001	0.4582	0.5727
NC_005101.4_56700001	2	56700001	0.4653	0.5746
NC_005101.4_56800001	2	56800001	0.4434	0.53
NC_005101.4_56900001	2	56900001	0.392	0.5076
NC_005101.4_57000001	2	57000001	0.4131	0.5869
NC_005101.4_57100001	2	57100001	0.4086	0.6435
NC_005101.4_57200001	2	57200001	0.4471	0.6304
NC_005101.4_57300001	2	57300001	0.493	0.6524
NC_005101.4_57400001	2	57400001	0.476	0.6553
NC_005101.4_57500001	2	57500001	0.4276	0.6014
NC_005101.4_57600001	2	57600001	0.538	0.6762
NC_005101.4_57700001	2	57700001	0.5356	0.7033
NC_005101.4_57800001	2	57800001	0.5122	0.6865
NC_005101.4_57900001	2	57900001	0.5535	0.729
NC_005101.4_58000001	2	58000001	0.5575	0.7124
NC_005101.4_58100001	2	58100001	0.4672	0.674
NC_005101.4_58200001	2	58200001	0.3863	0.5779
NC_005101.4_58900001	2	58900001	0.4147	0.8201
NC_005101.4_59000001	2	59000001	0.5785	0.8049
NC_005101.4_59100001	2	59100001	0.6618	0.8119
NC_005101.4_59200001	2	59200001	0.6298	0.8182
NC_005101.4_59300001	2	59300001	0.6752	0.7805
NC_005101.4_59400001	2	59400001	0.6251	0.7382
NC_005101.4_59500001	2	59500001	0.5289	0.6097
NC_005101.4_59600001	2	59600001	0.5446	0.6822
NC_005101.4_59700001	2	59700001	0.4746	0.6164
NC_005101.4_59800001	2	59800001	0.4776	0.6276
NC_005101.4_59900001	2	59900001	0.5727	0.714
NC_005101.4_60000001	2	60000001	0.6094	0.7967
NC_005101.4_60100001	2	60100001	0.5906	0.7531
NC_005101.4_60200001	2	60200001	0.6059	0.7415
NC_005101.4_60300001	2	60300001	0.6156	0.757

NC_005101.4_60400001	2	60400001	0.587	0.735
NC_005101.4_60500001	2	60500001	0.6243	0.7675
NC_005101.4_60600001	2	60600001	0.6393	0.7721
NC_005101.4_60700001	2	60700001	0.639	0.8041
NC_005101.4_60800001	2	60800001	0.5505	0.7745
NC_005101.4_60900001	2	60900001	0.5284	0.7595
NC_005101.4_61000001	2	61000001	0.4862	0.7005
NC_005101.4_61100001	2	61100001	0.5047	0.6926
NC_005101.4_61200001	2	61200001	0.5871	0.7294
NC_005101.4_61300001	2	61300001	0.5991	0.6925
NC_005101.4_61400001	2	61400001	0.5058	0.6144
NC_005101.4_61500001	2	61500001	0.4309	0.551
NC_005101.4_61600001	2	61600001	0.4177	0.5058
NC_005101.4_61700001	2	61700001	0.364	0.4462
NC_005101.4_61800001	2	61800001	0.3852	0.5026
NC_005101.4_61900001	2	61900001	0.4367	0.581
NC_005101.4_62000001	2	62000001	0.4234	0.5348
NC_005101.4_62100001	2	62100001	0.4355	0.5563
NC_005101.4_62200001	2	62200001	0.4577	0.5774
NC_005101.4_62300001	2	62300001	0.3913	0.482
NC_005101.4_62400001	2	62400001	0.3826	0.4463
NC_005101.4_62500001	2	62500001	0.4022	0.4655
NC_005101.4_62600001	2	62600001	0.4357	0.5374
NC_005101.4_62700001	2	62700001	0.4046	0.5194
NC_005101.4_62800001	2	62800001	0.4843	0.5946
NC_005101.4_62900001	2	62900001	0.4972	0.5842
NC_005101.4_63000001	2	63000001	0.4689	0.5547
NC_005101.4_63100001	2	63100001	0.4072	0.4394
NC_005101.4_63200001	2	63200001	0.4263	0.4205
NC_005101.4_63300001	2	63300001	0.3373	0.2972
NC_005101.4_63400001	2	63400001	0.2656	0.2836
NC_005101.4_63500001	2	63500001	0.2714	0.301
NC_005101.4_63600001	2	63600001	0.1671	0.197
NC_005101.4_63700001	2	63700001	0.1573	0.2133
NC_005101.4_63800001	2	63800001	0.185	0.253
NC_005101.4_64200001	2	64200001	0.2553	0.6597
NC_005101.4_64300001	2	64300001	0.2287	0.5506
NC_005101.4_64400001	2	64400001	0.3209	0.5593
NC_005101.4_64500001	2	64500001	0.4128	0.6509
NC_005101.4_64600001	2	64600001	0.5307	0.742
NC_005101.4_64700001	2	64700001	0.4689	0.6557
NC_005101.4_64800001	2	64800001	0.5265	0.6556
NC_005101.4_64900001	2	64900001	0.4235	0.6527
NC_005101.4_65000001	2	65000001	0.2794	0.4104
NC_005101.4_65100001	2	65100001	0.2277	0.2011
NC_005101.4_65200001	2	65200001	0.3516	0.3952
NC_005101.4_65300001	2	65300001	0.3502	0.3892
NC_005101.4_65400001	2	65400001	0.4095	0.4265
NC_005101.4_65500001	2	65500001	0.5867	0.5828
NC_005101.4_65600001	2	65600001	0.6617	0.6709
NC_005101.4_65700001	2	65700001	0.8203	0.7463
NC_005101.4_65800001	2	65800001	0.8203	0.7463
NC_005101.4_65900001	2	65900001	0.8333	0.7873
NC_005101.4_66000001	2	66000001	0.4822	0.5638
NC_005101.4_66300001	2	66300001	0.516	0.5387
NC_005101.4_66400001	2	66400001	0.516	0.5387
NC_005101.4_66600001	2	66600001	0.6085	0.8163
NC_005101.4_66700001	2	66700001	0.7053	0.921

NC_005101.4_66800001	2	66800001	0.4424	0.651
NC_005101.4_66900001	2	66900001	0.3292	0.5842
NC_005101.4_67000001	2	67000001	0.36	0.6347
NC_005101.4_67100001	2	67100001	0.44	0.641
NC_005101.4_67200001	2	67200001	0.4448	0.6467
NC_005101.4_67300001	2	67300001	0.4992	0.679
NC_005101.4_67400001	2	67400001	0.6031	0.6371
NC_005101.4_67500001	2	67500001	0.626	0.616
NC_005101.4_67600001	2	67600001	0.5152	0.5456
NC_005101.4_67700001	2	67700001	0.4436	0.4456
NC_005101.4_67800001	2	67800001	0.5208	0.5782
NC_005101.4_68000001	2	68000001	0.6441	0.8642
NC_005101.4_68100001	2	68100001	0.5237	0.7359
NC_005101.4_68200001	2	68200001	0.5107	0.7717
NC_005101.4_68300001	2	68300001	0.5395	0.7831
NC_005101.4_68400001	2	68400001	0.5382	0.78
NC_005101.4_68500001	2	68500001	0.4958	0.747
NC_005101.4_68600001	2	68600001	0.6204	0.8295
NC_005101.4_68700001	2	68700001	0.6185	0.7659
NC_005101.4_68800001	2	68800001	0.2846	0.3701
NC_005101.4_68900001	2	68900001	0.3468	0.4002
NC_005101.4_69000001	2	69000001	0.3663	0.4524
NC_005101.4_69100001	2	69100001	0.3554	0.3839
NC_005101.4_69200001	2	69200001	0.3815	0.3728
NC_005101.4_69300001	2	69300001	0.5034	0.4781
NC_005101.4_69400001	2	69400001	0.4807	0.4561
NC_005101.4_69500001	2	69500001	0.5203	0.4389
NC_005101.4_69600001	2	69600001	0.5081	0.4883
NC_005101.4_69700001	2	69700001	0.463	0.5129
NC_005101.4_69800001	2	69800001	0.3895	0.4562
NC_005101.4_69900001	2	69900001	0.4184	0.5184
NC_005101.4_70000001	2	70000001	0.3234	0.5281
NC_005101.4_70100001	2	70100001	0.2599	0.4987
NC_005101.4_70200001	2	70200001	0.3131	0.5509
NC_005101.4_70300001	2	70300001	0.3609	0.5779
NC_005101.4_71000001	2	71000001	0.4531	0.7597
NC_005101.4_71100001	2	71100001	0.4091	0.7251
NC_005101.4_71200001	2	71200001	0.4379	0.7635
NC_005101.4_71300001	2	71300001	0.4072	0.7519
NC_005101.4_71400001	2	71400001	0.3567	0.5828
NC_005101.4_71500001	2	71500001	0.321	0.4867
NC_005101.4_71600001	2	71600001	0.2958	0.3995
NC_005101.4_71700001	2	71700001	0.2958	0.3995
NC_005101.4_71800001	2	71800001	0.3261	0.4088
NC_005101.4_72000001	2	72000001	0.2643	0.4505
NC_005101.4_72100001	2	72100001	0.3811	0.6133
NC_005101.4_72200001	2	72200001	0.3811	0.6133
NC_005101.4_72300001	2	72300001	0.3811	0.6133
NC_005101.4_72400001	2	72400001	0.4413	0.6288
NC_005101.4_72500001	2	72500001	0.7453	0.8078
NC_005101.4_72600001	2	72600001	0.7389	0.776
NC_005101.4_72700001	2	72700001	0.7389	0.776
NC_005101.4_72800001	2	72800001	0.6639	0.7906
NC_005101.4_72900001	2	72900001	0.6143	0.7821
NC_005101.4_73000001	2	73000001	0.492	0.6886
NC_005101.4_73100001	2	73100001	0.3842	0.6549
NC_005101.4_73200001	2	73200001	0.3705	0.6201
NC_005101.4_73300001	2	73300001	0.4196	0.6441

NC_005101.4_73400001	2	73400001	0.4257	0.5748
NC_005101.4_73500001	2	73500001	0.4114	0.5855
NC_005101.4_73600001	2	73600001	0.4972	0.6806
NC_005101.4_73700001	2	73700001	0.5969	0.7261
NC_005101.4_74700001	2	74700001	0.3654	0.2934
NC_005101.4_74800001	2	74800001	0.3654	0.2934
NC_005101.4_74900001	2	74900001	0.452	0.4077
NC_005101.4_75000001	2	75000001	0.4583	0.4072
NC_005101.4_75100001	2	75100001	0.4583	0.4072
NC_005101.4_75200001	2	75200001	0.4982	0.4214
NC_005101.4_75300001	2	75300001	0.4982	0.4214
NC_005101.4_75400001	2	75400001	0.3787	0.2525
NC_005101.4_75800001	2	75800001	0.6447	0.8856
NC_005101.4_75900001	2	75900001	0.6178	0.8073
NC_005101.4_76000001	2	76000001	0.583	0.7976
NC_005101.4_76100001	2	76100001	0.5848	0.7552
NC_005101.4_76200001	2	76200001	0.5848	0.7552
NC_005101.4_76300001	2	76300001	0.5274	0.6239
NC_005101.4_77100001	2	77100001	0.6437	0.8782
NC_005101.4_77200001	2	77200001	0.6437	0.8782
NC_005101.4_77300001	2	77300001	0.62	0.7383
NC_005101.4_77400001	2	77400001	0.4491	0.6527
NC_005101.4_77500001	2	77500001	0.4003	0.5277
NC_005101.4_77600001	2	77600001	0.4558	0.63
NC_005101.4_77700001	2	77700001	0.5407	0.666
NC_005101.4_77800001	2	77800001	0.5419	0.716
NC_005101.4_77900001	2	77900001	0.6806	0.7704
NC_005101.4_78000001	2	78000001	0.7103	0.8049
NC_005101.4_78100001	2	78100001	0.9021	0.8197
NC_005101.4_78200001	2	78200001	0.9481	0.9066
NC_005101.4_78300001	2	78300001	0.6802	0.7378
NC_005101.4_78400001	2	78400001	0.6121	0.6941
NC_005101.4_78500001	2	78500001	0.5828	0.6926
NC_005101.4_78600001	2	78600001	0.4861	0.5883
NC_005101.4_78700001	2	78700001	0.3867	0.5095
NC_005101.4_78800001	2	78800001	0.3599	0.514
NC_005101.4_78900001	2	78900001	0.4002	0.6767
NC_005101.4_79000001	2	79000001	0.4775	0.7152
NC_005101.4_79100001	2	79100001	0.5211	0.7677
NC_005101.4_79200001	2	79200001	0.5295	0.741
NC_005101.4_79300001	2	79300001	0.5312	0.7409
NC_005101.4_79400001	2	79400001	0.542	0.6855
NC_005101.4_79500001	2	79500001	0.419	0.5184
NC_005101.4_79600001	2	79600001	0.3271	0.4632
NC_005101.4_79700001	2	79700001	0.3211	0.4376
NC_005101.4_79800001	2	79800001	0.3845	0.5265
NC_005101.4_79900001	2	79900001	0.313	0.4685
NC_005101.4_80000001	2	80000001	0.3953	0.5979
NC_005101.4_80100001	2	80100001	0.4562	0.6463
NC_005101.4_80200001	2	80200001	0.5131	0.6784
NC_005101.4_80300001	2	80300001	0.5673	0.6885
NC_005101.4_80400001	2	80400001	0.5581	0.6608
NC_005101.4_80500001	2	80500001	0.5514	0.6323
NC_005101.4_80600001	2	80600001	0.568	0.6062
NC_005101.4_80700001	2	80700001	0.4938	0.5263
NC_005101.4_80800001	2	80800001	0.3488	0.3649
NC_005101.4_81200001	2	81200001	0.2244	0.4595
NC_005101.4_81300001	2	81300001	0.2144	0.3948

NC_005101.4_81400001	2	81400001	0.2912	0.5775
NC_005101.4_81500001	2	81500001	0.3364	0.6506
NC_005101.4_81600001	2	81600001	0.3509	0.6221
NC_005101.4_81700001	2	81700001	0.3621	0.4382
NC_005101.4_81800001	2	81800001	0.4039	0.4648
NC_005101.4_81900001	2	81900001	0.428	0.4497
NC_005101.4_82000001	2	82000001	0.4492	0.3657
NC_005101.4_82100001	2	82100001	0.437	0.3677
NC_005101.4_82400001	2	82400001	0.3387	0.7037
NC_005101.4_82500001	2	82500001	0.4237	0.6283
NC_005101.4_82600001	2	82600001	0.4131	0.5844
NC_005101.4_82700001	2	82700001	0.4137	0.5867
NC_005101.4_82800001	2	82800001	0.5163	0.6801
NC_005101.4_82900001	2	82900001	0.5918	0.6242
NC_005101.4_83000001	2	83000001	0.6297	0.7069
NC_005101.4_83100001	2	83100001	0.6663	0.7308
NC_005101.4_83200001	2	83200001	0.6167	0.7544
NC_005101.4_83300001	2	83300001	0.5922	0.7444
NC_005101.4_83400001	2	83400001	0.584	0.7605
NC_005101.4_83500001	2	83500001	0.5934	0.7376
NC_005101.4_83600001	2	83600001	0.5739	0.7299
NC_005101.4_83700001	2	83700001	0.5977	0.7023
NC_005101.4_83800001	2	83800001	0.574	0.6892
NC_005101.4_83900001	2	83900001	0.6073	0.6978
NC_005101.4_84000001	2	84000001	0.5881	0.6623
NC_005101.4_84100001	2	84100001	0.6411	0.6809
NC_005101.4_84200001	2	84200001	0.6346	0.6954
NC_005101.4_84300001	2	84300001	0.5747	0.6439
NC_005101.4_84400001	2	84400001	0.5357	0.611
NC_005101.4_84500001	2	84500001	0.4652	0.594
NC_005101.4_84600001	2	84600001	0.321	0.4752
NC_005101.4_84700001	2	84700001	0.3468	0.4386
NC_005101.4_84800001	2	84800001	0.372	0.4875
NC_005101.4_84900001	2	84900001	0.4047	0.5246
NC_005101.4_85000001	2	85000001	0.3826	0.4492
NC_005101.4_85100001	2	85100001	0.4739	0.5382
NC_005101.4_85200001	2	85200001	0.5482	0.6691
NC_005101.4_85300001	2	85300001	0.5806	0.6702
NC_005101.4_85400001	2	85400001	0.5629	0.676
NC_005101.4_85500001	2	85500001	0.5237	0.7241
NC_005101.4_85600001	2	85600001	0.5178	0.7178
NC_005101.4_85700001	2	85700001	0.4263	0.6247
NC_005101.4_85800001	2	85800001	0.3642	0.5397
NC_005101.4_85900001	2	85900001	0.3692	0.558
NC_005101.4_86000001	2	86000001	0.3749	0.4118
NC_005101.4_86100001	2	86100001	0.299	0.2417
NC_005101.4_86200001	2	86200001	0.427	0.4222
NC_005101.4_86400001	2	86400001	0.3783	0.3408
NC_005101.4_86500001	2	86500001	0.3846	0.3202
NC_005101.4_86600001	2	86600001	0.4316	0.3456
NC_005101.4_86700001	2	86700001	0.3479	0.2067
NC_005101.4_86800001	2	86800001	0.3479	0.2067
NC_005101.4_87400001	2	87400001	0.3958	0.2854
NC_005101.4_87500001	2	87500001	0.4014	0.3503
NC_005101.4_87600001	2	87600001	0.3765	0.3319
NC_005101.4_87700001	2	87700001	0.28	0.2874
NC_005101.4_87800001	2	87800001	0.3216	0.4123
NC_005101.4_87900001	2	87900001	0.3151	0.4645

NC_005101.4_88000001	2	88000001	0.2907	0.454
NC_005101.4_88100001	2	88100001	0.3059	0.5
NC_005101.4_88600001	2	88600001	0.4589	0.3393
NC_005101.4_88700001	2	88700001	0.4489	0.3395
NC_005101.4_88800001	2	88800001	0.5087	0.4159
NC_005101.4_88900001	2	88900001	0.6658	0.5912
NC_005101.4_89000001	2	89000001	0.6658	0.5912
NC_005101.4_89200001	2	89200001	0.5062	0.67
NC_005101.4_89300001	2	89300001	0.2949	0.5482
NC_005101.4_89400001	2	89400001	0.2908	0.5354
NC_005101.4_89500001	2	89500001	0.235	0.5113
NC_005101.4_89600001	2	89600001	0.341	0.6628
NC_005101.4_89700001	2	89700001	0.3773	0.6537
NC_005101.4_89800001	2	89800001	0.4709	0.7007
NC_005101.4_89900001	2	89900001	0.3846	0.6042
NC_005101.4_90000001	2	90000001	0.491	0.6192
NC_005101.4_90800001	2	90800001	0.5415	0.6983
NC_005101.4_90900001	2	90900001	0.4651	0.6787
NC_005101.4_91000001	2	91000001	0.5323	0.7423
NC_005101.4_91100001	2	91100001	0.5323	0.7423
NC_005101.4_91200001	2	91200001	0.6376	0.8869
NC_005101.4_91700001	2	91700001	0.5907	0.9008
NC_005101.4_91800001	2	91800001	0.2772	0.401
NC_005101.4_91900001	2	91900001	0.2487	0.3538
NC_005101.4_92000001	2	92000001	0.2487	0.3538
NC_005101.4_92100001	2	92100001	0.4279	0.5808
NC_005101.4_92200001	2	92200001	0.47	0.5406
NC_005101.4_92300001	2	92300001	0.5547	0.6754
NC_005101.4_92400001	2	92400001	0.5035	0.6731
NC_005101.4_92500001	2	92500001	0.4111	0.6051
NC_005101.4_92600001	2	92600001	0.284	0.4245
NC_005101.4_92700001	2	92700001	0.3071	0.5737
NC_005101.4_92800001	2	92800001	0.3363	0.6383
NC_005101.4_92900001	2	92900001	0.391	0.7141
NC_005101.4_93000001	2	93000001	0.4103	0.7607
NC_005101.4_93100001	2	93100001	0.3882	0.7341
NC_005101.4_93200001	2	93200001	0.5176	0.8049
NC_005101.4_93300001	2	93300001	0.4659	0.718
NC_005101.4_93400001	2	93400001	0.4844	0.6955
NC_005101.4_93500001	2	93500001	0.4973	0.6924
NC_005101.4_93600001	2	93600001	0.5647	0.7259
NC_005101.4_93700001	2	93700001	0.457	0.6361
NC_005101.4_93800001	2	93800001	0.4791	0.6198
NC_005101.4_93900001	2	93900001	0.4887	0.6473
NC_005101.4_94000001	2	94000001	0.5414	0.6717
NC_005101.4_94100001	2	94100001	0.5067	0.6641
NC_005101.4_94200001	2	94200001	0.5773	0.7117
NC_005101.4_94300001	2	94300001	0.6504	0.7875
NC_005101.4_94400001	2	94400001	0.5791	0.6824
NC_005101.4_94500001	2	94500001	0.492	0.6048
NC_005101.4_94600001	2	94600001	0.5574	0.6112
NC_005101.4_94700001	2	94700001	0.5284	0.5938
NC_005101.4_94800001	2	94800001	0.5614	0.6146
NC_005101.4_94900001	2	94900001	0.592	0.6499
NC_005101.4_95000001	2	95000001	0.6004	0.6928
NC_005101.4_95100001	2	95100001	0.5471	0.6398
NC_005101.4_95200001	2	95200001	0.5482	0.6243
NC_005101.4_95300001	2	95300001	0.4576	0.5186

NC_005101.4_95400001	2	95400001	0.4351	0.4977
NC_005101.4_95500001	2	95500001	0.3908	0.2894
NC_005101.4_96000001	2	96000001	0.5575	0.9376
NC_005101.4_96100001	2	96100001	0.5575	0.9376
NC_005101.4_96200001	2	96200001	0.5455	0.8171
NC_005101.4_96300001	2	96300001	0.4601	0.7304
NC_005101.4_96400001	2	96400001	0.5075	0.7355
NC_005101.4_96500001	2	96500001	0.5751	0.7002
NC_005101.4_96600001	2	96600001	0.5571	0.6391
NC_005101.4_96700001	2	96700001	0.5012	0.6071
NC_005101.4_96800001	2	96800001	0.6098	0.7205
NC_005101.4_96900001	2	96900001	0.5914	0.7288
NC_005101.4_97000001	2	97000001	0.4512	0.5789
NC_005101.4_97100001	2	97100001	0.4296	0.5525
NC_005101.4_97200001	2	97200001	0.487	0.6114
NC_005101.4_97300001	2	97300001	0.4285	0.5362
NC_005101.4_97400001	2	97400001	0.5782	0.6446
NC_005101.4_97500001	2	97500001	0.5782	0.6446
NC_005101.4_97600001	2	97600001	0.8945	0.8375
NC_005101.4_97700001	2	97700001	0.9104	0.8637
NC_005101.4_97800001	2	97800001	0.8416	0.8574
NC_005101.4_97900001	2	97900001	0.7102	0.7685
NC_005101.4_98000001	2	98000001	0.7255	0.7698
NC_005101.4_98100001	2	98100001	0.6978	0.7724
NC_005101.4_98200001	2	98200001	0.5816	0.7242
NC_005101.4_98300001	2	98300001	0.4177	0.5356
NC_005101.4_98400001	2	98400001	0.4037	0.6047
NC_005101.4_98500001	2	98500001	0.2554	0.4336
NC_005101.4_98600001	2	98600001	0.3601	0.6083
NC_005101.4_99000001	2	99000001	0.8295	0.7575
NC_005101.4_99100001	2	99100001	0.6246	0.6248
NC_005101.4_99200001	2	99200001	0.6246	0.6248
NC_005101.4_99300001	2	99300001	0.548	0.6166
NC_005101.4_99400001	2	99400001	0.5567	0.6326
NC_005101.4_99500001	2	99500001	0.4742	0.6326
NC_005101.4_99600001	2	99600001	0.569	0.7389
NC_005101.4_99700001	2	99700001	0.6469	0.8028
NC_005101.4_99800001	2	99800001	0.6179	0.8186
NC_005101.4_99900001	2	99900001	0.5265	0.8076
NC_005101.4_100000001	2	100000001	0.686	0.8501
NC_005101.4_100100001	2	100100001	0.5701	0.712
NC_005101.4_100200001	2	100200001	0.4535	0.6253
NC_005101.4_100300001	2	100300001	0.4865	0.6378
NC_005101.4_100400001	2	100400001	0.5631	0.6058
NC_005101.4_100500001	2	100500001	0.4478	0.35
NC_005101.4_100600001	2	100600001	0.5377	0.4203
NC_005101.4_100700001	2	100700001	0.5008	0.3884
NC_005101.4_100800001	2	100800001	0.5465	0.5015
NC_005101.4_100900001	2	100900001	0.5443	0.5294
NC_005101.4_101000001	2	101000001	0.3995	0.6633
NC_005101.4_101100001	2	101100001	0.4806	0.7454
NC_005101.4_101200001	2	101200001	0.6025	0.8392
NC_005101.4_101300001	2	101300001	0.6997	0.8163
NC_005101.4_101400001	2	101400001	0.761	0.8246
NC_005101.4_101500001	2	101500001	0.8147	0.7954
NC_005101.4_101600001	2	101600001	0.7806	0.784
NC_005101.4_101700001	2	101700001	0.7441	0.7729
NC_005101.4_101800001	2	101800001	0.7221	0.7756

NC_005101.4_101900001	2	101900001	0.6281	0.7279
NC_005101.4_102000001	2	102000001	0.6926	0.8296
NC_005101.4_102100001	2	102100001	0.6123	0.82
NC_005101.4_102200001	2	102200001	0.5365	0.767
NC_005101.4_102300001	2	102300001	0.54	0.7212
NC_005101.4_102400001	2	102400001	0.4838	0.6846
NC_005101.4_102500001	2	102500001	0.2659	0.2947
NC_005101.4_102600001	2	102600001	0.3304	0.3017
NC_005101.4_102700001	2	102700001	0.3348	0.3406
NC_005101.4_102800001	2	102800001	0.2651	0.3328
NC_005101.4_102900001	2	102900001	0.3134	0.5065
NC_005101.4_103000001	2	103000001	0.3717	0.5153
NC_005101.4_103100001	2	103100001	0.4572	0.5726
NC_005101.4_103200001	2	103200001	0.4598	0.5634
NC_005101.4_103300001	2	103300001	0.4824	0.5362
NC_005101.4_103400001	2	103400001	0.5149	0.5622
NC_005101.4_103500001	2	103500001	0.471	0.5575
NC_005101.4_103600001	2	103600001	0.5397	0.6751
NC_005101.4_103700001	2	103700001	0.5297	0.6765
NC_005101.4_103800001	2	103800001	0.5803	0.7115
NC_005101.4_103900001	2	103900001	0.6609	0.7625
NC_005101.4_104000001	2	104000001	0.626	0.7647
NC_005101.4_104100001	2	104100001	0.5349	0.7117
NC_005101.4_104200001	2	104200001	0.5798	0.7353
NC_005101.4_104300001	2	104300001	0.5773	0.7313
NC_005101.4_104400001	2	104400001	0.4506	0.6182
NC_005101.4_104500001	2	104500001	0.5481	0.5929
NC_005101.4_104600001	2	104600001	0.6728	0.6595
NC_005101.4_104700001	2	104700001	0.6401	0.6563
NC_005101.4_104800001	2	104800001	0.6442	0.7023
NC_005101.4_104900001	2	104900001	0.736	0.7639
NC_005101.4_105000001	2	105000001	0.6851	0.8071
NC_005101.4_105700001	2	105700001	0.6388	0.7528
NC_005101.4_105800001	2	105800001	0.554	0.6685
NC_005101.4_105900001	2	105900001	0.527	0.6843
NC_005101.4_106000001	2	106000001	0.5293	0.6548
NC_005101.4_106100001	2	106100001	0.5718	0.6768
NC_005101.4_106200001	2	106200001	0.6528	0.7148
NC_005101.4_106300001	2	106300001	0.7028	0.7159
NC_005101.4_106400001	2	106400001	0.7289	0.7647
NC_005101.4_106500001	2	106500001	0.7282	0.7749
NC_005101.4_106600001	2	106600001	0.6944	0.7525
NC_005101.4_106700001	2	106700001	0.6944	0.7525
NC_005101.4_106800001	2	106800001	0.6355	0.7536
NC_005101.4_106900001	2	106900001	0.5414	0.6709
NC_005101.4_107000001	2	107000001	0.693	0.829
NC_005101.4_107100001	2	107100001	0.693	0.829
NC_005101.4_107200001	2	107200001	0.693	0.829
NC_005101.4_107400001	2	107400001	0.9921	0.9887
NC_005101.4_107600001	2	107600001	0.4022	0.7505
NC_005101.4_107700001	2	107700001	0.4022	0.7505
NC_005101.4_107800001	2	107800001	0.4967	0.7474
NC_005101.4_107900001	2	107900001	0.3433	0.628
NC_005101.4_108000001	2	108000001	0.4021	0.7091
NC_005101.4_108100001	2	108100001	0.4872	0.6631
NC_005101.4_108200001	2	108200001	0.4872	0.6631
NC_005101.4_108300001	2	108300001	0.2831	0.3434
NC_005101.4_108400001	2	108400001	0.365	0.3815

NC_005101.4_108500001	2	108500001	0.4007	0.4976
NC_005101.4_108600001	2	108600001	0.3754	0.4851
NC_005101.4_108700001	2	108700001	0.4534	0.5468
NC_005101.4_108800001	2	108800001	0.544	0.6355
NC_005101.4_108900001	2	108900001	0.7317	0.7808
NC_005101.4_109000001	2	109000001	0.8014	0.7689
NC_005101.4_109100001	2	109100001	0.7709	0.7888
NC_005101.4_109200001	2	109200001	0.7272	0.8096
NC_005101.4_109300001	2	109300001	0.7658	0.8348
NC_005101.4_109400001	2	109400001	0.5562	0.7642
NC_005101.4_109500001	2	109500001	0.4137	0.5904
NC_005101.4_109600001	2	109600001	0.3567	0.5011
NC_005101.4_109700001	2	109700001	0.3586	0.5355
NC_005101.4_109800001	2	109800001	0.4175	0.642
NC_005101.4_109900001	2	109900001	0.412	0.6362
NC_005101.4_110000001	2	110000001	0.5351	0.7311
NC_005101.4_110100001	2	110100001	0.5279	0.7438
NC_005101.4_110200001	2	110200001	0.4382	0.6803
NC_005101.4_110300001	2	110300001	0.415	0.6246
NC_005101.4_110400001	2	110400001	0.5113	0.6626
NC_005101.4_110500001	2	110500001	0.4784	0.6995
NC_005101.4_110600001	2	110600001	0.4911	0.7293
NC_005101.4_110700001	2	110700001	0.5493	0.7458
NC_005101.4_110800001	2	110800001	0.5119	0.774
NC_005101.4_110900001	2	110900001	0.3797	0.6929
NC_005101.4_111000001	2	111000001	0.3907	0.6572
NC_005101.4_111100001	2	111100001	0.3159	0.4534
NC_005101.4_111200001	2	111200001	0.3324	0.3774
NC_005101.4_111300001	2	111300001	0.3338	0.3367
NC_005101.4_111400001	2	111400001	0.4311	0.5019
NC_005101.4_111500001	2	111500001	0.5116	0.5381
NC_005101.4_111600001	2	111600001	0.5334	0.5572
NC_005101.4_111700001	2	111700001	0.5906	0.6414
NC_005101.4_111800001	2	111800001	0.6359	0.6315
NC_005101.4_111900001	2	111900001	0.622	0.6089
NC_005101.4_112000001	2	112000001	0.6052	0.6235
NC_005101.4_112100001	2	112100001	0.6558	0.684
NC_005101.4_112200001	2	112200001	0.5292	0.6342
NC_005101.4_112300001	2	112300001	0.4698	0.6589
NC_005101.4_112400001	2	112400001	0.3489	0.6774
NC_005101.4_112500001	2	112500001	0.3489	0.6774
NC_005101.4_112600001	2	112600001	0.3289	0.6557
NC_005101.4_112700001	2	112700001	0.8552	0.8867
NC_005101.4_112800001	2	112800001	0.919	0.935
NC_005101.4_112900001	2	112900001	0.9071	0.9501
NC_005101.4_113000001	2	113000001	0.9103	0.9518
NC_005101.4_113100001	2	113100001	0.8685	0.9117
NC_005101.4_113200001	2	113200001	0.7833	0.9105
NC_005101.4_113300001	2	113300001	0.7244	0.8144
NC_005101.4_113400001	2	113400001	0.6241	0.6977
NC_005101.4_113500001	2	113500001	0.5777	0.6808
NC_005101.4_113600001	2	113600001	0.4898	0.6502
NC_005101.4_113700001	2	113700001	0.4988	0.6282
NC_005101.4_113800001	2	113800001	0.5738	0.6893
NC_005101.4_113900001	2	113900001	0.4834	0.6685
NC_005101.4_114000001	2	114000001	0.5105	0.6398
NC_005101.4_114100001	2	114100001	0.5543	0.6522
NC_005101.4_114200001	2	114200001	0.5518	0.6534

NC_005101.4_114300001	2	114300001	0.4304	0.6093
NC_005101.4_115000001	2	115000001	0.4758	0.6238
NC_005101.4_115100001	2	115100001	0.402	0.5479
NC_005101.4_115200001	2	115200001	0.4083	0.5696
NC_005101.4_115300001	2	115300001	0.3981	0.5211
NC_005101.4_115400001	2	115400001	0.4059	0.5568
NC_005101.4_115500001	2	115500001	0.3872	0.5433
NC_005101.4_115600001	2	115600001	0.4743	0.652
NC_005101.4_115700001	2	115700001	0.5249	0.7049
NC_005101.4_115800001	2	115800001	0.6973	0.9032
NC_005101.4_115900001	2	115900001	0.6576	0.8419
NC_005101.4_116000001	2	116000001	0.6991	0.8036
NC_005101.4_116100001	2	116100001	0.6864	0.7861
NC_005101.4_116200001	2	116200001	0.6023	0.725
NC_005101.4_116300001	2	116300001	0.4475	0.5456
NC_005101.4_116400001	2	116400001	0.5492	0.5887
NC_005101.4_116500001	2	116500001	0.4348	0.6108
NC_005101.4_116600001	2	116600001	0.4523	0.6648
NC_005101.4_116700001	2	116700001	0.4996	0.6932
NC_005101.4_116800001	2	116800001	0.5307	0.7193
NC_005101.4_116900001	2	116900001	0.456	0.692
NC_005101.4_117000001	2	117000001	0.6021	0.7953
NC_005101.4_117100001	2	117100001	0.6051	0.7772
NC_005101.4_117200001	2	117200001	0.7047	0.8433
NC_005101.4_117300001	2	117300001	0.7388	0.8238
NC_005101.4_117400001	2	117400001	0.8727	0.8491
NC_005101.4_117500001	2	117500001	0.724	0.7341
NC_005101.4_117600001	2	117600001	0.6421	0.727
NC_005101.4_117700001	2	117700001	0.5836	0.6638
NC_005101.4_117800001	2	117800001	0.5415	0.6521
NC_005101.4_117900001	2	117900001	0.6144	0.7416
NC_005101.4_118000001	2	118000001	0.5742	0.7126
NC_005101.4_118100001	2	118100001	0.6401	0.7212
NC_005101.4_118200001	2	118200001	0.6283	0.7417
NC_005101.4_118300001	2	118300001	0.6679	0.7758
NC_005101.4_118400001	2	118400001	0.5525	0.6113
NC_005101.4_118500001	2	118500001	0.5407	0.6001
NC_005101.4_118600001	2	118600001	0.522	0.6326
NC_005101.4_118700001	2	118700001	0.5131	0.6447
NC_005101.4_118800001	2	118800001	0.5138	0.643
NC_005101.4_118900001	2	118900001	0.5244	0.7131
NC_005101.4_119000001	2	119000001	0.4667	0.7352
NC_005101.4_119100001	2	119100001	0.4808	0.7494
NC_005101.4_119200001	2	119200001	0.4721	0.7288
NC_005101.4_119300001	2	119300001	0.4246	0.5912
NC_005101.4_119400001	2	119400001	0.4116	0.6207
NC_005101.4_119500001	2	119500001	0.4547	0.5578
NC_005101.4_119600001	2	119600001	0.4074	0.4846
NC_005101.4_119700001	2	119700001	0.3919	0.3782
NC_005101.4_119800001	2	119800001	0.408	0.5224
NC_005101.4_119900001	2	119900001	0.5431	0.6572
NC_005101.4_120000001	2	120000001	0.5619	0.6622
NC_005101.4_120100001	2	120100001	0.463	0.6283
NC_005101.4_120200001	2	120200001	0.507	0.7104
NC_005101.4_120300001	2	120300001	0.5223	0.6793
NC_005101.4_120400001	2	120400001	0.4962	0.6792
NC_005101.4_120500001	2	120500001	0.5528	0.7069
NC_005101.4_120600001	2	120600001	0.6227	0.7458

NC_005101.4_120700001	2	120700001	0.6333	0.6769
NC_005101.4_120800001	2	120800001	0.6389	0.6794
NC_005101.4_120900001	2	120900001	0.6501	0.6338
NC_005101.4_121000001	2	121000001	0.5751	0.6228
NC_005101.4_121100001	2	121100001	0.5121	0.5845
NC_005101.4_121200001	2	121200001	0.4798	0.6392
NC_005101.4_121300001	2	121300001	0.4491	0.6552
NC_005101.4_121400001	2	121400001	0.5166	0.6964
NC_005101.4_121500001	2	121500001	0.4879	0.7105
NC_005101.4_121600001	2	121600001	0.4811	0.6618
NC_005101.4_121700001	2	121700001	0.435	0.5852
NC_005101.4_121800001	2	121800001	0.4471	0.5732
NC_005101.4_121900001	2	121900001	0.3349	0.4758
NC_005101.4_122000001	2	122000001	0.4963	0.6817
NC_005101.4_122100001	2	122100001	0.5113	0.7639
NC_005101.4_122200001	2	122200001	0.5704	0.8054
NC_005101.4_122300001	2	122300001	0.5677	0.7703
NC_005101.4_122400001	2	122400001	0.5104	0.7595
NC_005101.4_122500001	2	122500001	0.4699	0.6377
NC_005101.4_122600001	2	122600001	0.4942	0.6105
NC_005101.4_122700001	2	122700001	0.4265	0.5355
NC_005101.4_122800001	2	122800001	0.4629	0.5632
NC_005101.4_122900001	2	122900001	0.4574	0.5128
NC_005101.4_123000001	2	123000001	0.5	0.5481
NC_005101.4_123100001	2	123100001	0.5242	0.5966
NC_005101.4_123200001	2	123200001	0.5286	0.6145
NC_005101.4_123300001	2	123300001	0.547	0.6587
NC_005101.4_123400001	2	123400001	0.6179	0.7549
NC_005101.4_123500001	2	123500001	0.6551	0.826
NC_005101.4_123600001	2	123600001	0.6564	0.8602
NC_005101.4_123700001	2	123700001	0.6707	0.8502
NC_005101.4_123800001	2	123800001	0.676	0.8613
NC_005101.4_123900001	2	123900001	0.704	0.8218
NC_005101.4_124000001	2	124000001	0.6319	0.7632
NC_005101.4_124100001	2	124100001	0.632	0.7176
NC_005101.4_124200001	2	124200001	0.5874	0.6139
NC_005101.4_124300001	2	124300001	0.5807	0.6365
NC_005101.4_124400001	2	124400001	0.6431	0.7363
NC_005101.4_124500001	2	124500001	0.6555	0.7738
NC_005101.4_124600001	2	124600001	0.6429	0.7931
NC_005101.4_124700001	2	124700001	0.6451	0.8666
NC_005101.4_124800001	2	124800001	0.6042	0.8112
NC_005101.4_124900001	2	124900001	0.4882	0.7007
NC_005101.4_125000001	2	125000001	0.522	0.7122
NC_005101.4_125100001	2	125100001	0.5207	0.6699
NC_005101.4_125200001	2	125200001	0.5375	0.6501
NC_005101.4_125300001	2	125300001	0.545	0.6537
NC_005101.4_125400001	2	125400001	0.549	0.6275
NC_005101.4_125500001	2	125500001	0.4443	0.5428
NC_005101.4_125600001	2	125600001	0.3856	0.4528
NC_005101.4_125700001	2	125700001	0.3791	0.4668
NC_005101.4_125800001	2	125800001	0.3776	0.396
NC_005101.4_125900001	2	125900001	0.5712	0.653
NC_005101.4_126000001	2	126000001	0.7303	0.684
NC_005101.4_126100001	2	126100001	0.6841	0.676
NC_005101.4_126200001	2	126200001	0.7035	0.7264
NC_005101.4_126300001	2	126300001	0.6997	0.7381
NC_005101.4_126400001	2	126400001	0.6225	0.6807

NC_005101.4_126500001	2	126500001	0.6239	0.6949
NC_005101.4_126600001	2	126600001	0.6222	0.6959
NC_005101.4_126700001	2	126700001	0.4935	0.6273
NC_005101.4_126800001	2	126800001	0.4049	0.5689
NC_005101.4_126900001	2	126900001	0.3402	0.4963
NC_005101.4_127000001	2	127000001	0.2576	0.4275
NC_005101.4_127100001	2	127100001	0.2903	0.3892
NC_005101.4_127200001	2	127200001	0.3858	0.4725
NC_005101.4_127300001	2	127300001	0.4978	0.6656
NC_005101.4_127400001	2	127400001	0.603	0.72
NC_005101.4_127500001	2	127500001	0.7195	0.7695
NC_005101.4_127600001	2	127600001	0.7055	0.8238
NC_005101.4_127700001	2	127700001	0.6458	0.7873
NC_005101.4_127800001	2	127800001	0.6255	0.7234
NC_005101.4_127900001	2	127900001	0.5403	0.6668
NC_005101.4_128000001	2	128000001	0.4937	0.6129
NC_005101.4_128100001	2	128100001	0.4537	0.5693
NC_005101.4_128200001	2	128200001	0.4102	0.4924
NC_005101.4_128300001	2	128300001	0.3824	0.4919
NC_005101.4_128400001	2	128400001	0.4628	0.5543
NC_005101.4_128700001	2	128700001	0.6753	0.6982
NC_005101.4_128800001	2	128800001	0.6753	0.6982
NC_005101.4_128900001	2	128900001	0.5816	0.5967
NC_005101.4_129000001	2	129000001	0.6611	0.6904
NC_005101.4_129100001	2	129100001	0.662	0.6535
NC_005101.4_129200001	2	129200001	0.6611	0.69
NC_005101.4_129300001	2	129300001	0.6611	0.69
NC_005101.4_129400001	2	129400001	0.6088	0.6777
NC_005101.4_130000001	2	130000001	0.6694	0.8834
NC_005101.4_130100001	2	130100001	0.7262	0.8843
NC_005101.4_130200001	2	130200001	0.559	0.7934
NC_005101.4_130300001	2	130300001	0.559	0.7934
NC_005101.4_130400001	2	130400001	0.5085	0.6669
NC_005101.4_130500001	2	130500001	0.5287	0.6277
NC_005101.4_130600001	2	130600001	0.3422	0.4384
NC_005101.4_131200001	2	131200001	0.4498	0.424
NC_005101.4_131300001	2	131300001	0.4498	0.424
NC_005101.4_131400001	2	131400001	0.5781	0.5341
NC_005101.4_131500001	2	131500001	0.5667	0.4734
NC_005101.4_131600001	2	131600001	0.5667	0.4734
NC_005101.4_131700001	2	131700001	0.5647	0.5582
NC_005101.4_131800001	2	131800001	0.6048	0.5911
NC_005101.4_131900001	2	131900001	0.4803	0.513
NC_005101.4_132000001	2	132000001	0.4803	0.513
NC_005101.4_132100001	2	132100001	0.4803	0.513
NC_005101.4_132900001	2	132900001	0.282	0.5838
NC_005101.4_133000001	2	133000001	0.3389	0.4754
NC_005101.4_133100001	2	133100001	0.3389	0.4754
NC_005101.4_133200001	2	133200001	0.4085	0.5148
NC_005101.4_133300001	2	133300001	0.2329	0.2149
NC_005101.4_133400001	2	133400001	0.3176	0.4384
NC_005101.4_133500001	2	133500001	0.3134	0.5363
NC_005101.4_133600001	2	133600001	0.3134	0.5363
NC_005101.4_133700001	2	133700001	0.3134	0.5363
NC_005101.4_133800001	2	133800001	0.4186	0.6862
NC_005101.4_134100001	2	134100001	0.6615	0.6645
NC_005101.4_134200001	2	134200001	0.6615	0.6645
NC_005101.4_134300001	2	134300001	0.5953	0.5813

NC_005101.4_134400001	2	134400001	0.2901	0.209
NC_005101.4_134500001	2	134500001	0.3671	0.3564
NC_005101.4_134600001	2	134600001	0.3677	0.5059
NC_005101.4_134700001	2	134700001	0.3677	0.5059
NC_005101.4_134800001	2	134800001	0.3677	0.5059
NC_005101.4_134900001	2	134900001	0.5557	0.7412
NC_005101.4_135000001	2	135000001	0.4931	0.6487
NC_005101.4_135700001	2	135700001	0.7468	0.7014
NC_005101.4_135800001	2	135800001	0.7042	0.703
NC_005101.4_135900001	2	135900001	0.797	0.8805
NC_005101.4_136000001	2	136000001	0.736	0.8373
NC_005101.4_136100001	2	136100001	0.6673	0.8506
NC_005101.4_136200001	2	136200001	0.5673	0.7231
NC_005101.4_136300001	2	136300001	0.5617	0.7219
NC_005101.4_136400001	2	136400001	0.4649	0.6311
NC_005101.4_136500001	2	136500001	0.4649	0.6311
NC_005101.4_136800001	2	136800001	0.5703	0.4798
NC_005101.4_136900001	2	136900001	0.5703	0.4798
NC_005101.4_137000001	2	137000001	0.5573	0.4375
NC_005101.4_137100001	2	137100001	0.5573	0.4375
NC_005101.4_137200001	2	137200001	0.5573	0.4375
NC_005101.4_137500001	2	137500001	0.7878	0.7963
NC_005101.4_137600001	2	137600001	0.8384	0.8489
NC_005101.4_137700001	2	137700001	0.814	0.7933
NC_005101.4_137800001	2	137800001	0.7165	0.7141
NC_005101.4_137900001	2	137900001	0.7165	0.7141
NC_005101.4_138000001	2	138000001	0.6495	0.7635
NC_005101.4_138100001	2	138100001	0.4755	0.505
NC_005101.4_138200001	2	138200001	0.4785	0.6103
NC_005101.4_138300001	2	138300001	0.5748	0.7216
NC_005101.4_138400001	2	138400001	0.5126	0.6579
NC_005101.4_138500001	2	138500001	0.5085	0.6598
NC_005101.4_138600001	2	138600001	0.4652	0.6666
NC_005101.4_138700001	2	138700001	0.4473	0.634
NC_005101.4_138800001	2	138800001	0.3424	0.5373
NC_005101.4_138900001	2	138900001	0.4449	0.686
NC_005101.4_139000001	2	139000001	0.4922	0.6673
NC_005101.4_139100001	2	139100001	0.5571	0.6881
NC_005101.4_139200001	2	139200001	0.5256	0.6537
NC_005101.4_139300001	2	139300001	0.5678	0.6599
NC_005101.4_139400001	2	139400001	0.5965	0.5637
NC_005101.4_139500001	2	139500001	0.555	0.5075
NC_005101.4_139600001	2	139600001	0.5729	0.6052
NC_005101.4_139700001	2	139700001	0.6238	0.6626
NC_005101.4_139800001	2	139800001	0.5577	0.6172
NC_005101.4_139900001	2	139900001	0.5514	0.6749
NC_005101.4_140000001	2	140000001	0.5116	0.6508
NC_005101.4_140100001	2	140100001	0.531	0.6365
NC_005101.4_140200001	2	140200001	0.4778	0.5569
NC_005101.4_140300001	2	140300001	0.5704	0.6213
NC_005101.4_140400001	2	140400001	0.6664	0.6932
NC_005101.4_140500001	2	140500001	0.7575	0.7934
NC_005101.4_140600001	2	140600001	0.6521	0.7368
NC_005101.4_140700001	2	140700001	0.5983	0.7322
NC_005101.4_140800001	2	140800001	0.5728	0.7057
NC_005101.4_140900001	2	140900001	0.5121	0.6256
NC_005101.4_141000001	2	141000001	0.5017	0.5638
NC_005101.4_141100001	2	141100001	0.4278	0.5181

NC_005101.4_141200001	2	141200001	0.5067	0.5944
NC_005101.4_141300001	2	141300001	0.483	0.5828
NC_005101.4_141400001	2	141400001	0.4678	0.6017
NC_005101.4_141500001	2	141500001	0.4968	0.6923
NC_005101.4_141600001	2	141600001	0.666	0.7281
NC_005101.4_141700001	2	141700001	0.6069	0.644
NC_005101.4_141800001	2	141800001	0.5888	0.6614
NC_005101.4_141900001	2	141900001	0.5826	0.696
NC_005101.4_142000001	2	142000001	0.5479	0.6323
NC_005101.4_142100001	2	142100001	0.4611	0.6036
NC_005101.4_142200001	2	142200001	0.4665	0.6395
NC_005101.4_142300001	2	142300001	0.4812	0.6034
NC_005101.4_142400001	2	142400001	0.4663	0.5635
NC_005101.4_142500001	2	142500001	0.5016	0.6001
NC_005101.4_142600001	2	142600001	0.5455	0.6404
NC_005101.4_142700001	2	142700001	0.5023	0.6328
NC_005101.4_142800001	2	142800001	0.4439	0.6238
NC_005101.4_142900001	2	142900001	0.4095	0.5753
NC_005101.4_143000001	2	143000001	0.2882	0.5378
NC_005101.4_143100001	2	143100001	0.1736	0.322
NC_005101.4_143200001	2	143200001	0.2531	0.411
NC_005101.4_143300001	2	143300001	0.3673	0.5189
NC_005101.4_143400001	2	143400001	0.4232	0.5703
NC_005101.4_143500001	2	143500001	0.5792	0.694
NC_005101.4_143600001	2	143600001	0.6283	0.7309
NC_005101.4_143700001	2	143700001	0.6491	0.7728
NC_005101.4_143800001	2	143800001	0.614	0.7437
NC_005101.4_143900001	2	143900001	0.6593	0.7615
NC_005101.4_144000001	2	144000001	0.6322	0.6792
NC_005101.4_144100001	2	144100001	0.6411	0.6576
NC_005101.4_144200001	2	144200001	0.6907	0.695
NC_005101.4_144300001	2	144300001	0.6638	0.725
NC_005101.4_144400001	2	144400001	0.6117	0.6806
NC_005101.4_144500001	2	144500001	0.533	0.6491
NC_005101.4_144600001	2	144600001	0.5211	0.6367
NC_005101.4_144700001	2	144700001	0.5353	0.6734
NC_005101.4_144800001	2	144800001	0.5621	0.6967
NC_005101.4_144900001	2	144900001	0.5669	0.7398
NC_005101.4_145000001	2	145000001	0.6343	0.7938
NC_005101.4_145100001	2	145100001	0.7718	0.9086
NC_005101.4_145200001	2	145200001	0.7807	0.8809
NC_005101.4_145300001	2	145300001	0.5908	0.7636
NC_005101.4_145400001	2	145400001	0.5979	0.7392
NC_005101.4_145500001	2	145500001	0.5673	0.7175
NC_005101.4_145600001	2	145600001	0.4565	0.5906
NC_005101.4_145700001	2	145700001	0.4785	0.6418
NC_005101.4_145800001	2	145800001	0.5119	0.6918
NC_005101.4_145900001	2	145900001	0.5004	0.6915
NC_005101.4_146000001	2	146000001	0.5924	0.7703
NC_005101.4_146100001	2	146100001	0.5147	0.6715
NC_005101.4_146200001	2	146200001	0.4685	0.6152
NC_005101.4_146300001	2	146300001	0.5598	0.672
NC_005101.4_146400001	2	146400001	0.6567	0.7579
NC_005101.4_146500001	2	146500001	0.5217	0.73
NC_005101.4_146600001	2	146600001	0.6021	0.8649
NC_005101.4_146700001	2	146700001	0.5815	0.8028
NC_005101.4_146800001	2	146800001	0.6026	0.8174
NC_005101.4_146900001	2	146900001	0.551	0.7793

NC_005101.4_147100001	2	147100001	0.6827	0.7891
NC_005101.4_147200001	2	147200001	0.6864	0.8246
NC_005101.4_147300001	2	147300001	0.5792	0.787
NC_005101.4_147400001	2	147400001	0.5216	0.7833
NC_005101.4_147500001	2	147500001	0.5224	0.7746
NC_005101.4_147600001	2	147600001	0.4565	0.7426
NC_005101.4_147700001	2	147700001	0.2965	0.6663
NC_005101.4_147800001	2	147800001	0.3294	0.7826
NC_005101.4_147900001	2	147900001	0.2444	0.4789
NC_005101.4_148000001	2	148000001	0.3503	0.5576
NC_005101.4_148100001	2	148100001	0.3643	0.5534
NC_005101.4_148200001	2	148200001	0.4194	0.5281
NC_005101.4_148300001	2	148300001	0.4421	0.53
NC_005101.4_148400001	2	148400001	0.5989	0.6222
NC_005101.4_148500001	2	148500001	0.605	0.6457
NC_005101.4_148600001	2	148600001	0.6968	0.7199
NC_005101.4_148700001	2	148700001	0.6087	0.7564
NC_005101.4_148800001	2	148800001	0.5073	0.7704
NC_005101.4_148900001	2	148900001	0.456	0.813
NC_005101.4_149000001	2	149000001	0.4831	0.802
NC_005101.4_149100001	2	149100001	0.5229	0.7786
NC_005101.4_149200001	2	149200001	0.582	0.8076
NC_005101.4_149300001	2	149300001	0.6553	0.7942
NC_005101.4_149400001	2	149400001	0.627	0.7391
NC_005101.4_149500001	2	149500001	0.6716	0.6771
NC_005101.4_149600001	2	149600001	0.702	0.6608
NC_005101.4_149700001	2	149700001	0.7354	0.7065
NC_005101.4_149800001	2	149800001	0.7178	0.748
NC_005101.4_149900001	2	149900001	0.7737	0.8103
NC_005101.4_150000001	2	150000001	0.7253	0.7963
NC_005101.4_150100001	2	150100001	0.5931	0.7458
NC_005101.4_150200001	2	150200001	0.5229	0.6824
NC_005101.4_150300001	2	150300001	0.437	0.6765
NC_005101.4_150400001	2	150400001	0.402	0.6144
NC_005101.4_150500001	2	150500001	0.4102	0.6435
NC_005101.4_150600001	2	150600001	0.4413	0.6455
NC_005101.4_150700001	2	150700001	0.4313	0.6933
NC_005101.4_150800001	2	150800001	0.3288	0.5511
NC_005101.4_150900001	2	150900001	0.3324	0.6083
NC_005101.4_151000001	2	151000001	0.2982	0.5784
NC_005101.4_151100001	2	151100001	0.2629	0.5394
NC_005101.4_151200001	2	151200001	0.283	0.5425
NC_005101.4_151300001	2	151300001	0.3712	0.6062
NC_005101.4_151400001	2	151400001	0.3323	0.5249
NC_005101.4_151500001	2	151500001	0.4066	0.5616
NC_005101.4_151600001	2	151600001	0.8588	0.8361
NC_005101.4_151700001	2	151700001	0.707	0.6323
NC_005101.4_151800001	2	151800001	0.6868	0.6347
NC_005101.4_151900001	2	151900001	0.5691	0.5697
NC_005101.4_152000001	2	152000001	0.5691	0.5697
NC_005101.4_152100001	2	152100001	0.277	0.2055
NC_005101.4_152200001	2	152200001	0.2202	0.2027
NC_005101.4_152300001	2	152300001	0.2208	0.2267
NC_005101.4_152400001	2	152400001	0.21	0.2147
NC_005101.4_152500001	2	152500001	0.2032	0.1974
NC_005101.4_153000001	2	153000001	0.6108	0.9138
NC_005101.4_153100001	2	153100001	0.5465	0.825
NC_005101.4_153200001	2	153200001	0.5465	0.825

NC_005101.4_153300001	2	153300001	0.6113	0.8589
NC_005101.4_153400001	2	153400001	0.6801	0.795
NC_005101.4_153500001	2	153500001	0.6527	0.6921
NC_005101.4_153600001	2	153600001	0.6985	0.7727
NC_005101.4_153700001	2	153700001	0.637	0.6945
NC_005101.4_153800001	2	153800001	0.5182	0.6225
NC_005101.4_153900001	2	153900001	0.5036	0.6537
NC_005101.4_154000001	2	154000001	0.4527	0.5844
NC_005101.4_154100001	2	154100001	0.419	0.5436
NC_005101.4_154200001	2	154200001	0.4853	0.6753
NC_005101.4_154300001	2	154300001	0.6101	0.7876
NC_005101.4_154400001	2	154400001	0.6429	0.7636
NC_005101.4_154500001	2	154500001	0.7225	0.9328
NC_005101.4_154900001	2	154900001	0.4589	0.6006
NC_005101.4_155000001	2	155000001	0.3962	0.4955
NC_005101.4_155100001	2	155100001	0.3962	0.4955
NC_005101.4_155200001	2	155200001	0.4839	0.5899
NC_005101.4_155300001	2	155300001	0.5057	0.6333
NC_005101.4_155400001	2	155400001	0.5346	0.6921
NC_005101.4_155500001	2	155500001	0.5569	0.6869
NC_005101.4_155600001	2	155600001	0.5802	0.7109
NC_005101.4_155700001	2	155700001	0.5532	0.6913
NC_005101.4_155800001	2	155800001	0.5476	0.6739
NC_005101.4_155900001	2	155900001	0.5522	0.6446
NC_005101.4_156900001	2	156900001	0.6176	0.9049
NC_005101.4_157000001	2	157000001	0.5992	0.897
NC_005101.4_157100001	2	157100001	0.5747	0.769
NC_005101.4_157200001	2	157200001	0.6483	0.8009
NC_005101.4_157300001	2	157300001	0.676	0.806
NC_005101.4_157400001	2	157400001	0.604	0.7353
NC_005101.4_157500001	2	157500001	0.5814	0.6928
NC_005101.4_157600001	2	157600001	0.5672	0.697
NC_005101.4_157700001	2	157700001	0.3945	0.56
NC_005101.4_157800001	2	157800001	0.4848	0.6511
NC_005101.4_157900001	2	157900001	0.5377	0.7296
NC_005101.4_158000001	2	158000001	0.6364	0.8317
NC_005101.4_158100001	2	158100001	0.5696	0.7349
NC_005101.4_158200001	2	158200001	0.5725	0.7373
NC_005101.4_158300001	2	158300001	0.5189	0.6964
NC_005101.4_158400001	2	158400001	0.4599	0.6321
NC_005101.4_158500001	2	158500001	0.3676	0.5087
NC_005101.4_158600001	2	158600001	0.379	0.5903
NC_005101.4_158700001	2	158700001	0.4529	0.615
NC_005101.4_158800001	2	158800001	0.4529	0.615
NC_005101.4_158900001	2	158900001	0.4529	0.615
NC_005101.4_163800001	2	163800001	0.5622	0.7366
NC_005101.4_163900001	2	163900001	0.5919	0.7768
NC_005101.4_164000001	2	164000001	0.5575	0.757
NC_005101.4_164100001	2	164100001	0.6089	0.7967
NC_005101.4_164200001	2	164200001	0.5678	0.7732
NC_005101.4_164300001	2	164300001	0.5854	0.7883
NC_005101.4_164400001	2	164400001	0.6148	0.7821
NC_005101.4_164500001	2	164500001	0.6095	0.7774
NC_005101.4_164600001	2	164600001	0.6483	0.7501
NC_005101.4_164700001	2	164700001	0.7302	0.7763
NC_005101.4_164800001	2	164800001	0.6856	0.7556
NC_005101.4_164900001	2	164900001	0.567	0.5902
NC_005101.4_165000001	2	165000001	0.2929	0.386

NC_005101.4_165100001	2	165100001	0.3411	0.383
NC_005101.4_165200001	2	165200001	0.3583	0.3842
NC_005101.4_165300001	2	165300001	0.4494	0.4958
NC_005101.4_165400001	2	165400001	0.4568	0.5138
NC_005101.4_165500001	2	165500001	0.6296	0.5886
NC_005101.4_165600001	2	165600001	0.7041	0.6788
NC_005101.4_165700001	2	165700001	0.6445	0.6475
NC_005101.4_165800001	2	165800001	0.5527	0.5826
NC_005101.4_165900001	2	165900001	0.5311	0.5644
NC_005101.4_166000001	2	166000001	0.4992	0.56
NC_005101.4_166100001	2	166100001	0.4288	0.4695
NC_005101.4_166200001	2	166200001	0.5076	0.5578
NC_005101.4_166300001	2	166300001	0.5316	0.5206
NC_005101.4_166400001	2	166400001	0.5254	0.5149
NC_005101.4_166500001	2	166500001	0.4876	0.4541
NC_005101.4_166600001	2	166600001	0.4876	0.4541
NC_005101.4_166700001	2	166700001	0.43	0.3899
NC_005101.4_166800001	2	166800001	0.5125	0.5416
NC_005101.4_166900001	2	166900001	0.4455	0.581
NC_005101.4_167000001	2	167000001	0.4	0.5458
NC_005101.4_167100001	2	167100001	0.4794	0.6459
NC_005101.4_167200001	2	167200001	0.4794	0.6459
NC_005101.4_167300001	2	167300001	0.4247	0.6236
NC_005101.4_167400001	2	167400001	0.4241	0.5013
NC_005101.4_167500001	2	167500001	0.5119	0.6878
NC_005101.4_167600001	2	167600001	0.3193	0.416
NC_005101.4_167700001	2	167700001	0.2934	0.3887
NC_005101.4_167800001	2	167800001	0.3302	0.4728
NC_005101.4_167900001	2	167900001	0.3443	0.5889
NC_005101.4_168000001	2	168000001	0.316	0.4724
NC_005101.4_168100001	2	168100001	0.3001	0.5284
NC_005101.4_168200001	2	168200001	0.4688	0.6217
NC_005101.4_168400001	2	168400001	0.3378	0.5459
NC_005101.4_168500001	2	168500001	0.3538	0.4962
NC_005101.4_168600001	2	168600001	0.4951	0.6314
NC_005101.4_168800001	2	168800001	0.3659	0.6941
NC_005101.4_168900001	2	168900001	0.4909	0.7435
NC_005101.4_169000001	2	169000001	0.6515	0.816
NC_005101.4_169100001	2	169100001	0.6014	0.8033
NC_005101.4_169200001	2	169200001	0.506	0.7855
NC_005101.4_169300001	2	169300001	0.477	0.6098
NC_005101.4_169400001	2	169400001	0.4836	0.5572
NC_005101.4_169600001	2	169600001	0.2594	0.2127
NC_005101.4_169700001	2	169700001	0.3051	0.2166
NC_005101.4_169900001	2	169900001	0.2024	0.1992
NC_005101.4_170000001	2	170000001	0.3233	0.3553
NC_005101.4_170100001	2	170100001	0.4035	0.4294
NC_005101.4_170200001	2	170200001	0.4035	0.4294
NC_005101.4_170300001	2	170300001	0.4035	0.4294
NC_005101.4_170400001	2	170400001	0.482	0.5374
NC_005101.4_170500001	2	170500001	0.4888	0.5384
NC_005101.4_170600001	2	170600001	0.4959	0.6006
NC_005101.4_170700001	2	170700001	0.4959	0.6006
NC_005101.4_170800001	2	170800001	0.5252	0.6072
NC_005101.4_171100001	2	171100001	0.5261	0.4896
NC_005101.4_171200001	2	171200001	0.5249	0.5611
NC_005101.4_171300001	2	171300001	0.4847	0.5398
NC_005101.4_171400001	2	171400001	0.4753	0.3947

NC_005101.4_171500001	2	171500001	0.3852	0.3641
NC_005101.4_171600001	2	171600001	0.3577	0.5048
NC_005101.4_171700001	2	171700001	0.3655	0.4415
NC_005101.4_171800001	2	171800001	0.4559	0.5956
NC_005101.4_171900001	2	171900001	0.4094	0.585
NC_005101.4_172000001	2	172000001	0.4542	0.6454
NC_005101.4_172100001	2	172100001	0.441	0.5958
NC_005101.4_172200001	2	172200001	0.416	0.4832
NC_005101.4_172300001	2	172300001	0.4188	0.449
NC_005101.4_172400001	2	172400001	0.4485	0.4857
NC_005101.4_172500001	2	172500001	0.4643	0.4595
NC_005101.4_172600001	2	172600001	0.5046	0.4819
NC_005101.4_172700001	2	172700001	0.6163	0.6145
NC_005101.4_172800001	2	172800001	0.5498	0.5722
NC_005101.4_172900001	2	172900001	0.5464	0.5983
NC_005101.4_173000001	2	173000001	0.5344	0.6176
NC_005101.4_173100001	2	173100001	0.4727	0.6025
NC_005101.4_173200001	2	173200001	0.3503	0.5485
NC_005101.4_173300001	2	173300001	0.5474	0.7939
NC_005101.4_173400001	2	173400001	0.4539	0.7111
NC_005101.4_173500001	2	173500001	0.5374	0.7519
NC_005101.4_173600001	2	173600001	0.5402	0.7165
NC_005101.4_173700001	2	173700001	0.6042	0.6953
NC_005101.4_173800001	2	173800001	0.539	0.547
NC_005101.4_173900001	2	173900001	0.5381	0.4991
NC_005101.4_174000001	2	174000001	0.4408	0.392
NC_005101.4_174100001	2	174100001	0.4519	0.4352
NC_005101.4_174200001	2	174200001	0.4083	0.3619
NC_005101.4_174300001	2	174300001	0.3942	0.4342
NC_005101.4_174400001	2	174400001	0.4163	0.407
NC_005101.4_174500001	2	174500001	0.3384	0.4238
NC_005101.4_174600001	2	174600001	0.229	0.2144
NC_005101.4_174700001	2	174700001	0.229	0.2144
NC_005101.4_174800001	2	174800001	0.2868	0.5325
NC_005101.4_175700001	2	175700001	0.532	0.7167
NC_005101.4_175800001	2	175800001	0.532	0.7167
NC_005101.4_176400001	2	176400001	0.3473	0.2876
NC_005101.4_176500001	2	176500001	0.3016	0.2783
NC_005101.4_176600001	2	176600001	0.3207	0.3792
NC_005101.4_176700001	2	176700001	0.3347	0.411
NC_005101.4_176800001	2	176800001	0.3822	0.4374
NC_005101.4_176900001	2	176900001	0.3216	0.5429
NC_005101.4_177000001	2	177000001	0.3901	0.4181
NC_005101.4_177100001	2	177100001	0.3727	0.3257
NC_005101.4_177200001	2	177200001	0.3386	0.3564
NC_005101.4_177300001	2	177300001	0.3303	0.3831
NC_005101.4_177400001	2	177400001	0.3564	0.3758
NC_005101.4_177500001	2	177500001	0.3567	0.4415
NC_005101.4_177600001	2	177600001	0.4118	0.5361
NC_005101.4_177700001	2	177700001	0.5451	0.6722
NC_005101.4_177800001	2	177800001	0.6356	0.7295
NC_005101.4_177900001	2	177900001	0.5654	0.7327
NC_005101.4_178000001	2	178000001	0.5838	0.7052
NC_005101.4_178100001	2	178100001	0.5938	0.6887
NC_005101.4_178200001	2	178200001	0.5127	0.661
NC_005101.4_178300001	2	178300001	0.4882	0.6203
NC_005101.4_178400001	2	178400001	0.5506	0.6541
NC_005101.4_178500001	2	178500001	0.5514	0.6677

NC_005101.4_178600001	2	178600001	0.5698	0.7126
NC_005101.4_178700001	2	178700001	0.6105	0.6844
NC_005101.4_178800001	2	178800001	0.591	0.7012
NC_005101.4_178900001	2	178900001	0.5743	0.6787
NC_005101.4_179000001	2	179000001	0.5953	0.7535
NC_005101.4_179100001	2	179100001	0.5528	0.6584
NC_005101.4_179200001	2	179200001	0.6106	0.6594
NC_005101.4_179300001	2	179300001	0.595	0.6811
NC_005101.4_179400001	2	179400001	0.5115	0.6509
NC_005101.4_179500001	2	179500001	0.477	0.6509
NC_005101.4_179600001	2	179600001	0.4644	0.5972
NC_005101.4_179700001	2	179700001	0.4184	0.5726
NC_005101.4_179800001	2	179800001	0.4207	0.5845
NC_005101.4_179900001	2	179900001	0.5319	0.6783
NC_005101.4_180000001	2	180000001	0.4751	0.6242
NC_005101.4_180100001	2	180100001	0.5084	0.825
NC_005101.4_180200001	2	180200001	0.5084	0.825
NC_005101.4_180300001	2	180300001	0.3795	0.6184
NC_005101.4_180400001	2	180400001	0.2474	0.3533
NC_005101.4_180500001	2	180500001	0.3495	0.5051
NC_005101.4_180600001	2	180600001	0.3495	0.5051
NC_005101.4_180700001	2	180700001	0.3644	0.454
NC_005101.4_180800001	2	180800001	0.4575	0.5694
NC_005101.4_180900001	2	180900001	0.6128	0.6647
NC_005101.4_181000001	2	181000001	0.5827	0.7009
NC_005101.4_181100001	2	181100001	0.5708	0.7116
NC_005101.4_181200001	2	181200001	0.5844	0.7519
NC_005101.4_181300001	2	181300001	0.5973	0.767
NC_005101.4_181400001	2	181400001	0.6069	0.8075
NC_005101.4_181500001	2	181500001	0.5773	0.7968
NC_005101.4_181600001	2	181600001	0.6059	0.6969
NC_005101.4_181700001	2	181700001	0.6305	0.698
NC_005101.4_181800001	2	181800001	0.5468	0.6583
NC_005101.4_181900001	2	181900001	0.5196	0.6003
NC_005101.4_182000001	2	182000001	0.5183	0.5191
NC_005101.4_182100001	2	182100001	0.5183	0.6283
NC_005101.4_182200001	2	182200001	0.5097	0.6414
NC_005101.4_182300001	2	182300001	0.5023	0.6132
NC_005101.4_182400001	2	182400001	0.5197	0.6295
NC_005101.4_182500001	2	182500001	0.6517	0.793
NC_005101.4_182600001	2	182600001	0.612	0.739
NC_005101.4_182700001	2	182700001	0.6182	0.7341
NC_005101.4_182800001	2	182800001	0.6873	0.7559
NC_005101.4_182900001	2	182900001	0.6155	0.7175
NC_005101.4_183000001	2	183000001	0.5712	0.6491
NC_005101.4_183100001	2	183100001	0.5412	0.6767
NC_005101.4_183200001	2	183200001	0.5007	0.6587
NC_005101.4_183300001	2	183300001	0.5616	0.6994
NC_005101.4_183400001	2	183400001	0.6118	0.7425
NC_005101.4_183500001	2	183500001	0.5645	0.7194
NC_005101.4_183600001	2	183600001	0.4844	0.6511
NC_005101.4_183700001	2	183700001	0.5453	0.6355
NC_005101.4_183800001	2	183800001	0.4637	0.627
NC_005101.4_183900001	2	183900001	0.4764	0.5936
NC_005101.4_184000001	2	184000001	0.4837	0.5974
NC_005101.4_184100001	2	184100001	0.5245	0.5909
NC_005101.4_184200001	2	184200001	0.4521	0.5792
NC_005101.4_184300001	2	184300001	0.5094	0.5845

NC_005101.4_184400001	2	184400001	0.4639	0.5839
NC_005101.4_184500001	2	184500001	0.4161	0.5754
NC_005101.4_184600001	2	184600001	0.4779	0.6295
NC_005101.4_184700001	2	184700001	0.62	0.7409
NC_005101.4_184800001	2	184800001	0.4993	0.6624
NC_005101.4_184900001	2	184900001	0.5383	0.7032
NC_005101.4_185000001	2	185000001	0.49	0.5946
NC_005101.4_185100001	2	185100001	0.4566	0.6006
NC_005101.4_185200001	2	185200001	0.4545	0.6017
NC_005101.4_185300001	2	185300001	0.4402	0.5624
NC_005101.4_185400001	2	185400001	0.433	0.6141
NC_005101.4_185500001	2	185500001	0.4665	0.7004
NC_005101.4_185600001	2	185600001	0.5856	0.7932
NC_005101.4_185700001	2	185700001	0.6603	0.7775
NC_005101.4_185800001	2	185800001	0.5782	0.6502
NC_005101.4_185900001	2	185900001	0.5246	0.563
NC_005101.4_186000001	2	186000001	0.4547	0.5408
NC_005101.4_186100001	2	186100001	0.4582	0.5438
NC_005101.4_186200001	2	186200001	0.4588	0.5333
NC_005101.4_186300001	2	186300001	0.4812	0.5965
NC_005101.4_186400001	2	186400001	0.5527	0.6649
NC_005101.4_186500001	2	186500001	0.6604	0.7251
NC_005101.4_186600001	2	186600001	0.6556	0.7191
NC_005101.4_186700001	2	186700001	0.6696	0.8185
NC_005101.4_186800001	2	186800001	0.6458	0.7794
NC_005101.4_186900001	2	186900001	0.6054	0.7662
NC_005101.4_187000001	2	187000001	0.5453	0.7044
NC_005101.4_187100001	2	187100001	0.5587	0.7327
NC_005101.4_187200001	2	187200001	0.527	0.7062
NC_005101.4_187300001	2	187300001	0.5662	0.7791
NC_005101.4_187400001	2	187400001	0.5191	0.7506
NC_005101.4_187500001	2	187500001	0.5228	0.7728
NC_005101.4_187600001	2	187600001	0.43	0.6242
NC_005101.4_187700001	2	187700001	0.5194	0.7181
NC_005101.4_187800001	2	187800001	0.4854	0.6762
NC_005101.4_187900001	2	187900001	0.5237	0.7197
NC_005101.4_188000001	2	188000001	0.595	0.7818
NC_005101.4_188100001	2	188100001	0.6359	0.8761
NC_005101.4_188200001	2	188200001	0.7081	0.8799
NC_005101.4_188300001	2	188300001	0.6403	0.8313
NC_005101.4_188400001	2	188400001	0.6749	0.8255
NC_005101.4_188500001	2	188500001	0.6868	0.8407
NC_005101.4_188600001	2	188600001	0.6807	0.7946
NC_005101.4_188700001	2	188700001	0.5751	0.7037
NC_005101.4_188800001	2	188800001	0.6262	0.7519
NC_005101.4_188900001	2	188900001	0.6335	0.7671
NC_005101.4_189000001	2	189000001	0.5481	0.688
NC_005101.4_189100001	2	189100001	0.5338	0.7063
NC_005101.4_189200001	2	189200001	0.7251	0.8883
NC_005101.4_189300001	2	189300001	0.7792	0.8851
NC_005101.4_189400001	2	189400001	0.7212	0.8481
NC_005101.4_189500001	2	189500001	0.6703	0.7552
NC_005101.4_189600001	2	189600001	0.701	0.7522
NC_005101.4_189700001	2	189700001	0.6998	0.7469
NC_005101.4_189800001	2	189800001	0.6403	0.6922
NC_005101.4_189900001	2	189900001	0.6454	0.6827
NC_005101.4_190000001	2	190000001	0.783	0.801
NC_005101.4_190800001	2	190800001	0.4104	0.4994

NC_005101.4_190900001	2	190900001	0.2658	0.2101
NC_005101.4_191000001	2	191000001	0.3386	0.2528
NC_005101.4_191100001	2	191100001	0.2859	0.2394
NC_005101.4_191200001	2	191200001	0.2859	0.2394
NC_005101.4_191600001	2	191600001	0.375	0.7405
NC_005101.4_191700001	2	191700001	0.5101	0.8386
NC_005101.4_191800001	2	191800001	0.5101	0.8386
NC_005101.4_191900001	2	191900001	0.5101	0.8386
NC_005101.4_192000001	2	192000001	0.5151	0.7388
NC_005101.4_192100001	2	192100001	0.4756	0.5904
NC_005101.4_192200001	2	192200001	0.2557	0.2489
NC_005101.4_192300001	2	192300001	0.3271	0.3634
NC_005101.4_192400001	2	192400001	0.3271	0.3634
NC_005101.4_192500001	2	192500001	0.2979	0.3877
NC_005101.4_192600001	2	192600001	0.3346	0.5258
NC_005101.4_192700001	2	192700001	0.3964	0.5702
NC_005101.4_192800001	2	192800001	0.4259	0.5063
NC_005101.4_192900001	2	192900001	0.3938	0.5269
NC_005101.4_193000001	2	193000001	0.4384	0.5559
NC_005101.4_193100001	2	193100001	0.3982	0.5163
NC_005101.4_193200001	2	193200001	0.4292	0.5657
NC_005101.4_193300001	2	193300001	0.4208	0.7174
NC_005101.4_193400001	2	193400001	0.454	0.7276
NC_005101.4_193500001	2	193500001	0.3984	0.6997
NC_005101.4_193600001	2	193600001	0.4112	0.6654
NC_005101.4_193700001	2	193700001	0.3726	0.6197
NC_005101.4_193800001	2	193800001	0.2795	0.4121
NC_005101.4_193900001	2	193900001	0.2773	0.2933
NC_005101.4_194000001	2	194000001	0.3772	0.3601
NC_005101.4_195200001	2	195200001	0.6952	0.881
NC_005101.4_195300001	2	195300001	0.6496	0.8523
NC_005101.4_195400001	2	195400001	0.6089	0.792
NC_005101.4_195500001	2	195500001	0.6093	0.793
NC_005101.4_195600001	2	195600001	0.5629	0.7641
NC_005101.4_195700001	2	195700001	0.5816	0.773
NC_005101.4_195800001	2	195800001	0.6178	0.8011
NC_005101.4_195900001	2	195900001	0.716	0.8501
NC_005101.4_196000001	2	196000001	0.7045	0.8408
NC_005101.4_196100001	2	196100001	0.7566	0.8609
NC_005101.4_196200001	2	196200001	0.7635	0.8643
NC_005101.4_196300001	2	196300001	0.7517	0.8353
NC_005101.4_196400001	2	196400001	0.727	0.8545
NC_005101.4_197400001	2	197400001	0.854	0.9183
NC_005101.4_197500001	2	197500001	0.8352	0.8632
NC_005101.4_197600001	2	197600001	0.6521	0.8402
NC_005101.4_197700001	2	197700001	0.657	0.8668
NC_005101.4_197800001	2	197800001	0.6154	0.7888
NC_005101.4_197900001	2	197900001	0.4211	0.6035
NC_005101.4_198000001	2	198000001	0.3971	0.6421
NC_005101.4_198100001	2	198100001	0.4127	0.6399
NC_005101.4_198200001	2	198200001	0.4393	0.6331
NC_005101.4_198300001	2	198300001	0.4919	0.7107
NC_005101.4_198400001	2	198400001	0.5488	0.7751
NC_005101.4_198500001	2	198500001	0.5704	0.7416
NC_005101.4_198600001	2	198600001	0.6885	0.8028
NC_005101.4_198700001	2	198700001	0.6259	0.7921
NC_005101.4_198800001	2	198800001	0.622	0.7722
NC_005101.4_198900001	2	198900001	0.6208	0.7615

NC_005101.4_199000001	2	199000001	0.605	0.7797
NC_005101.4_199100001	2	199100001	0.5472	0.7297
NC_005101.4_199200001	2	199200001	0.5271	0.6539
NC_005101.4_199300001	2	199300001	0.5302	0.6847
NC_005101.4_199400001	2	199400001	0.5514	0.7937
NC_005101.4_199500001	2	199500001	0.4994	0.7844
NC_005101.4_199600001	2	199600001	0.5784	0.7582
NC_005101.4_199700001	2	199700001	0.6239	0.7886
NC_005101.4_199800001	2	199800001	0.5756	0.7455
NC_005101.4_199900001	2	199900001	0.5433	0.7284
NC_005101.4_200000001	2	200000001	0.5435	0.7102
NC_005101.4_200100001	2	200100001	0.4933	0.717
NC_005101.4_200200001	2	200200001	0.464	0.7051
NC_005101.4_200300001	2	200300001	0.4839	0.7121
NC_005101.4_200400001	2	200400001	0.5096	0.6071
NC_005101.4_200500001	2	200500001	0.6894	0.7353
NC_005101.4_200600001	2	200600001	0.6462	0.6494
NC_005101.4_200700001	2	200700001	0.5622	0.5398
NC_005101.4_200800001	2	200800001	0.5177	0.5744
NC_005101.4_200900001	2	200900001	0.4072	0.4167
NC_005101.4_201000001	2	201000001	0.398	0.395
NC_005101.4_201100001	2	201100001	0.374	0.3364
NC_005101.4_201200001	2	201200001	0.3747	0.3918
NC_005101.4_201300001	2	201300001	0.447	0.3997
NC_005101.4_201400001	2	201400001	0.5596	0.5928
NC_005101.4_201500001	2	201500001	0.7016	0.6797
NC_005101.4_201600001	2	201600001	0.5292	0.6054
NC_005101.4_201700001	2	201700001	0.5441	0.6234
NC_005101.4_201800001	2	201800001	0.5665	0.6487
NC_005101.4_201900001	2	201900001	0.6032	0.6967
NC_005101.4_202000001	2	202000001	0.6381	0.7735
NC_005101.4_202100001	2	202100001	0.6399	0.7581
NC_005101.4_202200001	2	202200001	0.6421	0.7745
NC_005101.4_202300001	2	202300001	0.6195	0.7242
NC_005101.4_202400001	2	202400001	0.6799	0.778
NC_005101.4_202500001	2	202500001	0.6471	0.7525
NC_005101.4_202600001	2	202600001	0.7337	0.7977
NC_005101.4_202700001	2	202700001	0.736	0.7589
NC_005101.4_202800001	2	202800001	0.7657	0.7697
NC_005101.4_202900001	2	202900001	0.5583	0.6065
NC_005101.4_203000001	2	203000001	0.4803	0.5534
NC_005101.4_203100001	2	203100001	0.4087	0.4784
NC_005101.4_203200001	2	203200001	0.3378	0.4211
NC_005101.4_203300001	2	203300001	0.3087	0.4026
NC_005101.4_203400001	2	203400001	0.3854	0.5185
NC_005101.4_203500001	2	203500001	0.3839	0.5221
NC_005101.4_203600001	2	203600001	0.4248	0.598
NC_005101.4_203700001	2	203700001	0.4886	0.6577
NC_005101.4_203800001	2	203800001	0.477	0.6247
NC_005101.4_203900001	2	203900001	0.4696	0.5666
NC_005101.4_204000001	2	204000001	0.5106	0.5873
NC_005101.4_204100001	2	204100001	0.5292	0.5726
NC_005101.4_204200001	2	204200001	0.4899	0.5506
NC_005101.4_204300001	2	204300001	0.4979	0.602
NC_005101.4_204400001	2	204400001	0.4894	0.5663
NC_005101.4_204500001	2	204500001	0.4602	0.5617
NC_005101.4_204600001	2	204600001	0.401	0.5034
NC_005101.4_204700001	2	204700001	0.4518	0.5559

NC_005101.4_204800001	2	204800001	0.4892	0.5559
NC_005101.4_204900001	2	204900001	0.4765	0.5875
NC_005101.4_205000001	2	205000001	0.5425	0.6285
NC_005101.4_205100001	2	205100001	0.5975	0.7024
NC_005101.4_205200001	2	205200001	0.6383	0.763
NC_005101.4_205300001	2	205300001	0.4504	0.696
NC_005101.4_205400001	2	205400001	0.4314	0.6212
NC_005101.4_205500001	2	205500001	0.3826	0.5602
NC_005101.4_205600001	2	205600001	0.3903	0.5161
NC_005101.4_205700001	2	205700001	0.4082	0.5276
NC_005101.4_205800001	2	205800001	0.4375	0.5603
NC_005101.4_205900001	2	205900001	0.4288	0.549
NC_005101.4_206000001	2	206000001	0.4142	0.5341
NC_005101.4_206100001	2	206100001	0.5591	0.705
NC_005101.4_206200001	2	206200001	0.4927	0.749
NC_005101.4_206300001	2	206300001	0.5593	0.6742
NC_005101.4_206400001	2	206400001	0.5446	0.6087
NC_005101.4_206500001	2	206500001	0.5852	0.6686
NC_005101.4_206600001	2	206600001	0.5064	0.5849
NC_005101.4_206700001	2	206700001	0.4963	0.5461
NC_005101.4_206800001	2	206800001	0.5081	0.5881
NC_005101.4_206900001	2	206900001	0.5617	0.708
NC_005101.4_207000001	2	207000001	0.6034	0.69
NC_005101.4_207100001	2	207100001	0.5875	0.7131
NC_005101.4_207200001	2	207200001	0.6129	0.7333
NC_005101.4_207300001	2	207300001	0.615	0.7291
NC_005101.4_207400001	2	207400001	0.5672	0.6687
NC_005101.4_207500001	2	207500001	0.5951	0.6873
NC_005101.4_207600001	2	207600001	0.5957	0.6875
NC_005101.4_207700001	2	207700001	0.6086	0.6795
NC_005101.4_207800001	2	207800001	0.5641	0.6351
NC_005101.4_207900001	2	207900001	0.615	0.695
NC_005101.4_208000001	2	208000001	0.5157	0.6093
NC_005101.4_208100001	2	208100001	0.4801	0.5587
NC_005101.4_208200001	2	208200001	0.5293	0.668
NC_005101.4_208300001	2	208300001	0.5863	0.7256
NC_005101.4_208400001	2	208400001	0.4826	0.6846
NC_005101.4_208500001	2	208500001	0.4826	0.6846
NC_005101.4_208600001	2	208600001	0.6299	0.7657
NC_005101.4_208700001	2	208700001	0.4864	0.602
NC_005101.4_208800001	2	208800001	0.48	0.5514
NC_005101.4_208900001	2	208900001	0.439	0.4477
NC_005101.4_209000001	2	209000001	0.439	0.4477
NC_005101.4_209100001	2	209100001	0.3843	0.4359
NC_005101.4_209200001	2	209200001	0.4013	0.4586
NC_005101.4_209300001	2	209300001	0.4228	0.652
NC_005101.4_209400001	2	209400001	0.4548	0.6592
NC_005101.4_209500001	2	209500001	0.5164	0.6316
NC_005101.4_209600001	2	209600001	0.5857	0.6923
NC_005101.4_209700001	2	209700001	0.5765	0.7081
NC_005101.4_209800001	2	209800001	0.6116	0.7079
NC_005101.4_209900001	2	209900001	0.5888	0.7354
NC_005101.4_210000001	2	210000001	0.5845	0.8113
NC_005101.4_210100001	2	210100001	0.6043	0.8186
NC_005101.4_210200001	2	210200001	0.5549	0.7288
NC_005101.4_210300001	2	210300001	0.5583	0.6851
NC_005101.4_210400001	2	210400001	0.5821	0.6613
NC_005101.4_210500001	2	210500001	0.5343	0.585

NC_005101.4_210600001	2	210600001	0.5486	0.5884
NC_005101.4_210700001	2	210700001	0.5641	0.65
NC_005101.4_210800001	2	210800001	0.5682	0.6604
NC_005101.4_210900001	2	210900001	0.5784	0.6553
NC_005101.4_211000001	2	211000001	0.642	0.7194
NC_005101.4_211100001	2	211100001	0.6538	0.739
NC_005101.4_211200001	2	211200001	0.6816	0.7342
NC_005101.4_211300001	2	211300001	0.6877	0.7451
NC_005101.4_211400001	2	211400001	0.7267	0.8046
NC_005101.4_211500001	2	211500001	0.6581	0.7816
NC_005101.4_211600001	2	211600001	0.6792	0.8177
NC_005101.4_211700001	2	211700001	0.7429	0.801
NC_005101.4_211800001	2	211800001	0.8173	0.859
NC_005101.4_211900001	2	211900001	0.799	0.8419
NC_005101.4_212000001	2	212000001	0.6698	0.7601
NC_005101.4_212100001	2	212100001	0.5566	0.6829
NC_005101.4_212200001	2	212200001	0.5973	0.7496
NC_005101.4_212300001	2	212300001	0.4231	0.6261
NC_005101.4_212400001	2	212400001	0.3548	0.542
NC_005101.4_213100001	2	213100001	0.4631	0.6981
NC_005101.4_213200001	2	213200001	0.4631	0.6981
NC_005101.4_213300001	2	213300001	0.4127	0.658
NC_005101.4_213400001	2	213400001	0.4141	0.6357
NC_005101.4_213500001	2	213500001	0.5085	0.7365
NC_005101.4_213600001	2	213600001	0.5364	0.6434
NC_005101.4_213700001	2	213700001	0.5364	0.6434
NC_005101.4_213800001	2	213800001	0.4884	0.6035
NC_005101.4_213900001	2	213900001	0.5198	0.6526
NC_005101.4_214000001	2	214000001	0.4068	0.5054
NC_005101.4_214200001	2	214200001	0.3567	0.5747
NC_005101.4_214300001	2	214300001	0.494	0.7433
NC_005101.4_214500001	2	214500001	0.4081	0.5358
NC_005101.4_214600001	2	214600001	0.3979	0.4791
NC_005101.4_214700001	2	214700001	0.5331	0.539
NC_005101.4_214800001	2	214800001	0.5316	0.4886
NC_005101.4_214900001	2	214900001	0.5957	0.5582
NC_005101.4_215000001	2	215000001	0.6029	0.5627
NC_005101.4_215100001	2	215100001	0.6242	0.5878
NC_005101.4_215200001	2	215200001	0.6368	0.5979
NC_005101.4_215300001	2	215300001	0.6368	0.5979
NC_005101.4_215400001	2	215400001	0.5418	0.5032
NC_005101.4_215600001	2	215600001	0.7812	0.7517
NC_005101.4_216000001	2	216000001	0.7675	0.8154
NC_005101.4_216300001	2	216300001	0.5746	0.803
NC_005101.4_216400001	2	216400001	0.6484	0.8425
NC_005101.4_216500001	2	216500001	0.6305	0.9563
NC_005101.4_216600001	2	216600001	0.6777	0.8263
NC_005101.4_216700001	2	216700001	0.6649	0.7452
NC_005101.4_216800001	2	216800001	0.4871	0.5337
NC_005101.4_216900001	2	216900001	0.4871	0.5337
NC_005101.4_217000001	2	217000001	0.3556	0.4529
NC_005101.4_217100001	2	217100001	0.3085	0.4306
NC_005101.4_217200001	2	217200001	0.339	0.5304
NC_005101.4_217300001	2	217300001	0.3502	0.5835
NC_005101.4_217400001	2	217400001	0.3356	0.5661
NC_005101.4_217500001	2	217500001	0.3806	0.62
NC_005101.4_217600001	2	217600001	0.4193	0.6665
NC_005101.4_217700001	2	217700001	0.4284	0.7571

NC_005101.4_217800001	2	217800001	0.3965	0.7151
NC_005101.4_217900001	2	217900001	0.4517	0.774
NC_005101.4_218000001	2	218000001	0.5646	0.7846
NC_005101.4_218100001	2	218100001	0.504	0.7336
NC_005101.4_218200001	2	218200001	0.4078	0.4869
NC_005101.4_218300001	2	218300001	0.4282	0.4985
NC_005101.4_218400001	2	218400001	0.3978	0.5219
NC_005101.4_218500001	2	218500001	0.3225	0.3582
NC_005101.4_218600001	2	218600001	0.3526	0.4111
NC_005101.4_218700001	2	218700001	0.3847	0.4657
NC_005101.4_218800001	2	218800001	0.4226	0.5189
NC_005101.4_218900001	2	218900001	0.537	0.614
NC_005101.4_219000001	2	219000001	0.6636	0.7527
NC_005101.4_219100001	2	219100001	0.6076	0.6702
NC_005101.4_219200001	2	219200001	0.5952	0.6785
NC_005101.4_219300001	2	219300001	0.482	0.5918
NC_005101.4_219400001	2	219400001	0.3754	0.4435
NC_005101.4_219500001	2	219500001	0.5511	0.6894
NC_005101.4_219600001	2	219600001	0.5707	0.7726
NC_005101.4_219700001	2	219700001	0.5375	0.678
NC_005101.4_219800001	2	219800001	0.6454	0.7613
NC_005101.4_219900001	2	219900001	0.6427	0.7588
NC_005101.4_220000001	2	220000001	0.5716	0.7043
NC_005101.4_220100001	2	220100001	0.5716	0.7043
NC_005101.4_220200001	2	220200001	0.587	0.757
NC_005101.4_220300001	2	220300001	0.7677	0.7666
NC_005101.4_220400001	2	220400001	0.7114	0.6704
NC_005101.4_220500001	2	220500001	0.6763	0.6927
NC_005101.4_220600001	2	220600001	0.6506	0.6188
NC_005101.4_220700001	2	220700001	0.703	0.6438
NC_005101.4_220800001	2	220800001	0.5048	0.3887
NC_005101.4_220900001	2	220900001	0.4966	0.475
NC_005101.4_221000001	2	221000001	0.437	0.4634
NC_005101.4_221100001	2	221100001	0.4124	0.5154
NC_005101.4_221200001	2	221200001	0.3733	0.5046
NC_005101.4_221300001	2	221300001	0.4136	0.6995
NC_005101.4_221400001	2	221400001	0.3479	0.528
NC_005101.4_221500001	2	221500001	0.3448	0.4757
NC_005101.4_221600001	2	221600001	0.3753	0.5441
NC_005101.4_221700001	2	221700001	0.3992	0.5512
NC_005101.4_221800001	2	221800001	0.3924	0.5853
NC_005101.4_221900001	2	221900001	0.4837	0.7505
NC_005101.4_222000001	2	222000001	0.4837	0.7505
NC_005101.4_222100001	2	222100001	0.4135	0.6767
NC_005101.4_222200001	2	222200001	0.4787	0.6083
NC_005101.4_222300001	2	222300001	0.4668	0.5455
NC_005101.4_222400001	2	222400001	0.4719	0.5544
NC_005101.4_222500001	2	222500001	0.4719	0.5544
NC_005101.4_222600001	2	222600001	0.5127	0.5608
NC_005101.4_222700001	2	222700001	0.5028	0.551
NC_005101.4_222800001	2	222800001	0.5075	0.6263
NC_005101.4_222900001	2	222900001	0.5865	0.7146
NC_005101.4_223000001	2	223000001	0.5794	0.6946
NC_005101.4_223100001	2	223100001	0.5584	0.6718
NC_005101.4_223200001	2	223200001	0.5344	0.6875
NC_005101.4_223300001	2	223300001	0.5354	0.6676
NC_005101.4_223400001	2	223400001	0.503	0.6305
NC_005101.4_223500001	2	223500001	0.5479	0.5995

NC_005101.4_223600001	2	223600001	0.535	0.6412
NC_005101.4_223700001	2	223700001	0.5483	0.6644
NC_005101.4_223800001	2	223800001	0.5612	0.6761
NC_005101.4_223900001	2	223900001	0.5196	0.6213
NC_005101.4_224000001	2	224000001	0.462	0.6747
NC_005101.4_224100001	2	224100001	0.4198	0.6097
NC_005101.4_224200001	2	224200001	0.4172	0.6446
NC_005101.4_224300001	2	224300001	0.3339	0.5368
NC_005101.4_224400001	2	224400001	0.3977	0.5746
NC_005101.4_224500001	2	224500001	0.4478	0.6111
NC_005101.4_224600001	2	224600001	0.4114	0.5722
NC_005101.4_224700001	2	224700001	0.4038	0.4869
NC_005101.4_224800001	2	224800001	0.4855	0.579
NC_005101.4_224900001	2	224900001	0.5439	0.6466
NC_005101.4_225000001	2	225000001	0.5337	0.654
NC_005101.4_225100001	2	225100001	0.6095	0.6952
NC_005101.4_225200001	2	225200001	0.6764	0.7589
NC_005101.4_225300001	2	225300001	0.6875	0.742
NC_005101.4_225400001	2	225400001	0.5251	0.6994
NC_005101.4_225500001	2	225500001	0.5811	0.7129
NC_005101.4_225600001	2	225600001	0.5811	0.7129
NC_005101.4_225700001	2	225700001	0.6166	0.7538
NC_005101.4_225800001	2	225800001	0.5249	0.6892
NC_005101.4_225900001	2	225900001	0.6264	0.7253
NC_005101.4_226100001	2	226100001	0.5635	0.6951
NC_005101.4_226400001	2	226400001	0.5938	0.7042
NC_005101.4_226500001	2	226500001	0.6144	0.6809
NC_005101.4_226600001	2	226600001	0.5343	0.5489
NC_005101.4_226700001	2	226700001	0.528	0.5835
NC_005101.4_226800001	2	226800001	0.5264	0.588
NC_005101.4_226900001	2	226900001	0.5907	0.6729
NC_005101.4_227000001	2	227000001	0.5846	0.7006
NC_005101.4_227100001	2	227100001	0.5768	0.7403
NC_005101.4_227200001	2	227200001	0.5515	0.7124
NC_005101.4_227300001	2	227300001	0.569	0.7232
NC_005101.4_227400001	2	227400001	0.5538	0.6996
NC_005101.4_227500001	2	227500001	0.4404	0.621
NC_005101.4_227600001	2	227600001	0.4726	0.6322
NC_005101.4_227700001	2	227700001	0.5093	0.6385
NC_005101.4_227800001	2	227800001	0.4211	0.5922
NC_005101.4_227900001	2	227900001	0.3762	0.5417
NC_005101.4_228000001	2	228000001	0.5271	0.6659
NC_005101.4_228100001	2	228100001	0.6181	0.7271
NC_005101.4_228200001	2	228200001	0.6066	0.7252
NC_005101.4_228300001	2	228300001	0.6852	0.7111
NC_005101.4_228400001	2	228400001	0.7046	0.7002
NC_005101.4_228500001	2	228500001	0.6556	0.6751
NC_005101.4_228600001	2	228600001	0.6138	0.6136
NC_005101.4_228700001	2	228700001	0.6012	0.6462
NC_005101.4_228800001	2	228800001	0.5463	0.7114
NC_005101.4_228900001	2	228900001	0.4246	0.7107
NC_005101.4_229000001	2	229000001	0.4373	0.6623
NC_005101.4_229100001	2	229100001	0.3997	0.6426
NC_005101.4_229200001	2	229200001	0.3819	0.5793
NC_005101.4_229300001	2	229300001	0.4093	0.5796
NC_005101.4_229400001	2	229400001	0.4607	0.6093
NC_005101.4_229500001	2	229500001	0.4286	0.5567
NC_005101.4_229600001	2	229600001	0.3984	0.5396

NC_005101.4_229700001	2	229700001	0.4217	0.5638
NC_005101.4_229800001	2	229800001	0.3435	0.4655
NC_005101.4_229900001	2	229900001	0.483	0.5962
NC_005101.4_230000001	2	230000001	0.5071	0.6257
NC_005101.4_230100001	2	230100001	0.4974	0.5738
NC_005101.4_230200001	2	230200001	0.4881	0.5393
NC_005101.4_230300001	2	230300001	0.5178	0.5418
NC_005101.4_230400001	2	230400001	0.51	0.5262
NC_005101.4_230500001	2	230500001	0.4911	0.4995
NC_005101.4_230600001	2	230600001	0.647	0.6389
NC_005101.4_230700001	2	230700001	0.7236	0.6897
NC_005101.4_230800001	2	230800001	0.7869	0.7311
NC_005101.4_230900001	2	230900001	0.6689	0.6373
NC_005101.4_231000001	2	231000001	0.5997	0.5921
NC_005101.4_231100001	2	231100001	0.53	0.5822
NC_005101.4_231200001	2	231200001	0.4843	0.5111
NC_005101.4_231300001	2	231300001	0.4502	0.4882
NC_005101.4_231400001	2	231400001	0.427	0.5197
NC_005101.4_231500001	2	231500001	0.3928	0.5444
NC_005101.4_231600001	2	231600001	0.3672	0.4683
NC_005101.4_231700001	2	231700001	0.3865	0.5474
NC_005101.4_231800001	2	231800001	0.5129	0.7185
NC_005101.4_231900001	2	231900001	0.5483	0.718
NC_005101.4_232000001	2	232000001	0.6609	0.6892
NC_005101.4_232100001	2	232100001	0.6499	0.687
NC_005101.4_232200001	2	232200001	0.6565	0.6901
NC_005101.4_232300001	2	232300001	0.5734	0.5794
NC_005101.4_232400001	2	232400001	0.5677	0.6053
NC_005101.4_232500001	2	232500001	0.5991	0.6411
NC_005101.4_232600001	2	232600001	0.6358	0.6944
NC_005101.4_232700001	2	232700001	0.6297	0.7198
NC_005101.4_232800001	2	232800001	0.5731	0.7169
NC_005101.4_232900001	2	232900001	0.5766	0.6362
NC_005101.4_233000001	2	233000001	0.5024	0.6344
NC_005101.4_233100001	2	233100001	0.454	0.5788
NC_005101.4_233200001	2	233200001	0.4591	0.6127
NC_005101.4_233300001	2	233300001	0.425	0.5652
NC_005101.4_233400001	2	233400001	0.4112	0.6111
NC_005101.4_233500001	2	233500001	0.3943	0.5917
NC_005101.4_233600001	2	233600001	0.3801	0.6167
NC_005101.4_234300001	2	234300001	0.3856	0.4659
NC_005101.4_234400001	2	234400001	0.4766	0.568
NC_005101.4_234500001	2	234500001	0.4766	0.568
NC_005101.4_234600001	2	234600001	0.4766	0.568
NC_005101.4_234700001	2	234700001	0.4733	0.5194
NC_005101.4_234800001	2	234800001	0.5103	0.5114
NC_005101.4_234900001	2	234900001	0.2539	0.2729
NC_005101.4_235000001	2	235000001	0.3901	0.5102
NC_005101.4_235100001	2	235100001	0.3501	0.4845
NC_005101.4_235200001	2	235200001	0.3337	0.4648
NC_005101.4_235300001	2	235300001	0.2752	0.4722
NC_005101.4_235400001	2	235400001	0.3509	0.5544
NC_005101.4_235500001	2	235500001	0.2787	0.4146
NC_005101.4_235600001	2	235600001	0.4083	0.5506
NC_005101.4_235700001	2	235700001	0.4422	0.5577
NC_005101.4_235800001	2	235800001	0.5238	0.5877
NC_005101.4_235900001	2	235900001	0.4819	0.56
NC_005101.4_236000001	2	236000001	0.4631	0.6038

NC_005101.4_236100001	2	236100001	0.473	0.6578
NC_005101.4_236200001	2	236200001	0.4367	0.6804
NC_005101.4_236300001	2	236300001	0.4471	0.7429
NC_005101.4_236400001	2	236400001	0.4645	0.7165
NC_005101.4_236500001	2	236500001	0.57	0.8243
NC_005101.4_236600001	2	236600001	0.4917	0.5929
NC_005101.4_236700001	2	236700001	0.5373	0.619
NC_005101.4_236800001	2	236800001	0.4768	0.5452
NC_005101.4_236900001	2	236900001	0.441	0.5171
NC_005101.4_237000001	2	237000001	0.4261	0.4654
NC_005101.4_237100001	2	237100001	0.4121	0.4749
NC_005101.4_237200001	2	237200001	0.4169	0.4463
NC_005101.4_237300001	2	237300001	0.444	0.4619
NC_005101.4_237400001	2	237400001	0.4357	0.5205
NC_005101.4_237500001	2	237500001	0.4157	0.5278
NC_005101.4_237600001	2	237600001	0.3859	0.581
NC_005101.4_237700001	2	237700001	0.3085	0.5124
NC_005101.4_237800001	2	237800001	0.3085	0.5124
NC_005101.4_237900001	2	237900001	0.4061	0.5401
NC_005101.4_238000001	2	238000001	0.3315	0.5342
NC_005101.4_238100001	2	238100001	0.6009	0.6912
NC_005101.4_238200001	2	238200001	0.7211	0.7612
NC_005101.4_238300001	2	238300001	0.7199	0.8081
NC_005101.4_238400001	2	238400001	0.7247	0.7887
NC_005101.4_238500001	2	238500001	0.7882	0.7695
NC_005101.4_238600001	2	238600001	0.6962	0.7155
NC_005101.4_238700001	2	238700001	0.703	0.746
NC_005101.4_238800001	2	238800001	0.7119	0.7273
NC_005101.4_238900001	2	238900001	0.6832	0.6855
NC_005101.4_239000001	2	239000001	0.6736	0.7033
NC_005101.4_239100001	2	239100001	0.7118	0.7393
NC_005101.4_239200001	2	239200001	0.6438	0.6058
NC_005101.4_239300001	2	239300001	0.5889	0.5275
NC_005101.4_239400001	2	239400001	0.4443	0.5822
NC_005101.4_239500001	2	239500001	0.377	0.4265
NC_005101.4_239600001	2	239600001	0.3344	0.3517
NC_005101.4_239700001	2	239700001	0.3365	0.3768
NC_005101.4_239800001	2	239800001	0.3565	0.3763
NC_005101.4_239900001	2	239900001	0.3444	0.3288
NC_005101.4_240000001	2	240000001	0.3711	0.428
NC_005101.4_240100001	2	240100001	0.4163	0.5413
NC_005101.4_240200001	2	240200001	0.4515	0.5889
NC_005101.4_240300001	2	240300001	0.551	0.8063
NC_005101.4_240400001	2	240400001	0.5649	0.7911
NC_005101.4_240500001	2	240500001	0.5171	0.7608
NC_005101.4_240600001	2	240600001	0.4895	0.7501
NC_005101.4_240700001	2	240700001	0.4694	0.7489
NC_005101.4_240800001	2	240800001	0.3526	0.6145
NC_005101.4_240900001	2	240900001	0.2371	0.5151
NC_005101.4_241000001	2	241000001	0.2267	0.3497
NC_005101.4_241100001	2	241100001	0.2647	0.3567
NC_005101.4_241200001	2	241200001	0.3566	0.4472
NC_005101.4_241300001	2	241300001	0.4488	0.5297
NC_005101.4_241400001	2	241400001	0.532	0.6109
NC_005101.4_241500001	2	241500001	0.6653	0.7475
NC_005101.4_241600001	2	241600001	0.7314	0.8586
NC_005101.4_241700001	2	241700001	0.5311	0.6878
NC_005101.4_241800001	2	241800001	0.5534	0.6939

NC_005101.4_241900001	2	241900001	0.5648	0.6933
NC_005101.4_242000001	2	242000001	0.57	0.7024
NC_005101.4_242100001	2	242100001	0.4283	0.5346
NC_005101.4_242200001	2	242200001	0.4369	0.7486
NC_005101.4_242300001	2	242300001	0.4769	0.7608
NC_005101.4_242400001	2	242400001	0.4344	0.6557
NC_005101.4_242500001	2	242500001	0.4887	0.6716
NC_005101.4_242600001	2	242600001	0.4468	0.5965
NC_005101.4_242700001	2	242700001	0.4811	0.5657
NC_005101.4_242800001	2	242800001	0.4816	0.5438
NC_005101.4_242900001	2	242900001	0.4631	0.5678
NC_005101.4_243000001	2	243000001	0.4168	0.5191
NC_005101.4_243100001	2	243100001	0.5701	0.6556
NC_005101.4_243200001	2	243200001	0.6646	0.7393
NC_005101.4_243300001	2	243300001	0.6611	0.7739
NC_005101.4_243400001	2	243400001	0.7293	0.7969
NC_005101.4_243500001	2	243500001	0.7124	0.7791
NC_005101.4_243600001	2	243600001	0.725	0.8233
NC_005101.4_243700001	2	243700001	0.6252	0.7579
NC_005101.4_243800001	2	243800001	0.5807	0.6868
NC_005101.4_243900001	2	243900001	0.5207	0.606
NC_005101.4_244000001	2	244000001	0.5117	0.6222
NC_005101.4_244100001	2	244100001	0.4495	0.528
NC_005101.4_244200001	2	244200001	0.5109	0.624
NC_005101.4_244300001	2	244300001	0.4944	0.5615
NC_005101.4_244400001	2	244400001	0.4976	0.6392
NC_005101.4_244500001	2	244500001	0.4963	0.6106
NC_005101.4_244600001	2	244600001	0.4295	0.5379
NC_005101.4_244700001	2	244700001	0.3407	0.3971
NC_005101.4_244800001	2	244800001	0.4308	0.5947
NC_005101.4_244900001	2	244900001	0.4517	0.5374
NC_005101.4_245000001	2	245000001	0.4107	0.5073
NC_005101.4_245100001	2	245100001	0.465	0.5157
NC_005101.4_245200001	2	245200001	0.4775	0.6056
NC_005101.4_245300001	2	245300001	0.3011	0.3934
NC_005101.4_245400001	2	245400001	0.3836	0.5194
NC_005101.4_245500001	2	245500001	0.424	0.5276
NC_005101.4_245600001	2	245600001	0.4589	0.5345
NC_005101.4_245700001	2	245700001	0.4633	0.4439
NC_005101.4_245800001	2	245800001	0.5677	0.5124
NC_005101.4_245900001	2	245900001	0.4947	0.3957
NC_005101.4_246000001	2	246000001	0.5038	0.4007
NC_005101.4_246200001	2	246200001	0.5779	0.5431
NC_005101.4_246300001	2	246300001	0.5905	0.65
NC_005101.4_246400001	2	246400001	0.6208	0.7213
NC_005101.4_246500001	2	246500001	0.5885	0.7565
NC_005101.4_246600001	2	246600001	0.6005	0.764
NC_005101.4_246700001	2	246700001	0.5603	0.8353
NC_005101.4_246800001	2	246800001	0.5172	0.8125
NC_005101.4_246900001	2	246900001	0.5172	0.8125
NC_005101.4_247000001	2	247000001	0.3531	0.617
NC_005101.4_247100001	2	247100001	0.2981	0.5425
NC_005101.4_247200001	2	247200001	0.2666	0.4525
NC_005101.4_247300001	2	247300001	0.274	0.4189
NC_005101.4_247400001	2	247400001	0.309	0.4563
NC_005101.4_247500001	2	247500001	0.3741	0.5082
NC_005101.4_247600001	2	247600001	0.3303	0.3384
NC_005101.4_247700001	2	247700001	0.3032	0.3162

NC_005101.4_247800001	2	247800001	0.29	0.3002
NC_005101.4_247900001	2	247900001	0.2741	0.3097
NC_005101.4_248000001	2	248000001	0.234	0.2886
NC_005101.4_248100001	2	248100001	0.2671	0.3025
NC_005101.4_248200001	2	248200001	0.2971	0.3339
NC_005101.4_248300001	2	248300001	0.3853	0.5594
NC_005101.4_248400001	2	248400001	0.3101	0.4649
NC_005101.4_248500001	2	248500001	0.4903	0.5293
NC_005101.4_248600001	2	248600001	0.5429	0.6568
NC_005101.4_248700001	2	248700001	0.476	0.6275
NC_005101.4_248800001	2	248800001	0.6214	0.7199
NC_005101.4_248900001	2	248900001	0.5942	0.6923
NC_005101.4_249000001	2	249000001	0.5421	0.7456
NC_005101.4_249100001	2	249100001	0.4961	0.7024
NC_005101.4_249200001	2	249200001	0.5507	0.7193
NC_005101.4_249300001	2	249300001	0.2707	0.3827
NC_005101.4_249400001	2	249400001	0.4011	0.6507
NC_005101.4_249500001	2	249500001	0.4573	0.7228
NC_005101.4_249600001	2	249600001	0.4627	0.7085
NC_005101.4_249700001	2	249700001	0.5054	0.7555
NC_005101.4_249800001	2	249800001	0.5435	0.7023
NC_005101.4_249900001	2	249900001	0.5076	0.6772
NC_005101.4_250000001	2	250000001	0.4522	0.6038
NC_005101.4_250100001	2	250100001	0.5572	0.6854
NC_005101.4_250200001	2	250200001	0.5104	0.6454
NC_005101.4_250300001	2	250300001	0.4907	0.6266
NC_005101.4_250400001	2	250400001	0.5985	0.5555
NC_005101.4_250500001	2	250500001	0.6522	0.5828
NC_005101.4_250600001	2	250600001	0.5492	0.4767
NC_005101.4_250700001	2	250700001	0.5531	0.4734
NC_005101.4_250800001	2	250800001	0.5518	0.5166
NC_005101.4_250900001	2	250900001	0.4051	0.5114
NC_005101.4_251000001	2	251000001	0.3927	0.5296
NC_005101.4_251100001	2	251100001	0.4371	0.5779
NC_005101.4_251200001	2	251200001	0.4416	0.56
NC_005101.4_251300001	2	251300001	0.4346	0.5182
NC_005101.4_251400001	2	251400001	0.5057	0.5906
NC_005101.4_251500001	2	251500001	0.5599	0.6893
NC_005101.4_251600001	2	251600001	0.5589	0.6506
NC_005101.4_251700001	2	251700001	0.5419	0.626
NC_005101.4_251800001	2	251800001	0.5818	0.7114
NC_005101.4_251900001	2	251900001	0.6047	0.7186
NC_005101.4_252000001	2	252000001	0.4851	0.5633
NC_005101.4_252100001	2	252100001	0.4424	0.5648
NC_005101.4_252200001	2	252200001	0.4508	0.5877
NC_005101.4_252300001	2	252300001	0.4333	0.551
NC_005101.4_252400001	2	252400001	0.4679	0.5746
NC_005101.4_252500001	2	252500001	0.5593	0.6362
NC_005101.4_252600001	2	252600001	0.5578	0.6397
NC_005101.4_252700001	2	252700001	0.6235	0.7154
NC_005101.4_252800001	2	252800001	0.5557	0.6631
NC_005101.4_252900001	2	252900001	0.4799	0.5721
NC_005101.4_253000001	2	253000001	0.4479	0.5218
NC_005101.4_253100001	2	253100001	0.498	0.565
NC_005101.4_253200001	2	253200001	0.4285	0.4748
NC_005101.4_253300001	2	253300001	0.4914	0.5341
NC_005101.4_253400001	2	253400001	0.5323	0.6102
NC_005101.4_253500001	2	253500001	0.4817	0.6846

NC_005101.4_253600001	2	253600001	0.4255	0.6495
NC_005101.4_253700001	2	253700001	0.4351	0.7154
NC_005101.4_253800001	2	253800001	0.4233	0.6663
NC_005101.4_253900001	2	253900001	0.3874	0.5079
NC_005101.4_254000001	2	254000001	0.3572	0.3323
NC_005101.4_254100001	2	254100001	0.3008	0.2948
NC_005101.4_254200001	2	254200001	0.3338	0.3576
NC_005101.4_254300001	2	254300001	0.2772	0.2672
NC_005101.4_254400001	2	254400001	0.236	0.2572
NC_005101.4_254500001	2	254500001	0.3655	0.5602
NC_005101.4_254600001	2	254600001	0.4827	0.6895
NC_005101.4_254700001	2	254700001	0.4392	0.7029
NC_005101.4_254800001	2	254800001	0.4881	0.7767
NC_005101.4_254900001	2	254900001	0.5061	0.7804
NC_005101.4_255000001	2	255000001	0.4481	0.6729
NC_005101.4_255100001	2	255100001	0.398	0.5631
NC_005101.4_255200001	2	255200001	0.5945	0.7172
NC_005101.4_255300001	2	255300001	0.559	0.611
NC_005101.4_255400001	2	255400001	0.682	0.6831
NC_005101.4_255500001	2	255500001	0.5387	0.6165
NC_005101.4_255600001	2	255600001	0.5528	0.7186
NC_005101.4_255700001	2	255700001	0.5514	0.7081
NC_005101.4_255800001	2	255800001	0.6104	0.7286
NC_005101.4_255900001	2	255900001	0.523	0.6593
NC_005101.4_256000001	2	256000001	0.4626	0.5875
NC_005101.4_256100001	2	256100001	0.4945	0.5704
NC_005101.4_256200001	2	256200001	0.3779	0.4397
NC_005101.4_256300001	2	256300001	0.3948	0.5019
NC_005101.4_256400001	2	256400001	0.4971	0.6516
NC_005101.4_256500001	2	256500001	0.6175	0.7769
NC_005101.4_256600001	2	256600001	0.5484	0.7907
NC_005101.4_256700001	2	256700001	0.5179	0.7248
NC_005101.4_256800001	2	256800001	0.4822	0.719
NC_005101.4_256900001	2	256900001	0.3677	0.636
NC_005101.4_257000001	2	257000001	0.436	0.7113
NC_005101.4_257100001	2	257100001	0.4694	0.7793
NC_005101.4_257200001	2	257200001	0.5121	0.8566
NC_005101.4_257300001	2	257300001	0.5086	0.7923
NC_005101.4_257400001	2	257400001	0.5215	0.8083
NC_005101.4_257500001	2	257500001	0.5006	0.7689
NC_005101.4_257600001	2	257600001	0.4535	0.673
NC_005101.4_257700001	2	257700001	0.4525	0.6733
NC_005101.4_257800001	2	257800001	0.4735	0.6076
NC_005101.4_257900001	2	257900001	0.533	0.6161
NC_005101.4_258000001	2	258000001	0.4939	0.5671
NC_005101.4_258100001	2	258100001	0.5414	0.588
NC_005101.4_258200001	2	258200001	0.5638	0.586
NC_005101.4_258300001	2	258300001	0.634	0.6875
NC_005101.4_258400001	2	258400001	0.6621	0.6591
NC_005101.4_258500001	2	258500001	0.6556	0.6685
NC_005101.4_258600001	2	258600001	0.5974	0.6997
NC_005101.4_258700001	2	258700001	0.5539	0.6043
NC_005101.4_258800001	2	258800001	0.4849	0.5419
NC_005101.4_258900001	2	258900001	0.3506	0.4272
NC_005101.4_259000001	2	259000001	0.3973	0.4805
NC_005101.4_259100001	2	259100001	0.4279	0.4442
NC_005101.4_259200001	2	259200001	0.3982	0.4746
NC_005101.4_259300001	2	259300001	0.4408	0.5301

NC_005101.4_259400001	2	259400001	0.4548	0.5358
NC_005101.4_259500001	2	259500001	0.4173	0.5113
NC_005101.4_259600001	2	259600001	0.405	0.551
NC_005101.4_259700001	2	259700001	0.4497	0.5964
NC_005101.4_259800001	2	259800001	0.4	0.5671
NC_005101.4_259900001	2	259900001	0.4354	0.553
NC_005101.4_260000001	2	260000001	0.5208	0.6352
NC_005101.4_260100001	2	260100001	0.5447	0.6339
NC_005101.4_260200001	2	260200001	0.5526	0.5622
NC_005101.4_260300001	2	260300001	0.5195	0.559
NC_005101.4_260400001	2	260400001	0.5796	0.6314
NC_005101.4_260500001	2	260500001	0.4855	0.5782
NC_005101.4_260600001	2	260600001	0.443	0.5601
NC_005101.4_260700001	2	260700001	0.4278	0.6856
NC_005101.4_260800001	2	260800001	0.5605	0.7469
NC_005101.4_260900001	2	260900001	0.4176	0.7205
NC_005101.4_261000001	2	261000001	0.4894	0.7796
NC_005101.4_261100001	2	261100001	0.4792	0.755
NC_005101.4_261200001	2	261200001	0.5259	0.7181
NC_005101.4_261300001	2	261300001	0.3714	0.5512
NC_005101.4_261400001	2	261400001	0.5398	0.6321
NC_005101.4_261500001	2	261500001	0.6519	0.6682
NC_005101.4_261600001	2	261600001	0.6436	0.6868
NC_005101.4_261700001	2	261700001	0.7142	0.7461
NC_005101.4_261800001	2	261800001	0.7531	0.7708
NC_005101.4_261900001	2	261900001	0.7144	0.7811
NC_005101.4_262000001	2	262000001	0.6203	0.6797
NC_005101.4_262100001	2	262100001	0.6291	0.6656
NC_005101.4_262200001	2	262200001	0.534	0.6495
NC_005101.4_262300001	2	262300001	0.544	0.7196
NC_005101.4_262400001	2	262400001	0.5467	0.6655
NC_005101.4_262500001	2	262500001	0.5725	0.7149
NC_005101.4_262600001	2	262600001	0.5815	0.7437
NC_005101.4_262700001	2	262700001	0.5849	0.6389
NC_005101.4_262800001	2	262800001	0.4502	0.444
NC_005101.4_262900001	2	262900001	0.4379	0.5034
NC_005101.4_263000001	2	263000001	0.3651	0.4022
NC_005101.4_263100001	2	263100001	0.3877	0.5113
NC_005101.4_263200001	2	263200001	0.454	0.5971
NC_005101.4_263300001	2	263300001	0.5304	0.6771
NC_005101.4_263400001	2	263400001	0.5984	0.7072
NC_005101.4_263500001	2	263500001	0.6225	0.7075
NC_005101.4_263600001	2	263600001	0.7279	0.7025
NC_005101.4_263700001	2	263700001	0.6618	0.6505
NC_005101.4_263800001	2	263800001	0.6821	0.6035
NC_005101.4_263900001	2	263900001	0.6487	0.5637
NC_005101.4_264000001	2	264000001	0.668	0.5924
NC_005101.4_264100001	2	264100001	0.4762	0.4817
NC_005101.4_264200001	2	264200001	0.4176	0.5024
NC_005101.4_264300001	2	264300001	0.2514	0.4148
NC_005101.4_264400001	2	264400001	0.3245	0.5418
NC_005101.4_264500001	2	264500001	0.1764	0.3783
NC_005101.4_264600001	2	264600001	0.3582	0.7303
NC_005101.4_264700001	2	264700001	0.4513	0.7665
NC_005101.4_264800001	2	264800001	0.3613	0.7044
NC_005101.4_264900001	2	264900001	0.3204	0.6651
NC_005101.4_265000001	2	265000001	0.4224	0.7533
NC_005101.4_265100001	2	265100001	0.1874	0.4786

NC_005101.4_265200001	2	265200001	0.1874	0.4786
NC_005101.4_265300001	2	265300001	0.2384	0.5878
NC_005101.4_265400001	2	265400001	0.406	0.4791
NC_005101.4_265500001	2	265500001	0.4499	0.5129
NC_005101.4_265600001	2	265600001	0.5361	0.5275
NC_005101.4_265700001	2	265700001	0.5814	0.5644
NC_005101.4_265800001	2	265800001	0.6238	0.6179
NC_005102.4_300001	3	300001	0.7666	0.5421
NC_005102.4_400001	3	400001	0.8386	0.6831
NC_005102.4_500001	3	500001	0.7415	0.6666
NC_005102.4_600001	3	600001	0.8334	0.9418
NC_005102.4_700001	3	700001	0.8334	0.9418
NC_005102.4_800001	3	800001	0.6805	0.8175
NC_005102.4_900001	3	900001	0.5837	0.734
NC_005102.4_1000001	3	1000001	0.5563	0.6059
NC_005102.4_1100001	3	1100001	0.6616	0.774
NC_005102.4_1200001	3	1200001	0.5942	0.7208
NC_005102.4_1300001	3	1300001	0.5626	0.7068
NC_005102.4_1400001	3	1400001	0.5366	0.6677
NC_005102.4_1500001	3	1500001	0.5324	0.699
NC_005102.4_1600001	3	1600001	0.4457	0.5977
NC_005102.4_1700001	3	1700001	0.4716	0.5689
NC_005102.4_1800001	3	1800001	0.5107	0.6226
NC_005102.4_1900001	3	1900001	0.5342	0.6585
NC_005102.4_2000001	3	2000001	0.6064	0.7256
NC_005102.4_2100001	3	2100001	0.5595	0.7187
NC_005102.4_2200001	3	2200001	0.5261	0.7231
NC_005102.4_2300001	3	2300001	0.5162	0.7026
NC_005102.4_2400001	3	2400001	0.543	0.728
NC_005102.4_2500001	3	2500001	0.5116	0.6833
NC_005102.4_2600001	3	2600001	0.5254	0.6685
NC_005102.4_2700001	3	2700001	0.5231	0.7036
NC_005102.4_2800001	3	2800001	0.549	0.6948
NC_005102.4_2900001	3	2900001	0.6166	0.7631
NC_005102.4_3000001	3	3000001	0.6882	0.776
NC_005102.4_3100001	3	3100001	0.6783	0.7469
NC_005102.4_3200001	3	3200001	0.6456	0.7419
NC_005102.4_3300001	3	3300001	0.5916	0.7112
NC_005102.4_3400001	3	3400001	0.5901	0.6972
NC_005102.4_3500001	3	3500001	0.5567	0.6816
NC_005102.4_3600001	3	3600001	0.5681	0.701
NC_005102.4_3700001	3	3700001	0.5831	0.6897
NC_005102.4_3800001	3	3800001	0.644	0.7147
NC_005102.4_3900001	3	3900001	0.6208	0.6967
NC_005102.4_4000001	3	4000001	0.6629	0.7305
NC_005102.4_4100001	3	4100001	0.6517	0.7078
NC_005102.4_4200001	3	4200001	0.6782	0.7552
NC_005102.4_4700001	3	4700001	0.3725	0.3627
NC_005102.4_4800001	3	4800001	0.3128	0.3425
NC_005102.4_4900001	3	4900001	0.5442	0.5731
NC_005102.4_5000001	3	5000001	0.445	0.428
NC_005102.4_5100001	3	5100001	0.5977	0.629
NC_005102.4_5200001	3	5200001	0.5373	0.7288
NC_005102.4_5300001	3	5300001	0.5819	0.7439
NC_005102.4_5400001	3	5400001	0.5125	0.7054
NC_005102.4_5500001	3	5500001	0.4836	0.7111
NC_005102.4_5600001	3	5600001	0.4275	0.7005
NC_005102.4_5700001	3	5700001	0.4046	0.6125

NC_005102.4_5800001	3	5800001	0.3694	0.5664
NC_005102.4_5900001	3	5900001	0.3856	0.5283
NC_005102.4_6000001	3	6000001	0.4294	0.5475
NC_005102.4_6100001	3	6100001	0.4551	0.5427
NC_005102.4_6200001	3	6200001	0.4268	0.5353
NC_005102.4_6300001	3	6300001	0.4369	0.6224
NC_005102.4_6400001	3	6400001	0.3903	0.6595
NC_005102.4_6500001	3	6500001	0.4275	0.6421
NC_005102.4_6600001	3	6600001	0.4789	0.659
NC_005102.4_6700001	3	6700001	0.5323	0.7188
NC_005102.4_6800001	3	6800001	0.5313	0.7062
NC_005102.4_6900001	3	6900001	0.6116	0.7347
NC_005102.4_7000001	3	7000001	0.6744	0.8051
NC_005102.4_7100001	3	7100001	0.7105	0.845
NC_005102.4_7200001	3	7200001	0.7128	0.8401
NC_005102.4_7300001	3	7300001	0.8119	0.8351
NC_005102.4_7400001	3	7400001	0.587	0.7564
NC_005102.4_7500001	3	7500001	0.5663	0.7051
NC_005102.4_7600001	3	7600001	0.476	0.6818
NC_005102.4_7700001	3	7700001	0.4775	0.6862
NC_005102.4_7800001	3	7800001	0.4065	0.6532
NC_005102.4_7900001	3	7900001	0.5221	0.658
NC_005102.4_8000001	3	8000001	0.5102	0.6767
NC_005102.4_8100001	3	8100001	0.5375	0.6623
NC_005102.4_8200001	3	8200001	0.5029	0.6071
NC_005102.4_8300001	3	8300001	0.516	0.5956
NC_005102.4_8400001	3	8400001	0.4497	0.6173
NC_005102.4_8500001	3	8500001	0.4771	0.6838
NC_005102.4_8600001	3	8600001	0.5108	0.7086
NC_005102.4_8700001	3	8700001	0.5088	0.7152
NC_005102.4_8800001	3	8800001	0.5141	0.7636
NC_005102.4_8900001	3	8900001	0.528	0.8033
NC_005102.4_9000001	3	9000001	0.5697	0.7176
NC_005102.4_9100001	3	9100001	0.4648	0.5777
NC_005102.4_9200001	3	9200001	0.4656	0.5667
NC_005102.4_9300001	3	9300001	0.4511	0.5698
NC_005102.4_9400001	3	9400001	0.5286	0.6443
NC_005102.4_9500001	3	9500001	0.523	0.6521
NC_005102.4_9600001	3	9600001	0.5624	0.7131
NC_005102.4_9700001	3	9700001	0.5449	0.7482
NC_005102.4_9800001	3	9800001	0.6401	0.8079
NC_005102.4_9900001	3	9900001	0.4476	0.6819
NC_005102.4_10000001	3	10000001	0.4422	0.5992
NC_005102.4_10100001	3	10100001	0.4446	0.621
NC_005102.4_10200001	3	10200001	0.4493	0.5972
NC_005102.4_10300001	3	10300001	0.3778	0.4901
NC_005102.4_10400001	3	10400001	0.4333	0.5462
NC_005102.4_10500001	3	10500001	0.4379	0.6316
NC_005102.4_10600001	3	10600001	0.5811	0.7942
NC_005102.4_10700001	3	10700001	0.7402	0.8526
NC_005102.4_10800001	3	10800001	0.7193	0.8005
NC_005102.4_10900001	3	10900001	0.6216	0.8028
NC_005102.4_11000001	3	11000001	0.5848	0.7835
NC_005102.4_11100001	3	11100001	0.5636	0.7332
NC_005102.4_11200001	3	11200001	0.4754	0.6791
NC_005102.4_11300001	3	11300001	0.4769	0.7546
NC_005102.4_11400001	3	11400001	0.5405	0.7037
NC_005102.4_11500001	3	11500001	0.5936	0.7345

NC_005102.4_11600001	3	11600001	0.5001	0.7533
NC_005102.4_11700001	3	11700001	0.5082	0.7535
NC_005102.4_11800001	3	11800001	0.4905	0.7527
NC_005102.4_11900001	3	11900001	0.5331	0.7547
NC_005102.4_12000001	3	12000001	0.5683	0.781
NC_005102.4_12100001	3	12100001	0.6607	0.7856
NC_005102.4_12200001	3	12200001	0.7124	0.8068
NC_005102.4_12300001	3	12300001	0.7096	0.7823
NC_005102.4_12400001	3	12400001	0.6232	0.7418
NC_005102.4_12500001	3	12500001	0.5914	0.7047
NC_005102.4_12600001	3	12600001	0.4976	0.5908
NC_005102.4_12700001	3	12700001	0.4031	0.4892
NC_005102.4_12800001	3	12800001	0.4705	0.569
NC_005102.4_12900001	3	12900001	0.4934	0.6167
NC_005102.4_13000001	3	13000001	0.4575	0.607
NC_005102.4_13100001	3	13100001	0.5161	0.7381
NC_005102.4_13200001	3	13200001	0.6776	0.8795
NC_005102.4_13300001	3	13300001	0.6017	0.8794
NC_005102.4_13400001	3	13400001	0.6115	0.8752
NC_005102.4_13500001	3	13500001	0.5325	0.8186
NC_005102.4_13600001	3	13600001	0.5495	0.7542
NC_005102.4_13700001	3	13700001	0.4362	0.624
NC_005102.4_13800001	3	13800001	0.4002	0.4587
NC_005102.4_13900001	3	13900001	0.3547	0.3817
NC_005102.4_14000001	3	14000001	0.5176	0.5822
NC_005102.4_14100001	3	14100001	0.5171	0.6468
NC_005102.4_14200001	3	14200001	0.4885	0.6005
NC_005102.4_14300001	3	14300001	0.4855	0.6212
NC_005102.4_14400001	3	14400001	0.4562	0.5723
NC_005102.4_14500001	3	14500001	0.4856	0.5753
NC_005102.4_14600001	3	14600001	0.5027	0.5719
NC_005102.4_14700001	3	14700001	0.5514	0.5803
NC_005102.4_14800001	3	14800001	0.541	0.5641
NC_005102.4_14900001	3	14900001	0.5707	0.6301
NC_005102.4_15000001	3	15000001	0.5575	0.6402
NC_005102.4_15100001	3	15100001	0.5538	0.6454
NC_005102.4_15200001	3	15200001	0.5749	0.6838
NC_005102.4_15300001	3	15300001	0.5882	0.7392
NC_005102.4_15400001	3	15400001	0.5712	0.727
NC_005102.4_15500001	3	15500001	0.5674	0.6972
NC_005102.4_16000001	3	16000001	0.4607	0.2864
NC_005102.4_16100001	3	16100001	0.5374	0.3728
NC_005102.4_16200001	3	16200001	0.5374	0.3728
NC_005102.4_16300001	3	16300001	0.5374	0.3728
NC_005102.4_16400001	3	16400001	0.5562	0.3963
NC_005102.4_16700001	3	16700001	0.1791	0.0861
NC_005102.4_16800001	3	16800001	0.1791	0.0861
NC_005102.4_16900001	3	16900001	0.3323	0.4711
NC_005102.4_17000001	3	17000001	0.3323	0.4711
NC_005102.4_17100001	3	17100001	0.3538	0.7264
NC_005102.4_19700001	3	19700001	0.1639	0.1374
NC_005102.4_19800001	3	19800001	0.3417	0.4537
NC_005102.4_19900001	3	19900001	0.3376	0.3879
NC_005102.4_20000001	3	20000001	0.3376	0.3879
NC_005102.4_20700001	3	20700001	0.482	0.5784
NC_005102.4_20800001	3	20800001	0.4588	0.5106
NC_005102.4_20900001	3	20900001	0.5017	0.7146
NC_005102.4_21000001	3	21000001	0.5095	0.6845

NC_005102.4_21100001	3	21100001	0.5095	0.6845
NC_005102.4_21200001	3	21200001	0.6353	0.7454
NC_005102.4_21300001	3	21300001	0.7462	0.8622
NC_005102.4_21400001	3	21400001	0.8751	0.9005
NC_005102.4_21500001	3	21500001	0.9603	0.9385
NC_005102.4_21600001	3	21600001	0.9695	0.9525
NC_005102.4_21700001	3	21700001	0.9339	0.9638
NC_005102.4_21800001	3	21800001	0.7929	0.8479
NC_005102.4_21900001	3	21900001	0.7291	0.7585
NC_005102.4_22000001	3	22000001	0.7058	0.7136
NC_005102.4_22100001	3	22100001	0.6387	0.6831
NC_005102.4_22200001	3	22200001	0.5656	0.5729
NC_005102.4_22300001	3	22300001	0.5574	0.5895
NC_005102.4_22400001	3	22400001	0.5616	0.6767
NC_005102.4_22500001	3	22500001	0.5835	0.6974
NC_005102.4_22600001	3	22600001	0.6185	0.7101
NC_005102.4_22700001	3	22700001	0.724	0.8719
NC_005102.4_22800001	3	22800001	0.6989	0.8526
NC_005102.4_22900001	3	22900001	0.728	0.7971
NC_005102.4_23000001	3	23000001	0.6849	0.8028
NC_005102.4_23100001	3	23100001	0.5611	0.6769
NC_005102.4_23200001	3	23200001	0.5533	0.684
NC_005102.4_23300001	3	23300001	0.6123	0.6506
NC_005102.4_23400001	3	23400001	0.5905	0.6865
NC_005102.4_23500001	3	23500001	0.5905	0.6865
NC_005102.4_23600001	3	23600001	0.555	0.6999
NC_005102.4_23700001	3	23700001	0.3689	0.553
NC_005102.4_24000001	3	24000001	0.2996	0.4696
NC_005102.4_24100001	3	24100001	0.4415	0.6011
NC_005102.4_24200001	3	24200001	0.4408	0.5851
NC_005102.4_24300001	3	24300001	0.5626	0.6517
NC_005102.4_24400001	3	24400001	0.5025	0.6223
NC_005102.4_24500001	3	24500001	0.5712	0.5638
NC_005102.4_24600001	3	24600001	0.4722	0.4695
NC_005102.4_24700001	3	24700001	0.4605	0.4298
NC_005102.4_24800001	3	24800001	0.4605	0.4298
NC_005102.4_24900001	3	24900001	0.5159	0.4479
NC_005102.4_25100001	3	25100001	0.5181	0.661
NC_005102.4_25300001	3	25300001	0.4295	0.6508
NC_005102.4_25400001	3	25400001	0.3347	0.531
NC_005102.4_25500001	3	25500001	0.3347	0.531
NC_005102.4_25600001	3	25600001	0.2637	0.411
NC_005102.4_25700001	3	25700001	0.3481	0.4222
NC_005102.4_25800001	3	25800001	0.4544	0.552
NC_005102.4_25900001	3	25900001	0.4377	0.4994
NC_005102.4_26000001	3	26000001	0.3858	0.442
NC_005102.4_26100001	3	26100001	0.3967	0.3959
NC_005102.4_26200001	3	26200001	0.357	0.4266
NC_005102.4_26300001	3	26300001	0.3422	0.3641
NC_005102.4_26400001	3	26400001	0.3845	0.4019
NC_005102.4_26500001	3	26500001	0.377	0.4121
NC_005102.4_26600001	3	26600001	0.3463	0.5331
NC_005102.4_26700001	3	26700001	0.364	0.5609
NC_005102.4_26800001	3	26800001	0.3344	0.5225
NC_005102.4_26900001	3	26900001	0.3251	0.5196
NC_005102.4_27000001	3	27000001	0.3803	0.5809
NC_005102.4_27100001	3	27100001	0.4241	0.6031
NC_005102.4_27200001	3	27200001	0.4251	0.5351

NC_005102.4_27300001	3	27300001	0.4588	0.5691
NC_005102.4_27400001	3	27400001	0.6414	0.6819
NC_005102.4_27500001	3	27500001	0.6202	0.6301
NC_005102.4_27600001	3	27600001	0.5766	0.5868
NC_005102.4_27700001	3	27700001	0.5546	0.6032
NC_005102.4_27800001	3	27800001	0.4568	0.5909
NC_005102.4_27900001	3	27900001	0.3643	0.4848
NC_005102.4_28000001	3	28000001	0.2351	0.467
NC_005102.4_28100001	3	28100001	0.2752	0.5436
NC_005102.4_28200001	3	28200001	0.3575	0.6317
NC_005102.4_28300001	3	28300001	0.4071	0.6631
NC_005102.4_28400001	3	28400001	0.4004	0.6411
NC_005102.4_28500001	3	28500001	0.4538	0.6547
NC_005102.4_28600001	3	28600001	0.4298	0.5128
NC_005102.4_28700001	3	28700001	0.3096	0.3712
NC_005102.4_28800001	3	28800001	0.4649	0.5349
NC_005102.4_28900001	3	28900001	0.7001	0.7569
NC_005102.4_29000001	3	29000001	0.6978	0.7852
NC_005102.4_29100001	3	29100001	0.6994	0.8052
NC_005102.4_29200001	3	29200001	0.7314	0.8201
NC_005102.4_29300001	3	29300001	0.7055	0.8177
NC_005102.4_29400001	3	29400001	0.5275	0.7104
NC_005102.4_29500001	3	29500001	0.4983	0.7111
NC_005102.4_29600001	3	29600001	0.4639	0.7466
NC_005102.4_29700001	3	29700001	0.3942	0.5313
NC_005102.4_29800001	3	29800001	0.4192	0.5402
NC_005102.4_29900001	3	29900001	0.4173	0.464
NC_005102.4_30000001	3	30000001	0.3776	0.3221
NC_005102.4_30100001	3	30100001	0.3745	0.3206
NC_005102.4_30200001	3	30200001	0.3708	0.3989
NC_005102.4_30300001	3	30300001	0.3209	0.3501
NC_005102.4_30400001	3	30400001	0.2875	0.4055
NC_005102.4_30500001	3	30500001	0.3313	0.3921
NC_005102.4_30600001	3	30600001	0.3093	0.4104
NC_005102.4_30700001	3	30700001	0.3342	0.4284
NC_005102.4_30800001	3	30800001	0.3342	0.4284
NC_005102.4_30900001	3	30900001	0.4213	0.4891
NC_005102.4_31000001	3	31000001	0.3322	0.4719
NC_005102.4_31200001	3	31200001	0.3301	0.3823
NC_005102.4_31300001	3	31300001	0.3704	0.354
NC_005102.4_31400001	3	31400001	0.3112	0.2913
NC_005102.4_31500001	3	31500001	0.3522	0.3405
NC_005102.4_31600001	3	31600001	0.4204	0.4945
NC_005102.4_31700001	3	31700001	0.4639	0.5592
NC_005102.4_31800001	3	31800001	0.4434	0.5757
NC_005102.4_31900001	3	31900001	0.5274	0.7075
NC_005102.4_32000001	3	32000001	0.477	0.7365
NC_005102.4_32100001	3	32100001	0.4703	0.6598
NC_005102.4_32200001	3	32200001	0.5091	0.7119
NC_005102.4_32300001	3	32300001	0.5851	0.7465
NC_005102.4_32400001	3	32400001	0.6375	0.7776
NC_005102.4_32500001	3	32500001	0.6402	0.7169
NC_005102.4_32600001	3	32600001	0.6667	0.6877
NC_005102.4_32700001	3	32700001	0.7882	0.7753
NC_005102.4_32800001	3	32800001	0.7606	0.8176
NC_005102.4_32900001	3	32900001	0.7174	0.7924
NC_005102.4_33000001	3	33000001	0.7366	0.8846
NC_005102.4_33100001	3	33100001	0.6447	0.8047

NC_005102.4_33200001	3	33200001	0.4335	0.5955
NC_005102.4_33300001	3	33300001	0.6258	0.7934
NC_005102.4_33400001	3	33400001	0.5693	0.7644
NC_005102.4_33500001	3	33500001	0.5651	0.6641
NC_005102.4_33600001	3	33600001	0.5516	0.6775
NC_005102.4_33700001	3	33700001	0.5516	0.6775
NC_005102.4_33800001	3	33800001	0.426	0.5453
NC_005102.4_33900001	3	33900001	0.4084	0.5151
NC_005102.4_34000001	3	34000001	0.3818	0.5451
NC_005102.4_34100001	3	34100001	0.4715	0.6086
NC_005102.4_34200001	3	34200001	0.5515	0.6999
NC_005102.4_34300001	3	34300001	0.5547	0.6672
NC_005102.4_34400001	3	34400001	0.6012	0.6838
NC_005102.4_34500001	3	34500001	0.8435	0.9204
NC_005102.4_34600001	3	34600001	0.8534	0.9565
NC_005102.4_34700001	3	34700001	0.6445	0.8228
NC_005102.4_34800001	3	34800001	0.5477	0.7628
NC_005102.4_34900001	3	34900001	0.5717	0.778
NC_005102.4_35000001	3	35000001	0.6044	0.7958
NC_005102.4_35100001	3	35100001	0.5695	0.7942
NC_005102.4_35200001	3	35200001	0.4502	0.7295
NC_005102.4_35300001	3	35300001	0.472	0.77
NC_005102.4_35400001	3	35400001	0.465	0.7604
NC_005102.4_35500001	3	35500001	0.4604	0.7317
NC_005102.4_35600001	3	35600001	0.4167	0.6902
NC_005102.4_35700001	3	35700001	0.5169	0.7017
NC_005102.4_35800001	3	35800001	0.4998	0.6285
NC_005102.4_35900001	3	35900001	0.5066	0.6166
NC_005102.4_36000001	3	36000001	0.5129	0.5863
NC_005102.4_36100001	3	36100001	0.5301	0.5821
NC_005102.4_36200001	3	36200001	0.6355	0.7045
NC_005102.4_36300001	3	36300001	0.643	0.7502
NC_005102.4_36400001	3	36400001	0.5758	0.7601
NC_005102.4_36500001	3	36500001	0.5529	0.7425
NC_005102.4_36600001	3	36600001	0.539	0.7566
NC_005102.4_36700001	3	36700001	0.4587	0.6001
NC_005102.4_36800001	3	36800001	0.4163	0.5203
NC_005102.4_36900001	3	36900001	0.4364	0.467
NC_005102.4_37000001	3	37000001	0.4128	0.4332
NC_005102.4_37100001	3	37100001	0.4205	0.4659
NC_005102.4_37200001	3	37200001	0.4886	0.639
NC_005102.4_37300001	3	37300001	0.4836	0.6186
NC_005102.4_37400001	3	37400001	0.4655	0.5865
NC_005102.4_37500001	3	37500001	0.5181	0.6187
NC_005102.4_37600001	3	37600001	0.5254	0.6028
NC_005102.4_37700001	3	37700001	0.4597	0.494
NC_005102.4_37800001	3	37800001	0.4945	0.5488
NC_005102.4_37900001	3	37900001	0.4783	0.5572
NC_005102.4_38000001	3	38000001	0.3977	0.5177
NC_005102.4_38100001	3	38100001	0.3997	0.5086
NC_005102.4_38200001	3	38200001	0.3897	0.5764
NC_005102.4_38300001	3	38300001	0.4181	0.6692
NC_005102.4_38400001	3	38400001	0.4543	0.7128
NC_005102.4_38500001	3	38500001	0.5941	0.7545
NC_005102.4_38600001	3	38600001	0.5446	0.7596
NC_005102.4_38700001	3	38700001	0.552	0.6854
NC_005102.4_38800001	3	38800001	0.4449	0.5553
NC_005102.4_38900001	3	38900001	0.5418	0.6668

NC_005102.4_39000001	3	39000001	0.4729	0.6231
NC_005102.4_39100001	3	39100001	0.6038	0.6641
NC_005102.4_39200001	3	39200001	0.5114	0.5919
NC_005102.4_39300001	3	39300001	0.467	0.5622
NC_005102.4_39400001	3	39400001	0.338	0.3841
NC_005102.4_39500001	3	39500001	0.4582	0.5143
NC_005102.4_39600001	3	39600001	0.4982	0.6527
NC_005102.4_39700001	3	39700001	0.5779	0.7336
NC_005102.4_39800001	3	39800001	0.5942	0.7662
NC_005102.4_39900001	3	39900001	0.5514	0.6703
NC_005102.4_40000001	3	40000001	0.5157	0.6478
NC_005102.4_40100001	3	40100001	0.3833	0.488
NC_005102.4_40200001	3	40200001	0.3673	0.4691
NC_005102.4_40300001	3	40300001	0.4112	0.4213
NC_005102.4_40400001	3	40400001	0.4462	0.3754
NC_005102.4_40500001	3	40500001	0.4525	0.4376
NC_005102.4_40600001	3	40600001	0.5026	0.4315
NC_005102.4_40700001	3	40700001	0.4241	0.3679
NC_005102.4_40800001	3	40800001	0.513	0.492
NC_005102.4_40900001	3	40900001	0.618	0.6741
NC_005102.4_41000001	3	41000001	0.7444	0.7717
NC_005102.4_41100001	3	41100001	0.7062	0.7994
NC_005102.4_41200001	3	41200001	0.8091	0.8182
NC_005102.4_41300001	3	41300001	0.7658	0.8215
NC_005102.4_41400001	3	41400001	0.6852	0.743
NC_005102.4_41500001	3	41500001	0.4897	0.512
NC_005102.4_41600001	3	41600001	0.8517	0.8336
NC_005102.4_41700001	3	41700001	0.7129	0.7716
NC_005102.4_41800001	3	41800001	0.7599	0.8434
NC_005102.4_41900001	3	41900001	0.647	0.7296
NC_005102.4_42000001	3	42000001	0.5976	0.6521
NC_005102.4_42100001	3	42100001	0.4036	0.4008
NC_005102.4_42200001	3	42200001	0.2681	0.2149
NC_005102.4_42300001	3	42300001	0.2681	0.2149
NC_005102.4_42400001	3	42400001	0.2244	0.1487
NC_005102.4_42600001	3	42600001	0.3331	0.4787
NC_005102.4_42800001	3	42800001	0.4569	0.7362
NC_005102.4_42900001	3	42900001	0.5231	0.7897
NC_005102.4_43000001	3	43000001	0.5185	0.6952
NC_005102.4_43100001	3	43100001	0.5229	0.7022
NC_005102.4_43200001	3	43200001	0.4435	0.6898
NC_005102.4_43300001	3	43300001	0.6227	0.7442
NC_005102.4_43400001	3	43400001	0.6398	0.686
NC_005102.4_43600001	3	43600001	0.6063	0.7695
NC_005102.4_43700001	3	43700001	0.5608	0.7467
NC_005102.4_43800001	3	43800001	0.5551	0.7654
NC_005102.4_43900001	3	43900001	0.4842	0.7193
NC_005102.4_44000001	3	44000001	0.521	0.6768
NC_005102.4_44100001	3	44100001	0.5466	0.6642
NC_005102.4_44200001	3	44200001	0.5903	0.6765
NC_005102.4_44300001	3	44300001	0.5646	0.6264
NC_005102.4_44400001	3	44400001	0.5671	0.667
NC_005102.4_44500001	3	44500001	0.5185	0.7445
NC_005102.4_44600001	3	44600001	0.3695	0.6883
NC_005102.4_44700001	3	44700001	0.3852	0.6316
NC_005102.4_44800001	3	44800001	0.4754	0.7693
NC_005102.4_44900001	3	44900001	0.4626	0.7091
NC_005102.4_45000001	3	45000001	0.3974	0.5551

NC_005102.4_45100001	3	45100001	0.4987	0.6319
NC_005102.4_45200001	3	45200001	0.5041	0.6698
NC_005102.4_45300001	3	45300001	0.4995	0.5911
NC_005102.4_45400001	3	45400001	0.5084	0.6207
NC_005102.4_45500001	3	45500001	0.5607	0.6675
NC_005102.4_45600001	3	45600001	0.497	0.6173
NC_005102.4_45700001	3	45700001	0.4936	0.6005
NC_005102.4_45800001	3	45800001	0.5481	0.6594
NC_005102.4_45900001	3	45900001	0.5637	0.6012
NC_005102.4_46000001	3	46000001	0.5144	0.5591
NC_005102.4_46100001	3	46100001	0.5421	0.5581
NC_005102.4_46200001	3	46200001	0.5295	0.5611
NC_005102.4_46300001	3	46300001	0.3884	0.4451
NC_005102.4_46400001	3	46400001	0.3903	0.501
NC_005102.4_46500001	3	46500001	0.4089	0.5148
NC_005102.4_46600001	3	46600001	0.4188	0.4958
NC_005102.4_46700001	3	46700001	0.3935	0.4645
NC_005102.4_46800001	3	46800001	0.3735	0.4478
NC_005102.4_46900001	3	46900001	0.4403	0.5873
NC_005102.4_47000001	3	47000001	0.4153	0.6219
NC_005102.4_47100001	3	47100001	0.3386	0.6225
NC_005102.4_47200001	3	47200001	0.3836	0.665
NC_005102.4_47300001	3	47300001	0.4017	0.6796
NC_005102.4_47400001	3	47400001	0.3634	0.6443
NC_005102.4_47500001	3	47500001	0.411	0.6092
NC_005102.4_47600001	3	47600001	0.548	0.6363
NC_005102.4_47700001	3	47700001	0.6197	0.624
NC_005102.4_47800001	3	47800001	0.6197	0.624
NC_005102.4_47900001	3	47900001	0.5063	0.6088
NC_005102.4_48000001	3	48000001	0.4001	0.5899
NC_005102.4_48100001	3	48100001	0.5018	0.7161
NC_005102.4_48200001	3	48200001	0.3255	0.763
NC_005102.4_48300001	3	48300001	0.3255	0.763
NC_005102.4_48800001	3	48800001	0.495	0.3816
NC_005102.4_48900001	3	48900001	0.4181	0.3509
NC_005102.4_49000001	3	49000001	0.4651	0.4555
NC_005102.4_49100001	3	49100001	0.5427	0.6486
NC_005102.4_49200001	3	49200001	0.479	0.6368
NC_005102.4_49300001	3	49300001	0.5277	0.7317
NC_005102.4_49400001	3	49400001	0.5633	0.7714
NC_005102.4_49500001	3	49500001	0.5568	0.7702
NC_005102.4_49600001	3	49600001	0.5302	0.7637
NC_005102.4_49700001	3	49700001	0.6603	0.8236
NC_005102.4_49800001	3	49800001	0.5627	0.7696
NC_005102.4_49900001	3	49900001	0.4793	0.7511
NC_005102.4_50000001	3	50000001	0.4922	0.7146
NC_005102.4_50100001	3	50100001	0.5241	0.686
NC_005102.4_50200001	3	50200001	0.4253	0.5497
NC_005102.4_50300001	3	50300001	0.4106	0.5491
NC_005102.4_50400001	3	50400001	0.4866	0.5502
NC_005102.4_50500001	3	50500001	0.478	0.5624
NC_005102.4_50600001	3	50600001	0.411	0.5104
NC_005102.4_50700001	3	50700001	0.4167	0.5509
NC_005102.4_50800001	3	50800001	0.4835	0.6375
NC_005102.4_50900001	3	50900001	0.5439	0.7733
NC_005102.4_51000001	3	51000001	0.5641	0.7755
NC_005102.4_51100001	3	51100001	0.6017	0.7811
NC_005102.4_51200001	3	51200001	0.6033	0.7689

NC_005102.4_51300001	3	51300001	0.6518	0.7866
NC_005102.4_51400001	3	51400001	0.6109	0.7035
NC_005102.4_51500001	3	51500001	0.6148	0.706
NC_005102.4_51600001	3	51600001	0.6371	0.7351
NC_005102.4_51700001	3	51700001	0.6517	0.7049
NC_005102.4_51800001	3	51800001	0.5894	0.6677
NC_005102.4_51900001	3	51900001	0.5327	0.5884
NC_005102.4_52000001	3	52000001	0.4451	0.5256
NC_005102.4_52100001	3	52100001	0.4536	0.5119
NC_005102.4_52200001	3	52200001	0.4354	0.4758
NC_005102.4_52300001	3	52300001	0.3665	0.3984
NC_005102.4_52400001	3	52400001	0.3655	0.4273
NC_005102.4_52500001	3	52500001	0.3642	0.4277
NC_005102.4_52600001	3	52600001	0.3493	0.3802
NC_005102.4_52700001	3	52700001	0.3034	0.3179
NC_005102.4_52800001	3	52800001	0.306	0.2276
NC_005102.4_52900001	3	52900001	0.3352	0.2439
NC_005102.4_53000001	3	53000001	0.3559	0.245
NC_005102.4_53100001	3	53100001	0.3531	0.2359
NC_005102.4_53200001	3	53200001	0.3113	0.215
NC_005102.4_53300001	3	53300001	0.3829	0.3644
NC_005102.4_53400001	3	53400001	0.5685	0.6524
NC_005102.4_53500001	3	53500001	0.5378	0.596
NC_005102.4_53600001	3	53600001	0.4626	0.5448
NC_005102.4_53700001	3	53700001	0.5136	0.5527
NC_005102.4_53800001	3	53800001	0.3654	0.4311
NC_005102.4_53900001	3	53900001	0.2742	0.2736
NC_005102.4_54000001	3	54000001	0.4511	0.5554
NC_005102.4_54100001	3	54100001	0.4985	0.6597
NC_005102.4_54200001	3	54200001	0.5451	0.6356
NC_005102.4_54300001	3	54300001	0.6116	0.6834
NC_005102.4_54400001	3	54400001	0.5652	0.6906
NC_005102.4_54500001	3	54500001	0.4818	0.6485
NC_005102.4_54600001	3	54600001	0.4748	0.651
NC_005102.4_54700001	3	54700001	0.4427	0.7245
NC_005102.4_54800001	3	54800001	0.3486	0.5639
NC_005102.4_54900001	3	54900001	0.3478	0.5311
NC_005102.4_55000001	3	55000001	0.4483	0.5369
NC_005102.4_55100001	3	55100001	0.4498	0.494
NC_005102.4_55200001	3	55200001	0.4719	0.5167
NC_005102.4_55300001	3	55300001	0.5102	0.5363
NC_005102.4_55400001	3	55400001	0.5644	0.6001
NC_005102.4_55500001	3	55500001	0.508	0.6266
NC_005102.4_55600001	3	55600001	0.5151	0.6551
NC_005102.4_55700001	3	55700001	0.4417	0.5795
NC_005102.4_55800001	3	55800001	0.4446	0.5983
NC_005102.4_55900001	3	55900001	0.3952	0.5474
NC_005102.4_56000001	3	56000001	0.4175	0.5906
NC_005102.4_56100001	3	56100001	0.407	0.5461
NC_005102.4_56200001	3	56200001	0.3988	0.5108
NC_005102.4_56300001	3	56300001	0.4341	0.5461
NC_005102.4_56400001	3	56400001	0.4085	0.5109
NC_005102.4_56500001	3	56500001	0.3439	0.3861
NC_005102.4_56600001	3	56600001	0.3447	0.4099
NC_005102.4_56700001	3	56700001	0.3386	0.4775
NC_005102.4_56800001	3	56800001	0.4072	0.5587
NC_005102.4_56900001	3	56900001	0.4116	0.5543
NC_005102.4_57000001	3	57000001	0.4327	0.6057

NC_005102.4_57100001	3	57100001	0.4102	0.6526
NC_005102.4_57200001	3	57200001	0.5576	0.842
NC_005102.4_57300001	3	57300001	0.5094	0.8216
NC_005102.4_57400001	3	57400001	0.495	0.6842
NC_005102.4_57500001	3	57500001	0.5656	0.6985
NC_005102.4_57600001	3	57600001	0.6004	0.6923
NC_005102.4_57700001	3	57700001	0.6211	0.6934
NC_005102.4_57800001	3	57800001	0.6751	0.6866
NC_005102.4_57900001	3	57900001	0.7206	0.7434
NC_005102.4_58000001	3	58000001	0.6776	0.7259
NC_005102.4_58100001	3	58100001	0.6454	0.7284
NC_005102.4_58200001	3	58200001	0.5419	0.6442
NC_005102.4_58300001	3	58300001	0.427	0.5682
NC_005102.4_58400001	3	58400001	0.4301	0.5724
NC_005102.4_58500001	3	58500001	0.3995	0.5381
NC_005102.4_58600001	3	58600001	0.4243	0.5372
NC_005102.4_58700001	3	58700001	0.5213	0.6392
NC_005102.4_58800001	3	58800001	0.6623	0.7445
NC_005102.4_58900001	3	58900001	0.5612	0.7295
NC_005102.4_59000001	3	59000001	0.5514	0.7585
NC_005102.4_59100001	3	59100001	0.4885	0.7434
NC_005102.4_59200001	3	59200001	0.395	0.6935
NC_005102.4_59300001	3	59300001	0.3676	0.5828
NC_005102.4_59400001	3	59400001	0.3785	0.5828
NC_005102.4_59500001	3	59500001	0.3983	0.5831
NC_005102.4_59600001	3	59600001	0.4	0.6011
NC_005102.4_59700001	3	59700001	0.4267	0.5291
NC_005102.4_59800001	3	59800001	0.4527	0.6167
NC_005102.4_59900001	3	59900001	0.4754	0.6424
NC_005102.4_60000001	3	60000001	0.4361	0.6132
NC_005102.4_60100001	3	60100001	0.5415	0.6909
NC_005102.4_60200001	3	60200001	0.6081	0.7827
NC_005102.4_60300001	3	60300001	0.6118	0.7712
NC_005102.4_60400001	3	60400001	0.5433	0.6737
NC_005102.4_60500001	3	60500001	0.5028	0.6227
NC_005102.4_60600001	3	60600001	0.4799	0.5836
NC_005102.4_60700001	3	60700001	0.4451	0.5422
NC_005102.4_60800001	3	60800001	0.4214	0.5308
NC_005102.4_60900001	3	60900001	0.4857	0.5872
NC_005102.4_61000001	3	61000001	0.5624	0.6651
NC_005102.4_61100001	3	61100001	0.5748	0.6528
NC_005102.4_61200001	3	61200001	0.6197	0.7184
NC_005102.4_61300001	3	61300001	0.6807	0.7311
NC_005102.4_61400001	3	61400001	0.64	0.7641
NC_005102.4_61500001	3	61500001	0.6556	0.7304
NC_005102.4_61600001	3	61600001	0.6062	0.7166
NC_005102.4_61700001	3	61700001	0.618	0.6889
NC_005102.4_61800001	3	61800001	0.5732	0.6509
NC_005102.4_61900001	3	61900001	0.5404	0.5784
NC_005102.4_62000001	3	62000001	0.5344	0.626
NC_005102.4_62100001	3	62100001	0.5033	0.5888
NC_005102.4_62200001	3	62200001	0.4934	0.6232
NC_005102.4_62300001	3	62300001	0.4938	0.6553
NC_005102.4_62400001	3	62400001	0.5332	0.6801
NC_005102.4_62500001	3	62500001	0.4905	0.6244
NC_005102.4_62600001	3	62600001	0.5406	0.6665
NC_005102.4_62700001	3	62700001	0.5385	0.627
NC_005102.4_62800001	3	62800001	0.53	0.6378

NC_005102.4_62900001	3	62900001	0.5758	0.6672
NC_005102.4_63000001	3	63000001	0.6725	0.7442
NC_005102.4_63100001	3	63100001	0.6363	0.7598
NC_005102.4_63200001	3	63200001	0.5848	0.7315
NC_005102.4_63300001	3	63300001	0.6433	0.7871
NC_005102.4_63400001	3	63400001	0.6176	0.8551
NC_005102.4_63500001	3	63500001	0.5542	0.7741
NC_005102.4_63600001	3	63600001	0.6506	0.7892
NC_005102.4_63700001	3	63700001	0.7173	0.8387
NC_005102.4_63800001	3	63800001	0.637	0.7205
NC_005102.4_63900001	3	63900001	0.5336	0.63
NC_005102.4_64000001	3	64000001	0.5197	0.6011
NC_005102.4_64100001	3	64100001	0.4804	0.5845
NC_005102.4_64200001	3	64200001	0.4515	0.5178
NC_005102.4_64300001	3	64300001	0.4821	0.5947
NC_005102.4_64400001	3	64400001	0.5296	0.6261
NC_005102.4_64500001	3	64500001	0.5638	0.6762
NC_005102.4_64600001	3	64600001	0.5604	0.632
NC_005102.4_64700001	3	64700001	0.5893	0.6801
NC_005102.4_64800001	3	64800001	0.5395	0.5879
NC_005102.4_64900001	3	64900001	0.3833	0.5544
NC_005102.4_65000001	3	65000001	0.3972	0.5036
NC_005102.4_65100001	3	65100001	0.4227	0.6781
NC_005102.4_65200001	3	65200001	0.4607	0.6429
NC_005102.4_65300001	3	65300001	0.5214	0.7017
NC_005102.4_65400001	3	65400001	0.4947	0.6752
NC_005102.4_65500001	3	65500001	0.5048	0.7599
NC_005102.4_65600001	3	65600001	0.5056	0.7456
NC_005102.4_65700001	3	65700001	0.5292	0.8165
NC_005102.4_65800001	3	65800001	0.4771	0.7795
NC_005102.4_65900001	3	65900001	0.5994	0.756
NC_005102.4_66000001	3	66000001	0.6016	0.788
NC_005102.4_66100001	3	66100001	0.6386	0.7901
NC_005102.4_66200001	3	66200001	0.6069	0.7167
NC_005102.4_66300001	3	66300001	0.7266	0.7837
NC_005102.4_66400001	3	66400001	0.7085	0.8573
NC_005102.4_66500001	3	66500001	0.7967	0.8231
NC_005102.4_66600001	3	66600001	0.7543	0.7845
NC_005102.4_66700001	3	66700001	0.8901	0.8827
NC_005102.4_67100001	3	67100001	0.7266	0.724
NC_005102.4_67200001	3	67200001	0.6339	0.6523
NC_005102.4_67300001	3	67300001	0.6674	0.692
NC_005102.4_67400001	3	67400001	0.5756	0.6882
NC_005102.4_67500001	3	67500001	0.5279	0.6777
NC_005102.4_67600001	3	67600001	0.3133	0.514
NC_005102.4_67700001	3	67700001	0.3714	0.6299
NC_005102.4_67800001	3	67800001	0.2583	0.4148
NC_005102.4_67900001	3	67900001	0.3021	0.4414
NC_005102.4_68000001	3	68000001	0.335	0.4571
NC_005102.4_68100001	3	68100001	0.4633	0.5715
NC_005102.4_68200001	3	68200001	0.4867	0.621
NC_005102.4_68300001	3	68300001	0.6901	0.7759
NC_005102.4_68400001	3	68400001	0.7458	0.8223
NC_005102.4_68500001	3	68500001	0.7941	0.8958
NC_005102.4_68600001	3	68600001	0.8945	0.9865
NC_005102.4_68700001	3	68700001	0.8216	0.9056
NC_005102.4_68800001	3	68800001	0.6485	0.8487
NC_005102.4_69000001	3	69000001	0.49	0.6426

NC_005102.4_69100001	3	69100001	0.5611	0.6986
NC_005102.4_69200001	3	69200001	0.5652	0.7768
NC_005102.4_69300001	3	69300001	0.6694	0.7943
NC_005102.4_69400001	3	69400001	0.5146	0.7357
NC_005102.4_69500001	3	69500001	0.4963	0.6448
NC_005102.4_69600001	3	69600001	0.3688	0.3689
NC_005102.4_69700001	3	69700001	0.3688	0.3689
NC_005102.4_69800001	3	69800001	0.4689	0.5246
NC_005102.4_69900001	3	69900001	0.4078	0.4734
NC_005102.4_70000001	3	70000001	0.4432	0.5668
NC_005102.4_70100001	3	70100001	0.4722	0.7611
NC_005102.4_70200001	3	70200001	0.4442	0.62
NC_005102.4_70300001	3	70300001	0.4415	0.5849
NC_005102.4_70400001	3	70400001	0.5191	0.6285
NC_005102.4_70500001	3	70500001	0.5507	0.6676
NC_005102.4_70600001	3	70600001	0.5101	0.661
NC_005102.4_70700001	3	70700001	0.5433	0.7488
NC_005102.4_70800001	3	70800001	0.4941	0.7677
NC_005102.4_70900001	3	70900001	0.5691	0.851
NC_005102.4_71000001	3	71000001	0.5295	0.7544
NC_005102.4_71100001	3	71100001	0.6125	0.786
NC_005102.4_71200001	3	71200001	0.5756	0.743
NC_005102.4_71300001	3	71300001	0.5349	0.6445
NC_005102.4_71400001	3	71400001	0.4464	0.5935
NC_005102.4_71500001	3	71500001	0.4546	0.7318
NC_005102.4_71600001	3	71600001	0.5078	0.7598
NC_005102.4_71700001	3	71700001	0.5729	0.8257
NC_005102.4_71800001	3	71800001	0.6083	0.8315
NC_005102.4_71900001	3	71900001	0.6064	0.8015
NC_005102.4_72000001	3	72000001	0.636	0.8275
NC_005102.4_72100001	3	72100001	0.6037	0.7823
NC_005102.4_72200001	3	72200001	0.5598	0.7554
NC_005102.4_72300001	3	72300001	0.547	0.7682
NC_005102.4_72400001	3	72400001	0.5479	0.7645
NC_005102.4_72500001	3	72500001	0.5021	0.6425
NC_005102.4_72600001	3	72600001	0.4992	0.7073
NC_005102.4_72700001	3	72700001	0.4916	0.6391
NC_005102.4_72900001	3	72900001	0.7124	0.5876
NC_005102.4_73000001	3	73000001	0.6681	0.5524
NC_005102.4_73100001	3	73100001	0.5827	0.5495
NC_005102.4_73200001	3	73200001	0.5827	0.5495
NC_005102.4_73300001	3	73300001	0.4968	0.4961
NC_005102.4_74200001	3	74200001	0.4567	0.4689
NC_005102.4_74300001	3	74300001	0.4567	0.4689
NC_005102.4_74400001	3	74400001	0.4567	0.4689
NC_005102.4_75300001	3	75300001	0.8159	0.7982
NC_005102.4_75400001	3	75400001	0.7562	0.7589
NC_005102.4_75500001	3	75500001	0.7562	0.7589
NC_005102.4_75600001	3	75600001	0.5432	0.6664
NC_005102.4_75700001	3	75700001	0.3889	0.4986
NC_005102.4_75800001	3	75800001	0.1931	0.1604
NC_005102.4_75900001	3	75900001	0.1908	0.1733
NC_005102.4_76000001	3	76000001	0.2087	0.1681
NC_005102.4_76100001	3	76100001	0.2576	0.18
NC_005102.4_76200001	3	76200001	0.2349	0.163
NC_005102.4_76700001	3	76700001	0.6934	0.8041
NC_005102.4_76800001	3	76800001	0.7703	0.8772
NC_005102.4_77000001	3	77000001	0.5104	0.6667

NC_005102.4_77100001	3	77100001	0.4327	0.4769
NC_005102.4_77200001	3	77200001	0.4179	0.3549
NC_005102.4_77300001	3	77300001	0.4179	0.3549
NC_005102.4_77400001	3	77400001	0.4148	0.3438
NC_005102.4_77500001	3	77500001	0.5036	0.4555
NC_005102.4_77600001	3	77600001	0.5193	0.4692
NC_005102.4_77700001	3	77700001	0.4648	0.4539
NC_005102.4_77800001	3	77800001	0.4673	0.4004
NC_005102.4_77900001	3	77900001	0.4827	0.427
NC_005102.4_78000001	3	78000001	0.4464	0.3323
NC_005102.4_78200001	3	78200001	0.4641	0.3797
NC_005102.4_78300001	3	78300001	0.4292	0.481
NC_005102.4_78400001	3	78400001	0.4475	0.6251
NC_005102.4_78500001	3	78500001	0.3938	0.6115
NC_005102.4_78600001	3	78600001	0.4574	0.679
NC_005102.4_78700001	3	78700001	0.4484	0.7037
NC_005102.4_78800001	3	78800001	0.4508	0.7393
NC_005102.4_78900001	3	78900001	0.4492	0.7229
NC_005102.4_79000001	3	79000001	0.4378	0.679
NC_005102.4_79100001	3	79100001	0.3555	0.6233
NC_005102.4_79200001	3	79200001	0.4409	0.7404
NC_005102.4_79300001	3	79300001	0.4599	0.7604
NC_005102.4_79400001	3	79400001	0.4703	0.7189
NC_005102.4_79500001	3	79500001	0.5643	0.7686
NC_005102.4_79600001	3	79600001	0.5432	0.7641
NC_005102.4_79700001	3	79700001	0.5471	0.7395
NC_005102.4_79800001	3	79800001	0.518	0.7353
NC_005102.4_79900001	3	79900001	0.5167	0.7587
NC_005102.4_80000001	3	80000001	0.3954	0.734
NC_005102.4_80100001	3	80100001	0.573	0.8181
NC_005102.4_80200001	3	80200001	0.6073	0.8389
NC_005102.4_80300001	3	80300001	0.5471	0.7665
NC_005102.4_80400001	3	80400001	0.6525	0.8178
NC_005102.4_80500001	3	80500001	0.622	0.7839
NC_005102.4_80600001	3	80600001	0.6208	0.7568
NC_005102.4_80700001	3	80700001	0.6389	0.7734
NC_005102.4_80800001	3	80800001	0.6389	0.8046
NC_005102.4_80900001	3	80900001	0.5738	0.761
NC_005102.4_81000001	3	81000001	0.6682	0.8384
NC_005102.4_81100001	3	81100001	0.6302	0.8302
NC_005102.4_81200001	3	81200001	0.6138	0.8192
NC_005102.4_81300001	3	81300001	0.5972	0.7563
NC_005102.4_81400001	3	81400001	0.5767	0.7315
NC_005102.4_81500001	3	81500001	0.5351	0.7223
NC_005102.4_81600001	3	81600001	0.5046	0.6772
NC_005102.4_81700001	3	81700001	0.4668	0.6084
NC_005102.4_81800001	3	81800001	0.4444	0.6444
NC_005102.4_81900001	3	81900001	0.4572	0.5785
NC_005102.4_82000001	3	82000001	0.4947	0.6346
NC_005102.4_82100001	3	82100001	0.5594	0.7097
NC_005102.4_82200001	3	82200001	0.6034	0.7456
NC_005102.4_82300001	3	82300001	0.6046	0.7228
NC_005102.4_82400001	3	82400001	0.5907	0.7644
NC_005102.4_82500001	3	82500001	0.5767	0.7299
NC_005102.4_82600001	3	82600001	0.5646	0.7096
NC_005102.4_82700001	3	82700001	0.5017	0.693
NC_005102.4_82800001	3	82800001	0.6179	0.7741
NC_005102.4_82900001	3	82900001	0.6973	0.8304

NC_005102.4_83000001	3	83000001	0.7256	0.8352
NC_005102.4_83100001	3	83100001	0.7019	0.7614
NC_005102.4_83200001	3	83200001	0.5488	0.6215
NC_005102.4_83300001	3	83300001	0.4642	0.4091
NC_005102.4_83400001	3	83400001	0.4044	0.3959
NC_005102.4_83500001	3	83500001	0.5569	0.5862
NC_005102.4_83600001	3	83600001	0.4658	0.4488
NC_005102.4_83700001	3	83700001	0.3879	0.438
NC_005102.4_83800001	3	83800001	0.3813	0.47
NC_005102.4_83900001	3	83900001	0.4018	0.4743
NC_005102.4_84000001	3	84000001	0.2552	0.2839
NC_005102.4_84100001	3	84100001	0.214	0.4287
NC_005102.4_84200001	3	84200001	0.4285	0.5486
NC_005102.4_84300001	3	84300001	0.4285	0.5486
NC_005102.4_84400001	3	84400001	0.4285	0.5486
NC_005102.4_84900001	3	84900001	0.3417	0.3336
NC_005102.4_85000001	3	85000001	0.4818	0.4896
NC_005102.4_85100001	3	85100001	0.4818	0.4896
NC_005102.4_85200001	3	85200001	0.5594	0.5322
NC_005102.4_85500001	3	85500001	0.3973	0.6711
NC_005102.4_85600001	3	85600001	0.3317	0.6024
NC_005102.4_85700001	3	85700001	0.3608	0.6227
NC_005102.4_85800001	3	85800001	0.3392	0.6464
NC_005102.4_85900001	3	85900001	0.375	0.6106
NC_005102.4_86000001	3	86000001	0.4172	0.6931
NC_005102.4_86100001	3	86100001	0.4864	0.6906
NC_005102.4_86200001	3	86200001	0.5235	0.6763
NC_005102.4_86300001	3	86300001	0.5342	0.6444
NC_005102.4_86400001	3	86400001	0.5941	0.6839
NC_005102.4_86500001	3	86500001	0.5321	0.5948
NC_005102.4_86600001	3	86600001	0.5607	0.5904
NC_005102.4_86700001	3	86700001	0.5962	0.6313
NC_005102.4_86800001	3	86800001	0.678	0.6605
NC_005102.4_86900001	3	86900001	0.6352	0.6342
NC_005102.4_87000001	3	87000001	0.6368	0.695
NC_005102.4_87100001	3	87100001	0.6646	0.741
NC_005102.4_87200001	3	87200001	0.4384	0.6572
NC_005102.4_87300001	3	87300001	0.4384	0.6572
NC_005102.4_87400001	3	87400001	0.4193	0.5983
NC_005102.4_88300001	3	88300001	0.3716	0.663
NC_005102.4_88400001	3	88400001	0.4559	0.8546
NC_005102.4_88500001	3	88500001	0.4559	0.8546
NC_005102.4_88600001	3	88600001	0.4559	0.8546
NC_005102.4_88700001	3	88700001	0.683	0.9281
NC_005102.4_88900001	3	88900001	0.5233	0.6917
NC_005102.4_89000001	3	89000001	0.5197	0.7441
NC_005102.4_89100001	3	89100001	0.5843	0.793
NC_005102.4_89200001	3	89200001	0.5893	0.785
NC_005102.4_89300001	3	89300001	0.5793	0.7866
NC_005102.4_89400001	3	89400001	0.663	0.9135
NC_005102.4_89500001	3	89500001	0.774	0.9292
NC_005102.4_89700001	3	89700001	0.4653	0.6361
NC_005102.4_89800001	3	89800001	0.6081	0.7132
NC_005102.4_89900001	3	89900001	0.546	0.6979
NC_005102.4_90000001	3	90000001	0.4459	0.5928
NC_005102.4_90100001	3	90100001	0.4362	0.6125
NC_005102.4_90200001	3	90200001	0.4639	0.6902
NC_005102.4_90300001	3	90300001	0.4618	0.6141

NC_005102.4_90400001	3	90400001	0.5084	0.5818
NC_005102.4_90500001	3	90500001	0.4413	0.5885
NC_005102.4_90600001	3	90600001	0.4297	0.5486
NC_005102.4_90700001	3	90700001	0.5246	0.6215
NC_005102.4_90800001	3	90800001	0.444	0.6188
NC_005102.4_90900001	3	90900001	0.5064	0.68
NC_005102.4_91000001	3	91000001	0.5657	0.6927
NC_005102.4_91100001	3	91100001	0.6012	0.7164
NC_005102.4_91200001	3	91200001	0.5988	0.707
NC_005102.4_91300001	3	91300001	0.6153	0.7196
NC_005102.4_91400001	3	91400001	0.5962	0.714
NC_005102.4_91500001	3	91500001	0.6187	0.7109
NC_005102.4_91600001	3	91600001	0.6274	0.7052
NC_005102.4_91700001	3	91700001	0.5826	0.6913
NC_005102.4_91800001	3	91800001	0.4852	0.6205
NC_005102.4_91900001	3	91900001	0.475	0.6194
NC_005102.4_92000001	3	92000001	0.439	0.586
NC_005102.4_92100001	3	92100001	0.3627	0.5095
NC_005102.4_92200001	3	92200001	0.4058	0.5214
NC_005102.4_92300001	3	92300001	0.3823	0.4937
NC_005102.4_92400001	3	92400001	0.3942	0.4867
NC_005102.4_92500001	3	92500001	0.4448	0.6256
NC_005102.4_92600001	3	92600001	0.3993	0.5816
NC_005102.4_92700001	3	92700001	0.459	0.617
NC_005102.4_92800001	3	92800001	0.5235	0.639
NC_005102.4_92900001	3	92900001	0.479	0.6565
NC_005102.4_93000001	3	93000001	0.5303	0.64
NC_005102.4_93100001	3	93100001	0.5733	0.6773
NC_005102.4_93200001	3	93200001	0.5726	0.7073
NC_005102.4_93300001	3	93300001	0.5787	0.697
NC_005102.4_93400001	3	93400001	0.577	0.6947
NC_005102.4_93500001	3	93500001	0.4889	0.6106
NC_005102.4_93600001	3	93600001	0.4889	0.6106
NC_005102.4_93700001	3	93700001	0.4363	0.5033
NC_005102.4_93800001	3	93800001	0.3996	0.5008
NC_005102.4_93900001	3	93900001	0.4269	0.5016
NC_005102.4_94000001	3	94000001	0.4808	0.5526
NC_005102.4_94100001	3	94100001	0.4406	0.5494
NC_005102.4_94200001	3	94200001	0.4033	0.6015
NC_005102.4_94300001	3	94300001	0.326	0.5155
NC_005102.4_94400001	3	94400001	0.2939	0.4514
NC_005102.4_94500001	3	94500001	0.1278	0.1131
NC_005102.4_94600001	3	94600001	0.3125	0.4483
NC_005102.4_94700001	3	94700001	0.3552	0.5197
NC_005102.4_94800001	3	94800001	0.3564	0.5689
NC_005102.4_94900001	3	94900001	0.435	0.6645
NC_005102.4_95000001	3	95000001	0.4665	0.7081
NC_005102.4_95100001	3	95100001	0.4986	0.7724
NC_005102.4_95200001	3	95200001	0.499	0.7577
NC_005102.4_95300001	3	95300001	0.5566	0.7145
NC_005102.4_95400001	3	95400001	0.5361	0.6727
NC_005102.4_95500001	3	95500001	0.5222	0.678
NC_005102.4_95600001	3	95600001	0.4314	0.6302
NC_005102.4_95700001	3	95700001	0.3986	0.5805
NC_005102.4_95800001	3	95800001	0.3115	0.5546
NC_005102.4_95900001	3	95900001	0.198	0.3958
NC_005102.4_96000001	3	96000001	0.1043	0.1034
NC_005102.4_96800001	3	96800001	0.6263	0.7996

NC_005102.4_96900001	3	96900001	0.6278	0.8248
NC_005102.4_97000001	3	97000001	0.6044	0.801
NC_005102.4_97100001	3	97100001	0.5641	0.788
NC_005102.4_97200001	3	97200001	0.5636	0.8083
NC_005102.4_97300001	3	97300001	0.5118	0.7791
NC_005102.4_97400001	3	97400001	0.4676	0.6876
NC_005102.4_97500001	3	97500001	0.4787	0.6969
NC_005102.4_97600001	3	97600001	0.4787	0.6595
NC_005102.4_97700001	3	97700001	0.4471	0.5955
NC_005102.4_97800001	3	97800001	0.535	0.6707
NC_005102.4_97900001	3	97900001	0.6144	0.7972
NC_005102.4_98000001	3	98000001	0.6694	0.831
NC_005102.4_98100001	3	98100001	0.7579	0.8969
NC_005102.4_98200001	3	98200001	0.7874	0.9
NC_005102.4_98300001	3	98300001	0.7214	0.8458
NC_005102.4_98400001	3	98400001	0.7041	0.7894
NC_005102.4_98500001	3	98500001	0.6363	0.7508
NC_005102.4_98600001	3	98600001	0.5569	0.6723
NC_005102.4_98700001	3	98700001	0.5569	0.6723
NC_005102.4_98800001	3	98800001	0.5678	0.6661
NC_005102.4_99000001	3	99000001	0.5256	0.6532
NC_005102.4_99100001	3	99100001	0.5568	0.6408
NC_005102.4_99200001	3	99200001	0.5427	0.6824
NC_005102.4_99300001	3	99300001	0.6146	0.8006
NC_005102.4_99400001	3	99400001	0.6175	0.8232
NC_005102.4_99500001	3	99500001	0.682	0.8847
NC_005102.4_99600001	3	99600001	0.6984	0.9335
NC_005102.4_99700001	3	99700001	0.7425	0.9163
NC_005102.4_99800001	3	99800001	0.651	0.8803
NC_005102.4_99900001	3	99900001	0.5329	0.7309
NC_005102.4_100000001	3	100000001	0.4318	0.6035
NC_005102.4_100100001	3	100100001	0.3581	0.4407
NC_005102.4_100200001	3	100200001	0.4252	0.5276
NC_005102.4_100300001	3	100300001	0.524	0.5754
NC_005102.4_100400001	3	100400001	0.5565	0.5945
NC_005102.4_100500001	3	100500001	0.5688	0.6147
NC_005102.4_100600001	3	100600001	0.6113	0.6587
NC_005102.4_100700001	3	100700001	0.5535	0.6713
NC_005102.4_100800001	3	100800001	0.5286	0.6848
NC_005102.4_100900001	3	100900001	0.6088	0.7734
NC_005102.4_101000001	3	101000001	0.6424	0.7972
NC_005102.4_101100001	3	101100001	0.6801	0.8269
NC_005102.4_101200001	3	101200001	0.8498	0.8016
NC_005102.4_101300001	3	101300001	0.9012	0.9029
NC_005102.4_101400001	3	101400001	0.6373	0.7892
NC_005102.4_101500001	3	101500001	0.5798	0.7393
NC_005102.4_101600001	3	101600001	0.5849	0.7277
NC_005102.4_101700001	3	101700001	0.518	0.7165
NC_005102.4_101800001	3	101800001	0.48	0.6788
NC_005102.4_101900001	3	101900001	0.4418	0.6247
NC_005102.4_102000001	3	102000001	0.4671	0.6628
NC_005102.4_102100001	3	102100001	0.3863	0.6581
NC_005102.4_102200001	3	102200001	0.5191	0.6988
NC_005102.4_102300001	3	102300001	0.5997	0.7703
NC_005102.4_102500001	3	102500001	0.6516	0.634
NC_005102.4_102600001	3	102600001	0.512	0.5882
NC_005102.4_102700001	3	102700001	0.5075	0.5823
NC_005102.4_102800001	3	102800001	0.4184	0.458

NC_005102.4_103400001	3	103400001	0.6149	0.7165
NC_005102.4_103500001	3	103500001	0.6294	0.6804
NC_005102.4_103600001	3	103600001	0.6294	0.6804
NC_005102.4_103700001	3	103700001	0.5668	0.7054
NC_005102.4_103800001	3	103800001	0.5003	0.6682
NC_005102.4_103900001	3	103900001	0.344	0.5704
NC_005102.4_104000001	3	104000001	0.2447	0.4872
NC_005102.4_104100001	3	104100001	0.4102	0.6944
NC_005102.4_104200001	3	104200001	0.4201	0.7004
NC_005102.4_104300001	3	104300001	0.5158	0.738
NC_005102.4_104400001	3	104400001	0.5423	0.6751
NC_005102.4_104500001	3	104500001	0.6324	0.7234
NC_005102.4_104600001	3	104600001	0.5175	0.5444
NC_005102.4_104700001	3	104700001	0.7197	0.7169
NC_005102.4_104800001	3	104800001	0.732	0.7267
NC_005102.4_104900001	3	104900001	0.7516	0.7527
NC_005102.4_105000001	3	105000001	0.7426	0.7125
NC_005102.4_105100001	3	105100001	0.6712	0.6778
NC_005102.4_105200001	3	105200001	0.6019	0.6246
NC_005102.4_105300001	3	105300001	0.5456	0.6091
NC_005102.4_105400001	3	105400001	0.5232	0.5986
NC_005102.4_105500001	3	105500001	0.3849	0.5545
NC_005102.4_105600001	3	105600001	0.3463	0.5945
NC_005102.4_105700001	3	105700001	0.3767	0.6069
NC_005102.4_105800001	3	105800001	0.291	0.4697
NC_005102.4_105900001	3	105900001	0.4435	0.6583
NC_005102.4_106000001	3	106000001	0.5698	0.7973
NC_005102.4_106100001	3	106100001	0.5776	0.8083
NC_005102.4_106200001	3	106200001	0.546	0.7692
NC_005102.4_106300001	3	106300001	0.477	0.7341
NC_005102.4_106400001	3	106400001	0.3975	0.6487
NC_005102.4_106500001	3	106500001	0.4496	0.671
NC_005102.4_106600001	3	106600001	0.4668	0.7212
NC_005102.4_106700001	3	106700001	0.5268	0.7213
NC_005102.4_106800001	3	106800001	0.586	0.7303
NC_005102.4_106900001	3	106900001	0.6715	0.7648
NC_005102.4_107000001	3	107000001	0.6295	0.7082
NC_005102.4_107100001	3	107100001	0.6309	0.6957
NC_005102.4_107200001	3	107200001	0.5639	0.6991
NC_005102.4_107300001	3	107300001	0.684	0.7159
NC_005102.4_107400001	3	107400001	0.6465	0.7337
NC_005102.4_107500001	3	107500001	0.6333	0.7554
NC_005102.4_107600001	3	107600001	0.6946	0.7802
NC_005102.4_107700001	3	107700001	0.7419	0.7858
NC_005102.4_107800001	3	107800001	0.7125	0.8589
NC_005102.4_107900001	3	107900001	0.6253	0.7527
NC_005102.4_108000001	3	108000001	0.6588	0.7585
NC_005102.4_108100001	3	108100001	0.5929	0.6822
NC_005102.4_108200001	3	108200001	0.5211	0.655
NC_005102.4_108300001	3	108300001	0.5736	0.6904
NC_005102.4_108400001	3	108400001	0.6246	0.7506
NC_005102.4_108500001	3	108500001	0.5736	0.7415
NC_005102.4_108600001	3	108600001	0.5576	0.7699
NC_005102.4_108700001	3	108700001	0.6002	0.8127
NC_005102.4_108800001	3	108800001	0.6362	0.8235
NC_005102.4_108900001	3	108900001	0.6461	0.8108
NC_005102.4_109000001	3	109000001	0.7273	0.7861
NC_005102.4_109100001	3	109100001	0.7249	0.7301

NC_005102.4_109200001	3	109200001	0.6952	0.763
NC_005102.4_109300001	3	109300001	0.5471	0.7066
NC_005102.4_109400001	3	109400001	0.5823	0.744
NC_005102.4_109500001	3	109500001	0.5776	0.7751
NC_005102.4_109600001	3	109600001	0.4176	0.6977
NC_005102.4_109700001	3	109700001	0.4985	0.6766
NC_005102.4_109800001	3	109800001	0.4973	0.6372
NC_005102.4_109900001	3	109900001	0.4788	0.6095
NC_005102.4_110000001	3	110000001	0.5348	0.6869
NC_005102.4_110100001	3	110100001	0.632	0.7619
NC_005102.4_110200001	3	110200001	0.7123	0.8497
NC_005102.4_110300001	3	110300001	0.8179	0.9058
NC_005102.4_110400001	3	110400001	0.7957	0.8923
NC_005102.4_110500001	3	110500001	0.7049	0.8666
NC_005102.4_110600001	3	110600001	0.7915	0.9187
NC_005102.4_110700001	3	110700001	0.7279	0.8469
NC_005102.4_110800001	3	110800001	0.6113	0.8472
NC_005102.4_110900001	3	110900001	0.6457	0.8325
NC_005102.4_111000001	3	111000001	0.6557	0.8058
NC_005102.4_111100001	3	111100001	0.5884	0.7452
NC_005102.4_111200001	3	111200001	0.5343	0.7437
NC_005102.4_111300001	3	111300001	0.5318	0.7271
NC_005102.4_111400001	3	111400001	0.5914	0.7781
NC_005102.4_111500001	3	111500001	0.585	0.787
NC_005102.4_111600001	3	111600001	0.5639	0.6944
NC_005102.4_111700001	3	111700001	0.5897	0.7076
NC_005102.4_111800001	3	111800001	0.5944	0.7142
NC_005102.4_111900001	3	111900001	0.5261	0.6506
NC_005102.4_112000001	3	112000001	0.5469	0.6136
NC_005102.4_112100001	3	112100001	0.5758	0.7472
NC_005102.4_112200001	3	112200001	0.6107	0.7876
NC_005102.4_112300001	3	112300001	0.7828	0.8896
NC_005102.4_112400001	3	112400001	0.6862	0.838
NC_005102.4_112500001	3	112500001	0.6474	0.885
NC_005102.4_112600001	3	112600001	0.5983	0.7698
NC_005102.4_112700001	3	112700001	0.5511	0.7268
NC_005102.4_112800001	3	112800001	0.5255	0.7068
NC_005102.4_112900001	3	112900001	0.4679	0.7012
NC_005102.4_113000001	3	113000001	0.5033	0.7141
NC_005102.4_113100001	3	113100001	0.5497	0.7833
NC_005102.4_113200001	3	113200001	0.5792	0.7573
NC_005102.4_113300001	3	113300001	0.653	0.7339
NC_005102.4_113400001	3	113400001	0.7985	0.7356
NC_005102.4_113500001	3	113500001	0.76	0.681
NC_005102.4_113600001	3	113600001	0.7409	0.6828
NC_005102.4_113700001	3	113700001	0.7594	0.7101
NC_005102.4_113800001	3	113800001	0.8267	0.7949
NC_005102.4_113900001	3	113900001	0.5717	0.6949
NC_005102.4_114000001	3	114000001	0.5749	0.7471
NC_005102.4_114100001	3	114100001	0.5497	0.772
NC_005102.4_114200001	3	114200001	0.4935	0.722
NC_005102.4_114300001	3	114300001	0.4841	0.6384
NC_005102.4_114400001	3	114400001	0.7033	0.7578
NC_005102.4_114500001	3	114500001	0.7033	0.7578
NC_005102.4_114600001	3	114600001	0.6233	0.6907
NC_005102.4_114700001	3	114700001	0.5954	0.6482
NC_005102.4_114800001	3	114800001	0.4408	0.3978
NC_005102.4_114900001	3	114900001	0.4533	0.4798

NC_005102.4_115000001	3	115000001	0.4418	0.5059
NC_005102.4_115100001	3	115100001	0.4699	0.5392
NC_005102.4_115200001	3	115200001	0.5238	0.6182
NC_005102.4_115300001	3	115300001	0.6111	0.7415
NC_005102.4_115400001	3	115400001	0.6135	0.6989
NC_005102.4_115500001	3	115500001	0.7377	0.7244
NC_005102.4_115600001	3	115600001	0.7377	0.7244
NC_005102.4_115700001	3	115700001	0.6816	0.669
NC_005102.4_116100001	3	116100001	0.7451	0.7327
NC_005102.4_116200001	3	116200001	0.6527	0.6004
NC_005102.4_116300001	3	116300001	0.5948	0.5671
NC_005102.4_116400001	3	116400001	0.5558	0.5545
NC_005102.4_116500001	3	116500001	0.5002	0.5309
NC_005102.4_116600001	3	116600001	0.469	0.5477
NC_005102.4_116700001	3	116700001	0.4956	0.617
NC_005102.4_116800001	3	116800001	0.5235	0.6563
NC_005102.4_116900001	3	116900001	0.5514	0.6702
NC_005102.4_117000001	3	117000001	0.6252	0.7129
NC_005102.4_117100001	3	117100001	0.6184	0.7064
NC_005102.4_117200001	3	117200001	0.5617	0.6497
NC_005102.4_117300001	3	117300001	0.5155	0.6798
NC_005102.4_117400001	3	117400001	0.4587	0.6737
NC_005102.4_117500001	3	117500001	0.4623	0.6664
NC_005102.4_117600001	3	117600001	0.4508	0.6309
NC_005102.4_117700001	3	117700001	0.5156	0.7388
NC_005102.4_117800001	3	117800001	0.5318	0.726
NC_005102.4_117900001	3	117900001	0.6511	0.7715
NC_005102.4_118000001	3	118000001	0.6288	0.785
NC_005102.4_118100001	3	118100001	0.5544	0.7775
NC_005102.4_118200001	3	118200001	0.5289	0.7238
NC_005102.4_118300001	3	118300001	0.5046	0.708
NC_005102.4_118400001	3	118400001	0.399	0.5009
NC_005102.4_118500001	3	118500001	0.4138	0.4828
NC_005102.4_118600001	3	118600001	0.45	0.5206
NC_005102.4_118700001	3	118700001	0.438	0.5066
NC_005102.4_118800001	3	118800001	0.4172	0.4368
NC_005102.4_118900001	3	118900001	0.5947	0.7122
NC_005102.4_119000001	3	119000001	0.5123	0.6469
NC_005102.4_119100001	3	119100001	0.46	0.6253
NC_005102.4_119200001	3	119200001	0.5004	0.679
NC_005102.4_119300001	3	119300001	0.5229	0.7125
NC_005102.4_119400001	3	119400001	0.3713	0.5518
NC_005102.4_119500001	3	119500001	0.4544	0.6116
NC_005102.4_119600001	3	119600001	0.4461	0.5571
NC_005102.4_119700001	3	119700001	0.4321	0.5375
NC_005102.4_119800001	3	119800001	0.4022	0.4745
NC_005102.4_119900001	3	119900001	0.4826	0.5945
NC_005102.4_120000001	3	120000001	0.5017	0.6118
NC_005102.4_120100001	3	120100001	0.5675	0.7327
NC_005102.4_120200001	3	120200001	0.6144	0.7225
NC_005102.4_120300001	3	120300001	0.6296	0.7466
NC_005102.4_120400001	3	120400001	0.6139	0.732
NC_005102.4_120500001	3	120500001	0.5859	0.7085
NC_005102.4_120600001	3	120600001	0.5652	0.6766
NC_005102.4_120700001	3	120700001	0.514	0.6634
NC_005102.4_120800001	3	120800001	0.5733	0.735
NC_005102.4_120900001	3	120900001	0.502	0.7058
NC_005102.4_121000001	3	121000001	0.4894	0.7209

NC_005102.4_121100001	3	121100001	0.4922	0.7778
NC_005102.4_121200001	3	121200001	0.4771	0.7794
NC_005102.4_121300001	3	121300001	0.3578	0.693
NC_005102.4_121400001	3	121400001	0.4324	0.729
NC_005102.4_121500001	3	121500001	0.5036	0.7872
NC_005102.4_121600001	3	121600001	0.5341	0.7776
NC_005102.4_121700001	3	121700001	0.5356	0.7121
NC_005102.4_121800001	3	121800001	0.5256	0.7162
NC_005102.4_121900001	3	121900001	0.5548	0.7055
NC_005102.4_122000001	3	122000001	0.4965	0.6607
NC_005102.4_122100001	3	122100001	0.4863	0.6661
NC_005102.4_122200001	3	122200001	0.5023	0.6877
NC_005102.4_122300001	3	122300001	0.5174	0.6909
NC_005102.4_122400001	3	122400001	0.4842	0.6634
NC_005102.4_122500001	3	122500001	0.5205	0.7069
NC_005102.4_122600001	3	122600001	0.5651	0.6991
NC_005102.4_122700001	3	122700001	0.5131	0.7008
NC_005102.4_122800001	3	122800001	0.5061	0.6888
NC_005102.4_122900001	3	122900001	0.5152	0.7309
NC_005102.4_123000001	3	123000001	0.5631	0.7659
NC_005102.4_123100001	3	123100001	0.5333	0.7984
NC_005102.4_123200001	3	123200001	0.6019	0.7938
NC_005102.4_123300001	3	123300001	0.569	0.8017
NC_005102.4_123400001	3	123400001	0.5683	0.7993
NC_005102.4_123500001	3	123500001	0.5675	0.7416
NC_005102.4_123600001	3	123600001	0.6178	0.7825
NC_005102.4_123700001	3	123700001	0.6405	0.7406
NC_005102.4_123800001	3	123800001	0.6439	0.6283
NC_005102.4_123900001	3	123900001	0.6121	0.6212
NC_005102.4_124000001	3	124000001	0.5043	0.5599
NC_005102.4_124100001	3	124100001	0.472	0.5406
NC_005102.4_124200001	3	124200001	0.4916	0.596
NC_005102.4_124300001	3	124300001	0.5258	0.6723
NC_005102.4_124400001	3	124400001	0.5002	0.6413
NC_005102.4_124500001	3	124500001	0.5469	0.662
NC_005102.4_124600001	3	124600001	0.4774	0.5744
NC_005102.4_124700001	3	124700001	0.4003	0.4808
NC_005102.4_124800001	3	124800001	0.3885	0.5399
NC_005102.4_124900001	3	124900001	0.4499	0.5894
NC_005102.4_125000001	3	125000001	0.4612	0.5612
NC_005102.4_125100001	3	125100001	0.5018	0.6394
NC_005102.4_125200001	3	125200001	0.453	0.5712
NC_005102.4_125300001	3	125300001	0.5134	0.5734
NC_005102.4_125400001	3	125400001	0.425	0.493
NC_005102.4_125500001	3	125500001	0.3769	0.517
NC_005102.4_125600001	3	125600001	0.465	0.5965
NC_005102.4_125700001	3	125700001	0.5452	0.7491
NC_005102.4_125900001	3	125900001	0.317	0.4445
NC_005102.4_126000001	3	126000001	0.3145	0.4385
NC_005102.4_126100001	3	126100001	0.348	0.4871
NC_005102.4_126200001	3	126200001	0.4226	0.5884
NC_005102.4_126300001	3	126300001	0.4137	0.5985
NC_005102.4_126400001	3	126400001	0.3592	0.5672
NC_005102.4_126500001	3	126500001	0.4518	0.7104
NC_005102.4_126600001	3	126600001	0.4901	0.7583
NC_005102.4_126700001	3	126700001	0.4709	0.8223
NC_005102.4_126800001	3	126800001	0.4732	0.6782
NC_005102.4_126900001	3	126900001	0.6079	0.7562

NC_005102.4_127000001	3	127000001	0.5624	0.7247
NC_005102.4_127100001	3	127100001	0.5633	0.7284
NC_005102.4_127200001	3	127200001	0.6249	0.769
NC_005102.4_127300001	3	127300001	0.7078	0.7685
NC_005102.4_127400001	3	127400001	0.5951	0.7007
NC_005102.4_127500001	3	127500001	0.6023	0.6972
NC_005102.4_127600001	3	127600001	0.6312	0.6524
NC_005102.4_127700001	3	127700001	0.5677	0.5873
NC_005102.4_127800001	3	127800001	0.5697	0.6451
NC_005102.4_127900001	3	127900001	0.6091	0.6701
NC_005102.4_128000001	3	128000001	0.6457	0.7008
NC_005102.4_128100001	3	128100001	0.5579	0.7133
NC_005102.4_128200001	3	128200001	0.4896	0.6689
NC_005102.4_128300001	3	128300001	0.5275	0.6764
NC_005102.4_128400001	3	128400001	0.5344	0.6513
NC_005102.4_128500001	3	128500001	0.5823	0.6765
NC_005102.4_128600001	3	128600001	0.5919	0.6701
NC_005102.4_128700001	3	128700001	0.6773	0.6946
NC_005102.4_128800001	3	128800001	0.6451	0.6563
NC_005102.4_128900001	3	128900001	0.6448	0.6536
NC_005102.4_129000001	3	129000001	0.5218	0.5543
NC_005102.4_129100001	3	129100001	0.5713	0.5916
NC_005102.4_129200001	3	129200001	0.5738	0.6258
NC_005102.4_129300001	3	129300001	0.4804	0.6403
NC_005102.4_129400001	3	129400001	0.535	0.7259
NC_005102.4_129500001	3	129500001	0.5403	0.7484
NC_005102.4_129600001	3	129600001	0.5381	0.7691
NC_005102.4_129700001	3	129700001	0.5379	0.8431
NC_005102.4_129800001	3	129800001	0.5816	0.8344
NC_005102.4_129900001	3	129900001	0.4886	0.7521
NC_005102.4_130000001	3	130000001	0.4713	0.7416
NC_005102.4_130100001	3	130100001	0.5187	0.6672
NC_005102.4_130200001	3	130200001	0.5264	0.6165
NC_005102.4_130300001	3	130300001	0.5764	0.6153
NC_005102.4_130400001	3	130400001	0.6776	0.6887
NC_005102.4_130500001	3	130500001	0.7118	0.6855
NC_005102.4_130600001	3	130600001	0.7291	0.7353
NC_005102.4_130700001	3	130700001	0.6877	0.7389
NC_005102.4_130800001	3	130800001	0.6084	0.7233
NC_005102.4_130900001	3	130900001	0.5692	0.6985
NC_005102.4_131000001	3	131000001	0.4616	0.656
NC_005102.4_131100001	3	131100001	0.4205	0.6363
NC_005102.4_131200001	3	131200001	0.3746	0.5344
NC_005102.4_131300001	3	131300001	0.4551	0.5988
NC_005102.4_131400001	3	131400001	0.3656	0.5089
NC_005102.4_131500001	3	131500001	0.4491	0.569
NC_005102.4_131600001	3	131600001	0.3741	0.5044
NC_005102.4_131700001	3	131700001	0.4013	0.5141
NC_005102.4_131800001	3	131800001	0.1933	0.1622
NC_005102.4_131900001	3	131900001	0.3014	0.3038
NC_005102.4_132000001	3	132000001	0.253	0.2702
NC_005102.4_132100001	3	132100001	0.2948	0.2523
NC_005102.4_132200001	3	132200001	0.4995	0.6158
NC_005102.4_132300001	3	132300001	0.5068	0.6791
NC_005102.4_132400001	3	132400001	0.5316	0.643
NC_005102.4_132500001	3	132500001	0.5112	0.6372
NC_005102.4_132600001	3	132600001	0.5418	0.6725
NC_005102.4_132700001	3	132700001	0.5507	0.6717

NC_005102.4_132800001	3	132800001	0.617	0.6893
NC_005102.4_132900001	3	132900001	0.647	0.7467
NC_005102.4_133000001	3	133000001	0.7445	0.7742
NC_005102.4_133100001	3	133100001	0.7646	0.7798
NC_005102.4_133200001	3	133200001	0.7846	0.8032
NC_005102.4_133900001	3	133900001	0.7716	0.8667
NC_005102.4_134000001	3	134000001	0.7828	0.8582
NC_005102.4_134100001	3	134100001	0.7462	0.8553
NC_005102.4_134200001	3	134200001	0.5946	0.7962
NC_005102.4_134300001	3	134300001	0.4594	0.6517
NC_005102.4_134400001	3	134400001	0.366	0.5055
NC_005102.4_134500001	3	134500001	0.3581	0.4808
NC_005102.4_134600001	3	134600001	0.3495	0.4748
NC_005102.4_134700001	3	134700001	0.4799	0.5642
NC_005102.4_134800001	3	134800001	0.5688	0.6843
NC_005102.4_134900001	3	134900001	0.4275	0.6367
NC_005102.4_135000001	3	135000001	0.4529	0.6602
NC_005102.4_135100001	3	135100001	0.4508	0.6639
NC_005102.4_135200001	3	135200001	0.4199	0.6218
NC_005102.4_135300001	3	135300001	0.4318	0.5952
NC_005102.4_135400001	3	135400001	0.6588	0.7245
NC_005102.4_135500001	3	135500001	0.5919	0.6681
NC_005102.4_135600001	3	135600001	0.5896	0.5622
NC_005102.4_135700001	3	135700001	0.5612	0.5477
NC_005102.4_135800001	3	135800001	0.5843	0.532
NC_005102.4_135900001	3	135900001	0.4679	0.4652
NC_005102.4_136000001	3	136000001	0.5375	0.5728
NC_005102.4_136100001	3	136100001	0.5776	0.6547
NC_005102.4_136200001	3	136200001	0.6041	0.741
NC_005102.4_136300001	3	136300001	0.573	0.6976
NC_005102.4_136400001	3	136400001	0.6324	0.7207
NC_005102.4_136500001	3	136500001	0.59	0.6921
NC_005102.4_136600001	3	136600001	0.569	0.6691
NC_005102.4_136700001	3	136700001	0.4983	0.6115
NC_005102.4_136800001	3	136800001	0.556	0.6991
NC_005102.4_136900001	3	136900001	0.5054	0.7564
NC_005102.4_137000001	3	137000001	0.4505	0.7762
NC_005102.4_137100001	3	137100001	0.4995	0.7597
NC_005102.4_137200001	3	137200001	0.5917	0.7374
NC_005102.4_137300001	3	137300001	0.5617	0.649
NC_005102.4_137400001	3	137400001	0.5627	0.6183
NC_005102.4_137500001	3	137500001	0.5571	0.5767
NC_005102.4_137600001	3	137600001	0.4886	0.5928
NC_005102.4_137700001	3	137700001	0.4891	0.6329
NC_005102.4_137800001	3	137800001	0.4711	0.6315
NC_005102.4_137900001	3	137900001	0.4245	0.5672
NC_005102.4_138000001	3	138000001	0.4236	0.6133
NC_005102.4_138100001	3	138100001	0.4406	0.5618
NC_005102.4_138200001	3	138200001	0.441	0.4992
NC_005102.4_138300001	3	138300001	0.3867	0.4613
NC_005102.4_138400001	3	138400001	0.4132	0.5142
NC_005102.4_138500001	3	138500001	0.4831	0.5759
NC_005102.4_138600001	3	138600001	0.4542	0.5968
NC_005102.4_138700001	3	138700001	0.409	0.6304
NC_005102.4_138800001	3	138800001	0.4294	0.6327
NC_005102.4_138900001	3	138900001	0.4962	0.6372
NC_005102.4_139000001	3	139000001	0.3918	0.5189
NC_005102.4_139100001	3	139100001	0.3789	0.4572

NC_005102.4_139200001	3	139200001	0.5205	0.6036
NC_005102.4_139300001	3	139300001	0.5379	0.6546
NC_005102.4_139400001	3	139400001	0.5464	0.6862
NC_005102.4_139500001	3	139500001	0.5055	0.6793
NC_005102.4_139600001	3	139600001	0.6145	0.7598
NC_005102.4_139700001	3	139700001	0.5205	0.7051
NC_005102.4_139800001	3	139800001	0.54	0.6309
NC_005102.4_139900001	3	139900001	0.5574	0.6193
NC_005102.4_140000001	3	140000001	0.5536	0.6093
NC_005102.4_140100001	3	140100001	0.5927	0.6299
NC_005102.4_140200001	3	140200001	0.6428	0.6665
NC_005102.4_140300001	3	140300001	0.6363	0.7264
NC_005102.4_140400001	3	140400001	0.6111	0.7285
NC_005102.4_140500001	3	140500001	0.6833	0.7506
NC_005102.4_140600001	3	140600001	0.6058	0.717
NC_005102.4_140700001	3	140700001	0.5285	0.6578
NC_005102.4_140800001	3	140800001	0.5683	0.6733
NC_005102.4_140900001	3	140900001	0.4934	0.5137
NC_005102.4_141000001	3	141000001	0.4045	0.4277
NC_005102.4_141100001	3	141100001	0.317	0.2797
NC_005102.4_141200001	3	141200001	0.3258	0.3194
NC_005102.4_141300001	3	141300001	0.2743	0.2636
NC_005102.4_141400001	3	141400001	0.2712	0.2634
NC_005102.4_141500001	3	141500001	0.3058	0.3182
NC_005102.4_141600001	3	141600001	0.3942	0.4732
NC_005102.4_141700001	3	141700001	0.4675	0.5622
NC_005102.4_141800001	3	141800001	0.5496	0.619
NC_005102.4_141900001	3	141900001	0.6265	0.7464
NC_005102.4_142000001	3	142000001	0.6165	0.7619
NC_005102.4_142100001	3	142100001	0.5079	0.6934
NC_005102.4_142200001	3	142200001	0.4392	0.623
NC_005102.4_142300001	3	142300001	0.3896	0.5189
NC_005102.4_142400001	3	142400001	0.3104	0.3419
NC_005102.4_142500001	3	142500001	0.3981	0.4714
NC_005102.4_142600001	3	142600001	0.3667	0.4444
NC_005102.4_142700001	3	142700001	0.4826	0.5793
NC_005102.4_142800001	3	142800001	0.4415	0.5828
NC_005102.4_142900001	3	142900001	0.4399	0.6346
NC_005102.4_143000001	3	143000001	0.3576	0.5436
NC_005102.4_143100001	3	143100001	0.5224	0.6729
NC_005102.4_143700001	3	143700001	0.5889	0.7428
NC_005102.4_143800001	3	143800001	0.5889	0.7428
NC_005102.4_143900001	3	143900001	0.6542	0.7478
NC_005102.4_144000001	3	144000001	0.6909	0.7357
NC_005102.4_144100001	3	144100001	0.659	0.7105
NC_005102.4_144200001	3	144200001	0.5733	0.6521
NC_005102.4_144300001	3	144300001	0.568	0.67
NC_005102.4_144400001	3	144400001	0.5188	0.6421
NC_005102.4_144500001	3	144500001	0.4654	0.6012
NC_005102.4_144600001	3	144600001	0.5274	0.7124
NC_005102.4_144700001	3	144700001	0.6979	0.7465
NC_005102.4_144800001	3	144800001	0.8617	0.8296
NC_005102.4_144900001	3	144900001	0.7478	0.7627
NC_005102.4_145000001	3	145000001	0.7893	0.8109
NC_005102.4_145100001	3	145100001	0.7606	0.7828
NC_005102.4_145200001	3	145200001	0.7289	0.7981
NC_005102.4_145600001	3	145600001	0.677	0.7494
NC_005102.4_145700001	3	145700001	0.5518	0.7363

NC_005102.4_145800001	3	145800001	0.5883	0.7525
NC_005102.4_145900001	3	145900001	0.5528	0.7426
NC_005102.4_146000001	3	146000001	0.6157	0.6858
NC_005102.4_146100001	3	146100001	0.6006	0.6668
NC_005102.4_146200001	3	146200001	0.6785	0.6903
NC_005102.4_146300001	3	146300001	0.5414	0.6648
NC_005102.4_146400001	3	146400001	0.5588	0.6683
NC_005102.4_146500001	3	146500001	0.4559	0.6418
NC_005102.4_146600001	3	146600001	0.4057	0.5163
NC_005102.4_146700001	3	146700001	0.4574	0.6105
NC_005102.4_146800001	3	146800001	0.4882	0.5655
NC_005102.4_146900001	3	146900001	0.5078	0.6004
NC_005102.4_147000001	3	147000001	0.5238	0.5915
NC_005102.4_147100001	3	147100001	0.5256	0.6594
NC_005102.4_147200001	3	147200001	0.4879	0.5895
NC_005102.4_147300001	3	147300001	0.5118	0.6626
NC_005102.4_147400001	3	147400001	0.4589	0.5872
NC_005102.4_147500001	3	147500001	0.6129	0.6681
NC_005102.4_147600001	3	147600001	0.6865	0.7272
NC_005102.4_147700001	3	147700001	0.5883	0.7214
NC_005102.4_147800001	3	147800001	0.6228	0.7398
NC_005102.4_147900001	3	147900001	0.6446	0.7594
NC_005102.4_148000001	3	148000001	0.5924	0.719
NC_005102.4_148100001	3	148100001	0.6267	0.7561
NC_005102.4_148200001	3	148200001	0.7079	0.7599
NC_005102.4_148300001	3	148300001	0.6534	0.7417
NC_005102.4_148400001	3	148400001	0.6619	0.7524
NC_005102.4_148500001	3	148500001	0.6421	0.8048
NC_005102.4_148600001	3	148600001	0.5356	0.6648
NC_005102.4_148700001	3	148700001	0.4568	0.6123
NC_005102.4_148800001	3	148800001	0.4733	0.6094
NC_005102.4_148900001	3	148900001	0.4217	0.5653
NC_005102.4_149000001	3	149000001	0.4544	0.5777
NC_005102.4_149100001	3	149100001	0.4583	0.661
NC_005102.4_149200001	3	149200001	0.4753	0.6695
NC_005102.4_149300001	3	149300001	0.5501	0.735
NC_005102.4_149400001	3	149400001	0.5808	0.7027
NC_005102.4_149500001	3	149500001	0.6715	0.8
NC_005102.4_149600001	3	149600001	0.7416	0.8007
NC_005102.4_149700001	3	149700001	0.7502	0.8238
NC_005102.4_149800001	3	149800001	0.7124	0.7786
NC_005102.4_149900001	3	149900001	0.7469	0.8301
NC_005102.4_150000001	3	150000001	0.7305	0.7731
NC_005102.4_150100001	3	150100001	0.5915	0.6921
NC_005102.4_150200001	3	150200001	0.5289	0.6337
NC_005102.4_150300001	3	150300001	0.5569	0.715
NC_005102.4_150400001	3	150400001	0.575	0.7186
NC_005102.4_150500001	3	150500001	0.5796	0.7388
NC_005102.4_150600001	3	150600001	0.6516	0.7667
NC_005102.4_150700001	3	150700001	0.6461	0.7385
NC_005102.4_150800001	3	150800001	0.5551	0.6568
NC_005102.4_150900001	3	150900001	0.4767	0.6029
NC_005102.4_151000001	3	151000001	0.4824	0.5928
NC_005102.4_151100001	3	151100001	0.3823	0.4702
NC_005102.4_151200001	3	151200001	0.3904	0.5086
NC_005102.4_151300001	3	151300001	0.4668	0.5747
NC_005102.4_151400001	3	151400001	0.489	0.5565
NC_005102.4_151500001	3	151500001	0.421	0.4712

NC_005102.4_151800001	3	151800001	0.3181	0.2189
NC_005102.4_151900001	3	151900001	0.3455	0.361
NC_005102.4_152000001	3	152000001	0.4171	0.503
NC_005102.4_152100001	3	152100001	0.4712	0.5716
NC_005102.4_152200001	3	152200001	0.5087	0.6029
NC_005102.4_152300001	3	152300001	0.4993	0.5944
NC_005102.4_152400001	3	152400001	0.5161	0.6126
NC_005102.4_152500001	3	152500001	0.4817	0.5917
NC_005102.4_152600001	3	152600001	0.5057	0.5984
NC_005102.4_152700001	3	152700001	0.4679	0.5683
NC_005102.4_152800001	3	152800001	0.4759	0.6645
NC_005102.4_152900001	3	152900001	0.4812	0.7156
NC_005102.4_153000001	3	153000001	0.4761	0.6993
NC_005102.4_153100001	3	153100001	0.3756	0.6552
NC_005102.4_153200001	3	153200001	0.3638	0.6295
NC_005102.4_153300001	3	153300001	0.4098	0.6649
NC_005102.4_153400001	3	153400001	0.4728	0.655
NC_005102.4_153500001	3	153500001	0.5026	0.6641
NC_005102.4_153600001	3	153600001	0.5858	0.7276
NC_005102.4_153700001	3	153700001	0.4909	0.6575
NC_005102.4_153800001	3	153800001	0.5056	0.6603
NC_005102.4_153900001	3	153900001	0.4634	0.6355
NC_005102.4_154000001	3	154000001	0.4719	0.6319
NC_005102.4_154100001	3	154100001	0.4985	0.6223
NC_005102.4_154200001	3	154200001	0.5753	0.656
NC_005102.4_154300001	3	154300001	0.576	0.6709
NC_005102.4_154400001	3	154400001	0.5784	0.6613
NC_005102.4_154500001	3	154500001	0.6164	0.6742
NC_005102.4_154600001	3	154600001	0.5081	0.5622
NC_005102.4_154700001	3	154700001	0.5652	0.6458
NC_005102.4_154800001	3	154800001	0.5129	0.5566
NC_005102.4_154900001	3	154900001	0.5647	0.5805
NC_005102.4_155000001	3	155000001	0.429	0.5431
NC_005102.4_155100001	3	155100001	0.4086	0.576
NC_005102.4_155200001	3	155200001	0.3464	0.4556
NC_005102.4_155300001	3	155300001	0.3464	0.4463
NC_005102.4_155400001	3	155400001	0.3172	0.4144
NC_005102.4_155500001	3	155500001	0.3366	0.4248
NC_005102.4_155600001	3	155600001	0.4503	0.5706
NC_005102.4_155700001	3	155700001	0.4148	0.5434
NC_005102.4_155800001	3	155800001	0.4747	0.5767
NC_005102.4_155900001	3	155900001	0.5382	0.6432
NC_005102.4_156000001	3	156000001	0.5974	0.6577
NC_005102.4_156100001	3	156100001	0.5088	0.6008
NC_005102.4_156200001	3	156200001	0.5494	0.6307
NC_005102.4_156300001	3	156300001	0.5609	0.6886
NC_005102.4_156400001	3	156400001	0.5437	0.6629
NC_005102.4_156500001	3	156500001	0.4861	0.6025
NC_005102.4_156600001	3	156600001	0.5799	0.6798
NC_005102.4_156700001	3	156700001	0.5112	0.5852
NC_005102.4_156800001	3	156800001	0.5122	0.5609
NC_005102.4_156900001	3	156900001	0.4809	0.5724
NC_005102.4_157000001	3	157000001	0.5429	0.6494
NC_005102.4_157100001	3	157100001	0.5088	0.6356
NC_005102.4_157200001	3	157200001	0.5927	0.7515
NC_005102.4_157300001	3	157300001	0.543	0.7302
NC_005102.4_157400001	3	157400001	0.5587	0.6634
NC_005102.4_157500001	3	157500001	0.555	0.6471

NC_005102.4_157600001	3	157600001	0.5975	0.6842
NC_005102.4_157700001	3	157700001	0.5676	0.6784
NC_005102.4_157800001	3	157800001	0.5556	0.6953
NC_005102.4_157900001	3	157900001	0.5775	0.7267
NC_005102.4_158000001	3	158000001	0.5621	0.6998
NC_005102.4_158100001	3	158100001	0.532	0.6668
NC_005102.4_158200001	3	158200001	0.5563	0.6629
NC_005102.4_158300001	3	158300001	0.5245	0.6378
NC_005102.4_158400001	3	158400001	0.4938	0.6089
NC_005102.4_158500001	3	158500001	0.4684	0.6415
NC_005102.4_158600001	3	158600001	0.4406	0.588
NC_005102.4_158700001	3	158700001	0.3629	0.4048
NC_005102.4_158800001	3	158800001	0.3441	0.1987
NC_005102.4_158900001	3	158900001	0.4174	0.393
NC_005102.4_159000001	3	159000001	0.4285	0.5112
NC_005102.4_159100001	3	159100001	0.4661	0.5847
NC_005102.4_159200001	3	159200001	0.5325	0.6767
NC_005102.4_159300001	3	159300001	0.5648	0.687
NC_005102.4_159400001	3	159400001	0.5499	0.683
NC_005102.4_159500001	3	159500001	0.5738	0.6844
NC_005102.4_159600001	3	159600001	0.5955	0.6934
NC_005102.4_159700001	3	159700001	0.5619	0.6501
NC_005102.4_159800001	3	159800001	0.6199	0.7309
NC_005102.4_159900001	3	159900001	0.6128	0.7502
NC_005102.4_160000001	3	160000001	0.6729	0.7678
NC_005102.4_160100001	3	160100001	0.6406	0.7347
NC_005102.4_160200001	3	160200001	0.6638	0.7489
NC_005102.4_160300001	3	160300001	0.6015	0.7015
NC_005102.4_160400001	3	160400001	0.6055	0.5709
NC_005102.4_160500001	3	160500001	0.4608	0.4621
NC_005102.4_160600001	3	160600001	0.5006	0.5253
NC_005102.4_160700001	3	160700001	0.5929	0.631
NC_005102.4_160800001	3	160800001	0.6103	0.6523
NC_005102.4_160900001	3	160900001	0.5662	0.7389
NC_005102.4_161000001	3	161000001	0.5953	0.7584
NC_005102.4_161100001	3	161100001	0.5458	0.6834
NC_005102.4_161200001	3	161200001	0.4658	0.6293
NC_005102.4_161300001	3	161300001	0.4221	0.5588
NC_005102.4_161400001	3	161400001	0.4585	0.5633
NC_005102.4_161500001	3	161500001	0.462	0.5173
NC_005102.4_161600001	3	161600001	0.5492	0.6526
NC_005102.4_161700001	3	161700001	0.5368	0.5982
NC_005102.4_161800001	3	161800001	0.5472	0.5796
NC_005102.4_161900001	3	161900001	0.4924	0.5817
NC_005102.4_162000001	3	162000001	0.485	0.5698
NC_005102.4_162100001	3	162100001	0.4537	0.5372
NC_005102.4_162200001	3	162200001	0.4529	0.546
NC_005102.4_162300001	3	162300001	0.4348	0.5892
NC_005102.4_162400001	3	162400001	0.4795	0.5974
NC_005102.4_162500001	3	162500001	0.4941	0.6297
NC_005102.4_162600001	3	162600001	0.5187	0.6447
NC_005102.4_162700001	3	162700001	0.5416	0.6973
NC_005102.4_162800001	3	162800001	0.6068	0.7309
NC_005102.4_162900001	3	162900001	0.5888	0.7486
NC_005102.4_163000001	3	163000001	0.5697	0.7456
NC_005102.4_163100001	3	163100001	0.5945	0.7682
NC_005102.4_163200001	3	163200001	0.6081	0.7809
NC_005102.4_163300001	3	163300001	0.6266	0.7893

NC_005102.4_163400001	3	163400001	0.6455	0.7181
NC_005102.4_163500001	3	163500001	0.6896	0.7519
NC_005102.4_163600001	3	163600001	0.6843	0.733
NC_005102.4_163700001	3	163700001	0.6633	0.7383
NC_005102.4_163800001	3	163800001	0.6172	0.7259
NC_005102.4_163900001	3	163900001	0.6256	0.7958
NC_005102.4_164000001	3	164000001	0.597	0.7567
NC_005102.4_164100001	3	164100001	0.4551	0.6566
NC_005102.4_164200001	3	164200001	0.412	0.5753
NC_005102.4_164300001	3	164300001	0.3658	0.4976
NC_005102.4_164400001	3	164400001	0.4009	0.5103
NC_005102.4_164500001	3	164500001	0.4587	0.5908
NC_005102.4_164600001	3	164600001	0.4928	0.6032
NC_005102.4_164700001	3	164700001	0.4933	0.5973
NC_005102.4_164800001	3	164800001	0.5573	0.6451
NC_005102.4_164900001	3	164900001	0.5855	0.6931
NC_005102.4_165000001	3	165000001	0.5504	0.6827
NC_005102.4_165100001	3	165100001	0.6275	0.7909
NC_005102.4_165200001	3	165200001	0.595	0.7729
NC_005102.4_165300001	3	165300001	0.5526	0.7769
NC_005102.4_165400001	3	165400001	0.4767	0.7516
NC_005102.4_165500001	3	165500001	0.4414	0.6965
NC_005102.4_165600001	3	165600001	0.4258	0.6659
NC_005102.4_165700001	3	165700001	0.477	0.7462
NC_005102.4_165800001	3	165800001	0.4957	0.695
NC_005102.4_165900001	3	165900001	0.4774	0.6182
NC_005102.4_166000001	3	166000001	0.5484	0.6751
NC_005102.4_166100001	3	166100001	0.5407	0.6568
NC_005102.4_166200001	3	166200001	0.5601	0.6699
NC_005102.4_166300001	3	166300001	0.5926	0.6952
NC_005102.4_166400001	3	166400001	0.6725	0.7702
NC_005102.4_166500001	3	166500001	0.6775	0.82
NC_005102.4_166600001	3	166600001	0.6707	0.8493
NC_005102.4_166700001	3	166700001	0.5886	0.7675
NC_005102.4_166800001	3	166800001	0.5583	0.7593
NC_005102.4_166900001	3	166900001	0.5376	0.7181
NC_005102.4_167000001	3	167000001	0.4312	0.495
NC_005102.4_167100001	3	167100001	0.468	0.4648
NC_005102.4_167500001	3	167500001	0.4204	0.5568
NC_005102.4_167600001	3	167600001	0.6725	0.7408
NC_005102.4_167700001	3	167700001	0.7073	0.7311
NC_005102.4_167800001	3	167800001	0.6299	0.7218
NC_005102.4_167900001	3	167900001	0.6365	0.743
NC_005102.4_168000001	3	168000001	0.6225	0.7124
NC_005102.4_168100001	3	168100001	0.4981	0.6063
NC_005102.4_168200001	3	168200001	0.464	0.5211
NC_005102.4_168300001	3	168300001	0.481	0.5109
NC_005102.4_168400001	3	168400001	0.4725	0.4948
NC_005102.4_168500001	3	168500001	0.5202	0.545
NC_005102.4_168600001	3	168600001	0.5271	0.5561
NC_005102.4_168700001	3	168700001	0.5062	0.6044
NC_005102.4_168800001	3	168800001	0.5756	0.6644
NC_005102.4_168900001	3	168900001	0.5478	0.6319
NC_005102.4_169000001	3	169000001	0.4221	0.5151
NC_005102.4_169100001	3	169100001	0.4463	0.4812
NC_005102.4_169200001	3	169200001	0.3654	0.4243
NC_005102.4_169300001	3	169300001	0.4017	0.4943
NC_005102.4_169400001	3	169400001	0.4079	0.5077

NC_005102.4_169500001	3	169500001	0.4515	0.5282
NC_005102.4_169600001	3	169600001	0.4222	0.514
NC_005102.4_169700001	3	169700001	0.63	0.6867
NC_005102.4_169800001	3	169800001	0.6267	0.6479
NC_005102.4_169900001	3	169900001	0.535	0.6465
NC_005102.4_170000001	3	170000001	0.5648	0.6896
NC_005102.4_170100001	3	170100001	0.6109	0.7209
NC_005102.4_170200001	3	170200001	0.5216	0.6839
NC_005102.4_170300001	3	170300001	0.514	0.7044
NC_005102.4_170400001	3	170400001	0.5366	0.6453
NC_005102.4_170500001	3	170500001	0.4954	0.5971
NC_005102.4_170600001	3	170600001	0.4292	0.5474
NC_005102.4_170700001	3	170700001	0.4898	0.5787
NC_005102.4_170800001	3	170800001	0.4759	0.5655
NC_005102.4_170900001	3	170900001	0.5194	0.5924
NC_005102.4_171000001	3	171000001	0.486	0.5609
NC_005102.4_171100001	3	171100001	0.5581	0.5667
NC_005102.4_171200001	3	171200001	0.5792	0.6374
NC_005102.4_171300001	3	171300001	0.6343	0.7087
NC_005102.4_171400001	3	171400001	0.6509	0.7659
NC_005102.4_171500001	3	171500001	0.6872	0.8052
NC_005102.4_171600001	3	171600001	0.6806	0.9104
NC_005102.4_171700001	3	171700001	0.7384	0.9243
NC_005102.4_171800001	3	171800001	0.7134	0.8937
NC_005102.4_171900001	3	171900001	0.8116	0.9179
NC_005102.4_172000001	3	172000001	0.8451	0.96
NC_005102.4_172100001	3	172100001	0.8741	0.9338
NC_005102.4_172200001	3	172200001	0.8673	0.9311
NC_005102.4_172300001	3	172300001	0.8739	0.9331
NC_005102.4_172400001	3	172400001	0.7462	0.8349
NC_005102.4_172500001	3	172500001	0.7552	0.8026
NC_005102.4_172600001	3	172600001	0.6519	0.6962
NC_005102.4_172700001	3	172700001	0.5825	0.6152
NC_005102.4_172800001	3	172800001	0.5568	0.584
NC_005102.4_172900001	3	172900001	0.6081	0.6028
NC_005102.4_173000001	3	173000001	0.4967	0.5214
NC_005102.4_173200001	3	173200001	0.3898	0.8046
NC_005102.4_173300001	3	173300001	0.4878	0.776
NC_005102.4_173400001	3	173400001	0.4424	0.6707
NC_005102.4_173500001	3	173500001	0.4717	0.6935
NC_005102.4_173600001	3	173600001	0.4669	0.6699
NC_005102.4_173700001	3	173700001	0.4676	0.5794
NC_005102.4_173800001	3	173800001	0.451	0.529
NC_005102.4_173900001	3	173900001	0.3986	0.5826
NC_005102.4_174000001	3	174000001	0.3563	0.5348
NC_005102.4_174100001	3	174100001	0.3751	0.528
NC_005102.4_174200001	3	174200001	0.3975	0.5604
NC_005102.4_174300001	3	174300001	0.4869	0.6644
NC_005102.4_174400001	3	174400001	0.5731	0.6784
NC_005102.4_174500001	3	174500001	0.6088	0.6908
NC_005102.4_174600001	3	174600001	0.7003	0.7332
NC_005102.4_174700001	3	174700001	0.6437	0.7464
NC_005102.4_174800001	3	174800001	0.5937	0.664
NC_005102.4_174900001	3	174900001	0.5884	0.6379
NC_005102.4_175000001	3	175000001	0.5769	0.6358
NC_005102.4_175100001	3	175100001	0.4884	0.6015
NC_005102.4_175200001	3	175200001	0.5666	0.6274
NC_005102.4_175300001	3	175300001	0.5735	0.6789

NC_005102.4_175400001	3	175400001	0.5745	0.6599
NC_005102.4_175500001	3	175500001	0.5798	0.6464
NC_005102.4_175600001	3	175600001	0.6047	0.6449
NC_005102.4_175700001	3	175700001	0.5774	0.6482
NC_005102.4_175800001	3	175800001	0.6267	0.7077
NC_005102.4_175900001	3	175900001	0.5785	0.6462
NC_005102.4_176000001	3	176000001	0.5976	0.703
NC_005102.4_176100001	3	176100001	0.6251	0.7206
NC_005102.4_176200001	3	176200001	0.6783	0.7522
NC_005102.4_176300001	3	176300001	0.6486	0.7222
NC_005102.4_176400001	3	176400001	0.7055	0.847
NC_005102.4_176500001	3	176500001	0.7208	0.8509
NC_005102.4_176600001	3	176600001	0.7551	0.895
NC_005102.4_176700001	3	176700001	0.7144	0.8952
NC_005102.4_176800001	3	176800001	0.6415	0.8849
NC_005103.4_1	4	1	0.6313	0.6781
NC_005103.4_100001	4	100001	0.6581	0.7076
NC_005103.4_200001	4	200001	0.6581	0.7076
NC_005103.4_300001	4	300001	0.6455	0.7264
NC_005103.4_400001	4	400001	0.6594	0.7029
NC_005103.4_500001	4	500001	0.7016	0.8079
NC_005103.4_600001	4	600001	0.5843	0.7589
NC_005103.4_700001	4	700001	0.5752	0.679
NC_005103.4_800001	4	800001	0.6604	0.7633
NC_005103.4_900001	4	900001	0.6372	0.7416
NC_005103.4_1000001	4	1000001	0.5424	0.645
NC_005103.4_1100001	4	1100001	0.5798	0.6348
NC_005103.4_1200001	4	1200001	0.6034	0.7603
NC_005103.4_1400001	4	1400001	0.2924	0.2579
NC_005103.4_1500001	4	1500001	0.4559	0.551
NC_005103.4_1600001	4	1600001	0.4663	0.5766
NC_005103.4_1700001	4	1700001	0.538	0.6932
NC_005103.4_1800001	4	1800001	0.538	0.6932
NC_005103.4_1900001	4	1900001	0.6791	0.8508
NC_005103.4_2000001	4	2000001	0.6473	0.7572
NC_005103.4_2100001	4	2100001	0.6979	0.7699
NC_005103.4_2200001	4	2200001	0.52	0.6028
NC_005103.4_2300001	4	2300001	0.5756	0.6434
NC_005103.4_2400001	4	2400001	0.5127	0.62
NC_005103.4_2500001	4	2500001	0.5009	0.6362
NC_005103.4_2600001	4	2600001	0.5009	0.6362
NC_005103.4_2700001	4	2700001	0.6178	0.7324
NC_005103.4_2800001	4	2800001	0.6651	0.8745
NC_005103.4_2900001	4	2900001	0.5436	0.8144
NC_005103.4_3000001	4	3000001	0.5436	0.8144
NC_005103.4_3100001	4	3100001	0.5436	0.8144
NC_005103.4_3200001	4	3200001	0.4774	0.7601
NC_005103.4_3300001	4	3300001	0.2971	0.5437
NC_005103.4_3600001	4	3600001	0.4233	0.5739
NC_005103.4_3700001	4	3700001	0.4065	0.5302
NC_005103.4_3800001	4	3800001	0.4808	0.5981
NC_005103.4_3900001	4	3900001	0.489	0.5837
NC_005103.4_4000001	4	4000001	0.4439	0.5335
NC_005103.4_4100001	4	4100001	0.4984	0.6151
NC_005103.4_4200001	4	4200001	0.5448	0.6466
NC_005103.4_4300001	4	4300001	0.4921	0.5604
NC_005103.4_4400001	4	4400001	0.5314	0.5829
NC_005103.4_4500001	4	4500001	0.5118	0.5449

NC_005103.4_4600001	4	4600001	0.4971	0.4894
NC_005103.4_4700001	4	4700001	0.5277	0.5132
NC_005103.4_4800001	4	4800001	0.591	0.5944
NC_005103.4_4900001	4	4900001	0.5529	0.6464
NC_005103.4_5000001	4	5000001	0.6534	0.752
NC_005103.4_5100001	4	5100001	0.6769	0.808
NC_005103.4_5200001	4	5200001	0.5911	0.7069
NC_005103.4_5300001	4	5300001	0.4928	0.6458
NC_005103.4_5400001	4	5400001	0.4512	0.5752
NC_005103.4_5500001	4	5500001	0.4755	0.5941
NC_005103.4_5600001	4	5600001	0.4785	0.5953
NC_005103.4_5700001	4	5700001	0.4588	0.6096
NC_005103.4_5800001	4	5800001	0.5351	0.6682
NC_005103.4_5900001	4	5900001	0.5716	0.7037
NC_005103.4_6000001	4	6000001	0.4569	0.6545
NC_005103.4_6100001	4	6100001	0.443	0.5427
NC_005103.4_6200001	4	6200001	0.435	0.5752
NC_005103.4_6300001	4	6300001	0.4908	0.6409
NC_005103.4_6400001	4	6400001	0.477	0.6238
NC_005103.4_6500001	4	6500001	0.4871	0.6353
NC_005103.4_6600001	4	6600001	0.4471	0.6515
NC_005103.4_6700001	4	6700001	0.4826	0.6711
NC_005103.4_6800001	4	6800001	0.3999	0.6135
NC_005103.4_6900001	4	6900001	0.5288	0.7575
NC_005103.4_7000001	4	7000001	0.5537	0.7716
NC_005103.4_7100001	4	7100001	0.6036	0.822
NC_005103.4_7200001	4	7200001	0.6401	0.823
NC_005103.4_7300001	4	7300001	0.5502	0.7553
NC_005103.4_7400001	4	7400001	0.4467	0.5678
NC_005103.4_7500001	4	7500001	0.3679	0.4998
NC_005103.4_7600001	4	7600001	0.411	0.5707
NC_005103.4_7700001	4	7700001	0.3978	0.5423
NC_005103.4_7800001	4	7800001	0.5009	0.6372
NC_005103.4_7900001	4	7900001	0.5221	0.7104
NC_005103.4_8000001	4	8000001	0.5321	0.684
NC_005103.4_8100001	4	8100001	0.5404	0.6567
NC_005103.4_8200001	4	8200001	0.6335	0.7784
NC_005103.4_8300001	4	8300001	0.5961	0.7161
NC_005103.4_8400001	4	8400001	0.5523	0.7063
NC_005103.4_8500001	4	8500001	0.6506	0.7479
NC_005103.4_8600001	4	8600001	0.5391	0.8403
NC_005103.4_8700001	4	8700001	0.4397	0.7373
NC_005103.4_8800001	4	8800001	0.5141	0.793
NC_005103.4_8900001	4	8900001	0.4872	0.7605
NC_005103.4_9000001	4	9000001	0.4536	0.7248
NC_005103.4_9100001	4	9100001	0.5413	0.7589
NC_005103.4_9200001	4	9200001	0.5869	0.7633
NC_005103.4_9300001	4	9300001	0.5091	0.7167
NC_005103.4_9400001	4	9400001	0.5327	0.7134
NC_005103.4_9500001	4	9500001	0.4951	0.6497
NC_005103.4_9600001	4	9600001	0.4389	0.5745
NC_005103.4_9700001	4	9700001	0.4588	0.5519
NC_005103.4_9800001	4	9800001	0.469	0.5538
NC_005103.4_9900001	4	9900001	0.452	0.5032
NC_005103.4_10000001	4	10000001	0.4823	0.5177
NC_005103.4_10100001	4	10100001	0.554	0.5645
NC_005103.4_10200001	4	10200001	0.6052	0.6786
NC_005103.4_10300001	4	10300001	0.5716	0.6207

NC_005103.4_10400001	4	10400001	0.6261	0.707
NC_005103.4_10500001	4	10500001	0.6572	0.7744
NC_005103.4_10600001	4	10600001	0.666	0.7904
NC_005103.4_10700001	4	10700001	0.6677	0.7594
NC_005103.4_10800001	4	10800001	0.7106	0.7888
NC_005103.4_10900001	4	10900001	0.6181	0.6912
NC_005103.4_11000001	4	11000001	0.6662	0.7263
NC_005103.4_11100001	4	11100001	0.6401	0.6999
NC_005103.4_11200001	4	11200001	0.4916	0.6341
NC_005103.4_11300001	4	11300001	0.4237	0.5879
NC_005103.4_11400001	4	11400001	0.2415	0.4145
NC_005103.4_11500001	4	11500001	0.2658	0.4693
NC_005103.4_11600001	4	11600001	0.3896	0.6292
NC_005103.4_11700001	4	11700001	0.4273	0.6474
NC_005103.4_11800001	4	11800001	0.4064	0.595
NC_005103.4_11900001	4	11900001	0.4976	0.6825
NC_005103.4_12000001	4	12000001	0.483	0.6524
NC_005103.4_12100001	4	12100001	0.4199	0.5992
NC_005103.4_12200001	4	12200001	0.3628	0.5405
NC_005103.4_12300001	4	12300001	0.439	0.6577
NC_005103.4_12400001	4	12400001	0.4209	0.5588
NC_005103.4_12500001	4	12500001	0.4862	0.6193
NC_005103.4_12600001	4	12600001	0.4862	0.6193
NC_005103.4_12700001	4	12700001	0.6283	0.6657
NC_005103.4_12800001	4	12800001	0.641	0.7049
NC_005103.4_12900001	4	12900001	0.7221	0.8463
NC_005103.4_13000001	4	13000001	0.4952	0.5822
NC_005103.4_13100001	4	13100001	0.4941	0.5548
NC_005103.4_13200001	4	13200001	0.4611	0.5307
NC_005103.4_13300001	4	13300001	0.4518	0.4531
NC_005103.4_13400001	4	13400001	0.4239	0.4676
NC_005103.4_13500001	4	13500001	0.4705	0.489
NC_005103.4_13600001	4	13600001	0.5211	0.597
NC_005103.4_13700001	4	13700001	0.5211	0.597
NC_005103.4_13800001	4	13800001	0.545	0.7154
NC_005103.4_14000001	4	14000001	0.4945	0.5959
NC_005103.4_14100001	4	14100001	0.4386	0.5979
NC_005103.4_14200001	4	14200001	0.5002	0.5754
NC_005103.4_14300001	4	14300001	0.433	0.5534
NC_005103.4_14400001	4	14400001	0.4804	0.6143
NC_005103.4_14500001	4	14500001	0.5678	0.6777
NC_005103.4_14600001	4	14600001	0.588	0.6344
NC_005103.4_14700001	4	14700001	0.7323	0.8553
NC_005103.4_14800001	4	14800001	0.9175	0.8888
NC_005103.4_14900001	4	14900001	0.7723	0.8516
NC_005103.4_15000001	4	15000001	0.8341	0.8991
NC_005103.4_15100001	4	15100001	0.7052	0.8385
NC_005103.4_15200001	4	15200001	0.4876	0.6065
NC_005103.4_15300001	4	15300001	0.4736	0.6631
NC_005103.4_15400001	4	15400001	0.4689	0.6121
NC_005103.4_15500001	4	15500001	0.4114	0.5455
NC_005103.4_15600001	4	15600001	0.4245	0.5471
NC_005103.4_15700001	4	15700001	0.4182	0.5193
NC_005103.4_15800001	4	15800001	0.4664	0.5414
NC_005103.4_15900001	4	15900001	0.4365	0.5664
NC_005103.4_16000001	4	16000001	0.3849	0.5351
NC_005103.4_16100001	4	16100001	0.4387	0.6152
NC_005103.4_16200001	4	16200001	0.5097	0.7939

NC_005103.4_16300001	4	16300001	0.4165	0.7459
NC_005103.4_16400001	4	16400001	0.5548	0.7665
NC_005103.4_16500001	4	16500001	0.5804	0.7294
NC_005103.4_16600001	4	16600001	0.4294	0.5752
NC_005103.4_17300001	4	17300001	0.3797	0.4675
NC_005103.4_17400001	4	17400001	0.469	0.5996
NC_005103.4_17500001	4	17500001	0.4981	0.619
NC_005103.4_17600001	4	17600001	0.4981	0.619
NC_005103.4_17700001	4	17700001	0.5176	0.6863
NC_005103.4_17900001	4	17900001	0.5443	0.6144
NC_005103.4_18000001	4	18000001	0.5558	0.6174
NC_005103.4_18100001	4	18100001	0.5837	0.6563
NC_005103.4_18200001	4	18200001	0.52	0.6254
NC_005103.4_18300001	4	18300001	0.5653	0.6815
NC_005103.4_18400001	4	18400001	0.5141	0.6826
NC_005103.4_18500001	4	18500001	0.4854	0.6895
NC_005103.4_18600001	4	18600001	0.5706	0.7587
NC_005103.4_18700001	4	18700001	0.5947	0.6032
NC_005103.4_18800001	4	18800001	0.5488	0.5517
NC_005103.4_18900001	4	18900001	0.5527	0.525
NC_005103.4_19000001	4	19000001	0.5527	0.525
NC_005103.4_19100001	4	19100001	0.3129	0.1807
NC_005103.4_19300001	4	19300001	0.1653	0.1162
NC_005103.4_19400001	4	19400001	0.1631	0.1251
NC_005103.4_19500001	4	19500001	0.1529	0.1155
NC_005103.4_19600001	4	19600001	0.2822	0.4298
NC_005103.4_19700001	4	19700001	0.2822	0.4298
NC_005103.4_19900001	4	19900001	0.2982	0.6162
NC_005103.4_20000001	4	20000001	0.4747	0.8118
NC_005103.4_20100001	4	20100001	0.4506	0.7064
NC_005103.4_20200001	4	20200001	0.4782	0.7381
NC_005103.4_20300001	4	20300001	0.5106	0.7461
NC_005103.4_20400001	4	20400001	0.6195	0.7478
NC_005103.4_20500001	4	20500001	0.5891	0.7143
NC_005103.4_20600001	4	20600001	0.5691	0.7063
NC_005103.4_20700001	4	20700001	0.5718	0.6531
NC_005103.4_20800001	4	20800001	0.5461	0.5468
NC_005103.4_20900001	4	20900001	0.6673	0.6768
NC_005103.4_21000001	4	21000001	0.6183	0.6253
NC_005103.4_21100001	4	21100001	0.6681	0.6714
NC_005103.4_21200001	4	21200001	0.6099	0.6519
NC_005103.4_21300001	4	21300001	0.6526	0.7515
NC_005103.4_21400001	4	21400001	0.562	0.6389
NC_005103.4_21500001	4	21500001	0.6847	0.7687
NC_005103.4_21600001	4	21600001	0.6522	0.7325
NC_005103.4_21700001	4	21700001	0.6795	0.7845
NC_005103.4_21800001	4	21800001	0.6237	0.7531
NC_005103.4_21900001	4	21900001	0.517	0.6761
NC_005103.4_22000001	4	22000001	0.4788	0.6442
NC_005103.4_22100001	4	22100001	0.4916	0.5673
NC_005103.4_22200001	4	22200001	0.5346	0.5715
NC_005103.4_22300001	4	22300001	0.5893	0.6024
NC_005103.4_22400001	4	22400001	0.6487	0.6458
NC_005103.4_22500001	4	22500001	0.5735	0.6397
NC_005103.4_22600001	4	22600001	0.4761	0.6024
NC_005103.4_22700001	4	22700001	0.4342	0.5584
NC_005103.4_22800001	4	22800001	0.3998	0.5147
NC_005103.4_22900001	4	22900001	0.3867	0.5364

NC_005103.4_23000001	4	23000001	0.417	0.5028
NC_005103.4_23100001	4	23100001	0.655	0.8074
NC_005103.4_23200001	4	23200001	0.5649	0.7857
NC_005103.4_23300001	4	23300001	0.3853	0.5897
NC_005103.4_23400001	4	23400001	0.3294	0.5219
NC_005103.4_23500001	4	23500001	0.2824	0.3647
NC_005103.4_23600001	4	23600001	0.293	0.3758
NC_005103.4_23700001	4	23700001	0.4021	0.484
NC_005103.4_23800001	4	23800001	0.5156	0.5586
NC_005103.4_23900001	4	23900001	0.5273	0.6019
NC_005103.4_24000001	4	24000001	0.4709	0.5893
NC_005103.4_24100001	4	24100001	0.4624	0.5605
NC_005103.4_24200001	4	24200001	0.377	0.5233
NC_005103.4_24300001	4	24300001	0.4061	0.5571
NC_005103.4_24400001	4	24400001	0.3358	0.4553
NC_005103.4_24500001	4	24500001	0.5955	0.6343
NC_005103.4_24600001	4	24600001	0.4788	0.5773
NC_005103.4_24700001	4	24700001	0.4865	0.4853
NC_005103.4_24800001	4	24800001	0.4743	0.4396
NC_005103.4_24900001	4	24900001	0.4743	0.4396
NC_005103.4_25000001	4	25000001	0.3871	0.3369
NC_005103.4_25100001	4	25100001	0.4573	0.3659
NC_005103.4_25200001	4	25200001	0.407	0.4088
NC_005103.4_25300001	4	25300001	0.3752	0.4985
NC_005103.4_25400001	4	25400001	0.4497	0.6001
NC_005103.4_25500001	4	25500001	0.4665	0.6114
NC_005103.4_25600001	4	25600001	0.5617	0.6972
NC_005103.4_25700001	4	25700001	0.554	0.7178
NC_005103.4_25800001	4	25800001	0.5413	0.6828
NC_005103.4_25900001	4	25900001	0.5458	0.6566
NC_005103.4_26000001	4	26000001	0.5953	0.6905
NC_005103.4_26100001	4	26100001	0.5051	0.6509
NC_005103.4_26200001	4	26200001	0.5254	0.6382
NC_005103.4_26300001	4	26300001	0.5695	0.6826
NC_005103.4_26400001	4	26400001	0.5589	0.6964
NC_005103.4_26500001	4	26500001	0.4143	0.6769
NC_005103.4_26600001	4	26600001	0.3438	0.5854
NC_005103.4_26700001	4	26700001	0.3693	0.6313
NC_005103.4_26800001	4	26800001	0.3167	0.5432
NC_005103.4_26900001	4	26900001	0.2774	0.4651
NC_005103.4_27000001	4	27000001	0.2582	0.3467
NC_005103.4_27100001	4	27100001	0.3121	0.4275
NC_005103.4_27200001	4	27200001	0.37	0.4942
NC_005103.4_27300001	4	27300001	0.3918	0.4987
NC_005103.4_27400001	4	27400001	0.4956	0.6497
NC_005103.4_27500001	4	27500001	0.5143	0.673
NC_005103.4_27600001	4	27600001	0.5855	0.7128
NC_005103.4_27700001	4	27700001	0.5603	0.7072
NC_005103.4_27800001	4	27800001	0.6485	0.7856
NC_005103.4_27900001	4	27900001	0.4194	0.5854
NC_005103.4_28000001	4	28000001	0.2781	0.512
NC_005103.4_28100001	4	28100001	0.3162	0.5615
NC_005103.4_28200001	4	28200001	0.385	0.6973
NC_005103.4_28300001	4	28300001	0.3886	0.6044
NC_005103.4_28400001	4	28400001	0.4492	0.6212
NC_005103.4_28500001	4	28500001	0.6824	0.6674
NC_005103.4_28600001	4	28600001	0.5086	0.585
NC_005103.4_28700001	4	28700001	0.4756	0.5068

NC_005103.4_28800001	4	28800001	0.485	0.597
NC_005103.4_28900001	4	28900001	0.5225	0.6644
NC_005103.4_29000001	4	29000001	0.502	0.5947
NC_005103.4_29100001	4	29100001	0.5603	0.6161
NC_005103.4_29200001	4	29200001	0.5639	0.6718
NC_005103.4_29300001	4	29300001	0.6054	0.7038
NC_005103.4_29400001	4	29400001	0.6361	0.7301
NC_005103.4_29500001	4	29500001	0.7636	0.8621
NC_005103.4_29600001	4	29600001	0.7241	0.8553
NC_005103.4_29700001	4	29700001	0.7681	0.8739
NC_005103.4_29800001	4	29800001	0.7068	0.8455
NC_005103.4_29900001	4	29900001	0.6983	0.8405
NC_005103.4_30000001	4	30000001	0.7119	0.8682
NC_005103.4_30100001	4	30100001	0.6589	0.8426
NC_005103.4_30200001	4	30200001	0.6168	0.7727
NC_005103.4_30300001	4	30300001	0.6253	0.7543
NC_005103.4_30400001	4	30400001	0.509	0.6002
NC_005103.4_30500001	4	30500001	0.3949	0.4512
NC_005103.4_30600001	4	30600001	0.4398	0.4531
NC_005103.4_30700001	4	30700001	0.3101	0.2554
NC_005103.4_30800001	4	30800001	0.39	0.3682
NC_005103.4_30900001	4	30900001	0.4332	0.4627
NC_005103.4_31200001	4	31200001	0.4173	0.3889
NC_005103.4_31300001	4	31300001	0.3845	0.4176
NC_005103.4_31400001	4	31400001	0.4028	0.4409
NC_005103.4_31500001	4	31500001	0.4283	0.4674
NC_005103.4_31600001	4	31600001	0.3813	0.4844
NC_005103.4_31700001	4	31700001	0.4203	0.6024
NC_005103.4_31800001	4	31800001	0.3608	0.5829
NC_005103.4_31900001	4	31900001	0.4746	0.7648
NC_005103.4_32000001	4	32000001	0.4558	0.8207
NC_005103.4_32100001	4	32100001	0.5365	0.7369
NC_005103.4_32200001	4	32200001	0.4419	0.6055
NC_005103.4_32300001	4	32300001	0.5123	0.6294
NC_005103.4_32400001	4	32400001	0.2686	0.2357
NC_005103.4_32500001	4	32500001	0.2686	0.2357
NC_005103.4_32600001	4	32600001	0.2219	0.2108
NC_005103.4_33000001	4	33000001	0.3214	0.3335
NC_005103.4_33100001	4	33100001	0.2376	0.2881
NC_005103.4_33200001	4	33200001	0.3103	0.33
NC_005103.4_33300001	4	33300001	0.3494	0.3635
NC_005103.4_33400001	4	33400001	0.3403	0.4334
NC_005103.4_33500001	4	33500001	0.4338	0.5495
NC_005103.4_33600001	4	33600001	0.5042	0.5748
NC_005103.4_33700001	4	33700001	0.5017	0.6466
NC_005103.4_33800001	4	33800001	0.6058	0.7315
NC_005103.4_33900001	4	33900001	0.6812	0.7467
NC_005103.4_34000001	4	34000001	0.6268	0.6963
NC_005103.4_34100001	4	34100001	0.645	0.7215
NC_005103.4_34200001	4	34200001	0.6542	0.7187
NC_005103.4_34300001	4	34300001	0.4648	0.6228
NC_005103.4_34400001	4	34400001	0.4552	0.6019
NC_005103.4_34500001	4	34500001	0.4244	0.5719
NC_005103.4_34600001	4	34600001	0.3777	0.5092
NC_005103.4_34700001	4	34700001	0.4114	0.5309
NC_005103.4_34800001	4	34800001	0.5008	0.5643
NC_005103.4_34900001	4	34900001	0.4996	0.6039
NC_005103.4_35000001	4	35000001	0.5765	0.7499

NC_005103.4_35100001	4	35100001	0.7367	0.8206
NC_005103.4_35200001	4	35200001	0.7823	0.9587
NC_005103.4_35300001	4	35300001	0.5988	0.9129
NC_005103.4_35400001	4	35400001	0.5312	0.8868
NC_005103.4_35500001	4	35500001	0.3676	0.8102
NC_005103.4_35600001	4	35600001	0.0519	0.0209
NC_005103.4_35700001	4	35700001	0.1942	0.5285
NC_005103.4_35800001	4	35800001	0.3599	0.747
NC_005103.4_35900001	4	35900001	0.3599	0.747
NC_005103.4_36000001	4	36000001	0.4471	0.789
NC_005103.4_36100001	4	36100001	0.5917	0.8419
NC_005103.4_36200001	4	36200001	0.5907	0.877
NC_005103.4_36400001	4	36400001	0.6635	0.8599
NC_005103.4_36500001	4	36500001	0.6635	0.8599
NC_005103.4_36600001	4	36600001	0.7451	0.9057
NC_005103.4_36700001	4	36700001	0.7117	0.8961
NC_005103.4_36800001	4	36800001	0.6918	0.918
NC_005103.4_37000001	4	37000001	0.8694	0.944
NC_005103.4_37100001	4	37100001	0.8289	0.9267
NC_005103.4_37200001	4	37200001	0.8411	0.8832
NC_005103.4_37300001	4	37300001	0.7128	0.8516
NC_005103.4_37400001	4	37400001	0.6351	0.8443
NC_005103.4_37500001	4	37500001	0.4652	0.6908
NC_005103.4_37600001	4	37600001	0.5694	0.8119
NC_005103.4_37700001	4	37700001	0.511	0.8314
NC_005103.4_37800001	4	37800001	0.6337	0.8688
NC_005103.4_37900001	4	37900001	0.6298	0.7506
NC_005103.4_38000001	4	38000001	0.6497	0.7835
NC_005103.4_38100001	4	38100001	0.6119	0.6713
NC_005103.4_38200001	4	38200001	0.5173	0.6409
NC_005103.4_38300001	4	38300001	0.5012	0.6162
NC_005103.4_38400001	4	38400001	0.4928	0.6386
NC_005103.4_38500001	4	38500001	0.411	0.5943
NC_005103.4_38600001	4	38600001	0.4142	0.6222
NC_005103.4_38700001	4	38700001	0.4613	0.6477
NC_005103.4_38800001	4	38800001	0.5882	0.8151
NC_005103.4_38900001	4	38900001	0.5461	0.7099
NC_005103.4_39000001	4	39000001	0.6945	0.7404
NC_005103.4_39100001	4	39100001	0.7602	0.7297
NC_005103.4_39200001	4	39200001	0.6303	0.6669
NC_005103.4_39300001	4	39300001	0.5974	0.6295
NC_005103.4_39400001	4	39400001	0.5541	0.5615
NC_005103.4_39500001	4	39500001	0.4596	0.4848
NC_005103.4_39600001	4	39600001	0.5274	0.5679
NC_005103.4_39700001	4	39700001	0.5483	0.6036
NC_005103.4_39800001	4	39800001	0.5503	0.6351
NC_005103.4_39900001	4	39900001	0.5784	0.7233
NC_005103.4_40000001	4	40000001	0.5503	0.7424
NC_005103.4_40100001	4	40100001	0.3941	0.7069
NC_005103.4_40200001	4	40200001	0.407	0.6661
NC_005103.4_40500001	4	40500001	0.2737	0.4173
NC_005103.4_40600001	4	40600001	0.2737	0.4173
NC_005103.4_40800001	4	40800001	0.248	0.4041
NC_005103.4_40900001	4	40900001	0.5165	0.5696
NC_005103.4_41000001	4	41000001	0.5813	0.6316
NC_005103.4_41100001	4	41100001	0.5513	0.5838
NC_005103.4_41200001	4	41200001	0.5561	0.6232
NC_005103.4_41300001	4	41300001	0.6199	0.6868

NC_005103.4_41400001	4	41400001	0.5747	0.6578
NC_005103.4_41500001	4	41500001	0.6038	0.7002
NC_005103.4_41600001	4	41600001	0.7196	0.8088
NC_005103.4_41700001	4	41700001	0.752	0.7756
NC_005103.4_41800001	4	41800001	0.6601	0.7294
NC_005103.4_41900001	4	41900001	0.7141	0.7825
NC_005103.4_42000001	4	42000001	0.6826	0.7809
NC_005103.4_42100001	4	42100001	0.5279	0.6655
NC_005103.4_42200001	4	42200001	0.4558	0.6505
NC_005103.4_42300001	4	42300001	0.3823	0.4885
NC_005103.4_42400001	4	42400001	0.3966	0.508
NC_005103.4_42500001	4	42500001	0.5034	0.7102
NC_005103.4_42600001	4	42600001	0.5537	0.7229
NC_005103.4_42700001	4	42700001	0.513	0.7199
NC_005103.4_42800001	4	42800001	0.5485	0.784
NC_005103.4_42900001	4	42900001	0.5113	0.6717
NC_005103.4_43000001	4	43000001	0.4312	0.5773
NC_005103.4_43100001	4	43100001	0.3901	0.5341
NC_005103.4_43200001	4	43200001	0.4665	0.5623
NC_005103.4_43300001	4	43300001	0.4606	0.5206
NC_005103.4_43400001	4	43400001	0.4997	0.5979
NC_005103.4_43500001	4	43500001	0.5151	0.5516
NC_005103.4_43600001	4	43600001	0.4956	0.4804
NC_005103.4_43700001	4	43700001	0.5247	0.5524
NC_005103.4_43800001	4	43800001	0.5036	0.5447
NC_005103.4_43900001	4	43900001	0.3833	0.4294
NC_005103.4_44000001	4	44000001	0.4229	0.4873
NC_005103.4_44100001	4	44100001	0.4175	0.5019
NC_005103.4_44200001	4	44200001	0.3383	0.4104
NC_005103.4_44300001	4	44300001	0.4132	0.5093
NC_005103.4_44400001	4	44400001	0.5009	0.5936
NC_005103.4_44500001	4	44500001	0.4335	0.5495
NC_005103.4_44600001	4	44600001	0.4913	0.6089
NC_005103.4_44700001	4	44700001	0.6885	0.737
NC_005103.4_44800001	4	44800001	0.6368	0.6661
NC_005103.4_44900001	4	44900001	0.5917	0.7294
NC_005103.4_45000001	4	45000001	0.6458	0.7593
NC_005103.4_45100001	4	45100001	0.6511	0.7664
NC_005103.4_45200001	4	45200001	0.528	0.6537
NC_005103.4_45300001	4	45300001	0.4853	0.6409
NC_005103.4_45400001	4	45400001	0.3613	0.4776
NC_005103.4_45500001	4	45500001	0.2419	0.3117
NC_005103.4_45600001	4	45600001	0.2659	0.4601
NC_005103.4_45700001	4	45700001	0.3228	0.73
NC_005103.4_45800001	4	45800001	0.3998	0.6753
NC_005103.4_45900001	4	45900001	0.5345	0.706
NC_005103.4_46000001	4	46000001	0.6049	0.6972
NC_005103.4_46100001	4	46100001	0.4792	0.5814
NC_005103.4_46200001	4	46200001	0.4422	0.4427
NC_005103.4_46300001	4	46300001	0.3462	0.3436
NC_005103.4_47000001	4	47000001	0.4522	0.7604
NC_005103.4_47100001	4	47100001	0.6125	0.9562
NC_005103.4_47200001	4	47200001	0.5768	0.8215
NC_005103.4_47300001	4	47300001	0.5768	0.8215
NC_005103.4_47400001	4	47400001	0.6412	0.8332
NC_005103.4_47500001	4	47500001	0.6222	0.7675
NC_005103.4_47600001	4	47600001	0.6008	0.7071
NC_005103.4_47700001	4	47700001	0.3914	0.6151

NC_005103.4_47800001	4	47800001	0.4884	0.7046
NC_005103.4_47900001	4	47900001	0.5095	0.6862
NC_005103.4_48000001	4	48000001	0.5226	0.6896
NC_005103.4_48100001	4	48100001	0.4737	0.6287
NC_005103.4_48200001	4	48200001	0.5977	0.698
NC_005103.4_48300001	4	48300001	0.5652	0.6365
NC_005103.4_48400001	4	48400001	0.5807	0.6053
NC_005103.4_48500001	4	48500001	0.5661	0.6197
NC_005103.4_48600001	4	48600001	0.5881	0.6315
NC_005103.4_48700001	4	48700001	0.4966	0.5696
NC_005103.4_48800001	4	48800001	0.4486	0.5695
NC_005103.4_48900001	4	48900001	0.4112	0.6106
NC_005103.4_49000001	4	49000001	0.3807	0.5583
NC_005103.4_49100001	4	49100001	0.4224	0.6641
NC_005103.4_49200001	4	49200001	0.4517	0.6698
NC_005103.4_49300001	4	49300001	0.4683	0.6954
NC_005103.4_49400001	4	49400001	0.519	0.6524
NC_005103.4_49500001	4	49500001	0.5887	0.6364
NC_005103.4_49600001	4	49600001	0.5184	0.5452
NC_005103.4_49700001	4	49700001	0.4319	0.5232
NC_005103.4_49800001	4	49800001	0.4546	0.5336
NC_005103.4_49900001	4	49900001	0.4914	0.6014
NC_005103.4_50000001	4	50000001	0.4985	0.6721
NC_005103.4_50100001	4	50100001	0.4847	0.6837
NC_005103.4_50200001	4	50200001	0.6882	0.8143
NC_005103.4_50300001	4	50300001	0.6367	0.8068
NC_005103.4_50400001	4	50400001	0.5581	0.7459
NC_005103.4_50500001	4	50500001	0.51	0.7275
NC_005103.4_50600001	4	50600001	0.51	0.7275
NC_005103.4_50700001	4	50700001	0.5747	0.7629
NC_005103.4_50800001	4	50800001	0.6529	0.787
NC_005103.4_50900001	4	50900001	0.5662	0.7116
NC_005103.4_51000001	4	51000001	0.5777	0.697
NC_005103.4_51100001	4	51100001	0.5777	0.697
NC_005103.4_51200001	4	51200001	0.4369	0.5424
NC_005103.4_51300001	4	51300001	0.4847	0.6041
NC_005103.4_51400001	4	51400001	0.912	0.9084
NC_005103.4_51500001	4	51500001	0.8517	0.9842
NC_005103.4_51600001	4	51600001	0.867	0.9793
NC_005103.4_51700001	4	51700001	0.6071	0.8479
NC_005103.4_51800001	4	51800001	0.5718	0.8276
NC_005103.4_51900001	4	51900001	0.2397	0.4581
NC_005103.4_52000001	4	52000001	0.3109	0.3653
NC_005103.4_52100001	4	52100001	0.2915	0.2981
NC_005103.4_52200001	4	52200001	0.3986	0.3587
NC_005103.4_52300001	4	52300001	0.3079	0.3044
NC_005103.4_52400001	4	52400001	0.3187	0.2906
NC_005103.4_52500001	4	52500001	0.3753	0.4411
NC_005103.4_52600001	4	52600001	0.3901	0.5479
NC_005103.4_52700001	4	52700001	0.3901	0.5479
NC_005103.4_52800001	4	52800001	0.4259	0.5966
NC_005103.4_52900001	4	52900001	0.4249	0.7124
NC_005103.4_53000001	4	53000001	0.4882	0.7965
NC_005103.4_53100001	4	53100001	0.4051	0.7158
NC_005103.4_53200001	4	53200001	0.4051	0.7158
NC_005103.4_53300001	4	53300001	0.4498	0.736
NC_005103.4_53400001	4	53400001	0.5128	0.7359
NC_005103.4_53900001	4	53900001	0.5543	0.5383

NC_005103.4_54000001	4	54000001	0.631	0.6793
NC_005103.4_54100001	4	54100001	0.6067	0.6343
NC_005103.4_54200001	4	54200001	0.5773	0.6306
NC_005103.4_54300001	4	54300001	0.5279	0.6504
NC_005103.4_54400001	4	54400001	0.501	0.6442
NC_005103.4_54500001	4	54500001	0.4182	0.5009
NC_005103.4_54600001	4	54600001	0.4639	0.5857
NC_005103.4_54700001	4	54700001	0.4691	0.5325
NC_005103.4_54800001	4	54800001	0.4276	0.497
NC_005103.4_54900001	4	54900001	0.4954	0.5749
NC_005103.4_55000001	4	55000001	0.5316	0.6243
NC_005103.4_55100001	4	55100001	0.4425	0.5609
NC_005103.4_55200001	4	55200001	0.4118	0.6112
NC_005103.4_55300001	4	55300001	0.4601	0.5862
NC_005103.4_55400001	4	55400001	0.4143	0.5521
NC_005103.4_55500001	4	55500001	0.5083	0.6804
NC_005103.4_55600001	4	55600001	0.6743	0.818
NC_005103.4_55700001	4	55700001	0.8045	0.8882
NC_005103.4_55800001	4	55800001	0.7669	0.9201
NC_005103.4_55900001	4	55900001	0.6941	0.8274
NC_005103.4_56000001	4	56000001	0.6465	0.7825
NC_005103.4_56100001	4	56100001	0.5876	0.7334
NC_005103.4_56200001	4	56200001	0.4699	0.6356
NC_005103.4_56300001	4	56300001	0.4843	0.5888
NC_005103.4_56400001	4	56400001	0.5165	0.6779
NC_005103.4_56500001	4	56500001	0.502	0.6453
NC_005103.4_56600001	4	56600001	0.4743	0.6261
NC_005103.4_56700001	4	56700001	0.5302	0.6636
NC_005103.4_56800001	4	56800001	0.6037	0.7581
NC_005103.4_56900001	4	56900001	0.5304	0.6395
NC_005103.4_57000001	4	57000001	0.6647	0.7395
NC_005103.4_57100001	4	57100001	0.6369	0.7371
NC_005103.4_57200001	4	57200001	0.5848	0.7019
NC_005103.4_57300001	4	57300001	0.5742	0.722
NC_005103.4_57400001	4	57400001	0.6365	0.7402
NC_005103.4_57500001	4	57500001	0.595	0.7601
NC_005103.4_57600001	4	57600001	0.5975	0.7616
NC_005103.4_57700001	4	57700001	0.6277	0.7837
NC_005103.4_57800001	4	57800001	0.6214	0.7325
NC_005103.4_57900001	4	57900001	0.5507	0.7699
NC_005103.4_58000001	4	58000001	0.4759	0.6707
NC_005103.4_58100001	4	58100001	0.4253	0.5682
NC_005103.4_58200001	4	58200001	0.4009	0.55
NC_005103.4_58300001	4	58300001	0.3468	0.4815
NC_005103.4_58400001	4	58400001	0.3706	0.5157
NC_005103.4_58500001	4	58500001	0.4074	0.4898
NC_005103.4_58600001	4	58600001	0.5118	0.6316
NC_005103.4_58700001	4	58700001	0.5733	0.625
NC_005103.4_58800001	4	58800001	0.5126	0.6509
NC_005103.4_58900001	4	58900001	0.4714	0.5962
NC_005103.4_59000001	4	59000001	0.4138	0.6428
NC_005103.4_59100001	4	59100001	0.3985	0.6272
NC_005103.4_59200001	4	59200001	0.384	0.6171
NC_005103.4_59300001	4	59300001	0.3849	0.6003
NC_005103.4_59400001	4	59400001	0.455	0.6984
NC_005103.4_59500001	4	59500001	0.425	0.6767
NC_005103.4_59600001	4	59600001	0.4213	0.7059
NC_005103.4_59700001	4	59700001	0.4666	0.7879

NC_005103.4_59800001	4	59800001	0.4952	0.8076
NC_005103.4_59900001	4	59900001	0.5087	0.7926
NC_005103.4_60000001	4	60000001	0.5832	0.7668
NC_005103.4_60100001	4	60100001	0.5647	0.7531
NC_005103.4_60200001	4	60200001	0.5641	0.7313
NC_005103.4_60300001	4	60300001	0.5464	0.7021
NC_005103.4_60400001	4	60400001	0.5085	0.6954
NC_005103.4_60500001	4	60500001	0.5649	0.8032
NC_005103.4_60600001	4	60600001	0.8492	0.8767
NC_005103.4_60700001	4	60700001	0.7405	0.796
NC_005103.4_60800001	4	60800001	0.7502	0.7581
NC_005103.4_60900001	4	60900001	0.6316	0.6935
NC_005103.4_61000001	4	61000001	0.6352	0.7339
NC_005103.4_61100001	4	61100001	0.5488	0.7085
NC_005103.4_61200001	4	61200001	0.5454	0.7268
NC_005103.4_61300001	4	61300001	0.43	0.6798
NC_005103.4_61400001	4	61400001	0.453	0.7265
NC_005103.4_61500001	4	61500001	0.398	0.527
NC_005103.4_61600001	4	61600001	0.3604	0.4844
NC_005103.4_61700001	4	61700001	0.3883	0.5196
NC_005103.4_61800001	4	61800001	0.49	0.6349
NC_005103.4_61900001	4	61900001	0.5359	0.631
NC_005103.4_62000001	4	62000001	0.6106	0.7754
NC_005103.4_62100001	4	62100001	0.6722	0.7651
NC_005103.4_62200001	4	62200001	0.64	0.7357
NC_005103.4_62300001	4	62300001	0.541	0.6902
NC_005103.4_62400001	4	62400001	0.5738	0.7642
NC_005103.4_62500001	4	62500001	0.6034	0.8218
NC_005103.4_62600001	4	62600001	0.6157	0.7822
NC_005103.4_62700001	4	62700001	0.5538	0.761
NC_005103.4_62800001	4	62800001	0.5791	0.7204
NC_005103.4_62900001	4	62900001	0.5145	0.6061
NC_005103.4_63000001	4	63000001	0.432	0.4424
NC_005103.4_63100001	4	63100001	0.4135	0.4341
NC_005103.4_63200001	4	63200001	0.4733	0.4292
NC_005103.4_63300001	4	63300001	0.5345	0.4671
NC_005103.4_63400001	4	63400001	0.5165	0.513
NC_005103.4_63500001	4	63500001	0.5851	0.6251
NC_005103.4_63600001	4	63600001	0.5928	0.6646
NC_005103.4_63700001	4	63700001	0.5983	0.6718
NC_005103.4_63800001	4	63800001	0.5785	0.6917
NC_005103.4_63900001	4	63900001	0.6177	0.7042
NC_005103.4_64000001	4	64000001	0.59	0.6771
NC_005103.4_64100001	4	64100001	0.6067	0.6779
NC_005103.4_64200001	4	64200001	0.5112	0.5754
NC_005103.4_64300001	4	64300001	0.4791	0.5347
NC_005103.4_64400001	4	64400001	0.4322	0.5266
NC_005103.4_64500001	4	64500001	0.4533	0.5515
NC_005103.4_64600001	4	64600001	0.4664	0.5783
NC_005103.4_64700001	4	64700001	0.5229	0.7029
NC_005103.4_64800001	4	64800001	0.4946	0.6931
NC_005103.4_64900001	4	64900001	0.5241	0.6704
NC_005103.4_65000001	4	65000001	0.5198	0.6926
NC_005103.4_65100001	4	65100001	0.4456	0.6309
NC_005103.4_65200001	4	65200001	0.516	0.6588
NC_005103.4_65300001	4	65300001	0.5467	0.6385
NC_005103.4_65400001	4	65400001	0.5134	0.6136
NC_005103.4_65500001	4	65500001	0.4654	0.5731

NC_005103.4_65600001	4	65600001	0.5119	0.6288
NC_005103.4_65700001	4	65700001	0.5785	0.7104
NC_005103.4_65800001	4	65800001	0.6512	0.7774
NC_005103.4_65900001	4	65900001	0.8116	0.8994
NC_005103.4_66000001	4	66000001	0.7139	0.7903
NC_005103.4_66100001	4	66100001	0.7272	0.8078
NC_005103.4_66200001	4	66200001	0.6644	0.7533
NC_005103.4_66300001	4	66300001	0.6435	0.7316
NC_005103.4_66400001	4	66400001	0.5852	0.7208
NC_005103.4_66500001	4	66500001	0.5916	0.7599
NC_005103.4_66600001	4	66600001	0.5505	0.673
NC_005103.4_66700001	4	66700001	0.5835	0.704
NC_005103.4_66800001	4	66800001	0.5636	0.6662
NC_005103.4_66900001	4	66900001	0.6544	0.677
NC_005103.4_67000001	4	67000001	0.6471	0.6596
NC_005103.4_67100001	4	67100001	0.6735	0.701
NC_005103.4_67200001	4	67200001	0.5635	0.5991
NC_005103.4_67300001	4	67300001	0.5506	0.6108
NC_005103.4_67400001	4	67400001	0.5437	0.6234
NC_005103.4_67500001	4	67500001	0.463	0.5786
NC_005103.4_67600001	4	67600001	0.4261	0.58
NC_005103.4_67700001	4	67700001	0.4779	0.6906
NC_005103.4_67800001	4	67800001	0.4719	0.6652
NC_005103.4_67900001	4	67900001	0.3544	0.624
NC_005103.4_68000001	4	68000001	0.4933	0.6075
NC_005103.4_68100001	4	68100001	0.6191	0.6658
NC_005103.4_68200001	4	68200001	0.6479	0.6877
NC_005103.4_68300001	4	68300001	0.6479	0.6877
NC_005103.4_68400001	4	68400001	0.6335	0.6993
NC_005103.4_68500001	4	68500001	0.5962	0.7298
NC_005103.4_68600001	4	68600001	0.5301	0.6419
NC_005103.4_68700001	4	68700001	0.3444	0.4703
NC_005103.4_68800001	4	68800001	0.509	0.6002
NC_005103.4_68900001	4	68900001	0.4889	0.5766
NC_005103.4_69000001	4	69000001	0.4617	0.521
NC_005103.4_69100001	4	69100001	0.4713	0.5619
NC_005103.4_69200001	4	69200001	0.5639	0.6346
NC_005103.4_69300001	4	69300001	0.4628	0.6236
NC_005103.4_69400001	4	69400001	0.5252	0.6495
NC_005103.4_69500001	4	69500001	0.7361	0.7887
NC_005103.4_69600001	4	69600001	0.7897	0.7676
NC_005103.4_69700001	4	69700001	0.7486	0.7213
NC_005103.4_69800001	4	69800001	0.6085	0.5479
NC_005103.4_70100001	4	70100001	0.5543	0.6064
NC_005103.4_70200001	4	70200001	0.5305	0.5703
NC_005103.4_70300001	4	70300001	0.5207	0.5214
NC_005103.4_70400001	4	70400001	0.5465	0.5827
NC_005103.4_70500001	4	70500001	0.6297	0.6125
NC_005103.4_70600001	4	70600001	0.5581	0.5322
NC_005103.4_70700001	4	70700001	0.5543	0.4933
NC_005103.4_70800001	4	70800001	0.5949	0.5438
NC_005103.4_70900001	4	70900001	0.5698	0.573
NC_005103.4_71000001	4	71000001	0.4616	0.4877
NC_005103.4_71100001	4	71100001	0.4073	0.5488
NC_005103.4_71200001	4	71200001	0.4156	0.7102
NC_005103.4_71300001	4	71300001	0.3889	0.6975
NC_005103.4_71400001	4	71400001	0.3507	0.5826
NC_005103.4_71500001	4	71500001	0.3643	0.6189

NC_005103.4_71600001	4	71600001	0.3573	0.4966
NC_005103.4_71700001	4	71700001	0.1908	0.2038
NC_005103.4_71800001	4	71800001	0.1617	0.2073
NC_005103.4_72000001	4	72000001	0.2445	0.4315
NC_005103.4_72100001	4	72100001	0.3896	0.7163
NC_005103.4_72200001	4	72200001	0.3496	0.7355
NC_005103.4_72300001	4	72300001	0.2888	0.478
NC_005103.4_72400001	4	72400001	0.2486	0.435
NC_005103.4_72500001	4	72500001	0.3031	0.4615
NC_005103.4_72600001	4	72600001	0.2422	0.3707
NC_005103.4_72700001	4	72700001	0.2608	0.3645
NC_005103.4_72800001	4	72800001	0.3016	0.42
NC_005103.4_72900001	4	72900001	0.5225	0.6803
NC_005103.4_73000001	4	73000001	0.374	0.6775
NC_005103.4_73100001	4	73100001	0.4893	0.7075
NC_005103.4_73200001	4	73200001	0.4766	0.677
NC_005103.4_73300001	4	73300001	0.4953	0.7218
NC_005103.4_73400001	4	73400001	0.4144	0.5904
NC_005103.4_73500001	4	73500001	0.4991	0.6239
NC_005103.4_73600001	4	73600001	0.3338	0.4651
NC_005103.4_73700001	4	73700001	0.3284	0.4855
NC_005103.4_73800001	4	73800001	0.3492	0.3899
NC_005103.4_73900001	4	73900001	0.3782	0.3952
NC_005103.4_74000001	4	74000001	0.3479	0.3594
NC_005103.4_74100001	4	74100001	0.3969	0.3776
NC_005103.4_74200001	4	74200001	0.4906	0.4716
NC_005103.4_74300001	4	74300001	0.6711	0.6382
NC_005103.4_74400001	4	74400001	0.5915	0.6046
NC_005103.4_74500001	4	74500001	0.6154	0.623
NC_005103.4_74600001	4	74600001	0.6216	0.6349
NC_005103.4_74700001	4	74700001	0.605	0.6032
NC_005103.4_74800001	4	74800001	0.605	0.6032
NC_005103.4_74900001	4	74900001	0.6273	0.5211
NC_005103.4_75000001	4	75000001	0.6111	0.4907
NC_005103.4_75100001	4	75100001	0.7509	0.6474
NC_005103.4_75200001	4	75200001	0.7811	0.7136
NC_005103.4_75300001	4	75300001	0.8075	0.756
NC_005103.4_75400001	4	75400001	0.9292	0.9077
NC_005103.4_75500001	4	75500001	0.9931	0.9864
NC_005103.4_75700001	4	75700001	0.5567	0.9016
NC_005103.4_75800001	4	75800001	0.56	0.8263
NC_005103.4_75900001	4	75900001	0.5902	0.7369
NC_005103.4_76000001	4	76000001	0.5685	0.7341
NC_005103.4_76100001	4	76100001	0.5518	0.7144
NC_005103.4_76200001	4	76200001	0.6231	0.6907
NC_005103.4_76300001	4	76300001	0.5996	0.655
NC_005103.4_76400001	4	76400001	0.576	0.6579
NC_005103.4_76500001	4	76500001	0.6158	0.5856
NC_005103.4_76600001	4	76600001	0.5708	0.5741
NC_005103.4_76700001	4	76700001	0.614	0.6463
NC_005103.4_76800001	4	76800001	0.6628	0.7196
NC_005103.4_76900001	4	76900001	0.5776	0.7028
NC_005103.4_77000001	4	77000001	0.5571	0.7925
NC_005103.4_77100001	4	77100001	0.6421	0.8719
NC_005103.4_77200001	4	77200001	0.508	0.7261
NC_005103.4_77300001	4	77300001	0.4712	0.7079
NC_005103.4_77400001	4	77400001	0.4464	0.6738
NC_005103.4_77500001	4	77500001	0.4528	0.6757

NC_005103.4_77600001	4	77600001	0.5412	0.7517
NC_005103.4_77700001	4	77700001	0.5749	0.7683
NC_005103.4_77800001	4	77800001	0.6192	0.7639
NC_005103.4_77900001	4	77900001	0.594	0.7407
NC_005103.4_78000001	4	78000001	0.5495	0.7265
NC_005103.4_78100001	4	78100001	0.4445	0.5771
NC_005103.4_78200001	4	78200001	0.4449	0.6136
NC_005103.4_78300001	4	78300001	0.4065	0.5746
NC_005103.4_78400001	4	78400001	0.5064	0.6345
NC_005103.4_78500001	4	78500001	0.4949	0.6197
NC_005103.4_78600001	4	78600001	0.4716	0.6556
NC_005103.4_78700001	4	78700001	0.4637	0.6334
NC_005103.4_78800001	4	78800001	0.4515	0.5651
NC_005103.4_78900001	4	78900001	0.4554	0.5964
NC_005103.4_79000001	4	79000001	0.5895	0.6992
NC_005103.4_79100001	4	79100001	0.5861	0.6626
NC_005103.4_79200001	4	79200001	0.5554	0.6252
NC_005103.4_79300001	4	79300001	0.606	0.6922
NC_005103.4_79400001	4	79400001	0.5212	0.6214
NC_005103.4_79500001	4	79500001	0.4368	0.522
NC_005103.4_79600001	4	79600001	0.4535	0.4953
NC_005103.4_79700001	4	79700001	0.5437	0.5951
NC_005103.4_79800001	4	79800001	0.5258	0.5801
NC_005103.4_79900001	4	79900001	0.5788	0.6257
NC_005103.4_80000001	4	80000001	0.5854	0.6402
NC_005103.4_80100001	4	80100001	0.5793	0.6594
NC_005103.4_80200001	4	80200001	0.5857	0.7067
NC_005103.4_80300001	4	80300001	0.6554	0.7477
NC_005103.4_80400001	4	80400001	0.6367	0.7012
NC_005103.4_80500001	4	80500001	0.5997	0.686
NC_005103.4_80600001	4	80600001	0.5648	0.6981
NC_005103.4_80700001	4	80700001	0.5431	0.6717
NC_005103.4_80800001	4	80800001	0.4915	0.6312
NC_005103.4_80900001	4	80900001	0.3902	0.5884
NC_005103.4_81000001	4	81000001	0.4984	0.6991
NC_005103.4_81100001	4	81100001	0.5887	0.718
NC_005103.4_81200001	4	81200001	0.6157	0.7524
NC_005103.4_81300001	4	81300001	0.5921	0.7964
NC_005103.4_81400001	4	81400001	0.6432	0.7584
NC_005103.4_81500001	4	81500001	0.4657	0.6466
NC_005103.4_81600001	4	81600001	0.5372	0.7125
NC_005103.4_81700001	4	81700001	0.5372	0.7125
NC_005103.4_81800001	4	81800001	0.5768	0.7072
NC_005103.4_81900001	4	81900001	0.5757	0.7642
NC_005103.4_82000001	4	82000001	0.5115	0.6131
NC_005103.4_82100001	4	82100001	0.3792	0.3724
NC_005103.4_82200001	4	82200001	0.3346	0.3835
NC_005103.4_82300001	4	82300001	0.3907	0.4347
NC_005103.4_82400001	4	82400001	0.4012	0.4482
NC_005103.4_82500001	4	82500001	0.4896	0.5984
NC_005103.4_82600001	4	82600001	0.5037	0.6646
NC_005103.4_82700001	4	82700001	0.5121	0.6868
NC_005103.4_82800001	4	82800001	0.5066	0.7195
NC_005103.4_82900001	4	82900001	0.5016	0.7753
NC_005103.4_83000001	4	83000001	0.4364	0.7232
NC_005103.4_83100001	4	83100001	0.4687	0.7191
NC_005103.4_83200001	4	83200001	0.6078	0.7728
NC_005103.4_83300001	4	83300001	0.5725	0.7242

NC_005103.4_83400001	4	83400001	0.6048	0.7191
NC_005103.4_83500001	4	83500001	0.5855	0.7293
NC_005103.4_83600001	4	83600001	0.6089	0.7366
NC_005103.4_83700001	4	83700001	0.562	0.6855
NC_005103.4_83800001	4	83800001	0.599	0.7247
NC_005103.4_83900001	4	83900001	0.5389	0.6854
NC_005103.4_84000001	4	84000001	0.5878	0.6863
NC_005103.4_84100001	4	84100001	0.4809	0.5762
NC_005103.4_84200001	4	84200001	0.4627	0.5774
NC_005103.4_84300001	4	84300001	0.4397	0.5722
NC_005103.4_84400001	4	84400001	0.4666	0.6115
NC_005103.4_84500001	4	84500001	0.4171	0.5688
NC_005103.4_84600001	4	84600001	0.4745	0.6539
NC_005103.4_84700001	4	84700001	0.5101	0.6289
NC_005103.4_84800001	4	84800001	0.4897	0.5754
NC_005103.4_84900001	4	84900001	0.4889	0.5812
NC_005103.4_85000001	4	85000001	0.5749	0.645
NC_005103.4_85100001	4	85100001	0.5256	0.6178
NC_005103.4_85200001	4	85200001	0.4911	0.6466
NC_005103.4_85300001	4	85300001	0.5449	0.7451
NC_005103.4_85400001	4	85400001	0.5491	0.7203
NC_005103.4_85500001	4	85500001	0.5103	0.6669
NC_005103.4_85600001	4	85600001	0.5494	0.6595
NC_005103.4_85700001	4	85700001	0.5	0.7164
NC_005103.4_85800001	4	85800001	0.4833	0.6411
NC_005103.4_85900001	4	85900001	0.504	0.6571
NC_005103.4_86000001	4	86000001	0.4588	0.698
NC_005103.4_86100001	4	86100001	0.4569	0.6885
NC_005103.4_86200001	4	86200001	0.536	0.7154
NC_005103.4_86300001	4	86300001	0.4602	0.6389
NC_005103.4_86400001	4	86400001	0.4325	0.6181
NC_005103.4_86500001	4	86500001	0.4247	0.5632
NC_005103.4_86600001	4	86600001	0.5189	0.6888
NC_005103.4_86700001	4	86700001	0.4739	0.6272
NC_005103.4_86800001	4	86800001	0.5354	0.6926
NC_005103.4_86900001	4	86900001	0.5936	0.7274
NC_005103.4_87000001	4	87000001	0.6179	0.723
NC_005103.4_87100001	4	87100001	0.5017	0.5862
NC_005103.4_87200001	4	87200001	0.5109	0.6211
NC_005103.4_87400001	4	87400001	0.4562	0.4632
NC_005103.4_87600001	4	87600001	0.2165	0.2009
NC_005103.4_87700001	4	87700001	0.359	0.5393
NC_005103.4_87800001	4	87800001	0.3638	0.6153
NC_005103.4_87900001	4	87900001	0.3696	0.6539
NC_005103.4_88000001	4	88000001	0.4368	0.6409
NC_005103.4_88100001	4	88100001	0.5391	0.6723
NC_005103.4_88200001	4	88200001	0.6012	0.6423
NC_005103.4_88300001	4	88300001	0.6029	0.6587
NC_005103.4_88400001	4	88400001	0.5685	0.6657
NC_005103.4_88500001	4	88500001	0.5408	0.6396
NC_005103.4_88600001	4	88600001	0.4998	0.5815
NC_005103.4_88700001	4	88700001	0.3968	0.5228
NC_005103.4_88800001	4	88800001	0.295	0.358
NC_005103.4_88900001	4	88900001	0.3689	0.3876
NC_005103.4_89000001	4	89000001	0.5823	0.6604
NC_005103.4_90100001	4	90100001	0.4252	0.3958
NC_005103.4_90200001	4	90200001	0.4693	0.5055
NC_005103.4_90300001	4	90300001	0.4699	0.532

NC_005103.4_90400001	4	90400001	0.4882	0.5852
NC_005103.4_90500001	4	90500001	0.4599	0.5699
NC_005103.4_90600001	4	90600001	0.4725	0.6502
NC_005103.4_90700001	4	90700001	0.4478	0.6634
NC_005103.4_90800001	4	90800001	0.4713	0.6889
NC_005103.4_90900001	4	90900001	0.4259	0.5968
NC_005103.4_91000001	4	91000001	0.4152	0.5574
NC_005103.4_91100001	4	91100001	0.4854	0.5805
NC_005103.4_91200001	4	91200001	0.5014	0.5871
NC_005103.4_91300001	4	91300001	0.52	0.5229
NC_005103.4_91400001	4	91400001	0.475	0.5549
NC_005103.4_91500001	4	91500001	0.5354	0.6466
NC_005103.4_91600001	4	91600001	0.4442	0.5876
NC_005103.4_91700001	4	91700001	0.5417	0.6801
NC_005103.4_91800001	4	91800001	0.5165	0.6857
NC_005103.4_91900001	4	91900001	0.6165	0.7614
NC_005103.4_92000001	4	92000001	0.6536	0.752
NC_005103.4_92100001	4	92100001	0.7187	0.8005
NC_005103.4_92200001	4	92200001	0.6788	0.7831
NC_005103.4_92300001	4	92300001	0.815	0.8404
NC_005103.4_93100001	4	93100001	0.3464	0.4298
NC_005103.4_93200001	4	93200001	0.3464	0.4298
NC_005103.4_93300001	4	93300001	0.3777	0.476
NC_005103.4_93900001	4	93900001	0.4011	0.4778
NC_005103.4_94000001	4	94000001	0.3427	0.4616
NC_005103.4_94100001	4	94100001	0.3779	0.5114
NC_005103.4_94200001	4	94200001	0.4526	0.6201
NC_005103.4_94300001	4	94300001	0.4637	0.6777
NC_005103.4_94400001	4	94400001	0.5191	0.7923
NC_005103.4_94500001	4	94500001	0.5815	0.738
NC_005103.4_94600001	4	94600001	0.5224	0.6886
NC_005103.4_94700001	4	94700001	0.583	0.6851
NC_005103.4_94800001	4	94800001	0.6292	0.7047
NC_005103.4_94900001	4	94900001	0.6489	0.7337
NC_005103.4_95000001	4	95000001	0.6667	0.7663
NC_005103.4_95100001	4	95100001	0.7297	0.8034
NC_005103.4_95200001	4	95200001	0.6852	0.78
NC_005103.4_95300001	4	95300001	0.657	0.7406
NC_005103.4_95400001	4	95400001	0.6202	0.713
NC_005103.4_95500001	4	95500001	0.6121	0.727
NC_005103.4_95600001	4	95600001	0.5584	0.6815
NC_005103.4_95700001	4	95700001	0.5272	0.6728
NC_005103.4_95800001	4	95800001	0.5281	0.7107
NC_005103.4_95900001	4	95900001	0.6765	0.8231
NC_005103.4_96000001	4	96000001	0.5272	0.5788
NC_005103.4_96100001	4	96100001	0.5461	0.5928
NC_005103.4_96200001	4	96200001	0.5929	0.6467
NC_005103.4_96300001	4	96300001	0.6398	0.671
NC_005103.4_96400001	4	96400001	0.5713	0.5871
NC_005103.4_96500001	4	96500001	0.7632	0.7406
NC_005103.4_96600001	4	96600001	0.6712	0.7281
NC_005103.4_96700001	4	96700001	0.4566	0.4746
NC_005103.4_96800001	4	96800001	0.3713	0.3668
NC_005103.4_96900001	4	96900001	0.3841	0.3363
NC_005103.4_97000001	4	97000001	0.3875	0.3381
NC_005103.4_97100001	4	97100001	0.3925	0.3326
NC_005103.4_97200001	4	97200001	0.587	0.5781
NC_005103.4_97300001	4	97300001	0.5716	0.5908

NC_005103.4_97400001	4	97400001	0.593	0.6555
NC_005103.4_97500001	4	97500001	0.6053	0.6918
NC_005103.4_97600001	4	97600001	0.6485	0.7137
NC_005103.4_97700001	4	97700001	0.5112	0.554
NC_005103.4_97900001	4	97900001	0.3319	0.2681
NC_005103.4_98000001	4	98000001	0.3319	0.2681
NC_005103.4_98100001	4	98100001	0.3513	0.3991
NC_005103.4_98200001	4	98200001	0.394	0.4534
NC_005103.4_98300001	4	98300001	0.4524	0.5282
NC_005103.4_98400001	4	98400001	0.5233	0.6719
NC_005103.4_98500001	4	98500001	0.4938	0.6672
NC_005103.4_98600001	4	98600001	0.4624	0.6035
NC_005103.4_98700001	4	98700001	0.4917	0.6046
NC_005103.4_98800001	4	98800001	0.519	0.6348
NC_005103.4_98900001	4	98900001	0.5026	0.6346
NC_005103.4_99000001	4	99000001	0.5049	0.6283
NC_005103.4_99100001	4	99100001	0.5473	0.6891
NC_005103.4_99200001	4	99200001	0.467	0.5167
NC_005103.4_99300001	4	99300001	0.4653	0.5219
NC_005103.4_99400001	4	99400001	0.4636	0.4946
NC_005103.4_99500001	4	99500001	0.4644	0.4787
NC_005103.4_99600001	4	99600001	0.4859	0.4853
NC_005103.4_99700001	4	99700001	0.6083	0.7665
NC_005103.4_99800001	4	99800001	0.6178	0.7695
NC_005103.4_99900001	4	99900001	0.5382	0.7351
NC_005103.4_100000001	4	100000001	0.5722	0.7585
NC_005103.4_100100001	4	100100001	0.5593	0.7476
NC_005103.4_100200001	4	100200001	0.4648	0.6973
NC_005103.4_100300001	4	100300001	0.485	0.7103
NC_005103.4_100400001	4	100400001	0.5173	0.7366
NC_005103.4_100500001	4	100500001	0.4807	0.7209
NC_005103.4_100600001	4	100600001	0.4539	0.7142
NC_005103.4_100700001	4	100700001	0.4926	0.6972
NC_005103.4_100800001	4	100800001	0.5569	0.7324
NC_005103.4_100900001	4	100900001	0.5705	0.6943
NC_005103.4_101000001	4	101000001	0.6359	0.7169
NC_005103.4_101100001	4	101100001	0.7082	0.6965
NC_005103.4_101200001	4	101200001	0.8295	0.7298
NC_005103.4_101700001	4	101700001	0.3601	0.5037
NC_005103.4_101800001	4	101800001	0.3601	0.5037
NC_005103.4_101900001	4	101900001	0.3601	0.5037
NC_005103.4_102000001	4	102000001	0.3601	0.5037
NC_005103.4_102100001	4	102100001	0.3601	0.5037
NC_005103.4_103700001	4	103700001	0.3136	0.3599
NC_005103.4_103800001	4	103800001	0.3136	0.3599
NC_005103.4_103900001	4	103900001	0.4826	0.6146
NC_005103.4_104000001	4	104000001	0.5673	0.6877
NC_005103.4_104100001	4	104100001	0.5692	0.7133
NC_005103.4_104200001	4	104200001	0.7288	0.8092
NC_005103.4_104300001	4	104300001	0.5974	0.7567
NC_005103.4_104400001	4	104400001	0.5522	0.7138
NC_005103.4_104500001	4	104500001	0.5172	0.6358
NC_005103.4_104600001	4	104600001	0.4275	0.6436
NC_005103.4_104700001	4	104700001	0.4635	0.7709
NC_005103.4_104800001	4	104800001	0.5533	0.8407
NC_005103.4_104900001	4	104900001	0.453	0.7498
NC_005103.4_105000001	4	105000001	0.4137	0.6947
NC_005103.4_105100001	4	105100001	0.4585	0.6594

NC_005103.4_105200001	4	105200001	0.3792	0.5437
NC_005103.4_105300001	4	105300001	0.3102	0.4602
NC_005103.4_105400001	4	105400001	0.3249	0.4553
NC_005103.4_105500001	4	105500001	0.3452	0.4849
NC_005103.4_105600001	4	105600001	0.3393	0.4327
NC_005103.4_105700001	4	105700001	0.4329	0.4961
NC_005103.4_105800001	4	105800001	0.4356	0.4879
NC_005103.4_105900001	4	105900001	0.4836	0.5172
NC_005103.4_106000001	4	106000001	0.5258	0.4732
NC_005103.4_106100001	4	106100001	0.5674	0.5835
NC_005103.4_106300001	4	106300001	0.379	0.3839
NC_005103.4_106400001	4	106400001	0.3566	0.4066
NC_005103.4_106500001	4	106500001	0.5671	0.6418
NC_005103.4_106600001	4	106600001	0.4118	0.5747
NC_005103.4_106700001	4	106700001	0.4031	0.5345
NC_005103.4_106800001	4	106800001	0.556	0.6413
NC_005103.4_106900001	4	106900001	0.556	0.6413
NC_005103.4_107000001	4	107000001	0.4277	0.5655
NC_005103.4_107100001	4	107100001	0.5029	0.6015
NC_005103.4_107200001	4	107200001	0.5555	0.6849
NC_005103.4_107300001	4	107300001	0.4597	0.6998
NC_005103.4_107400001	4	107400001	0.4674	0.7151
NC_005103.4_107500001	4	107500001	0.5836	0.8015
NC_005103.4_107600001	4	107600001	0.6151	0.8619
NC_005103.4_107700001	4	107700001	0.5785	0.7538
NC_005103.4_107800001	4	107800001	0.6205	0.7387
NC_005103.4_107900001	4	107900001	0.6342	0.7295
NC_005103.4_108000001	4	108000001	0.5434	0.7078
NC_005103.4_108100001	4	108100001	0.5481	0.7085
NC_005103.4_108200001	4	108200001	0.3911	0.5737
NC_005103.4_108300001	4	108300001	0.5266	0.6558
NC_005103.4_108400001	4	108400001	0.4899	0.611
NC_005103.4_108500001	4	108500001	0.5501	0.6526
NC_005103.4_108600001	4	108600001	0.5075	0.6276
NC_005103.4_108700001	4	108700001	0.5799	0.6982
NC_005103.4_108800001	4	108800001	0.5138	0.7255
NC_005103.4_108900001	4	108900001	0.6322	0.8234
NC_005103.4_109000001	4	109000001	0.6035	0.8049
NC_005103.4_109100001	4	109100001	0.6873	0.8572
NC_005103.4_109200001	4	109200001	0.4785	0.7272
NC_005103.4_109300001	4	109300001	0.487	0.7248
NC_005103.4_109400001	4	109400001	0.3577	0.6251
NC_005103.4_109500001	4	109500001	0.3577	0.6251
NC_005103.4_109600001	4	109600001	0.2772	0.5202
NC_005103.4_109700001	4	109700001	0.399	0.708
NC_005103.4_109800001	4	109800001	0.328	0.6297
NC_005103.4_109900001	4	109900001	0.4504	0.7967
NC_005103.4_110000001	4	110000001	0.3472	0.7681
NC_005103.4_110100001	4	110100001	0.4309	0.8307
NC_005103.4_110200001	4	110200001	0.2221	0.4024
NC_005103.4_110300001	4	110300001	0.3381	0.5744
NC_005103.4_110400001	4	110400001	0.4121	0.6755
NC_005103.4_110500001	4	110500001	0.3988	0.6632
NC_005103.4_110600001	4	110600001	0.293	0.5216
NC_005103.4_110700001	4	110700001	0.3947	0.6369
NC_005103.4_110800001	4	110800001	0.3824	0.5869
NC_005103.4_110900001	4	110900001	0.4107	0.6066
NC_005103.4_111000001	4	111000001	0.4736	0.6205

NC_005103.4_111100001	4	111100001	0.527	0.5798
NC_005103.4_111200001	4	111200001	0.5157	0.6282
NC_005103.4_111300001	4	111300001	0.5699	0.6125
NC_005103.4_111400001	4	111400001	0.5475	0.6248
NC_005103.4_111500001	4	111500001	0.5978	0.6803
NC_005103.4_111600001	4	111600001	0.6376	0.7518
NC_005103.4_111700001	4	111700001	0.6435	0.7389
NC_005103.4_111800001	4	111800001	0.7033	0.8624
NC_005103.4_111900001	4	111900001	0.9589	0.9202
NC_005103.4_112000001	4	112000001	0.7607	0.7482
NC_005103.4_112100001	4	112100001	0.7738	0.7585
NC_005103.4_112200001	4	112200001	0.6791	0.7103
NC_005103.4_112300001	4	112300001	0.6299	0.6402
NC_005103.4_112400001	4	112400001	0.5379	0.5723
NC_005103.4_112500001	4	112500001	0.4767	0.5679
NC_005103.4_112600001	4	112600001	0.3643	0.525
NC_005103.4_112700001	4	112700001	0.3463	0.5548
NC_005103.4_112800001	4	112800001	0.3254	0.6371
NC_005103.4_112900001	4	112900001	0.3735	0.677
NC_005103.4_113000001	4	113000001	0.464	0.4888
NC_005103.4_113100001	4	113100001	0.5528	0.5127
NC_005103.4_113200001	4	113200001	0.6358	0.6241
NC_005103.4_113300001	4	113300001	0.6432	0.6353
NC_005103.4_113400001	4	113400001	0.5895	0.5858
NC_005103.4_113500001	4	113500001	0.6575	0.7136
NC_005103.4_113600001	4	113600001	0.6451	0.6622
NC_005103.4_113700001	4	113700001	0.587	0.5919
NC_005103.4_113800001	4	113800001	0.5847	0.6119
NC_005103.4_114000001	4	114000001	0.6498	0.6619
NC_005103.4_114100001	4	114100001	0.6711	0.6696
NC_005103.4_114200001	4	114200001	0.6173	0.6036
NC_005103.4_114300001	4	114300001	0.5284	0.4859
NC_005103.4_114400001	4	114400001	0.6859	0.7294
NC_005103.4_114500001	4	114500001	0.7023	0.6853
NC_005103.4_114600001	4	114600001	0.7367	0.756
NC_005103.4_114700001	4	114700001	0.7006	0.7013
NC_005103.4_114800001	4	114800001	0.6749	0.7197
NC_005103.4_114900001	4	114900001	0.6955	0.7362
NC_005103.4_115000001	4	115000001	0.6217	0.7518
NC_005103.4_115100001	4	115100001	0.5605	0.7306
NC_005103.4_115200001	4	115200001	0.5843	0.7914
NC_005103.4_115300001	4	115300001	0.5738	0.792
NC_005103.4_115400001	4	115400001	0.4877	0.6905
NC_005103.4_115500001	4	115500001	0.6717	0.78
NC_005103.4_115600001	4	115600001	0.7459	0.8075
NC_005103.4_115700001	4	115700001	0.7297	0.7769
NC_005103.4_115800001	4	115800001	0.7452	0.7597
NC_005103.4_115900001	4	115900001	0.7356	0.7823
NC_005103.4_116000001	4	116000001	0.6877	0.7234
NC_005103.4_116100001	4	116100001	0.5384	0.6698
NC_005103.4_116200001	4	116200001	0.5291	0.7116
NC_005103.4_116300001	4	116300001	0.6029	0.7763
NC_005103.4_116400001	4	116400001	0.6452	0.8434
NC_005103.4_116500001	4	116500001	0.5701	0.8334
NC_005103.4_116600001	4	116600001	0.5285	0.8055
NC_005103.4_116700001	4	116700001	0.5238	0.7976
NC_005103.4_116800001	4	116800001	0.5446	0.8361
NC_005103.4_116900001	4	116900001	0.5585	0.8495

NC_005103.4_117000001	4	117000001	0.5463	0.8461
NC_005103.4_117100001	4	117100001	0.5585	0.8206
NC_005103.4_117200001	4	117200001	0.5868	0.8159
NC_005103.4_117300001	4	117300001	0.5788	0.7398
NC_005103.4_117400001	4	117400001	0.5627	0.6805
NC_005103.4_117500001	4	117500001	0.5784	0.6798
NC_005103.4_117600001	4	117600001	0.6122	0.7183
NC_005103.4_117700001	4	117700001	0.6001	0.7222
NC_005103.4_117800001	4	117800001	0.566	0.744
NC_005103.4_117900001	4	117900001	0.5809	0.7469
NC_005103.4_118000001	4	118000001	0.6768	0.8228
NC_005103.4_118100001	4	118100001	0.6415	0.8117
NC_005103.4_118200001	4	118200001	0.5999	0.7864
NC_005103.4_118300001	4	118300001	0.6236	0.8069
NC_005103.4_118400001	4	118400001	0.6738	0.864
NC_005103.4_118500001	4	118500001	0.604	0.8285
NC_005103.4_118600001	4	118600001	0.6311	0.8159
NC_005103.4_118700001	4	118700001	0.64	0.7765
NC_005103.4_118800001	4	118800001	0.5472	0.6833
NC_005103.4_118900001	4	118900001	0.4201	0.4865
NC_005103.4_119000001	4	119000001	0.4475	0.4763
NC_005103.4_119100001	4	119100001	0.4831	0.5161
NC_005103.4_119200001	4	119200001	0.5351	0.5831
NC_005103.4_119300001	4	119300001	0.5842	0.5796
NC_005103.4_119400001	4	119400001	0.6177	0.6818
NC_005103.4_119500001	4	119500001	0.6202	0.7107
NC_005103.4_119600001	4	119600001	0.6178	0.7479
NC_005103.4_119700001	4	119700001	0.5735	0.7384
NC_005103.4_119800001	4	119800001	0.5565	0.7445
NC_005103.4_119900001	4	119900001	0.5748	0.757
NC_005103.4_120000001	4	120000001	0.5255	0.7672
NC_005103.4_120100001	4	120100001	0.4708	0.7313
NC_005103.4_120200001	4	120200001	0.4047	0.6862
NC_005103.4_120300001	4	120300001	0.3871	0.6466
NC_005103.4_120400001	4	120400001	0.3904	0.6304
NC_005103.4_120500001	4	120500001	0.4077	0.6425
NC_005103.4_120600001	4	120600001	0.3708	0.5666
NC_005103.4_120700001	4	120700001	0.4441	0.5846
NC_005103.4_120800001	4	120800001	0.4475	0.6264
NC_005103.4_120900001	4	120900001	0.4593	0.5935
NC_005103.4_121000001	4	121000001	0.4589	0.5911
NC_005103.4_121100001	4	121100001	0.5164	0.681
NC_005103.4_121200001	4	121200001	0.499	0.7223
NC_005103.4_121300001	4	121300001	0.4752	0.6443
NC_005103.4_121400001	4	121400001	0.4187	0.6647
NC_005103.4_121500001	4	121500001	0.434	0.6501
NC_005103.4_121600001	4	121600001	0.3987	0.5241
NC_005103.4_121700001	4	121700001	0.6095	0.584
NC_005103.4_121800001	4	121800001	0.5987	0.5232
NC_005103.4_121900001	4	121900001	0.4407	0.5316
NC_005103.4_122000001	4	122000001	0.3898	0.4849
NC_005103.4_122100001	4	122100001	0.439	0.5592
NC_005103.4_122200001	4	122200001	0.4106	0.4967
NC_005103.4_122300001	4	122300001	0.521	0.6186
NC_005103.4_122400001	4	122400001	0.6052	0.6827
NC_005103.4_122500001	4	122500001	0.6365	0.7399
NC_005103.4_122600001	4	122600001	0.6269	0.7429
NC_005103.4_122700001	4	122700001	0.6199	0.7422

NC_005103.4_122800001	4	122800001	0.5733	0.7384
NC_005103.4_122900001	4	122900001	0.5515	0.699
NC_005103.4_123000001	4	123000001	0.518	0.6734
NC_005103.4_123100001	4	123100001	0.5	0.6678
NC_005103.4_123200001	4	123200001	0.5071	0.6728
NC_005103.4_123300001	4	123300001	0.5354	0.6912
NC_005103.4_123400001	4	123400001	0.5814	0.7531
NC_005103.4_123500001	4	123500001	0.6111	0.7812
NC_005103.4_123600001	4	123600001	0.6645	0.8163
NC_005103.4_123700001	4	123700001	0.6851	0.8507
NC_005103.4_123800001	4	123800001	0.6092	0.8028
NC_005103.4_123900001	4	123900001	0.5497	0.7638
NC_005103.4_124000001	4	124000001	0.5391	0.6695
NC_005103.4_124100001	4	124100001	0.4496	0.6132
NC_005103.4_124200001	4	124200001	0.454	0.6128
NC_005103.4_124300001	4	124300001	0.5423	0.6789
NC_005103.4_124400001	4	124400001	0.5111	0.6711
NC_005103.4_124500001	4	124500001	0.53	0.7012
NC_005103.4_124600001	4	124600001	0.6068	0.6777
NC_005103.4_124700001	4	124700001	0.5387	0.5872
NC_005103.4_124800001	4	124800001	0.4604	0.5364
NC_005103.4_124900001	4	124900001	0.4994	0.5437
NC_005103.4_125000001	4	125000001	0.4493	0.5444
NC_005103.4_125100001	4	125100001	0.4456	0.5467
NC_005103.4_125200001	4	125200001	0.4165	0.5504
NC_005103.4_125300001	4	125300001	0.4244	0.5847
NC_005103.4_125400001	4	125400001	0.4246	0.594
NC_005103.4_125500001	4	125500001	0.4541	0.6064
NC_005103.4_125600001	4	125600001	0.4458	0.5904
NC_005103.4_125700001	4	125700001	0.5111	0.6533
NC_005103.4_125800001	4	125800001	0.5039	0.6176
NC_005103.4_125900001	4	125900001	0.4848	0.6015
NC_005103.4_126000001	4	126000001	0.4962	0.5835
NC_005103.4_126100001	4	126100001	0.5499	0.6414
NC_005103.4_126200001	4	126200001	0.5499	0.6879
NC_005103.4_126300001	4	126300001	0.6174	0.7408
NC_005103.4_126400001	4	126400001	0.6171	0.7567
NC_005103.4_126500001	4	126500001	0.6036	0.7905
NC_005103.4_126600001	4	126600001	0.5145	0.6775
NC_005103.4_126700001	4	126700001	0.4259	0.509
NC_005103.4_126800001	4	126800001	0.3801	0.5439
NC_005103.4_126900001	4	126900001	0.4081	0.5238
NC_005103.4_127000001	4	127000001	0.4137	0.5032
NC_005103.4_127100001	4	127100001	0.4667	0.6304
NC_005103.4_127200001	4	127200001	0.542	0.701
NC_005103.4_127300001	4	127300001	0.6245	0.7231
NC_005103.4_127400001	4	127400001	0.6322	0.6802
NC_005103.4_127500001	4	127500001	0.631	0.6584
NC_005103.4_127600001	4	127600001	0.6556	0.6478
NC_005103.4_127700001	4	127700001	0.6072	0.5847
NC_005103.4_127800001	4	127800001	0.6098	0.5453
NC_005103.4_127900001	4	127900001	0.6786	0.6087
NC_005103.4_128000001	4	128000001	0.6203	0.7213
NC_005103.4_128100001	4	128100001	0.5364	0.6444
NC_005103.4_128200001	4	128200001	0.5364	0.6444
NC_005103.4_128300001	4	128300001	0.4317	0.5644
NC_005103.4_128400001	4	128400001	0.3938	0.6506
NC_005103.4_128500001	4	128500001	0.4904	0.6957

NC_005103.4_128600001	4	128600001	0.4296	0.6857
NC_005103.4_128700001	4	128700001	0.4154	0.6195
NC_005103.4_128800001	4	128800001	0.4566	0.6634
NC_005103.4_128900001	4	128900001	0.4826	0.6752
NC_005103.4_129000001	4	129000001	0.4347	0.6209
NC_005103.4_129100001	4	129100001	0.4652	0.6664
NC_005103.4_129200001	4	129200001	0.4572	0.6412
NC_005103.4_129300001	4	129300001	0.4671	0.6203
NC_005103.4_129400001	4	129400001	0.4805	0.5853
NC_005103.4_129500001	4	129500001	0.4876	0.6028
NC_005103.4_129600001	4	129600001	0.4445	0.5686
NC_005103.4_129700001	4	129700001	0.5345	0.6338
NC_005103.4_129800001	4	129800001	0.526	0.6439
NC_005103.4_129900001	4	129900001	0.5	0.6589
NC_005103.4_130000001	4	130000001	0.5629	0.6811
NC_005103.4_130100001	4	130100001	0.571	0.6737
NC_005103.4_130200001	4	130200001	0.4969	0.6685
NC_005103.4_130300001	4	130300001	0.5055	0.6444
NC_005103.4_130400001	4	130400001	0.4351	0.6154
NC_005103.4_130500001	4	130500001	0.5005	0.6676
NC_005103.4_130600001	4	130600001	0.6067	0.6921
NC_005103.4_130700001	4	130700001	0.5826	0.6839
NC_005103.4_130800001	4	130800001	0.5387	0.6699
NC_005103.4_130900001	4	130900001	0.5534	0.6505
NC_005103.4_131000001	4	131000001	0.5128	0.5878
NC_005103.4_131100001	4	131100001	0.5378	0.5887
NC_005103.4_131200001	4	131200001	0.5832	0.5605
NC_005103.4_131300001	4	131300001	0.634	0.5981
NC_005103.4_131400001	4	131400001	0.6319	0.622
NC_005103.4_131500001	4	131500001	0.6068	0.6164
NC_005103.4_131600001	4	131600001	0.5533	0.6302
NC_005103.4_131700001	4	131700001	0.5555	0.6907
NC_005103.4_131800001	4	131800001	0.48	0.6247
NC_005103.4_131900001	4	131900001	0.3514	0.5488
NC_005103.4_132000001	4	132000001	0.4005	0.4531
NC_005103.4_132100001	4	132100001	0.4577	0.4774
NC_005103.4_132200001	4	132200001	0.472	0.5345
NC_005103.4_132300001	4	132300001	0.5083	0.5847
NC_005103.4_132400001	4	132400001	0.6025	0.6739
NC_005103.4_132500001	4	132500001	0.6728	0.8013
NC_005103.4_132600001	4	132600001	0.6613	0.8257
NC_005103.4_132700001	4	132700001	0.6682	0.7918
NC_005103.4_132800001	4	132800001	0.6713	0.7884
NC_005103.4_132900001	4	132900001	0.6138	0.7233
NC_005103.4_133000001	4	133000001	0.5145	0.6271
NC_005103.4_133100001	4	133100001	0.396	0.3784
NC_005103.4_133200001	4	133200001	0.3762	0.4039
NC_005103.4_133300001	4	133300001	0.4395	0.5254
NC_005103.4_133400001	4	133400001	0.4333	0.5412
NC_005103.4_133500001	4	133500001	0.4884	0.6245
NC_005103.4_133600001	4	133600001	0.4719	0.6515
NC_005103.4_133700001	4	133700001	0.4646	0.6358
NC_005103.4_133800001	4	133800001	0.4573	0.5949
NC_005103.4_133900001	4	133900001	0.5365	0.6613
NC_005103.4_134000001	4	134000001	0.6494	0.7339
NC_005103.4_134100001	4	134100001	0.7981	0.8229
NC_005103.4_134200001	4	134200001	0.7508	0.8034
NC_005103.4_134300001	4	134300001	0.7217	0.8185

NC_005103.4_134400001	4	134400001	0.6649	0.7565
NC_005103.4_134500001	4	134500001	0.5707	0.689
NC_005103.4_134600001	4	134600001	0.4859	0.6244
NC_005103.4_134700001	4	134700001	0.4751	0.5694
NC_005103.4_134800001	4	134800001	0.4428	0.5022
NC_005103.4_134900001	4	134900001	0.4541	0.566
NC_005103.4_135000001	4	135000001	0.4745	0.543
NC_005103.4_135100001	4	135100001	0.4989	0.5039
NC_005103.4_135200001	4	135200001	0.4599	0.4506
NC_005103.4_135300001	4	135300001	0.4875	0.5126
NC_005103.4_135400001	4	135400001	0.4875	0.5126
NC_005103.4_135500001	4	135500001	0.3608	0.4342
NC_005103.4_135600001	4	135600001	0.322	0.336
NC_005103.4_135700001	4	135700001	0.3372	0.3896
NC_005103.4_135800001	4	135800001	0.1809	0.1813
NC_005103.4_135900001	4	135900001	0.3586	0.5409
NC_005103.4_136000001	4	136000001	0.4128	0.6894
NC_005103.4_136100001	4	136100001	0.3305	0.6578
NC_005103.4_136200001	4	136200001	0.3305	0.6578
NC_005103.4_136300001	4	136300001	0.3135	0.5389
NC_005103.4_136400001	4	136400001	0.2402	0.3973
NC_005103.4_136500001	4	136500001	0.3659	0.5996
NC_005103.4_136600001	4	136600001	0.3413	0.5172
NC_005103.4_136700001	4	136700001	0.4152	0.6331
NC_005103.4_136800001	4	136800001	0.4957	0.6889
NC_005103.4_136900001	4	136900001	0.5276	0.7154
NC_005103.4_137000001	4	137000001	0.4965	0.6837
NC_005103.4_137100001	4	137100001	0.624	0.8396
NC_005103.4_137200001	4	137200001	0.5819	0.8074
NC_005103.4_137300001	4	137300001	0.6083	0.7837
NC_005103.4_137400001	4	137400001	0.4375	0.5779
NC_005103.4_137500001	4	137500001	0.5268	0.7104
NC_005103.4_137600001	4	137600001	0.4536	0.5256
NC_005103.4_137700001	4	137700001	0.4538	0.4931
NC_005103.4_137800001	4	137800001	0.4497	0.5166
NC_005103.4_137900001	4	137900001	0.5014	0.5895
NC_005103.4_138100001	4	138100001	0.4135	0.4721
NC_005103.4_138200001	4	138200001	0.4129	0.5078
NC_005103.4_138300001	4	138300001	0.4883	0.6226
NC_005103.4_138400001	4	138400001	0.5875	0.7261
NC_005103.4_138500001	4	138500001	0.5952	0.7064
NC_005103.4_138600001	4	138600001	0.5882	0.7026
NC_005103.4_138700001	4	138700001	0.5941	0.7581
NC_005103.4_138800001	4	138800001	0.5645	0.6491
NC_005103.4_138900001	4	138900001	0.5412	0.6253
NC_005103.4_139000001	4	139000001	0.5249	0.5905
NC_005103.4_139100001	4	139100001	0.5391	0.6375
NC_005103.4_139200001	4	139200001	0.5431	0.6038
NC_005103.4_139300001	4	139300001	0.525	0.6524
NC_005103.4_139400001	4	139400001	0.3843	0.4853
NC_005103.4_139500001	4	139500001	0.3556	0.4995
NC_005103.4_139600001	4	139600001	0.3096	0.4306
NC_005103.4_139700001	4	139700001	0.2458	0.3402
NC_005103.4_139800001	4	139800001	0.2899	0.4032
NC_005103.4_139900001	4	139900001	0.4775	0.6158
NC_005103.4_140000001	4	140000001	0.5239	0.6108
NC_005103.4_140100001	4	140100001	0.568	0.6188
NC_005103.4_140200001	4	140200001	0.6869	0.6655

NC_005103.4_140300001	4	140300001	0.6704	0.6579
NC_005103.4_140400001	4	140400001	0.5783	0.6058
NC_005103.4_140500001	4	140500001	0.6042	0.6755
NC_005103.4_140600001	4	140600001	0.6105	0.6269
NC_005103.4_140700001	4	140700001	0.5612	0.6417
NC_005103.4_140800001	4	140800001	0.5005	0.6493
NC_005103.4_140900001	4	140900001	0.512	0.6561
NC_005103.4_141000001	4	141000001	0.4862	0.6018
NC_005103.4_141100001	4	141100001	0.5396	0.7202
NC_005103.4_141200001	4	141200001	0.4542	0.6783
NC_005103.4_141300001	4	141300001	0.5018	0.6879
NC_005103.4_141400001	4	141400001	0.5398	0.6145
NC_005103.4_141500001	4	141500001	0.5692	0.6162
NC_005103.4_141600001	4	141600001	0.5002	0.5238
NC_005103.4_141700001	4	141700001	0.5738	0.6135
NC_005103.4_141800001	4	141800001	0.5606	0.5495
NC_005103.4_141900001	4	141900001	0.7948	0.8407
NC_005103.4_142000001	4	142000001	0.7038	0.8281
NC_005103.4_142100001	4	142100001	0.6774	0.7707
NC_005103.4_142200001	4	142200001	0.6992	0.7207
NC_005103.4_142300001	4	142300001	0.6881	0.7769
NC_005103.4_142400001	4	142400001	0.4687	0.6263
NC_005103.4_142500001	4	142500001	0.4798	0.6234
NC_005103.4_142600001	4	142600001	0.4604	0.6525
NC_005103.4_142700001	4	142700001	0.3673	0.6083
NC_005103.4_142800001	4	142800001	0.3338	0.5484
NC_005103.4_142900001	4	142900001	0.3756	0.4511
NC_005103.4_143000001	4	143000001	0.3756	0.4511
NC_005103.4_143100001	4	143100001	0.3729	0.5076
NC_005103.4_143200001	4	143200001	0.4788	0.5977
NC_005103.4_143300001	4	143300001	0.5318	0.5628
NC_005103.4_143400001	4	143400001	0.5568	0.6285
NC_005103.4_143500001	4	143500001	0.5762	0.6364
NC_005103.4_143600001	4	143600001	0.6229	0.6547
NC_005103.4_143700001	4	143700001	0.5218	0.5865
NC_005103.4_143800001	4	143800001	0.4803	0.5974
NC_005103.4_143900001	4	143900001	0.4537	0.5677
NC_005103.4_144000001	4	144000001	0.4163	0.5208
NC_005103.4_144100001	4	144100001	0.4207	0.5018
NC_005103.4_144200001	4	144200001	0.4789	0.6397
NC_005103.4_144300001	4	144300001	0.5412	0.6683
NC_005103.4_144400001	4	144400001	0.5794	0.7036
NC_005103.4_144500001	4	144500001	0.5455	0.6978
NC_005103.4_144600001	4	144600001	0.4996	0.6996
NC_005103.4_144700001	4	144700001	0.5159	0.632
NC_005103.4_144800001	4	144800001	0.311	0.4299
NC_005103.4_144900001	4	144900001	0.3145	0.475
NC_005103.4_145000001	4	145000001	0.3493	0.519
NC_005103.4_145100001	4	145100001	0.3775	0.5027
NC_005103.4_145200001	4	145200001	0.4237	0.6294
NC_005103.4_145300001	4	145300001	0.575	0.74
NC_005103.4_145400001	4	145400001	0.586	0.6995
NC_005103.4_145500001	4	145500001	0.6149	0.6952
NC_005103.4_145600001	4	145600001	0.5373	0.6594
NC_005103.4_145700001	4	145700001	0.4975	0.6272
NC_005103.4_145800001	4	145800001	0.4559	0.5507
NC_005103.4_145900001	4	145900001	0.4821	0.5725
NC_005103.4_146000001	4	146000001	0.4465	0.5346

NC_005103.4_146100001	4	146100001	0.5254	0.5553
NC_005103.4_146200001	4	146200001	0.5215	0.6407
NC_005103.4_146300001	4	146300001	0.6407	0.7791
NC_005103.4_146400001	4	146400001	0.5795	0.7303
NC_005103.4_146500001	4	146500001	0.6389	0.8041
NC_005103.4_146600001	4	146600001	0.6229	0.7924
NC_005103.4_146700001	4	146700001	0.7933	0.826
NC_005103.4_146800001	4	146800001	0.7061	0.8059
NC_005103.4_146900001	4	146900001	0.7986	0.8756
NC_005103.4_147000001	4	147000001	0.6642	0.7865
NC_005103.4_147100001	4	147100001	0.6814	0.7764
NC_005103.4_147200001	4	147200001	0.5461	0.7012
NC_005103.4_147300001	4	147300001	0.6107	0.7511
NC_005103.4_147400001	4	147400001	0.5652	0.7177
NC_005103.4_147500001	4	147500001	0.6356	0.7868
NC_005103.4_147600001	4	147600001	0.5956	0.773
NC_005103.4_147700001	4	147700001	0.6303	0.7873
NC_005103.4_147800001	4	147800001	0.5231	0.6883
NC_005103.4_147900001	4	147900001	0.6264	0.7825
NC_005103.4_148000001	4	148000001	0.4395	0.5915
NC_005103.4_148100001	4	148100001	0.4776	0.5763
NC_005103.4_148200001	4	148200001	0.4365	0.5751
NC_005103.4_148300001	4	148300001	0.4671	0.6135
NC_005103.4_148400001	4	148400001	0.5206	0.6406
NC_005103.4_148500001	4	148500001	0.4881	0.6125
NC_005103.4_148600001	4	148600001	0.4509	0.6189
NC_005103.4_148700001	4	148700001	0.4777	0.6003
NC_005103.4_148800001	4	148800001	0.4609	0.5467
NC_005103.4_148900001	4	148900001	0.375	0.431
NC_005103.4_149000001	4	149000001	0.3911	0.4262
NC_005103.4_149100001	4	149100001	0.4595	0.5265
NC_005103.4_149200001	4	149200001	0.4667	0.6123
NC_005103.4_149300001	4	149300001	0.5449	0.6884
NC_005103.4_149400001	4	149400001	0.5894	0.7507
NC_005103.4_149500001	4	149500001	0.618	0.7872
NC_005103.4_149600001	4	149600001	0.6665	0.7514
NC_005103.4_149700001	4	149700001	0.5635	0.6403
NC_005103.4_149800001	4	149800001	0.4846	0.6027
NC_005103.4_149900001	4	149900001	0.4672	0.557
NC_005103.4_150000001	4	150000001	0.4238	0.5204
NC_005103.4_150100001	4	150100001	0.387	0.486
NC_005103.4_150200001	4	150200001	0.4319	0.5316
NC_005103.4_150300001	4	150300001	0.3778	0.442
NC_005103.4_150400001	4	150400001	0.367	0.4412
NC_005103.4_150500001	4	150500001	0.4381	0.5019
NC_005103.4_150600001	4	150600001	0.4202	0.5726
NC_005103.4_150700001	4	150700001	0.5178	0.702
NC_005103.4_150800001	4	150800001	0.603	0.7866
NC_005103.4_150900001	4	150900001	0.5951	0.8049
NC_005103.4_151000001	4	151000001	0.5821	0.8226
NC_005103.4_151100001	4	151100001	0.6047	0.7745
NC_005103.4_151200001	4	151200001	0.5447	0.695
NC_005103.4_151300001	4	151300001	0.5131	0.6451
NC_005103.4_151400001	4	151400001	0.5083	0.5019
NC_005103.4_151500001	4	151500001	0.6717	0.6702
NC_005103.4_151600001	4	151600001	0.5246	0.7048
NC_005103.4_151700001	4	151700001	0.5715	0.7624
NC_005103.4_151800001	4	151800001	0.5902	0.7629

NC_005103.4_151900001	4	151900001	0.6063	0.7686
NC_005103.4_152000001	4	152000001	0.5122	0.7393
NC_005103.4_152100001	4	152100001	0.8046	0.8538
NC_005103.4_152200001	4	152200001	0.5417	0.7755
NC_005103.4_152300001	4	152300001	0.5197	0.7949
NC_005103.4_152400001	4	152400001	0.5117	0.7872
NC_005103.4_152500001	4	152500001	0.5488	0.8119
NC_005103.4_152600001	4	152600001	0.5853	0.8197
NC_005103.4_152700001	4	152700001	0.7074	0.8347
NC_005103.4_152800001	4	152800001	0.7183	0.8359
NC_005103.4_152900001	4	152900001	0.751	0.8154
NC_005103.4_153000001	4	153000001	0.6874	0.7704
NC_005103.4_153100001	4	153100001	0.5943	0.7291
NC_005103.4_153200001	4	153200001	0.5904	0.7235
NC_005103.4_153300001	4	153300001	0.5811	0.702
NC_005103.4_153400001	4	153400001	0.5531	0.6573
NC_005103.4_153500001	4	153500001	0.5114	0.6334
NC_005103.4_153600001	4	153600001	0.5519	0.663
NC_005103.4_153700001	4	153700001	0.4659	0.6136
NC_005103.4_153800001	4	153800001	0.4958	0.6436
NC_005103.4_153900001	4	153900001	0.4858	0.6898
NC_005103.4_154000001	4	154000001	0.4944	0.7255
NC_005103.4_154100001	4	154100001	0.2893	0.5126
NC_005103.4_154200001	4	154200001	0.4628	0.7772
NC_005103.4_154500001	4	154500001	0.2307	0.5792
NC_005103.4_154800001	4	154800001	0.5336	0.8687
NC_005103.4_154900001	4	154900001	0.4864	0.6897
NC_005103.4_155000001	4	155000001	0.5128	0.6827
NC_005103.4_155100001	4	155100001	0.5827	0.7311
NC_005103.4_155200001	4	155200001	0.5406	0.7303
NC_005103.4_155300001	4	155300001	0.4995	0.6717
NC_005103.4_155400001	4	155400001	0.5499	0.793
NC_005103.4_155500001	4	155500001	0.6514	0.8697
NC_005103.4_155600001	4	155600001	0.5799	0.8728
NC_005103.4_155800001	4	155800001	0.9653	0.9386
NC_005103.4_155900001	4	155900001	0.9729	0.9527
NC_005103.4_156500001	4	156500001	0.2708	0.1561
NC_005103.4_156700001	4	156700001	0.4357	0.5985
NC_005103.4_156800001	4	156800001	0.4881	0.6618
NC_005103.4_156900001	4	156900001	0.5115	0.6909
NC_005103.4_157000001	4	157000001	0.5488	0.8037
NC_005103.4_157100001	4	157100001	0.6206	0.8506
NC_005103.4_157200001	4	157200001	0.7608	0.8903
NC_005103.4_157300001	4	157300001	0.7755	0.8513
NC_005103.4_157400001	4	157400001	0.6586	0.8287
NC_005103.4_157500001	4	157500001	0.6335	0.8366
NC_005103.4_157600001	4	157600001	0.5792	0.8032
NC_005103.4_157700001	4	157700001	0.4494	0.7312
NC_005103.4_157800001	4	157800001	0.3548	0.6721
NC_005103.4_157900001	4	157900001	0.3597	0.6307
NC_005103.4_158000001	4	158000001	0.3531	0.5848
NC_005103.4_158100001	4	158100001	0.3695	0.5615
NC_005103.4_158200001	4	158200001	0.4123	0.5664
NC_005103.4_158300001	4	158300001	0.4823	0.6123
NC_005103.4_158400001	4	158400001	0.5082	0.6428
NC_005103.4_158500001	4	158500001	0.5719	0.6312
NC_005103.4_158600001	4	158600001	0.5231	0.6389
NC_005103.4_158700001	4	158700001	0.4911	0.5701

NC_005103.4_158800001	4	158800001	0.5319	0.6406
NC_005103.4_158900001	4	158900001	0.5672	0.6579
NC_005103.4_159000001	4	159000001	0.5182	0.6638
NC_005103.4_159100001	4	159100001	0.5286	0.6762
NC_005103.4_159200001	4	159200001	0.5787	0.7839
NC_005103.4_159300001	4	159300001	0.4735	0.6591
NC_005103.4_159400001	4	159400001	0.4033	0.6369
NC_005103.4_159500001	4	159500001	0.4173	0.6685
NC_005103.4_159600001	4	159600001	0.4233	0.6527
NC_005103.4_159700001	4	159700001	0.3875	0.6093
NC_005103.4_159800001	4	159800001	0.4515	0.7303
NC_005103.4_159900001	4	159900001	0.4181	0.724
NC_005103.4_160000001	4	160000001	0.4733	0.7351
NC_005103.4_160100001	4	160100001	0.5027	0.6827
NC_005103.4_160200001	4	160200001	0.4892	0.644
NC_005103.4_160300001	4	160300001	0.3982	0.5327
NC_005103.4_160400001	4	160400001	0.4207	0.5388
NC_005103.4_160500001	4	160500001	0.3747	0.4956
NC_005103.4_160600001	4	160600001	0.3859	0.4998
NC_005103.4_160700001	4	160700001	0.3538	0.4423
NC_005103.4_161200001	4	161200001	0.3201	0.3526
NC_005103.4_161300001	4	161300001	0.4848	0.6026
NC_005103.4_161400001	4	161400001	0.5009	0.6123
NC_005103.4_161500001	4	161500001	0.4768	0.6044
NC_005103.4_161600001	4	161600001	0.4673	0.6029
NC_005103.4_161700001	4	161700001	0.4715	0.6326
NC_005103.4_161800001	4	161800001	0.3823	0.5036
NC_005103.4_161900001	4	161900001	0.2538	0.3055
NC_005103.4_162000001	4	162000001	0.2892	0.3223
NC_005103.4_162300001	4	162300001	0.2276	0.3339
NC_005103.4_162400001	4	162400001	0.2309	0.3093
NC_005103.4_162500001	4	162500001	0.2309	0.3093
NC_005103.4_162600001	4	162600001	0.243	0.3955
NC_005103.4_162700001	4	162700001	0.3171	0.3742
NC_005103.4_162800001	4	162800001	0.3732	0.5396
NC_005103.4_162900001	4	162900001	0.3894	0.4929
NC_005103.4_163000001	4	163000001	0.3798	0.4613
NC_005103.4_163100001	4	163100001	0.4072	0.4818
NC_005103.4_163200001	4	163200001	0.3925	0.5164
NC_005103.4_163300001	4	163300001	0.4062	0.3692
NC_005103.4_163900001	4	163900001	0.478	0.2534
NC_005103.4_164000001	4	164000001	0.478	0.2534
NC_005103.4_164100001	4	164100001	0.478	0.2534
NC_005103.4_164200001	4	164200001	0.478	0.2534
NC_005103.4_164300001	4	164300001	0.3491	0.1743
NC_005103.4_164600001	4	164600001	0.3636	0.2296
NC_005103.4_164700001	4	164700001	0.3636	0.2296
NC_005103.4_164800001	4	164800001	0.4379	0.2361
NC_005103.4_164900001	4	164900001	0.4175	0.2406
NC_005103.4_165000001	4	165000001	0.4253	0.2532
NC_005103.4_165100001	4	165100001	0.4606	0.3938
NC_005103.4_165200001	4	165200001	0.4638	0.4541
NC_005103.4_165300001	4	165300001	0.4504	0.4697
NC_005103.4_165400001	4	165400001	0.4419	0.4405
NC_005103.4_165500001	4	165500001	0.4402	0.4687
NC_005103.4_165600001	4	165600001	0.2722	0.5434
NC_005103.4_165700001	4	165700001	0.271	0.2095
NC_005103.4_165800001	4	165800001	0.271	0.2095

NC_005103.4_166600001	4	166600001	0.3799	0.5931
NC_005103.4_166700001	4	166700001	0.3799	0.5931
NC_005103.4_167300001	4	167300001	0.3347	0.3626
NC_005103.4_167400001	4	167400001	0.3347	0.3626
NC_005103.4_167500001	4	167500001	0.2927	0.4318
NC_005103.4_167600001	4	167600001	0.331	0.4035
NC_005103.4_167700001	4	167700001	0.34	0.4141
NC_005103.4_167800001	4	167800001	0.4332	0.5448
NC_005103.4_167900001	4	167900001	0.4535	0.5499
NC_005103.4_168000001	4	168000001	0.4723	0.4817
NC_005103.4_168100001	4	168100001	0.4956	0.6164
NC_005103.4_168200001	4	168200001	0.4606	0.5307
NC_005103.4_168300001	4	168300001	0.4133	0.4474
NC_005103.4_168400001	4	168400001	0.3732	0.4059
NC_005103.4_168500001	4	168500001	0.4108	0.4387
NC_005103.4_168600001	4	168600001	0.3754	0.3464
NC_005103.4_168700001	4	168700001	0.507	0.511
NC_005103.4_168800001	4	168800001	0.5293	0.5194
NC_005103.4_168900001	4	168900001	0.5216	0.557
NC_005103.4_169000001	4	169000001	0.567	0.5921
NC_005103.4_169100001	4	169100001	0.6088	0.6288
NC_005103.4_169200001	4	169200001	0.6064	0.6481
NC_005103.4_169300001	4	169300001	0.5678	0.7212
NC_005103.4_169400001	4	169400001	0.5537	0.7234
NC_005103.4_169500001	4	169500001	0.5319	0.7325
NC_005103.4_169600001	4	169600001	0.5742	0.7596
NC_005103.4_169700001	4	169700001	0.5608	0.7718
NC_005103.4_169800001	4	169800001	0.4881	0.6716
NC_005103.4_169900001	4	169900001	0.4593	0.6445
NC_005103.4_170000001	4	170000001	0.3523	0.5425
NC_005103.4_170100001	4	170100001	0.2522	0.353
NC_005103.4_170200001	4	170200001	0.3264	0.4885
NC_005103.4_170300001	4	170300001	0.4337	0.558
NC_005103.4_170400001	4	170400001	0.4228	0.4978
NC_005103.4_170500001	4	170500001	0.4466	0.5037
NC_005103.4_170600001	4	170600001	0.4747	0.5336
NC_005103.4_170700001	4	170700001	0.4762	0.518
NC_005103.4_170800001	4	170800001	0.4685	0.5107
NC_005103.4_170900001	4	170900001	0.5153	0.5885
NC_005103.4_171000001	4	171000001	0.5434	0.6319
NC_005103.4_171100001	4	171100001	0.5611	0.6529
NC_005103.4_171200001	4	171200001	0.5395	0.6525
NC_005103.4_171300001	4	171300001	0.545	0.6992
NC_005103.4_171400001	4	171400001	0.6528	0.8234
NC_005103.4_171500001	4	171500001	0.5858	0.822
NC_005103.4_171600001	4	171600001	0.617	0.8281
NC_005103.4_171700001	4	171700001	0.7406	0.8946
NC_005103.4_171800001	4	171800001	0.7252	0.8643
NC_005103.4_171900001	4	171900001	0.6727	0.8094
NC_005103.4_172000001	4	172000001	0.61	0.8108
NC_005103.4_172100001	4	172100001	0.5271	0.8124
NC_005103.4_172200001	4	172200001	0.4461	0.7236
NC_005103.4_172300001	4	172300001	0.4003	0.6891
NC_005103.4_172400001	4	172400001	0.3375	0.6362
NC_005103.4_172500001	4	172500001	0.4289	0.7225
NC_005103.4_172600001	4	172600001	0.3938	0.6787
NC_005103.4_172700001	4	172700001	0.3491	0.6282
NC_005103.4_172800001	4	172800001	0.4675	0.707

NC_005103.4_172900001	4	172900001	0.411	0.6691
NC_005103.4_173000001	4	173000001	0.3672	0.581
NC_005103.4_173100001	4	173100001	0.4512	0.6809
NC_005103.4_173200001	4	173200001	0.4914	0.6844
NC_005103.4_173300001	4	173300001	0.4613	0.6601
NC_005103.4_173400001	4	173400001	0.5308	0.6022
NC_005103.4_173500001	4	173500001	0.6387	0.6217
NC_005103.4_173600001	4	173600001	0.5682	0.5588
NC_005103.4_173700001	4	173700001	0.5137	0.5447
NC_005103.4_173800001	4	173800001	0.5111	0.5935
NC_005103.4_173900001	4	173900001	0.5584	0.6718
NC_005103.4_174000001	4	174000001	0.4455	0.6375
NC_005103.4_174100001	4	174100001	0.3797	0.6369
NC_005103.4_174200001	4	174200001	0.4996	0.7407
NC_005103.4_174300001	4	174300001	0.5134	0.7462
NC_005103.4_174400001	4	174400001	0.446	0.7116
NC_005103.4_174500001	4	174500001	0.4669	0.6522
NC_005103.4_174600001	4	174600001	0.4963	0.6295
NC_005103.4_174700001	4	174700001	0.4592	0.6203
NC_005103.4_174800001	4	174800001	0.4765	0.5373
NC_005103.4_174900001	4	174900001	0.5077	0.558
NC_005103.4_175000001	4	175000001	0.4965	0.5647
NC_005103.4_175100001	4	175100001	0.4822	0.5656
NC_005103.4_175200001	4	175200001	0.4759	0.5025
NC_005103.4_175300001	4	175300001	0.3848	0.4843
NC_005103.4_175400001	4	175400001	0.397	0.4732
NC_005103.4_175500001	4	175500001	0.3619	0.4457
NC_005103.4_175600001	4	175600001	0.3844	0.4532
NC_005103.4_175700001	4	175700001	0.4117	0.5344
NC_005103.4_175800001	4	175800001	0.5499	0.6499
NC_005103.4_175900001	4	175900001	0.5662	0.6967
NC_005103.4_176000001	4	176000001	0.6284	0.6811
NC_005103.4_176100001	4	176100001	0.6173	0.6945
NC_005103.4_176200001	4	176200001	0.5451	0.6204
NC_005103.4_176300001	4	176300001	0.6112	0.6863
NC_005103.4_176400001	4	176400001	0.5702	0.6828
NC_005103.4_176500001	4	176500001	0.5005	0.6901
NC_005103.4_176600001	4	176600001	0.474	0.6423
NC_005103.4_176700001	4	176700001	0.4989	0.7552
NC_005103.4_176800001	4	176800001	0.3645	0.5888
NC_005103.4_176900001	4	176900001	0.5694	0.6955
NC_005103.4_177000001	4	177000001	0.5751	0.6562
NC_005103.4_177100001	4	177100001	0.549	0.6112
NC_005103.4_177200001	4	177200001	0.5345	0.5529
NC_005103.4_177300001	4	177300001	0.5214	0.5345
NC_005103.4_177400001	4	177400001	0.5057	0.5151
NC_005103.4_177500001	4	177500001	0.5677	0.5733
NC_005103.4_177600001	4	177600001	0.6015	0.583
NC_005103.4_177700001	4	177700001	0.6167	0.5951
NC_005103.4_177800001	4	177800001	0.6363	0.6306
NC_005103.4_177900001	4	177900001	0.5191	0.5711
NC_005103.4_178000001	4	178000001	0.4315	0.4873
NC_005103.4_178100001	4	178100001	0.3843	0.4654
NC_005103.4_178200001	4	178200001	0.4771	0.5951
NC_005103.4_178300001	4	178300001	0.5101	0.6053
NC_005103.4_178400001	4	178400001	0.5379	0.6312
NC_005103.4_178500001	4	178500001	0.5476	0.6656
NC_005103.4_178600001	4	178600001	0.5227	0.6474

NC_005103.4_178700001	4	178700001	0.4496	0.5845
NC_005103.4_178800001	4	178800001	0.4185	0.5671
NC_005103.4_178900001	4	178900001	0.4769	0.6289
NC_005103.4_179000001	4	179000001	0.5054	0.6681
NC_005103.4_179100001	4	179100001	0.5254	0.7212
NC_005103.4_179200001	4	179200001	0.5119	0.6974
NC_005103.4_179300001	4	179300001	0.5098	0.7136
NC_005103.4_179400001	4	179400001	0.5415	0.7085
NC_005103.4_179500001	4	179500001	0.5479	0.7135
NC_005103.4_179600001	4	179600001	0.5724	0.7479
NC_005103.4_179700001	4	179700001	0.6338	0.7767
NC_005103.4_179800001	4	179800001	0.6498	0.7831
NC_005103.4_179900001	4	179900001	0.5657	0.7447
NC_005103.4_180000001	4	180000001	0.5418	0.7149
NC_005103.4_180100001	4	180100001	0.5353	0.664
NC_005103.4_180200001	4	180200001	0.462	0.6165
NC_005103.4_180300001	4	180300001	0.4683	0.6394
NC_005103.4_180400001	4	180400001	0.4881	0.6472
NC_005103.4_180500001	4	180500001	0.4773	0.6314
NC_005103.4_180600001	4	180600001	0.5388	0.6931
NC_005103.4_180700001	4	180700001	0.5852	0.7234
NC_005103.4_180800001	4	180800001	0.5921	0.72
NC_005103.4_180900001	4	180900001	0.6273	0.7613
NC_005103.4_181000001	4	181000001	0.7504	0.8817
NC_005103.4_181100001	4	181100001	0.7467	0.8877
NC_005103.4_181200001	4	181200001	0.673	0.8421
NC_005103.4_181300001	4	181300001	0.6084	0.8292
NC_005103.4_181400001	4	181400001	0.6168	0.7722
NC_005103.4_181500001	4	181500001	0.5805	0.7175
NC_005103.4_181600001	4	181600001	0.4508	0.6371
NC_005103.4_181700001	4	181700001	0.4492	0.6313
NC_005103.4_181800001	4	181800001	0.4926	0.6535
NC_005103.4_181900001	4	181900001	0.4305	0.599
NC_005103.4_182000001	4	182000001	0.4066	0.4826
NC_005103.4_182100001	4	182100001	0.4643	0.4701
NC_005103.4_182200001	4	182200001	0.5227	0.5302
NC_005103.4_182300001	4	182300001	0.4788	0.4914
NC_005103.4_182400001	4	182400001	0.4925	0.5521
NC_005103.4_182500001	4	182500001	0.5871	0.7174
NC_005103.4_182600001	4	182600001	0.5821	0.7097
NC_005103.4_182700001	4	182700001	0.5332	0.6437
NC_005103.4_182800001	4	182800001	0.6061	0.6871
NC_005103.4_182900001	4	182900001	0.682	0.6909
NC_005103.4_183000001	4	183000001	0.5246	0.5618
NC_005103.4_183100001	4	183100001	0.6062	0.7315
NC_005103.4_183200001	4	183200001	0.6758	0.7645
NC_005103.4_183300001	4	183300001	0.5214	0.6846
NC_005103.4_183400001	4	183400001	0.5246	0.6487
NC_005103.4_183500001	4	183500001	0.5925	0.6628
NC_005103.4_183600001	4	183600001	0.5215	0.5786
NC_005103.4_183700001	4	183700001	0.517	0.7197
NC_005104.4_100001	5	100001	0.3511	0.476
NC_005104.4_200001	5	200001	0.4633	0.625
NC_005104.4_300001	5	300001	0.4633	0.625
NC_005104.4_400001	5	400001	0.4108	0.6059
NC_005104.4_500001	5	500001	0.5866	0.7958
NC_005104.4_600001	5	600001	0.5818	0.6932
NC_005104.4_700001	5	700001	0.6753	0.7788

NC_005104.4_800001	5	800001	0.6753	0.7788
NC_005104.4_900001	5	900001	0.7397	0.8062
NC_005104.4_1000001	5	1000001	0.7664	0.815
NC_005104.4_1100001	5	1100001	0.8728	0.9293
NC_005104.4_1900001	5	1900001	0.6923	0.5905
NC_005104.4_2000001	5	2000001	0.7335	0.6513
NC_005104.4_2100001	5	2100001	0.5108	0.651
NC_005104.4_2200001	5	2200001	0.5435	0.7117
NC_005104.4_2300001	5	2300001	0.604	0.783
NC_005104.4_2400001	5	2400001	0.5855	0.8021
NC_005104.4_2500001	5	2500001	0.5444	0.7728
NC_005104.4_2600001	5	2600001	0.6492	0.7887
NC_005104.4_2700001	5	2700001	0.612	0.7607
NC_005104.4_3000001	5	3000001	0.5999	0.6134
NC_005104.4_3100001	5	3100001	0.5999	0.6134
NC_005104.4_3200001	5	3200001	0.6684	0.6197
NC_005104.4_3300001	5	3300001	0.4785	0.5279
NC_005104.4_3400001	5	3400001	0.5046	0.5788
NC_005104.4_3500001	5	3500001	0.356	0.4619
NC_005104.4_3600001	5	3600001	0.3708	0.5195
NC_005104.4_3700001	5	3700001	0.3887	0.5438
NC_005104.4_3800001	5	3800001	0.5487	0.6994
NC_005104.4_3900001	5	3900001	0.5611	0.7016
NC_005104.4_4000001	5	4000001	0.5453	0.6693
NC_005104.4_4100001	5	4100001	0.5869	0.6758
NC_005104.4_4200001	5	4200001	0.5934	0.6416
NC_005104.4_4300001	5	4300001	0.5249	0.6483
NC_005104.4_4400001	5	4400001	0.5135	0.6359
NC_005104.4_4500001	5	4500001	0.5371	0.6292
NC_005104.4_4600001	5	4600001	0.5287	0.6035
NC_005104.4_4700001	5	4700001	0.5334	0.6203
NC_005104.4_4800001	5	4800001	0.7326	0.714
NC_005104.4_4900001	5	4900001	0.7717	0.6732
NC_005104.4_5000001	5	5000001	0.7481	0.7696
NC_005104.4_5100001	5	5100001	0.7569	0.8212
NC_005104.4_5200001	5	5200001	0.7387	0.8074
NC_005104.4_5300001	5	5300001	0.6799	0.7618
NC_005104.4_5400001	5	5400001	0.5341	0.7305
NC_005104.4_5500001	5	5500001	0.4499	0.6563
NC_005104.4_5600001	5	5600001	0.4349	0.6283
NC_005104.4_5700001	5	5700001	0.4892	0.6763
NC_005104.4_5800001	5	5800001	0.498	0.663
NC_005104.4_5900001	5	5900001	0.6023	0.7008
NC_005104.4_6000001	5	6000001	0.6632	0.7075
NC_005104.4_6100001	5	6100001	0.583	0.6053
NC_005104.4_6200001	5	6200001	0.4815	0.4814
NC_005104.4_6300001	5	6300001	0.2973	0.3394
NC_005104.4_6400001	5	6400001	0.2687	0.3336
NC_005104.4_6500001	5	6500001	0.2213	0.1465
NC_005104.4_6600001	5	6600001	0.1984	0.1043
NC_005104.4_6700001	5	6700001	0.1854	0.1021
NC_005104.4_6800001	5	6800001	0.3292	0.4101
NC_005104.4_6900001	5	6900001	0.3458	0.4361
NC_005104.4_7000001	5	7000001	0.3881	0.626
NC_005104.4_7100001	5	7100001	0.4616	0.7205
NC_005104.4_7200001	5	7200001	0.5941	0.7192
NC_005104.4_7300001	5	7300001	0.5772	0.701
NC_005104.4_7400001	5	7400001	0.5291	0.6758

NC_005104.4_7500001	5	7500001	0.4995	0.6701
NC_005104.4_7600001	5	7600001	0.4689	0.6095
NC_005104.4_7700001	5	7700001	0.4035	0.612
NC_005104.4_7800001	5	7800001	0.4574	0.6038
NC_005104.4_7900001	5	7900001	0.5257	0.6896
NC_005104.4_8000001	5	8000001	0.5997	0.7049
NC_005104.4_8100001	5	8100001	0.5897	0.741
NC_005104.4_8200001	5	8200001	0.6449	0.6842
NC_005104.4_8300001	5	8300001	0.583	0.7303
NC_005104.4_8400001	5	8400001	0.5759	0.7054
NC_005104.4_8500001	5	8500001	0.5601	0.7212
NC_005104.4_8600001	5	8600001	0.6662	0.7485
NC_005104.4_8700001	5	8700001	0.6414	0.8425
NC_005104.4_8800001	5	8800001	0.6745	0.8066
NC_005104.4_8900001	5	8900001	0.6632	0.7792
NC_005104.4_9000001	5	9000001	0.6646	0.783
NC_005104.4_9100001	5	9100001	0.6327	0.7876
NC_005104.4_9200001	5	9200001	0.5412	0.7137
NC_005104.4_9300001	5	9300001	0.5556	0.7111
NC_005104.4_9400001	5	9400001	0.4269	0.6386
NC_005104.4_9500001	5	9500001	0.239	0.3669
NC_005104.4_9600001	5	9600001	0.239	0.3669
NC_005104.4_9700001	5	9700001	0.3144	0.4706
NC_005104.4_9800001	5	9800001	0.1563	0.243
NC_005104.4_9900001	5	9900001	0.17	0.1802
NC_005104.4_10000001	5	10000001	0.1584	0.1235
NC_005104.4_10100001	5	10100001	0.1584	0.1235
NC_005104.4_10200001	5	10200001	0.2199	0.1414
NC_005104.4_10300001	5	10300001	0.2462	0.1461
NC_005104.4_10600001	5	10600001	0.298	0.3446
NC_005104.4_10700001	5	10700001	0.2286	0.5624
NC_005104.4_10800001	5	10800001	0.2286	0.5624
NC_005104.4_10900001	5	10900001	0.2286	0.5624
NC_005104.4_11000001	5	11000001	0.2286	0.5624
NC_005104.4_11200001	5	11200001	0.6211	0.6054
NC_005104.4_11300001	5	11300001	0.5344	0.5109
NC_005104.4_11400001	5	11400001	0.5912	0.5917
NC_005104.4_11500001	5	11500001	0.5912	0.5917
NC_005104.4_11600001	5	11600001	0.729	0.7253
NC_005104.4_12000001	5	12000001	0.4152	0.6319
NC_005104.4_12100001	5	12100001	0.4152	0.6319
NC_005104.4_12200001	5	12200001	0.5926	0.6476
NC_005104.4_12300001	5	12300001	0.6087	0.6336
NC_005104.4_12400001	5	12400001	0.5772	0.6284
NC_005104.4_12500001	5	12500001	0.5136	0.5479
NC_005104.4_12600001	5	12600001	0.4412	0.5939
NC_005104.4_12700001	5	12700001	0.3945	0.657
NC_005104.4_12800001	5	12800001	0.4748	0.7735
NC_005104.4_12900001	5	12900001	0.5568	0.7822
NC_005104.4_13000001	5	13000001	0.5703	0.7559
NC_005104.4_13100001	5	13100001	0.763	0.7607
NC_005104.4_13200001	5	13200001	0.6997	0.7244
NC_005104.4_13300001	5	13300001	0.4581	0.511
NC_005104.4_13400001	5	13400001	0.3005	0.3841
NC_005104.4_13500001	5	13500001	0.2831	0.4002
NC_005104.4_13600001	5	13600001	0.3144	0.4828
NC_005104.4_13700001	5	13700001	0.3678	0.5409
NC_005104.4_13800001	5	13800001	0.4303	0.6421

NC_005104.4_13900001	5	13900001	0.486	0.6476
NC_005104.4_14000001	5	14000001	0.568	0.7242
NC_005104.4_14100001	5	14100001	0.55	0.6829
NC_005104.4_14200001	5	14200001	0.4857	0.6328
NC_005104.4_14300001	5	14300001	0.5308	0.6596
NC_005104.4_14400001	5	14400001	0.5076	0.6579
NC_005104.4_14500001	5	14500001	0.4776	0.5894
NC_005104.4_14600001	5	14600001	0.4491	0.5837
NC_005104.4_14700001	5	14700001	0.4664	0.6563
NC_005104.4_14800001	5	14800001	0.28	0.4839
NC_005104.4_14900001	5	14900001	0.5528	0.7466
NC_005104.4_15000001	5	15000001	0.485	0.6929
NC_005104.4_15100001	5	15100001	0.5408	0.7377
NC_005104.4_15200001	5	15200001	0.613	0.7215
NC_005104.4_15300001	5	15300001	0.5799	0.6711
NC_005104.4_15400001	5	15400001	0.4306	0.5018
NC_005104.4_15500001	5	15500001	0.4076	0.4622
NC_005104.4_15600001	5	15600001	0.4443	0.4794
NC_005104.4_15700001	5	15700001	0.3329	0.3572
NC_005104.4_15800001	5	15800001	0.3645	0.385
NC_005104.4_15900001	5	15900001	0.2992	0.3142
NC_005104.4_16000001	5	16000001	0.3516	0.474
NC_005104.4_16100001	5	16100001	0.4109	0.5295
NC_005104.4_16200001	5	16200001	0.4572	0.5542
NC_005104.4_16300001	5	16300001	0.4073	0.5631
NC_005104.4_16400001	5	16400001	0.4665	0.6317
NC_005104.4_16500001	5	16500001	0.4325	0.6013
NC_005104.4_16600001	5	16600001	0.3983	0.5168
NC_005104.4_16700001	5	16700001	0.4264	0.555
NC_005104.4_16800001	5	16800001	0.5963	0.6346
NC_005104.4_16900001	5	16900001	0.5589	0.6146
NC_005104.4_17000001	5	17000001	0.5757	0.5989
NC_005104.4_17100001	5	17100001	0.5553	0.6236
NC_005104.4_17200001	5	17200001	0.5287	0.6464
NC_005104.4_17300001	5	17300001	0.3586	0.5202
NC_005104.4_17400001	5	17400001	0.3021	0.5018
NC_005104.4_17500001	5	17500001	0.2891	0.5269
NC_005104.4_17600001	5	17600001	0.5087	0.7383
NC_005104.4_17700001	5	17700001	0.4718	0.7031
NC_005104.4_17800001	5	17800001	0.6391	0.7956
NC_005104.4_17900001	5	17900001	0.8373	0.912
NC_005104.4_18000001	5	18000001	0.852	0.919
NC_005104.4_18100001	5	18100001	0.827	0.9296
NC_005104.4_18200001	5	18200001	0.887	0.9512
NC_005104.4_18300001	5	18300001	0.8645	0.8936
NC_005104.4_18400001	5	18400001	0.8185	0.9022
NC_005104.4_18500001	5	18500001	0.8292	0.9076
NC_005104.4_18600001	5	18600001	0.7705	0.8016
NC_005104.4_18700001	5	18700001	0.7473	0.779
NC_005104.4_18800001	5	18800001	0.5684	0.6315
NC_005104.4_18900001	5	18900001	0.5455	0.523
NC_005104.4_19000001	5	19000001	0.4913	0.4534
NC_005104.4_19100001	5	19100001	0.4962	0.5643
NC_005104.4_19200001	5	19200001	0.5187	0.6041
NC_005104.4_19300001	5	19300001	0.5556	0.6431
NC_005104.4_19400001	5	19400001	0.5416	0.6847
NC_005104.4_19500001	5	19500001	0.6391	0.7625
NC_005104.4_19600001	5	19600001	0.6102	0.7673

NC_005104.4_19700001	5	19700001	0.5101	0.6668
NC_005104.4_19800001	5	19800001	0.531	0.6674
NC_005104.4_19900001	5	19900001	0.531	0.6674
NC_005104.4_20000001	5	20000001	0.5194	0.6857
NC_005104.4_20100001	5	20100001	0.629	0.7021
NC_005104.4_20200001	5	20200001	0.7101	0.6811
NC_005104.4_20300001	5	20300001	0.7265	0.7351
NC_005104.4_20400001	5	20400001	0.7048	0.7097
NC_005104.4_20500001	5	20500001	0.615	0.657
NC_005104.4_20600001	5	20600001	0.5818	0.6471
NC_005104.4_20700001	5	20700001	0.5346	0.6548
NC_005104.4_20800001	5	20800001	0.4789	0.5721
NC_005104.4_20900001	5	20900001	0.4708	0.5512
NC_005104.4_21000001	5	21000001	0.488	0.5392
NC_005104.4_21100001	5	21100001	0.4606	0.4878
NC_005104.4_21200001	5	21200001	0.4952	0.5692
NC_005104.4_21300001	5	21300001	0.4823	0.5679
NC_005104.4_21400001	5	21400001	0.5238	0.6274
NC_005104.4_21500001	5	21500001	0.533	0.6255
NC_005104.4_21600001	5	21600001	0.5873	0.6963
NC_005104.4_21700001	5	21700001	0.5949	0.6881
NC_005104.4_21800001	5	21800001	0.6196	0.7261
NC_005104.4_21900001	5	21900001	0.583	0.6783
NC_005104.4_22000001	5	22000001	0.5272	0.6387
NC_005104.4_22100001	5	22100001	0.4685	0.5752
NC_005104.4_22200001	5	22200001	0.4002	0.4844
NC_005104.4_22300001	5	22300001	0.3563	0.4392
NC_005104.4_22400001	5	22400001	0.349	0.4589
NC_005104.4_22500001	5	22500001	0.3553	0.4758
NC_005104.4_22600001	5	22600001	0.3188	0.4475
NC_005104.4_22700001	5	22700001	0.4093	0.5019
NC_005104.4_22800001	5	22800001	0.4026	0.3935
NC_005104.4_22900001	5	22900001	0.4026	0.3935
NC_005104.4_23000001	5	23000001	0.4026	0.3935
NC_005104.4_23100001	5	23100001	0.4219	0.4341
NC_005104.4_23200001	5	23200001	0.3822	0.3942
NC_005104.4_23300001	5	23300001	0.3706	0.4609
NC_005104.4_23400001	5	23400001	0.4227	0.5509
NC_005104.4_23500001	5	23500001	0.4851	0.6
NC_005104.4_23600001	5	23600001	0.5507	0.6655
NC_005104.4_23700001	5	23700001	0.563	0.7195
NC_005104.4_23800001	5	23800001	0.546	0.7066
NC_005104.4_23900001	5	23900001	0.5007	0.6254
NC_005104.4_24000001	5	24000001	0.4055	0.5438
NC_005104.4_24100001	5	24100001	0.3628	0.4344
NC_005104.4_24200001	5	24200001	0.3935	0.4676
NC_005104.4_24300001	5	24300001	0.3867	0.4391
NC_005104.4_24400001	5	24400001	0.4026	0.4601
NC_005104.4_24500001	5	24500001	0.4559	0.5055
NC_005104.4_24600001	5	24600001	0.4119	0.4305
NC_005104.4_24700001	5	24700001	0.3457	0.31
NC_005104.4_24800001	5	24800001	0.3457	0.31
NC_005104.4_25000001	5	25000001	0.4738	0.7094
NC_005104.4_25100001	5	25100001	0.4275	0.6176
NC_005104.4_25200001	5	25200001	0.5569	0.7388
NC_005104.4_25300001	5	25300001	0.5569	0.7388
NC_005104.4_25400001	5	25400001	0.4999	0.663
NC_005104.4_25500001	5	25500001	0.5201	0.5984

NC_005104.4_25600001	5	25600001	0.6457	0.6742
NC_005104.4_25700001	5	25700001	0.5355	0.5723
NC_005104.4_25800001	5	25800001	0.5716	0.6187
NC_005104.4_25900001	5	25900001	0.5881	0.6515
NC_005104.4_26000001	5	26000001	0.6006	0.6944
NC_005104.4_26100001	5	26100001	0.5477	0.6838
NC_005104.4_26200001	5	26200001	0.4685	0.613
NC_005104.4_26300001	5	26300001	0.4732	0.6826
NC_005104.4_26400001	5	26400001	0.4667	0.6409
NC_005104.4_26500001	5	26500001	0.5542	0.7035
NC_005104.4_26600001	5	26600001	0.5661	0.6921
NC_005104.4_26700001	5	26700001	0.545	0.6757
NC_005104.4_26800001	5	26800001	0.603	0.6751
NC_005104.4_26900001	5	26900001	0.6886	0.739
NC_005104.4_27000001	5	27000001	0.6281	0.7096
NC_005104.4_27100001	5	27100001	0.6155	0.7604
NC_005104.4_27200001	5	27200001	0.6414	0.791
NC_005104.4_27300001	5	27300001	0.6012	0.7623
NC_005104.4_27400001	5	27400001	0.5175	0.6778
NC_005104.4_27500001	5	27500001	0.5463	0.6542
NC_005104.4_27600001	5	27600001	0.4733	0.5737
NC_005104.4_27700001	5	27700001	0.5219	0.6031
NC_005104.4_27800001	5	27800001	0.449	0.5161
NC_005104.4_27900001	5	27900001	0.4514	0.5351
NC_005104.4_28000001	5	28000001	0.3635	0.4662
NC_005104.4_28100001	5	28100001	0.3728	0.4656
NC_005104.4_28200001	5	28200001	0.2885	0.3864
NC_005104.4_28300001	5	28300001	0.2422	0.3973
NC_005104.4_28400001	5	28400001	0.5173	0.6487
NC_005104.4_28500001	5	28500001	0.5422	0.6929
NC_005104.4_28600001	5	28600001	0.5326	0.6358
NC_005104.4_28700001	5	28700001	0.5617	0.6178
NC_005104.4_28800001	5	28800001	0.6431	0.6365
NC_005104.4_28900001	5	28900001	0.4485	0.4899
NC_005104.4_29000001	5	29000001	0.4582	0.4669
NC_005104.4_29100001	5	29100001	0.4469	0.4939
NC_005104.4_29200001	5	29200001	0.4222	0.4356
NC_005104.4_29300001	5	29300001	0.4952	0.5301
NC_005104.4_29400001	5	29400001	0.6251	0.6699
NC_005104.4_29500001	5	29500001	0.6931	0.7238
NC_005104.4_29600001	5	29600001	0.7921	0.7645
NC_005104.4_29700001	5	29700001	0.8783	0.8834
NC_005104.4_29800001	5	29800001	0.846	0.8734
NC_005104.4_30000001	5	30000001	0.3258	0.5234
NC_005104.4_30100001	5	30100001	0.485	0.7372
NC_005104.4_30200001	5	30200001	0.3528	0.6234
NC_005104.4_30300001	5	30300001	0.3882	0.639
NC_005104.4_30400001	5	30400001	0.3976	0.603
NC_005104.4_30500001	5	30500001	0.4813	0.7373
NC_005104.4_30600001	5	30600001	0.3753	0.6508
NC_005104.4_30700001	5	30700001	0.3753	0.6508
NC_005104.4_30800001	5	30800001	0.3435	0.5621
NC_005104.4_30900001	5	30900001	0.4203	0.608
NC_005104.4_31000001	5	31000001	0.4281	0.5216
NC_005104.4_31100001	5	31100001	0.3951	0.4038
NC_005104.4_31200001	5	31200001	0.3951	0.4038
NC_005104.4_31300001	5	31300001	0.6017	0.6729
NC_005104.4_31400001	5	31400001	0.5939	0.6717

NC_005104.4_31600001	5	31600001	0.641	0.8104
NC_005104.4_31700001	5	31700001	0.641	0.8104
NC_005104.4_32200001	5	32200001	0.5126	0.7001
NC_005104.4_32300001	5	32300001	0.7101	0.8506
NC_005104.4_32400001	5	32400001	0.6564	0.7869
NC_005104.4_32500001	5	32500001	0.6559	0.7752
NC_005104.4_32600001	5	32600001	0.6698	0.7833
NC_005104.4_32700001	5	32700001	0.689	0.7855
NC_005104.4_32800001	5	32800001	0.4643	0.5714
NC_005104.4_32900001	5	32900001	0.5383	0.6047
NC_005104.4_33100001	5	33100001	0.1582	0.0901
NC_005104.4_33200001	5	33200001	0.5024	0.7181
NC_005104.4_33300001	5	33300001	0.6433	0.7822
NC_005104.4_33400001	5	33400001	0.667	0.7556
NC_005104.4_33500001	5	33500001	0.7254	0.7429
NC_005104.4_33600001	5	33600001	0.7419	0.7433
NC_005104.4_33700001	5	33700001	0.606	0.6529
NC_005104.4_33800001	5	33800001	0.5927	0.6687
NC_005104.4_33900001	5	33900001	0.4915	0.5611
NC_005104.4_34000001	5	34000001	0.3824	0.4876
NC_005104.4_34100001	5	34100001	0.3241	0.4908
NC_005104.4_34200001	5	34200001	0.2937	0.3887
NC_005104.4_34300001	5	34300001	0.2937	0.3887
NC_005104.4_34400001	5	34400001	0.2995	0.4207
NC_005104.4_34500001	5	34500001	0.449	0.6279
NC_005104.4_34600001	5	34600001	0.5549	0.7009
NC_005104.4_34700001	5	34700001	0.5549	0.7009
NC_005104.4_34800001	5	34800001	0.5549	0.7009
NC_005104.4_34900001	5	34900001	0.6783	0.767
NC_005104.4_35000001	5	35000001	0.646	0.7311
NC_005104.4_35300001	5	35300001	0.3778	0.5175
NC_005104.4_35400001	5	35400001	0.3775	0.5653
NC_005104.4_35500001	5	35500001	0.3185	0.4956
NC_005104.4_35600001	5	35600001	0.3785	0.533
NC_005104.4_35700001	5	35700001	0.434	0.5898
NC_005104.4_35800001	5	35800001	0.4805	0.6016
NC_005104.4_35900001	5	35900001	0.5075	0.6443
NC_005104.4_36000001	5	36000001	0.5351	0.5869
NC_005104.4_36100001	5	36100001	0.4916	0.5714
NC_005104.4_36200001	5	36200001	0.4397	0.4321
NC_005104.4_36300001	5	36300001	0.4852	0.4565
NC_005104.4_36400001	5	36400001	0.4773	0.3297
NC_005104.4_36900001	5	36900001	0.453	0.7426
NC_005104.4_37000001	5	37000001	0.5265	0.7559
NC_005104.4_37100001	5	37100001	0.5113	0.7965
NC_005104.4_37200001	5	37200001	0.5222	0.7487
NC_005104.4_37300001	5	37300001	0.5233	0.723
NC_005104.4_37400001	5	37400001	0.4855	0.6982
NC_005104.4_37500001	5	37500001	0.4267	0.6502
NC_005104.4_37600001	5	37600001	0.4671	0.5908
NC_005104.4_37700001	5	37700001	0.5002	0.5655
NC_005104.4_37800001	5	37800001	0.4482	0.554
NC_005104.4_37900001	5	37900001	0.4241	0.3902
NC_005104.4_38000001	5	38000001	0.3581	0.3571
NC_005104.4_38100001	5	38100001	0.3738	0.3742
NC_005104.4_38200001	5	38200001	0.3262	0.4542
NC_005104.4_38300001	5	38300001	0.3073	0.4346
NC_005104.4_38400001	5	38400001	0.2748	0.5586

NC_005104.4_38500001	5	38500001	0.3233	0.4537
NC_005104.4_38600001	5	38600001	0.3898	0.5891
NC_005104.4_38700001	5	38700001	0.3711	0.5345
NC_005104.4_38800001	5	38800001	0.4101	0.5788
NC_005104.4_38900001	5	38900001	0.445	0.5495
NC_005104.4_39000001	5	39000001	0.5344	0.6674
NC_005104.4_39100001	5	39100001	0.5313	0.5705
NC_005104.4_39200001	5	39200001	0.4428	0.5283
NC_005104.4_39300001	5	39300001	0.4468	0.4781
NC_005104.4_39400001	5	39400001	0.3901	0.4679
NC_005104.4_39500001	5	39500001	0.3776	0.437
NC_005104.4_39600001	5	39600001	0.2588	0.3115
NC_005104.4_39700001	5	39700001	0.3803	0.537
NC_005104.4_39800001	5	39800001	0.3767	0.6102
NC_005104.4_39900001	5	39900001	0.3767	0.6102
NC_005104.4_40000001	5	40000001	0.3815	0.6582
NC_005104.4_40100001	5	40100001	0.4482	0.7106
NC_005104.4_40200001	5	40200001	0.4151	0.6602
NC_005104.4_40300001	5	40300001	0.4052	0.6537
NC_005104.4_40400001	5	40400001	0.4064	0.5723
NC_005104.4_40500001	5	40500001	0.4426	0.5856
NC_005104.4_40600001	5	40600001	0.3756	0.4927
NC_005104.4_40700001	5	40700001	0.3404	0.4948
NC_005104.4_40800001	5	40800001	0.4251	0.5252
NC_005104.4_40900001	5	40900001	0.5182	0.6187
NC_005104.4_41000001	5	41000001	0.5602	0.6619
NC_005104.4_41100001	5	41100001	0.5602	0.6619
NC_005104.4_41200001	5	41200001	0.609	0.6257
NC_005104.4_41300001	5	41300001	0.6141	0.653
NC_005104.4_41600001	5	41600001	0.2137	0.2248
NC_005104.4_41700001	5	41700001	0.366	0.7195
NC_005104.4_41800001	5	41800001	0.3618	0.5992
NC_005104.4_41900001	5	41900001	0.3618	0.5992
NC_005104.4_42000001	5	42000001	0.4031	0.5172
NC_005104.4_42100001	5	42100001	0.4418	0.5245
NC_005104.4_42200001	5	42200001	0.4002	0.4166
NC_005104.4_42300001	5	42300001	0.5396	0.6317
NC_005104.4_42400001	5	42400001	0.5396	0.6317
NC_005104.4_42500001	5	42500001	0.693	0.8483
NC_005104.4_42600001	5	42600001	0.6284	0.7657
NC_005104.4_42700001	5	42700001	0.7348	0.7712
NC_005104.4_42800001	5	42800001	0.7086	0.6283
NC_005104.4_42900001	5	42900001	0.7086	0.6283
NC_005104.4_43000001	5	43000001	0.6138	0.5963
NC_005104.4_43100001	5	43100001	0.7762	0.7668
NC_005104.4_43200001	5	43200001	0.5101	0.5672
NC_005104.4_43300001	5	43300001	0.6379	0.6906
NC_005104.4_43400001	5	43400001	0.5203	0.595
NC_005104.4_43500001	5	43500001	0.4777	0.6032
NC_005104.4_43600001	5	43600001	0.3995	0.5451
NC_005104.4_43700001	5	43700001	0.3798	0.6067
NC_005104.4_43800001	5	43800001	0.2762	0.6073
NC_005104.4_43900001	5	43900001	0.3175	0.6892
NC_005104.4_44000001	5	44000001	0.3583	0.6629
NC_005104.4_44100001	5	44100001	0.3438	0.6156
NC_005104.4_44200001	5	44200001	0.4411	0.6654
NC_005104.4_44300001	5	44300001	0.4773	0.5675
NC_005104.4_44400001	5	44400001	0.6012	0.6916

NC_005104.4_44500001	5	44500001	0.6648	0.7324
NC_005104.4_44600001	5	44600001	0.5996	0.7299
NC_005104.4_44700001	5	44700001	0.5966	0.7382
NC_005104.4_44800001	5	44800001	0.5725	0.7534
NC_005104.4_44900001	5	44900001	0.5103	0.7379
NC_005104.4_45000001	5	45000001	0.533	0.726
NC_005104.4_45100001	5	45100001	0.5317	0.6661
NC_005104.4_45200001	5	45200001	0.5941	0.6868
NC_005104.4_45300001	5	45300001	0.5418	0.6692
NC_005104.4_45400001	5	45400001	0.5324	0.7357
NC_005104.4_45500001	5	45500001	0.5406	0.739
NC_005104.4_45600001	5	45600001	0.4335	0.6149
NC_005104.4_45700001	5	45700001	0.3647	0.5394
NC_005104.4_45800001	5	45800001	0.3857	0.5534
NC_005104.4_45900001	5	45900001	0.3825	0.497
NC_005104.4_46000001	5	46000001	0.3743	0.4171
NC_005104.4_46100001	5	46100001	0.481	0.5862
NC_005104.4_46200001	5	46200001	0.4923	0.5184
NC_005104.4_46300001	5	46300001	0.4923	0.5184
NC_005104.4_46400001	5	46400001	0.4744	0.4604
NC_005104.4_46500001	5	46500001	0.4541	0.4789
NC_005104.4_46600001	5	46600001	0.467	0.4123
NC_005104.4_46700001	5	46700001	0.4648	0.4339
NC_005104.4_46800001	5	46800001	0.5099	0.5344
NC_005104.4_46900001	5	46900001	0.4929	0.5414
NC_005104.4_47000001	5	47000001	0.4826	0.5633
NC_005104.4_47100001	5	47100001	0.4584	0.563
NC_005104.4_47200001	5	47200001	0.4623	0.5704
NC_005104.4_47300001	5	47300001	0.4455	0.5529
NC_005104.4_47400001	5	47400001	0.4464	0.5659
NC_005104.4_47500001	5	47500001	0.5101	0.6221
NC_005104.4_47600001	5	47600001	0.5717	0.6322
NC_005104.4_47700001	5	47700001	0.5778	0.6839
NC_005104.4_47800001	5	47800001	0.5345	0.6371
NC_005104.4_47900001	5	47900001	0.5155	0.6555
NC_005104.4_48000001	5	48000001	0.5015	0.6441
NC_005104.4_48100001	5	48100001	0.5445	0.6948
NC_005104.4_48200001	5	48200001	0.4919	0.6415
NC_005104.4_48300001	5	48300001	0.5819	0.7137
NC_005104.4_48400001	5	48400001	0.499	0.6612
NC_005104.4_48500001	5	48500001	0.4373	0.603
NC_005104.4_48600001	5	48600001	0.4165	0.5575
NC_005104.4_48700001	5	48700001	0.4987	0.647
NC_005104.4_48800001	5	48800001	0.4892	0.6095
NC_005104.4_48900001	5	48900001	0.6418	0.6701
NC_005104.4_49000001	5	49000001	0.7063	0.7045
NC_005104.4_49100001	5	49100001	0.7541	0.7881
NC_005104.4_49200001	5	49200001	0.6727	0.7506
NC_005104.4_49300001	5	49300001	0.5568	0.7447
NC_005104.4_49400001	5	49400001	0.505	0.673
NC_005104.4_49500001	5	49500001	0.5352	0.6678
NC_005104.4_49600001	5	49600001	0.5366	0.6478
NC_005104.4_49700001	5	49700001	0.5532	0.6459
NC_005104.4_49800001	5	49800001	0.6117	0.6532
NC_005104.4_49900001	5	49900001	0.5711	0.7
NC_005104.4_50000001	5	50000001	0.4771	0.6695
NC_005104.4_50100001	5	50100001	0.3533	0.4054
NC_005104.4_50200001	5	50200001	0.4713	0.5422

NC_005104.4_50300001	5	50300001	0.5033	0.521
NC_005104.4_50400001	5	50400001	0.5623	0.5039
NC_005104.4_50500001	5	50500001	0.5097	0.5151
NC_005104.4_50600001	5	50600001	0.5265	0.5991
NC_005104.4_50700001	5	50700001	0.3942	0.4704
NC_005104.4_50800001	5	50800001	0.484	0.6956
NC_005104.4_50900001	5	50900001	0.4336	0.5331
NC_005104.4_51100001	5	51100001	0.4717	0.5894
NC_005104.4_51200001	5	51200001	0.5472	0.6746
NC_005104.4_51300001	5	51300001	0.4749	0.6263
NC_005104.4_51400001	5	51400001	0.5255	0.6731
NC_005104.4_51500001	5	51500001	0.6386	0.7854
NC_005104.4_51600001	5	51600001	0.6912	0.7452
NC_005104.4_51700001	5	51700001	0.6434	0.7133
NC_005104.4_51800001	5	51800001	0.6452	0.5983
NC_005104.4_51900001	5	51900001	0.7059	0.6786
NC_005104.4_52000001	5	52000001	0.7013	0.6755
NC_005104.4_52100001	5	52100001	0.5592	0.6163
NC_005104.4_52200001	5	52200001	0.5172	0.5578
NC_005104.4_52300001	5	52300001	0.4472	0.6305
NC_005104.4_52400001	5	52400001	0.3326	0.5622
NC_005104.4_52500001	5	52500001	0.3337	0.5615
NC_005104.4_52600001	5	52600001	0.3527	0.6404
NC_005104.4_52700001	5	52700001	0.3419	0.6565
NC_005104.4_52800001	5	52800001	0.4753	0.7907
NC_005104.4_52900001	5	52900001	0.4574	0.799
NC_005104.4_53000001	5	53000001	0.3754	0.7305
NC_005104.4_53100001	5	53100001	0.4434	0.7568
NC_005104.4_53800001	5	53800001	0.5196	0.6548
NC_005104.4_53900001	5	53900001	0.5382	0.6192
NC_005104.4_54000001	5	54000001	0.5846	0.6388
NC_005104.4_54100001	5	54100001	0.5359	0.6064
NC_005104.4_54200001	5	54200001	0.5359	0.6064
NC_005104.4_54300001	5	54300001	0.5204	0.5961
NC_005104.4_54400001	5	54400001	0.548	0.7221
NC_005104.4_54500001	5	54500001	0.4851	0.6946
NC_005104.4_54600001	5	54600001	0.4953	0.7827
NC_005104.4_54700001	5	54700001	0.5624	0.8303
NC_005104.4_54800001	5	54800001	0.6263	0.7785
NC_005104.4_54900001	5	54900001	0.6072	0.7109
NC_005104.4_55000001	5	55000001	0.5392	0.7207
NC_005104.4_55100001	5	55100001	0.7159	0.8419
NC_005104.4_55200001	5	55200001	0.6965	0.8291
NC_005104.4_55300001	5	55300001	0.7807	0.9019
NC_005104.4_55400001	5	55400001	0.7807	0.9019
NC_005104.4_55500001	5	55500001	0.7574	0.8064
NC_005104.4_55700001	5	55700001	0.348	0.3982
NC_005104.4_55800001	5	55800001	0.4355	0.5548
NC_005104.4_55900001	5	55900001	0.4058	0.5115
NC_005104.4_56000001	5	56000001	0.4746	0.6371
NC_005104.4_56100001	5	56100001	0.5332	0.6451
NC_005104.4_56200001	5	56200001	0.5703	0.673
NC_005104.4_56300001	5	56300001	0.5266	0.6334
NC_005104.4_56400001	5	56400001	0.4725	0.623
NC_005104.4_56500001	5	56500001	0.4895	0.617
NC_005104.4_56600001	5	56600001	0.5155	0.6881
NC_005104.4_56700001	5	56700001	0.558	0.7751
NC_005104.4_56800001	5	56800001	0.5731	0.7589

NC_005104.4_56900001	5	56900001	0.6173	0.7841
NC_005104.4_57000001	5	57000001	0.6418	0.8411
NC_005104.4_57100001	5	57100001	0.5542	0.7782
NC_005104.4_57200001	5	57200001	0.5165	0.7253
NC_005104.4_57300001	5	57300001	0.4813	0.7297
NC_005104.4_57400001	5	57400001	0.5052	0.7695
NC_005104.4_57500001	5	57500001	0.5475	0.7141
NC_005104.4_57600001	5	57600001	0.5003	0.6429
NC_005104.4_57700001	5	57700001	0.4574	0.632
NC_005104.4_57800001	5	57800001	0.5542	0.6854
NC_005104.4_57900001	5	57900001	0.5189	0.6042
NC_005104.4_58000001	5	58000001	0.5235	0.6401
NC_005104.4_58100001	5	58100001	0.5429	0.6867
NC_005104.4_58200001	5	58200001	0.5586	0.6944
NC_005104.4_58300001	5	58300001	0.5045	0.6624
NC_005104.4_58400001	5	58400001	0.597	0.8113
NC_005104.4_58500001	5	58500001	0.5391	0.7721
NC_005104.4_58600001	5	58600001	0.5299	0.6958
NC_005104.4_58700001	5	58700001	0.5302	0.7083
NC_005104.4_58800001	5	58800001	0.4592	0.5854
NC_005104.4_58900001	5	58900001	0.4933	0.5831
NC_005104.4_59000001	5	59000001	0.5118	0.6248
NC_005104.4_59100001	5	59100001	0.5343	0.6421
NC_005104.4_59200001	5	59200001	0.5341	0.6289
NC_005104.4_59300001	5	59300001	0.5922	0.7009
NC_005104.4_59400001	5	59400001	0.5808	0.7093
NC_005104.4_59500001	5	59500001	0.6178	0.6424
NC_005104.4_59600001	5	59600001	0.686	0.7056
NC_005104.4_59700001	5	59700001	0.6759	0.692
NC_005104.4_59800001	5	59800001	0.7102	0.6942
NC_005104.4_59900001	5	59900001	0.6992	0.7193
NC_005104.4_60000001	5	60000001	0.6882	0.7653
NC_005104.4_60100001	5	60100001	0.6017	0.6932
NC_005104.4_60200001	5	60200001	0.5027	0.7359
NC_005104.4_60300001	5	60300001	0.522	0.7085
NC_005104.4_60400001	5	60400001	0.531	0.6979
NC_005104.4_60500001	5	60500001	0.5208	0.6761
NC_005104.4_60600001	5	60600001	0.5894	0.7727
NC_005104.4_60700001	5	60700001	0.6286	0.7721
NC_005104.4_60800001	5	60800001	0.6644	0.7994
NC_005104.4_60900001	5	60900001	0.514	0.7225
NC_005104.4_61000001	5	61000001	0.6321	0.8314
NC_005104.4_61100001	5	61100001	0.5508	0.7941
NC_005104.4_61200001	5	61200001	0.5827	0.7857
NC_005104.4_61300001	5	61300001	0.5619	0.7618
NC_005104.4_61400001	5	61400001	0.7043	0.8062
NC_005104.4_61500001	5	61500001	0.6057	0.7531
NC_005104.4_61600001	5	61600001	0.6166	0.7455
NC_005104.4_61700001	5	61700001	0.6158	0.7784
NC_005104.4_61800001	5	61800001	0.5321	0.7718
NC_005104.4_61900001	5	61900001	0.5112	0.8023
NC_005104.4_62000001	5	62000001	0.4877	0.7143
NC_005104.4_62100001	5	62100001	0.5097	0.6855
NC_005104.4_62200001	5	62200001	0.5021	0.6371
NC_005104.4_62300001	5	62300001	0.4843	0.6048
NC_005104.4_62400001	5	62400001	0.4441	0.5535
NC_005104.4_62500001	5	62500001	0.4522	0.5663
NC_005104.4_62600001	5	62600001	0.4231	0.5561

NC_005104.4_62700001	5	62700001	0.3186	0.5043
NC_005104.4_62800001	5	62800001	0.4466	0.7224
NC_005104.4_62900001	5	62900001	0.4219	0.6343
NC_005104.4_63000001	5	63000001	0.4719	0.6367
NC_005104.4_63100001	5	63100001	0.4526	0.5837
NC_005104.4_63200001	5	63200001	0.5113	0.6447
NC_005104.4_63300001	5	63300001	0.4561	0.6079
NC_005104.4_63400001	5	63400001	0.5113	0.6664
NC_005104.4_63500001	5	63500001	0.5077	0.7199
NC_005104.4_63600001	5	63600001	0.598	0.8531
NC_005104.4_63700001	5	63700001	0.5825	0.8272
NC_005104.4_63800001	5	63800001	0.6112	0.8172
NC_005104.4_63900001	5	63900001	0.5983	0.7581
NC_005104.4_64000001	5	64000001	0.5822	0.7327
NC_005104.4_64100001	5	64100001	0.5387	0.6901
NC_005104.4_64200001	5	64200001	0.513	0.5925
NC_005104.4_64300001	5	64300001	0.532	0.4891
NC_005104.4_64400001	5	64400001	0.3316	0.3856
NC_005104.4_64500001	5	64500001	0.4641	0.6
NC_005104.4_64600001	5	64600001	0.4659	0.613
NC_005104.4_64700001	5	64700001	0.4581	0.6306
NC_005104.4_64800001	5	64800001	0.4407	0.5653
NC_005104.4_64900001	5	64900001	0.4715	0.5936
NC_005104.4_65000001	5	65000001	0.4553	0.5401
NC_005104.4_65100001	5	65100001	0.5054	0.573
NC_005104.4_65200001	5	65200001	0.576	0.6163
NC_005104.4_65300001	5	65300001	0.7334	0.7264
NC_005104.4_65400001	5	65400001	0.7306	0.6965
NC_005104.4_65500001	5	65500001	0.6815	0.686
NC_005104.4_65600001	5	65600001	0.6204	0.6355
NC_005104.4_65700001	5	65700001	0.6107	0.651
NC_005104.4_65800001	5	65800001	0.4842	0.628
NC_005104.4_65900001	5	65900001	0.4017	0.6765
NC_005104.4_66000001	5	66000001	0.5971	0.7841
NC_005104.4_66100001	5	66100001	0.5636	0.8366
NC_005104.4_66200001	5	66200001	0.3902	0.7326
NC_005104.4_66300001	5	66300001	0.3265	0.6072
NC_005104.4_66400001	5	66400001	0.3763	0.68
NC_005104.4_66500001	5	66500001	0.2142	0.393
NC_005104.4_66600001	5	66600001	0.239	0.4042
NC_005104.4_66700001	5	66700001	0.2267	0.3048
NC_005104.4_66800001	5	66800001	0.2683	0.3189
NC_005104.4_66900001	5	66900001	0.1801	0.0992
NC_005104.4_67000001	5	67000001	0.1907	0.1717
NC_005104.4_67100001	5	67100001	0.1737	0.1591
NC_005104.4_67200001	5	67200001	0.2484	0.2765
NC_005104.4_67300001	5	67300001	0.2515	0.293
NC_005104.4_67400001	5	67400001	0.2918	0.3193
NC_005104.4_67500001	5	67500001	0.3301	0.3192
NC_005104.4_67600001	5	67600001	0.3058	0.3186
NC_005104.4_67700001	5	67700001	0.2553	0.2424
NC_005104.4_67800001	5	67800001	0.2553	0.2424
NC_005104.4_67900001	5	67900001	0.2952	0.3545
NC_005104.4_68000001	5	68000001	0.3344	0.5136
NC_005104.4_68100001	5	68100001	0.4471	0.6049
NC_005104.4_68200001	5	68200001	0.5682	0.7659
NC_005104.4_68300001	5	68300001	0.5682	0.7659
NC_005104.4_68400001	5	68400001	0.6287	0.775

NC_005104.4_68500001	5	68500001	0.6124	0.7186
NC_005104.4_68600001	5	68600001	0.6735	0.7235
NC_005104.4_68700001	5	68700001	0.6405	0.6919
NC_005104.4_68800001	5	68800001	0.6405	0.6919
NC_005104.4_68900001	5	68900001	0.5852	0.625
NC_005104.4_69400001	5	69400001	0.6339	0.6874
NC_005104.4_69500001	5	69500001	0.5344	0.6266
NC_005104.4_69600001	5	69600001	0.4566	0.5627
NC_005104.4_69700001	5	69700001	0.4369	0.5565
NC_005104.4_69800001	5	69800001	0.3543	0.4351
NC_005104.4_69900001	5	69900001	0.3459	0.4328
NC_005104.4_70000001	5	70000001	0.2595	0.349
NC_005104.4_70100001	5	70100001	0.3871	0.413
NC_005104.4_70200001	5	70200001	0.4058	0.3953
NC_005104.4_70300001	5	70300001	0.3675	0.376
NC_005104.4_70400001	5	70400001	0.4227	0.5255
NC_005104.4_70500001	5	70500001	0.3967	0.4938
NC_005104.4_70600001	5	70600001	0.3456	0.5462
NC_005104.4_70700001	5	70700001	0.4278	0.6945
NC_005104.4_70800001	5	70800001	0.5573	0.8139
NC_005104.4_70900001	5	70900001	0.5246	0.796
NC_005104.4_71000001	5	71000001	0.4916	0.7166
NC_005104.4_71100001	5	71100001	0.5132	0.723
NC_005104.4_71200001	5	71200001	0.4933	0.6841
NC_005104.4_71300001	5	71300001	0.4465	0.6017
NC_005104.4_71400001	5	71400001	0.4822	0.6154
NC_005104.4_71500001	5	71500001	0.4732	0.6069
NC_005104.4_71600001	5	71600001	0.4445	0.4915
NC_005104.4_71700001	5	71700001	0.5127	0.5432
NC_005104.4_71800001	5	71800001	0.6123	0.6669
NC_005104.4_71900001	5	71900001	0.5346	0.6275
NC_005104.4_72000001	5	72000001	0.561	0.6374
NC_005104.4_72100001	5	72100001	0.5489	0.6605
NC_005104.4_72200001	5	72200001	0.505	0.6795
NC_005104.4_72300001	5	72300001	0.378	0.5223
NC_005104.4_72400001	5	72400001	0.507	0.6712
NC_005104.4_72500001	5	72500001	0.5107	0.7504
NC_005104.4_72600001	5	72600001	0.6258	0.8301
NC_005104.4_72700001	5	72700001	0.5981	0.6633
NC_005104.4_72800001	5	72800001	0.6649	0.7327
NC_005104.4_72900001	5	72900001	0.6138	0.6809
NC_005104.4_73000001	5	73000001	0.7113	0.7092
NC_005104.4_73100001	5	73100001	0.6226	0.6468
NC_005104.4_73200001	5	73200001	0.6447	0.6929
NC_005104.4_73300001	5	73300001	0.6148	0.6803
NC_005104.4_73400001	5	73400001	0.5619	0.7146
NC_005104.4_73500001	5	73500001	0.5288	0.6967
NC_005104.4_73600001	5	73600001	0.5851	0.6743
NC_005104.4_73700001	5	73700001	0.5962	0.6729
NC_005104.4_73800001	5	73800001	0.5594	0.618
NC_005104.4_73900001	5	73900001	0.5588	0.67
NC_005104.4_74000001	5	74000001	0.5581	0.6271
NC_005104.4_74100001	5	74100001	0.5002	0.6314
NC_005104.4_74200001	5	74200001	0.5777	0.6722
NC_005104.4_74300001	5	74300001	0.6034	0.7213
NC_005104.4_74400001	5	74400001	0.5759	0.6347
NC_005104.4_74500001	5	74500001	0.5959	0.677
NC_005104.4_74600001	5	74600001	0.6178	0.6984

NC_005104.4_74700001	5	74700001	0.5198	0.6628
NC_005104.4_74800001	5	74800001	0.4858	0.6055
NC_005104.4_74900001	5	74900001	0.5332	0.6051
NC_005104.4_75000001	5	75000001	0.5763	0.6692
NC_005104.4_75100001	5	75100001	0.6139	0.6797
NC_005104.4_75200001	5	75200001	0.5969	0.7179
NC_005104.4_75300001	5	75300001	0.591	0.7539
NC_005104.4_75400001	5	75400001	0.5069	0.7555
NC_005104.4_75500001	5	75500001	0.4038	0.6907
NC_005104.4_75600001	5	75600001	0.4147	0.6273
NC_005104.4_75700001	5	75700001	0.4834	0.6616
NC_005104.4_75800001	5	75800001	0.4793	0.625
NC_005104.4_75900001	5	75900001	0.5939	0.7154
NC_005104.4_76000001	5	76000001	0.5892	0.722
NC_005104.4_76100001	5	76100001	0.5062	0.621
NC_005104.4_76200001	5	76200001	0.3604	0.3804
NC_005104.4_76300001	5	76300001	0.3737	0.4021
NC_005104.4_76400001	5	76400001	0.3226	0.3044
NC_005104.4_76500001	5	76500001	0.3224	0.285
NC_005104.4_76600001	5	76600001	0.4983	0.5781
NC_005104.4_76700001	5	76700001	0.5449	0.6853
NC_005104.4_76800001	5	76800001	0.5842	0.6989
NC_005104.4_76900001	5	76900001	0.6368	0.764
NC_005104.4_77000001	5	77000001	0.6129	0.7424
NC_005104.4_77100001	5	77100001	0.5274	0.7083
NC_005104.4_77200001	5	77200001	0.6676	0.726
NC_005104.4_77700001	5	77700001	0.7737	0.744
NC_005104.4_77800001	5	77800001	0.7705	0.7321
NC_005104.4_77900001	5	77900001	0.73	0.7496
NC_005104.4_78000001	5	78000001	0.7242	0.7624
NC_005104.4_78100001	5	78100001	0.7206	0.7743
NC_005104.4_78200001	5	78200001	0.6607	0.7485
NC_005104.4_78300001	5	78300001	0.5526	0.6756
NC_005104.4_78400001	5	78400001	0.4819	0.6067
NC_005104.4_78500001	5	78500001	0.4163	0.5429
NC_005104.4_78600001	5	78600001	0.3225	0.476
NC_005104.4_78700001	5	78700001	0.2987	0.436
NC_005104.4_78800001	5	78800001	0.3104	0.4865
NC_005104.4_78900001	5	78900001	0.3248	0.516
NC_005104.4_79000001	5	79000001	0.3583	0.5655
NC_005104.4_79100001	5	79100001	0.3954	0.5903
NC_005104.4_79200001	5	79200001	0.4704	0.6929
NC_005104.4_79300001	5	79300001	0.5544	0.6773
NC_005104.4_79400001	5	79400001	0.6746	0.7142
NC_005104.4_79500001	5	79500001	0.5705	0.7108
NC_005104.4_79600001	5	79600001	0.6295	0.725
NC_005104.4_79700001	5	79700001	0.5571	0.6907
NC_005104.4_79800001	5	79800001	0.5641	0.7497
NC_005104.4_79900001	5	79900001	0.5108	0.7413
NC_005104.4_80000001	5	80000001	0.5853	0.7341
NC_005104.4_80100001	5	80100001	0.537	0.7259
NC_005104.4_80200001	5	80200001	0.5262	0.7494
NC_005104.4_80300001	5	80300001	0.6123	0.888
NC_005104.4_80400001	5	80400001	0.6646	0.8862
NC_005104.4_80500001	5	80500001	0.6667	0.8665
NC_005104.4_80600001	5	80600001	0.7115	0.8368
NC_005104.4_80700001	5	80700001	0.787	0.8685
NC_005104.4_80800001	5	80800001	0.7721	0.8368

NC_005104.4_80900001	5	80900001	0.7868	0.8161
NC_005104.4_81000001	5	81000001	0.8357	0.8421
NC_005104.4_81100001	5	81100001	0.6146	0.7991
NC_005104.4_81200001	5	81200001	0.4426	0.6622
NC_005104.4_81300001	5	81300001	0.5046	0.6969
NC_005104.4_81400001	5	81400001	0.5149	0.694
NC_005104.4_81500001	5	81500001	0.4522	0.6365
NC_005104.4_81600001	5	81600001	0.5107	0.6501
NC_005104.4_81700001	5	81700001	0.5367	0.6283
NC_005104.4_81800001	5	81800001	0.5616	0.6375
NC_005104.4_81900001	5	81900001	0.5385	0.5968
NC_005104.4_82000001	5	82000001	0.6563	0.643
NC_005104.4_82100001	5	82100001	0.6513	0.6627
NC_005104.4_82200001	5	82200001	0.6322	0.6298
NC_005104.4_82300001	5	82300001	0.4589	0.4642
NC_005104.4_82400001	5	82400001	0.4589	0.4642
NC_005104.4_82500001	5	82500001	0.4229	0.4377
NC_005104.4_82600001	5	82600001	0.4233	0.4763
NC_005104.4_82700001	5	82700001	0.3726	0.4325
NC_005104.4_82800001	5	82800001	0.6188	0.6433
NC_005104.4_82900001	5	82900001	0.6188	0.6433
NC_005104.4_83000001	5	83000001	0.6741	0.6985
NC_005104.4_83100001	5	83100001	0.698	0.6883
NC_005104.4_83200001	5	83200001	0.698	0.6883
NC_005104.4_83300001	5	83300001	0.5	0.6503
NC_005104.4_83400001	5	83400001	0.4328	0.6169
NC_005104.4_83500001	5	83500001	0.2783	0.5426
NC_005104.4_83600001	5	83600001	0.3118	0.4963
NC_005104.4_83700001	5	83700001	0.3057	0.4017
NC_005104.4_83800001	5	83800001	0.2288	0.2317
NC_005104.4_83900001	5	83900001	0.353	0.4819
NC_005104.4_84000001	5	84000001	0.4924	0.598
NC_005104.4_84100001	5	84100001	0.5881	0.7447
NC_005104.4_84200001	5	84200001	0.6093	0.9088
NC_005104.4_84300001	5	84300001	0.6093	0.9088
NC_005104.4_84400001	5	84400001	0.3975	0.63
NC_005104.4_84500001	5	84500001	0.3256	0.5388
NC_005104.4_84600001	5	84600001	0.3361	0.4946
NC_005104.4_84700001	5	84700001	0.3594	0.4932
NC_005104.4_84800001	5	84800001	0.3594	0.4932
NC_005104.4_84900001	5	84900001	0.3665	0.5192
NC_005104.4_85000001	5	85000001	0.3785	0.4691
NC_005104.4_85100001	5	85100001	0.4192	0.4971
NC_005104.4_85200001	5	85200001	0.3985	0.4597
NC_005104.4_85300001	5	85300001	0.535	0.6095
NC_005104.4_85400001	5	85400001	0.6027	0.6422
NC_005104.4_85500001	5	85500001	0.6076	0.7346
NC_005104.4_85600001	5	85600001	0.6339	0.8101
NC_005104.4_85700001	5	85700001	0.6121	0.8465
NC_005104.4_85800001	5	85800001	0.4705	0.706
NC_005104.4_85900001	5	85900001	0.4501	0.6502
NC_005104.4_86000001	5	86000001	0.4435	0.6017
NC_005104.4_86100001	5	86100001	0.4391	0.6355
NC_005104.4_86200001	5	86200001	0.4642	0.6292
NC_005104.4_86300001	5	86300001	0.4555	0.6768
NC_005104.4_86400001	5	86400001	0.4958	0.7953
NC_005104.4_86500001	5	86500001	0.5687	0.9261
NC_005104.4_86600001	5	86600001	0.6002	0.8505

NC_005104.4_86700001	5	86700001	0.4914	0.8225
NC_005104.4_86800001	5	86800001	0.5782	0.8162
NC_005104.4_86900001	5	86900001	0.5782	0.8162
NC_005104.4_87000001	5	87000001	0.5996	0.7758
NC_005104.4_87100001	5	87100001	0.5353	0.7793
NC_005104.4_87200001	5	87200001	0.717	0.7638
NC_005104.4_87300001	5	87300001	0.7752	0.8223
NC_005104.4_87400001	5	87400001	0.7997	0.8415
NC_005104.4_87500001	5	87500001	0.8308	0.8691
NC_005104.4_87600001	5	87600001	0.762	0.7802
NC_005104.4_87700001	5	87700001	0.5778	0.6355
NC_005104.4_87800001	5	87800001	0.5277	0.5551
NC_005104.4_87900001	5	87900001	0.4729	0.511
NC_005104.4_88000001	5	88000001	0.3715	0.3224
NC_005104.4_88100001	5	88100001	0.4701	0.5368
NC_005104.4_88200001	5	88200001	0.4416	0.6149
NC_005104.4_88300001	5	88300001	0.5508	0.7596
NC_005104.4_88400001	5	88400001	0.5524	0.738
NC_005104.4_88500001	5	88500001	0.4902	0.7081
NC_005104.4_88600001	5	88600001	0.3847	0.5622
NC_005104.4_88700001	5	88700001	0.5835	0.7449
NC_005104.4_88800001	5	88800001	0.5163	0.6995
NC_005104.4_88900001	5	88900001	0.6404	0.8016
NC_005104.4_89100001	5	89100001	0.831	0.9556
NC_005104.4_89200001	5	89200001	0.5955	0.7742
NC_005104.4_89300001	5	89300001	0.5296	0.7216
NC_005104.4_89400001	5	89400001	0.4186	0.5773
NC_005104.4_89500001	5	89500001	0.3762	0.5408
NC_005104.4_89600001	5	89600001	0.4132	0.5448
NC_005104.4_89700001	5	89700001	0.625	0.7728
NC_005104.4_89800001	5	89800001	0.5659	0.6925
NC_005104.4_89900001	5	89900001	0.4787	0.7633
NC_005104.4_90000001	5	90000001	0.5276	0.8228
NC_005104.4_90100001	5	90100001	0.4021	0.7448
NC_005104.4_90200001	5	90200001	0.2876	0.6201
NC_005104.4_90300001	5	90300001	0.2908	0.715
NC_005104.4_90400001	5	90400001	0.2801	0.4365
NC_005104.4_90900001	5	90900001	0.5035	0.7398
NC_005104.4_91000001	5	91000001	0.4804	0.5722
NC_005104.4_91100001	5	91100001	0.4899	0.6264
NC_005104.4_91200001	5	91200001	0.4899	0.6264
NC_005104.4_91300001	5	91300001	0.4728	0.6599
NC_005104.4_91400001	5	91400001	0.4844	0.5972
NC_005104.4_91500001	5	91500001	0.5694	0.8313
NC_005104.4_91600001	5	91600001	0.5377	0.7637
NC_005104.4_91700001	5	91700001	0.5377	0.7637
NC_005104.4_91800001	5	91800001	0.4624	0.7482
NC_005104.4_91900001	5	91900001	0.4615	0.7165
NC_005104.4_92000001	5	92000001	0.3031	0.5596
NC_005104.4_92100001	5	92100001	0.3484	0.5709
NC_005104.4_92200001	5	92200001	0.3484	0.5709
NC_005104.4_92300001	5	92300001	0.4499	0.5981
NC_005104.4_92400001	5	92400001	0.3856	0.5107
NC_005104.4_92500001	5	92500001	0.473	0.5308
NC_005104.4_92600001	5	92600001	0.5071	0.5769
NC_005104.4_92700001	5	92700001	0.5937	0.6753
NC_005104.4_92800001	5	92800001	0.6522	0.7524
NC_005104.4_92900001	5	92900001	0.6383	0.8144

NC_005104.4_93000001	5	93000001	0.6599	0.8264
NC_005104.4_93100001	5	93100001	0.6022	0.801
NC_005104.4_93200001	5	93200001	0.5625	0.7197
NC_005104.4_93300001	5	93300001	0.5664	0.6668
NC_005104.4_93400001	5	93400001	0.5945	0.6637
NC_005104.4_93500001	5	93500001	0.4884	0.5604
NC_005104.4_93600001	5	93600001	0.5334	0.4997
NC_005104.4_93700001	5	93700001	0.5416	0.5834
NC_005104.4_93800001	5	93800001	0.5319	0.7222
NC_005104.4_93900001	5	93900001	0.471	0.6901
NC_005104.4_94000001	5	94000001	0.5873	0.7284
NC_005104.4_94100001	5	94100001	0.6552	0.8532
NC_005104.4_94200001	5	94200001	0.6552	0.8532
NC_005104.4_94500001	5	94500001	0.5931	0.7797
NC_005104.4_94600001	5	94600001	0.5931	0.7797
NC_005104.4_94700001	5	94700001	0.4442	0.7325
NC_005104.4_94800001	5	94800001	0.4442	0.7325
NC_005104.4_94900001	5	94900001	0.5021	0.7832
NC_005104.4_95000001	5	95000001	0.4732	0.8401
NC_005104.4_95100001	5	95100001	0.4972	0.8191
NC_005104.4_95200001	5	95200001	0.5822	0.8868
NC_005104.4_95300001	5	95300001	0.5822	0.8868
NC_005104.4_95400001	5	95400001	0.327	0.513
NC_005104.4_95500001	5	95500001	0.327	0.513
NC_005104.4_95600001	5	95600001	0.2895	0.3514
NC_005104.4_95700001	5	95700001	0.2456	0.1868
NC_005104.4_95800001	5	95800001	0.2238	0.1805
NC_005104.4_95900001	5	95900001	0.1808	0.1671
NC_005104.4_96000001	5	96000001	0.1743	0.1391
NC_005104.4_96100001	5	96100001	0.092	0.0434
NC_005104.4_96200001	5	96200001	0.195	0.4142
NC_005104.4_96300001	5	96300001	0.2287	0.3761
NC_005104.4_96400001	5	96400001	0.3399	0.517
NC_005104.4_96500001	5	96500001	0.4062	0.6238
NC_005104.4_96600001	5	96600001	0.4062	0.6238
NC_005104.4_97300001	5	97300001	0.3931	0.1833
NC_005104.4_97400001	5	97400001	0.3931	0.1833
NC_005104.4_97500001	5	97500001	0.3931	0.1833
NC_005104.4_97600001	5	97600001	0.6085	0.646
NC_005104.4_97700001	5	97700001	0.6169	0.7196
NC_005104.4_97800001	5	97800001	0.659	0.7707
NC_005104.4_97900001	5	97900001	0.5362	0.6939
NC_005104.4_98000001	5	98000001	0.5019	0.6776
NC_005104.4_98100001	5	98100001	0.39	0.546
NC_005104.4_98200001	5	98200001	0.39	0.546
NC_005104.4_98600001	5	98600001	0.7043	0.8173
NC_005104.4_98700001	5	98700001	0.6889	0.7381
NC_005104.4_98800001	5	98800001	0.6889	0.7381
NC_005104.4_98900001	5	98900001	0.6363	0.7468
NC_005104.4_99000001	5	99000001	0.6911	0.8284
NC_005104.4_99100001	5	99100001	0.5331	0.688
NC_005104.4_99200001	5	99200001	0.5189	0.7214
NC_005104.4_99300001	5	99300001	0.4609	0.7165
NC_005104.4_99400001	5	99400001	0.4876	0.7371
NC_005104.4_99500001	5	99500001	0.4924	0.7241
NC_005104.4_99600001	5	99600001	0.5162	0.7272
NC_005104.4_99700001	5	99700001	0.5447	0.7487
NC_005104.4_99800001	5	99800001	0.5888	0.7575

NC_005104.4_99900001	5	99900001	0.5917	0.7127
NC_005104.4_100000001	5	100000001	0.6262	0.7739
NC_005104.4_100100001	5	100100001	0.6889	0.8615
NC_005104.4_100200001	5	100200001	0.592	0.7331
NC_005104.4_100300001	5	100300001	0.5288	0.7029
NC_005104.4_100400001	5	100400001	0.5231	0.6577
NC_005104.4_100500001	5	100500001	0.4895	0.6012
NC_005104.4_100600001	5	100600001	0.5062	0.6367
NC_005104.4_100700001	5	100700001	0.4965	0.669
NC_005104.4_100800001	5	100800001	0.5565	0.625
NC_005104.4_100900001	5	100900001	0.4973	0.64
NC_005104.4_101000001	5	101000001	0.5114	0.6221
NC_005104.4_101100001	5	101100001	0.4701	0.5746
NC_005104.4_101200001	5	101200001	0.4935	0.5665
NC_005104.4_101300001	5	101300001	0.4368	0.5579
NC_005104.4_101400001	5	101400001	0.4508	0.5425
NC_005104.4_101500001	5	101500001	0.3461	0.4559
NC_005104.4_101600001	5	101600001	0.3454	0.4662
NC_005104.4_101700001	5	101700001	0.4469	0.5985
NC_005104.4_101800001	5	101800001	0.6513	0.7737
NC_005104.4_101900001	5	101900001	0.5744	0.7557
NC_005104.4_102000001	5	102000001	0.6221	0.7903
NC_005104.4_102100001	5	102100001	0.6386	0.782
NC_005104.4_102200001	5	102200001	0.6081	0.7994
NC_005104.4_102300001	5	102300001	0.5176	0.7704
NC_005104.4_102400001	5	102400001	0.585	0.7743
NC_005104.4_102500001	5	102500001	0.5985	0.7496
NC_005104.4_102600001	5	102600001	0.5254	0.7516
NC_005104.4_102700001	5	102700001	0.5356	0.6231
NC_005104.4_102800001	5	102800001	0.5356	0.6231
NC_005104.4_102900001	5	102900001	0.5628	0.5599
NC_005104.4_103000001	5	103000001	0.4951	0.4484
NC_005104.4_103100001	5	103100001	0.5039	0.4443
NC_005104.4_103200001	5	103200001	0.4557	0.4678
NC_005104.4_103300001	5	103300001	0.4557	0.4678
NC_005104.4_103400001	5	103400001	0.4578	0.5576
NC_005104.4_103500001	5	103500001	0.5041	0.7147
NC_005104.4_103600001	5	103600001	0.6162	0.7984
NC_005104.4_103700001	5	103700001	0.6719	0.8048
NC_005104.4_103800001	5	103800001	0.5784	0.7506
NC_005104.4_103900001	5	103900001	0.52	0.7472
NC_005104.4_104000001	5	104000001	0.4884	0.7149
NC_005104.4_104100001	5	104100001	0.4162	0.6011
NC_005104.4_104200001	5	104200001	0.3779	0.5614
NC_005104.4_104300001	5	104300001	0.4198	0.6573
NC_005104.4_104400001	5	104400001	0.4493	0.6928
NC_005104.4_104500001	5	104500001	0.4407	0.6996
NC_005104.4_104600001	5	104600001	0.4574	0.7253
NC_005104.4_104700001	5	104700001	0.4803	0.7696
NC_005104.4_104800001	5	104800001	0.4965	0.784
NC_005104.4_104900001	5	104900001	0.5914	0.7193
NC_005104.4_105000001	5	105000001	0.4846	0.7118
NC_005104.4_105100001	5	105100001	0.5267	0.7995
NC_005104.4_105200001	5	105200001	0.5674	0.7736
NC_005104.4_105300001	5	105300001	0.5304	0.6676
NC_005104.4_105400001	5	105400001	0.5202	0.7165
NC_005104.4_105500001	5	105500001	0.6066	0.746
NC_005104.4_105600001	5	105600001	0.5065	0.5678

NC_005104.4_105700001	5	105700001	0.4398	0.5111
NC_005104.4_105800001	5	105800001	0.5004	0.5594
NC_005104.4_105900001	5	105900001	0.4713	0.5341
NC_005104.4_106000001	5	106000001	0.4623	0.4937
NC_005104.4_106100001	5	106100001	0.5869	0.6628
NC_005104.4_106200001	5	106200001	0.7298	0.7845
NC_005104.4_106300001	5	106300001	0.7745	0.8712
NC_005104.4_106400001	5	106400001	0.745	0.8549
NC_005104.4_106500001	5	106500001	0.7337	0.8882
NC_005104.4_106600001	5	106600001	0.683	0.8686
NC_005104.4_106700001	5	106700001	0.5685	0.7347
NC_005104.4_106800001	5	106800001	0.5839	0.68
NC_005104.4_106900001	5	106900001	0.6451	0.6986
NC_005104.4_107000001	5	107000001	0.6942	0.661
NC_005104.4_107100001	5	107100001	0.6942	0.661
NC_005104.4_107200001	5	107200001	0.7478	0.749
NC_005104.4_107300001	5	107300001	0.7396	0.837
NC_005104.4_107400001	5	107400001	0.6277	0.8677
NC_005104.4_107500001	5	107500001	0.5483	0.6693
NC_005104.4_107600001	5	107600001	0.5483	0.6693
NC_005104.4_107700001	5	107700001	0.5262	0.6174
NC_005104.4_107800001	5	107800001	0.4987	0.59
NC_005104.4_107900001	5	107900001	0.5193	0.5744
NC_005104.4_108000001	5	108000001	0.5683	0.6757
NC_005104.4_108100001	5	108100001	0.6293	0.6875
NC_005104.4_108200001	5	108200001	0.6755	0.7165
NC_005104.4_108400001	5	108400001	0.7583	0.696
NC_005104.4_108500001	5	108500001	0.5949	0.5801
NC_005104.4_108600001	5	108600001	0.4048	0.3641
NC_005104.4_108700001	5	108700001	0.5019	0.4416
NC_005104.4_108800001	5	108800001	0.3926	0.3975
NC_005104.4_108900001	5	108900001	0.3414	0.3224
NC_005104.4_109000001	5	109000001	0.3312	0.3312
NC_005104.4_109100001	5	109100001	0.3593	0.4269
NC_005104.4_109200001	5	109200001	0.2932	0.4403
NC_005104.4_109300001	5	109300001	0.4111	0.4789
NC_005104.4_109400001	5	109400001	0.4287	0.4874
NC_005104.4_109500001	5	109500001	0.4557	0.5458
NC_005104.4_109600001	5	109600001	0.438	0.4959
NC_005104.4_109700001	5	109700001	0.475	0.5062
NC_005104.4_109800001	5	109800001	0.5659	0.6635
NC_005104.4_109900001	5	109900001	0.5373	0.6834
NC_005104.4_110000001	5	110000001	0.5779	0.7297
NC_005104.4_110100001	5	110100001	0.5907	0.7731
NC_005104.4_110200001	5	110200001	0.5157	0.7545
NC_005104.4_110300001	5	110300001	0.4158	0.721
NC_005104.4_110400001	5	110400001	0.4408	0.794
NC_005104.4_110500001	5	110500001	0.4149	0.7322
NC_005104.4_110600001	5	110600001	0.443	0.7606
NC_005104.4_110700001	5	110700001	0.352	0.4287
NC_005104.4_110800001	5	110800001	0.324	0.3878
NC_005104.4_110900001	5	110900001	0.5016	0.6382
NC_005104.4_111000001	5	111000001	0.5016	0.6382
NC_005104.4_111100001	5	111100001	0.5608	0.7012
NC_005104.4_111200001	5	111200001	0.6906	0.8685
NC_005104.4_111300001	5	111300001	0.8117	0.941
NC_005104.4_111400001	5	111400001	0.5441	0.6307
NC_005104.4_111500001	5	111500001	0.5127	0.6264

NC_005104.4_111600001	5	111600001	0.5386	0.6186
NC_005104.4_111700001	5	111700001	0.5054	0.5783
NC_005104.4_111800001	5	111800001	0.4127	0.4501
NC_005104.4_111900001	5	111900001	0.6516	0.9612
NC_005104.4_112000001	5	112000001	0.7842	0.977
NC_005104.4_112100001	5	112100001	0.7224	0.8839
NC_005104.4_112200001	5	112200001	0.7224	0.8839
NC_005104.4_112300001	5	112300001	0.7224	0.8839
NC_005104.4_112500001	5	112500001	0.6046	0.7856
NC_005104.4_112600001	5	112600001	0.6256	0.8078
NC_005104.4_112700001	5	112700001	0.6872	0.8069
NC_005104.4_112800001	5	112800001	0.6518	0.8022
NC_005104.4_112900001	5	112900001	0.5969	0.7713
NC_005104.4_113000001	5	113000001	0.6626	0.762
NC_005104.4_113100001	5	113100001	0.6139	0.7092
NC_005104.4_113200001	5	113200001	0.4716	0.6293
NC_005104.4_113300001	5	113300001	0.5394	0.6409
NC_005104.4_113400001	5	113400001	0.6041	0.6836
NC_005104.4_113500001	5	113500001	0.6069	0.6751
NC_005104.4_113600001	5	113600001	0.675	0.7378
NC_005104.4_113700001	5	113700001	0.6805	0.7337
NC_005104.4_113800001	5	113800001	0.5864	0.6907
NC_005104.4_113900001	5	113900001	0.5686	0.6934
NC_005104.4_114000001	5	114000001	0.5587	0.7077
NC_005104.4_114100001	5	114100001	0.5751	0.7442
NC_005104.4_114200001	5	114200001	0.5567	0.7152
NC_005104.4_114300001	5	114300001	0.6201	0.7609
NC_005104.4_114400001	5	114400001	0.63	0.7888
NC_005104.4_114500001	5	114500001	0.6093	0.781
NC_005104.4_114600001	5	114600001	0.5258	0.6802
NC_005104.4_114700001	5	114700001	0.458	0.6779
NC_005104.4_114800001	5	114800001	0.4358	0.6694
NC_005104.4_114900001	5	114900001	0.2907	0.3806
NC_005104.4_115000001	5	115000001	0.4285	0.624
NC_005104.4_115100001	5	115100001	0.5419	0.766
NC_005104.4_115200001	5	115200001	0.5162	0.8094
NC_005104.4_115300001	5	115300001	0.4117	0.7003
NC_005104.4_115400001	5	115400001	0.4274	0.7153
NC_005104.4_115500001	5	115500001	0.3871	0.6063
NC_005104.4_115600001	5	115600001	0.3527	0.5105
NC_005104.4_115700001	5	115700001	0.4014	0.5436
NC_005104.4_115800001	5	115800001	0.4242	0.5855
NC_005104.4_115900001	5	115900001	0.5185	0.6673
NC_005104.4_116000001	5	116000001	0.5427	0.7166
NC_005104.4_116100001	5	116100001	0.5833	0.7272
NC_005104.4_116200001	5	116200001	0.5941	0.7525
NC_005104.4_116300001	5	116300001	0.5991	0.76
NC_005104.4_116400001	5	116400001	0.5526	0.6674
NC_005104.4_116500001	5	116500001	0.549	0.701
NC_005104.4_116600001	5	116600001	0.531	0.7579
NC_005104.4_116700001	5	116700001	0.5146	0.6769
NC_005104.4_116800001	5	116800001	0.5878	0.6834
NC_005104.4_116900001	5	116900001	0.5412	0.6185
NC_005104.4_117000001	5	117000001	0.5236	0.5301
NC_005104.4_117100001	5	117100001	0.5381	0.5772
NC_005104.4_117200001	5	117200001	0.5715	0.6395
NC_005104.4_117300001	5	117300001	0.5581	0.6452
NC_005104.4_117400001	5	117400001	0.5632	0.7329

NC_005104.4_117500001	5	117500001	0.5863	0.7915
NC_005104.4_117600001	5	117600001	0.5886	0.7871
NC_005104.4_117700001	5	117700001	0.6128	0.7672
NC_005104.4_117800001	5	117800001	0.5192	0.7202
NC_005104.4_117900001	5	117900001	0.5672	0.7046
NC_005104.4_118000001	5	118000001	0.5402	0.6999
NC_005104.4_118100001	5	118100001	0.5295	0.6711
NC_005104.4_118200001	5	118200001	0.5202	0.6597
NC_005104.4_118300001	5	118300001	0.5685	0.6756
NC_005104.4_118400001	5	118400001	0.5823	0.6962
NC_005104.4_118500001	5	118500001	0.6391	0.6402
NC_005104.4_118600001	5	118600001	0.6218	0.55
NC_005104.4_118700001	5	118700001	0.6568	0.6057
NC_005104.4_118800001	5	118800001	0.581	0.6464
NC_005104.4_118900001	5	118900001	0.4998	0.59
NC_005104.4_119000001	5	119000001	0.5376	0.6345
NC_005104.4_119100001	5	119100001	0.5775	0.7342
NC_005104.4_119200001	5	119200001	0.5117	0.6779
NC_005104.4_119300001	5	119300001	0.5474	0.657
NC_005104.4_119400001	5	119400001	0.531	0.7093
NC_005104.4_119500001	5	119500001	0.4726	0.6662
NC_005104.4_119600001	5	119600001	0.5538	0.7718
NC_005104.4_119700001	5	119700001	0.6275	0.7772
NC_005104.4_119800001	5	119800001	0.5719	0.7479
NC_005104.4_119900001	5	119900001	0.6496	0.7166
NC_005104.4_120000001	5	120000001	0.6857	0.7414
NC_005104.4_120100001	5	120100001	0.6204	0.6675
NC_005104.4_120200001	5	120200001	0.5869	0.6714
NC_005104.4_120300001	5	120300001	0.6576	0.697
NC_005104.4_120400001	5	120400001	0.7354	0.6977
NC_005104.4_120500001	5	120500001	0.6501	0.6659
NC_005104.4_120600001	5	120600001	0.6409	0.671
NC_005104.4_120700001	5	120700001	0.6409	0.671
NC_005104.4_120800001	5	120800001	0.6409	0.671
NC_005104.4_120900001	5	120900001	0.5938	0.7294
NC_005104.4_121200001	5	121200001	0.6005	0.6312
NC_005104.4_121300001	5	121300001	0.5365	0.5956
NC_005104.4_121400001	5	121400001	0.6139	0.6858
NC_005104.4_121500001	5	121500001	0.6404	0.6989
NC_005104.4_121600001	5	121600001	0.6271	0.7085
NC_005104.4_121700001	5	121700001	0.4908	0.6507
NC_005104.4_121800001	5	121800001	0.5036	0.6879
NC_005104.4_121900001	5	121900001	0.418	0.5125
NC_005104.4_122000001	5	122000001	0.4606	0.5792
NC_005104.4_122100001	5	122100001	0.4431	0.5778
NC_005104.4_122200001	5	122200001	0.5343	0.6136
NC_005104.4_122300001	5	122300001	0.5527	0.6579
NC_005104.4_122400001	5	122400001	0.6008	0.7894
NC_005104.4_122500001	5	122500001	0.554	0.7089
NC_005104.4_122600001	5	122600001	0.5703	0.6769
NC_005104.4_122700001	5	122700001	0.5144	0.611
NC_005104.4_122800001	5	122800001	0.4791	0.5513
NC_005104.4_122900001	5	122900001	0.5105	0.5737
NC_005104.4_123000001	5	123000001	0.4902	0.599
NC_005104.4_123100001	5	123100001	0.4849	0.6172
NC_005104.4_123200001	5	123200001	0.4681	0.6156
NC_005104.4_123300001	5	123300001	0.4966	0.6283
NC_005104.4_123400001	5	123400001	0.5022	0.5979

NC_005104.4_123500001	5	123500001	0.5864	0.6307
NC_005104.4_123600001	5	123600001	0.5925	0.6697
NC_005104.4_123700001	5	123700001	0.6409	0.7079
NC_005104.4_123800001	5	123800001	0.6646	0.7702
NC_005104.4_123900001	5	123900001	0.6218	0.7677
NC_005104.4_124000001	5	124000001	0.5954	0.7718
NC_005104.4_124100001	5	124100001	0.5796	0.7832
NC_005104.4_124200001	5	124200001	0.5837	0.8134
NC_005104.4_124300001	5	124300001	0.5769	0.7637
NC_005104.4_124400001	5	124400001	0.607	0.7534
NC_005104.4_124500001	5	124500001	0.5893	0.7376
NC_005104.4_124600001	5	124600001	0.613	0.7457
NC_005104.4_124700001	5	124700001	0.601	0.699
NC_005104.4_124800001	5	124800001	0.5578	0.6757
NC_005104.4_124900001	5	124900001	0.4422	0.5453
NC_005104.4_125000001	5	125000001	0.403	0.526
NC_005104.4_125100001	5	125100001	0.3541	0.4626
NC_005104.4_125200001	5	125200001	0.2788	0.4064
NC_005104.4_125300001	5	125300001	0.3328	0.471
NC_005104.4_125400001	5	125400001	0.3805	0.5611
NC_005104.4_125500001	5	125500001	0.4389	0.5475
NC_005104.4_125600001	5	125600001	0.4629	0.6273
NC_005104.4_125700001	5	125700001	0.5027	0.6668
NC_005104.4_125800001	5	125800001	0.5792	0.7448
NC_005104.4_125900001	5	125900001	0.5628	0.738
NC_005104.4_126000001	5	126000001	0.5587	0.7219
NC_005104.4_126100001	5	126100001	0.6045	0.712
NC_005104.4_126200001	5	126200001	0.6443	0.7359
NC_005104.4_126300001	5	126300001	0.5743	0.6388
NC_005104.4_126400001	5	126400001	0.5239	0.6463
NC_005104.4_126500001	5	126500001	0.5058	0.6764
NC_005104.4_126600001	5	126600001	0.4972	0.6642
NC_005104.4_126700001	5	126700001	0.3826	0.538
NC_005104.4_126800001	5	126800001	0.3462	0.5638
NC_005104.4_126900001	5	126900001	0.3957	0.5432
NC_005104.4_127000001	5	127000001	0.3943	0.5212
NC_005104.4_127100001	5	127100001	0.3984	0.5347
NC_005104.4_127200001	5	127200001	0.4675	0.6085
NC_005104.4_127300001	5	127300001	0.5391	0.6027
NC_005104.4_127400001	5	127400001	0.4529	0.5888
NC_005104.4_127500001	5	127500001	0.4766	0.6108
NC_005104.4_127600001	5	127600001	0.4532	0.581
NC_005104.4_127700001	5	127700001	0.4092	0.5133
NC_005104.4_127800001	5	127800001	0.4533	0.6193
NC_005104.4_127900001	5	127900001	0.6379	0.6244
NC_005104.4_128000001	5	128000001	0.6249	0.6579
NC_005104.4_128100001	5	128100001	0.6774	0.7192
NC_005104.4_128200001	5	128200001	0.6419	0.7602
NC_005104.4_128300001	5	128300001	0.6675	0.755
NC_005104.4_128400001	5	128400001	0.5953	0.7885
NC_005104.4_128500001	5	128500001	0.6318	0.7924
NC_005104.4_128600001	5	128600001	0.5897	0.8034
NC_005104.4_128700001	5	128700001	0.6207	0.74
NC_005104.4_128800001	5	128800001	0.5302	0.5776
NC_005104.4_128900001	5	128900001	0.6178	0.6265
NC_005104.4_129000001	5	129000001	0.6178	0.6265
NC_005104.4_129100001	5	129100001	0.7357	0.6984
NC_005104.4_129200001	5	129200001	0.7973	0.7458

NC_005104.4_129300001	5	129300001	0.8058	0.8483
NC_005104.4_129400001	5	129400001	0.85	0.8876
NC_005104.4_129500001	5	129500001	0.8388	0.852
NC_005104.4_129600001	5	129600001	0.6778	0.8074
NC_005104.4_129700001	5	129700001	0.593	0.7684
NC_005104.4_129800001	5	129800001	0.5435	0.7513
NC_005104.4_129900001	5	129900001	0.4727	0.6834
NC_005104.4_130000001	5	130000001	0.4391	0.6927
NC_005104.4_130100001	5	130100001	0.4163	0.6022
NC_005104.4_130200001	5	130200001	0.2493	0.3799
NC_005104.4_130300001	5	130300001	0.4467	0.6554
NC_005104.4_130400001	5	130400001	0.4545	0.6296
NC_005104.4_130500001	5	130500001	0.5212	0.6535
NC_005104.4_130600001	5	130600001	0.6277	0.7414
NC_005104.4_130700001	5	130700001	0.7772	0.8027
NC_005104.4_130800001	5	130800001	0.6622	0.7661
NC_005104.4_130900001	5	130900001	0.6355	0.7905
NC_005104.4_131000001	5	131000001	0.6386	0.8176
NC_005104.4_131100001	5	131100001	0.6348	0.8288
NC_005104.4_131200001	5	131200001	0.6007	0.8163
NC_005104.4_131300001	5	131300001	0.6249	0.8464
NC_005104.4_131400001	5	131400001	0.625	0.7632
NC_005104.4_131500001	5	131500001	0.5535	0.7085
NC_005104.4_131600001	5	131600001	0.4869	0.6351
NC_005104.4_131700001	5	131700001	0.3483	0.4358
NC_005104.4_131800001	5	131800001	0.3921	0.444
NC_005104.4_132700001	5	132700001	0.5173	0.6913
NC_005104.4_132800001	5	132800001	0.6052	0.7433
NC_005104.4_132900001	5	132900001	0.4964	0.661
NC_005104.4_133000001	5	133000001	0.4793	0.668
NC_005104.4_133100001	5	133100001	0.4909	0.6543
NC_005104.4_133200001	5	133200001	0.4764	0.5995
NC_005104.4_133300001	5	133300001	0.404	0.5378
NC_005104.4_133400001	5	133400001	0.4833	0.6227
NC_005104.4_133500001	5	133500001	0.4477	0.5922
NC_005104.4_133600001	5	133600001	0.4164	0.6149
NC_005104.4_133700001	5	133700001	0.3686	0.7517
NC_005104.4_133800001	5	133800001	0.445	0.7936
NC_005104.4_133900001	5	133900001	0.2632	0.5931
NC_005104.4_134000001	5	134000001	0.4541	0.6293
NC_005104.4_134100001	5	134100001	0.703	0.7906
NC_005104.4_134200001	5	134200001	0.6641	0.7751
NC_005104.4_134300001	5	134300001	0.5884	0.6957
NC_005104.4_134400001	5	134400001	0.5937	0.6997
NC_005104.4_134500001	5	134500001	0.5936	0.6808
NC_005104.4_134600001	5	134600001	0.5228	0.6077
NC_005104.4_134700001	5	134700001	0.5686	0.6448
NC_005104.4_134800001	5	134800001	0.629	0.7511
NC_005104.4_134900001	5	134900001	0.6683	0.7726
NC_005104.4_135000001	5	135000001	0.7685	0.8863
NC_005104.4_135100001	5	135100001	0.7497	0.9249
NC_005104.4_135200001	5	135200001	0.6125	0.8822
NC_005104.4_135300001	5	135300001	0.644	0.833
NC_005104.4_135400001	5	135400001	0.6211	0.849
NC_005104.4_135500001	5	135500001	0.5271	0.7974
NC_005104.4_135600001	5	135600001	0.5694	0.7284
NC_005104.4_135700001	5	135700001	0.6176	0.6991
NC_005104.4_135800001	5	135800001	0.5889	0.7047

NC_005104.4_135900001	5	135900001	0.5649	0.6513
NC_005104.4_136000001	5	136000001	0.6442	0.7177
NC_005104.4_136100001	5	136100001	0.6489	0.773
NC_005104.4_136200001	5	136200001	0.6229	0.7303
NC_005104.4_136300001	5	136300001	0.6411	0.7051
NC_005104.4_136400001	5	136400001	0.6958	0.7391
NC_005104.4_136500001	5	136500001	0.6591	0.6691
NC_005104.4_136600001	5	136600001	0.6215	0.6092
NC_005104.4_136700001	5	136700001	0.6338	0.6455
NC_005104.4_136800001	5	136800001	0.628	0.6561
NC_005104.4_136900001	5	136900001	0.5738	0.6204
NC_005104.4_137000001	5	137000001	0.5446	0.5584
NC_005104.4_137100001	5	137100001	0.5671	0.644
NC_005104.4_137200001	5	137200001	0.487	0.579
NC_005104.4_137300001	5	137300001	0.4963	0.5877
NC_005104.4_137400001	5	137400001	0.5134	0.6042
NC_005104.4_137500001	5	137500001	0.5265	0.7036
NC_005104.4_137600001	5	137600001	0.4826	0.5923
NC_005104.4_137700001	5	137700001	0.5427	0.6374
NC_005104.4_137800001	5	137800001	0.5493	0.6759
NC_005104.4_137900001	5	137900001	0.5723	0.6547
NC_005104.4_138000001	5	138000001	0.5713	0.6725
NC_005104.4_138100001	5	138100001	0.6098	0.6946
NC_005104.4_138200001	5	138200001	0.5921	0.6586
NC_005104.4_138300001	5	138300001	0.5926	0.5901
NC_005104.4_138400001	5	138400001	0.5475	0.5858
NC_005104.4_138500001	5	138500001	0.5438	0.5474
NC_005104.4_138600001	5	138600001	0.5785	0.564
NC_005104.4_138700001	5	138700001	0.4949	0.5458
NC_005104.4_138800001	5	138800001	0.47	0.5838
NC_005104.4_138900001	5	138900001	0.4427	0.5785
NC_005104.4_139000001	5	139000001	0.4956	0.6405
NC_005104.4_139100001	5	139100001	0.4482	0.6067
NC_005104.4_139200001	5	139200001	0.4712	0.676
NC_005104.4_139300001	5	139300001	0.4535	0.6558
NC_005104.4_139400001	5	139400001	0.4619	0.6394
NC_005104.4_139500001	5	139500001	0.4095	0.5762
NC_005104.4_139600001	5	139600001	0.4706	0.7027
NC_005104.4_139700001	5	139700001	0.5297	0.6698
NC_005104.4_139800001	5	139800001	0.6009	0.7378
NC_005104.4_139900001	5	139900001	0.6544	0.8344
NC_005104.4_140000001	5	140000001	0.607	0.8358
NC_005104.4_140100001	5	140100001	0.549	0.8239
NC_005104.4_140200001	5	140200001	0.5821	0.7645
NC_005104.4_140300001	5	140300001	0.6108	0.7813
NC_005104.4_140400001	5	140400001	0.5434	0.6431
NC_005104.4_140500001	5	140500001	0.4748	0.595
NC_005104.4_140600001	5	140600001	0.4834	0.6003
NC_005104.4_140700001	5	140700001	0.447	0.6231
NC_005104.4_140800001	5	140800001	0.456	0.6382
NC_005104.4_140900001	5	140900001	0.4876	0.6994
NC_005104.4_141000001	5	141000001	0.4928	0.6885
NC_005104.4_141100001	5	141100001	0.4742	0.6697
NC_005104.4_141200001	5	141200001	0.4118	0.5671
NC_005104.4_141300001	5	141300001	0.4257	0.5634
NC_005104.4_141400001	5	141400001	0.4857	0.6312
NC_005104.4_141500001	5	141500001	0.5447	0.6567
NC_005104.4_141600001	5	141600001	0.5999	0.6947

NC_005104.4_141700001	5	141700001	0.6402	0.7293
NC_005104.4_141800001	5	141800001	0.6281	0.7397
NC_005104.4_141900001	5	141900001	0.6222	0.7378
NC_005104.4_142000001	5	142000001	0.5866	0.6856
NC_005104.4_142100001	5	142100001	0.5419	0.662
NC_005104.4_142200001	5	142200001	0.4981	0.6342
NC_005104.4_142300001	5	142300001	0.4514	0.5775
NC_005104.4_142400001	5	142400001	0.4514	0.5775
NC_005104.4_142500001	5	142500001	0.5103	0.7383
NC_005104.4_142600001	5	142600001	0.5056	0.6394
NC_005104.4_142700001	5	142700001	0.5078	0.5978
NC_005104.4_142800001	5	142800001	0.5168	0.5275
NC_005104.4_142900001	5	142900001	0.4802	0.4831
NC_005104.4_143000001	5	143000001	0.4562	0.4826
NC_005104.4_143100001	5	143100001	0.4391	0.5114
NC_005104.4_143200001	5	143200001	0.4506	0.508
NC_005104.4_143300001	5	143300001	0.477	0.5974
NC_005104.4_143400001	5	143400001	0.4897	0.6393
NC_005104.4_143500001	5	143500001	0.4859	0.6648
NC_005104.4_143600001	5	143600001	0.4597	0.6237
NC_005104.4_143700001	5	143700001	0.487	0.6846
NC_005104.4_143800001	5	143800001	0.4715	0.6283
NC_005104.4_143900001	5	143900001	0.4492	0.5868
NC_005104.4_144000001	5	144000001	0.42	0.5151
NC_005104.4_144100001	5	144100001	0.4271	0.5065
NC_005104.4_144200001	5	144200001	0.4469	0.5278
NC_005104.4_144300001	5	144300001	0.3935	0.4205
NC_005104.4_144400001	5	144400001	0.5041	0.5476
NC_005104.4_144500001	5	144500001	0.5639	0.5848
NC_005104.4_144600001	5	144600001	0.6623	0.6477
NC_005104.4_144700001	5	144700001	0.672	0.6356
NC_005104.4_144800001	5	144800001	0.7469	0.7549
NC_005104.4_144900001	5	144900001	0.6363	0.6618
NC_005104.4_145000001	5	145000001	0.6675	0.709
NC_005104.4_145100001	5	145100001	0.6458	0.7213
NC_005104.4_145200001	5	145200001	0.591	0.6942
NC_005104.4_145300001	5	145300001	0.5832	0.7008
NC_005104.4_145400001	5	145400001	0.5563	0.705
NC_005104.4_145500001	5	145500001	0.4918	0.6277
NC_005104.4_145600001	5	145600001	0.4984	0.6049
NC_005104.4_145700001	5	145700001	0.4937	0.5927
NC_005104.4_145800001	5	145800001	0.5063	0.6227
NC_005104.4_145900001	5	145900001	0.5584	0.6389
NC_005104.4_146000001	5	146000001	0.5373	0.6313
NC_005104.4_146100001	5	146100001	0.5321	0.6132
NC_005104.4_146200001	5	146200001	0.547	0.6361
NC_005104.4_146300001	5	146300001	0.5897	0.6869
NC_005104.4_146400001	5	146400001	0.6057	0.7321
NC_005104.4_146500001	5	146500001	0.6475	0.7874
NC_005104.4_146600001	5	146600001	0.6045	0.83
NC_005104.4_146700001	5	146700001	0.5341	0.7627
NC_005104.4_146800001	5	146800001	0.4933	0.7017
NC_005104.4_146900001	5	146900001	0.5071	0.7174
NC_005104.4_147000001	5	147000001	0.494	0.6886
NC_005104.4_147100001	5	147100001	0.5654	0.6832
NC_005104.4_147200001	5	147200001	0.6862	0.7689
NC_005104.4_147300001	5	147300001	0.7231	0.7654
NC_005104.4_147400001	5	147400001	0.6277	0.6916

NC_005104.4_147500001	5	147500001	0.5608	0.6445
NC_005104.4_147600001	5	147600001	0.5405	0.6684
NC_005104.4_147700001	5	147700001	0.5016	0.6452
NC_005104.4_147800001	5	147800001	0.5113	0.6466
NC_005104.4_147900001	5	147900001	0.503	0.6268
NC_005104.4_148000001	5	148000001	0.5277	0.5999
NC_005104.4_148100001	5	148100001	0.5276	0.592
NC_005104.4_148200001	5	148200001	0.5	0.5395
NC_005104.4_148300001	5	148300001	0.4646	0.5478
NC_005104.4_148400001	5	148400001	0.4823	0.595
NC_005104.4_148500001	5	148500001	0.493	0.6461
NC_005104.4_148600001	5	148600001	0.3423	0.5563
NC_005104.4_148700001	5	148700001	0.3259	0.5638
NC_005104.4_148800001	5	148800001	0.3253	0.478
NC_005104.4_148900001	5	148900001	0.3088	0.4574
NC_005104.4_149000001	5	149000001	0.3286	0.4787
NC_005104.4_149100001	5	149100001	0.3956	0.4819
NC_005104.4_149200001	5	149200001	0.4282	0.5304
NC_005104.4_149300001	5	149300001	0.4035	0.5374
NC_005104.4_149400001	5	149400001	0.5129	0.6054
NC_005104.4_149500001	5	149500001	0.4652	0.594
NC_005104.4_149600001	5	149600001	0.4797	0.6215
NC_005104.4_149700001	5	149700001	0.4911	0.6263
NC_005104.4_149800001	5	149800001	0.5268	0.6517
NC_005104.4_149900001	5	149900001	0.3319	0.5197
NC_005104.4_150000001	5	150000001	0.3813	0.554
NC_005104.4_150100001	5	150100001	0.4046	0.6077
NC_005104.4_150200001	5	150200001	0.3877	0.5668
NC_005104.4_150300001	5	150300001	0.4307	0.6497
NC_005104.4_150400001	5	150400001	0.562	0.7386
NC_005104.4_150500001	5	150500001	0.5292	0.6805
NC_005104.4_150600001	5	150600001	0.4892	0.6547
NC_005104.4_150700001	5	150700001	0.5657	0.697
NC_005104.4_150800001	5	150800001	0.6036	0.7386
NC_005104.4_150900001	5	150900001	0.6166	0.7497
NC_005104.4_151000001	5	151000001	0.6412	0.796
NC_005104.4_151100001	5	151100001	0.6995	0.7958
NC_005104.4_151200001	5	151200001	0.6013	0.7522
NC_005104.4_151300001	5	151300001	0.6041	0.7428
NC_005104.4_151400001	5	151400001	0.6198	0.7283
NC_005104.4_151500001	5	151500001	0.7364	0.811
NC_005104.4_151600001	5	151600001	0.7175	0.8361
NC_005104.4_151700001	5	151700001	0.7763	0.8179
NC_005104.4_151800001	5	151800001	0.7344	0.8065
NC_005104.4_151900001	5	151900001	0.6977	0.8347
NC_005104.4_152000001	5	152000001	0.4984	0.674
NC_005104.4_152100001	5	152100001	0.4871	0.5871
NC_005104.4_152200001	5	152200001	0.4692	0.6097
NC_005104.4_152300001	5	152300001	0.481	0.5664
NC_005104.4_152400001	5	152400001	0.5118	0.5628
NC_005104.4_152500001	5	152500001	0.5906	0.6208
NC_005104.4_152600001	5	152600001	0.6548	0.6786
NC_005104.4_152700001	5	152700001	0.6733	0.6691
NC_005104.4_152800001	5	152800001	0.6408	0.6828
NC_005104.4_152900001	5	152900001	0.4523	0.5461
NC_005104.4_153000001	5	153000001	0.4714	0.5416
NC_005104.4_153100001	5	153100001	0.441	0.5384
NC_005104.4_153200001	5	153200001	0.4405	0.5599

NC_005104.4_153300001	5	153300001	0.5344	0.652
NC_005104.4_153400001	5	153400001	0.5684	0.6832
NC_005104.4_153500001	5	153500001	0.584	0.7441
NC_005104.4_153600001	5	153600001	0.5941	0.7555
NC_005104.4_153700001	5	153700001	0.6534	0.7566
NC_005104.4_153800001	5	153800001	0.6054	0.7302
NC_005104.4_153900001	5	153900001	0.6308	0.7617
NC_005104.4_154000001	5	154000001	0.5804	0.736
NC_005104.4_154100001	5	154100001	0.6246	0.752
NC_005104.4_154200001	5	154200001	0.5481	0.7007
NC_005104.4_154300001	5	154300001	0.4657	0.6844
NC_005104.4_154400001	5	154400001	0.3587	0.5342
NC_005104.4_154500001	5	154500001	0.3905	0.4728
NC_005104.4_154600001	5	154600001	0.3971	0.4966
NC_005104.4_154700001	5	154700001	0.3829	0.5094
NC_005104.4_154800001	5	154800001	0.4303	0.5178
NC_005104.4_154900001	5	154900001	0.5055	0.598
NC_005104.4_155000001	5	155000001	0.4574	0.6067
NC_005104.4_155100001	5	155100001	0.4527	0.5485
NC_005104.4_155200001	5	155200001	0.5317	0.5954
NC_005104.4_155300001	5	155300001	0.5649	0.6225
NC_005104.4_155400001	5	155400001	0.4812	0.5634
NC_005104.4_155500001	5	155500001	0.5486	0.5951
NC_005104.4_155600001	5	155600001	0.6033	0.6873
NC_005104.4_155700001	5	155700001	0.5347	0.6807
NC_005104.4_155800001	5	155800001	0.3669	0.6313
NC_005104.4_155900001	5	155900001	0.4174	0.6855
NC_005104.4_156000001	5	156000001	0.5315	0.7422
NC_005104.4_156100001	5	156100001	0.5407	0.7097
NC_005104.4_156200001	5	156200001	0.547	0.6967
NC_005104.4_156300001	5	156300001	0.63	0.7341
NC_005104.4_156400001	5	156400001	0.685	0.7797
NC_005104.4_156500001	5	156500001	0.6606	0.7134
NC_005104.4_156600001	5	156600001	0.6212	0.7285
NC_005104.4_156700001	5	156700001	0.5963	0.689
NC_005104.4_156800001	5	156800001	0.5121	0.6308
NC_005104.4_156900001	5	156900001	0.4754	0.5865
NC_005104.4_157000001	5	157000001	0.4418	0.547
NC_005104.4_157100001	5	157100001	0.4227	0.5766
NC_005104.4_157200001	5	157200001	0.4342	0.6115
NC_005104.4_157300001	5	157300001	0.4972	0.6921
NC_005104.4_157400001	5	157400001	0.5188	0.7161
NC_005104.4_157500001	5	157500001	0.6051	0.8162
NC_005104.4_157600001	5	157600001	0.6305	0.7322
NC_005104.4_157700001	5	157700001	0.5943	0.7212
NC_005104.4_157800001	5	157800001	0.592	0.7073
NC_005104.4_157900001	5	157900001	0.5432	0.6898
NC_005104.4_158000001	5	158000001	0.5065	0.6531
NC_005104.4_158100001	5	158100001	0.5343	0.7078
NC_005104.4_158200001	5	158200001	0.4887	0.623
NC_005104.4_158300001	5	158300001	0.5026	0.6581
NC_005104.4_158400001	5	158400001	0.5416	0.6873
NC_005104.4_158500001	5	158500001	0.5331	0.6406
NC_005104.4_158600001	5	158600001	0.4689	0.5984
NC_005104.4_158700001	5	158700001	0.5196	0.6775
NC_005104.4_158800001	5	158800001	0.5076	0.6451
NC_005104.4_158900001	5	158900001	0.4514	0.5899
NC_005104.4_159000001	5	159000001	0.4538	0.6308

NC_005104.4_159100001	5	159100001	0.4734	0.66
NC_005104.4_159200001	5	159200001	0.4458	0.6957
NC_005104.4_159300001	5	159300001	0.3921	0.6697
NC_005104.4_159400001	5	159400001	0.4365	0.6965
NC_005104.4_159500001	5	159500001	0.4704	0.7037
NC_005104.4_159600001	5	159600001	0.5121	0.7116
NC_005104.4_159700001	5	159700001	0.5193	0.6591
NC_005104.4_159800001	5	159800001	0.5557	0.6646
NC_005104.4_159900001	5	159900001	0.6316	0.7363
NC_005104.4_160000001	5	160000001	0.6143	0.7086
NC_005104.4_160100001	5	160100001	0.5574	0.7165
NC_005104.4_160200001	5	160200001	0.5481	0.733
NC_005104.4_160300001	5	160300001	0.5279	0.7281
NC_005104.4_160400001	5	160400001	0.4759	0.688
NC_005104.4_160500001	5	160500001	0.4353	0.6812
NC_005104.4_160600001	5	160600001	0.4566	0.6971
NC_005104.4_160700001	5	160700001	0.5018	0.6574
NC_005104.4_160800001	5	160800001	0.5389	0.6686
NC_005104.4_160900001	5	160900001	0.6358	0.7165
NC_005104.4_161000001	5	161000001	0.5637	0.6466
NC_005104.4_161100001	5	161100001	0.5902	0.6371
NC_005104.4_161200001	5	161200001	0.6271	0.7092
NC_005104.4_161300001	5	161300001	0.5736	0.6815
NC_005104.4_161400001	5	161400001	0.4899	0.6449
NC_005104.4_161500001	5	161500001	0.6066	0.7954
NC_005104.4_161600001	5	161600001	0.5686	0.7407
NC_005104.4_161700001	5	161700001	0.4707	0.6449
NC_005104.4_161800001	5	161800001	0.5077	0.6766
NC_005104.4_161900001	5	161900001	0.3877	0.5567
NC_005104.4_162000001	5	162000001	0.4529	0.5871
NC_005104.4_162100001	5	162100001	0.4186	0.5987
NC_005104.4_162200001	5	162200001	0.396	0.5608
NC_005104.4_162300001	5	162300001	0.4202	0.598
NC_005104.4_162400001	5	162400001	0.5773	0.678
NC_005104.4_162500001	5	162500001	0.6015	0.741
NC_005104.4_162600001	5	162600001	0.701	0.8029
NC_005104.4_162700001	5	162700001	0.7344	0.8139
NC_005104.4_162800001	5	162800001	0.6952	0.7657
NC_005104.4_162900001	5	162900001	0.5885	0.7357
NC_005104.4_163000001	5	163000001	0.5641	0.6925
NC_005104.4_163100001	5	163100001	0.4948	0.6128
NC_005104.4_163200001	5	163200001	0.4997	0.6372
NC_005104.4_164300001	5	164300001	0.3253	0.3876
NC_005104.4_164400001	5	164400001	0.1575	0.1322
NC_005104.4_164500001	5	164500001	0.1175	0.0997
NC_005104.4_164600001	5	164600001	0.1928	0.2365
NC_005104.4_164700001	5	164700001	0.2447	0.3019
NC_005104.4_164800001	5	164800001	0.2588	0.3467
NC_005104.4_164900001	5	164900001	0.2969	0.3587
NC_005104.4_165000001	5	165000001	0.4323	0.4975
NC_005104.4_165100001	5	165100001	0.4237	0.504
NC_005104.4_165200001	5	165200001	0.4631	0.5314
NC_005104.4_165300001	5	165300001	0.5068	0.5955
NC_005104.4_165400001	5	165400001	0.5806	0.6976
NC_005104.4_165500001	5	165500001	0.4637	0.6281
NC_005104.4_165600001	5	165600001	0.5199	0.6933
NC_005104.4_165700001	5	165700001	0.5059	0.7398
NC_005104.4_165800001	5	165800001	0.5225	0.7751

NC_005104.4_165900001	5	165900001	0.4717	0.7474
NC_005104.4_166000001	5	166000001	0.4932	0.6534
NC_005104.4_166100001	5	166100001	0.4275	0.5116
NC_005104.4_166200001	5	166200001	0.4732	0.5374
NC_005104.4_166300001	5	166300001	0.4721	0.5064
NC_005104.4_166400001	5	166400001	0.4771	0.5522
NC_005104.4_166500001	5	166500001	0.4936	0.6201
NC_005104.4_166600001	5	166600001	0.5102	0.7187
NC_005104.4_166700001	5	166700001	0.4396	0.7047
NC_005104.4_166800001	5	166800001	0.495	0.7747
NC_005104.4_166900001	5	166900001	0.5348	0.7956
NC_005104.4_167000001	5	167000001	0.5948	0.8769
NC_005104.4_167100001	5	167100001	0.6918	0.8911
NC_005104.4_167200001	5	167200001	0.8	0.8537
NC_005104.4_167300001	5	167300001	0.6435	0.7241
NC_005104.4_167400001	5	167400001	0.6078	0.6826
NC_005104.4_167500001	5	167500001	0.581	0.6775
NC_005104.4_167600001	5	167600001	0.486	0.5461
NC_005104.4_167700001	5	167700001	0.5602	0.671
NC_005104.4_167800001	5	167800001	0.6355	0.7053
NC_005104.4_167900001	5	167900001	0.6604	0.7284
NC_005104.4_168000001	5	168000001	0.664	0.7671
NC_005104.4_168100001	5	168100001	0.6406	0.8139
NC_005104.4_168200001	5	168200001	0.5713	0.7575
NC_005104.4_168300001	5	168300001	0.5251	0.7841
NC_005104.4_168400001	5	168400001	0.517	0.739
NC_005104.4_168500001	5	168500001	0.5144	0.7008
NC_005104.4_168600001	5	168600001	0.5462	0.6698
NC_005104.4_168700001	5	168700001	0.6055	0.7181
NC_005104.4_168800001	5	168800001	0.6766	0.7843
NC_005104.4_168900001	5	168900001	0.6831	0.7575
NC_005104.4_169000001	5	169000001	0.7122	0.7632
NC_005104.4_169100001	5	169100001	0.7816	0.817
NC_005104.4_169200001	5	169200001	0.6793	0.7513
NC_005104.4_169300001	5	169300001	0.5856	0.6665
NC_005104.4_169400001	5	169400001	0.5412	0.6553
NC_005104.4_169500001	5	169500001	0.5431	0.6529
NC_005104.4_169600001	5	169600001	0.4694	0.5876
NC_005104.4_169700001	5	169700001	0.491	0.6057
NC_005104.4_169800001	5	169800001	0.5089	0.5945
NC_005104.4_169900001	5	169900001	0.5209	0.6065
NC_005104.4_170000001	5	170000001	0.5273	0.5964
NC_005104.4_170100001	5	170100001	0.5665	0.6238
NC_005104.4_170200001	5	170200001	0.5529	0.6915
NC_005104.4_170300001	5	170300001	0.5521	0.6898
NC_005104.4_170400001	5	170400001	0.5364	0.6503
NC_005104.4_170500001	5	170500001	0.5271	0.6239
NC_005104.4_170600001	5	170600001	0.526	0.6097
NC_005104.4_170700001	5	170700001	0.5389	0.5935
NC_005104.4_170800001	5	170800001	0.4987	0.5438
NC_005104.4_170900001	5	170900001	0.485	0.5756
NC_005104.4_171000001	5	171000001	0.4983	0.6303
NC_005104.4_171100001	5	171100001	0.4803	0.6608
NC_005104.4_171200001	5	171200001	0.4745	0.6395
NC_005104.4_171300001	5	171300001	0.4971	0.619
NC_005104.4_171400001	5	171400001	0.4995	0.5993
NC_005104.4_171500001	5	171500001	0.4553	0.5688
NC_005104.4_171600001	5	171600001	0.3889	0.5142

NC_005104.4_171700001	5	171700001	0.4734	0.575
NC_005104.4_171800001	5	171800001	0.5061	0.6572
NC_005104.4_171900001	5	171900001	0.5406	0.6921
NC_005104.4_172000001	5	172000001	0.5833	0.6812
NC_005104.4_172100001	5	172100001	0.6739	0.7042
NC_005104.4_172200001	5	172200001	0.6613	0.7231
NC_005104.4_172300001	5	172300001	0.6557	0.6951
NC_005104.4_172400001	5	172400001	0.6583	0.7023
NC_005104.4_172500001	5	172500001	0.6232	0.6938
NC_005104.4_172600001	5	172600001	0.6154	0.7728
NC_005104.4_172700001	5	172700001	0.5853	0.7286
NC_005104.4_172800001	5	172800001	0.6458	0.8284
NC_005104.4_172900001	5	172900001	0.6832	0.8345
NC_005104.4_173000001	5	173000001	0.7772	0.8669
NC_005104.4_173100001	5	173100001	0.7059	0.7756
NC_005104.4_173200001	5	173200001	0.6934	0.7708
NC_005105.4_100001	6	100001	0.322	0.5641
NC_005105.4_200001	6	200001	0.3243	0.4902
NC_005105.4_300001	6	300001	0.4685	0.6208
NC_005105.4_400001	6	400001	0.5651	0.7088
NC_005105.4_500001	6	500001	0.5283	0.7473
NC_005105.4_600001	6	600001	0.5393	0.7079
NC_005105.4_700001	6	700001	0.5487	0.6881
NC_005105.4_800001	6	800001	0.545	0.703
NC_005105.4_900001	6	900001	0.5157	0.664
NC_005105.4_1000001	6	1000001	0.5282	0.6513
NC_005105.4_1100001	6	1100001	0.5938	0.7241
NC_005105.4_1200001	6	1200001	0.6221	0.84
NC_005105.4_1300001	6	1300001	0.524	0.7196
NC_005105.4_1400001	6	1400001	0.4493	0.5407
NC_005105.4_1500001	6	1500001	0.4925	0.479
NC_005105.4_1600001	6	1600001	0.5107	0.5514
NC_005105.4_1700001	6	1700001	0.4865	0.557
NC_005105.4_1800001	6	1800001	0.5721	0.637
NC_005105.4_1900001	6	1900001	0.6254	0.7205
NC_005105.4_2000001	6	2000001	0.611	0.7752
NC_005105.4_2100001	6	2100001	0.5821	0.7147
NC_005105.4_2200001	6	2200001	0.6438	0.7542
NC_005105.4_2300001	6	2300001	0.5884	0.7746
NC_005105.4_2400001	6	2400001	0.4982	0.6039
NC_005105.4_2500001	6	2500001	0.552	0.6704
NC_005105.4_2600001	6	2600001	0.5691	0.685
NC_005105.4_2700001	6	2700001	0.6656	0.7426
NC_005105.4_2800001	6	2800001	0.6088	0.712
NC_005105.4_2900001	6	2900001	0.6861	0.8684
NC_005105.4_3000001	6	3000001	0.71	0.8595
NC_005105.4_3100001	6	3100001	0.6096	0.7613
NC_005105.4_3200001	6	3200001	0.5876	0.6981
NC_005105.4_3300001	6	3300001	0.6147	0.7226
NC_005105.4_3400001	6	3400001	0.6406	0.715
NC_005105.4_3500001	6	3500001	0.5474	0.644
NC_005105.4_3600001	6	3600001	0.6134	0.7255
NC_005105.4_3700001	6	3700001	0.5419	0.7712
NC_005105.4_3800001	6	3800001	0.3895	0.5323
NC_005105.4_3900001	6	3900001	0.3068	0.4928
NC_005105.4_4000001	6	4000001	0.3846	0.5805
NC_005105.4_4200001	6	4200001	0.389	0.4427
NC_005105.4_4300001	6	4300001	0.3759	0.4815

NC_005105.4_4400001	6	4400001	0.5559	0.5488
NC_005105.4_4500001	6	4500001	0.5559	0.5488
NC_005105.4_4600001	6	4600001	0.5006	0.516
NC_005105.4_4700001	6	4700001	0.5024	0.5754
NC_005105.4_4800001	6	4800001	0.4772	0.5343
NC_005105.4_4900001	6	4900001	0.3657	0.3903
NC_005105.4_5000001	6	5000001	0.3386	0.3587
NC_005105.4_5100001	6	5100001	0.3657	0.3653
NC_005105.4_5200001	6	5200001	0.4373	0.4301
NC_005105.4_5300001	6	5300001	0.5085	0.4682
NC_005105.4_5400001	6	5400001	0.6025	0.5407
NC_005105.4_5500001	6	5500001	0.6863	0.5704
NC_005105.4_5600001	6	5600001	0.7196	0.6054
NC_005105.4_5700001	6	5700001	0.578	0.5269
NC_005105.4_5800001	6	5800001	0.475	0.5416
NC_005105.4_5900001	6	5900001	0.5479	0.618
NC_005105.4_6000001	6	6000001	0.497	0.5948
NC_005105.4_6100001	6	6100001	0.4757	0.6032
NC_005105.4_6200001	6	6200001	0.4809	0.6191
NC_005105.4_6300001	6	6300001	0.5103	0.6099
NC_005105.4_6400001	6	6400001	0.4895	0.6061
NC_005105.4_6500001	6	6500001	0.5606	0.5866
NC_005105.4_6600001	6	6600001	0.5687	0.5979
NC_005105.4_6700001	6	6700001	0.5947	0.5926
NC_005105.4_6800001	6	6800001	0.6049	0.619
NC_005105.4_6900001	6	6900001	0.4886	0.5512
NC_005105.4_7000001	6	7000001	0.484	0.5893
NC_005105.4_7100001	6	7100001	0.4723	0.5745
NC_005105.4_7200001	6	7200001	0.5438	0.6701
NC_005105.4_7300001	6	7300001	0.519	0.7116
NC_005105.4_7400001	6	7400001	0.6389	0.7583
NC_005105.4_7500001	6	7500001	0.5776	0.7079
NC_005105.4_7600001	6	7600001	0.6102	0.7674
NC_005105.4_7700001	6	7700001	0.4755	0.6416
NC_005105.4_7800001	6	7800001	0.4719	0.6103
NC_005105.4_7900001	6	7900001	0.4686	0.5798
NC_005105.4_8000001	6	8000001	0.5412	0.7112
NC_005105.4_8100001	6	8100001	0.5434	0.7441
NC_005105.4_8200001	6	8200001	0.6061	0.7667
NC_005105.4_8300001	6	8300001	0.6071	0.8013
NC_005105.4_8400001	6	8400001	0.4924	0.7737
NC_005105.4_8500001	6	8500001	0.5235	0.7648
NC_005105.4_8600001	6	8600001	0.4627	0.6884
NC_005105.4_8700001	6	8700001	0.4976	0.7329
NC_005105.4_8800001	6	8800001	0.4871	0.6703
NC_005105.4_8900001	6	8900001	0.5701	0.6847
NC_005105.4_9000001	6	9000001	0.6034	0.7174
NC_005105.4_9100001	6	9100001	0.6673	0.7418
NC_005105.4_9200001	6	9200001	0.6177	0.6923
NC_005105.4_9300001	6	9300001	0.5653	0.6933
NC_005105.4_9400001	6	9400001	0.497	0.627
NC_005105.4_9500001	6	9500001	0.4988	0.6493
NC_005105.4_9600001	6	9600001	0.5016	0.6652
NC_005105.4_9700001	6	9700001	0.4805	0.6339
NC_005105.4_9800001	6	9800001	0.4987	0.5865
NC_005105.4_9900001	6	9900001	0.6375	0.688
NC_005105.4_10000001	6	10000001	0.4949	0.5768
NC_005105.4_10100001	6	10100001	0.594	0.6733

NC_005105.4_10200001	6	10200001	0.6735	0.7656
NC_005105.4_10300001	6	10300001	0.7639	0.849
NC_005105.4_10400001	6	10400001	0.699	0.8395
NC_005105.4_10500001	6	10500001	0.6472	0.8372
NC_005105.4_10600001	6	10600001	0.611	0.7946
NC_005105.4_10700001	6	10700001	0.412	0.6059
NC_005105.4_10800001	6	10800001	0.4108	0.6011
NC_005105.4_10900001	6	10900001	0.4953	0.7114
NC_005105.4_11000001	6	11000001	0.5754	0.712
NC_005105.4_11100001	6	11100001	0.5827	0.7406
NC_005105.4_11200001	6	11200001	0.7024	0.8384
NC_005105.4_11300001	6	11300001	0.6157	0.8047
NC_005105.4_11400001	6	11400001	0.625	0.7918
NC_005105.4_11500001	6	11500001	0.602	0.7575
NC_005105.4_11600001	6	11600001	0.5191	0.7179
NC_005105.4_11700001	6	11700001	0.4499	0.6261
NC_005105.4_11800001	6	11800001	0.4801	0.547
NC_005105.4_11900001	6	11900001	0.4794	0.5396
NC_005105.4_12000001	6	12000001	0.4662	0.5732
NC_005105.4_12100001	6	12100001	0.5166	0.6374
NC_005105.4_12200001	6	12200001	0.5365	0.626
NC_005105.4_12300001	6	12300001	0.5155	0.6399
NC_005105.4_12400001	6	12400001	0.5315	0.647
NC_005105.4_12500001	6	12500001	0.5107	0.6361
NC_005105.4_12600001	6	12600001	0.5234	0.7032
NC_005105.4_12700001	6	12700001	0.4699	0.7132
NC_005105.4_12800001	6	12800001	0.5834	0.7897
NC_005105.4_12900001	6	12900001	0.5092	0.8504
NC_005105.4_13000001	6	13000001	0.6484	0.8746
NC_005105.4_13100001	6	13100001	0.6354	0.8608
NC_005105.4_13200001	6	13200001	0.609	0.7556
NC_005105.4_13300001	6	13300001	0.5139	0.6323
NC_005105.4_13400001	6	13400001	0.6002	0.6965
NC_005105.4_13500001	6	13500001	0.6829	0.7131
NC_005105.4_13600001	6	13600001	0.6829	0.7131
NC_005105.4_13700001	6	13700001	0.7651	0.7896
NC_005105.4_13900001	6	13900001	0.8654	0.9304
NC_005105.4_14000001	6	14000001	0.69	0.814
NC_005105.4_14100001	6	14100001	0.69	0.814
NC_005105.4_14200001	6	14200001	0.6517	0.7982
NC_005105.4_14300001	6	14300001	0.6517	0.7982
NC_005105.4_14400001	6	14400001	0.2879	0.4524
NC_005105.4_14500001	6	14500001	0.3655	0.7831
NC_005105.4_14600001	6	14600001	0.4073	0.7941
NC_005105.4_14700001	6	14700001	0.4766	0.8085
NC_005105.4_14800001	6	14800001	0.5538	0.8512
NC_005105.4_14900001	6	14900001	0.6143	0.8759
NC_005105.4_15000001	6	15000001	0.7249	0.8814
NC_005105.4_15100001	6	15100001	0.5994	0.8428
NC_005105.4_15200001	6	15200001	0.645	0.8937
NC_005105.4_15300001	6	15300001	0.5101	0.7828
NC_005105.4_15400001	6	15400001	0.5101	0.7828
NC_005105.4_15500001	6	15500001	0.5101	0.7828
NC_005105.4_15600001	6	15600001	0.7718	0.8588
NC_005105.4_15700001	6	15700001	0.7107	0.8179
NC_005105.4_17100001	6	17100001	0.9957	0.9935
NC_005105.4_17200001	6	17200001	0.7568	0.9427
NC_005105.4_17300001	6	17300001	0.7568	0.9427

NC_005105.4_17400001	6	17400001	0.7568	0.9427
NC_005105.4_17500001	6	17500001	0.7568	0.9427
NC_005105.4_19200001	6	19200001	0.405	0.8198
NC_005105.4_19300001	6	19300001	0.3254	0.6732
NC_005105.4_19400001	6	19400001	0.3254	0.6732
NC_005105.4_19500001	6	19500001	0.3254	0.6732
NC_005105.4_19600001	6	19600001	0.312	0.5893
NC_005105.4_19700001	6	19700001	0.228	0.2234
NC_005105.4_19800001	6	19800001	0.4167	0.4258
NC_005105.4_19900001	6	19900001	0.5527	0.6158
NC_005105.4_20000001	6	20000001	0.632	0.7095
NC_005105.4_20100001	6	20100001	0.6806	0.7211
NC_005105.4_20200001	6	20200001	0.7046	0.7927
NC_005105.4_20300001	6	20300001	0.6888	0.7731
NC_005105.4_20400001	6	20400001	0.6126	0.7256
NC_005105.4_20500001	6	20500001	0.5611	0.6421
NC_005105.4_20600001	6	20600001	0.5889	0.6863
NC_005105.4_20700001	6	20700001	0.5737	0.6405
NC_005105.4_20800001	6	20800001	0.5271	0.6797
NC_005105.4_20900001	6	20900001	0.5542	0.6962
NC_005105.4_21000001	6	21000001	0.5938	0.7356
NC_005105.4_21100001	6	21100001	0.5078	0.6451
NC_005105.4_21200001	6	21200001	0.514	0.6445
NC_005105.4_21300001	6	21300001	0.5392	0.6242
NC_005105.4_21400001	6	21400001	0.4997	0.6038
NC_005105.4_21500001	6	21500001	0.5037	0.6266
NC_005105.4_21600001	6	21600001	0.5906	0.74
NC_005105.4_21700001	6	21700001	0.6079	0.7507
NC_005105.4_21800001	6	21800001	0.6043	0.7471
NC_005105.4_21900001	6	21900001	0.5477	0.7656
NC_005105.4_22000001	6	22000001	0.4778	0.7342
NC_005105.4_22100001	6	22100001	0.4161	0.644
NC_005105.4_22200001	6	22200001	0.2578	0.604
NC_005105.4_22300001	6	22300001	0.3621	0.6528
NC_005105.4_22400001	6	22400001	0.4393	0.6194
NC_005105.4_22500001	6	22500001	0.4559	0.594
NC_005105.4_22600001	6	22600001	0.4633	0.6101
NC_005105.4_22700001	6	22700001	0.5005	0.5741
NC_005105.4_22800001	6	22800001	0.4816	0.584
NC_005105.4_22900001	6	22900001	0.4556	0.5754
NC_005105.4_23000001	6	23000001	0.4352	0.5782
NC_005105.4_23100001	6	23100001	0.4046	0.5787
NC_005105.4_23200001	6	23200001	0.4155	0.616
NC_005105.4_23300001	6	23300001	0.3837	0.501
NC_005105.4_23400001	6	23400001	0.4099	0.497
NC_005105.4_23500001	6	23500001	0.4677	0.5179
NC_005105.4_23600001	6	23600001	0.4574	0.4971
NC_005105.4_23700001	6	23700001	0.4517	0.4979
NC_005105.4_23800001	6	23800001	0.4787	0.5563
NC_005105.4_23900001	6	23900001	0.4997	0.5684
NC_005105.4_24000001	6	24000001	0.4596	0.5833
NC_005105.4_24100001	6	24100001	0.5115	0.614
NC_005105.4_24200001	6	24200001	0.5354	0.6654
NC_005105.4_24300001	6	24300001	0.4946	0.6338
NC_005105.4_24400001	6	24400001	0.4847	0.6662
NC_005105.4_24500001	6	24500001	0.4506	0.59
NC_005105.4_24600001	6	24600001	0.4245	0.5927
NC_005105.4_24700001	6	24700001	0.4399	0.6044

NC_005105.4_24800001	6	24800001	0.4729	0.6108
NC_005105.4_24900001	6	24900001	0.476	0.5883
NC_005105.4_25000001	6	25000001	0.5154	0.64
NC_005105.4_25100001	6	25100001	0.5146	0.6311
NC_005105.4_25200001	6	25200001	0.5137	0.6223
NC_005105.4_25300001	6	25300001	0.536	0.6751
NC_005105.4_25400001	6	25400001	0.5627	0.7263
NC_005105.4_25500001	6	25500001	0.5666	0.7405
NC_005105.4_25600001	6	25600001	0.6865	0.8022
NC_005105.4_25700001	6	25700001	0.7302	0.8337
NC_005105.4_25800001	6	25800001	0.7003	0.7498
NC_005105.4_25900001	6	25900001	0.6856	0.6863
NC_005105.4_26000001	6	26000001	0.7084	0.6811
NC_005105.4_26100001	6	26100001	0.713	0.6865
NC_005105.4_26200001	6	26200001	0.6395	0.7029
NC_005105.4_26300001	6	26300001	0.5951	0.7039
NC_005105.4_26400001	6	26400001	0.6228	0.82
NC_005105.4_26500001	6	26500001	0.6729	0.8416
NC_005105.4_26600001	6	26600001	0.5518	0.7279
NC_005105.4_26700001	6	26700001	0.6003	0.6655
NC_005105.4_26800001	6	26800001	0.4864	0.638
NC_005105.4_26900001	6	26900001	0.5251	0.637
NC_005105.4_27000001	6	27000001	0.5018	0.6452
NC_005105.4_27100001	6	27100001	0.5032	0.6532
NC_005105.4_27200001	6	27200001	0.5032	0.6616
NC_005105.4_27300001	6	27300001	0.5826	0.722
NC_005105.4_27400001	6	27400001	0.6085	0.7789
NC_005105.4_27500001	6	27500001	0.5152	0.726
NC_005105.4_27600001	6	27600001	0.549	0.7335
NC_005105.4_27700001	6	27700001	0.5306	0.6911
NC_005105.4_27800001	6	27800001	0.5013	0.6415
NC_005105.4_27900001	6	27900001	0.438	0.5836
NC_005105.4_28000001	6	28000001	0.5578	0.6747
NC_005105.4_28100001	6	28100001	0.5658	0.677
NC_005105.4_28200001	6	28200001	0.6137	0.6754
NC_005105.4_28300001	6	28300001	0.7569	0.8187
NC_005105.4_28400001	6	28400001	0.7531	0.7485
NC_005105.4_28500001	6	28500001	0.7389	0.7342
NC_005105.4_28600001	6	28600001	0.749	0.7905
NC_005105.4_28700001	6	28700001	0.7629	0.8748
NC_005105.4_28800001	6	28800001	0.6575	0.7896
NC_005105.4_28900001	6	28900001	0.6582	0.8349
NC_005105.4_29000001	6	29000001	0.5972	0.7224
NC_005105.4_29100001	6	29100001	0.6597	0.7611
NC_005105.4_29200001	6	29200001	0.6496	0.7223
NC_005105.4_29300001	6	29300001	0.5766	0.614
NC_005105.4_29400001	6	29400001	0.578	0.6429
NC_005105.4_29500001	6	29500001	0.5621	0.6686
NC_005105.4_29600001	6	29600001	0.479	0.5699
NC_005105.4_29700001	6	29700001	0.5673	0.6265
NC_005105.4_29800001	6	29800001	0.6041	0.6823
NC_005105.4_29900001	6	29900001	0.6129	0.6693
NC_005105.4_30000001	6	30000001	0.7544	0.7638
NC_005105.4_30100001	6	30100001	0.7334	0.7418
NC_005105.4_30800001	6	30800001	0.686	0.7482
NC_005105.4_30900001	6	30900001	0.5227	0.665
NC_005105.4_31000001	6	31000001	0.5227	0.665
NC_005105.4_31100001	6	31100001	0.4584	0.5923

NC_005105.4_31200001	6	31200001	0.4584	0.5923
NC_005105.4_31300001	6	31300001	0.2332	0.2852
NC_005105.4_31500001	6	31500001	0.3728	0.4661
NC_005105.4_31600001	6	31600001	0.536	0.6335
NC_005105.4_31700001	6	31700001	0.536	0.6335
NC_005105.4_31800001	6	31800001	0.6342	0.7309
NC_005105.4_31900001	6	31900001	0.6623	0.7232
NC_005105.4_32000001	6	32000001	0.771	0.7727
NC_005105.4_32100001	6	32100001	0.6862	0.729
NC_005105.4_32200001	6	32200001	0.638	0.7251
NC_005105.4_32300001	6	32300001	0.5822	0.7371
NC_005105.4_32400001	6	32400001	0.5023	0.7429
NC_005105.4_32500001	6	32500001	0.404	0.5989
NC_005105.4_32600001	6	32600001	0.4524	0.6677
NC_005105.4_32700001	6	32700001	0.4263	0.6547
NC_005105.4_32800001	6	32800001	0.4114	0.5824
NC_005105.4_32900001	6	32900001	0.4159	0.5377
NC_005105.4_33000001	6	33000001	0.5026	0.6717
NC_005105.4_33100001	6	33100001	0.4321	0.5691
NC_005105.4_33200001	6	33200001	0.4774	0.6104
NC_005105.4_33300001	6	33300001	0.4883	0.6625
NC_005105.4_33400001	6	33400001	0.5076	0.7059
NC_005105.4_33500001	6	33500001	0.3811	0.4948
NC_005105.4_33600001	6	33600001	0.4036	0.5252
NC_005105.4_33700001	6	33700001	0.4103	0.4989
NC_005105.4_33800001	6	33800001	0.3556	0.3789
NC_005105.4_33900001	6	33900001	0.3853	0.439
NC_005105.4_34000001	6	34000001	0.3847	0.5322
NC_005105.4_34100001	6	34100001	0.4066	0.557
NC_005105.4_34200001	6	34200001	0.3821	0.5203
NC_005105.4_34300001	6	34300001	0.4748	0.6043
NC_005105.4_34400001	6	34400001	0.4102	0.5653
NC_005105.4_34500001	6	34500001	0.4968	0.5965
NC_005105.4_34600001	6	34600001	0.5207	0.6276
NC_005105.4_34700001	6	34700001	0.5876	0.6847
NC_005105.4_34800001	6	34800001	0.5809	0.7188
NC_005105.4_34900001	6	34900001	0.5898	0.7068
NC_005105.4_35000001	6	35000001	0.5268	0.6676
NC_005105.4_35100001	6	35100001	0.5285	0.6771
NC_005105.4_35200001	6	35200001	0.5007	0.6643
NC_005105.4_35300001	6	35300001	0.4759	0.654
NC_005105.4_35400001	6	35400001	0.4892	0.7155
NC_005105.4_35500001	6	35500001	0.5871	0.8402
NC_005105.4_35600001	6	35600001	0.536	0.6941
NC_005105.4_35700001	6	35700001	0.5145	0.6936
NC_005105.4_35800001	6	35800001	0.4697	0.624
NC_005105.4_35900001	6	35900001	0.4868	0.6177
NC_005105.4_36000001	6	36000001	0.4958	0.5956
NC_005105.4_36100001	6	36100001	0.5366	0.6954
NC_005105.4_36200001	6	36200001	0.4769	0.6042
NC_005105.4_36300001	6	36300001	0.5697	0.7053
NC_005105.4_36400001	6	36400001	0.6655	0.772
NC_005105.4_36500001	6	36500001	0.6888	0.8084
NC_005105.4_36600001	6	36600001	0.7214	0.8046
NC_005105.4_36700001	6	36700001	0.7624	0.817
NC_005105.4_36800001	6	36800001	0.7701	0.8089
NC_005105.4_36900001	6	36900001	0.772	0.7865
NC_005105.4_37000001	6	37000001	0.7035	0.7387

NC_005105.4_37100001	6	37100001	0.5911	0.5973
NC_005105.4_37200001	6	37200001	0.6419	0.6909
NC_005105.4_37300001	6	37300001	0.5974	0.6418
NC_005105.4_37400001	6	37400001	0.447	0.5737
NC_005105.4_37500001	6	37500001	0.4642	0.6613
NC_005105.4_37600001	6	37600001	0.4148	0.5581
NC_005105.4_37700001	6	37700001	0.3538	0.437
NC_005105.4_37800001	6	37800001	0.3688	0.4865
NC_005105.4_37900001	6	37900001	0.4389	0.5333
NC_005105.4_38000001	6	38000001	0.4704	0.5037
NC_005105.4_38100001	6	38100001	0.5141	0.5868
NC_005105.4_38200001	6	38200001	0.5329	0.628
NC_005105.4_38300001	6	38300001	0.5189	0.6285
NC_005105.4_38400001	6	38400001	0.4974	0.6214
NC_005105.4_38500001	6	38500001	0.4472	0.6164
NC_005105.4_38600001	6	38600001	0.3508	0.5091
NC_005105.4_38700001	6	38700001	0.286	0.4866
NC_005105.4_38800001	6	38800001	0.3186	0.4076
NC_005105.4_38900001	6	38900001	0.3708	0.4949
NC_005105.4_39000001	6	39000001	0.3451	0.49
NC_005105.4_39100001	6	39100001	0.437	0.5818
NC_005105.4_39200001	6	39200001	0.4714	0.6488
NC_005105.4_39300001	6	39300001	0.3959	0.6327
NC_005105.4_39400001	6	39400001	0.3524	0.5757
NC_005105.4_39500001	6	39500001	0.3443	0.5232
NC_005105.4_39600001	6	39600001	0.3744	0.4695
NC_005105.4_39700001	6	39700001	0.3378	0.326
NC_005105.4_39800001	6	39800001	0.3294	0.3798
NC_005105.4_39900001	6	39900001	0.4074	0.5721
NC_005105.4_40000001	6	40000001	0.4915	0.6705
NC_005105.4_40100001	6	40100001	0.4959	0.7293
NC_005105.4_40200001	6	40200001	0.4959	0.7293
NC_005105.4_40300001	6	40300001	0.5463	0.6662
NC_005105.4_40400001	6	40400001	0.4621	0.4882
NC_005105.4_40500001	6	40500001	0.4919	0.4838
NC_005105.4_40600001	6	40600001	0.4574	0.4637
NC_005105.4_40700001	6	40700001	0.4595	0.4739
NC_005105.4_40800001	6	40800001	0.4026	0.5088
NC_005105.4_40900001	6	40900001	0.4776	0.6893
NC_005105.4_41000001	6	41000001	0.4892	0.6652
NC_005105.4_41100001	6	41100001	0.5527	0.7258
NC_005105.4_41200001	6	41200001	0.525	0.7567
NC_005105.4_41300001	6	41300001	0.6157	0.7805
NC_005105.4_41400001	6	41400001	0.7371	0.8362
NC_005105.4_41500001	6	41500001	0.6992	0.7965
NC_005105.4_41600001	6	41600001	0.616	0.73
NC_005105.4_41700001	6	41700001	0.6343	0.6937
NC_005105.4_41800001	6	41800001	0.6173	0.6716
NC_005105.4_41900001	6	41900001	0.5145	0.553
NC_005105.4_42000001	6	42000001	0.5235	0.5559
NC_005105.4_42100001	6	42100001	0.5126	0.5099
NC_005105.4_42200001	6	42200001	0.4904	0.4974
NC_005105.4_42300001	6	42300001	0.4725	0.5221
NC_005105.4_42400001	6	42400001	0.4364	0.4496
NC_005105.4_42500001	6	42500001	0.4616	0.506
NC_005105.4_42600001	6	42600001	0.4025	0.4682
NC_005105.4_42700001	6	42700001	0.449	0.5244
NC_005105.4_42800001	6	42800001	0.506	0.5417

NC_005105.4_42900001	6	42900001	0.5971	0.6691
NC_005105.4_43000001	6	43000001	0.566	0.6238
NC_005105.4_43100001	6	43100001	0.6206	0.6705
NC_005105.4_43200001	6	43200001	0.5508	0.6144
NC_005105.4_43300001	6	43300001	0.5536	0.6406
NC_005105.4_43400001	6	43400001	0.4061	0.4962
NC_005105.4_43500001	6	43500001	0.3367	0.4738
NC_005105.4_43600001	6	43600001	0.3766	0.4418
NC_005105.4_43700001	6	43700001	0.466	0.5641
NC_005105.4_43800001	6	43800001	0.4729	0.6123
NC_005105.4_43900001	6	43900001	0.5614	0.627
NC_005105.4_44000001	6	44000001	0.5599	0.6103
NC_005105.4_44100001	6	44100001	0.5424	0.6162
NC_005105.4_44200001	6	44200001	0.5984	0.6974
NC_005105.4_44300001	6	44300001	0.6175	0.6808
NC_005105.4_44400001	6	44400001	0.607	0.6701
NC_005105.4_44500001	6	44500001	0.5899	0.6285
NC_005105.4_44600001	6	44600001	0.6035	0.6606
NC_005105.4_44700001	6	44700001	0.5273	0.5658
NC_005105.4_44800001	6	44800001	0.5231	0.5139
NC_005105.4_44900001	6	44900001	0.525	0.5093
NC_005105.4_45000001	6	45000001	0.5394	0.5089
NC_005105.4_45100001	6	45100001	0.439	0.4048
NC_005105.4_45200001	6	45200001	0.4278	0.4133
NC_005105.4_45300001	6	45300001	0.3284	0.3062
NC_005105.4_45400001	6	45400001	0.2955	0.2935
NC_005105.4_45500001	6	45500001	0.3517	0.3905
NC_005105.4_45600001	6	45600001	0.3868	0.444
NC_005105.4_45700001	6	45700001	0.4476	0.5267
NC_005105.4_45800001	6	45800001	0.4593	0.4852
NC_005105.4_45900001	6	45900001	0.5658	0.5997
NC_005105.4_46000001	6	46000001	0.5375	0.6809
NC_005105.4_46100001	6	46100001	0.56	0.6689
NC_005105.4_46200001	6	46200001	0.5816	0.7081
NC_005105.4_46300001	6	46300001	0.5706	0.7681
NC_005105.4_46400001	6	46400001	0.5533	0.6642
NC_005105.4_46500001	6	46500001	0.5481	0.6658
NC_005105.4_46600001	6	46600001	0.5191	0.6497
NC_005105.4_46700001	6	46700001	0.5007	0.5976
NC_005105.4_46800001	6	46800001	0.5485	0.5981
NC_005105.4_46900001	6	46900001	0.5103	0.6507
NC_005105.4_47000001	6	47000001	0.4659	0.5952
NC_005105.4_47100001	6	47100001	0.5402	0.6511
NC_005105.4_47200001	6	47200001	0.5451	0.6514
NC_005105.4_47300001	6	47300001	0.5339	0.6807
NC_005105.4_47400001	6	47400001	0.5424	0.6819
NC_005105.4_47500001	6	47500001	0.5404	0.628
NC_005105.4_47600001	6	47600001	0.5063	0.5938
NC_005105.4_47700001	6	47700001	0.4779	0.6048
NC_005105.4_47800001	6	47800001	0.4444	0.5525
NC_005105.4_47900001	6	47900001	0.421	0.5232
NC_005105.4_48000001	6	48000001	0.4239	0.5917
NC_005105.4_48100001	6	48100001	0.4898	0.6885
NC_005105.4_48200001	6	48200001	0.6147	0.7474
NC_005105.4_48300001	6	48300001	0.696	0.7804
NC_005105.4_48400001	6	48400001	0.6601	0.7694
NC_005105.4_48500001	6	48500001	0.6986	0.7923
NC_005105.4_48600001	6	48600001	0.6747	0.7162

NC_005105.4_48700001	6	48700001	0.6177	0.7099
NC_005105.4_48800001	6	48800001	0.5188	0.6869
NC_005105.4_48900001	6	48900001	0.6021	0.733
NC_005105.4_49000001	6	49000001	0.5506	0.7118
NC_005105.4_49100001	6	49100001	0.5436	0.7341
NC_005105.4_49200001	6	49200001	0.501	0.6964
NC_005105.4_49300001	6	49300001	0.5385	0.6791
NC_005105.4_49400001	6	49400001	0.4149	0.533
NC_005105.4_49500001	6	49500001	0.4479	0.5195
NC_005105.4_49600001	6	49600001	0.4198	0.5215
NC_005105.4_49700001	6	49700001	0.4138	0.4954
NC_005105.4_49800001	6	49800001	0.3999	0.5069
NC_005105.4_49900001	6	49900001	0.4137	0.5345
NC_005105.4_50000001	6	50000001	0.4715	0.5776
NC_005105.4_50100001	6	50100001	0.5463	0.6105
NC_005105.4_50200001	6	50200001	0.5246	0.6094
NC_005105.4_50300001	6	50300001	0.5506	0.6041
NC_005105.4_50400001	6	50400001	0.5806	0.6197
NC_005105.4_50500001	6	50500001	0.562	0.6391
NC_005105.4_50600001	6	50600001	0.5126	0.6016
NC_005105.4_50700001	6	50700001	0.5931	0.6507
NC_005105.4_50800001	6	50800001	0.5561	0.6926
NC_005105.4_50900001	6	50900001	0.4832	0.6906
NC_005105.4_51000001	6	51000001	0.5804	0.7755
NC_005105.4_51100001	6	51100001	0.5716	0.739
NC_005105.4_51200001	6	51200001	0.5648	0.7212
NC_005105.4_51300001	6	51300001	0.5888	0.6981
NC_005105.4_51400001	6	51400001	0.6269	0.7046
NC_005105.4_51500001	6	51500001	0.5565	0.6058
NC_005105.4_51600001	6	51600001	0.532	0.6551
NC_005105.4_51700001	6	51700001	0.5396	0.6309
NC_005105.4_51800001	6	51800001	0.5231	0.5498
NC_005105.4_51900001	6	51900001	0.5503	0.5493
NC_005105.4_52000001	6	52000001	0.5857	0.6606
NC_005105.4_52100001	6	52100001	0.5852	0.6571
NC_005105.4_52200001	6	52200001	0.5751	0.6814
NC_005105.4_52300001	6	52300001	0.574	0.7381
NC_005105.4_52400001	6	52400001	0.5568	0.6956
NC_005105.4_52500001	6	52500001	0.5124	0.6281
NC_005105.4_52600001	6	52600001	0.5807	0.6851
NC_005105.4_52700001	6	52700001	0.4941	0.5415
NC_005105.4_52800001	6	52800001	0.4574	0.5827
NC_005105.4_52900001	6	52900001	0.615	0.7746
NC_005105.4_53000001	6	53000001	0.6203	0.7582
NC_005105.4_53100001	6	53100001	0.6406	0.7632
NC_005105.4_53200001	6	53200001	0.7075	0.8346
NC_005105.4_53300001	6	53300001	0.6784	0.8292
NC_005105.4_53400001	6	53400001	0.6556	0.7803
NC_005105.4_53500001	6	53500001	0.5828	0.7512
NC_005105.4_53600001	6	53600001	0.5502	0.7199
NC_005105.4_53700001	6	53700001	0.5437	0.7355
NC_005105.4_53800001	6	53800001	0.5896	0.7656
NC_005105.4_53900001	6	53900001	0.5227	0.7908
NC_005105.4_54000001	6	54000001	0.6585	0.8621
NC_005105.4_54100001	6	54100001	0.6437	0.7906
NC_005105.4_54200001	6	54200001	0.6855	0.7964
NC_005105.4_54300001	6	54300001	0.6817	0.7255
NC_005105.4_54400001	6	54400001	0.6723	0.7018

NC_005105.4_54500001	6	54500001	0.6295	0.6787
NC_005105.4_54600001	6	54600001	0.5834	0.7195
NC_005105.4_54700001	6	54700001	0.5503	0.6875
NC_005105.4_54800001	6	54800001	0.507	0.6897
NC_005105.4_54900001	6	54900001	0.5961	0.7193
NC_005105.4_55000001	6	55000001	0.5348	0.6744
NC_005105.4_55100001	6	55100001	0.5721	0.6782
NC_005105.4_55200001	6	55200001	0.5333	0.6686
NC_005105.4_55300001	6	55300001	0.4279	0.5789
NC_005105.4_55400001	6	55400001	0.3645	0.4824
NC_005105.4_55500001	6	55500001	0.4103	0.4321
NC_005105.4_55600001	6	55600001	0.4014	0.4106
NC_005105.4_55700001	6	55700001	0.4279	0.4138
NC_005105.4_55800001	6	55800001	0.5414	0.5276
NC_005105.4_55900001	6	55900001	0.5693	0.505
NC_005105.4_56000001	6	56000001	0.5643	0.5435
NC_005105.4_56100001	6	56100001	0.5331	0.5765
NC_005105.4_56200001	6	56200001	0.4559	0.5359
NC_005105.4_56300001	6	56300001	0.4002	0.4326
NC_005105.4_56400001	6	56400001	0.3374	0.3909
NC_005105.4_56500001	6	56500001	0.3413	0.4717
NC_005105.4_56600001	6	56600001	0.2838	0.3833
NC_005105.4_56700001	6	56700001	0.3653	0.4998
NC_005105.4_56800001	6	56800001	0.3843	0.5559
NC_005105.4_56900001	6	56900001	0.4918	0.6991
NC_005105.4_57000001	6	57000001	0.5444	0.6791
NC_005105.4_57100001	6	57100001	0.7547	0.8627
NC_005105.4_57200001	6	57200001	0.7403	0.8389
NC_005105.4_57300001	6	57300001	0.6929	0.839
NC_005105.4_57400001	6	57400001	0.681	0.8299
NC_005105.4_57500001	6	57500001	0.5665	0.7463
NC_005105.4_57600001	6	57600001	0.5102	0.6602
NC_005105.4_57700001	6	57700001	0.485	0.6495
NC_005105.4_57800001	6	57800001	0.396	0.5501
NC_005105.4_57900001	6	57900001	0.3758	0.5011
NC_005105.4_58000001	6	58000001	0.4399	0.6194
NC_005105.4_58100001	6	58100001	0.3614	0.5281
NC_005105.4_58200001	6	58200001	0.3804	0.5702
NC_005105.4_58300001	6	58300001	0.3519	0.4897
NC_005105.4_58400001	6	58400001	0.4687	0.6462
NC_005105.4_58500001	6	58500001	0.4062	0.5755
NC_005105.4_58600001	6	58600001	0.4466	0.6145
NC_005105.4_58700001	6	58700001	0.4247	0.5387
NC_005105.4_58800001	6	58800001	0.5018	0.6912
NC_005105.4_58900001	6	58900001	0.4939	0.727
NC_005105.4_59000001	6	59000001	0.579	0.747
NC_005105.4_59100001	6	59100001	0.579	0.747
NC_005105.4_59200001	6	59200001	0.5114	0.6896
NC_005105.4_59300001	6	59300001	0.5466	0.7028
NC_005105.4_59400001	6	59400001	0.5054	0.5719
NC_005105.4_59500001	6	59500001	0.4474	0.4286
NC_005105.4_59600001	6	59600001	0.4574	0.4893
NC_005105.4_59700001	6	59700001	0.4836	0.4761
NC_005105.4_59800001	6	59800001	0.4751	0.4375
NC_005105.4_59900001	6	59900001	0.4484	0.383
NC_005105.4_60000001	6	60000001	0.4436	0.4592
NC_005105.4_60100001	6	60100001	0.3776	0.4097
NC_005105.4_60200001	6	60200001	0.3705	0.4525

NC_005105.4_60300001	6	60300001	0.4126	0.5316
NC_005105.4_60400001	6	60400001	0.4439	0.567
NC_005105.4_60500001	6	60500001	0.553	0.6703
NC_005105.4_60600001	6	60600001	0.6024	0.6511
NC_005105.4_60700001	6	60700001	0.5828	0.6553
NC_005105.4_60800001	6	60800001	0.6301	0.6546
NC_005105.4_60900001	6	60900001	0.5785	0.6932
NC_005105.4_61000001	6	61000001	0.5004	0.7572
NC_005105.4_61100001	6	61100001	0.5013	0.8501
NC_005105.4_61200001	6	61200001	0.5923	0.8141
NC_005105.4_61300001	6	61300001	0.5726	0.7684
NC_005105.4_61400001	6	61400001	0.5892	0.7424
NC_005105.4_61500001	6	61500001	0.5555	0.6898
NC_005105.4_61600001	6	61600001	0.5283	0.6815
NC_005105.4_61700001	6	61700001	0.4875	0.608
NC_005105.4_61800001	6	61800001	0.4578	0.5248
NC_005105.4_61900001	6	61900001	0.5763	0.6517
NC_005105.4_62000001	6	62000001	0.5099	0.5837
NC_005105.4_62100001	6	62100001	0.6013	0.6125
NC_005105.4_62200001	6	62200001	0.6358	0.6958
NC_005105.4_62300001	6	62300001	0.7154	0.8116
NC_005105.4_62400001	6	62400001	0.4957	0.7372
NC_005105.4_62500001	6	62500001	0.4357	0.6863
NC_005105.4_62600001	6	62600001	0.3913	0.6779
NC_005105.4_62700001	6	62700001	0.3586	0.6646
NC_005105.4_62800001	6	62800001	0.191	0.5066
NC_005105.4_62900001	6	62900001	0.4273	0.7721
NC_005105.4_63000001	6	63000001	0.4342	0.8352
NC_005105.4_63100001	6	63100001	0.4421	0.7553
NC_005105.4_63200001	6	63200001	0.4558	0.7627
NC_005105.4_63300001	6	63300001	0.4271	0.7413
NC_005105.4_63400001	6	63400001	0.4381	0.6245
NC_005105.4_63500001	6	63500001	0.4811	0.5995
NC_005105.4_63600001	6	63600001	0.4571	0.634
NC_005105.4_63700001	6	63700001	0.588	0.6923
NC_005105.4_63800001	6	63800001	0.7181	0.7817
NC_005105.4_63900001	6	63900001	0.7629	0.9166
NC_005105.4_64000001	6	64000001	0.6286	0.838
NC_005105.4_64100001	6	64100001	0.6655	0.786
NC_005105.4_64200001	6	64200001	0.5256	0.697
NC_005105.4_64300001	6	64300001	0.3363	0.4549
NC_005105.4_64400001	6	64400001	0.4278	0.5472
NC_005105.4_64500001	6	64500001	0.3919	0.4356
NC_005105.4_64600001	6	64600001	0.4843	0.6021
NC_005105.4_64700001	6	64700001	0.5389	0.6651
NC_005105.4_64800001	6	64800001	0.4731	0.5403
NC_005105.4_64900001	6	64900001	0.3782	0.4175
NC_005105.4_65000001	6	65000001	0.3822	0.4474
NC_005105.4_65100001	6	65100001	0.293	0.3017
NC_005105.4_65200001	6	65200001	0.2446	0.1964
NC_005105.4_65300001	6	65300001	0.1981	0.1862
NC_005105.4_65400001	6	65400001	0.3402	0.5124
NC_005105.4_65500001	6	65500001	0.5041	0.6925
NC_005105.4_65600001	6	65600001	0.6016	0.6575
NC_005105.4_65700001	6	65700001	0.6231	0.5835
NC_005105.4_65800001	6	65800001	0.618	0.5594
NC_005105.4_65900001	6	65900001	0.5653	0.4956
NC_005105.4_66000001	6	66000001	0.5685	0.5031

NC_005105.4_66100001	6	66100001	0.5702	0.5303
NC_005105.4_66200001	6	66200001	0.5596	0.6253
NC_005105.4_66300001	6	66300001	0.6107	0.7206
NC_005105.4_66400001	6	66400001	0.6918	0.8188
NC_005105.4_66500001	6	66500001	0.6936	0.8172
NC_005105.4_66600001	6	66600001	0.5466	0.6819
NC_005105.4_66700001	6	66700001	0.453	0.6832
NC_005105.4_66800001	6	66800001	0.2849	0.563
NC_005105.4_66900001	6	66900001	0.2033	0.3997
NC_005105.4_67000001	6	67000001	0.1096	0.093
NC_005105.4_67100001	6	67100001	0.1047	0.1088
NC_005105.4_67200001	6	67200001	0.2811	0.4532
NC_005105.4_67300001	6	67300001	0.3884	0.3814
NC_005105.4_67400001	6	67400001	0.3884	0.3814
NC_005105.4_67500001	6	67500001	0.4005	0.5833
NC_005105.4_67600001	6	67600001	0.4317	0.5031
NC_005105.4_67700001	6	67700001	0.3776	0.4572
NC_005105.4_67800001	6	67800001	0.4246	0.5939
NC_005105.4_67900001	6	67900001	0.4682	0.6394
NC_005105.4_68000001	6	68000001	0.5106	0.522
NC_005105.4_68200001	6	68200001	0.6453	0.8545
NC_005105.4_68300001	6	68300001	0.6546	0.5814
NC_005105.4_68400001	6	68400001	0.6114	0.5266
NC_005105.4_68500001	6	68500001	0.6778	0.6672
NC_005105.4_68600001	6	68600001	0.7018	0.6949
NC_005105.4_68700001	6	68700001	0.7258	0.6749
NC_005105.4_68800001	6	68800001	0.7759	0.8437
NC_005105.4_68900001	6	68900001	0.8004	0.8633
NC_005105.4_69000001	6	69000001	0.8092	0.8275
NC_005105.4_69100001	6	69100001	0.6958	0.7917
NC_005105.4_69200001	6	69200001	0.6958	0.7917
NC_005105.4_69400001	6	69400001	0.8048	0.9382
NC_005105.4_69500001	6	69500001	0.7425	0.8899
NC_005105.4_69600001	6	69600001	0.8472	0.9277
NC_005105.4_69700001	6	69700001	0.8056	0.9202
NC_005105.4_69800001	6	69800001	0.7846	0.8598
NC_005105.4_69900001	6	69900001	0.6992	0.7645
NC_005105.4_70000001	6	70000001	0.6542	0.6906
NC_005105.4_70100001	6	70100001	0.6599	0.6921
NC_005105.4_70200001	6	70200001	0.6472	0.6257
NC_005105.4_70300001	6	70300001	0.5698	0.6472
NC_005105.4_70400001	6	70400001	0.544	0.6505
NC_005105.4_70500001	6	70500001	0.4621	0.6764
NC_005105.4_70600001	6	70600001	0.3905	0.6
NC_005105.4_70700001	6	70700001	0.4306	0.5858
NC_005105.4_70800001	6	70800001	0.4414	0.5216
NC_005105.4_70900001	6	70900001	0.4298	0.4723
NC_005105.4_71000001	6	71000001	0.3654	0.3578
NC_005105.4_71100001	6	71100001	0.3048	0.32
NC_005105.4_71200001	6	71200001	0.3127	0.2726
NC_005105.4_71300001	6	71300001	0.3843	0.4003
NC_005105.4_71400001	6	71400001	0.4378	0.5336
NC_005105.4_71500001	6	71500001	0.5052	0.5173
NC_005105.4_71600001	6	71600001	0.5422	0.5302
NC_005105.4_71700001	6	71700001	0.4804	0.5499
NC_005105.4_71800001	6	71800001	0.4249	0.5236
NC_005105.4_71900001	6	71900001	0.4282	0.5236
NC_005105.4_72000001	6	72000001	0.4409	0.6323

NC_005105.4_72100001	6	72100001	0.4828	0.6829
NC_005105.4_72200001	6	72200001	0.5981	0.7509
NC_005105.4_72300001	6	72300001	0.6855	0.8179
NC_005105.4_72400001	6	72400001	0.6814	0.7954
NC_005105.4_72500001	6	72500001	0.6638	0.7176
NC_005105.4_72600001	6	72600001	0.6495	0.6999
NC_005105.4_72700001	6	72700001	0.5879	0.6294
NC_005105.4_72800001	6	72800001	0.4835	0.5384
NC_005105.4_72900001	6	72900001	0.4993	0.5513
NC_005105.4_73000001	6	73000001	0.4693	0.6386
NC_005105.4_73100001	6	73100001	0.368	0.5669
NC_005105.4_73200001	6	73200001	0.2356	0.4104
NC_005105.4_73300001	6	73300001	0.4602	0.6975
NC_005105.4_73400001	6	73400001	0.4559	0.6566
NC_005105.4_73500001	6	73500001	0.4943	0.6558
NC_005105.4_73600001	6	73600001	0.5847	0.6999
NC_005105.4_73700001	6	73700001	0.6062	0.7289
NC_005105.4_73800001	6	73800001	0.4666	0.6667
NC_005105.4_73900001	6	73900001	0.4603	0.7289
NC_005105.4_74000001	6	74000001	0.297	0.7074
NC_005105.4_74100001	6	74100001	0.3823	0.5804
NC_005105.4_74200001	6	74200001	0.4496	0.6011
NC_005105.4_74300001	6	74300001	0.5994	0.6924
NC_005105.4_74400001	6	74400001	0.631	0.6844
NC_005105.4_74500001	6	74500001	0.6478	0.6754
NC_005105.4_74600001	6	74600001	0.653	0.6863
NC_005105.4_74700001	6	74700001	0.7052	0.7202
NC_005105.4_74800001	6	74800001	0.5969	0.6431
NC_005105.4_74900001	6	74900001	0.5912	0.678
NC_005105.4_75000001	6	75000001	0.5699	0.687
NC_005105.4_75100001	6	75100001	0.5808	0.7105
NC_005105.4_75200001	6	75200001	0.5304	0.7017
NC_005105.4_75300001	6	75300001	0.5011	0.7537
NC_005105.4_75400001	6	75400001	0.4707	0.7347
NC_005105.4_75500001	6	75500001	0.5145	0.7622
NC_005105.4_75600001	6	75600001	0.5642	0.8276
NC_005105.4_75700001	6	75700001	0.4543	0.685
NC_005105.4_75800001	6	75800001	0.509	0.7204
NC_005105.4_75900001	6	75900001	0.5041	0.6864
NC_005105.4_76000001	6	76000001	0.5337	0.7267
NC_005105.4_76100001	6	76100001	0.502	0.6908
NC_005105.4_76200001	6	76200001	0.6149	0.8069
NC_005105.4_76300001	6	76300001	0.7302	0.8954
NC_005105.4_76400001	6	76400001	0.8294	0.9233
NC_005105.4_76500001	6	76500001	0.7492	0.7408
NC_005105.4_76600001	6	76600001	0.6514	0.6284
NC_005105.4_76700001	6	76700001	0.6129	0.6058
NC_005105.4_76800001	6	76800001	0.6512	0.6912
NC_005105.4_76900001	6	76900001	0.5852	0.6573
NC_005105.4_77000001	6	77000001	0.5877	0.6405
NC_005105.4_77100001	6	77100001	0.5739	0.6572
NC_005105.4_77200001	6	77200001	0.5733	0.696
NC_005105.4_77300001	6	77300001	0.4653	0.559
NC_005105.4_77400001	6	77400001	0.4926	0.5359
NC_005105.4_77500001	6	77500001	0.4474	0.5882
NC_005105.4_77600001	6	77600001	0.5327	0.7063
NC_005105.4_77700001	6	77700001	0.5919	0.735
NC_005105.4_77800001	6	77800001	0.7007	0.8014

NC_005105.4_77900001	6	77900001	0.7258	0.8142
NC_005105.4_78000001	6	78000001	0.6888	0.8171
NC_005105.4_78100001	6	78100001	0.7045	0.8063
NC_005105.4_78200001	6	78200001	0.5592	0.7487
NC_005105.4_78300001	6	78300001	0.466	0.6141
NC_005105.4_78400001	6	78400001	0.4147	0.6139
NC_005105.4_78500001	6	78500001	0.4139	0.6119
NC_005105.4_78600001	6	78600001	0.409	0.5839
NC_005105.4_78700001	6	78700001	0.4447	0.5893
NC_005105.4_78800001	6	78800001	0.5694	0.7792
NC_005105.4_78900001	6	78900001	0.6076	0.7421
NC_005105.4_79000001	6	79000001	0.6262	0.712
NC_005105.4_79100001	6	79100001	0.6424	0.6861
NC_005105.4_79200001	6	79200001	0.554	0.5996
NC_005105.4_79300001	6	79300001	0.4657	0.4642
NC_005105.4_79400001	6	79400001	0.4076	0.3929
NC_005105.4_79500001	6	79500001	0.4811	0.5082
NC_005105.4_79600001	6	79600001	0.5055	0.5225
NC_005105.4_79700001	6	79700001	0.6075	0.6206
NC_005105.4_79800001	6	79800001	0.6001	0.7027
NC_005105.4_79900001	6	79900001	0.6354	0.7633
NC_005105.4_80000001	6	80000001	0.5343	0.6807
NC_005105.4_80100001	6	80100001	0.4246	0.6252
NC_005105.4_80200001	6	80200001	0.4647	0.5839
NC_005105.4_80300001	6	80300001	0.5543	0.5705
NC_005105.4_80400001	6	80400001	0.4522	0.3131
NC_005105.4_80600001	6	80600001	0.498	0.3198
NC_005105.4_80700001	6	80700001	0.5689	0.4985
NC_005105.4_80800001	6	80800001	0.5659	0.5597
NC_005105.4_80900001	6	80900001	0.6023	0.669
NC_005105.4_81000001	6	81000001	0.6023	0.669
NC_005105.4_81100001	6	81100001	0.6059	0.7943
NC_005105.4_81200001	6	81200001	0.492	0.755
NC_005105.4_81300001	6	81300001	0.4495	0.7184
NC_005105.4_81400001	6	81400001	0.1333	0.0685
NC_005105.4_81500001	6	81500001	0.1333	0.0685
NC_005105.4_82300001	6	82300001	0.3716	0.6449
NC_005105.4_82400001	6	82400001	0.3822	0.5501
NC_005105.4_82500001	6	82500001	0.4833	0.7129
NC_005105.4_82600001	6	82600001	0.5366	0.6853
NC_005105.4_82700001	6	82700001	0.5366	0.6853
NC_005105.4_82800001	6	82800001	0.7063	0.7589
NC_005105.4_82900001	6	82900001	0.7453	0.8542
NC_005105.4_83100001	6	83100001	0.3497	0.3958
NC_005105.4_83200001	6	83200001	0.3497	0.3958
NC_005105.4_83300001	6	83300001	0.4009	0.5121
NC_005105.4_83400001	6	83400001	0.4009	0.5121
NC_005105.4_83500001	6	83500001	0.4252	0.6092
NC_005105.4_83600001	6	83600001	0.3081	0.557
NC_005105.4_83700001	6	83700001	0.3104	0.4873
NC_005105.4_83800001	6	83800001	0.2072	0.2843
NC_005105.4_83900001	6	83900001	0.2096	0.2565
NC_005105.4_84000001	6	84000001	0.1605	0.1446
NC_005105.4_84100001	6	84100001	0.3821	0.364
NC_005105.4_84300001	6	84300001	0.5168	0.5729
NC_005105.4_84400001	6	84400001	0.3949	0.5825
NC_005105.4_84500001	6	84500001	0.3859	0.5412
NC_005105.4_84600001	6	84600001	0.3753	0.5135

NC_005105.4_84700001	6	84700001	0.3809	0.4593
NC_005105.4_84800001	6	84800001	0.3329	0.3788
NC_005105.4_84900001	6	84900001	0.4177	0.3575
NC_005105.4_85300001	6	85300001	0.3624	0.3887
NC_005105.4_85400001	6	85400001	0.3624	0.3887
NC_005105.4_85500001	6	85500001	0.419	0.4095
NC_005105.4_85600001	6	85600001	0.419	0.4095
NC_005105.4_85700001	6	85700001	0.4076	0.4432
NC_005105.4_85800001	6	85800001	0.4869	0.5422
NC_005105.4_85900001	6	85900001	0.3891	0.4705
NC_005105.4_86000001	6	86000001	0.4493	0.6459
NC_005105.4_86100001	6	86100001	0.433	0.5969
NC_005105.4_86200001	6	86200001	0.4862	0.6157
NC_005105.4_86300001	6	86300001	0.5202	0.6551
NC_005105.4_86400001	6	86400001	0.7488	0.8041
NC_005105.4_86500001	6	86500001	0.6915	0.7542
NC_005105.4_86600001	6	86600001	0.7416	0.7903
NC_005105.4_86700001	6	86700001	0.6914	0.7773
NC_005105.4_86800001	6	86800001	0.5642	0.6943
NC_005105.4_86900001	6	86900001	0.4826	0.681
NC_005105.4_87000001	6	87000001	0.4826	0.681
NC_005105.4_87100001	6	87100001	0.4499	0.6633
NC_005105.4_87200001	6	87200001	0.4699	0.6726
NC_005105.4_87300001	6	87300001	0.4661	0.6516
NC_005105.4_87400001	6	87400001	0.4773	0.5718
NC_005105.4_87500001	6	87500001	0.4837	0.5602
NC_005105.4_87600001	6	87600001	0.5331	0.565
NC_005105.4_87700001	6	87700001	0.5126	0.5661
NC_005105.4_87800001	6	87800001	0.4935	0.561
NC_005105.4_87900001	6	87900001	0.5034	0.5379
NC_005105.4_88000001	6	88000001	0.4774	0.526
NC_005105.4_88100001	6	88100001	0.4795	0.5947
NC_005105.4_88200001	6	88200001	0.444	0.5285
NC_005105.4_88300001	6	88300001	0.5319	0.6164
NC_005105.4_88400001	6	88400001	0.5288	0.6074
NC_005105.4_88500001	6	88500001	0.5181	0.6218
NC_005105.4_88600001	6	88600001	0.3903	0.5047
NC_005105.4_88700001	6	88700001	0.5138	0.6835
NC_005105.4_88800001	6	88800001	0.3727	0.6498
NC_005105.4_88900001	6	88900001	0.3998	0.7481
NC_005105.4_89000001	6	89000001	0.4599	0.798
NC_005105.4_89100001	6	89100001	0.4927	0.7858
NC_005105.4_89200001	6	89200001	0.4231	0.7564
NC_005105.4_89300001	6	89300001	0.3441	0.5762
NC_005105.4_89400001	6	89400001	0.3741	0.6443
NC_005105.4_89500001	6	89500001	0.429	0.6499
NC_005105.4_89600001	6	89600001	0.5214	0.6439
NC_005105.4_89700001	6	89700001	0.5036	0.623
NC_005105.4_89800001	6	89800001	0.4956	0.6415
NC_005105.4_89900001	6	89900001	0.5237	0.5824
NC_005105.4_90000001	6	90000001	0.4903	0.5536
NC_005105.4_90100001	6	90100001	0.3181	0.4826
NC_005105.4_90200001	6	90200001	0.3701	0.5216
NC_005105.4_90300001	6	90300001	0.425	0.4665
NC_005105.4_90400001	6	90400001	0.3806	0.4707
NC_005105.4_90500001	6	90500001	0.3806	0.4707
NC_005105.4_90600001	6	90600001	0.4574	0.5169
NC_005105.4_90700001	6	90700001	0.4379	0.4771

NC_005105.4_90800001	6	90800001	0.4877	0.5619
NC_005105.4_90900001	6	90900001	0.5109	0.4861
NC_005105.4_91000001	6	91000001	0.5249	0.5103
NC_005105.4_91100001	6	91100001	0.4332	0.4968
NC_005105.4_91200001	6	91200001	0.4079	0.4747
NC_005105.4_91300001	6	91300001	0.5058	0.5758
NC_005105.4_91400001	6	91400001	0.5727	0.6859
NC_005105.4_91500001	6	91500001	0.5922	0.7553
NC_005105.4_91600001	6	91600001	0.8207	0.8462
NC_005105.4_91700001	6	91700001	0.8185	0.8685
NC_005105.4_91800001	6	91800001	0.7842	0.8948
NC_005105.4_91900001	6	91900001	0.7449	0.925
NC_005105.4_92000001	6	92000001	0.7914	0.9036
NC_005105.4_92100001	6	92100001	0.36	0.4568
NC_005105.4_92200001	6	92200001	0.5861	0.7319
NC_005105.4_92300001	6	92300001	0.5822	0.713
NC_005105.4_92400001	6	92400001	0.5822	0.713
NC_005105.4_92500001	6	92500001	0.6227	0.7064
NC_005105.4_92600001	6	92600001	0.7023	0.7645
NC_005105.4_92700001	6	92700001	0.6592	0.7271
NC_005105.4_92800001	6	92800001	0.6736	0.7521
NC_005105.4_92900001	6	92900001	0.6724	0.7773
NC_005105.4_93000001	6	93000001	0.6652	0.8892
NC_005105.4_93100001	6	93100001	0.752	0.9238
NC_005105.4_93200001	6	93200001	0.8385	0.9388
NC_005105.4_93300001	6	93300001	0.7722	0.8666
NC_005105.4_93400001	6	93400001	0.6506	0.7506
NC_005105.4_93500001	6	93500001	0.6508	0.7507
NC_005105.4_93600001	6	93600001	0.5197	0.6164
NC_005105.4_93700001	6	93700001	0.4238	0.5316
NC_005105.4_93800001	6	93800001	0.3646	0.52
NC_005105.4_93900001	6	93900001	0.4194	0.6302
NC_005105.4_94000001	6	94000001	0.3812	0.581
NC_005105.4_94100001	6	94100001	0.3638	0.636
NC_005105.4_94200001	6	94200001	0.4976	0.8032
NC_005105.4_94300001	6	94300001	0.5903	0.8294
NC_005105.4_94400001	6	94400001	0.5134	0.7532
NC_005105.4_94500001	6	94500001	0.519	0.7529
NC_005105.4_94600001	6	94600001	0.5307	0.7333
NC_005105.4_94700001	6	94700001	0.5289	0.6962
NC_005105.4_94800001	6	94800001	0.5768	0.7168
NC_005105.4_94900001	6	94900001	0.6579	0.7322
NC_005105.4_95000001	6	95000001	0.6988	0.7272
NC_005105.4_95100001	6	95100001	0.7548	0.8011
NC_005105.4_95200001	6	95200001	0.8247	0.8402
NC_005105.4_95300001	6	95300001	0.8093	0.825
NC_005105.4_95400001	6	95400001	0.7017	0.8553
NC_005105.4_95500001	6	95500001	0.7497	0.9357
NC_005105.4_95600001	6	95600001	0.7021	0.8766
NC_005105.4_95700001	6	95700001	0.7457	0.8975
NC_005105.4_95800001	6	95800001	0.6513	0.8577
NC_005105.4_95900001	6	95900001	0.6535	0.8408
NC_005105.4_96000001	6	96000001	0.5842	0.7107
NC_005105.4_96100001	6	96100001	0.6141	0.71
NC_005105.4_96200001	6	96200001	0.4533	0.5762
NC_005105.4_96300001	6	96300001	0.4668	0.5804
NC_005105.4_96400001	6	96400001	0.4672	0.5801
NC_005105.4_96500001	6	96500001	0.5542	0.7232

NC_005105.4_96600001	6	96600001	0.5247	0.7411
NC_005105.4_96700001	6	96700001	0.6515	0.8343
NC_005105.4_96800001	6	96800001	0.5614	0.7866
NC_005105.4_96900001	6	96900001	0.5863	0.7731
NC_005105.4_97000001	6	97000001	0.5346	0.7181
NC_005105.4_97100001	6	97100001	0.5088	0.6829
NC_005105.4_97200001	6	97200001	0.5001	0.6486
NC_005105.4_97300001	6	97300001	0.5795	0.6482
NC_005105.4_97400001	6	97400001	0.5315	0.6172
NC_005105.4_97500001	6	97500001	0.5334	0.6622
NC_005105.4_97600001	6	97600001	0.6442	0.7762
NC_005105.4_97700001	6	97700001	0.6385	0.7637
NC_005105.4_97800001	6	97800001	0.6549	0.8007
NC_005105.4_97900001	6	97900001	0.713	0.8467
NC_005105.4_98000001	6	98000001	0.7288	0.8489
NC_005105.4_98100001	6	98100001	0.6694	0.8457
NC_005105.4_98200001	6	98200001	0.6968	0.8796
NC_005105.4_98300001	6	98300001	0.6976	0.8776
NC_005105.4_98400001	6	98400001	0.6815	0.8269
NC_005105.4_98500001	6	98500001	0.645	0.7992
NC_005105.4_98600001	6	98600001	0.6896	0.7559
NC_005105.4_98700001	6	98700001	0.6321	0.6347
NC_005105.4_98800001	6	98800001	0.575	0.6289
NC_005105.4_98900001	6	98900001	0.5592	0.6183
NC_005105.4_99000001	6	99000001	0.5592	0.6183
NC_005105.4_99100001	6	99100001	0.5012	0.527
NC_005105.4_99200001	6	99200001	0.5706	0.6813
NC_005105.4_99300001	6	99300001	0.5427	0.6817
NC_005105.4_99400001	6	99400001	0.5669	0.7783
NC_005105.4_99500001	6	99500001	0.547	0.7486
NC_005105.4_99600001	6	99600001	0.5382	0.745
NC_005105.4_99700001	6	99700001	0.5264	0.7232
NC_005105.4_99800001	6	99800001	0.4996	0.6651
NC_005105.4_99900001	6	99900001	0.4773	0.5837
NC_005105.4_100000001	6	100000001	0.5122	0.6177
NC_005105.4_100100001	6	100100001	0.4519	0.6125
NC_005105.4_100200001	6	100200001	0.4825	0.6559
NC_005105.4_100300001	6	100300001	0.5108	0.7371
NC_005105.4_100400001	6	100400001	0.4991	0.7521
NC_005105.4_100500001	6	100500001	0.4761	0.7052
NC_005105.4_100600001	6	100600001	0.5391	0.6918
NC_005105.4_100700001	6	100700001	0.3959	0.5854
NC_005105.4_100800001	6	100800001	0.4879	0.6096
NC_005105.4_100900001	6	100900001	0.4512	0.5628
NC_005105.4_101000001	6	101000001	0.4609	0.6077
NC_005105.4_101100001	6	101100001	0.4861	0.7151
NC_005105.4_101200001	6	101200001	0.6107	0.6916
NC_005105.4_101600001	6	101600001	0.5091	0.5924
NC_005105.4_101700001	6	101700001	0.4729	0.615
NC_005105.4_101800001	6	101800001	0.5483	0.6608
NC_005105.4_101900001	6	101900001	0.5941	0.7081
NC_005105.4_102000001	6	102000001	0.5422	0.6553
NC_005105.4_102100001	6	102100001	0.539	0.6671
NC_005105.4_102200001	6	102200001	0.5656	0.686
NC_005105.4_102300001	6	102300001	0.5015	0.6582
NC_005105.4_102400001	6	102400001	0.465	0.6244
NC_005105.4_102500001	6	102500001	0.5316	0.6915
NC_005105.4_102600001	6	102600001	0.57	0.7366

NC_005105.4_102700001	6	102700001	0.5564	0.7024
NC_005105.4_102800001	6	102800001	0.5979	0.7308
NC_005105.4_102900001	6	102900001	0.6117	0.7495
NC_005105.4_103000001	6	103000001	0.5533	0.7019
NC_005105.4_103100001	6	103100001	0.5224	0.7018
NC_005105.4_103200001	6	103200001	0.5253	0.7144
NC_005105.4_103300001	6	103300001	0.4936	0.709
NC_005105.4_103400001	6	103400001	0.4401	0.6449
NC_005105.4_103500001	6	103500001	0.4417	0.6983
NC_005105.4_103900001	6	103900001	0.4485	0.5693
NC_005105.4_104000001	6	104000001	0.4511	0.6138
NC_005105.4_104100001	6	104100001	0.4292	0.5802
NC_005105.4_104200001	6	104200001	0.4567	0.6063
NC_005105.4_104300001	6	104300001	0.5478	0.6677
NC_005105.4_104400001	6	104400001	0.5397	0.7316
NC_005105.4_104500001	6	104500001	0.5664	0.7237
NC_005105.4_104600001	6	104600001	0.6315	0.7831
NC_005105.4_104700001	6	104700001	0.636	0.7937
NC_005105.4_104800001	6	104800001	0.5814	0.8579
NC_005105.4_104900001	6	104900001	0.6856	0.8405
NC_005105.4_105000001	6	105000001	0.6512	0.8477
NC_005105.4_105100001	6	105100001	0.6738	0.8287
NC_005105.4_105200001	6	105200001	0.6738	0.8287
NC_005105.4_105300001	6	105300001	0.7449	0.8272
NC_005105.4_105400001	6	105400001	0.7022	0.7686
NC_005105.4_105500001	6	105500001	0.5862	0.7652
NC_005105.4_105600001	6	105600001	0.4722	0.7148
NC_005105.4_105700001	6	105700001	0.4927	0.7323
NC_005105.4_105800001	6	105800001	0.431	0.7234
NC_005105.4_105900001	6	105900001	0.371	0.7356
NC_005105.4_106000001	6	106000001	0.4068	0.6045
NC_005105.4_106100001	6	106100001	0.4287	0.5553
NC_005105.4_106200001	6	106200001	0.5016	0.6525
NC_005105.4_106300001	6	106300001	0.5061	0.6712
NC_005105.4_106400001	6	106400001	0.5177	0.7011
NC_005105.4_106500001	6	106500001	0.5052	0.7406
NC_005105.4_106600001	6	106600001	0.494	0.7702
NC_005105.4_106700001	6	106700001	0.4122	0.7037
NC_005105.4_106800001	6	106800001	0.4295	0.6402
NC_005105.4_106900001	6	106900001	0.4045	0.5428
NC_005105.4_107000001	6	107000001	0.5347	0.6614
NC_005105.4_107100001	6	107100001	0.5316	0.6335
NC_005105.4_107200001	6	107200001	0.4889	0.5824
NC_005105.4_107300001	6	107300001	0.4991	0.6719
NC_005105.4_107400001	6	107400001	0.5435	0.7462
NC_005105.4_107500001	6	107500001	0.4427	0.7007
NC_005105.4_107600001	6	107600001	0.4854	0.7652
NC_005105.4_107700001	6	107700001	0.5161	0.7681
NC_005105.4_107800001	6	107800001	0.467	0.6203
NC_005105.4_107900001	6	107900001	0.4287	0.5662
NC_005105.4_108000001	6	108000001	0.4469	0.4656
NC_005105.4_108100001	6	108100001	0.3743	0.3806
NC_005105.4_108200001	6	108200001	0.3933	0.4311
NC_005105.4_108300001	6	108300001	0.4093	0.4844
NC_005105.4_108400001	6	108400001	0.49	0.6263
NC_005105.4_108500001	6	108500001	0.4565	0.6569
NC_005105.4_108600001	6	108600001	0.5443	0.7902
NC_005105.4_108700001	6	108700001	0.5663	0.7508

NC_005105.4_108800001	6	108800001	0.5332	0.7202
NC_005105.4_108900001	6	108900001	0.438	0.5769
NC_005105.4_109000001	6	109000001	0.5513	0.6635
NC_005105.4_109100001	6	109100001	0.5269	0.6076
NC_005105.4_109200001	6	109200001	0.5134	0.6204
NC_005105.4_109300001	6	109300001	0.6096	0.6956
NC_005105.4_109400001	6	109400001	0.7172	0.758
NC_005105.4_109500001	6	109500001	0.7119	0.7571
NC_005105.4_109600001	6	109600001	0.6858	0.8081
NC_005105.4_109700001	6	109700001	0.7488	0.8568
NC_005105.4_109800001	6	109800001	0.6671	0.8269
NC_005105.4_109900001	6	109900001	0.6392	0.7746
NC_005105.4_110000001	6	110000001	0.5451	0.6975
NC_005105.4_110100001	6	110100001	0.573	0.6278
NC_005105.4_110200001	6	110200001	0.554	0.6044
NC_005105.4_110300001	6	110300001	0.5281	0.5619
NC_005105.4_110400001	6	110400001	0.4218	0.4244
NC_005105.4_110500001	6	110500001	0.4318	0.4786
NC_005105.4_110600001	6	110600001	0.3261	0.5434
NC_005105.4_110700001	6	110700001	0.3201	0.5532
NC_005105.4_110800001	6	110800001	0.3973	0.6663
NC_005105.4_110900001	6	110900001	0.3801	0.6415
NC_005105.4_111000001	6	111000001	0.3554	0.5943
NC_005105.4_111100001	6	111100001	0.4082	0.6173
NC_005105.4_111200001	6	111200001	0.4053	0.6054
NC_005105.4_111300001	6	111300001	0.3019	0.5075
NC_005105.4_111400001	6	111400001	0.3345	0.57
NC_005105.4_111500001	6	111500001	0.3417	0.616
NC_005105.4_111600001	6	111600001	0.331	0.5474
NC_005105.4_111700001	6	111700001	0.4376	0.6038
NC_005105.4_111800001	6	111800001	0.4114	0.5848
NC_005105.4_111900001	6	111900001	0.4013	0.4514
NC_005105.4_112000001	6	112000001	0.4238	0.4549
NC_005105.4_112100001	6	112100001	0.4077	0.4705
NC_005105.4_112200001	6	112200001	0.3268	0.332
NC_005105.4_112300001	6	112300001	0.3416	0.3266
NC_005105.4_112400001	6	112400001	0.2734	0.2486
NC_005105.4_112500001	6	112500001	0.2734	0.2486
NC_005105.4_112600001	6	112600001	0.2643	0.1991
NC_005105.4_112700001	6	112700001	0.3405	0.3855
NC_005105.4_112800001	6	112800001	0.4085	0.4102
NC_005105.4_112900001	6	112900001	0.464	0.4953
NC_005105.4_113000001	6	113000001	0.4409	0.4774
NC_005105.4_113100001	6	113100001	0.5637	0.6567
NC_005105.4_113200001	6	113200001	0.5909	0.7062
NC_005105.4_113300001	6	113300001	0.7336	0.9139
NC_005105.4_113400001	6	113400001	0.7088	0.9056
NC_005105.4_113500001	6	113500001	0.7705	0.945
NC_005105.4_113600001	6	113600001	0.8272	0.9448
NC_005105.4_113900001	6	113900001	0.6784	0.7031
NC_005105.4_114000001	6	114000001	0.6151	0.5779
NC_005105.4_114100001	6	114100001	0.5014	0.5286
NC_005105.4_114200001	6	114200001	0.5018	0.5436
NC_005105.4_114300001	6	114300001	0.5393	0.5596
NC_005105.4_114400001	6	114400001	0.5326	0.5882
NC_005105.4_114500001	6	114500001	0.5641	0.6894
NC_005105.4_114600001	6	114600001	0.6357	0.7667
NC_005105.4_114700001	6	114700001	0.5843	0.718

NC_005105.4_114800001	6	114800001	0.5869	0.7471
NC_005105.4_114900001	6	114900001	0.5954	0.7467
NC_005105.4_115000001	6	115000001	0.5606	0.6605
NC_005105.4_115100001	6	115100001	0.5447	0.6258
NC_005105.4_115200001	6	115200001	0.6296	0.695
NC_005105.4_115300001	6	115300001	0.6067	0.675
NC_005105.4_115400001	6	115400001	0.6143	0.6466
NC_005105.4_115500001	6	115500001	0.6884	0.7632
NC_005105.4_115600001	6	115600001	0.5942	0.6554
NC_005105.4_115700001	6	115700001	0.6048	0.6532
NC_005105.4_115800001	6	115800001	0.6536	0.6681
NC_005105.4_115900001	6	115900001	0.5857	0.6381
NC_005105.4_116000001	6	116000001	0.5857	0.6381
NC_005105.4_116100001	6	116100001	0.6778	0.6537
NC_005105.4_116300001	6	116300001	0.589	0.6045
NC_005105.4_116400001	6	116400001	0.6752	0.7072
NC_005105.4_116500001	6	116500001	0.7141	0.7159
NC_005105.4_116600001	6	116600001	0.7659	0.7618
NC_005105.4_116700001	6	116700001	0.7269	0.7252
NC_005105.4_116800001	6	116800001	0.7839	0.7592
NC_005105.4_116900001	6	116900001	0.6098	0.5993
NC_005105.4_117000001	6	117000001	0.2843	0.2734
NC_005105.4_117100001	6	117100001	0.2941	0.2642
NC_005105.4_117200001	6	117200001	0.2662	0.242
NC_005105.4_117300001	6	117300001	0.2414	0.2336
NC_005105.4_117400001	6	117400001	0.2121	0.2027
NC_005105.4_117500001	6	117500001	0.3557	0.6083
NC_005105.4_117600001	6	117600001	0.354	0.6446
NC_005105.4_117700001	6	117700001	0.3961	0.7685
NC_005105.4_117800001	6	117800001	0.5115	0.8008
NC_005105.4_117900001	6	117900001	0.5809	0.8424
NC_005105.4_118000001	6	118000001	0.6483	0.7177
NC_005105.4_118100001	6	118100001	0.4975	0.5412
NC_005105.4_118200001	6	118200001	0.4328	0.4469
NC_005105.4_118300001	6	118300001	0.4012	0.4011
NC_005105.4_118400001	6	118400001	0.3861	0.4242
NC_005105.4_118500001	6	118500001	0.3721	0.4046
NC_005105.4_118600001	6	118600001	0.3904	0.4604
NC_005105.4_118700001	6	118700001	0.4493	0.6325
NC_005105.4_118800001	6	118800001	0.2828	0.4319
NC_005105.4_119000001	6	119000001	0.233	0.3759
NC_005105.4_119100001	6	119100001	0.3185	0.5095
NC_005105.4_119200001	6	119200001	0.3426	0.5009
NC_005105.4_119300001	6	119300001	0.3973	0.5309
NC_005105.4_119400001	6	119400001	0.3832	0.4667
NC_005105.4_119500001	6	119500001	0.4136	0.4794
NC_005105.4_119600001	6	119600001	0.4675	0.5571
NC_005105.4_119700001	6	119700001	0.5214	0.5917
NC_005105.4_119800001	6	119800001	0.5292	0.6011
NC_005105.4_120300001	6	120300001	0.4826	0.446
NC_005105.4_120400001	6	120400001	0.4471	0.4104
NC_005105.4_120500001	6	120500001	0.5706	0.5689
NC_005105.4_120600001	6	120600001	0.5376	0.5432
NC_005105.4_120700001	6	120700001	0.5897	0.615
NC_005105.4_120800001	6	120800001	0.6752	0.6905
NC_005105.4_120900001	6	120900001	0.6693	0.7081
NC_005105.4_121000001	6	121000001	0.57	0.6589
NC_005105.4_121100001	6	121100001	0.4522	0.582

NC_005105.4_121200001	6	121200001	0.4629	0.5973
NC_005105.4_121300001	6	121300001	0.4581	0.6238
NC_005105.4_121400001	6	121400001	0.4624	0.6561
NC_005105.4_121500001	6	121500001	0.4451	0.6126
NC_005105.4_121600001	6	121600001	0.5479	0.7426
NC_005105.4_121700001	6	121700001	0.55	0.8044
NC_005105.4_121800001	6	121800001	0.4883	0.7904
NC_005105.4_121900001	6	121900001	0.5102	0.8117
NC_005105.4_122000001	6	122000001	0.6161	0.896
NC_005105.4_122100001	6	122100001	0.6207	0.8455
NC_005105.4_122200001	6	122200001	0.6332	0.8076
NC_005105.4_122300001	6	122300001	0.6867	0.805
NC_005105.4_122400001	6	122400001	0.7023	0.8061
NC_005105.4_122500001	6	122500001	0.6622	0.7717
NC_005105.4_122600001	6	122600001	0.6889	0.7923
NC_005105.4_122700001	6	122700001	0.5993	0.7526
NC_005105.4_122800001	6	122800001	0.552	0.6912
NC_005105.4_122900001	6	122900001	0.5383	0.671
NC_005105.4_123000001	6	123000001	0.4962	0.6265
NC_005105.4_123100001	6	123100001	0.3551	0.4184
NC_005105.4_123200001	6	123200001	0.4953	0.523
NC_005105.4_123300001	6	123300001	0.6026	0.6172
NC_005105.4_123400001	6	123400001	0.5419	0.5275
NC_005105.4_123500001	6	123500001	0.5741	0.5587
NC_005105.4_123600001	6	123600001	0.5737	0.6247
NC_005105.4_123700001	6	123700001	0.5463	0.6477
NC_005105.4_123800001	6	123800001	0.4779	0.6007
NC_005105.4_123900001	6	123900001	0.5256	0.7383
NC_005105.4_124000001	6	124000001	0.483	0.7159
NC_005105.4_124100001	6	124100001	0.4977	0.7037
NC_005105.4_124200001	6	124200001	0.5215	0.7232
NC_005105.4_124300001	6	124300001	0.5891	0.7692
NC_005105.4_124400001	6	124400001	0.5619	0.7514
NC_005105.4_124500001	6	124500001	0.6261	0.8247
NC_005105.4_124600001	6	124600001	0.6679	0.8069
NC_005105.4_124700001	6	124700001	0.6568	0.7807
NC_005105.4_124800001	6	124800001	0.5887	0.7476
NC_005105.4_124900001	6	124900001	0.6853	0.7885
NC_005105.4_125000001	6	125000001	0.6284	0.7438
NC_005105.4_125200001	6	125200001	0.5753	0.5607
NC_005105.4_125300001	6	125300001	0.7408	0.7459
NC_005105.4_125400001	6	125400001	0.7513	0.771
NC_005105.4_125500001	6	125500001	0.7628	0.7717
NC_005105.4_125600001	6	125600001	0.7022	0.7369
NC_005105.4_125700001	6	125700001	0.6818	0.764
NC_005105.4_125800001	6	125800001	0.5035	0.5906
NC_005105.4_125900001	6	125900001	0.4672	0.4912
NC_005105.4_126000001	6	126000001	0.4261	0.464
NC_005105.4_126100001	6	126100001	0.4286	0.4602
NC_005105.4_126200001	6	126200001	0.478	0.5175
NC_005105.4_126300001	6	126300001	0.6271	0.6922
NC_005105.4_126400001	6	126400001	0.6228	0.7931
NC_005105.4_126500001	6	126500001	0.6433	0.8144
NC_005105.4_126600001	6	126600001	0.6166	0.8311
NC_005105.4_126700001	6	126700001	0.5523	0.7171
NC_005105.4_126800001	6	126800001	0.4313	0.5528
NC_005105.4_126900001	6	126900001	0.5169	0.6508
NC_005105.4_127000001	6	127000001	0.5236	0.6558

NC_005105.4_127100001	6	127100001	0.6133	0.7164
NC_005105.4_127200001	6	127200001	0.575	0.7583
NC_005105.4_127300001	6	127300001	0.5971	0.7962
NC_005105.4_127400001	6	127400001	0.5733	0.786
NC_005105.4_127500001	6	127500001	0.5747	0.7743
NC_005105.4_127600001	6	127600001	0.4326	0.6779
NC_005105.4_127700001	6	127700001	0.5668	0.7372
NC_005105.4_127800001	6	127800001	0.5495	0.6555
NC_005105.4_127900001	6	127900001	0.5472	0.6664
NC_005105.4_128000001	6	128000001	0.5885	0.7204
NC_005105.4_128100001	6	128100001	0.5544	0.6627
NC_005105.4_128200001	6	128200001	0.5407	0.6347
NC_005105.4_128300001	6	128300001	0.5094	0.6251
NC_005105.4_128400001	6	128400001	0.4905	0.571
NC_005105.4_128500001	6	128500001	0.4467	0.4983
NC_005105.4_128600001	6	128600001	0.4709	0.5221
NC_005105.4_128700001	6	128700001	0.3623	0.4302
NC_005105.4_128800001	6	128800001	0.5711	0.6615
NC_005105.4_128900001	6	128900001	0.5386	0.6884
NC_005105.4_129000001	6	129000001	0.4281	0.6978
NC_005105.4_129100001	6	129100001	0.3943	0.6325
NC_005105.4_129200001	6	129200001	0.3874	0.6581
NC_005105.4_129300001	6	129300001	0.3835	0.665
NC_005105.4_129400001	6	129400001	0.3765	0.6396
NC_005105.4_129500001	6	129500001	0.479	0.6489
NC_005105.4_129600001	6	129600001	0.5618	0.7024
NC_005105.4_129700001	6	129700001	0.6244	0.6901
NC_005105.4_129800001	6	129800001	0.6092	0.6104
NC_005105.4_129900001	6	129900001	0.6011	0.642
NC_005105.4_130000001	6	130000001	0.5514	0.6006
NC_005105.4_130100001	6	130100001	0.5297	0.6238
NC_005105.4_130200001	6	130200001	0.5309	0.5931
NC_005105.4_130300001	6	130300001	0.5262	0.5977
NC_005105.4_130400001	6	130400001	0.5829	0.6351
NC_005105.4_130500001	6	130500001	0.5706	0.6539
NC_005105.4_130600001	6	130600001	0.6018	0.6606
NC_005105.4_130700001	6	130700001	0.5655	0.6549
NC_005105.4_130800001	6	130800001	0.5807	0.6648
NC_005105.4_130900001	6	130900001	0.5122	0.615
NC_005105.4_131000001	6	131000001	0.5309	0.6243
NC_005105.4_131100001	6	131100001	0.5486	0.6527
NC_005105.4_131200001	6	131200001	0.5292	0.6837
NC_005105.4_131300001	6	131300001	0.5196	0.681
NC_005105.4_131400001	6	131400001	0.475	0.6167
NC_005105.4_131500001	6	131500001	0.4678	0.6008
NC_005105.4_131600001	6	131600001	0.4721	0.5606
NC_005105.4_131700001	6	131700001	0.4667	0.5397
NC_005105.4_131800001	6	131800001	0.4952	0.5628
NC_005105.4_131900001	6	131900001	0.5928	0.6221
NC_005105.4_132000001	6	132000001	0.6213	0.6495
NC_005105.4_132100001	6	132100001	0.564	0.6095
NC_005105.4_132200001	6	132200001	0.5835	0.6197
NC_005105.4_132300001	6	132300001	0.6254	0.6625
NC_005105.4_132400001	6	132400001	0.6187	0.688
NC_005105.4_132500001	6	132500001	0.5861	0.6576
NC_005105.4_132600001	6	132600001	0.6344	0.6869
NC_005105.4_132700001	6	132700001	0.6121	0.6967
NC_005105.4_132800001	6	132800001	0.5457	0.6282

NC_005105.4_132900001	6	132900001	0.4975	0.5353
NC_005105.4_133100001	6	133100001	0.5943	0.7791
NC_005105.4_133200001	6	133200001	0.6707	0.8819
NC_005105.4_133300001	6	133300001	0.6979	0.8767
NC_005105.4_133400001	6	133400001	0.6831	0.8228
NC_005105.4_133500001	6	133500001	0.6718	0.7764
NC_005105.4_133600001	6	133600001	0.6385	0.7522
NC_005105.4_133700001	6	133700001	0.5642	0.6713
NC_005105.4_133800001	6	133800001	0.5771	0.6616
NC_005105.4_133900001	6	133900001	0.5756	0.6749
NC_005105.4_134000001	6	134000001	0.5503	0.682
NC_005105.4_134100001	6	134100001	0.5199	0.6776
NC_005105.4_134200001	6	134200001	0.5556	0.7294
NC_005105.4_134300001	6	134300001	0.4982	0.7128
NC_005105.4_134400001	6	134400001	0.501	0.7245
NC_005105.4_134500001	6	134500001	0.569	0.7789
NC_005105.4_134600001	6	134600001	0.594	0.7772
NC_005105.4_134700001	6	134700001	0.7259	0.8543
NC_005105.4_134800001	6	134800001	0.7522	0.8978
NC_005105.4_134900001	6	134900001	0.8663	0.9416
NC_005105.4_135000001	6	135000001	0.8447	0.8973
NC_005105.4_135100001	6	135100001	0.8514	0.9021
NC_005105.4_135200001	6	135200001	0.8239	0.8867
NC_005105.4_135300001	6	135300001	0.6956	0.8351
NC_005105.4_135400001	6	135400001	0.678	0.7969
NC_005105.4_135500001	6	135500001	0.645	0.8131
NC_005105.4_135600001	6	135600001	0.6347	0.8165
NC_005105.4_135700001	6	135700001	0.62	0.8083
NC_005105.4_135800001	6	135800001	0.6824	0.8176
NC_005105.4_135900001	6	135900001	0.6887	0.8685
NC_005105.4_136000001	6	136000001	0.6891	0.8551
NC_005105.4_136100001	6	136100001	0.73	0.8498
NC_005105.4_136200001	6	136200001	0.6999	0.7654
NC_005105.4_136300001	6	136300001	0.5966	0.6917
NC_005105.4_136400001	6	136400001	0.6071	0.72
NC_005105.4_136500001	6	136500001	0.5404	0.6556
NC_005105.4_136600001	6	136600001	0.551	0.651
NC_005105.4_136700001	6	136700001	0.5549	0.6415
NC_005105.4_136800001	6	136800001	0.5906	0.681
NC_005105.4_136900001	6	136900001	0.5547	0.6152
NC_005105.4_137000001	6	137000001	0.5607	0.6199
NC_005105.4_137100001	6	137100001	0.5282	0.5662
NC_005105.4_137200001	6	137200001	0.5089	0.5604
NC_005105.4_137300001	6	137300001	0.5089	0.5654
NC_005105.4_137400001	6	137400001	0.492	0.5775
NC_005105.4_137500001	6	137500001	0.4967	0.5601
NC_005105.4_137600001	6	137600001	0.5414	0.5791
NC_005105.4_137700001	6	137700001	0.531	0.5191
NC_005105.4_137800001	6	137800001	0.5018	0.4795
NC_005105.4_137900001	6	137900001	0.5454	0.4757
NC_005105.4_138000001	6	138000001	0.6042	0.5652
NC_005105.4_138100001	6	138100001	0.5943	0.6109
NC_005105.4_138200001	6	138200001	0.6212	0.7235
NC_005105.4_140400001	6	140400001	0.4036	0.5366
NC_005105.4_140500001	6	140500001	0.4036	0.5366
NC_005105.4_140600001	6	140600001	0.3703	0.4972
NC_005105.4_140700001	6	140700001	0.295	0.4201
NC_005105.4_140800001	6	140800001	0.2465	0.4287

NC_005105.4_140900001	6	140900001	0.1738	0.19
NC_005105.4_141000001	6	141000001	0.1738	0.19
NC_005105.4_141500001	6	141500001	0.3063	0.4793
NC_005105.4_141600001	6	141600001	0.2851	0.4605
NC_005105.4_141700001	6	141700001	0.2758	0.3967
NC_005105.4_141800001	6	141800001	0.2971	0.3788
NC_005105.4_141900001	6	141900001	0.269	0.3039
NC_005105.4_142000001	6	142000001	0.2515	0.1561
NC_005105.4_142100001	6	142100001	0.2866	0.1642
NC_005105.4_142500001	6	142500001	0.1161	0.1264
NC_005105.4_142600001	6	142600001	0.1161	0.1264
NC_005105.4_142700001	6	142700001	0.1161	0.1264
NC_005105.4_142900001	6	142900001	0.2942	0.2791
NC_005105.4_143500001	6	143500001	0.2796	0.3371
NC_005105.4_143600001	6	143600001	0.5018	0.6686
NC_005105.4_143700001	6	143700001	0.5423	0.6765
NC_005105.4_143800001	6	143800001	0.5586	0.6939
NC_005105.4_143900001	6	143900001	0.5763	0.7207
NC_005105.4_144000001	6	144000001	0.5822	0.7464
NC_005105.4_144100001	6	144100001	0.4532	0.5673
NC_005105.4_144200001	6	144200001	0.4184	0.5199
NC_005105.4_144300001	6	144300001	0.3617	0.4638
NC_005105.4_144400001	6	144400001	0.2591	0.3168
NC_005105.4_144500001	6	144500001	0.3931	0.5218
NC_005105.4_144600001	6	144600001	0.4452	0.6108
NC_005105.4_144700001	6	144700001	0.48	0.6482
NC_005105.4_144800001	6	144800001	0.4638	0.6233
NC_005105.4_144900001	6	144900001	0.4935	0.6408
NC_005105.4_145000001	6	145000001	0.4531	0.6122
NC_005105.4_145100001	6	145100001	0.3979	0.5995
NC_005105.4_145200001	6	145200001	0.3206	0.5526
NC_005105.4_145300001	6	145300001	0.4658	0.642
NC_005105.4_145400001	6	145400001	0.5315	0.6698
NC_005105.4_145500001	6	145500001	0.5238	0.6409
NC_005105.4_145600001	6	145600001	0.5452	0.6284
NC_005105.4_145700001	6	145700001	0.5891	0.6216
NC_005105.4_145800001	6	145800001	0.5997	0.6566
NC_005105.4_145900001	6	145900001	0.6076	0.6353
NC_005105.4_146000001	6	146000001	0.5477	0.6241
NC_005105.4_146100001	6	146100001	0.4742	0.6103
NC_005105.4_146200001	6	146200001	0.3972	0.5788
NC_005105.4_146300001	6	146300001	0.3296	0.4722
NC_005105.4_146400001	6	146400001	0.3551	0.5264
NC_005105.4_146500001	6	146500001	0.3944	0.5543
NC_005105.4_146600001	6	146600001	0.4275	0.62
NC_005105.4_146700001	6	146700001	0.4454	0.6565
NC_005105.4_146800001	6	146800001	0.4436	0.7108
NC_005105.4_146900001	6	146900001	0.3682	0.5569
NC_005105.4_147000001	6	147000001	0.3666	0.4942
NC_005105.4_147100001	6	147100001	0.389	0.403
NC_005105.4_147200001	6	147200001	0.4306	0.4659
NC_005105.4_147300001	6	147300001	0.4567	0.4694
NC_005105.4_147400001	6	147400001	0.3936	0.4077
NC_005106.4_900001	7	900001	0.6298	0.8698
NC_005106.4_1000001	7	1000001	0.6298	0.8698
NC_005106.4_1100001	7	1100001	0.6298	0.8698
NC_005106.4_2100001	7	2100001	0.7484	0.9608
NC_005106.4_2200001	7	2200001	0.6919	0.8259

NC_005106.4_2300001	7	2300001	0.7305	0.8868
NC_005106.4_2400001	7	2400001	0.7183	0.8655
NC_005106.4_2500001	7	2500001	0.612	0.7815
NC_005106.4_2600001	7	2600001	0.5398	0.6844
NC_005106.4_2700001	7	2700001	0.5648	0.7398
NC_005106.4_2800001	7	2800001	0.5102	0.6792
NC_005106.4_2900001	7	2900001	0.4766	0.6374
NC_005106.4_3000001	7	3000001	0.5924	0.7289
NC_005106.4_3100001	7	3100001	0.6338	0.8035
NC_005106.4_3200001	7	3200001	0.6105	0.7727
NC_005106.4_3300001	7	3300001	0.6609	0.8266
NC_005106.4_3400001	7	3400001	0.6251	0.9521
NC_005106.4_4200001	7	4200001	0.6079	0.3836
NC_005106.4_4300001	7	4300001	0.6079	0.3836
NC_005106.4_4400001	7	4400001	0.6079	0.3836
NC_005106.4_4500001	7	4500001	0.6079	0.3836
NC_005106.4_4600001	7	4600001	0.6079	0.3836
NC_005106.4_7300001	7	7300001	0.782	0.8041
NC_005106.4_7400001	7	7400001	0.782	0.8041
NC_005106.4_7500001	7	7500001	0.782	0.8041
NC_005106.4_8100001	7	8100001	0.5907	0.5273
NC_005106.4_8200001	7	8200001	0.5907	0.5273
NC_005106.4_8300001	7	8300001	0.5907	0.5273
NC_005106.4_9200001	7	9200001	0.8326	0.8568
NC_005106.4_9300001	7	9300001	0.8605	0.8846
NC_005106.4_9400001	7	9400001	0.663	0.6578
NC_005106.4_9500001	7	9500001	0.6503	0.637
NC_005106.4_9600001	7	9600001	0.6222	0.603
NC_005106.4_9700001	7	9700001	0.5871	0.543
NC_005106.4_9800001	7	9800001	0.5372	0.4696
NC_005106.4_10300001	7	10300001	0.207	0.3026
NC_005106.4_10400001	7	10400001	0.314	0.4022
NC_005106.4_10500001	7	10500001	0.4519	0.6035
NC_005106.4_10600001	7	10600001	0.4908	0.6669
NC_005106.4_10700001	7	10700001	0.5755	0.7411
NC_005106.4_10800001	7	10800001	0.7654	0.7969
NC_005106.4_10900001	7	10900001	0.8234	0.8754
NC_005106.4_11000001	7	11000001	0.7197	0.8372
NC_005106.4_11100001	7	11100001	0.7131	0.7894
NC_005106.4_11200001	7	11200001	0.6667	0.7096
NC_005106.4_11300001	7	11300001	0.5844	0.6902
NC_005106.4_11400001	7	11400001	0.5265	0.6563
NC_005106.4_11500001	7	11500001	0.5796	0.687
NC_005106.4_11600001	7	11600001	0.6283	0.7586
NC_005106.4_11700001	7	11700001	0.6499	0.7722
NC_005106.4_11800001	7	11800001	0.756	0.8261
NC_005106.4_11900001	7	11900001	0.7536	0.8285
NC_005106.4_12000001	7	12000001	0.6736	0.8065
NC_005106.4_12100001	7	12100001	0.6036	0.7497
NC_005106.4_12200001	7	12200001	0.6234	0.8364
NC_005106.4_12300001	7	12300001	0.4893	0.7732
NC_005106.4_12400001	7	12400001	0.532	0.8139
NC_005106.4_12500001	7	12500001	0.6082	0.8395
NC_005106.4_12600001	7	12600001	0.6745	0.8611
NC_005106.4_12700001	7	12700001	0.6361	0.8394
NC_005106.4_12800001	7	12800001	0.7222	0.8702
NC_005106.4_12900001	7	12900001	0.6004	0.806
NC_005106.4_13000001	7	13000001	0.5279	0.7167

NC_005106.4_13100001	7	13100001	0.3928	0.6416
NC_005106.4_13200001	7	13200001	0.3928	0.6416
NC_005106.4_13300001	7	13300001	0.3707	0.4682
NC_005106.4_13400001	7	13400001	0.4433	0.5001
NC_005106.4_13500001	7	13500001	0.4293	0.5261
NC_005106.4_13600001	7	13600001	0.4285	0.5309
NC_005106.4_13700001	7	13700001	0.4234	0.4896
NC_005106.4_13800001	7	13800001	0.4669	0.5764
NC_005106.4_13900001	7	13900001	0.4885	0.6049
NC_005106.4_14000001	7	14000001	0.5394	0.6107
NC_005106.4_14100001	7	14100001	0.5677	0.6031
NC_005106.4_14200001	7	14200001	0.7051	0.7283
NC_005106.4_14300001	7	14300001	0.7432	0.6916
NC_005106.4_14400001	7	14400001	0.5857	0.6274
NC_005106.4_14500001	7	14500001	0.6408	0.7162
NC_005106.4_14600001	7	14600001	0.6307	0.7947
NC_005106.4_14700001	7	14700001	0.661	0.8444
NC_005106.4_14800001	7	14800001	0.6855	0.8459
NC_005106.4_14900001	7	14900001	0.7971	0.9032
NC_005106.4_15000001	7	15000001	0.7402	0.8694
NC_005106.4_15100001	7	15100001	0.7494	0.795
NC_005106.4_15200001	7	15200001	0.7497	0.7212
NC_005106.4_15300001	7	15300001	0.5211	0.6077
NC_005106.4_15400001	7	15400001	0.4275	0.4893
NC_005106.4_15500001	7	15500001	0.4214	0.4774
NC_005106.4_15600001	7	15600001	0.3532	0.4488
NC_005106.4_15700001	7	15700001	0.318	0.4381
NC_005106.4_16500001	7	16500001	0.2446	0.1621
NC_005106.4_16600001	7	16600001	0.2278	0.209
NC_005106.4_16700001	7	16700001	0.3704	0.4245
NC_005106.4_16800001	7	16800001	0.3704	0.4245
NC_005106.4_16900001	7	16900001	0.3753	0.3923
NC_005106.4_17000001	7	17000001	0.5679	0.6277
NC_005106.4_17100001	7	17100001	0.618	0.624
NC_005106.4_17900001	7	17900001	0.4936	0.7548
NC_005106.4_18000001	7	18000001	0.5884	0.7785
NC_005106.4_18100001	7	18100001	0.5322	0.758
NC_005106.4_18200001	7	18200001	0.5518	0.7266
NC_005106.4_18300001	7	18300001	0.5518	0.7266
NC_005106.4_18400001	7	18400001	0.5241	0.7186
NC_005106.4_18500001	7	18500001	0.4048	0.6403
NC_005106.4_23100001	7	23100001	0.6214	0.6453
NC_005106.4_23200001	7	23200001	0.596	0.6304
NC_005106.4_23300001	7	23300001	0.6147	0.6514
NC_005106.4_23400001	7	23400001	0.5803	0.6362
NC_005106.4_23500001	7	23500001	0.5149	0.6081
NC_005106.4_23600001	7	23600001	0.3963	0.4889
NC_005106.4_23700001	7	23700001	0.3885	0.5337
NC_005106.4_23800001	7	23800001	0.3921	0.5106
NC_005106.4_23900001	7	23900001	0.483	0.6148
NC_005106.4_24000001	7	24000001	0.4733	0.6345
NC_005106.4_24100001	7	24100001	0.5247	0.6978
NC_005106.4_24200001	7	24200001	0.583	0.7392
NC_005106.4_24300001	7	24300001	0.5536	0.7366
NC_005106.4_24400001	7	24400001	0.4367	0.6261
NC_005106.4_24500001	7	24500001	0.3865	0.5865
NC_005106.4_24600001	7	24600001	0.3498	0.574
NC_005106.4_24700001	7	24700001	0.286	0.4519

NC_005106.4_24800001	7	24800001	0.2458	0.3926
NC_005106.4_24900001	7	24900001	0.2976	0.5572
NC_005106.4_25000001	7	25000001	0.3349	0.4994
NC_005106.4_25100001	7	25100001	0.4181	0.5275
NC_005106.4_25200001	7	25200001	0.5166	0.668
NC_005106.4_25300001	7	25300001	0.5528	0.7122
NC_005106.4_25400001	7	25400001	0.5668	0.6826
NC_005106.4_25500001	7	25500001	0.6131	0.764
NC_005106.4_25600001	7	25600001	0.6034	0.8027
NC_005106.4_25700001	7	25700001	0.5284	0.7487
NC_005106.4_25800001	7	25800001	0.5498	0.7358
NC_005106.4_25900001	7	25900001	0.4948	0.7155
NC_005106.4_26000001	7	26000001	0.5273	0.7293
NC_005106.4_26100001	7	26100001	0.5155	0.7242
NC_005106.4_26200001	7	26200001	0.5617	0.7054
NC_005106.4_26300001	7	26300001	0.482	0.6345
NC_005106.4_26400001	7	26400001	0.4374	0.573
NC_005106.4_26500001	7	26500001	0.3802	0.4431
NC_005106.4_26600001	7	26600001	0.4301	0.5046
NC_005106.4_26700001	7	26700001	0.4438	0.5472
NC_005106.4_26800001	7	26800001	0.5216	0.5863
NC_005106.4_26900001	7	26900001	0.6248	0.6367
NC_005106.4_27000001	7	27000001	0.6905	0.7537
NC_005106.4_27100001	7	27100001	0.6598	0.737
NC_005106.4_27200001	7	27200001	0.6527	0.7307
NC_005106.4_27300001	7	27300001	0.6443	0.7867
NC_005106.4_27400001	7	27400001	0.6943	0.8625
NC_005106.4_27500001	7	27500001	0.6573	0.8125
NC_005106.4_27600001	7	27600001	0.6296	0.7751
NC_005106.4_27700001	7	27700001	0.5995	0.786
NC_005106.4_27800001	7	27800001	0.5699	0.759
NC_005106.4_27900001	7	27900001	0.4408	0.5963
NC_005106.4_28000001	7	28000001	0.5248	0.6792
NC_005106.4_28100001	7	28100001	0.5523	0.7075
NC_005106.4_28200001	7	28200001	0.5921	0.7046
NC_005106.4_28300001	7	28300001	0.5894	0.6691
NC_005106.4_28400001	7	28400001	0.6654	0.7404
NC_005106.4_28500001	7	28500001	0.5931	0.695
NC_005106.4_28600001	7	28600001	0.5561	0.7515
NC_005106.4_28700001	7	28700001	0.5507	0.7549
NC_005106.4_28800001	7	28800001	0.5483	0.7955
NC_005106.4_28900001	7	28900001	0.514	0.7054
NC_005106.4_29000001	7	29000001	0.4982	0.6372
NC_005106.4_29100001	7	29100001	0.4752	0.5342
NC_005106.4_29200001	7	29200001	0.5061	0.5275
NC_005106.4_29300001	7	29300001	0.5076	0.5256
NC_005106.4_29400001	7	29400001	0.4778	0.5002
NC_005106.4_29500001	7	29500001	0.4327	0.4416
NC_005106.4_29600001	7	29600001	0.419	0.4276
NC_005106.4_29700001	7	29700001	0.3278	0.3082
NC_005106.4_29800001	7	29800001	0.479	0.5046
NC_005106.4_29900001	7	29900001	0.5148	0.5632
NC_005106.4_30000001	7	30000001	0.6032	0.6425
NC_005106.4_30100001	7	30100001	0.6967	0.7032
NC_005106.4_30200001	7	30200001	0.7082	0.7197
NC_005106.4_30300001	7	30300001	0.6662	0.6973
NC_005106.4_30400001	7	30400001	0.6858	0.6785
NC_005106.4_30500001	7	30500001	0.7359	0.7369

NC_005106.4_30600001	7	30600001	0.4689	0.6026
NC_005106.4_30700001	7	30700001	0.4379	0.5552
NC_005106.4_30800001	7	30800001	0.4692	0.5939
NC_005106.4_30900001	7	30900001	0.5281	0.6846
NC_005106.4_31000001	7	31000001	0.4938	0.6188
NC_005106.4_31100001	7	31100001	0.524	0.6436
NC_005106.4_31200001	7	31200001	0.5233	0.696
NC_005106.4_31300001	7	31300001	0.4969	0.6849
NC_005106.4_31400001	7	31400001	0.4595	0.6109
NC_005106.4_31500001	7	31500001	0.4069	0.5922
NC_005106.4_31600001	7	31600001	0.4324	0.5805
NC_005106.4_31700001	7	31700001	0.4558	0.5452
NC_005106.4_31800001	7	31800001	0.5283	0.602
NC_005106.4_31900001	7	31900001	0.4545	0.5722
NC_005106.4_32000001	7	32000001	0.5218	0.6292
NC_005106.4_32100001	7	32100001	0.5281	0.6936
NC_005106.4_32200001	7	32200001	0.5005	0.6605
NC_005106.4_32300001	7	32300001	0.3676	0.4947
NC_005106.4_32400001	7	32400001	0.5141	0.6117
NC_005106.4_32500001	7	32500001	0.4475	0.5786
NC_005106.4_32600001	7	32600001	0.4446	0.4509
NC_005106.4_32700001	7	32700001	0.5604	0.618
NC_005106.4_32800001	7	32800001	0.561	0.6501
NC_005106.4_32900001	7	32900001	0.5887	0.6244
NC_005106.4_33000001	7	33000001	0.5872	0.6685
NC_005106.4_33100001	7	33100001	0.6283	0.7216
NC_005106.4_33200001	7	33200001	0.6681	0.7458
NC_005106.4_33300001	7	33300001	0.7012	0.7881
NC_005106.4_33400001	7	33400001	0.7806	0.8803
NC_005106.4_33500001	7	33500001	0.7225	0.8465
NC_005106.4_33600001	7	33600001	0.6821	0.809
NC_005106.4_33700001	7	33700001	0.5924	0.7765
NC_005106.4_33800001	7	33800001	0.5672	0.7356
NC_005106.4_33900001	7	33900001	0.4426	0.5697
NC_005106.4_34000001	7	34000001	0.483	0.5428
NC_005106.4_34100001	7	34100001	0.4023	0.5026
NC_005106.4_34200001	7	34200001	0.4135	0.4676
NC_005106.4_34300001	7	34300001	0.4656	0.5465
NC_005106.4_34400001	7	34400001	0.4268	0.5485
NC_005106.4_34500001	7	34500001	0.4111	0.5266
NC_005106.4_34600001	7	34600001	0.5048	0.5878
NC_005106.4_34700001	7	34700001	0.5145	0.5994
NC_005106.4_34800001	7	34800001	0.4864	0.5944
NC_005106.4_34900001	7	34900001	0.4766	0.5748
NC_005106.4_35000001	7	35000001	0.5045	0.6222
NC_005106.4_35100001	7	35100001	0.4519	0.5947
NC_005106.4_35200001	7	35200001	0.4253	0.5465
NC_005106.4_35300001	7	35300001	0.4663	0.558
NC_005106.4_35400001	7	35400001	0.5458	0.6034
NC_005106.4_35500001	7	35500001	0.5324	0.6176
NC_005106.4_35600001	7	35600001	0.5362	0.6231
NC_005106.4_35700001	7	35700001	0.5345	0.6569
NC_005106.4_35800001	7	35800001	0.4976	0.5946
NC_005106.4_35900001	7	35900001	0.4721	0.5988
NC_005106.4_36000001	7	36000001	0.5153	0.6242
NC_005106.4_36100001	7	36100001	0.5419	0.6356
NC_005106.4_36200001	7	36200001	0.555	0.6159
NC_005106.4_36300001	7	36300001	0.5759	0.6313

NC_005106.4_36400001	7	36400001	0.5931	0.6211
NC_005106.4_36500001	7	36500001	0.4849	0.5279
NC_005106.4_36600001	7	36600001	0.5077	0.5747
NC_005106.4_36700001	7	36700001	0.5826	0.6016
NC_005106.4_36800001	7	36800001	0.5697	0.6272
NC_005106.4_36900001	7	36900001	0.459	0.568
NC_005106.4_37000001	7	37000001	0.5033	0.5774
NC_005106.4_37100001	7	37100001	0.4766	0.5509
NC_005106.4_37200001	7	37200001	0.4834	0.6104
NC_005106.4_37300001	7	37300001	0.4314	0.578
NC_005106.4_37400001	7	37400001	0.5269	0.6609
NC_005106.4_37500001	7	37500001	0.5223	0.7011
NC_005106.4_37600001	7	37600001	0.5366	0.7677
NC_005106.4_37700001	7	37700001	0.4303	0.5685
NC_005106.4_37800001	7	37800001	0.5218	0.6579
NC_005106.4_37900001	7	37900001	0.4291	0.4737
NC_005106.4_38000001	7	38000001	0.4539	0.4975
NC_005106.4_38100001	7	38100001	0.4557	0.4917
NC_005106.4_38200001	7	38200001	0.4304	0.4577
NC_005106.4_38300001	7	38300001	0.4077	0.4086
NC_005106.4_38400001	7	38400001	0.3133	0.3004
NC_005106.4_38500001	7	38500001	0.2763	0.2299
NC_005106.4_38600001	7	38600001	0.3491	0.3719
NC_005106.4_38700001	7	38700001	0.5025	0.5176
NC_005106.4_38900001	7	38900001	0.5417	0.7649
NC_005106.4_39000001	7	39000001	0.7129	0.9498
NC_005106.4_39100001	7	39100001	0.5047	0.7491
NC_005106.4_39200001	7	39200001	0.5163	0.7418
NC_005106.4_39300001	7	39300001	0.5309	0.6925
NC_005106.4_39400001	7	39400001	0.5213	0.6017
NC_005106.4_39500001	7	39500001	0.5079	0.5606
NC_005106.4_39600001	7	39600001	0.5814	0.6101
NC_005106.4_39700001	7	39700001	0.5528	0.508
NC_005106.4_39800001	7	39800001	0.5832	0.5997
NC_005106.4_39900001	7	39900001	0.6825	0.7025
NC_005106.4_40000001	7	40000001	0.6583	0.718
NC_005106.4_40100001	7	40100001	0.6583	0.718
NC_005106.4_40200001	7	40200001	0.6583	0.718
NC_005106.4_40300001	7	40300001	0.5974	0.6492
NC_005106.4_40500001	7	40500001	0.47	0.8218
NC_005106.4_40600001	7	40600001	0.5303	0.8576
NC_005106.4_40700001	7	40700001	0.5326	0.7435
NC_005106.4_40800001	7	40800001	0.436	0.6502
NC_005106.4_40900001	7	40900001	0.5002	0.6068
NC_005106.4_41000001	7	41000001	0.4484	0.5586
NC_005106.4_41100001	7	41100001	0.4084	0.4995
NC_005106.4_41200001	7	41200001	0.3618	0.4452
NC_005106.4_41300001	7	41300001	0.4683	0.5054
NC_005106.4_41400001	7	41400001	0.4081	0.4676
NC_005106.4_41500001	7	41500001	0.425	0.4602
NC_005106.4_41600001	7	41600001	0.414	0.4668
NC_005106.4_41700001	7	41700001	0.414	0.4668
NC_005106.4_41800001	7	41800001	0.4251	0.534
NC_005106.4_41900001	7	41900001	0.4204	0.5234
NC_005106.4_42000001	7	42000001	0.4998	0.6329
NC_005106.4_42100001	7	42100001	0.5072	0.6181
NC_005106.4_42200001	7	42200001	0.5072	0.6181
NC_005106.4_42300001	7	42300001	0.436	0.5127

NC_005106.4_42900001	7	42900001	0.6902	0.6929
NC_005106.4_43000001	7	43000001	0.7315	0.7259
NC_005106.4_43100001	7	43100001	0.7356	0.7784
NC_005106.4_43200001	7	43200001	0.7627	0.8526
NC_005106.4_43300001	7	43300001	0.7134	0.8301
NC_005106.4_43400001	7	43400001	0.6712	0.8412
NC_005106.4_43500001	7	43500001	0.5542	0.7497
NC_005106.4_43600001	7	43600001	0.4287	0.6261
NC_005106.4_43700001	7	43700001	0.5177	0.7196
NC_005106.4_43800001	7	43800001	0.6518	0.7279
NC_005106.4_43900001	7	43900001	0.7481	0.7695
NC_005106.4_44000001	7	44000001	0.8053	0.8114
NC_005106.4_44100001	7	44100001	0.6759	0.7964
NC_005106.4_44200001	7	44200001	0.6507	0.7758
NC_005106.4_44300001	7	44300001	0.4828	0.5045
NC_005106.4_44400001	7	44400001	0.436	0.4365
NC_005106.4_44500001	7	44500001	0.436	0.4365
NC_005106.4_44600001	7	44600001	0.5256	0.409
NC_005106.4_44700001	7	44700001	0.4205	0.3735
NC_005106.4_44900001	7	44900001	0.2973	0.5586
NC_005106.4_45000001	7	45000001	0.3693	0.6156
NC_005106.4_45100001	7	45100001	0.2722	0.4613
NC_005106.4_45200001	7	45200001	0.4616	0.5756
NC_005106.4_45300001	7	45300001	0.4513	0.5152
NC_005106.4_45400001	7	45400001	0.7472	0.6433
NC_005106.4_45600001	7	45600001	0.646	0.5079
NC_005106.4_45700001	7	45700001	0.6285	0.4946
NC_005106.4_45800001	7	45800001	0.5726	0.5081
NC_005106.4_45900001	7	45900001	0.5114	0.4144
NC_005106.4_46000001	7	46000001	0.4169	0.3972
NC_005106.4_46100001	7	46100001	0.4586	0.6195
NC_005106.4_46200001	7	46200001	0.4006	0.6402
NC_005106.4_46300001	7	46300001	0.3334	0.612
NC_005106.4_46400001	7	46400001	0.3646	0.5803
NC_005106.4_46500001	7	46500001	0.5854	0.7722
NC_005106.4_46600001	7	46600001	0.4191	0.7017
NC_005106.4_46700001	7	46700001	0.441	0.7454
NC_005106.4_46800001	7	46800001	0.4608	0.6679
NC_005106.4_46900001	7	46900001	0.3695	0.6586
NC_005106.4_47000001	7	47000001	0.4781	0.7499
NC_005106.4_47100001	7	47100001	0.6175	0.7484
NC_005106.4_47200001	7	47200001	0.6097	0.7316
NC_005106.4_47300001	7	47300001	0.6389	0.7296
NC_005106.4_47400001	7	47400001	0.6578	0.6992
NC_005106.4_47500001	7	47500001	0.5679	0.6085
NC_005106.4_47600001	7	47600001	0.4469	0.5277
NC_005106.4_47700001	7	47700001	0.4604	0.4798
NC_005106.4_47800001	7	47800001	0.4862	0.5696
NC_005106.4_47900001	7	47900001	0.5883	0.6902
NC_005106.4_48000001	7	48000001	0.5151	0.6205
NC_005106.4_48100001	7	48100001	0.5773	0.688
NC_005106.4_48200001	7	48200001	0.5075	0.7083
NC_005106.4_48300001	7	48300001	0.5075	0.7083
NC_005106.4_48400001	7	48400001	0.4703	0.6574
NC_005106.4_48500001	7	48500001	0.36	0.4943
NC_005106.4_48600001	7	48600001	0.2939	0.4456
NC_005106.4_48700001	7	48700001	0.3164	0.4643
NC_005106.4_48800001	7	48800001	0.3525	0.4807

NC_005106.4_48900001	7	48900001	0.3626	0.4506
NC_005106.4_49000001	7	49000001	0.4004	0.503
NC_005106.4_49100001	7	49100001	0.5139	0.5636
NC_005106.4_49200001	7	49200001	0.5513	0.5863
NC_005106.4_49300001	7	49300001	0.4534	0.5195
NC_005106.4_49400001	7	49400001	0.4859	0.5582
NC_005106.4_49500001	7	49500001	0.4446	0.5688
NC_005106.4_49600001	7	49600001	0.3597	0.4574
NC_005106.4_49700001	7	49700001	0.5342	0.6218
NC_005106.4_49800001	7	49800001	0.5582	0.6353
NC_005106.4_49900001	7	49900001	0.5986	0.7058
NC_005106.4_50000001	7	50000001	0.6492	0.726
NC_005106.4_50100001	7	50100001	0.5532	0.657
NC_005106.4_50200001	7	50200001	0.4863	0.6121
NC_005106.4_50300001	7	50300001	0.478	0.6247
NC_005106.4_50400001	7	50400001	0.3194	0.5567
NC_005106.4_50500001	7	50500001	0.3853	0.6246
NC_005106.4_50600001	7	50600001	0.3407	0.7175
NC_005106.4_50700001	7	50700001	0.3206	0.7014
NC_005106.4_50800001	7	50800001	0.3405	0.5357
NC_005106.4_50900001	7	50900001	0.3801	0.5463
NC_005106.4_51000001	7	51000001	0.3346	0.4822
NC_005106.4_51100001	7	51100001	0.4756	0.5924
NC_005106.4_51200001	7	51200001	0.4526	0.5755
NC_005106.4_51300001	7	51300001	0.4634	0.8111
NC_005106.4_51400001	7	51400001	0.2386	0.5988
NC_005106.4_51500001	7	51500001	0.2386	0.5988
NC_005106.4_51600001	7	51600001	0.2434	0.6666
NC_005106.4_51700001	7	51700001	0.2484	0.6184
NC_005106.4_51800001	7	51800001	0.485	0.7693
NC_005106.4_51900001	7	51900001	0.5796	0.7363
NC_005106.4_52000001	7	52000001	0.623	0.7613
NC_005106.4_52100001	7	52100001	0.6113	0.7289
NC_005106.4_52200001	7	52200001	0.695	0.7937
NC_005106.4_52300001	7	52300001	0.6982	0.7994
NC_005106.4_52400001	7	52400001	0.7707	0.8736
NC_005106.4_52500001	7	52500001	0.8082	0.8796
NC_005106.4_52600001	7	52600001	0.7283	0.8575
NC_005106.4_52700001	7	52700001	0.6409	0.7758
NC_005106.4_52800001	7	52800001	0.5436	0.7369
NC_005106.4_52900001	7	52900001	0.6309	0.768
NC_005106.4_53000001	7	53000001	0.5962	0.766
NC_005106.4_53100001	7	53100001	0.6404	0.7808
NC_005106.4_53200001	7	53200001	0.5232	0.7893
NC_005106.4_53300001	7	53300001	0.5651	0.7115
NC_005106.4_53400001	7	53400001	0.4106	0.5833
NC_005106.4_53500001	7	53500001	0.3774	0.5437
NC_005106.4_53600001	7	53600001	0.3384	0.438
NC_005106.4_53700001	7	53700001	0.4117	0.4348
NC_005106.4_53800001	7	53800001	0.4427	0.5288
NC_005106.4_53900001	7	53900001	0.4861	0.5839
NC_005106.4_54000001	7	54000001	0.4804	0.5654
NC_005106.4_54100001	7	54100001	0.5189	0.6225
NC_005106.4_54200001	7	54200001	0.4933	0.6732
NC_005106.4_54300001	7	54300001	0.5494	0.6336
NC_005106.4_54400001	7	54400001	0.4685	0.5282
NC_005106.4_54500001	7	54500001	0.5273	0.6097
NC_005106.4_54600001	7	54600001	0.5801	0.6183

NC_005106.4_54700001	7	54700001	0.6414	0.6644
NC_005106.4_54800001	7	54800001	0.536	0.5598
NC_005106.4_54900001	7	54900001	0.5359	0.5971
NC_005106.4_55000001	7	55000001	0.5462	0.5675
NC_005106.4_55100001	7	55100001	0.5403	0.5778
NC_005106.4_55200001	7	55200001	0.5549	0.6126
NC_005106.4_55300001	7	55300001	0.5479	0.6217
NC_005106.4_55400001	7	55400001	0.585	0.6697
NC_005106.4_55500001	7	55500001	0.4369	0.6441
NC_005106.4_55600001	7	55600001	0.5593	0.6898
NC_005106.4_55700001	7	55700001	0.5269	0.6877
NC_005106.4_55800001	7	55800001	0.4017	0.665
NC_005106.4_55900001	7	55900001	0.4197	0.6889
NC_005106.4_56000001	7	56000001	0.4878	0.7171
NC_005106.4_56100001	7	56100001	0.324	0.6737
NC_005106.4_56200001	7	56200001	0.284	0.5898
NC_005106.4_56300001	7	56300001	0.3228	0.5767
NC_005106.4_56400001	7	56400001	0.2173	0.5076
NC_005106.4_56500001	7	56500001	0.2206	0.4547
NC_005106.4_56600001	7	56600001	0.2391	0.4791
NC_005106.4_56700001	7	56700001	0.3009	0.6035
NC_005106.4_56800001	7	56800001	0.3996	0.7017
NC_005106.4_56900001	7	56900001	0.4974	0.7271
NC_005106.4_57000001	7	57000001	0.4267	0.7441
NC_005106.4_57100001	7	57100001	0.4267	0.7441
NC_005106.4_57200001	7	57200001	0.3992	0.709
NC_005106.4_57300001	7	57300001	0.3971	0.6908
NC_005106.4_57400001	7	57400001	0.4904	0.7496
NC_005106.4_57500001	7	57500001	0.5984	0.7959
NC_005106.4_57600001	7	57600001	0.5713	0.7014
NC_005106.4_57700001	7	57700001	0.5795	0.69
NC_005106.4_57800001	7	57800001	0.6129	0.7139
NC_005106.4_57900001	7	57900001	0.6145	0.7166
NC_005106.4_58000001	7	58000001	0.6949	0.7878
NC_005106.4_58100001	7	58100001	0.737	0.8792
NC_005106.4_58200001	7	58200001	0.7432	0.9234
NC_005106.4_58300001	7	58300001	0.783	0.9671
NC_005106.4_58400001	7	58400001	0.6105	0.8785
NC_005106.4_58500001	7	58500001	0.4452	0.7312
NC_005106.4_58600001	7	58600001	0.5253	0.7289
NC_005106.4_58700001	7	58700001	0.616	0.7693
NC_005106.4_58800001	7	58800001	0.5577	0.6994
NC_005106.4_58900001	7	58900001	0.591	0.6955
NC_005106.4_59000001	7	59000001	0.5523	0.6868
NC_005106.4_59100001	7	59100001	0.507	0.6463
NC_005106.4_59200001	7	59200001	0.5263	0.6198
NC_005106.4_59300001	7	59300001	0.5696	0.6778
NC_005106.4_59400001	7	59400001	0.5955	0.6706
NC_005106.4_59500001	7	59500001	0.6131	0.664
NC_005106.4_59600001	7	59600001	0.5996	0.6539
NC_005106.4_59700001	7	59700001	0.6429	0.7289
NC_005106.4_59800001	7	59800001	0.5419	0.6737
NC_005106.4_59900001	7	59900001	0.5724	0.7531
NC_005106.4_60000001	7	60000001	0.6113	0.7852
NC_005106.4_60100001	7	60100001	0.6273	0.7564
NC_005106.4_60200001	7	60200001	0.5203	0.6796
NC_005106.4_60300001	7	60300001	0.6067	0.7198
NC_005106.4_60400001	7	60400001	0.5833	0.6783

NC_005106.4_60500001	7	60500001	0.5341	0.6466
NC_005106.4_60600001	7	60600001	0.4755	0.6086
NC_005106.4_60700001	7	60700001	0.4407	0.5475
NC_005106.4_60800001	7	60800001	0.4855	0.5422
NC_005106.4_60900001	7	60900001	0.4781	0.5201
NC_005106.4_61000001	7	61000001	0.4774	0.5063
NC_005106.4_61100001	7	61100001	0.4993	0.5137
NC_005106.4_61200001	7	61200001	0.4401	0.4378
NC_005106.4_61300001	7	61300001	0.4422	0.4681
NC_005106.4_61400001	7	61400001	0.4089	0.422
NC_005106.4_61500001	7	61500001	0.4278	0.522
NC_005106.4_61600001	7	61600001	0.4322	0.5615
NC_005106.4_61700001	7	61700001	0.4905	0.6304
NC_005106.4_61800001	7	61800001	0.4221	0.5666
NC_005106.4_61900001	7	61900001	0.4535	0.6272
NC_005106.4_62000001	7	62000001	0.4452	0.6093
NC_005106.4_62100001	7	62100001	0.4435	0.6275
NC_005106.4_62200001	7	62200001	0.4249	0.6063
NC_005106.4_62300001	7	62300001	0.5008	0.6914
NC_005106.4_62400001	7	62400001	0.4808	0.6379
NC_005106.4_62500001	7	62500001	0.5417	0.6677
NC_005106.4_62600001	7	62600001	0.5903	0.7004
NC_005106.4_62700001	7	62700001	0.6685	0.7552
NC_005106.4_62800001	7	62800001	0.6192	0.7213
NC_005106.4_62900001	7	62900001	0.5873	0.7624
NC_005106.4_63000001	7	63000001	0.6669	0.8344
NC_005106.4_63100001	7	63100001	0.5656	0.747
NC_005106.4_63200001	7	63200001	0.5062	0.7372
NC_005106.4_63300001	7	63300001	0.5431	0.7715
NC_005106.4_63400001	7	63400001	0.5665	0.7972
NC_005106.4_63500001	7	63500001	0.4036	0.5243
NC_005106.4_63600001	7	63600001	0.4255	0.5495
NC_005106.4_63700001	7	63700001	0.5244	0.6431
NC_005106.4_63800001	7	63800001	0.5425	0.6578
NC_005106.4_63900001	7	63900001	0.4502	0.5836
NC_005106.4_64000001	7	64000001	0.506	0.6869
NC_005106.4_64100001	7	64100001	0.4917	0.6399
NC_005106.4_64200001	7	64200001	0.3613	0.4473
NC_005106.4_64300001	7	64300001	0.3504	0.3804
NC_005106.4_64400001	7	64400001	0.4512	0.5012
NC_005106.4_64500001	7	64500001	0.446	0.4983
NC_005106.4_64600001	7	64600001	0.4956	0.604
NC_005106.4_64700001	7	64700001	0.5812	0.7262
NC_005106.4_64800001	7	64800001	0.5928	0.7604
NC_005106.4_64900001	7	64900001	0.5959	0.7379
NC_005106.4_65000001	7	65000001	0.5702	0.6881
NC_005106.4_65100001	7	65100001	0.5409	0.6468
NC_005106.4_65200001	7	65200001	0.5108	0.5783
NC_005106.4_65300001	7	65300001	0.4861	0.5411
NC_005106.4_65400001	7	65400001	0.5643	0.6149
NC_005106.4_65500001	7	65500001	0.5409	0.6259
NC_005106.4_65600001	7	65600001	0.6031	0.6958
NC_005106.4_65700001	7	65700001	0.5797	0.6351
NC_005106.4_65800001	7	65800001	0.556	0.624
NC_005106.4_65900001	7	65900001	0.5495	0.5836
NC_005106.4_66000001	7	66000001	0.5862	0.6483
NC_005106.4_66100001	7	66100001	0.5234	0.6434
NC_005106.4_66200001	7	66200001	0.5204	0.6915

NC_005106.4_66300001	7	66300001	0.5914	0.6983
NC_005106.4_66400001	7	66400001	0.4824	0.6498
NC_005106.4_66500001	7	66500001	0.4977	0.6408
NC_005106.4_66600001	7	66600001	0.6112	0.7023
NC_005106.4_66700001	7	66700001	0.5735	0.6639
NC_005106.4_66800001	7	66800001	0.4903	0.5845
NC_005106.4_66900001	7	66900001	0.5587	0.6476
NC_005106.4_67000001	7	67000001	0.5107	0.5755
NC_005106.4_67100001	7	67100001	0.4031	0.4046
NC_005106.4_67200001	7	67200001	0.4398	0.447
NC_005106.4_67300001	7	67300001	0.4908	0.6934
NC_005106.4_67400001	7	67400001	0.4804	0.6865
NC_005106.4_67500001	7	67500001	0.5103	0.7561
NC_005106.4_67600001	7	67600001	0.4374	0.648
NC_005106.4_67700001	7	67700001	0.4374	0.648
NC_005106.4_67800001	7	67800001	0.4684	0.6215
NC_005106.4_67900001	7	67900001	0.4839	0.6399
NC_005106.4_68000001	7	68000001	0.503	0.5866
NC_005106.4_68100001	7	68100001	0.4711	0.6264
NC_005106.4_68200001	7	68200001	0.4316	0.6208
NC_005106.4_68300001	7	68300001	0.3011	0.5992
NC_005106.4_68400001	7	68400001	0.1883	0.3805
NC_005106.4_68500001	7	68500001	0.2208	0.3686
NC_005106.4_68600001	7	68600001	0.2182	0.4144
NC_005106.4_68700001	7	68700001	0.2251	0.4083
NC_005106.4_68800001	7	68800001	0.3354	0.52
NC_005106.4_68900001	7	68900001	0.351	0.5603
NC_005106.4_69000001	7	69000001	0.3724	0.546
NC_005106.4_69100001	7	69100001	0.4145	0.5242
NC_005106.4_69200001	7	69200001	0.4018	0.5211
NC_005106.4_69300001	7	69300001	0.3703	0.4649
NC_005106.4_69400001	7	69400001	0.3442	0.4227
NC_005106.4_69500001	7	69500001	0.2866	0.3719
NC_005106.4_69600001	7	69600001	0.3607	0.53
NC_005106.4_69700001	7	69700001	0.4029	0.626
NC_005106.4_69800001	7	69800001	0.4146	0.6972
NC_005106.4_69900001	7	69900001	0.4955	0.7712
NC_005106.4_70000001	7	70000001	0.5243	0.7775
NC_005106.4_70100001	7	70100001	0.6752	0.8333
NC_005106.4_70200001	7	70200001	0.6738	0.8148
NC_005106.4_70300001	7	70300001	0.6961	0.8061
NC_005106.4_70400001	7	70400001	0.5733	0.6863
NC_005106.4_70500001	7	70500001	0.5358	0.6981
NC_005106.4_70600001	7	70600001	0.4272	0.6468
NC_005106.4_70700001	7	70700001	0.4398	0.6241
NC_005106.4_70800001	7	70800001	0.4566	0.6388
NC_005106.4_70900001	7	70900001	0.4764	0.6726
NC_005106.4_71000001	7	71000001	0.5236	0.6479
NC_005106.4_71100001	7	71100001	0.4588	0.6118
NC_005106.4_71200001	7	71200001	0.4893	0.6209
NC_005106.4_71300001	7	71300001	0.4663	0.5617
NC_005106.4_71400001	7	71400001	0.4731	0.5414
NC_005106.4_71500001	7	71500001	0.4438	0.5104
NC_005106.4_71600001	7	71600001	0.4468	0.4173
NC_005106.4_71700001	7	71700001	0.3882	0.3136
NC_005106.4_71800001	7	71800001	0.4025	0.3563
NC_005106.4_71900001	7	71900001	0.4742	0.4582
NC_005106.4_72000001	7	72000001	0.5118	0.4837

NC_005106.4_72100001	7	72100001	0.5678	0.534
NC_005106.4_72200001	7	72200001	0.5886	0.5599
NC_005106.4_72300001	7	72300001	0.743	0.7953
NC_005106.4_72500001	7	72500001	0.2925	0.508
NC_005106.4_72600001	7	72600001	0.2576	0.4132
NC_005106.4_72700001	7	72700001	0.3448	0.5187
NC_005106.4_72800001	7	72800001	0.2942	0.4075
NC_005106.4_72900001	7	72900001	0.3654	0.4986
NC_005106.4_73000001	7	73000001	0.4096	0.5236
NC_005106.4_73100001	7	73100001	0.4224	0.5373
NC_005106.4_73200001	7	73200001	0.3645	0.4632
NC_005106.4_73300001	7	73300001	0.4098	0.5743
NC_005106.4_73400001	7	73400001	0.3808	0.6223
NC_005106.4_73500001	7	73500001	0.461	0.7281
NC_005106.4_73600001	7	73600001	0.5863	0.8912
NC_005106.4_73700001	7	73700001	0.6064	0.8336
NC_005106.4_73800001	7	73800001	0.7193	0.8392
NC_005106.4_73900001	7	73900001	0.6738	0.81
NC_005106.4_74000001	7	74000001	0.6938	0.7552
NC_005106.4_74100001	7	74100001	0.6141	0.6507
NC_005106.4_74200001	7	74200001	0.479	0.6413
NC_005106.4_74300001	7	74300001	0.4839	0.6028
NC_005106.4_74400001	7	74400001	0.4971	0.6415
NC_005106.4_74500001	7	74500001	0.4878	0.6491
NC_005106.4_74600001	7	74600001	0.5779	0.7006
NC_005106.4_74700001	7	74700001	0.6138	0.6871
NC_005106.4_74800001	7	74800001	0.5749	0.6779
NC_005106.4_74900001	7	74900001	0.4883	0.6
NC_005106.4_75000001	7	75000001	0.4933	0.5958
NC_005106.4_75100001	7	75100001	0.4114	0.5156
NC_005106.4_75200001	7	75200001	0.4866	0.6044
NC_005106.4_75300001	7	75300001	0.5271	0.6325
NC_005106.4_75400001	7	75400001	0.6115	0.6759
NC_005106.4_75500001	7	75500001	0.5822	0.6631
NC_005106.4_75600001	7	75600001	0.5533	0.6609
NC_005106.4_75700001	7	75700001	0.4764	0.621
NC_005106.4_75800001	7	75800001	0.3964	0.5977
NC_005106.4_75900001	7	75900001	0.4704	0.586
NC_005106.4_76000001	7	76000001	0.4514	0.5804
NC_005106.4_76100001	7	76100001	0.479	0.6033
NC_005106.4_76200001	7	76200001	0.4798	0.6278
NC_005106.4_76300001	7	76300001	0.4452	0.5848
NC_005106.4_76400001	7	76400001	0.4887	0.6417
NC_005106.4_76500001	7	76500001	0.5092	0.6632
NC_005106.4_76600001	7	76600001	0.4987	0.6939
NC_005106.4_76700001	7	76700001	0.5082	0.652
NC_005106.4_76800001	7	76800001	0.5706	0.6846
NC_005106.4_76900001	7	76900001	0.4655	0.6295
NC_005106.4_77000001	7	77000001	0.4966	0.6849
NC_005106.4_77100001	7	77100001	0.5132	0.66
NC_005106.4_77200001	7	77200001	0.507	0.6839
NC_005106.4_77300001	7	77300001	0.4771	0.7082
NC_005106.4_77400001	7	77400001	0.3848	0.6206
NC_005106.4_77500001	7	77500001	0.3804	0.5941
NC_005106.4_77600001	7	77600001	0.3804	0.5941
NC_005106.4_77700001	7	77700001	0.3997	0.6214
NC_005106.4_77800001	7	77800001	0.4464	0.6717
NC_005106.4_77900001	7	77900001	0.743	0.887

NC_005106.4_78000001	7	78000001	0.6856	0.9459
NC_005106.4_78100001	7	78100001	0.4953	0.7557
NC_005106.4_78200001	7	78200001	0.434	0.6849
NC_005106.4_78300001	7	78300001	0.3217	0.5818
NC_005106.4_78400001	7	78400001	0.3749	0.59
NC_005106.4_78500001	7	78500001	0.4089	0.5722
NC_005106.4_78600001	7	78600001	0.4824	0.633
NC_005106.4_78700001	7	78700001	0.5846	0.7462
NC_005106.4_78800001	7	78800001	0.609	0.7148
NC_005106.4_78900001	7	78900001	0.6561	0.7416
NC_005106.4_79000001	7	79000001	0.703	0.7621
NC_005106.4_79100001	7	79100001	0.7211	0.7703
NC_005106.4_79200001	7	79200001	0.6501	0.6914
NC_005106.4_79300001	7	79300001	0.4291	0.6631
NC_005106.4_79400001	7	79400001	0.4858	0.7212
NC_005106.4_79500001	7	79500001	0.6038	0.8102
NC_005106.4_79600001	7	79600001	0.6038	0.8102
NC_005106.4_79700001	7	79700001	0.3928	0.6597
NC_005106.4_79800001	7	79800001	0.4798	0.6738
NC_005106.4_79900001	7	79900001	0.4571	0.665
NC_005106.4_80000001	7	80000001	0.4906	0.7041
NC_005106.4_80100001	7	80100001	0.4758	0.6808
NC_005106.4_80200001	7	80200001	0.5492	0.6671
NC_005106.4_80300001	7	80300001	0.5771	0.6993
NC_005106.4_80400001	7	80400001	0.6162	0.7152
NC_005106.4_80500001	7	80500001	0.5344	0.6486
NC_005106.4_80600001	7	80600001	0.5483	0.6678
NC_005106.4_80700001	7	80700001	0.6668	0.7923
NC_005106.4_80800001	7	80800001	0.6765	0.8175
NC_005106.4_80900001	7	80900001	0.702	0.819
NC_005106.4_81000001	7	81000001	0.7398	0.8152
NC_005106.4_81100001	7	81100001	0.7668	0.849
NC_005106.4_81200001	7	81200001	0.5761	0.7322
NC_005106.4_81300001	7	81300001	0.5176	0.7365
NC_005106.4_81400001	7	81400001	0.4794	0.7046
NC_005106.4_81500001	7	81500001	0.5634	0.8087
NC_005106.4_81600001	7	81600001	0.5071	0.694
NC_005106.4_81700001	7	81700001	0.5492	0.7275
NC_005106.4_81800001	7	81800001	0.5483	0.6405
NC_005106.4_81900001	7	81900001	0.4641	0.6287
NC_005106.4_82000001	7	82000001	0.314	0.4186
NC_005106.4_82100001	7	82100001	0.4004	0.574
NC_005106.4_82200001	7	82200001	0.4888	0.6768
NC_005106.4_82300001	7	82300001	0.4761	0.727
NC_005106.4_82400001	7	82400001	0.6637	0.7553
NC_005106.4_82500001	7	82500001	0.5569	0.6978
NC_005106.4_82600001	7	82600001	0.5244	0.7095
NC_005106.4_82700001	7	82700001	0.4783	0.5306
NC_005106.4_82800001	7	82800001	0.564	0.5607
NC_005106.4_82900001	7	82900001	0.5096	0.5564
NC_005106.4_83000001	7	83000001	0.6038	0.5992
NC_005106.4_83100001	7	83100001	0.5706	0.5016
NC_005106.4_83200001	7	83200001	0.5326	0.4091
NC_005106.4_83300001	7	83300001	0.4161	0.2991
NC_005106.4_83400001	7	83400001	0.4679	0.3877
NC_005106.4_83500001	7	83500001	0.4172	0.4351
NC_005106.4_83600001	7	83600001	0.4466	0.5643
NC_005106.4_83700001	7	83700001	0.4616	0.6663

NC_005106.4_83800001	7	83800001	0.5061	0.8716
NC_005106.4_83900001	7	83900001	0.5298	0.72
NC_005106.4_84000001	7	84000001	0.5671	0.6947
NC_005106.4_84100001	7	84100001	0.4899	0.6074
NC_005106.4_84200001	7	84200001	0.3737	0.5536
NC_005106.4_84300001	7	84300001	0.3111	0.4692
NC_005106.4_84400001	7	84400001	0.3023	0.69
NC_005106.4_84500001	7	84500001	0.3798	0.7639
NC_005106.4_84600001	7	84600001	0.379	0.7134
NC_005106.4_84700001	7	84700001	0.4449	0.6723
NC_005106.4_84800001	7	84800001	0.5198	0.6733
NC_005106.4_84900001	7	84900001	0.4423	0.5766
NC_005106.4_85000001	7	85000001	0.4423	0.5766
NC_005106.4_85500001	7	85500001	0.5343	0.6139
NC_005106.4_85600001	7	85600001	0.6402	0.6509
NC_005106.4_85700001	7	85700001	0.6402	0.6509
NC_005106.4_86600001	7	86600001	0.5563	0.7364
NC_005106.4_86700001	7	86700001	0.5714	0.7542
NC_005106.4_86800001	7	86800001	0.5714	0.7542
NC_005106.4_86900001	7	86900001	0.5714	0.7542
NC_005106.4_87000001	7	87000001	0.4048	0.594
NC_005106.4_87100001	7	87100001	0.4445	0.6411
NC_005106.4_87200001	7	87200001	0.269	0.2992
NC_005106.4_87300001	7	87300001	0.3665	0.4567
NC_005106.4_87400001	7	87400001	0.3665	0.4567
NC_005106.4_87500001	7	87500001	0.3884	0.5231
NC_005106.4_88400001	7	88400001	0.5273	0.5905
NC_005106.4_88500001	7	88500001	0.5675	0.6737
NC_005106.4_88600001	7	88600001	0.5675	0.6737
NC_005106.4_88700001	7	88700001	0.5957	0.8195
NC_005106.4_88800001	7	88800001	0.5209	0.7882
NC_005106.4_88900001	7	88900001	0.4022	0.7701
NC_005106.4_89100001	7	89100001	0.6443	0.9155
NC_005106.4_89200001	7	89200001	0.6552	0.8425
NC_005106.4_89300001	7	89300001	0.7813	0.871
NC_005106.4_89400001	7	89400001	0.6541	0.797
NC_005106.4_89500001	7	89500001	0.6276	0.7714
NC_005106.4_89600001	7	89600001	0.4425	0.6322
NC_005106.4_89700001	7	89700001	0.456	0.6712
NC_005106.4_89800001	7	89800001	0.4059	0.6175
NC_005106.4_89900001	7	89900001	0.4439	0.6028
NC_005106.4_90000001	7	90000001	0.5134	0.6748
NC_005106.4_90100001	7	90100001	0.547	0.6881
NC_005106.4_90200001	7	90200001	0.4549	0.6279
NC_005106.4_90300001	7	90300001	0.5466	0.7302
NC_005106.4_90400001	7	90400001	0.5073	0.7752
NC_005106.4_90500001	7	90500001	0.4441	0.7571
NC_005106.4_90600001	7	90600001	0.6284	0.8286
NC_005106.4_90700001	7	90700001	0.7107	0.8404
NC_005106.4_90800001	7	90800001	0.717	0.8305
NC_005106.4_90900001	7	90900001	0.7294	0.832
NC_005106.4_91000001	7	91000001	0.7469	0.8062
NC_005106.4_91100001	7	91100001	0.6559	0.7296
NC_005106.4_91200001	7	91200001	0.6112	0.7279
NC_005106.4_91300001	7	91300001	0.6037	0.685
NC_005106.4_91400001	7	91400001	0.6662	0.7058
NC_005106.4_91500001	7	91500001	0.5985	0.7
NC_005106.4_91600001	7	91600001	0.6085	0.7202

NC_005106.4_91700001	7	91700001	0.5243	0.6568
NC_005106.4_91800001	7	91800001	0.5413	0.6821
NC_005106.4_91900001	7	91900001	0.4732	0.6362
NC_005106.4_92000001	7	92000001	0.4889	0.6369
NC_005106.4_92100001	7	92100001	0.3637	0.4733
NC_005106.4_92200001	7	92200001	0.4241	0.4744
NC_005106.4_92300001	7	92300001	0.3962	0.4444
NC_005106.4_92400001	7	92400001	0.3524	0.4398
NC_005106.4_92500001	7	92500001	0.2988	0.3653
NC_005106.4_92600001	7	92600001	0.3606	0.4518
NC_005106.4_92700001	7	92700001	0.4074	0.4211
NC_005106.4_92800001	7	92800001	0.3397	0.2778
NC_005106.4_92900001	7	92900001	0.3222	0.275
NC_005106.4_93000001	7	93000001	0.3656	0.3397
NC_005106.4_93100001	7	93100001	0.3558	0.4089
NC_005106.4_93200001	7	93200001	0.3163	0.4796
NC_005106.4_93300001	7	93300001	0.2821	0.4924
NC_005106.4_93400001	7	93400001	0.3322	0.5251
NC_005106.4_93500001	7	93500001	0.3168	0.5902
NC_005106.4_93600001	7	93600001	0.3827	0.5706
NC_005106.4_93700001	7	93700001	0.4465	0.5723
NC_005106.4_93800001	7	93800001	0.479	0.5546
NC_005106.4_93900001	7	93900001	0.5114	0.5735
NC_005106.4_94000001	7	94000001	0.4751	0.5493
NC_005106.4_94100001	7	94100001	0.5552	0.6827
NC_005106.4_94200001	7	94200001	0.604	0.7581
NC_005106.4_94300001	7	94300001	0.5809	0.7717
NC_005106.4_94400001	7	94400001	0.5789	0.7789
NC_005106.4_94500001	7	94500001	0.6143	0.8133
NC_005106.4_94600001	7	94600001	0.5684	0.7418
NC_005106.4_94700001	7	94700001	0.4098	0.6519
NC_005106.4_94800001	7	94800001	0.658	0.7527
NC_005106.4_94900001	7	94900001	0.586	0.7555
NC_005106.4_95000001	7	95000001	0.6494	0.8106
NC_005106.4_95100001	7	95100001	0.6684	0.7623
NC_005106.4_95200001	7	95200001	0.6687	0.7154
NC_005106.4_95300001	7	95300001	0.6103	0.6829
NC_005106.4_95400001	7	95400001	0.6532	0.6817
NC_005106.4_95500001	7	95500001	0.5928	0.6431
NC_005106.4_95600001	7	95600001	0.5602	0.6348
NC_005106.4_95700001	7	95700001	0.5647	0.6567
NC_005106.4_95800001	7	95800001	0.6455	0.8114
NC_005106.4_95900001	7	95900001	0.6734	0.8669
NC_005106.4_96000001	7	96000001	0.8285	0.8958
NC_005106.4_96100001	7	96100001	0.7709	0.8353
NC_005106.4_96200001	7	96200001	0.7272	0.7328
NC_005106.4_96300001	7	96300001	0.5874	0.5611
NC_005106.4_96400001	7	96400001	0.5297	0.4809
NC_005106.4_96500001	7	96500001	0.5461	0.4784
NC_005106.4_96600001	7	96600001	0.5661	0.4878
NC_005106.4_96700001	7	96700001	0.571	0.5543
NC_005106.4_96800001	7	96800001	0.5637	0.5669
NC_005106.4_96900001	7	96900001	0.5148	0.5783
NC_005106.4_97000001	7	97000001	0.4433	0.5124
NC_005106.4_97100001	7	97100001	0.4887	0.5905
NC_005106.4_97200001	7	97200001	0.4642	0.5951
NC_005106.4_97300001	7	97300001	0.4772	0.6524
NC_005106.4_97400001	7	97400001	0.5384	0.6903

NC_005106.4_97500001	7	97500001	0.5065	0.6946
NC_005106.4_97600001	7	97600001	0.5046	0.6704
NC_005106.4_97700001	7	97700001	0.5417	0.6449
NC_005106.4_97800001	7	97800001	0.5172	0.5871
NC_005106.4_97900001	7	97900001	0.4649	0.5332
NC_005106.4_98000001	7	98000001	0.521	0.491
NC_005106.4_98100001	7	98100001	0.4683	0.4539
NC_005106.4_98200001	7	98200001	0.4744	0.4693
NC_005106.4_98300001	7	98300001	0.597	0.6272
NC_005106.4_98400001	7	98400001	0.6181	0.7333
NC_005106.4_98500001	7	98500001	0.5889	0.7729
NC_005106.4_98600001	7	98600001	0.5673	0.7058
NC_005106.4_98700001	7	98700001	0.5582	0.7348
NC_005106.4_98800001	7	98800001	0.5441	0.7004
NC_005106.4_98900001	7	98900001	0.4694	0.5881
NC_005106.4_99000001	7	99000001	0.4177	0.4374
NC_005106.4_99200001	7	99200001	0.6688	0.8957
NC_005106.4_99300001	7	99300001	0.6196	0.8686
NC_005106.4_99400001	7	99400001	0.7012	0.8824
NC_005106.4_99500001	7	99500001	0.6342	0.8677
NC_005106.4_99600001	7	99600001	0.6214	0.8448
NC_005106.4_99700001	7	99700001	0.5421	0.7406
NC_005106.4_99800001	7	99800001	0.5652	0.7313
NC_005106.4_99900001	7	99900001	0.4265	0.62
NC_005106.4_100000001	7	100000001	0.5063	0.5925
NC_005106.4_100100001	7	100100001	0.3974	0.5182
NC_005106.4_100200001	7	100200001	0.477	0.6583
NC_005106.4_100300001	7	100300001	0.4957	0.5955
NC_005106.4_100400001	7	100400001	0.6712	0.7492
NC_005106.4_100500001	7	100500001	0.6327	0.7654
NC_005106.4_100600001	7	100600001	0.7018	0.7862
NC_005106.4_100700001	7	100700001	0.6585	0.7059
NC_005106.4_100800001	7	100800001	0.6434	0.7114
NC_005106.4_100900001	7	100900001	0.6038	0.6714
NC_005106.4_101000001	7	101000001	0.5627	0.5955
NC_005106.4_101100001	7	101100001	0.5979	0.6686
NC_005106.4_101200001	7	101200001	0.5773	0.7133
NC_005106.4_101300001	7	101300001	0.6247	0.7613
NC_005106.4_101400001	7	101400001	0.5518	0.726
NC_005106.4_101500001	7	101500001	0.5307	0.6991
NC_005106.4_101600001	7	101600001	0.4503	0.5995
NC_005106.4_101700001	7	101700001	0.4335	0.5799
NC_005106.4_101800001	7	101800001	0.4335	0.5895
NC_005106.4_101900001	7	101900001	0.429	0.5442
NC_005106.4_102000001	7	102000001	0.4492	0.6314
NC_005106.4_102100001	7	102100001	0.4733	0.667
NC_005106.4_102200001	7	102200001	0.4818	0.6953
NC_005106.4_102300001	7	102300001	0.3811	0.549
NC_005106.4_102400001	7	102400001	0.2932	0.6039
NC_005106.4_102500001	7	102500001	0.5251	0.7967
NC_005106.4_102600001	7	102600001	0.617	0.8329
NC_005106.4_102700001	7	102700001	0.5576	0.7273
NC_005106.4_102800001	7	102800001	0.4716	0.6068
NC_005106.4_102900001	7	102900001	0.536	0.6252
NC_005106.4_103000001	7	103000001	0.5526	0.6363
NC_005106.4_103100001	7	103100001	0.4872	0.5329
NC_005106.4_103200001	7	103200001	0.5913	0.6514
NC_005106.4_103300001	7	103300001	0.6417	0.7009

NC_005106.4_103400001	7	103400001	0.6417	0.7009
NC_005106.4_103500001	7	103500001	0.6076	0.6351
NC_005106.4_103600001	7	103600001	0.8324	0.8441
NC_005106.4_103700001	7	103700001	0.7505	0.7341
NC_005106.4_103800001	7	103800001	0.7039	0.6884
NC_005106.4_103900001	7	103900001	0.6673	0.6545
NC_005106.4_104000001	7	104000001	0.554	0.6073
NC_005106.4_104100001	7	104100001	0.5603	0.6054
NC_005106.4_104200001	7	104200001	0.5739	0.6251
NC_005106.4_104300001	7	104300001	0.5502	0.6643
NC_005106.4_104400001	7	104400001	0.5969	0.7363
NC_005106.4_104500001	7	104500001	0.5903	0.7317
NC_005106.4_104600001	7	104600001	0.5626	0.7385
NC_005106.4_104700001	7	104700001	0.5507	0.7773
NC_005106.4_104800001	7	104800001	0.5664	0.7427
NC_005106.4_104900001	7	104900001	0.518	0.7007
NC_005106.4_105000001	7	105000001	0.9961	0.9929
NC_005106.4_105100001	7	105100001	0.6135	0.6991
NC_005106.4_105200001	7	105200001	0.6135	0.6991
NC_005106.4_105300001	7	105300001	0.6781	0.7637
NC_005106.4_105400001	7	105400001	0.6776	0.7688
NC_005106.4_105500001	7	105500001	0.4879	0.6027
NC_005106.4_105600001	7	105600001	0.6047	0.7592
NC_005106.4_105700001	7	105700001	0.6047	0.7592
NC_005106.4_105800001	7	105800001	0.554	0.6632
NC_005106.4_105900001	7	105900001	0.4546	0.5355
NC_005106.4_106100001	7	106100001	0.6127	0.5904
NC_005106.4_106200001	7	106200001	0.5987	0.6473
NC_005106.4_106300001	7	106300001	0.5997	0.7293
NC_005106.4_106400001	7	106400001	0.4928	0.6106
NC_005106.4_106500001	7	106500001	0.4463	0.5626
NC_005106.4_106600001	7	106600001	0.4319	0.5547
NC_005106.4_106700001	7	106700001	0.4188	0.5234
NC_005106.4_106800001	7	106800001	0.267	0.2855
NC_005106.4_106900001	7	106900001	0.3418	0.4532
NC_005106.4_107000001	7	107000001	0.3684	0.5813
NC_005106.4_107100001	7	107100001	0.5224	0.9017
NC_005106.4_107200001	7	107200001	0.4292	0.8734
NC_005106.4_107300001	7	107300001	0.4456	0.6791
NC_005106.4_107400001	7	107400001	0.4924	0.674
NC_005106.4_107500001	7	107500001	0.519	0.6812
NC_005106.4_107600001	7	107600001	0.5327	0.6842
NC_005106.4_107700001	7	107700001	0.558	0.7088
NC_005106.4_107800001	7	107800001	0.5482	0.7033
NC_005106.4_107900001	7	107900001	0.5478	0.7026
NC_005106.4_108000001	7	108000001	0.5254	0.6796
NC_005106.4_108100001	7	108100001	0.4505	0.558
NC_005106.4_108200001	7	108200001	0.4502	0.4919
NC_005106.4_108300001	7	108300001	0.5509	0.59
NC_005106.4_108400001	7	108400001	0.5108	0.5766
NC_005106.4_108500001	7	108500001	0.5423	0.555
NC_005106.4_108600001	7	108600001	0.6395	0.7087
NC_005106.4_108700001	7	108700001	0.5532	0.6296
NC_005106.4_108800001	7	108800001	0.6007	0.6592
NC_005106.4_108900001	7	108900001	0.5727	0.625
NC_005106.4_109000001	7	109000001	0.5338	0.5629
NC_005106.4_109100001	7	109100001	0.4064	0.4359
NC_005106.4_109200001	7	109200001	0.482	0.5323

NC_005106.4_109300001	7	109300001	0.3744	0.352
NC_005106.4_109400001	7	109400001	0.3744	0.352
NC_005106.4_109500001	7	109500001	0.4891	0.6192
NC_005106.4_109600001	7	109600001	0.5356	0.5913
NC_005106.4_109700001	7	109700001	0.4794	0.5724
NC_005106.4_109800001	7	109800001	0.4528	0.6761
NC_005106.4_109900001	7	109900001	0.3984	0.6254
NC_005106.4_110000001	7	110000001	0.3409	0.543
NC_005106.4_110100001	7	110100001	0.3353	0.5276
NC_005106.4_110200001	7	110200001	0.3043	0.4788
NC_005106.4_110300001	7	110300001	0.3086	0.409
NC_005106.4_110400001	7	110400001	0.4013	0.5114
NC_005106.4_110500001	7	110500001	0.3663	0.2809
NC_005106.4_110800001	7	110800001	0.516	0.7765
NC_005106.4_110900001	7	110900001	0.516	0.7765
NC_005106.4_111000001	7	111000001	0.5417	0.8463
NC_005106.4_111100001	7	111100001	0.5526	0.82
NC_005106.4_111200001	7	111200001	0.5289	0.6802
NC_005106.4_111300001	7	111300001	0.594	0.6445
NC_005106.4_111400001	7	111400001	0.5267	0.6908
NC_005106.4_111500001	7	111500001	0.4994	0.6587
NC_005106.4_111600001	7	111600001	0.5066	0.664
NC_005106.4_111700001	7	111700001	0.5236	0.7623
NC_005106.4_111800001	7	111800001	0.4848	0.7791
NC_005106.4_111900001	7	111900001	0.553	0.6877
NC_005106.4_112000001	7	112000001	0.6545	0.7418
NC_005106.4_112100001	7	112100001	0.6972	0.7932
NC_005106.4_112200001	7	112200001	0.6052	0.7018
NC_005106.4_112300001	7	112300001	0.3559	0.5083
NC_005106.4_112400001	7	112400001	0.3301	0.5192
NC_005106.4_112500001	7	112500001	0.3014	0.4955
NC_005106.4_112600001	7	112600001	0.314	0.472
NC_005106.4_112700001	7	112700001	0.3503	0.5191
NC_005106.4_112800001	7	112800001	0.4199	0.5817
NC_005106.4_112900001	7	112900001	0.4526	0.6028
NC_005106.4_113000001	7	113000001	0.4793	0.6323
NC_005106.4_113100001	7	113100001	0.4555	0.5865
NC_005106.4_113200001	7	113200001	0.4505	0.5326
NC_005106.4_113300001	7	113300001	0.4504	0.5766
NC_005106.4_113400001	7	113400001	0.4128	0.5829
NC_005106.4_113500001	7	113500001	0.3198	0.3951
NC_005106.4_113600001	7	113600001	0.3843	0.5629
NC_005106.4_113700001	7	113700001	0.4342	0.6643
NC_005106.4_113800001	7	113800001	0.4765	0.6139
NC_005106.4_113900001	7	113900001	0.6298	0.712
NC_005106.4_114000001	7	114000001	0.6322	0.7886
NC_005106.4_114100001	7	114100001	0.5871	0.7541
NC_005106.4_114200001	7	114200001	0.5824	0.7591
NC_005106.4_114300001	7	114300001	0.5538	0.7693
NC_005106.4_114400001	7	114400001	0.3857	0.6269
NC_005106.4_114500001	7	114500001	0.5797	0.7449
NC_005106.4_114600001	7	114600001	0.5459	0.664
NC_005106.4_114700001	7	114700001	0.5544	0.6016
NC_005106.4_114800001	7	114800001	0.5616	0.6452
NC_005106.4_114900001	7	114900001	0.6029	0.6524
NC_005106.4_115000001	7	115000001	0.5793	0.6181
NC_005106.4_115100001	7	115100001	0.6338	0.6798
NC_005106.4_115200001	7	115200001	0.571	0.6989

NC_005106.4_115300001	7	115300001	0.5892	0.6664
NC_005106.4_115400001	7	115400001	0.5514	0.6601
NC_005106.4_115500001	7	115500001	0.5091	0.6141
NC_005106.4_115600001	7	115600001	0.5026	0.6115
NC_005106.4_115700001	7	115700001	0.6394	0.6383
NC_005106.4_115800001	7	115800001	0.6882	0.7146
NC_005106.4_115900001	7	115900001	0.7823	0.7562
NC_005106.4_116000001	7	116000001	0.8668	0.8008
NC_005106.4_116100001	7	116100001	0.7271	0.6485
NC_005106.4_116200001	7	116200001	0.6965	0.7294
NC_005106.4_116300001	7	116300001	0.5959	0.649
NC_005106.4_116400001	7	116400001	0.5784	0.6307
NC_005106.4_116500001	7	116500001	0.5578	0.6223
NC_005106.4_116600001	7	116600001	0.564	0.6559
NC_005106.4_116700001	7	116700001	0.5617	0.6918
NC_005106.4_116800001	7	116800001	0.5412	0.6866
NC_005106.4_116900001	7	116900001	0.5824	0.7309
NC_005106.4_117000001	7	117000001	0.6493	0.7978
NC_005106.4_117100001	7	117100001	0.6683	0.8076
NC_005106.4_117200001	7	117200001	0.6414	0.7936
NC_005106.4_117300001	7	117300001	0.6953	0.8318
NC_005106.4_117400001	7	117400001	0.6619	0.8441
NC_005106.4_117500001	7	117500001	0.5512	0.7689
NC_005106.4_117600001	7	117600001	0.5904	0.8334
NC_005106.4_117700001	7	117700001	0.6467	0.7698
NC_005106.4_117800001	7	117800001	0.6442	0.7841
NC_005106.4_117900001	7	117900001	0.6796	0.834
NC_005106.4_118000001	7	118000001	0.6792	0.802
NC_005106.4_118100001	7	118100001	0.6194	0.7525
NC_005106.4_118200001	7	118200001	0.5417	0.742
NC_005106.4_118300001	7	118300001	0.482	0.6617
NC_005106.4_118400001	7	118400001	0.4095	0.4737
NC_005106.4_118500001	7	118500001	0.3173	0.2564
NC_005106.4_118600001	7	118600001	0.3173	0.2564
NC_005106.4_118700001	7	118700001	0.4729	0.5323
NC_005106.4_118800001	7	118800001	0.5218	0.5932
NC_005106.4_118900001	7	118900001	0.5521	0.6735
NC_005106.4_119000001	7	119000001	0.6007	0.7531
NC_005106.4_119100001	7	119100001	0.5952	0.7631
NC_005106.4_119200001	7	119200001	0.5959	0.685
NC_005106.4_119300001	7	119300001	0.5479	0.6383
NC_005106.4_119400001	7	119400001	0.5261	0.6055
NC_005106.4_119500001	7	119500001	0.5447	0.6198
NC_005106.4_119600001	7	119600001	0.5711	0.6143
NC_005106.4_119700001	7	119700001	0.5664	0.6491
NC_005106.4_119800001	7	119800001	0.5425	0.6369
NC_005106.4_119900001	7	119900001	0.5503	0.6373
NC_005106.4_120000001	7	120000001	0.5033	0.589
NC_005106.4_120100001	7	120100001	0.4306	0.5417
NC_005106.4_120200001	7	120200001	0.4339	0.5643
NC_005106.4_120300001	7	120300001	0.5706	0.6942
NC_005106.4_120400001	7	120400001	0.5792	0.6898
NC_005106.4_120500001	7	120500001	0.6482	0.7541
NC_005106.4_120600001	7	120600001	0.6669	0.744
NC_005106.4_120700001	7	120700001	0.6715	0.6951
NC_005106.4_120800001	7	120800001	0.6713	0.6934
NC_005106.4_120900001	7	120900001	0.6815	0.6999
NC_005106.4_121000001	7	121000001	0.6191	0.6306

NC_005106.4_121100001	7	121100001	0.5782	0.6558
NC_005106.4_121200001	7	121200001	0.6412	0.783
NC_005106.4_121300001	7	121300001	0.5797	0.7631
NC_005106.4_121400001	7	121400001	0.5933	0.7818
NC_005106.4_121500001	7	121500001	0.6041	0.7511
NC_005106.4_121600001	7	121600001	0.6444	0.7616
NC_005106.4_121700001	7	121700001	0.5961	0.7219
NC_005106.4_121800001	7	121800001	0.6629	0.719
NC_005106.4_121900001	7	121900001	0.6505	0.7605
NC_005106.4_122000001	7	122000001	0.6446	0.8451
NC_005106.4_122100001	7	122100001	0.7088	0.814
NC_005106.4_122200001	7	122200001	0.7616	0.8174
NC_005106.4_122300001	7	122300001	0.717	0.7851
NC_005106.4_122400001	7	122400001	0.6758	0.7375
NC_005106.4_122500001	7	122500001	0.7309	0.7591
NC_005106.4_122600001	7	122600001	0.5858	0.7051
NC_005106.4_122700001	7	122700001	0.4917	0.6242
NC_005106.4_122800001	7	122800001	0.5352	0.6885
NC_005106.4_122900001	7	122900001	0.652	0.7632
NC_005106.4_123000001	7	123000001	0.567	0.6956
NC_005106.4_123100001	7	123100001	0.5893	0.6912
NC_005106.4_123200001	7	123200001	0.6471	0.7429
NC_005106.4_123300001	7	123300001	0.6555	0.7546
NC_005106.4_123400001	7	123400001	0.58	0.6708
NC_005106.4_123500001	7	123500001	0.6649	0.7448
NC_005106.4_123600001	7	123600001	0.636	0.7041
NC_005106.4_123700001	7	123700001	0.5692	0.6336
NC_005106.4_123800001	7	123800001	0.5399	0.588
NC_005106.4_123900001	7	123900001	0.5684	0.6133
NC_005106.4_124000001	7	124000001	0.5601	0.5953
NC_005106.4_124100001	7	124100001	0.526	0.5413
NC_005106.4_124200001	7	124200001	0.5495	0.6038
NC_005106.4_124300001	7	124300001	0.5379	0.5755
NC_005106.4_124400001	7	124400001	0.4516	0.5244
NC_005106.4_124500001	7	124500001	0.4661	0.5581
NC_005106.4_124600001	7	124600001	0.4931	0.6342
NC_005106.4_124700001	7	124700001	0.4914	0.6265
NC_005106.4_124800001	7	124800001	0.4973	0.7255
NC_005106.4_124900001	7	124900001	0.5242	0.7197
NC_005106.4_125000001	7	125000001	0.5015	0.6872
NC_005106.4_125100001	7	125100001	0.4628	0.6336
NC_005106.4_125200001	7	125200001	0.4244	0.5841
NC_005106.4_125300001	7	125300001	0.3322	0.4771
NC_005106.4_125400001	7	125400001	0.4472	0.6473
NC_005106.4_125500001	7	125500001	0.4321	0.6411
NC_005106.4_125600001	7	125600001	0.4086	0.6243
NC_005106.4_125700001	7	125700001	0.4133	0.6041
NC_005106.4_125800001	7	125800001	0.5545	0.7256
NC_005106.4_125900001	7	125900001	0.5643	0.6481
NC_005106.4_126000001	7	126000001	0.5707	0.6265
NC_005106.4_126100001	7	126100001	0.578	0.6487
NC_005106.4_126200001	7	126200001	0.5995	0.6458
NC_005106.4_126300001	7	126300001	0.5529	0.5981
NC_005106.4_126400001	7	126400001	0.5023	0.5345
NC_005106.4_126500001	7	126500001	0.4825	0.5369
NC_005106.4_126600001	7	126600001	0.452	0.5101
NC_005106.4_126700001	7	126700001	0.4742	0.591
NC_005106.4_126800001	7	126800001	0.5106	0.6438

NC_005106.4_126900001	7	126900001	0.5486	0.8
NC_005106.4_127000001	7	127000001	0.6939	0.8954
NC_005106.4_127100001	7	127100001	0.7634	0.9003
NC_005106.4_127200001	7	127200001	0.6868	0.8472
NC_005106.4_127300001	7	127300001	0.6307	0.7675
NC_005106.4_127400001	7	127400001	0.6335	0.6924
NC_005106.4_127500001	7	127500001	0.5381	0.6356
NC_005106.4_127600001	7	127600001	0.4627	0.5583
NC_005106.4_127700001	7	127700001	0.4417	0.5243
NC_005106.4_127800001	7	127800001	0.3883	0.5226
NC_005106.4_127900001	7	127900001	0.3003	0.4984
NC_005106.4_128000001	7	128000001	0.3447	0.4815
NC_005106.4_128100001	7	128100001	0.3574	0.5385
NC_005106.4_128200001	7	128200001	0.3676	0.5536
NC_005106.4_128300001	7	128300001	0.4046	0.5737
NC_005106.4_128400001	7	128400001	0.4771	0.6149
NC_005106.4_128500001	7	128500001	0.4561	0.648
NC_005106.4_128600001	7	128600001	0.5321	0.6745
NC_005106.4_128700001	7	128700001	0.629	0.7111
NC_005106.4_128800001	7	128800001	0.6167	0.6828
NC_005106.4_128900001	7	128900001	0.5409	0.6277
NC_005106.4_129000001	7	129000001	0.49	0.5948
NC_005106.4_129100001	7	129100001	0.4714	0.5719
NC_005106.4_129200001	7	129200001	0.4183	0.5438
NC_005106.4_129300001	7	129300001	0.465	0.6138
NC_005106.4_129400001	7	129400001	0.482	0.6354
NC_005106.4_129500001	7	129500001	0.5031	0.6555
NC_005106.4_129600001	7	129600001	0.5611	0.7544
NC_005106.4_129700001	7	129700001	0.5105	0.7436
NC_005106.4_129800001	7	129800001	0.4429	0.666
NC_005106.4_129900001	7	129900001	0.4874	0.7196
NC_005106.4_130000001	7	130000001	0.5027	0.6599
NC_005106.4_130100001	7	130100001	0.4494	0.6106
NC_005106.4_130200001	7	130200001	0.5758	0.6666
NC_005106.4_130300001	7	130300001	0.5761	0.6123
NC_005106.4_130400001	7	130400001	0.5276	0.5798
NC_005106.4_130500001	7	130500001	0.6216	0.7229
NC_005106.4_130600001	7	130600001	0.5924	0.6351
NC_005106.4_130700001	7	130700001	0.3735	0.3282
NC_005106.4_131000001	7	131000001	0.178	0.2288
NC_005106.4_131100001	7	131100001	0.4712	0.6225
NC_005106.4_131200001	7	131200001	0.4909	0.6874
NC_005106.4_131300001	7	131300001	0.4178	0.6479
NC_005106.4_131400001	7	131400001	0.4312	0.6342
NC_005106.4_131500001	7	131500001	0.52	0.6703
NC_005106.4_131600001	7	131600001	0.5556	0.7489
NC_005106.4_131700001	7	131700001	0.5579	0.7308
NC_005106.4_131800001	7	131800001	0.6106	0.7768
NC_005106.4_131900001	7	131900001	0.5976	0.7995
NC_005106.4_132000001	7	132000001	0.5566	0.8182
NC_005106.4_132100001	7	132100001	0.4773	0.7175
NC_005106.4_132200001	7	132200001	0.4321	0.6588
NC_005106.4_132300001	7	132300001	0.4637	0.6562
NC_005106.4_132400001	7	132400001	0.5183	0.6334
NC_005106.4_132500001	7	132500001	0.535	0.5895
NC_005106.4_132600001	7	132600001	0.4154	0.5029
NC_005106.4_132800001	7	132800001	0.5459	0.5663
NC_005106.4_132900001	7	132900001	0.4189	0.509

NC_005106.4_133000001	7	133000001	0.4131	0.466
NC_005106.4_133100001	7	133100001	0.4165	0.4941
NC_005106.4_133200001	7	133200001	0.4415	0.5396
NC_005106.4_133300001	7	133300001	0.3895	0.5124
NC_005106.4_133400001	7	133400001	0.4414	0.6153
NC_005106.4_133500001	7	133500001	0.4562	0.711
NC_005106.4_133600001	7	133600001	0.4266	0.6276
NC_005106.4_133700001	7	133700001	0.3667	0.5113
NC_005106.4_133800001	7	133800001	0.4534	0.5398
NC_005106.4_133900001	7	133900001	0.5083	0.552
NC_005106.4_134000001	7	134000001	0.4888	0.5714
NC_005106.4_134100001	7	134100001	0.5958	0.6863
NC_005106.4_134200001	7	134200001	0.6448	0.7594
NC_005106.4_134300001	7	134300001	0.6499	0.7901
NC_005106.4_134400001	7	134400001	0.597	0.7635
NC_005106.4_134500001	7	134500001	0.6246	0.7592
NC_005106.4_134600001	7	134600001	0.509	0.7143
NC_005106.4_134700001	7	134700001	0.4716	0.6593
NC_005106.4_134800001	7	134800001	0.496	0.7
NC_005106.4_134900001	7	134900001	0.5476	0.7388
NC_005106.4_135000001	7	135000001	0.4947	0.733
NC_005106.4_135100001	7	135100001	0.5955	0.7079
NC_005106.4_135200001	7	135200001	0.5821	0.7049
NC_005106.4_135300001	7	135300001	0.5721	0.677
NC_005106.4_135400001	7	135400001	0.5172	0.5938
NC_005106.4_135500001	7	135500001	0.5778	0.5921
NC_005106.4_135600001	7	135600001	0.547	0.5851
NC_005106.4_135700001	7	135700001	0.5123	0.4762
NC_005106.4_135800001	7	135800001	0.4374	0.4351
NC_005106.4_135900001	7	135900001	0.3354	0.3559
NC_005106.4_136000001	7	136000001	0.425	0.4399
NC_005106.4_136100001	7	136100001	0.3731	0.3806
NC_005106.4_136200001	7	136200001	0.3961	0.4683
NC_005106.4_136300001	7	136300001	0.5192	0.6593
NC_005106.4_136400001	7	136400001	0.5513	0.6833
NC_005106.4_136500001	7	136500001	0.69	0.8508
NC_005106.4_136600001	7	136600001	0.7257	0.8869
NC_005106.4_136700001	7	136700001	0.6863	0.8454
NC_005106.4_136800001	7	136800001	0.7045	0.8339
NC_005106.4_136900001	7	136900001	0.6357	0.723
NC_005106.4_137000001	7	137000001	0.4089	0.4226
NC_005106.4_137100001	7	137100001	0.4412	0.4602
NC_005106.4_137200001	7	137200001	0.5234	0.5747
NC_005106.4_137300001	7	137300001	0.5234	0.5747
NC_005106.4_137400001	7	137400001	0.5287	0.6181
NC_005106.4_137500001	7	137500001	0.5581	0.6548
NC_005106.4_137600001	7	137600001	0.5533	0.6846
NC_005106.4_137700001	7	137700001	0.5145	0.6453
NC_005106.4_137800001	7	137800001	0.4551	0.63
NC_005106.4_137900001	7	137900001	0.5027	0.6812
NC_005106.4_138000001	7	138000001	0.4925	0.6682
NC_005106.4_138100001	7	138100001	0.3931	0.5783
NC_005106.4_138200001	7	138200001	0.3069	0.4204
NC_005106.4_138300001	7	138300001	0.3478	0.4348
NC_005106.4_138400001	7	138400001	0.3294	0.4549
NC_005106.4_138500001	7	138500001	0.2882	0.3803
NC_005106.4_138600001	7	138600001	0.391	0.4728
NC_005106.4_138700001	7	138700001	0.5404	0.6088

NC_005106.4_138800001	7	138800001	0.4259	0.6043
NC_005106.4_138900001	7	138900001	0.4102	0.529
NC_005106.4_139000001	7	139000001	0.4575	0.6071
NC_005106.4_139100001	7	139100001	0.4618	0.6279
NC_005106.4_139200001	7	139200001	0.4742	0.6665
NC_005106.4_139300001	7	139300001	0.5895	0.7446
NC_005106.4_139400001	7	139400001	0.6739	0.8157
NC_005106.4_139500001	7	139500001	0.586	0.7489
NC_005106.4_139600001	7	139600001	0.5286	0.6948
NC_005106.4_139700001	7	139700001	0.5115	0.6616
NC_005106.4_139800001	7	139800001	0.4561	0.5482
NC_005106.4_139900001	7	139900001	0.4408	0.5391
NC_005106.4_140000001	7	140000001	0.5513	0.6376
NC_005106.4_140100001	7	140100001	0.6191	0.6484
NC_005106.4_140200001	7	140200001	0.6191	0.6484
NC_005106.4_140300001	7	140300001	0.5338	0.6719
NC_005106.4_140400001	7	140400001	0.5548	0.6042
NC_005106.4_140500001	7	140500001	0.4759	0.4696
NC_005106.4_140600001	7	140600001	0.4899	0.5683
NC_005106.4_140700001	7	140700001	0.5624	0.6736
NC_005106.4_140800001	7	140800001	0.5988	0.7123
NC_005106.4_140900001	7	140900001	0.619	0.7403
NC_005106.4_141000001	7	141000001	0.7005	0.8825
NC_005106.4_141100001	7	141100001	0.6999	0.8514
NC_005106.4_141200001	7	141200001	0.6581	0.7971
NC_005106.4_141300001	7	141300001	0.6371	0.7423
NC_005106.4_141400001	7	141400001	0.5996	0.7
NC_005106.4_141500001	7	141500001	0.5819	0.6331
NC_005106.4_141600001	7	141600001	0.577	0.5809
NC_005106.4_141700001	7	141700001	0.5739	0.5962
NC_005106.4_141800001	7	141800001	0.5581	0.6246
NC_005106.4_141900001	7	141900001	0.5795	0.666
NC_005106.4_142000001	7	142000001	0.5439	0.6425
NC_005106.4_142100001	7	142100001	0.5782	0.6997
NC_005106.4_142200001	7	142200001	0.6313	0.7492
NC_005106.4_142300001	7	142300001	0.5738	0.6737
NC_005106.4_142400001	7	142400001	0.5855	0.7067
NC_005106.4_142500001	7	142500001	0.6045	0.7146
NC_005106.4_142600001	7	142600001	0.5647	0.7139
NC_005106.4_142700001	7	142700001	0.513	0.6506
NC_005106.4_142800001	7	142800001	0.556	0.708
NC_005106.4_142900001	7	142900001	0.5789	0.708
NC_005106.4_143000001	7	143000001	0.5726	0.7127
NC_005106.4_143100001	7	143100001	0.6554	0.7289
NC_005106.4_143200001	7	143200001	0.6782	0.7617
NC_005106.4_143300001	7	143300001	0.678	0.7603
NC_005106.4_143400001	7	143400001	0.5897	0.7027
NC_005106.4_143500001	7	143500001	0.5648	0.6878
NC_005106.4_143600001	7	143600001	0.4845	0.6764
NC_005106.4_143700001	7	143700001	0.4316	0.6548
NC_005106.4_143800001	7	143800001	0.4128	0.6584
NC_005106.4_143900001	7	143900001	0.3868	0.6415
NC_005106.4_144000001	7	144000001	0.443	0.7486
NC_005106.4_144100001	7	144100001	0.3661	0.6705
NC_005106.4_144200001	7	144200001	0.487	0.6323
NC_005106.4_144300001	7	144300001	0.5001	0.6604
NC_005106.4_144400001	7	144400001	0.5376	0.7094
NC_005106.4_144500001	7	144500001	0.54	0.6892

NC_005106.4_144600001	7	144600001	0.5831	0.6782
NC_005106.4_144700001	7	144700001	0.4687	0.6852
NC_005106.4_144800001	7	144800001	0.4792	0.7199
NC_005106.4_144900001	7	144900001	0.4503	0.6647
NC_005106.4_145000001	7	145000001	0.3651	0.5925
NC_005107.4_400001	8	400001	0.4659	0.6073
NC_005107.4_500001	8	500001	0.6029	0.7174
NC_005107.4_600001	8	600001	0.6029	0.7174
NC_005107.4_700001	8	700001	0.5352	0.6074
NC_005107.4_800001	8	800001	0.651	0.6631
NC_005107.4_900001	8	900001	0.7802	0.7145
NC_005107.4_1300001	8	1300001	0.6256	0.7137
NC_005107.4_1400001	8	1400001	0.6648	0.7518
NC_005107.4_1500001	8	1500001	0.5635	0.6865
NC_005107.4_1600001	8	1600001	0.423	0.6506
NC_005107.4_1700001	8	1700001	0.4692	0.6815
NC_005107.4_1800001	8	1800001	0.3982	0.6109
NC_005107.4_1900001	8	1900001	0.3446	0.55
NC_005107.4_2000001	8	2000001	0.3407	0.5622
NC_005107.4_2100001	8	2100001	0.3742	0.5827
NC_005107.4_2200001	8	2200001	0.3059	0.4268
NC_005107.4_2300001	8	2300001	0.3587	0.4695
NC_005107.4_2400001	8	2400001	0.3265	0.3221
NC_005107.4_2500001	8	2500001	0.3119	0.3371
NC_005107.4_2600001	8	2600001	0.2602	0.1867
NC_005107.4_2700001	8	2700001	0.2907	0.3833
NC_005107.4_2800001	8	2800001	0.4289	0.5027
NC_005107.4_2900001	8	2900001	0.4343	0.5805
NC_005107.4_3000001	8	3000001	0.5827	0.6675
NC_005107.4_3100001	8	3100001	0.5235	0.662
NC_005107.4_3200001	8	3200001	0.6735	0.7223
NC_005107.4_3500001	8	3500001	0.458	0.5267
NC_005107.4_3600001	8	3600001	0.5564	0.5342
NC_005107.4_4200001	8	4200001	0.5172	0.4616
NC_005107.4_4300001	8	4300001	0.4564	0.421
NC_005107.4_4400001	8	4400001	0.3933	0.3999
NC_005107.4_4500001	8	4500001	0.4411	0.4717
NC_005107.4_4600001	8	4600001	0.4675	0.5173
NC_005107.4_4700001	8	4700001	0.4014	0.5035
NC_005107.4_4800001	8	4800001	0.423	0.5821
NC_005107.4_4900001	8	4900001	0.4987	0.6596
NC_005107.4_5000001	8	5000001	0.5041	0.6922
NC_005107.4_5100001	8	5100001	0.4058	0.7138
NC_005107.4_5200001	8	5200001	0.4752	0.6961
NC_005107.4_5300001	8	5300001	0.4827	0.6131
NC_005107.4_5400001	8	5400001	0.5896	0.7247
NC_005107.4_5500001	8	5500001	0.4695	0.6396
NC_005107.4_5600001	8	5600001	0.5031	0.6726
NC_005107.4_5700001	8	5700001	0.4915	0.7082
NC_005107.4_5800001	8	5800001	0.4962	0.7804
NC_005107.4_5900001	8	5900001	0.4122	0.6887
NC_005107.4_6000001	8	6000001	0.4894	0.838
NC_005107.4_6100001	8	6100001	0.4139	0.8373
NC_005107.4_6200001	8	6200001	0.2652	0.6195
NC_005107.4_6300001	8	6300001	0.298	0.5544
NC_005107.4_6400001	8	6400001	0.3438	0.4974
NC_005107.4_6500001	8	6500001	0.3901	0.5401
NC_005107.4_6600001	8	6600001	0.4672	0.5674

NC_005107.4_6700001	8	6700001	0.4566	0.5943
NC_005107.4_6800001	8	6800001	0.4195	0.6062
NC_005107.4_6900001	8	6900001	0.3941	0.7131
NC_005107.4_7000001	8	7000001	0.4103	0.7448
NC_005107.4_7100001	8	7100001	0.3654	0.7742
NC_005107.4_7200001	8	7200001	0.4567	0.8038
NC_005107.4_7300001	8	7300001	0.6166	0.8393
NC_005107.4_7400001	8	7400001	0.6866	0.8846
NC_005107.4_7500001	8	7500001	0.4245	0.5962
NC_005107.4_7600001	8	7600001	0.4245	0.5962
NC_005107.4_7700001	8	7700001	0.4639	0.7099
NC_005107.4_7800001	8	7800001	0.4239	0.6529
NC_005107.4_7900001	8	7900001	0.4578	0.5925
NC_005107.4_8000001	8	8000001	0.5004	0.6332
NC_005107.4_8100001	8	8100001	0.4732	0.5087
NC_005107.4_8200001	8	8200001	0.4679	0.4486
NC_005107.4_8300001	8	8300001	0.4448	0.3765
NC_005107.4_8400001	8	8400001	0.5373	0.4983
NC_005107.4_8500001	8	8500001	0.5114	0.5561
NC_005107.4_8600001	8	8600001	0.6177	0.8619
NC_005107.4_8700001	8	8700001	0.5825	0.85
NC_005107.4_8800001	8	8800001	0.5825	0.85
NC_005107.4_8900001	8	8900001	0.4807	0.782
NC_005107.4_9000001	8	9000001	0.4963	0.6021
NC_005107.4_9100001	8	9100001	0.6633	0.7357
NC_005107.4_9200001	8	9200001	0.7973	0.7552
NC_005107.4_9300001	8	9300001	0.8182	0.7818
NC_005107.4_9400001	8	9400001	0.8182	0.7818
NC_005107.4_9500001	8	9500001	0.9329	0.8772
NC_005107.4_9600001	8	9600001	0.7185	0.7678
NC_005107.4_9700001	8	9700001	0.4541	0.5048
NC_005107.4_9800001	8	9800001	0.363	0.364
NC_005107.4_9900001	8	9900001	0.363	0.364
NC_005107.4_10000001	8	10000001	0.363	0.364
NC_005107.4_10100001	8	10100001	0.4081	0.4157
NC_005107.4_10600001	8	10600001	0.6996	0.6828
NC_005107.4_10700001	8	10700001	0.5949	0.6955
NC_005107.4_10800001	8	10800001	0.5949	0.6955
NC_005107.4_10900001	8	10900001	0.5875	0.7049
NC_005107.4_11000001	8	11000001	0.5631	0.7476
NC_005107.4_11100001	8	11100001	0.5682	0.7615
NC_005107.4_11200001	8	11200001	0.5157	0.7503
NC_005107.4_11300001	8	11300001	0.5157	0.7503
NC_005107.4_11400001	8	11400001	0.4765	0.7696
NC_005107.4_11500001	8	11500001	0.2146	0.3204
NC_005107.4_11600001	8	11600001	0.1913	0.2942
NC_005107.4_11700001	8	11700001	0.2618	0.4819
NC_005107.4_11800001	8	11800001	0.4563	0.6775
NC_005107.4_11900001	8	11900001	0.4907	0.7193
NC_005107.4_12000001	8	12000001	0.4994	0.7244
NC_005107.4_12100001	8	12100001	0.481	0.7302
NC_005107.4_12200001	8	12200001	0.5086	0.6955
NC_005107.4_12300001	8	12300001	0.4719	0.6629
NC_005107.4_12400001	8	12400001	0.4477	0.6401
NC_005107.4_12500001	8	12500001	0.5746	0.7261
NC_005107.4_12600001	8	12600001	0.6658	0.7898
NC_005107.4_12700001	8	12700001	0.629	0.7716
NC_005107.4_12800001	8	12800001	0.6702	0.7899

NC_005107.4_12900001	8	12900001	0.6425	0.741
NC_005107.4_13000001	8	13000001	0.6396	0.7508
NC_005107.4_13100001	8	13100001	0.5535	0.6697
NC_005107.4_13200001	8	13200001	0.5547	0.7185
NC_005107.4_13300001	8	13300001	0.3936	0.6353
NC_005107.4_13400001	8	13400001	0.3793	0.6567
NC_005107.4_13500001	8	13500001	0.3716	0.6399
NC_005107.4_13600001	8	13600001	0.3838	0.6213
NC_005107.4_13700001	8	13700001	0.4323	0.6248
NC_005107.4_13800001	8	13800001	0.5149	0.6585
NC_005107.4_13900001	8	13900001	0.5874	0.6791
NC_005107.4_14000001	8	14000001	0.5895	0.6269
NC_005107.4_14100001	8	14100001	0.5836	0.6254
NC_005107.4_14200001	8	14200001	0.6354	0.6635
NC_005107.4_14300001	8	14300001	0.6251	0.6695
NC_005107.4_14400001	8	14400001	0.6297	0.6891
NC_005107.4_14500001	8	14500001	0.6657	0.8465
NC_005107.4_14600001	8	14600001	0.8195	0.9013
NC_005107.4_14700001	8	14700001	0.8855	0.9198
NC_005107.4_14800001	8	14800001	1	1
NC_005107.4_15300001	8	15300001	0.4948	0.4583
NC_005107.4_15400001	8	15400001	0.5599	0.574
NC_005107.4_15500001	8	15500001	0.5599	0.574
NC_005107.4_15600001	8	15600001	0.5599	0.574
NC_005107.4_15700001	8	15700001	0.5599	0.574
NC_005107.4_15800001	8	15800001	0.5311	0.6609
NC_005107.4_16300001	8	16300001	0.2552	0.147
NC_005107.4_16400001	8	16400001	0.2552	0.147
NC_005107.4_16500001	8	16500001	0.2552	0.147
NC_005107.4_16600001	8	16600001	0.2972	0.1945
NC_005107.4_16700001	8	16700001	0.3776	0.4304
NC_005107.4_17100001	8	17100001	0.5782	0.6777
NC_005107.4_17200001	8	17200001	0.6009	0.6434
NC_005107.4_17300001	8	17300001	0.55	0.5715
NC_005107.4_17400001	8	17400001	0.6164	0.6513
NC_005107.4_17500001	8	17500001	0.6164	0.6513
NC_005107.4_18200001	8	18200001	0.4177	0.6493
NC_005107.4_18300001	8	18300001	0.5524	0.7703
NC_005107.4_18400001	8	18400001	0.5524	0.7703
NC_005107.4_18500001	8	18500001	0.5459	0.8023
NC_005107.4_18600001	8	18600001	0.5459	0.8023
NC_005107.4_19000001	8	19000001	0.5981	0.6075
NC_005107.4_19100001	8	19100001	0.521	0.5741
NC_005107.4_19200001	8	19200001	0.4543	0.562
NC_005107.4_19300001	8	19300001	0.3774	0.4806
NC_005107.4_19400001	8	19400001	0.307	0.3907
NC_005107.4_19500001	8	19500001	0.1452	0.1446
NC_005107.4_19600001	8	19600001	0.1567	0.1537
NC_005107.4_19700001	8	19700001	0.1784	0.1577
NC_005107.4_21200001	8	21200001	0.4349	0.6213
NC_005107.4_21300001	8	21300001	0.4807	0.7222
NC_005107.4_21400001	8	21400001	0.4999	0.6814
NC_005107.4_21500001	8	21500001	0.4296	0.5629
NC_005107.4_21600001	8	21600001	0.4707	0.5753
NC_005107.4_21700001	8	21700001	0.5545	0.6603
NC_005107.4_21800001	8	21800001	0.5449	0.6387
NC_005107.4_21900001	8	21900001	0.5544	0.6349
NC_005107.4_22000001	8	22000001	0.775	0.834

NC_005107.4_22100001	8	22100001	0.6633	0.8348
NC_005107.4_22200001	8	22200001	0.4959	0.6824
NC_005107.4_22300001	8	22300001	0.6251	0.7927
NC_005107.4_22400001	8	22400001	0.6162	0.7441
NC_005107.4_22500001	8	22500001	0.5737	0.6984
NC_005107.4_22600001	8	22600001	0.6063	0.6932
NC_005107.4_22700001	8	22700001	0.6093	0.6873
NC_005107.4_22800001	8	22800001	0.5407	0.6349
NC_005107.4_22900001	8	22900001	0.4908	0.6444
NC_005107.4_23000001	8	23000001	0.5518	0.7026
NC_005107.4_23100001	8	23100001	0.5496	0.7248
NC_005107.4_23200001	8	23200001	0.6329	0.8034
NC_005107.4_23300001	8	23300001	0.5562	0.6139
NC_005107.4_23400001	8	23400001	0.5755	0.6179
NC_005107.4_23500001	8	23500001	0.3915	0.4441
NC_005107.4_23600001	8	23600001	0.403	0.4743
NC_005107.4_23700001	8	23700001	0.4	0.4299
NC_005107.4_23800001	8	23800001	0.4819	0.5815
NC_005107.4_23900001	8	23900001	0.4845	0.5044
NC_005107.4_24000001	8	24000001	0.5037	0.528
NC_005107.4_24100001	8	24100001	0.5527	0.5675
NC_005107.4_24200001	8	24200001	0.5801	0.6132
NC_005107.4_24300001	8	24300001	0.4975	0.536
NC_005107.4_24400001	8	24400001	0.5132	0.6009
NC_005107.4_24500001	8	24500001	0.5495	0.5991
NC_005107.4_24600001	8	24600001	0.4945	0.5315
NC_005107.4_24700001	8	24700001	0.5134	0.5942
NC_005107.4_24800001	8	24800001	0.5295	0.6321
NC_005107.4_24900001	8	24900001	0.5634	0.6677
NC_005107.4_25000001	8	25000001	0.5545	0.6999
NC_005107.4_25100001	8	25100001	0.5513	0.7488
NC_005107.4_25200001	8	25200001	0.6885	0.8126
NC_005107.4_25300001	8	25300001	0.669	0.7267
NC_005107.4_25400001	8	25400001	0.4719	0.6352
NC_005107.4_25500001	8	25500001	0.4935	0.6372
NC_005107.4_25600001	8	25600001	0.3911	0.5551
NC_005107.4_25700001	8	25700001	0.3473	0.4375
NC_005107.4_25800001	8	25800001	0.3208	0.3988
NC_005107.4_25900001	8	25900001	0.4073	0.5171
NC_005107.4_26000001	8	26000001	0.4146	0.5269
NC_005107.4_26100001	8	26100001	0.5453	0.5823
NC_005107.4_26200001	8	26200001	0.5189	0.6137
NC_005107.4_26300001	8	26300001	0.5469	0.6659
NC_005107.4_26400001	8	26400001	0.5662	0.6009
NC_005107.4_26500001	8	26500001	0.6237	0.6605
NC_005107.4_26600001	8	26600001	0.635	0.7302
NC_005107.4_26700001	8	26700001	0.7205	0.7225
NC_005107.4_26800001	8	26800001	0.5992	0.6203
NC_005107.4_26900001	8	26900001	0.5887	0.6182
NC_005107.4_27000001	8	27000001	0.4912	0.5299
NC_005107.4_27100001	8	27100001	0.4481	0.478
NC_005107.4_27200001	8	27200001	0.4437	0.4937
NC_005107.4_27300001	8	27300001	0.5906	0.6747
NC_005107.4_27400001	8	27400001	0.5328	0.5826
NC_005107.4_27500001	8	27500001	0.5422	0.5843
NC_005107.4_27600001	8	27600001	0.5273	0.5998
NC_005107.4_27700001	8	27700001	0.5488	0.6636
NC_005107.4_27800001	8	27800001	0.4463	0.5489

NC_005107.4_27900001	8	27900001	0.4415	0.6338
NC_005107.4_28000001	8	28000001	0.4669	0.6269
NC_005107.4_28100001	8	28100001	0.4685	0.6116
NC_005107.4_28200001	8	28200001	0.3502	0.4624
NC_005107.4_28300001	8	28300001	0.3157	0.4748
NC_005107.4_28400001	8	28400001	0.3493	0.5025
NC_005107.4_28500001	8	28500001	0.3275	0.5027
NC_005107.4_28600001	8	28600001	0.4189	0.6158
NC_005107.4_28700001	8	28700001	0.4845	0.681
NC_005107.4_28800001	8	28800001	0.5141	0.6751
NC_005107.4_28900001	8	28900001	0.5323	0.687
NC_005107.4_29000001	8	29000001	0.5113	0.6777
NC_005107.4_29100001	8	29100001	0.4523	0.6192
NC_005107.4_29200001	8	29200001	0.4567	0.5029
NC_005107.4_29300001	8	29300001	0.5168	0.5664
NC_005107.4_29400001	8	29400001	0.5153	0.5886
NC_005107.4_29500001	8	29500001	0.5381	0.58
NC_005107.4_29600001	8	29600001	0.5508	0.609
NC_005107.4_29700001	8	29700001	0.5235	0.6263
NC_005107.4_29800001	8	29800001	0.5711	0.7205
NC_005107.4_29900001	8	29900001	0.5846	0.7032
NC_005107.4_30000001	8	30000001	0.5412	0.6921
NC_005107.4_30100001	8	30100001	0.4064	0.5912
NC_005107.4_30200001	8	30200001	0.4493	0.6644
NC_005107.4_30300001	8	30300001	0.2724	0.3831
NC_005107.4_30400001	8	30400001	0.3984	0.5628
NC_005107.4_30500001	8	30500001	0.4321	0.6819
NC_005107.4_30600001	8	30600001	0.5417	0.7469
NC_005107.4_30700001	8	30700001	0.4971	0.6378
NC_005107.4_30800001	8	30800001	0.5101	0.6461
NC_005107.4_30900001	8	30900001	0.4612	0.6045
NC_005107.4_31000001	8	31000001	0.4539	0.5325
NC_005107.4_31100001	8	31100001	0.3923	0.4764
NC_005107.4_31200001	8	31200001	0.4618	0.5632
NC_005107.4_31300001	8	31300001	0.4724	0.5282
NC_005107.4_31400001	8	31400001	0.4116	0.461
NC_005107.4_31500001	8	31500001	0.4696	0.5343
NC_005107.4_31600001	8	31600001	0.4781	0.5124
NC_005107.4_31700001	8	31700001	0.4221	0.4975
NC_005107.4_31800001	8	31800001	0.5121	0.605
NC_005107.4_31900001	8	31900001	0.4924	0.5824
NC_005107.4_32000001	8	32000001	0.4389	0.4983
NC_005107.4_32100001	8	32100001	0.5454	0.5962
NC_005107.4_32200001	8	32200001	0.4966	0.5407
NC_005107.4_32300001	8	32300001	0.4533	0.5487
NC_005107.4_32400001	8	32400001	0.4947	0.609
NC_005107.4_32500001	8	32500001	0.4788	0.6205
NC_005107.4_32600001	8	32600001	0.4096	0.5544
NC_005107.4_32700001	8	32700001	0.4125	0.6032
NC_005107.4_32800001	8	32800001	0.4682	0.576
NC_005107.4_32900001	8	32900001	0.406	0.4423
NC_005107.4_33000001	8	33000001	0.5567	0.5862
NC_005107.4_33100001	8	33100001	0.5068	0.6083
NC_005107.4_33200001	8	33200001	0.5033	0.5903
NC_005107.4_33300001	8	33300001	0.4658	0.5807
NC_005107.4_33400001	8	33400001	0.4933	0.6236
NC_005107.4_33500001	8	33500001	0.4193	0.5955
NC_005107.4_33600001	8	33600001	0.3607	0.5296

NC_005107.4_33700001	8	33700001	0.4261	0.6467
NC_005107.4_33800001	8	33800001	0.4873	0.7186
NC_005107.4_33900001	8	33900001	0.4242	0.5654
NC_005107.4_34000001	8	34000001	0.4415	0.5426
NC_005107.4_34100001	8	34100001	0.617	0.6802
NC_005107.4_34200001	8	34200001	0.5611	0.622
NC_005107.4_34300001	8	34300001	0.4871	0.528
NC_005107.4_34400001	8	34400001	0.5407	0.6401
NC_005107.4_34500001	8	34500001	0.6031	0.6165
NC_005107.4_34600001	8	34600001	0.323	0.2023
NC_005107.4_34700001	8	34700001	0.2084	0.1919
NC_005107.4_34800001	8	34800001	0.2357	0.2299
NC_005107.4_34900001	8	34900001	0.2126	0.2058
NC_005107.4_35000001	8	35000001	0.1853	0.1912
NC_005107.4_35100001	8	35100001	0.1657	0.1675
NC_005107.4_35200001	8	35200001	0.251	0.2808
NC_005107.4_35300001	8	35300001	0.2614	0.268
NC_005107.4_35400001	8	35400001	0.3408	0.4294
NC_005107.4_35500001	8	35500001	0.3217	0.42
NC_005107.4_35600001	8	35600001	0.3139	0.3947
NC_005107.4_35700001	8	35700001	0.3536	0.4611
NC_005107.4_35800001	8	35800001	0.3772	0.5279
NC_005107.4_35900001	8	35900001	0.4506	0.6055
NC_005107.4_36000001	8	36000001	0.5266	0.6864
NC_005107.4_36100001	8	36100001	0.705	0.8009
NC_005107.4_36200001	8	36200001	0.7586	0.8637
NC_005107.4_36300001	8	36300001	0.7684	0.8691
NC_005107.4_36400001	8	36400001	0.7523	0.8882
NC_005107.4_36500001	8	36500001	0.7635	0.879
NC_005107.4_36600001	8	36600001	0.6764	0.9227
NC_005107.4_36700001	8	36700001	0.5898	0.8616
NC_005107.4_36800001	8	36800001	0.5707	0.8784
NC_005107.4_36900001	8	36900001	0.4741	0.8304
NC_005107.4_37000001	8	37000001	0.4741	0.8304
NC_005107.4_37100001	8	37100001	0.529	0.8713
NC_005107.4_37600001	8	37600001	0.3509	0.4942
NC_005107.4_37700001	8	37700001	0.3509	0.4942
NC_005107.4_37800001	8	37800001	0.3509	0.4942
NC_005107.4_38900001	8	38900001	0.3842	0.4821
NC_005107.4_39000001	8	39000001	0.2995	0.3983
NC_005107.4_39100001	8	39100001	0.3544	0.4312
NC_005107.4_39200001	8	39200001	0.4016	0.4926
NC_005107.4_39300001	8	39300001	0.3656	0.4652
NC_005107.4_39400001	8	39400001	0.3855	0.5148
NC_005107.4_39500001	8	39500001	0.4537	0.5855
NC_005107.4_39600001	8	39600001	0.5261	0.7007
NC_005107.4_39700001	8	39700001	0.5217	0.7455
NC_005107.4_39800001	8	39800001	0.5391	0.7383
NC_005107.4_39900001	8	39900001	0.5201	0.7153
NC_005107.4_40000001	8	40000001	0.4951	0.6607
NC_005107.4_40100001	8	40100001	0.3672	0.5308
NC_005107.4_40200001	8	40200001	0.3438	0.4104
NC_005107.4_40300001	8	40300001	0.3442	0.4441
NC_005107.4_40400001	8	40400001	0.2262	0.1121
NC_005107.4_42900001	8	42900001	0.6543	0.6859
NC_005107.4_43000001	8	43000001	0.6543	0.6859
NC_005107.4_43100001	8	43100001	0.6543	0.6859
NC_005107.4_43200001	8	43200001	0.7037	0.8867

NC_005107.4_43300001	8	43300001	0.6362	0.766
NC_005107.4_43400001	8	43400001	0.7653	0.8733
NC_005107.4_43500001	8	43500001	0.8482	0.9227
NC_005107.4_43600001	8	43600001	0.6613	0.8528
NC_005107.4_43700001	8	43700001	0.589	0.7981
NC_005107.4_43800001	8	43800001	0.5488	0.7789
NC_005107.4_43900001	8	43900001	0.5259	0.7172
NC_005107.4_44000001	8	44000001	0.4568	0.7007
NC_005107.4_44100001	8	44100001	0.4687	0.7001
NC_005107.4_44200001	8	44200001	0.4634	0.7085
NC_005107.4_44300001	8	44300001	0.4749	0.7391
NC_005107.4_44400001	8	44400001	0.449	0.8732
NC_005107.4_44500001	8	44500001	0.6146	0.8492
NC_005107.4_44600001	8	44600001	0.7589	0.8745
NC_005107.4_44700001	8	44700001	0.7367	0.8567
NC_005107.4_44800001	8	44800001	0.6278	0.7501
NC_005107.4_44900001	8	44900001	0.5794	0.7248
NC_005107.4_45000001	8	45000001	0.5604	0.7026
NC_005107.4_45100001	8	45100001	0.4929	0.6305
NC_005107.4_45200001	8	45200001	0.5148	0.62
NC_005107.4_45300001	8	45300001	0.5068	0.625
NC_005107.4_45400001	8	45400001	0.5241	0.6131
NC_005107.4_45500001	8	45500001	0.5357	0.6065
NC_005107.4_45600001	8	45600001	0.5509	0.6135
NC_005107.4_45700001	8	45700001	0.4527	0.5963
NC_005107.4_45800001	8	45800001	0.4557	0.5786
NC_005107.4_45900001	8	45900001	0.4594	0.5908
NC_005107.4_46000001	8	46000001	0.4264	0.5245
NC_005107.4_46100001	8	46100001	0.4749	0.5903
NC_005107.4_46200001	8	46200001	0.5607	0.6522
NC_005107.4_46300001	8	46300001	0.5922	0.6433
NC_005107.4_46400001	8	46400001	0.6153	0.6423
NC_005107.4_46500001	8	46500001	0.5932	0.6537
NC_005107.4_46600001	8	46600001	0.539	0.6118
NC_005107.4_46700001	8	46700001	0.5074	0.5326
NC_005107.4_46800001	8	46800001	0.5696	0.6136
NC_005107.4_46900001	8	46900001	0.5577	0.6359
NC_005107.4_47000001	8	47000001	0.5981	0.6419
NC_005107.4_47100001	8	47100001	0.6629	0.6737
NC_005107.4_47200001	8	47200001	0.6949	0.7018
NC_005107.4_47300001	8	47300001	0.5507	0.5734
NC_005107.4_47400001	8	47400001	0.5602	0.6041
NC_005107.4_47500001	8	47500001	0.4974	0.5618
NC_005107.4_47600001	8	47600001	0.4007	0.568
NC_005107.4_47700001	8	47700001	0.3725	0.5823
NC_005107.4_47800001	8	47800001	0.4136	0.6422
NC_005107.4_47900001	8	47900001	0.365	0.593
NC_005107.4_48000001	8	48000001	0.3795	0.6443
NC_005107.4_48100001	8	48100001	0.4674	0.6831
NC_005107.4_48200001	8	48200001	0.5606	0.7107
NC_005107.4_48300001	8	48300001	0.4958	0.7097
NC_005107.4_48400001	8	48400001	0.5415	0.708
NC_005107.4_48500001	8	48500001	0.5681	0.7404
NC_005107.4_48600001	8	48600001	0.5612	0.7524
NC_005107.4_48700001	8	48700001	0.5965	0.8054
NC_005107.4_48800001	8	48800001	0.6767	0.8223
NC_005107.4_48900001	8	48900001	0.6684	0.8288
NC_005107.4_49000001	8	49000001	0.6003	0.7189

NC_005107.4_49100001	8	49100001	0.5843	0.6825
NC_005107.4_49200001	8	49200001	0.5229	0.6242
NC_005107.4_49300001	8	49300001	0.5147	0.6076
NC_005107.4_49400001	8	49400001	0.5249	0.6069
NC_005107.4_49500001	8	49500001	0.505	0.6009
NC_005107.4_49600001	8	49600001	0.4656	0.5784
NC_005107.4_49700001	8	49700001	0.4821	0.6049
NC_005107.4_49800001	8	49800001	0.4943	0.6359
NC_005107.4_49900001	8	49900001	0.4775	0.6414
NC_005107.4_50000001	8	50000001	0.5466	0.7539
NC_005107.4_50100001	8	50100001	0.5431	0.788
NC_005107.4_50200001	8	50200001	0.5154	0.7813
NC_005107.4_50300001	8	50300001	0.4893	0.7421
NC_005107.4_50400001	8	50400001	0.4655	0.6724
NC_005107.4_50500001	8	50500001	0.3983	0.5761
NC_005107.4_50600001	8	50600001	0.4302	0.5798
NC_005107.4_50700001	8	50700001	0.4615	0.6494
NC_005107.4_50800001	8	50800001	0.4397	0.6358
NC_005107.4_50900001	8	50900001	0.4832	0.7213
NC_005107.4_51000001	8	51000001	0.5333	0.7439
NC_005107.4_51100001	8	51100001	0.5573	0.7802
NC_005107.4_51200001	8	51200001	0.6542	0.7819
NC_005107.4_51300001	8	51300001	0.6654	0.7919
NC_005107.4_51400001	8	51400001	0.6924	0.7828
NC_005107.4_51500001	8	51500001	0.6954	0.8082
NC_005107.4_51600001	8	51600001	0.7674	0.843
NC_005107.4_51700001	8	51700001	0.7539	0.8687
NC_005107.4_51800001	8	51800001	0.8027	0.9083
NC_005107.4_51900001	8	51900001	0.7386	0.8922
NC_005107.4_52000001	8	52000001	0.7081	0.7938
NC_005107.4_52100001	8	52100001	0.6493	0.7561
NC_005107.4_52200001	8	52200001	0.6388	0.7481
NC_005107.4_52300001	8	52300001	0.5215	0.6047
NC_005107.4_52400001	8	52400001	0.5613	0.6732
NC_005107.4_52500001	8	52500001	0.5732	0.7031
NC_005107.4_52600001	8	52600001	0.4639	0.6536
NC_005107.4_52700001	8	52700001	0.4442	0.664
NC_005107.4_52800001	8	52800001	0.4734	0.681
NC_005107.4_52900001	8	52900001	0.5258	0.7258
NC_005107.4_53000001	8	53000001	0.4251	0.6725
NC_005107.4_53100001	8	53100001	0.4881	0.7096
NC_005107.4_53200001	8	53200001	0.4926	0.7027
NC_005107.4_53300001	8	53300001	0.5349	0.7585
NC_005107.4_53400001	8	53400001	0.4497	0.6807
NC_005107.4_53500001	8	53500001	0.5569	0.8155
NC_005107.4_53600001	8	53600001	0.5299	0.7581
NC_005107.4_53700001	8	53700001	0.5402	0.7084
NC_005107.4_53800001	8	53800001	0.5188	0.658
NC_005107.4_53900001	8	53900001	0.5446	0.676
NC_005107.4_54000001	8	54000001	0.4924	0.5669
NC_005107.4_54100001	8	54100001	0.5598	0.6926
NC_005107.4_54200001	8	54200001	0.5046	0.6827
NC_005107.4_54300001	8	54300001	0.4911	0.702
NC_005107.4_54400001	8	54400001	0.4723	0.7089
NC_005107.4_54500001	8	54500001	0.51	0.7034
NC_005107.4_54600001	8	54600001	0.5209	0.7084
NC_005107.4_54700001	8	54700001	0.5762	0.7921
NC_005107.4_54800001	8	54800001	0.5506	0.7774

NC_005107.4_54900001	8	54900001	0.5388	0.773
NC_005107.4_55000001	8	55000001	0.5958	0.764
NC_005107.4_55100001	8	55100001	0.4968	0.7196
NC_005107.4_55200001	8	55200001	0.5142	0.7429
NC_005107.4_55300001	8	55300001	0.5002	0.634
NC_005107.4_55400001	8	55400001	0.5588	0.6523
NC_005107.4_55500001	8	55500001	0.5164	0.628
NC_005107.4_55600001	8	55600001	0.543	0.5917
NC_005107.4_55700001	8	55700001	0.5124	0.5297
NC_005107.4_55800001	8	55800001	0.549	0.5939
NC_005107.4_55900001	8	55900001	0.5246	0.5942
NC_005107.4_56000001	8	56000001	0.5547	0.5971
NC_005107.4_56100001	8	56100001	0.5837	0.6837
NC_005107.4_56200001	8	56200001	0.6261	0.7387
NC_005107.4_56300001	8	56300001	0.6086	0.7204
NC_005107.4_56400001	8	56400001	0.5202	0.6746
NC_005107.4_56500001	8	56500001	0.5288	0.6775
NC_005107.4_56600001	8	56600001	0.5088	0.6089
NC_005107.4_56700001	8	56700001	0.4702	0.6116
NC_005107.4_56800001	8	56800001	0.4602	0.5472
NC_005107.4_56900001	8	56900001	0.5002	0.5433
NC_005107.4_57000001	8	57000001	0.4751	0.5525
NC_005107.4_57100001	8	57100001	0.3636	0.5246
NC_005107.4_57200001	8	57200001	0.3267	0.4729
NC_005107.4_57300001	8	57300001	0.3469	0.5679
NC_005107.4_57400001	8	57400001	0.4098	0.6943
NC_005107.4_57500001	8	57500001	0.4179	0.7583
NC_005107.4_57600001	8	57600001	0.5902	0.8418
NC_005107.4_57700001	8	57700001	0.5909	0.8451
NC_005107.4_57800001	8	57800001	0.5526	0.8528
NC_005107.4_57900001	8	57900001	0.482	0.6841
NC_005107.4_58000001	8	58000001	0.5339	0.6811
NC_005107.4_58100001	8	58100001	0.5478	0.6653
NC_005107.4_58200001	8	58200001	0.5434	0.6619
NC_005107.4_58300001	8	58300001	0.5404	0.6186
NC_005107.4_58400001	8	58400001	0.5474	0.6291
NC_005107.4_58500001	8	58500001	0.5682	0.649
NC_005107.4_58600001	8	58600001	0.5446	0.6357
NC_005107.4_58700001	8	58700001	0.4471	0.5464
NC_005107.4_58800001	8	58800001	0.459	0.6077
NC_005107.4_58900001	8	58900001	0.4532	0.5723
NC_005107.4_59000001	8	59000001	0.415	0.5243
NC_005107.4_59100001	8	59100001	0.394	0.5174
NC_005107.4_59200001	8	59200001	0.4979	0.6839
NC_005107.4_59300001	8	59300001	0.508	0.6869
NC_005107.4_59400001	8	59400001	0.5305	0.7305
NC_005107.4_59500001	8	59500001	0.5455	0.6818
NC_005107.4_59600001	8	59600001	0.5836	0.722
NC_005107.4_59700001	8	59700001	0.6017	0.6865
NC_005107.4_59800001	8	59800001	0.6069	0.7032
NC_005107.4_59900001	8	59900001	0.5662	0.7088
NC_005107.4_60000001	8	60000001	0.5642	0.7894
NC_005107.4_60100001	8	60100001	0.5661	0.7774
NC_005107.4_60200001	8	60200001	0.593	0.7805
NC_005107.4_60300001	8	60300001	0.5074	0.7051
NC_005107.4_60400001	8	60400001	0.6306	0.759
NC_005107.4_60500001	8	60500001	0.6498	0.7524
NC_005107.4_60600001	8	60600001	0.5837	0.7478

NC_005107.4_60700001	8	60700001	0.5227	0.6757
NC_005107.4_60800001	8	60800001	0.5265	0.7126
NC_005107.4_60900001	8	60900001	0.4662	0.6414
NC_005107.4_61000001	8	61000001	0.4477	0.635
NC_005107.4_61100001	8	61100001	0.5611	0.7061
NC_005107.4_61200001	8	61200001	0.6078	0.7436
NC_005107.4_61300001	8	61300001	0.6689	0.7255
NC_005107.4_61400001	8	61400001	0.7793	0.8105
NC_005107.4_61500001	8	61500001	0.7168	0.8036
NC_005107.4_61600001	8	61600001	0.6184	0.7079
NC_005107.4_61700001	8	61700001	0.5603	0.7191
NC_005107.4_61800001	8	61800001	0.4581	0.6191
NC_005107.4_61900001	8	61900001	0.4599	0.6235
NC_005107.4_62000001	8	62000001	0.4221	0.6025
NC_005107.4_62100001	8	62100001	0.4292	0.6524
NC_005107.4_62200001	8	62200001	0.4192	0.6098
NC_005107.4_62300001	8	62300001	0.4559	0.638
NC_005107.4_62400001	8	62400001	0.4379	0.6218
NC_005107.4_62500001	8	62500001	0.5436	0.6737
NC_005107.4_62600001	8	62600001	0.5414	0.6455
NC_005107.4_62700001	8	62700001	0.5332	0.6714
NC_005107.4_62800001	8	62800001	0.578	0.7117
NC_005107.4_62900001	8	62900001	0.5665	0.6738
NC_005107.4_63000001	8	63000001	0.5246	0.6234
NC_005107.4_63100001	8	63100001	0.5292	0.5532
NC_005107.4_63200001	8	63200001	0.475	0.4783
NC_005107.4_63300001	8	63300001	0.4593	0.5529
NC_005107.4_63400001	8	63400001	0.5302	0.6874
NC_005107.4_63500001	8	63500001	0.4319	0.6105
NC_005107.4_63600001	8	63600001	0.4612	0.6938
NC_005107.4_63700001	8	63700001	0.5161	0.7287
NC_005107.4_63800001	8	63800001	0.5117	0.7072
NC_005107.4_63900001	8	63900001	0.4281	0.5986
NC_005107.4_64000001	8	64000001	0.5381	0.6901
NC_005107.4_64100001	8	64100001	0.5508	0.6941
NC_005107.4_64200001	8	64200001	0.5575	0.651
NC_005107.4_64300001	8	64300001	0.568	0.6531
NC_005107.4_64400001	8	64400001	0.6843	0.7021
NC_005107.4_64500001	8	64500001	0.6875	0.7179
NC_005107.4_64600001	8	64600001	0.6282	0.7059
NC_005107.4_64700001	8	64700001	0.6193	0.7211
NC_005107.4_64800001	8	64800001	0.5788	0.6962
NC_005107.4_64900001	8	64900001	0.4722	0.6064
NC_005107.4_65000001	8	65000001	0.5167	0.6629
NC_005107.4_65100001	8	65100001	0.5284	0.6577
NC_005107.4_65200001	8	65200001	0.4941	0.6054
NC_005107.4_65300001	8	65300001	0.4962	0.6324
NC_005107.4_65400001	8	65400001	0.602	0.6995
NC_005107.4_65500001	8	65500001	0.5565	0.6438
NC_005107.4_65600001	8	65600001	0.5203	0.6282
NC_005107.4_65700001	8	65700001	0.532	0.6878
NC_005107.4_65800001	8	65800001	0.4678	0.639
NC_005107.4_65900001	8	65900001	0.4975	0.6816
NC_005107.4_66000001	8	66000001	0.4572	0.6403
NC_005107.4_66100001	8	66100001	0.5083	0.7211
NC_005107.4_66200001	8	66200001	0.4815	0.6835
NC_005107.4_66300001	8	66300001	0.4761	0.6227
NC_005107.4_66400001	8	66400001	0.4657	0.5785

NC_005107.4_66500001	8	66500001	0.5066	0.6039
NC_005107.4_66600001	8	66600001	0.5294	0.6341
NC_005107.4_66700001	8	66700001	0.542	0.6539
NC_005107.4_66800001	8	66800001	0.612	0.6987
NC_005107.4_66900001	8	66900001	0.5267	0.7064
NC_005107.4_67000001	8	67000001	0.4731	0.6229
NC_005107.4_67100001	8	67100001	0.3984	0.553
NC_005107.4_67200001	8	67200001	0.394	0.5263
NC_005107.4_67300001	8	67300001	0.437	0.5912
NC_005107.4_67400001	8	67400001	0.4793	0.598
NC_005107.4_67500001	8	67500001	0.5579	0.6831
NC_005107.4_67600001	8	67600001	0.5404	0.6829
NC_005107.4_67700001	8	67700001	0.6058	0.7381
NC_005107.4_67800001	8	67800001	0.5738	0.7121
NC_005107.4_67900001	8	67900001	0.5494	0.7235
NC_005107.4_68000001	8	68000001	0.5429	0.8115
NC_005107.4_68100001	8	68100001	0.7204	0.87
NC_005107.4_68200001	8	68200001	0.5751	0.7501
NC_005107.4_68300001	8	68300001	0.5677	0.7695
NC_005107.4_68400001	8	68400001	0.6149	0.7921
NC_005107.4_68500001	8	68500001	0.5974	0.7701
NC_005107.4_68600001	8	68600001	0.5465	0.6953
NC_005107.4_68700001	8	68700001	0.5783	0.7127
NC_005107.4_68800001	8	68800001	0.5477	0.6597
NC_005107.4_68900001	8	68900001	0.539	0.6375
NC_005107.4_69000001	8	69000001	0.4803	0.5798
NC_005107.4_69100001	8	69100001	0.5258	0.6713
NC_005107.4_69200001	8	69200001	0.4855	0.7769
NC_005107.4_69300001	8	69300001	0.6803	0.5866
NC_005107.4_69400001	8	69400001	0.6671	0.5682
NC_005107.4_69500001	8	69500001	0.6843	0.6048
NC_005107.4_69600001	8	69600001	0.5898	0.5482
NC_005107.4_69700001	8	69700001	0.671	0.6777
NC_005107.4_69800001	8	69800001	0.6396	0.7704
NC_005107.4_69900001	8	69900001	0.6329	0.7863
NC_005107.4_70000001	8	70000001	0.6932	0.775
NC_005107.4_70100001	8	70100001	0.7581	0.8276
NC_005107.4_70200001	8	70200001	0.6001	0.7093
NC_005107.4_70300001	8	70300001	0.5884	0.6772
NC_005107.4_70400001	8	70400001	0.577	0.6756
NC_005107.4_70500001	8	70500001	0.4891	0.6255
NC_005107.4_70600001	8	70600001	0.4702	0.5917
NC_005107.4_70700001	8	70700001	0.5316	0.6616
NC_005107.4_70800001	8	70800001	0.6311	0.7593
NC_005107.4_70900001	8	70900001	0.6524	0.766
NC_005107.4_71000001	8	71000001	0.701	0.7939
NC_005107.4_71100001	8	71100001	0.7444	0.8541
NC_005107.4_71200001	8	71200001	0.8373	0.9031
NC_005107.4_71300001	8	71300001	0.6946	0.7994
NC_005107.4_71400001	8	71400001	0.6958	0.7756
NC_005107.4_71500001	8	71500001	0.6873	0.7961
NC_005107.4_71600001	8	71600001	0.6719	0.7856
NC_005107.4_71700001	8	71700001	0.6857	0.7915
NC_005107.4_71800001	8	71800001	0.7337	0.8256
NC_005107.4_71900001	8	71900001	0.7177	0.7723
NC_005107.4_72000001	8	72000001	0.689	0.7053
NC_005107.4_72100001	8	72100001	0.7172	0.7179
NC_005107.4_72200001	8	72200001	0.684	0.6772

NC_005107.4_72300001	8	72300001	0.6266	0.6594
NC_005107.4_72400001	8	72400001	0.5815	0.7155
NC_005107.4_72500001	8	72500001	0.4564	0.6197
NC_005107.4_72600001	8	72600001	0.4126	0.5101
NC_005107.4_72700001	8	72700001	0.4863	0.5574
NC_005107.4_72800001	8	72800001	0.4645	0.5407
NC_005107.4_72900001	8	72900001	0.5302	0.5928
NC_005107.4_73000001	8	73000001	0.6182	0.6806
NC_005107.4_73100001	8	73100001	0.6449	0.7498
NC_005107.4_73200001	8	73200001	0.6149	0.7941
NC_005107.4_73300001	8	73300001	0.6436	0.7982
NC_005107.4_73400001	8	73400001	0.5901	0.7387
NC_005107.4_73500001	8	73500001	0.595	0.7156
NC_005107.4_73600001	8	73600001	0.5812	0.7038
NC_005107.4_73700001	8	73700001	0.555	0.6642
NC_005107.4_73800001	8	73800001	0.485	0.6345
NC_005107.4_73900001	8	73900001	0.4165	0.6478
NC_005107.4_74000001	8	74000001	0.3693	0.597
NC_005107.4_74100001	8	74100001	0.3592	0.5722
NC_005107.4_74200001	8	74200001	0.2981	0.4751
NC_005107.4_74300001	8	74300001	0.3467	0.5451
NC_005107.4_74400001	8	74400001	0.4585	0.5509
NC_005107.4_75100001	8	75100001	0.8328	0.8453
NC_005107.4_75200001	8	75200001	0.8052	0.9017
NC_005107.4_75300001	8	75300001	0.6617	0.7694
NC_005107.4_75400001	8	75400001	0.6079	0.7616
NC_005107.4_75500001	8	75500001	0.5806	0.7355
NC_005107.4_75600001	8	75600001	0.4667	0.6653
NC_005107.4_75700001	8	75700001	0.3825	0.5271
NC_005107.4_75800001	8	75800001	0.3902	0.5861
NC_005107.4_75900001	8	75900001	0.3269	0.4729
NC_005107.4_76000001	8	76000001	0.2847	0.4528
NC_005107.4_76100001	8	76100001	0.3883	0.4835
NC_005107.4_76200001	8	76200001	0.3388	0.4374
NC_005107.4_76300001	8	76300001	0.3289	0.4052
NC_005107.4_76400001	8	76400001	0.399	0.5239
NC_005107.4_76500001	8	76500001	0.3686	0.5062
NC_005107.4_76600001	8	76600001	0.4392	0.686
NC_005107.4_76700001	8	76700001	0.4966	0.7455
NC_005107.4_76800001	8	76800001	0.5486	0.8102
NC_005107.4_76900001	8	76900001	0.4839	0.7857
NC_005107.4_77000001	8	77000001	0.6783	0.7649
NC_005107.4_77100001	8	77100001	0.6532	0.7374
NC_005107.4_77200001	8	77200001	0.6241	0.6513
NC_005107.4_77300001	8	77300001	0.545	0.6365
NC_005107.4_77400001	8	77400001	0.5469	0.6159
NC_005107.4_77500001	8	77500001	0.4688	0.6365
NC_005107.4_77600001	8	77600001	0.4767	0.6093
NC_005107.4_77700001	8	77700001	0.4853	0.7122
NC_005107.4_77800001	8	77800001	0.5076	0.7259
NC_005107.4_77900001	8	77900001	0.5337	0.7497
NC_005107.4_78000001	8	78000001	0.5337	0.7497
NC_005107.4_78100001	8	78100001	0.5325	0.7526
NC_005107.4_78200001	8	78200001	0.5256	0.8096
NC_005107.4_78600001	8	78600001	0.5253	0.7276
NC_005107.4_78700001	8	78700001	0.524	0.7536
NC_005107.4_78800001	8	78800001	0.4661	0.6802
NC_005107.4_78900001	8	78900001	0.5162	0.7019

NC_005107.4_79000001	8	79000001	0.554	0.7283
NC_005107.4_79100001	8	79100001	0.5522	0.7147
NC_005107.4_79200001	8	79200001	0.5535	0.6497
NC_005107.4_79300001	8	79300001	0.6369	0.7413
NC_005107.4_79400001	8	79400001	0.682	0.7616
NC_005107.4_79500001	8	79500001	0.6601	0.7376
NC_005107.4_79600001	8	79600001	0.6346	0.7145
NC_005107.4_79700001	8	79700001	0.4704	0.6322
NC_005107.4_79800001	8	79800001	0.3501	0.3756
NC_005107.4_79900001	8	79900001	0.3317	0.4205
NC_005107.4_80000001	8	80000001	0.3654	0.3916
NC_005107.4_80100001	8	80100001	0.4174	0.4619
NC_005107.4_80200001	8	80200001	0.4875	0.4253
NC_005107.4_80300001	8	80300001	0.4907	0.464
NC_005107.4_80400001	8	80400001	0.5779	0.423
NC_005107.4_80500001	8	80500001	0.6091	0.5006
NC_005107.4_80600001	8	80600001	0.4998	0.3709
NC_005107.4_80700001	8	80700001	0.5266	0.4691
NC_005107.4_80800001	8	80800001	0.4887	0.4401
NC_005107.4_80900001	8	80900001	0.515	0.5234
NC_005107.4_81000001	8	81000001	0.5226	0.543
NC_005107.4_81100001	8	81100001	0.553	0.5906
NC_005107.4_81200001	8	81200001	0.4488	0.5541
NC_005107.4_81300001	8	81300001	0.4181	0.56
NC_005107.4_81400001	8	81400001	0.3647	0.5586
NC_005107.4_81500001	8	81500001	0.4626	0.6428
NC_005107.4_81600001	8	81600001	0.4337	0.635
NC_005107.4_81700001	8	81700001	0.5111	0.6863
NC_005107.4_81800001	8	81800001	0.5087	0.7053
NC_005107.4_81900001	8	81900001	0.485	0.6667
NC_005107.4_82000001	8	82000001	0.3957	0.6215
NC_005107.4_82100001	8	82100001	0.4111	0.6403
NC_005107.4_82200001	8	82200001	0.4743	0.7431
NC_005107.4_82300001	8	82300001	0.5281	0.7451
NC_005107.4_82400001	8	82400001	0.5584	0.8627
NC_005107.4_82500001	8	82500001	0.5917	0.8958
NC_005107.4_82600001	8	82600001	0.5259	0.7016
NC_005107.4_82700001	8	82700001	0.4033	0.4976
NC_005107.4_82800001	8	82800001	0.3894	0.568
NC_005107.4_82900001	8	82900001	0.332	0.5143
NC_005107.4_83000001	8	83000001	0.386	0.4831
NC_005107.4_83100001	8	83100001	0.3254	0.4877
NC_005107.4_83200001	8	83200001	0.3557	0.5687
NC_005107.4_83300001	8	83300001	0.3979	0.5556
NC_005107.4_83400001	8	83400001	0.5189	0.6426
NC_005107.4_83500001	8	83500001	0.512	0.6516
NC_005107.4_83600001	8	83600001	0.529	0.6688
NC_005107.4_83700001	8	83700001	0.5321	0.5757
NC_005107.4_83800001	8	83800001	0.5018	0.5522
NC_005107.4_83900001	8	83900001	0.3271	0.4062
NC_005107.4_84000001	8	84000001	0.3784	0.5713
NC_005107.4_84100001	8	84100001	0.4122	0.5289
NC_005107.4_84200001	8	84200001	0.5594	0.6746
NC_005107.4_84300001	8	84300001	0.5994	0.7704
NC_005107.4_84400001	8	84400001	0.6497	0.7728
NC_005107.4_84500001	8	84500001	0.6225	0.7429
NC_005107.4_84600001	8	84600001	0.5789	0.7138
NC_005107.4_84700001	8	84700001	0.5054	0.6366

NC_005107.4_84800001	8	84800001	0.4397	0.5284
NC_005107.4_84900001	8	84900001	0.373	0.416
NC_005107.4_85000001	8	85000001	0.4216	0.4551
NC_005107.4_85100001	8	85100001	0.4617	0.4747
NC_005107.4_85200001	8	85200001	0.4805	0.5184
NC_005107.4_85300001	8	85300001	0.5241	0.5277
NC_005107.4_85400001	8	85400001	0.7226	0.73
NC_005107.4_85500001	8	85500001	0.615	0.6251
NC_005107.4_85600001	8	85600001	0.6346	0.6452
NC_005107.4_85700001	8	85700001	0.5463	0.6309
NC_005107.4_85800001	8	85800001	0.4727	0.5369
NC_005107.4_85900001	8	85900001	0.4599	0.4658
NC_005107.4_86000001	8	86000001	0.4618	0.5512
NC_005107.4_86100001	8	86100001	0.4729	0.6124
NC_005107.4_86200001	8	86200001	0.5742	0.6782
NC_005107.4_86300001	8	86300001	0.5742	0.6782
NC_005107.4_86400001	8	86400001	0.5118	0.6783
NC_005107.4_86500001	8	86500001	0.5223	0.6203
NC_005107.4_86600001	8	86600001	0.5007	0.6002
NC_005107.4_86700001	8	86700001	0.4279	0.5019
NC_005107.4_86800001	8	86800001	0.4003	0.4879
NC_005107.4_86900001	8	86900001	0.475	0.6201
NC_005107.4_87000001	8	87000001	0.4702	0.6486
NC_005107.4_87100001	8	87100001	0.5395	0.7019
NC_005107.4_87200001	8	87200001	0.6027	0.7146
NC_005107.4_87300001	8	87300001	0.6082	0.701
NC_005107.4_87400001	8	87400001	0.6068	0.6475
NC_005107.4_87500001	8	87500001	0.5936	0.6639
NC_005107.4_87600001	8	87600001	0.5623	0.5834
NC_005107.4_87800001	8	87800001	0.7521	0.6317
NC_005107.4_87900001	8	87900001	0.6034	0.6655
NC_005107.4_88000001	8	88000001	0.593	0.6597
NC_005107.4_88100001	8	88100001	0.567	0.6298
NC_005107.4_88200001	8	88200001	0.5822	0.667
NC_005107.4_88300001	8	88300001	0.588	0.6961
NC_005107.4_88400001	8	88400001	0.6298	0.6889
NC_005107.4_88500001	8	88500001	0.72	0.7669
NC_005107.4_88600001	8	88600001	0.7668	0.76
NC_005107.4_88700001	8	88700001	0.6491	0.6964
NC_005107.4_88800001	8	88800001	0.5849	0.6105
NC_005107.4_88900001	8	88900001	0.5849	0.6105
NC_005107.4_89000001	8	89000001	0.4451	0.4573
NC_005107.4_89100001	8	89100001	0.3696	0.4677
NC_005107.4_89200001	8	89200001	0.4794	0.5295
NC_005107.4_89300001	8	89300001	0.4984	0.5485
NC_005107.4_89400001	8	89400001	0.4984	0.5485
NC_005107.4_89500001	8	89500001	0.5505	0.5775
NC_005107.4_89600001	8	89600001	0.6522	0.777
NC_005107.4_89700001	8	89700001	0.5501	0.8464
NC_005107.4_89800001	8	89800001	0.5079	0.6777
NC_005107.4_89900001	8	89900001	0.5309	0.7231
NC_005107.4_90000001	8	90000001	0.5558	0.7446
NC_005107.4_90100001	8	90100001	0.5087	0.6377
NC_005107.4_90200001	8	90200001	0.4727	0.5996
NC_005107.4_90300001	8	90300001	0.4423	0.6959
NC_005107.4_90400001	8	90400001	0.3384	0.5112
NC_005107.4_90500001	8	90500001	0.2842	0.3924
NC_005107.4_90600001	8	90600001	0.2705	0.2876

NC_005107.4_90700001	8	90700001	0.3568	0.4131
NC_005107.4_90800001	8	90800001	0.3787	0.4396
NC_005107.4_90900001	8	90900001	0.4575	0.5032
NC_005107.4_91000001	8	91000001	0.5216	0.5886
NC_005107.4_91100001	8	91100001	0.5132	0.5987
NC_005107.4_91200001	8	91200001	0.4908	0.6047
NC_005107.4_91300001	8	91300001	0.4949	0.6027
NC_005107.4_91400001	8	91400001	0.492	0.629
NC_005107.4_91500001	8	91500001	0.4356	0.5505
NC_005107.4_91600001	8	91600001	0.4458	0.5748
NC_005107.4_91700001	8	91700001	0.4728	0.5395
NC_005107.4_91800001	8	91800001	0.4719	0.5455
NC_005107.4_91900001	8	91900001	0.4425	0.517
NC_005107.4_92000001	8	92000001	0.4656	0.5184
NC_005107.4_92100001	8	92100001	0.4563	0.5261
NC_005107.4_92200001	8	92200001	0.5213	0.6655
NC_005107.4_92300001	8	92300001	0.5164	0.6409
NC_005107.4_92400001	8	92400001	0.6258	0.7422
NC_005107.4_92500001	8	92500001	0.596	0.72
NC_005107.4_92600001	8	92600001	0.6445	0.7269
NC_005107.4_92700001	8	92700001	0.4994	0.5623
NC_005107.4_92800001	8	92800001	0.478	0.4827
NC_005107.4_92900001	8	92900001	0.3306	0.2325
NC_005107.4_93000001	8	93000001	0.4585	0.5384
NC_005107.4_93100001	8	93100001	0.476	0.6053
NC_005107.4_93200001	8	93200001	0.6901	0.8302
NC_005107.4_93300001	8	93300001	0.6957	0.9344
NC_005107.4_93400001	8	93400001	0.7055	0.8532
NC_005107.4_93500001	8	93500001	0.674	0.8108
NC_005107.4_93600001	8	93600001	0.71	0.7981
NC_005107.4_93700001	8	93700001	0.6329	0.7041
NC_005107.4_93800001	8	93800001	0.5393	0.5915
NC_005107.4_93900001	8	93900001	0.5388	0.6011
NC_005107.4_94000001	8	94000001	0.5188	0.5486
NC_005107.4_94100001	8	94100001	0.4209	0.5065
NC_005107.4_94200001	8	94200001	0.4748	0.5301
NC_005107.4_94300001	8	94300001	0.5687	0.6384
NC_005107.4_94400001	8	94400001	0.5268	0.5975
NC_005107.4_94500001	8	94500001	0.5629	0.6573
NC_005107.4_94600001	8	94600001	0.6483	0.6916
NC_005107.4_94700001	8	94700001	0.6992	0.8234
NC_005107.4_94800001	8	94800001	0.5616	0.7116
NC_005107.4_94900001	8	94900001	0.6033	0.7049
NC_005107.4_95000001	8	95000001	0.6011	0.6543
NC_005107.4_95100001	8	95100001	0.6101	0.6668
NC_005107.4_95200001	8	95200001	0.5829	0.6164
NC_005107.4_95300001	8	95300001	0.6601	0.6522
NC_005107.4_95400001	8	95400001	0.6426	0.6765
NC_005107.4_95500001	8	95500001	0.5117	0.6418
NC_005107.4_95600001	8	95600001	0.4615	0.5909
NC_005107.4_95700001	8	95700001	0.4349	0.6369
NC_005107.4_95800001	8	95800001	0.3406	0.6173
NC_005107.4_95900001	8	95900001	0.3344	0.5312
NC_005107.4_96000001	8	96000001	0.4978	0.7455
NC_005107.4_96100001	8	96100001	0.435	0.7145
NC_005107.4_96200001	8	96200001	0.3733	0.4677
NC_005107.4_96300001	8	96300001	0.3733	0.4677
NC_005107.4_96400001	8	96400001	0.3383	0.4993

NC_005107.4_96500001	8	96500001	0.3278	0.4972
NC_005107.4_96600001	8	96600001	0.3897	0.5528
NC_005107.4_96700001	8	96700001	0.4946	0.7914
NC_005107.4_96800001	8	96800001	0.5466	0.7692
NC_005107.4_96900001	8	96900001	0.5451	0.7488
NC_005107.4_97000001	8	97000001	0.5577	0.7306
NC_005107.4_97100001	8	97100001	0.5851	0.6929
NC_005107.4_97200001	8	97200001	0.58	0.6469
NC_005107.4_97300001	8	97300001	0.56	0.6129
NC_005107.4_97400001	8	97400001	0.5853	0.5964
NC_005107.4_97500001	8	97500001	0.6052	0.5834
NC_005107.4_97600001	8	97600001	0.7236	0.7752
NC_005107.4_97700001	8	97700001	0.6001	0.8629
NC_005107.4_97800001	8	97800001	0.4498	0.6668
NC_005107.4_97900001	8	97900001	0.521	0.7001
NC_005107.4_98000001	8	98000001	0.5482	0.7243
NC_005107.4_98100001	8	98100001	0.3664	0.4485
NC_005107.4_98200001	8	98200001	0.4052	0.4027
NC_005107.4_98300001	8	98300001	0.5039	0.5289
NC_005107.4_98400001	8	98400001	0.4313	0.4948
NC_005107.4_98500001	8	98500001	0.4185	0.5041
NC_005107.4_98600001	8	98600001	0.3913	0.488
NC_005107.4_98700001	8	98700001	0.4022	0.582
NC_005107.4_98800001	8	98800001	0.267	0.4913
NC_005107.4_98900001	8	98900001	0.4812	0.5956
NC_005107.4_99000001	8	99000001	0.3958	0.4489
NC_005107.4_99100001	8	99100001	0.6072	0.6441
NC_005107.4_99200001	8	99200001	0.5324	0.495
NC_005107.4_99300001	8	99300001	0.6196	0.6398
NC_005107.4_99400001	8	99400001	0.5491	0.6236
NC_005107.4_99500001	8	99500001	0.5733	0.6559
NC_005107.4_99600001	8	99600001	0.5627	0.6608
NC_005107.4_99700001	8	99700001	0.6294	0.7928
NC_005107.4_99800001	8	99800001	0.5247	0.7087
NC_005107.4_99900001	8	99900001	0.5697	0.7583
NC_005107.4_100000001	8	100000001	0.5781	0.7297
NC_005107.4_100100001	8	100100001	0.5891	0.6964
NC_005107.4_100200001	8	100200001	0.4276	0.4406
NC_005107.4_100300001	8	100300001	0.6698	0.6909
NC_005107.4_100400001	8	100400001	0.7009	0.694
NC_005107.4_100500001	8	100500001	0.6852	0.702
NC_005107.4_100600001	8	100600001	0.669	0.7241
NC_005107.4_100700001	8	100700001	0.7428	0.7759
NC_005107.4_100800001	8	100800001	0.6044	0.6996
NC_005107.4_100900001	8	100900001	0.5748	0.6426
NC_005107.4_101000001	8	101000001	0.5215	0.5985
NC_005107.4_101100001	8	101100001	0.461	0.5623
NC_005107.4_101200001	8	101200001	0.4225	0.5525
NC_005107.4_101300001	8	101300001	0.3935	0.483
NC_005107.4_101400001	8	101400001	0.3123	0.4499
NC_005107.4_101500001	8	101500001	0.2662	0.41
NC_005107.4_101600001	8	101600001	0.3999	0.5111
NC_005107.4_101700001	8	101700001	0.3829	0.3865
NC_005107.4_101800001	8	101800001	0.5447	0.5816
NC_005107.4_101900001	8	101900001	0.636	0.6921
NC_005107.4_102000001	8	102000001	0.5595	0.6129
NC_005107.4_102100001	8	102100001	0.6018	0.6296
NC_005107.4_102200001	8	102200001	0.5721	0.679

NC_005107.4_102300001	8	102300001	0.4995	0.6095
NC_005107.4_102400001	8	102400001	0.4034	0.5204
NC_005107.4_102500001	8	102500001	0.4023	0.5835
NC_005107.4_102600001	8	102600001	0.4305	0.5636
NC_005107.4_102700001	8	102700001	0.3756	0.5172
NC_005107.4_102800001	8	102800001	0.4109	0.5632
NC_005107.4_102900001	8	102900001	0.4369	0.5327
NC_005107.4_103000001	8	103000001	0.4531	0.5203
NC_005107.4_103100001	8	103100001	0.4563	0.5438
NC_005107.4_103200001	8	103200001	0.5264	0.6078
NC_005107.4_103300001	8	103300001	0.4323	0.4852
NC_005107.4_103400001	8	103400001	0.3124	0.4071
NC_005107.4_103500001	8	103500001	0.3861	0.5219
NC_005107.4_103600001	8	103600001	0.387	0.529
NC_005107.4_103700001	8	103700001	0.5915	0.698
NC_005107.4_103800001	8	103800001	0.6842	0.8094
NC_005107.4_103900001	8	103900001	0.6372	0.7461
NC_005107.4_104000001	8	104000001	0.5788	0.7509
NC_005107.4_104100001	8	104100001	0.5235	0.6914
NC_005107.4_104200001	8	104200001	0.4029	0.5612
NC_005107.4_104300001	8	104300001	0.3732	0.527
NC_005107.4_104400001	8	104400001	0.3736	0.5175
NC_005107.4_104500001	8	104500001	0.3739	0.5169
NC_005107.4_104600001	8	104600001	0.2666	0.3936
NC_005107.4_104700001	8	104700001	0.3205	0.418
NC_005107.4_104800001	8	104800001	0.3864	0.4708
NC_005107.4_104900001	8	104900001	0.4371	0.557
NC_005107.4_105000001	8	105000001	0.4868	0.5776
NC_005107.4_105100001	8	105100001	0.4634	0.5513
NC_005107.4_105200001	8	105200001	0.4431	0.5701
NC_005107.4_105300001	8	105300001	0.3795	0.5272
NC_005107.4_105400001	8	105400001	0.2718	0.3924
NC_005107.4_105500001	8	105500001	0.3181	0.395
NC_005107.4_105600001	8	105600001	0.3884	0.5201
NC_005107.4_105700001	8	105700001	0.3959	0.515
NC_005107.4_105800001	8	105800001	0.4758	0.5934
NC_005107.4_105900001	8	105900001	0.4998	0.623
NC_005107.4_106000001	8	106000001	0.4934	0.665
NC_005107.4_106100001	8	106100001	0.5763	0.7122
NC_005107.4_106200001	8	106200001	0.5903	0.7333
NC_005107.4_106300001	8	106300001	0.5936	0.7534
NC_005107.4_106400001	8	106400001	0.6149	0.7985
NC_005107.4_106500001	8	106500001	0.7484	0.8948
NC_005107.4_106700001	8	106700001	0.4291	0.4664
NC_005107.4_106800001	8	106800001	0.5367	0.5868
NC_005107.4_106900001	8	106900001	0.6846	0.7822
NC_005107.4_107000001	8	107000001	0.6367	0.7368
NC_005107.4_107100001	8	107100001	0.627	0.7539
NC_005107.4_107200001	8	107200001	0.6054	0.7549
NC_005107.4_107300001	8	107300001	0.5732	0.6897
NC_005107.4_107400001	8	107400001	0.4647	0.5388
NC_005107.4_107500001	8	107500001	0.572	0.6315
NC_005107.4_107600001	8	107600001	0.6166	0.6351
NC_005107.4_107700001	8	107700001	0.6524	0.6448
NC_005107.4_107800001	8	107800001	0.6269	0.6344
NC_005107.4_107900001	8	107900001	0.5841	0.5992
NC_005107.4_108000001	8	108000001	0.5733	0.6183
NC_005107.4_108100001	8	108100001	0.5782	0.6534

NC_005107.4_108200001	8	108200001	0.5782	0.6534
NC_005107.4_108300001	8	108300001	0.5746	0.6943
NC_005107.4_108400001	8	108400001	0.6077	0.7159
NC_005107.4_108500001	8	108500001	0.4342	0.6155
NC_005107.4_108600001	8	108600001	0.3744	0.5416
NC_005107.4_108700001	8	108700001	0.3744	0.5416
NC_005107.4_108800001	8	108800001	0.4049	0.5328
NC_005107.4_108900001	8	108900001	0.4004	0.6118
NC_005107.4_109000001	8	109000001	0.5969	0.7216
NC_005107.4_109100001	8	109100001	0.5833	0.7413
NC_005107.4_109200001	8	109200001	0.5702	0.675
NC_005107.4_109300001	8	109300001	0.5586	0.6637
NC_005107.4_109400001	8	109400001	0.5567	0.6325
NC_005107.4_109500001	8	109500001	0.4957	0.5523
NC_005107.4_109600001	8	109600001	0.4917	0.4994
NC_005107.4_109700001	8	109700001	0.5896	0.6337
NC_005107.4_109800001	8	109800001	0.5329	0.5939
NC_005107.4_109900001	8	109900001	0.5083	0.5872
NC_005107.4_110000001	8	110000001	0.5347	0.5828
NC_005107.4_110100001	8	110100001	0.5882	0.6369
NC_005107.4_110200001	8	110200001	0.5332	0.5948
NC_005107.4_110300001	8	110300001	0.5249	0.6362
NC_005107.4_110400001	8	110400001	0.5219	0.6151
NC_005107.4_110500001	8	110500001	0.4632	0.5964
NC_005107.4_110600001	8	110600001	0.3945	0.5737
NC_005107.4_110700001	8	110700001	0.389	0.5513
NC_005107.4_110800001	8	110800001	0.3863	0.5554
NC_005107.4_110900001	8	110900001	0.359	0.5594
NC_005107.4_111000001	8	111000001	0.4401	0.6642
NC_005107.4_111100001	8	111100001	0.4936	0.6918
NC_005107.4_111200001	8	111200001	0.552	0.7701
NC_005107.4_111300001	8	111300001	0.6012	0.7807
NC_005107.4_111400001	8	111400001	0.7136	0.8221
NC_005107.4_111500001	8	111500001	0.6987	0.8406
NC_005107.4_111600001	8	111600001	0.6119	0.7539
NC_005107.4_111700001	8	111700001	0.4756	0.5904
NC_005107.4_111800001	8	111800001	0.4335	0.5137
NC_005107.4_111900001	8	111900001	0.3731	0.4863
NC_005107.4_112000001	8	112000001	0.3351	0.3899
NC_005107.4_112100001	8	112100001	0.3849	0.436
NC_005107.4_112200001	8	112200001	0.3676	0.3867
NC_005107.4_112300001	8	112300001	0.3665	0.4057
NC_005107.4_112400001	8	112400001	0.4239	0.4131
NC_005107.4_112500001	8	112500001	0.4301	0.4241
NC_005107.4_112600001	8	112600001	0.3513	0.3935
NC_005107.4_112700001	8	112700001	0.3707	0.5359
NC_005107.4_112800001	8	112800001	0.3557	0.5324
NC_005107.4_112900001	8	112900001	0.4193	0.5755
NC_005107.4_113000001	8	113000001	0.5453	0.6996
NC_005107.4_113100001	8	113100001	0.6041	0.7472
NC_005107.4_113200001	8	113200001	0.6993	0.7807
NC_005107.4_113300001	8	113300001	0.6931	0.7548
NC_005107.4_113400001	8	113400001	0.6053	0.7065
NC_005107.4_113500001	8	113500001	0.5114	0.6066
NC_005107.4_113600001	8	113600001	0.4534	0.4881
NC_005107.4_113700001	8	113700001	0.4335	0.4902
NC_005107.4_113800001	8	113800001	0.3933	0.4852
NC_005107.4_113900001	8	113900001	0.4234	0.5488

NC_005107.4_114000001	8	114000001	0.3931	0.5815
NC_005107.4_114100001	8	114100001	0.4371	0.6355
NC_005107.4_114200001	8	114200001	0.4118	0.5961
NC_005107.4_114300001	8	114300001	0.3697	0.4899
NC_005107.4_114400001	8	114400001	0.3516	0.4577
NC_005107.4_114500001	8	114500001	0.3724	0.485
NC_005107.4_114600001	8	114600001	0.4063	0.5725
NC_005107.4_114700001	8	114700001	0.4696	0.668
NC_005107.4_114800001	8	114800001	0.5642	0.7772
NC_005107.4_114900001	8	114900001	0.561	0.7415
NC_005107.4_115000001	8	115000001	0.6265	0.7786
NC_005107.4_115100001	8	115100001	0.6607	0.797
NC_005107.4_115200001	8	115200001	0.7488	0.8418
NC_005107.4_115300001	8	115300001	0.7476	0.8281
NC_005107.4_115400001	8	115400001	0.8167	0.9026
NC_005107.4_115500001	8	115500001	0.7187	0.8601
NC_005107.4_115600001	8	115600001	0.6302	0.792
NC_005107.4_115700001	8	115700001	0.5031	0.7373
NC_005107.4_115800001	8	115800001	0.5021	0.6871
NC_005107.4_115900001	8	115900001	0.5476	0.654
NC_005107.4_116000001	8	116000001	0.5752	0.6775
NC_005107.4_116100001	8	116100001	0.6252	0.7099
NC_005107.4_116200001	8	116200001	0.6539	0.7204
NC_005107.4_116300001	8	116300001	0.6527	0.7725
NC_005107.4_116400001	8	116400001	0.6431	0.828
NC_005107.4_116500001	8	116500001	0.6287	0.7938
NC_005107.4_116600001	8	116600001	0.5932	0.7682
NC_005107.4_116700001	8	116700001	0.5677	0.787
NC_005107.4_116800001	8	116800001	0.6048	0.7601
NC_005107.4_116900001	8	116900001	0.6678	0.8287
NC_005107.4_117000001	8	117000001	0.6389	0.8719
NC_005107.4_117100001	8	117100001	0.6253	0.8658
NC_005107.4_117200001	8	117200001	0.6738	0.8116
NC_005107.4_117300001	8	117300001	0.6256	0.8125
NC_005107.4_117400001	8	117400001	0.5515	0.7348
NC_005107.4_117500001	8	117500001	0.681	0.7653
NC_005107.4_117600001	8	117600001	0.6627	0.7413
NC_005107.4_117700001	8	117700001	0.6593	0.7603
NC_005107.4_117800001	8	117800001	0.7418	0.794
NC_005107.4_117900001	8	117900001	0.6904	0.761
NC_005107.4_118000001	8	118000001	0.6449	0.7398
NC_005107.4_118100001	8	118100001	0.7066	0.7496
NC_005107.4_118200001	8	118200001	0.62	0.6588
NC_005107.4_118300001	8	118300001	0.5646	0.5947
NC_005107.4_118400001	8	118400001	0.6212	0.6237
NC_005107.4_118500001	8	118500001	0.5453	0.5549
NC_005107.4_118600001	8	118600001	0.4777	0.4957
NC_005107.4_118700001	8	118700001	0.5247	0.5328
NC_005107.4_118800001	8	118800001	0.5448	0.5295
NC_005107.4_118900001	8	118900001	0.5193	0.5097
NC_005107.4_119000001	8	119000001	0.5405	0.5554
NC_005107.4_119100001	8	119100001	0.5644	0.6145
NC_005107.4_119200001	8	119200001	0.6002	0.6715
NC_005107.4_119300001	8	119300001	0.5393	0.7205
NC_005107.4_119400001	8	119400001	0.4734	0.6657
NC_005107.4_119500001	8	119500001	0.4799	0.6658
NC_005107.4_119600001	8	119600001	0.5068	0.6436
NC_005107.4_119700001	8	119700001	0.4343	0.5821

NC_005107.4_119800001	8	119800001	0.4311	0.539
NC_005107.4_119900001	8	119900001	0.5213	0.6086
NC_005107.4_120000001	8	120000001	0.6003	0.7079
NC_005107.4_120100001	8	120100001	0.553	0.7166
NC_005107.4_120200001	8	120200001	0.5241	0.732
NC_005107.4_120300001	8	120300001	0.5206	0.7405
NC_005107.4_120400001	8	120400001	0.5286	0.7454
NC_005107.4_120500001	8	120500001	0.4333	0.6189
NC_005107.4_120600001	8	120600001	0.3849	0.5627
NC_005107.4_120700001	8	120700001	0.4571	0.6237
NC_005107.4_120800001	8	120800001	0.4493	0.5823
NC_005107.4_120900001	8	120900001	0.4436	0.6285
NC_005107.4_121000001	8	121000001	0.4468	0.6555
NC_005107.4_121100001	8	121100001	0.5167	0.7469
NC_005107.4_121200001	8	121200001	0.4958	0.7342
NC_005107.4_121300001	8	121300001	0.4536	0.7236
NC_005107.4_121400001	8	121400001	0.4384	0.6637
NC_005107.4_121500001	8	121500001	0.3937	0.5929
NC_005107.4_121600001	8	121600001	0.3689	0.559
NC_005107.4_121700001	8	121700001	0.3218	0.5167
NC_005107.4_121800001	8	121800001	0.424	0.5327
NC_005107.4_121900001	8	121900001	0.5002	0.6261
NC_005107.4_122000001	8	122000001	0.5566	0.704
NC_005107.4_122100001	8	122100001	0.5344	0.7134
NC_005107.4_122200001	8	122200001	0.5706	0.6977
NC_005107.4_122300001	8	122300001	0.6091	0.7874
NC_005107.4_122400001	8	122400001	0.6758	0.806
NC_005107.4_122500001	8	122500001	0.6496	0.7971
NC_005107.4_122600001	8	122600001	0.5797	0.7135
NC_005107.4_122700001	8	122700001	0.5907	0.7447
NC_005107.4_122800001	8	122800001	0.4934	0.6371
NC_005107.4_122900001	8	122900001	0.3112	0.5149
NC_005107.4_123000001	8	123000001	0.3953	0.5948
NC_005107.4_123100001	8	123100001	0.3661	0.5879
NC_005107.4_123200001	8	123200001	0.3365	0.5308
NC_005107.4_123300001	8	123300001	0.3318	0.4901
NC_005107.4_123400001	8	123400001	0.3209	0.4435
NC_005107.4_123500001	8	123500001	0.2797	0.3438
NC_005107.4_123600001	8	123600001	0.4088	0.5393
NC_005107.4_123700001	8	123700001	0.4014	0.5293
NC_005107.4_123800001	8	123800001	0.4569	0.6382
NC_005107.4_123900001	8	123900001	0.5098	0.6835
NC_005107.4_124000001	8	124000001	0.4604	0.6021
NC_005107.4_124100001	8	124100001	0.5035	0.6363
NC_005107.4_124200001	8	124200001	0.4912	0.6008
NC_005107.4_124300001	8	124300001	0.5207	0.5555
NC_005107.4_124400001	8	124400001	0.5911	0.6334
NC_005107.4_124500001	8	124500001	0.6165	0.6719
NC_005107.4_124600001	8	124600001	0.5996	0.6187
NC_005107.4_124700001	8	124700001	0.6211	0.6404
NC_005107.4_124800001	8	124800001	0.6239	0.6667
NC_005107.4_124900001	8	124900001	0.5515	0.6021
NC_005107.4_125000001	8	125000001	0.5941	0.6196
NC_005107.4_125100001	8	125100001	0.4829	0.5681
NC_005107.4_125200001	8	125200001	0.4408	0.5397
NC_005107.4_125300001	8	125300001	0.3389	0.4624
NC_005107.4_125400001	8	125400001	0.3812	0.5458
NC_005107.4_125500001	8	125500001	0.378	0.5756

NC_005107.4_125600001	8	125600001	0.4063	0.5836
NC_005107.4_125700001	8	125700001	0.5463	0.6892
NC_005107.4_125800001	8	125800001	0.6711	0.7453
NC_005107.4_125900001	8	125900001	0.6525	0.6714
NC_005107.4_126000001	8	126000001	0.7097	0.7035
NC_005107.4_126100001	8	126100001	0.7076	0.7348
NC_005107.4_126200001	8	126200001	0.6451	0.7513
NC_005107.4_126300001	8	126300001	0.5504	0.7172
NC_005107.4_126400001	8	126400001	0.554	0.8165
NC_005107.4_126500001	8	126500001	0.4661	0.7589
NC_005107.4_126600001	8	126600001	0.3691	0.6472
NC_005107.4_126700001	8	126700001	0.451	0.5666
NC_005107.4_126800001	8	126800001	0.4894	0.5962
NC_005107.4_126900001	8	126900001	0.5351	0.6438
NC_005107.4_127000001	8	127000001	0.5499	0.6384
NC_005107.4_127100001	8	127100001	0.5431	0.6319
NC_005107.4_127200001	8	127200001	0.5652	0.6124
NC_005107.4_127300001	8	127300001	0.6517	0.677
NC_005107.4_127400001	8	127400001	0.682	0.6895
NC_005107.4_127500001	8	127500001	0.724	0.7733
NC_005107.4_127600001	8	127600001	0.7682	0.7995
NC_005107.4_127700001	8	127700001	0.6486	0.7869
NC_005107.4_127800001	8	127800001	0.5197	0.7088
NC_005107.4_127900001	8	127900001	0.4327	0.6553
NC_005107.4_128000001	8	128000001	0.4005	0.5456
NC_005107.4_128100001	8	128100001	0.3176	0.4521
NC_005107.4_128200001	8	128200001	0.3729	0.5304
NC_005107.4_128300001	8	128300001	0.4052	0.5997
NC_005107.4_128400001	8	128400001	0.44	0.5974
NC_005107.4_128500001	8	128500001	0.4168	0.6257
NC_005107.4_128600001	8	128600001	0.4504	0.6682
NC_005107.4_128700001	8	128700001	0.4662	0.6777
NC_005107.4_128800001	8	128800001	0.4356	0.6021
NC_005107.4_128900001	8	128900001	0.4198	0.5834
NC_005107.4_129000001	8	129000001	0.4555	0.6314
NC_005107.4_129100001	8	129100001	0.4325	0.5687
NC_005107.4_129200001	8	129200001	0.428	0.5946
NC_005107.4_129300001	8	129300001	0.4754	0.6473
NC_005107.4_129400001	8	129400001	0.5346	0.7599
NC_005107.4_129500001	8	129500001	0.5772	0.717
NC_005107.4_129600001	8	129600001	0.5501	0.7063
NC_005107.4_129700001	8	129700001	0.4598	0.5957
NC_005107.4_129800001	8	129800001	0.474	0.5873
NC_005107.4_129900001	8	129900001	0.4673	0.5911
NC_005107.4_130000001	8	130000001	0.4723	0.6326
NC_005107.4_130100001	8	130100001	0.6039	0.6929
NC_005107.4_130200001	8	130200001	0.7144	0.7763
NC_005107.4_130300001	8	130300001	0.6824	0.7637
NC_005107.4_130400001	8	130400001	0.7137	0.7792
NC_005107.4_130500001	8	130500001	0.6521	0.7446
NC_005107.4_130600001	8	130600001	0.5778	0.7727
NC_005107.4_130700001	8	130700001	0.5429	0.742
NC_005107.4_130800001	8	130800001	0.5641	0.7725
NC_005107.4_130900001	8	130900001	0.5155	0.7491
NC_005107.4_131000001	8	131000001	0.5734	0.6638
NC_005107.4_131100001	8	131100001	0.5315	0.6569
NC_005107.4_131200001	8	131200001	0.5446	0.6936
NC_005107.4_131300001	8	131300001	0.6048	0.7432

NC_005107.4_131400001	8	131400001	0.5997	0.8031
NC_005107.4_131500001	8	131500001	0.6	0.8245
NC_005107.4_131600001	8	131600001	0.6825	0.8114
NC_005107.4_131700001	8	131700001	0.6356	0.7863
NC_005107.4_131800001	8	131800001	0.5279	0.7115
NC_005107.4_131900001	8	131900001	0.4962	0.6968
NC_005107.4_132000001	8	132000001	0.4844	0.7028
NC_005107.4_132100001	8	132100001	0.4209	0.7053
NC_005107.4_132200001	8	132200001	0.4624	0.7242
NC_005107.4_132300001	8	132300001	0.5377	0.8111
NC_005107.4_132400001	8	132400001	0.6079	0.7758
NC_005107.4_132500001	8	132500001	0.6328	0.699
NC_005107.4_132600001	8	132600001	0.6443	0.6918
NC_005108.4_1	9	1	0.3372	0.4165
NC_005108.4_100001	9	100001	0.2923	0.4856
NC_005108.4_1300001	9	1300001	0.7233	0.8462
NC_005108.4_1400001	9	1400001	0.7458	0.8356
NC_005108.4_1500001	9	1500001	0.7498	0.8464
NC_005108.4_1600001	9	1600001	0.7014	0.8424
NC_005108.4_1700001	9	1700001	0.6447	0.7916
NC_005108.4_1800001	9	1800001	0.6496	0.7968
NC_005108.4_1900001	9	1900001	0.5984	0.7152
NC_005108.4_2000001	9	2000001	0.5798	0.6585
NC_005108.4_2100001	9	2100001	0.4881	0.5385
NC_005108.4_2200001	9	2200001	0.4059	0.4192
NC_005108.4_2300001	9	2300001	0.4387	0.4539
NC_005108.4_2400001	9	2400001	0.4144	0.4986
NC_005108.4_2500001	9	2500001	0.4242	0.5292
NC_005108.4_2600001	9	2600001	0.4783	0.5912
NC_005108.4_2700001	9	2700001	0.483	0.6036
NC_005108.4_2800001	9	2800001	0.4965	0.666
NC_005108.4_2900001	9	2900001	0.5322	0.7034
NC_005108.4_3000001	9	3000001	0.4142	0.5211
NC_005108.4_3100001	9	3100001	0.527	0.6387
NC_005108.4_3200001	9	3200001	0.6451	0.6875
NC_005108.4_3300001	9	3300001	0.6172	0.6234
NC_005108.4_3400001	9	3400001	0.7506	0.7561
NC_005108.4_3500001	9	3500001	0.7108	0.823
NC_005108.4_3600001	9	3600001	0.7067	0.8429
NC_005108.4_3700001	9	3700001	0.7067	0.8429
NC_005108.4_3800001	9	3800001	0.6914	0.8805
NC_005108.4_4000001	9	4000001	0.53	0.5782
NC_005108.4_4100001	9	4100001	0.4581	0.5516
NC_005108.4_4200001	9	4200001	0.4581	0.5516
NC_005108.4_4300001	9	4300001	0.4581	0.5516
NC_005108.4_4400001	9	4400001	0.4688	0.5996
NC_005108.4_4500001	9	4500001	0.3079	0.4455
NC_005108.4_4600001	9	4600001	0.4491	0.5578
NC_005108.4_4700001	9	4700001	0.4145	0.5296
NC_005108.4_4800001	9	4800001	0.4335	0.6017
NC_005108.4_4900001	9	4900001	0.4109	0.5298
NC_005108.4_5000001	9	5000001	0.494	0.5752
NC_005108.4_5100001	9	5100001	0.4011	0.4566
NC_005108.4_5200001	9	5200001	0.4229	0.4255
NC_005108.4_5300001	9	5300001	0.3757	0.3696
NC_005108.4_5400001	9	5400001	0.5101	0.569
NC_005108.4_5500001	9	5500001	0.5224	0.6317
NC_005108.4_5600001	9	5600001	0.6148	0.6242

NC_005108.4_5700001	9	5700001	0.6618	0.6765
NC_005108.4_5800001	9	5800001	0.7515	0.6899
NC_005108.4_5900001	9	5900001	0.6604	0.5989
NC_005108.4_6000001	9	6000001	0.7055	0.6063
NC_005108.4_6100001	9	6100001	0.552	0.6337
NC_005108.4_6200001	9	6200001	0.4752	0.532
NC_005108.4_6300001	9	6300001	0.444	0.481
NC_005108.4_6400001	9	6400001	0.4404	0.4982
NC_005108.4_6500001	9	6500001	0.4722	0.5267
NC_005108.4_6600001	9	6600001	0.4782	0.5415
NC_005108.4_6700001	9	6700001	0.4667	0.5236
NC_005108.4_6800001	9	6800001	0.5044	0.5751
NC_005108.4_7300001	9	7300001	0.404	0.363
NC_005108.4_7400001	9	7400001	0.5251	0.5943
NC_005108.4_7500001	9	7500001	0.5263	0.6355
NC_005108.4_7600001	9	7600001	0.4763	0.5775
NC_005108.4_7700001	9	7700001	0.4132	0.5614
NC_005108.4_7800001	9	7800001	0.4363	0.5523
NC_005108.4_7900001	9	7900001	0.3405	0.3729
NC_005108.4_8000001	9	8000001	0.2868	0.2372
NC_005108.4_8100001	9	8100001	0.2935	0.2278
NC_005108.4_9000001	9	9000001	0.2874	0.4282
NC_005108.4_9100001	9	9100001	0.2874	0.4282
NC_005108.4_9200001	9	9200001	0.4234	0.5109
NC_005108.4_9300001	9	9300001	0.4294	0.4992
NC_005108.4_9400001	9	9400001	0.4567	0.5342
NC_005108.4_9500001	9	9500001	0.5447	0.6083
NC_005108.4_9600001	9	9600001	0.6025	0.6872
NC_005108.4_9700001	9	9700001	0.6379	0.7274
NC_005108.4_9800001	9	9800001	0.6753	0.7639
NC_005108.4_9900001	9	9900001	0.6586	0.7703
NC_005108.4_10000001	9	10000001	0.7097	0.81
NC_005108.4_10100001	9	10100001	0.615	0.8023
NC_005108.4_10200001	9	10200001	0.6175	0.8395
NC_005108.4_10300001	9	10300001	0.689	0.8672
NC_005108.4_10400001	9	10400001	0.6744	0.8586
NC_005108.4_10500001	9	10500001	0.6685	0.8672
NC_005108.4_10600001	9	10600001	0.7795	0.8829
NC_005108.4_10700001	9	10700001	0.7016	0.8917
NC_005108.4_10800001	9	10800001	0.6128	0.8949
NC_005108.4_10900001	9	10900001	0.6891	0.9325
NC_005108.4_11000001	9	11000001	0.5872	0.8892
NC_005108.4_12400001	9	12400001	0.3852	0.6007
NC_005108.4_12500001	9	12500001	0.4136	0.702
NC_005108.4_12600001	9	12600001	0.3581	0.6275
NC_005108.4_12700001	9	12700001	0.4875	0.6549
NC_005108.4_12800001	9	12800001	0.5176	0.658
NC_005108.4_12900001	9	12900001	0.5426	0.6817
NC_005108.4_13000001	9	13000001	0.6027	0.6869
NC_005108.4_13100001	9	13100001	0.5621	0.6438
NC_005108.4_13200001	9	13200001	0.5498	0.6444
NC_005108.4_13300001	9	13300001	0.5068	0.6048
NC_005108.4_13400001	9	13400001	0.5029	0.6032
NC_005108.4_13500001	9	13500001	0.4354	0.5671
NC_005108.4_13600001	9	13600001	0.4986	0.6682
NC_005108.4_13700001	9	13700001	0.5037	0.6802
NC_005108.4_13800001	9	13800001	0.5115	0.7158
NC_005108.4_13900001	9	13900001	0.4981	0.6925

NC_005108.4_14000001	9	14000001	0.504	0.6666
NC_005108.4_14100001	9	14100001	0.4622	0.558
NC_005108.4_14200001	9	14200001	0.4121	0.5075
NC_005108.4_14300001	9	14300001	0.4287	0.5173
NC_005108.4_14400001	9	14400001	0.4137	0.5173
NC_005108.4_14500001	9	14500001	0.5175	0.6093
NC_005108.4_14600001	9	14600001	0.3631	0.5506
NC_005108.4_14700001	9	14700001	0.5643	0.7714
NC_005108.4_14800001	9	14800001	0.5181	0.5786
NC_005108.4_14900001	9	14900001	0.4928	0.6174
NC_005108.4_15000001	9	15000001	0.5305	0.6458
NC_005108.4_15100001	9	15100001	0.619	0.7159
NC_005108.4_15200001	9	15200001	0.5666	0.6911
NC_005108.4_15300001	9	15300001	0.6024	0.7834
NC_005108.4_15400001	9	15400001	0.584	0.7456
NC_005108.4_15500001	9	15500001	0.5663	0.7456
NC_005108.4_15600001	9	15600001	0.4713	0.6652
NC_005108.4_15700001	9	15700001	0.52	0.6895
NC_005108.4_15800001	9	15800001	0.4702	0.6128
NC_005108.4_15900001	9	15900001	0.648	0.7361
NC_005108.4_16000001	9	16000001	0.5456	0.6884
NC_005108.4_16100001	9	16100001	0.5834	0.7122
NC_005108.4_16200001	9	16200001	0.5489	0.6846
NC_005108.4_16300001	9	16300001	0.5977	0.7423
NC_005108.4_16400001	9	16400001	0.5893	0.7411
NC_005108.4_16500001	9	16500001	0.6819	0.797
NC_005108.4_16600001	9	16600001	0.6617	0.83
NC_005108.4_16700001	9	16700001	0.7207	0.8918
NC_005108.4_16800001	9	16800001	0.6212	0.8073
NC_005108.4_16900001	9	16900001	0.5712	0.7723
NC_005108.4_17000001	9	17000001	0.5118	0.7401
NC_005108.4_17100001	9	17100001	0.5188	0.7217
NC_005108.4_17200001	9	17200001	0.4722	0.6431
NC_005108.4_17300001	9	17300001	0.4181	0.5759
NC_005108.4_17400001	9	17400001	0.4717	0.5717
NC_005108.4_17500001	9	17500001	0.472	0.5445
NC_005108.4_17600001	9	17600001	0.4128	0.4919
NC_005108.4_17700001	9	17700001	0.3579	0.4005
NC_005108.4_17800001	9	17800001	0.39	0.4813
NC_005108.4_17900001	9	17900001	0.3284	0.4665
NC_005108.4_18000001	9	18000001	0.4355	0.5927
NC_005108.4_18100001	9	18100001	0.5899	0.7651
NC_005108.4_18200001	9	18200001	0.6745	0.8644
NC_005108.4_18300001	9	18300001	0.6985	0.764
NC_005108.4_18400001	9	18400001	0.6433	0.7614
NC_005108.4_18500001	9	18500001	0.6003	0.695
NC_005108.4_18600001	9	18600001	0.4645	0.5452
NC_005108.4_18700001	9	18700001	0.5592	0.6618
NC_005108.4_18800001	9	18800001	0.6306	0.7569
NC_005108.4_18900001	9	18900001	0.6387	0.6075
NC_005108.4_19000001	9	19000001	0.6115	0.6631
NC_005108.4_19100001	9	19100001	0.5609	0.5775
NC_005108.4_19200001	9	19200001	0.4543	0.4432
NC_005108.4_19300001	9	19300001	0.4647	0.4601
NC_005108.4_19400001	9	19400001	0.4502	0.4864
NC_005108.4_19500001	9	19500001	0.441	0.4362
NC_005108.4_19600001	9	19600001	0.4684	0.4708
NC_005108.4_19700001	9	19700001	0.435	0.4917

NC_005108.4_19800001	9	19800001	0.4075	0.4796
NC_005108.4_19900001	9	19900001	0.4228	0.4871
NC_005108.4_20000001	9	20000001	0.3049	0.448
NC_005108.4_20100001	9	20100001	0.2873	0.3659
NC_005108.4_20200001	9	20200001	0.4294	0.5441
NC_005108.4_20300001	9	20300001	0.4181	0.5301
NC_005108.4_20400001	9	20400001	0.4113	0.5366
NC_005108.4_20500001	9	20500001	0.5049	0.6109
NC_005108.4_20600001	9	20600001	0.5502	0.6677
NC_005108.4_20700001	9	20700001	0.5294	0.6566
NC_005108.4_20800001	9	20800001	0.5442	0.6652
NC_005108.4_20900001	9	20900001	0.6516	0.74
NC_005108.4_21000001	9	21000001	0.6759	0.7401
NC_005108.4_21100001	9	21100001	0.7058	0.7951
NC_005108.4_21200001	9	21200001	0.7852	0.7984
NC_005108.4_21300001	9	21300001	0.8531	0.8342
NC_005108.4_22300001	9	22300001	0.6725	0.6187
NC_005108.4_22400001	9	22400001	0.6439	0.6213
NC_005108.4_22500001	9	22500001	0.6534	0.5719
NC_005108.4_22600001	9	22600001	0.6534	0.5719
NC_005108.4_22700001	9	22700001	0.527	0.5244
NC_005108.4_22800001	9	22800001	0.5516	0.5499
NC_005108.4_22900001	9	22900001	0.5009	0.5103
NC_005108.4_23000001	9	23000001	0.5054	0.5444
NC_005108.4_23100001	9	23100001	0.5354	0.5612
NC_005108.4_23200001	9	23200001	0.4811	0.5115
NC_005108.4_23300001	9	23300001	0.4811	0.5115
NC_005108.4_23400001	9	23400001	0.5157	0.5506
NC_005108.4_23500001	9	23500001	0.5503	0.6237
NC_005108.4_23600001	9	23600001	0.3179	0.509
NC_005108.4_23700001	9	23700001	0.6174	0.8216
NC_005108.4_23800001	9	23800001	0.6174	0.8216
NC_005108.4_23900001	9	23900001	0.6174	0.8216
NC_005108.4_24000001	9	24000001	0.4399	0.7086
NC_005108.4_24100001	9	24100001	0.521	0.8313
NC_005108.4_24200001	9	24200001	0.4364	0.7081
NC_005108.4_24300001	9	24300001	0.4364	0.7081
NC_005108.4_24400001	9	24400001	0.5454	0.7505
NC_005108.4_24500001	9	24500001	0.624	0.7392
NC_005108.4_24600001	9	24600001	0.5882	0.6775
NC_005108.4_24700001	9	24700001	0.3942	0.5313
NC_005108.4_24800001	9	24800001	0.3337	0.4916
NC_005108.4_24900001	9	24900001	0.1883	0.2116
NC_005108.4_25000001	9	25000001	0.1559	0.2182
NC_005108.4_25100001	9	25100001	0.2332	0.2574
NC_005108.4_25200001	9	25200001	0.2427	0.213
NC_005108.4_25300001	9	25300001	0.4218	0.4492
NC_005108.4_25400001	9	25400001	0.4618	0.4492
NC_005108.4_25500001	9	25500001	0.5292	0.5647
NC_005108.4_25600001	9	25600001	0.4617	0.5603
NC_005108.4_25700001	9	25700001	0.5061	0.608
NC_005108.4_25800001	9	25800001	0.4734	0.5657
NC_005108.4_25900001	9	25900001	0.5051	0.7005
NC_005108.4_26000001	9	26000001	0.6005	0.7006
NC_005108.4_26100001	9	26100001	0.6953	0.7591
NC_005108.4_26200001	9	26200001	0.7273	0.826
NC_005108.4_26300001	9	26300001	0.693	0.802
NC_005108.4_26400001	9	26400001	0.6851	0.8004

NC_005108.4_26500001	9	26500001	0.5342	0.7885
NC_005108.4_26600001	9	26600001	0.5175	0.7083
NC_005108.4_26700001	9	26700001	0.3256	0.4911
NC_005108.4_26800001	9	26800001	0.3249	0.4702
NC_005108.4_26900001	9	26900001	0.3865	0.5306
NC_005108.4_27000001	9	27000001	0.4622	0.5433
NC_005108.4_27100001	9	27100001	0.4421	0.5818
NC_005108.4_27200001	9	27200001	0.4567	0.5742
NC_005108.4_27300001	9	27300001	0.5639	0.7306
NC_005108.4_27400001	9	27400001	0.547	0.6839
NC_005108.4_27500001	9	27500001	0.4978	0.7246
NC_005108.4_27600001	9	27600001	0.5857	0.7705
NC_005108.4_27700001	9	27700001	0.6635	0.7693
NC_005108.4_27800001	9	27800001	0.6171	0.6561
NC_005108.4_27900001	9	27900001	0.6399	0.7027
NC_005108.4_28000001	9	28000001	0.7039	0.6969
NC_005108.4_28100001	9	28100001	0.6237	0.6453
NC_005108.4_28200001	9	28200001	0.6132	0.6557
NC_005108.4_28300001	9	28300001	0.61	0.7564
NC_005108.4_28400001	9	28400001	0.5303	0.7474
NC_005108.4_28500001	9	28500001	0.4983	0.8166
NC_005108.4_28600001	9	28600001	0.7105	0.8405
NC_005108.4_28700001	9	28700001	0.6444	0.7755
NC_005108.4_28800001	9	28800001	0.5684	0.7248
NC_005108.4_28900001	9	28900001	0.4583	0.6034
NC_005108.4_29000001	9	29000001	0.3969	0.5602
NC_005108.4_29100001	9	29100001	0.3135	0.4868
NC_005108.4_29200001	9	29200001	0.3493	0.5443
NC_005108.4_29300001	9	29300001	0.3043	0.4526
NC_005108.4_29400001	9	29400001	0.3961	0.5632
NC_005108.4_29500001	9	29500001	0.5069	0.6393
NC_005108.4_29600001	9	29600001	0.5205	0.6338
NC_005108.4_29700001	9	29700001	0.4451	0.543
NC_005108.4_29800001	9	29800001	0.4678	0.5804
NC_005108.4_29900001	9	29900001	0.4572	0.5853
NC_005108.4_30000001	9	30000001	0.4879	0.6301
NC_005108.4_30100001	9	30100001	0.5017	0.6299
NC_005108.4_30200001	9	30200001	0.6497	0.7755
NC_005108.4_30300001	9	30300001	0.6731	0.7872
NC_005108.4_30400001	9	30400001	0.6121	0.7518
NC_005108.4_30500001	9	30500001	0.4746	0.6363
NC_005108.4_30600001	9	30600001	0.3585	0.4926
NC_005108.4_30700001	9	30700001	0.3388	0.4846
NC_005108.4_30800001	9	30800001	0.2997	0.4254
NC_005108.4_30900001	9	30900001	0.2513	0.3494
NC_005108.4_31000001	9	31000001	0.3151	0.4497
NC_005108.4_31100001	9	31100001	0.4035	0.6637
NC_005108.4_31200001	9	31200001	0.4457	0.6816
NC_005108.4_31300001	9	31300001	0.4259	0.6342
NC_005108.4_31400001	9	31400001	0.4644	0.6123
NC_005108.4_31500001	9	31500001	0.5125	0.6423
NC_005108.4_31600001	9	31600001	0.4796	0.6041
NC_005108.4_31700001	9	31700001	0.4569	0.5333
NC_005108.4_31800001	9	31800001	0.5143	0.5405
NC_005108.4_31900001	9	31900001	0.5909	0.5929
NC_005108.4_32000001	9	32000001	0.5772	0.5144
NC_005108.4_32100001	9	32100001	0.5772	0.5144
NC_005108.4_32700001	9	32700001	0.5709	0.65

NC_005108.4_32800001	9	32800001	0.5546	0.6297
NC_005108.4_32900001	9	32900001	0.3553	0.3753
NC_005108.4_33000001	9	33000001	0.3562	0.42
NC_005108.4_33100001	9	33100001	0.3837	0.4947
NC_005108.4_33200001	9	33200001	0.4369	0.5494
NC_005108.4_33300001	9	33300001	0.4625	0.57
NC_005108.4_33400001	9	33400001	0.4954	0.6329
NC_005108.4_33500001	9	33500001	0.5575	0.6454
NC_005108.4_33600001	9	33600001	0.5679	0.6334
NC_005108.4_33700001	9	33700001	0.5311	0.6654
NC_005108.4_33800001	9	33800001	0.4883	0.6126
NC_005108.4_33900001	9	33900001	0.601	0.6761
NC_005108.4_34000001	9	34000001	0.6376	0.7635
NC_005108.4_34100001	9	34100001	0.7349	0.6954
NC_005108.4_34400001	9	34400001	0.7074	0.63
NC_005108.4_34500001	9	34500001	0.6289	0.6271
NC_005108.4_34600001	9	34600001	0.5664	0.5601
NC_005108.4_34700001	9	34700001	0.6437	0.6183
NC_005108.4_34800001	9	34800001	0.6436	0.6007
NC_005108.4_34900001	9	34900001	0.5975	0.6173
NC_005108.4_35000001	9	35000001	0.7427	0.7104
NC_005108.4_35100001	9	35100001	0.7704	0.7598
NC_005108.4_35200001	9	35200001	0.7317	0.8014
NC_005108.4_35300001	9	35300001	0.7373	0.8057
NC_005108.4_35400001	9	35400001	0.7024	0.6946
NC_005108.4_35500001	9	35500001	0.618	0.5885
NC_005108.4_35600001	9	35600001	0.6372	0.5867
NC_005108.4_35700001	9	35700001	0.6667	0.6665
NC_005108.4_35800001	9	35800001	0.6121	0.6461
NC_005108.4_35900001	9	35900001	0.6961	0.7808
NC_005108.4_36000001	9	36000001	0.705	0.7253
NC_005108.4_36100001	9	36100001	0.6397	0.7392
NC_005108.4_36200001	9	36200001	0.596	0.618
NC_005108.4_36300001	9	36300001	0.5815	0.5967
NC_005108.4_36400001	9	36400001	0.543	0.5674
NC_005108.4_36500001	9	36500001	0.5012	0.5648
NC_005108.4_36600001	9	36600001	0.4685	0.5089
NC_005108.4_36700001	9	36700001	0.4959	0.5801
NC_005108.4_36800001	9	36800001	0.5142	0.6165
NC_005108.4_36900001	9	36900001	0.601	0.6972
NC_005108.4_37000001	9	37000001	0.5644	0.6593
NC_005108.4_37100001	9	37100001	0.7454	0.7732
NC_005108.4_37300001	9	37300001	0.5439	0.6595
NC_005108.4_37400001	9	37400001	0.4504	0.54
NC_005108.4_37500001	9	37500001	0.3578	0.5564
NC_005108.4_37600001	9	37600001	0.3357	0.4982
NC_005108.4_37700001	9	37700001	0.3673	0.5446
NC_005108.4_37800001	9	37800001	0.3425	0.5124
NC_005108.4_37900001	9	37900001	0.2989	0.4979
NC_005108.4_38000001	9	38000001	0.3635	0.5622
NC_005108.4_38100001	9	38100001	0.438	0.7325
NC_005108.4_38200001	9	38200001	0.4564	0.6995
NC_005108.4_38300001	9	38300001	0.4579	0.7488
NC_005108.4_38400001	9	38400001	0.5063	0.7795
NC_005108.4_38500001	9	38500001	0.4923	0.6917
NC_005108.4_38600001	9	38600001	0.6225	0.6788
NC_005108.4_38700001	9	38700001	0.5896	0.6652
NC_005108.4_38800001	9	38800001	0.6336	0.5829

NC_005108.4_38900001	9	38900001	0.6176	0.6187
NC_005108.4_39000001	9	39000001	0.6448	0.6511
NC_005108.4_39100001	9	39100001	0.5193	0.5607
NC_005108.4_39200001	9	39200001	0.5119	0.5388
NC_005108.4_39300001	9	39300001	0.4699	0.5862
NC_005108.4_39400001	9	39400001	0.5553	0.6495
NC_005108.4_39500001	9	39500001	0.5969	0.6948
NC_005108.4_39600001	9	39600001	0.6618	0.7509
NC_005108.4_39700001	9	39700001	0.6897	0.8185
NC_005108.4_39800001	9	39800001	0.7076	0.8413
NC_005108.4_39900001	9	39900001	0.6338	0.8678
NC_005108.4_40000001	9	40000001	0.3666	0.8323
NC_005108.4_40100001	9	40100001	0.4195	0.8326
NC_005108.4_40200001	9	40200001	0.29	0.6846
NC_005108.4_40300001	9	40300001	0.296	0.6893
NC_005108.4_40400001	9	40400001	0.2051	0.5884
NC_005108.4_40500001	9	40500001	0.352	0.7147
NC_005108.4_40600001	9	40600001	0.2758	0.5511
NC_005108.4_40700001	9	40700001	0.3857	0.619
NC_005108.4_40800001	9	40800001	0.3938	0.615
NC_005108.4_40900001	9	40900001	0.4466	0.6332
NC_005108.4_41000001	9	41000001	0.4386	0.5969
NC_005108.4_41100001	9	41100001	0.5433	0.6419
NC_005108.4_41200001	9	41200001	0.477	0.5961
NC_005108.4_41300001	9	41300001	0.5183	0.589
NC_005108.4_41400001	9	41400001	0.509	0.5813
NC_005108.4_41500001	9	41500001	0.4821	0.6059
NC_005108.4_41600001	9	41600001	0.4083	0.6043
NC_005108.4_41700001	9	41700001	0.5013	0.7376
NC_005108.4_41800001	9	41800001	0.494	0.7356
NC_005108.4_41900001	9	41900001	0.4928	0.6588
NC_005108.4_42000001	9	42000001	0.4922	0.6652
NC_005108.4_42100001	9	42100001	0.5125	0.67
NC_005108.4_42200001	9	42200001	0.5197	0.6807
NC_005108.4_42300001	9	42300001	0.618	0.7693
NC_005108.4_42400001	9	42400001	0.6515	0.8493
NC_005108.4_42500001	9	42500001	0.6781	0.8425
NC_005108.4_42600001	9	42600001	0.7351	0.89
NC_005108.4_42700001	9	42700001	0.7448	0.877
NC_005108.4_42800001	9	42800001	0.6208	0.785
NC_005108.4_42900001	9	42900001	0.6046	0.7364
NC_005108.4_43000001	9	43000001	0.5823	0.7569
NC_005108.4_43100001	9	43100001	0.5586	0.7516
NC_005108.4_43200001	9	43200001	0.5889	0.8069
NC_005108.4_43300001	9	43300001	0.6701	0.8617
NC_005108.4_43400001	9	43400001	0.6702	0.8939
NC_005108.4_43500001	9	43500001	0.7074	0.8859
NC_005108.4_43600001	9	43600001	0.6766	0.82
NC_005108.4_43700001	9	43700001	0.6608	0.7705
NC_005108.4_43800001	9	43800001	0.5697	0.6867
NC_005108.4_43900001	9	43900001	0.5877	0.6611
NC_005108.4_44000001	9	44000001	0.5571	0.6329
NC_005108.4_44100001	9	44100001	0.5442	0.6249
NC_005108.4_44200001	9	44200001	0.4441	0.5151
NC_005108.4_44300001	9	44300001	0.4608	0.5636
NC_005108.4_44400001	9	44400001	0.4977	0.5892
NC_005108.4_44500001	9	44500001	0.4453	0.5491
NC_005108.4_44600001	9	44600001	0.462	0.5694

NC_005108.4_44700001	9	44700001	0.5148	0.6289
NC_005108.4_44800001	9	44800001	0.5033	0.6104
NC_005108.4_44900001	9	44900001	0.4903	0.6576
NC_005108.4_45000001	9	45000001	0.5219	0.6777
NC_005108.4_45100001	9	45100001	0.4982	0.6352
NC_005108.4_45200001	9	45200001	0.4419	0.5893
NC_005108.4_45300001	9	45300001	0.4872	0.6153
NC_005108.4_45400001	9	45400001	0.4852	0.5642
NC_005108.4_45500001	9	45500001	0.5135	0.5744
NC_005108.4_45600001	9	45600001	0.5386	0.6361
NC_005108.4_45700001	9	45700001	0.6311	0.7048
NC_005108.4_45800001	9	45800001	0.6187	0.7117
NC_005108.4_45900001	9	45900001	0.6677	0.7896
NC_005108.4_46000001	9	46000001	0.689	0.7789
NC_005108.4_46100001	9	46100001	0.6887	0.778
NC_005108.4_46200001	9	46200001	0.521	0.7095
NC_005108.4_46300001	9	46300001	0.5141	0.7266
NC_005108.4_46400001	9	46400001	0.5668	0.7578
NC_005108.4_46500001	9	46500001	0.5855	0.8224
NC_005108.4_46600001	9	46600001	0.5868	0.8406
NC_005108.4_46700001	9	46700001	0.709	0.8521
NC_005108.4_46800001	9	46800001	0.7227	0.841
NC_005108.4_46900001	9	46900001	0.6626	0.8275
NC_005108.4_47000001	9	47000001	0.5866	0.7616
NC_005108.4_47100001	9	47100001	0.5334	0.6736
NC_005108.4_47200001	9	47200001	0.4508	0.5777
NC_005108.4_47300001	9	47300001	0.4286	0.5528
NC_005108.4_47400001	9	47400001	0.3635	0.417
NC_005108.4_47500001	9	47500001	0.4164	0.4757
NC_005108.4_47600001	9	47600001	0.4627	0.564
NC_005108.4_47700001	9	47700001	0.5305	0.6246
NC_005108.4_47800001	9	47800001	0.4917	0.5878
NC_005108.4_47900001	9	47900001	0.4477	0.572
NC_005108.4_48000001	9	48000001	0.517	0.6204
NC_005108.4_48100001	9	48100001	0.6099	0.6435
NC_005108.4_48200001	9	48200001	0.547	0.6618
NC_005108.4_48300001	9	48300001	0.5417	0.7021
NC_005108.4_48400001	9	48400001	0.5918	0.737
NC_005108.4_48500001	9	48500001	0.5223	0.6765
NC_005108.4_48600001	9	48600001	0.41	0.5215
NC_005108.4_48700001	9	48700001	0.3758	0.5086
NC_005108.4_48800001	9	48800001	0.3734	0.4521
NC_005108.4_48900001	9	48900001	0.4577	0.5379
NC_005108.4_49000001	9	49000001	0.4497	0.5689
NC_005108.4_49100001	9	49100001	0.5188	0.6622
NC_005108.4_49200001	9	49200001	0.5604	0.6929
NC_005108.4_49300001	9	49300001	0.5926	0.7204
NC_005108.4_49400001	9	49400001	0.5495	0.7023
NC_005108.4_49500001	9	49500001	0.5711	0.7313
NC_005108.4_49600001	9	49600001	0.5742	0.7073
NC_005108.4_49700001	9	49700001	0.5161	0.6563
NC_005108.4_49800001	9	49800001	0.5001	0.6491
NC_005108.4_49900001	9	49900001	0.5269	0.6583
NC_005108.4_50000001	9	50000001	0.5688	0.6471
NC_005108.4_50100001	9	50100001	0.6029	0.7041
NC_005108.4_50200001	9	50200001	0.7199	0.7424
NC_005108.4_50300001	9	50300001	0.7399	0.7479
NC_005108.4_50400001	9	50400001	0.7449	0.7666

NC_005108.4_50500001	9	50500001	0.73	0.8531
NC_005108.4_50600001	9	50600001	0.618	0.8289
NC_005108.4_50700001	9	50700001	0.54	0.6523
NC_005108.4_50800001	9	50800001	0.54	0.6523
NC_005108.4_50900001	9	50900001	0.5974	0.6608
NC_005108.4_51000001	9	51000001	0.4707	0.4403
NC_005108.4_51100001	9	51100001	0.4707	0.4403
NC_005108.4_51200001	9	51200001	0.4869	0.4648
NC_005108.4_51300001	9	51300001	0.5331	0.4916
NC_005108.4_51400001	9	51400001	0.4964	0.4634
NC_005108.4_51500001	9	51500001	0.4096	0.3985
NC_005108.4_51600001	9	51600001	0.42	0.4265
NC_005108.4_51700001	9	51700001	0.38	0.4521
NC_005108.4_51800001	9	51800001	0.3527	0.5052
NC_005108.4_51900001	9	51900001	0.3751	0.5595
NC_005108.4_52000001	9	52000001	0.376	0.5019
NC_005108.4_52100001	9	52100001	0.3494	0.4999
NC_005108.4_52200001	9	52200001	0.416	0.5194
NC_005108.4_52500001	9	52500001	0.7053	0.7998
NC_005108.4_52600001	9	52600001	0.7053	0.7998
NC_005108.4_52700001	9	52700001	0.5933	0.7611
NC_005108.4_52800001	9	52800001	0.5923	0.7607
NC_005108.4_52900001	9	52900001	0.6248	0.7881
NC_005108.4_53000001	9	53000001	0.621	0.817
NC_005108.4_53100001	9	53100001	0.5535	0.8021
NC_005108.4_53300001	9	53300001	0.2697	0.4647
NC_005108.4_53400001	9	53400001	0.2201	0.2343
NC_005108.4_53500001	9	53500001	0.3036	0.3489
NC_005108.4_53600001	9	53600001	0.3782	0.4121
NC_005108.4_53700001	9	53700001	0.3666	0.396
NC_005108.4_53800001	9	53800001	0.4154	0.4274
NC_005108.4_53900001	9	53900001	0.4596	0.5168
NC_005108.4_54000001	9	54000001	0.5046	0.6112
NC_005108.4_54100001	9	54100001	0.5089	0.6225
NC_005108.4_54200001	9	54200001	0.5636	0.6666
NC_005108.4_54300001	9	54300001	0.6119	0.6892
NC_005108.4_54400001	9	54400001	0.5432	0.6377
NC_005108.4_54500001	9	54500001	0.5113	0.6264
NC_005108.4_54600001	9	54600001	0.5066	0.6175
NC_005108.4_54700001	9	54700001	0.5728	0.7008
NC_005108.4_54800001	9	54800001	0.5265	0.677
NC_005108.4_54900001	9	54900001	0.5061	0.6933
NC_005108.4_55000001	9	55000001	0.4797	0.6506
NC_005108.4_55100001	9	55100001	0.4804	0.6272
NC_005108.4_55200001	9	55200001	0.3497	0.4651
NC_005108.4_55300001	9	55300001	0.3534	0.5111
NC_005108.4_55400001	9	55400001	0.3445	0.4025
NC_005108.4_55500001	9	55500001	0.3774	0.4094
NC_005108.4_55600001	9	55600001	0.4794	0.5393
NC_005108.4_55700001	9	55700001	0.5008	0.6104
NC_005108.4_55800001	9	55800001	0.5447	0.6146
NC_005108.4_55900001	9	55900001	0.5889	0.7087
NC_005108.4_56000001	9	56000001	0.4945	0.6581
NC_005108.4_56100001	9	56100001	0.3379	0.4533
NC_005108.4_56200001	9	56200001	0.4391	0.4956
NC_005108.4_56300001	9	56300001	0.4391	0.4956
NC_005108.4_56400001	9	56400001	0.4391	0.4956
NC_005108.4_56500001	9	56500001	0.51	0.4847

NC_005108.4_57500001	9	57500001	0.2475	0.2381
NC_005108.4_57600001	9	57600001	0.1462	0.1063
NC_005108.4_57700001	9	57700001	0.1462	0.1063
NC_005108.4_57800001	9	57800001	0.1856	0.2005
NC_005108.4_57900001	9	57900001	0.2315	0.3252
NC_005108.4_58000001	9	58000001	0.2515	0.3602
NC_005108.4_58100001	9	58100001	0.2296	0.3951
NC_005108.4_58200001	9	58200001	0.2296	0.3951
NC_005108.4_58300001	9	58300001	0.2122	0.4448
NC_005108.4_58800001	9	58800001	0.3692	0.4958
NC_005108.4_58900001	9	58900001	0.3692	0.4958
NC_005108.4_59000001	9	59000001	0.261	0.5157
NC_005108.4_59100001	9	59100001	0.4034	0.7182
NC_005108.4_59200001	9	59200001	0.3734	0.5394
NC_005108.4_59300001	9	59300001	0.317	0.3369
NC_005108.4_59400001	9	59400001	0.3298	0.3547
NC_005108.4_59500001	9	59500001	0.4004	0.4305
NC_005108.4_59600001	9	59600001	0.3569	0.3672
NC_005108.4_59700001	9	59700001	0.3695	0.4355
NC_005108.4_59800001	9	59800001	0.6372	0.8304
NC_005108.4_59900001	9	59900001	0.7686	0.8112
NC_005108.4_60000001	9	60000001	0.7342	0.879
NC_005108.4_60100001	9	60100001	0.6523	0.8518
NC_005108.4_60200001	9	60200001	0.5933	0.818
NC_005108.4_60300001	9	60300001	0.5284	0.6447
NC_005108.4_60400001	9	60400001	0.4997	0.6168
NC_005108.4_60500001	9	60500001	0.4721	0.5372
NC_005108.4_60600001	9	60600001	0.5409	0.6229
NC_005108.4_60700001	9	60700001	0.6043	0.6112
NC_005108.4_60800001	9	60800001	0.6848	0.7073
NC_005108.4_60900001	9	60900001	0.6841	0.7358
NC_005108.4_61000001	9	61000001	0.6507	0.7263
NC_005108.4_61100001	9	61100001	0.655	0.7021
NC_005108.4_61200001	9	61200001	0.6521	0.7087
NC_005108.4_61300001	9	61300001	0.6344	0.7618
NC_005108.4_61400001	9	61400001	0.6641	0.8066
NC_005108.4_61500001	9	61500001	0.6851	0.8039
NC_005108.4_61600001	9	61600001	0.6182	0.7377
NC_005108.4_61700001	9	61700001	0.62	0.726
NC_005108.4_61800001	9	61800001	0.6799	0.7178
NC_005108.4_61900001	9	61900001	0.6711	0.7276
NC_005108.4_62000001	9	62000001	0.6668	0.7251
NC_005108.4_62100001	9	62100001	0.7301	0.7741
NC_005108.4_62200001	9	62200001	0.7625	0.7946
NC_005108.4_62300001	9	62300001	0.6362	0.6899
NC_005108.4_62400001	9	62400001	0.4706	0.5067
NC_005108.4_62500001	9	62500001	0.4282	0.4614
NC_005108.4_62600001	9	62600001	0.4331	0.477
NC_005108.4_62700001	9	62700001	0.4309	0.4596
NC_005108.4_62800001	9	62800001	0.4138	0.4278
NC_005108.4_62900001	9	62900001	0.4488	0.4592
NC_005108.4_63000001	9	63000001	0.4782	0.5473
NC_005108.4_63100001	9	63100001	0.4451	0.5247
NC_005108.4_63200001	9	63200001	0.4118	0.5866
NC_005108.4_63300001	9	63300001	0.4246	0.704
NC_005108.4_63400001	9	63400001	0.3788	0.6307
NC_005108.4_63500001	9	63500001	0.3231	0.5586
NC_005108.4_63600001	9	63600001	0.3118	0.5443

NC_005108.4_63700001	9	63700001	0.2994	0.4696
NC_005108.4_63800001	9	63800001	0.2745	0.3883
NC_005108.4_63900001	9	63900001	0.2327	0.3334
NC_005108.4_64000001	9	64000001	0.1676	0.0981
NC_005108.4_64100001	9	64100001	0.169	0.1053
NC_005108.4_64200001	9	64200001	0.3419	0.4267
NC_005108.4_64300001	9	64300001	0.5345	0.6633
NC_005108.4_64400001	9	64400001	0.6204	0.7001
NC_005108.4_64500001	9	64500001	0.6957	0.752
NC_005108.4_64600001	9	64600001	0.5626	0.647
NC_005108.4_64700001	9	64700001	0.5738	0.6758
NC_005108.4_64800001	9	64800001	0.537	0.64
NC_005108.4_64900001	9	64900001	0.528	0.658
NC_005108.4_65000001	9	65000001	0.5192	0.6679
NC_005108.4_65100001	9	65100001	0.6298	0.7896
NC_005108.4_65200001	9	65200001	0.4541	0.6332
NC_005108.4_65300001	9	65300001	0.5081	0.6691
NC_005108.4_65400001	9	65400001	0.5401	0.6972
NC_005108.4_65500001	9	65500001	0.435	0.608
NC_005108.4_65600001	9	65600001	0.4716	0.6292
NC_005108.4_65700001	9	65700001	0.4147	0.6625
NC_005108.4_65800001	9	65800001	0.4602	0.6752
NC_005108.4_65900001	9	65900001	0.4786	0.6796
NC_005108.4_66000001	9	66000001	0.546	0.7194
NC_005108.4_66100001	9	66100001	0.5379	0.729
NC_005108.4_66200001	9	66200001	0.7604	0.8012
NC_005108.4_66300001	9	66300001	0.7753	0.7867
NC_005108.4_66400001	9	66400001	0.648	0.7297
NC_005108.4_66500001	9	66500001	0.5962	0.6987
NC_005108.4_66600001	9	66600001	0.553	0.6432
NC_005108.4_66700001	9	66700001	0.5187	0.6037
NC_005108.4_66800001	9	66800001	0.5482	0.6843
NC_005108.4_66900001	9	66900001	0.6288	0.6932
NC_005108.4_67000001	9	67000001	0.5226	0.7289
NC_005108.4_67100001	9	67100001	0.5358	0.7372
NC_005108.4_67200001	9	67200001	0.4967	0.6889
NC_005108.4_67300001	9	67300001	0.44	0.6529
NC_005108.4_67400001	9	67400001	0.5548	0.739
NC_005108.4_67500001	9	67500001	0.4729	0.5979
NC_005108.4_67600001	9	67600001	0.5139	0.6569
NC_005108.4_67700001	9	67700001	0.558	0.7929
NC_005108.4_67800001	9	67800001	0.5527	0.7814
NC_005108.4_67900001	9	67900001	0.4599	0.7428
NC_005108.4_68000001	9	68000001	0.5221	0.8295
NC_005108.4_68100001	9	68100001	0.519	0.8239
NC_005108.4_68200001	9	68200001	0.5764	0.7861
NC_005108.4_68300001	9	68300001	0.6327	0.847
NC_005108.4_68400001	9	68400001	0.7284	0.8701
NC_005108.4_68500001	9	68500001	0.7977	0.8907
NC_005108.4_68600001	9	68600001	0.7705	0.8956
NC_005108.4_68700001	9	68700001	0.7101	0.8412
NC_005108.4_68800001	9	68800001	0.721	0.8162
NC_005108.4_68900001	9	68900001	0.711	0.7883
NC_005108.4_69000001	9	69000001	0.5026	0.5699
NC_005108.4_69100001	9	69100001	0.4642	0.5296
NC_005108.4_69200001	9	69200001	0.3947	0.5137
NC_005108.4_69300001	9	69300001	0.3401	0.4612
NC_005108.4_69400001	9	69400001	0.3497	0.4846

NC_005108.4_69500001	9	69500001	0.3634	0.5173
NC_005108.4_69600001	9	69600001	0.3741	0.5741
NC_005108.4_69700001	9	69700001	0.4666	0.6726
NC_005108.4_69800001	9	69800001	0.6737	0.8311
NC_005108.4_69900001	9	69900001	0.6617	0.7616
NC_005108.4_70000001	9	70000001	0.6283	0.6816
NC_005108.4_70100001	9	70100001	0.604	0.6225
NC_005108.4_70200001	9	70200001	0.6358	0.5806
NC_005108.4_70300001	9	70300001	0.5641	0.5298
NC_005108.4_70400001	9	70400001	0.65	0.6101
NC_005108.4_70500001	9	70500001	0.593	0.6036
NC_005108.4_70600001	9	70600001	0.5434	0.5514
NC_005108.4_70700001	9	70700001	0.5455	0.5447
NC_005108.4_70800001	9	70800001	0.5548	0.5433
NC_005108.4_70900001	9	70900001	0.5286	0.5993
NC_005108.4_71000001	9	71000001	0.5956	0.6743
NC_005108.4_71100001	9	71100001	0.637	0.7741
NC_005108.4_71200001	9	71200001	0.6391	0.7826
NC_005108.4_71300001	9	71300001	0.6094	0.76
NC_005108.4_71400001	9	71400001	0.6477	0.708
NC_005108.4_71500001	9	71500001	0.5368	0.6155
NC_005108.4_71600001	9	71600001	0.5874	0.5545
NC_005108.4_71700001	9	71700001	0.574	0.5583
NC_005108.4_71800001	9	71800001	0.7949	0.716
NC_005108.4_71900001	9	71900001	0.615	0.6818
NC_005108.4_72000001	9	72000001	0.5385	0.6694
NC_005108.4_72100001	9	72100001	0.4002	0.5373
NC_005108.4_72200001	9	72200001	0.327	0.4753
NC_005108.4_72300001	9	72300001	0.2623	0.2797
NC_005108.4_72400001	9	72400001	0.291	0.2966
NC_005108.4_72500001	9	72500001	0.3153	0.3011
NC_005108.4_72600001	9	72600001	0.2254	0.2169
NC_005108.4_72700001	9	72700001	0.3359	0.3648
NC_005108.4_72800001	9	72800001	0.6071	0.7011
NC_005108.4_72900001	9	72900001	0.5692	0.6781
NC_005108.4_73000001	9	73000001	0.5099	0.6065
NC_005108.4_73100001	9	73100001	0.6369	0.6735
NC_005108.4_73200001	9	73200001	0.6015	0.6633
NC_005108.4_73300001	9	73300001	0.458	0.5348
NC_005108.4_73400001	9	73400001	0.4401	0.5485
NC_005108.4_73500001	9	73500001	0.5501	0.7133
NC_005108.4_73600001	9	73600001	0.6131	0.7428
NC_005108.4_73700001	9	73700001	0.6323	0.7348
NC_005108.4_73800001	9	73800001	0.6636	0.7678
NC_005108.4_73900001	9	73900001	0.6979	0.7709
NC_005108.4_74000001	9	74000001	0.6679	0.7484
NC_005108.4_74100001	9	74100001	0.6064	0.6833
NC_005108.4_74200001	9	74200001	0.6742	0.7637
NC_005108.4_74300001	9	74300001	0.4925	0.6099
NC_005108.4_74400001	9	74400001	0.3697	0.4529
NC_005108.4_74500001	9	74500001	0.3548	0.4461
NC_005108.4_74600001	9	74600001	0.3185	0.4457
NC_005108.4_74700001	9	74700001	0.2508	0.2988
NC_005108.4_74800001	9	74800001	0.3473	0.3738
NC_005108.4_74900001	9	74900001	0.4342	0.4324
NC_005108.4_75000001	9	75000001	0.5152	0.5048
NC_005108.4_75100001	9	75100001	0.5491	0.5187
NC_005108.4_75200001	9	75200001	0.6799	0.5874

NC_005108.4_75300001	9	75300001	0.6866	0.7392
NC_005108.4_75400001	9	75400001	0.4802	0.8422
NC_005108.4_75500001	9	75500001	0.2736	0.7164
NC_005108.4_75600001	9	75600001	0.2736	0.7164
NC_005108.4_75700001	9	75700001	0.4276	0.8503
NC_005108.4_75800001	9	75800001	0.4914	0.7324
NC_005108.4_75900001	9	75900001	0.5544	0.764
NC_005108.4_76000001	9	76000001	0.6021	0.778
NC_005108.4_76100001	9	76100001	0.6274	0.7851
NC_005108.4_76200001	9	76200001	0.6189	0.7176
NC_005108.4_76300001	9	76300001	0.6194	0.7749
NC_005108.4_76400001	9	76400001	0.6951	0.756
NC_005108.4_76500001	9	76500001	0.6794	0.7409
NC_005108.4_76600001	9	76600001	0.647	0.7111
NC_005108.4_76700001	9	76700001	0.7034	0.7974
NC_005108.4_76800001	9	76800001	0.5921	0.7252
NC_005108.4_76900001	9	76900001	0.5306	0.7087
NC_005108.4_77000001	9	77000001	0.6105	0.7474
NC_005108.4_77100001	9	77100001	0.6342	0.7338
NC_005108.4_77200001	9	77200001	0.5687	0.6996
NC_005108.4_77300001	9	77300001	0.601	0.6864
NC_005108.4_77400001	9	77400001	0.5355	0.6961
NC_005108.4_77500001	9	77500001	0.4285	0.583
NC_005108.4_77600001	9	77600001	0.281	0.49
NC_005108.4_77700001	9	77700001	0.2223	0.3553
NC_005108.4_77800001	9	77800001	0.1922	0.3395
NC_005108.4_77900001	9	77900001	0.2788	0.511
NC_005108.4_78000001	9	78000001	0.3121	0.647
NC_005108.4_78100001	9	78100001	0.4522	0.5929
NC_005108.4_78200001	9	78200001	0.4277	0.5247
NC_005108.4_78300001	9	78300001	0.5281	0.6419
NC_005108.4_78400001	9	78400001	0.5259	0.5834
NC_005108.4_78500001	9	78500001	0.5642	0.646
NC_005108.4_78600001	9	78600001	0.5961	0.7023
NC_005108.4_78700001	9	78700001	0.6395	0.7243
NC_005108.4_78800001	9	78800001	0.5744	0.7384
NC_005108.4_78900001	9	78900001	0.5554	0.7138
NC_005108.4_79000001	9	79000001	0.5566	0.6898
NC_005108.4_79100001	9	79100001	0.591	0.7242
NC_005108.4_79200001	9	79200001	0.5936	0.7039
NC_005108.4_79300001	9	79300001	0.6372	0.6341
NC_005108.4_79400001	9	79400001	0.6689	0.6315
NC_005108.4_79500001	9	79500001	0.5869	0.6405
NC_005108.4_79600001	9	79600001	0.4237	0.5297
NC_005108.4_79700001	9	79700001	0.3321	0.4532
NC_005108.4_79800001	9	79800001	0.4189	0.6216
NC_005108.4_79900001	9	79900001	0.4547	0.6687
NC_005108.4_80000001	9	80000001	0.4924	0.6023
NC_005108.4_80100001	9	80100001	0.5441	0.603
NC_005108.4_80200001	9	80200001	0.5306	0.5957
NC_005108.4_80300001	9	80300001	0.5194	0.573
NC_005108.4_80400001	9	80400001	0.5262	0.5963
NC_005108.4_80500001	9	80500001	0.5278	0.5879
NC_005108.4_80600001	9	80600001	0.5734	0.6133
NC_005108.4_80700001	9	80700001	0.5859	0.6982
NC_005108.4_80800001	9	80800001	0.54	0.6369
NC_005108.4_80900001	9	80900001	0.5261	0.6476
NC_005108.4_81000001	9	81000001	0.5096	0.649

NC_005108.4_81100001	9	81100001	0.4951	0.6577
NC_005108.4_81200001	9	81200001	0.5355	0.6537
NC_005108.4_81300001	9	81300001	0.5856	0.7303
NC_005108.4_81400001	9	81400001	0.5803	0.6752
NC_005108.4_81500001	9	81500001	0.6171	0.7568
NC_005108.4_81600001	9	81600001	0.7078	0.7964
NC_005108.4_81700001	9	81700001	0.7124	0.8241
NC_005108.4_81800001	9	81800001	0.7003	0.8009
NC_005108.4_81900001	9	81900001	0.7541	0.8342
NC_005108.4_82000001	9	82000001	0.7253	0.8287
NC_005108.4_82100001	9	82100001	0.6375	0.7531
NC_005108.4_82200001	9	82200001	0.6008	0.749
NC_005108.4_82300001	9	82300001	0.5884	0.7096
NC_005108.4_82400001	9	82400001	0.4751	0.6521
NC_005108.4_82500001	9	82500001	0.4299	0.6
NC_005108.4_82600001	9	82600001	0.4312	0.6413
NC_005108.4_82700001	9	82700001	0.448	0.6382
NC_005108.4_82800001	9	82800001	0.4475	0.7042
NC_005108.4_82900001	9	82900001	0.4774	0.712
NC_005108.4_83000001	9	83000001	0.497	0.6731
NC_005108.4_83100001	9	83100001	0.4847	0.6151
NC_005108.4_83200001	9	83200001	0.4602	0.5781
NC_005108.4_83300001	9	83300001	0.4517	0.5113
NC_005108.4_83400001	9	83400001	0.4041	0.459
NC_005108.4_83500001	9	83500001	0.3605	0.4918
NC_005108.4_83600001	9	83600001	0.4857	0.6874
NC_005108.4_83700001	9	83700001	0.4315	0.5977
NC_005108.4_83800001	9	83800001	0.5198	0.6637
NC_005108.4_83900001	9	83900001	0.5354	0.6523
NC_005108.4_84000001	9	84000001	0.553	0.6524
NC_005108.4_84100001	9	84100001	0.5056	0.6419
NC_005108.4_84200001	9	84200001	0.5823	0.6622
NC_005108.4_84300001	9	84300001	0.3688	0.5058
NC_005108.4_84400001	9	84400001	0.3705	0.5684
NC_005108.4_84500001	9	84500001	0.4345	0.624
NC_005108.4_84600001	9	84600001	0.3641	0.5288
NC_005108.4_84700001	9	84700001	0.3271	0.5811
NC_005108.4_84800001	9	84800001	0.5312	0.7079
NC_005108.4_84900001	9	84900001	0.4686	0.6581
NC_005108.4_85000001	9	85000001	0.5081	0.7027
NC_005108.4_85100001	9	85100001	0.517	0.6485
NC_005108.4_85200001	9	85200001	0.5449	0.6644
NC_005108.4_85300001	9	85300001	0.5287	0.6929
NC_005108.4_85400001	9	85400001	0.6104	0.7199
NC_005108.4_85500001	9	85500001	0.5177	0.6348
NC_005108.4_85600001	9	85600001	0.5376	0.7251
NC_005108.4_85700001	9	85700001	0.5787	0.7348
NC_005108.4_85800001	9	85800001	0.5612	0.669
NC_005108.4_85900001	9	85900001	0.5115	0.6319
NC_005108.4_86000001	9	86000001	0.6409	0.765
NC_005108.4_86100001	9	86100001	0.6409	0.765
NC_005108.4_86200001	9	86200001	0.568	0.6907
NC_005108.4_86300001	9	86300001	0.5439	0.5227
NC_005108.4_86400001	9	86400001	0.6054	0.5975
NC_005108.4_86500001	9	86500001	0.5501	0.4959
NC_005108.4_86600001	9	86600001	0.5501	0.4959
NC_005108.4_86700001	9	86700001	0.5087	0.535
NC_005108.4_86800001	9	86800001	0.4779	0.5954

NC_005108.4_86900001	9	86900001	0.4289	0.5502
NC_005108.4_87000001	9	87000001	0.5048	0.6758
NC_005108.4_87100001	9	87100001	0.5048	0.6758
NC_005108.4_87200001	9	87200001	0.6501	0.7669
NC_005108.4_87300001	9	87300001	0.5375	0.6713
NC_005108.4_87400001	9	87400001	0.6086	0.6986
NC_005108.4_87500001	9	87500001	0.5818	0.7124
NC_005108.4_87600001	9	87600001	0.5328	0.706
NC_005108.4_87700001	9	87700001	0.4924	0.673
NC_005108.4_87800001	9	87800001	0.5408	0.731
NC_005108.4_87900001	9	87900001	0.4983	0.7545
NC_005108.4_88000001	9	88000001	0.4269	0.6906
NC_005108.4_88100001	9	88100001	0.4712	0.712
NC_005108.4_88200001	9	88200001	0.4028	0.6756
NC_005108.4_88300001	9	88300001	0.2428	0.537
NC_005108.4_88400001	9	88400001	0.2667	0.5569
NC_005108.4_88500001	9	88500001	0.3336	0.5203
NC_005108.4_88600001	9	88600001	0.2134	0.318
NC_005108.4_88700001	9	88700001	0.2387	0.3099
NC_005108.4_88800001	9	88800001	0.2506	0.3125
NC_005108.4_88900001	9	88900001	0.3333	0.4693
NC_005108.4_89000001	9	89000001	0.33	0.4805
NC_005108.4_89100001	9	89100001	0.4723	0.5901
NC_005108.4_89200001	9	89200001	0.6848	0.8247
NC_005108.4_89300001	9	89300001	0.7061	0.8141
NC_005108.4_89400001	9	89400001	0.5632	0.6825
NC_005108.4_89500001	9	89500001	0.5634	0.6441
NC_005108.4_89600001	9	89600001	0.5699	0.6693
NC_005108.4_89700001	9	89700001	0.5152	0.5996
NC_005108.4_89800001	9	89800001	0.4427	0.5331
NC_005108.4_89900001	9	89900001	0.5023	0.6083
NC_005108.4_90000001	9	90000001	0.438	0.6277
NC_005108.4_90100001	9	90100001	0.4257	0.5856
NC_005108.4_90200001	9	90200001	0.3761	0.5814
NC_005108.4_90300001	9	90300001	0.225	0.4222
NC_005108.4_90400001	9	90400001	0.225	0.4222
NC_005108.4_90500001	9	90500001	0.2554	0.5207
NC_005108.4_90600001	9	90600001	0.2965	0.5532
NC_005108.4_90800001	9	90800001	0.6442	0.7077
NC_005108.4_90900001	9	90900001	0.5396	0.6815
NC_005108.4_91000001	9	91000001	0.5356	0.6454
NC_005108.4_91100001	9	91100001	0.4747	0.5866
NC_005108.4_91200001	9	91200001	0.4527	0.5577
NC_005108.4_91300001	9	91300001	0.3475	0.4152
NC_005108.4_91400001	9	91400001	0.3863	0.5163
NC_005108.4_91500001	9	91500001	0.4146	0.5253
NC_005108.4_91600001	9	91600001	0.3951	0.4963
NC_005108.4_91700001	9	91700001	0.5017	0.6183
NC_005108.4_91800001	9	91800001	0.4875	0.6042
NC_005108.4_91900001	9	91900001	0.5849	0.6412
NC_005108.4_92000001	9	92000001	0.5653	0.6585
NC_005108.4_92100001	9	92100001	0.5687	0.6314
NC_005108.4_92200001	9	92200001	0.4031	0.4694
NC_005108.4_92300001	9	92300001	0.4863	0.5939
NC_005108.4_92400001	9	92400001	0.5009	0.6149
NC_005108.4_92500001	9	92500001	0.5286	0.621
NC_005108.4_92600001	9	92600001	0.6611	0.7833
NC_005108.4_92700001	9	92700001	0.7205	0.8234

NC_005108.4_92800001	9	92800001	0.7079	0.8029
NC_005108.4_92900001	9	92900001	0.6332	0.7498
NC_005108.4_93000001	9	93000001	0.6298	0.7543
NC_005108.4_93100001	9	93100001	0.5442	0.6606
NC_005108.4_93200001	9	93200001	0.5224	0.6189
NC_005108.4_93300001	9	93300001	0.5214	0.663
NC_005108.4_93400001	9	93400001	0.6139	0.7599
NC_005108.4_93500001	9	93500001	0.6943	0.843
NC_005108.4_93600001	9	93600001	0.7211	0.882
NC_005108.4_93700001	9	93700001	0.5986	0.8201
NC_005108.4_93800001	9	93800001	0.4842	0.7177
NC_005108.4_93900001	9	93900001	0.3872	0.5868
NC_005108.4_94000001	9	94000001	0.2802	0.364
NC_005108.4_94100001	9	94100001	0.304	0.4085
NC_005108.4_94200001	9	94200001	0.3485	0.4526
NC_005108.4_94300001	9	94300001	0.3658	0.4995
NC_005108.4_94400001	9	94400001	0.4188	0.495
NC_005108.4_94500001	9	94500001	0.4475	0.5695
NC_005108.4_94600001	9	94600001	0.3515	0.4298
NC_005108.4_94700001	9	94700001	0.337	0.4111
NC_005108.4_94800001	9	94800001	0.4463	0.4953
NC_005108.4_94900001	9	94900001	0.4185	0.5683
NC_005108.4_95000001	9	95000001	0.5056	0.6232
NC_005108.4_95100001	9	95100001	0.5207	0.5822
NC_005108.4_95200001	9	95200001	0.5428	0.5864
NC_005108.4_95300001	9	95300001	0.5073	0.5656
NC_005108.4_95400001	9	95400001	0.5298	0.6246
NC_005108.4_95500001	9	95500001	0.5118	0.5774
NC_005108.4_95600001	9	95600001	0.5802	0.6534
NC_005108.4_95700001	9	95700001	0.5727	0.6652
NC_005108.4_95800001	9	95800001	0.6043	0.7007
NC_005108.4_95900001	9	95900001	0.619	0.6472
NC_005108.4_96000001	9	96000001	0.5906	0.6347
NC_005108.4_96100001	9	96100001	0.4396	0.5554
NC_005108.4_96200001	9	96200001	0.4237	0.5575
NC_005108.4_96300001	9	96300001	0.44	0.5607
NC_005108.4_96400001	9	96400001	0.375	0.557
NC_005108.4_96500001	9	96500001	0.4206	0.6524
NC_005108.4_96600001	9	96600001	0.529	0.6717
NC_005108.4_96700001	9	96700001	0.4819	0.6561
NC_005108.4_96800001	9	96800001	0.4672	0.6117
NC_005108.4_96900001	9	96900001	0.4884	0.6205
NC_005108.4_97000001	9	97000001	0.4853	0.5969
NC_005108.4_97100001	9	97100001	0.4588	0.5568
NC_005108.4_97200001	9	97200001	0.5329	0.587
NC_005108.4_97300001	9	97300001	0.4726	0.5333
NC_005108.4_97400001	9	97400001	0.4709	0.5185
NC_005108.4_97500001	9	97500001	0.4514	0.5315
NC_005108.4_97600001	9	97600001	0.4654	0.5964
NC_005108.4_97700001	9	97700001	0.4802	0.5787
NC_005108.4_97800001	9	97800001	0.518	0.6281
NC_005108.4_97900001	9	97900001	0.5322	0.6752
NC_005108.4_98000001	9	98000001	0.5938	0.6857
NC_005108.4_98100001	9	98100001	0.5694	0.6776
NC_005108.4_98200001	9	98200001	0.5753	0.7476
NC_005108.4_98300001	9	98300001	0.5899	0.6886
NC_005108.4_98400001	9	98400001	0.6736	0.7368
NC_005108.4_98500001	9	98500001	0.6606	0.7551

NC_005108.4_98600001	9	98600001	0.6721	0.7487
NC_005108.4_98700001	9	98700001	0.6674	0.7254
NC_005108.4_98800001	9	98800001	0.7058	0.7994
NC_005108.4_98900001	9	98900001	0.6064	0.7653
NC_005108.4_99000001	9	99000001	0.6382	0.7567
NC_005108.4_99100001	9	99100001	0.6372	0.7566
NC_005108.4_99200001	9	99200001	0.6158	0.7411
NC_005108.4_99300001	9	99300001	0.543	0.6636
NC_005108.4_99400001	9	99400001	0.54	0.6663
NC_005108.4_99500001	9	99500001	0.484	0.6255
NC_005108.4_99600001	9	99600001	0.4576	0.6067
NC_005108.4_99700001	9	99700001	0.4056	0.6405
NC_005108.4_99800001	9	99800001	0.4151	0.6028
NC_005108.4_99900001	9	99900001	0.4447	0.5894
NC_005108.4_100000001	9	100000001	0.428	0.5703
NC_005108.4_100100001	9	100100001	0.4359	0.5902
NC_005108.4_100200001	9	100200001	0.4685	0.5907
NC_005108.4_100300001	9	100300001	0.5224	0.7147
NC_005108.4_100400001	9	100400001	0.5558	0.7928
NC_005108.4_100500001	9	100500001	0.5412	0.7363
NC_005108.4_100600001	9	100600001	0.559	0.7434
NC_005108.4_100700001	9	100700001	0.5502	0.7114
NC_005108.4_100800001	9	100800001	0.5235	0.5892
NC_005108.4_100900001	9	100900001	0.4101	0.4881
NC_005108.4_101500001	9	101500001	0.3498	0.5088
NC_005108.4_102200001	9	102200001	0.5979	0.6291
NC_005108.4_102300001	9	102300001	0.4434	0.589
NC_005108.4_102400001	9	102400001	0.4645	0.5505
NC_005108.4_102500001	9	102500001	0.397	0.5942
NC_005108.4_102600001	9	102600001	0.5394	0.7758
NC_005108.4_102700001	9	102700001	0.4708	0.6914
NC_005108.4_102800001	9	102800001	0.5949	0.7728
NC_005108.4_102900001	9	102900001	0.5519	0.8148
NC_005108.4_103000001	9	103000001	0.5924	0.6912
NC_005108.4_103100001	9	103100001	0.4577	0.4922
NC_005108.4_103200001	9	103200001	0.5773	0.6153
NC_005108.4_103300001	9	103300001	0.5357	0.501
NC_005108.4_103400001	9	103400001	0.594	0.5449
NC_005108.4_103500001	9	103500001	0.684	0.689
NC_005108.4_103600001	9	103600001	0.7247	0.7376
NC_005108.4_103700001	9	103700001	0.6518	0.695
NC_005108.4_103800001	9	103800001	0.6296	0.686
NC_005108.4_103900001	9	103900001	0.5952	0.7367
NC_005108.4_104000001	9	104000001	0.554	0.7498
NC_005108.4_104100001	9	104100001	0.5108	0.7017
NC_005108.4_104200001	9	104200001	0.4944	0.7705
NC_005108.4_104300001	9	104300001	0.5467	0.8369
NC_005108.4_104400001	9	104400001	0.6361	0.8516
NC_005108.4_104500001	9	104500001	0.6333	0.7225
NC_005108.4_104600001	9	104600001	0.7027	0.749
NC_005108.4_104700001	9	104700001	0.6377	0.7041
NC_005108.4_104800001	9	104800001	0.6089	0.6736
NC_005108.4_104900001	9	104900001	0.6089	0.6736
NC_005108.4_105000001	9	105000001	0.5551	0.6856
NC_005108.4_105100001	9	105100001	0.5218	0.6619
NC_005108.4_105200001	9	105200001	0.5587	0.686
NC_005108.4_105300001	9	105300001	0.5222	0.6546
NC_005108.4_105400001	9	105400001	0.5541	0.7243

NC_005108.4_105500001	9	105500001	0.4968	0.7025
NC_005108.4_105600001	9	105600001	0.4399	0.8124
NC_005108.4_106500001	9	106500001	0.2916	0.3279
NC_005108.4_106600001	9	106600001	0.3321	0.4014
NC_005108.4_106700001	9	106700001	0.3922	0.4636
NC_005108.4_106800001	9	106800001	0.3922	0.4636
NC_005108.4_106900001	9	106900001	0.3606	0.4576
NC_005108.4_107000001	9	107000001	0.3128	0.3948
NC_005108.4_107100001	9	107100001	0.36	0.4722
NC_005108.4_107200001	9	107200001	0.3735	0.4467
NC_005108.4_107300001	9	107300001	0.4397	0.5419
NC_005108.4_107400001	9	107400001	0.4882	0.5736
NC_005108.4_107500001	9	107500001	0.5341	0.6382
NC_005108.4_107600001	9	107600001	0.5489	0.6262
NC_005108.4_107700001	9	107700001	0.6046	0.7104
NC_005108.4_107800001	9	107800001	0.4186	0.5699
NC_005108.4_107900001	9	107900001	0.3259	0.4532
NC_005108.4_108000001	9	108000001	0.4507	0.4719
NC_005108.4_108100001	9	108100001	0.464	0.5046
NC_005108.4_108200001	9	108200001	0.3995	0.458
NC_005108.4_108300001	9	108300001	0.4274	0.4311
NC_005108.4_108400001	9	108400001	0.4182	0.4028
NC_005108.4_108500001	9	108500001	0.2092	0.1097
NC_005108.4_108600001	9	108600001	0.3243	0.2876
NC_005108.4_108700001	9	108700001	0.3864	0.3198
NC_005108.4_108800001	9	108800001	0.5338	0.599
NC_005108.4_108900001	9	108900001	0.5377	0.6624
NC_005108.4_109000001	9	109000001	0.5436	0.6654
NC_005108.4_109100001	9	109100001	0.4963	0.5992
NC_005108.4_109200001	9	109200001	0.4904	0.6116
NC_005108.4_109300001	9	109300001	0.4361	0.5216
NC_005108.4_109400001	9	109400001	0.4101	0.498
NC_005108.4_109500001	9	109500001	0.3713	0.4664
NC_005108.4_109600001	9	109600001	0.3481	0.5075
NC_005108.4_109700001	9	109700001	0.3908	0.5837
NC_005108.4_109800001	9	109800001	0.423	0.5836
NC_005108.4_109900001	9	109900001	0.432	0.5723
NC_005108.4_110000001	9	110000001	0.4865	0.6491
NC_005108.4_110100001	9	110100001	0.5421	0.6876
NC_005108.4_110200001	9	110200001	0.5406	0.6894
NC_005108.4_110300001	9	110300001	0.5607	0.7635
NC_005108.4_110400001	9	110400001	0.5656	0.7827
NC_005108.4_110500001	9	110500001	0.5866	0.8033
NC_005108.4_110600001	9	110600001	0.5103	0.7141
NC_005108.4_110700001	9	110700001	0.5061	0.7052
NC_005108.4_110800001	9	110800001	0.5338	0.7278
NC_005108.4_110900001	9	110900001	0.588	0.7645
NC_005108.4_111000001	9	111000001	0.5795	0.7179
NC_005108.4_111100001	9	111100001	0.6472	0.7803
NC_005108.4_111200001	9	111200001	0.6525	0.7874
NC_005108.4_111300001	9	111300001	0.633	0.7471
NC_005108.4_111400001	9	111400001	0.5351	0.6413
NC_005108.4_111500001	9	111500001	0.5077	0.7151
NC_005108.4_111600001	9	111600001	0.5532	0.7379
NC_005108.4_111700001	9	111700001	0.5899	0.6982
NC_005108.4_111800001	9	111800001	0.5229	0.6619
NC_005108.4_111900001	9	111900001	0.4735	0.6374
NC_005108.4_112000001	9	112000001	0.4354	0.599

NC_005108.4_112100001	9	112100001	0.4348	0.6052
NC_005108.4_112200001	9	112200001	0.42	0.6063
NC_005108.4_112300001	9	112300001	0.4727	0.6499
NC_005108.4_112400001	9	112400001	0.5228	0.6938
NC_005108.4_112500001	9	112500001	0.5729	0.7045
NC_005108.4_112600001	9	112600001	0.4979	0.6413
NC_005108.4_112700001	9	112700001	0.4171	0.5933
NC_005108.4_112800001	9	112800001	0.31	0.4884
NC_005108.4_112900001	9	112900001	0.3202	0.5009
NC_005108.4_113000001	9	113000001	0.3663	0.5489
NC_005108.4_113100001	9	113100001	0.4869	0.7318
NC_005108.4_113200001	9	113200001	0.5011	0.7449
NC_005108.4_113300001	9	113300001	0.5224	0.7667
NC_005108.4_113400001	9	113400001	0.515	0.7567
NC_005108.4_113500001	9	113500001	0.4805	0.7625
NC_005108.4_113600001	9	113600001	0.3131	0.5375
NC_005108.4_113700001	9	113700001	0.3786	0.6
NC_005108.4_113800001	9	113800001	0.4284	0.6157
NC_005108.4_113900001	9	113900001	0.5064	0.7075
NC_005108.4_114000001	9	114000001	0.7784	0.7826
NC_005108.4_114100001	9	114100001	0.63	0.7054
NC_005108.4_114200001	9	114200001	0.6018	0.6472
NC_005108.4_114300001	9	114300001	0.6193	0.6684
NC_005108.4_114400001	9	114400001	0.5735	0.612
NC_005108.4_114500001	9	114500001	0.5579	0.59
NC_005108.4_114600001	9	114600001	0.5691	0.5998
NC_005108.4_114700001	9	114700001	0.5812	0.673
NC_005108.4_114800001	9	114800001	0.5833	0.6651
NC_005108.4_114900001	9	114900001	0.5794	0.6745
NC_005108.4_115000001	9	115000001	0.5318	0.6782
NC_005108.4_115100001	9	115100001	0.5252	0.6784
NC_005108.4_115200001	9	115200001	0.5307	0.6705
NC_005108.4_115300001	9	115300001	0.4905	0.6642
NC_005108.4_115400001	9	115400001	0.4807	0.6299
NC_005108.4_115500001	9	115500001	0.4694	0.6064
NC_005108.4_115600001	9	115600001	0.4559	0.5854
NC_005108.4_115700001	9	115700001	0.4057	0.5295
NC_005108.4_115800001	9	115800001	0.4078	0.4983
NC_005108.4_115900001	9	115900001	0.4205	0.5394
NC_005108.4_116000001	9	116000001	0.508	0.6069
NC_005108.4_116100001	9	116100001	0.4923	0.5492
NC_005108.4_116200001	9	116200001	0.5043	0.5366
NC_005108.4_116300001	9	116300001	0.5052	0.5584
NC_005108.4_116400001	9	116400001	0.5249	0.6626
NC_005108.4_116500001	9	116500001	0.5443	0.7367
NC_005108.4_116600001	9	116600001	0.5502	0.7899
NC_005108.4_116700001	9	116700001	0.5816	0.8099
NC_005108.4_116800001	9	116800001	0.4964	0.7942
NC_005108.4_116900001	9	116900001	0.4596	0.8266
NC_005108.4_117000001	9	117000001	0.2913	0.7111
NC_005108.4_117100001	9	117100001	0.3991	0.8881
NC_005108.4_117200001	9	117200001	0.3991	0.8881
NC_005108.4_117300001	9	117300001	0.4203	0.7566
NC_005108.4_117400001	9	117400001	0.3323	0.6735
NC_005108.4_117500001	9	117500001	0.3104	0.5686
NC_005108.4_117600001	9	117600001	0.2586	0.4718
NC_005108.4_117700001	9	117700001	0.2499	0.458
NC_005108.4_117800001	9	117800001	0.2932	0.3491

NC_005108.4_117900001	9	117900001	0.4438	0.5142
NC_005108.4_118000001	9	118000001	0.471	0.5707
NC_005108.4_118100001	9	118100001	0.497	0.6333
NC_005108.4_118200001	9	118200001	0.4553	0.5641
NC_005108.4_118300001	9	118300001	0.435	0.5988
NC_005108.4_118400001	9	118400001	0.4245	0.6034
NC_005108.4_118500001	9	118500001	0.5101	0.6407
NC_005108.4_118600001	9	118600001	0.49	0.5344
NC_005108.4_118700001	9	118700001	0.589	0.6467
NC_005108.4_118800001	9	118800001	0.6584	0.6993
NC_005108.4_118900001	9	118900001	0.5702	0.6334
NC_005108.4_119000001	9	119000001	0.5356	0.5933
NC_005108.4_119100001	9	119100001	0.5495	0.6882
NC_005108.4_119200001	9	119200001	0.483	0.5728
NC_005108.4_119300001	9	119300001	0.3431	0.4268
NC_005108.4_119400001	9	119400001	0.3489	0.4223
NC_005108.4_119500001	9	119500001	0.3445	0.396
NC_005108.4_119600001	9	119600001	0.3841	0.4341
NC_005108.4_119700001	9	119700001	0.303	0.3265
NC_005108.4_119800001	9	119800001	0.3221	0.3205
NC_005108.4_119900001	9	119900001	0.4043	0.4092
NC_005108.4_120000001	9	120000001	0.486	0.5863
NC_005108.4_120100001	9	120100001	0.4973	0.5989
NC_005108.4_120200001	9	120200001	0.5955	0.6749
NC_005108.4_120300001	9	120300001	0.6818	0.7303
NC_005108.4_120400001	9	120400001	0.6813	0.7303
NC_005108.4_120500001	9	120500001	0.6456	0.6853
NC_005108.4_120600001	9	120600001	0.5737	0.6655
NC_005108.4_120700001	9	120700001	0.5311	0.7119
NC_005108.4_121500001	9	121500001	0.8155	0.7261
NC_005109.4_1	10	1	0.5798	0.6534
NC_005109.4_100001	10	100001	0.5798	0.6534
NC_005109.4_300001	10	300001	0.6241	0.9216
NC_005109.4_400001	10	400001	0.6681	0.8654
NC_005109.4_500001	10	500001	0.6681	0.8654
NC_005109.4_600001	10	600001	0.5998	0.8452
NC_005109.4_700001	10	700001	0.6521	0.8433
NC_005109.4_800001	10	800001	0.7073	0.8576
NC_005109.4_900001	10	900001	0.6203	0.8788
NC_005109.4_1000001	10	1000001	0.6203	0.8788
NC_005109.4_1100001	10	1100001	0.7058	0.902
NC_005109.4_1600001	10	1600001	0.6967	0.784
NC_005109.4_1700001	10	1700001	0.7212	0.77
NC_005109.4_1800001	10	1800001	0.7952	0.8406
NC_005109.4_1900001	10	1900001	0.7627	0.8586
NC_005109.4_2000001	10	2000001	0.6966	0.7484
NC_005109.4_2100001	10	2100001	0.739	0.7426
NC_005109.4_2200001	10	2200001	0.7057	0.75
NC_005109.4_2300001	10	2300001	0.6901	0.728
NC_005109.4_2400001	10	2400001	0.6727	0.7257
NC_005109.4_2500001	10	2500001	0.582	0.7656
NC_005109.4_2600001	10	2600001	0.5541	0.7682
NC_005109.4_2700001	10	2700001	0.5328	0.7626
NC_005109.4_2800001	10	2800001	0.4843	0.7253
NC_005109.4_2900001	10	2900001	0.4463	0.6608
NC_005109.4_3000001	10	3000001	0.474	0.6677
NC_005109.4_3100001	10	3100001	0.4798	0.7237
NC_005109.4_3200001	10	3200001	0.533	0.7599

NC_005109.4_3300001	10	3300001	0.5379	0.749
NC_005109.4_3400001	10	3400001	0.5252	0.7333
NC_005109.4_3500001	10	3500001	0.5157	0.6847
NC_005109.4_3600001	10	3600001	0.5468	0.6796
NC_005109.4_3700001	10	3700001	0.5272	0.6865
NC_005109.4_3800001	10	3800001	0.5209	0.7034
NC_005109.4_3900001	10	3900001	0.5247	0.7111
NC_005109.4_4000001	10	4000001	0.5355	0.7582
NC_005109.4_4100001	10	4100001	0.4668	0.7406
NC_005109.4_4200001	10	4200001	0.4343	0.6903
NC_005109.4_4300001	10	4300001	0.3648	0.5657
NC_005109.4_4400001	10	4400001	0.5143	0.6915
NC_005109.4_4500001	10	4500001	0.5763	0.7484
NC_005109.4_4600001	10	4600001	0.65	0.8057
NC_005109.4_4700001	10	4700001	0.6651	0.8143
NC_005109.4_4800001	10	4800001	0.6654	0.7909
NC_005109.4_4900001	10	4900001	0.656	0.7785
NC_005109.4_5000001	10	5000001	0.6477	0.7538
NC_005109.4_5100001	10	5100001	0.5474	0.6985
NC_005109.4_5200001	10	5200001	0.493	0.6758
NC_005109.4_5300001	10	5300001	0.4813	0.7283
NC_005109.4_5400001	10	5400001	0.5147	0.7547
NC_005109.4_5500001	10	5500001	0.4227	0.6852
NC_005109.4_5600001	10	5600001	0.4454	0.7004
NC_005109.4_5700001	10	5700001	0.5099	0.6912
NC_005109.4_5800001	10	5800001	0.5537	0.6564
NC_005109.4_5900001	10	5900001	0.5673	0.6578
NC_005109.4_6000001	10	6000001	0.5619	0.639
NC_005109.4_6100001	10	6100001	0.5755	0.632
NC_005109.4_6200001	10	6200001	0.5483	0.6157
NC_005109.4_6300001	10	6300001	0.484	0.6042
NC_005109.4_6400001	10	6400001	0.5075	0.648
NC_005109.4_6500001	10	6500001	0.5699	0.7345
NC_005109.4_6600001	10	6600001	0.5762	0.746
NC_005109.4_6700001	10	6700001	0.5923	0.7608
NC_005109.4_6800001	10	6800001	0.7455	0.8144
NC_005109.4_6900001	10	6900001	0.7132	0.7958
NC_005109.4_7000001	10	7000001	0.6273	0.733
NC_005109.4_7100001	10	7100001	0.6136	0.7186
NC_005109.4_7200001	10	7200001	0.6617	0.7189
NC_005109.4_7300001	10	7300001	0.5219	0.7071
NC_005109.4_7400001	10	7400001	0.4983	0.6498
NC_005109.4_7500001	10	7500001	0.5598	0.6981
NC_005109.4_7600001	10	7600001	0.61	0.7452
NC_005109.4_7700001	10	7700001	0.6043	0.756
NC_005109.4_7800001	10	7800001	0.6823	0.751
NC_005109.4_7900001	10	7900001	0.6855	0.7817
NC_005109.4_8000001	10	8000001	0.5999	0.7075
NC_005109.4_8100001	10	8100001	0.5132	0.6119
NC_005109.4_8200001	10	8200001	0.4314	0.6064
NC_005109.4_8300001	10	8300001	0.4356	0.6414
NC_005109.4_8400001	10	8400001	0.4735	0.6767
NC_005109.4_8500001	10	8500001	0.4885	0.7227
NC_005109.4_8600001	10	8600001	0.4665	0.6444
NC_005109.4_8700001	10	8700001	0.6278	0.6919
NC_005109.4_8800001	10	8800001	0.5018	0.5955
NC_005109.4_8900001	10	8900001	0.4118	0.4976
NC_005109.4_9000001	10	9000001	0.4066	0.5092

NC_005109.4_9100001	10	9100001	0.4682	0.5866
NC_005109.4_9200001	10	9200001	0.461	0.611
NC_005109.4_9300001	10	9300001	0.4873	0.5913
NC_005109.4_9400001	10	9400001	0.4811	0.5804
NC_005109.4_9500001	10	9500001	0.5124	0.6164
NC_005109.4_9600001	10	9600001	0.4375	0.6109
NC_005109.4_9700001	10	9700001	0.4222	0.5566
NC_005109.4_9800001	10	9800001	0.4485	0.5818
NC_005109.4_9900001	10	9900001	0.4814	0.6335
NC_005109.4_10000001	10	10000001	0.5271	0.6766
NC_005109.4_10100001	10	10100001	0.5833	0.6475
NC_005109.4_10200001	10	10200001	0.5653	0.6278
NC_005109.4_10300001	10	10300001	0.6128	0.682
NC_005109.4_10400001	10	10400001	0.6391	0.6707
NC_005109.4_10500001	10	10500001	0.5999	0.6428
NC_005109.4_10600001	10	10600001	0.596	0.6705
NC_005109.4_10700001	10	10700001	0.6563	0.7374
NC_005109.4_10800001	10	10800001	0.5791	0.6212
NC_005109.4_10900001	10	10900001	0.6396	0.6418
NC_005109.4_11000001	10	11000001	0.6106	0.6075
NC_005109.4_11100001	10	11100001	0.5742	0.5406
NC_005109.4_11200001	10	11200001	0.5335	0.5407
NC_005109.4_11300001	10	11300001	0.6148	0.6922
NC_005109.4_11400001	10	11400001	0.5455	0.7086
NC_005109.4_11500001	10	11500001	0.5258	0.6983
NC_005109.4_11600001	10	11600001	0.525	0.7492
NC_005109.4_11700001	10	11700001	0.5292	0.7324
NC_005109.4_11800001	10	11800001	0.4129	0.6484
NC_005109.4_11900001	10	11900001	0.3223	0.4378
NC_005109.4_12000001	10	12000001	0.3157	0.5102
NC_005109.4_12200001	10	12200001	0.2351	0.2878
NC_005109.4_12300001	10	12300001	0.2351	0.2878
NC_005109.4_12400001	10	12400001	0.2617	0.2487
NC_005109.4_12500001	10	12500001	0.4325	0.4366
NC_005109.4_12600001	10	12600001	0.4805	0.4848
NC_005109.4_12700001	10	12700001	0.4717	0.5061
NC_005109.4_12800001	10	12800001	0.4717	0.5061
NC_005109.4_12900001	10	12900001	0.507	0.549
NC_005109.4_13000001	10	13000001	0.4729	0.5754
NC_005109.4_13100001	10	13100001	0.5685	0.6944
NC_005109.4_13200001	10	13200001	0.5893	0.7348
NC_005109.4_13300001	10	13300001	0.5532	0.7237
NC_005109.4_13400001	10	13400001	0.522	0.6974
NC_005109.4_13500001	10	13500001	0.5225	0.6964
NC_005109.4_13600001	10	13600001	0.4194	0.6101
NC_005109.4_13700001	10	13700001	0.3553	0.5
NC_005109.4_13800001	10	13800001	0.4145	0.5557
NC_005109.4_13900001	10	13900001	0.4337	0.5841
NC_005109.4_14000001	10	14000001	0.4352	0.5627
NC_005109.4_14100001	10	14100001	0.5923	0.7112
NC_005109.4_14200001	10	14200001	0.5333	0.6808
NC_005109.4_14300001	10	14300001	0.4495	0.6248
NC_005109.4_14400001	10	14400001	0.4652	0.6447
NC_005109.4_14500001	10	14500001	0.4186	0.5921
NC_005109.4_14600001	10	14600001	0.3634	0.5228
NC_005109.4_14700001	10	14700001	0.4737	0.675
NC_005109.4_14800001	10	14800001	0.5826	0.7595
NC_005109.4_14900001	10	14900001	0.6188	0.7751

NC_005109.4_15000001	10	15000001	0.6527	0.8008
NC_005109.4_15100001	10	15100001	0.7078	0.8515
NC_005109.4_15200001	10	15200001	0.6549	0.8137
NC_005109.4_15300001	10	15300001	0.5666	0.7462
NC_005109.4_15400001	10	15400001	0.4946	0.6824
NC_005109.4_15500001	10	15500001	0.517	0.7136
NC_005109.4_15600001	10	15600001	0.4513	0.6139
NC_005109.4_15700001	10	15700001	0.4083	0.5834
NC_005109.4_15800001	10	15800001	0.4076	0.5972
NC_005109.4_15900001	10	15900001	0.4349	0.6324
NC_005109.4_16000001	10	16000001	0.3381	0.4745
NC_005109.4_16100001	10	16100001	0.4225	0.5504
NC_005109.4_16200001	10	16200001	0.3659	0.5028
NC_005109.4_16300001	10	16300001	0.4371	0.566
NC_005109.4_16400001	10	16400001	0.4535	0.5933
NC_005109.4_16500001	10	16500001	0.5003	0.6427
NC_005109.4_16600001	10	16600001	0.525	0.6788
NC_005109.4_16700001	10	16700001	0.6403	0.7526
NC_005109.4_16800001	10	16800001	0.4863	0.6541
NC_005109.4_16900001	10	16900001	0.4517	0.6164
NC_005109.4_17000001	10	17000001	0.4546	0.608
NC_005109.4_17100001	10	17100001	0.4497	0.6129
NC_005109.4_17200001	10	17200001	0.4513	0.5975
NC_005109.4_17300001	10	17300001	0.59	0.7074
NC_005109.4_17400001	10	17400001	0.5756	0.7269
NC_005109.4_17500001	10	17500001	0.5406	0.7193
NC_005109.4_17600001	10	17600001	0.4682	0.6509
NC_005109.4_17700001	10	17700001	0.502	0.6654
NC_005109.4_17800001	10	17800001	0.523	0.6897
NC_005109.4_17900001	10	17900001	0.5442	0.7192
NC_005109.4_18000001	10	18000001	0.5273	0.7319
NC_005109.4_18100001	10	18100001	0.5982	0.783
NC_005109.4_18200001	10	18200001	0.4915	0.7309
NC_005109.4_18300001	10	18300001	0.4591	0.6575
NC_005109.4_18400001	10	18400001	0.4994	0.6366
NC_005109.4_18500001	10	18500001	0.5014	0.5836
NC_005109.4_18600001	10	18600001	0.536	0.6141
NC_005109.4_18700001	10	18700001	0.5683	0.6024
NC_005109.4_18800001	10	18800001	0.5591	0.6002
NC_005109.4_18900001	10	18900001	0.5332	0.5866
NC_005109.4_19000001	10	19000001	0.5917	0.6042
NC_005109.4_19100001	10	19100001	0.5377	0.597
NC_005109.4_19200001	10	19200001	0.5151	0.6314
NC_005109.4_19300001	10	19300001	0.5076	0.6166
NC_005109.4_19400001	10	19400001	0.4651	0.5271
NC_005109.4_19500001	10	19500001	0.3962	0.5186
NC_005109.4_19600001	10	19600001	0.3212	0.3832
NC_005109.4_19700001	10	19700001	0.3484	0.3718
NC_005109.4_19800001	10	19800001	0.4133	0.4676
NC_005109.4_19900001	10	19900001	0.4373	0.5005
NC_005109.4_20000001	10	20000001	0.4232	0.4914
NC_005109.4_20100001	10	20100001	0.4832	0.6213
NC_005109.4_20200001	10	20200001	0.4634	0.6101
NC_005109.4_20300001	10	20300001	0.4668	0.5687
NC_005109.4_20400001	10	20400001	0.4902	0.6432
NC_005109.4_20500001	10	20500001	0.5549	0.6897
NC_005109.4_20600001	10	20600001	0.5837	0.6868
NC_005109.4_20700001	10	20700001	0.6301	0.7349

NC_005109.4_20800001	10	20800001	0.5752	0.8141
NC_005109.4_20900001	10	20900001	0.5495	0.7533
NC_005109.4_21000001	10	21000001	0.5216	0.7369
NC_005109.4_21100001	10	21100001	0.5366	0.7034
NC_005109.4_21200001	10	21200001	0.551	0.7369
NC_005109.4_21300001	10	21300001	0.5558	0.7583
NC_005109.4_21400001	10	21400001	0.3908	0.6306
NC_005109.4_21500001	10	21500001	0.3908	0.6306
NC_005109.4_21600001	10	21600001	0.6055	0.8314
NC_005109.4_21700001	10	21700001	0.5716	0.8248
NC_005109.4_21800001	10	21800001	0.513	0.7188
NC_005109.4_21900001	10	21900001	0.4416	0.6518
NC_005109.4_22000001	10	22000001	0.4306	0.6274
NC_005109.4_22100001	10	22100001	0.2084	0.1988
NC_005109.4_22200001	10	22200001	0.2319	0.1962
NC_005109.4_22300001	10	22300001	0.3311	0.3103
NC_005109.4_22400001	10	22400001	0.3628	0.406
NC_005109.4_22500001	10	22500001	0.3898	0.4565
NC_005109.4_22600001	10	22600001	0.4975	0.5766
NC_005109.4_22700001	10	22700001	0.508	0.6042
NC_005109.4_22800001	10	22800001	0.4595	0.6704
NC_005109.4_22900001	10	22900001	0.471	0.6535
NC_005109.4_23000001	10	23000001	0.577	0.572
NC_005109.4_23100001	10	23100001	0.4965	0.489
NC_005109.4_23200001	10	23200001	0.4259	0.4237
NC_005109.4_23300001	10	23300001	0.4374	0.4413
NC_005109.4_23400001	10	23400001	0.575	0.5661
NC_005109.4_23500001	10	23500001	0.4589	0.561
NC_005109.4_23600001	10	23600001	0.4327	0.5627
NC_005109.4_23700001	10	23700001	0.5182	0.6824
NC_005109.4_23800001	10	23800001	0.5493	0.6584
NC_005109.4_23900001	10	23900001	0.4544	0.5839
NC_005109.4_24000001	10	24000001	0.5019	0.6115
NC_005109.4_24100001	10	24100001	0.5302	0.6482
NC_005109.4_24200001	10	24200001	0.6025	0.6968
NC_005109.4_24300001	10	24300001	0.5997	0.7001
NC_005109.4_24400001	10	24400001	0.5252	0.6185
NC_005109.4_24500001	10	24500001	0.5357	0.5672
NC_005109.4_24600001	10	24600001	0.4525	0.4015
NC_005109.4_24700001	10	24700001	0.5181	0.5446
NC_005109.4_24800001	10	24800001	0.4453	0.472
NC_005109.4_24900001	10	24900001	0.4305	0.4499
NC_005109.4_25000001	10	25000001	0.3818	0.4532
NC_005109.4_25100001	10	25100001	0.4487	0.5603
NC_005109.4_25200001	10	25200001	0.3392	0.4468
NC_005109.4_25300001	10	25300001	0.385	0.6511
NC_005109.4_25400001	10	25400001	0.4842	0.7125
NC_005109.4_25500001	10	25500001	0.5388	0.7187
NC_005109.4_25600001	10	25600001	0.4946	0.6791
NC_005109.4_25700001	10	25700001	0.5275	0.5694
NC_005109.4_25800001	10	25800001	0.4486	0.5303
NC_005109.4_25900001	10	25900001	0.3088	0.4542
NC_005109.4_26000001	10	26000001	0.3985	0.6025
NC_005109.4_26100001	10	26100001	0.4717	0.7051
NC_005109.4_26200001	10	26200001	0.503	0.8009
NC_005109.4_26300001	10	26300001	0.5564	0.8723
NC_005109.4_26400001	10	26400001	0.6694	0.9154
NC_005109.4_26500001	10	26500001	0.7407	0.9336

NC_005109.4_26600001	10	26600001	0.6835	0.8961
NC_005109.4_26700001	10	26700001	0.6964	0.8832
NC_005109.4_26800001	10	26800001	0.7094	0.8929
NC_005109.4_26900001	10	26900001	0.7263	0.8373
NC_005109.4_27000001	10	27000001	0.6235	0.7521
NC_005109.4_27100001	10	27100001	0.7514	0.7485
NC_005109.4_27300001	10	27300001	0.5585	0.6397
NC_005109.4_27400001	10	27400001	0.3614	0.6399
NC_005109.4_27500001	10	27500001	0.4517	0.7281
NC_005109.4_27600001	10	27600001	0.4788	0.6277
NC_005109.4_27700001	10	27700001	0.525	0.6135
NC_005109.4_27800001	10	27800001	0.5621	0.5955
NC_005109.4_27900001	10	27900001	0.6288	0.606
NC_005109.4_28000001	10	28000001	0.5456	0.5467
NC_005109.4_28100001	10	28100001	0.5572	0.5502
NC_005109.4_28200001	10	28200001	0.4744	0.4761
NC_005109.4_28300001	10	28300001	0.6205	0.7612
NC_005109.4_28400001	10	28400001	0.5959	0.7928
NC_005109.4_28500001	10	28500001	0.565	0.7419
NC_005109.4_28600001	10	28600001	0.6215	0.8074
NC_005109.4_28700001	10	28700001	0.5475	0.7654
NC_005109.4_28800001	10	28800001	0.4101	0.5991
NC_005109.4_28900001	10	28900001	0.4427	0.6022
NC_005109.4_29000001	10	29000001	0.5206	0.6983
NC_005109.4_29100001	10	29100001	0.4679	0.6464
NC_005109.4_29200001	10	29200001	0.5434	0.7101
NC_005109.4_29300001	10	29300001	0.552	0.7603
NC_005109.4_29400001	10	29400001	0.4699	0.6271
NC_005109.4_29500001	10	29500001	0.4443	0.5839
NC_005109.4_29600001	10	29600001	0.4421	0.5676
NC_005109.4_29700001	10	29700001	0.4019	0.5296
NC_005109.4_29800001	10	29800001	0.3953	0.5299
NC_005109.4_29900001	10	29900001	0.3954	0.59
NC_005109.4_30000001	10	30000001	0.347	0.522
NC_005109.4_30100001	10	30100001	0.3202	0.4297
NC_005109.4_30200001	10	30200001	0.3505	0.4232
NC_005109.4_30300001	10	30300001	0.3412	0.3625
NC_005109.4_30400001	10	30400001	0.3819	0.4111
NC_005109.4_30500001	10	30500001	0.4251	0.4566
NC_005109.4_30600001	10	30600001	0.425	0.4972
NC_005109.4_30700001	10	30700001	0.4042	0.5364
NC_005109.4_30800001	10	30800001	0.4635	0.6339
NC_005109.4_30900001	10	30900001	0.4554	0.6467
NC_005109.4_31000001	10	31000001	0.4472	0.6382
NC_005109.4_31100001	10	31100001	0.4726	0.6489
NC_005109.4_31200001	10	31200001	0.5143	0.6473
NC_005109.4_31300001	10	31300001	0.4918	0.5505
NC_005109.4_31400001	10	31400001	0.4942	0.4838
NC_005109.4_31500001	10	31500001	0.5002	0.4619
NC_005109.4_31600001	10	31600001	0.4799	0.4092
NC_005109.4_31700001	10	31700001	0.4156	0.36
NC_005109.4_31800001	10	31800001	0.4833	0.5175
NC_005109.4_31900001	10	31900001	0.4629	0.5418
NC_005109.4_32000001	10	32000001	0.4946	0.6085
NC_005109.4_32100001	10	32100001	0.4912	0.6557
NC_005109.4_32200001	10	32200001	0.552	0.7192
NC_005109.4_32300001	10	32300001	0.4745	0.6359
NC_005109.4_32400001	10	32400001	0.4554	0.6522

NC_005109.4_32500001	10	32500001	0.5173	0.6799
NC_005109.4_32600001	10	32600001	0.5126	0.6666
NC_005109.4_32700001	10	32700001	0.568	0.7404
NC_005109.4_32800001	10	32800001	0.597	0.7127
NC_005109.4_32900001	10	32900001	0.6973	0.7316
NC_005109.4_33000001	10	33000001	0.5702	0.7026
NC_005109.4_33100001	10	33100001	0.5545	0.6717
NC_005109.4_33200001	10	33200001	0.5202	0.6227
NC_005109.4_33300001	10	33300001	0.4599	0.6092
NC_005109.4_33400001	10	33400001	0.39	0.5659
NC_005109.4_33500001	10	33500001	0.4631	0.5801
NC_005109.4_33600001	10	33600001	0.5088	0.5793
NC_005109.4_33700001	10	33700001	0.2041	0.1217
NC_005109.4_33800001	10	33800001	0.2391	0.1434
NC_005109.4_33900001	10	33900001	0.234	0.1387
NC_005109.4_34000001	10	34000001	0.3412	0.3791
NC_005109.4_34100001	10	34100001	0.4001	0.423
NC_005109.4_34200001	10	34200001	0.4441	0.4421
NC_005109.4_34300001	10	34300001	0.3999	0.4649
NC_005109.4_34400001	10	34400001	0.4153	0.4624
NC_005109.4_34500001	10	34500001	0.3522	0.3344
NC_005109.4_34600001	10	34600001	0.3085	0.3241
NC_005109.4_34700001	10	34700001	0.4513	0.7095
NC_005109.4_34800001	10	34800001	0.55	0.7806
NC_005109.4_34900001	10	34900001	0.5903	0.7001
NC_005109.4_35000001	10	35000001	0.5611	0.7106
NC_005109.4_35100001	10	35100001	0.5386	0.707
NC_005109.4_35200001	10	35200001	0.5151	0.5697
NC_005109.4_35300001	10	35300001	0.496	0.562
NC_005109.4_35400001	10	35400001	0.5666	0.6987
NC_005109.4_35500001	10	35500001	0.5908	0.6883
NC_005109.4_35600001	10	35600001	0.5171	0.6182
NC_005109.4_35700001	10	35700001	0.532	0.6398
NC_005109.4_35800001	10	35800001	0.5221	0.6541
NC_005109.4_35900001	10	35900001	0.3593	0.5065
NC_005109.4_36000001	10	36000001	0.4367	0.5487
NC_005109.4_36100001	10	36100001	0.5245	0.609
NC_005109.4_36200001	10	36200001	0.5015	0.6207
NC_005109.4_36300001	10	36300001	0.4842	0.5682
NC_005109.4_36400001	10	36400001	0.5133	0.5778
NC_005109.4_36500001	10	36500001	0.4306	0.522
NC_005109.4_36600001	10	36600001	0.3783	0.475
NC_005109.4_36700001	10	36700001	0.3727	0.4524
NC_005109.4_36800001	10	36800001	0.451	0.6452
NC_005109.4_36900001	10	36900001	0.4687	0.7488
NC_005109.4_37000001	10	37000001	0.4777	0.7957
NC_005109.4_37100001	10	37100001	0.4838	0.8053
NC_005109.4_37200001	10	37200001	0.5177	0.7742
NC_005109.4_37300001	10	37300001	0.5196	0.7184
NC_005109.4_37400001	10	37400001	0.4043	0.5629
NC_005109.4_37500001	10	37500001	0.5278	0.626
NC_005109.4_37600001	10	37600001	0.5257	0.6073
NC_005109.4_37700001	10	37700001	0.5404	0.6625
NC_005109.4_37800001	10	37800001	0.5609	0.6804
NC_005109.4_37900001	10	37900001	0.7189	0.7716
NC_005109.4_38000001	10	38000001	0.5925	0.7133
NC_005109.4_38100001	10	38100001	0.5713	0.6512
NC_005109.4_38200001	10	38200001	0.56	0.6352

NC_005109.4_38300001	10	38300001	0.5577	0.6396
NC_005109.4_38400001	10	38400001	0.5097	0.6028
NC_005109.4_38500001	10	38500001	0.5934	0.6517
NC_005109.4_38600001	10	38600001	0.6293	0.7281
NC_005109.4_38700001	10	38700001	0.5977	0.7111
NC_005109.4_38800001	10	38800001	0.6286	0.7404
NC_005109.4_38900001	10	38900001	0.6187	0.6998
NC_005109.4_39000001	10	39000001	0.602	0.702
NC_005109.4_39100001	10	39100001	0.6117	0.6735
NC_005109.4_39200001	10	39200001	0.6864	0.7043
NC_005109.4_39300001	10	39300001	0.4487	0.5569
NC_005109.4_39400001	10	39400001	0.5164	0.6459
NC_005109.4_39500001	10	39500001	0.5363	0.7085
NC_005109.4_39600001	10	39600001	0.5237	0.7623
NC_005109.4_39700001	10	39700001	0.5611	0.8053
NC_005109.4_39800001	10	39800001	0.6592	0.8154
NC_005109.4_39900001	10	39900001	0.6289	0.8302
NC_005109.4_40000001	10	40000001	0.6407	0.7906
NC_005109.4_40100001	10	40100001	0.6401	0.638
NC_005109.4_40200001	10	40200001	0.5755	0.5582
NC_005109.4_40300001	10	40300001	0.4533	0.499
NC_005109.4_40400001	10	40400001	0.4714	0.4994
NC_005109.4_40500001	10	40500001	0.4361	0.471
NC_005109.4_40600001	10	40600001	0.3669	0.4753
NC_005109.4_40700001	10	40700001	0.29	0.4191
NC_005109.4_40800001	10	40800001	0.2587	0.3187
NC_005109.4_40900001	10	40900001	0.1423	0.1098
NC_005109.4_41000001	10	41000001	0.1707	0.1294
NC_005109.4_41100001	10	41100001	0.1898	0.1346
NC_005109.4_41200001	10	41200001	0.1775	0.1409
NC_005109.4_41300001	10	41300001	0.2359	0.2892
NC_005109.4_41400001	10	41400001	0.2589	0.3355
NC_005109.4_41500001	10	41500001	0.3459	0.5508
NC_005109.4_41600001	10	41600001	0.3505	0.5452
NC_005109.4_41700001	10	41700001	0.4092	0.5484
NC_005109.4_41800001	10	41800001	0.3924	0.536
NC_005109.4_41900001	10	41900001	0.44	0.5886
NC_005109.4_42000001	10	42000001	0.4294	0.5517
NC_005109.4_42100001	10	42100001	0.4814	0.6283
NC_005109.4_42200001	10	42200001	0.5174	0.6796
NC_005109.4_42300001	10	42300001	0.5214	0.6602
NC_005109.4_42400001	10	42400001	0.4778	0.6366
NC_005109.4_42500001	10	42500001	0.4726	0.6421
NC_005109.4_42600001	10	42600001	0.3964	0.4639
NC_005109.4_42700001	10	42700001	0.3324	0.3471
NC_005109.4_42800001	10	42800001	0.4357	0.5039
NC_005109.4_42900001	10	42900001	0.4135	0.4724
NC_005109.4_43000001	10	43000001	0.4135	0.4724
NC_005109.4_43100001	10	43100001	0.4647	0.5789
NC_005109.4_43200001	10	43200001	0.5553	0.7086
NC_005109.4_43300001	10	43300001	0.4618	0.6363
NC_005109.4_43400001	10	43400001	0.4856	0.6829
NC_005109.4_43500001	10	43500001	0.4741	0.6938
NC_005109.4_43600001	10	43600001	0.4569	0.6811
NC_005109.4_43700001	10	43700001	0.3828	0.6495
NC_005109.4_43800001	10	43800001	0.3473	0.5702
NC_005109.4_43900001	10	43900001	0.2582	0.4159
NC_005109.4_44000001	10	44000001	0.1788	0.1009

NC_005109.4_44100001	10	44100001	0.1305	0.084
NC_005109.4_44200001	10	44200001	0.3471	0.5529
NC_005109.4_44300001	10	44300001	0.3394	0.5419
NC_005109.4_44400001	10	44400001	0.4136	0.6369
NC_005109.4_44500001	10	44500001	0.4297	0.6524
NC_005109.4_44600001	10	44600001	0.5129	0.6836
NC_005109.4_44700001	10	44700001	0.4268	0.5879
NC_005109.4_44800001	10	44800001	0.7833	0.7518
NC_005109.4_44900001	10	44900001	0.4241	0.4318
NC_005109.4_45000001	10	45000001	0.4229	0.4345
NC_005109.4_45100001	10	45100001	0.408	0.4221
NC_005109.4_45200001	10	45200001	0.4804	0.5064
NC_005109.4_45300001	10	45300001	0.4406	0.484
NC_005109.4_45400001	10	45400001	0.5178	0.5717
NC_005109.4_45500001	10	45500001	0.5753	0.638
NC_005109.4_45600001	10	45600001	0.6425	0.7123
NC_005109.4_45700001	10	45700001	0.6322	0.7203
NC_005109.4_45800001	10	45800001	0.7082	0.7677
NC_005109.4_45900001	10	45900001	0.6064	0.7317
NC_005109.4_46000001	10	46000001	0.6312	0.7475
NC_005109.4_46100001	10	46100001	0.5782	0.7129
NC_005109.4_46200001	10	46200001	0.5622	0.7411
NC_005109.4_46300001	10	46300001	0.5804	0.7559
NC_005109.4_46400001	10	46400001	0.6162	0.7575
NC_005109.4_46500001	10	46500001	0.5499	0.7208
NC_005109.4_46600001	10	46600001	0.5933	0.7304
NC_005109.4_46700001	10	46700001	0.5991	0.7214
NC_005109.4_46800001	10	46800001	0.5568	0.6873
NC_005109.4_46900001	10	46900001	0.6104	0.7209
NC_005109.4_47000001	10	47000001	0.636	0.674
NC_005109.4_47100001	10	47100001	0.5987	0.5894
NC_005109.4_47200001	10	47200001	0.6079	0.6503
NC_005109.4_47300001	10	47300001	0.5602	0.6122
NC_005109.4_47400001	10	47400001	0.5774	0.6365
NC_005109.4_47500001	10	47500001	0.5741	0.687
NC_005109.4_47600001	10	47600001	0.6284	0.7524
NC_005109.4_47700001	10	47700001	0.6234	0.7202
NC_005109.4_47800001	10	47800001	0.7581	0.8341
NC_005109.4_47900001	10	47900001	0.6658	0.6993
NC_005109.4_48000001	10	48000001	0.6591	0.6727
NC_005109.4_48100001	10	48100001	0.6724	0.7037
NC_005109.4_48200001	10	48200001	0.6402	0.6626
NC_005109.4_48300001	10	48300001	0.6724	0.6842
NC_005109.4_48400001	10	48400001	0.6738	0.8067
NC_005109.4_48500001	10	48500001	0.6673	0.8139
NC_005109.4_48600001	10	48600001	0.6332	0.7635
NC_005109.4_48700001	10	48700001	0.6126	0.774
NC_005109.4_48800001	10	48800001	0.4957	0.707
NC_005109.4_48900001	10	48900001	0.4564	0.6987
NC_005109.4_49000001	10	49000001	0.3684	0.6764
NC_005109.4_49100001	10	49100001	0.4798	0.7029
NC_005109.4_49200001	10	49200001	0.4744	0.6859
NC_005109.4_49300001	10	49300001	0.511	0.6064
NC_005109.4_49400001	10	49400001	0.6995	0.6918
NC_005109.4_49500001	10	49500001	0.6583	0.6504
NC_005109.4_49600001	10	49600001	0.6645	0.6697
NC_005109.4_49700001	10	49700001	0.525	0.6493
NC_005109.4_49800001	10	49800001	0.5151	0.6909

NC_005109.4_49900001	10	49900001	0.4316	0.5533
NC_005109.4_50000001	10	50000001	0.4116	0.5377
NC_005109.4_50100001	10	50100001	0.3877	0.4943
NC_005109.4_50200001	10	50200001	0.4259	0.4731
NC_005109.4_50300001	10	50300001	0.4357	0.4638
NC_005109.4_50400001	10	50400001	0.5047	0.5391
NC_005109.4_50500001	10	50500001	0.5501	0.6057
NC_005109.4_50600001	10	50600001	0.5379	0.6299
NC_005109.4_50700001	10	50700001	0.5487	0.6434
NC_005109.4_50800001	10	50800001	0.574	0.7052
NC_005109.4_50900001	10	50900001	0.5318	0.6747
NC_005109.4_51000001	10	51000001	0.4363	0.6303
NC_005109.4_51100001	10	51100001	0.5143	0.6434
NC_005109.4_51200001	10	51200001	0.5391	0.6817
NC_005109.4_51300001	10	51300001	0.5594	0.7063
NC_005109.4_51400001	10	51400001	0.5778	0.7455
NC_005109.4_51500001	10	51500001	0.658	0.7691
NC_005109.4_51600001	10	51600001	0.6713	0.8114
NC_005109.4_51700001	10	51700001	0.6711	0.7902
NC_005109.4_51800001	10	51800001	0.5994	0.7511
NC_005109.4_51900001	10	51900001	0.661	0.7817
NC_005109.4_52000001	10	52000001	0.6189	0.7965
NC_005109.4_52100001	10	52100001	0.5873	0.7479
NC_005109.4_52200001	10	52200001	0.5237	0.6103
NC_005109.4_52300001	10	52300001	0.5783	0.6376
NC_005109.4_52400001	10	52400001	0.5376	0.5811
NC_005109.4_52500001	10	52500001	0.5093	0.6064
NC_005109.4_52600001	10	52600001	0.4374	0.535
NC_005109.4_52700001	10	52700001	0.4273	0.6493
NC_005109.4_52800001	10	52800001	0.4178	0.6095
NC_005109.4_52900001	10	52900001	0.4064	0.6513
NC_005109.4_53000001	10	53000001	0.4505	0.5869
NC_005109.4_53100001	10	53100001	0.4819	0.6144
NC_005109.4_53200001	10	53200001	0.4108	0.597
NC_005109.4_53300001	10	53300001	0.3674	0.5405
NC_005109.4_53400001	10	53400001	0.4077	0.5215
NC_005109.4_53500001	10	53500001	0.4022	0.5769
NC_005109.4_53600001	10	53600001	0.4139	0.5893
NC_005109.4_53700001	10	53700001	0.5242	0.647
NC_005109.4_53800001	10	53800001	0.5469	0.6815
NC_005109.4_53900001	10	53900001	0.5202	0.6855
NC_005109.4_54000001	10	54000001	0.4856	0.6374
NC_005109.4_54100001	10	54100001	0.4394	0.616
NC_005109.4_54200001	10	54200001	0.4348	0.6359
NC_005109.4_54300001	10	54300001	0.4697	0.6847
NC_005109.4_54400001	10	54400001	0.4386	0.6577
NC_005109.4_54500001	10	54500001	0.4292	0.7374
NC_005109.4_54600001	10	54600001	0.4397	0.7409
NC_005109.4_54700001	10	54700001	0.3848	0.6231
NC_005109.4_54800001	10	54800001	0.3095	0.5239
NC_005109.4_54900001	10	54900001	0.3822	0.6392
NC_005109.4_55000001	10	55000001	0.4062	0.6471
NC_005109.4_55100001	10	55100001	0.4999	0.6918
NC_005109.4_55200001	10	55200001	0.5473	0.7851
NC_005109.4_55300001	10	55300001	0.4976	0.7355
NC_005109.4_55400001	10	55400001	0.4979	0.7399
NC_005109.4_55500001	10	55500001	0.5843	0.7542
NC_005109.4_55600001	10	55600001	0.5888	0.7533

NC_005109.4_55700001	10	55700001	0.6689	0.7707
NC_005109.4_55800001	10	55800001	0.7472	0.8141
NC_005109.4_55900001	10	55900001	0.6995	0.7573
NC_005109.4_56000001	10	56000001	0.6567	0.6928
NC_005109.4_56100001	10	56100001	0.662	0.6965
NC_005109.4_56200001	10	56200001	0.5457	0.6197
NC_005109.4_56300001	10	56300001	0.5827	0.6769
NC_005109.4_56400001	10	56400001	0.6559	0.7756
NC_005109.4_56500001	10	56500001	0.6545	0.8379
NC_005109.4_56600001	10	56600001	0.5633	0.7852
NC_005109.4_56700001	10	56700001	0.5699	0.8074
NC_005109.4_56800001	10	56800001	0.5149	0.7611
NC_005109.4_56900001	10	56900001	0.48	0.7241
NC_005109.4_57000001	10	57000001	0.4804	0.7047
NC_005109.4_57100001	10	57100001	0.553	0.7619
NC_005109.4_57200001	10	57200001	0.6146	0.7094
NC_005109.4_57300001	10	57300001	0.6588	0.7226
NC_005109.4_57400001	10	57400001	0.6433	0.6929
NC_005109.4_57500001	10	57500001	0.6159	0.7376
NC_005109.4_57600001	10	57600001	0.529	0.6003
NC_005109.4_57700001	10	57700001	0.525	0.602
NC_005109.4_57800001	10	57800001	0.4747	0.5889
NC_005109.4_57900001	10	57900001	0.546	0.6658
NC_005109.4_58000001	10	58000001	0.542	0.6202
NC_005109.4_58100001	10	58100001	0.6424	0.7563
NC_005109.4_58200001	10	58200001	0.5691	0.7402
NC_005109.4_58300001	10	58300001	0.6181	0.7549
NC_005109.4_58400001	10	58400001	0.5157	0.7139
NC_005109.4_58500001	10	58500001	0.5241	0.7355
NC_005109.4_58600001	10	58600001	0.5183	0.7595
NC_005109.4_58700001	10	58700001	0.5857	0.8008
NC_005109.4_58800001	10	58800001	0.5696	0.7953
NC_005109.4_58900001	10	58900001	0.6422	0.7918
NC_005109.4_59000001	10	59000001	0.6696	0.7868
NC_005109.4_59100001	10	59100001	0.6096	0.771
NC_005109.4_59200001	10	59200001	0.6215	0.7858
NC_005109.4_59300001	10	59300001	0.6221	0.7978
NC_005109.4_59400001	10	59400001	0.6679	0.8485
NC_005109.4_59500001	10	59500001	0.6414	0.8484
NC_005109.4_59600001	10	59600001	0.7077	0.8623
NC_005109.4_59700001	10	59700001	0.6772	0.8207
NC_005109.4_59800001	10	59800001	0.5652	0.6303
NC_005109.4_59900001	10	59900001	0.3562	0.3925
NC_005109.4_60000001	10	60000001	0.3425	0.435
NC_005109.4_60100001	10	60100001	0.3594	0.4823
NC_005109.4_60200001	10	60200001	0.3594	0.4823
NC_005109.4_60300001	10	60300001	0.3679	0.5907
NC_005109.4_60400001	10	60400001	0.2824	0.4894
NC_005109.4_60500001	10	60500001	0.3607	0.5368
NC_005109.4_60600001	10	60600001	0.4608	0.5887
NC_005109.4_60700001	10	60700001	0.5187	0.6092
NC_005109.4_60800001	10	60800001	0.475	0.5807
NC_005109.4_60900001	10	60900001	0.5479	0.6825
NC_005109.4_61000001	10	61000001	0.6047	0.7162
NC_005109.4_61100001	10	61100001	0.6356	0.7517
NC_005109.4_61200001	10	61200001	0.6477	0.803
NC_005109.4_61300001	10	61300001	0.7458	0.86
NC_005109.4_61400001	10	61400001	0.7107	0.8472

NC_005109.4_61500001	10	61500001	0.6897	0.8573
NC_005109.4_61600001	10	61600001	0.6634	0.8227
NC_005109.4_61700001	10	61700001	0.6216	0.8266
NC_005109.4_61800001	10	61800001	0.6462	0.8273
NC_005109.4_61900001	10	61900001	0.7201	0.8207
NC_005109.4_62000001	10	62000001	0.7006	0.8009
NC_005109.4_62100001	10	62100001	0.6072	0.7712
NC_005109.4_62200001	10	62200001	0.7211	0.8181
NC_005109.4_62300001	10	62300001	0.7418	0.8213
NC_005109.4_62400001	10	62400001	0.6304	0.8325
NC_005109.4_62500001	10	62500001	0.6877	0.8608
NC_005109.4_62600001	10	62600001	0.701	0.9026
NC_005109.4_62700001	10	62700001	0.6469	0.8209
NC_005109.4_62800001	10	62800001	0.59	0.7802
NC_005109.4_62900001	10	62900001	0.5761	0.7477
NC_005109.4_63000001	10	63000001	0.5529	0.6892
NC_005109.4_63100001	10	63100001	0.4993	0.6083
NC_005109.4_63200001	10	63200001	0.5481	0.6835
NC_005109.4_63300001	10	63300001	0.5703	0.7182
NC_005109.4_63400001	10	63400001	0.6216	0.785
NC_005109.4_63500001	10	63500001	0.6101	0.8026
NC_005109.4_63600001	10	63600001	0.6321	0.8092
NC_005109.4_63700001	10	63700001	0.5862	0.7693
NC_005109.4_63800001	10	63800001	0.6403	0.7914
NC_005109.4_63900001	10	63900001	0.6154	0.7577
NC_005109.4_64000001	10	64000001	0.6088	0.7723
NC_005109.4_64100001	10	64100001	0.6178	0.8071
NC_005109.4_64200001	10	64200001	0.6037	0.7608
NC_005109.4_64300001	10	64300001	0.5166	0.6924
NC_005109.4_64400001	10	64400001	0.5082	0.6556
NC_005109.4_64500001	10	64500001	0.5841	0.6837
NC_005109.4_64600001	10	64600001	0.6401	0.6972
NC_005109.4_64700001	10	64700001	0.6429	0.6758
NC_005109.4_64800001	10	64800001	0.7139	0.7072
NC_005109.4_64900001	10	64900001	0.7461	0.7438
NC_005109.4_65000001	10	65000001	0.6344	0.6932
NC_005109.4_65100001	10	65100001	0.4719	0.5607
NC_005109.4_65200001	10	65200001	0.3293	0.5039
NC_005109.4_65300001	10	65300001	0.398	0.5712
NC_005109.4_65400001	10	65400001	0.3725	0.5298
NC_005109.4_65500001	10	65500001	0.3732	0.512
NC_005109.4_65600001	10	65600001	0.44	0.5785
NC_005109.4_65700001	10	65700001	0.5605	0.6488
NC_005109.4_65800001	10	65800001	0.6307	0.6499
NC_005109.4_65900001	10	65900001	0.6664	0.7072
NC_005109.4_66000001	10	66000001	0.5716	0.6234
NC_005109.4_66100001	10	66100001	0.5384	0.6007
NC_005109.4_66200001	10	66200001	0.5093	0.5834
NC_005109.4_66300001	10	66300001	0.5466	0.6299
NC_005109.4_66400001	10	66400001	0.5186	0.5882
NC_005109.4_66500001	10	66500001	0.6719	0.7982
NC_005109.4_66600001	10	66600001	0.6492	0.7417
NC_005109.4_66700001	10	66700001	0.6061	0.6913
NC_005109.4_66800001	10	66800001	0.5873	0.6628
NC_005109.4_66900001	10	66900001	0.603	0.6695
NC_005109.4_67000001	10	67000001	0.5499	0.5843
NC_005109.4_67100001	10	67100001	0.5185	0.6197
NC_005109.4_67200001	10	67200001	0.5957	0.7275

NC_005109.4_67300001	10	67300001	0.6097	0.804
NC_005109.4_67400001	10	67400001	0.3853	0.7017
NC_005109.4_67500001	10	67500001	0.3958	0.681
NC_005109.4_67600001	10	67600001	0.3901	0.6566
NC_005109.4_67700001	10	67700001	0.4102	0.6337
NC_005109.4_67800001	10	67800001	0.453	0.6269
NC_005109.4_67900001	10	67900001	0.5625	0.6547
NC_005109.4_68000001	10	68000001	0.5574	0.6545
NC_005109.4_68100001	10	68100001	0.5137	0.583
NC_005109.4_68200001	10	68200001	0.5215	0.5686
NC_005109.4_68300001	10	68300001	0.5288	0.585
NC_005109.4_68400001	10	68400001	0.4455	0.5701
NC_005109.4_68500001	10	68500001	0.4305	0.5207
NC_005109.4_68600001	10	68600001	0.4544	0.5368
NC_005109.4_68700001	10	68700001	0.4328	0.5157
NC_005109.4_68800001	10	68800001	0.3704	0.442
NC_005109.4_68900001	10	68900001	0.4813	0.4332
NC_005109.4_69000001	10	69000001	0.5303	0.5122
NC_005109.4_69100001	10	69100001	0.5174	0.5764
NC_005109.4_69200001	10	69200001	0.4853	0.5681
NC_005109.4_69300001	10	69300001	0.4432	0.5256
NC_005109.4_69400001	10	69400001	0.415	0.5291
NC_005109.4_69500001	10	69500001	0.4114	0.528
NC_005109.4_69600001	10	69600001	0.4424	0.5866
NC_005109.4_69700001	10	69700001	0.4531	0.6445
NC_005109.4_69800001	10	69800001	0.4908	0.7257
NC_005109.4_69900001	10	69900001	0.5226	0.7056
NC_005109.4_70000001	10	70000001	0.476	0.6848
NC_005109.4_70100001	10	70100001	0.4659	0.6425
NC_005109.4_70200001	10	70200001	0.4501	0.5676
NC_005109.4_70300001	10	70300001	0.557	0.5724
NC_005109.4_70400001	10	70400001	0.6347	0.6973
NC_005109.4_70500001	10	70500001	0.808	0.8248
NC_005109.4_70600001	10	70600001	0.6898	0.7755
NC_005109.4_70700001	10	70700001	0.6017	0.7567
NC_005109.4_70800001	10	70800001	0.5757	0.7674
NC_005109.4_70900001	10	70900001	0.5541	0.7343
NC_005109.4_71000001	10	71000001	0.5392	0.6931
NC_005109.4_71100001	10	71100001	0.5721	0.756
NC_005109.4_71200001	10	71200001	0.6423	0.7744
NC_005109.4_71300001	10	71300001	0.6374	0.7357
NC_005109.4_71400001	10	71400001	0.5879	0.7619
NC_005109.4_71500001	10	71500001	0.4981	0.7062
NC_005109.4_71600001	10	71600001	0.4437	0.601
NC_005109.4_71700001	10	71700001	0.4564	0.6432
NC_005109.4_71800001	10	71800001	0.4308	0.6058
NC_005109.4_71900001	10	71900001	0.4317	0.5899
NC_005109.4_72000001	10	72000001	0.3997	0.6004
NC_005109.4_72100001	10	72100001	0.381	0.6324
NC_005109.4_72200001	10	72200001	0.3379	0.5359
NC_005109.4_72300001	10	72300001	0.3208	0.6122
NC_005109.4_72400001	10	72400001	0.4317	0.7592
NC_005109.4_72500001	10	72500001	0.7125	0.891
NC_005109.4_72600001	10	72600001	0.7767	0.927
NC_005109.4_72700001	10	72700001	0.7712	0.926
NC_005109.4_72800001	10	72800001	0.7291	0.9019
NC_005109.4_72900001	10	72900001	0.618	0.8142
NC_005109.4_73000001	10	73000001	0.5802	0.7806

NC_005109.4_73100001	10	73100001	0.5405	0.7498
NC_005109.4_73200001	10	73200001	0.5099	0.7277
NC_005109.4_73300001	10	73300001	0.4621	0.661
NC_005109.4_73400001	10	73400001	0.5979	0.7687
NC_005109.4_73500001	10	73500001	0.5997	0.7562
NC_005109.4_73600001	10	73600001	0.5886	0.7484
NC_005109.4_73700001	10	73700001	0.5027	0.618
NC_005109.4_73800001	10	73800001	0.5093	0.6298
NC_005109.4_73900001	10	73900001	0.5455	0.7158
NC_005109.4_74000001	10	74000001	0.564	0.7463
NC_005109.4_74100001	10	74100001	0.6216	0.7848
NC_005109.4_74200001	10	74200001	0.6343	0.81
NC_005109.4_74300001	10	74300001	0.6485	0.8068
NC_005109.4_74400001	10	74400001	0.67	0.8307
NC_005109.4_74500001	10	74500001	0.7009	0.8271
NC_005109.4_74600001	10	74600001	0.7271	0.8167
NC_005109.4_74700001	10	74700001	0.6019	0.7767
NC_005109.4_74800001	10	74800001	0.5723	0.7257
NC_005109.4_74900001	10	74900001	0.6031	0.7455
NC_005109.4_75000001	10	75000001	0.5859	0.7502
NC_005109.4_75100001	10	75100001	0.5283	0.6519
NC_005109.4_75200001	10	75200001	0.5407	0.6201
NC_005109.4_75300001	10	75300001	0.6433	0.7107
NC_005109.4_75400001	10	75400001	0.5387	0.6211
NC_005109.4_75500001	10	75500001	0.4994	0.5939
NC_005109.4_75600001	10	75600001	0.4791	0.6258
NC_005109.4_75700001	10	75700001	0.495	0.6143
NC_005109.4_75800001	10	75800001	0.3981	0.5063
NC_005109.4_75900001	10	75900001	0.415	0.5334
NC_005109.4_76000001	10	76000001	0.4179	0.5429
NC_005109.4_76100001	10	76100001	0.4037	0.4717
NC_005109.4_76200001	10	76200001	0.4052	0.4635
NC_005109.4_76300001	10	76300001	0.452	0.5427
NC_005109.4_76400001	10	76400001	0.4205	0.489
NC_005109.4_76500001	10	76500001	0.4642	0.5788
NC_005109.4_76600001	10	76600001	0.5059	0.6167
NC_005109.4_76700001	10	76700001	0.5251	0.643
NC_005109.4_76800001	10	76800001	0.5478	0.6604
NC_005109.4_76900001	10	76900001	0.5321	0.6615
NC_005109.4_77000001	10	77000001	0.5085	0.5767
NC_005109.4_77100001	10	77100001	0.4342	0.5191
NC_005109.4_77200001	10	77200001	0.3731	0.4308
NC_005109.4_77300001	10	77300001	0.2693	0.285
NC_005109.4_77400001	10	77400001	0.3982	0.4632
NC_005109.4_77500001	10	77500001	0.4616	0.6089
NC_005109.4_77600001	10	77600001	0.4836	0.5934
NC_005109.4_77700001	10	77700001	0.4972	0.63
NC_005109.4_77800001	10	77800001	0.548	0.6443
NC_005109.4_77900001	10	77900001	0.5596	0.6516
NC_005109.4_78000001	10	78000001	0.5897	0.6885
NC_005109.4_78100001	10	78100001	0.6795	0.7369
NC_005109.4_78200001	10	78200001	0.6679	0.7889
NC_005109.4_78300001	10	78300001	0.6199	0.7421
NC_005109.4_78400001	10	78400001	0.6425	0.7796
NC_005109.4_78500001	10	78500001	0.5796	0.7308
NC_005109.4_78600001	10	78600001	0.5157	0.6461
NC_005109.4_78700001	10	78700001	0.4844	0.4991
NC_005109.4_78800001	10	78800001	0.4186	0.5415

NC_005109.4_78900001	10	78900001	0.4622	0.5996
NC_005109.4_79000001	10	79000001	0.4552	0.5906
NC_005109.4_79100001	10	79100001	0.4288	0.7503
NC_005109.4_79200001	10	79200001	0.452	0.7432
NC_005109.4_79300001	10	79300001	0.5079	0.7915
NC_005109.4_79400001	10	79400001	0.3709	0.6671
NC_005109.4_79500001	10	79500001	0.2442	0.5341
NC_005109.4_79600001	10	79600001	0.2031	0.3403
NC_005109.4_79700001	10	79700001	0.1866	0.2004
NC_005109.4_79800001	10	79800001	0.1864	0.207
NC_005109.4_79900001	10	79900001	0.2461	0.4453
NC_005109.4_80000001	10	80000001	0.4488	0.5529
NC_005109.4_80100001	10	80100001	0.5366	0.6526
NC_005109.4_80200001	10	80200001	0.5519	0.7032
NC_005109.4_80300001	10	80300001	0.5399	0.6059
NC_005109.4_80400001	10	80400001	0.577	0.5876
NC_005109.4_80500001	10	80500001	0.5754	0.6545
NC_005109.4_80600001	10	80600001	0.4941	0.6053
NC_005109.4_80700001	10	80700001	0.5181	0.6096
NC_005109.4_80800001	10	80800001	0.5999	0.7553
NC_005109.4_80900001	10	80900001	0.5769	0.6988
NC_005109.4_81000001	10	81000001	0.5727	0.6923
NC_005109.4_81100001	10	81100001	0.6612	0.7509
NC_005109.4_81200001	10	81200001	0.6981	0.786
NC_005109.4_81300001	10	81300001	0.7029	0.81
NC_005109.4_81400001	10	81400001	0.7342	0.8973
NC_005109.4_81500001	10	81500001	0.7909	0.8922
NC_005109.4_81600001	10	81600001	0.6475	0.8429
NC_005109.4_81700001	10	81700001	0.5797	0.719
NC_005109.4_81800001	10	81800001	0.601	0.7291
NC_005109.4_81900001	10	81900001	0.6396	0.7539
NC_005109.4_82000001	10	82000001	0.616	0.7135
NC_005109.4_82100001	10	82100001	0.6006	0.6758
NC_005109.4_82200001	10	82200001	0.6191	0.7677
NC_005109.4_82300001	10	82300001	0.4989	0.7188
NC_005109.4_82400001	10	82400001	0.4625	0.6977
NC_005109.4_82500001	10	82500001	0.4576	0.7339
NC_005109.4_82600001	10	82600001	0.5671	0.7439
NC_005109.4_82700001	10	82700001	0.6162	0.7644
NC_005109.4_82800001	10	82800001	0.6173	0.7158
NC_005109.4_82900001	10	82900001	0.6301	0.7009
NC_005109.4_83000001	10	83000001	0.699	0.7302
NC_005109.4_83100001	10	83100001	0.71	0.761
NC_005109.4_83200001	10	83200001	0.6852	0.7335
NC_005109.4_83300001	10	83300001	0.6797	0.7905
NC_005109.4_83400001	10	83400001	0.6772	0.79
NC_005109.4_83500001	10	83500001	0.6611	0.7935
NC_005109.4_83600001	10	83600001	0.6387	0.7527
NC_005109.4_83700001	10	83700001	0.6932	0.7986
NC_005109.4_83800001	10	83800001	0.6895	0.7711
NC_005109.4_83900001	10	83900001	0.6861	0.7356
NC_005109.4_84000001	10	84000001	0.5998	0.6822
NC_005109.4_84100001	10	84100001	0.5732	0.6717
NC_005109.4_84200001	10	84200001	0.4408	0.5422
NC_005109.4_84300001	10	84300001	0.5481	0.6618
NC_005109.4_84400001	10	84400001	0.5553	0.7434
NC_005109.4_84500001	10	84500001	0.6692	0.7716
NC_005109.4_84600001	10	84600001	0.7212	0.7902

NC_005109.4_84700001	10	84700001	0.7566	0.793
NC_005109.4_84800001	10	84800001	0.6378	0.7339
NC_005109.4_84900001	10	84900001	0.6532	0.7388
NC_005109.4_85000001	10	85000001	0.5884	0.7625
NC_005109.4_85100001	10	85100001	0.5501	0.7155
NC_005109.4_85200001	10	85200001	0.6638	0.8112
NC_005109.4_85300001	10	85300001	0.7365	0.8447
NC_005109.4_85400001	10	85400001	0.6293	0.7867
NC_005109.4_85500001	10	85500001	0.6778	0.7071
NC_005109.4_85600001	10	85600001	0.7168	0.7231
NC_005109.4_85700001	10	85700001	0.6744	0.6966
NC_005109.4_85800001	10	85800001	0.6353	0.6761
NC_005109.4_85900001	10	85900001	0.6203	0.691
NC_005109.4_86000001	10	86000001	0.5437	0.6675
NC_005109.4_86100001	10	86100001	0.4879	0.6424
NC_005109.4_86200001	10	86200001	0.4721	0.6008
NC_005109.4_86300001	10	86300001	0.4131	0.614
NC_005109.4_86400001	10	86400001	0.4513	0.647
NC_005109.4_86500001	10	86500001	0.5091	0.7514
NC_005109.4_86600001	10	86600001	0.5204	0.7633
NC_005109.4_86700001	10	86700001	0.506	0.8005
NC_005109.4_86800001	10	86800001	0.5972	0.7701
NC_005109.4_86900001	10	86900001	0.6336	0.7693
NC_005109.4_87000001	10	87000001	0.5489	0.6676
NC_005109.4_87100001	10	87100001	0.5128	0.6291
NC_005109.4_87200001	10	87200001	0.3887	0.4715
NC_005109.4_87300001	10	87300001	0.3679	0.4607
NC_005109.4_87400001	10	87400001	0.3239	0.3868
NC_005109.4_87500001	10	87500001	0.4299	0.5152
NC_005109.4_87600001	10	87600001	0.5864	0.6403
NC_005109.4_87700001	10	87700001	0.6366	0.6689
NC_005109.4_87800001	10	87800001	0.6318	0.6932
NC_005109.4_87900001	10	87900001	0.6052	0.694
NC_005109.4_88000001	10	88000001	0.5965	0.7088
NC_005109.4_88100001	10	88100001	0.4969	0.6714
NC_005109.4_88200001	10	88200001	0.4632	0.6537
NC_005109.4_88300001	10	88300001	0.4664	0.6624
NC_005109.4_88400001	10	88400001	0.4918	0.6413
NC_005109.4_88500001	10	88500001	0.4996	0.6325
NC_005109.4_88600001	10	88600001	0.5435	0.6906
NC_005109.4_88700001	10	88700001	0.522	0.6709
NC_005109.4_88800001	10	88800001	0.5386	0.6669
NC_005109.4_88900001	10	88900001	0.4885	0.6764
NC_005109.4_89000001	10	89000001	0.4902	0.6605
NC_005109.4_89100001	10	89100001	0.4766	0.6446
NC_005109.4_89200001	10	89200001	0.4896	0.6849
NC_005109.4_89300001	10	89300001	0.4457	0.6475
NC_005109.4_89400001	10	89400001	0.5165	0.6149
NC_005109.4_89500001	10	89500001	0.4961	0.6024
NC_005109.4_89600001	10	89600001	0.4951	0.5307
NC_005109.4_89700001	10	89700001	0.4192	0.4395
NC_005109.4_89800001	10	89800001	0.5556	0.6073
NC_005109.4_89900001	10	89900001	0.5593	0.613
NC_005109.4_90000001	10	90000001	0.5784	0.6374
NC_005109.4_90100001	10	90100001	0.6136	0.7118
NC_005109.4_90200001	10	90200001	0.6937	0.7585
NC_005109.4_90300001	10	90300001	0.661	0.7324
NC_005109.4_90400001	10	90400001	0.7056	0.8167

NC_005109.4_90500001	10	90500001	0.6416	0.7884
NC_005109.4_90600001	10	90600001	0.5406	0.6345
NC_005109.4_90700001	10	90700001	0.495	0.5555
NC_005109.4_90800001	10	90800001	0.4344	0.4524
NC_005109.4_90900001	10	90900001	0.454	0.4764
NC_005109.4_91000001	10	91000001	0.464	0.4829
NC_005109.4_91100001	10	91100001	0.4221	0.5466
NC_005109.4_91200001	10	91200001	0.5261	0.6741
NC_005109.4_91300001	10	91300001	0.5605	0.6857
NC_005109.4_91400001	10	91400001	0.614	0.7321
NC_005109.4_91500001	10	91500001	0.6725	0.7837
NC_005109.4_91600001	10	91600001	0.7685	0.7825
NC_005109.4_91700001	10	91700001	0.6637	0.7245
NC_005109.4_91800001	10	91800001	0.6549	0.7826
NC_005109.4_91900001	10	91900001	0.5919	0.72
NC_005109.4_92000001	10	92000001	0.5457	0.6785
NC_005109.4_92100001	10	92100001	0.5588	0.698
NC_005109.4_92200001	10	92200001	0.6256	0.7354
NC_005109.4_92300001	10	92300001	0.615	0.6868
NC_005109.4_92400001	10	92400001	0.5263	0.6111
NC_005109.4_92500001	10	92500001	0.497	0.5745
NC_005109.4_92600001	10	92600001	0.4636	0.5907
NC_005109.4_92700001	10	92700001	0.5268	0.6289
NC_005109.4_92800001	10	92800001	0.5267	0.687
NC_005109.4_92900001	10	92900001	0.511	0.7162
NC_005109.4_93000001	10	93000001	0.5344	0.7542
NC_005109.4_93100001	10	93100001	0.5744	0.7687
NC_005109.4_93200001	10	93200001	0.4697	0.6903
NC_005109.4_93300001	10	93300001	0.5673	0.7122
NC_005109.4_93400001	10	93400001	0.6484	0.7432
NC_005109.4_93500001	10	93500001	0.6541	0.7473
NC_005109.4_93600001	10	93600001	0.6205	0.7431
NC_005109.4_93700001	10	93700001	0.7073	0.7741
NC_005109.4_93800001	10	93800001	0.6559	0.7632
NC_005109.4_93900001	10	93900001	0.6123	0.7817
NC_005109.4_94000001	10	94000001	0.5539	0.7078
NC_005109.4_94100001	10	94100001	0.6787	0.7231
NC_005109.4_94200001	10	94200001	0.6263	0.7098
NC_005109.4_94300001	10	94300001	0.5454	0.6595
NC_005109.4_94400001	10	94400001	0.5268	0.6391
NC_005109.4_94500001	10	94500001	0.5444	0.6638
NC_005109.4_94600001	10	94600001	0.432	0.6031
NC_005109.4_94700001	10	94700001	0.4095	0.5491
NC_005109.4_94800001	10	94800001	0.5074	0.6067
NC_005109.4_94900001	10	94900001	0.5287	0.6215
NC_005109.4_95000001	10	95000001	0.6233	0.6657
NC_005109.4_95100001	10	95100001	0.6384	0.7185
NC_005109.4_95200001	10	95200001	0.7243	0.7653
NC_005109.4_95300001	10	95300001	0.7103	0.7809
NC_005109.4_95400001	10	95400001	0.7389	0.792
NC_005109.4_95500001	10	95500001	0.7083	0.7804
NC_005109.4_95600001	10	95600001	0.7517	0.7582
NC_005109.4_95700001	10	95700001	0.6675	0.7568
NC_005109.4_95800001	10	95800001	0.6171	0.7245
NC_005109.4_95900001	10	95900001	0.4666	0.6489
NC_005109.4_96000001	10	96000001	0.481	0.6379
NC_005109.4_96100001	10	96100001	0.4338	0.6533
NC_005109.4_96200001	10	96200001	0.4387	0.6202

NC_005109.4_96300001	10	96300001	0.5486	0.7365
NC_005109.4_96400001	10	96400001	0.6644	0.7886
NC_005109.4_96500001	10	96500001	0.7119	0.8318
NC_005109.4_96600001	10	96600001	0.6761	0.7458
NC_005109.4_96700001	10	96700001	0.6942	0.8395
NC_005109.4_96800001	10	96800001	0.5408	0.7434
NC_005109.4_96900001	10	96900001	0.4463	0.6647
NC_005109.4_97000001	10	97000001	0.435	0.5905
NC_005109.4_97100001	10	97100001	0.5049	0.6485
NC_005109.4_97200001	10	97200001	0.5013	0.6164
NC_005109.4_97300001	10	97300001	0.5457	0.6314
NC_005109.4_97400001	10	97400001	0.5421	0.6519
NC_005109.4_97500001	10	97500001	0.5544	0.6828
NC_005109.4_97600001	10	97600001	0.4548	0.5684
NC_005109.4_97700001	10	97700001	0.387	0.4807
NC_005109.4_97800001	10	97800001	0.3567	0.4525
NC_005109.4_97900001	10	97900001	0.32	0.3672
NC_005109.4_98000001	10	98000001	0.3758	0.4959
NC_005109.4_98100001	10	98100001	0.4095	0.5182
NC_005109.4_98200001	10	98200001	0.4934	0.6211
NC_005109.4_98300001	10	98300001	0.4287	0.5254
NC_005109.4_98400001	10	98400001	0.5232	0.5974
NC_005109.4_98500001	10	98500001	0.4799	0.5467
NC_005109.4_98600001	10	98600001	0.4654	0.5621
NC_005109.4_98700001	10	98700001	0.4833	0.5917
NC_005109.4_98800001	10	98800001	0.667	0.7802
NC_005109.4_98900001	10	98900001	0.5059	0.769
NC_005109.4_99000001	10	99000001	0.4218	0.7477
NC_005109.4_99100001	10	99100001	0.3791	0.6991
NC_005109.4_99200001	10	99200001	0.3241	0.5953
NC_005109.4_99300001	10	99300001	0.225	0.4675
NC_005109.4_99400001	10	99400001	0.3456	0.4721
NC_005109.4_99500001	10	99500001	0.4591	0.5864
NC_005109.4_99600001	10	99600001	0.5676	0.6846
NC_005109.4_99700001	10	99700001	0.5529	0.6664
NC_005109.4_99800001	10	99800001	0.5304	0.5986
NC_005109.4_99900001	10	99900001	0.5055	0.5828
NC_005109.4_100000001	10	100000001	0.485	0.5471
NC_005109.4_100100001	10	100100001	0.5108	0.5447
NC_005109.4_100200001	10	100200001	0.5147	0.5425
NC_005109.4_100300001	10	100300001	0.4833	0.5849
NC_005109.4_100400001	10	100400001	0.5219	0.6492
NC_005109.4_100500001	10	100500001	0.5672	0.7075
NC_005109.4_100600001	10	100600001	0.5249	0.6825
NC_005109.4_100700001	10	100700001	0.4543	0.6606
NC_005109.4_100800001	10	100800001	0.4895	0.6747
NC_005109.4_100900001	10	100900001	0.5002	0.7026
NC_005109.4_101000001	10	101000001	0.5133	0.7118
NC_005109.4_101100001	10	101100001	0.5003	0.7013
NC_005109.4_101200001	10	101200001	0.5244	0.7446
NC_005109.4_101300001	10	101300001	0.5249	0.77
NC_005109.4_101400001	10	101400001	0.5174	0.7249
NC_005109.4_101500001	10	101500001	0.4814	0.6609
NC_005109.4_101600001	10	101600001	0.4746	0.6704
NC_005109.4_101700001	10	101700001	0.4719	0.6504
NC_005109.4_101800001	10	101800001	0.4994	0.6398
NC_005109.4_101900001	10	101900001	0.4282	0.5844
NC_005109.4_102000001	10	102000001	0.3833	0.583

NC_005109.4_102100001	10	102100001	0.3838	0.5532
NC_005109.4_102200001	10	102200001	0.4125	0.5977
NC_005109.4_102300001	10	102300001	0.368	0.5567
NC_005109.4_102400001	10	102400001	0.4442	0.6503
NC_005109.4_102500001	10	102500001	0.5061	0.6706
NC_005109.4_102600001	10	102600001	0.5994	0.6917
NC_005109.4_102700001	10	102700001	0.5536	0.6
NC_005109.4_102800001	10	102800001	0.4825	0.6575
NC_005109.4_102900001	10	102900001	0.413	0.584
NC_005109.4_103000001	10	103000001	0.4176	0.6031
NC_005109.4_103100001	10	103100001	0.3486	0.5856
NC_005109.4_103200001	10	103200001	0.3504	0.5492
NC_005109.4_103300001	10	103300001	0.4012	0.5144
NC_005109.4_103400001	10	103400001	0.453	0.5479
NC_005109.4_103500001	10	103500001	0.4663	0.5398
NC_005109.4_103600001	10	103600001	0.4717	0.5607
NC_005109.4_103700001	10	103700001	0.5332	0.6642
NC_005109.4_103800001	10	103800001	0.5694	0.6945
NC_005109.4_103900001	10	103900001	0.651	0.7609
NC_005109.4_104000001	10	104000001	0.6873	0.8068
NC_005109.4_104100001	10	104100001	0.7101	0.8316
NC_005109.4_104200001	10	104200001	0.6693	0.7867
NC_005109.4_104300001	10	104300001	0.6975	0.8118
NC_005109.4_104400001	10	104400001	0.6238	0.7732
NC_005109.4_104500001	10	104500001	0.6071	0.7541
NC_005109.4_104600001	10	104600001	0.5083	0.6431
NC_005109.4_104700001	10	104700001	0.4669	0.6342
NC_005109.4_104800001	10	104800001	0.3344	0.5153
NC_005109.4_104900001	10	104900001	0.5639	0.719
NC_005109.4_105000001	10	105000001	0.4464	0.6729
NC_005109.4_105100001	10	105100001	0.5301	0.7383
NC_005109.4_105200001	10	105200001	0.5384	0.7044
NC_005109.4_105300001	10	105300001	0.5807	0.7654
NC_005109.4_105400001	10	105400001	0.5442	0.7197
NC_005109.4_105500001	10	105500001	0.5476	0.7153
NC_005109.4_105600001	10	105600001	0.5503	0.6713
NC_005109.4_105700001	10	105700001	0.5936	0.7237
NC_005109.4_105800001	10	105800001	0.5666	0.6757
NC_005109.4_105900001	10	105900001	0.5461	0.6575
NC_005109.4_106000001	10	106000001	0.5754	0.6637
NC_005109.4_106100001	10	106100001	0.5278	0.6314
NC_005109.4_106200001	10	106200001	0.4853	0.5792
NC_005109.4_106300001	10	106300001	0.4239	0.4963
NC_005109.4_106400001	10	106400001	0.4034	0.5412
NC_005109.4_106500001	10	106500001	0.4377	0.6276
NC_005109.4_106600001	10	106600001	0.55	0.7514
NC_005109.4_106700001	10	106700001	0.5126	0.6553
NC_005109.4_106800001	10	106800001	0.5788	0.7251
NC_005109.4_106900001	10	106900001	0.5627	0.7358
NC_005109.4_107000001	10	107000001	0.5288	0.7026
NC_005109.4_107100001	10	107100001	0.4604	0.6799
NC_005109.4_107200001	10	107200001	0.4639	0.715
NC_005109.4_107300001	10	107300001	0.3961	0.6413
NC_005109.4_107400001	10	107400001	0.3993	0.5894
NC_005109.4_107500001	10	107500001	0.3895	0.5512
NC_005109.4_107600001	10	107600001	0.4058	0.5632
NC_005109.4_107700001	10	107700001	0.4265	0.5946
NC_005109.4_107800001	10	107800001	0.4498	0.5778

NC_005109.4_107900001	10	107900001	0.4457	0.5905
NC_005109.4_108000001	10	108000001	0.4454	0.5682
NC_005109.4_108100001	10	108100001	0.4353	0.5823
NC_005109.4_108200001	10	108200001	0.4173	0.573
NC_005109.4_108300001	10	108300001	0.4676	0.5994
NC_005109.4_108400001	10	108400001	0.5007	0.6324
NC_005109.4_108500001	10	108500001	0.599	0.7623
NC_005109.4_108600001	10	108600001	0.6978	0.7994
NC_005109.4_108700001	10	108700001	0.7706	0.7996
NC_005109.4_108800001	10	108800001	0.7419	0.8975
NC_005109.4_108900001	10	108900001	0.6611	0.8472
NC_005109.4_109000001	10	109000001	0.5592	0.7382
NC_005109.4_109100001	10	109100001	0.5184	0.7167
NC_005109.4_109200001	10	109200001	0.5323	0.7205
NC_005109.4_109300001	10	109300001	0.5287	0.6957
NC_005109.4_109400001	10	109400001	0.5596	0.7078
NC_005109.4_109500001	10	109500001	0.6231	0.7357
NC_005109.4_109600001	10	109600001	0.6699	0.7497
NC_005109.4_109700001	10	109700001	0.7186	0.7859
NC_005109.4_109800001	10	109800001	0.6389	0.6544
NC_005109.4_109900001	10	109900001	0.526	0.6111
NC_005109.4_110000001	10	110000001	0.48	0.612
NC_005109.4_110100001	10	110100001	0.4171	0.5609
NC_005109.4_110200001	10	110200001	0.5074	0.6746
NC_005109.4_110300001	10	110300001	0.5948	0.8127
NC_005109.4_110400001	10	110400001	0.6707	0.8435
NC_005109.4_110500001	10	110500001	0.6417	0.8389
NC_005109.4_110600001	10	110600001	0.5889	0.8106
NC_005110.4_1	11	1	0.7143	0.6544
NC_005110.4_100001	11	100001	0.7742	0.6185
NC_005110.4_200001	11	200001	0.738	0.5532
NC_005110.4_500001	11	500001	0.5949	0.8064
NC_005110.4_600001	11	600001	0.6054	0.6901
NC_005110.4_700001	11	700001	0.6054	0.6901
NC_005110.4_800001	11	800001	0.7202	0.7494
NC_005110.4_900001	11	900001	0.7313	0.6935
NC_005110.4_1000001	11	1000001	0.7561	0.7402
NC_005110.4_1200001	11	1200001	0.7664	0.9457
NC_005110.4_1300001	11	1300001	0.7238	0.8997
NC_005110.4_1400001	11	1400001	0.6471	0.8822
NC_005110.4_1500001	11	1500001	0.6477	0.8837
NC_005110.4_1600001	11	1600001	0.6242	0.8389
NC_005110.4_1700001	11	1700001	0.6732	0.8031
NC_005110.4_1800001	11	1800001	0.6945	0.8603
NC_005110.4_1900001	11	1900001	0.7293	0.847
NC_005110.4_2000001	11	2000001	0.7072	0.8326
NC_005110.4_2100001	11	2100001	0.7409	0.8409
NC_005110.4_2200001	11	2200001	0.6116	0.7676
NC_005110.4_2300001	11	2300001	0.5785	0.7051
NC_005110.4_2400001	11	2400001	0.4617	0.6056
NC_005110.4_2500001	11	2500001	0.4527	0.5719
NC_005110.4_2600001	11	2600001	0.3958	0.5062
NC_005110.4_2700001	11	2700001	0.3703	0.5509
NC_005110.4_2800001	11	2800001	0.2959	0.4886
NC_005110.4_2900001	11	2900001	0.5315	0.6819
NC_005110.4_3000001	11	3000001	0.5341	0.6938
NC_005110.4_3100001	11	3100001	0.5341	0.6938
NC_005110.4_3200001	11	3200001	0.5532	0.6747

NC_005110.4_3300001	11	3300001	0.5655	0.6869
NC_005110.4_3400001	11	3400001	0.4961	0.678
NC_005110.4_3500001	11	3500001	0.5033	0.7847
NC_005110.4_3600001	11	3600001	0.5033	0.7847
NC_005110.4_3700001	11	3700001	0.4806	0.7578
NC_005110.4_3800001	11	3800001	0.4209	0.688
NC_005110.4_3900001	11	3900001	0.4858	0.7129
NC_005110.4_4000001	11	4000001	0.4678	0.6868
NC_005110.4_4100001	11	4100001	0.4871	0.6669
NC_005110.4_4200001	11	4200001	0.5528	0.6869
NC_005110.4_4300001	11	4300001	0.577	0.6656
NC_005110.4_4400001	11	4400001	0.6076	0.669
NC_005110.4_4500001	11	4500001	0.5547	0.5939
NC_005110.4_4600001	11	4600001	0.5817	0.6411
NC_005110.4_4700001	11	4700001	0.5836	0.6353
NC_005110.4_4800001	11	4800001	0.6509	0.6549
NC_005110.4_4900001	11	4900001	0.577	0.5918
NC_005110.4_5000001	11	5000001	0.6741	0.7817
NC_005110.4_5100001	11	5100001	0.5371	0.7432
NC_005110.4_5200001	11	5200001	0.4161	0.5689
NC_005110.4_5300001	11	5300001	0.4161	0.5689
NC_005110.4_5400001	11	5400001	0.4546	0.6062
NC_005110.4_5500001	11	5500001	0.4352	0.5436
NC_005110.4_5600001	11	5600001	0.4662	0.5703
NC_005110.4_5800001	11	5800001	0.5346	0.839
NC_005110.4_5900001	11	5900001	0.5296	0.7689
NC_005110.4_6000001	11	6000001	0.4481	0.6483
NC_005110.4_6100001	11	6100001	0.4885	0.6509
NC_005110.4_6200001	11	6200001	0.5442	0.7044
NC_005110.4_6300001	11	6300001	0.5527	0.6696
NC_005110.4_6400001	11	6400001	0.5807	0.7456
NC_005110.4_6500001	11	6500001	0.6682	0.7434
NC_005110.4_6600001	11	6600001	0.642	0.692
NC_005110.4_6700001	11	6700001	0.5552	0.6585
NC_005110.4_6800001	11	6800001	0.4922	0.5915
NC_005110.4_6900001	11	6900001	0.4951	0.5966
NC_005110.4_7000001	11	7000001	0.4684	0.6366
NC_005110.4_7100001	11	7100001	0.4153	0.6297
NC_005110.4_7200001	11	7200001	0.3942	0.5981
NC_005110.4_7300001	11	7300001	0.5893	0.8364
NC_005110.4_7400001	11	7400001	0.5061	0.7125
NC_005110.4_7500001	11	7500001	0.542	0.7194
NC_005110.4_7600001	11	7600001	0.4649	0.6565
NC_005110.4_7700001	11	7700001	0.4391	0.5853
NC_005110.4_7800001	11	7800001	0.3466	0.4525
NC_005110.4_7900001	11	7900001	0.31	0.4212
NC_005110.4_8000001	11	8000001	0.3908	0.6248
NC_005110.4_8100001	11	8100001	0.3915	0.6496
NC_005110.4_8200001	11	8200001	0.3898	0.8223
NC_005110.4_8300001	11	8300001	0.4307	0.7953
NC_005110.4_8400001	11	8400001	0.4157	0.7268
NC_005110.4_8500001	11	8500001	0.3619	0.5923
NC_005110.4_8600001	11	8600001	0.3541	0.5175
NC_005110.4_8700001	11	8700001	0.3878	0.6002
NC_005110.4_8800001	11	8800001	0.3116	0.4757
NC_005110.4_8900001	11	8900001	0.4117	0.6965
NC_005110.4_9000001	11	9000001	0.345	0.6014
NC_005110.4_9100001	11	9100001	0.6918	0.8351

NC_005110.4_9200001	11	9200001	0.7606	0.8396
NC_005110.4_9300001	11	9300001	0.6782	0.7385
NC_005110.4_9400001	11	9400001	0.6011	0.7036
NC_005110.4_9500001	11	9500001	0.6992	0.7522
NC_005110.4_9600001	11	9600001	0.6091	0.683
NC_005110.4_9700001	11	9700001	0.5425	0.5802
NC_005110.4_9800001	11	9800001	0.4795	0.5962
NC_005110.4_9900001	11	9900001	0.6576	0.6918
NC_005110.4_10000001	11	10000001	0.6589	0.6998
NC_005110.4_10100001	11	10100001	0.6462	0.6871
NC_005110.4_10200001	11	10200001	0.6254	0.7115
NC_005110.4_10300001	11	10300001	0.6314	0.7086
NC_005110.4_10400001	11	10400001	0.478	0.6222
NC_005110.4_10500001	11	10500001	0.3685	0.5638
NC_005110.4_10600001	11	10600001	0.3685	0.5638
NC_005110.4_10700001	11	10700001	0.3855	0.6185
NC_005110.4_10800001	11	10800001	0.3939	0.6891
NC_005110.4_10900001	11	10900001	0.417	0.6307
NC_005110.4_11000001	11	11000001	0.4627	0.553
NC_005110.4_11100001	11	11100001	0.5145	0.6198
NC_005110.4_11200001	11	11200001	0.5039	0.6149
NC_005110.4_11300001	11	11300001	0.4775	0.5922
NC_005110.4_11400001	11	11400001	0.4783	0.608
NC_005110.4_11500001	11	11500001	0.4774	0.619
NC_005110.4_11600001	11	11600001	0.4348	0.5735
NC_005110.4_11700001	11	11700001	0.4014	0.5244
NC_005110.4_11800001	11	11800001	0.3715	0.5141
NC_005110.4_11900001	11	11900001	0.4576	0.6252
NC_005110.4_12000001	11	12000001	0.4106	0.6821
NC_005110.4_12100001	11	12100001	0.4106	0.6821
NC_005110.4_12200001	11	12200001	0.5223	0.8789
NC_005110.4_12300001	11	12300001	0.6941	0.9296
NC_005110.4_12400001	11	12400001	0.6759	0.9007
NC_005110.4_12500001	11	12500001	0.6123	0.9343
NC_005110.4_12600001	11	12600001	0.581	0.8108
NC_005110.4_12700001	11	12700001	0.6093	0.8121
NC_005110.4_12800001	11	12800001	0.4797	0.7409
NC_005110.4_12900001	11	12900001	0.456	0.7343
NC_005110.4_13000001	11	13000001	0.5836	0.655
NC_005110.4_13100001	11	13100001	0.7424	0.814
NC_005110.4_13200001	11	13200001	0.728	0.7946
NC_005110.4_13300001	11	13300001	0.885	0.8226
NC_005110.4_13400001	11	13400001	0.87	0.7999
NC_005110.4_13500001	11	13500001	0.8395	0.832
NC_005110.4_13600001	11	13600001	0.6721	0.7056
NC_005110.4_13700001	11	13700001	0.5026	0.6386
NC_005110.4_13800001	11	13800001	0.5426	0.6694
NC_005110.4_13900001	11	13900001	0.5434	0.6841
NC_005110.4_14000001	11	14000001	0.4453	0.5834
NC_005110.4_14100001	11	14100001	0.4368	0.615
NC_005110.4_14200001	11	14200001	0.4718	0.6044
NC_005110.4_14300001	11	14300001	0.4189	0.5511
NC_005110.4_14400001	11	14400001	0.4139	0.5381
NC_005110.4_14500001	11	14500001	0.4304	0.6347
NC_005110.4_14600001	11	14600001	0.4159	0.6074
NC_005110.4_14700001	11	14700001	0.5185	0.7266
NC_005110.4_14800001	11	14800001	0.5702	0.7227
NC_005110.4_14900001	11	14900001	0.6221	0.762

NC_005110.4_15000001	11	15000001	0.4857	0.5559
NC_005110.4_15100001	11	15100001	0.5713	0.6117
NC_005110.4_15200001	11	15200001	0.5169	0.5442
NC_005110.4_15300001	11	15300001	0.5137	0.5776
NC_005110.4_15400001	11	15400001	0.5497	0.6208
NC_005110.4_15500001	11	15500001	0.6867	0.7298
NC_005110.4_15600001	11	15600001	0.6278	0.7099
NC_005110.4_15700001	11	15700001	0.6481	0.6956
NC_005110.4_15800001	11	15800001	0.6635	0.7549
NC_005110.4_15900001	11	15900001	0.6088	0.6154
NC_005110.4_16000001	11	16000001	0.6088	0.6154
NC_005110.4_16100001	11	16100001	0.5825	0.6053
NC_005110.4_16200001	11	16200001	0.6602	0.7066
NC_005110.4_16300001	11	16300001	0.5649	0.5217
NC_005110.4_16400001	11	16400001	0.5798	0.5707
NC_005110.4_16500001	11	16500001	0.62	0.5852
NC_005110.4_16600001	11	16600001	0.5978	0.5815
NC_005110.4_16700001	11	16700001	0.5291	0.523
NC_005110.4_16800001	11	16800001	0.5176	0.5398
NC_005110.4_16900001	11	16900001	0.4827	0.5467
NC_005110.4_17000001	11	17000001	0.4072	0.4987
NC_005110.4_17100001	11	17100001	0.5036	0.5969
NC_005110.4_17200001	11	17200001	0.5226	0.6701
NC_005110.4_17300001	11	17300001	0.5362	0.6545
NC_005110.4_17400001	11	17400001	0.5476	0.6283
NC_005110.4_17500001	11	17500001	0.5624	0.6135
NC_005110.4_17600001	11	17600001	0.5599	0.64
NC_005110.4_17700001	11	17700001	0.5483	0.6227
NC_005110.4_17800001	11	17800001	0.5187	0.6028
NC_005110.4_17900001	11	17900001	0.4851	0.5907
NC_005110.4_18000001	11	18000001	0.4683	0.6492
NC_005110.4_18100001	11	18100001	0.2669	0.3336
NC_005110.4_18200001	11	18200001	0.3047	0.5222
NC_005110.4_18300001	11	18300001	0.6296	0.8136
NC_005110.4_18400001	11	18400001	0.5344	0.744
NC_005110.4_18500001	11	18500001	0.5775	0.7772
NC_005110.4_18600001	11	18600001	0.5061	0.741
NC_005110.4_18700001	11	18700001	0.5607	0.7583
NC_005110.4_18800001	11	18800001	0.4054	0.55
NC_005110.4_18900001	11	18900001	0.3648	0.5047
NC_005110.4_19000001	11	19000001	0.2784	0.3449
NC_005110.4_19100001	11	19100001	0.3361	0.3855
NC_005110.4_19200001	11	19200001	0.4733	0.6291
NC_005110.4_19300001	11	19300001	0.3923	0.6149
NC_005110.4_19400001	11	19400001	0.3993	0.6333
NC_005110.4_19500001	11	19500001	0.4255	0.6485
NC_005110.4_19600001	11	19600001	0.4196	0.5359
NC_005110.4_19700001	11	19700001	0.4377	0.5144
NC_005110.4_19800001	11	19800001	0.5544	0.6253
NC_005110.4_19900001	11	19900001	0.536	0.6056
NC_005110.4_20000001	11	20000001	0.4925	0.5813
NC_005110.4_20100001	11	20100001	0.486	0.6716
NC_005110.4_20200001	11	20200001	0.3483	0.551
NC_005110.4_20300001	11	20300001	0.1624	0.1481
NC_005110.4_20400001	11	20400001	0.4885	0.7097
NC_005110.4_20500001	11	20500001	0.6443	0.7959
NC_005110.4_20600001	11	20600001	0.6438	0.7516
NC_005110.4_20700001	11	20700001	0.6638	0.7513

NC_005110.4_20800001	11	20800001	0.6219	0.7354
NC_005110.4_20900001	11	20900001	0.6183	0.6244
NC_005110.4_21000001	11	21000001	0.5305	0.5076
NC_005110.4_21100001	11	21100001	0.6128	0.5925
NC_005110.4_21200001	11	21200001	0.3963	0.5056
NC_005110.4_21300001	11	21300001	0.406	0.5267
NC_005110.4_21400001	11	21400001	0.3762	0.6742
NC_005110.4_21500001	11	21500001	0.4058	0.7028
NC_005110.4_21600001	11	21600001	0.4729	0.75
NC_005110.4_21700001	11	21700001	0.5232	0.7695
NC_005110.4_21800001	11	21800001	0.6603	0.889
NC_005110.4_21900001	11	21900001	0.5925	0.7705
NC_005110.4_22000001	11	22000001	0.6506	0.8282
NC_005110.4_22100001	11	22100001	0.5611	0.7855
NC_005110.4_22200001	11	22200001	0.5611	0.7855
NC_005110.4_22300001	11	22300001	0.454	0.6354
NC_005110.4_22400001	11	22400001	0.6136	0.7844
NC_005110.4_22500001	11	22500001	0.5916	0.6809
NC_005110.4_22600001	11	22600001	0.5916	0.6809
NC_005110.4_22700001	11	22700001	0.6544	0.7474
NC_005110.4_23100001	11	23100001	0.7104	0.7563
NC_005110.4_23200001	11	23200001	0.6552	0.7008
NC_005110.4_23300001	11	23300001	0.5434	0.7499
NC_005110.4_23400001	11	23400001	0.4753	0.6744
NC_005110.4_23500001	11	23500001	0.5861	0.7734
NC_005110.4_23600001	11	23600001	0.517	0.8482
NC_005110.4_23700001	11	23700001	0.5416	0.8175
NC_005110.4_23800001	11	23800001	0.5071	0.6661
NC_005110.4_23900001	11	23900001	0.4854	0.6429
NC_005110.4_24000001	11	24000001	0.3744	0.4615
NC_005110.4_24100001	11	24100001	0.3857	0.4226
NC_005110.4_24200001	11	24200001	0.3504	0.3932
NC_005110.4_24300001	11	24300001	0.3035	0.3277
NC_005110.4_24400001	11	24400001	0.3124	0.3459
NC_005110.4_24500001	11	24500001	0.2827	0.3639
NC_005110.4_24600001	11	24600001	0.2891	0.3791
NC_005110.4_24700001	11	24700001	0.2736	0.3195
NC_005110.4_24800001	11	24800001	0.279	0.3717
NC_005110.4_24900001	11	24900001	0.2338	0.3164
NC_005110.4_25000001	11	25000001	0.3216	0.3878
NC_005110.4_25100001	11	25100001	0.3798	0.398
NC_005110.4_25200001	11	25200001	0.3859	0.4059
NC_005110.4_25300001	11	25300001	0.3795	0.3661
NC_005110.4_25400001	11	25400001	0.3971	0.387
NC_005110.4_25500001	11	25500001	0.2879	0.2038
NC_005110.4_25600001	11	25600001	0.1374	0.1341
NC_005110.4_25700001	11	25700001	0.2471	0.2632
NC_005110.4_25800001	11	25800001	0.3067	0.4386
NC_005110.4_25900001	11	25900001	0.374	0.5515
NC_005110.4_26000001	11	26000001	0.4089	0.5412
NC_005110.4_26100001	11	26100001	0.4294	0.5437
NC_005110.4_26200001	11	26200001	0.4688	0.637
NC_005110.4_26300001	11	26300001	0.4248	0.6048
NC_005110.4_26400001	11	26400001	0.4505	0.5603
NC_005110.4_26500001	11	26500001	0.4499	0.6016
NC_005110.4_26600001	11	26600001	0.563	0.7148
NC_005110.4_26700001	11	26700001	0.5052	0.6584
NC_005110.4_26800001	11	26800001	0.6055	0.7764

NC_005110.4_26900001	11	26900001	0.5843	0.9249
NC_005110.4_27000001	11	27000001	0.5843	0.9249
NC_005110.4_27300001	11	27300001	0.2702	0.3215
NC_005110.4_27400001	11	27400001	0.4538	0.5886
NC_005110.4_27500001	11	27500001	0.4913	0.5713
NC_005110.4_27600001	11	27600001	0.5253	0.6032
NC_005110.4_27700001	11	27700001	0.5429	0.5837
NC_005110.4_27800001	11	27800001	0.5896	0.5813
NC_005110.4_27900001	11	27900001	0.5287	0.4863
NC_005110.4_28000001	11	28000001	0.4322	0.3964
NC_005110.4_28100001	11	28100001	0.3028	0.2098
NC_005110.4_28200001	11	28200001	0.3134	0.2212
NC_005110.4_28300001	11	28300001	0.2929	0.2428
NC_005110.4_28400001	11	28400001	0.3977	0.3523
NC_005110.4_28500001	11	28500001	0.4246	0.3469
NC_005110.4_28600001	11	28600001	0.4722	0.4318
NC_005110.4_28700001	11	28700001	0.4722	0.4318
NC_005110.4_28800001	11	28800001	0.4874	0.5155
NC_005110.4_28900001	11	28900001	0.4478	0.6438
NC_005110.4_29000001	11	29000001	0.4464	0.7264
NC_005110.4_29100001	11	29100001	0.4321	0.6758
NC_005110.4_29200001	11	29200001	0.3418	0.5176
NC_005110.4_29300001	11	29300001	0.3224	0.4663
NC_005110.4_29400001	11	29400001	0.3332	0.3791
NC_005110.4_29500001	11	29500001	0.3939	0.4788
NC_005110.4_29600001	11	29600001	0.3934	0.4841
NC_005110.4_29700001	11	29700001	0.4563	0.5648
NC_005110.4_29800001	11	29800001	0.5242	0.6164
NC_005110.4_29900001	11	29900001	0.5429	0.6511
NC_005110.4_30000001	11	30000001	0.5604	0.6732
NC_005110.4_30100001	11	30100001	0.5688	0.7
NC_005110.4_30200001	11	30200001	0.5481	0.6642
NC_005110.4_30300001	11	30300001	0.5479	0.6992
NC_005110.4_30400001	11	30400001	0.4874	0.6504
NC_005110.4_30500001	11	30500001	0.433	0.6018
NC_005110.4_30600001	11	30600001	0.4106	0.5847
NC_005110.4_30700001	11	30700001	0.4428	0.662
NC_005110.4_30800001	11	30800001	0.3228	0.5433
NC_005110.4_30900001	11	30900001	0.4061	0.637
NC_005110.4_31000001	11	31000001	0.4878	0.745
NC_005110.4_31100001	11	31100001	0.5405	0.7425
NC_005110.4_31200001	11	31200001	0.5401	0.7187
NC_005110.4_31300001	11	31300001	0.6129	0.769
NC_005110.4_31400001	11	31400001	0.6453	0.8067
NC_005110.4_31500001	11	31500001	0.6122	0.7566
NC_005110.4_31600001	11	31600001	0.6201	0.782
NC_005110.4_31700001	11	31700001	0.6548	0.804
NC_005110.4_31800001	11	31800001	0.6601	0.823
NC_005110.4_31900001	11	31900001	0.6512	0.749
NC_005110.4_32000001	11	32000001	0.5816	0.6579
NC_005110.4_32100001	11	32100001	0.5284	0.6729
NC_005110.4_32200001	11	32200001	0.5542	0.7152
NC_005110.4_32300001	11	32300001	0.4975	0.6692
NC_005110.4_32400001	11	32400001	0.4416	0.6294
NC_005110.4_32500001	11	32500001	0.5062	0.7082
NC_005110.4_32600001	11	32600001	0.5191	0.659
NC_005110.4_32700001	11	32700001	0.4625	0.5828
NC_005110.4_32800001	11	32800001	0.4863	0.586

NC_005110.4_32900001	11	32900001	0.4284	0.574
NC_005110.4_33000001	11	33000001	0.3658	0.4967
NC_005110.4_33100001	11	33100001	0.331	0.4606
NC_005110.4_33200001	11	33200001	0.3226	0.4628
NC_005110.4_33300001	11	33300001	0.3579	0.4969
NC_005110.4_33400001	11	33400001	0.4203	0.5286
NC_005110.4_33500001	11	33500001	0.5136	0.6738
NC_005110.4_33600001	11	33600001	0.5673	0.7372
NC_005110.4_33700001	11	33700001	0.6163	0.7435
NC_005110.4_33800001	11	33800001	0.698	0.8707
NC_005110.4_33900001	11	33900001	0.6658	0.8041
NC_005110.4_34000001	11	34000001	0.5932	0.7104
NC_005110.4_34100001	11	34100001	0.6238	0.719
NC_005110.4_34200001	11	34200001	0.5747	0.6962
NC_005110.4_34300001	11	34300001	0.4995	0.6023
NC_005110.4_34400001	11	34400001	0.4796	0.6163
NC_005110.4_34500001	11	34500001	0.4636	0.5856
NC_005110.4_34600001	11	34600001	0.4064	0.5067
NC_005110.4_34700001	11	34700001	0.5427	0.6297
NC_005110.4_34800001	11	34800001	0.6008	0.6923
NC_005110.4_34900001	11	34900001	0.5897	0.6782
NC_005110.4_35000001	11	35000001	0.5968	0.6968
NC_005110.4_35100001	11	35100001	0.5753	0.7042
NC_005110.4_35200001	11	35200001	0.5001	0.6732
NC_005110.4_35300001	11	35300001	0.5221	0.6507
NC_005110.4_35400001	11	35400001	0.4967	0.6532
NC_005110.4_35500001	11	35500001	0.5043	0.638
NC_005110.4_35600001	11	35600001	0.3923	0.5252
NC_005110.4_35700001	11	35700001	0.4132	0.5308
NC_005110.4_35800001	11	35800001	0.3465	0.477
NC_005110.4_35900001	11	35900001	0.4201	0.5201
NC_005110.4_36000001	11	36000001	0.4264	0.5459
NC_005110.4_36100001	11	36100001	0.5702	0.6853
NC_005110.4_36200001	11	36200001	0.5679	0.6662
NC_005110.4_36300001	11	36300001	0.7384	0.7381
NC_005110.4_36400001	11	36400001	0.6448	0.7325
NC_005110.4_36500001	11	36500001	0.5937	0.6471
NC_005110.4_36600001	11	36600001	0.3722	0.4341
NC_005110.4_36700001	11	36700001	0.4299	0.5399
NC_005110.4_36800001	11	36800001	0.43	0.5544
NC_005110.4_36900001	11	36900001	0.4006	0.4964
NC_005110.4_37000001	11	37000001	0.4408	0.5449
NC_005110.4_37100001	11	37100001	0.4499	0.6075
NC_005110.4_37200001	11	37200001	0.3693	0.4815
NC_005110.4_37300001	11	37300001	0.4191	0.5596
NC_005110.4_37400001	11	37400001	0.4499	0.5538
NC_005110.4_37500001	11	37500001	0.3833	0.5067
NC_005110.4_37600001	11	37600001	0.4079	0.5251
NC_005110.4_37700001	11	37700001	0.4169	0.5442
NC_005110.4_37800001	11	37800001	0.4119	0.4934
NC_005110.4_37900001	11	37900001	0.4699	0.6002
NC_005110.4_38000001	11	38000001	0.5324	0.5904
NC_005110.4_38100001	11	38100001	0.6273	0.5579
NC_005110.4_38200001	11	38200001	0.6261	0.5682
NC_005110.4_38300001	11	38300001	0.6326	0.5472
NC_005110.4_38400001	11	38400001	0.5614	0.4184
NC_005110.4_39400001	11	39400001	0.5761	0.6956
NC_005110.4_39500001	11	39500001	0.5121	0.6368

NC_005110.4_39600001	11	39600001	0.5121	0.6368
NC_005110.4_39700001	11	39700001	0.5121	0.6368
NC_005110.4_39800001	11	39800001	0.4254	0.5705
NC_005110.4_40000001	11	40000001	0.4081	0.501
NC_005110.4_40100001	11	40100001	0.4997	0.5748
NC_005110.4_40200001	11	40200001	0.4997	0.5748
NC_005110.4_40300001	11	40300001	0.6561	0.7104
NC_005110.4_40400001	11	40400001	0.6232	0.6754
NC_005110.4_40500001	11	40500001	0.6048	0.6079
NC_005110.4_40600001	11	40600001	0.5251	0.5454
NC_005110.4_40700001	11	40700001	0.4606	0.4798
NC_005110.4_40800001	11	40800001	0.3364	0.3294
NC_005110.4_40900001	11	40900001	0.3628	0.3416
NC_005110.4_41000001	11	41000001	0.3929	0.4332
NC_005110.4_41100001	11	41100001	0.3815	0.4996
NC_005110.4_41200001	11	41200001	0.5249	0.6302
NC_005110.4_41300001	11	41300001	0.584	0.6571
NC_005110.4_41400001	11	41400001	0.6248	0.7022
NC_005110.4_41500001	11	41500001	0.6674	0.7085
NC_005110.4_41600001	11	41600001	0.6649	0.7165
NC_005110.4_41700001	11	41700001	0.6835	0.7804
NC_005110.4_41800001	11	41800001	0.6599	0.7594
NC_005110.4_41900001	11	41900001	0.6399	0.7746
NC_005110.4_42000001	11	42000001	0.6026	0.7759
NC_005110.4_42100001	11	42100001	0.651	0.7069
NC_005110.4_42200001	11	42200001	0.4233	0.5605
NC_005110.4_42300001	11	42300001	0.4167	0.5786
NC_005110.4_42400001	11	42400001	0.5348	0.6768
NC_005110.4_42500001	11	42500001	0.5287	0.7541
NC_005110.4_42600001	11	42600001	0.5624	0.8232
NC_005110.4_42700001	11	42700001	0.6788	0.8747
NC_005110.4_42800001	11	42800001	0.7157	0.8864
NC_005110.4_42900001	11	42900001	0.642	0.9071
NC_005110.4_43000001	11	43000001	0.6035	0.6652
NC_005110.4_43100001	11	43100001	0.4808	0.4207
NC_005110.4_43800001	11	43800001	0.7451	0.7713
NC_005110.4_43900001	11	43900001	0.6749	0.7628
NC_005110.4_44000001	11	44000001	0.6966	0.783
NC_005110.4_44100001	11	44100001	0.6666	0.7476
NC_005110.4_44200001	11	44200001	0.6789	0.8133
NC_005110.4_44300001	11	44300001	0.5197	0.7992
NC_005110.4_44400001	11	44400001	0.5656	0.8399
NC_005110.4_44500001	11	44500001	0.5529	0.8101
NC_005110.4_44600001	11	44600001	0.5243	0.8165
NC_005110.4_44700001	11	44700001	0.5421	0.8084
NC_005110.4_44800001	11	44800001	0.5707	0.7917
NC_005110.4_44900001	11	44900001	0.5722	0.7939
NC_005110.4_45000001	11	45000001	0.5939	0.8413
NC_005110.4_45100001	11	45100001	0.7008	0.8754
NC_005110.4_45200001	11	45200001	0.6173	0.8165
NC_005110.4_45300001	11	45300001	0.6726	0.8786
NC_005110.4_45400001	11	45400001	0.5986	0.8607
NC_005110.4_45500001	11	45500001	0.4406	0.6896
NC_005110.4_45600001	11	45600001	0.315	0.5146
NC_005110.4_45800001	11	45800001	0.4304	0.5765
NC_005110.4_45900001	11	45900001	0.5029	0.6567
NC_005110.4_46000001	11	46000001	0.5537	0.5999
NC_005110.4_46100001	11	46100001	0.5337	0.6125

NC_005110.4_46200001	11	46200001	0.5719	0.6524
NC_005110.4_46300001	11	46300001	0.5814	0.7072
NC_005110.4_46400001	11	46400001	0.5641	0.6693
NC_005110.4_46500001	11	46500001	0.6164	0.7917
NC_005110.4_46600001	11	46600001	0.793	0.8793
NC_005110.4_46700001	11	46700001	0.582	0.69
NC_005110.4_46800001	11	46800001	0.5666	0.6011
NC_005110.4_46900001	11	46900001	0.614	0.6232
NC_005110.4_47000001	11	47000001	0.6249	0.6387
NC_005110.4_47100001	11	47100001	0.6068	0.6436
NC_005110.4_47200001	11	47200001	0.6357	0.6924
NC_005110.4_47300001	11	47300001	0.6942	0.7512
NC_005110.4_47400001	11	47400001	0.6306	0.7903
NC_005110.4_47500001	11	47500001	0.567	0.812
NC_005110.4_47600001	11	47600001	0.6539	0.859
NC_005110.4_47700001	11	47700001	0.59	0.8731
NC_005110.4_47800001	11	47800001	0.6646	0.9262
NC_005110.4_47900001	11	47900001	0.7443	0.9345
NC_005110.4_48000001	11	48000001	0.702	0.8628
NC_005110.4_48100001	11	48100001	0.6262	0.8273
NC_005110.4_48200001	11	48200001	0.4944	0.7215
NC_005110.4_48300001	11	48300001	0.32	0.4359
NC_005110.4_48400001	11	48400001	0.1919	0.1478
NC_005110.4_48500001	11	48500001	0.1504	0.1106
NC_005110.4_48600001	11	48600001	0.1867	0.1481
NC_005110.4_49500001	11	49500001	0.2273	0.2132
NC_005110.4_49600001	11	49600001	0.2685	0.2841
NC_005110.4_49700001	11	49700001	0.35	0.3149
NC_005110.4_49800001	11	49800001	0.3421	0.3474
NC_005110.4_49900001	11	49900001	0.374	0.3597
NC_005110.4_50000001	11	50000001	0.3131	0.3439
NC_005110.4_50100001	11	50100001	0.2069	0.2044
NC_005110.4_50200001	11	50200001	0.2069	0.2044
NC_005110.4_50300001	11	50300001	0.2095	0.209
NC_005110.4_50400001	11	50400001	0.2215	0.3472
NC_005110.4_50500001	11	50500001	0.4148	0.6268
NC_005110.4_50600001	11	50600001	0.4998	0.7156
NC_005110.4_50700001	11	50700001	0.5314	0.744
NC_005110.4_50800001	11	50800001	0.6086	0.7854
NC_005110.4_50900001	11	50900001	0.5958	0.7654
NC_005110.4_51000001	11	51000001	0.5375	0.7152
NC_005110.4_51100001	11	51100001	0.5324	0.7137
NC_005110.4_51200001	11	51200001	0.5261	0.6845
NC_005110.4_51300001	11	51300001	0.5505	0.7039
NC_005110.4_51400001	11	51400001	0.5165	0.6552
NC_005110.4_51500001	11	51500001	0.5608	0.6851
NC_005110.4_51600001	11	51600001	0.6033	0.6352
NC_005110.4_51700001	11	51700001	0.5696	0.6501
NC_005110.4_51800001	11	51800001	0.5169	0.5593
NC_005110.4_51900001	11	51900001	0.4996	0.554
NC_005110.4_52000001	11	52000001	0.4993	0.5536
NC_005110.4_52100001	11	52100001	0.4587	0.5636
NC_005110.4_52200001	11	52200001	0.3942	0.4779
NC_005110.4_52300001	11	52300001	0.3552	0.5329
NC_005110.4_52400001	11	52400001	0.5632	0.622
NC_005110.4_52500001	11	52500001	0.5798	0.6461
NC_005110.4_52600001	11	52600001	0.6953	0.7231
NC_005110.4_52700001	11	52700001	0.8005	0.7776

NC_005110.4_52800001	11	52800001	0.8272	0.8397
NC_005110.4_52900001	11	52900001	0.8117	0.9081
NC_005110.4_53000001	11	53000001	0.8003	0.904
NC_005110.4_53100001	11	53100001	0.7764	0.89
NC_005110.4_53200001	11	53200001	0.6206	0.8028
NC_005110.4_53300001	11	53300001	0.5163	0.6969
NC_005110.4_53400001	11	53400001	0.4853	0.6258
NC_005110.4_53500001	11	53500001	0.4199	0.5847
NC_005110.4_53600001	11	53600001	0.3857	0.5526
NC_005110.4_53700001	11	53700001	0.4828	0.6562
NC_005110.4_53800001	11	53800001	0.4265	0.6851
NC_005110.4_53900001	11	53900001	0.3725	0.5208
NC_005110.4_54000001	11	54000001	0.5349	0.6111
NC_005110.4_54100001	11	54100001	0.5008	0.5875
NC_005110.4_54200001	11	54200001	0.4271	0.4898
NC_005110.4_54300001	11	54300001	0.4608	0.4901
NC_005110.4_54400001	11	54400001	0.3321	0.4261
NC_005110.4_54500001	11	54500001	0.3321	0.4261
NC_005110.4_54600001	11	54600001	0.3779	0.5261
NC_005110.4_54700001	11	54700001	0.399	0.6145
NC_005110.4_54800001	11	54800001	0.447	0.5573
NC_005110.4_54900001	11	54900001	0.6502	0.7258
NC_005110.4_55000001	11	55000001	0.5876	0.6781
NC_005110.4_55100001	11	55100001	0.5536	0.6424
NC_005110.4_55200001	11	55200001	0.5855	0.6754
NC_005110.4_55300001	11	55300001	0.5801	0.6578
NC_005110.4_55400001	11	55400001	0.4845	0.4564
NC_005110.4_55600001	11	55600001	0.4386	0.4566
NC_005110.4_55700001	11	55700001	0.2158	0.2124
NC_005110.4_55800001	11	55800001	0.2289	0.4641
NC_005110.4_55900001	11	55900001	0.2119	0.3772
NC_005110.4_56000001	11	56000001	0.258	0.506
NC_005110.4_56100001	11	56100001	0.3036	0.4702
NC_005110.4_56200001	11	56200001	0.3519	0.4975
NC_005110.4_56300001	11	56300001	0.3766	0.451
NC_005110.4_56400001	11	56400001	0.3736	0.4248
NC_005110.4_56500001	11	56500001	0.4254	0.4553
NC_005110.4_56600001	11	56600001	0.4656	0.5388
NC_005110.4_56700001	11	56700001	0.4854	0.5449
NC_005110.4_56800001	11	56800001	0.4685	0.5102
NC_005110.4_56900001	11	56900001	0.5788	0.6144
NC_005110.4_57000001	11	57000001	0.5798	0.6005
NC_005110.4_57100001	11	57100001	0.6	0.622
NC_005110.4_57200001	11	57200001	0.6434	0.6778
NC_005110.4_57300001	11	57300001	0.6704	0.689
NC_005110.4_57400001	11	57400001	0.6072	0.6823
NC_005110.4_57500001	11	57500001	0.587	0.7809
NC_005110.4_57600001	11	57600001	0.596	0.7562
NC_005110.4_57700001	11	57700001	0.6278	0.7511
NC_005110.4_57800001	11	57800001	0.6003	0.8032
NC_005110.4_57900001	11	57900001	0.5764	0.7795
NC_005110.4_58000001	11	58000001	0.5442	0.7408
NC_005110.4_58100001	11	58100001	0.6043	0.7453
NC_005110.4_58200001	11	58200001	0.5164	0.6624
NC_005110.4_58300001	11	58300001	0.4989	0.6607
NC_005110.4_58400001	11	58400001	0.5579	0.7267
NC_005110.4_58500001	11	58500001	0.5579	0.7267
NC_005110.4_58600001	11	58600001	0.5049	0.6629

NC_005110.4_58700001	11	58700001	0.4831	0.7371
NC_005110.4_58800001	11	58800001	0.5358	0.7532
NC_005110.4_58900001	11	58900001	0.4278	0.6235
NC_005110.4_59000001	11	59000001	0.4307	0.6113
NC_005110.4_59100001	11	59100001	0.4427	0.6588
NC_005110.4_59200001	11	59200001	0.4552	0.5951
NC_005110.4_59300001	11	59300001	0.4111	0.5534
NC_005110.4_59400001	11	59400001	0.4928	0.6266
NC_005110.4_59500001	11	59500001	0.3625	0.5148
NC_005110.4_59600001	11	59600001	0.2727	0.3526
NC_005110.4_59700001	11	59700001	0.514	0.6536
NC_005110.4_59800001	11	59800001	0.523	0.6255
NC_005110.4_59900001	11	59900001	0.5639	0.667
NC_005110.4_60000001	11	60000001	0.6203	0.6823
NC_005110.4_60100001	11	60100001	0.6419	0.7086
NC_005110.4_60200001	11	60200001	0.4121	0.4564
NC_005110.4_60300001	11	60300001	0.4721	0.6456
NC_005110.4_60400001	11	60400001	0.4016	0.5329
NC_005110.4_60500001	11	60500001	0.4409	0.5894
NC_005110.4_60600001	11	60600001	0.4617	0.6084
NC_005110.4_60700001	11	60700001	0.4892	0.6421
NC_005110.4_60800001	11	60800001	0.5189	0.6061
NC_005110.4_60900001	11	60900001	0.5318	0.6131
NC_005110.4_61000001	11	61000001	0.5925	0.6591
NC_005110.4_61100001	11	61100001	0.5976	0.6462
NC_005110.4_61200001	11	61200001	0.5273	0.5972
NC_005110.4_61300001	11	61300001	0.5232	0.638
NC_005110.4_61400001	11	61400001	0.5785	0.7383
NC_005110.4_61500001	11	61500001	0.508	0.6706
NC_005110.4_61600001	11	61600001	0.5915	0.7088
NC_005110.4_61700001	11	61700001	0.7026	0.7664
NC_005110.4_61800001	11	61800001	0.7282	0.783
NC_005110.4_61900001	11	61900001	0.7567	0.7857
NC_005110.4_62000001	11	62000001	0.7807	0.8104
NC_005110.4_62100001	11	62100001	0.7226	0.8
NC_005110.4_62200001	11	62200001	0.7766	0.8465
NC_005110.4_62300001	11	62300001	0.7605	0.8795
NC_005110.4_62400001	11	62400001	0.7632	0.8429
NC_005110.4_62500001	11	62500001	0.7144	0.8033
NC_005110.4_62600001	11	62600001	0.6975	0.8291
NC_005110.4_62700001	11	62700001	0.6063	0.73
NC_005110.4_62800001	11	62800001	0.5468	0.69
NC_005110.4_62900001	11	62900001	0.463	0.6296
NC_005110.4_63000001	11	63000001	0.4596	0.5452
NC_005110.4_63100001	11	63100001	0.3837	0.4305
NC_005110.4_63200001	11	63200001	0.3687	0.4353
NC_005110.4_63300001	11	63300001	0.3116	0.336
NC_005110.4_63400001	11	63400001	0.2884	0.2621
NC_005110.4_63500001	11	63500001	0.2972	0.3281
NC_005110.4_63600001	11	63600001	0.3326	0.4298
NC_005110.4_63700001	11	63700001	0.4046	0.4803
NC_005110.4_63800001	11	63800001	0.4257	0.5298
NC_005110.4_63900001	11	63900001	0.4078	0.4824
NC_005110.4_64000001	11	64000001	0.4288	0.5184
NC_005110.4_64100001	11	64100001	0.4401	0.4944
NC_005110.4_64200001	11	64200001	0.4048	0.5107
NC_005110.4_64300001	11	64300001	0.4176	0.5045
NC_005110.4_64400001	11	64400001	0.4074	0.4805

NC_005110.4_64500001	11	64500001	0.2921	0.3612
NC_005110.4_64600001	11	64600001	0.3689	0.4931
NC_005110.4_64700001	11	64700001	0.3753	0.5183
NC_005110.4_64800001	11	64800001	0.4127	0.5155
NC_005110.4_64900001	11	64900001	0.4292	0.585
NC_005110.4_65000001	11	65000001	0.6189	0.7132
NC_005110.4_65100001	11	65100001	0.6385	0.6947
NC_005110.4_65200001	11	65200001	0.6615	0.595
NC_005110.4_65300001	11	65300001	0.6157	0.6202
NC_005110.4_65400001	11	65400001	0.67	0.7446
NC_005110.4_65500001	11	65500001	0.6756	0.7669
NC_005110.4_65600001	11	65600001	0.5022	0.648
NC_005110.4_65700001	11	65700001	0.5235	0.6928
NC_005110.4_65800001	11	65800001	0.5477	0.685
NC_005110.4_65900001	11	65900001	0.4635	0.5748
NC_005110.4_66000001	11	66000001	0.4261	0.5248
NC_005110.4_66100001	11	66100001	0.657	0.6911
NC_005110.4_66200001	11	66200001	0.7146	0.6466
NC_005110.4_66300001	11	66300001	0.3501	0.5492
NC_005110.4_66400001	11	66400001	0.4449	0.59
NC_005110.4_66500001	11	66500001	0.4738	0.6247
NC_005110.4_66600001	11	66600001	0.4635	0.6238
NC_005110.4_66700001	11	66700001	0.4712	0.6382
NC_005110.4_66800001	11	66800001	0.5336	0.5912
NC_005110.4_66900001	11	66900001	0.5074	0.5931
NC_005110.4_67000001	11	67000001	0.5082	0.587
NC_005110.4_67100001	11	67100001	0.4578	0.519
NC_005110.4_67200001	11	67200001	0.472	0.5793
NC_005110.4_67300001	11	67300001	0.5548	0.7103
NC_005110.4_67400001	11	67400001	0.5495	0.7019
NC_005110.4_67500001	11	67500001	0.5321	0.7173
NC_005110.4_67600001	11	67600001	0.6561	0.8306
NC_005110.4_67700001	11	67700001	0.4299	0.651
NC_005110.4_67800001	11	67800001	0.1781	0.1505
NC_005110.4_67900001	11	67900001	0.5325	0.6105
NC_005110.4_68000001	11	68000001	0.56	0.6262
NC_005110.4_68100001	11	68100001	0.5661	0.6257
NC_005110.4_68200001	11	68200001	0.6539	0.6719
NC_005110.4_68300001	11	68300001	0.6851	0.7545
NC_005110.4_68400001	11	68400001	0.6532	0.8407
NC_005110.4_68500001	11	68500001	0.6347	0.8678
NC_005110.4_68600001	11	68600001	0.5468	0.7953
NC_005110.4_68700001	11	68700001	0.5237	0.7614
NC_005110.4_68800001	11	68800001	0.4701	0.6924
NC_005110.4_68900001	11	68900001	0.4765	0.6243
NC_005110.4_69000001	11	69000001	0.4862	0.6498
NC_005110.4_69100001	11	69100001	0.5544	0.6603
NC_005110.4_69200001	11	69200001	0.5447	0.6462
NC_005110.4_69300001	11	69300001	0.5779	0.6587
NC_005110.4_69400001	11	69400001	0.5281	0.6367
NC_005110.4_69500001	11	69500001	0.555	0.6418
NC_005110.4_69600001	11	69600001	0.4632	0.6385
NC_005110.4_69700001	11	69700001	0.4448	0.6458
NC_005110.4_69800001	11	69800001	0.4677	0.6825
NC_005110.4_69900001	11	69900001	0.5042	0.7126
NC_005110.4_70000001	11	70000001	0.4663	0.64
NC_005110.4_70100001	11	70100001	0.5119	0.6648
NC_005110.4_70200001	11	70200001	0.5735	0.6957

NC_005110.4_70300001	11	70300001	0.5935	0.6409
NC_005110.4_70400001	11	70400001	0.5558	0.6448
NC_005110.4_70500001	11	70500001	0.5748	0.7007
NC_005110.4_70600001	11	70600001	0.5868	0.6987
NC_005110.4_70700001	11	70700001	0.5249	0.6722
NC_005110.4_70800001	11	70800001	0.5309	0.7501
NC_005110.4_70900001	11	70900001	0.4738	0.7381
NC_005110.4_71000001	11	71000001	0.5169	0.8029
NC_005110.4_71100001	11	71100001	0.5785	0.8403
NC_005110.4_71200001	11	71200001	0.6364	0.8669
NC_005110.4_71300001	11	71300001	0.6152	0.8543
NC_005110.4_71400001	11	71400001	0.6803	0.8404
NC_005110.4_71500001	11	71500001	0.6346	0.7669
NC_005110.4_71600001	11	71600001	0.5723	0.6781
NC_005110.4_71700001	11	71700001	0.5703	0.7075
NC_005110.4_71800001	11	71800001	0.5027	0.6615
NC_005110.4_71900001	11	71900001	0.4773	0.5612
NC_005110.4_72000001	11	72000001	0.4338	0.5556
NC_005110.4_72100001	11	72100001	0.5305	0.6524
NC_005110.4_72200001	11	72200001	0.5453	0.6151
NC_005110.4_72300001	11	72300001	0.5134	0.5644
NC_005110.4_72400001	11	72400001	0.5331	0.6066
NC_005110.4_72500001	11	72500001	0.6079	0.6459
NC_005110.4_72600001	11	72600001	0.4635	0.4919
NC_005110.4_72700001	11	72700001	0.3776	0.4058
NC_005110.4_72800001	11	72800001	0.4496	0.471
NC_005110.4_72900001	11	72900001	0.4464	0.4496
NC_005110.4_73000001	11	73000001	0.4374	0.4786
NC_005110.4_73100001	11	73100001	0.4502	0.5193
NC_005110.4_73200001	11	73200001	0.4461	0.573
NC_005110.4_73300001	11	73300001	0.4687	0.6219
NC_005110.4_73400001	11	73400001	0.4949	0.8482
NC_005110.4_73500001	11	73500001	0.5258	0.8591
NC_005110.4_73600001	11	73600001	0.6206	0.8297
NC_005110.4_73700001	11	73700001	0.5808	0.6806
NC_005110.4_73800001	11	73800001	0.6004	0.6311
NC_005110.4_73900001	11	73900001	0.5702	0.5869
NC_005110.4_74000001	11	74000001	0.5564	0.6207
NC_005110.4_74100001	11	74100001	0.556	0.6254
NC_005110.4_74200001	11	74200001	0.5446	0.6125
NC_005110.4_74300001	11	74300001	0.5507	0.6935
NC_005110.4_74400001	11	74400001	0.5848	0.7433
NC_005110.4_74500001	11	74500001	0.607	0.7242
NC_005110.4_74600001	11	74600001	0.5628	0.6443
NC_005110.4_74700001	11	74700001	0.5897	0.7357
NC_005110.4_74800001	11	74800001	0.5729	0.7006
NC_005110.4_74900001	11	74900001	0.5423	0.7569
NC_005110.4_75000001	11	75000001	0.5264	0.7102
NC_005110.4_75100001	11	75100001	0.5674	0.775
NC_005110.4_75200001	11	75200001	0.5804	0.7547
NC_005110.4_75300001	11	75300001	0.4995	0.6966
NC_005110.4_75400001	11	75400001	0.5004	0.6
NC_005110.4_75500001	11	75500001	0.5215	0.6601
NC_005110.4_75600001	11	75600001	0.4279	0.541
NC_005110.4_75700001	11	75700001	0.4273	0.5547
NC_005110.4_75800001	11	75800001	0.4329	0.5403
NC_005110.4_75900001	11	75900001	0.4067	0.5081
NC_005110.4_76000001	11	76000001	0.3531	0.5083

NC_005110.4_76100001	11	76100001	0.3862	0.5265
NC_005110.4_76200001	11	76200001	0.4645	0.6515
NC_005110.4_76300001	11	76300001	0.5369	0.6608
NC_005110.4_76400001	11	76400001	0.5371	0.6757
NC_005110.4_76500001	11	76500001	0.5614	0.6471
NC_005110.4_76600001	11	76600001	0.5981	0.6709
NC_005110.4_76700001	11	76700001	0.6251	0.6247
NC_005110.4_76800001	11	76800001	0.7111	0.6896
NC_005110.4_76900001	11	76900001	0.7795	0.7798
NC_005110.4_77000001	11	77000001	0.6427	0.7321
NC_005110.4_77100001	11	77100001	0.5919	0.7381
NC_005110.4_77200001	11	77200001	0.594	0.7564
NC_005110.4_77300001	11	77300001	0.585	0.7431
NC_005110.4_77400001	11	77400001	0.5301	0.6888
NC_005110.4_77500001	11	77500001	0.553	0.6634
NC_005110.4_77600001	11	77600001	0.5818	0.6435
NC_005110.4_77700001	11	77700001	0.6057	0.6585
NC_005110.4_77800001	11	77800001	0.5969	0.6418
NC_005110.4_77900001	11	77900001	0.6474	0.7037
NC_005110.4_78000001	11	78000001	0.6205	0.7151
NC_005110.4_78100001	11	78100001	0.5694	0.7149
NC_005110.4_78200001	11	78200001	0.483	0.6879
NC_005110.4_78300001	11	78300001	0.4876	0.6956
NC_005110.4_78400001	11	78400001	0.4242	0.6709
NC_005110.4_78500001	11	78500001	0.4548	0.6504
NC_005110.4_78600001	11	78600001	0.4603	0.6533
NC_005110.4_78700001	11	78700001	0.4238	0.5772
NC_005110.4_78800001	11	78800001	0.4716	0.593
NC_005110.4_78900001	11	78900001	0.4581	0.5775
NC_005110.4_79000001	11	79000001	0.4736	0.6535
NC_005110.4_79100001	11	79100001	0.4885	0.6867
NC_005110.4_79200001	11	79200001	0.5505	0.7555
NC_005110.4_79300001	11	79300001	0.5774	0.8509
NC_005110.4_79400001	11	79400001	0.5945	0.76
NC_005110.4_79500001	11	79500001	0.5841	0.7112
NC_005110.4_79600001	11	79600001	0.5692	0.6807
NC_005110.4_79700001	11	79700001	0.4635	0.6006
NC_005110.4_79800001	11	79800001	0.4033	0.5265
NC_005110.4_79900001	11	79900001	0.405	0.5115
NC_005110.4_80000001	11	80000001	0.4487	0.5697
NC_005110.4_80100001	11	80100001	0.5093	0.5652
NC_005110.4_80200001	11	80200001	0.5203	0.5428
NC_005110.4_80300001	11	80300001	0.5708	0.5946
NC_005110.4_80400001	11	80400001	0.5494	0.6399
NC_005110.4_80500001	11	80500001	0.5316	0.6324
NC_005110.4_80600001	11	80600001	0.5549	0.7034
NC_005110.4_80700001	11	80700001	0.5958	0.7407
NC_005110.4_80800001	11	80800001	0.5614	0.6779
NC_005110.4_80900001	11	80900001	0.6448	0.6974
NC_005110.4_81000001	11	81000001	0.5551	0.6287
NC_005110.4_81100001	11	81100001	0.4683	0.553
NC_005110.4_81200001	11	81200001	0.4871	0.5799
NC_005110.4_81300001	11	81300001	0.4919	0.5813
NC_005110.4_81400001	11	81400001	0.3663	0.4981
NC_005110.4_81500001	11	81500001	0.4174	0.4591
NC_005110.4_81600001	11	81600001	0.4755	0.5924
NC_005110.4_81700001	11	81700001	0.4482	0.5891
NC_005110.4_81800001	11	81800001	0.5081	0.6455

NC_005110.4_81900001	11	81900001	0.5371	0.6566
NC_005110.4_82000001	11	82000001	0.5402	0.7537
NC_005110.4_82100001	11	82100001	0.4995	0.6737
NC_005110.4_82200001	11	82200001	0.6231	0.6933
NC_005110.4_82300001	11	82300001	0.3256	0.3525
NC_005110.4_82400001	11	82400001	0.4833	0.5353
NC_005110.4_82500001	11	82500001	0.4618	0.5157
NC_005110.4_82600001	11	82600001	0.4307	0.502
NC_005110.4_82700001	11	82700001	0.3516	0.4284
NC_005110.4_82800001	11	82800001	0.3485	0.5812
NC_005110.4_82900001	11	82900001	0.3524	0.4727
NC_005110.4_83000001	11	83000001	0.379	0.4735
NC_005110.4_83100001	11	83100001	0.3958	0.4852
NC_005110.4_83200001	11	83200001	0.395	0.4839
NC_005110.4_83300001	11	83300001	0.3852	0.452
NC_005110.4_83400001	11	83400001	0.3383	0.4256
NC_005110.4_83500001	11	83500001	0.4525	0.6218
NC_005110.4_83600001	11	83600001	0.4769	0.6073
NC_005110.4_83700001	11	83700001	0.492	0.6047
NC_005110.4_83800001	11	83800001	0.5804	0.6613
NC_005110.4_83900001	11	83900001	0.5754	0.6467
NC_005110.4_84000001	11	84000001	0.5371	0.5764
NC_005110.4_84100001	11	84100001	0.5835	0.7054
NC_005110.4_84200001	11	84200001	0.5563	0.6914
NC_005110.4_84300001	11	84300001	0.5754	0.6788
NC_005110.4_84400001	11	84400001	0.6322	0.7288
NC_005110.4_84500001	11	84500001	0.6228	0.7678
NC_005110.4_84600001	11	84600001	0.6294	0.6372
NC_005110.4_84700001	11	84700001	0.7852	0.6801
NC_005110.4_85100001	11	85100001	0.392	0.4124
NC_005110.4_85200001	11	85200001	0.3982	0.4992
NC_005110.4_85300001	11	85300001	0.3982	0.4992
NC_005110.4_85900001	11	85900001	0.4925	0.7266
NC_005110.4_86000001	11	86000001	0.4649	0.7733
NC_005110.4_86100001	11	86100001	0.5867	0.8022
NC_005110.4_86200001	11	86200001	0.5729	0.7821
NC_005110.4_86300001	11	86300001	0.6492	0.8337
NC_005110.4_86400001	11	86400001	0.7041	0.8575
NC_005110.4_86500001	11	86500001	0.7276	0.8404
NC_005110.4_86600001	11	86600001	0.6775	0.8323
NC_005110.4_86700001	11	86700001	0.6593	0.794
NC_005110.4_86800001	11	86800001	0.5691	0.7255
NC_005110.4_86900001	11	86900001	0.5356	0.7193
NC_005110.4_87000001	11	87000001	0.5448	0.7414
NC_005110.4_87100001	11	87100001	0.6555	0.8278
NC_005110.4_87200001	11	87200001	0.744	0.9125
NC_005110.4_87300001	11	87300001	0.8169	0.9207
NC_005110.4_87400001	11	87400001	0.7527	0.8428
NC_005110.4_87500001	11	87500001	0.6697	0.7681
NC_005110.4_87600001	11	87600001	0.5997	0.6946
NC_005110.4_87700001	11	87700001	0.6141	0.7266
NC_005110.4_87800001	11	87800001	0.6374	0.718
NC_005110.4_87900001	11	87900001	0.6414	0.7565
NC_005110.4_88000001	11	88000001	0.655	0.7872
NC_005110.4_88100001	11	88100001	0.6743	0.7868
NC_005110.4_88200001	11	88200001	0.5924	0.6948
NC_005110.4_88300001	11	88300001	0.5347	0.6834
NC_005110.4_88400001	11	88400001	0.4988	0.5871

NC_005110.4_88500001	11	88500001	0.4918	0.5917
NC_005110.4_88600001	11	88600001	0.4885	0.6215
NC_005110.4_88700001	11	88700001	0.4615	0.6457
NC_005110.4_88800001	11	88800001	0.4596	0.5901
NC_005110.4_88900001	11	88900001	0.4563	0.5771
NC_005110.4_89000001	11	89000001	0.4855	0.5804
NC_005110.4_89100001	11	89100001	0.4565	0.4594
NC_005110.4_89200001	11	89200001	0.4565	0.4594
NC_005110.4_89300001	11	89300001	0.4499	0.4378
NC_005110.4_89400001	11	89400001	0.3686	0.4218
NC_005110.4_89500001	11	89500001	0.3194	0.3558
NC_005110.4_89600001	11	89600001	0.3008	0.3516
NC_005110.4_89700001	11	89700001	0.3008	0.3516
NC_005110.4_89800001	11	89800001	0.2964	0.3787
NC_005110.4_89900001	11	89900001	0.3814	0.4239
NC_005111.4_100001	12	100001	0.8981	0.9217
NC_005111.4_200001	12	200001	0.64	0.7972
NC_005111.4_300001	12	300001	0.6463	0.8067
NC_005111.4_400001	12	400001	0.6597	0.8077
NC_005111.4_500001	12	500001	0.6492	0.8112
NC_005111.4_600001	12	600001	0.5996	0.7965
NC_005111.4_700001	12	700001	0.6899	0.8425
NC_005111.4_800001	12	800001	0.7298	0.8523
NC_005111.4_900001	12	900001	0.7266	0.8367
NC_005111.4_1000001	12	1000001	0.7098	0.8169
NC_005111.4_1100001	12	1100001	0.7331	0.81
NC_005111.4_1200001	12	1200001	0.7351	0.7711
NC_005111.4_1300001	12	1300001	0.6257	0.8062
NC_005111.4_1400001	12	1400001	0.6373	0.7884
NC_005111.4_1500001	12	1500001	0.6332	0.6965
NC_005111.4_1600001	12	1600001	0.6663	0.7341
NC_005111.4_1700001	12	1700001	0.6725	0.7507
NC_005111.4_1800001	12	1800001	0.6297	0.7031
NC_005111.4_1900001	12	1900001	0.6001	0.7065
NC_005111.4_2000001	12	2000001	0.6088	0.7941
NC_005111.4_2100001	12	2100001	0.5885	0.7785
NC_005111.4_2200001	12	2200001	0.5744	0.7111
NC_005111.4_2300001	12	2300001	0.6372	0.6919
NC_005111.4_2400001	12	2400001	0.7443	0.7477
NC_005111.4_2500001	12	2500001	0.7704	0.7218
NC_005111.4_2600001	12	2600001	0.7276	0.6686
NC_005111.4_2700001	12	2700001	0.6614	0.7077
NC_005111.4_2800001	12	2800001	0.6581	0.7238
NC_005111.4_3700001	12	3700001	0.1898	0.2071
NC_005111.4_4000001	12	4000001	0.4461	0.657
NC_005111.4_4100001	12	4100001	0.5188	0.7594
NC_005111.4_4200001	12	4200001	0.5515	0.7856
NC_005111.4_4300001	12	4300001	0.5499	0.8181
NC_005111.4_4400001	12	4400001	0.5329	0.7919
NC_005111.4_4500001	12	4500001	0.5904	0.8759
NC_005111.4_4600001	12	4600001	0.5026	0.79
NC_005111.4_4700001	12	4700001	0.5605	0.805
NC_005111.4_5100001	12	5100001	0.3175	0.3284
NC_005111.4_5200001	12	5200001	0.5958	0.6448
NC_005111.4_5300001	12	5300001	0.6049	0.6491
NC_005111.4_5400001	12	5400001	0.636	0.6559
NC_005111.4_5500001	12	5500001	0.6393	0.6895
NC_005111.4_5600001	12	5600001	0.712	0.7113

NC_005111.4_5700001	12	5700001	0.6994	0.6658
NC_005111.4_5800001	12	5800001	0.6855	0.7093
NC_005111.4_5900001	12	5900001	0.6804	0.7586
NC_005111.4_6000001	12	6000001	0.6404	0.74
NC_005111.4_6100001	12	6100001	0.6465	0.7259
NC_005111.4_6200001	12	6200001	0.5882	0.7383
NC_005111.4_6300001	12	6300001	0.5349	0.668
NC_005111.4_6400001	12	6400001	0.4941	0.6148
NC_005111.4_6500001	12	6500001	0.5876	0.6745
NC_005111.4_6600001	12	6600001	0.6111	0.6972
NC_005111.4_6700001	12	6700001	0.676	0.692
NC_005111.4_6800001	12	6800001	0.7675	0.7797
NC_005111.4_6900001	12	6900001	0.7216	0.7283
NC_005111.4_7000001	12	7000001	0.724	0.6808
NC_005111.4_7200001	12	7200001	0.5823	0.6262
NC_005111.4_7300001	12	7300001	0.6521	0.6377
NC_005111.4_7400001	12	7400001	0.6606	0.6386
NC_005111.4_7500001	12	7500001	0.55	0.5998
NC_005111.4_7600001	12	7600001	0.5451	0.5605
NC_005111.4_7700001	12	7700001	0.5469	0.5743
NC_005111.4_7800001	12	7800001	0.5702	0.658
NC_005111.4_7900001	12	7900001	0.5589	0.634
NC_005111.4_8000001	12	8000001	0.6095	0.59
NC_005111.4_8100001	12	8100001	0.6188	0.6199
NC_005111.4_8200001	12	8200001	0.6208	0.6026
NC_005111.4_8300001	12	8300001	0.5608	0.482
NC_005111.4_8400001	12	8400001	0.5337	0.441
NC_005111.4_8800001	12	8800001	0.809	0.8391
NC_005111.4_8900001	12	8900001	0.8107	0.8034
NC_005111.4_9000001	12	9000001	0.8107	0.7945
NC_005111.4_9100001	12	9100001	0.793	0.7709
NC_005111.4_9200001	12	9200001	0.6435	0.6782
NC_005111.4_9300001	12	9300001	0.5774	0.6315
NC_005111.4_9400001	12	9400001	0.5408	0.6122
NC_005111.4_9500001	12	9500001	0.4816	0.5584
NC_005111.4_9600001	12	9600001	0.471	0.5834
NC_005111.4_9700001	12	9700001	0.6059	0.7616
NC_005111.4_9800001	12	9800001	0.5821	0.7213
NC_005111.4_9900001	12	9900001	0.5325	0.6155
NC_005111.4_10000001	12	10000001	0.6093	0.6648
NC_005111.4_10100001	12	10100001	0.5856	0.6511
NC_005111.4_10200001	12	10200001	0.5457	0.5681
NC_005111.4_10300001	12	10300001	0.5712	0.5836
NC_005111.4_10400001	12	10400001	0.6347	0.6664
NC_005111.4_10500001	12	10500001	0.477	0.5764
NC_005111.4_10600001	12	10600001	0.4606	0.4937
NC_005111.4_11000001	12	11000001	0.6513	0.7103
NC_005111.4_11100001	12	11100001	0.8336	0.8366
NC_005111.4_11200001	12	11200001	0.8449	0.8484
NC_005111.4_11300001	12	11300001	0.8045	0.8665
NC_005111.4_11400001	12	11400001	0.6585	0.8043
NC_005111.4_11500001	12	11500001	0.595	0.7419
NC_005111.4_11600001	12	11600001	0.4698	0.6264
NC_005111.4_11700001	12	11700001	0.4415	0.5792
NC_005111.4_11800001	12	11800001	0.514	0.6663
NC_005111.4_11900001	12	11900001	0.5235	0.6452
NC_005111.4_12000001	12	12000001	0.6208	0.725
NC_005111.4_12100001	12	12100001	0.6569	0.7622

NC_005111.4_12200001	12	12200001	0.6346	0.8049
NC_005111.4_12300001	12	12300001	0.604	0.7407
NC_005111.4_12400001	12	12400001	0.6406	0.7666
NC_005111.4_12500001	12	12500001	0.6272	0.7922
NC_005111.4_12600001	12	12600001	0.6352	0.7409
NC_005111.4_12700001	12	12700001	0.6809	0.6986
NC_005111.4_12800001	12	12800001	0.6569	0.6895
NC_005111.4_12900001	12	12900001	0.641	0.7224
NC_005111.4_13000001	12	13000001	0.6047	0.6552
NC_005111.4_13100001	12	13100001	0.5689	0.6276
NC_005111.4_13200001	12	13200001	0.5581	0.6265
NC_005111.4_13300001	12	13300001	0.5799	0.6609
NC_005111.4_13400001	12	13400001	0.4885	0.6033
NC_005111.4_13500001	12	13500001	0.473	0.6231
NC_005111.4_13600001	12	13600001	0.5066	0.6939
NC_005111.4_13700001	12	13700001	0.5562	0.7275
NC_005111.4_13800001	12	13800001	0.5435	0.6743
NC_005111.4_13900001	12	13900001	0.6274	0.7297
NC_005111.4_14000001	12	14000001	0.5653	0.7049
NC_005111.4_14100001	12	14100001	0.5711	0.684
NC_005111.4_14200001	12	14200001	0.5007	0.5901
NC_005111.4_14300001	12	14300001	0.5328	0.6273
NC_005111.4_14400001	12	14400001	0.4851	0.546
NC_005111.4_14500001	12	14500001	0.5319	0.5456
NC_005111.4_14600001	12	14600001	0.4594	0.548
NC_005111.4_14700001	12	14700001	0.4143	0.5654
NC_005111.4_14800001	12	14800001	0.4336	0.5992
NC_005111.4_14900001	12	14900001	0.4238	0.579
NC_005111.4_15000001	12	15000001	0.4176	0.5375
NC_005111.4_15100001	12	15100001	0.4126	0.5061
NC_005111.4_15200001	12	15200001	0.4366	0.5378
NC_005111.4_15300001	12	15300001	0.3934	0.4441
NC_005111.4_15400001	12	15400001	0.4946	0.538
NC_005111.4_15500001	12	15500001	0.4913	0.5803
NC_005111.4_15600001	12	15600001	0.5148	0.5806
NC_005111.4_15700001	12	15700001	0.6018	0.7053
NC_005111.4_15800001	12	15800001	0.5839	0.7329
NC_005111.4_15900001	12	15900001	0.5769	0.7586
NC_005111.4_16000001	12	16000001	0.5519	0.7387
NC_005111.4_16100001	12	16100001	0.5639	0.6973
NC_005111.4_16200001	12	16200001	0.5726	0.6907
NC_005111.4_16300001	12	16300001	0.6111	0.7469
NC_005111.4_16400001	12	16400001	0.5739	0.7264
NC_005111.4_16500001	12	16500001	0.6231	0.7526
NC_005111.4_16600001	12	16600001	0.652	0.8368
NC_005111.4_16700001	12	16700001	0.5724	0.7339
NC_005111.4_16800001	12	16800001	0.4868	0.6448
NC_005111.4_16900001	12	16900001	0.5076	0.6637
NC_005111.4_17000001	12	17000001	0.5036	0.6779
NC_005111.4_17100001	12	17100001	0.5326	0.6868
NC_005111.4_17200001	12	17200001	0.5632	0.7732
NC_005111.4_17300001	12	17300001	0.6174	0.7945
NC_005111.4_17400001	12	17400001	0.6344	0.8159
NC_005111.4_17500001	12	17500001	0.5896	0.7433
NC_005111.4_17600001	12	17600001	0.5237	0.6853
NC_005111.4_17700001	12	17700001	0.5944	0.7086
NC_005111.4_17800001	12	17800001	0.5373	0.6518
NC_005111.4_17900001	12	17900001	0.4651	0.5164

NC_005111.4_18000001	12	18000001	0.3916	0.5471
NC_005111.4_18100001	12	18100001	0.2973	0.397
NC_005111.4_18200001	12	18200001	0.2973	0.397
NC_005111.4_18300001	12	18300001	0.3618	0.5992
NC_005111.4_18400001	12	18400001	0.3401	0.5585
NC_005111.4_18500001	12	18500001	0.3261	0.5515
NC_005111.4_18600001	12	18600001	0.3261	0.5515
NC_005111.4_18700001	12	18700001	0.4107	0.6433
NC_005111.4_18800001	12	18800001	0.51	0.6311
NC_005111.4_18900001	12	18900001	0.5423	0.6946
NC_005111.4_19000001	12	19000001	0.5423	0.6946
NC_005111.4_19100001	12	19100001	0.4501	0.6395
NC_005111.4_19200001	12	19200001	0.4382	0.643
NC_005111.4_19300001	12	19300001	0.399	0.6603
NC_005111.4_19400001	12	19400001	0.3857	0.6245
NC_005111.4_19500001	12	19500001	0.3857	0.6245
NC_005111.4_19600001	12	19600001	0.4652	0.6952
NC_005111.4_21700001	12	21700001	0.5807	0.8273
NC_005111.4_21800001	12	21800001	0.6268	0.8741
NC_005111.4_21900001	12	21900001	0.4867	0.7851
NC_005111.4_22000001	12	22000001	0.4867	0.7851
NC_005111.4_22100001	12	22100001	0.5886	0.7784
NC_005111.4_22200001	12	22200001	0.4722	0.6747
NC_005111.4_22300001	12	22300001	0.4416	0.569
NC_005111.4_22400001	12	22400001	0.5306	0.5694
NC_005111.4_22500001	12	22500001	0.482	0.5139
NC_005111.4_22600001	12	22600001	0.4619	0.5187
NC_005111.4_22700001	12	22700001	0.4821	0.5279
NC_005111.4_22800001	12	22800001	0.477	0.5415
NC_005111.4_22900001	12	22900001	0.4785	0.5352
NC_005111.4_23000001	12	23000001	0.5096	0.6126
NC_005111.4_23100001	12	23100001	0.5097	0.6025
NC_005111.4_23200001	12	23200001	0.5386	0.6215
NC_005111.4_23300001	12	23300001	0.4743	0.5869
NC_005111.4_23400001	12	23400001	0.4109	0.5337
NC_005111.4_23500001	12	23500001	0.4864	0.6169
NC_005111.4_23600001	12	23600001	0.4163	0.5751
NC_005111.4_23700001	12	23700001	0.4284	0.6261
NC_005111.4_23800001	12	23800001	0.4608	0.6226
NC_005111.4_23900001	12	23900001	0.5478	0.6668
NC_005111.4_24000001	12	24000001	0.5216	0.636
NC_005111.4_24100001	12	24100001	0.6116	0.6709
NC_005111.4_24200001	12	24200001	0.516	0.592
NC_005111.4_24300001	12	24300001	0.5251	0.6385
NC_005111.4_24400001	12	24400001	0.5226	0.6721
NC_005111.4_24500001	12	24500001	0.4852	0.6401
NC_005111.4_24600001	12	24600001	0.4649	0.6548
NC_005111.4_24700001	12	24700001	0.5355	0.6861
NC_005111.4_24800001	12	24800001	0.5227	0.6653
NC_005111.4_24900001	12	24900001	0.4434	0.6172
NC_005111.4_25000001	12	25000001	0.4416	0.6438
NC_005111.4_25100001	12	25100001	0.4344	0.588
NC_005111.4_25200001	12	25200001	0.394	0.5763
NC_005111.4_25300001	12	25300001	0.3554	0.5598
NC_005111.4_25400001	12	25400001	0.3758	0.5764
NC_005111.4_25500001	12	25500001	0.4189	0.6117
NC_005111.4_25600001	12	25600001	0.45	0.6004
NC_005111.4_25700001	12	25700001	0.4272	0.5941

NC_005111.4_25800001	12	25800001	0.4202	0.5653
NC_005111.4_25900001	12	25900001	0.4582	0.5738
NC_005111.4_26000001	12	26000001	0.4508	0.5558
NC_005111.4_26100001	12	26100001	0.463	0.6009
NC_005111.4_26200001	12	26200001	0.4521	0.632
NC_005111.4_26300001	12	26300001	0.5372	0.6861
NC_005111.4_26400001	12	26400001	0.4953	0.6241
NC_005111.4_26500001	12	26500001	0.448	0.5262
NC_005111.4_26600001	12	26600001	0.4445	0.513
NC_005111.4_26700001	12	26700001	0.4937	0.5283
NC_005111.4_26800001	12	26800001	0.4618	0.4752
NC_005111.4_26900001	12	26900001	0.5	0.5409
NC_005111.4_27000001	12	27000001	0.4411	0.526
NC_005111.4_27100001	12	27100001	0.384	0.4972
NC_005111.4_27200001	12	27200001	0.3845	0.4871
NC_005111.4_27300001	12	27300001	0.3909	0.4903
NC_005111.4_27400001	12	27400001	0.3854	0.476
NC_005111.4_27500001	12	27500001	0.4058	0.4822
NC_005111.4_27600001	12	27600001	0.4074	0.4713
NC_005111.4_27700001	12	27700001	0.362	0.4249
NC_005111.4_27800001	12	27800001	0.3709	0.443
NC_005111.4_27900001	12	27900001	0.3181	0.3474
NC_005111.4_28000001	12	28000001	0.3176	0.3038
NC_005111.4_28100001	12	28100001	0.3255	0.3042
NC_005111.4_28200001	12	28200001	0.3782	0.3087
NC_005111.4_28300001	12	28300001	0.3416	0.2001
NC_005111.4_28400001	12	28400001	0.3834	0.2953
NC_005111.4_28500001	12	28500001	0.4056	0.3678
NC_005111.4_28600001	12	28600001	0.4056	0.3678
NC_005111.4_28700001	12	28700001	0.4926	0.5374
NC_005111.4_28800001	12	28800001	0.4373	0.6081
NC_005111.4_28900001	12	28900001	0.4344	0.5361
NC_005111.4_29000001	12	29000001	0.4039	0.5231
NC_005111.4_29100001	12	29100001	0.4412	0.5849
NC_005111.4_29200001	12	29200001	0.4454	0.5512
NC_005111.4_29300001	12	29300001	0.4683	0.5598
NC_005111.4_29400001	12	29400001	0.5087	0.6239
NC_005111.4_29500001	12	29500001	0.555	0.6686
NC_005111.4_29600001	12	29600001	0.5677	0.6476
NC_005111.4_29700001	12	29700001	0.4918	0.6773
NC_005111.4_29800001	12	29800001	0.4624	0.657
NC_005111.4_29900001	12	29900001	0.4218	0.6496
NC_005111.4_30000001	12	30000001	0.3843	0.6158
NC_005111.4_30100001	12	30100001	0.3796	0.6836
NC_005111.4_30200001	12	30200001	0.3872	0.7097
NC_005111.4_30300001	12	30300001	0.5439	0.7764
NC_005111.4_30400001	12	30400001	0.563	0.7796
NC_005111.4_30500001	12	30500001	0.5075	0.716
NC_005111.4_30600001	12	30600001	0.4253	0.6215
NC_005111.4_30700001	12	30700001	0.4396	0.6205
NC_005111.4_30800001	12	30800001	0.4479	0.6198
NC_005111.4_30900001	12	30900001	0.4602	0.616
NC_005111.4_31000001	12	31000001	0.5213	0.6438
NC_005111.4_31100001	12	31100001	0.5573	0.652
NC_005111.4_31200001	12	31200001	0.5542	0.6347
NC_005111.4_31300001	12	31300001	0.5105	0.585
NC_005111.4_31400001	12	31400001	0.4868	0.5506
NC_005111.4_31500001	12	31500001	0.4179	0.4876

NC_005111.4_31600001	12	31600001	0.4024	0.516
NC_005111.4_31700001	12	31700001	0.4504	0.524
NC_005111.4_31800001	12	31800001	0.4377	0.5046
NC_005111.4_31900001	12	31900001	0.4657	0.553
NC_005111.4_32000001	12	32000001	0.4895	0.5683
NC_005111.4_32100001	12	32100001	0.4776	0.5782
NC_005111.4_32200001	12	32200001	0.4219	0.5474
NC_005111.4_32300001	12	32300001	0.4417	0.6147
NC_005111.4_32400001	12	32400001	0.4419	0.6055
NC_005111.4_32500001	12	32500001	0.4694	0.6166
NC_005111.4_32600001	12	32600001	0.5097	0.6026
NC_005111.4_32700001	12	32700001	0.5692	0.6775
NC_005111.4_32800001	12	32800001	0.4976	0.5946
NC_005111.4_32900001	12	32900001	0.417	0.5107
NC_005111.4_33000001	12	33000001	0.3928	0.5022
NC_005111.4_33100001	12	33100001	0.4123	0.5537
NC_005111.4_33200001	12	33200001	0.3781	0.4886
NC_005111.4_33300001	12	33300001	0.4851	0.6067
NC_005111.4_33400001	12	33400001	0.6407	0.7391
NC_005111.4_33500001	12	33500001	0.7202	0.7173
NC_005111.4_33600001	12	33600001	0.6819	0.6774
NC_005111.4_33700001	12	33700001	0.6345	0.6503
NC_005111.4_33800001	12	33800001	0.454	0.5886
NC_005111.4_33900001	12	33900001	0.3577	0.4931
NC_005111.4_34000001	12	34000001	0.3507	0.5806
NC_005111.4_34100001	12	34100001	0.3143	0.4893
NC_005111.4_34200001	12	34200001	0.3498	0.5361
NC_005111.4_34300001	12	34300001	0.3826	0.5371
NC_005111.4_34400001	12	34400001	0.4026	0.4938
NC_005111.4_34500001	12	34500001	0.3133	0.3674
NC_005111.4_35800001	12	35800001	0.5886	0.7218
NC_005111.4_35900001	12	35900001	0.4463	0.6184
NC_005111.4_36000001	12	36000001	0.4305	0.623
NC_005111.4_36100001	12	36100001	0.4023	0.6195
NC_005111.4_36200001	12	36200001	0.433	0.6223
NC_005111.4_36300001	12	36300001	0.3611	0.5265
NC_005111.4_36400001	12	36400001	0.419	0.6168
NC_005111.4_36500001	12	36500001	0.4527	0.6062
NC_005111.4_36600001	12	36600001	0.4785	0.5727
NC_005111.4_36700001	12	36700001	0.4789	0.567
NC_005111.4_36800001	12	36800001	0.5487	0.6161
NC_005111.4_36900001	12	36900001	0.5455	0.6152
NC_005111.4_37000001	12	37000001	0.5841	0.6625
NC_005111.4_37100001	12	37100001	0.6303	0.7309
NC_005111.4_37200001	12	37200001	0.678	0.7922
NC_005111.4_37300001	12	37300001	0.6824	0.8021
NC_005111.4_37400001	12	37400001	0.6696	0.7359
NC_005111.4_37500001	12	37500001	0.5751	0.7133
NC_005111.4_37600001	12	37600001	0.5439	0.6672
NC_005111.4_37700001	12	37700001	0.5081	0.6125
NC_005111.4_37800001	12	37800001	0.4935	0.6332
NC_005111.4_37900001	12	37900001	0.5247	0.7036
NC_005111.4_38000001	12	38000001	0.5853	0.7053
NC_005111.4_38100001	12	38100001	0.6283	0.7154
NC_005111.4_38200001	12	38200001	0.6225	0.7111
NC_005111.4_38300001	12	38300001	0.6115	0.6551
NC_005111.4_38400001	12	38400001	0.6066	0.6175
NC_005111.4_38500001	12	38500001	0.5114	0.5827

NC_005111.4_38600001	12	38600001	0.5186	0.6222
NC_005111.4_38700001	12	38700001	0.4919	0.6165
NC_005111.4_38800001	12	38800001	0.5024	0.6027
NC_005111.4_38900001	12	38900001	0.4098	0.5749
NC_005111.4_39000001	12	39000001	0.4796	0.6486
NC_005111.4_39100001	12	39100001	0.4546	0.6373
NC_005111.4_39200001	12	39200001	0.4619	0.6449
NC_005111.4_39300001	12	39300001	0.4528	0.6445
NC_005111.4_39400001	12	39400001	0.5972	0.7257
NC_005111.4_39500001	12	39500001	0.5758	0.7172
NC_005111.4_39600001	12	39600001	0.5771	0.712
NC_005111.4_39700001	12	39700001	0.5903	0.7299
NC_005111.4_39800001	12	39800001	0.6251	0.7927
NC_005111.4_39900001	12	39900001	0.6245	0.7621
NC_005111.4_40000001	12	40000001	0.586	0.738
NC_005111.4_40100001	12	40100001	0.6129	0.7349
NC_005111.4_40200001	12	40200001	0.6264	0.741
NC_005111.4_40300001	12	40300001	0.6187	0.7549
NC_005111.4_40400001	12	40400001	0.6303	0.8063
NC_005111.4_40500001	12	40500001	0.6849	0.8151
NC_005111.4_40600001	12	40600001	0.558	0.8044
NC_005111.4_40700001	12	40700001	0.4976	0.7439
NC_005111.4_40800001	12	40800001	0.4383	0.6832
NC_005111.4_40900001	12	40900001	0.3321	0.5314
NC_005111.4_41000001	12	41000001	0.308	0.5196
NC_005111.4_41100001	12	41100001	0.465	0.6049
NC_005111.4_41200001	12	41200001	0.5138	0.6687
NC_005111.4_41300001	12	41300001	0.5112	0.6466
NC_005111.4_41400001	12	41400001	0.5858	0.6935
NC_005111.4_41500001	12	41500001	0.5648	0.6685
NC_005111.4_41600001	12	41600001	0.5398	0.6457
NC_005111.4_41700001	12	41700001	0.532	0.6459
NC_005111.4_41800001	12	41800001	0.5716	0.6796
NC_005111.4_41900001	12	41900001	0.5494	0.6358
NC_005111.4_42000001	12	42000001	0.5288	0.6376
NC_005111.4_42100001	12	42100001	0.5642	0.6563
NC_005111.4_42200001	12	42200001	0.5525	0.6431
NC_005111.4_42300001	12	42300001	0.551	0.6508
NC_005111.4_42400001	12	42400001	0.5535	0.6869
NC_005111.4_42500001	12	42500001	0.6135	0.7076
NC_005111.4_42600001	12	42600001	0.5277	0.6802
NC_005111.4_42700001	12	42700001	0.4549	0.6276
NC_005111.4_42800001	12	42800001	0.4535	0.6288
NC_005111.4_42900001	12	42900001	0.4659	0.6242
NC_005111.4_43000001	12	43000001	0.4665	0.6585
NC_005111.4_43100001	12	43100001	0.555	0.7554
NC_005111.4_43200001	12	43200001	0.6727	0.8266
NC_005111.4_43300001	12	43300001	0.6649	0.8254
NC_005111.4_43400001	12	43400001	0.6793	0.8674
NC_005111.4_43500001	12	43500001	0.673	0.8713
NC_005111.4_43600001	12	43600001	0.5434	0.7736
NC_005111.4_43700001	12	43700001	0.5862	0.7707
NC_005111.4_43800001	12	43800001	0.5902	0.7111
NC_005111.4_43900001	12	43900001	0.5724	0.6837
NC_005111.4_44000001	12	44000001	0.5002	0.5932
NC_005111.4_44100001	12	44100001	0.5908	0.7077
NC_005111.4_44200001	12	44200001	0.5166	0.6488
NC_005111.4_44300001	12	44300001	0.4355	0.6303

NC_005111.4_44400001	12	44400001	0.3757	0.5963
NC_005111.4_44500001	12	44500001	0.3968	0.6297
NC_005111.4_44600001	12	44600001	0.3592	0.5266
NC_005111.4_44700001	12	44700001	0.411	0.5877
NC_005111.4_44800001	12	44800001	0.4544	0.6071
NC_005111.4_44900001	12	44900001	0.4853	0.5975
NC_005111.4_45000001	12	45000001	0.5117	0.6585
NC_005111.4_45100001	12	45100001	0.4977	0.6437
NC_005111.4_45200001	12	45200001	0.4481	0.6173
NC_005111.4_45300001	12	45300001	0.4202	0.5739
NC_005111.4_45400001	12	45400001	0.4153	0.5485
NC_005111.4_45500001	12	45500001	0.4081	0.5149
NC_005111.4_45600001	12	45600001	0.4113	0.5153
NC_005111.4_45700001	12	45700001	0.4289	0.5805
NC_005111.4_45800001	12	45800001	0.4539	0.6307
NC_005111.4_45900001	12	45900001	0.4678	0.6721
NC_005111.4_46000001	12	46000001	0.4799	0.7036
NC_005111.4_46100001	12	46100001	0.4313	0.6539
NC_005111.4_46200001	12	46200001	0.4957	0.7009
NC_005111.4_46300001	12	46300001	0.5064	0.6717
NC_005111.4_46400001	12	46400001	0.5194	0.6863
NC_005111.4_46500001	12	46500001	0.6181	0.7599
NC_005111.4_46600001	12	46600001	0.6788	0.796
NC_005111.4_46700001	12	46700001	0.649	0.748
NC_005111.4_46800001	12	46800001	0.6565	0.7806
NC_005111.4_46900001	12	46900001	0.6725	0.774
NC_005111.4_47000001	12	47000001	0.5772	0.6715
NC_005111.4_47100001	12	47100001	0.615	0.7126
NC_005111.4_47200001	12	47200001	0.6617	0.7301
NC_005111.4_47300001	12	47300001	0.6057	0.6553
NC_005111.4_47400001	12	47400001	0.6038	0.6801
NC_005111.4_47500001	12	47500001	0.6976	0.7681
NC_005111.4_47600001	12	47600001	0.6069	0.6999
NC_005111.4_47700001	12	47700001	0.5614	0.6617
NC_005111.4_47800001	12	47800001	0.6126	0.7199
NC_005111.4_47900001	12	47900001	0.5861	0.7049
NC_005111.4_48000001	12	48000001	0.5269	0.6659
NC_005111.4_48100001	12	48100001	0.6128	0.738
NC_005111.4_48200001	12	48200001	0.7335	0.8549
NC_005111.4_48300001	12	48300001	0.6371	0.7671
NC_005111.4_48400001	12	48400001	0.6213	0.7417
NC_005111.4_48500001	12	48500001	0.5363	0.7014
NC_005111.4_48600001	12	48600001	0.538	0.705
NC_005111.4_48700001	12	48700001	0.4113	0.5548
NC_005111.4_48800001	12	48800001	0.3699	0.5527
NC_005111.4_48900001	12	48900001	0.5918	0.7782
NC_005111.4_49000001	12	49000001	0.7094	0.8233
NC_005111.4_49100001	12	49100001	0.7181	0.8483
NC_005111.4_49200001	12	49200001	0.6162	0.7151
NC_005111.4_49300001	12	49300001	0.5785	0.6725
NC_005111.4_49400001	12	49400001	0.5961	0.6998
NC_005111.4_49500001	12	49500001	0.5865	0.7094
NC_005111.4_49600001	12	49600001	0.5346	0.6562
NC_005111.4_49700001	12	49700001	0.5525	0.7095
NC_005111.4_49800001	12	49800001	0.5654	0.7449
NC_005111.4_49900001	12	49900001	0.5523	0.7228
NC_005111.4_50000001	12	50000001	0.5223	0.6935
NC_005111.4_50100001	12	50100001	0.5232	0.7111

NC_005111.4_50200001	12	50200001	0.5223	0.6789
NC_005111.4_50300001	12	50300001	0.49	0.6172
NC_005111.4_50400001	12	50400001	0.3875	0.5135
NC_005111.4_50500001	12	50500001	0.3928	0.5039
NC_005111.4_50600001	12	50600001	0.4338	0.526
NC_005111.4_50700001	12	50700001	0.4728	0.5581
NC_005111.4_50800001	12	50800001	0.5182	0.6604
NC_005111.4_50900001	12	50900001	0.5501	0.7151
NC_005111.4_51000001	12	51000001	0.5653	0.7319
NC_005111.4_51100001	12	51100001	0.5607	0.7409
NC_005111.4_51200001	12	51200001	0.5531	0.7899
NC_005111.4_51300001	12	51300001	0.5315	0.7578
NC_005111.4_51400001	12	51400001	0.6152	0.7752
NC_005111.4_51500001	12	51500001	0.6467	0.8101
NC_005111.4_51600001	12	51600001	0.7316	0.8722
NC_005111.4_51700001	12	51700001	0.6727	0.8397
NC_005111.4_51800001	12	51800001	0.7043	0.8613
NC_005111.4_51900001	12	51900001	0.6474	0.8664
NC_005111.4_52000001	12	52000001	0.6226	0.7416
NC_005111.4_52100001	12	52100001	0.5754	0.6984
NC_005111.4_52200001	12	52200001	0.5362	0.6034
NC_005112.4_1600001	13	1600001	0.6361	0.6949
NC_005112.4_1700001	13	1700001	0.6361	0.6949
NC_005112.4_1800001	13	1800001	0.6611	0.6826
NC_005112.4_1900001	13	1900001	0.6281	0.7287
NC_005112.4_2000001	13	2000001	0.3645	0.6108
NC_005112.4_2100001	13	2100001	0.1928	0.2991
NC_005112.4_2200001	13	2200001	0.2092	0.2823
NC_005112.4_2300001	13	2300001	0.1505	0.1147
NC_005112.4_2400001	13	2400001	0.1505	0.1147
NC_005112.4_2500001	13	2500001	0.2124	0.1566
NC_005112.4_2600001	13	2600001	0.4224	0.4875
NC_005112.4_2700001	13	2700001	0.3885	0.4979
NC_005112.4_2800001	13	2800001	0.505	0.6775
NC_005112.4_2900001	13	2900001	0.4501	0.6603
NC_005112.4_3000001	13	3000001	0.55	0.7271
NC_005112.4_3100001	13	3100001	0.4711	0.707
NC_005112.4_3200001	13	3200001	0.4711	0.7573
NC_005112.4_3300001	13	3300001	0.4346	0.7281
NC_005112.4_3400001	13	3400001	0.4	0.7175
NC_005112.4_3500001	13	3500001	0.3054	0.6281
NC_005112.4_3600001	13	3600001	0.3563	0.6553
NC_005112.4_3700001	13	3700001	0.3354	0.6327
NC_005112.4_4600001	13	4600001	0.5092	0.7388
NC_005112.4_4700001	13	4700001	0.3084	0.5918
NC_005112.4_4900001	13	4900001	0.205	0.2236
NC_005112.4_5000001	13	5000001	0.3245	0.4038
NC_005112.4_5100001	13	5100001	0.3567	0.5794
NC_005112.4_5200001	13	5200001	0.4761	0.6138
NC_005112.4_5300001	13	5300001	0.4761	0.6138
NC_005112.4_5400001	13	5400001	0.4669	0.7215
NC_005112.4_5900001	13	5900001	0.3098	0.8555
NC_005112.4_6000001	13	6000001	0.3098	0.8555
NC_005112.4_6300001	13	6300001	0.7253	0.7857
NC_005112.4_6400001	13	6400001	0.64	0.7214
NC_005112.4_6500001	13	6500001	0.64	0.7214
NC_005112.4_6600001	13	6600001	0.5339	0.704
NC_005112.4_6700001	13	6700001	0.562	0.7205

NC_005112.4_6800001	13	6800001	0.5261	0.7211
NC_005112.4_6900001	13	6900001	0.5887	0.7778
NC_005112.4_7000001	13	7000001	0.6222	0.801
NC_005112.4_7100001	13	7100001	0.74	0.8175
NC_005112.4_7200001	13	7200001	0.7012	0.8657
NC_005112.4_7300001	13	7300001	0.6587	0.9015
NC_005112.4_7400001	13	7400001	0.5739	0.7826
NC_005112.4_7500001	13	7500001	0.4888	0.711
NC_005112.4_7600001	13	7600001	0.4233	0.6961
NC_005112.4_8200001	13	8200001	0.433	0.6665
NC_005112.4_8300001	13	8300001	0.4207	0.549
NC_005112.4_8400001	13	8400001	0.4001	0.565
NC_005112.4_8500001	13	8500001	0.4159	0.5804
NC_005112.4_8600001	13	8600001	0.4134	0.6589
NC_005112.4_8700001	13	8700001	0.4848	0.6589
NC_005112.4_8800001	13	8800001	0.5946	0.8247
NC_005112.4_8900001	13	8900001	0.7913	0.9348
NC_005112.4_9000001	13	9000001	0.767	0.8643
NC_005112.4_9100001	13	9100001	0.8275	0.8268
NC_005112.4_9500001	13	9500001	0.8032	0.9595
NC_005112.4_9600001	13	9600001	0.6371	0.8076
NC_005112.4_9700001	13	9700001	0.6119	0.8271
NC_005112.4_9800001	13	9800001	0.5583	0.8104
NC_005112.4_9900001	13	9900001	0.5885	0.799
NC_005112.4_10000001	13	10000001	0.4478	0.6804
NC_005112.4_10100001	13	10100001	0.5812	0.8591
NC_005112.4_10200001	13	10200001	0.5848	0.8399
NC_005112.4_10300001	13	10300001	0.622	0.8622
NC_005112.4_10400001	13	10400001	0.5926	0.8982
NC_005112.4_10500001	13	10500001	0.7105	0.9096
NC_005112.4_10600001	13	10600001	0.6773	0.8954
NC_005112.4_10700001	13	10700001	0.7633	0.9234
NC_005112.4_10800001	13	10800001	0.7992	0.9433
NC_005112.4_10900001	13	10900001	0.7992	0.9433
NC_005112.4_11000001	13	11000001	0.6879	0.8854
NC_005112.4_11100001	13	11100001	0.6879	0.8854
NC_005112.4_12000001	13	12000001	0.7413	0.8714
NC_005112.4_12100001	13	12100001	0.7413	0.8714
NC_005112.4_12200001	13	12200001	0.6009	0.7802
NC_005112.4_12300001	13	12300001	0.4855	0.6746
NC_005112.4_12400001	13	12400001	0.4855	0.6746
NC_005112.4_14200001	13	14200001	0.5997	0.4911
NC_005112.4_14300001	13	14300001	0.5997	0.4911
NC_005112.4_14400001	13	14400001	0.601	0.5465
NC_005112.4_14500001	13	14500001	0.499	0.5819
NC_005112.4_14600001	13	14600001	0.451	0.5551
NC_005112.4_14700001	13	14700001	0.5545	0.7205
NC_005112.4_14800001	13	14800001	0.6088	0.7539
NC_005112.4_14900001	13	14900001	0.5897	0.7882
NC_005112.4_15000001	13	15000001	0.5707	0.7707
NC_005112.4_15100001	13	15100001	0.5996	0.7844
NC_005112.4_15200001	13	15200001	0.55	0.686
NC_005112.4_15300001	13	15300001	0.5152	0.6646
NC_005112.4_15400001	13	15400001	0.6402	0.7689
NC_005112.4_15500001	13	15500001	0.6883	0.8049
NC_005112.4_15600001	13	15600001	0.7065	0.7452
NC_005112.4_15700001	13	15700001	0.748	0.8093
NC_005112.4_15800001	13	15800001	0.5894	0.7639

NC_005112.4_15900001	13	15900001	0.3812	0.6162
NC_005112.4_16000001	13	16000001	0.4179	0.6216
NC_005112.4_16100001	13	16100001	0.1947	0.5704
NC_005112.4_16200001	13	16200001	0.2041	0.6719
NC_005112.4_16300001	13	16300001	0.2777	0.7759
NC_005112.4_16500001	13	16500001	0.6247	0.9302
NC_005112.4_16600001	13	16600001	0.7389	0.9521
NC_005112.4_16700001	13	16700001	0.8551	0.9613
NC_005112.4_16800001	13	16800001	0.733	0.9443
NC_005112.4_16900001	13	16900001	0.5656	0.8502
NC_005112.4_17000001	13	17000001	0.5543	0.8455
NC_005112.4_17100001	13	17100001	0.485	0.7995
NC_005112.4_17200001	13	17200001	0.4298	0.4898
NC_005112.4_17300001	13	17300001	0.4769	0.4985
NC_005112.4_17400001	13	17400001	0.584	0.4518
NC_005112.4_17500001	13	17500001	0.3863	0.2453
NC_005112.4_17600001	13	17600001	0.5173	0.4784
NC_005112.4_17700001	13	17700001	0.5581	0.674
NC_005112.4_17800001	13	17800001	0.5581	0.674
NC_005112.4_17900001	13	17900001	0.6182	0.7744
NC_005112.4_18000001	13	18000001	0.6215	0.7508
NC_005112.4_18100001	13	18100001	0.5825	0.7909
NC_005112.4_18200001	13	18200001	0.5825	0.7909
NC_005112.4_18300001	13	18300001	0.6541	0.8415
NC_005112.4_18400001	13	18400001	0.4075	0.6269
NC_005112.4_18600001	13	18600001	0.4438	0.8164
NC_005112.4_18700001	13	18700001	0.383	0.7742
NC_005112.4_18800001	13	18800001	0.3673	0.7484
NC_005112.4_19500001	13	19500001	0.5531	0.7049
NC_005112.4_19600001	13	19600001	0.7458	0.8155
NC_005112.4_19700001	13	19700001	0.73	0.8766
NC_005112.4_19800001	13	19800001	0.679	0.849
NC_005112.4_19900001	13	19900001	0.7011	0.8708
NC_005112.4_20000001	13	20000001	0.674	0.8463
NC_005112.4_20100001	13	20100001	0.5777	0.8297
NC_005112.4_20200001	13	20200001	0.5929	0.7762
NC_005112.4_20300001	13	20300001	0.6345	0.8197
NC_005112.4_20400001	13	20400001	0.6349	0.8345
NC_005112.4_20500001	13	20500001	0.6957	0.8869
NC_005112.4_20600001	13	20600001	0.6601	0.8791
NC_005112.4_20700001	13	20700001	0.6313	0.9296
NC_005112.4_20800001	13	20800001	0.6673	0.9369
NC_005112.4_20900001	13	20900001	0.6673	0.9369
NC_005112.4_21100001	13	21100001	0.8881	0.9808
NC_005112.4_21200001	13	21200001	0.8003	0.9698
NC_005112.4_21300001	13	21300001	0.8339	0.9465
NC_005112.4_21400001	13	21400001	0.8653	0.9567
NC_005112.4_21500001	13	21500001	0.756	0.915
NC_005112.4_21600001	13	21600001	0.6722	0.8893
NC_005112.4_21700001	13	21700001	0.6216	0.8767
NC_005112.4_21800001	13	21800001	0.6553	0.9056
NC_005112.4_21900001	13	21900001	0.6042	0.8855
NC_005112.4_22000001	13	22000001	0.5778	0.8114
NC_005112.4_22100001	13	22100001	0.6316	0.8107
NC_005112.4_22200001	13	22200001	0.736	0.8001
NC_005112.4_22300001	13	22300001	0.5653	0.5976
NC_005112.4_22400001	13	22400001	0.6483	0.6708
NC_005112.4_22500001	13	22500001	0.8777	0.8433

NC_005112.4_22600001	13	22600001	0.8742	0.8324
NC_005112.4_22700001	13	22700001	0.8742	0.8324
NC_005112.4_22800001	13	22800001	0.9053	0.8841
NC_005112.4_23200001	13	23200001	0.5433	0.7484
NC_005112.4_23300001	13	23300001	0.5442	0.6682
NC_005112.4_23400001	13	23400001	0.5442	0.6682
NC_005112.4_23500001	13	23500001	0.5442	0.6682
NC_005112.4_23600001	13	23600001	0.5442	0.6682
NC_005112.4_24000001	13	24000001	0.6135	0.7703
NC_005112.4_24100001	13	24100001	0.6233	0.7896
NC_005112.4_24200001	13	24200001	0.6939	0.8411
NC_005112.4_24300001	13	24300001	0.6672	0.8866
NC_005112.4_24400001	13	24400001	0.6277	0.8344
NC_005112.4_24500001	13	24500001	0.6323	0.8527
NC_005112.4_24600001	13	24600001	0.6962	0.8461
NC_005112.4_24700001	13	24700001	0.5627	0.7976
NC_005112.4_24800001	13	24800001	0.6189	0.7603
NC_005112.4_24900001	13	24900001	0.6723	0.8188
NC_005112.4_25000001	13	25000001	0.6234	0.8049
NC_005112.4_25100001	13	25100001	0.5339	0.7855
NC_005112.4_25200001	13	25200001	0.6027	0.8187
NC_005112.4_25300001	13	25300001	0.6015	0.8986
NC_005112.4_25400001	13	25400001	0.6093	0.8773
NC_005112.4_25500001	13	25500001	0.639	0.8749
NC_005112.4_25600001	13	25600001	0.629	0.8233
NC_005112.4_25700001	13	25700001	0.6255	0.8459
NC_005112.4_25800001	13	25800001	0.5999	0.8408
NC_005112.4_25900001	13	25900001	0.5716	0.8402
NC_005112.4_26000001	13	26000001	0.6415	0.879
NC_005112.4_26100001	13	26100001	0.661	0.9129
NC_005112.4_26200001	13	26200001	0.605	0.8424
NC_005112.4_26300001	13	26300001	0.6173	0.8046
NC_005112.4_26400001	13	26400001	0.5505	0.732
NC_005112.4_26500001	13	26500001	0.553	0.7468
NC_005112.4_26600001	13	26600001	0.5243	0.7161
NC_005112.4_26700001	13	26700001	0.5898	0.7573
NC_005112.4_26800001	13	26800001	0.6075	0.801
NC_005112.4_26900001	13	26900001	0.7662	0.9252
NC_005112.4_27000001	13	27000001	0.5693	0.8802
NC_005112.4_27100001	13	27100001	0.6563	0.9694
NC_005112.4_27200001	13	27200001	0.593	0.9601
NC_005112.4_27400001	13	27400001	0.4618	0.6245
NC_005112.4_27500001	13	27500001	0.653	0.6922
NC_005112.4_27600001	13	27600001	0.6515	0.6889
NC_005112.4_27700001	13	27700001	0.5832	0.6476
NC_005112.4_27800001	13	27800001	0.5673	0.6691
NC_005112.4_27900001	13	27900001	0.6128	0.7313
NC_005112.4_28000001	13	28000001	0.5231	0.6354
NC_005112.4_28100001	13	28100001	0.5919	0.7476
NC_005112.4_28200001	13	28200001	0.5962	0.7758
NC_005112.4_28300001	13	28300001	0.5804	0.7132
NC_005112.4_28400001	13	28400001	0.6149	0.7679
NC_005112.4_28500001	13	28500001	0.6179	0.813
NC_005112.4_28600001	13	28600001	0.3802	0.5486
NC_005112.4_28700001	13	28700001	0.4108	0.5139
NC_005112.4_28800001	13	28800001	0.4249	0.5641
NC_005112.4_28900001	13	28900001	0.2442	0.2292
NC_005112.4_29000001	13	29000001	0.2784	0.2438

NC_005112.4_29800001	13	29800001	0.8819	0.7919
NC_005112.4_29900001	13	29900001	0.8819	0.7919
NC_005112.4_30000001	13	30000001	0.9031	0.83
NC_005112.4_30100001	13	30100001	0.9186	0.8562
NC_005112.4_30200001	13	30200001	0.9155	0.8454
NC_005112.4_30300001	13	30300001	0.8458	0.9444
NC_005112.4_30400001	13	30400001	0.8458	0.9444
NC_005112.4_30500001	13	30500001	0.7246	0.7483
NC_005112.4_30600001	13	30600001	0.7532	0.7767
NC_005112.4_30700001	13	30700001	0.7045	0.7436
NC_005112.4_30800001	13	30800001	0.6376	0.6979
NC_005112.4_30900001	13	30900001	0.6376	0.6979
NC_005112.4_31100001	13	31100001	0.3289	0.4689
NC_005112.4_31200001	13	31200001	0.3289	0.4689
NC_005112.4_31300001	13	31300001	0.3923	0.4794
NC_005112.4_31400001	13	31400001	0.4879	0.6061
NC_005112.4_31500001	13	31500001	0.465	0.5754
NC_005112.4_31900001	13	31900001	0.3778	0.5612
NC_005112.4_32100001	13	32100001	0.5373	0.5254
NC_005112.4_32200001	13	32200001	0.472	0.5456
NC_005112.4_32300001	13	32300001	0.4779	0.5881
NC_005112.4_32400001	13	32400001	0.5047	0.6723
NC_005112.4_32500001	13	32500001	0.5086	0.6794
NC_005112.4_32600001	13	32600001	0.3996	0.7201
NC_005112.4_32700001	13	32700001	0.392	0.7136
NC_005112.4_32800001	13	32800001	0.2981	0.6366
NC_005112.4_33900001	13	33900001	0.8002	0.944
NC_005112.4_34000001	13	34000001	0.6728	0.9215
NC_005112.4_34100001	13	34100001	0.7427	0.9503
NC_005112.4_34200001	13	34200001	0.7169	0.9499
NC_005112.4_34300001	13	34300001	0.6502	0.8782
NC_005112.4_34400001	13	34400001	0.6444	0.8652
NC_005112.4_34500001	13	34500001	0.6806	0.8453
NC_005112.4_34600001	13	34600001	0.6638	0.8214
NC_005112.4_34700001	13	34700001	0.6868	0.8074
NC_005112.4_34800001	13	34800001	0.6343	0.8291
NC_005112.4_34900001	13	34900001	0.506	0.7456
NC_005112.4_35000001	13	35000001	0.5051	0.7446
NC_005112.4_35100001	13	35100001	0.523	0.7167
NC_005112.4_35200001	13	35200001	0.4988	0.7237
NC_005112.4_35300001	13	35300001	0.5576	0.6237
NC_005112.4_35400001	13	35400001	0.6933	0.6633
NC_005112.4_35500001	13	35500001	0.6073	0.6595
NC_005112.4_35600001	13	35600001	0.6152	0.7035
NC_005112.4_35700001	13	35700001	0.5413	0.6311
NC_005112.4_35800001	13	35800001	0.4978	0.6002
NC_005112.4_35900001	13	35900001	0.4578	0.573
NC_005112.4_36000001	13	36000001	0.4259	0.4931
NC_005112.4_36100001	13	36100001	0.3646	0.4048
NC_005112.4_36200001	13	36200001	0.4837	0.5809
NC_005112.4_36300001	13	36300001	0.5306	0.7171
NC_005112.4_36400001	13	36400001	0.6119	0.7016
NC_005112.4_36500001	13	36500001	0.6429	0.7433
NC_005112.4_36600001	13	36600001	0.5592	0.7201
NC_005112.4_36700001	13	36700001	0.4321	0.6306
NC_005112.4_36800001	13	36800001	0.3951	0.5953
NC_005112.4_36900001	13	36900001	0.3648	0.662
NC_005112.4_37000001	13	37000001	0.441	0.6982

NC_005112.4_37100001	13	37100001	0.4663	0.6975
NC_005112.4_37200001	13	37200001	0.5542	0.7329
NC_005112.4_37300001	13	37300001	0.6563	0.7719
NC_005112.4_37400001	13	37400001	0.5874	0.7344
NC_005112.4_37500001	13	37500001	0.4606	0.6003
NC_005112.4_37600001	13	37600001	0.4659	0.5771
NC_005112.4_37700001	13	37700001	0.4524	0.5274
NC_005112.4_37800001	13	37800001	0.3158	0.3303
NC_005112.4_37900001	13	37900001	0.3218	0.3086
NC_005112.4_38000001	13	38000001	0.3556	0.3314
NC_005112.4_38100001	13	38100001	0.3042	0.2429
NC_005112.4_38200001	13	38200001	0.3504	0.4024
NC_005112.4_38300001	13	38300001	0.3469	0.3785
NC_005112.4_38400001	13	38400001	0.3918	0.5392
NC_005112.4_38500001	13	38500001	0.3918	0.5392
NC_005112.4_38600001	13	38600001	0.3863	0.4656
NC_005112.4_38700001	13	38700001	0.3326	0.397
NC_005112.4_38800001	13	38800001	0.3986	0.503
NC_005112.4_38900001	13	38900001	0.5071	0.5459
NC_005112.4_39000001	13	39000001	0.4686	0.5194
NC_005112.4_39100001	13	39100001	0.4465	0.5331
NC_005112.4_39200001	13	39200001	0.4653	0.5182
NC_005112.4_39300001	13	39300001	0.4623	0.5184
NC_005112.4_39400001	13	39400001	0.4596	0.5138
NC_005112.4_39500001	13	39500001	0.4459	0.5722
NC_005112.4_39600001	13	39600001	0.4796	0.6153
NC_005112.4_39700001	13	39700001	0.4923	0.6598
NC_005112.4_39800001	13	39800001	0.4398	0.592
NC_005112.4_39900001	13	39900001	0.4788	0.6139
NC_005112.4_40000001	13	40000001	0.4921	0.6283
NC_005112.4_40100001	13	40100001	0.503	0.6661
NC_005112.4_40200001	13	40200001	0.5122	0.6777
NC_005112.4_40300001	13	40300001	0.4823	0.6555
NC_005112.4_40400001	13	40400001	0.3895	0.5176
NC_005112.4_40500001	13	40500001	0.4191	0.5602
NC_005112.4_40600001	13	40600001	0.4179	0.5462
NC_005112.4_40700001	13	40700001	0.4322	0.5474
NC_005112.4_40800001	13	40800001	0.5205	0.5842
NC_005112.4_40900001	13	40900001	0.5067	0.5927
NC_005112.4_41000001	13	41000001	0.5602	0.6252
NC_005112.4_41100001	13	41100001	0.6126	0.6843
NC_005112.4_41200001	13	41200001	0.6618	0.7061
NC_005112.4_41300001	13	41300001	0.6326	0.7645
NC_005112.4_41400001	13	41400001	0.6331	0.7897
NC_005112.4_41500001	13	41500001	0.5739	0.7346
NC_005112.4_41600001	13	41600001	0.5483	0.6827
NC_005112.4_41700001	13	41700001	0.5005	0.6547
NC_005112.4_41800001	13	41800001	0.4849	0.5746
NC_005112.4_41900001	13	41900001	0.5405	0.58
NC_005112.4_42000001	13	42000001	0.5235	0.5623
NC_005112.4_42100001	13	42100001	0.5055	0.5779
NC_005112.4_42200001	13	42200001	0.4786	0.5983
NC_005112.4_42300001	13	42300001	0.4959	0.6573
NC_005112.4_42400001	13	42400001	0.4703	0.6447
NC_005112.4_42500001	13	42500001	0.5061	0.6164
NC_005112.4_42600001	13	42600001	0.4789	0.5827
NC_005112.4_42700001	13	42700001	0.4663	0.5073
NC_005112.4_42800001	13	42800001	0.4148	0.4736

NC_005112.4_42900001	13	42900001	0.3922	0.4377
NC_005112.4_43000001	13	43000001	0.317	0.3674
NC_005112.4_43100001	13	43100001	0.3041	0.3185
NC_005112.4_43200001	13	43200001	0.3545	0.46
NC_005112.4_43300001	13	43300001	0.4005	0.4665
NC_005112.4_43400001	13	43400001	0.42	0.5483
NC_005112.4_43600001	13	43600001	0.7106	0.8222
NC_005112.4_43700001	13	43700001	0.7315	0.7324
NC_005112.4_43800001	13	43800001	0.5802	0.6769
NC_005112.4_43900001	13	43900001	0.5799	0.6465
NC_005112.4_44000001	13	44000001	0.5765	0.6331
NC_005112.4_44100001	13	44100001	0.5317	0.5889
NC_005112.4_44200001	13	44200001	0.3674	0.4008
NC_005112.4_44300001	13	44300001	0.4773	0.4394
NC_005112.4_44400001	13	44400001	0.5414	0.567
NC_005112.4_44500001	13	44500001	0.5406	0.626
NC_005112.4_44600001	13	44600001	0.5207	0.6334
NC_005112.4_44700001	13	44700001	0.4426	0.5676
NC_005112.4_44800001	13	44800001	0.4188	0.5434
NC_005112.4_44900001	13	44900001	0.3916	0.4961
NC_005112.4_45000001	13	45000001	0.403	0.4483
NC_005112.4_45100001	13	45100001	0.4196	0.4359
NC_005112.4_45200001	13	45200001	0.4596	0.4543
NC_005112.4_45300001	13	45300001	0.5289	0.5041
NC_005112.4_45400001	13	45400001	0.5596	0.49
NC_005112.4_45500001	13	45500001	0.5467	0.5064
NC_005112.4_45600001	13	45600001	0.555	0.5294
NC_005112.4_45700001	13	45700001	0.542	0.6495
NC_005112.4_45800001	13	45800001	0.5783	0.8083
NC_005112.4_45900001	13	45900001	0.589	0.8868
NC_005112.4_46000001	13	46000001	0.5957	0.9016
NC_005112.4_46100001	13	46100001	0.5723	0.9032
NC_005112.4_46200001	13	46200001	0.6186	0.9056
NC_005112.4_46300001	13	46300001	0.5055	0.8212
NC_005112.4_46400001	13	46400001	0.4593	0.7256
NC_005112.4_46500001	13	46500001	0.4392	0.6115
NC_005112.4_46600001	13	46600001	0.4606	0.6139
NC_005112.4_46700001	13	46700001	0.4712	0.6222
NC_005112.4_46800001	13	46800001	0.4251	0.6417
NC_005112.4_46900001	13	46900001	0.4837	0.694
NC_005112.4_47000001	13	47000001	0.5495	0.8055
NC_005112.4_47100001	13	47100001	0.5458	0.7298
NC_005112.4_47200001	13	47200001	0.4498	0.679
NC_005112.4_47300001	13	47300001	0.4907	0.6806
NC_005112.4_47400001	13	47400001	0.4471	0.6255
NC_005112.4_47500001	13	47500001	0.3791	0.5671
NC_005112.4_47600001	13	47600001	0.2824	0.4594
NC_005112.4_47700001	13	47700001	0.3727	0.4505
NC_005112.4_47800001	13	47800001	0.4335	0.6126
NC_005112.4_47900001	13	47900001	0.4694	0.6468
NC_005112.4_48000001	13	48000001	0.5288	0.613
NC_005112.4_48100001	13	48100001	0.5465	0.6168
NC_005112.4_48200001	13	48200001	0.5464	0.6195
NC_005112.4_48300001	13	48300001	0.6029	0.6145
NC_005112.4_48400001	13	48400001	0.5648	0.5915
NC_005112.4_48500001	13	48500001	0.6037	0.656
NC_005112.4_48600001	13	48600001	0.5521	0.6867
NC_005112.4_48700001	13	48700001	0.5299	0.6511

NC_005112.4_48800001	13	48800001	0.5108	0.6442
NC_005112.4_48900001	13	48900001	0.5213	0.65
NC_005112.4_49000001	13	49000001	0.4078	0.6006
NC_005112.4_49100001	13	49100001	0.454	0.604
NC_005112.4_49200001	13	49200001	0.5488	0.7008
NC_005112.4_49300001	13	49300001	0.5002	0.6726
NC_005112.4_49400001	13	49400001	0.4835	0.6419
NC_005112.4_49500001	13	49500001	0.4718	0.605
NC_005112.4_49600001	13	49600001	0.5115	0.6473
NC_005112.4_49700001	13	49700001	0.3391	0.4949
NC_005112.4_49800001	13	49800001	0.4288	0.5684
NC_005112.4_49900001	13	49900001	0.5103	0.6914
NC_005112.4_50000001	13	50000001	0.5774	0.7627
NC_005112.4_50100001	13	50100001	0.5629	0.7568
NC_005112.4_50200001	13	50200001	0.666	0.8322
NC_005112.4_50300001	13	50300001	0.6733	0.8209
NC_005112.4_50400001	13	50400001	0.621	0.8137
NC_005112.4_50500001	13	50500001	0.5981	0.8061
NC_005112.4_50600001	13	50600001	0.5852	0.7465
NC_005112.4_50700001	13	50700001	0.52	0.6995
NC_005112.4_50800001	13	50800001	0.4858	0.6453
NC_005112.4_50900001	13	50900001	0.5855	0.7328
NC_005112.4_51000001	13	51000001	0.5705	0.6989
NC_005112.4_51100001	13	51100001	0.5117	0.6179
NC_005112.4_51200001	13	51200001	0.5281	0.6196
NC_005112.4_51300001	13	51300001	0.5273	0.6081
NC_005112.4_51400001	13	51400001	0.4787	0.5511
NC_005112.4_51500001	13	51500001	0.4795	0.5581
NC_005112.4_51600001	13	51600001	0.5114	0.6149
NC_005112.4_51700001	13	51700001	0.4977	0.5833
NC_005112.4_51800001	13	51800001	0.4662	0.5951
NC_005112.4_51900001	13	51900001	0.4334	0.5984
NC_005112.4_52000001	13	52000001	0.4605	0.5982
NC_005112.4_52100001	13	52100001	0.4185	0.5462
NC_005112.4_52200001	13	52200001	0.416	0.55
NC_005112.4_52300001	13	52300001	0.5108	0.6219
NC_005112.4_52400001	13	52400001	0.5554	0.6478
NC_005112.4_52500001	13	52500001	0.5782	0.6894
NC_005112.4_52600001	13	52600001	0.5862	0.7008
NC_005112.4_52700001	13	52700001	0.6121	0.7275
NC_005112.4_52800001	13	52800001	0.545	0.7001
NC_005112.4_52900001	13	52900001	0.5747	0.7351
NC_005112.4_53000001	13	53000001	0.5385	0.694
NC_005112.4_53100001	13	53100001	0.5274	0.6704
NC_005112.4_53200001	13	53200001	0.4329	0.5419
NC_005112.4_53300001	13	53300001	0.5138	0.5965
NC_005112.4_53400001	13	53400001	0.5508	0.6191
NC_005112.4_53500001	13	53500001	0.5272	0.6335
NC_005112.4_53600001	13	53600001	0.529	0.6163
NC_005112.4_53700001	13	53700001	0.579	0.6584
NC_005112.4_53800001	13	53800001	0.5759	0.6565
NC_005112.4_53900001	13	53900001	0.505	0.6034
NC_005112.4_54000001	13	54000001	0.547	0.5959
NC_005112.4_54100001	13	54100001	0.4468	0.5813
NC_005112.4_54200001	13	54200001	0.3672	0.56
NC_005112.4_54300001	13	54300001	0.3418	0.5449
NC_005112.4_54400001	13	54400001	0.2899	0.4674
NC_005112.4_54500001	13	54500001	0.2697	0.452

NC_005112.4_54600001	13	54600001	0.3965	0.5728
NC_005112.4_54700001	13	54700001	0.4124	0.5465
NC_005112.4_54800001	13	54800001	0.4282	0.5963
NC_005112.4_54900001	13	54900001	0.4821	0.6741
NC_005112.4_55000001	13	55000001	0.4347	0.6379
NC_005112.4_55100001	13	55100001	0.4359	0.5972
NC_005112.4_55200001	13	55200001	0.5027	0.6881
NC_005112.4_55300001	13	55300001	0.4286	0.5896
NC_005112.4_55400001	13	55400001	0.5027	0.6681
NC_005112.4_55500001	13	55500001	0.5867	0.7158
NC_005112.4_55600001	13	55600001	0.5571	0.755
NC_005112.4_55700001	13	55700001	0.5573	0.7533
NC_005112.4_55800001	13	55800001	0.5824	0.7715
NC_005112.4_55900001	13	55900001	0.6149	0.7596
NC_005112.4_56000001	13	56000001	0.5577	0.7245
NC_005112.4_56100001	13	56100001	0.5884	0.7449
NC_005112.4_56200001	13	56200001	0.4401	0.5693
NC_005112.4_56300001	13	56300001	0.4732	0.5957
NC_005112.4_56400001	13	56400001	0.2943	0.3922
NC_005112.4_56600001	13	56600001	0.5843	0.7038
NC_005112.4_56700001	13	56700001	0.6335	0.7498
NC_005112.4_56800001	13	56800001	0.5012	0.6606
NC_005112.4_56900001	13	56900001	0.4677	0.5553
NC_005112.4_57000001	13	57000001	0.4448	0.495
NC_005112.4_57100001	13	57100001	0.3183	0.307
NC_005112.4_57200001	13	57200001	0.3984	0.3505
NC_005112.4_57300001	13	57300001	0.4349	0.4657
NC_005112.4_57400001	13	57400001	0.5716	0.6443
NC_005112.4_57500001	13	57500001	0.5716	0.6443
NC_005112.4_57600001	13	57600001	0.5888	0.6321
NC_005112.4_57700001	13	57700001	0.6035	0.6723
NC_005112.4_57800001	13	57800001	0.7523	0.6293
NC_005112.4_57900001	13	57900001	0.5052	0.3852
NC_005112.4_58000001	13	58000001	0.3796	0.3601
NC_005112.4_58100001	13	58100001	0.3312	0.3524
NC_005112.4_58200001	13	58200001	0.3391	0.4225
NC_005112.4_58300001	13	58300001	0.4093	0.5084
NC_005112.4_58400001	13	58400001	0.4695	0.6557
NC_005112.4_58500001	13	58500001	0.5475	0.5854
NC_005112.4_58600001	13	58600001	0.4811	0.5089
NC_005112.4_58700001	13	58700001	0.3886	0.4596
NC_005112.4_58800001	13	58800001	0.3311	0.3754
NC_005112.4_58900001	13	58900001	0.2163	0.2054
NC_005112.4_59000001	13	59000001	0.1749	0.1985
NC_005112.4_59100001	13	59100001	0.0696	0.0366
NC_005112.4_59500001	13	59500001	0.428	0.6089
NC_005112.4_59600001	13	59600001	0.4646	0.6419
NC_005112.4_59700001	13	59700001	0.4661	0.6649
NC_005112.4_59800001	13	59800001	0.4408	0.6073
NC_005112.4_59900001	13	59900001	0.3694	0.5388
NC_005112.4_60000001	13	60000001	0.6506	0.726
NC_005112.4_60100001	13	60100001	0.6483	0.7108
NC_005112.4_60200001	13	60200001	0.6321	0.62
NC_005112.4_60400001	13	60400001	0.7636	0.8133
NC_005112.4_60500001	13	60500001	0.4513	0.7573
NC_005112.4_60600001	13	60600001	0.3573	0.6818
NC_005112.4_60700001	13	60700001	0.4182	0.7094
NC_005112.4_60800001	13	60800001	0.4545	0.6788

NC_005112.4_60900001	13	60900001	0.4253	0.6577
NC_005112.4_61000001	13	61000001	0.5208	0.6839
NC_005112.4_61100001	13	61100001	0.526	0.6933
NC_005112.4_61200001	13	61200001	0.5416	0.6614
NC_005112.4_61300001	13	61300001	0.448	0.7107
NC_005112.4_61400001	13	61400001	0.5034	0.6973
NC_005112.4_61500001	13	61500001	0.4693	0.706
NC_005112.4_61600001	13	61600001	0.5429	0.6759
NC_005112.4_61700001	13	61700001	0.5521	0.6871
NC_005112.4_61800001	13	61800001	0.6102	0.6275
NC_005112.4_61900001	13	61900001	0.5108	0.5217
NC_005112.4_62000001	13	62000001	0.4754	0.4826
NC_005112.4_62100001	13	62100001	0.4828	0.5189
NC_005112.4_62200001	13	62200001	0.4712	0.5207
NC_005112.4_62300001	13	62300001	0.4527	0.6205
NC_005112.4_62400001	13	62400001	0.542	0.729
NC_005112.4_62500001	13	62500001	0.5884	0.7758
NC_005112.4_62600001	13	62600001	0.5338	0.7275
NC_005112.4_62700001	13	62700001	0.4632	0.6531
NC_005112.4_62900001	13	62900001	0.447	0.6588
NC_005112.4_63000001	13	63000001	0.4634	0.6658
NC_005112.4_63100001	13	63100001	0.3895	0.5688
NC_005112.4_63200001	13	63200001	0.3622	0.5351
NC_005112.4_63300001	13	63300001	0.3751	0.5193
NC_005112.4_63400001	13	63400001	0.2966	0.3467
NC_005112.4_63500001	13	63500001	0.2442	0.3334
NC_005112.4_63600001	13	63600001	0.2877	0.4442
NC_005112.4_63700001	13	63700001	0.3641	0.5533
NC_005112.4_63800001	13	63800001	0.3168	0.4407
NC_005112.4_63900001	13	63900001	0.3501	0.4681
NC_005112.4_64000001	13	64000001	0.4251	0.5652
NC_005112.4_64100001	13	64100001	0.451	0.6144
NC_005112.4_64200001	13	64200001	0.4659	0.6288
NC_005112.4_64300001	13	64300001	0.6407	0.8804
NC_005112.4_64400001	13	64400001	0.6407	0.8804
NC_005112.4_64500001	13	64500001	0.6365	0.6241
NC_005112.4_64600001	13	64600001	0.4442	0.3827
NC_005112.4_64700001	13	64700001	0.4442	0.3827
NC_005112.4_64800001	13	64800001	0.3512	0.2247
NC_005112.4_64900001	13	64900001	0.3252	0.2224
NC_005112.4_65000001	13	65000001	0.3671	0.3511
NC_005112.4_65100001	13	65100001	0.5616	0.6602
NC_005112.4_65200001	13	65200001	0.5616	0.6602
NC_005112.4_65300001	13	65300001	0.4946	0.6504
NC_005112.4_65400001	13	65400001	0.5833	0.7539
NC_005112.4_65500001	13	65500001	0.6595	0.8799
NC_005112.4_65600001	13	65600001	0.6212	0.928
NC_005112.4_65700001	13	65700001	0.6212	0.928
NC_005112.4_65900001	13	65900001	0.6284	0.7788
NC_005112.4_66000001	13	66000001	0.563	0.7069
NC_005112.4_66100001	13	66100001	0.5978	0.7414
NC_005112.4_66200001	13	66200001	0.4886	0.7392
NC_005112.4_66300001	13	66300001	0.4954	0.7409
NC_005112.4_66400001	13	66400001	0.4357	0.7058
NC_005112.4_66500001	13	66500001	0.4849	0.6846
NC_005112.4_66600001	13	66600001	0.4878	0.6881
NC_005112.4_66700001	13	66700001	0.514	0.5477
NC_005112.4_66800001	13	66800001	0.5099	0.5285

NC_005112.4_66900001	13	66900001	0.5419	0.545
NC_005112.4_67000001	13	67000001	0.5418	0.5249
NC_005112.4_67100001	13	67100001	0.5409	0.4965
NC_005112.4_67200001	13	67200001	0.5197	0.583
NC_005112.4_67300001	13	67300001	0.5668	0.621
NC_005112.4_67400001	13	67400001	0.567	0.6121
NC_005112.4_67500001	13	67500001	0.5651	0.598
NC_005112.4_67600001	13	67600001	0.4889	0.5556
NC_005112.4_67700001	13	67700001	0.5	0.5369
NC_005112.4_67800001	13	67800001	0.5112	0.5684
NC_005112.4_67900001	13	67900001	0.5463	0.6241
NC_005112.4_68000001	13	68000001	0.515	0.6621
NC_005112.4_68100001	13	68100001	0.5518	0.7453
NC_005112.4_68200001	13	68200001	0.6252	0.7626
NC_005112.4_68300001	13	68300001	0.617	0.7778
NC_005112.4_68400001	13	68400001	0.5776	0.7485
NC_005112.4_68500001	13	68500001	0.6055	0.7515
NC_005112.4_68600001	13	68600001	0.5403	0.6957
NC_005112.4_68700001	13	68700001	0.5275	0.7661
NC_005112.4_68800001	13	68800001	0.5606	0.6747
NC_005112.4_68900001	13	68900001	0.6092	0.6852
NC_005112.4_69000001	13	69000001	0.6053	0.6852
NC_005112.4_69100001	13	69100001	0.6185	0.6867
NC_005112.4_69200001	13	69200001	0.6101	0.6683
NC_005112.4_69300001	13	69300001	0.4928	0.605
NC_005112.4_69400001	13	69400001	0.4501	0.6075
NC_005112.4_69500001	13	69500001	0.5666	0.7289
NC_005112.4_69600001	13	69600001	0.6161	0.7639
NC_005112.4_69700001	13	69700001	0.6423	0.7996
NC_005112.4_69800001	13	69800001	0.6652	0.8389
NC_005112.4_69900001	13	69900001	0.6221	0.8013
NC_005112.4_70000001	13	70000001	0.5607	0.7106
NC_005112.4_70100001	13	70100001	0.5256	0.6202
NC_005112.4_70200001	13	70200001	0.5004	0.6119
NC_005112.4_70300001	13	70300001	0.5373	0.638
NC_005112.4_70400001	13	70400001	0.5913	0.6529
NC_005112.4_70500001	13	70500001	0.5263	0.6083
NC_005112.4_70600001	13	70600001	0.4823	0.6384
NC_005112.4_70700001	13	70700001	0.4414	0.5673
NC_005112.4_70800001	13	70800001	0.3617	0.4733
NC_005112.4_70900001	13	70900001	0.5092	0.6692
NC_005112.4_71000001	13	71000001	0.4825	0.7093
NC_005112.4_71100001	13	71100001	0.5708	0.7665
NC_005112.4_71200001	13	71200001	0.6059	0.8063
NC_005112.4_71300001	13	71300001	0.6296	0.8166
NC_005112.4_71400001	13	71400001	0.5103	0.7599
NC_005112.4_71500001	13	71500001	0.6406	0.7821
NC_005112.4_71600001	13	71600001	0.5951	0.7873
NC_005112.4_71700001	13	71700001	0.6517	0.8178
NC_005112.4_71800001	13	71800001	0.7404	0.8426
NC_005112.4_71900001	13	71900001	0.7261	0.8044
NC_005112.4_72000001	13	72000001	0.731	0.8015
NC_005112.4_72100001	13	72100001	0.6887	0.7911
NC_005112.4_72200001	13	72200001	0.5611	0.7388
NC_005112.4_72300001	13	72300001	0.5998	0.7196
NC_005112.4_72400001	13	72400001	0.5474	0.7249
NC_005112.4_72500001	13	72500001	0.5461	0.7189
NC_005112.4_72600001	13	72600001	0.5195	0.6804

NC_005112.4_72700001	13	72700001	0.5247	0.648
NC_005112.4_72800001	13	72800001	0.5157	0.6275
NC_005112.4_72900001	13	72900001	0.5508	0.6594
NC_005112.4_73000001	13	73000001	0.5201	0.644
NC_005112.4_73100001	13	73100001	0.6089	0.7027
NC_005112.4_73200001	13	73200001	0.534	0.7021
NC_005112.4_73300001	13	73300001	0.3912	0.7083
NC_005112.4_73400001	13	73400001	0.3728	0.665
NC_005112.4_73500001	13	73500001	0.4403	0.7353
NC_005112.4_73600001	13	73600001	0.4512	0.7825
NC_005112.4_73700001	13	73700001	0.5304	0.8536
NC_005112.4_73800001	13	73800001	0.6601	0.8608
NC_005112.4_73900001	13	73900001	0.6543	0.8271
NC_005112.4_74000001	13	74000001	0.6142	0.7628
NC_005112.4_74100001	13	74100001	0.6314	0.7339
NC_005112.4_74200001	13	74200001	0.6585	0.7577
NC_005112.4_74300001	13	74300001	0.6057	0.7493
NC_005112.4_74400001	13	74400001	0.574	0.7449
NC_005112.4_74500001	13	74500001	0.4735	0.7158
NC_005112.4_74600001	13	74600001	0.4294	0.6957
NC_005112.4_74700001	13	74700001	0.2542	0.47
NC_005112.4_74800001	13	74800001	0.2737	0.4105
NC_005112.4_74900001	13	74900001	0.343	0.496
NC_005112.4_75000001	13	75000001	0.4873	0.5996
NC_005112.4_75100001	13	75100001	0.5082	0.5753
NC_005112.4_75200001	13	75200001	0.6448	0.6807
NC_005112.4_75300001	13	75300001	0.5604	0.764
NC_005112.4_75400001	13	75400001	0.4861	0.7017
NC_005112.4_75500001	13	75500001	0.5117	0.7241
NC_005112.4_75600001	13	75600001	0.5723	0.7521
NC_005112.4_75700001	13	75700001	0.6156	0.7764
NC_005112.4_75800001	13	75800001	0.7395	0.8394
NC_005112.4_75900001	13	75900001	0.7773	0.8629
NC_005112.4_76000001	13	76000001	0.7525	0.8606
NC_005112.4_76100001	13	76100001	0.7327	0.8609
NC_005112.4_76200001	13	76200001	0.6784	0.8517
NC_005112.4_76300001	13	76300001	0.6598	0.828
NC_005112.4_76400001	13	76400001	0.5593	0.7314
NC_005112.4_76500001	13	76500001	0.4692	0.6742
NC_005112.4_76600001	13	76600001	0.3607	0.5644
NC_005112.4_76700001	13	76700001	0.4839	0.6887
NC_005112.4_76800001	13	76800001	0.454	0.6937
NC_005112.4_76900001	13	76900001	0.433	0.7647
NC_005112.4_77000001	13	77000001	0.4885	0.6263
NC_005112.4_77100001	13	77100001	0.5169	0.6487
NC_005112.4_77200001	13	77200001	0.4263	0.5476
NC_005112.4_77300001	13	77300001	0.433	0.5751
NC_005112.4_77400001	13	77400001	0.4027	0.5094
NC_005112.4_77500001	13	77500001	0.4077	0.5517
NC_005112.4_77600001	13	77600001	0.3427	0.4752
NC_005112.4_77700001	13	77700001	0.3589	0.4685
NC_005112.4_77800001	13	77800001	0.3985	0.498
NC_005112.4_77900001	13	77900001	0.5062	0.591
NC_005112.4_78000001	13	78000001	0.5848	0.691
NC_005112.4_78100001	13	78100001	0.6823	0.7435
NC_005112.4_78200001	13	78200001	0.72	0.6934
NC_005112.4_78300001	13	78300001	0.5912	0.6613
NC_005112.4_78400001	13	78400001	0.5054	0.5353

NC_005112.4_78500001	13	78500001	0.5192	0.578
NC_005112.4_78600001	13	78600001	0.5342	0.5715
NC_005112.4_78700001	13	78700001	0.4458	0.5624
NC_005112.4_78800001	13	78800001	0.4401	0.5028
NC_005112.4_78900001	13	78900001	0.434	0.5452
NC_005112.4_79000001	13	79000001	0.435	0.509
NC_005112.4_79100001	13	79100001	0.4169	0.5248
NC_005112.4_79200001	13	79200001	0.5025	0.5631
NC_005112.4_79300001	13	79300001	0.5076	0.6466
NC_005112.4_79400001	13	79400001	0.5319	0.653
NC_005112.4_79500001	13	79500001	0.5126	0.6744
NC_005112.4_79600001	13	79600001	0.4493	0.5921
NC_005112.4_79700001	13	79700001	0.4179	0.5704
NC_005112.4_79800001	13	79800001	0.3765	0.4953
NC_005112.4_79900001	13	79900001	0.3425	0.4649
NC_005112.4_80000001	13	80000001	0.3243	0.398
NC_005112.4_80100001	13	80100001	0.3385	0.4096
NC_005112.4_80200001	13	80200001	0.4017	0.5378
NC_005112.4_80300001	13	80300001	0.4706	0.5906
NC_005112.4_80400001	13	80400001	0.5121	0.607
NC_005112.4_80500001	13	80500001	0.6209	0.725
NC_005112.4_80600001	13	80600001	0.6691	0.7493
NC_005112.4_80700001	13	80700001	0.739	0.7667
NC_005112.4_80800001	13	80800001	0.7827	0.7726
NC_005112.4_80900001	13	80900001	0.823	0.7725
NC_005112.4_81000001	13	81000001	0.7425	0.7942
NC_005112.4_81100001	13	81100001	0.7166	0.864
NC_005112.4_81200001	13	81200001	0.5035	0.6446
NC_005112.4_81300001	13	81300001	0.4756	0.6262
NC_005112.4_81400001	13	81400001	0.4701	0.6623
NC_005112.4_81500001	13	81500001	0.5061	0.642
NC_005112.4_81600001	13	81600001	0.4711	0.6245
NC_005112.4_81700001	13	81700001	0.6058	0.7828
NC_005112.4_81800001	13	81800001	0.6841	0.8171
NC_005112.4_81900001	13	81900001	0.6422	0.746
NC_005112.4_82000001	13	82000001	0.5829	0.6886
NC_005112.4_82100001	13	82100001	0.58	0.6643
NC_005112.4_82200001	13	82200001	0.5311	0.6357
NC_005112.4_82300001	13	82300001	0.4868	0.6205
NC_005112.4_82400001	13	82400001	0.4404	0.6271
NC_005112.4_82500001	13	82500001	0.4633	0.66
NC_005112.4_82600001	13	82600001	0.4538	0.6431
NC_005112.4_82700001	13	82700001	0.5193	0.5949
NC_005112.4_82800001	13	82800001	0.4904	0.5289
NC_005112.4_82900001	13	82900001	0.5314	0.5532
NC_005112.4_83000001	13	83000001	0.5474	0.5965
NC_005112.4_83100001	13	83100001	0.5638	0.6241
NC_005112.4_83200001	13	83200001	0.5702	0.7272
NC_005112.4_83300001	13	83300001	0.7132	0.8769
NC_005112.4_83400001	13	83400001	0.6375	0.7052
NC_005112.4_83500001	13	83500001	0.5839	0.6287
NC_005112.4_83600001	13	83600001	0.5464	0.6028
NC_005112.4_83700001	13	83700001	0.6494	0.7165
NC_005112.4_83800001	13	83800001	0.6317	0.6979
NC_005112.4_83900001	13	83900001	0.6428	0.7691
NC_005112.4_84000001	13	84000001	0.6766	0.7829
NC_005112.4_84100001	13	84100001	0.7494	0.8205
NC_005112.4_84200001	13	84200001	0.6795	0.7571

NC_005112.4_84300001	13	84300001	0.6329	0.738
NC_005112.4_84400001	13	84400001	0.6267	0.7432
NC_005112.4_84500001	13	84500001	0.5898	0.7875
NC_005112.4_84600001	13	84600001	0.5182	0.7614
NC_005112.4_84700001	13	84700001	0.4975	0.8085
NC_005112.4_84800001	13	84800001	0.469	0.8199
NC_005112.4_84900001	13	84900001	0.6112	0.8327
NC_005112.4_85000001	13	85000001	0.5706	0.7839
NC_005112.4_85100001	13	85100001	0.5024	0.727
NC_005112.4_85200001	13	85200001	0.4795	0.6948
NC_005112.4_85300001	13	85300001	0.61	0.7233
NC_005112.4_85400001	13	85400001	0.5109	0.6441
NC_005112.4_85500001	13	85500001	0.574	0.7389
NC_005112.4_85600001	13	85600001	0.5886	0.7386
NC_005112.4_85700001	13	85700001	0.6184	0.7523
NC_005112.4_85800001	13	85800001	0.594	0.7554
NC_005112.4_85900001	13	85900001	0.5644	0.7266
NC_005112.4_86000001	13	86000001	0.547	0.6609
NC_005112.4_86100001	13	86100001	0.4974	0.6491
NC_005112.4_86200001	13	86200001	0.4956	0.6448
NC_005112.4_86300001	13	86300001	0.4918	0.6284
NC_005112.4_86400001	13	86400001	0.4194	0.526
NC_005112.4_86500001	13	86500001	0.4419	0.5544
NC_005112.4_86600001	13	86600001	0.499	0.6064
NC_005112.4_86700001	13	86700001	0.4702	0.5704
NC_005112.4_86800001	13	86800001	0.4292	0.5276
NC_005112.4_86900001	13	86900001	0.4952	0.6071
NC_005112.4_87000001	13	87000001	0.4965	0.6116
NC_005112.4_87100001	13	87100001	0.4811	0.5934
NC_005112.4_87200001	13	87200001	0.4915	0.6205
NC_005112.4_87300001	13	87300001	0.5121	0.6051
NC_005112.4_87400001	13	87400001	0.5473	0.6393
NC_005112.4_87500001	13	87500001	0.5534	0.6531
NC_005112.4_87600001	13	87600001	0.5231	0.606
NC_005112.4_87700001	13	87700001	0.4928	0.5782
NC_005112.4_87800001	13	87800001	0.5316	0.6045
NC_005112.4_87900001	13	87900001	0.4852	0.5668
NC_005112.4_88000001	13	88000001	0.5112	0.5759
NC_005112.4_88100001	13	88100001	0.4897	0.5778
NC_005112.4_88200001	13	88200001	0.4374	0.5249
NC_005112.4_88300001	13	88300001	0.4861	0.6633
NC_005112.4_88400001	13	88400001	0.4673	0.6624
NC_005112.4_88500001	13	88500001	0.4481	0.5673
NC_005112.4_88600001	13	88600001	0.5202	0.6652
NC_005112.4_88700001	13	88700001	0.5414	0.6662
NC_005112.4_88800001	13	88800001	0.4858	0.6331
NC_005112.4_88900001	13	88900001	0.5254	0.6394
NC_005112.4_89000001	13	89000001	0.4898	0.6513
NC_005112.4_89100001	13	89100001	0.4427	0.5902
NC_005112.4_89200001	13	89200001	0.5003	0.6305
NC_005112.4_89300001	13	89300001	0.6367	0.6951
NC_005112.4_89400001	13	89400001	0.6023	0.6432
NC_005112.4_89500001	13	89500001	0.6583	0.7283
NC_005112.4_89600001	13	89600001	0.4944	0.7225
NC_005112.4_89700001	13	89700001	0.4093	0.6952
NC_005112.4_89800001	13	89800001	0.2939	0.59
NC_005112.4_89900001	13	89900001	0.3793	0.6945
NC_005112.4_90000001	13	90000001	0.3571	0.6076

NC_005112.4_90100001	13	90100001	0.446	0.6657
NC_005112.4_90200001	13	90200001	0.4745	0.687
NC_005112.4_90300001	13	90300001	0.5177	0.6738
NC_005112.4_90400001	13	90400001	0.4171	0.5717
NC_005112.4_90500001	13	90500001	0.5061	0.6724
NC_005112.4_90600001	13	90600001	0.4464	0.5894
NC_005112.4_90700001	13	90700001	0.4561	0.568
NC_005112.4_90800001	13	90800001	0.426	0.5465
NC_005112.4_90900001	13	90900001	0.464	0.6057
NC_005112.4_91000001	13	91000001	0.2682	0.4295
NC_005112.4_91100001	13	91100001	0.4345	0.6276
NC_005112.4_91200001	13	91200001	0.4032	0.6148
NC_005112.4_91300001	13	91300001	0.3853	0.6449
NC_005112.4_91400001	13	91400001	0.4199	0.6403
NC_005112.4_91500001	13	91500001	0.4819	0.6225
NC_005112.4_91600001	13	91600001	0.4466	0.575
NC_005112.4_91700001	13	91700001	0.528	0.6325
NC_005112.4_91800001	13	91800001	0.5625	0.6049
NC_005112.4_91900001	13	91900001	0.5705	0.6001
NC_005112.4_92100001	13	92100001	0.6039	0.6259
NC_005112.4_92200001	13	92200001	0.3809	0.3919
NC_005112.4_92300001	13	92300001	0.5707	0.6408
NC_005112.4_92400001	13	92400001	0.5014	0.5949
NC_005112.4_92500001	13	92500001	0.4678	0.6471
NC_005112.4_92600001	13	92600001	0.4869	0.7122
NC_005112.4_92700001	13	92700001	0.5187	0.7998
NC_005112.4_92800001	13	92800001	0.4533	0.7822
NC_005112.4_92900001	13	92900001	0.4645	0.8173
NC_005112.4_93000001	13	93000001	0.4668	0.7887
NC_005112.4_93100001	13	93100001	0.4831	0.727
NC_005112.4_93200001	13	93200001	0.4963	0.6021
NC_005112.4_93300001	13	93300001	0.6395	0.7185
NC_005112.4_93400001	13	93400001	0.6076	0.6518
NC_005112.4_93500001	13	93500001	0.6767	0.6606
NC_005112.4_93600001	13	93600001	0.6156	0.6362
NC_005112.4_93700001	13	93700001	0.6155	0.6424
NC_005112.4_93800001	13	93800001	0.5224	0.5367
NC_005112.4_93900001	13	93900001	0.5345	0.569
NC_005112.4_94000001	13	94000001	0.298	0.3786
NC_005112.4_94100001	13	94100001	0.3399	0.3936
NC_005112.4_94200001	13	94200001	0.3355	0.4213
NC_005112.4_94300001	13	94300001	0.3356	0.4352
NC_005112.4_94400001	13	94400001	0.3704	0.4947
NC_005112.4_94500001	13	94500001	0.5445	0.6833
NC_005112.4_94600001	13	94600001	0.596	0.7904
NC_005112.4_94700001	13	94700001	0.7789	0.8931
NC_005112.4_94800001	13	94800001	0.6657	0.7742
NC_005112.4_94900001	13	94900001	0.6387	0.696
NC_005112.4_95000001	13	95000001	0.5468	0.6239
NC_005112.4_95100001	13	95100001	0.4863	0.5548
NC_005112.4_95200001	13	95200001	0.5014	0.5761
NC_005112.4_95300001	13	95300001	0.5617	0.6739
NC_005112.4_95400001	13	95400001	0.5805	0.7635
NC_005112.4_95500001	13	95500001	0.5039	0.7516
NC_005112.4_95600001	13	95600001	0.5333	0.7561
NC_005112.4_95700001	13	95700001	0.4741	0.7153
NC_005112.4_95800001	13	95800001	0.4256	0.7245
NC_005112.4_95900001	13	95900001	0.3688	0.6311

NC_005112.4_96000001	13	96000001	0.445	0.617
NC_005112.4_96100001	13	96100001	0.3831	0.5373
NC_005112.4_96200001	13	96200001	0.456	0.6583
NC_005112.4_96300001	13	96300001	0.3994	0.5594
NC_005112.4_96400001	13	96400001	0.3968	0.5505
NC_005112.4_96500001	13	96500001	0.5025	0.6604
NC_005112.4_96600001	13	96600001	0.5259	0.6517
NC_005112.4_96700001	13	96700001	0.5105	0.5904
NC_005112.4_96800001	13	96800001	0.5818	0.6382
NC_005112.4_96900001	13	96900001	0.5386	0.5708
NC_005112.4_97000001	13	97000001	0.5275	0.5544
NC_005112.4_97100001	13	97100001	0.5546	0.5858
NC_005112.4_97200001	13	97200001	0.5672	0.5957
NC_005112.4_97300001	13	97300001	0.559	0.5792
NC_005112.4_97400001	13	97400001	0.6621	0.7308
NC_005112.4_97500001	13	97500001	0.4743	0.6328
NC_005112.4_97600001	13	97600001	0.5166	0.6889
NC_005112.4_97700001	13	97700001	0.4337	0.658
NC_005112.4_97800001	13	97800001	0.3733	0.6309
NC_005112.4_97900001	13	97900001	0.3609	0.6053
NC_005112.4_98000001	13	98000001	0.4099	0.6666
NC_005112.4_98100001	13	98100001	0.3459	0.6284
NC_005112.4_98200001	13	98200001	0.3545	0.5884
NC_005112.4_98300001	13	98300001	0.5164	0.7613
NC_005112.4_98400001	13	98400001	0.5081	0.7236
NC_005112.4_98500001	13	98500001	0.491	0.6684
NC_005112.4_98600001	13	98600001	0.4546	0.6357
NC_005112.4_98700001	13	98700001	0.5628	0.6884
NC_005112.4_98800001	13	98800001	0.5528	0.642
NC_005112.4_98900001	13	98900001	0.5533	0.644
NC_005112.4_99000001	13	99000001	0.57	0.6622
NC_005112.4_99100001	13	99100001	0.6666	0.742
NC_005112.4_99200001	13	99200001	0.5316	0.666
NC_005112.4_99300001	13	99300001	0.453	0.6074
NC_005112.4_99400001	13	99400001	0.4453	0.5343
NC_005112.4_99500001	13	99500001	0.4064	0.5362
NC_005112.4_99600001	13	99600001	0.3446	0.4454
NC_005112.4_99700001	13	99700001	0.3275	0.4362
NC_005112.4_99800001	13	99800001	0.361	0.4764
NC_005112.4_99900001	13	99900001	0.3179	0.5273
NC_005112.4_100000001	13	100000001	0.3195	0.5067
NC_005112.4_100100001	13	100100001	0.4575	0.6154
NC_005112.4_100200001	13	100200001	0.478	0.7188
NC_005112.4_100300001	13	100300001	0.5365	0.6791
NC_005112.4_100400001	13	100400001	0.6151	0.7407
NC_005112.4_100500001	13	100500001	0.6141	0.7122
NC_005112.4_100600001	13	100600001	0.599	0.6644
NC_005112.4_100700001	13	100700001	0.6108	0.6692
NC_005112.4_100800001	13	100800001	0.6281	0.6853
NC_005112.4_100900001	13	100900001	0.5717	0.6555
NC_005112.4_101000001	13	101000001	0.5743	0.6609
NC_005112.4_101100001	13	101100001	0.5899	0.7464
NC_005112.4_101200001	13	101200001	0.6035	0.726
NC_005112.4_101300001	13	101300001	0.5746	0.7835
NC_005112.4_101400001	13	101400001	0.5421	0.7595
NC_005112.4_101500001	13	101500001	0.5107	0.7653
NC_005112.4_101600001	13	101600001	0.4026	0.6506
NC_005112.4_101700001	13	101700001	0.4467	0.727

NC_005112.4_101800001	13	101800001	0.5181	0.7473
NC_005112.4_101900001	13	101900001	0.6009	0.7574
NC_005112.4_102000001	13	102000001	0.5947	0.7438
NC_005112.4_102100001	13	102100001	0.575	0.7355
NC_005112.4_102200001	13	102200001	0.541	0.6294
NC_005112.4_102300001	13	102300001	0.5353	0.6078
NC_005112.4_102400001	13	102400001	0.5148	0.5992
NC_005112.4_102500001	13	102500001	0.5398	0.634
NC_005112.4_102600001	13	102600001	0.5821	0.6518
NC_005112.4_102700001	13	102700001	0.6381	0.7053
NC_005112.4_102800001	13	102800001	0.5706	0.7482
NC_005112.4_102900001	13	102900001	0.5782	0.7732
NC_005112.4_103000001	13	103000001	0.5253	0.74
NC_005112.4_103100001	13	103100001	0.4671	0.671
NC_005112.4_103200001	13	103200001	0.4616	0.6777
NC_005112.4_103300001	13	103300001	0.4538	0.581
NC_005112.4_103400001	13	103400001	0.4472	0.5333
NC_005112.4_103500001	13	103500001	0.4487	0.509
NC_005112.4_103600001	13	103600001	0.5262	0.5915
NC_005112.4_103700001	13	103700001	0.4933	0.5789
NC_005112.4_103800001	13	103800001	0.4649	0.5877
NC_005112.4_103900001	13	103900001	0.4484	0.5784
NC_005112.4_104000001	13	104000001	0.4735	0.6083
NC_005112.4_104100001	13	104100001	0.4482	0.6047
NC_005112.4_104200001	13	104200001	0.419	0.6235
NC_005112.4_104300001	13	104300001	0.4896	0.6618
NC_005112.4_104400001	13	104400001	0.446	0.6249
NC_005112.4_104500001	13	104500001	0.4471	0.6702
NC_005112.4_104600001	13	104600001	0.3932	0.6437
NC_005112.4_104700001	13	104700001	0.3767	0.6266
NC_005112.4_104800001	13	104800001	0.368	0.6535
NC_005112.4_104900001	13	104900001	0.3762	0.6332
NC_005112.4_105000001	13	105000001	0.4452	0.6213
NC_005112.4_105100001	13	105100001	0.4995	0.6734
NC_005112.4_105200001	13	105200001	0.5284	0.6542
NC_005112.4_105300001	13	105300001	0.5577	0.6918
NC_005112.4_105400001	13	105400001	0.6254	0.7567
NC_005112.4_105500001	13	105500001	0.6166	0.7635
NC_005112.4_105600001	13	105600001	0.618	0.6817
NC_005112.4_105700001	13	105700001	0.5554	0.6291
NC_005112.4_105800001	13	105800001	0.4923	0.5607
NC_005112.4_105900001	13	105900001	0.4439	0.4397
NC_005112.4_106000001	13	106000001	0.4601	0.4834
NC_005112.4_106100001	13	106100001	0.5109	0.5756
NC_005112.4_106200001	13	106200001	0.6185	0.6626
NC_005112.4_106300001	13	106300001	0.6505	0.6927
NC_005112.4_106400001	13	106400001	0.6228	0.7356
NC_005112.4_106500001	13	106500001	0.5406	0.7385
NC_005112.4_106600001	13	106600001	0.4295	0.6591
NC_005112.4_106700001	13	106700001	0.3577	0.5659
NC_005112.4_106800001	13	106800001	0.2484	0.375
NC_005112.4_106900001	13	106900001	0.2989	0.4458
NC_005112.4_107000001	13	107000001	0.3832	0.549
NC_005112.4_107100001	13	107100001	0.4036	0.5688
NC_005112.4_107200001	13	107200001	0.4495	0.5868
NC_005112.4_107300001	13	107300001	0.4801	0.6257
NC_005112.4_107400001	13	107400001	0.5037	0.6339
NC_005112.4_107500001	13	107500001	0.4778	0.5798

NC_005112.4_107600001	13	107600001	0.4401	0.6016
NC_005112.4_107700001	13	107700001	0.5383	0.7679
NC_005112.4_107800001	13	107800001	0.568	0.7621
NC_005112.4_107900001	13	107900001	0.6048	0.7946
NC_005112.4_108000001	13	108000001	0.6195	0.8031
NC_005112.4_108100001	13	108100001	0.7438	0.8342
NC_005112.4_108200001	13	108200001	0.6652	0.7689
NC_005112.4_108300001	13	108300001	0.6249	0.7521
NC_005112.4_108400001	13	108400001	0.6405	0.7741
NC_005112.4_108500001	13	108500001	0.6429	0.7644
NC_005112.4_108600001	13	108600001	0.5613	0.6931
NC_005112.4_108700001	13	108700001	0.5092	0.642
NC_005112.4_108800001	13	108800001	0.4923	0.6475
NC_005112.4_108900001	13	108900001	0.5128	0.6446
NC_005112.4_109000001	13	109000001	0.5502	0.6799
NC_005112.4_109100001	13	109100001	0.621	0.7482
NC_005112.4_109200001	13	109200001	0.7	0.8103
NC_005112.4_109300001	13	109300001	0.6915	0.8085
NC_005112.4_109400001	13	109400001	0.6844	0.8544
NC_005112.4_109500001	13	109500001	0.671	0.8074
NC_005112.4_109600001	13	109600001	0.809	0.852
NC_005112.4_109700001	13	109700001	0.5924	0.7776
NC_005112.4_109800001	13	109800001	0.6683	0.8303
NC_005112.4_109900001	13	109900001	0.7032	0.8459
NC_005112.4_110000001	13	110000001	0.6366	0.8006
NC_005112.4_110100001	13	110100001	0.5041	0.6655
NC_005112.4_110200001	13	110200001	0.5654	0.7065
NC_005112.4_110300001	13	110300001	0.4534	0.5891
NC_005112.4_110400001	13	110400001	0.4139	0.5182
NC_005112.4_110500001	13	110500001	0.3379	0.4127
NC_005112.4_110600001	13	110600001	0.3893	0.5057
NC_005112.4_110700001	13	110700001	0.289	0.3922
NC_005112.4_110800001	13	110800001	0.3465	0.4761
NC_005112.4_110900001	13	110900001	0.4344	0.6295
NC_005112.4_111000001	13	111000001	0.5073	0.7162
NC_005112.4_111100001	13	111100001	0.5064	0.7101
NC_005112.4_111200001	13	111200001	0.6468	0.7866
NC_005112.4_111300001	13	111300001	0.6468	0.7936
NC_005112.4_111400001	13	111400001	0.6197	0.768
NC_005112.4_111500001	13	111500001	0.5302	0.7103
NC_005112.4_111600001	13	111600001	0.4833	0.6911
NC_005112.4_111700001	13	111700001	0.3804	0.5621
NC_005112.4_111800001	13	111800001	0.3804	0.5671
NC_005112.4_111900001	13	111900001	0.4382	0.6079
NC_005112.4_112000001	13	112000001	0.4713	0.6162
NC_005112.4_112100001	13	112100001	0.5314	0.6325
NC_005112.4_112200001	13	112200001	0.5854	0.6669
NC_005112.4_112300001	13	112300001	0.554	0.5884
NC_005112.4_112400001	13	112400001	0.5525	0.5819
NC_005112.4_112500001	13	112500001	0.5515	0.5494
NC_005112.4_112600001	13	112600001	0.5938	0.6014
NC_005112.4_112700001	13	112700001	0.5253	0.5578
NC_005112.4_112800001	13	112800001	0.5043	0.611
NC_005112.4_112900001	13	112900001	0.4552	0.5617
NC_005112.4_113000001	13	113000001	0.4911	0.5826
NC_005112.4_113100001	13	113100001	0.4263	0.5551
NC_005112.4_113200001	13	113200001	0.4896	0.5671
NC_005112.4_113300001	13	113300001	0.5421	0.632

NC_005112.4_113400001	13	113400001	0.55	0.6231
NC_005112.4_113500001	13	113500001	0.5231	0.6412
NC_005112.4_113600001	13	113600001	0.5241	0.5852
NC_005113.4_900001	14	900001	0.3547	0.3354
NC_005113.4_1000001	14	1000001	0.3599	0.4746
NC_005113.4_1100001	14	1100001	0.3301	0.4552
NC_005113.4_1200001	14	1200001	0.2735	0.3974
NC_005113.4_1300001	14	1300001	0.2937	0.4472
NC_005113.4_1400001	14	1400001	0.294	0.5721
NC_005113.4_1600001	14	1600001	0.4205	0.7801
NC_005113.4_1700001	14	1700001	0.394	0.7261
NC_005113.4_1800001	14	1800001	0.4555	0.6936
NC_005113.4_1900001	14	1900001	0.4182	0.6876
NC_005113.4_2000001	14	2000001	0.4298	0.7026
NC_005113.4_2100001	14	2100001	0.3921	0.5916
NC_005113.4_2200001	14	2200001	0.4226	0.6396
NC_005113.4_2300001	14	2300001	0.3214	0.5472
NC_005113.4_2400001	14	2400001	0.4637	0.5942
NC_005113.4_2500001	14	2500001	0.5101	0.6724
NC_005113.4_2600001	14	2600001	0.5422	0.6325
NC_005113.4_2700001	14	2700001	0.5272	0.6655
NC_005113.4_2800001	14	2800001	0.5338	0.73
NC_005113.4_2900001	14	2900001	0.5592	0.7725
NC_005113.4_3000001	14	3000001	0.637	0.8107
NC_005113.4_3100001	14	3100001	0.6541	0.8679
NC_005113.4_3200001	14	3200001	0.6774	0.7806
NC_005113.4_3300001	14	3300001	0.5997	0.7167
NC_005113.4_3400001	14	3400001	0.565	0.6532
NC_005113.4_3500001	14	3500001	0.4737	0.5556
NC_005113.4_3600001	14	3600001	0.5128	0.611
NC_005113.4_3700001	14	3700001	0.5182	0.6169
NC_005113.4_3800001	14	3800001	0.4648	0.5769
NC_005113.4_3900001	14	3900001	0.4464	0.6129
NC_005113.4_4000001	14	4000001	0.5009	0.6524
NC_005113.4_4100001	14	4100001	0.441	0.5822
NC_005113.4_4200001	14	4200001	0.3706	0.504
NC_005113.4_4300001	14	4300001	0.4661	0.6337
NC_005113.4_4400001	14	4400001	0.4704	0.6537
NC_005113.4_4500001	14	4500001	0.4227	0.6087
NC_005113.4_4600001	14	4600001	0.4279	0.6391
NC_005113.4_4700001	14	4700001	0.5173	0.7052
NC_005113.4_4800001	14	4800001	0.54	0.6885
NC_005113.4_4900001	14	4900001	0.5409	0.6392
NC_005113.4_5000001	14	5000001	0.5464	0.6782
NC_005113.4_5100001	14	5100001	0.5383	0.6533
NC_005113.4_5200001	14	5200001	0.4843	0.6295
NC_005113.4_5300001	14	5300001	0.3656	0.5349
NC_005113.4_5400001	14	5400001	0.4254	0.6688
NC_005113.4_5700001	14	5700001	0.3534	0.2845
NC_005113.4_5800001	14	5800001	0.3534	0.2845
NC_005113.4_5900001	14	5900001	0.4068	0.3858
NC_005113.4_6000001	14	6000001	0.4007	0.4163
NC_005113.4_6100001	14	6100001	0.3607	0.4557
NC_005113.4_6200001	14	6200001	0.3964	0.5107
NC_005113.4_6300001	14	6300001	0.4492	0.5316
NC_005113.4_6400001	14	6400001	0.4961	0.5206
NC_005113.4_6500001	14	6500001	0.5212	0.5705
NC_005113.4_6600001	14	6600001	0.6122	0.5952

NC_005113.4_6700001	14	6700001	0.5053	0.5385
NC_005113.4_6800001	14	6800001	0.4514	0.5525
NC_005113.4_6900001	14	6900001	0.438	0.6269
NC_005113.4_7000001	14	7000001	0.4326	0.611
NC_005113.4_7100001	14	7100001	0.4223	0.6171
NC_005113.4_7200001	14	7200001	0.4885	0.6923
NC_005113.4_7300001	14	7300001	0.5776	0.7917
NC_005113.4_7400001	14	7400001	0.5978	0.7587
NC_005113.4_7500001	14	7500001	0.5852	0.7289
NC_005113.4_7600001	14	7600001	0.6291	0.7683
NC_005113.4_7700001	14	7700001	0.6123	0.7187
NC_005113.4_7800001	14	7800001	0.6016	0.7145
NC_005113.4_7900001	14	7900001	0.4921	0.6115
NC_005113.4_8000001	14	8000001	0.4817	0.5354
NC_005113.4_8100001	14	8100001	0.4517	0.4684
NC_005113.4_8200001	14	8200001	0.4421	0.4737
NC_005113.4_8300001	14	8300001	0.4285	0.4188
NC_005113.4_8400001	14	8400001	0.4912	0.4611
NC_005113.4_8500001	14	8500001	0.5442	0.5559
NC_005113.4_8600001	14	8600001	0.5218	0.5848
NC_005113.4_8700001	14	8700001	0.4618	0.5376
NC_005113.4_8800001	14	8800001	0.4565	0.5174
NC_005113.4_8900001	14	8900001	0.4199	0.4689
NC_005113.4_9000001	14	9000001	0.2967	0.2623
NC_005113.4_9100001	14	9100001	0.3208	0.2655
NC_005113.4_9200001	14	9200001	0.3036	0.3034
NC_005113.4_9300001	14	9300001	0.3881	0.4592
NC_005113.4_9400001	14	9400001	0.449	0.5512
NC_005113.4_9500001	14	9500001	0.4193	0.5795
NC_005113.4_9600001	14	9600001	0.4364	0.6209
NC_005113.4_9700001	14	9700001	0.4424	0.5615
NC_005113.4_9800001	14	9800001	0.3889	0.4991
NC_005113.4_9900001	14	9900001	0.2933	0.4135
NC_005113.4_10000001	14	10000001	0.3497	0.3927
NC_005113.4_10100001	14	10100001	0.2739	0.2918
NC_005113.4_10200001	14	10200001	0.2893	0.3719
NC_005113.4_10300001	14	10300001	0.3525	0.4762
NC_005113.4_10400001	14	10400001	0.3284	0.4504
NC_005113.4_10500001	14	10500001	0.3945	0.5744
NC_005113.4_10600001	14	10600001	0.4277	0.5934
NC_005113.4_10700001	14	10700001	0.5073	0.5713
NC_005113.4_10800001	14	10800001	0.4537	0.5339
NC_005113.4_10900001	14	10900001	0.5533	0.6025
NC_005113.4_11000001	14	11000001	0.5615	0.6071
NC_005113.4_11100001	14	11100001	0.5724	0.6043
NC_005113.4_11200001	14	11200001	0.545	0.6525
NC_005113.4_11300001	14	11300001	0.5528	0.6605
NC_005113.4_11400001	14	11400001	0.3787	0.5116
NC_005113.4_11500001	14	11500001	0.3765	0.4655
NC_005113.4_11600001	14	11600001	0.3292	0.3919
NC_005113.4_11700001	14	11700001	0.3505	0.4416
NC_005113.4_11800001	14	11800001	0.3773	0.4355
NC_005113.4_11900001	14	11900001	0.3356	0.3791
NC_005113.4_12000001	14	12000001	0.4153	0.5249
NC_005113.4_12100001	14	12100001	0.5108	0.6415
NC_005113.4_12200001	14	12200001	0.5296	0.641
NC_005113.4_12300001	14	12300001	0.4501	0.6356
NC_005113.4_12400001	14	12400001	0.5188	0.6915

NC_005113.4_12500001	14	12500001	0.5089	0.6093
NC_005113.4_12600001	14	12600001	0.4714	0.6051
NC_005113.4_12700001	14	12700001	0.4693	0.604
NC_005113.4_12800001	14	12800001	0.595	0.6319
NC_005113.4_12900001	14	12900001	0.5948	0.6294
NC_005113.4_13000001	14	13000001	0.5487	0.6748
NC_005113.4_13100001	14	13100001	0.5815	0.6728
NC_005113.4_13200001	14	13200001	0.5623	0.6241
NC_005113.4_13300001	14	13300001	0.5443	0.6188
NC_005113.4_13400001	14	13400001	0.5005	0.6066
NC_005113.4_13500001	14	13500001	0.4126	0.5037
NC_005113.4_13600001	14	13600001	0.3945	0.4546
NC_005113.4_13700001	14	13700001	0.3429	0.4441
NC_005113.4_13800001	14	13800001	0.3332	0.4511
NC_005113.4_13900001	14	13900001	0.3437	0.4241
NC_005113.4_14000001	14	14000001	0.4186	0.524
NC_005113.4_14100001	14	14100001	0.395	0.5236
NC_005113.4_14200001	14	14200001	0.4828	0.5803
NC_005113.4_14300001	14	14300001	0.4515	0.5255
NC_005113.4_14400001	14	14400001	0.4746	0.595
NC_005113.4_14500001	14	14500001	0.4814	0.5452
NC_005113.4_14600001	14	14600001	0.5673	0.6026
NC_005113.4_14700001	14	14700001	0.4985	0.5529
NC_005113.4_14800001	14	14800001	0.5743	0.6033
NC_005113.4_14900001	14	14900001	0.5886	0.5996
NC_005113.4_15000001	14	15000001	0.6338	0.7229
NC_005113.4_15100001	14	15100001	0.6165	0.7388
NC_005113.4_15200001	14	15200001	0.6861	0.7509
NC_005113.4_15300001	14	15300001	0.6149	0.8101
NC_005113.4_15400001	14	15400001	0.475	0.7775
NC_005113.4_15500001	14	15500001	0.341	0.5939
NC_005113.4_15700001	14	15700001	0.4036	0.5411
NC_005113.4_15800001	14	15800001	0.3653	0.5515
NC_005113.4_15900001	14	15900001	0.4332	0.5362
NC_005113.4_16000001	14	16000001	0.362	0.4188
NC_005113.4_16100001	14	16100001	0.3625	0.4022
NC_005113.4_16200001	14	16200001	0.3969	0.523
NC_005113.4_16300001	14	16300001	0.3957	0.4749
NC_005113.4_16400001	14	16400001	0.3709	0.4359
NC_005113.4_16500001	14	16500001	0.3808	0.4617
NC_005113.4_16600001	14	16600001	0.3744	0.4479
NC_005113.4_16700001	14	16700001	0.3515	0.3649
NC_005113.4_16800001	14	16800001	0.3781	0.4864
NC_005113.4_16900001	14	16900001	0.5611	0.7004
NC_005113.4_17000001	14	17000001	0.5827	0.722
NC_005113.4_17100001	14	17100001	0.6278	0.7661
NC_005113.4_17200001	14	17200001	0.6136	0.7419
NC_005113.4_17300001	14	17300001	0.6448	0.7179
NC_005113.4_17400001	14	17400001	0.4796	0.5435
NC_005113.4_17500001	14	17500001	0.4667	0.5025
NC_005113.4_17600001	14	17600001	0.4442	0.4816
NC_005113.4_17700001	14	17700001	0.4757	0.49
NC_005113.4_17800001	14	17800001	0.4356	0.444
NC_005113.4_17900001	14	17900001	0.4864	0.4564
NC_005113.4_18000001	14	18000001	0.4796	0.4213
NC_005113.4_18100001	14	18100001	0.4311	0.4384
NC_005113.4_18200001	14	18200001	0.4452	0.4968
NC_005113.4_18300001	14	18300001	0.5031	0.6165

NC_005113.4_18400001	14	18400001	0.4917	0.6277
NC_005113.4_18500001	14	18500001	0.4319	0.6299
NC_005113.4_18600001	14	18600001	0.4619	0.6035
NC_005113.4_18700001	14	18700001	0.4377	0.6351
NC_005113.4_18800001	14	18800001	0.4417	0.6456
NC_005113.4_18900001	14	18900001	0.4892	0.697
NC_005113.4_19000001	14	19000001	0.5917	0.7706
NC_005113.4_19100001	14	19100001	0.605	0.859
NC_005113.4_19200001	14	19200001	0.7805	0.9372
NC_005113.4_19300001	14	19300001	0.8464	0.9741
NC_005113.4_19400001	14	19400001	0.5598	0.8785
NC_005113.4_19500001	14	19500001	0.6326	0.8267
NC_005113.4_19600001	14	19600001	0.679	0.8084
NC_005113.4_19700001	14	19700001	0.5825	0.6318
NC_005113.4_19800001	14	19800001	0.6095	0.6414
NC_005113.4_19900001	14	19900001	0.6685	0.6713
NC_005113.4_20000001	14	20000001	0.6742	0.6906
NC_005113.4_20100001	14	20100001	0.6013	0.6284
NC_005113.4_20200001	14	20200001	0.498	0.5986
NC_005113.4_20300001	14	20300001	0.4995	0.5547
NC_005113.4_20400001	14	20400001	0.4934	0.5636
NC_005113.4_20500001	14	20500001	0.477	0.5364
NC_005113.4_20600001	14	20600001	0.5223	0.5965
NC_005113.4_20700001	14	20700001	0.6452	0.7113
NC_005113.4_20800001	14	20800001	0.6531	0.7773
NC_005113.4_20900001	14	20900001	0.737	0.8154
NC_005113.4_21000001	14	21000001	0.8819	0.9034
NC_005113.4_21100001	14	21100001	0.852	0.874
NC_005113.4_21600001	14	21600001	0.0986	0.0659
NC_005113.4_21700001	14	21700001	0.1594	0.1662
NC_005113.4_21800001	14	21800001	0.2328	0.2139
NC_005113.4_21900001	14	21900001	0.2882	0.2986
NC_005113.4_22000001	14	22000001	0.3071	0.3017
NC_005113.4_22100001	14	22100001	0.4271	0.3346
NC_005113.4_22200001	14	22200001	0.3925	0.3449
NC_005113.4_22300001	14	22300001	0.3316	0.361
NC_005113.4_22400001	14	22400001	0.429	0.5521
NC_005113.4_22500001	14	22500001	0.3997	0.5798
NC_005113.4_22600001	14	22600001	0.5588	0.6564
NC_005113.4_22700001	14	22700001	0.5827	0.6349
NC_005113.4_22800001	14	22800001	0.6775	0.6951
NC_005113.4_22900001	14	22900001	0.6173	0.7138
NC_005113.4_23000001	14	23000001	0.5904	0.6703
NC_005113.4_23100001	14	23100001	0.4923	0.6345
NC_005113.4_23200001	14	23200001	0.523	0.6674
NC_005113.4_23300001	14	23300001	0.4593	0.6141
NC_005113.4_23400001	14	23400001	0.471	0.5515
NC_005113.4_23500001	14	23500001	0.5861	0.6554
NC_005113.4_23600001	14	23600001	0.7122	0.6759
NC_005113.4_23700001	14	23700001	0.7565	0.7734
NC_005113.4_23800001	14	23800001	0.6787	0.6928
NC_005113.4_23900001	14	23900001	0.5696	0.6486
NC_005113.4_24000001	14	24000001	0.5947	0.6197
NC_005113.4_24100001	14	24100001	0.5858	0.6193
NC_005113.4_24200001	14	24200001	0.5267	0.5424
NC_005113.4_24300001	14	24300001	0.403	0.5224
NC_005113.4_24400001	14	24400001	0.3749	0.4526
NC_005113.4_24500001	14	24500001	0.2728	0.3508

NC_005113.4_24600001	14	24600001	0.398	0.5342
NC_005113.4_24700001	14	24700001	0.4179	0.5046
NC_005113.4_24800001	14	24800001	0.6462	0.5911
NC_005113.4_24900001	14	24900001	0.5585	0.5836
NC_005113.4_25000001	14	25000001	0.5306	0.577
NC_005113.4_25100001	14	25100001	0.2842	0.3871
NC_005113.4_25200001	14	25200001	0.3495	0.5647
NC_005113.4_25300001	14	25300001	0.422	0.6744
NC_005113.4_25400001	14	25400001	0.4225	0.7109
NC_005113.4_25500001	14	25500001	0.4225	0.7109
NC_005113.4_25600001	14	25600001	0.7059	0.9008
NC_005113.4_25700001	14	25700001	0.5714	0.7321
NC_005113.4_25800001	14	25800001	0.4521	0.4504
NC_005113.4_25900001	14	25900001	0.5087	0.5376
NC_005113.4_26000001	14	26000001	0.6256	0.6514
NC_005113.4_26100001	14	26100001	0.6263	0.666
NC_005113.4_26200001	14	26200001	0.6739	0.7536
NC_005113.4_26300001	14	26300001	0.6904	0.769
NC_005113.4_26400001	14	26400001	0.6296	0.6753
NC_005113.4_26500001	14	26500001	0.5533	0.5633
NC_005113.4_26600001	14	26600001	0.5038	0.4813
NC_005113.4_26700001	14	26700001	0.4016	0.3015
NC_005113.4_26800001	14	26800001	0.4467	0.3629
NC_005113.4_26900001	14	26900001	0.5005	0.4574
NC_005113.4_27000001	14	27000001	0.4578	0.4522
NC_005113.4_27100001	14	27100001	0.4489	0.4598
NC_005113.4_27200001	14	27200001	0.5201	0.6294
NC_005113.4_27300001	14	27300001	0.4264	0.5531
NC_005113.4_27400001	14	27400001	0.3945	0.4104
NC_005113.4_27500001	14	27500001	0.3786	0.4027
NC_005113.4_27600001	14	27600001	0.4222	0.4443
NC_005113.4_28100001	14	28100001	0.2504	0.7933
NC_005113.4_28200001	14	28200001	0.4244	0.8017
NC_005113.4_28300001	14	28300001	0.652	0.8685
NC_005113.4_28400001	14	28400001	0.5412	0.8197
NC_005113.4_28500001	14	28500001	0.513	0.8182
NC_005113.4_28600001	14	28600001	0.5413	0.8195
NC_005113.4_28700001	14	28700001	0.5555	0.8529
NC_005113.4_28800001	14	28800001	0.4266	0.7665
NC_005113.4_28900001	14	28900001	0.5089	0.7034
NC_005113.4_29000001	14	29000001	0.4372	0.6259
NC_005113.4_29100001	14	29100001	0.4718	0.6318
NC_005113.4_29200001	14	29200001	0.3473	0.5052
NC_005113.4_29300001	14	29300001	0.349	0.548
NC_005113.4_29400001	14	29400001	0.1238	0.2038
NC_005113.4_29700001	14	29700001	0.6082	0.7047
NC_005113.4_29800001	14	29800001	0.6082	0.7047
NC_005113.4_29900001	14	29900001	0.6321	0.6901
NC_005113.4_30000001	14	30000001	0.6785	0.74
NC_005113.4_30100001	14	30100001	0.6785	0.74
NC_005113.4_30200001	14	30200001	0.5417	0.5979
NC_005113.4_30300001	14	30300001	0.4957	0.5529
NC_005113.4_30400001	14	30400001	0.3354	0.4273
NC_005113.4_30500001	14	30500001	0.3243	0.4012
NC_005113.4_30600001	14	30600001	0.3243	0.4012
NC_005113.4_30700001	14	30700001	0.2981	0.3256
NC_005113.4_30800001	14	30800001	0.2712	0.2817
NC_005113.4_30900001	14	30900001	0.3537	0.3933

NC_005113.4_31000001	14	31000001	0.4099	0.4613
NC_005113.4_31100001	14	31100001	0.3879	0.4561
NC_005113.4_31200001	14	31200001	0.4278	0.5428
NC_005113.4_31300001	14	31300001	0.5583	0.7018
NC_005113.4_31400001	14	31400001	0.5496	0.6803
NC_005113.4_31500001	14	31500001	0.6168	0.6576
NC_005113.4_31600001	14	31600001	0.5587	0.58
NC_005113.4_31700001	14	31700001	0.4828	0.5686
NC_005113.4_31800001	14	31800001	0.472	0.5404
NC_005113.4_31900001	14	31900001	0.479	0.5777
NC_005113.4_32000001	14	32000001	0.3928	0.5503
NC_005113.4_32100001	14	32100001	0.4774	0.6149
NC_005113.4_32200001	14	32200001	0.418	0.4687
NC_005113.4_32300001	14	32300001	0.4134	0.4866
NC_005113.4_32400001	14	32400001	0.434	0.5398
NC_005113.4_32500001	14	32500001	0.4391	0.5624
NC_005113.4_32600001	14	32600001	0.3947	0.5106
NC_005113.4_32700001	14	32700001	0.4714	0.6236
NC_005113.4_32800001	14	32800001	0.4553	0.6145
NC_005113.4_32900001	14	32900001	0.4804	0.6089
NC_005113.4_33000001	14	33000001	0.4901	0.5934
NC_005113.4_33100001	14	33100001	0.6058	0.7358
NC_005113.4_33200001	14	33200001	0.5121	0.6681
NC_005113.4_33300001	14	33300001	0.6056	0.6821
NC_005113.4_33400001	14	33400001	0.5634	0.6694
NC_005113.4_33500001	14	33500001	0.5754	0.6914
NC_005113.4_33600001	14	33600001	0.5181	0.6293
NC_005113.4_33700001	14	33700001	0.5764	0.735
NC_005113.4_33800001	14	33800001	0.5772	0.8274
NC_005113.4_33900001	14	33900001	0.4919	0.7403
NC_005113.4_34000001	14	34000001	0.5177	0.7073
NC_005113.4_34100001	14	34100001	0.5378	0.7397
NC_005113.4_34200001	14	34200001	0.5474	0.6794
NC_005113.4_34300001	14	34300001	0.507	0.6189
NC_005113.4_34400001	14	34400001	0.6291	0.6529
NC_005113.4_34500001	14	34500001	0.5208	0.6226
NC_005113.4_34600001	14	34600001	0.4794	0.557
NC_005113.4_34700001	14	34700001	0.3623	0.472
NC_005113.4_34800001	14	34800001	0.3322	0.4432
NC_005113.4_34900001	14	34900001	0.3559	0.4907
NC_005113.4_35000001	14	35000001	0.3618	0.5128
NC_005113.4_35100001	14	35100001	0.394	0.556
NC_005113.4_35200001	14	35200001	0.4473	0.5853
NC_005113.4_35300001	14	35300001	0.4297	0.5488
NC_005113.4_35400001	14	35400001	0.44	0.5458
NC_005113.4_35500001	14	35500001	0.5195	0.6
NC_005113.4_35600001	14	35600001	0.4371	0.5595
NC_005113.4_35700001	14	35700001	0.4589	0.6179
NC_005113.4_35800001	14	35800001	0.5246	0.6808
NC_005113.4_35900001	14	35900001	0.4984	0.7277
NC_005113.4_36000001	14	36000001	0.4705	0.7091
NC_005113.4_36100001	14	36100001	0.5675	0.6861
NC_005113.4_36200001	14	36200001	0.4652	0.62
NC_005113.4_36300001	14	36300001	0.4579	0.6065
NC_005113.4_36400001	14	36400001	0.5044	0.6032
NC_005113.4_36500001	14	36500001	0.459	0.5593
NC_005113.4_36600001	14	36600001	0.4589	0.5161
NC_005113.4_36700001	14	36700001	0.6602	0.6447

NC_005113.4_36800001	14	36800001	0.6147	0.6158
NC_005113.4_36900001	14	36900001	0.6342	0.6803
NC_005113.4_37000001	14	37000001	0.5467	0.633
NC_005113.4_37100001	14	37100001	0.553	0.662
NC_005113.4_37200001	14	37200001	0.5527	0.6366
NC_005113.4_37300001	14	37300001	0.558	0.6439
NC_005113.4_37400001	14	37400001	0.5244	0.6043
NC_005113.4_37500001	14	37500001	0.581	0.6425
NC_005113.4_37600001	14	37600001	0.6298	0.6618
NC_005113.4_37700001	14	37700001	0.5568	0.6077
NC_005113.4_37800001	14	37800001	0.5166	0.5971
NC_005113.4_37900001	14	37900001	0.5425	0.6438
NC_005113.4_38000001	14	38000001	0.5134	0.6371
NC_005113.4_38100001	14	38100001	0.4035	0.5245
NC_005113.4_38200001	14	38200001	0.4037	0.5389
NC_005113.4_38300001	14	38300001	0.4221	0.6218
NC_005113.4_38400001	14	38400001	0.4209	0.6187
NC_005113.4_38500001	14	38500001	0.4421	0.7163
NC_005113.4_38600001	14	38600001	0.4415	0.8352
NC_005113.4_38700001	14	38700001	0.5375	0.8186
NC_005113.4_38800001	14	38800001	0.5179	0.7607
NC_005113.4_38900001	14	38900001	0.4375	0.6672
NC_005113.4_39000001	14	39000001	0.4748	0.6671
NC_005113.4_39100001	14	39100001	0.5034	0.6452
NC_005113.4_39200001	14	39200001	0.5122	0.6741
NC_005113.4_39300001	14	39300001	0.5851	0.7355
NC_005113.4_39500001	14	39500001	0.6809	0.803
NC_005113.4_39600001	14	39600001	0.5588	0.7867
NC_005113.4_39700001	14	39700001	0.4937	0.7828
NC_005113.4_39800001	14	39800001	0.4937	0.7828
NC_005113.4_39900001	14	39900001	0.4937	0.7828
NC_005113.4_40000001	14	40000001	0.6101	0.8214
NC_005113.4_40100001	14	40100001	0.6677	0.7635
NC_005113.4_40200001	14	40200001	0.605	0.7078
NC_005113.4_40300001	14	40300001	0.605	0.7078
NC_005113.4_40400001	14	40400001	0.6386	0.74
NC_005113.4_40500001	14	40500001	0.4646	0.5514
NC_005113.4_40600001	14	40600001	0.5682	0.5004
NC_005113.4_40700001	14	40700001	0.5988	0.5735
NC_005113.4_40800001	14	40800001	0.5047	0.517
NC_005113.4_40900001	14	40900001	0.4655	0.4606
NC_005113.4_41000001	14	41000001	0.5324	0.5304
NC_005113.4_41100001	14	41100001	0.4884	0.563
NC_005113.4_41200001	14	41200001	0.5267	0.5692
NC_005113.4_41300001	14	41300001	0.6463	0.636
NC_005113.4_41400001	14	41400001	0.6052	0.6291
NC_005113.4_41500001	14	41500001	0.5453	0.5694
NC_005113.4_41600001	14	41600001	0.6278	0.614
NC_005113.4_41700001	14	41700001	0.6064	0.6154
NC_005113.4_41800001	14	41800001	0.5858	0.6661
NC_005113.4_41900001	14	41900001	0.5374	0.6345
NC_005113.4_42000001	14	42000001	0.5166	0.6471
NC_005113.4_42100001	14	42100001	0.5242	0.7143
NC_005113.4_42200001	14	42200001	0.4463	0.7265
NC_005113.4_42300001	14	42300001	0.3746	0.5535
NC_005113.4_42400001	14	42400001	0.4078	0.5579
NC_005113.4_42500001	14	42500001	0.4297	0.5867
NC_005113.4_42600001	14	42600001	0.44	0.5687

NC_005113.4_42700001	14	42700001	0.4857	0.5653
NC_005113.4_42800001	14	42800001	0.5414	0.6382
NC_005113.4_42900001	14	42900001	0.5412	0.645
NC_005113.4_43000001	14	43000001	0.5672	0.6412
NC_005113.4_43100001	14	43100001	0.5346	0.6518
NC_005113.4_43200001	14	43200001	0.4095	0.6242
NC_005113.4_43300001	14	43300001	0.4282	0.6241
NC_005113.4_43400001	14	43400001	0.4478	0.7217
NC_005113.4_43500001	14	43500001	0.4059	0.7032
NC_005113.4_43600001	14	43600001	0.4273	0.6741
NC_005113.4_43700001	14	43700001	0.5056	0.7082
NC_005113.4_43800001	14	43800001	0.5041	0.7267
NC_005113.4_43900001	14	43900001	0.4309	0.6183
NC_005113.4_44000001	14	44000001	0.6526	0.7485
NC_005113.4_44100001	14	44100001	0.609	0.6634
NC_005113.4_44200001	14	44200001	0.6427	0.6871
NC_005113.4_44300001	14	44300001	0.7087	0.7552
NC_005113.4_44400001	14	44400001	0.7684	0.7797
NC_005113.4_44500001	14	44500001	0.6755	0.7175
NC_005113.4_44600001	14	44600001	0.6759	0.7741
NC_005113.4_44700001	14	44700001	0.5852	0.7041
NC_005113.4_44800001	14	44800001	0.4902	0.6507
NC_005113.4_44900001	14	44900001	0.4276	0.6276
NC_005113.4_45000001	14	45000001	0.3988	0.6018
NC_005113.4_45100001	14	45100001	0.3993	0.6023
NC_005113.4_45200001	14	45200001	0.4527	0.6697
NC_005113.4_45300001	14	45300001	0.5403	0.6927
NC_005113.4_45400001	14	45400001	0.4972	0.6825
NC_005113.4_45500001	14	45500001	0.4935	0.7255
NC_005113.4_45600001	14	45600001	0.5027	0.6869
NC_005113.4_45700001	14	45700001	0.4959	0.7175
NC_005113.4_45800001	14	45800001	0.4335	0.7056
NC_005113.4_45900001	14	45900001	0.5037	0.752
NC_005113.4_46000001	14	46000001	0.6323	0.7738
NC_005113.4_46100001	14	46100001	0.7542	0.9601
NC_005113.4_47100001	14	47100001	0.8293	0.9248
NC_005113.4_47200001	14	47200001	0.6588	0.9078
NC_005113.4_48200001	14	48200001	0.7939	0.7941
NC_005113.4_48300001	14	48300001	0.6678	0.7825
NC_005113.4_48400001	14	48400001	0.7438	0.7938
NC_005113.4_48500001	14	48500001	0.8206	0.9098
NC_005113.4_48600001	14	48600001	0.8206	0.9098
NC_005113.4_48700001	14	48700001	0.8252	0.9079
NC_005113.4_48800001	14	48800001	0.9725	0.9333
NC_005113.4_48900001	14	48900001	0.8304	0.97
NC_005113.4_49000001	14	49000001	0.8305	0.8643
NC_005113.4_49100001	14	49100001	0.8305	0.8643
NC_005113.4_49200001	14	49200001	0.7315	0.7202
NC_005113.4_49300001	14	49300001	0.6516	0.6429
NC_005113.4_49400001	14	49400001	0.7714	0.6226
NC_005113.4_49600001	14	49600001	0.7304	0.6584
NC_005113.4_49700001	14	49700001	0.6485	0.7168
NC_005113.4_49800001	14	49800001	0.6103	0.7053
NC_005113.4_49900001	14	49900001	0.6794	0.8286
NC_005113.4_50000001	14	50000001	0.6868	0.8522
NC_005113.4_50100001	14	50100001	0.6912	0.9149
NC_005113.4_50200001	14	50200001	0.7216	0.8849
NC_005113.4_50300001	14	50300001	0.809	0.9179

NC_005113.4_50400001	14	50400001	0.7584	0.894
NC_005113.4_50700001	14	50700001	0.964	0.9238
NC_005113.4_51100001	14	51100001	0.9612	0.9176
NC_005113.4_51200001	14	51200001	0.6776	0.8746
NC_005113.4_51300001	14	51300001	0.6265	0.7502
NC_005113.4_51400001	14	51400001	0.616	0.7213
NC_005113.4_51500001	14	51500001	0.6956	0.7923
NC_005113.4_51600001	14	51600001	0.6318	0.7511
NC_005113.4_51700001	14	51700001	0.6839	0.7583
NC_005113.4_51800001	14	51800001	0.7776	0.8442
NC_005113.4_51900001	14	51900001	0.693	0.6771
NC_005113.4_52000001	14	52000001	0.3475	0.2244
NC_005113.4_52200001	14	52200001	0.3649	0.2327
NC_005113.4_52300001	14	52300001	0.3649	0.2327
NC_005113.4_52400001	14	52400001	0.504	0.5205
NC_005113.4_52500001	14	52500001	0.5951	0.6421
NC_005113.4_52600001	14	52600001	0.5788	0.7019
NC_005113.4_52700001	14	52700001	0.817	0.9692
NC_005113.4_52800001	14	52800001	0.6631	0.9346
NC_005113.4_52900001	14	52900001	0.3589	0.5763
NC_005113.4_53000001	14	53000001	0.3589	0.5763
NC_005113.4_53100001	14	53100001	0.3037	0.4467
NC_005113.4_53200001	14	53200001	0.2761	0.3933
NC_005113.4_53300001	14	53300001	0.2881	0.4141
NC_005113.4_53400001	14	53400001	0.475	0.8011
NC_005113.4_53500001	14	53500001	0.5941	0.8706
NC_005113.4_53600001	14	53600001	0.5091	0.8132
NC_005113.4_53700001	14	53700001	0.4823	0.8504
NC_005113.4_53800001	14	53800001	0.5129	0.8043
NC_005113.4_53900001	14	53900001	0.5314	0.7834
NC_005113.4_54000001	14	54000001	0.4278	0.6926
NC_005113.4_54100001	14	54100001	0.4474	0.7496
NC_005113.4_54200001	14	54200001	0.5028	0.7655
NC_005113.4_54300001	14	54300001	0.4267	0.6278
NC_005113.4_54500001	14	54500001	0.1914	0.1793
NC_005113.4_54600001	14	54600001	0.2473	0.3469
NC_005113.4_54700001	14	54700001	0.2473	0.3469
NC_005113.4_54800001	14	54800001	0.2431	0.41
NC_005113.4_54900001	14	54900001	0.2431	0.41
NC_005113.4_55000001	14	55000001	0.3914	0.7159
NC_005113.4_55200001	14	55200001	0.3256	0.5132
NC_005113.4_55300001	14	55300001	0.4547	0.6087
NC_005113.4_55400001	14	55400001	0.4101	0.57
NC_005113.4_55500001	14	55500001	0.3084	0.4185
NC_005113.4_55600001	14	55600001	0.5237	0.7061
NC_005113.4_55700001	14	55700001	0.5242	0.7299
NC_005113.4_55800001	14	55800001	0.4034	0.752
NC_005113.4_55900001	14	55900001	0.5507	0.7421
NC_005113.4_56000001	14	56000001	0.5507	0.7421
NC_005113.4_56100001	14	56100001	0.3921	0.5906
NC_005113.4_56200001	14	56200001	0.4882	0.622
NC_005113.4_56300001	14	56300001	0.5026	0.6507
NC_005113.4_56400001	14	56400001	0.4864	0.6715
NC_005113.4_56500001	14	56500001	0.4864	0.6715
NC_005113.4_56600001	14	56600001	0.5577	0.6445
NC_005113.4_56700001	14	56700001	0.5191	0.6346
NC_005113.4_56800001	14	56800001	0.5251	0.5432
NC_005113.4_56900001	14	56900001	0.5316	0.5507

NC_005113.4_57000001	14	57000001	0.5916	0.5873
NC_005113.4_57100001	14	57100001	0.7682	0.781
NC_005113.4_57200001	14	57200001	0.7131	0.7148
NC_005113.4_57300001	14	57300001	0.6975	0.7389
NC_005113.4_57400001	14	57400001	0.5683	0.6971
NC_005113.4_57500001	14	57500001	0.4453	0.6715
NC_005113.4_57600001	14	57600001	0.3883	0.6055
NC_005113.4_57700001	14	57700001	0.1816	0.5273
NC_005113.4_57800001	14	57800001	0.2024	0.5615
NC_005113.4_58200001	14	58200001	0.1953	0.5153
NC_005113.4_58300001	14	58300001	0.3551	0.6705
NC_005113.4_58400001	14	58400001	0.4644	0.7727
NC_005113.4_58500001	14	58500001	0.555	0.7895
NC_005113.4_58600001	14	58600001	0.5694	0.7664
NC_005113.4_58700001	14	58700001	0.5329	0.7298
NC_005113.4_58800001	14	58800001	0.5044	0.7458
NC_005113.4_58900001	14	58900001	0.4418	0.6368
NC_005113.4_59000001	14	59000001	0.432	0.669
NC_005113.4_59100001	14	59100001	0.4111	0.6324
NC_005113.4_59200001	14	59200001	0.5256	0.7242
NC_005113.4_59300001	14	59300001	0.5745	0.6388
NC_005113.4_59400001	14	59400001	0.5265	0.6263
NC_005113.4_59500001	14	59500001	0.5554	0.6813
NC_005113.4_59600001	14	59600001	0.5689	0.683
NC_005113.4_59700001	14	59700001	0.5283	0.6389
NC_005113.4_59800001	14	59800001	0.5008	0.6403
NC_005113.4_59900001	14	59900001	0.5245	0.6449
NC_005113.4_60000001	14	60000001	0.5541	0.6414
NC_005113.4_60100001	14	60100001	0.5729	0.6798
NC_005113.4_60200001	14	60200001	0.6097	0.7487
NC_005113.4_60300001	14	60300001	0.7086	0.827
NC_005113.4_60400001	14	60400001	0.7402	0.8427
NC_005113.4_60500001	14	60500001	0.6552	0.8281
NC_005113.4_60600001	14	60600001	0.5494	0.7213
NC_005113.4_60700001	14	60700001	0.5743	0.7149
NC_005113.4_60800001	14	60800001	0.5673	0.7076
NC_005113.4_60900001	14	60900001	0.5447	0.6818
NC_005113.4_61000001	14	61000001	0.558	0.6657
NC_005113.4_61100001	14	61100001	0.698	0.715
NC_005113.4_61200001	14	61200001	0.73	0.7315
NC_005113.4_61300001	14	61300001	0.6873	0.6831
NC_005113.4_61400001	14	61400001	0.522	0.5166
NC_005113.4_61500001	14	61500001	0.3881	0.3618
NC_005113.4_61600001	14	61600001	0.3756	0.3872
NC_005113.4_61700001	14	61700001	0.3269	0.3266
NC_005113.4_61800001	14	61800001	0.3269	0.3266
NC_005113.4_61900001	14	61900001	0.3363	0.3926
NC_005113.4_62300001	14	62300001	0.4663	0.7205
NC_005113.4_62400001	14	62400001	0.5582	0.7959
NC_005113.4_62500001	14	62500001	0.5729	0.7692
NC_005113.4_62600001	14	62600001	0.5066	0.6653
NC_005113.4_62700001	14	62700001	0.346	0.5301
NC_005113.4_62800001	14	62800001	0.4041	0.6369
NC_005113.4_62900001	14	62900001	0.3831	0.5627
NC_005113.4_63000001	14	63000001	0.3374	0.5203
NC_005113.4_63100001	14	63100001	0.5263	0.8078
NC_005113.4_63200001	14	63200001	0.7195	0.8805
NC_005113.4_63300001	14	63300001	0.795	0.8816

NC_005113.4_63400001	14	63400001	0.7219	0.9585
NC_005113.4_63500001	14	63500001	0.657	0.8983
NC_005113.4_63600001	14	63600001	0.4658	0.8183
NC_005113.4_63700001	14	63700001	0.5154	0.6642
NC_005113.4_63800001	14	63800001	0.5154	0.6642
NC_005113.4_63900001	14	63900001	0.5098	0.608
NC_005113.4_64000001	14	64000001	0.5142	0.5884
NC_005113.4_64100001	14	64100001	0.6281	0.6085
NC_005113.4_64200001	14	64200001	0.721	0.7275
NC_005113.4_64300001	14	64300001	0.721	0.6874
NC_005113.4_64400001	14	64400001	0.6337	0.6543
NC_005113.4_64500001	14	64500001	0.6706	0.6919
NC_005113.4_64600001	14	64600001	0.6163	0.6554
NC_005113.4_64700001	14	64700001	0.5351	0.5752
NC_005113.4_64800001	14	64800001	0.3841	0.5183
NC_005113.4_65100001	14	65100001	0.3896	0.5231
NC_005113.4_65200001	14	65200001	0.4583	0.5729
NC_005113.4_65600001	14	65600001	0.5592	0.5765
NC_005113.4_65700001	14	65700001	0.4301	0.5601
NC_005113.4_65800001	14	65800001	0.5863	0.6629
NC_005113.4_65900001	14	65900001	0.6119	0.6635
NC_005113.4_66000001	14	66000001	0.603	0.6837
NC_005113.4_66100001	14	66100001	0.5852	0.6987
NC_005113.4_66200001	14	66200001	0.5245	0.6724
NC_005113.4_66300001	14	66300001	0.5075	0.6432
NC_005113.4_66400001	14	66400001	0.4074	0.6145
NC_005113.4_66500001	14	66500001	0.4452	0.6451
NC_005113.4_66600001	14	66600001	0.5471	0.7161
NC_005113.4_66700001	14	66700001	0.5499	0.6549
NC_005113.4_66800001	14	66800001	0.5284	0.6627
NC_005113.4_66900001	14	66900001	0.5389	0.6504
NC_005113.4_67000001	14	67000001	0.4803	0.5799
NC_005113.4_67100001	14	67100001	0.3432	0.4139
NC_005113.4_67200001	14	67200001	0.3776	0.5952
NC_005113.4_67300001	14	67300001	0.3168	0.4272
NC_005113.4_67500001	14	67500001	0.7492	0.745
NC_005113.4_67600001	14	67600001	0.7756	0.743
NC_005113.4_67700001	14	67700001	0.6321	0.6115
NC_005113.4_67800001	14	67800001	0.5218	0.5487
NC_005113.4_67900001	14	67900001	0.5137	0.5693
NC_005113.4_68000001	14	68000001	0.3373	0.3416
NC_005113.4_68100001	14	68100001	0.2871	0.2887
NC_005113.4_68200001	14	68200001	0.3857	0.4768
NC_005113.4_68300001	14	68300001	0.5708	0.7327
NC_005113.4_68400001	14	68400001	0.6655	0.7414
NC_005113.4_68500001	14	68500001	0.5708	0.6706
NC_005113.4_68600001	14	68600001	0.4944	0.5586
NC_005113.4_68700001	14	68700001	0.507	0.5983
NC_005113.4_68800001	14	68800001	0.4412	0.4859
NC_005113.4_68900001	14	68900001	0.3969	0.4483
NC_005113.4_69000001	14	69000001	0.4264	0.499
NC_005113.4_69100001	14	69100001	0.4897	0.5193
NC_005113.4_69200001	14	69200001	0.3836	0.5115
NC_005113.4_69300001	14	69300001	0.5862	0.7357
NC_005113.4_69400001	14	69400001	0.5862	0.7357
NC_005113.4_69500001	14	69500001	0.6828	0.7466
NC_005113.4_69600001	14	69600001	0.7998	0.9605
NC_005113.4_69700001	14	69700001	0.6629	0.7384

NC_005113.4_69800001	14	69800001	0.543	0.5904
NC_005113.4_69900001	14	69900001	0.4675	0.5812
NC_005113.4_70000001	14	70000001	0.4418	0.5144
NC_005113.4_70100001	14	70100001	0.429	0.5376
NC_005113.4_70200001	14	70200001	0.4339	0.5114
NC_005113.4_70300001	14	70300001	0.437	0.5106
NC_005113.4_70400001	14	70400001	0.4637	0.4958
NC_005113.4_70500001	14	70500001	0.4227	0.4747
NC_005113.4_70600001	14	70600001	0.392	0.4172
NC_005113.4_70700001	14	70700001	0.3958	0.4707
NC_005113.4_70800001	14	70800001	0.3706	0.4727
NC_005113.4_70900001	14	70900001	0.3625	0.434
NC_005113.4_71000001	14	71000001	0.4589	0.6058
NC_005113.4_71100001	14	71100001	0.4388	0.5679
NC_005113.4_71200001	14	71200001	0.4093	0.5108
NC_005113.4_71300001	14	71300001	0.4172	0.4875
NC_005113.4_71400001	14	71400001	0.4531	0.5545
NC_005113.4_71500001	14	71500001	0.4089	0.4915
NC_005113.4_71600001	14	71600001	0.4136	0.468
NC_005113.4_71700001	14	71700001	0.3901	0.4706
NC_005113.4_71800001	14	71800001	0.3702	0.4593
NC_005113.4_71900001	14	71900001	0.3319	0.4822
NC_005113.4_72000001	14	72000001	0.2921	0.3986
NC_005113.4_72100001	14	72100001	0.3266	0.4845
NC_005113.4_72200001	14	72200001	0.4277	0.6163
NC_005113.4_72300001	14	72300001	0.411	0.6006
NC_005113.4_72400001	14	72400001	0.4858	0.6372
NC_005113.4_72500001	14	72500001	0.5149	0.6766
NC_005113.4_72600001	14	72600001	0.6098	0.7065
NC_005113.4_72700001	14	72700001	0.4528	0.5158
NC_005113.4_72800001	14	72800001	0.4283	0.4956
NC_005113.4_72900001	14	72900001	0.3834	0.4143
NC_005113.4_73000001	14	73000001	0.4235	0.4365
NC_005113.4_73100001	14	73100001	0.3764	0.3897
NC_005113.4_73200001	14	73200001	0.4459	0.4736
NC_005113.4_73300001	14	73300001	0.5518	0.5162
NC_005113.4_73400001	14	73400001	0.5774	0.4961
NC_005113.4_73500001	14	73500001	0.5102	0.369
NC_005113.4_73600001	14	73600001	0.3859	0.3309
NC_005113.4_73700001	14	73700001	0.3521	0.3222
NC_005113.4_73800001	14	73800001	0.2742	0.2533
NC_005113.4_73900001	14	73900001	0.4304	0.5362
NC_005113.4_74000001	14	74000001	0.385	0.5527
NC_005113.4_74100001	14	74100001	0.4208	0.5332
NC_005113.4_74200001	14	74200001	0.5477	0.6197
NC_005113.4_74300001	14	74300001	0.6599	0.7056
NC_005113.4_74400001	14	74400001	0.5444	0.5555
NC_005113.4_74500001	14	74500001	0.5685	0.5339
NC_005113.4_74600001	14	74600001	0.554	0.6038
NC_005113.4_74700001	14	74700001	0.4439	0.5153
NC_005113.4_74800001	14	74800001	0.3492	0.3656
NC_005113.4_74900001	14	74900001	0.4082	0.5197
NC_005113.4_75000001	14	75000001	0.429	0.5992
NC_005113.4_75100001	14	75100001	0.488	0.574
NC_005113.4_75200001	14	75200001	0.5771	0.6162
NC_005113.4_75300001	14	75300001	0.5771	0.6162
NC_005113.4_75400001	14	75400001	0.4028	0.36
NC_005113.4_75500001	14	75500001	0.3426	0.4041

NC_005113.4_75600001	14	75600001	0.3426	0.4041
NC_005113.4_75700001	14	75700001	0.4725	0.5329
NC_005113.4_75800001	14	75800001	0.5071	0.5859
NC_005113.4_75900001	14	75900001	0.5999	0.73
NC_005113.4_76000001	14	76000001	0.6178	0.7131
NC_005113.4_76100001	14	76100001	0.6479	0.7412
NC_005113.4_76200001	14	76200001	0.5733	0.7552
NC_005113.4_76300001	14	76300001	0.6133	0.7688
NC_005113.4_76400001	14	76400001	0.6062	0.7764
NC_005113.4_76500001	14	76500001	0.6309	0.768
NC_005113.4_76600001	14	76600001	0.5296	0.6984
NC_005113.4_76700001	14	76700001	0.5467	0.6497
NC_005113.4_76800001	14	76800001	0.5699	0.6607
NC_005113.4_76900001	14	76900001	0.5621	0.6419
NC_005113.4_77000001	14	77000001	0.5771	0.6404
NC_005113.4_77100001	14	77100001	0.5224	0.6192
NC_005113.4_77200001	14	77200001	0.4998	0.6593
NC_005113.4_77300001	14	77300001	0.4229	0.6294
NC_005113.4_77400001	14	77400001	0.4128	0.6292
NC_005113.4_77500001	14	77500001	0.3726	0.6198
NC_005113.4_77600001	14	77600001	0.4114	0.6677
NC_005113.4_77700001	14	77700001	0.4202	0.6308
NC_005113.4_77800001	14	77800001	0.4427	0.6096
NC_005113.4_77900001	14	77900001	0.3926	0.5397
NC_005113.4_78000001	14	78000001	0.3542	0.5019
NC_005113.4_78100001	14	78100001	0.3484	0.4709
NC_005113.4_78200001	14	78200001	0.4044	0.5622
NC_005113.4_78300001	14	78300001	0.3983	0.5572
NC_005113.4_78400001	14	78400001	0.4174	0.5783
NC_005113.4_78500001	14	78500001	0.4661	0.5909
NC_005113.4_78600001	14	78600001	0.5602	0.6887
NC_005113.4_78700001	14	78700001	0.4601	0.5587
NC_005113.4_78800001	14	78800001	0.4601	0.5587
NC_005113.4_78900001	14	78900001	0.5626	0.6743
NC_005113.4_79000001	14	79000001	0.3576	0.678
NC_005113.4_79100001	14	79100001	0.2155	0.4802
NC_005113.4_79200001	14	79200001	0.2765	0.5999
NC_005113.4_79300001	14	79300001	0.3491	0.5735
NC_005113.4_79400001	14	79400001	0.3809	0.573
NC_005113.4_79500001	14	79500001	0.4669	0.6194
NC_005113.4_79600001	14	79600001	0.4814	0.6479
NC_005113.4_79700001	14	79700001	0.4835	0.5863
NC_005113.4_79800001	14	79800001	0.4833	0.5911
NC_005113.4_79900001	14	79900001	0.4681	0.5929
NC_005113.4_80000001	14	80000001	0.4336	0.5625
NC_005113.4_80100001	14	80100001	0.5126	0.6056
NC_005113.4_80200001	14	80200001	0.5429	0.6476
NC_005113.4_80300001	14	80300001	0.5167	0.6684
NC_005113.4_80400001	14	80400001	0.4768	0.6059
NC_005113.4_80500001	14	80500001	0.5145	0.6172
NC_005113.4_80600001	14	80600001	0.4436	0.5249
NC_005113.4_80700001	14	80700001	0.486	0.5962
NC_005113.4_80800001	14	80800001	0.6038	0.6784
NC_005113.4_80900001	14	80900001	0.6705	0.8001
NC_005113.4_81000001	14	81000001	0.662	0.8246
NC_005113.4_81100001	14	81100001	0.7324	0.906
NC_005113.4_81200001	14	81200001	0.8103	0.9141
NC_005113.4_81300001	14	81300001	0.7419	0.8378

NC_005113.4_81400001	14	81400001	0.6707	0.7724
NC_005113.4_81500001	14	81500001	0.6739	0.7113
NC_005113.4_81600001	14	81600001	0.5619	0.6213
NC_005113.4_81700001	14	81700001	0.493	0.5418
NC_005113.4_81800001	14	81800001	0.472	0.5544
NC_005113.4_81900001	14	81900001	0.4652	0.5171
NC_005113.4_82000001	14	82000001	0.4367	0.5381
NC_005113.4_82100001	14	82100001	0.4906	0.5713
NC_005113.4_82200001	14	82200001	0.525	0.6289
NC_005113.4_82300001	14	82300001	0.5502	0.6576
NC_005113.4_82400001	14	82400001	0.563	0.6704
NC_005113.4_82500001	14	82500001	0.5383	0.6732
NC_005113.4_82600001	14	82600001	0.5362	0.7682
NC_005113.4_82700001	14	82700001	0.4091	0.6577
NC_005113.4_82800001	14	82800001	0.3875	0.6648
NC_005113.4_82900001	14	82900001	0.4503	0.7347
NC_005113.4_83000001	14	83000001	0.4624	0.7434
NC_005113.4_83100001	14	83100001	0.4164	0.6607
NC_005113.4_83200001	14	83200001	0.4602	0.6923
NC_005113.4_83300001	14	83300001	0.4903	0.6975
NC_005113.4_83400001	14	83400001	0.4663	0.6307
NC_005113.4_83500001	14	83500001	0.4452	0.6245
NC_005113.4_83600001	14	83600001	0.5156	0.7136
NC_005113.4_83700001	14	83700001	0.5322	0.7063
NC_005113.4_83800001	14	83800001	0.4299	0.6241
NC_005113.4_83900001	14	83900001	0.4062	0.6084
NC_005113.4_84000001	14	84000001	0.5106	0.6799
NC_005113.4_84100001	14	84100001	0.4498	0.5909
NC_005113.4_84200001	14	84200001	0.5708	0.7386
NC_005113.4_84300001	14	84300001	0.6744	0.8222
NC_005113.4_84400001	14	84400001	0.6806	0.8404
NC_005113.4_84500001	14	84500001	0.6905	0.8955
NC_005113.4_84600001	14	84600001	0.7054	0.8908
NC_005113.4_84700001	14	84700001	0.7052	0.8719
NC_005113.4_84800001	14	84800001	0.7315	0.8545
NC_005113.4_84900001	14	84900001	0.8031	0.91
NC_005113.4_85000001	14	85000001	0.8545	0.8928
NC_005113.4_85100001	14	85100001	0.8365	0.9153
NC_005113.4_85200001	14	85200001	0.7406	0.846
NC_005113.4_85300001	14	85300001	0.723	0.8798
NC_005113.4_85400001	14	85400001	0.6207	0.7975
NC_005113.4_85500001	14	85500001	0.596	0.7214
NC_005113.4_85600001	14	85600001	0.6382	0.7335
NC_005113.4_85700001	14	85700001	0.5682	0.7044
NC_005113.4_85800001	14	85800001	0.5271	0.6417
NC_005113.4_85900001	14	85900001	0.5257	0.6265
NC_005113.4_86000001	14	86000001	0.5275	0.6694
NC_005113.4_86100001	14	86100001	0.4823	0.6409
NC_005113.4_86200001	14	86200001	0.5526	0.664
NC_005113.4_86300001	14	86300001	0.6282	0.6947
NC_005113.4_86400001	14	86400001	0.5784	0.6793
NC_005113.4_86500001	14	86500001	0.5917	0.64
NC_005113.4_86600001	14	86600001	0.5826	0.628
NC_005113.4_86700001	14	86700001	0.532	0.5909
NC_005113.4_86800001	14	86800001	0.4964	0.6118
NC_005113.4_86900001	14	86900001	0.4829	0.5944
NC_005113.4_87000001	14	87000001	0.4964	0.6107
NC_005113.4_87100001	14	87100001	0.4828	0.6064

NC_005113.4_87200001	14	87200001	0.4645	0.5875
NC_005113.4_87300001	14	87300001	0.5061	0.6002
NC_005113.4_87400001	14	87400001	0.5629	0.6241
NC_005113.4_87500001	14	87500001	0.5294	0.695
NC_005113.4_87600001	14	87600001	0.6699	0.8125
NC_005113.4_87700001	14	87700001	0.6939	0.8232
NC_005113.4_87800001	14	87800001	0.753	0.8649
NC_005113.4_87900001	14	87900001	0.7434	0.8539
NC_005113.4_88000001	14	88000001	0.7696	0.8254
NC_005113.4_88100001	14	88100001	0.7042	0.7749
NC_005113.4_88200001	14	88200001	0.4867	0.6007
NC_005113.4_88300001	14	88300001	0.4361	0.534
NC_005113.4_88400001	14	88400001	0.4495	0.5819
NC_005113.4_88500001	14	88500001	0.4172	0.5705
NC_005113.4_88600001	14	88600001	0.4318	0.6038
NC_005113.4_88700001	14	88700001	0.4755	0.6428
NC_005113.4_88800001	14	88800001	0.5024	0.6862
NC_005113.4_88900001	14	88900001	0.4765	0.6575
NC_005113.4_89000001	14	89000001	0.5009	0.6919
NC_005113.4_89100001	14	89100001	0.5134	0.7061
NC_005113.4_89200001	14	89200001	0.5799	0.785
NC_005113.4_89300001	14	89300001	0.5025	0.7473
NC_005113.4_89400001	14	89400001	0.469	0.747
NC_005113.4_89500001	14	89500001	0.5229	0.7362
NC_005113.4_89600001	14	89600001	0.5275	0.7496
NC_005113.4_89700001	14	89700001	0.4325	0.7137
NC_005113.4_89800001	14	89800001	0.3246	0.608
NC_005113.4_89900001	14	89900001	0.5544	0.7407
NC_005113.4_90000001	14	90000001	0.5093	0.7311
NC_005113.4_90100001	14	90100001	0.5086	0.6921
NC_005113.4_90200001	14	90200001	0.6218	0.7805
NC_005113.4_90300001	14	90300001	0.7862	0.8246
NC_005113.4_90400001	14	90400001	0.6097	0.8301
NC_005113.4_90500001	14	90500001	0.6097	0.8301
NC_005113.4_90600001	14	90600001	0.4532	0.6633
NC_005113.4_90700001	14	90700001	0.1545	0.1544
NC_005113.4_90800001	14	90800001	0.3158	0.4927
NC_005113.4_90900001	14	90900001	0.3923	0.5131
NC_005113.4_91000001	14	91000001	0.3857	0.5034
NC_005113.4_91100001	14	91100001	0.4911	0.6529
NC_005113.4_91200001	14	91200001	0.4732	0.5673
NC_005113.4_91300001	14	91300001	0.4993	0.5945
NC_005113.4_91400001	14	91400001	0.5251	0.6618
NC_005113.4_91500001	14	91500001	0.5696	0.7077
NC_005113.4_91600001	14	91600001	0.5575	0.6701
NC_005113.4_91700001	14	91700001	0.6307	0.7618
NC_005113.4_91800001	14	91800001	0.6258	0.7403
NC_005113.4_91900001	14	91900001	0.5336	0.7065
NC_005113.4_92000001	14	92000001	0.4792	0.6729
NC_005113.4_92100001	14	92100001	0.4253	0.5885
NC_005113.4_92200001	14	92200001	0.3704	0.5092
NC_005113.4_92300001	14	92300001	0.377	0.469
NC_005113.4_92400001	14	92400001	0.4141	0.4842
NC_005113.4_92500001	14	92500001	0.4733	0.4948
NC_005113.4_92600001	14	92600001	0.5165	0.5035
NC_005113.4_92700001	14	92700001	0.6867	0.6304
NC_005113.4_92800001	14	92800001	0.67	0.6546
NC_005113.4_92900001	14	92900001	0.592	0.5698

NC_005113.4_93000001	14	93000001	0.417	0.5075
NC_005113.4_93100001	14	93100001	0.3233	0.4364
NC_005113.4_93200001	14	93200001	0.2919	0.2655
NC_005113.4_93300001	14	93300001	0.2972	0.2782
NC_005113.4_93400001	14	93400001	0.3248	0.2981
NC_005113.4_93500001	14	93500001	0.3819	0.3419
NC_005113.4_93600001	14	93600001	0.5325	0.4481
NC_005113.4_93700001	14	93700001	0.5985	0.5504
NC_005113.4_93800001	14	93800001	0.5985	0.5504
NC_005113.4_97300001	14	97300001	0.6512	0.7073
NC_005113.4_97400001	14	97400001	0.4962	0.6485
NC_005113.4_97500001	14	97500001	0.6135	0.7875
NC_005113.4_97600001	14	97600001	0.6135	0.7875
NC_005113.4_97700001	14	97700001	0.3935	0.6861
NC_005113.4_98200001	14	98200001	0.5666	0.7942
NC_005113.4_98300001	14	98300001	0.5666	0.7942
NC_005113.4_98400001	14	98400001	0.5598	0.7885
NC_005113.4_98500001	14	98500001	0.5703	0.645
NC_005113.4_98600001	14	98600001	0.5703	0.645
NC_005113.4_98700001	14	98700001	0.5963	0.6181
NC_005113.4_98800001	14	98800001	0.5181	0.5738
NC_005113.4_98900001	14	98900001	0.5307	0.6001
NC_005113.4_99000001	14	99000001	0.5647	0.7196
NC_005113.4_99100001	14	99100001	0.5737	0.7787
NC_005113.4_99200001	14	99200001	0.5516	0.7472
NC_005113.4_99300001	14	99300001	0.5654	0.8114
NC_005113.4_99400001	14	99400001	0.4902	0.7852
NC_005113.4_99500001	14	99500001	0.4274	0.6923
NC_005113.4_99600001	14	99600001	0.4069	0.5602
NC_005113.4_99700001	14	99700001	0.4124	0.5704
NC_005113.4_99800001	14	99800001	0.4071	0.5241
NC_005113.4_99900001	14	99900001	0.3945	0.4406
NC_005113.4_100000001	14	100000001	0.4205	0.4707
NC_005113.4_100100001	14	100100001	0.4315	0.4815
NC_005113.4_100200001	14	100200001	0.4908	0.4377
NC_005113.4_100300001	14	100300001	0.4933	0.4501
NC_005113.4_100400001	14	100400001	0.5322	0.4773
NC_005113.4_100500001	14	100500001	0.4684	0.4414
NC_005113.4_100600001	14	100600001	0.4308	0.4389
NC_005113.4_100700001	14	100700001	0.3466	0.5043
NC_005113.4_100800001	14	100800001	0.3466	0.5043
NC_005113.4_100900001	14	100900001	0.3466	0.5043
NC_005113.4_102200001	14	102200001	0.4847	0.4603
NC_005113.4_102300001	14	102300001	0.4281	0.4535
NC_005113.4_102400001	14	102400001	0.3537	0.4058
NC_005113.4_102500001	14	102500001	0.3086	0.3684
NC_005113.4_102600001	14	102600001	0.2574	0.2643
NC_005113.4_102700001	14	102700001	0.2107	0.2525
NC_005113.4_102800001	14	102800001	0.3516	0.4893
NC_005113.4_102900001	14	102900001	0.4044	0.532
NC_005113.4_103000001	14	103000001	0.5231	0.6238
NC_005113.4_103100001	14	103100001	0.5958	0.6551
NC_005113.4_103200001	14	103200001	0.6703	0.7394
NC_005113.4_103300001	14	103300001	0.6167	0.7196
NC_005113.4_103400001	14	103400001	0.5561	0.6371
NC_005113.4_103500001	14	103500001	0.5001	0.5557
NC_005113.4_103600001	14	103600001	0.4378	0.491
NC_005113.4_103700001	14	103700001	0.408	0.4649

NC_005113.4_103800001	14	103800001	0.4066	0.4559
NC_005113.4_103900001	14	103900001	0.3875	0.4306
NC_005113.4_104000001	14	104000001	0.4471	0.5102
NC_005113.4_104100001	14	104100001	0.4812	0.5519
NC_005113.4_104200001	14	104200001	0.4981	0.5652
NC_005113.4_104300001	14	104300001	0.4837	0.5172
NC_005113.4_104400001	14	104400001	0.5228	0.5654
NC_005113.4_104500001	14	104500001	0.513	0.6106
NC_005113.4_104600001	14	104600001	0.5858	0.6758
NC_005113.4_104700001	14	104700001	0.5543	0.6649
NC_005113.4_104800001	14	104800001	0.584	0.7504
NC_005113.4_104900001	14	104900001	0.527	0.7208
NC_005113.4_105000001	14	105000001	0.479	0.6123
NC_005113.4_105100001	14	105100001	0.5598	0.7608
NC_005113.4_105200001	14	105200001	0.6204	0.762
NC_005113.4_105300001	14	105300001	0.5486	0.6321
NC_005113.4_105400001	14	105400001	0.5598	0.6407
NC_005113.4_105500001	14	105500001	0.5538	0.6622
NC_005113.4_105600001	14	105600001	0.5481	0.621
NC_005113.4_105700001	14	105700001	0.4954	0.5408
NC_005113.4_105800001	14	105800001	0.584	0.7816
NC_005113.4_105900001	14	105900001	0.5717	0.7316
NC_005113.4_106000001	14	106000001	0.6324	0.7363
NC_005113.4_106100001	14	106100001	0.5542	0.7006
NC_005113.4_106200001	14	106200001	0.4894	0.635
NC_005113.4_106300001	14	106300001	0.5356	0.6564
NC_005113.4_106400001	14	106400001	0.5501	0.6591
NC_005113.4_106500001	14	106500001	0.542	0.6889
NC_005113.4_106600001	14	106600001	0.5115	0.7182
NC_005113.4_106700001	14	106700001	0.4848	0.5799
NC_005113.4_106800001	14	106800001	0.4555	0.5551
NC_005113.4_106900001	14	106900001	0.426	0.488
NC_005113.4_107000001	14	107000001	0.4813	0.5447
NC_005113.4_107100001	14	107100001	0.4426	0.49
NC_005113.4_107200001	14	107200001	0.4544	0.5557
NC_005113.4_107300001	14	107300001	0.4324	0.5632
NC_005113.4_107400001	14	107400001	0.4563	0.639
NC_005113.4_107500001	14	107500001	0.3834	0.5949
NC_005113.4_107600001	14	107600001	0.3875	0.631
NC_005113.4_107700001	14	107700001	0.4722	0.6627
NC_005113.4_107800001	14	107800001	0.6835	0.7923
NC_005113.4_107900001	14	107900001	0.6361	0.7987
NC_005113.4_108000001	14	108000001	0.6333	0.7854
NC_005113.4_108100001	14	108100001	0.614	0.7514
NC_005113.4_108200001	14	108200001	0.6172	0.7661
NC_005113.4_108300001	14	108300001	0.4872	0.6307
NC_005113.4_108400001	14	108400001	0.4983	0.6031
NC_005113.4_108500001	14	108500001	0.5076	0.6161
NC_005113.4_108600001	14	108600001	0.5999	0.6975
NC_005113.4_108700001	14	108700001	0.5972	0.7037
NC_005113.4_108800001	14	108800001	0.6559	0.7124
NC_005113.4_108900001	14	108900001	0.6891	0.72
NC_005113.4_109000001	14	109000001	0.6918	0.7378
NC_005113.4_109100001	14	109100001	0.6071	0.6894
NC_005113.4_109200001	14	109200001	0.5705	0.6493
NC_005113.4_109300001	14	109300001	0.5731	0.687
NC_005113.4_109400001	14	109400001	0.5256	0.6579
NC_005113.4_109500001	14	109500001	0.6145	0.7183

NC_005113.4_109600001	14	109600001	0.5825	0.7035
NC_005113.4_109700001	14	109700001	0.6182	0.7315
NC_005113.4_109800001	14	109800001	0.6217	0.7342
NC_005113.4_109900001	14	109900001	0.7025	0.7944
NC_005113.4_110000001	14	110000001	0.604	0.7275
NC_005113.4_110100001	14	110100001	0.6671	0.7267
NC_005113.4_110200001	14	110200001	0.6881	0.7713
NC_005113.4_110300001	14	110300001	0.7496	0.8315
NC_005113.4_110400001	14	110400001	0.696	0.7603
NC_005113.4_110500001	14	110500001	0.7509	0.8364
NC_005113.4_110600001	14	110600001	0.6633	0.7894
NC_005113.4_110700001	14	110700001	0.5322	0.7477
NC_005113.4_110800001	14	110800001	0.2664	0.4017
NC_005113.4_110900001	14	110900001	0.3175	0.5015
NC_005113.4_111000001	14	111000001	0.3657	0.4974
NC_005113.4_111100001	14	111100001	0.3896	0.4726
NC_005113.4_111200001	14	111200001	0.4009	0.4315
NC_005113.4_111300001	14	111300001	0.3778	0.394
NC_005113.4_111400001	14	111400001	0.3373	0.3575
NC_005113.4_111500001	14	111500001	0.2385	0.1447
NC_005113.4_112100001	14	112100001	0.3704	0.2239
NC_005113.4_112200001	14	112200001	0.414	0.2733
NC_005113.4_112300001	14	112300001	0.4668	0.399
NC_005113.4_112400001	14	112400001	0.5047	0.4099
NC_005113.4_112500001	14	112500001	0.5043	0.4392
NC_005113.4_112600001	14	112600001	0.6419	0.669
NC_005113.4_112700001	14	112700001	0.6972	0.7766
NC_005113.4_112800001	14	112800001	0.653	0.7197
NC_005113.4_112900001	14	112900001	0.6951	0.8002
NC_005113.4_113000001	14	113000001	0.7251	0.8241
NC_005113.4_113100001	14	113100001	0.5327	0.6958
NC_005113.4_113200001	14	113200001	0.5327	0.6958
NC_005113.4_113400001	14	113400001	0.4636	0.7964
NC_005113.4_113800001	14	113800001	0.1103	0.184
NC_005113.4_113900001	14	113900001	0.4268	0.6245
NC_005113.4_114000001	14	114000001	0.3108	0.516
NC_005113.4_114100001	14	114100001	0.3968	0.5883
NC_005113.4_114200001	14	114200001	0.365	0.5526
NC_005113.4_114300001	14	114300001	0.3916	0.5581
NC_005113.4_114400001	14	114400001	0.3505	0.543
NC_005113.4_114500001	14	114500001	0.3828	0.5964
NC_005113.4_114600001	14	114600001	0.313	0.4285
NC_005113.4_114700001	14	114700001	0.5186	0.6792
NC_005113.4_114800001	14	114800001	0.5186	0.6792
NC_005113.4_114900001	14	114900001	0.4837	0.6598
NC_005113.4_115000001	14	115000001	0.5972	0.6288
NC_005114.4_1	15	1	0.4752	0.4973
NC_005114.4_100001	15	100001	0.577	0.5834
NC_005114.4_200001	15	200001	0.6124	0.6068
NC_005114.4_300001	15	300001	0.6731	0.6346
NC_005114.4_400001	15	400001	0.6497	0.5804
NC_005114.4_500001	15	500001	0.6686	0.6244
NC_005114.4_600001	15	600001	0.5641	0.6013
NC_005114.4_700001	15	700001	0.4843	0.582
NC_005114.4_800001	15	800001	0.3989	0.5209
NC_005114.4_900001	15	900001	0.3072	0.4649
NC_005114.4_1000001	15	1000001	0.4016	0.5877
NC_005114.4_1100001	15	1100001	0.4058	0.6345

NC_005114.4_1200001	15	1200001	0.4338	0.6658
NC_005114.4_1300001	15	1300001	0.5383	0.7595
NC_005114.4_1400001	15	1400001	0.5233	0.7175
NC_005114.4_1500001	15	1500001	0.5705	0.7316
NC_005114.4_1600001	15	1600001	0.602	0.7549
NC_005114.4_1700001	15	1700001	0.5485	0.7238
NC_005114.4_1800001	15	1800001	0.45	0.6516
NC_005114.4_1900001	15	1900001	0.4472	0.6723
NC_005114.4_2000001	15	2000001	0.4126	0.646
NC_005114.4_2100001	15	2100001	0.3776	0.5926
NC_005114.4_2200001	15	2200001	0.4365	0.6421
NC_005114.4_2300001	15	2300001	0.4991	0.6724
NC_005114.4_2400001	15	2400001	0.5364	0.7451
NC_005114.4_2500001	15	2500001	0.6306	0.814
NC_005114.4_2600001	15	2600001	0.6265	0.7939
NC_005114.4_2700001	15	2700001	0.6427	0.7676
NC_005114.4_2800001	15	2800001	0.7831	0.8295
NC_005114.4_2900001	15	2900001	0.737	0.7948
NC_005114.4_3000001	15	3000001	0.6445	0.7184
NC_005114.4_3100001	15	3100001	0.4977	0.616
NC_005114.4_3200001	15	3200001	0.4112	0.5964
NC_005114.4_3300001	15	3300001	0.2973	0.4231
NC_005114.4_3400001	15	3400001	0.3179	0.4597
NC_005114.4_3500001	15	3500001	0.3235	0.4792
NC_005114.4_3600001	15	3600001	0.2459	0.4732
NC_005114.4_3700001	15	3700001	0.1141	0.0849
NC_005114.4_3800001	15	3800001	0.2348	0.4137
NC_005114.4_3900001	15	3900001	0.3597	0.6583
NC_005114.4_4000001	15	4000001	0.3737	0.6845
NC_005114.4_4100001	15	4100001	0.5244	0.7989
NC_005114.4_4200001	15	4200001	0.4666	0.7599
NC_005114.4_4300001	15	4300001	0.4649	0.738
NC_005114.4_4400001	15	4400001	0.4297	0.7006
NC_005114.4_4500001	15	4500001	0.453	0.6619
NC_005114.4_4600001	15	4600001	0.4123	0.6238
NC_005114.4_4700001	15	4700001	0.4911	0.6421
NC_005114.4_4800001	15	4800001	0.476	0.6501
NC_005114.4_4900001	15	4900001	0.5233	0.6619
NC_005114.4_5800001	15	5800001	0.4843	0.7819
NC_005114.4_5900001	15	5900001	0.4811	0.7331
NC_005114.4_6000001	15	6000001	0.6076	0.8047
NC_005114.4_6100001	15	6100001	0.5691	0.7469
NC_005114.4_6200001	15	6200001	0.6335	0.7657
NC_005114.4_6300001	15	6300001	0.5982	0.6773
NC_005114.4_6400001	15	6400001	0.5757	0.6557
NC_005114.4_6500001	15	6500001	0.3401	0.3883
NC_005114.4_6600001	15	6600001	0.2967	0.326
NC_005114.4_6700001	15	6700001	0.308	0.3454
NC_005114.4_6800001	15	6800001	0.5411	0.6731
NC_005114.4_6900001	15	6900001	0.598	0.735
NC_005114.4_7000001	15	7000001	0.7091	0.7531
NC_005114.4_7100001	15	7100001	0.7372	0.7807
NC_005114.4_7200001	15	7200001	0.7573	0.7917
NC_005114.4_7300001	15	7300001	0.6937	0.7114
NC_005114.4_7400001	15	7400001	0.6689	0.749
NC_005114.4_7500001	15	7500001	0.6473	0.7697
NC_005114.4_7600001	15	7600001	0.573	0.7137
NC_005114.4_7700001	15	7700001	0.6361	0.772

NC_005114.4_7800001	15	7800001	0.6294	0.8038
NC_005114.4_7900001	15	7900001	0.6576	0.7574
NC_005114.4_8000001	15	8000001	0.6493	0.7607
NC_005114.4_8100001	15	8100001	0.6891	0.8114
NC_005114.4_8200001	15	8200001	0.5093	0.7148
NC_005114.4_8300001	15	8300001	0.4903	0.697
NC_005114.4_8400001	15	8400001	0.4985	0.6531
NC_005114.4_8500001	15	8500001	0.4813	0.6023
NC_005114.4_8600001	15	8600001	0.4983	0.5824
NC_005114.4_8700001	15	8700001	0.5536	0.6313
NC_005114.4_8800001	15	8800001	0.5322	0.6045
NC_005114.4_8900001	15	8900001	0.4778	0.5439
NC_005114.4_9000001	15	9000001	0.4355	0.5291
NC_005114.4_9100001	15	9100001	0.4292	0.5337
NC_005114.4_9200001	15	9200001	0.3531	0.4438
NC_005114.4_9300001	15	9300001	0.3391	0.4145
NC_005114.4_9400001	15	9400001	0.3364	0.4567
NC_005114.4_9500001	15	9500001	0.3586	0.4624
NC_005114.4_9600001	15	9600001	0.4217	0.56
NC_005114.4_9700001	15	9700001	0.4746	0.5914
NC_005114.4_9800001	15	9800001	0.5502	0.6791
NC_005114.4_9900001	15	9900001	0.5588	0.6906
NC_005114.4_10000001	15	10000001	0.6573	0.7621
NC_005114.4_10100001	15	10100001	0.6033	0.7064
NC_005114.4_10200001	15	10200001	0.5941	0.7335
NC_005114.4_10300001	15	10300001	0.4872	0.652
NC_005114.4_10400001	15	10400001	0.4681	0.6184
NC_005114.4_10500001	15	10500001	0.4154	0.5326
NC_005114.4_10600001	15	10600001	0.4159	0.5128
NC_005114.4_10700001	15	10700001	0.4894	0.5505
NC_005114.4_10800001	15	10800001	0.5032	0.5269
NC_005114.4_10900001	15	10900001	0.5252	0.5349
NC_005114.4_11000001	15	11000001	0.5492	0.5612
NC_005114.4_11100001	15	11100001	0.5334	0.5736
NC_005114.4_11200001	15	11200001	0.4553	0.4875
NC_005114.4_11300001	15	11300001	0.4437	0.5063
NC_005114.4_11400001	15	11400001	0.4035	0.5231
NC_005114.4_11500001	15	11500001	0.4421	0.5542
NC_005114.4_11600001	15	11600001	0.4023	0.5133
NC_005114.4_11700001	15	11700001	0.4633	0.6671
NC_005114.4_11800001	15	11800001	0.4485	0.8001
NC_005114.4_11900001	15	11900001	0.2719	0.4539
NC_005114.4_12000001	15	12000001	0.6782	0.8862
NC_005114.4_12100001	15	12100001	0.8292	0.9334
NC_005114.4_12200001	15	12200001	0.8387	0.9377
NC_005114.4_12300001	15	12300001	0.8209	0.9047
NC_005114.4_12400001	15	12400001	0.7291	0.8056
NC_005114.4_12500001	15	12500001	0.5823	0.6672
NC_005114.4_12600001	15	12600001	0.4486	0.5162
NC_005114.4_12700001	15	12700001	0.4592	0.5477
NC_005114.4_12800001	15	12800001	0.487	0.5813
NC_005114.4_12900001	15	12900001	0.6448	0.7442
NC_005114.4_13000001	15	13000001	0.7051	0.7894
NC_005114.4_13100001	15	13100001	0.7101	0.8027
NC_005114.4_13200001	15	13200001	0.7299	0.8017
NC_005114.4_13300001	15	13300001	0.717	0.8056
NC_005114.4_13400001	15	13400001	0.6298	0.7434
NC_005114.4_13500001	15	13500001	0.5418	0.7086

NC_005114.4_13600001	15	13600001	0.6567	0.7354
NC_005114.4_13700001	15	13700001	0.6201	0.693
NC_005114.4_13800001	15	13800001	0.6591	0.7099
NC_005114.4_13900001	15	13900001	0.6709	0.697
NC_005114.4_14000001	15	14000001	0.6532	0.6644
NC_005114.4_14100001	15	14100001	0.5086	0.5688
NC_005114.4_14200001	15	14200001	0.4603	0.5428
NC_005114.4_14300001	15	14300001	0.5379	0.6307
NC_005114.4_14400001	15	14400001	0.5011	0.6024
NC_005114.4_14500001	15	14500001	0.5808	0.6905
NC_005114.4_14600001	15	14600001	0.6931	0.7135
NC_005114.4_14700001	15	14700001	0.6603	0.6865
NC_005114.4_14800001	15	14800001	0.5221	0.6168
NC_005114.4_14900001	15	14900001	0.5811	0.7221
NC_005114.4_15000001	15	15000001	0.5852	0.7412
NC_005114.4_15100001	15	15100001	0.565	0.6767
NC_005114.4_15200001	15	15200001	0.6096	0.7115
NC_005114.4_15300001	15	15300001	0.6039	0.6973
NC_005114.4_15400001	15	15400001	0.5187	0.637
NC_005114.4_15500001	15	15500001	0.4678	0.5559
NC_005114.4_15600001	15	15600001	0.4039	0.5332
NC_005114.4_15700001	15	15700001	0.3678	0.4824
NC_005114.4_15800001	15	15800001	0.413	0.5865
NC_005114.4_15900001	15	15900001	0.4745	0.5876
NC_005114.4_16000001	15	16000001	0.5388	0.6381
NC_005114.4_16100001	15	16100001	0.5238	0.6664
NC_005114.4_16200001	15	16200001	0.514	0.6682
NC_005114.4_16300001	15	16300001	0.4426	0.5487
NC_005114.4_16400001	15	16400001	0.4946	0.6204
NC_005114.4_16500001	15	16500001	0.4702	0.5977
NC_005114.4_16600001	15	16600001	0.5189	0.6304
NC_005114.4_16700001	15	16700001	0.511	0.6296
NC_005114.4_16800001	15	16800001	0.5492	0.5878
NC_005114.4_16900001	15	16900001	0.5211	0.5733
NC_005114.4_17000001	15	17000001	0.4532	0.5161
NC_005114.4_17100001	15	17100001	0.4282	0.4766
NC_005114.4_17200001	15	17200001	0.4704	0.511
NC_005114.4_17300001	15	17300001	0.4892	0.6013
NC_005114.4_17400001	15	17400001	0.4917	0.632
NC_005114.4_17500001	15	17500001	0.5064	0.6531
NC_005114.4_17600001	15	17600001	0.5453	0.7153
NC_005114.4_17700001	15	17700001	0.5625	0.8357
NC_005114.4_17800001	15	17800001	0.4595	0.7119
NC_005114.4_17900001	15	17900001	0.1257	0.0836
NC_005114.4_18000001	15	18000001	0.2081	0.3694
NC_005114.4_18100001	15	18100001	0.3115	0.5126
NC_005114.4_18200001	15	18200001	0.3364	0.4394
NC_005114.4_18300001	15	18300001	0.3377	0.4384
NC_005114.4_18400001	15	18400001	0.4659	0.5102
NC_005114.4_18500001	15	18500001	0.4742	0.4866
NC_005114.4_18600001	15	18600001	0.5058	0.4701
NC_005114.4_18700001	15	18700001	0.6796	0.5749
NC_005114.4_18800001	15	18800001	0.7486	0.6004
NC_005114.4_18900001	15	18900001	0.5483	0.5352
NC_005114.4_19000001	15	19000001	0.5077	0.5384
NC_005114.4_19100001	15	19100001	0.5001	0.5468
NC_005114.4_19200001	15	19200001	0.4535	0.5427
NC_005114.4_19300001	15	19300001	0.4408	0.5273

NC_005114.4_19400001	15	19400001	0.38	0.5845
NC_005114.4_19500001	15	19500001	0.3776	0.5721
NC_005114.4_19600001	15	19600001	0.3333	0.4967
NC_005114.4_19700001	15	19700001	0.3964	0.5601
NC_005114.4_19800001	15	19800001	0.4824	0.6653
NC_005114.4_19900001	15	19900001	0.5562	0.7042
NC_005114.4_20000001	15	20000001	0.5688	0.7365
NC_005114.4_20100001	15	20100001	0.6617	0.8682
NC_005114.4_20200001	15	20200001	0.5946	0.7334
NC_005114.4_20300001	15	20300001	0.3895	0.5238
NC_005114.4_20400001	15	20400001	0.3044	0.4056
NC_005114.4_20500001	15	20500001	0.3282	0.3902
NC_005114.4_20600001	15	20600001	0.2989	0.3761
NC_005114.4_20700001	15	20700001	0.2724	0.4464
NC_005114.4_20800001	15	20800001	0.3325	0.5396
NC_005114.4_23100001	15	23100001	0.5659	0.6528
NC_005114.4_23200001	15	23200001	0.5739	0.6487
NC_005114.4_23300001	15	23300001	0.5519	0.6539
NC_005114.4_23400001	15	23400001	0.5619	0.6753
NC_005114.4_23500001	15	23500001	0.5409	0.6777
NC_005114.4_23600001	15	23600001	0.5181	0.6419
NC_005114.4_23700001	15	23700001	0.5123	0.6717
NC_005114.4_23800001	15	23800001	0.5142	0.6236
NC_005114.4_23900001	15	23900001	0.4977	0.6162
NC_005114.4_24000001	15	24000001	0.4977	0.6162
NC_005114.4_24100001	15	24100001	0.509	0.6307
NC_005114.4_24200001	15	24200001	0.4192	0.556
NC_005114.4_24300001	15	24300001	0.4467	0.6849
NC_005114.4_24400001	15	24400001	0.4546	0.6113
NC_005114.4_24500001	15	24500001	0.438	0.606
NC_005114.4_24600001	15	24600001	0.4714	0.688
NC_005114.4_24700001	15	24700001	0.6048	0.7316
NC_005114.4_24800001	15	24800001	0.6399	0.7343
NC_005114.4_24900001	15	24900001	0.6664	0.7876
NC_005114.4_25000001	15	25000001	0.6532	0.715
NC_005114.4_25100001	15	25100001	0.6571	0.7055
NC_005114.4_25200001	15	25200001	0.615	0.6897
NC_005114.4_25300001	15	25300001	0.5877	0.6811
NC_005114.4_25400001	15	25400001	0.4906	0.6049
NC_005114.4_25500001	15	25500001	0.5835	0.7684
NC_005114.4_25600001	15	25600001	0.5734	0.7455
NC_005114.4_25700001	15	25700001	0.5422	0.7083
NC_005114.4_25800001	15	25800001	0.5907	0.7483
NC_005114.4_25900001	15	25900001	0.6579	0.7804
NC_005114.4_26000001	15	26000001	0.6056	0.7372
NC_005114.4_26100001	15	26100001	0.6439	0.7424
NC_005114.4_26200001	15	26200001	0.6544	0.7517
NC_005114.4_26300001	15	26300001	0.4966	0.6171
NC_005114.4_26400001	15	26400001	0.5404	0.6256
NC_005114.4_26500001	15	26500001	0.4263	0.5159
NC_005114.4_26600001	15	26600001	0.2108	0.1847
NC_005114.4_26700001	15	26700001	0.3705	0.4708
NC_005114.4_26800001	15	26800001	0.4292	0.4911
NC_005114.4_26900001	15	26900001	0.3538	0.42
NC_005114.4_27000001	15	27000001	0.3538	0.42
NC_005114.4_27100001	15	27100001	0.369	0.4389
NC_005114.4_27200001	15	27200001	0.1495	0.1199
NC_005114.4_27300001	15	27300001	0.4616	0.6042

NC_005114.4_27400001	15	27400001	0.4929	0.5954
NC_005114.4_27500001	15	27500001	0.5187	0.6256
NC_005114.4_27600001	15	27600001	0.539	0.6416
NC_005114.4_27700001	15	27700001	0.5987	0.6502
NC_005114.4_27800001	15	27800001	0.4924	0.603
NC_005114.4_27900001	15	27900001	0.5885	0.7125
NC_005114.4_28000001	15	28000001	0.5794	0.6318
NC_005114.4_28100001	15	28100001	0.555	0.6422
NC_005114.4_28200001	15	28200001	0.5103	0.6091
NC_005114.4_28300001	15	28300001	0.5297	0.6114
NC_005114.4_28400001	15	28400001	0.5391	0.5882
NC_005114.4_28500001	15	28500001	0.5211	0.6463
NC_005114.4_28600001	15	28600001	0.4474	0.4697
NC_005114.4_28700001	15	28700001	0.5649	0.5416
NC_005114.4_28800001	15	28800001	0.5649	0.5416
NC_005114.4_32200001	15	32200001	0.5338	0.8917
NC_005114.4_32300001	15	32300001	0.4343	0.8026
NC_005114.4_32400001	15	32400001	0.3554	0.6436
NC_005114.4_32500001	15	32500001	0.2686	0.4892
NC_005114.4_32600001	15	32600001	0.3111	0.5232
NC_005114.4_32700001	15	32700001	0.3155	0.4561
NC_005114.4_32800001	15	32800001	0.4026	0.5227
NC_005114.4_32900001	15	32900001	0.4634	0.5655
NC_005114.4_33000001	15	33000001	0.5046	0.6009
NC_005114.4_33100001	15	33100001	0.5229	0.6283
NC_005114.4_33200001	15	33200001	0.5438	0.6776
NC_005114.4_33300001	15	33300001	0.519	0.6866
NC_005114.4_33400001	15	33400001	0.5329	0.7114
NC_005114.4_33500001	15	33500001	0.5504	0.7474
NC_005114.4_33600001	15	33600001	0.4954	0.6666
NC_005114.4_33700001	15	33700001	0.5277	0.6958
NC_005114.4_33800001	15	33800001	0.5294	0.7305
NC_005114.4_33900001	15	33900001	0.5535	0.7445
NC_005114.4_34000001	15	34000001	0.5481	0.7143
NC_005114.4_34100001	15	34100001	0.6114	0.8027
NC_005114.4_34200001	15	34200001	0.6096	0.793
NC_005114.4_34300001	15	34300001	0.5989	0.7571
NC_005114.4_34400001	15	34400001	0.5487	0.7257
NC_005114.4_34500001	15	34500001	0.3955	0.6913
NC_005114.4_34600001	15	34600001	0.3197	0.5395
NC_005114.4_34700001	15	34700001	0.328	0.4296
NC_005114.4_34800001	15	34800001	0.3604	0.4515
NC_005114.4_34900001	15	34900001	0.3115	0.3639
NC_005114.4_35000001	15	35000001	0.3635	0.4116
NC_005114.4_35100001	15	35100001	0.3371	0.3274
NC_005114.4_36400001	15	36400001	0.348	0.3815
NC_005114.4_36500001	15	36500001	0.4652	0.6204
NC_005114.4_36600001	15	36600001	0.5129	0.6605
NC_005114.4_36700001	15	36700001	0.5801	0.6649
NC_005114.4_36800001	15	36800001	0.6238	0.729
NC_005114.4_36900001	15	36900001	0.5443	0.7087
NC_005114.4_37000001	15	37000001	0.4359	0.6252
NC_005114.4_37100001	15	37100001	0.4309	0.6376
NC_005114.4_37200001	15	37200001	0.3797	0.61
NC_005114.4_37300001	15	37300001	0.382	0.5505
NC_005114.4_37400001	15	37400001	0.4069	0.6177
NC_005114.4_37500001	15	37500001	0.5173	0.7098
NC_005114.4_37600001	15	37600001	0.5762	0.7387

NC_005114.4_37700001	15	37700001	0.6366	0.755
NC_005114.4_37800001	15	37800001	0.6132	0.752
NC_005114.4_37900001	15	37900001	0.6193	0.7207
NC_005114.4_38000001	15	38000001	0.5664	0.6799
NC_005114.4_38100001	15	38100001	0.542	0.6817
NC_005114.4_38200001	15	38200001	0.5071	0.6565
NC_005114.4_38300001	15	38300001	0.4453	0.6403
NC_005114.4_38400001	15	38400001	0.3743	0.6155
NC_005114.4_38500001	15	38500001	0.4244	0.6527
NC_005114.4_38600001	15	38600001	0.3773	0.5873
NC_005114.4_38700001	15	38700001	0.4548	0.6859
NC_005114.4_38800001	15	38800001	0.4963	0.6673
NC_005114.4_38900001	15	38900001	0.5172	0.6608
NC_005114.4_39000001	15	39000001	0.4878	0.6518
NC_005114.4_39100001	15	39100001	0.5739	0.6753
NC_005114.4_39200001	15	39200001	0.5021	0.5564
NC_005114.4_39300001	15	39300001	0.5516	0.6483
NC_005114.4_39400001	15	39400001	0.5126	0.7773
NC_005114.4_39500001	15	39500001	0.4955	0.745
NC_005114.4_39600001	15	39600001	0.3983	0.6617
NC_005114.4_39700001	15	39700001	0.2982	0.5724
NC_005114.4_39800001	15	39800001	0.287	0.6007
NC_005114.4_39900001	15	39900001	0.3155	0.5347
NC_005114.4_40000001	15	40000001	0.4066	0.6604
NC_005114.4_40100001	15	40100001	0.4182	0.6931
NC_005114.4_40200001	15	40200001	0.4838	0.6975
NC_005114.4_40300001	15	40300001	0.4912	0.6499
NC_005114.4_40400001	15	40400001	0.4733	0.5982
NC_005114.4_40500001	15	40500001	0.4756	0.5136
NC_005114.4_40600001	15	40600001	0.5045	0.5258
NC_005114.4_40700001	15	40700001	0.4957	0.5463
NC_005114.4_40800001	15	40800001	0.4887	0.527
NC_005114.4_40900001	15	40900001	0.4884	0.5449
NC_005114.4_41000001	15	41000001	0.4611	0.541
NC_005114.4_41100001	15	41100001	0.463	0.5206
NC_005114.4_41200001	15	41200001	0.4268	0.4648
NC_005114.4_41300001	15	41300001	0.4645	0.5266
NC_005114.4_41400001	15	41400001	0.4663	0.5392
NC_005114.4_41500001	15	41500001	0.5276	0.6227
NC_005114.4_41600001	15	41600001	0.529	0.7007
NC_005114.4_41700001	15	41700001	0.5672	0.755
NC_005114.4_41800001	15	41800001	0.6031	0.8167
NC_005114.4_41900001	15	41900001	0.5528	0.6551
NC_005114.4_42000001	15	42000001	0.5096	0.5741
NC_005114.4_42100001	15	42100001	0.5345	0.5853
NC_005114.4_42200001	15	42200001	0.617	0.6568
NC_005114.4_42300001	15	42300001	0.6283	0.6763
NC_005114.4_42400001	15	42400001	0.7369	0.8109
NC_005114.4_42500001	15	42500001	0.6976	0.7901
NC_005114.4_42600001	15	42600001	0.6386	0.7811
NC_005114.4_42700001	15	42700001	0.5478	0.7268
NC_005114.4_42800001	15	42800001	0.5994	0.7712
NC_005114.4_42900001	15	42900001	0.6169	0.789
NC_005114.4_43000001	15	43000001	0.6293	0.8471
NC_005114.4_43100001	15	43100001	0.6598	0.8588
NC_005114.4_43200001	15	43200001	0.7426	0.9035
NC_005114.4_43300001	15	43300001	0.7036	0.8443
NC_005114.4_43400001	15	43400001	0.6547	0.7799

NC_005114.4_43500001	15	43500001	0.6807	0.6956
NC_005114.4_43600001	15	43600001	0.6725	0.6961
NC_005114.4_43700001	15	43700001	0.6787	0.7397
NC_005114.4_43800001	15	43800001	0.6207	0.7446
NC_005114.4_43900001	15	43900001	0.651	0.7992
NC_005114.4_44000001	15	44000001	0.6215	0.7617
NC_005114.4_44100001	15	44100001	0.5969	0.7691
NC_005114.4_44200001	15	44200001	0.5516	0.7074
NC_005114.4_44300001	15	44300001	0.5505	0.6917
NC_005114.4_44400001	15	44400001	0.5498	0.6972
NC_005114.4_44500001	15	44500001	0.5725	0.7539
NC_005114.4_44600001	15	44600001	0.6276	0.7001
NC_005114.4_44700001	15	44700001	0.5662	0.5739
NC_005114.4_44800001	15	44800001	0.5746	0.6408
NC_005114.4_44900001	15	44900001	0.515	0.6176
NC_005114.4_45000001	15	45000001	0.5281	0.6167
NC_005114.4_45100001	15	45100001	0.4714	0.5813
NC_005114.4_45200001	15	45200001	0.4455	0.6169
NC_005114.4_45300001	15	45300001	0.4126	0.5467
NC_005114.4_45400001	15	45400001	0.467	0.6165
NC_005114.4_45500001	15	45500001	0.4547	0.5972
NC_005114.4_45600001	15	45600001	0.5471	0.6133
NC_005114.4_45700001	15	45700001	0.611	0.6326
NC_005114.4_45800001	15	45800001	0.5712	0.6337
NC_005114.4_45900001	15	45900001	0.5686	0.6791
NC_005114.4_46000001	15	46000001	0.506	0.6782
NC_005114.4_46100001	15	46100001	0.4436	0.5955
NC_005114.4_46200001	15	46200001	0.4702	0.5943
NC_005114.4_46300001	15	46300001	0.4787	0.6066
NC_005114.4_46400001	15	46400001	0.4428	0.5035
NC_005114.4_46500001	15	46500001	0.4487	0.4139
NC_005114.4_46600001	15	46600001	0.5523	0.5707
NC_005114.4_46700001	15	46700001	0.6178	0.7293
NC_005114.4_46800001	15	46800001	0.606	0.6639
NC_005114.4_46900001	15	46900001	0.5421	0.6295
NC_005114.4_47000001	15	47000001	0.5731	0.643
NC_005114.4_47100001	15	47100001	0.5872	0.667
NC_005114.4_47200001	15	47200001	0.5153	0.635
NC_005114.4_47300001	15	47300001	0.5294	0.695
NC_005114.4_47400001	15	47400001	0.5576	0.7016
NC_005114.4_47500001	15	47500001	0.5563	0.6838
NC_005114.4_47600001	15	47600001	0.5527	0.7155
NC_005114.4_47700001	15	47700001	0.5438	0.7099
NC_005114.4_47800001	15	47800001	0.5922	0.6958
NC_005114.4_47900001	15	47900001	0.615	0.7145
NC_005114.4_48000001	15	48000001	0.4811	0.6661
NC_005114.4_48100001	15	48100001	0.441	0.631
NC_005114.4_48200001	15	48200001	0.4937	0.649
NC_005114.4_48300001	15	48300001	0.4318	0.6107
NC_005114.4_48400001	15	48400001	0.4288	0.6013
NC_005114.4_48500001	15	48500001	0.5332	0.7257
NC_005114.4_48600001	15	48600001	0.5579	0.7075
NC_005114.4_48700001	15	48700001	0.5048	0.6763
NC_005114.4_48800001	15	48800001	0.5048	0.6763
NC_005114.4_48900001	15	48900001	0.4642	0.6697
NC_005114.4_49000001	15	49000001	0.5044	0.6681
NC_005114.4_49100001	15	49100001	0.4532	0.6172
NC_005114.4_49200001	15	49200001	0.6171	0.6245

NC_005114.4_49300001	15	49300001	0.6171	0.6245
NC_005114.4_50000001	15	50000001	0.5481	0.5129
NC_005114.4_50100001	15	50100001	0.4395	0.4969
NC_005114.4_50200001	15	50200001	0.4395	0.4969
NC_005114.4_50300001	15	50300001	0.5859	0.6779
NC_005114.4_50400001	15	50400001	0.599	0.7182
NC_005114.4_50500001	15	50500001	0.6428	0.9171
NC_005114.4_50600001	15	50600001	0.7841	0.93
NC_005114.4_50700001	15	50700001	0.6626	0.9256
NC_005114.4_50800001	15	50800001	0.5298	0.8009
NC_005114.4_50900001	15	50900001	0.5388	0.8159
NC_005114.4_51000001	15	51000001	0.5909	0.787
NC_005114.4_51100001	15	51100001	0.583	0.7922
NC_005114.4_51200001	15	51200001	0.6127	0.7567
NC_005114.4_51300001	15	51300001	0.6697	0.8271
NC_005114.4_51400001	15	51400001	0.6662	0.8114
NC_005114.4_51500001	15	51500001	0.7099	0.8494
NC_005114.4_51600001	15	51600001	0.7479	0.8604
NC_005114.4_51700001	15	51700001	0.7366	0.8618
NC_005114.4_51800001	15	51800001	0.6177	0.8089
NC_005114.4_51900001	15	51900001	0.5825	0.8174
NC_005114.4_52000001	15	52000001	0.5874	0.8121
NC_005114.4_52100001	15	52100001	0.5113	0.6998
NC_005114.4_52200001	15	52200001	0.4864	0.6654
NC_005114.4_52300001	15	52300001	0.501	0.6486
NC_005114.4_52400001	15	52400001	0.4638	0.601
NC_005114.4_52500001	15	52500001	0.3524	0.4692
NC_005114.4_52600001	15	52600001	0.3334	0.5579
NC_005114.4_52700001	15	52700001	0.3168	0.6146
NC_005114.4_52800001	15	52800001	0.3258	0.631
NC_005114.4_52900001	15	52900001	0.3274	0.6329
NC_005114.4_53000001	15	53000001	0.5152	0.8019
NC_005114.4_53100001	15	53100001	0.523	0.8436
NC_005114.4_53200001	15	53200001	0.5258	0.7617
NC_005114.4_53300001	15	53300001	0.518	0.6979
NC_005114.4_53400001	15	53400001	0.5634	0.7105
NC_005114.4_53500001	15	53500001	0.4177	0.4684
NC_005114.4_53600001	15	53600001	0.4546	0.5604
NC_005114.4_53700001	15	53700001	0.4851	0.5871
NC_005114.4_53800001	15	53800001	0.5171	0.6619
NC_005114.4_53900001	15	53900001	0.5406	0.6875
NC_005114.4_54000001	15	54000001	0.6153	0.7561
NC_005114.4_54100001	15	54100001	0.6014	0.7225
NC_005114.4_54200001	15	54200001	0.5765	0.7202
NC_005114.4_54300001	15	54300001	0.6321	0.7224
NC_005114.4_54400001	15	54400001	0.6775	0.7307
NC_005114.4_54500001	15	54500001	0.455	0.5823
NC_005114.4_54600001	15	54600001	0.5308	0.67
NC_005114.4_54700001	15	54700001	0.5378	0.7034
NC_005114.4_54800001	15	54800001	0.5437	0.753
NC_005114.4_54900001	15	54900001	0.4765	0.7212
NC_005114.4_55000001	15	55000001	0.6518	0.8161
NC_005114.4_55100001	15	55100001	0.5786	0.7201
NC_005114.4_55200001	15	55200001	0.6102	0.7184
NC_005114.4_55300001	15	55300001	0.5501	0.6925
NC_005114.4_55400001	15	55400001	0.5745	0.6499
NC_005114.4_55500001	15	55500001	0.4962	0.5601
NC_005114.4_55600001	15	55600001	0.5416	0.6653

NC_005114.4_55700001	15	55700001	0.5119	0.5977
NC_005114.4_55800001	15	55800001	0.4786	0.5075
NC_005114.4_55900001	15	55900001	0.5044	0.5463
NC_005114.4_56000001	15	56000001	0.4779	0.5167
NC_005114.4_56100001	15	56100001	0.5202	0.5353
NC_005114.4_56200001	15	56200001	0.4299	0.4826
NC_005114.4_56300001	15	56300001	0.4051	0.4809
NC_005114.4_56400001	15	56400001	0.3087	0.3156
NC_005114.4_56500001	15	56500001	0.3379	0.3446
NC_005114.4_56600001	15	56600001	0.3701	0.4173
NC_005114.4_56700001	15	56700001	0.3572	0.4169
NC_005114.4_56800001	15	56800001	0.3727	0.4464
NC_005114.4_56900001	15	56900001	0.4035	0.5233
NC_005114.4_57000001	15	57000001	0.4432	0.6168
NC_005114.4_57100001	15	57100001	0.483	0.672
NC_005114.4_57200001	15	57200001	0.7041	0.8071
NC_005114.4_57300001	15	57300001	0.6197	0.758
NC_005114.4_57400001	15	57400001	0.6339	0.7684
NC_005114.4_57500001	15	57500001	0.6295	0.78
NC_005114.4_57600001	15	57600001	0.5324	0.7108
NC_005114.4_57700001	15	57700001	0.4753	0.7301
NC_005114.4_57800001	15	57800001	0.4944	0.7247
NC_005114.4_57900001	15	57900001	0.4536	0.6774
NC_005114.4_58000001	15	58000001	0.4833	0.7079
NC_005114.4_58100001	15	58100001	0.5334	0.7209
NC_005114.4_58200001	15	58200001	0.5319	0.6472
NC_005114.4_58300001	15	58300001	0.5785	0.6628
NC_005114.4_58400001	15	58400001	0.529	0.6214
NC_005114.4_58500001	15	58500001	0.5154	0.632
NC_005114.4_58600001	15	58600001	0.5327	0.6522
NC_005114.4_58700001	15	58700001	0.5248	0.6101
NC_005114.4_58800001	15	58800001	0.4101	0.545
NC_005114.4_58900001	15	58900001	0.484	0.6133
NC_005114.4_59000001	15	59000001	0.5308	0.6252
NC_005114.4_59100001	15	59100001	0.5057	0.5572
NC_005114.4_59200001	15	59200001	0.5105	0.6066
NC_005114.4_59300001	15	59300001	0.5211	0.6161
NC_005114.4_59400001	15	59400001	0.4688	0.557
NC_005114.4_59500001	15	59500001	0.4345	0.491
NC_005114.4_59600001	15	59600001	0.4209	0.4918
NC_005114.4_59700001	15	59700001	0.3582	0.4563
NC_005114.4_59800001	15	59800001	0.3819	0.5234
NC_005114.4_59900001	15	59900001	0.4042	0.5211
NC_005114.4_60000001	15	60000001	0.3676	0.4489
NC_005114.4_60100001	15	60100001	0.3714	0.4665
NC_005114.4_60200001	15	60200001	0.4821	0.4758
NC_005114.4_60300001	15	60300001	0.5153	0.482
NC_005114.4_60400001	15	60400001	0.5316	0.5339
NC_005114.4_60500001	15	60500001	0.6083	0.6447
NC_005114.4_60600001	15	60600001	0.582	0.633
NC_005114.4_60700001	15	60700001	0.5655	0.6478
NC_005114.4_60800001	15	60800001	0.6238	0.7315
NC_005114.4_60900001	15	60900001	0.6646	0.7072
NC_005114.4_61000001	15	61000001	0.6391	0.6653
NC_005114.4_61100001	15	61100001	0.653	0.6876
NC_005114.4_61200001	15	61200001	0.5996	0.6555
NC_005114.4_61300001	15	61300001	0.5442	0.5833
NC_005114.4_61400001	15	61400001	0.5136	0.5668

NC_005114.4_61500001	15	61500001	0.553	0.6296
NC_005114.4_61600001	15	61600001	0.5811	0.5932
NC_005114.4_61700001	15	61700001	0.692	0.6422
NC_005114.4_61800001	15	61800001	0.5953	0.6449
NC_005114.4_61900001	15	61900001	0.4785	0.6856
NC_005114.4_62000001	15	62000001	0.3524	0.5292
NC_005114.4_62100001	15	62100001	0.3498	0.6248
NC_005114.4_62200001	15	62200001	0.333	0.5952
NC_005114.4_62300001	15	62300001	0.4058	0.6549
NC_005114.4_62400001	15	62400001	0.4338	0.6459
NC_005114.4_62500001	15	62500001	0.4112	0.6865
NC_005114.4_62600001	15	62600001	0.4274	0.683
NC_005114.4_62700001	15	62700001	0.4949	0.7017
NC_005114.4_62800001	15	62800001	0.2251	0.4251
NC_005114.4_62900001	15	62900001	0.3398	0.4028
NC_005114.4_63000001	15	63000001	0.4283	0.4812
NC_005114.4_63100001	15	63100001	0.3691	0.3983
NC_005114.4_63200001	15	63200001	0.3866	0.4089
NC_005114.4_63300001	15	63300001	0.3702	0.3929
NC_005114.4_63400001	15	63400001	0.3598	0.4042
NC_005114.4_63500001	15	63500001	0.3788	0.4199
NC_005114.4_63600001	15	63600001	0.3819	0.4271
NC_005114.4_63700001	15	63700001	0.3686	0.4569
NC_005114.4_63800001	15	63800001	0.5154	0.5861
NC_005114.4_63900001	15	63900001	0.5153	0.6101
NC_005114.4_64100001	15	64100001	0.5771	0.748
NC_005114.4_64200001	15	64200001	0.5771	0.748
NC_005114.4_64300001	15	64300001	0.6296	0.7547
NC_005114.4_64400001	15	64400001	0.5654	0.7586
NC_005114.4_64500001	15	64500001	0.5272	0.7457
NC_005114.4_64600001	15	64600001	0.4012	0.5621
NC_005114.4_64700001	15	64700001	0.4012	0.5621
NC_005114.4_65300001	15	65300001	0.6382	0.695
NC_005114.4_65400001	15	65400001	0.6382	0.695
NC_005114.4_65500001	15	65500001	0.6382	0.695
NC_005114.4_65600001	15	65600001	0.4901	0.6683
NC_005114.4_65700001	15	65700001	0.4585	0.7274
NC_005114.4_65900001	15	65900001	0.297	0.543
NC_005114.4_66000001	15	66000001	0.297	0.543
NC_005114.4_66100001	15	66100001	0.5747	0.6885
NC_005114.4_66200001	15	66200001	0.5747	0.6885
NC_005114.4_66300001	15	66300001	0.6942	0.7101
NC_005114.4_66700001	15	66700001	0.6403	0.7497
NC_005114.4_66800001	15	66800001	0.6827	0.7883
NC_005114.4_66900001	15	66900001	0.7375	0.8158
NC_005114.4_67000001	15	67000001	0.714	0.75
NC_005114.4_67100001	15	67100001	0.6694	0.6736
NC_005114.4_67200001	15	67200001	0.8296	0.7656
NC_005114.4_67300001	15	67300001	0.7984	0.7257
NC_005114.4_67400001	15	67400001	0.7745	0.697
NC_005114.4_67500001	15	67500001	0.7475	0.7317
NC_005114.4_67600001	15	67600001	0.618	0.7185
NC_005114.4_67700001	15	67700001	0.4694	0.6854
NC_005114.4_67800001	15	67800001	0.3245	0.6503
NC_005114.4_67900001	15	67900001	0.3829	0.7611
NC_005114.4_68000001	15	68000001	0.4126	0.7743
NC_005114.4_68100001	15	68100001	0.4955	0.8467
NC_005114.4_68200001	15	68200001	0.5454	0.826

NC_005114.4_68300001	15	68300001	0.6271	0.9094
NC_005114.4_68400001	15	68400001	0.7022	0.8612
NC_005114.4_68500001	15	68500001	0.7022	0.8612
NC_005114.4_68600001	15	68600001	0.4896	0.7508
NC_005114.4_68700001	15	68700001	0.4185	0.7119
NC_005114.4_68800001	15	68800001	0.3824	0.6728
NC_005114.4_68900001	15	68900001	0.3279	0.6281
NC_005114.4_69000001	15	69000001	0.3621	0.5919
NC_005114.4_69100001	15	69100001	0.4476	0.6765
NC_005114.4_69200001	15	69200001	0.663	0.7292
NC_005114.4_69300001	15	69300001	0.7398	0.8005
NC_005114.4_69400001	15	69400001	0.6779	0.7605
NC_005114.4_69500001	15	69500001	0.7306	0.8558
NC_005114.4_69600001	15	69600001	0.7904	0.8474
NC_005114.4_69700001	15	69700001	0.8039	0.8334
NC_005114.4_69800001	15	69800001	0.7411	0.7855
NC_005114.4_69900001	15	69900001	0.8007	0.8233
NC_005114.4_70000001	15	70000001	0.6839	0.8165
NC_005114.4_70100001	15	70100001	0.5461	0.7732
NC_005114.4_70200001	15	70200001	0.4628	0.7477
NC_005114.4_70300001	15	70300001	0.4878	0.7978
NC_005114.4_70400001	15	70400001	0.3729	0.7038
NC_005114.4_70500001	15	70500001	0.4431	0.6506
NC_005114.4_70600001	15	70600001	0.3886	0.5778
NC_005114.4_70700001	15	70700001	0.3973	0.589
NC_005114.4_70800001	15	70800001	0.3308	0.5039
NC_005114.4_70900001	15	70900001	0.5306	0.7142
NC_005114.4_71000001	15	71000001	0.4888	0.7905
NC_005114.4_71100001	15	71100001	0.5891	0.8697
NC_005114.4_71200001	15	71200001	0.4252	0.6977
NC_005114.4_71300001	15	71300001	0.4755	0.7075
NC_005114.4_71400001	15	71400001	0.1463	0.0757
NC_005114.4_71500001	15	71500001	0.1463	0.0757
NC_005114.4_71600001	15	71600001	0.2023	0.3713
NC_005114.4_71700001	15	71700001	0.2727	0.6197
NC_005114.4_71800001	15	71800001	0.4054	0.7559
NC_005114.4_71900001	15	71900001	0.4871	0.7775
NC_005114.4_72000001	15	72000001	0.4871	0.7775
NC_005114.4_72100001	15	72100001	0.6077	0.7989
NC_005114.4_72200001	15	72200001	0.7326	0.802
NC_005114.4_72500001	15	72500001	0.5227	0.5226
NC_005114.4_72600001	15	72600001	0.462	0.5113
NC_005114.4_72700001	15	72700001	0.4046	0.4874
NC_005114.4_72800001	15	72800001	0.4875	0.6019
NC_005114.4_72900001	15	72900001	0.5601	0.7803
NC_005114.4_73000001	15	73000001	0.5092	0.735
NC_005114.4_73100001	15	73100001	0.5432	0.7068
NC_005114.4_73200001	15	73200001	0.5834	0.723
NC_005114.4_73300001	15	73300001	0.5401	0.6799
NC_005114.4_73400001	15	73400001	0.4864	0.543
NC_005114.4_73500001	15	73500001	0.4828	0.5901
NC_005114.4_73700001	15	73700001	0.4843	0.672
NC_005114.4_73800001	15	73800001	0.6245	0.7762
NC_005114.4_73900001	15	73900001	0.7002	0.8799
NC_005114.4_74000001	15	74000001	0.8042	0.8737
NC_005114.4_74100001	15	74100001	0.736	0.8616
NC_005114.4_74200001	15	74200001	0.77	0.8774
NC_005114.4_74300001	15	74300001	0.6587	0.7818

NC_005114.4_74400001	15	74400001	0.6149	0.7158
NC_005114.4_74700001	15	74700001	0.3006	0.4316
NC_005114.4_74800001	15	74800001	0.359	0.5031
NC_005114.4_74900001	15	74900001	0.359	0.5031
NC_005114.4_75000001	15	75000001	0.3289	0.4973
NC_005114.4_75100001	15	75100001	0.3289	0.4973
NC_005114.4_75200001	15	75200001	0.3422	0.4869
NC_005114.4_75400001	15	75400001	0.3038	0.5973
NC_005114.4_75500001	15	75500001	0.6173	0.8285
NC_005114.4_75600001	15	75600001	0.657	0.8529
NC_005114.4_75700001	15	75700001	0.5413	0.7781
NC_005114.4_75800001	15	75800001	0.4271	0.6986
NC_005114.4_75900001	15	75900001	0.5118	0.6174
NC_005114.4_76000001	15	76000001	0.3842	0.433
NC_005114.4_76100001	15	76100001	0.3419	0.3614
NC_005114.4_76200001	15	76200001	0.3301	0.2441
NC_005114.4_76300001	15	76300001	0.3608	0.2581
NC_005114.4_76400001	15	76400001	0.3134	0.2373
NC_005114.4_76600001	15	76600001	0.379	0.3753
NC_005114.4_76700001	15	76700001	0.2716	0.3589
NC_005114.4_76800001	15	76800001	0.3357	0.3658
NC_005114.4_76900001	15	76900001	0.3886	0.5217
NC_005114.4_77000001	15	77000001	0.3886	0.5217
NC_005114.4_77100001	15	77100001	0.3289	0.4658
NC_005114.4_77200001	15	77200001	0.3868	0.5129
NC_005114.4_77300001	15	77300001	0.2717	0.5234
NC_005114.4_77400001	15	77400001	0.1117	0.0893
NC_005114.4_77500001	15	77500001	0.1117	0.0893
NC_005114.4_77600001	15	77600001	0.3394	0.4881
NC_005114.4_77700001	15	77700001	0.3751	0.4929
NC_005114.4_77800001	15	77800001	0.4769	0.5323
NC_005114.4_78000001	15	78000001	0.7047	0.7378
NC_005114.4_78100001	15	78100001	0.6162	0.6497
NC_005114.4_78200001	15	78200001	0.543	0.6306
NC_005114.4_78300001	15	78300001	0.5422	0.6587
NC_005114.4_78400001	15	78400001	0.5687	0.5959
NC_005114.4_78500001	15	78500001	0.5333	0.5572
NC_005114.4_78600001	15	78600001	0.4705	0.5947
NC_005114.4_78700001	15	78700001	0.4815	0.5688
NC_005114.4_78800001	15	78800001	0.4657	0.5201
NC_005114.4_78900001	15	78900001	0.3326	0.5135
NC_005114.4_79000001	15	79000001	0.5039	0.6603
NC_005114.4_79100001	15	79100001	0.5414	0.6311
NC_005114.4_79200001	15	79200001	0.5375	0.6831
NC_005114.4_79300001	15	79300001	0.5036	0.655
NC_005114.4_79400001	15	79400001	0.5187	0.6799
NC_005114.4_79500001	15	79500001	0.4664	0.5816
NC_005114.4_79600001	15	79600001	0.4038	0.5046
NC_005114.4_79700001	15	79700001	0.4083	0.4404
NC_005114.4_79900001	15	79900001	0.2878	0.1635
NC_005114.4_80100001	15	80100001	0.2864	0.3421
NC_005114.4_80200001	15	80200001	0.2864	0.3421
NC_005114.4_80300001	15	80300001	0.2454	0.3316
NC_005114.4_80400001	15	80400001	0.2251	0.4007
NC_005114.4_81100001	15	81100001	0.3515	0.4102
NC_005114.4_81200001	15	81200001	0.3191	0.3587
NC_005114.4_81300001	15	81300001	0.2897	0.3531
NC_005114.4_81400001	15	81400001	0.2553	0.3245

NC_005114.4_81500001	15	81500001	0.3279	0.421
NC_005114.4_81600001	15	81600001	0.3971	0.5093
NC_005114.4_81700001	15	81700001	0.4991	0.6189
NC_005114.4_81800001	15	81800001	0.5328	0.6238
NC_005114.4_81900001	15	81900001	0.6466	0.6282
NC_005114.4_82000001	15	82000001	0.6297	0.6964
NC_005114.4_82100001	15	82100001	0.5445	0.6986
NC_005114.4_82200001	15	82200001	0.4627	0.5986
NC_005114.4_82300001	15	82300001	0.4669	0.5554
NC_005114.4_82400001	15	82400001	0.5373	0.6347
NC_005114.4_82500001	15	82500001	0.496	0.576
NC_005114.4_82600001	15	82600001	0.5233	0.5785
NC_005114.4_82700001	15	82700001	0.5532	0.5746
NC_005114.4_82800001	15	82800001	0.3759	0.5395
NC_005114.4_82900001	15	82900001	0.3728	0.5757
NC_005114.4_83000001	15	83000001	0.3611	0.6055
NC_005114.4_83100001	15	83100001	0.3717	0.6207
NC_005114.4_83200001	15	83200001	0.3722	0.6665
NC_005114.4_83300001	15	83300001	0.4308	0.6876
NC_005114.4_83400001	15	83400001	0.3394	0.4997
NC_005114.4_83500001	15	83500001	0.3965	0.5347
NC_005114.4_83600001	15	83600001	0.4122	0.5393
NC_005114.4_83700001	15	83700001	0.4499	0.5605
NC_005114.4_83800001	15	83800001	0.5112	0.6897
NC_005114.4_83900001	15	83900001	0.6304	0.8367
NC_005114.4_84000001	15	84000001	0.6855	0.8399
NC_005114.4_84100001	15	84100001	0.6543	0.8165
NC_005114.4_84200001	15	84200001	0.5877	0.7163
NC_005114.4_84300001	15	84300001	0.5617	0.6692
NC_005114.4_84400001	15	84400001	0.577	0.6303
NC_005114.4_84500001	15	84500001	0.4615	0.5009
NC_005114.4_84600001	15	84600001	0.4849	0.5219
NC_005114.4_84700001	15	84700001	0.3967	0.4177
NC_005114.4_84800001	15	84800001	0.4233	0.4662
NC_005114.4_84900001	15	84900001	0.3951	0.4383
NC_005114.4_85000001	15	85000001	0.4282	0.4869
NC_005114.4_85100001	15	85100001	0.3556	0.4161
NC_005114.4_85200001	15	85200001	0.5031	0.6422
NC_005114.4_85300001	15	85300001	0.3979	0.5093
NC_005114.4_85400001	15	85400001	0.3909	0.5121
NC_005114.4_85500001	15	85500001	0.4777	0.6248
NC_005114.4_85600001	15	85600001	0.5553	0.6637
NC_005114.4_85700001	15	85700001	0.4777	0.5812
NC_005114.4_85800001	15	85800001	0.5117	0.6137
NC_005114.4_85900001	15	85900001	0.5704	0.5908
NC_005114.4_86000001	15	86000001	0.4798	0.4586
NC_005114.4_86100001	15	86100001	0.4272	0.4012
NC_005114.4_86200001	15	86200001	0.4149	0.4487
NC_005114.4_86300001	15	86300001	0.4335	0.5096
NC_005114.4_86400001	15	86400001	0.4334	0.5783
NC_005114.4_86500001	15	86500001	0.3066	0.6054
NC_005114.4_86600001	15	86600001	0.4134	0.6431
NC_005114.4_86700001	15	86700001	0.4443	0.6118
NC_005114.4_86800001	15	86800001	0.4712	0.5661
NC_005114.4_86900001	15	86900001	0.3622	0.4683
NC_005114.4_87000001	15	87000001	0.4736	0.5133
NC_005114.4_87200001	15	87200001	0.4379	0.4435
NC_005114.4_87300001	15	87300001	0.4013	0.3995

NC_005114.4_87400001	15	87400001	0.4474	0.4734
NC_005114.4_87500001	15	87500001	0.4611	0.5051
NC_005114.4_87600001	15	87600001	0.4533	0.5196
NC_005114.4_87700001	15	87700001	0.413	0.5158
NC_005114.4_87800001	15	87800001	0.4848	0.622
NC_005114.4_87900001	15	87900001	0.4523	0.6134
NC_005114.4_88000001	15	88000001	0.4079	0.6642
NC_005114.4_88100001	15	88100001	0.4232	0.7275
NC_005114.4_88200001	15	88200001	0.4499	0.7483
NC_005114.4_88300001	15	88300001	0.426	0.7175
NC_005114.4_88400001	15	88400001	0.4229	0.6736
NC_005114.4_88500001	15	88500001	0.3972	0.542
NC_005114.4_88600001	15	88600001	0.3712	0.4553
NC_005114.4_88700001	15	88700001	0.3677	0.4012
NC_005114.4_88800001	15	88800001	0.2688	0.2531
NC_005114.4_88900001	15	88900001	0.3356	0.3467
NC_005114.4_89000001	15	89000001	0.3754	0.5031
NC_005114.4_89100001	15	89100001	0.4463	0.6096
NC_005114.4_89200001	15	89200001	0.481	0.704
NC_005114.4_89300001	15	89300001	0.542	0.7194
NC_005114.4_89400001	15	89400001	0.5304	0.724
NC_005114.4_89500001	15	89500001	0.5165	0.6064
NC_005114.4_89600001	15	89600001	0.3983	0.4837
NC_005114.4_89700001	15	89700001	0.4111	0.5014
NC_005114.4_89800001	15	89800001	0.4469	0.5187
NC_005114.4_89900001	15	89900001	0.4799	0.5764
NC_005114.4_90000001	15	90000001	0.5214	0.6918
NC_005114.4_90100001	15	90100001	0.715	0.8051
NC_005114.4_90200001	15	90200001	0.7828	0.773
NC_005114.4_90300001	15	90300001	0.7688	0.7476
NC_005114.4_90400001	15	90400001	0.7336	0.7144
NC_005114.4_90500001	15	90500001	0.6957	0.7244
NC_005114.4_90600001	15	90600001	0.6648	0.695
NC_005114.4_90700001	15	90700001	0.5694	0.6543
NC_005114.4_90800001	15	90800001	0.4877	0.6663
NC_005114.4_90900001	15	90900001	0.4753	0.6039
NC_005114.4_91000001	15	91000001	0.3842	0.4399
NC_005114.4_91100001	15	91100001	0.3842	0.4399
NC_005114.4_91200001	15	91200001	0.4323	0.451
NC_005114.4_91300001	15	91300001	0.2981	0.308
NC_005114.4_91800001	15	91800001	0.4478	0.5966
NC_005114.4_91900001	15	91900001	0.4955	0.5876
NC_005114.4_92000001	15	92000001	0.5275	0.626
NC_005114.4_92100001	15	92100001	0.5275	0.626
NC_005114.4_92200001	15	92200001	0.5602	0.6379
NC_005114.4_92300001	15	92300001	0.533	0.5773
NC_005114.4_92400001	15	92400001	0.5246	0.5725
NC_005114.4_92500001	15	92500001	0.3838	0.3393
NC_005114.4_92600001	15	92600001	0.3632	0.3245
NC_005114.4_92700001	15	92700001	0.3993	0.4114
NC_005114.4_92800001	15	92800001	0.4324	0.5028
NC_005114.4_92900001	15	92900001	0.4324	0.5028
NC_005114.4_93000001	15	93000001	0.4658	0.602
NC_005114.4_93100001	15	93100001	0.4869	0.7046
NC_005114.4_93200001	15	93200001	0.5731	0.7447
NC_005114.4_93300001	15	93300001	0.6281	0.7855
NC_005114.4_93400001	15	93400001	0.6281	0.7855
NC_005114.4_93500001	15	93500001	0.6639	0.8087

NC_005114.4_93600001	15	93600001	0.8063	0.8918
NC_005114.4_93800001	15	93800001	0.5086	0.6393
NC_005114.4_93900001	15	93900001	0.3669	0.5619
NC_005114.4_94000001	15	94000001	0.3717	0.5722
NC_005114.4_94100001	15	94100001	0.2509	0.3458
NC_005114.4_94200001	15	94200001	0.2289	0.3408
NC_005114.4_94300001	15	94300001	0.205	0.3893
NC_005114.4_94400001	15	94400001	0.3094	0.5058
NC_005114.4_94500001	15	94500001	0.2638	0.3938
NC_005114.4_94600001	15	94600001	0.3236	0.3746
NC_005114.4_94700001	15	94700001	0.3424	0.3766
NC_005114.4_94800001	15	94800001	0.3688	0.3889
NC_005114.4_94900001	15	94900001	0.3388	0.3568
NC_005114.4_95000001	15	95000001	0.3386	0.308
NC_005114.4_95200001	15	95200001	0.2317	0.3428
NC_005114.4_95300001	15	95300001	0.6244	0.7681
NC_005114.4_95400001	15	95400001	0.6682	0.7865
NC_005114.4_95500001	15	95500001	0.5749	0.6789
NC_005114.4_95600001	15	95600001	0.6722	0.7191
NC_005114.4_95700001	15	95700001	0.7118	0.7748
NC_005114.4_95800001	15	95800001	0.6175	0.6514
NC_005114.4_95900001	15	95900001	0.5958	0.6338
NC_005114.4_96000001	15	96000001	0.5224	0.6187
NC_005114.4_96100001	15	96100001	0.5377	0.5916
NC_005114.4_96200001	15	96200001	0.4499	0.4935
NC_005114.4_96300001	15	96300001	0.3243	0.2523
NC_005114.4_96400001	15	96400001	0.3243	0.2523
NC_005114.4_96500001	15	96500001	0.5004	0.3596
NC_005114.4_96600001	15	96600001	0.4462	0.3013
NC_005114.4_97000001	15	97000001	0.393	0.4607
NC_005114.4_97100001	15	97100001	0.382	0.4371
NC_005114.4_97200001	15	97200001	0.4721	0.5992
NC_005114.4_97300001	15	97300001	0.4903	0.6759
NC_005114.4_97400001	15	97400001	0.5821	0.7788
NC_005114.4_97500001	15	97500001	0.5775	0.8104
NC_005114.4_97600001	15	97600001	0.5928	0.7969
NC_005114.4_97700001	15	97700001	0.5515	0.6981
NC_005114.4_97800001	15	97800001	0.5781	0.7157
NC_005114.4_97900001	15	97900001	0.5096	0.5296
NC_005114.4_98000001	15	98000001	0.51	0.486
NC_005114.4_98100001	15	98100001	0.4665	0.4686
NC_005114.4_98200001	15	98200001	0.4704	0.5147
NC_005114.4_98300001	15	98300001	0.4407	0.4237
NC_005114.4_98500001	15	98500001	0.2572	0.2574
NC_005114.4_98600001	15	98600001	0.3002	0.2996
NC_005114.4_98700001	15	98700001	0.2606	0.2252
NC_005114.4_98800001	15	98800001	0.1958	0.2221
NC_005114.4_98900001	15	98900001	0.1784	0.2107
NC_005114.4_99000001	15	99000001	0.2592	0.2915
NC_005114.4_99100001	15	99100001	0.2458	0.2365
NC_005114.4_99200001	15	99200001	0.3233	0.4052
NC_005114.4_99300001	15	99300001	0.4721	0.5287
NC_005114.4_99400001	15	99400001	0.5774	0.6125
NC_005114.4_99500001	15	99500001	0.6779	0.7858
NC_005114.4_99600001	15	99600001	0.618	0.7805
NC_005114.4_99700001	15	99700001	0.556	0.7421
NC_005114.4_99800001	15	99800001	0.5718	0.766
NC_005114.4_99900001	15	99900001	0.569	0.763

NC_005114.4_100000001	15	100000001	0.5683	0.7023
NC_005114.4_100100001	15	100100001	0.6321	0.6907
NC_005114.4_100200001	15	100200001	0.6558	0.6492
NC_005114.4_100300001	15	100300001	0.5997	0.6156
NC_005114.4_100400001	15	100400001	0.5707	0.5787
NC_005114.4_100500001	15	100500001	0.3923	0.4164
NC_005114.4_100600001	15	100600001	0.3505	0.377
NC_005114.4_100700001	15	100700001	0.2711	0.2704
NC_005114.4_100800001	15	100800001	0.4472	0.5363
NC_005114.4_100900001	15	100900001	0.4116	0.5281
NC_005114.4_101000001	15	101000001	0.4951	0.6348
NC_005114.4_101100001	15	101100001	0.5622	0.7368
NC_005114.4_101200001	15	101200001	0.6223	0.8198
NC_005114.4_101300001	15	101300001	0.5446	0.7271
NC_005114.4_101400001	15	101400001	0.5525	0.6772
NC_005114.4_101500001	15	101500001	0.5128	0.516
NC_005114.4_101600001	15	101600001	0.4532	0.4894
NC_005114.4_101700001	15	101700001	0.44	0.4593
NC_005114.4_101800001	15	101800001	0.4932	0.522
NC_005114.4_101900001	15	101900001	0.5186	0.5322
NC_005114.4_102000001	15	102000001	0.5656	0.6187
NC_005114.4_102100001	15	102100001	0.5583	0.5822
NC_005114.4_102200001	15	102200001	0.5853	0.6349
NC_005114.4_102300001	15	102300001	0.5057	0.6016
NC_005114.4_102400001	15	102400001	0.481	0.6053
NC_005114.4_102500001	15	102500001	0.4584	0.5528
NC_005114.4_102600001	15	102600001	0.4096	0.5424
NC_005114.4_102700001	15	102700001	0.3917	0.4807
NC_005114.4_102800001	15	102800001	0.4314	0.5132
NC_005114.4_102900001	15	102900001	0.4189	0.5158
NC_005114.4_103000001	15	103000001	0.3978	0.55
NC_005114.4_103100001	15	103100001	0.4282	0.5573
NC_005114.4_103200001	15	103200001	0.3704	0.5041
NC_005114.4_103300001	15	103300001	0.4003	0.5336
NC_005114.4_103400001	15	103400001	0.4479	0.5278
NC_005114.4_103500001	15	103500001	0.4155	0.4931
NC_005114.4_103600001	15	103600001	0.4102	0.4868
NC_005114.4_103700001	15	103700001	0.4538	0.5489
NC_005114.4_103800001	15	103800001	0.4786	0.5716
NC_005114.4_103900001	15	103900001	0.5058	0.6878
NC_005114.4_104000001	15	104000001	0.6932	0.7825
NC_005114.4_104100001	15	104100001	0.7352	0.7887
NC_005114.4_104200001	15	104200001	0.6037	0.7091
NC_005114.4_104300001	15	104300001	0.5832	0.6483
NC_005114.4_104400001	15	104400001	0.5646	0.6087
NC_005114.4_104500001	15	104500001	0.6045	0.6843
NC_005114.4_104600001	15	104600001	0.6006	0.7017
NC_005114.4_104700001	15	104700001	0.6254	0.6998
NC_005114.4_104800001	15	104800001	0.5645	0.7245
NC_005114.4_104900001	15	104900001	0.563	0.7347
NC_005114.4_105000001	15	105000001	0.5245	0.6984
NC_005114.4_105100001	15	105100001	0.4973	0.6488
NC_005114.4_105200001	15	105200001	0.5252	0.6706
NC_005114.4_105300001	15	105300001	0.552	0.6621
NC_005114.4_105400001	15	105400001	0.5553	0.6723
NC_005114.4_105500001	15	105500001	0.5718	0.5847
NC_005114.4_105600001	15	105600001	0.5782	0.6178
NC_005114.4_105700001	15	105700001	0.5046	0.5729

NC_005114.4_105800001	15	105800001	0.5175	0.5411
NC_005114.4_105900001	15	105900001	0.4961	0.5085
NC_005114.4_106000001	15	106000001	0.4162	0.4931
NC_005114.4_106100001	15	106100001	0.5397	0.6224
NC_005114.4_106200001	15	106200001	0.6813	0.7501
NC_005114.4_106300001	15	106300001	0.6608	0.7944
NC_005114.4_106400001	15	106400001	0.6996	0.8423
NC_005114.4_106500001	15	106500001	0.7817	0.9101
NC_005114.4_106600001	15	106600001	0.7779	0.9391
NC_005114.4_106700001	15	106700001	0.5716	0.8694
NC_005114.4_107600001	15	107600001	0.4106	0.7623
NC_005114.4_107700001	15	107700001	0.3978	0.5929
NC_005114.4_107800001	15	107800001	0.412	0.6222
NC_005114.4_107900001	15	107900001	0.4782	0.7374
NC_005114.4_108000001	15	108000001	0.4446	0.6547
NC_005114.4_108100001	15	108100001	0.4819	0.6578
NC_005114.4_108200001	15	108200001	0.538	0.7251
NC_005114.4_108300001	15	108300001	0.5615	0.7119
NC_005114.4_108400001	15	108400001	0.4972	0.6048
NC_005114.4_108500001	15	108500001	0.637	0.7555
NC_005114.4_108600001	15	108600001	0.6158	0.7382
NC_005114.4_108700001	15	108700001	0.5907	0.77
NC_005114.4_108800001	15	108800001	0.6853	0.8477
NC_005114.4_108900001	15	108900001	0.8382	0.9317
NC_005114.4_109000001	15	109000001	0.7954	0.8924
NC_005114.4_109100001	15	109100001	0.7582	0.8631
NC_005114.4_109200001	15	109200001	0.7991	0.874
NC_005114.4_109300001	15	109300001	0.6569	0.7756
NC_005114.4_109400001	15	109400001	0.5424	0.6118
NC_005114.4_109500001	15	109500001	0.5478	0.6168
NC_005114.4_109600001	15	109600001	0.6114	0.684
NC_005114.4_109700001	15	109700001	0.5433	0.6517
NC_005114.4_109800001	15	109800001	0.6032	0.7676
NC_005114.4_109900001	15	109900001	0.6865	0.8931
NC_005114.4_110000001	15	110000001	0.6865	0.8931
NC_005114.4_110100001	15	110100001	0.6515	0.8793
NC_005114.4_110200001	15	110200001	0.7838	0.9165
NC_005114.4_110300001	15	110300001	0.8787	0.9116
NC_005114.4_110400001	15	110400001	0.9067	0.8488
NC_005114.4_110500001	15	110500001	0.9067	0.8488
NC_005114.4_110600001	15	110600001	0.7924	0.7736
NC_005115.4_1	16	1	0.4333	0.41
NC_005115.4_100001	16	100001	0.4333	0.41
NC_005115.4_300001	16	300001	0.4621	0.6672
NC_005115.4_400001	16	400001	0.412	0.5524
NC_005115.4_500001	16	500001	0.4506	0.5828
NC_005115.4_600001	16	600001	0.526	0.6171
NC_005115.4_700001	16	700001	0.508	0.6044
NC_005115.4_800001	16	800001	0.3702	0.5148
NC_005115.4_900001	16	900001	0.359	0.4642
NC_005115.4_1000001	16	1000001	0.3601	0.4874
NC_005115.4_1100001	16	1100001	0.4106	0.5248
NC_005115.4_1200001	16	1200001	0.4013	0.5176
NC_005115.4_1300001	16	1300001	0.4439	0.5257
NC_005115.4_1400001	16	1400001	0.4782	0.5572
NC_005115.4_1500001	16	1500001	0.4548	0.5034
NC_005115.4_1600001	16	1600001	0.3828	0.4331
NC_005115.4_1700001	16	1700001	0.4382	0.4987

NC_005115.4_1800001	16	1800001	0.4376	0.5334
NC_005115.4_1900001	16	1900001	0.4243	0.5586
NC_005115.4_2000001	16	2000001	0.5722	0.8114
NC_005115.4_2100001	16	2100001	0.6265	0.8637
NC_005115.4_2200001	16	2200001	0.4366	0.727
NC_005115.4_2300001	16	2300001	0.5266	0.7714
NC_005115.4_2400001	16	2400001	0.5045	0.7621
NC_005115.4_2500001	16	2500001	0.4739	0.7875
NC_005115.4_2600001	16	2600001	0.4575	0.7694
NC_005115.4_2700001	16	2700001	0.5126	0.7825
NC_005115.4_2800001	16	2800001	0.4739	0.7534
NC_005115.4_2900001	16	2900001	0.4703	0.7404
NC_005115.4_3000001	16	3000001	0.4662	0.6633
NC_005115.4_3100001	16	3100001	0.4946	0.6602
NC_005115.4_3200001	16	3200001	0.5331	0.7328
NC_005115.4_3300001	16	3300001	0.5351	0.6728
NC_005115.4_3400001	16	3400001	0.5501	0.6823
NC_005115.4_3500001	16	3500001	0.6266	0.7352
NC_005115.4_3600001	16	3600001	0.6027	0.7065
NC_005115.4_3700001	16	3700001	0.5131	0.6449
NC_005115.4_3800001	16	3800001	0.525	0.7022
NC_005115.4_3900001	16	3900001	0.5119	0.6907
NC_005115.4_4000001	16	4000001	0.49	0.6735
NC_005115.4_4100001	16	4100001	0.4581	0.6799
NC_005115.4_4200001	16	4200001	0.4801	0.7502
NC_005115.4_4300001	16	4300001	0.4416	0.7092
NC_005115.4_4400001	16	4400001	0.4549	0.7083
NC_005115.4_4500001	16	4500001	0.5287	0.7204
NC_005115.4_4600001	16	4600001	0.4549	0.6727
NC_005115.4_4700001	16	4700001	0.5188	0.6146
NC_005115.4_4800001	16	4800001	0.4794	0.5687
NC_005115.4_4900001	16	4900001	0.4664	0.5653
NC_005115.4_5000001	16	5000001	0.4515	0.5934
NC_005115.4_5100001	16	5100001	0.5486	0.6527
NC_005115.4_5200001	16	5200001	0.5418	0.6759
NC_005115.4_5300001	16	5300001	0.5843	0.733
NC_005115.4_5400001	16	5400001	0.5692	0.6534
NC_005115.4_5500001	16	5500001	0.52	0.6212
NC_005115.4_5600001	16	5600001	0.3924	0.4997
NC_005115.4_5700001	16	5700001	0.4193	0.5658
NC_005115.4_5800001	16	5800001	0.4516	0.5864
NC_005115.4_5900001	16	5900001	0.5542	0.6893
NC_005115.4_6000001	16	6000001	0.5109	0.6045
NC_005115.4_6100001	16	6100001	0.5746	0.6614
NC_005115.4_6200001	16	6200001	0.6281	0.6801
NC_005115.4_6300001	16	6300001	0.6626	0.7144
NC_005115.4_6400001	16	6400001	0.6307	0.6998
NC_005115.4_6500001	16	6500001	0.7706	0.7878
NC_005115.4_6600001	16	6600001	0.7576	0.7767
NC_005115.4_6700001	16	6700001	0.7349	0.6996
NC_005115.4_6800001	16	6800001	0.8284	0.8097
NC_005115.4_6900001	16	6900001	0.8119	0.8113
NC_005115.4_7000001	16	7000001	0.7	0.7901
NC_005115.4_7100001	16	7100001	0.6976	0.8082
NC_005115.4_7200001	16	7200001	0.6937	0.8409
NC_005115.4_7300001	16	7300001	0.558	0.7044
NC_005115.4_7400001	16	7400001	0.586	0.7539
NC_005115.4_7500001	16	7500001	0.6776	0.8009

NC_005115.4_7600001	16	7600001	0.6121	0.7214
NC_005115.4_7700001	16	7700001	0.6009	0.6664
NC_005115.4_7800001	16	7800001	0.6186	0.6958
NC_005115.4_7900001	16	7900001	0.5525	0.6655
NC_005115.4_8000001	16	8000001	0.5482	0.6779
NC_005115.4_8100001	16	8100001	0.6265	0.7315
NC_005115.4_8200001	16	8200001	0.6217	0.7414
NC_005115.4_8300001	16	8300001	0.7001	0.8168
NC_005115.4_8400001	16	8400001	0.7168	0.7757
NC_005115.4_8500001	16	8500001	0.6009	0.658
NC_005115.4_8600001	16	8600001	0.5248	0.5825
NC_005115.4_8700001	16	8700001	0.5241	0.5817
NC_005115.4_8800001	16	8800001	0.4309	0.5078
NC_005115.4_8900001	16	8900001	0.4241	0.5062
NC_005115.4_9000001	16	9000001	0.3999	0.534
NC_005115.4_9100001	16	9100001	0.4374	0.5872
NC_005115.4_9200001	16	9200001	0.4118	0.5774
NC_005115.4_9300001	16	9300001	0.4745	0.6203
NC_005115.4_9400001	16	9400001	0.4563	0.6153
NC_005115.4_9500001	16	9500001	0.5128	0.6145
NC_005115.4_9600001	16	9600001	0.4863	0.5637
NC_005115.4_9700001	16	9700001	0.4772	0.5456
NC_005115.4_9800001	16	9800001	0.3749	0.4803
NC_005115.4_9900001	16	9900001	0.41	0.5794
NC_005115.4_10000001	16	10000001	0.3939	0.607
NC_005115.4_10100001	16	10100001	0.4042	0.6229
NC_005115.4_10200001	16	10200001	0.4302	0.7032
NC_005115.4_10300001	16	10300001	0.4898	0.7635
NC_005115.4_10400001	16	10400001	0.5217	0.7352
NC_005115.4_10500001	16	10500001	0.5572	0.6659
NC_005115.4_10600001	16	10600001	0.5431	0.6017
NC_005115.4_10700001	16	10700001	0.4978	0.5745
NC_005115.4_10800001	16	10800001	0.5702	0.6485
NC_005115.4_10900001	16	10900001	0.5277	0.6079
NC_005115.4_11000001	16	11000001	0.5064	0.6154
NC_005115.4_11100001	16	11100001	0.4985	0.612
NC_005115.4_11200001	16	11200001	0.4574	0.5785
NC_005115.4_11300001	16	11300001	0.3611	0.4599
NC_005115.4_11400001	16	11400001	0.367	0.4948
NC_005115.4_11500001	16	11500001	0.3893	0.4918
NC_005115.4_11600001	16	11600001	0.4081	0.6121
NC_005115.4_11700001	16	11700001	0.5604	0.6909
NC_005115.4_11800001	16	11800001	0.5749	0.7875
NC_005115.4_11900001	16	11900001	0.5749	0.7875
NC_005115.4_12500001	16	12500001	0.413	0.5474
NC_005115.4_12600001	16	12600001	0.4522	0.5824
NC_005115.4_12700001	16	12700001	0.4082	0.5656
NC_005115.4_12800001	16	12800001	0.4635	0.7062
NC_005115.4_12900001	16	12900001	0.5319	0.7038
NC_005115.4_13000001	16	13000001	0.5351	0.6627
NC_005115.4_13100001	16	13100001	0.5575	0.6874
NC_005115.4_13200001	16	13200001	0.7755	0.8563
NC_005115.4_13500001	16	13500001	0.4934	0.6865
NC_005115.4_13600001	16	13600001	0.5349	0.7397
NC_005115.4_13700001	16	13700001	0.4324	0.6283
NC_005115.4_13800001	16	13800001	0.3935	0.667
NC_005115.4_13900001	16	13900001	0.3783	0.5018
NC_005115.4_14000001	16	14000001	0.4882	0.6226

NC_005115.4_14100001	16	14100001	0.48	0.6029
NC_005115.4_14200001	16	14200001	0.5178	0.6475
NC_005115.4_14300001	16	14300001	0.5561	0.6033
NC_005115.4_14400001	16	14400001	0.5955	0.756
NC_005115.4_14500001	16	14500001	0.6227	0.6959
NC_005115.4_14600001	16	14600001	0.6921	0.7788
NC_005115.4_14700001	16	14700001	0.7219	0.7872
NC_005115.4_14800001	16	14800001	0.7199	0.7887
NC_005115.4_14900001	16	14900001	0.6807	0.7624
NC_005115.4_15000001	16	15000001	0.6004	0.6822
NC_005115.4_15100001	16	15100001	0.4986	0.5034
NC_005115.4_15200001	16	15200001	0.4986	0.5034
NC_005115.4_15300001	16	15300001	0.5083	0.4793
NC_005115.4_15400001	16	15400001	0.4146	0.3703
NC_005115.4_15500001	16	15500001	0.2435	0.252
NC_005115.4_15600001	16	15600001	0.213	0.2525
NC_005115.4_15700001	16	15700001	0.213	0.2525
NC_005115.4_15800001	16	15800001	0.2957	0.5625
NC_005115.4_15900001	16	15900001	0.2858	0.449
NC_005115.4_16000001	16	16000001	0.4391	0.6388
NC_005115.4_16100001	16	16100001	0.4813	0.6499
NC_005115.4_16200001	16	16200001	0.442	0.5721
NC_005115.4_16300001	16	16300001	0.409	0.4679
NC_005115.4_16400001	16	16400001	0.543	0.6234
NC_005115.4_16500001	16	16500001	0.3307	0.4008
NC_005115.4_16600001	16	16600001	0.3621	0.4074
NC_005115.4_16700001	16	16700001	0.3933	0.4765
NC_005115.4_16800001	16	16800001	0.4194	0.5223
NC_005115.4_17100001	16	17100001	0.3491	0.291
NC_005115.4_17200001	16	17200001	0.4428	0.5349
NC_005115.4_17300001	16	17300001	0.4299	0.5632
NC_005115.4_17400001	16	17400001	0.4299	0.5632
NC_005115.4_17500001	16	17500001	0.4415	0.5588
NC_005115.4_17600001	16	17600001	0.463	0.6468
NC_005115.4_17700001	16	17700001	0.3884	0.4907
NC_005115.4_17900001	16	17900001	0.5872	0.5506
NC_005115.4_18000001	16	18000001	0.6206	0.616
NC_005115.4_18100001	16	18100001	0.6206	0.616
NC_005115.4_18200001	16	18200001	0.7714	0.7146
NC_005115.4_18300001	16	18300001	0.8303	0.8098
NC_005115.4_18400001	16	18400001	0.812	0.789
NC_005115.4_18500001	16	18500001	0.7688	0.7755
NC_005115.4_18600001	16	18600001	0.6431	0.7369
NC_005115.4_18700001	16	18700001	0.57	0.7405
NC_005115.4_18800001	16	18800001	0.4284	0.6723
NC_005115.4_18900001	16	18900001	0.3771	0.6167
NC_005115.4_19000001	16	19000001	0.4078	0.6765
NC_005115.4_19100001	16	19100001	0.398	0.6064
NC_005115.4_19200001	16	19200001	0.4281	0.5819
NC_005115.4_19300001	16	19300001	0.5768	0.6777
NC_005115.4_19400001	16	19400001	0.6053	0.7196
NC_005115.4_19500001	16	19500001	0.5988	0.7155
NC_005115.4_19600001	16	19600001	0.6404	0.7499
NC_005115.4_19700001	16	19700001	0.6054	0.7229
NC_005115.4_19800001	16	19800001	0.5555	0.6956
NC_005115.4_19900001	16	19900001	0.5534	0.6259
NC_005115.4_20000001	16	20000001	0.4326	0.5165
NC_005115.4_20100001	16	20100001	0.4576	0.5223

NC_005115.4_20200001	16	20200001	0.5472	0.6101
NC_005115.4_20300001	16	20300001	0.5608	0.6584
NC_005115.4_20400001	16	20400001	0.5965	0.7046
NC_005115.4_20500001	16	20500001	0.5809	0.6628
NC_005115.4_20600001	16	20600001	0.6107	0.7176
NC_005115.4_20700001	16	20700001	0.5866	0.7235
NC_005115.4_20800001	16	20800001	0.6545	0.7622
NC_005115.4_20900001	16	20900001	0.6051	0.7542
NC_005115.4_21000001	16	21000001	0.6619	0.7495
NC_005115.4_21100001	16	21100001	0.6574	0.7799
NC_005115.4_21200001	16	21200001	0.6321	0.7455
NC_005115.4_21300001	16	21300001	0.5272	0.6473
NC_005115.4_21400001	16	21400001	0.504	0.6034
NC_005115.4_21800001	16	21800001	0.3142	0.5108
NC_005115.4_21900001	16	21900001	0.5511	0.5962
NC_005115.4_22000001	16	22000001	0.5778	0.6426
NC_005115.4_22100001	16	22100001	0.5119	0.5964
NC_005115.4_22200001	16	22200001	0.5593	0.6371
NC_005115.4_22300001	16	22300001	0.5808	0.6413
NC_005115.4_22400001	16	22400001	0.5588	0.6565
NC_005115.4_22500001	16	22500001	0.636	0.7032
NC_005115.4_22600001	16	22600001	0.7342	0.7563
NC_005115.4_22700001	16	22700001	0.607	0.6733
NC_005115.4_22800001	16	22800001	0.5924	0.6545
NC_005115.4_22900001	16	22900001	0.6091	0.6596
NC_005115.4_23000001	16	23000001	0.5056	0.5616
NC_005115.4_23100001	16	23100001	0.523	0.572
NC_005115.4_23200001	16	23200001	0.551	0.6524
NC_005115.4_23300001	16	23300001	0.5703	0.6855
NC_005115.4_23400001	16	23400001	0.5792	0.6427
NC_005115.4_23500001	16	23500001	0.5742	0.6281
NC_005115.4_23600001	16	23600001	0.5659	0.6266
NC_005115.4_23700001	16	23700001	0.7275	0.6892
NC_005115.4_23800001	16	23800001	0.7527	0.6482
NC_005115.4_23900001	16	23900001	0.7376	0.7017
NC_005115.4_24000001	16	24000001	0.5837	0.685
NC_005115.4_24100001	16	24100001	0.6055	0.6967
NC_005115.4_24200001	16	24200001	0.5365	0.6568
NC_005115.4_24300001	16	24300001	0.5184	0.6674
NC_005115.4_24400001	16	24400001	0.5322	0.6806
NC_005115.4_24500001	16	24500001	0.5305	0.7026
NC_005115.4_24600001	16	24600001	0.51	0.693
NC_005115.4_24700001	16	24700001	0.3729	0.5404
NC_005115.4_24800001	16	24800001	0.252	0.4387
NC_005115.4_24900001	16	24900001	0.3264	0.4174
NC_005115.4_25000001	16	25000001	0.4129	0.4411
NC_005115.4_25100001	16	25100001	0.5018	0.5373
NC_005115.4_25200001	16	25200001	0.6298	0.5969
NC_005115.4_25300001	16	25300001	0.5145	0.5641
NC_005115.4_25400001	16	25400001	0.4064	0.4478
NC_005115.4_25500001	16	25500001	0.3968	0.4368
NC_005115.4_25600001	16	25600001	0.4307	0.5148
NC_005115.4_25700001	16	25700001	0.4741	0.5328
NC_005115.4_25800001	16	25800001	0.5018	0.5306
NC_005115.4_25900001	16	25900001	0.6669	0.6742
NC_005115.4_26000001	16	26000001	0.7176	0.7021
NC_005115.4_26100001	16	26100001	0.5597	0.5487
NC_005115.4_26200001	16	26200001	0.4162	0.443

NC_005115.4_26300001	16	26300001	0.6015	0.6821
NC_005115.4_26400001	16	26400001	0.4841	0.5767
NC_005115.4_26500001	16	26500001	0.4912	0.615
NC_005115.4_26600001	16	26600001	0.469	0.6037
NC_005115.4_26700001	16	26700001	0.515	0.6701
NC_005115.4_26800001	16	26800001	0.4739	0.605
NC_005115.4_26900001	16	26900001	0.5166	0.6335
NC_005115.4_27000001	16	27000001	0.5292	0.5853
NC_005115.4_27100001	16	27100001	0.585	0.6124
NC_005115.4_27200001	16	27200001	0.5242	0.5359
NC_005115.4_27700001	16	27700001	0.4529	0.7594
NC_005115.4_27800001	16	27800001	0.423	0.6917
NC_005115.4_27900001	16	27900001	0.4025	0.6697
NC_005115.4_28000001	16	28000001	0.386	0.6209
NC_005115.4_28100001	16	28100001	0.4314	0.708
NC_005115.4_28200001	16	28200001	0.3872	0.6519
NC_005115.4_28300001	16	28300001	0.537	0.7809
NC_005115.4_28400001	16	28400001	0.5546	0.824
NC_005115.4_28500001	16	28500001	0.6088	0.8295
NC_005115.4_28600001	16	28600001	0.5936	0.7347
NC_005115.4_28700001	16	28700001	0.6275	0.7476
NC_005115.4_28800001	16	28800001	0.6057	0.7332
NC_005115.4_28900001	16	28900001	0.7112	0.7406
NC_005115.4_29000001	16	29000001	0.6085	0.6374
NC_005115.4_29100001	16	29100001	0.683	0.7791
NC_005115.4_29400001	16	29400001	0.4056	0.4093
NC_005115.4_29500001	16	29500001	0.4562	0.4206
NC_005115.4_29600001	16	29600001	0.4562	0.4206
NC_005115.4_29700001	16	29700001	0.3832	0.2928
NC_005115.4_31200001	16	31200001	0.2862	0.349
NC_005115.4_31300001	16	31300001	0.2855	0.3129
NC_005115.4_31400001	16	31400001	0.3732	0.5384
NC_005115.4_31500001	16	31500001	0.4483	0.609
NC_005115.4_31600001	16	31600001	0.4645	0.6311
NC_005115.4_31700001	16	31700001	0.55	0.7227
NC_005115.4_31800001	16	31800001	0.5493	0.7156
NC_005115.4_31900001	16	31900001	0.5839	0.7168
NC_005115.4_32000001	16	32000001	0.577	0.7162
NC_005115.4_32100001	16	32100001	0.6328	0.7327
NC_005115.4_32200001	16	32200001	0.6089	0.6982
NC_005115.4_32300001	16	32300001	0.666	0.7304
NC_005115.4_32400001	16	32400001	0.6296	0.7079
NC_005115.4_32500001	16	32500001	0.5954	0.6264
NC_005115.4_32600001	16	32600001	0.5144	0.5141
NC_005115.4_32700001	16	32700001	0.4553	0.4455
NC_005115.4_32800001	16	32800001	0.3889	0.3386
NC_005115.4_32900001	16	32900001	0.4078	0.3555
NC_005115.4_33000001	16	33000001	0.5231	0.5418
NC_005115.4_33100001	16	33100001	0.5482	0.6135
NC_005115.4_33200001	16	33200001	0.5545	0.5869
NC_005115.4_33300001	16	33300001	0.4593	0.5163
NC_005115.4_33400001	16	33400001	0.427	0.4864
NC_005115.4_33500001	16	33500001	0.2793	0.2386
NC_005115.4_33600001	16	33600001	0.3241	0.3311
NC_005115.4_33700001	16	33700001	0.3973	0.4749
NC_005115.4_33800001	16	33800001	0.4224	0.5027
NC_005115.4_33900001	16	33900001	0.4063	0.5191
NC_005115.4_34000001	16	34000001	0.4422	0.5863

NC_005115.4_34100001	16	34100001	0.395	0.4922
NC_005115.4_34200001	16	34200001	0.35	0.3234
NC_005115.4_34300001	16	34300001	0.4816	0.5292
NC_005115.4_34400001	16	34400001	0.5569	0.5831
NC_005115.4_34500001	16	34500001	0.5899	0.6003
NC_005115.4_34600001	16	34600001	0.6897	0.6874
NC_005115.4_34700001	16	34700001	0.674	0.7945
NC_005115.4_34800001	16	34800001	0.462	0.6024
NC_005115.4_34900001	16	34900001	0.3502	0.4847
NC_005115.4_35000001	16	35000001	0.5262	0.7528
NC_005115.4_35100001	16	35100001	0.5153	0.8015
NC_005115.4_35200001	16	35200001	0.5455	0.8108
NC_005115.4_35300001	16	35300001	0.5696	0.798
NC_005115.4_35400001	16	35400001	0.5806	0.8092
NC_005115.4_35500001	16	35500001	0.5252	0.7436
NC_005115.4_35600001	16	35600001	0.5538	0.7234
NC_005115.4_35700001	16	35700001	0.5388	0.6936
NC_005115.4_35800001	16	35800001	0.5312	0.7032
NC_005115.4_35900001	16	35900001	0.5695	0.7286
NC_005115.4_36000001	16	36000001	0.5842	0.7068
NC_005115.4_36100001	16	36100001	0.6752	0.7736
NC_005115.4_36200001	16	36200001	0.6078	0.7783
NC_005115.4_36300001	16	36300001	0.6451	0.8021
NC_005115.4_36400001	16	36400001	0.6451	0.8021
NC_005115.4_36500001	16	36500001	0.6451	0.8021
NC_005115.4_36600001	16	36600001	0.6602	0.7693
NC_005115.4_36700001	16	36700001	0.5943	0.7582
NC_005115.4_36800001	16	36800001	0.5435	0.7257
NC_005115.4_36900001	16	36900001	0.6634	0.7886
NC_005115.4_37000001	16	37000001	0.5613	0.7417
NC_005115.4_37100001	16	37100001	0.4716	0.7292
NC_005115.4_37200001	16	37200001	0.5236	0.743
NC_005115.4_37300001	16	37300001	0.5236	0.743
NC_005115.4_39000001	16	39000001	0.8662	0.9435
NC_005115.4_39100001	16	39100001	0.8662	0.9435
NC_005115.4_39200001	16	39200001	0.8869	0.9527
NC_005115.4_39400001	16	39400001	0.8545	0.9275
NC_005115.4_39600001	16	39600001	0.6513	0.7541
NC_005115.4_39700001	16	39700001	0.6565	0.73
NC_005115.4_39800001	16	39800001	0.6565	0.73
NC_005115.4_39900001	16	39900001	0.608	0.6385
NC_005115.4_40000001	16	40000001	0.6614	0.6594
NC_005115.4_40100001	16	40100001	0.6919	0.6704
NC_005115.4_40200001	16	40200001	0.6584	0.6362
NC_005115.4_40300001	16	40300001	0.6343	0.6604
NC_005115.4_40400001	16	40400001	0.5918	0.6522
NC_005115.4_40500001	16	40500001	0.6393	0.7052
NC_005115.4_40600001	16	40600001	0.4478	0.6853
NC_005115.4_40700001	16	40700001	0.4	0.673
NC_005115.4_40800001	16	40800001	0.442	0.676
NC_005115.4_40900001	16	40900001	0.3998	0.5977
NC_005115.4_41000001	16	41000001	0.421	0.6481
NC_005115.4_41100001	16	41100001	0.5217	0.7198
NC_005115.4_41200001	16	41200001	0.5929	0.7256
NC_005115.4_41300001	16	41300001	0.5407	0.6861
NC_005115.4_41400001	16	41400001	0.4652	0.6201
NC_005115.4_41500001	16	41500001	0.3926	0.4858
NC_005115.4_41600001	16	41600001	0.5027	0.6355

NC_005115.4_41700001	16	41700001	0.3773	0.4954
NC_005115.4_41800001	16	41800001	0.3326	0.4577
NC_005115.4_41900001	16	41900001	0.3374	0.456
NC_005115.4_42000001	16	42000001	0.3627	0.4408
NC_005115.4_42100001	16	42100001	0.2918	0.3389
NC_005115.4_42200001	16	42200001	0.3065	0.3639
NC_005115.4_42300001	16	42300001	0.3703	0.4071
NC_005115.4_42400001	16	42400001	0.4016	0.433
NC_005115.4_42500001	16	42500001	0.353	0.5556
NC_005115.4_42600001	16	42600001	0.4451	0.6964
NC_005115.4_42700001	16	42700001	0.4674	0.7598
NC_005115.4_42800001	16	42800001	0.4674	0.7598
NC_005115.4_42900001	16	42900001	0.473	0.702
NC_005115.4_43000001	16	43000001	0.5197	0.7229
NC_005115.4_43100001	16	43100001	0.4745	0.6774
NC_005115.4_43200001	16	43200001	0.4557	0.6438
NC_005115.4_43300001	16	43300001	0.461	0.6025
NC_005115.4_43400001	16	43400001	0.4863	0.6463
NC_005115.4_43500001	16	43500001	0.5432	0.615
NC_005115.4_43600001	16	43600001	0.6176	0.6309
NC_005115.4_43700001	16	43700001	0.6805	0.6372
NC_005115.4_43800001	16	43800001	0.5579	0.6756
NC_005115.4_43900001	16	43900001	0.5835	0.7384
NC_005115.4_44000001	16	44000001	0.5441	0.7333
NC_005115.4_44100001	16	44100001	0.5378	0.7291
NC_005115.4_44200001	16	44200001	0.4274	0.7406
NC_005115.4_44300001	16	44300001	0.4721	0.6155
NC_005115.4_44400001	16	44400001	0.4186	0.4915
NC_005115.4_44500001	16	44500001	0.4181	0.3628
NC_005115.4_44600001	16	44600001	0.3967	0.3723
NC_005115.4_44700001	16	44700001	0.5801	0.5564
NC_005115.4_44800001	16	44800001	0.688	0.6513
NC_005115.4_44900001	16	44900001	0.6476	0.7699
NC_005115.4_45000001	16	45000001	0.601	0.7898
NC_005115.4_45100001	16	45100001	0.6335	0.7707
NC_005115.4_45200001	16	45200001	0.4647	0.6784
NC_005115.4_45300001	16	45300001	0.4647	0.6784
NC_005115.4_45400001	16	45400001	0.3972	0.553
NC_005115.4_45500001	16	45500001	0.3733	0.5851
NC_005115.4_45600001	16	45600001	0.3874	0.6127
NC_005115.4_45700001	16	45700001	0.5537	0.7354
NC_005115.4_45800001	16	45800001	0.5915	0.7404
NC_005115.4_45900001	16	45900001	0.573	0.726
NC_005115.4_46000001	16	46000001	0.5931	0.7266
NC_005115.4_46100001	16	46100001	0.5927	0.7212
NC_005115.4_46200001	16	46200001	0.6121	0.7431
NC_005115.4_46300001	16	46300001	0.5096	0.6098
NC_005115.4_46400001	16	46400001	0.5106	0.5982
NC_005115.4_46500001	16	46500001	0.509	0.5744
NC_005115.4_46600001	16	46600001	0.4733	0.5451
NC_005115.4_46700001	16	46700001	0.3999	0.4021
NC_005115.4_46800001	16	46800001	0.4905	0.5621
NC_005115.4_46900001	16	46900001	0.342	0.5429
NC_005115.4_47000001	16	47000001	0.5292	0.7358
NC_005115.4_47100001	16	47100001	0.524	0.7174
NC_005115.4_47200001	16	47200001	0.5187	0.6955
NC_005115.4_47300001	16	47300001	0.535	0.7241
NC_005115.4_47400001	16	47400001	0.5754	0.6787

NC_005115.4_47500001	16	47500001	0.5131	0.6263
NC_005115.4_47600001	16	47600001	0.5229	0.6014
NC_005115.4_47700001	16	47700001	0.5318	0.618
NC_005115.4_47800001	16	47800001	0.4562	0.5301
NC_005115.4_47900001	16	47900001	0.4254	0.5586
NC_005115.4_48000001	16	48000001	0.5013	0.6631
NC_005115.4_48100001	16	48100001	0.532	0.6721
NC_005115.4_48200001	16	48200001	0.497	0.6551
NC_005115.4_48300001	16	48300001	0.5509	0.733
NC_005115.4_48400001	16	48400001	0.4819	0.7174
NC_005115.4_48500001	16	48500001	0.4323	0.6342
NC_005115.4_48600001	16	48600001	0.4198	0.6535
NC_005115.4_48700001	16	48700001	0.4029	0.6747
NC_005115.4_48800001	16	48800001	0.3631	0.621
NC_005115.4_48900001	16	48900001	0.605	0.723
NC_005115.4_49000001	16	49000001	0.6015	0.7504
NC_005115.4_49100001	16	49100001	0.5681	0.7378
NC_005115.4_49200001	16	49200001	0.5507	0.7013
NC_005115.4_49300001	16	49300001	0.5051	0.6686
NC_005115.4_49400001	16	49400001	0.4716	0.5831
NC_005115.4_49500001	16	49500001	0.4069	0.4503
NC_005115.4_49600001	16	49600001	0.3893	0.4331
NC_005115.4_49700001	16	49700001	0.4274	0.4749
NC_005115.4_49800001	16	49800001	0.4767	0.4782
NC_005115.4_49900001	16	49900001	0.4299	0.4945
NC_005115.4_50000001	16	50000001	0.4558	0.6097
NC_005115.4_50100001	16	50100001	0.4008	0.6317
NC_005115.4_50200001	16	50200001	0.3809	0.6938
NC_005115.4_50300001	16	50300001	0.3809	0.6938
NC_005115.4_50400001	16	50400001	0.2851	0.734
NC_005115.4_50500001	16	50500001	0.3088	0.6393
NC_005115.4_50600001	16	50600001	0.4355	0.6749
NC_005115.4_50700001	16	50700001	0.4148	0.6177
NC_005115.4_50800001	16	50800001	0.3879	0.5937
NC_005115.4_50900001	16	50900001	0.4147	0.5271
NC_005115.4_51000001	16	51000001	0.3392	0.4589
NC_005115.4_51100001	16	51100001	0.2826	0.3673
NC_005115.4_51200001	16	51200001	0.328	0.3759
NC_005115.4_51300001	16	51300001	0.3774	0.4086
NC_005115.4_51400001	16	51400001	0.4874	0.4944
NC_005115.4_51500001	16	51500001	0.5183	0.4686
NC_005115.4_51600001	16	51600001	0.5409	0.5147
NC_005115.4_51700001	16	51700001	0.544	0.5188
NC_005115.4_51800001	16	51800001	0.5363	0.5336
NC_005115.4_51900001	16	51900001	0.5341	0.5953
NC_005115.4_52000001	16	52000001	0.5646	0.629
NC_005115.4_52100001	16	52100001	0.5572	0.7073
NC_005115.4_52200001	16	52200001	0.4148	0.6132
NC_005115.4_52300001	16	52300001	0.4059	0.5932
NC_005115.4_52400001	16	52400001	0.3475	0.5406
NC_005115.4_52500001	16	52500001	0.3176	0.526
NC_005115.4_52600001	16	52600001	0.3093	0.4413
NC_005115.4_52700001	16	52700001	0.409	0.594
NC_005115.4_52800001	16	52800001	0.4572	0.68
NC_005115.4_52900001	16	52900001	0.6111	0.7703
NC_005115.4_53000001	16	53000001	0.598	0.7569
NC_005115.4_53100001	16	53100001	0.68	0.8781
NC_005115.4_53200001	16	53200001	0.6271	0.8601

NC_005115.4_53300001	16	53300001	0.684	0.8446
NC_005115.4_53400001	16	53400001	0.4732	0.7558
NC_005115.4_53500001	16	53500001	0.573	0.8141
NC_005115.4_53600001	16	53600001	0.5224	0.7772
NC_005115.4_53700001	16	53700001	0.5954	0.7617
NC_005115.4_53800001	16	53800001	0.5061	0.7229
NC_005115.4_53900001	16	53900001	0.6031	0.7134
NC_005115.4_54000001	16	54000001	0.5724	0.6716
NC_005115.4_54100001	16	54100001	0.5724	0.6716
NC_005115.4_54200001	16	54200001	0.5191	0.6476
NC_005115.4_54300001	16	54300001	0.566	0.7236
NC_005115.4_54400001	16	54400001	0.521	0.7257
NC_005115.4_54500001	16	54500001	0.4955	0.7971
NC_005115.4_54600001	16	54600001	0.4885	0.7163
NC_005115.4_54700001	16	54700001	0.4895	0.7124
NC_005115.4_54800001	16	54800001	0.479	0.6546
NC_005115.4_54900001	16	54900001	0.4971	0.6985
NC_005115.4_55000001	16	55000001	0.5172	0.6738
NC_005115.4_55100001	16	55100001	0.5411	0.7538
NC_005115.4_55200001	16	55200001	0.3954	0.6453
NC_005115.4_55300001	16	55300001	0.4112	0.6785
NC_005115.4_55400001	16	55400001	0.3697	0.6177
NC_005115.4_55500001	16	55500001	0.3746	0.6822
NC_005115.4_55600001	16	55600001	0.3746	0.6822
NC_005115.4_55700001	16	55700001	0.5923	0.835
NC_005115.4_55800001	16	55800001	0.5774	0.7734
NC_005115.4_55900001	16	55900001	0.4582	0.5373
NC_005115.4_56000001	16	56000001	0.4115	0.5217
NC_005115.4_56100001	16	56100001	0.4922	0.6406
NC_005115.4_56200001	16	56200001	0.586	0.721
NC_005115.4_56300001	16	56300001	0.5736	0.7237
NC_005115.4_56400001	16	56400001	0.6224	0.8222
NC_005115.4_56500001	16	56500001	0.6768	0.8392
NC_005115.4_56600001	16	56600001	0.6641	0.8192
NC_005115.4_57300001	16	57300001	0.1187	0.2247
NC_005115.4_57400001	16	57400001	0.1076	0.2118
NC_005115.4_57500001	16	57500001	0.1917	0.4213
NC_005115.4_57600001	16	57600001	0.1931	0.3645
NC_005115.4_57700001	16	57700001	0.2384	0.3986
NC_005115.4_57800001	16	57800001	0.2518	0.4402
NC_005115.4_57900001	16	57900001	0.275	0.455
NC_005115.4_58000001	16	58000001	0.3528	0.3559
NC_005115.4_58100001	16	58100001	0.5261	0.6617
NC_005115.4_58200001	16	58200001	0.536	0.5708
NC_005115.4_58300001	16	58300001	0.5711	0.6748
NC_005115.4_58400001	16	58400001	0.5774	0.6624
NC_005115.4_58500001	16	58500001	0.6081	0.7214
NC_005115.4_58600001	16	58600001	0.4377	0.5842
NC_005115.4_58700001	16	58700001	0.4125	0.6523
NC_005115.4_58800001	16	58800001	0.3527	0.5486
NC_005115.4_58900001	16	58900001	0.3467	0.5246
NC_005115.4_59000001	16	59000001	0.3522	0.554
NC_005115.4_59100001	16	59100001	0.4086	0.6071
NC_005115.4_59200001	16	59200001	0.4902	0.6642
NC_005115.4_59300001	16	59300001	0.4833	0.6749
NC_005115.4_59400001	16	59400001	0.4938	0.7278
NC_005115.4_59500001	16	59500001	0.493	0.6221
NC_005115.4_59600001	16	59600001	0.5084	0.6304

NC_005115.4_59700001	16	59700001	0.4895	0.6464
NC_005115.4_59800001	16	59800001	0.5405	0.7025
NC_005115.4_59900001	16	59900001	0.4976	0.718
NC_005115.4_60000001	16	60000001	0.4709	0.6921
NC_005115.4_60100001	16	60100001	0.4422	0.7087
NC_005115.4_60200001	16	60200001	0.5099	0.6965
NC_005115.4_60300001	16	60300001	0.5213	0.67
NC_005115.4_60400001	16	60400001	0.5732	0.6423
NC_005115.4_60500001	16	60500001	0.5463	0.6502
NC_005115.4_60600001	16	60600001	0.5923	0.6671
NC_005115.4_60700001	16	60700001	0.538	0.6357
NC_005115.4_60800001	16	60800001	0.4603	0.6037
NC_005115.4_60900001	16	60900001	0.4707	0.7115
NC_005115.4_61000001	16	61000001	0.5116	0.7881
NC_005115.4_61100001	16	61100001	0.4687	0.7251
NC_005115.4_61200001	16	61200001	0.4791	0.6139
NC_005115.4_61300001	16	61300001	0.5507	0.669
NC_005115.4_61400001	16	61400001	0.5243	0.6016
NC_005115.4_61500001	16	61500001	0.5303	0.5397
NC_005115.4_61600001	16	61600001	0.5964	0.6099
NC_005115.4_61700001	16	61700001	0.7857	0.9048
NC_005115.4_61800001	16	61800001	0.5939	0.8435
NC_005115.4_61900001	16	61900001	0.5774	0.8968
NC_005115.4_62000001	16	62000001	0.4997	0.8315
NC_005115.4_62100001	16	62100001	0.5575	0.8553
NC_005115.4_62200001	16	62200001	0.4142	0.7101
NC_005115.4_62300001	16	62300001	0.3892	0.5749
NC_005115.4_62400001	16	62400001	0.4275	0.531
NC_005115.4_62500001	16	62500001	0.5784	0.6284
NC_005115.4_62600001	16	62600001	0.508	0.5599
NC_005115.4_62700001	16	62700001	0.4566	0.5633
NC_005115.4_62800001	16	62800001	0.4619	0.58
NC_005115.4_62900001	16	62900001	0.4513	0.6023
NC_005115.4_63000001	16	63000001	0.4595	0.6499
NC_005115.4_63100001	16	63100001	0.4912	0.6907
NC_005115.4_63200001	16	63200001	0.4865	0.7466
NC_005115.4_63300001	16	63300001	0.4841	0.6855
NC_005115.4_63400001	16	63400001	0.5391	0.7127
NC_005115.4_63500001	16	63500001	0.5202	0.6812
NC_005115.4_63600001	16	63600001	0.4672	0.5741
NC_005115.4_63700001	16	63700001	0.5321	0.5775
NC_005115.4_63800001	16	63800001	0.4875	0.5836
NC_005115.4_63900001	16	63900001	0.4328	0.524
NC_005115.4_64000001	16	64000001	0.448	0.5358
NC_005115.4_64100001	16	64100001	0.4625	0.5568
NC_005115.4_64200001	16	64200001	0.4708	0.5325
NC_005115.4_64300001	16	64300001	0.5406	0.5584
NC_005115.4_64400001	16	64400001	0.5782	0.5573
NC_005115.4_64500001	16	64500001	0.584	0.5229
NC_005115.4_64600001	16	64600001	0.6237	0.623
NC_005115.4_64700001	16	64700001	0.634	0.6573
NC_005115.4_64800001	16	64800001	0.6165	0.622
NC_005115.4_64900001	16	64900001	0.6538	0.6527
NC_005115.4_65000001	16	65000001	0.6611	0.6467
NC_005115.4_65100001	16	65100001	0.5882	0.5354
NC_005115.4_65200001	16	65200001	0.651	0.6074
NC_005115.4_65300001	16	65300001	0.6634	0.6843
NC_005115.4_65400001	16	65400001	0.6606	0.7089

NC_005115.4_65500001	16	65500001	0.58	0.7134
NC_005115.4_65600001	16	65600001	0.6059	0.7715
NC_005115.4_65700001	16	65700001	0.468	0.7947
NC_005115.4_65800001	16	65800001	0.264	0.6303
NC_005115.4_66400001	16	66400001	0.4999	0.6561
NC_005115.4_66500001	16	66500001	0.5431	0.6828
NC_005115.4_66600001	16	66600001	0.6356	0.7698
NC_005115.4_66700001	16	66700001	0.6028	0.7432
NC_005115.4_66800001	16	66800001	0.5758	0.768
NC_005115.4_66900001	16	66900001	0.528	0.6722
NC_005115.4_67000001	16	67000001	0.5448	0.7403
NC_005115.4_67100001	16	67100001	0.4709	0.7083
NC_005115.4_67200001	16	67200001	0.4329	0.706
NC_005115.4_67300001	16	67300001	0.4614	0.7336
NC_005115.4_67400001	16	67400001	0.4763	0.7181
NC_005115.4_67500001	16	67500001	0.4046	0.6606
NC_005115.4_67600001	16	67600001	0.4205	0.6281
NC_005115.4_67700001	16	67700001	0.4417	0.5233
NC_005115.4_67800001	16	67800001	0.3667	0.4613
NC_005115.4_67900001	16	67900001	0.3564	0.4642
NC_005115.4_68000001	16	68000001	0.3548	0.471
NC_005115.4_68100001	16	68100001	0.347	0.4834
NC_005115.4_68200001	16	68200001	0.2678	0.4902
NC_005115.4_68300001	16	68300001	0.2678	0.4902
NC_005115.4_68400001	16	68400001	0.3904	0.6292
NC_005115.4_68500001	16	68500001	0.3932	0.5711
NC_005115.4_68600001	16	68600001	0.4164	0.6066
NC_005115.4_68700001	16	68700001	0.55	0.7057
NC_005115.4_68800001	16	68800001	0.5281	0.7036
NC_005115.4_68900001	16	68900001	0.5309	0.6742
NC_005115.4_69000001	16	69000001	0.6109	0.7125
NC_005115.4_69100001	16	69100001	0.6436	0.7475
NC_005115.4_69200001	16	69200001	0.4975	0.6194
NC_005115.4_69300001	16	69300001	0.5171	0.6128
NC_005115.4_69400001	16	69400001	0.5007	0.6301
NC_005115.4_69500001	16	69500001	0.546	0.7143
NC_005115.4_69600001	16	69600001	0.295	0.3998
NC_005115.4_69900001	16	69900001	0.3662	0.4333
NC_005115.4_70000001	16	70000001	0.3319	0.4199
NC_005115.4_70100001	16	70100001	0.3414	0.3807
NC_005115.4_70200001	16	70200001	0.461	0.5291
NC_005115.4_70300001	16	70300001	0.4618	0.56
NC_005115.4_70400001	16	70400001	0.4943	0.6516
NC_005115.4_70500001	16	70500001	0.5031	0.6145
NC_005115.4_70600001	16	70600001	0.6712	0.8004
NC_005115.4_70700001	16	70700001	0.6359	0.8386
NC_005115.4_70800001	16	70800001	0.6936	0.851
NC_005115.4_70900001	16	70900001	0.794	0.8982
NC_005115.4_71000001	16	71000001	0.76	0.9292
NC_005115.4_71100001	16	71100001	0.7167	0.8869
NC_005115.4_71200001	16	71200001	0.6584	0.8276
NC_005115.4_71300001	16	71300001	0.5627	0.7903
NC_005115.4_71400001	16	71400001	0.3795	0.583
NC_005115.4_71500001	16	71500001	0.4127	0.611
NC_005115.4_71600001	16	71600001	0.4137	0.6263
NC_005115.4_71700001	16	71700001	0.3701	0.6006
NC_005115.4_71800001	16	71800001	0.4556	0.697
NC_005115.4_71900001	16	71900001	0.4557	0.7363

NC_005115.4_72000001	16	72000001	0.4602	0.7058
NC_005115.4_72100001	16	72100001	0.3668	0.5897
NC_005115.4_72200001	16	72200001	0.3384	0.5558
NC_005115.4_72300001	16	72300001	0.3305	0.4291
NC_005115.4_72400001	16	72400001	0.3824	0.4803
NC_005115.4_72500001	16	72500001	0.404	0.5107
NC_005115.4_72600001	16	72600001	0.4245	0.5501
NC_005115.4_72700001	16	72700001	0.4927	0.5897
NC_005115.4_72800001	16	72800001	0.4948	0.6627
NC_005115.4_72900001	16	72900001	0.4594	0.5986
NC_005115.4_73000001	16	73000001	0.4573	0.5845
NC_005115.4_73100001	16	73100001	0.4916	0.587
NC_005115.4_73200001	16	73200001	0.4918	0.5938
NC_005115.4_73300001	16	73300001	0.4815	0.5501
NC_005115.4_73400001	16	73400001	0.4484	0.5528
NC_005115.4_73500001	16	73500001	0.468	0.5943
NC_005115.4_73600001	16	73600001	0.4225	0.5939
NC_005115.4_73700001	16	73700001	0.4513	0.6339
NC_005115.4_73800001	16	73800001	0.4663	0.6257
NC_005115.4_73900001	16	73900001	0.5206	0.6888
NC_005115.4_74000001	16	74000001	0.4711	0.6311
NC_005115.4_74100001	16	74100001	0.5604	0.7266
NC_005115.4_74200001	16	74200001	0.4879	0.6356
NC_005115.4_74300001	16	74300001	0.6323	0.7352
NC_005115.4_74400001	16	74400001	0.4864	0.6411
NC_005115.4_74500001	16	74500001	0.525	0.7188
NC_005115.4_74600001	16	74600001	0.4805	0.6225
NC_005115.4_74700001	16	74700001	0.4766	0.6267
NC_005115.4_74800001	16	74800001	0.4265	0.5874
NC_005115.4_74900001	16	74900001	0.5118	0.7245
NC_005115.4_75000001	16	75000001	0.4183	0.5382
NC_005115.4_75100001	16	75100001	0.2947	0.5238
NC_005115.4_75200001	16	75200001	0.3334	0.5517
NC_005115.4_75300001	16	75300001	0.4232	0.6586
NC_005115.4_75400001	16	75400001	0.5673	0.6949
NC_005115.4_75500001	16	75500001	0.5569	0.6503
NC_005115.4_75600001	16	75600001	0.6137	0.7042
NC_005115.4_75700001	16	75700001	0.5619	0.6805
NC_005115.4_75800001	16	75800001	0.5435	0.6357
NC_005115.4_75900001	16	75900001	0.5022	0.59
NC_005115.4_76000001	16	76000001	0.5344	0.6191
NC_005115.4_76100001	16	76100001	0.5721	0.6301
NC_005115.4_76200001	16	76200001	0.5903	0.6379
NC_005115.4_76300001	16	76300001	0.5947	0.675
NC_005115.4_76400001	16	76400001	0.5947	0.675
NC_005115.4_76500001	16	76500001	0.598	0.7286
NC_005115.4_76600001	16	76600001	0.4069	0.5684
NC_005115.4_76700001	16	76700001	0.5595	0.5449
NC_005115.4_76800001	16	76800001	0.4116	0.5839
NC_005115.4_76900001	16	76900001	0.4834	0.6298
NC_005115.4_77000001	16	77000001	0.4367	0.613
NC_005115.4_77100001	16	77100001	0.4798	0.7006
NC_005115.4_77200001	16	77200001	0.4914	0.7819
NC_005115.4_77300001	16	77300001	0.5769	0.7984
NC_005115.4_77400001	16	77400001	0.5525	0.8619
NC_005115.4_77500001	16	77500001	0.6165	0.86
NC_005115.4_77600001	16	77600001	0.5815	0.7714
NC_005115.4_77700001	16	77700001	0.5781	0.7179

NC_005115.4_77800001	16	77800001	0.6001	0.7027
NC_005115.4_77900001	16	77900001	0.5607	0.6093
NC_005115.4_78000001	16	78000001	0.5398	0.5939
NC_005115.4_78100001	16	78100001	0.5436	0.5939
NC_005115.4_78200001	16	78200001	0.3698	0.4625
NC_005115.4_78300001	16	78300001	0.3896	0.5315
NC_005115.4_78400001	16	78400001	0.4168	0.5737
NC_005115.4_78500001	16	78500001	0.3664	0.5558
NC_005115.4_78600001	16	78600001	0.3567	0.5839
NC_005115.4_78700001	16	78700001	0.404	0.6763
NC_005115.4_78800001	16	78800001	0.3335	0.6213
NC_005115.4_78900001	16	78900001	0.2505	0.5519
NC_005115.4_79000001	16	79000001	0.3424	0.6621
NC_005115.4_79100001	16	79100001	0.3399	0.6291
NC_005115.4_79200001	16	79200001	0.3217	0.6353
NC_005115.4_79300001	16	79300001	0.3522	0.4918
NC_005115.4_79400001	16	79400001	0.3561	0.4857
NC_005115.4_79500001	16	79500001	0.4005	0.6064
NC_005115.4_79600001	16	79600001	0.4717	0.6978
NC_005115.4_79700001	16	79700001	0.454	0.6661
NC_005115.4_79800001	16	79800001	0.465	0.7301
NC_005115.4_79900001	16	79900001	0.461	0.7442
NC_005115.4_80000001	16	80000001	0.4623	0.7331
NC_005115.4_80100001	16	80100001	0.47	0.7256
NC_005115.4_80200001	16	80200001	0.5014	0.7801
NC_005115.4_80300001	16	80300001	0.5085	0.778
NC_005115.4_80400001	16	80400001	0.5736	0.8043
NC_005115.4_80500001	16	80500001	0.6426	0.822
NC_005115.4_80600001	16	80600001	0.5043	0.7566
NC_005115.4_80700001	16	80700001	0.3951	0.6007
NC_005115.4_80800001	16	80800001	0.4193	0.578
NC_005115.4_80900001	16	80900001	0.4294	0.5978
NC_005115.4_81000001	16	81000001	0.4274	0.6036
NC_005115.4_81100001	16	81100001	0.4392	0.6112
NC_005115.4_81200001	16	81200001	0.4874	0.6666
NC_005115.4_81300001	16	81300001	0.5545	0.7519
NC_005115.4_81400001	16	81400001	0.5787	0.7416
NC_005115.4_81500001	16	81500001	0.6657	0.7487
NC_005115.4_81600001	16	81600001	0.6634	0.7356
NC_005115.4_81700001	16	81700001	0.6662	0.7399
NC_005115.4_81800001	16	81800001	0.639	0.7433
NC_005115.4_81900001	16	81900001	0.5576	0.7117
NC_005115.4_82000001	16	82000001	0.515	0.6998
NC_005115.4_82100001	16	82100001	0.4967	0.712
NC_005115.4_82200001	16	82200001	0.5147	0.7105
NC_005115.4_82300001	16	82300001	0.516	0.7012
NC_005115.4_82400001	16	82400001	0.5294	0.6995
NC_005115.4_82500001	16	82500001	0.5612	0.7157
NC_005115.4_82600001	16	82600001	0.5885	0.7367
NC_005115.4_82700001	16	82700001	0.5934	0.7476
NC_005115.4_82800001	16	82800001	0.6001	0.7383
NC_005115.4_82900001	16	82900001	0.6234	0.7836
NC_005115.4_83000001	16	83000001	0.5344	0.7786
NC_005115.4_83100001	16	83100001	0.4528	0.7129
NC_005115.4_83200001	16	83200001	0.4212	0.6602
NC_005115.4_83300001	16	83300001	0.4605	0.6964
NC_005115.4_83400001	16	83400001	0.4436	0.6143
NC_005115.4_83500001	16	83500001	0.4778	0.6262

NC_005115.4_83600001	16	83600001	0.4468	0.5728
NC_005115.4_83700001	16	83700001	0.4642	0.6015
NC_005115.4_83800001	16	83800001	0.4373	0.5394
NC_005115.4_83900001	16	83900001	0.4548	0.5143
NC_005115.4_84000001	16	84000001	0.4374	0.5297
NC_005115.4_84100001	16	84100001	0.4426	0.5439
NC_005115.4_84200001	16	84200001	0.3988	0.4688
NC_005115.4_84300001	16	84300001	0.4579	0.606
NC_005115.4_84400001	16	84400001	0.4377	0.5913
NC_005115.4_84500001	16	84500001	0.4339	0.5566
NC_005115.4_84600001	16	84600001	0.4268	0.5266
NC_005115.4_84700001	16	84700001	0.4473	0.5329
NC_005115.4_84800001	16	84800001	0.3811	0.3396
NC_005115.4_84900001	16	84900001	0.3912	0.3245
NC_005115.4_85000001	16	85000001	0.3738	0.2985
NC_005115.4_85100001	16	85100001	0.402	0.3389
NC_005115.4_85200001	16	85200001	0.5472	0.5178
NC_005115.4_85300001	16	85300001	0.5368	0.6431
NC_005115.4_85400001	16	85400001	0.6313	0.7375
NC_005115.4_85500001	16	85500001	0.5635	0.6627
NC_005115.4_85600001	16	85600001	0.5144	0.6638
NC_005115.4_85700001	16	85700001	0.5178	0.649
NC_005115.4_85800001	16	85800001	0.5988	0.7221
NC_005115.4_85900001	16	85900001	0.5362	0.6507
NC_005115.4_86000001	16	86000001	0.5525	0.6823
NC_005115.4_86100001	16	86100001	0.5564	0.7179
NC_005115.4_86200001	16	86200001	0.5258	0.6917
NC_005115.4_86300001	16	86300001	0.3708	0.5112
NC_005115.4_86400001	16	86400001	0.4231	0.6
NC_005115.4_86500001	16	86500001	0.3975	0.5212
NC_005115.4_86600001	16	86600001	0.381	0.4445
NC_005115.4_86700001	16	86700001	0.3786	0.4575
NC_005115.4_86800001	16	86800001	0.4198	0.5207
NC_005115.4_86900001	16	86900001	0.4367	0.5084
NC_005115.4_87000001	16	87000001	0.4923	0.5656
NC_005115.4_87100001	16	87100001	0.5513	0.6167
NC_005115.4_87200001	16	87200001	0.4893	0.5713
NC_005115.4_87300001	16	87300001	0.5862	0.6953
NC_005115.4_87400001	16	87400001	0.4938	0.5945
NC_005115.4_87500001	16	87500001	0.4058	0.5241
NC_005115.4_87600001	16	87600001	0.4275	0.5778
NC_005115.4_87700001	16	87700001	0.4882	0.5679
NC_005115.4_87800001	16	87800001	0.3869	0.4209
NC_005115.4_87900001	16	87900001	0.4628	0.5257
NC_005115.4_88000001	16	88000001	0.4928	0.59
NC_005115.4_88100001	16	88100001	0.4598	0.539
NC_005115.4_88200001	16	88200001	0.446	0.5902
NC_005115.4_88300001	16	88300001	0.5497	0.6988
NC_005115.4_88400001	16	88400001	0.4582	0.7002
NC_005115.4_88500001	16	88500001	0.4896	0.6628
NC_005115.4_88600001	16	88600001	0.4861	0.6957
NC_005115.4_88700001	16	88700001	0.4055	0.645
NC_005115.4_88800001	16	88800001	0.3244	0.5106
NC_005115.4_88900001	16	88900001	0.379	0.5728
NC_005115.4_89000001	16	89000001	0.3255	0.5825
NC_005115.4_89100001	16	89100001	0.3195	0.5241
NC_005115.4_89200001	16	89200001	0.3327	0.467
NC_005115.4_89300001	16	89300001	0.4483	0.6716

NC_005115.4_89400001	16	89400001	0.43	0.5998
NC_005115.4_89500001	16	89500001	0.4685	0.6613
NC_005115.4_89600001	16	89600001	0.4916	0.6986
NC_005115.4_89700001	16	89700001	0.4813	0.7149
NC_005115.4_89800001	16	89800001	0.3503	0.4613
NC_005115.4_89900001	16	89900001	0.3988	0.4474
NC_005115.4_90000001	16	90000001	0.3353	0.2841
NC_005115.4_90100001	16	90100001	0.3013	0.2595
NC_005115.4_90200001	16	90200001	0.4162	0.4016
NC_005116.4_100001	17	100001	0.6053	0.7565
NC_005116.4_200001	17	200001	0.5964	0.7648
NC_005116.4_300001	17	300001	0.6017	0.7555
NC_005116.4_400001	17	400001	0.6435	0.7431
NC_005116.4_500001	17	500001	0.6287	0.7608
NC_005116.4_600001	17	600001	0.6294	0.7731
NC_005116.4_700001	17	700001	0.6003	0.7374
NC_005116.4_800001	17	800001	0.6047	0.7521
NC_005116.4_900001	17	900001	0.5447	0.7631
NC_005116.4_1000001	17	1000001	0.522	0.7082
NC_005116.4_1100001	17	1100001	0.5005	0.638
NC_005116.4_1200001	17	1200001	0.4503	0.63
NC_005116.4_1300001	17	1300001	0.4828	0.6897
NC_005116.4_1400001	17	1400001	0.4652	0.6907
NC_005116.4_1500001	17	1500001	0.4344	0.7167
NC_005116.4_1600001	17	1600001	0.4435	0.743
NC_005116.4_1700001	17	1700001	0.4789	0.7794
NC_005116.4_1800001	17	1800001	0.3501	0.5631
NC_005116.4_1900001	17	1900001	0.3813	0.5349
NC_005116.4_2000001	17	2000001	0.4331	0.5522
NC_005116.4_2200001	17	2200001	0.3759	0.4574
NC_005116.4_2300001	17	2300001	0.6293	0.709
NC_005116.4_2400001	17	2400001	0.5188	0.5921
NC_005116.4_2500001	17	2500001	0.5188	0.5921
NC_005116.4_2600001	17	2600001	0.5188	0.5921
NC_005116.4_3300001	17	3300001	0.3668	0.3368
NC_005116.4_3400001	17	3400001	0.5164	0.532
NC_005116.4_3500001	17	3500001	0.4592	0.5114
NC_005116.4_3600001	17	3600001	0.3554	0.445
NC_005116.4_3700001	17	3700001	0.3804	0.6437
NC_005116.4_3800001	17	3800001	0.4313	0.633
NC_005116.4_3900001	17	3900001	0.3334	0.5453
NC_005116.4_4000001	17	4000001	0.4332	0.6159
NC_005116.4_4100001	17	4100001	0.5091	0.6974
NC_005116.4_4200001	17	4200001	0.461	0.6303
NC_005116.4_4300001	17	4300001	0.5375	0.6775
NC_005116.4_4400001	17	4400001	0.5984	0.7026
NC_005116.4_4500001	17	4500001	0.5105	0.672
NC_005116.4_4600001	17	4600001	0.5329	0.6563
NC_005116.4_4700001	17	4700001	0.6055	0.7157
NC_005116.4_4800001	17	4800001	0.6079	0.7531
NC_005116.4_4900001	17	4900001	0.6705	0.7523
NC_005116.4_5000001	17	5000001	0.7652	0.8
NC_005116.4_5100001	17	5100001	0.7569	0.8036
NC_005116.4_5200001	17	5200001	0.7645	0.8086
NC_005116.4_5300001	17	5300001	0.7018	0.7839
NC_005116.4_5400001	17	5400001	0.6742	0.7847
NC_005116.4_5500001	17	5500001	0.6762	0.7962
NC_005116.4_5600001	17	5600001	0.6663	0.7772

NC_005116.4_5700001	17	5700001	0.6144	0.7291
NC_005116.4_5800001	17	5800001	0.6644	0.7127
NC_005116.4_5900001	17	5900001	0.6784	0.7421
NC_005116.4_6000001	17	6000001	0.6573	0.6765
NC_005116.4_6100001	17	6100001	0.6171	0.6479
NC_005116.4_6200001	17	6200001	0.6452	0.672
NC_005116.4_6300001	17	6300001	0.5796	0.6346
NC_005116.4_6400001	17	6400001	0.555	0.6065
NC_005116.4_6500001	17	6500001	0.5688	0.6676
NC_005116.4_6600001	17	6600001	0.6391	0.7187
NC_005116.4_6700001	17	6700001	0.58	0.6943
NC_005116.4_6800001	17	6800001	0.4832	0.6174
NC_005116.4_6900001	17	6900001	0.4489	0.5748
NC_005116.4_7000001	17	7000001	0.4296	0.5486
NC_005116.4_7100001	17	7100001	0.4336	0.5411
NC_005116.4_7200001	17	7200001	0.4539	0.565
NC_005116.4_7300001	17	7300001	0.463	0.5844
NC_005116.4_7400001	17	7400001	0.4391	0.5432
NC_005116.4_7500001	17	7500001	0.4694	0.5417
NC_005116.4_7600001	17	7600001	0.3889	0.4791
NC_005116.4_7700001	17	7700001	0.3303	0.3829
NC_005116.4_7800001	17	7800001	0.3984	0.4644
NC_005116.4_7900001	17	7900001	0.4352	0.5597
NC_005116.4_8000001	17	8000001	0.3618	0.4873
NC_005116.4_8100001	17	8100001	0.358	0.5145
NC_005116.4_8200001	17	8200001	0.4162	0.5988
NC_005116.4_8300001	17	8300001	0.3893	0.5583
NC_005116.4_8400001	17	8400001	0.4591	0.5901
NC_005116.4_8500001	17	8500001	0.533	0.6824
NC_005116.4_8600001	17	8600001	0.6053	0.7214
NC_005116.4_8700001	17	8700001	0.6052	0.7247
NC_005116.4_8800001	17	8800001	0.6317	0.7524
NC_005116.4_8900001	17	8900001	0.6162	0.7715
NC_005116.4_9000001	17	9000001	0.6091	0.7479
NC_005116.4_9100001	17	9100001	0.533	0.7158
NC_005116.4_9200001	17	9200001	0.4452	0.6848
NC_005116.4_9300001	17	9300001	0.3931	0.6136
NC_005116.4_9400001	17	9400001	0.4081	0.6182
NC_005116.4_9500001	17	9500001	0.5106	0.7217
NC_005116.4_9600001	17	9600001	0.5475	0.7361
NC_005116.4_9700001	17	9700001	0.6164	0.77
NC_005116.4_9800001	17	9800001	0.6686	0.8259
NC_005116.4_9900001	17	9900001	0.6844	0.816
NC_005116.4_10000001	17	10000001	0.5823	0.7698
NC_005116.4_10100001	17	10100001	0.5131	0.7378
NC_005116.4_10200001	17	10200001	0.5506	0.7551
NC_005116.4_10300001	17	10300001	0.4808	0.6515
NC_005116.4_10400001	17	10400001	0.4654	0.6637
NC_005116.4_10500001	17	10500001	0.4546	0.62
NC_005116.4_10600001	17	10600001	0.4839	0.593
NC_005116.4_10700001	17	10700001	0.4528	0.5629
NC_005116.4_10800001	17	10800001	0.4978	0.6322
NC_005116.4_10900001	17	10900001	0.5046	0.6052
NC_005116.4_11000001	17	11000001	0.5194	0.5345
NC_005116.4_11100001	17	11100001	0.5502	0.5947
NC_005116.4_11200001	17	11200001	0.4732	0.5685
NC_005116.4_11300001	17	11300001	0.4704	0.5496
NC_005116.4_11400001	17	11400001	0.4912	0.5616

NC_005116.4_11500001	17	11500001	0.4498	0.6065
NC_005116.4_11600001	17	11600001	0.3964	0.6089
NC_005116.4_11700001	17	11700001	0.5078	0.6193
NC_005116.4_11800001	17	11800001	0.4879	0.6129
NC_005116.4_11900001	17	11900001	0.5309	0.6683
NC_005116.4_12000001	17	12000001	0.5823	0.7181
NC_005116.4_12100001	17	12100001	0.5833	0.7322
NC_005116.4_12200001	17	12200001	0.5388	0.6877
NC_005116.4_12300001	17	12300001	0.5893	0.7261
NC_005116.4_12400001	17	12400001	0.5507	0.6702
NC_005116.4_12500001	17	12500001	0.515	0.5553
NC_005116.4_12600001	17	12600001	0.4793	0.5234
NC_005116.4_12700001	17	12700001	0.4865	0.6148
NC_005116.4_12800001	17	12800001	0.5531	0.6797
NC_005116.4_12900001	17	12900001	0.5663	0.7127
NC_005116.4_13000001	17	13000001	0.5813	0.7373
NC_005116.4_13100001	17	13100001	0.6452	0.7978
NC_005116.4_13200001	17	13200001	0.6534	0.8017
NC_005116.4_13300001	17	13300001	0.5619	0.7453
NC_005116.4_13400001	17	13400001	0.5225	0.685
NC_005116.4_13500001	17	13500001	0.5032	0.6333
NC_005116.4_13600001	17	13600001	0.4096	0.4867
NC_005116.4_13700001	17	13700001	0.4282	0.4604
NC_005116.4_13800001	17	13800001	0.5324	0.5417
NC_005116.4_13900001	17	13900001	0.4727	0.5065
NC_005116.4_14000001	17	14000001	0.455	0.54
NC_005116.4_14100001	17	14100001	0.4117	0.5058
NC_005116.4_14200001	17	14200001	0.4378	0.5243
NC_005116.4_14300001	17	14300001	0.3011	0.3969
NC_005116.4_14400001	17	14400001	0.3622	0.4604
NC_005116.4_14500001	17	14500001	0.412	0.5307
NC_005116.4_14600001	17	14600001	0.3907	0.4705
NC_005116.4_14700001	17	14700001	0.3907	0.4705
NC_005116.4_14800001	17	14800001	0.4901	0.5235
NC_005116.4_14900001	17	14900001	0.4332	0.504
NC_005116.4_15000001	17	15000001	0.4332	0.504
NC_005116.4_15100001	17	15100001	0.4888	0.618
NC_005116.4_15200001	17	15200001	0.502	0.6545
NC_005116.4_15300001	17	15300001	0.3752	0.6403
NC_005116.4_15400001	17	15400001	0.4296	0.7225
NC_005116.4_15500001	17	15500001	0.4043	0.6378
NC_005116.4_15600001	17	15600001	0.3895	0.6158
NC_005116.4_15700001	17	15700001	0.4404	0.678
NC_005116.4_15800001	17	15800001	0.4717	0.6786
NC_005116.4_15900001	17	15900001	0.4632	0.6135
NC_005116.4_16000001	17	16000001	0.4968	0.665
NC_005116.4_16100001	17	16100001	0.5677	0.7092
NC_005116.4_16200001	17	16200001	0.481	0.6232
NC_005116.4_16300001	17	16300001	0.4951	0.6198
NC_005116.4_16400001	17	16400001	0.4955	0.6444
NC_005116.4_16500001	17	16500001	0.4997	0.6464
NC_005116.4_16600001	17	16600001	0.4846	0.5916
NC_005116.4_16700001	17	16700001	0.5306	0.6373
NC_005116.4_16800001	17	16800001	0.5367	0.5933
NC_005116.4_16900001	17	16900001	0.5995	0.6307
NC_005116.4_17000001	17	17000001	0.5238	0.6128
NC_005116.4_17100001	17	17100001	0.4866	0.662
NC_005116.4_17200001	17	17200001	0.4528	0.6372

NC_005116.4_17300001	17	17300001	0.4962	0.7285
NC_005116.4_17400001	17	17400001	0.4939	0.719
NC_005116.4_17500001	17	17500001	0.5286	0.7397
NC_005116.4_17600001	17	17600001	0.6188	0.7627
NC_005116.4_17700001	17	17700001	0.6839	0.7733
NC_005116.4_17800001	17	17800001	0.6981	0.7799
NC_005116.4_17900001	17	17900001	0.6965	0.7902
NC_005116.4_18000001	17	18000001	0.7185	0.7739
NC_005116.4_18100001	17	18100001	0.6318	0.7046
NC_005116.4_18200001	17	18200001	0.561	0.6243
NC_005116.4_18300001	17	18300001	0.4766	0.5458
NC_005116.4_18400001	17	18400001	0.4545	0.4893
NC_005116.4_18500001	17	18500001	0.4383	0.4676
NC_005116.4_18600001	17	18600001	0.3447	0.4544
NC_005116.4_18700001	17	18700001	0.3736	0.5698
NC_005116.4_18800001	17	18800001	0.4374	0.5794
NC_005116.4_18900001	17	18900001	0.4462	0.6157
NC_005116.4_19000001	17	19000001	0.4225	0.6057
NC_005116.4_19100001	17	19100001	0.4707	0.5919
NC_005116.4_19200001	17	19200001	0.4501	0.585
NC_005116.4_19300001	17	19300001	0.431	0.5617
NC_005116.4_19400001	17	19400001	0.4083	0.5319
NC_005116.4_19500001	17	19500001	0.4548	0.5518
NC_005116.4_19600001	17	19600001	0.4986	0.5964
NC_005116.4_19700001	17	19700001	0.5445	0.6508
NC_005116.4_19800001	17	19800001	0.583	0.7094
NC_005116.4_19900001	17	19900001	0.6027	0.7203
NC_005116.4_20000001	17	20000001	0.6426	0.8237
NC_005116.4_20100001	17	20100001	0.5738	0.7201
NC_005116.4_20200001	17	20200001	0.5148	0.6639
NC_005116.4_20300001	17	20300001	0.4693	0.5761
NC_005116.4_20400001	17	20400001	0.5127	0.5813
NC_005116.4_20500001	17	20500001	0.5075	0.5664
NC_005116.4_20600001	17	20600001	0.5006	0.5612
NC_005116.4_20700001	17	20700001	0.4349	0.5028
NC_005116.4_20800001	17	20800001	0.4434	0.5196
NC_005116.4_20900001	17	20900001	0.4196	0.5429
NC_005116.4_21000001	17	21000001	0.4163	0.657
NC_005116.4_21100001	17	21100001	0.3791	0.6282
NC_005116.4_21200001	17	21200001	0.4517	0.7287
NC_005116.4_21300001	17	21300001	0.489	0.6612
NC_005116.4_21400001	17	21400001	0.4454	0.6374
NC_005116.4_21500001	17	21500001	0.5183	0.6638
NC_005116.4_21600001	17	21600001	0.5627	0.7135
NC_005116.4_21700001	17	21700001	0.599	0.7225
NC_005116.4_21800001	17	21800001	0.577	0.7688
NC_005116.4_21900001	17	21900001	0.6087	0.7649
NC_005116.4_22000001	17	22000001	0.5654	0.7092
NC_005116.4_22100001	17	22100001	0.5397	0.6587
NC_005116.4_22200001	17	22200001	0.5359	0.6436
NC_005116.4_22300001	17	22300001	0.5526	0.6307
NC_005116.4_22400001	17	22400001	0.5535	0.6241
NC_005116.4_22500001	17	22500001	0.528	0.6086
NC_005116.4_22600001	17	22600001	0.5305	0.6059
NC_005116.4_22700001	17	22700001	0.5081	0.5367
NC_005116.4_22800001	17	22800001	0.5099	0.5379
NC_005116.4_22900001	17	22900001	0.4924	0.4753
NC_005116.4_23000001	17	23000001	0.4526	0.4957

NC_005116.4_23100001	17	23100001	0.4833	0.5814
NC_005116.4_23200001	17	23200001	0.465	0.5621
NC_005116.4_23300001	17	23300001	0.4317	0.5169
NC_005116.4_23400001	17	23400001	0.4795	0.6188
NC_005116.4_23500001	17	23500001	0.4732	0.6639
NC_005116.4_23600001	17	23600001	0.4154	0.6198
NC_005116.4_23700001	17	23700001	0.4055	0.689
NC_005116.4_23800001	17	23800001	0.4444	0.6551
NC_005116.4_23900001	17	23900001	0.3495	0.5327
NC_005116.4_24000001	17	24000001	0.3879	0.4586
NC_005116.4_24100001	17	24100001	0.4887	0.4904
NC_005116.4_24200001	17	24200001	0.4996	0.5393
NC_005116.4_24300001	17	24300001	0.514	0.6171
NC_005116.4_24400001	17	24400001	0.5385	0.6553
NC_005116.4_24500001	17	24500001	0.6084	0.7192
NC_005116.4_24600001	17	24600001	0.682	0.7951
NC_005116.4_24700001	17	24700001	0.7152	0.8044
NC_005116.4_24800001	17	24800001	0.7244	0.7726
NC_005116.4_24900001	17	24900001	0.6366	0.7668
NC_005116.4_25000001	17	25000001	0.5921	0.7621
NC_005116.4_25100001	17	25100001	0.4695	0.6519
NC_005116.4_25200001	17	25200001	0.4788	0.6566
NC_005116.4_25300001	17	25300001	0.4215	0.6204
NC_005116.4_25400001	17	25400001	0.4623	0.5646
NC_005116.4_25500001	17	25500001	0.4696	0.5659
NC_005116.4_25600001	17	25600001	0.3913	0.5825
NC_005116.4_25700001	17	25700001	0.3734	0.5659
NC_005116.4_25800001	17	25800001	0.3654	0.5166
NC_005116.4_25900001	17	25900001	0.3495	0.5578
NC_005116.4_26000001	17	26000001	0.3994	0.6129
NC_005116.4_26100001	17	26100001	0.5257	0.6458
NC_005116.4_26200001	17	26200001	0.4527	0.587
NC_005116.4_26300001	17	26300001	0.4972	0.6905
NC_005116.4_26400001	17	26400001	0.5188	0.7148
NC_005116.4_26500001	17	26500001	0.4422	0.6477
NC_005116.4_26600001	17	26600001	0.4076	0.6038
NC_005116.4_26700001	17	26700001	0.4317	0.6666
NC_005116.4_26800001	17	26800001	0.3442	0.5134
NC_005116.4_26900001	17	26900001	0.3035	0.3983
NC_005116.4_27000001	17	27000001	0.3481	0.3407
NC_005116.4_27100001	17	27100001	0.4457	0.5368
NC_005116.4_27200001	17	27200001	0.4754	0.5833
NC_005116.4_27300001	17	27300001	0.4817	0.5954
NC_005116.4_27400001	17	27400001	0.505	0.6775
NC_005116.4_27500001	17	27500001	0.4962	0.7018
NC_005116.4_27600001	17	27600001	0.4273	0.613
NC_005116.4_27700001	17	27700001	0.3692	0.5292
NC_005116.4_27800001	17	27800001	0.381	0.4597
NC_005116.4_27900001	17	27900001	0.4508	0.5249
NC_005116.4_28000001	17	28000001	0.4516	0.5392
NC_005116.4_28100001	17	28100001	0.4847	0.6225
NC_005116.4_28200001	17	28200001	0.5328	0.6461
NC_005116.4_28300001	17	28300001	0.5659	0.7271
NC_005116.4_28400001	17	28400001	0.555	0.6849
NC_005116.4_28500001	17	28500001	0.5649	0.6868
NC_005116.4_28600001	17	28600001	0.5172	0.6581
NC_005116.4_28700001	17	28700001	0.5367	0.6916
NC_005116.4_28800001	17	28800001	0.4525	0.6266

NC_005116.4_28900001	17	28900001	0.3819	0.5309
NC_005116.4_29000001	17	29000001	0.3664	0.4926
NC_005116.4_29100001	17	29100001	0.4078	0.4901
NC_005116.4_29200001	17	29200001	0.4061	0.4803
NC_005116.4_29300001	17	29300001	0.4499	0.4972
NC_005116.4_29400001	17	29400001	0.4788	0.5245
NC_005116.4_29500001	17	29500001	0.5418	0.5853
NC_005116.4_29600001	17	29600001	0.5552	0.6427
NC_005116.4_29700001	17	29700001	0.5315	0.6529
NC_005116.4_29800001	17	29800001	0.5458	0.6994
NC_005116.4_29900001	17	29900001	0.6	0.7959
NC_005116.4_30000001	17	30000001	0.4717	0.7144
NC_005116.4_30100001	17	30100001	0.3912	0.5822
NC_005116.4_30200001	17	30200001	0.3673	0.5803
NC_005116.4_30300001	17	30300001	0.3853	0.6444
NC_005116.4_30400001	17	30400001	0.3408	0.5285
NC_005116.4_30500001	17	30500001	0.4168	0.6104
NC_005116.4_30600001	17	30600001	0.4432	0.6298
NC_005116.4_30700001	17	30700001	0.4334	0.5729
NC_005116.4_30800001	17	30800001	0.439	0.5712
NC_005116.4_30900001	17	30900001	0.4917	0.6213
NC_005116.4_31000001	17	31000001	0.4488	0.5981
NC_005116.4_31100001	17	31100001	0.4389	0.6134
NC_005116.4_31200001	17	31200001	0.4562	0.6424
NC_005116.4_31300001	17	31300001	0.4213	0.5855
NC_005116.4_31400001	17	31400001	0.2532	0.4783
NC_005116.4_32000001	17	32000001	0.2746	0.2034
NC_005116.4_32100001	17	32100001	0.2746	0.2034
NC_005116.4_32200001	17	32200001	0.2056	0.1763
NC_005116.4_32300001	17	32300001	0.1567	0.1004
NC_005116.4_32400001	17	32400001	0.1565	0.1007
NC_005116.4_32500001	17	32500001	0.3522	0.6132
NC_005116.4_32600001	17	32600001	0.3588	0.5379
NC_005116.4_32700001	17	32700001	0.4418	0.6196
NC_005116.4_32800001	17	32800001	0.4624	0.5853
NC_005116.4_32900001	17	32900001	0.4679	0.6195
NC_005116.4_33000001	17	33000001	0.4733	0.6117
NC_005116.4_33100001	17	33100001	0.4962	0.6421
NC_005116.4_33200001	17	33200001	0.4705	0.6023
NC_005116.4_33300001	17	33300001	0.4085	0.6821
NC_005116.4_33400001	17	33400001	0.5198	0.6034
NC_005116.4_33500001	17	33500001	0.452	0.5141
NC_005116.4_33600001	17	33600001	0.5234	0.6049
NC_005116.4_33700001	17	33700001	0.6629	0.7482
NC_005116.4_33800001	17	33800001	0.6706	0.6873
NC_005116.4_33900001	17	33900001	0.7043	0.7351
NC_005116.4_34000001	17	34000001	0.6874	0.7428
NC_005116.4_34100001	17	34100001	0.6443	0.7145
NC_005116.4_34200001	17	34200001	0.6324	0.7261
NC_005116.4_34300001	17	34300001	0.6428	0.7431
NC_005116.4_34400001	17	34400001	0.641	0.7186
NC_005116.4_34500001	17	34500001	0.6552	0.719
NC_005116.4_34600001	17	34600001	0.6504	0.7276
NC_005116.4_34700001	17	34700001	0.6216	0.6536
NC_005116.4_34800001	17	34800001	0.5813	0.6802
NC_005116.4_34900001	17	34900001	0.5747	0.7322
NC_005116.4_35000001	17	35000001	0.554	0.7494
NC_005116.4_35100001	17	35100001	0.5622	0.778

NC_005116.4_35200001	17	35200001	0.472	0.6506
NC_005116.4_35300001	17	35300001	0.4794	0.5884
NC_005116.4_35400001	17	35400001	0.3995	0.4394
NC_005116.4_35500001	17	35500001	0.4142	0.4741
NC_005116.4_35600001	17	35600001	0.4243	0.5102
NC_005116.4_35700001	17	35700001	0.4767	0.5572
NC_005116.4_35800001	17	35800001	0.4737	0.5771
NC_005116.4_35900001	17	35900001	0.476	0.5877
NC_005116.4_36000001	17	36000001	0.4352	0.5198
NC_005116.4_36100001	17	36100001	0.4681	0.5722
NC_005116.4_36200001	17	36200001	0.4887	0.5909
NC_005116.4_36300001	17	36300001	0.5051	0.6172
NC_005116.4_36400001	17	36400001	0.5231	0.6489
NC_005116.4_36500001	17	36500001	0.6453	0.814
NC_005116.4_36600001	17	36600001	0.6243	0.7199
NC_005116.4_36700001	17	36700001	0.42	0.6094
NC_005116.4_36800001	17	36800001	0.4171	0.5974
NC_005116.4_36900001	17	36900001	0.4442	0.6191
NC_005116.4_37000001	17	37000001	0.3949	0.5772
NC_005116.4_37100001	17	37100001	0.4598	0.6151
NC_005116.4_37200001	17	37200001	0.5688	0.6984
NC_005116.4_37300001	17	37300001	0.6592	0.7638
NC_005116.4_37400001	17	37400001	0.6543	0.7061
NC_005116.4_37500001	17	37500001	0.5969	0.6526
NC_005116.4_37600001	17	37600001	0.5532	0.6667
NC_005116.4_37700001	17	37700001	0.5659	0.6525
NC_005116.4_37800001	17	37800001	0.5137	0.6228
NC_005116.4_37900001	17	37900001	0.4962	0.6962
NC_005116.4_38000001	17	38000001	0.7475	0.8879
NC_005116.4_38100001	17	38100001	0.6986	0.7672
NC_005116.4_38200001	17	38200001	0.6361	0.7101
NC_005116.4_38300001	17	38300001	0.7295	0.725
NC_005116.4_38400001	17	38400001	0.7295	0.725
NC_005116.4_38500001	17	38500001	0.688	0.7573
NC_005116.4_38700001	17	38700001	0.7733	0.9023
NC_005116.4_38800001	17	38800001	0.6334	0.8555
NC_005116.4_38900001	17	38900001	0.6334	0.8555
NC_005116.4_39000001	17	39000001	0.3951	0.6981
NC_005116.4_39100001	17	39100001	0.3547	0.6522
NC_005116.4_39200001	17	39200001	0.0802	0.0405
NC_005116.4_39400001	17	39400001	0.5202	0.8452
NC_005116.4_39500001	17	39500001	0.6155	0.8248
NC_005116.4_39600001	17	39600001	0.6176	0.8451
NC_005116.4_39700001	17	39700001	0.6176	0.8451
NC_005116.4_39800001	17	39800001	0.6176	0.8451
NC_005116.4_40100001	17	40100001	0.522	0.6409
NC_005116.4_40200001	17	40200001	0.5576	0.587
NC_005116.4_40300001	17	40300001	0.5923	0.6604
NC_005116.4_40400001	17	40400001	0.5984	0.6753
NC_005116.4_40500001	17	40500001	0.6042	0.7036
NC_005116.4_40600001	17	40600001	0.6252	0.6656
NC_005116.4_40700001	17	40700001	0.6039	0.692
NC_005116.4_40800001	17	40800001	0.5374	0.595
NC_005116.4_40900001	17	40900001	0.4394	0.57
NC_005116.4_41000001	17	41000001	0.5308	0.6972
NC_005116.4_41100001	17	41100001	0.5218	0.7193
NC_005116.4_41200001	17	41200001	0.5118	0.7686
NC_005116.4_41300001	17	41300001	0.4976	0.7447

NC_005116.4_41400001	17	41400001	0.6087	0.7813
NC_005116.4_41500001	17	41500001	0.5332	0.7231
NC_005116.4_41600001	17	41600001	0.5224	0.7209
NC_005116.4_41700001	17	41700001	0.5266	0.6995
NC_005116.4_41800001	17	41800001	0.5495	0.7341
NC_005116.4_41900001	17	41900001	0.4024	0.6139
NC_005116.4_42000001	17	42000001	0.4906	0.6976
NC_005116.4_42100001	17	42100001	0.5089	0.7109
NC_005116.4_42200001	17	42200001	0.5061	0.7288
NC_005116.4_42300001	17	42300001	0.517	0.7285
NC_005116.4_42400001	17	42400001	0.6474	0.8262
NC_005116.4_42500001	17	42500001	0.5308	0.7329
NC_005116.4_42600001	17	42600001	0.2546	0.4887
NC_005116.4_42700001	17	42700001	0.3515	0.5511
NC_005116.4_42800001	17	42800001	0.3749	0.4731
NC_005116.4_42900001	17	42900001	0.3813	0.4578
NC_005116.4_43000001	17	43000001	0.3735	0.4197
NC_005116.4_43100001	17	43100001	0.4673	0.4581
NC_005116.4_43200001	17	43200001	0.3022	0.2555
NC_005116.4_43300001	17	43300001	0.3258	0.3906
NC_005116.4_43400001	17	43400001	0.3317	0.3621
NC_005116.4_43500001	17	43500001	0.3377	0.4202
NC_005116.4_43600001	17	43600001	0.3377	0.4202
NC_005116.4_43700001	17	43700001	0.4286	0.4548
NC_005116.4_44400001	17	44400001	0.8406	0.9098
NC_005116.4_44500001	17	44500001	0.8406	0.9098
NC_005116.4_44600001	17	44600001	0.822	0.8978
NC_005116.4_44700001	17	44700001	0.8015	0.8799
NC_005116.4_44800001	17	44800001	0.8593	0.9195
NC_005116.4_44900001	17	44900001	0.9985	0.9971
NC_005116.4_45000001	17	45000001	0.8797	0.9821
NC_005116.4_45100001	17	45100001	0.797	0.9288
NC_005116.4_45200001	17	45200001	0.6417	0.7617
NC_005116.4_45300001	17	45300001	0.4724	0.5849
NC_005116.4_45400001	17	45400001	0.4699	0.5828
NC_005116.4_45500001	17	45500001	0.4353	0.5227
NC_005116.4_45600001	17	45600001	0.4474	0.5187
NC_005116.4_45700001	17	45700001	0.4709	0.5764
NC_005116.4_45800001	17	45800001	0.563	0.7295
NC_005116.4_45900001	17	45900001	0.4813	0.6706
NC_005116.4_46000001	17	46000001	0.5487	0.7718
NC_005116.4_46100001	17	46100001	0.494	0.6851
NC_005116.4_46200001	17	46200001	0.4943	0.6398
NC_005116.4_46300001	17	46300001	0.4297	0.6143
NC_005116.4_46400001	17	46400001	0.4414	0.6201
NC_005116.4_46500001	17	46500001	0.4528	0.6256
NC_005116.4_46600001	17	46600001	0.4673	0.6621
NC_005116.4_46700001	17	46700001	0.4477	0.7537
NC_005116.4_46800001	17	46800001	0.4782	0.6812
NC_005116.4_46900001	17	46900001	0.5096	0.6697
NC_005116.4_47000001	17	47000001	0.3865	0.5147
NC_005116.4_47100001	17	47100001	0.3027	0.3585
NC_005116.4_47200001	17	47200001	0.3586	0.4244
NC_005116.4_47300001	17	47300001	0.3607	0.4318
NC_005116.4_47400001	17	47400001	0.4098	0.5569
NC_005116.4_47500001	17	47500001	0.446	0.5823
NC_005116.4_47600001	17	47600001	0.5723	0.7355
NC_005116.4_47700001	17	47700001	0.642	0.88

NC_005116.4_47800001	17	47800001	0.642	0.88
NC_005116.4_48200001	17	48200001	0.5955	0.8156
NC_005116.4_48300001	17	48300001	0.6707	0.8588
NC_005116.4_48400001	17	48400001	0.6707	0.8588
NC_005116.4_48500001	17	48500001	0.601	0.5977
NC_005116.4_48600001	17	48600001	0.6614	0.6382
NC_005116.4_48700001	17	48700001	0.5917	0.5152
NC_005116.4_48800001	17	48800001	0.5463	0.4788
NC_005116.4_48900001	17	48900001	0.4988	0.4543
NC_005116.4_49000001	17	49000001	0.5279	0.5616
NC_005116.4_49100001	17	49100001	0.5085	0.5573
NC_005116.4_49200001	17	49200001	0.5425	0.6037
NC_005116.4_49300001	17	49300001	0.6241	0.6988
NC_005116.4_49400001	17	49400001	0.7342	0.7728
NC_005116.4_49500001	17	49500001	0.7694	0.7883
NC_005116.4_49600001	17	49600001	0.73	0.7522
NC_005116.4_49700001	17	49700001	0.7354	0.7879
NC_005116.4_49900001	17	49900001	0.5264	0.6111
NC_005116.4_50000001	17	50000001	0.5379	0.5434
NC_005116.4_50200001	17	50200001	0.6218	0.6931
NC_005116.4_50300001	17	50300001	0.5893	0.682
NC_005116.4_50400001	17	50400001	0.5863	0.6738
NC_005116.4_50500001	17	50500001	0.5841	0.7349
NC_005116.4_50600001	17	50600001	0.558	0.6943
NC_005116.4_50700001	17	50700001	0.4922	0.6201
NC_005116.4_50800001	17	50800001	0.4836	0.7053
NC_005116.4_50900001	17	50900001	0.6626	0.8034
NC_005116.4_51000001	17	51000001	0.6463	0.7234
NC_005116.4_51100001	17	51100001	0.7219	0.7812
NC_005116.4_51200001	17	51200001	0.7659	0.822
NC_005116.4_51300001	17	51300001	0.7953	0.7759
NC_005116.4_51400001	17	51400001	0.6562	0.6869
NC_005116.4_51500001	17	51500001	0.7698	0.8766
NC_005116.4_51600001	17	51600001	0.7357	0.8548
NC_005116.4_51700001	17	51700001	0.5833	0.6631
NC_005116.4_51800001	17	51800001	0.6033	0.6779
NC_005116.4_51900001	17	51900001	0.7405	0.7238
NC_005116.4_52000001	17	52000001	0.6878	0.7359
NC_005116.4_52100001	17	52100001	0.6863	0.7306
NC_005116.4_52200001	17	52200001	0.6719	0.7365
NC_005116.4_52300001	17	52300001	0.6795	0.7568
NC_005116.4_52400001	17	52400001	0.6249	0.7473
NC_005116.4_52500001	17	52500001	0.5585	0.6908
NC_005116.4_52600001	17	52600001	0.5382	0.6945
NC_005116.4_52700001	17	52700001	0.6731	0.8793
NC_005116.4_52800001	17	52800001	0.6429	0.7394
NC_005116.4_52900001	17	52900001	0.6032	0.7298
NC_005116.4_53000001	17	53000001	0.5484	0.7187
NC_005116.4_53100001	17	53100001	0.4929	0.7265
NC_005116.4_53200001	17	53200001	0.4512	0.6848
NC_005116.4_53300001	17	53300001	0.4038	0.7501
NC_005116.4_53400001	17	53400001	0.4313	0.7752
NC_005116.4_53500001	17	53500001	0.5759	0.9141
NC_005116.4_53600001	17	53600001	0.8275	0.9785
NC_005116.4_53700001	17	53700001	0.8343	0.9782
NC_005116.4_53800001	17	53800001	0.7671	0.9235
NC_005116.4_53900001	17	53900001	0.7671	0.9235
NC_005116.4_54000001	17	54000001	0.7098	0.9206

NC_005116.4_54100001	17	54100001	0.6369	0.9084
NC_005116.4_54200001	17	54200001	0.5659	0.8046
NC_005116.4_54300001	17	54300001	0.5925	0.8518
NC_005116.4_54400001	17	54400001	0.6112	0.8246
NC_005116.4_54500001	17	54500001	0.5194	0.7202
NC_005116.4_54600001	17	54600001	0.5988	0.7135
NC_005116.4_54700001	17	54700001	0.4529	0.5227
NC_005116.4_54800001	17	54800001	0.4586	0.5752
NC_005116.4_54900001	17	54900001	0.4334	0.5642
NC_005116.4_55000001	17	55000001	0.4974	0.632
NC_005116.4_55100001	17	55100001	0.4776	0.6241
NC_005116.4_55200001	17	55200001	0.4323	0.5943
NC_005116.4_55300001	17	55300001	0.3852	0.5534
NC_005116.4_55400001	17	55400001	0.3598	0.5153
NC_005116.4_55500001	17	55500001	0.3567	0.511
NC_005116.4_55600001	17	55600001	0.3293	0.481
NC_005116.4_55700001	17	55700001	0.3736	0.5496
NC_005116.4_55800001	17	55800001	0.5638	0.6955
NC_005116.4_55900001	17	55900001	0.6148	0.7544
NC_005116.4_56000001	17	56000001	0.5471	0.6778
NC_005116.4_56100001	17	56100001	0.612	0.7146
NC_005116.4_56200001	17	56200001	0.6134	0.7341
NC_005116.4_56300001	17	56300001	0.4398	0.6597
NC_005116.4_56400001	17	56400001	0.3573	0.5175
NC_005116.4_56500001	17	56500001	0.4771	0.6655
NC_005116.4_56600001	17	56600001	0.4947	0.689
NC_005116.4_56700001	17	56700001	0.5125	0.6866
NC_005116.4_56800001	17	56800001	0.5152	0.6705
NC_005116.4_56900001	17	56900001	0.708	0.8143
NC_005116.4_57000001	17	57000001	0.6856	0.8496
NC_005116.4_57100001	17	57100001	0.6878	0.8196
NC_005116.4_57200001	17	57200001	0.6717	0.8675
NC_005116.4_57300001	17	57300001	0.8312	0.9081
NC_005116.4_57600001	17	57600001	0.6308	0.7115
NC_005116.4_57700001	17	57700001	0.5758	0.7599
NC_005116.4_57800001	17	57800001	0.5524	0.7597
NC_005116.4_57900001	17	57900001	0.5089	0.7153
NC_005116.4_58000001	17	58000001	0.4818	0.6852
NC_005116.4_58100001	17	58100001	0.5443	0.7808
NC_005116.4_58200001	17	58200001	0.4967	0.7377
NC_005116.4_58300001	17	58300001	0.5166	0.6843
NC_005116.4_58400001	17	58400001	0.5194	0.6759
NC_005116.4_58500001	17	58500001	0.5717	0.7364
NC_005116.4_58600001	17	58600001	0.4908	0.6269
NC_005116.4_58700001	17	58700001	0.5156	0.6339
NC_005116.4_58800001	17	58800001	0.4958	0.649
NC_005116.4_58900001	17	58900001	0.571	0.6856
NC_005116.4_59000001	17	59000001	0.5745	0.6378
NC_005116.4_59100001	17	59100001	0.6274	0.6438
NC_005116.4_59200001	17	59200001	0.7088	0.6973
NC_005116.4_59300001	17	59300001	0.6896	0.6978
NC_005116.4_59400001	17	59400001	0.6863	0.7483
NC_005116.4_59500001	17	59500001	0.543	0.7214
NC_005116.4_59600001	17	59600001	0.5203	0.7121
NC_005116.4_59700001	17	59700001	0.4866	0.6648
NC_005116.4_59800001	17	59800001	0.4371	0.643
NC_005116.4_59900001	17	59900001	0.3846	0.587
NC_005116.4_60000001	17	60000001	0.4035	0.6171

NC_005116.4_60100001	17	60100001	0.2993	0.469
NC_005116.4_60200001	17	60200001	0.2674	0.4826
NC_005116.4_60300001	17	60300001	0.3091	0.4631
NC_005116.4_60400001	17	60400001	0.1512	0.1453
NC_005116.4_60500001	17	60500001	0.3245	0.463
NC_005116.4_60600001	17	60600001	0.4479	0.5415
NC_005116.4_60700001	17	60700001	0.4479	0.5415
NC_005116.4_60800001	17	60800001	0.5376	0.6542
NC_005116.4_60900001	17	60900001	0.5735	0.6571
NC_005116.4_61000001	17	61000001	0.6144	0.6638
NC_005116.4_61100001	17	61100001	0.551	0.7022
NC_005116.4_61200001	17	61200001	0.5703	0.6797
NC_005116.4_61300001	17	61300001	0.5156	0.6707
NC_005116.4_61400001	17	61400001	0.4725	0.5997
NC_005116.4_61500001	17	61500001	0.4119	0.4731
NC_005116.4_61600001	17	61600001	0.3994	0.4509
NC_005116.4_61700001	17	61700001	0.3487	0.4016
NC_005116.4_61800001	17	61800001	0.4105	0.502
NC_005116.4_61900001	17	61900001	0.4651	0.5948
NC_005116.4_62000001	17	62000001	0.5003	0.7612
NC_005116.4_62100001	17	62100001	0.5682	0.8282
NC_005116.4_62200001	17	62200001	0.5682	0.8282
NC_005116.4_62300001	17	62300001	0.6143	0.8274
NC_005116.4_62400001	17	62400001	0.3889	0.7044
NC_005116.4_62800001	17	62800001	0.5776	0.8358
NC_005116.4_62900001	17	62900001	0.6216	0.7536
NC_005116.4_63000001	17	63000001	0.685	0.7725
NC_005116.4_63100001	17	63100001	0.6617	0.7874
NC_005116.4_63200001	17	63200001	0.6773	0.7679
NC_005116.4_63300001	17	63300001	0.6116	0.7329
NC_005116.4_63400001	17	63400001	0.5768	0.8101
NC_005116.4_63500001	17	63500001	0.4973	0.7662
NC_005116.4_63600001	17	63600001	0.4983	0.7319
NC_005116.4_63700001	17	63700001	0.4927	0.7164
NC_005116.4_63800001	17	63800001	0.4854	0.7288
NC_005116.4_63900001	17	63900001	0.5043	0.6962
NC_005116.4_64000001	17	64000001	0.4642	0.6734
NC_005116.4_64100001	17	64100001	0.4479	0.611
NC_005116.4_64200001	17	64200001	0.3874	0.4983
NC_005116.4_64300001	17	64300001	0.4223	0.5196
NC_005116.4_64400001	17	64400001	0.363	0.428
NC_005116.4_64500001	17	64500001	0.339	0.3758
NC_005116.4_64600001	17	64600001	0.3488	0.4387
NC_005116.4_64700001	17	64700001	0.3582	0.5161
NC_005116.4_64800001	17	64800001	0.5531	0.6936
NC_005116.4_64900001	17	64900001	0.6122	0.7497
NC_005116.4_65000001	17	65000001	0.6862	0.791
NC_005116.4_65100001	17	65100001	0.6893	0.7786
NC_005116.4_65200001	17	65200001	0.7198	0.7827
NC_005116.4_65300001	17	65300001	0.6293	0.7697
NC_005116.4_65400001	17	65400001	0.5818	0.7231
NC_005116.4_65500001	17	65500001	0.55	0.6815
NC_005116.4_65600001	17	65600001	0.546	0.6585
NC_005116.4_65700001	17	65700001	0.526	0.6313
NC_005116.4_65800001	17	65800001	0.5521	0.5898
NC_005116.4_65900001	17	65900001	0.5581	0.6272
NC_005116.4_66000001	17	66000001	0.5191	0.6148
NC_005116.4_66100001	17	66100001	0.4987	0.5842

NC_005116.4_66200001	17	66200001	0.5148	0.6021
NC_005116.4_66300001	17	66300001	0.3923	0.5595
NC_005116.4_66400001	17	66400001	0.2734	0.3067
NC_005116.4_66500001	17	66500001	0.2472	0.2731
NC_005116.4_66600001	17	66600001	0.3422	0.5173
NC_005116.4_66700001	17	66700001	0.3504	0.4822
NC_005116.4_66800001	17	66800001	0.3968	0.5534
NC_005116.4_66900001	17	66900001	0.4787	0.6634
NC_005116.4_67000001	17	67000001	0.5622	0.7012
NC_005116.4_67100001	17	67100001	0.4645	0.5574
NC_005116.4_67200001	17	67200001	0.4578	0.6
NC_005116.4_67300001	17	67300001	0.512	0.6559
NC_005116.4_67400001	17	67400001	0.467	0.6203
NC_005116.4_67500001	17	67500001	0.498	0.6534
NC_005116.4_67600001	17	67600001	0.4535	0.7032
NC_005116.4_67700001	17	67700001	0.4544	0.6666
NC_005116.4_67800001	17	67800001	0.3536	0.5004
NC_005116.4_67900001	17	67900001	0.3291	0.4706
NC_005116.4_68000001	17	68000001	0.317	0.4694
NC_005116.4_68100001	17	68100001	0.4769	0.5997
NC_005116.4_68200001	17	68200001	0.5082	0.6495
NC_005116.4_68300001	17	68300001	0.5181	0.6861
NC_005116.4_68400001	17	68400001	0.5699	0.714
NC_005116.4_68500001	17	68500001	0.7253	0.7767
NC_005116.4_68600001	17	68600001	0.6192	0.8359
NC_005116.4_68700001	17	68700001	0.5498	0.8115
NC_005116.4_68800001	17	68800001	0.4989	0.739
NC_005116.4_68900001	17	68900001	0.5319	0.7062
NC_005116.4_69000001	17	69000001	0.4783	0.65
NC_005116.4_69100001	17	69100001	0.6129	0.7108
NC_005116.4_69200001	17	69200001	0.5172	0.5793
NC_005116.4_69300001	17	69300001	0.5041	0.601
NC_005116.4_69400001	17	69400001	0.5556	0.6825
NC_005116.4_69500001	17	69500001	0.5028	0.6987
NC_005116.4_69600001	17	69600001	0.4643	0.7302
NC_005116.4_69700001	17	69700001	0.475	0.7211
NC_005116.4_69800001	17	69800001	0.4861	0.687
NC_005116.4_69900001	17	69900001	0.4405	0.6361
NC_005116.4_70000001	17	70000001	0.5128	0.6445
NC_005116.4_70100001	17	70100001	0.4603	0.5472
NC_005116.4_70200001	17	70200001	0.4562	0.5691
NC_005116.4_70300001	17	70300001	0.5301	0.6122
NC_005116.4_70400001	17	70400001	0.5479	0.6175
NC_005116.4_70500001	17	70500001	0.4884	0.5628
NC_005116.4_70600001	17	70600001	0.6327	0.6578
NC_005116.4_70700001	17	70700001	0.669	0.6396
NC_005116.4_70800001	17	70800001	0.5503	0.6392
NC_005116.4_70900001	17	70900001	0.4266	0.562
NC_005116.4_71000001	17	71000001	0.5044	0.7177
NC_005116.4_71100001	17	71100001	0.4563	0.728
NC_005116.4_71200001	17	71200001	0.4334	0.6631
NC_005116.4_71300001	17	71300001	0.5463	0.7818
NC_005116.4_71400001	17	71400001	0.671	0.8638
NC_005116.4_71500001	17	71500001	0.7053	0.8596
NC_005116.4_71600001	17	71600001	0.746	0.8615
NC_005116.4_71700001	17	71700001	0.8906	0.9337
NC_005116.4_71800001	17	71800001	0.8545	0.9067
NC_005116.4_71900001	17	71900001	0.7874	0.7911

NC_005116.4_72000001	17	72000001	0.7314	0.7697
NC_005116.4_72100001	17	72100001	0.6566	0.7621
NC_005116.4_72200001	17	72200001	0.5874	0.7356
NC_005116.4_72300001	17	72300001	0.5875	0.7233
NC_005116.4_72400001	17	72400001	0.5631	0.7752
NC_005116.4_72500001	17	72500001	0.4512	0.7446
NC_005116.4_72600001	17	72600001	0.4969	0.7657
NC_005116.4_72700001	17	72700001	0.6391	0.7728
NC_005116.4_72800001	17	72800001	0.6312	0.7797
NC_005116.4_72900001	17	72900001	0.6337	0.7826
NC_005116.4_73000001	17	73000001	0.6303	0.7309
NC_005116.4_73100001	17	73100001	0.6303	0.7309
NC_005116.4_73200001	17	73200001	0.509	0.6797
NC_005116.4_73300001	17	73300001	0.509	0.6797
NC_005116.4_73900001	17	73900001	0.2277	0.2421
NC_005116.4_74000001	17	74000001	0.3143	0.5168
NC_005116.4_74100001	17	74100001	0.2768	0.4918
NC_005116.4_74200001	17	74200001	0.2919	0.481
NC_005116.4_74300001	17	74300001	0.3434	0.4223
NC_005116.4_74400001	17	74400001	0.3916	0.4454
NC_005116.4_74500001	17	74500001	0.406	0.5224
NC_005116.4_74600001	17	74600001	0.336	0.4331
NC_005116.4_74700001	17	74700001	0.3751	0.5511
NC_005116.4_74800001	17	74800001	0.3706	0.5376
NC_005116.4_74900001	17	74900001	0.3706	0.5376
NC_005116.4_75000001	17	75000001	0.4009	0.553
NC_005116.4_75100001	17	75100001	0.5286	0.6349
NC_005116.4_75200001	17	75200001	0.5723	0.6774
NC_005116.4_75300001	17	75300001	0.6013	0.738
NC_005116.4_75400001	17	75400001	0.5442	0.7407
NC_005116.4_75500001	17	75500001	0.5774	0.7486
NC_005116.4_75600001	17	75600001	0.6075	0.7833
NC_005116.4_75700001	17	75700001	0.6426	0.7767
NC_005116.4_75800001	17	75800001	0.6541	0.7791
NC_005116.4_75900001	17	75900001	0.7297	0.793
NC_005116.4_76000001	17	76000001	0.7596	0.8147
NC_005116.4_76100001	17	76100001	0.7269	0.7711
NC_005116.4_76200001	17	76200001	0.7225	0.7399
NC_005116.4_76300001	17	76300001	0.6865	0.749
NC_005116.4_76400001	17	76400001	0.6602	0.7295
NC_005116.4_76500001	17	76500001	0.5922	0.6766
NC_005116.4_76600001	17	76600001	0.5629	0.6748
NC_005116.4_76700001	17	76700001	0.481	0.6315
NC_005116.4_76800001	17	76800001	0.4756	0.6207
NC_005116.4_76900001	17	76900001	0.4788	0.6201
NC_005116.4_77000001	17	77000001	0.4704	0.5911
NC_005116.4_77100001	17	77100001	0.4485	0.5671
NC_005116.4_77200001	17	77200001	0.3618	0.5561
NC_005116.4_77300001	17	77300001	0.3499	0.514
NC_005116.4_77400001	17	77400001	0.3653	0.5215
NC_005116.4_77500001	17	77500001	0.3795	0.5484
NC_005116.4_77600001	17	77600001	0.3387	0.5
NC_005116.4_77700001	17	77700001	0.4156	0.4973
NC_005116.4_77800001	17	77800001	0.4942	0.6257
NC_005116.4_77900001	17	77900001	0.5608	0.7073
NC_005116.4_78000001	17	78000001	0.4956	0.6223
NC_005116.4_78100001	17	78100001	0.5616	0.7032
NC_005116.4_78200001	17	78200001	0.5822	0.7083

NC_005116.4_78300001	17	78300001	0.524	0.6664
NC_005116.4_78400001	17	78400001	0.483	0.6137
NC_005116.4_78500001	17	78500001	0.5488	0.7062
NC_005116.4_78600001	17	78600001	0.5247	0.6725
NC_005116.4_78700001	17	78700001	0.5222	0.6848
NC_005116.4_78800001	17	78800001	0.6032	0.7345
NC_005116.4_78900001	17	78900001	0.639	0.782
NC_005116.4_79000001	17	79000001	0.609	0.7497
NC_005116.4_79100001	17	79100001	0.6066	0.7581
NC_005116.4_79200001	17	79200001	0.5741	0.7795
NC_005116.4_79300001	17	79300001	0.4819	0.6825
NC_005116.4_79400001	17	79400001	0.3737	0.6115
NC_005116.4_79500001	17	79500001	0.2544	0.413
NC_005116.4_79600001	17	79600001	0.264	0.427
NC_005116.4_79700001	17	79700001	0.2336	0.3271
NC_005116.4_79800001	17	79800001	0.3811	0.5197
NC_005116.4_79900001	17	79900001	0.461	0.5696
NC_005116.4_80000001	17	80000001	0.6188	0.6451
NC_005116.4_80100001	17	80100001	0.6176	0.6581
NC_005116.4_80200001	17	80200001	0.6382	0.6819
NC_005116.4_80300001	17	80300001	0.5637	0.672
NC_005116.4_80400001	17	80400001	0.4787	0.6092
NC_005116.4_80500001	17	80500001	0.4646	0.6296
NC_005116.4_80600001	17	80600001	0.3849	0.5608
NC_005116.4_80700001	17	80700001	0.3554	0.4704
NC_005116.4_80800001	17	80800001	0.3841	0.4824
NC_005116.4_80900001	17	80900001	0.4458	0.5983
NC_005116.4_81000001	17	81000001	0.5115	0.6798
NC_005116.4_81100001	17	81100001	0.5868	0.7361
NC_005116.4_81200001	17	81200001	0.5529	0.7445
NC_005116.4_81300001	17	81300001	0.52	0.7653
NC_005116.4_81400001	17	81400001	0.5502	0.7194
NC_005116.4_81500001	17	81500001	0.5768	0.7125
NC_005116.4_81600001	17	81600001	0.5604	0.6938
NC_005116.4_81700001	17	81700001	0.582	0.7094
NC_005116.4_81800001	17	81800001	0.629	0.6921
NC_005116.4_81900001	17	81900001	0.5565	0.7113
NC_005116.4_82000001	17	82000001	0.4817	0.6703
NC_005116.4_82100001	17	82100001	0.3795	0.6094
NC_005116.4_82200001	17	82200001	0.6098	0.8795
NC_005116.4_82300001	17	82300001	0.6098	0.8795
NC_005116.4_82400001	17	82400001	0.6264	0.8422
NC_005116.4_82500001	17	82500001	0.6899	0.8755
NC_005116.4_82600001	17	82600001	0.725	0.8641
NC_005116.4_82700001	17	82700001	0.5784	0.7454
NC_005116.4_82800001	17	82800001	0.6517	0.7383
NC_005116.4_82900001	17	82900001	0.6573	0.7186
NC_005116.4_83000001	17	83000001	0.4864	0.5974
NC_005116.4_83100001	17	83100001	0.5093	0.5879
NC_005116.4_83200001	17	83200001	0.4861	0.5182
NC_005116.4_83300001	17	83300001	0.4248	0.4622
NC_005116.4_83400001	17	83400001	0.4032	0.4377
NC_005116.4_83500001	17	83500001	0.4974	0.5285
NC_005116.4_83600001	17	83600001	0.4657	0.4977
NC_005116.4_83700001	17	83700001	0.5196	0.6028
NC_005116.4_83800001	17	83800001	0.634	0.6971
NC_005116.4_83900001	17	83900001	0.681	0.7234
NC_005116.4_84000001	17	84000001	0.6332	0.6686

NC_005116.4_84100001	17	84100001	0.6093	0.6758
NC_005116.4_84200001	17	84200001	0.4921	0.66
NC_005116.4_84300001	17	84300001	0.4459	0.6152
NC_005116.4_84400001	17	84400001	0.4316	0.638
NC_005116.4_84500001	17	84500001	0.4896	0.7599
NC_005116.4_84600001	17	84600001	0.5847	0.8212
NC_005116.4_84700001	17	84700001	0.7302	0.8825
NC_005116.4_84800001	17	84800001	0.6806	0.8772
NC_005116.4_84900001	17	84900001	0.7265	0.8911
NC_005116.4_85000001	17	85000001	0.7227	0.8384
NC_005116.4_85100001	17	85100001	0.6431	0.8022
NC_005116.4_85200001	17	85200001	0.6185	0.788
NC_005116.4_85300001	17	85300001	0.5281	0.8004
NC_005116.4_85400001	17	85400001	0.427	0.7072
NC_005116.4_85500001	17	85500001	0.3525	0.6552
NC_005116.4_85600001	17	85600001	0.3254	0.6154
NC_005116.4_85700001	17	85700001	0.3298	0.5872
NC_005116.4_85800001	17	85800001	0.4498	0.6811
NC_005116.4_85900001	17	85900001	0.4997	0.7256
NC_005116.4_86000001	17	86000001	0.5139	0.7221
NC_005116.4_86100001	17	86100001	0.5701	0.7607
NC_005116.4_86200001	17	86200001	0.5794	0.8054
NC_005116.4_86300001	17	86300001	0.4946	0.746
NC_005116.4_86400001	17	86400001	0.5013	0.7518
NC_005116.4_86500001	17	86500001	0.5218	0.7041
NC_005116.4_86600001	17	86600001	0.3871	0.5776
NC_005116.4_86700001	17	86700001	0.3027	0.5199
NC_005116.4_86800001	17	86800001	0.2311	0.3343
NC_005116.4_86900001	17	86900001	0.2204	0.2841
NC_005116.4_87000001	17	87000001	0.2008	0.271
NC_005116.4_87100001	17	87100001	0.3139	0.4337
NC_005116.4_87200001	17	87200001	0.3777	0.5186
NC_005116.4_87300001	17	87300001	0.4427	0.5973
NC_005116.4_87400001	17	87400001	0.4766	0.6155
NC_005116.4_87500001	17	87500001	0.5184	0.6392
NC_005116.4_87600001	17	87600001	0.6047	0.7095
NC_005116.4_87700001	17	87700001	0.5703	0.6707
NC_005116.4_87800001	17	87800001	0.5799	0.7053
NC_005116.4_87900001	17	87900001	0.6004	0.7298
NC_005116.4_88000001	17	88000001	0.4926	0.6483
NC_005116.4_88100001	17	88100001	0.4062	0.6082
NC_005116.4_88200001	17	88200001	0.3845	0.5449
NC_005116.4_88300001	17	88300001	0.4147	0.5499
NC_005116.4_88400001	17	88400001	0.4271	0.5957
NC_005116.4_88500001	17	88500001	0.4513	0.6072
NC_005116.4_88600001	17	88600001	0.4252	0.5635
NC_005116.4_88700001	17	88700001	0.4674	0.5742
NC_005116.4_88800001	17	88800001	0.3688	0.3939
NC_005116.4_88900001	17	88900001	0.305	0.2903
NC_005116.4_89000001	17	89000001	0.3413	0.3906
NC_005116.4_89100001	17	89100001	0.4008	0.4746
NC_005116.4_89200001	17	89200001	0.4394	0.5026
NC_005116.4_89300001	17	89300001	0.4808	0.5803
NC_005116.4_89400001	17	89400001	0.5746	0.6594
NC_005116.4_89500001	17	89500001	0.7455	0.8631
NC_005116.4_89600001	17	89600001	0.7633	0.8901
NC_005116.4_89700001	17	89700001	0.7906	0.9699
NC_005116.4_89800001	17	89800001	0.731	0.9123

NC_005116.4_89900001	17	89900001	0.7788	0.8979
NC_005116.4_90000001	17	90000001	0.7236	0.8114
NC_005116.4_90100001	17	90100001	0.4913	0.7162
NC_005116.4_90200001	17	90200001	0.419	0.6581
NC_005116.4_90300001	17	90300001	0.3898	0.6629
NC_005117.4_1	18	1	0.4473	0.7447
NC_005117.4_100001	18	100001	0.4732	0.7505
NC_005117.4_200001	18	200001	0.3427	0.6842
NC_005117.4_300001	18	300001	0.3191	0.6308
NC_005117.4_400001	18	400001	0.3091	0.5093
NC_005117.4_500001	18	500001	0.3203	0.4374
NC_005117.4_600001	18	600001	0.3448	0.4723
NC_005117.4_700001	18	700001	0.4331	0.5415
NC_005117.4_800001	18	800001	0.5122	0.6122
NC_005117.4_900001	18	900001	0.5159	0.5521
NC_005117.4_1000001	18	1000001	0.5588	0.6279
NC_005117.4_1100001	18	1100001	0.5999	0.6085
NC_005117.4_1200001	18	1200001	0.466	0.5542
NC_005117.4_1300001	18	1300001	0.4506	0.4928
NC_005117.4_1400001	18	1400001	0.3765	0.4507
NC_005117.4_1500001	18	1500001	0.4632	0.529
NC_005117.4_1600001	18	1600001	0.4313	0.5432
NC_005117.4_1700001	18	1700001	0.4492	0.6345
NC_005117.4_1800001	18	1800001	0.3931	0.5474
NC_005117.4_1900001	18	1900001	0.3925	0.5884
NC_005117.4_2000001	18	2000001	0.3502	0.6047
NC_005117.4_2100001	18	2100001	0.4706	0.6876
NC_005117.4_2200001	18	2200001	0.4681	0.6314
NC_005117.4_2300001	18	2300001	0.5383	0.7086
NC_005117.4_2400001	18	2400001	0.5271	0.6798
NC_005117.4_2500001	18	2500001	0.5313	0.6788
NC_005117.4_2600001	18	2600001	0.3734	0.4985
NC_005117.4_2700001	18	2700001	0.3872	0.4964
NC_005117.4_2800001	18	2800001	0.4312	0.5398
NC_005117.4_2900001	18	2900001	0.4968	0.6123
NC_005117.4_3000001	18	3000001	0.5348	0.5955
NC_005117.4_3100001	18	3100001	0.5982	0.7011
NC_005117.4_3200001	18	3200001	0.5919	0.7558
NC_005117.4_3300001	18	3300001	0.5812	0.7944
NC_005117.4_3400001	18	3400001	0.5449	0.7757
NC_005117.4_3500001	18	3500001	0.4819	0.7569
NC_005117.4_3600001	18	3600001	0.5077	0.7648
NC_005117.4_3700001	18	3700001	0.5334	0.7529
NC_005117.4_3800001	18	3800001	0.594	0.7832
NC_005117.4_3900001	18	3900001	0.6629	0.8393
NC_005117.4_4000001	18	4000001	0.7857	0.9061
NC_005117.4_4100001	18	4100001	0.7	0.8369
NC_005117.4_4200001	18	4200001	0.6765	0.7938
NC_005117.4_4300001	18	4300001	0.5361	0.638
NC_005117.4_4400001	18	4400001	0.4798	0.5781
NC_005117.4_4500001	18	4500001	0.4772	0.5288
NC_005117.4_4600001	18	4600001	0.5023	0.5605
NC_005117.4_4700001	18	4700001	0.4725	0.5331
NC_005117.4_4800001	18	4800001	0.5029	0.5804
NC_005117.4_4900001	18	4900001	0.4632	0.5256
NC_005117.4_5000001	18	5000001	0.4513	0.5467
NC_005117.4_5100001	18	5100001	0.395	0.5256
NC_005117.4_5200001	18	5200001	0.3876	0.5544

NC_005117.4_5300001	18	5300001	0.3815	0.5782
NC_005117.4_5400001	18	5400001	0.4371	0.6361
NC_005117.4_5500001	18	5500001	0.4672	0.6762
NC_005117.4_5600001	18	5600001	0.5468	0.7091
NC_005117.4_5700001	18	5700001	0.5847	0.7585
NC_005117.4_5800001	18	5800001	0.5748	0.7343
NC_005117.4_5900001	18	5900001	0.5532	0.8161
NC_005117.4_6000001	18	6000001	0.6137	0.8693
NC_005117.4_6100001	18	6100001	0.6207	0.7707
NC_005117.4_6200001	18	6200001	0.6035	0.7349
NC_005117.4_6300001	18	6300001	0.5744	0.6906
NC_005117.4_6400001	18	6400001	0.5902	0.6626
NC_005117.4_6500001	18	6500001	0.5758	0.62
NC_005117.4_6600001	18	6600001	0.4911	0.6333
NC_005117.4_6700001	18	6700001	0.5694	0.6553
NC_005117.4_6800001	18	6800001	0.5541	0.5998
NC_005117.4_6900001	18	6900001	0.4725	0.6376
NC_005117.4_7000001	18	7000001	0.4131	0.6167
NC_005117.4_7100001	18	7100001	0.4706	0.6382
NC_005117.4_7200001	18	7200001	0.539	0.7193
NC_005117.4_7300001	18	7300001	0.5771	0.8466
NC_005117.4_7400001	18	7400001	0.5961	0.8194
NC_005117.4_7500001	18	7500001	0.6873	0.8374
NC_005117.4_7600001	18	7600001	0.5846	0.8208
NC_005117.4_7700001	18	7700001	0.4246	0.7487
NC_005117.4_7800001	18	7800001	0.3678	0.5697
NC_005117.4_7900001	18	7900001	0.3872	0.4791
NC_005117.4_8000001	18	8000001	0.3565	0.4322
NC_005117.4_8100001	18	8100001	0.3364	0.3729
NC_005117.4_8200001	18	8200001	0.3913	0.4186
NC_005117.4_8300001	18	8300001	0.3609	0.4093
NC_005117.4_8400001	18	8400001	0.3488	0.4844
NC_005117.4_8500001	18	8500001	0.4018	0.6345
NC_005117.4_8600001	18	8600001	0.4446	0.6061
NC_005117.4_8700001	18	8700001	0.4609	0.6656
NC_005117.4_8800001	18	8800001	0.5537	0.723
NC_005117.4_8900001	18	8900001	0.5181	0.705
NC_005117.4_9000001	18	9000001	0.5175	0.6793
NC_005117.4_9100001	18	9100001	0.6054	0.6563
NC_005117.4_9200001	18	9200001	0.5596	0.5226
NC_005117.4_9300001	18	9300001	0.514	0.4532
NC_005117.4_9400001	18	9400001	0.5396	0.4983
NC_005117.4_9500001	18	9500001	0.4934	0.4941
NC_005117.4_9600001	18	9600001	0.4649	0.5554
NC_005117.4_9700001	18	9700001	0.3754	0.5094
NC_005117.4_9800001	18	9800001	0.3754	0.5094
NC_005117.4_10500001	18	10500001	0.2948	0.4691
NC_005117.4_10600001	18	10600001	0.4206	0.583
NC_005117.4_10700001	18	10700001	0.4383	0.6442
NC_005117.4_10800001	18	10800001	0.3106	0.5901
NC_005117.4_10900001	18	10900001	0.3216	0.659
NC_005117.4_11000001	18	11000001	0.3713	0.6867
NC_005117.4_11100001	18	11100001	0.326	0.663
NC_005117.4_11200001	18	11200001	0.3184	0.5107
NC_005117.4_11300001	18	11300001	0.3499	0.5203
NC_005117.4_11400001	18	11400001	0.3471	0.4287
NC_005117.4_11500001	18	11500001	0.3466	0.4035
NC_005117.4_11600001	18	11600001	0.3357	0.3594

NC_005117.4_11700001	18	11700001	0.327	0.3563
NC_005117.4_11800001	18	11800001	0.3688	0.407
NC_005117.4_11900001	18	11900001	0.4807	0.5137
NC_005117.4_12000001	18	12000001	0.4807	0.5137
NC_005117.4_12100001	18	12100001	0.5461	0.6346
NC_005117.4_12200001	18	12200001	0.5635	0.657
NC_005117.4_12300001	18	12300001	0.6132	0.6697
NC_005117.4_12400001	18	12400001	0.57	0.685
NC_005117.4_12500001	18	12500001	0.5808	0.7353
NC_005117.4_12600001	18	12600001	0.5848	0.7182
NC_005117.4_12700001	18	12700001	0.5405	0.6521
NC_005117.4_12800001	18	12800001	0.4705	0.6153
NC_005117.4_12900001	18	12900001	0.5709	0.701
NC_005117.4_13000001	18	13000001	0.4722	0.5279
NC_005117.4_13100001	18	13100001	0.4724	0.5402
NC_005117.4_13200001	18	13200001	0.5452	0.6352
NC_005117.4_13300001	18	13300001	0.5704	0.6599
NC_005117.4_13400001	18	13400001	0.5679	0.6558
NC_005117.4_13500001	18	13500001	0.6882	0.7566
NC_005117.4_13600001	18	13600001	0.7378	0.7863
NC_005117.4_13700001	18	13700001	0.6402	0.754
NC_005117.4_13800001	18	13800001	0.5572	0.6751
NC_005117.4_13900001	18	13900001	0.4727	0.5635
NC_005117.4_14000001	18	14000001	0.5368	0.6228
NC_005117.4_14100001	18	14100001	0.4852	0.5831
NC_005117.4_14200001	18	14200001	0.5025	0.5669
NC_005117.4_14300001	18	14300001	0.5157	0.6208
NC_005117.4_14400001	18	14400001	0.5645	0.6857
NC_005117.4_14500001	18	14500001	0.4926	0.6364
NC_005117.4_14600001	18	14600001	0.3722	0.5641
NC_005117.4_14700001	18	14700001	0.3757	0.6671
NC_005117.4_14800001	18	14800001	0.4223	0.6092
NC_005117.4_14900001	18	14900001	0.4464	0.6201
NC_005117.4_15000001	18	15000001	0.4265	0.5809
NC_005117.4_15100001	18	15100001	0.5391	0.6318
NC_005117.4_15200001	18	15200001	0.5561	0.5925
NC_005117.4_15300001	18	15300001	0.5561	0.6107
NC_005117.4_15400001	18	15400001	0.5775	0.5978
NC_005117.4_15500001	18	15500001	0.6299	0.686
NC_005117.4_15600001	18	15600001	0.6462	0.7565
NC_005117.4_15700001	18	15700001	0.6497	0.8177
NC_005117.4_15800001	18	15800001	0.6332	0.8531
NC_005117.4_15900001	18	15900001	0.6381	0.818
NC_005117.4_16000001	18	16000001	0.6203	0.7683
NC_005117.4_16100001	18	16100001	0.5637	0.7187
NC_005117.4_16200001	18	16200001	0.4792	0.6169
NC_005117.4_16300001	18	16300001	0.4497	0.5477
NC_005117.4_16400001	18	16400001	0.4145	0.5122
NC_005117.4_16500001	18	16500001	0.3828	0.445
NC_005117.4_16600001	18	16600001	0.3435	0.2966
NC_005117.4_16700001	18	16700001	0.4617	0.4714
NC_005117.4_16800001	18	16800001	0.5756	0.5973
NC_005117.4_16900001	18	16900001	0.5971	0.6238
NC_005117.4_17000001	18	17000001	0.6541	0.6969
NC_005117.4_17100001	18	17100001	0.6489	0.8009
NC_005117.4_17200001	18	17200001	0.5986	0.8037
NC_005117.4_17300001	18	17300001	0.4036	0.6741
NC_005117.4_17400001	18	17400001	0.4177	0.6249

NC_005117.4_17500001	18	17500001	0.4158	0.5453
NC_005117.4_17600001	18	17600001	0.4579	0.5812
NC_005117.4_17700001	18	17700001	0.4939	0.5939
NC_005117.4_17800001	18	17800001	0.5336	0.6033
NC_005117.4_17900001	18	17900001	0.5088	0.6078
NC_005117.4_18000001	18	18000001	0.5248	0.6419
NC_005117.4_18100001	18	18100001	0.5064	0.6007
NC_005117.4_18200001	18	18200001	0.4758	0.5352
NC_005117.4_18300001	18	18300001	0.4259	0.5983
NC_005117.4_18400001	18	18400001	0.4	0.5167
NC_005117.4_18500001	18	18500001	0.5884	0.7808
NC_005117.4_18600001	18	18600001	0.5103	0.7479
NC_005117.4_18700001	18	18700001	0.5111	0.7657
NC_005117.4_18800001	18	18800001	0.5293	0.7572
NC_005117.4_18900001	18	18900001	0.5293	0.7572
NC_005117.4_19000001	18	19000001	0.4475	0.7413
NC_005117.4_19100001	18	19100001	0.4787	0.6831
NC_005117.4_19200001	18	19200001	0.5616	0.7145
NC_005117.4_19300001	18	19300001	0.8602	0.8676
NC_005117.4_19400001	18	19400001	0.835	0.8831
NC_005117.4_19500001	18	19500001	0.7658	0.8692
NC_005117.4_19600001	18	19600001	0.812	0.8987
NC_005117.4_19700001	18	19700001	0.7187	0.833
NC_005117.4_19800001	18	19800001	0.6739	0.8009
NC_005117.4_19900001	18	19900001	0.5396	0.6889
NC_005117.4_20000001	18	20000001	0.4848	0.5334
NC_005117.4_20100001	18	20100001	0.4832	0.5963
NC_005117.4_20200001	18	20200001	0.5123	0.6162
NC_005117.4_20300001	18	20300001	0.4441	0.5921
NC_005117.4_20400001	18	20400001	0.5998	0.7302
NC_005117.4_20500001	18	20500001	0.6116	0.7801
NC_005117.4_20600001	18	20600001	0.6219	0.7928
NC_005117.4_20700001	18	20700001	0.6446	0.8073
NC_005117.4_20800001	18	20800001	0.5937	0.7556
NC_005117.4_20900001	18	20900001	0.5215	0.7065
NC_005117.4_21000001	18	21000001	0.5546	0.6918
NC_005117.4_21100001	18	21100001	0.541	0.7131
NC_005117.4_21200001	18	21200001	0.5361	0.6477
NC_005117.4_21300001	18	21300001	0.5839	0.6664
NC_005117.4_21400001	18	21400001	0.5516	0.6416
NC_005117.4_21500001	18	21500001	0.4963	0.5958
NC_005117.4_21600001	18	21600001	0.503	0.5525
NC_005117.4_21700001	18	21700001	0.5506	0.6357
NC_005117.4_21800001	18	21800001	0.6002	0.7697
NC_005117.4_21900001	18	21900001	0.6236	0.8437
NC_005117.4_22000001	18	22000001	0.6236	0.8437
NC_005117.4_22100001	18	22100001	0.5687	0.8198
NC_005117.4_22200001	18	22200001	0.4939	0.7861
NC_005117.4_22300001	18	22300001	0.4779	0.7209
NC_005117.4_22400001	18	22400001	0.4772	0.5905
NC_005117.4_22500001	18	22500001	0.5345	0.6303
NC_005117.4_22600001	18	22600001	0.5632	0.6152
NC_005117.4_22700001	18	22700001	0.6216	0.634
NC_005117.4_22800001	18	22800001	0.5383	0.5487
NC_005117.4_22900001	18	22900001	0.5722	0.6473
NC_005117.4_23000001	18	23000001	0.5338	0.6999
NC_005117.4_23100001	18	23100001	0.4872	0.6497
NC_005117.4_23200001	18	23200001	0.4549	0.5442

NC_005117.4_23300001	18	23300001	0.4754	0.5779
NC_005117.4_23400001	18	23400001	0.426	0.4943
NC_005117.4_23500001	18	23500001	0.421	0.4081
NC_005117.4_23600001	18	23600001	0.4673	0.443
NC_005117.4_23700001	18	23700001	0.4581	0.5466
NC_005117.4_23800001	18	23800001	0.4797	0.5298
NC_005117.4_23900001	18	23900001	0.5189	0.589
NC_005117.4_24000001	18	24000001	0.4774	0.5738
NC_005117.4_24100001	18	24100001	0.5863	0.7324
NC_005117.4_24200001	18	24200001	0.7866	0.8369
NC_005117.4_24300001	18	24300001	0.6864	0.8543
NC_005117.4_24400001	18	24400001	0.6864	0.8543
NC_005117.4_24500001	18	24500001	0.7114	0.8164
NC_005117.4_24600001	18	24600001	0.5673	0.736
NC_005117.4_24700001	18	24700001	0.5034	0.5666
NC_005117.4_24800001	18	24800001	0.5345	0.5531
NC_005117.4_24900001	18	24900001	0.5376	0.5472
NC_005117.4_25000001	18	25000001	0.4897	0.5008
NC_005117.4_25100001	18	25100001	0.5581	0.5479
NC_005117.4_25200001	18	25200001	0.6272	0.667
NC_005117.4_25300001	18	25300001	0.5713	0.6386
NC_005117.4_25400001	18	25400001	0.5134	0.6428
NC_005117.4_25500001	18	25500001	0.5232	0.6225
NC_005117.4_25600001	18	25600001	0.4842	0.6124
NC_005117.4_25700001	18	25700001	0.4645	0.576
NC_005117.4_25800001	18	25800001	0.4482	0.584
NC_005117.4_25900001	18	25900001	0.5089	0.6451
NC_005117.4_26000001	18	26000001	0.5225	0.6822
NC_005117.4_26100001	18	26100001	0.5278	0.6416
NC_005117.4_26200001	18	26200001	0.4613	0.5925
NC_005117.4_26300001	18	26300001	0.4902	0.5644
NC_005117.4_26400001	18	26400001	0.4991	0.5676
NC_005117.4_26500001	18	26500001	0.4983	0.5518
NC_005117.4_26600001	18	26600001	0.5095	0.5783
NC_005117.4_26700001	18	26700001	0.6173	0.6229
NC_005117.4_26800001	18	26800001	0.6687	0.7041
NC_005117.4_26900001	18	26900001	0.6963	0.6906
NC_005117.4_27000001	18	27000001	0.7838	0.7667
NC_005117.4_27100001	18	27100001	0.7841	0.7669
NC_005117.4_27200001	18	27200001	0.7241	0.8127
NC_005117.4_27300001	18	27300001	0.596	0.7398
NC_005117.4_27400001	18	27400001	0.5125	0.8077
NC_005117.4_27500001	18	27500001	0.3763	0.687
NC_005117.4_27600001	18	27600001	0.3034	0.6077
NC_005117.4_27700001	18	27700001	0.4609	0.5473
NC_005117.4_27800001	18	27800001	0.4616	0.5425
NC_005117.4_27900001	18	27900001	0.4972	0.5969
NC_005117.4_28000001	18	28000001	0.5485	0.6221
NC_005117.4_28100001	18	28100001	0.6028	0.6542
NC_005117.4_28200001	18	28200001	0.6706	0.757
NC_005117.4_28300001	18	28300001	0.6704	0.7576
NC_005117.4_28400001	18	28400001	0.698	0.7488
NC_005117.4_28500001	18	28500001	0.5855	0.7637
NC_005117.4_28600001	18	28600001	0.522	0.6838
NC_005117.4_28700001	18	28700001	0.4764	0.6361
NC_005117.4_28800001	18	28800001	0.4269	0.5601
NC_005117.4_28900001	18	28900001	0.3887	0.5433
NC_005117.4_29000001	18	29000001	0.3089	0.3276

NC_005117.4_29100001	18	29100001	0.3034	0.3171
NC_005117.4_29200001	18	29200001	0.3287	0.3695
NC_005117.4_29300001	18	29300001	0.3414	0.3984
NC_005117.4_29400001	18	29400001	0.3267	0.3521
NC_005117.4_29500001	18	29500001	0.411	0.3803
NC_005117.4_29600001	18	29600001	0.4283	0.4217
NC_005117.4_29700001	18	29700001	0.6647	0.6191
NC_005117.4_29800001	18	29800001	0.4626	0.5625
NC_005117.4_29900001	18	29900001	0.4292	0.4551
NC_005117.4_30000001	18	30000001	0.3596	0.4428
NC_005117.4_30100001	18	30100001	0.3596	0.4428
NC_005117.4_30200001	18	30200001	0.244	0.2178
NC_005117.4_30300001	18	30300001	0.3163	0.2383
NC_005117.4_30400001	18	30400001	0.2993	0.4372
NC_005117.4_30500001	18	30500001	0.4334	0.6324
NC_005117.4_30600001	18	30600001	0.4133	0.5726
NC_005117.4_30700001	18	30700001	0.4592	0.5981
NC_005117.4_30800001	18	30800001	0.4866	0.6353
NC_005117.4_30900001	18	30900001	0.5675	0.671
NC_005117.4_31000001	18	31000001	0.5613	0.6564
NC_005117.4_31100001	18	31100001	0.5985	0.6686
NC_005117.4_31200001	18	31200001	0.6334	0.7437
NC_005117.4_31300001	18	31300001	0.6138	0.7248
NC_005117.4_31400001	18	31400001	0.6191	0.7183
NC_005117.4_31500001	18	31500001	0.6095	0.7068
NC_005117.4_31600001	18	31600001	0.6718	0.7712
NC_005117.4_31700001	18	31700001	0.695	0.7763
NC_005117.4_31800001	18	31800001	0.6766	0.7602
NC_005117.4_31900001	18	31900001	0.6937	0.7715
NC_005117.4_32000001	18	32000001	0.6852	0.7693
NC_005117.4_32100001	18	32100001	0.6648	0.8072
NC_005117.4_32200001	18	32200001	0.6727	0.8045
NC_005117.4_32300001	18	32300001	0.6999	0.7474
NC_005117.4_32400001	18	32400001	0.6774	0.7569
NC_005117.4_32500001	18	32500001	0.689	0.7658
NC_005117.4_32600001	18	32600001	0.618	0.5141
NC_005117.4_32700001	18	32700001	0.6501	0.5359
NC_005117.4_32800001	18	32800001	0.6968	0.709
NC_005117.4_32900001	18	32900001	0.5144	0.6125
NC_005117.4_33000001	18	33000001	0.547	0.6839
NC_005117.4_33100001	18	33100001	0.556	0.7384
NC_005117.4_33200001	18	33200001	0.4475	0.6773
NC_005117.4_33300001	18	33300001	0.3588	0.5837
NC_005117.4_33400001	18	33400001	0.5974	0.754
NC_005117.4_33500001	18	33500001	0.535	0.6216
NC_005117.4_33600001	18	33600001	0.5568	0.5966
NC_005117.4_33700001	18	33700001	0.5911	0.6259
NC_005117.4_33800001	18	33800001	0.6029	0.6473
NC_005117.4_33900001	18	33900001	0.3885	0.3892
NC_005117.4_34000001	18	34000001	0.4451	0.4665
NC_005117.4_34100001	18	34100001	0.4152	0.4711
NC_005117.4_34200001	18	34200001	0.4633	0.5428
NC_005117.4_34300001	18	34300001	0.5018	0.552
NC_005117.4_34400001	18	34400001	0.6259	0.6123
NC_005117.4_34500001	18	34500001	0.5774	0.8066
NC_005117.4_34600001	18	34600001	0.5095	0.7379
NC_005117.4_35600001	18	35600001	0.7427	0.7581
NC_005117.4_35700001	18	35700001	0.5365	0.6746

NC_005117.4_35800001	18	35800001	0.4428	0.6003
NC_005117.4_35900001	18	35900001	0.556	0.7077
NC_005117.4_36000001	18	36000001	0.5887	0.7229
NC_005117.4_36100001	18	36100001	0.598	0.7481
NC_005117.4_36200001	18	36200001	0.6644	0.8013
NC_005117.4_36300001	18	36300001	0.6732	0.8388
NC_005117.4_36400001	18	36400001	0.5718	0.7704
NC_005117.4_36500001	18	36500001	0.4686	0.7756
NC_005117.4_36600001	18	36600001	0.3425	0.5828
NC_005117.4_36700001	18	36700001	0.3213	0.5115
NC_005117.4_36800001	18	36800001	0.4784	0.6471
NC_005117.4_36900001	18	36900001	0.4557	0.63
NC_005117.4_37000001	18	37000001	0.4892	0.6742
NC_005117.4_37100001	18	37100001	0.5293	0.7001
NC_005117.4_37200001	18	37200001	0.529	0.7059
NC_005117.4_37300001	18	37300001	0.4242	0.6025
NC_005117.4_37400001	18	37400001	0.4934	0.7154
NC_005117.4_37500001	18	37500001	0.4914	0.6909
NC_005117.4_37600001	18	37600001	0.3675	0.6446
NC_005117.4_37700001	18	37700001	0.3675	0.6446
NC_005117.4_37800001	18	37800001	0.3675	0.6446
NC_005117.4_37900001	18	37900001	0.2852	0.4457
NC_005117.4_38300001	18	38300001	0.4936	0.6876
NC_005117.4_38500001	18	38500001	0.6928	0.7891
NC_005117.4_38900001	18	38900001	0.5714	0.6732
NC_005117.4_39200001	18	39200001	0.4928	0.7077
NC_005117.4_39300001	18	39300001	0.6515	0.8321
NC_005117.4_39400001	18	39400001	0.6038	0.7455
NC_005117.4_39500001	18	39500001	0.6128	0.7543
NC_005117.4_39600001	18	39600001	0.6128	0.7543
NC_005117.4_39700001	18	39700001	0.6764	0.7795
NC_005117.4_39800001	18	39800001	0.6333	0.7085
NC_005117.4_39900001	18	39900001	0.6612	0.7029
NC_005117.4_40000001	18	40000001	0.6414	0.6804
NC_005117.4_40100001	18	40100001	0.6777	0.7201
NC_005117.4_40200001	18	40200001	0.7232	0.7235
NC_005117.4_40300001	18	40300001	0.7445	0.7557
NC_005117.4_40500001	18	40500001	0.8566	0.9208
NC_005117.4_40600001	18	40600001	0.712	0.8679
NC_005117.4_40700001	18	40700001	0.5238	0.7798
NC_005117.4_40800001	18	40800001	0.5238	0.7798
NC_005117.4_40900001	18	40900001	0.5973	0.7838
NC_005117.4_41000001	18	41000001	0.5733	0.8179
NC_005117.4_41100001	18	41100001	0.5602	0.7169
NC_005117.4_41200001	18	41200001	0.6644	0.7735
NC_005117.4_41300001	18	41300001	0.6581	0.7363
NC_005117.4_41400001	18	41400001	0.7185	0.7691
NC_005117.4_41500001	18	41500001	0.5919	0.5427
NC_005117.4_41600001	18	41600001	0.6077	0.5926
NC_005117.4_41700001	18	41700001	0.6347	0.6241
NC_005117.4_41800001	18	41800001	0.639	0.6421
NC_005117.4_41900001	18	41900001	0.4577	0.4862
NC_005117.4_42300001	18	42300001	0.4248	0.6254
NC_005117.4_42400001	18	42400001	0.6792	0.8068
NC_005117.4_42500001	18	42500001	0.5983	0.7767
NC_005117.4_42600001	18	42600001	0.6502	0.8208
NC_005117.4_42700001	18	42700001	0.7041	0.8737
NC_005117.4_42800001	18	42800001	0.6366	0.8323

NC_005117.4_42900001	18	42900001	0.5023	0.7268
NC_005117.4_43000001	18	43000001	0.5674	0.683
NC_005117.4_43100001	18	43100001	0.4131	0.5243
NC_005117.4_43200001	18	43200001	0.4246	0.5497
NC_005117.4_43300001	18	43300001	0.4689	0.6001
NC_005117.4_43400001	18	43400001	0.4655	0.5875
NC_005117.4_43500001	18	43500001	0.4504	0.627
NC_005117.4_43600001	18	43600001	0.6143	0.7776
NC_005117.4_43700001	18	43700001	0.5319	0.665
NC_005117.4_43800001	18	43800001	0.5205	0.5937
NC_005117.4_43900001	18	43900001	0.6496	0.6575
NC_005117.4_44000001	18	44000001	0.6022	0.6391
NC_005117.4_44100001	18	44100001	0.5301	0.616
NC_005117.4_44200001	18	44200001	0.5922	0.6244
NC_005117.4_44300001	18	44300001	0.4733	0.5884
NC_005117.4_44400001	18	44400001	0.4141	0.5413
NC_005117.4_44500001	18	44500001	0.4313	0.5399
NC_005117.4_44600001	18	44600001	0.4306	0.5072
NC_005117.4_44700001	18	44700001	0.4025	0.5018
NC_005117.4_44800001	18	44800001	0.5196	0.5842
NC_005117.4_44900001	18	44900001	0.5546	0.6047
NC_005117.4_45000001	18	45000001	0.5156	0.5834
NC_005117.4_45100001	18	45100001	0.5243	0.5564
NC_005117.4_45200001	18	45200001	0.5384	0.5941
NC_005117.4_45300001	18	45300001	0.5491	0.5401
NC_005117.4_45400001	18	45400001	0.451	0.5283
NC_005117.4_45500001	18	45500001	0.2998	0.3691
NC_005117.4_45600001	18	45600001	0.3802	0.5441
NC_005117.4_45700001	18	45700001	0.3212	0.5359
NC_005117.4_45800001	18	45800001	0.3212	0.5359
NC_005117.4_45900001	18	45900001	0.2941	0.4696
NC_005117.4_46000001	18	46000001	0.3308	0.558
NC_005117.4_46100001	18	46100001	0.3991	0.6602
NC_005117.4_46200001	18	46200001	0.6538	0.6756
NC_005117.4_46300001	18	46300001	0.5899	0.6431
NC_005117.4_46400001	18	46400001	0.5392	0.6245
NC_005117.4_46500001	18	46500001	0.5128	0.603
NC_005117.4_46600001	18	46600001	0.536	0.6244
NC_005117.4_46700001	18	46700001	0.484	0.639
NC_005117.4_46800001	18	46800001	0.4953	0.6595
NC_005117.4_46900001	18	46900001	0.5322	0.7154
NC_005117.4_47000001	18	47000001	0.629	0.7627
NC_005117.4_47100001	18	47100001	0.544	0.7153
NC_005117.4_47200001	18	47200001	0.5589	0.6576
NC_005117.4_47300001	18	47300001	0.5981	0.6721
NC_005117.4_47400001	18	47400001	0.4945	0.6405
NC_005117.4_47500001	18	47500001	0.4007	0.5436
NC_005117.4_47600001	18	47600001	0.4256	0.5561
NC_005117.4_47700001	18	47700001	0.4243	0.5576
NC_005117.4_47800001	18	47800001	0.3892	0.508
NC_005117.4_47900001	18	47900001	0.3778	0.4262
NC_005117.4_48000001	18	48000001	0.4686	0.5411
NC_005117.4_48100001	18	48100001	0.3737	0.4546
NC_005117.4_48200001	18	48200001	0.3627	0.4752
NC_005117.4_48300001	18	48300001	0.4081	0.5602
NC_005117.4_48400001	18	48400001	0.4618	0.6126
NC_005117.4_48500001	18	48500001	0.398	0.5503
NC_005117.4_48600001	18	48600001	0.3828	0.521

NC_005117.4_48700001	18	48700001	0.39	0.5824
NC_005117.4_48800001	18	48800001	0.3863	0.5213
NC_005117.4_48900001	18	48900001	0.3172	0.4314
NC_005117.4_49000001	18	49000001	0.3151	0.462
NC_005117.4_49100001	18	49100001	0.3351	0.5068
NC_005117.4_49200001	18	49200001	0.3912	0.504
NC_005117.4_49300001	18	49300001	0.3887	0.4986
NC_005117.4_49400001	18	49400001	0.4241	0.5468
NC_005117.4_49500001	18	49500001	0.5158	0.6172
NC_005117.4_49600001	18	49600001	0.508	0.6329
NC_005117.4_49700001	18	49700001	0.4126	0.575
NC_005117.4_49800001	18	49800001	0.4127	0.5695
NC_005117.4_49900001	18	49900001	0.4699	0.6355
NC_005117.4_50000001	18	50000001	0.5447	0.6674
NC_005117.4_50100001	18	50100001	0.6464	0.7382
NC_005117.4_50200001	18	50200001	0.6911	0.7775
NC_005117.4_50300001	18	50300001	0.7929	0.8707
NC_005117.4_50400001	18	50400001	0.744	0.8335
NC_005117.4_50500001	18	50500001	0.675	0.7951
NC_005117.4_50800001	18	50800001	0.5576	0.632
NC_005117.4_50900001	18	50900001	0.6481	0.71
NC_005117.4_51000001	18	51000001	0.5859	0.6553
NC_005117.4_51100001	18	51100001	0.6183	0.652
NC_005117.4_51200001	18	51200001	0.5351	0.6087
NC_005117.4_51300001	18	51300001	0.5162	0.6002
NC_005117.4_51400001	18	51400001	0.499	0.5731
NC_005117.4_51500001	18	51500001	0.4999	0.6024
NC_005117.4_51600001	18	51600001	0.4705	0.5717
NC_005117.4_51700001	18	51700001	0.5279	0.6319
NC_005117.4_51800001	18	51800001	0.4814	0.5936
NC_005117.4_51900001	18	51900001	0.4316	0.6001
NC_005117.4_52000001	18	52000001	0.4345	0.5908
NC_005117.4_52100001	18	52100001	0.4059	0.6292
NC_005117.4_52200001	18	52200001	0.4292	0.6121
NC_005117.4_52300001	18	52300001	0.5362	0.7065
NC_005117.4_52400001	18	52400001	0.7255	0.8002
NC_005117.4_52500001	18	52500001	0.6539	0.7664
NC_005117.4_52600001	18	52600001	0.634	0.704
NC_005117.4_52700001	18	52700001	0.6391	0.7077
NC_005117.4_52800001	18	52800001	0.6267	0.6886
NC_005117.4_52900001	18	52900001	0.4507	0.5437
NC_005117.4_53000001	18	53000001	0.5421	0.6403
NC_005117.4_53100001	18	53100001	0.5067	0.5892
NC_005117.4_53200001	18	53200001	0.5067	0.5892
NC_005117.4_53300001	18	53300001	0.4619	0.5472
NC_005117.4_53400001	18	53400001	0.4596	0.5445
NC_005117.4_53500001	18	53500001	0.4001	0.3295
NC_005117.4_53600001	18	53600001	0.4402	0.4078
NC_005117.4_53700001	18	53700001	0.5172	0.5671
NC_005117.4_53800001	18	53800001	0.5042	0.6207
NC_005117.4_53900001	18	53900001	0.5201	0.6178
NC_005117.4_54000001	18	54000001	0.5738	0.7502
NC_005117.4_54100001	18	54100001	0.5784	0.7795
NC_005117.4_54200001	18	54200001	0.5559	0.7382
NC_005117.4_54500001	18	54500001	0.539	0.6955
NC_005117.4_54600001	18	54600001	0.4619	0.5078
NC_005117.4_54700001	18	54700001	0.4093	0.5449
NC_005117.4_54800001	18	54800001	0.3915	0.5372

NC_005117.4_54900001	18	54900001	0.3914	0.4695
NC_005117.4_55000001	18	55000001	0.3823	0.4468
NC_005117.4_55100001	18	55100001	0.3876	0.4999
NC_005117.4_55200001	18	55200001	0.3486	0.3219
NC_005117.4_55300001	18	55300001	0.476	0.4103
NC_005117.4_55400001	18	55400001	0.4965	0.57
NC_005117.4_55500001	18	55500001	0.445	0.5575
NC_005117.4_55600001	18	55600001	0.4287	0.552
NC_005117.4_55700001	18	55700001	0.5141	0.6387
NC_005117.4_55800001	18	55800001	0.488	0.6157
NC_005117.4_55900001	18	55900001	0.4796	0.5967
NC_005117.4_56000001	18	56000001	0.5714	0.6999
NC_005117.4_56100001	18	56100001	0.6675	0.7508
NC_005117.4_56200001	18	56200001	0.6078	0.7379
NC_005117.4_56300001	18	56300001	0.6082	0.7453
NC_005117.4_56400001	18	56400001	0.6365	0.7867
NC_005117.4_56500001	18	56500001	0.6577	0.7687
NC_005117.4_56600001	18	56600001	0.5577	0.6606
NC_005117.4_56700001	18	56700001	0.4978	0.5833
NC_005117.4_56800001	18	56800001	0.504	0.5793
NC_005117.4_56900001	18	56900001	0.5003	0.5829
NC_005117.4_57000001	18	57000001	0.4725	0.5464
NC_005117.4_57100001	18	57100001	0.5175	0.6165
NC_005117.4_57200001	18	57200001	0.6579	0.752
NC_005117.4_57300001	18	57300001	0.6649	0.7821
NC_005117.4_57400001	18	57400001	0.5102	0.7127
NC_005117.4_57500001	18	57500001	0.5048	0.7018
NC_005117.4_57600001	18	57600001	0.4632	0.6941
NC_005117.4_57700001	18	57700001	0.3496	0.5615
NC_005117.4_57800001	18	57800001	0.3272	0.569
NC_005117.4_57900001	18	57900001	0.4359	0.618
NC_005117.4_58000001	18	58000001	0.379	0.4902
NC_005117.4_58100001	18	58100001	0.3896	0.4835
NC_005117.4_58200001	18	58200001	0.4396	0.5249
NC_005117.4_58300001	18	58300001	0.4304	0.4912
NC_005117.4_58400001	18	58400001	0.3779	0.4861
NC_005117.4_58500001	18	58500001	0.4128	0.6458
NC_005117.4_58600001	18	58600001	0.4347	0.7033
NC_005117.4_58700001	18	58700001	0.4223	0.6511
NC_005117.4_58800001	18	58800001	0.4558	0.6728
NC_005117.4_58900001	18	58900001	0.4974	0.5981
NC_005117.4_59000001	18	59000001	0.5923	0.6039
NC_005117.4_59100001	18	59100001	0.5623	0.5672
NC_005117.4_59200001	18	59200001	0.5713	0.5863
NC_005117.4_59300001	18	59300001	0.556	0.5586
NC_005117.4_59400001	18	59400001	0.5672	0.6761
NC_005117.4_59500001	18	59500001	0.5204	0.6157
NC_005117.4_59600001	18	59600001	0.5598	0.6551
NC_005117.4_59700001	18	59700001	0.5433	0.674
NC_005117.4_59800001	18	59800001	0.5429	0.7142
NC_005117.4_59900001	18	59900001	0.5182	0.6272
NC_005117.4_60000001	18	60000001	0.5846	0.6881
NC_005117.4_60100001	18	60100001	0.4563	0.5478
NC_005117.4_60200001	18	60200001	0.4731	0.505
NC_005117.4_60300001	18	60300001	0.4123	0.4156
NC_005117.4_60400001	18	60400001	0.4123	0.4156
NC_005117.4_60500001	18	60500001	0.3654	0.3525
NC_005117.4_60600001	18	60600001	0.3349	0.3413

NC_005117.4_60700001	18	60700001	0.3423	0.416
NC_005117.4_60800001	18	60800001	0.3806	0.4644
NC_005117.4_60900001	18	60900001	0.3825	0.4642
NC_005117.4_61000001	18	61000001	0.297	0.3812
NC_005117.4_61100001	18	61100001	0.3812	0.4985
NC_005117.4_61200001	18	61200001	0.3848	0.4521
NC_005117.4_61300001	18	61300001	0.4959	0.5465
NC_005117.4_61400001	18	61400001	0.5238	0.5649
NC_005117.4_61500001	18	61500001	0.6745	0.6592
NC_005117.4_61600001	18	61600001	0.6767	0.6764
NC_005117.4_61700001	18	61700001	0.6859	0.6795
NC_005117.4_61800001	18	61800001	0.5802	0.6858
NC_005117.4_61900001	18	61900001	0.5736	0.6952
NC_005117.4_62000001	18	62000001	0.5662	0.6316
NC_005117.4_62100001	18	62100001	0.5825	0.653
NC_005117.4_62200001	18	62200001	0.6389	0.7013
NC_005117.4_62300001	18	62300001	0.6433	0.7062
NC_005117.4_62400001	18	62400001	0.6456	0.6848
NC_005117.4_62500001	18	62500001	0.6275	0.7303
NC_005117.4_62600001	18	62600001	0.6966	0.8102
NC_005117.4_62700001	18	62700001	0.4972	0.7071
NC_005117.4_62800001	18	62800001	0.539	0.7447
NC_005117.4_62900001	18	62900001	0.4403	0.7328
NC_005117.4_63000001	18	63000001	0.4161	0.7331
NC_005117.4_63100001	18	63100001	0.327	0.6454
NC_005117.4_63200001	18	63200001	0.375	0.7352
NC_005117.4_63300001	18	63300001	0.3096	0.6934
NC_005117.4_63400001	18	63400001	0.6934	0.7551
NC_005117.4_63500001	18	63500001	0.6441	0.7109
NC_005117.4_63600001	18	63600001	0.6878	0.7566
NC_005117.4_63700001	18	63700001	0.6456	0.7421
NC_005117.4_63800001	18	63800001	0.5988	0.7164
NC_005117.4_63900001	18	63900001	0.4176	0.6534
NC_005117.4_64000001	18	64000001	0.403	0.6805
NC_005117.4_64100001	18	64100001	0.5167	0.7212
NC_005117.4_64200001	18	64200001	0.5029	0.7194
NC_005117.4_64300001	18	64300001	0.4744	0.7134
NC_005117.4_64400001	18	64400001	0.6052	0.7657
NC_005117.4_64500001	18	64500001	0.5917	0.7549
NC_005117.4_64600001	18	64600001	0.4377	0.6803
NC_005117.4_64700001	18	64700001	0.4219	0.5974
NC_005117.4_64800001	18	64800001	0.5003	0.67
NC_005117.4_64900001	18	64900001	0.469	0.6476
NC_005117.4_65000001	18	65000001	0.5438	0.6937
NC_005117.4_65100001	18	65100001	0.6346	0.6786
NC_005117.4_65200001	18	65200001	0.6977	0.7581
NC_005117.4_65300001	18	65300001	0.7096	0.754
NC_005117.4_65400001	18	65400001	0.6636	0.752
NC_005117.4_65500001	18	65500001	0.644	0.6948
NC_005117.4_65600001	18	65600001	0.6571	0.745
NC_005117.4_65700001	18	65700001	0.5306	0.6898
NC_005117.4_65800001	18	65800001	0.5164	0.6028
NC_005117.4_65900001	18	65900001	0.5598	0.6598
NC_005117.4_66000001	18	66000001	0.5342	0.7206
NC_005117.4_66100001	18	66100001	0.5324	0.6153
NC_005117.4_66200001	18	66200001	0.5771	0.6394
NC_005117.4_66300001	18	66300001	0.4655	0.5819
NC_005117.4_66400001	18	66400001	0.4562	0.5045

NC_005117.4_66500001	18	66500001	0.471	0.4862
NC_005117.4_66600001	18	66600001	0.4566	0.5206
NC_005117.4_66700001	18	66700001	0.3623	0.4333
NC_005117.4_66800001	18	66800001	0.3263	0.3476
NC_005117.4_66900001	18	66900001	0.3233	0.3166
NC_005117.4_67000001	18	67000001	0.3618	0.3638
NC_005117.4_67100001	18	67100001	0.2796	0.3036
NC_005117.4_67200001	18	67200001	0.3271	0.3777
NC_005117.4_67300001	18	67300001	0.4272	0.518
NC_005117.4_67400001	18	67400001	0.4448	0.6289
NC_005117.4_67500001	18	67500001	0.542	0.7747
NC_005117.4_67600001	18	67600001	0.6621	0.8277
NC_005117.4_67700001	18	67700001	0.5417	0.7349
NC_005117.4_67800001	18	67800001	0.5034	0.614
NC_005117.4_67900001	18	67900001	0.47	0.5989
NC_005117.4_68000001	18	68000001	0.4197	0.4718
NC_005117.4_68100001	18	68100001	0.4515	0.5623
NC_005117.4_68200001	18	68200001	0.4934	0.5518
NC_005117.4_68300001	18	68300001	0.5045	0.5609
NC_005117.4_68400001	18	68400001	0.4756	0.5376
NC_005117.4_68500001	18	68500001	0.3825	0.4766
NC_005117.4_68600001	18	68600001	0.3947	0.3712
NC_005117.4_68700001	18	68700001	0.4302	0.4496
NC_005117.4_68800001	18	68800001	0.3999	0.4652
NC_005117.4_68900001	18	68900001	0.3942	0.4677
NC_005117.4_69000001	18	69000001	0.4452	0.5312
NC_005117.4_69100001	18	69100001	0.4879	0.6842
NC_005117.4_69200001	18	69200001	0.5377	0.6955
NC_005117.4_69300001	18	69300001	0.5358	0.6903
NC_005117.4_69400001	18	69400001	0.5666	0.6927
NC_005117.4_69500001	18	69500001	0.5291	0.6372
NC_005117.4_69600001	18	69600001	0.5529	0.6475
NC_005117.4_69700001	18	69700001	0.4908	0.5962
NC_005117.4_69800001	18	69800001	0.536	0.6072
NC_005117.4_69900001	18	69900001	0.5828	0.6456
NC_005117.4_70000001	18	70000001	0.6065	0.6629
NC_005117.4_70100001	18	70100001	0.5758	0.6561
NC_005117.4_70200001	18	70200001	0.5701	0.6601
NC_005117.4_70300001	18	70300001	0.4442	0.6224
NC_005117.4_70400001	18	70400001	0.4	0.551
NC_005117.4_70500001	18	70500001	0.3913	0.5569
NC_005117.4_70600001	18	70600001	0.3559	0.5267
NC_005117.4_70700001	18	70700001	0.4732	0.6762
NC_005117.4_70800001	18	70800001	0.5603	0.681
NC_005117.4_70900001	18	70900001	0.5943	0.755
NC_005117.4_71000001	18	71000001	0.6477	0.8009
NC_005117.4_71100001	18	71100001	0.6922	0.8064
NC_005117.4_71200001	18	71200001	0.5325	0.7053
NC_005117.4_71300001	18	71300001	0.4108	0.6136
NC_005117.4_71400001	18	71400001	0.3042	0.3656
NC_005117.4_71500001	18	71500001	0.2314	0.3626
NC_005117.4_71600001	18	71600001	0.2314	0.3626
NC_005117.4_71700001	18	71700001	0.2314	0.3626
NC_005117.4_71800001	18	71800001	0.3895	0.5685
NC_005117.4_71900001	18	71900001	0.511	0.6887
NC_005117.4_72000001	18	72000001	0.4875	0.6333
NC_005117.4_72100001	18	72100001	0.4412	0.6618
NC_005117.4_72200001	18	72200001	0.4905	0.7073

NC_005117.4_72300001	18	72300001	0.4629	0.6825
NC_005117.4_72400001	18	72400001	0.457	0.6899
NC_005117.4_72500001	18	72500001	0.4488	0.6854
NC_005117.4_72600001	18	72600001	0.4714	0.6959
NC_005117.4_72700001	18	72700001	0.3991	0.6046
NC_005117.4_72800001	18	72800001	0.4152	0.655
NC_005117.4_72900001	18	72900001	0.3989	0.668
NC_005117.4_73000001	18	73000001	0.4386	0.6839
NC_005117.4_73100001	18	73100001	0.4527	0.6433
NC_005117.4_73200001	18	73200001	0.4878	0.5789
NC_005117.4_73300001	18	73300001	0.4511	0.5353
NC_005117.4_73400001	18	73400001	0.4367	0.4567
NC_005117.4_73500001	18	73500001	0.4438	0.4456
NC_005117.4_73600001	18	73600001	0.5016	0.4924
NC_005117.4_73700001	18	73700001	0.518	0.5052
NC_005117.4_73800001	18	73800001	0.5618	0.5075
NC_005117.4_73900001	18	73900001	0.5364	0.5279
NC_005117.4_74000001	18	74000001	0.5569	0.6531
NC_005117.4_74100001	18	74100001	0.5166	0.63
NC_005117.4_74200001	18	74200001	0.508	0.7036
NC_005117.4_74300001	18	74300001	0.4974	0.7199
NC_005117.4_74400001	18	74400001	0.5405	0.7465
NC_005117.4_74500001	18	74500001	0.5399	0.7021
NC_005117.4_74600001	18	74600001	0.3608	0.6392
NC_005117.4_74700001	18	74700001	0.4425	0.7213
NC_005117.4_74800001	18	74800001	0.5082	0.7698
NC_005117.4_74900001	18	74900001	0.5546	0.7398
NC_005117.4_75000001	18	75000001	0.5584	0.7409
NC_005117.4_75100001	18	75100001	0.8583	0.7789
NC_005117.4_75200001	18	75200001	0.7981	0.6776
NC_005117.4_75300001	18	75300001	0.6057	0.4815
NC_005117.4_75400001	18	75400001	0.4536	0.4126
NC_005117.4_75500001	18	75500001	0.5045	0.4314
NC_005117.4_75600001	18	75600001	0.4906	0.4496
NC_005117.4_75700001	18	75700001	0.49	0.45
NC_005117.4_75800001	18	75800001	0.5015	0.4836
NC_005117.4_75900001	18	75900001	0.5336	0.4813
NC_005117.4_76000001	18	76000001	0.4942	0.4877
NC_005117.4_76100001	18	76100001	0.5145	0.4891
NC_005117.4_76300001	18	76300001	0.3254	0.4732
NC_005117.4_76400001	18	76400001	0.3412	0.3433
NC_005117.4_76500001	18	76500001	0.426	0.4517
NC_005117.4_76600001	18	76600001	0.4146	0.439
NC_005117.4_76700001	18	76700001	0.5014	0.5681
NC_005117.4_76800001	18	76800001	0.598	0.6652
NC_005117.4_76900001	18	76900001	0.696	0.742
NC_005117.4_77000001	18	77000001	0.7831	0.8462
NC_005117.4_77100001	18	77100001	0.8129	0.8497
NC_005117.4_77200001	18	77200001	0.7966	0.8435
NC_005117.4_77300001	18	77300001	0.5912	0.6543
NC_005117.4_77400001	18	77400001	0.48	0.6264
NC_005117.4_77500001	18	77500001	0.3404	0.4704
NC_005117.4_77600001	18	77600001	0.3168	0.4358
NC_005117.4_77700001	18	77700001	0.3496	0.4646
NC_005117.4_77800001	18	77800001	0.45	0.6213
NC_005117.4_77900001	18	77900001	0.4842	0.6131
NC_005117.4_78000001	18	78000001	0.5134	0.6551
NC_005117.4_78100001	18	78100001	0.502	0.6606

NC_005117.4_78200001	18	78200001	0.4751	0.6498
NC_005117.4_78300001	18	78300001	0.399	0.5793
NC_005117.4_78400001	18	78400001	0.511	0.6945
NC_005117.4_78500001	18	78500001	0.5981	0.7155
NC_005117.4_78600001	18	78600001	0.6052	0.7365
NC_005117.4_78700001	18	78700001	0.602	0.7149
NC_005117.4_78800001	18	78800001	0.6131	0.7218
NC_005117.4_78900001	18	78900001	0.6459	0.7122
NC_005117.4_79000001	18	79000001	0.7235	0.7694
NC_005117.4_79100001	18	79100001	0.6871	0.7523
NC_005117.4_79200001	18	79200001	0.6678	0.7277
NC_005117.4_79300001	18	79300001	0.6688	0.7689
NC_005117.4_79400001	18	79400001	0.6014	0.7442
NC_005117.4_79500001	18	79500001	0.505	0.5942
NC_005117.4_79600001	18	79600001	0.4947	0.57
NC_005117.4_79700001	18	79700001	0.4743	0.5719
NC_005117.4_79800001	18	79800001	0.5017	0.5722
NC_005117.4_79900001	18	79900001	0.5148	0.5931
NC_005117.4_80000001	18	80000001	0.5054	0.6485
NC_005117.4_80100001	18	80100001	0.5441	0.6514
NC_005117.4_80200001	18	80200001	0.5452	0.6256
NC_005117.4_80300001	18	80300001	0.5558	0.6239
NC_005117.4_80400001	18	80400001	0.5314	0.5958
NC_005117.4_80500001	18	80500001	0.5135	0.5829
NC_005117.4_80600001	18	80600001	0.4901	0.5976
NC_005117.4_80700001	18	80700001	0.5036	0.6684
NC_005117.4_80800001	18	80800001	0.5409	0.7218
NC_005117.4_80900001	18	80900001	0.6171	0.7998
NC_005117.4_81000001	18	81000001	0.6604	0.84
NC_005117.4_81100001	18	81100001	0.6559	0.7858
NC_005117.4_81200001	18	81200001	0.6329	0.7642
NC_005117.4_81300001	18	81300001	0.5704	0.7088
NC_005117.4_81400001	18	81400001	0.5568	0.695
NC_005117.4_81500001	18	81500001	0.5837	0.6956
NC_005117.4_81600001	18	81600001	0.5882	0.7182
NC_005117.4_81700001	18	81700001	0.5287	0.6275
NC_005117.4_81800001	18	81800001	0.5343	0.6282
NC_005117.4_81900001	18	81900001	0.4851	0.5666
NC_005117.4_82000001	18	82000001	0.4403	0.5039
NC_005117.4_82100001	18	82100001	0.4786	0.5219
NC_005117.4_82200001	18	82200001	0.4843	0.462
NC_005117.4_82300001	18	82300001	0.4843	0.462
NC_005117.4_82400001	18	82400001	0.4843	0.462
NC_005117.4_82500001	18	82500001	0.3421	0.4286
NC_005117.4_82900001	18	82900001	0.24	0.3087
NC_005117.4_83000001	18	83000001	0.4818	0.6051
NC_005117.4_83100001	18	83100001	0.5139	0.644
NC_005117.4_83200001	18	83200001	0.5285	0.6719
NC_005117.4_83300001	18	83300001	0.5346	0.6514
NC_005117.4_83400001	18	83400001	0.7131	0.7528
NC_005117.4_83500001	18	83500001	0.6603	0.7387
NC_005117.4_83600001	18	83600001	0.6296	0.7063
NC_005117.4_83700001	18	83700001	0.571	0.659
NC_005117.4_83800001	18	83800001	0.645	0.7678
NC_005117.4_83900001	18	83900001	0.6402	0.6631
NC_005117.4_84000001	18	84000001	0.63	0.5946
NC_005117.4_84100001	18	84100001	0.6006	0.5336
NC_005117.4_84200001	18	84200001	0.5401	0.4122

NC_005117.4_84300001	18	84300001	0.4805	0.3821
NC_005117.4_84400001	18	84400001	0.417	0.3673
NC_005117.4_84500001	18	84500001	0.417	0.3673
NC_005117.4_84600001	18	84600001	0.4024	0.4913
NC_005117.4_84700001	18	84700001	0.5251	0.7219
NC_005117.4_84800001	18	84800001	0.4172	0.5705
NC_005117.4_84900001	18	84900001	0.2907	0.4478
NC_005117.4_85000001	18	85000001	0.3884	0.6118
NC_005117.4_85100001	18	85100001	0.3532	0.5972
NC_005117.4_85200001	18	85200001	0.2986	0.5341
NC_005117.4_85300001	18	85300001	0.3221	0.6287
NC_005117.4_85400001	18	85400001	0.4587	0.6774
NC_005117.4_85500001	18	85500001	0.3349	0.4499
NC_005117.4_85600001	18	85600001	0.3945	0.4604
NC_005117.4_85700001	18	85700001	0.3945	0.4604
NC_005117.4_85800001	18	85800001	0.4041	0.3382
NC_005117.4_85900001	18	85900001	0.6129	0.4947
NC_005117.4_86000001	18	86000001	0.5714	0.5034
NC_005117.4_86100001	18	86100001	0.4047	0.4512
NC_005117.4_86200001	18	86200001	0.4272	0.4537
NC_005117.4_86300001	18	86300001	0.4268	0.5425
NC_005117.4_86400001	18	86400001	0.1591	0.2624
NC_005117.4_86500001	18	86500001	0.1783	0.2777
NC_005117.4_86600001	18	86600001	0.5123	0.6246
NC_005118.4_100001	19	100001	0.2648	0.409
NC_005118.4_200001	19	200001	0.3486	0.3584
NC_005118.4_300001	19	300001	0.416	0.4991
NC_005118.4_400001	19	400001	0.5132	0.6269
NC_005118.4_500001	19	500001	0.5532	0.6315
NC_005118.4_600001	19	600001	0.5848	0.6114
NC_005118.4_700001	19	700001	0.5897	0.6779
NC_005118.4_800001	19	800001	0.5733	0.6542
NC_005118.4_900001	19	900001	0.5185	0.6135
NC_005118.4_1000001	19	1000001	0.4224	0.5434
NC_005118.4_1100001	19	1100001	0.4912	0.6997
NC_005118.4_1200001	19	1200001	0.5385	0.7038
NC_005118.4_1300001	19	1300001	0.5711	0.7178
NC_005118.4_1400001	19	1400001	0.4881	0.658
NC_005118.4_1500001	19	1500001	0.54	0.6971
NC_005118.4_1600001	19	1600001	0.495	0.6522
NC_005118.4_1700001	19	1700001	0.4681	0.6411
NC_005118.4_1800001	19	1800001	0.3892	0.5723
NC_005118.4_1900001	19	1900001	0.5342	0.6135
NC_005118.4_2000001	19	2000001	0.5116	0.6278
NC_005118.4_2100001	19	2100001	0.6283	0.7907
NC_005118.4_2200001	19	2200001	0.5526	0.7316
NC_005118.4_2300001	19	2300001	0.5817	0.706
NC_005118.4_2400001	19	2400001	0.6242	0.7577
NC_005118.4_2500001	19	2500001	0.5556	0.6927
NC_005118.4_2600001	19	2600001	0.4441	0.5438
NC_005118.4_2700001	19	2700001	0.5383	0.5982
NC_005118.4_2800001	19	2800001	0.5739	0.683
NC_005118.4_2900001	19	2900001	0.4999	0.6464
NC_005118.4_3000001	19	3000001	0.5051	0.5612
NC_005118.4_3100001	19	3100001	0.4116	0.4996
NC_005118.4_3200001	19	3200001	0.4116	0.4996
NC_005118.4_3300001	19	3300001	0.3736	0.4364
NC_005118.4_3400001	19	3400001	0.3736	0.4364

NC_005118.4_3700001	19	3700001	0.6021	0.7173
NC_005118.4_3800001	19	3800001	0.5639	0.6792
NC_005118.4_3900001	19	3900001	0.5639	0.6792
NC_005118.4_4000001	19	4000001	0.5639	0.6792
NC_005118.4_4100001	19	4100001	0.7613	0.8109
NC_005118.4_4200001	19	4200001	0.5457	0.6286
NC_005118.4_4300001	19	4300001	0.6412	0.7166
NC_005118.4_4400001	19	4400001	0.6412	0.7166
NC_005118.4_4500001	19	4500001	0.6788	0.7365
NC_005118.4_4600001	19	4600001	0.7101	0.7649
NC_005118.4_4700001	19	4700001	0.9218	0.9127
NC_005118.4_4800001	19	4800001	0.8005	0.824
NC_005118.4_4900001	19	4900001	0.7319	0.8191
NC_005118.4_5000001	19	5000001	0.6508	0.7998
NC_005118.4_5100001	19	5100001	0.5781	0.7059
NC_005118.4_5200001	19	5200001	0.5781	0.7059
NC_005118.4_5300001	19	5300001	0.6094	0.8146
NC_005118.4_5500001	19	5500001	0.5073	0.5942
NC_005118.4_5600001	19	5600001	0.3156	0.2434
NC_005118.4_5700001	19	5700001	0.4724	0.4773
NC_005118.4_5800001	19	5800001	0.4399	0.4602
NC_005118.4_5900001	19	5900001	0.4865	0.5055
NC_005118.4_6000001	19	6000001	0.5978	0.5911
NC_005118.4_6100001	19	6100001	0.616	0.6425
NC_005118.4_6200001	19	6200001	0.5494	0.578
NC_005118.4_6300001	19	6300001	0.5721	0.6115
NC_005118.4_6400001	19	6400001	0.5425	0.6164
NC_005118.4_6500001	19	6500001	0.4584	0.589
NC_005118.4_6600001	19	6600001	0.4584	0.589
NC_005118.4_6700001	19	6700001	0.418	0.4573
NC_005118.4_6800001	19	6800001	0.4105	0.3948
NC_005118.4_6900001	19	6900001	0.5044	0.4807
NC_005118.4_7000001	19	7000001	0.6009	0.5715
NC_005118.4_7100001	19	7100001	0.6706	0.6711
NC_005118.4_7200001	19	7200001	0.8293	0.8211
NC_005118.4_7300001	19	7300001	0.7268	0.7799
NC_005118.4_7400001	19	7400001	0.6474	0.7662
NC_005118.4_7500001	19	7500001	0.5954	0.7944
NC_005118.4_7600001	19	7600001	0.5263	0.7528
NC_005118.4_7700001	19	7700001	0.425	0.5614
NC_005118.4_7800001	19	7800001	0.3985	0.5095
NC_005118.4_7900001	19	7900001	0.4491	0.518
NC_005118.4_8000001	19	8000001	0.2562	0.2295
NC_005118.4_8100001	19	8100001	0.2768	0.3026
NC_005118.4_8200001	19	8200001	0.3987	0.4267
NC_005118.4_8300001	19	8300001	0.3673	0.4469
NC_005118.4_8400001	19	8400001	0.3673	0.4469
NC_005118.4_8500001	19	8500001	0.4296	0.4528
NC_005118.4_8600001	19	8600001	0.4141	0.448
NC_005118.4_8700001	19	8700001	0.3523	0.4781
NC_005118.4_8800001	19	8800001	0.3768	0.4545
NC_005118.4_8900001	19	8900001	0.3933	0.5086
NC_005118.4_9000001	19	9000001	0.3875	0.513
NC_005118.4_9100001	19	9100001	0.5116	0.6719
NC_005118.4_9200001	19	9200001	0.6024	0.7394
NC_005118.4_9300001	19	9300001	0.7012	0.8168
NC_005118.4_9400001	19	9400001	0.6925	0.7822
NC_005118.4_9500001	19	9500001	0.7192	0.7847

NC_005118.4_9600001	19	9600001	0.6073	0.6753
NC_005118.4_9700001	19	9700001	0.5948	0.7016
NC_005118.4_9800001	19	9800001	0.534	0.6127
NC_005118.4_9900001	19	9900001	0.5215	0.6281
NC_005118.4_10000001	19	10000001	0.4396	0.5942
NC_005118.4_10100001	19	10100001	0.4522	0.6765
NC_005118.4_10200001	19	10200001	0.4269	0.5492
NC_005118.4_10300001	19	10300001	0.479	0.6196
NC_005118.4_10400001	19	10400001	0.5074	0.6624
NC_005118.4_10500001	19	10500001	0.545	0.6441
NC_005118.4_10600001	19	10600001	0.5245	0.6061
NC_005118.4_10700001	19	10700001	0.5988	0.7593
NC_005118.4_10800001	19	10800001	0.4775	0.6692
NC_005118.4_10900001	19	10900001	0.4444	0.603
NC_005118.4_11000001	19	11000001	0.4584	0.6868
NC_005118.4_11100001	19	11100001	0.6092	0.7861
NC_005118.4_11200001	19	11200001	0.4979	0.6492
NC_005118.4_11300001	19	11300001	0.5111	0.6624
NC_005118.4_11400001	19	11400001	0.5462	0.6831
NC_005118.4_11500001	19	11500001	0.5988	0.6735
NC_005118.4_11600001	19	11600001	0.5487	0.5509
NC_005118.4_11700001	19	11700001	0.5412	0.544
NC_005118.4_11800001	19	11800001	0.6332	0.588
NC_005118.4_11900001	19	11900001	0.6332	0.588
NC_005118.4_12000001	19	12000001	0.616	0.5978
NC_005118.4_12100001	19	12100001	0.4174	0.5073
NC_005118.4_12200001	19	12200001	0.4904	0.6282
NC_005118.4_12300001	19	12300001	0.4826	0.6489
NC_005118.4_12400001	19	12400001	0.4435	0.6183
NC_005118.4_12500001	19	12500001	0.455	0.6093
NC_005118.4_12600001	19	12600001	0.4854	0.6244
NC_005118.4_12700001	19	12700001	0.492	0.6277
NC_005118.4_12800001	19	12800001	0.4016	0.5409
NC_005118.4_12900001	19	12900001	0.41	0.5353
NC_005118.4_13000001	19	13000001	0.3152	0.4525
NC_005118.4_13100001	19	13100001	0.3329	0.4787
NC_005118.4_13200001	19	13200001	0.3004	0.3578
NC_005118.4_13300001	19	13300001	0.5219	0.5852
NC_005118.4_13400001	19	13400001	0.5991	0.636
NC_005118.4_13500001	19	13500001	0.612	0.623
NC_005118.4_13600001	19	13600001	0.6777	0.6869
NC_005118.4_13700001	19	13700001	0.6609	0.7061
NC_005118.4_13800001	19	13800001	0.5907	0.6363
NC_005118.4_13900001	19	13900001	0.5918	0.6416
NC_005118.4_14000001	19	14000001	0.5954	0.6704
NC_005118.4_14100001	19	14100001	0.5528	0.6528
NC_005118.4_14200001	19	14200001	0.5006	0.6328
NC_005118.4_14300001	19	14300001	0.4899	0.7121
NC_005118.4_14400001	19	14400001	0.4511	0.7267
NC_005118.4_14500001	19	14500001	0.3063	0.5026
NC_005118.4_14600001	19	14600001	0.1645	0.1501
NC_005118.4_14900001	19	14900001	0.457	0.55
NC_005118.4_15000001	19	15000001	0.4396	0.4959
NC_005118.4_15100001	19	15100001	0.5022	0.5745
NC_005118.4_15200001	19	15200001	0.446	0.5589
NC_005118.4_15300001	19	15300001	0.4383	0.5919
NC_005118.4_15400001	19	15400001	0.4258	0.5903
NC_005118.4_15500001	19	15500001	0.51	0.708

NC_005118.4_15600001	19	15600001	0.479	0.6909
NC_005118.4_15700001	19	15700001	0.4778	0.6507
NC_005118.4_15800001	19	15800001	0.5423	0.6523
NC_005118.4_15900001	19	15900001	0.5584	0.6894
NC_005118.4_16000001	19	16000001	0.5313	0.6563
NC_005118.4_16100001	19	16100001	0.5119	0.6548
NC_005118.4_16200001	19	16200001	0.4698	0.6416
NC_005118.4_16300001	19	16300001	0.433	0.6375
NC_005118.4_16400001	19	16400001	0.4298	0.6103
NC_005118.4_16500001	19	16500001	0.4021	0.5921
NC_005118.4_16600001	19	16600001	0.4622	0.657
NC_005118.4_16700001	19	16700001	0.553	0.7307
NC_005118.4_16800001	19	16800001	0.5364	0.7213
NC_005118.4_16900001	19	16900001	0.4971	0.6953
NC_005118.4_17000001	19	17000001	0.5042	0.6834
NC_005118.4_17100001	19	17100001	0.5449	0.6407
NC_005118.4_17200001	19	17200001	0.6003	0.7081
NC_005118.4_17300001	19	17300001	0.6039	0.6766
NC_005118.4_17400001	19	17400001	0.6065	0.72
NC_005118.4_17500001	19	17500001	0.5694	0.6799
NC_005118.4_17600001	19	17600001	0.5192	0.645
NC_005118.4_17700001	19	17700001	0.4342	0.5421
NC_005118.4_17800001	19	17800001	0.4105	0.5678
NC_005118.4_17900001	19	17900001	0.4138	0.5343
NC_005118.4_18000001	19	18000001	0.4714	0.5842
NC_005118.4_18100001	19	18100001	0.5501	0.6544
NC_005118.4_18200001	19	18200001	0.5279	0.6186
NC_005118.4_18300001	19	18300001	0.5208	0.5878
NC_005118.4_18400001	19	18400001	0.4776	0.5221
NC_005118.4_18500001	19	18500001	0.475	0.5274
NC_005118.4_18600001	19	18600001	0.3766	0.4187
NC_005118.4_18700001	19	18700001	0.4899	0.536
NC_005118.4_18800001	19	18800001	0.5176	0.5783
NC_005118.4_18900001	19	18900001	0.5618	0.6466
NC_005118.4_19000001	19	19000001	0.5284	0.6666
NC_005118.4_19100001	19	19100001	0.5459	0.7366
NC_005118.4_19200001	19	19200001	0.5628	0.7758
NC_005118.4_19300001	19	19300001	0.5696	0.7139
NC_005118.4_19400001	19	19400001	0.5759	0.7256
NC_005118.4_19500001	19	19500001	0.6086	0.725
NC_005118.4_19600001	19	19600001	0.5601	0.6327
NC_005118.4_19700001	19	19700001	0.5756	0.6436
NC_005118.4_19800001	19	19800001	0.4937	0.6452
NC_005118.4_19900001	19	19900001	0.4253	0.5358
NC_005118.4_20000001	19	20000001	0.4338	0.5215
NC_005118.4_20100001	19	20100001	0.4395	0.548
NC_005118.4_20200001	19	20200001	0.4186	0.541
NC_005118.4_20300001	19	20300001	0.4684	0.5665
NC_005118.4_20400001	19	20400001	0.5589	0.6602
NC_005118.4_20500001	19	20500001	0.5631	0.693
NC_005118.4_20600001	19	20600001	0.6098	0.715
NC_005118.4_20700001	19	20700001	0.6094	0.7037
NC_005118.4_20800001	19	20800001	0.6172	0.7167
NC_005118.4_20900001	19	20900001	0.5801	0.7341
NC_005118.4_21000001	19	21000001	0.6153	0.7588
NC_005118.4_21100001	19	21100001	0.5997	0.8395
NC_005118.4_21200001	19	21200001	0.5601	0.7927
NC_005118.4_21300001	19	21300001	0.6035	0.7959

NC_005118.4_21400001	19	21400001	0.6362	0.7591
NC_005118.4_21500001	19	21500001	0.6525	0.7633
NC_005118.4_21600001	19	21600001	0.6821	0.7432
NC_005118.4_21700001	19	21700001	0.7161	0.7698
NC_005118.4_21800001	19	21800001	0.8093	0.7572
NC_005118.4_21900001	19	21900001	0.9278	0.9116
NC_005118.4_22000001	19	22000001	0.5817	0.8793
NC_005118.4_22100001	19	22100001	0.735	0.9068
NC_005118.4_22200001	19	22200001	0.6901	0.8957
NC_005118.4_22300001	19	22300001	0.6901	0.8957
NC_005118.4_22400001	19	22400001	0.6599	0.872
NC_005118.4_22500001	19	22500001	0.6518	0.8003
NC_005118.4_22600001	19	22600001	0.3711	0.5321
NC_005118.4_22700001	19	22700001	0.4078	0.4565
NC_005118.4_22800001	19	22800001	0.3743	0.4333
NC_005118.4_22900001	19	22900001	0.3783	0.4305
NC_005118.4_23000001	19	23000001	0.4578	0.5156
NC_005118.4_23100001	19	23100001	0.3932	0.4976
NC_005118.4_23200001	19	23200001	0.3932	0.5376
NC_005118.4_23300001	19	23300001	0.4011	0.5543
NC_005118.4_23400001	19	23400001	0.4406	0.5752
NC_005118.4_23500001	19	23500001	0.3624	0.4856
NC_005118.4_23600001	19	23600001	0.4978	0.59
NC_005118.4_23700001	19	23700001	0.4943	0.5809
NC_005118.4_23800001	19	23800001	0.5333	0.61
NC_005118.4_23900001	19	23900001	0.6555	0.6995
NC_005118.4_24000001	19	24000001	0.6803	0.7219
NC_005118.4_24100001	19	24100001	0.6644	0.6767
NC_005118.4_24200001	19	24200001	0.7238	0.723
NC_005118.4_24300001	19	24300001	0.7395	0.7456
NC_005118.4_24400001	19	24400001	0.7989	0.8234
NC_005118.4_24500001	19	24500001	0.7444	0.7833
NC_005118.4_24600001	19	24600001	0.7461	0.817
NC_005118.4_24700001	19	24700001	0.7252	0.8511
NC_005118.4_24800001	19	24800001	0.7321	0.8858
NC_005118.4_24900001	19	24900001	0.6382	0.7735
NC_005118.4_25000001	19	25000001	0.5939	0.7558
NC_005118.4_25100001	19	25100001	0.5801	0.7365
NC_005118.4_25200001	19	25200001	0.5268	0.6489
NC_005118.4_25300001	19	25300001	0.5745	0.7249
NC_005118.4_25400001	19	25400001	0.5426	0.7711
NC_005118.4_25500001	19	25500001	0.6261	0.8452
NC_005118.4_25600001	19	25600001	0.5891	0.8047
NC_005118.4_25700001	19	25700001	0.6536	0.8457
NC_005118.4_25800001	19	25800001	0.6529	0.8379
NC_005118.4_25900001	19	25900001	0.7083	0.8544
NC_005118.4_26000001	19	26000001	0.7104	0.8619
NC_005118.4_26100001	19	26100001	0.8718	0.9813
NC_005118.4_27100001	19	27100001	0.6832	0.8928
NC_005118.4_27200001	19	27200001	0.6832	0.8928
NC_005118.4_27300001	19	27300001	0.6832	0.8928
NC_005118.4_27400001	19	27400001	0.6832	0.8928
NC_005118.4_29100001	19	29100001	0.4286	0.6292
NC_005118.4_29200001	19	29200001	0.5608	0.7125
NC_005118.4_29300001	19	29300001	0.5522	0.7556
NC_005118.4_29400001	19	29400001	0.5019	0.723
NC_005118.4_29500001	19	29500001	0.5019	0.723
NC_005118.4_29600001	19	29600001	0.4974	0.7326

NC_005118.4_29700001	19	29700001	0.5401	0.7826
NC_005118.4_29800001	19	29800001	0.5357	0.7386
NC_005118.4_29900001	19	29900001	0.5323	0.7
NC_005118.4_30000001	19	30000001	0.5429	0.7042
NC_005118.4_30100001	19	30100001	0.6082	0.701
NC_005118.4_30200001	19	30200001	0.5362	0.6269
NC_005118.4_30300001	19	30300001	0.504	0.5731
NC_005118.4_30400001	19	30400001	0.4761	0.5393
NC_005118.4_30500001	19	30500001	0.5011	0.5492
NC_005118.4_30600001	19	30600001	0.5055	0.5908
NC_005118.4_30700001	19	30700001	0.5384	0.6063
NC_005118.4_30800001	19	30800001	0.5374	0.6506
NC_005118.4_30900001	19	30900001	0.5994	0.7049
NC_005118.4_31000001	19	31000001	0.5416	0.6593
NC_005118.4_31100001	19	31100001	0.5232	0.6177
NC_005118.4_31200001	19	31200001	0.4696	0.5657
NC_005118.4_31300001	19	31300001	0.5796	0.6729
NC_005118.4_31400001	19	31400001	0.5695	0.7108
NC_005118.4_31500001	19	31500001	0.6047	0.7245
NC_005118.4_31600001	19	31600001	0.5886	0.7183
NC_005118.4_31700001	19	31700001	0.5659	0.7458
NC_005118.4_31800001	19	31800001	0.4232	0.6371
NC_005118.4_31900001	19	31900001	0.4089	0.6313
NC_005118.4_32000001	19	32000001	0.5017	0.7503
NC_005118.4_32100001	19	32100001	0.4972	0.7388
NC_005118.4_32200001	19	32200001	0.5554	0.7439
NC_005118.4_32300001	19	32300001	0.6067	0.7518
NC_005118.4_32400001	19	32400001	0.5592	0.6818
NC_005118.4_32500001	19	32500001	0.4822	0.5746
NC_005118.4_32600001	19	32600001	0.5481	0.5825
NC_005118.4_32700001	19	32700001	0.3674	0.5171
NC_005118.4_32800001	19	32800001	0.3109	0.4652
NC_005118.4_32900001	19	32900001	0.3623	0.5207
NC_005118.4_33000001	19	33000001	0.4628	0.6032
NC_005118.4_33100001	19	33100001	0.4377	0.5688
NC_005118.4_33200001	19	33200001	0.5478	0.6285
NC_005118.4_33300001	19	33300001	0.6565	0.692
NC_005118.4_33400001	19	33400001	0.683	0.7119
NC_005118.4_33500001	19	33500001	0.7309	0.7839
NC_005118.4_33600001	19	33600001	0.7882	0.8328
NC_005118.4_33700001	19	33700001	0.6947	0.7695
NC_005118.4_33800001	19	33800001	0.7555	0.7945
NC_005118.4_33900001	19	33900001	0.6772	0.7872
NC_005118.4_34000001	19	34000001	0.5789	0.7076
NC_005118.4_34100001	19	34100001	0.5445	0.6776
NC_005118.4_34200001	19	34200001	0.5046	0.66
NC_005118.4_34300001	19	34300001	0.4018	0.5976
NC_005118.4_34400001	19	34400001	0.4121	0.5787
NC_005118.4_34500001	19	34500001	0.3271	0.4492
NC_005118.4_34600001	19	34600001	0.2645	0.4355
NC_005118.4_34700001	19	34700001	0.3871	0.4861
NC_005118.4_34800001	19	34800001	0.4234	0.5161
NC_005118.4_34900001	19	34900001	0.5103	0.5585
NC_005118.4_35000001	19	35000001	0.482	0.5952
NC_005118.4_35100001	19	35100001	0.5087	0.6014
NC_005118.4_35200001	19	35200001	0.4289	0.5919
NC_005118.4_35300001	19	35300001	0.3631	0.4987
NC_005118.4_35400001	19	35400001	0.2983	0.3888

NC_005118.4_35500001	19	35500001	0.2705	0.2367
NC_005118.4_35600001	19	35600001	0.2615	0.2167
NC_005118.4_35700001	19	35700001	0.321	0.2405
NC_005118.4_35800001	19	35800001	0.3214	0.2421
NC_005118.4_36300001	19	36300001	0.3798	0.4229
NC_005118.4_36400001	19	36400001	0.3331	0.4863
NC_005118.4_36500001	19	36500001	0.3831	0.5144
NC_005118.4_36600001	19	36600001	0.3302	0.4378
NC_005118.4_36700001	19	36700001	0.3081	0.4929
NC_005118.4_36800001	19	36800001	0.3651	0.5898
NC_005118.4_36900001	19	36900001	0.5592	0.759
NC_005118.4_37000001	19	37000001	0.6399	0.8014
NC_005118.4_37100001	19	37100001	0.6935	0.8557
NC_005118.4_37200001	19	37200001	0.7999	0.8438
NC_005118.4_37300001	19	37300001	0.8105	0.8473
NC_005118.4_37400001	19	37400001	0.7573	0.8065
NC_005118.4_37500001	19	37500001	0.7201	0.8192
NC_005118.4_37600001	19	37600001	0.6711	0.7897
NC_005118.4_37700001	19	37700001	0.6179	0.772
NC_005118.4_37800001	19	37800001	0.5664	0.7039
NC_005118.4_37900001	19	37900001	0.5525	0.6783
NC_005118.4_38000001	19	38000001	0.5058	0.5888
NC_005118.4_38100001	19	38100001	0.5146	0.5805
NC_005118.4_38200001	19	38200001	0.5349	0.6066
NC_005118.4_38300001	19	38300001	0.5573	0.6569
NC_005118.4_38400001	19	38400001	0.536	0.6484
NC_005118.4_38500001	19	38500001	0.5914	0.7065
NC_005118.4_38600001	19	38600001	0.6071	0.7238
NC_005118.4_38700001	19	38700001	0.554	0.7656
NC_005118.4_38800001	19	38800001	0.6546	0.8576
NC_005118.4_38900001	19	38900001	0.6768	0.8707
NC_005118.4_39000001	19	39000001	0.6978	0.8933
NC_005118.4_39100001	19	39100001	0.6902	0.9019
NC_005118.4_39200001	19	39200001	0.7326	0.8408
NC_005118.4_39300001	19	39300001	0.6503	0.7281
NC_005118.4_39400001	19	39400001	0.6274	0.6793
NC_005118.4_39500001	19	39500001	0.4852	0.5195
NC_005118.4_39600001	19	39600001	0.5477	0.5724
NC_005118.4_39700001	19	39700001	0.5192	0.6296
NC_005118.4_39800001	19	39800001	0.4809	0.6122
NC_005118.4_39900001	19	39900001	0.4677	0.6206
NC_005118.4_40000001	19	40000001	0.4493	0.5989
NC_005118.4_40100001	19	40100001	0.422	0.6057
NC_005118.4_40200001	19	40200001	0.383	0.5035
NC_005118.4_40300001	19	40300001	0.3939	0.5246
NC_005118.4_40400001	19	40400001	0.31	0.3991
NC_005118.4_40500001	19	40500001	0.3418	0.492
NC_005118.4_40600001	19	40600001	0.2658	0.3891
NC_005118.4_40700001	19	40700001	0.3704	0.5057
NC_005118.4_40800001	19	40800001	0.4486	0.6188
NC_005118.4_40900001	19	40900001	0.5044	0.6136
NC_005118.4_41000001	19	41000001	0.5226	0.5711
NC_005118.4_41100001	19	41100001	0.5761	0.6322
NC_005118.4_41200001	19	41200001	0.5872	0.6776
NC_005118.4_41300001	19	41300001	0.5422	0.597
NC_005118.4_41400001	19	41400001	0.6794	0.7562
NC_005118.4_41500001	19	41500001	0.6266	0.7412
NC_005118.4_41600001	19	41600001	0.5719	0.7099

NC_005118.4_41700001	19	41700001	0.5832	0.7421
NC_005118.4_41800001	19	41800001	0.6312	0.7822
NC_005118.4_41900001	19	41900001	0.5086	0.6568
NC_005118.4_42000001	19	42000001	0.555	0.722
NC_005118.4_42100001	19	42100001	0.6154	0.7186
NC_005118.4_42200001	19	42200001	0.5906	0.6501
NC_005118.4_42300001	19	42300001	0.394	0.4908
NC_005118.4_42400001	19	42400001	0.5094	0.6253
NC_005118.4_42500001	19	42500001	0.5413	0.6836
NC_005118.4_42600001	19	42600001	0.4295	0.6475
NC_005118.4_42700001	19	42700001	0.3971	0.5341
NC_005118.4_42800001	19	42800001	0.4398	0.6142
NC_005118.4_42900001	19	42900001	0.4601	0.5841
NC_005118.4_43000001	19	43000001	0.4115	0.5107
NC_005118.4_43100001	19	43100001	0.4548	0.5511
NC_005118.4_43200001	19	43200001	0.4956	0.6029
NC_005118.4_43300001	19	43300001	0.5307	0.5615
NC_005118.4_43400001	19	43400001	0.5368	0.606
NC_005118.4_43500001	19	43500001	0.5552	0.6381
NC_005118.4_43600001	19	43600001	0.5978	0.6899
NC_005118.4_43700001	19	43700001	0.5793	0.7059
NC_005118.4_43800001	19	43800001	0.5034	0.7322
NC_005118.4_43900001	19	43900001	0.426	0.6537
NC_005118.4_44000001	19	44000001	0.4295	0.6817
NC_005118.4_44100001	19	44100001	0.3583	0.5031
NC_005118.4_44200001	19	44200001	0.3322	0.4655
NC_005118.4_44300001	19	44300001	0.4642	0.5702
NC_005118.4_44400001	19	44400001	0.4885	0.5889
NC_005118.4_44500001	19	44500001	0.5369	0.5915
NC_005118.4_44600001	19	44600001	0.5405	0.6056
NC_005118.4_44700001	19	44700001	0.5536	0.6104
NC_005118.4_44800001	19	44800001	0.5176	0.5877
NC_005118.4_44900001	19	44900001	0.5925	0.6179
NC_005118.4_45000001	19	45000001	0.4111	0.5817
NC_005118.4_45100001	19	45100001	0.4007	0.657
NC_005118.4_45200001	19	45200001	0.3291	0.6092
NC_005118.4_45300001	19	45300001	0.4006	0.646
NC_005118.4_45400001	19	45400001	0.3801	0.5653
NC_005118.4_45500001	19	45500001	0.4071	0.5354
NC_005118.4_45600001	19	45600001	0.351	0.4299
NC_005118.4_45700001	19	45700001	0.3744	0.4243
NC_005118.4_45800001	19	45800001	0.3676	0.3735
NC_005118.4_45900001	19	45900001	0.3766	0.365
NC_005118.4_46000001	19	46000001	0.4207	0.4683
NC_005118.4_46100001	19	46100001	0.3929	0.4711
NC_005118.4_46200001	19	46200001	0.3907	0.4902
NC_005118.4_46300001	19	46300001	0.3977	0.4868
NC_005118.4_46400001	19	46400001	0.4075	0.5177
NC_005118.4_46500001	19	46500001	0.4272	0.5504
NC_005118.4_46600001	19	46600001	0.4856	0.5869
NC_005118.4_46700001	19	46700001	0.5716	0.6624
NC_005118.4_46800001	19	46800001	0.5373	0.6015
NC_005118.4_46900001	19	46900001	0.5055	0.5909
NC_005118.4_47000001	19	47000001	0.4814	0.5421
NC_005118.4_47100001	19	47100001	0.3985	0.4793
NC_005118.4_47200001	19	47200001	0.3734	0.4485
NC_005118.4_47300001	19	47300001	0.3969	0.5532
NC_005118.4_47400001	19	47400001	0.4454	0.5683

NC_005118.4_47500001	19	47500001	0.4556	0.5963
NC_005118.4_47600001	19	47600001	0.5307	0.6701
NC_005118.4_47700001	19	47700001	0.5591	0.6575
NC_005118.4_47800001	19	47800001	0.5977	0.6703
NC_005118.4_47900001	19	47900001	0.5564	0.6775
NC_005118.4_48000001	19	48000001	0.6066	0.6635
NC_005118.4_48100001	19	48100001	0.6495	0.7225
NC_005118.4_48200001	19	48200001	0.5578	0.6254
NC_005118.4_48300001	19	48300001	0.432	0.5552
NC_005118.4_48400001	19	48400001	0.4616	0.586
NC_005118.4_48500001	19	48500001	0.4277	0.5825
NC_005118.4_48600001	19	48600001	0.3215	0.3897
NC_005118.4_48700001	19	48700001	0.3263	0.4621
NC_005118.4_48800001	19	48800001	0.5092	0.5519
NC_005118.4_48900001	19	48900001	0.4602	0.5151
NC_005118.4_49000001	19	49000001	0.4384	0.5173
NC_005118.4_49100001	19	49100001	0.5056	0.6428
NC_005118.4_49200001	19	49200001	0.5356	0.6564
NC_005118.4_49300001	19	49300001	0.5147	0.6631
NC_005118.4_49400001	19	49400001	0.5161	0.6434
NC_005118.4_49500001	19	49500001	0.5788	0.6562
NC_005118.4_49600001	19	49600001	0.5532	0.5916
NC_005118.4_49700001	19	49700001	0.5374	0.608
NC_005118.4_49800001	19	49800001	0.5341	0.6216
NC_005118.4_49900001	19	49900001	0.5679	0.6635
NC_005118.4_50000001	19	50000001	0.4675	0.6107
NC_005118.4_50100001	19	50100001	0.4597	0.6318
NC_005118.4_50200001	19	50200001	0.4506	0.6146
NC_005118.4_50300001	19	50300001	0.3902	0.5788
NC_005118.4_50400001	19	50400001	0.4761	0.5917
NC_005118.4_50500001	19	50500001	0.5927	0.6587
NC_005118.4_50600001	19	50600001	0.5664	0.6407
NC_005118.4_50700001	19	50700001	0.5361	0.6034
NC_005118.4_50800001	19	50800001	0.576	0.6489
NC_005118.4_50900001	19	50900001	0.5815	0.6808
NC_005118.4_51000001	19	51000001	0.5501	0.6351
NC_005118.4_51100001	19	51100001	0.5724	0.6769
NC_005118.4_51200001	19	51200001	0.5337	0.6475
NC_005118.4_51300001	19	51300001	0.4983	0.6192
NC_005118.4_51400001	19	51400001	0.4658	0.5454
NC_005118.4_51500001	19	51500001	0.4314	0.5285
NC_005118.4_51600001	19	51600001	0.3809	0.4472
NC_005118.4_51700001	19	51700001	0.466	0.5559
NC_005118.4_51800001	19	51800001	0.4929	0.5524
NC_005118.4_51900001	19	51900001	0.4281	0.5395
NC_005118.4_52000001	19	52000001	0.4599	0.5943
NC_005118.4_52100001	19	52100001	0.4234	0.5467
NC_005118.4_52200001	19	52200001	0.4032	0.5164
NC_005118.4_52300001	19	52300001	0.416	0.5506
NC_005118.4_52400001	19	52400001	0.4284	0.5901
NC_005118.4_52500001	19	52500001	0.4392	0.5685
NC_005118.4_52600001	19	52600001	0.4801	0.6173
NC_005118.4_52700001	19	52700001	0.4986	0.5951
NC_005118.4_52800001	19	52800001	0.5373	0.5698
NC_005118.4_52900001	19	52900001	0.5988	0.5974
NC_005118.4_53000001	19	53000001	0.5828	0.6127
NC_005118.4_53100001	19	53100001	0.5932	0.5972
NC_005118.4_53200001	19	53200001	0.6298	0.7025

NC_005118.4_53300001	19	53300001	0.669	0.774
NC_005118.4_53400001	19	53400001	0.6466	0.772
NC_005118.4_53500001	19	53500001	0.5686	0.7209
NC_005118.4_53600001	19	53600001	0.5806	0.7305
NC_005118.4_53700001	19	53700001	0.5554	0.6928
NC_005118.4_53800001	19	53800001	0.5101	0.6715
NC_005118.4_53900001	19	53900001	0.4995	0.6688
NC_005118.4_54000001	19	54000001	0.69	0.8032
NC_005118.4_54100001	19	54100001	0.6424	0.7459
NC_005118.4_54200001	19	54200001	0.557	0.6614
NC_005118.4_54300001	19	54300001	0.5311	0.63
NC_005118.4_54400001	19	54400001	0.5372	0.6106
NC_005118.4_54500001	19	54500001	0.4648	0.5311
NC_005118.4_54600001	19	54600001	0.4473	0.5702
NC_005118.4_54700001	19	54700001	0.4048	0.5378
NC_005118.4_54800001	19	54800001	0.3678	0.5036
NC_005118.4_54900001	19	54900001	0.4168	0.5414
NC_005118.4_55000001	19	55000001	0.4025	0.5111
NC_005118.4_55100001	19	55100001	0.4199	0.482
NC_005118.4_55200001	19	55200001	0.4844	0.5229
NC_005118.4_55300001	19	55300001	0.5222	0.5801
NC_005118.4_55400001	19	55400001	0.4396	0.5472
NC_005118.4_55500001	19	55500001	0.4262	0.5732
NC_005118.4_55600001	19	55600001	0.422	0.6255
NC_005118.4_55700001	19	55700001	0.4685	0.6868
NC_005118.4_55800001	19	55800001	0.5303	0.717
NC_005118.4_55900001	19	55900001	0.5736	0.7186
NC_005118.4_56000001	19	56000001	0.651	0.7787
NC_005118.4_56100001	19	56100001	0.5938	0.7515
NC_005118.4_56200001	19	56200001	0.5273	0.7292
NC_005118.4_56300001	19	56300001	0.604	0.7929
NC_005118.4_56400001	19	56400001	0.6234	0.8184
NC_005118.4_56500001	19	56500001	0.535	0.7603
NC_005118.4_56600001	19	56600001	0.5683	0.7078
NC_005118.4_56700001	19	56700001	0.5823	0.6561
NC_005118.4_56800001	19	56800001	0.4471	0.4953
NC_005118.4_56900001	19	56900001	0.4469	0.4762
NC_005118.4_57000001	19	57000001	0.501	0.4901
NC_005118.4_57100001	19	57100001	0.4529	0.4564
NC_005118.4_57200001	19	57200001	0.4856	0.5697
NC_005118.4_57300001	19	57300001	0.503	0.6374
NC_005118.4_57400001	19	57400001	0.4899	0.6214
NC_005118.4_57500001	19	57500001	0.5107	0.6209
NC_005118.4_57600001	19	57600001	0.4798	0.614
NC_005118.4_57700001	19	57700001	0.4621	0.6159
NC_005118.4_57800001	19	57800001	0.4883	0.6314
NC_005118.4_57900001	19	57900001	0.5782	0.7638
NC_005118.4_58000001	19	58000001	0.5111	0.7422
NC_005118.4_58100001	19	58100001	0.4825	0.7247
NC_005118.4_58200001	19	58200001	0.4507	0.6784
NC_005118.4_58300001	19	58300001	0.4707	0.6931
NC_005118.4_58400001	19	58400001	0.3541	0.4948
NC_005118.4_58500001	19	58500001	0.4043	0.5256
NC_005118.4_58600001	19	58600001	0.4767	0.5813
NC_005118.4_58700001	19	58700001	0.5089	0.603
NC_005118.4_58800001	19	58800001	0.4548	0.5719
NC_005118.4_58900001	19	58900001	0.4615	0.6242
NC_005118.4_59000001	19	59000001	0.466	0.6093

NC_005118.4_59100001	19	59100001	0.4201	0.5736
NC_005118.4_59200001	19	59200001	0.4637	0.6164
NC_005118.4_59300001	19	59300001	0.4878	0.608
NC_005118.4_59400001	19	59400001	0.5429	0.674
NC_005118.4_59500001	19	59500001	0.5536	0.7105
NC_005118.4_59600001	19	59600001	0.6001	0.7428
NC_005118.4_59700001	19	59700001	0.4719	0.6201
NC_005118.4_59800001	19	59800001	0.4405	0.5512
NC_005118.4_59900001	19	59900001	0.3278	0.4177
NC_005118.4_60000001	19	60000001	0.3363	0.45
NC_005118.4_60100001	19	60100001	0.3679	0.4528
NC_005118.4_60200001	19	60200001	0.43	0.5274
NC_005118.4_60300001	19	60300001	0.3879	0.5344
NC_005118.4_60400001	19	60400001	0.4633	0.557
NC_005118.4_60500001	19	60500001	0.4263	0.5013
NC_005118.4_60600001	19	60600001	0.603	0.8051
NC_005118.4_60700001	19	60700001	0.5648	0.8173
NC_005118.4_60800001	19	60800001	0.5722	0.8286
NC_005118.4_60900001	19	60900001	0.5327	0.8053
NC_005118.4_61000001	19	61000001	0.5581	0.7704
NC_005118.4_61100001	19	61100001	0.5466	0.7388
NC_005118.4_61200001	19	61200001	0.5256	0.6915
NC_005118.4_61300001	19	61300001	0.5687	0.6996
NC_005118.4_61400001	19	61400001	0.5792	0.6989
NC_005118.4_61500001	19	61500001	0.577	0.7279
NC_005118.4_61600001	19	61600001	0.4338	0.6103
NC_005119.4_1	20	1	0.2326	0.3712
NC_005119.4_100001	20	100001	0.2298	0.3251
NC_005119.4_200001	20	200001	0.2298	0.3251
NC_005119.4_300001	20	300001	0.2476	0.3859
NC_005119.4_400001	20	400001	0.3108	0.4783
NC_005119.4_700001	20	700001	0.4198	0.4322
NC_005119.4_800001	20	800001	0.4198	0.4322
NC_005119.4_900001	20	900001	0.3711	0.4655
NC_005119.4_1000001	20	1000001	0.3711	0.4655
NC_005119.4_1100001	20	1100001	0.344	0.5562
NC_005119.4_1300001	20	1300001	0.5868	0.6654
NC_005119.4_1400001	20	1400001	0.4697	0.5625
NC_005119.4_1500001	20	1500001	0.3574	0.4508
NC_005119.4_1600001	20	1600001	0.3954	0.4756
NC_005119.4_1700001	20	1700001	0.3965	0.4849
NC_005119.4_1800001	20	1800001	0.3574	0.4502
NC_005119.4_1900001	20	1900001	0.4488	0.5539
NC_005119.4_2000001	20	2000001	0.5179	0.5957
NC_005119.4_2100001	20	2100001	0.5438	0.653
NC_005119.4_2200001	20	2200001	0.5761	0.6669
NC_005119.4_2300001	20	2300001	0.8042	0.812
NC_005119.4_2900001	20	2900001	0.5694	0.5659
NC_005119.4_3000001	20	3000001	0.547	0.6763
NC_005119.4_3100001	20	3100001	0.524	0.6151
NC_005119.4_3200001	20	3200001	0.4938	0.5961
NC_005119.4_3300001	20	3300001	0.4497	0.5667
NC_005119.4_3400001	20	3400001	0.399	0.5601
NC_005119.4_3500001	20	3500001	0.3539	0.4343
NC_005119.4_3600001	20	3600001	0.2816	0.3474
NC_005119.4_3700001	20	3700001	0.2447	0.2681
NC_005119.4_3800001	20	3800001	0.3642	0.453
NC_005119.4_3900001	20	3900001	0.4331	0.4364

NC_005119.4_4000001	20	4000001	0.3794	0.3849
NC_005119.4_4100001	20	4100001	0.4795	0.5536
NC_005119.4_4200001	20	4200001	0.5232	0.5915
NC_005119.4_4300001	20	4300001	0.4234	0.5204
NC_005119.4_4400001	20	4400001	0.4763	0.6379
NC_005119.4_4500001	20	4500001	0.5047	0.7219
NC_005119.4_4600001	20	4600001	0.4709	0.7871
NC_005119.4_4700001	20	4700001	0.4996	0.7403
NC_005119.4_4800001	20	4800001	0.5388	0.7624
NC_005119.4_4900001	20	4900001	0.5215	0.7306
NC_005119.4_5000001	20	5000001	0.4715	0.6866
NC_005119.4_5100001	20	5100001	0.353	0.6641
NC_005119.4_5200001	20	5200001	0.3159	0.7474
NC_005119.4_5300001	20	5300001	0.3119	0.7217
NC_005119.4_5400001	20	5400001	0.4205	0.7185
NC_005119.4_5500001	20	5500001	0.5266	0.786
NC_005119.4_5600001	20	5600001	0.6451	0.8174
NC_005119.4_5700001	20	5700001	0.6066	0.7979
NC_005119.4_5800001	20	5800001	0.6199	0.8051
NC_005119.4_5900001	20	5900001	0.6387	0.8166
NC_005119.4_6000001	20	6000001	0.5458	0.7539
NC_005119.4_6100001	20	6100001	0.4517	0.5708
NC_005119.4_6200001	20	6200001	0.6015	0.685
NC_005119.4_6300001	20	6300001	0.5761	0.6797
NC_005119.4_6400001	20	6400001	0.556	0.6868
NC_005119.4_6500001	20	6500001	0.6143	0.712
NC_005119.4_6600001	20	6600001	0.5752	0.7533
NC_005119.4_6700001	20	6700001	0.5049	0.7138
NC_005119.4_6800001	20	6800001	0.5254	0.7032
NC_005119.4_6900001	20	6900001	0.514	0.7182
NC_005119.4_7000001	20	7000001	0.4922	0.725
NC_005119.4_7100001	20	7100001	0.4488	0.6666
NC_005119.4_7200001	20	7200001	0.4238	0.639
NC_005119.4_7300001	20	7300001	0.4987	0.6937
NC_005119.4_7400001	20	7400001	0.4618	0.6603
NC_005119.4_7500001	20	7500001	0.4537	0.6934
NC_005119.4_7600001	20	7600001	0.61	0.8164
NC_005119.4_7700001	20	7700001	0.5965	0.807
NC_005119.4_7800001	20	7800001	0.5395	0.8293
NC_005119.4_7900001	20	7900001	0.6096	0.8348
NC_005119.4_8000001	20	8000001	0.6133	0.7395
NC_005119.4_8100001	20	8100001	0.4857	0.6579
NC_005119.4_8200001	20	8200001	0.437	0.6062
NC_005119.4_8300001	20	8300001	0.4543	0.6335
NC_005119.4_8400001	20	8400001	0.4855	0.6763
NC_005119.4_8500001	20	8500001	0.4942	0.7251
NC_005119.4_8600001	20	8600001	0.4971	0.683
NC_005119.4_8700001	20	8700001	0.6552	0.7609
NC_005119.4_8800001	20	8800001	0.6515	0.7528
NC_005119.4_8900001	20	8900001	0.6345	0.7163
NC_005119.4_9000001	20	9000001	0.6527	0.7585
NC_005119.4_9100001	20	9100001	0.7102	0.8112
NC_005119.4_9200001	20	9200001	0.657	0.7802
NC_005119.4_9300001	20	9300001	0.666	0.788
NC_005119.4_9400001	20	9400001	0.5404	0.7162
NC_005119.4_9500001	20	9500001	0.5034	0.6598
NC_005119.4_9600001	20	9600001	0.3978	0.5249
NC_005119.4_9700001	20	9700001	0.4861	0.6793

NC_005119.4_9800001	20	9800001	0.5186	0.7017
NC_005119.4_9900001	20	9900001	0.5843	0.7508
NC_005119.4_10000001	20	10000001	0.5087	0.6887
NC_005119.4_10100001	20	10100001	0.5245	0.7088
NC_005119.4_10200001	20	10200001	0.5092	0.6494
NC_005119.4_10300001	20	10300001	0.4569	0.5715
NC_005119.4_10400001	20	10400001	0.4599	0.5298
NC_005119.4_10500001	20	10500001	0.5439	0.5806
NC_005119.4_10600001	20	10600001	0.5177	0.5576
NC_005119.4_10700001	20	10700001	0.4813	0.547
NC_005119.4_10800001	20	10800001	0.5601	0.6296
NC_005119.4_10900001	20	10900001	0.5596	0.6093
NC_005119.4_11000001	20	11000001	0.4733	0.5906
NC_005119.4_11100001	20	11100001	0.5125	0.5939
NC_005119.4_11200001	20	11200001	0.5158	0.556
NC_005119.4_11300001	20	11300001	0.5027	0.5394
NC_005119.4_11400001	20	11400001	0.4309	0.5227
NC_005119.4_11500001	20	11500001	0.411	0.513
NC_005119.4_11600001	20	11600001	0.3181	0.4834
NC_005119.4_11700001	20	11700001	0.2474	0.4086
NC_005119.4_11800001	20	11800001	0.2306	0.3785
NC_005119.4_11900001	20	11900001	0.2646	0.4455
NC_005119.4_12000001	20	12000001	0.2454	0.4117
NC_005119.4_12100001	20	12100001	0.2853	0.4566
NC_005119.4_12200001	20	12200001	0.3872	0.6407
NC_005119.4_12300001	20	12300001	0.4154	0.6446
NC_005119.4_12400001	20	12400001	0.4511	0.6768
NC_005119.4_12500001	20	12500001	0.4512	0.6597
NC_005119.4_12600001	20	12600001	0.5056	0.7172
NC_005119.4_12700001	20	12700001	0.5428	0.7488
NC_005119.4_12800001	20	12800001	0.6421	0.812
NC_005119.4_12900001	20	12900001	0.6242	0.8131
NC_005119.4_13000001	20	13000001	0.7654	0.8963
NC_005119.4_13100001	20	13100001	0.8101	0.8313
NC_005119.4_13500001	20	13500001	0.5949	0.8536
NC_005119.4_13600001	20	13600001	0.6796	0.801
NC_005119.4_13700001	20	13700001	0.6391	0.7937
NC_005119.4_13800001	20	13800001	0.607	0.7326
NC_005119.4_13900001	20	13900001	0.5833	0.7188
NC_005119.4_14000001	20	14000001	0.5431	0.6466
NC_005119.4_14100001	20	14100001	0.5315	0.5868
NC_005119.4_14200001	20	14200001	0.5509	0.5937
NC_005119.4_14300001	20	14300001	0.5306	0.5699
NC_005119.4_14400001	20	14400001	0.5539	0.5275
NC_005119.4_14500001	20	14500001	0.6151	0.5462
NC_005119.4_14600001	20	14600001	0.5919	0.6683
NC_005119.4_14700001	20	14700001	0.5474	0.6213
NC_005119.4_14800001	20	14800001	0.566	0.679
NC_005119.4_14900001	20	14900001	0.5394	0.6513
NC_005119.4_15000001	20	15000001	0.503	0.6093
NC_005119.4_15100001	20	15100001	0.503	0.6093
NC_005119.4_15200001	20	15200001	0.3305	0.5343
NC_005119.4_15400001	20	15400001	0.5977	0.7446
NC_005119.4_15500001	20	15500001	0.6707	0.7627
NC_005119.4_15600001	20	15600001	0.6707	0.7627
NC_005119.4_15700001	20	15700001	0.617	0.7403
NC_005119.4_15800001	20	15800001	0.7073	0.7644
NC_005119.4_15900001	20	15900001	0.6126	0.6534

NC_005119.4_16000001	20	16000001	0.3939	0.4354
NC_005119.4_16100001	20	16100001	0.4472	0.5412
NC_005119.4_16200001	20	16200001	0.4882	0.5392
NC_005119.4_16300001	20	16300001	0.3875	0.4558
NC_005119.4_16400001	20	16400001	0.3609	0.4967
NC_005119.4_16500001	20	16500001	0.3223	0.529
NC_005119.4_16600001	20	16600001	0.3124	0.4429
NC_005119.4_16700001	20	16700001	0.3678	0.5112
NC_005119.4_16800001	20	16800001	0.4003	0.5465
NC_005119.4_16900001	20	16900001	0.4108	0.5844
NC_005119.4_17000001	20	17000001	0.5052	0.6498
NC_005119.4_17100001	20	17100001	0.4315	0.5746
NC_005119.4_17200001	20	17200001	0.3841	0.5314
NC_005119.4_17300001	20	17300001	0.405	0.4645
NC_005119.4_17400001	20	17400001	0.3745	0.4166
NC_005119.4_17500001	20	17500001	0.3531	0.374
NC_005119.4_17600001	20	17600001	0.4041	0.438
NC_005119.4_17700001	20	17700001	0.5167	0.5052
NC_005119.4_17800001	20	17800001	0.5254	0.6529
NC_005119.4_17900001	20	17900001	0.5596	0.6678
NC_005119.4_18000001	20	18000001	0.5463	0.6826
NC_005119.4_18100001	20	18100001	0.5213	0.7207
NC_005119.4_18200001	20	18200001	0.4912	0.6873
NC_005119.4_18300001	20	18300001	0.4499	0.6583
NC_005119.4_18400001	20	18400001	0.4015	0.629
NC_005119.4_18500001	20	18500001	0.3271	0.5167
NC_005119.4_18600001	20	18600001	0.2744	0.4058
NC_005119.4_18700001	20	18700001	0.2934	0.5001
NC_005119.4_18800001	20	18800001	0.3682	0.5427
NC_005119.4_18900001	20	18900001	0.3682	0.5031
NC_005119.4_19000001	20	19000001	0.3856	0.4974
NC_005119.4_19100001	20	19100001	0.4051	0.5174
NC_005119.4_19200001	20	19200001	0.4427	0.5301
NC_005119.4_19300001	20	19300001	0.4	0.4914
NC_005119.4_19400001	20	19400001	0.4486	0.5725
NC_005119.4_19500001	20	19500001	0.4571	0.5731
NC_005119.4_19600001	20	19600001	0.4566	0.5513
NC_005119.4_19700001	20	19700001	0.4592	0.5526
NC_005119.4_19800001	20	19800001	0.4878	0.5324
NC_005119.4_19900001	20	19900001	0.483	0.5165
NC_005119.4_20000001	20	20000001	0.4433	0.5059
NC_005119.4_20100001	20	20100001	0.4418	0.5243
NC_005119.4_20200001	20	20200001	0.4242	0.4604
NC_005119.4_20300001	20	20300001	0.3555	0.4138
NC_005119.4_20400001	20	20400001	0.3735	0.4097
NC_005119.4_20500001	20	20500001	0.4346	0.4542
NC_005119.4_20600001	20	20600001	0.4672	0.5038
NC_005119.4_20700001	20	20700001	0.4232	0.5106
NC_005119.4_20800001	20	20800001	0.4455	0.5998
NC_005119.4_20900001	20	20900001	0.4673	0.6359
NC_005119.4_21000001	20	21000001	0.4541	0.6486
NC_005119.4_21100001	20	21100001	0.4688	0.6845
NC_005119.4_21200001	20	21200001	0.47	0.6789
NC_005119.4_21300001	20	21300001	0.5063	0.7059
NC_005119.4_21400001	20	21400001	0.5019	0.6836
NC_005119.4_21500001	20	21500001	0.4978	0.7001
NC_005119.4_21600001	20	21600001	0.4772	0.599
NC_005119.4_21700001	20	21700001	0.4695	0.5973

NC_005119.4_21800001	20	21800001	0.4095	0.5742
NC_005119.4_21900001	20	21900001	0.3636	0.5574
NC_005119.4_22000001	20	22000001	0.3603	0.4905
NC_005119.4_22100001	20	22100001	0.4511	0.6194
NC_005119.4_22200001	20	22200001	0.5046	0.66
NC_005119.4_22300001	20	22300001	0.5869	0.6948
NC_005119.4_22400001	20	22400001	0.6344	0.742
NC_005119.4_22500001	20	22500001	0.5752	0.7207
NC_005119.4_22600001	20	22600001	0.5259	0.7329
NC_005119.4_22700001	20	22700001	0.5011	0.7268
NC_005119.4_22800001	20	22800001	0.4156	0.6491
NC_005119.4_22900001	20	22900001	0.3287	0.5357
NC_005119.4_23000001	20	23000001	0.2383	0.3412
NC_005119.4_23100001	20	23100001	0.1636	0.1168
NC_005119.4_23200001	20	23200001	0.3743	0.4527
NC_005119.4_23300001	20	23300001	0.4597	0.535
NC_005119.4_23400001	20	23400001	0.4611	0.5397
NC_005119.4_23500001	20	23500001	0.505	0.618
NC_005119.4_23600001	20	23600001	0.4525	0.5581
NC_005119.4_23700001	20	23700001	0.4056	0.493
NC_005119.4_23800001	20	23800001	0.405	0.4645
NC_005119.4_23900001	20	23900001	0.4377	0.499
NC_005119.4_24000001	20	24000001	0.3757	0.3842
NC_005119.4_24100001	20	24100001	0.497	0.5683
NC_005119.4_24200001	20	24200001	0.525	0.6505
NC_005119.4_24300001	20	24300001	0.5124	0.6821
NC_005119.4_24400001	20	24400001	0.5045	0.6727
NC_005119.4_24500001	20	24500001	0.6635	0.8348
NC_005119.4_24600001	20	24600001	0.5871	0.7292
NC_005119.4_24700001	20	24700001	0.5508	0.7313
NC_005119.4_24800001	20	24800001	0.5686	0.7268
NC_005119.4_24900001	20	24900001	0.5258	0.753
NC_005119.4_25000001	20	25000001	0.5157	0.7697
NC_005119.4_25100001	20	25100001	0.4852	0.75
NC_005119.4_25200001	20	25200001	0.4147	0.7183
NC_005119.4_25300001	20	25300001	0.3446	0.6545
NC_005119.4_25400001	20	25400001	0.3545	0.5312
NC_005119.4_25500001	20	25500001	0.3164	0.471
NC_005119.4_25600001	20	25600001	0.4146	0.6001
NC_005119.4_25700001	20	25700001	0.4522	0.6441
NC_005119.4_25800001	20	25800001	0.4764	0.6747
NC_005119.4_25900001	20	25900001	0.5245	0.8374
NC_005119.4_26000001	20	26000001	0.5229	0.8414
NC_005119.4_26100001	20	26100001	0.4878	0.7561
NC_005119.4_26200001	20	26200001	0.5485	0.7296
NC_005119.4_26300001	20	26300001	0.5886	0.7482
NC_005119.4_26400001	20	26400001	0.6863	0.7474
NC_005119.4_26500001	20	26500001	0.6436	0.7218
NC_005119.4_26600001	20	26600001	0.7439	0.8152
NC_005119.4_26700001	20	26700001	0.6566	0.8121
NC_005119.4_26800001	20	26800001	0.6665	0.8089
NC_005119.4_26900001	20	26900001	0.6543	0.8242
NC_005119.4_27000001	20	27000001	0.6916	0.8486
NC_005119.4_27100001	20	27100001	0.6248	0.8418
NC_005119.4_27200001	20	27200001	0.6553	0.8189
NC_005119.4_27300001	20	27300001	0.6345	0.7378
NC_005119.4_27400001	20	27400001	0.4759	0.5333
NC_005119.4_27500001	20	27500001	0.2882	0.2685

NC_005119.4_27600001	20	27600001	0.3335	0.4035
NC_005119.4_27700001	20	27700001	0.5157	0.6564
NC_005119.4_27800001	20	27800001	0.6407	0.8618
NC_005119.4_27900001	20	27900001	0.6621	0.7973
NC_005119.4_28000001	20	28000001	0.5972	0.7086
NC_005119.4_28100001	20	28100001	0.5399	0.6319
NC_005119.4_28200001	20	28200001	0.4825	0.5589
NC_005119.4_28300001	20	28300001	0.4006	0.4128
NC_005119.4_28400001	20	28400001	0.4347	0.4918
NC_005119.4_28500001	20	28500001	0.6084	0.7121
NC_005119.4_28600001	20	28600001	0.6387	0.7284
NC_005119.4_28700001	20	28700001	0.5797	0.6831
NC_005119.4_28800001	20	28800001	0.5977	0.7182
NC_005119.4_28900001	20	28900001	0.5684	0.6796
NC_005119.4_29000001	20	29000001	0.4997	0.5816
NC_005119.4_29100001	20	29100001	0.5016	0.5669
NC_005119.4_29200001	20	29200001	0.5657	0.5678
NC_005119.4_29300001	20	29300001	0.5745	0.5974
NC_005119.4_29400001	20	29400001	0.5913	0.6151
NC_005119.4_29500001	20	29500001	0.5583	0.5764
NC_005119.4_29600001	20	29600001	0.5882	0.6481
NC_005119.4_29700001	20	29700001	0.5374	0.7197
NC_005119.4_29800001	20	29800001	0.4517	0.6187
NC_005119.4_29900001	20	29900001	0.4476	0.5574
NC_005119.4_30000001	20	30000001	0.5295	0.614
NC_005119.4_30100001	20	30100001	0.4615	0.5502
NC_005119.4_30200001	20	30200001	0.4934	0.6083
NC_005119.4_30300001	20	30300001	0.4801	0.5719
NC_005119.4_30400001	20	30400001	0.5004	0.6057
NC_005119.4_30500001	20	30500001	0.518	0.6027
NC_005119.4_30600001	20	30600001	0.5818	0.6461
NC_005119.4_30700001	20	30700001	0.5737	0.6032
NC_005119.4_30800001	20	30800001	0.6247	0.6527
NC_005119.4_30900001	20	30900001	0.6636	0.6714
NC_005119.4_31000001	20	31000001	0.6051	0.6851
NC_005119.4_31100001	20	31100001	0.5516	0.718
NC_005119.4_31200001	20	31200001	0.5298	0.7023
NC_005119.4_31300001	20	31300001	0.5489	0.7315
NC_005119.4_31400001	20	31400001	0.5482	0.7018
NC_005119.4_31500001	20	31500001	0.5743	0.7361
NC_005119.4_31600001	20	31600001	0.5697	0.6976
NC_005119.4_31700001	20	31700001	0.606	0.723
NC_005119.4_31800001	20	31800001	0.5845	0.687
NC_005119.4_31900001	20	31900001	0.6185	0.7487
NC_005119.4_32000001	20	32000001	0.5817	0.6564
NC_005119.4_32100001	20	32100001	0.6736	0.6383
NC_005119.4_32400001	20	32400001	0.5191	0.5657
NC_005119.4_32500001	20	32500001	0.293	0.5231
NC_005119.4_32600001	20	32600001	0.266	0.5028
NC_005119.4_32700001	20	32700001	0.302	0.6001
NC_005119.4_32800001	20	32800001	0.244	0.5533
NC_005119.4_32900001	20	32900001	0.4275	0.7947
NC_005119.4_33000001	20	33000001	0.5214	0.7209
NC_005119.4_33100001	20	33100001	0.5514	0.6714
NC_005119.4_33200001	20	33200001	0.468	0.6427
NC_005119.4_33300001	20	33300001	0.443	0.6339
NC_005119.4_33400001	20	33400001	0.4552	0.6094
NC_005119.4_33500001	20	33500001	0.4376	0.6092

NC_005119.4_33600001	20	33600001	0.4968	0.6623
NC_005119.4_33700001	20	33700001	0.6635	0.6579
NC_005119.4_33800001	20	33800001	0.7707	0.7327
NC_005119.4_33900001	20	33900001	0.7824	0.8067
NC_005119.4_34000001	20	34000001	0.7515	0.8281
NC_005119.4_34100001	20	34100001	0.7565	0.8546
NC_005119.4_34200001	20	34200001	0.6324	0.7995
NC_005119.4_34300001	20	34300001	0.5925	0.7342
NC_005119.4_34400001	20	34400001	0.5868	0.6967
NC_005119.4_34500001	20	34500001	0.5212	0.6199
NC_005119.4_34600001	20	34600001	0.5352	0.6602
NC_005119.4_34700001	20	34700001	0.5854	0.6782
NC_005119.4_34800001	20	34800001	0.5839	0.6976
NC_005119.4_34900001	20	34900001	0.6563	0.8285
NC_005119.4_35000001	20	35000001	0.6298	0.7831
NC_005119.4_35100001	20	35100001	0.5566	0.6387
NC_005119.4_35200001	20	35200001	0.5814	0.6663
NC_005119.4_35300001	20	35300001	0.558	0.5981
NC_005119.4_35400001	20	35400001	0.4436	0.4605
NC_005119.4_35500001	20	35500001	0.4869	0.5068
NC_005119.4_35600001	20	35600001	0.4035	0.459
NC_005119.4_35700001	20	35700001	0.2911	0.2891
NC_005119.4_35800001	20	35800001	0.2869	0.3686
NC_005119.4_35900001	20	35900001	0.3236	0.4138
NC_005119.4_36000001	20	36000001	0.3543	0.511
NC_005119.4_36100001	20	36100001	0.388	0.5164
NC_005119.4_36200001	20	36200001	0.4306	0.5747
NC_005119.4_36300001	20	36300001	0.4125	0.4851
NC_005119.4_36400001	20	36400001	0.4217	0.5083
NC_005119.4_36500001	20	36500001	0.4055	0.4229
NC_005119.4_36600001	20	36600001	0.4379	0.4356
NC_005119.4_36700001	20	36700001	0.5082	0.4412
NC_005119.4_37100001	20	37100001	0.4612	0.5896
NC_005119.4_37200001	20	37200001	0.3671	0.4561
NC_005119.4_37300001	20	37300001	0.4072	0.4244
NC_005119.4_37400001	20	37400001	0.5827	0.6181
NC_005119.4_37500001	20	37500001	0.5664	0.6122
NC_005119.4_37600001	20	37600001	0.4798	0.5761
NC_005119.4_37700001	20	37700001	0.5377	0.6932
NC_005119.4_37800001	20	37800001	0.5063	0.6415
NC_005119.4_37900001	20	37900001	0.406	0.5622
NC_005119.4_38000001	20	38000001	0.3423	0.4742
NC_005119.4_38100001	20	38100001	0.3699	0.4993
NC_005119.4_38200001	20	38200001	0.2828	0.3695
NC_005119.4_38300001	20	38300001	0.2814	0.4422
NC_005119.4_38400001	20	38400001	0.2501	0.3438
NC_005119.4_38500001	20	38500001	0.4515	0.8097
NC_005119.4_38600001	20	38600001	0.4515	0.8097
NC_005119.4_38800001	20	38800001	0.584	0.6759
NC_005119.4_38900001	20	38900001	0.584	0.6759
NC_005119.4_39000001	20	39000001	0.5882	0.6962
NC_005119.4_39100001	20	39100001	0.5685	0.7366
NC_005119.4_39200001	20	39200001	0.516	0.7202
NC_005119.4_39300001	20	39300001	0.5186	0.786
NC_005119.4_39400001	20	39400001	0.5186	0.786
NC_005119.4_39500001	20	39500001	0.4444	0.7475
NC_005119.4_39600001	20	39600001	0.4035	0.71
NC_005119.4_39700001	20	39700001	0.5686	0.8249

NC_005119.4_39800001	20	39800001	0.4679	0.7366
NC_005119.4_39900001	20	39900001	0.4326	0.6718
NC_005119.4_40000001	20	40000001	0.4531	0.6117
NC_005119.4_40100001	20	40100001	0.4752	0.6608
NC_005119.4_40200001	20	40200001	0.467	0.6404
NC_005119.4_40300001	20	40300001	0.4936	0.6542
NC_005119.4_40400001	20	40400001	0.5386	0.7022
NC_005119.4_40500001	20	40500001	0.4965	0.7027
NC_005119.4_40700001	20	40700001	0.5686	0.7124
NC_005119.4_40800001	20	40800001	0.5951	0.7031
NC_005119.4_40900001	20	40900001	0.5747	0.7143
NC_005119.4_41000001	20	41000001	0.5415	0.7145
NC_005119.4_41100001	20	41100001	0.5315	0.7111
NC_005119.4_41200001	20	41200001	0.4999	0.7044
NC_005119.4_41300001	20	41300001	0.3932	0.7058
NC_005119.4_41400001	20	41400001	0.2484	0.5966
NC_005119.4_41500001	20	41500001	0.1951	0.4178
NC_005119.4_41600001	20	41600001	0.3833	0.7134
NC_005119.4_41700001	20	41700001	0.4801	0.7671
NC_005119.4_41800001	20	41800001	0.4775	0.6522
NC_005119.4_41900001	20	41900001	0.6327	0.79
NC_005119.4_42000001	20	42000001	0.7308	0.7815
NC_005119.4_42100001	20	42100001	0.5058	0.6582
NC_005119.4_42200001	20	42200001	0.5585	0.7114
NC_005119.4_42300001	20	42300001	0.6906	0.8171
NC_005119.4_42400001	20	42400001	0.4696	0.7094
NC_005119.4_42500001	20	42500001	0.4444	0.7039
NC_005119.4_42600001	20	42600001	0.5757	0.7893
NC_005119.4_42700001	20	42700001	0.5223	0.6793
NC_005119.4_42800001	20	42800001	0.4063	0.5876
NC_005119.4_42900001	20	42900001	0.5858	0.6919
NC_005119.4_43000001	20	43000001	0.6164	0.6935
NC_005119.4_43100001	20	43100001	0.5996	0.6756
NC_005119.4_43200001	20	43200001	0.6319	0.7337
NC_005119.4_43300001	20	43300001	0.705	0.742
NC_005119.4_43400001	20	43400001	0.3805	0.4838
NC_005119.4_43500001	20	43500001	0.416	0.5063
NC_005119.4_43600001	20	43600001	0.4541	0.5424
NC_005119.4_43700001	20	43700001	0.4906	0.5538
NC_005119.4_43800001	20	43800001	0.5487	0.6124
NC_005119.4_43900001	20	43900001	0.5703	0.601
NC_005119.4_44000001	20	44000001	0.5452	0.5631
NC_005119.4_44100001	20	44100001	0.533	0.5453
NC_005119.4_44200001	20	44200001	0.5	0.5038
NC_005119.4_44300001	20	44300001	0.4915	0.4782
NC_005119.4_44400001	20	44400001	0.5452	0.5999
NC_005119.4_44500001	20	44500001	0.6001	0.7068
NC_005119.4_44600001	20	44600001	0.5619	0.6951
NC_005119.4_44700001	20	44700001	0.5539	0.7208
NC_005119.4_44800001	20	44800001	0.468	0.6966
NC_005119.4_44900001	20	44900001	0.3873	0.5807
NC_005119.4_45000001	20	45000001	0.3552	0.4638
NC_005119.4_45100001	20	45100001	0.4837	0.6111
NC_005119.4_45200001	20	45200001	0.4743	0.6277
NC_005119.4_45300001	20	45300001	0.5021	0.6321
NC_005119.4_45400001	20	45400001	0.5819	0.6922
NC_005119.4_45500001	20	45500001	0.5492	0.7055
NC_005119.4_45600001	20	45600001	0.4389	0.5917

NC_005119.4_45700001	20	45700001	0.5065	0.6426
NC_005119.4_45800001	20	45800001	0.5718	0.7329
NC_005119.4_45900001	20	45900001	0.4799	0.6863
NC_005119.4_46000001	20	46000001	0.5102	0.7136
NC_005119.4_46100001	20	46100001	0.5517	0.6953
NC_005119.4_46200001	20	46200001	0.5439	0.7199
NC_005119.4_46300001	20	46300001	0.5684	0.6823
NC_005119.4_46400001	20	46400001	0.513	0.6687
NC_005119.4_46500001	20	46500001	0.5177	0.6699
NC_005119.4_46600001	20	46600001	0.6035	0.7978
NC_005119.4_46700001	20	46700001	0.6278	0.8154
NC_005119.4_46800001	20	46800001	0.5936	0.833
NC_005119.4_46900001	20	46900001	0.7112	0.8285
NC_005119.4_47000001	20	47000001	0.7458	0.8401
NC_005119.4_47100001	20	47100001	0.5735	0.7725
NC_005119.4_47200001	20	47200001	0.4598	0.6378
NC_005119.4_47300001	20	47300001	0.4629	0.628
NC_005119.4_47400001	20	47400001	0.4678	0.6063
NC_005119.4_47500001	20	47500001	0.4684	0.5949
NC_005119.4_47600001	20	47600001	0.4664	0.5318
NC_005119.4_47700001	20	47700001	0.4682	0.5485
NC_005119.4_47800001	20	47800001	0.5068	0.5977
NC_005119.4_47900001	20	47900001	0.434	0.5332
NC_005119.4_48000001	20	48000001	0.4679	0.5822
NC_005119.4_48100001	20	48100001	0.4912	0.6071
NC_005119.4_48200001	20	48200001	0.4981	0.6209
NC_005119.4_48300001	20	48300001	0.4541	0.5808
NC_005119.4_48400001	20	48400001	0.4636	0.6115
NC_005119.4_48500001	20	48500001	0.3806	0.5101
NC_005119.4_48600001	20	48600001	0.3287	0.3988
NC_005119.4_48700001	20	48700001	0.4091	0.4581
NC_005119.4_48800001	20	48800001	0.4435	0.4656
NC_005119.4_48900001	20	48900001	0.4995	0.5165
NC_005119.4_49000001	20	49000001	0.475	0.5205
NC_005119.4_49100001	20	49100001	0.5352	0.5872
NC_005119.4_49200001	20	49200001	0.5242	0.5561
NC_005119.4_49300001	20	49300001	0.5137	0.5557
NC_005119.4_49400001	20	49400001	0.5266	0.5609
NC_005119.4_49500001	20	49500001	0.6069	0.6418
NC_005119.4_49600001	20	49600001	0.6032	0.6158
NC_005119.4_49700001	20	49700001	0.6348	0.6481
NC_005119.4_49800001	20	49800001	0.645	0.6549
NC_005119.4_49900001	20	49900001	0.5988	0.6769
NC_005119.4_50000001	20	50000001	0.5572	0.6446
NC_005119.4_50100001	20	50100001	0.5298	0.6467
NC_005119.4_50200001	20	50200001	0.5584	0.7033
NC_005119.4_50300001	20	50300001	0.5595	0.6919
NC_005119.4_50400001	20	50400001	0.5345	0.6075
NC_005119.4_50500001	20	50500001	0.542	0.5792
NC_005119.4_50600001	20	50600001	0.6391	0.6727
NC_005119.4_50700001	20	50700001	0.5736	0.6138
NC_005119.4_50800001	20	50800001	0.5556	0.6198
NC_005119.4_50900001	20	50900001	0.5806	0.6414
NC_005119.4_51000001	20	51000001	0.6941	0.7444
NC_005119.4_51100001	20	51100001	0.7064	0.7941
NC_005119.4_51200001	20	51200001	0.7316	0.7062
NC_005119.4_51300001	20	51300001	0.6865	0.6881
NC_005119.4_51400001	20	51400001	0.6733	0.6735

NC_005119.4_51500001	20	51500001	0.5486	0.5034
NC_005119.4_51600001	20	51600001	0.5043	0.509
NC_005119.4_51700001	20	51700001	0.5671	0.6511
NC_005119.4_51800001	20	51800001	0.5701	0.6482
NC_005119.4_51900001	20	51900001	0.579	0.6736
NC_005119.4_52000001	20	52000001	0.6038	0.8117
NC_005119.4_52100001	20	52100001	0.6438	0.7789
NC_005119.4_52200001	20	52200001	0.4834	0.7643
NC_005119.4_52300001	20	52300001	0.5858	0.8581
NC_005119.4_52400001	20	52400001	0.626	0.7722
NC_005119.4_52500001	20	52500001	0.5555	0.7003
NC_005119.4_52600001	20	52600001	0.5218	0.6645
NC_005119.4_52700001	20	52700001	0.5823	0.6404
NC_005119.4_52800001	20	52800001	0.5837	0.6414
NC_005119.4_52900001	20	52900001	0.6802	0.756
NC_005119.4_53000001	20	53000001	0.9429	0.8941
NC_005119.4_53100001	20	53100001	0.8201	0.8378
NC_005119.4_53200001	20	53200001	0.8088	0.8826
NC_005119.4_53300001	20	53300001	0.8016	0.8768
NC_005119.4_53400001	20	53400001	0.6695	0.8735
NC_005119.4_53500001	20	53500001	0.6695	0.8735
NC_005119.4_53600001	20	53600001	0.445	0.7462
NC_005119.4_53700001	20	53700001	0.456	0.6392
NC_005119.4_53800001	20	53800001	0.4225	0.5999
NC_005119.4_53900001	20	53900001	0.4663	0.5793
NC_005119.4_54000001	20	54000001	0.5317	0.6481
NC_005119.4_54100001	20	54100001	0.599	0.7221
NC_005119.4_54200001	20	54200001	0.5719	0.8267
NC_005119.4_54300001	20	54300001	0.5848	0.7349
NC_005119.4_54400001	20	54400001	0.4995	0.7093
NC_005119.4_54500001	20	54500001	0.3568	0.4666
NC_005119.4_54600001	20	54600001	0.4109	0.4945
NC_005119.4_54700001	20	54700001	0.403	0.4695
NC_005119.4_54800001	20	54800001	0.4097	0.547
NC_005119.4_54900001	20	54900001	0.3826	0.4977
NC_005119.4_55000001	20	55000001	0.3917	0.6359
NC_005119.4_55100001	20	55100001	0.3822	0.5656
NC_005119.4_55200001	20	55200001	0.5488	0.6784
NC_005119.4_55300001	20	55300001	0.515	0.5811
NC_005119.4_55400001	20	55400001	0.3657	0.47
NC_005119.4_55500001	20	55500001	0.4086	0.5056
NC_005119.4_55600001	20	55600001	0.3729	0.4327
NC_005120.4_500001	X	500001	0.7281	0.7864
NC_005120.4_600001	X	600001	0.7281	0.7864
NC_005120.4_700001	X	700001	0.7281	0.7864
NC_005120.4_800001	X	800001	0.7281	0.7864
NC_005120.4_1300001	X	1300001	0.8292	0.9746
NC_005120.4_1400001	X	1400001	0.8523	0.9755
NC_005120.4_1500001	X	1500001	0.8259	0.975
NC_005120.4_3200001	X	3200001	0.3385	0.5274
NC_005120.4_3300001	X	3300001	0.5036	0.6845
NC_005120.4_3400001	X	3400001	0.5515	0.7754
NC_005120.4_3500001	X	3500001	0.6126	0.7836
NC_005120.4_3600001	X	3600001	0.4658	0.7883
NC_005120.4_3700001	X	3700001	0.5244	0.8428
NC_005120.4_3800001	X	3800001	0.3762	0.7051
NC_005120.4_5100001	X	5100001	0.7801	0.9586
NC_005120.4_5200001	X	5200001	0.7801	0.9586

NC_005120.4_5400001	X	5400001	0.7804	0.9461
NC_005120.4_6100001	X	6100001	0.5704	0.6863
NC_005120.4_6200001	X	6200001	0.5016	0.6191
NC_005120.4_6300001	X	6300001	0.5016	0.6191
NC_005120.4_6400001	X	6400001	0.4977	0.7378
NC_005120.4_6500001	X	6500001	0.4977	0.7378
NC_005120.4_7100001	X	7100001	0.5264	0.4937
NC_005120.4_7200001	X	7200001	0.5894	0.5569
NC_005120.4_7300001	X	7300001	0.5064	0.5448
NC_005120.4_7400001	X	7400001	0.5064	0.5448
NC_005120.4_8300001	X	8300001	0.9383	0.8728
NC_005120.4_8400001	X	8400001	0.8653	0.8167
NC_005120.4_8500001	X	8500001	0.7764	0.7171
NC_005120.4_8600001	X	8600001	0.7564	0.7016
NC_005120.4_8700001	X	8700001	0.6769	0.5904
NC_005120.4_8800001	X	8800001	0.7101	0.6691
NC_005120.4_8900001	X	8900001	0.506	0.6236
NC_005120.4_9000001	X	9000001	0.531	0.7132
NC_005120.4_9100001	X	9100001	0.531	0.7132
NC_005120.4_9200001	X	9200001	0.6715	0.9249
NC_005120.4_9300001	X	9300001	0.5021	0.8869
NC_005120.4_9400001	X	9400001	0.7993	0.9762
NC_005120.4_9500001	X	9500001	0.8491	0.9732
NC_005120.4_9600001	X	9600001	0.8491	0.9732
NC_005120.4_9700001	X	9700001	0.7555	0.9528
NC_005120.4_10200001	X	10200001	0.6312	0.8257
NC_005120.4_10300001	X	10300001	0.608	0.8398
NC_005120.4_10400001	X	10400001	0.6899	0.8608
NC_005120.4_10500001	X	10500001	0.646	0.8321
NC_005120.4_10600001	X	10600001	0.646	0.8321
NC_005120.4_10700001	X	10700001	0.5791	0.7809
NC_005120.4_10800001	X	10800001	0.6114	0.6913
NC_005120.4_10900001	X	10900001	0.4918	0.7104
NC_005120.4_11000001	X	11000001	0.4736	0.7096
NC_005120.4_11100001	X	11100001	0.4978	0.7318
NC_005120.4_11200001	X	11200001	0.5635	0.7961
NC_005120.4_11300001	X	11300001	0.6126	0.8414
NC_005120.4_11400001	X	11400001	0.8056	0.8501
NC_005120.4_11500001	X	11500001	0.863	0.8116
NC_005120.4_11600001	X	11600001	0.8893	0.8484
NC_005120.4_11700001	X	11700001	0.8719	0.8303
NC_005120.4_11800001	X	11800001	0.8279	0.8119
NC_005120.4_11900001	X	11900001	0.7418	0.8075
NC_005120.4_12000001	X	12000001	0.8446	0.923
NC_005120.4_12100001	X	12100001	0.7499	0.8695
NC_005120.4_12200001	X	12200001	0.77	0.8674
NC_005120.4_12300001	X	12300001	0.8298	0.9407
NC_005120.4_12400001	X	12400001	0.863	0.8943
NC_005120.4_12500001	X	12500001	0.861	0.8788
NC_005120.4_12600001	X	12600001	0.861	0.8788
NC_005120.4_12700001	X	12700001	0.8952	0.8864
NC_005120.4_12800001	X	12800001	0.8548	0.8376
NC_005120.4_13300001	X	13300001	0.7838	0.86
NC_005120.4_13400001	X	13400001	0.7726	0.8375
NC_005120.4_13500001	X	13500001	0.6861	0.8395
NC_005120.4_13600001	X	13600001	0.7456	0.8766
NC_005120.4_13700001	X	13700001	0.7111	0.8565
NC_005120.4_13800001	X	13800001	0.7931	0.9319

NC_005120.4_13900001	X	13900001	0.7722	0.9028
NC_005120.4_14000001	X	14000001	0.6833	0.8125
NC_005120.4_14100001	X	14100001	0.6876	0.8187
NC_005120.4_14200001	X	14200001	0.6081	0.7506
NC_005120.4_14300001	X	14300001	0.5243	0.6493
NC_005120.4_14400001	X	14400001	0.5134	0.6677
NC_005120.4_14500001	X	14500001	0.6691	0.8124
NC_005120.4_14600001	X	14600001	0.6266	0.7896
NC_005120.4_14700001	X	14700001	0.8299	0.9378
NC_005120.4_14800001	X	14800001	0.8299	0.9378
NC_005120.4_14900001	X	14900001	0.8299	0.9378
NC_005120.4_15100001	X	15100001	0.716	0.8747
NC_005120.4_15200001	X	15200001	0.639	0.8097
NC_005120.4_15300001	X	15300001	0.6519	0.8418
NC_005120.4_15400001	X	15400001	0.6358	0.8457
NC_005120.4_15500001	X	15500001	0.5901	0.8183
NC_005120.4_15600001	X	15600001	0.5474	0.7735
NC_005120.4_15700001	X	15700001	0.5081	0.7677
NC_005120.4_15800001	X	15800001	0.4385	0.672
NC_005120.4_15900001	X	15900001	0.4863	0.6909
NC_005120.4_16000001	X	16000001	0.4381	0.669
NC_005120.4_16100001	X	16100001	0.4381	0.669
NC_005120.4_16500001	X	16500001	0.4343	0.5257
NC_005120.4_16600001	X	16600001	0.4343	0.5257
NC_005120.4_16700001	X	16700001	0.3971	0.5455
NC_005120.4_16800001	X	16800001	0.4458	0.6236
NC_005120.4_16900001	X	16900001	0.3322	0.5838
NC_005120.4_17100001	X	17100001	0.5854	0.6828
NC_005120.4_17200001	X	17200001	0.6559	0.7984
NC_005120.4_17300001	X	17300001	0.7087	0.8278
NC_005120.4_17400001	X	17400001	0.6572	0.752
NC_005120.4_17500001	X	17500001	0.5812	0.6071
NC_005120.4_17600001	X	17600001	0.6061	0.6171
NC_005120.4_17700001	X	17700001	0.5578	0.5453
NC_005120.4_17800001	X	17800001	0.5482	0.5285
NC_005120.4_17900001	X	17900001	0.5617	0.5337
NC_005120.4_18000001	X	18000001	0.8182	0.936
NC_005120.4_18100001	X	18100001	0.8182	0.936
NC_005120.4_18400001	X	18400001	0.7854	0.9779
NC_005120.4_18800001	X	18800001	0.7811	0.9657
NC_005120.4_18900001	X	18900001	0.5956	0.8419
NC_005120.4_19000001	X	19000001	0.6545	0.8761
NC_005120.4_19100001	X	19100001	0.6545	0.8761
NC_005120.4_19200001	X	19200001	0.7255	0.9045
NC_005120.4_19300001	X	19300001	0.5927	0.8467
NC_005120.4_19500001	X	19500001	0.9158	0.8396
NC_005120.4_19600001	X	19600001	0.939	0.8826
NC_005120.4_19700001	X	19700001	0.9185	0.8465
NC_005120.4_19800001	X	19800001	0.9185	0.8465
NC_005120.4_19900001	X	19900001	0.9593	0.9252
NC_005120.4_20800001	X	20800001	0.711	0.795
NC_005120.4_21400001	X	21400001	0.9519	0.9432
NC_005120.4_21500001	X	21500001	0.9519	0.9432
NC_005120.4_21600001	X	21600001	0.9519	0.9432
NC_005120.4_21700001	X	21700001	0.9344	0.9235
NC_005120.4_21800001	X	21800001	0.8421	0.8405
NC_005120.4_21900001	X	21900001	0.5904	0.6509
NC_005120.4_22000001	X	22000001	0.5904	0.6509

NC_005120.4_22100001	X	22100001	0.5904	0.6509
NC_005120.4_22200001	X	22200001	0.5096	0.6222
NC_005120.4_25300001	X	25300001	0.5902	0.705
NC_005120.4_25400001	X	25400001	0.5504	0.6915
NC_005120.4_25500001	X	25500001	0.5542	0.6985
NC_005120.4_25600001	X	25600001	0.5825	0.7322
NC_005120.4_25700001	X	25700001	0.5825	0.7322
NC_005120.4_26500001	X	26500001	0.2422	0.3216
NC_005120.4_26600001	X	26600001	0.267	0.3045
NC_005120.4_26700001	X	26700001	0.392	0.4717
NC_005120.4_26800001	X	26800001	0.3605	0.5361
NC_005120.4_26900001	X	26900001	0.4021	0.5531
NC_005120.4_27000001	X	27000001	0.4968	0.5553
NC_005120.4_27200001	X	27200001	0.6236	0.7296
NC_005120.4_27300001	X	27300001	0.624	0.7635
NC_005120.4_27400001	X	27400001	0.6671	0.8008
NC_005120.4_27500001	X	27500001	0.769	0.8973
NC_005120.4_27600001	X	27600001	0.896	0.9333
NC_005120.4_27700001	X	27700001	0.8099	0.8657
NC_005120.4_27800001	X	27800001	0.7631	0.8622
NC_005120.4_27900001	X	27900001	0.7492	0.8524
NC_005120.4_28000001	X	28000001	0.7466	0.8107
NC_005120.4_28100001	X	28100001	0.5699	0.6466
NC_005120.4_28200001	X	28200001	0.4831	0.6872
NC_005120.4_28300001	X	28300001	0.6102	0.6204
NC_005120.4_28400001	X	28400001	0.6102	0.6204
NC_005120.4_28900001	X	28900001	0.6136	0.6576
NC_005120.4_29000001	X	29000001	0.7129	0.7529
NC_005120.4_29100001	X	29100001	0.6798	0.7135
NC_005120.4_29200001	X	29200001	0.6983	0.7215
NC_005120.4_29300001	X	29300001	0.7029	0.6957
NC_005120.4_29400001	X	29400001	0.8506	0.8181
NC_005120.4_29600001	X	29600001	0.7652	0.777
NC_005120.4_30100001	X	30100001	0.7903	0.9469
NC_005120.4_30200001	X	30200001	0.7903	0.9469
NC_005120.4_30300001	X	30300001	0.5682	0.8947
NC_005120.4_30400001	X	30400001	0.5682	0.8947
NC_005120.4_30500001	X	30500001	0.399	0.8084
NC_005120.4_30600001	X	30600001	0.4898	0.596
NC_005120.4_30700001	X	30700001	0.6471	0.6428
NC_005120.4_30800001	X	30800001	0.6711	0.6515
NC_005120.4_30900001	X	30900001	0.6711	0.6515
NC_005120.4_31000001	X	31000001	0.6711	0.6515
NC_005120.4_31100001	X	31100001	0.6259	0.6511
NC_005120.4_31200001	X	31200001	0.2539	0.4919
NC_005120.4_31300001	X	31300001	0.4018	0.6661
NC_005120.4_31400001	X	31400001	0.4018	0.6661
NC_005120.4_31500001	X	31500001	0.4711	0.5874
NC_005120.4_31600001	X	31600001	0.4247	0.5609
NC_005120.4_31700001	X	31700001	0.5796	0.6723
NC_005120.4_31800001	X	31800001	0.5368	0.5853
NC_005120.4_31900001	X	31900001	0.5695	0.6206
NC_005120.4_32000001	X	32000001	0.5485	0.7163
NC_005120.4_32100001	X	32100001	0.548	0.7484
NC_005120.4_32200001	X	32200001	0.5786	0.7545
NC_005120.4_32300001	X	32300001	0.6241	0.8004
NC_005120.4_32400001	X	32400001	0.6023	0.7895
NC_005120.4_32500001	X	32500001	0.6623	0.7993

NC_005120.4_32600001	X	32600001	0.6991	0.695
NC_005120.4_32700001	X	32700001	0.7262	0.7246
NC_005120.4_32800001	X	32800001	0.6623	0.6525
NC_005120.4_32900001	X	32900001	0.6039	0.6748
NC_005120.4_33000001	X	33000001	0.5742	0.6412
NC_005120.4_33100001	X	33100001	0.583	0.7259
NC_005120.4_33200001	X	33200001	0.5485	0.6916
NC_005120.4_33300001	X	33300001	0.5717	0.9159
NC_005120.4_33400001	X	33400001	0.6771	0.7822
NC_005120.4_33500001	X	33500001	0.6771	0.7822
NC_005120.4_33600001	X	33600001	0.805	0.8103
NC_005120.4_33700001	X	33700001	0.7869	0.8579
NC_005120.4_33800001	X	33800001	0.6939	0.7759
NC_005120.4_33900001	X	33900001	0.7243	0.8386
NC_005120.4_34000001	X	34000001	0.6791	0.781
NC_005120.4_34100001	X	34100001	0.6374	0.7265
NC_005120.4_34200001	X	34200001	0.5408	0.5964
NC_005120.4_34300001	X	34300001	0.6211	0.6556
NC_005120.4_34400001	X	34400001	0.6211	0.6556
NC_005120.4_34500001	X	34500001	0.5199	0.6012
NC_005120.4_34600001	X	34600001	0.6869	0.7846
NC_005120.4_34700001	X	34700001	0.7846	0.874
NC_005120.4_34800001	X	34800001	0.7332	0.8896
NC_005120.4_34900001	X	34900001	0.6124	0.8741
NC_005120.4_35000001	X	35000001	0.6498	0.8777
NC_005120.4_35700001	X	35700001	0.3094	0.4883
NC_005120.4_35800001	X	35800001	0.3094	0.4883
NC_005120.4_36000001	X	36000001	0.3791	0.4763
NC_005120.4_36100001	X	36100001	0.4273	0.6666
NC_005120.4_36200001	X	36200001	0.549	0.7285
NC_005120.4_36300001	X	36300001	0.549	0.7285
NC_005120.4_36400001	X	36400001	0.6671	0.7356
NC_005120.4_36500001	X	36500001	0.6515	0.726
NC_005120.4_36600001	X	36600001	0.656	0.7219
NC_005120.4_36700001	X	36700001	0.61	0.674
NC_005120.4_36800001	X	36800001	0.5395	0.6668
NC_005120.4_36900001	X	36900001	0.3087	0.5222
NC_005120.4_37100001	X	37100001	0.5918	0.93
NC_005120.4_37200001	X	37200001	0.657	0.9463
NC_005120.4_37300001	X	37300001	0.794	0.9584
NC_005120.4_37400001	X	37400001	0.6712	0.9308
NC_005120.4_37500001	X	37500001	0.5912	0.7252
NC_005120.4_37600001	X	37600001	0.4639	0.5194
NC_005120.4_37700001	X	37700001	0.4021	0.4562
NC_005120.4_37800001	X	37800001	0.4021	0.4562
NC_005120.4_37900001	X	37900001	0.5301	0.6298
NC_005120.4_38000001	X	38000001	0.5757	0.7816
NC_005120.4_38100001	X	38100001	0.5275	0.7431
NC_005120.4_38200001	X	38200001	0.5181	0.7584
NC_005120.4_38300001	X	38300001	0.5994	0.8205
NC_005120.4_38400001	X	38400001	0.7987	0.85
NC_005120.4_38500001	X	38500001	0.8261	0.852
NC_005120.4_38600001	X	38600001	0.8794	0.8769
NC_005120.4_38700001	X	38700001	0.8463	0.8616
NC_005120.4_38800001	X	38800001	0.8367	0.8525
NC_005120.4_38900001	X	38900001	0.7546	0.8026
NC_005120.4_39400001	X	39400001	0.7453	0.8595
NC_005120.4_39500001	X	39500001	0.7453	0.8595

NC_005120.4_39600001	X	39600001	0.6589	0.7719
NC_005120.4_39700001	X	39700001	0.6589	0.7719
NC_005120.4_39800001	X	39800001	0.6064	0.7455
NC_005120.4_39900001	X	39900001	0.4366	0.5198
NC_005120.4_40000001	X	40000001	0.4366	0.5198
NC_005120.4_41900001	X	41900001	0.9893	0.9763
NC_005120.4_42000001	X	42000001	0.8095	0.9489
NC_005120.4_42100001	X	42100001	0.8151	0.9496
NC_005120.4_42200001	X	42200001	0.7804	0.941
NC_005120.4_43100001	X	43100001	0.8206	0.7555
NC_005120.4_43200001	X	43200001	0.8206	0.7555
NC_005120.4_44000001	X	44000001	0.8969	0.8594
NC_005120.4_44100001	X	44100001	0.8253	0.9079
NC_005120.4_44200001	X	44200001	0.8346	0.9861
NC_005120.4_44300001	X	44300001	0.8275	0.9852
NC_005120.4_44400001	X	44400001	0.8275	0.9852
NC_005120.4_44500001	X	44500001	0.8231	0.9679
NC_005120.4_44600001	X	44600001	0.8689	0.926
NC_005120.4_44700001	X	44700001	0.8863	0.9363
NC_005120.4_44800001	X	44800001	0.9408	0.9053
NC_005120.4_44900001	X	44900001	0.9408	0.9053
NC_005120.4_45000001	X	45000001	0.9595	0.9419
NC_005120.4_45100001	X	45100001	0.9985	0.9967
NC_005120.4_46300001	X	46300001	0.7777	0.7203
NC_005120.4_46400001	X	46400001	0.673	0.6966
NC_005120.4_46500001	X	46500001	0.5313	0.6804
NC_005120.4_46600001	X	46600001	0.4745	0.625
NC_005120.4_46700001	X	46700001	0.3839	0.5063
NC_005120.4_46800001	X	46800001	0.2832	0.709
NC_005120.4_47100001	X	47100001	0.0886	0.0771
NC_005120.4_47200001	X	47200001	0.4238	0.7522
NC_005120.4_47300001	X	47300001	0.4173	0.7491
NC_005120.4_47400001	X	47400001	0.4762	0.7836
NC_005120.4_47500001	X	47500001	0.4762	0.7836
NC_005120.4_47600001	X	47600001	0.8542	0.9634
NC_005120.4_47800001	X	47800001	0.9914	0.9816
NC_005120.4_48800001	X	48800001	0.5811	0.6804
NC_005120.4_48900001	X	48900001	0.5811	0.6804
NC_005120.4_49000001	X	49000001	0.5811	0.6804
NC_005120.4_49100001	X	49100001	0.5811	0.6804
NC_005120.4_49200001	X	49200001	0.5719	0.7418
NC_005120.4_50200001	X	50200001	0.7973	0.7056
NC_005120.4_50300001	X	50300001	0.7561	0.6477
NC_005120.4_50400001	X	50400001	0.7993	0.7046
NC_005120.4_50500001	X	50500001	0.7554	0.6717
NC_005120.4_50600001	X	50600001	0.7016	0.7084
NC_005120.4_50700001	X	50700001	0.6948	0.8977
NC_005120.4_50800001	X	50800001	0.7283	0.9084
NC_005120.4_50900001	X	50900001	0.7659	0.9229
NC_005120.4_51000001	X	51000001	0.7207	0.9197
NC_005120.4_51100001	X	51100001	0.7679	0.9176
NC_005120.4_51200001	X	51200001	0.8478	0.9268
NC_005120.4_51300001	X	51300001	0.7365	0.9113
NC_005120.4_51400001	X	51400001	0.4272	0.7711
NC_005120.4_51600001	X	51600001	0.6724	0.9418
NC_005120.4_51700001	X	51700001	0.708	0.9542
NC_005120.4_51800001	X	51800001	0.7897	0.9666
NC_005120.4_51900001	X	51900001	0.997	0.9934

NC_005120.4_52000001	X	52000001	0.9964	0.993
NC_005120.4_52100001	X	52100001	0.998	0.9966
NC_005120.4_52400001	X	52400001	0.6123	0.845
NC_005120.4_52700001	X	52700001	0.3868	0.5477
NC_005120.4_52800001	X	52800001	0.4764	0.6508
NC_005120.4_53100001	X	53100001	0.6668	0.7631
NC_005120.4_54000001	X	54000001	0.5863	0.8082
NC_005120.4_54100001	X	54100001	0.5316	0.7129
NC_005120.4_54200001	X	54200001	0.5316	0.7129
NC_005120.4_54300001	X	54300001	0.5367	0.7325
NC_005120.4_54400001	X	54400001	0.4706	0.6649
NC_005120.4_54500001	X	54500001	0.4764	0.6527
NC_005120.4_54600001	X	54600001	0.5212	0.764
NC_005120.4_54700001	X	54700001	0.6136	0.8311
NC_005120.4_54900001	X	54900001	0.5275	0.7705
NC_005120.4_55700001	X	55700001	0.6295	0.5462
NC_005120.4_55800001	X	55800001	0.6295	0.5462
NC_005120.4_55900001	X	55900001	0.6273	0.5491
NC_005120.4_56800001	X	56800001	0.6516	0.7625
NC_005120.4_56900001	X	56900001	0.6517	0.838
NC_005120.4_57000001	X	57000001	0.6559	0.8403
NC_005120.4_57100001	X	57100001	0.5703	0.7823
NC_005120.4_57200001	X	57200001	0.7616	0.8609
NC_005120.4_57300001	X	57300001	0.996	0.9937
NC_005120.4_57400001	X	57400001	0.996	0.9937
NC_005120.4_58400001	X	58400001	0.4681	0.7272
NC_005120.4_58500001	X	58500001	0.6807	0.8459
NC_005120.4_58600001	X	58600001	0.6807	0.8459
NC_005120.4_58700001	X	58700001	0.619	0.8018
NC_005120.4_60200001	X	60200001	0.6815	0.8551
NC_005120.4_60300001	X	60300001	0.6815	0.8551
NC_005120.4_60400001	X	60400001	0.7024	0.8043
NC_005120.4_60600001	X	60600001	0.6719	0.7865
NC_005120.4_61000001	X	61000001	0.5057	0.94
NC_005120.4_61100001	X	61100001	0.4215	0.9169
NC_005120.4_61200001	X	61200001	0.4215	0.9169
NC_005120.4_61300001	X	61300001	0.4215	0.9169
NC_005120.4_61400001	X	61400001	0.582	0.9525
NC_005120.4_61900001	X	61900001	0.6419	0.6733
NC_005120.4_62000001	X	62000001	0.7414	0.7772
NC_005120.4_62100001	X	62100001	0.7784	0.815
NC_005120.4_62200001	X	62200001	0.7784	0.815
NC_005120.4_62300001	X	62300001	0.8649	0.9681
NC_005120.4_62400001	X	62400001	0.8286	0.9602
NC_005120.4_62500001	X	62500001	0.648	0.8775
NC_005120.4_62600001	X	62600001	0.7069	0.9052
NC_005120.4_62700001	X	62700001	0.7448	0.9174
NC_005120.4_62800001	X	62800001	0.6744	0.9053
NC_005120.4_62900001	X	62900001	0.6747	0.902
NC_005120.4_63000001	X	63000001	0.7632	0.9482
NC_005120.4_63100001	X	63100001	0.6975	0.93
NC_005120.4_63200001	X	63200001	0.4862	0.9053
NC_005120.4_63300001	X	63300001	0.5621	0.9166
NC_005120.4_63400001	X	63400001	0.4974	0.9151
NC_005120.4_63600001	X	63600001	0.3214	0.603
NC_005120.4_63700001	X	63700001	0.7547	0.8331
NC_005120.4_63800001	X	63800001	0.7547	0.8331
NC_005120.4_63900001	X	63900001	0.8003	0.835

NC_005120.4_64000001	X	64000001	0.8003	0.835
NC_005120.4_64100001	X	64100001	0.8704	0.8864
NC_005120.4_64200001	X	64200001	0.4574	0.7251
NC_005120.4_64300001	X	64300001	0.4574	0.7251
NC_005120.4_64500001	X	64500001	0.0602	0.0976
NC_005120.4_64600001	X	64600001	0.3986	0.8276
NC_005120.4_64700001	X	64700001	0.5362	0.843
NC_005120.4_64800001	X	64800001	0.5362	0.843
NC_005120.4_64900001	X	64900001	0.6972	0.9013
NC_005120.4_65000001	X	65000001	0.7799	0.9094
NC_005120.4_65100001	X	65100001	0.7641	0.891
NC_005120.4_65200001	X	65200001	0.7495	0.8898
NC_005120.4_65300001	X	65300001	0.815	0.9239
NC_005120.4_65400001	X	65400001	0.7139	0.8702
NC_005120.4_67000001	X	67000001	0.79	0.8863
NC_005120.4_67100001	X	67100001	0.79	0.8863
NC_005120.4_67200001	X	67200001	0.838	0.9185
NC_005120.4_67300001	X	67300001	0.8312	0.9144
NC_005120.4_67400001	X	67400001	0.8492	0.9885
NC_005120.4_67600001	X	67600001	0.9979	0.9967
NC_005120.4_68100001	X	68100001	0.6702	0.8426
NC_005120.4_68200001	X	68200001	0.6702	0.8426
NC_005120.4_68300001	X	68300001	0.6368	0.7577
NC_005120.4_68400001	X	68400001	0.6812	0.795
NC_005120.4_68500001	X	68500001	0.7319	0.8178
NC_005120.4_68700001	X	68700001	0.81	0.7973
NC_005120.4_68800001	X	68800001	0.6792	0.8783
NC_005120.4_68900001	X	68900001	0.6116	0.842
NC_005120.4_69000001	X	69000001	0.6565	0.9121
NC_005120.4_69100001	X	69100001	0.6088	0.9162
NC_005120.4_69200001	X	69200001	0.5917	0.9042
NC_005120.4_69300001	X	69300001	0.6435	0.8361
NC_005120.4_69400001	X	69400001	0.6654	0.8421
NC_005120.4_69500001	X	69500001	0.6654	0.8421
NC_005120.4_69600001	X	69600001	0.6729	0.7409
NC_005120.4_69700001	X	69700001	0.7205	0.7935
NC_005120.4_69800001	X	69800001	0.6994	0.8682
NC_005120.4_69900001	X	69900001	0.6994	0.8682
NC_005120.4_70000001	X	70000001	0.7129	0.8419
NC_005120.4_70100001	X	70100001	0.75	0.8857
NC_005120.4_70200001	X	70200001	0.7816	0.88
NC_005120.4_70300001	X	70300001	0.8536	0.8788
NC_005120.4_70400001	X	70400001	0.8536	0.8788
NC_005120.4_70600001	X	70600001	0.8871	0.9776
NC_005120.4_70700001	X	70700001	0.8851	0.9772
NC_005120.4_70800001	X	70800001	0.8607	0.9597
NC_005120.4_70900001	X	70900001	0.8271	0.9606
NC_005120.4_71000001	X	71000001	0.8367	0.9634
NC_005120.4_71100001	X	71100001	0.8294	0.9572
NC_005120.4_71200001	X	71200001	0.842	0.9577
NC_005120.4_71300001	X	71300001	0.7325	0.9402
NC_005120.4_71400001	X	71400001	0.5308	0.6877
NC_005120.4_71500001	X	71500001	0.4401	0.5975
NC_005120.4_71600001	X	71600001	0.4243	0.5588
NC_005120.4_71700001	X	71700001	0.3689	0.506
NC_005120.4_71800001	X	71800001	0.3505	0.4455
NC_005120.4_71900001	X	71900001	0.7091	0.8275
NC_005120.4_72000001	X	72000001	0.7833	0.8503

NC_005120.4_72100001	X	72100001	0.835	0.8922
NC_005120.4_72200001	X	72200001	0.9059	0.8876
NC_005120.4_72300001	X	72300001	0.9059	0.8876
NC_005120.4_72900001	X	72900001	0.4026	0.8575
NC_005120.4_73000001	X	73000001	0.524	0.5906
NC_005120.4_73100001	X	73100001	0.4621	0.5101
NC_005120.4_73200001	X	73200001	0.5181	0.5812
NC_005120.4_73300001	X	73300001	0.5181	0.5812
NC_005120.4_73400001	X	73400001	0.6761	0.6053
NC_005120.4_73900001	X	73900001	0.7565	0.8297
NC_005120.4_74000001	X	74000001	0.7134	0.7605
NC_005120.4_74100001	X	74100001	0.7655	0.7738
NC_005120.4_74200001	X	74200001	0.785	0.7841
NC_005120.4_74300001	X	74300001	0.7379	0.7788
NC_005120.4_74400001	X	74400001	0.6574	0.6893
NC_005120.4_75100001	X	75100001	0.7009	0.6941
NC_005120.4_75200001	X	75200001	0.7009	0.6941
NC_005120.4_75300001	X	75300001	0.4844	0.5924
NC_005120.4_75400001	X	75400001	0.595	0.6928
NC_005120.4_75500001	X	75500001	0.6258	0.7492
NC_005120.4_75600001	X	75600001	0.7042	0.8429
NC_005120.4_75700001	X	75700001	0.7042	0.8429
NC_005120.4_75800001	X	75800001	0.9333	0.901
NC_005120.4_75900001	X	75900001	0.9312	0.8881
NC_005120.4_76000001	X	76000001	0.7872	0.802
NC_005120.4_76100001	X	76100001	0.6223	0.6995
NC_005120.4_76200001	X	76200001	0.682	0.7582
NC_005120.4_76300001	X	76300001	0.7044	0.7799
NC_005120.4_76400001	X	76400001	0.5814	0.7048
NC_005120.4_76500001	X	76500001	0.7718	0.8649
NC_005120.4_76600001	X	76600001	0.9011	0.9618
NC_005120.4_76700001	X	76700001	0.8992	0.962
NC_005120.4_76800001	X	76800001	0.8997	0.9642
NC_005120.4_76900001	X	76900001	0.7814	0.908
NC_005120.4_77000001	X	77000001	0.6722	0.8181
NC_005120.4_77100001	X	77100001	0.6545	0.8155
NC_005120.4_77200001	X	77200001	0.5788	0.7587
NC_005120.4_77300001	X	77300001	0.4515	0.6294
NC_005120.4_77400001	X	77400001	0.5003	0.6862
NC_005120.4_77500001	X	77500001	0.5084	0.7488
NC_005120.4_78100001	X	78100001	0.5131	0.6309
NC_005120.4_78300001	X	78300001	0.4132	0.5344
NC_005120.4_78400001	X	78400001	0.3852	0.5599
NC_005120.4_78500001	X	78500001	0.2909	0.5092
NC_005120.4_78600001	X	78600001	0.2352	0.5706
NC_005120.4_78700001	X	78700001	0.2135	0.5559
NC_005120.4_78800001	X	78800001	0.2271	0.5626
NC_005120.4_78900001	X	78900001	0.4145	0.7237
NC_005120.4_79000001	X	79000001	0.4145	0.7237
NC_005120.4_79100001	X	79100001	0.5202	0.7405
NC_005120.4_79200001	X	79200001	0.6005	0.7543
NC_005120.4_79300001	X	79300001	0.6938	0.77
NC_005120.4_80300001	X	80300001	0.9937	0.9881
NC_005120.4_80400001	X	80400001	0.8059	0.8484
NC_005120.4_80500001	X	80500001	0.8019	0.845
NC_005120.4_80600001	X	80600001	0.6876	0.8196
NC_005120.4_80700001	X	80700001	0.6876	0.8196
NC_005120.4_80800001	X	80800001	0.5709	0.7274

NC_005120.4_81400001	X	81400001	0.5835	0.8718
NC_005120.4_81500001	X	81500001	0.5615	0.866
NC_005120.4_81600001	X	81600001	0.5615	0.866
NC_005120.4_81700001	X	81700001	0.7942	0.9572
NC_005120.4_81800001	X	81800001	0.7942	0.9572
NC_005120.4_81900001	X	81900001	0.8506	0.964
NC_005120.4_82000001	X	82000001	0.8527	0.9655
NC_005120.4_82100001	X	82100001	0.8674	0.9694
NC_005120.4_82200001	X	82200001	0.6814	0.9722
NC_005120.4_82300001	X	82300001	0.6814	0.9722
NC_005120.4_82400001	X	82400001	0.6264	0.9148
NC_005120.4_82500001	X	82500001	0.5708	0.8932
NC_005120.4_82600001	X	82600001	0.5809	0.8874
NC_005120.4_83700001	X	83700001	0.6824	0.8506
NC_005120.4_83800001	X	83800001	0.7647	0.8956
NC_005120.4_83900001	X	83900001	0.7952	0.9107
NC_005120.4_84000001	X	84000001	0.7952	0.9107
NC_005120.4_84100001	X	84100001	0.7657	0.8353
NC_005120.4_84300001	X	84300001	0.7785	0.7354
NC_005120.4_84500001	X	84500001	0.5883	0.6459
NC_005120.4_84600001	X	84600001	0.733	0.8518
NC_005120.4_84700001	X	84700001	0.6775	0.7976
NC_005120.4_84800001	X	84800001	0.6317	0.8075
NC_005120.4_84900001	X	84900001	0.644	0.7611
NC_005120.4_85000001	X	85000001	0.7247	0.7683
NC_005120.4_85100001	X	85100001	0.6217	0.6557
NC_005120.4_85500001	X	85500001	0.4143	0.707
NC_005120.4_85600001	X	85600001	0.6416	0.8705
NC_005120.4_85700001	X	85700001	0.6425	0.8589
NC_005120.4_85800001	X	85800001	0.6928	0.8846
NC_005120.4_85900001	X	85900001	0.7472	0.9387
NC_005120.4_86000001	X	86000001	0.8899	0.9554
NC_005120.4_86100001	X	86100001	0.8223	0.9256
NC_005120.4_86400001	X	86400001	0.7294	0.865
NC_005120.4_86500001	X	86500001	0.6638	0.8054
NC_005120.4_86600001	X	86600001	0.6638	0.8054
NC_005120.4_86700001	X	86700001	0.6143	0.8374
NC_005120.4_86800001	X	86800001	0.5356	0.7804
NC_005120.4_86900001	X	86900001	0.3618	0.7084
NC_005120.4_87000001	X	87000001	0.3616	0.7881
NC_005120.4_87100001	X	87100001	0.4501	0.8361
NC_005120.4_88700001	X	88700001	0.5259	0.6835
NC_005120.4_88800001	X	88800001	0.5135	0.7275
NC_005120.4_88900001	X	88900001	0.5135	0.7275
NC_005120.4_89000001	X	89000001	0.5498	0.6922
NC_005120.4_89100001	X	89100001	0.4745	0.5395
NC_005120.4_89200001	X	89200001	0.4967	0.4759
NC_005120.4_89300001	X	89300001	0.5246	0.4887
NC_005120.4_89400001	X	89400001	0.5246	0.4887
NC_005120.4_89500001	X	89500001	0.5891	0.5736
NC_005120.4_89600001	X	89600001	0.7306	0.6982
NC_005120.4_89900001	X	89900001	0.8905	0.8274
NC_005120.4_90100001	X	90100001	0.8934	0.8366
NC_005120.4_90200001	X	90200001	0.8172	0.9371
NC_005120.4_90300001	X	90300001	0.8172	0.9371
NC_005120.4_90600001	X	90600001	0.5292	0.7894
NC_005120.4_90800001	X	90800001	0.6373	0.6907
NC_005120.4_90900001	X	90900001	0.6373	0.6907

NC_005120.4_91000001	X	91000001	0.6373	0.6907
NC_005120.4_91300001	X	91300001	0.9199	0.9064
NC_005120.4_91400001	X	91400001	0.9006	0.8743
NC_005120.4_91500001	X	91500001	0.9125	0.8896
NC_005120.4_91600001	X	91600001	0.7593	0.8521
NC_005120.4_91700001	X	91700001	0.7405	0.8633
NC_005120.4_91800001	X	91800001	0.6552	0.8443
NC_005120.4_91900001	X	91900001	0.7468	0.9182
NC_005120.4_92000001	X	92000001	0.7682	0.9244
NC_005120.4_92100001	X	92100001	0.8405	0.9644
NC_005120.4_92200001	X	92200001	0.7836	0.8564
NC_005120.4_92300001	X	92300001	0.7508	0.8337
NC_005120.4_92400001	X	92400001	0.6178	0.7171
NC_005120.4_92500001	X	92500001	0.4906	0.5736
NC_005120.4_92600001	X	92600001	0.5748	0.5842
NC_005120.4_92700001	X	92700001	0.6397	0.8448
NC_005120.4_92900001	X	92900001	0.8508	0.9296
NC_005120.4_93000001	X	93000001	0.8508	0.9296
NC_005120.4_93200001	X	93200001	0.6273	0.9307
NC_005120.4_93300001	X	93300001	0.6273	0.9307
NC_005120.4_94600001	X	94600001	0.5569	0.7356
NC_005120.4_94700001	X	94700001	0.5773	0.9102
NC_005120.4_94800001	X	94800001	0.5085	0.8908
NC_005120.4_94900001	X	94900001	0.619	0.9362
NC_005120.4_95000001	X	95000001	0.619	0.9362
NC_005120.4_95500001	X	95500001	0.5261	0.7707
NC_005120.4_95600001	X	95600001	0.4644	0.7107
NC_005120.4_95900001	X	95900001	0.6495	0.8561
NC_005120.4_96100001	X	96100001	0.7567	0.8462
NC_005120.4_96200001	X	96200001	0.7567	0.8462
NC_005120.4_96300001	X	96300001	0.7567	0.8462
NC_005120.4_97400001	X	97400001	0.6766	0.929
NC_005120.4_97500001	X	97500001	0.5289	0.8643
NC_005120.4_97600001	X	97600001	0.6005	0.8818
NC_005120.4_97700001	X	97700001	0.5094	0.9011
NC_005120.4_97800001	X	97800001	0.5578	0.9126
NC_005120.4_97900001	X	97900001	0.5067	0.8795
NC_005120.4_98900001	X	98900001	0.8612	0.9681
NC_005120.4_99000001	X	99000001	0.8612	0.9681
NC_005120.4_99100001	X	99100001	0.8612	0.9681
NC_005120.4_99200001	X	99200001	0.8443	0.966
NC_005120.4_99300001	X	99300001	0.8104	0.9573
NC_005120.4_101600001	X	101600001	0.3426	0.4219
NC_005120.4_101700001	X	101700001	0.2987	0.4128
NC_005120.4_101800001	X	101800001	0.2987	0.4128
NC_005120.4_101900001	X	101900001	0.3265	0.3971
NC_005120.4_102000001	X	102000001	0.4832	0.7279
NC_005120.4_102400001	X	102400001	0.6771	0.835
NC_005120.4_102500001	X	102500001	0.5583	0.8009
NC_005120.4_102600001	X	102600001	0.5188	0.7481
NC_005120.4_102700001	X	102700001	0.5202	0.8453
NC_005120.4_102800001	X	102800001	0.5754	0.8728
NC_005120.4_103900001	X	103900001	0.7516	0.8645
NC_005120.4_104000001	X	104000001	0.6015	0.8042
NC_005120.4_104100001	X	104100001	0.5959	0.7035
NC_005120.4_104200001	X	104200001	0.5396	0.6319
NC_005120.4_104300001	X	104300001	0.5383	0.667
NC_005120.4_104400001	X	104400001	0.6218	0.7635

NC_005120.4_104500001	X	104500001	0.6869	0.7889
NC_005120.4_104600001	X	104600001	0.6921	0.8677
NC_005120.4_104700001	X	104700001	0.6337	0.855
NC_005120.4_104800001	X	104800001	0.6533	0.777
NC_005120.4_104900001	X	104900001	0.578	0.6753
NC_005120.4_105000001	X	105000001	0.6076	0.6972
NC_005120.4_105100001	X	105100001	0.5875	0.6446
NC_005120.4_105200001	X	105200001	0.6649	0.6627
NC_005120.4_105300001	X	105300001	0.5719	0.6599
NC_005120.4_105400001	X	105400001	0.6484	0.7498
NC_005120.4_105500001	X	105500001	0.5473	0.6467
NC_005120.4_105600001	X	105600001	0.6931	0.8585
NC_005120.4_105700001	X	105700001	0.6931	0.8585
NC_005120.4_105800001	X	105800001	0.8256	0.9499
NC_005120.4_105900001	X	105900001	0.7844	0.9579
NC_005120.4_106000001	X	106000001	0.7844	0.9579
NC_005120.4_106100001	X	106100001	0.5807	0.8827
NC_005120.4_106200001	X	106200001	0.6568	0.9117
NC_005120.4_106300001	X	106300001	0.4636	0.8165
NC_005120.4_106400001	X	106400001	0.4855	0.8334
NC_005120.4_106500001	X	106500001	0.4855	0.8334
NC_005120.4_106600001	X	106600001	0.6127	0.8872
NC_005120.4_106700001	X	106700001	0.4519	0.7999
NC_005120.4_107200001	X	107200001	0.6642	0.8609
NC_005120.4_107300001	X	107300001	0.7624	0.9106
NC_005120.4_107400001	X	107400001	0.7121	0.886
NC_005120.4_107500001	X	107500001	0.8218	0.9856
NC_005120.4_107600001	X	107600001	0.6478	0.9061
NC_005120.4_107700001	X	107700001	0.6917	0.8969
NC_005120.4_108000001	X	108000001	0.5958	0.8495
NC_005120.4_108100001	X	108100001	0.5056	0.7835
NC_005120.4_108200001	X	108200001	0.5086	0.7965
NC_005120.4_108300001	X	108300001	0.462	0.789
NC_005120.4_108400001	X	108400001	0.3812	0.6805
NC_005120.4_108500001	X	108500001	0.2903	0.5617
NC_005120.4_109300001	X	109300001	0.5337	0.6317
NC_005120.4_109400001	X	109400001	0.5337	0.6317
NC_005120.4_109500001	X	109500001	0.5849	0.64
NC_005120.4_109600001	X	109600001	0.5849	0.64
NC_005120.4_109700001	X	109700001	0.3782	0.6188
NC_005120.4_109800001	X	109800001	0.4853	0.7956
NC_005120.4_109900001	X	109900001	0.4656	0.6701
NC_005120.4_110000001	X	110000001	0.5699	0.8309
NC_005120.4_110100001	X	110100001	0.5324	0.7811
NC_005120.4_110200001	X	110200001	0.6402	0.8058
NC_005120.4_110300001	X	110300001	0.6754	0.8301
NC_005120.4_110400001	X	110400001	0.7082	0.8939
NC_005120.4_110500001	X	110500001	0.6618	0.833
NC_005120.4_110700001	X	110700001	0.9833	0.9592
NC_005120.4_110900001	X	110900001	0.8272	0.8664
NC_005120.4_111000001	X	111000001	0.6786	0.7274
NC_005120.4_111100001	X	111100001	0.6786	0.7274
NC_005120.4_111300001	X	111300001	0.429	0.5358
NC_005120.4_111400001	X	111400001	0.4583	0.5862
NC_005120.4_111500001	X	111500001	0.5642	0.7201
NC_005120.4_111600001	X	111600001	0.5642	0.7201
NC_005120.4_111700001	X	111700001	0.6146	0.7646
NC_005120.4_111800001	X	111800001	0.6344	0.8556

NC_005120.4_111900001	X	111900001	0.722	0.8879
NC_005120.4_112000001	X	112000001	0.7991	0.9402
NC_005120.4_112100001	X	112100001	0.8144	0.9419
NC_005120.4_112200001	X	112200001	0.7978	0.9374
NC_005120.4_112300001	X	112300001	0.9619	0.9307
NC_005120.4_112400001	X	112400001	0.9484	0.9123
NC_005120.4_112500001	X	112500001	0.5098	0.5099
NC_005120.4_112600001	X	112600001	0.5151	0.5089
NC_005120.4_112700001	X	112700001	0.6357	0.7424
NC_005120.4_112800001	X	112800001	0.592	0.724
NC_005120.4_112900001	X	112900001	0.5665	0.6964
NC_005120.4_113000001	X	113000001	0.554	0.772
NC_005120.4_113100001	X	113100001	0.5171	0.7065
NC_005120.4_113200001	X	113200001	0.1582	0.0973
NC_005120.4_113300001	X	113300001	0.1718	0.1467
NC_005120.4_113400001	X	113400001	0.1759	0.1751
NC_005120.4_113500001	X	113500001	0.485	0.5743
NC_005120.4_113600001	X	113600001	0.5403	0.6461
NC_005120.4_113700001	X	113700001	0.6248	0.7236
NC_005120.4_113800001	X	113800001	0.8206	0.8214
NC_005120.4_113900001	X	113900001	0.7555	0.7975
NC_005120.4_114000001	X	114000001	0.7326	0.8204
NC_005120.4_114100001	X	114100001	0.7395	0.8475
NC_005120.4_114200001	X	114200001	0.5898	0.764
NC_005120.4_114300001	X	114300001	0.4994	0.6566
NC_005120.4_114400001	X	114400001	0.547	0.7038
NC_005120.4_114500001	X	114500001	0.3674	0.4801
NC_005120.4_114600001	X	114600001	0.664	0.7969
NC_005120.4_114700001	X	114700001	0.8527	0.8972
NC_005120.4_114800001	X	114800001	0.9887	0.9787
NC_005120.4_114900001	X	114900001	0.8013	0.9381
NC_005120.4_115000001	X	115000001	0.8013	0.9381
NC_005120.4_115100001	X	115100001	0.6263	0.8796
NC_005120.4_115800001	X	115800001	0.6325	0.7569
NC_005120.4_115900001	X	115900001	0.6557	0.8424
NC_005120.4_116000001	X	116000001	0.6557	0.8424
NC_005120.4_116100001	X	116100001	0.701	0.8211
NC_005120.4_116200001	X	116200001	0.701	0.8211
NC_005120.4_116300001	X	116300001	0.7765	0.863
NC_005120.4_116400001	X	116400001	0.503	0.6194
NC_005120.4_116500001	X	116500001	0.5158	0.6505
NC_005120.4_116600001	X	116600001	0.4781	0.6183
NC_005120.4_116700001	X	116700001	0.4781	0.6183
NC_005120.4_116800001	X	116800001	0.4307	0.5586
NC_005120.4_117700001	X	117700001	0.479	0.7496
NC_005120.4_118400001	X	118400001	0.7062	0.933
NC_005120.4_118500001	X	118500001	0.7062	0.933
NC_005120.4_118600001	X	118600001	0.8247	0.9554
NC_005120.4_118700001	X	118700001	0.6078	0.875
NC_005120.4_118800001	X	118800001	0.6078	0.875
NC_005120.4_118900001	X	118900001	0.3883	0.6069
NC_005120.4_119000001	X	119000001	0.3883	0.6069
NC_005120.4_119100001	X	119100001	0.3883	0.6069
NC_005120.4_119300001	X	119300001	0.3151	0.5
NC_005120.4_119700001	X	119700001	0.5666	0.8069
NC_005120.4_119800001	X	119800001	0.5854	0.7696
NC_005120.4_119900001	X	119900001	0.7688	0.9443
NC_005120.4_120000001	X	120000001	0.6703	0.8364

NC_005120.4_120100001	X	120100001	0.6695	0.8155
NC_005120.4_120200001	X	120200001	0.6395	0.7511
NC_005120.4_120300001	X	120300001	0.7459	0.8264
NC_005120.4_120400001	X	120400001	0.696	0.7833
NC_005120.4_120500001	X	120500001	0.8576	0.8838
NC_005120.4_121200001	X	121200001	0.7977	0.6932
NC_005120.4_121300001	X	121300001	0.6301	0.6575
NC_005120.4_121400001	X	121400001	0.577	0.652
NC_005120.4_121500001	X	121500001	0.5667	0.6889
NC_005120.4_121600001	X	121600001	0.5994	0.7222
NC_005120.4_121700001	X	121700001	0.526	0.8939
NC_005120.4_121800001	X	121800001	0.6823	0.9606
NC_005120.4_122100001	X	122100001	0.4784	0.4625
NC_005120.4_122200001	X	122200001	0.576	0.5472
NC_005120.4_122300001	X	122300001	0.6208	0.6003
NC_005120.4_122400001	X	122400001	0.6974	0.6869
NC_005120.4_122500001	X	122500001	0.7809	0.7454
NC_005120.4_122600001	X	122600001	0.8089	0.867
NC_005120.4_122700001	X	122700001	0.866	0.9612
NC_005120.4_122800001	X	122800001	0.7496	0.8646
NC_005120.4_122900001	X	122900001	0.7596	0.8292
NC_005120.4_123000001	X	123000001	0.7192	0.7976
NC_005120.4_123100001	X	123100001	0.6742	0.7829
NC_005120.4_123200001	X	123200001	0.5051	0.6715
NC_005120.4_123300001	X	123300001	0.573	0.7571
NC_005120.4_123400001	X	123400001	0.5622	0.7858
NC_005120.4_123500001	X	123500001	0.6032	0.8133
NC_005120.4_123600001	X	123600001	0.6014	0.8492
NC_005120.4_123700001	X	123700001	0.8318	0.9772
NC_005120.4_124300001	X	124300001	0.6557	0.7354
NC_005120.4_124400001	X	124400001	0.702	0.7666
NC_005120.4_124500001	X	124500001	0.6389	0.7418
NC_005120.4_124600001	X	124600001	0.6389	0.7418
NC_005120.4_124700001	X	124700001	0.7511	0.8522
NC_005120.4_124800001	X	124800001	0.6983	0.822
NC_005120.4_124900001	X	124900001	0.761	0.8668
NC_005120.4_125100001	X	125100001	0.771	0.8547
NC_005120.4_125200001	X	125200001	0.771	0.8547
NC_005120.4_125300001	X	125300001	0.771	0.8547
NC_005120.4_125400001	X	125400001	0.5836	0.6549
NC_005120.4_125500001	X	125500001	0.6944	0.7398
NC_005120.4_125600001	X	125600001	0.6745	0.7044
NC_005120.4_125700001	X	125700001	0.6745	0.7044
NC_005120.4_125800001	X	125800001	0.6745	0.7044
NC_005120.4_126700001	X	126700001	0.6224	0.8523
NC_005120.4_126800001	X	126800001	0.6224	0.8523
NC_005120.4_126900001	X	126900001	0.6224	0.8523
NC_005120.4_127100001	X	127100001	0.6795	0.9275
NC_005120.4_127200001	X	127200001	0.5935	0.6867
NC_005120.4_127300001	X	127300001	0.7031	0.7805
NC_005120.4_127400001	X	127400001	0.6917	0.7429
NC_005120.4_127500001	X	127500001	0.6917	0.7429
NC_005120.4_127600001	X	127600001	0.7142	0.7318
NC_005120.4_127700001	X	127700001	0.77	0.7772
NC_005120.4_128300001	X	128300001	0.629	0.7811
NC_005120.4_128400001	X	128400001	0.5572	0.7163
NC_005120.4_128500001	X	128500001	0.5572	0.7163
NC_005120.4_128600001	X	128600001	0.5105	0.736

NC_005120.4_128700001	X	128700001	0.5964	0.7765
NC_005120.4_128800001	X	128800001	0.5188	0.7718
NC_005120.4_128900001	X	128900001	0.5188	0.7718
NC_005120.4_129000001	X	129000001	0.5188	0.7718
NC_005120.4_129500001	X	129500001	0.4514	0.776
NC_005120.4_129600001	X	129600001	0.3953	0.7634
NC_005120.4_129700001	X	129700001	0.3953	0.7634
NC_005120.4_130400001	X	130400001	0.6212	0.6939
NC_005120.4_130500001	X	130500001	0.6584	0.7649
NC_005120.4_130600001	X	130600001	0.6584	0.7649
NC_005120.4_130700001	X	130700001	0.7266	0.8195
NC_005120.4_130800001	X	130800001	0.7424	0.9326
NC_005120.4_130900001	X	130900001	0.8584	0.9483
NC_005120.4_132100001	X	132100001	0.535	0.6476
NC_005120.4_132200001	X	132200001	0.558	0.787
NC_005120.4_132300001	X	132300001	0.673	0.7326
NC_005120.4_132400001	X	132400001	0.673	0.7326
NC_005120.4_132500001	X	132500001	0.5073	0.6263
NC_005120.4_132600001	X	132600001	0.425	0.4951
NC_005120.4_132700001	X	132700001	0.5605	0.638
NC_005120.4_132800001	X	132800001	0.5373	0.7982
NC_005120.4_132900001	X	132900001	0.5631	0.763
NC_005120.4_133000001	X	133000001	0.5495	0.7813
NC_005120.4_133100001	X	133100001	0.5495	0.7813
NC_005120.4_133200001	X	133200001	0.5219	0.7155
NC_005120.4_133300001	X	133300001	0.4702	0.6599
NC_005120.4_133400001	X	133400001	0.5558	0.7876
NC_005120.4_133500001	X	133500001	0.7878	0.8617
NC_005120.4_133600001	X	133600001	0.7895	0.8011
NC_005120.4_133700001	X	133700001	0.898	0.8168
NC_005120.4_133800001	X	133800001	0.7681	0.708
NC_005120.4_133900001	X	133900001	0.6356	0.5611
NC_005120.4_134000001	X	134000001	0.7001	0.6456
NC_005120.4_134100001	X	134100001	0.6125	0.6309
NC_005120.4_134200001	X	134200001	0.6323	0.6865
NC_005120.4_134300001	X	134300001	0.7562	0.8358
NC_005120.4_134400001	X	134400001	0.8158	0.8785
NC_005120.4_134500001	X	134500001	0.7396	0.8364
NC_005120.4_134600001	X	134600001	0.7412	0.8258
NC_005120.4_134700001	X	134700001	0.649	0.7886
NC_005120.4_134800001	X	134800001	0.5115	0.7347
NC_005120.4_134900001	X	134900001	0.4982	0.774
NC_005120.4_135000001	X	135000001	0.4283	0.7849
NC_005120.4_135100001	X	135100001	0.4651	0.8207
NC_005120.4_135200001	X	135200001	0.5143	0.828
NC_005120.4_135300001	X	135300001	0.7339	0.9158
NC_005120.4_135400001	X	135400001	0.8727	0.9791
NC_005120.4_135500001	X	135500001	0.6877	0.7136
NC_005120.4_135600001	X	135600001	0.6877	0.7136
NC_005120.4_135700001	X	135700001	0.6868	0.7139
NC_005120.4_135800001	X	135800001	0.4891	0.3946
NC_005120.4_135900001	X	135900001	0.5993	0.5594
NC_005120.4_136000001	X	136000001	0.6998	0.7664
NC_005120.4_136100001	X	136100001	0.6998	0.7664
NC_005120.4_136200001	X	136200001	0.7044	0.7829
NC_005120.4_136300001	X	136300001	0.7728	0.7744
NC_005120.4_137800001	X	137800001	0.8666	0.8229
NC_005120.4_137900001	X	137900001	0.8889	0.8525

NC_005120.4_138000001	X	138000001	0.97	0.9355
NC_005120.4_138100001	X	138100001	0.8844	0.8543
NC_005120.4_138200001	X	138200001	0.8312	0.8799
NC_005120.4_138300001	X	138300001	0.6649	0.851
NC_005120.4_138400001	X	138400001	0.5668	0.8275
NC_005120.4_138500001	X	138500001	0.5942	0.8446
NC_005120.4_138600001	X	138600001	0.567	0.9146
NC_005120.4_138700001	X	138700001	0.5725	0.8679
NC_005120.4_138800001	X	138800001	0.6278	0.8314
NC_005120.4_138900001	X	138900001	0.7078	0.8325
NC_005120.4_139000001	X	139000001	0.6799	0.8107
NC_005120.4_139100001	X	139100001	0.7081	0.8322
NC_005120.4_139200001	X	139200001	0.851	0.9109
NC_005120.4_139300001	X	139300001	0.9911	0.9787
NC_005120.4_139400001	X	139400001	0.9911	0.9787
NC_005120.4_139500001	X	139500001	0.879	0.9742
NC_005120.4_139600001	X	139600001	0.8901	0.9753
NC_005120.4_139700001	X	139700001	0.7726	0.9407
NC_005120.4_139800001	X	139800001	0.7726	0.9407
NC_005120.4_139900001	X	139900001	0.7971	0.9421
NC_005120.4_140000001	X	140000001	0.7357	0.7718
NC_005120.4_140100001	X	140100001	0.6039	0.7261
NC_005120.4_140200001	X	140200001	0.6027	0.715
NC_005120.4_140300001	X	140300001	0.6894	0.8247
NC_005120.4_140400001	X	140400001	0.6694	0.8137
NC_005120.4_140500001	X	140500001	0.7074	0.8803
NC_005120.4_140600001	X	140600001	0.8249	0.9004
NC_005120.4_140700001	X	140700001	0.8423	0.9123
NC_005120.4_140900001	X	140900001	0.6257	0.638
NC_005120.4_141000001	X	141000001	0.6683	0.7243
NC_005120.4_141100001	X	141100001	0.6683	0.7243
NC_005120.4_141200001	X	141200001	0.6743	0.7219
NC_005120.4_141300001	X	141300001	0.6291	0.6668
NC_005120.4_141400001	X	141400001	0.9012	0.9105
NC_005120.4_141500001	X	141500001	0.8042	0.8967
NC_005120.4_141600001	X	141600001	0.8042	0.8967
NC_005120.4_141700001	X	141700001	0.6815	0.8681
NC_005120.4_141800001	X	141800001	0.7649	0.9595
NC_005120.4_141900001	X	141900001	0.6894	0.9452
NC_005120.4_142000001	X	142000001	0.6878	0.9588
NC_005120.4_142100001	X	142100001	0.6878	0.9588
NC_005120.4_142200001	X	142200001	0.8177	0.9859
NC_005120.4_143300001	X	143300001	0.9896	0.9782
NC_005120.4_143400001	X	143400001	0.8365	0.9521
NC_005120.4_143500001	X	143500001	0.8783	0.9657
NC_005120.4_143600001	X	143600001	0.8925	0.9701
NC_005120.4_143700001	X	143700001	0.7176	0.9445
NC_005120.4_143800001	X	143800001	0.6793	0.9385
NC_005120.4_143900001	X	143900001	0.6624	0.922
NC_005120.4_144000001	X	144000001	0.5896	0.8946
NC_005120.4_144100001	X	144100001	0.4687	0.834
NC_005120.4_144900001	X	144900001	0.5856	0.8834
NC_005120.4_145000001	X	145000001	0.5856	0.8834
NC_005120.4_145100001	X	145100001	0.7395	0.9245
NC_005120.4_145200001	X	145200001	0.7839	0.9511
NC_005120.4_145300001	X	145300001	0.7839	0.9511
NC_005120.4_145400001	X	145400001	0.8942	0.9822
NC_005120.4_145500001	X	145500001	0.789	0.9348

NC_005120.4_145600001	X	145600001	0.7483	0.9214
NC_005120.4_145700001	X	145700001	0.7648	0.8977
NC_005120.4_145800001	X	145800001	0.8008	0.9148
NC_005120.4_145900001	X	145900001	0.7067	0.7725
NC_005120.4_146000001	X	146000001	0.7521	0.761
NC_005120.4_146100001	X	146100001	0.7564	0.7649
NC_005120.4_146200001	X	146200001	0.6965	0.6437
NC_005120.4_146300001	X	146300001	0.6951	0.6416
NC_005120.4_147500001	X	147500001	0.2383	0.3245
NC_005120.4_147600001	X	147600001	0.3625	0.5467
NC_005120.4_147700001	X	147700001	0.4261	0.5702
NC_005120.4_147800001	X	147800001	0.4261	0.5702
NC_005120.4_151000001	X	151000001	0.2651	0.6065
NC_005120.4_151100001	X	151100001	0.2508	0.5096
NC_005120.4_151200001	X	151200001	0.2508	0.5096
NC_005120.4_151900001	X	151900001	0.8023	0.9107
NC_005120.4_152000001	X	152000001	0.8549	0.9844
NC_005120.4_152100001	X	152100001	0.8723	0.9867
NC_005120.4_152200001	X	152200001	0.8869	0.9882
NC_005120.4_152300001	X	152300001	0.817	0.9791
NC_005120.4_152500001	X	152500001	0.9982	0.9972
NC_005120.4_152600001	X	152600001	0.9979	0.9967
NC_005120.4_152700001	X	152700001	0.6324	0.8818
NC_005120.4_152800001	X	152800001	0.6324	0.8818
NC_005120.4_152900001	X	152900001	0.4761	0.6569
NC_005120.4_153100001	X	153100001	0.6104	0.8238
NC_005120.4_153200001	X	153200001	0.7923	0.8846
NC_005120.4_153300001	X	153300001	0.7923	0.8846
NC_005120.4_153400001	X	153400001	0.8699	0.9868
NC_005120.4_153500001	X	153500001	0.8699	0.9868
NC_005120.4_154300001	X	154300001	0.5561	0.6268
NC_005120.4_154500001	X	154500001	0.4053	0.4237
NC_005120.4_154600001	X	154600001	0.4053	0.4237
NC_005120.4_154700001	X	154700001	0.7198	0.7157
NC_005120.4_154900001	X	154900001	0.6982	0.7626
NC_005120.4_155000001	X	155000001	0.8697	0.9008
NC_005120.4_155100001	X	155100001	0.7776	0.9085
NC_005120.4_155200001	X	155200001	0.6103	0.8569
NC_005120.4_155300001	X	155300001	0.6103	0.8569
NC_005120.4_155400001	X	155400001	0.7126	0.9613
NC_005120.4_155500001	X	155500001	0.5953	0.9336
NC_005120.4_155600001	X	155600001	0.5927	0.784
NC_005120.4_155800001	X	155800001	0.7455	0.8406
NC_005120.4_155900001	X	155900001	0.8026	0.8819
NC_005120.4_156000001	X	156000001	0.8263	0.8614
NC_005120.4_156100001	X	156100001	0.9398	0.9162
NC_005120.4_156200001	X	156200001	0.8005	0.9142
NC_005120.4_156300001	X	156300001	0.7846	0.9073
NC_005120.4_156400001	X	156400001	0.7626	0.8974
NC_005120.4_156500001	X	156500001	0.6911	0.967
NC_005120.4_156600001	X	156600001	0.7396	0.9659
NC_005120.4_156700001	X	156700001	0.9869	0.9695
NC_005120.4_156800001	X	156800001	0.9886	0.9738
NC_005120.4_156900001	X	156900001	0.9881	0.9715
NC_005120.4_157000001	X	157000001	0.9881	0.9715
NC_005120.4_157100001	X	157100001	0.8238	0.9292
NC_005120.4_157200001	X	157200001	0.76	0.8434
NC_005120.4_158500001	X	158500001	0.6766	0.7261

NC_005120.4_158600001	X	158600001	0.7356	0.7468
NC_005120.4_158700001	X	158700001	0.6838	0.7423
NC_005120.4_158800001	X	158800001	0.782	0.7839
NC_005120.4_158900001	X	158900001	0.8129	0.8656
NC_005120.4_159000001	X	159000001	0.7959	0.8656
NC_005120.4_159100001	X	159100001	0.8314	0.9043
NC_005120.4_159200001	X	159200001	0.8375	0.8986
NC_005120.4_159300001	X	159300001	0.8265	0.8921
NC_005120.4_159400001	X	159400001	0.8407	0.9016
NC_005120.4_159500001	X	159500001	0.8631	0.9056
