# Supplementary material for: Comparative phylogeography of two commensal rat species (Rattus tanezumi and Rattus norvegicus) in China: Insights from mitochondrial DNA, microsatellite, and 2b‐RAD data
Source: Ecol Evol. 2022 Oct 13;12(10):e9409. doi: 10.1002/ece3.9409 (PMC9557235; doi:10.1002/ece3.9409)
Supplement: Supplementary file 4 — Figure S4 [file ECE3-12-e9409-s014.pdf]

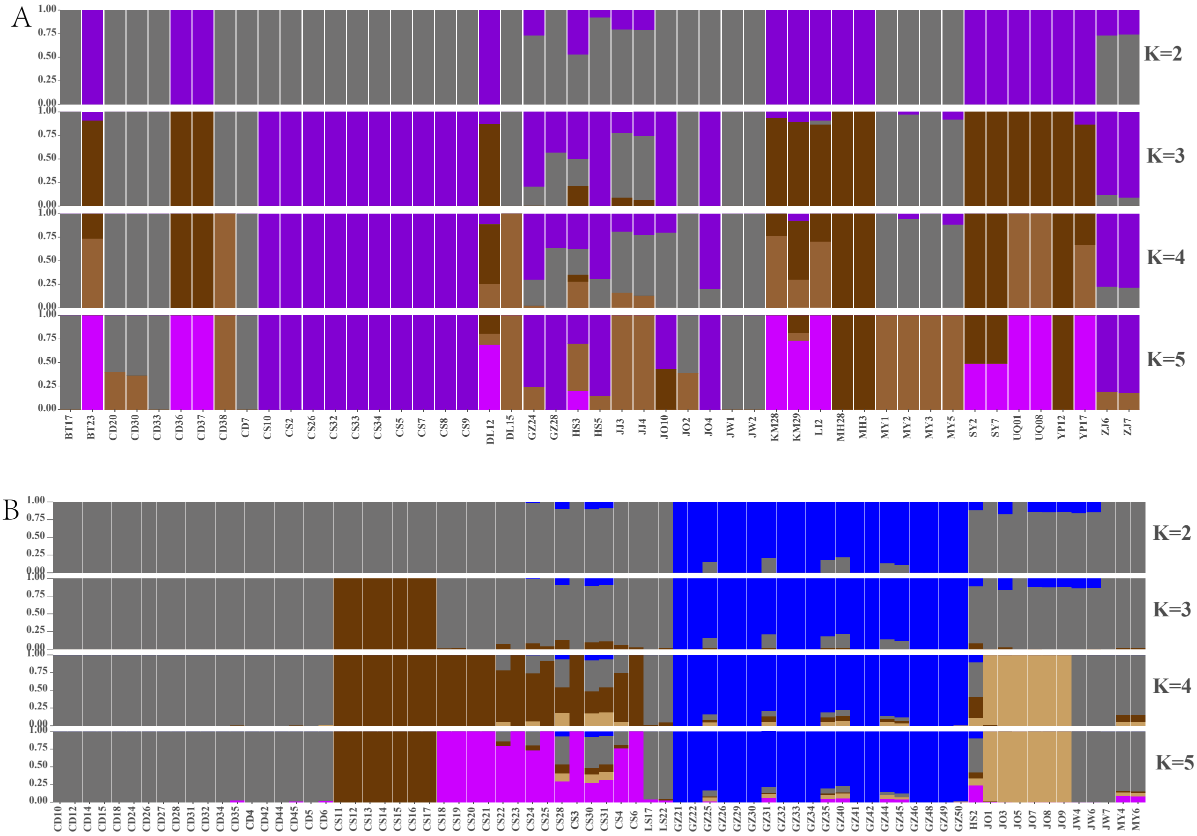
A
B
1.00
0.75
0.50
0.25
0.00
K=2
K=3
K=4
K=5
BT17 BT23 CD20 CD30 CD33 CD36 CD37 CD38 CD7 CS10 CS2 CS26 CS32 CS33 CS34 CS5 CS7 CS8 CS9 DL12 DL15 GZ24 GZ28 HS3 HS5 JJ3 JJ4 JO10 JO2 JO4 JW1 JW2 KM28 KM29 LI2 MH28 MH3 MY1 MY2 MY3 MY5 SY2 SY7 UQ01 UQ08 YP12 YP17 ZJ6 ZJ7
CD10 CD12 CD14 CD15 CD18 CD24 CD26 CD27 CD28 CD31 CD32 CD34 CD35 CD4 CD42 CD44 CD45 CD5 CD6 CS11 CS12 CS13 CS14 CS15 CS16 CS17 CS18 CS19 CS20 CS21 CS22 CS23 CS24 CS25 CS28 CS3 CS30 CS31 CS4 CS6 LS17 LS22 GZ21 GZ22 GZ25 GZ26 GZ29 GZ30 GZ31 GZ32 GZ33 GZ34 GZ35 GZ40 GZ41 GZ42 GZ44 GZ45 GZ46 GZ48 GZ49 GZ50 HS2 JO1 JO3 JO5 JO7 JO8 JO9 JW4 JW6 JW7 MY4 MY6
