# Supplementary material for: Comparative phylogeography of two commensal rat species (Rattus tanezumi and Rattus norvegicus) in China: Insights from mitochondrial DNA, microsatellite, and 2b‐RAD data
Source: Ecol Evol. 2022 Oct 13;12(10):e9409. doi: 10.1002/ece3.9409 (PMC9557235; doi:10.1002/ece3.9409)
Supplement: Supplementary file 16 — Table S10 [file ECE3-12-e9409-s010.pdf]

Ref_ID	Ref_Seq	Loc	Ref_Base	Ho	He
NC_005100.4-1182365	CTTCACCCAACCAGACCTCCTCACTTC	12	C	0.1833	0.1665
NC_005100.4-1182365	CTTCACCCAACCAGACCTCCTCACTTC	14	G	0.1917	0.1733
NC_005100.4-1382897	AGCTGAGGCACTGCAGCTCCCAGGGGA	27	A	0.0492	0.048
NC_005100.4-1429785	ACACAGCACACGCACTCTCCTTTCACT	4	C	0.0417	0.0408
NC_005100.4-1782188	GGGGGTCCGACACCCTCTCCACCTTCA	13	C	0	0.4779
NC_005100.4-1816249	CTCCCCACAACCGTGTCTCCTCAGGAC	12	C	0.3252	0.427
NC_005100.4-1816249	CTCCCCACAACCGTGTCTCCTCAGGAC	25	G	0	0.4793
NC_005100.4-1820845	GGTGTTACCACATCAGCTCCTAATGGA	6	T	0.0504	0.4285
NC_005100.4-1846694	GAGCTCTGTACCTCAGCTCCGGGAATC	24	A	0	0.4688
NC_005100.4-1876980	AAAACACGAACTTAATCTCCAGTTAAC	2	A	0.0256	0.4714
NC_005100.4-1876980	AAAACACGAACTTAATCTCCAGTTAAC	14	A	0	0.4772
NC_005100.4-1895272	TGATCCTTAACTGATTCTCCTGCTTCC	21	T	0.1789	0.2017
NC_005100.4-1973018	CAATTTTCCACACTCTCTCCGTTGGTG	21	G	0	0.4793
NC_005100.4-1978068	CTGTAACTAACTCGAGCTCCAGGGAGT	2	T	0.0083	0.4769
NC_005100.4-1978068	CTGTAACTAACTCGAGCTCCAGGGAGT	24	G	0.0082	0.479
NC_005100.4-1978422	TATTAGAGAACTTACACTCCACAAGAC	9	A	0	0.4861
NC_005100.4-1982212	AGATAGCGGACATGAGCTCCTATCCCT	6	G	0.3415	0.4
NC_005100.4-1982212	AGATAGCGGACATGAGCTCCTATCCCT	8	G	0.0488	0.0476
NC_005100.4-2140039	CTTTATTCCACCTGTGCTCCAACCCAG	22	A	0	0.4826
NC_005100.4-2175310	CACACACACACACACACTCCTGCCAAT	4	A	0.087	0.2005
NC_005100.4-2194473	CATTCCACAACAAGCCCTCCCAGTTGG	13	A	0.0088	0.4735
NC_005100.4-2206635	AAACCGAGGACTCCCTCTCCCATGGGA	13	C	0.0877	0.0839
NC_005100.4-2206635	AAACCGAGGACTCCCTCTCCCATGGGA	22	A	0	0.4875
NC_005100.4-2251415	ACCTTCACCACGGTAGCTCCAGCTTTT	6	C	0.157	0.2355
NC_005100.4-2289736	TTTCTTCAGACTCAATCTCCAGCTTAC	15	A	0.3554	0.4004
NC_005100.4-2289736	TTTCTTCAGACTCAATCTCCAGCTTAC	26	A	0.2333	0.32
NC_005100.4-2328484	TTATGCTCTACTGCAACTCCGGGAGTC	1	T	0.122	0.208
NC_005100.4-2328484	TTATGCTCTACTGCAACTCCGGGAGTC	4	T	0.3033	0.4822
NC_005100.4-2368801	TCAGGTCTAACATTGGCTCCTGCTTTA	12	A	0.1102	0.2282
NC_005100.4-2433850	TGAATTGGGACAGGACCTCCTGAGTAG	27	G	0.0492	0.0634
NC_005100.4-2454845	CAATGCTATACAATTCCTCCCATTCCT	23	T	0.1081	0.1182
NC_005100.4-2472059	GGAAGCCAAACTCAGCCTCCATCACTG	23	C	0.1667	0.1528
NC_005100.4-2509834	ATGTCCTCAACTGAGGCTCCTTTTCTG	2	T	0.0901	0.4967
NC_005100.4-2561851	GTATGGTACACAGTCTCTCCTTGATGC	26	G	0.2353	0.4957
NC_005100.4-2564268	GAGGCGTGGACCGGCCCTCCCATGCAC	22	A	0	0.4793
NC_005100.4-2568202	CTGACCAATACCAGAACTCCCCAGCTG	1	C	0.1638	0.1646
NC_005100.4-2603655	TACAGCCACACACTGGCTCCTACGTTC	21	T	0.0579	0.4951
NC_005100.4-2603655	TACAGCCACACACTGGCTCCTACGTTC	27	C	0.1653	0.318
NC_005100.4-2629859	CTCAGAACAACGGGAACTCCTGCTGAA	12	G	0.0917	0.4983
NC_005100.4-2629859	CTCAGAACAACGGGAACTCCTGCTGAA	21	T	0	0.48
NC_005100.4-2641423	CATTTCCAAACATCAGCTCCTGACTGG	24	C	0.1074	0.4999
NC_005100.4-2712868	AATGGCTTAACCATCTCTCCAGCTCCC	5	G	0.0917	0.4972
NC_005100.4-2790395	CCGAGGAAGACCTGGGCTCCCGGGGAG	2	C	0.0259	0.0255
NC_005100.4-2797247	CAAAAACAAACACCACCTCCTCTTTTG	13	C	0.0248	0.0868
NC_005100.4-2797247	CAAAAACAAACACCACCTCCTCTTTTG	26	T	0.0083	0.4847
NC_005100.4-2800284	ACGAAGTTAACCTTGGCTCCGGCAAAA	26	A	0.082	0.3947
NC_005100.4-2862790	ATCATGAACACAGGACCTCCCGGCACC	22	G	0.1624	0.4823
NC_005100.4-2866343	ATGAATGACACATGAACTCCCACATCC	12	A	0.1429	0.232
NC_005100.4-2886618	CCGTGACCAACTCTTGCTCCTCTAGAA	2	C	0.0492	0.0786
NC_005100.4-2886618	CCGTGACCAACTCTTGCTCCTCTAGAA	16	G	0.1157	0.3079

NC_005100.4-2914088	TCTTTATTCACTGGAACTCCAAGCTCT	7	T	0	0.4793
NC_005100.4-2914527	CTGTTTCACACTGTATCTCCCTTTTTC	6	T	0	0.4786
NC_005100.4-2938535	AATAAACAAACTGGTCCTCCATGTGTA	6	A	0.0086	0.4688
NC_005100.4-2947759	GCCACACTGACCATCTCTCCTGTTTCA	22	G	0	0.4677
NC_005100.4-2947759	GCCACACTGACCATCTCTCCTGTTTCA	23	T	0	0.4677
NC_005100.4-2966075	CCCTGGACTACAGATCCTCCTGCCTTT	7	A	0	0.4693
NC_005100.4-3046051	GCACAAGCCACCGTGGCTCCTAGCTTT	5	A	0.0164	0.4891
NC_005100.4-3067988	ATTCCGCACACATGCACTCCCACAAGT	1	A	0.0086	0.4836
NC_005100.4-3082741	ATGACAAATACACACACTCCCTTCTAG	9	T	0.0504	0.0805
NC_005100.4-3091434	TCACCACCAACATCATCTCCAGCATGA	3	A	0	0.4773
NC_005100.4-3128713	TTCAGGCTTACCGTACCTCCTGCCGTA	21	T	0.0992	0.1653
NC_005100.4-3141928	TCCCTTCACACAGATTCTCCCCCACAC	12	A	0.0531	0.4793
NC_005100.4-3141928	TCCCTTCACACAGATTCTCCCCCACAC	27	C	0	0.4574
NC_005100.4-3198745	AGACATCTCACTCTGCCTCCTTGCTCA	9	C	0.1301	0.2612
NC_005100.4-3209281	AGTTGAAGTACCAAGACTCCTTGGCAG	12	C	0.0593	0.4919
NC_005100.4-3212866	TGGGAGTTGACTGTTTCTCCCTTGCCT	9	G	0	0.4851
NC_005100.4-3246757	GTGTAGAGCACTATGTCTCCCTCTGAT	3	G	0.0084	0.4797
NC_005100.4-3333444	GGGACTACCACTTTTGCTCCAAGAGAC	9	C	0.0488	0.078
NC_005100.4-3382427	AGGCCATCGACTCCGACTCCCTCATTT	9	G	0.1382	0.208
NC_005100.4-3382427	AGGCCATCGACTCCGACTCCCTCATTT	24	A	0	0.4807
NC_005100.4-3391937	TTATTTCTAACTGTGACTCCAACGCAA	8	T	0.0083	0.4747
NC_005100.4-3391937	TTATTTCTAACTGTGACTCCAACGCAA	23	C	0.0083	0.4747
NC_005100.4-3426120	CTCACTACCACCTGTGCTCCATGCTAC	27	C	0.025	0.0247
NC_005100.4-3474806	ATACGCTTGACCCCACCTCCCACACAC	24	A	0	0.4793
NC_005100.4-3514483	GAATGCTTCACTGGGGCTCCTTTTAAA	27	A	0.1901	0.4889
NC_005100.4-3518502	CAAGCTCTTACCCACACTCCTGTAAGT	1	C	0.0492	0.0786
NC_005100.4-3518502	CAAGCTCTTACCCACACTCCTGTAAGT	21	T	0.0492	0.0786
NC_005100.4-3554260	AGGAAATGCACAAAAGCTCCCAGAGAT	27	T	0.0769	0.4527
NC_005100.4-3637969	AATTTAAATACAGAGTCTCCACTTGTC	26	T	0.0174	0.4764
NC_005100.4-3640428	CTGTCTGTAACTCTAGCTCCAGAGAAT	13	C	0.0738	0.071
NC_005100.4-3645945	AACCTCACTACTCTAGCTCCATGCTTT	21	A	0.0174	0.4833
NC_005100.4-3660169	GTAACCTGGACCACAGCTCCAAATGCT	26	C	0.1071	0.1172
NC_005100.4-3674641	TCCATCAGGACATCCTCTCCCCGGGGG	23	G	0.1864	0.4977
NC_005100.4-3677561	ATGACATGGACAGCTGCTCCGGGTCCA	27	A	0.0696	0.0671
NC_005100.4-3700479	TTGTGGTATACTTAGGCTCCAACTGCA	13	T	0.1504	0.3495
NC_005100.4-3700479	TTGTGGTATACTTAGGCTCCAACTGCA	22	A	0.1416	0.3446
NC_005100.4-3711248	TCATCTGCCACACCCACTCCCTGAATG	21	C	0.0902	0.4959
NC_005100.4-3725008	TAAACACAGACTTCCCCTCCAGGTACA	27	A	0.1416	0.4624
NC_005100.4-3778007	GATAAGTACACACCCACTCCCTCAAAT	3	T	0.0085	0.4842
NC_005100.4-3778007	GATAAGTACACACCCACTCCCTCAAAT	22	T	0.0085	0.4842
NC_005100.4-3780420	CTTACAACCACCACACCTCCCAAACCT	3	T	0.1607	0.2698
NC_005100.4-3780420	CTTACAACCACCACACCTCCCAAACCT	9	C	0.0783	0.0752
NC_005100.4-3801041	AGCCTGCCCACCCCGACTCCATTCTCA	13	C	0.05	0.0799
NC_005100.4-3805496	ACCATCATTACGGCATCTCCTGTCAGG	4	A	0.1311	0.2956
NC_005100.4-3805496	ACCATCATTACGGCATCTCCTGTCAGG	15	A	0.0656	0.4952
NC_005100.4-3807683	TTCCCACCAACGATGCCTCCTAGGCTG	6	A	0	0.4793
NC_005100.4-3807683	TTCCCACCAACGATGCCTCCTAGGCTG	8	C	0.1545	0.2556
NC_005100.4-3830254	TACGTTTGAACCTAGTCTCCGTGGTAG	3	C	0.125	0.375
NC_005100.4-3830254	TACGTTTGAACCTAGTCTCCGTGGTAG	21	G	0.1842	0.2719
NC_005100.4-3849735	AGGTTTCACACTCAGGCTCCAGGGAGA	22	G	0.0488	0.078
NC_005100.4-3905661	AAAGTGTCCACCATAGCTCCTTCTCCC	4	G	0.319	0.4108

NC_005100.4-3905661	AAAGTGTCCACCATAGCTCCTTCTCCC	8	C	0.0413	0.0716
NC_005100.4-3956467	GTGAGATTCACTTGATCTCCGTGGCCA	13	T	0.1626	0.2382
NC_005100.4-3981090	GAGTGCTGCACACTGGCTCCTCTTGGC	8	G	0.0488	0.4926
NC_005100.4-3984260	TTTCGAATGACACCCTCTCCGAGCAGA	21	G	0	0.4772
NC_005100.4-4015155	TGTTCATTAACTCAGGCTCCTTTTGGA	12	T	0.0084	0.4761
NC_005100.4-4092766	TCTGCTGTGACTGGATCTCCAAGTGCA	7	G	0.2787	0.3947
NC_005100.4-4107385	GGTTGGCCTACTTGGACTCCATGGTTT	4	T	0.0522	0.0832
NC_005100.4-4107385	GGTTGGCCTACTTGGACTCCATGGTTT	22	T	0	0.4764
NC_005100.4-4109212	TGGGGTAACACACCTTCTCCCATCATG	5	G	0.1593	0.3446
NC_005100.4-4109212	TGGGGTAACACACCTTCTCCCATCATG	16	T	0	0.4871
NC_005100.4-4121993	GCTAGTCCAACCACGTCTCCACGCTAC	14	C	0.0081	0.0398
NC_005100.4-4145491	AGAGTGGAAACAAGTTCTCCGATGAGG	3	A	0.0175	0.4846
NC_005100.4-4167928	TCTCTTACTACCCCGTCTCCTTCTGAG	15	G	0.2895	0.3661
NC_005100.4-4167928	TCTCTTACTACCCCGTCTCCTTCTGAG	23	C	0.2261	0.32
NC_005100.4-4199710	AAGCCCTGAACTATCTCTCCAGCCCCA	22	G	0.1429	0.1626
NC_005100.4-4199710	AAGCCCTGAACTATCTCTCCAGCCCCA	25	C	0.1416	0.1613
NC_005100.4-4222167	TACTTCCCAACTGAGCCTCCCCTTCCC	21	C	0.0088	0.4889
NC_005100.4-4247429	ACCACTGGCACTGATGCTCCTAGGGCA	3	C	0.0261	0.0752
NC_005100.4-4284764	ATTTCCAGGACAAATACTCCTTTGCTT	2	T	0	0.4814
NC_005100.4-4284764	ATTTCCAGGACAAATACTCCTTTGCTT	26	T	0	0.4814
NC_005100.4-4342362	GAACCTTACACAGTGTCTCCAAGATCA	3	A	0.0492	0.4934
NC_005100.4-4342362	GAACCTTACACAGTGTCTCCAAGATCA	9	C	0.0492	0.4934
NC_005100.4-4347825	ACAAACAATACAGTCACTCCACAGGCA	9	T	0.0536	0.0853
NC_005100.4-4382822	AGCCTGACCACAAGAGCTCCTCCTCCA	3	C	0.0522	0.0832
NC_005100.4-4390032	ACTTACAACACCATCTCTCCTCATCAT	13	A	0.0569	0.4915
NC_005100.4-4403530	CTTGTACCTACCACGGCTCCAAATGAC	15	G	0.0496	0.0792
NC_005100.4-4453158	AGACTTTCTACAACACCTCCATGGAAG	13	A	0.1301	0.1629
NC_005100.4-4454446	CCTGTAATCACTATCCCTCCCCACACC	21	C	0.1311	0.4995
NC_005100.4-4454446	CCTGTAATCACTATCCCTCCCCACACC	27	C	0.1311	0.4995
NC_005100.4-4479920	TGCCTTGACACATGTGCTCCCATACTC	22	A	0	0.4793
NC_005100.4-4496202	GGCCAGACCACATCAACTCCTTCAGAC	16	A	0.0085	0.4753
NC_005100.4-4613406	AGTCATCCCACAACTCCTCCAAAAGAT	8	C	0.2602	0.4497
NC_005100.4-4613406	AGTCATCCCACAACTCCTCCAAAAGAT	9	C	0.1545	0.2885
NC_005100.4-4622081	CACCAACCAACCAGAGCTCCCTGGGAC	8	C	0.313	0.4234
NC_005100.4-4632003	AGTTGCACCACTATGTCTCCTTGGACA	13	A	0	0.4649
NC_005100.4-4632003	AGTTGCACCACTATGTCTCCTTGGACA	15	G	0.0598	0.1634
NC_005100.4-4643481	GGACCAATCACCCTGCCTCCAGTAGAT	25	G	0.2114	0.3239
NC_005100.4-4751837	CATGAATCTACTCTTACTCCTTCACAG	26	A	0.0268	0.0605
NC_005100.4-4757204	CCCAAGCACACTGGCCCTCCACCACCA	22	C	0	0.4787
NC_005100.4-4814763	AGTCAGATCACACTATCTCCATGTGTG	16	T	0	0.4793
NC_005100.4-4826514	GACTGGCAGACCCAGCCTCCTCTCCCC	1	G	0.0345	0.0339
NC_005100.4-4826514	GACTGGCAGACCCAGCCTCCTCTCCCC	23	T	0.0517	0.0825
NC_005100.4-4850359	ATAGTGAGCACATACCCTCCCCTGCTT	16	C	0	0.4839
NC_005100.4-4884779	CACTACATCACGGCAACTCCAAATGTT	5	A	0	0.4588
NC_005100.4-4884779	CACTACATCACGGCAACTCCAAATGTT	16	A	0	0.4606
NC_005100.4-4897691	TTTCAATGCACTGCATCTCCTGCTTAG	5	A	0.0169	0.4757
NC_005100.4-4981118	CAGCTTGAAACTCCAGCTCCAGGGCCC	25	C	0.2869	0.4435
NC_005100.4-4983617	TCTTCATAGACAGACTCTCCAAAGGCT	15	C	0.2869	0.3984
NC_005100.4-5009652	TTCCCATGAACCACACCTCCATGCATG	3	C	0	0.48
NC_005100.4-5014176	TCAATACATACTTTCACTCCTGTATCA	21	T	0.0083	0.4803
NC_005100.4-5024499	ACGATCGTGACCCCTACTCCACTCTCT	7	G	0.2951	0.416

NC_005100.4-5149442	AGGCAGAGAACACAGCCTCCCCACATA	27	A	0.0417	0.4622
NC_005100.4-5212794	CCCTAAGCCACCTCCTCTCCCCCTCTT	2	C	0.2719	0.4796
NC_005100.4-5212794	CCCTAAGCCACCTCCTCTCCCCCTCTT	16	T	0	0.474
NC_005100.4-5257629	TTAATTGAAACATTAGCTCCTCTGAGT	4	A	0.0413	0.0562
NC_005100.4-5257629	TTAATTGAAACATTAGCTCCTCTGAGT	21	T	0.1092	0.5
NC_005100.4-5266626	CTCTGCCTGACACCTGCTCCTCTCTGC	23	T	0.0254	0.0251
NC_005100.4-5336962	GGTTTCCCCACCACCCCTCCCTGATTT	3	T	0	0.4677
NC_005100.4-5336962	GGTTTCCCCACCACCCCTCCCTGATTT	16	C	0.0508	0.4856
NC_005100.4-5461102	AGGTTAAGTACCAGGACTCCAGAGTGG	12	C	0.1795	0.2041
NC_005100.4-5495975	GGAGTGGGGACTGTCTCTCCTTTGGCT	3	A	0.115	0.4923
NC_005100.4-5495975	GGAGTGGGGACTGTCTCTCCTTTGGCT	4	G	0.1593	0.5
NC_005100.4-5499605	ATGCTCCTCACTCGGTCTCCGTTGGTA	14	G	0.15	0.18
NC_005100.4-5499819	TCACCTTCCACCCGGTCTCCAGGGATA	12	C	0.1545	0.5
NC_005100.4-5499819	TCACCTTCCACCCGGTCTCCAGGGATA	22	G	0.1545	0.5
NC_005100.4-5503034	CTACCAACAACATTCCCTCCTCACAGT	1	C	0.1552	0.4987
NC_005100.4-5503034	CTACCAACAACATTCCCTCCTCACAGT	26	G	0.0517	0.0825
NC_005100.4-5548782	CACATCAGCACAATTGCTCCCCTAAAT	25	A	0.1917	0.4941
NC_005100.4-5717761	CTAAGTGGAACCATGGCTCCAAGGTTC	22	A	0.0492	0.4914
NC_005100.4-5717761	CTAAGTGGAACCATGGCTCCAAGGTTC	23	G	0.0492	0.4914
NC_005100.4-5722846	TTCCAGTATACTCTCCCTCCTTTCCCC	13	C	0.0714	0.496
NC_005100.4-5722846	TTCCAGTATACTCTCCCTCCTTTCCCC	16	C	0.045	0.0611
NC_005100.4-5896333	TTTGCAGTGACTTCAGCTCCAGGAAAT	26	A	0.25	0.4483
NC_005100.4-5989126	CTCCTGGGCACTACATCTCCTTGTACC	1	C	0.042	0.0571
NC_005100.4-5998887	CAGACAAGCACCCTAGCTCCTGGAGAA	3	G	0	0.4773
NC_005100.4-5998887	CAGACAAGCACCCTAGCTCCTGGAGAA	16	G	0.2131	0.4952
NC_005100.4-6060749	GGATCTGTCACGCCTGCTCCGCAAGCA	4	T	0	0.4931
NC_005100.4-6104869	GAAATCAAGACAGAAGCTCCTTGCATG	6	C	0.2541	0.4943
NC_005100.4-6182339	TAGATAAGAACACAGCCTCCCTAGGCA	8	G	0.05	0.0799
NC_005100.4-6189220	GTAACTTTGACGCCATCTCCACCATCA	12	G	0.1639	0.1904
NC_005100.4-6189220	GTAACTTTGACGCCATCTCCACCATCA	24	A	0.1653	0.1918
NC_005100.4-6250706	TAGAAAGTCACCCTTTCTCCACAACTC	7	G	0.193	0.2538
NC_005100.4-6254049	TATATGTGAACACATTCTCCCATGAAC	3	T	0	0.0351
NC_005100.4-6254049	TATATGTGAACACATTCTCCCATGAAC	14	A	0	0.0351
NC_005100.4-6272394	TGCATCCCTACTGCTCCTCCCAGCCCC	21	C	0.2049	0.2457
NC_005100.4-6272394	TGCATCCCTACTGCTCCTCCCAGCCCC	25	C	0	0.4819
NC_005100.4-6272447	CAGAAATCCACCCCTCCTCCATTTCCC	13	C	0	0.4898
NC_005100.4-6301663	CCCACTGCTACAGATCCTCCTTCATTT	14	A	0.065	0.0629
NC_005100.4-6329211	CATGGCAAAACTGTCTCTCCTTCTTTG	1	C	0.2288	0.4454
NC_005100.4-6396352	CCCGGGCTCACACAGACTCCTTGAACA	9	C	0.0625	0.0605
NC_005100.4-6396352	CCCGGGCTCACACAGACTCCTTGAACA	22	T	0.0625	0.0605
NC_005100.4-6421438	GAAGATCACACCATTTCTCCTTTCCCC	4	G	0.2435	0.4864
NC_005100.4-6427587	AATTGATCAACAGTAGCTCCTAGCCTA	21	T	0.1583	0.1733
NC_005100.4-6475188	ATGACTTCCACAAATACTCCCACACTC	14	A	0	0.4743
NC_005100.4-6564653	ACTGCCTAAACACCTTCTCCCTTGGTA	23	T	0	0.4779
NC_005100.4-6589997	CAAGGTTCTACCTAGCCTCCACATGGA	1	C	0.1532	0.492
NC_005100.4-6603720	CTCTGATCCACTGCCACTCCTGTGTCA	24	G	0.1626	0.4973
NC_005100.4-6621664	ATGGAAGCCACATCTTCTCCAACATGA	26	G	0.161	0.2282
NC_005100.4-6626990	GTGGCGCTCACTGTAACTCCGTTTCAG	16	A	0	0.4793
NC_005100.4-6626990	GTGGCGCTCACTGTAACTCCGTTTCAG	21	G	0.1707	0.2323
NC_005100.4-6656953	TAATGGCCCACACAAACTCCTGTCTTT	15	A	0.0667	0.4965
NC_005100.4-6696613	TACGGGTTTACCTAAGCTCCAAAGGCA	3	C	0.069	0.1134

NC_005100.4-6696613	TACGGGTTTACCTAAGCTCCAAAGGCA	4	G	0	0.4786
NC_005100.4-6760727	TTCGAACTTACTGAAGCTCCGTCTGTG	4	G	0.1053	0.1744
NC_005100.4-6760727	TTCGAACTTACTGAAGCTCCGTCTGTG	5	A	0.0619	0.4547
NC_005100.4-6781597	CTACACCAAACTACAGCTCCAACGGTG	16	G	0.2523	0.3178
NC_005100.4-6781597	CTACACCAAACTACAGCTCCAACGGTG	23	C	0.0265	0.0262
NC_005100.4-6825900	CTGTGCATTACATGACCTCCAGCCCCA	4	T	0.0513	0.05
NC_005100.4-6825900	CTGTGCATTACATGACCTCCAGCCCCA	13	T	0.0513	0.0818
NC_005100.4-6877878	AGTCCAGGCACTCGGCCTCCATCATGA	13	C	0.0522	0.0832
NC_005100.4-6877878	AGTCCAGGCACTCGGCCTCCATCATGA	16	C	0.3043	0.4055
NC_005100.4-6884604	CGTATCTCCACACAGACTCCACAGGAG	2	G	0.2931	0.4144
NC_005100.4-6903902	CATGGAGAAACCCTGTCTCCGAACACC	21	G	0	0.4793
NC_005100.4-6915407	TTGTTTATAACAATTGCTCCAAAGAGT	22	A	0.1966	0.4987
NC_005100.4-6932142	CAGGATTTTACTGGCTCTCCCAGGACT	22	A	0.0492	0.0634
NC_005100.4-6947523	TGCTGCAGGACCGTGCCTCCCTGCTAC	22	T	0.2869	0.3909
NC_005100.4-6947523	TGCTGCAGGACCGTGCCTCCCTGCTAC	27	C	0.0082	0.0243
NC_005100.4-6960677	CTTAGGAGAACACAGCCTCCTCAGAGT	1	C	0.2261	0.3856
NC_005100.4-6960677	CTTAGGAGAACACAGCCTCCTCAGAGT	13	C	0	0.482
NC_005100.4-6975247	GTGAACATCACAAGTCCTCCCAAGTGT	2	T	0	0.4851
NC_005100.4-6987947	TGTACCTGTACCTGACCTCCGAAGGGT	24	G	0.0569	0.0553
NC_005100.4-7035224	CTCTCCCCCACTCCACCTCCCTTCAGA	6	C	0.1593	0.2302
NC_005100.4-7041471	CACTAGCAAACACATCCTCCTGTACAT	13	C	0.05	0.0799
NC_005100.4-7056209	ATTCCACCCACTGCACCTCCCTACCAT	1	A	0.0603	0.4034
NC_005100.4-7056209	ATTCCACCCACTGCACCTCCCTACCAT	12	T	0.1282	0.2041
NC_005100.4-7099976	TCAAATCCCACTCTACCTCCAAGCCCT	14	T	0.0083	0.4747
NC_005100.4-7239607	GGCCTCAAAACAGCATCTCCTTCCCCC	7	A	0.0656	0.4612
NC_005100.4-7239607	GGCCTCAAAACAGCATCTCCTTCCCCC	24	C	0.065	0.078
NC_005100.4-7286027	CACAGTTTCACTTTCCCTCCCCATGCT	12	T	0.0488	0.078
NC_005100.4-7295373	AAGCCCGAAACAAAACCTCCCTTCTTG	7	G	0.4696	0.3593
NC_005100.4-7295373	AAGCCCGAAACAAAACCTCCCTTCTTG	15	A	0.1513	0.2203
NC_005100.4-7319218	TTACGTGCTACATTGACTCCGTTTGTA	5	G	0.0488	0.078
NC_005100.4-7319218	TTACGTGCTACATTGACTCCGTTTGTA	21	G	0.0569	0.0854
NC_005100.4-7326344	TTTGGCTCTACCGTCTCTCCTGCACGG	9	T	0	0.4864
NC_005100.4-7326344	TTTGGCTCTACCGTCTCTCCTGCACGG	12	C	0	0.4851
NC_005100.4-7332296	CACGTCTACACCGCTGCTCCCGTGGCC	12	C	0	0.4807
NC_005100.4-7332296	CACGTCTACACCGCTGCTCCCGTGGCC	15	T	0	0.4807
NC_005100.4-7356961	AGACCTGTTACACTCCCTCCTCTGTAC	9	T	0	0.4856
NC_005100.4-7356961	AGACCTGTTACACTCCCTCCTCTGTAC	12	A	0	0.4856
NC_005100.4-7363932	GACTCCCGGACTCTGTCTCCCCAGCAG	15	G	0.0081	0.4777
NC_005100.4-7363932	GACTCCCGGACTCTGTCTCCCCAGCAG	27	G	0	0.4793
NC_005100.4-7439750	CTGTCCTAGACCAAGACTCCTCACTAG	8	A	0.0492	0.0786
NC_005100.4-7477694	GAAAACATTACACCAACTCCAAGGAGC	4	A	0.0531	0.4934
NC_005100.4-7510811	GGATCGCAGACCTGTCCTCCATTTCTG	6	G	0.0084	0.4797
NC_005100.4-7510811	GGATCGCAGACCTGTCCTCCATTTCTG	12	C	0.084	0.3415
NC_005100.4-7519043	TATAATCCAACAATCACTCCTTGGCAT	4	A	0.0504	0.0805
NC_005100.4-7521452	GGTTGAATCACCTTAGCTCCTTTCCCT	3	T	0.0083	0.4816
NC_005100.4-7523235	CCCGGTGACACACCTCCTCCAATAGAA	2	C	0.1217	0.1295
NC_005100.4-7523235	CCCGGTGACACACCTCCTCCAATAGAA	4	G	0.1217	0.4482
NC_005100.4-7536282	CCTTCTAGAACTCTCTCTCCTAAGAAC	4	T	0.0901	0.4909
NC_005100.4-7536282	CCTTCTAGAACTCTCTCTCCTAAGAAC	14	T	0.0531	0.4859
NC_005100.4-7626214	TCAGCTTTTACACCGACTCCAGGGGTT	3	A	0.094	0.4928
NC_005100.4-7626214	TCAGCTTTTACACCGACTCCAGGGGTT	14	C	0.094	0.0896

NC_005100.4-7637239	CACACACACACACACACTCCATGAGTG	1	C	0.0413	0.0405
NC_005100.4-7666511	CAGAACGACACCGTCACTCCTGACGTG	6	C	0.1707	0.5
NC_005100.4-7666511	CAGAACGACACCGTCACTCCTGACGTG	14	T	0.0976	0.4944
NC_005100.4-7670861	GCCAAGCACACTAATCCTCCATGCTTT	2	C	0.0504	0.0805
NC_005100.4-7670861	GCCAAGCACACTAATCCTCCATGCTTT	9	C	0.0504	0.0805
NC_005100.4-7721827	ACTGCTTGTACCTTATCTCCATAACAT	12	C	0.0348	0.0342
NC_005100.4-7727982	TTTCTCTCAACTGCCACTCCATTTGCT	6	C	0.125	0.4228
NC_005100.4-7765165	CTCCCTCACACCTATTCTCCTATGCTC	3	C	0.0171	0.05
NC_005100.4-7765165	CTCCCTCACACCTATTCTCCTATGCTC	5	C	0.0259	0.4893
NC_005100.4-7802433	TCCTCTTGGACCCCTACTCCCCACCCA	23	A	0	0.4787
NC_005100.4-7827438	CATCCCTACACTCTGACTCCTGTGCAG	5	C	0.0082	0.4822
NC_005100.4-7886519	CATTGAGCAACATCAACTCCACGTTGA	22	C	0	0.4813
NC_005100.4-7886519	CATTGAGCAACATCAACTCCACGTTGA	24	T	0.1513	0.1678
NC_005100.4-7889743	TCTCTAGCTACACCCACTCCTCTCCCT	4	C	0.0536	0.0853
NC_005100.4-7908047	TTTAGAGCAACTTGTTCTCCTGGTGTC	3	T	0.2759	0.2965
NC_005100.4-7908047	TTTAGAGCAACTTGTTCTCCTGGTGTC	8	C	0.0435	0.059
NC_005100.4-7910005	TTACTCATCACCGTTCCTCCGGTTGGC	27	C	0.0268	0.4617
NC_005100.4-7933000	AAACAAGCTACGAGTCCTCCATCCCAG	9	T	0	0.4773
NC_005100.4-7933000	AAACAAGCTACGAGTCCTCCATCCCAG	13	A	0	0.4773
NC_005100.4-7955082	TGGCCATGCACAGCTGCTCCCTAGATT	7	T	0.122	0.1695
NC_005100.4-7973244	ACGATGTTCACACAAACTCCATGCAAG	22	T	0.113	0.2204
NC_005100.4-7973244	ACGATGTTCACACAAACTCCATGCAAG	24	C	0.0696	0.1295
NC_005100.4-7979691	TGCTAAAGCACCCAAGCTCCTTCTCTG	6	A	0.1322	0.1653
NC_005100.4-7979691	TGCTAAAGCACCCAAGCTCCTTCTCTG	7	A	0.1322	0.1653
NC_005100.4-7981633	AGACAATTTACAGCAACTCCATAGCCA	22	T	0.05	0.0799
NC_005100.4-7981633	AGACAATTTACAGCAACTCCATAGCCA	23	A	0.05	0.0799
NC_005100.4-8044034	TGCCACCTCACAGTCCCTCCTTGAGAC	5	A	0	0.0322
NC_005100.4-8044034	TGCCACCTCACAGTCCCTCCTTGAGAC	24	A	0.0492	0.4934
NC_005100.4-8082102	CAGTCCCACACACCTTCTCCCCGCCCC	7	C	0.0574	0.4924
NC_005100.4-8105405	AACCATTGAACCATCTCTCCTTTGCCT	15	C	0.2195	0.3091
NC_005100.4-8138109	AGTTTTAACACACCAACTCCTCCATGG	6	T	0	0.4793
NC_005100.4-8157768	GGACTGCGTACTACCACTCCAAGCCTT	12	T	0	0.4819
NC_005100.4-8175993	AGTGCTTCAACATCTGCTCCACACCTC	8	C	0	0.4793
NC_005100.4-8175993	AGTGCTTCAACATCTGCTCCACACCTC	9	A	0	0.4793
NC_005100.4-8274491	AAGCAGGTAACGAGACCTCCATGTATG	12	G	0.05	0.0799
NC_005100.4-8274491	AAGCAGGTAACGAGACCTCCATGTATG	25	A	0.05	0.4888
NC_005100.4-8277740	GTCTTTCTGACTTTGTCTCCCTGTCTC	13	T	0.5085	0.3792
NC_005100.4-8277740	GTCTTTCTGACTTTGTCTCCCTGTCTC	21	C	0.5085	0.3792
NC_005100.4-8315661	AGTAAATCCACTGAGGCTCCGCCCACT	27	T	0.0342	0.05
NC_005100.4-8319868	ATTTCCCAAACCAGGTCTCCATCCTGT	21	A	0.0496	0.4942
NC_005100.4-8341242	TCACTTGGGACCTATGCTCCTTGTAAT	24	T	0.125	0.3622
NC_005100.4-8355489	GATTTATTTACGACATCTCCTGCTGTA	4	T	0.0609	0.0911
NC_005100.4-8416616	GCATGCTTTACTTTACCTCCACACATG	6	C	0.0536	0.0853
NC_005100.4-8423289	TCCCAAACGACAGGATCTCCACAACAC	9	G	0	0.4682
NC_005100.4-8442879	CCATTCAGCACCCATCCTCCAATGAAG	2	C	0.0531	0.4934
NC_005100.4-8442879	CCATTCAGCACCCATCCTCCAATGAAG	3	A	0.0893	0.4539
NC_005100.4-8454714	ATTCTCCTTACTCTGTCTCCCCAACTC	9	T	0.069	0.4463
NC_005100.4-8461938	CATAGAGTAACCTGCTCTCCCTTCTTC	9	A	0.0862	0.4927
NC_005100.4-8562903	GGAACTTGAACTGTGACTCCCTAACAC	5	C	0	0.4851
NC_005100.4-8577052	ATGCTTGAAACCATGACTCCAAAGAAC	3	G	0.0259	0.0255
NC_005100.4-8684166	AGACTGTAAACATAGTCTCCATAAATT	12	A	0.0348	0.0342

NC_005100.4-8773110	AACTGCCCAACTTCCTCTCCAGGAGGT	2	A	0.0488	0.078
NC_005100.4-8773110	AACTGCCCAACTTCCTCTCCAGGAGGT	26	G	0.0244	0.0241
NC_005100.4-8851821	CTGTATTTGACTGGCTCTCCAGTTTTC	23	T	0.0168	0.4703
NC_005100.4-8951288	AATGTACAAACAAGTTCTCCACAGTAT	3	T	0.05	0.0799
NC_005100.4-8951288	AATGTACAAACAAGTTCTCCACAGTAT	4	G	0.05	0.0799
NC_005100.4-9035520	CTCACCAGGACTTGAACTCCACTGCAG	15	A	0	0.4833
NC_005100.4-9049878	CACATGTGCACATGGGCTCCTCACATA	4	A	0.0086	0.4893
NC_005100.4-9221799	CAATGGCCAACTCTTCCTCCTGTGTTT	22	G	0.0084	0.4761
NC_005100.4-9340010	TGGGCTGTCACTGGACCTCCTTCCCCT	12	T	0.1026	0.0973
NC_005100.4-9340010	TGGGCTGTCACTGGACCTCCTTCCCCT	25	C	0.0932	0.0889
NC_005100.4-9376102	TCCTTTCTCACGCTCTCTCCATGTGCT	21	A	0.0992	0.1235
NC_005100.4-9391687	ACCTGGGGCACCAAGCCTCCACAGGAC	12	C	0.0488	0.0928
NC_005100.4-9566884	GAATGGAGTACATTTGCTCCGGTTTAG	5	G	0.0536	0.0853
NC_005100.4-9566884	GAATGGAGTACATTTGCTCCGGTTTAG	21	G	0.2321	0.3367
NC_005100.4-9614177	GTGTCTATGACTCGATCTCCTTCCTAT	14	G	0	0.4778
NC_005100.4-9648148	CCAGACCCTACCAGAGCTCCTAGGGAC	13	A	0.2353	0.2076
NC_005100.4-9660427	ATCAGAGCCACATATCCTCCTACTATT	2	T	0.0087	0.4782
NC_005100.4-9660427	ATCAGAGCCACATATCCTCCTACTATT	21	T	0.069	0.1134
NC_005100.4-9818447	CTGTTCTATACATTGTCTCCATATTCT	1	C	0.2787	0.3537
NC_005100.4-9827064	ACAAAACGGACTCAAGCTCCAACCAAG	8	G	0.0488	0.078
NC_005100.4-9833444	AGACAAGCCACCCTTCCTCCTTGATTT	21	T	0.3417	0.4472
NC_005100.4-9895992	TCATCTTAGACAGTCCCTCCTATGCCT	23	T	0	0.4751
NC_005100.4-9946474	GGTATCTGAACATTGGCTCCAGGGAAG	13	T	0.0714	0.0689
NC_005100.4-10247530	CCTTTACGAACCTCTTCTCCATACTAT	2	C	0.2119	0.298
NC_005100.4-10277025	CACCTTCCTACCTCACCTCCCATCCAC	5	T	0.2627	0.4776
NC_005100.4-10277025	CACCTTCCTACCTCACCTCCCATCCAC	27	C	0.2119	0.2871
NC_005100.4-10367928	AGAAAAACAACTGTCTCTCCAGACAAC	6	A	0	0.4746
NC_005100.4-10378191	TACATCCCCACAACCTCTCCTACTAAT	23	C	0.2768	0.3612
NC_005100.4-10378191	TACATCCCCACAACCTCTCCTACTAAT	25	A	0	0.4793
NC_005100.4-10410052	AATCACGAAACCATTTCTCCACACCTC	6	C	0	0.4734
NC_005100.4-10452249	GTTGTACCTACTTGATCTCCTTTAACT	14	G	0.2759	0.4666
NC_005100.4-10503036	ATCTCTCATACTTATTCTCCTTTGATC	25	A	0	0.4793
NC_005100.4-10589544	CTGAACTTAACACTCACTCCACAGGAT	8	T	0.0667	0.095
NC_005100.4-10589544	CTGAACTTAACACTCACTCCACAGGAT	22	C	0.0168	0.4813
NC_005100.4-10630617	TTCACCAGAACCACCTCTCCATTAAAT	25	A	0.5041	0.4548
NC_005100.4-10733362	CCCACCCAAACACCCACTCCTGCCTCA	8	A	0.4098	0.3259
NC_005100.4-10733362	CCCACCCAAACACCCACTCCTGCCTCA	22	G	0.4098	0.3259
NC_005100.4-10744308	CATAGCATCACTGACTCTCCAAAGGTC	26	T	0.2414	0.4786
NC_005100.4-10754718	CTGTCCTAGACGCTCGCTCCACCTTAA	7	T	0.0083	0.4847
NC_005100.4-10754718	CTGTCCTAGACGCTCGCTCCACCTTAA	12	G	0.0083	0.4847
NC_005100.4-10873966	CCTCAGCCTACTGTGTCTCCTGTGCTC	7	C	0	0.4734
NC_005100.4-10873966	CCTCAGCCTACTGTGTCTCCTGTGCTC	12	T	0.0508	0.0812
NC_005100.4-10906687	GCATTGATGACTCCACCTCCTAATCGA	9	G	0.1842	0.295
NC_005100.4-10906687	GCATTGATGACTCCACCTCCTAATCGA	22	A	0.1638	0.2681
NC_005100.4-10962664	ACAGAGATAACCTTATCTCCAGAGTCA	24	G	0	0.4839
NC_005100.4-10994299	TCCTATAAGACAACACCTCCTAACAGT	16	C	0.2212	0.2738
NC_005100.4-11171134	CTTCTCACAACCATCCCTCCAGATTCC	14	T	0	0.4764
NC_005100.4-11182685	CCTCCCTCCACCATGCCTCCTTCTTTT	5	C	0	0.4891
NC_005100.4-11182685	CCTCCCTCCACCATGCCTCCTTCTTTT	14	T	0	0.4875
NC_005100.4-11229545	CTGGTCATCACAGTGCCTCCCATGATC	9	C	0.0175	0.4778
NC_005100.4-11383546	CATGTCCTTACTAGATCTCCAGTATCC	26	C	0.1789	0.189

NC_005100.4-11383546	CATGTCCTTACTAGATCTCCAGTATCC	27	C	0.1789	0.189
NC_005100.4-11388257	TATCTAACAACTTGAACTCCTGCGTAA	23	C	0	0.4755
NC_005100.4-11423213	CCAAAATTGACTATACCTCCACCCTAC	22	C	0.0085	0.4776
NC_005100.4-11609119	GGGCCTGTAACTGGACCTCCTTTTCTT	1	G	0.0261	0.0425
NC_005100.4-11609119	GGGCCTGTAACTGGACCTCCTTTTCTT	4	C	0.0609	0.0911
NC_005100.4-11646610	CCTCTGTCTACTGAGCCTCCTCACCAG	12	T	0	0.4793
NC_005100.4-11646610	CCTCTGTCTACTGAGCCTCCTCACCAG	13	G	0.123	0.1154
NC_005100.4-11654841	CCAAGTTTCACGCCTGCTCCCTGGAGA	13	C	0.0085	0.4882
NC_005100.4-11654841	CCAAGTTTCACGCCTGCTCCCTGGAGA	16	G	0.0254	0.0251
NC_005100.4-11733736	TCACCCTCCACTGTCTCTCCTTCTTTC	14	T	0.0492	0.4934
NC_005100.4-11998572	AACACAAATACCCAGGCTCCTTACAAG	9	T	0.0684	0.066
NC_005100.4-12126242	TTATGAGCAACTGTTACTCCAGCTGAC	27	C	0.0084	0.4682
NC_005100.4-12369606	TACCTACCGACCAGAGCTCCCAGGGAC	27	C	0.0088	0.472
NC_005100.4-12406508	CAGAGCTGCACCATTCCTCCTGGTCTC	13	A	0	0.4743
NC_005100.4-12430352	GTGACCTATACACAGGCTCCATCACAG	12	A	0.1597	0.2852
NC_005100.4-12594476	GCCACTCCAACATCGTCTCCCCTTTAA	14	C	0.0504	0.0805
NC_005100.4-12625666	TAAAGGGCAACCGCGCCTCCCACCCTA	16	C	0.0424	0.4919
NC_005100.4-12636665	GACTACAATACACCAACTCCTGGGTTT	1	G	0	0.4827
NC_005100.4-12748167	GCACCCCAGACATGTACTCCACCACTC	13	T	0	0.4765
NC_005100.4-12767958	TGTGCCCTTACCTTTACTCCAGGCTCA	2	G	0.0488	0.078
NC_005100.4-12802241	ATAAGTCACACAGTCACTCCTGGTGTC	1	A	0.009	0.4869
NC_005100.4-12802241	ATAAGTCACACAGTCACTCCTGGTGTC	5	G	0.0089	0.4885
NC_005100.4-12817178	AGCTAGAAAACCACCCCTCCAGCAAGA	5	A	0	0.4787
NC_005100.4-12820670	CTGCCTCCAACCTGGACTCCCTGTTTC	7	C	0.0254	0.0251
NC_005100.4-12832825	AATCCTATTACGGCATCTCCTTACTTT	12	G	0	0.4832
NC_005100.4-12838157	TTCAAGAGTACCCTGGCTCCAGGGCCT	2	T	0.0609	0.0911
NC_005100.4-12867790	CCCCACAACACTGTTTCTCCCCCATAC	1	C	0.2895	0.3568
NC_005100.4-12949655	CACAGAGGGACTCTGGCTCCACTGTTG	15	G	0	0.4846
NC_005100.4-13046431	AACGGTGCCACCTACCCTCCCCTGCTT	3	C	0	0.4779
NC_005100.4-13072752	AGCGCAGCCACCCACCCTCCTTCACAA	2	G	0	0.4793
NC_005100.4-13072752	AGCGCAGCCACCCACCCTCCTTCACAA	12	C	0.1138	0.4973
NC_005100.4-13099872	ACCCCTGACACCCATTCTCCCCACACC	22	C	0.1148	0.1225
NC_005100.4-13115063	AGTAAACAAACACAAACTCCAACTACT	15	A	0.0085	0.481
NC_005100.4-13140873	AGTAGGGTCACCCACTCTCCTGCCAGT	4	A	0.1171	0.2403
NC_005100.4-13179314	TCCCCTCTTACCATCTCTCCTTAATTT	7	C	0.0496	0.0792
NC_005100.4-13179314	TCCCCTCTTACCATCTCTCCTTAATTT	23	A	0.0083	0.4803
NC_005100.4-13179903	CCACAGCCTACAAGCTCTCCTACTGTC	23	C	0.0431	0.0746
NC_005100.4-13179903	CCACAGCCTACAAGCTCTCCTACTGTC	25	G	0.0862	0.1284
NC_005100.4-13203240	CTTTTTGTCACAGTGTCTCCCTACACA	8	T	0.0496	0.4942
NC_005100.4-13203500	CACTGAGCTACTCAGTCTCCTCCTACT	9	T	0	0.48
NC_005100.4-13205334	CAATGTCTGACGGATGCTCCATCAGCA	15	T	0.042	0.0728
NC_005100.4-13205334	CAATGTCTGACGGATGCTCCATCAGCA	16	G	0.0169	0.0496
NC_005100.4-13265319	AATCCCGCCACGCAGCCTCCTACTCTT	23	C	0.0244	0.0553
NC_005100.4-13354368	TCATAACCAACTCTGGCTCCAGGGGAT	22	G	0.0508	0.4718
NC_005100.4-13467161	CCTCCTGTCACCACCTCTCCTGTACTA	27	A	0	0.4807
NC_005100.4-13471853	GGATATTGGACTCAGCCTCCTTAAATT	16	C	0	0.4764
NC_005100.4-13496383	CATCCTCTCACCCTGACTCCAAGGGTT	5	C	0.2195	0.4741
NC_005100.4-13496383	CATCCTCTCACCCTGACTCCAAGGGTT	15	G	0.1138	0.189
NC_005100.4-13526414	TCGATGTCAACCTTAGCTCCCCATTCT	15	A	0.157	0.4557
NC_005100.4-13526414	TCGATGTCAACCTTAGCTCCCCATTCT	21	C	0.0082	0.4822
NC_005100.4-13604207	GTACCGAGCACCATCTCTCCCTTCCTC	7	A	0.2627	0.487

NC_005100.4-13670867	CGCCTCCAAACCCTGCCTCCCCGTGTC	22	C	0.0417	0.0722
NC_005100.4-13690447	GGGCCTCGGACTGTGGCTCCATGCTAG	4	C	0	0.4746
NC_005100.4-13690447	GGGCCTCGGACTGTGGCTCCATGCTAG	15	G	0	0.4755
NC_005100.4-13695021	TGTGGAGTCACTCGGACTCCCTGACCC	13	C	0.0081	0.4777
NC_005100.4-13698255	TTGACCTCCACCACCACTCCCAACGTG	24	C	0.0508	0.0812
NC_005100.4-13881208	TCCCAGCACACGGTTGCTCCTAGCCGT	13	G	0.0333	0.0328
NC_005100.4-13898934	ATTCCTGCCACACCCTCTCCAACTCCT	12	A	0.1176	0.3014
NC_005100.4-13899495	CCGAAAGCCACCTGGACTCCAACTGCA	9	C	0.0435	0.0752
NC_005100.4-13987535	CAGTACACAACCAGCCCTCCTGAGCTG	6	C	0.05	0.0799
NC_005100.4-14011195	GCTGTCCTGACTCACACTCCTCTCCAA	27	A	0.0169	0.4718
NC_005100.4-14026266	AGGAGTTACACACAACCTCCAACAACA	1	A	0.0504	0.065
NC_005100.4-14026266	AGGAGTTACACACAACCTCCAACAACA	23	C	0.0252	0.0411
NC_005100.4-14037532	ATGCACTTCACATGGTCTCCATTAGAA	12	A	0	0.4864
NC_005100.4-14040963	AGGAGACTGACTCCGTCTCCTTAACTG	12	T	0.0488	0.0476
NC_005100.4-14040963	AGGAGACTGACTCCGTCTCCTTAACTG	14	C	0.0909	0.1163
NC_005100.4-14055332	CCTCACTAAACTACAGCTCCACTCTTC	25	T	0.0569	0.4915
NC_005100.4-14071070	AGACTATATACATGGACTCCAGCTGCA	15	G	0.0536	0.0689
NC_005100.4-14086154	TGTACATGGACTGTCTCTCCCTATAGG	6	A	0.0614	0.0918
NC_005100.4-14086154	TGTACATGGACTGTCTCTCCCTATAGG	14	T	0.0526	0.0839
NC_005100.4-14131545	CATCCCCTTACTCGTCCTCCATAAGTA	25	G	0.0248	0.4863
NC_005100.4-14138641	GAATCTGAGACTTGGTCTCCATAGAAG	9	G	0.0261	0.0257
NC_005100.4-14260195	GAGAGTGACACACTGTCTCCAAACAGG	1	G	0.0513	0.4868
NC_005100.4-14309552	ATCCTGTTCACCTACTCTCCTCTTTCT	22	C	0.1696	0.4952
NC_005100.4-14395290	GGAAGGTTCACCTCACCTCCAACCTGC	22	A	0	0.4671
NC_005100.4-14395290	GGAAGGTTCACCTCACCTCCAACCTGC	26	G	0	0.4715
NC_005100.4-14413829	ACTGTCCTCACAGACCCTCCTTGGGCA	8	T	0.1301	0.3334
NC_005100.4-14435341	CTTAATAGAACGGAAGCTCCCTGTAGA	8	G	0	0.4793
NC_005100.4-14489116	GAGGTGGGGACATTAACTCCACCCATA	5	T	0.0991	0.2656
NC_005100.4-14489116	GAGGTGGGGACATTAACTCCACCCATA	13	T	0	0.4807
NC_005100.4-14490748	GTGATGCGTACACAGACTCCAGATGAA	6	G	0.0598	0.058
NC_005100.4-14490748	GTGATGCGTACACAGACTCCAGATGAA	8	G	0.0769	0.4987
NC_005100.4-14512014	AAAGCCAGAACAGAGGCTCCAACTCAG	22	A	0.0536	0.4898
NC_005100.4-14689196	TGTCTACAGACAGGAGCTCCATGATGA	24	A	0.0492	0.0786
NC_005100.4-14689196	TGTCTACAGACAGGAGCTCCATGATGA	25	T	0.0496	0.4901
NC_005100.4-14718460	TAGTTTATAACTATCTCTCCACCACTT	1	T	0.0877	0.16
NC_005100.4-14753597	CTCAGGGAAACAAGAACTCCTGGGCTG	9	A	0.2564	0.4693
NC_005100.4-14757056	TGTATCCCAACCTTATCTCCACATCCG	15	A	0	0.4793
NC_005100.4-14802894	AGCTTCAGCACCCGCGCTCCGGGTTTG	21	G	0.0179	0.1014
NC_005100.4-14806478	ACAGCTCATACTAGGTCTCCTCACAAG	21	T	0	0.4827
NC_005100.4-14806478	ACAGCTCATACTAGGTCTCCTCACAAG	22	C	0.1754	0.16
NC_005100.4-14813587	GAAAGGACTACAAAGACTCCTAAAGAT	12	A	0	0.4749
NC_005100.4-14847138	CCCCGGAGCACAGAGTCTCCTTGATGT	1	C	0.0165	0.0943
NC_005100.4-14847138	CCCCGGAGCACAGAGTCTCCTTGATGT	16	T	0.0165	0.0943
NC_005100.4-14903046	GAGGACAGCACTCAGCCTCCCAGTTTC	24	T	0.0086	0.4595
NC_005100.4-14920377	CCGTTGTAAACTTTTCCTCCACAGGAC	21	A	0	0.4851
NC_005100.4-14930822	AGAATCACTACACGGCCTCCATGTCTT	4	A	0.0088	0.4861
NC_005100.4-14930822	AGAATCACTACACGGCCTCCATGTCTT	22	T	0.0526	0.4975
NC_005100.4-14945895	TGTTCATTCACTCTGTCTCCTCACTTT	6	A	0.0855	0.4693
NC_005100.4-14945895	TGTTCATTCACTCTGTCTCCTCACTTT	24	C	0	0.4868
NC_005100.4-14951913	ATTGCAGGCACGCATGCTCCATCAAGT	24	A	0.181	0.3231
NC_005100.4-14952109	TGGTATCAGACAGCGCCTCCTCTGGCT	3	G	0.0583	0.49

NC_005100.4-14952109	TGGTATCAGACAGCGCCTCCTCTGGCT	8	A	0.0083	0.4732
NC_005100.4-14953921	TCATTTAGTACACTTCCTCCCCCATCT	23	C	0.1789	0.2382
NC_005100.4-14953921	TCATTTAGTACACTTCCTCCCCCATCT	24	A	0.1789	0.2382
NC_005100.4-14982064	GAGTCTGTTACTGAGTCTCCACACTGA	4	T	0	0.4704
NC_005100.4-15012796	GGGAACAAAACTAACTCTCCTGGGTTT	8	A	0.0088	0.4774
NC_005100.4-15031085	ATTTCAGTCACTCCGGCTCCTGCTGAG	7	G	0.2314	0.3467
NC_005100.4-15060263	CCTCAGAGGACACAAGCTCCATGGGGG	21	A	0.2479	0.4714
NC_005100.4-15106227	GCGAACTACACTGCCTCTCCTAAGCAA	3	G	0.1736	0.2704
NC_005100.4-15117500	TGTATGGCCACTGAGTCTCCAAGCGTC	21	A	0.1405	0.1982
NC_005100.4-15117500	TGTATGGCCACTGAGTCTCCAAGCGTC	24	C	0.0496	0.4942
NC_005100.4-15159754	AACGTGCAGACTGAGGCTCCCCTCAGC	21	C	0	0.4724
NC_005100.4-15159754	AACGTGCAGACTGAGGCTCCCCTCAGC	24	C	0	0.4724
NC_005100.4-15185558	CTGATAAGCACGTGGTCTCCAGAGAAA	4	A	0.0526	0.0997
NC_005100.4-15230184	TGTAGAAACACAATTTCTCCTTTGGAA	3	T	0	0.4786
NC_005100.4-15257275	AACTTATGTACCTCTTCTCCTGTGAAC	7	T	0.0413	0.4835
NC_005100.4-15257275	AACTTATGTACCTCTTCTCCTGTGAAC	22	G	0.0579	0.0562
NC_005100.4-15271014	CCAAACATCACGTGTACTCCCAAGAGT	12	G	0.0531	0.0846
NC_005100.4-15271146	ACAGATACAACTGAGTCTCCAACAAAA	8	C	0	0.4807
NC_005100.4-15445407	AACCGCTGAACTATTCCTCCAACTGAG	13	A	0.0508	0.4826
NC_005100.4-15445407	AACCGCTGAACTATTCCTCCAACTGAG	15	T	0.2017	0.2568
NC_005100.4-15459226	GTATCTAAGACCGGGACTCCTGGTATT	5	C	0.0504	0.0805
NC_005100.4-15459226	GTATCTAAGACCGGGACTCCTGGTATT	16	A	0	0.4743
NC_005100.4-15486674	TGAGAGAGCACGTGCTCTCCATGTCCT	21	A	0	0.4749
NC_005100.4-15486674	TGAGAGAGCACGTGCTCTCCATGTCCT	22	T	0	0.4749
NC_005100.4-15505091	TGGCCTGCTACCCTGGCTCCTTCAGCT	21	T	0	0.4751
NC_005100.4-15505091	TGGCCTGCTACCCTGGCTCCTTCAGCT	23	C	0.0492	0.0786
NC_005100.4-15524451	TCCTCCGCCACAGCATCTCCACACAGA	7	G	0	0.488
NC_005100.4-15530793	TAGCCTTGGACCTCCTCTCCCCACCAC	13	T	0.0492	0.0786
NC_005100.4-15530793	TAGCCTTGGACCTCCTCTCCCCACCAC	24	C	0.1066	0.1154
NC_005100.4-15532896	GATAGGAGTACCTGACCTCCGTCCAGT	16	C	0.1333	0.255
NC_005100.4-15532896	GATAGGAGTACCTGACCTCCGTCCAGT	21	G	0.1833	0.4932
NC_005100.4-15535324	GCAATTAAGACAAGGCCTCCTGCCCTT	2	C	0	0.4922
NC_005100.4-15535324	GCAATTAAGACAAGGCCTCCTGCCCTT	23	C	0	0.4922
NC_005100.4-15662538	CGATGTTACACTCCTACTCCCAGCCCC	27	C	0.094	0.2041
NC_005100.4-15683523	CACATAGGGACCAGGCCTCCAACATTT	2	A	0	0.4682
NC_005100.4-15683523	CACATAGGGACCAGGCCTCCAACATTT	12	C	0.0087	0.466
NC_005100.4-15695737	TGTTCTAAGACAAGCTCTCCTTCGTGT	3	T	0	0.4773
NC_005100.4-15722843	GTGGCACACACCTTTACTCCCAACATT	12	C	0.178	0.298
NC_005100.4-15722843	GTGGCACACACCTTTACTCCCAACATT	14	T	0.1897	0.482
NC_005100.4-15787327	AACTTCTGAACCATCTCTCCAGCATTT	2	A	0.4472	0.4703
NC_005100.4-15787327	AACTTCTGAACCATCTCTCCAGCATTT	26	T	0.4426	0.3447
NC_005100.4-15854762	GCTGTCCTTACAGGCTCTCCTACTGTT	26	T	0.0084	0.4829
NC_005100.4-15886299	TGATCCCTGACAGAATCTCCATGACCT	1	T	0	0.4851
NC_005100.4-15901518	TGATCGCTAACAGTGGCTCCTTTGAGT	1	T	0.0656	0.0786
NC_005100.4-15908097	CACTGCAACACCCCCTCTCCCGTCCCT	6	C	0.0163	0.032
NC_005100.4-15908097	CACTGCAACACCCCCTCTCCCGTCCCT	22	G	0.065	0.1073
NC_005100.4-15966819	TGTCCCAAGACAAGAGCTCCTCACTTC	24	C	0.1545	0.3287
NC_005100.4-15977493	CTACTTTTTACACTGGCTCCTCAGCAG	5	T	0	0.4793
NC_005100.4-15999236	CTCATTGTCACCCCGGCTCCTCCTGCA	5	T	0.2623	0.4515
NC_005100.4-16103024	CCGCAGGTGACTACAGCTCCCACAAAT	13	A	0	0.4677
NC_005100.4-16180166	CCTGGCCCCACCTTCTCTCCCACTCTC	12	C	0.0268	0.0264

NC_005100.4-16227096	TAAACAGAGACGGAGTCTCCTGAGCGT	12	G	0.161	0.4896
NC_005100.4-16251566	CTCACACACACAGACACTCCCACATAT	2	T	0	0.4793
NC_005100.4-16251566	CTCACACACACAGACACTCCCACATAT	4	A	0.065	0.1073
NC_005100.4-16346039	TGACGGTGCACCCCTCCTCCCCCACCA	5	G	0	0.4909
NC_005100.4-16346039	TGACGGTGCACCCCTCCTCCCCCACCA	21	C	0.0087	0.0425
NC_005100.4-16347604	TCACAAATTACTATGGCTCCCTCTACG	26	C	0.0526	0.0839
NC_005100.4-16351348	CTTACCAGGACATTCTCTCCATTAAAG	4	A	0.0536	0.0853
NC_005100.4-16351348	CTTACCAGGACATTCTCTCCATTAAAG	14	T	0	0.4731
NC_005100.4-16356638	AGATGATACACAGTAGCTCCTTGTATA	21	T	0.0179	0.4841
NC_005100.4-16376395	TCATACCGGACTCGATCTCCCTGGGCT	8	G	0	0.4779
NC_005100.4-16445723	TCAGACGTGACCTCTCCTCCAGGCATT	6	C	0.0804	0.1093
NC_005100.4-16445723	TCAGACGTGACCTCTCCTCCAGGCATT	7	G	0.0804	0.1093
NC_005100.4-16445772	ATAGCCCGGACTCTGTCTCCGTGAGCA	7	C	0.0885	0.1316
NC_005100.4-16550917	CCCTTTCCCACCCTTTCTCCTGTATCT	1	C	0	0.4841
NC_005100.4-16555056	AGGCTCCTTACGGGGACTCCAGCTTCA	15	G	0.2105	0.4846
NC_005100.4-16631212	TTCCACAGAACACCTCCTCCCTACGTC	25	G	0.0488	0.078
NC_005100.4-16643841	TTTTCTTGCACTGTGACTCCTGACTAC	8	G	0.1545	0.3646
NC_005100.4-16649071	TAAACACAAACACTTCCTCCATAATGA	25	T	0	0.4864
NC_005100.4-16654018	CTCTTCCTCACATCTTCTCCAGACACC	27	C	0	0.0342
NC_005100.4-16665993	CTTGATAAAACAGAATCTCCTCGAGGG	22	C	0	0.48
NC_005100.4-16667881	TGCATGAGGACTGGCCCTCCTCTCCCA	6	G	0.0417	0.0722
NC_005100.4-16667881	TGCATGAGGACTGGCCCTCCTCTCCCA	16	C	0.1271	0.2026
NC_005100.4-16720423	CTGGTTTTAACTTTCACTCCTTTCGTT	4	G	0.0569	0.0553
NC_005100.4-16720423	CTGGTTTTAACTTTCACTCCTTTCGTT	24	C	0.0569	0.0553
NC_005100.4-16735794	GTGGTGGCCACAGCCCCTCCCTTGGGC	5	T	0.2389	0.4373
NC_005100.4-16739761	GGGATCGAGACATAGACTCCAGCGCTA	5	T	0.0439	0.138
NC_005100.4-16739761	GGGATCGAGACATAGACTCCAGCGCTA	23	C	0	0.4814
NC_005100.4-16767996	GTTACACACACGTGCACTCCGTGATTG	2	T	0.0536	0.0853
NC_005100.4-16767996	GTTACACACACGTGCACTCCGTGATTG	8	A	0.0531	0.0846
NC_005100.4-16801992	TCCTTCTCTACCACACCTCCCACGGGC	14	C	0.0526	0.0839
NC_005100.4-16801992	TCCTTCTCTACCACACCTCCCACGGGC	24	G	0	0.4778
NC_005100.4-16824651	CCGATGTGCACTACTACTCCGAGCAGG	2	C	0	0.4813
NC_005100.4-16824651	CCGATGTGCACTACTACTCCGAGCAGG	12	T	0.0504	0.494
NC_005100.4-16827464	TGAGCTATAACCACATCTCCTTTATCT	15	A	0.0333	0.0488
NC_005100.4-17060611	CACATTCATACATACACTCCCATACAT	9	T	0.1062	0.4953
NC_005100.4-17060611	CACATTCATACATACACTCCCATACAT	15	C	0.0259	0.0255
NC_005100.4-17147796	ATAGTCTGTACACAAACTCCTTATAAC	15	A	0.181	0.4768
NC_005100.4-17255999	AGTAGGATCACCGTTCCTCCAACAGCT	13	G	0.1333	0.2994
NC_005100.4-17263733	TTTCAGCTCACATCCCCTCCATTCTCA	25	T	0.1351	0.1565
NC_005100.4-17350127	TGATAAGGCACACTTGCTCCCTTTAGT	3	A	0.0583	0.0566
NC_005100.4-17350127	TGATAAGGCACACTTGCTCCCTTTAGT	12	A	0.0084	0.4761
NC_005100.4-17355735	CTGCCTCTCACTGTGGCTCCTTTCCGC	6	T	0.0684	0.066
NC_005100.4-17475387	TCCTTAAACACGACCACTCCTTTTCGT	5	T	0	0.4773
NC_005100.4-17475387	TCCTTAAACACGACCACTCCTTTTCGT	26	G	0.0492	0.0786
NC_005100.4-17512282	GGTCCAGTAACACAGCCTCCTTGCAAC	1	G	0.0492	0.0786
NC_005100.4-17522307	AGGACTTTGACCTTTCCTCCTTCTACT	2	G	0.1261	0.4999
NC_005100.4-17522307	AGGACTTTGACCTTTCCTCCTTCTACT	26	C	0.084	0.1254
NC_005100.4-17535021	GAAGCTACGACCTTAACTCCTGATTTA	7	A	0.0331	0.4819
NC_005100.4-17535021	GAAGCTACGACCTTAACTCCTGATTTA	27	A	0.0331	0.4819
NC_005100.4-17543328	CTCCCTTTCACACACCCTCCACCCCAT	12	A	0.0342	0.0336
NC_005100.4-17543328	CTCCCTTTCACACACCCTCCACCCCAT	15	C	0.0862	0.4946

NC_005100.4-17665145	CGCTGCAAAACGCAGTCTCCATTCCAT	2	G	0.0081	0.0398
NC_005100.4-17712942	TTATAAAACACCAAGTCTCCCAAAGAA	4	T	0.0603	0.0585
NC_005100.4-17712942	TTATAAAACACCAAGTCTCCCAAAGAA	24	A	0.1842	0.2599
NC_005100.4-17717151	TAATGTCTAACCATCTCTCCACCCTCC	7	C	0.0496	0.4942
NC_005100.4-17798337	AAGCTACTGACACCAGCTCCCATCTCT	25	T	0	0.032
NC_005100.4-17866534	CCTCTGAAGACAGCAGCTCCGTAAGTC	21	G	0	0.48
NC_005100.4-17866534	CCTCTGAAGACAGCAGCTCCGTAAGTC	22	T	0.0496	0.0792
NC_005100.4-17869202	CTGATGTGCACGCACTCTCCAACGGCG	27	G	0	0.4868
NC_005100.4-17885855	AATCTTCCCACACTTTCTCCATGGACA	15	T	0.0496	0.0792
NC_005100.4-17974062	CAGAGACCGACTTGCTCTCCACGGTTA	9	G	0.2295	0.387
NC_005100.4-18001705	CTACACCCAACACACCCTCCGGCAATG	25	A	0.0504	0.0805
NC_005100.4-18018421	CAACATGTAACTCCAGCTCCAGGGGGT	4	C	0.0531	0.0846
NC_005100.4-18033246	CTGTAGCAAACGTCTGCTCCCTAGGCT	5	A	0	0.4728
NC_005100.4-18127904	TGAGTTATTACCCATACTCCTGGACTC	6	T	0	0.4757
NC_005100.4-18127904	TGAGTTATTACCCATACTCCTGGACTC	27	C	0	0.4772
NC_005100.4-18128418	CTACTGCCGACACTTGCTCCACCATCT	12	A	0.0336	0.033
NC_005100.4-18161421	CACATGTAAACTACATCTCCTTGAAGT	3	C	0.0328	0.0322
NC_005100.4-18226218	ATTATAACTACTGTCTCTCCCCAGCCT	9	T	0	0.4819
NC_005100.4-18236297	CTAACAGGCACATCACCTCCTTAAATA	9	C	0.2459	0.2279
NC_005100.4-18252079	GAGGATGATACCTGAGCTCCCTTAATT	15	A	0.0085	0.479
NC_005100.4-18252079	GAGGATGATACCTGAGCTCCCTTAATT	23	T	0.0342	0.0336
NC_005100.4-18614794	TGTTGCGAGACTGGGTCTCCTTGCAAT	5	G	0.2155	0.3956
NC_005100.4-18614794	TGTTGCGAGACTGGGTCTCCTTGCAAT	6	C	0.2261	0.4017
NC_005100.4-18624188	CATAACTGAACCATCTCTCCGTTACCC	16	T	0	0.48
NC_005100.4-18681554	CTGCTGCCAACTTGCCCTCCAATAGTC	3	G	0.0325	0.0476
NC_005100.4-18681554	CTGCTGCCAACTTGCCCTCCAATAGTC	22	A	0.0328	0.4656
NC_005100.4-18710701	GTCATCAACACCATGACTCCTGTGTCC	1	G	0	0.4728
NC_005100.4-18779224	TTACACACAACTCTGGCTCCAGGGGAT	9	A	0.1261	0.4977
NC_005100.4-18896811	TATCTACCAACAAGACCTCCTTGGGAC	5	T	0.0083	0.4803
NC_005100.4-18930868	CTCCTTCTAACTACCACTCCCTCCGTC	25	G	0	0.4755
NC_005100.4-18963695	CACATTCAAACATCACCTCCCTGTTTT	8	A	0	0.4846
NC_005100.4-18963695	CACATTCAAACATCACCTCCCTGTTTT	24	T	0	0.4846
NC_005100.4-19074828	ACCCATTAAACACAGGCTCCATTTAAT	4	C	0	0.48
NC_005100.4-19074828	ACCCATTAAACACAGGCTCCATTTAAT	6	T	0.2101	0.2852
NC_005100.4-19138593	TGATCTAAAACATACCCTCCTTGCCAT	13	T	0.0174	0.4537
NC_005100.4-19147474	CATAGTGACACACTTCCTCCACAAGGC	26	G	0.0256	0.0253
NC_005100.4-19164228	GGAGTTGGAACATGGCCTCCTTACTCT	5	T	0.0684	0.4938
NC_005100.4-19209789	CCAGTGCACACACAGACTCCTCTGATG	16	A	0.0268	0.0264
NC_005100.4-19222043	AACGCATTCACCTCAGCTCCAGCCCAC	21	A	0	0.48
NC_005100.4-19272157	GAAACTCAGACATAGGCTCCATGCACT	15	G	0.1638	0.4997
NC_005100.4-19298069	ACTCCAAGGACCCATCCTCCCTAGTAC	5	C	0.0413	0.1017
NC_005100.4-19298069	ACTCCAAGGACCCATCCTCCCTAGTAC	24	G	0.0331	0.0639
NC_005100.4-19354041	CCAACTCAGACCTCCTCTCCTCAGAAT	2	C	0.1138	0.2017
NC_005100.4-19358771	TGGTCTTAAACTCTTTCTCCCAAGGCT	25	G	0.123	0.2903
NC_005100.4-19388359	CTTGTCTGGACCCCATCTCCTCTTCCC	9	G	0.2149	0.4849
NC_005100.4-19417233	ATATGCATCACATAGGCTCCTATATAC	6	C	0.0348	0.4482
NC_005100.4-19438367	CTGTTTGAGACCGGATCTCCTTGTGCA	13	G	0.2623	0.3354
NC_005100.4-19451611	GAATCAGACACACCCCCTCCAAGACAA	8	A	0	0.4793
NC_005100.4-19675010	AGGTAGACTACCTAGACTCCTTTGTTG	6	G	0	0.4832
NC_005100.4-19675010	AGGTAGACTACCTAGACTCCTTTGTTG	15	G	0.0583	0.0722
NC_005100.4-19678598	TACTTCTATACTGCTCCTCCAAGCTTT	1	T	0.0531	0.4934

NC_005100.4-19678598	TACTTCTATACTGCTCCTCCAAGCTTT	2	A	0.0531	0.4934
NC_005100.4-19868345	CTACCTGTCACCCCAGCTCCAATAGAT	8	T	0.0084	0.4723
NC_005100.4-19868345	CTACCTGTCACCCCAGCTCCAATAGAT	24	A	0.0588	0.4859
NC_005100.4-19909870	AAGATGAACACCTTATCTCCTTGCAGA	7	A	0.0488	0.4926
NC_005100.4-19909870	AAGATGAACACCTTATCTCCTTGCAGA	16	T	0.0492	0.0786
NC_005100.4-19911474	TCTCTCAGAACCCTAACTCCTCAAGCA	21	T	0.0085	0.487
NC_005100.4-20011535	CAGGCAGAGACCTCCACTCCTGTACTC	16	A	0	0.482
NC_005100.4-20103944	GATGAGGACACCAGACCTCCTAGTCAT	7	G	0.0492	0.048
NC_005100.4-20106052	ATCTGGAGCACATCGACTCCAAGAAAT	15	G	0.2683	0.2778
NC_005100.4-20133360	GTGTCCCTGACTCTCTCTCCATGTGTG	6	C	0.0328	0.048
NC_005100.4-20191853	GAATGAGGCACAGAACCTCCTTTGTGT	3	A	0	0.4826
NC_005100.4-20191853	GAATGAGGCACAGAACCTCCTTTGTGT	14	A	0	0.4839
NC_005100.4-20234231	ACATTCCAGACGTTATCTCCCTCCCAG	15	A	0.9831	0.4999
NC_005100.4-20234231	ACATTCCAGACGTTATCTCCCTCCCAG	23	C	0.0256	0.0253
NC_005100.4-20269361	CTAATGTTTACAGCTTCTCCCAACCAG	22	A	0.1475	0.316
NC_005100.4-20274943	GGTGCCCCTACTTCCCCTCCTTTCCTG	16	C	0.0579	0.0868
NC_005100.4-20304119	CTCTTTCAGACCGACTCTCCTACCTCC	25	T	0.113	0.2332
NC_005100.4-20343513	ATACCTTCCACCTGTGCTCCATTGGTC	21	A	0.0081	0.481
NC_005100.4-20404586	ATCTTGAAAACACAAACTCCTTTAGCA	7	A	0.1148	0.402
NC_005100.4-20424511	AGTAGCCAAACAGGACCTCCAGTAGCA	14	G	0.1475	0.1505
NC_005100.4-20471233	ATCAGGGACACAAAGTCTCCTAGAACA	25	A	0.0261	0.4994
NC_005100.4-20471233	ATCAGGGACACAAAGTCTCCTAGAACA	26	C	0.0261	0.4994
NC_005100.4-20525673	TGCTAAGAAACCAATTCTCCCACCTCC	1	T	0.3274	0.2738
NC_005100.4-20526442	CACACACACACACACACTCCCTCATTC	2	A	0.3636	0.2975
NC_005100.4-20539871	CTAGCTATCACAGCACCTCCATTCTTC	13	G	0.0442	0.0433
NC_005100.4-20539871	CTAGCTATCACAGCACCTCCATTCTTC	15	A	0	0.4793
NC_005100.4-20602177	CACACACACACCTCTACTCCATTCCAC	7	C	0.2295	0.263
NC_005100.4-20631568	TATAGGGCAACGGGAACTCCCAGGAAT	3	T	0.0339	0.4964
NC_005100.4-20631568	TATAGGGCAACGGGAACTCCCAGGAAT	12	G	0.1379	0.4214
NC_005100.4-20632683	CTGGCTGCCACTGTTACTCCCCTCTAG	4	G	0.1913	0.32
NC_005100.4-20692461	TGCTTCCCTACTTCACCTCCAGACATT	15	A	0.25	0.2698
NC_005100.4-20882026	TGTCCCCCAACCTGGTCTCCCCAGCAT	14	G	0	0.4819
NC_005100.4-20892768	ACGCAGTCCACGGGGGCTCCTCAGCCA	3	G	0.0082	0.4822
NC_005100.4-20892768	ACGCAGTCCACGGGGGCTCCTCAGCCA	23	A	0.0082	0.4822
NC_005100.4-20944314	AGCATGCACACTATGCCTCCTCCCATG	2	G	0	0.4751
NC_005100.4-20980272	CCTCCTGACACCATCGCTCCCACAGCA	14	T	0.0588	0.4949
NC_005100.4-21037700	CAGTGAAGTACGCTCGCTCCTAGGGAG	21	T	0.0488	0.078
NC_005100.4-21054736	TTATTTGAAACAGGATCTCCTGTAGCC	15	A	0.05	0.4911
NC_005100.4-21054736	TTATTTGAAACAGGATCTCCTGTAGCC	21	T	0.05	0.4911
NC_005100.4-21055047	AAAGTCTGCACAGCCCCTCCATTGTTA	15	C	0	0.4832
NC_005100.4-21055047	AAAGTCTGCACAGCCCCTCCATTGTTA	23	T	0	0.4832
NC_005100.4-21066318	GAGTTCAAAACCCCACCTCCCACGGCC	3	G	0	0.4728
NC_005100.4-21139003	CCACCCCATACCCTGCCTCCTTGGCTC	14	T	0.4083	0.325
NC_005100.4-21151815	TGTGTCTAGACTGACACTCCGGGAGTG	1	T	0	0.4731
NC_005100.4-21154381	GTCACGTGTACTTTTCCTCCTTGTCTT	5	C	0.0813	0.496
NC_005100.4-21154381	GTCACGTGTACTTTTCCTCCTTGTCTT	7	T	0.0488	0.0476
NC_005100.4-21159836	CAGGATAACACAGACACTCCACAATCA	22	C	0	0.4832
NC_005100.4-21205534	AATAAGCAAACAAAACCTCCATTGGCT	7	C	0.0504	0.0805
NC_005100.4-21205534	AATAAGCAAACAAAACCTCCATTGGCT	27	T	0.0508	0.4908
NC_005100.4-21240384	CTGCCTGTAACTTCAGCTCCTGGGAAA	1	C	0.0508	0.0812
NC_005100.4-21262474	TTGGTCTTAACCCATCCTCCCTTTCAT	9	A	0.1951	0.2263

NC_005100.4-21366549	GAGCTCAGAACTATAGCTCCCGTGCTT	24	G	0.1463	0.464
NC_005100.4-21366549	GAGCTCAGAACTATAGCTCCCGTGCTT	27	T	0	0.4793
NC_005100.4-21405345	CACCGTGATACATTTCCTCCAACAAGA	5	G	0.0417	0.0408
NC_005100.4-21433649	GACACATTCACACCAACTCCCCCATAC	2	A	0.0085	0.4823
NC_005100.4-21433649	GACACATTCACACCAACTCCCCCATAC	27	C	0.1111	0.4999
NC_005100.4-21542455	ACCTCACCCACAGACGCTCCAGGCATG	21	A	0	0.4773
NC_005100.4-21550145	TTGTGGTTCACTATTCCTCCTGCTAGG	22	G	0.0536	0.0853
NC_005100.4-21658954	AACACAGTCACAGTTCCTCCTGCAGAG	22	G	0.0508	0.0812
NC_005100.4-21700817	GTCTAGACCACAATGGCTCCTTTGTGC	6	G	0.0351	0.4654
NC_005100.4-21734704	GGTCCCGCAACCACATCTCCTTATACA	21	T	0	0.4807
NC_005100.4-21734704	GGTCCCGCAACCACATCTCCTTATACA	23	A	0	0.4807
NC_005100.4-21747820	CTCTGGGACACCAGGTCTCCTGCCTTA	7	G	0.0496	0.0792
NC_005100.4-21899734	TTGTATATCACACCATCTCCAGTCCTT	5	A	0.2288	0.4273
NC_005100.4-21993890	TCAGATTCCACTATGTCTCCATATGAA	1	T	0.0492	0.0786
NC_005100.4-21994012	TCCATTTCCACTTGGACTCCTCTGCCT	14	G	0	0.477
NC_005100.4-21997170	AAGGCAGATACTGGGACTCCTCAGAGC	25	A	0.0496	0.4901
NC_005100.4-22051017	TACTGTCCCACACAGACTCCCTTGTCT	2	A	0.1803	0.4952
NC_005100.4-22051017	TACTGTCCCACACAGACTCCCTTGTCT	6	T	0.0569	0.0705
NC_005100.4-22076981	AGGCTGATAACGTTTTCTCCACTGTTG	2	G	0.0085	0.481
NC_005100.4-22076981	AGGCTGATAACGTTTTCTCCACTGTTG	5	T	0.0085	0.481
NC_005100.4-22077995	AGAGCAGACACTCAGACTCCAAGTGTG	24	T	0.1176	0.3014
NC_005100.4-22079945	ATCCTGGGTACCACAGCTCCATACAAG	7	G	0.1736	0.4995
NC_005100.4-22079945	ATCCTGGGTACCACAGCTCCATACAAG	21	A	0	0.4751
NC_005100.4-22096563	CCCAAGAGCACCTTGTCTCCCCCATCT	12	C	0.0244	0.0398
NC_005100.4-22115068	TTCTCAATAACAAAGACTCCTGCATTC	21	T	0.2846	0.4174
NC_005100.4-22115068	TTCTCAATAACAAAGACTCCTGCATTC	23	C	0.0488	0.078
NC_005100.4-22135163	TCCATGTGCACATAACCTCCCCCATAC	9	C	0.1709	0.497
NC_005100.4-22141421	GAATGTAGCACCACTTCTCCTTTTCAA	1	G	0.0488	0.078
NC_005100.4-22141421	GAATGTAGCACCACTTCTCCTTTTCAA	3	A	0.0488	0.4926
NC_005100.4-22146584	CCCTCAGATACTGAAACTCCTGGAAAT	2	C	0.0588	0.4949
NC_005100.4-22146584	CCCTCAGATACTGAAACTCCTGGAAAT	8	A	0	0.4826
NC_005100.4-22154602	AGTTAGTCAACCTTGTCTCCTCAGTTT	15	G	0.0086	0.4836
NC_005100.4-22154602	AGTTAGTCAACCTTGTCTCCTCAGTTT	27	T	0.0345	0.488
NC_005100.4-22263552	GTTGTATGAACAGAGTCTCCCGAGATC	3	T	0	0.4793
NC_005100.4-22263552	GTTGTATGAACAGAGTCTCCCGAGATC	4	G	0	0.4793
NC_005100.4-22273340	CAGAGTGCTACAGTCACTCCTTAGCCT	22	T	0.0083	0.4803
NC_005100.4-22289869	CGCTTGAGAACCAGCCCTCCCAACCAC	2	G	0.1653	0.4959
NC_005100.4-22454850	GAATGCTTAACCTTCCCTCCTTCATGT	4	T	0.0488	0.078
NC_005100.4-22524187	ACCCCTGTAACCCCATCTCCAGGGAAC	12	C	0.1271	0.1894
NC_005100.4-22528104	GCAGTCATTACGTCATCTCCAGAAGAG	5	T	0	0.4724
NC_005100.4-22528104	GCAGTCATTACGTCATCTCCAGAAGAG	23	A	0	0.4724
NC_005100.4-22542508	TCCAAGCAGACCAAACCTCCCATTCAT	14	A	0	0.4793
NC_005100.4-22542508	TCCAAGCAGACCAAACCTCCCATTCAT	24	T	0.122	0.4979
NC_005100.4-22559688	ATGGTCATCACTATCGCTCCGATCATG	2	T	0.122	0.1826
NC_005100.4-22569071	CCCAGCCACACCCCTTCTCCTCCAGTA	3	C	0.1552	0.1855
NC_005100.4-22569071	CCCAGCCACACCCCTTCTCCTCCAGTA	25	G	0.0517	0.0825
NC_005100.4-22614063	TTCTGTAATACCAGTTCTCCTGTAGCT	1	T	0	0.4624
NC_005100.4-22745336	CTCTTCTCGACATCTTCTCCTTAAAGA	9	G	0.1583	0.3708
NC_005100.4-22760720	AAATTTTGGACTCAGGCTCCCTGGGGG	14	A	0.0541	0.086
NC_005100.4-22760720	AAATTTTGGACTCAGGCTCCCTGGGGG	21	C	0.2857	0.4362
NC_005100.4-22855096	TGCCTGACTACGTGGGCTCCACCTTAT	13	T	0.0431	0.0903

NC_005100.4-22855096	TGCCTGACTACGTGGGCTCCACCTTAT	15	G	0.0086	0.4836
NC_005100.4-22897580	CTGTCTGTAACTCCAGCTCCAGTGTCT	7	G	0.281	0.418
NC_005100.4-22911627	CTGCACATAACCAATGCTCCAAACCGC	25	C	0	0.4887
NC_005100.4-23015334	TTAGAGTAGACATACTCTCCAGGTTTG	7	T	0.0087	0.4744
NC_005100.4-23035726	ACAAGGTGAACATGTGCTCCACATGTG	6	G	0	0.4779
NC_005100.4-23058173	GGATCCTACACACTCTCTCCTAGTCTT	9	C	0.1739	0.1869
NC_005100.4-23284716	ATCCTGAAGACCATTTCTCCTTTCCAC	13	A	0.1858	0.3189
NC_005100.4-23317523	TACACATGCACACACACTCCTTCTGAC	27	C	0.187	0.4952
NC_005100.4-23329265	AAGAGAAGAACCATTCCTCCTCAGTCT	25	T	0	0.4703
NC_005100.4-23332833	CCTCTCTCAACCATTTCTCCCAGATAC	13	A	0.193	0.2538
NC_005100.4-23438857	AGTAAGAAGACCAGAACTCCCTGGTAG	9	G	0.0333	0.0328
NC_005100.4-23611722	TAACATGATACAGATTCTCCTCTCATT	13	G	0.0579	0.0716
NC_005100.4-23641246	AGGGCTGCAACAGAACCTCCAAAGATA	1	A	0	0.4819
NC_005100.4-23643621	CCCAATCCCACCCTATCTCCTGTGGTG	24	G	0.2562	0.4769
NC_005100.4-23647872	AACAAGTGCACAGGTGCTCCTCAAGGC	16	G	0.0168	0.033
NC_005100.4-23647872	AACAAGTGCACAGGTGCTCCTCAAGGC	23	A	0.1652	0.4903
NC_005100.4-23675197	CATCTCTAGACTTCACCTCCCCGATCT	23	G	0.1864	0.4425
NC_005100.4-23678017	TGCTAGAAGACACTGTCTCCTTATAGT	14	T	0.1949	0.375
NC_005100.4-23687265	GAAAGCAGAACGCGTACTCCATAAAAC	12	G	0.1157	0.389
NC_005100.4-23687265	GAAAGCAGAACGCGTACTCCATAAAAC	27	C	0	0.4743
NC_005100.4-23700711	CTTCTTGCCACATGGCCTCCTAGGTGG	24	G	0.0536	0.4943
NC_005100.4-23716106	CCGAGGCCAACAAGGACTCCGGCAATG	2	C	0.0574	0.0861
NC_005100.4-23795312	TGTATCCTAACCGTCTCTCCATATAAT	27	T	0.05	0.095
NC_005100.4-23847491	CGAAGTGAGACACTGTCTCCAAAACAA	13	C	0.0085	0.479
NC_005100.4-23865435	CCGCACCAAACGGTGGCTCCCAACTGT	2	C	0.0976	0.2382
NC_005100.4-23865435	CCGCACCAAACGGTGGCTCCCAACTGT	23	A	0.1789	0.4926
NC_005100.4-23882709	AACTCCCTGACCCTCCCTCCGGTTTCC	13	C	0.0982	0.0934
NC_005100.4-23900774	CTGAAAGCCACTGACCCTCCCCCACCT	14	A	0.0513	0.0818
NC_005100.4-23906205	GTAAAATAAACCTCTCCTCCTCGTCTT	2	T	0.0492	0.0786
NC_005100.4-23906205	GTAAAATAAACCTCTCCTCCTCGTCTT	23	G	0.0492	0.4914
NC_005100.4-23916910	TTTACTAAAACCCATACTCCATCTTTG	1	T	0.1626	0.2723
NC_005100.4-23934603	CTCCATTCCACTCTCCCTCCCCAATAC	5	A	0.0536	0.496
NC_005100.4-23957971	CAAGCCCTAACTCTGTCTCCCTTTCAC	21	C	0.0488	0.078
NC_005100.4-23966385	CTGTGTATGACACCAACTCCGTCTCAA	15	A	0.0179	0.4641
NC_005100.4-23966385	CTGTGTATGACACCAACTCCGTCTCAA	21	G	0.0179	0.4641
NC_005100.4-23988505	CACAGTTTGACCCATTCTCCATCTCCA	6	T	0.0354	0.0517
NC_005100.4-24004484	CGTTTGAGAACCAGGCCTCCTGTCGTG	1	C	0.0901	0.1786
NC_005100.4-24004484	CGTTTGAGAACCAGGCCTCCTGTCGTG	26	T	0.0531	0.0846
NC_005100.4-24044337	AACTCCGAAACCTGACCTCCCTTGTCA	6	C	0	0.4832
NC_005100.4-24044337	AACTCCGAAACCTGACCTCCCTTGTCA	7	G	0.0083	0.0245
NC_005100.4-24075590	AGCAGGCAGACCCAACCTCCAACTCAC	13	C	0.1351	0.1414
NC_005100.4-24076618	ATATTCTTCACTGTCACTCCTGCGCAT	24	G	0.1382	0.1425
NC_005100.4-24082724	CACACCCCCACCTTCCCTCCATGTGCT	21	A	0.122	0.1425
NC_005100.4-24122301	ATTTTCTTGACTAAGGCTCCTTCCTCT	14	A	0.2353	0.4983
NC_005100.4-24130871	AATTAACTGACACCAGCTCCTTGCCTG	27	G	0.0804	0.1093
NC_005100.4-24132308	TGGATGTCCACCTGTCCTCCAGTGACG	23	T	0.009	0.4766
NC_005100.4-24167914	CCTCTGAGGACCTGACCTCCTCCGAGT	23	C	0	0.4827
NC_005100.4-24173288	CTCAGACACACATTTGCTCCTGGGGAA	21	T	0.0508	0.0812
NC_005100.4-24173288	CTCAGACACACATTTGCTCCTGGGGAA	22	G	0.0517	0.4851
NC_005100.4-24254626	GGTAATGCTACTTCCTCTCCTGTTGTA	2	G	0.0513	0.4734
NC_005100.4-24254626	GGTAATGCTACTTCCTCTCCTGTTGTA	7	G	0.0427	0.058

NC_005100.4-24267477	CCTAGACCCACAATTTCTCCTGCTTAT	2	C	0.1593	0.3242
NC_005100.4-24267477	CCTAGACCCACAATTTCTCCTGCTTAT	6	A	0	0.4731
NC_005100.4-24288475	GTGTTTATTACCAAACCTCCTAGAGGC	27	C	0.0357	0.0351
NC_005100.4-24293301	TTTCTAAACACTGAGTCTCCTAACACG	21	T	0.0342	0.0336
NC_005100.4-24313549	ATTCTCCCAACTTACCCTCCTGAATGC	13	T	0	0.4555
NC_005100.4-24313549	ATTCTCCCAACTTACCCTCCTGAATGC	27	C	0	0.4555
NC_005100.4-24346437	CCCCGACCCACCCTACCTCCTATTTCC	4	C	0.027	0.0611
NC_005100.4-24350531	GTTAATAGGACCCCCTCTCCCTTGTAT	26	A	0.0431	0.0903
NC_005100.4-24350531	GTTAATAGGACCCCCTCTCCCTTGTAT	27	T	0.386	0.4555
NC_005100.4-24390577	GCCATGTGCACTGGCGCTCCGGGGTGG	21	G	0.1356	0.4632
NC_005100.4-24400207	CCCAGGGAGACACTGACTCCATCAAGC	23	C	0.1565	0.4833
NC_005100.4-24400484	AACCAAACCACAAGCCCTCCAACACTG	4	C	0	0.4793
NC_005100.4-24421318	CACAGGTTCACACCGCCTCCCACCATA	15	G	0	0.4793
NC_005100.4-24440431	TCCTATAACACATTGCCTCCAGTCTTC	5	A	0	0.4931
NC_005100.4-24440431	TCCTATAACACATTGCCTCCAGTCTTC	27	C	0.036	0.1182
NC_005100.4-24463374	CCCCATCATACCCCATCTCCCCCTGAC	15	A	0	0.4859
NC_005100.4-24517491	GGTTATTCTACAGGGTCTCCAACAGTG	2	G	0	0.4793
NC_005100.4-24517491	GGTTATTCTACAGGGTCTCCAACAGTG	5	A	0	0.4793
NC_005100.4-24541667	TGTAAAAGAACTACAACTCCCAGCATT	1	T	0.1721	0.1839
NC_005100.4-24546983	TCTGATGGTACTGAAACTCCAAACAAC	26	A	0.0496	0.0792
NC_005100.4-24557998	AAAGAAAGAACACTTGCTCCAAACCCA	9	A	0	0.4718
NC_005100.4-24575784	ATCAGAGCCACCTACCCTCCAACATCA	16	C	0.0252	0.0249
NC_005100.4-24619020	AAGGGGGGCACTGTTCCTCCCTGCAAC	1	A	0	0.4787
NC_005100.4-24619020	AAGGGGGGCACTGTTCCTCCCTGCAAC	3	G	0	0.4787
NC_005100.4-24640284	ACTACCTGCACATTAACTCCCATGGCA	1	A	0	0.4827
NC_005100.4-24644969	ACTGCCACTACAACACCTCCCTGCAGC	14	C	0.0088	0.4796
NC_005100.4-24657833	CTGGTTTGGACCAGACCTCCAGATCTG	5	T	0.2105	0.2021
NC_005100.4-24726499	TCCTGCTGCACTCTGGCTCCAGTGATG	14	T	0.0738	0.1154
NC_005100.4-24810829	AGCCTTACTACCATTGCTCCTTTGCCA	12	C	0.0804	0.4824
NC_005100.4-24810829	AGCCTTACTACCATTGCTCCTTTGCCA	13	A	0.0804	0.4824
NC_005100.4-25266552	TTGTGTGATACATGGTCTCCTTACAGT	14	G	0.2213	0.3831
NC_005100.4-25267938	ATTGCAAGAACAAGGTCTCCCTGGATT	3	T	0.1951	0.4
NC_005100.4-25281686	TCCCATCCCACCAGGCCTCCCTATTCC	26	C	0.0244	0.0705
NC_005100.4-25317699	ACTTTCCCTACCTACCCTCCTTCTCAT	6	C	0	0.4887
NC_005100.4-25317699	ACTTTCCCTACCTACCCTCCTTCTCAT	9	T	0	0.4887
NC_005100.4-25416831	GCAGGTGCGACATGGGCTCCTGTGTTG	8	C	0.1504	0.2367
NC_005100.4-25419401	CAATCCTTTACTTAATCTCCTGCTAAT	23	C	0.0088	0.481
NC_005100.4-25548193	CTGCTAGAAACCTGGTCTCCAGGAGTG	27	G	0.2269	0.4761
NC_005100.4-25646676	TTAACAGACACCTGGCCTCCAATTATG	3	A	0.2881	0.3792
NC_005100.4-25646676	TTAACAGACACCTGGCCTCCAATTATG	12	C	0.0339	0.0655
NC_005100.4-27284688	TTTCCTTGCACTAAATCTCCGCCTTCA	9	C	0.0089	0.491
NC_005100.4-27290543	CTCATATCAACAGGACCTCCCTTGGTA	23	T	0.1083	0.1597
NC_005100.4-27409789	GTAGTTATAACACTGGCTCCCATTCAG	1	G	0.1475	0.1774
NC_005100.4-27409789	GTAGTTATAACACTGGCTCCCATTCAG	4	G	0	0.48
NC_005100.4-27516285	CATCTATGTACAGATTCTCCTGGAACT	24	A	0.0522	0.0832
NC_005100.4-27571598	TATGTATGTACCTGAACTCCTTCATTG	1	T	0	0.4773
NC_005100.4-27588515	ATTCTGTCTACTTTGTCTCCATACTGG	21	A	0	0.4755
NC_005100.4-27597679	AAGCTAAATACAGCCCCTCCCTCCTCA	4	C	0.2541	0.479
NC_005100.4-27597679	AAGCTAAATACAGCCCCTCCCTCCTCA	7	A	0	0.4793
NC_005100.4-27666397	GAGTTAGGCACCTCATCTCCCACTGAG	9	C	0.0984	0.1367
NC_005100.4-27760572	CACTGTGGAACTTTTGCTCCATATTTA	15	T	0.0169	0.4632

NC_005100.4-27760572	CACTGTGGAACTTTTGCTCCATATTTA	24	T	0.0169	0.4632
NC_005100.4-27810023	GAATCCCAAACGTCTTCTCCTAGAGAG	1	G	0.0088	0.4861
NC_005100.4-27810023	GAATCCCAAACGTCTTCTCCTAGAGAG	24	A	0.0522	0.0832
NC_005100.4-27810808	ATTTAAGAGACATTCTCTCCCTACCCC	9	G	0.0431	0.0746
NC_005100.4-27832312	TTAGACTACACAGGGACTCCAGCTGCA	14	G	0.1404	0.4875
NC_005100.4-27881344	TTAAGACATACATCTTCTCCTCATCAT	22	C	0.1712	0.2271
NC_005100.4-27989333	CTCAGTTTCACTTTCACTCCCAAGATG	14	T	0.0536	0.0853
NC_005100.4-28039013	TCCAGGGAAACTCTGCCTCCTATGCCT	23	T	0	0.4807
NC_005100.4-28119500	CCTGACTTCACACTGTCTCCTCTGCAT	9	C	0	0.4841
NC_005100.4-28162905	CAAAGGTCCACAGCATCTCCACTGCAT	7	T	0	0.4819
NC_005100.4-28162905	CAAAGGTCCACAGCATCTCCACTGCAT	22	C	0	0.4819
NC_005100.4-28184820	AATGTTCACACCTTCTCTCCAGCATTC	4	G	0.0492	0.0634
NC_005100.4-28376442	TCAGGCTGAACTGGAACTCCTGGGTTC	24	G	0.0172	0.4709
NC_005100.4-28396242	CTTGTGTATACCAATACTCCTGTATCT	2	T	0.065	0.496
NC_005100.4-28416693	AGCCTGTTGACATGAGCTCCACCTGCT	7	T	0	0.4641
NC_005100.4-28416693	AGCCTGTTGACATGAGCTCCACCTGCT	22	C	0	0.0345
NC_005100.4-28518145	TTCTGCTCCACCTCCCCTCCAACCTCA	15	C	0	0.4807
NC_005100.4-28558908	TATTTTGAGACAGAATCTCCTGCATAT	23	C	0.0488	0.078
NC_005100.4-28570341	ATTTGATTAACCAGTACTCCCTATCCT	15	T	0.1111	0.1492
NC_005100.4-28703538	GCCCTACTAACTTTCTCTCCCTTCCTT	6	A	0	0.0325
NC_005100.4-28735310	CAACACTCCACATGTTCTCCACCCCAT	22	C	0.1475	0.1904
NC_005100.4-28735310	CAACACTCCACATGTTCTCCACCCCAT	23	C	0.1475	0.1904
NC_005100.4-28811632	CTCATGATGACTTGGTCTCCATCTGCT	3	C	0.0083	0.4708
NC_005100.4-28821733	ATACGTTGGACACCAACTCCCATGAGT	5	G	0	0.4807
NC_005100.4-28868421	AAAATAAGAACCCACCCTCCTCTGGCT	23	T	0	0.4807
NC_005100.4-28868421	AAAATAAGAACCCACCCTCCTCTGGCT	24	G	0.1311	0.3791
NC_005100.4-28989333	AAAATACCAACCTGCTCTCCACAGACC	16	T	0.2083	0.225
NC_005100.4-28989333	AAAATACCAACCTGCTCTCCACAGACC	26	C	0.0917	0.2608
NC_005100.4-29136412	ACCAGTCCAACAGTGTCTCCGTGCAAA	9	A	0.2301	0.4934
NC_005100.4-29185381	TTCAGCAGAACACCAGCTCCTTCTCTT	13	C	0.1538	0.4956
NC_005100.4-29194368	GCCACTTTCACAGCAGCTCCTCAGACA	15	A	0.15	0.2061
NC_005100.4-29229254	GACTGTTTAACATCTCCTCCGTTGTGT	9	A	0	0.48
NC_005100.4-29253860	TACAGTGGGACCCAGTCTCCAAAAGAA	4	A	0.0708	0.1162
NC_005100.4-29253860	TACAGTGGGACCCAGTCTCCAAAAGAA	21	A	0.1239	0.4934
NC_005100.4-29256576	TATTACCCTACTCTTACTCCTTAGCAT	14	T	0.1417	0.1733
NC_005100.4-29290740	TTTGAAAGAACCCACGCTCCATTGTGT	15	C	0	0.4839
NC_005100.4-29419012	ATGCTTGTAACTCCAGCTCCAGGAGAT	15	A	0.0256	0.4356
NC_005100.4-29419012	ATGCTTGTAACTCCAGCTCCAGGAGAT	25	G	0	0.4724
NC_005100.4-29465475	ACGCAGCACACAAAGACTCCACTCCTT	16	A	0.0084	0.4859
NC_005100.4-29590526	CTCACAAACACTCATGCTCCGGAGAGA	15	T	0.1607	0.2575
NC_005100.4-29832739	GTATCTATAACTCTAGCTCCTGAGGGA	6	T	0	0.4793
NC_005100.4-29832739	GTATCTATAACTCTAGCTCCTGAGGGA	7	A	0.042	0.0571
NC_005100.4-29982790	TATCTTCTAACTCAGTCTCCTAAGTAA	7	C	0.3103	0.3381
NC_005100.4-29982790	TATCTTCTAACTCAGTCTCCTAAGTAA	16	T	0	0.4814
NC_005100.4-30205267	AACCTCTTGACTTTATCTCCTAAGATC	25	A	0	0.4773
NC_005100.4-30206669	CATCTGAAAACTTGGACTCCAACTGGG	6	G	0.1062	0.4981
NC_005100.4-30360576	GAGGTTGAGACCTGTTCTCCCAATATA	5	T	0.0508	0.0812
NC_005100.4-30360576	GAGGTTGAGACCTGTTCTCCCAATATA	26	T	0.0508	0.0812
NC_005100.4-30372111	GTATCTCATACTCCTACTCCTTCTTCT	6	T	0.069	0.1134
NC_005100.4-30420443	GCTCTACCCACCTTTGCTCCGGGAGGT	25	G	0.5135	0.3985
NC_005100.4-30465212	GAAGACATAACATTGACTCCTCTAAGA	6	C	0.0855	0.1274

NC_005100.4-30465212	GAAGACATAACATTGACTCCTCTAAGA	7	A	0.0603	0.0903
NC_005100.4-30483859	TCACAACCTACTGTCACTCCAGTGTCA	9	T	0.0086	0.4729
NC_005100.4-30483859	TCACAACCTACTGTCACTCCAGTGTCA	14	T	0.0087	0.4744
NC_005100.4-30493795	CACTTTGTGACTTCCTCTCCTTGCCTG	24	C	0.0488	0.078
NC_005100.4-30493795	CACTTTGTGACTTCCTCTCCTTGCCTG	25	C	0.0244	0.0241
NC_005100.4-30542467	CTGACCTCTACACAATCTCCATGGAGC	15	A	0.113	0.258
NC_005100.4-30649341	TTTTAACAAACCCATCCTCCATTAAGG	1	T	0.0965	0.1075
NC_005100.4-30650122	GGAATAAAGACCCAGGCTCCCAAGCAC	6	A	0	0.4757
NC_005100.4-30650122	GGAATAAAGACCCAGGCTCCCAAGCAC	26	A	0	0.4757
NC_005100.4-30662589	TGTGTAAAAACCAACTCTCCTCCAAAC	16	T	0.1066	0.1154
NC_005100.4-30673640	GAAAGCCTTACAACTTCTCCCAAGATG	16	T	0	0.4807
NC_005100.4-30682485	AGTTCAGGCACCGGCTCTCCTACCCCA	22	A	0.05	0.0799
NC_005100.4-30711372	AGTCTAACCACTCTTTCTCCCTCTGAT	26	A	0	0.482
NC_005100.4-30714170	CATGTGTACACAGGGCCTCCTGATTCC	2	A	0.0517	0.4946
NC_005100.4-30714170	CATGTGTACACAGGGCCTCCTGATTCC	24	T	0.0603	0.0903
NC_005100.4-30743787	TTTAGCTAAACCTTCTCTCCCTAACTA	3	T	0	0.4688
NC_005100.4-30743787	TTTAGCTAAACCTTCTCTCCCTAACTA	6	C	0	0.4688
NC_005100.4-30785209	CCCCTTCCCACCTCCACTCCCTGACAT	1	C	0.0268	0.0264
NC_005100.4-30824998	GTGTTTCTAACACCCCCTCCTTCACGT	7	C	0.0536	0.0689
NC_005100.4-30867533	TTAGTGTGTACTCCTACTCCATAGCCA	8	G	0.0488	0.4926
NC_005100.4-30914853	ATACCTACAACACCAACTCCTGCACCT	27	T	0.0174	0.0342
NC_005100.4-30923271	CACTTTGGTACAGTGGCTCCTAGAGAA	27	A	0	0.4773
NC_005100.4-31120950	GAGGCCCGCACTTCCCCTCCCCCATCC	25	T	0	0.4839
NC_005100.4-31443498	GCAGCCCTAACCATCTCTCCCTGAGTG	27	G	0.0504	0.4957
NC_005100.4-31444948	TATTTGTGGACACTTTCTCCAGAAGGA	5	T	0.0684	0.066
NC_005100.4-31528339	ATAGAAACAACCAGGCCTCCTCCCCAT	12	C	0.0504	0.0805
NC_005100.4-31535309	ACCTCCGTGACACCTCCTCCCACAGGA	14	C	0.0268	0.0264
NC_005100.4-31594491	CCCCTAGGAACACTGTCTCCATTTTCT	12	A	0	0.4787
NC_005100.4-31649038	CTGGGAAAGACTACCCCTCCCGTTCTC	9	G	0.0991	0.126
NC_005100.4-31649038	CTGGGAAAGACTACCCCTCCCGTTCTC	12	T	0.0536	0.0853
NC_005100.4-31656078	TATGTTGAGACCAGGGCTCCCATGGTC	23	T	0.1583	0.2833
NC_005100.4-31657055	AATATGTTAACTTCATCTCCTAGTCCA	5	T	0	0.4786
NC_005100.4-31684772	ACAAGAGCAACATATGCTCCTCCAACT	3	A	0.0492	0.0786
NC_005100.4-31685103	ACAGGAAAGACGATCTCTCCTTGTTAC	6	A	0.2946	0.4193
NC_005100.4-31685103	ACAGGAAAGACGATCTCTCCTTGTTAC	21	T	0	0.4827
NC_005100.4-31715354	GTATTCTTCACAGTGACTCCCTGGGAC	2	T	0	0.4773
NC_005100.4-31751288	CTTTAGGACACCCATACTCCTTGTATT	16	A	0.1311	0.1774
NC_005100.4-31751288	CTTTAGGACACCCATACTCCTTGTATT	23	G	0.0488	0.0476
NC_005100.4-31770852	GCTCACTTCACCGTATCTCCTCAGGCA	12	C	0.113	0.1219
NC_005100.4-31773862	TGTGCATGGACTCTTCCTCCTGGTAAG	24	T	0.1513	0.4997
NC_005100.4-31854826	AGAGGGCTGACGTGCCCTCCAGCCTTG	12	G	0	0.4773
NC_005100.4-31854826	AGAGGGCTGACGTGCCCTCCAGCCTTG	13	T	0	0.4773
NC_005100.4-31862269	AGAGTTTAGACTGCTTCTCCCTAAATC	7	T	0	0.4765
NC_005100.4-31896894	CCAGAAGGCACGTTTTCTCCCTGGCTG	8	G	0	0.4813
NC_005100.4-31926418	GTGTCAGACACTGGGACTCCTACTCAC	12	T	0.0165	0.4849
NC_005100.4-31926418	GTGTCAGACACTGGGACTCCTACTCAC	23	C	0.0492	0.4612
NC_005100.4-31931951	ACCATCCTCACCCTGTCTCCCCTAGAC	1	A	0.0085	0.4882
NC_005100.4-31968397	CATGATCCGACTTTACCTCCATCCCAC	3	T	0.1849	0.4957
NC_005100.4-31979512	GTCACTTGCACTGTGCCTCCCTGAGCA	16	C	0.1707	0.2441
NC_005100.4-31981032	CACGGACCTACTGTCCCTCCTACCATG	1	C	0.1789	0.4944
NC_005100.4-31981032	CACGGACCTACTGTCCCTCCTACCATG	3	C	0.0081	0.4777

NC_005100.4-31981473	CCTGTCATCACCCTCACTCCCACTCAG	4	G	0.0086	0.4893
NC_005100.4-31981473	CCTGTCATCACCCTCACTCCCACTCAG	7	A	0.0086	0.4893
NC_005100.4-32023501	GTATCAAACACAGGTCCTCCTGCTTTT	6	A	0	0.4773
NC_005100.4-32023501	GTATCAAACACAGGTCCTCCTGCTTTT	27	T	0	0.4773
NC_005100.4-32064674	TGGACATAGACCACTTCTCCCTACAGG	2	G	0.0531	0.0846
NC_005100.4-32083564	CCAAGCCCCACGTGGACTCCGTGGGCT	3	A	0	0.4773
NC_005100.4-32084725	TGCGCCAGGACGGTGTCTCCATGGCAG	3	C	0	0.48
NC_005100.4-32087677	GTTTGTCACACTGTGGCTCCCGGTAGT	22	G	0	0.474
NC_005100.4-32111629	CTTGACCTCACCCTGGCTCCCTCCCTG	12	C	0	0.4786
NC_005100.4-32174518	AAGTCTTGAACCTCATCTCCTAGTTCC	7	T	0	0.4807
NC_005100.4-32263229	CTGAATAGGACCCAGCCTCCAGAGGGA	14	A	0.0984	0.1774
NC_005100.4-32281059	TAAACGACAACCTGAGCTCCTAGGATG	12	C	0	0.4819
NC_005100.4-32286331	TCACCGGCCACTCCTGCTCCTGTTCTA	1	T	0.0894	0.1287
NC_005100.4-32286331	TCACCGGCCACTCCTGCTCCTGTTCTA	8	C	0	0.4793
NC_005100.4-32300663	CCATGATGTACTGTCCCTCCATCTTGG	9	T	0.1478	0.2204
NC_005100.4-32330210	GTCCATTAAACTTGAGCTCCCATCCCA	24	C	0.1803	0.2157
NC_005100.4-32360125	TGGTGGTGAACCTCACCTCCTTGCCTG	8	G	0.0813	0.0928
NC_005100.4-32370922	TTTTGTTCCACGTCCTCTCCAGCATGT	24	A	0.2072	0.4351
NC_005100.4-32380064	AGGAAACAAACTAAACCTCCAGTCGCT	22	G	0.0526	0.0839
NC_005100.4-32424506	ACATCAGGAACTCTAGCTCCGGAGTGG	14	T	0	0.4807
NC_005100.4-32426860	CCCCCTCCTACAAACCCTCCCTGTCCA	26	C	0.0087	0.0257
NC_005100.4-32487127	GAAATATCCACCAGGCCTCCTTTACTG	1	G	0.1111	0.1634
NC_005100.4-32530369	CTTTGACACACACACGCTCCTTTTAAT	23	T	0.1478	0.5
NC_005100.4-32533804	CACAAGCACACTGTGACTCCCAAGGCC	5	A	0.069	0.0825
NC_005100.4-32533804	CACAAGCACACTGTGACTCCCAAGGCC	8	A	0	0.4851
NC_005100.4-32540246	CCATCACACACCGTATCTCCCTCTCAG	12	C	0.0081	0.481
NC_005100.4-32611920	CCTTCCCTCACCTTCTCTCCCTCTATT	1	C	0.0088	0.4648
NC_005100.4-32629659	CACACCCTGACCTATGCTCCGGAAGAC	9	G	0.0261	0.0257
NC_005100.4-32629659	CACACCCTGACCTATGCTCCGGAAGAC	21	G	0.0603	0.0903
NC_005100.4-32680933	GGGACTTTAACAAGAACTCCATTTGAT	2	G	0	0.4814
NC_005100.4-32815132	TTGGTCCTCACACTTGCTCCTGTGGAA	25	G	0.0488	0.078
NC_005100.4-32826072	TTCCCATCTACCCCTTCTCCTCAAGCT	12	C	0.0504	0.0805
NC_005100.4-32853142	ATTTCATGTACTTTTCCTCCATGATAT	24	A	0.2743	0.4547
NC_005100.4-32867863	GCTTCCTGGACCAGTCCTCCTGATCTG	25	C	0.0492	0.0786
NC_005100.4-32908679	ACACCTAGCACCGTATCTCCATTCAAG	12	C	0.1304	0.2204
NC_005100.4-32978312	TCCTCTAACACTCAGCCTCCCTGTGGC	5	C	0	0.4868
NC_005100.4-32985554	CCTGACCTGACCCTCACTCCCTATGAT	3	T	0.0179	0.4641
NC_005100.4-33015153	AACAAGAGCACTCTATCTCCCGTTGCC	22	G	0.2566	0.3081
NC_005100.4-33036099	CATTCTACCACCGAGCCTCCCCAGTGA	12	C	0	0.4839
NC_005100.4-33057344	AACAGTAGAACTAATTCTCCAAAGTTC	25	T	0	0.4757
NC_005100.4-33065162	AGACATTGCACAACTGCTCCCACATCT	6	T	0.2975	0.4369
NC_005100.4-33144694	TTCTTTGACACAGTCTCTCCTCAAGAC	6	T	0	0.4807
NC_005100.4-33144694	TTCTTTGACACAGTCTCTCCTCAAGAC	14	T	0.0328	0.0322
NC_005100.4-33164286	GGTCAACTGACTGAGACTCCCTCTCTC	3	T	0	0.48
NC_005100.4-33174097	TACAGTGACACACCTACTCCTTGGATG	21	T	0.0179	0.4841
NC_005100.4-33192039	AACCTCACCACAACTCCTCCTGATTAT	1	A	0	0.4787
NC_005100.4-33192039	AACCTCACCACAACTCCTCCTGATTAT	2	A	0	0.4787
NC_005100.4-33222177	AACCCATGCACAGTGACTCCCATGACA	15	G	0.094	0.4987
NC_005100.4-33223399	GGTGACTACACTAGTTCTCCACAGAGA	15	T	0.0579	0.0562
NC_005100.4-33231644	TCCTCCTGCACTATGCCTCCTTAGACA	13	A	0	0.48
NC_005100.4-33231644	TCCTCCTGCACTATGCCTCCTTAGACA	25	A	0.0417	0.4922

NC_005100.4-33232640	TAAGTCATGACACTCTCTCCTGTGACC	21	T	0	0.4751
NC_005100.4-33278062	GTGTGCTTGACTTTGTCTCCTTGTGCC	22	T	0.0082	0.4755
NC_005100.4-33278062	GTGTGCTTGACTTTGTCTCCTTGTGCC	26	C	0.0574	0.4903
NC_005100.4-33289846	TGAGATTTGACTCTGACTCCTTGGTAA	22	T	0.0833	0.4999
NC_005100.4-33311274	GCCTAGTGCACCTCTACTCCTGGTTTG	3	C	0	0.477
NC_005100.4-33313992	AAAGCCAGTACCCTGGCTCCAGTAGGT	5	C	0.0439	0.0595
NC_005100.4-33313992	AAAGCCAGTACCCTGGCTCCAGTAGGT	24	A	0.0088	0.483
NC_005100.4-33322156	ATCAGAGACACATGTCCTCCTTGCTTG	27	G	0.1453	0.1908
NC_005100.4-33340121	CTGGGAAGCACTGTGCCTCCTGTGTAT	1	C	0.0488	0.078
NC_005100.4-33340121	CTGGGAAGCACTGTGCCTCCTGTGTAT	25	T	0.1463	0.2499
NC_005100.4-33344426	GAGTAGACAACGTATGCTCCTTCAGTA	13	T	0.1301	0.3604
NC_005100.4-33481189	ATGGTCTACACAGTGACTCCCTGACTC	24	A	0	0.4751
NC_005100.4-33494982	ACATACATCACATGTGCTCCAGATGCA	6	C	0.0847	0.1827
NC_005100.4-33528663	GAACTGAACACCAAGACTCCCAGTCTG	27	G	0.1667	0.295
NC_005100.4-33657434	CTGGTTGGTACTTCTGCTCCATGTGTT	3	G	0	0.4787
NC_005100.4-33665091	GACTTGGCCACATGGGCTCCACCCCCT	23	C	0.161	0.375
NC_005100.4-33676482	GCTAGGTCGACTTCTACTCCTTCATCT	16	A	0	0.4793
NC_005100.4-33676482	GCTAGGTCGACTTCTACTCCTTCATCT	22	T	0.0488	0.4926
NC_005100.4-33710702	CAGTAATGGACTGAGCCTCCGAAACTG	15	G	0.3083	0.2608
NC_005100.4-33734277	AATACCCTCACTGATGCTCCTTAGAGG	16	G	0	0.4833
NC_005100.4-33837498	CTTGCTGGGACCCGGCCTCCCTCTCAA	14	G	0.0531	0.4953
NC_005100.4-33849937	CCATATGCAACTGCAGCTCCAGGAGAT	4	T	0.3077	0.2604
NC_005100.4-33849937	CCATATGCAACTGCAGCTCCAGGAGAT	6	T	0.3077	0.2604
NC_005100.4-33892885	ATTCCTACCACAGCTGCTCCGAGGTCG	5	C	0.0488	0.4926
NC_005100.4-33943871	GTCACCAGAACTCTGCCTCCTTCAGGT	27	T	0.1	0.1244
NC_005100.4-33948617	CCATGGTCCACGGTGGCTCCCTAGTAG	22	T	0.0492	0.4934
NC_005100.4-33960002	TGATGGGTGACTTCATCTCCAAGAGAG	24	A	0.0763	0.0889
NC_005100.4-33960002	TGATGGGTGACTTCATCTCCAAGAGAG	25	G	0.1504	0.49
NC_005100.4-33964082	AAACCTGCTACCTTCTCTCCCGGAGAA	22	G	0.0783	0.0752
NC_005100.4-34018992	CACTCAGCCACTTTGTCTCCAGCCTTA	7	G	0.1885	0.4755
NC_005100.4-34033127	TGGCCAGTCACTGTCTCTCCACCAGCA	22	C	0	0.48
NC_005100.4-34038608	TGGTGGGTCACACCTGCTCCTTTGCAG	7	G	0	0.4883
NC_005100.4-34038608	TGGTGGGTCACACCTGCTCCTTTGCAG	24	G	0.0541	0.4951
NC_005100.4-34136591	CATCTTTCTACAGGCTCTCCTGTGCCT	3	T	0.1429	0.214
NC_005100.4-34136591	CATCTTTCTACAGGCTCTCCTGTGCCT	9	T	0.1429	0.214
NC_005100.4-34180006	TGATCTGTCACTCTCTCTCCTCTGCAC	9	C	0.2231	0.4277
NC_005100.4-34180006	TGATCTGTCACTCTCTCTCCTCTGCAC	27	C	0.0083	0.4747
NC_005100.4-34215595	CAGAACAGAACGACTCCTCCTTTCCTA	6	C	0.1721	0.2457
NC_005100.4-34215595	CAGAACAGAACGACTCCTCCTTTCCTA	25	C	0.0083	0.4769
NC_005100.4-34231022	AAGATTGCCACAGATGCTCCTTAGTTA	12	A	0.2583	0.4525
NC_005100.4-34231022	AAGATTGCCACAGATGCTCCTTAGTTA	21	T	0.2479	0.3811
NC_005100.4-34316572	TGCTCCTACACCTTGCCTCCATCTTAC	12	C	0	0.4793
NC_005100.4-34350288	GTCTGTGCCACCTCACCTCCAACCCTA	26	T	0	0.4819
NC_005100.4-34352540	TACAGCACAACAGGAACTCCTCAAGGA	1	T	0	0.482
NC_005100.4-34352540	TACAGCACAACAGGAACTCCTCAAGGA	2	A	0	0.482
NC_005100.4-34363046	GAGGAGTTGACTCTTCCTCCATTACCA	7	T	0.0083	0.4708
NC_005100.4-34429417	ACCACTACCACATTCTCTCCTCCTGTG	7	A	0.0496	0.0792
NC_005100.4-34431017	ACCGCACAAACTCCACCTCCAAAGTTA	4	G	0.0492	0.0786
NC_005100.4-34471712	AACAGTGACACTGCCACTCCAATTTGT	1	A	0	0.4773
NC_005100.4-34506549	AGAAGAGAAACGTCCCCTCCTTAATGA	12	G	0.0756	0.0728
NC_005100.4-34506549	AGAAGAGAAACGTCCCCTCCTTAATGA	21	T	0	0.4793

NC_005100.4-34533337	GTTGTCTCCACCTCATCTCCTTATAGG	23	A	0.3571	0.4228
NC_005100.4-34543345	GCAGGATAGACAGGAGCTCCATATCAT	6	A	0	0.4807
NC_005100.4-34543345	GCAGGATAGACAGGAGCTCCATATCAT	24	T	0	0.4807
NC_005100.4-34550147	GTACGGACCACAAATGCTCCTGACCAG	4	C	0.0569	0.0553
NC_005100.4-34550147	GTACGGACCACAAATGCTCCTGACCAG	7	A	0.3252	0.3604
NC_005100.4-34580526	GACATTTATACCCACTCTCCCTTCCTT	25	C	0.0492	0.4914
NC_005100.4-34605673	GGGATGGTTACATACGCTCCCTTCCAT	16	G	0	0.4819
NC_005100.4-34605673	GGGATGGTTACATACGCTCCCTTCCAT	27	T	0.2602	0.2832
NC_005100.4-34632155	CAACAGGACACCCCTTCTCCCAGAGCT	1	C	0	0.4772
NC_005100.4-34726285	GAACAACTCACCCTTTCTCCATGTCTG	1	G	0.0488	0.078
NC_005100.4-34726285	GAACAACTCACCCTTTCTCCATGTCTG	14	T	0.2276	0.2832
NC_005100.4-34755512	TCCACATAAACAGTTACTCCCAAAGTG	27	G	0.2051	0.2107
NC_005100.4-34783972	AAGGATAGAACTGCAGCTCCGGATATC	21	G	0.1789	0.4881
NC_005100.4-34913896	ACCAACGTAACTCCATCTCCATGGTAT	14	C	0.2773	0.2852
NC_005100.4-34913896	ACCAACGTAACTCCATCTCCATGGTAT	16	T	0.05	0.0799
NC_005100.4-34914973	GGCAGTGGCACACTCTCTCCCTGTTGC	12	A	0.3033	0.3401
NC_005100.4-34954745	AGAATAGTGACTCTCCCTCCCTCACCG	27	G	0.0932	0.119
NC_005100.4-34960316	CTCCCAGGGACAAGATCTCCTTTTTTA	1	C	0.1316	0.1229
NC_005100.4-34962582	AGGTTCTCTACAACAGCTCCCTTTGAA	3	G	0.0177	0.4887
NC_005100.4-34962582	AGGTTCTCTACAACAGCTCCCTTTGAA	14	C	0.0177	0.4887
NC_005100.4-34965386	TCTAAAATAACTCAATCTCCAAACTTC	2	C	0.0614	0.0595
NC_005100.4-34982273	GGCATCTTGACATACCCTCCTCCACTC	22	C	0.0087	0.4563
NC_005100.4-34982273	GGCATCTTGACATACCCTCCTCCACTC	24	A	0.0087	0.4563
NC_005100.4-34986937	TCTTCTAACACATTCTCTCCAGGTAGG	26	G	0.0087	0.466
NC_005100.4-35034533	AGAAGCTTTACAGGTGCTCCGCACAGA	9	T	0	0.4807
NC_005100.4-35034533	AGAAGCTTTACAGGTGCTCCGCACAGA	27	A	0.1066	0.432
NC_005100.4-35081309	CCTCTAATAACCGTGACTCCCATAGCT	13	G	0.1293	0.302
NC_005100.4-35173507	AAGTCTTCCACACACCCTCCCTTTCCA	15	C	0.2314	0.418
NC_005100.4-35187747	AAACAGCGTACAGCTCCTCCAGGCAGT	12	A	0.1983	0.4375
NC_005100.4-35210222	CAGATTCAGACCCTGTCTCCAAAATGA	5	T	0.0177	0.4755
NC_005100.4-35210222	CAGATTCAGACCCTGTCTCCAAAATGA	25	T	0.0088	0.026
NC_005100.4-35225981	CACTCACCCACATGGTCTCCTTAGAAA	24	G	0.3	0.3832
NC_005100.4-35250233	CCATTGCCAACATTAACTCCGTTCGTG	15	A	0.0085	0.4823
NC_005100.4-35250233	CCATTGCCAACATTAACTCCGTTCGTG	22	T	0.0085	0.4823
NC_005100.4-35262750	CAATCAGCGACAGCAGCTCCAGAGTGC	4	T	0.2358	0.4618
NC_005100.4-35262750	CAATCAGCGACAGCAGCTCCAGAGTGC	9	G	0.0813	0.078
NC_005100.4-35271487	ATAGCAGCCACCCACACTCCCAAGGGT	13	C	0.065	0.0629
NC_005100.4-35294121	AACTTAGAGACTAGACCTCCATGAAGT	2	A	0.25	0.32
NC_005100.4-35320568	CACGGTGGGACCTAGGCTCCAGAGGTA	7	G	0.3033	0.3667
NC_005100.4-35327221	CCAAGGAATACAACACCTCCAGCTTCC	3	A	0.0325	0.032
NC_005100.4-35327221	CCAAGGAATACAACACCTCCAGCTTCC	8	A	0.0165	0.4787
NC_005100.4-35333022	CAGACAGACACGGCTGCTCCCATGGTG	1	C	0.3109	0.449
NC_005100.4-35333022	CAGACAGACACGGCTGCTCCCATGGTG	2	A	0.3109	0.449
NC_005100.4-35388556	TGGTTTCTGACACTGGCTCCTTGGCCT	25	C	0.1074	0.1017
NC_005100.4-35420298	CTAACCAAGACATCTCCTCCACTTCTG	4	A	0	0.4773
NC_005100.4-35420298	CTAACCAAGACATCTCCTCCACTTCTG	26	T	0.0488	0.078
NC_005100.4-35519337	GATATGTGAACATTCTCTCCATATCTT	23	A	0.0089	0.4566
NC_005100.4-35564814	TTCAGTGGCACTTCCCCTCCCTGCCAG	14	C	0	0.4793
NC_005100.4-35583571	AGGTTGCCAACCCATCCTCCTAGCTCA	16	C	0.0504	0.0805
NC_005100.4-35594890	TAAAAAGGAACTTCTTCTCCTGTAACT	3	A	0.0087	0.466
NC_005100.4-35594890	TAAAAAGGAACTTCTTCTCCTGTAACT	12	T	0.0088	0.463

NC_005100.4-35634718	TGGCTGGTGACTGCCACTCCAAGTGGA	22	A	0.2348	0.4333
NC_005100.4-35655181	ACTTTCTGTACACACCCTCCATTTGTG	7	T	0	0.4765
NC_005100.4-35655181	ACTTTCTGTACACACCCTCCATTTGTG	8	G	0.157	0.3027
NC_005100.4-35698034	GTGTGTTGGACGCCTCCTCCAGGAAAT	12	G	0.124	0.1306
NC_005100.4-35698034	GTGTGTTGGACGCCTCCTCCAGGAAAT	14	C	0	0.4787
NC_005100.4-35714634	CACTGAGATACATATCCTCCTTATTCC	16	C	0.1429	0.4987
NC_005100.4-35718773	GTGCAGCCCACAACCACTCCAACAGAA	16	A	0.0083	0.4747
NC_005100.4-35722327	TGAGGCTTCACAAAGCCTCCCTATCCT	15	G	0.0982	0.125
NC_005100.4-35722453	CTGTGATACACCTGCCCTCCTCCAACC	7	T	0	0.4751
NC_005100.4-35755469	ACCCACTGAACTATCACTCCCTACACA	8	G	0.0526	0.0839
NC_005100.4-35770955	TCCACACCCACAATGGCTCCATACCCA	3	C	0.0174	0.4637
NC_005100.4-35770955	TCCACACCCACAATGGCTCCATACCCA	5	C	0.0598	0.074
NC_005100.4-35778235	CAGGGTCCTACCTTTACTCCTCTTGGT	21	T	0	0.4773
NC_005100.4-35803188	TACACTAGAACACATGCTCCAAGCACA	5	C	0	0.4793
NC_005100.4-35803188	TACACTAGAACACATGCTCCAAGCACA	25	A	0.187	0.208
NC_005100.4-35804973	CATGTTTACACAGGCCCTCCCAGGCAC	16	C	0.0084	0.4682
NC_005100.4-35835065	CACGTCAGTACTCATTCTCCTGTGCAT	3	C	0.0354	0.4715
NC_005100.4-35835065	CACGTCAGTACTCATTCTCCTGTGCAT	16	T	0.0522	0.0832
NC_005100.4-35836993	CCCTGCCACACCACCTCTCCCACAATC	1	C	0	0.4688
NC_005100.4-35836993	CCCTGCCACACCACCTCTCCCACAATC	15	C	0.0442	0.4873
NC_005100.4-35854473	TTCCCCTTCACCCTCTCTCCCTTTTTA	6	C	0.1017	0.4367
NC_005100.4-35875645	ACTTACATTACCTCTGCTCCCTCTCTG	2	C	0.1833	0.255
NC_005100.4-35926406	CCACCTTCAACTCTCCCTCCTTCTTTC	7	T	0.0508	0.4908
NC_005100.4-35926406	CCACCTTCAACTCTCCCTCCTTCTTTC	15	C	0.0256	0.12
NC_005100.4-35929784	GACCAGACCACGGCAACTCCTTTCCTC	8	C	0.05	0.4932
NC_005100.4-35952678	TCACCCACTACCAGAGCTCCCTGGTTA	5	C	0.0522	0.0832
NC_005100.4-36002593	CTTCTGGGCACCACTGCTCCCAGCCAT	3	T	0.0488	0.078
NC_005100.4-36002593	CTTCTGGGCACCACTGCTCCCAGCCAT	26	A	0.1789	0.2263
NC_005100.4-36014050	CAGATCCTCACAGCATCTCCCTGGGCA	3	G	0.1789	0.4984
NC_005100.4-36014050	CAGATCCTCACAGCATCTCCCTGGGCA	5	T	0.1626	0.3041
NC_005100.4-36020238	GTACGCAGCACCTGTGCTCCAGAGAAC	3	A	0	0.48
NC_005100.4-36020238	GTACGCAGCACCTGTGCTCCAGAGAAC	4	C	0.177	0.4981
NC_005100.4-36022498	GATCATGAAACGTAACCTCCCTTGGCA	13	T	0	0.4891
NC_005100.4-36022498	GATCATGAAACGTAACCTCCCTTGGCA	23	T	0	0.4891
NC_005100.4-36024112	CTTTTGTTGACTTTGTCTCCTTCCCTG	23	C	0	0.4772
NC_005100.4-36030666	CATCAAGAGACCCTTGCTCCCAGATTG	15	T	0.041	0.071
NC_005100.4-36032884	GTTTCCAGGACGAGAGCTCCAGAGCAC	12	G	0.0531	0.0846
NC_005100.4-36058118	ATTTACACAACTCTAGCTCCCTCATGG	5	A	0	0.4728
NC_005100.4-36085661	TGACTGTTTACCACTGCTCCTGGATAC	16	G	0.0167	0.0328
NC_005100.4-36085661	TGACTGTTTACCACTGCTCCTGGATAC	21	T	0.0413	0.0716
NC_005100.4-36112055	CTGGGTTGGACCTCGGCTCCCTTCAGA	14	C	0.2613	0.3124
NC_005100.4-36130221	GGCTCACTCACTTTTCCTCCATTTGCA	4	T	0.1138	0.1356
NC_005100.4-36140194	CCATCTGGAACCGTCCCTCCAATTGCT	5	C	0.0164	0.1225
NC_005100.4-36140194	CCATCTGGAACCGTCCCTCCAATTGCT	16	C	0	0.4765
NC_005100.4-36140411	GCCATGGATACAGGGCCTCCTCTGTGA	2	C	0.2917	0.4472
NC_005100.4-36147965	TCCTTCAGCACCCAGGCTCCTAGCCTT	4	T	0	0.4793
NC_005100.4-36152514	CACTTGGTTACTCTGGCTCCGGTTGTG	21	G	0.0813	0.1761
NC_005100.4-36154544	TCTCCAACTACTAGGCCTCCCCACCAT	3	T	0.1441	0.1338
NC_005100.4-36155318	CTTAAAGGGACCATGGCTCCACCACAG	2	T	0.1826	0.4986
NC_005100.4-36155477	TGTCAGGGCACAGTTGCTCCAGTTGGA	6	G	0.0435	0.0752
NC_005100.4-36155477	TGTCAGGGCACAGTTGCTCCAGTTGGA	15	T	0	0.4749

NC_005100.4-36155795	GCAAACAGCACCATTTCTCCGCAGGCT	21	G	0	0.4773
NC_005100.4-36197550	GTGGCAGTTACTCTACCTCCTCTTGTA	9	T	0	0.4779
NC_005100.4-36247558	TCTTCTAATACCACCACTCCCTGGGTA	15	C	0.0522	0.0508
NC_005100.4-36247558	TCTTCTAATACCACCACTCCCTGGGTA	27	A	0	0.4833
NC_005100.4-36257681	TACTTGCCCACTCCTCCTCCTCTCTGT	5	T	0	0.4793
NC_005100.4-36257681	TACTTGCCCACTCCTCCTCCTCTCTGT	27	T	0.0244	0.0241
NC_005100.4-36305048	AGTGGACACACTGCAACTCCACTCTTA	23	T	0	0.4793
NC_005100.4-36337645	TTCTCAGTCACATGCCCTCCTCTGTGT	25	T	0	0.4793
NC_005100.4-36345979	CTTTCGGCCACCCTTTCTCCCTCCTTC	3	T	0	0.4677
NC_005100.4-36345979	CTTTCGGCCACCCTTTCTCCCTCCTTC	7	G	0.0508	0.4856
NC_005100.4-36347436	GGAGGACGGACCGGTGCTCCAGGCGCA	27	A	0	0.488
NC_005100.4-36406240	ACCCTGCCTACCTACACTCCTAGCACC	21	T	0	0.4814
NC_005100.4-36493911	TCTCCCCTGACTCTCTCTCCTCTGACT	1	T	0.5508	0.4776
NC_005100.4-36493911	TCTCCCCTGACTCTCTCTCCTCTGACT	21	T	0.7	0.4978
NC_005100.4-36511031	AGATGTTCTACTCTCTCTCCAATTAGA	16	T	0.0513	0.0818
NC_005100.4-36586044	CTGGAAAAGACTGAGTCTCCTTCTCTC	9	G	0.0667	0.095
NC_005100.4-36599111	TATTTTGTGACAAGGTCTCCCTCACTG	2	A	0	0.4832
NC_005100.4-36610561	TGCAGACACACTTTGTCTCCTCCTGTT	25	G	0.0168	0.4844
NC_005100.4-36610561	TGCAGACACACTTTGTCTCCTCCTGTT	26	T	0.0168	0.4844
NC_005100.4-36705382	TGAGATGTCACATGGCCTCCAGAAGGA	12	A	0.0248	0.0245
NC_005100.4-36705382	TGAGATGTCACATGGCCTCCAGAAGGA	15	G	0.0744	0.1163
NC_005100.4-36780184	CAGTTGCTCACAGTTACTCCTCGAGAA	5	T	0	0.4757
NC_005100.4-36780184	CAGTTGCTCACAGTTACTCCTCGAGAA	23	G	0.0084	0.0249
NC_005100.4-36808272	AGGAAACAAACTGTAACTCCTTTTGAG	8	A	0.1488	0.3729
NC_005100.4-36837024	TTGCTGAACACTTACTCTCCCAGAGTA	14	A	0.0088	0.0262
NC_005100.4-36838066	GTGGAGTTCACAGAGTCTCCATGGGTG	14	A	0	0.4793
NC_005100.4-36838066	GTGGAGTTCACAGAGTCTCCATGGGTG	21	A	0.0163	0.032
NC_005100.4-36893633	AGTACCAAGACAGTCTCTCCAGAGGAA	1	A	0	0.4765
NC_005100.4-36951043	TCCATAGCAACCGTACCTCCGCAATTC	12	C	0.0492	0.4934
NC_005100.4-36966433	CGTTGCTCCACATCCTCTCCAACATGT	24	A	0.0504	0.0805
NC_005100.4-36988520	ACACACATTACCTGGTCTCCCATGATG	1	A	0	0.4841
NC_005100.4-37039076	CTGTCCCCAACTCCATCTCCATCTCTC	6	C	0.0417	0.0408
NC_005100.4-37039076	CTGTCCCCAACTCCATCTCCATCTCTC	9	A	0.0333	0.0328
NC_005100.4-37042098	CCCAGGTCAACCTTGCCTCCAAGGAGG	22	A	0.0082	0.4755
NC_005100.4-37106454	ATGTAAACAACGGGATCTCCATCACTG	12	G	0.0082	0.0243
NC_005100.4-37106454	ATGTAAACAACGGGATCTCCATCACTG	27	G	0	0.4773
NC_005100.4-37120430	TACAGAGCCACTCCTGCTCCCACAGGC	24	A	0.0088	0.4599
NC_005100.4-37125550	TGAAAACAAACAGCATCTCCCACGTGG	23	C	0.0488	0.0476
NC_005100.4-37125550	TGAAAACAAACAGCATCTCCCACGTGG	27	G	0.0488	0.0476
NC_005100.4-37248762	GAACTGCACACTCGGTCTCCTTTTACC	9	C	0	0.4864
NC_005100.4-37248762	GAACTGCACACTCGGTCTCCTTTTACC	14	G	0.3043	0.4563
NC_005100.4-37358407	AGGACTATCACAGTGGCTCCCTATCTC	21	C	0.1607	0.2188
NC_005100.4-37411125	ATGTCCAATACAGGACCTCCTTGGAAG	25	A	0	0.4793
NC_005100.4-37510945	TCCCATTAAACAACAGCTCCTCACATA	21	T	0.0083	0.4835
NC_005100.4-37534287	AGCAATTATACACATTCTCCCCATGTG	25	G	0.287	0.4055
NC_005100.4-37537203	GGTCTCTGTACCAACCCTCCCTCTAAA	14	A	0.0496	0.0792
NC_005100.4-37563022	GACTGACTGACCCTGGCTCCAACTGCG	26	C	0.0261	0.0752
NC_005100.4-37575265	CTCCCACCCACCCCGACTCCTGCCTCA	8	C	0	0.4832
NC_005100.4-37746865	TCCTACCACACGCTCACTCCTTTGTCA	6	C	0.0259	0.0255
NC_005100.4-37779873	ATCTTCTTCACTGTCTCTCCTCCCCCA	24	C	0.0168	0.4779
NC_005100.4-37815263	CCAAACATTACAGAGTCTCCATTCATA	26	T	0.0087	0.4849

NC_005100.4-37816344	AGAGTATGGACTTGCACTCCGATGACT	21	G	0	0.4779
NC_005100.4-37816344	AGAGTATGGACTTGCACTCCGATGACT	23	T	0	0.4779
NC_005100.4-37895434	TGACATGTTACTTACTCTCCTGCTCAC	9	T	0.065	0.078
NC_005100.4-37937842	CTCTGATCCACCTCAGCTCCGTTCCCT	3	C	0	0.4773
NC_005100.4-37937842	CTCTGATCCACCTCAGCTCCGTTCCCT	6	A	0	0.4773
NC_005100.4-38522477	AGGCCAAAGACTGATGCTCCAAGTCTT	8	A	0.1818	0.2975
NC_005100.4-38596213	GTATGCAAGACTATTTCTCCTCCCTAG	25	T	0.0174	0.4864
NC_005100.4-38618206	TTTGCGAAAACTACACCTCCGTATATT	5	C	0.0513	0.066
NC_005100.4-38618206	TTTGCGAAAACTACACCTCCGTATATT	21	G	0	0.4786
NC_005100.4-38973905	TTGAACCCCACTAGACCTCCCAGGCAC	16	C	0.0435	0.1067
NC_005100.4-39047824	GAAGGCCTCACACTGACTCCAGTCTTA	8	T	0.3967	0.318
NC_005100.4-39747985	CCAAAGCAGACCTGGCCTCCAGTTTCT	21	A	0.0357	0.0351
NC_005100.4-39765285	AGTAACCACACTGACCCTCCATATTGT	15	C	0.0492	0.0786
NC_005100.4-39836879	CTCCAGTCTACCCAGACTCCAAGGAAG	2	T	0	0.4793
NC_005100.4-39931391	TACAACAGGACCACAGCTCCTAACAGT	7	A	0.0492	0.048
NC_005100.4-39931391	TACAACAGGACCACAGCTCCTAACAGT	8	G	0	0.4751
NC_005100.4-40073574	AGTTCACCCACCATGCCTCCCCCCATC	8	C	0.0088	0.49
NC_005100.4-40100283	GTGACGGTCACTACCACTCCCCTCAAC	15	C	0.0588	0.4859
NC_005100.4-40144428	CATGGAGAAACCATTCCTCCTAGAATA	26	T	0.0082	0.479
NC_005100.4-40236486	CCCCTGTCTACAATCTCTCCTTAGCTA	3	C	0.0333	0.0328
NC_005100.4-40236486	CCCCTGTCTACAATCTCTCCTTAGCTA	13	A	0.2605	0.4859
NC_005100.4-40240332	AGCTACTACACAGTAGCTCCTTCACTA	26	T	0	0.4793
NC_005100.4-40261261	CACATGCACACATGATCTCCCTCCCCA	26	C	0.0083	0.0408
NC_005100.4-40261289	CGCATGTACACATGAACTCCCTCCCCA	26	C	0.1429	0.1469
NC_005100.4-40360976	CCGCTTTCTACTCCTCCTCCCTATCCT	5	T	0	0.4718
NC_005100.4-40369218	CTCTAATTGACTGTGCCTCCTCCTGTG	1	C	0.2869	0.4489
NC_005100.4-40434869	CACACACACACACACACTCCAGTTGTG	14	A	0.569	0.4281
NC_005100.4-40434869	CACACACACACACACACTCCAGTTGTG	16	A	0.5641	0.426
NC_005100.4-40444667	GCCAGTTAAACAGTAACTCCAGAGGCC	15	A	0	0.4898
NC_005100.4-40528239	ATCCCCAGAACAATCCCTCCGCCTACC	9	A	0.0348	0.0342
NC_005100.4-40537828	TCCATGGAGACATGATCTCCTTTCCCT	12	A	0.0625	0.1093
NC_005100.4-40597072	TCTCTGTCCACAGTGCCTCCCATGTGG	4	C	0	0.4839
NC_005100.4-40659225	GCATTGGCCACACCCCCTCCCACAGAT	3	A	0	0.4764
NC_005100.4-40662843	ATAACAGTGACAGCGGCTCCCAGGACA	7	G	0	0.48
NC_005100.4-40833241	CTGTTCATAACATCAGCTCCAGGAGAT	4	T	0.1405	0.4213
NC_005100.4-40849499	CCCTCCCCCACATCTGCTCCCCCAGTG	27	G	0.0333	0.0488
NC_005100.4-40894958	ATCATTCTCACAAACACTCCCTACCAG	3	C	0	0.4793
NC_005100.4-40918930	AGCAGTCCCACCTAAGCTCCGAGATGC	9	C	0.0992	0.0943
NC_005100.4-40918930	AGCAGTCCCACCTAAGCTCCGAGATGC	24	A	0	0.4773
NC_005100.4-40931424	GGCAGGAAAACACCCCCTCCTCCACAG	13	C	0.0508	0.1409
NC_005100.4-40962376	GTTTGCGTCACACCAACTCCCTTGTAA	6	C	0	0.4793
NC_005100.4-40988087	CACAGTGAGACCCTCTCTCCAAGCAAA	1	C	0.2049	0.2796
NC_005100.4-40998122	CTCAACTGCACTCTTACTCCTGACCAG	8	G	0.2222	0.3261
NC_005100.4-40998122	CTCAACTGCACTCTTACTCCTGACCAG	16	A	0.0424	0.1894
NC_005100.4-41021794	GAATTTCAAACGTTACCTCCAACTTCT	21	A	0	0.4793
NC_005100.4-41021794	GAATTTCAAACGTTACCTCCAACTTCT	27	T	0.1197	0.497
NC_005100.4-41186814	TTCCTTATGACTGTAGCTCCTTGTCCT	1	T	0	0.4793
NC_005100.4-41336050	ACTCACCTCACACCAGCTCCTCTCAGA	9	C	0.009	0.4959
NC_005100.4-41336050	ACTCACCTCACACCAGCTCCTCTCAGA	22	C	0.1667	0.4529
NC_005100.4-41351877	TAGTGTGTGACTATGTCTCCATAGGCA	1	T	0	0.4751
NC_005100.4-41357002	TGGTATCACACATCCTCTCCTAAGGCT	26	C	0.0333	0.0644

NC_005100.4-41403412	ATCCCAAGCACCATCCCTCCACCTTTT	8	G	0.0084	0.4761
NC_005100.4-41403412	ATCCCAAGCACCATCCCTCCACCTTTT	9	C	0.0084	0.4761
NC_005100.4-41502059	TGTCATTATACACTCACTCCATCTCGG	12	A	0	0.4793
NC_005100.4-41508460	TAGACAGAGACTCTCCCTCCTCCATCC	27	C	0.1282	0.1908
NC_005100.4-41512564	GACATACCTACTTGCTCTCCATTCTTC	4	A	0.0325	0.032
NC_005100.4-41512564	GACATACCTACTTGCTCTCCATTCTTC	6	A	0.0325	0.032
NC_005100.4-41518114	AAGAGATTGACTAAGTCTCCAGAGGGC	1	A	0	0.474
NC_005100.4-41548710	ACTAAGCATACTTAAGCTCCTTCTCCT	9	T	0.1488	0.2533
NC_005100.4-41566289	AACTAAATGACAAAATCTCCAACTGTG	27	G	0	0.477
NC_005100.4-41581388	TCTAGCAAGACCACACCTCCTTAGCCT	21	T	0.1964	0.3264
NC_005100.4-41604189	GCTACACCCACAGCTCCTCCCAGGTTT	27	T	0	0.4793
NC_005100.4-41619305	AAGCAGAGTACATGCGCTCCTCAGTGT	22	C	0.1491	0.235
NC_005100.4-41653186	GTGGGAAGAACTCTGACTCCTAACCAG	5	G	0	0.4826
NC_005100.4-41653186	GTGGGAAGAACTCTGACTCCTAACCAG	27	G	0	0.4826
NC_005100.4-41667091	ACTCTGAAAACTGAGGCTCCATCATTG	26	T	0.0089	0.4856
NC_005100.4-41680649	ACAAGTGATACCTCTGCTCCTTTTAGG	2	C	0.2735	0.3641
NC_005100.4-41680649	ACAAGTGATACCTCTGCTCCTTTTAGG	3	A	0	0.4844
NC_005100.4-41704584	AAGATTGAGACTCTGTCTCCAAATGTA	15	G	0.2261	0.4588
NC_005100.4-41726181	GATACAAGAACCCTACCTCCAACCCTT	23	C	0.0583	0.0722
NC_005100.4-41726181	GATACAAGAACCCTACCTCCAACCCTT	27	T	0	0.4728
NC_005100.4-41736273	AAGTGCATAACAGACACTCCACTACAC	16	A	0.0738	0.071
NC_005100.4-41780040	TTTTCAAACACTTTGCCTCCACCAGAG	26	A	0.0609	0.1219
NC_005100.4-41868657	CTGTGTTTGACCTCTCCTCCATTTCGT	26	G	0.0488	0.0476
NC_005100.4-41913802	TTGTACTGAACGCATTCTCCGAAGTTG	12	G	0.1696	0.3316
NC_005100.4-41939021	CATTGATACACTAACCCTCCGAGGCAA	21	G	0.115	0.1968
NC_005100.4-41939021	CATTGATACACTAACCCTCCGAGGCAA	25	C	0	0.4731
NC_005100.4-41986468	AGAGTAGTCACCATGACTCCACCCCAG	23	C	0.0442	0.1239
NC_005100.4-42011110	CTGATGCCTACCTTCTCTCCACCCTGT	15	C	0.0424	0.0576
NC_005100.4-42045370	CCTCCTTCTACCAACACTCCTCCATTG	4	C	0.2066	0.2474
NC_005100.4-42113841	CAACCTATAACTTCAACTCCAGGGGAT	4	C	0	0.4902
NC_005100.4-42143434	TTATTGACGACTTAGTCTCCAGACAAT	9	G	0.2683	0.381
NC_005100.4-42143434	TTATTGACGACTTAGTCTCCAGACAAT	14	A	0	0.4793
NC_005100.4-42174358	TGAAGAAATACTTGAACTCCATTCTAA	26	A	0.1083	0.4233
NC_005100.4-42433765	CATGACACAACTCAGTCTCCAATGAAA	4	G	0.2759	0.4281
NC_005100.4-42433765	CATGACACAACTCAGTCTCCAATGAAA	6	C	0.25	0.3876
NC_005100.4-42553074	GATATGTTCACTATTGCTCCTGGATTT	5	T	0.1417	0.4958
NC_005100.4-42635618	TCCCCCCTCACACCCACTCCTGCTTCA	9	C	0.0796	0.0926
NC_005100.4-42657043	AGATCAAAAACTCATGCTCCACTATGT	5	C	0.2353	0.4008
NC_005100.4-42673949	CACAGCATCACATAGGCTCCTTTGTTA	8	T	0.0536	0.0521
NC_005100.4-42711734	CGCAATGGGACAAATCCTCCTTTCCTG	15	T	0	0.4793
NC_005100.4-42745770	AGTTCTGTCACCTGGCCTCCCTCTCCT	16	C	0	0.4912
NC_005100.4-42754637	ACTGGTCACACACTCCCTCCCTATGTA	6	T	0	0.4846
NC_005100.4-42754637	ACTGGTCACACACTCCCTCCCTATGTA	15	C	0.287	0.3898
NC_005100.4-42829232	ACTTGTAAAACATTTTCTCCTAAGGTG	27	G	0.1304	0.1938
NC_005100.4-42882040	GTGGATGTCACTCTTTCTCCCAGGTTG	23	G	0.2231	0.4913
NC_005100.4-43102660	AGCAGTGACACATTTTCTCCGCTGTTG	21	G	0.2353	0.2449
NC_005100.4-43106629	CTGCACGGCACTCTCGCTCCCGACCTG	4	C	0	0.4637
NC_005100.4-43106629	CTGCACGGCACTCTCGCTCCCGACCTG	16	G	0.1053	0.1744
NC_005100.4-43149286	TTCTTTGAGACTGCTGCTCCTCAGCTG	2	T	0.0083	0.4769
NC_005100.4-43149286	TTCTTTGAGACTGCTGCTCCTCAGCTG	6	T	0.0083	0.4769
NC_005100.4-43423585	ATGTCCACCACCATCACTCCTTTGTCC	5	C	0	0.4807

NC_005100.4-43423585	ATGTCCACCACCATCACTCCTTTGTCC	13	A	0.1721	0.2686
NC_005100.4-43442566	CTCACAACCACTGTAACTCCATCGTTC	23	C	0.2162	0.2594
NC_005100.4-43540994	ATATTGTCAACATGGTCTCCCTTTCTA	2	T	0.1148	0.416
NC_005100.4-43549734	ATACTCTTCACAGATTCTCCTAGTTTG	13	G	0.0261	0.0257
NC_005100.4-43576956	ATGACTTGTACAGTTTCTCCATCTCAT	1	A	0.1707	0.2556
NC_005100.4-43604478	TCTTGCATCACTTGAGCTCCTACAGTG	5	G	0	0.4793
NC_005100.4-43604478	TCTTGCATCACTTGAGCTCCTACAGTG	7	A	0.2049	0.2573
NC_005100.4-43605735	TACTGATGGACTTGCTCTCCATGCTTT	26	T	0.0083	0.4769
NC_005100.4-43618733	CATGCATCTACATATGCTCCTTTATGA	5	C	0.1795	0.4906
NC_005100.4-43618733	CATGCATCTACATATGCTCCTTTATGA	16	G	0.0085	0.4823
NC_005100.4-43626340	CTTCTTTTTACCTCTTCTCCCTTTGTC	3	T	0.0517	0.0504
NC_005100.4-43630327	CGCCAGGTCACCTGACCTCCTCCTGCA	15	A	0.0325	0.0629
NC_005100.4-43707965	CAGGATTTAACATTACCTCCATCTGCT	16	C	0.0531	0.0517
NC_005100.4-43759540	GTTGAGTTCACAGTAGCTCCTCACTAT	15	A	0.0083	0.4835
NC_005100.4-43773217	AGCCAACTCACCTTCTCTCCATAAGAA	21	A	0.2083	0.4997
NC_005100.4-43781758	ACAGAAGCTACCTAAGCTCCCCACGTT	24	C	0	0.4734
NC_005100.4-43792528	TCTATCAGAACAAGAACTCCCAGACCT	2	C	0.041	0.0401
NC_005100.4-43794815	GGCCAATGTACTCAGGCTCCATTAGCA	26	C	0.1271	0.2282
NC_005100.4-43822022	TGCCCTGACACCTTCCCTCCGGTGTCC	2	G	0.0325	0.032
NC_005100.4-43826308	CCTCAGAAGACAGAGGCTCCACCTGCA	6	G	0.0885	0.1006
NC_005100.4-43826308	CCTCAGAAGACAGAGGCTCCACCTGCA	21	A	0	0.4793
NC_005100.4-43844021	AAAAGCTAAACCAGCTCTCCTAAGCTG	15	C	0.1053	0.1305
NC_005100.4-43980651	TCCATTTATACCCCCACTCCTCCCCTG	6	T	0	0.4709
NC_005100.4-43980651	TCCATTTATACCCCCACTCCTCCCCTG	27	G	0.0678	0.0965
NC_005100.4-44027336	TTCAGGACAACATCCTCTCCTCCTTTG	7	A	0.0508	0.0496
NC_005100.4-44046042	AAAGTAAGTACATGTGCTCCTTCCTAC	23	C	0	0.4846
NC_005100.4-44062790	CTCAACTCTACCTGTCCTCCTGTGCTC	12	C	0.0169	0.4757
NC_005100.4-44115295	CATGCCGGGACATCATCTCCACTTGTT	2	A	0.0744	0.1017
NC_005100.4-44147034	CACATCAGCACTTGGTCTCCACCAGTA	13	T	0	0.4807
NC_005100.4-44179128	CCAGTCCTCACCCAGGCTCCAGGTCCT	25	C	0.15	0.2432
NC_005100.4-44187814	GGCATGCACACCTGGTCTCCTCTTCAG	7	C	0.1597	0.274
NC_005100.4-44187814	GGCATGCACACCTGGTCTCCTCTTCAG	8	A	0.0256	0.479
NC_005100.4-44192774	GCCTTATCTACATAGTCTCCTGGAGGA	9	T	0	0.4765
NC_005100.4-44225106	CAACACTCAACTCCAACTCCACTGATC	8	C	0.1416	0.1757
NC_005100.4-44225106	CAACACTCAACTCCAACTCCACTGATC	13	C	0.2544	0.3999
NC_005100.4-44267230	AACATGAGCACACATACTCCCATACAT	25	C	0.2951	0.4565
NC_005100.4-44267230	AACATGAGCACACATACTCCCATACAT	26	A	0.2951	0.4565
NC_005100.4-44269432	GGAAAGTGCACAGACCCTCCCAGGGTA	2	G	0.1071	0.1478
NC_005100.4-44274409	TTCATTCACACTGAGGCTCCAGTCAGC	5	T	0.0354	0.0348
NC_005100.4-44274409	TTCATTCACACTGAGGCTCCAGTCAGC	24	C	0.0357	0.0853
NC_005100.4-44330233	GGGAGAGCCACATCACCTCCAGGGACT	3	G	0.0163	0.032
NC_005100.4-44332555	CTGCTGACCACCATGCCTCCTCACGCA	24	C	0.2787	0.316
NC_005100.4-44367389	AGTTGCTTTACGGCCACTCCTGAGTTA	8	T	0.2105	0.3115
NC_005100.4-44396952	TGATGATAGACTTCAGCTCCCTGGGGA	7	T	0.2119	0.4896
NC_005100.4-44420729	CTCACTGCCACCTTCCCTCCTCACATT	26	T	0.2459	0.4934
NC_005100.4-44423387	GCTGACTTGACCTTCTCTCCTCCCTTC	15	C	0.1429	0.4994
NC_005100.4-44427281	TAAACACTAACATGGACTCCTTAGATT	5	C	0.0631	0.0611
NC_005100.4-44427281	TAAACACTAACATGGACTCCTTAGATT	6	A	0	0.4746
NC_005100.4-44472594	ATTCCTACAACTGGACCTCCTTGGATG	5	C	0	0.4778
NC_005100.4-44472594	ATTCCTACAACTGGACCTCCTTGGATG	26	T	0.0877	0.4255
NC_005100.4-44516406	CTCACACATACTCACTCTCCCTCATAC	8	A	0.0902	0.1573

NC_005100.4-44516406	CTCACACATACTCACTCTCCCTCATAC	12	T	0	0.4807
NC_005100.4-44528649	CACACCCTGACCTTCACTCCTAAGTTT	4	A	0.3083	0.4166
NC_005100.4-44574414	GAATCAGAGACCAGATCTCCCAGGGAC	8	A	0.0431	0.0585
NC_005100.4-44575220	ATGGCCAGCACCCACTCTCCTAGTGAA	2	T	0.0084	0.491
NC_005100.4-44575220	ATGGCCAGCACCCACTCTCCTAGTGAA	21	T	0.1261	0.4987
NC_005100.4-44664332	TTGTTACTTACTCATCCTCCATGAGCT	2	T	0.1311	0.263
NC_005100.4-44674917	AGAAGGCAAACTCAGGCTCCAGCCAGC	23	C	0.1053	0.1153
NC_005100.4-44708340	GACTGTGAGACAGGGTCTCCCTGAGTC	15	G	0.094	0.4947
NC_005100.4-44715186	TGCAGAACAACATGGCCTCCTGAAAGG	3	C	0.0268	0.0264
NC_005100.4-44721833	TGGTCAAACACAATTCCTCCCTATAAA	8	A	0.1917	0.225
NC_005100.4-44732725	AGAGTTGTTACATTAGCTCCTGTGTTA	3	A	0.3077	0.4734
NC_005100.4-44732725	AGAGTTGTTACATTAGCTCCTGTGTTA	24	G	0.3077	0.3457
NC_005100.4-44844882	GTTAGCCGTACAGATGCTCCGCATTCC	8	G	0.0268	0.0264
NC_005100.4-44867966	CACACACACACACACACTCCTTAGTCC	3	C	0.0339	0.0333
NC_005100.4-44893478	ATTCTTCACACGCCTGCTCCTCCAGAC	12	G	0.0446	0.0436
NC_005100.4-45002066	CATGGAGCAACCTATACTCCTGTGGGT	1	C	0.0901	0.1338
NC_005100.4-45002066	CATGGAGCAACCTATACTCCTGTGGGT	15	T	0.009	0.4682
NC_005100.4-45020101	ACCCTGGTGACTCCAGCTCCAGGGTAT	2	C	0.1121	0.1209
NC_005100.4-45022936	CACTTTCGCACATTACCTCCAGTCAGA	7	C	0.1565	0.3094
NC_005100.4-45077430	CCATTTTCCACACCGGCTCCAGACAAT	3	A	0.0351	0.0345
NC_005100.4-45077430	CCATTTTCCACACCGGCTCCAGACAAT	8	C	0	0.4859
NC_005100.4-45129487	CATGAATAGACTGCACCTCCCCCTGGA	3	T	0.1964	0.4943
NC_005100.4-45150650	GAGTGTGGGACTGTGCCTCCATTCCAT	3	G	0.0254	0.0251
NC_005100.4-45168892	ACAGAAGATACCCCCTCTCCCGTCAGT	9	T	0.0325	0.032
NC_005100.4-45168892	ACAGAAGATACCCCCTCTCCCGTCAGT	22	G	0.0407	0.0398
NC_005100.4-45190206	CTCCTATAAACACAAGCTCCTTAGTCA	6	A	0.0085	0.4776
NC_005100.4-45203344	GAGCTCGGCACACTCTCTCCTTTCCAT	6	C	0.0407	0.0553
NC_005100.4-45203344	GAGCTCGGCACACTCTCTCCTTTCCAT	7	G	0	0.4793
NC_005100.4-45237155	TTAAGTCTCACCTTTTCTCCACCTGTG	3	A	0.0333	0.0328
NC_005100.4-45251204	ATGTCTATCACCATGCCTCCATTGTGT	26	G	0.3	0.4265
NC_005100.4-45290270	GGAGGCAGTACTGTCCCTCCGTGGCTT	24	G	0.2768	0.4193
NC_005100.4-45303578	GGGAAAAGAACAGGTGCTCCCAAGGTC	8	G	0.0259	0.0255
NC_005100.4-45375645	CTACCGTGCACTCAGCCTCCTTCAAGC	6	G	0	0.4827
NC_005100.4-45384532	GAATTTACAACAGAGTCTCCTTTCCTG	15	G	0.036	0.0526
NC_005100.4-45393063	CTCATGCAGACAATCCCTCCAGTCCCT	4	A	0.0328	0.0322
NC_005100.4-45431360	ATATCGGGTACACCTTCTCCAGCACCC	5	C	0.2589	0.4968
NC_005100.4-45431360	ATATCGGGTACACCTTCTCCAGCACCC	9	T	0.1875	0.5
NC_005100.4-45449294	AATGGCATCACTGCATCTCCTGTATTT	2	A	0.0085	0.058
NC_005100.4-45449294	AATGGCATCACTGCATCTCCTGTATTT	22	G	0.2735	0.3054
NC_005100.4-45462598	TCCCTGTCAACCTCCCCTCCTCTCTGT	2	C	0.1053	0.1153
NC_005100.4-45462598	TCCCTGTCAACCTCCCCTCCTCTCTGT	22	C	0.0609	0.059
NC_005100.4-45491529	CAATCTCAAACCCCTGCTCCTACCCAG	25	C	0.1239	0.1316
NC_005100.4-45520678	CCAAAATAAACACTTCCTCCCACCTCT	16	C	0.1071	0.2052
NC_005100.4-45576290	TTTAAGGCAACCAAGTCTCCCAACACC	6	G	0.0179	0.477
NC_005100.4-45576675	CCACCTCTAACTCTAGCTCCAGGCACT	5	C	0.175	0.2491
NC_005100.4-45576675	CCACCTCTAACTCTAGCTCCAGGCACT	12	T	0.0417	0.0408
NC_005100.4-45639659	CCAGCCTGAACAAGCTCTCCTGCTATC	6	C	0.225	0.4025
NC_005100.4-45639659	CCAGCCTGAACAAGCTCTCCTGCTATC	23	C	0.2417	0.4097
NC_005100.4-45648131	CGGTGATAAACCCAGTCTCCCAGTGTG	2	G	0.1478	0.2204
NC_005100.4-45648131	CGGTGATAAACCCAGTCTCCCAGTGTG	16	T	0.0702	0.0839
NC_005100.4-45648846	TGGCACATGACAGTCACTCCGCTACAT	12	A	0.0085	0.4671

NC_005100.4-45669925	CTCAGCTACACTGGACCTCCCTCATGA	21	C	0.2927	0.4331
NC_005100.4-45711418	ACAATCCAGACCTATGCTCCTTGGCCT	5	T	0.082	0.1082
NC_005100.4-45739007	AGCTGCCAAACCAAGCCTCCTGCACTC	24	A	0.188	0.5
NC_005100.4-45757865	CCAGGGATGACTAAATCTCCCTTCTAG	5	G	0.0696	0.0989
NC_005100.4-45757865	CCAGGGATGACTAAATCTCCCTTCTAG	23	T	0	0.4793
NC_005100.4-45758576	CAGTCCACCACTCTATCTCCTCCCTCA	8	C	0.1171	0.1857
NC_005100.4-45771017	CCTGGCTGGACCGCCCCTCCCCCACTG	13	G	0.0656	0.0786
NC_005100.4-45796596	CTGGGTGTTACAGCCACTCCCTCAGTA	4	G	0.0081	0.481
NC_005100.4-45796596	CTGGGTGTTACAGCCACTCCCTCAGTA	7	G	0.1382	0.3381
NC_005100.4-45824439	ACCAAAGAAACCATATCTCCTCGTTCA	12	C	0.0254	0.0251
NC_005100.4-45824439	ACCAAAGAAACCATATCTCCTCGTTCA	14	T	0.075	0.0722
NC_005100.4-45835347	GGAGGCTGAACCTCCCCTCCTGTGATT	15	C	0	0.4814
NC_005100.4-45843687	GGAAAGCCGACCCTACCTCCATTTTCT	9	G	0.122	0.1425
NC_005100.4-45843687	GGAAAGCCGACCCTACCTCCATTTTCT	13	C	0.1557	0.4997
NC_005100.4-45867610	GTCCTGTTGACTAGAACTCCAGCTATT	9	G	0.045	0.044
NC_005100.4-45931253	GTGAGTAAAACCGTCCCTCCCTCTCTG	7	A	0	0.4827
NC_005100.4-45931253	GTGAGTAAAACCGTCCCTCCCTCTCTG	26	T	0.0708	0.4934
NC_005100.4-45967701	AGAACAATGACAGCAACTCCTTCGATC	23	C	0.0957	0.4926
NC_005100.4-46016330	CCATCACCAACACACACTCCTCATGCC	13	C	0.0965	0.4953
NC_005100.4-46020274	TGATCAAGTACATCAGCTCCCTAGTGT	8	G	0.0357	0.0351
NC_005100.4-46031264	AGGGAAGCAACTCAGCCTCCAGGTATA	12	T	0.0492	0.048
NC_005100.4-46048566	CATGACTGGACCCCAGCTCCACATCTG	2	A	0.0885	0.4981
NC_005100.4-46051403	GGGCTTGGGACAGTGTCTCCTGTCCCC	25	C	0	0.4734
NC_005100.4-46060437	AGCTGGCACACCCGAGCTCCACACCCT	2	G	0.0333	0.0328
NC_005100.4-46060437	AGCTGGCACACCCGAGCTCCACACCCT	21	A	0	0.4826
NC_005100.4-46093724	TCAATTCCCACAGCCCCTCCCAGATGC	3	A	0	0.4819
NC_005100.4-46093724	TCAATTCCCACAGCCCCTCCCAGATGC	5	T	0	0.4819
NC_005100.4-46115819	CTCCTGAGCACATGAACTCCCCTTTCA	12	A	0	0.4844
NC_005100.4-46171949	ATCAATACCACCATTCCTCCCAGAGAG	4	A	0.0348	0.0342
NC_005100.4-46171949	ATCAATACCACCATTCCTCCCAGAGAG	21	C	0.1316	0.499
NC_005100.4-46180509	CCAGGTCACACCCTGGCTCCCTCAGTT	25	G	0.0614	0.0595
NC_005100.4-46181118	CGAAAGCCCACCGCCCCTCCGTCCCAG	2	G	0	0.4793
NC_005100.4-46184607	GAGGCTGGCACAGCTGCTCCATTGCGA	22	T	0.0407	0.0398
NC_005100.4-46184607	GAGGCTGGCACAGCTGCTCCATTGCGA	26	G	0.0407	0.0398
NC_005100.4-46186403	GCCTGGAAGACGGCACCTCCTCCTTAA	12	G	0	0.4859
NC_005100.4-46225071	AGTCAGAGCACCCTGGCTCCTAGGGTA	23	G	0.1301	0.4984
NC_005100.4-46230007	GTCTATAAAACCCTCGCTCCTGCTCCT	3	C	0.0268	0.0264
NC_005100.4-46230007	GTCTATAAAACCCTCGCTCCTGCTCCT	25	C	0.0357	0.0351
NC_005100.4-46239579	CGCTGAAATACTGCAGCTCCCTTTAAC	15	A	0.0168	0.4779
NC_005100.4-46240571	GTAAGTGCGACTCTCCCTCCAACTGCT	21	A	0	0.4773
NC_005100.4-46273443	GATCAGGGAACCCTCTCTCCTGTTTGC	7	G	0.0508	0.0496
NC_005100.4-46273443	GATCAGGGAACCCTCTCTCCTGTTTGC	22	G	0.1983	0.2057
NC_005100.4-46288073	AAGGAGACTACAGGCACTCCTGCTTCC	5	A	0.3109	0.397
NC_005100.4-46292817	AGCCTGTGCACTGAGGCTCCCGCCAGT	12	T	0.042	0.0571
NC_005100.4-46292817	AGCCTGTGCACTGAGGCTCCCGCCAGT	16	G	0.1197	0.1563
NC_005100.4-46303070	CTTAGGAAAACATTCCCTCCATGTGCC	4	A	0.0488	0.1073
NC_005100.4-46322232	CTGAGCCCGACACCTGCTCCTCTCATG	8	C	0.0593	0.119
NC_005100.4-46322232	CTGAGCCCGACACCTGCTCCTCTCATG	27	G	0.0593	0.0734
NC_005100.4-46322883	AAACTGATCACGCCAACTCCACACCAG	12	G	0.0763	0.0889
NC_005100.4-46370931	GATCCCTGCACTTCACCTCCATTAACT	27	T	0.0246	0.0243
NC_005100.4-46415422	AAGGTATCTACTGCTCCTCCCACTGCT	14	C	0.0948	0.1504

NC_005100.4-46415422	AAGGTATCTACTGCTCCTCCCACTGCT	26	C	0.1795	0.1773
NC_005100.4-46453713	GCTCCCGCCACCTTAGCTCCCTGCAGA	1	G	0.1081	0.4854
NC_005100.4-46453713	GCTCCCGCCACCTTAGCTCCCTGCAGA	4	C	0.1081	0.4854
NC_005100.4-46505578	TAGGAAAACACTGACGCTCCCACGGTT	5	A	0.0325	0.0476
NC_005100.4-46505578	TAGGAAAACACTGACGCTCCCACGGTT	24	G	0.1301	0.1494
NC_005100.4-46513266	CTTGGCCGCACAGCCCCTCCCACAGTT	7	C	0.0407	0.0398
NC_005100.4-46513266	CTTGGCCGCACAGCCCCTCCCACAGTT	8	G	0.0894	0.1001
NC_005100.4-46527667	AGAAGCCAAACAACCACTCCAAGGTTA	15	C	0.1967	0.2032
NC_005100.4-46528141	CAGACATTCACTATCACTCCTAGACCA	12	T	0.1071	0.4974
NC_005100.4-46528141	CAGACATTCACTATCACTCCTAGACCA	22	A	0.1217	0.1443
NC_005100.4-46540387	GACTTAAAGACCTCATCTCCTACAGCA	12	C	0.0909	0.0868
NC_005100.4-46543205	GCCCAAGTCACCCCTCCTCCGCTGCCA	2	C	0.0847	0.0965
NC_005100.4-46579130	ACCCTCAGAACCATGTCTCCCTCAGAA	25	G	0.0684	0.066
NC_005100.4-46596336	TTTGGTCTAACACATCCTCCTTGAACC	14	A	0.0417	0.4875
NC_005100.4-46615907	CCACCAATGACACTGCCTCCTAATGGA	9	G	0	0.4772
NC_005100.4-46619732	AACCTCAGCACTGGTCCTCCAGACATT	4	C	0.1157	0.2172
NC_005100.4-46619732	AACCTCAGCACTGGTCCTCCAGACATT	7	A	0.0413	0.4889
NC_005100.4-46636781	GACCTGTCTACGGTCCCTCCCACCTTA	1	G	0.0513	0.1125
NC_005100.4-46637848	GTTTTCACTACCTACTCTCCCAGTACT	1	G	0.2456	0.396
NC_005100.4-46637848	GTTTTCACTACCTACTCTCCCAGTACT	26	C	0	0.4764
NC_005100.4-46680793	TCAAATGCAACTGGGTCTCCACAGAAC	5	A	0.1161	0.1553
NC_005100.4-46680793	TCAAATGCAACTGGGTCTCCACAGAAC	22	C	0.0268	0.4824
NC_005100.4-46712743	TCGCCTGACACACATGCTCCAATTACC	2	C	0.1463	0.189
NC_005100.4-46712743	TCGCCTGACACACATGCTCCAATTACC	3	G	0.0325	0.032
NC_005100.4-46719927	TTTATTGGAACTTAATCTCCCGACAAT	22	G	0.1453	0.1908
NC_005100.4-46737210	TTCATGATCACCCAATCTCCTGTTCCA	1	T	0.0086	0.4729
NC_005100.4-46763889	AACATCCAAACCCCAGCTCCTGTGAGG	14	C	0.0325	0.078
NC_005100.4-46784840	GCACTCGGCACCAACACTCCCTCAGTG	7	G	0.0656	0.0935
NC_005100.4-46784840	GCACTCGGCACCAACACTCCCTCAGTG	23	C	0.1901	0.4769
NC_005100.4-46787662	GGACCTGGAACTTGGGCTCCAAAGTTG	9	A	0.0084	0.4723
NC_005100.4-46791938	TCAGTACTGACACCATCTCCTTGGAAC	27	C	0.0246	0.0243
NC_005100.4-46794769	CTCCGCTCTACACTTGCTCCCCGGGTT	5	G	0.0574	0.0861
NC_005100.4-46794769	CTCCGCTCTACACTTGCTCCCCGGGTT	12	A	0	0.48
NC_005100.4-46823930	TCATCCATAACCCCAGCTCCAGGGACC	4	T	0.0488	0.4926
NC_005100.4-46836820	GACTCTAAGACACTTGCTCCAGGCAAT	13	C	0.0579	0.1017
NC_005100.4-46896038	CTTGGAGCAACTGCCCCTCCATCTTGG	12	T	0	0.4844
NC_005100.4-46953370	CTGTTGGCCACAGTGTCTCCCCTGGCT	13	G	0	0.4839
NC_005100.4-47015287	ATAATTTAGACTCACTCTCCACAGGGA	14	A	0.0177	0.4793
NC_005100.4-47031983	GTGAAGAGAACAGCTTCTCCATCATCA	12	A	0	0.4813
NC_005100.4-47119070	GTGTAACTCACCTCTCCTCCTCCTTCT	26	C	0.1488	0.5
NC_005100.4-47167416	TCCAGCAAGACCACACCTCCTAACCTG	8	A	0.0574	0.071
NC_005100.4-47167416	TCCAGCAAGACCACACCTCCTAACCTG	26	T	0.1393	0.1296
NC_005100.4-47174459	CTACAGACAACGCCTCCTCCTGGCTGT	15	T	0.0446	0.0436
NC_005100.4-47174459	CTACAGACAACGCCTCCTCCTGGCTGT	23	G	0.0431	0.0585
NC_005100.4-47179504	GTGTCCATCACACACACTCCATAACAC	1	G	0.0924	0.4977
NC_005100.4-47179504	GTGTCCATCACACACACTCCATAACAC	25	C	0.0252	0.0249
NC_005100.4-47188927	CCTCCCGCGACCCAAGCTCCAAGGGAT	8	C	0.0263	0.026
NC_005100.4-47199539	ATTGGTTAGACTTGATCTCCAGTTCCT	2	T	0.2241	0.4963
NC_005100.4-47206053	GTAGCTTCTACTAAAACTCCAGGAACC	2	T	0.0345	0.4905
NC_005100.4-47221717	TCTGCTTTGACCTCCCCTCCATTATGG	14	C	0.2261	0.4891
NC_005100.4-47221717	TCTGCTTTGACCTCCCCTCCATTATGG	15	C	0.0833	0.095

NC_005100.4-47246370	CACACGGGCACATGCTCTCCTGTGTAC	5	C	0.0246	0.0243
NC_005100.4-47246370	CACACGGGCACATGCTCTCCTGTGTAC	6	G	0.1311	0.4995
NC_005100.4-47256962	GAACTTAGAACCTCCTCTCCCCCTCCT	3	A	0.0427	0.058
NC_005100.4-47264835	AGAGAGGTGACACTTGCTCCCGTGAAC	21	C	0.0583	0.49
NC_005100.4-47264835	AGAGAGGTGACACTTGCTCCCGTGAAC	26	A	0	0.4765
NC_005100.4-47277545	TGCTGCCAGACAGGTCCTCCCAGGGAA	8	A	0	0.4773
NC_005100.4-47306098	CACATCCCCACACCCTCTCCCACTCTC	22	A	0.0909	0.4933
NC_005100.4-47314540	AAGGATTCCACGCGTGCTCCGCCATAC	14	G	0.0336	0.033
NC_005100.4-47314540	AAGGATTCCACGCGTGCTCCGCCATAC	21	G	0.2269	0.4045
NC_005100.4-47337370	CCTGTCCACACAAACTCTCCATTCTCT	13	A	0	0.4444
NC_005100.4-47339728	GGTCTGCCCACCAGTTCTCCAGCTCTG	1	G	0.0427	0.058
NC_005100.4-47339728	GGTCTGCCCACCAGTTCTCCAGCTCTG	22	G	0.1356	0.1264
NC_005100.4-47357707	AACCTCTGTACCACATCTCCAAAGCCT	2	A	0.1949	0.4957
NC_005100.4-47372425	ATCTTCTTGACCTCAGCTCCTGTTGTG	14	C	0.2276	0.4926
NC_005100.4-47470652	ATGTTGGGAACTATTACTCCTACAGAA	3	G	0.0598	0.0896
NC_005100.4-47481093	GCAGGATGGACAGCTCCTCCATAGTCA	7	T	0.2276	0.3239
NC_005100.4-47505945	AGGGGTCACACCAGATCTCCTGCATAT	3	G	0.1707	0.1826
NC_005100.4-47553419	GGGAATCCAACCATTCCTCCAAGTCAC	21	A	0.1709	0.3159
NC_005100.4-47575913	AGTGTGCCAACCCTGGCTCCTTAGGGA	12	C	0.0172	0.4749
NC_005100.4-47584002	CAAACCACCACACTAGCTCCATGTGAT	27	T	0	0.482
NC_005100.4-47654575	AAAGTCCTCACCAGGTCTCCTTCGCTG	14	G	0.0342	0.05
NC_005100.4-47654575	AAAGTCCTCACCAGGTCTCCTTCGCTG	16	T	0.1207	0.4987
NC_005100.4-47662056	ACTACCACTACCACCACTCCGGAACCT	21	G	0.0172	0.482
NC_005100.4-47664605	TACAGAATCACAGAAACTCCTGGGTCT	8	T	0.0684	0.4649
NC_005100.4-47745364	TGGTTCCATACCACAGCTCCAACATCA	7	C	0.1301	0.1216
NC_005100.4-47751052	CTTGGTCCTACTGATGCTCCAGCCAGA	4	G	0.2459	0.3791
NC_005100.4-47885767	TCACCTCTTACCCACACTCCTGTCAGT	24	C	0.2397	0.4863
NC_005100.4-47903372	AGATCTCACACAGCAGCTCCGTTCCAC	3	A	0.3	0.4265
NC_005100.4-47908294	CCTCCTCTCACTCTCACTCCTTCATCA	24	A	0.0325	0.032
NC_005100.4-47982927	TATGAAGAAACATGATCTCCAGGGGTA	13	T	0.0083	0.4747
NC_005100.4-48039865	GGGCACCGCACCGAAACTCCCACCCAC	25	C	0	0.4793
NC_005100.4-48059456	GCAGTGTCCACACAGCCTCCCGACCCT	4	G	0.0862	0.0825
NC_005100.4-48059456	GCAGTGTCCACACAGCCTCCCGACCCT	21	C	0	0.4693
NC_005100.4-48067537	GGAACAGGCACAAAGCCTCCGAATGCT	12	A	0.0407	0.0398
NC_005100.4-48067537	GGAACAGGCACAAAGCCTCCGAATGCT	21	G	0.0407	0.0398
NC_005100.4-48078860	TAAGCTTTCACTGAAGCTCCCCAGGCC	26	C	0.2288	0.4939
NC_005100.4-48186874	ATCTTCCTGACTCAGCCTCCAGTGCAG	22	G	0	0.4773
NC_005100.4-48225355	TGCTTAGAGACAGATTCTCCAGCAATC	14	A	0.0088	0.4843
NC_005100.4-48245653	CCACCCTTCACCTGTGCTCCCACGTTC	13	T	0.0263	0.4473
NC_005100.4-48245653	CCACCCTTCACCTGTGCTCCCACGTTC	27	C	0.0263	0.4473
NC_005100.4-48267841	CATCCCCTTACCTTGGCTCCAGAAGGT	23	A	0.0328	0.4656
NC_005100.4-48271748	GTGGCCTCAACACCAGCTCCTGCCTCT	1	G	0.1513	0.4997
NC_005100.4-48271748	GTGGCCTCAACACCAGCTCCTGCCTCT	27	T	0.1417	0.1316
NC_005100.4-48288002	CCGGCATCCACGCCACCTCCCTGGGCC	12	G	0	0.4743
NC_005100.4-48288002	CCGGCATCCACGCCACCTCCCTGGGCC	27	C	0.084	0.1254
NC_005100.4-48288287	TCTACATGTACACGCGCTCCAGGCACC	3	T	0	0.4693
NC_005100.4-48288287	TCTACATGTACACGCGCTCCAGGCACC	16	G	0.0684	0.4868
NC_005100.4-48295007	TATAATTAGACCCTACCTCCTAAAGCA	2	A	0	0.4731
NC_005100.4-48295007	TATAATTAGACCCTACCTCCTAAAGCA	3	T	0	0.4731
NC_005100.4-48346233	GTATCCCTCACAGGCTCTCCCACACTG	1	G	0.094	0.1492
NC_005100.4-48346233	GTATCCCTCACAGGCTCTCCCACACTG	2	T	0.094	0.1492

NC_005100.4-48350433	GGGCAGCAGACCGAGACTCCAGGAATA	27	A	0.1626	0.1629
NC_005100.4-48367276	CAAACTAAAACAACCTCTCCTGCAACA	15	C	0.1653	0.1653
NC_005100.4-48438254	CATCTATGCACAAAAGCTCCTAAGTAG	9	C	0	0.4637
NC_005100.4-48508952	ATGGTACTCACTTCCCCTCCTGACTGG	5	T	0.1803	0.4979
NC_005100.4-48508952	ATGGTACTCACTTCCCCTCCTGACTGG	23	A	0	0.4773
NC_005100.4-48538559	ACATCAGTAACACTTCCTCCTCTGGGT	3	A	0.0569	0.0705
NC_005100.4-48538665	GATTCCGCAACACCCCCTCCTGTCTCC	12	A	0.0583	0.0722
NC_005100.4-48551426	CATATCCAAACAGGAGCTCCTCTGTCC	7	C	0.0244	0.0241
NC_005100.4-48564513	GAAGCACTTACTGCTGCTCCAGAGAAC	4	G	0	0.4764
NC_005100.4-48566299	GTGCTGGGCACCGCTCCTCCTTGAGGA	6	G	0.0083	0.4769
NC_005100.4-48570290	TGGGTTACAACTCTTCCTCCTCTCACC	26	C	0.2017	0.4921
NC_005100.4-48581791	TTTACATGCACATGTGCTCCCACCCAT	8	G	0	0.4793
NC_005100.4-48588567	TCCTCTCTGACTGCTCCTCCTGTCTCT	27	T	0.2066	0.4966
NC_005100.4-48590379	AAAGAGAAGACTGTGCCTCCACAAAGT	1	A	0	0.48
NC_005100.4-48590379	AAAGAGAAGACTGTGCCTCCACAAAGT	13	G	0.084	0.0805
NC_005100.4-48600191	CGGAGTGCCACAAAGCCTCCCAGATCC	22	A	0.0714	0.4539
NC_005100.4-48649188	GTCCTCTCCACGTGCTCTCCTTTGTCT	6	C	0	0.4793
NC_005100.4-48649188	GTCCTCTCCACGTGCTCTCCTTTGTCT	12	G	0	0.4793
NC_005100.4-48671244	TATTGTCTAACATGGCCTCCTTTGGAG	9	A	0	0.4751
NC_005100.4-48686385	GTTGTTGGAACTAATGCTCCTCCCTCA	1	G	0.025	0.0247
NC_005100.4-48698956	AGGTCTCATACGTTAGCTCCTGTGACC	12	G	0.0348	0.0342
NC_005100.4-48722359	GGGGAGGGGACTCCAGCTCCATGTCGT	1	G	0.0263	0.0429
NC_005100.4-48722359	GGGGAGGGGACTCCAGCTCCATGTCGT	26	G	0.0087	0.0257
NC_005100.4-48966811	CTTCCTGCCACTTTCTCTCCATGTAAG	16	T	0	0.4841
NC_005100.4-48966811	CTTCCTGCCACTTTCTCTCCATGTAAG	22	T	0.0089	0.4856
NC_005100.4-48966987	GCTGACATCACTCCTGCTCCTAAGGAC	12	T	0	0.4807
NC_005100.4-49004291	TCTGCCTACACTATGCCTCCATCAGGG	24	A	0.0084	0.4797
NC_005100.4-49018645	TGCCTGTGGACCTTCTCTCCTTTATAA	22	T	0.0339	0.0333
NC_005100.4-49038498	CGTGTCCATACATAGGCTCCCAGCAGA	12	A	0.122	0.4036
NC_005100.4-49115531	ACTGCAGGCACCTGATCTCCTCAGCTC	9	C	0.0248	0.0245
NC_005100.4-49115531	ACTGCAGGCACCTGATCTCCTCAGCTC	27	C	0.1653	0.2172
NC_005100.4-49132903	GAATGTTTAACAGCCTCTCCTGGTCAA	2	A	0.041	0.0861
NC_005100.4-49159947	CTACTTGTAACTCCAACTCCAGGAGTT	2	T	0.0678	0.0812
NC_005100.4-49166974	CATGCCCAAACTCACCCTCCCCCCAAT	9	A	0	0.4779
NC_005100.4-49173901	CAGGAAGTAACAGAGTCTCCCTCAGCC	13	G	0.0089	0.4617
NC_005100.4-49187307	GTGGTCACCACTGGGACTCCTGATGAC	5	T	0.1327	0.3495
NC_005100.4-49187307	GTGGTCACCACTGGGACTCCTGATGAC	23	A	0.0354	0.0348
NC_005100.4-49200504	TTGGAAACAACATATGCTCCGTGAGCA	26	C	0.0345	0.0339
NC_005100.4-49203055	CAAGACAGAACTGCTGCTCCTAGGGGA	7	A	0.0083	0.4783
NC_005100.4-49208724	AAGAACTGGACTGTCCCTCCCTTTCTC	6	C	0	0.4859
NC_005100.4-49208724	AAGAACTGGACTGTCCCTCCCTTTCTC	8	G	0	0.4859
NC_005100.4-49237480	GATTACAGGACTGTTGCTCCTGGCTCA	8	G	0.0909	0.1163
NC_005100.4-49289621	TAGCACTGGACCATCTCTCCCATACAA	24	A	0.0442	0.0433
NC_005100.4-49338835	AGGACACACACCTTTGCTCCTCACCAG	4	A	0.1	0.4911
NC_005100.4-49338835	AGGACACACACCTTTGCTCCTCACCAG	21	T	0.2167	0.375
NC_005100.4-49358688	AAAATAAGAACCCAAACTCCTACTCAT	12	C	0.0446	0.0436
NC_005100.4-49370116	GCCACTCTCACCCCCGCTCCCTGTGAG	12	C	0.0513	0.05
NC_005100.4-49377422	TGTGGGGCAACGTAAGCTCCTCCATCC	12	G	0.0982	0.125
NC_005100.4-49435298	GACCATGAGACACAACCTCCATTATCT	16	C	0	0.4868
NC_005100.4-49435298	GACCATGAGACACAACCTCCATTATCT	24	A	0	0.4868
NC_005100.4-49470337	GGCATATAGACAGAGTCTCCTGTGCAC	1	G	0.2241	0.2252

NC_005100.4-49470337	GGCATATAGACAGAGTCTCCTGTGCAC	6	A	0.2328	0.244
NC_005100.4-49496987	TCTCTTGCAACACAGTCTCCTCTTCTG	9	A	0.1951	0.4881
NC_005100.4-49497041	GAAGGACCCACAGATGCTCCAGCACGT	22	G	0.2414	0.4405
NC_005100.4-49497041	GAAGGACCCACAGATGCTCCAGCACGT	25	C	0.0256	0.0253
NC_005100.4-49501254	TAACAACATACTCCACCTCCATCAATT	8	A	0.1917	0.4816
NC_005100.4-49501254	TAACAACATACTCCACCTCCATCAATT	25	A	0.1983	0.2415
NC_005100.4-49504112	TAGCATCCCACTGTGACTCCCTTGCCC	13	G	0.1818	0.2869
NC_005100.4-49504112	TAGCATCCCACTGTGACTCCCTTGCCC	24	G	0	0.4793
NC_005100.4-49541514	TATATAAAAACTCACCCTCCTTGCGAT	24	C	0.0439	0.0595
NC_005100.4-49545308	AGTCAGCTCACGTAATCTCCAGCTCAC	2	G	0.042	0.0571
NC_005100.4-49631210	CAAAGCAGCACAGGGCCTCCTTTCTAC	13	G	0	0.4807
NC_005100.4-49631210	CAAAGCAGCACAGGGCCTCCTTTCTAC	14	G	0.0894	0.3888
NC_005100.4-49643389	GCCTACACAACCACCACTCCCCTTTAG	1	G	0	0.4704
NC_005100.4-49644569	GATCTGGTTACACAACCTCCATACACC	9	T	0	0.4807
NC_005100.4-49644569	GATCTGGTTACACAACCTCCATACACC	15	A	0	0.4807
NC_005100.4-49676455	GTTTCATTCACACCAACTCCAGATACC	1	G	0.2679	0.3264
NC_005100.4-49676455	GTTTCATTCACACCAACTCCAGATACC	7	T	0.2432	0.4804
NC_005100.4-49681487	GTCCTTAACACCCCAGCTCCTCAAAGG	2	T	0.0256	0.0253
NC_005100.4-49681487	GTCCTTAACACCCCAGCTCCTCAAAGG	4	C	0.0965	0.4953
NC_005100.4-49695495	TCAGTCAATACGTACTCTCCTGCTGTG	15	C	0.0579	0.0562
NC_005100.4-49715413	GTATTTAGAACCAGGTCTCCAAACCCA	12	C	0.0541	0.4659
NC_005100.4-49793430	AGGGGAGCCACAGAGGCTCCCAAATGT	5	G	0.0427	0.4823
NC_005100.4-49806023	ACTTAAACAACCCTCTCTCCCTCCTTT	1	A	0.0087	0.4903
NC_005100.4-49816926	TGTCCCTGCACTCAAGCTCCTCCACCA	27	A	0.1322	0.1653
NC_005100.4-49829631	TGCTGCAACACCGTGGCTCCTCCCATG	8	A	0	0.4773
NC_005100.4-49829631	TGCTGCAACACCGTGGCTCCTCCCATG	13	G	0.082	0.1367
NC_005100.4-49864126	TACAATGTGACCTCCACTCCCGCCAGG	14	C	0	0.4826
NC_005100.4-49864126	TACAATGTGACCTCCACTCCCGCCAGG	21	C	0.0339	0.0496
NC_005100.4-49877296	GGTAAGTAGACGCTACCTCCTTAGAGT	4	A	0.252	0.4174
NC_005100.4-49877296	GGTAAGTAGACGCTACCTCCTTAGAGT	12	G	0.0813	0.4331
NC_005100.4-49949005	CACATTCAAACCAACTCTCCCATTACT	15	C	0.2114	0.4984
NC_005100.4-49982362	TTGACAGTTACACCTACTCCCTCATTT	7	G	0.0088	0.4735
NC_005100.4-50014835	CTGTTGACTACGGACACTCCCTTGTCT	15	C	0.1949	0.3388
NC_005100.4-50098804	CATTCATTGACAAAAGCTCCATTATCT	16	G	0	0.4839
NC_005100.4-50123338	GAGTAAGATACTTGCCCTCCTTTCTAC	4	T	0.0976	0.1494
NC_005100.4-50123338	GAGTAAGATACTTGCCCTCCTTTCTAC	9	T	0.0407	0.4662
NC_005100.4-50155401	CCACCTTTAACATTGTCTCCTTCAGGA	23	C	0	0.4743
NC_005100.4-50262201	CTGGAGAAGACTAGGTCTCCAGCTCCA	2	T	0.0424	0.0415
NC_005100.4-50262201	CTGGAGAAGACTAGGTCTCCAGCTCCA	22	G	0.0424	0.0415
NC_005100.4-50264760	TCTTGATCTACATGTGCTCCAGATGTG	14	G	0.0342	0.0336
NC_005100.4-50325425	GGCCATGCAACGGTCTCTCCACTCACT	9	A	0.2348	0.4962
NC_005100.4-50325425	GGCCATGCAACGGTCTCTCCACTCACT	12	G	0	0.4703
NC_005100.4-50339461	GCTGCAGAGACACTCTCTCCAAGGTCA	14	T	0	0.4807
NC_005100.4-50360565	TGTTTGAGCACACACACTCCCAGCCAT	8	G	0.0088	0.4774
NC_005100.4-50382048	CTGTTTCCCACCCCGTCTCCCACCATG	15	G	0	0.4807
NC_005100.4-50382048	CTGTTTCCCACCCCGTCTCCCACCATG	26	T	0.1228	0.1454
NC_005100.4-50420577	CCGTCTCTGACACGTTCTCCTGTTGAC	3	G	0.1138	0.2499
NC_005100.4-50446767	GGCTGTGAAACTTGCCCTCCTGACTCT	2	G	0.2185	0.2328
NC_005100.4-50446767	GGCTGTGAAACTTGCCCTCCTGACTCT	23	A	0.025	0.0875
NC_005100.4-50484744	GCCAGGCTCACTGTGTCTCCTGGTGAT	4	A	0.0254	0.487
NC_005100.4-50511369	TCACCTCGCACTGCTGCTCCATTAGCT	1	T	0.1391	0.3094

NC_005100.4-50511369	TCACCTCGCACTGCTGCTCCATTAGCT	22	T	0.1478	0.3814
NC_005100.4-50521680	CTCAGCCCCACCTCAACTCCTGTCCCT	13	T	0	0.4807
NC_005100.4-50552155	AGGAGCAGGACTTGAACTCCTGCCTTG	8	G	0.027	0.0267
NC_005100.4-50565290	ATGAAAATTACTCCCCCTCCTCCCCAT	5	A	0.0084	0.4723
NC_005100.4-50565290	ATGAAAATTACTCCCCCTCCTCCCCAT	14	C	0.124	0.1306
NC_005100.4-51127263	AGCAGAGGGACCCTGTCTCCTCACCAT	12	C	0.0172	0.0339
NC_005100.4-51185051	AGGCTCATAACTAATTCTCCACAAACA	25	A	0	0.474
NC_005100.4-51202942	TGGATAAGCACAGGGGCTCCTGACAGA	2	G	0.125	0.225
NC_005100.4-51242142	TCAGCTTTGACGATCTCTCCATTGATA	23	T	0.2917	0.4025
NC_005100.4-51253301	TCAGTTCAGACTCTGGCTCCCTGTTCT	14	T	0	0.4807
NC_005100.4-51253301	TCAGTTCAGACTCTGGCTCCCTGTTCT	24	T	0	0.4807
NC_005100.4-51276331	TATTTTAAGACAGGATCTCCTTGAACT	27	T	0.1	0.095
NC_005100.4-51390143	CTGCACAAAACGGGAGCTCCTCTACGT	2	T	0.1818	0.3557
NC_005100.4-51390143	CTGCACAAAACGGGAGCTCCTCTACGT	27	T	0.1818	0.1653
NC_005100.4-51425860	AAGCACAGAACGCTCACTCCATCTTAC	4	C	0.2609	0.264
NC_005100.4-51450857	GGCACACAGACCTGTCCTCCTTCCTGG	2	G	0.065	0.078
NC_005100.4-51450857	GGCACACAGACCTGTCCTCCTTCCTGG	8	A	0.3361	0.454
NC_005100.4-51453881	GCATCCCTTACCCCACCTCCACCACAC	9	T	0.0574	0.0557
NC_005100.4-51453881	GCATCCCTTACCCCACCTCCACCACAC	15	A	0.0082	0.0243
NC_005100.4-51469914	CTATTCTTGACTCTAACTCCTTGCAAT	7	T	0.082	0.1082
NC_005100.4-51469914	CTATTCTTGACTCTAACTCCTTGCAAT	26	A	0.0246	0.0401
NC_005100.4-51561323	TTGTTATAGACGTTCACTCCACAGTTT	21	A	0.2295	0.4656
NC_005100.4-51563629	CCTGGGTGTACTCAGCCTCCCCACTGC	16	C	0.2544	0.3919
NC_005100.4-51563629	CCTGGGTGTACTCAGCCTCCCCACTGC	21	C	0	0.4698
NC_005100.4-51564472	AGAAAGAGCACAAGGGCTCCATGAGTG	13	A	0	0.4718
NC_005100.4-51564472	AGAAAGAGCACAAGGGCTCCATGAGTG	14	G	0.1102	0.119
NC_005100.4-51611469	AGCACACAGACAAATGCTCCCTGACTT	24	A	0.1681	0.2856
NC_005100.4-51611469	AGCACACAGACAAATGCTCCCTGACTT	25	C	0.1858	0.4873
NC_005100.4-51630833	TCTTAGATAACTCAGCCTCCAGAACTA	27	A	0.0661	0.0639
NC_005100.4-51632893	CACACACACACACACCCTCCTGGATAG	14	A	0.0345	0.0504
NC_005100.4-51632893	CACACACACACACACCCTCCTGGATAG	27	G	0.2301	0.3728
NC_005100.4-51632940	TGTTTGTCAACACCATCTCCCTATTTG	7	T	0.1186	0.1264
NC_005100.4-51632940	TGTTTGTCAACACCATCTCCCTATTTG	21	C	0.0593	0.0889
NC_005100.4-51774459	TCAATCCTTACTCTAACTCCTCCTATT	23	C	0	0.4793
NC_005100.4-51844936	GTGTGTCAAACCATCTCTCCTTTGTCT	2	T	0.1951	0.2142
NC_005100.4-51844936	GTGTGTCAAACCATCTCTCCTTTGTCT	22	T	0.0244	0.0241
NC_005100.4-51850000	CACTTGAATACTGTCACTCCTAAGCAT	4	T	0	0.4793
NC_005100.4-51855473	TCTCAGTGCACACTAACTCCACTGAGG	1	T	0	0.4734
NC_005100.4-51855473	TCTCAGTGCACACTAACTCCACTGAGG	8	G	0.0339	0.0333
NC_005100.4-51928864	TTTCCCAAGACTACAGCTCCCAGCATT	1	T	0.2566	0.2494
NC_005100.4-51979479	CTCTGAGCTACCTTACCTCCAACCTCT	12	C	0.1382	0.4988
NC_005100.4-51979479	CTCTGAGCTACCTTACCTCCAACCTCT	14	T	0	0.4793
NC_005100.4-51989722	TGGACGGTGACAAGACCTCCTTGGAGA	5	C	0	0.4833
NC_005100.4-52021664	TCAAAGTAAACTCAAACTCCAACAGTG	1	T	0.1652	0.451
NC_005100.4-52028986	CCACTGGAAACAGTTTCTCCTGTTAAG	2	C	0.0598	0.058
NC_005100.4-52028986	CCACTGGAAACAGTTTCTCCTGTTAAG	15	T	0.0342	0.0336
NC_005100.4-52038587	CTGGAGCAAACTGGTTCTCCTCTCTTA	9	A	0.0244	0.0241
NC_005100.4-52074351	TTTGTCATGACTCTAACTCCCAGGGGT	4	G	0.2054	0.1843
NC_005100.4-52120619	TACACTTGCACAAAAGCTCCAGAGCCA	24	G	0.1795	0.4753
NC_005100.4-52120619	TACACTTGCACAAAAGCTCCAGAGCCA	25	C	0.0333	0.0644
NC_005100.4-52129050	ACCAAAGCCACCCACGCTCCATTCTCC	3	C	0.0333	0.0488

NC_005100.4-52129050	ACCAAAGCCACCCACGCTCCATTCTCC	13	C	0.2773	0.4253
NC_005100.4-52148786	TCATATTCTACCCCCACTCCCACCCCC	3	A	0.0625	0.0934
NC_005100.4-52148786	TCATATTCTACCCCCACTCCCACCCCC	26	C	0.0089	0.4709
NC_005100.4-52220421	AGCTACAGCACTGTTTCTCCACTTGAG	21	A	0.0083	0.4732
NC_005100.4-52220421	AGCTACAGCACTGTTTCTCCACTTGAG	25	G	0.2787	0.3947
NC_005100.4-52220667	GATGAAAGTACATCAGCTCCTAATTAG	14	C	0.2609	0.3772
NC_005100.4-52220667	GATGAAAGTACATCAGCTCCTAATTAG	15	A	0.0086	0.4836
NC_005100.4-52252814	TCCTGGTCCACTTCATCTCCCTGCCCT	13	T	0	0.477
NC_005100.4-52275678	AGAAGGCTTACTGTGCCTCCATCTACA	2	G	0.3333	0.3487
NC_005100.4-52276769	TGATTCCTCACTAAGTCTCCCTCCGAC	25	G	0.0268	0.0264
NC_005100.4-52277167	CATGCTTAAACCTTTGCTCCATAATCT	3	T	0	0.4793
NC_005100.4-52277167	CATGCTTAAACCTTTGCTCCATAATCT	5	C	0	0.4793
NC_005100.4-52316722	TTCATGTGTACTGCTTCTCCCACATAC	2	T	0	0.4728
NC_005100.4-52316722	TTCATGTGTACTGCTTCTCCCACATAC	4	A	0.3636	0.4112
NC_005100.4-52334689	CACAGGTACACTGGAACTCCAACCATA	5	G	0.0826	0.4942
NC_005100.4-52381263	ACAATGACAACATTGTCTCCATGGAGT	2	C	0	0.4773
NC_005100.4-52386606	AATCCTGGAACCAAGCCTCCATCTTGA	7	G	0.1833	0.18
NC_005100.4-52459274	GTTACAACCACAGGGCCTCCATCACAG	27	G	0.1707	0.4988
NC_005100.4-52460013	CTGCATGTAACTGCAGCTCCAAGAGAC	8	T	0.1724	0.4994
NC_005100.4-52464442	TGATCATGCACAGAGCCTCCCCGACAA	23	G	0.188	0.4997
NC_005100.4-52474223	CTCTTCTACACCCCATCTCCTCATCAG	8	A	0.0087	0.4744
NC_005100.4-52474223	CTCTTCTACACCCCATCTCCTCATCAG	12	C	0.0789	0.1953
NC_005100.4-52474256	CTGTTCTGCACCCCATCTCCCATAAGT	9	C	0.1583	0.5
NC_005100.4-52474256	CTGTTCTGCACCCCATCTCCCATAAGT	14	C	0.1583	0.2125
NC_005100.4-52476743	TTCCACATAACGACACCTCCATTCAAG	8	T	0	0.4819
NC_005100.4-52487093	CTGCCCACAACTCCAGCTCCCTATGCC	25	G	0.2035	0.4923
NC_005100.4-52506634	GGCAGGATTACTGACTCTCCTAGTCAA	13	G	0.2846	0.4174
NC_005100.4-52508236	TGTTAATGAACAATTTCTCCGGAGTTT	13	A	0	0.4637
NC_005100.4-52508236	TGTTAATGAACAATTTCTCCGGAGTTT	21	G	0.2783	0.4364
NC_005100.4-52508630	CTTTTAACCACCATCTCTCCTCTGTAC	27	C	0.314	0.4481
NC_005100.4-52554278	CATTTCCAAACTCCAACTCCATACCGC	27	C	0	0.4807
NC_005100.4-52554872	TTCTTATAAACACCATCTCCAGGTCAG	25	C	0.0085	0.4823
NC_005100.4-52599532	AGGACAATAACAGTGCCTCCCTCTTGC	16	C	0.2276	0.4984
NC_005100.4-52599532	AGGACAATAACAGTGCCTCCCTCTTGC	23	C	0.2276	0.4984
NC_005100.4-52632237	ACTCAGGTCACAGCAACTCCATTACAT	25	C	0.175	0.2833
NC_005100.4-52643691	CCACCAAAGACATGAGCTCCTAGCTTC	21	T	0.0522	0.0671
NC_005100.4-52647290	GCTCTGCAAACTGCACCTCCACTTTCA	9	A	0	0.4765
NC_005100.4-52647290	GCTCTGCAAACTGCACCTCCACTTTCA	24	T	0.1	0.495
NC_005100.4-52672216	CAGAGCTGCACTTGGACTCCACAGAGT	9	C	0.0325	0.032
NC_005100.4-52672216	CAGAGCTGCACTTGGACTCCACAGAGT	12	T	0.0407	0.0398
NC_005100.4-52675761	GCCATGAGCACTGATCCTCCCTCACCA	1	G	0.0984	0.1225
NC_005100.4-52675761	GCCATGAGCACTGATCCTCCCTCACCA	12	T	0.0574	0.4943
NC_005100.4-52690364	TAGGTTCATACGCCTCCTCCACAGACA	5	T	0	0.4793
NC_005100.4-52709569	ACTGCTTCCACATCCGCTCCGTCCCCG	21	G	0.1525	0.1961
NC_005100.4-52746100	CCACACGATACCTTCCCTCCTCTAATT	14	T	0.1391	0.2758
NC_005100.4-52762586	AGACATACCACACCCTCTCCTGGGGAA	3	A	0.0902	0.0861
NC_005100.4-52762586	AGACATACCACACCCTCTCCTGGGGAA	22	G	0.0328	0.0322
NC_005100.4-52774857	GTCTCTTGTACCAAGGCTCCAACTTGG	7	T	0.0678	0.4948
NC_005100.4-52804328	CAAAACCTCACGAGCCCTCCTTTTCAC	5	A	0.0089	0.4824
NC_005100.4-52809777	CCTCCCGTGACCATGCCTCCCACTGTG	6	C	0	0.4757
NC_005100.4-52809777	CCTCCCGTGACCATGCCTCCCACTGTG	9	G	0.0254	0.0251

NC_005100.4-52815542	ACTCCAGCCACGCTTTCTCCTGCCAAG	12	G	0.0776	0.0903
NC_005100.4-52815542	ACTCCAGCCACGCTTTCTCCTGCCAAG	13	C	0.0517	0.0504
NC_005100.4-52817177	ATCTATTCTACCTGCTCTCCTGTTGGT	15	C	0.2203	0.2344
NC_005100.4-52855004	ACCATAAACACCCTACCTCCATCTCCA	8	A	0.1538	0.1563
NC_005100.4-52855004	ACCATAAACACCCTACCTCCATCTCCA	22	T	0.1026	0.1704
NC_005100.4-52886222	CACAGGGTGACACCTGCTCCTACTCAG	15	T	0.0583	0.0875
NC_005100.4-52893823	CATTGTCTTACCTGTCCTCCTCCATTG	15	T	0.0244	0.0398
NC_005100.4-52895316	TAAACTCCCACGCGCACTCCGCACTCT	2	A	0	0.4793
NC_005100.4-52895316	TAAACTCCCACGCGCACTCCGCACTCT	3	A	0	0.4793
NC_005100.4-52900958	GTCATACACACTACTCCTCCAGGCTCC	8	A	0	0.4734
NC_005100.4-52906975	GTACCTTCCACAGCTTCTCCACCATCG	2	T	0	0.4773
NC_005100.4-52906975	GTACCTTCCACAGCTTCTCCACCATCG	26	C	0.0738	0.1009
NC_005100.4-52915630	GGCTGTCACACAAGGCCTCCTAGAGGT	27	T	0	0.488
NC_005100.4-52921527	CACTAGGTTACCAGCCCTCCTCTGAGT	4	T	0.0325	0.032
NC_005100.4-52921527	CACTAGGTTACCAGCCCTCCTCTGAGT	21	T	0.1967	0.416
NC_005100.4-52934379	CCAGCACTCACTGTGACTCCAGCTCTA	8	T	0.0492	0.4891
NC_005100.4-52949383	TATGTTAAGACAGAGGCTCCGTGTTTG	8	A	0.0084	0.4797
NC_005100.4-52949383	TATGTTAAGACAGAGGCTCCGTGTTTG	21	G	0.2797	0.4273
NC_005100.4-52957963	ATCCCAGCAACACTGTCTCCAGCTCAT	3	C	0.1638	0.4937
NC_005100.4-53012519	CCTTGTGACACGCGCTCTCCAGACATA	12	G	0.3636	0.4983
NC_005100.4-53013337	CTCTACACAACTTTCTCTCCTCCAGAA	5	A	0.1441	0.3389
NC_005100.4-53022951	GGACAGGACACACAGCCTCCTTCCCAC	1	G	0.0331	0.0484
NC_005100.4-53022951	GGACAGGACACACAGCCTCCTTCCCAC	5	A	0.0847	0.4948
NC_005100.4-53060174	TTCTCACACACTTAACCTCCCTCTATG	9	C	0.0082	0.4822
NC_005100.4-53060174	TTCTCACACACTTAACCTCCCTCTATG	26	T	0.1138	0.3926
NC_005100.4-53067668	GGAGGATTCACACGGCCTCCTTATAAA	13	C	0.1339	0.1843
NC_005100.4-53067668	GGAGGATTCACACGGCCTCCTTATAAA	24	T	0.0268	0.0264
NC_005100.4-53072829	GGTTGCACTACATTGTCTCCCTGCACT	4	T	0.0714	0.0853
NC_005100.4-53072853	ACTTCGGCCACTCAGCCTCCACACTTA	23	A	0.1008	0.4898
NC_005100.4-53072853	ACTTCGGCCACTCAGCCTCCACACTTA	27	A	0.0813	0.0928
NC_005100.4-53086930	ACAACTGAGACTCAGACTCCTTTCCTG	4	A	0	0.4773
NC_005100.4-53090414	GGCTGCAGGACTTGCTCTCCACAAACA	14	G	0.0246	0.0401
NC_005100.4-53090414	GGCTGCAGGACTTGCTCTCCACAAACA	27	A	0.0246	0.0243
NC_005100.4-53097101	AATGAGTACACTCGGTCTCCAAACTAG	14	G	0.1525	0.4102
NC_005100.4-53106274	AGCAGCCGCACCTGGGCTCCTCGGGGA	7	C	0.2049	0.3667
NC_005100.4-53106274	AGCAGCCGCACCTGGGCTCCTCGGGGA	12	C	0.0248	0.0245
NC_005100.4-53150799	TTTAGGTTAACATTATCTCCAGCAGGT	26	G	0.0265	0.0262
NC_005100.4-53165376	TCCGTAGCAACCAAAACTCCCTCTCTT	21	C	0.0086	0.0255
NC_005100.4-53248095	AACAAAACAACAATTGCTCCTGACAGT	7	A	0.0086	0.4768
NC_005100.4-53253423	GATGACTACACCATTTCTCCTTGTGTC	9	C	0.1913	0.3499
NC_005100.4-53271760	CCAGATATAACAATGACTCCTTAAGTT	9	A	0.175	0.3622
NC_005100.4-53283525	TCTCTGAGTACTCTTTCTCCACACAGA	2	C	0.0333	0.4832
NC_005100.4-53283525	TCTCTGAGTACTCTTTCTCCACACAGA	27	A	0.0333	0.4832
NC_005100.4-53308768	AGAGCACGAACCTGTTCTCCAACTGTG	2	G	0.0504	0.0491
NC_005100.4-53335602	CCGGTCCTGACTCAGTCTCCTTTCCCC	6	C	0.0163	0.0476
NC_005100.4-53335602	CCGGTCCTGACTCAGTCTCCTTTCCCC	24	C	0	0.4793
NC_005100.4-53343555	TTAGGGTTCACAGTGCCTCCAGAATCT	1	T	0.0603	0.0585
NC_005100.4-53343555	TTAGGGTTCACAGTGCCTCCAGAATCT	14	T	0.1724	0.199
NC_005100.4-53367680	ATGGGCGGGACACCCCCTCCTGAGCAT	23	A	0.0259	0.4768
NC_005100.4-53374678	AAGAGTCCCACAAAACCTCCAAGCTAA	14	A	0.1707	0.4988
NC_005100.4-53393991	TCCTGAAGTACCCCAGCTCCTCAGCTC	13	C	0.1167	0.1528

NC_005100.4-53400387	TGTACCATCACACCACCTCCTCCATGT	8	T	0	0.4912
NC_005100.4-53463353	AATGTAGCAACTCTGGCTCCCTCATGA	24	A	0.065	0.078
NC_005100.4-53494350	ACCCTCAGTACCGCCTCTCCCCCTGCT	12	C	0.0413	0.0562
NC_005100.4-53494584	AGCGCTGTGACCAAAACTCCTGCCATA	3	C	0.2797	0.4508
NC_005100.4-53494584	AGCGCTGTGACCAAAACTCCTGCCATA	6	T	0.2797	0.375
NC_005100.4-53517786	TGTGACAGGACTGGCTCTCCATCACCA	26	C	0	0.4734
NC_005100.4-53522046	AGGAATATGACAGATCCTCCTTTACTC	25	C	0.0254	0.0576
NC_005100.4-53584254	CAAGGACAAACACGGCCTCCCATCATC	23	T	0.1382	0.2441
NC_005100.4-53757403	CCCAGCCAAACCTAAGCTCCAATAGCC	22	A	0.0087	0.4782
NC_005100.4-53918731	ACTTCCCCAACTGTACCTCCAATCAGG	24	C	0.0492	0.4866
NC_005100.4-53938637	AATGGGGAGACTCTCACTCCAGTCTCG	26	C	0.0702	0.1454
NC_005100.4-53944595	CTACCCAAGACCCTGCCTCCTGCATCG	14	T	0.1311	0.1641
NC_005100.4-53948197	CAATTTCAGACAGTAGCTCCAAGTTAA	9	G	0	0.4751
NC_005100.4-53951694	CACATTGGTACTGAATCTCCTATAACT	5	T	0.0177	0.0348
NC_005100.4-53951694	CACATTGGTACTGAATCTCCTATAACT	24	A	0.0442	0.0433
NC_005100.4-53957858	GTCTATCAGACCATGGCTCCCTCCTCC	5	A	0.1083	0.1025
NC_005100.4-53957858	GTCTATCAGACCATGGCTCCCTCCTCC	26	C	0.0672	0.065
NC_005100.4-53980780	TCTTCTCTCACCGAATCTCCTTCTCTC	9	C	0.0407	0.1001
NC_005100.4-53980780	TCTTCTCTCACCGAATCTCCTTCTCTC	26	T	0.1707	0.5
NC_005100.4-53982764	CCTGTTTGCACCCCTTCTCCATCTTTT	8	G	0.0763	0.0889
NC_005100.4-53982764	CCTGTTTGCACCCCTTCTCCATCTTTT	22	T	0.0756	0.0728
NC_005100.4-54024933	AGCAGCTTGACTGGATCTCCGAAGAAT	5	G	0.563	0.4045
NC_005100.4-54041939	GACCGCCTCACAGTCGCTCCTCATGAG	13	G	0.3967	0.318
NC_005100.4-54076667	ACTGTCATCACCATTTCTCCATGCATC	26	T	0.0407	0.0398
NC_005100.4-54076667	ACTGTCATCACCATTTCTCCATGCATC	27	C	0.3607	0.4773
NC_005100.4-54080982	CCCTTCTGGACCAGGGCTCCTTCTGTG	15	G	0.4146	0.4703
NC_005100.4-54082832	AGCAAAGCCACACCTCCTCCAACAAGG	9	C	0.1157	0.109
NC_005100.4-54082832	AGCAAAGCCACACCTCCTCCAACAAGG	27	G	0.1356	0.1264
NC_005100.4-54083702	GCCCCCTCAACAGGCACTCCTCACTCT	9	A	0.0348	0.0342
NC_005100.4-54097551	GATGTATTTACAGTTTCTCCTTGTTCG	4	G	0.0265	0.0262
NC_005100.4-54155700	TGTCCTTGTACAACCCCTCCTTGATCA	15	C	0.9328	0.4977
NC_005100.4-54193237	CTCCTCCCCACGCCTTCTCCCTCTTCT	1	C	0.0248	0.4835
NC_005100.4-54193237	CTCCTCCCCACGCCTTCTCCCTCTTCT	12	G	0.0248	0.4835
NC_005100.4-54333135	CACCGTCACACACAGTCTCCCTGAGTA	5	G	0.3008	0.436
NC_005100.4-54344243	TGTCTACAGACACTGCCTCCAATCACA	15	G	0.3	0.3394
NC_005100.4-54344243	TGTCTACAGACACTGCCTCCAATCACA	21	A	0.3	0.4728
NC_005100.4-54350924	TTCACTGTCACAGCAACTCCACTGTCA	2	T	0.0083	0.4816
NC_005100.4-54350924	TTCACTGTCACAGCAACTCCACTGTCA	27	A	0.0083	0.4816
NC_005100.4-54391483	GGGCTGCTCACCTATCCTCCTCTCTCC	21	T	0.042	0.491
NC_005100.4-54392676	CCTCTCTCCACACCTCCTCCTTGAAAA	24	A	0	0.4724
NC_005100.4-54399964	TCATGTTCCACTCACGCTCCCTACAAT	4	T	0.0488	0.4904
NC_005100.4-54399964	TCATGTTCCACTCACGCTCCCTACAAT	5	G	0.0163	0.4825
NC_005100.4-54628317	GAGATTGGGACTGCCGCTCCTGAAGTA	13	G	0.0261	0.4782
NC_005100.4-54628317	GAGATTGGGACTGCCGCTCCTGAAGTA	24	A	0.0261	0.4782
NC_005100.4-54647910	AAAGTACCCACTTGTCCTCCGCGGCAG	21	G	0.4545	0.3512
NC_005100.4-54647910	AAAGTACCCACTTGTCCTCCGCGGCAG	23	G	0	0.4751
NC_005100.4-54663848	CAGCTCTGCACTTACCCTCCTGTGACA	13	T	0.5656	0.432
NC_005100.4-54664807	ATCTTTCTCACCAATTCTCCTTCCTGT	7	C	0.314	0.2647
NC_005100.4-54824010	GTTTTACCCACAACCCCTCCTTCTGGC	15	C	0.475	0.3708
NC_005100.4-54979922	GGTTTTGCTACACAGGCTCCCTCTAGA	14	A	0.1739	0.1588
NC_005100.4-55363922	ACAACTGTCACAGGATCTCCAGTGTCT	15	A	0.2768	0.2385

NC_005100.4-55470785	TTAACTAACACAGGACCTCCACTGTTG	3	A	0.547	0.3974
NC_005100.4-55470785	TTAACTAACACAGGACCTCCACTGTTG	23	T	0.3509	0.2893
NC_005100.4-55474946	TCCACTGTCACCAGAACTCCACTGTCT	5	C	0.3784	0.3068
NC_005100.4-55661769	GACTGGTGCACTTCCACTCCTCTTTAG	7	T	0.0488	0.0629
NC_005100.4-55708072	TGGGAGAAAACTCTTCCTCCACCCCCT	26	C	0.0893	0.0853
NC_005100.4-55914465	TATGGGAATACATTTTCTCCTTCTACA	15	T	0.0085	0.4776
NC_005100.4-56236255	TGGATCTACACGACTCCTCCCCAGATG	12	G	0.2301	0.4827
NC_005100.4-56275020	AGCCTCCTCACCAATCCTCCCAACTCT	23	A	0.2231	0.4951
NC_005100.4-56320408	ACAACAGGCACCGGCTCTCCAGGCACA	12	C	0.1138	0.1494
NC_005100.4-56320408	ACAACAGGCACCGGCTCTCCAGGCACA	21	A	0.1138	0.4984
NC_005100.4-56328926	GTCAGTTAAACTCTTTCTCCCGGCAAT	21	C	0.0083	0.4783
NC_005100.4-56365074	ACCAGTCCCACCACTTCTCCTTCATTC	24	A	0.2521	0.4347
NC_005100.4-56368074	AGTGATAACACACGTCCTCCACCTCAG	3	T	0.1545	0.1562
NC_005100.4-56368074	AGTGATAACACACGTCCTCCACCTCAG	13	C	0.0407	0.0398
NC_005100.4-56377409	CTGCAAAGTACAGACCCTCCTAGCAGC	15	C	0	0.4787
NC_005100.4-56418260	ATCACAGGCACTCTCTCTCCAGGTCCC	16	T	0.1803	0.4995
NC_005100.4-56466513	TTCCTAGGTACTTGACCTCCAACATTC	21	A	0.0084	0.4797
NC_005100.4-56467816	GAGGGGGAGACCTTGTCTCCATCTCCC	3	G	0.0901	0.4909
NC_005100.4-56467816	GAGGGGGAGACCTTGTCTCCATCTCCC	9	G	0.0541	0.0526
NC_005100.4-56471396	AATAAATGCACCCCACCTCCCAGGCGC	26	G	0.0261	0.0257
NC_005100.4-56474705	TCATATTCTACCCCAACTCCTCTCTTA	24	C	0.0439	0.0429
NC_005100.4-56489542	ACCGGCATTACAGAGGCTCCACTCACA	3	C	0.1057	0.1145
NC_005100.4-56524584	TCTATCACCACTCGGACTCCGTCCGTC	14	G	0	0.4832
NC_005100.4-56546996	ATCACTGAAACACTCTCTCCTGCAATG	5	C	0	0.4793
NC_005100.4-56546996	ATCACTGAAACACTCTCTCCTGCAATG	6	T	0.2195	0.2556
NC_005100.4-56568920	ACTCTTAGCACGTGAGCTCCTTGGCCC	5	T	0.0672	0.0805
NC_005100.4-56650310	TCAACTTTCACAATATCTCCTCTTTTA	24	T	0.0625	0.0934
NC_005100.4-56657468	CCTTTAGAAACACTAACTCCTGTCGTG	25	G	0.0085	0.4882
NC_005100.4-56657468	CCTTTAGAAACACTAACTCCTGTCGTG	26	T	0.1391	0.2138
NC_005100.4-56671757	GTCAGCCCCACTCTGTCTCCACAGTGT	7	C	0.075	0.1025
NC_005100.4-56671757	GTCAGCCCCACTCTGTCTCCACAGTGT	9	C	0.1583	0.225
NC_005100.4-56727459	ATCAAGACCACCTTCACTCCAGTGCTT	16	A	0.2213	0.375
NC_005100.4-56727459	ATCAAGACCACCTTCACTCCAGTGCTT	27	T	0.0826	0.4942
NC_005100.4-56825422	CTGGGGACCACTTTCACTCCCTCACTC	9	C	0	0.4778
NC_005100.4-56825422	CTGGGGACCACTTTCACTCCCTCACTC	24	A	0	0.4814
NC_005100.4-56906992	GGCACACTAACAGACTCTCCCCAACAC	14	A	0	0.4718
NC_005100.4-56906992	GGCACACTAACAGACTCTCCCCAACAC	16	T	0.0417	0.0408
NC_005100.4-56913700	CACAATGTTACAGTGACTCCTGATGAC	25	G	0.2348	0.4926
NC_005100.4-56932480	TGCAGGAACACCAGTGCTCCTAACCAT	24	C	0.0259	0.0422
NC_005100.4-56933612	AGCCATGACACAGCCTCTCCATCCCCA	9	C	0.1345	0.1813
NC_005100.4-56937867	CTTAATAAGACCACACCTCCTAGTCCT	9	G	0.2212	0.481
NC_005100.4-56947356	AGCTGTAGCACCTACTCTCCTGCAGAC	2	G	0.1	0.1244
NC_005100.4-56955828	AGCCCGACAACTGTCACTCCCACTGGC	13	G	0.0783	0.0911
NC_005100.4-56964420	CAGAAAGGAACCTCACCTCCCATCACA	3	G	0.025	0.0247
NC_005100.4-56984281	TACCAGTGGACAACTTCTCCAGAGGTT	21	A	0.1579	0.4444
NC_005100.4-57048205	ATGCAGACAACTCCTGCTCCAGCCATG	7	A	0.2333	0.4999
NC_005100.4-57048205	ATGCAGACAACTCCTGCTCCAGCCATG	8	C	0.1653	0.1516
NC_005100.4-57098763	CATCCTTGCACCTACGCTCCCAGGTAG	9	C	0.1382	0.1562
NC_005100.4-57098763	CATCCTTGCACCTACGCTCCCAGGTAG	15	C	0	0.4793
NC_005100.4-57103385	CTATTATCCACTGAGTCTCCCACCCTA	15	G	0.0833	0.1244
NC_005100.4-57107463	GTTGGCTATACTTTCCCTCCAACACAG	4	G	0.1721	0.4677

NC_005100.4-57110831	CCGCTCCCTACACTTCCTCCAGTACCT	2	C	0.082	0.4226
NC_005100.4-57110831	CCGCTCCCTACACTTCCTCCAGTACCT	3	G	0.2683	0.4572
NC_005100.4-57117833	GTGGAAGGCACAGCAGCTCCACACTTT	3	G	0	0.4773
NC_005100.4-57119156	AGCACAAAAACACCAGCTCCAGGAGAC	8	A	0.0431	0.4643
NC_005100.4-57119156	AGCACAAAAACACCAGCTCCAGGAGAC	23	G	0.0948	0.1646
NC_005100.4-57193900	CTCATGAACACATTGCCTCCCAAAGTT	1	C	0.1626	0.2017
NC_005100.4-57208605	GGTTCTCAGACAAGAACTCCTGGGGAA	7	C	0.2479	0.4416
NC_005100.4-57208605	GGTTCTCAGACAAGAACTCCTGGGGAA	8	A	0.0088	0.4796
NC_005100.4-57219260	TCCAGAGCTACACGTCCTCCAACCAAG	3	C	0.1391	0.1443
NC_005100.4-57219260	TCCAGAGCTACACGTCCTCCAACCAAG	14	G	0.2632	0.396
NC_005100.4-57229831	TGCAGATGAACTCACTCTCCACTAGCT	8	G	0.1565	0.1588
NC_005100.4-57279200	CTTGCTAGCACGCAGCCTCCTGAGCCG	3	T	0.0541	0.4704
NC_005100.4-57279200	CTTGCTAGCACGCAGCCTCCTGAGCCG	5	C	0.1667	0.1953
NC_005100.4-57308081	CACCGCTCCACCTCAGCTCCCAGGAAC	6	C	0.0984	0.4999
NC_005100.4-57308081	CACCGCTCCACCTCAGCTCCCAGGAAC	23	G	0.1818	0.5
NC_005100.4-57318385	GGAGAAATCACAAGCTCTCCAATGATA	24	G	0	0.4839
NC_005100.4-57331376	GTCTGCAGCACATCAACTCCCTTTTTA	23	T	0.1789	0.2499
NC_005100.4-57363359	GGGAACCCCACTGTAGCTCCCTAGACA	16	G	0.0163	0.032
NC_005100.4-57384841	ATGACAGGAACTCATCCTCCTGTGGAA	6	A	0.2066	0.4966
NC_005100.4-57384841	ATGACAGGAACTCATCCTCCTGTGGAA	25	G	0.2353	0.3853
NC_005100.4-57387747	AATTCTAAGACTACAACTCCAGTGGGT	14	C	0.0086	0.4866
NC_005100.4-57397965	GTCCCTCTGACCCTTTCTCCACGTGAT	14	T	0	0.4827
NC_005100.4-57403583	ATACAGAGAACATAGCCTCCAGAACCA	14	A	0	0.4864
NC_005100.4-57547043	TGCACACTCACCAGTGCTCCCTGAGTG	1	T	0	0.4718
NC_005100.4-57547043	TGCACACTCACCAGTGCTCCCTGAGTG	2	G	0	0.4718
NC_005100.4-57578230	CAGAAGTCTACCATGTCTCCTGCGGGA	16	T	0	0.4772
NC_005100.4-57578230	CAGAAGTCTACCATGTCTCCTGCGGGA	23	C	0	0.4757
NC_005100.4-57625548	CTGCAACAGACAGATTCTCCTGTAGCC	15	T	0.0656	0.0786
NC_005100.4-57680173	CACAGAGACACCGGTTCTCCACAGGAT	14	G	0	0.4793
NC_005100.4-57834260	AACACAAGAACACTTACTCCACTATGC	16	A	0.0351	0.0345
NC_005100.4-57859177	GCCTAGGCTACATGAACTCCCACCTCA	23	C	0.1579	0.4986
NC_005100.4-57978665	ATGGAAAGGACCACCTCTCCCTGCTTT	1	A	0.1161	0.1403
NC_005100.4-57998973	ACCTGGGGGACACAAGCTCCTGATTAC	5	G	0.0357	0.4974
NC_005100.4-57998973	ACCTGGGGGACACAAGCTCCTGATTAC	23	A	0.0357	0.4974
NC_005100.4-58012825	ATGTACACCACAGAATCTCCAGTAACA	12	A	0.025	0.4847
NC_005100.4-58012825	ATGTACACCACAGAATCTCCAGTAACA	22	G	0.2479	0.4819
NC_005100.4-58016224	AACTGCTGAACCCTTCCTCCAGCACCA	5	G	0.0531	0.0517
NC_005100.4-58016224	AACTGCTGAACCCTTCCTCCAGCACCA	16	C	0.0536	0.0521
NC_005100.4-58018556	GGCGGCACAACAAAACCTCCCTGGGAG	13	A	0.1228	0.4925
NC_005100.4-58018914	AGGTAGATCACTCTTTCTCCAACCATA	9	C	0.2	0.2072
NC_005100.4-58018914	AGGTAGATCACTCTTTCTCCAACCATA	13	C	0.1652	0.1516
NC_005100.4-58043674	CCATTAGTAACAGGGCCTCCAGGGGAA	7	G	0.0084	0.4829
NC_005100.4-58091177	TTGCTGCCTACGAGCTCTCCGCGGCGG	14	G	0.3393	0.2817
NC_005100.4-58271557	TACCACCAAACTATGTCTCCCACTACA	5	A	0.0672	0.065
NC_005100.4-58271557	TACCACCAAACTATGTCTCCCACTACA	15	G	0.0672	0.065
NC_005100.4-58281252	CATCTAGACACTTCCTCTCCCTCCCAG	21	C	0.0789	0.4889
NC_005100.4-58310555	GATGTCCCCACCACCCCTCCACCCACC	7	C	0	0.4649
NC_005100.4-58440183	CAGAAGCGGACTTACCCTCCTTATCTA	1	C	0.0085	0.487
NC_005100.4-58440183	CAGAAGCGGACTTACCCTCCTTATCTA	9	G	0.0087	0.4878
NC_005100.4-58444115	CTGTTCCTGACATTCACTCCTCCACAG	6	C	0.1681	0.4898
NC_005100.4-58452372	GGTAATAGCACTTTATCTCCTGCAATC	24	A	0.0948	0.1058

NC_005100.4-58716356	TAGGAGTGCACAAGACCTCCTCCAACC	26	C	0.0614	0.0595
NC_005100.4-58741829	GCAAGCCTGACTTGAACTCCACTGGTA	22	C	0.0087	0.4613
NC_005100.4-58850983	ACTTCTCAGACTTCACCTCCTATTTCC	1	A	0.1933	0.3462
NC_005100.4-58850983	ACTTCTCAGACTTCACCTCCTATTTCC	25	T	0	0.4787
NC_005100.4-59090679	ACTGGCTTGACATTTCCTCCATCAAAT	5	G	0.2411	0.2876
NC_005100.4-59090679	ACTGGCTTGACATTTCCTCCATCAAAT	9	G	0.0625	0.0934
NC_005100.4-59142999	AGCTGCTCCACAATCCCTCCAGTAAGT	9	C	0.1545	0.4988
NC_005100.4-59152150	TTAAGAGCCACTTATTCTCCTTTCTAG	13	T	0	0.482
NC_005100.4-59152150	TTAAGAGCCACTTATTCTCCTTTCTAG	23	T	0	0.482
NC_005100.4-59434852	TTCAGAGTTACTGTTGCTCCTCCTGTT	16	G	0.2946	0.4996
NC_005100.4-59445526	ACTGTTTAAACATGCACTCCCTTTCAG	7	T	0.0354	0.4934
NC_005100.4-59445526	ACTGTTTAAACATGCACTCCCTTTCAG	12	A	0.0177	0.0348
NC_005100.4-59687465	CTGTCACTTACGACCTCTCCTGTGTCA	5	C	0.1453	0.1773
NC_005100.4-59715559	AGTCCTGATACCAATTCTCCTTAGAAA	1	A	0.027	0.0267
NC_005100.4-59724162	CAGTTAGAGACATTGTCTCCTCAAAGC	13	T	0.0789	0.1229
NC_005100.4-59725476	AGGATTCTCACATCTCCTCCAACAGCA	6	T	0.0684	0.066
NC_005100.4-59733207	ACAGAGTATACCCCAGCTCCTTGGGGC	8	A	0.2773	0.4377
NC_005100.4-59733670	CTCTCACTCACTTCTCCTCCTCTGACT	22	C	0.0598	0.058
NC_005100.4-59851761	CTCAATGACACCTGTGCTCCAACAAAA	8	A	0	0.4814
NC_005100.4-59851761	CTCAATGACACCTGTGCTCCAACAAAA	23	C	0.0702	0.0677
NC_005100.4-59988354	ATGATGACTACTGGGACTCCTTGCTGG	16	A	0	0.4793
NC_005100.4-59988354	ATGATGACTACTGGGACTCCTTGCTGG	26	G	0.065	0.4944
NC_005100.4-60054549	ACCCCTGCCACTTTGTCTCCCCCTTCT	7	G	0	0.4844
NC_005100.4-60115569	CTCTTTGTGACTGACTCTCCAGTAATT	25	A	0.0351	0.4654
NC_005100.4-60250083	GTACAGCTCACTTATGCTCCAGGACAT	16	G	0.0574	0.0557
NC_005100.4-60252127	GCATTACACACCTTTGCTCCAATGGCT	2	C	0.2583	0.225
NC_005100.4-60252127	GCATTACACACCTTTGCTCCAATGGCT	9	C	0.0588	0.0571
NC_005100.4-60253929	TTAAAAAGTACATATGCTCCCCTATGT	13	T	0.5652	0.4454
NC_005100.4-60258396	TTGTCACTTACAAGGTCTCCTGTATTC	12	A	0.5462	0.397
NC_005100.4-60452303	TTTAGTAACACCCCACCTCCAATGCCA	13	C	0.2241	0.3916
NC_005100.4-60466180	GTGTTGCTGACCATCACTCCCAAATCA	3	G	0.3445	0.2961
NC_005100.4-60466180	GTGTTGCTGACCATCACTCCCAAATCA	9	G	0.025	0.0247
NC_005100.4-61061600	CTGCTTTGCACCCTTCCTCCACTCAAA	26	A	0	0.4751
NC_005100.4-61133668	TGCTCATGAACCACTACTCCATAGCAT	12	C	0.0569	0.0553
NC_005100.4-61311478	CAATCTGTAACCTCGACTCCAGGACAT	27	T	0	0.4787
NC_005100.4-61380520	CAGCAAACTACTTGGTCTCCTGCCTCT	3	G	0.1311	0.2157
NC_005100.4-61406240	ATCACCCAGACCTGCTCTCCTGTGGAA	13	T	0.2054	0.4617
NC_005100.4-61406240	ATCACCCAGACCTGCTCTCCTGTGGAA	14	G	0.2054	0.4617
NC_005100.4-61452358	TCATTAGGAACACAAGCTCCAGAGTGT	26	G	0.0259	0.4836
NC_005100.4-61461272	CCGTCTGTAACCTCAACTCCAGGTCAT	2	C	0	0.4793
NC_005100.4-61461272	CCGTCTGTAACCTCAACTCCAGGTCAT	7	G	0.1102	0.119
NC_005100.4-61710394	GGTGTATGTACCTGTTCTCCAAGATAT	27	T	0.0083	0.4803
NC_005100.4-61878657	AGTGTTGACACAGCATCTCCACACTAA	5	T	0	0.4728
NC_005100.4-62060998	CTCCTCTGAACTTTGCCTCCATATCCA	3	C	0.0439	0.0429
NC_005100.4-62275352	TTCATATTTACTCTGTCTCCAACAGGA	24	A	0.6387	0.4406
NC_005100.4-62285386	GCCAGGAGCACTCTTACTCCTAAGGAC	16	A	0.1849	0.1678
NC_005100.4-62368076	GGGATCCAAACACTAACTCCTGCAATA	5	T	0.1707	0.2556
NC_005100.4-62392176	CCCCTCCCAACCCAGTCTCCTCTGTTC	6	C	0.0248	0.0245
NC_005100.4-62469843	ATCACAGGAACTCTTTCTCCTTCCTCA	14	T	0.453	0.4714
NC_005100.4-62553974	CTGCCCTGGACCTCCTCTCCACAGCCT	9	G	0.0263	0.026
NC_005100.4-62566400	TTCTACAGTACCATGTCTCCTGTGCAC	27	C	0.0656	0.0786

NC_005100.4-62570374	AAACCTTATACAATACCTCCAGATCGT	25	C	0.2522	0.4055
NC_005100.4-62570374	AAACCTTATACAATACCTCCAGATCGT	26	G	0.0522	0.0671
NC_005100.4-62670054	CCTACTCACACGCCTACTCCTGCCTCA	12	G	0	0.4779
NC_005100.4-62670054	CCTACTCACACGCCTACTCCTGCCTCA	14	C	0.4831	0.375
NC_005100.4-63680608	ACAAGACACACAATATCTCCCTATGTG	5	G	0.1	0.095
NC_005100.4-63717942	GTGTCTAATACCACAGCTCCAGAGAAC	4	T	0.0261	0.0257
NC_005100.4-63735174	GGAAGGATCACAGAAGCTCCTCTGGAG	2	G	0.1639	0.1505
NC_005100.4-64174481	TAACAGGGCACCCCCACTCCCTATCTC	7	G	0.0769	0.0896
NC_005100.4-64174481	TAACAGGGCACCCCCACTCCCTATCTC	25	C	0.0769	0.0896
NC_005100.4-64188315	AACTGCTAAACCACCTCTCCAGGGGCC	3	C	0.0909	0.0868
NC_005100.4-64287567	GCACCCATAACATCAGCTCCAGGGGAT	14	C	0.0175	0.0345
NC_005100.4-64287567	GCACCCATAACATCAGCTCCAGGGGAT	15	A	0.1652	0.4976
NC_005100.4-64303257	GTGCATCACACCCAGCCTCCTTTTATT	7	C	0.0336	0.065
NC_005100.4-64303257	GTGCATCACACCCAGCCTCCTTTTATT	16	C	0.1345	0.1678
NC_005100.4-64345604	GCCTTCCCTACTATGTCTCCAGCAGTC	2	C	0	0.4826
NC_005100.4-64345604	GCCTTCCCTACTATGTCTCCAGCAGTC	23	C	0	0.4826
NC_005100.4-64351619	CAGACTCTCACAGTCCCTCCTGTACCT	2	A	0	0.4765
NC_005100.4-64370967	GACTTGTTAACCCTTCCTCCCGATACA	2	A	0.0083	0.4732
NC_005100.4-64370967	GACTTGTTAACCCTTCCTCCCGATACA	3	C	0.0902	0.4924
NC_005100.4-64376246	ATTGCACCCACAGTCCCTCCACCTTCC	4	G	0	0.4793
NC_005100.4-64376246	ATTGCACCCACAGTCCCTCCACCTTCC	14	T	0	0.4793
NC_005100.4-64381345	CCTTAAGAAACACCTCCTCCAAAGCCA	7	G	0.1293	0.2315
NC_005100.4-64399917	CCCTCTCCCACTCTTCCTCCTTCCTTC	6	T	0.839	0.487
NC_005100.4-64399917	CCCTCTCCCACTCTTCCTCCTTCCTTC	9	C	0.839	0.487
NC_005100.4-64435169	CAACGGAGAACTACTGCTCCCGACGTG	23	A	0.0088	0.4796
NC_005100.4-64439611	AGGGACACTACATGGCCTCCTTCCCAA	2	G	0.0348	0.0342
NC_005100.4-64439611	AGGGACACTACATGGCCTCCTTCCCAA	4	G	0	0.4764
NC_005100.4-64449184	AAACCCATCACTACGACTCCCCGTCCA	15	G	0	0.4819
NC_005100.4-64449184	AAACCCATCACTACGACTCCCCGTCCA	16	A	0.0826	0.4959
NC_005100.4-64450267	TCAATTGTAACTCTCACTCCAGGAGAT	16	A	0	0.0348
NC_005100.4-64451338	CCTAGGTTGACTTGAACTCCAAATCCT	12	T	0.5804	0.4119
NC_005100.4-64454760	AACAAAAGAACCCAAGCTCCTGTGCTT	15	A	0.0702	0.0839
NC_005100.4-64454760	AACAAAAGAACCCAAGCTCCTGTGCTT	27	T	0.0531	0.1006
NC_005100.4-64550789	ACATGAAAAACTGTGTCTCCTTGCTCT	1	A	0	0.48
NC_005100.4-64598790	ACCACTCACACCTGCTCTCCTGCAGTA	3	C	0.0256	0.0418
NC_005100.4-64608112	AAGCACCACACTCGGTCTCCTTGTATG	13	C	0.05	0.4861
NC_005100.4-64625470	ATGATTTTTACTCTTGCTCCTCATGGC	16	G	0	0.4709
NC_005100.4-64716015	CAATCTGTAACCTCAACTCCAAGACAT	6	T	0.0172	0.4749
NC_005100.4-64716015	CAATCTGTAACCTCAACTCCAAGACAT	7	G	0.0172	0.4749
NC_005100.4-64757792	GCTACTCTAACAACACCTCCTGGGGAA	1	G	0.0513	0.066
NC_005100.4-64757792	GCTACTCTAACAACACCTCCTGGGGAA	26	A	0.0336	0.0491
NC_005100.4-64831421	GAGCTCTCAACAGAGACTCCAGGGCAT	9	A	0.0744	0.0716
NC_005100.4-64831421	GAGCTCTCAACAGAGACTCCAGGGCAT	26	A	0.0826	0.0792
NC_005100.4-64839849	TTCCCCCTAACCCCTCCTCCTCCCCTT	9	A	0.0261	0.0257
NC_005100.4-65687472	CCCGCGCTCACCGGGTCTCCGGAACTC	15	G	0.0261	0.0257
NC_005100.4-65716245	AACCACTAAACCATCCCTCCAGCACAA	7	T	0.2797	0.329
NC_005100.4-65735854	CCTTTCTTCACTATCTCTCCCAATTCC	27	C	0.3036	0.3367
NC_005100.4-65782095	ATCTATCTAACTGCCCCTCCCTACCTC	1	A	0	0.4793
NC_005100.4-65782095	ATCTATCTAACTGCCCCTCCCTACCTC	21	C	0.2735	0.4602
NC_005100.4-65783984	GGGAGATAAACGCTTTCTCCACAAGCT	8	A	0	0.4751
NC_005100.4-65783984	GGGAGATAAACGCTTTCTCCACAAGCT	12	G	0.3471	0.4112

NC_005100.4-65859514	CATCCTGGTACTCTACCTCCAGGTTCT	14	T	0.1949	0.3991
NC_005100.4-65939368	GAATCTGTAACTCCATCTCCAGACATG	13	C	0.3982	0.3189
NC_005100.4-65947053	TGCTAGTGGACACATTCTCCTCCTCTG	22	C	0.0088	0.0262
NC_005100.4-65947053	TGCTAGTGGACACATTCTCCTCCTCTG	26	T	0.0354	0.0348
NC_005100.4-65993487	AGTACCAGGACCTAAGCTCCTATTCTT	6	C	0.0083	0.4747
NC_005100.4-65993487	AGTACCAGGACCTAAGCTCCTATTCTT	21	T	0.042	0.0411
NC_005100.4-66019599	GCCTTTATTACAACATCTCCAGTTAAC	21	A	0.5929	0.4242
NC_005100.4-66230696	GCGGTAGTAACAATGCCTCCTTCATAG	24	A	0.2479	0.321
NC_005100.4-66230696	GCGGTAGTAACAATGCCTCCTTCATAG	25	T	0.2735	0.3457
NC_005100.4-66237172	CCTAGTCCCACTGTAACTCCTCGTTAC	15	A	0	0.4807
NC_005100.4-66255766	AATATCAAGACAGCCACTCCTTTTAGA	5	T	0.2703	0.349
NC_005100.4-66313444	TGTAATGACACAAAGCCTCCTCATCTT	15	G	0.042	0.0411
NC_005100.4-66315073	GGAGAAAGGACCACTCCTCCATTGCTG	16	C	0.4103	0.3261
NC_005100.4-66429443	ACCAGACCTACTGGAGCTCCCAAGGAG	26	A	0.1858	0.1686
NC_005100.4-66575212	ATCTTCACCACTCTTTCTCCTCCTCCA	25	C	0.4324	0.3389
NC_005100.4-66695621	CCTGACACCACCAGAGCTCCCAGGGAC	6	C	0	0.4875
NC_005100.4-66695621	CCTGACACCACCAGAGCTCCCAGGGAC	26	A	0.0088	0.4889
NC_005100.4-66876784	GTGGTCTCTACAGGTCCTCCCTCCCAT	5	T	0.5492	0.4258
NC_005100.4-66876784	GTGGTCTCTACAGGTCCTCCCTCCCAT	13	G	0.5492	0.4258
NC_005100.4-66935434	AATTCCATGACAGCATCTCCACAATGG	24	A	0.0348	0.4915
NC_005100.4-66999974	ATATGGGACACACATGCTCCTCTCTAC	12	A	0	0.4832
NC_005100.4-66999974	ATATGGGACACACATGCTCCTCTCTAC	24	C	0.1983	0.4819
NC_005100.4-67042936	ACTTGCTCAACCAAGCCTCCAGTGGTG	2	C	0.2373	0.3953
NC_005100.4-67207970	TGTAGTGTTACTGAAACTCCCACATCT	7	G	0.1849	0.2328
NC_005100.4-67252620	CACTGGTTGACCAGTCCTCCAATCTTT	23	T	0.0248	0.0245
NC_005100.4-67252620	CACTGGTTGACCAGTCCTCCAATCTTT	24	C	0.0083	0.4783
NC_005100.4-67342586	GTGTGGAACACTCCCCCTCCCACCAAC	9	C	0.0164	0.0322
NC_005100.4-67342586	GTGTGGAACACTCCCCCTCCCACCAAC	16	C	0.0732	0.0705
NC_005100.4-67571228	GACTCTGTGACCTCATCTCCCTGCAGA	16	T	0	0.4793
NC_005100.4-67757112	ACAGATCCCACCAGATCTCCCAGGGAC	14	G	0.0175	0.0345
NC_005100.4-67767528	TCCTTAGCTACATCATCTCCATGGTGA	2	C	0.045	0.0611
NC_005100.4-67966597	CTTTGTTGTACTTCCCCTCCAGCTTGG	8	G	0	0.4779
NC_005100.4-68102445	ATAAAAAAGACACGTGCTCCACTATTC	2	T	0.3739	0.3147
NC_005100.4-69421268	ATAGCAAGGACCCATACTCCACTAAGT	4	G	0	0.4887
NC_005100.4-69426547	AAGATATTTACCAATCCTCCAACTGAT	26	A	0.6018	0.4276
NC_005100.4-69434871	GCTCTAGAAACACAGTCTCCTGTTTTG	4	C	0.0325	0.032
NC_005100.4-69494524	TCTTGTTATACCAGGACTCCCTTGTGT	6	T	0	0.4807
NC_005100.4-69503331	GTGCTCTAAACTTTGCCTCCTTTTCCC	24	T	0.2241	0.3074
NC_005100.4-69609464	CACCCATAGACGTGTCCTCCAGAGTAT	12	G	0	0.4751
NC_005100.4-69741744	TCCAACAGTACCTAACCTCCTAATCCT	9	T	0.1652	0.42
NC_005100.4-69742963	ACCCAAATCACCCCCACTCCACACTTG	13	C	0.0081	0.4777
NC_005100.4-69860685	CTGGCTCTTACAATCCCTCCTCACCCT	23	A	0.0435	0.0425
NC_005100.4-69860685	CTGGCTCTTACAATCCCTCCTCACCCT	26	C	0.0435	0.0425
NC_005100.4-69948804	AACCACTAAACAAACCCTCCTTCCCTG	2	A	0.1765	0.3067
NC_005100.4-69948804	AACCACTAAACAAACCCTCCTTCCCTG	4	C	0.0333	0.0328
NC_005100.4-70053610	GTCTGCGCCACCACCTCTCCTCTGAGT	6	C	0	0.4649
NC_005100.4-70065663	CTACTTTTAACTGACTCTCCACTCACA	2	T	0.1157	0.109
NC_005100.4-70065663	CTACTTTTAACTGACTCTCCACTCACA	23	T	0.0244	0.0241
NC_005100.4-70081097	AATGTAGGAACACCCGCTCCACTGTGG	1	A	0	0.4841
NC_005100.4-70081097	AATGTAGGAACACCCGCTCCACTGTGG	5	T	0.0268	0.0264
NC_005100.4-70099563	TAAGATCATACTCTCTCTCCTCGAGTT	9	T	0.0738	0.4973

NC_005100.4-70106502	AATGAATAAACAAGAACTCCATGCACA	23	G	0	0.4793
NC_005100.4-70119699	ATGCCTGTAACTGTGGCTCCAAAGGAT	25	G	0.0087	0.0257
NC_005100.4-70191413	AAAACCCTCACCAAGGCTCCATGGCTT	4	A	0.0603	0.0585
NC_005100.4-70219100	GCATTTCAAACTTGAGCTCCTCATCTA	27	A	0.2	0.3665
NC_005100.4-70231272	GAAAGGAATACAAGGGCTCCATCATCC	21	A	0	0.4793
NC_005100.4-70288436	CTGACTAAGACACCATCTCCCTAGGCC	3	G	0.0254	0.4776
NC_005100.4-70288436	CTGACTAAGACACCATCTCCCTAGGCC	21	C	0.0254	0.4776
NC_005100.4-70306957	AATAGGAGTACTATGTCTCCTAGTTAA	22	A	0.0089	0.4789
NC_005100.4-70351242	CTCATGTAAACAGCATCTCCGACCTGG	21	G	0	0.4793
NC_005100.4-70424703	CAATGAGAGACTCTGCCTCCATAAATG	3	A	0	0.482
NC_005100.4-70430361	TTCATCCTCACCATAGCTCCATGAAAC	13	A	0.0084	0.4859
NC_005100.4-70433402	GTACTAGTCACCTCGCCTCCTTATGTC	14	C	0.1789	0.2017
NC_005100.4-70513314	CAAAGCTACACCCTTGCTCCAGGAGTT	1	C	0.0656	0.1225
NC_005100.4-70513314	CAAAGCTACACCCTTGCTCCAGGAGTT	6	C	0.0656	0.1225
NC_005100.4-71094681	TGACTGCAAACTCACACTCCTTTTACA	12	T	0.122	0.4988
NC_005100.4-71103062	GTGCTGCTCACATCCACTCCAGTGCAG	21	A	0.1681	0.2568
NC_005100.4-71169912	TCACCTGTAACTGCAGCTCCGGGGATC	2	C	0.075	0.0722
NC_005100.4-71224010	ACCTATAACACCAGTACTCCAAGAGCC	1	A	0.0847	0.493
NC_005100.4-71224010	ACCTATAACACCAGTACTCCAAGAGCC	15	T	0.1379	0.1431
NC_005100.4-71237112	CACAGTGACACACCTTCTCCTACAAGA	23	C	0.0789	0.0918
NC_005100.4-71272207	TGCACTTAAACTGCTTCTCCCCACTAT	6	T	0.1475	0.1904
NC_005100.4-71296104	GAAAAGGGTACCTGGTCTCCTGTAGCT	26	C	0.1453	0.4227
NC_005100.4-71328205	CTACTGCGCACATGCTCTCCCATAATG	14	G	0.0661	0.0639
NC_005100.4-71399409	TGAGGTCCAACTTCCACTCCACTCTCA	3	A	0	0.4868
NC_005100.4-71459450	TTTAACATGACAGCAGCTCCATTGTTT	8	T	0	0.4734
NC_005100.4-71459450	TTTAACATGACAGCAGCTCCATTGTTT	23	T	0	0.4718
NC_005100.4-71477240	GACATCGAAACAGAACCTCCACAGTCT	22	C	0.0164	0.4837
NC_005100.4-71568181	CACTCTTCAACTGATCCTCCTTCCTGT	9	A	0.1148	0.4773
NC_005100.4-71691257	GGGGCTTTCACCTTCACTCCTCACTCC	9	C	0.065	0.1073
NC_005100.4-71691257	GGGGCTTTCACCTTCACTCCTCACTCC	16	A	0.0407	0.0398
NC_005100.4-71705851	GGAGTGGAGACTGCAGCTCCATAATGG	8	A	0.0087	0.4782
NC_005100.4-71705851	GGAGTGGAGACTGCAGCTCCATAATGG	22	T	0	0.4764
NC_005100.4-71720313	CTGCTCCAAACTTTGCCTCCATTATCC	4	C	0.1356	0.1264
NC_005100.4-71720313	CTGCTCCAAACTTTGCCTCCATTATCC	6	C	0.042	0.0411
NC_005100.4-71742499	AATGTCAACACTTCTCCTCCTCTTCCT	23	T	0	0.4793
NC_005100.4-71742499	AATGTCAACACTTCTCCTCCTCTTCCT	26	C	0.0081	0.481
NC_005100.4-71941409	CATGGTGAAACTGATCCTCCTGACTCA	5	G	0.2627	0.3664
NC_005100.4-71943557	ACCCTTTTCACCCAAACTCCAGTGGAT	6	T	0.0431	0.0422
NC_005100.4-72210877	AGCTTCCCCACCATTTCTCCCTTAATA	27	A	0	0.4807
NC_005100.4-72306976	AGCAACCAGACTGACGCTCCACAATCA	4	A	0.0244	0.0241
NC_005100.4-72324121	CTACGGAAGACATTCCCTCCTCCAGCA	4	C	0	0.4813
NC_005100.4-72337765	GAAAGAGAAACATGGACTCCATTTGGG	13	T	0	0.4765
NC_005100.4-72337765	GAAAGAGAAACATGGACTCCATTTGGG	21	A	0	0.4765
NC_005100.4-72340658	CGTTCAGAAACGCACGCTCCACAGACG	3	T	0.094	0.1049
NC_005100.4-72340658	CGTTCAGAAACGCACGCTCCACAGACG	24	G	0.0088	0.4599
NC_005100.4-72359444	GGCCTTGGCACCCCTGCTCCGGCACCA	13	C	0	0.4773
NC_005100.4-72396001	TGGGATTTTACTCAGTCTCCACCTCCT	4	G	0.0088	0.4873
NC_005100.4-72407479	CAGCCACATACAGCATCTCCCTATATC	9	T	0.1066	0.1436
NC_005100.4-72407479	CAGCCACATACAGCATCTCCCTATATC	25	A	0.0083	0.4835
NC_005100.4-72417203	ATCTTCCCAACTCGGCCTCCTGAATTC	13	C	0	0.032
NC_005100.4-72417203	ATCTTCCCAACTCGGCCTCCTGAATTC	25	T	0.1901	0.4995

NC_005100.4-72469830	CAGCCTTGTACACACTCTCCCTGGTAC	23	G	0.6066	0.4226
NC_005100.4-72470463	TCCCCACGTACCCCAGCTCCTTGCTGC	25	T	0.0081	0.481
NC_005100.4-72478404	GGCCTGGCCACCAGCTCTCCTCAAGGA	2	G	0.0084	0.0249
NC_005100.4-72487018	TCTCAACCCACCCTCACTCCCACAGAA	12	C	0.0248	0.0245
NC_005100.4-72491536	ACCATGCAGACATGGCCTCCGTTAGCC	12	A	0	0.4765
NC_005100.4-72524337	TAGTTCTCCACCCTCCCTCCGGGTTAG	2	A	0	0.4773
NC_005100.4-72527269	CAGACACTCACTGGCCCTCCAGGAAAT	15	C	0	0.48
NC_005100.4-72546984	CTCTCTTTTACTTCCCCTCCTAATCCC	21	T	0	0.4793
NC_005100.4-72554264	TGGGAAATAACTGGAGCTCCATGACAG	22	T	0.1301	0.2499
NC_005100.4-72663394	TCATGCTGTACCTCTACTCCATCCCTC	5	G	0.1138	0.4997
NC_005100.4-72715687	AGTGCACACACCTCCTCTCCTCAAATA	13	T	0	0.4778
NC_005100.4-72726105	AGGTTCCTGACCCCTCCTCCTCCTATA	9	G	0.0265	0.0262
NC_005100.4-72745310	CCTGTGCCCACACAGTCTCCCTCCTTA	16	T	0	0.4787
NC_005100.4-72784096	GTTGGGTGAACATGCTCTCCATGGCCT	21	A	0.2541	0.2796
NC_005100.4-72804843	TAATTGTGAACAATTTCTCCAGCCCCC	8	G	0	0.4793
NC_005100.4-72804843	TAATTGTGAACAATTTCTCCAGCCCCC	12	A	0.0081	0.481
NC_005100.4-72852299	TGCATCACCACCGTGGCTCCTGTATTA	14	T	0.0244	0.0241
NC_005100.4-72863691	TTAACGCATACTTCCCCTCCTTGCTCA	5	C	0.0083	0.0245
NC_005100.4-72863763	GGTACTGGAACCCAGCCTCCATTCCTA	16	C	0.0354	0.0517
NC_005100.4-72885276	AGAGGGAAGACGCGGTCTCCTTGCCCC	13	C	0	0.4786
NC_005100.4-72891483	AGGGCTGAGACCTGATCTCCTGGGTCT	14	G	0.1351	0.1414
NC_005100.4-72937884	TATTACCCCACACCCTCTCCTGGACCA	15	C	0.0678	0.1264
NC_005100.4-72937884	TATTACCCCACACCCTCTCCTGGACCA	25	C	0.0847	0.1116
NC_005100.4-72979664	TTATTTGAGACAGCATCTCCTCCTTGA	16	T	0.1897	0.488
NC_005100.4-73064953	ATGTTAGGCACATGCTCTCCCACTGAT	8	G	0.0246	0.0243
NC_005100.4-73064953	ATGTTAGGCACATGCTCTCCCACTGAT	15	C	0.0413	0.0868
NC_005100.4-73149770	AAGCACAAGACCCTGTCTCCCAAAAAG	24	A	0.5877	0.463
NC_005100.4-73149770	AAGCACAAGACCCTGTCTCCCAAAAAG	25	A	0.5877	0.463
NC_005100.4-73233566	ACAAGTCTCACCTTTTCTCCTTCCTTT	2	C	0.0087	0.4849
NC_005100.4-73233566	ACAAGTCTCACCTTTTCTCCTTCCTTT	9	C	0.0087	0.4849
NC_005100.4-73343546	TATCAACCAACCAGAGCTCCCTGGGAG	1	T	0.2174	0.3978
NC_005100.4-73366776	CCATCTTGGACTCCGGCTCCATCTTAG	9	G	0.1803	0.2741
NC_005100.4-73366776	CCATCTTGGACTCCGGCTCCATCTTAG	15	G	0	0.4793
NC_005100.4-73510572	AAGACCCTGACTGTTCCTCCAGTTCAA	2	A	0.1875	0.4952
NC_005100.4-73638953	GTAACAAGGACACCTACTCCACTATGT	6	A	0	0.4787
NC_005100.4-73714649	CCCAGCCTGACCTGGCCTCCTTGGAGC	22	T	0	0.4724
NC_005100.4-73719408	CCAATGCCCACTGCTGCTCCAATGCTG	8	C	0.0569	0.0553
NC_005100.4-73727185	CAAACCCACACACCTTCTCCGGAAGTG	4	A	0.0667	0.0644
NC_005100.4-73727185	CAAACCCACACACCTTCTCCGGAAGTG	5	C	0.161	0.3991
NC_005100.4-73737325	GGGGACCCTACCCTCTCTCCCTTGCTG	22	T	0.0088	0.476
NC_005100.4-73778934	GTTTCTATAACTTGGACTCCTCATATA	4	T	0.114	0.4953
NC_005100.4-73778934	GTTTCTATAACTTGGACTCCTCATATA	6	T	0.114	0.4953
NC_005100.4-73831100	AGACTGGTTACTGGAACTCCTTTCCTT	1	A	0	0.4765
NC_005100.4-73831100	AGACTGGTTACTGGAACTCCTTTCCTT	27	T	0	0.4765
NC_005100.4-73838400	CCAGGATCTACCTCTCCTCCCTACTCT	12	C	0.0087	0.4817
NC_005100.4-73839379	TCCGAGAAAACTGACCCTCCCTTCCCC	24	C	0.1795	0.3855
NC_005100.4-73865680	TCTCTGTCGACCCTGTCTCCATGAGGG	3	T	0	0.4827
NC_005100.4-73866264	CTGCAGGTAACCAAGACTCCATGGCAG	15	G	0.0259	0.4916
NC_005100.4-73979748	AAATGAACTACGTTGTCTCCTTCCACA	9	T	0	0.4778
NC_005100.4-74271704	TCATTTGCAACAAGGGCTCCATTGTGG	3	A	0	0.4787
NC_005100.4-74417383	TCCCCATCTACCCACACTCCAATACCT	23	T	0	0.4764

NC_005100.4-74601253	GACCAAGGAACACCCCCTCCAGTGATG	8	G	0	0.4819
NC_005100.4-74601253	GACCAAGGAACACCCCCTCCAGTGATG	9	A	0	0.4819
NC_005100.4-74818179	ATCCAAGAGACTCTCCCTCCCACCCTG	13	C	0	0.4592
NC_005100.4-74988582	TTCACACAAACACTGACTCCCTCCCAG	5	C	0.0894	0.0854
NC_005100.4-75032257	CCCAATTGAACTATATCTCCATTGCTG	13	A	0.359	0.355
NC_005100.4-75164791	AGTTGTTATACAGCAACTCCACACACA	14	C	0.2479	0.2172
NC_005100.4-75334769	GGCATATGTACTTCAGCTCCCAGTTCT	2	G	0.0902	0.0861
NC_005100.4-75700613	CAATCCACAACTCCAACTCCAGGAGAA	14	C	0.2203	0.4535
NC_005100.4-75791173	ATGGTTTGCACTGAGCCTCCTCACTCA	23	A	0.5833	0.4132
NC_005100.4-75833652	TATTAACCAACCACAGCTCCCAAGGTC	4	T	0	0.4749
NC_005100.4-75876822	ATAACAAGGACACATGCTCCCCTTTAA	21	C	0.2321	0.4539
NC_005100.4-75882985	GGAAAGAGGACCACTCCTCCATTGCTG	7	A	0.1964	0.4998
NC_005100.4-75882985	GGAAAGAGGACCACTCCTCCATTGCTG	24	G	0.0603	0.0746
NC_005100.4-75924991	AATGTCAGTACCTCTCCTCCCCATGCT	3	T	0	0.4859
NC_005100.4-75937899	GGCCTCTAAACCTGGCCTCCAAATACA	5	T	0	0.48
NC_005100.4-75991358	TACTGTGACACCCTCTCTCCTACAGTG	1	T	0.5984	0.4193
NC_005100.4-75991358	TACTGTGACACCCTCTCTCCTACAGTG	27	G	0.0246	0.0243
NC_005100.4-76157427	TGACCCCCAACCCCAACTCCAGCAGAC	2	G	0.0847	0.4948
NC_005100.4-76391549	ACTGATTGGACCTAAGCTCCCTATACA	4	G	0.2035	0.1968
NC_005100.4-76391549	ACTGATTGGACCTAAGCTCCCTATACA	15	A	0.1681	0.3294
NC_005100.4-76737024	AATAGTAGAACATGTGCTCCTATGACA	7	A	0.0714	0.0689
NC_005100.4-77202093	CTAGCCATGACCTTTTCTCCAAGAGAT	12	C	0.4793	0.3645
NC_005100.4-77304302	AGGTCCCTCACCCTGACTCCCATGATT	2	G	0.5	0.4281
NC_005100.4-77304302	AGGTCCCTCACCCTGACTCCCATGATT	16	A	0	0.48
NC_005100.4-77343725	CTTTCTACAACTCCAGCTCCAGGGCAT	1	C	0.5966	0.4316
NC_005100.4-77343725	CTTTCTACAACTCCAGCTCCAGGGCAT	5	C	0.0336	0.033
NC_005100.4-77596787	AACAGAATCACAGTAACTCCACAATGT	9	C	0.1913	0.2758
NC_005100.4-77700661	TGGGATGTGACCCTCTCTCCTGGTGTG	23	G	0.2	0.2816
NC_005100.4-77743170	CACATAGCCACTTACACTCCTTAGTAA	16	A	0.2033	0.2203
NC_005100.4-77748344	TCTTGGCAAACAGGGCCTCCAGCTCCT	8	A	0.4602	0.4135
NC_005100.4-77748344	TCTTGGCAAACAGGGCCTCCAGCTCCT	25	C	0.4435	0.3451
NC_005100.4-77749209	TTATCAATAACCATGACTCCTGCTTAT	7	A	0.2308	0.4947
NC_005100.4-77773442	GTCAACCTCACCCTACCTCCGGGTCAC	14	T	0.1453	0.2171
NC_005100.4-77773442	GTCAACCTCACCCTACCTCCGGGTCAC	21	G	0	0.482
NC_005100.4-77828326	CCTCCCTTCACCTCTTCTCCTCTCCTC	16	T	0.0413	0.0405
NC_005100.4-77828326	CCTCCCTTCACCTCTTCTCCTCTCCTC	21	T	0.0488	0.0476
NC_005100.4-77837996	TCTCCCGAAACACGGACTCCTAGAGAG	14	G	0.1983	0.3707
NC_005100.4-77839907	TTCCATAGCACCTGCCCTCCAGCAGCC	15	C	0.2054	0.3316
NC_005100.4-77893921	CTAGGGAACACATGCGCTCCGGCCTCC	15	C	0.2807	0.3793
NC_005100.4-77916570	TCTGACTGCACACTTGCTCCAGAGCAC	15	T	0	0.48
NC_005100.4-77929765	TGTGCTATGACATGCACTCCCTCTTCA	12	A	0	0.4793
NC_005100.4-77945706	AGAAACACAACTGTGGCTCCCAAAAGG	6	C	0.2231	0.2234
NC_005100.4-77945706	AGAAACACAACTGTGGCTCCCAAAAGG	7	A	0	0.4819
NC_005100.4-77956116	CCTTCTGCCACAGAATCTCCAACGGCT	23	C	0	0.4779
NC_005100.4-78012038	TTACTGACAACTCGTACTCCAAGCAGA	15	T	0	0.4793
NC_005100.4-78016645	CAGAGTACAACCTGGACTCCATTGCAG	27	G	0.027	0.0267
NC_005100.4-78027926	CACACACACACACACACTCCACTCACT	15	C	0.6228	0.4415
NC_005100.4-78033299	GCGCTGGCAACTGAATCTCCTGGGTCC	2	C	0.0855	0.0818
NC_005100.4-78066717	CTGGCTGCCACACCCACTCCTGTCCCA	12	A	0.0339	0.0333
NC_005100.4-78073414	ACAGACCATACAGTCACTCCTGCACTG	15	C	0.1681	0.481
NC_005100.4-78073414	ACAGACCATACAGTCACTCCTGCACTG	27	G	0.1593	0.2036

NC_005100.4-78092087	CTCCTGTCCACACATCCTCCATCTGTC	1	C	0.2602	0.4497
NC_005100.4-78092087	CTCCTGTCCACACATCCTCCATCTGTC	2	T	0.2602	0.4497
NC_005100.4-78209887	CTTCGAGTCACCCACACTCCCCTATAA	16	A	0.2143	0.3047
NC_005100.4-78212463	CTTCCACCAACTCCAGCTCCGCACACT	13	C	0	0.4779
NC_005100.4-78212463	CTTCCACCAACTCCAGCTCCGCACACT	21	G	0	0.4757
NC_005100.4-78281647	CCCTCACAGACAGCAGCTCCTTCCTTT	27	T	0	0.4807
NC_005100.4-78292853	TGCATAGGCACACTCACTCCATAAACA	8	G	0.0631	0.0611
NC_005100.4-78292853	TGCATAGGCACACTCACTCCATAAACA	15	C	0	0.4659
NC_005100.4-78305897	TACTTTGGGACACAGTCTCCGTTTCCA	8	G	0	0.4819
NC_005100.4-78323694	GCTTGGTCTACACAGGCTCCCAGCCAA	13	C	0.1712	0.4414
NC_005100.4-78379158	GTGTGTGCCACCATATCTCCCGTTATT	21	C	0	0.4779
NC_005100.4-78439142	TGGCAGCACACCTGTCCTCCCAGCATT	4	C	0	0.4773
NC_005100.4-78550987	TTCCTCCTGACAGCCTCTCCTTCCCAC	9	G	0.0263	0.026
NC_005100.4-78578050	TGCCATTTAACATTGCCTCCTTCCTCT	5	A	0	0.48
NC_005100.4-78578050	TGCCATTTAACATTGCCTCCTTCCTCT	12	A	0.0342	0.0336
NC_005100.4-78582169	ATTCTCTCAACTTCATCTCCCTCATGC	13	T	0.1417	0.1597
NC_005100.4-78585006	TCCAACAAGACCACACCTCCAAGACTC	21	A	0.0413	0.0405
NC_005100.4-78606754	GTAGCCTCTACTCACCCTCCAAAGGTT	4	G	0.0667	0.0644
NC_005100.4-78614833	CCCCGCCATACAGACACTCCCACAGTT	21	C	0.1074	0.1447
NC_005100.4-78614833	CCCCGCCATACAGACACTCCCACAGTT	26	T	0.1885	0.4973
NC_005100.4-78651789	GATGAGCGCACTTAAACTCCCCGAGAT	1	G	0.1417	0.4983
NC_005100.4-78673176	CTACTAAATACTCACCCTCCTTCTGTC	6	A	0	0.4793
NC_005100.4-78673176	CTACTAAATACTCACCCTCCTTCTGTC	9	T	0.122	0.356
NC_005100.4-78676109	CCCCACTCCACCCTGCCTCCACTCATC	25	A	0.2241	0.199
NC_005100.4-78676109	CCCCACTCCACCCTGCCTCCACTCATC	27	C	0.2222	0.1975
NC_005100.4-78705708	CTCTGGGAGACTCAGGCTCCATTAGTG	25	G	0	0.4793
NC_005100.4-78713609	AGAACAAGGACAAACTCTCCCTTGAGA	9	G	0.2	0.4965
NC_005100.4-78713609	AGAACAAGGACAAACTCTCCCTTGAGA	14	A	0.0574	0.071
NC_005100.4-78743032	GCAGCTGACACTTATTCTCCCAATCAT	8	A	0	0.4779
NC_005100.4-78779079	AGTACCTGCACTTTCTCTCCCACAGCT	5	C	0.0427	0.0418
NC_005100.4-78784147	TTTGACACCACTATGTCTCCAGTACTC	13	A	0	0.4734
NC_005100.4-78784147	TTTGACACCACTATGTCTCCAGTACTC	24	A	0.0345	0.0339
NC_005100.4-78842758	ACTGATGCTACCTGGGCTCCGTTGGTG	13	T	0.1301	0.4997
NC_005100.4-78844930	AGGCTTGTCACTGAGGCTCCTCTGTCA	7	G	0.0732	0.0854
NC_005100.4-78863080	TCCACAGCTACAGTGACTCCTCTAGAT	5	C	0	0.4819
NC_005100.4-78863080	TCCACAGCTACAGTGACTCCTCTAGAT	21	T	0.2066	0.323
NC_005100.4-78868521	TATCCTGCAACTCTCTCTCCCTATAGA	25	A	0	0.4793
NC_005100.4-78917127	ACTTCTGCCACACCCTCTCCCTCCAAG	26	A	0.175	0.1733
NC_005100.4-78932788	GCAAGGGCAACCCATGCTCCTAACCAT	15	T	0.0082	0.479
NC_005100.4-78935961	ACTGACCACACCTTTCCTCCCCTTTCC	5	A	0.0082	0.4822
NC_005100.4-78943327	TCTCTGGCCACGAAAGCTCCTGCAGTC	14	A	0.0894	0.4952
NC_005100.4-78963704	GTAGGTCTGACCCCTCCTCCCAAGGAT	21	C	0	0.4813
NC_005100.4-78964212	TGTACAAAGACCTGCCCTCCAGGGACT	7	A	0	0.4773
NC_005100.4-78964212	TGTACAAAGACCTGCCCTCCAGGGACT	9	G	0.0082	0.0401
NC_005100.4-78988481	GGAGGGGTCACACGGCCTCCCCAGTTC	26	T	0.1826	0.466
NC_005100.4-79039142	CCCCAAGGCACAGGTCCTCCTCTGCAA	3	C	0	0.4641
NC_005100.4-79077263	GAGACCCTCACCCACACTCCTCAACTG	24	A	0.0893	0.4922
NC_005100.4-79111133	CTGACAAACACCTGCCCTCCTAACCTG	23	A	0	0.4787
NC_005100.4-79122673	CTTGCTGGAACGAAAGCTCCTCTCCTA	12	G	0	0.48
NC_005100.4-79122673	CTTGCTGGAACGAAAGCTCCTCTCCTA	13	A	0	0.48
NC_005100.4-79206367	GTCCTTTCTACCTGGGCTCCACCACAA	2	T	0.027	0.0267

NC_005100.4-79361653	CCTCCCAGCACCTTCCCTCCCTCTACT	2	C	0.0488	0.078
NC_005100.4-79368121	GCCAGAAACACTCTCCCTCCTAAGCCC	2	C	0.5902	0.416
NC_005100.4-79368121	GCCAGAAACACTCTCCCTCCTAAGCCC	13	C	0.122	0.2989
NC_005100.4-79401070	GAGGCTATGACTTTTACTCCATTTCTC	23	T	0.1951	0.2723
NC_005100.4-79412498	CCTGCCTCAACTGACTCTCCAATGAGT	8	C	0.0583	0.0722
NC_005100.4-79477839	AGGATACTCACCTACACTCCAAGGCTG	3	G	0.1301	0.1629
NC_005100.4-79492285	CCTGCCTCAACTGGCTCTCCAATGAGT	26	G	0.0769	0.074
NC_005100.4-79505209	TATGCCCCCACTCCCTCTCCTATCTCC	8	C	0.1301	0.1494
NC_005100.4-79537894	TATTTGGTGACACATGCTCCTGGATAC	1	T	0.0254	0.0251
NC_005100.4-79537894	TATTTGGTGACACATGCTCCTGGATAC	15	T	0.2119	0.4991
NC_005100.4-79563840	TAGAAAATGACCTGCACTCCATACTTC	21	A	0.0348	0.0671
NC_005100.4-79565420	GACAAAGCCACTCCCTCTCCTTCATAA	24	A	0.0086	0.4866
NC_005100.4-79565420	GACAAAGCCACTCCCTCTCCTTCATAA	26	A	0.0086	0.4866
NC_005100.4-79627691	CTGCACCTTACACATCCTCCTTTCTTC	25	T	0.2439	0.2499
NC_005100.4-79703021	AAGTCAGCAACATGGCCTCCATTCCCT	2	A	0.2049	0.4852
NC_005100.4-79745081	TCTGACTTCACCACCTCTCCACTGCAT	27	T	0.1293	0.2797
NC_005100.4-79791716	CCATCTGTCACACCTGCTCCAGTGTCA	14	C	0.0631	0.4635
NC_005100.4-79804156	CATAGCAAAACTTGGTCTCCAAGGAAA	24	G	0	0.4807
NC_005100.4-79808039	TAACTCATGACAGTCACTCCTGCAGAC	25	G	0	0.4703
NC_005100.4-79808039	TAACTCATGACAGTCACTCCTGCAGAC	27	C	0	0.4703
NC_005100.4-79812886	GACCATAAAACTGAGGCTCCCACAGTT	7	A	0	0.4807
NC_005100.4-79836611	GAGATCCAAACTCCAGCTCCTTGCTGG	26	G	0.0351	0.0345
NC_005100.4-79874976	CAGTTTTGCACGCACTCTCCCTGTGTG	13	C	0.0248	0.0245
NC_005100.4-79874976	CAGTTTTGCACGCACTCTCCCTGTGTG	25	G	0.0833	0.095
NC_005100.4-79890182	AGGGGGCTCACCAGCTCTCCAACAACA	5	G	0.1721	0.5
NC_005100.4-79890182	AGGGGGCTCACCAGCTCTCCAACAACA	26	C	0.0407	0.1425
NC_005100.4-79961632	TCTAGTGACACCAACCCTCCATCTCTC	3	T	0.0085	0.479
NC_005100.4-79961632	TCTAGTGACACCAACCCTCCATCTCTC	15	C	0.161	0.2282
NC_005100.4-79992747	CACCCTTCTACGCCGACTCCACAGCCG	14	C	0.122	0.1287
NC_005100.4-79997912	GCACCATCTACCAGCTCTCCCTCATCT	6	A	0.1545	0.4999
NC_005100.4-80014100	GAATTACAGACATGAGCTCCATACCCA	22	T	0.0435	0.059
NC_005100.4-80018046	AAGATGAGGACGTCCTCTCCCACATTA	12	G	0.0085	0.4714
NC_005100.4-80055518	TCACCCACCACCACTACTCCCCCACAC	15	T	0	0.4715
NC_005100.4-80097058	GGATTGTACACACGCACTCCTGTACCT	13	C	0	0.4807
NC_005100.4-80121724	TTGTTTGAGACCGAGTCTCCCATCATA	13	G	0	0.4821
NC_005100.4-80126436	TGCCTCCCCACACCTGCTCCTCCTCTG	3	C	0.1626	0.2017
NC_005100.4-80126436	TGCCTCCCCACACCTGCTCCTCCTCTG	5	T	0.1707	0.4995
NC_005100.4-80127858	ATTAGCTCCACCCGGTCTCCTGACCCT	14	G	0	0.4787
NC_005100.4-80159104	TGATGCCCCACTCTCTCTCCTCATTCT	2	G	0.5868	0.4146
NC_005100.4-80170520	CTGGCAGCAACCCCGTCTCCTGGTGGG	15	G	0.0088	0.4843
NC_005100.4-80181207	CACAGTGAGACCCGGTCTCCAAGGAAT	14	G	0.2155	0.1923
NC_005100.4-80181207	CACAGTGAGACCCGGTCTCCAAGGAAT	24	G	0.2155	0.1923
NC_005100.4-80198098	TTAATAAAAACCATTTCTCCAGCTTCT	6	A	0	0.4734
NC_005100.4-80198098	TTAATAAAAACCATTTCTCCAGCTTCT	13	A	0	0.4749
NC_005100.4-80204779	CATAGTGAGACCCTGTCTCCAATAATT	1	C	0.065	0.078
NC_005100.4-80266571	CCAGCTCTAACGCCTCCTCCTACAGGA	1	C	0.0526	0.0677
NC_005100.4-80266571	CCAGCTCTAACGCCTCCTCCTACAGGA	12	G	0	0.4846
NC_005100.4-80299315	CCTCACCCCACCCAGCCTCCGGCCCTC	8	C	0.0909	0.0868
NC_005100.4-80303476	ATGGAAGAGACAACCACTCCACTTGTT	22	C	0.0175	0.4814
NC_005100.4-80324783	CATCAGGCAACTTGGGCTCCTGATATT	25	A	0	0.4807
NC_005100.4-80334537	GCCTGCCATACCATGACTCCCTACTAT	2	C	0	0.4773

NC_005100.4-80334537	GCCTGCCATACCATGACTCCCTACTAT	6	C	0	0.4773
NC_005100.4-80338640	AGAAAAGGCACCAACACTCCCACATAG	12	C	0.0085	0.4655
NC_005100.4-80338640	AGAAAAGGCACCAACACTCCCACATAG	16	A	0.0252	0.0249
NC_005100.4-80344199	GCAGCTGTGACTTCTCCTCCAGGACAG	27	G	0	0.4698
NC_005100.4-80381446	TGTTGGAACACTCCTACTCCTCCATCC	2	G	0.2358	0.3964
NC_005100.4-80389219	TGACCTGTGACTCCAGCTCCAGGGGTG	12	T	0	0.4698
NC_005100.4-80417095	GAAGGCCATACCCGGTCTCCGAAGCTC	13	C	0.0263	0.472
NC_005100.4-80467082	ACTGCCCTTACCACCTCTCCCTTACTC	9	T	0	0.4773
NC_005100.4-80467840	AGATAGGCAACTAGAGCTCCAGACACC	4	T	0	0.4793
NC_005100.4-80467840	AGATAGGCAACTAGAGCTCCAGACACC	16	G	0.122	0.1954
NC_005100.4-80470934	GCCACAGCTACCGTGTCTCCAGGGGTC	3	C	0	0.4779
NC_005100.4-80473377	GCACCTGTAACCCTAGCTCCTGCAGAG	24	A	0.0085	0.4842
NC_005100.4-80492440	GGCGGCTGTACTGGCGCTCCCATTCTT	23	T	0	0.4773
NC_005100.4-80506509	CCAAGGCCCACCATGCCTCCCTGAGTA	21	C	0.0328	0.0322
NC_005100.4-80506509	CCAAGGCCCACCATGCCTCCCTGAGTA	23	G	0	0.4807
NC_005100.4-80531620	CTTGCAACAACGTGCCCTCCCTTCCTC	12	G	0.3017	0.4545
NC_005100.4-80542197	TGTTTGAATACTTAATCTCCAGTTGGT	12	T	0.5983	0.426
NC_005100.4-80543588	TCCCTCTCCACAGGAGCTCCTCCAATC	15	A	0	0.4793
NC_005100.4-80551546	ACCGCACAGACAGCCCCTCCAGAGACT	8	A	0.0084	0.4797
NC_005100.4-80551546	ACCGCACAGACAGCCCCTCCAGAGACT	23	A	0.0833	0.1244
NC_005100.4-80572997	TCAGCACATACAGGGCCTCCCTGTCTC	25	C	0	0.4688
NC_005100.4-80577439	ATTTTTGTTACCGTGTCTCCACCTATG	12	C	0.0084	0.4797
NC_005100.4-80721151	TATGTGAGCACACAGGCTCCTTGGGCT	3	T	0	0.4868
NC_005100.4-80721151	TATGTGAGCACACAGGCTCCTTGGGCT	4	G	0	0.0336
NC_005100.4-80736844	AGGAAGGCCACATTAGCTCCACCATTC	7	G	0.1217	0.1443
NC_005100.4-80736844	AGGAAGGCCACATTAGCTCCACCATTC	25	T	0.0086	0.4893
NC_005100.4-80741349	CCAGCCTCTACTTAGCCTCCCCAGAAG	1	C	0.0333	0.0488
NC_005100.4-80776331	ATGATTGGAACTTCTCCTCCCACACAG	14	C	0.0339	0.0333
NC_005100.4-80788270	AATCCTCCCACCCCCACTCCCGAGACT	6	T	0.181	0.244
NC_005100.4-80788270	AATCCTCCCACCCCCACTCCCGAGACT	21	C	0.1858	0.4986
NC_005100.4-80789082	CTGGCCACAACCTTGACTCCTGACTCT	1	C	0	0.4728
NC_005100.4-80810867	ACTACAGTCACCCCCACTCCCACTGAG	12	C	0.1157	0.2046
NC_005100.4-80816991	TTAAAGGTCACAAAGACTCCGTCTGAC	21	G	0.1304	0.4986
NC_005100.4-80825238	TCCTCGCGTACCTCCCCTCCTTGTCCG	6	G	0	0.4871
NC_005100.4-80832685	CTTCCTCATACCTCTTCTCCATGTCCC	14	C	0.3186	0.4624
NC_005100.4-80864027	CCGGGGATGACTTGAGCTCCTGATCCT	2	C	0.0435	0.059
NC_005100.4-80868571	AGTCAGGCAACCTCAGCTCCATCCATG	21	A	0.25	0.3525
NC_005100.4-80877363	CAAAACAACACAGCGCCTCCATCAGAT	9	C	0.0992	0.1787
NC_005100.4-80932425	AATCAGAACACTGCTGCTCCTCTGGAG	8	A	0	0.4793
NC_005100.4-80961369	CATAGGGGAACAGTGCCTCCAGGCTAG	12	A	0.0088	0.4861
NC_005100.4-80981612	AATCCGTGAACCATCTCTCCAGCCCAA	9	A	0.0328	0.0322
NC_005100.4-81155631	GGAAGACTCACACTGACTCCCATCAAT	2	G	0.0684	0.4918
NC_005100.4-81155949	CCATCTGTAACCACAACTCCACAGGAC	12	C	0.0357	0.0351
NC_005100.4-81215413	TTAAGTCACACGGAGCCTCCCTCACAG	5	G	0.2213	0.4193
NC_005100.4-81223359	CAACAGCTAACCATGCCTCCTGCTCTG	12	C	0.2276	0.496
NC_005100.4-81275531	AGGGGAGTCACCCTGCCTCCCTGCGTG	27	G	0.2439	0.4072
NC_005100.4-81330501	CCACCCTTGACTATGTCTCCTCTTCTC	1	C	0.0088	0.4923
NC_005100.4-81330501	CCACCCTTGACTATGTCTCCTCTTCTC	24	T	0.0088	0.4923
NC_005100.4-81347707	ATACCTGTAACTCCAGCTCCAAGGGAC	5	C	0.1967	0.2956
NC_005100.4-81347707	ATACCTGTAACTCCAGCTCCAAGGGAC	9	A	0.5798	0.4186
NC_005100.4-81358247	CTTGCAGGAACAGGTTCTCCTACGATA	7	G	0.2807	0.3615

NC_005100.4-81398423	CCATCCCCCACCCCCTCTCCCAACTCC	27	C	0.3362	0.4375
NC_005100.4-81407069	ATTTCTGAGACATCCCCTCCCTCGGTC	23	C	0.0342	0.0336
NC_005100.4-81430989	AACTGAGCTACACCCTCTCCCTTCCCT	22	T	0	0.4844
NC_005100.4-81456350	CACGGGAGTACACACCCTCCTGTAATT	6	G	0	0.4773
NC_005100.4-81494217	AAATCAACCACAGGGCCTCCTGCTCCC	13	G	0	0.4773
NC_005100.4-81503083	TGCAGAAATACAGTCCCTCCTGAGCAG	15	C	0.1826	0.293
NC_005100.4-81503083	TGCAGAAATACAGTCCCTCCTGAGCAG	26	A	0.1652	0.258
NC_005100.4-81781942	AAGTACTCTACCGCACCTCCCCAGCCT	1	A	0	0.4813
NC_005100.4-81781942	AAGTACTCTACCGCACCTCCCCAGCCT	13	G	0	0.4813
NC_005100.4-81803830	AGATCTTCTACAGACACTCCACACACC	3	A	0	0.4793
NC_005100.4-81803830	AGATCTTCTACAGACACTCCACACACC	23	A	0	0.4793
NC_005100.4-81819734	GTATCGGATACTCCCCCTCCTCTACGG	5	C	0.2295	0.4226
NC_005100.4-81882927	ATACACAGCACCCACTCTCCTTCACAT	2	T	0	0.48
NC_005100.4-81882927	ATACACAGCACCCACTCTCCTTCACAT	22	T	0	0.48
NC_005100.4-81901387	GTTTCCAACACTGTGCCTCCAAGTGTG	21	A	0	0.4856
NC_005100.4-81901387	GTTTCCAACACTGTGCCTCCAAGTGTG	24	T	0	0.4856
NC_005100.4-81920448	CTGTTCTGCACATCAGCTCCTCTTGTC	24	T	0.0083	0.4708
NC_005100.4-81939100	AGGCCCTGCACATCTCCTCCGGCTCTG	21	G	0	0.4813
NC_005100.4-81949168	ATGGTTTGCACTTTGGCTCCTACACAA	27	A	0.0083	0.4816
NC_005100.4-81966865	TCCTTCGTCACTCCTGCTCCGAGGATC	6	C	0.0081	0.0241
NC_005100.4-81978202	TCTGGCCACACAACCTCTCCTTGTCAC	13	A	0.1789	0.4997
NC_005100.4-82046032	TCCAAGCCCACCCAGCCTCCCACCTTC	22	A	0	0.4751
NC_005100.4-82076215	TCCTTCTTTACCAACTCTCCCAACCAC	8	T	0	0.4764
NC_005100.4-82079932	CACACTATCACGTAAACTCCTGGGGGC	12	G	0.1607	0.496
NC_005100.4-82079932	CACACTATCACGTAAACTCCTGGGGGC	27	C	0	0.4503
NC_005100.4-82092568	ATGGGATGGACTGTGGCTCCTGTTGTG	14	T	0.2358	0.3381
NC_005100.4-82092568	ATGGGATGGACTGTGGCTCCTGTTGTG	21	T	0.2358	0.4741
NC_005100.4-82140762	ACTGCCACCACCCCAGCTCCTGTCACC	7	A	0	0.4688
NC_005100.4-82158075	ATCCGTTTCACTCTCCCTCCGGGCACC	12	T	0	0.4773
NC_005100.4-82163366	TTACCTGAAACTTAACCTCCTCGTGCA	9	A	0	0.4864
NC_005100.4-82165829	ATCCTCAGCACCGCAGCTCCTGGGGAT	22	G	0.0574	0.0861
NC_005100.4-82177323	GCCCTGTCTACCGCCCCTCCCCACAGA	12	C	0	0.477
NC_005100.4-82194758	GTGTCTCCCACTGAGACTCCCTGGGGT	16	A	0	0.4786
NC_005100.4-82202740	GTACCAGTTACCCCATCTCCCTCTTCT	25	T	0.1463	0.1356
NC_005100.4-82215877	CTACTTAACACAGACTCTCCTGCTGTC	4	C	0	0.4887
NC_005100.4-82232981	CATGAGCTGACAGTGTCTCCTGCCCTG	12	A	0.0164	0.0322
NC_005100.4-82232981	CATGAGCTGACAGTGTCTCCTGCCCTG	16	T	0	0.4773
NC_005100.4-82309006	GACATATAAACATGTCCTCCTTTTCTT	27	T	0.1532	0.1414
NC_005100.4-82314749	CCTTCCCTTACCCCTTCTCCGTACAAC	1	C	0.0813	0.078
NC_005100.4-82328379	AGGCTGTAAACAATGCCTCCGGTATGA	23	T	0	0.4734
NC_005100.4-82347478	CGCTGTGTTACTATCCCTCCCTCCCCA	23	C	0.0984	0.2157
NC_005100.4-82347478	CGCTGTGTTACTATCCCTCCCTCCCCA	25	C	0.065	0.0629
NC_005100.4-82395349	TCCCCCATCACCAGAGCTCCCAGGGAC	2	C	0.0526	0.0512
NC_005100.4-82395349	TCCCCCATCACCAGAGCTCCCAGGGAC	26	A	0.114	0.4353
NC_005100.4-82414024	AAGTGAGAGACCCTACCTCCATACAGA	24	C	0	0.4868
NC_005100.4-82428454	CCAAAGGGGACAGGGACTCCATAGGAA	22	T	0.5492	0.3984
NC_005100.4-82456449	TGCTGAGACACATGGCCTCCTGCTGCT	12	A	0.0082	0.4822
NC_005100.4-82456449	TGCTGAGACACATGGCCTCCTGCTGCT	13	T	0.1721	0.4258
NC_005100.4-82465035	CTATGTGGAACCTTCTCTCCAGCCTAC	16	T	0.2328	0.4729
NC_005100.4-82472475	CTGCCTGCAACTCCATCTCCAGGACGC	7	G	0.0089	0.4709
NC_005100.4-82498388	CTCATAGCCACACTTGCTCCCTCCTTC	5	T	0	0.4773

NC_005100.4-82563867	TCCAGGTCCACAGAGCCTCCCCGATGG	6	G	0.0259	0.0255
NC_005100.4-82563967	GCGGCGGCCACAAGTGCTCCCAGCAGT	2	C	0.187	0.2323
NC_005100.4-82563967	GCGGCGGCCACAAGTGCTCCCAGCAGT	3	G	0.0976	0.0928
NC_005100.4-82582474	TCCATATCCACCACTCCTCCTTCGACA	23	C	0.3197	0.4634
NC_005100.4-82639309	TGCTTTCTCACTAGCTCTCCCCTGCTC	14	G	0.2727	0.4213
NC_005100.4-82653963	CTCTGGGCCACTCTTGCTCCTCTGATG	16	G	0	0.4793
NC_005100.4-82785542	GGTCAGTTTACCGGAACTCCACAGCTG	7	T	0.0082	0.4755
NC_005100.4-82785542	GGTCAGTTTACCGGAACTCCACAGCTG	13	G	0.1311	0.263
NC_005100.4-82916361	ACCCCACCCACATTCTCTCCATCTTGT	14	T	0.0167	0.4832
NC_005100.4-82916361	ACCCCACCCACATTCTCTCCATCTTGT	27	T	0.0167	0.4832
NC_005100.4-82977011	CTTTCTGCCACCCCTTCTCCCACTCAT	12	C	0.0569	0.0553
NC_005100.4-83161575	AGCAATAGCACCCTGGCTCCCACTCTG	6	T	0	0.4813
NC_005100.4-83275091	CTATTCCTGACTCTGTCTCCATTTTTT	16	T	0.2411	0.2991
NC_005100.4-83281593	GACAATGTCACCACTGCTCCCACTAGC	2	A	0	0.4787
NC_005100.4-83358500	GGCAGTATCACCCCTGCTCCCATTAAG	8	T	0	0.4793
NC_005100.4-83391541	AGTCTCAACACAATGTCTCCTTCTCTG	4	C	0.1545	0.1826
NC_005100.4-83391541	AGTCTCAACACAATGTCTCCTTCTCTG	6	C	0.0164	0.4837
NC_005100.4-83412453	TGCACTGTAACCACATCTCCATATTTG	9	A	0	0.4765
NC_005100.4-83414267	ATTGGCATCACCCCTCCTCCTCACTGT	22	C	0.1282	0.2041
NC_005100.4-83414267	ATTGGCATCACCCCTCCTCCTCACTGT	26	G	0.0508	0.0965
NC_005100.4-83464627	TCGTATGTAACTCTAGCTCCATCGATT	5	A	0.0442	0.0926
NC_005100.4-83676376	AACAGGCCAACTAAAGCTCCAGGACTT	4	A	0.0917	0.0875
NC_005100.4-83676376	AACAGGCCAACTAAAGCTCCAGGACTT	22	G	0.225	0.4958
NC_005100.4-83802834	ACACCCAGGACCTTTGCTCCTGCCTCC	4	C	0.5366	0.3926
NC_005100.4-83802834	ACACCCAGGACCTTTGCTCCTGCCTCC	12	C	0.2869	0.2457
NC_005100.4-83946236	ACACCGTGCACTATGACTCCGACCAAT	23	C	0.2241	0.2739
NC_005100.4-83946236	ACACCGTGCACTATGACTCCGACCAAT	26	A	0.2735	0.426
NC_005100.4-83947404	TGTTCATATACCTCACCTCCATTCTCC	6	A	0	0.4844
NC_005100.4-83947404	TGTTCATATACCTCACCTCCATTCTCC	15	A	0	0.4844
NC_005100.4-83963704	CTAGCTGACACGCAGGCTCCTGTGGCA	23	T	0	0.4793
NC_005100.4-83963704	CTAGCTGACACGCAGGCTCCTGTGGCA	26	C	0	0.4793
NC_005100.4-84013018	TTAACTCCCACCTATTCTCCCCATCTG	5	C	0.0588	0.0728
NC_005100.4-84094219	TCCCATCCCACTCTAACTCCCCACCCC	14	T	0.2203	0.1961
NC_005100.4-84094219	TCCCATCCCACTCTAACTCCCCACCCC	16	A	0.2203	0.1961
NC_005100.4-84116377	TACATCGGGACGGCAGCTCCCTCCCTC	25	C	0	0.4773
NC_005100.4-84125855	GGTGTCTAGACAATCACTCCACCACCT	4	G	0.2975	0.4713
NC_005100.4-84143065	CATGGGCTTACTCTGCCTCCTCCCTCC	9	T	0.0171	0.4839
NC_005100.4-84167821	GTGGGATTCACGCATTCTCCTTTGGCA	6	A	0	0.4887
NC_005100.4-84181842	CCAAAGATAACTGGAACTCCACCTGCA	27	A	0	0.4772
NC_005100.4-84230319	CCTGGCGTAACTCTCACTCCTCAACTC	4	G	0	0.4731
NC_005100.4-84263339	GTATTCCTAACCATCTCTCCCCAACCC	3	A	0	0.4826
NC_005100.4-84302361	CCGAACATAACAGGCCCTCCTGAAGGC	21	T	0.0085	0.4776
NC_005100.4-84305146	TACCAGGAGACTTTGTCTCCAACGTAC	1	T	0	0.4807
NC_005100.4-84305146	TACCAGGAGACTTTGTCTCCAACGTAC	16	T	0.025	0.0247
NC_005100.4-84327808	TCTCTCTACACATCCTCTCCATGTTAC	27	C	0	0.4793
NC_005100.4-84348121	TCGTATAACACAAGGTCTCCTTATACT	23	A	0.1066	0.1154
NC_005100.4-84348121	TCGTATAACACAAGGTCTCCTTATACT	24	T	0.0246	0.0243
NC_005100.4-84352799	CACTTGTAAACTTTAGCTCCTGAGAAT	13	T	0	0.4793
NC_005100.4-84374125	GTCTAGCTGACAGAGTCTCCATGTCTC	12	A	0.0325	0.032
NC_005100.4-84376448	AGTAACTGCACTCCTGCTCCTCCTGCC	2	G	0.1795	0.1634
NC_005100.4-84395911	TACAAAGTTACCTTGTCTCCCACGCCC	5	A	0	0.4698

NC_005100.4-84395911	TACAAAGTTACCTTGTCTCCCACGCCC	24	G	0.2105	0.2413
NC_005100.4-84397485	CCTGGGGGTACCTACACTCCATCATTA	7	G	0	0.4757
NC_005100.4-84397485	CCTGGGGGTACCTACACTCCATCATTA	22	T	0	0.4757
NC_005100.4-84399166	TCTAGTCCTACACGGGCTCCAATTACT	13	C	0.0088	0.472
NC_005100.4-84420977	TAGCAGAACACTCTGTCTCCCCTGCTG	3	G	0.0254	0.0251
NC_005100.4-84421391	TACGCGTTAACAAGTGCTCCGAGTCCC	4	G	0	0.4807
NC_005100.4-84440544	CTGGGACTGACTTCCTCTCCAGCAGCT	14	C	0.0531	0.0517
NC_005100.4-84486336	CCACATGTAACTTCAGCTCCAGAGGAT	14	C	0.225	0.3047
NC_005100.4-84502453	CTCACATCAACCGTCCCTCCTGAGACA	9	A	0	0.4724
NC_005100.4-84502453	CTCACATCAACCGTCCCTCCTGAGACA	12	C	0.0351	0.0345
NC_005100.4-84528278	ATGCTGCACACCCGGCCTCCGCCCCTC	14	G	0.2321	0.4688
NC_005100.4-84528278	ATGCTGCACACCCGGCCTCCGCCCCTC	21	G	0	0.477
NC_005100.4-84540633	TCCTTGGTCACTTATCCTCCTTCCATT	22	T	0	0.4773
NC_005100.4-84586978	CTGACTCCCACCCATGCTCCTGCTACG	26	C	0.0342	0.0336
NC_005100.4-84659408	AAACAGCTGACTTGGGCTCCAGCCCTA	8	T	0.1802	0.2337
NC_005100.4-84674067	TATGGTAGGACCAGTCCTCCTTTTGCT	1	T	0.0086	0.4803
NC_005100.4-84674067	TATGGTAGGACCAGTCCTCCTTTTGCT	2	A	0.0086	0.4803
NC_005100.4-84740595	CAGCAATAAACTTTCCCTCCATATGCC	8	A	0.1607	0.3157
NC_005100.4-84740595	CAGCAATAAACTTTCCCTCCATATGCC	15	C	0	0.48
NC_005100.4-84810770	GACCAGGCAACAATGTCTCCAACTCTA	24	T	0.0087	0.466
NC_005100.4-84897969	CTCTGGGCTACTTCTGCTCCAGCAGTC	26	T	0.1488	0.4983
NC_005100.4-84897969	CTCTGGGCTACTTCTGCTCCAGCAGTC	27	C	0.1488	0.4983
NC_005100.4-84904733	CTGCCAGTAACTTAAACTCCAGGAGAT	27	T	0.0417	0.0408
NC_005100.4-84965589	CTGCCTATCACTTCACCTCCAAGAGAT	25	G	0.0488	0.0476
NC_005100.4-84967658	TAAAGCCGAACCTTCACTCCAGAACTC	7	C	0	0.482
NC_005100.4-84968124	CTATGGATGACCATGGCTCCTGCATGT	8	T	0.0756	0.1033
NC_005100.4-84968124	CTATGGATGACCATGGCTCCTGCATGT	13	A	0.1333	0.2061
NC_005100.4-84984843	TGCTTCCGAACAGCAGCTCCAAACCCA	7	C	0.0407	0.0398
NC_005100.4-85001580	CAGAGTCTCACCCACGCTCCACTTGTC	3	G	0.0331	0.0639
NC_005100.4-85001580	CAGAGTCTCACCCACGCTCCACTTGTC	16	G	0.0579	0.0868
NC_005100.4-85019241	GTGTTTAGTACGGGGCCTCCAGATAAG	16	C	0	0.4813
NC_005100.4-85040527	ATGTGACCCACTGGGCCTCCTCTCCCT	8	C	0.0574	0.0557
NC_005100.4-85090959	GCCACACTCACTGTGCCTCCTCTCCCT	14	T	0.0325	0.032
NC_005100.4-85098621	GACATCTCCACAGGGACTCCCACGCTG	4	A	0.0081	0.481
NC_005100.4-85099884	AGCTAGTCGACGGGTTCTCCAGGGCAG	12	G	0.0256	0.0253
NC_005100.4-85104899	GCCTCCGACACAGCTTCTCCAGCGGCA	24	G	0	0.4779
NC_005100.4-85149188	ACACTCAGCACCCACCCTCCTTTTTTT	16	C	0.2544	0.295
NC_005100.4-85153721	GATAGTAACACTTTTACTCCTTATCCC	1	G	0.2373	0.2926
NC_005100.4-85153721	GATAGTAACACTTTTACTCCTTATCCC	3	T	0.2373	0.2926
NC_005100.4-85173167	ATAGTAGCCACCTCTGCTCCAGGCTTC	25	T	0	0.4922
NC_005100.4-85173167	ATAGTAGCCACCTCTGCTCCAGGCTTC	27	C	0	0.4922
NC_005100.4-85179904	CCTCCCTTGACCTTATCTCCTCAGGAT	25	G	0	0.4734
NC_005100.4-85217010	GACCCTTACACGCCCCCTCCTACTTCT	16	C	0.0252	0.0249
NC_005100.4-85217010	GACCCTTACACGCCCCCTCCTACTTCT	21	T	0.2066	0.2814
NC_005100.4-85231096	GCCCATAAAACCTCAGCTCCTGGAATA	13	T	0.0082	0.4822
NC_005100.4-85240921	ATGAAAACAACTCTCCCTCCCCAGGGA	2	T	0	0.4765
NC_005100.4-85244271	GGTTAGGGCACCCAAGCTCCAGGCCAT	4	T	0.0667	0.0799
NC_005100.4-85270664	AACCCTGTAACTCCAGCTCCAGAGGAT	22	G	0.1066	0.1009
NC_005100.4-85281701	TCCACATGCACTCACCCTCCACCTCCT	7	T	0	0.4793
NC_005100.4-85281701	TCCACATGCACTCACCCTCCACCTCCT	24	T	0.1301	0.3516
NC_005100.4-85320875	AAACAAAGAACCCTGTCTCCAGCTTGG	15	G	0.0348	0.0342

NC_005100.4-85325080	GTGCCCTAAACCTTTTCTCCTGAGCCC	26	C	0.041	0.0401
NC_005100.4-85325080	GTGCCCTAAACCTTTTCTCCTGAGCCC	27	C	0	0.4773
NC_005100.4-85394773	TGAATTCTGACCATCTCTCCCACAGCT	5	T	0.0738	0.4435
NC_005100.4-85398621	GCCATTGTCACCTGACCTCCCAGACAT	23	G	0.0696	0.0671
NC_005100.4-85402032	CCTTACCCCACCCCAGCTCCTATCTGT	1	C	0	0.477
NC_005100.4-85402032	CCTTACCCCACCCCAGCTCCTATCTGT	3	T	0	0.477
NC_005100.4-85402458	CTCAGGGACACCCCACCTCCCCTTCCC	15	A	0.0252	0.4797
NC_005100.4-85440289	TGCACTGTCACTACAGCTCCCTGAATT	2	G	0.161	0.3664
NC_005100.4-85440289	TGCACTGTCACTACAGCTCCCTGAATT	14	C	0.0339	0.0496
NC_005100.4-85444993	CAGCCTGTAACCAGACCTCCATCTGCA	21	A	0	0.4839
NC_005100.4-85444993	CAGCCTGTAACCAGACCTCCATCTGCA	27	A	0	0.4851
NC_005100.4-85472897	TGTACACCAACCCCTCCTCCGCCTATG	24	T	0.1721	0.3581
NC_005100.4-85490669	TCAAGGCCAACTTGGCCTCCGGGGAAC	26	A	0	0.4688
NC_005100.4-85499964	CACATCTGGACTCTTTCTCCTTCATAC	22	T	0	0.4773
NC_005100.4-85540362	CCGCTCCCTACACCTCCTCCTAGCTCC	2	C	0.0083	0.4803
NC_005100.4-85637926	CCCCACTCCACACTCACTCCCATCTCA	3	C	0.1333	0.2778
NC_005100.4-85654039	CAAGATGACACCTCACCTCCATAATAC	25	T	0	0.4728
NC_005100.4-85665876	TCTGAGGTCACATGGGCTCCATGGGTC	25	G	0.0492	0.4914
NC_005100.4-86026798	TGACAATGAACTCATCCTCCTTCTTCT	3	A	0.2155	0.3525
NC_005100.4-86026798	TGACAATGAACTCATCCTCCTTCTTCT	12	T	0.2308	0.4626
NC_005100.4-86112283	AAGACCATCACTTCTGCTCCCACAGAC	2	A	0.0261	0.4878
NC_005100.4-86158750	TAGCAATCAACCAGATCTCCGGAGCTC	27	C	0.2893	0.4557
NC_005100.4-86275646	GCTGCTAACACAGTCACTCCTCTTCTA	13	G	0	0.4819
NC_005100.4-86275646	GCTGCTAACACAGTCACTCCTCTTCTA	27	A	0	0.4819
NC_005100.4-86390076	ACACCAATGACTTATGCTCCCAGATCA	2	C	0.3153	0.2896
NC_005100.4-86390076	ACACCAATGACTTATGCTCCCAGATCA	3	A	0.625	0.4297
NC_005100.4-86459451	ACACTCTATACAACACCTCCTTCTCCT	4	C	0.2689	0.3014
NC_005100.4-86473870	ACACTCTATACAACACCTCCATCTCCT	6	C	0.2101	0.317
NC_005100.4-86473870	ACACTCTATACAACACCTCCATCTCCT	26	C	0.0084	0.4859
NC_005100.4-86828879	AGCACTTATACGTCAGCTCCCAGCCAT	12	G	0.0488	0.4904
NC_005100.4-86840981	GGGGTGGTCACCGAGTCTCCTGAGGTT	4	G	0.2439	0.2832
NC_005100.4-86855820	TTAACCACCACCATCTCTCCAGCCCCC	14	T	0.2167	0.255
NC_005100.4-86859121	AACAAAACCACCCTCACTCCACCCCTA	16	A	0.2541	0.2903
NC_005100.4-86859121	AACAAAACCACCCTCACTCCACCCCTA	27	A	0.2541	0.4903
NC_005100.4-86874303	TTTCAGAAGACTGAGCCTCCTGACTTG	13	G	0.0992	0.1653
NC_005100.4-86875453	GCCATTGTCACATCCCCTCCTCCACCC	4	A	0	0.4787
NC_005100.4-86877950	GAGTCAAGCACTGTCCCTCCCTGGTTC	15	C	0	0.4807
NC_005100.4-86910283	CTTCTTGAGACCAGCTCTCCCTGTGTA	2	T	0.2833	0.48
NC_005100.4-86910283	CTTCTTGAGACCAGCTCTCCCTGTGTA	7	G	0.0333	0.0799
NC_005100.4-86931699	CCCTCCCTGACCCAGTCTCCCACCCTC	15	G	0.0517	0.0666
NC_005100.4-86934372	GTCCCCAAGACAGCCTCTCCATCACTC	8	A	0.252	0.381
NC_005100.4-86934372	GTCCCCAAGACAGCCTCTCCATCACTC	13	G	0.3415	0.4444
NC_005100.4-86977739	GAATGGTTGACTTAGTCTCCAGCCTTG	3	A	0	0.4773
NC_005100.4-86977739	GAATGGTTGACTTAGTCTCCAGCCTTG	24	C	0.0738	0.4943
NC_005100.4-86980333	CCACTCCACACCGGGTCTCCTGGGTGG	8	A	0.0909	0.4978
NC_005100.4-87035898	TGATTAGCTACTGCGTCTCCAGGGTAT	14	C	0.0082	0.4822
NC_005100.4-87035898	TGATTAGCTACTGCGTCTCCAGGGTAT	23	G	0.0328	0.048
NC_005100.4-87086666	TGCATACATACTTGAGCTCCTGGGCAA	8	A	0.0488	0.0629
NC_005100.4-87094008	TACCATTTGACCCTAACTCCTGGGCTG	6	T	0	0.4807
NC_005100.4-87123636	GATGCTCACACAGATCCTCCTTCTCCA	3	T	0.0407	0.0553
NC_005100.4-87134939	GGCAGTGAGACACTGTCTCCAAAGCCG	2	G	0	0.48

NC_005100.4-87134939	GGCAGTGAGACACTGTCTCCAAAGCCG	4	A	0	0.4764
NC_005100.4-87140682	GATGACTCAACTCTTTCTCCCTCTGCC	12	T	0	0.4819
NC_005100.4-87145918	CATCCTCACACCCTCTCTCCCATCACT	12	C	0.0342	0.0336
NC_005100.4-87145918	CATCCTCACACCCTCTCTCCCATCACT	13	C	0.1008	0.1107
NC_005100.4-87146083	AAAGTACTTACCTACTCTCCCAGGACA	8	T	0.1161	0.499
NC_005100.4-87146083	AAAGTACTTACCTACTCTCCCAGGACA	15	C	0.1228	0.1305
NC_005100.4-87239070	CAGAGTTCAACAAGGCCTCCAGTGGGA	5	G	0.0089	0.4824
NC_005100.4-87257979	CCCCTTGCCACCAAGTCTCCTTTATTG	1	C	0.0087	0.0257
NC_005100.4-87299902	TCTTCCTCCACACACACTCCTACAGAC	3	T	0	0.4832
NC_005100.4-87300775	TGAGAATTAACTGGAACTCCAGACTGT	5	A	0	0.4793
NC_005100.4-87313240	TCCCTCGCCACCCTCCCTCCAGCTCTC	1	T	0.0732	0.4915
NC_005100.4-87330818	GTCTTCCCAACTTAGCCTCCTAAGTGC	9	A	0.0085	0.481
NC_005100.4-87346991	AATGAACCCACTGCTTCTCCTGCCCAG	7	C	0	0.4793
NC_005100.4-87394354	AATTCGATCACATTCCCTCCAGCGTAA	4	T	0	0.4844
NC_005100.4-87394354	AATTCGATCACATTCCCTCCAGCGTAA	23	C	0	0.4844
NC_005100.4-87431411	CTCAGTGAGACCCTATCTCCCAACAGC	2	T	0.0089	0.4617
NC_005100.4-87431411	CTCAGTGAGACCCTATCTCCCAACAGC	27	C	0	0.4641
NC_005100.4-87472866	TGTGGGCGCACTTCAGCTCCTTAGCTG	7	C	0.1345	0.1539
NC_005100.4-87654319	AGTCTCCTAACATTTTCTCCACTAACT	1	A	0.1356	0.4977
NC_005100.4-87654319	AGTCTCCTAACATTTTCTCCACTAACT	13	T	0.0085	0.4776
NC_005100.4-87716381	TTAGTTTCAACACACCCTCCCTGATTT	16	C	0	0.4787
NC_005100.4-87785500	TAGCGGTTAACAGCGGCTCCACTGAGG	23	T	0.0609	0.1516
NC_005100.4-87947506	GCAGCTTCCACCGTCCCTCCTCTTCTC	13	G	0	0.4698
NC_005100.4-87958556	GACACAAACACACTTTCTCCACAGCCG	5	C	0.0496	0.0484
NC_005100.4-87958556	GACACAAACACACTTTCTCCACAGCCG	7	A	0	0.48
NC_005100.4-87984891	CAAAGGAACACTTGGGCTCCTCCACCA	23	C	0.0086	0.4866
NC_005100.4-87984891	CAAAGGAACACTTGGGCTCCTCCACCA	24	A	0.0086	0.4866
NC_005100.4-87987642	AGACACAGAACACAGACTCCCACAACA	12	A	0.1695	0.334
NC_005100.4-88000772	TCCCAGACCACAGCCCCTCCGGGTTCT	21	G	0.0513	0.4918
NC_005100.4-88010856	CAACTCTCAACAGTGCCTCCTCCTCTG	12	A	0	0.4883
NC_005100.4-88013019	CTGTGTGCGACACCAGCTCCTGCAAGG	2	T	0.2435	0.4537
NC_005100.4-88043992	GAACACAGTACAACACCTCCAGGATGC	14	C	0.2301	0.2556
NC_005100.4-88057891	CCGCATTAGACTCCATCTCCACCTCAG	3	G	0	0.4765
NC_005100.4-88080042	TGGCCGATGACTATAACTCCAGTTCTA	13	A	0.0339	0.0333
NC_005100.4-88080777	CCTGCCCACACACACCCTCCATGTCCC	5	C	0.0407	0.0398
NC_005100.4-88081771	ATCTCAGCAACCGTGTCTCCTCCCACT	13	G	0	0.4819
NC_005100.4-88117528	GGGTACCACACTCCTCCTCCACGGGTC	22	C	0	0.4807
NC_005100.4-88119594	TATGTATCAACCCCATCTCCCACTACA	12	C	0.0541	0.0695
NC_005100.4-88127774	TTACATGAGACCCTCGCTCCACAGAAG	25	A	0	0.4922
NC_005100.4-88149794	GTGTGTGCTACCCCATCTCCCGGGTCT	9	T	0	0.4773
NC_005100.4-88588668	TCTTCCAAGACATCGCCTCCCACTAGG	26	G	0	0.4688
NC_005100.4-88820090	TCACCCAGTACCCATGCTCCACCCGTC	15	T	0.2893	0.3601
NC_005100.4-88820090	TCACCCAGTACCCATGCTCCACCCGTC	25	G	0.2727	0.3687
NC_005100.4-88820251	CATCCTCCCACACAAACTCCCTATTTT	16	A	0	0.4693
NC_005100.4-88845102	TCTGCCTGGACAGTCTCTCCTATTTCT	12	A	0.161	0.4991
NC_005100.4-88850037	AGGTGGCACACTCAAGCTCCATGTAAT	14	A	0.0569	0.0854
NC_005100.4-88855460	CCACTACACACAGCCACTCCGGCATGA	4	C	0.0246	0.0243
NC_005100.4-88889031	CCAGCGCTTACAGTGGCTCCATCATCC	1	C	0.0082	0.4822
NC_005100.4-88889031	CCAGCGCTTACAGTGGCTCCATCATCC	9	T	0.0082	0.4822
NC_005100.4-88890381	ACACATGTTACAAGGCCTCCCTTAGCC	2	C	0.0513	0.05
NC_005100.4-88939630	CAGAGATGCACTGCCACTCCAGCTAGA	6	A	0	0.4793

NC_005100.4-88955151	TGCCGCTGTACGGCCACTCCAAGCCCT	8	G	0.1066	0.1296
NC_005100.4-88966210	GAGCATGGAACTGTGGCTCCATTGGGC	3	G	0.1301	0.1356
NC_005100.4-88966390	GCATGTGTGACCACGTCTCCCTGGCAT	21	C	0.0081	0.481
NC_005100.4-88977092	GCAGCGCCAACCTTTCCTCCTGCAGAA	6	G	0	0.4773
NC_005100.4-88982800	TATCGCTGAACAGGACCTCCACGTGGG	23	G	0.0492	0.0634
NC_005100.4-89038164	TCACTGCATACCGAGGCTCCATCTCTC	13	G	0.025	0.0408
NC_005100.4-89097260	GCTTACTCCACCTAGCCTCCTCCATCC	1	G	0.0081	0.481
NC_005100.4-89097260	GCTTACTCCACCTAGCCTCCTCCATCC	12	C	0.0407	0.0398
NC_005100.4-89149690	TGGTCCTGCACCATGCCTCCAGCCCCT	5	C	0.0084	0.4797
NC_005100.4-89153177	TAGCCTGTAACTCCAGCTCCAGGAGAT	22	G	0.1466	0.4997
NC_005100.4-89212870	CACACACACACACACACTCCAGGAACT	6	A	0.1453	0.1908
NC_005100.4-89212870	CACACACACACACACACTCCAGGAACT	14	A	0.1754	0.3324
NC_005100.4-89221918	GACCTGGTGACAGCAGCTCCCTTTCCG	23	T	0	0.4887
NC_005100.4-89221918	GACCTGGTGACAGCAGCTCCCTTTCCG	27	G	0	0.4887
NC_005100.4-89283072	ACCTTCCACACAGCAACTCCATCTTCA	1	A	0.0083	0.4803
NC_005100.4-89295502	TATGCACCCACACACGCTCCTCCGAAA	15	C	0.0427	0.074
NC_005100.4-89295502	TATGCACCCACACACGCTCCTCCGAAA	24	G	0	0.4814
NC_005100.4-89315399	AGCCCTCCCACTGGGTCTCCATTGTCC	1	A	0.2232	0.4968
NC_005100.4-89315399	AGCCCTCCCACTGGGTCTCCATTGTCC	2	G	0.3036	0.4592
NC_005100.4-89318379	TCCCACGATACTGCCACTCCGACCTCT	6	C	0.0894	0.4417
NC_005100.4-89347086	GACTCCTGGACACCCTCTCCACACACC	26	C	0.1008	0.4971
NC_005100.4-89371635	ATGTCTTTGACCCCAGCTCCCATACCC	6	T	0.0082	0.4822
NC_005100.4-89398412	GATCGGGAGACCCTTGCTCCTGCCCAC	5	G	0.1339	0.1983
NC_005100.4-89410896	CTTTTTGAGACAGGGTCTCCCTATGTT	9	G	0.2035	0.1828
NC_005100.4-89410896	CTTTTTGAGACAGGGTCTCCCTATGTT	22	T	0.2035	0.1828
NC_005100.4-89419947	CACAAGTAAACTTCCTCTCCTGTTAAC	21	T	0	0.4728
NC_005100.4-89419947	CACAAGTAAACTTCCTCTCCTGTTAAC	23	T	0.15	0.4988
NC_005100.4-89423055	GCCCAGGGCACTGAATCTCCTGGGACT	14	A	0	0.4902
NC_005100.4-89436288	CAAACTCACACTGCCCCTCCCTTAGCA	13	G	0.2301	0.2913
NC_005100.4-89436288	CAAACTCACACTGCCCCTCCCTTAGCA	16	C	0.2301	0.2913
NC_005100.4-89451149	CACACACACACACAACCTCCCAACATA	13	C	0.0328	0.048
NC_005100.4-89464321	CCAGAGCCCACCCTTCCTCCATTGGAC	14	T	0	0.4912
NC_005100.4-89464321	CCAGAGCCCACCCTTCCTCCATTGGAC	22	T	0.1062	0.1898
NC_005100.4-89464609	CTGGTTATAACCCAGGCTCCTGAACAG	23	A	0	0.4773
NC_005100.4-89485483	CTCGCCCCCACCGCCTCTCCACTGCGG	27	G	0.0526	0.0512
NC_005100.4-89513697	CCCATCTGAACCACCTCTCCAGCTCCT	2	C	0	0.4793
NC_005100.4-89513697	CCCATCTGAACCACCTCTCCAGCTCCT	3	C	0	0.4793
NC_005100.4-89516379	CATGTCCTAACTTAACCTCCTGCATAT	9	A	0	0.4821
NC_005100.4-89519726	TGCAGACAGACAGGGCCTCCACCAACC	13	G	0.0336	0.033
NC_005100.4-89534851	TTTCCCATTACCCTGGCTCCTTCTCTA	22	T	0.0085	0.058
NC_005100.4-89550516	CCTGTCCCGACTCTTACTCCTTGAAAT	21	T	0	0.4778
NC_005100.4-89572853	TTTGGGTCAACTGCTCCTCCTCACTCC	2	T	0.0081	0.481
NC_005100.4-89572853	TTTGGGTCAACTGCTCCTCCTCACTCC	5	G	0.0081	0.481
NC_005100.4-89594288	TGCTAATGGACTAAACCTCCGAAACTG	24	A	0.3814	0.4776
NC_005100.4-89603086	TGCCTTGAGACCCTGTCTCCAAAAAGA	9	G	0.0268	0.0264
NC_005100.4-89603086	TGCCTTGAGACCCTGTCTCCAAAAAGA	24	A	0.0268	0.4617
NC_005100.4-89721560	AGGTAGGACACAATTTCTCCATTAAGT	26	G	0.5798	0.4117
NC_005100.4-89742250	TTGAGGCCAACCTCATCTCCAGAGGGA	26	G	0	0.4793
NC_005100.4-89748274	ACCGCGCCAACTAAGACTCCATACATG	6	G	0.0083	0.4816
NC_005100.4-89748274	ACCGCGCCAACTAAGACTCCATACATG	25	A	0.0083	0.4816
NC_005100.4-89917379	AGTACTCTAACAATGTCTCCCTTTCCC	25	C	0.1624	0.1908

NC_005100.4-89967562	CTCTCACCAACTCCAACTCCAGTTCTG	16	A	0.009	0.4942
NC_005100.4-89967562	CTCTCACCAACTCCAACTCCAGTTCTG	27	G	0.0631	0.4994
NC_005100.4-90005900	TTGCCTGTAACCCAGGCTCCATTGAAA	15	G	0	0.4807
NC_005100.4-90005900	TTGCCTGTAACCCAGGCTCCATTGAAA	22	T	0	0.4807
NC_005100.4-90027487	ACAATTATGACTCACACTCCACAATTC	14	A	0	0.4734
NC_005100.4-90042658	TTGTGTGTCACTCTGGCTCCTGGAAGG	5	G	0.0593	0.0734
NC_005100.4-90072854	AATTTAGCCACGGCTCCTCCAGCTTCT	3	T	0.0083	0.4803
NC_005100.4-90167127	AATGACCTCACATGGGCTCCCTAGGCA	1	A	0	0.4693
NC_005100.4-90167127	AATGACCTCACATGGGCTCCCTAGGCA	4	G	0	0.4693
NC_005100.4-90171426	GGACTATGCACCACCACTCCTGGTGAG	15	C	0.1557	0.2095
NC_005100.4-90171426	GGACTATGCACCACCACTCCTGGTGAG	27	G	0.1475	0.1774
NC_005100.4-90171460	CACAGAGAAACCCTCACTCCACCTAAG	16	A	0	0.4751
NC_005100.4-90171460	CACAGAGAAACCCTCACTCCACCTAAG	22	C	0	0.4751
NC_005100.4-90199220	AGCAGTGCCACCTGGGCTCCTGTAAGA	14	G	0	0.477
NC_005100.4-90257785	AGGCAAGGCACGTCCCCTCCACTACAA	12	G	0	0.4856
NC_005100.4-90257785	AGGCAAGGCACGTCCCCTCCACTACAA	23	T	0.0508	0.4757
NC_005100.4-90259102	CCCATAGTCACAAACGCTCCAGCTATT	5	T	0	0.4827
NC_005100.4-90259102	CCCATAGTCACAAACGCTCCAGCTATT	15	C	0.2143	0.3565
NC_005100.4-90270966	TCACTTAGGACTGAGCCTCCAGTCCTG	7	A	0.123	0.4973
NC_005100.4-90270966	TCACTTAGGACTGAGCCTCCAGTCCTG	8	G	0.1885	0.5
NC_005100.4-90274449	TACCACTGAACTGAATCTCCAAGCCCA	14	A	0.0177	0.4755
NC_005100.4-90301989	CTTCTCTCCACCTTCTCTCCTCCTCTG	13	T	0.0081	0.481
NC_005100.4-90301989	CTTCTCTCCACCTTCTCTCCTCCTCTG	14	T	0.0081	0.481
NC_005100.4-90427720	CTCTCAAGTACTGGGACTCCAGATGTA	26	T	0.0508	0.0655
NC_005100.4-90427720	CTCTCAAGTACTGGGACTCCAGATGTA	27	A	0.188	0.2484
NC_005100.4-90461203	GTTCTAGAAACTGACTCTCCTCTCTCC	5	T	0.113	0.1369
NC_005100.4-90500706	CTTCCCTCCACAGGGACTCCCCTCCTC	2	T	0.1858	0.481
NC_005100.4-90506564	CTCCCACCGACGGCTGCTCCTTCCTCC	8	C	0.1786	0.3047
NC_005100.4-90508651	TGTGGGCCGACATGGCCTCCTGAATTT	12	A	0.2185	0.4008
NC_005100.4-90528797	CCCGGATCCACAGGGACTCCATAGCTG	2	C	0.025	0.0247
NC_005100.4-90531460	CTGATGCAGACTCAGACTCCAGTCTTG	4	A	0.042	0.0571
NC_005100.4-90531460	CTGATGCAGACTCAGACTCCAGTCTTG	21	A	0.2101	0.2961
NC_005100.4-90547791	AGGAGCATCACGCAGACTCCTCCCACA	12	G	0.1765	0.3067
NC_005100.4-90551466	AAGCAGTCCACACTGTCTCCTCACGTG	25	G	0.1707	0.1954
NC_005100.4-90554210	CTCTTTTGCACCCCACCTCCTTTTCTC	12	C	0	0.4844
NC_005100.4-90556237	AAGCACCAGACTTTATCTCCCAGGGTT	7	C	0.0179	0.0351
NC_005100.4-90556237	AAGCACCAGACTTTATCTCCCAGGGTT	15	A	0.1518	0.1699
NC_005100.4-90580377	GAAGTAGCCACACAAACTCCCCAGTCT	15	A	0.125	0.225
NC_005100.4-90609639	ACCTCAGCCACAGCTACTCCATTCTGG	2	C	0.094	0.1049
NC_005100.4-90631314	GATGTTAGGACATCACCTCCAGCCAGG	9	G	0.0732	0.0854
NC_005100.4-90631314	GATGTTAGGACATCACCTCCAGCCAGG	24	C	0.0738	0.4943
NC_005100.4-90646220	CTTCCACCCACGTGTGCTCCAGGAGTC	2	T	0	0.48
NC_005100.4-90680599	TGCAGGCTCACTGTAGCTCCGTTCTCA	21	G	0	0.4749
NC_005100.4-90699093	TACAGATTCACAGCCCCTCCCACCATC	27	C	0.1429	0.232
NC_005100.4-90720958	GTCCTTCTAACTTCTCCTCCAAAGTCC	14	C	0	0.4778
NC_005100.4-90720958	GTCCTTCTAACTTCTCCTCCAAAGTCC	15	T	0.0517	0.0504
NC_005100.4-90741183	GAGTTCAGGACACCTCCTCCCACCCTG	14	C	0.1062	0.4827
NC_005100.4-90748557	CTGTGAGTAACCTTGGCTCCAAGGCAG	26	A	0.2832	0.4715
NC_005100.4-90817817	TGTCTGGCCACTTGCTCTCCAGATGAT	12	T	0.2456	0.2659
NC_005100.4-90826917	CTTGTGCCCACCAAACCTCCCACTCAG	12	C	0.0345	0.0666
NC_005100.4-90875787	TCTTCCTACACTCAGTCTCCAGTGTGG	6	C	0.3097	0.4171

NC_005100.4-91230378	GATGTCCCTACCCCTGCTCCACTCTGG	2	A	0.1176	0.1539
NC_005100.4-91242212	CACCATAGCACACCACCTCCCTTCACT	26	C	0.065	0.0629
NC_005100.4-91244687	TCTTTCTCCACATCGTCTCCCTGAAGG	27	G	0.2397	0.2355
NC_005100.4-91279124	CAAGTGCCAACACCCACTCCCCCTTAG	6	G	0.0992	0.0943
NC_005100.4-91279124	CAAGTGCCAACACCCACTCCCCCTTAG	15	C	0.1	0.495
NC_005100.4-91311525	ATACAGTCTACAGCCCCTCCGCCCTGG	1	A	0.1405	0.465
NC_005100.4-91311525	ATACAGTCTACAGCCCCTCCGCCCTGG	23	C	0	0.4819
NC_005100.4-91328478	GCATGCACCACCACGCCTCCCTCAACA	14	C	0.0492	0.0634
NC_005100.4-91328478	GCATGCACCACCACGCCTCCCTCAACA	15	G	0.0246	0.071
NC_005100.4-91338369	CTGTGTGGAACTCCACCTCCAGATTTG	4	T	0.0744	0.0716
NC_005100.4-91338734	CCAGTGAACACCAGCTCTCCCTCCTCC	3	A	0	0.4814
NC_005100.4-91338734	CCAGTGAACACCAGCTCTCCCTCCTCC	12	C	0.2895	0.4796
NC_005100.4-91354272	AGCCATCTCACCAGCTCTCCTCTAGTT	6	T	0	0.4764
NC_005100.4-91369069	GCTGTGCCCACATCCACTCCCCGTGTG	23	G	0	0.4703
NC_005100.4-91377215	CAGATGACAACAGCCTCTCCTCAAATT	1	C	0.0763	0.0889
NC_005100.4-91377215	CAGATGACAACAGCCTCTCCTCAAATT	26	T	0.2759	0.3916
NC_005100.4-91384054	CCACTCCCCACCGAGCCTCCCTAACCT	12	C	0.181	0.3231
NC_005100.4-91410261	TCCATAGGCACTCAGACTCCAGGGTGT	7	G	0.2	0.304
NC_005100.4-91423555	CCACACAGAACAAGAACTCCAGGGGTT	25	G	0.1081	0.5
NC_005100.4-91427430	CCGTGACTGACTCTTACTCCTCTGACA	7	C	0.1207	0.1431
NC_005100.4-91435559	TTTGGACTTACCTTACCTCCTTTGATC	2	T	0.1864	0.3034
NC_005100.4-91442635	TCCTGGAACACTCGGGCTCCGTGGGTT	3	C	0.0702	0.0839
NC_005100.4-91442635	TCCTGGAACACTCGGGCTCCGTGGGTT	14	G	0.0526	0.0997
NC_005100.4-91498889	TCAAATGTCACTTGTCCTCCATGGGTC	8	T	0.2195	0.4868
NC_005100.4-91502594	ATCAGAGTCACACAGGCTCCTTGGTTT	22	T	0	0.4773
NC_005100.4-91509818	TTTCTTGGAACACATGCTCCATGAGGG	21	A	0.2288	0.3388
NC_005100.4-91510404	GGACGGAAGACATTGCCTCCTGCTTGA	4	C	0	0.4793
NC_005100.4-91510404	GGACGGAAGACATTGCCTCCTGCTTGA	5	G	0.122	0.1826
NC_005100.4-91527258	CTGTTTAGTACTCTTCCTCCTCTTCTT	25	C	0.0574	0.0861
NC_005100.4-91541452	CCATCTGCCACTGGCTCTCCCAAGTGC	26	G	0.0088	0.463
NC_005100.4-91572840	AAACTTGGTACACACACTCCATTACTT	15	C	0.1765	0.4592
NC_005100.4-91600655	AGTAGTAAAACCCTGTCTCCGAATGCA	15	G	0.1803	0.4891
NC_005100.4-91600655	AGTAGTAAAACCCTGTCTCCGAATGCA	22	A	0.0488	0.0629
NC_005100.4-91638003	GTCCAGTCCACGTCACCTCCAGGCTAA	15	A	0.025	0.0247
NC_005100.4-91638003	GTCCAGTCCACGTCACCTCCAGGCTAA	27	A	0.075	0.0875
NC_005100.4-91664850	GGAATCATGACACACACTCCATTCCTC	8	T	0	0.4807
NC_005100.4-91664850	GGAATCATGACACACACTCCATTCCTC	9	G	0.041	0.0401
NC_005100.4-91696090	GACACCAGCACATCCCCTCCCACCTTA	2	A	0.042	0.0411
NC_005100.4-91720635	CATCAGCGGACTGGTCCTCCTGGCGCA	7	C	0.1626	0.2017
NC_005100.4-91720635	CATCAGCGGACTGGTCCTCCTGGCGCA	25	G	0.2602	0.3604
NC_005100.4-91732228	CTCCTCATAACCACATCTCCCCTGTCC	25	T	0	0.4813
NC_005100.4-91734332	GGATTCCCCACAAAGCCTCCTTTCCAG	21	T	0.0603	0.0585
NC_005100.4-91734332	GGATTCCCCACAAAGCCTCCTTTCCAG	22	T	0.0086	0.4836
NC_005100.4-91744732	CCACAGAGAACTGGTCCTCCCTCCCCA	27	A	0.0492	0.048
NC_005100.4-91745603	AACCTGCCCACTCATGCTCCATCTACT	9	C	0.2893	0.4146
NC_005100.4-91841041	AGATAGTGTACTTAACCTCCCTAGAAA	2	G	0.287	0.4268
NC_005100.4-91841041	AGATAGTGTACTTAACCTCCCTAGAAA	22	T	0.0085	0.4842
NC_005100.4-91861815	GGCTCCTCTACACCCCCTCCTCCCATG	5	C	0.2544	0.4076
NC_005100.4-91861815	GGCTCCTCTACACCCCCTCCTCCCATG	23	C	0.0261	0.0425
NC_005100.4-91879039	CACTCTCCCACCCGTCCTCCCTCTCTT	2	A	0	0.4851
NC_005100.4-91879039	CACTCTCCCACCCGTCCTCCCTCTCTT	13	C	0.0924	0.1181

NC_005100.4-91999864	GATTCTGAAACAAGGTCTCCATGTTGC	1	G	0	0.4793
NC_005100.4-92016898	GTGGGAGAAACCTGGGCTCCACTGTAA	4	G	0	0.4807
NC_005100.4-92016898	GTGGGAGAAACCTGGGCTCCACTGTAA	13	T	0.1261	0.3682
NC_005100.4-92037754	GGAGAGGGCACTGCATCTCCTAGAACT	25	A	0.2222	0.4325
NC_005100.4-92054760	AGCCTGTCAACTCCTCCTCCTGTGCTT	6	G	0.1532	0.1414
NC_005100.4-92075455	GCACCAGCAACGGATACTCCAGCAAAC	12	G	0.2314	0.389
NC_005100.4-92127324	TTTTTTGAGACAAGATCTCCATGCTAC	27	C	0.177	0.2302
NC_005100.4-92170788	TTCCTGGCTACCTCTTCTCCTCTTCCC	24	T	0.0603	0.0585
NC_005100.4-92170788	TTCCTGGCTACCTCTTCTCCTCTTCCC	27	C	0.0603	0.0585
NC_005100.4-92180431	GGCTGCATCACACACGCTCCTCTCAGT	8	T	0.0246	0.0243
NC_005100.4-92214384	CCACATCCGACTGGACCTCCTACTGCC	8	C	0.0081	0.4777
NC_005100.4-92214384	CCACATCCGACTGGACCTCCTACTGCC	23	C	0.2131	0.4979
NC_005100.4-92246284	ATCCTATCCACTCTCCCTCCTCACAAC	3	C	0.0976	0.4973
NC_005100.4-92296254	CTCACTATCACTGTGGCTCCTGTCCAC	25	C	0.3008	0.381
NC_005100.4-92346016	CATAACATTACGTCCTCTCCAGGGCTA	1	C	0.3077	0.3729
NC_005100.4-92346016	CATAACATTACGTCCTCTCCAGGGCTA	3	T	0.2308	0.4356
NC_005100.4-92352597	TCCACGGTCACAGGGCCTCCTCTGAGA	7	G	0	0.4724
NC_005100.4-92352597	TCCACGGTCACAGGGCCTCCTCTGAGA	23	T	0.2143	0.3047
NC_005100.4-92375980	GAATGAGTCACACTACCTCCTTGGGCC	25	G	0.157	0.172
NC_005100.4-92375980	GAATGAGTCACACTACCTCCTTGGGCC	26	C	0.0492	0.048
NC_005100.4-92397646	CTTACCCCAACTGCCTCTCCCACACTC	6	C	0.0357	0.0351
NC_005100.4-92418479	GAGACTCTAACCTTTGCTCCAGATGAT	26	A	0.0163	0.0476
NC_005100.4-92418479	GAGACTCTAACCTTTGCTCCAGATGAT	27	T	0.2764	0.4
NC_005100.4-92462223	GTAACATTGACTTGGCCTCCATCTCCA	7	T	0.0088	0.4889
NC_005100.4-92462223	GTAACATTGACTTGGCCTCCATCTCCA	8	T	0.0088	0.4913
NC_005100.4-92510779	CAAGAGGCTACTCATGCTCCAGTAGAT	6	G	0.3214	0.4483
NC_005100.4-92562476	ATATCACTTACAACATCTCCCAGGTAC	12	A	0.2	0.4782
NC_005100.4-92575246	AGCTCTTACACTGCTGCTCCAGTTTGC	5	C	0.1026	0.1704
NC_005100.4-92575246	AGCTCTTACACTGCTGCTCCAGTTTGC	9	C	0.1795	0.2299
NC_005100.4-92578127	TTCCCTCGGACCTGTGCTCCCTGCAGA	4	C	0.2683	0.3091
NC_005100.4-92578127	TTCCCTCGGACCTGTGCTCCCTGCAGA	13	T	0	0.4793
NC_005100.4-92644101	AGTAAATTCACTAAGGCTCCTCCCTCC	16	G	0.0508	0.0655
NC_005100.4-92700377	TCTGTCTTCACAAAGGCTCCATCAACA	26	C	0	0.4832
NC_005100.4-92709980	ACCCTTAAGACTCTCTCTCCACTTGCT	23	T	0.2602	0.4072
NC_005100.4-92721264	CCCACCGGCACGGGCCCTCCTAACCTG	12	G	0	0.4827
NC_005100.4-92721264	CCCACCGGCACGGGCCCTCCTAACCTG	14	G	0.0522	0.0508
NC_005100.4-92722115	AGAGTTCAAACCCTTGCTCCACAGGAT	12	C	0.2269	0.2389
NC_005100.4-92722115	AGAGTTCAAACCCTTGCTCCACAGGAT	15	T	0.2645	0.318
NC_005100.4-92778153	GAATGCGGGACCTTAACTCCCTCAACC	9	G	0.0254	0.0251
NC_005100.4-92843084	AATGCCTCAACTGAAGCTCCCTTTTCT	23	T	0.5593	0.4029
NC_005100.4-92845574	TTGTATCTCACTTTCCCTCCCTTTACA	9	C	0.1754	0.4555
NC_005100.4-92845574	TTGTATCTCACTTTCCCTCCCTTTACA	15	C	0.1754	0.4555
NC_005100.4-92859931	CAAAATGCCACCGCGTCTCCAAATGAA	15	G	0.2124	0.2797
NC_005100.4-92870465	AACTCAGTAACCCTGCCTCCAGTAGGC	1	A	0	0.4793
NC_005100.4-92909162	AGTCTTCCCACTCTGCCTCCCTAGCGA	14	T	0.2131	0.4565
NC_005100.4-92909162	AGTCTTCCCACTCTGCCTCCCTAGCGA	25	C	0.0826	0.2415
NC_005100.4-92924224	CACTAGTGCACTCATTCTCCTCCACCA	5	A	0	0.4772
NC_005100.4-92925622	TGCCACCGCACTTGGGCTCCTTAATGT	13	T	0	0.4793
NC_005100.4-92925622	TGCCACCGCACTTGGGCTCCTTAATGT	24	A	0.2439	0.2937
NC_005100.4-92928913	CTATCTGCCACCCTCTCTCCACTAGCT	23	T	0	0.4793
NC_005100.4-92930678	GGGACATGCACGGGCACTCCCAAAGAG	12	G	0.2586	0.2854

NC_005100.4-92946084	GGAGGCCAGACAACAGCTCCCATGGCT	4	G	0.2083	0.49
NC_005100.4-92976943	CACTGATCCACCCACCCTCCATCTACT	1	C	0.0082	0.479
NC_005100.4-92976943	CACTGATCCACCCACCCTCCATCTACT	27	T	0.0164	0.4807
NC_005100.4-92986193	TACTGAGACACGAGCTCTCCTATTCTG	12	G	0.3058	0.3421
NC_005100.4-93004591	ACCAGACACACTAGAGCTCCCAGGGAC	2	C	0.2797	0.4957
NC_005100.4-93031395	CGGTTCCCCACCCCCTCTCCTAATGAT	13	C	0.1518	0.2637
NC_005100.4-93053233	TCACCGCGTACCCTATCTCCCACTTGT	5	C	0.0082	0.4822
NC_005100.4-93053233	TCACCGCGTACCCTATCTCCCACTTGT	7	C	0.0082	0.4822
NC_005100.4-93057195	ACTTCCTCCACCAAAGCTCCACCTCCT	27	T	0.5667	0.42
NC_005100.4-93087395	GTCCAGGCCACTTGTCCTCCGAAGCTC	13	T	0.0328	0.4612
NC_005100.4-93087395	GTCCAGGCCACTTGTCCTCCGAAGCTC	21	G	0.0244	0.0241
NC_005100.4-93105506	GGTGCCCAGACAGCACCTCCTGTCCCA	3	T	0.1802	0.4026
NC_005100.4-93121106	CCTCTCTTGACTCTATCTCCCCTCTTT	13	C	0.0522	0.0508
NC_005100.4-93126133	AGTGACTCAACATAGCCTCCGCCATGT	7	T	0.0407	0.0398
NC_005100.4-93143169	GACATAAGGACTAAGCCTCCACCCCAT	8	G	0.0976	0.1073
NC_005100.4-93149066	AAAAGGGGAACATTGCCTCCTTGGATG	3	A	0.0738	0.4903
NC_005100.4-93151940	TCTTCCCAAACACCATCTCCACAGCTT	3	T	0.0569	0.0553
NC_005100.4-93176140	ATAATTAACACAGTTCCTCCACAAGTG	4	A	0.124	0.1447
NC_005100.4-93195989	TACGAGACAACTCACTCTCCAGAGCCA	8	C	0.0246	0.0243
NC_005100.4-93198772	CAAGACATCACTTGGACTCCAAGACGG	25	C	0.0504	0.4873
NC_005100.4-93198863	CCCAGATGCACTTCATCTCCTAGGCCA	26	C	0	0.4846
NC_005100.4-93198863	CCCAGATGCACTTCATCTCCTAGGCCA	27	A	0.0087	0.4849
NC_005100.4-93256318	TCCTTGGTCACACTGCCTCCCTAGTAA	2	C	0.0656	0.0786
NC_005100.4-93256318	TCCTTGGTCACACTGCCTCCCTAGTAA	12	A	0.0902	0.1968
NC_005100.4-93269866	AAATTTGTGACACCTCCTCCACCCATC	7	G	0.0089	0.4824
NC_005100.4-93311090	CTGCTAACAACTGTAACTCCAGGGGAC	21	A	0.0417	0.0566
NC_005100.4-93328069	TCGTTTCTGACACAGGCTCCTCACATC	3	G	0	0.4773
NC_005100.4-93397736	CGTGCAGCGACCAAGCCTCCTTTCTGT	2	G	0.161	0.1481
NC_005100.4-93431548	CTGGGTGACACTGACTCTCCATTGTCT	6	T	0.3115	0.4226
NC_005100.4-93442957	ATAACCACTACTTCCTCTCCTAGCCAC	1	A	0.3043	0.4055
NC_005100.4-93445800	CTGTAGAACACTGTCTCTCCTGGTTAC	14	T	0.3083	0.4097
NC_005100.4-93452490	CATGACTGCACCTCACCTCCTGTCCTT	5	A	0.0328	0.0322
NC_005100.4-93460625	AATTATATGACAGCATCTCCAGGAGCA	1	A	0	0.4743
NC_005100.4-93483026	GGATAGCCCACTTGTACTCCTAAGGTG	12	T	0.0661	0.0639
NC_005100.4-93483204	CTACTGCGTACACACCCTCCCGGGCCA	21	C	0.3008	0.3888
NC_005100.4-93487985	GACAGTTGCACAGTCCCTCCCAACACC	8	G	0	0.4793
NC_005100.4-93527982	AATCATCACACCACTGCTCCCACACGC	25	C	0	0.4718
NC_005100.4-93541145	CCACAGGCAACACTTCCTCCCTTAAGT	16	C	0.2377	0.375
NC_005100.4-93541145	CCACAGGCAACACTTCCTCCCTTAAGT	21	C	0.252	0.4301
NC_005100.4-93549420	AGGTGATTAACCAAATCTCCCTTCAAT	4	T	0	0.4807
NC_005100.4-93562148	CAGCTTGTAACTCCACCTCCAAGGGAT	7	G	0.2602	0.4548
NC_005100.4-93562148	CAGCTTGTAACTCCACCTCCAAGGGAT	25	G	0.252	0.4523
NC_005100.4-93562593	CTGCATGCAACTCCAGCTCCATAGGAT	16	G	0.2602	0.377
NC_005100.4-93596621	AGGCACAGGACCTCATCTCCACCTGTC	23	C	0	0.4807
NC_005100.4-93663517	CCCATAAACACCCAGGCTCCCCCACCC	23	C	0.2167	0.2994
NC_005100.4-93668390	AGTTGTCTTACAGGGTCTCCCTAACCT	14	G	0.2314	0.4877
NC_005100.4-93668390	AGTTGTCTTACAGGGTCTCCCTAACCT	25	C	0	0.4807
NC_005100.4-93676586	TGTAATGTGACAGATTCTCCCTGCCCA	6	T	0.1933	0.4987
NC_005100.4-93688624	GATCTGACAACCTCTTCTCCCCTCTGT	26	G	0.094	0.1049
NC_005100.4-93711195	TGCAGGTTAACTGGCGCTCCATGCCTT	16	G	0.1271	0.298
NC_005100.4-93727050	GTGAGAATCACTGTCACTCCGCTGGCC	1	G	0.1652	0.3547

NC_005100.4-93844947	TGGTAGCACACTCTTCCTCCCCTCTCC	25	T	0	0.4755
NC_005100.4-93872258	GCCTCTTCCACCTCGGCTCCTTGCTAT	2	C	0.0081	0.0241
NC_005100.4-93901701	ATACGAAGGACCTAGGCTCCTTGCAAG	2	T	0	0.4793
NC_005100.4-93901701	ATACGAAGGACCTAGGCTCCTTGCAAG	5	G	0.122	0.5
NC_005100.4-93924894	CACATGCACACCATGGCTCCTGTATGT	9	C	0.0244	0.0241
NC_005100.4-93953748	CAGAAAACCACATTTTCTCCTTGAGCT	8	C	0.0407	0.0398
NC_005100.4-93953748	CAGAAAACCACATTTTCTCCTTGAGCT	12	A	0	0.4773
NC_005100.4-93967952	CAGCCACGCACAGCAGCTCCCAGAGAC	24	A	0	0.4793
NC_005100.4-93986075	CAGCATCCTACGAACTCTCCACTAGCA	21	A	0.0088	0.483
NC_005100.4-93999660	AGGAAAAGGACATTGTCTCCACACTCT	7	A	0.0354	0.0846
NC_005100.4-93999660	AGGAAAAGGACATTGTCTCCACACTCT	24	C	0.0431	0.0903
NC_005100.4-94081946	AGCTGCAGTACCCACGCTCCCCGGTCC	9	T	0	0.4773
NC_005100.4-94081946	AGCTGCAGTACCCACGCTCCCCGGTCC	22	C	0.0246	0.0243
NC_005100.4-94106126	TGCTGCCCTACTCAGACTCCTTGATAG	4	T	0.2437	0.397
NC_005100.4-94146759	CATTCAAGAACTCATCCTCCCCGAAAT	15	T	0	0.4793
NC_005100.4-94146759	CATTCAAGAACTCATCCTCCCCGAAAT	22	C	0	0.4793
NC_005100.4-94159973	ATCTCCCTGACTCTGCCTCCTGAGCTC	7	C	0.1466	0.1646
NC_005100.4-94159973	ATCTCCCTGACTCTGCCTCCTGAGCTC	23	A	0.0427	0.0418
NC_005100.4-94201588	CACTCGTGCACAGTAGCTCCCCTTCCA	8	G	0.2083	0.4997
NC_005100.4-94214706	CAAATCGCCACCCAATCTCCTTCTGGA	7	G	0.2397	0.4277
NC_005100.4-94260926	GGAACAGAAACACCCACTCCAAAGCCT	8	A	0	0.4793
NC_005100.4-94272077	CACCTTTTCACTGCTCCTCCCTGTTCT	1	C	0.0163	0.1073
NC_005100.4-94272077	CACCTTTTCACTGCTCCTCCCTGTTCT	14	C	0.2602	0.3926
NC_005100.4-94286374	CAACGTGGTACACCTCCTCCATTCAAG	4	C	0.1707	0.1954
NC_005100.4-94341073	GGGCCAACCACACTGGCTCCAAGGTTA	21	A	0	0.4779
NC_005100.4-94367991	CACAGCAGAACAAATTCTCCTGAAGCA	16	T	0	0.4787
NC_005100.4-94391700	TTCATTGCCACATAAACTCCTAGCTAG	3	C	0.1652	0.1516
NC_005100.4-94391700	TTCATTGCCACATAAACTCCTAGCTAG	8	C	0	0.4764
NC_005100.4-94496132	TTCCCCAGGACGTCCTCTCCAGTGTCC	27	C	0.009	0.4682
NC_005100.4-94503727	GGGATTGTTACCCACGCTCCCTATAAT	5	T	0.0244	0.484
NC_005100.4-94503727	GGGATTGTTACCCACGCTCCCTATAAT	16	G	0.0325	0.4854
NC_005100.4-94514109	CAGCTCTGAACAGCATCTCCGTTTGGA	9	A	0.1333	0.2888
NC_005100.4-94514109	CAGCTCTGAACAGCATCTCCGTTTGGA	12	A	0.1333	0.2888
NC_005100.4-94524235	TTCCCTGACACACAGACTCCTGTTTCT	2	T	0	0.4765
NC_005100.4-94578721	TATGGTGGTACTATCTCTCCTGTTAGC	26	G	0.0427	0.4473
NC_005100.4-94596235	AACCAGCGCACTGCCCCTCCACCCAGT	8	G	0.2523	0.4178
NC_005100.4-94601991	GTCTGCCCAACTGAAGCTCCCCCAGCA	14	A	0	0.4773
NC_005100.4-94601991	GTCTGCCCAACTGAAGCTCCCCCAGCA	26	C	0.0574	0.071
NC_005100.4-94603988	ATTGTGCGCACTTCTGCTCCAGCCTGG	3	T	0	0.4693
NC_005100.4-94603988	ATTGTGCGCACTTCTGCTCCAGCCTGG	7	C	0	0.4693
NC_005100.4-94665404	GAGGCTAGGACAGAAGCTCCATGATGA	21	A	0.0268	0.4751
NC_005100.4-94691672	TGGAATGTCACTGTTGCTCCTTAGGTT	12	T	0.0738	0.4258
NC_005100.4-94691672	TGGAATGTCACTGTTGCTCCTTAGGTT	13	G	0.0492	0.048
NC_005100.4-94748371	CAGAGAGGCACATCAGCTCCTTGTAGA	1	C	0.2314	0.4246
NC_005100.4-94748371	CAGAGAGGCACATCAGCTCCTTGTAGA	8	G	0.2314	0.418
NC_005100.4-94917631	GCCCATGAGACAGTCTCTCCTGGCTGG	12	A	0.193	0.5
NC_005100.4-94991823	ACAGACATCACTGGAACTCCAGTGGCA	14	G	0.0244	0.0854
NC_005100.4-95003052	CACCTCCCCACAGCCTCTCCAAACAGT	7	C	0.1488	0.4426
NC_005100.4-95003052	CACCTCCCCACAGCCTCTCCAAACAGT	13	G	0.157	0.4398
NC_005100.4-95007526	TAACACTCTACTTTATCTCCTGCTGTT	16	T	0.1552	0.2622
NC_005100.4-95017620	GGGCTGGTAACCCTCTCTCCTATTCTT	12	C	0.2087	0.4537

NC_005100.4-95056718	ATTTGCATCACTCCTTCTCCCTTAGGT	8	T	0.0488	0.0476
NC_005100.4-95057950	AGCCACCGAACTATCTCTCCAGTCTCC	27	C	0	0.4644
NC_005100.4-95149903	ATCTCAGGGACCTTGTCTCCATGGGGG	6	A	0.2797	0.3483
NC_005100.4-95149903	ATCTCAGGGACCTTGTCTCCATGGGGG	24	G	0.094	0.1347
NC_005100.4-95153534	GATGAAGGTACCTGACCTCCCCAGTTC	22	C	0	0.4751
NC_005100.4-95153534	GATGAAGGTACCTGACCTCCCCAGTTC	24	G	0	0.4751
NC_005100.4-95172468	TATCAACCAACCAGAGCTCCTAGAGAC	6	A	0.6083	0.4233
NC_005100.4-95175542	GAATTCAACACCTCAACTCCCACACAT	13	T	0.1017	0.1116
NC_005100.4-95175542	GAATTCAACACCTCAACTCCCACACAT	25	C	0.0339	0.0333
NC_005100.4-95200198	TCTCAGACAACTCTAGCTCCTATCAAG	4	C	0.0246	0.0243
NC_005100.4-95200198	TCTCAGACAACTCTAGCTCCTATCAAG	22	A	0.0083	0.4732
NC_005100.4-95247568	TCCATCCAAACCCATACTCCATCATTT	15	T	0	0.48
NC_005100.4-95247568	TCCATCCAAACCCATACTCCATCATTT	24	A	0	0.48
NC_005100.4-95262688	TATGAAACAACCATGTCTCCAACAAAG	13	A	0.0504	0.4898
NC_005100.4-95262688	TATGAAACAACCATGTCTCCAACAAAG	14	T	0.0496	0.0639
NC_005100.4-95279813	ACATCAGGGACCTTTTCTCCTTTGCTC	13	T	0.0244	0.4868
NC_005100.4-95307408	ACTAATGAGACTTTGACTCCTCCAATG	8	A	0.1724	0.199
NC_005100.4-95308232	CTCACATGCACACACTCTCCAATACAC	23	T	0.0244	0.0241
NC_005100.4-95309733	GAGAGGAAGACTAAACCTCCTACTTAT	5	G	0.2627	0.4454
NC_005100.4-95370169	AGCAAGCTTACTAGTGCTCCTCATAGC	5	A	0.0088	0.0262
NC_005100.4-96730687	GTCAAAGACACACGAGCTCCATGTTTA	13	C	0.1538	0.4997
NC_005100.4-96730687	GTCAAAGACACACGAGCTCCATGTTTA	14	G	0.15	0.1932
NC_005100.4-96793573	GAGGGGATGACCATGGCTCCAGCTGCA	12	C	0.1552	0.2378
NC_005100.4-96793573	GAGGGGATGACCATGGCTCCAGCTGCA	23	C	0.0259	0.0422
NC_005100.4-96794805	TTCTTCAGCACATTAGCTCCTCATAGT	12	A	0	0.4807
NC_005100.4-96794805	TTCTTCAGCACATTAGCTCCTCATAGT	14	T	0.0246	0.4717
NC_005100.4-96805031	AAGCTCACAACTCCAGCTCCAGAGATA	16	G	0.009	0.0267
NC_005100.4-96881485	CATGGCCATACAGATACTCCCACTGCT	5	G	0.0171	0.0336
NC_005100.4-96881485	CATGGCCATACAGATACTCCCACTGCT	23	C	0.1207	0.4994
NC_005100.4-96887496	CATGTGAACACTACTTCTCCCTTCCCG	25	C	0.0769	0.074
NC_005100.4-96888196	AGAAGAGGCACCCATACTCCAGCCTTC	7	G	0.2667	0.3099
NC_005100.4-96889044	ACTTTTATGACCACTCCTCCCATGGAG	7	A	0	0.48
NC_005100.4-96889044	ACTTTTATGACCACTCCTCCCATGGAG	13	A	0.3017	0.343
NC_005100.4-96953058	AATATCTATACCCTGTCTCCAATACAC	12	C	0.2288	0.2406
NC_005100.4-96953058	AATATCTATACCCTGTCTCCAATACAC	26	A	0.1695	0.1827
NC_005100.4-96965868	ATGCTAAGGACTGTTCCTCCAATGCAT	27	T	0.0417	0.0722
NC_005100.4-96977183	GGGAAAATCACTGCCTCTCCTTTAGGC	24	A	0.0333	0.4832
NC_005100.4-96993947	ATAGAGAATACTGAACCTCCATAGTGC	21	A	0.1652	0.2458
NC_005100.4-97002872	CTGCAGGCTACTCATACTCCACAGATA	22	C	0.1565	0.1869
NC_005100.4-97190357	TCTATACAAACACAGCCTCCATAAAGG	13	C	0.2252	0.3232
NC_005100.4-97243306	GTGTTGGGAACTTAATCTCCAACCATG	3	G	0.0164	0.4737
NC_005100.4-97243306	GTGTTGGGAACTTAATCTCCAACCATG	7	G	0.2705	0.4588
NC_005100.4-97255462	TGTGGTACCACACCACCTCCATCAACC	2	G	0.2439	0.4595
NC_005100.4-97280596	CGAGGCTCCACTTCATCTCCAGATGCC	2	G	0.1696	0.3612
NC_005100.4-97280596	CGAGGCTCCACTTCATCTCCAGATGCC	4	G	0.0268	0.0264
NC_005100.4-97298830	GCAGCTACCACTGTTCCTCCTACATTT	6	T	0.25	0.3707
NC_005100.4-97339201	AGCCTTACGACTTAGCCTCCACAACCC	21	A	0	0.4875
NC_005100.4-97340155	TTAGATATTACCACATCTCCTTCCTGC	4	G	0.2623	0.4698
NC_005100.4-97340155	TTAGATATTACCACATCTCCTTCCTGC	14	C	0.2623	0.4698
NC_005100.4-97390583	TATTGTGTCACTGTCTCTCCTTGAGTT	25	G	0.0579	0.0562
NC_005100.4-97431117	TCCTGTTGTACACACTCTCCTCTAGGG	6	T	0.177	0.4464

NC_005100.4-97485292	AATCCAGACACTGCTTCTCCATGCCTG	13	G	0.082	0.0935
NC_005100.4-97524061	CCTTTGCACACAATAACTCCATCATTG	13	A	0.2018	0.3273
NC_005100.4-97524061	CCTTTGCACACAATAACTCCATCATTG	25	T	0.0175	0.0345
NC_005100.4-97532349	ATCTACCTTACTATTACTCCACGTCAC	13	A	0.2137	0.2423
NC_005100.4-97532349	ATCTACCTTACTATTACTCCACGTCAC	22	C	0	0.4807
NC_005100.4-97546584	TGAATTGAAACATTGCCTCCAAAAGAT	13	T	0.1327	0.4998
NC_005100.4-97552871	ACAGCTACAACTGGGACTCCAGTCTGG	22	G	0.0413	0.0405
NC_005100.4-97585481	TTTTGTAAGACAGTTGCTCCCTAGTCA	14	T	0.2034	0.493
NC_005100.4-97602015	CTCTACCACACCCCATCTCCTCATTTG	3	C	0.0081	0.481
NC_005100.4-97622272	CAACAGACCACTGGAGCTCCTTTCAAA	23	T	0.1491	0.4997
NC_005100.4-97622923	CTGAAGAGGACGTAGCCTCCAAACAGT	12	G	0.0084	0.4723
NC_005100.4-97622923	CTGAAGAGGACGTAGCCTCCAAACAGT	26	G	0.0167	0.0328
NC_005100.4-97662778	GGGACAGATACTTCATCTCCTTGCATG	23	G	0.0909	0.0868
NC_005100.4-97673957	CTATTCACAACTTCCACTCCACCAGCC	12	T	0.1081	0.4558
NC_005100.4-97673957	CTATTCACAACTTCCACTCCACCAGCC	27	C	0	0.4898
NC_005100.4-97684116	AATGTTTTCACTACATCTCCAAATTCT	7	T	0.0083	0.4747
NC_005100.4-97876580	TGCCAAAGTACAGTGGCTCCTGTTGCT	14	T	0.3103	0.3662
NC_005100.4-97943826	GCCCCCTCAACATTCTCTCCATTCTTG	5	C	0.1681	0.2076
NC_005100.4-97943826	GCCCCCTCAACATTCTCTCCATTCTTG	7	T	0.2857	0.3686
NC_005100.4-97946289	CGGATCATTACCACCTCTCCCATGCAT	23	T	0.0248	0.0245
NC_005100.4-97981356	TCGTTGGTCACTTCCACTCCCTTCACA	6	G	0.2035	0.1828
NC_005100.4-97981356	TCGTTGGTCACTTCCACTCCCTTCACA	9	C	0.2389	0.2237
NC_005100.4-97997173	TTTCTGGCAACAGTGGCTCCCAGACTG	1	T	0.4701	0.4473
NC_005100.4-97997173	TTTCTGGCAACAGTGGCTCCCAGACTG	8	C	0.0256	0.0253
NC_005100.4-98186784	CTGGTCCTCACCATCTCTCCAGCAGTG	14	T	0	0.48
NC_005100.4-98387337	ATCCCTCTTACCACCCCTCCCTGACCT	14	C	0.181	0.4836
NC_005100.4-98387337	ATCCCTCTTACCACCCCTCCCTGACCT	26	C	0.1466	0.4916
NC_005100.4-98432102	CCATCCTGCACATGACCTCCACCACGC	26	G	0.0244	0.0241
NC_005100.4-98472207	ACGAGGCCCACACTGGCTCCAAGTTCA	13	C	0.0345	0.0339
NC_005100.4-98472207	ACGAGGCCCACACTGGCTCCAAGTTCA	27	A	0.0877	0.1744
NC_005100.4-98485371	TGTCTGTAAACCCTAGCTCCAGGGTAT	22	G	0.0252	0.0249
NC_005100.4-98488437	CCAAGACCCACTCCTTCTCCTCTTCAG	25	C	0.2393	0.4868
NC_005100.4-98488437	CCAAGACCCACTCCTTCTCCTCTTCAG	26	A	0.0769	0.3409
NC_005100.4-98491149	TGTGTTAGAACTGATACTCCTTTGGCT	12	T	0.05	0.0799
NC_005100.4-98497624	GGACATAATACAGTCACTCCTGCGCTG	9	T	0	0.4807
NC_005100.4-98497733	TACACCCATACTCATCCTCCAAAGCCT	21	A	0.0407	0.0553
NC_005100.4-98546619	GAGGCAGTCACCTTTTCTCCTTCTCCC	25	C	0.1271	0.2759
NC_005100.4-98546619	GAGGCAGTCACCTTTTCTCCTTCTCCC	27	C	0.0667	0.0644
NC_005100.4-98562747	ACAATAAACACATTCTCTCCTTGGTAC	15	C	0.1441	0.2717
NC_005100.4-98570473	CAAAGACGCACAGACCCTCCTGCACCG	26	C	0.1301	0.1216
NC_005100.4-98572911	GACCAGTATACCCAAACTCCCTCTATA	15	A	0.2586	0.488
NC_005100.4-98715779	AACCTGAGAACCATGTCTCCTTTATTC	16	T	0.0439	0.0429
NC_005100.4-98730464	GGACTCACCACACTAGCTCCACACTGT	3	A	0.0084	0.4682
NC_005100.4-98730464	GGACTCACCACACTAGCTCCACACTGT	24	C	0.3729	0.3034
NC_005100.4-98767028	AACCTCACAACTGACTCTCCCTACCAC	8	C	0.0246	0.0243
NC_005100.4-99104396	CCTGTGACCACTACTGCTCCGGGACTG	12	T	0.3361	0.2796
NC_005100.4-99480677	AGTACTGTTACACGGACTCCATTTGTC	3	T	0.1017	0.2219
NC_005100.4-99506370	CATAGATACACATTGGCTCCTCAAGGC	2	A	0.0265	0.0262
NC_005100.4-99506370	CATAGATACACATTGGCTCCTCAAGGC	27	C	0.1947	0.3637
NC_005100.4-99544651	GCCCCTGCGACTCCAGCTCCAAGGGAT	1	G	0.0351	0.0512
NC_005100.4-99544651	GCCCCTGCGACTCCAGCTCCAAGGGAT	27	T	0.2105	0.4186

NC_005100.4-99592783	ATTGAAACCACTACAGCTCCCGAACAA	22	G	0	0.4787
NC_005100.4-99601664	GAAGGACAGACCTGAGCTCCACTGCAG	13	T	0.0492	0.1082
NC_005100.4-99608734	CATCCTGTAACTCTAACTCCAAGATCT	14	T	0	0.4734
NC_005100.4-99660362	TTCACAGCAACATTCCCTCCACCAGCC	14	T	0.1026	0.4938
NC_005100.4-99682665	GTCTGCAAGACCTTTACTCCAGTACAT	6	C	0.1754	0.4994
NC_005100.4-99682665	GTCTGCAAGACCTTTACTCCAGTACAT	9	G	0.0087	0.4782
NC_005100.4-99686004	CTAAACATCACGTGTCCTCCACAGGTT	3	A	0.082	0.0786
NC_005100.4-99686004	CTAAACATCACGTGTCCTCCACAGGTT	23	A	0.082	0.4934
NC_005100.4-99711598	AACTGCCATACCAGCTCTCCAGCCTCA	8	A	0.1217	0.2395
NC_005100.4-99730972	AAACTCTTAACTCCTACTCCTCCATGT	1	A	0	0.4793
NC_005100.4-99730972	AAACTCTTAACTCCTACTCCTCCATGT	16	A	0.0569	0.0553
NC_005100.4-99732263	TCATCTCCTACCACCTCTCCTCTGTTC	14	C	0.1271	0.2526
NC_005100.4-99732263	TCATCTCCTACCACCTCTCCTCTGTTC	24	G	0	0.4772
NC_005100.4-99733490	TGATTATACACCAGGCCTCCTGGGTAT	1	T	0.0086	0.4893
NC_005100.4-99733490	TGATTATACACCAGGCCTCCTGGGTAT	9	C	0.0087	0.4878
NC_005100.4-99745754	CACCACCTCACAGTTCCTCCTTCGGTA	6	C	0.0248	0.0562
NC_005100.4-99753915	CCTGGCTTTACCTTTCCTCCAGGACAT	25	C	0.0826	0.1235
NC_005100.4-99755850	CATAGTGAGACCCTATCTCCAAAGGAT	25	G	0.082	0.1225
NC_005100.4-99797237	CGGGTCAGCACAGAGCCTCCTATTCTG	2	G	0.1565	0.173
NC_005100.4-99820047	ACTCATGTGACCTTTGCTCCTCCGACC	12	C	0.0446	0.0436
NC_005100.4-99955072	TGACACCTGACACTTCCTCCCCAAACA	14	T	0.0086	0.4893
NC_005100.4-99955072	TGACACCTGACACTTCCTCCCCAAACA	26	C	0.0847	0.0965
NC_005100.4-100020942	GTGTTTACCACAGTCTCTCCTGCTGCA	3	G	0.5574	0.4463
NC_005100.4-100116031	TCTCTGGGGACTGCAGCTCCTGCAGTA	25	G	0.0492	0.048
NC_005100.4-100116264	TCCCTCAGAACCAAAACTCCAGACCCA	16	A	0	0.474
NC_005100.4-100116264	TCCCTCAGAACCAAAACTCCAGACCCA	23	A	0.3448	0.2854
NC_005100.4-100132024	ATCATGACAACTGGTCCTCCATCTTCT	4	A	0.0976	0.0928
NC_005100.4-100242860	GTGATATCAACAGGGCCTCCTATGGCT	7	T	0.1897	0.3282
NC_005100.4-100243735	CTCAGTCGGACAAGGTCTCCATCACCA	15	G	0.0175	0.0677
NC_005100.4-100268231	ATGATATCTACAGGTTCTCCCTCCCCT	5	T	0.0708	0.2036
NC_005100.4-100287981	GCTGCCACCACCTCTTCTCCCTGGCAT	4	G	0.0254	0.0251
NC_005100.4-100287981	GCTGCCACCACCTCTTCTCCCTGGCAT	24	G	0	0.4764
NC_005100.4-100331338	GCCCCCACCACTGGCTCTCCCTCCATT	5	C	0.0244	0.0241
NC_005100.4-100331338	GCCCCCACCACTGGCTCTCCCTCCATT	27	T	0.0081	0.481
NC_005100.4-100331544	CATAAGGAGACCTTGCCTCCACATGAA	24	T	0	0.4793
NC_005100.4-100335118	TCCATTACCACAGTCCCTCCTCCATCA	1	T	0	0.4793
NC_005100.4-100335118	TCCATTACCACAGTCCCTCCTCCATCA	23	C	0.1057	0.1695
NC_005100.4-100356488	CCTTCAAACACTGCATCTCCTGGAGCA	8	A	0	0.032
NC_005100.4-100356488	CCTTCAAACACTGCATCTCCTGGAGCA	25	G	0.2213	0.3008
NC_005100.4-100410124	CTACCTGTAACACCAGCTCCAAGGGAT	14	C	0.123	0.1296
NC_005100.4-100417523	ACCAAAGCAACACCACCTCCCTGGTTA	26	T	0	0.4793
NC_005100.4-100484445	CTGCTGTCTACTCTCCCTCCTTCGCAT	6	G	0.0165	0.4819
NC_005100.4-100484445	CTGCTGTCTACTCTCCCTCCTTCGCAT	15	C	0	0.4819
NC_005100.4-100502348	CAGCTCCTAACCTGGTCTCCACTAGTT	14	G	0	0.4779
NC_005100.4-100541511	AAACAGGTGACTGCTGCTCCAAAGTGT	23	A	0.0263	0.4796
NC_005100.4-100562929	CTGAAGACTACTTCAGCTCCTGTTGAG	24	T	0.178	0.5
NC_005100.4-100564525	ACATCTGTAACTACCACTCCAGGGATC	27	C	0.0619	0.06
NC_005100.4-100572239	CCCGACCCTACACTTCCTCCAGCTCAG	4	G	0	0.4819
NC_005100.4-100583120	TGTAACAAGACCTGGTCTCCGTCCCCA	21	G	0	0.4704
NC_005100.4-100593830	TGCTTCTCTACCAAGGCTCCGCCCCCA	4	T	0.15	0.2311
NC_005100.4-100593830	TGCTTCTCTACCAAGGCTCCGCCCCCA	13	A	0.0084	0.4829

NC_005100.4-100597279	AGAACCATAACCCCCTCTCCATCTACA	22	T	0.0413	0.0716
NC_005100.4-100601176	CTCGGTCACACTCGCCCTCCCTGCCTC	4	G	0.0569	0.0553
NC_005100.4-100601176	CTCGGTCACACTCGCCCTCCCTGCCTC	14	G	0.1382	0.208
NC_005100.4-100604798	TGGATGCTGACTGACTCTCCGATTTCT	9	G	0.0081	0.0241
NC_005100.4-100613518	GCTGAATTGACCCTTTCTCCCTCAAGC	8	T	0	0.4751
NC_005100.4-100614641	TCCCTCCTCACCTGCTCTCCTGCTCCA	9	C	0.2241	0.3995
NC_005100.4-100616966	TCTCTCATCACTGGGCCTCCTCCTACC	7	A	0	0.4746
NC_005100.4-100626441	CTGCCACCTACTCACCCTCCGTGCTTG	6	A	0.1967	0.306
NC_005100.4-100631222	CTTCCCCACACTCCCACTCCTTCTGTG	6	C	0.1301	0.2723
NC_005100.4-100631489	GGGAAAAATACAGTAGCTCCAGAAAGG	3	G	0.045	0.044
NC_005100.4-100632436	GATTCCCCAACTCCACCTCCACAGCTC	4	T	0	0.4807
NC_005100.4-100645560	GGGGGTCTTACATCTTCTCCTGTCCTC	6	T	0.0164	0.4837
NC_005100.4-100763301	GTAGCAAGTACCTTTACTCCAGCCATC	21	A	0	0.4793
NC_005100.4-100766048	GGAAAGACTACTGCTACTCCAACTGAA	1	G	0.1933	0.2961
NC_005100.4-100818631	GGAAGCCTGACACTACCTCCTGGGACT	25	A	0.0082	0.4822
NC_005100.4-100833666	TCCAGTGTCACCCATACTCCCACCCAG	3	C	0.0413	0.0716
NC_005100.4-100833666	TCCAGTGTCACCCATACTCCCACCCAG	7	G	0.0083	0.0245
NC_005100.4-100860196	CTGTCAATAACTGTGGCTCCCCAGGGT	2	T	0.0513	0.05
NC_005100.4-100887396	TGGCAAGGAACAGGGCCTCCCAACTTC	13	G	0	0.4773
NC_005100.4-100887396	TGGCAAGGAACAGGGCCTCCCAACTTC	23	A	0.2231	0.2814
NC_005100.4-100923193	GATTGAAGTACCATCTCTCCATAGGGT	2	A	0	0.4787
NC_005100.4-100966105	GGGCACATCACTGCAACTCCTCCGTAG	16	A	0.0082	0.4755
NC_005100.4-100972926	TTTCTTTTGACCTAATCTCCATAGGCA	6	T	0	0.4807
NC_005100.4-101020139	ACATCTATGACCCTTTCTCCCCTGTCC	21	C	0.0893	0.1626
NC_005100.4-101020139	ACATCTATGACCCTTTCTCCCCTGTCC	27	C	0.0354	0.0348
NC_005100.4-101023698	CTGGCCAGCACCCCTCCTCCTGCGCGC	26	G	0	0.4755
NC_005100.4-101023698	CTGGCCAGCACCCCTCCTCCTGCGCGC	27	C	0.2281	0.4902
NC_005100.4-101024650	CCTAGTAAGACCCAGTCTCCACAAAAC	6	T	0.1765	0.2961
NC_005100.4-101024650	CCTAGTAAGACCCAGTCTCCACAAAAC	21	A	0	0.4743
NC_005100.4-101040236	CGGCCTCAAACTCGTACTCCTGCGCAT	23	C	0.2276	0.4548
NC_005100.4-101076675	ACCCGAAACACAGCTCCTCCTGTTGTG	2	C	0.1239	0.2171
NC_005100.4-101114603	GGAGTTACAACCCCAGCTCCTCACTGT	26	G	0.0522	0.1143
NC_005100.4-101116935	CTAAGACTAACACAAACTCCAGCTTCT	27	T	0	0.4833
NC_005100.4-101164876	CATTCTCTGACCCTCTCTCCCTAGGTT	13	C	0.0256	0.0253
NC_005100.4-101165322	CCAGCTATCACTCCCCCTCCCTGCCCC	14	C	0.0328	0.0322
NC_005100.4-101170474	CTTCCAATAACTCCAGCTCCAGTTGGT	24	T	0	0.482
NC_005100.4-101177300	CCAGGCCCCACTCCACCTCCCTTGCCC	14	C	0	0.4773
NC_005100.4-101210878	TTCTTCCTGACCACACCTCCACCTGAC	16	C	0.2541	0.2686
NC_005100.4-101210878	TTCTTCCTGACCACACCTCCACCTGAC	25	G	0.041	0.0401
NC_005100.4-101263993	GGTCTCATGACGACATCTCCTCTCATG	4	C	0.3417	0.2833
NC_005100.4-101263993	GGTCTCATGACGACATCTCCTCTCATG	15	A	0.025	0.0247
NC_005100.4-101288894	TTGTGGCTGACTCTCCCTCCCTGACCA	3	G	0	0.4793
NC_005100.4-101288894	TTGTGGCTGACTCTCCCTCCCTGACCA	25	C	0.065	0.0928
NC_005100.4-101306082	AACCAGTGAACCATTCCTCCAGGCCCA	5	A	0.0574	0.0557
NC_005100.4-101306082	AACCAGTGAACCATTCCTCCAGGCCCA	23	G	0.0574	0.0557
NC_005100.4-101307340	CACACGCTCACCTGTACTCCATCCTGG	5	C	0	0.482
NC_005100.4-101311640	CACAGCTGCACTGAAGCTCCATCCTAC	3	C	0.036	0.0354
NC_005100.4-101313897	CCCGGTGTCACCCTCTCTCCTCCTGCA	2	C	0.036	0.0526
NC_005100.4-101319864	CAATAAAGGACGCGACCTCCTCCTCCA	1	C	0.0894	0.1562
NC_005100.4-101333125	CAGTCAGAGACTCCATCTCCTCGTGTG	2	A	0	0.4807
NC_005100.4-101359783	TGTTGAGATACGGGCACTCCAGCCAGG	27	G	0.1652	0.2458

NC_005100.4-101417575	GACTGCCTCACTCTGCCTCCTGAGTAT	21	T	0.2605	0.3812
NC_005100.4-101421275	CCTGGCAGAACCTTGGCTCCTCCCACC	12	C	0	0.477
NC_005100.4-101422792	ACCTAACACACCCTGCCTCCCTGACAT	4	T	0	0.4902
NC_005100.4-101422792	ACCTAACACACCCTGCCTCCCTGACAT	21	C	0	0.4902
NC_005100.4-101433179	CCAGCCCCAACAGAGTCTCCATTGAAC	14	A	0	0.4793
NC_005100.4-101433643	AGGGACTCAACCATTTCTCCCTGTGTG	6	C	0	0.4749
NC_005100.4-101442749	ATTCCCATTACTAGGCCTCCTCTGGTT	6	C	0.0661	0.4901
NC_005100.4-101581494	CATACCTCAACCCTTCCTCCTCTTCCT	5	C	0.0336	0.033
NC_005100.4-101581494	CATACCTCAACCCTTCCTCCTCTTCCT	23	T	0.0756	0.0882
NC_005100.4-101581685	CTTCTACCCACCCCTTCTCCAGGTCCT	22	G	0.0336	0.4813
NC_005100.4-101581685	CTTCTACCCACCCCTTCTCCAGGTCCT	27	T	0.0083	0.0247
NC_005100.4-101604041	CTTTGATTAACGATATCTCCTTTTACT	14	T	0.0354	0.0846
NC_005100.4-101666990	GGCTGTGTCACCTCTGCTCCACCATCC	24	A	0	0.4793
NC_005100.4-101705276	CAAGGCTGGACTCCTGCTCCTCTGGGT	13	C	0.187	0.1826
NC_005100.4-101731025	AGGTATGACACACTTCCTCCGACATTG	5	A	0	0.4793
NC_005100.4-101738454	GGAATGCTCACTGAGCCTCCTGGCCAG	13	G	0.0569	0.0553
NC_005100.4-101769017	AGACACAGTACCTCTTCTCCCCTTGAC	5	A	0	0.4793
NC_005100.4-101780791	GACTGGCACACCTGCACTCCCACAACT	8	A	0	0.4793
NC_005100.4-101791474	CCTACTCTCACAGATACTCCCGCTAGG	22	G	0.0265	0.0262
NC_005100.4-101798552	CAGCCTGTGACTTCCCCTCCAACCGGG	26	G	0.0345	0.0339
NC_005100.4-101838356	TTACATTCTACCCAACCTCCAGGCGTA	16	C	0.0252	0.0249
NC_005100.4-101838356	TTACATTCTACCCAACCTCCAGGCGTA	25	G	0.0084	0.4859
NC_005100.4-101857125	GACAGACAGACCCCTCCTCCCTCAGCT	6	A	0.0325	0.032
NC_005100.4-101883643	GGGCCGAGGACCAGTGCTCCTCTCTGG	15	T	0.1008	0.3686
NC_005100.4-101883643	GGGCCGAGGACCAGTGCTCCTCTCTGG	23	T	0	0.4703
NC_005100.4-101884934	GGGCCTTGAACAGGCACTCCGGGACAT	3	G	0.0169	0.4718
NC_005100.4-101921726	ACCTCTCCCACCTGTCCTCCTGATTAT	12	C	0.0088	0.4843
NC_005100.4-101963063	GGGACGAATACCCCTTCTCCTCTCCCT	26	C	0.027	0.0267
NC_005100.4-101981950	GCCTGAAGCACACATTCTCCACCACAG	21	A	0.2069	0.2501
NC_005100.4-101988652	CAATCATTCACTCTTCCTCCTTTTCAG	6	A	0.3223	0.4557
NC_005100.4-101988652	CAATCATTCACTCTTCCTCCTTTTCAG	24	T	0.1066	0.1296
NC_005100.4-101988977	GCGTCACAAACGCCTTCTCCGCATCCA	2	C	0.0083	0.4769
NC_005100.4-101988977	GCGTCACAAACGCCTTCTCCGCATCCA	12	G	0.041	0.0401
NC_005100.4-102001263	GATGGACTGACAATCTCTCCAAGTGTG	4	G	0.1217	0.1869
NC_005100.4-102031088	TAGAGTGAGACCCTGTCTCCAAACAAA	9	G	0.187	0.2203
NC_005100.4-102039897	GAAAAGGGTACCAGGTCTCCCGGAGTT	25	G	0.2231	0.4077
NC_005100.4-102045449	TGCAGTGTAACAGGCCCTCCTCAGCCG	9	A	0.2281	0.4945
NC_005100.4-102061800	AGGAAAATGACTCTCTCTCCCCCAACT	13	C	0.2049	0.311
NC_005100.4-102108971	GACAAGCTCACTTCCACTCCTCCACTG	22	C	0.0082	0.4755
NC_005100.4-102111703	CTCCTACCTACACCCACTCCTATCTCT	7	C	0.0088	0.4843
NC_005100.4-102111703	CTCCTACCTACACCCACTCCTATCTCT	22	A	0.3158	0.4555
NC_005100.4-102118407	CCATTGCCCACAGATGCTCCCAGCCCA	2	C	0.2667	0.3911
NC_005100.4-102118407	CCATTGCCCACAGATGCTCCCAGCCCA	3	A	0	0.4832
NC_005100.4-102136820	CTTCTCTACACTCTCTCTCCCTCTGCT	13	C	0.2069	0.2501
NC_005100.4-102157376	AGAATGATGACAGCCGCTCCAATCAAG	21	A	0	0.4746
NC_005100.4-102161381	CTCTTGGACACGGTTGCTCCGCACTCT	21	G	0.2931	0.3835
NC_005100.4-102161381	CTCTTGGACACGGTTGCTCCGCACTCT	22	C	0.1525	0.1827
NC_005100.4-102184349	CAGCTGTTGACCACATCTCCAGCCCTA	15	A	0.2087	0.3593
NC_005100.4-102196896	GCCAGAGGGACCTTAGCTCCGGTTTCC	5	G	0.1111	0.2423
NC_005100.4-102217967	TGCCTTGAAACCATTGCTCCTCTGCTT	24	G	0.125	0.1997
NC_005100.4-102217967	TGCCTTGAAACCATTGCTCCTCTGCTT	25	C	0.125	0.1997

NC_005100.4-102227104	CTGTGTCCCACCCTGACTCCTAAGGTC	21	T	0	0.4793
NC_005100.4-102233763	AGGTCACTCACTGACGCTCCATCTCCT	4	T	0.0179	0.4807
NC_005100.4-102233763	AGGTCACTCACTGACGCTCCATCTCCT	5	C	0.1327	0.4998
NC_005100.4-102246877	CACACACACACCCCACCTCCACTTAGT	22	C	0.1626	0.1629
NC_005100.4-102246877	CACACACACACCCCACCTCCACTTAGT	23	T	0	0.4793
NC_005100.4-102263547	CTCTTATAGACTTGTTCTCCTGCTTCA	6	A	0	0.4773
NC_005100.4-102304084	GTGAGCCACACCGTTGCTCCCTTGGTG	12	C	0.1157	0.1918
NC_005100.4-102312934	TCTACCATCACCACCACTCCTACCACC	2	C	0	0.4693
NC_005100.4-102324058	ATTAGACCGACCACTCCTCCATGGGCT	24	G	0.2288	0.329
NC_005100.4-102327306	TCCTTCATCACCCCAACTCCCACCCAG	13	C	0.1261	0.1609
NC_005100.4-102327306	TCCTTCATCACCCCAACTCCCACCCAG	26	A	0.084	0.1398
NC_005100.4-102336741	TGACAGTGGACTAAACCTCCGAACTAT	26	A	0.2328	0.2057
NC_005100.4-102336741	TGACAGTGGACTAAACCTCCGAACTAT	27	T	0.2018	0.235
NC_005100.4-102351887	AGGATGTCCACAGCAGCTCCCTGATTC	24	A	0.2586	0.375
NC_005100.4-102353189	TGCACAGGGACAGCAGCTCCAAGGCTG	22	A	0.2672	0.4435
NC_005100.4-102355509	TGTGATTTAACTCAAGCTCCAGCAAAG	14	A	0.1833	0.4999
NC_005100.4-102375229	TGCTACGAGACTGTGTCTCCTAGCAAT	21	T	0.0625	0.0934
NC_005100.4-102388931	GTCGTTCACACCGTTTCTCCCATCTGA	2	T	0.3361	0.3508
NC_005100.4-102396064	CTGGGTGTCACCATTTCTCCGATCTTC	21	G	0.0085	0.481
NC_005100.4-102402326	TGTTTTAGCACAGGGTCTCCCTTTATA	7	A	0	0.48
NC_005100.4-102417380	AAATTTGAAACAGTCCCTCCCCACTTA	24	C	0.0089	0.4789
NC_005100.4-102417724	ATAGAGAGCACTTGGTCTCCCATGTTG	1	A	0.0082	0.4755
NC_005100.4-102419572	GTCTGACTGACGGCTTCTCCCTGCGTG	25	G	0.0179	0.4841
NC_005100.4-102425455	CTGGGAATCACTTCCTCTCCCCTACTG	2	T	0.1983	0.418
NC_005100.4-102425455	CTGGGAATCACTTCCTCTCCCCTACTG	14	C	0.0083	0.0245
NC_005100.4-102439618	GCCGTGCACACACACACTCCAGCTCAT	4	G	0.0328	0.0634
NC_005100.4-102439618	GCCGTGCACACACACACTCCAGCTCAT	16	A	0	0.4773
NC_005100.4-102441096	TGTGGTTCCACCACTACTCCATGTGCA	22	T	0.0081	0.4777
NC_005100.4-102444401	CCAAGCAATACAGGGACTCCTAGAGCA	6	C	0.0174	0.0342
NC_005100.4-102450990	CTCCAGCCCACAAACCCTCCACGAGGG	23	G	0.1197	0.1274
NC_005100.4-102457491	CTTCCTGGTACCCAGGCTCCACCGTAA	24	G	0.0325	0.032
NC_005100.4-102458226	CAAAGGACCACTAAGTCTCCTCACCCA	5	G	0	0.4813
NC_005100.4-102496233	CTTCCTGTTACATGTTCTCCTCAATCA	3	T	0.1557	0.3307
NC_005100.4-102496233	CTTCCTGTTACATGTTCTCCTCAATCA	13	T	0.1901	0.4913
NC_005100.4-102561552	CCACTTGTCACTCCAGCTCCATGGATC	9	C	0	0.4819
NC_005100.4-102564801	CCAAATCCTACCCGGTCTCCTGGCCAG	7	C	0.0855	0.4918
NC_005100.4-102578861	TCAGCGACAACTGCTCCTCCACACAAG	6	G	0.1043	0.1143
NC_005100.4-102599746	TAGTGTTTCACTGCTTCTCCATGAGGG	8	T	0.0813	0.0928
NC_005100.4-102605036	ACGTGTTAGACAAGGACTCCTCTACTA	3	G	0.094	0.4947
NC_005100.4-102605976	GTGTATCAGACCTCAGCTCCAACCCCA	5	A	0.1382	0.1826
NC_005100.4-102605976	GTGTATCAGACCTCAGCTCCAACCCCA	25	C	0.1382	0.1826
NC_005100.4-102670475	AAGCCAGTGACTGATTCTCCAGCATCA	16	T	0.0625	0.4933
NC_005100.4-102671494	GCTGACACCACACGTGCTCCAATCCTT	6	C	0	0.4757
NC_005100.4-102671494	GCTGACACCACACGTGCTCCAATCCTT	13	C	0	0.4757
NC_005100.4-102677460	CTTCTACACACCACATCTCCCATCGTG	14	C	0.1951	0.2612
NC_005100.4-102677460	CTTCTACACACCACATCTCCCATCGTG	24	C	0.1707	0.373
NC_005100.4-102716548	TTACCCAGGACATGCTCTCCTCGAAGT	21	T	0.0492	0.048
NC_005100.4-102716548	TTACCCAGGACATGCTCTCCTCGAAGT	24	A	0.0082	0.4822
NC_005100.4-102738180	TAAAGGCATACCTGCTCTCCTTATATA	25	A	0.025	0.0408
NC_005100.4-102783062	TGGGTACTTACTTTGTCTCCAGTGACA	9	T	0	0.4773
NC_005100.4-102783062	TGGGTACTTACTTTGTCTCCAGTGACA	12	T	0	0.4773

NC_005100.4-102801664	TTTCATTCCACTTTGGCTCCTCTTCTG	27	G	0.0667	0.1665
NC_005100.4-102802518	TTATGAGCGACGCTGCCTCCTTATCCA	24	T	0.0583	0.2125
NC_005100.4-102822261	CACAACAGGACAGTTCCTCCATTCCCT	1	C	0.1311	0.316
NC_005100.4-102822261	CACAACAGGACAGTTCCTCCATTCCCT	16	C	0.0246	0.0401
NC_005100.4-102822940	CAGTCTGACACCTGAGCTCCATCCTAG	1	C	0	0.4819
NC_005100.4-102834138	ATGTCTGTAACTCCAGCTCCAGGAGGT	27	T	0.1583	0.2722
NC_005100.4-102835133	CGTGATCATACTGAGGCTCCGAGCTAG	2	G	0.252	0.3646
NC_005100.4-102908067	CCCAGCCAGACCGTACCTCCTAATCAC	14	T	0	0.4864
NC_005100.4-102908067	CCCAGCCAGACCGTACCTCCTAATCAC	22	A	0.0261	0.0425
NC_005100.4-102912500	GCAAAAGCAACAAGTCCTCCTTTGAGC	2	C	0.1176	0.1254
NC_005100.4-102940215	CACGCAACTACTAGAACTCCAGTATAA	3	C	0	0.4833
NC_005100.4-102956102	GAAGAGGGAACTGGATCTCCCAGAAGT	4	G	0.0164	0.0322
NC_005100.4-102999775	AGGTATGCTACTGCTCCTCCTGAGTTT	14	C	0	0.4807
NC_005100.4-103018965	GCTGGGCCTACATTGCCTCCTACCCTC	24	C	0.2261	0.4637
NC_005100.4-103031079	GGGGGCCAGACCTACCCTCCTTGCTTC	22	T	0.281	0.3811
NC_005100.4-103369934	CCCCTCAGAACCCATGCTCCTTTTCTG	1	C	0.1393	0.1968
NC_005100.4-103370454	GCTCTAGCCACCATGCCTCCTGCCATC	13	A	0.2066	0.4863
NC_005100.4-103393489	AAATCCTTCACTGAAGCTCCTGGAAAT	15	A	0.0083	0.4732
NC_005100.4-103393489	AAATCCTTCACTGAAGCTCCTGGAAAT	24	A	0.1653	0.3079
NC_005100.4-103513589	CTCTAATCTACACCCACTCCCAAGCTG	5	A	0.1207	0.1134
NC_005100.4-103513589	CTCTAATCTACACCCACTCCCAAGCTG	13	C	0.2241	0.2122
NC_005100.4-103690056	ATGCTGAAGACTGGTTCTCCAATGTTG	9	G	0.2087	0.3403
NC_005100.4-103690056	ATGCTGAAGACTGGTTCTCCAATGTTG	12	T	0.1491	0.1814
NC_005100.4-103807974	GGCTTCCATACAAGGACTCCTTACTCC	23	A	0.0619	0.0765
NC_005100.4-103827050	CTTCCCTCTACCTTTACTCCCTGATTG	24	A	0.2054	0.4968
NC_005100.4-103908084	CCAGACACCACCAGAGCTCCTATGGTC	16	G	0	0.4787
NC_005100.4-104009798	GAGAATCTTACCTTCCCTCCATTCTTC	14	T	0	0.4793
NC_005100.4-104009798	GAGAATCTTACCTTCCCTCCATTCTTC	15	C	0.0261	0.0257
NC_005100.4-104056378	ACCAGAAAAACATTTCCTCCCATCACA	14	T	0.0446	0.0436
NC_005100.4-104067498	ATGAAGGGTACCAGCCCTCCCTTTCTA	1	A	0.0165	0.4819
NC_005100.4-104067498	ATGAAGGGTACCAGCCCTCCCTTTCTA	4	A	0	0.4787
NC_005100.4-104068705	GAAGCTGGGACAACACCTCCATAAACC	22	T	0.1333	0.1932
NC_005100.4-104096585	GTCCCTGTCACTTCGTCTCCATAACAA	6	T	0.0083	0.4783
NC_005100.4-104163310	CACAAATCAACTGTTTCTCCAGCATTT	27	T	0.2759	0.457
NC_005100.4-104267789	GTGTCAGGGACAGCATCTCCATCCCGT	26	G	0.0763	0.4982
NC_005100.4-104268182	CCAGCCAAAACCACCTCTCCTGGGAGG	12	C	0.0427	0.0418
NC_005100.4-104277138	GTTATAGCCACCTCCTCTCCCATCGTC	3	T	0.0256	0.0253
NC_005100.4-104277138	GTTATAGCCACCTCCTCTCCCATCGTC	25	G	0.1552	0.3179
NC_005100.4-104285910	AGCTCTGTTACCAACTCTCCTCTCTGC	2	G	0.0088	0.026
NC_005100.4-104285910	AGCTCTGTTACCAACTCTCCTCTCTGC	27	C	0.0901	0.1338
NC_005100.4-104286080	CTGGATCTGACCACAGCTCCCCCGGAG	24	G	0.0672	0.4957
NC_005100.4-104286546	TCCTGCCACACACCATCTCCCACAGCA	24	A	0.0163	0.4825
NC_005100.4-104306364	AAGTAATAGACCGCCACTCCATACACA	13	G	0.0244	0.0241
NC_005100.4-104313708	AGGCAATCGACTTGTTCTCCAGCAGTC	26	T	0.0171	0.4868
NC_005100.4-104338112	GGCTCCTCCACACCATCTCCTATTGCA	12	A	0.1393	0.4056
NC_005100.4-104341423	GGGAAAGTCACCCACCCTCCCTTGCTG	5	A	0.1057	0.4979
NC_005100.4-104350710	AAGCACTTTACGGAGCCTCCTCTCTAA	12	G	0.2212	0.3772
NC_005100.4-104350710	AAGCACTTTACGGAGCCTCCTCTCTAA	22	C	0.0625	0.0771
NC_005100.4-104387120	GCAAGCGGTACTCTTCCTCCCTGTGAA	7	G	0.0603	0.1058
NC_005100.4-104387120	GCAAGCGGTACTCTTCCTCCCTGTGAA	27	A	0.2328	0.2681
NC_005100.4-104420838	GCCCAGCGTACACATGCTCCATGGGCT	8	G	0.2261	0.3499

NC_005100.4-104420838	GCCCAGCGTACACATGCTCCATGGGCT	12	A	0.0342	0.0336
NC_005100.4-104477613	ATAGCAAGAACTTTACCTCCCCAGCCA	4	G	0.0085	0.0251
NC_005100.4-104477613	ATAGCAAGAACTTTACCTCCCCAGCCA	9	A	0.0259	0.4768
NC_005100.4-104477860	GATTCAGGAACGCAGGCTCCTTCAGCC	12	G	0.2432	0.3232
NC_005100.4-104477860	GATTCAGGAACGCAGGCTCCTTCAGCC	16	G	0.2411	0.3418
NC_005100.4-104484155	GAAAACAAAACTCACCCTCCCCACGTG	1	G	0.2377	0.4126
NC_005100.4-104484155	GAAAACAAAACTCACCCTCCCCACGTG	9	A	0.2131	0.4737
NC_005100.4-104535282	CTAAGGCCAACCCTCCCTCCTCCTTTG	12	C	0.1092	0.1609
NC_005100.4-104549381	GAACAGGATACTCCTTCTCCGGTCTGA	1	G	0.0087	0.4704
NC_005100.4-104549381	GAACAGGATACTCCTTCTCCGGTCTGA	21	G	0.3684	0.4975
NC_005100.4-104559482	ACTGAAGTCACAAGCACTCCGTGATGA	21	G	0.0738	0.1154
NC_005100.4-104559512	TGGTCTCTGACAGCGGCTCCTCCTTGC	15	G	0.1707	0.1954
NC_005100.4-104566669	CAAGGGAGCACAGTCCCTCCCTCAGGG	24	A	0.1557	0.3581
NC_005100.4-104574334	GGTCCCATCACGAGGGCTCCTCGGTCA	12	G	0.0088	0.481
NC_005100.4-104574334	GGTCCCATCACGAGGGCTCCTCGGTCA	23	G	0	0.4841
NC_005100.4-104581516	GCTTTTCACACTTTCTCTCCGACCACG	22	A	0	0.4772
NC_005100.4-104581516	GCTTTTCACACTTTCTCTCCGACCACG	26	C	0.1724	0.3381
NC_005100.4-104597424	TGGGGGCCCACACCCCCTCCACTGCAA	4	G	0	0.4737
NC_005100.4-104621084	TAATTATTAACTGCTCCTCCTGCCACA	14	C	0.1789	0.2832
NC_005100.4-104634362	ACCTGTGTAACTTCCACTCCAGGCCAC	1	A	0.0442	0.06
NC_005100.4-104644664	TCTTACCACACCTACCCTCCTTGGAGA	24	G	0.0348	0.0342
NC_005100.4-104651396	CAGCAGCGCACAGTAGCTCCAGTCTCT	2	A	0.0342	0.4602
NC_005100.4-104651396	CAGCAGCGCACAGTAGCTCCAGTCTCT	21	A	0.0508	0.4826
NC_005100.4-104698140	GTGGCTGGCACTGAGCCTCCTTTAAGC	12	T	0.3277	0.4435
NC_005100.4-104752313	CTGCAGAAGACCCAAGCTCCAATCCCC	22	A	0	0.4841
NC_005100.4-104752313	CTGCAGAAGACCCAAGCTCCAATCCCC	24	C	0.2719	0.3568
NC_005100.4-104772808	CTTCTAAAAACTGTGACTCCCCCACCT	8	A	0.0598	0.058
NC_005100.4-104808728	CCAGATTGTACTTTCCCTCCGCCTGCC	6	T	0.2562	0.4339
NC_005100.4-104808728	CCAGATTGTACTTTCCCTCCGCCTGCC	22	C	0.2562	0.4339
NC_005100.4-104815866	ATGTGTTGCACTAAAACTCCAGGGCAG	1	A	0.2131	0.4979
NC_005100.4-104815866	ATGTGTTGCACTAAAACTCCAGGGCAG	6	T	0.3279	0.4515
NC_005100.4-104848754	TTCTGTAGGACATAGTCTCCAGTATGT	12	A	0	0.4765
NC_005100.4-104905714	ACTCCTCCGACAGTGTCTCCAGTATCA	8	C	0.0492	0.4866
NC_005100.4-104969726	TCCCTTTTTACTCCCGCTCCACTCATC	6	T	0.2696	0.293
NC_005100.4-104969726	TCCCTTTTTACTCCCGCTCCACTCATC	15	C	0.2522	0.2816
NC_005100.4-104985569	GGCTGGTGAACTCAGGCTCCCTGGATG	23	G	0	0.4851
NC_005100.4-104985569	GGCTGGTGAACTCAGGCTCCCTGGATG	27	G	0.2931	0.4463
NC_005100.4-104987136	ACTTATTAGACAGGGTCTCCTATATCC	24	A	0.0174	0.4833
NC_005100.4-104991374	GCTGCGGTGACCCCCTCTCCCACTATA	2	C	0	0.4786
NC_005100.4-104991374	GCTGCGGTGACCCCCTCTCCCACTATA	5	C	0.0431	0.0585
NC_005100.4-104997284	TCCCATGGGACTTATTCTCCATCAACA	14	A	0.0088	0.476
NC_005100.4-105084139	GTGCTCTTAACCATCTCTCCATCTCTC	4	C	0.1311	0.2157
NC_005100.4-105084139	GTGCTCTTAACCATCTCTCCATCTCTC	13	A	0.1311	0.1225
NC_005100.4-105091749	ATGCTCTTAACCATCTCTCCATCTCTC	27	C	0.541	0.4092
NC_005100.4-105116079	ACACATCACACTCTCACTCCCCTACAT	16	A	0.0083	0.4769
NC_005100.4-105130601	CCTTTCAGAACCCGCCCTCCTTGTTTG	13	C	0	0.4793
NC_005100.4-105130601	CCTTTCAGAACCCGCCCTCCTTGTTTG	15	C	0.0407	0.0398
NC_005100.4-105172975	CTGTTCGGGACGCTGTCTCCTGTGCAG	13	C	0.1557	0.2095
NC_005100.4-105172975	CTGTTCGGGACGCTGTCTCCTGTGCAG	22	G	0.0246	0.0401
NC_005100.4-105211517	CCAGGTTAAACTACCCCTCCCCTTATA	9	A	0.0179	0.4841
NC_005100.4-105274726	GGCCTGGGAACAGGAACTCCAAGGTCC	5	T	0.0339	0.0333

NC_005100.4-105274726	GGCCTGGGAACAGGAACTCCAAGGTCC	25	T	0.0678	0.0655
NC_005100.4-105299376	GCAATTTCAACTCAGTCTCCACAACTT	2	C	0.0924	0.1033
NC_005100.4-105324635	CCCACAGACACTGTCTCTCCTGCACGT	1	C	0	0.4891
NC_005100.4-105324635	CCCACAGACACTGTCTCTCCTGCACGT	26	G	0.0783	0.2072
NC_005100.4-105329558	CTTAAAGTCACCACTGCTCCCTTTGTT	15	T	0.2521	0.3014
NC_005100.4-105342951	TCCTCAGAAACCTGGTCTCCTCCTGAG	16	T	0.1639	0.1774
NC_005100.4-105342951	TCCTCAGAAACCTGGTCTCCTCCTGAG	21	T	0.1639	0.1774
NC_005100.4-105395314	GCATCTCTAACATGAGCTCCAGTGTAT	9	A	0.0744	0.465
NC_005100.4-105433580	GGCAACACAACATTTGCTCCAGGGGCT	7	A	0	0.4757
NC_005100.4-105469328	GTTGGTCACACTGCATCTCCATTCAGG	22	T	0.0244	0.0241
NC_005100.4-105472818	ACCTATTGGACCATCTCTCCAGTCCCC	15	C	0.0893	0.1327
NC_005100.4-105479998	GACCTTTGAACCATCTCTCCAGGCTCA	4	C	0.1066	0.4984
NC_005100.4-105512791	TCCAACCAAACCAGACCTCCTCAGAGC	3	C	0.0088	0.4889
NC_005100.4-105513036	GCTGTTCTGACTCCAACTCCTCACCAA	14	C	0.0164	0.4737
NC_005100.4-105684522	GGAATTGGAACCTCGCCTCCTGTACAG	15	G	0.0583	0.49
NC_005100.4-105717859	CTCCTCTTTACGTTGTCTCCACAGTAG	12	G	0	0.4827
NC_005100.4-105727330	CATTGGCACACTCCCTCTCCATTAAAT	1	C	0.1525	0.1691
NC_005100.4-105727330	CATTGGCACACTCCCTCTCCATTAAAT	9	C	0	0.4891
NC_005100.4-105737884	GAGAGTCTCACTTTGTCTCCTGTGAGA	12	T	0.1803	0.263
NC_005100.4-105740398	CTGTCCTTGACTGCAGCTCCTGTGGGA	16	G	0.0167	0.0328
NC_005100.4-105771045	CAAAGCCAAACTTTGGCTCCTTGGGAA	1	C	0.1858	0.4843
NC_005100.4-105771045	CAAAGCCAAACTTTGGCTCCTTGGGAA	2	A	0.0088	0.4889
NC_005100.4-105799581	CTCTAACAAACTGGCTCTCCATCTTGT	15	C	0.0171	0.05
NC_005100.4-105799581	CTCTAACAAACTGGCTCTCCATCTTGT	21	A	0.0256	0.0253
NC_005100.4-105822281	CCCTATGGTACCACACCTCCTTGTGTG	2	C	0.2846	0.3091
NC_005100.4-105822281	CCCTATGGTACCACACCTCCTTGTGTG	27	G	0	0.4793
NC_005100.4-105843568	CATGGCTAGACATTTGCTCCAAACCAG	9	G	0	0.4793
NC_005100.4-105843568	CATGGCTAGACATTTGCTCCAAACCAG	27	G	0.2602	0.2723
NC_005100.4-105865342	AACCAGCCCACTTCCTCTCCTTTAATA	6	G	0.0086	0.4836
NC_005100.4-105865342	AACCAGCCCACTTCCTCTCCTTTAATA	21	T	0.0086	0.4836
NC_005100.4-105871636	ACTGAAGGAACAGTGACTCCCCATGGA	23	A	0.0088	0.4774
NC_005100.4-105893016	GGTAGACCAACTGACACTCCTTTAGAA	5	G	0.1111	0.1049
NC_005100.4-105893016	GGTAGACCAACTGACACTCCTTTAGAA	27	A	0	0.4772
NC_005100.4-105894606	TTCCATGGAACAACCTCTCCTAGGGGA	13	A	0.0085	0.4882
NC_005100.4-105903603	ACTGCTTTTACCATCCCTCCATCTTGA	12	C	0.3675	0.4473
NC_005100.4-105903603	ACTGCTTTTACCATCCCTCCATCTTGA	15	C	0.2137	0.2423
NC_005100.4-105996543	AAAAATGCAACCCTCGCTCCTGCTCTC	15	C	0	0.4864
NC_005100.4-105996543	AAAAATGCAACCCTCGCTCCTGCTCTC	22	G	0.1207	0.1431
NC_005100.4-106011446	GGCGGCTCTACTGCTTCTCCCTTCACT	2	G	0.2281	0.3793
NC_005100.4-106011446	GGCGGCTCTACTGCTTCTCCCTTCACT	3	C	0	0.4749
NC_005100.4-106015843	CCCACTGAAACCCTTCCTCCCAACATG	24	C	0.3109	0.4377
NC_005100.4-106030332	GTAAGCAACACTTCTGCTCCAGTTCCT	22	G	0.0435	0.0425
NC_005100.4-106058001	AACTGCTGAACCATGTCTCCAGCCTCA	3	C	0.1322	0.1653
NC_005100.4-106058001	AACTGCTGAACCATGTCTCCAGCCTCA	23	C	0.0496	0.0484
NC_005100.4-106071899	AATTCTCCCACTGAGACTCCTTTCACT	2	A	0.0081	0.0241
NC_005100.4-106071899	AATTCTCCCACTGAGACTCCTTTCACT	9	C	0.2927	0.4793
NC_005100.4-106154382	ACAAAGCTAACCAGGACTCCTGCCTCT	23	C	0.1624	0.1773
NC_005100.4-106207962	GTGCCCAGCACTAATACTCCTCATACT	24	T	0.0081	0.481
NC_005100.4-106222368	AGCAGTGTGACAAAGTCTCCAGATACA	13	A	0	0.4592
NC_005100.4-106251249	CTCTTTTGTACCCGATCTCCTCAAGCA	13	C	0.1017	0.1116
NC_005100.4-106251249	CTCTTTTGTACCCGATCTCCTCAAGCA	14	G	0	0.482

NC_005100.4-106528980	GCTGCACAGACAAATTCTCCAGACATC	7	C	0.0357	0.0351
NC_005100.4-106553159	ACTCACCTCACTGTCCCTCCGTTTACT	5	A	0.042	0.0571
NC_005100.4-106599980	AACAACTCCACAAATCCTCCCTCTTTA	25	T	0.0083	0.4803
NC_005100.4-106643990	CCAAACCCTACCAGAGCTCCAAGGGAC	8	C	0.1282	0.12
NC_005100.4-106708405	AGGAACTGGACACACTCTCCTGGCCTC	9	G	0.0342	0.05
NC_005100.4-106756626	ACTCCAATAACTGTTACTCCTTATGAG	12	T	0.0427	0.074
NC_005100.4-106756626	ACTCCAATAACTGTTACTCCTTATGAG	23	A	0.0517	0.0504
NC_005100.4-106790504	CTGGGTCTAACTCCAGCTCCAAGATAT	6	T	0.2846	0.4703
NC_005100.4-106790504	CTGGGTCTAACTCCAGCTCCAAGATAT	24	A	0.3659	0.4301
NC_005100.4-106870175	TAAAAAGTTACAGCATCTCCTCGATAA	23	G	0.0992	0.0943
NC_005100.4-106875558	GTTTGCCTCACCCCAGCTCCTGTTATT	3	T	0.0261	0.0257
NC_005100.4-106902698	CATGCAATCACTCTGTCTCCTTTAGTC	23	T	0	0.4677
NC_005100.4-106915241	CAACCATCCACCATGTCTCCTGCCTCT	13	A	0	0.4819
NC_005100.4-106959888	TTCATTTGGACGATATCTCCTATTGTG	13	A	0	0.4779
NC_005100.4-106961405	AAAGCCCACACTGTCTCTCCCTCAGGT	14	T	0.125	0.232
NC_005100.4-107012378	ATGTCATGTACATAGACTCCTGTGGGC	27	C	0.0333	0.0328
NC_005100.4-107075573	ATATCCTGCACAGGGTCTCCACACTGA	24	C	0.1311	0.1505
NC_005100.4-107109370	GCCCGCAGTACACAATCTCCCTGGATG	2	C	0.0177	0.0517
NC_005100.4-107221764	AGTTCAGGAACATGCTCTCCTTCACAT	13	T	0.1066	0.4973
NC_005100.4-107221764	AGTTCAGGAACATGCTCTCCTTCACAT	26	A	0.2377	0.4634
NC_005100.4-107243254	AACAAATTTACCCATTCTCCATGAGTG	9	T	0	0.4751
NC_005100.4-107283857	CCCCACCCTACCCCAGCTCCTCTTTAT	26	A	0.0435	0.0425
NC_005100.4-107326211	CTACTGTCAACAGGAACTCCTTGGACT	13	G	0.0407	0.0398
NC_005100.4-107333251	ATTAGTAACACCTTGCCTCCCCATCTG	7	A	0	0.4793
NC_005100.4-107333251	ATTAGTAACACCTTGCCTCCCCATCTG	23	A	0.0813	0.1494
NC_005100.4-107338746	GTATCTGTCACAGCTACTCCATTGTCT	3	A	0	0.4688
NC_005100.4-107405031	ACTGGACCCACAGTATCTCCTGGGGAA	15	A	0	0.4839
NC_005100.4-107405031	ACTGGACCCACAGTATCTCCTGGGGAA	16	T	0.0339	0.0333
NC_005100.4-107556225	AAATAAAGCACATTGTCTCCTTCATAT	2	A	0	0.4841
NC_005100.4-107556225	AAATAAAGCACATTGTCTCCTTCATAT	24	A	0.0531	0.0517
NC_005100.4-107760803	TGCCAAGCAACCACAGCTCCCAGGGAC	4	C	0.5082	0.4515
NC_005100.4-107877479	CAGAAACAAACCCTGCCTCCCATGTTA	7	C	0.1345	0.2203
NC_005100.4-107897556	ATTGCTAACACCTCGACTCCCCTTATA	16	A	0.0796	0.0926
NC_005100.4-107980205	TAGCCTGTCACTCTCCCTCCTCCTGTG	5	C	0.4174	0.3403
NC_005100.4-108002035	TTATGTTCCACTATGACTCCTGAGCCT	8	C	0.1138	0.1216
NC_005100.4-108028259	AACTTAGGAACATCTACTCCATGCTTA	15	T	0.1161	0.125
NC_005100.4-108121732	AATAAAAACACATGGTCTCCTTCACAC	6	A	0.0439	0.0595
NC_005100.4-108121732	AATAAAAACACATGGTCTCCTTCACAC	9	C	0.0439	0.0429
NC_005100.4-108181965	AACACACTCACCCGCTCTCCACAAGTC	3	C	0.1475	0.1505
NC_005100.4-108181965	AACACACTCACCCGCTCTCCACAAGTC	13	C	0.1901	0.4988
NC_005100.4-108204991	TAAGAAGGTACTCCATCTCCACTCCTG	8	G	0.0569	0.0553
NC_005100.4-108305724	CAGCTGTCTACCATCACTCCTGCCTCT	22	G	0.2411	0.3211
NC_005100.4-108634631	CACATCTTCACCTTTTCTCCCTCGGAA	23	C	0.2562	0.2814
NC_005100.4-108644713	CTAGATTCTACAAGTACTCCCAACTAA	24	C	0.1709	0.1841
NC_005100.4-108681070	CTGTTCTAAACATTGCCTCCCTATTAC	15	G	0.1712	0.4065
NC_005100.4-108909745	ATGTCTGCAACAGGACCTCCAAGAACA	5	C	0.0351	0.0839
NC_005100.4-109008998	GGAAGTAAAACCAGAGCTCCTCTTCAG	5	G	0.0574	0.1154
NC_005100.4-109008998	GGAAGTAAAACCAGAGCTCCTCTTCAG	14	G	0	0.4787
NC_005100.4-109105283	ATAGACTCTACATGTTCTCCCTCCCCT	24	C	0.087	0.4954
NC_005100.4-109105283	ATAGACTCTACATGTTCTCCCTCCCCT	25	C	0.0877	0.4945
NC_005100.4-109938127	TCCTACTCCACCAGACCTCCCCACTCT	3	C	0.1491	0.3568

NC_005100.4-109938127	TCCTACTCCACCAGACCTCCCCACTCT	22	C	0.1565	0.4234
NC_005100.4-110018769	CCAATTGGAACTCTGTCTCCTCCAATA	7	G	0.0614	0.0918
NC_005100.4-110018769	CCAATTGGAACTCTGTCTCCTCCAATA	15	G	0.2018	0.3273
NC_005100.4-110066684	TTCAATCAGACCATGCCTCCTAATGGT	2	T	0	0.4846
NC_005100.4-110066684	TTCAATCAGACCATGCCTCCTAATGGT	13	A	0.1913	0.2395
NC_005100.4-110181565	TACATACCTACAAACACTCCTACACAC	5	T	0	0.4814
NC_005100.4-110254676	ATTGGTAATACCATGCCTCCCAATGTG	23	A	0.1416	0.3136
NC_005100.4-110254676	ATTGGTAATACCATGCCTCCCAATGTG	26	T	0.1504	0.297
NC_005100.4-110264293	CACTGCTCAACTGGTGCTCCTGGTTAA	22	G	0.0333	0.4832
NC_005100.4-110273147	GACTCCCTCACAACCCCTCCTCTCTCT	5	C	0.0248	0.0245
NC_005100.4-110273147	GACTCCCTCACAACCCCTCCTCTCTCT	27	T	0.075	0.0722
NC_005100.4-110342765	TGTCCTCCTACAGATTCTCCCTCCTGC	24	C	0.125	0.1316
NC_005100.4-110823300	TTCTTTCTCACTCTTTCTCCATTCCTC	1	T	0.2288	0.298
NC_005100.4-110823300	TTCTTTCTCACTCTTTCTCCATTCCTC	27	C	0.25	0.2778
NC_005100.4-110994291	ACACCTGTAACAGCAGCTCCATGTGAT	5	C	0.1765	0.214
NC_005100.4-110994291	ACACCTGTAACAGCAGCTCCATGTGAT	22	T	0.1261	0.4117
NC_005100.4-111046256	CAAGGGCCAACATGCACTCCTTAGGTG	22	T	0.0957	0.0911
NC_005100.4-111046256	CAAGGGCCAACATGCACTCCTTAGGTG	25	G	0	0.4902
NC_005100.4-111058161	GCTCCTGGCACTCTTTCTCCTGGTGAG	6	T	0.0667	0.1244
NC_005100.4-111058671	TTATATTTCACAAACTCTCCTAAAATA	4	T	0.0948	0.3876
NC_005100.4-111153432	GAGAGCCTCACCACCTCTCCTCTGGTA	13	A	0.0661	0.2295
NC_005100.4-111153432	GAGAGCCTCACCACCTCTCCTCTGGTA	14	C	0.0248	0.0245
NC_005100.4-111348974	ATCAGCCCTACCTTTCCTCCCAGTACC	1	A	0.0248	0.0245
NC_005100.4-111348974	ATCAGCCCTACCTTTCCTCCCAGTACC	8	C	0.1405	0.1447
NC_005100.4-111390736	AACCAGATAACAGCACCTCCTGTAGCC	13	G	0	0.4807
NC_005100.4-111437502	TGTCCCCCAACCCTCTCTCCTTCCCTT	12	C	0.275	0.4025
NC_005100.4-111555017	AAGGGTCCCACCCAGTCTCCATTTCCC	25	C	0.0082	0.4822
NC_005100.4-111555370	GATTCTCCCACCAACACTCCCCTCATT	12	C	0.042	0.0882
NC_005100.4-111894252	TTCCCTTCCACAACACCTCCATCACAT	7	T	0.2793	0.3124
NC_005100.4-112035112	AGGTTCTTGACAATCTCTCCTTCCATC	15	C	0.1525	0.2091
NC_005100.4-112102297	AATTGGGGCACTGGATCTCCATCTACA	15	A	0.1967	0.2157
NC_005100.4-112115978	GCGAGGCTTACTACTCCTCCATCTGGT	26	G	0	0.4827
NC_005100.4-112127671	GTTAAAAACACTTTTCCTCCTTTAACA	27	A	0.0174	0.0342
NC_005100.4-112289054	ATTGCTCTTACAGTGTCTCCCTGTTTT	7	C	0.1488	0.3278
NC_005100.4-112289054	ATTGCTCTTACAGTGTCTCCCTGTTTT	21	C	0.0246	0.0557
NC_005100.4-112302774	GAGCTCCACACACAAGCTCCACACAGC	8	A	0.2672	0.4729
NC_005100.4-112302774	GAGCTCCACACACAAGCTCCACACAGC	24	C	0.0254	0.0251
NC_005100.4-112328839	ATTCACGTCACATCCTCTCCCCATAAA	6	C	0	0.4833
NC_005100.4-112328839	ATTCACGTCACATCCTCTCCCCATAAA	15	C	0.2783	0.4682
NC_005100.4-112364002	CTGGGGTCCACTATGGCTCCTTTACTT	27	T	0	0.4844
NC_005100.4-112388698	CATTACCCTACCCATCCTCCTTGCTCA	13	C	0.1754	0.3221
NC_005100.4-112399067	CCAAGAAGGACCAACACTCCCATAAGA	26	G	0.0263	0.026
NC_005100.4-112417632	CTGACAGACACGAAGTCTCCCTTGCTG	23	T	0	0.4765
NC_005100.4-112417632	CTGACAGACACGAAGTCTCCCTTGCTG	24	G	0	0.4765
NC_005100.4-112432368	CCATCTGTAACTCTAGCTCCATGCGAT	15	A	0.0826	0.1235
NC_005100.4-112482109	TCTTTCTCCACATCCTCTCCAATGTAT	4	T	0.2035	0.4774
NC_005100.4-112558255	AAAGAGTTAACTGGGTCTCCTTTCTAG	14	G	0.2232	0.4193
NC_005100.4-112561304	AGCATATAAACTGTGGCTCCTCTGGAT	4	A	0.0756	0.0882
NC_005100.4-112561304	AGCATATAAACTGTGGCTCCTCTGGAT	7	T	0.2051	0.3159
NC_005100.4-112588496	TTCCAGTGAACTTCCCCTCCTCTCTGT	21	T	0.1008	0.4921
NC_005100.4-112653214	TAGATATATACAAGGTCTCCTTGGACC	27	C	0.2167	0.3487

NC_005100.4-112669584	TGCCTCCAAACCACATCTCCTGCTTTA	23	C	0.1949	0.4698
NC_005100.4-112681037	AGCTTAGCAACCTAGGCTCCCAGAGTA	4	T	0.0088	0.4923
NC_005100.4-112681037	AGCTTAGCAACCTAGGCTCCCAGAGTA	25	G	0.0088	0.4923
NC_005100.4-112774326	ATGGGCAGAACCATGGCTCCAGCTATA	6	C	0.0625	0.0605
NC_005100.4-112774326	ATGGGCAGAACCATGGCTCCAGCTATA	15	G	0.0531	0.0517
NC_005100.4-112796779	TACCACTGCACCACACCTCCAGTGTCT	24	G	0.0614	0.499
NC_005100.4-112829893	CCCTGACACACCTCATCTCCCCCAGAT	12	C	0.0085	0.4842
NC_005100.4-112829893	CCCTGACACACCTCATCTCCCCCAGAT	23	C	0.0085	0.4842
NC_005100.4-112870417	GTGGCATAGACCAAATCTCCCACATGA	26	G	0.1393	0.4997
NC_005100.4-112893012	CTGGTGTACACTTTTTCTCCATTGCTG	14	T	0	0.4649
NC_005100.4-112907119	TATGATACTACTCACTCTCCTATGTAC	25	T	0.0625	0.0605
NC_005100.4-112927739	AAAACAAGAACCACTCCTCCTTATATG	14	C	0.1066	0.4973
NC_005100.4-112937704	GCAGAAATGACTGCGTCTCCCTTCCTA	5	A	0.3058	0.4004
NC_005100.4-112937704	GCAGAAATGACTGCGTCTCCCTTCCTA	14	C	0.0165	0.4751
NC_005100.4-112972257	GTAGAATTCACAGCAGCTCCTGTTTGT	4	G	0.0085	0.4753
NC_005100.4-112974583	AGAGGCAAGACCTGCTCTCCAATCCTC	5	G	0.0517	0.0825
NC_005100.4-113077760	AGGGAACCAACTCGTTCTCCAGATGTC	13	C	0.0252	0.0249
NC_005100.4-113078404	CAGTGTGCAACTCACACTCCTGGCGAC	24	C	0.0909	0.4507
NC_005100.4-113086808	AGATTTCCCACACATCCTCCTTGGCTG	13	C	0.0413	0.0562
NC_005100.4-113095682	AACGAAGCCACTCGTACTCCAGCAAAT	3	C	0.0877	0.0839
NC_005100.4-113095682	AACGAAGCCACTCGTACTCCAGCAAAT	14	G	0.1228	0.1454
NC_005100.4-113099486	AGAATTGTGACACCTTCTCCTCTTCCT	8	T	0.0328	0.0322
NC_005100.4-113099486	AGAATTGTGACACCTTCTCCTCTTCCT	22	C	0.3697	0.3771
NC_005100.4-113109681	ATAAATCCCACTTCTCCTCCAACTGCT	2	T	0.0244	0.0241
NC_005100.4-113109681	ATAAATCCCACTTCTCCTCCAACTGCT	25	G	0.3577	0.377
NC_005100.4-113110216	CCCTCCACAACACCCTCTCCTTGCTCT	16	T	0.036	0.4883
NC_005100.4-113110216	CCCTCCACAACACCCTCTCCTTGCTCT	22	T	0	0.4807
NC_005100.4-113135502	ACCTGATTCACAGGAGCTCCCTTAAAT	15	A	0.115	0.3294
NC_005100.4-113179082	CACCCATTCACTCATCCTCCAGTGATT	7	T	0	0.4793
NC_005100.4-113180875	GAGATGAAAACTGGTACTCCTCTATGT	6	G	0.1429	0.1626
NC_005100.4-113185656	GTCAACAGAACAGACTCTCCTCCGACC	7	A	0.0833	0.1099
NC_005100.4-113185656	GTCAACAGAACAGACTCTCCTCCGACC	23	C	0.3167	0.3487
NC_005100.4-113203540	CCAAAGGCCACTACATCTCCATTGGAT	22	T	0.0081	0.4777
NC_005100.4-113229214	ATTGAAGTGACAGTGTCTCCTATCCAC	9	G	0.025	0.0247
NC_005100.4-113255682	CGGCTTATAACTGAGTCTCCCCTTAAT	8	T	0.0536	0.1014
NC_005100.4-113273976	GTTGGCTGTACCCTTGCTCCATCTCTA	21	A	0.0336	0.0491
NC_005100.4-113320501	ATGGCTGTGACAGATGCTCCAATGTTA	7	G	0	0.4833
NC_005100.4-113320501	ATGGCTGTGACAGATGCTCCAATGTTA	25	T	0.0087	0.0257
NC_005100.4-113366979	CTGCCTGACACTTTGTCTCCATATTTC	15	G	0.2281	0.4186
NC_005100.4-113456408	CACTGTAAAACAGGCTCTCCTCCTGAG	6	T	0.0862	0.1284
NC_005100.4-113456408	CACTGTAAAACAGGCTCTCCTCCTGAG	7	A	0.181	0.4982
NC_005100.4-113515331	TCCTATTCCACCCCCTCTCCTTCTACT	15	C	0.2314	0.3645
NC_005100.4-113566183	AATACATACACACACCCTCCTTGTGCG	4	A	0	0.4773
NC_005100.4-113566183	AATACATACACACACCCTCCTTGTGCG	16	C	0.0244	0.0241
NC_005100.4-113639850	TATATGCACACTGCTTCTCCTCCATGC	2	A	0.0084	0.4723
NC_005100.4-113639850	TATATGCACACTGCTTCTCCTCCATGC	26	G	0.0084	0.4723
NC_005100.4-113646968	ATTCCTCCCACTACCCCTCCTCTCCCA	5	C	0.1017	0.0965
NC_005100.4-113646968	ATTCCTCCCACTACCCCTCCTCTCCCA	6	T	0.1356	0.2344
NC_005100.4-113647344	AGCTCTTTCACTCTATCTCCTCTTCCT	2	G	0.1066	0.1436
NC_005100.4-113820615	ATCTGTACCACACTTTCTCCTTCGTCC	23	C	0.2174	0.3147
NC_005100.4-113880129	AAACAGGCCACACCAGCTCCCCCACAT	5	A	0.0256	0.1049

NC_005100.4-113965061	CTCCCAACAACTCTTACTCCTTGATCC	12	T	0	0.4654
NC_005100.4-114071957	AGAGCACTGACTGTTCCTCCAGAGGCC	16	C	0.1983	0.1786
NC_005100.4-114078615	TGGTCTCGAACCCATCCTCCAGCTAGT	25	A	0	0.4773
NC_005100.4-114078615	TGGTCTCGAACCCATCCTCCAGCTAGT	26	G	0.2131	0.3709
NC_005100.4-114143482	CTAAACCACACTCCACCTCCCAAGGTT	14	C	0.2846	0.4301
NC_005100.4-114143482	CTAAACCACACTCCACCTCCCAAGGTT	22	A	0.1707	0.2556
NC_005100.4-114196176	AAGTAGCTGACAGGACCTCCGTTGTTT	21	G	0.1875	0.2637
NC_005100.4-114289953	CACAGTGGCACACTTTCTCCCGCAAGC	21	C	0.0789	0.1229
NC_005100.4-114309106	AGTTTGCTAACATTAACTCCCAGGTCA	24	G	0.1504	0.154
NC_005100.4-114355377	CAAAGGAGGACTTGAGCTCCCCTGGGA	26	G	0.0086	0.4866
NC_005100.4-114395973	CACAAGGCCACATCACCTCCCACCACA	25	A	0.0089	0.4856
NC_005100.4-114409081	TTGAGGCCCACCCGACCTCCATATTCT	15	A	0.0579	0.0716
NC_005100.4-114513347	TGGCGCTGAACTGTCTCTCCAGTGGTG	1	T	0	0.4793
NC_005100.4-114647709	ATAAACACCACCTTGCCTCCAGCCCTG	23	C	0.0894	0.1145
NC_005100.4-114736457	TGAACCTATACGCTAGCTCCAATGAAA	12	G	0.0862	0.0981
NC_005100.4-114745136	ACAGCGAGGACCTCACCTCCTTCCTTA	4	G	0.2114	0.2832
NC_005100.4-114794556	ATCTACCATACTTCCTCTCCCTCTTCC	8	A	0.2328	0.2797
NC_005100.4-114794556	ATCTACCATACTTCCTCTCCCTCTTCC	14	C	0	0.4764
NC_005100.4-114797614	AGATATGAAACCATATCTCCTCTGGCT	12	C	0.1696	0.212
NC_005100.4-114924918	ATACTTTCCACTTTTCCTCCTCCATGC	26	G	0.225	0.4622
NC_005100.4-114989008	ATCATGATCACTTTTGCTCCTCTCCAT	26	A	0.2314	0.2975
NC_005100.4-115055396	TCCAAAGATACATGATCTCCATGACAC	5	A	0.0678	0.1409
NC_005100.4-115055396	TCCAAAGATACATGATCTCCATGACAC	27	C	0.1681	0.3771
NC_005100.4-115125358	TAAATAATAACAGGGCCTCCATTCTCC	3	A	0.6207	0.4281
NC_005100.4-115156164	GTGGTGCTTACATGTTCTCCACATCAG	24	T	0.0084	0.4829
NC_005100.4-115193952	CACTCTCACACGGTCACTCCAACTTGC	12	G	0	0.4793
NC_005100.4-115193952	CACTCTCACACGGTCACTCCAACTTGC	13	G	0	0.4793
NC_005100.4-115306574	GGCTTACCTACATACTCTCCACCAGGA	2	G	0.1034	0.1284
NC_005100.4-115308609	TATGAAAGTACTTAACCTCCATAGGTT	7	A	0	0.4787
NC_005100.4-115308609	TATGAAAGTACTTAACCTCCATAGGTT	16	C	0.2049	0.3667
NC_005100.4-115436692	CAGTTGAAGACAAATTCTCCACACTGT	13	A	0.0086	0.4729
NC_005100.4-115453804	CCTCTCTCGACCTCCTCTCCCCATACC	6	C	0.0847	0.1116
NC_005100.4-115472476	TGCAACACCACCTTCACTCCTCCAATG	8	C	0.1579	0.3424
NC_005100.4-115601630	GAGGGCATTACCCCCTCTCCTGCTCCA	5	G	0.069	0.1134
NC_005100.4-115604000	TCTTTAGAAACATGTCCTCCATTGCCT	14	G	0.2411	0.2876
NC_005100.4-115604000	TCTTTAGAAACATGTCCTCCATTGCCT	16	C	0.1786	0.4974
NC_005100.4-115604370	ATCATGCAGACCCTGTCTCCTACAGGT	8	A	0.0973	0.0926
NC_005100.4-115604370	ATCATGCAGACCCTGTCTCCTACAGGT	13	C	0.2212	0.2738
NC_005100.4-115648973	GACTTGTTTACAGCAGCTCCTACACTG	2	A	0	0.4773
NC_005100.4-115648973	GACTTGTTTACAGCAGCTCCTACACTG	7	T	0	0.4773
NC_005100.4-115745368	AAACTGCCCACTAAGCCTCCATACACT	15	G	0.0504	0.065
NC_005100.4-115745368	AAACTGCCCACTAAGCCTCCATACACT	22	T	0.0084	0.4761
NC_005100.4-115753035	TGCCACAAAACCCTGGCTCCAGGCCCT	6	C	0	0.4787
NC_005100.4-115753035	TGCCACAAAACCCTGGCTCCAGGCCCT	22	G	0.1074	0.1163
NC_005100.4-115959425	GAACTTCAAACAAAGTCTCCCTTAGAA	4	C	0.0631	0.0611
NC_005100.4-115983644	CGCTTGATCACCATCTCTCCCTTATTC	2	G	0.2018	0.2719
NC_005100.4-115983644	CGCTTGATCACCATCTCTCCCTTATTC	13	A	0.0526	0.0677
NC_005100.4-116039196	TGTATAACAACTATGCCTCCTTGCTTT	22	T	0.1062	0.1757
NC_005100.4-116055356	AGGTTGGGAACATTATCTCCTCAAAGG	6	G	0.1532	0.1857
NC_005100.4-116097596	CAGCCTAAAACGTCTTCTCCCAGAGAA	16	T	0.1351	0.1857
NC_005100.4-116097596	CAGCCTAAAACGTCTTCTCCCAGAGAA	22	A	0.1351	0.1857

NC_005100.4-116098107	CCAGTACTCACATGTCCTCCCTCATAC	12	A	0.1653	0.4959
NC_005100.4-116098107	CCAGTACTCACATGTCCTCCCTCATAC	26	A	0.0833	0.18
NC_005100.4-116116197	GCCACCATCACAGCCCCTCCCCCACTA	22	C	0.0508	0.0812
NC_005100.4-116117713	ATGACAGGGACTTCTACTCCTCTGCTG	7	G	0.0588	0.4542
NC_005100.4-116117713	ATGACAGGGACTTCTACTCCTCTGCTG	9	G	0.0254	0.0251
NC_005100.4-116133861	CTGTCCCTCACCAGAACTCCAGTGTCA	7	C	0.0407	0.0398
NC_005100.4-116153193	ACACAGTACACATCCCCTCCAGAAACT	23	A	0.0088	0.4774
NC_005100.4-116162312	CCATCTGCTACTCCAGCTCCATGGATT	16	G	0.2541	0.3984
NC_005100.4-116170921	AATAAAGTAACTGAGCCTCCTAAACTC	13	G	0.0088	0.4873
NC_005100.4-116663388	GTGCCCTCCACCCCCGCTCCCCATTCT	16	G	0.0085	0.0253
NC_005100.4-118524179	AGGAATGGTACATGATCTCCTACCCTT	14	G	0	0.482
NC_005100.4-119547656	CTCACAGACACAGGCACTCCTTTGGGT	4	A	0	0.4846
NC_005100.4-119547656	CTCACAGACACAGGCACTCCTTTGGGT	24	G	0	0.4846
NC_005100.4-119561085	ATGCTCTAAACTTTGCCTCCCTATGCA	1	A	0.5897	0.4227
NC_005100.4-119561085	ATGCTCTAAACTTTGCCTCCCTATGCA	25	G	0.5948	0.4248
NC_005100.4-119675176	TCTAATATAACATATGCTCCATACTTC	1	T	0.2212	0.1968
NC_005100.4-119913943	CCCTCTCTCACGCCCTCTCCTTCTCCC	5	C	0.1008	0.1254
NC_005100.4-119913943	CCCTCTCTCACGCCCTCTCCTTCTCCC	12	G	0.0924	0.0882
NC_005100.4-120237294	CACACACACACAAACTCTCCATTCAAT	15	C	0.4622	0.3554
NC_005100.4-120237294	CACACACACACAAACTCTCCATTCAAT	23	T	0.4831	0.4776
NC_005100.4-120744352	ATGGAGAGAACATTGACTCCAACCACA	8	G	0	0.48
NC_005100.4-120848795	TTGGACAATACCTGAGCTCCTTTCTAT	22	T	0	0.4765
NC_005100.4-120871361	GTGAGAGCTACCCACACTCCCACTTTT	8	C	0	0.4773
NC_005100.4-120898254	TGTTGAAAAACCAGAGCTCCCAGGGAA	1	T	0.0265	0.0262
NC_005100.4-120898254	TGTTGAAAAACCAGAGCTCCCAGGGAA	6	A	0.0796	0.1239
NC_005100.4-120958940	TTCGAATCTACCTTCTCTCCATTCCTC	3	C	0.0351	0.0512
NC_005100.4-121163597	CACATCTCCACTCTTACTCCTTGACCC	26	C	0	0.4654
NC_005100.4-121239378	GGGTTGACTACTCTGTCTCCTGTGTTG	5	T	0	0.4793
NC_005100.4-121354631	TTGGAATTTACCACAGCTCCGTAACAT	4	G	0.042	0.0411
NC_005100.4-122300277	CCTTCTGTCACCACTCCTCCTCATAAA	1	C	0.0569	0.0854
NC_005100.4-122349887	GGGTATGTCACTAGAACTCCTTTTGCT	1	G	0.1667	0.5
NC_005100.4-122359983	CCCTACCTTACCAATCCTCCCGACTTG	9	T	0	0.4819
NC_005100.4-122395027	AGATTGTGCACAGGGACTCCAATGGAG	24	G	0.0248	0.0245
NC_005100.4-122668560	CTAATTGCAACGTCCTCTCCCTCCACT	15	C	0.1062	0.3346
NC_005100.4-122799530	GGCTCTGTAACCAATTCTCCTCTGAGT	8	T	0	0.4765
NC_005100.4-122817733	CTTCTCTATACATCCACTCCAACTCAC	15	C	0.1696	0.1553
NC_005100.4-122817733	CTTCTCTATACATCCACTCCAACTCAC	21	A	0	0.474
NC_005100.4-122830400	ACCATTCATACAGCGTCTCCACAAGTC	13	G	0	0.4793
NC_005100.4-122830400	ACCATTCATACAGCGTCTCCACAAGTC	25	G	0.0328	0.0322
NC_005100.4-122844356	GGACCAAGCACCTGCCCTCCCAGTGAT	12	C	0.2672	0.2315
NC_005100.4-122902045	CCTGCAGTTACCTTATCTCCAGCATAA	4	G	0	0.4688
NC_005100.4-122927902	CGATCCAGCACTCACACTCCTAGTTAT	1	C	0.0708	0.406
NC_005100.4-122927902	CGATCCAGCACTCACACTCCTAGTTAT	22	A	0	0.4755
NC_005100.4-122929654	CCACTGCACACTTCCCCTCCCAGCTTT	2	C	0	0.4793
NC_005100.4-122929654	CCACTGCACACTTCCCCTCCCAGCTTT	3	A	0	0.4793
NC_005100.4-123035699	TCTGCTCCTACTTAAGCTCCTGACCTG	14	A	0	0.477
NC_005100.4-123035699	TCTGCTCCTACTTAAGCTCCTGACCTG	21	T	0	0.477
NC_005100.4-123066661	TTAAACATAACAGGTACTCCACAATCA	14	G	0.0446	0.0436
NC_005100.4-123067234	ACTCTGAACACCGTGTCTCCTCAAGCT	12	C	0.1057	0.2556
NC_005100.4-123094906	TCAGCCCATACCACCTCTCCTTTGGCT	8	A	0.0672	0.0805
NC_005100.4-123123866	GAGACACTGACATTCACTCCATTTAAA	12	A	0	0.4846

NC_005100.4-123248580	CATTCTACAACCATCTCTCCTCCATAT	2	A	0.1057	0.3287
NC_005100.4-123305710	TACCAACCAACCAGAGCTCCCAGCCAC	1	T	0.0088	0.0429
NC_005100.4-123305710	TACCAACCAACCAGAGCTCCCAGCCAC	23	G	0.1316	0.1672
NC_005100.4-123540291	CATACTTAAACCTGCTCTCCCTTTCTT	26	T	0.0084	0.4723
NC_005100.4-123665114	CCCAGGGACACAGGAACTCCCAACCTG	7	G	0.0625	0.1093
NC_005100.4-123703690	AATTTATTGACTCTACCTCCAGTACAA	6	A	0.1875	0.4824
NC_005100.4-123703690	AATTTATTGACTCTACCTCCAGTACAA	25	C	0.0536	0.0853
NC_005100.4-123740036	TTTCTCCTAACACATGCTCCCTTCATG	2	T	0.0089	0.4824
NC_005100.4-123783773	CTTCCTTCTACTTGAGCTCCATATGAC	21	A	0.027	0.0267
NC_005100.4-123826037	TTGCTACAGACACACTCTCCTTTATCT	4	C	0.0087	0.4704
NC_005100.4-123826037	TTGCTACAGACACACTCTCCTTTATCT	5	T	0.1565	0.4017
NC_005100.4-124037765	AGCCTTTACACCTCCGCTCCCTCCCTG	15	C	0.2203	0.334
NC_005100.4-124037765	AGCCTTTACACCTCCGCTCCCTCCCTG	27	G	0.2881	0.3792
NC_005100.4-124040480	AGTCATCAGACTGTTGCTCCTGTGGGA	4	C	0.0417	0.0408
NC_005100.4-124116168	ATGCCCTCTACTCTCCCTCCTCTTACT	3	G	0.2562	0.313
NC_005100.4-124201930	AGTCCCCCTACCTGTCCTCCTTCACAT	6	C	0.0413	0.0405
NC_005100.4-124210768	TGGGGAAAAACTGAGTCTCCTTAGGGG	3	G	0	0.4793
NC_005100.4-124210768	TGGGGAAAAACTGAGTCTCCTTAGGGG	6	A	0.2155	0.4435
NC_005100.4-124218849	ACTTTCTACACAGCCTCTCCAGCCTGT	8	A	0.1967	0.4988
NC_005100.4-124218849	ACTTTCTACACAGCCTCTCCAGCCTGT	9	C	0.187	0.208
NC_005100.4-124286085	GACCGTCTAACAGAAACTCCAACACCA	5	G	0.2982	0.4555
NC_005100.4-124317712	AAGAATCAGACTGTGTCTCCTTCCGTG	4	A	0.1736	0.2355
NC_005100.4-124317712	AAGAATCAGACTGTGTCTCCTTCCGTG	24	C	0.0083	0.4835
NC_005100.4-124322162	TCCAACAAGACCCTCCCTCCTCCATTG	7	A	0.0244	0.0241
NC_005100.4-124341462	CCCACTGACACAGATACTCCTTTCTTC	14	A	0.0345	0.0666
NC_005100.4-124341462	CCCACTGACACAGATACTCCTTTCTTC	16	A	0	0.4637
NC_005100.4-124344080	CAGTCATCCACACATCCTCCCCTAGTC	2	A	0.0244	0.0241
NC_005100.4-124370380	TGGCTTTTTACTTTAGCTCCTAGAGCC	9	T	0	0.4751
NC_005100.4-124370380	TGGCTTTTTACTTTAGCTCCTAGAGCC	24	A	0.1074	0.2109
NC_005100.4-124383434	TGAGCCAAAACAAACACTCCCACCCTT	5	C	0	0.4693
NC_005100.4-124393374	GGAACTCCTACAGGGCCTCCTTGTAAC	25	A	0.0244	0.484
NC_005100.4-124433595	ATCCTCCCTACTATCTCTCCACCCACC	14	T	0.0345	0.482
NC_005100.4-124496249	CTGACTGATACCTTTCCTCCCCCATCC	24	A	0	0.4819
NC_005100.4-124512530	GTGGGTGTCACCCGTCCTCCTTCCTTC	13	C	0	0.4793
NC_005100.4-124551087	ACAAGTTGGACTAGCTCTCCAAATGCC	2	C	0.0086	0.4643
NC_005100.4-124564977	CCTGGGTAGACATTTACTCCTCGATGA	12	A	0	0.4813
NC_005100.4-124565168	CTTCCGTGGACACAGCCTCCTTGGTGG	5	C	0.0244	0.0241
NC_005100.4-124630869	ACAGATATTACGTGTGCTCCTAGCACA	12	G	0.1557	0.2903
NC_005100.4-124642130	AAGAGGGACACTTGCCCTCCAAAGGCA	4	A	0.0086	0.4595
NC_005100.4-124642130	AAGAGGGACACTTGCCCTCCAAAGGCA	23	A	0.0086	0.4595
NC_005100.4-124650303	GACCCAGCTACTCTGTCTCCTAGTCCA	5	C	0.0424	0.481
NC_005100.4-124650303	GACCCAGCTACTCTGTCTCCTAGTCCA	16	T	0.0847	0.1551
NC_005100.4-124665159	GCCCTGACCACTGTGCCTCCATCCCCT	9	C	0	0.4649
NC_005100.4-124665159	GCCCTGACCACTGTGCCTCCATCCCCT	24	C	0	0.4649
NC_005100.4-124671225	GTGGCTTATACCTTGGCTCCCAACACT	26	C	0.0619	0.4774
NC_005100.4-124739094	ATTGCACATACGTTAGCTCCTCCATAG	23	C	0	0.4807
NC_005100.4-124739094	ATTGCACATACGTTAGCTCCTCCATAG	25	T	0	0.4807
NC_005100.4-124741854	ACCTTCTTTACCCATTCTCCCAGCCAC	24	C	0.027	0.0267
NC_005100.4-124760927	ATCAATGAAACCTGCTCTCCTTTAAGA	23	T	0.1897	0.5
NC_005100.4-124765071	AGCCTGCTGACACCTCCTCCTACTGTC	12	A	0	0.4787
NC_005100.4-124771652	AATTTTCGTACGGCACCTCCCAGAGCA	8	G	0.0619	0.4435

NC_005100.4-124771652	AATTTTCGTACGGCACCTCCCAGAGCA	15	A	0	0.4846
NC_005100.4-124775425	TGTGAAGGCACTGGCCCTCCCAAGTAA	2	G	0	0.4793
NC_005100.4-124775425	TGTGAAGGCACTGGCCCTCCCAAGTAA	3	T	0	0.4793
NC_005100.4-124824842	CAGATCATTACTGATGCTCCCTTGACC	26	C	0.0569	0.0854
NC_005100.4-124847429	TGTCCTTTGACCTTCACTCCTGCACCA	14	T	0	0.4757
NC_005100.4-124869930	GGGGCCAACACCCCTTCTCCAGCCGCG	26	C	0.0957	0.1369
NC_005100.4-124893978	CTGCACGGGACCCAGACTCCTGGGGAG	21	T	0.123	0.2218
NC_005100.4-124897152	GCCTTTACCACCAGGGCTCCTGTGCAG	6	T	0.1475	0.4979
NC_005100.4-124897152	GCCTTTACCACCAGGGCTCCTGTGCAG	27	G	0.0246	0.4717
NC_005100.4-124960976	ATGGGTTTCACAGTACCTCCTCTTACT	16	C	0	0.4751
NC_005100.4-124969396	TCCTGTCAAACTCTTCCTCCAGGGCTG	4	T	0.1441	0.1759
NC_005100.4-124969396	TCCTGTCAAACTCTTCCTCCAGGGCTG	8	A	0.0339	0.0333
NC_005100.4-125021264	AGGTCTGCAACTGCAGCTCCCAAAAGT	3	G	0.2957	0.3499
NC_005100.4-125021264	AGGTCTGCAACTGCAGCTCCCAAAAGT	9	A	0.3009	0.2797
NC_005100.4-125032758	GAGGGATCTACTGAGCCTCCACCTATA	4	G	0.1228	0.1153
NC_005100.4-125046575	GTGGGAGGCACAAGTCCTCCCTGCCTC	24	C	0.1545	0.4995
NC_005100.4-125055281	AGCAGGCCTACATCTCCTCCTTGCCAA	27	A	0.1695	0.2344
NC_005100.4-125078052	AAAAAAACAACACACACTCCTCGTATC	14	A	0.0163	0.4825
NC_005100.4-125132716	GTGCCAAGCACCTGACCTCCTTTCATA	9	C	0.0325	0.032
NC_005100.4-125150841	AATCAGCGAACCCTGACTCCCTAGTGG	7	C	0.0407	0.4868
NC_005100.4-125155500	GGTCTAGCCACCATGTCTCCCAGCCAG	1	G	0.187	0.1954
NC_005100.4-125164285	AGCAAAGCGACATGACCTCCTCTATGT	13	T	0.2368	0.3473
NC_005100.4-125186233	CACAATTCAACAATGGCTCCAGGATCA	5	A	0.2683	0.3381
NC_005100.4-125239043	AGACAAATAACCGCCCCTCCAACTGCA	9	A	0.2768	0.3705
NC_005100.4-125239043	AGACAAATAACCGCCCCTCCAACTGCA	15	C	0.2522	0.3547
NC_005100.4-125274505	GGTCTCTGGACTCTTCCTCCTCCAGCA	13	C	0.2072	0.1999
NC_005100.4-125305541	ATGTGTATTACAGTTACTCCACTGTCT	1	A	0.0342	0.0336
NC_005100.4-125347403	GGGATGGCTACCTCGCCTCCAGTTGTT	15	G	0.0179	0.477
NC_005100.4-125347403	GGGATGGCTACCTCGCCTCCAGTTGTT	16	C	0.0625	0.0605
NC_005100.4-125349031	CATTAAGCCACACCCTCTCCCATTTAA	5	A	0.0357	0.0351
NC_005100.4-125360345	GACATGAGTACTAGGCCTCCTGGCTGG	4	A	0.1463	0.377
NC_005100.4-125371566	CTGCTTACTACCTGCCCTCCCAGGGTC	16	C	0.1217	0.4234
NC_005100.4-125462111	CCGTGGATGACCCAAGCTCCATCCTTG	3	G	0.0574	0.0557
NC_005100.4-125462111	CCGTGGATGACCCAAGCTCCATCCTTG	23	C	0.3471	0.4582
NC_005100.4-125483233	CTCTGGTTAACGTTCCCTCCCTTGGAC	24	G	0.0984	0.0935
NC_005100.4-125492278	AGGTTCATCACAGCATCTCCTAAGCGA	2	G	0.0167	0.4832
NC_005100.4-125492278	AGGTTCATCACAGCATCTCCTAAGCGA	13	G	0.3025	0.3118
NC_005100.4-125495195	TGACAGGCCACACGATCTCCGATTATT	13	C	0.05	0.4832
NC_005100.4-125495195	TGACAGGCCACACGATCTCCGATTATT	25	A	0.0583	0.0566
NC_005100.4-125523593	TTGGCTCTGACCTTTCCTCCTCTGCCA	12	C	0.15	0.2061
NC_005100.4-125523593	TTGGCTCTGACCTTTCCTCCTCTGCCA	27	A	0.0083	0.4847
NC_005100.4-125539482	CAGAGGAAGACCCAAACTCCCCCAGCA	16	A	0	0.4718
NC_005100.4-125559957	TGTCCTAAAACCTGTCCTCCAGAAATG	24	A	0.0084	0.4682
NC_005100.4-125575909	CACACAAACACAGACCCTCCCTACATA	1	C	0.0492	0.048
NC_005100.4-125575909	CACACAAACACAGACCCTCCCTACATA	13	G	0.0492	0.048
NC_005100.4-125605800	TCAGGCACCACTGTGTCTCCATCCTCT	21	A	0.0431	0.0422
NC_005100.4-125616952	TGATTCTGTACCCCAACTCCTTCTAGA	4	T	0.2049	0.4852
NC_005100.4-125626757	AATGGCTCTACTCTCTCTCCCAACTCC	14	T	0.1034	0.0981
NC_005100.4-125626757	AATGGCTCTACTCTCTCTCCCAACTCC	27	C	0	0.4826
NC_005100.4-125636722	GTCTCTATGACTTGACCTCCTCTGAGA	22	C	0	0.4832
NC_005100.4-125636722	GTCTCTATGACTTGACCTCCTCTGAGA	23	T	0	0.4832

NC_005100.4-125647134	AGGTACACCACACTGCCTCCGGTTTTC	21	G	0.2137	0.1908
NC_005100.4-125710303	TGACTCAGGACTTGGTCTCCAGGACAT	27	T	0.1441	0.1621
NC_005100.4-125713736	AACAAGACTACCTAATCTCCTTTTGTC	8	C	0.0446	0.4933
NC_005100.4-125720508	CAGATGATCACCAACACTCCCAGCTTA	5	T	0.0526	0.4875
NC_005100.4-125796746	ACATGCACAACCACACCTCCCTTCCAT	21	C	0.2195	0.3287
NC_005100.4-125798928	CACAGAGATACCACGTCTCCAAGGTGA	25	T	0.0431	0.0422
NC_005100.4-125847658	ACCAAATGCACCCAGGCTCCCATTGAT	9	C	0.1261	0.188
NC_005100.4-125871489	GTGTGCATAACCACTTCTCCCTCCGCC	1	G	0.0345	0.0504
NC_005100.4-125871489	GTGTGCATAACCACTTCTCCCTCCGCC	25	G	0.1504	0.4994
NC_005100.4-125940622	TGTCCTATCACATGATCTCCTCTCATA	26	T	0.0081	0.481
NC_005100.4-126012939	TACACAAAGACACTCGCTCCACCATGT	8	A	0	0.4793
NC_005100.4-126097026	CTGTTGGATACTTCAGCTCCAATGTCC	22	A	0.0855	0.0818
NC_005100.4-126099419	GCCATATTTACCCATTCTCCTGCTAGG	24	T	0	0.4814
NC_005100.4-126139725	TCATGATGGACTGTATCTCCTGAAACT	2	C	0.0084	0.4797
NC_005100.4-126139725	TCATGATGGACTGTATCTCCTGAAACT	4	T	0.1583	0.1458
NC_005100.4-126148675	AAGCTGCTGACTCTTCCTCCCTTTATA	13	C	0	0.4793
NC_005100.4-126148675	AAGCTGCTGACTCTTCCTCCCTTTATA	26	T	0	0.4793
NC_005100.4-126184381	CTCATATACACACTATCTCCACCACGC	5	T	0	0.4693
NC_005100.4-126265864	CCAAGCAGGACAAAGACTCCTTGCACA	9	G	0.2269	0.3893
NC_005100.4-126275754	GGAGCCGTGACTCCTTCTCCTTCATTT	7	G	0.2276	0.3688
NC_005100.4-126293097	TGCCCTGCCACACGCCCTCCCTTCCTG	13	C	0.1639	0.4698
NC_005100.4-126336226	GACTCCCATACCTTTTCTCCAACTCCC	12	C	0.065	0.0629
NC_005100.4-126347953	ACAGTGTAAACATGGCCTCCACCTCTC	24	T	0.0082	0.479
NC_005100.4-126384123	ATCAGGTTCACTAAAGCTCCTCCTTCC	23	C	0.0164	0.4807
NC_005100.4-126395605	ACACTGTATACCGCCTCTCCCTCTCCC	9	T	0	0.4793
NC_005100.4-126395605	ACACTGTATACCGCCTCTCCCTCTCCC	27	C	0.0488	0.0476
NC_005100.4-126401310	GTGGTGTTGACATCTCCTCCCTGACCC	14	C	0.1607	0.5
NC_005100.4-126401310	GTGGTGTTGACATCTCCTCCCTGACCC	15	T	0.1504	0.154
NC_005100.4-126404190	AGCATGGGGACCCCAGCTCCATCCCCA	8	G	0.1707	0.2441
NC_005100.4-126434693	AAACAGGATACTAGGCCTCCATGTCTG	3	A	0.0081	0.4741
NC_005100.4-126434693	AAACAGGATACTAGGCCTCCATGTCTG	16	C	0.1789	0.4992
NC_005100.4-126462299	ACAGTATTTACAGAAACTCCCTGATCT	9	T	0	0.4749
NC_005100.4-126491747	TATATGGAAACTTTGCCTCCAATAGAA	13	T	0.3058	0.4769
NC_005100.4-126492639	CTGACCGCAACCACGTCTCCTGCCATG	7	G	0.065	0.078
NC_005100.4-126509065	CTGTAAGCTACAGGGTCTCCTCTCAGG	1	C	0.0261	0.0425
NC_005100.4-126514317	GTAGCATGCACAAAGCCTCCAGTCCCT	15	G	0.0982	0.4751
NC_005100.4-126519858	AGTGTTTCCACCTTTGCTCCACCAAGA	16	G	0.0424	0.0415
NC_005100.4-126579050	ATATTTTATACTGCTCCTCCCGTGGAG	22	G	0.313	0.4637
NC_005100.4-126579050	ATATTTTATACTGCTCCTCCCGTGGAG	25	G	0.0254	0.0251
NC_005100.4-126581161	TCCCATGTTACATGTGCTCCTGTCCAT	4	C	0.2439	0.3141
NC_005100.4-126581161	TCCCATGTTACATGTGCTCCTGTCCAT	5	A	0.0082	0.4822
NC_005100.4-126689806	ATGCTTAGTACTGTCCCTCCATTCTGC	21	A	0.075	0.0875
NC_005100.4-126692708	GGTTCCATTACTGAGGCTCCTGGGCAG	13	G	0.1951	0.189
NC_005100.4-126706991	TCCCTCCAAACATGGCCTCCTTTCTTT	3	C	0.0081	0.0553
NC_005100.4-126714874	CCCTACAGAACTACAGCTCCAGTCAGT	5	A	0	0.4856
NC_005100.4-126758996	GGAAACCTTACCCGTTCTCCTTCCTTA	16	T	0.0348	0.0671
NC_005100.4-126815318	GAATCCATGACACTCTCTCCCTGAGTC	5	C	0.0435	0.059
NC_005100.4-126830284	ATGTCATCCACATGGTCTCCCTGATCC	15	G	0.0087	0.4782
NC_005100.4-126842252	GAGCGGGCAACACCCACTCCTACTGTT	5	G	0.1322	0.4942
NC_005100.4-126847387	CATGACTCAACCCCCACTCCCACTGTT	16	A	0	0.4839
NC_005100.4-126854156	AGGTTAGCAACCTTGACTCCATGTATA	26	T	0.1217	0.4981

NC_005100.4-126862131	CCCTTTGTGACAGGGCCTCCATCAGCT	21	A	0	0.4851
NC_005100.4-126862131	CCCTTTGTGACAGGGCCTCCATCAGCT	26	C	0.1652	0.4903
NC_005100.4-126879133	CCGTGTGGAACAGCTCCTCCTCCCCTG	12	A	0.0776	0.1209
NC_005100.4-126879133	CCGTGTGGAACAGCTCCTCCTCCCCTG	26	T	0.1593	0.4996
NC_005100.4-126886243	TTTGCTTCCACACTCTCTCCAGTACAA	3	T	0.2213	0.2457
NC_005100.4-126898940	TGCAGAGCAACCTGTCCTCCAGGTTGT	2	G	0.0609	0.059
NC_005100.4-126898940	TGCAGAGCAACCTGTCCTCCAGGTTGT	22	G	0.0696	0.0989
NC_005100.4-126909497	TGTAATCCAACAGAACCTCCTCTCTCC	23	T	0.2544	0.295
NC_005100.4-126925976	CTGCACAGCACACTGACTCCGGAAGCC	6	C	0.0083	0.4769
NC_005100.4-126972438	CACAGAATAACAAACTCTCCAGTGGTG	13	A	0.2	0.27
NC_005100.4-126974523	ATAGACATTACTCCTGCTCCTGAAATA	22	G	0.1949	0.3388
NC_005100.4-126984475	CAAGCATACACCCTCCCTCCTAACGCT	6	A	0	0.4749
NC_005100.4-126984475	CAAGCATACACCCTCCCTCCTAACGCT	25	G	0.0517	0.0504
NC_005100.4-127009066	ATTCAGGCCACTTCAGCTCCATGAGCA	4	C	0.0407	0.0398
NC_005100.4-127009066	ATTCAGGCCACTTCAGCTCCATGAGCA	16	G	0.0244	0.0241
NC_005100.4-127095062	GACACAGAAACATAAGCTCCGAGGATA	5	C	0.0244	0.0241
NC_005100.4-127096286	ATCACCATCACCACATCTCCTCTCCGG	25	C	0.1681	0.4983
NC_005100.4-127130364	CTGCAATGGACGCATTCTCCCAACATC	6	A	0.3051	0.324
NC_005100.4-127130364	CTGCAATGGACGCATTCTCCCAACATC	23	A	0	0.4693
NC_005100.4-127139380	CACACACACACACACACTCCAGAGTGA	12	A	0.0488	0.0476
NC_005100.4-127139380	CACACACACACACACACTCCAGAGTGA	27	A	0.1057	0.1001
NC_005100.4-127152440	TCCTTCGAAACCCCAGCTCCTACATCT	7	G	0.0732	0.4471
NC_005100.4-127158998	AGGGATCTGACACTCTCTCCAGCCTTT	15	C	0	0.4807
NC_005100.4-127177095	GAAAAGATTACCTTGACTCCTCCTTCC	9	T	0.0917	0.1597
NC_005100.4-127177095	GAAAAGATTACCTTGACTCCTCCTTCC	14	T	0	0.4819
NC_005100.4-127186965	CGGGCATCAACCCTTGCTCCAAAAGAA	2	G	0.2456	0.4986
NC_005100.4-127197994	GTCGCCTGCACCAAAACTCCCCACTAT	4	G	0	0.4654
NC_005100.4-127197994	GTCGCCTGCACCAAAACTCCCCACTAT	6	C	0.2478	0.4827
NC_005100.4-127202113	CCGAAGTACACGTGCTCTCCTCTAAGT	2	C	0.3197	0.311
NC_005100.4-127202113	CCGAAGTACACGTGCTCTCCTCTAAGT	12	G	0	0.4773
NC_005100.4-127241912	CCAGTGTGCACTCTGCCTCCTGCTTGC	4	G	0.0082	0.0243
NC_005100.4-127254113	TGAGGCTTTACCCCTCCTCCCCACCTC	26	T	0	0.4898
NC_005100.4-127284003	CAAGTTCTTACCATTGCTCCTGGGCAC	3	A	0	0.4807
NC_005100.4-127321385	CTGGAATTCACCATGCCTCCTGCCGAA	13	A	0.2759	0.375
NC_005100.4-127345224	CAAATCAGAACCGCTGCTCCATGGGCT	2	A	0	0.488
NC_005100.4-127377408	AAACTACTGACACCCGCTCCGCTTCGT	2	A	0.0083	0.4803
NC_005100.4-127397652	AAGGCAATGACTGCTCCTCCCTGAATC	7	A	0	0.4755
NC_005100.4-127397652	AAGGCAATGACTGCTCCTCCCTGAATC	15	T	0	0.4755
NC_005100.4-127403773	AGTAAGTCTACATCGTCTCCCATGGAC	14	C	0	0.4773
NC_005100.4-127461652	TATACAGCAACCTAGCCTCCAACTCAT	24	T	0.0407	0.0398
NC_005100.4-127470739	ATCATTCAAACTCACTCTCCTACATCC	22	A	0.1875	0.498
NC_005100.4-127475827	TCACTGCTCACTCAGTCTCCTGTTCCC	16	T	0.0894	0.1001
NC_005100.4-127497965	TTCTCTTATACTGCCCCTCCCCCTTGT	9	T	0.0169	0.0496
NC_005100.4-127513507	AGGAGCAAGACAACTCCTCCCAAATTA	8	A	0.2288	0.4991
NC_005100.4-127579050	GATAGTTTGACTTTCTCTCCCTTCTTG	15	C	0.1786	0.1626
NC_005100.4-127602731	AGGTCCTGCACAAGGTCTCCAGTGCTC	8	G	0.0248	0.0245
NC_005100.4-127680960	AGTTGTATAACTTTTGCTCCTACATTT	23	C	0.1504	0.2494
NC_005100.4-127739673	AGCCTGGGAACTTCAGCTCCTCTCCAT	7	G	0.1157	0.1235
NC_005100.4-127739673	AGCCTGGGAACTTCAGCTCCTCTCCAT	24	C	0.124	0.2109
NC_005100.4-127760564	CACATCCAGACTTTCTCTCCTGTCTCC	16	T	0	0.4807
NC_005100.4-127765059	AGCTGTGGAACTCTGTCTCCTTTTCTC	1	A	0.0254	0.487

NC_005100.4-127765059	AGCTGTGGAACTCTGTCTCCTTTTCTC	3	C	0.0424	0.0415
NC_005100.4-127808139	ATGCTTGCTACGCTATCTCCCCGCTAA	23	G	0	0.4859
NC_005100.4-127820350	TGTTCCCCCACCACCACTCCCACATCT	12	C	0.3115	0.4515
NC_005100.4-127820786	TGACAAGACACCAGCGCTCCAATGCCC	16	G	0.2269	0.4638
NC_005100.4-127847275	CATCAGCCAACCCCGCCTCCTACCCTC	15	G	0.0417	0.4922
NC_005100.4-127850585	GGAGACATTACTAGGTCTCCCTCCATG	23	C	0.1607	0.3659
NC_005100.4-127850585	GGAGACATTACTAGGTCTCCCTCCATG	26	T	0.1786	0.4362
NC_005100.4-127861829	ATATGTCACACCCAGACTCCCAAGGCA	1	A	0.082	0.1082
NC_005100.4-127861829	ATATGTCACACCCAGACTCCCAAGGCA	8	A	0.0902	0.4943
NC_005100.4-127876156	TTCTGGTGAACCCCGGCTCCTCTGCCT	15	G	0.3033	0.4634
NC_005100.4-127876156	TTCTGGTGAACCCCGGCTCCTCTGCCT	26	C	0.1066	0.4943
NC_005100.4-127925109	CCGAGACACACTACAGCTCCCATGAGG	1	C	0.2727	0.4605
NC_005100.4-127925109	CCGAGACACACTACAGCTCCCATGAGG	15	A	0.1653	0.1653
NC_005100.4-127965883	CCAATCGTCACCTCTTCTCCTGGACTG	7	G	0.2195	0.2885
NC_005100.4-127965883	CCAATCGTCACCTCTTCTCCTGGACTG	9	C	0.2114	0.2017
NC_005100.4-127986679	GCACTGCCCACTTGGACTCCTAGCAAG	14	G	0.25	0.2797
NC_005100.4-127995779	TACATCTTGACCTGCCCTCCTGCTGCC	23	C	0.3167	0.4688
NC_005100.4-128002513	CAAGCTTTGACATACACTCCCACATTT	7	T	0.2951	0.4891
NC_005100.4-128002513	CAAGCTTTGACATACACTCCCACATTT	14	A	0.2459	0.3791
NC_005100.4-128032500	GGGCGAGGCACTGAGACTCCACAAGAG	4	C	0.2185	0.3686
NC_005100.4-128032932	CGAGATCCAACCCCTACTCCCCAGTTA	2	G	0.0909	0.5
NC_005100.4-128032932	CGAGATCCAACCCCTACTCCCCAGTTA	26	T	0	0.4819
NC_005100.4-128066873	GGGGCTAGAACCAAGTCTCCTGGACCT	22	G	0	0.4773
NC_005100.4-128066873	GGGGCTAGAACCAAGTCTCCTGGACCT	27	T	0	0.4773
NC_005100.4-128078739	CAACTCCACACTGTTCCTCCTTCACAC	3	A	0.0336	0.4873
NC_005100.4-128098143	GGATAGTTAACCTGGCCTCCTCCGTAG	24	G	0.0965	0.235
NC_005100.4-128098143	GGATAGTTAACCTGGCCTCCTCCGTAG	26	A	0.0708	0.4464
NC_005100.4-128290663	CAAACGCAAACCTCCTCTCCACACAAC	7	C	0.1818	0.4787
NC_005100.4-128356131	TACTCGTGTACATTCTCTCCAACAATG	22	A	0	0.4807
NC_005100.4-128373313	CACCACCTAACACTCCCTCCATGCACA	1	C	0.0259	0.0255
NC_005100.4-128412396	CTGCAGTTTACATTGGCTCCATGCAGT	16	G	0.0164	0.0322
NC_005100.4-128417656	GAATTTGCCACCCACCCTCCAGGCCTC	23	G	0.3162	0.4158
NC_005100.4-128419332	TGTCCTTGGACTTTAACTCCTTCCTTA	27	A	0	0.4779
NC_005100.4-128445730	CCTGAGGTCACCCAGTCTCCAGCAGCT	22	G	0.0504	0.0491
NC_005100.4-128447967	AGCCCCTCCACCTTCCCTCCCAGTTAA	14	T	0.2377	0.4943
NC_005100.4-128472642	GCCAGGGGTACTGTACCTCCTCCTTTC	8	G	0.187	0.4935
NC_005100.4-128472642	GCCAGGGGTACTGTACCTCCTCCTTTC	15	A	0.2114	0.4881
NC_005100.4-128480520	TATAATCACACCCCTGCTCCCACAATA	2	A	0.0087	0.4782
NC_005100.4-128480520	TATAATCACACCCCTGCTCCCACAATA	14	C	0.0522	0.4864
NC_005100.4-128485125	TGAGGCGTTACCCTTGCTCCAGTGATT	6	C	0	0.4807
NC_005100.4-128513998	CCTAAACATACATTGTCTCCACTCCTT	5	A	0.0833	0.0799
NC_005100.4-128528026	CAGCTAACAACTGCAACTCCCAGATCT	6	A	0	0.4793
NC_005100.4-128528026	CAGCTAACAACTGCAACTCCCAGATCT	13	G	0.2712	0.3707
NC_005100.4-128552678	GTCAGGACCACTCCCGCTCCCTACTTT	27	T	0	0.4773
NC_005100.4-128565609	GGAGGCATCACTGTGGCTCCCACATGG	13	G	0.1	0.1244
NC_005100.4-128565609	GGAGGCATCACTGTGGCTCCCACATGG	26	G	0.0496	0.0484
NC_005100.4-128587726	CTCACACACACACATACTCCCCCAAGC	8	A	0.2137	0.3
NC_005100.4-128587726	CTCACACACACACATACTCCCCCAAGC	22	C	0.2414	0.4709
NC_005100.4-128593342	GAAAGTCACACCTGCGCTCCCTGACAG	16	G	0	0.4793
NC_005100.4-128657969	TTCCCTGAGACATCTGCTCCGCTCTGT	21	G	0	0.4751
NC_005100.4-128667624	GGTCTGGGGACAGTTTCTCCTTTCTCA	26	C	0.042	0.0411

NC_005100.4-128680804	ACACTACTCACAGCCCCTCCTCAGCGG	4	C	0.2288	0.3483
NC_005100.4-128680804	ACACTACTCACAGCCCCTCCTCAGCGG	6	A	0	0.4793
NC_005100.4-128687329	TTTCTGTCTACAGGATCTCCGAGACTC	14	G	0.2397	0.2814
NC_005100.4-128687329	TTTCTGTCTACAGGATCTCCGAGACTC	24	A	0	0.4751
NC_005100.4-128708352	GACTATATCACGGAAGCTCCAGTGACA	12	G	0.2459	0.285
NC_005100.4-128708352	GACTATATCACGGAAGCTCCAGTGACA	13	G	0.0813	0.0928
NC_005100.4-128729661	ACATTGCCTACTTCCACTCCTAAGACT	7	C	0.1311	0.1367
NC_005100.4-128753190	TATCTATTGACTGTGTCTCCTTTTCAT	5	T	0	0.4807
NC_005100.4-128753190	TATCTATTGACTGTGTCTCCTTTTCAT	16	T	0	0.4807
NC_005100.4-128760173	TATCTGCACACCAGAGCTCCCTCACCT	24	A	0.1368	0.1274
NC_005100.4-128760173	TATCTGCACACCAGAGCTCCCTCACCT	27	T	0.2759	0.4345
NC_005100.4-128776676	TATGTGTGCACGTGTACTCCAGGTGAA	2	A	0.1709	0.45
NC_005100.4-128788886	CACTCAGCCACTGTGACTCCCTCCGTG	2	A	0.0083	0.4803
NC_005100.4-128788886	CACTCAGCCACTGTGACTCCCTCCGTG	3	C	0.0083	0.4803
NC_005100.4-128810763	GTCTCACACACACTCTCTCCATTCCCA	23	T	0.2869	0.3492
NC_005100.4-128829885	CAGGATGCCACCTGCCCTCCCTTCCCT	5	A	0.082	0.0786
NC_005100.4-128843188	AAGATTTACACTTGAACTCCAATGGAC	25	G	0.094	0.0896
NC_005100.4-128866536	CCATGTTAGACCACATCTCCCTGGGAA	13	A	0	0.4793
NC_005100.4-128866536	CCATGTTAGACCACATCTCCCTGGGAA	23	G	0.2893	0.3421
NC_005100.4-128893159	CTGACTGTCACCTCCACTCCCTAGCCA	4	A	0.0345	0.0339
NC_005100.4-128939403	ACTGCAGTAACCGTGGCTCCCAGTGAC	5	C	0.0619	0.06
NC_005100.4-128958433	ATTTCTCCAACTCTTCCTCCATCGCTG	6	T	0.1885	0.1968
NC_005100.4-128958433	ATTTCTCCAACTCTTCCTCCATCGCTG	21	A	0.2314	0.4959
NC_005100.4-128983209	TACTGTGTCACTCCGCCTCCCTCCTAC	6	T	0.0492	0.4891
NC_005100.4-128987409	AGCATTTGAACTGGGTCTCCCGCTGCC	3	C	0.05	0.0488
NC_005100.4-128987409	AGCATTTGAACTGGGTCTCCCGCTGCC	4	A	0	0.4743
NC_005100.4-129000694	TGTTAGAGGACAGTGCCTCCTTTCAGC	15	G	0.0261	0.0257
NC_005100.4-129049727	CGGGCTTTAACTGAAGCTCCTTGAGGG	1	C	0.0179	0.0351
NC_005100.4-129049727	CGGGCTTTAACTGAAGCTCCTTGAGGG	12	T	0.009	0.4682
NC_005100.4-129089748	TTACTCAGCACTTTGACTCCACCAGAA	3	A	0	0.4875
NC_005100.4-129100480	TTCCATTCGACTTTTGCTCCTCTAAAG	16	G	0.0268	0.0264
NC_005100.4-129123172	TGGACCACCACAGCCCCTCCATAACCT	21	A	0.2143	0.4986
NC_005100.4-129123644	CAGTCTCCCACTAAGCCTCCAACTTAT	2	A	0.0331	0.4901
NC_005100.4-129127871	TTCACTCTAACACCGGCTCCAACTGTA	21	A	0	0.4751
NC_005100.4-129132951	TTTCTGTGGACTCTCTCTCCTGCCCTT	6	G	0.0427	0.0418
NC_005100.4-129132951	TTTCTGTGGACTCTCTCTCCTGCCCTT	13	C	0	0.4734
NC_005100.4-129136369	TGCTCAAGGACTGCCCCTCCCCCTTCC	16	C	0.0261	0.059
NC_005100.4-129178878	TCCTCGTACACACCATCTCCCCAATAC	24	A	0.292	0.3081
NC_005100.4-129179268	CTGATAAAGACCGATTCTCCTTCTCTC	21	T	0.114	0.138
NC_005100.4-129179268	CTGATAAAGACCGATTCTCCTTCTCTC	22	T	0.0263	0.026
NC_005100.4-129187861	CTATAGCCTACCACAACTCCAGCTGCC	4	T	0	0.4786
NC_005100.4-129216593	ACCTTCAGCACAGTAGCTCCCTTACTG	15	A	0	0.4773
NC_005100.4-129264311	AAACAAACTACCACCCCTCCCTCCATC	6	A	0.2368	0.2719
NC_005100.4-129264311	AAACAAACTACCACCCCTCCCTCCATC	23	C	0.0351	0.0345
NC_005100.4-129296947	AACTCTATAACCCTTACTCCAGGCTGG	2	A	0.1639	0.316
NC_005100.4-129296947	AACTCTATAACCCTTACTCCAGGCTGG	23	G	0.0976	0.1216
NC_005100.4-129316358	TGATGCTGGACTGCATCTCCATTGCAT	14	C	0.094	0.0896
NC_005100.4-129320170	CAGCTGCCGACTGTACCTCCCTTCTTG	7	C	0.2087	0.2758
NC_005100.4-129328741	CATATTCTAACTCTTGCTCCCGTATTT	23	T	0.1681	0.1946
NC_005100.4-129328741	CATATTCTAACTCTTGCTCCCGTATTT	26	T	0.1681	0.1946
NC_005100.4-129330902	GAAGAGGACACGGACACTCCCAGGCAG	12	G	0.1217	0.3772

NC_005100.4-129438849	GCTTGTTCAACTCTATCTCCTACTCTT	25	C	0.0508	0.0965
NC_005100.4-129446086	TTTGAATCAACAGACACTCCTGTGCTC	2	T	0.036	0.4931
NC_005100.4-129446086	TTTGAATCAACAGACACTCCTGTGCTC	4	G	0.1607	0.4539
NC_005100.4-129461109	TTGTTTTAAACCCACTCTCCATACCCC	23	A	0	0.4807
NC_005100.4-129476682	AGAAAGGGGACCGTCTCTCCTGACCTT	12	C	0.122	0.4935
NC_005100.4-129480212	TGGCAGTTGACCCAAGCTCCTCAGGAG	16	G	0.0252	0.0249
NC_005100.4-129535560	GGGAATGAAACAAGTCCTCCTTTGTTT	5	A	0	0.48
NC_005100.4-129609624	TCACTTTGAACTTTGTCTCCTGTTGAG	24	T	0.1696	0.1843
NC_005100.4-129620976	ACTGTGCTCACTTCTCCTCCTATATGT	13	T	0	0.4841
NC_005100.4-129635763	GGTTATGTCACTCACACTCCAGGACAA	16	A	0.1111	0.1634
NC_005100.4-129740275	CTTTGAACAACATCGTCTCCACATACA	15	G	0.2072	0.4804
NC_005100.4-129740275	CTTTGAACAACATCGTCTCCACATACA	23	A	0.125	0.3367
NC_005100.4-129769733	GCAGCACTGACGGGGCCTCCGCTCAGC	12	G	0.1696	0.2991
NC_005100.4-129770528	GCTCATGTAACAAACACTCCTCATATC	4	C	0.1897	0.4071
NC_005100.4-129770528	GCTCATGTAACAAACACTCCTCATATC	5	A	0.0088	0.4796
NC_005100.4-129950368	TGGGTGTTTACAGTAGCTCCGTTCATT	21	G	0	0.48
NC_005100.4-129955088	TCAACTTTTACTGTCCCTCCCGCTCTT	21	C	0.0085	0.0253
NC_005100.4-129978067	AAATTCCTTACCCAGCCTCCAAGTCCT	26	C	0.2411	0.3102
NC_005100.4-129982230	TTCCTGACTACAGACACTCCTTTATTC	16	A	0.0526	0.4875
NC_005100.4-130111623	CTGATTCAGACTCCCTCTCCTCATTAC	5	T	0.1057	0.1145
NC_005100.4-130111623	CTGATTCAGACTCCCTCTCCTCATTAC	21	T	0.2195	0.2989
NC_005100.4-130157543	ACTCTTATTACTAACTCTCCTCTCACT	1	A	0.0345	0.0504
NC_005100.4-130157543	ACTCTTATTACTAACTCTCCTCTCACT	2	C	0.0342	0.05
NC_005100.4-130248761	GAATACAGAACAGGGCCTCCTGCTCTG	23	C	0.1795	0.1908
NC_005100.4-130296411	ACCTTTTACACCAGACCTCCTCATTTC	4	T	0.0446	0.0436
NC_005100.4-130406982	GCCAGATGTACTTTTCCTCCCAGACAT	23	G	0.0244	0.0241
NC_005100.4-130415731	AGTTCATTTACAGTGGCTCCCTTACAG	4	T	0.0263	0.026
NC_005100.4-130477905	GGTCCTCCTACAGAGTCTCCCTCCTCC	4	C	0.124	0.1306
NC_005100.4-130539119	AGAAGCTGAACATTACCTCCCCTTAGT	22	C	0.0714	0.1771
NC_005100.4-130639233	CACATATAAACATTGTCTCCACGCTGC	6	A	0.0244	0.0241
NC_005100.4-130656098	AACAGAATAACAGGAACTCCTTGGTAA	14	G	0.084	0.4991
NC_005100.4-130697557	TGCCATTCTACCATTCCTCCTGGCAAG	4	C	0.2437	0.2961
NC_005100.4-130713579	TTCCAGAGGACCACAGCTCCTAGCATG	26	T	0.0909	0.0868
NC_005100.4-130713652	ATCTGATGCACCTGGCCTCCAACAGTA	26	T	0	0.4833
NC_005100.4-130798535	AAGAGTATAACGGAATCTCCTCATATT	24	T	0.0667	0.0644
NC_005100.4-130808053	CTCCCCTGCACTTCTCCTCCACCCTCA	4	C	0	0.4773
NC_005100.4-130837876	AGTTATCTTACAATGCCTCCATCCATG	5	A	0.0088	0.4843
NC_005100.4-130837876	AGTTATCTTACAATGCCTCCATCCATG	6	T	0.0088	0.4843
NC_005100.4-130877460	TTAATGGGCACTCAATCTCCCAGACAA	1	T	0.027	0.0267
NC_005100.4-130877460	TTAATGGGCACTCAATCTCCCAGACAA	16	T	0.027	0.0267
NC_005100.4-130894938	GGTGAAGACACCCAGCCTCCTCACCCC	4	G	0.0526	0.0677
NC_005100.4-130929572	AAACTTTAGACCCAACCTCCCTGCTAT	13	C	0.0085	0.4823
NC_005100.4-130933280	ATTTCGCATACTTTGTCTCCACCATGG	5	C	0.1083	0.1172
NC_005100.4-130940272	GTCCAGTCTACTCTTTCTCCCCCAACA	9	T	0.0263	0.026
NC_005100.4-130946051	TTTCAGCTAACGTCATCTCCATTGGAT	4	C	0.4956	0.3815
NC_005100.4-130946051	TTTCAGCTAACGTCATCTCCATTGGAT	12	G	0.4741	0.3876
NC_005100.4-131003450	AAATGCAGGACTAAGTCTCCGTTCAAG	6	C	0.0081	0.4777
NC_005100.4-131045657	AACCACCGAACCATCACTCCAGCCAGC	7	C	0.0342	0.0336
NC_005100.4-131086101	GTCTCCTTCACTCCCTCTCCAAAGTAT	6	C	0.2627	0.3575
NC_005100.4-131086101	GTCTCCTTCACTCCCTCTCCAAAGTAT	8	T	0.2627	0.3575
NC_005100.4-131124626	GGGGCATCCACCTTCTCTCCTTGCTTG	2	G	0.2195	0.2668

NC_005100.4-131164852	TTAAGGAAAACTGACTCTCCCTATCCC	8	A	0.2609	0.3684
NC_005100.4-131164852	TTAAGGAAAACTGACTCTCCCTATCCC	15	C	0.0431	0.0422
NC_005100.4-131166465	CCGATTCTCACAGTGGCTCCCCGAGTC	2	C	0	0.4839
NC_005100.4-131166465	CCGATTCTCACAGTGGCTCCCCGAGTC	22	C	0	0.4839
NC_005100.4-131249646	ATGTTGAATACATTCCCTCCTTGCTGT	23	G	0.0984	0.1082
NC_005100.4-131286457	GTGCTCTCAACATGGCCTCCTGGCATT	9	A	0.0172	0.488
NC_005100.4-131344561	ACAGAGAACACAAGCTCTCCTGTCCCA	4	G	0.1301	0.1761
NC_005100.4-131380864	CCTTCTCACACTCCTCCTCCGGCTGTG	1	C	0.0083	0.4835
NC_005100.4-131383866	CCTTTCACAACCCTCTCTCCATCAGTA	21	A	0	0.4807
NC_005100.4-131400663	CGCTCCCTAACCTCAGCTCCCACCTCC	1	C	0.0085	0.4882
NC_005100.4-131414362	CCTTTGGACACTCTTCCTCCTGTACGA	2	C	0.0175	0.474
NC_005100.4-131417084	TCCATCTCAACCATTTCTCCTCCTCTG	1	T	0.2149	0.2869
NC_005100.4-131434108	CCCTTGACAACTGTGGCTCCTGTGGCT	26	C	0.0165	0.0484
NC_005100.4-131463317	ATCCTAGCCACCTGGACTCCAGCCCCC	3	C	0.0609	0.059
NC_005100.4-131533688	TTTAGTGCGACTTTCTCTCCTGTGCTG	13	T	0.0442	0.0433
NC_005100.4-131569522	ACCCATGGCACAAGACCTCCTGGCAAT	24	C	0.0413	0.0716
NC_005100.4-131587253	GAGGATCAAACCCAGCCTCCACGCCGT	22	C	0.1652	0.1938
NC_005100.4-131587253	GAGGATCAAACCCAGCCTCCACGCCGT	25	C	0.2321	0.4004
NC_005100.4-131590857	GGGGAAGCCACAGAAGCTCCAGGCCTG	13	G	0.1967	0.4226
NC_005100.4-131663312	TCCTAGCAGACAATGCCTCCATAGGAT	5	A	0.0446	0.0436
NC_005100.4-131690062	TAAACCCTCACCCATACTCCCACAAGT	14	A	0.2131	0.4979
NC_005100.4-131705090	CACACACACACACACACTCCCACACAG	12	A	0.4065	0.3334
NC_005100.4-131724138	GCCATAAGTACCATCACTCCATCTATA	8	G	0.0342	0.0336
NC_005100.4-131729834	CATGAATGAACAGGAGCTCCACCCTGA	16	G	0.1949	0.4982
NC_005100.4-131852340	CACACACACACACACACTCCAAGACAT	15	C	0.4508	0.4717
NC_005100.4-131855519	CAATGCAAAACAGCAACTCCCTTCCCT	1	C	0.2276	0.3688
NC_005100.4-131856098	ACCAACTGAACTACATCTCCGGGCCAG	25	C	0.082	0.1082
NC_005100.4-131875589	CTGGACAGAACTGTGACTCCTATTCAG	16	A	0.0776	0.4916
NC_005100.4-131878481	GAACTTGATACTCAGCCTCCCTCACCC	12	T	0.0826	0.0792
NC_005100.4-131878481	GAACTTGATACTCAGCCTCCCTCACCC	21	C	0.124	0.1585
NC_005100.4-131898645	AAACCCCATACCTCTCCTCCCCTCCTC	8	A	0.2222	0.4807
NC_005100.4-131914301	AAGAACTCAACAATGTCTCCTTCCACA	15	G	0.0696	0.0671
NC_005100.4-131914301	AAGAACTCAACAATGTCTCCTTCCACA	26	C	0	0.4833
NC_005100.4-131957475	GGCTCTGAAACAATGGCTCCACCAAGT	12	A	0.2689	0.2796
NC_005100.4-131988698	AGCTCCAGCACGTCTTCTCCACATCTA	9	C	0.2542	0.2702
NC_005100.4-131995976	CCTGCTGCCACTGCTGCTCCAGTTAAT	24	T	0.0083	0.0247
NC_005100.4-132046023	ATTAGATCAACAAATCCTCCCCGTCCC	21	C	0.0354	0.0348
NC_005100.4-132202994	GGGACACAAACCTCTGCTCCTTTCCAC	22	T	0.1607	0.4986
NC_005100.4-132276301	CTCTCGGGGACAGCAGCTCCAGAGTGG	2	T	0	0.4734
NC_005100.4-132276301	CTCTCGGGGACAGCAGCTCCAGAGTGG	6	G	0	0.4734
NC_005100.4-132287447	TGAGTGTGGACTGTCGCTCCTTTTCAG	9	G	0.1197	0.1704
NC_005100.4-132287447	TGAGTGTGGACTGTCGCTCCTTTTCAG	16	G	0.2308	0.4947
NC_005100.4-132341955	GGATATATCACATGTCCTCCTTATCAA	2	G	0.042	0.0571
NC_005100.4-132341955	GGATATATCACATGTCCTCCTTATCAA	6	T	0.2564	0.3641
NC_005100.4-132422255	CATGGTCTTACGGTGACTCCTGTCTAT	12	G	0.2114	0.2723
NC_005100.4-132422255	CATGGTCTTACGGTGACTCCTGTCTAT	27	T	0	0.4793
NC_005100.4-132424133	CTGCCAAGAACCATGCCTCCATTGGGA	15	G	0.1	0.095
NC_005100.4-132496917	CAGACTGTTACTGACCCTCCCATCATT	16	C	0.2542	0.4173
NC_005100.4-132496917	CAGACTGTTACTGACCCTCCCATCATT	26	T	0	0.4856
NC_005100.4-132497918	TGGGCACGGACTGCCCCTCCCGTGATT	8	G	0.1293	0.291
NC_005100.4-132497918	TGGGCACGGACTGCCCCTCCCGTGATT	12	T	0	0.4813

NC_005100.4-132626202	TGTACACATACCTGCACTCCTCAGATC	1	T	0.1721	0.3401
NC_005100.4-132699016	GAAACTTCCACTCACCCTCCACCCTAG	5	C	0.0885	0.1757
NC_005100.4-132699016	GAAACTTCCACTCACCCTCCACCCTAG	23	C	0.0619	0.06
NC_005100.4-132717654	CTCGTTTGCACCTTACCTCCTTCCCTG	1	C	0	0.4793
NC_005100.4-132717654	CTCGTTTGCACCTTACCTCCTTCCCTG	4	G	0	0.4793
NC_005100.4-132725079	TTTGTCTTCACCTGCTCTCCTATTTGT	2	T	0	0.4793
NC_005100.4-132728154	ATGACCTGTACTTTTCCTCCGCATGCT	2	T	0.123	0.1708
NC_005100.4-132728154	ATGACCTGTACTTTTCCTCCGCATGCT	21	G	0	0.4751
NC_005100.4-132745155	AACACGCACACATCTTCTCCAGGAGCT	5	C	0.0702	0.0677
NC_005100.4-132771248	TTCAGTTGCACTTAAGCTCCTCATGTC	21	T	0	0.4793
NC_005100.4-132803236	TTACTTCATACTTAGTCTCCACCAATT	9	T	0	0.4773
NC_005100.4-132813405	GATGCTGTAACAGTGTCTCCTCTCAAA	23	T	0.0168	0.4661
NC_005100.4-132813405	GATGCTGTAACAGTGTCTCCTCTCAAA	24	C	0.0417	0.0408
NC_005100.4-132839283	AGTAGGTTTACATGGCCTCCTCCGTCC	24	G	0	0.4832
NC_005100.4-132863101	AGAACTAGAACACAGCCTCCTAACAGA	4	A	0.0172	0.4749
NC_005100.4-132863101	AGAACTAGAACACAGCCTCCTAACAGA	24	C	0.0517	0.0504
NC_005100.4-132882017	ATCCGAAGTACATCTTCTCCAGAGCTC	5	G	0.1	0.2188
NC_005100.4-132882017	ATCCGAAGTACATCTTCTCCAGAGCTC	8	G	0.0167	0.0328
NC_005100.4-132906176	CTGGGAAAGACAGTTACTCCAGCTTCT	5	G	0.0082	0.4755
NC_005100.4-132930360	GACTATCGAACAACAACTCCAGTGCCA	7	C	0.0531	0.4968
NC_005100.4-132930360	GACTATCGAACAACAACTCCAGTGCCA	26	C	0.0526	0.0677
NC_005100.4-133205557	TTACAACTCACTGTCCCTCCTACATTG	21	T	0	0.4807
NC_005100.4-133250258	GGCAGGAGTACATCAGCTCCATCTCAC	24	T	0.1207	0.1717
NC_005100.4-133250258	GGCAGGAGTACATCAGCTCCATCTCAC	27	C	0.0256	0.0253
NC_005100.4-133255433	CAAGTACAGACCCACTCTCCTGACTAT	25	T	0	0.4749
NC_005100.4-133260946	AAAGGTAGAACTCTAGCTCCTCCAGCA	5	G	0.1092	0.1033
NC_005100.4-133329994	ACTATGATCACATGTGCTCCCTATTTT	14	G	0.2833	0.2778
NC_005100.4-133329994	ACTATGATCACATGTGCTCCCTATTTT	15	T	0.275	0.2722
NC_005100.4-133369871	TCTGGGTTCACACAGCCTCCATATCCT	4	G	0	0.4773
NC_005100.4-133369871	TCTGGGTTCACACAGCCTCCATATCCT	22	T	0.2623	0.4612
NC_005100.4-133437288	GGTGTCAGCACTGCTCCTCCAGAGACA	22	G	0.1405	0.1585
NC_005100.4-133443887	TGTTAGAACACATTGTCTCCCTGTGTA	14	T	0	0.4813
NC_005100.4-133454625	GTCCACTGCACCATCACTCCAGCCTTC	9	C	0.2066	0.313
NC_005100.4-133468738	CTAATTCACACTTACACTCCAGGGCAG	24	G	0.0088	0.476
NC_005100.4-133493836	TTATCTGCAACCCCAGCTCCTAGGGAG	1	T	0	0.4819
NC_005100.4-133493836	TTATCTGCAACCCCAGCTCCTAGGGAG	14	C	0.2149	0.4532
NC_005100.4-133507574	GTAACTAAGACCCATGCTCCACTATGT	27	T	0.1102	0.2759
NC_005100.4-133521918	CAGAATTGTACCCAGCCTCCCTCTCAG	12	C	0	0.4734
NC_005100.4-133521918	CAGAATTGTACCCAGCCTCCCTCTCAG	21	C	0	0.4743
NC_005100.4-133553747	TTATGTTCGACCTGATCTCCTCTTGTC	27	C	0.0163	0.032
NC_005100.4-133574349	CACTAAGCAACATTGCCTCCCCAGGTG	14	T	0.1897	0.2739
NC_005100.4-133600385	CTAGAAATCACTCAAGCTCCATGCTAA	6	A	0	0.1014
NC_005100.4-133677146	CAAAAGTAGACACAAGCTCCTTAATCC	9	G	0.0427	0.058
NC_005100.4-133737919	GCTGTGCCCACTGCACCTCCAGCCTGC	6	G	0.0081	0.481
NC_005100.4-133844812	AATCTGTGGACCCCTACTCCATTCTCT	23	T	0.018	0.0695
NC_005100.4-133854957	CTGCTTAAAACTAAGTCTCCTTTCTAA	12	T	0	0.4765
NC_005100.4-133902908	GAAGAGGGCACTGGATCTCCTAAAGCT	4	G	0.2951	0.2515
NC_005100.4-133902908	GAAGAGGGCACTGGATCTCCTAAAGCT	14	G	0.6116	0.4308
NC_005100.4-133932623	CTACACCACACTCAGGCTCCCTGCAGG	15	G	0	0.4814
NC_005100.4-133932623	CTACACCACACTCAGGCTCCCTGCAGG	22	T	0	0.4814
NC_005100.4-133970454	CCACACACCACTCTAGCTCCAAGAAGT	8	C	0	0.4841

NC_005100.4-133970454	CCACACACCACTCTAGCTCCAAGAAGT	22	A	0.0268	0.491
NC_005100.4-134063564	TCTCCTTCCACTGCATCTCCCTTTTAC	21	C	0.1491	0.4889
NC_005100.4-134122309	ACAAATGCTACTTAAGCTCCAAGGACA	7	G	0.0089	0.4856
NC_005100.4-134122309	ACAAATGCTACTTAAGCTCCAAGGACA	9	T	0.0089	0.4856
NC_005100.4-134132043	CTACCTATGACTCCAACTCCAGGGATT	23	G	0.125	0.1327
NC_005100.4-134132043	CTACCTATGACTCCAACTCCAGGGATT	26	T	0.1504	0.2237
NC_005100.4-134139570	CAGCAAAACACAGATGCTCCTTCATTT	23	C	0.0169	0.4856
NC_005100.4-134148130	TTTGCTGTCACAGAACCTCCGCATCGT	21	G	0.1339	0.1699
NC_005100.4-134148130	TTTGCTGTCACAGAACCTCCGCATCGT	26	G	0.2411	0.3211
NC_005100.4-134200473	ATTCACTGCACATCTCCTCCTTGAGTC	3	T	0.0087	0.4782
NC_005100.4-134200473	ATTCACTGCACATCTCCTCCTTGAGTC	5	A	0.1739	0.4234
NC_005100.4-134219316	AGAATGAAAACAGGGACTCCCAGAGGA	8	A	0	0.4728
NC_005100.4-134242873	GATGGGAAGACCCTCTCTCCTTTTGGA	7	A	0.2764	0.4854
NC_005100.4-134354779	TGGGAGAGCACCGCTGCTCCTGAGTGA	12	C	0.0513	0.0818
NC_005100.4-134354779	TGGGAGAGCACCGCTGCTCCTGAGTGA	25	T	0.0609	0.4946
NC_005100.4-134430429	GCCCCTGCCACTTCCTCTCCATGTTTC	2	C	0.2832	0.4276
NC_005100.4-134467484	GACTCCAATACTAGCCCTCCAAAGCCT	5	C	0.0244	0.0553
NC_005100.4-134488841	AGGGAAAAGACATAGCCTCCTAGGGCA	2	G	0.0492	0.0786
NC_005100.4-134496409	AAATAAAGCACAGTCACTCCTCCCAAG	16	A	0	0.4787
NC_005100.4-134508819	AGCCAGCAAACACCCTCTCCCCAAGCT	9	A	0.0082	0.0401
NC_005100.4-134508819	AGCCAGCAAACACCCTCTCCCCAAGCT	23	A	0	0.4807
NC_005100.4-134516880	CTGGATGTGACTCCCGCTCCTAGCGCT	25	G	0.0446	0.0934
NC_005100.4-134531071	CTGCTCTACACGTTGTCTCCATATCTC	27	C	0.2185	0.4285
NC_005100.4-134535118	TATCTTTCTACCTAAGCTCCCAGTGCT	26	C	0.2689	0.4463
NC_005100.4-134549854	AGCCTACGTACAACAGCTCCTCTTGTA	3	C	0.0513	0.142
NC_005100.4-134549872	CCTCTTGTAACCGTTCCTCCCATCTTC	13	G	0.1949	0.4655
NC_005100.4-134572304	ATCGAGAGCACTTTCCCTCCCGGGATT	3	C	0.0325	0.4722
NC_005100.4-134572304	ATCGAGAGCACTTTCCCTCCCGGGATT	21	C	0.0244	0.0241
NC_005100.4-134606786	TCCAGCTAGACCCTACCTCCCCACACC	16	C	0.1261	0.4994
NC_005100.4-134635849	TGGGCTGTGACCCTACCTCCCAGGATG	9	G	0.025	0.0247
NC_005100.4-134663119	TCAAACACCACGTGTCCTCCATGTGCC	8	C	0	0.4909
NC_005100.4-134701029	GTGAAGGGCACTCTGGCTCCTTGGAGA	22	T	0.0163	0.4825
NC_005100.4-134711747	TTGTAAACGACTGTCACTCCAGGTTCA	2	T	0.0333	0.4888
NC_005100.4-134711747	TTGTAAACGACTGTCACTCCAGGTTCA	7	A	0	0.4765
NC_005100.4-134729877	CCCAAACAGACCGCACCTCCTGATCCT	13	G	0.1161	0.125
NC_005100.4-134737528	TCACTCATCACATGGCCTCCTGACCCC	16	C	0.1736	0.259
NC_005100.4-134738799	CCCTTTACTACAGATCCTCCATGTTAT	14	A	0.1525	0.2219
NC_005100.4-134770406	TGAGCCCATACGCTGACTCCTGTGAGT	9	T	0	0.4793
NC_005100.4-134771306	TCTTTTACCACTGCACCTCCCTGTCTC	21	C	0.0086	0.4729
NC_005100.4-134792963	TATTCAGTAACCCTGTCTCCCTGTTCA	2	A	0.0492	0.048
NC_005100.4-134792963	TATTCAGTAACCCTGTCTCCCTGTTCA	4	T	0.0492	0.048
NC_005100.4-134826596	CCTGTAGACACCAGCTCTCCTTCCTGT	25	T	0	0.4764
NC_005100.4-134858173	TCTCATTCTACTTCTGCTCCCAATGAC	23	A	0	0.48
NC_005100.4-134858173	TCTCATTCTACTTCTGCTCCCAATGAC	25	G	0	0.4786
NC_005100.4-134931529	TGGGTGGAGACAGGGCCTCCCTATGCT	3	G	0	0.4606
NC_005100.4-134963407	TCACCTCCCACCTGTGCTCCTTTACCT	26	C	0.0339	0.0333
NC_005100.4-134970941	GACCACTGGACTTTCTCTCCAGTCACT	24	C	0.0948	0.1209
NC_005100.4-135037409	CCATTTCCAACAGCGTCTCCTTCAGCC	12	A	0.2602	0.3427
NC_005100.4-135112255	AGCATTGTGACTGTGGCTCCTGGAGAA	4	A	0	0.48
NC_005100.4-135112255	AGCATTGTGACTGTGGCTCCTGGAGAA	27	A	0	0.48
NC_005100.4-135145235	CAACTCTGAACCTGTTCTCCACTCTCC	16	T	0.2562	0.4454

NC_005100.4-135176616	CTGCCAGAGACTTCACCTCCCTTCTCT	25	T	0.0174	0.0508
NC_005100.4-135182702	GAATGAGTTACCTCAACTCCGGCCTCA	21	G	0	0.4772
NC_005100.4-135249828	TGAGGTTTGACCTGGGCTCCTTTTTTT	5	G	0	0.4649
NC_005100.4-135249828	TGAGGTTTGACCTGGGCTCCTTTTTTT	26	T	0	0.4649
NC_005100.4-135251119	GCCCAGCCCACTGCTCCTCCCGAGACC	21	C	0	0.4786
NC_005100.4-135251119	GCCCAGCCCACTGCTCCTCCCGAGACC	22	G	0.0783	0.1067
NC_005100.4-135310701	CCAGGAATAACCCTGTCTCCACCTCCC	6	A	0.1217	0.1143
NC_005100.4-135310701	CCAGGAATAACCCTGTCTCCACCTCCC	9	A	0.1207	0.1134
NC_005100.4-135379541	TCTCTTGCCACCCCTTCTCCTGTTTTA	7	G	0.2857	0.3922
NC_005100.4-135421247	TTCTCAGGAACACTGCCTCCATTAGGT	5	C	0.1583	0.2125
NC_005100.4-135540205	TACTTTGTAACCTTGACTCCATCAGTT	1	T	0.0082	0.4755
NC_005100.4-135540205	TACTTTGTAACCTTGACTCCATCAGTT	25	G	0.0082	0.4755
NC_005100.4-135556606	ATCTATAATACCAGTACTCCCGTGAGT	21	C	0.2333	0.255
NC_005100.4-135707220	CAGAGAAGGACCTGATCTCCAGTATTA	8	G	0.113	0.5
NC_005100.4-135716026	TCTTGTGCTACCCGGGCTCCACCCATT	15	G	0	0.4734
NC_005100.4-135725873	CTGGGAGCCACATTTTCTCCATCAAAG	24	A	0	0.4757
NC_005100.4-135726895	ACACACACCACCCCTCCTCCTGACACA	24	C	0.15	0.2311
NC_005100.4-135761758	CTGCCTGTAACTCTGGCTCCATGAGGC	21	A	0.1121	0.1209
NC_005100.4-135775172	TGCCCTTTGACATCCCCTCCCCACACT	6	T	0.1356	0.4994
NC_005100.4-135775172	TGCCCTTTGACATCCCCTCCCCACACT	24	C	0.2373	0.4481
NC_005100.4-135787361	TTTGCTTCCACACCTCCTCCAGGAGCC	3	T	0.0331	0.4751
NC_005100.4-135787361	TTTGCTTCCACACCTCCTCCAGGAGCC	21	A	0.0164	0.4773
NC_005100.4-135813831	AATGATCTAACACGCTCTCCAGCGTGT	13	C	0.0086	0.4866
NC_005100.4-135813831	AATGATCTAACACGCTCTCCAGCGTGT	23	C	0.0431	0.4803
NC_005100.4-135845595	AGTCCTTACACTTGTACTCCTTCAGTA	6	T	0.0168	0.0491
NC_005100.4-135856907	GTCTCATTGACCCATCCTCCCCTCTAT	16	C	0.0982	0.0934
NC_005100.4-135906106	AGTATGTGCACACAGCCTCCCCAAAGA	13	C	0.0333	0.0328
NC_005100.4-135906106	AGTATGTGCACACAGCCTCCCCAAAGA	16	C	0.2583	0.4297
NC_005100.4-135976841	CTACAACACACACCCCCTCCTGCCTCC	13	C	0.3089	0.4206
NC_005100.4-135989724	CATCCTCAAACTCTCTCTCCATTGGCC	22	T	0	0.4787
NC_005100.4-136022598	CACTGGCATACACGGTCTCCATGCTTC	12	A	0.1207	0.1855
NC_005100.4-136022598	CACTGGCATACACGGTCTCCATGCTTC	27	C	0	0.4868
NC_005100.4-136029056	GCCCTCTGAACATCTTCTCCTCCCTCC	9	A	0.1405	0.1982
NC_005100.4-136042967	ATCAGAAACACCCTAGCTCCAAATAAT	27	T	0.0179	0.0521
NC_005100.4-136060709	ACTCAAGACACTCAGCCTCCCCACCTG	8	A	0.0163	0.0476
NC_005100.4-136060709	ACTCAAGACACTCAGCCTCCCCACCTG	25	C	0	0.032
NC_005100.4-136169709	TTTTGCATGACCTCTGCTCCTCAGTGA	12	C	0.1724	0.3478
NC_005100.4-136169709	TTTTGCATGACCTCTGCTCCTCAGTGA	27	A	0.2328	0.4375
NC_005100.4-136170496	CACAGGGAAACCCCATCTCCCAGAATG	26	T	0.0619	0.0765
NC_005100.4-136196158	CTGAGTTTTACTCAAGCTCCTAGAGTT	6	T	0.0169	0.0496
NC_005100.4-136196158	CTGAGTTTTACTCAAGCTCCTAGAGTT	23	G	0.0339	0.0333
NC_005100.4-136321435	GGTTCTTTGACATTTTCTCCACATGGC	2	G	0.0088	0.4843
NC_005100.4-136349548	ACTGACAAGACTAAAGCTCCCAGTACA	21	C	0.025	0.4847
NC_005100.4-136349548	ACTGACAAGACTAAAGCTCCCAGTACA	25	A	0.025	0.4847
NC_005100.4-136368445	GCAGCCCAAACCAGTCCTCCTTATTGA	4	G	0.2743	0.3858
NC_005100.4-136370646	GAGCAGGAAACAGCCTCTCCTCATCCT	16	T	0	0.4715
NC_005100.4-136376794	GAGCTTCCCACCTTCACTCCCCCAATG	23	C	0	0.4833
NC_005100.4-136531903	CTTCCACCTACAGTGTCTCCAAAGGAT	23	A	0	0.4793
NC_005100.4-136541426	TAGAGCATCACGACACCTCCTTACACT	12	G	0.187	0.2203
NC_005100.4-136541426	TAGAGCATCACGACACCTCCTTACACT	27	T	0.065	0.4944
NC_005100.4-136668665	ACCATTGGAACATGGACTCCAAGAAGA	4	A	0.082	0.0786

NC_005100.4-136755151	GTAGGGCCAACCACACCTCCCATATAT	6	G	0.2797	0.3086
NC_005100.4-136782873	AAACAGGAAACATTTCCTCCTGGGCTG	26	T	0.3051	0.4173
NC_005100.4-136803932	AACCTAGCCACAGGATCTCCACAAACC	12	A	0.2857	0.4463
NC_005100.4-136848224	ACAATTGTTACTAATTCTCCATGCCAG	24	C	0.0579	0.0562
NC_005100.4-136858084	GGACCAACCACTTATGCTCCTACACGA	26	G	0.2437	0.4592
NC_005100.4-136893415	TCTATTACAACCCATGCTCCCTGTCAG	1	T	0.2193	0.472
NC_005100.4-136941555	TCCATGTCCACCTTCACTCCGAGATGT	21	G	0.2241	0.3478
NC_005100.4-136979033	CTTGCTGAGACATCGTCTCCTGTGAGC	13	T	0.0088	0.483
NC_005100.4-137006000	ACTTTCTCAACCTTTCCTCCTACCCTA	21	T	0.1382	0.1425
NC_005100.4-137036397	CTGCTCCAAACTCTGCCTCCATGTCTC	21	A	0	0.4827
NC_005100.4-137069606	TGGATCCTCACCAGCACTCCTCTTGGA	25	G	0.3509	0.4875
NC_005100.4-137072121	CTGACCTCTACCTGTACTCCAGGGCAT	24	G	0.075	0.0722
NC_005100.4-137106040	GAATAGAGAACTGCCTCTCCTCCTCAG	26	A	0.0625	0.0605
NC_005100.4-137113890	GCCAAATGAACCCTTCCTCCCTTAAGT	3	C	0.0086	0.4768
NC_005100.4-137147014	GACTATTAAACCCAGGCTCCATGTTTG	3	C	0	0.4827
NC_005100.4-137147363	CTGCCTGCAACTCTGGCTCCCTCTTCT	14	T	0.0082	0.479
NC_005100.4-137158430	CTGACCTAGACCCAACCTCCAGCTCTG	14	A	0	0.4793
NC_005100.4-137247984	GACTGGGCGACAGGCGCTCCCTTCCCG	8	C	0	0.4844
NC_005100.4-137258191	GAGGGTAACACTGTCCCTCCCTTGCCC	7	A	0.0579	0.0868
NC_005100.4-137264980	TGCATGTGCACATGTGCTCCATGTCTC	24	T	0.0885	0.0846
NC_005100.4-137266782	GTGAGAAGCACCTCTTCTCCCTCAGTC	6	A	0	0.4807
NC_005100.4-137312258	CCCTCAGCCACAGGTCCTCCTCACATA	4	T	0	0.4856
NC_005100.4-137312258	CCCTCAGCCACAGGTCCTCCTCACATA	25	A	0.0333	0.0328
NC_005100.4-137344620	GCCCCTCCCACCATCCCTCCTGCTGGT	1	G	0.1545	0.1425
NC_005100.4-137344620	GCCCCTCCCACCATCCCTCCTGCTGGT	13	A	0.0163	0.4825
NC_005100.4-137355213	TCGACTTCGACATTGTCTCCCGCACTG	21	C	0	0.4688
NC_005100.4-137364059	ACCACCCCAACCTCTACTCCATCTATG	27	G	0.0339	0.0333
NC_005100.4-137366768	GAGGAAGTAACTTTCCCTCCATCTCTA	7	G	0.0656	0.0634
NC_005100.4-137438571	AGATTACCAACAAAGACTCCTGGGGGC	27	C	0.0254	0.0251
NC_005100.4-137450589	TACTCTTCTACACCACCTCCAGCATAG	3	C	0.1626	0.189
NC_005100.4-137450589	TACTCTTCTACACCACCTCCAGCATAG	8	C	0.0163	0.032
NC_005100.4-137500538	TCATGGCAAACTGTTCCTCCAACTTTA	22	A	0	0.4749
NC_005100.4-137529068	CCCACACTCACTCGCCCTCCCCCTAGA	7	C	0.2411	0.4263
NC_005100.4-137529068	CCCACACTCACTCGCCCTCCCCCTAGA	14	G	0.0265	0.0262
NC_005100.4-137530266	CTCTCTCTAACAGTCTCTCCAGCGCAC	8	T	0.3089	0.427
NC_005100.4-137583385	GGCAGGTAGACAGACCCTCCCTTCACT	2	G	0.0169	0.0333
NC_005100.4-137621589	AGTGTACACACAGAGTCTCCTGCTCTC	7	C	0.2417	0.4666
NC_005100.4-137658218	CCAGCTCCCACTGATGCTCCTGTCTCA	6	T	0.0084	0.0249
NC_005100.4-137658218	CCAGCTCCCACTGATGCTCCTGTCTCA	7	C	0	0.4743
NC_005100.4-137670915	CACCTTCCTACATGAGCTCCTCCTTCC	6	T	0.0084	0.4761
NC_005100.4-137828264	CATGTTGTCACATCCCCTCCCCACCCA	9	C	0.0885	0.4859
NC_005100.4-137828264	CATGTTGTCACATCCCCTCCCCACCCA	13	T	0	0.4624
NC_005100.4-137929364	GAGCTTGTGACCTCCCCTCCAGGAGAA	26	A	0.0263	0.0595
NC_005100.4-138074027	GATGAGGCTACACATGCTCCTTTGTAA	26	A	0.1379	0.4963
NC_005100.4-138187897	GAAGACAAGACCAGGGCTCCAACAGCT	13	A	0.1597	0.317
NC_005100.4-138236453	ATAACATGTACCTATTCTCCCCTTCAT	8	G	0.0083	0.0247
NC_005100.4-138237768	GACAAACACACAAACCCTCCTCACTTC	6	A	0.1301	0.5
NC_005100.4-138237768	GACAAACACACAAACCCTCCTCACTTC	23	A	0.1138	0.1073
NC_005100.4-138251078	CTGTTGATTACCTATGCTCCTGCACCA	9	T	0.1466	0.1646
NC_005100.4-138251078	CTGTTGATTACCTATGCTCCTGCACCA	23	C	0.1466	0.1646
NC_005100.4-138259502	ATGGTGAATACACATCCTCCGTGTGTG	21	G	0.0268	0.4824

NC_005100.4-138259502	ATGGTGAATACACATCCTCCGTGTGTG	27	G	0	0.4898
NC_005100.4-138287239	GGTGCCCTCACCATTCCTCCTACAATC	13	A	0	0.462
NC_005100.4-138295616	TGTCCCTGAACTTGATCTCCATGGAGT	23	G	0.1967	0.2741
NC_005100.4-138351609	CTCTGCTTCACTTCATCTCCGTATTTC	27	C	0.0714	0.0689
NC_005100.4-138397944	AGACTACGCACAACCTCTCCAACCAAC	7	C	0.0082	0.4755
NC_005100.4-138397944	AGACTACGCACAACCTCTCCAACCAAC	24	C	0.0492	0.4914
NC_005100.4-138410878	ACGAAGTTAACCATAGCTCCCAACCTC	2	C	0.1901	0.4951
NC_005100.4-138472884	TGTTCTGGAACACTACCTCCAGCGGTA	12	A	0.1441	0.4609
NC_005100.4-138473671	TAGCCATGCACTTTAGCTCCAACAGCA	9	C	0	0.4779
NC_005100.4-138473671	TAGCCATGCACTTTAGCTCCAACAGCA	16	G	0.1008	0.4971
NC_005100.4-138516139	GTGTCCAGGACTTTCCCTCCTGATGCA	14	T	0	0.4724
NC_005100.4-138556401	TGTCCATCCACAGCTTCTCCAGGCCTG	14	C	0	0.4807
NC_005100.4-138556401	TGTCCATCCACAGCTTCTCCAGGCCTG	15	T	0.0492	0.048
NC_005100.4-138628843	CATTATGTGACCTTCTCTCCTCTTTAA	1	C	0.1897	0.2739
NC_005100.4-138645368	TTAGTTGTAACACATGCTCCCGAGAAT	22	G	0.2417	0.2608
NC_005100.4-138645368	TTAGTTGTAACACATGCTCCCGAGAAT	27	T	0.0672	0.4567
NC_005100.4-138658256	GGAGCCATCACCACATCTCCTTTTTAA	26	A	0.1624	0.1908
NC_005100.4-138660417	TCATTCTAGACCTCTGCTCCGAAGCTG	6	C	0.1382	0.1287
NC_005100.4-138672003	ACATAAAACACAGGCACTCCGTAATCT	1	A	0	0.482
NC_005100.4-138672003	ACATAAAACACAGGCACTCCGTAATCT	21	G	0.0086	0.0422
NC_005100.4-138706661	GGCTTGAGCACAGTCACTCCATATACC	26	C	0	0.48
NC_005100.4-138798225	TGTTTCATGACGGGCCCTCCTGTAGAG	27	G	0.0603	0.0585
NC_005100.4-138868619	AGAACAAACACCACCTCTCCTTTCATT	22	T	0.0085	0.4776
NC_005100.4-138868619	AGAACAAACACCACCTCTCCTTTCATT	26	T	0.1525	0.2585
NC_005100.4-138921493	ACCACAAGGACACATGCTCCACTATTT	7	A	0.5812	0.4444
NC_005100.4-138921493	ACCACAAGGACACATGCTCCACTATTT	12	A	0.4237	0.362
NC_005100.4-139001128	TGCCTGTGTACATGAGCTCCTGGATCT	5	T	0.2358	0.2668
NC_005100.4-139024034	TGCTCAGTCACCTTTACTCCTAGTTTA	9	C	0.0957	0.0911
NC_005100.4-139024034	TGCTCAGTCACCTTTACTCCTAGTTTA	24	T	0.3043	0.4878
NC_005100.4-139148396	TCTGGCTTGACTAAACCTCCAGCCATT	1	T	0.1964	0.2575
NC_005100.4-139285857	CAGCTAGACACAATGGCTCCATTGATA	26	T	0.2034	0.4977
NC_005100.4-139293371	TACTCGCTGACTCTATCTCCGGTGCAT	15	A	0.0246	0.0243
NC_005100.4-139328523	ACTCTGCCCACACAGACTCCAACCTCT	2	C	0	0.48
NC_005100.4-139462480	AATTCTGCCACTGCTTCTCCCTGATTA	8	C	0.175	0.4783
NC_005100.4-139475169	CTTGCTGGAACCATGTCTCCCTTTCTT	15	G	0.125	0.1458
NC_005100.4-139497452	AAACCTGTTACCTGCCCTCCTTCCTAA	12	C	0.1207	0.4976
NC_005100.4-139507157	CCTGCTCTAACCCTAGCTCCTCATTTT	25	T	0.0089	0.4751
NC_005100.4-139531957	CTCCGTGGCACATGTCCTCCTCCCTCT	1	C	0.0246	0.4822
NC_005100.4-139544118	GACATCAATACCACAGCTCCTCTTCCC	8	A	0.0492	0.0634
NC_005100.4-139647699	CTAGGTCCAACCCATGCTCCATCTTCG	27	G	0.1849	0.2683
NC_005100.4-139716860	TCCATCTCCACATTTCCTCCTCTGATA	26	T	0.0924	0.397
NC_005100.4-139733044	ACAGTACCGACATGACCTCCAAAGAGT	8	C	0.0893	0.1014
NC_005100.4-139775928	TTTCCACTGACTTTCTCTCCCTCTGTT	6	A	0.2759	0.4786
NC_005100.4-139888037	CCACCTCAAACCCTTTCTCCACCTTCT	8	A	0	0.4793
NC_005100.4-139899645	AATAGTACTACTGCTGCTCCTACTAAC	22	A	0	0.4779
NC_005100.4-139968621	TGCCTCCTCACTTCCTCTCCTACCCTC	3	C	0.0894	0.1001
NC_005100.4-139968621	TGCCTCCTCACTTCCTCTCCTACCCTC	24	C	0.0894	0.1001
NC_005100.4-139984108	AGATGGAGGACTGAGCCTCCTGTTCGG	25	C	0.0976	0.1356
NC_005100.4-140013007	ACTCTGCCCACCATTGCTCCCGTATCA	21	C	0.187	0.2778
NC_005100.4-140013007	ACTCTGCCCACCATTGCTCCCGTATCA	22	G	0.1066	0.2573
NC_005100.4-140037660	TGTGAAAGTACGGAAACTCCAGGCATG	12	G	0.0847	0.1264

NC_005100.4-140086870	CCATGAGAGACTACTCCTCCAAGAACT	12	T	0.0244	0.481
NC_005100.4-140086870	CCATGAGAGACTACTCCTCCAAGAACT	13	A	0.0081	0.4777
NC_005100.4-140115733	GCATTGAGGACTATCTCTCCCATTTGT	14	T	0.1739	0.2985
NC_005100.4-140115733	GCATTGAGGACTATCTCTCCCATTTGT	15	C	0.1293	0.2057
NC_005100.4-140149725	AGCCATGCTACTTCCACTCCATGCAGT	22	T	0.2377	0.3307
NC_005100.4-140162117	CTCCATCCCACTCCTCCTCCACCCATC	6	T	0.0165	0.4819
NC_005100.4-140171311	ATGCCCCTCACCTGACCTCCTTCGATT	25	A	0	0.48
NC_005100.4-140203615	TCCATGACAACACAAACTCCAGGAGCC	15	A	0	0.4772
NC_005100.4-140203807	GGACCCAGGACCAATGCTCCTGATTCA	15	T	0	0.4772
NC_005100.4-140220096	TTGTACTGGACAGATCCTCCCACTGGC	9	G	0.2895	0.3273
NC_005100.4-140220096	TTGTACTGGACAGATCCTCCCACTGGC	15	T	0	0.4833
NC_005100.4-140227062	TTCTACTCTACAATTCCTCCTCTGTCA	15	T	0.0984	0.1505
NC_005100.4-140227062	TTCTACTCTACAATTCCTCCTCTGTCA	24	G	0.1074	0.1447
NC_005100.4-140230066	TGGGGGAATACTGGGTCTCCACCCCAG	8	A	0.1639	0.2032
NC_005100.4-140230066	TGGGGGAATACTGGGTCTCCACCCCAG	23	C	0.0496	0.0484
NC_005100.4-140234927	GTCATTCCTACTTTTCCTCCCTTCTAT	7	C	0.0351	0.0345
NC_005100.4-140234927	GTCATTCCTACTTTTCCTCCCTTCTAT	8	C	0.0089	0.4856
NC_005100.4-140261734	CTATCTGTCACCTTCCCTCCTCAGCTA	6	T	0.1966	0.3107
NC_005100.4-140261734	CTATCTGTCACCTTCCCTCCTCAGCTA	7	G	0.1966	0.3107
NC_005100.4-140268828	ATCACCCACACCTACTCTCCACTACAG	24	A	0.1463	0.1494
NC_005100.4-140301016	CCGGGACTGACAGAGTCTCCTATAATC	25	A	0.1626	0.464
NC_005100.4-140308279	GGATTCTTTACTGTACCTCCCTCCGGG	24	C	0.1607	0.1913
NC_005100.4-140313844	TTATAAGGGACTCTCACTCCCACATCA	7	G	0.0625	0.0771
NC_005100.4-140324062	TTTATTGAAACTTAACCTCCCACTGGG	14	A	0.1681	0.4493
NC_005100.4-140338910	CAAGCCAGAACCACTGCTCCATAGGTG	4	G	0.0536	0.0689
NC_005100.4-140354150	GGCTCTCTTACGTTTCCTCCACCGCAC	24	G	0.0732	0.1001
NC_005100.4-140362034	TAGATCCTCACTGGCACTCCTCCTGGT	22	C	0.0661	0.0943
NC_005100.4-140362210	GGTCATCATACCACATCTCCCTTATCC	4	C	0.065	0.0928
NC_005100.4-140362210	GGTCATCATACCACATCTCCCTTATCC	9	T	0.1789	0.377
NC_005100.4-140367566	CCATTCTGCACAGCCTCTCCCACAACA	26	C	0.1849	0.2568
NC_005100.4-140367566	CCATTCTGCACAGCCTCTCCCACAACA	27	A	0.1849	0.2568
NC_005100.4-140379358	CCCTGGCTAACCCCATCTCCCTGAGTG	25	G	0	0.48
NC_005100.4-140381707	AACCAAGAAACTGAGGCTCCCGGAATA	21	C	0.0259	0.4803
NC_005100.4-140393132	TCATAGGAAACTGATTCTCCCTCTCCT	26	C	0.1053	0.474
NC_005100.4-140399302	AATCATGAGACTCGGCCTCCTTGTCAG	14	G	0.3089	0.3516
NC_005100.4-140399302	AATCATGAGACTCGGCCTCCTTGTCAG	23	G	0.0976	0.1073
NC_005100.4-140446841	GTTAGCAACACCAACACTCCCAATGCT	22	A	0.0614	0.0758
NC_005100.4-140493896	GTTGAGCCCACACTCCCTCCTCCCTCC	2	T	0.065	0.078
NC_005100.4-140554907	GTCTGTGCTACATGTACTCCATGACTC	13	T	0.1765	0.2012
NC_005100.4-140554907	GTCTGTGCTACATGTACTCCATGACTC	22	T	0.0932	0.0889
NC_005100.4-140570364	ACCCCCTCCACCTCTCCTCCGAAGCTG	1	A	0	0.4793
NC_005100.4-140570364	ACCCCCTCCACCTCTCCTCCGAAGCTG	2	C	0	0.4793
NC_005100.4-140609119	ACTGTTCTGACTCCGCCTCCTGAGTGC	14	C	0.2787	0.387
NC_005100.4-140705073	GTGCCTGAAACCGAAGCTCCAGGCCAT	14	A	0	0.4698
NC_005100.4-140713158	GGTGTTACAACAACCCCTCCTGCAACA	2	G	0.1545	0.208
NC_005100.4-140731700	CAGCTCCTCACTCCTCCTCCTTCTCAC	26	A	0.2623	0.3791
NC_005100.4-140765538	GATAATATCACAGAGCCTCCCATGGAG	5	A	0.2308	0.289
NC_005100.4-140769404	CATCATGTCACCCCACCTCCACCCTGA	22	C	0	0.4666
NC_005100.4-140769404	CATCATGTCACCCCACCTCCACCCTGA	23	C	0	0.4666
NC_005100.4-140773409	AGGTCTTGTACCCAGGCTCCCTACATT	23	A	0.1545	0.1826
NC_005100.4-140801330	GTGCGAGTGACCATCTCTCCAGGTGCA	12	C	0.045	0.044

NC_005100.4-140808550	CCTCTGGCGACCTTGACTCCAGTGGTC	8	C	0.1239	0.1466
NC_005100.4-140813615	ATCTCCCTCACTCTGCCTCCACTGCTC	6	C	0.1157	0.1377
NC_005100.4-140824760	TTGTTGGTTACTTCACCTCCAGGGAGA	2	T	0	0.4592
NC_005100.4-140844390	AGAGGTGTTACTCCCTCTCCTGTGTAT	24	G	0.2459	0.316
NC_005100.4-140899068	CCCATCTTTACAATCTCTCCATCACTG	22	T	0.0174	0.48
NC_005100.4-140968146	CATGGTCACACCCTACCTCCAAGAGAA	3	T	0.2288	0.375
NC_005100.4-140968146	CATGGTCACACCCTACCTCCAAGAGAA	21	A	0.2119	0.3833
NC_005100.4-140991922	CTCCCTACTACCTCTCCTCCTGTCCTA	6	T	0	0.474
NC_005100.4-140991922	CTCCCTACTACCTCTCCTCCTGTCCTA	7	A	0.0268	0.0264
NC_005100.4-141030550	AGGTACATCACAGAGGCTCCTGTGATG	7	A	0.2231	0.3027
NC_005100.4-141046521	CATATTAGGACTGTAACTCCCAGTGTA	12	T	0.2295	0.4914
NC_005100.4-141046521	CATATTAGGACTGTAACTCCCAGTGTA	21	C	0.1322	0.1377
NC_005100.4-141067821	CAGTTTTGTACAAATGCTCCATTAGAT	9	T	0	0.4827
NC_005100.4-141067821	CAGTTTTGTACAAATGCTCCATTAGAT	14	A	0	0.4827
NC_005100.4-141117945	TCATTACCTACAACTGCTCCTTTATAA	26	A	0	0.4751
NC_005100.4-141130920	GGCTTTCAAACGTCTGCTCCACAGGGA	12	G	0.2	0.2311
NC_005100.4-141139150	GGGACTGGAACCTGTCCTCCATGACGA	9	A	0.1102	0.1041
NC_005100.4-141177520	CGGTGAGCCACGTGGGCTCCACAGCGC	26	G	0.1949	0.2526
NC_005100.4-141212505	GGGAAAGGCACCTCAGCTCCTGGGATG	4	A	0	0.4773
NC_005100.4-141274069	TGAGGCGGAACAAAAACTCCCCGAGAT	22	C	0.161	0.2026
NC_005100.4-141282768	AGCCCGCAGACGTCATCTCCGGCAGAG	5	C	0.1405	0.1585
NC_005100.4-141282768	AGCCCGCAGACGTCATCTCCGGCAGAG	13	T	0.0246	0.0243
NC_005100.4-141302466	GTTCCATCAACACTAACTCCCCAGTTG	22	C	0.1026	0.0973
NC_005100.4-141322310	CCTTCGAAAACTCCATCTCCCAGGTCC	6	G	0.0333	0.0328
NC_005100.4-141325794	TGTACCAGAACATTCACTCCCAGTCTA	5	C	0.0351	0.0677
NC_005100.4-141328202	AGGCCAGTGACCCCGGCTCCAAGAGGA	14	C	0.2389	0.3294
NC_005100.4-141337671	GGACTCCAGACATTGACTCCAAGCCAG	21	A	0.2066	0.313
NC_005100.4-141337671	GGACTCCAGACATTGACTCCAAGCCAG	22	A	0	0.4751
NC_005100.4-141340736	CTCATGTCCACCCATACTCCCACCTCC	26	C	0	0.4819
NC_005100.4-141348834	GTGGATCCCACCATCACTCCGTAGAGA	24	G	0.0965	0.4861
NC_005100.4-141363054	AAAGCCCTGACCCCCACTCCATTACCC	21	A	0.1393	0.2796
NC_005100.4-141363054	AAAGCCCTGACCCCCACTCCATTACCC	27	C	0.1138	0.189
NC_005100.4-141376882	AGGGTGGCAACTCTTACTCCTCCCCTG	4	G	0	0.4751
NC_005100.4-141386915	GTGGGAGCTACCCCCACTCCTCAGCCC	15	C	0	0.4659
NC_005100.4-141386915	GTGGGAGCTACCCCCACTCCTCAGCCC	22	C	0	0.4641
NC_005100.4-141439840	CTGGGCAGAACTGTCTCTCCCCACACT	1	C	0.0088	0.4599
NC_005100.4-141439840	CTGGGCAGAACTGTCTCTCCCCACACT	4	G	0.0088	0.463
NC_005100.4-141463529	TGGGGCAGGACCACCTCTCCGTTTGCG	24	T	0	0.4773
NC_005100.4-141519542	AGGAAAAGGACTTTCCCTCCCATGGTA	24	G	0.0732	0.0705
NC_005100.4-141519542	AGGAAAAGGACTTTCCCTCCCATGGTA	25	G	0.0163	0.032
NC_005100.4-141545828	CAGCCTAGAACCTGGGCTCCCACACCA	4	C	0.2821	0.4671
NC_005100.4-141546103	CCTTGACTTACCAACTCTCCTACAGTC	15	C	0.2288	0.2526
NC_005100.4-141595145	GTGTCTATCACAGAGGCTCCAGCATAC	6	T	0.0583	0.0566
NC_005100.4-141595145	GTGTCTATCACAGAGGCTCCAGCATAC	25	T	0.1	0.1099
NC_005100.4-141610007	TAAGGATGGACGCACTCTCCTGTGTAC	25	T	0	0.4793
NC_005100.4-141641881	ATCTTCTCAACTGAGGCTCCCTCTTTC	9	A	0.2124	0.4859
NC_005100.4-141644475	ATGACCTGTACATCAGCTCCTGATTCC	1	A	0.2333	0.4328
NC_005100.4-141673382	CATAATATTACTTTGCCTCCCACTTCC	6	T	0	0.48
NC_005100.4-141681782	CCCCAGGAGACTTGAGCTCCAAGGCTG	24	G	0.1917	0.395
NC_005100.4-141734184	CCCAGAACTACCTGAACTCCATGCTGG	12	C	0	0.4555
NC_005100.4-141764408	CTATGTACAACTGTGCCTCCCTCTATT	25	A	0	0.4793

NC_005100.4-141790764	GTGTTTGTCACGCTACCTCCGTCTGCC	12	G	0.0508	0.0496
NC_005100.4-141790764	GTGTTTGTCACGCTACCTCCGTCTGCC	13	C	0.1186	0.3792
NC_005100.4-141843990	TACCTTCCTACTGAGGCTCCCCGAGGG	22	C	0.2479	0.4582
NC_005100.4-141848412	CCCCAGTAAACCCAGCCTCCTACCGTG	22	A	0.1653	0.2046
NC_005100.4-141934995	CCATCTGCAACTCCAGCTCCAAGGGAT	14	C	0.3471	0.2975
NC_005100.4-141934995	CCATCTGCAACTCCAGCTCCAAGGGAT	15	A	0.3802	0.3278
NC_005100.4-141943841	TTCAAGGGAACAAAGACTCCAGGTGCT	1	T	0.0168	0.033
NC_005100.4-141944347	GTGTCCCGCACAAAGCCTCCAGGGAAG	7	C	0	0.4793
NC_005100.4-141944347	GTGTCCCGCACAAAGCCTCCAGGGAAG	24	G	0.065	0.4595
NC_005100.4-141974699	ATGATACACACGTTCACTCCCTCCCAT	2	T	0	0.4807
NC_005100.4-141992663	AAATGTGAAACAGTGTCTCCTGAGCCT	15	G	0.1803	0.2515
NC_005100.4-142093620	AAAGTACCCACTGCAACTCCATGATAT	3	A	0	0.4751
NC_005100.4-142113086	GACATCTGCACAGACACTCCACCTATA	15	C	0	0.4813
NC_005100.4-142150142	GAGTTTAGGACCGTCCCTCCTCAGCCT	13	G	0.225	0.4233
NC_005100.4-142160655	AGAGCGCTCACCCACCCTCCTTTCTCT	1	A	0.1557	0.1839
NC_005100.4-142160655	AGAGCGCTCACCCACCCTCCTTTCTCT	15	C	0	0.4807
NC_005100.4-142180294	GCTCCTCGCACTGGAGCTCCCGCTGCA	8	G	0.0569	0.0553
NC_005100.4-142261058	AGGAGCCCCACTGTACCTCCATGGTAT	5	G	0.0488	0.0629
NC_005100.4-142280866	CTACTTTTAACAAATCCTCCAATGCCT	4	C	0	0.4827
NC_005100.4-142317289	ATTCTTCTGACTCTAGCTCCTGAGAGC	1	A	0.0086	0.4803
NC_005100.4-142419869	GGGGCAGAGACAGGCTCTCCGGAGCAA	3	G	0.0256	0.0418
NC_005100.4-142419869	GGGGCAGAGACAGGCTCTCCGGAGCAA	21	G	0.2478	0.2913
NC_005100.4-142420756	ACCAGTGAGACAAATGCTCCTCCTGCT	8	A	0.0083	0.0245
NC_005100.4-142433259	CTGCATTGCACATCGGCTCCAGGCTTC	1	C	0.0325	0.032
NC_005100.4-142470831	CAGGAGGCAACTGTAGCTCCCTGCCAG	1	C	0.0244	0.0241
NC_005100.4-142484180	AGACATCTTACTGTTCCTCCCCATCTG	15	T	0.0088	0.4796
NC_005100.4-142552051	CAGGCTCAGACCATGCCTCCCTGGAAT	24	G	0	0.4793
NC_005100.4-142731744	TGTGCAGCAACTGTATCTCCTGGACTG	16	T	0	0.4779
NC_005100.4-142736960	ACGTTCTGTACCAACTCTCCTGCCAGT	3	G	0.2137	0.2662
NC_005100.4-142813725	AAGCAGGACACTGGCTCTCCCGAGCTG	9	C	0.1043	0.4981
NC_005100.4-142859987	GGAGATCCAACTCCCTCTCCTAACCTC	4	G	0.0442	0.0926
NC_005100.4-142890722	TTGGGCCCCACCCCTCCTCCTCTTCAC	23	T	0	0.4833
NC_005100.4-142895713	ATGCACGAAACCCTGGCTCCAAGACAA	16	G	0.1964	0.4641
NC_005100.4-142896747	TCACTTGCCACATAACCTCCATGACCC	4	C	0.1463	0.189
NC_005100.4-142896747	TCACTTGCCACATAACCTCCATGACCC	14	A	0	0.4793
NC_005100.4-142913328	CTATCACAAACCACATCTCCAACTTCT	24	T	0.0252	0.0249
NC_005100.4-142940097	GGTTCACCTACATGCTCTCCTAGTCAC	27	C	0.0177	0.4574
NC_005100.4-142968254	CCCTAGATCACACTCCCTCCGCCTCTC	27	C	0.1074	0.1982
NC_005100.4-142977829	AGAACTGTGACCATCACTCCCACAGCA	6	T	0.1138	0.4973
NC_005100.4-142990303	GATTTGCCAACTTCAGCTCCATTGGCA	5	T	0.2397	0.4988
NC_005100.4-143018847	CCTCTCTCTACTCCACCTCCCTCTCTT	12	T	0.0924	0.1327
NC_005100.4-143018847	CCTCTCTCTACTCCACCTCCCTCTCTT	13	C	0.042	0.0411
NC_005100.4-143078909	ATTCAGGTGACGCCCTCTCCCAACCCA	12	G	0	0.4749
NC_005100.4-143094094	GTCCTGATAACATCGTCTCCATACCCC	13	T	0	0.488
NC_005100.4-143094094	GTCCTGATAACATCGTCTCCATACCCC	23	A	0	0.488
NC_005100.4-143145799	GCTGTCTCCACTGAGACTCCCACTTCA	6	C	0.0504	0.1107
NC_005100.4-143150491	CCCCGAGGCACAAGCTCTCCAGCACAT	5	G	0.2541	0.4489
NC_005100.4-143166173	ACTCAGATCACTGAGGCTCCACTGGGG	27	G	0	0.4827
NC_005100.4-143195384	CACACACACACCAGTCCTCCATTGTCC	2	A	0.042	0.0882
NC_005100.4-143195384	CACACACACACCAGTCCTCCATTGTCC	4	A	0.0252	0.0411
NC_005100.4-143218100	CCAGCACATACTCCCACTCCTCTTCAC	24	T	0	0.4793

NC_005100.4-143218100	CCAGCACATACTCCCACTCCTCTTCAC	27	C	0	0.4793
NC_005100.4-143248824	TGCATGTTAACTGTAGCTCCAGGGAAT	16	G	0.0254	0.0251
NC_005100.4-143248824	TGCATGTTAACTGTAGCTCCAGGGAAT	23	G	0.0417	0.0408
NC_005100.4-143279131	CTGCTTCCGACGCTCCCTCCAGATAGG	27	G	0.0435	0.059
NC_005100.4-143286403	AGCAAAGCTACTGGGCCTCCCTCTGTC	15	G	0.0246	0.0243
NC_005100.4-143297371	AGAAAGGTCACGCTGCCTCCTCCCTCC	12	G	0.2917	0.315
NC_005100.4-143305305	TGGATCTCCACCATCCCTCCACTTAGG	13	A	0.0625	0.0934
NC_005100.4-143365872	ACTGACTTGACTCATGCTCCTACGGTC	12	T	0.0169	0.0333
NC_005100.4-143390813	CCCCATCCCACCCCAACTCCCAGGACA	1	C	0.1966	0.2171
NC_005100.4-143428844	CTGTTCTCCACGAGATCTCCATTAGGT	2	T	0	0.4832
NC_005100.4-143428844	CTGTTCTCCACGAGATCTCCATTAGGT	4	T	0.1	0.4988
NC_005100.4-143453567	CCGGACTTCACAGTGTCTCCAGACTAT	3	G	0.2193	0.235
NC_005100.4-143453567	CCGGACTTCACAGTGTCTCCAGACTAT	14	T	0.0877	0.0839
NC_005100.4-143454772	GCAAGAAATACAGCTTCTCCACAGAAG	24	G	0.0862	0.1284
NC_005100.4-143472431	GACTGAAGTACACCTACTCCATCCCAG	26	A	0.0902	0.1009
NC_005100.4-143494053	TGGTTAAAAACAGGAGCTCCGTGCTTA	16	G	0.0263	0.026
NC_005100.4-143494053	TGGTTAAAAACAGGAGCTCCGTGCTTA	21	G	0.114	0.4981
NC_005100.4-143502088	TGTCTCTCCACTGTCACTCCTGGGCAG	7	T	0.1565	0.3094
NC_005100.4-143521373	AAGCTGATGACCCAGCCTCCCACTCGT	22	A	0.0732	0.0705
NC_005100.4-143521607	GCTACTCTCACTGGAGCTCCCTTGTAT	23	T	0.0504	0.2076
NC_005100.4-143524532	CTCCAGCCCACTCTTGCTCCTTCCTGC	1	C	0.0083	0.4816
NC_005100.4-143524806	CATCCCCCAACCCTCGCTCCTTATATG	15	C	0.2083	0.3347
NC_005100.4-143524806	CATCCCCCAACCCTCGCTCCTTATATG	25	A	0	0.4813
NC_005100.4-143528140	AGGAAGAACACTGGGTCTCCGTCCACT	21	G	0.0087	0.0257
NC_005100.4-143528725	TCCCTCTTCACTGTTACTCCATGGTAC	4	C	0	0.4757
NC_005100.4-143528725	TCCCTCTTCACTGTTACTCCATGGTAC	5	T	0	0.4757
NC_005100.4-143600396	CACAACACCACTGAGTCTCCAACAAAA	5	A	0.2377	0.479
NC_005100.4-143600396	CACAACACCACTGAGTCTCCAACAAAA	21	A	0.2295	0.4698
NC_005100.4-143677167	AGCACAGGGACCTAGGCTCCTACTGTG	24	T	0	0.4765
NC_005100.4-143708497	CAACATTCCACTGTGGCTCCCTCTTCA	25	T	0	0.4793
NC_005100.4-143755959	GCAGTATCCACTCAAGCTCCGACTGTG	13	C	0	0.4773
NC_005100.4-143827619	ATACTTCGGACCACTGCTCCAGTTTCC	27	C	0.1382	0.1954
NC_005100.4-143889883	AACACACATACCCACACTCCTCCCCAC	13	C	0.0174	0.4682
NC_005100.4-143898065	CCTATCGTCACTTCCTCTCCACCCCGG	26	G	0.0574	0.0557
NC_005100.4-143902444	GTCAGCCATACATTTCCTCCCAGTCTG	1	G	0.0776	0.2315
NC_005100.4-144081042	GACGGATGAACTAACTCTCCACTCAGC	1	G	0.042	0.0411
NC_005100.4-144081042	GACGGATGAACTAACTCTCCACTCAGC	3	C	0	0.4703
NC_005100.4-144100158	TGCATCTACACTCCTGCTCCACATCTG	8	A	0	0.4773
NC_005100.4-144107548	TGACACTAGACAAAGACTCCTGTAGGC	8	A	0.2054	0.388
NC_005100.4-144109563	GTTCAAGCTACAGACTCTCCCTAGTGG	9	T	0	0.4826
NC_005100.4-144110684	GCCTAGGGCACTCAGTCTCCTTTCGTA	9	C	0.0248	0.0245
NC_005100.4-144110684	GCCTAGGGCACTCAGTCTCCTTTCGTA	25	G	0.0413	0.0405
NC_005100.4-144120020	CTCAGCGACACATCTCCTCCTACAAGG	27	G	0.1754	0.2538
NC_005100.4-144123248	CAAACACACACATATACTCCTACACAC	5	C	0.1565	0.2519
NC_005100.4-144123248	CAAACACACACATATACTCCTACACAC	24	A	0.0261	0.0257
NC_005100.4-144191856	AGCACGCAGACCTGTGCTCCAAAGACA	25	A	0.1322	0.1787
NC_005100.4-144235199	CAATCTGACACAGTGGCTCCTTCTATG	12	A	0.0083	0.0247
NC_005100.4-144328723	AGGAGCTGGACTGGAACTCCAGACAGC	15	A	0.281	0.2975
NC_005100.4-144335160	CAAAAGCAAACATGTTCTCCTTCCTTC	14	G	0.2917	0.4233
NC_005100.4-144342639	GTGCTCACCACCATTACTCCTTAACGT	4	C	0.0248	0.0245
NC_005100.4-144342639	GTGCTCACCACCATTACTCCTTAACGT	8	C	0.1885	0.1839

NC_005100.4-144355392	GACAAGGGTACTAGATCTCCTAAAGCT	7	G	0.0246	0.0243
NC_005100.4-144364513	AGGAGTCTCACAGAAACTCCAAGCTAA	2	G	0.2439	0.4904
NC_005100.4-144390632	CTGACTACCACACGTGCTCCCTGATAC	13	C	0.2521	0.3686
NC_005100.4-144492136	CACACACACACTGTTGCTCCTTTCTGA	12	T	0	0.4778
NC_005100.4-144532367	GTCTCCAGTACCATTGCTCCATCGATG	24	G	0	0.4807
NC_005100.4-144553854	CGTGTGCTCACAACAGCTCCAACTCTG	8	T	0	0.4751
NC_005100.4-144594136	TCTAGTGATACTATTGCTCCCAATCCA	16	G	0.1171	0.1414
NC_005100.4-144728524	GAAAGCTCAACTAATTCTCCTTTATTA	2	A	0.0179	0.4641
NC_005100.4-144745017	TGAAGGAAGACCCGTGCTCCAGTAAGT	23	T	0	0.4606
NC_005100.4-144791809	ATGCACACCACACACACTCCATGAATA	1	A	0.2231	0.4454
NC_005100.4-144791934	CCACACACCACACATTCTCCATAAATA	1	C	0.0714	0.4943
NC_005100.4-144791934	CCACACACCACACATTCTCCATAAATA	26	T	0.0354	0.0348
NC_005100.4-144811770	CTAGACTGCACATTCGCTCCCGCTCCC	16	G	0	0.4833
NC_005100.4-144811770	CTAGACTGCACATTCGCTCCCGCTCCC	23	C	0.1217	0.1869
NC_005100.4-144832924	CTCCCTTGAACCCTGGCTCCCAGGTTG	1	C	0.1667	0.4861
NC_005100.4-144832924	CTCCCTTGAACCCTGGCTCCCAGGTTG	13	C	0.0417	0.0566
NC_005100.4-144871034	TTTCTTCTTACCTGTGCTCCCAGGACA	6	T	0.2787	0.3624
NC_005100.4-144872067	CTGAGTCACACGTTGCCTCCTATGTTG	5	G	0.041	0.4903
NC_005100.4-144895983	TTTAGTCTCACATCAGCTCCTCCAGCT	12	A	0	0.4793
NC_005100.4-144895983	TTTAGTCTCACATCAGCTCCTCCAGCT	16	G	0.0325	0.032
NC_005100.4-144905644	TACCAACTAACATGATCTCCCAGGGAT	14	G	0.0932	0.2156
NC_005100.4-144905644	TACCAACTAACATGATCTCCCAGGGAT	27	T	0.0085	0.4842
NC_005100.4-145213210	TGACTTCCCACCCCTACTCCATGTAGA	27	A	0	0.4773
NC_005100.4-145311215	CATTGGGTCACTTCTGCTCCAGCAGTC	23	C	0.041	0.0557
NC_005100.4-145411617	ACTTTAGAGACAGAGACTCCTTGTATA	16	A	0.0965	0.4529
NC_005100.4-145411617	ACTTTAGAGACAGAGACTCCTTGTATA	23	G	0.2105	0.4875
NC_005100.4-145450084	AATGCTAAGACTTTGCCTCCCCCTGCT	6	T	0.1864	0.4964
NC_005100.4-145450084	AATGCTAAGACTTTGCCTCCCCCTGCT	7	A	0.0508	0.0812
NC_005100.4-145453019	AGCATACTGACTACATCTCCTTTGTCT	13	A	0.1626	0.5
NC_005100.4-145453019	AGCATACTGACTACATCTCCTTTGTCT	22	T	0.122	0.4979
NC_005100.4-145467761	GTGGCCTCTACATCAGCTCCTGTCCCC	27	C	0.2397	0.4277
NC_005100.4-145469697	CCATCTGTAACATCAGCTCCAAGAATG	6	T	0.0442	0.06
NC_005100.4-145482159	CTGCCTGTAACTCCAGCTCCTGGCATC	22	G	0.0517	0.0825
NC_005100.4-145549184	CCGAGTATAACTCCACCTCCAGCCACC	26	C	0.1724	0.2501
NC_005100.4-145564554	CTTCCTATAACTCCAGCTCCAAGGAAT	8	T	0	0.4709
NC_005100.4-145599844	GAACCTCAGACCAGATCTCCTGAACGT	26	G	0.1513	0.322
NC_005100.4-145624494	TTAGATTCCACACCAGCTCCTCATTCT	14	C	0.0855	0.0818
NC_005100.4-145666922	AATACCAACACCTCTACTCCAACAATT	2	A	0.1429	0.1327
NC_005100.4-145697180	ATTCCCTAAACCATTGCTCCTAGAATT	8	A	0.1593	0.4953
NC_005100.4-145714533	ACTGCCCTGACCTCTGCTCCCTGAGAT	9	G	0.0261	0.0257
NC_005100.4-145714533	ACTGCCCTGACCTCTGCTCCCTGAGAT	12	C	0.0254	0.0251
NC_005100.4-145717920	CAGGTTTTTACATTGTCTCCAAACTCA	4	G	0.1053	0.4501
NC_005100.4-145717920	CAGGTTTTTACATTGTCTCCAAACTCA	27	A	0.0789	0.0758
NC_005100.4-145732453	CCAGGTCGCACAAAGGCTCCATCTCAG	15	G	0.2231	0.4693
NC_005100.4-145754010	GTTGTGAGTACTACACCTCCCAGCCTG	21	C	0.1161	0.2254
NC_005100.4-145798809	CCCACTGGCACTCATTCTCCCTCTCTT	4	A	0.1176	0.1539
NC_005100.4-145812432	TGACGCCCCACTAATTCTCCAAACTCA	24	C	0.2321	0.4731
NC_005100.4-145854904	GTGCTCTGCACTTTCTCTCCTTAACTC	6	C	0.2114	0.2499
NC_005100.4-145856635	CTGTGCCACACACCCCCTCCTCAGTCC	1	C	0.2689	0.4779
NC_005100.4-145856635	CTGTGCCACACACCCCCTCCTCAGTCC	14	C	0.2605	0.4761
NC_005100.4-145868065	GTAGCAGGCACCTCAGCTCCTGATTCT	2	T	0.1345	0.1539

NC_005100.4-145883712	CCCTTTAATACAGTTCCTCCTGTTGTT	24	T	0.3675	0.3
NC_005100.4-145885027	TCAAGAAAGACCTTTCCTCCTCAGTTA	6	A	0	0.482
NC_005100.4-145906743	GAAGAGGGCACCAGCTCTCCTGGAGCT	23	G	0.1849	0.1678
NC_005100.4-145931968	GAGATCTTGACTCTACCTCCCAATGGT	26	G	0	0.4832
NC_005100.4-145968254	CTGGCTATCACAGCTCCTCCACTCTTA	8	T	0.0526	0.2021
NC_005100.4-145979701	AAAGCAGATACAGGGACTCCCGTGGCA	22	G	0.0086	0.4688
NC_005100.4-145979701	AAAGCAGATACAGGGACTCCCGTGGCA	25	G	0.1624	0.4987
NC_005100.4-146020905	ACCAGCACCACACAAGCTCCTGACTTA	26	T	0.2542	0.4677
NC_005100.4-146053641	CTCCTGTCAACTGATGCTCCATTCTAT	1	C	0.2017	0.3118
NC_005100.4-146053641	CTCCTGTCAACTGATGCTCCATTCTAT	25	T	0.1176	0.2796
NC_005100.4-146067115	GTAATGAGGACAGACTCTCCTAAGCGC	12	A	0.1795	0.2299
NC_005100.4-146070557	GACTCTGGGACTAAGGCTCCTTGACAC	3	C	0.0331	0.0325
NC_005100.4-146078697	TACCACATGACCTCGACTCCCATCAAT	1	T	0.1271	0.3575
NC_005100.4-146086183	TTCCCACCCACGATCTCTCCTCTCCTC	12	G	0.0163	0.0629
NC_005100.4-146093812	GTAGCCAGGACCATGACTCCCTTTTCA	7	A	0.0252	0.0249
NC_005100.4-146093812	GTAGCCAGGACCATGACTCCCTTTTCA	8	G	0.0254	0.0415
NC_005100.4-146108345	GCCCCATGTACTTCAGCTCCACACCCT	2	C	0.2114	0.4389
NC_005100.4-146115365	AGAAGTCTAACATTGTCTCCCTTCATA	15	G	0.1478	0.258
NC_005100.4-146146956	TAATGGAAAACATCAGCTCCAGGCTTG	3	A	0.1463	0.2142
NC_005100.4-146178091	CTGACTCCCACCCAACCTCCTGTCCCA	15	A	0.0339	0.0333
NC_005100.4-146219927	CAGGAGTACACTGCATCTCCAGCTCCC	21	A	0.0656	0.0786
NC_005100.4-146271050	ATCACATGTACACAGTCTCCTCATGAG	9	T	0.2541	0.321
NC_005100.4-146271050	ATCACATGTACACAGTCTCCTCATGAG	13	C	0.1311	0.1774
NC_005100.4-146281693	GCATCTGGTACAGCAACTCCCAACAGT	23	A	0.2358	0.381
NC_005100.4-146282308	CTGCTCTCTACCATCTCTCCCAGGCTT	14	T	0.0354	0.0348
NC_005100.4-146286065	ATCACTTCTACCAAAGCTCCACTTCTG	21	A	0.0246	0.0243
NC_005100.4-146304570	AAATGTTGAACTTCCACTCCCCTGCCC	4	T	0	0.4755
NC_005100.4-146331794	GAGACAAAAACAGATTCTCCCTCACAG	6	A	0.0504	0.065
NC_005100.4-146331794	GAGACAAAAACAGATTCTCCCTCACAG	9	A	0	0.4793
NC_005100.4-146413553	TCTAATCATACACTCTCTCCTCTGACC	8	A	0.0902	0.1573
NC_005100.4-146419268	TGACGAACCACCTTTTCTCCTTGCACT	5	G	0.225	0.2125
NC_005100.4-146438926	TATGCCGAGACAGGAACTCCGTGGGCA	7	G	0.065	0.0629
NC_005100.4-146462854	CCTTTCAAGACAGGCTCTCCACACCAG	24	C	0.2241	0.488
NC_005100.4-146467133	TTTAATGTGACATGCCCTCCACAGGCT	21	A	0.2241	0.4281
NC_005100.4-146475012	GTTGTGGGTACCCGAACTCCTATACAA	16	A	0.0714	0.0689
NC_005100.4-146499119	CACCACGTGACTAAGCCTCCTCTCATC	7	G	0.0855	0.2107
NC_005100.4-146499119	CACCACGTGACTAAGCCTCCTCTCATC	9	G	0.0678	0.0812
NC_005100.4-146551512	AAGACTTCAACCTGATCTCCCACAGTA	23	C	0.2883	0.349
NC_005100.4-146556974	CCCAAGGCCACCGTCCCTCCAAGGCAC	12	C	0.0588	0.0571
NC_005100.4-146600436	AGCACGCTCACAATCACTCCAGCACTC	2	G	0.0086	0.4836
NC_005100.4-146650022	GCATTGGGAACCTCTTCTCCCAGTAAC	21	C	0.1391	0.1588
NC_005100.4-146650022	GCATTGGGAACCTCTTCTCCCAGTAAC	26	A	0.0435	0.0425
NC_005100.4-146655167	CAGTGTCCCACCCCTCCTCCTCCTCCT	15	T	0.0811	0.0942
NC_005100.4-146667439	CCTTGCTTGACCTAAACTCCATATCAT	7	T	0.1538	0.497
NC_005100.4-146674454	GAGAGAAAGACCTGATCTCCTTTAGAT	7	A	0.0427	0.4671
NC_005100.4-146674454	GAGAGAAAGACCTGATCTCCTTTAGAT	9	G	0	0.4749
NC_005100.4-146706367	CCCCACCCTACTTCAACTCCTGTTTCC	13	T	0.1696	0.1843
NC_005100.4-146706367	CCCCACCCTACTTCAACTCCTGTTTCC	16	A	0.0088	0.0262
NC_005100.4-146744839	GCAGTCTGGACGACATCTCCAGCATTA	1	G	0.0169	0.4793
NC_005100.4-146856080	TTGTTGGAAACAGGATCTCCCAGTCAG	23	G	0.1518	0.1843
NC_005100.4-146876453	AAGGACTGTACGGTCCCTCCCCAAACT	5	A	0.2101	0.2852

NC_005100.4-146876453	AAGGACTGTACGGTCCCTCCCCAAACT	6	C	0.2101	0.2852
NC_005100.4-146952851	AGCAGGTCAACAATCACTCCTCTGCAT	7	T	0.0085	0.4714
NC_005100.4-147022593	CTGCAGGAAACCATCTCTCCCTCGGGA	6	G	0.0175	0.4925
NC_005100.4-147685645	ACAAGTGGAACACATACTCCTGTAGCA	9	A	0.5902	0.4226
NC_005100.4-147706414	TTCTACATTACCAAACCTCCTGGCATG	9	T	0.3719	0.4863
NC_005100.4-147714847	TTCGGTACTACACAGTCTCCACACTTG	3	C	0.437	0.3415
NC_005100.4-147714847	TTCGGTACTACACAGTCTCCACACTTG	24	C	0.0256	0.4906
NC_005100.4-147789146	TCCTCTAACACTAAACCTCCTGTGATT	9	C	0.2975	0.2533
NC_005100.4-148110084	CACACACACACACACACTCCATTATCA	16	A	0.0661	0.0639
NC_005100.4-148268898	GTAGGGTACACCCAACCTCCAACATCC	27	C	0.425	0.3347
NC_005100.4-148269598	ATGAAACACACCTTCACTCCTTTGCCT	3	G	0.5398	0.4021
NC_005100.4-148269598	ATGAAACACACCTTCACTCCTTTGCCT	9	C	0.5398	0.4021
NC_005100.4-149130848	CGCTCAGACACTGGACCTCCAACATGG	16	C	0.1441	0.3575
NC_005100.4-149179031	CACCTTTCTACACATCCTCCACAGAAT	8	C	0.0492	0.0634
NC_005100.4-149179031	CACCTTTCTACACATCCTCCACAGAAT	21	A	0.0082	0.4822
NC_005100.4-150075642	AAGATCTGAACCCAGACTCCTACAACA	1	A	0	0.4793
NC_005100.4-150226826	GGAACGTGAACCTCATCTCCTTCCCAC	13	T	0.3577	0.2937
NC_005100.4-150311683	AACTCTAGGACCACATCTCCTTAGATC	13	A	0.1148	0.4515
NC_005100.4-150313541	TTCCTGTCAACATGCACTCCTTAGCCT	8	C	0.0526	0.0677
NC_005100.4-150362807	CTCCAGCCCACCAGACCTCCCCATGAC	25	G	0.125	0.4483
NC_005100.4-150368456	ATACCAACAACCATAGCTCCCAGGGTC	27	C	0	0.4786
NC_005100.4-150399928	ACTCTACCCACCGCCACTCCCTGCTCT	4	C	0.0488	0.0476
NC_005100.4-150399928	ACTCTACCCACCGCCACTCCCTGCTCT	12	C	0.0244	0.0398
NC_005100.4-150519418	GACTATTTTACAATTACTCCTACCAAT	12	A	0	0.4772
NC_005100.4-150519418	GACTATTTTACAATTACTCCTACCAAT	16	A	0.1026	0.1125
NC_005100.4-150687162	TATCTACCCACCCATTCTCCACTGAAT	23	T	0.0833	0.1099
NC_005100.4-150846170	CACATCTCCACTTGCTCTCCCTGGACC	24	G	0	0.4539
NC_005100.4-150846170	CACATCTCCACTTGCTCTCCCTGGACC	27	C	0	0.4539
NC_005100.4-150927298	CATTGGTTAACAGAGACTCCATGTGGA	1	C	0.2764	0.4595
NC_005100.4-150927298	CATTGGTTAACAGAGACTCCATGTGGA	7	T	0.2195	0.2203
NC_005100.4-150951614	ATAACCCATACCTCCTCTCCCCCTCTC	2	T	0	0.4698
NC_005100.4-150998787	CTGCTCGACACTATGTCTCCTTATTTC	27	C	0.0708	0.4887
NC_005100.4-151102680	TTACTTTAAACACATCCTCCTGCTGTT	13	C	0.1393	0.1708
NC_005100.4-151148659	TCAAGCTATACCACTTCTCCTTCCCTC	6	C	0.0902	0.0861
NC_005100.4-151148659	TCAAGCTATACCACTTCTCCTTCCCTC	9	T	0.1393	0.1573
NC_005100.4-151230648	CTCACCAGAACTGACTCTCCATTCAAT	3	C	0.2712	0.4718
NC_005100.4-151231249	TCTTTTCTCACCCTAACTCCTCCACCT	23	C	0.0088	0.4873
NC_005100.4-151231249	TCTTTTCTCACCCTAACTCCTCCACCT	27	T	0.0088	0.4873
NC_005100.4-151248183	TTTTACTGCACACTTCCTCCATGATTG	5	A	0.2605	0.3367
NC_005100.4-151248183	TTTTACTGCACACTTCCTCCATGATTG	22	T	0.2941	0.274
NC_005100.4-151409238	AAATGTCAGACTTTATCTCCTTGTAGT	5	G	0.1171	0.1565
NC_005100.4-151409238	AAATGTCAGACTTTATCTCCTTGTAGT	13	T	0.045	0.0611
NC_005100.4-151420576	GTGCCAAGCACCTCCCCTCCCTTTGAT	3	G	0	0.4793
NC_005100.4-151426971	TCCTGCCAAACACCAGCTCCACTTCCC	27	C	0.0583	0.0566
NC_005100.4-151501446	CTAGTGTCAACTGTGCCTCCTGGGTAC	13	G	0.1917	0.2372
NC_005100.4-151519007	GTCCTGATCACTCTTGCTCCTTGTTCA	3	C	0.2049	0.4997
NC_005100.4-151519007	GTCCTGATCACTCTTGCTCCTTGTTCA	14	T	0.2049	0.4997
NC_005100.4-151748630	CCTCCCACAACCTGCACTCCTACCCTA	16	A	0.1875	0.4751
NC_005100.4-151772138	GTTCTGAGCACAGGAGCTCCTGCACTC	1	G	0.0165	0.4713
NC_005100.4-151772138	GTTCTGAGCACAGGAGCTCCTGCACTC	12	A	0.0083	0.4732
NC_005100.4-151774752	CCTTATCACACACCGTCTCCAGCAATG	15	G	0.0171	0.05

NC_005100.4-151775334	TCCCTCAGTACTGAGGCTCCATCTGGC	13	G	0.1933	0.3729
NC_005100.4-151794039	GTCAACATGACCAAACCTCCTACGAAC	23	C	0.1947	0.3543
NC_005100.4-151794039	GTCAACATGACCAAACCTCCTACGAAC	24	G	0.0442	0.0765
NC_005100.4-151809608	GGCACATGAACTCTTACTCCCTGTCAG	2	G	0.1062	0.1316
NC_005100.4-151828004	CATGGAGATACCTTTGCTCCCTGTGAC	23	G	0.1478	0.2072
NC_005100.4-151887104	TACCAACCAACTAGATCTCCCTACCCC	7	C	0	0.0322
NC_005100.4-151892510	ATCCAAACCACTTCACCTCCTTGAGTT	3	C	0.0339	0.0333
NC_005100.4-151908963	ATATTCTTCACTGTCTCTCCCTGGAAC	23	G	0.042	0.0411
NC_005100.4-151910852	ACAGCCGGCACCACTACTCCTGGTTTG	7	G	0.1532	0.3338
NC_005100.4-151979418	TGGAGAACAACGAACACTCCTCCATTG	26	T	0.0246	0.0243
NC_005100.4-152026068	ACCTATCTTACTTTACCTCCTTCCTAC	6	T	0	0.4718
NC_005100.4-152026068	ACCTATCTTACTTTACCTCCTTCCTAC	25	T	0	0.4718
NC_005100.4-152083956	TCATTATGCACCTTTGCTCCAGGCTCT	13	T	0.2066	0.4978
NC_005100.4-152087066	TCTTCCCTCACAGATACTCCCTCACAG	16	A	0.1545	0.5
NC_005100.4-152143417	ACCACTCCCACCTAACCTCCCTGGCAT	4	A	0.1597	0.5
NC_005100.4-152189813	AGGGGTCCCACAGGAACTCCTAGCTAT	21	T	0	0.4755
NC_005100.4-152391365	CACACATGCACACTCACTCCTTAGGTC	9	C	0	0.4819
NC_005100.4-152410003	CAGAGTTACACTAGGACTCCACAGGCA	12	T	0.0084	0.4723
NC_005100.4-152415882	CTCCTTCAAACTGGACCTCCTTTCCAG	8	A	0	0.477
NC_005100.4-152473264	CACCATCTCACCTCTCCTCCCCGTCAT	5	A	0	0.4786
NC_005100.4-152488170	GAAATCATCACATTTCCTCCTTCCTTC	4	A	0.1513	0.1813
NC_005100.4-152676809	ATCACTTCTACTCAGGCTCCTTCCTAA	13	C	0.1653	0.1516
NC_005100.4-152751877	TGCTCTAACACTGTGGCTCCTGCACCT	12	T	0.2893	0.4507
NC_005100.4-152751877	TGCTCTAACACTGTGGCTCCTGCACCT	26	C	0.0246	0.0401
NC_005100.4-152798040	AGAGAGGAAACCTGATCTCCACCCTCT	7	G	0.1057	0.1145
NC_005100.4-152831099	GGATGTGTGACTGTCTCTCCTTCTCCC	27	C	0.0407	0.0398
NC_005100.4-152837261	TGGAACAAAACCACTGCTCCTAGAGTC	6	C	0.2018	0.463
NC_005100.4-152855467	GAATTGAGCACCTTGTCTCCCTTTACA	25	A	0	0.4693
NC_005100.4-152855467	GAATTGAGCACCTTGTCTCCCTTTACA	27	A	0	0.4693
NC_005100.4-152892773	TCTTTCGATACTTGGGCTCCTAAATAT	5	T	0.2174	0.27
NC_005100.4-152916949	GAGTCTGAAACAGAGGCTCCAGCCTCT	8	A	0.0088	0.4796
NC_005100.4-152916949	GAGTCTGAAACAGAGGCTCCAGCCTCT	23	C	0.0259	0.0255
NC_005100.4-152924412	AAATGGAAAACAGAAGCTCCCTTGGTC	7	A	0.1885	0.3008
NC_005100.4-152962993	TGGGTACCGACCTGACCTCCAGTCCTC	16	C	0.0446	0.0605
NC_005100.4-152962993	TGGGTACCGACCTGACCTCCAGTCCTC	23	T	0.2143	0.4871
NC_005100.4-152982827	TGAGGACTTACTAACCCTCCACTAGTC	4	G	0.0083	0.4747
NC_005100.4-152984194	AGGCTTACTACTGCTTCTCCAGACAGA	3	G	0.045	0.4896
NC_005100.4-152999164	ACCCTTTGTACCTGTTCTCCATCCTTG	25	T	0	0.4773
NC_005100.4-153022922	CTGACACTGACACGGCCTCCACGTTCC	23	G	0.0336	0.033
NC_005100.4-153093131	GAAGCAATTACATCAACTCCTGTCATT	8	T	0	0.4773
NC_005100.4-153169705	CACTGCCTCACTAGAGCTCCAATAGCC	24	A	0.1917	0.225
NC_005100.4-153169705	CACTGCCTCACTAGAGCTCCAATAGCC	26	C	0.0756	0.1469
NC_005100.4-153173181	CAACTCTACACTTACACTCCAACATTC	9	C	0.0902	0.1009
NC_005100.4-153230894	ATTTTTACTACCCTGCCTCCTGGCCTG	7	A	0.1789	0.414
NC_005100.4-153230894	ATTTTTACTACCCTGCCTCCTGGCCTG	23	G	0.0732	0.1826
NC_005100.4-153305936	CCAGCAGAGACATTTCCTCCGCGCAGA	21	G	0.1593	0.3136
NC_005100.4-153331120	TGGAAGGTCACTTACCCTCCCTGGGCC	24	G	0.1786	0.3047
NC_005100.4-153416851	GTATTGTTTACCTTTCCTCCTCACTGT	3	A	0.0847	0.4481
NC_005100.4-153431578	GGAGTTGCAACATGACCTCCTCATCTC	2	G	0.0488	0.0476
NC_005100.4-153517664	GCTATTCAAACTCTATCTCCCTTTGAT	13	C	0.1786	0.2698
NC_005100.4-153553969	TTGCCTGCCACATCTCCTCCCACAGAC	21	C	0.0488	0.496

NC_005100.4-153553969	TTGCCTGCCACATCTCCTCCCACAGAC	22	A	0.0163	0.032
NC_005100.4-153669924	CCTGGGTTCACGTTATCTCCCTTCTGT	12	G	0.1282	0.4994
NC_005100.4-153675686	TGTTTCTTAACATTAGCTCCAACAATG	25	A	0.0684	0.4918
NC_005100.4-153709423	TACCTTCTTACCTCTCCTCCCATCACC	22	A	0.0328	0.4737
NC_005100.4-153757543	AGAGAGGGGACCACTTCTCCCTTAATT	21	C	0.1947	0.2431
NC_005100.4-153770639	GAATGCTGTACATTTCCTCCTGAGGAC	2	A	0.1983	0.4983
NC_005100.4-153772957	CTGAATTGCACGCGAGCTCCATGGCAT	8	G	0.0336	0.065
NC_005100.4-153789585	CTTATACCCACTGGTGCTCCCTTCTGA	3	T	0.1043	0.4997
NC_005100.4-153789585	CTTATACCCACTGGTGCTCCCTTCTGA	14	G	0.2155	0.2797
NC_005100.4-153791402	TTCTATTGGACATGCTCTCCTACCTAT	23	C	0.0089	0.4751
NC_005100.4-153859211	GGTCTTTCTACCCAGCCTCCTAACATG	6	T	0	0.4833
NC_005100.4-153859211	GGTCTTTCTACCCAGCCTCCTAACATG	27	G	0.1913	0.1869
NC_005100.4-153876216	TCCAAGGCCACACTTCCTCCAACAAGT	13	C	0.1053	0.1884
NC_005100.4-153884713	GCAAACGACACAGGTTCTCCACAGTCT	6	C	0.2437	0.327
NC_005100.4-153930013	AGAATGTCAACATGGCCTCCTTCAGAG	1	A	0	0.4832
NC_005100.4-154018512	AAATGAAGCACATGTGCTCCAGACTTC	7	A	0	0.4793
NC_005100.4-154030239	GGGGCCACTACTTTCCCTCCAGCCTTA	15	C	0.0924	0.1327
NC_005100.4-154056505	GAAGGTCTGACGCCATCTCCAGAGATT	15	A	0.0084	0.4829
NC_005100.4-154149039	CGGTGGTGCACACATTCTCCCAGCACT	1	C	0.0089	0.4789
NC_005100.4-154160899	TTCTATTTCACTAGCCCTCCCAAGGAA	22	A	0	0.474
NC_005100.4-154190155	CTTAGAGAAACCTAAGCTCCAGCCTTG	8	A	0.122	0.1425
NC_005100.4-154228962	GCTTGTGTTACAAAACCTCCATAAATA	15	A	0.0901	0.1182
NC_005100.4-154246142	TGGACATTAACAGTGTCTCCAACAGTT	15	G	0.3008	0.373
NC_005100.4-154287619	CAATTTCCCACATCCACTCCAGCTGGT	13	T	0.188	0.3729
NC_005100.4-154315205	TTTGTTCTCACTGGCCCTCCTTCCTTT	14	G	0	0.4793
NC_005100.4-154315205	TTTGTTCTCACTGGCCCTCCTTCCTTT	26	T	0.187	0.4952
NC_005100.4-154331752	ATCTACCAAACCATTTCTCCGGCCTTA	21	G	0.1463	0.4992
NC_005100.4-154347959	AGCAGAGCAACCACCGCTCCAGTTTCC	15	C	0.1333	0.1932
NC_005100.4-154352471	AGCATTGTCACCCCTCCTCCATTATTT	5	T	0.2821	0.3935
NC_005100.4-154409580	AACCCATGAACCATCTCTCCAGCCCCT	3	C	0.115	0.154
NC_005100.4-154498151	TCCTAGTTCACCACAACTCCATATCAT	2	C	0.1441	0.4842
NC_005100.4-154503574	ATACTTCTCACCCCGCCTCCCAATTCT	15	G	0.0763	0.0889
NC_005100.4-154748388	CACCCAGAGACATGTCCTCCAGTGCAC	27	C	0.0976	0.0928
NC_005100.4-154768337	GGTCAATCTACTGGCTCTCCTACTTAC	21	T	0.1057	0.1287
NC_005100.4-154971184	TCTGTGGCCACTGTCTCTCCTCCCCAG	12	T	0.2521	0.3932
NC_005100.4-154971184	TCTGTGGCCACTGTCTCTCCTCCCCAG	13	G	0.1008	0.0958
NC_005100.4-154978780	TATTTGTGGACAGATACTCCAAAACAT	15	T	0.114	0.499
NC_005100.4-154978780	TATTTGTGGACAGATACTCCAAAACAT	27	T	0.114	0.499
NC_005100.4-154997131	TCCTGGTTGACCTATCCTCCTATTCTG	26	T	0.1504	0.5
NC_005100.4-155016885	TAGATCAGCACTCTCTCTCCTTCAGCT	4	A	0.1008	0.0958
NC_005100.4-155016885	TAGATCAGCACTCTCTCTCCTTCAGCT	26	C	0.1167	0.1665
NC_005100.4-155034570	TACGTTGATACGTCATCTCCATACTTG	9	T	0.1071	0.1014
NC_005100.4-155034570	TACGTTGATACGTCATCTCCATACTTG	25	T	0.0089	0.4751
NC_005100.4-155135220	TTTTCAGAAACCCCACCTCCTATCCCT	2	T	0	0.4807
NC_005100.4-155194656	AATGACCAGACTCTATCTCCATTACCC	3	T	0	0.4839
NC_005100.4-155222653	ACAGATAGCACCCTTTCTCCTCTTTTT	27	T	0	0.4772
NC_005100.4-155451803	GCATGAGACACAGCTCCTCCAAACAGA	7	G	0.0331	0.0325
NC_005100.4-155451803	GCATGAGACACAGCTCCTCCAAACAGA	12	A	0.157	0.4999
NC_005100.4-155454043	TGCTTACTGACTCTTACTCCAAGCTGG	9	G	0.0333	0.0328
NC_005100.4-155454043	TGCTTACTGACTCTTACTCCAAGCTGG	13	C	0	0.4832
NC_005100.4-155454353	TTTAACTATACACTTGCTCCATCTGTT	16	G	0.1083	0.4525

NC_005100.4-155466311	GAAACACGCACAGCATCTCCAGCCAGT	7	C	0.2478	0.4276
NC_005100.4-155487341	CTAGAGTTGACTTCATCTCCTTATGGG	4	G	0.25	0.375
NC_005100.4-155487341	CTAGAGTTGACTTCATCTCCTTATGGG	26	G	0.0417	0.0408
NC_005100.4-155517312	AGGTTAAAGACACTGTCTCCAGGAGTG	25	G	0.0328	0.0322
NC_005100.4-155596483	GAAGGTTGCACCGCCTCTCCTGAACCT	16	T	0.0488	0.0476
NC_005100.4-155599980	TAATGTGCAACTCAGACTCCTCGGTCA	23	G	0.124	0.1585
NC_005100.4-155601046	GATGCTCAGACTGGAACTCCCCTCAGT	5	C	0.045	0.044
NC_005100.4-155601046	GATGCTCAGACTGGAACTCCCCTCAGT	16	A	0	0.4731
NC_005100.4-155936968	CTTGGATCTACCATTACTCCTTAAATA	7	T	0.1034	0.0981
NC_005100.4-155936968	CTTGGATCTACCATTACTCCTTAAATA	14	T	0.0435	0.0425
NC_005100.4-155981331	CCTCCTCCTACTCTGCCTCCCTGAATG	9	T	0	0.4773
NC_005100.4-155981331	CCTCCTCCTACTCTGCCTCCCTGAATG	16	C	0.0732	0.0854
NC_005100.4-156060146	ATAATCCTTACCCTATCTCCCTATCCT	4	A	0	0.4757
NC_005100.4-156060146	ATAATCCTTACCCTATCTCCCTATCCT	14	T	0	0.4757
NC_005100.4-156090742	CTTTAATGTACTCCTGCTCCCAGTGTG	21	C	0.0446	0.4824
NC_005100.4-156422016	CTGCGACGCACCCAGGCTCCCACCTCG	5	G	0.2149	0.2759
NC_005100.4-156422016	CTGCGACGCACCCAGGCTCCCACCTCG	8	G	0.2149	0.2759
NC_005100.4-156458835	GTCCAGAGAACCTGGGCTCCAATCCCA	21	A	0.2389	0.4098
NC_005100.4-156463100	TGCATCCAGACCTGGGCTCCCAGCCTC	21	C	0.0976	0.0928
NC_005100.4-156559357	AGGATGGAGACCACCCCTCCAGGTATC	12	C	0.1947	0.2556
NC_005100.4-156573510	AATGAACTGACCCTTACTCCCAGTAAG	16	A	0	0.48
NC_005100.4-156650866	GGAACCATTACATCAGCTCCCTCTTCC	1	G	0.042	0.0411
NC_005100.4-156653056	GAGTGTCACACAGAGACTCCAGCAACT	15	G	0.094	0.0896
NC_005100.4-156713774	AGGGCTATCACTTTTCCTCCCATCCTA	5	C	0.0345	0.0666
NC_005100.4-156770662	CTAGTGCGTACTCTTCCTCCTGCCTCT	9	T	0.0333	0.0328
NC_005100.4-156808941	GCCAAACATACTCTTCCTCCTACAAAA	8	A	0.0086	0.4729
NC_005100.4-156827538	AAAGGCATTACTTAATCTCCTCTAAAT	12	T	0.0924	0.1033
NC_005100.4-156827538	AAAGGCATTACTTAATCTCCTCTAAAT	13	T	0.0336	0.4844
NC_005100.4-156867500	ATCTTTTCCACATTGCCTCCATTATAG	14	T	0.2542	0.4908
NC_005100.4-156870116	ACACAGCAAACCAAACCTCCACAAGAA	26	A	0	0.4854
NC_005100.4-156891195	CACCTCTGGACCTTAGCTCCTTCCTGT	27	T	0.0976	0.0928
NC_005100.4-157000989	CCTGTGCTAACACATGCTCCTTCATTG	9	A	0.0082	0.4822
NC_005100.4-157027049	TAGCTCATCACTCTGCCTCCTCCATCA	26	C	0.0625	0.491
NC_005100.4-157027049	TAGCTCATCACTCTGCCTCCTCCATCA	27	A	0	0.4807
NC_005100.4-157056422	CCTGCTCCTACTGTACCTCCACTCGAC	21	A	0.0259	0.0422
NC_005100.4-157056422	CCTGCTCCTACTGTACCTCCACTCGAC	24	C	0.0259	0.0585
NC_005100.4-157059581	ATAGCTGTAACAATTCCTCCATTTTAT	21	A	0	0.0348
NC_005100.4-157059581	ATAGCTGTAACAATTCCTCCATTTTAT	22	T	0.2035	0.2738
NC_005100.4-157100999	TTCACTCTGACAAGCCCTCCTATCACA	23	T	0	0.4637
NC_005100.4-157157469	CTTGAACTAACATGTTCTCCTACAAGA	5	A	0.0086	0.4803
NC_005100.4-157157469	CTTGAACTAACATGTTCTCCTACAAGA	21	T	0.1368	0.3457
NC_005100.4-157163223	AATCCAAATACGTGTCCTCCCTACCCC	1	A	0	0.4851
NC_005100.4-157213573	GACTAGCCCACACAAACTCCAGCAAAA	3	C	0.0446	0.0436
NC_005100.4-157216567	TGTTCAGTCACACAGCCTCCTTACAAT	2	G	0.0086	0.0255
NC_005100.4-157216567	TGTTCAGTCACACAGCCTCCTTACAAT	4	T	0.0086	0.4836
NC_005100.4-157300136	ATACACAGCACAGAAGCTCCGTGGTCT	21	G	0	0.4779
NC_005100.4-157301344	CAGAGGTTTACCTCCTCTCCTTGAGCT	21	T	0.0246	0.0243
NC_005100.4-157383553	CTAAGCAGCACAGTTTCTCCAGGAACT	12	A	0.0259	0.0585
NC_005100.4-157391156	GCCACCTCCACCTCCACTCCCTCCATC	1	G	0	0.4624
NC_005100.4-157391297	ATGTTTATAACCAAATCTCCCTCCACC	12	C	0.0847	0.0812
NC_005100.4-157503127	CCAGGCCTTACACCGTCTCCCGTTCCC	15	G	0	0.4773

NC_005100.4-157503127	CCAGGCCTTACACCGTCTCCCGTTCCC	22	G	0.0246	0.0243
NC_005100.4-157505354	CCTACAGAGACCTTGTCTCCTCAGAAA	2	C	0.0083	0.0405
NC_005100.4-157579973	GCCTGGTCTACAAGAGCTCCAGATAGC	12	A	0.1228	0.1153
NC_005100.4-157592399	CCCTGAGAGACCTTGTCTCCTTGTCCT	3	C	0.2131	0.2515
NC_005100.4-157592399	CCCTGAGAGACCTTGTCTCCTTGTCCT	4	T	0.2459	0.4934
NC_005100.4-157765131	AGTGAGTCAACAGGTTCTCCAGGACAG	22	G	0.1695	0.2091
NC_005100.4-157855599	AGCTTATTGACTAAGCCTCCAGGCCTT	25	C	0.5508	0.4609
NC_005100.4-157880336	CACTGCTGAACCATCTCTCCAACTCAG	24	T	0.0085	0.481
NC_005100.4-157902894	AGGCACCAGACAAGGTCTCCCAGAAGT	1	A	0.0965	0.4953
NC_005100.4-157902894	AGGCACCAGACAAGGTCTCCCAGAAGT	3	G	0.0965	0.4953
NC_005100.4-157919478	CCCAAAGACACTTCAACTCCCACAGCA	6	A	0	0.4731
NC_005100.4-157919478	CCCAAAGACACTTCAACTCCCACAGCA	26	C	0.0177	0.0348
NC_005100.4-157933175	TTCCTAAATACGTTTCCTCCCTGGAAA	16	C	0.2174	0.258
NC_005100.4-157958803	TGTCTCCTTACCCAGTCTCCATTGGTT	25	G	0.0427	0.058
NC_005100.4-158000853	TTCTGAGCCACCGTTGCTCCAGCGCAT	23	C	0.1368	0.4193
NC_005100.4-158019211	GAGTTGGAGACATGAGCTCCTTATGAA	23	A	0	0.4757
NC_005100.4-158056680	GCCTCCAAGACTAGCTCTCCTAGGATG	22	A	0	0.4909
NC_005100.4-158227191	CTCAACAAGACCACACCTCCTTTTTTT	6	C	0.1858	0.3683
NC_005100.4-158227191	CTCAACAAGACCACACCTCCTTTTTTT	27	T	0.2212	0.4021
NC_005100.4-158285919	ATTTAACTTACACCCACTCCAGGAGAT	27	T	0.2589	0.3705
NC_005100.4-158395597	TACCGACCAACCAGAACTCCCAGAGAT	27	T	0	0.0322
NC_005100.4-158429827	ATCTGTAGTACTTGCACTCCACTGAAA	24	G	0.0256	0.0253
NC_005100.4-158527406	CCTCTTACCACCAATACTCCTTAACTC	1	C	0.1017	0.1116
NC_005100.4-158551682	ATGCCTTTGACCTTACCTCCACTTGTC	1	A	0.0574	0.1154
NC_005100.4-158551682	ATGCCTTTGACCTTACCTCCACTTGTC	21	A	0	0.4773
NC_005100.4-158588126	TGGACAGTGACATTATCTCCAACCCTC	1	T	0.0246	0.0243
NC_005100.4-158588126	TGGACAGTGACATTATCTCCAACCCTC	4	A	0.0246	0.0401
NC_005100.4-158701225	GTACTTTCTACAGGGCCTCCCCATCTT	8	C	0.0439	0.0429
NC_005100.4-158701225	GTACTTTCTACAGGGCCTCCCCATCTT	25	C	0.0609	0.059
NC_005100.4-158731397	ACCACAAAAACATTTGCTCCACTCTTG	4	A	0.075	0.1172
NC_005100.4-158731397	ACCACAAAAACATTTGCTCCACTCTTG	24	C	0	0.4787
NC_005100.4-158796032	AAGTCCACAACTGCCTCTCCCTCATAG	8	C	0.0885	0.0846
NC_005100.4-158829111	TCTAAATTGACCCAGTCTCCAGAGATG	2	C	0	0.4764
NC_005100.4-158896904	TTAACATGAACTCAACCTCCCGCCATT	21	C	0.0083	0.4803
NC_005100.4-158896904	TTAACATGAACTCAACCTCCCGCCATT	23	C	0.0083	0.4816
NC_005100.4-159145075	CTCCCCTCCACATCTCCTCCTTGGGTA	5	C	0	0.4807
NC_005100.4-159287598	GAGGCAAGAACAGGAACTCCAAGGACA	6	A	0.0254	0.0251
NC_005100.4-159287598	GAGGCAAGAACAGGAACTCCAAGGACA	26	C	0	0.0333
NC_005100.4-159297967	CATGTGTGTACTTTTGCTCCTTCCGCT	9	T	0.0169	0.4884
NC_005100.4-159368169	TGGAATATCACGTTTTCTCCCTTAATG	12	G	0.0342	0.0336
NC_005100.4-159416172	GAATGAATCACACTTTCTCCAGCAATG	22	G	0.0259	0.0255
NC_005100.4-159547928	AGAATGCATACATCTCCTCCAAAGGTC	6	G	0.0667	0.0799
NC_005100.4-159547928	AGAATGCATACATCTCCTCCAAAGGTC	27	C	0.2417	0.2722
NC_005100.4-159569317	CCAGACCTCACTAGAGCTCCCAGGGAC	3	A	0.0609	0.059
NC_005100.4-159569317	CCAGACCTCACTAGAGCTCCCAGGGAC	6	C	0.386	0.3115
NC_005100.4-159583265	CTCTCCCCAACCCCATCTCCTCAAGTA	6	C	0.0351	0.4698
NC_005100.4-159596296	ACTCTTTCAACATCTTCTCCATGTCTC	6	T	0.2605	0.4829
NC_005100.4-159596296	ACTCTTTCAACATCTTCTCCATGTCTC	26	T	0.0336	0.033
NC_005100.4-159596888	TATATACCCACTTAGACTCCCTTGAAG	26	A	0.0354	0.0348
NC_005100.4-159804943	AACCACGGAACTCTGTCTCCAGCCTTA	7	G	0.0407	0.0553
NC_005100.4-159843627	ACAACAAAAACACAGGCTCCATTATGT	13	C	0.2051	0.2834

NC_005100.4-159857337	CAGACACCCACAGGCCCTCCTTTGCAG	22	T	0	0.4793
NC_005100.4-159857337	CAGACACCCACAGGCCCTCCTTTGCAG	23	T	0.065	0.0629
NC_005100.4-159893739	AGATCTGTGACTAAGGCTCCATAAAGC	13	A	0	0.4743
NC_005100.4-159901767	TATCAACCAACCAACCCTCCTCCCACA	1	T	0.1765	0.2389
NC_005100.4-159901767	TATCAACCAACCAACCCTCCTCCCACA	7	C	0.1765	0.2389
NC_005100.4-160089701	AAAAAGAGAACACCTCCTCCTAGGATA	14	C	0.0488	0.1073
NC_005100.4-160123691	ATTCCCTGCACTTCATCTCCTCACCCT	24	C	0.1707	0.3287
NC_005100.4-160134377	TGTATCTTTACTGAGACTCCTGCATGT	3	T	0.0976	0.1629
NC_005100.4-160138826	ATGAGTTTGACTCTGCCTCCTTCAGCA	12	T	0.1441	0.3833
NC_005100.4-160155009	CTACCTTTAACTAAAGCTCCAAGCCAT	2	T	0.2479	0.4877
NC_005100.4-160352007	CCTTTTCCCACAGTATCTCCTTCTTCC	2	C	0.2437	0.214
NC_005100.4-160352007	CCTTTTCCCACAGTATCTCCTTCTTCC	13	G	0.7565	0.4704
NC_005100.4-160398039	TGCATGTACACAAAATCTCCCCATCAC	26	A	0.2232	0.433
NC_005100.4-160429159	GAATGAATCACTGAACCTCCCCATACT	2	A	0	0.4765
NC_005100.4-160460164	AACACCCCCACCTCATCTCCACCTAAA	6	C	0	0.4743
NC_005100.4-160460164	AACACCCCCACCTCATCTCCACCTAAA	15	A	0.1653	0.2869
NC_005100.4-160498230	AGAAGCCAAACACCCCCTCCCCCATAG	27	G	0.2353	0.3319
NC_005100.4-160546052	GGGTTCTCAACCTGTGCTCCATGACCC	1	G	0.0431	0.0422
NC_005100.4-160546052	GGGTTCTCAACCTGTGCTCCATGACCC	25	C	0.0431	0.0422
NC_005100.4-160553950	CTGCTGCCAACTCTTCCTCCTTTTCCC	27	C	0.1491	0.5
NC_005100.4-160662070	ACGCTGAAAACAGTGCCTCCATCTTTT	2	C	0.2787	0.316
NC_005100.4-160754726	AGATTTGCCACCATTTCTCCAGTACAC	24	A	0	0.4728
NC_005100.4-160766742	TTTGACGAAACACTAGCTCCCTAGTCC	6	C	0.1624	0.2041
NC_005100.4-160866369	TAGATACCTACCTGACCTCCTGAGAGA	21	T	0.2645	0.2533
NC_005100.4-160866369	TAGATACCTACCTGACCTCCTGAGAGA	23	A	0.0083	0.0247
NC_005100.4-160869449	CAGACTGGAACTGTGTCTCCAATGGAC	8	G	0.0833	0.1244
NC_005100.4-160872385	GAATGAGGGACTCCAGCTCCGAATTTA	8	G	0.0165	0.4787
NC_005100.4-160877612	CACTTAGACACTTGACCTCCATCCACA	24	C	0.2479	0.418
NC_005100.4-160884538	ACCACATTCACTCACTCTCCACAGACC	14	A	0.0088	0.4774
NC_005100.4-160891066	CAAGACCCCACCAGGGCTCCCAAGAAC	14	G	0.2458	0.4939
NC_005100.4-160891066	CAAGACCCCACCAGGGCTCCCAAGAAC	25	A	0	0.4757
NC_005100.4-161002758	AAGATGACCACATGGCCTCCTTAAGTG	21	T	0	0.4819
NC_005100.4-161002758	AAGATGACCACATGGCCTCCTTAAGTG	25	G	0.0826	0.4942
NC_005100.4-161004794	TATTACTTCACTTAATCTCCACAGTAA	7	T	0	0.4704
NC_005100.4-161071816	CTAGACATCACTGGCTCTCCTTCTATC	22	T	0	0.4832
NC_005100.4-161075304	TGCCTATGCACCCACCCTCCACAGTCC	24	G	0.2764	0.4881
NC_005100.4-161123541	GTTTCTTCTACCCGACCTCCTAGGGAT	12	C	0	0.4813
NC_005100.4-161123541	GTTTCTTCTACCCGACCTCCTAGGGAT	14	G	0	0.4832
NC_005100.4-161127703	AGACATCAAACCCAGCCTCCATGTGAC	13	C	0.2195	0.3381
NC_005100.4-161127703	AGACATCAAACCCAGCCTCCATGTGAC	27	C	0.0163	0.4759
NC_005100.4-161152220	GAAGCAAGAACACACGCTCCCTGACAA	14	A	0	0.4793
NC_005100.4-161160270	GGTGCCTGCACACTTTCTCCTCTCACT	2	G	0.041	0.0557
NC_005100.4-161187397	GGCAAGGAAACCCTTTCTCCTAGTGGG	8	A	0.2917	0.4847
NC_005100.4-161228042	CATCTTCCTACTGTACCTCCTCAGTGC	26	G	0.065	0.078
NC_005100.4-161235801	TTCAGGACAACCTTGTCTCCAGTTGTT	13	T	0.0085	0.4753
NC_005100.4-161235801	TTCAGGACAACCTTGTCTCCAGTTGTT	22	G	0.0256	0.0253
NC_005100.4-161238059	AAGATTTGAACTCAGGCTCCTGCTAGC	26	G	0.0265	0.0262
NC_005100.4-161268600	GGTTTTCTTACAGGTCCTCCTACAACT	8	T	0.1282	0.4158
NC_005100.4-161278325	GAAGGGCCCACCGCATCTCCATCCCAC	13	G	0	0.4793
NC_005100.4-161343829	AGAATATATACTTCTGCTCCACACATG	24	C	0.0804	0.125
NC_005100.4-161411114	ATTTCCGTCACTGTTTCTCCTAACTCT	22	A	0.2231	0.3929

NC_005100.4-161452534	TGTGGCTGCACATCTGCTCCCACCTCA	1	T	0.1148	0.1367
NC_005100.4-161452534	TGTGGCTGCACATCTGCTCCCACCTCA	6	C	0.2066	0.2814
NC_005100.4-161452702	CCCTGCATCACATACTCTCCTCAGAGA	13	T	0.2149	0.2869
NC_005100.4-161467664	CAGAGCTGTACAAACTCTCCTACCCAA	13	A	0.2195	0.3381
NC_005100.4-161467664	CAGAGCTGTACAAACTCTCCTACCCAA	26	A	0	0.4793
NC_005100.4-161474402	TGAGATGCTACAATCTCTCCAACCTTG	5	A	0.2368	0.295
NC_005100.4-161474402	TGAGATGCTACAATCTCTCCAACCTTG	21	A	0	0.4778
NC_005100.4-161484210	TGCTCTCTAACCCATTCTCCTTCTAAA	9	A	0.084	0.1539
NC_005100.4-161484210	TGCTCTCTAACCCATTCTCCTTCTAAA	23	C	0.0333	0.0328
NC_005100.4-161485503	GAAACCACAACATTTGCTCCAGAGGCC	21	A	0.0708	0.1006
NC_005100.4-161502589	TGTGTGTTAACACCGACTCCCAGGGTT	12	A	0	0.4839
NC_005100.4-161502589	TGTGTGTTAACACCGACTCCCAGGGTT	22	A	0.1429	0.2626
NC_005100.4-161532571	GCCTGTGGAACCGGTCCTCCCTTCTCT	12	C	0.0265	0.4873
NC_005100.4-161536378	AGCAGGGCCACAGGGACTCCTGACCCC	27	C	0.0504	0.0491
NC_005100.4-161539223	ACCTTTGAAACTATGCCTCCTGGTTAG	14	T	0.0492	0.048
NC_005100.4-161565573	CATTGTAATACCAAATCTCCCTTCATG	16	T	0.1405	0.1447
NC_005100.4-161579282	CTAGTACCCACATCAACTCCACATGCA	23	A	0.1624	0.1908
NC_005100.4-161588456	ACTTGCCTAACTTGACCTCCTTTCCTG	3	T	0.0992	0.1377
NC_005100.4-161598924	GCAGAAGGCACTTTGTCTCCTGATTCC	12	T	0.0248	0.0245
NC_005100.4-161614818	GTGGGGAGAACAAAGGCTCCATGGACA	26	C	0	0.4844
NC_005100.4-161649204	TTAGGAAAGACCATTGCTCCATGCCCT	4	G	0	0.4839
NC_005100.4-161649204	TTAGGAAAGACCATTGCTCCATGCCCT	15	T	0	0.4839
NC_005100.4-161650238	ATGGGTTCCACCAGATCTCCCAAGCAC	8	C	0.0328	0.048
NC_005100.4-161660422	CAGGTTCAAACGTCCTCTCCCCTTCTA	2	A	0.0826	0.4942
NC_005100.4-161679552	ACTGGGATCACGCTGACTCCCTTGCAA	23	T	0.0732	0.4935
NC_005100.4-161713649	CGTACTTGCACTGAACCTCCGATAATT	2	G	0.1557	0.1708
NC_005100.4-161730112	ACCTGCATAACTCCAGCTCCTGGAGAT	3	C	0.0244	0.0241
NC_005100.4-161748961	CCCGCCAGAACCCTGGCTCCTTCCCCA	1	C	0.2	0.4926
NC_005100.4-161748961	CCCGCCAGAACCCTGGCTCCTTCCCCA	13	C	0.0261	0.0257
NC_005100.4-161783541	ATTCTACTCACAGGCACTCCAAGGCCA	16	A	0	0.4757
NC_005100.4-161816843	AGTAACAGCACCAGCGCTCCACAGCAG	1	A	0.2627	0.456
NC_005100.4-161816843	AGTAACAGCACCAGCGCTCCACAGCAG	16	G	0.188	0.1704
NC_005100.4-161818291	GATGAGCAAACTGCATCTCCCTGAGCA	15	A	0.1709	0.4938
NC_005100.4-161837464	TTCTAACAAACCAGCGCTCCATCTCCT	8	A	0	0.4821
NC_005100.4-161837464	TTCTAACAAACCAGCGCTCCATCTCCT	24	T	0	0.4841
NC_005100.4-161851480	TGCTGAAGTACAGTGTCTCCCTTTAGC	7	A	0	0.48
NC_005100.4-161851480	TGCTGAAGTACAGTGTCTCCCTTTAGC	12	A	0.2295	0.306
NC_005100.4-161852277	TGGATTTAGACCCCGGCTCCAGCATTA	2	G	0.0336	0.033
NC_005100.4-161871551	GGGAACGTCACCTTTACTCCTCTGAAA	1	G	0.0684	0.1274
NC_005100.4-161871551	GGGAACGTCACCTTTACTCCTCTGAAA	22	C	0.0254	0.0415
NC_005100.4-161888284	ACAGCCTTGACTTCCCCTCCTTTTCTC	1	A	0.0089	0.4933
NC_005100.4-161888284	ACAGCCTTGACTTCCCCTCCTTTTCTC	13	T	0.0089	0.4933
NC_005100.4-161901530	AGGACTCTCACACTGCCTCCGGTGTTT	12	A	0.2276	0.3334
NC_005100.4-161911616	ATGCTCTCAACCACCTCTCCAGCCCTA	4	C	0.0446	0.0436
NC_005100.4-161940483	CATGTGGTGACCACCCCTCCCTCCAAA	7	G	0.2231	0.3687
NC_005100.4-161940864	AGACTCTTGACAGTTCCTCCTCAGTTC	23	A	0	0.4807
NC_005100.4-161957665	TGATGGCTCACCAGCTCTCCCTTAGCC	13	A	0	0.4773
NC_005100.4-162043202	CACTCTCCCACATTTCCTCCTCTCTAT	3	C	0.0246	0.0401
NC_005100.4-162058607	TACAGCAAGACACTGTCTCCAAAGACA	26	C	0.0513	0.066
NC_005100.4-162096436	GGACCAACAACCTCCCCTCCCATTACT	12	C	0.0331	0.0484
NC_005100.4-162140741	CATCCCAGTACTTCATCTCCGTCTTCA	21	G	0.0163	0.0476

NC_005100.4-162221472	TGTTATGGAACTAGTTCTCCACAAGCC	15	T	0.0083	0.4747
NC_005100.4-162242053	TATCAGAAGACTTTCTCTCCTTCTGAC	4	C	0.0088	0.4774
NC_005100.4-162242053	TATCAGAAGACTTTCTCTCCTTCTGAC	5	A	0.0088	0.4774
NC_005100.4-162286954	TGCCTCCGAACATTCTCTCCACAGATG	8	G	0	0.4793
NC_005100.4-162296351	CTGTCACCTACCCAGGCTCCGTCATTC	25	T	0	0.4807
NC_005100.4-162296390	ATGGGAGTCACACGTCCTCCTGTGCTA	23	T	0.3628	0.4735
NC_005100.4-162298764	AGGCGCTGGACGGTGGCTCCTCCTCTG	8	G	0.2735	0.405
NC_005100.4-162302313	GAATGCCTCACCTGCCCTCCCTATCTG	14	G	0	0.4793
NC_005100.4-162302313	GAATGCCTCACCTGCCCTCCCTATCTG	15	C	0.3577	0.3427
NC_005100.4-162360917	TCACTGTGTACAGTATCTCCTCGCTGA	5	T	0	0.4826
NC_005100.4-162365668	TGGTTCACAACTCCTGCTCCACAGGAT	4	T	0	0.4786
NC_005100.4-162366639	TGTGCTGACACAAGAGCTCCCTATGTT	8	A	0.2069	0.488
NC_005100.4-162366639	TGTGCTGACACAAGAGCTCCCTATGTT	26	T	0.181	0.4937
NC_005100.4-162385011	TGAGAGCTCACTTGGCCTCCTTCTACT	15	G	0.1557	0.5
NC_005100.4-162385011	TGAGAGCTCACTTGGCCTCCTTCTACT	16	C	0	0.48
NC_005100.4-162400279	AATTCTTGCACCACCACTCCCCGCTTT	16	A	0.1583	0.4972
NC_005100.4-162400279	AATTCTTGCACCACCACTCCCCGCTTT	23	G	0.0244	0.0398
NC_005100.4-162409824	CGTGCTCTCACTCCATCTCCTTGCTTA	14	C	0.1197	0.2235
NC_005100.4-162424999	CTACTTATAACTTCAGCTCCAGAAGTT	23	A	0.065	0.078
NC_005100.4-162441333	TGAGATCTTACATATGCTCCTTACACC	13	T	0.2895	0.4076
NC_005100.4-162441333	TGAGATCTTACATATGCTCCTTACACC	26	C	0.0174	0.4915
NC_005100.4-162463043	CTTTATACCACCTGCCCTCCACCCCCA	1	C	0.0088	0.4676
NC_005100.4-162463043	CTTTATACCACCTGCCCTCCACCCCCA	22	C	0.0088	0.4676
NC_005100.4-162535535	CACACACACACACACACTCCCTGATAT	1	C	0.1197	0.142
NC_005100.4-162535535	CACACACACACACACACTCCCTGATAT	2	A	0.1261	0.1469
NC_005100.4-162611061	GTCCCTCTCACTGCACCTCCGTGCAGG	27	G	0.0085	0.4714
NC_005100.4-162682517	TTGTTTGAGACCTGGACTCCCTATAGA	1	T	0.1695	0.493
NC_005100.4-162751821	ACCTCTCTTACTTGGTCTCCTCACAGA	25	A	0.1121	0.244
NC_005100.4-162771766	CTTCTCTTCACATGCTCTCCTTCCAGT	15	C	0.0089	0.4856
NC_005100.4-162771766	CTTCTCTTCACATGCTCTCCTTCCAGT	21	T	0.0089	0.4856
NC_005100.4-162793403	TGATAAACAACTTGTCCTCCTTGTTTT	8	C	0	0.4859
NC_005100.4-162804058	TAGCTTTCAACTTAATCTCCAATAACC	26	C	0.0431	0.0422
NC_005100.4-162873315	GGCTCACACACCTCAACTCCTGTAAGA	1	G	0.2521	0.4463
NC_005100.4-162873315	GGCTCACACACCTCAACTCCTGTAAGA	9	C	0.2167	0.3578
NC_005100.4-162878038	CCACAAATAACACATCCTCCTCTGCCT	6	A	0	0.4773
NC_005100.4-162878038	CCACAAATAACACATCCTCCTCTGCCT	14	A	0	0.4773
NC_005100.4-162883728	CATCCTAATACTGCCACTCCCTACAAA	25	A	0.1429	0.4994
NC_005100.4-162883977	CTTATGCCAACCCCAACTCCCAGCAAC	8	C	0.0435	0.0425
NC_005100.4-162900130	TTTTTGTCAACATTACCTCCATCGGTG	23	C	0.0924	0.0882
NC_005100.4-162921937	AGAGCTTATACCCACACTCCTGTCACT	8	A	0.0085	0.4776
NC_005100.4-162946033	TTCCTGTGCACGCTGCCTCCGTCTCTG	1	T	0.0811	0.0778
NC_005100.4-162946033	TTCCTGTGCACGCTGCCTCCGTCTCTG	12	G	0.0811	0.0778
NC_005100.4-162981644	ACCAACTGAACCATTTCTCCAACCCTT	5	A	0.1475	0.4988
NC_005100.4-162981644	ACCAACTGAACCATTTCTCCAACCCTT	7	T	0.0082	0.479
NC_005100.4-163007860	ACTGGCCCCACCCTGACTCCCATCTTC	23	T	0	0.4807
NC_005100.4-163011050	GCTCTGGGGACAGAGCCTCCAGCCCCT	23	C	0	0.0339
NC_005100.4-163028911	CATGCTCAGACCTGAGCTCCGGCAGAG	1	C	0.1066	0.1296
NC_005100.4-163055372	GTGCTGAGAACCACCTCTCCAGGGAAA	3	G	0.2522	0.3353
NC_005100.4-163057219	TAAGAAACAACTCCACCTCCTATGTGG	21	T	0.1197	0.142
NC_005100.4-163057219	TAAGAAACAACTCCACCTCCTATGTGG	22	A	0.2759	0.4463
NC_005100.4-163063593	CACGACAGGACGCAGGCTCCTAATTCA	3	C	0.0088	0.026

NC_005100.4-163063593	CACGACAGGACGCAGGCTCCTAATTCA	7	A	0.0179	0.4688
NC_005100.4-163081648	ATGTTGCCAACAACACCTCCAGGAGTG	6	G	0.1557	0.4852
NC_005100.4-163081648	ATGTTGCCAACAACACCTCCAGGAGTG	21	A	0.0492	0.048
NC_005100.4-163093922	GGGTGGGCCACAGTGACTCCTGCTCAT	4	T	0.0517	0.0825
NC_005100.4-163093922	GGGTGGGCCACAGTGACTCCTGCTCAT	6	G	0.1466	0.4179
NC_005100.4-163136583	CACTCCTGCACCAAACCTCCTCCTCCA	1	C	0.0088	0.026
NC_005100.4-163136583	CACTCCTGCACCAAACCTCCTCCTCCA	13	A	0.1416	0.2797
NC_005100.4-163149036	CAGGCCATCACTCCTCCTCCTCAGGGG	27	G	0.1951	0.2263
NC_005100.4-163160656	GCAAGTACGACAAATGCTCCTTATTGC	9	G	0	0.4807
NC_005100.4-163314164	ATAGGATAAACATTTGCTCCCCAGTAA	23	A	0	0.4793
NC_005100.4-163315919	TGTGCCCTCACCTTCACTCCAGGAGCT	4	G	0.1293	0.4997
NC_005100.4-163381138	CCAGTGAGTACCCAGCCTCCGGCTCAG	2	C	0.2114	0.4548
NC_005100.4-163411571	GGTCCGTACACACCAGCTCCTTCTCAG	5	C	0.0407	0.0398
NC_005100.4-163411571	GGTCCGTACACACCAGCTCCTTCTCAG	22	T	0	0.4793
NC_005100.4-163456057	CTGGGCTAGACTGCAGCTCCACAGAAC	27	C	0	0.4909
NC_005100.4-163515338	ATCACCCCCACCAAGACTCCCACATCC	8	C	0.2066	0.4277
NC_005100.4-163523285	GTTCATCCTACTAGGTCTCCAAAGCAC	8	C	0.1967	0.2399
NC_005100.4-163523285	GTTCATCCTACTAGGTCTCCAAAGCAC	9	T	0	0.4751
NC_005100.4-163546469	TCAAATGGCACCTCTGCTCCTAATCCT	24	T	0.065	0.4904
NC_005100.4-163600439	ACAAATGTTACATGAGCTCCATCTTAG	6	T	0.05	0.0488
NC_005100.4-163600439	ACAAATGTTACATGAGCTCCATCTTAG	7	G	0.05	0.0488
NC_005100.4-163652079	GATCTCTGCACCCAAACTCCCGGTGGT	3	T	0	0.4743
NC_005100.4-163652079	GATCTCTGCACCCAAACTCCCGGTGGT	21	C	0.1849	0.2449
NC_005100.4-163707627	CATCTGTCCACCCCTCCTCCCATCCCT	5	T	0.0579	0.0716
NC_005100.4-163709425	CAAAACAAAACACCGACTCCCGACTCC	15	G	0.0804	0.498
NC_005100.4-163732228	GTTCAATTTACATTCCCTCCTTTGGGC	27	C	0.0268	0.0264
NC_005100.4-163747598	CTCTGAGGCACCAAGCCTCCACAGGAC	4	T	0.1826	0.1659
NC_005100.4-163747598	CTCTGAGGCACCAAGCCTCCACAGGAC	24	G	0.4174	0.3303
NC_005100.4-163787423	GGTGACATCACCCCACCTCCTCCTGTT	26	T	0.1057	0.1287
NC_005100.4-163802549	GAGCTGTCCACGGTGGCTCCCCCGCCT	1	G	0.0909	0.1163
NC_005100.4-163835524	CTGAAACCAACCCTTCCTCCCTCTGTG	5	A	0.2417	0.2833
NC_005100.4-163870425	ACATGCAGTACCACACCTCCCTACATC	12	C	0	0.4787
NC_005100.4-163907010	TCTAGTAAGACTGACCCTCCTGTAGAA	5	G	0	0.4807
NC_005100.4-163907010	TCTAGTAAGACTGACCCTCCTGTAGAA	15	C	0.0246	0.0243
NC_005100.4-163915500	GTGTTTAAGACAGCCCCTCCGATGCGG	5	T	0	0.4856
NC_005100.4-163942182	CCCAAGACCACTGAGCCTCCAATGGGA	2	C	0.0336	0.033
NC_005100.4-163988357	GGACGAAGAACTGCAGCTCCTGCTCAC	27	C	0.0244	0.0553
NC_005100.4-164055624	CACAAGGCAACTGTACCTCCAGTACCA	13	G	0.0492	0.4837
NC_005100.4-164055624	CACAAGGCAACTGTACCTCCAGTACCA	24	A	0.0083	0.4803
NC_005100.4-164076041	CTTAGAAACACAAAAGCTCCAAAGTAG	3	T	0.0965	0.1229
NC_005100.4-164100711	CCATGACTGACAGTTTCTCCAAGTCCT	22	A	0.0333	0.0488
NC_005100.4-164102643	ACAGCAGCCACTTTGCCTCCCCGAATT	23	G	0.042	0.491
NC_005100.4-164114022	TGTTTTTAAACGTGGCCTCCGTCTCTT	12	G	0	0.4833
NC_005100.4-164127945	TGAATCACCACAGCCTCTCCATTAATT	7	A	0.2231	0.4913
NC_005100.4-164170626	AGTAGACTTACCTTGTCTCCCTTGGTA	14	T	0.0492	0.4612
NC_005100.4-164191578	AGCCTGGGCACCCTAGCTCCAAACGCA	25	G	0.339	0.4425
NC_005100.4-164215895	GGTACCCAAACTCTTCCTCCTTCCTTA	2	G	0.0177	0.0348
NC_005100.4-164215895	GGTACCCAAACTCTTCCTCCTTCCTTA	16	C	0.0796	0.0926
NC_005100.4-164218813	AGCACCATGACATCATCTCCATTATCA	7	A	0.0167	0.4832
NC_005100.4-164218813	AGCACCATGACATCATCTCCATTATCA	23	T	0	0.48
NC_005100.4-164228098	TGCCAGACCACGCCCTCTCCAGCCATC	5	A	0.124	0.3327

NC_005100.4-164228098	TGCCAGACCACGCCCTCTCCAGCCATC	23	C	0.0083	0.0245
NC_005100.4-164242432	CATAGGAAAACGTTGTCTCCTTTTGCA	3	T	0.1833	0.2888
NC_005100.4-164252451	GCTGCCAACACTCAGGCTCCAGGGATG	2	C	0	0.4851
NC_005100.4-164252451	GCTGCCAACACTCAGGCTCCAGGGATG	12	T	0	0.4851
NC_005100.4-164272044	ACGAATCTTACCTTTCCTCCTCAGCAC	5	A	0.1393	0.1708
NC_005100.4-164272923	TTCCCGGGAACCTCAGCTCCAAGCCAA	6	G	0.027	0.044
NC_005100.4-164310759	AGAAGGTAAACTGGGGCTCCCTTTGTT	25	G	0.2066	0.1982
NC_005100.4-164311113	AAGAGGCTGACTCAATCTCCTTGGAAA	13	C	0.1475	0.1904
NC_005100.4-164326193	TGAGTGTGGACCCAGCCTCCCACTCCC	7	T	0.1712	0.2271
NC_005100.4-164326193	TGAGTGTGGACCCAGCCTCCCACTCCC	25	C	0.1696	0.2254
NC_005100.4-164339718	GAAGAAGCTACTCTGCCTCCTGATCCA	16	C	0.2232	0.388
NC_005100.4-164366773	CTGAGGCCAACTGCAGCTCCCAGCAGG	12	T	0.0508	0.0655
NC_005100.4-164413518	TCACTGCTGACCTGATCTCCTTCAGCC	8	T	0.1721	0.4984
NC_005100.4-164504612	AGACTAGCTACACAAACTCCACCTGTC	3	A	0.1667	0.2188
NC_005100.4-164504612	AGACTAGCTACACAAACTCCACCTGTC	4	C	0.1333	0.2188
NC_005100.4-164510402	GTGTGCTCGACTAATGCTCCACCGCTG	23	C	0	0.4819
NC_005100.4-164523014	TGAGCTACAACCCTAGCTCCAGTTTTA	27	A	0.0342	0.0336
NC_005100.4-164528856	GTAAGAGTCACCTATACTCCTTGGTTG	14	A	0.2295	0.4891
NC_005100.4-164572096	AAAAGAACCACCACAGCTCCTTGGAGA	8	C	0.2703	0.4444
NC_005100.4-164572096	AAAAGAACCACCACAGCTCCTTGGAGA	14	C	0.0893	0.1913
NC_005100.4-164585123	AAGTCGGGGACTTGATCTCCAGCATCC	6	G	0.0894	0.1695
NC_005100.4-164585123	AAGTCGGGGACTTGATCTCCAGCATCC	7	G	0.1951	0.2832
NC_005100.4-164586210	GACAGTTACACACACACTCCTCCCCTC	12	A	0.0167	0.4832
NC_005100.4-164586210	GACAGTTACACACACACTCCTCCCCTC	26	T	0.0167	0.4832
NC_005100.4-164604682	TGACACCTCACCTCTGCTCCCCAGTGC	5	A	0.2241	0.4345
NC_005100.4-164619032	AAGCTGGCCACAAAGTCTCCATAGCAG	9	C	0.0492	0.0634
NC_005100.4-164619200	ACAACCCTTACCTATCCTCCTTTCTAC	15	T	0.2087	0.4364
NC_005100.4-164627642	ATGCTGGCCACTGCTGCTCCCAGGCTG	12	T	0.187	0.3091
NC_005100.4-164627642	ATGCTGGCCACTGCTGCTCCCAGGCTG	21	C	0.122	0.1287
NC_005100.4-164633801	GTCCCTCCCACTCTCTCTCCCTCCTCC	16	T	0.122	0.4979
NC_005100.4-164676508	CACTCCAGAACATGCCCTCCATTGAGC	24	G	0.1721	0.3401
NC_005100.4-164687078	ACTGAGGCCACTGCCACTCCTCTGGGC	12	T	0.0081	0.481
NC_005100.4-164712528	GTGGATGATACTGGATCTCCTTCCTCA	2	T	0.0992	0.4942
NC_005100.4-164779642	AATACAGGGACCTAAACTCCACAAGCA	3	T	0.0357	0.0351
NC_005100.4-164782566	GTAGTGGGGACAGGGGCTCCAATGGCA	16	G	0.0536	0.0853
NC_005100.4-164803959	AGTGAGCACACAGCCTCTCCTTTTAAT	12	A	0.2975	0.418
NC_005100.4-164803959	AGTGAGCACACAGCCTCTCCTTTTAAT	24	T	0.1157	0.1377
NC_005100.4-164830638	AAAGGCCAAACTCAGGCTCCTATGCTT	1	A	0.0085	0.4776
NC_005100.4-164857320	GCTTGCCTTACTCTACCTCCCTCTCCC	5	G	0	0.4819
NC_005100.4-164881722	CATAGTGAGACCTTATCTCCCATCAAA	15	A	0.2333	0.255
NC_005100.4-164903099	GCCAAGCTGACCCTTCCTCCCCCAAAT	5	A	0	0.4793
NC_005100.4-164964334	GCCAGCTACACCCTCTCTCCTTCCTGT	24	C	0.0684	0.066
NC_005100.4-164974552	TCTGTTGGGACAGAGCCTCCTATAGCC	22	A	0	0.4743
NC_005100.4-164994426	GCATTGAACACTTCTGCTCCTGAGAGG	1	G	0.2155	0.2562
NC_005100.4-165025250	GTCTGAAGTACTTAGACTCCTTGTGCC	14	A	0.0492	0.048
NC_005100.4-165042348	CCCCTTCTTACCTCTCCTCCCCTTTAC	24	T	0	0.4724
NC_005100.4-165042348	CCCCTTCTTACCTCTCCTCCCCTTTAC	25	T	0.2174	0.3147
NC_005100.4-165042722	CAGTGTCCAACAGCACCTCCTTATCCT	16	C	0.0325	0.0476
NC_005100.4-165050921	GGGTATGACACAGATGCTCCTTGGCTT	24	G	0.3089	0.4497
NC_005100.4-165062142	GTGTGTGATACCCTTTCTCCTTGTCCA	16	T	0	0.4902
NC_005100.4-165067573	TGCATGCAAACTGGATCTCCACCTCCT	25	C	0.2114	0.2723

NC_005100.4-165067573	TGCATGCAAACTGGATCTCCACCTCCT	26	C	0.2114	0.2723
NC_005100.4-165088638	GCAACCAACACAATGTCTCCACACGCT	24	C	0	0.4773
NC_005100.4-165158378	GTATTAATGACTCAACCTCCATCTTGT	16	C	0.2479	0.3079
NC_005100.4-165192927	GGGCAGCCAACCCAGTCTCCTAAGATC	1	G	0.1393	0.1573
NC_005100.4-165201224	TGGCCCAGGACAATGACTCCAACCACT	3	G	0	0.4793
NC_005100.4-165201823	CAAAGTGCTACCGAGACTCCTGAACAC	1	C	0	0.4786
NC_005100.4-165201823	CAAAGTGCTACCGAGACTCCTGAACAC	2	A	0	0.4786
NC_005100.4-165222199	CTGGTGTTCACCGTGGCTCCAACACCA	2	T	0.3008	0.4036
NC_005100.4-165246876	AAGTTTTGAACATGAGCTCCTCATCAG	27	G	0.275	0.395
NC_005100.4-165251646	TGAAAGAAAACTCTCTCTCCTCACAGC	22	C	0.009	0.0267
NC_005100.4-165256571	AGAGTTTGTACATAGGCTCCCTTTAAT	26	A	0.0982	0.0934
NC_005100.4-165312463	AACCATTGAACCATCTCTCCAGCCTCC	5	A	0.0769	0.4963
NC_005100.4-165312463	AACCATTGAACCATCTCTCCAGCCTCC	8	G	0.0175	0.4902
NC_005100.4-165331731	CACCTACGCACCCTGCCTCCTCTGAAG	7	C	0.2845	0.3707
NC_005100.4-165413690	CCCCTGAGAACTCTGTCTCCCCTTGCT	13	C	0.0593	0.0734
NC_005100.4-165414792	TGTGGATAGACTGGCTCTCCTGGGAAG	2	G	0.0081	0.0398
NC_005100.4-165424992	CTGCAAACAACTCTTTCTCCAGGCTGG	4	C	0.0413	0.0562
NC_005100.4-165439810	CACCCCCAGACTCTGGCTCCCTCTCCA	22	T	0.1333	0.495
NC_005100.4-165468671	TCAGAGCAGACTGCGCCTCCAGAGGAG	2	C	0.0492	0.4891
NC_005100.4-165495570	CACCTGCATACACTGCCTCCTGCTCTC	12	A	0	0.4807
NC_005100.4-165500612	ACCTGGCCCACTTGGGCTCCAGTGGTG	5	G	0.0744	0.0868
NC_005100.4-165500612	ACCTGGCCCACTTGGGCTCCAGTGGTG	21	A	0	0.4793
NC_005100.4-165518949	CCAGGTTGAACTCAAACTCCTAAGGCT	14	A	0	0.4793
NC_005100.4-165519530	CAAATTGACACTTGCTCTCCATCTTAT	22	T	0.1795	0.3685
NC_005100.4-165552024	AAGGAGTGAACTCAGTCTCCAGCAGTG	5	A	0	0.4793
NC_005100.4-165554856	GTGGCACACACCTTTACTCCCAGGGAC	23	G	0.0083	0.4803
NC_005100.4-165561245	GTCAGTGCCACAAGTGCTCCAAAGTGA	5	G	0.1478	0.4998
NC_005100.4-165632259	AACTGCTGAACCATCTCTCCAGTCAAT	16	T	0.1463	0.1356
NC_005100.4-165632259	AACTGCTGAACCATCTCTCCAGTCAAT	26	A	0.2602	0.2263
NC_005100.4-165639592	CTCTGAGGTACCATATCTCCAGCTTCA	15	A	0.0244	0.0241
NC_005100.4-165687918	TATCCTGTAACTCCAGCTCCAGGGAAT	15	A	0.0163	0.4825
NC_005100.4-165687918	TATCCTGTAACTCCAGCTCCAGGGAAT	24	G	0.0325	0.0629
NC_005100.4-165718508	TTACCTATAACCCCAGCTCCAGGGAAT	16	G	0.0603	0.0585
NC_005100.4-165718508	TTACCTATAACCCCAGCTCCAGGGAAT	27	T	0.0609	0.4817
NC_005100.4-165721065	ACTTTTCTGACATAGCCTCCTGGGTCC	2	C	0.0672	0.494
NC_005100.4-165765321	AGACTTTATACTGCACCTCCCTTTAGG	25	A	0.0179	0.4641
NC_005100.4-165783748	GGTACCACCACGACGTCTCCAGTTGTG	5	C	0	0.4773
NC_005100.4-165852104	CTCAGCTGGACTCTGCCTCCCTACCCT	7	T	0	0.48
NC_005100.4-165890334	ACAGTCCCTACCCAGACTCCGGGGTAT	21	G	0.2478	0.3026
NC_005100.4-165903320	ATAGAGACAACCAGATCTCCATGAGTT	1	A	0	0.4841
NC_005100.4-165903320	ATAGAGACAACCAGATCTCCATGAGTT	15	A	0.1858	0.4774
NC_005100.4-165915391	TGCCCACCCACAGCAGCTCCTCACACA	26	C	0.2295	0.4737
NC_005100.4-165948398	TCTGGTGCCACTCCGGCTCCTTCTGCA	14	C	0.0661	0.0639
NC_005100.4-165951698	GAACTACAGACCTCTTCTCCAGAGCAG	9	G	0.0847	0.1116
NC_005100.4-165955215	GACCCCATCACTTCCCCTCCAGAACTC	25	C	0.0244	0.0241
NC_005100.4-165968936	GTAGGCAGAACTCAGCCTCCTTGGGAA	22	T	0.1382	0.1954
NC_005100.4-165998539	ACAGGAATTACTAGGTCTCCCTTTCAA	7	A	0	0.4751
NC_005100.4-165998539	ACAGGAATTACTAGGTCTCCCTTTCAA	27	A	0	0.4751
NC_005100.4-166019557	TTTTCATTTACACTGGCTCCATTTAGC	6	A	0.3017	0.4248
NC_005100.4-166022246	CACGGGGACACACTTCCTCCCACAAGA	5	G	0.0526	0.0677
NC_005100.4-166025503	AACAGCCGAACCCGCTCTCCATCCCCC	13	C	0.0164	0.0322

NC_005100.4-166035820	AACGGCAGTACTAAATCTCCCTCTGCA	3	C	0.0574	0.0557
NC_005100.4-166035820	AACGGCAGTACTAAATCTCCCTCTGCA	9	T	0.0328	0.0322
NC_005100.4-166053537	TGGCCTTGAACTCTGTCTCCCAGATGC	21	C	0.0574	0.0557
NC_005100.4-166078417	GGAAAGCAGACGGCCCCTCCATTCTCC	8	A	0.0504	0.0491
NC_005100.4-166128458	CCCCCCCCCACCGGACCTCCCCACTCT	8	C	0.0427	0.0418
NC_005100.4-166128458	CCCCCCCCCACCGGACCTCCCCACTCT	12	C	0.0862	0.0981
NC_005100.4-166132673	GTGTCAGCCACTGCCTCTCCTCTCAAG	23	T	0	0.4773
NC_005100.4-166133533	TGGCGACCGACAGCCACTCCAGGCATT	1	T	0	0.4814
NC_005100.4-166133533	TGGCGACCGACAGCCACTCCAGGCATT	8	C	0.0522	0.0508
NC_005100.4-166237183	CAGAGACTCACTCGTCCTCCGAGTCAG	13	C	0.1379	0.1717
NC_005100.4-166317112	TCTTTGTGGACGTAGTCTCCTACTGTA	12	G	0.0087	0.4878
NC_005100.4-166317112	TCTTTGTGGACGTAGTCTCCTACTGTA	25	G	0.1043	0.1869
NC_005100.4-166336528	TCATAACCAACTCTACCTCCTGGTCCA	2	C	0.181	0.497
NC_005100.4-166336528	TCATAACCAACTCTACCTCCTGGTCCA	25	C	0.1638	0.4982
NC_005100.4-166377797	AGGAGCTGCACTGTAGCTCCTTCACTG	13	G	0.0084	0.4829
NC_005100.4-166379492	CCACTTGTAACTCCAACTCCTGACTTC	7	G	0	0.4793
NC_005100.4-166399064	CAGAGGTTCACATGCACTCCCTCAGGA	15	C	0.0252	0.0249
NC_005100.4-166407416	TGGACACACACATGAACTCCCATGCTT	12	A	0.1707	0.2441
NC_005100.4-166407416	TGGACACACACATGAACTCCCATGCTT	22	A	0	0.4793
NC_005100.4-166409141	CACACACTGACTCAGTCTCCATAGCAT	14	A	0.1513	0.4008
NC_005100.4-166410031	GTGCACCCCACCACCACTCCAGCTCAT	15	C	0.161	0.2156
NC_005100.4-166431011	GATTAGCTGACGCTTCCTCCCACACCG	27	G	0.0796	0.5
NC_005100.4-166455861	TGCTAACATACTCAGCCTCCTGTTCTC	9	T	0.0088	0.4861
NC_005100.4-166455861	TGCTAACATACTCAGCCTCCTGTTCTC	15	G	0.0175	0.0345
NC_005100.4-166485702	CCTCTTTCTACAGGCTCTCCACTTCTT	14	G	0.2114	0.4497
NC_005100.4-166485702	CCTCTTTCTACAGGCTCTCCACTTCTT	26	T	0.1545	0.1826
NC_005100.4-166497333	ACAGTCTCCACCCCAACTCCCAAGCTT	13	C	0.3008	0.4572
NC_005100.4-166502488	CCTAAGATGACACCTCCTCCTGACCCT	3	T	0	0.488
NC_005100.4-166502488	CCTAAGATGACACCTCCTCCTGACCCT	8	T	0.0172	0.4946
NC_005100.4-166509123	CCAAGAGCTACCTACCCTCCTAGGGCA	6	A	0.0081	0.4777
NC_005100.4-166524599	GTGCTCCCTACAGCTCCTCCGTCACGT	25	C	0.0407	0.0553
NC_005100.4-166528404	CATGCACACACTCATGCTCCGCACACA	21	G	0.265	0.2299
NC_005100.4-166551400	TGGGCTCTGACCAGCACTCCTCTCACC	15	C	0.2083	0.2722
NC_005100.4-166569616	GGGAAGAAGACTGCCACTCCCGTGTCC	9	G	0.1053	0.2155
NC_005100.4-166592274	CTATACACCACAGGTTCTCCCCTGGAG	26	A	0	0.4793
NC_005100.4-166592726	TTCCCATTCACAGCTGCTCCTTCCCCC	21	T	0.2521	0.4971
NC_005100.4-166623453	GGACCTGGCACACCACCTCCGCATCTT	1	G	0.2459	0.3447
NC_005100.4-166645193	CAGTCGACAACCCTTTCTCCCTTAGAT	3	G	0.1803	0.2399
NC_005100.4-166773481	TAGGAGTCTACCCTGGCTCCAGGATCA	15	G	0	0.4773
NC_005100.4-166773481	TAGGAGTCTACCCTGGCTCCAGGATCA	16	G	0	0.4751
NC_005100.4-166797809	ATAACTCAGACCCTGTCTCCAACAAAA	7	C	0	0.4787
NC_005100.4-166797809	ATAACTCAGACCCTGTCTCCAACAAAA	15	G	0.0328	0.0322
NC_005100.4-166810803	AGCCTGGAAACCCAGGCTCCCTTGGGT	22	T	0.1261	0.4977
NC_005100.4-166814854	ATTTTACAGACAGCATCTCCTCGGCCC	1	A	0.0614	0.0758
NC_005100.4-166814854	ATTTTACAGACAGCATCTCCTCGGCCC	12	A	0.0089	0.4454
NC_005100.4-166844020	TCACCTGTAACTGGGCCTCCAGTCCCA	5	C	0.113	0.1067
NC_005100.4-166845134	ATGTTCAGAACTAGGCCTCCAATCACT	1	A	0.1513	0.4991
NC_005100.4-166845134	ATGTTCAGAACTAGGCCTCCAATCACT	4	T	0.0924	0.1033
NC_005100.4-166867509	CATAGTAAAACACTGTCTCCAAGGGTC	12	A	0	0.4832
NC_005100.4-166867509	CATAGTAAAACACTGTCTCCAAGGGTC	15	G	0.2833	0.4265
NC_005100.4-166868169	AGCCTCCAAACCACAGCTCCCATTTCT	6	C	0.281	0.4308

NC_005100.4-166871372	TCAAACATCACATGTCCTCCACGGGTC	16	C	0.094	0.0896
NC_005100.4-166893218	TCCATGCCCACTTCCCCTCCCTGTCCC	12	T	0	0.474
NC_005100.4-166894736	TTTATTTCCACATTCCCTCCATCCCAG	26	A	0.0268	0.491
NC_005100.4-166933610	AGCACCCACACAGCAGCTCCCAAAGGA	13	G	0.0579	0.0562
NC_005100.4-166933610	AGCACCCACACAGCAGCTCCCAAAGGA	25	G	0.0579	0.0562
NC_005100.4-166957796	AGGTGTACAACACCACCTCCCTGCAAT	7	A	0	0.4728
NC_005100.4-166965354	TGGAAGAGGACATCAACTCCTCTGGAT	14	C	0.2314	0.4972
NC_005100.4-167078536	AGCCCCAGAACACCCTCTCCTTTCTAG	3	C	0	0.4773
NC_005100.4-167107525	GGGAGTCCAACTCACTCTCCTAGCTTA	14	A	0.0672	0.065
NC_005100.4-167107525	GGGAGTCCAACTCACTCTCCTAGCTTA	24	C	0	0.4856
NC_005100.4-167117527	GTGCACTGGACATTAGCTCCTGTGCCA	3	G	0.0342	0.0336
NC_005100.4-167124908	AAAAACAAGACCTGAGCTCCTAAACTA	1	A	0.0351	0.0345
NC_005100.4-167124908	AAAAACAAGACCTGAGCTCCTAAACTA	14	G	0.0088	0.4843
NC_005100.4-167140269	AATCGCTTAACAAGACCTCCAGGTGTT	4	C	0.0345	0.0339
NC_005100.4-167155005	CCATCCATAACCACAGCTCCAAGGGAT	13	A	0	0.4807
NC_005100.4-167155005	CCATCCATAACCACAGCTCCAAGGGAT	16	G	0.0813	0.078
NC_005100.4-167184404	GAGAGGGCCACTCAGGCTCCCGACTGG	6	G	0.0446	0.0436
NC_005100.4-167184404	GAGAGGGCCACTCAGGCTCCCGACTGG	23	A	0.1712	0.4942
NC_005100.4-167187666	TGATCTAAAACTGATGCTCCTGTCTCT	8	A	0.0085	0.4655
NC_005100.4-167201902	TGACTCAAGACTGGGCCTCCTTCTTAG	7	A	0.0407	0.0553
NC_005100.4-167201902	TGACTCAAGACTGGGCCTCCTTCTTAG	15	G	0.0244	0.0854
NC_005100.4-167334071	TCATCATCAACTTCATCTCCTTCTTCT	5	C	0.6016	0.4206
NC_005100.4-167346153	AATGGCCCAACACACTCTCCCCACTCC	24	C	0.2087	0.4954
NC_005100.4-167368462	GGTTAGCGCACGCCACCTCCTGTTTGG	7	C	0.0088	0.4735
NC_005100.4-167415776	CAGTATCTCACCTCAGCTCCTTAGTGG	5	A	0.0086	0.4643
NC_005100.4-167473061	CCCCAAATAACATTGTCTCCTTATGTC	23	A	0	0.4856
NC_005100.4-167473061	CCCCAAATAACATTGTCTCCTTATGTC	27	C	0.1695	0.4757
NC_005100.4-167494102	ATATGAGTCACAAGTTCTCCTGTGTCT	3	A	0.042	0.0728
NC_005100.4-167579172	CAAGCCATGACCCCTGCTCCTGCTACA	15	T	0.1626	0.4997
NC_005100.4-167579172	CAAGCCATGACCCCTGCTCCTGCTACA	16	G	0.2358	0.4741
NC_005100.4-167580660	ACCCTGGTAACTCCCTCTCCCCTCTCA	14	C	0.1754	0.4902
NC_005100.4-167582284	TAGGAGTTCACCAGAGCTCCAAGCACA	1	T	0.087	0.4234
NC_005100.4-167715287	TCCTAAAGCACAGGAGCTCCCTTTGGT	3	C	0	0.4922
NC_005100.4-167715287	TCCTAAAGCACAGGAGCTCCCTTTGGT	5	A	0	0.4912
NC_005100.4-167723367	TTGTCTCCCACCCTCCCTCCTTCATTT	13	C	0	0.4793
NC_005100.4-167723367	TTGTCTCCCACCCTCCCTCCTTCATTT	15	C	0	0.4793
NC_005100.4-167789788	GGTGCCAGGACTCCCCCTCCCATGGTT	6	C	0.0427	0.0418
NC_005100.4-167789788	GGTGCCAGGACTCCCCCTCCCATGGTT	13	C	0.2672	0.497
NC_005100.4-167841571	TGCACAGTTACTGTTGCTCCTGCCGCT	4	A	0.0614	0.0595
NC_005100.4-167841571	TGCACAGTTACTGTTGCTCCTGCCGCT	24	C	0.0708	0.1006
NC_005100.4-167866650	ACTCTAAGAACCTTCTCTCCCACATAT	27	T	0.1818	0.2533
NC_005100.4-167940012	CCGGGATCCACATCATCTCCATCGGGG	8	C	0.2035	0.4309
NC_005100.4-167940012	CCGGGATCCACATCATCTCCATCGGGG	24	G	0	0.4715
NC_005100.4-168038588	CCTTGGCATACCACAGCTCCTCACTAT	6	G	0.2602	0.385
NC_005100.4-168038588	CCTTGGCATACCACAGCTCCTCACTAT	7	C	0.0325	0.032
NC_005100.4-168199217	CATGAGGGGACCAGGCCTCCAGCAGAC	8	G	0	0.4864
NC_005100.4-168199217	CATGAGGGGACCAGGCCTCCAGCAGAC	26	A	0.3097	0.4843
NC_005100.4-168448006	TGGGATTTTACCTGTTCTCCTCAAGCG	13	T	0.1186	0.4102
NC_005100.4-168498504	ATCAACCAAACCGACCCTCCACAGCTA	12	C	0.0976	0.1761
NC_005100.4-168611586	CCCAGATGTACACTGTCTCCAACCCTG	22	A	0.1368	0.272
NC_005100.4-168611586	CCCAGATGTACACTGTCTCCAACCCTG	26	T	0.1197	0.142

NC_005100.4-168623457	TCTTTATTCACACTCTCTCCTCTGTAG	14	T	0	0.4807
NC_005100.4-168847078	GCTGGTAGCACAGCAGCTCCCAGGCAC	3	T	0	0.4743
NC_005100.4-168991232	CATCTTTCTACTCAGTCTCCCTGGTAA	16	T	0.0508	0.1264
NC_005100.4-168991232	CATCTTTCTACTCAGTCTCCCTGGTAA	24	G	0.0756	0.0728
NC_005100.4-169034531	ACCTGTGTAACTCCTGCTCCATGGGCT	26	C	0	0.4807
NC_005100.4-169072092	AACTTACCAACAACTCCTCCCTCCACT	14	C	0.2869	0.4435
NC_005100.4-169109773	TGTTGTTCTACATCCTCTCCTAACCCA	3	T	0	0.4793
NC_005100.4-169111045	ATGTAGAACACACTGTCTCCTCACTCC	13	C	0.2895	0.3919
NC_005100.4-169122992	AATCTCTTTACCAAACCTCCATCCCTC	13	A	0	0.4833
NC_005100.4-169122992	AATCTCTTTACCAAACCTCCATCCCTC	22	T	0	0.4833
NC_005100.4-169269872	CGAAGAAAAACACACACTCCCATCCAC	6	A	0.1579	0.3793
NC_005100.4-169269872	CGAAGAAAAACACACACTCCCATCCAC	27	C	0.0259	0.0255
NC_005100.4-169319182	CTAGAACACACAGAAGCTCCAAGCAAT	1	C	0	0.4807
NC_005100.4-169319182	CTAGAACACACAGAAGCTCCAAGCAAT	14	A	0	0.4807
NC_005100.4-169362907	CCCTACCCTACATCTCCTCCACTTAAG	22	C	0.2321	0.3367
NC_005100.4-169389914	CCCACCCACACACCCGCTCCCATCTCA	15	C	0.0932	0.0889
NC_005100.4-169389914	CCCACCCACACACCCGCTCCCATCTCA	16	G	0.3983	0.4066
NC_005100.4-169395547	ATGCTGTGGACAGGCCCTCCACTATAG	6	G	0.2645	0.4112
NC_005100.4-169395547	ATGCTGTGGACAGGCCCTCCACTATAG	26	A	0.2645	0.418
NC_005100.4-169420401	GGCTTGAGAACCGGCTCTCCAGACGCT	13	G	0.1875	0.212
NC_005100.4-169434036	GGAGTCTTTACCCCCTCTCCCCCGTTG	23	C	0.0345	0.0339
NC_005100.4-169456349	CACTCTCTTACCACCTCTCCTCTACCT	22	C	0.2377	0.4984
NC_005100.4-169502444	TTGCACAAAACCTCTCCTCCCATCCAG	6	C	0.1947	0.2431
NC_005100.4-169588607	GCTGATGAAACTATAGCTCCTATTGTA	13	A	0.0901	0.086
NC_005100.4-169598528	GATTGTACTACTGAAGCTCCATGAATG	9	T	0.065	0.0629
NC_005100.4-169598528	GATTGTACTACTGAAGCTCCATGAATG	21	A	0.0569	0.4868
NC_005100.4-169599619	TATGAAGTCACTGCTTCTCCTCATCTC	15	T	0.0625	0.4968
NC_005100.4-169599619	TATGAAGTCACTGCTTCTCCTCATCTC	16	T	0.2162	0.4821
NC_005100.4-169618217	GACCACCAAACCACCCCTCCAGACCCT	6	C	0.041	0.0557
NC_005100.4-169618217	GACCACCAAACCACCCCTCCAGACCCT	23	A	0.0579	0.0562
NC_005100.4-169634191	TACCATTGTACAAGCTCTCCACTCCCA	23	T	0.1681	0.2367
NC_005100.4-169641619	CCCGTACCCACTCTGACTCCTCAGGAT	22	C	0.3482	0.2876
NC_005100.4-169925864	GGATAGTGCACCCCAGCTCCATGAAAG	9	C	0.1092	0.2852
NC_005100.4-169925864	GGATAGTGCACCCCAGCTCCATGAAAG	12	C	0.05	0.0488
NC_005100.4-169942606	AGATGATCAACTTTCCCTCCAGCTTCT	23	C	0.0331	0.0325
NC_005100.4-169995083	AGCCTACCTACCCACACTCCCACATTC	24	A	0.1057	0.2989
NC_005100.4-170029063	AGAGTCATCACCCTGGCTCCCTGGACT	1	A	0	0.4819
NC_005100.4-170067775	CGCTCAATAACCCTGTCTCCCAAGTTC	8	T	0.0598	0.4823
NC_005100.4-170137603	TTTACAAACACTCTCACTCCAGAAGGG	21	A	0.2768	0.3316
NC_005100.4-170137603	TTTACAAACACTCTCACTCCAGAAGGG	26	G	0.25	0.4731
NC_005100.4-170166491	CACAACACAACTGAGTCTCCAATGAAA	4	A	0	0.4779
NC_005100.4-170204197	CATCTACTCACTGTGCCTCCTCCGTCA	12	T	0.0984	0.0935
NC_005100.4-170270403	CAGATGGCTACGCTCCCTCCCTAAAGC	12	G	0.0504	0.494
NC_005100.4-170332934	CCCAGTCAGACTTGTGCTCCTTGTTTC	6	T	0	0.4793
NC_005100.4-170386045	CCTTCATATACTCTGTCTCCTGCCACT	8	A	0	0.4793
NC_005100.4-170388856	GGACTCCTGACCCTTTCTCCTGTCTCA	3	A	0	0.4641
NC_005100.4-170388856	GGACTCCTGACCCTTTCTCCTGTCTCA	27	A	0.0089	0.4617
NC_005100.4-170398203	CCTGCCTCCACCCTCCCTCCTCGCATC	3	T	0.0171	0.4839
NC_005100.4-170403514	GAGGAGAAGACAAGTCCTCCTCCTCCT	26	C	0	0.4773
NC_005100.4-170405533	TCTAACTTAACACCGTCTCCTTATTCT	26	C	0.2439	0.4389
NC_005100.4-170410623	GACTCGGGGACCCGGACTCCTGTGAAG	22	G	0.0354	0.0348

NC_005100.4-170410623	GACTCGGGGACCCGGACTCCTGTGAAG	23	T	0	0.477
NC_005100.4-170441603	ACAGTAGATACCAATACTCCAACCCAT	2	C	0	0.4859
NC_005100.4-170441603	ACAGTAGATACCAATACTCCAACCCAT	26	A	0	0.4859
NC_005100.4-170456724	TCTCTCCCCACCATATCTCCTGCAGAG	14	T	0.0086	0.4688
NC_005100.4-170464450	ATCTCTCAGACCCCGCCTCCAGCCCCT	12	C	0.0446	0.0605
NC_005100.4-170512389	GGCTGTACAACAGGCTCTCCTGGAGGT	15	C	0.075	0.4958
NC_005100.4-170513782	ATCAGGCCAACCATGGCTCCTATTGCC	6	G	0	0.4793
NC_005100.4-170633758	TGGTCTATGACAGTTTCTCCATCTCTC	3	G	0.0517	0.488
NC_005100.4-170633758	TGGTCTATGACAGTTTCTCCATCTCTC	8	T	0.0086	0.4768
NC_005100.4-170639484	GTTACAGTAACACCTTCTCCACTTGCA	7	G	0	0.4757
NC_005100.4-170651922	TGATAAGACACACACGCTCCTGTAAGT	16	G	0.0492	0.048
NC_005100.4-170651922	TGATAAGACACACACGCTCCTGTAAGT	27	T	0.0492	0.048
NC_005100.4-171124019	AAAACGCACACCTAATCTCCAGCTGTA	5	C	0.0948	0.1209
NC_005100.4-171124019	AAAACGCACACCTAATCTCCAGCTGTA	25	G	0	0.4786
NC_005100.4-171430929	GGACCAAACACCTCCTCTCCTACTGGT	16	T	0.1186	0.1409
NC_005100.4-171548078	ACGTAAACAACAATGTCTCCTGCTACA	2	C	0	0.48
NC_005100.4-171657111	GAGTGTATAACACCCTCTCCTCTGGCT	12	A	0	0.4793
NC_005100.4-171664057	AGAATCACAACTTAGGCTCCATAGTGA	9	A	0.027	0.4726
NC_005100.4-171664057	AGAATCACAACTTAGGCTCCATAGTGA	16	G	0.1239	0.1316
NC_005100.4-171717149	TACTGACTGACATTCACTCCAGCATGT	15	C	0.0088	0.4774
NC_005100.4-171732191	AAACCATCCACATCGGCTCCAGCTTTT	1	A	0.0488	0.0476
NC_005100.4-171784118	GCAGCCCTCACATTAGCTCCTGAGTCC	5	C	0.0424	0.0415
NC_005100.4-171784118	GCAGCCCTCACATTAGCTCCTGAGTCC	7	C	0.2393	0.497
NC_005100.4-171809180	GCCCAGTGCACACCTTCTCCAACAGGA	7	T	0.1897	0.462
NC_005100.4-171857322	TCATTACTCACGTGTCCTCCAATCCAG	2	C	0.1721	0.1708
NC_005100.4-171857322	TCATTACTCACGTGTCCTCCAATCCAG	3	A	0.1707	0.1695
NC_005100.4-171864612	AGTCTGCATACAGTTCCTCCTCCCTAC	12	A	0.2114	0.4944
NC_005100.4-171867075	AGTAACAGCACACAGCCTCCACTGCCT	2	G	0.1983	0.4991
NC_005100.4-171948662	GTACTCCCGACTTGGTCTCCTGTGGAA	8	C	0	0.4787
NC_005100.4-171948662	GTACTCCCGACTTGGTCTCCTGTGGAA	24	G	0	0.4787
NC_005100.4-171960666	AGGGATTAAACACCCACTCCATAGGAA	22	T	0.05	0.0488
NC_005100.4-172003172	TCTCAACTAACCCAGACTCCAGGGAGT	24	G	0.2946	0.2512
NC_005100.4-172064866	CTACAACTAACTGCAACTCCAGGAGAT	1	C	0.0333	0.0328
NC_005100.4-172067392	CAAATGTTCACACATCCTCCAATGCAG	23	T	0.2833	0.3394
NC_005100.4-172067392	CAAATGTTCACACATCCTCCAATGCAG	24	G	0.1417	0.2125
NC_005100.4-172097423	ATTGGAAGAACTCAGGCTCCTCATCAG	8	G	0	0.4814
NC_005100.4-172103342	ATTGCAGCCACTCTTCCTCCTCTCCTC	15	T	0.6	0.42
NC_005100.4-172103342	ATTGCAGCCACTCTTCCTCCTCTCCTC	24	C	0.6	0.42
NC_005100.4-172246148	CCACCTGTAACTCTAGCTCCAGAAATA	13	C	0.0536	0.0521
NC_005100.4-172246148	CCACCTGTAACTCTAGCTCCAGAAATA	23	A	0.0536	0.0521
NC_005100.4-172389014	AGAGGAAGCACCTAGCCTCCTAGAGAC	12	C	0.2522	0.3147
NC_005100.4-172558629	AATCTTTATACCTCCCCTCCTAGCATC	3	T	0	0.4827
NC_005100.4-172696920	AGAACAAGGACACATGCTCCACCATGT	24	A	0.5167	0.4499
NC_005100.4-172775515	TGGACTATGACAGACCCTCCTCTTTTA	8	T	0.1607	0.2052
NC_005100.4-172871585	ACTGAAGATACCTCATCTCCAGCCAAG	21	A	0.1681	0.2076
NC_005100.4-173035978	TTCTTTTCTACCTCCCCTCCTCTCCAG	7	T	0.605	0.422
NC_005100.4-173035978	TTCTTTTCTACCTCCCCTCCTCTCCAG	21	T	0.605	0.422
NC_005100.4-173183274	AGGAAACCCACAGAGTCTCCTAACTTG	26	T	0	0.4832
NC_005100.4-173237285	TCCTCTGCTACATAAGCTCCATGGACG	27	G	0.1161	0.1553
NC_005100.4-173240984	TACTATCCTACACACTCTCCAATGTGT	5	A	0.0917	0.1458
NC_005100.4-173240984	TACTATCCTACACACTCTCCAATGTGT	21	A	0	0.4773

NC_005100.4-173318417	GGACTAGGCACATCAACTCCCACTGAT	6	A	0.1789	0.2499
NC_005100.4-173387733	CGCCCTATAACCCTGACTCCTCGTCCT	2	G	0.1271	0.4957
NC_005100.4-173405561	CCTGTAAGCACCATCCCTCCATGGCCT	15	C	0.0085	0.4698
NC_005100.4-173440951	AAGAGCTGCACTGTATCTCCCTCCCTT	14	T	0	0.4807
NC_005100.4-173524918	TGCATCCCCACCCCTTCTCCAACATAG	4	A	0.1083	0.1866
NC_005100.4-173552005	GGTTTGCCCACGTTCTCTCCTCTCTTG	25	T	0.0427	0.058
NC_005100.4-173591348	CTCCAAAGGACGTGCCCTCCCGCCTGG	12	G	0.0167	0.48
NC_005100.4-173591348	CTCCAAAGGACGTGCCCTCCCGCCTGG	21	C	0.1736	0.2474
NC_005100.4-173598114	CCACACAGCACATCTGCTCCTTCCTGT	25	T	0.0574	0.0861
NC_005100.4-173682866	TGTTAAAAAACCTTGGCTCCCTGCCAC	23	G	0.1964	0.2449
NC_005100.4-173700630	AACCCCTCAACTGAGACTCCCTCTTTC	12	T	0.0738	0.071
NC_005100.4-173713142	CTGTGGTGGACACCCCCTCCAGATGCA	23	A	0	0.4827
NC_005100.4-173718450	GCCGTAAGGACTGTGTCTCCCAGTCCT	3	C	0.1176	0.2203
NC_005100.4-173718450	GCCGTAAGGACTGTGTCTCCCAGTCCT	4	G	0.1271	0.3914
NC_005100.4-173737497	CCGGGAAGCACTATCCCTCCATGGCCT	1	C	0.125	0.1866
NC_005100.4-173737497	CCGGGAAGCACTATCCCTCCATGGCCT	5	G	0	0.4813
NC_005100.4-173744036	CAGCAGAGCACGGCACCTCCTAGCTAC	8	G	0.1624	0.2171
NC_005100.4-173761030	TGAAAGGTAACTGGTGCTCCAGACAAT	23	A	0.0813	0.0928
NC_005100.4-173775793	AGTATGCACACCGTCTCTCCCGGCCCC	13	G	0.0976	0.1216
NC_005100.4-173780595	GTCCTGTAAACCTTCCCTCCCTCCTTT	7	T	0.2321	0.4898
NC_005100.4-173780595	GTCCTGTAAACCTTCCCTCCCTCCTTT	26	T	0.0088	0.4774
NC_005100.4-173839232	GTGGGAGAAACCTGGGCTCCCTACCTA	24	C	0.0721	0.1023
NC_005100.4-173849191	ATTCTGCCCACTAACTCTCCTCCGCCC	4	C	0.0089	0.4751
NC_005100.4-173900898	CCCCCAAACACCCTGTCTCCAAGAAGA	5	C	0.0082	0.0243
NC_005100.4-173919944	CTTTCATTCACTCTTCCTCCCATATCC	23	T	0.2124	0.4464
NC_005100.4-173964860	CAAACAACCACCAGAACTCCAGCTTCA	6	A	0.1475	0.2157
NC_005100.4-173968711	GTCAGAGTCACACAGCCTCCTTCATCT	8	T	0	0.4765
NC_005100.4-173980524	ATTCTCCTAACTCAGCCTCCTTAGTGG	7	C	0.0614	0.0595
NC_005100.4-174046525	TGTGAAGGTACAGATACTCCATTGGCT	9	T	0.1803	0.2279
NC_005100.4-174070038	AGGGTGCACACCCATTCTCCCCTGCAC	27	C	0.0333	0.0328
NC_005100.4-174072017	CACTGTCCTACTCACTCTCCTTGGGCA	14	A	0.2033	0.4741
NC_005100.4-174072017	CACTGTCCTACTCACTCTCCTTGGGCA	21	T	0.2033	0.4741
NC_005100.4-174099641	ATGTCTCTCACATCCTCTCCACAGTAT	22	C	0.2295	0.4515
NC_005100.4-174099641	ATGTCTCTCACATCCTCTCCACAGTAT	23	A	0	0.4787
NC_005100.4-174110189	ACACGACCTACCTCTTCTCCAACCACC	4	C	0.0504	0.0491
NC_005100.4-174144745	GCACTAGGGACCAGATCTCCCATGGAT	16	T	0.115	0.1391
NC_005100.4-174147000	AGTGACAAGACATTCTCTCCTTCTTAT	6	C	0	0.4807
NC_005100.4-174147000	AGTGACAAGACATTCTCTCCTTCTTAT	13	T	0	0.4807
NC_005100.4-174208624	GTCTGCGCCACAACCACTCCACCTTCC	7	G	0.0164	0.0322
NC_005100.4-174250118	ACAGAGCACACAGAACCTCCCGTCAGT	16	C	0.1301	0.1356
NC_005100.4-174250118	ACAGAGCACACAGAACCTCCCGTCAGT	21	C	0	0.4793
NC_005100.4-174276383	CTAGCCACGACAGTATCTCCACAGCAG	5	C	0.3089	0.4
NC_005100.4-174276383	CTAGCCACGACAGTATCTCCACAGCAG	9	G	0	0.4793
NC_005100.4-174347586	AAGGAGAACACTGACTCTCCGCGTGCG	21	G	0	0.4832
NC_005100.4-174354307	AATAGCTGGACTATCTCTCCAGCCCCC	14	T	0.2167	0.3578
NC_005100.4-174387746	GCACATTAGACAAGCACTCCACCACTA	27	A	0	0.4666
NC_005100.4-174395905	CTTTGACTGACTCTTTCTCCTGCTAAC	1	C	0.0536	0.0521
NC_005100.4-174395905	CTTTGACTGACTCTTTCTCCTGCTAAC	3	T	0.0536	0.0521
NC_005100.4-174411286	CAACTACTAACCTCCACTCCATCACCC	21	A	0.025	0.0247
NC_005100.4-174411286	CAACTACTAACCTCCACTCCATCACCC	27	C	0.1949	0.2026
NC_005100.4-174435172	GGAAGACAGACATTTTCTCCAGTTGTG	6	A	0.2034	0.3436

NC_005100.4-174437135	GTCTGAGGAACTGCGACTCCTGATGTC	14	C	0.1951	0.2612
NC_005100.4-174437135	GTCTGAGGAACTGCGACTCCTGATGTC	15	G	0.1707	0.1695
NC_005100.4-174445566	AGGGACACTACCTAAGCTCCCTGTGCT	5	A	0.0081	0.481
NC_005100.4-174445566	AGGGACACTACCTAAGCTCCCTGTGCT	8	C	0.0488	0.0476
NC_005100.4-174448101	TCGAACGACACTGAAGCTCCTTACAGT	6	C	0.0531	0.0517
NC_005100.4-174487060	CAGTCGTGGACACAGCCTCCATTGAGA	12	A	0.2018	0.4415
NC_005100.4-174493016	AGTTCAAGTACCTCTCCTCCGTTCTAT	8	G	0	0.4793
NC_005100.4-174493016	AGTTCAAGTACCTCTCCTCCGTTCTAT	21	G	0.0976	0.1356
NC_005100.4-174512414	ACATGTTAAACATGTCCTCCACCACTG	6	T	0.0351	0.0345
NC_005100.4-174512414	ACATGTTAAACATGTCCTCCACCACTG	16	C	0.1404	0.1884
NC_005100.4-174520124	TCAGGGCTGACCCGGGCTCCCTTACTG	14	G	0.0522	0.4891
NC_005100.4-174554329	AATCCTCAAACATCCACTCCCACAACT	16	A	0	0.482
NC_005100.4-174727925	AACCACTGAACCATCTCTCCAGCTGCT	6	C	0.374	0.3041
NC_005100.4-174793502	GCTTTTGAAACACAGTCTCCCTATATA	25	A	0	0.4826
NC_005100.4-174797502	ACCTCCCCGACAGTGACTCCTTCACTG	9	G	0.041	0.071
NC_005100.4-174812607	GATTTGAGTACTTGTTCTCCAGCTGAG	15	T	0	0.4765
NC_005100.4-174921217	TGGGTAGTGACAGCTCCTCCTTACAGT	2	G	0.2119	0.4896
NC_005100.4-174921217	TGGGTAGTGACAGCTCCTCCTTACAGT	22	T	0.15	0.2311
NC_005100.4-175015585	AAGGTGCTAACATCTGCTCCTAATGAG	5	T	0.0504	0.4844
NC_005100.4-175148550	TTTTTTGCTACTGTGGCTCCTAGTGTA	1	T	0	0.4764
NC_005100.4-175148550	TTTTTTGCTACTGTGGCTCCTAGTGTA	5	T	0	0.474
NC_005100.4-175182091	TTTGTTATTACATTCACTCCTGCCTCA	2	T	0.0083	0.4769
NC_005100.4-175182091	TTTGTTATTACATTCACTCCTGCCTCA	5	T	0.0083	0.4769
NC_005100.4-175213738	CACAATAGCACAGCCTCTCCAGCTGTG	26	T	0.2459	0.4807
NC_005100.4-175216632	ATCTTCAGAACAGAAGCTCCACTTCCC	21	A	0	0.4814
NC_005100.4-175216632	ATCTTCAGAACAGAAGCTCCACTTCCC	27	C	0.0526	0.0512
NC_005100.4-175418670	GAAAACATAACTGATTCTCCACACTGG	8	T	0.0089	0.4709
NC_005100.4-175469470	GTGCAGTTGACTTCATCTCCACTTAGT	25	A	0.0862	0.4927
NC_005100.4-175487517	CTGCCTGTAACTCCAGCTCCAAGAGGT	14	C	0.2821	0.4356
NC_005100.4-175497643	CCTGAAGGGACACGTGCTCCGGCCTCT	9	G	0	0.4698
NC_005100.4-175531665	CTTAGTGCCACCGTCTCTCCCTCTAAA	12	C	0.0656	0.0935
NC_005100.4-175531665	CTTAGTGCCACCGTCTCTCCCTCTAAA	13	G	0.0492	0.0786
NC_005100.4-175541666	GGGATCAAAACTCAGACTCCCAAGTTT	22	A	0	0.48
NC_005100.4-175591534	GATGCTATCACCATCGCTCCTCAGCTT	15	C	0.0417	0.0722
NC_005100.4-175594277	AGCCAGTACACTGAACCTCCAGTACAG	24	A	0	0.4793
NC_005100.4-175627049	CACACACACACACACCCTCCATCCTCC	24	C	0.6847	0.498
NC_005100.4-175628062	AGCACCGACACTTAGGCTCCATGGGAA	14	A	0.0163	0.032
NC_005100.4-175628062	AGCACCGACACTTAGGCTCCATGGGAA	22	T	0.2358	0.373
NC_005100.4-175639064	CAATCTCCAACTCCTCCTCCCAACCAC	21	C	0	0.4778
NC_005100.4-175639064	CAATCTCCAACTCCTCCTCCCAACCAC	24	C	0.1062	0.4981
NC_005100.4-175648026	AGACCCAAGACTGCAGCTCCCTTTGTT	5	C	0.122	0.356
NC_005100.4-175680128	ATTCAGCTCACCATTTCTCCCTCTTGC	13	A	0	0.4751
NC_005100.4-175684221	CTGTGCCAAACAGCAGCTCCAACCTGG	25	T	0.0086	0.4768
NC_005100.4-175723420	AGAGTTTCAACCCAAGCTCCACACGAA	5	T	0	0.4807
NC_005100.4-175738586	TTCCCACTGACGCTTGCTCCAGAGGTC	26	T	0	0.4814
NC_005100.4-175739026	CACAAACTCACAGGCTCTCCGAGGTGA	24	G	0.0348	0.0342
NC_005100.4-175785568	CTGCTCTGCACGGCATCTCCGAGCACT	2	T	0.0894	0.4979
NC_005100.4-175785568	CTGCTCTGCACGGCATCTCCGAGCACT	3	G	0.2377	0.4717
NC_005100.4-175823001	CAATATATCACCTGGACTCCCACCATC	8	T	0	0.4743
NC_005100.4-175829598	GTTACCCCCACAGTCACTCCAGAAGGA	1	G	0.1626	0.2017
NC_005100.4-175829598	GTTACCCCCACAGTCACTCCAGAAGGA	14	T	0.0244	0.0398

NC_005100.4-175841013	GCCCCCGGCACCATGTCTCCTTATGAT	9	C	0.2368	0.2836
NC_005100.4-175903840	TCAAAGGACACAGAGTCTCCTTCCCAG	7	G	0.2683	0.4301
NC_005100.4-175903840	TCAAAGGACACAGAGTCTCCTTCCCAG	9	C	0.0081	0.4777
NC_005100.4-175974654	ACCTCTCCAACATTCCCTCCACTCATC	23	T	0.2232	0.498
NC_005100.4-176034657	TGTCTGGTTACCTACTCTCCAGCTGGT	13	T	0.0088	0.4774
NC_005100.4-176043882	ACCACACCCACAAAGGCTCCTTTATGT	4	A	0.0732	0.1001
NC_005100.4-176043882	ACCACACCCACAAAGGCTCCTTTATGT	16	G	0.1545	0.4893
NC_005100.4-176058646	ACGTCTGTAACTTTAGCTCCAAGGGAT	9	A	0.0894	0.0854
NC_005100.4-176058713	ATATGGTATACACTTTCTCCCCACTAC	7	T	0.0446	0.0436
NC_005100.4-176132587	GGTATGTTGACCTAGACTCCTGGAGAT	22	G	0.075	0.0875
NC_005100.4-176132587	GGTATGTTGACCTAGACTCCTGGAGAT	23	G	0.075	0.0875
NC_005100.4-176164538	CCCACTGCCACAGCTGCTCCTGTCACC	21	T	0.0246	0.4852
NC_005100.4-176164538	CCCACTGCCACAGCTGCTCCTGTCACC	22	G	0.1066	0.1009
NC_005100.4-176186598	CAGCTCTCCACCCTCTCTCCCTGTCTC	14	T	0.0165	0.0325
NC_005100.4-176186598	CAGCTCTCCACCCTCTCTCCCTGTCTC	23	G	0.1322	0.1377
NC_005100.4-176310894	GCTTCAGTTACTTAGTCTCCTGAATGT	9	T	0	0.4773
NC_005100.4-176320646	TACAAGTCCACTGCTTCTCCTACTGCC	21	T	0.0082	0.4822
NC_005100.4-176325032	AGGTGAGGCACCTGGTCTCCATGGACA	16	T	0.0177	0.0348
NC_005100.4-176346243	TCTGAGAGAACACACACTCCTCCAAGC	13	C	0.1271	0.1481
NC_005100.4-176359657	TTAACTTCCACAGGTACTCCACATGTG	1	T	0	0.4728
NC_005100.4-176380093	GGTAGCGGCACCACAGCTCCCAGTCGA	8	G	0.0504	0.0491
NC_005100.4-176380093	GGTAGCGGCACCACAGCTCCCAGTCGA	26	G	0.0924	0.0882
NC_005100.4-176400110	AAGCTCTGTACCATATCTCCAGTCAGG	9	T	0	0.4772
NC_005100.4-176408579	ACTTAATATACCTGACCTCCAGCCCAG	8	A	0.1475	0.1774
NC_005100.4-176436287	TTCATGGCTACCTAGACTCCTTTCAAG	1	T	0	0.4851
NC_005100.4-176455240	CCTGTCTATACTTTTTCTCCAAGACTG	5	T	0.0756	0.4435
NC_005100.4-176491969	GACATGGGGACATGTCCTCCCCACAGT	26	G	0.1333	0.4994
NC_005100.4-176491969	GACATGGGGACATGTCCTCCCCACAGT	27	T	0.1583	0.4983
NC_005100.4-176508758	CCCATAATGACATCAACTCCTCTGTCA	7	A	0.1453	0.3
NC_005100.4-176511670	TCACCTCTAACTCCAGCTCCCAATCTC	23	A	0.0086	0.4729
NC_005100.4-176511670	TCACCTCTAACTCCAGCTCCCAATCTC	24	T	0.0087	0.4744
NC_005100.4-176532430	CAGCTCACCACAGGCCCTCCTTAGAGA	7	A	0.0656	0.0935
NC_005100.4-176542946	GGCACTAACACAAGAGCTCCATTGTCA	27	A	0.1802	0.2594
NC_005100.4-176565432	AACAAGGACACCTTTTCTCCAAGTGTG	2	A	0	0.48
NC_005100.4-176565432	AACAAGGACACCTTTTCTCCAAGTGTG	16	T	0.0744	0.1017
NC_005100.4-176600575	AGTACAATGACCCAACCTCCCACGGCT	4	A	0.0172	0.4786
NC_005100.4-176606520	GACACGTGCACATCGCCTCCCCATATT	1	G	0.05	0.0644
NC_005100.4-176606520	GACACGTGCACATCGCCTCCCCATATT	2	A	0.05	0.0644
NC_005100.4-176617679	CCGCTTCCCACCACCCCTCCCTCAGTG	3	G	0.0244	0.0705
NC_005100.4-176657173	GCTCTCCTCACCTCATCTCCAGCATGG	2	C	0.0492	0.0935
NC_005100.4-176657173	GCTCTCCTCACCTCATCTCCAGCATGG	27	G	0.0164	0.0322
NC_005100.4-176719129	ATGCTGCTCACTGCCCCTCCTCCCGGT	23	C	0.0984	0.1641
NC_005100.4-176719129	ATGCTGCTCACTGCCCCTCCTCCCGGT	24	C	0.1148	0.1367
NC_005100.4-176745662	GTAATGGATACAGTGTCTCCTAAACTG	6	G	0.0631	0.0611
NC_005100.4-176755078	TTGTTTATCACCCTTTCTCCTTGGTCT	15	T	0	0.4793
NC_005100.4-176766404	AATGGAATGACTCTGACTCCACCTATG	5	G	0.1795	0.2778
NC_005100.4-176767094	CCCAGAGTCACTTCTCCTCCAGTCACA	21	A	0.2797	0.4138
NC_005100.4-176767094	CCCAGAGTCACTTCTCCTCCAGTCACA	23	T	0.0083	0.4708
NC_005100.4-176835796	GGATAGGAAACTGAGTCTCCAGCATAC	22	G	0.1739	0.3403
NC_005100.4-176836596	CTGACTGTAACTCTACCTCCAAGGGAC	14	T	0.0252	0.0249
NC_005100.4-176845516	CATAGAGATACTTGTACTCCATGTTCA	21	A	0.161	0.3575

NC_005100.4-176930084	TGAAGGCGCACCTGCTCTCCGGGAGCC	7	C	0.0351	0.0345
NC_005100.4-176940811	TGGGCAGTCACCTCCACTCCTGCAAAA	3	G	0	0.4773
NC_005100.4-176940811	TGGGCAGTCACCTCCACTCCTGCAAAA	26	A	0	0.4773
NC_005100.4-176962903	ATTGGTACCACGAAATCTCCCTGGGGA	1	A	0.1301	0.1629
NC_005100.4-176962903	ATTGGTACCACGAAATCTCCCTGGGGA	2	T	0	0.4807
NC_005100.4-176963525	AGCTGTCCAACAGATCCTCCAAACCCA	5	G	0.2222	0.4868
NC_005100.4-176963525	AGCTGTCCAACAGATCCTCCAAACCCA	23	A	0.0256	0.4854
NC_005100.4-177021973	TACCTAGTAACAATGCCTCCCTCCTCC	12	A	0.124	0.1853
NC_005100.4-177029994	CCATGGGCCACTTTTTCTCCACCATGA	27	A	0.2478	0.3242
NC_005100.4-177111354	TACTTTCATACAACAGCTCCTTTCCAG	1	T	0.0081	0.481
NC_005100.4-177111354	TACTTTCATACAACAGCTCCTTTCCAG	9	T	0.0081	0.481
NC_005100.4-177137040	CATGTGTGCACTTTCACTCCAGCTTTA	2	A	0.3276	0.375
NC_005100.4-177137040	CATGTGTGCACTTTCACTCCAGCTTTA	13	T	0.2241	0.3835
NC_005100.4-177138534	ACCAGGACAACCTGCTCTCCTATGCCA	23	T	0.0569	0.4893
NC_005100.4-177152994	CGTGAAACCACAGAACCTCCAGTGCAG	1	C	0.2185	0.4898
NC_005100.4-177155313	CTCCTCCACACTGTACCTCCTCCTCCC	1	C	0.2222	0.336
NC_005100.4-177194295	AGAGACACCACCGATCCTCCCATCTCA	13	G	0.0331	0.0325
NC_005100.4-177219137	GTAATGTTAACCCTGTCTCCAAATAGA	6	G	0.0357	0.0351
NC_005100.4-177242828	GAGATGCCAACTTTCTCTCCATTATCT	25	T	0.1417	0.1458
NC_005100.4-177260484	TGTCATAGCACACCCTCTCCATGACCA	9	C	0.15	0.1932
NC_005100.4-177260484	TGTCATAGCACACCCTCTCCATGACCA	22	T	0.15	0.1932
NC_005100.4-177261793	GGCAAGCACACTCCAGCTCCCTGCAGT	2	G	0.1681	0.5
NC_005100.4-177261793	GGCAAGCACACTCCAGCTCCCTGCAGT	13	C	0.1681	0.5
NC_005100.4-177290281	GGCTTGACTACTTGCTCTCCCCGCCCT	6	G	0	0.4813
NC_005100.4-177290281	GGCTTGACTACTTGCTCTCCCCGCCCT	12	T	0.2857	0.4082
NC_005100.4-177314987	TCCATCCTGACGGTTCCTCCACCACTT	22	C	0.1967	0.4979
NC_005100.4-177322311	ATTTCACCCACTCCACCTCCAATGTCT	12	T	0.2927	0.3604
NC_005100.4-177328583	CTGCTCTGCACGGTGTCTCCAACTTCC	2	T	0.2705	0.321
NC_005100.4-177336961	CCAAACAAAACTCTTCCTCCACTGTCT	12	T	0	0.4793
NC_005100.4-177375792	AGAAGCTGCACGGACTCTCCAGTGTAC	25	T	0	0.4793
NC_005100.4-177388144	AACTTTGGGACAGAGGCTCCAGAAGTC	3	C	0	0.4793
NC_005100.4-177388144	AACTTTGGGACAGAGGCTCCAGAAGTC	12	A	0	0.4793
NC_005100.4-177394377	AGCATATCTACCCCGACTCCCTCATCC	15	G	0.1983	0.2533
NC_005100.4-177411345	AAACGTCTAACTCTACCTCCCCTACAC	1	A	0	0.48
NC_005100.4-177411345	AAACGTCTAACTCTACCTCCCCTACAC	24	A	0	0.48
NC_005100.4-177417651	ACATCCAAAACAAACCCTCCTCTGTTG	16	C	0.2167	0.4911
NC_005100.4-177437842	AGGTTCATAACTGTAACTCCAGGTCCA	5	T	0.2167	0.255
NC_005100.4-177445073	ATCTACATCACACTCACTCCTTCAGGG	1	A	0.0259	0.0255
NC_005100.4-177477506	GTTGCAAGCACTCTTACTCCTTGGGCT	2	T	0.0407	0.0398
NC_005100.4-177492841	CACATTCACACTCCCCCTCCATATGCA	4	A	0.193	0.4654
NC_005100.4-177512791	ACTCCAAACACCAAACCTCCTTTCCCA	8	A	0	0.4606
NC_005100.4-177593775	AGTATGTGTACCTGTCCTCCTTTCACA	6	G	0.265	0.3935
NC_005100.4-177615293	AAGACCTTCACCTTTCCTCCTAAGGGA	14	T	0	0.4749
NC_005100.4-177628574	TGCCTGTTGACTCCAGCTCCAGGGGAT	2	G	0.0259	0.0255
NC_005100.4-177667163	CAAGGCCTGACCATTGCTCCATCCATA	24	C	0	0.4773
NC_005100.4-177673855	AGAGCCTACACAGGCCCTCCCCTTCAT	4	G	0.0508	0.0496
NC_005100.4-177676520	ACCCACAGCACAACCCCTCCCTGTCAG	24	T	0.0991	0.4974
NC_005100.4-177679698	TCAGAGCCCACCTCAGCTCCAGTAGTG	4	G	0.0431	0.0422
NC_005100.4-177685293	AAGATTCCCACTCATGCTCCATTCACA	14	A	0	0.4793
NC_005100.4-177685293	AAGATTCCCACTCATGCTCCATTCACA	16	G	0	0.4793
NC_005100.4-177759798	AACTACCGAACTATCTCTCCAGTCCGT	26	G	0.0417	0.49

NC_005100.4-177784496	TCTAGTCTCACTGCGTCTCCTGGTCTG	15	G	0	0.4779
NC_005100.4-177784496	TCTAGTCTCACTGCGTCTCCTGGTCTG	26	T	0.1681	0.4991
NC_005100.4-177820528	CCCTAGAGGACTGGCCCTCCTAGGTTT	9	G	0.2167	0.3394
NC_005100.4-177820528	CCCTAGAGGACTGGCCCTCCTAGGTTT	23	G	0.0413	0.0562
NC_005100.4-177830588	TAATCTTTCACGTCAGCTCCATGGAGG	22	T	0.0172	0.4851
NC_005100.4-177830703	TAAGGACAAACTTTGGCTCCGTAGTTT	1	T	0.2845	0.3876
NC_005100.4-177867844	AAGGCTCCCACGCATTCTCCATCCACT	21	A	0	0.4728
NC_005100.4-177884495	TGCACCATTACTGATGCTCCTTCATGT	3	C	0	0.482
NC_005100.4-177885486	GGAATTAAGACACTTGCTCCAGGACAA	5	T	0.0084	0.0249
NC_005100.4-177901678	GTTGAACAGACACTGTCTCCAAAGACA	1	G	0.0593	0.0734
NC_005100.4-177901678	GTTGAACAGACACTGTCTCCAAAGACA	15	G	0.265	0.4714
NC_005100.4-177935334	TTGAACACAACCATCTCTCCACCATTT	3	G	0.2562	0.4732
NC_005100.4-177935334	TTGAACACAACCATCTCTCCACCATTT	22	C	0.025	0.0247
NC_005100.4-177955937	AACCACTTAACCAACGCTCCAGCCCCT	15	C	0.0508	0.0496
NC_005100.4-177956875	AGTTTGAATACCTCTTCTCCAGTTGGT	23	T	0.0826	0.4877
NC_005100.4-178017192	TATGGAGTAACTGCAGCTCCTTCCTGG	12	T	0	0.4787
NC_005100.4-178017192	TATGGAGTAACTGCAGCTCCTTCCTGG	16	G	0.0413	0.0405
NC_005100.4-178027381	CAGCTCCACACACACTCTCCTCTTGGC	5	T	0.027	0.0267
NC_005100.4-178041169	CTAGCTCCAACTGGAACTCCGGTGGCT	3	A	0.0342	0.4894
NC_005100.4-178082804	TGCTCCTTGACGTCCCCTCCTGCAGAG	12	G	0.0328	0.0322
NC_005100.4-178087076	GGAAGATGCACCTGATCTCCTCTTTTC	15	A	0.0569	0.4915
NC_005100.4-178093239	GGCTTGTCCACCTGCTCTCCCTCAGGT	1	G	0.0413	0.0562
NC_005100.4-178121073	AAGATAAGGACTTCGCCTCCACCTGTT	2	A	0	0.4844
NC_005100.4-178132231	CCCGTCCCTACCTTAGCTCCTTATTCT	2	C	0.0732	0.1145
NC_005100.4-178132231	CCCGTCCCTACCTTAGCTCCTTATTCT	3	C	0	0.4807
NC_005100.4-178221318	TGCACACAAACAAAAACTCCTTTGACA	13	A	0.0083	0.4769
NC_005100.4-178305473	TTTCCAGTCACCTTTGCTCCTGCGTTT	24	G	0	0.4832
NC_005100.4-178389200	AGGTAGTCAACTTTAGCTCCTGTGGAT	3	G	0	0.4755
NC_005100.4-178453645	AGAAGAGTAACAGTCCCTCCCTCTGTC	9	A	0.0328	0.0322
NC_005100.4-178483554	GGGGCCTCTACATCAGCTCCTGCTTTA	25	T	0.2609	0.4234
NC_005100.4-178556133	GTGCTCTCTACTGTTTCTCCTTTCAAA	15	T	0	0.4779
NC_005100.4-178560863	TGCTCTGTAACTGACCCTCCCCCATCC	16	C	0.0413	0.0405
NC_005100.4-178560863	TGCTCTGTAACTGACCCTCCCCCATCC	24	A	0	0.4807
NC_005100.4-178620508	GTGGTTCCCACCCCAGCTCCAAGGGGC	14	C	0.1043	0.173
NC_005100.4-178642678	TAAGCGCCCACCCAAGCTCCATGTAGC	1	T	0.1138	0.1216
NC_005100.4-178642678	TAAGCGCCCACCCAAGCTCCATGTAGC	6	G	0	0.4793
NC_005100.4-178661037	TTCCATCTAACCTAGGCTCCTGGTTAA	2	T	0.0877	0.0997
NC_005100.4-178661037	TTCCATCTAACCTAGGCTCCTGGTTAA	4	C	0.0877	0.0997
NC_005100.4-178663573	GTCCCTTCCACCATGCCTCCCACGAGA	14	T	0.1138	0.1073
NC_005100.4-178665065	CAACTTGTCACTCCAGCTCCCAGTCAT	26	A	0.1393	0.2218
NC_005100.4-178669924	CAACATTGCACCAGTGCTCCATCTCAG	15	T	0.1653	0.4923
NC_005100.4-178688177	GTCCTTGCTACTTCTGCTCCTGTAATT	5	T	0.0756	0.1746
NC_005100.4-178737528	AGGCTGGGGACATGTGCTCCATTCCTG	3	G	0.2759	0.4214
NC_005100.4-178737528	AGGCTGGGGACATGTGCTCCATTCCTG	22	T	0	0.482
NC_005100.4-178748698	CAAGATCATACATAACCTCCACCTCTA	22	C	0.0569	0.0705
NC_005100.4-178748698	CAAGATCATACATAACCTCCACCTCTA	27	A	0.0569	0.0705
NC_005100.4-178767149	TGCAGCTGTACTGCGTCTCCTTTATGC	14	C	0.0339	0.0333
NC_005100.4-178767149	TGCAGCTGTACTGCGTCTCCTTTATGC	15	G	0.0339	0.0333
NC_005100.4-178786569	GGCCTATGTACAAAACCTCCCAGGGTG	13	A	0.1579	0.4998
NC_005100.4-178786569	GGCCTATGTACAAAACCTCCCAGGGTG	22	A	0.0696	0.0671
NC_005100.4-178796992	CCAGCCTTCACCCCTCCTCCAATTGAA	2	C	0.265	0.4671

NC_005100.4-178823828	TCATTGGAAACTGCCGCTCCTGAGAAT	15	C	0.1488	0.2046
NC_005100.4-178840204	ATAACCCAAACTGATCCTCCTGCCTCA	7	C	0.0263	0.0429
NC_005100.4-178848580	ACGTGTACTACTACTTCTCCAGAGGAC	3	G	0.0089	0.4789
NC_005100.4-178848580	ACGTGTACTACTACTTCTCCAGAGGAC	23	A	0.0696	0.0989
NC_005100.4-178857192	CTGCTTGTGACTACAGCTCCAGGAGGA	3	G	0.25	0.3578
NC_005100.4-178857192	CTGCTTGTGACTACAGCTCCAGGAGGA	9	G	0.0336	0.033
NC_005100.4-178875471	AGCCTGGAAACATGTGCTCCCTCAGGT	24	A	0.0522	0.0832
NC_005100.4-178924646	GAGGATCTAACGCCCTCTCCTAACTGG	12	G	0.1967	0.435
NC_005100.4-178937620	GTCACATCCACAGAAGCTCCTACTAAG	25	A	0.0574	0.071
NC_005100.4-178937620	GTCACATCCACAGAAGCTCCTACTAAG	27	G	0.0574	0.071
NC_005100.4-178938150	GCCAATCACACTGCAGCTCCTACCCAG	5	A	0.1557	0.2218
NC_005100.4-178945546	TCTCAGGCCACAGTACCTCCTTGTTGT	14	T	0	0.4755
NC_005100.4-178945546	TCTCAGGCCACAGTACCTCCTTGTTGT	23	G	0.0177	0.4793
NC_005100.4-179001043	GCAAGCAAAACAAGATCTCCACCATTT	24	A	0	0.4624
NC_005100.4-179008705	ATGACATGAACATCCTCTCCGAATACA	1	A	0.0246	0.0557
NC_005100.4-179008705	ATGACATGAACATCCTCTCCGAATACA	8	G	0	0.4819
NC_005100.4-182086898	CATGCGTACACGCTCCCTCCCCCCAAC	7	T	0	0.4703
NC_005100.4-182086898	CATGCGTACACGCTCCCTCCCCCCAAC	27	C	0	0.4703
NC_005100.4-183639606	GTCACATATACTTGTCCTCCATATATA	2	T	0.1008	0.1107
NC_005100.4-183639606	GTCACATATACTTGTCCTCCATATATA	24	T	0.1933	0.4723
NC_005100.4-183642829	TTTAGGTACACAATACCTCCTAAGCTC	26	T	0.0088	0.4873
NC_005100.4-183696008	CACAGCCTAACTACGCCTCCATTGGAT	14	C	0	0.4793
NC_005100.4-183696008	CACAGCCTAACTACGCCTCCATTGGAT	16	C	0.0244	0.0241
NC_005100.4-183755565	AACAGTTACACACCTACTCCAACAACA	22	A	0.1913	0.2519
NC_005100.4-183979376	ACTTTGTCAACAGCTTCTCCTCAGGAC	6	G	0.2439	0.3604
NC_005100.4-184049126	ACACACAGGACATTTCCTCCAATACAC	6	C	0.2583	0.4708
NC_005100.4-184115257	GTAACCTCCACATGGTCTCCCACCTTT	24	C	0.0333	0.0328
NC_005100.4-184199580	GGGTTGGTGACCCACGCTCCAGTCTTA	6	G	0.0168	0.033
NC_005100.4-184199580	GGGTTGGTGACCCACGCTCCAGTCTTA	15	C	0.084	0.1107
NC_005100.4-184269657	CTTAACATCACAGAGTCTCCTTTGCAG	7	A	0	0.4868
NC_005100.4-184269657	CTTAACATCACAGAGTCTCCTTTGCAG	9	C	0.1453	0.1908
NC_005100.4-184321746	AGAAAAGGGACATTCCCTCCTTCACCT	9	G	0.2764	0.4926
NC_005100.4-184373414	AAGTCACTGACTGCCGCTCCTTCCTCT	14	C	0.1161	0.4824
NC_005100.4-184377459	ACTCAAATCACTGCATCTCCAAGCAGA	1	A	0.1736	0.3327
NC_005100.4-184377459	ACTCAAATCACTGCATCTCCAAGCAGA	14	C	0.0909	0.1017
NC_005100.4-184381752	CCAGGACCAACCTCATCTCCACATATG	14	C	0.1667	0.1528
NC_005100.4-184388592	CTCCCTTGTACCTACTCTCCACCCTTG	2	T	0	0.4832
NC_005100.4-184389291	AGTAGCAGAACCAACTCTCCCATGTTC	4	A	0.082	0.4979
NC_005100.4-184392437	AAAAACAGTACCAAGACTCCCACCCAC	6	C	0.1453	0.1347
NC_005100.4-184397496	TCTGAACCTACAAATCCTCCCTATCCT	16	C	0	0.4724
NC_005100.4-184433928	GCTTCAGACACAGTTTCTCCTAGTCTG	16	T	0.181	0.4982
NC_005100.4-184489896	TCATATTTAACCTTAACTCCGTTCTTA	21	G	0.0085	0.4842
NC_005100.4-184497840	TTTAGTGAAACCCTAGCTCCCATCAAA	16	G	0.1429	0.2449
NC_005100.4-184513343	TATTCACTTACCCCATCTCCAATTGTC	12	C	0.1552	0.1576
NC_005100.4-184545090	TGGTCCTTCACCCACACTCCTGTAAGT	2	G	0	0.4813
NC_005100.4-184545090	TGGTCCTTCACCCACACTCCTGTAAGT	12	C	0.0424	0.0415
NC_005100.4-184593822	GTTCAGTCAACCCGTGCTCCCTTTTAT	12	C	0.0244	0.0241
NC_005100.4-184656724	GGAAGTACTACTCCATCTCCACTGCCC	27	C	0.065	0.078
NC_005100.4-184679084	TGCTCCTTTACTCTTCCTCCCTCCTTT	1	T	0.0336	0.033
NC_005100.4-184679084	TGCTCCTTTACTCTTCCTCCCTCCTTT	5	C	0.2991	0.3596
NC_005100.4-184706515	ATTTGTCTCACTTGGACTCCGTCTGAT	24	T	0.2869	0.479

NC_005100.4-184791796	GGTTTTCCTACCCCCACTCCTCCCTGT	15	C	0.1917	0.3347
NC_005100.4-184792431	ATATCAGGTACCTAGTCTCCCCAAGTC	23	A	0	0.4807
NC_005100.4-184800522	CTCTGTGCCACTGCCTCTCCTATGGTT	26	T	0.0583	0.4958
NC_005100.4-184824984	CTCTAAGGGACTGGGACTCCAACTTCG	4	T	0	0.4841
NC_005100.4-184824984	CTCTAAGGGACTGGGACTCCAACTTCG	12	T	0.2124	0.2797
NC_005100.4-184875120	GAAAGTGTAACTCTGTCTCCCTGTGCG	14	T	0	0.48
NC_005100.4-184891368	GCAATATAGACTTACCCTCCACCTGTG	1	G	0.2869	0.4126
NC_005100.4-184944112	ATCGGGGCTACATTAACTCCTCAAACA	5	G	0.1429	0.1609
NC_005100.4-184944112	ATCGGGGCTACATTAACTCCTCAAACA	16	A	0.075	0.4922
NC_005100.4-184946220	ACTCTTACAACTCTGTCTCCTGCTCCC	13	C	0.0619	0.0926
NC_005100.4-184986325	CCCCAGCCAACAGCACCTCCCTGCCTT	26	T	0	0.4793
NC_005100.4-184988558	TGAGAACCAACTGTACCTCCTCACACA	12	T	0.0574	0.071
NC_005100.4-184998745	CATTCTTCCACCATTCCTCCTGGATGC	4	T	0	0.4787
NC_005100.4-184998745	CATTCTTCCACCATTCCTCCTGGATGC	23	G	0	0.4787
NC_005100.4-185046917	GAGAGAAAAACTTGGTCTCCAATGTTG	3	G	0.0342	0.0336
NC_005100.4-185054214	TTTTCCACAACTTGGGCTCCGTGGCTG	4	T	0	0.4677
NC_005100.4-185054214	TTTTCCACAACTTGGGCTCCGTGGCTG	6	C	0.0508	0.0496
NC_005100.4-185058919	AGTTTGCGAACAGAGGCTCCACCTGTG	21	A	0.113	0.4333
NC_005100.4-185068633	GTGCACCCCACCCTACCTCCAGGAGCT	22	G	0.3033	0.432
NC_005100.4-185102635	GATGTGGCCACAGTCTCTCCTTCTTCA	9	C	0.2627	0.3388
NC_005100.4-185113209	TGGTCTGTAACTCTAGCTCCAGGAAAC	3	G	0.0531	0.1006
NC_005100.4-185120747	CAGCTCTTGACTCAGCCTCCCGCTCTG	22	G	0.265	0.3685
NC_005100.4-185124474	TAATACTTCACAATCTCTCCACTGGCT	13	A	0.5946	0.4444
NC_005100.4-185141543	GTAAACGCAACAGGGCCTCCACCACAA	16	C	0	0.4749
NC_005100.4-185182686	GCATGAAGGACAGCAGCTCCAGAGCAG	13	G	0.1966	0.4012
NC_005100.4-185191464	ACATGCTTGACACTCTCTCCAAGGCCC	3	A	0.065	0.4904
NC_005100.4-185191464	ACATGCTTGACACTCTCTCCAAGGCCC	22	A	0	0.4793
NC_005100.4-185202381	TGACTCACAACTATAACTCCAGTTATG	27	G	0.2119	0.4971
NC_005100.4-185350683	TGGTCTGTAACTCCAGCTCCAGGGAGT	1	T	0.4153	0.329
NC_005100.4-185350683	TGGTCTGTAACTCCAGCTCCAGGGAGT	27	T	0.4083	0.325
NC_005100.4-185364402	GCTCTCTAAACAGGCCCTCCAAACACC	27	C	0	0.4839
NC_005100.4-185389270	GGAGCTTAGACATCTACTCCAACCCAC	27	C	0.0261	0.0257
NC_005100.4-185399413	GGAACTGAAACAAAAGCTCCCTGGATT	21	C	0	0.48
NC_005100.4-185404391	CTGGTCCTCACTTTCACTCCCATGGTG	15	C	0.3	0.42
NC_005100.4-185418532	GTACTAGTCACAGCATCTCCTGAAGTG	12	A	0	0.4807
NC_005100.4-185422163	GTCAGGAGCACACTAGCTCCATTTGAA	2	T	0.0357	0.3659
NC_005100.4-185422163	GTCAGGAGCACACTAGCTCCATTTGAA	16	G	0	0.4659
NC_005100.4-185435553	AGGCCCCAGACCCTCCCTCCTGCACCT	25	C	0.0833	0.1099
NC_005100.4-185444563	AACTGCTCAACTATCTCTCCAGTCGTG	24	C	0.2542	0.3436
NC_005100.4-185470956	AGCACTGTTACAGGGCCTCCTTGCTCA	22	T	0.1382	0.4301
NC_005100.4-185472312	AAAACCAGCACCTTCTCTCCAGACACT	12	C	0.2397	0.323
NC_005100.4-185510558	TCAGTTCACACAGCGGCTCCCAAGCAA	26	A	0	0.4793
NC_005100.4-185533679	AGCATCTGAACTCACCCTCCACCCTAC	7	T	0.1121	0.2797
NC_005100.4-185578184	TCATCTATAACTCCAGCTCCAGGGGAT	8	T	0.4336	0.3396
NC_005100.4-185592741	GGTTTCACAACACTCTCTCCCAGCCCT	24	C	0.0684	0.1125
NC_005100.4-185598427	TCCCCTTAGACCTCTTCTCCTCTCCAG	25	C	0	0.4793
NC_005100.4-185630814	CCCCCTGTAACCGTTGCTCCACTCTCT	1	C	0.2066	0.465
NC_005100.4-185651079	TATACCATAACATTACCTCCATGTAGA	5	C	0.0783	0.1219
NC_005100.4-185651079	TATACCATAACATTACCTCCATGTAGA	16	C	0.2368	0.4529
NC_005100.4-185653680	ACATCATATACATTAACTCCAAATGTG	16	A	0.2479	0.3811
NC_005100.4-185692341	GTCTCAGCGACCTTTGCTCCCACCCCT	9	G	0.2411	0.4566

NC_005100.4-185693352	GCAGTCAGGACCTTGCCTCCAAAGCAA	2	C	0.0569	0.0705
NC_005100.4-185704830	CCACAGTTTACCCAGTCTCCTGCTGGC	7	T	0	0.4765
NC_005100.4-185704830	CCACAGTTTACCCAGTCTCCTGCTGGC	24	T	0	0.4765
NC_005100.4-185723024	AGCCACCTTACAAGTTCTCCTGCATCC	9	T	0	0.4807
NC_005100.4-185725729	AGAACGCCCACCTGACCTCCAGTGTAC	27	C	0.0085	0.4626
NC_005100.4-185735756	CAAGAACAAACATGGACTCCAGGAGCT	14	G	0.0877	0.4945
NC_005100.4-185766577	AATGCCTTCACCTGGCCTCCATAGCAC	25	C	0	0.4793
NC_005100.4-185853341	AGCTTGTATACGGTAACTCCAGGGTAC	22	G	0	0.4772
NC_005100.4-186040786	CAGAGGGTTACAGGCACTCCTAGCCCC	2	A	0	0.4833
NC_005100.4-186065421	AGCTCATAAACTCCCTCTCCTCCATGC	8	A	0.0174	0.4833
NC_005100.4-186065421	AGCTCATAAACTCCCTCTCCTCCATGC	9	A	0.0265	0.0262
NC_005100.4-186067872	TTCTTGGTAACATCTGCTCCCTGTCTG	2	T	0	0.4773
NC_005100.4-186107310	ACTTGGGGGACTGTATCTCCTTTCAAT	1	A	0.0087	0.4878
NC_005100.4-186136289	CGTGGTGTCACTCTCACTCCCTGATCA	2	G	0.0085	0.0418
NC_005100.4-186143090	CACTCCCACACTGAAGCTCCAGTCTAA	1	C	0.0084	0.4797
NC_005100.4-186187973	AGAGGTGGTACATATCCTCCTCCACAG	14	A	0	0.4731
NC_005100.4-186212582	CACACAGACACACACACTCCCACACAG	8	A	0.2212	0.3081
NC_005100.4-186212582	CACACAGACACACACACTCCCACACAG	24	A	0.2212	0.3081
NC_005100.4-186260470	CAAATGTCTACTCTCCCTCCCTTGGTG	21	C	0.1721	0.1839
NC_005100.4-186260470	CAAATGTCTACTCTCCCTCCCTTGGTG	24	G	0.1736	0.1853
NC_005100.4-186280935	AACAGTTCTACCAAGGCTCCATTACAG	21	A	0.1382	0.4988
NC_005100.4-186287640	CATTTGGCAACCTCCTCTCCTTATCTC	6	G	0.1712	0.4414
NC_005100.4-186287640	CATTTGGCAACCTCCTCTCCTTATCTC	23	A	0.1171	0.1713
NC_005100.4-186384977	GGCTTTAAAACCTCAACTCCTATCCCC	2	G	0.0531	0.0517
NC_005100.4-186384977	GGCTTTAAAACCTCAACTCCTATCCCC	27	C	0.1351	0.4974
NC_005100.4-186415705	AGCACCAGTACCCTACCTCCTGTACAG	6	C	0	0.4793
NC_005100.4-186417178	GCCAGAGAAACTTCCCCTCCAAAGGCC	8	A	0.0593	0.0734
NC_005100.4-186524457	TAGGACCACACATCCCCTCCTGGTGTG	14	C	0.041	0.0401
NC_005100.4-186574401	AGATTTAAGACATGTGCTCCCCATGTA	24	T	0.0917	0.4922
NC_005100.4-186582279	CACAACTGTACTTTAACTCCTAAGGGT	1	C	0.0894	0.1001
NC_005100.4-186582279	CACAACTGTACTTTAACTCCTAAGGGT	26	G	0.1138	0.1356
NC_005100.4-186583818	AACAGCCTCACAGAGGCTCCGGGGTCA	24	G	0.0696	0.2519
NC_005100.4-186652083	AAATAAGGTACGATGACTCCTAAACTA	4	T	0.2542	0.324
NC_005100.4-186652083	AAATAAGGTACGATGACTCCTAAACTA	12	G	0.3051	0.362
NC_005100.4-186666950	AAGCGTTGCACACCTACTCCTCCAGTC	4	C	0.0325	0.0476
NC_005100.4-186701369	CATCCATCCACTCTAACTCCATCATTG	21	A	0.0259	0.0255
NC_005100.4-186701369	CATCCATCCACTCTAACTCCATCATTG	22	T	0.0086	0.0255
NC_005100.4-186719648	TCCCTTCACACTGGTCCTCCCTGCCAC	3	C	0.1504	0.4961
NC_005100.4-186740141	TGAGGATAAACTTGAGCTCCTGATCCT	23	A	0.3089	0.4595
NC_005100.4-186741186	AATAGGTTCACTGTGGCTCCTGGTTTC	1	A	0	0.4844
NC_005100.4-186741186	AATAGGTTCACTGTGGCTCCTGGTTTC	14	T	0.2627	0.3664
NC_005100.4-186762716	TTCATAATTACTATAGCTCCCGCTCCA	7	A	0.2846	0.484
NC_005100.4-186762716	TTCATAATTACTATAGCTCCCGCTCCA	22	G	0.2358	0.2441
NC_005100.4-186803717	TGTTGGCTGACACGTTCTCCTACATTA	7	C	0.2101	0.2852
NC_005100.4-186803717	TGTTGGCTGACACGTTCTCCTACATTA	13	C	0.0087	0.4782
NC_005100.4-186815410	GTCACTGATACACCACCTCCAACAATA	16	C	0.2927	0.4389
NC_005100.4-186827001	ATGCAAACAACAGGGCCTCCTCTTGTT	16	C	0.0492	0.048
NC_005100.4-186885513	CACAAGGACACCTGCACTCCCATGTTC	23	T	0.2149	0.4819
NC_005100.4-186914956	GAAGTGGCTACCTTTACTCCTCAATGT	7	G	0.2411	0.3517
NC_005100.4-186917122	CAGAACCAGACTCAGGCTCCCCAGTCA	14	A	0.0513	0.05
NC_005100.4-186924675	GTGTTCCCTACAAGTGCTCCTTATCCC	7	C	0.0348	0.0342

NC_005100.4-186941583	ATTGGGACTACCAAGGCTCCAGCCTCA	12	C	0.2358	0.436
NC_005100.4-186941583	ATTGGGACTACCAAGGCTCCAGCCTCA	13	A	0.0328	0.048
NC_005100.4-186965365	GAGGGCACTACTTTGCCTCCATGCCCA	9	T	0	0.4841
NC_005100.4-186979824	CAGTGATGCACAACCCCTCCATGTGTG	4	T	0.0351	0.0345
NC_005100.4-186987529	CATCCATCCACTCATCCTCCCACCCAC	2	A	0.5122	0.381
NC_005100.4-186987529	CATCCATCCACTCATCCTCCCACCCAC	3	T	0.5122	0.381
NC_005100.4-187026224	TAACTTACAACTAAAACTCCAGTTCTA	27	A	0.0172	0.4749
NC_005100.4-187041219	TCACCCATTACAGCAGCTCCCCCTTCT	14	C	0.1849	0.2203
NC_005100.4-187050322	GGGGTTCTGACCCTATCTCCTGGCATC	23	G	0	0.4793
NC_005100.4-187098954	AGTGCATTCACGCATCCTCCTGTTGCG	8	T	0.2439	0.2382
NC_005100.4-187098954	AGTGCATTCACGCATCCTCCTGTTGCG	12	G	0.2705	0.375
NC_005100.4-187104242	GGCATGGTCACGCTACCTCCCCTGCAC	25	C	0.0088	0.4735
NC_005100.4-187116067	CTCCCTCCAACCCTTGCTCCCTATAAT	1	C	0.0248	0.0405
NC_005100.4-187116067	CTCCCTCCAACCCTTGCTCCCTATAAT	22	T	0.2479	0.4112
NC_005100.4-187133340	CTGTCTGTCACCCAGCCTCCTTTGGAC	8	T	0.0081	0.481
NC_005100.4-187154066	CGCACAACAACTCTCCCTCCCTTAGGT	2	G	0.1707	0.2556
NC_005100.4-187160831	TCTGCCATAACATGAGCTCCTCTGCCT	16	G	0.3063	0.3285
NC_005100.4-187168648	GCAGATGCTACTCACTCTCCTTGGTAG	6	T	0	0.4793
NC_005100.4-187168648	GCAGATGCTACTCACTCTCCTTGGTAG	7	G	0.122	0.1695
NC_005100.4-187184420	CTGGCTATGACCTTGTCTCCTGTGTTG	23	T	0.2101	0.491
NC_005100.4-187193513	GTTTTTTACACTCCCTCTCCAGTGTTT	27	T	0.1391	0.4997
NC_005100.4-187211457	CCTCTATTCACAGTTCCTCCCCTGTTA	7	T	0.0496	0.4849
NC_005100.4-187235297	AGCTACATCACATAATCTCCCCGTCAT	14	A	0	0.4773
NC_005100.4-187277421	ATTTCATATACTGCCTCTCCTGCTCCT	24	T	0.0348	0.0342
NC_005100.4-187285033	CAGCTTGTCACAGACCCTCCCCAAGCA	7	G	0.0439	0.0429
NC_005100.4-187285033	CAGCTTGTCACAGACCCTCCCCAAGCA	27	A	0.2807	0.3793
NC_005100.4-187328562	TGGTTTCAAACATAAACTCCAGGACTT	25	C	0.0348	0.0508
NC_005100.4-187359979	AGTTGCACCACTATTGCTCCAGTGAGC	26	G	0.1339	0.3211
NC_005100.4-187366405	GTTTTCATTACTCTAACTCCCAGCTGT	13	C	0.0268	0.0264
NC_005100.4-187366405	GTTTTCATTACTCTAACTCCCAGCTGT	21	C	0.0446	0.0436
NC_005100.4-187374993	AGAAGGGAAACCATCTCTCCACCTTCA	8	A	0.1271	0.1894
NC_005100.4-187377479	CTGAAGGGGACAAAGACTCCTGGAGGA	6	G	0	0.477
NC_005100.4-187382242	TGAAGACAAACAGTAACTCCACCCTGA	4	A	0.0248	0.4732
NC_005100.4-187434961	CATAATAAGACCCTGCCTCCAAACAAC	12	C	0.0348	0.0342
NC_005100.4-187450849	AGATACACCACGTTTCCTCCATTCATT	16	C	0	0.4832
NC_005100.4-187454034	TGAAGACAGACCCTACCTCCGGGCCAC	21	G	0.0256	0.0253
NC_005100.4-187454034	TGAAGACAGACCCTACCTCCGGGCCAC	27	C	0.0855	0.0818
NC_005100.4-187471868	ATGGAGTGAACGTCTTCTCCATAACCA	13	T	0.1121	0.1058
NC_005100.4-187478034	AGCTACGGCACTGCTACTCCTCCAGTG	6	C	0.045	0.0611
NC_005100.4-187479196	GGTCCCAGCACTCAAGCTCCAGGGCTT	24	G	0.2951	0.4289
NC_005100.4-187479196	GGTCCCAGCACTCAAGCTCCAGGGCTT	25	C	0.2951	0.4289
NC_005100.4-187514796	TGCCCTTAAACTCTGCCTCCTCCACAG	7	T	0	0.4779
NC_005100.4-187516142	CTTCCCTGCACCCCAGCTCCTAAACTT	3	T	0	0.4793
NC_005100.4-187529710	GGTCCTATTACCTCTCCTCCATTAGTA	13	T	0	0.4779
NC_005100.4-187560918	GTCTACTGCACTCTTTCTCCACAGCAT	27	T	0.1557	0.1436
NC_005100.4-187585325	TAAAAGAACACCATTCCTCCTTCCTAT	15	T	0.075	0.0722
NC_005100.4-187585325	TAAAAGAACACCATTCCTCCTTCCTAT	23	C	0.1933	0.4931
NC_005100.4-187632500	GAACAGGACACCAGATCTCCTCCCATG	3	A	0.0435	0.0425
NC_005100.4-187721824	CCATGTCTAACCCTTCCTCCAGCTCCC	12	C	0.0656	0.0634
NC_005100.4-187734322	ACTGGCCCTACTTCCTCTCCTTTATGT	9	T	0.0088	0.463
NC_005100.4-187734322	ACTGGCCCTACTTCCTCTCCTTTATGT	14	C	0.0088	0.463

NC_005100.4-187828876	GTGCATTCAACAAACTCTCCAGACTCG	6	T	0.0084	0.0249
NC_005100.4-187834471	TCACCTTCTACCCTCCCTCCACAGGAT	9	T	0	0.4793
NC_005100.4-187834471	TCACCTTCTACCCTCCCTCCACAGGAT	13	C	0.0244	0.0398
NC_005100.4-187868542	TGTTTTGAGACAGGGTCTCCTGATGTA	12	A	0.1391	0.1295
NC_005100.4-187876849	GTAGTGAGAACCCTGTCTCCAAATTAG	25	T	0	0.4793
NC_005100.4-187887386	AGCCTGGCCACCCGAGCTCCATCCCAG	24	C	0.084	0.1946
NC_005100.4-187908451	ATAAACACCACCCCAGCTCCAAACCCT	1	A	0.1754	0.396
NC_005100.4-187916606	CAGTGAAAAACAGACTCTCCAGTCAAG	3	G	0.2069	0.3916
NC_005100.4-187935866	AAGGTGAACACCACCTCTCCTGACTGT	13	A	0.2353	0.4921
NC_005100.4-187935866	AAGGTGAACACCACCTCTCCTGACTGT	22	G	0.3109	0.4045
NC_005100.4-187976326	CTGTGTGTGACAGGTTCTCCATCAGAA	24	A	0	0.4793
NC_005100.4-187985707	CCCTCACAAACCTCAACTCCACATGAA	24	T	0	0.4728
NC_005100.4-187993418	TCTCTTCAGACTCTTCCTCCTTAGGTT	13	C	0	0.4819
NC_005100.4-188065415	CCATCATCCACCCATCCTCCCATAATC	5	C	0.2131	0.4737
NC_005100.4-188067204	TAATCTGGGACCATACCTCCTTCCTTC	14	T	0	0.4793
NC_005100.4-188067204	TAATCTGGGACCATACCTCCTTCCTTC	16	C	0.1933	0.327
NC_005100.4-188102824	TTAGGGGTCACCCACGCTCCTGTAAGT	7	G	0.2583	0.395
NC_005100.4-188102824	TTAGGGGTCACCCACGCTCCTGTAAGT	26	G	0	0.0328
NC_005100.4-188131112	GCCAGTTGTACGTAAGCTCCAGTTACC	27	C	0.0407	0.0398
NC_005100.4-188156007	GAGGGCCTGACTTGATCTCCAGCACCT	25	C	0.1983	0.302
NC_005100.4-188175785	CTGCGTGTAACTCCAGCTCCATGGGAC	22	T	0.2787	0.3624
NC_005100.4-188265679	AGTTCCAACACCCCACCTCCACATGGA	24	T	0.1803	0.3354
NC_005100.4-188278653	ATCCAGACTACCACCACTCCCTAGCAG	3	C	0.2314	0.4751
NC_005100.4-188290629	AACAGGGAAACAAAAGCTCCTCCTTTT	12	A	0.0325	0.032
NC_005100.4-188470120	CCAAACCTCACATGTCCTCCGTGGGTC	8	T	0	0.4682
NC_005100.4-188473769	CTGGACTCTACGGTTACTCCCAGTTAT	3	G	0	0.0328
NC_005100.4-188499653	TTTGTGTCCACCCTGCCTCCTGGATTC	9	C	0.1322	0.2759
NC_005100.4-188509358	AGCCTGGCTACCTATCCTCCTGCCGGA	24	C	0	0.4666
NC_005100.4-188528992	CACCACAGCACTTATCCTCCAGCCTCA	21	A	0	0.4793
NC_005100.4-188585800	TCCCATGATACGCCTTCTCCAGCAAGT	9	T	0	0.4773
NC_005100.4-188585800	TCCCATGATACGCCTTCTCCAGCAAGT	12	G	0	0.4773
NC_005100.4-188587058	AGAAAAGTCACTCAAGCTCCTTGGAAG	1	A	0.0268	0.0436
NC_005100.4-188616546	CATCCACTCACACGCCCTCCTAGATCA	7	C	0	0.4734
NC_005100.4-188618670	CTCTACCCAACTCCTGCTCCATTACCT	27	T	0.2435	0.3593
NC_005100.4-188620551	ATGTACTACACCCAGACTCCAGATCAG	8	A	0.1345	0.2076
NC_005100.4-188646037	ACCAAATAAACCCTCCCTCCCCACATT	25	A	0.0088	0.476
NC_005100.4-188679217	GAGTCATGCACTTTAACTCCACATACG	27	G	0.0268	0.0264
NC_005100.4-188685742	TAGCAGCACACAGGAACTCCTATAATA	4	C	0	0.4813
NC_005100.4-188685742	TAGCAGCACACAGGAACTCCTATAATA	5	A	0	0.4813
NC_005100.4-188718649	CTGACTTCCACATGCACTCCATAATGG	7	T	0.0721	0.149
NC_005100.4-188730572	AATCACATAACATGGTCTCCACTTTAA	21	A	0.157	0.3851
NC_005100.4-188810904	ACAGTGATGACAGCTTCTCCTTACCCT	7	A	0.2222	0.3457
NC_005100.4-188810904	ACAGTGATGACAGCTTCTCCTTACCCT	9	G	0.0261	0.0257
NC_005100.4-188818030	TGCCATTCCACAGAGCCTCCCGATCCA	25	C	0.0984	0.1641
NC_005100.4-188866848	TTTCAAATGACAATAGCTCCTCAGGTA	4	C	0.1786	0.2052
NC_005100.4-188866848	TTTCAAATGACAATAGCTCCTCAGGTA	25	G	0.0172	0.4709
NC_005100.4-188895343	TGGCTGCGCACCTGCCCTCCTAGATAG	6	G	0.0085	0.4698
NC_005100.4-188932132	CCCTATCTTACAGCGTCTCCATCTGCA	13	G	0.2035	0.2237
NC_005100.4-189064981	GCCTGCTTCACCCCTTCTCCCCCTCCC	21	C	0.0177	0.0348
NC_005100.4-189073691	TGAGCAAGAACTTTACCTCCTTTTGCA	12	T	0.2373	0.4977
NC_005100.4-189074215	TGAATGCCCACCTCAACTCCGACCTCC	3	A	0.0179	0.4807

NC_005100.4-189074215	TGAATGCCCACCTCAACTCCGACCTCC	14	C	0.0179	0.4807
NC_005100.4-189092612	CCTAGGTTCACTTCACCTCCTTTGCAA	1	C	0.0268	0.491
NC_005100.4-189105008	TCCTCCTCCACTGTTTCTCCTCCACGT	16	T	0.0082	0.479
NC_005100.4-189105008	TCCTCCTCCACTGTTTCTCCTCCACGT	26	G	0.0656	0.0935
NC_005100.4-189145197	GGTCCATGCACACCTTCTCCTGTGCTG	6	A	0.0574	0.4903
NC_005100.4-189145197	GGTCCATGCACACCTTCTCCTGTGCTG	8	G	0.0244	0.0241
NC_005100.4-189148192	TGGGCAAGAACCTGTGCTCCTCATCAG	24	T	0.1667	0.4999
NC_005100.4-189149007	CTCCTTGTGACCCTTTCTCCTGAGGCT	3	C	0	0.4787
NC_005100.4-189149007	CTCCTTGTGACCCTTTCTCCTGAGGCT	21	T	0.0413	0.0405
NC_005100.4-189163788	ATCAGTGCAACTCAAGCTCCGAGGCCC	15	A	0.0354	0.0348
NC_005100.4-189201678	CAGAGCCATACCCCAACTCCTGTCTCC	1	C	0.0431	0.0585
NC_005100.4-189201678	CAGAGCCATACCCCAACTCCTGTCTCC	9	T	0.0431	0.0585
NC_005100.4-189237095	AAGACTGCAACCCTTCCTCCCAGCCAT	14	T	0.0169	0.0333
NC_005100.4-189279996	AAAGTGTGGACCTACTCTCCCAAGTAC	2	A	0.0083	0.4747
NC_005100.4-189335562	GTGTCTTGTACTCTCTCTCCCAGAGGA	21	C	0.2212	0.2738
NC_005100.4-189375606	AAGCCCATCACCTCCACTCCTTACCCT	12	C	0.0721	0.1023
NC_005100.4-189375606	AAGCCCATCACCTCCACTCCTTACCCT	24	C	0	0.4688
NC_005100.4-189390178	TGTCACCAGACTTAGCCTCCAATAAGC	23	T	0.1339	0.4885
NC_005100.4-189395548	TATCATTTGACCCTACCTCCACTAATA	1	T	0.2672	0.4375
NC_005100.4-189395548	TATCATTTGACCCTACCTCCACTAATA	27	A	0.0171	0.4868
NC_005100.4-189408560	CTGACAGAAACCCTGACTCCACAGGCT	2	T	0.2797	0.4273
NC_005100.4-189422754	CTGAACTACACCTGGGCTCCACAGTAT	5	A	0	0.4807
NC_005100.4-189532099	GGACACCATACCCACCCTCCCTCTTTA	1	G	0	0.474
NC_005100.4-189661052	CTCCCCAACACACTGTCTCCTAGACTA	22	A	0.0082	0.479
NC_005100.4-189818520	ATATCAAAGACAGCTCCTCCCTGAAGA	6	A	0	0.4749
NC_005100.4-189877131	CGAGGATTGACATCTCCTCCAACAAAT	1	C	0.1466	0.2315
NC_005100.4-189877131	CGAGGATTGACATCTCCTCCAACAAAT	2	G	0.1552	0.2122
NC_005100.4-189945044	TCACTGGGGACTAGGCCTCCTTTGATC	3	A	0	0.4793
NC_005100.4-189993748	ACTTCATTGACTAGGTCTCCTGCACCC	23	C	0.0088	0.4843
NC_005100.4-190080777	CTCCTAACCACACAAGCTCCCTGCTCC	12	A	0.2458	0.3664
NC_005100.4-190099362	CCTGTGCACACAGGCACTCCAGGCATT	24	C	0	0.4772
NC_005100.4-190478188	ACCTTAAGTACCAGCCCTCCGAAAGCA	22	A	0.1261	0.4045
NC_005100.4-190484159	CTTCTGGTTACTGTGCCTCCCACACGC	26	G	0.0813	0.1629
NC_005100.4-190504100	ACAGGTAGAACAGACTCTCCCTTCAAT	26	A	0.2087	0.3856
NC_005100.4-190515670	GTCCCTCCCACAGTCCCTCCTTTACCC	4	C	0.1667	0.1953
NC_005100.4-190515670	GTCCCTCCCACAGTCCCTCCTTTACCC	15	C	0.0265	0.0262
NC_005100.4-190604195	GGCGTCCCTACATTCGCTCCCCCTCCC	23	C	0.0167	0.0488
NC_005100.4-190604195	GGCGTCCCTACATTCGCTCCCCCTCCC	24	T	0.0167	0.0488
NC_005100.4-190621727	GTCAGAGACACTCTGCCTCCCAAAACA	24	A	0.1488	0.2295
NC_005100.4-190679224	CACATTCCTACCTCCCCTCCTTTGTCT	22	T	0.0261	0.4926
NC_005100.4-190679224	CACATTCCTACCTCCCCTCCTTTGTCT	24	G	0.0261	0.4926
NC_005100.4-190697883	AGTTGTTCTACCCTTCCTCCCAAGCTT	5	G	0	0.4793
NC_005100.4-190712098	TTCCCGGTAACATCAGCTCCAAAGGAT	12	A	0	0.4778
NC_005100.4-190733164	GCTTAGCCAACCGGTTCTCCTTATACT	7	C	0.0488	0.0476
NC_005100.4-190756653	TTGGCTGCAACCTGACCTCCCTCTACC	24	T	0.2301	0.4953
NC_005100.4-190777699	AACTGGAAAACTGCAACTCCAGGAGAC	16	A	0	0.4709
NC_005100.4-190951439	ACCCCTAACACCCCATCTCCAAAAGTA	14	C	0	0.4898
NC_005100.4-190982904	AACCATCTTACTGCCTCTCCTCCCTTG	2	A	0	0.4826
NC_005100.4-190992566	GTCGTCATGACAGCTCCTCCCTCTGTA	4	G	0	0.4819
NC_005100.4-190998892	CCAGTAAGTACTGAGACTCCATGTACA	27	A	0.1161	0.125
NC_005100.4-191027486	ACTGCCGAGACAGGCTCTCCCACTTCC	9	G	0.0522	0.0508

NC_005100.4-191027486	ACTGCCGAGACAGGCTCTCCCACTTCC	26	C	0.0172	0.4851
NC_005100.4-191035708	TCCTCAATGACTGACTCTCCCACGCCC	15	C	0.0902	0.1154
NC_005100.4-191053037	CCCATGAACACACATGCTCCTCCCCAT	7	A	0	0.4743
NC_005100.4-191053037	CCCATGAACACACATGCTCCTCCCCAT	23	C	0.0744	0.0868
NC_005100.4-191075017	TCCAATTAGACCCCGCCTCCTTCCTTT	1	T	0.0357	0.0689
NC_005100.4-191078516	CGTGACATAACAGAGTCTCCTTTCCAA	1	C	0	0.4773
NC_005100.4-191078516	CGTGACATAACAGAGTCTCCTTTCCAA	15	G	0.041	0.0401
NC_005100.4-191087692	TGTCCCATGACCTATGCTCCTCCTGAG	22	C	0.0081	0.481
NC_005100.4-191181084	CCAGTCTTTACTGCCTCTCCACAGAAT	3	A	0.1552	0.2739
NC_005100.4-191181084	CCAGTCTTTACTGCCTCTCCACAGAAT	21	A	0.1379	0.4709
NC_005100.4-191181245	CAGTGAGGGACAGCGGCTCCTAGGAAG	15	G	0.1983	0.4959
NC_005100.4-191217647	TCTACTCTCACGACATCTCCAACACAT	6	T	0.2348	0.4817
NC_005100.4-191263248	AATCCCAGAACATGTCCTCCACAGAAA	3	T	0.0517	0.0825
NC_005100.4-191298990	TCACAGCATACAGACGCTCCTCAGATC	16	G	0.1138	0.1494
NC_005100.4-191298990	TCACAGCATACAGACGCTCCTCAGATC	23	A	0.0081	0.481
NC_005100.4-191341463	AGAACAGGCACTGCCACTCCTGTTCCC	4	A	0.178	0.2282
NC_005100.4-191341463	AGAACAGGCACTGCCACTCCTGTTCCC	21	T	0	0.4813
NC_005100.4-191434047	AGAGCAAGCACTGGGCCTCCTGTTTGG	23	T	0.1404	0.2021
NC_005100.4-191455164	AACAGTTCAACATGTACTCCCATCACA	2	A	0	0.4793
NC_005100.4-191456233	AGCTTTCTCACAGAGACTCCTGGACCC	12	A	0	0.4677
NC_005100.4-191456233	AGCTTTCTCACAGAGACTCCTGGACCC	16	A	0	0.4677
NC_005100.4-191493575	CCTTTCCAAACAGTAACTCCAAGGGCA	24	G	0.1624	0.3409
NC_005100.4-191519825	AGCGGTTGTACTGTGCCTCCGCTTTCT	4	G	0.0086	0.4893
NC_005100.4-191521739	TCGTGTTCCACCATGCCTCCCCACCAT	2	C	0.0246	0.0557
NC_005100.4-191521739	TCGTGTTCCACCATGCCTCCCCACCAT	4	T	0.1736	0.172
NC_005100.4-191522953	CTACCTACGACTCCCACTCCCAGCTCC	23	G	0	0.4827
NC_005100.4-191533619	TGGGTGGATACTGAGTCTCCATCTCCA	4	G	0.0088	0.0429
NC_005100.4-191537162	TCACTCTGGACATTAGCTCCAACGCAG	13	T	0.0082	0.4822
NC_005100.4-191537162	TCACTCTGGACATTAGCTCCAACGCAG	16	G	0.0574	0.4943
NC_005100.4-191550343	GTCCTCCCCACCTCTCCTCCATGCAGA	9	C	0.2149	0.2975
NC_005100.4-191550343	GTCCTCCCCACCTCTCCTCCATGCAGA	23	G	0.1849	0.4971
NC_005100.4-191576758	GCACTGCATACTCCCTCTCCCAAGCCT	2	C	0.2035	0.359
NC_005100.4-191576758	GCACTGCATACTCCCTCTCCCAAGCCT	15	C	0.0598	0.074
NC_005100.4-191619928	CAGCTCTGCACACCAACTCCTTTGAGT	1	C	0.0763	0.1041
NC_005100.4-191621837	CTCCCCTTAACCTTCTCTCCCTCCCTA	1	C	0.25	0.2188
NC_005100.4-191621837	CTCCCCTTAACCTTCTCTCCCTCCCTA	9	A	0.2542	0.2219
NC_005100.4-191623161	GACCTCCACACACAAACTCCACAAATA	2	A	0	0.477
NC_005100.4-191680237	AGATATCTCACTGCCCCTCCCTTGCAC	6	T	0.0246	0.0243
NC_005100.4-191710740	AGCAAATACACTAGGGCTCCCAGGAGT	8	A	0	0.4637
NC_005100.4-191723927	TATCACTTAACTTTTGCTCCTTACGGT	24	C	0	0.4793
NC_005100.4-191739428	ATGACCTGAACTTGACCTCCAGAAAAC	23	A	0.275	0.4875
NC_005100.4-191739428	ATGACCTGAACTTGACCTCCAGAAAAC	25	A	0	0.4703
NC_005100.4-191745022	CGTAAGACCACTAGGTCTCCATCAACC	1	C	0.0678	0.0655
NC_005100.4-191745022	CGTAAGACCACTAGGTCTCCATCAACC	7	A	0.2017	0.4991
NC_005100.4-191750862	TGATCTCACACCAGGACTCCACAGCAA	21	A	0.1724	0.1576
NC_005100.4-191822413	CACAGCTGAACTGTGGCTCCAGCTCCT	8	G	0.2479	0.2662
NC_005100.4-191822413	CACAGCTGAACTGTGGCTCCAGCTCCT	27	T	0.2414	0.2622
NC_005100.4-191829041	TCAAGTGCCACCTCTTCTCCTCTTAAA	2	C	0	0.4764
NC_005100.4-191895215	CACACACACACACCGGCTCCACCACCA	1	C	0.0244	0.0398
NC_005100.4-191895215	CACACACACACACCGGCTCCACCACCA	2	A	0.0244	0.0398
NC_005100.4-191903773	CAGAGCAGCACCCAACCTCCCTAGCTC	12	C	0.0087	0.4817

NC_005100.4-191907416	ACATGGGGAACCCAGCCTCCCTGTGTC	1	A	0.188	0.272
NC_005100.4-191908250	AGGATGCAAACTGCAGCTCCTAGCTCA	21	T	0	0.4807
NC_005100.4-191908250	AGGATGCAAACTGCAGCTCCTAGCTCA	23	G	0.0248	0.0405
NC_005100.4-191913516	TATACAACCACTCAACCTCCTTTACAA	15	A	0.2333	0.3987
NC_005100.4-191931681	CCCTGACCCACTAGCACTCCCAAGTAA	12	T	0.0088	0.4774
NC_005100.4-191934252	AGTAAGGAAACGGCACCTCCAGCTTCA	4	A	0.1478	0.3639
NC_005100.4-191983568	CTGTCTCCAACAGCTTCTCCTACACGT	25	C	0.2623	0.4837
NC_005100.4-191993092	AATTCTAATACAGGCCCTCCTAGCCCA	13	G	0.0619	0.06
NC_005100.4-191999727	AAAAGAAATACAAAGACTCCAAACTCC	1	A	0.0804	0.0771
NC_005100.4-191999727	AAAAGAAATACAAAGACTCCAAACTCC	5	G	0.0804	0.0771
NC_005100.4-192009783	CCTTAGTCAACTAACTCTCCAGTTCCC	4	T	0.0252	0.0411
NC_005100.4-192029971	CTCTGTCCTACTGTCTCTCCCTTTCTC	22	T	0.3058	0.465
NC_005100.4-192029971	CTCTGTCCTACTGTCTCTCCCTTTCTC	27	C	0.1138	0.1073
NC_005100.4-192069009	CAGTCTAAAACGTGTGCTCCTCCAGCC	12	G	0.161	0.298
NC_005100.4-192095061	CAAACCTTAACCTAAGCTCCATCACCT	12	C	0.1488	0.5
NC_005100.4-192099252	CCTAGAGAAACCCTGTCTCCTTAAACA	2	C	0.0625	0.0934
NC_005100.4-192099252	CCTAGAGAAACCCTGTCTCCTTAAACA	21	T	0.0893	0.0853
NC_005100.4-192105740	ACTGATAGCACACCTCCTCCTGGTTCT	14	C	0.0345	0.0339
NC_005100.4-192136615	ATCCCTGTCACAGGCACTCCCAGGTCC	7	G	0.0932	0.0889
NC_005100.4-192136615	ATCCCTGTCACAGGCACTCCCAGGTCC	24	G	0	0.4743
NC_005100.4-192180063	ACTACTCCCACATCTTCTCCATTTGAC	3	T	0.2281	0.474
NC_005100.4-192207874	CAAGAAGTCACAAATACTCCAGGGGCC	13	A	0.0085	0.4714
NC_005100.4-192218127	TTAGCCAAGACAGGTCCTCCTCTTGTC	6	C	0.1207	0.1134
NC_005100.4-192218127	TTAGCCAAGACAGGTCCTCCTCTTGTC	9	G	0	0.4709
NC_005100.4-192384251	CAGAACGAAACTTTGTCTCCTCCTCAC	6	C	0.178	0.319
NC_005100.4-192402752	GGCAGGTTGACTGCTGCTCCTACCACA	1	G	0.187	0.319
NC_005100.4-192482349	AAACTGTGGACCAAGCCTCCAGCACGG	16	C	0.0407	0.0398
NC_005100.4-192520802	ACAGCCTCTACCCTGTCTCCTTCACCA	1	A	0.0081	0.481
NC_005100.4-192571770	AGGCCAATGACACAAGCTCCATACTTG	2	G	0.0982	0.4933
NC_005100.4-192575683	GCTCTTACAACCTCTGCTCCCCAAGAG	22	C	0.2696	0.4744
NC_005100.4-192576030	GGTCAACCCACCAGCTCTCCCACACCC	12	C	0.5656	0.4126
NC_005100.4-192576030	GGTCAACCCACCAGCTCTCCCACACCC	22	A	0.5656	0.4126
NC_005100.4-192576444	CTTGTGCTCACCCATGCTCCCACCACC	7	C	0.122	0.1287
NC_005100.4-192576444	CTTGTGCTCACCCATGCTCCCACCACC	21	C	0.122	0.1287
NC_005100.4-192587794	GGGAATCCGACAGCCCCTCCCTGCCCA	2	G	0.1148	0.1225
NC_005100.4-192659705	CTCCTGTCCACCTCAACTCCCAACACA	13	T	0.0254	0.481
NC_005100.4-192668035	CTGGCTCGAACGTTGCCTCCTCCCTCA	8	G	0	0.4793
NC_005100.4-192668923	TTAAAGTCCACCCAACCTCCAGGTATC	7	T	0.0088	0.49
NC_005100.4-192689464	GGAGCAGTGACATCATCTCCTTGCAAT	4	G	0.0976	0.4984
NC_005100.4-192689464	GGAGCAGTGACATCATCTCCTTGCAAT	12	A	0.0976	0.4984
NC_005100.4-192701699	CTGTCAGGTACTGGGCCTCCAGATCAT	27	T	0	0.4793
NC_005100.4-192717335	TGTGGTGTGACATGCTCTCCTGCTGTC	15	C	0.0083	0.4783
NC_005100.4-192717335	TGTGGTGTGACATGCTCTCCTGCTGTC	26	T	0.0083	0.4783
NC_005100.4-192728377	TCCAACAAGACCACATCTCCTGATCCT	6	C	0.0407	0.0398
NC_005100.4-192729450	TGGAAAACAACCAAATCTCCATCATCA	27	A	0.2727	0.4693
NC_005100.4-192775272	TTCATATGAACCAAGCCTCCTGGATGG	16	C	0.1933	0.2389
NC_005100.4-192775872	CCACATGGGACCCTCCCTCCTCTTTGA	27	A	0	0.4688
NC_005100.4-192791169	ATTACAGTAACAAAAGCTCCATTATAT	4	A	0.2479	0.4823
NC_005100.4-192800171	TGATAGTGAACTAAGCCTCCGAAACTG	4	T	0.0847	0.0965
NC_005100.4-192810259	CAGAAATACACAGCCTCTCCCCACATT	15	C	0	0.4819
NC_005100.4-192839347	AAAGCTGTTACTCCTTCTCCACCTCAT	8	T	0.0083	0.4803

NC_005100.4-192847558	ATCCTTCAAACACACTCTCCATCATGA	27	A	0	0.4793
NC_005100.4-192858859	GTCCTTGAGACAGGGTCTCCTGCTATG	27	G	0.0769	0.074
NC_005100.4-192864655	TTATGAAGGACCTTGTCTCCAGCATTT	5	G	0.087	0.0832
NC_005100.4-192864655	TTATGAAGGACCTTGTCTCCAGCATTT	13	T	0.2	0.2458
NC_005100.4-192989094	AGAAAAACGACAAATACTCCTACCTTG	1	A	0.0893	0.1014
NC_005100.4-192994828	TATAACATCACTGTGTCTCCCCAGATC	12	T	0	0.4773
NC_005100.4-193040383	GTGACGGCCACAATTTCTCCCAGACAG	5	C	0.0252	0.0249
NC_005100.4-193063240	CAAACAATAACATGGGCTCCTTCCCGT	4	A	0	0.4807
NC_005100.4-193063240	CAAACAATAACATGGGCTCCTTCCCGT	26	G	0.2295	0.316
NC_005100.4-193107558	TGTGTCTCCACAATGTCTCCGTCATCC	21	G	0	0.4807
NC_005100.4-193128959	TCCGCGTTTACTCTAGCTCCAGGGTGT	1	T	0.0084	0.4723
NC_005100.4-193128959	TCCGCGTTTACTCTAGCTCCAGGGTGT	24	G	0.0167	0.4765
NC_005100.4-193176567	GGCCCTCACACTCACCCTCCTGCCCAT	5	C	0.2353	0.3932
NC_005100.4-193176567	GGCCCTCACACTCACCCTCCTGCCCAT	22	G	0.0504	0.0491
NC_005100.4-193191937	GCTTTTCAAACGTTCTCTCCCTTCTGT	3	T	0.2787	0.402
NC_005100.4-193191937	GCTTTTCAAACGTTCTCTCCCTTCTGT	5	T	0	0.4751
NC_005100.4-193234314	TATCTCCCAACTCGAGCTCCACCTCCC	14	G	0.124	0.1163
NC_005100.4-193255676	GTGGAAGTGACCAACTCTCCACGCACC	23	G	0.2232	0.4394
NC_005100.4-193255676	GTGGAAGTGACCAACTCTCCACGCACC	24	C	0.0089	0.4789
NC_005100.4-193297634	ACCTGGTCCACAGCAGCTCCGACGGGG	7	T	0.0331	0.0325
NC_005100.4-193297634	ACCTGGTCCACAGCAGCTCCGACGGGG	24	G	0.0661	0.0792
NC_005100.4-193348044	GGAATTCAAACCTGGACTCCATTAATT	26	T	0.0084	0.4797
NC_005100.4-193365598	AATGTCTGAACCATCTCTCCAACACCA	4	G	0	0.4773
NC_005100.4-193383431	CCCAAGTCCACAGTAGCTCCTCATATA	15	A	0	0.4641
NC_005100.4-193392242	CTACCTGTTACCCCAGCTCCAGGGAAA	8	T	0.0265	0.0262
NC_005100.4-193403504	TAATGATATACAGGGACTCCTCGGATT	22	C	0.0333	0.0328
NC_005100.4-193403504	TAATGATATACAGGGACTCCTCGGATT	23	G	0.0333	0.0328
NC_005100.4-193407327	CTATGCTGAACCTTTACTCCTAGCACT	24	C	0.0357	0.0689
NC_005100.4-193423213	CTAAGGGTGACTCTGACTCCTCCTGCA	12	T	0	0.4779
NC_005100.4-193426771	TAATTCCCTACTGGGTCTCCCAGCTGT	8	C	0.0424	0.0415
NC_005100.4-193430524	CACTTTGCTACGGAGCCTCCTGTTCTC	23	T	0.1197	0.3457
NC_005100.4-193437358	GACTGTTGCACAGGGACTCCTGTGGAT	25	G	0.3089	0.4595
NC_005100.4-193437358	GACTGTTGCACAGGGACTCCTGTGGAT	26	A	0.3089	0.4595
NC_005100.4-193472486	CCGCTTGCCACCTCTGCTCCAGCACCG	2	C	0.0678	0.1827
NC_005100.4-193472486	CCGCTTGCCACCTCTGCTCCAGCACCG	3	G	0.1724	0.1717
NC_005100.4-193490412	TCCCTGGTAACAATCCCTCCAATGATG	14	T	0	0.4819
NC_005100.4-193490412	TCCCTGGTAACAATCCCTCCAATGATG	22	A	0	0.4819
NC_005100.4-193497002	TCCACTCAGACATCGCCTCCACCCTCC	14	C	0.0328	0.0322
NC_005100.4-193537852	GAGACTTCAACAGCAACTCCTACTTTT	14	C	0.1967	0.4463
NC_005100.4-193547616	GCAGCATCAACTCTACCTCCTTACTCC	3	A	0.0894	0.1001
NC_005100.4-193596794	CCAGACCTCACTAGAGCTCCCATGATC	6	C	0.0259	0.0585
NC_005100.4-193608637	GTTACATCAACCTGTTCTCCAGATTGC	8	C	0.0909	0.1163
NC_005100.4-193616208	CAAGGCCAAACATTGCCTCCAGCTACT	1	C	0.1463	0.1356
NC_005100.4-193616208	CAAGGCCAAACATTGCCTCCAGCTACT	16	C	0.1463	0.4984
NC_005100.4-193618700	TTCGCTCCCACTCCAACTCCTCCCATT	3	C	0.1466	0.5
NC_005100.4-193618700	TTCGCTCCCACTCCAACTCCTCCCATT	6	T	0.0517	0.4946
NC_005100.4-193629239	AGAGGGGAAACATAATCTCCTTAAAAG	12	A	0	0.4728
NC_005100.4-193797616	TTTGTTACCACATCAGCTCCTTGCTGT	12	A	0.1967	0.4966
NC_005100.4-193797616	TTTGTTACCACATCAGCTCCTTGCTGT	24	C	0.2623	0.4837
NC_005100.4-193799445	TCTGAGGAAACCTTTCCTCCACATAGA	1	T	0.0776	0.1504
NC_005100.4-193799445	TCTGAGGAAACCTTTCCTCCACATAGA	6	G	0	0.4839

NC_005100.4-193824465	TACACACCTACTACCACTCCTTAGCAA	5	C	0.0244	0.0398
NC_005100.4-193829504	GATAGACTCACTGTGGCTCCAAGCTTC	8	T	0.0508	0.4884
NC_005100.4-193853938	TCCAGCAGCACTGCCTCTCCTCTGTAT	2	C	0.1057	0.1001
NC_005100.4-193853938	TCCAGCAGCACTGCCTCTCCTCTGTAT	3	C	0.1057	0.1001
NC_005100.4-193882975	CTCTTTGGCACTCCTCCTCCATATGAA	12	T	0.1008	0.0958
NC_005100.4-193914543	CTAATACCTACCCATCCTCCCAGGATC	15	T	0.1261	0.4987
NC_005100.4-193932317	AAAACACAAACTCTGTCTCCTTGATCT	1	A	0.0163	0.032
NC_005100.4-193932317	AAAACACAAACTCTGTCTCCTTGATCT	2	A	0.0163	0.032
NC_005100.4-193978786	TCATGGAGAACAGCAGCTCCCAGGCTG	1	T	0.3125	0.3612
NC_005100.4-193978786	TCATGGAGAACAGCAGCTCCCAGGCTG	26	T	0.1696	0.1843
NC_005100.4-193985513	TATCCACAGACGATGTCTCCAAACCAG	25	C	0.1111	0.4963
NC_005100.4-193989203	TATATGAATACCCCAGCTCCTCCTTTC	6	G	0.0246	0.4879
NC_005100.4-193989203	TATATGAATACCCCAGCTCCTCCTTTC	16	G	0.2927	0.2832
NC_005100.4-194069084	ATTTGGTGCACACAGACTCCTGTTTGA	1	A	0	0.4793
NC_005100.4-194069084	ATTTGGTGCACACAGACTCCTGTTTGA	13	C	0	0.4793
NC_005100.4-194100240	CATGATGGAACTGTTCCTCCTACAGGA	6	T	0.2957	0.3094
NC_005100.4-194100240	CATGATGGAACTGTTCCTCCTACAGGA	7	G	0.0435	0.0425
NC_005100.4-194133042	GTCCAAAGGACTCCATCTCCAACCACA	4	C	0	0.4793
NC_005100.4-194146219	CTGTACAGCACCTGGACTCCTCAAGGA	21	T	0.0656	0.4952
NC_005100.4-194146219	CTGTACAGCACCTGGACTCCTCAAGGA	24	A	0.0164	0.0322
NC_005100.4-194156473	GGTTCTGGAACATGGACTCCAATGTGA	24	G	0.0175	0.0345
NC_005100.4-194163049	CAGCCCACCACAGTCCCTCCCTGGAAG	22	T	0.0354	0.4827
NC_005100.4-194176456	CCTATTCGAACTGCCCCTCCAACTCCA	8	G	0.1062	0.452
NC_005100.4-194202243	TGAGAAAATACAAGTCCTCCATCAGTC	15	T	0	0.4875
NC_005100.4-194214579	CCCCATAGAACTCCTTCTCCTCAATAC	8	G	0.05	0.0488
NC_005100.4-194350212	TTCTCTTTAACCCCCACTCCTCATCCT	26	C	0.1157	0.1653
NC_005100.4-194358763	TCCTCACCCACAGTTCCTCCCTAGTTT	21	C	0.0957	0.0911
NC_005100.4-194358763	TCCTCACCCACAGTTCCTCCCTAGTTT	26	T	0.0261	0.0425
NC_005100.4-194407979	GTTACATGTACACATACTCCAGGGACA	13	C	0.0796	0.0765
NC_005100.4-194435266	GAGGCAGATACGTGACCTCCCTTCTCC	5	C	0.0536	0.0521
NC_005100.4-194435266	GAGGCAGATACGTGACCTCCCTTCTCC	12	G	0.0446	0.0436
NC_005100.4-194445212	ACCAATCTCACAGTGGCTCCTTATTCT	14	T	0.123	0.1154
NC_005100.4-194701738	AACCATCACACCATCTCTCCATCCCTC	7	C	0.0763	0.0889
NC_005100.4-194701738	AACCATCACACCATCTCTCCATCCCTC	9	C	0.0763	0.0889
NC_005100.4-195047295	CAGGAGCCTACCATGGCTCCCCTCTGA	5	A	0.0855	0.0818
NC_005100.4-195047295	CAGGAGCCTACCATGGCTCCCCTCTGA	16	G	0.0862	0.0825
NC_005100.4-195063561	GTGTGGCACACAGTTTCTCCTTGCTGA	5	G	0.2672	0.2562
NC_005100.4-195063561	GTGTGGCACACAGTTTCTCCTTGCTGA	15	T	0.2672	0.2562
NC_005100.4-195102325	AGTGCCTGAACATTAGCTCCCTGATCT	12	A	0	0.4793
NC_005100.4-195105128	TTGATAAAAACATCCCCTCCCAAGGAC	13	T	0.2458	0.4207
NC_005100.4-195163624	AACCAGAGCACCATAGCTCCCAGGGAC	6	G	0.1	0.1099
NC_005100.4-195163624	AACCAGAGCACCATAGCTCCCAGGGAC	8	G	0	0.4832
NC_005100.4-195279810	GAACTGAAAACAGGACCTCCTTTGGGG	4	C	0.2348	0.27
NC_005100.4-195279810	GAACTGAAAACAGGACCTCCTTTGGGG	5	T	0.2368	0.2719
NC_005100.4-195314638	GTCCAATATACAATTGCTCCTAAAGGA	14	T	0	0.474
NC_005100.4-195314638	GTCCAATATACAATTGCTCCTAAAGGA	25	G	0.177	0.1613
NC_005100.4-195328652	ATCAATGTCACCAATGCTCCCTGTCAC	13	A	0.6316	0.4321
NC_005100.4-195398486	GAACTGAGAACAGGACCTCCTTTGGGA	24	G	0.0678	0.0655
NC_005100.4-195398486	GAACTGAGAACAGGACCTCCTTTGGGA	25	G	0.0678	0.0655
NC_005100.4-196013488	CATGGATGGACCAGAGCTCCTCATCTC	14	G	0.1271	0.3991
NC_005100.4-196093929	TACCATGACACAGTAACTCCAAGAGTC	1	T	0.0446	0.0436

NC_005100.4-196093929	TACCATGACACAGTAACTCCAAGAGTC	13	G	0.1071	0.4986
NC_005100.4-196106192	CAGTCTAGCACCCAAGCTCCTTTACTC	4	T	0.0513	0.4602
NC_005100.4-196106192	CAGTCTAGCACCCAAGCTCCTTTACTC	16	G	0.0508	0.4793
NC_005100.4-196132424	CTTGCTAGAACCCACACTCCCAATAGT	21	C	0.0342	0.0336
NC_005100.4-196132424	CTTGCTAGAACCCACACTCCCAATAGT	22	A	0	0.4772
NC_005100.4-196138487	AGCAACTTCACTTTGTCTCCAGGGATA	22	G	0.2114	0.2142
NC_005100.4-196226754	CAATCTGTCACTCCAGCTCCACAGGGT	4	T	0	0.4807
NC_005100.4-196229189	ACCACTCCCACACGGACTCCTTCCTGC	13	C	0.0492	0.0634
NC_005100.4-196243927	GGTGCTGAAACAACAGCTCCGGAGGTA	21	G	0	0.4833
NC_005100.4-196340668	CTGGAGCTCACAAAAGCTCCTAAAGAG	2	T	0	0.48
NC_005100.4-196345124	GGGAGCTGGACTATAACTCCTCAACCA	13	A	0.0261	0.4782
NC_005100.4-196345982	ACATCAAGGACTGAATCTCCGTTCATT	23	T	0.2458	0.4738
NC_005100.4-196379751	AGCTGTTGAACCATCTCTCCAGCCCAC	22	G	0.065	0.0629
NC_005100.4-196414811	TCCATCTAGACTTTACCTCCTAAAGAT	23	A	0.2373	0.324
NC_005100.4-196492046	AAGGATGGGACTGAGACTCCATCAACA	8	G	0.0965	0.1528
NC_005100.4-196511965	CTCAGTAAAACTCCCTCTCCCTTCTCT	21	C	0.0531	0.0683
NC_005100.4-196540391	TTCTCTAGTACCATGTCTCCTTAGATA	15	G	0.0496	0.0639
NC_005100.4-196540391	TTCTCTAGTACCATGTCTCCTTAGATA	26	T	0.0083	0.4769
NC_005100.4-196561834	GAACCTTCCACAACAGCTCCACCCCTC	22	C	0.1475	0.2157
NC_005100.4-196588576	TTCCCATAGACTCCGACTCCCTCACCT	6	A	0.2276	0.2723
NC_005100.4-196588576	TTCCCATAGACTCCGACTCCCTCACCT	15	G	0.2276	0.2723
NC_005100.4-196595659	ACTCCTGTTACTGTAGCTCCTTGGGAG	13	G	0.045	0.0778
NC_005100.4-196613189	GGGAGGCTGACCTCATCTCCAACTTCA	3	G	0.0407	0.0398
NC_005100.4-196639878	ACTGCTGGTACTGGTGCTCCCAACAAC	13	G	0.122	0.1695
NC_005100.4-196642423	TGATGTCCAACCCCAACTCCTAAGCTT	8	C	0	0.4868
NC_005100.4-196654190	CTAGGCATTACACCATCTCCAACTTGA	8	T	0.0081	0.4777
NC_005100.4-196660663	CCCCTCCCCACTGCTTCTCCTTCTCTT	4	C	0.2321	0.3157
NC_005100.4-196721929	GACTTTTCCACACCACCTCCTATTTTC	24	T	0.0177	0.4671
NC_005100.4-196723251	GTCTCCCACACCACCTCTCCTTCATCT	23	C	0.0081	0.481
NC_005100.4-196737566	TCCCTTACAACTAGAACTCCCACTATG	23	C	0.2821	0.4012
NC_005100.4-196737666	CTCCCACCTACCCTGCCTCCACCTCAC	9	T	0.1311	0.2157
NC_005100.4-196789077	TCTTGAGACACGATCTCTCCTTGAACC	21	T	0.0259	0.0422
NC_005100.4-196799129	CTCTTTCAAACTGACTCTCCAACGCCT	1	C	0	0.4793
NC_005100.4-196799129	CTCTTTCAAACTGACTCTCCAACGCCT	22	A	0	0.4793
NC_005100.4-196799662	TGTGTCTGCACCACCCCTCCCCAACAT	8	G	0.0407	0.4703
NC_005100.4-196800239	CCACTAGAGACCAATCCTCCTGCACAT	1	C	0.187	0.1826
NC_005100.4-196801933	ACTCACTCTACTCCTTCTCCACCACAG	22	C	0.3008	0.4301
NC_005100.4-196806269	GCAGGTGTCACATCTGCTCCACAGCCC	1	G	0.122	0.1826
NC_005100.4-196816456	CAAGCGTGGACCCTGGCTCCCCTCTCC	3	A	0	0.474
NC_005100.4-196816456	CAAGCGTGGACCCTGGCTCCCCTCTCC	5	C	0.0862	0.0981
NC_005100.4-196816879	TCCCAGCTAACCCACCCTCCTCTCGCT	12	C	0.0265	0.0262
NC_005100.4-196839905	GGCCACTTAACCAGCCCTCCCATGCAG	4	C	0.0732	0.0705
NC_005100.4-196868894	GCATGAATGACGGGGCCTCCTGCACTG	22	G	0.1026	0.1125
NC_005100.4-196959448	TGCCCTGTGACTCCTCCTCCTCATACT	14	C	0.036	0.0354
NC_005100.4-196970752	TTTTCTCCCACAAATGCTCCTGCTCTG	12	A	0.1121	0.1786
NC_005100.4-196972557	TATGGACAGACCCTTCCTCCTTCCCTT	22	T	0.1322	0.3729
NC_005100.4-197019286	TACATCTCCACCCTGACTCCTTCCCTA	8	C	0.1102	0.2026
NC_005100.4-197019286	TACATCTCCACCCTGACTCCTTCCCTA	26	T	0.0508	0.4884
NC_005100.4-197049749	GCATTAGTAACCCATCCTCCTGCATGG	8	T	0.0088	0.472
NC_005100.4-197056334	AACCCAGCCACAGTCCCTCCTCTAGGT	3	C	0	0.4746
NC_005100.4-197075421	TGCAGCAACACCATCCCTCCTGATGAT	8	A	0	0.4793

NC_005100.4-197075421	TGCAGCAACACCATCCCTCCTGATGAT	21	T	0	0.4793
NC_005100.4-197075746	GAAATCCTGACCACCTCTCCTTCAACA	12	C	0	0.4779
NC_005100.4-197082973	CCTGATTTAACTGTTTCTCCAGAGTCT	13	G	0.0667	0.0644
NC_005100.4-197200652	ACCCTTGTAACTTCCCCTCCAACACAG	7	G	0.2212	0.297
NC_005100.4-197201113	AAAGACATGACCAATTCTCCCATTCTC	2	A	0.2269	0.2961
NC_005100.4-197218847	ATCAGAGTCACCAGCCCTCCTCCCCTG	2	T	0.094	0.2041
NC_005100.4-197218847	ATCAGAGTCACCAGCCCTCCTCCCCTG	22	C	0.0336	0.033
NC_005100.4-197218952	ATATTGTCTACCCCACCTCCCAGTATT	3	A	0.0336	0.033
NC_005100.4-197218952	ATATTGTCTACCCCACCTCCCAGTATT	26	T	0.0336	0.033
NC_005100.4-197244956	CTCCTGTCAACCTTCCCTCCTCCATCT	13	T	0.0336	0.033
NC_005100.4-197244956	CTCCTGTCAACCTTCCCTCCTCCATCT	16	C	0.1949	0.2759
NC_005100.4-197247343	TCCAAGAGTACAAATCCTCCCCATACC	12	A	0	0.4807
NC_005100.4-197247343	TCCAAGAGTACAAATCCTCCCCATACC	21	C	0.0413	0.0562
NC_005100.4-197281132	GATCCCTGCACCTGCCCTCCTCCAGAG	8	G	0.0325	0.0476
NC_005100.4-197281132	GATCCCTGCACCTGCCCTCCTCCAGAG	27	G	0.1057	0.1695
NC_005100.4-197286697	TTTCCTCCCACACTCCCTCCATCATTT	5	C	0.0083	0.4803
NC_005100.4-197297904	ATTAACTAAACCTTTACTCCTACTCTG	21	T	0.0087	0.466
NC_005100.4-197302128	CTTTAAGACACAATCTCTCCCTGAACA	27	A	0.0179	0.4943
NC_005100.4-197319101	CTAGTCAGGACCCTCCCTCCTGTCCAT	14	T	0.2846	0.373
NC_005100.4-197319101	CTAGTCAGGACCCTCCCTCCTGTCCAT	26	A	0.2114	0.3334
NC_005100.4-197328836	GGTCTTCTCACTCTAGCTCCTGTTGAC	1	G	0.0331	0.0325
NC_005100.4-197329365	TCTTACAAGACAAGCGCTCCACTACTG	16	G	0	0.474
NC_005100.4-197338039	TGGAATGTCACCCAGCCTCCTACAGCC	8	T	0.0085	0.479
NC_005100.4-197361961	GGTGCTGTAACACGTGCTCCAGAGATA	15	T	0.1138	0.3041
NC_005100.4-197382688	GAGGGACCCACAGAGGCTCCTGTTCTC	23	T	0.2602	0.4926
NC_005100.4-197440023	TATCGGTGCACTCTCCCTCCTCCGCAG	13	C	0	0.4787
NC_005100.4-197446701	TACTGTGTAACAGAACCTCCCACAGAT	13	G	0.065	0.4904
NC_005100.4-197464725	ATTCCCAACACCTCCACTCCACTGCCT	13	T	0.0244	0.0241
NC_005100.4-197464725	ATTCCCAACACCTCCACTCCACTGCCT	22	C	0.065	0.4944
NC_005100.4-197466901	ACCCCACACACTCCACCTCCTGCTGCA	9	C	0.1311	0.1505
NC_005100.4-197493099	CCCCCTCCCACACCGTCTCCTAGACGG	25	C	0.2857	0.4743
NC_005100.4-197502554	AAACACCAAACCAGAACTCCCTCGGTG	9	A	0.0261	0.0257
NC_005100.4-197541833	TTGAGGGTCACCCGTGCTCCTGTAAAT	22	G	0.2982	0.4814
NC_005100.4-197543904	TTTAGATCAACTTTTTCTCCTCATCCT	6	A	0	0.4807
NC_005100.4-197543904	TTTAGATCAACTTTTTCTCCTCATCCT	14	T	0	0.4807
NC_005100.4-197553470	AGGTGTCCCACTCCCACTCCAACCCAG	15	C	0	0.4832
NC_005100.4-197553702	CACTTTCACACTTACCCTCCTGGCCAT	15	C	0.0085	0.4776
NC_005100.4-197563303	TTTTCCTCCACTCCTCCTCCTTCTCCT	2	T	0.0811	0.0778
NC_005100.4-197575899	CAAGAGAATACCAGAGCTCCTACCCAC	12	C	0.0088	0.49
NC_005100.4-197632642	ACCCGGGTGACTTCTGCTCCAGCAGTC	12	T	0.2105	0.4846
NC_005100.4-197637345	ACCAGGAATACCCTGGCTCCCCGCCAT	23	G	0	0.4839
NC_005100.4-197641620	AAAACCTACACACAGACTCCATTAAGA	6	C	0.2759	0.3835
NC_005100.4-197653319	CCCATCAGAACCCTTCCTCCCAATCCC	25	C	0.1721	0.4997
NC_005100.4-197736970	TTCATAGGCACCAACACTCCCTGTAAC	8	G	0.041	0.071
NC_005100.4-197748660	AGTCCTTCTACCCCACCTCCCTATTAC	4	C	0.124	0.1306
NC_005100.4-197755831	GGTGGCCTGACAAAGGCTCCGTCTGAA	4	G	0	0.4807
NC_005100.4-197764266	GACCACACAACACACACTCCAACTCAA	1	G	0	0.4785
NC_005100.4-197794382	GGGATCCACACCTGATCTCCCGGGCTC	6	C	0	0.4773
NC_005100.4-197794382	GGGATCCACACCTGATCTCCCGGGCTC	8	A	0	0.4773
NC_005100.4-197862337	CACCACAGTACCTGAGCTCCGCACTAC	23	A	0	0.4819
NC_005100.4-197868061	ATGACAGATACAGACTCTCCTGTGGAG	15	C	0.075	0.0722

NC_005100.4-197883765	TCAAGGCCCACTGTACCTCCCTCCATT	24	C	0.2	0.2061
NC_005100.4-197883765	TCAAGGCCCACTGTACCTCCCTCCATT	25	A	0	0.4751
NC_005100.4-197884206	CTCTGTATCACTCCTGCTCCGTCTCTC	21	G	0.0081	0.0241
NC_005100.4-197885526	CCAGCCTCAACCACAGCTCCTACTGCT	15	A	0.157	0.4988
NC_005100.4-197914505	TAATTGTGCACCAAAGCTCCCGGCCAG	21	C	0.0268	0.0264
NC_005100.4-197914505	TAATTGTGCACCAAAGCTCCCGGCCAG	22	G	0.018	0.4785
NC_005100.4-197953015	ATTCTCAATACCACCACTCCCAATGCT	2	T	0.4701	0.3685
NC_005100.4-197953015	ATTCTCAATACCACCACTCCCAATGCT	7	A	0.4741	0.3707
NC_005100.4-198127532	GACTGAGACACAGTGCCTCCTTCCTAG	27	G	0.2213	0.2903
NC_005100.4-198151512	CAAGTAAGAACTCGAGCTCCTCACCTC	13	C	0.0328	0.0322
NC_005100.4-198153108	GAGCAGCGTACATTGACTCCTGTTATT	12	A	0.1157	0.1235
NC_005100.4-198153108	GAGCAGCGTACATTGACTCCTGTTATT	25	A	0.1322	0.1918
NC_005100.4-198163839	CTAGCACGAACTAATGCTCCTCCCTCA	16	G	0.0417	0.0408
NC_005100.4-198202936	GCCTGCACCACTACAGCTCCTGCTGGC	1	G	0.1964	0.2575
NC_005100.4-198202936	GCCTGCACCACTACAGCTCCTGCTGGC	22	G	0.0526	0.0677
NC_005100.4-198209094	AACAAAGAGACTCAGCCTCCTCAGTCT	25	T	0	0.4844
NC_005100.4-198248208	TGGAGACACACAGGTACTCCTGGCACG	2	G	0	0.4772
NC_005100.4-198285121	TCTCACGTTACTCTCTCTCCTCTCGCT	1	T	0	0.4807
NC_005100.4-198302021	GACCGAAAGACCCCTGCTCCTTCTGGA	1	G	0.5714	0.4082
NC_005100.4-198302021	GACCGAAAGACCCCTGCTCCTTCTGGA	5	G	0.1504	0.1391
NC_005100.4-198319340	CAAGTACAAACGCACACTCCAACTAAA	3	A	0.1917	0.2491
NC_005100.4-198388733	AATCAGATGACCAAGTCTCCTCCTCAC	24	T	0	0.4724
NC_005100.4-198429570	TGCAGCATGACAAAGGCTCCAGAACAT	27	T	0	0.4772
NC_005100.4-198450199	CATGGCCTCACTTTTCCTCCTCCTCCT	3	T	0.0167	0.1244
NC_005100.4-198515719	AGGTGCCCTACAAATACTCCTTGGATT	4	T	0.1754	0.4875
NC_005100.4-198516782	ATGCTTTACACCCCACCTCCTGGTAAA	27	A	0	0.4718
NC_005100.4-198528598	CACACACACACACACACTCCCGGCATG	14	A	0.2857	0.375
NC_005100.4-198541509	TGCATTGATACGTAACCTCCAGCGATG	4	A	0	0.4793
NC_005100.4-198570457	CTGCCTATCACTCGAGCTCCAAGAGCT	21	A	0.1639	0.1505
NC_005100.4-198627602	ATTCGTGTAACAATCCCTCCTCACCAC	4	C	0	0.4819
NC_005100.4-198627602	ATTCGTGTAACAATCCCTCCTCACCAC	16	C	0.0252	0.0249
NC_005100.4-198637630	GCCTCAAGCACCCGGTCTCCTGCCCAC	27	C	0	0.474
NC_005100.4-198644402	TTCCATAGCACTGTCCCTCCTCTGGAA	15	C	0.0333	0.0644
NC_005100.4-198695892	GTGCCTAAGACCCCTTCTCCTGGGCTG	27	G	0.0081	0.0553
NC_005100.4-198721376	GCATGATTGACAGTCGCTCCATCCAAT	3	A	0	0.4786
NC_005100.4-198723068	ATCCTTATCACTCCAGCTCCAGGGTAT	1	A	0.187	0.2323
NC_005100.4-198733057	TTCTCTAAGACAGCGTCTCCTTATCTC	6	T	0.0089	0.4885
NC_005100.4-198744661	CCTTTGGAGACCTGCCCTCCCTTTACC	9	G	0	0.4819
NC_005100.4-198747388	CAGGTTCTAACCATACCTCCAAATACT	23	A	0.25	0.42
NC_005100.4-198754395	ATCTGCCTGACTCTGCCTCCCAAGTAG	23	A	0.5299	0.3974
NC_005100.4-198754395	ATCTGCCTGACTCTGCCTCCCAAGTAG	24	G	0.5299	0.3974
NC_005100.4-198828914	TAATTACCCACATGCCCTCCTTGATTC	1	T	0	0.4793
NC_005100.4-198837704	TGTGAGTGTACAGGTGCTCCACCAGTT	27	T	0.2541	0.4755
NC_005100.4-198837833	GGTCCTTGGACAGCAGCTCCAGGTAGC	5	C	0.0336	0.033
NC_005100.4-198861664	ACTCTATAAACAGGGACTCCTTAAGAC	27	C	0.2017	0.494
NC_005100.4-198870372	CCTGTGGGGACAGCTTCTCCTGAGATG	15	T	0	0.4787
NC_005100.4-198984613	CCAGGCTACACTTTATCTCCCGTTGCT	9	C	0.0085	0.4823
NC_005100.4-198987842	CACACACTGACTTTGGCTCCAGCAGCA	16	G	0.2114	0.2937
NC_005100.4-199017388	GAGTATGGAACCACACCTCCCAGCTTA	2	A	0	0.4819
NC_005100.4-199017388	GAGTATGGAACCACACCTCCCAGCTTA	26	T	0	0.4807
NC_005100.4-199069957	CCCCTGACAACGATGTCTCCTTCAGCC	12	G	0.1983	0.4849

NC_005100.4-199097289	CAGACTTTTACCGCCTCTCCTACAACT	15	C	0.1707	0.208
NC_005100.4-199099945	TTCCCTAGTACCCACCCTCCACTCTTG	8	G	0.1138	0.4389
NC_005100.4-199099945	TTCCCTAGTACCCACCCTCCACTCTTG	9	T	0	0.4793
NC_005100.4-199180175	AAGTTTGCCACAGCCTCTCCAGGGCTG	5	T	0	0.4793
NC_005100.4-199189769	CCTAGTGACACACCTCCTCCCCAGTGA	3	T	0.5877	0.4353
NC_005100.4-199245922	GTTGCTACCACTGCCACTCCCCCAGGG	1	G	0	0.4773
NC_005100.4-199247148	GTGGCTCACACTGTCCCTCCTGCAGCC	7	C	0	0.4728
NC_005100.4-199263098	CTTCATCCCACACTCCCTCCTCCCACT	16	C	0.0261	0.0257
NC_005100.4-199315345	AACATCCACACAGCTGCTCCTAGGATC	22	A	0.0424	0.0576
NC_005100.4-199324262	TGATGGAGCACCACCGCTCCCCACCGC	7	A	0	0.4693
NC_005100.4-199324262	TGATGGAGCACCACCGCTCCCCACCGC	25	C	0	0.4693
NC_005100.4-199340132	CAGCTCCTAACACGTGCTCCACCAGCC	14	G	0.1017	0.1409
NC_005100.4-199342748	CAGCACAGCACTGCAGCTCCTAGGAGA	22	A	0.1356	0.4977
NC_005100.4-199377540	CAAGTGCCTACTTGTGCTCCCGGGTGC	1	C	0.0083	0.4803
NC_005100.4-199377540	CAAGTGCCTACTTGTGCTCCCGGGTGC	21	C	0.0407	0.0553
NC_005100.4-199449823	CTGCTGACTACTCACACTCCACATTTA	15	C	0	0.4773
NC_005100.4-199454331	CACTCAAGAACCTCCCCTCCCTGCCTC	2	A	0	0.48
NC_005100.4-199454331	CACTCAAGAACCTCCCCTCCCTGCCTC	6	A	0	0.48
NC_005100.4-199461851	TTCCCTCCTACCCCCTCTCCCACCCAG	14	C	0.5929	0.4547
NC_005100.4-199461851	TTCCCTCCTACCCCCTCTCCCACCCAG	23	C	0.5929	0.4547
NC_005100.4-199505214	GCTGCTACTACCGTCCCTCCACAGCTG	12	C	0.0413	0.0405
NC_005100.4-199516955	AGCTAAAGGACATGACCTCCGCTTGAA	21	G	0.0569	0.0553
NC_005100.4-199527518	CAGGTGCTTACCCACCCTCCTTCTTGC	12	C	0.2655	0.3815
NC_005100.4-199544984	TTCCCTTTTACATTTTCTCCTGGCCCC	5	C	0	0.4833
NC_005100.4-199544984	TTCCCTTTTACATTTTCTCCTGGCCCC	6	T	0	0.4833
NC_005100.4-199549687	CTCCCTGTGACACAATCTCCGTGTGAC	21	G	0.0348	0.0342
NC_005100.4-199549687	CTCCCTGTGACACAATCTCCGTGTGAC	27	C	0.0348	0.0342
NC_005100.4-199558460	GTTAACAGCACCCGTACTCCAGAGGGT	25	G	0.0488	0.0476
NC_005100.4-199566840	CCCTTCCTTACAAAACCTCCAACCCAT	2	C	0.0569	0.0854
NC_005100.4-199566840	CCCTTCCTTACAAAACCTCCAACCCAT	3	C	0	0.4793
NC_005100.4-199575797	TCACTGCGGACTCTAGCTCCTCTCTTC	12	T	0	0.4757
NC_005100.4-199575797	TCACTGCGGACTCTAGCTCCTCTCTTC	27	C	0.2542	0.4367
NC_005100.4-199575984	CTTCTTTGAACCTGCCCTCCTGAGAGG	7	T	0	0.4813
NC_005100.4-199613307	TGGTGTCCCACTTTTTCTCCTGCCCCT	3	G	0.036	0.0695
NC_005100.4-199613307	TGGTGTCCCACTTTTTCTCCTGCCCCT	27	T	0	0.4704
NC_005100.4-199618994	CCAGAGTGGACTGTTACTCCATGGAGT	25	A	0	0.48
NC_005100.4-199619761	CACGGTGGCACACGCCCTCCTCCAGAT	2	A	0.1167	0.2311
NC_005100.4-199677583	CTTAACCAGACAAGTCCTCCTGATTCA	6	C	0.1339	0.1553
NC_005100.4-199696387	CTGACTTGCACTGGGCCTCCTGCATCC	23	C	0	0.4793
NC_005100.4-199696387	CTGACTTGCACTGGGCCTCCTGCATCC	27	C	0.0813	0.0928
NC_005100.4-199701614	CATGCTTACACTCTACCTCCTTAGACG	3	T	0.1639	0.1774
NC_005100.4-199705873	CAGGTGGCTACCCCATCTCCATTCTAC	15	A	0	0.4807
NC_005100.4-199717147	CAGCAGTTCACATTCTCTCCAGCAGCG	25	G	0.1339	0.1553
NC_005100.4-199717147	CAGCAGTTCACATTCTCTCCAGCAGCG	27	G	0.2679	0.4943
NC_005100.4-199722576	ATCCTTGTGACCAGTCCTCCAATGCTC	8	T	0.2119	0.4896
NC_005100.4-199726204	CACACACACACACACACTCCTGATCCC	1	C	0.1083	0.1025
NC_005100.4-199771359	GACATGAACACCGGGCCTCCTGACCAA	12	C	0.0348	0.0671
NC_005100.4-199775947	GAAAACTGAACCATCTCTCCAGCTTCC	21	A	0	0.4764
NC_005100.4-199775947	GAAAACTGAACCATCTCTCCAGCTTCC	22	G	0.0696	0.4915
NC_005100.4-199776506	ATGAGAAGAACTCGGTCTCCAAGGTCA	4	A	0.124	0.4988
NC_005100.4-199785066	GTCTGTGTCACAAGTGCTCCCACCTAC	1	G	0.2213	0.2218

NC_005100.4-199785680	TGGATGGCAACGGCTGCTCCACTCTGT	1	T	0.3033	0.3307
NC_005100.4-199788124	CATTCTAGAACATTCCCTCCCCACTCC	16	C	0.1913	0.2005
NC_005100.4-199788124	CATTCTAGAACATTCCCTCCCCACTCC	25	T	0	0.4868
NC_005100.4-199794459	TGAGCCGAGACTGCCCCTCCACATTGA	23	A	0.0083	0.4783
NC_005100.4-199815605	CTACTTACGACTCGTTCTCCAAGGTGT	8	C	0.0976	0.1494
NC_005100.4-199845504	GGTCATCACACCTTACCTCCAGTCTGC	1	G	0.0169	0.4677
NC_005100.4-199845504	GGTCATCACACCTTACCTCCAGTCTGC	9	C	0.0252	0.0411
NC_005100.4-199880638	TGTTTGAATACCTGGTCTCCGGTAGAT	1	T	0	0.4875
NC_005100.4-199996825	ACAACTGAAACAATGCCTCCTATCACC	6	T	0.1933	0.4994
NC_005100.4-200008014	CTGTGCTTAACACCAGCTCCCCCTGGT	22	C	0.2397	0.4398
NC_005100.4-200009543	ACAGCCATAACACCAGCTCCCCCTCCC	9	A	0.0087	0.4849
NC_005100.4-200010853	TCAAGGACCACCTGTGCTCCAGAGCAA	3	A	0.0702	0.0997
NC_005100.4-200010853	TCAAGGACCACCTGTGCTCCAGAGCAA	15	T	0	0.4698
NC_005100.4-200011438	TGTTCTGAGACCAGGCCTCCCTGCGTC	24	C	0.1333	0.3299
NC_005100.4-200015287	TCCTAAGCTACGTGGGCTCCTTAGCCT	24	G	0.1057	0.1695
NC_005100.4-200015287	TCCTAAGCTACGTGGGCTCCTTAGCCT	27	T	0	0.4793
NC_005100.4-200020999	TCCACAAGGACATACTCTCCCCACACC	7	A	0	0.4832
NC_005100.4-200020999	TCCACAAGGACATACTCTCCCCACACC	23	A	0.1176	0.4082
NC_005100.4-200025541	TCCTGGAGCACGTGGTCTCCTATCTCT	21	T	0.1345	0.3014
NC_005100.4-200104194	AACAGGTGGACAGTATCTCCCACTAAG	2	A	0.2773	0.274
NC_005100.4-200166613	CGTGGCCGAACACTCACTCCCTTTTAA	21	C	0.1917	0.1733
NC_005100.4-200233726	TTCACTGGTACAAGCTCTCCCTCACTC	21	C	0	0.4826
NC_005100.4-200251916	AGGCTGCTCACATTAACTCCCTTATAC	4	C	0.0442	0.0765
NC_005100.4-200251916	AGGCTGCTCACATTAACTCCCTTATAC	14	T	0.1504	0.4994
NC_005100.4-200404806	GCATGGAGGACATCTTCTCCCACTTAG	4	T	0.2212	0.4873
NC_005100.4-200405922	TAGTGAACAACTGGTTCTCCACGTGGT	4	T	0.2411	0.2876
NC_005100.4-200442069	AAGTAATTTACTCTGCCTCCGTGGAGT	21	G	0.2137	0.2544
NC_005100.4-200442069	AAGTAATTTACTCTGCCTCCGTGGAGT	25	A	0.2035	0.4373
NC_005100.4-200454319	AGAACTGTCACCCCAGCTCCCTCTTCA	4	A	0	0.4677
NC_005100.4-200472569	CTGGCAGATACAACCCCTCCAGGAATA	1	C	0	0.4807
NC_005100.4-200542773	CCCCCATGAACCATTGCTCCCTGCATC	1	C	0.0678	0.0965
NC_005100.4-200542773	CCCCCATGAACCATTGCTCCCTGCATC	14	T	0.0513	0.4938
NC_005100.4-200580636	CAAATTCACACACAAGCTCCAGCCACT	13	C	0.0413	0.0562
NC_005100.4-200620208	CTCCAGTAAACTGGTTCTCCGCCCACC	14	G	0	0.4827
NC_005100.4-200620208	CTCCAGTAAACTGGTTCTCCGCCCACC	21	G	0	0.4827
NC_005100.4-200623566	CTCACAACTACCTGGACTCCAGTTCTG	23	T	0.075	0.0722
NC_005100.4-200623566	CTCACAACTACCTGGACTCCAGTTCTG	26	T	0.0756	0.4931
NC_005100.4-200632320	ATCTTCTTCACAGCGGCTCCATCACTA	2	T	0	0.4671
NC_005100.4-200659569	AGGAGCCACACGCACACTCCACAAACC	24	A	0.0083	0.4803
NC_005100.4-200659569	AGGAGCCACACGCACACTCCACAAACC	25	A	0.0083	0.4803
NC_005100.4-200665284	CACTCAATGACACCAACTCCGTAACCA	1	C	0	0.4832
NC_005100.4-200665284	CACTCAATGACACCAACTCCGTAACCA	2	A	0	0.4832
NC_005100.4-200672778	CACACGCACACCCGAGCTCCCACACTG	14	G	0.2743	0.3396
NC_005100.4-200672778	CACACGCACACCCGAGCTCCCACACTG	15	A	0.1239	0.4968
NC_005100.4-200691983	TTCAGAACCACTGTTTCTCCTAAACAA	12	T	0	0.4859
NC_005100.4-200691983	TTCAGAACCACTGTTTCTCCTAAACAA	13	G	0	0.4859
NC_005100.4-200699803	AGGATTTTTACCCCCTCTCCCTTCTTC	13	C	0.0331	0.0325
NC_005100.4-200701578	TCACTGCCGACACGCCCTCCCACTCCA	13	C	0.0328	0.0322
NC_005100.4-200731461	AAACTGATTACTTCATCTCCTCAGTAT	7	A	0	0.4773
NC_005100.4-200731461	AAACTGATTACTTCATCTCCTCAGTAT	16	T	0	0.4773
NC_005100.4-200749861	GGCTTGCAAACCCTGCCTCCACAGCTG	14	T	0.0976	0.4984

NC_005100.4-200749861	GGCTTGCAAACCCTGCCTCCACAGCTG	24	G	0.0407	0.4868
NC_005100.4-200751305	TACAGAAAAACTTACCCTCCATAAGTC	24	A	0.1217	0.4981
NC_005100.4-201163097	CCGTGAAATACAGCACCTCCAACTCTT	9	T	0.1739	0.2005
NC_005100.4-201163097	CCGTGAAATACAGCACCTCCAACTCTT	21	A	0	0.4778
NC_005100.4-201166408	GGAATAAGGACAAGTCCTCCTCACAGC	13	A	0.1951	0.2142
NC_005100.4-201201006	CTTTGAGACACAGTCTCTCCCTGAACA	14	T	0.5133	0.3982
NC_005100.4-201243244	CAGGAGCATACAACATCTCCCTCCCGT	3	G	0	0.4773
NC_005100.4-201243244	CAGGAGCATACAACATCTCCCTCCCGT	6	G	0.2623	0.3791
NC_005100.4-201251003	TGTGGGTCTACCCAGCCTCCTCACTGA	2	G	0.0163	0.032
NC_005100.4-201270688	CTGTTTCATACTCATCCTCCTGGATTT	6	T	0.1239	0.3982
NC_005100.4-201270688	CTGTTTCATACTCATCCTCCTGGATTT	9	T	0.1062	0.4968
NC_005100.4-201274744	TGTTCATGGACCACGGCTCCTGACCGT	15	G	0.2049	0.3984
NC_005100.4-201293281	TAAGTGACCACTTTCTCTCCATTTCCT	21	A	0.0957	0.4998
NC_005100.4-201299141	GGAACCAGGACCTTAGCTCCAGGCATG	25	A	0.084	0.0958
NC_005100.4-201322999	TGTAGACCCACAGGGTCTCCAGTGTAG	1	T	0	0.4773
NC_005100.4-201329306	CCGAGACCTACCAGGACTCCTTGCTAT	2	C	0.082	0.0935
NC_005100.4-201336147	AGCGTGTGCACACAGCCTCCCCATCGG	22	C	0.1391	0.173
NC_005100.4-201356285	TTACCCAGTACCGTGCCTCCAATATTG	1	T	0.0082	0.4755
NC_005100.4-201392819	GCCAGCCTCACTCAGACTCCCTCACCC	14	A	0.1466	0.4866
NC_005100.4-201401441	TCCACTTTTACCTCACCTCCCTGAGTT	5	C	0.188	0.1975
NC_005100.4-201401441	TCCACTTTTACCTCACCTCCCTGAGTT	22	T	0.0086	0.4893
NC_005100.4-201496577	ATAAGGGCTACCTGGGCTCCAACTGCA	4	A	0.252	0.3091
NC_005100.4-201552840	GCTCCCACAACCCATTCTCCTCACTGT	2	C	0.1545	0.4995
NC_005100.4-201554956	GACAGAGCCACACCCACTCCAAGAAGT	23	G	0.0598	0.058
NC_005100.4-201710026	TTCTTAGGAACCAAGTCTCCTTCTCAT	5	T	0	0.4743
NC_005100.4-201771004	ACCCACGGAACCCTCTCTCCAGCCCCT	7	G	0.2368	0.3568
NC_005100.4-201774229	TGATGAGCGACCCCTGCTCCATCACCT	3	A	0.122	0.1826
NC_005100.4-201774229	TGATGAGCGACCCCTGCTCCATCACCT	8	C	0.3197	0.3909
NC_005100.4-201826076	TGCAGCCTCACATAAGCTCCTCTTGTA	22	C	0.025	0.0408
NC_005100.4-201826076	TGCAGCCTCACATAAGCTCCTCTTGTA	23	T	0.0084	0.4797
NC_005100.4-201835696	TGTGCAGCTACTCCAGCTCCTGAGGAA	23	A	0.1327	0.1391
NC_005100.4-201862611	TTTTACTTGACTGCCTCTCCTGGGATT	14	C	0.0087	0.4817
NC_005100.4-201868565	TCCCTCAGCACCACCCCTCCTTAAGGC	2	C	0.0177	0.452
NC_005100.4-201868565	TCCCTCAGCACCACCCCTCCTTAAGGC	23	A	0.2321	0.4688
NC_005100.4-201884786	CGGTTATGTACAGAATCTCCACAGTTG	2	G	0.0179	0.0351
NC_005100.4-201913901	CAACCACAAACACACCCTCCTACTCAC	25	C	0.25	0.4265
NC_005100.4-201932129	AACCATTCAACACCAACTCCAGACACT	24	C	0.0504	0.0491
NC_005100.4-202030707	CAGAGTCACACATTTACTCCAAGGAGT	2	A	0.0885	0.1162
NC_005100.4-202056305	ATCTCTTTGACTTTAACTCCATGGCAA	7	T	0.122	0.4174
NC_005100.4-202057887	TGCTTGGCCACTTGTTCTCCATCTGCT	16	T	0.1681	0.4998
NC_005100.4-202079611	ATGGGGCCCACAAAGACTCCCTGGTCA	24	G	0.1466	0.1923
NC_005100.4-202095106	AGGTACAACACTTAGCCTCCCTACAGG	1	A	0	0.48
NC_005100.4-202104156	AGTAGAATGACCCTTTCTCCCTCAGCA	12	C	0.069	0.3074
NC_005100.4-202111991	GGCAAGTACACCCCCACTCCCACTCAC	8	A	0	0.4793
NC_005100.4-202112018	TCACCTCTCACACCAACTCCAGAGTAA	14	C	0.1351	0.126
NC_005100.4-202131809	CTATGTTCTACGTTGTCTCCTTTGCCC	6	T	0.0796	0.4923
NC_005100.4-202149155	ACACTTAATACATGACCTCCCTGAAGT	26	G	0.0342	0.0336
NC_005100.4-202152074	CATATTTACACTTATTCTCCTGGCCCT	22	G	0.0513	0.4918
NC_005100.4-202152703	TAGCGATCTACTCAGGCTCCAGAGCCT	1	T	0.0325	0.032
NC_005100.4-202152703	TAGCGATCTACTCAGGCTCCAGAGCCT	4	C	0.0488	0.0476
NC_005100.4-202155201	GTGTTGTCCACTATCCCTCCCGAGGGT	21	C	0.1138	0.1073

NC_005100.4-202178734	CATGACACAACAGAGTCTCCAAAGGAA	22	A	0.1739	0.3593
NC_005100.4-202285297	CATGAAGGAACTCCCACTCCAATGTCA	15	C	0.2982	0.4038
NC_005100.4-202295232	GGCTTGATGACCCAAGCTCCATCCCTG	4	T	0.5128	0.3813
NC_005100.4-202295232	GGCTTGATGACCCAAGCTCCATCCCTG	8	T	0.5128	0.3813
NC_005100.4-202303830	GTCATGGATACCTCAACTCCAGAGTTA	9	T	0.1518	0.3316
NC_005100.4-202314595	AACCCATATACCAAGTCTCCATGGCAC	24	G	0	0.4793
NC_005100.4-202338557	AAAGGTACCACATTGGCTCCTGTGTGG	16	G	0.2432	0.2656
NC_005100.4-202379156	ATTGACTGAACCATTTCTCCAGCCCCT	24	C	0.5902	0.416
NC_005100.4-202410494	CTCTACTCTACCTTTGCTCCATGTTTC	22	T	0.0407	0.0398
NC_005100.4-202435571	AAGGTGTTTACCAGAACTCCTTGTTAG	6	G	0.2	0.2432
NC_005100.4-202447250	GTGCTGGACACATAATCTCCAGGGTAA	4	C	0.2521	0.2796
NC_005100.4-202468335	ATCTTCTCCACACATTCTCCATCCCAC	1	A	0.084	0.1678
NC_005100.4-202468335	ATCTTCTCCACACATTCTCCATCCCAC	21	A	0.1008	0.4983
NC_005100.4-202556578	AATACTGGGACTTCATCTCCATCTGAG	3	T	0.0826	0.2869
NC_005100.4-202561682	CTGCTTGGCACTCATGCTCCACGGAAT	12	T	0.1301	0.1216
NC_005100.4-202561682	CTGCTTGGCACTCATGCTCCACGGAAT	21	A	0.0407	0.0705
NC_005100.4-202602074	TAAGTGGGAACCACTGCTCCCTGTGAA	13	A	0.2807	0.3115
NC_005100.4-202631617	GCTTTCTCTACCCTGTCTCCTGTTTAA	8	C	0.0088	0.4843
NC_005100.4-202631617	GCTTTCTCTACCCTGTCTCCTGTTTAA	12	C	0.2281	0.4975
NC_005100.4-202654244	ATGCTCCTTACCTAGCCTCCTCCCTTT	5	T	0.1721	0.375
NC_005100.4-202697293	TAATGATTCACTTTTCCTCCATTCTCA	14	T	0.0721	0.1023
NC_005100.4-202719511	AACAATTACACGCATCCTCCTGAGAAA	25	A	0.1071	0.4922
NC_005100.4-202743258	ACTTTGTCCACGGCACCTCCTCCCACA	12	G	0	0.4787
NC_005100.4-202743258	ACTTTGTCCACGGCACCTCCTCCCACA	22	C	0.0083	0.0245
NC_005100.4-202831434	AATTTACCAACCCTCACTCCCATGTGT	9	A	0	0.4814
NC_005100.4-202831752	CTCCTCTCCACGGTGTCTCCCCCTCCC	21	C	0	0.4807
NC_005100.4-202874760	GATTTTCACACCTATGCTCCAATCCCA	15	T	0	0.4786
NC_005100.4-202879382	GGTCTGGCTACTCCCTCTCCTTTAGAA	13	C	0.0248	0.0245
NC_005100.4-202902196	AGGAGATAAACAGACTCTCCTCAGGTT	14	A	0	0.4793
NC_005100.4-202909613	ACCATGACCACCATCACTCCATCCACC	25	A	0.5913	0.4165
NC_005100.4-202909969	GCCATGGCCACTACCACTCCACTTCCA	24	T	0	0.4773
NC_005100.4-202957876	CCCTATTCCACAGGGCCTCCCAATCTC	24	T	0	0.4819
NC_005100.4-202978385	ACCATTCTTACACAGTCTCCATTCTTA	16	T	0.2833	0.4499
NC_005100.4-202978385	ACCATTCTTACACAGTCTCCATTCTTA	24	C	0.1597	0.5
NC_005100.4-202993205	CTGCAGAGCACTTTGCCTCCCTGTCAG	14	T	0	0.4793
NC_005100.4-202993205	CTGCAGAGCACTTTGCCTCCCTGTCAG	15	G	0.065	0.4904
NC_005100.4-203010629	TTATTTCAAACAACCACTCCAAAGGCG	13	A	0	0.4772
NC_005100.4-203010629	TTATTTCAAACAACCACTCCAAAGGCG	27	G	0.0756	0.0728
NC_005100.4-203073894	TCACTATGAACCTCCACTCCTTACACA	5	T	0.2035	0.3081
NC_005100.4-203075823	CATGGTTCAACCTGCACTCCAAAACAA	2	A	0.0796	0.0926
NC_005100.4-203075823	CATGGTTCAACCTGCACTCCAAAACAA	15	C	0.2193	0.3568
NC_005100.4-203114959	GGAGCTTGCACTCTATCTCCCAGGCTA	27	A	0.1917	0.4922
NC_005100.4-203122954	GTCGGATACACCGGGTCTCCAAGTGGC	21	A	0	0.4793
NC_005100.4-203122954	GTCGGATACACCGGGTCTCCAAGTGGC	26	G	0.0407	0.0398
NC_005100.4-203123657	CTCTGAGAAACAACCACTCCCTCTCAT	12	A	0	0.4832
NC_005100.4-203124462	AGGACATCAACACGGCCTCCAGAGCAA	12	A	0.0732	0.0854
NC_005100.4-203376148	CCACATCCAACAGTGCCTCCTTCAGCT	14	T	0.1795	0.2171
NC_005100.4-203376148	CCACATCCAACAGTGCCTCCTTCAGCT	15	G	0.1795	0.2171
NC_005100.4-203376283	ATATCAGGGACACTATCTCCTTTAGAA	1	A	0.2397	0.2355
NC_005100.4-203434533	CTAATTGAAACATTTACTCCTTGGGAG	25	G	0.0826	0.1235
NC_005100.4-203464961	TTGGTCTCTACGATGTCTCCCAAGGAA	12	G	0.0446	0.0436

NC_005100.4-203494894	GAATGTTTAACTTCCACTCCCTGCTTC	24	C	0	0.4786
NC_005100.4-203508577	TGCGAGAACACCGCTACTCCGACAGTT	1	T	0.2295	0.4515
NC_005100.4-203533236	ATATGATATACTGAGTCTCCTTCCGGC	23	C	0.0089	0.4824
NC_005100.4-203533236	ATATGATATACTGAGTCTCCTTCCGGC	25	G	0.2478	0.3982
NC_005100.4-203576022	TGATGAAGTACATTTTCTCCCTTTGCC	12	A	0.0667	0.095
NC_005100.4-203606157	GATAAAACGACATGCTCTCCTCCTGTG	15	C	0.2951	0.2956
NC_005100.4-203621091	TGTCTCTCCACACAGGCTCCAGGGAGC	3	T	0.0254	0.0251
NC_005100.4-203621091	TGTCTCTCCACACAGGCTCCAGGGAGC	22	G	0.0261	0.0257
NC_005100.4-203666275	CCCAGTGACACACTTTCTCCAACACAG	9	C	0	0.4765
NC_005100.4-203686202	ACTACTTCTACAGGCCCTCCATCTCGG	4	A	0	0.4793
NC_005100.4-203686202	ACTACTTCTACAGGCCCTCCATCTCGG	5	C	0	0.4793
NC_005100.4-203712715	TCTATGGAGACCACGCCTCCTAATTCT	3	T	0	0.4773
NC_005100.4-203739687	ATGGGTCCCACGCTGGCTCCAGTGCTC	12	G	0.2069	0.199
NC_005100.4-203739687	ATGGGTCCCACGCTGGCTCCAGTGCTC	24	G	0.0171	0.0336
NC_005100.4-203741623	ATGCTTTTCACTAAGCCTCCCAAGGTA	8	T	0.2033	0.319
NC_005100.4-203756701	GCACTCTGCACAGAACCTCCTTTACAT	15	A	0	0.4773
NC_005100.4-203768798	CTCCTTGACACCGTGTCTCCTTTCCAC	13	G	0.2667	0.2665
NC_005100.4-203773562	CTGAGCTAGACATGGCCTCCAGGCTCC	16	C	0.042	0.0411
NC_005100.4-203847294	ACAATGGAAACTCTTCCTCCAGTATGG	16	C	0.2261	0.2985
NC_005100.4-203878520	AAAAAGAAAACATCGGCTCCCTCTCAG	15	G	0.188	0.4938
NC_005100.4-203878520	AAAAAGAAAACATCGGCTCCCTCTCAG	25	C	0.1121	0.1504
NC_005100.4-203944530	AGCTCTCTAACCTTGCCTCCCCTCATT	15	G	0.036	0.4883
NC_005100.4-203980133	TTCACTGTCACCCAGTCTCCTCTTCCC	2	T	0.0354	0.0348
NC_005100.4-204057191	ACCCATGGCACCTCCCCTCCTTGTGAC	1	A	0.0085	0.4776
NC_005100.4-204095837	TGCCTCCGGACCTTCTCTCCAACCCTG	6	C	0.0246	0.4924
NC_005100.4-204146725	GTGCACTCAACACACGCTCCCAAGGGT	7	T	0.0932	0.4982
NC_005100.4-204166900	AGCCCCAGCACACACGCTCCTTAGACT	9	C	0.0263	0.4913
NC_005100.4-204183504	ACAGCTCTAACCTTTTCTCCTTTCTAT	1	A	0.0492	0.048
NC_005100.4-204183504	ACAGCTCTAACCTTTTCTCCTTTCTAT	13	T	0.0738	0.4924
NC_005100.4-204287032	ATCACTGGGACACTGGCTCCTCCACTC	14	T	0.0244	0.484
NC_005100.4-204318760	CCTTGCTTCACTCTGACTCCCAGGCCT	2	C	0.0763	0.487
NC_005100.4-204318760	CCTTGCTTCACTCTGACTCCCAGGCCT	3	T	0.0763	0.487
NC_005100.4-204348329	TCTCTATCGACTGAAACTCCTGTTCAC	21	T	0.15	0.2061
NC_005100.4-204367347	TGTGTGTTTACCCATCCTCCCTGTCCA	24	T	0.0984	0.0935
NC_005100.4-204383011	GGGCTCCCCACCTACACTCCCTGATCC	15	C	0.2439	0.4
NC_005100.4-204383011	GGGCTCCCCACCTACACTCCCTGATCC	16	A	0	0.4793
NC_005100.4-204385636	ACAATAAATACTGCAGCTCCATGTCTG	22	T	0.1818	0.4997
NC_005100.4-204401579	CAGCCAGACACAGTCCCTCCCAATAGG	16	C	0.0246	0.0401
NC_005100.4-204419899	GTGTCTGTCACTGGTCCTCCACATCTG	1	G	0.1695	0.4994
NC_005100.4-204419899	GTGTCTGTCACTGGTCCTCCACATCTG	12	T	0.1597	0.1746
NC_005100.4-204452534	ACACAGGGAACTTGACCTCCCTAGCTC	8	G	0.0328	0.0322
NC_005100.4-204453055	TGCTCCACGACTGTTACTCCGGACATG	9	G	0.1138	0.1494
NC_005100.4-204453055	TGCTCCACGACTGTTACTCCGGACATG	21	G	0	0.4793
NC_005100.4-204463662	GGGTGAGCAACCCCTTCTCCAAGGTAG	5	G	0.252	0.3091
NC_005100.4-204586146	ATATTTGTAACTCCAGCTCCAGGAAAC	27	C	0	0.4698
NC_005100.4-204616389	CTCTTGCAAACTGATTCTCCAGTAAGA	2	T	0	0.4757
NC_005100.4-204616389	CTCTTGCAAACTGATTCTCCAGTAAGA	12	T	0.0339	0.0333
NC_005100.4-204629017	CTCCCTCTCACGGTTCCTCCAGGATAC	4	C	0	0.4765
NC_005100.4-204629017	CTCCCTCTCACGGTTCCTCCAGGATAC	12	G	0.2917	0.4666
NC_005100.4-204632670	CATGCATTCACATGATCTCCTTGCTTT	13	T	0.0894	0.1145
NC_005100.4-204683456	TTGAGGTAAACATGCCCTCCTCTCTGT	12	A	0	0.4793

NC_005100.4-204686182	ACAGATGACACCAGGACTCCAGAAATA	8	A	0.0177	0.4574
NC_005100.4-204690661	TTTGTGCCAACTGTCACTCCTCCAGCC	9	A	0.2689	0.4898
NC_005100.4-204706864	GAATTCAGGACTCGGCCTCCTCGAGGG	6	C	0.1463	0.2142
NC_005100.4-204721197	GGGTGAGGAACCACATCTCCCCCTTTG	5	G	0.0893	0.1172
NC_005100.4-204731688	TGGCAACAGACCCCAACTCCACTTGTC	23	T	0.0081	0.4777
NC_005100.4-204754924	AACCACTGGACTATCTCTCCAGGCCTC	7	T	0	0.4773
NC_005100.4-204754924	AACCACTGGACTATCTCTCCAGGCCTC	12	T	0	0.4773
NC_005100.4-204787088	CGTGCGCGCACAGGAACTCCAGGCTAC	3	T	0	0.4778
NC_005100.4-204787088	CGTGCGCGCACAGGAACTCCAGGCTAC	7	C	0	0.4778
NC_005100.4-204800576	TTGACTTCCACATGTGCTCCATAGCAT	8	C	0.0574	0.071
NC_005100.4-204826345	CTTTCCTCCACTCTTCCTCCCTGTGTC	24	T	0	0.48
NC_005100.4-204826345	CTTTCCTCCACTCTTCCTCCCTGTGTC	25	G	0.1083	0.1316
NC_005100.4-204906572	GCCCTTACCACCTCCTCTCCCCCACCC	2	C	0	0.4773
NC_005100.4-204906572	GCCCTTACCACCTCCTCTCCCCCACCC	25	C	0.0732	0.0854
NC_005100.4-204964600	TACAGTGAGACCTTGTCTCCAAAAATG	7	G	0	0.48
NC_005100.4-205055428	AATGTACTCACCTGCTCTCCAGAGGTT	24	G	0.2377	0.3492
NC_005100.4-205073455	TAAGGCTGCACTCGTCCTCCACAGCCC	6	C	0.1463	0.2142
NC_005100.4-205073455	TAAGGCTGCACTCGTCCTCCACAGCCC	14	G	0.0081	0.481
NC_005100.4-205075271	CTCTCCCCTACACAGTCTCCTTTAGTT	1	C	0	0.4793
NC_005100.4-205075271	CTCTCCCCTACACAGTCTCCTTTAGTT	26	T	0.2439	0.377
NC_005100.4-205111336	AACCAACCAACTCACCCTCCAGTCTTC	4	C	0.0246	0.0243
NC_005100.4-205111336	AACCAACCAACTCACCCTCCAGTCTTC	16	C	0.0256	0.0253
NC_005100.4-205112977	AGAAGCTCGACATAATCTCCAAGGAGA	14	A	0.187	0.1695
NC_005100.4-205115325	AGCTCTAGCACAATTACTCCCATGAAC	1	A	0	0.4793
NC_005100.4-205212314	AGGCTTGGGACTCTTTCTCCAGTTTCC	3	G	0.1429	0.2389
NC_005100.4-205221087	ACAACATGCACCCTTTCTCCAAAGGCA	5	C	0	0.4772
NC_005100.4-205221087	ACAACATGCACCCTTTCTCCAAAGGCA	26	C	0.1111	0.4947
NC_005100.4-205256838	GGACCGGAGACATTGGCTCCTTTTTGG	5	C	0.0593	0.0576
NC_005100.4-205256838	GGACCGGAGACATTGGCTCCTTTTTGG	22	T	0	0.4807
NC_005100.4-205270970	TGATTCATGACCTTTACTCCTCATTTT	25	T	0.0088	0.026
NC_005100.4-205287739	ATAGCACAAACTGAAGCTCCCAGAAAT	7	C	0	0.4839
NC_005100.4-205287739	ATAGCACAAACTGAAGCTCCCAGAAAT	26	A	0.0259	0.4893
NC_005100.4-205308427	ACATTCTTGACTCTGCCTCCTCCAAAT	8	T	0.1949	0.4207
NC_005100.4-205376860	CTAGTCCCCACCATCACTCCTCCCTCT	12	C	0.0254	0.0251
NC_005100.4-205406341	TCATCCAGGACAGGAGCTCCAACAGAG	15	A	0.2893	0.4339
NC_005100.4-205406341	TCATCCAGGACAGGAGCTCCAACAGAG	16	G	0.2397	0.323
NC_005100.4-205417961	CCTGCTCCCACCGCCTCTCCTGGCCTC	2	C	0.1197	0.1841
NC_005100.4-205417961	CCTGCTCCCACCGCCTCTCCTGGCCTC	13	G	0.3419	0.4325
NC_005100.4-205428420	CAACTTCCAACCATTCCTCCTTTTCCC	6	T	0.0246	0.0243
NC_005100.4-205428420	CAACTTCCAACCATTCCTCCTTTTCCC	26	C	0.0165	0.4849
NC_005100.4-205429653	GTAGTAAGCACGCCTGCTCCTTTCCCC	3	A	0.0672	0.065
NC_005100.4-205429653	GTAGTAAGCACGCCTGCTCCTTTCCCC	13	C	0	0.4793
NC_005100.4-205442142	TTGACCATGACGCTTGCTCCTGCTTTT	12	G	0.0609	0.4926
NC_005100.4-205463227	TAGAGTCCCACAGAGCCTCCTTCCAGG	8	C	0.2432	0.2656
NC_005100.4-205463227	TAGAGTCCCACAGAGCCTCCTTCCAGG	23	C	0.1712	0.2271
NC_005100.4-205492507	CACCAGAAGACAGTTGCTCCTCCAGCC	26	C	0	0.4757
NC_005100.4-205542987	CAGTAGAAAACCTTGTCTCCCTTCCTA	27	A	0.2119	0.3914
NC_005100.4-205558384	TCTTTCTCTACCGTCTCTCCCTGAACT	13	G	0.0492	0.048
NC_005100.4-205558384	TCTTTCTCTACCGTCTCTCCCTGAACT	26	C	0.0902	0.0861
NC_005100.4-205591229	TCCAACTCAACCAATCCTCCCCAGCAG	2	C	0.0345	0.0504
NC_005100.4-205594031	ACCATCTACACTGTCTCTCCCCACACC	27	C	0.0732	0.4952

NC_005100.4-205736124	CATGGTAGAACGTAGCCTCCAGTTTCT	24	T	0	0.4759
NC_005100.4-205760284	AGTGGAGACACCTCAGCTCCTGGAGTT	12	C	0.1463	0.2382
NC_005100.4-205760284	AGTGGAGACACCTCAGCTCCTGGAGTT	14	C	0.1463	0.2382
NC_005100.4-205768827	TAAAGTACAACATAAACTCCATTAGTG	15	A	0	0.4826
NC_005100.4-205779047	CCGAGCCACACTCCCACTCCCTCTAAT	7	C	0.041	0.0557
NC_005100.4-205790669	TTTACCCTCACCTTCCCTCCTCCTCTG	3	T	0	0.4807
NC_005100.4-205795108	ATAAAGGCTACACCAACTCCTGATGTG	23	A	0	0.4793
NC_005100.4-205799331	TGGTTTCGGACTAGGCCTCCAGGTTCT	8	G	0.0488	0.0476
NC_005100.4-205801500	AACAGTCAGACACGAGCTCCTGTCACG	14	G	0	0.48
NC_005100.4-205801500	AACAGTCAGACACGAGCTCCTGTCACG	26	C	0.1345	0.4983
NC_005100.4-205809740	CCGTTAATCACTAGAACTCCTGCACAC	5	T	0	0.4793
NC_005100.4-205809740	CCGTTAATCACTAGAACTCCTGCACAC	15	A	0.0894	0.2778
NC_005100.4-205814619	AGCTTAAGCACTCTCCCTCCTCCTCAT	16	C	0.123	0.1154
NC_005100.4-205830187	ATGTACCACACAAACACTCCACCACTG	26	T	0.0171	0.4839
NC_005100.4-205865853	ATGGGACTCACCAGGGCTCCATCTCAG	21	A	0	0.4703
NC_005100.4-205949677	GGTCTCGTCACTGGGTCTCCAAAGAAC	15	G	0.1951	0.3239
NC_005100.4-205960189	ACAGCGATAACAGGACCTCCTAAGCCC	5	C	0.0569	0.0553
NC_005100.4-205981566	AGACTCAGTACCTCTGCTCCCTTCCCT	22	T	0.0248	0.0245
NC_005100.4-205990094	CTGTCCTCAACTTGTGCTCCAGTGTGC	8	C	0.0089	0.4824
NC_005100.4-205997003	GCAGTGTTTACATTTGCTCCTACCATG	5	T	0.1951	0.2499
NC_005100.4-206006561	AAATACATCACTCTGTCTCCTAGACTG	3	A	0	0.4883
NC_005100.4-206031108	ATTGTGAGTACTGTGACTCCAGACTTC	1	A	0	0.4787
NC_005100.4-206033502	GCACTACTCACCCACGCTCCACCCTGC	24	C	0	0.4728
NC_005100.4-206034733	AGACCTCAGACTGAAACTCCCAAAGCC	7	C	0.114	0.1075
NC_005100.4-206034733	AGACCTCAGACTGAAACTCCCAAAGCC	26	C	0.2193	0.3836
NC_005100.4-206038466	TTCTCTCCAACCCTCTCTCCCCCAGTA	3	C	0.0089	0.0264
NC_005100.4-206038466	TTCTCTCCAACCCTCTCTCCCCCAGTA	25	G	0	0.4641
NC_005100.4-206043573	TCAATGCCAACCTTGTCTCCCTTAAAT	8	C	0.3125	0.3102
NC_005100.4-206053437	ATAACTGTTACCAGTGCTCCCTCGTGG	24	G	0.0885	0.1757
NC_005100.4-206055605	CAACTGGGGACCAAAGCTCCAGCATAT	15	A	0	0.4844
NC_005100.4-206149640	AATAACCTTACTCTCTCTCCCTTTAAA	13	C	0.2414	0.4405
NC_005100.4-206160977	ATTCCCTCAACTAAGGCTCCTTCTTCT	6	C	0.2689	0.3319
NC_005100.4-206180967	ATCTGCCAAACGCAATCTCCACACTAG	6	C	0.0256	0.0253
NC_005100.4-206180967	ATCTGCCAAACGCAATCTCCACACTAG	24	C	0.1949	0.4939
NC_005100.4-206197335	ATAAACTCCACCATACCTCCAAGGAGC	4	A	0.2066	0.3327
NC_005100.4-206197335	ATAAACTCCACCATACCTCCAAGGAGC	13	A	0.1138	0.1494
NC_005100.4-206206381	CACTCAGCCACACTTTCTCCAATGATG	1	C	0.0588	0.0571
NC_005100.4-206206381	CACTCAGCCACACTTTCTCCAATGATG	3	C	0.2269	0.317
NC_005100.4-206213235	CACAAACCCACTATCTCTCCAGGAGCT	3	C	0	0.488
NC_005100.4-206213235	CACAAACCCACTATCTCTCCAGGAGCT	4	A	0.0598	0.0896
NC_005100.4-206354354	ATAAAGAGAACTGCTTCTCCTGGGTAG	1	A	0	0.4787
NC_005100.4-206395539	TTAAAGGTCACATCTACTCCCTGGGAT	3	A	0	0.4826
NC_005100.4-206395539	TTAAAGGTCACATCTACTCCCTGGGAT	9	C	0.0252	0.0249
NC_005100.4-206432626	GACACATAAACTCTGCCTCCAAAACAA	15	G	0	0.4827
NC_005100.4-206432626	GACACATAAACTCTGCCTCCAAAACAA	24	A	0	0.4827
NC_005100.4-206448752	GTGAGGGCAACTCCGGCTCCATTGCTG	15	G	0.025	0.0247
NC_005100.4-206467588	CAGTTAAGAACGCTGGCTCCGCTCTAC	27	C	0.065	0.078
NC_005100.4-206468958	GGGAAACACACTGTGTCTCCTATTTCT	25	T	0.0085	0.479
NC_005100.4-206667141	TGTCAAACGACCCTGACTCCTTGTTAT	8	C	0.115	0.4021
NC_005100.4-206667141	TGTCAAACGACCCTGACTCCTTGTTAT	14	T	0.114	0.1814
NC_005100.4-206693931	AGAAGGCTTACTTTGGCTCCTGATTTA	7	C	0	0.4751

NC_005100.4-206707647	AGTGTTCTTACCTGCTCTCCAAGTCTG	6	T	0.0424	0.0415
NC_005100.4-206707683	TTCTTCACCACCTCATCTCCAACCTCC	4	T	0	0.4807
NC_005100.4-206709664	AAAGGGTCTACCATAACTCCATCTTCA	13	A	0.2542	0.4757
NC_005100.4-206728452	GTCAGCTGGACCAAGGCTCCTCTGTTC	1	G	0.2033	0.2323
NC_005100.4-206756012	GCCATTCCCACTCAAACTCCTGGGGTT	12	T	0.2478	0.3346
NC_005100.4-206771598	GACAAGGTTACCATGACTCCAAAGACT	21	A	0.0254	0.0251
NC_005100.4-206781459	TACTCACAAACCTTCTCTCCCTTCCAC	24	C	0.0526	0.0512
NC_005100.4-206797130	CTTATCAACACAGTTTCTCCAGTGAAG	22	G	0.2066	0.4966
NC_005100.4-206830166	TGCGCACCCACTTCAGCTCCCATTCCC	4	G	0.1121	0.1504
NC_005100.4-206873423	GTGCTGAAAACAGGCACTCCAACTCTG	9	A	0	0.4931
NC_005100.4-206910000	GAAGGTGTCACTGTGTCTCCGAAAGTG	5	G	0.0345	0.0666
NC_005100.4-206912610	GACTTGTTCACTGCTGCTCCTTCTATT	22	T	0	0.4807
NC_005100.4-206914791	CCACAATGCACTTCTCCTCCAATCCCT	6	A	0.0847	0.0812
NC_005100.4-206914791	CCACAATGCACTTCTCCTCCAATCCCT	16	C	0.2222	0.3729
NC_005100.4-206936370	CCTCCCTCTACCCTCTCTCCCCCTTAG	12	C	0.0265	0.0262
NC_005100.4-206936370	CCTCCCTCTACCCTCTCTCCCCCTTAG	15	C	0.036	0.0526
NC_005100.4-206971148	CTACATGTGACTATGTCTCCAAACAAC	5	A	0.5268	0.388
NC_005100.4-206971431	CTCTGCTGAACCACCTCTCCCTCTCTC	13	A	0.1391	0.1588
NC_005100.4-206971431	CTCTGCTGAACCACCTCTCCCTCTCTC	26	T	0	0.4755
NC_005100.4-206971717	ATGTCGTAGACTGGAGCTCCTTTTTAG	12	T	0.0796	0.4435
NC_005100.4-206981279	AATCACGGGACAGAAACTCCAGCCTCT	2	A	0.2389	0.4493
NC_005100.4-206981279	AATCACGGGACAGAAACTCCAGCCTCT	5	A	0.2212	0.4843
NC_005100.4-206996012	GCCTGCAAAACCTAAACTCCATCTTTT	25	T	0.0439	0.4796
NC_005100.4-207001028	GATGCAATTACTTCCTCTCCTCCATTG	25	T	0.1441	0.1621
NC_005100.4-207040172	ATACTCCCAACAGGTCCTCCATTAACT	23	T	0.0796	0.297
NC_005100.4-207083348	GCAGCAAGTACCATTACTCCACTAAGT	1	G	0.0083	0.4732
NC_005100.4-207125304	CACATGCCCACGTTGACTCCATCGTTC	2	A	0	0.4793
NC_005100.4-207125304	CACATGCCCACGTTGACTCCATCGTTC	12	G	0.0678	0.4908
NC_005100.4-207179426	AAACGTCAAACGGGTTCTCCAAGCCTC	12	G	0.0177	0.4827
NC_005100.4-207184278	TTGACATCAACAAGAACTCCATCAAAA	26	A	0.287	0.4849
NC_005100.4-207193329	CTCGCCCTCACTGCACCTCCTTTCTAT	6	C	0	0.4793
NC_005100.4-207217723	CATGTGCTCACCCTTCCTCCCAGATGA	7	C	0.2541	0.375
NC_005100.4-207217723	CATGTGCTCACCCTTCCTCCCAGATGA	8	T	0	0.4773
NC_005100.4-207260123	CCCTGAAGCACATGGGCTCCGTGTGGA	16	G	0.1721	0.2573
NC_005100.4-207265355	GACCTTGGCACCTAAACTCCCTAACAA	21	C	0.1626	0.2017
NC_005100.4-207265355	GACCTTGGCACCTAAACTCCCTAACAA	22	T	0.2602	0.3239
NC_005100.4-207285683	AAATCCCTTACTCAGGCTCCCTGAAAC	14	A	0	0.4731
NC_005100.4-207355137	TGTTAATCCACTGTATCTCCATCCTTA	3	T	0	0.4787
NC_005100.4-207355137	TGTTAATCCACTGTATCTCCATCCTTA	16	T	0	0.4765
NC_005100.4-207360534	TCTCATGCTACAAGATCTCCCAGGCTT	3	T	0	0.48
NC_005100.4-207360534	TCTCATGCTACAAGATCTCCCAGGCTT	13	A	0	0.48
NC_005100.4-207398186	AGTATAGATACCAAGTCTCCAGGTATC	2	G	0.0924	0.1327
NC_005100.4-207422950	GCTCACAGCACCCAGACTCCTCAGCTG	8	G	0.2231	0.1982
NC_005100.4-207437509	AGGTCCCCTACCTCTACTCCTGCCCTC	12	C	0.1453	0.1492
NC_005100.4-207437509	AGGTCCCCTACCTCTACTCCTGCCCTC	15	T	0.0427	0.0418
NC_005100.4-207440338	CAGCCACACACTGAGACTCCTGGCTCC	25	T	0.0776	0.1504
NC_005100.4-207448967	GTCCCCATTACTCTCTCTCCTTCTCTC	12	T	0.0175	0.4875
NC_005100.4-207448967	GTCCCCATTACTCTCTCTCCTTCTCTC	26	T	0	0.4846
NC_005100.4-207492170	GCTCATCCCACCCAACCTCCCTCATGG	21	C	0.1475	0.2279
NC_005100.4-207499706	TCTGGTCCGACACAGCCTCCTACCATG	5	G	0.1008	0.4406
NC_005100.4-207524340	TCAGGTTGCACCCTTTCTCCTTCTTGA	5	G	0.0086	0.4866

NC_005100.4-207524340	TCAGGTTGCACCCTTTCTCCTTCTTGA	23	C	0.4035	0.4384
NC_005100.4-207574863	AACATCTGTACGGTTTCTCCTCAGACT	12	G	0.3058	0.4557
NC_005100.4-207581766	CACCTTGTTACTGAGCCTCCTCTCTCA	13	G	0.0083	0.49
NC_005100.4-207581766	CACCTTGTTACTGAGCCTCCTCTCTCA	25	T	0.0083	0.49
NC_005100.4-207596089	CCAGAACCCACAATGACTCCAAAGACC	9	C	0.0351	0.0839
NC_005100.4-207601714	GCAGAACACACTGATCCTCCCTGCCCT	23	G	0.1197	0.1975
NC_005100.4-207651090	TTTTATTTAACTTGACCTCCCGTCCAT	22	G	0.0431	0.4836
NC_005100.4-207651090	TTTTATTTAACTTGACCTCCCGTCCAT	26	A	0.3051	0.324
NC_005100.4-207720013	CCATTTACAACGTTCACTCCTCAAACC	12	G	0.0579	0.0716
NC_005100.4-207720013	CCATTTACAACGTTCACTCCTCAAACC	26	C	0.1092	0.4949
NC_005100.4-207732005	TCTCACACAACCGATTCTCCCAAGAGA	27	A	0.0085	0.4842
NC_005100.4-207757495	TGGGTCCAAACTCTAACTCCCTGGGCT	3	G	0.2301	0.4981
NC_005100.4-207757495	TGGGTCCAAACTCTAACTCCCTGGGCT	26	C	0.0259	0.0585
NC_005100.4-207852721	CCCAGCAACACACCTCCTCCAAGGCCT	24	G	0.2185	0.4957
NC_005100.4-207853015	GCAAGGAGCACATACCCTCCCTCTGCG	23	C	0.0165	0.4819
NC_005100.4-207853015	GCAAGGAGCACATACCCTCCCTCTGCG	27	G	0.0165	0.4819
NC_005100.4-207898045	TCTTCCACAACCCAAACTCCTGGTCAG	2	C	0.2562	0.3027
NC_005100.4-207942871	CCCAGGATGACACTGCCTCCTGCAGGT	12	A	0.0583	0.0722
NC_005100.4-207961698	TGAAGCCAGACCCTAACTCCCAGCACC	14	T	0.0976	0.4973
NC_005100.4-207962189	CTGCTCCACACTGATACTCCTAGGCCG	27	G	0.0244	0.0241
NC_005100.4-207975300	AAACAGGCCACCTTCTCTCCAGAAGCA	2	A	0.1138	0.4992
NC_005100.4-208023571	CCTCTACCTACCTGACCTCCTTCCTTT	7	C	0.1074	0.1447
NC_005100.4-208023571	CCTCTACCTACCTGACCTCCTTCCTTT	15	A	0	0.4832
NC_005100.4-208026237	CATGTTCTAACAAGGACTCCACGATTA	24	A	0.1707	0.1826
NC_005100.4-208037764	ATCCAGGGGACCACAACTCCATTCATA	21	A	0	0.4773
NC_005100.4-208037764	ATCCAGGGGACCACAACTCCATTCATA	25	A	0	0.4773
NC_005100.4-208077877	CAGCAGCCCACCACATCTCCAGGGTAA	22	G	0	0.4856
NC_005100.4-208077877	CAGCAGCCCACCACATCTCCAGGGTAA	26	A	0.0508	0.0655
NC_005100.4-208082593	GACCTGGCCACTGGCACTCCCTTATAG	24	A	0.1157	0.4983
NC_005100.4-208117588	GACACAGCAACACTGGCTCCAGGGTCT	7	G	0.0171	0.4693
NC_005100.4-208136222	TCGTATCATACAGGTTCTCCACACCAG	14	G	0	0.4846
NC_005100.4-208180312	TTGGCTTTCACCTATCCTCCACCCTGC	24	C	0.0333	0.0328
NC_005100.4-208188770	CTCTCTATCACACTGGCTCCTGGAGTT	6	T	0.0087	0.4782
NC_005100.4-208202001	TAAATCAGAACTACAGCTCCTCAACAA	24	A	0.252	0.4239
NC_005100.4-208208247	ATGGATTGAACTTGGCCTCCTTTCAGG	3	G	0.0541	0.0526
NC_005100.4-208208247	ATGGATTGAACTTGGCCTCCTTTCAGG	8	G	0.0625	0.0605
NC_005100.4-208219776	CACACAGACACATACACTCCCATACAT	5	C	0.25	0.3578
NC_005100.4-208219776	CACACAGACACATACACTCCCATACAT	14	A	0.1074	0.1853
NC_005100.4-208226418	GTTACCTACACATACTCTCCACCTTTC	21	A	0	0.0325
NC_005100.4-208226418	GTTACCTACACATACTCTCCACCTTTC	26	T	0.1803	0.5
NC_005100.4-208263424	TGTGACACTACCCTTCCTCCATGCATG	1	T	0.084	0.4921
NC_005100.4-208263424	TGTGACACTACCCTTCCTCCATGCATG	2	G	0.0084	0.4761
NC_005100.4-208285470	TTAACTACCACCCTCCCTCCAAGCCAC	15	C	0	0.4793
NC_005100.4-208285470	TTAACTACCACCCTCCCTCCAAGCCAC	16	C	0	0.4793
NC_005100.4-208306741	CATACTAAAACCCTTCCTCCGCCCTTC	16	C	0.1261	0.1786
NC_005100.4-208306741	CATACTAAAACCCTTCCTCCGCCCTTC	21	G	0.0631	0.126
NC_005100.4-208316171	CTCCCCAACACAGACCCTCCACAGGCT	8	A	0.187	0.2556
NC_005100.4-208320849	TATTCAGTCACATGACCTCCTGTGGAC	27	C	0.2479	0.4112
NC_005100.4-208344046	CAAACAACAACAAAAGCTCCCAGAGAT	1	C	0.0336	0.033
NC_005100.4-208344046	CAAACAACAACAAAAGCTCCCAGAGAT	2	A	0.0336	0.033
NC_005100.4-208372618	GTTTGGCTGACTCTGTCTCCTCCTGTA	15	G	0.2105	0.3324

NC_005100.4-208372618	GTTTGGCTGACTCTGTCTCCTCCTGTA	27	A	0.0089	0.4789
NC_005100.4-208378703	ATTGATTCCACCATGACTCCATCCATC	12	C	0.1849	0.4661
NC_005100.4-208378703	ATTGATTCCACCATGACTCCATCCATC	21	A	0.1849	0.4661
NC_005100.4-208443025	CACAGGCTGACTGGAACTCCATGCACT	16	A	0.113	0.1369
NC_005100.4-208443025	CACAGGCTGACTGGAACTCCATGCACT	24	C	0.2261	0.4891
NC_005100.4-208556022	GCATGTCCCACATGCTCTCCCGCTCTC	21	C	0.1228	0.1305
NC_005100.4-208561452	AGTGAGGTAACCCAGACTCCAGACACA	21	A	0.0847	0.0812
NC_005100.4-208576308	TTGAAACTCACTGACCCTCCCCCTTCT	26	C	0	0.4698
NC_005100.4-208576308	TTGAAACTCACTGACCCTCCCCCTTCT	27	T	0	0.4698
NC_005100.4-208600838	CAAGCTGTCACAGCACCTCCCAGCGTC	13	G	0	0.4793
NC_005100.4-208600838	CAAGCTGTCACAGCACCTCCCAGCGTC	25	G	0.1789	0.5
NC_005100.4-208601994	GTCATATGAACTGGACCTCCTTACTTC	5	T	0.1736	0.1982
NC_005100.4-208628027	GATTCAAGGACTTGAACTCCAATCTTT	22	A	0.1186	0.1264
NC_005100.4-208685153	TTGAAAGACACAGAGCCTCCACTACCT	16	C	0	0.4731
NC_005100.4-208690906	TCACATGTTACTTCAGCTCCAGGGCAT	4	C	0.1382	0.1562
NC_005100.4-208722635	ACACACTCCACACACACTCCATACACA	7	T	0.0769	0.074
NC_005100.4-208722635	ACACACTCCACACACACTCCATACACA	8	C	0.3445	0.3367
NC_005100.4-208726065	GCATGGGACACAGCTTCTCCGTCCTTT	3	A	0	0.4743
NC_005100.4-208728534	ATTCAGGAAACATATGCTCCACACTCT	21	A	0.2069	0.3282
NC_005100.4-208732143	ACGATCTCCACTTACCCTCCTCCATCT	9	C	0.0488	0.4904
NC_005100.4-208732143	ACGATCTCCACTTACCCTCCTCCATCT	13	T	0.0081	0.481
NC_005100.4-208756579	TCATGAATTACCATGACTCCATTTTGA	13	A	0.0088	0.0429
NC_005100.4-208756579	TCATGAATTACCATGACTCCATTTTGA	21	A	0.0526	0.0512
NC_005100.4-208772817	CACAGCAATACATCTGCTCCTGGTGCC	13	T	0.0084	0.4829
NC_005100.4-208783831	TGAACCTCCACTGTGTCTCCCTGCCCA	8	C	0.2222	0.3457
NC_005100.4-208797234	GCTTAAGTAACTGGCCCTCCAGTAATT	3	T	0.0667	0.095
NC_005100.4-208800045	CTGAACCTCACCTTTCCTCCACTGTTG	14	T	0	0.4819
NC_005100.4-208800045	CTGAACCTCACCTTTCCTCCACTGTTG	23	T	0	0.4819
NC_005100.4-208813504	ACGTAAGGCACACACACTCCATCGTGG	23	C	0.0656	0.0786
NC_005100.4-208836721	ATGCTGTTTACATGTCCTCCCACAGTT	26	T	0.2035	0.3495
NC_005100.4-208840502	CCATGATCCACACTTCCTCCCAGCGTG	3	A	0	0.4825
NC_005100.4-208850615	ACCTCCTGCACCCCTGCTCCTGGGCCC	6	C	0.2705	0.4379
NC_005100.4-208878644	ATCCAAGACACCCTCCCTCCAAACAAA	7	G	0.2	0.42
NC_005100.4-208884236	GACTCTGAAACTTTACCTCCAACTAAC	25	A	0.0598	0.4854
NC_005100.4-208905640	CAACTGTGGACATACCCTCCTATTGTT	25	G	0.25	0.3468
NC_005100.4-208910848	CCTCTAAGCACTCTGGCTCCAGGCCCT	16	G	0.0085	0.481
NC_005100.4-208910894	TGCCTCTACACTGCAGCTCCCAAAGTC	3	C	0.0336	0.033
NC_005100.4-208944024	TGAGCCAATACATTCCCTCCTTTCCTC	8	A	0	0.4807
NC_005100.4-209013920	ACAGAAGTCACACATGCTCCTGGAGAT	21	T	0.0661	0.0639
NC_005100.4-209046958	TTGCATCACACCTATACTCCCATGTAC	14	A	0.2114	0.2017
NC_005100.4-209058126	TGCACACACACGGTACCTCCTTACACT	8	A	0	0.4844
NC_005100.4-209058126	TGCACACACACGGTACCTCCTTACACT	23	A	0	0.4844
NC_005100.4-209061217	TGGCCAGAGACCCTGCCTCCTCAGGAA	5	C	0.0083	0.4732
NC_005100.4-209084462	GCCTAAGTAACAAGAGCTCCTCAGCCT	8	T	0	0.4793
NC_005100.4-209135472	ATTCTCTTCACACCAGCTCCCTGAATA	16	G	0.0333	0.0328
NC_005100.4-209136905	AGGATTTCTACCTGGTCTCCCTGACAG	25	C	0.0325	0.032
NC_005100.4-209144741	TAGACGAGTACCATGTCTCCCCACTTT	21	C	0.0167	0.4765
NC_005100.4-209155642	CTGAAATAAACCCTTCCTCCTCGAGCT	22	C	0.1892	0.2137
NC_005100.4-209178158	GCTCCAGACACCTGAACTCCAGTATTG	16	A	0	0.4743
NC_005100.4-209206282	ACACTTGTCACTAATCCTCCGGTTCAG	21	G	0	0.4757
NC_005100.4-209209277	ATCCGTTGAACCCTAGCTCCTCCCATC	4	C	0.1393	0.1573

NC_005100.4-209209277	ATCCGTTGAACCCTAGCTCCTCCCATC	5	G	0.0328	0.048
NC_005100.4-209267731	AGGGAAGCCACACTCTCTCCTTTTCTA	6	A	0.0089	0.4885
NC_005100.4-209267731	AGGGAAGCCACACTCTCTCCTTTTCTA	9	C	0.0089	0.4885
NC_005100.4-209272096	AGGTGAGGAACAGCCACTCCACAGAGG	7	G	0.0083	0.4803
NC_005100.4-209272096	AGGTGAGGAACAGCCACTCCACAGAGG	8	G	0.0083	0.4803
NC_005100.4-209284064	GAGGCAGAGACTCCTCCTCCCTCTGTG	21	C	0.1724	0.4994
NC_005100.4-209320391	TTTATGGCCACCCTTCCTCCACGGGAG	3	T	0	0.4819
NC_005100.4-209320391	TTTATGGCCACCCTTCCTCCACGGGAG	24	G	0	0.4819
NC_005100.4-209321843	AATCAAACCACCCAGCCTCCTCTCTCC	7	A	0.1667	0.18
NC_005100.4-209321843	AATCAAACCACCCAGCCTCCTCTCTCC	25	T	0.1157	0.109
NC_005100.4-209326393	GAGGTTTTCACAGGCTCTCCTCATTCA	8	T	0.0569	0.4662
NC_005100.4-209391090	TGGAATGAGACATGACCTCCACGGGCT	14	G	0.1057	0.1145
NC_005100.4-209423467	TGCAGCCCCACAGAGGCTCCAGCCTGT	24	C	0.0175	0.4778
NC_005100.4-209423467	TGCAGCCCCACAGAGGCTCCAGCCTGT	25	T	0.0175	0.4778
NC_005100.4-209457434	AATAATACCACCCTTTCTCCCTGGGTC	5	A	0	0.4864
NC_005100.4-209457434	AATAATACCACCCTTTCTCCCTGGGTC	7	A	0	0.4864
NC_005100.4-209457769	AGAGATTCCACACTGCCTCCAGAGTAC	1	A	0	0.4832
NC_005100.4-209457769	AGAGATTCCACACTGCCTCCAGAGTAC	23	A	0	0.4832
NC_005100.4-209461972	CAGCACATAACACACGCTCCTTGCCTG	16	G	0.041	0.0861
NC_005100.4-209544248	GCAGGTAGCACAGGTGCTCCAGCAAGA	22	G	0	0.4807
NC_005100.4-209567230	TGATAGGGTACTTGTCCTCCTGCCAAG	14	G	0	0.4793
NC_005100.4-209567230	TGATAGGGTACTTGTCCTCCTGCCAAG	25	A	0	0.4793
NC_005100.4-209590924	GGTCGACCCACCCCTCCTCCTGGCCTA	14	C	0.2393	0.2945
NC_005100.4-209593873	AACCCCTTCACTGCAGCTCCTCAGGTG	25	G	0.2869	0.4755
NC_005100.4-209596831	CTGGCCTGCACAGCTACTCCCACCTCC	8	G	0	0.4821
NC_005100.4-209596831	CTGGCCTGCACAGCTACTCCCACCTCC	21	C	0	0.4821
NC_005100.4-209598361	GTCTCAGACACTGTGACTCCAGGTTTT	22	G	0	0.4671
NC_005100.4-209647291	GATGCTGTAACTGCGACTCCTAAGCTG	1	G	0.0569	0.0705
NC_005100.4-209647291	GATGCTGTAACTGCGACTCCTAAGCTG	8	T	0	0.4793
NC_005100.4-209687112	TCATTTGGCACCAGTCCTCCCGTGGAT	21	C	0.1513	0.4997
NC_005100.4-209687112	TCATTTGGCACCAGTCCTCCCGTGGAT	23	T	0.1429	0.214
NC_005100.4-209691185	GGAGATTGGACTGCAGCTCCCCAGCCC	27	C	0.2222	0.405
NC_005100.4-209708227	TGTGGACCAACTAACCCTCCATGTGAA	12	T	0.0631	0.1713
NC_005100.4-209719057	GGATCCTTGACACTCACTCCTGTGCCT	16	A	0.1429	0.3264
NC_005100.4-209735846	GGTGGCCAAACAGCAGCTCCACCACGG	25	C	0.1071	0.1478
NC_005100.4-209831720	CTTGTCATTACTGGCCCTCCATGGTTT	15	C	0	0.4846
NC_005100.4-209831720	CTTGTCATTACTGGCCCTCCATGGTTT	22	T	0.1913	0.2519
NC_005100.4-209834933	TCCTCTCCCACATCCTCTCCCAGTGAT	25	G	0.0531	0.0683
NC_005100.4-209866971	TCTGTTTTGACCAGACCTCCATTTCTG	26	T	0.1826	0.2204
NC_005100.4-209929627	CTGTTTGAGACTTAGTCTCCAAGCCAG	14	A	0.157	0.172
NC_005100.4-209994918	GTTTATTGTACAAAGTCTCCCCCAGGA	7	T	0.193	0.4994
NC_005100.4-209995479	TGATCCCTGACAGCTTCTCCAGACCCC	27	C	0	0.4844
NC_005100.4-209998382	TTCAAGCCCACATGCCCTCCAATGCCC	5	A	0.1983	0.5
NC_005100.4-210037953	TCTTCCTAGACCCCAGCTCCACCTTTG	14	C	0.1983	0.4991
NC_005100.4-210039298	ATGCCTTTGACAGTAGCTCCGTCCATA	2	T	0	0.4773
NC_005100.4-210039298	ATGCCTTTGACAGTAGCTCCGTCCATA	3	G	0	0.4773
NC_005100.4-210050310	TGATAATGGACTGAATCTCCAAACCAA	21	A	0.0756	0.4592
NC_005100.4-210081386	GACGCCATCACCCCACCTCCCTGCCAT	3	C	0.124	0.4213
NC_005100.4-210081386	GACGCCATCACCCCACCTCCCTGCCAT	21	C	0.0744	0.4966
NC_005100.4-210102288	TTCCTATGCACAAAGGCTCCACTTAGC	13	A	0.2	0.2072
NC_005100.4-210102288	TTCCTATGCACAAAGGCTCCACTTAGC	16	G	0.0348	0.0342

NC_005100.4-210115914	GGGAGGGACACTATGTCTCCTCAGCAG	1	G	0	0.4779
NC_005100.4-210115914	GGGAGGGACACTATGTCTCCTCAGCAG	25	C	0	0.4779
NC_005100.4-210122797	GATTGATTGACTCATCCTCCAAATGTT	2	A	0.0263	0.026
NC_005100.4-210122797	GATTGATTGACTCATCCTCCAAATGTT	5	G	0.0263	0.026
NC_005100.4-210161029	GGCTGCCATACGCCAGCTCCCATCCCA	22	A	0.225	0.4783
NC_005100.4-210221524	CATCTTGGAACCCAGTCTCCAATTTCA	26	C	0.0982	0.0934
NC_005100.4-210236961	GAGGATCCCACCTGCTCTCCATGCGAT	21	A	0	0.4751
NC_005100.4-210236961	GAGGATCCCACCTGCTCTCCATGCGAT	25	G	0.1074	0.2109
NC_005100.4-210299663	CTCCCCTCCACCCCGACTCCAGCAATC	14	C	0.069	0.488
NC_005100.4-210300571	AACTCCTGAACAGTCTCTCCAGGCCCC	23	G	0.018	0.0354
NC_005100.4-210354366	CTGCTTGATACAGAGTCTCCCTTGTCC	5	T	0.0826	0.0792
NC_005100.4-210361682	GTCTGCTACACATGGCCTCCATTCCCC	15	G	0.0488	0.0629
NC_005100.4-210368607	TTGACTTCCACTTCCACTCCAAAAGCA	22	A	0.1304	0.18
NC_005100.4-210372190	GGATGCTGGACAGGGTCTCCTGGTGTC	27	C	0.1652	0.1659
NC_005100.4-210412982	CTGGGTAAAACCTTGACTCCTCTAGGC	15	G	0.0536	0.1172
NC_005100.4-210421278	AGCTGTCTCACTATGCCTCCTTCTTGG	12	T	0.2114	0.4944
NC_005100.4-210430400	AATAGGAAGACAGAAGCTCCTGACATG	9	G	0.2456	0.3115
NC_005100.4-210438038	TTGTCATCGACCAGAGCTCCAATGTCA	9	G	0.2609	0.264
NC_005100.4-210474909	TTACTTACTACCAACCCTCCGCCCACC	21	G	0	0.4891
NC_005100.4-210496884	AGCCAACTCACACCCTCTCCAGCTTCC	3	C	0.1339	0.125
NC_005100.4-210505132	CTGTGCCAAACTGAAACTCCAATTGGT	9	A	0.0357	0.0351
NC_005100.4-210542279	AACCTCCTAACTCCATCTCCACCTTAC	25	T	0.1639	0.4999
NC_005100.4-210552867	CTGGAAGTCACACTGTCTCCCTTAAAC	25	A	0	0.4793
NC_005100.4-210597097	ATCCTGAGAACTCCCACTCCCTTGTTC	27	C	0	0.4807
NC_005100.4-210605349	CTGCTGGGCACATGGTCTCCAGTGGAG	9	C	0.2696	0.4817
NC_005100.4-210607152	GTGTGCCTAACCTGGGCTCCAACCCTG	2	T	0.314	0.4787
NC_005100.4-210607152	GTGTGCCTAACCTGGGCTCCAACCCTG	24	C	0	0.4751
NC_005100.4-210610211	AAGAGATGCACTATTGCTCCTGCACCT	1	A	0.0488	0.4926
NC_005100.4-210610211	AAGAGATGCACTATTGCTCCTGCACCT	24	A	0.2927	0.2937
NC_005100.4-210625669	AGGCCTGGAACTAGATCTCCAGTACTG	13	A	0	0.4786
NC_005100.4-210625669	AGGCCTGGAACTAGATCTCCAGTACTG	24	A	0.0517	0.0666
NC_005100.4-210630926	GCCCAATCTACTGATCCTCCAGCTGTC	9	T	0.0813	0.078
NC_005100.4-210652207	ATGTTTACCACAGTTGCTCCTGGGTGT	5	T	0.0081	0.481
NC_005100.4-210653943	TGCTTCCTGACTTCCCCTCCCATGCCA	13	T	0.0088	0.472
NC_005100.4-210654046	CCTTTCCCAACCTCATCTCCCATTTAC	4	T	0.0965	0.4953
NC_005100.4-210654046	CCTTTCCCAACCTCATCTCCCATTTAC	24	T	0.0696	0.4954
NC_005100.4-210671976	GGTTGTGTCACAGCTTCTCCTGTATGG	23	T	0.122	0.2203
NC_005100.4-210671976	GGTTGTGTCACAGCTTCTCCTGTATGG	27	G	0.122	0.2203
NC_005100.4-210675485	CTCTAGCCCACCTCCTCTCCTTCCTTC	9	C	0.0348	0.0508
NC_005100.4-210675485	CTCTAGCCCACCTCCTCTCCTTCCTTC	13	T	0.113	0.4994
NC_005100.4-210679280	GTCCCTATGACACTAACTCCACGGAGA	14	T	0.1102	0.1759
NC_005100.4-210741509	CCTGCAGGAACTCTAGCTCCTGGAATG	8	G	0	0.4773
NC_005100.4-210750027	ACACCATTTACTGCTCCTCCAGAGAAA	4	C	0	0.4793
NC_005100.4-210760957	TCCACTACAACACCGACTCCAGGAAGA	15	G	0	0.4859
NC_005100.4-210775229	CCCATATGTACCATGTCTCCTGTCTAA	14	T	0	0.4743
NC_005100.4-210802812	TTCCTCCAGACACTAACTCCTGAGATT	7	C	0.3248	0.4325
NC_005100.4-210802812	TTCCTCCAGACACTAACTCCTGAGATT	25	A	0	0.4786
NC_005100.4-210879271	ACTACCAACACTTCCTCTCCTCCCTCC	14	C	0.3554	0.4277
NC_005100.4-210920556	CAGTAAAAGACAATGGCTCCTCTCCTC	1	C	0.2034	0.362
NC_005100.4-210920556	CAGTAAAAGACAATGGCTCCTCTCCTC	24	C	0.2051	0.45
NC_005100.4-210942277	GGTATCGCAACACTATCTCCTCAGTGA	7	G	0.0244	0.0241

NC_005100.4-210944069	ATGGTCATTACTCTACCTCCACACTTG	13	C	0	0.4856
NC_005100.4-211019069	CTATGTCTAACCAGGACTCCCAAGGAA	1	C	0.2479	0.4158
NC_005100.4-211019069	CTATGTCTAACCAGGACTCCCAAGGAA	21	C	0.0256	0.4823
NC_005100.4-211024110	CTGCTATCCACTGTAACTCCACTTCTC	9	C	0.2764	0.4206
NC_005100.4-211024110	CTGCTATCCACTGTAACTCCACTTCTC	15	A	0	0.4793
NC_005100.4-211061590	CCTCTCAATACCCGGACTCCACTACTA	13	C	0.2358	0.381
NC_005100.4-211075659	CAATACCTCACCACATCTCCATAGAAC	7	C	0	0.4793
NC_005100.4-211076157	TACCAATCAACTGAGGCTCCCTCCGCA	24	C	0.2241	0.4463
NC_005100.4-211078795	ATAGGCAACACTGTCACTCCCTCCATA	9	C	0.0259	0.0255
NC_005100.4-211078795	ATAGGCAACACTGTCACTCCCTCCATA	23	C	0	0.4851
NC_005100.4-211085267	TGGCAAGTTACTTACGCTCCAGCAGGT	13	T	0	0.4764
NC_005100.4-211085267	TGGCAAGTTACTTACGCTCCAGCAGGT	21	A	0.2609	0.3938
NC_005100.4-211119509	GGTTATTCCACACTCCCTCCAGCAGTG	13	C	0.2353	0.3686
NC_005100.4-211127799	CTCCCCAAGACCCTTGCTCCCCATGTA	24	T	0.0087	0.4744
NC_005100.4-211127799	CTCCCCAAGACCCTTGCTCCCCATGTA	25	G	0.0431	0.0585
NC_005100.4-211133751	CCGTATCCCACTGGCCCTCCTCTTGTC	2	C	0.0738	0.4943
NC_005100.4-211133751	CCGTATCCCACTGGCCCTCCTCTTGTC	23	T	0.3197	0.4193
NC_005100.4-211140257	ACCAAAGAAACTCTTTCTCCCTCAAGT	12	T	0.2252	0.4414
NC_005100.4-211149136	AAGCCTACAACAAGCCCTCCCTTAGCA	6	T	0	0.48
NC_005100.4-211149136	AAGCCTACAACAAGCCCTCCCTTAGCA	12	A	0	0.4813
NC_005100.4-211172061	GGTCCCCTAACACTGCCTCCCACAACA	24	A	0.0732	0.0705
NC_005100.4-211173488	TCTCAGAGAACACGCTCTCCCTCCCTC	5	A	0.2353	0.3599
NC_005100.4-211201173	AGGATAGGAACTGTTGCTCCTAACTCT	24	C	0	0.4871
NC_005100.4-211302079	CCTCTCCCTACCTTCCCTCCCTTTAGA	21	C	0.1043	0.4969
NC_005100.4-211306125	TTCTGTTGTACTAGCCCTCCACCCTTG	1	T	0.0541	0.0526
NC_005100.4-211340955	GGTGTCTATACCCATACTCCCTCAACC	15	T	0.1736	0.4978
NC_005100.4-211370674	ATAACACAGACACTTCCTCCTCTCCCA	22	C	0.1951	0.2832
NC_005100.4-211375185	CTGGGAACCACACCAGCTCCCTGGAGA	25	A	0	0.4793
NC_005100.4-211383340	GGCAACACAACACCAGCTCCAGTGGTT	16	G	0.1228	0.2659
NC_005100.4-211383534	GTCTTGGGAACTACCTCTCCATCAGCA	5	T	0.0574	0.1154
NC_005100.4-211384427	GTAGGTTATACAGAGGCTCCGGCTGTC	21	G	0.0574	0.0557
NC_005100.4-211391764	ATGGGGTCAACTGTAGCTCCCAGGAAG	1	A	0.2564	0.2834
NC_005100.4-211391764	ATGGGGTCAACTGTAGCTCCCAGGAAG	23	G	0.0583	0.0722
NC_005100.4-211392935	CTGCCACTCACATATCCTCCTGTCCCT	25	C	0.0813	0.078
NC_005100.4-211394987	GGCATCGGGACAAGACCTCCAGGGGTC	7	G	0.2034	0.2466
NC_005100.4-211398000	GGTCCCAGCACAAGCCCTCCAGAGGCA	26	C	0.041	0.0401
NC_005100.4-211409822	CAGACAGACACTCCCACTCCTTACCTA	12	T	0.0171	0.4807
NC_005100.4-211409822	CAGACAGACACTCCCACTCCTTACCTA	27	A	0.0248	0.0245
NC_005100.4-211422463	GCCTCTTCAACTTGTCCTCCCCCTCCT	8	C	0.0992	0.4983
NC_005100.4-211425980	GTACAAGACACCTTGCCTCCATCTGTC	7	G	0.0354	0.0348
NC_005100.4-211425980	GTACAAGACACCTTGCCTCCATCTGTC	8	A	0.0442	0.0433
NC_005100.4-211432927	GTCTCAGTCACTACACCTCCCAAAGAC	1	G	0.0991	0.4942
NC_005100.4-211445887	ACACAAGCAACCAATGCTCCCAGCAGT	12	C	0.0087	0.4849
NC_005100.4-211445887	ACACAAGCAACCAATGCTCCCAGCAGT	13	A	0.1228	0.1305
NC_005100.4-211453086	CCACCATGAACATGTCCTCCTCATCTT	26	T	0.0965	0.0918
NC_005100.4-211465979	CTGATGCTGACCTCACCTCCTGACCTT	27	T	0.1157	0.4923
NC_005100.4-211477991	ACAGTACGGACTCTCTCTCCTGGTAAC	6	A	0.1197	0.4997
NC_005100.4-211533305	GGAACTGCCACACCGACTCCCATACTG	25	C	0	0.4819
NC_005100.4-211538031	TTCCTAGGAACCATGGCTCCAGGTCCA	21	A	0.0826	0.1235
NC_005100.4-211546369	ACTCTGCCCACGTCTTCTCCAAGGCCT	9	C	0	0.4793
NC_005100.4-211583032	CAGAGCAGAACCCTTCCTCCTTTGACA	12	C	0.0256	0.0253

NC_005100.4-211619902	CCTGGCCCTACCTCTGCTCCATCAACA	3	T	0.1074	0.1163
NC_005100.4-211619902	CCTGGCCCTACCTCTGCTCCATCAACA	12	C	0.1074	0.1163
NC_005100.4-211626602	TTCCTATGCACACATTCTCCACCAGCC	3	C	0.084	0.0805
NC_005100.4-211629913	TTCTCCTGTACACACACTCCCACCACC	2	T	0.2917	0.49
NC_005100.4-211629913	TTCTCCTGTACACACACTCCCACCACC	3	C	0.2917	0.49
NC_005100.4-211653593	GTGCAGTTCACATCAGCTCCTCCAAGT	6	G	0.1062	0.1006
NC_005100.4-211670135	TCCCCTGTCACAAAGTCTCCAGAAAGA	4	C	0	0.4844
NC_005100.4-211677387	CTAAATCAAACGCCATCTCCATCTGTC	12	G	0.1057	0.1145
NC_005100.4-211696126	ACATGCCAAACACATTCTCCAAAGTAG	25	T	0.082	0.1225
NC_005100.4-211696355	TAGGCGCACACAGCTGCTCCTGGGTCT	5	C	0.2397	0.3512
NC_005100.4-211733543	TAATTCTGCACCCCACCTCCCATCCCT	14	C	0.2521	0.3599
NC_005100.4-211750035	GAGGTTAAGACCTAAGCTCCCAGAGGA	26	G	0.1597	0.188
NC_005100.4-211784088	CTAAATGTCACGTGTCCTCCACCTGTG	12	G	0.2437	0.3462
NC_005100.4-211784088	CTAAATGTCACGTGTCCTCCACCTGTG	23	C	0	0.4813
NC_005100.4-211804742	CTTTAGGTCACTTCTGCTCCTATGAGT	6	G	0.0085	0.479
NC_005100.4-211839529	CCAGCTTGGACTAGGTCTCCTCGATTG	22	C	0.0244	0.0398
NC_005100.4-211922864	CAAGCGGGCACCGAGGCTCCCCTCACC	2	A	0.2195	0.2668
NC_005100.4-211922864	CAAGCGGGCACCGAGGCTCCCCTCACC	13	G	0.065	0.078
NC_005100.4-211933475	CACCCTAACACCCTGACTCCTTAGGTT	26	T	0	0.4902
NC_005100.4-211933475	CACCCTAACACCCTGACTCCTTAGGTT	27	T	0	0.4902
NC_005100.4-211976019	CTGGCCAGCACCCTATCTCCAGCCTTC	9	C	0.2667	0.4644
NC_005100.4-211976019	CTGGCCAGCACCCTATCTCCAGCCTTC	23	C	0.0917	0.1316
NC_005100.4-212026586	AAACCCTCTACTGCTCCTCCATCTCCA	8	C	0.0531	0.0517
NC_005100.4-212042520	CTTCCCTACACCCATCCTCCCTTCATC	5	C	0.0089	0.4856
NC_005100.4-212046670	TACCTACCTACCCTCTCTCCCATCCAT	3	C	0.0696	0.4954
NC_005100.4-212108823	CTGCCGGTCACCCAGTCTCCACCCTCA	8	T	0.2276	0.4072
NC_005100.4-212191279	AGTCTACAGACACAGCCTCCTTTCTAT	6	A	0.0579	0.0562
NC_005100.4-212207044	CGGGGAGCTACTATTTCTCCTCTATGT	2	G	0.0168	0.033
NC_005100.4-212207044	CGGGGAGCTACTATTTCTCCTCTATGT	3	G	0.1695	0.2091
NC_005100.4-212220965	GCGCCTACAACTGTAACTCCATTTGAT	1	G	0.0976	0.0928
NC_005100.4-212229305	CAATTACAAACCCACCCTCCTTCACTC	6	A	0.1053	0.4925
NC_005100.4-212231896	ACAACAAAAACTTAGTCTCCAAACTAG	27	G	0.009	0.0267
NC_005100.4-212236449	GACTGCCATACACTCCCTCCTGAGCCA	25	C	0.0732	0.4967
NC_005100.4-212241632	CACGCACACACACCCTCTCCTCTTCTG	4	G	0.0259	0.4803
NC_005100.4-212250576	TCCCCTGTAACCCCAGCTCCCGGTTAT	3	C	0	0.4793
NC_005100.4-212263619	CGGCACTTCACTGTGCCTCCCCTTTCA	3	G	0	0.4677
NC_005100.4-212263619	CGGCACTTCACTGTGCCTCCCCTTTCA	5	A	0	0.4677
NC_005100.4-212305604	CATAAGTGCACAAGCACTCCGAGGTCC	3	T	0	0.477
NC_005100.4-212305604	CATAAGTGCACAAGCACTCCGAGGTCC	21	G	0.1071	0.1478
NC_005100.4-212316843	CGGCACCGTACCTAGACTCCAGGACAG	7	C	0.0488	0.0928
NC_005100.4-212319284	CACACATATACAAGTTCTCCAGCATCA	9	T	0	0.4793
NC_005100.4-212319284	CACACATATACAAGTTCTCCAGCATCA	22	G	0	0.4807
NC_005100.4-212331780	TGTCCAGGGACATGGGCTCCTGGAGAC	15	G	0.0804	0.1403
NC_005100.4-212343754	GAGTGAGTGACACACGCTCCTCAGCTC	1	G	0.0769	0.0896
NC_005100.4-212512375	AGTATGCTCACCTGCACTCCAATAAGG	13	T	0.1545	0.4868
NC_005100.4-212512375	AGTATGCTCACCTGCACTCCAATAAGG	16	A	0	0.4793
NC_005100.4-212521104	TGTTTTGAGACACCATCTCCTTACATG	27	G	0.0088	0.4889
NC_005100.4-212598735	GATACCCTCACTCTGGCTCCCTGAAAG	3	T	0.1818	0.4713
NC_005100.4-212605267	CCTTTTGAGACAACATCTCCCCGTGGT	12	A	0.1826	0.2204
NC_005100.4-212605267	CCTTTTGAGACAACATCTCCCCGTGGT	22	C	0.0796	0.4171
NC_005100.4-212625441	AACCTGCCAACACGTGCTCCTGCTTGA	12	A	0.2869	0.4852

NC_005100.4-212634217	GTCCATCTGACTTCACCTCCACATCCG	1	G	0	0.4773
NC_005100.4-212658529	CATTGTAACACTCAGGCTCCTGGCCTG	8	A	0.0325	0.0476
NC_005100.4-212658814	TTTGTTCTGACTTTCCCTCCCTGTCAC	14	T	0	0.4749
NC_005100.4-212658814	TTTGTTCTGACTTTCCCTCCCTGTCAC	16	C	0.0259	0.0255
NC_005100.4-212680960	CAGCACAGAACCCAGCCTCCACCCACT	22	C	0.2054	0.212
NC_005100.4-212695975	GGCTCTGGGACAAGATCTCCTTTTTTG	3	C	0.1081	0.4383
NC_005100.4-212717935	ATAGTCTGCACTTAAGCTCCTGCCTCC	5	T	0	0.4839
NC_005100.4-212744931	GATCAGCCCACATATGCTCCTTGGTTG	3	T	0.4595	0.3727
NC_005100.4-212806956	TTTGACATGACCAGCCCTCCCTAACTG	14	G	0.2301	0.2302
NC_005100.4-212813368	ATCACAAGAACACGCACTCCATTATCT	14	G	0.1463	0.1494
NC_005100.4-212833812	CTGTGGATGACTGCTGCTCCAGGTCAA	27	A	0	0.4765
NC_005100.4-212842147	ACCACCAAGACAAGCTCTCCAGAACTC	5	C	0.0261	0.0257
NC_005100.4-212885523	CCTTCGGGTACCACTTCTCCTCAGCTT	2	C	0.0325	0.032
NC_005100.4-212885523	CCTTCGGGTACCACTTCTCCTCAGCTT	22	C	0.0325	0.032
NC_005100.4-212912673	TGTTTTTTTACCTGGCCTCCTGACTTC	1	T	0.1441	0.1759
NC_005100.4-212912673	TGTTTTTTTACCTGGCCTCCTGACTTC	2	G	0.1453	0.1773
NC_005100.4-212917379	TGTCTTAGCACTGGGACTCCCTCTTGC	2	G	0.0342	0.05
NC_005100.4-212917379	TGTCTTAGCACTGGGACTCCCTCTTGC	24	T	0	0.4839
NC_005100.4-212927718	CCATGGTTTACATTGACTCCATGTGTT	24	T	0.2174	0.4395
NC_005100.4-212941652	AAAGTAATGACAGGCTCTCCAGAGATA	14	G	0.0088	0.0262
NC_005100.4-212941652	AAAGTAATGACAGGCTCTCCAGAGATA	15	C	0.0088	0.0262
NC_005100.4-213039185	ATTTTAGTGACCACAGCTCCCTCTCTT	8	T	0.1391	0.1588
NC_005100.4-213039710	ACAAGCGTGACAATCTCTCCAGCCTTG	6	C	0.0488	0.0476
NC_005100.4-213039710	ACAAGCGTGACAATCTCTCCAGCCTTG	7	G	0.2358	0.4662
NC_005100.4-213058615	GACCAAATGACCCAAGCTCCCTGTAGA	6	A	0.6033	0.4213
NC_005100.4-213058615	GACCAAATGACCCAAGCTCCCTGTAGA	9	G	0.6167	0.4265
NC_005100.4-213091739	ACCACAAGGACACAGACTCCAGCATAT	23	C	0.0672	0.4567
NC_005100.4-213335492	TGAGATCCCACCACTCCTCCTGCTCTC	23	C	0.082	0.0786
NC_005100.4-213341631	AAACAACCAACCAGAGCTCCCAGGGAT	16	G	0.5738	0.416
NC_005100.4-213390781	TCTGGTAGGACAGAGGCTCCTTATTCA	5	G	0	0.4728
NC_005100.4-213390781	TCTGGTAGGACAGAGGCTCCTTATTCA	14	A	0.0083	0.4747
NC_005100.4-213404275	GATGGAAATACATTCCCTCCTTCTCTT	3	T	0.0085	0.4698
NC_005100.4-213426334	GGTAGTAGAACAACAACTCCAGATATC	14	C	0	0.4826
NC_005100.4-213451593	GGAGGAAATACATTCACTCCTTCCCTT	2	G	0.0614	0.0758
NC_005100.4-213461623	CTGTAGACAACTTCCACTCCAATACAA	7	A	0	0.48
NC_005100.4-213461623	CTGTAGACAACTTCCACTCCAATACAA	8	C	0.2632	0.2893
NC_005100.4-213513633	ATCTTGTTCACCTGTTCTCCCCTGCAG	22	C	0.042	0.0728
NC_005100.4-213537161	CAAACTATCACTTCCACTCCCTGGGCC	12	T	0.0083	0.4769
NC_005100.4-213546280	GGCCCAGCAACAGAAGCTCCATCACAG	2	G	0.1639	0.2032
NC_005100.4-213564012	AAGGGCATAACCAACCCTCCAACCATG	8	T	0.0894	0.0854
NC_005100.4-213564012	AAGGGCATAACCAACCCTCCAACCATG	12	C	0.2602	0.3604
NC_005100.4-213580171	CTTCCAGGCACCAGAACTCCTGCTGTC	6	A	0.0169	0.4856
NC_005100.4-213580171	CTTCCAGGCACCAGAACTCCTGCTGTC	9	C	0.1765	0.2012
NC_005100.4-213596241	TTGTTGGTTACCAGCTCTCCTGGTCCC	9	T	0	0.4793
NC_005100.4-213779689	CCAGCCCCAACACCTTCTCCACTAAGC	25	A	0.0171	0.4868
NC_005100.4-213787469	CTTTGAGGCACTTTCTCTCCCTCTGTT	3	T	0	0.4832
NC_005100.4-213787469	CTTTGAGGCACTTTCTCTCCCTCTGTT	23	C	0.0333	0.4888
NC_005100.4-213825629	CACACCTCCACCTGGACTCCTGCTATG	22	G	0.0702	0.0677
NC_005100.4-213841831	AGTGTGGGGACAGACCCTCCTAATACT	14	A	0	0.4793
NC_005100.4-213877485	CCACGGTGGACTTGAACTCCTCAGTGG	9	G	0.0424	0.0415
NC_005100.4-213890171	TCCTCTTTCACCTTCTCTCCCACCAAT	7	T	0	0.4922

NC_005100.4-213890171	TCCTCTTTCACCTTCTCTCCCACCAAT	26	A	0	0.4922
NC_005100.4-213905193	CGCCAAAACACAAGAGCTCCGATGTAG	2	G	0.0348	0.0671
NC_005100.4-213905193	CGCCAAAACACAAGAGCTCCGATGTAG	26	A	0.115	0.154
NC_005100.4-213918495	TACAGAGCCACCTTCTCTCCAAAGTAT	5	G	0.1538	0.1563
NC_005100.4-213931496	CTGCCCAGGACACCTGCTCCCCTCTCC	24	C	0	0.48
NC_005100.4-214131730	ATGGTGTGCACGTGTGCTCCCAGATAC	21	C	0.0348	0.0671
NC_005100.4-214131730	ATGGTGTGCACGTGTGCTCCCAGATAC	26	A	0	0.4864
NC_005100.4-214131943	TGGTCTGTAACTCCAGCTCCAGGGGAT	13	C	0.525	0.4025
NC_005100.4-214133432	GATCCTAAAACCAGCACTCCACGGATA	7	A	0	0.4765
NC_005100.4-214166441	AACCAGAACACACCTGCTCCCAGCACT	3	C	0	0.4807
NC_005100.4-214174525	CCGTCACAGACTAGCCCTCCTCCGTAG	2	C	0.2333	0.2994
NC_005100.4-214177621	GCATAGCAGACCCTATCTCCAACCCAC	12	C	0.0256	0.0253
NC_005100.4-214293108	CAGGTCTGTACCAAGCCTCCAGACTCT	6	C	0.0413	0.0716
NC_005100.4-214296310	AGGGATGTCACATCCCCTCCCTGAACA	15	C	0	0.4677
NC_005100.4-214303719	TGTCACAGCACTATTTCTCCCAGCCCA	14	T	0	0.4793
NC_005100.4-214303719	TGTCACAGCACTATTTCTCCCAGCCCA	22	A	0.2439	0.385
NC_005100.4-214347679	CAGGAAGCCACAGTCCCTCCCTGAGGA	27	A	0.0082	0.4822
NC_005100.4-214579503	TGAGCCGTGACCTCTGCTCCTTAGACT	1	T	0.3391	0.3639
NC_005100.4-214647142	TTCTTGACCACAAATACTCCGGTTCTC	23	T	0.1404	0.2021
NC_005100.4-214647142	TTCTTGACCACAAATACTCCGGTTCTC	24	T	0.1964	0.2449
NC_005100.4-214654475	GAGGCAGATACTCACACTCCTGAGCTG	23	A	0.1368	0.2235
NC_005100.4-214660373	CTATATACCACCCACTCTCCCACTCAC	7	A	0.1626	0.1761
NC_005100.4-214675055	CGAAGCCTGACCATGTCTCCATCCTCT	2	G	0.1057	0.4471
NC_005100.4-214685424	TAAATGAACACAGAAACTCCCTCTGTG	4	A	0	0.4864
NC_005100.4-214685424	TAAATGAACACAGAAACTCCCTCTGTG	8	A	0.0789	0.0918
NC_005100.4-214686392	GAAAACTGCACTGTGTCTCCTGTCTGT	4	A	0	0.4833
NC_005100.4-214686392	GAAAACTGCACTGTGTCTCCTGTCTGT	5	A	0	0.4833
NC_005100.4-214701945	AGATGTGCCACAGGGTCTCCTCAATGC	8	C	0.0244	0.0241
NC_005100.4-214716746	TTGGTGAAAACCAATGCTCCACTCACT	21	A	0.1966	0.2778
NC_005100.4-214716746	TTGGTGAAAACCAATGCTCCACTCACT	27	T	0.0769	0.12
NC_005100.4-214741282	ACAGTGCCTACAAGATCTCCGTGGCCT	3	A	0.0082	0.479
NC_005100.4-214744447	CGATGGAGGACACAACCTCCTCAGTCC	2	G	0	0.4751
NC_005100.4-214750555	CCCAGGTCTACACCTACTCCGTGCTCA	1	C	0.0081	0.0241
NC_005100.4-214759956	TCTACTCACACTCTGGCTCCATTCTCT	4	A	0.2149	0.2647
NC_005100.4-214785837	GAGGCCATCACTCCCTCTCCAAGCCTG	7	A	0	0.4793
NC_005100.4-214808767	AGAGACCTGACCACCTCTCCTGTCCTC	16	T	0.0328	0.0634
NC_005100.4-214817018	CTTAGTGACACACCTCCTCCAACAAAG	12	A	0.0354	0.0348
NC_005100.4-214820635	TTACACAGCACTGGGTCTCCAACTTCC	6	C	0.0083	0.4783
NC_005100.4-214820635	TTACACAGCACTGGGTCTCCAACTTCC	24	T	0.1167	0.495
NC_005100.4-214854089	AGAATGAGGACAATGTCTCCTTATTCT	1	A	0	0.4779
NC_005100.4-214892440	GTGGTTTCTACATGAGCTCCCACAACT	13	T	0.2114	0.2499
NC_005100.4-214908934	CTCTTCCTGACCCTTCCTCCTGTATCA	24	A	0	0.4793
NC_005100.4-214909230	CTCAGTTGGACATTAGCTCCATGGGAC	13	T	0	0.4765
NC_005100.4-214916700	ACCAAAGTGACATTCTCTCCCATCTGT	3	C	0	0.4827
NC_005100.4-214930466	GCTCATCTGACCCTTCCTCCCCAGCCA	6	T	0	0.4793
NC_005100.4-214955260	GTAGTGGGCACTCAGGCTCCATGTGCT	23	G	0.4118	0.327
NC_005100.4-214963836	ACCCTTCTTACTCTAGCTCCTTTTCTT	12	T	0	0.4671
NC_005100.4-214991585	TCTGTGTGCACGGCAACTCCTTAACAG	2	C	0	0.4856
NC_005100.4-215007919	CACCTGCATACAACTGCTCCAAGTGTA	25	G	0.2083	0.315
NC_005100.4-215007919	CACCTGCATACAACTGCTCCAAGTGTA	26	T	0	0.4832
NC_005100.4-215018279	GGAGACAAAACGATGCCTCCCTCTCAC	16	C	0.0328	0.048

NC_005100.4-215020589	CATGCTGCCACCCACCCTCCGATGCTT	12	C	0.0259	0.0255
NC_005100.4-215023574	TTGGTCAAGACTGGCTCTCCTTCTGGT	1	T	0	0.4793
NC_005100.4-215033511	TGGCCTAGCACCTGGACTCCTGCTGGT	27	T	0.2459	0.3447
NC_005100.4-215040559	TGCCAGGCAACGGCTGCTCCTACTGCA	24	T	0.2212	0.2618
NC_005100.4-215063809	GCAAAATCCACCCCCACTCCCCCACCC	26	C	0.1197	0.142
NC_005100.4-215159780	CTGGTTTGGACTCAAGCTCCTTACAAT	15	A	0.0089	0.4665
NC_005100.4-215333724	ATGTAGCCTACTTGCTCTCCTTGGCGT	25	C	0.2719	0.4581
NC_005100.4-215449473	TTGCTTGGGACACTGGCTCCTCATAAA	25	A	0.1947	0.2036
NC_005100.4-215514827	GGAGGCTTCACTTTGCCTCCTGCTACA	1	G	0.0082	0.0243
NC_005100.4-215514827	GGAGGCTTCACTTTGCCTCCTGCTACA	21	T	0.1885	0.311
NC_005100.4-215543648	TAGGCTCAAACAGATTCTCCTGCCTTT	12	A	0	0.48
NC_005100.4-215547999	GAACGCTTGACTGTCCCTCCAGTTTGG	6	C	0.1709	0.4918
NC_005100.4-215573648	TGATCGCTGACCACCTCTCCATTTCCT	6	G	0.0583	0.0722
NC_005100.4-215592117	CACACCTCCACACTGTCTCCTAAGTGG	4	A	0.0168	0.4661
NC_005100.4-215592117	CACACCTCCACACTGTCTCCTAAGTGG	13	C	0.0168	0.4703
NC_005100.4-215594216	CGGAAAGGCACTTACCCTCCACACATT	1	C	0.1695	0.4908
NC_005100.4-215597432	CCTGTGCATACAGCTGCTCCTGCTGGC	21	T	0.1739	0.3938
NC_005100.4-215598832	CAGGACTGAACCCAGCCTCCGATGGCG	26	C	0.0085	0.4753
NC_005100.4-215603527	AGGAACAACACCTTAGCTCCAGCCCCT	2	G	0	0.4793
NC_005100.4-215604550	CAGCAAGTCACAGTGGCTCCCAGCCCC	25	C	0.2589	0.3418
NC_005100.4-215637486	CTGTGGTACACACGGTCTCCCTTAATA	25	A	0.2017	0.3508
NC_005100.4-215638307	CTGCTCTACACCCTGGCTCCATTTCCT	6	C	0	0.4891
NC_005100.4-215689669	CATTGTGAGACTTTGTCTCCTTTCTTC	4	T	0	0.4764
NC_005100.4-215689669	CATTGTGAGACTTTGTCTCCTTTCTTC	23	T	0	0.4764
NC_005100.4-215694832	TTTCAGAGGACCTCCGCTCCAACCCCA	14	C	0.2712	0.3874
NC_005100.4-215696390	ATAGTGTGCACCACCTCTCCCCAACCC	1	A	0.0081	0.481
NC_005100.4-215696390	ATAGTGTGCACCACCTCTCCCCAACCC	22	C	0.1463	0.5
NC_005100.4-215699494	GCCATTAGGACCCCAGCTCCCAGGGCC	6	T	0	0.477
NC_005100.4-215738792	GAATCAAGAACTTCAACTCCTTCCTAT	26	A	0	0.4787
NC_005100.4-215771574	AGGAAGCCCACCTCTTCTCCAAGTTGC	16	T	0	0.482
NC_005100.4-215774592	GGTGTTCCCACTGTCTCTCCTTCCCCA	8	C	0.0776	0.1358
NC_005100.4-215798686	ATTCCACCCACTCTTGCTCCCCATAAG	15	T	0.2389	0.3081
NC_005100.4-215799020	ATTTTGTATACCTCTTCTCCTTTGTGG	15	T	0.2288	0.481
NC_005100.4-215802396	CTGGCATCAACAACCCCTCCCGGCACT	8	C	0.065	0.078
NC_005100.4-215829210	CACTGCCCCACGCCCCCTCCACTCCTC	12	G	0.0325	0.032
NC_005100.4-215846207	CAGGGTCCTACCCACCCTCCAGTATCC	14	A	0	0.4787
NC_005100.4-216068275	AGGCCTTCCACAGGTGCTCCCTGGAGT	14	G	0.2623	0.387
NC_005100.4-216078977	CAGCTTTGAACCAGTACTCCTAATCTC	21	T	0.0957	0.1369
NC_005100.4-216084848	GCTTCCTCTACCCTGGCTCCCCACTTC	4	T	0	0.4773
NC_005100.4-216091824	GTATCTGCAACAGCCACTCCTTGACTC	15	C	0.1138	0.1761
NC_005100.4-216103229	TGCAGTTGGACTCTGCCTCCAAGGATG	21	A	0.0246	0.0243
NC_005100.4-216115413	TGTCCATGTACACCTCCTCCATACTTC	22	T	0.1026	0.1563
NC_005100.4-216117066	GTCCACCTGACATATCCTCCTTTTCTT	9	G	0.1043	0.0989
NC_005100.4-216173572	CCAGGAGCAACTGCTGCTCCCCATGCT	1	C	0.1947	0.2913
NC_005100.4-216173572	CCAGGAGCAACTGCTGCTCCCCATGCT	12	T	0.2054	0.4789
NC_005100.4-216216228	GAAGGTGCCACACTGGCTCCACGGCAA	8	C	0	0.4868
NC_005100.4-216216228	GAAGGTGCCACACTGGCTCCACGGCAA	12	A	0.1795	0.479
NC_005100.4-216221593	TTGCACATGACCCATGCTCCTCAAACA	25	A	0.0328	0.4837
NC_005100.4-216230514	CAACTTGTAACTCCAGCTCCAGAAGCT	9	A	0.0569	0.0854
NC_005100.4-216230514	CAACTTGTAACTCCAGCTCCAGAAGCT	24	A	0	0.4773
NC_005100.4-216237153	CCTTCCCAAACGCACTCTCCAGAACTG	12	G	0.1504	0.4994

NC_005100.4-216237153	CCTTCCCAAACGCACTCTCCAGAACTG	15	C	0.0087	0.4849
NC_005100.4-216249043	GGGGCCAGAACCTGGTCTCCTTCGAGG	5	C	0	0.4844
NC_005100.4-216259470	CTCCTCCTCACTGTCCCTCCTCCGTTT	6	C	0.0174	0.0342
NC_005100.4-216285836	AGGGAGCTCACAAAAGCTCCCACACAC	26	A	0.0339	0.4977
NC_005100.4-216368851	AGAACCAAGACCAGGCCTCCTGACATC	1	A	0.1681	0.2618
NC_005100.4-216368851	AGAACCAAGACCAGGCCTCCTGACATC	24	C	0.1081	0.1338
NC_005100.4-216499013	CACATGGCAACACGTACTCCACGCCCG	13	C	0	0.4859
NC_005100.4-216523425	GAAATGTCCACCCAGGCTCCTGACTCA	1	G	0.1217	0.4997
NC_005100.4-216562745	CATCCACCCACATACTCTCCAGCTGCC	12	A	0	0.4773
NC_005100.4-216562745	CATCCACCCACATACTCTCCAGCTGCC	23	C	0.0732	0.0705
NC_005100.4-216579152	AGGCCCTCCACAGCAGCTCCTACAGGG	12	A	0.0339	0.0333
NC_005100.4-216581695	CTGTCCCTGACAAGGCCTCCAGATGGG	4	T	0.0088	0.472
NC_005100.4-216656789	AAACATAGCACCAAGTCTCCAGATTGC	9	C	0.2655	0.406
NC_005100.4-216656789	AAACATAGCACCAAGTCTCCAGATTGC	27	C	0.2589	0.4119
NC_005100.4-216680559	GTGGTGACAACAAACACTCCAAACTTG	16	A	0.0089	0.4751
NC_005100.4-216737098	TCATATTCCACCACGTCTCCCACACTA	13	A	0.2231	0.465
NC_005100.4-216737675	AGGCTGCATACTTCCTCTCCAGGTCAT	2	G	0.0885	0.4996
NC_005100.4-216747333	AACACCTAAACCGGCACTCCTGATAGA	5	C	0.0732	0.0705
NC_005100.4-216747333	AACACCTAAACCGGCACTCCTGATAGA	13	G	0.1057	0.1287
NC_005100.4-216757046	TTTCCCTTAACTCAGGCTCCTGCAAGC	9	A	0.1681	0.4998
NC_005100.4-216760099	ACTCCTTGTACACTTTCTCCTGAGCTT	23	A	0.0696	0.0832
NC_005100.4-216761214	CATACTCCCACCCTATCTCCTCTACCA	12	C	0	0.4709
NC_005100.4-216768277	TGCAGCCAGACCCTATCTCCAACCTTC	16	T	0.2328	0.3617
NC_005100.4-216868642	TGGCTTCATACACTGACTCCAGGTTCT	22	G	0.0902	0.1154
NC_005100.4-216873075	TGTATGGTCACTTTACCTCCCAGCTCC	2	G	0	0.4898
NC_005100.4-216873075	TGTATGGTCACTTTACCTCCCAGCTCC	24	C	0.2054	0.3418
NC_005100.4-216880314	TCTCCTCTGACACTGTCTCCACCTGGG	23	C	0.1074	0.1306
NC_005100.4-216884996	GAGCCTGGGACAGTCCCTCCTTCCCCT	5	C	0.1736	0.4769
NC_005100.4-216884996	GAGCCTGGGACAGTCCCTCCTTCCCCT	21	T	0	0.4819
NC_005100.4-216904147	CTCTCTTTTACCCTCTCTCCCACTGTC	16	T	0.0847	0.493
NC_005100.4-216910390	CAGAGTGAGACCCTGTCTCCCAACAGC	6	T	0.0336	0.033
NC_005100.4-216910390	CAGAGTGAGACCCTGTCTCCCAACAGC	16	T	0.0169	0.0333
NC_005100.4-216917907	GATACCAACACTTCTGCTCCTGTGACC	3	T	0	0.4826
NC_005100.4-216973309	TGGAGTAAGACCCATCCTCCTTTCCCG	26	C	0.1739	0.4764
NC_005100.4-216988892	CTCTGATCCACCATATCTCCTCTTTCC	4	T	0.1301	0.3334
NC_005100.4-216988892	CTCTGATCCACCATATCTCCTCTTTCC	26	C	0.0488	0.4904
NC_005100.4-217020761	CTGACCTCCACACGTGCTCCCTTCTGT	9	C	0.0776	0.1209
NC_005100.4-217020761	CTGACCTCCACACGTGCTCCCTTCTGT	25	T	0	0.4755
NC_005100.4-217043747	GTCCTGGAGACCCGATCTCCTCTGATG	13	C	0.075	0.0722
NC_005100.4-217052623	GAACAAACCACTTGCTCTCCTCAGCCC	2	A	0	0.4819
NC_005100.4-217075108	GGAATATGTACCCTTCCTCCCTCCATC	24	C	0.2541	0.3909
NC_005100.4-217075380	GCAACTAAAACACTTGCTCCTCTCCTT	2	C	0.1709	0.3641
NC_005100.4-217075380	GCAACTAAAACACTTGCTCCTCTCCTT	3	A	0.1709	0.3641
NC_005100.4-217088144	TGAGCTCAGACAATAACTCCCAACCTG	23	A	0	0.48
NC_005100.4-217103074	TTTGTCGCCACCCTGTCTCCGTGGTGA	6	C	0.2295	0.4565
NC_005100.4-217119343	CATGGTTTTACAAAGCCTCCTCTGTTC	3	T	0.1721	0.4822
NC_005100.4-217129538	CCCCTAAGAACACATGCTCCAACCGTG	25	G	0.0246	0.0243
NC_005100.4-217164252	AGTCTAAAAACAGTGACTCCTCTGCCA	3	T	0.1593	0.1898
NC_005100.4-217164252	AGTCTAAAAACAGTGACTCCTCTGCCA	16	A	0.0268	0.4789
NC_005100.4-217182959	CAGCAGAGAACAGCATCTCCCAAGGGA	2	A	0.3214	0.4297
NC_005100.4-217182959	CAGCAGAGAACAGCATCTCCCAAGGGA	21	C	0.3214	0.4157

NC_005100.4-217185383	GTCCAGAGCACACCACCTCCTCCACAG	25	C	0.0804	0.0934
NC_005100.4-217187981	GCAGGTATAACATGTTCTCCCTGACAC	22	T	0	0.4793
NC_005100.4-217187981	GCAGGTATAACATGTTCTCCCTGACAC	23	G	0.2358	0.3091
NC_005100.4-217240550	CACATTTGGACTTCCGCTCCAGGGATG	8	G	0.0569	0.0553
NC_005100.4-217268242	CGGGAATGGACATGGCCTCCAGGCAAG	2	G	0.0574	0.071
NC_005100.4-217322799	TGACTCCTTACCTGTTCTCCCACTCAA	15	T	0.125	0.1866
NC_005100.4-217322799	TGACTCCTTACCTGTTCTCCCACTCAA	27	A	0.157	0.172
NC_005100.4-217339943	TTGTCCACCACCTTTCCTCCTGATGAC	24	T	0.3186	0.406
NC_005100.4-217339943	TTGTCCACCACCTTTCCTCCTGATGAC	26	A	0	0.4778
NC_005100.4-217342254	CTGCCTGCCACAGCCACTCCACAGGGT	25	G	0.2087	0.2519
NC_005100.4-217359241	GTCCAGCAAACGTGTCCTCCCCAGACT	6	G	0.0756	0.0882
NC_005100.4-217359241	GTCCAGCAAACGTGTCCTCCCCAGACT	7	C	0.0756	0.0882
NC_005100.4-217394295	GAGCTCATCACCGAGCCTCCCTCTGCT	12	C	0.3115	0.416
NC_005100.4-217394295	GAGCTCATCACCGAGCCTCCCTCTGCT	15	G	0.0656	0.0634
NC_005100.4-217422689	AGAGAAAATACCAACTCTCCAGTGAGG	9	T	0	0.4793
NC_005100.4-217450481	AGTGACAACACCGTGGCTCCTGAAGAC	14	T	0.2018	0.3061
NC_005100.4-217467398	TTGGCTTATACTTCTGCTCCCTGAGTA	8	A	0.1111	0.1347
NC_005100.4-217472012	TGGGCTAAAACATACACTCCAGAGCTG	26	T	0.0252	0.0249
NC_005100.4-217472110	CTGCCTCTCACACCAGCTCCAGAGGAC	24	G	0.0579	0.0562
NC_005100.4-217474529	CACCCTTCCACCCACCCTCCCATCCAT	24	C	0.065	0.0629
NC_005100.4-217474651	CACCTATCCACCCATCCTCCTATCCAT	6	A	0.2033	0.2989
NC_005100.4-217474651	CACCTATCCACCCATCCTCCTATCCAT	25	C	0.0732	0.1145
NC_005100.4-217474706	CACCCTCTCACCCATCCTCCTATCCAT	21	T	0.0833	0.1099
NC_005100.4-217489035	TTATGTCCCACCCCTTCTCCCCACACC	23	A	0	0.4813
NC_005100.4-217496972	TAAACAAGCACTCTGCCTCCTGAGCCC	9	C	0.0738	0.1968
NC_005100.4-217526980	CAGAGCTCCACTCTTCCTCCCTTGGTG	23	T	0.0082	0.479
NC_005100.4-217535728	GGACATGGGACATTCGCTCCAGCCCAG	16	G	0.2119	0.3086
NC_005100.4-217543755	GGTGCTGAGACAGGGGCTCCGGGTCAT	12	A	0.1327	0.2104
NC_005100.4-217544129	AAGTCTCTCACTTGCCCTCCCAGCACG	12	T	0	0.4751
NC_005100.4-217545604	TCTTAATCAACTGTGCCTCCCTCAAAG	6	A	0.0331	0.0792
NC_005100.4-217666351	GCTCTGCAGACCAGGTCTCCATGCTGG	15	G	0.0504	0.0805
NC_005100.4-217666978	GAGACAGACACTTGAGCTCCACATTGA	26	G	0.0265	0.0262
NC_005100.4-217666978	GAGACAGACACTTGAGCTCCACATTGA	27	A	0.0265	0.0262
NC_005100.4-217680542	GGCTCCTCTACCATCCCTCCCCAGCAA	4	T	0	0.4807
NC_005100.4-217682661	CTTGATGTAACTTGAGCTCCAGAGGCT	1	C	0	0.4887
NC_005100.4-217684262	ATCTCTACAACTAAAGCTCCCGATATG	6	T	0	0.4743
NC_005100.4-217691503	CTACGGCTGACTGTACCTCCTATACAT	5	G	0.1864	0.324
NC_005100.4-217700819	CACAGAGGAACTGCAGCTCCATAAAGG	1	C	0.1951	0.4759
NC_005100.4-217700819	CACAGAGGAACTGCAGCTCCATAAAGG	3	C	0.1951	0.4759
NC_005100.4-217702245	CACACTTCCACAGGCGCTCCTAGGACC	22	A	0.0087	0.0425
NC_005100.4-217723342	GTCCATCCAACCACCACTCCTTTTTTG	2	T	0.0083	0.4816
NC_005100.4-217729599	GGCCCAGAGACCCCTTCTCCTGAATCC	22	G	0.122	0.1425
NC_005100.4-217729599	GGCCCAGAGACCCCTTCTCCTGAATCC	23	A	0	0.4751
NC_005100.4-217735008	AAATCTCACACCTCCCCTCCAGCAGGC	8	A	0.1327	0.154
NC_005100.4-217747505	CTTTGGTCCACATGCCCTCCAAATGCT	22	A	0	0.4793
NC_005100.4-217747505	CTTTGGTCCACATGCCCTCCAAATGCT	23	A	0.0569	0.4417
NC_005100.4-217756688	TCCACCTACACACTACCTCCTGTCTAC	21	T	0.2101	0.4117
NC_005100.4-217763613	TGAGCACAGACCTCACCTCCTGGGCTT	12	C	0.025	0.0247
NC_005100.4-217773196	GACTGTAAAACAGTCCCTCCCCTGTCA	5	G	0.0164	0.0322
NC_005100.4-217786136	CACAGTGTAACTTCTTCTCCCTGTCCC	4	A	0.069	0.0666
NC_005100.4-217788752	AGCTCTGTAACCCCAGCTCCCAGGAGG	7	G	0.0504	0.4921

NC_005100.4-217790666	ACGCAACATACATACACTCCAGTGTGT	2	C	0.075	0.1316
NC_005100.4-217790666	ACGCAACATACATACACTCCAGTGTGT	26	G	0.0667	0.0644
NC_005100.4-217816609	TGGTGCAGGACTGATCCTCCTGGCCTT	27	T	0	0.4793
NC_005100.4-217826691	CATGTTCCCACTGCTCCTCCACCCACA	6	T	0.0325	0.0476
NC_005100.4-217849493	GTTGAGCAGACAGCCTCTCCTCCCCGT	6	G	0.1157	0.1918
NC_005100.4-217849493	GTTGAGCAGACAGCCTCTCCTCCCCGT	25	C	0.0082	0.479
NC_005100.4-217854916	TTGTCTCACACAAACTCTCCACAGGTG	12	A	0.1239	0.5
NC_005100.4-217873254	CACATGCTCACACACGCTCCCGTGCCC	5	T	0.1892	0.3727
NC_005100.4-217884303	AGATGGACCACTATCGCTCCCAGCTCA	4	T	0.124	0.1585
NC_005100.4-217900819	CCAGACATTACAGCTGCTCCACCAGTC	25	G	0.0667	0.095
NC_005100.4-217914641	CACTCAGGCACCTGTCCTCCTTGTCCC	12	C	0.1167	0.1528
NC_005100.4-217914641	CACTCAGGCACCTGTCCTCCTTGTCCC	13	T	0	0.4832
NC_005100.4-217925086	CTCTCAGGAACTTACTCTCCTCAACCA	16	T	0	0.488
NC_005100.4-217925086	CTCTCAGGAACTTACTCTCCTCAACCA	26	C	0.0862	0.0981
NC_005100.4-217966288	GCTTGGCAGACCCTTACTCCTGTGACA	24	G	0.2589	0.2991
NC_005100.4-218021612	CTGTGTGTCACAGCAGCTCCAAGGGAG	9	C	0.124	0.1447
NC_005100.4-218072175	TGGTCTGCCACTGACCCTCCCCAGCAC	13	G	0.1736	0.2234
NC_005100.4-218091654	CAAAATAAAACTGACCCTCCAATAGCA	6	T	0.0086	0.4643
NC_005100.4-218095386	ACCGCATCAACTCTGGCTCCCATGGAG	3	C	0.2846	0.2989
NC_005100.4-218129097	TTCTAACCTACCTCCCCTCCACCCGTC	24	C	0.082	0.1082
NC_005100.4-218133681	CTGCTTGAAACTCCAGCTCCAGGAGAC	23	G	0.2	0.2432
NC_005100.4-218149828	CCCACTCACACCCCCCCTCCTGGTGGT	21	T	0.0261	0.4926
NC_005100.4-218178059	CAGGCACGGACTGGGGCTCCCAGGAAT	6	A	0.2358	0.4036
NC_005100.4-218178059	CAGGCACGGACTGGGGCTCCCAGGAAT	23	G	0.0488	0.0476
NC_005100.4-218189250	CTCAGATGAACTTTCCCTCCTTGGATA	16	C	0	0.4819
NC_005100.4-218251067	CCTTGGAGGACCCTAGCTCCAACTCTA	26	T	0.1579	0.4962
NC_005100.4-218264402	TGAATTCCTACTCATCCTCCAAGGCCT	5	T	0	0.4793
NC_005100.4-218267956	ATAGTTGTGACTAGGACTCCATCATAC	5	T	0.1301	0.4984
NC_005100.4-218267956	ATAGTTGTGACTAGGACTCCATCATAC	21	A	0	0.4793
NC_005100.4-218282034	GTGGGCCCCACCTACCCTCCGTGTGCA	23	G	0.0976	0.1494
NC_005100.4-218308162	CCCCCATCCACTCTGCCTCCATGGTTC	5	C	0	0.4787
NC_005100.4-218313112	TCATCTGCCACTCCAGCTCCAAGGGGT	26	G	0.1545	0.1954
NC_005100.4-218316864	TCAGTAATAACTAAAGCTCCCACAGTT	4	G	0	0.4846
NC_005100.4-218320534	CTGAGAGAGACACTATCTCCAAAACAA	27	A	0.0246	0.0243
NC_005100.4-218354468	GCCACTTTGACCTTTGCTCCCAAAGTG	5	C	0	0.4826
NC_005100.4-218354468	GCCACTTTGACCTTTGCTCCCAAAGTG	25	G	0.0254	0.0251
NC_005100.4-218357737	CTGGGGGGAACTTCCTCTCCCTCCCAT	5	G	0.0248	0.4835
NC_005100.4-218372500	TCCCACTGCACTGGGCCTCCCCGCCTG	8	G	0.1709	0.3895
NC_005100.4-218394035	TTTGACTCTACCATCCCTCCTGTCAGG	8	C	0.1327	0.4735
NC_005100.4-218394035	TTTGACTCTACCATCCCTCCTGTCAGG	9	T	0	0.4827
NC_005100.4-218400437	CTGGCCAAGACCAGCTCTCCCTGTGGC	13	A	0	0.4755
NC_005100.4-218411205	ATGTGTGACACCAGCACTCCTGCTGAG	27	G	0.0325	0.0476
NC_005100.4-218446343	CCATGAACCACAAAAGCTCCTTCCATC	27	C	0.0171	0.4693
NC_005100.4-218472490	ACTCCACCCACAGGTCCTCCAAACCCT	5	C	0	0.4793
NC_005100.4-218479623	GGCCTCGAAACAATCTCTCCCCATTAG	6	C	0.1724	0.4405
NC_005100.4-218479623	GGCCTCGAAACAATCTCTCCCCATTAG	14	T	0.0172	0.4905
NC_005100.4-218494362	CCAGTCTGCACCCCAGCTCCAGTCCCT	21	A	0.1429	0.3554
NC_005100.4-218505593	TGAAGCTTGACTGGTACTCCTGGCAGC	7	T	0.2845	0.4768
NC_005100.4-218544676	CCCAGACCTACAGCAGCTCCCCAAGAT	8	C	0.3089	0.4854
NC_005100.4-218551425	ACACCTCAAACAGGAACTCCATGGGTT	2	C	0.2893	0.4077
NC_005100.4-218581948	GGACACTTAACACTCTCTCCATCTTGT	12	A	0.1475	0.285

NC_005100.4-218600576	CAGCTCTCCACCGTTCCTCCAGAGACT	4	C	0.2288	0.4454
NC_005100.4-218600576	CAGCTCTCCACCGTTCCTCCAGAGACT	13	G	0.0847	0.1264
NC_005100.4-218650633	CTTAGTAGTACCTCCACTCCGTTCAAG	7	A	0.0894	0.4979
NC_005100.4-218650633	CTTAGTAGTACCTCCACTCCGTTCAAG	21	G	0.0732	0.1287
NC_005100.4-218667178	TTCCTCACCACCTTTGCTCCCTGGTGC	1	T	0.1062	0.4981
NC_005100.4-218668603	TCTACGCAAACTGGACCTCCAAGGAAA	5	C	0.0924	0.1181
NC_005100.4-218675376	TGGCCTTTCACTGAGACTCCATCCTCC	21	A	0	0.4709
NC_005100.4-218690721	GATGCTTGCACCTTTTCTCCACAAACT	27	T	0.0084	0.4723
NC_005100.4-218692835	AGATGTGGAACATGCCCTCCTTCATCG	14	G	0.0259	0.1209
NC_005100.4-218715802	TCAAGTGCCACCACACCTCCTCACTCT	13	A	0	0.4779
NC_005100.4-218715802	TCAAGTGCCACCACACCTCCTCACTCT	15	A	0	0.4779
NC_005100.4-218747688	AACTAGGATACCCCTCCTCCATCTTCA	21	A	0.0684	0.0973
NC_005100.4-218879182	CTCATCCCCACAGGCCCTCCTAGTCCT	4	A	0	0.4773
NC_005100.4-218879182	CTCATCCCCACAGGCCCTCCTAGTCCT	5	T	0.1721	0.3307
NC_005100.4-218885852	GAGTAGGGAACACTCACTCCTGCAATA	5	A	0	0.4844
NC_005100.4-218888864	CACATGTGTACAAACTCTCCTGCATAC	22	G	0	0.4826
NC_005100.4-218888954	CATTTGCACACAAGCACTCCTGCATAC	3	T	0.0259	0.0422
NC_005100.4-218906884	ATGTTTCTAACTAAAGCTCCACCATTC	22	C	0.2241	0.3995
NC_005100.4-218958318	TGTGGCAGAACCTTTGCTCCTTCTTCC	23	C	0.1463	0.1629
NC_005100.4-218998992	TGCCTGTTAACTCCAGCTCCAGGGCAT	8	T	0.6218	0.4285
NC_005100.4-219028369	CATAGAAAAACCTTGTCTCCAAAACAA	3	T	0.825	0.4997
NC_005100.4-219028369	CATAGAAAAACCTTGTCTCCAAAACAA	24	A	0.825	0.4997
NC_005100.4-219053936	CATGAGGCCACTGCTCCTCCCCAGTAC	5	A	0	0.4786
NC_005100.4-219053936	CATGAGGCCACTGCTCCTCCCCAGTAC	8	C	0	0.4786
NC_005100.4-219118291	GTAGGCCCAACCACCCCTCCCTCTTTC	23	C	0.1967	0.2279
NC_005100.4-219135458	ACGGCCCCAACCCTGGCTCCTGCCAGC	8	C	0	0.4826
NC_005100.4-219135458	ACGGCCCCAACCCTGGCTCCTGCCAGC	14	T	0.0254	0.0251
NC_005100.4-219141440	CCTTGTTGTACAGCAACTCCTCATGCG	23	A	0	0.4773
NC_005100.4-219141440	CCTTGTTGTACAGCAACTCCTCATGCG	26	C	0	0.4773
NC_005100.4-219161635	CCCCCAGCCACTCTGGCTCCTGTAACT	25	A	0	0.4749
NC_005100.4-219194480	TCTCACAGGACCTTCTCTCCCTTCTGA	21	C	0	0.48
NC_005100.4-219197657	TGTAAGACTACTGAGGCTCCAAGAGGC	4	A	0.0504	0.065
NC_005100.4-219197686	GCTGTCCCGACAGGAGCTCCACTCCAA	5	T	0.0721	0.086
NC_005100.4-219203905	ATCACCACCACTGGCCCTCCATCTGTC	15	C	0.1892	0.3124
NC_005100.4-219203905	ATCACCACCACTGGCCCTCCATCTGTC	22	T	0	0.4785
NC_005100.4-219231212	GCTTAGCCAACTGTCACTCCCAGCCCT	8	C	0.0702	0.0839
NC_005100.4-219231212	GCTTAGCCAACTGTCACTCCCAGCCCT	9	A	0	0.4755
NC_005100.4-219311070	GGGTAACCCACTTGCCCTCCAGCCAGG	2	G	0	0.4826
NC_005100.4-219335499	GTCACATGTACTCCTGCTCCAGCCTGG	25	T	0.1786	0.1626
NC_005100.4-219354920	ATTCCCCTCACTGGTTCTCCATTGTCT	5	C	0.0407	0.0705
NC_005100.4-219360112	GCAGTGGGTACTCGGGCTCCTCCTGTG	5	T	0	0.4833
NC_005100.4-219360112	GCAGTGGGTACTCGGGCTCCTCCTGTG	6	G	0.2174	0.258
NC_005100.4-219363032	TTCCTGGACACGACCCCTCCCTGCTGG	2	T	0	0.4654
NC_005100.4-219363032	TTCCTGGACACGACCCCTCCCTGCTGG	12	G	0	0.4682
NC_005100.4-219405301	CATCAGGTGACAACTCCTCCCTACTGT	6	G	0	0.4787
NC_005100.4-219405301	CATCAGGTGACAACTCCTCCCTACTGT	13	A	0.2881	0.4102
NC_005100.4-219423102	ACTCCAGGCACTCTGCCTCCCAGACAG	24	A	0	0.4793
NC_005100.4-219466687	AGGCTCCTGACTATATCTCCTGTTATA	12	T	0.0909	0.1306
NC_005100.4-219466687	AGGCTCCTGACTATATCTCCTGTTATA	13	A	0.1983	0.4877
NC_005100.4-219476361	TGGCCTGTAACTCCAGCTCCAGGGGGA	4	C	0.1429	0.1327
NC_005100.4-219477607	AGGCAAGTCACCATGCCTCCCTCACCA	12	C	0.1951	0.4881

NC_005100.4-219493667	CCCTTCTCCACCTGCCCTCCCCCCTTT	27	T	0	0.452
NC_005100.4-219648198	TTCAAGGACACATAATCTCCTCAGCTT	15	A	0	0.4807
NC_005100.4-219664565	TAGCTCACAACCGTTACTCCAGTACCC	8	C	0.0574	0.0557
NC_005100.4-219664565	TAGCTCACAACCGTTACTCCAGTACCC	12	C	0.0902	0.4959
NC_005100.4-219677141	GACTTGTCCACTGGACCTCCCTGGCTC	2	A	0	0.4793
NC_005100.4-219694518	GGAGCATCCACAGACCCTCCTTACTCT	3	A	0.2623	0.4773
NC_005100.4-219694518	GGAGCATCCACAGACCCTCCTTACTCT	4	G	0.2623	0.4773
NC_005100.4-219711905	ATAGCAGACACTTTTCCTCCTGAGTCA	9	C	0.0086	0.4866
NC_005100.4-219715439	GTAGAAGGCACCTGCACTCCCAGCAAT	24	C	0.2397	0.2814
NC_005100.4-219725953	CACACACACACACACACTCCACATGCC	15	C	0.3306	0.2759
NC_005100.4-219734978	AGAGCTCAGACCCAGCCTCCCAATCTG	25	C	0.2308	0.3504
NC_005100.4-219766115	GACTGTGGCACCTCCTCTCCTGATGGA	6	T	0.0325	0.032
NC_005100.4-219766115	GACTGTGGCACCTCCTCTCCTGATGGA	16	T	0.0082	0.4822
NC_005100.4-219796613	TGCCTGGTTACCCTCTCTCCTCCTTTA	6	G	0.0244	0.0241
NC_005100.4-219807249	AGGCTTCCTACTACCTCTCCAGGCCCT	25	C	0	0.4773
NC_005100.4-219976009	TTACAGGCTACACCTTCTCCCAGTCCA	22	A	0.0085	0.479
NC_005100.4-220002634	CTCCTGGCCACAGAGCCTCCTAACTCC	4	C	0	0.4793
NC_005100.4-220002634	CTCCTGGCCACAGAGCCTCCTAACTCC	15	G	0.1951	0.2612
NC_005100.4-220005188	AGCCTCCCTACGCAGTCTCCCCATGGT	12	G	0.0168	0.4743
NC_005100.4-220033140	CCCTCCCAAACCTCTGCTCCCATGTGC	1	C	0.1624	0.2041
NC_005100.4-220050935	TTAGTTACTACCAGCCCTCCAGATAGA	16	C	0.1913	0.2268
NC_005100.4-220063490	TGGCTCACAACCATCCCTCCGATTTCA	21	G	0.0083	0.4769
NC_005100.4-220092430	GGAGACCACACAGGCTCTCCGCAAGAG	21	G	0.2946	0.4263
NC_005100.4-220130389	AAGGATGTCACAACTGCTCCTTGTCCT	2	A	0.1379	0.3572
NC_005100.4-220130389	AAGGATGTCACAACTGCTCCTTGTCCT	23	G	0	0.48
NC_005100.4-220139712	GCTTCCTGAACCCTTCCTCCTGACCTT	22	G	0.0446	0.0605
NC_005100.4-220163922	AAAGTCATTACCTGACCTCCACACTTC	3	A	0	0.4773
NC_005100.4-220163922	AAAGTCATTACCTGACCTCCACACTTC	7	A	0	0.4773
NC_005100.4-220192528	CATCATCCTACAGTCCCTCCTCTGACA	2	A	0.1826	0.3814
NC_005100.4-220507590	TGCCTCTGAACTTATTCTCCACTCTTC	23	T	0	0.4755
NC_005100.4-220525519	CATGAGGACACACACACTCCTCAACTC	24	A	0	0.4787
NC_005100.4-220526016	ATGAGCCCCACCATTCCTCCCAACATG	14	T	0	0.4793
NC_005100.4-220555260	ATATGTGGAACCCACTCTCCAGCAATC	22	G	0	0.4773
NC_005100.4-220600465	CAGATAATCACGTATGCTCCATCTTCT	12	G	0.0342	0.0336
NC_005100.4-220725603	CCAGGTGGCACTCGGCCTCCGTTGCCT	14	G	0.1951	0.2723
NC_005100.4-220725603	CCAGGTGGCACTCGGCCTCCGTTGCCT	15	G	0.1463	0.1761
NC_005100.4-220733664	CCCAGAGAAACCAACTCTCCCTTAAGA	2	C	0.275	0.4747
NC_005100.4-220733664	CCCAGAGAAACCAACTCTCCCTTAAGA	3	C	0.2397	0.3421
NC_005100.4-220743601	CATAGGAACACAGGCTCTCCAACACTG	25	C	0.2195	0.4662
NC_005100.4-220762839	AAATGCAGCACCCCCACTCCCCTCCCA	27	A	0	0.4793
NC_005100.4-220794131	AGGCTGGCGACTCAAACTCCATCCCAG	2	G	0.1557	0.2686
NC_005100.4-220823418	AGAACATAGACATCACCTCCAGCTCCC	7	T	0.0083	0.4747
NC_005100.4-220824373	TTAAACACCACCGCTCCTCCCGCTTCC	3	A	0	0.4666
NC_005100.4-220824373	TTAAACACCACCGCTCCTCCCGCTTCC	27	C	0.009	0.4584
NC_005100.4-220827235	GATCAGAGGACTCCATCTCCAATTGAT	21	A	0	0.4833
NC_005100.4-220840998	TCTGCTTCCACCCCAGCTCCATTCCAG	3	T	0.25	0.302
NC_005100.4-220881098	GAAGACCCAACAACCTCTCCTGGCATT	27	T	0	0.482
NC_005100.4-220883704	AAACTCTTCACGTTGCCTCCATCTGCC	22	T	0	0.4793
NC_005100.4-220941923	CTGTGTCTCACATGCACTCCTGGTTTT	9	C	0.0813	0.1216
NC_005100.4-220956404	AAGGACATCACCCGAACTCCGTAAGAC	3	G	0.0522	0.0989
NC_005100.4-220976228	GAACTAATCACTGAGGCTCCATAGCAG	25	C	0.0085	0.4823

NC_005100.4-220979340	CCAAACATCACCTGTCCTCCACGTGCC	15	T	0	0.4807
NC_005100.4-221054445	GTCCACCTGACCTGCCCTCCACCCCGC	2	T	0	0.4793
NC_005100.4-221054445	GTCCACCTGACCTGCCCTCCACCCCGC	26	G	0	0.4793
NC_005100.4-221058453	GACATCACAACTAGGGCTCCCCACACA	3	C	0	0.032
NC_005100.4-221076398	GCTTCTATCACAAGTACTCCATGACCT	23	G	0.1333	0.1387
NC_005100.4-221090563	AAGTAAATTACCAGTGCTCCTCATAAT	3	G	0	0.4841
NC_005100.4-221097888	ATTCATATCACCTCTTCTCCCATATCA	5	A	0	0.4746
NC_005100.4-221113629	AAGAGGGATACACCCACTCCTTCCCAG	22	T	0.0721	0.0695
NC_005100.4-221141689	AGCTCACCAACACTTCCTCCCACACTC	27	C	0.1803	0.4934
NC_005100.4-221230874	AATGACGACACTGAGCCTCCTTTTGTC	1	A	0	0.4786
NC_005100.4-221278127	GCCTTCCCCACCCTCACTCCATCACAC	9	C	0	0.4868
NC_005100.4-221312327	AAACTTCTAACCCTCCCTCCTGCTGTG	21	T	0.2358	0.4893
NC_005100.4-221320766	ACCACGGGGACCATCTCTCCAGCACCA	9	G	0.2213	0.4193
NC_005100.4-221328704	AGGACACTGACCTGCTCTCCATAGCAG	9	G	0.0894	0.4952
NC_005100.4-221362948	CACACCCCTACCCTGTCTCCCAACCAC	12	C	0	0.4773
NC_005100.4-221362948	CACACCCCTACCCTGTCTCCCAACCAC	24	C	0.0492	0.4891
NC_005100.4-221504834	TTAAGGTGTACCATGGCTCCAAGGCTG	4	A	0	0.4718
NC_005100.4-221504834	TTAAGGTGTACCATGGCTCCAAGGCTG	8	G	0.0169	0.0333
NC_005100.4-221518289	TATTGTGAGACAGGGTCTCCTGTAGTC	2	A	0.3739	0.304
NC_005100.4-221518289	TATTGTGAGACAGGGTCTCCTGTAGTC	15	G	0.3739	0.304
NC_005100.4-221535716	CTGCCCGCCACTCCCTCTCCCTTCTGA	4	C	0.0826	0.0943
NC_005100.4-221569826	CTGCCTATGACTATAGCTCCAGGGGAT	15	A	0.0862	0.0825
NC_005100.4-221569826	CTGCCTATGACTATAGCTCCAGGGGAT	27	T	0.094	0.0896
NC_005100.4-221592964	TCCCTGGAGACAGGGCCTCCCTTTGTA	21	C	0.0333	0.0644
NC_005100.4-221631064	GTGGCTCTAACTCCAGCTCCAAGGGAT	21	A	0.1148	0.1904
NC_005100.4-221644209	ATCCAGCGCACTTCTCCTCCCTTATCA	22	T	0.0083	0.4803
NC_005100.4-221684958	AGCAGGAGGACCCAGCCTCCATCTCTC	1	A	0.0085	0.4823
NC_005100.4-221684958	AGCAGGAGGACCCAGCCTCCATCTCTC	3	C	0.0252	0.0249
NC_005100.4-221708169	TCTGGGTGAACAAGGACTCCAGCCTCT	3	T	0	0.4757
NC_005100.4-221791337	CTGCAGAAGACCTGCACTCCAGGACCA	23	G	0.2193	0.2088
NC_005100.4-221794030	ATCCACATGACAGCTACTCCTGAGACG	16	A	0	0.4764
NC_005100.4-221794030	ATCCACATGACAGCTACTCCTGAGACG	26	C	0	0.4718
NC_005100.4-221794262	CAGCAAACCACCCACGCTCCCCGCCCA	24	C	0.0081	0.0398
NC_005100.4-221794262	CAGCAAACCACCCACGCTCCCCGCCCA	27	A	0.0081	0.0398
NC_005100.4-221800177	TTCCTCTCCACCTGTCCTCCGCTCCTC	22	C	0.0732	0.1562
NC_005100.4-221804382	CCCCTCCTTACCTAGCCTCCCTACGAA	24	C	0.0513	0.05
NC_005100.4-221824402	TCTCCCCCAACGTGGTCTCCTCACCCT	12	G	0.125	0.1597
NC_005100.4-221836071	GCCGCATAAACTGCTTCTCCATACCTA	6	A	0.0089	0.4789
NC_005100.4-221844481	GTGATTATGACCAATGCTCCCCTCAAA	4	A	0.2281	0.4778
NC_005100.4-221852111	CCACCTGTAACGTCAGCTCCAGGGATC	8	T	0	0.48
NC_005100.4-221852111	CCACCTGTAACGTCAGCTCCAGGGATC	21	A	0.2975	0.389
NC_005100.4-221882216	CCCAGTGACACATCAGCTCCCACTGCA	24	T	0.0417	0.0566
NC_005100.4-221886736	CACACACACACACACCCTCCTTACTTG	14	A	0.1966	0.4947
NC_005100.4-221899857	AGGAGTCACACCTTTCCTCCTCACCTC	22	C	0.0789	0.0758
NC_005100.4-221905152	AAGGGACAAACCCCAGCTCCTGTGTGT	21	T	0.114	0.1953
NC_005100.4-221911336	CTCACTTGTACTCTCTCTCCCTCCCCT	7	T	0	0.4832
NC_005100.4-221933820	ACATGGCAAACAGGTTCTCCCCTTTTA	9	A	0.1083	0.4972
NC_005100.4-221935392	TATTGTGTAACAGTTTCTCCTAGATTG	14	T	0.0357	0.477
NC_005100.4-221935392	TATTGTGTAACAGTTTCTCCTAGATTG	24	A	0.0357	0.0521
NC_005100.4-221959210	GAGCAGGGAACACTCCCTCCATGGTGG	27	G	0.1261	0.1609
NC_005100.4-221992626	CTGCTGTGGACTCAGACTCCATTTCTC	12	T	0.2411	0.388

NC_005100.4-221992788	CTACCTTTAACTCCAGCTCCAGGGGAT	24	G	0.0174	0.4915
NC_005100.4-222022125	CAAATCCTAACTCCAGCTCCAGGGGCT	9	A	0.0084	0.4797
NC_005100.4-222072562	GCATCTCTAACCCCAGCTCCAGAAAGT	16	G	0.177	0.4981
NC_005100.4-222111338	CCAGCCCGGACCATAGCTCCCTGCACC	2	C	0.0083	0.4803
NC_005100.4-222111338	CCAGCCCGGACCATAGCTCCCTGCACC	7	C	0.0083	0.4803
NC_005100.4-222130913	GGCATCTCCACAGAGACTCCCTCCTTC	15	G	0.2389	0.3772
NC_005100.4-222133276	GGCTGTCTAACTCCCGCTCCAAGGGAT	5	G	0.2049	0.3667
NC_005100.4-222133276	GGCTGTCTAACTCCCGCTCCAAGGGAT	15	C	0.2459	0.3947
NC_005100.4-222136410	GCTAGTCCTACAAAACCTCCGTGCCCA	6	T	0.0252	0.4859
NC_005100.4-222142067	GGCTGTGAAACATTAGCTCCCTGGATC	7	G	0.25	0.32
NC_005100.4-222176208	CATATTCTGACATGCTCTCCTGGGTGA	3	T	0.0087	0.4817
NC_005100.4-222176208	CATATTCTGACATGCTCTCCTGGGTGA	4	A	0.0087	0.4817
NC_005100.4-222189822	GGTACTAACACCTAGCCTCCCTCTTAC	21	C	0.2566	0.4547
NC_005100.4-222222267	CGCCGACACACAGGTGCTCCTGGAGAC	1	C	0.0246	0.0243
NC_005100.4-222244056	CCACTCTTGACCCACCCTCCACCCAGT	13	C	0	0.4778
NC_005100.4-222263799	TTTCTTAGGACGTTAACTCCATCCCCA	12	G	0	0.482
NC_005100.4-222281769	CACTCCCAGACCCTGACTCCTTTTCCC	1	C	0.0901	0.086
NC_005100.4-222294032	CGCCAATGCACACCCTCTCCATCAGGT	24	A	0	0.4718
NC_005100.4-222329699	ATGCCAACCACTCTTTCTCCTTACAGT	16	T	0.1917	0.4991
NC_005100.4-222336558	AACACCCAGACTGTAGCTCCTTTGATC	12	T	0	0.4755
NC_005100.4-222336558	AACACCCAGACTGTAGCTCCTTTGATC	26	T	0.0531	0.0517
NC_005100.4-222350962	CACACACACACATGTTCTCCACTTACA	2	A	0.0328	0.0322
NC_005100.4-222363460	GGTAGCACAACATGGGCTCCAGATGCT	3	T	0	0.4871
NC_005100.4-222388583	ATCAGGGGAACTCCACCTCCCCAGCTG	2	T	0.05	0.0488
NC_005100.4-222416470	TTTGCGTACACCCACGCTCCCATCGAG	12	C	0.0667	0.0644
NC_005100.4-222420167	CAACCAAAGACTGTAACTCCAGGACAG	16	A	0	0.4793
NC_005100.4-222507726	GAATCTCACACAGGCTCTCCTTCAACT	8	A	0.1983	0.5
NC_005100.4-222583351	TGCAGCCCAACGCCTGCTCCACCTACT	2	G	0	0.4773
NC_005100.4-222620201	TCCTTATGGACTTCTGCTCCAGTGTTA	26	T	0.175	0.4983
NC_005100.4-222641457	TTGATCCTGACCCATCCTCCAGTTGCT	26	C	0.042	0.0411
NC_005100.4-222685913	AAGCTCATAACCTGTACTCCAGGGATC	14	G	0	0.4787
NC_005100.4-222700528	AATACCACTACGCTACCTCCCTAGCCC	4	A	0	0.4749
NC_005100.4-222701554	GGAAAGAGCACTGGACCTCCCGAGCTG	2	G	0	0.4779
NC_005100.4-222701554	GGAAAGAGCACTGGACCTCCCGAGCTG	6	G	0.0588	0.0571
NC_005100.4-222779617	TGTTTATGGACTGCTGCTCCTTGTTAG	25	T	0.1074	0.2474
NC_005100.4-222840837	CAGTCCTAGACGTGCCCTCCATCTCAC	26	A	0.0413	0.4889
NC_005100.4-222866725	TATAATAAAACCTCTTCTCCGTTCCCT	24	C	0.0598	0.074
NC_005100.4-222877807	TGCTTATTGACTTGCTCTCCGTGGCAT	3	C	0.2541	0.2218
NC_005100.4-222881054	GCAGTGGCAACTGTACCTCCAGCAGCC	3	A	0.0325	0.032
NC_005100.4-222881054	GCAGTGGCAACTGTACCTCCAGCAGCC	7	G	0.1301	0.1356
NC_005100.4-222928153	ATCATTGTTACATCTGCTCCTGTGTCT	25	T	0.2212	0.3081
NC_005100.4-222928153	ATCATTGTTACATCTGCTCCTGTGTCT	27	T	0.2212	0.3081
NC_005100.4-222935191	ACTAATACAACACTCTCTCCTCCAACA	22	C	0	0.4785
NC_005100.4-223490549	CTGCCCACAACTCTGACTCCAGAGGAA	3	G	0.0246	0.0243
NC_005100.4-223490549	CTGCCCACAACTCTGACTCCAGAGGAA	7	A	0.0246	0.0243
NC_005100.4-223570953	GGGCCTGTCACTGCACCTCCATTTCCG	16	C	0.0085	0.0253
NC_005100.4-223570953	GGGCCTGTCACTGCACCTCCATTTCCG	26	C	0	0.4734
NC_005100.4-224472754	CTAGCTCCAACAGTCTCTCCTCTTCCA	6	T	0	0.4832
NC_005100.4-224472754	CTAGCTCCAACAGTCTCTCCTCTTCCA	12	A	0.15	0.2665
NC_005100.4-224500560	TACCAACCAACTAGAGCTCCTCGGCAT	27	T	0	0.4856
NC_005100.4-224547297	GACCCAGTAACCTAATCTCCCCAGCTC	13	T	0.115	0.4944

NC_005100.4-224659493	AGTGTAGCAACACTGGCTCCTGAGCAA	16	G	0.0984	0.4934
NC_005100.4-224782397	TTGACGTCCACTTCACCTCCAGCAGCT	5	C	0.025	0.0247
NC_005100.4-224782397	TTGACGTCCACTTCACCTCCAGCAGCT	25	G	0.0667	0.4932
NC_005100.4-224799864	ATGGCAGGAACGGTGCCTCCCATGCCC	12	G	0.1057	0.1425
NC_005100.4-224806883	TCTCACAGAACAACAACTCCCATGTTT	16	A	0	0.4787
NC_005100.4-224829741	TACACAGTAACCTGTCCTCCCTCAGCC	24	A	0.1478	0.4994
NC_005100.4-224834583	GGGATGTCTACACCAACTCCTTGGAAC	23	G	0	0.4832
NC_005100.4-224845338	TGTTCCTGGACCTTGGCTCCTGTTATT	2	G	0.0261	0.0257
NC_005100.4-224845338	TGTTCCTGGACCTTGGCTCCTGTTATT	8	G	0.0265	0.0262
NC_005100.4-224890205	GAGAGAGACACTTGAGCTCCTACAATC	3	G	0.287	0.3814
NC_005100.4-224890205	GAGAGAGACACTTGAGCTCCTACAATC	12	T	0.287	0.3814
NC_005100.4-224894737	TGGAGGCAAACACCATCTCCATCCCAG	25	C	0.181	0.1786
NC_005100.4-224907661	AATCCATAAACCTGTACTCCGATCCCT	25	C	0	0.4746
NC_005100.4-224910033	CTGTCTGTAACTTCAGCTCCAAGGGAC	9	A	0.0088	0.4861
NC_005100.4-225008946	TTATGGGAAACTAACTCTCCCTCTTCT	1	T	0	0.4793
NC_005100.4-225008946	TTATGGGAAACTAACTCTCCCTCTTCT	7	G	0	0.4793
NC_005100.4-225067951	CACCCGTCCACCCTGTCTCCACGTGAC	12	C	0	0.4793
NC_005100.4-225076950	ATAGAACCCACGCCACCTCCCGCGCCT	5	A	0.4054	0.3232
NC_005100.4-225076950	ATAGAACCCACGCCACCTCCCGCGCCT	16	C	0	0.4671
NC_005100.4-225084854	AAAACGTTAACTATCACTCCGGGGGCA	23	G	0.0431	0.4803
NC_005100.4-225100804	TGCTCTCTGACCTCACCTCCTACCTGT	23	C	0	0.4793
NC_005100.4-225165553	TTCTAAGCTACATGTTCTCCCTTTGCA	12	A	0.2051	0.3261
NC_005100.4-225226572	AATGAAGCAACCTTGTCTCCAAATAGA	12	C	0.0924	0.188
NC_005100.4-225242940	CTTCAAGTTACATCTTCTCCCACCTTG	3	T	0.3125	0.3316
NC_005100.4-225259866	GTCCTTCTGACTTGACCTCCTGACTAG	26	A	0.2541	0.4056
NC_005100.4-225269500	CTGCTCGGAACTGTAGCTCCCGGGTAT	14	T	0.1513	0.1539
NC_005100.4-225269500	CTGCTCGGAACTGTAGCTCCCGGGTAT	21	C	0.1513	0.1539
NC_005100.4-225300077	AGTGTAAAGACAGAGACTCCGTCCAGT	21	G	0.1624	0.4626
NC_005100.4-225300077	AGTGTAAAGACAGAGACTCCGTCCAGT	22	T	0	0.4864
NC_005100.4-225310205	GCAACTCCTACACACTCTCCTGCCACA	22	G	0.0342	0.05
NC_005100.4-225613725	ATAAGAAGGACACACTCTCCACTATGT	25	T	0.0517	0.0825
NC_005100.4-225624899	ATATATGGTACAGATTCTCCCATAGCC	1	A	0.2288	0.2026
NC_005100.4-225668984	ATGTGCATAACACCCACTCCTTGTCAA	23	G	0.225	0.4297
NC_005100.4-225947972	CCAGAGGGCACCACGTCTCCAGACACC	13	A	0	0.4765
NC_005100.4-225951227	GGCCCTTGCACTGAGTCTCCCTATCCC	8	G	0.0085	0.479
NC_005100.4-225965197	TCTGGCTAGACTGAAACTCCCACTGTA	26	T	0.0522	0.0671
NC_005100.4-226054111	TTTTGGTATACTAGGTCTCCTATACAC	22	A	0.0246	0.4717
NC_005100.4-226098825	CCTGATAACACATAGTCTCCATGCCCA	8	A	0.1667	0.2719
NC_005100.4-226140953	AGGTGATAGACCTGCACTCCCACCCTT	12	C	0.1696	0.2385
NC_005100.4-226198630	GTCTTGGGTACTTCTGCTCCATGTCTG	7	G	0	0.0342
NC_005100.4-226210733	CCAAGGCTCACAGTTTCTCCCAGTTTA	21	C	0.2119	0.4971
NC_005100.4-226211402	AGGAGGACAACCATGACTCCTAATCCT	2	G	0.065	0.078
NC_005100.4-226211402	AGGAGGACAACCATGACTCCTAATCCT	22	A	0	0.4793
NC_005100.4-226213235	CATATGCATACACTCACTCCAGTGAGT	5	T	0.2174	0.4946
NC_005100.4-226218456	GCACTGGAGACCTGAACTCCCATCTGT	3	A	0.2906	0.4602
NC_005100.4-226218456	GCACTGGAGACCTGAACTCCCATCTGT	22	A	0.2906	0.3729
NC_005100.4-226227523	CACCTCCACACTAGATCTCCTCAGGAC	4	C	0.2523	0.349
NC_005100.4-226232292	ATGGCGATCACGTCAACTCCCACCGGG	4	G	0.0259	0.0255
NC_005100.4-226232292	ATGGCGATCACGTCAACTCCCACCGGG	24	C	0.2845	0.343
NC_005100.4-226236781	GCTCCCATCACCAGTACTCCCATCAGT	2	C	0.386	0.3115
NC_005100.4-226240259	GAGCTCCAGACTTGGGCTCCATCTGTC	6	C	0	0.4728

NC_005100.4-226242183	TTATTACCCACTTCATCTCCTCAGTTG	7	C	0.2276	0.4944
NC_005100.4-226242183	TTATTACCCACTTCATCTCCTCAGTTG	25	T	0.0163	0.4825
NC_005100.4-226252533	GCTCACTCCACTTCAGCTCCACAGACT	5	A	0.2602	0.3604
NC_005100.4-226276105	ACCCGTCTCACCCAATCTCCCCAGGCA	15	A	0.0667	0.4911
NC_005100.4-226276105	ACCCGTCTCACCCAATCTCCCCAGGCA	22	C	0	0.4765
NC_005100.4-226295000	TGGGCACTAACCACAGCTCCCTCTTTC	9	A	0.0082	0.4822
NC_005100.4-226314348	TAGCTGCTGACCCTTCCTCCACCTTCC	27	C	0	0.4793
NC_005100.4-226324115	ACCTCCCCAACATCCCCTCCAGCTTCC	6	C	0.0661	0.0639
NC_005100.4-226333172	GTAGAGTTCACCTCAGCTCCTATTCCT	5	A	0	0.4718
NC_005100.4-226333172	GTAGAGTTCACCTCAGCTCCTATTCCT	12	C	0.0259	0.0255
NC_005100.4-226339434	TTTGACACCACCACAACTCCAGAAGAT	15	A	0.1066	0.1839
NC_005100.4-226355099	CAGGATACCACACAAGCTCCTTACACA	21	T	0.0175	0.0345
NC_005100.4-226355099	CAGGATACCACACAAGCTCCTTACACA	22	T	0.0789	0.138
NC_005100.4-226367794	AGCATGAGGACCAGACCTCCTGAACTG	22	G	0.0569	0.0705
NC_005100.4-226371042	ATCTCCTGGACTCCCTCTCCCTGTGAG	7	T	0.252	0.319
NC_005100.4-226395356	GGGAGTCAAACCGGGGCTCCACACATG	6	T	0.0488	0.0476
NC_005100.4-226395356	GGGAGTCAAACCGGGGCTCCACACATG	15	G	0.0569	0.0705
NC_005100.4-226400712	TTGCCAACAACCCCACCTCCACCCCGG	4	C	0.0833	0.095
NC_005100.4-226400712	TTGCCAACAACCCCACCTCCACCCCGG	13	C	0.2689	0.3118
NC_005100.4-226402705	CACACACACACACATCCTCCCACCACC	15	T	0	0.4807
NC_005100.4-226404635	GTGTGTTGGACATTTGCTCCCAGCTTT	3	G	0.0813	0.0928
NC_005100.4-226429601	TGGGCCATTACAAGCTCTCCACTATTA	23	T	0	0.4682
NC_005100.4-226452848	GAAGACAGGACATTACCTCCATAGCCT	15	A	0.15	0.4978
NC_005100.4-226465894	AACCACTGAACCGCCTCTCCAGCTCTC	23	C	0.2727	0.4557
NC_005100.4-226465894	AACCACTGAACCGCCTCTCCAGCTCTC	27	C	0.0492	0.048
NC_005100.4-226478205	CAGCATTTCACCTTTGCTCCTTGGGAT	3	G	0.2562	0.4213
NC_005100.4-226489854	ACCTTAATTACTTCAGCTCCTAGCTGA	2	C	0.0179	0.0689
NC_005100.4-226489854	ACCTTAATTACTTCAGCTCCTAGCTGA	23	G	0.0268	0.0264
NC_005100.4-226499456	TAGATCTATACCCAGCCTCCTCCTTAC	2	A	0.1681	0.4844
NC_005100.4-226499456	TAGATCTATACCCAGCCTCCTCCTTAC	21	T	0	0.4813
NC_005100.4-226513300	CATACACTCACATCCTCTCCCTTTAGC	26	G	0.0574	0.1009
NC_005100.4-226522655	CCCAGGGGCACAGCTTCTCCCTCAAGT	8	G	0.0169	0.0333
NC_005100.4-226524840	TACAGCTGCACATTCCCTCCACACTTA	21	A	0	0.4755
NC_005100.4-226611519	GACGTTAGGACCCTTGCTCCAGAGGCC	7	A	0.0833	0.095
NC_005100.4-226814482	GGGCCTTGTACATCATCTCCCATTGTG	1	G	0.1885	0.2218
NC_005100.4-226814482	GGGCCTTGTACATCATCTCCCATTGTG	15	A	0.0163	0.032
NC_005100.4-226848125	GTGATAAGGACTTGGGCTCCTCTACAC	1	G	0	0.4864
NC_005100.4-226848125	GTGATAAGGACTTGGGCTCCTCTACAC	6	A	0.1034	0.4987
NC_005100.4-226850749	CTGTCCTGTACCCCAACTCCTGCCGTG	15	A	0.2358	0.4572
NC_005100.4-226850749	CTGTCCTGTACCCCAACTCCTGCCGTG	24	C	0.122	0.1826
NC_005100.4-226912462	CTCGTCAGGACCCAGACTCCCAGAACT	14	A	0.2759	0.3835
NC_005100.4-226912462	CTCGTCAGGACCCAGACTCCCAGAACT	25	A	0.0086	0.4729
NC_005100.4-226916669	GAACTTACAACTTAAGCTCCTCGGTTC	23	G	0.1795	0.3771
NC_005100.4-226916669	GAACTTACAACTTAAGCTCCTCGGTTC	26	T	0.0086	0.4866
NC_005100.4-226922605	CAGCACGCCACGTGGGCTCCACACTGT	6	C	0.1322	0.1653
NC_005100.4-226979538	GAAAAGGACACTGGGACTCCGGTCTCT	21	G	0.3033	0.4379
NC_005100.4-227006542	GTGACTTTAACTCAGACTCCTCAGTTT	25	T	0.2679	0.4004
NC_005100.4-227015582	CACACAGACACCCACACTCCCCCACAG	4	A	0.2927	0.4206
NC_005100.4-227020462	GTTGCTTCCACCATGCCTCCAGAGGGG	24	G	0	0.4779
NC_005100.4-227020462	GTTGCTTCCACCATGCCTCCAGAGGGG	26	G	0.1345	0.1539
NC_005100.4-227026371	TTGACCGACACTCTTCCTCCATCAAGT	6	C	0.1062	0.2302

NC_005100.4-227032379	TTAGCCACAACCTCTGCTCCGCATCCT	21	G	0.0957	0.1516
NC_005100.4-227307804	TTTCAAAGTACATGAGCTCCTCACTAT	24	C	0.114	0.1229
NC_005100.4-227307804	TTTCAAAGTACATGAGCTCCTCACTAT	25	T	0.114	0.1229
NC_005100.4-227329382	CTCTGTTTGACCACATCTCCAAAAGGA	26	G	0.0175	0.0677
NC_005100.4-227410462	AACCACAACACGGTCTCTCCAGTCCTT	27	T	0	0.4787
NC_005100.4-227462351	AACCTTTCTACATACTCTCCCACAGTA	12	A	0.1261	0.4026
NC_005100.4-227535692	ACACATCATACAGATTCTCCAGTTATT	26	T	0	0.4715
NC_005100.4-227544953	TTCTGATGCACATCAGCTCCAACAACA	14	C	0.1709	0.1841
NC_005100.4-227544953	TTCTGATGCACATCAGCTCCAACAACA	15	A	0.2696	0.3252
NC_005100.4-227650736	GTTTCATCCACCAAGCCTCCCAAGCTC	3	T	0.0517	0.0504
NC_005100.4-227687958	GTATGTGCTACGAGCTCTCCTTGCATT	12	G	0.0796	0.0926
NC_005100.4-227837489	TGACCAACAACAATAGCTCCTAACTAC	2	G	0.0992	0.0943
NC_005100.4-227866906	TGTTTCACCACATTTGCTCCCTAAAGT	15	T	0.0431	0.0903
NC_005100.4-227867536	ACCAAGAGCACTTTCCCTCCTAAACCC	3	C	0.0678	0.0965
NC_005100.4-227867748	GGCAGAAGCACTCTCACTCCAAAGATC	12	T	0.1301	0.1216
NC_005100.4-227867748	GGCAGAAGCACTCTCACTCCAAAGATC	25	A	0	0.4751
NC_005100.4-227967362	CAAGTCCTTACAGATCCTCCTTAGTTC	1	C	0.0574	0.071
NC_005100.4-227967362	CAAGTCCTTACAGATCCTCCTTAGTTC	9	T	0	0.4751
NC_005100.4-228059114	TCAGATGAAACACTCTCTCCAGGAGGT	25	G	0.1197	0.4325
NC_005100.4-228159317	ACACACACCACCAGCACTCCTGCTCAC	7	A	0.0593	0.4919
NC_005100.4-228159317	ACACACACCACCAGCACTCCTGCTCAC	8	C	0.0593	0.4919
NC_005100.4-228189292	ACATGTGCCACCACCTCTCCTTGTAGA	5	G	0	0.4793
NC_005100.4-228189292	ACATGTGCCACCACCTCTCCTTGTAGA	7	G	0	0.4793
NC_005100.4-228221784	TACCAATGAACCAGAGCTCCCAGAGAC	23	G	0.125	0.1172
NC_005100.4-228299828	CCTCCCTGCACCTCTCCTCCCCTCATG	7	T	0	0.4773
NC_005100.4-228336023	CTTTGCCACACACTCCCTCCCACTCCA	12	A	0.0769	0.0896
NC_005100.4-228350106	TGGCCCAGGACCTCACCTCCAGAACCT	1	T	0.3193	0.4082
NC_005100.4-228435509	ACCACTATTACACAGCCTCCTGTTCAT	15	G	0	0.4807
NC_005100.4-228481011	AAACTCTGCACATGAGCTCCCTGCACT	8	G	0.225	0.3441
NC_005100.4-228504743	AGAGAAAACACATCCACTCCCCCAATA	15	C	0.0702	0.0677
NC_005100.4-228510175	AGAGACCAGACTGCTTCTCCCTCCACA	26	C	0.1333	0.2888
NC_005100.4-228681418	AGGATCTAAACTCAATCTCCAGCACCT	3	G	0.1858	0.4493
NC_005100.4-228681418	AGGATCTAAACTCAATCTCCAGCACCT	26	C	0.0619	0.06
NC_005100.4-228688794	AGATATGCCACTTAGACTCCTGCAGAT	2	G	0.0083	0.0245
NC_005100.4-228750911	AGTCCTTTCACTCTTCCTCCCATGATT	16	C	0.0732	0.0705
NC_005100.4-228754623	TGAAATGGGACAATGTCTCCAGAGTCA	13	A	0.041	0.0557
NC_005100.4-228772836	AGAAATCTGACGTGGCCTCCCTGGCCC	26	C	0.0435	0.059
NC_005100.4-228772836	AGAAATCTGACGTGGCCTCCCTGGCCC	27	C	0.2414	0.4518
NC_005100.4-228785571	TCAAAATACACTGTATCTCCGTGTGTG	21	G	0.319	0.3617
NC_005100.4-228822971	TTCATCTTCACAGGTGCTCCAGATTGT	3	C	0.0088	0.476
NC_005100.4-228822971	TTCATCTTCACAGGTGCTCCAGATTGT	25	T	0.0175	0.4814
NC_005100.4-228964155	AACCCTTTCACTCTGGCTCCACCCATT	12	T	0	0.4718
NC_005100.4-229021946	AACCCTGGAACCAACTCTCCAGCTTGT	16	T	0.0336	0.0491
NC_005100.4-229107110	TCTGCATTTACAGAAGCTCCTTTTGCC	6	A	0.0446	0.0436
NC_005100.4-229108298	TATACCACCACAATGACTCCAACTACT	23	C	0.0246	0.0243
NC_005100.4-229225489	AGACATAAGACTTGGTCTCCAAGTGTG	4	C	0.0171	0.4918
NC_005100.4-229225489	AGACATAAGACTTGGTCTCCAAGTGTG	5	A	0.0513	0.4868
NC_005100.4-229402311	CTGGCATGGACTGCCACTCCCAGCAGT	16	A	0.2672	0.3793
NC_005100.4-229456949	AGACAATCTACTTGCTCTCCTTCTTTG	2	G	0.0407	0.0398
NC_005100.4-229483862	TTCCTTAGCACAACTCCTCCCAGACTA	24	A	0	0.482
NC_005100.4-229538241	TAGATATATACAATGTCTCCATTCCCT	3	G	0.2034	0.334

NC_005100.4-229644298	GGCTTCCATACTGATGCTCCAGAGGTC	27	C	0	0.4773
NC_005100.4-230015019	CACATAGGTACAGGCCCTCCACACCTT	24	C	0.2602	0.4
NC_005100.4-230087280	ATCACAGAAACCAGTTCTCCAACCAAA	25	A	0.0417	0.0722
NC_005100.4-230107172	AGTGCACAAACCTCAACTCCAAGTCAA	2	G	0	0.4786
NC_005100.4-230256934	TGCCAACAAACCCGAGCTCCCAGGGAT	4	C	0.2373	0.4677
NC_005100.4-230356487	ACCCATTCCACCAGACCTCCCCAATCT	1	A	0.1197	0.2107
NC_005100.4-230356487	ACCCATTCCACCAGACCTCCCCAATCT	22	C	0.2542	0.3138
NC_005100.4-230374838	TCCCTATGCACACTTCCTCCCCTTCAT	23	T	0.2683	0.4777
NC_005100.4-230498915	CCCATTCCTACCTTCACTCCAACCAAA	15	C	0.0088	0.026
NC_005100.4-230683355	TCCAACAAGACCACACCTCCTGCAACA	2	C	0.0708	0.0846
NC_005100.4-230710270	ACAGGACTAACAAGTTCTCCTCCTATT	22	C	0.2544	0.3836
NC_005100.4-230736158	ACCATTGGAACAAGTTCTCCCACCACA	8	G	0.0087	0.4817
NC_005100.4-230802147	AATGTGGACACTCCCACTCCTTCTTAA	6	G	0.0246	0.0243
NC_005100.4-230802147	AATGTGGACACTCCCACTCCTTCTTAA	26	A	0.0333	0.4888
NC_005100.4-230804829	TAGACTGATACTCACTCTCCTTGTTTT	2	A	0	0.4859
NC_005100.4-230804829	TAGACTGATACTCACTCTCCTTGTTTT	3	G	0	0.4859
NC_005100.4-230877526	TAAACATGCACATACACTCCATCATGT	1	T	0.2017	0.3771
NC_005100.4-230877526	TAAACATGCACATACACTCCATCATGT	2	A	0.2017	0.3771
NC_005100.4-230933181	AACAACAACACTACATCTCCATTACCA	2	A	0	0.4891
NC_005100.4-230933181	AACAACAACACTACATCTCCATTACCA	8	A	0	0.4891
NC_005100.4-230978492	TGGACTTTAACTATAGCTCCAGGATAT	4	A	0.0082	0.4755
NC_005100.4-230994570	CTGTCCTACACCCATCCTCCCCTGTTA	12	C	0.188	0.272
NC_005100.4-231082496	ACCACAGAAACACAGTCTCCACTTCCT	5	C	0.1736	0.259
NC_005100.4-231082496	ACCACAGAAACACAGTCTCCACTTCCT	12	A	0.1736	0.4933
NC_005100.4-231151815	GACCTGAACACAGAGTCTCCTAGGTTT	13	G	0.0598	0.2171
NC_005100.4-231151815	GACCTGAACACAGAGTCTCCTAGGTTT	16	T	0.1379	0.4851
NC_005100.4-231158584	AGCTAAAGAACTTCAGCTCCTCAGAGT	14	C	0.0517	0.0504
NC_005100.4-231158584	AGCTAAAGAACTTCAGCTCCTCAGAGT	22	C	0.0517	0.0504
NC_005100.4-231204070	CCTGCCCTCACACAGACTCCTCAGCAC	15	G	0.0656	0.4979
NC_005100.4-231248928	ATGGTGCTGACCACAACTCCACCAGCT	25	G	0.0265	0.0765
NC_005100.4-231292639	ACCATGACTACCATGGCTCCCCATTCA	27	A	0.1167	0.5
NC_005100.4-231316265	TAAATCACCACACAGGCTCCAGGTGTG	25	G	0.15	0.1387
NC_005100.4-231358288	CAGTATATCACACTCCCTCCATCCTAC	6	T	0.0732	0.0854
NC_005100.4-231358288	CAGTATATCACACTCCCTCCATCCTAC	8	T	0.0732	0.4935
NC_005100.4-231369245	TTGCGCTGTACGTATGCTCCTGGTTTA	4	C	0	0.4793
NC_005100.4-231369245	TTGCGCTGTACGTATGCTCCTGGTTTA	16	G	0.0085	0.4776
NC_005100.4-231381727	CATTTGCTTACTATTGCTCCTGGGGAT	7	C	0.0325	0.4854
NC_005100.4-231381727	CATTTGCTTACTATTGCTCCTGGGGAT	15	T	0.0488	0.0629
NC_005100.4-231467727	AACAGAGCAACAAGTCCTCCTAACCAT	27	T	0.1803	0.4934
NC_005100.4-231550677	TATTTGATTACACTCCCTCCCATCCTG	22	A	0	0.4787
NC_005100.4-231550677	TATTTGATTACACTCCCTCCCATCCTG	27	G	0.0583	0.0722
NC_005100.4-231585045	GGCCACACTACTGTCACTCCCTGAGAC	7	A	0.1593	0.2797
NC_005100.4-231585045	GGCCACACTACTGTCACTCCCTGAGAC	13	G	0.1593	0.4934
NC_005100.4-231599588	CCATCAGGCACCCATTCTCCCTGTCTC	24	T	0.0667	0.0644
NC_005100.4-231614736	TTTCTGTCCACTTGAGCTCCTAGGCAC	27	C	0.2119	0.2871
NC_005100.4-231639068	CTGTGCACTACAACCTCTCCTGAGAGA	16	T	0.2833	0.4444
NC_005100.4-231639068	CTGTGCACTACAACCTCTCCTGAGAGA	27	A	0.2	0.2994
NC_005100.4-231703503	CAGCCTGCAACTTCCGCTCCAAGGCAT	14	C	0.0614	0.0595
NC_005100.4-231703503	CAGCCTGCAACTTCCGCTCCAAGGCAT	15	C	0	0.4887
NC_005100.4-231843888	CTGATGTGTACCCCACCTCCCAATGGG	24	T	0.0442	0.06
NC_005100.4-231843888	CTGATGTGTACCCCACCTCCCAATGGG	25	G	0.2301	0.2913

NC_005100.4-231851427	CCCTCTCTGACGCAGCCTCCTGATTCT	13	C	0.0593	0.0576
NC_005100.4-231851427	CCCTCTCTGACGCAGCCTCCTGATTCT	21	T	0.0847	0.0965
NC_005100.4-231869498	ACCTATGCCACCCTTGCTCCTTCCTGT	3	C	0.1858	0.3683
NC_005100.4-231919065	CAGGAAGGTACTTGTTCTCCCAATGTG	21	C	0.1453	0.4977
NC_005100.4-231947880	AAACCAGGAACATCATCTCCCTTTATT	24	T	0.2845	0.4313
NC_005100.4-231973580	AAGAGTGAAACCACGCCTCCATGAAGA	3	G	0.1148	0.402
NC_005100.4-231981037	TAATATCTCACAATTGCTCCATCTCAG	8	T	0.0348	0.4425
NC_005100.4-231981037	TAATATCTCACAATTGCTCCATCTCAG	14	T	0.1379	0.1855
NC_005100.4-231986264	TATGCTATAACTCTGCCTCCCTGTTCA	13	C	0.3136	0.2644
NC_005100.4-232042303	CTCCCCCAAACCCTGCCTCCTGACACT	27	T	0.025	0.0247
NC_005100.4-232065869	AGATCTTTCACTGGTTCTCCCATTGGT	8	T	0.0696	0.0989
NC_005100.4-232143027	TCCAATAAGACCAAACCTCCTCCAACA	13	A	0.1379	0.5
NC_005100.4-232227631	AGAACAAGGACACATGCTCCACTACAT	23	T	0.0504	0.0491
NC_005100.4-232288820	AAATGTACTACAGTATCTCCTCTTTTC	25	T	0.1161	0.1699
NC_005100.4-232335569	ATCCCTCACACCTCATCTCCTTCATTG	1	A	0	0.4728
NC_005100.4-232386487	GTACCTGCAACTCTGTCTCCTTGTCTG	6	T	0.0738	0.1296
NC_005100.4-232386487	GTACCTGCAACTCTGTCTCCTTGTCTG	9	A	0.2541	0.3008
NC_005100.4-232556377	AAGACAATGACTGCATCTCCTCAAGTC	25	G	0.1417	0.1866
NC_005100.4-232611997	AATATGGTCACAAAGTCTCCACAGATT	14	A	0.0172	0.4709
NC_005100.4-232611997	AATATGGTCACAAAGTCTCCACAGATT	15	G	0.0172	0.4709
NC_005100.4-232614115	TATCTTTATACAGGATCTCCAAACAAA	3	T	0.2212	0.2618
NC_005100.4-232628307	CACATTTGCACTTATTCTCCAGGCAAT	9	C	0.2917	0.395
NC_005100.4-232634937	CTCCCACCAACAGTGGCTCCTCTTTCC	7	C	0.0656	0.0786
NC_005100.4-232634937	CTCCCACCAACAGTGGCTCCTCTTTCC	14	T	0.0328	0.0322
NC_005100.4-232697491	TCTCCTTCAACCCACCCTCCACGAGTT	5	C	0.094	0.1049
NC_005100.4-232745050	AACACATGAACATCCTCTCCTGTGATG	14	C	0.2231	0.4835
NC_005100.4-232936133	ATATGGACTACAGACCCTCCAACAAAA	4	T	0.1695	0.3436
NC_005100.4-232936133	ATATGGACTACAGACCCTCCAACAAAA	7	A	0.0932	0.2156
NC_005100.4-232963580	TACCAACCAACTAGAGCTCCCAGTGAC	22	A	0.2655	0.2302
NC_005100.4-232982125	ATCCTAATCACCTTGGCTCCTATGTAG	6	A	0.1293	0.2057
NC_005100.4-232982125	ATCCTAATCACCTTGGCTCCTATGTAG	24	G	0	0.488
NC_005100.4-233001825	ATTTGTGCCACACTGCCTCCGTGTAAT	13	C	0.1271	0.4336
NC_005100.4-233001825	ATTTGTGCCACACTGCCTCCGTGTAAT	21	G	0.1695	0.4173
NC_005100.4-233004213	CTAGAAGACACCAGCTCTCCCGGAGCT	21	C	0.1207	0.1576
NC_005100.4-233004213	CTAGAAGACACCAGCTCTCCCGGAGCT	22	G	0.0171	0.0336
NC_005100.4-233026470	AAAAGACAGACTACCACTCCAACGGAA	8	A	0.2759	0.4463
NC_005100.4-233026470	AAAAGACAGACTACCACTCCAACGGAA	24	G	0	0.482
NC_005100.4-233034835	CAAGGATGCACTTAACCTCCACCTCCA	1	C	0.1207	0.1431
NC_005100.4-233050401	ATCCACTGGACCTGGGCTCCCCCTATC	21	C	0.2564	0.3813
NC_005100.4-233050401	ATCCACTGGACCTGGGCTCCCCCTATC	22	C	0.2479	0.321
NC_005100.4-233131843	CTATGAACCACAGAACCTCCCAGGCTA	24	G	0.041	0.0401
NC_005100.4-233139656	AAATGTGTAACTCCAGCTCCAGAGTAT	2	A	0.1138	0.1073
NC_005100.4-233170831	GGTCTGAGGACAGCTTCTCCTCCATCT	16	T	0.0087	0.4878
NC_005100.4-233189200	ATACATTTGACCCTCTCTCCTCGGCTT	2	T	0.1261	0.1469
NC_005100.4-233189200	ATACATTTGACCCTCTCTCCTCGGCTT	23	G	0.157	0.4339
NC_005100.4-233191131	AGCCTGACCACTTACGCTCCATACACA	6	G	0	0.4637
NC_005100.4-233191131	AGCCTGACCACTTACGCTCCATACACA	13	T	0	0.4654
NC_005100.4-233234796	GTTTGGCTCACCTGGCCTCCTTCCAGA	8	T	0	0.4659
NC_005100.4-233248824	CATGAGATCACTGTCTCTCCACACCCC	7	A	0	0.4793
NC_005100.4-233260978	CACATGTAAACACTCGCTCCTGAATAC	12	A	0.041	0.0557
NC_005100.4-233369113	AGAAGCAAGACTCTGCCTCCTCCTGAG	27	G	0.1368	0.2235

NC_005100.4-233422784	CCAGCCTAAACCCAGCCTCCCTGTGAC	2	C	0.1228	0.1744
NC_005100.4-233422784	CCAGCCTAAACCCAGCCTCCCTGTGAC	8	A	0.0789	0.0758
NC_005100.4-233498512	CCTCTACCTACAGTAGCTCCTTCTCCT	5	T	0	0.4787
NC_005100.4-233517090	GAACAACTGACATGTTCTCCTCTGGAT	1	G	0.0656	0.0786
NC_005100.4-233549008	GTTTTCAGTACTTTTCCTCCCTGTTAT	5	T	0.0526	0.0839
NC_005100.4-233563646	TCCTGTTTTACATGTGCTCCAGTATGG	27	G	0.1062	0.1613
NC_005100.4-233595690	AGAAAAGCAACTATATCTCCCCTAAGA	16	T	0	0.4807
NC_005100.4-233681201	TTCCTGGATACTACCCCTCCATGATAT	13	A	0.0083	0.4783
NC_005100.4-233681201	TTCCTGGATACTACCCCTCCATGATAT	27	T	0.025	0.0247
NC_005100.4-233684327	CTCCAGTAGACAGGGCCTCCAGCTCAG	25	C	0.2017	0.4615
NC_005100.4-233762841	GACACCTTCACCTTATCTCCTGCTTAA	1	G	0	0.0351
NC_005100.4-233773582	CACTACCTGACCTTGTCTCCTCGCACA	23	G	0.1579	0.1454
NC_005100.4-234132090	AGACCAAGGACTTTTCCTCCTGTTGAT	2	G	0.4667	0.3578
NC_005100.4-234132090	AGACCAAGGACTTTTCCTCCTGTTGAT	5	C	0.4583	0.3533
NC_005100.4-234192080	ACCTCACTAACCTCTGCTCCATCATCT	7	C	0.1157	0.1377
NC_005100.4-234192080	ACCTCACTAACCTCTGCTCCATCATCT	8	T	0.1417	0.2491
NC_005100.4-234238195	ATTCCTACCACCTCACCTCCAACCCCC	7	A	0.0569	0.4471
NC_005100.4-234238195	ATTCCTACCACCTCACCTCCAACCCCC	16	C	0.0407	0.1145
NC_005100.4-234265726	ACCTAGGACACCCACGCTCCTGATAGT	6	G	0	0.4793
NC_005100.4-234265726	ACCTAGGACACCCACGCTCCTGATAGT	15	C	0.2927	0.377
NC_005100.4-234332493	TTATCAGGGACAGCAGCTCCAGCAGCT	1	T	0.1062	0.4968
NC_005100.4-234332493	TTATCAGGGACAGCAGCTCCAGCAGCT	2	T	0.0351	0.4925
NC_005100.4-234362575	ACCATTGATACCTTTTCTCCCTTGGCA	9	T	0.1795	0.3596
NC_005100.4-234362575	ACCATTGATACCTTTTCTCCCTTGGCA	26	C	0.178	0.3664
NC_005100.4-234374059	AAACCGAGGACGCTTTCTCCCTCATAA	5	C	0.0631	0.4584
NC_005100.4-234374059	AAACCGAGGACGCTTTCTCCCTCATAA	12	G	0.0536	0.0853
NC_005100.4-234396099	ACCTAGACTACTTGAGCTCCAGAACAG	24	A	0.0325	0.0629
NC_005100.4-234433095	CAACCCCAAACCCTTGCTCCTCTACTG	27	G	0.0609	0.0911
NC_005100.4-234440315	AAATCTGTGACACTCTCTCCCATTCTC	15	C	0.0259	0.0255
NC_005100.4-234461427	GAATCTCAGACTTGAACTCCTCCACAG	26	A	0.0991	0.4942
NC_005100.4-234491579	ACTTGTCTCACCTGAGCTCCTTAACGA	25	C	0.1171	0.4896
NC_005100.4-234491851	TAATCACTGACATAGACTCCGGTTCTT	21	G	0.0261	0.4744
NC_005100.4-234540203	AAAGAATGCACCGACACTCCCAGCCAC	9	C	0.0246	0.0243
NC_005100.4-234556619	AGAAGATGCACCTAACCTCCAAGAGAT	5	G	0.0902	0.1436
NC_005100.4-234577076	TAATCAACAACTCACCCTCCATCCTCC	4	T	0.1478	0.1369
NC_005100.4-234577076	TAATCAACAACTCACCCTCCATCCTCC	27	C	0.113	0.1067
NC_005100.4-234598655	ATTCTGAACACTGGGACTCCTGATAAT	3	T	0.2966	0.4273
NC_005100.4-234603115	GTTTCCTGTACATAAGCTCCCATTCCC	14	A	0.1441	0.1338
NC_005100.4-234623791	CAGATGCTAACAACATCTCCCAGCCCT	24	C	0.0696	0.4833
NC_005100.4-234639220	ATCCACTCCACCCACCCTCCCCAAATC	1	A	0.1207	0.1431
NC_005100.4-234674059	TTATGTAGAACATAGCCTCCTTGCTAC	27	C	0.0783	0.1219
NC_005100.4-234708727	AGAGAAGCCACATGTTCTCCAGCCGGG	22	G	0.1913	0.2395
NC_005100.4-234708727	AGAGAAGCCACATGTTCTCCAGCCGGG	25	G	0.2957	0.4301
NC_005100.4-234838317	GTTCTGTGAACTGGCCCTCCTCCTGTG	5	T	0.281	0.3967
NC_005100.4-234852329	AATTACAGCACAAATGCTCCACCTGTC	6	C	0.2917	0.4783
NC_005100.4-234862686	CGTCCTGTCACCCTACCTCCTGTGCTT	1	C	0	0.4891
NC_005100.4-234862686	CGTCCTGTCACCCTACCTCCTGTGCTT	16	C	0	0.4902
NC_005100.4-234867601	AAGTGACTAACACATTCTCCATAGTCA	5	G	0.2946	0.4566
NC_005100.4-234920734	CTGGGGAAGACTAATTCTCCCTCCTTA	3	G	0.0265	0.0262
NC_005100.4-234968464	AGACCGAAAACACAAACTCCCAGGCTT	12	A	0	0.4749
NC_005100.4-235006377	ATCCGTGTTACGTAGTCTCCTGCATTT	12	G	0.0167	0.0488

NC_005100.4-235022380	TCAGACCACACGAGCTCTCCAGCATAT	25	T	0	0.4765
NC_005100.4-235022534	ATTAAATATACCGAGTCTCCCAGGGAA	12	C	0.0917	0.0875
NC_005100.4-235022534	ATTAAATATACCGAGTCTCCCAGGGAA	22	A	0.0583	0.0875
NC_005100.4-235025884	AAACCTGCAACCCTGACTCCTACCCAG	21	T	0	0.4773
NC_005100.4-235025884	AAACCTGCAACCCTGACTCCTACCCAG	24	C	0.2705	0.4634
NC_005100.4-235035682	GTCTACTCCACCCATGCTCCTTGGTTG	6	C	0	0.4787
NC_005100.4-235035682	GTCTACTCCACCCATGCTCCTTGGTTG	12	C	0	0.4787
NC_005100.4-235048560	GATCTGCTCACTTTCTCTCCCTCTTTG	3	T	0.0179	0.496
NC_005100.4-235048560	GATCTGCTCACTTTCTCTCCCTCTTTG	7	C	0.0089	0.4933
NC_005100.4-235071466	GCAGTGGGTACAGCTGCTCCTTCCACT	13	G	0.075	0.4991
NC_005100.4-235109149	AACCAACTAACTCCCCCTCCAAACGAT	13	C	0.1356	0.4029
NC_005100.4-235109149	AACCAACTAACTCCCCCTCCAAACGAT	24	C	0.0504	0.0491
NC_005100.4-235147834	GTCTGTCCAACCCCTTCTCCCTTCCTT	2	T	0.0354	0.0348
NC_005100.4-235147834	GTCTGTCCAACCCCTTCTCCCTTCCTT	27	T	0.0357	0.0351
NC_005100.4-235183880	CTGTTGGTCACTGGAGCTCCAAGGTCA	16	G	0.0088	0.481
NC_005100.4-235183880	CTGTTGGTCACTGGAGCTCCAAGGTCA	24	G	0.0088	0.481
NC_005100.4-235188105	AAATTCGTGACTAATGCTCCGTTTATG	7	G	0	0.4813
NC_005100.4-235202557	GTGTCTACTACTGGGCCTCCAACCTCA	6	T	0.1382	0.1695
NC_005100.4-235225981	CCTTCGCTGACTGCCCCTCCTGTCCTA	1	C	0.2602	0.3688
NC_005100.4-235261155	CCTTGATTGACCTCCCCTCCACTCACA	8	T	0.3802	0.4923
NC_005100.4-235264063	GCATTTCTAACAGGTCCTCCCGGTCCA	21	C	0.2417	0.325
NC_005100.4-235296474	TGCTCTCTGACAGTTCCTCCTCCCATT	5	C	0	0.4793
NC_005100.4-235296474	TGCTCTCTGACAGTTCCTCCTCCCATT	8	T	0	0.4793
NC_005100.4-235317529	TGCTCTGGGACTCTGCCTCCTCATTTA	27	A	0.065	0.078
NC_005100.4-235342438	GGTGAGGAAACAGACCCTCCTTCTCTG	1	G	0.0081	0.481
NC_005100.4-235342438	GGTGAGGAAACAGACCCTCCTTCTCTG	12	A	0.0081	0.481
NC_005100.4-235353623	TGAGGCAGTACAAGTGCTCCTTCTCCA	27	A	0.0174	0.0342
NC_005100.4-235441974	CTGACCACCACCATTCCTCCAGGAATT	23	G	0.0244	0.0241
NC_005100.4-235463410	CAAATAACCACCCTCACTCCAGTCCCC	15	C	0.1712	0.2271
NC_005100.4-235520582	AATCTCTCGACCTGTACTCCTTGCTAG	8	C	0	0.4807
NC_005100.4-235636212	ATAAGTAGGACAAGTGCTCCAAGTGCT	24	T	0.0082	0.4755
NC_005100.4-235636212	ATAAGTAGGACAAGTGCTCCAAGTGCT	25	G	0.041	0.0401
NC_005100.4-235747838	ACCCTCACTACAAACCCTCCAGTCTAC	9	T	0.124	0.1447
NC_005100.4-235773547	CACCAATGGACTTGAACTCCAATTAAA	14	G	0.0085	0.481
NC_005100.4-235838380	CAAAGGGAAACAAAGACTCCATTAACA	25	A	0.0087	0.4744
NC_005100.4-235935553	ACCTATTAAACCTTGTCTCCCAAAAGG	3	C	0.0254	0.0251
NC_005100.4-235945131	AAATATGTCACCCTCTCTCCACGTCCC	23	G	0.0446	0.0436
NC_005100.4-235966027	TTCTGGACGACCTTTACTCCAGCTCAT	9	G	0	0.4592
NC_005100.4-235980739	TATCCAAAGACAGACTCTCCAGGAGTG	2	A	0.1092	0.1033
NC_005100.4-235980739	TATCCAAAGACAGACTCTCCAGGAGTG	16	T	0.1207	0.1134
NC_005100.4-236008256	GTAGAGTTCACTATCTCTCCACTCCCT	6	G	0.0331	0.0792
NC_005100.4-236011990	CTACCTGTAACTCCAGCTCCAGTGGCA	16	G	0.0744	0.1163
NC_005100.4-236063683	TTCTTCCCCACCCATGCTCCCTGCTGT	7	C	0.2353	0.4517
NC_005100.4-236063683	TTCTTCCCCACCCATGCTCCCTGCTGT	8	C	0.1405	0.1982
NC_005100.4-236144292	GGAAAGTGAACCACAGCTCCTGGGCAG	2	G	0.0833	0.4994
NC_005100.4-236144292	GGAAAGTGAACCACAGCTCCTGGGCAG	22	G	0.2185	0.4844
NC_005100.4-236187108	TGATCACTCACTGAGACTCCTGTGCTA	15	G	0.1803	0.4979
NC_005100.4-236187108	TGATCACTCACTGAGACTCCTGTGCTA	26	T	0.0164	0.0322
NC_005100.4-236228254	GAAAATTCCACTCAGCCTCCTGTGGCA	24	G	0.0164	0.0634
NC_005100.4-236231528	ACACACTAAACTGATTCTCCATTATTT	7	T	0.0084	0.4761
NC_005100.4-236231528	ACACACTAAACTGATTCTCCATTATTT	21	A	0.2941	0.4117

NC_005100.4-236284819	CAGGAGGGTACCCACCCTCCAAAGGAA	16	C	0.041	0.071
NC_005100.4-236284819	CAGGAGGGTACCCACCCTCCAAAGGAA	25	G	0.123	0.2095
NC_005100.4-236297739	AACCTCAGGACCTGGTCTCCATTGCCT	6	C	0.1626	0.2382
NC_005100.4-236297739	AACCTCAGGACCTGGTCTCCATTGCCT	16	T	0	0.4773
NC_005100.4-236299857	GATCACCCAACAGCCGCTCCTCTTTAA	7	C	0.0407	0.0398
NC_005100.4-236299857	GATCACCCAACAGCCGCTCCTCTTTAA	16	G	0.2114	0.2612
NC_005100.4-236327360	ACATGGGTTACAGACACTCCTTGGGAT	26	A	0	0.4891
NC_005100.4-236424707	AACCATTAAACCATCTCTCCATCCCCA	27	A	0.1478	0.1369
NC_005100.4-236454399	TCTGAACCAACCCTGCCTCCTGCCTCT	12	C	0.1111	0.1049
NC_005100.4-236466638	AGCTTCTCCACTAATACTCCCACAGCA	7	T	0.0085	0.481
NC_005100.4-236477734	CAAACTCCTACATGTCCTCCTTCACTT	24	A	0.1356	0.4964
NC_005100.4-236477734	CAAACTCCTACATGTCCTCCTTCACTT	25	C	0.1333	0.2432
NC_005100.4-236483082	CTTTAATGCACCAAAGCTCCCAGGAAG	13	A	0.1518	0.2512
NC_005100.4-236483082	CTTTAATGCACCAAAGCTCCCAGGAAG	25	A	0.1518	0.499
NC_005100.4-236496388	AAGTACCATACGCATGCTCCTATCGTG	24	C	0.1379	0.4927
NC_005100.4-236535214	TGACTCATAACCATCTCTCCAGGGCTG	14	T	0.1885	0.4755
NC_005100.4-236543056	AGTTAAAGCACAATTTCTCCTGTGTGA	23	T	0	0.4688
NC_005100.4-236581117	TGACCCGGGACTGCGCCTCCTTCATCA	14	C	0.114	0.499
NC_005100.4-236601569	GACATGTACACAGCAGCTCCTCTATTC	5	T	0.0328	0.048
NC_005100.4-236601569	GACATGTACACAGCAGCTCCTCTATTC	13	G	0	0.4773
NC_005100.4-236711389	ATAAATGGCACTGGGACTCCTGGGCAT	24	G	0	0.4793
NC_005100.4-236711611	CAGAAGGAGACATTCCCTCCCACACTG	5	A	0.0168	0.4844
NC_005100.4-236739278	GATTCACCTACCCTCTCTCCATCTCTA	15	C	0.1795	0.2171
NC_005100.4-236739535	CCATGATGGACTTCACCTCCTTAAAAC	3	A	0.1739	0.2138
NC_005100.4-236739535	CCATGATGGACTTCACCTCCTTAAAAC	14	C	0	0.4833
NC_005100.4-236751194	ACACAACCAACACAATCTCCACCCTCT	3	A	0.0179	0.4731
NC_005100.4-236899577	CATCTATCTACCTTACCTCCTCTTTCC	27	C	0.1327	0.4843
NC_005100.4-236899780	GATAGTCTCACAGTCACTCCAGGTCAG	24	T	0.0171	0.0336
NC_005100.4-236924618	GAGCACATCACAGGGTCTCCTAGATCT	22	A	0.1917	0.3347
NC_005100.4-236935252	GATAAGCCCACAGGTTCTCCACAGTCT	3	T	0.0932	0.1041
NC_005100.4-236935252	GATAAGCCCACAGGTTCTCCACAGTCT	25	T	0	0.4856
NC_005100.4-236948542	AGCTTGCTCACCATGCCTCCCCGACAC	22	C	0.2124	0.4934
NC_005100.4-236952323	TCACACCTTACACACTCTCCCCCTTCA	25	T	0.2544	0.2836
NC_005100.4-236996528	AGCTCAGGCACACTTTCTCCAGAAGCC	8	G	0.0877	0.1454
NC_005100.4-236996528	AGCTCAGGCACACTTTCTCCAGAAGCC	25	G	0	0.4755
NC_005100.4-237007712	ACCCTTTCTACAGTTTCTCCTGTCGTG	25	G	0.3136	0.3914
NC_005100.4-237024414	ATCCTCCTGACTACACCTCCCAAGTAC	14	C	0.0598	0.4473
NC_005100.4-237024414	ATCCTCCTGACTACACCTCCCAAGTAC	21	C	0.0336	0.0805
NC_005100.4-237163017	GACTTGTTCACTGAACCTCCTTCCCCT	23	C	0	0.4854
NC_005100.4-237163017	GACTTGTTCACTGAACCTCCTTCCCCT	24	C	0.1171	0.1857
NC_005100.4-237265843	TTCCACACCACTTCCCCTCCCATCTCA	22	A	0.2213	0.4634
NC_005100.4-237448882	GAGTCTCTCACTGGACCTCCTTTTCCT	22	T	0.3274	0.4242
NC_005100.4-237505570	CGTCTACTCACGGTGGCTCCTTGTGGG	2	G	0.1043	0.1295
NC_005100.4-237616596	ACTCTTCCAACTCCCCCTCCCCACACA	6	T	0	0.4793
NC_005100.4-237616596	ACTCTTCCAACTCCCCCTCCCCACACA	27	A	0.1545	0.1425
NC_005100.4-237617895	CTCCACACCACCTGACCTCCTGACTCC	27	C	0.2314	0.3729
NC_005100.4-237699609	TGTTCCCAAACTTCGCCTCCTTGCATA	14	C	0.1311	0.1225
NC_005100.4-237703068	ATCATGAACACGGATCCTCCCCCATCT	12	G	0.1947	0.4827
NC_005100.4-237703068	ATCATGAACACGGATCCTCCCCCATCT	24	A	0	0.4793
NC_005100.4-237713341	GCATTAATGACAGCCTCTCCAGTTCTG	14	C	0.1947	0.2556
NC_005100.4-237725791	AAAGATCCCACTCCCTCTCCTCACTTC	2	A	0.1074	0.4146

NC_005100.4-237787444	ACGGGACCTACCAGAGCTCCCTTTTAG	2	C	0.0175	0.0345
NC_005100.4-237787444	ACGGGACCTACCAGAGCTCCCTTTTAG	7	C	0.0174	0.0342
NC_005100.4-237958711	CTGTTTTACACTTATCCTCCTCCCATT	1	C	0.009	0.4531
NC_005100.4-237993868	ACTGTCTCCACCCTATCTCCCTACTCC	9	C	0.018	0.0354
NC_005100.4-238185105	CATCAGGTGACTACCCCTCCCATCTCT	1	C	0.0174	0.0508
NC_005100.4-238185105	CATCAGGTGACTACCCCTCCCATCTCT	24	C	0.1913	0.4891
NC_005100.4-238380889	TAACAAGTCACCCAAGCTCCATGACGA	26	G	0	0.48
NC_005100.4-238437830	TAATACTAAACCCTTACTCCCCATGCA	12	C	0.2435	0.4425
NC_005100.4-238452804	CCCCTAGTTACTCATCCTCCACTGTTA	22	C	0.0783	0.1219
NC_005100.4-238587006	AACAGTAAGACAACATCTCCCATCCAT	24	C	0.188	0.2361
NC_005100.4-238589566	AGTCAATAAACTATGGCTCCTGGACTT	15	G	0.0085	0.4738
NC_005100.4-238625421	TATCTGTCAACTGTCTCTCCATCCTAC	3	T	0.2368	0.3374
NC_005100.4-238625421	TATCTGTCAACTGTCTCTCCATCCTAC	22	T	0.2368	0.3999
NC_005100.4-238706619	GATGGTGATACAGTCACTCCTGACCAC	21	T	0.2542	0.4757
NC_005100.4-238765769	AGCTTCACCACCAGGCCTCCTCCATTC	16	C	0.065	0.1356
NC_005100.4-238795363	AGAGACAGGACAGGTTCTCCCTCCCCT	3	A	0.1667	0.1665
NC_005100.4-238812959	CAGGGTCATACAAACACTCCTGTCCAG	9	T	0	0.4786
NC_005100.4-238834521	AGCTGATTAACCATGTCTCCAGTATCA	1	A	0.0328	0.0322
NC_005100.4-238834521	AGCTGATTAACCATGTCTCCAGTATCA	6	A	0.1803	0.3354
NC_005100.4-238901770	TGTCGGTACACTCATCCTCCAGTGGAT	25	G	0.0087	0.4878
NC_005100.4-238930101	AAATGAGTCACAGACTCTCCCAGTGTT	24	T	0.0492	0.0786
NC_005100.4-239019582	ACTCTGAACACAGGTCCTCCCTAATCA	25	T	0.2667	0.4832
NC_005100.4-239022914	TACTACATCACCCCTGCTCCCATCCAG	4	T	0	0.4793
NC_005100.4-239202021	CACACACACACACACACTCCTGTGCTC	5	C	0.4553	0.3604
NC_005100.4-239202021	CACACACACACACACACTCCTGTGCTC	16	A	0.3008	0.2556
NC_005100.4-239227902	CAGGATGCCACGGTATCTCCATGTTGG	12	G	0.0496	0.0484
NC_005100.4-239227902	CAGGATGCCACGGTATCTCCATGTTGG	25	T	0.05	0.4832
NC_005100.4-239268235	CTGAATTGAACTACAACTCCAAGCATG	8	G	0	0.4793
NC_005100.4-239289016	GTGGTAGAAACATATTCTCCACATGTA	7	G	0	0.4773
NC_005100.4-239326657	AGCTTCTTCACTGCACCTCCCTGAATG	1	A	0.027	0.492
NC_005100.4-239326657	AGCTTCTTCACTGCACCTCCCTGAATG	4	T	0.1786	0.1913
NC_005100.4-239398204	CCGGCCCGAACTGCTTCTCCTACGGCG	9	A	0.009	0.4804
NC_005100.4-239405757	GGCCTGTGTACTCTGCCTCCTTTTGCT	13	C	0.0656	0.0634
NC_005100.4-239488816	GTCAGTGGTACTTATGCTCCCGATGTT	12	T	0.1833	0.3394
NC_005100.4-239527394	CCTTGCAGTACAGGACCTCCCCTTCTC	8	G	0.2542	0.4481
NC_005100.4-239618840	ACCACTATCACAACTGCTCCATTCCTC	9	C	0.0354	0.0348
NC_005100.4-239703498	ACTATTTCTACCTTATCTCCTCCTGTC	4	A	0	0.4624
NC_005100.4-239704472	CAAGATAAGACACCATCTCCCCTTTTG	4	G	0.0583	0.0722
NC_005100.4-239704472	CAAGATAAGACACCATCTCCCCTTTTG	13	C	0.0248	0.0245
NC_005100.4-239741099	AAATCCATAACTCTTCCTCCTAGAGAG	24	A	0.0087	0.4903
NC_005100.4-239814585	TCTTACATGACAGCTCCTCCTCCCTCC	21	T	0	0.4709
NC_005100.4-239814585	TCTTACATGACAGCTCCTCCTCCCTCC	26	C	0.181	0.2315
NC_005100.4-239840904	GGAAGGATCACTTGTGCTCCGGTCCAC	5	G	0	0.477
NC_005100.4-239841121	TGTGTGAGAACCTGATCTCCAAGTACT	15	A	0	0.4807
NC_005100.4-239884496	ACAAGTCCAACCCAACCTCCACTGATA	9	A	0.0252	0.0571
NC_005100.4-239884496	ACAAGTCCAACCCAACCTCCACTGATA	22	C	0.0252	0.0571
NC_005100.4-239903340	ATGGAATGGACCTTCCCTCCCCTTGAG	23	T	0.0984	0.4979
NC_005100.4-239917526	GGCTCTCAGACCTCTCCTCCCTCTGCC	6	T	0	0.4773
NC_005100.4-240017283	CAATTAAACACTCTTCCTCCTCACTTC	13	C	0.0569	0.0854
NC_005100.4-240031619	ATTATGTCTACTGCCCCTCCTTATTCC	15	C	0.1282	0.2041
NC_005100.4-240066934	GCACTGGCAACGACATCTCCACCTCAC	12	G	0.1322	0.4991

NC_005100.4-240070928	CCATGTCTTACATCCCCTCCCTCCCTG	25	C	0.0407	0.0398
NC_005100.4-240108844	CGGGAGCACACTGTCTCTCCTCATCCT	12	T	0.0083	0.4783
NC_005100.4-240133887	TGATGTAAGACGTTTCCTCCTCCCATT	24	C	0.0407	0.0398
NC_005100.4-240186571	GGAAGAGATACAAGGTCTCCCAACAGC	4	A	0	0.48
NC_005100.4-240186571	GGAAGAGATACAAGGTCTCCCAACAGC	7	G	0.1	0.4988
NC_005100.4-240193297	CTTTCCTTCACATCCCCTCCTCTGTCC	12	A	0.124	0.4988
NC_005100.4-240269163	GTATGGTCCACCTTTTCTCCATCAGGC	12	C	0.1913	0.4997
NC_005100.4-240289951	ACCTGGCATACCCTGGCTCCCAAACCC	4	T	0.1405	0.1306
NC_005100.4-240294408	CATGACCTGACACACCCTCCCTATCTA	21	C	0.1197	0.1563
NC_005100.4-240309820	CTCCACAGCACTCCCCCTCCTCTCCTG	1	C	0.0268	0.1093
NC_005100.4-240309820	CTCCACAGCACTCCCCCTCCTCTCCTG	4	C	0.0357	0.0351
NC_005100.4-240370737	GTCTAACTCACATCTTCTCCTTATCAG	4	T	0.0678	0.0655
NC_005100.4-240433939	ATACACTTCACTGGACCTCCATGAAGG	6	C	0.0619	0.0765
NC_005100.4-240482513	ACCGAAACAACTGTCCCTCCACCTGTC	4	G	0.187	0.4893
NC_005100.4-240499034	CCTGAAGGCACCCGTGCTCCCAGTGTC	8	G	0.05	0.4932
NC_005100.4-240582816	ATCCATATGACCATACCTCCAAAGGTC	16	C	0.5126	0.4045
NC_005100.4-240608471	TTGAGAAGAACGTGTTCTCCCTCTAGT	7	A	0.0265	0.4693
NC_005100.4-240608471	TTGAGAAGAACGTGTTCTCCCTCTAGT	15	T	0.5752	0.4242
NC_005100.4-240671404	AGAAAAGGCACACAGGCTCCCATTAGT	13	C	0.0504	0.0491
NC_005100.4-240671404	AGAAAAGGCACACAGGCTCCCATTAGT	15	G	0.0168	0.4743
NC_005100.4-240705320	CAGCACCCCACTCTCCCTCCTCAGTCC	24	G	0.0574	0.071
NC_005100.4-240742195	ACTTCTAGAACTCACTCTCCTTGCTCC	23	G	0.0574	0.0861
NC_005100.4-240755338	CCCTGGGACACAGACCCTCCAATGTAC	21	A	0.1789	0.2142
NC_005100.4-240827326	TGGGGTCACACAGCCTCTCCTCAAAAG	14	C	0.0619	0.0926
NC_005100.4-240876998	GCTTCCTCTACTGCCCCTCCTGGTGAC	13	G	0	0.4786
NC_005100.4-240876998	GCTTCCTCTACTGCCCCTCCTGGTGAC	25	G	0	0.4786
NC_005100.4-240954137	ACTGCCTTAACGCCAGCTCCAGGCAAT	8	T	0.0328	0.048
NC_005100.4-240966950	CACCCATATACTCTGTCTCCTTTACAT	13	C	0	0.4751
NC_005100.4-240968458	CTCCCACTAACCTTACCTCCTAAGACT	26	C	0	0.48
NC_005100.4-240994927	CCCAGAACAACAGAAGCTCCACCTGTA	12	A	0.1057	0.1826
NC_005100.4-241046631	CTGGTGCTCACATGACCTCCAAAGAAC	3	G	0.0261	0.0257
NC_005100.4-241047273	GGATTAGGCACTTCCCCTCCCACTGAG	9	C	0.0174	0.0342
NC_005100.4-241060816	ATTCCAGGCACTTGAACTCCTAGTGTA	3	T	0	0.4813
NC_005100.4-241060816	ATTCCAGGCACTTGAACTCCTAGTGTA	21	T	0.1345	0.5
NC_005100.4-241090970	TTGATTTTCACATCGTCTCCATCAATC	14	C	0.0331	0.0325
NC_005100.4-241090970	TTGATTTTCACATCGTCTCCATCAATC	24	A	0.0328	0.0322
NC_005100.4-241112188	GCTGTCTCCACATATCCTCCATGGTAC	2	C	0.2727	0.4769
NC_005100.4-241112188	GCTGTCTCCACATATCCTCCATGGTAC	27	C	0.2397	0.4213
NC_005100.4-241134315	TCCAGGGCGACATAGCCTCCTTTGGGA	9	G	0.123	0.3831
NC_005100.4-241138672	CATGTGATCACTCCAGCTCCCTCCCTC	16	G	0.0536	0.0521
NC_005100.4-241146017	CTGAACCCCACTATGCCTCCAGAACCC	7	C	0.0345	0.0666
NC_005100.4-241146395	GCCAATGACACTTGAACTCCATCCAAA	12	T	0.2155	0.244
NC_005100.4-241192038	AGTCCTCACACACCCACTCCACACAAT	13	C	0.1327	0.5
NC_005100.4-241192337	AGAGCACCCACTGTCTCTCCAGGTACA	7	C	0.2131	0.4226
NC_005100.4-241192337	AGAGCACCCACTGTCTCTCCAGGTACA	8	C	0	0.4773
NC_005100.4-241194555	TCCTGAAGAACTGCATCTCCCACTTTA	4	T	0.0614	0.0595
NC_005100.4-241194555	TCCTGAAGAACTGCATCTCCCACTTTA	15	A	0.1391	0.2873
NC_005100.4-241196642	TTCCTTCATACCTAGTCTCCTATTGTG	14	A	0.0763	0.4997
NC_005100.4-241202006	CTCAAGATCACCAAATCTCCATTCTTC	2	T	0.2583	0.3347
NC_005100.4-241217655	AGCCACTGAACCATCTCTCCAGCTCCT	21	A	0.1789	0.2017
NC_005100.4-241256871	AGGAAAACCACACACGCTCCCCTGCTC	16	G	0.0083	0.4816

NC_005100.4-241347173	TGCTCTCTGACCCCATCTCCAGCAAAT	5	C	0	0.4832
NC_005100.4-241347173	TGCTCTCTGACCCCATCTCCAGCAAAT	7	C	0.1849	0.3014
NC_005100.4-241361468	TCCATAGCCACTTCTGCTCCAGACCCA	14	C	0.2397	0.4213
NC_005100.4-241361468	TCCATAGCCACTTCTGCTCCAGACCCA	26	C	0.0744	0.0868
NC_005100.4-241461178	TTTGTTACCACCACATCTCCACTCACA	4	G	0.0894	0.1145
NC_005100.4-241478225	CCATGGCCTACGTAGCCTCCAAGGAGT	3	A	0.1239	0.1466
NC_005100.4-241478225	CCATGGCCTACGTAGCCTCCAAGGAGT	6	G	0.0088	0.4796
NC_005100.4-241478740	GAACAGCAAACAGAGACTCCTTCACTC	26	T	0	0.4868
NC_005100.4-241479050	TACATTATTACAGCTGCTCCTTTGCAA	8	T	0.2696	0.4849
NC_005100.4-241495694	CTCCCCACTACCCCATCTCCAAGAGCA	13	C	0.0982	0.1699
NC_005100.4-241495694	CTCCCCACTACCCCATCTCCAAGAGCA	25	G	0.2679	0.4539
NC_005100.4-241514253	TGCTGCAGTACAGAGGCTCCTTTCTAA	26	A	0	0.4751
NC_005100.4-241576200	AGCAAGAATACCAACTCTCCCGAGGGA	4	A	0.0259	0.0255
NC_005100.4-241578926	AGACTAAACACAGATGCTCCAACGTTT	15	T	0.2768	0.3612
NC_005100.4-241693101	GCAAAAGCCACTGAAACTCCGTCCCAT	21	G	0	0.4749
NC_005100.4-241693101	GCAAAAGCCACTGAAACTCCGTCCCAT	25	C	0.1111	0.1347
NC_005100.4-241695703	TGCACAACCACTGTAACTCCAGTTCCG	13	G	0.2377	0.4056
NC_005100.4-241807246	AATAACCACACTGGCACTCCTCCCTCT	2	A	0	0.4773
NC_005100.4-241816030	CTCTCACCAACCTACACTCCACTACAG	1	C	0.0342	0.05
NC_005100.4-241816030	CTCTCACCAACCTACACTCCACTACAG	26	A	0.1304	0.4976
NC_005100.4-241817850	ACCACCACCACACGCCCTCCTACAAAT	2	C	0.177	0.2797
NC_005100.4-241817850	ACCACCACCACACGCCCTCCTACAAAT	14	G	0.0354	0.4827
NC_005100.4-241829249	GCTCTGTACACTGGCCCTCCCATGACA	21	C	0.2308	0.3311
NC_005100.4-241831970	ACCTGGCTGACACCCCCTCCCTTGGCT	3	C	0.0268	0.0264
NC_005100.4-241831970	ACCTGGCTGACACCCCCTCCCTTGGCT	25	G	0.1607	0.2817
NC_005100.4-241845074	ATGTGCCTAACCAGCTCTCCTTTCTCT	25	T	0.2432	0.2137
NC_005100.4-241850962	ACCAACCGAACTACATCTCCAATCCCA	13	A	0.0427	0.0418
NC_005100.4-241874737	GAGCACAAAACGGGCACTCCCTGCAGA	8	A	0.2342	0.2467
NC_005100.4-241874737	GAGCACAAAACGGGCACTCCCTGCAGA	24	C	0	0.4854
NC_005100.4-241887238	ATTGCTAATACCTTGGCTCCAACACAA	13	T	0.0261	0.4744
NC_005100.4-241887238	ATTGCTAATACCTTGGCTCCAACACAA	27	A	0.0261	0.4744
NC_005100.4-241947459	AGTCTGCTCACGTGCCCTCCTAACACA	3	T	0.0342	0.0336
NC_005100.4-241947459	AGTCTGCTCACGTGCCCTCCTAACACA	12	G	0.0431	0.0585
NC_005100.4-241952635	ACATCAACTACTCAAGCTCCGAGTCAG	26	A	0.087	0.4537
NC_005100.4-241962775	AGCATTCCCACCGCCCCTCCTCTGTCA	12	C	0.25	0.343
NC_005100.4-241966212	GTTCCAGACACCAGTGCTCCATCAGCT	15	T	0.0086	0.4768
NC_005100.4-241972891	GACAAACAGACCTGTGCTCCTTACATT	2	A	0.2951	0.416
NC_005100.4-241972891	GACAAACAGACCTGTGCTCCTTACATT	12	C	0.0413	0.0562
NC_005100.4-241977983	TCTAGCCCAACGGTGTCTCCTTAAATT	12	G	0.1083	0.225
NC_005100.4-241994098	AATTGCTGAACCATCTCTCCAGCTCAA	4	T	0.4793	0.389
NC_005100.4-242015190	TGCCTGTTAACTCCAGCTCCAGGGACT	5	T	0.0579	0.0716
NC_005100.4-242015190	TGCCTGTTAACTCCAGCTCCAGGGACT	24	G	0.1826	0.4817
NC_005100.4-242022474	TATTTCTAGACCTCACCTCCCTTACCA	2	A	0.0598	0.074
NC_005100.4-242022474	TATTTCTAGACCTCACCTCCCTTACCA	13	T	0.0345	0.0339
NC_005100.4-242071844	AATCATGCCACTGCTACTCCAGCCCTG	5	A	0.1345	0.2076
NC_005100.4-242098946	TGTTGCCTCACACCTACTCCATTGTCC	6	C	0.1803	0.4988
NC_005100.4-242098946	TGTTGCCTCACACCTACTCCATTGTCC	16	A	0.0656	0.0935
NC_005100.4-242158810	GGCCACAGGACTCTGTCTCCCTTGCTG	23	T	0.1167	0.4999
NC_005100.4-242190745	CTAGGAACCACCCCCACTCCCTACCCC	27	C	0.1491	0.1814
NC_005100.4-242194230	AACCATGGAACCGTCTCTCCAGCTCCA	3	C	0.0348	0.0508
NC_005100.4-242194230	AACCATGGAACCGTCTCTCCAGCTCCA	6	T	0.0354	0.4912

NC_005100.4-242198119	ACATAGGAGACATCAACTCCTTTTAGA	8	A	0.0804	0.125
NC_005100.4-242202026	CCTACTAGGACAATCCCTCCTTTGTAA	2	C	0.2521	0.4873
NC_005100.4-242220844	CACACACTCACTCCTCCTCCTTTTTGG	27	G	0.1161	0.1093
NC_005100.4-242264949	CCACCCCCAACATCCTCTCCTTCTTCC	23	C	0.0732	0.0705
NC_005100.4-242284486	AACTACTGAACCATTGCTCCCCACCTC	5	A	0.0588	0.0728
NC_005100.4-242306471	GAAGTCTCTACTCTCCCTCCAAGCTTG	3	A	0.1545	0.1954
NC_005100.4-242327818	TTTCCAGGTACGGCGTCTCCATTTTCA	15	G	0.1186	0.1409
NC_005100.4-242364454	ACAGTTCTCACTTTCACTCCAAGTATT	21	A	0.0504	0.0491
NC_005100.4-242370195	AGTGATTCAACACAGGCTCCAAGAAAT	8	C	0	0.4793
NC_005100.4-242394954	GCACAAGTCACACATGCTCCGTCATAC	15	T	0	0.4703
NC_005100.4-242394954	GCACAAGTCACACATGCTCCGTCATAC	21	G	0.3025	0.3932
NC_005100.4-242412944	CCACCCTCTACATTAGCTCCGTTCGCC	16	G	0.1466	0.2797
NC_005100.4-242424247	CTATCTGTAACTCCAGCTCCAGGGAGC	24	G	0.5345	0.3916
NC_005100.4-242459619	ATGCAAACAACAGGGGCTCCCAGAATC	9	A	0.0902	0.1009
NC_005100.4-242466308	TCAAGACCAACGGTTTCTCCACAGCCA	12	G	0.2101	0.4931
NC_005100.4-242504374	TGTTAAGAAACTCGCCCTCCTTTAATG	13	C	0.1681	0.2203
NC_005100.4-242552068	CCCATTGATACACCTCCTCCAACAATG	4	A	0.0084	0.4797
NC_005100.4-242552068	CCCATTGATACACCTCCTCCAACAATG	27	G	0.0084	0.4797
NC_005100.4-242580208	AACGCCTTCACTCGCCCTCCAGCCGTC	3	C	0	0.4793
NC_005100.4-242580208	AACGCCTTCACTCGCCCTCCAGCCGTC	14	G	0	0.4793
NC_005100.4-242594876	ATGATCTGAACCTGGCCTCCACTCCGT	4	A	0	0.4793
NC_005100.4-242594876	ATGATCTGAACCTGGCCTCCACTCCGT	21	A	0.0894	0.3964
NC_005100.4-242651631	CTCAGCCCTACAGCGTCTCCCACAATC	14	C	0.1391	0.4991
NC_005100.4-242651631	CTCAGCCCTACAGCGTCTCCCACAATC	25	A	0.1379	0.1576
NC_005100.4-242671611	GGGTCAAATACAGGGACTCCCTCATAC	3	G	0	0.4773
NC_005100.4-242702286	CTGAAGAAGACTGATCCTCCCCCTCTC	21	C	0.0973	0.1828
NC_005100.4-242717277	GCCCCATGCACACGAGCTCCAGATTCT	6	A	0.0168	0.033
NC_005100.4-242736513	TCCACAGTAACCCAGCCTCCTGGAGTT	12	C	0.0086	0.4866
NC_005100.4-242736513	TCCACAGTAACCCAGCCTCCTGGAGTT	14	A	0.0085	0.4882
NC_005100.4-242746787	ATTATCACTACTTCAGCTCCTGCCTTG	8	C	0.1102	0.1337
NC_005100.4-242746787	ATTATCACTACTTCAGCTCCTGCCTTG	25	T	0.0427	0.058
NC_005100.4-242788305	ATGTATTCTACCGCTCCTCCCCCACTC	13	G	0.1466	0.3707
NC_005100.4-242788305	ATGTATTCTACCGCTCCTCCCCCACTC	15	T	0	0.4868
NC_005100.4-242788376	GCTTTACACACACGCACTCCCACATAT	13	C	0.0661	0.0792
NC_005100.4-242803703	GCAATTCCCACAATGACTCCATAGGAT	26	A	0.1858	0.4242
NC_005100.4-242810741	CTTGCCTCCACTCAAGCTCCACCGGAT	24	G	0	0.4718
NC_005100.4-242963610	AGCTAGTTTACACTACCTCCCTAACAG	23	A	0	0.4772
NC_005100.4-242989311	GACTGATGGACAGTTTCTCCATTGTCA	9	G	0.2314	0.318
NC_005100.4-243046732	CTGTCTCTAACGTCTCCTCCATACGTG	24	C	0.1416	0.4624
NC_005100.4-243072517	AACCGTGGAACCATTTCTCCGCACAGA	5	G	0.0171	0.4868
NC_005100.4-243160537	AACTCAGGAACTCGGCCTCCTTTCTAT	14	G	0.0541	0.1639
NC_005100.4-243161710	ACCTCATTTACATGCTCTCCCCATATA	1	A	0.0446	0.4617
NC_005100.4-243161710	ACCTCATTTACATGCTCTCCCCATATA	16	T	0.0088	0.4735
NC_005100.4-243192011	GTCACACACACCAGGTCTCCTCTGAGC	9	C	0.0081	0.481
NC_005100.4-243243220	CACACACACACACACACTCCAGCTGGG	7	C	0.0348	0.0342
NC_005100.4-243243220	CACACACACACACACACTCCAGCTGGG	9	C	0.0357	0.0351
NC_005100.4-243246241	GGCTGCATGACAAAGTCTCCCATGTTT	7	A	0.2521	0.3686
NC_005100.4-243246241	GGCTGCATGACAAAGTCTCCCATGTTT	25	T	0.0342	0.4649
NC_005100.4-243288849	GTTCCAATCACCCTTCCTCCACCACCT	23	C	0	0.4931
NC_005100.4-243330330	ACGATGCCAACTGCCTCTCCTTCCCCA	3	G	0.1043	0.0989
NC_005100.4-243330330	ACGATGCCAACTGCCTCTCCTTCCCCA	9	A	0.113	0.1516

NC_005100.4-243354217	GCCAGTGTGACTCCAGCTCCAGGTGAT	4	A	0.1765	0.1609
NC_005100.4-243385962	CTTAGCCCAACCACCTCTCCAGCCTAA	26	A	0.1967	0.1904
NC_005100.4-243414073	CTGTCTCTTACAGAAACTCCCTCATAG	2	T	0.275	0.4297
NC_005100.4-243414073	CTGTCTCTTACAGAAACTCCCTCATAG	26	A	0.2333	0.3911
NC_005100.4-243472933	CGGACATCCACCCAGCCTCCTGCATCA	12	C	0	0.48
NC_005100.4-243489477	GTTCTCTCCACTGCTTCTCCTTTGCTC	21	T	0.5798	0.4316
NC_005100.4-243489477	GTTCTCTCCACTGCTTCTCCTTTGCTC	22	T	0.5798	0.4316
NC_005100.4-243499599	TCTGATGCCACCAGCTCTCCTTTCCCC	16	T	0.0488	0.0476
NC_005100.4-243499599	TCTGATGCCACCAGCTCTCCTTTCCCC	27	C	0.0325	0.032
NC_005100.4-243510018	CACACACACACACACACTCCTTAAAGC	1	C	0.0261	0.0257
NC_005100.4-243510052	CACAGTGACACACTTCCTCCACCATGT	1	C	0.2195	0.2323
NC_005100.4-243571705	TTGTTAAGTACTTGCTCTCCTTGCTAA	1	T	0.1842	0.2836
NC_005100.4-243571705	TTGTTAAGTACTTGCTCTCCTTGCTAA	3	G	0.1842	0.2836
NC_005100.4-243575136	GTTACGTCCACACACCCTCCTCCTTAC	5	C	0.1074	0.1306
NC_005100.4-243575136	GTTACGTCCACACACCCTCCTCCTTAC	6	G	0.1157	0.1377
NC_005100.4-243597227	TTGAAATATACTTCTACTCCTCATGTT	15	T	0.1074	0.4999
NC_005100.4-243597227	TTGAAATATACTTCTACTCCTCATGTT	23	A	0.2149	0.2533
NC_005100.4-243749022	ACATGGCATACGGAACCTCCATCTTTG	14	A	0.2727	0.3421
NC_005100.4-243749022	ACATGGCATACGGAACCTCCATCTTTG	23	C	0.2667	0.4688
NC_005100.4-243753131	ACAGTGTGCACTCTTGCTCCTTGGCCT	14	T	0	0.4793
NC_005100.4-243759060	AGTGTCTCCACAGTCTCTCCTTTTCCA	6	C	0.0085	0.0418
NC_005100.4-243759060	AGTGTCTCCACAGTCTCTCCTTTTCCA	8	C	0.2906	0.4807
NC_005100.4-243807535	ATTTAAAGCACCCGTTCTCCCTCTCTA	12	C	0.0261	0.0257
NC_005100.4-243810993	TGATGTAACACTGTGGCTCCCACGATG	23	C	0.2764	0.427
NC_005100.4-243822693	GTGAAAACAACACAGACTCCAAGGGAA	3	G	0.0593	0.0576
NC_005100.4-243871271	GGCTTCTGCACCAGGTCTCCTGCACAG	9	C	0.0917	0.1172
NC_005100.4-243871271	GGCTTCTGCACCAGGTCTCCTGCACAG	27	G	0.3445	0.397
NC_005100.4-243923984	GCCTGTCCAACTTACACTCCATCACTG	24	A	0.027	0.2271
NC_005100.4-244006334	CCCTATGCCACAATCACTCCTTGCTAC	13	A	0.157	0.1853
NC_005100.4-244006334	CCCTATGCCACAATCACTCCTTGCTAC	27	C	0.1157	0.1377
NC_005100.4-244006703	GTCATAGCAACACAACCTCCAAGTTGA	24	T	0.0089	0.4751
NC_005100.4-244017985	ACCAGTCTGACACCAGCTCCTTCCCAG	1	A	0.1066	0.4984
NC_005100.4-244057195	CTCAAGTGCACTCTGTCTCCTTGTTGG	4	A	0.0088	0.476
NC_005100.4-244057195	CTCAAGTGCACTCTGTCTCCTTGTTGG	9	C	0.0088	0.476
NC_005100.4-244059393	GACCATTGGACTGGGACTCCAGCTCTG	13	G	0.1826	0.5
NC_005100.4-244059393	GACCATTGGACTGGGACTCCAGCTCTG	14	G	0.0609	0.0752
NC_005100.4-244070112	TGAATTCCTACACTAACTCCTCATTCA	23	A	0.2288	0.3086
NC_005100.4-244135429	CTGTCTATGACTCCCACTCCACAGATG	6	T	0.2696	0.3728
NC_005100.4-244135429	CTGTCTATGACTCCCACTCCACAGATG	23	A	0.2783	0.4537
NC_005100.4-244151107	TTGACTAACACAAGGACTCCGTGCCTT	14	G	0.0424	0.0415
NC_005100.4-244176799	ACTTAAGCCACTGACCCTCCTGGCCAT	4	T	0	0.4793
NC_005100.4-244176799	ACTTAAGCCACTGACCCTCCTGGCCAT	26	A	0.0325	0.0629
NC_005100.4-244177024	ATGTCTGTAACATGTGCTCCTCACAAA	6	T	0	0.4864
NC_005100.4-244177024	ATGTCTGTAACATGTGCTCCTCACAAA	7	G	0	0.4864
NC_005100.4-244189622	ATATCTGTTACCTTTGCTCCAATCCAG	2	T	0.1404	0.16
NC_005100.4-244189622	ATATCTGTTACCTTTGCTCCAATCCAG	23	T	0.1053	0.1884
NC_005100.4-244193761	AGAATAGACACCTCCCCTCCCCTAGGA	15	C	0.1322	0.1516
NC_005100.4-244221868	CCCCAGAACACAGGCACTCCCGAAGTG	3	C	0.0328	0.048
NC_005100.4-244221868	CCCCAGAACACAGGCACTCCCGAAGTG	16	A	0.0583	0.4708
NC_005100.4-244236527	ACCCATGTAACCTGCCCTCCCCCTCTC	27	C	0.0342	0.0336
NC_005100.4-244271055	TGTCCTCTGACTCCGACTCCAGGCTTA	14	C	0.0083	0.4783

NC_005100.4-244271055	TGTCCTCTGACTCCGACTCCAGGCTTA	21	A	0.1207	0.4999
NC_005100.4-244280057	AGTAGATATACTGCAGCTCCTTGATAA	9	T	0	0.4807
NC_005100.4-244284412	TTTCTCACAACTGCAGCTCCAATCTGT	2	T	0	0.4779
NC_005100.4-244284412	TTTCTCACAACTGCAGCTCCAATCTGT	21	A	0.0336	0.0491
NC_005100.4-244337839	TAAGGTTATACCAGGTCTCCTGTCCCT	12	C	0	0.4786
NC_005100.4-244337839	TAAGGTTATACCAGGTCTCCTGTCCCT	24	C	0.0517	0.0666
NC_005100.4-244358186	AGACAGAATACCATCACTCCCAACAAC	14	T	0.0442	0.4975
NC_005100.4-244364589	AACCCATGGACCATCTCTCCACACTCA	9	G	0.0407	0.0398
NC_005100.4-244367387	CATAGCAGAACAAAACCTCCTGGAGAT	2	A	0.069	0.4946
NC_005100.4-244367387	CATAGCAGAACAAAACCTCCTGGAGAT	13	A	0.0684	0.4918
NC_005100.4-244371038	TGATAACAGACTAACCCTCCGAAACTA	2	G	0	0.4807
NC_005100.4-244392489	AAGTGAATGACTCCAGCTCCTGCCTTC	7	A	0	0.4844
NC_005100.4-244394012	GGTACTAACACAACCCCTCCAACAGGC	4	A	0.1789	0.2612
NC_005100.4-244397020	GATAAAAGCACCATTACTCCTCTCCAA	27	A	0.009	0.4414
NC_005100.4-244402120	TACAATGTCACACTGCCTCCTACAGAG	26	A	0	0.4793
NC_005100.4-244402120	TACAATGTCACACTGCCTCCTACAGAG	27	G	0	0.4793
NC_005100.4-244431012	TGCGACTTTACTCTGACTCCTATGTTT	3	C	0.0789	0.3999
NC_005100.4-244445395	CCCAGTGACACACCTCCTCCTCCAATA	12	A	0.3534	0.4545
NC_005100.4-244445395	CCCAGTGACACACCTCCTCCTCCAATA	22	C	0.3478	0.4537
NC_005100.4-244616177	AATGCTCTAACCACAGCTCCGGGGTGG	21	G	0.0492	0.048
NC_005100.4-244626271	GAGCCCGATACAGTGTCTCCCTAGGAC	6	C	0.0348	0.0671
NC_005100.4-244631523	TGGGGATCCACTGTCCCTCCCCAGAAT	4	G	0.1261	0.1469
NC_005100.4-244632957	CTGTGTGTTACCAAACCTCCAGATGAC	2	T	0.1557	0.4984
NC_005100.4-244632957	CTGTGTGTTACCAAACCTCCAGATGAC	16	C	0.1311	0.1904
NC_005100.4-244682552	TGTCCGTCCACAGTGCCTCCGTACTTA	21	G	0.1167	0.1099
NC_005100.4-244715048	AATCACCGAACGCTCCCTCCGCACCAG	7	C	0	0.4891
NC_005100.4-244715048	AATCACCGAACGCTCCCTCCGCACCAG	21	G	0.0522	0.4864
NC_005100.4-244731491	TGTGTGGCCACCTCAGCTCCCTAGAAG	1	T	0.2439	0.4444
NC_005100.4-244738562	TGTAAGGCCACTTGCTCTCCAAGCTCC	14	G	0	0.4743
NC_005100.4-244738562	TGTAAGGCCACTTGCTCTCCAAGCTCC	27	C	0	0.4743
NC_005100.4-244751801	ATGTTCCAGACCCCATCTCCAACTGAT	23	C	0	0.4773
NC_005100.4-244751801	ATGTTCCAGACCCCATCTCCAACTGAT	27	T	0.041	0.0557
NC_005100.4-244800757	CCCAGTGACACATTTCCTCCAGCTCCA	1	C	0.0265	0.0433
NC_005100.4-244800757	CCCAGTGACACATTTCCTCCAGCTCCA	6	T	0.2232	0.388
NC_005100.4-244813675	CAATGGAGGACCACATCTCCAGTACAC	16	T	0.2276	0.377
NC_005100.4-244861906	GAATGCAAGACTTTTTCTCCAAGCCAT	13	T	0.0948	0.1209
NC_005100.4-244866449	CATAAATTCACTGTACCTCCATCAGTC	21	A	0.1626	0.2832
NC_005100.4-244866449	CATAAATTCACTGTACCTCCATCAGTC	22	T	0.2764	0.4
NC_005100.4-244902788	GAAACATCAACAAAGTCTCCAGTGGCA	13	A	0.2213	0.4634
NC_005100.4-244925968	TTTCCTGAGACCAGGTCTCCAATGAAT	16	T	0	0.4826
NC_005100.4-244947734	GCAGGTTGAACACTTCCTCCACTGGCA	9	A	0.0427	0.0418
NC_005100.4-244947734	GCAGGTTGAACACTTCCTCCACTGGCA	22	C	0.0085	0.4753
NC_005100.4-245181102	CAATGTAGCACACAGTCTCCTTCTGCC	5	G	0.1667	0.255
NC_005100.4-245182685	TTCTATGTGACTGAGTCTCCTCACTTG	5	A	0.1626	0.1494
NC_005100.4-245218308	GGGAAAAGCACCTGCCCTCCAAGCCTG	15	C	0	0.4813
NC_005100.4-245277787	GTCTGCCCCACACTATCTCCAGATCTG	2	T	0.225	0.2941
NC_005100.4-245279292	GTCAGTGACACGGGATCTCCTGCTGTC	4	A	0.2143	0.4641
NC_005100.4-245279292	GTCAGTGACACGGGATCTCCTGCTGTC	12	G	0.2143	0.375
NC_005100.4-245397378	GATGGCCACACTGACACTCCAACCCAG	22	A	0	0.4807
NC_005100.4-245397378	GATGGCCACACTGACACTCCAACCCAG	24	C	0.2033	0.2778
NC_005100.4-245402309	TAATGAAACACAGATTCTCCAACCCAT	8	A	0.0763	0.0734

NC_005100.4-245442305	CACTACCACACTGAGTCTCCATGGGAG	14	A	0	0.4787
NC_005100.4-245442305	CACTACCACACTGAGTCTCCATGGGAG	22	T	0	0.4787
NC_005100.4-245461389	TCTGAAGGGACAGTGGCTCCTCAGGAG	14	T	0.0684	0.1125
NC_005100.4-245481970	TTGAGAACAACCGACACTCCAAGCCCA	12	C	0	0.4819
NC_005100.4-245482959	CCCACAGTAACAGTGTCTCCTTTCCTT	9	A	0	0.4728
NC_005100.4-245501718	AATGATTGCACACTAACTCCCATCATT	2	A	0.2174	0.3451
NC_005100.4-245501718	AATGATTGCACACTAACTCCCATCATT	6	T	0.0254	0.0734
NC_005100.4-245525697	ATTCATGTTACCGTGGCTCCCGAAATT	27	T	0.1111	0.3596
NC_005100.4-245553169	CACTTTGCCACTAACACTCCATCTCTG	15	C	0.1429	0.188
NC_005100.4-245553169	CACTTTGCCACTAACACTCCATCTCTG	27	G	0.025	0.0247
NC_005100.4-245589231	GACAGCAAGACGCCACCTCCTTTTCAT	12	G	0.1197	0.142
NC_005100.4-245589231	GACAGCAAGACGCCACCTCCTTTTCAT	14	C	0.0598	0.058
NC_005100.4-245607720	GGTCATCTCACTCAATCTCCCCGTGGT	23	G	0.1062	0.2036
NC_005100.4-245622497	CATGGAGGGACCATGACTCCACCTACA	3	T	0.193	0.2155
NC_005100.4-245659585	AATTCTGCCACAACAGCTCCTCATAAG	26	A	0.0813	0.1216
NC_005100.4-245661468	TTCCCTTATACTTTACCTCCAGTGAAC	24	G	0.0708	0.0683
NC_005100.4-245717171	TAAATAACCACTTTCCCTCCAACTGAT	4	A	0.1504	0.1968
NC_005100.4-245725653	CCAAAGCCTACTTTGCCTCCATAGCCC	2	C	0	0.4856
NC_005100.4-245725653	CCAAAGCCTACTTTGCCTCCATAGCCC	3	A	0	0.4856
NC_005100.4-245731777	AAGAAAGTGACAAGACCTCCTATATGG	23	T	0.0776	0.0746
NC_005100.4-245741477	GACAGCCAGACTTACTCTCCCAACTAC	1	G	0.0439	0.0429
NC_005100.4-245742395	CTACCTGTAACTCCAGCTCCTAGGGTC	16	G	0.125	0.2941
NC_005100.4-245762530	TGGATCCCGACCCTAGCTCCTCTTGTT	27	T	0	0.4875
NC_005100.4-245767551	TCCACACCTACACCATCTCCTCCAGCT	12	A	0	0.4731
NC_005100.4-245767551	TCCACACCTACACCATCTCCTCCAGCT	25	G	0	0.4731
NC_005100.4-245783894	CCGCCTGTCACTCCAGCTCCAAGAGAT	8	T	0.1709	0.336
NC_005100.4-245899505	CTCTCAAAAACCTCCTCTCCATTTCCC	4	T	0.0252	0.0249
NC_005100.4-245910286	AAGCAGACCACAAACACTCCTAGGATA	14	A	0.0603	0.4893
NC_005100.4-245911035	CTCACTGGAACCAAGTCTCCAAATGAA	25	G	0.0593	0.4919
NC_005100.4-245967097	GGACTAGAAACAATGACTCCTCGGGTA	23	G	0	0.4773
NC_005100.4-245968493	TGGATGTCCACACCAGCTCCTGCACAT	26	A	0	0.4787
NC_005100.4-245977890	GGTGGAAAGACCATCCCTCCTAAAGCA	4	G	0.0522	0.0508
NC_005100.4-246002063	ATCACTACGACCTATCCTCCATGTCTG	8	C	0	0.4793
NC_005100.4-246002063	ATCACTACGACCTATCCTCCATGTCTG	24	T	0	0.4793
NC_005100.4-246086430	ACCATCTCTACAGCCCCTCCTTACACT	1	A	0.0081	0.4777
NC_005100.4-246118799	TTCTTAATTACCCTTTCTCCCGTGCCA	22	G	0.0088	0.481
NC_005100.4-246140932	TTATTTCAAACTGAATCTCCTACTGAA	15	A	0.0631	0.0611
NC_005100.4-246141420	GAAGATTAGACAGAGTCTCCAGGACTC	16	T	0.2523	0.4854
NC_005100.4-246246333	AGTAATAATACCATCTCTCCTATCTCC	1	A	0	0.4846
NC_005100.4-246305549	TTCTCTGCCACCGAGCCTCCTCCCTAG	12	C	0.2377	0.4755
NC_005100.4-246305549	TTCTCTGCCACCGAGCCTCCTCCCTAG	13	G	0.2623	0.316
NC_005100.4-246332725	CTTGCTAGCACACCCACTCCTTCACTA	8	G	0.1092	0.1181
NC_005100.4-246369016	ATGCTGAGTACATTCACTCCGGAGAGG	6	G	0	0.48
NC_005100.4-246369016	ATGCTGAGTACATTCACTCCGGAGAGG	21	G	0.2645	0.3557
NC_005100.4-246375033	CTGTTTCCCACTTTCACTCCCTCCCTC	3	G	0.0536	0.4986
NC_005100.4-246375033	CTGTTTCCCACTTTCACTCCCTCCCTC	5	T	0.0179	0.4871
NC_005100.4-246389908	TCTGTGGTTACAAAATCTCCAGCACTC	15	A	0.1167	0.4688
NC_005100.4-246415321	TGCTGTGTGACCTCATCTCCTCCGTAT	24	G	0.1393	0.321
NC_005100.4-246415321	TGCTGTGTGACCTCATCTCCTCCGTAT	26	A	0.1967	0.2157
NC_005100.4-246442161	ATCCTTTGAACATATGCTCCCCACTCT	2	T	0	0.477
NC_005100.4-246442161	ATCCTTTGAACATATGCTCCCCACTCT	21	C	0	0.477

NC_005100.4-246496685	CCAAGGACCACTTAGACTCCTTCCTCT	1	C	0.2389	0.4693
NC_005100.4-246517861	ACCTCTCGGACTTTGTCTCCCCATAAC	9	G	0.0261	0.0257
NC_005100.4-246554575	AAAGCAGGCACATATTCTCCAGAGCGT	21	A	0.0082	0.0243
NC_005100.4-246584534	CATCTCACTACCCACCCTCCTGCTACT	23	C	0.2124	0.4912
NC_005100.4-246591958	ACGCCTAAGACCTCTTCTCCAGCTCAG	2	C	0.0702	0.0997
NC_005100.4-246591958	ACGCCTAAGACCTCTTCTCCAGCTCAG	7	A	0.1842	0.4969
NC_005100.4-246619635	CCTCCATAGACGCCTTCTCCTTTCAAA	1	C	0.0254	0.0251
NC_005100.4-246619635	CCTCCATAGACGCCTTCTCCTTTCAAA	25	A	0.1017	0.0965
NC_005100.4-246653515	CCTAAATTGACAGTGGCTCCTAACTCT	22	A	0	0.477
NC_005100.4-246671777	GTACCTTTTACTTGGTCTCCTGGTCGA	24	T	0.1789	0.2263
NC_005100.4-246671777	GTACCTTTTACTTGGTCTCCTGGTCGA	26	G	0.0163	0.032
NC_005100.4-246677830	GGGTTAATAACTAAACCTCCCTCATCT	2	G	0.1121	0.1646
NC_005100.4-246683679	CCCGCTCTTACTCTCTCTCCAGCAGGA	3	C	0.2869	0.4379
NC_005100.4-246683679	CCCGCTCTTACTCTCTCTCCAGCAGGA	14	T	0.0325	0.032
NC_005100.4-246706637	ACGTTATTGACAAGCTCTCCATCCACA	3	G	0.0325	0.032
NC_005100.4-246724084	GTTGCCATTACCTGATCTCCTGATCTA	3	T	0.0833	0.0799
NC_005100.4-246724084	GTTGCCATTACCTGATCTCCTGATCTA	15	A	0.0917	0.0875
NC_005100.4-246732732	TCCCTAGTTACCATGGCTCCGAACACC	21	G	0.2087	0.4969
NC_005100.4-246739117	GACAGGCACACATGTTCTCCCTGTGTT	1	G	0.0603	0.0903
NC_005100.4-246739117	GACAGGCACACATGTTCTCCCTGTGTT	13	T	0	0.4764
NC_005100.4-246775784	GTTTTGATCACACCACCTCCCTCCCCC	14	C	0.0268	0.4751
NC_005100.4-246788919	CCCAATTCCACCTTCTCTCCAGGGCTC	4	A	0.1885	0.4984
NC_005100.4-246799445	CGTTCGAGCACACACGCTCCTTCCCAT	6	G	0.0855	0.4938
NC_005100.4-246812521	ACCCCCTACACACACACTCCTTCTGTA	8	A	0	0.482
NC_005100.4-246812521	ACCCCCTACACACACACTCCTTCTGTA	13	C	0.069	0.0981
NC_005100.4-246858472	TAGTTCTCAACCCTTCCTCCTAGGCCT	13	C	0	0.4807
NC_005100.4-246920898	GGCAAATATACTGAACCTCCAAGTTAT	13	G	0.2705	0.311
NC_005100.4-246920898	GGCAAATATACTGAACCTCCAAGTTAT	24	T	0.065	0.078
NC_005100.4-246957964	AGACACATTACTTAGTCTCCAGGACCC	1	A	0.2645	0.3467
NC_005100.4-246957964	AGACACATTACTTAGTCTCCAGGACCC	9	T	0.1818	0.1653
NC_005100.4-247000758	GGAGTCTCGACATGTCCTCCTTCCAGA	9	G	0.1239	0.1316
NC_005100.4-247017889	TCAGTGCTAACATAGTCTCCAGTTGTG	24	T	0.0531	0.0683
NC_005100.4-247045883	CACACACACACACAAGCTCCATCTAAT	3	C	0.0569	0.0553
NC_005100.4-247045883	CACACACACACACAAGCTCCATCTAAT	15	A	0.2846	0.2989
NC_005100.4-247061400	CGAGATACAACTGAGTCTCCCTCGGGC	24	G	0.0083	0.0247
NC_005100.4-247066373	GGCTCAACAACTCTCTCTCCCACTCTT	15	C	0.0085	0.487
NC_005100.4-247130426	CTAGTGGCTACATTGACTCCTTCACTC	1	C	0	0.4871
NC_005100.4-247130426	CTAGTGGCTACATTGACTCCTTCACTC	24	A	0.0536	0.0521
NC_005100.4-247141278	TCTCCAGATACGTGAACTCCTGTGGGA	12	G	0.0333	0.0328
NC_005100.4-247150154	CCACCTGTGACTCCTGCTCCAAGGGAT	6	T	0.0756	0.0882
NC_005100.4-247235147	CTGTCCCCTACCCTCTCTCCTGCAAGG	2	T	0.1593	0.4574
NC_005100.4-247264580	GAGGCTTGGACACGTGCTCCTTCCTCT	7	T	0.1053	0.1305
NC_005100.4-247337275	CACAGCACGACCTCTCCTCCATGACTG	7	A	0.0248	0.0245
NC_005100.4-247337275	CACAGCACGACCTCTCCTCCATGACTG	8	C	0.0168	0.4661
NC_005100.4-247376763	CCCCTTTCGACTACAGCTCCTGGGCAG	9	G	0.2941	0.3643
NC_005100.4-247413129	TAAGGCGGCACTGCCTCTCCAGCCCTG	7	G	0.178	0.3388
NC_005100.4-247413129	TAAGGCGGCACTGCCTCTCCAGCCCTG	9	C	0	0.4864
NC_005100.4-247468474	TTCCCATCCACCTTTGCTCCTGGAGGG	27	G	0.1368	0.2604
NC_005100.4-247481103	TCTTGCATGACAGACACTCCAGGGCCC	22	G	0	0.4864
NC_005100.4-247481103	TCTTGCATGACAGACACTCCAGGGCCC	25	C	0	0.4864
NC_005100.4-247497016	GGGTTTCCAACTCCTACTCCATTATCT	23	T	0.0084	0.4761

NC_005100.4-247534192	ACTGCCTGTACCACGTCTCCCACATAA	3	T	0.0569	0.1562
NC_005100.4-247534192	ACTGCCTGTACCACGTCTCCCACATAA	15	G	0.187	0.4967
NC_005100.4-247541901	TCTGCACGCACATGGGCTCCCTCCCTG	8	G	0.0086	0.0255
NC_005100.4-247541901	TCTGCACGCACATGGGCTCCCTCCCTG	26	T	0	0.4868
NC_005100.4-247556392	GTGGAATAAACCACTACTCCCTGGTAC	4	G	0.0656	0.0634
NC_005100.4-247588999	AAAAAAAGGACAGTATCTCCTACTGTG	1	A	0.0167	0.48
NC_005100.4-247597941	TGGGCTATCACTGGACCTCCTTTCCCT	8	T	0.3333	0.2778
NC_005100.4-247597941	TGGGCTATCACTGGACCTCCTTTCCCT	25	C	0.3333	0.2778
NC_005100.4-247657369	CTTGGTCCTACCAACCCTCCCCACTAC	1	C	0	0.4814
NC_005100.4-247663958	AGGCCTAGCACACCATCTCCAGAGTCC	7	A	0	0.4793
NC_005100.4-247696712	TTGTGTGCTACACTATCTCCCTGGATT	15	A	0	0.4765
NC_005100.4-247721511	TCATGATAAACTATACCTCCCTCATCA	13	A	0	0.4773
NC_005100.4-247744185	CCCTTTGAAACAGTGTCTCCCACTTGT	15	G	0.1441	0.1621
NC_005100.4-247781304	TATTGACTAACAGTATCTCCCGAGCCC	14	T	0.0583	0.0875
NC_005100.4-247815445	AAACCTCCCACTCATTCTCCCTACTCT	16	T	0	0.4765
NC_005100.4-247825178	ATACTTACCACAGCAGCTCCTGCCTCC	4	C	0.1404	0.4321
NC_005100.4-247872419	GACTTTTGCACGAGAGCTCCATCCTAC	12	G	0.0164	0.0322
NC_005100.4-247953184	CACTTCCCAACCTCCACTCCTGCCCTT	14	C	0	0.0325
NC_005100.4-248018755	CCTTTCTTTACCATTGCTCCCTCTCTC	9	T	0.122	0.2441
NC_005100.4-248071907	TTTTTGTGTACCCGGCCTCCATGACCT	13	C	0.3036	0.4974
NC_005100.4-248071907	TTTTTGTGTACCCGGCCTCCATGACCT	22	T	0.1947	0.2797
NC_005100.4-248078202	GGATATAATACCAAGTCTCCAGGGCTC	22	G	0.0776	0.4937
NC_005100.4-248122630	ATACACAGCACACTGTCTCCATCTACC	7	A	0.1167	0.495
NC_005100.4-248122630	ATACACAGCACACTGTCTCCATCTACC	21	A	0.1667	0.4994
NC_005100.4-248153861	CTACTCTCAACCTCACCTCCCTTGCCT	5	T	0.0089	0.4856
NC_005100.4-248153861	CTACTCTCAACCTCACCTCCCTTGCCT	15	A	0	0.4871
NC_005100.4-248161293	AGCATTCAAACAGCCCCTCCTCCTGTG	13	G	0.0082	0.479
NC_005100.4-248161293	AGCATTCAAACAGCCCCTCCTCCTGTG	14	C	0.2623	0.3537
NC_005100.4-248172386	AGCTACTGAACAGCACCTCCTTCAAGG	13	G	0.2439	0.4
NC_005100.4-248172866	ACCTTCTTTACTCAGGCTCCTCTTTGG	16	G	0.0325	0.0476
NC_005100.4-248172866	ACCTTCTTTACTCAGGCTCCTCTTTGG	22	C	0	0.032
NC_005100.4-248269325	ATTGCCTTCACTTCAGCTCCAGGCGAT	3	T	0	0.482
NC_005100.4-248291468	TTTAGCTACACACTGCCTCCCAAGTAT	22	A	0	0.4693
NC_005100.4-248310554	ATCCATCCTACTCATCCTCCATGCCTC	2	T	0.122	0.1695
NC_005100.4-248310554	ATCCATCCTACTCATCCTCCATGCCTC	22	T	0.0163	0.4682
NC_005100.4-248313241	GGAGGCAGGACTCAATCTCCTGGGGTC	13	C	0	0.4751
NC_005100.4-248313241	GGAGGCAGGACTCAATCTCCTGGGGTC	21	T	0.1639	0.2515
NC_005100.4-248393159	CAGAGGAGTACCTGGCCTCCAGGAGAT	15	G	0.0738	0.0861
NC_005100.4-248393159	CAGAGGAGTACCTGGCCTCCAGGAGAT	27	T	0	0.4819
NC_005100.4-248418575	CCAGTTCACACCTATACTCCCCAGCTC	6	T	0.122	0.1287
NC_005100.4-248418575	CCAGTTCACACCTATACTCCCCAGCTC	25	C	0.065	0.1073
NC_005100.4-248490535	GAGCACTTAACACCCTCTCCACCTCCT	4	C	0.122	0.2441
NC_005100.4-248490535	GAGCACTTAACACCCTCTCCACCTCCT	21	A	0.2623	0.306
NC_005100.4-248495516	ACTTCTGCCACCTCATCTCCTAGAACC	1	A	0.0259	0.4803
NC_005100.4-248495516	ACTTCTGCCACCTCATCTCCTAGAACC	2	C	0.0259	0.0422
NC_005100.4-248595184	AAAAGACACACAGTCACTCCTGGGGTT	7	C	0.1789	0.2263
NC_005100.4-248767973	GGTATGCAGACTCTTCCTCCTCCTGCA	24	T	0.1475	0.1505
NC_005100.4-248871953	AAAGGTGACACCAGTGCTCCAAAGAAA	1	A	0	0.4751
NC_005100.4-248871953	AAAGGTGACACCAGTGCTCCAAAGAAA	8	A	0.2083	0.225
NC_005100.4-249055522	CCCCACTGGACCATCCCTCCATCATGT	6	C	0.0738	0.4924
NC_005100.4-249062627	CTAAATAAAACCCATCCTCCACAAGCT	7	A	0.2314	0.4942

NC_005100.4-249251448	CAAGGACACACATCTCCTCCCAGCACA	7	C	0	0.4832
NC_005100.4-249260489	AGAAGTCCCACCCTTCCTCCTTGAATC	14	T	0.0769	0.3409
NC_005100.4-249312865	CTCCTAATCACATTCACTCCGGTAGTT	7	A	0.1404	0.16
NC_005100.4-249312865	CTCCTAATCACATTCACTCCGGTAGTT	13	T	0.1416	0.1613
NC_005100.4-249323266	AGGATCCCCACTTGACCTCCAAACTAT	6	C	0.1404	0.1454
NC_005100.4-249336734	TTTGGGCAAACCATTTCTCCACTGTTA	24	G	0.0328	0.048
NC_005100.4-249394569	CAAGGTGGGACTCCAACTCCTCAACTC	9	G	0.0488	0.0476
NC_005100.4-249394569	CAAGGTGGGACTCCAACTCCTCAACTC	26	T	0.3443	0.4565
NC_005100.4-249395464	AGTACCTAGACTTTACCTCCCAAGTGC	21	C	0.0965	0.1528
NC_005100.4-249395464	AGTACCTAGACTTTACCTCCCAAGTGC	22	A	0.0965	0.1528
NC_005100.4-249402541	TTGTAAAATACCCTCCCTCCAGCTGAC	12	C	0.0776	0.1058
NC_005100.4-249414570	TTGGCTGAGACAGGGGCTCCTGCTGCC	9	G	0.0885	0.4859
NC_005100.4-249414570	TTGGCTGAGACAGGGGCTCCTGCTGCC	26	C	0.0522	0.0508
NC_005100.4-249454091	AAAGGGAAGACACTGGCTCCAAAGCAT	14	T	0	0.4814
NC_005100.4-249497493	GTAGGAAGAACACAGTCTCCTTTTAGG	1	G	0.0894	0.1425
NC_005100.4-249497493	GTAGGAAGAACACAGTCTCCTTTTAGG	3	A	0.122	0.1287
NC_005100.4-249522652	CCACCAGAAACTCCAGCTCCATGGGAT	24	G	0	0.032
NC_005100.4-249545002	ACAAATGGAACAAACGCTCCATGTTCA	15	C	0.0265	0.0262
NC_005100.4-249568140	CAAATTTAGACTGGCACTCCTGAAGTA	6	T	0.042	0.0411
NC_005100.4-249568140	CAAATTTAGACTGGCACTCCTGAAGTA	9	G	0	0.4772
NC_005100.4-249571157	AGTGAAGTAACAGGAACTCCCTAGATG	25	A	0.0413	0.4863
NC_005100.4-249657078	AAAACACAAACCAGCTCTCCCTGTGTG	6	A	0	0.032
NC_005100.4-249663450	TGCTCCTGCACAGCCACTCCCACCTCA	13	G	0	0.4793
NC_005100.4-249663450	TGCTCCTGCACAGCCACTCCCACCTCA	26	C	0.2602	0.4072
NC_005100.4-249763307	CCATTTGTAACTTCAGCTCCCAGAACT	5	T	0.0088	0.483
NC_005100.4-249770315	ATCATTTAAACACCGTCTCCTGCAGTT	14	C	0.2479	0.289
NC_005100.4-249770315	ATCATTTAAACACCGTCTCCTGCAGTT	15	G	0.0593	0.0734
NC_005100.4-249851525	TACTGTTTTACAATGGCTCCAGCAGTC	16	G	0.0088	0.4861
NC_005100.4-249854550	ATGGGGCTCACTCAGGCTCCTCAGGAA	4	G	0.0081	0.4777
NC_005100.4-249879690	TCTTTCTCAACTCTATCTCCCCTCATG	4	T	0	0.4793
NC_005100.4-249947407	GCATCTTTAACGTTAGCTCCAGTCATA	12	G	0.2479	0.4012
NC_005100.4-249947407	GCATCTTTAACGTTAGCTCCAGTCATA	16	G	0.0085	0.0251
NC_005100.4-249968745	TGTCAAGTTACTTAAGCTCCTTGATCA	14	A	0.187	0.2323
NC_005100.4-249983065	TGGCTGAGAACAGCCACTCCCGTGATA	8	G	0	0.4793
NC_005100.4-249983065	TGGCTGAGAACAGCCACTCCCGTGATA	21	C	0	0.4793
NC_005100.4-250046882	ATGCCATAAACCCCATCTCCAATACCA	3	G	0.0902	0.1436
NC_005100.4-250066282	CTCATCATCACTCCAGCTCCAAGGGAT	1	C	0.025	0.0722
NC_005100.4-250066282	CTCATCATCACTCCAGCTCCAAGGGAT	9	C	0.0167	0.48
NC_005100.4-250069109	TGCCACCGTACCTTTGCTCCACTATCA	23	T	0.065	0.078
NC_005100.4-250069109	TGCCACCGTACCTTTGCTCCACTATCA	25	T	0.0976	0.1216
NC_005100.4-250099876	AGCTCCCTGACTCTGACTCCGCTATCA	21	G	0	0.4693
NC_005100.4-250122603	TCACTTTGGACTCTAGCTCCCCAAGTT	13	C	0.0446	0.0771
NC_005100.4-250185545	CCATCTCTGACTTCTTCTCCGCAGACC	21	G	0.0259	0.4893
NC_005100.4-250195268	ATTGCCCCAACTTCCACTCCTCTCTAT	13	T	0.0513	0.0818
NC_005100.4-250199290	TCTGATCCAACTCCAGCTCCAGCTCAT	26	A	0.0678	0.0655
NC_005100.4-250248957	CTATCTTCCACAGAGTCTCCAGGAGAC	2	T	0	0.4793
NC_005100.4-250248957	CTATCTTCCACAGAGTCTCCAGGAGAC	27	C	0.0081	0.4777
NC_005100.4-250253697	CAACGCAGAACCCAAGCTCCTGATCAC	1	C	0.1597	0.4965
NC_005100.4-250253697	CAACGCAGAACCCAAGCTCCTGATCAC	5	G	0.1597	0.4965
NC_005100.4-250281604	TGTGTCTTCACACTGCCTCCAGAAACC	3	T	0	0.4709
NC_005100.4-250281604	TGTGTCTTCACACTGCCTCCAGAAACC	16	C	0	0.4693

NC_005100.4-250343524	ATGGAGAGAACCATGGCTCCAGCTGCA	8	G	0.5537	0.4004
NC_005100.4-250344862	CTGAGTTTTACTGGAGCTCCTACATAA	4	A	0.0254	0.481
NC_005100.4-250344862	CTGAGTTTTACTGGAGCTCCTACATAA	22	A	0.2101	0.4435
NC_005100.4-250428368	CAGGCTCGAACACTCACTCCAAACCTC	7	C	0.1081	0.1786
NC_005100.4-250495037	GTAGGATCTACCTAGGCTCCTCTCCAT	6	A	0.1083	0.1172
NC_005100.4-250526328	CACAGCAGCACGAAAGCTCCTACTGAG	2	A	0.2231	0.465
NC_005100.4-250526328	CACAGCAGCACGAAAGCTCCTACTGAG	13	A	0.124	0.172
NC_005100.4-250531598	TAGAGCCGTACACTTCCTCCTTGAGCT	21	T	0.1552	0.2854
NC_005100.4-250531598	TAGAGCCGTACACTTCCTCCTTGAGCT	24	A	0.1034	0.4994
NC_005100.4-250532282	AGGCTTTGTACCTTGACTCCTGTGAGC	12	C	0.1053	0.16
NC_005100.4-250592224	CTACCAGCCACTGACTCTCCAATACTG	23	T	0.065	0.496
NC_005100.4-250614936	CTTGCCCCTACAGCACCTCCTTTCCCT	26	C	0.0588	0.1033
NC_005100.4-250621682	AAGGAACGCACCGTGTCTCCTTCCTCC	9	C	0.0847	0.0965
NC_005100.4-250625592	CAACAGACAACCCTTACTCCTTTGTAT	26	A	0.1864	0.4718
NC_005100.4-250686112	TTCCTTCTAACCTGTCCTCCGAAAGAG	13	T	0.0265	0.0262
NC_005100.4-250709840	GACAGATTTACCACCTCTCCATGAAGA	1	G	0.2609	0.4301
NC_005100.4-250709840	GACAGATTTACCACCTCTCCATGAAGA	6	A	0.2542	0.4029
NC_005100.4-250755038	CAACTGTGCACCTTAACTCCACTGCTG	26	T	0.0083	0.4816
NC_005100.4-250756362	CAAACCACAACACCAGCTCCTCACATG	12	A	0.0325	0.032
NC_005100.4-250765583	ACCTAGGCGACCTGGACTCCAAAGCAG	2	C	0.0488	0.1356
NC_005100.4-250765583	ACCTAGGCGACCTGGACTCCAAAGCAG	15	G	0	0.4807
NC_005100.4-250768069	CCCATGAGGACTCCCCCTCCCCCATAA	9	G	0.0167	0.48
NC_005100.4-250768069	CCCATGAGGACTCCCCCTCCCCCATAA	13	C	0.0084	0.4797
NC_005100.4-250777524	GAGCTCCCTACCCTCTCTCCTGCCAGG	25	A	0.2566	0.4171
NC_005100.4-250798114	GTTGAGAGAACTTATTCTCCTGAGCCT	2	T	0.2583	0.3622
NC_005100.4-250798114	GTTGAGAGAACTTATTCTCCTGAGCCT	14	A	0.0331	0.0325
NC_005100.4-250810427	CTGGTCATTACTGCCCCTCCTATCTGT	13	G	0	0.4765
NC_005100.4-250810487	TTGGTCATTACTAGCCCTCCTGACAGT	14	G	0	0.4751
NC_005100.4-250810487	TTGGTCATTACTAGCCCTCCTGACAGT	24	C	0.084	0.1539
NC_005100.4-250821351	TCATCACAGACATTCCCTCCTCTTCTG	1	T	0.1491	0.499
NC_005100.4-250835598	ACAGAAGACACAAACTCTCCCCTATAA	13	A	0.0087	0.4878
NC_005100.4-250835598	ACAGAAGACACAAACTCTCCCCTATAA	24	A	0.0087	0.4878
NC_005100.4-250846316	CTTTCACTGACTGCTTCTCCCTCTCAT	4	T	0.041	0.0401
NC_005100.4-250846526	CTCTACCCCACAAAGACTCCAGTCTCC	5	A	0.0973	0.1239
NC_005100.4-250846526	CTCTACCCCACAAAGACTCCAGTCTCC	13	A	0.0893	0.4974
NC_005100.4-250861088	CAGAATGCTACAGTTTCTCCCTCAGAA	14	T	0.0086	0.0255
NC_005100.4-250861088	CAGAATGCTACAGTTTCTCCCTCAGAA	23	C	0.0345	0.0339
NC_005100.4-250876544	AGTGTAAACACCTAAACTCCAGATGAA	9	C	0.1416	0.4968
NC_005100.4-250876544	AGTGTAAACACCTAAACTCCAGATGAA	12	C	0.1491	0.4969
NC_005100.4-250913884	TCAAGGCACACGTGAGCTCCTCCCACA	2	C	0.2679	0.375
NC_005100.4-250917511	CTATCTACAACTGTGACTCCATAAGCA	21	A	0.1102	0.1337
NC_005100.4-250922551	GCGGTCCGCACTGCAACTCCACAAGAC	6	C	0.292	0.3772
NC_005100.4-250924244	TTACAACTGACCATAACTCCATCTACA	7	C	0.1316	0.1814
NC_005100.4-250931059	CTGCCTGTGACTCTAGCTCCAGGATAT	26	A	0	0.4807
NC_005100.4-250934503	AGGATCTCCACGCAGCCTCCCTTACTA	13	C	0.275	0.4166
NC_005100.4-250948712	AATGCCTGGACATCCTCTCCCCTGCCT	1	A	0.0172	0.0339
NC_005100.4-250948712	AATGCCTGGACATCCTCTCCCCTGCCT	3	T	0.0172	0.0339
NC_005100.4-250989030	GGCATTTATACACCATCTCCTTACTTT	16	T	0.025	0.0247
NC_005100.4-250992943	TCCCTGCCTACTCACCCTCCAGCTACT	9	T	0	0.4793
NC_005100.4-251034037	AAAATCAGCACTTTGCCTCCACAAACA	5	T	0.0593	0.487
NC_005100.4-251075358	GCCCGTGACACATACACTCCAACAGGG	15	C	0.177	0.2036

NC_005100.4-251122173	TTAAATAAAACTGATTCTCCTTCCACT	21	T	0.1951	0.3604
NC_005100.4-251124881	AAAACCACTACCCTTCCTCCATGCGGA	8	C	0.0439	0.0429
NC_005100.4-251124881	AAAACCACTACCCTTCCTCCATGCGGA	23	G	0.0265	0.06
NC_005100.4-251128059	TGCATAGTCACCTAAGCTCCTCCTTCA	21	T	0.2035	0.4923
NC_005100.4-251145089	TGGGAGGTGACGAGTGCTCCAGCGGCC	3	G	0.0089	0.4665
NC_005100.4-251145089	TGGGAGGTGACGAGTGCTCCAGCGGCC	8	T	0.0536	0.0689
NC_005100.4-251244261	CCGGATCCCACCCAGTCTCCTAAGCCA	3	G	0.2167	0.4965
NC_005100.4-251245566	AGTGTGCACACCACTGCTCCTGTCATC	13	A	0.1803	0.2157
NC_005100.4-251307944	CCCACAGCAACAGGACCTCCTGCTTAC	8	C	0.0254	0.0251
NC_005100.4-251307944	CCCACAGCAACAGGACCTCCTGCTTAC	27	C	0.2288	0.329
NC_005100.4-251379609	GGTGAGCTCACCCCTCCTCCCCCATCT	12	C	0.0992	0.4923
NC_005100.4-251379609	GGTGAGCTCACCCCTCCTCCCCCATCT	24	A	0.1066	0.1573
NC_005100.4-251382990	CTACAAACCACACACGCTCCTTCCCAG	23	C	0.0804	0.0771
NC_005100.4-251393933	TAGAGTTTCACAAACCCTCCTACCTTC	12	A	0.1138	0.1356
NC_005100.4-251395294	AACCATCTCACTGGCTCTCCAGTTGCC	1	A	0.0083	0.0408
NC_005100.4-251395294	AACCATCTCACTGGCTCTCCAGTTGCC	4	C	0.2167	0.2778
NC_005100.4-251567886	AACACAGACACCACCCCTCCCCCCAAA	2	A	0.125	0.1997
NC_005100.4-251593105	AGCACAGTGACATCAGCTCCTGTGATG	25	A	0	0.4793
NC_005100.4-251668648	AGCGCTGTCACGTTTCCTCCGGTTTGC	4	G	0.1933	0.4994
NC_005100.4-251668648	AGCGCTGTCACGTTTCCTCCGGTTTGC	26	G	0.0168	0.4743
NC_005100.4-251711482	AGATGAGGCACACAAGCTCCATTAGTC	12	A	0.2114	0.464
NC_005100.4-251724558	TAAATAACAACTGTACCTCCACATCAT	9	A	0	0.4704
NC_005100.4-251741631	GGGAACATAACAGTGGCTCCATTCCTA	26	T	0.0081	0.0241
NC_005100.4-251741631	GGGAACATAACAGTGGCTCCATTCCTA	27	A	0.1057	0.4999
NC_005100.4-251746182	TGAATAGCAACATAGGCTCCTGAGTTC	7	G	0.2049	0.2796
NC_005100.4-251746182	TGAATAGCAACATAGGCTCCTGAGTTC	26	T	0.1311	0.2032
NC_005100.4-251761202	CTTGTAAAGACAACACCTCCATCTTCC	13	A	0.1311	0.435
NC_005100.4-251761202	CTTGTAAAGACAACACCTCCATCTTCC	15	A	0.0246	0.0401
NC_005100.4-251782743	TCTGCATACACTCCAGCTCCTGTGGAG	5	C	0.2368	0.415
NC_005100.4-251782743	TCTGCATACACTCCAGCTCCTGTGGAG	6	A	0.0088	0.481
NC_005100.4-251788229	CCTCTCCCCACCCATCCTCCTCCTCCA	4	C	0.0263	0.026
NC_005100.4-251805463	ATTGATTTGACAAGACCTCCCCACGGT	6	T	0.0177	0.4793
NC_005100.4-251856713	TATGTTCTGACACATGCTCCTGCCATG	22	G	0.2439	0.3041
NC_005100.4-251861195	GTTGTCATAACAACTCCTCCAACTCTG	13	A	0	0.4764
NC_005100.4-251906916	TGAGTGCGCACTTGGACTCCATAGGCA	5	T	0.0248	0.0405
NC_005100.4-251934242	AAATTCACGACCTCATCTCCATGTGTT	4	T	0.1138	0.1073
NC_005100.4-251934242	AAATTCACGACCTCATCTCCATGTGTT	8	C	0.122	0.1695
NC_005100.4-251961629	CCAGTGGTTACAGATTCTCCTCTGGCC	26	C	0	0.4793
NC_005100.4-251994380	ATCTTCATCACAGCCCCTCCAGGGAAT	22	G	0	0.482
NC_005100.4-252011337	AGAGGGGACACTCACGCTCCTGGTGAT	16	G	0.0442	0.06
NC_005100.4-252030665	TGTTTTTTCACCCCCACTCCCACCACA	6	T	0	0.4641
NC_005100.4-252037701	ACTTCATGTACCATTGCTCCTCATGTA	13	A	0.3217	0.3978
NC_005100.4-252163091	AGAGCCTCTACCAGGTCTCCTCCTGCT	5	C	0.0331	0.0325
NC_005100.4-252163091	AGAGCCTCTACCAGGTCTCCTCCTGCT	12	C	0.2917	0.3708
NC_005100.4-252178295	TGGGGGATCACCTATGCTCCTATAAGT	14	A	0	0.4793
NC_005100.4-252178295	TGGGGGATCACCTATGCTCCTATAAGT	22	A	0.0442	0.0765
NC_005100.4-252247620	CTCTCACCAACCCCCTCTCCTCCATCC	14	C	0.0769	0.0896
NC_005100.4-252303274	ACGAGATATACAATTCCTCCAACTGAC	8	A	0.2373	0.4964
NC_005100.4-252367586	AATCGTTCCACCTAAGCTCCTCATCCT	25	C	0.042	0.1327
NC_005100.4-252391935	AAAAGACGTACCTCCCCTCCGTTCTTT	8	G	0.2033	0.2441
NC_005100.4-252391935	AAAAGACGTACCTCCCCTCCGTTCTTT	21	G	0.1818	0.3557

NC_005100.4-252445350	TTAGTCCTGACACAGCCTCCCCTCATT	3	A	0.1416	0.1613
NC_005100.4-252445350	TTAGTCCTGACACAGCCTCCCCTCATT	15	G	0	0.4898
NC_005100.4-252495531	GGAGACACCACCGGTCCTCCACATTCT	2	G	0.0583	0.0566
NC_005100.4-252495531	GGAGACACCACCGGTCCTCCACATTCT	13	G	0.0083	0.4803
NC_005100.4-252556885	CCCTTTAAAACAGTGCCTCCCCACTCA	25	T	0.1849	0.2203
NC_005100.4-252558141	GGCCAGCCTACTTCCCCTCCCAACTGT	3	C	0.1897	0.2252
NC_005100.4-252558141	GGCCAGCCTACTTCCCCTCCCAACTGT	24	C	0.0513	0.05
NC_005100.4-252559767	ATAAGTGTCACCCTTGCTCCCAGCTTT	12	C	0.1429	0.2188
NC_005100.4-252559767	ATAAGTGTCACCCTTGCTCCCAGCTTT	24	C	0.0268	0.0264
NC_005100.4-252676868	GTGAACATCACGTAGGCTCCTTTCTTT	2	T	0.025	0.0566
NC_005100.4-252709312	TTAACAGTTACATGAGCTCCTGGTATT	2	T	0.1026	0.1125
NC_005100.4-252756658	TTTGCTTACACATAGACTCCCTTGTGG	12	A	0.0446	0.0436
NC_005100.4-252756658	TTTGCTTACACATAGACTCCCTTGTGG	14	A	0.0893	0.4943
NC_005100.4-252892430	ACCCTGAGTACAACGTCTCCATGTTCA	23	G	0.0167	0.0488
NC_005100.4-252916705	TATCAAGGAACTCAGTCTCCAAGATTT	7	G	0.1478	0.1369
NC_005100.4-252941193	CTGGAGAAGACCTCCTCTCCAAGCTTT	25	T	0	0.48
NC_005100.4-252954647	TATAATTCCACAGTTCCTCCACATGAT	1	T	0.2018	0.2088
NC_005100.4-252957773	TCAGGCCAAACTACAGCTCCTACCATG	23	C	0.0268	0.0264
NC_005100.4-252960776	CTTATGAAGACAGAAGCTCCATTTGAT	5	T	0.1864	0.334
NC_005100.4-252960776	CTTATGAAGACAGAAGCTCCATTTGAT	23	T	0	0.4749
NC_005100.4-252989078	AGTGGACCAACAGAGACTCCATGCCAA	7	C	0	0.4793
NC_005100.4-253016120	GGGTTTGATACCCATCCTCCTCTCTCT	2	G	0.1239	0.4981
NC_005100.4-253016120	GGGTTTGATACCCATCCTCCTCTCTCT	22	C	0.1504	0.4961
NC_005100.4-253035214	TAGGCAGGTACAGGAACTCCTTTGAGT	14	G	0.0714	0.0853
NC_005100.4-253035214	TAGGCAGGTACAGGAACTCCTTTGAGT	26	G	0.0265	0.0262
NC_005100.4-253069475	GTAAACTCAACATTTGCTCCATCTCCT	12	A	0.0248	0.0245
NC_005100.4-253069475	GTAAACTCAACATTTGCTCCATCTCCT	21	A	0.1583	0.2491
NC_005100.4-253085195	TAAGGAGTTACCCCATCTCCTAGCTCC	6	A	0	0.4868
NC_005100.4-253089186	GCTGCCACCACAGCTGCTCCTGTAGCC	16	G	0.0407	0.0553
NC_005100.4-253157185	AGGCCTTCCACTTACCCTCCACCACCA	21	A	0.1967	0.4914
NC_005100.4-253175905	GTGTAGACTACTGTCACTCCCAAGGAC	4	T	0.2051	0.497
NC_005100.4-253176160	AGTCTCTCCACCTTCTCTCCAAATGAC	9	C	0.2101	0.4977
NC_005100.4-253207809	CACACATGCACAAAAGCTCCCAGGCTT	4	A	0.0517	0.0666
NC_005100.4-253207809	CACACATGCACAAAAGCTCCCAGGCTT	27	T	0	0.4827
NC_005100.4-253241130	TCCCTCACTACCTCAGCTCCACTGTCC	14	C	0.0165	0.0325
NC_005100.4-253241130	TCCCTCACTACCTCAGCTCCACTGTCC	21	A	0	0.4751
NC_005100.4-253296239	CTTCTGGCCACAGTGACTCCTAGGCTT	2	T	0.0678	0.1116
NC_005100.4-253296239	CTTCTGGCCACAGTGACTCCTAGGCTT	7	G	0.0588	0.0571
NC_005100.4-253355901	GACATGAGCACAAACACTCCTTGCTCA	7	A	0.0721	0.086
NC_005100.4-253355901	GACATGAGCACAAACACTCCTTGCTCA	26	C	0.018	0.0526
NC_005100.4-253420890	AATACTATGACTCTACCTCCGGCCATG	2	A	0.0427	0.0418
NC_005100.4-253420890	AATACTATGACTCTACCTCCGGCCATG	21	G	0	0.4734
NC_005100.4-253504061	GGGCTTGTCACTGGACCTCCTTTTTCT	12	T	0.3451	0.2856
NC_005100.4-253504061	GGGCTTGTCACTGGACCTCCTTTTTCT	14	G	0.0702	0.0677
NC_005100.4-253543576	ATTATGCACACCTCTTCTCCCCACTCT	25	T	0.0526	0.0512
NC_005100.4-254130703	CGGGACAACACCCGATCTCCTTGATTT	24	A	0.1393	0.1968
NC_005100.4-254139924	TTAAACACCACCGTCACTCCTGCACAC	5	A	0.1667	0.2719
NC_005100.4-254139924	TTAAACACCACCGTCACTCCTGCACAC	13	G	0.2087	0.4915
NC_005100.4-254329064	TCCTTCCTGACTCTTTCTCCACCTTTC	25	T	0.1405	0.3421
NC_005100.4-254717662	AGCGCTCAGACCTGTGCTCCTTAGCTG	4	G	0.2586	0.3995
NC_005100.4-254717662	AGCGCTCAGACCTGTGCTCCTTAGCTG	8	A	0.25	0.4313

NC_005100.4-254890076	AGACCGTCAACTAGTTCTCCCAAAGTA	14	G	0.0086	0.4768
NC_005100.4-254900277	TGTTGGGGCACCAAACCTCCAAAGAAC	2	G	0.0325	0.032
NC_005100.4-254900277	TGTTGGGGCACCAAACCTCCAAAGAAC	6	G	0.2541	0.4258
NC_005100.4-254928913	TCAGAGCACACTCTTGCTCCTTCTCTC	12	T	0.0163	0.4793
NC_005100.4-254931874	ATAGTTTGTACTATCTCTCCTCACGCA	9	T	0.042	0.0411
NC_005100.4-254931874	ATAGTTTGTACTATCTCTCCTCACGCA	25	G	0.0593	0.0576
NC_005100.4-254937153	ATCATGTGAACGTTTTCTCCCATACAG	2	T	0.0769	0.074
NC_005100.4-254943477	AATTCGGTCACTCCCTCTCCTTCTTCA	27	A	0.1826	0.4055
NC_005100.4-255033909	TGGAGTTTCACAGGGTCTCCTCACTTA	23	A	0.2308	0.4977
NC_005100.4-255113780	TCATAGCTAACGGAGGCTCCTCCATTA	21	T	0.2034	0.4908
NC_005100.4-255129868	TTCTTGATTACAGTGGCTCCATGAATA	21	A	0.0541	0.0526
NC_005100.4-255141394	AATGTCACCACGTCCTCTCCTTTTGTC	8	C	0.0328	0.0322
NC_005100.4-255141394	AATGTCACCACGTCCTCTCCTTTTGTC	22	T	0.0328	0.0322
NC_005100.4-255190327	CAAAGCTGGACACCCCCTCCCCATTGT	8	G	0.0339	0.0333
NC_005100.4-255259780	CCTGCGTTTACTGCCCCTCCTCTGTGA	5	C	0.1696	0.2991
NC_005100.4-255266333	CTTTACCAAACCGCTCCTCCTCTTGTC	12	C	0.0252	0.0249
NC_005100.4-255278106	ATCCTGGGCACCAGCCCTCCTTATTGG	15	C	0.0407	0.0553
NC_005100.4-255323544	TATTCCTGGACTGTGCCTCCAGTTCAG	13	G	0.0492	0.048
NC_005100.4-255332393	TGTTCCATAACATATGCTCCTGAGTGA	4	T	0.0177	0.4934
NC_005100.4-255332393	TGTTCCATAACATATGCTCCTGAGTGA	13	T	0	0.4912
NC_005100.4-255369738	AGGGAGTGGACATGGGCTCCCACTCCT	6	G	0.5268	0.388
NC_005100.4-255369738	AGGGAGTGGACATGGGCTCCCACTCCT	25	C	0.5841	0.4135
NC_005100.4-255392463	CTGAAGATGACCATACCTCCTGATCTT	13	A	0	0.48
NC_005100.4-255412392	CCTGCCTGCACCCTGACTCCTATCTGT	7	T	0.0517	0.488
NC_005100.4-255417801	TAATCCCAGACTATGGCTCCATTTCCA	6	C	0.0424	0.0415
NC_005100.4-255446394	CACATCTTAACCCTGCCTCCTTGCTGA	12	C	0	0.4934
NC_005100.4-255561667	CAAGAGGTGACTCTGACTCCCAGGACT	16	A	0	0.4773
NC_005100.4-255787575	GGAGCATACACGTCACCTCCATCACCT	7	T	0	0.4807
NC_005100.4-255787575	GGAGCATACACGTCACCTCCATCACCT	14	C	0	0.4807
NC_005100.4-255799580	CTTACAACTACACTGGCTCCTCGCCAT	22	C	0	0.4787
NC_005100.4-255818006	ACAGGGTTCACACTAACTCCAGCCTTG	6	G	0.0088	0.472
NC_005100.4-255888705	CACTTGAACACTTCATCTCCAACTGAT	15	A	0	0.4854
NC_005100.4-255921450	TGCTTTGAGACACAGTCTCCTTTTGTG	3	C	0.0167	0.4861
NC_005100.4-255925511	TAGCTGAAGACAGAGACTCCAGTGGCC	6	G	0.0256	0.0253
NC_005100.4-255941858	CATGAGGAAACCCTATCTCCCTCTTTA	3	T	0	0.4749
NC_005100.4-255941858	CATGAGGAAACCCTATCTCCCTCTTTA	15	A	0	0.4749
NC_005100.4-255949727	GTAAGCCAAACACACTCTCCCTTCTGT	1	G	0	0.4786
NC_005100.4-255949727	GTAAGCCAAACACACTCTCCCTTCTGT	6	C	0.0259	0.0255
NC_005100.4-255968051	CTACGGATAACCCGTACTCCATCCACA	4	C	0	0.4787
NC_005100.4-255968051	CTACGGATAACCCGTACTCCATCCACA	9	A	0	0.4807
NC_005100.4-255968429	GTGGAACACACTAGAACTCCAGTTCAT	5	A	0	0.4743
NC_005100.4-256005637	CCTGGCAAAACACTAACTCCAAAAAGA	25	A	0.0083	0.4803
NC_005100.4-256026721	GCCTATAGAACTATATCTCCAGCCCTA	6	T	0	0.4793
NC_005100.4-256026721	GCCTATAGAACTATATCTCCAGCCCTA	14	T	0.122	0.1826
NC_005100.4-256162977	CCTTTTCAGACTCACCCTCCGGACCCT	3	T	0	0.4819
NC_005100.4-256188719	AGACAGGACACTGGATCTCCTGGAGCT	4	C	0	0.4773
NC_005100.4-256189548	TTCCCTGCAACGCTTCCTCCTTGCTCA	12	G	0.0081	0.0241
NC_005100.4-256189548	TTCCCTGCAACGCTTCCTCCTTGCTCA	22	T	0	0.4807
NC_005100.4-256277473	GCATCCCAAACAGAAACTCCACCTGTT	23	C	0.2301	0.39
NC_005100.4-256277473	GCATCCCAAACAGAAACTCCACCTGTT	26	T	0.2301	0.4464
NC_005100.4-256394547	AGTGCAGAGACAACCTCTCCCCTTGAA	15	C	0	0.4898

NC_005100.4-256443426	CAATCTGAGACTCAGCCTCCTCTCCTG	24	C	0	0.474
NC_005100.4-256468572	ATGAGGCTGACAGCATCTCCCAGGTAG	5	G	0.1157	0.4959
NC_005100.4-256498269	AGAAAACACACACAGGCTCCCCAATAG	23	A	0.0164	0.4837
NC_005100.4-256549500	CCCACCACCACCCAGTCTCCCTGGCAA	1	C	0.0413	0.0405
NC_005100.4-256549500	CCCACCACCACCCAGTCTCCCTGGCAA	14	A	0.1583	0.4747
NC_005100.4-256555976	AAAATCTTCACTTCTGCTCCATGGAGG	8	T	0.1017	0.3034
NC_005100.4-256586814	GGCTGTATGACCATGCCTCCAAACTCA	14	T	0.1463	0.3141
NC_005100.4-256586814	GGCTGTATGACCATGCCTCCAAACTCA	23	A	0.0325	0.032
NC_005100.4-256613717	ACAACTTAAACGCCAACTCCAGGGAAT	8	A	0.1391	0.4981
NC_005100.4-256627671	ACACTAGGAACTCTGCCTCCAAAGATA	16	C	0.0625	0.0605
NC_005100.4-256627671	ACACTAGGAACTCTGCCTCCAAAGATA	26	T	0.0625	0.491
NC_005100.4-256639355	GGGTCAACTACCCAGTCTCCTTCCCAT	9	T	0.0813	0.078
NC_005100.4-256659343	CTGTAGGCCACCCCTTCTCCCTGAAAT	9	C	0.1057	0.1425
NC_005100.4-256683959	GAGAGGCTGACAGCAGCTCCCAGTGTA	12	A	0.2564	0.4734
NC_005100.4-256683959	GAGAGGCTGACAGCAGCTCCCAGTGTA	23	G	0.025	0.0408
NC_005100.4-256709573	TTACCTGAGACCCTGCCTCCAAAGCAA	1	T	0.0603	0.0903
NC_005100.4-256732112	CACTTATAGACTGGACCTCCAAAGCCC	7	T	0.0417	0.0408
NC_005100.4-256733809	ATCCCCTCCACCACCGCTCCCTTCCAC	15	C	0.0504	0.0491
NC_005100.4-256775300	CTAAAATGGACGTGAGCTCCAACATGC	5	A	0	0.4728
NC_005100.4-256775300	CTAAAATGGACGTGAGCTCCAACATGC	12	G	0.084	0.0805
NC_005100.4-256790157	TTCCCAGCAACGCTAACTCCTCAACCC	9	A	0.161	0.1894
NC_005100.4-256798996	GGGAGAGAGACCTCTCCTCCCTCCTCT	15	T	0.1504	0.2494
NC_005100.4-256798996	GGGAGAGAGACCTCTCCTCCCTCCTCT	16	C	0	0.4859
NC_005100.4-256825810	GGAGAAAGAACTGCTTCTCCTTCCTGT	2	G	0.0804	0.1093
NC_005100.4-256840909	GCCCCTGTGACCTTTGCTCCTCTGCCA	26	C	0	0.4827
NC_005100.4-256893941	CAGGCATGCACACATGCTCCATTTATG	22	T	0.025	0.49
NC_005100.4-256896940	GACACTGCTACTTAGACTCCTAATCTA	8	C	0	0.4826
NC_005100.4-256897195	AAGAAATCAACTGCATCTCCACTGAAC	23	T	0.2083	0.2372
NC_005100.4-257003585	AGACACTGGACCCTGTCTCCTTCATTT	24	A	0.0738	0.0861
NC_005100.4-257017246	CTTCCTGGGACTCACGCTCCTGGTCAC	15	C	0.0083	0.4769
NC_005100.4-257044195	GGCACACAAACACTGGCTCCGTCATTC	22	T	0.0331	0.4819
NC_005100.4-257057467	GAGGTTGTCACTGGGGCTCCTCTCATT	25	A	0.2348	0.4962
NC_005100.4-257057467	GAGGTTGTCACTGGGGCTCCTCTCATT	27	T	0	0.4778
NC_005100.4-257177233	TCCCATTAAACAGCAGCTCCCTGCCAT	26	A	0.0088	0.4889
NC_005100.4-257177300	ATGTCATCTACGGAGCCTCCTCCCCGA	8	C	0.1339	0.125
NC_005100.4-257178968	GTGGTGGCGACTTAGACTCCTCCAGGT	7	G	0.0085	0.4842
NC_005100.4-257178968	GTGGTGGCGACTTAGACTCCTCCAGGT	9	G	0.1102	0.119
NC_005100.4-257183981	TAAGAAATGACTGGCACTCCACTCCAC	12	T	0.0082	0.4755
NC_005100.4-257187231	TAAACACGCACCTCATCTCCTCATTAT	7	C	0.2131	0.4914
NC_005100.4-257213615	ATAACACGGACTTGTGCTCCACTATGT	7	C	0.123	0.1296
NC_005100.4-257234857	AATACCAGAACTTTGTCTCCTTTGGTT	6	C	0.1034	0.4976
NC_005100.4-257239273	TGTGAACACACCTGTACTCCCAGAGCT	1	T	0.0328	0.048
NC_005100.4-257239273	TGTGAACACACCTGTACTCCCAGAGCT	5	A	0.0328	0.048
NC_005100.4-257248527	AGTTCCGCCACTAAGTCTCCAGGGCTG	2	G	0.1382	0.4995
NC_005100.4-257268459	AAGGAAGGGACGTTCCCTCCAGTGTTG	8	G	0.0248	0.0868
NC_005100.4-257273967	GATGGTCCCACCCTGCCTCCTTTATTT	13	C	0.2114	0.3604
NC_005100.4-257318451	CAACCACTAACTACCTCTCCAGCCCCT	27	T	0.6179	0.427
NC_005100.4-257334131	TGAGGACAGACTATTCCTCCAGAAAGC	5	G	0.0333	0.0488
NC_005100.4-257335526	TCTGAAAGTACAGCACCTCCTTCACAC	1	T	0	0.4728
NC_005100.4-257429030	CATATAGGTACCCAGCCTCCAGTCCAA	21	A	0.0877	0.474
NC_005100.4-257429030	CATATAGGTACCCAGCCTCCAGTCCAA	27	A	0.087	0.48

NC_005100.4-257438852	GGTGCAAACACGCTGGCTCCACTCGTG	7	A	0	0.4832
NC_005100.4-257438852	GGTGCAAACACGCTGGCTCCACTCGTG	12	G	0.0083	0.4816
NC_005100.4-257463338	AAGAAGTATACTTTACCTCCATCACAT	7	T	0	0.4773
NC_005100.4-257467105	CTAAAACACACTTAAGCTCCACAGACA	12	T	0.0877	0.1454
NC_005100.4-257480699	ATAGACCGGACTGAGGCTCCCAGCACA	2	T	0.1721	0.479
NC_005100.4-257480699	ATAGACCGGACTGAGGCTCCCAGCACA	22	A	0.0984	0.1505
NC_005100.4-257489713	TTCTCTCACACCCTCACTCCTGTGGCA	14	T	0.1681	0.322
NC_005100.4-257500478	AACATATCAACAGTTACTCCCAGGGCT	22	A	0.0246	0.0243
NC_005100.4-258188526	CACTCCTACACATGTACTCCCACCTCA	1	C	0.0331	0.0325
NC_005100.4-258726998	AAGGAGAATACTCCCACTCCCACCTAC	7	A	0.0174	0.4682
NC_005100.4-258769470	TCAGTGTTCACATTGGCTCCAGGCATG	9	C	0.4741	0.4545
NC_005100.4-258769470	TCAGTGTTCACATTGGCTCCAGGCATG	22	G	0.4522	0.3499
NC_005100.4-259219412	ACAACAAAGACACATACTCCACTAAGT	3	A	0.0333	0.0328
NC_005100.4-259219412	ACAACAAAGACACATACTCCACTAAGT	15	T	0.4872	0.3685
NC_005100.4-259282856	CAGGAAATAACACTGGCTCCTGTGCAG	13	C	0.2437	0.3462
NC_005100.4-259285813	TAACTGAACACAGCCCCTCCTGCATTA	22	G	0.252	0.3472
NC_005100.4-259285813	TAACTGAACACAGCCCCTCCTGCATTA	27	A	0	0.4793
NC_005100.4-259380611	TAAGTGACTACCTTTCCTCCTGTTTCT	7	A	0.0345	0.488
NC_005100.4-259386469	GCTCTAAGCACATGCGCTCCAAGGATG	4	C	0	0.4859
NC_005100.4-259429243	ACAGTTATCACAGCTGCTCCCGCTACC	12	A	0.3496	0.436
NC_005100.4-259429243	ACAGTTATCACAGCTGCTCCCGCTACC	27	C	0	0.4793
NC_005100.4-259477754	CTCTTCCCCACATACCCTCCAGTCTTG	15	C	0	0.48
NC_005100.4-259477754	CTCTTCCCCACATACCCTCCAGTCTTG	23	T	0	0.48
NC_005100.4-260114028	AGGGGAACTACAGGATCTCCACGACAC	15	A	0	0.4773
NC_005100.4-260125743	CAGTGTACTACTTTCTCTCCCTGCTCT	24	C	0.041	0.4879
NC_005100.4-260139290	TCCGTGGTCACTCTTCCTCCTAATCTT	3	C	0.041	0.4879
NC_005100.4-260139290	TCCGTGGTCACTCTTCCTCCTAATCTT	14	T	0.0082	0.4852
NC_005100.4-260141769	CAGTAAAGCACCATTCCTCCATGGCCT	8	G	0.0439	0.0595
NC_005100.4-260223838	TTTAAGACCACATCCACTCCAGGTGGT	3	T	0	0.4757
NC_005100.4-260225941	AGTTTCTTTACTGTGTCTCCCTGCATA	21	C	0.0086	0.4729
NC_005100.4-260279596	CCCATGGCAACAACCTCTCCCAACCAT	4	A	0.0894	0.1287
NC_005100.4-260279596	CCCATGGCAACAACCTCTCCCAACCAT	25	C	0.1803	0.2279
NC_005100.4-260304291	TACACCCCCACAAAGACTCCATGTGCA	8	C	0.0244	0.0241
NC_005100.4-260350869	TGCTTCCCAACAGCCTCTCCACAGTCT	12	A	0	0.4793
NC_005100.4-260350869	TGCTTCCCAACAGCCTCTCCACAGTCT	22	C	0.0407	0.0398
NC_005100.4-260444442	CAAGTGTGAACCGTGCCTCCTGGATCT	13	G	0.1789	0.2382
NC_005100.4-260446532	CGATAAGATACTGCCCCTCCACACCAC	12	T	0.0847	0.1551
NC_005100.4-260473764	AGCATCTACACGGCGGCTCCCACCCTC	12	G	0	0.4764
NC_005100.4-260481238	CCCGTGTCCACCGTGTCTCCATCTGTA	4	G	0	0.4734
NC_005100.4-260481238	CCCGTGTCCACCGTGTCTCCATCTGTA	13	G	0.0256	0.0253
NC_005100.4-260510982	ATCTAGGCTACAGAGTCTCCATCTTTT	26	T	0.0256	0.074
NC_005100.4-260510982	ATCTAGGCTACAGAGTCTCCATCTTTT	27	T	0.0256	0.074
NC_005100.4-260526736	GGCTACACCACTTTGGCTCCCGGAGGA	22	G	0.0086	0.0255
NC_005100.4-260527173	CATTTGGGCACTCAGACTCCTGATTGA	8	G	0.0522	0.0508
NC_005100.4-260536554	CCGGTTACAACTGGAGCTCCATCCACC	21	A	0	0.4773
NC_005100.4-260555929	TGAGTTACCACCTCCTCTCCCCTTTAG	26	A	0.1885	0.2218
NC_005100.4-260564245	CTGCAGCTTACAGCTCCTCCCACATCC	8	T	0.1463	0.496
NC_005100.4-260564245	CTGCAGCTTACAGCTCCTCCCACATCC	25	T	0.1463	0.496
NC_005100.4-260568083	GGTAGCATTACTCTGCCTCCCAGGCTG	3	T	0.2845	0.3876
NC_005100.4-260617596	CTCTCATGCACCACGTCTCCATTTGCC	6	A	0	0.4931
NC_005100.4-260633593	GCAAATACTACGCCTACTCCATAACCA	12	G	0	0.4807

NC_005100.4-260637680	TGGCTTGTAACATCTGCTCCCATCCTT	15	T	0.1327	0.3396
NC_005100.4-260637680	TGGCTTGTAACATCTGCTCCCATCCTT	16	G	0.0089	0.4751
NC_005100.4-260784611	CATGTTGTCACCTCTCCTCCCCACCCT	1	C	0	0.4682
NC_005100.4-260819787	GTGGTGGTGACCTTACCTCCCAGAGGT	6	G	0.1176	0.4991
NC_005100.4-260819787	GTGGTGGTGACCTTACCTCCCAGAGGT	9	G	0.0336	0.033
NC_005100.4-260874021	CCAACTTCTACTCTTGCTCCTTGTCTA	12	T	0	0.4793
NC_005100.4-260901177	ACCCAGATCACCAACTCTCCTTTTTGT	25	T	0.0268	0.0264
NC_005100.4-260910514	ACTACCGACACTAGGGCTCCTGTGTCC	2	C	0.0088	0.476
NC_005100.4-260910514	ACTACCGACACTAGGGCTCCTGTGTCC	6	C	0.0351	0.4814
NC_005100.4-260923432	CATCCGGTGACCATCTCTCCACCAGTG	5	C	0	0.4698
NC_005100.4-260944920	CCAAGCTCCACTTTGACTCCATGCAGC	23	G	0.0526	0.0677
NC_005100.4-260960709	CACCTCCCTACTTTGTCTCCTCTGGCT	25	G	0.025	0.4783
NC_005100.4-260977877	CCCCTTCCCACCCAGCCTCCTAGTGCT	15	G	0.2672	0.4643
NC_005100.4-260977877	CCCCTTCCCACCCAGCCTCCTAGTGCT	27	T	0.2672	0.4643
NC_005100.4-260978065	GCAGGAGTCACACTGACTCCTCTTCCC	1	G	0.0085	0.4626
NC_005100.4-260982148	GGAATGGAGACAAAACCTCCTTACCAT	5	T	0.0244	0.0241
NC_005100.4-260982148	GGAATGGAGACAAAACCTCCTTACCAT	26	A	0.252	0.4868
NC_005100.4-260990027	TTCTCCCACACATTACCTCCCATTGCC	12	A	0	0.4844
NC_005100.4-260994121	GATCACAGCACCTGCTCTCCATCCCAT	6	C	0	0.4819
NC_005100.4-261039288	GAATGCACTACTCCACCTCCTATAGCA	14	C	0.0261	0.0257
NC_005100.4-261062556	TCGATTGTAACACCAGCTCCAGAGGAT	16	G	0.0439	0.0429
NC_005100.4-261084001	AGTTTCCTAACCAACCCTCCCCACCAA	9	A	0.0256	0.074
NC_005100.4-261084001	AGTTTCCTAACCAACCCTCCCCACCAA	16	C	0.0259	0.0255
NC_005100.4-261087916	CCTCCCTGCACAGAGCCTCCTTGCCTT	7	T	0.225	0.4847
NC_005100.4-261096507	ATCCTCTTGACTCCACCTCCAGAGAAC	3	C	0.0574	0.0557
NC_005100.4-261096507	ATCCTCTTGACTCCACCTCCAGAGAAC	16	C	0.0248	0.0245
NC_005100.4-261100135	GGAGCCCCCACATTCTCTCCAGGGCTG	7	C	0.0164	0.0322
NC_005100.4-261100135	GGAGCCCCCACATTCTCTCCAGGGCTG	9	C	0.0492	0.048
NC_005100.4-261126263	GGTGGTAATACCTAGTCTCCTAGCACA	5	G	0.0268	0.4856
NC_005100.4-261126263	GGTGGTAATACCTAGTCTCCTAGCACA	27	A	0.0625	0.4968
NC_005100.4-261129523	AGCTCTCCAACTCCGGCTCCATCACAC	15	G	0	0.4793
NC_005100.4-261129523	AGCTCTCCAACTCCGGCTCCATCACAC	27	C	0.1138	0.4984
NC_005100.4-261176403	GTGCTGTTTACTGGCTCTCCTGGGCTC	27	C	0	0.4718
NC_005100.4-261210080	CTGTCTAGAACAATGCCTCCACATAGT	1	C	0.1138	0.1216
NC_005100.4-261210080	CTGTCTAGAACAATGCCTCCACATAGT	16	C	0	0.4773
NC_005100.4-261308027	CCACATACAACTCCAGCTCCAGAGGAT	22	G	0.122	0.1145
NC_005100.4-261308027	CCACATACAACTCCAGCTCCAGAGGAT	27	T	0.122	0.1145
NC_005100.4-261308516	ATGTGAGTGACTGCAACTCCCAGACTG	16	A	0	0.48
NC_005100.4-261370897	CAGAGCTAGACCAGAGCTCCATAGGAA	23	A	0	0.4807
NC_005100.4-261399701	AGGGTGTTTACTGGCTCTCCTGAAGCT	15	C	0.0984	0.1367
NC_005100.4-261410535	TGAGGATGGACCTCATCTCCGGAGACT	21	G	0.075	0.0722
NC_005100.4-261423798	GTGGCTGTGACTGAGACTCCAGACCCA	16	A	0.0082	0.479
NC_005100.4-261423798	GTGGCTGTGACTGAGACTCCAGACCCA	24	C	0.0082	0.479
NC_005100.4-261441204	GACGACCTCACCTGGTCTCCATAGTGA	3	C	0.0656	0.1367
NC_005100.4-261441204	GACGACCTCACCTGGTCTCCATAGTGA	4	G	0.041	0.4924
NC_005100.4-261470302	AGCCCGTGGACTCTGCCTCCTCTCCTG	1	A	0.0496	0.4901
NC_005100.4-261584947	CCGTTCCCCACAGGGACTCCAGGGATC	2	C	0.0917	0.1866
NC_005100.4-261584947	CCGTTCCCCACAGGGACTCCAGGGATC	4	T	0.0917	0.1866
NC_005100.4-261651733	ACATCCGTCACTGCCACTCCGTAGTTG	6	C	0.0965	0.2088
NC_005100.4-261719898	TCCTTTTGCACCCCAGCTCCCAACCAT	12	C	0.0087	0.4744
NC_005100.4-261730396	ATCTTCTTAACTCCCTCTCCCCATGCC	2	T	0.2583	0.2722

NC_005100.4-261730396	ATCTTCTTAACTCCCTCTCCCCATGCC	7	T	0.2583	0.2722
NC_005100.4-261750996	CGATCGCCCACATTTCCTCCACTGAAG	3	A	0.0446	0.0436
NC_005100.4-261761692	GCGTAGAGCACTGCAGCTCCAGTTATC	23	T	0.1333	0.3987
NC_005100.4-261821269	CTGTGAATGACAGTGACTCCCGCAGCC	8	T	0	0.4728
NC_005100.4-261857857	TCTCCCTTCACCACCCCTCCCCCAGCT	12	C	0.0526	0.4986
NC_005100.4-261880626	GACACACAGACAGTGCCTCCTCTCAGC	12	A	0.0714	0.2188
NC_005100.4-261944995	CTGGGTAGAACTCTGTCTCCGAGAAGC	26	G	0.125	0.1597
NC_005100.4-261949192	CCCAGAAAGACCAGGCCTCCTGTCTTG	26	T	0	0.48
NC_005100.4-261949192	CCCAGAAAGACCAGGCCTCCTGTCTTG	27	G	0.1724	0.199
NC_005100.4-261970077	ACCAGGGTCACTGGCTCTCCCTAGCAC	26	A	0.0244	0.0241
NC_005100.4-261982963	GAGCTGGGAACCTAGTCTCCAGATAGA	24	T	0.0631	0.0778
NC_005100.4-261988347	AAACTGTCCACGATCACTCCTGAAACC	2	A	0.1171	0.1713
NC_005100.4-262000323	GGAAGCCTCACAGAAACTCCTTTAAAC	27	C	0.1382	0.381
NC_005100.4-262025769	TCTTCTGAGACGACTTCTCCAACCCCC	25	C	0.1681	0.1968
NC_005100.4-262028976	AACTGCTGAACTGTCTCTCCAGCCCTT	7	T	0.2174	0.4926
NC_005100.4-262052574	AACAAGATCACACCTCCTCCAACATGG	8	T	0.094	0.1347
NC_005100.4-262075337	GCAGTCCTCACTGTCCCTCCTATGTGT	3	A	0.0086	0.4643
NC_005100.4-262075337	GCAGTCCTCACTGTCCCTCCTATGTGT	23	T	0.1826	0.4744
NC_005100.4-262075839	TCCATGCTCACATCGACTCCCACTTAG	6	G	0	0.4743
NC_005100.4-262076342	TGTGTTCTCACCTCTCCTCCAGCACTT	12	C	0.0083	0.4769
NC_005100.4-262076342	TGTGTTCTCACCTCTCCTCCAGCACTT	26	T	0.2314	0.4819
NC_005100.4-262087331	AACCCCAGCACCAAGTCTCCCCACCCT	4	C	0	0.4724
NC_005100.4-262120206	AGTGGCGGAACTCTCTCTCCATCCACA	16	T	0	0.4793
NC_005100.4-262129137	CTCGTGAGCACAGTTCCTCCGTCTTCT	4	G	0	0.4807
NC_005100.4-262129137	CTCGTGAGCACAGTTCCTCCGTCTTCT	21	G	0	0.4807
NC_005100.4-262154954	GTATTAAGGACGCTGTCTCCATGGAGG	12	G	0.2155	0.4643
NC_005100.4-262167672	AGAAGCAGCACTTAGTCTCCTATCATC	13	T	0.0248	0.0245
NC_005100.4-262667705	AATGTAAAGACAGTCTCTCCGAGCTTC	21	G	0	0.4793
NC_005100.4-262692706	GTCTGTAACACAGCGGCTCCAAGATAT	14	C	0.0333	0.0328
NC_005100.4-262786779	ACAATGCCGACTGCATCTCCATCTTTA	7	C	0	0.4807
NC_005100.4-263075879	AATAGGTCCACGGTGCCTCCACCCTGC	23	C	0	0.4778
NC_005100.4-263078751	TTCTACACCACGCTCCCTCCTCCCCTT	14	T	0	0.4898
NC_005100.4-263132154	CTCAAGAGAACATTCTCTCCATCATAC	25	T	0.0407	0.0553
NC_005100.4-263159760	AGAGTCCAGACATCAACTCCAGGCCTC	27	C	0	0.4677
NC_005100.4-263191890	TATTGCTCCACTGGGGCTCCTGCTTGG	27	G	0.0569	0.0705
NC_005100.4-263207960	GAGAACAGAACAGAGTCTCCTAAGTGG	7	A	0.0081	0.0398
NC_005100.4-263207960	GAGAACAGAACAGAGTCTCCTAAGTGG	15	G	0.1639	0.1904
NC_005100.4-263252564	GGCGAATCCACGCAGCCTCCTTGATGG	14	A	0.2049	0.2339
NC_005100.4-263252564	GGCGAATCCACGCAGCCTCCTTGATGG	25	T	0.0082	0.479
NC_005100.4-263362199	ATGAGAAGAACAGTCTCTCCGAAGATG	4	A	0.2222	0.3895
NC_005100.4-263393203	TGGACGAATACTTCGCCTCCAGTTGCC	6	G	0	0.4807
NC_005100.4-263422175	CTGATCCTGACTCTTGCTCCCAGAAAG	4	A	0.1017	0.4305
NC_005100.4-263422175	CTGATCCTGACTCTTGCTCCCAGAAAG	14	T	0	0.4826
NC_005100.4-263440332	ATCTGGGTCACACCGTCTCCTGGTAGT	8	T	0	0.48
NC_005100.4-263440332	ATCTGGGTCACACCGTCTCCTGGTAGT	27	T	0.0261	0.059
NC_005100.4-263455257	CTTCCTTCCACTCTCCCTCCTCATCTT	16	C	0.045	0.044
NC_005100.4-263508117	CACTGAGAAACCGTATCTCCAAGCATG	13	G	0.0488	0.0476
NC_005100.4-263601130	CATGTGGATACCTTGGCTCCAATAACA	23	T	0.1593	0.5
NC_005100.4-263616265	AGTAGCCACACTGATTCTCCTGAGCAA	7	C	0.0328	0.0634
NC_005100.4-263621745	ATTGTCTAGACGTAGACTCCTCACATC	25	A	0.1966	0.2544
NC_005100.4-263627999	ACGACACAGACTGTATCTCCTGACACC	3	G	0.2049	0.3008

NC_005100.4-263627999	ACGACACAGACTGTATCTCCTGACACC	4	A	0.0246	0.0243
NC_005100.4-263629783	AGAAAGGCCACCTGCACTCCTTTCCTC	22	T	0	0.4793
NC_005100.4-263689367	GCACCACGGACCCAGGCTCCATGCTTC	2	C	0.0536	0.4841
NC_005100.4-263715868	AACCCTCAGACTCAAACTCCTTGAACA	16	A	0.0569	0.0553
NC_005100.4-263719559	TCCCTTCGAACAGGGACTCCACTCCCA	8	G	0.15	0.2188
NC_005100.4-263720138	TGTTTTGAGACAGGGTCTCCTTTCTCT	22	T	0.0333	0.0644
NC_005100.4-263882371	CAATATGGAACATAGTCTCCTTCTGCA	16	T	0.2667	0.4265
NC_005100.4-263915856	AGCTGGCTTACGGCTCCTCCATGTGCT	12	G	0.2583	0.4416
NC_005100.4-263915856	AGCTGGCTTACGGCTCCTCCATGTGCT	24	T	0.2167	0.48
NC_005100.4-263924246	CACAGGGGAACAGAAACTCCTGCTGGA	21	T	0	0.48
NC_005100.4-263939771	GTGCCTGTCACATTTGCTCCTCATTAT	1	G	0.0894	0.0854
NC_005100.4-263939771	GTGCCTGTCACATTTGCTCCTCATTAT	22	C	0	0.4793
NC_005100.4-263957778	AACCTGGACACCAGAGCTCCCCAGCGA	25	C	0.1121	0.1209
NC_005100.4-263985148	AGGACTGTCACTCTGGCTCCCCTGTTA	23	T	0.2768	0.3316
NC_005100.4-263987905	AGGCATAGAACATTCTCTCCTCAATCC	6	T	0.0085	0.4854
NC_005100.4-263996520	ACTCCCACAACTCCAGCTCCAAAGGAT	21	A	0.2667	0.32
NC_005100.4-264078313	CCTCTGGAGACCACCCCTCCCACACTG	15	C	0	0.4813
NC_005100.4-264078313	CCTCTGGAGACCACCCCTCCCACACTG	26	T	0.0583	0.1316
NC_005100.4-264088889	ATCTTCTGAACACCTGCTCCTGAGCGT	26	G	0.2193	0.2599
NC_005100.4-264096294	CTATTAACAACTGAGCCTCCTAAGCCT	6	A	0.0569	0.0553
NC_005100.4-264096294	CTATTAACAACTGAGCCTCCTAAGCCT	27	T	0.0732	0.1425
NC_005100.4-264129357	GAGGCTCCTACCATGCCTCCCCCTCCC	1	G	0.2213	0.432
NC_005100.4-264129357	GAGGCTCCTACCATGCCTCCCCCTCCC	12	C	0.2213	0.4056
NC_005100.4-264140441	AGTCATAGGACAAAGGCTCCTTCCAAT	7	A	0.0348	0.4954
NC_005100.4-264140441	AGTCATAGGACAAAGGCTCCTTCCAAT	26	A	0	0.488
NC_005100.4-264142503	AACTGCTAAACGGTCTCTCCAGCACCA	24	A	0.2149	0.2046
NC_005100.4-264165226	AAGGAACAGACAAAGGCTCCTTCAGGG	27	G	0.2735	0.3974
NC_005100.4-264222695	CATGACACAACTGAGTCTCCAACAGAA	3	T	0.0583	0.1025
NC_005100.4-264223524	TACTTCCTCACCATACCTCCATTCCTC	6	C	0.094	0.1049
NC_005100.4-264248462	CTTCTTCACACTCAGACTCCCTGGTTC	4	C	0.1351	0.3012
NC_005100.4-264248647	TAATAATGGACTTTTCCTCCTCCTCCT	6	A	0.1121	0.1209
NC_005100.4-264248647	TAATAATGGACTTTTCCTCCTCCTCCT	26	C	0.1368	0.1841
NC_005100.4-264298733	CGAGAGCCCACGGAGCCTCCTCAGATC	1	C	0.188	0.3159
NC_005100.4-264345042	TCCCTGCAGACAGGCTCTCCCTCCCCT	4	C	0	0.4503
NC_005100.4-264345042	TCCCTGCAGACAGGCTCTCCCTCCCCT	25	C	0	0.4503
NC_005100.4-264354058	AAGATGCTGACTGGAGCTCCCGCATCC	4	A	0.0086	0.4893
NC_005100.4-264368775	GAGTACGTGACACTAACTCCTGTTAGA	8	T	0.1167	0.3578
NC_005100.4-264378022	TGTGCACACACTCTGTCTCCTGCTATC	1	T	0.1261	0.274
NC_005100.4-264385137	GTAATGCCCACTTGTCCTCCCTATCCC	24	T	0.0413	0.0405
NC_005100.4-264385137	GTAATGCCCACTTGTCCTCCCTATCCC	25	C	0	0.4819
NC_005100.4-264385737	CCTGTCCAGACTTCAGCTCCTCCCCTG	8	A	0.0085	0.0418
NC_005100.4-264385737	CCTGTCCAGACTTCAGCTCCTCCCCTG	22	C	0.0085	0.0418
NC_005100.4-264390103	GACTCCCCAACTTCTACTCCGCCTTCC	9	A	0.2411	0.3612
NC_005100.4-264390103	GACTCCCCAACTTCTACTCCGCCTTCC	15	T	0	0.477
NC_005100.4-264440285	TCCCAGGAGACCTCATCTCCGGTGATG	27	G	0.1721	0.4959
NC_005100.4-264458301	TGATCTAAGACTGACTCTCCTGCTGAT	15	C	0.1803	0.4952
NC_005100.4-264458301	TGATCTAAGACTGACTCTCCTGCTGAT	27	T	0	0.4773
NC_005100.4-264468459	TGTACCCCCACAGCCCCTCCCCACAAT	5	C	0.1	0.1665
NC_005100.4-264474992	GGTGGCCACACCGTTACTCCCAGGATT	25	A	0.1695	0.4987
NC_005100.4-264516694	ATGGGCTACACCACAGCTCCTGCTTAG	23	C	0.0244	0.0241
NC_005100.4-264533809	ACACATACCACCAGATCTCCAGTGACA	15	A	0	0.4832

NC_005100.4-264546855	CAAAGGTCAACGGGCTCTCCACGCTTT	22	C	0.2712	0.3707
NC_005100.4-264546855	CAAAGGTCAACGGGCTCTCCACGCTTT	26	T	0.2627	0.3664
NC_005100.4-264548038	TTCCATCCAACAGCCTCTCCAAGTCTG	2	T	0.2951	0.387
NC_005100.4-264548604	CTAATGTCCACACGGCCTCCCTGAGGA	13	C	0.2927	0.377
NC_005100.4-264588567	TAGCTGTTCACCAATGCTCCACCTTCA	2	A	0	0.4773
NC_005100.4-264588567	TAGCTGTTCACCAATGCTCCACCTTCA	6	G	0.0656	0.0786
NC_005100.4-264607974	CTTCACTCCACCAACTCTCCTCTCTTC	24	C	0.018	0.0354
NC_005100.4-264615739	CGAGGGCACACTTCGTCTCCAATTGTG	15	G	0.2522	0.3252
NC_005100.4-264628739	AAAAGTCCTACAGATGCTCCTTGTGGA	27	A	0.2542	0.3874
NC_005100.4-264633747	GATTCAGTCACTGGGGCTCCTGACTCT	25	T	0.0082	0.479
NC_005100.4-264680005	ATATATCTGACTGTGTCTCCTGCCGCC	25	G	0	0.4832
NC_005100.4-264695847	TTTAAAGTAACTCATTCTCCTCCTCCT	22	C	0.0804	0.4394
NC_005100.4-264724343	ATCTACTGAACCATCTCTCCAAACGCA	25	G	0.0088	0.4796
NC_005100.4-264751942	TCTGTTTGAACCTAGCCTCCTAAACAA	6	T	0.009	0.4682
NC_005100.4-264778040	CCCTGCTTAACCAGTGCTCCCAAGACT	13	A	0.0248	0.0245
NC_005100.4-264783956	TGCTAAGGAACCGTGACTCCTGCACTG	13	G	0.2605	0.4682
NC_005100.4-264783956	TGCTAAGGAACCGTGACTCCTGCACTG	22	G	0.0083	0.0247
NC_005100.4-264789666	CACGCTGAAACCCAGCCTCCCCGACGG	16	C	0.2417	0.4708
NC_005100.4-264789666	CACGCTGAAACCCAGCCTCCCCGACGG	22	C	0.2583	0.4575
NC_005100.4-264804611	GACACACCCACCTCAGCTCCTTCCTTT	16	G	0.0513	0.05
NC_005100.4-264806883	GCAGACAACACTTCGCCTCCTCAAACA	2	C	0	0.4868
NC_005100.4-264806883	GCAGACAACACTTCGCCTCCTCAAACA	27	A	0.1983	0.3876
NC_005100.4-264872894	GTATCCAAGACACTCTCTCCAACGGAA	21	A	0.1207	0.4999
NC_005100.4-264872894	GTATCCAAGACACTCTCTCCAACGGAA	23	C	0	0.4851
NC_005100.4-264887097	CTGGGATGTACCTGGTCTCCAGCCCCC	13	T	0	0.4793
NC_005100.4-264887097	CTGGGATGTACCTGGTCTCCAGCCCCC	16	T	0	0.4793
NC_005100.4-264898166	CTCTGGGATACTTCGGCTCCATTGATT	24	G	0.0087	0.4878
NC_005100.4-264933012	AACTGCATAACTTCAGCTCCAGGAGAG	2	A	0.0089	0.4856
NC_005100.4-264942446	TATTCTTGGACCTGAACTCCTGGAAGA	2	A	0	0.4793
NC_005100.4-264942446	TATTCTTGGACCTGAACTCCTGGAAGA	16	A	0	0.4793
NC_005100.4-264966182	GCACAGACCACCAGGCCTCCTGTGCAT	27	T	0.0492	0.0786
NC_005100.4-264969307	GCACAGAGCACGGACCCTCCTCTGCCT	14	A	0.2562	0.323
NC_005100.4-264995715	CTCTCTCACACCAGCACTCCTCAGACA	8	A	0	0.4793
NC_005100.4-264995715	CTCTCTCACACCAGCACTCCTCAGACA	14	G	0	0.4793
NC_005100.4-264996215	CTCCTGGAGACACATGCTCCAGTTCAA	12	A	0	0.4778
NC_005100.4-265033947	CATTTACCAACATCTCCTCCTCTGATT	26	T	0	0.477
NC_005100.4-265044050	GACCGCATCACTCTGCCTCCCGTTTCC	22	G	0.0244	0.0398
NC_005100.4-265075342	CACAGTGGCACACTTCCTCCAAACAAA	25	A	0.1626	0.2017
NC_005100.4-265092716	GGCTTCTTGACCTTGGCTCCTGTTGGT	12	C	0.0924	0.1327
NC_005100.4-265157091	CTTTTGGTTACTGGTGCTCCTTTTGAG	13	G	0.0088	0.4861
NC_005100.4-265160316	GGAGCCCAGACACCACCTCCTTGTAAC	4	G	0.1707	0.1954
NC_005100.4-265276865	GGCGGGGCAACACCCCCTCCACGCGAG	23	G	0.0877	0.2285
NC_005100.4-265280906	GCAGGTCCCACATCCGCTCCTACTTCA	6	T	0.0738	0.071
NC_005100.4-265280906	GCAGGTCCCACATCCGCTCCTACTTCA	21	T	0	0.4787
NC_005100.4-265283869	AGAGTCCCCACCCCACCTCCAACCCCC	8	C	0.1	0.095
NC_005100.4-265283869	AGAGTCCCCACCCCACCTCCAACCCCC	16	C	0.1271	0.119
NC_005100.4-265314899	GAGAATGGAACATATACTCCTCATGGA	15	T	0.0732	0.0705
NC_005100.4-265449620	GCAGCCCCTACAGGGACTCCAGTTCTT	22	G	0.0855	0.4991
NC_005100.4-265477989	AAATTCGACACCCAGCCTCCACATGTT	26	T	0	0.4864
NC_005100.4-265477989	AAATTCGACACCCAGCCTCCACATGTT	27	T	0	0.4864
NC_005100.4-265482702	TAGATGTCGACTCTGGCTCCCTTTCCT	21	C	0.1864	0.2091

NC_005100.4-265493451	CGAGGTGTTACAGAGCCTCCCTCCTAG	26	A	0	0.4807
NC_005100.4-265513249	TGGAGATCAACCTCTTCTCCCAGACTC	1	T	0	0.4677
NC_005100.4-265646111	CAGCAATAAACACACACTCCTGCCCTC	14	A	0	0.4703
NC_005100.4-265648234	CACAACAACACAACTACTCCCTGGATA	8	A	0	0.4787
NC_005100.4-265648234	CACAACAACACAACTACTCCCTGGATA	14	C	0	0.4787
NC_005100.4-265650707	CCTCCTGTGACTCCAGCTCCAGGGAAT	23	G	0.2672	0.244
NC_005100.4-265651211	AACCTCAAGACAGTGCCTCCTCTAACT	22	C	0	0.4839
NC_005100.4-265705182	CAGTCTGTAACTCTACCTCCAAGGAAT	1	C	0.2155	0.3127
NC_005100.4-265720074	ATCCTCCTGACTCTGCCTCCCAAGTGT	13	C	0.4917	0.4622
NC_005100.4-265720074	ATCCTCCTGACTCTGCCTCCCAAGTGT	15	G	0.4876	0.3771
NC_005100.4-265720795	CTCCAAGACACCTGCCCTCCTGAGCCC	4	C	0	0.4793
NC_005100.4-265720795	CTCCAAGACACCTGCCCTCCTGAGCCC	16	C	0	0.4793
NC_005100.4-265736612	CACACAAACACACACCCTCCATCACCC	26	C	0.1488	0.1918
NC_005100.4-265743638	ACATTATAGACAAGTGCTCCACCACTG	4	T	0.0084	0.4859
NC_005100.4-265760428	ACCACAACCACAGCTACTCCTATAGAA	13	G	0.1597	0.4987
NC_005100.4-265760428	ACCACAACCACAGCTACTCCTATAGAA	21	T	0.124	0.1163
NC_005100.4-265791574	TCTGTCTCTACTCAGCCTCCAAGACTC	3	T	0	0.482
NC_005100.4-265803842	TCGGTGCCAACTTCTGCTCCTCTTGTC	26	T	0.15	0.2188
NC_005100.4-265837260	CTGGTGGAGACAGCAGCTCCGACTCAG	8	A	0	0.4826
NC_005100.4-265889630	TCTAGTGCCACAGTGGCTCCATTTTGT	1	T	0.1513	0.3599
NC_005100.4-265889630	TCTAGTGCCACAGTGGCTCCATTTTGT	14	T	0	0.4839
NC_005100.4-265900683	CCTTTAAAGACCATCCCTCCTTTCCAT	15	C	0.1707	0.3472
NC_005100.4-266004109	GCGGGATCTACCTGAACTCCAAGGTTA	3	G	0.0163	0.032
NC_005100.4-266004109	GCGGGATCTACCTGAACTCCAAGGTTA	6	A	0.0813	0.1216
NC_005100.4-266010157	GGCTGAGTAACAAATACTCCATTGTGT	1	G	0.0172	0.482
NC_005100.4-266075329	TGGGGAGTCACTCTCCCTCCAATTAAT	5	G	0	0.4807
NC_005100.4-266081684	TTACCGACTACACCCTCTCCCCCTCCT	1	T	0.025	0.0247
NC_005100.4-266081684	TTACCGACTACACCCTCTCCCCCTCCT	15	C	0.0083	0.0247
NC_005100.4-266099728	GAACTGAACACGGATCCTCCTGAAGGA	24	A	0.1333	0.1387
NC_005100.4-266122641	GGGCCAGTTACTTCAGCTCCTTTCCTA	26	T	0	0.4821
NC_005100.4-266125694	ATGTGGAATACAGTCTCTCCTGGGGTG	8	A	0.2033	0.3091
NC_005100.4-266147819	GTATCTTGCACCATAGCTCCCTCTAGG	8	G	0	0.4819
NC_005100.4-266153451	TTAGTGTGGACTCAATCTCCTGCAGTT	25	G	0.1026	0.1975
NC_005100.4-266206260	AAAAAGACAACTTCAACTCCTCACCCA	8	C	0	0.4813
NC_005100.4-266206260	AAAAAGACAACTTCAACTCCTCACCCA	25	C	0	0.4813
NC_005100.4-266209652	AGCTTTGTCACACTGGCTCCTTTATAG	13	C	0.0976	0.4997
NC_005100.4-266232104	CACAGCTGTACCTGGTCTCCTTTGCAA	4	A	0	0.48
NC_005100.4-266350397	ACCCCAGAAACTGGCCCTCCGCGCCTT	21	G	0.0811	0.0778
NC_005100.4-266359963	AGAACTTTTACCTTTCCTCCTAGAGCT	7	T	0.0661	0.418
NC_005100.4-266387947	GTGACGTCAACACAGCCTCCACAGCTC	6	G	0.1429	0.4943
NC_005100.4-266392749	ACGTTGGCCACTATCTCTCCTTTGCTA	2	C	0.1593	0.2302
NC_005100.4-266398141	CTTGATTTGACCTTGCCTCCGGGTATG	25	A	0	0.4592
NC_005100.4-266494094	CCGGTGTCCACCTTGTCTCCTGAATAA	4	G	0.1466	0.4435
NC_005100.4-266527943	AACATCACCACCTTCTCTCCCTGGAAG	12	C	0.0244	0.0241
NC_005100.4-266553655	ACCTGTTGTACAAGACCTCCAGGAACG	9	T	0	0.4765
NC_005100.4-266573834	TCTTGGAGGACGTATCCTCCTGTATGC	15	T	0	0.4787
NC_005100.4-266573834	TCTTGGAGGACGTATCCTCCTGTATGC	25	T	0.0328	0.048
NC_005100.4-266635024	GCAAGAGCAACAGATGCTCCACACTGC	15	T	0	0.4793
NC_005100.4-266667586	CACAGACGCACCCCCTCTCCCATACTT	2	A	0	0.4793
NC_005100.4-266667586	CACAGACGCACCCCCTCTCCCATACTT	7	C	0	0.4793
NC_005100.4-266678579	CATAGAAACACCAAGTCTCCTTCCTCT	2	A	0.1356	0.4425

NC_005100.4-266739180	ATCACAGTGACAGAAACTCCTGAGGAC	21	T	0	0.4755
NC_005100.4-266746263	CTGCATGTAACTCGACCTCCAGGGGAT	23	G	0	0.4875
NC_005100.4-266768811	CTGAAGAAGACAGGCTCTCCATGTGAG	9	G	0.0246	0.0243
NC_005100.4-266782509	TTCAGAAAAACCTACTCTCCTTGGGGG	21	T	0	0.4909
NC_005100.4-266782509	TTCAGAAAAACCTACTCTCCTTGGGGG	22	T	0.1416	0.1466
NC_005100.4-266883242	ACTCCTGCCACAGACACTCCCTCAGCC	9	C	0.0976	0.2017
NC_005100.4-266899027	GCACTTCTAACTCTCTCTCCATGTTCT	8	T	0	0.4743
NC_005100.4-266904347	ACATGACTCACTCTCCCTCCTTCCGTA	13	C	0.1393	0.2457
NC_005100.4-266909415	CTTTAGCCCACGCACTCTCCTGTGCTC	12	G	0.0085	0.4842
NC_005100.4-266954552	TCTTTCATAACTTCTTCTCCACCCTGC	16	T	0.0678	0.1116
NC_005100.4-266985338	CCACTCACTACCAAGGCTCCCTCTGCG	26	C	0.1322	0.1235
NC_005100.4-266985338	CCACTCACTACCAAGGCTCCCTCTGCG	27	G	0.041	0.0557
NC_005100.4-266986486	ACCCACCCCACTCTATCTCCCTGTACC	5	A	0.115	0.5
NC_005100.4-266986486	ACCCACCCCACTCTATCTCCCTGTACC	25	A	0	0.4898
NC_005100.4-267075687	ACGACAATTACGACCACTCCAGCACCA	2	C	0.1983	0.4923
NC_005100.4-267078563	AAGCCACGGACAGTACCTCCTCTCACT	4	C	0	0.4793
NC_005100.4-267087593	GCATGTTAAACAAGCCCTCCACCACAG	1	G	0.1897	0.3916
NC_005100.4-267136300	GCCTACACAACCACCTCTCCCTGCCCC	14	C	0.2353	0.4008
NC_005100.4-267136300	GCCTACACAACCACCTCTCCCTGCCCC	21	C	0.0833	0.095
NC_005100.4-267137088	CAGGCAGACACGGAAGCTCCGTTACTC	12	G	0.0345	0.0339
NC_005100.4-267208658	ATTTTGAGCACACTCCCTCCCCTACGT	24	A	0.2213	0.3581
NC_005100.4-267228856	ACAGCTAACACAGTGTCTCCCTCATGC	21	C	0.0496	0.0639
NC_005100.4-267228856	ACAGCTAACACAGTGTCTCCCTCATGC	23	C	0.1322	0.2295
NC_005100.4-267240574	CCTGGAGCGACCCCCTCTCCCTTCTCT	7	G	0.1111	0.1492
NC_005100.4-267245651	CTAGTCGCCACCGAGACTCCCGACGTC	5	T	0.0678	0.0812
NC_005100.4-267264831	ATCTGCACCACACGTGCTCCTAGTACC	13	C	0.0082	0.479
NC_005100.4-267264831	ATCTGCACCACACGTGCTCCTAGTACC	14	G	0.0488	0.078
NC_005100.4-267285231	TACAGGTCCACTTCCTCTCCTTTACAG	2	A	0	0.48
NC_005100.4-267285231	TACAGGTCCACTTCCTCTCCTTTACAG	27	G	0.0583	0.1172
NC_005100.4-267333791	CCCAGAGACACTGGCTCTCCTGTAGGC	14	G	0.1933	0.4435
NC_005100.4-267341960	GAGAAAGGAACCCTTCCTCCCCTAAGC	16	C	0.0417	0.0566
NC_005100.4-267432725	GCTTGGAGCACATGGCCTCCTTGGTGG	22	T	0.0984	0.4934
NC_005100.4-267437127	GGCCCACAGACAACCCCTCCTCAGCAG	16	C	0.1545	0.1954
NC_005100.4-267449929	GTAGCCTGAACTTAAGCTCCTGATGAT	3	A	0.0348	0.4954
NC_005100.4-267473367	CACCTTTAAACTCAACCTCCCACAAAG	9	A	0.0982	0.1699
NC_005100.4-267473367	CACCTTTAAACTCAACCTCCCACAAAG	16	C	0.1327	0.1828
NC_005100.4-267484593	TCCCAAAGTACAGGATCTCCATGACAC	1	T	0.0684	0.066
NC_005100.4-267484593	TCCCAAAGTACAGGATCTCCATGACAC	23	G	0.1293	0.2188
NC_005100.4-267496871	TATATGTTTACTTTCTCTCCACAGACA	25	A	0.0169	0.4826
NC_005100.4-267497348	ATTTGAGCAACCTCCCCTCCCCCACTG	4	T	0.1311	0.1904
NC_005100.4-267497348	ATTTGAGCAACCTCCCCTCCCCCACTG	12	C	0.0328	0.0322
NC_005100.4-267497554	GATGGCGTCACACCCCCTCCTCAAAAT	7	G	0.1709	0.5
NC_005100.4-267498467	TACCCATTCACTCTACCTCCTGTTTGA	8	T	0.1157	0.1235
NC_005100.4-267518107	ATACAAAGCACCCAGGCTCCATGAAGA	1	A	0.177	0.2036
NC_005100.4-267518107	ATACAAAGCACCCAGGCTCCATGAAGA	4	C	0.2679	0.4157
NC_005100.4-267522204	TTTAAAATGACCCTGTCTCCGTAATAT	16	T	0.2143	0.3922
NC_005100.4-267522204	TTTAAAATGACCCTGTCTCCGTAATAT	23	A	0.307	0.3374
NC_005100.4-267522360	AGGACGAACACATCATCTCCAGGATTG	5	C	0.0081	0.4777
NC_005100.4-267522360	AGGACGAACACATCATCTCCAGGATTG	15	A	0.0325	0.032
NC_005100.4-267565857	ATCATTCTAACATCGGCTCCGACTATA	12	A	0.1416	0.3242
NC_005100.4-267601891	TCCTTGCTAACACTCCCTCCTCTTTGT	3	C	0.0168	0.033

NC_005100.4-267985972	ACTTCCCGCACGCCGGCTCCGGCTCCC	21	G	0.0082	0.4717
NC_005100.4-268080309	CTGAGATAAACTGAGGCTCCAAAGCCA	6	A	0.027	0.0267
NC_005100.4-268080309	CTGAGATAAACTGAGGCTCCAAAGCCA	26	C	0.027	0.0267
NC_005100.4-268277490	ATGAGTGTGACTCAGTCTCCTGGAACC	8	T	0.075	0.1172
NC_005100.4-268277490	ATGAGTGTGACTCAGTCTCCTGGAACC	27	C	0.0417	0.4847
NC_005100.4-268306850	TTTGGAAGGACAGTGCCTCCAAATCCC	2	T	0.0179	0.0351
NC_005100.4-268387689	CCATCCCTGACCCAGCCTCCCCTTTCC	23	T	0.1826	0.4926
NC_005100.4-268479004	ATAGCAAGGACATGTGCTCCACTATGT	2	T	0.2586	0.2252
NC_005100.4-268507379	TTAAACTAAACTGAATCTCCCTCTCCT	21	C	0.1261	0.3389
NC_005100.4-268507379	TTAAACTAAACTGAATCTCCCTCTCCT	27	T	0.1228	0.1884
NC_005100.4-268559899	ACCAGCCTCACATCCTCTCCATCACAG	6	C	0.0976	0.1073
NC_005100.4-268559899	ACCAGCCTCACATCCTCTCCATCACAG	12	A	0	0.4793
NC_005100.4-268584862	TTCTAGTGTACTTTACCTCCCTAACCA	4	T	0.2348	0.2816
NC_005100.4-268584862	TTCTAGTGTACTTTACCTCCCTAACCA	13	T	0.0085	0.4714
NC_005100.4-268588224	TTCAGAAAGACCATGTCTCCCAGGGAT	5	G	0.2155	0.4248
NC_005100.4-268588224	TTCAGAAAGACCATGTCTCCCAGGGAT	25	G	0.0504	0.0491
NC_005100.4-268633016	TGGAGAAATACAAACACTCCTCCATTG	5	G	0.0579	0.0562
NC_005100.4-268633016	TGGAGAAATACAAACACTCCTCCATTG	24	A	0.2833	0.2432
NC_005100.4-268640917	GGTCCCTCCACCTGAGCTCCATTCACC	3	T	0.1405	0.4995
NC_005100.4-268705129	GTTAGAGACACACAGGCTCCCCAAATG	7	G	0.1339	0.1699
NC_005100.4-268705129	GTTAGAGACACACAGGCTCCCCAAATG	22	C	0.1593	0.2036
NC_005100.4-268816967	AAGAGTATAACTGTAACTCCACAGACA	26	C	0.2185	0.3932
NC_005100.4-268839945	AGGGCTGCTACAACACCTCCCTGTTTT	13	A	0.0721	0.1023
NC_005100.4-268839945	AGGGCTGCTACAACACCTCCCTGTTTT	23	G	0.018	0.4883
NC_005100.4-268953419	GTTGGGCATACCATAGCTCCTTGTCAG	9	T	0	0.4844
NC_005100.4-268982782	ACTCAATCCACCACAACTCCTTCTGAT	2	C	0.1345	0.1946
NC_005100.4-269015283	CAGTTGATGACCGGACCTCCCAAGTTC	12	C	0.0265	0.0262
NC_005100.4-269015283	CAGTTGATGACCGGACCTCCCAAGTTC	13	G	0.2035	0.3189
NC_005100.4-269103468	TGAGTTCACACCTCAACTCCAGCGGAT	3	A	0.0081	0.4777
NC_005100.4-269103468	TGAGTTCACACCTCAACTCCAGCGGAT	24	G	0	0.4793
NC_005100.4-269138957	GGCCCACTCACTTGTTCTCCCACACTC	21	C	0.3248	0.405
NC_005100.4-269143114	GATACAAGAACCCAGACTCCTTAGTGC	2	A	0.1593	0.1898
NC_005100.4-269143114	GATACAAGAACCCAGACTCCTTAGTGC	27	C	0	0.4755
NC_005100.4-269176592	ACACTCCTGACACCACCTCCTCTGCTG	16	C	0	0.4839
NC_005100.4-269176592	ACACTCCTGACACCACCTCCTCTGCTG	25	C	0.0256	0.0253
NC_005100.4-269192094	CTCTTGGCAACTAGCACTCCATATCCG	27	G	0.025	0.0247
NC_005100.4-269207694	TGCTACAGTACCTGTGCTCCCAAGTAG	5	A	0	0.4765
NC_005100.4-269224908	ATGCCCTTAACTTTCACTCCTAGCTCA	1	A	0.0252	0.0249
NC_005100.4-269224908	ATGCCCTTAACTTTCACTCCTAGCTCA	21	T	0	0.4839
NC_005100.4-269244690	CCTCACAGAACAGGGACTCCAAAATTC	27	C	0.0085	0.4671
NC_005100.4-269259730	AGTGGAATTACCTCTGCTCCAATCTAA	2	G	0.1368	0.2107
NC_005100.4-269335590	TCACTAAATACAAGCACTCCACTGAGA	9	T	0.0342	0.0336
NC_005100.4-269341967	TCCAATAAGACTGCTTCTCCTAAGCCT	25	C	0.0333	0.4861
NC_005100.4-269364054	GAAAAGCCTACTTGGTCTCCTAGATCT	8	C	0.575	0.4097
NC_005100.4-269364054	GAAAAGCCTACTTGGTCTCCTAGATCT	25	T	0.575	0.4097
NC_005100.4-269427706	AAACAGTAGACCATTGCTCCTCACGTT	12	C	0	0.4819
NC_005100.4-269427706	AAACAGTAGACCATTGCTCCTCACGTT	15	T	0	0.4813
NC_005100.4-269482887	GAAGTGTGCACAGCTGCTCCGGGTCTG	7	T	0	0.4793
NC_005100.4-269482887	GAAGTGTGCACAGCTGCTCCGGGTCTG	21	G	0	0.4793
NC_005100.4-269605685	CATGAGTATACTTCACCTCCTAATAGT	9	T	0.0413	0.1163
NC_005100.4-269650488	CTGCAGATAACCACAGCTCCACTCAAT	26	A	0.2602	0.4759

NC_005100.4-269651558	TTTCATATCACTGTGTCTCCTCCCTCA	7	A	0.0732	0.0705
NC_005100.4-269710569	ATTAGGCTCACATACCCTCCTGAGATA	22	G	0.1597	0.5
NC_005100.4-269710569	ATTAGGCTCACATACCCTCCTGAGATA	24	G	0.0504	0.0491
NC_005100.4-269759946	AGTATGCACACCTCTACTCCAGACTCA	8	A	0.2564	0.3457
NC_005100.4-269777473	ATACCCCAAACCTCCTCTCCCTGTCTC	7	C	0.2435	0.4425
NC_005100.4-269953564	TTAAGCTACACCTAAGCTCCTGACCCA	9	C	0	0.4787
NC_005100.4-269961374	CACATTATCACTTGTGCTCCGGAATAA	23	A	0.2203	0.2344
NC_005100.4-269974201	CCTTTTATCACCCTGTCTCCACCCAAC	24	C	0.0333	0.4644
NC_005100.4-270039854	CCATAAAGTACTCTAGCTCCATCCTAT	1	C	0	0.4693
NC_005100.4-270068216	TCGCTTGATACCACTCCTCCAATGAAT	3	G	0	0.4724
NC_005100.4-270073009	TCTTGCTGTACAACAACTCCAGCCTTT	6	C	0.2348	0.4976
NC_005100.4-270073009	TCTTGCTGTACAACAACTCCAGCCTTT	9	T	0	0.4682
NC_005100.4-270093301	TCTTGTCCCACTTCCACTCCTCATACA	12	T	0	0.4787
NC_005100.4-270110119	TCCAGGCACACCCAAACTCCGTGTCCT	21	G	0.065	0.4904
NC_005100.4-270196229	TATGTAACAACATGGCCTCCTTCTCCA	5	T	0.1176	0.4983
NC_005100.4-270247382	CAACAGGCCACTCACTCTCCTTGGAGA	8	C	0.1171	0.2137
NC_005100.4-270267952	GACCATCCTACCTTGACTCCTTCTATG	26	T	0	0.4807
NC_005100.4-270308117	GGTTGTGATACACACACTCCGGCACAC	27	C	0.1311	0.1774
NC_005100.4-270349385	GTACATAACACTGTCGCTCCCTGAGTA	7	A	0.292	0.4648
NC_005100.4-270374029	ACTGAAAGAACCATCTCTCCAGCCTCA	7	A	0.065	0.1073
NC_005100.4-270374029	ACTGAAAGAACCATCTCTCCAGCCTCA	24	C	0.065	0.4497
NC_005100.4-270433624	GATAGCCTAACTGGCGCTCCCCACCCG	16	G	0.2348	0.4782
NC_005100.4-270433624	GATAGCCTAACTGGCGCTCCCCACCCG	21	C	0.0948	0.2057
NC_005100.4-270441789	ATCCTCCAAACCCCACCTCCAAGCACT	4	C	0	0.4793
NC_005100.4-270466675	TGGGGACGAACTTATTCTCCAGGTAGA	7	C	0	0.4813
NC_005100.4-270525209	GCAACTCAGACAAGAGCTCCATCTTCA	3	A	0.1441	0.4997
NC_005100.4-270525209	GCAACTCAGACAAGAGCTCCATCTTCA	4	A	0.0504	0.0805
NC_005100.4-270530585	TACCTCAAAACTTTGTCTCCTGCTTCT	12	T	0.042	0.0411
NC_005100.4-270692670	GAAGCAATCACTTTTGCTCCTTTGCAT	4	G	0.0991	0.4351
NC_005100.4-270692670	GAAGCAATCACTTTTGCTCCTTTGCAT	24	G	0.1171	0.2403
NC_005100.4-270751263	GTCTTTTTCACTGAAGCTCCAACAGCC	1	G	0	0.4778
NC_005100.4-270751263	GTCTTTTTCACTGAAGCTCCAACAGCC	13	G	0	0.4778
NC_005100.4-270779433	ATAACAATGACACAATCTCCACTATGT	13	C	0.1026	0.0973
NC_005100.4-270855101	TGCCCCACCACCTCGCCTCCCATCCAG	14	C	0.0496	0.0484
NC_005100.4-270855101	TGCCCCACCACCTCGCCTCCCATCCAG	15	G	0	0.4787
NC_005100.4-270866332	GTTCAATAAACTGAGTCTCCAAGGATG	9	A	0.2435	0.4425
NC_005100.4-270872418	CTGAAGAGTACTCTTTCTCCTTCTTAG	25	T	0.041	0.4677
NC_005100.4-270881586	GTACACATTACCAATACTCCATCAATG	2	T	0.0902	0.0861
NC_005100.4-270881586	GTACACATTACCAATACTCCATCAATG	9	T	0.0413	0.4889
NC_005100.4-270993240	ATCTTTATGACATTGCCTCCTCCAGTC	22	C	0.123	0.1436
NC_005100.4-271013813	GTTGTTGAGACAGTGTCTCCATAGTCC	14	T	0.0256	0.0253
NC_005100.4-271013813	GTTGTTGAGACAGTGTCTCCATAGTCC	26	C	0.0256	0.0253
NC_005100.4-271090467	TGGCTGAATACCATTCCTCCCTCTCTC	8	A	0.2602	0.3334
NC_005100.4-271943774	CTTGCCACCACTGACGCTCCTCCACTA	16	G	0	0.4844
NC_005100.4-271943774	CTTGCCACCACTGACGCTCCTCCACTA	27	A	0.0083	0.0247
NC_005100.4-271999469	GACTGGCGAACTCCACCTCCCTGTGCT	5	G	0.187	0.3091
NC_005100.4-271999469	GACTGGCGAACTCCACCTCCCTGTGCT	16	C	0.2683	0.356
NC_005100.4-272011179	AAGATCTTTACCAACCCTCCATTTGAT	7	T	0.0174	0.4833
NC_005100.4-272012249	AGCAGATAGACATGGCCTCCAGTGAAG	24	G	0.4167	0.4061
NC_005100.4-272028153	TCACAAGCAACCATGCCTCCAAAAGTC	1	T	0	0.4682
NC_005100.4-272028153	TCACAAGCAACCATGCCTCCAAAAGTC	4	C	0	0.4682

NC_005100.4-272262708	GCCAATTAGACCCTTACTCCACCCGTG	13	C	0.0265	0.0262
NC_005100.4-272320526	TCTTCAACAACACAATCTCCTCCTCTG	6	A	0.2793	0.4804
NC_005100.4-272400200	TTCTGAGTTACCCAGGCTCCCTGTTAG	13	C	0.0248	0.0245
NC_005100.4-272476958	GTGAATTTTACTGAGACTCCTAGCAAT	7	T	0.1488	0.2533
NC_005100.4-272501821	TAAGCAAGCACATATTCTCCCACCAAG	25	A	0.1583	0.1733
NC_005100.4-272604672	CCTCCTTCCACCCTAGCTCCATCAGTG	22	T	0.042	0.0882
NC_005100.4-272643022	CTGAGATGTACTTTAACTCCTTTATCT	2	T	0.2054	0.4933
NC_005100.4-272689046	GAGCCCATGACAAAATCTCCTATAACT	2	A	0.0973	0.4944
NC_005100.4-272789268	ACATCACAAACACTCGCTCCCCATTAC	15	C	0.1597	0.1746
NC_005100.4-272806026	CAAACCAGTACTCGAACTCCCACTGTC	25	G	0.2521	0.4567
NC_005100.4-272821090	TAAAGTGGCACAGAATCTCCTGGTACT	4	A	0	0.4731
NC_005100.4-272821090	TAAAGTGGCACAGAATCTCCTGGTACT	15	A	0	0.4731
NC_005100.4-272871542	CAAATGCCCACCTCTGCTCCTTATACA	4	A	0.0083	0.4783
NC_005100.4-272902948	GCTCCAAAAACTTTGTCTCCTTATCTC	4	C	0.1696	0.4996
NC_005100.4-272965111	CTGCCTATAACTCAAACTCCAAGTTAT	13	C	0.0917	0.1172
NC_005100.4-272971816	CTATTAATAACATAGGCTCCTTACCCA	13	T	0.1339	0.2385
NC_005100.4-272971816	CTATTAATAACATAGGCTCCTTACCCA	23	A	0.1947	0.4912
NC_005100.4-273025837	CACACACGCACACAGACTCCTATGACT	9	C	0.1121	0.1058
NC_005100.4-273094025	TGATCTTACACATTGGCTCCTGCCCTG	4	T	0.2231	0.3027
NC_005100.4-273110082	CTCTTCCGAACCCAATCTCCCAGCTCT	8	G	0	0.4793
NC_005100.4-273110082	CTCTTCCGAACCCAATCTCCCAGCTCT	26	C	0.0508	0.0812
NC_005100.4-273442041	TATGAAACCACATTTACTCCTTCTCAT	26	A	0.1176	0.2076
NC_005100.4-273467777	GATCTTCTCACCTCTGCTCCCAGCATC	15	T	0.0439	0.0429
NC_005100.4-273474953	GGTTACTCGACGCTGACTCCGTGGACT	8	C	0.0256	0.0253
NC_005100.4-273474953	GGTTACTCGACGCTGACTCCGTGGACT	12	G	0.0256	0.0253
NC_005100.4-273476797	TTCTCCCAGACAGACGCTCCTCCCTGT	1	T	0	0.48
NC_005100.4-273492520	ATTGTAGAGACCTACACTCCAGAATCC	23	A	0.1964	0.5
NC_005100.4-273540042	GCCAAAGAAACCCAGGCTCCCTCCATT	8	A	0	0.4773
NC_005100.4-273540042	GCCAAAGAAACCCAGGCTCCCTCCATT	15	G	0.2295	0.4914
NC_005100.4-273572047	CAGAGAGTAACCCTGACTCCTAACCGT	12	C	0.0263	0.026
NC_005100.4-273572047	CAGAGAGTAACCCTGACTCCTAACCGT	14	T	0.0086	0.4729
NC_005100.4-273649136	CTCCTCCTCACTCAGGCTCCTTCTCTC	15	G	0.1176	0.1678
NC_005100.4-273650817	GATCCTCCTACCCTACCTCCTGAACTA	6	T	0.0085	0.4823
NC_005100.4-273650817	GATCCTCCTACCCTACCTCCTGAACTA	25	C	0.0085	0.4823
NC_005100.4-273732879	GTGTCCAGAACAGAGTCTCCCTCCAGG	3	G	0.0656	0.0935
NC_005100.4-273732879	GTGTCCAGAACAGAGTCTCCCTCCAGG	12	A	0.0328	0.0322
NC_005100.4-273734448	CAGTCCTGAACCCTCCCTCCACACACA	5	C	0.0263	0.026
NC_005100.4-273734448	CAGTCCTGAACCCTCCCTCCACACACA	16	C	0.0696	0.0989
NC_005100.4-273745757	GAATGGCAGACTCAGCCTCCTGAGTTC	9	G	0.0088	0.4676
NC_005100.4-273750132	GCGTCTCCCACCTCAACTCCCCAATCA	4	T	0.045	0.0942
NC_005100.4-273781593	ACCGCCTGTACCTCGGCTCCAGGCAGT	3	C	0.1074	0.172
NC_005100.4-273796110	ATGTAAGAAACAGAGTCTCCTTATGAT	3	G	0.2821	0.3107
NC_005100.4-273819323	CACGTGTGCACATACACTCCATATATT	13	T	0.0246	0.0243
NC_005100.4-273823508	TCTTGTATCACATGTACTCCTGGTGTT	26	T	0.0417	0.0722
NC_005100.4-273848388	GGAAGGTAGACTCAGACTCCACAGGCA	23	A	0.2667	0.3832
NC_005100.4-273862976	CCAGTCCTCACAGCCCCTCCAGCTTAT	3	A	0.2114	0.2832
NC_005100.4-273867643	GTCACATCGACCTCTGCTCCCACAGTT	8	C	0.009	0.4869
NC_005100.4-273882856	TGCCCTCCCACCTCCACTCCATGGACA	25	A	0.0973	0.1239
NC_005100.4-273887237	ACAAAAGGCACTGAGGCTCCGTGCAGA	21	G	0.0431	0.0422
NC_005100.4-273944538	ATAAATTAAACCTCATCTCCAAGGGAA	12	C	0.0431	0.1058
NC_005100.4-273944538	ATAAATTAAACCTCATCTCCAAGGGAA	25	G	0.0087	0.4782

NC_005100.4-273947426	CTGAGAGAAACAGGGGCTCCTACACTT	15	G	0.2439	0.4331
NC_005100.4-273956254	AAACCTGGCACAAAGCCTCCGTGTGAG	21	G	0.1667	0.4999
NC_005100.4-273982960	TGCTGAAGAACGTGACCTCCCCTCTCT	1	T	0.2087	0.4891
NC_005100.4-274030281	GAGATGCTCACATCTCCTCCCTCTTCC	3	G	0.0435	0.4782
NC_005100.4-274071529	GCGTTTCTCACAGAGCCTCCTCATTAG	1	G	0.0504	0.4898
NC_005100.4-274106374	CAGTTAAGCACACAAACTCCTCTCTCT	26	C	0.0165	0.4819
NC_005100.4-274106374	CAGTTAAGCACACAAACTCCTCTCTCT	27	T	0.0163	0.4825
NC_005100.4-274119307	GAGAGAGTCACATCCTCTCCTTGGTCT	2	A	0.2167	0.4599
NC_005100.4-274132697	TCCTCTCATACACACACTCCACATTCA	12	A	0.15	0.3578
NC_005100.4-274132697	TCCTCTCATACACACACTCCACATTCA	23	A	0.0917	0.1733
NC_005100.4-274140683	ACACCACTCACCATTCCTCCGTGACCT	14	T	0	0.4793
NC_005100.4-274140683	ACACCACTCACCATTCCTCCGTGACCT	24	A	0	0.4793
NC_005100.4-274142822	GCCGGTGGGACAATGGCTCCATACCGG	26	G	0.2544	0.415
NC_005100.4-274144441	ACTTCCTGCACCCAGGCTCCATCTCGG	3	T	0.0336	0.033
NC_005100.4-274144441	ACTTCCTGCACCCAGGCTCCATCTCGG	26	G	0.2773	0.4377
NC_005100.4-274144842	TTCGTGGCTACACCATCTCCGTGAACA	4	G	0.0082	0.4755
NC_005100.4-274146520	AGTCAAGCCACTGCTTCTCCTTACCCT	7	G	0.0435	0.0425
NC_005100.4-274146520	AGTCAAGCCACTGCTTCTCCTTACCCT	16	T	0.0087	0.4817
NC_005100.4-274147603	CCAGACCACACCTGCTCTCCTAACCAG	14	G	0	0.4826
NC_005100.4-274147603	CCAGACCACACCTGCTCTCCTAACCAG	16	T	0.0172	0.488
NC_005100.4-274161688	CTTTTCAGAACCCTGGCTCCTTCGTAA	16	G	0.0348	0.0342
NC_005100.4-274162411	TGGCATGGTACCCTCCCTCCAGCTCTC	8	G	0	0.4773
NC_005100.4-274186812	TCCTCTAAGACGTGACCTCCTCGCTCC	7	A	0.1709	0.426
NC_005100.4-274186812	TCCTCTAAGACGTGACCTCCTCGCTCC	22	C	0.0085	0.0251
NC_005100.4-274195706	TGACCTATCACCTTTCCTCCCCTCCAT	26	A	0	0.4793
NC_005100.4-274219961	ATCTTCTCCACTTTCCCTCCTCCAGCC	12	T	0	0.4807
NC_005100.4-274230861	GACCATGCTACGTCTTCTCCACAACAC	25	C	0.2143	0.3047
NC_005100.4-274235002	ATAAATGAGACAGCTCCTCCTCCCTCA	3	A	0	0.4807
NC_005100.4-274242165	TGTGTCTACACCTCATCTCCCTCCTTC	4	G	0.1301	0.1356
NC_005100.4-274320037	AATGGAGAAACCCTGTCTCCAAAACAA	4	G	0.469	0.359
NC_005100.4-274320037	AATGGAGAAACCCTGTCTCCAAAACAA	16	T	0.469	0.359
NC_005100.4-274364004	AAGAATGAAACTGTAGCTCCACTGTGC	1	A	0.1513	0.1946
NC_005100.4-274364004	AAGAATGAAACTGTAGCTCCACTGTGC	3	G	0.1429	0.2012
NC_005100.4-274364747	TGGATTCTCACTTCCACTCCTGCAGTT	1	T	0	0.4793
NC_005100.4-274364747	TGGATTCTCACTTCCACTCCTGCAGTT	12	T	0.1545	0.208
NC_005100.4-274438994	CCCACGGAAACTCTTCCTCCCTGAAAT	5	C	0.1062	0.2036
NC_005100.4-274443149	CACAGAGAAACCTTGCCTCCCCATCCC	3	C	0.0488	0.0476
NC_005100.4-274467309	TCCTTGGGTACCTGCCCTCCCCCTTGG	9	T	0.5175	0.3836
NC_005100.4-274504148	CAAGACCAAACACAGCCTCCTAATTAG	1	C	0.1565	0.2519
NC_005100.4-274510658	CCAGCCTAGACCCTGTCTCCAAGGAGG	16	T	0.1789	0.4997
NC_005100.4-274515365	GGAATTCAAACACTTCCTCCAGGGACT	1	G	0.0254	0.0415
NC_005100.4-274585524	CCGCCCTCCACCTTCCCTCCTTCTGCC	14	T	0.0609	0.4946
NC_005100.4-274657686	CTGGTCTTCACCGACCCTCCGTTCCCC	15	C	0	0.4765
NC_005100.4-274693208	TGCCACTGCACTGCATCTCCAGCACAT	3	C	0	0.4793
NC_005100.4-274727624	GTGGTCACAACTACCTCTCCTGCTGAG	27	G	0.1951	0.3041
NC_005100.4-274785008	AGGAATTGTACCCCACCTCCAACACTC	13	C	0.0083	0.4847
NC_005100.4-274785008	AGGAATTGTACCCCACCTCCAACACTC	15	A	0.2667	0.2994
NC_005100.4-274786993	GGCTGGATGACCCAGCCTCCCTGTGTG	15	G	0.0492	0.048
NC_005100.4-274794942	CCCTTAGAGACAGGCCCTCCCACTTCT	5	T	0	0.4813
NC_005100.4-274794942	CCCTTAGAGACAGGCCCTCCCACTTCT	24	T	0	0.4813
NC_005100.4-274809528	CACTTCCAAACCCAGGCTCCCAGAGAA	25	G	0.2301	0.3982

NC_005100.4-274819634	CTTGATTGAACCTTCTCTCCAGCCCTG	15	C	0.2414	0.3835
NC_005100.4-274842318	TTGTCTGCAACAGTTTCTCCAGGTCTC	3	G	0.2797	0.4336
NC_005100.4-274842318	TTGTCTGCAACAGTTTCTCCAGGTCTC	15	T	0	0.4779
NC_005100.4-274848654	TGCTTTTGTACGGGGTCTCCTTGTATC	12	G	0.0248	0.0245
NC_005100.4-274848654	TGCTTTTGTACGGGGTCTCCTTGTATC	15	G	0.1736	0.4988
NC_005100.4-274867626	CTCTAGATGACCCCAACTCCTCAGTTG	25	T	0.041	0.0401
NC_005100.4-274879509	AACCTAAAAACCGAGGCTCCGGCTGCA	22	G	0.0614	0.4796
NC_005100.4-274887228	GCTCAATGCACTCAGCCTCCAAATATA	5	A	0.3025	0.3599
NC_005100.4-274890333	AGCTTGTGAACAGAATCTCCTGATAGG	22	G	0.0083	0.4783
NC_005100.4-274914870	AGTCTCCTTACTCTGTCTCCTGAGTGC	16	T	0	0.4922
NC_005100.4-274935955	CTGCCGAAGACTGAGACTCCAGGGATG	6	G	0.0333	0.0799
NC_005100.4-274981559	CTTCCTCCCACTATCCCTCCCTCATTC	4	C	0.187	0.3287
NC_005100.4-274982133	AAGCACCTTACATTTTCTCCTTCTGTG	6	C	0.0165	0.4751
NC_005100.4-275007757	CAGATTCAAACTCAGGCTCCTATGCTT	1	C	0	0.4833
NC_005100.4-275007757	CAGATTCAAACTCAGGCTCCTATGCTT	2	A	0	0.4833
NC_005100.4-275023758	GTACTGAGCACATGGTCTCCTTATAGG	13	T	0.05	0.0644
NC_005100.4-275029858	TGTAGTGATACAATGCCTCCTAATGAT	9	T	0.1901	0.4978
NC_005100.4-275029858	TGTAGTGATACAATGCCTCCTAATGAT	16	C	0.2	0.4965
NC_005100.4-275036847	GACTCATGCACCTTCTCTCCTTCCACA	2	A	0	0.4773
NC_005100.4-275103931	AATCAGTACACTCACCCTCCTCCAAAC	15	C	0.0087	0.0425
NC_005100.4-275200314	CCTATATAGACTGCTCCTCCCAACATT	21	C	0.0446	0.0605
NC_005100.4-275220058	CTTACCCATACACACCCTCCTACCTCC	14	A	0.0246	0.4755
NC_005100.4-275297656	AGTCATGCAACTTAACCTCCTTAATCT	26	C	0.1557	0.2457
NC_005100.4-275357015	GTGATGAGAACGACCACTCCAACACAA	1	G	0.0172	0.0339
NC_005100.4-275357015	GTGATGAGAACGACCACTCCAACACAA	22	A	0.1466	0.2057
NC_005100.4-275402818	GTCAGTCCCACAGCCCCTCCCCTCGCA	24	C	0.2705	0.4379
NC_005100.4-275405973	CATATCTAAACCAGTGCTCCCCACGCT	15	T	0.0531	0.1006
NC_005100.4-275405973	CATATCTAAACCAGTGCTCCCCACGCT	22	C	0.0531	0.1006
NC_005100.4-275417824	AGACGTTGGACAAATCCTCCCTGCTAT	26	A	0	0.4807
NC_005100.4-275475241	GAAGCTAAGACTGCAACTCCAGTGGTA	1	G	0	0.4825
NC_005100.4-275496201	CAGATCAGAACTACAGCTCCAAATACG	9	A	0	0.4807
NC_005100.4-275511640	CTTGTCACAACTTCCCCTCCCTCCTGG	16	C	0.0407	0.0398
NC_005100.4-275585721	TGAAAGTTCACACCTTCTCCATGATTG	7	T	0.1441	0.1621
NC_005100.4-275646430	TGGGGTTCCACAGCTTCTCCATCAGAG	14	C	0	0.482
NC_005100.4-275684554	ACTTGGAATACATGACCTCCTCCACAG	1	A	0.1607	0.2188
NC_005100.4-275701705	ACGTGACACACAGAAGCTCCTGTGACT	3	G	0.0496	0.0639
NC_005100.4-275732900	ACAATATACACAATAACTCCTACCTAT	8	A	0	0.4773
NC_005100.4-275747992	CTGGTTTCTACAAGAACTCCAAGTTAA	16	A	0	0.4773
NC_005100.4-275773897	GTGACGATTACTCTTGCTCCTGGCCAT	7	A	0.0081	0.481
NC_005100.4-275785117	ACGGCAAACACCTAAACTCCGAGAGGA	2	C	0.1966	0.4882
NC_005100.4-275785117	ACGGCAAACACCTAAACTCCGAGAGGA	21	G	0.2051	0.4894
NC_005100.4-275786700	GCAAGCCTAACAGAAACTCCATCCATA	1	G	0.1842	0.3168
NC_005100.4-275811798	GGAGCCTGGACATCGGCTCCAGAGGAT	15	G	0.0948	0.497
NC_005100.4-275826826	ATCTCTCCAACTTCTTCTCCCCAAACT	21	C	0.1034	0.3995
NC_005100.4-275837135	TTAGCCTGCACTAAGTCTCCAGCAAAG	13	A	0	0.4773
NC_005100.4-275839942	TTCCCTCAAACCTCGCCTCCACTTCTG	1	T	0.0265	0.06
NC_005100.4-275839942	TTCCCTCAAACCTCGCCTCCACTTCTG	27	G	0	0.4871
NC_005100.4-275846495	CACTCTCTGACGCTAGCTCCTTCTTCA	15	A	0.0083	0.4835
NC_005100.4-275850620	AGGCTCAGCACATGGTCTCCACATGGG	23	A	0	0.4773
NC_005100.4-275853349	GCCTGCCTGACCCTCCCTCCCATTTCC	24	T	0.125	0.1327
NC_005100.4-275893302	CAAAATAGTACAGAGTCTCCTTTACGG	26	G	0.1034	0.1134

NC_005100.4-275901354	GTCCATGTAACTACCCCTCCTCTGCAG	13	A	0.1368	0.4982
NC_005100.4-275916324	TCTGTAGGCACCCAAACTCCCCAGCCT	22	C	0.2857	0.4347
NC_005100.4-275959989	AATATCCAGACTTTTCCTCCGCATATA	26	T	0.1504	0.4873
NC_005100.4-275994767	CACACAAGCACGCCTGCTCCCCCACCA	15	T	0	0.4773
NC_005100.4-275994767	CACACAAGCACGCCTGCTCCCCCACCA	24	A	0.0902	0.0861
NC_005100.4-275995158	CAGTTCTGAACAAATCCTCCAGCCGTC	24	C	0.1379	0.1717
NC_005100.4-275997979	GAAAGACGAACTGCGCCTCCAAACAAG	23	A	0.075	0.0875
NC_005100.4-276000696	CACCAAAACACCAAGACTCCTCAAGTT	4	C	0	0.4819
NC_005100.4-276000696	CACCAAAACACCAAGACTCCTCAAGTT	27	T	0	0.4819
NC_005100.4-276034655	AGTTAAGGGACGTTAACTCCACAGGTA	12	G	0.0625	0.0605
NC_005100.4-276060639	CTTCCGGACACTGTCACTCCGTTCTGA	23	T	0.2124	0.4207
NC_005100.4-276065190	ACTGACTGAACTCTATCTCCAGTTCAT	12	T	0.0081	0.0398
NC_005100.4-276065190	ACTGACTGAACTCTATCTCCAGTTCAT	16	T	0.2439	0.4389
NC_005100.4-276114189	TGTTGGGTCACTGTGCCTCCTGGGGAG	27	G	0.1552	0.2122
NC_005100.4-276132724	CTCCTTGAGACAAGGCCTCCTTGGGGT	6	T	0.1681	0.2907
NC_005100.4-276147800	TCCCCACTGACAAGATCTCCTAAGGCA	26	C	0.0088	0.4861
NC_005100.4-276149582	AAAACGTTTACAGTCTCTCCTTTAAAA	6	G	0.0333	0.0328
NC_005100.4-276152990	CTGAGGACCACTGGGCCTCCTTAGGCC	15	G	0.2195	0.4868
NC_005100.4-276194977	GGCCTGCCCACCGTTGCTCCTGAATAC	13	G	0.25	0.4688
NC_005100.4-276211000	AGCCACCGAACCTTCTCTCCAACCCCA	8	G	0.0351	0.0345
NC_005100.4-276236657	GGGCACGTCACCCCAGCTCCTTCCTTT	7	G	0.0504	0.065
NC_005100.4-276287759	ATTGAGAACACTTGCTCTCCAGGTAGA	13	T	0	0.4765
NC_005100.4-276294563	CTGCACCACACTCTGTCTCCACAGTCT	9	C	0.0082	0.4822
NC_005100.4-276294563	CTGCACCACACTCTGTCTCCACAGTCT	24	G	0.0082	0.0243
NC_005100.4-276304186	CACACCTGAACAAGAGCTCCAACACTG	9	A	0	0.4793
NC_005100.4-276307941	ATGAAATTCACACTTCCTCCAGTTGTA	24	T	0.009	0.0267
NC_005100.4-276454329	CTTCATCTCACCTCACCTCCATCGTGG	2	T	0	0.488
NC_005100.4-276456129	GCATCTCCCACAAGTCCTCCCTGCTCC	15	T	0	0.4912
NC_005100.4-276467899	TGAGTTTGGACCAATGCTCCTGAGTAG	22	G	0	0.4779
NC_005100.4-276526009	CATGCCGTCACTTACTCTCCTGCTAGA	15	C	0.1858	0.4923
NC_005100.4-276533599	CTTCTCCTGACAACTGCTCCTCTTCCT	15	T	0.177	0.4574
NC_005100.4-276584600	TCAGGCGTTACCTCAGCTCCTGCCCTA	3	A	0	0.4773
NC_005100.4-276593735	GCCACTCGCACAGGCTCTCCTCTCCAG	4	A	0	0.4793
NC_005100.4-276663859	GGGTCGTTGACCATTGCTCCAAGCAAA	23	G	0.3826	0.4637
NC_005100.4-276706139	GGGAGGCTCACGGCCCCTCCGGCCCAT	12	G	0.1967	0.2399
NC_005100.4-276706139	GGGAGGCTCACGGCCCCTCCGGCCCAT	21	G	0.1967	0.4966
NC_005100.4-276732578	TCACTCTGTACTCTACCTCCTGACAAC	5	T	0	0.4846
NC_005100.4-276770349	TCCCTGCTTACCATCTCTCCAGGCTCC	27	C	0.0894	0.1145
NC_005100.4-276781945	AGATATCCTACCTCCTCTCCTTTGAAA	23	T	0	0.4688
NC_005100.4-276788881	CCCCTCTACACCCCCACTCCTATTCCT	6	C	0.2982	0.3115
NC_005100.4-276788881	CCCCTCTACACCCCCACTCCTATTCCT	8	A	0.0088	0.4676
NC_005100.4-276803761	ACAACCTCCACCCCCACTCCACAAACA	14	C	0.0909	0.1163
NC_005100.4-276810220	TTTCTGAAAACCGCCCCTCCATCTGTT	12	C	0.069	0.0666
NC_005100.4-276810220	TTTCTGAAAACCGCCCCTCCATCTGTT	13	G	0.069	0.1134
NC_005100.4-276896915	TGAGTACAGACCTTGACTCCATCCAAT	27	T	0.0086	0.0255
NC_005100.4-276950247	AGCCATTCTACTTGTTCTCCCCTTCTC	22	C	0.2232	0.2758
NC_005100.4-276955000	CAAGGCATGACCACATCTCCATGGTAC	7	A	0.0496	0.0484
NC_005100.4-276955000	CAAGGCATGACCACATCTCCATGGTAC	27	C	0.3583	0.4941
NC_005100.4-276967810	GAGTAGTGTACAGCTCCTCCTGTGCAT	9	T	0.3636	0.4246
NC_005100.4-276967810	GAGTAGTGTACAGCTCCTCCTGTGCAT	13	G	0.0248	0.0245
NC_005100.4-277024776	CAGGTGCACACCCACACTCCAGAAGTA	15	C	0.0163	0.4854

NC_005100.4-277024776	CAGGTGCACACCCACACTCCAGAAGTA	24	A	0	0.4793
NC_005100.4-277025290	CTACTAGTTACCAAGCCTCCCTTCCTT	21	C	0.0086	0.0255
NC_005100.4-277025290	CTACTAGTTACCAAGCCTCCCTTCCTT	27	T	0.0086	0.0255
NC_005100.4-277030963	CTGCCAGCAACTCCAGCTCCCGGGGAT	4	C	0.0684	0.1125
NC_005100.4-277038121	GGCTCTCAGACAAGGCCTCCCACACAT	23	C	0	0.4898
NC_005100.4-277059060	CAGGGGACGACTTAAACTCCAGAGACA	8	C	0.157	0.4951
NC_005100.4-277062044	CAGGGGGAGACGCATGCTCCCTGCCTT	8	A	0.0167	0.4861
NC_005100.4-277109478	ACGAGGTTCACGAGTTCTCCACACCCC	6	G	0.0526	0.0512
NC_005100.4-277116495	CTGCAAAGGACTGCCCCTCCGTGTCCA	4	C	0.2049	0.2686
NC_005100.4-277150293	CACTTACATACATTCTCTCCCTTTTAC	14	T	0.2846	0.319
NC_005100.4-277150293	CACTTACATACATTCTCTCCCTTTTAC	23	T	0.3279	0.435
NC_005100.4-277151777	GCTTTGTAGACACTACCTCCGAATCTT	15	A	0.0168	0.033
NC_005100.4-277153728	AGAACATACACAAAACCTCCATCCCGG	25	C	0.2768	0.4751
NC_005100.4-277153728	AGAACATACACAAAACCTCCATCCCGG	27	G	0.2768	0.4751
NC_005100.4-277187600	TGGTGTCATACAATGCCTCCCGGGGGG	3	G	0.0804	0.0934
NC_005100.4-277193187	AGGATTGAGACTTAGACTCCTGCATTT	3	G	0	0.4864
NC_005100.4-277193187	AGGATTGAGACTTAGACTCCTGCATTT	24	A	0	0.4864
NC_005100.4-277194991	CTTCCGTACACCCTCTCTCCTCGGGTT	25	G	0.069	0.1134
NC_005100.4-277220315	ATCGGAGGTACAGGGACTCCTTCCTTC	8	G	0.2975	0.389
NC_005100.4-277228312	GGAGATGGAACCGTGGCTCCGCAGTTA	12	C	0.2397	0.2814
NC_005100.4-277228312	GGAGATGGAACCGTGGCTCCGCAGTTA	15	G	0.2975	0.3967
NC_005100.4-277228442	ATGGCCCCCACAGACACTCCACATATG	27	G	0.2773	0.2961
NC_005100.4-277240862	GTGCTTTTGACAGGGCCTCCTTCTCAT	12	A	0.1864	0.3953
NC_005100.4-277296574	TTGGCAACTACCACATCTCCAGTGGCA	25	G	0	0.4677
NC_005100.4-277299559	AAACTGGACACCACAACTCCACAGTGC	24	G	0.0579	0.0562
NC_005100.4-277339723	TCTTTAAACACTGTATCTCCTTGAGCA	14	T	0.6066	0.4226
NC_005100.4-277339723	TCTTTAAACACTGTATCTCCTTGAGCA	24	A	0.6066	0.4226
NC_005100.4-277351465	ATAGAACGGACATGTTCTCCTCGATTC	7	C	0.4103	0.4839
NC_005100.4-277353484	ATAGTCAAAACTCATTCTCCATATAGT	4	G	0.0877	0.0997
NC_005100.4-277419113	CCATGGTCCACCTGTGCTCCTATGATG	26	T	0.1653	0.2533
NC_005100.4-277450274	CATGGCTGGACAGAGACTCCAAGTTGA	1	C	0.0579	0.1017
NC_005100.4-277450274	CATGGCTGGACAGAGACTCCAAGTTGA	4	G	0.0826	0.1377
NC_005100.4-277458272	GGCAGAGCAACACAGACTCCTGCTGTA	1	G	0	0.4743
NC_005100.4-277458272	GGCAGAGCAACACAGACTCCTGCTGTA	23	C	0.1176	0.1398
NC_005100.4-277516358	CTCCTACTGACCAAATCTCCTTCGCCT	8	T	0	0.4807
NC_005100.4-277517002	CCACGACAGACCCAGTCTCCAAACATA	4	C	0	0.4814
NC_005100.4-277517002	CCACGACAGACCCAGTCTCCAAACATA	22	A	0.3009	0.39
NC_005100.4-277562763	GTCACCCGGACAAGCTCTCCCGCCACA	21	C	0.2609	0.4364
NC_005100.4-277562763	GTCACCCGGACAAGCTCTCCCGCCACA	27	A	0	0.4793
NC_005100.4-277582587	AATGGTAGGACGGTTCCTCCGTTGTGT	3	T	0.178	0.1894
NC_005100.4-277591482	GGACAGGAGACAGCTGCTCCTGAGTTT	2	G	0.2018	0.5
NC_005100.4-277591482	GGACAGGAGACAGCTGCTCCTGAGTTT	14	C	0.3097	0.3941
NC_005100.4-277599340	TCACTCCTAACGCTCCCTCCACACTCC	4	C	0.009	0.4726
NC_005100.4-277632868	TTTACTGCCACACTTTCTCCAAACCAT	8	C	0.0991	0.4414
NC_005100.4-277664461	CCAGTGAATACATTCACTCCTTGGTTA	7	A	0	0.4846
NC_005100.4-277664461	CCAGTGAATACATTCACTCCTTGGTTA	13	T	0	0.4846
NC_005100.4-277682469	ATGCCAACCACAGCAGCTCCCTGGGGT	14	C	0	0.4728
NC_005100.4-277682469	ATGCCAACCACAGCAGCTCCCTGGGGT	26	G	0	0.4728
NC_005100.4-277696462	CAGATACACACCACTGCTCCCAGCTTT	12	C	0.025	0.0247
NC_005100.4-277696462	CAGATACACACCACTGCTCCCAGCTTT	25	T	0	0.4751
NC_005100.4-277724146	GGAGAACAAACTCATGCTCCTTTATAA	2	G	0.15	0.3578

NC_005100.4-277734101	TGCATCTTAACAGGGTCTCCTTGAGCT	14	G	0.1754	0.3006
NC_005100.4-277748740	GAGCTGCTCACAGCCTCTCCATCCTCT	21	A	0.1057	0.4952
NC_005100.4-277759081	CGGAGAACCACAGTTGCTCCCCTCTAC	7	A	0.0263	0.0429
NC_005100.4-277766037	CTAGCCAGCACGGTTCCTCCAAAAGGG	14	T	0.0508	0.0496
NC_005100.4-277792759	ATGGGTAGAACCTTGGCTCCCCAGGCA	3	G	0.0796	0.1968
NC_005100.4-277802896	TTCCTGTTCACTCTGCCTCCTGATGGC	24	T	0.1833	0.3578
NC_005100.4-277807550	CCTGGAAGAACAAACCCTCCAAGACCT	27	T	0	0.4841
NC_005100.4-277816992	CATGCGTGTACCCCATCTCCTCATATG	5	C	0.2541	0.4056
NC_005100.4-277816992	CATGCGTGTACCCCATCTCCTCATATG	13	C	0.2623	0.3447
NC_005100.4-277832365	ATCCAGAACACAGCCACTCCGTATAGT	13	G	0.0081	0.0241
NC_005100.4-277832365	ATCCAGAACACAGCCACTCCGTATAGT	15	C	0	0.4793
NC_005100.4-277838591	GGGAGTGAAACCCAGGCTCCTGGTTCT	7	G	0.0357	0.0853
NC_005100.4-277843186	CACTGAGGAACCCTGCCTCCAGTTGAT	23	T	0.0583	0.0875
NC_005100.4-277957178	GAGTTGAACACAATGACTCCTACATAC	16	A	0.2645	0.4787
NC_005100.4-277957178	GAGTTGAACACAATGACTCCTACATAC	24	A	0	0.4787
NC_005100.4-277985571	CCTAGCCCCACACAGCCTCCACAGCAG	1	C	0.0348	0.0342
NC_005100.4-277985571	CCTAGCCCCACACAGCCTCCACAGCAG	26	A	0.0088	0.4913
NC_005100.4-278017245	TCTGGCTAAACATGTGCTCCAACTCCC	4	G	0.2308	0.4928
NC_005100.4-278017245	TCTGGCTAAACATGTGCTCCAACTCCC	6	C	0.225	0.2722
NC_005100.4-278042192	CAGAGCTGCACAATTTCTCCAGGCACC	26	C	0.2301	0.2913
NC_005100.4-278045560	GCGCCTTTAACTCCAGCTCCGAGGGAT	3	G	0.1322	0.1918
NC_005100.4-278045560	GCGCCTTTAACTCCAGCTCCGAGGGAT	21	G	0.2295	0.3947
NC_005100.4-278058635	TGGACCTAGACTCTCCCTCCAGGAGGA	26	G	0.0082	0.4755
NC_005100.4-278068053	GTGGCGCCAACTGTCTCTCCACTGCTG	5	C	0.2893	0.4732
NC_005100.4-278108789	CTAGTTCCTACCCATCCTCCCGGGGGA	24	G	0.0619	0.0765
NC_005100.4-278127578	TATCCTGAGACAAGATCTCCGTGTGAA	2	A	0	0.4779
NC_005100.4-278127578	TATCCTGAGACAAGATCTCCGTGTGAA	21	G	0.042	0.0571
NC_005100.4-278132270	GACACTGCCACACCATCTCCTTATCCC	5	C	0.0082	0.4822
NC_005100.4-278132270	GACACTGCCACACCATCTCCTTATCCC	6	T	0.0082	0.4822
NC_005100.4-278153925	TCCTCTGCCACAGTCACTCCTGTCTTT	16	A	0	0.4856
NC_005100.4-278221521	TTAAGTTCCACGCTTCCTCCACACCGT	2	T	0.2051	0.4956
NC_005100.4-278298232	AGAGTCCCTACCCTCTCTCCTGGTCTG	1	A	0.0081	0.0241
NC_005100.4-278298232	AGAGTCCCTACCCTCTCTCCTGGTCTG	2	G	0.0081	0.0241
NC_005100.4-278335342	GGTTCTGGAACCCTCCCTCCCTGCCCG	26	C	0.1345	0.1398
NC_005100.4-278343962	GCATGGTAAACTCACTCTCCTCCTTAG	14	A	0	0.4746
NC_005100.4-278420955	CCACTGCCCACAACCTCTCCAGTTATG	2	C	0.0789	0.0918
NC_005100.4-278420955	CCACTGCCCACAACCTCTCCAGTTATG	6	G	0	0.4859
NC_005100.4-278428703	TAGAGGAGGACTTCAACTCCAACTTTT	8	G	0.2541	0.4379
NC_005100.4-278458867	ATGCATGCTACCAGAACTCCCTCCCTG	5	A	0.252	0.4301
NC_005100.4-278462338	AAGATCCTCACAGCCTCTCCAAGGCTC	7	C	0.0924	0.1033
NC_005100.4-278462338	AAGATCCTCACAGCCTCTCCAAGGCTC	8	T	0.0924	0.1033
NC_005100.4-278492996	AGCCCAGGTACCTTACCTCCCCACCAC	13	T	0.0339	0.0333
NC_005100.4-278494625	TATACAAAGACATGTGCTCCTACTAAC	3	T	0	0.4807
NC_005100.4-278494625	TATACAAAGACATGTGCTCCTACTAAC	6	A	0	0.4819
NC_005100.4-278613935	TCTTACTGCACCGGTTCTCCCTCAGCT	9	C	0.0081	0.0398
NC_005100.4-278613935	TCTTACTGCACCGGTTCTCCCTCAGCT	12	C	0.0732	0.1001
NC_005100.4-278623467	GCACATTCCACTCCCACTCCCAACAAT	27	T	0.1833	0.4988
NC_005100.4-278693644	CCCTGTCAGACTCTATCTCCTTCCTGC	6	T	0.2605	0.4542
NC_005100.4-278694996	CTGACCAACACCCTCACTCCAGCTGTC	7	A	0	0.4751
NC_005100.4-278697216	AAGGTACAGACACTTTCTCCCTTTCCC	14	T	0.0357	0.0689
NC_005100.4-278744433	GGCAGAGTCACTGTCCCTCCAATTACA	13	G	0.2719	0.3661

NC_005100.4-278744433	GGCAGAGTCACTGTCCCTCCAATTACA	15	C	0.0087	0.0257
NC_005100.4-278750963	TAGCTGGTCACTGTGCCTCCCTAACCA	4	C	0.2893	0.2922
NC_005100.4-278838044	GCACGTTTGACAGCCCCTCCTGAACCC	25	C	0.2623	0.4807
NC_005100.4-278867332	AAGCCTTCCACAGACGCTCCCACTGTG	3	G	0.042	0.0411
NC_005100.4-278867332	AAGCCTTCCACAGACGCTCCCACTGTG	14	A	0.0417	0.0408
NC_005100.4-278870887	CTTGATATCACTTCCGCTCCTGTCCCC	6	T	0.0087	0.0257
NC_005100.4-278893275	GTTCAGAGGACCTGTTCTCCTGCTGTA	26	T	0.0083	0.4769
NC_005100.4-278899080	GCGGAAGACACTGTAACTCCTAGATGA	1	G	0	0.48
NC_005100.4-278920169	CCCCCAAATACTGCCACTCCCTGAGCC	22	T	0.0088	0.4873
NC_005100.4-279015406	TTCTCAGTGACACACTCTCCTCTCAAG	25	A	0.287	0.3639
NC_005100.4-279037347	TTGAAGTCCACATACCCTCCTGATAAT	12	A	0.2458	0.4138
NC_005100.4-279038871	CTTGAAATCACAACCCCTCCAGATGTT	9	C	0.2411	0.3418
NC_005100.4-279038871	CTTGAAATCACAACCCCTCCAGATGTT	26	T	0.0089	0.4789
NC_005100.4-279065709	CCCATCGAGACATTTCCTCCAGGGTAG	22	G	0.0265	0.0262
NC_005100.4-279065709	CCCATCGAGACATTTCCTCCAGGGTAG	26	A	0	0.4912
NC_005100.4-279066554	CATTCCCTCACGCCTGCTCCCCTCATG	12	G	0.1282	0.1492
NC_005100.4-279088663	CTGGAGAGGACTGTTTCTCCCACTCAG	26	A	0.0081	0.0241
NC_005100.4-279129465	AATTGCCCTACCATCTCTCCAGCCCTT	3	T	0	0.4826
NC_005100.4-279152174	CCCTGTGTCACAGGTGCTCCGGGCGGG	21	G	0.0083	0.4769
NC_005100.4-279152174	CCCTGTGTCACAGGTGCTCCGGGCGGG	24	C	0.0744	0.0716
NC_005100.4-279159863	GCAAGGCTGACTTGGTCTCCTGCTGCC	21	T	0.0268	0.0264
NC_005100.4-279161779	GACTTTGTTACTAGAACTCCAGTGGTA	21	A	0.0254	0.0251
NC_005100.4-279170414	TACAGAGGGACCTGGGCTCCCGAAGGA	13	T	0	0.4793
NC_005100.4-279170414	TACAGAGGGACCTGGGCTCCCGAAGGA	27	A	0.0246	0.0243
NC_005100.4-279176037	AAGGGCATAACTGACCCTCCCTCGGGC	24	G	0	0.4922
NC_005100.4-279211386	AAAGCCCATACCAGACCTCCTTGTCTG	7	C	0.0339	0.0333
NC_005100.4-279211386	AAAGCCCATACCAGACCTCCTTGTCTG	9	T	0.0085	0.4842
NC_005100.4-279230736	AATGTTACGACAGATTCTCCAGCCCCC	9	G	0.0917	0.1597
NC_005100.4-279230736	AATGTTACGACAGATTCTCCAGCCCCC	23	C	0	0.4819
NC_005100.4-279231543	CTGTCTCTCACCATAGCTCCACAGCAC	22	C	0.0431	0.0746
NC_005100.4-279244336	GGATGTTACACTCTTACTCCTGCGGAT	6	T	0.1453	0.2662
NC_005100.4-279244336	GGATGTTACACTCTTACTCCTGCGGAT	23	C	0	0.488
NC_005100.4-279265122	CTGCAGCCCACCAGGCCTCCTGAGCTC	21	T	0.1138	0.1216
NC_005100.4-279625000	CTATAAAATACCGACACTCCCGCAGGT	12	C	0.1491	0.1672
NC_005100.4-279670083	GGAACCGACACGCTGTCTCCATCTTAC	5	C	0	0.0322
NC_005100.4-279670083	GGAACCGACACGCTGTCTCCATCTTAC	13	C	0.0328	0.048
NC_005100.4-279721117	CCACAGTGGACCCTGCCTCCTTTTAAA	14	T	0.0246	0.4755
NC_005100.4-279730986	CGGTGCAGGACTCCTCCTCCTCTCTAT	15	T	0.3443	0.3947
NC_005100.4-279747257	ATTTCCCCCACAGGTCCTCCTTTCACT	13	G	0	0.4832
NC_005100.4-279747591	CCTGATCCCACTCCCTCTCCTTTTTCC	21	T	0.0167	0.4861
NC_005100.4-279757940	GAAGCATCCACATTGTCTCCCATAATG	6	A	0.0783	0.4998
NC_005100.4-279843120	CACTGACTGACACCTCCTCCTATTCTT	23	T	0.1217	0.4991
NC_005100.4-279957603	GTCAGGTCTACCGTGCCTCCGCCACTG	24	A	0.1488	0.5
NC_005100.4-279961492	GAGCTTAAGACCCACCCTCCTGGCAGA	24	C	0.1171	0.4998
NC_005100.4-279962975	GTCCCAGTCACCTACCCTCCAGACTTG	2	T	0.2269	0.491
NC_005100.4-279982741	CCGGGAGCCACCCTCTCTCCACCTGCT	2	C	0.1933	0.4965
NC_005100.4-279990407	AGAACTCGGACACACCCTCCAACATTA	7	C	0	0.4793
NC_005100.4-280014134	TCTCCAAGAACCCTACCTCCTGCCTCA	7	A	0.0265	0.0262
NC_005100.4-280014134	TCTCCAAGAACCCTACCTCCTGCCTCA	12	C	0.0357	0.0351
NC_005100.4-280018777	CTCACAGCCACCCGGCCTCCTGTCCTG	13	C	0.1885	0.5
NC_005100.4-280018777	CTCACAGCCACCCGGCCTCCTGTCCTG	21	T	0.1311	0.1505

NC_005100.4-280021550	TGCAGCAGGACCAAGACTCCAACGTGC	23	C	0.0169	0.4718
NC_005100.4-280034825	ACCTTGGGCACTGTTCCTCCGGTGCCA	2	C	0.0435	0.4903
NC_005100.4-280034825	ACCTTGGGCACTGTTCCTCCGGTGCCA	5	T	0.0435	0.0425
NC_005100.4-280047549	CCTACAAATACCCTCTCTCCGTCCCCT	3	T	0.1504	0.4994
NC_005100.4-280047549	CCTACAAATACCCTCTCTCCGTCCCCT	16	T	0.1416	0.1316
NC_005100.4-280093971	ATGAGATGAACCCTGTCTCCCCACTCA	22	C	0.1709	0.4997
NC_005100.4-280137602	TATTTCCACACATACACTCCCACACAT	23	C	0.0175	0.4846
NC_005100.4-280157496	ATCCTTACAACAGCCTCTCCGAGTCAC	26	A	0.0661	0.0792
NC_005100.4-280169456	ATCACTACAACACAGCCTCCTGGATCT	14	A	0.0756	0.0882
NC_005100.4-280169456	ATCACTACAACACAGCCTCCTGGATCT	24	A	0.0756	0.0882
NC_005100.4-280173551	AGAACCCAGACTCATACTCCTTCCCTC	21	T	0	0.4793
NC_005100.4-280276590	AAACCACCAACATTTCCTCCTCGTCAT	23	G	0.0769	0.074
NC_005100.4-280323553	AGCAAGGCCACACCTCCTCCAATAAGG	8	C	0.3805	0.3081
NC_005100.4-280347512	AATCTTAGAACAGGGTCTCCTCTATCC	7	A	0	0.4819
NC_005100.4-280362138	CACACACACACACACACTCCTGCCTAC	16	A	0.1667	0.1528
NC_005100.4-280401427	GTGTTTCTCACGCAACCTCCCCTGTTC	6	T	0.0738	0.071
NC_005100.4-280402064	GAGGAGTATACACAGCCTCCTACATCC	9	T	0.1739	0.3403
NC_005100.4-280414601	CAAGCAGCCACCCAAGCTCCCAGGGCC	23	G	0.0325	0.032
NC_005100.4-280418513	TTCTGTTCCACTTGCCCTCCTCTCCCA	1	T	0.0708	0.0846
NC_005100.4-280431737	CATAACAACACAGTAGCTCCAAGCTTG	9	C	0.0244	0.0241
NC_005100.4-280431737	CATAACAACACAGTAGCTCCAAGCTTG	23	G	0.1951	0.4825
NC_005100.4-280463050	CCTGCTGCCACATCTTCTCCGTGATCC	3	T	0.0084	0.4797
NC_005100.4-280463050	CCTGCTGCCACATCTTCTCCGTGATCC	21	G	0.1653	0.2046
NC_005100.4-280494774	TTATTTTACACAATGGCTCCTCTAATG	22	C	0.0089	0.4751
NC_005100.4-280534904	GCAAAGCAGACCAGGCCTCCAGGTTCT	25	T	0.5517	0.3995
NC_005100.4-280561663	GCCTGTGACACCTTCTCTCCATGCACA	4	T	0.0268	0.4952
NC_005100.4-280561663	GCCTGTGACACCTTCTCTCCATGCACA	24	C	0.0089	0.4933
NC_005100.4-280566815	ACAATGCAAACGTCCACTCCCGTCTTT	14	C	0.069	0.0666
NC_005100.4-280566815	ACAATGCAAACGTCCACTCCCGTCTTT	21	C	0.2414	0.3282
NC_005100.4-280583260	CACTGGCCTACAGCCCCTCCCCAGACC	12	A	0.1043	0.1143
NC_005100.4-280583260	CACTGGCCTACAGCCCCTCCCCAGACC	27	C	0	0.4503
NC_005100.4-280585344	ATGTAGAGTACCCAACCTCCTGGTAGA	23	G	0.1983	0.244
NC_005100.4-280613725	CGGGGGTACACACTTCCTCCTCCTGCA	13	C	0.0992	0.1377
NC_005100.4-280613725	CGGGGGTACACACTTCCTCCTCCTGCA	27	A	0	0.4819
NC_005100.4-280643808	GTTTGGGAAACTTGGGCTCCTCCCCGT	25	C	0.0625	0.2254
NC_005100.4-280681175	TAGTATTGAACTGGAACTCCCCACACA	12	T	0.2951	0.3709
NC_005100.4-280712976	ACCAGTCAAACTCCCTCTCCAGTCCAG	13	C	0.2101	0.2389
NC_005100.4-280712976	ACCAGTCAAACTCCCTCTCCAGTCCAG	23	T	0.0504	0.4898
NC_005100.4-280749885	CTGTGTTAGACTCCCACTCCTGGAATA	5	G	0.1597	0.1746
NC_005100.4-280749885	CTGTGTTAGACTCCCACTCCTGGAATA	14	C	0.0083	0.0245
NC_005100.4-280773518	TGGGATCAAACCCTAGCTCCACTGATT	23	T	0.1802	0.1786
NC_005100.4-280786300	CTTGCAATAACGTCTCCTCCATCTAAA	12	G	0	0.4856
NC_005100.4-280786300	CTTGCAATAACGTCTCCTCCATCTAAA	22	T	0.1966	0.1908
NC_005100.4-280800286	CTTCCATACACTTGGTCTCCATGTATG	23	G	0.0244	0.0241
NC_005100.4-280843578	CTTCTTGGAACCTCAGCTCCCAAGCAC	27	C	0.2066	0.2704
NC_005100.4-280915135	ATATCATGCACATGCCCTCCCAGAAGC	7	T	0.0667	0.1387
NC_005100.4-280920482	AACACGGACACCTGAACTCCACAACTG	4	A	0.2459	0.4463
NC_005100.4-280920482	AACACGGACACCTGAACTCCACAACTG	5	C	0	0.4807
NC_005100.4-280936498	CCTGTCCTTACTCTCCCTCCTTCCCTT	27	T	0.1111	0.1347
NC_005100.4-280962864	CAGAGCAACACACAAACTCCACTATAA	22	C	0.0325	0.0629
NC_005100.4-280969355	GACATCATCACTCTGACTCCTAATACT	4	A	0.2203	0.4367

NC_005100.4-280980149	GCCACAAGGACCCGTGCTCCACCATGT	13	C	0.2683	0.436
NC_005100.4-281039309	TCTCTTCATACTTCCTCTCCAGTCTCT	6	T	0.0088	0.483
NC_005100.4-281039309	TCTCTTCATACTTCCTCTCCAGTCTCT	22	G	0.2411	0.3705
NC_005100.4-281128122	TCAGAGGACACCACGCCTCCAGCACCA	26	C	0.0976	0.496
NC_005100.4-281128122	TCAGAGGACACCACGCCTCCAGCACCA	27	A	0	0.4793
NC_005100.4-281142208	AGACTGACAACTCACGCTCCCTACTTA	15	C	0.0248	0.0245
NC_005100.4-281161118	ACCGGAATGACAGAACCTCCGGGACTT	4	G	0.1074	0.4978
NC_005100.4-281162041	AACAACCGAACCACCTCTCCAGCCTTC	4	A	0	0.4841
NC_005100.4-281236875	ATGGTAGAAACACGTGCTCCTTTAAAT	27	T	0	0.4821
NC_005100.4-281255831	CTATCTACCACTCATCCTCCAACCAAT	23	C	0.2149	0.3645
NC_005100.4-281272471	CTCTGTGCTACTGTCTCTCCCGGGTTT	24	G	0.0763	0.1759
NC_005100.4-281273495	GCCTCACATACCCTTCCTCCTGGGCTT	12	C	0.0248	0.0245
NC_005100.4-281278439	CATCCAGCAACACTCCCTCCGGTGCTT	21	G	0.3043	0.4055
NC_005100.4-281278439	CATCCAGCAACACTCCCTCCGGTGCTT	22	G	0.2696	0.3814
NC_005100.4-281299494	CCATCCTACACTCCAACTCCCTTTGGA	4	T	0.1557	0.5
NC_005100.4-281325537	TGCTGCGCCACCATGGCTCCTGGTGTC	7	G	0	0.4793
NC_005100.4-281325537	TGCTGCGCCACCATGGCTCCTGGTGTC	12	C	0.0163	0.032
NC_005100.4-281336031	CAACCAACCACTCATCCTCCGCCTCCA	3	A	0.0244	0.0241
NC_005100.4-281402938	TCCACTCTCACTTGCCCTCCATGGTCT	24	G	0	0.0328
NC_005100.4-281402938	TCCACTCTCACTTGCCCTCCATGGTCT	25	T	0.1092	0.1469
NC_005100.4-281492557	GGTGACAGAACAGTCTCTCCTTGAAAG	1	G	0	0.4909
NC_005100.4-281507946	ACCTCTCAGACCCTGGCTCCAAGCATC	8	A	0.1121	0.1058
NC_005100.4-281510295	AAATCATTCACCCCACCTCCTGGTCAA	6	A	0.0902	0.1154
NC_005100.4-281527171	CCTTCCACAACTGACTCTCCAACCAAT	4	T	0.0259	0.4937
NC_005100.4-281527171	CCTTCCACAACTGACTCTCCAACCAAT	6	C	0.0684	0.1125
NC_005100.4-281765599	AACCCAGATACATAAGCTCCTTCTGGT	8	A	0.1518	0.2254
NC_005100.4-281783401	AGGAATATCACGCTGGCTCCTAATGTA	16	G	0.0593	0.1041
NC_005100.4-281805195	ACTCTTTTCACAGCTGCTCCAAGATGG	1	A	0.0082	0.479
NC_005100.4-281818824	CCTCTAACCACATGGTCTCCATTATTA	3	T	0.0603	0.0585
NC_005100.4-281928672	CTCCAATAGACAGGGTCTCCCATATAT	24	A	0.595	0.4246
NC_005100.4-282065830	AATGGTCCCACCTGTCCTCCCTGAGCT	2	A	0.1301	0.2142
NC_005100.4-282065830	AATGGTCCCACCTGTCCTCCCTGAGCT	16	C	0.2927	0.3239
NC_005100.4-282071490	CACAGGTTCACTAGATCTCCATCAGAA	6	G	0.3333	0.4227
NC_005100.4-282113183	TCTCCCATTACCTCCCCTCCAGTGTCT	8	T	0.3419	0.4325
NC_005100.4-282113183	TCTCCCATTACCTCCCCTCCAGTGTCT	14	C	0.0948	0.1209
NC_005100.4-282184886	TAATATGTCACCAATTCTCCCAGTCCT	3	A	0.25	0.3231
NC_005100.4-282184886	TAATATGTCACCAATTCTCCCAGTCCT	21	C	0.0508	0.1116
NC_005100.4-282193581	TAAGCCATCACTCCACCTCCTTCATTT	14	C	0	0.4787
NC_005100.4-282247363	CAGAACCACACTAAAACTCCTCCATTA	27	A	0	0.4718
NC_005100.4-282260886	CTACATGAGACCCTGTCTCCAGAAATA	3	A	0.0261	0.0257
NC_005100.4-282260886	CTACATGAGACCCTGTCTCCAGAAATA	26	T	0.0261	0.0257
NC_005100.4-282295147	GGTTAGTTGACTCAAGCTCCATCCATT	6	G	0.0084	0.0249
NC_005100.4-282324736	GAGTATAAGACTGCAGCTCCAAGAGTG	21	A	0.0678	0.0655
NC_005100.4-282325865	CATGGATGGACCATGACTCCAGCTACA	5	G	0.1983	0.2057
NC_005100.4-282325865	CATGGATGGACCATGACTCCAGCTACA	12	C	0.0345	0.0339
NC_005100.4-282329139	TGGATCCTTACTGGGACTCCTTGAGTA	24	A	0.4831	0.3664
NC_005100.4-282329139	TGGATCCTTACTGGGACTCCTTGAGTA	25	G	0.4831	0.3664
NC_005100.4-282417252	AGGATCTGAACTGCATCTCCATAGGTA	26	T	0.0598	0.4626
NC_005100.4-282451584	CTATCACACACTGAGCCTCCCCAAGGG	9	C	0.1186	0.1409
NC_005100.4-282517083	TGTCTTTGGACCATCACTCCTAGCATG	9	G	0.2689	0.2907
NC_005100.4-282517083	TGTCTTTGGACCATCACTCCTAGCATG	27	G	0.2857	0.4008

NC_005100.4-282517589	AAAGGCCACACCTGTGCTCCTTCCCAA	6	C	0	0.0322
NC_005100.4-282517589	AAAGGCCACACCTGTGCTCCTTCCCAA	26	A	0.0492	0.048
NC_005100.4-282580528	CACCATTTCACATTTGCTCCAGCTATA	16	G	0.1933	0.2509
NC_005100.4-282580528	CACCATTTCACATTTGCTCCAGCTATA	25	A	0.0081	0.4777
NC_005100.4-282719687	TAAGTGCACACTGTCCCTCCAACCTTG	13	G	0.193	0.4945
NW_007906109.1-30489	CACTGGCCAACCATCCCTCCCACTGGC	14	T	0.0085	0.4753
NC_005101.4-1419346	CCTTGCTCTACATCATCTCCAGCATGT	4	T	0.0431	0.1058
NC_005101.4-1461067	ACTGATGGAACTTGGACTCCCTTGTTT	6	T	0	0.4827
NC_005101.4-1506874	AGTTTGCTAACTTCTTCTCCATGTGAT	7	C	0	0.4787
NC_005101.4-1557217	ACTGAATTAACTGGTTCTCCATCTCCA	25	C	0	0.4814
NC_005101.4-1635912	CTGCTCCAAACTATGTCTCCATATTTT	2	T	0.1081	0.4383
NC_005101.4-1715320	CACTGCAAAACCAGAACTCCAGAACTC	3	C	0.2982	0.2538
NC_005101.4-1715320	CACTGCAAAACCAGAACTCCAGAACTC	8	A	0.2982	0.2538
NC_005101.4-1779182	TGACACCCCACAACACCTCCTGGAGTA	13	A	0	0.4773
NC_005101.4-1868206	CATCCCGTTACCTGGGCTCCATTATAT	6	C	0	0.4773
NC_005101.4-1945053	TTATCACCCACCACCCCTCCTCTCCAT	14	C	0	0.4773
NC_005101.4-1991177	GGTGCTTGCACATGTACTCCCTGCTTG	14	G	0.5086	0.3793
NC_005101.4-2079609	GTTCAGGTAACCTGATCTCCTCAGCAA	6	G	0.0083	0.4732
NC_005101.4-2301528	CTCAAGTTCACAAGATCTCCCCTCCTC	5	A	0.0256	0.0896
NC_005101.4-2612983	GTGTGCTGAACCATCTCTCCAGCCCCA	12	C	0.113	0.4744
NC_005101.4-2663879	AAAACGAACACAAGACCTCCTAGCACT	6	G	0	0.48
NC_005101.4-2719418	AGGCAGAGGACCACATCTCCTGCTTTC	14	C	0.1157	0.4426
NC_005101.4-2719418	AGGCAGAGGACCACATCTCCTGCTTTC	15	A	0	0.4751
NC_005101.4-2812378	AGCACTTGGACAGTGACTCCTGCCCTG	9	G	0.0661	0.4672
NC_005101.4-2815446	GGATGTGTCACGTGGGCTCCTCTTTCT	15	G	0.0982	0.1093
NC_005101.4-3101119	CCCAGGAGCACTGTCACTCCTAAGTTC	27	C	0.0331	0.4849
NC_005101.4-3336340	CCTAGGACCACATCGTCTCCCTCCACT	14	C	0.0248	0.0245
NC_005101.4-3336340	CCTAGGACCACATCGTCTCCCTCCACT	26	C	0.025	0.0247
NC_005101.4-3559673	ATCATTTCTACCTATTCTCCATTTCTT	4	A	0.2973	0.4414
NC_005101.4-3645490	TTGATTGGCACTGACTCTCCTATAGGA	23	T	0.1491	0.3273
NC_005101.4-3649625	TTTCTCCTAACTGCTTCTCCATGCAAT	15	T	0.0965	0.1075
NC_005101.4-3673480	AACGAGGCCACATTTACTCCTCCTTGT	4	G	0.0087	0.4817
NC_005101.4-3736236	CAGGGGGTAACTGTGGCTCCAGCTGGA	5	G	0.2689	0.4703
NC_005101.4-3854016	AGGCCAAGTACAGCTCCTCCTTCTTGT	7	A	0.1463	0.3926
NC_005101.4-3854016	AGGCCAAGTACAGCTCCTCCTTCTTGT	25	T	0.0081	0.4777
NC_005101.4-3870067	ATCAGCAACACCAAAGCTCCTCACTGC	26	G	0.0081	0.0241
NC_005101.4-3888574	CATACTTTAACAATTGCTCCACCTATG	21	A	0.0254	0.481
NC_005101.4-3888574	CATACTTTAACAATTGCTCCACCTATG	27	G	0	0.4757
NC_005101.4-3937947	ATGGAAAGCACCATGGCTCCAGCTGCA	13	A	0.0333	0.4832
NC_005101.4-3952191	GCCAGTAGTACTAACTCTCCCTCAGAG	14	A	0	0.4731
NC_005101.4-4010220	AAAACAGTAACAGTCCCTCCCCCAGAA	3	A	0.0088	0.4796
NC_005101.4-4038878	TGACCTTGAACTGACACTCCCTGTCCT	21	C	0.065	0.0629
NC_005101.4-4039196	TTACCTCACACAATCCCTCCCCTCACA	26	C	0.1626	0.4973
NC_005101.4-4121835	CACGTCCCCACCATCCCTCCCCACACA	15	C	0.1597	0.4977
NC_005101.4-4177272	TAACCCATTACTTTTCCTCCTCGGCCA	15	T	0	0.4743
NC_005101.4-4418839	ATTGTAATCACGGTTTCTCCTCCTCCA	12	G	0.0085	0.479
NC_005101.4-4422548	TAGATATGAACAGAGGCTCCTCCTGTA	3	G	0.2155	0.4836
NC_005101.4-4512194	AATGAGGTCACGGTATCTCCATTCGTA	24	C	0.1951	0.3041
NC_005101.4-4512194	AATGAGGTCACGGTATCTCCATTCGTA	25	G	0.0813	0.078
NC_005101.4-4878795	ACAATAAAGACACAGGCTCCACTATGT	1	A	0.438	0.3421
NC_005101.4-4924395	GATTGTTCTACATCGGCTCCCTCACAG	15	G	0	0.4793

NC_005101.4-5372891	CAGGCTCTGACAGAGCCTCCTCTCAGG	3	G	0.1696	0.3963
NC_005101.4-5373638	CATCCTGCCACAGCTTCTCCCTCTCTC	14	C	0.0244	0.0241
NC_005101.4-5379780	CCATGTTGTACGGTGACTCCTGGTGTG	12	G	0	0.4773
NC_005101.4-5401382	TCTTTTTCCACATTCTCTCCAGCATCT	8	C	0.1043	0.0989
NC_005101.4-5494815	CTGCTCCATACTTTGTCTCCCTTAAGA	6	C	0.0088	0.472
NC_005101.4-5504073	TCCTCCCCTACTGTGGCTCCATTAATA	9	T	0	0.4813
NC_005101.4-5544674	GAACTCTGCACTGTACCTCCGGGCCAG	21	G	0	0.4793
NC_005101.4-5544674	GAACTCTGCACTGTACCTCCGGGCCAG	26	A	0.0325	0.032
NC_005101.4-5576836	GAAAGTACCACCCAACCTCCCAGCTAC	8	C	0.0085	0.4882
NC_005101.4-5592244	AGCGCTGAGACTGGGGCTCCTGTGCAA	16	G	0	0.4793
NC_005101.4-5608211	CTTGCTTCCACAGTTCCTCCTCCCTAC	13	G	0.1475	0.2032
NC_005101.4-5627009	GGAGTATCTACATTCCCTCCCATTCCC	23	T	0.1186	0.1551
NC_005101.4-5627009	GGAGTATCTACATTCCCTCCCATTCCC	24	T	0.0085	0.4753
NC_005101.4-5632665	TCCTCAAGCACATCCTCTCCCACGTAC	24	G	0.0357	0.0351
NC_005101.4-5718806	GCCCTTTGGACCCTCACTCCTGCTCTC	4	C	0.2377	0.2686
NC_005101.4-5779244	CTGCTTCAAACTTTGTCTCCATATACC	15	G	0.3707	0.343
NC_005101.4-5843580	AAATGTCACACCACTTCTCCACAGAGT	5	G	0.0244	0.0241
NC_005101.4-5854373	GGCAAGCCCACTAGCTCTCCCATACCC	12	T	0	0.4787
NC_005101.4-5935418	AGTCATTGTACTAATCCTCCCTTCTTT	4	C	0.0163	0.032
NC_005101.4-5935418	AGTCATTGTACTAATCCTCCCTTCTTT	25	T	0	0.4773
NC_005101.4-6099318	TGAGTCCTGACAGCATCTCCAAAAGAG	21	A	0	0.4772
NC_005101.4-6202686	ATAGCAACTACCCTTCCTCCTCTGCCC	6	A	0	0.4864
NC_005101.4-6202686	ATAGCAACTACCCTTCCTCCTCTGCCC	21	T	0	0.4864
NC_005101.4-6326301	CTGAAGAAAACTGGCTCTCCTTCTACT	15	C	0	0.4871
NC_005101.4-6423214	TAGTGGGGCACCTTTTCTCCCAGTCCT	24	T	0.1803	0.2399
NC_005101.4-6434182	AGCAAATGTACACTCGCTCCCTCTGCA	21	C	0	0.4793
NC_005101.4-6475831	AAAAAGCCTACATGGCCTCCAAGGAGT	2	A	0.0256	0.058
NC_005101.4-6567949	AGCTCCATCACTTTACCTCCTTTACCT	15	A	0	0.4787
NC_005101.4-6706439	CAGAAACAAACACACCCTCCAGTCTTT	1	C	0	0.4826
NC_005101.4-6706439	CAGAAACAAACACACCCTCCAGTCTTT	15	C	0.0254	0.0251
NC_005101.4-6763197	TGGCATTTGACCATGTCTCCTGTCCCT	12	C	0.181	0.4643
NC_005101.4-6862324	CTTTTGAACACATGGTCTCCATTGATG	15	G	0.1429	0.4228
NC_005101.4-6908419	AGTCCCCGAACTGCACCTCCCCTTTCA	9	A	0.0254	0.0251
NC_005101.4-7193355	CAACTCCTCACCATTTCTCCCATTTAT	23	T	0.0085	0.0253
NC_005101.4-7338356	TCCCAACTAACCAGAGCTCCTAATGTC	7	C	0	0.48
NC_005101.4-7346469	CCACAATGCACACTTCCTCCAATGATG	8	G	0.0244	0.0241
NC_005101.4-7346469	CCACAATGCACACTTCCTCCAATGATG	23	T	0	0.4765
NC_005101.4-7418970	CTCTCAGTAACAAAGTCTCCCATATTC	4	T	0.0855	0.0818
NC_005101.4-7423389	TGCTAAGAAACCAGTTCTCCCTCTTCC	1	T	0.0513	0.05
NC_005101.4-7423389	TGCTAAGAAACCAGTTCTCCCTCTTCC	7	G	0.0513	0.05
NC_005101.4-7640776	CATCCTGCCACGGCTTCTCCCTGTACG	27	G	0.0086	0.4595
NC_005101.4-7938202	CACACCCACACACCCACTCCTCTCTCT	6	C	0.0488	0.0476
NC_005101.4-8079787	ATCAGTATAACAAGAACTCCAATCTCT	4	A	0.0446	0.0605
NC_005101.4-8079787	ATCAGTATAACAAGAACTCCAATCTCT	22	A	0.0446	0.0605
NC_005101.4-8085721	TGGTTAGAAACAAGTTCTCCCACTGCA	24	T	0.0609	0.0911
NC_005101.4-8241860	ATAATTTTTACATTCTCTCCAAGCATG	7	T	0.0517	0.0504
NC_005101.4-8296966	AGGTCAATAACCTTTCCTCCCACAGTT	27	T	0.0085	0.4655
NC_005101.4-8336825	TTTCATCCTACCACCACTCCCACTTCA	15	C	0.2342	0.2717
NC_005101.4-8384504	CAGTGAGACACCACATCTCCAAAAGGA	25	G	0.1429	0.1609
NC_005101.4-8523067	GGAACCAAAACCCTGCCTCCCTCTCTT	27	T	0.2049	0.321
NC_005101.4-8548775	TTCCTGAATACCTTGTCTCCTGAAGAC	8	A	0.2393	0.355

NC_005101.4-8560101	GTCTGTTGTACATAGCCTCCTGAAACA	4	T	0.0902	0.4943
NC_005101.4-8560101	GTCTGTTGTACATAGCCTCCTGAAACA	6	T	0	0.4807
NC_005101.4-8560278	AAGAGGAAGACTATACCTCCTGTATGC	23	T	0	0.4807
NC_005101.4-8575957	CTGTAGGTAACTGGGCCTCCTGGTACA	6	G	0.0902	0.4943
NC_005101.4-8602371	TCTTTCACCACAGCCTCTCCAGCACAT	4	T	0	0.4856
NC_005101.4-8602371	TCTTTCACCACAGCCTCTCCAGCACAT	14	C	0.0256	0.4906
NC_005101.4-8765910	CACACACACACACACCCTCCACTGCTT	27	T	0.5537	0.4454
NC_005101.4-8789080	AGATTTGTCACCTCCCCTCCAACCCCC	21	A	0	0.4793
NC_005101.4-8789080	AGATTTGTCACCTCCCCTCCAACCCCC	26	C	0.0244	0.0241
NC_005101.4-8798328	TTGAAAGGCACAACGGCTCCACCCTTT	1	T	0.0407	0.4741
NC_005101.4-8798328	TTGAAAGGCACAACGGCTCCACCCTTT	14	C	0.0081	0.481
NC_005101.4-8815386	TCCAGGCTAACAATGCCTCCTACCAGA	13	A	0.0259	0.0255
NC_005101.4-8823208	CCAGGTAGCACAGCAGCTCCCATGGTG	14	C	0.2602	0.3141
NC_005101.4-8823208	CCAGGTAGCACAGCAGCTCCCATGGTG	24	G	0	0.4793
NC_005101.4-8823700	TCCCCTTCCACCTCCCCTCCCCATTCC	8	C	0.2586	0.4281
NC_005101.4-8888317	ATCCTCCTGACTCTACCTCCCACGTCC	15	A	0.1186	0.4994
NC_005101.4-8988059	TCCTTTAAAACTGTGGCTCCTAAAACA	15	G	0.0248	0.0245
NC_005101.4-9094546	AATGGCCAAACCATCTCTCCTGGCCTC	21	T	0.2276	0.3516
NC_005101.4-9094546	AATGGCCAAACCATCTCTCCTGGCCTC	27	C	0.2869	0.4379
NC_005101.4-9113093	CATATTTGCACATACCCTCCACTCCCA	16	C	0.1441	0.2156
NC_005101.4-9113093	CATATTTGCACATACCCTCCACTCCCA	23	T	0.0085	0.4882
NC_005101.4-9183715	AAGCACTTCACACATGCTCCATTATAA	3	G	0.0259	0.0255
NC_005101.4-9183715	AAGCACTTCACACATGCTCCATTATAA	15	T	0.1293	0.4997
NC_005101.4-9247259	AACTCACTAACCTGTCCTCCCTGTCTC	4	T	0.065	0.4904
NC_005101.4-9265816	TGCCCTCCCACTCCCCCTCCTTCTATG	25	A	0.0631	0.4214
NC_005101.4-9364115	CCAGGGCACACGGCCTCTCCTCAATTC	2	C	0	0.4851
NC_005101.4-9364115	CCAGGGCACACGGCCTCTCCTCAATTC	3	A	0	0.4851
NC_005101.4-9415817	TTTAACTCAACAAACTCTCCAGACAGT	25	A	0	0.4793
NC_005101.4-9415817	TTTAACTCAACAAACTCTCCAGACAGT	26	G	0	0.4793
NC_005101.4-9440997	CTGTATGTCACCACATCTCCGCAGTCC	6	T	0.0957	0.4998
NC_005101.4-9440997	CTGTATGTCACCACATCTCCGCAGTCC	15	A	0.0522	0.4864
NC_005101.4-9468683	CCTCACCTCACTGTCACTCCAAATGGA	7	C	0	0.4793
NC_005101.4-9472515	TCCAATATCACATCCCCTCCTCCCAGT	3	C	0.0439	0.0758
NC_005101.4-9472515	TCCAATATCACATCCCCTCCTCCCAGT	7	A	0.2368	0.476
NC_005101.4-9528309	CACGGTCACACACCCACTCCAACAAGG	5	G	0.0331	0.0325
NC_005101.4-9532275	AGGCACGCGACTGATGCTCCACACAAA	7	G	0.2602	0.4904
NC_005101.4-9541737	TTCCCAGCCACATTCTCTCCCACTTTG	22	A	0.0407	0.4893
NC_005101.4-9542366	ATAATCCTGACTCCACCTCCCAAGTAC	3	A	0	0.4793
NC_005101.4-9542366	ATAATCCTGACTCCACCTCCCAAGTAC	23	A	0.0488	0.0629
NC_005101.4-9544076	GAGAATATAACAAATACTCCACAGCTG	25	C	0.181	0.2057
NC_005101.4-9561654	GGTAAGCATACATGGCCTCCTAGGGAA	12	A	0.0424	0.0415
NC_005101.4-9623572	AGGAGCATTACAGTCTCTCCGCGGGAG	22	C	0.0085	0.481
NC_005101.4-9672143	CACTCTGTAACTCCCTCTCCAGGTATT	15	C	0.1667	0.2432
NC_005101.4-9769085	TGCATTGGTACAAGCTCTCCTTCCTAG	5	T	0.115	0.4435
NC_005101.4-9769085	TGCATTGGTACAAGCTCTCCTTCCTAG	25	T	0.0973	0.481
NC_005101.4-9770669	GGATCCAGCACTCCTGCTCCTCAGGAC	9	C	0.1765	0.214
NC_005101.4-9783020	ATGTTAGCGACTGAACCTCCCTTACAG	8	C	0.1639	0.4999
NC_005101.4-9796877	ATGTCAGTCACTTTAGCTCCATGCTTG	14	T	0	0.4793
NC_005101.4-9844502	TTTGTACCAACCCTCACTCCTTAATGC	26	G	0.1949	0.3483
NC_005101.4-9849737	GGCCAGGGCACTGCAGCTCCACTTCTC	21	A	0	0.48
NC_005101.4-9857439	AGATTCTTCACTCCAGCTCCTCCTGTT	7	T	0.2	0.3898

NC_005101.4-9863699	CATGACACAACTGAGTCTCCAAAGACT	21	A	0.0174	0.0508
NC_005101.4-9878148	ATTTACATCACTCTTCCTCCTTTCTTC	27	C	0	0.4715
NC_005101.4-9881382	GTATAGAAAACAGACTCTCCAAATAAT	13	G	0.1518	0.1983
NC_005101.4-9898912	ATCTCAACAACTCTCTCTCCTTCCTAC	16	T	0.1111	0.3596
NC_005101.4-9923467	TAACAACTAACGGGATCTCCCAGGGAC	9	A	0.0088	0.0429
NC_005101.4-9923467	TAACAACTAACGGGATCTCCCAGGGAC	12	G	0	0.474
NC_005101.4-10023320	GGCTTATTCACCTGAGCTCCAAGACAT	12	C	0.1565	0.2873
NC_005101.4-10043419	ATCCCTGGGACTAGCTCTCCCACTTAT	27	T	0.0813	0.1073
NC_005101.4-10056784	CATCTCTATACACTGCCTCCTTCCCTT	14	T	0.0901	0.4967
NC_005101.4-10056784	CATCTCTATACACTGCCTCCTTCCCTT	15	G	0.036	0.0354
NC_005101.4-10230476	GTTCTCATAACCTCTTCTCCACAGCAC	2	T	0.0357	0.1327
NC_005101.4-10383927	ACCCCTTGCACCAACTCTCCTGGGTCT	5	C	0.2645	0.4582
NC_005101.4-10494683	CCAGACCCCACCAGAGCTCCCCGAGAC	23	G	0.0702	0.0997
NC_005101.4-10503059	GATACTTAAACACTTGCTCCCTTGGGG	7	T	0.0086	0.4768
NC_005101.4-10503307	CCAAACTAAACTCATCCTCCCATACTT	16	C	0.2586	0.4214
NC_005101.4-10509346	ATCATATACACACACACTCCTTCCTAC	3	C	0.4454	0.3462
NC_005101.4-10509346	ATCATATACACACACACTCCTTCCTAC	5	T	0.4454	0.3462
NC_005101.4-10649775	GCCGCCACTACAGTTGCTCCAAGACTC	7	A	0.1833	0.2061
NC_005101.4-10741842	ATGAGATACACCAGAACTCCCAGGGAT	21	C	0.2602	0.4759
NC_005101.4-10807145	ATTTGGAGAACTTCTGCTCCAACACTT	21	A	0.3009	0.3815
NC_005101.4-10807145	ATTTGGAGAACTTCTGCTCCAACACTT	25	C	0	0.4846
NC_005101.4-10825788	CCTACCCACACCATCTCTCCAGGGGTC	21	A	0	0.4807
NC_005101.4-10866023	TTTGTTGCAACTCTACCTCCACTGGAC	7	G	0.0088	0.481
NC_005101.4-10866023	TTTGTTGCAACTCTACCTCCACTGGAC	15	A	0.0435	0.0425
NC_005101.4-10892771	CCAGGCTGCACCCTTTCTCCTGCTCAT	3	A	0.2605	0.3893
NC_005101.4-10892771	CCAGGCTGCACCCTTTCTCCTGCTCAT	8	G	0.2605	0.3893
NC_005101.4-11021124	AATGGCAGAACACTTCCTCCCATTTCA	5	G	0.2377	0.4489
NC_005101.4-11060872	TATGGAACCACAAGAACTCCGACAACA	21	G	0.0085	0.4842
NC_005101.4-11099651	ACAGGTTTAACCATGTCTCCATAAACT	14	T	0	0.4755
NC_005101.4-11127475	ACTTGGAAGACTATTGCTCCTAACTCT	15	T	0.0083	0.4803
NC_005101.4-11127475	ACTTGGAAGACTATTGCTCCTAACTCT	27	T	0.0246	0.0243
NC_005101.4-11194199	GAAAAAGAAACATTTCCTCCCACGTCT	15	T	0.1	0.1528
NC_005101.4-11203620	ACAACCCATACACTCCCTCCCACCTCC	1	A	0.5345	0.3916
NC_005101.4-11243419	GTCCCAGTGACTCAGGCTCCAGACTAC	1	G	0.0351	0.0345
NC_005101.4-11243419	GTCCCAGTGACTCAGGCTCCAGACTAC	22	G	0.0263	0.026
NC_005101.4-11332444	ATGGGTTAGACTGAAACTCCTTATGTT	5	G	0.3445	0.3893
NC_005101.4-11332444	ATGGGTTAGACTGAAACTCCTTATGTT	9	G	0.0342	0.0336
NC_005101.4-11473307	GGTGCCCTTACTTTTACTCCTAGCTCA	1	G	0.2667	0.2311
NC_005101.4-11543700	TGCCTCTCAACATGCTCTCCTCAGTCA	12	A	0	0.4728
NC_005101.4-11556723	CCGTCTCAGACTCGACCTCCTTCCTCC	14	G	0.0738	0.1154
NC_005101.4-11561196	TATCACATCACCTAAGCTCCTGTTTTC	14	A	0	0.4793
NC_005101.4-11712185	ATGAGTTGCACAGTATCTCCTCAACAC	9	C	0.0339	0.4908
NC_005101.4-11801619	GAGTGGGTCACTAGGCCTCCCTGTTCT	7	G	0.1429	0.3367
NC_005101.4-11808036	CCTGGAGGCACCAAAGCTCCAGTGCCT	3	T	0.0661	0.109
NC_005101.4-11808036	CCTGGAGGCACCAAAGCTCCAGTGCCT	23	T	0	0.4819
NC_005101.4-11814209	TGTCACTGTACCTGTTCTCCCTACCAC	7	T	0.1057	0.1145
NC_005101.4-11814209	TGTCACTGTACCTGTTCTCCCTACCAC	27	C	0	0.4787
NC_005101.4-11855686	CCCTCCAACACGTGTCCTCCATGGGTC	1	C	0.225	0.2941
NC_005101.4-11855686	CCCTCCAACACGTGTCCTCCATGGGTC	2	C	0.2185	0.2907
NC_005101.4-11872041	GGAGATAGAACTAGCTCTCCATTTAAT	6	T	0.2437	0.4117
NC_005101.4-11872959	TTCGCAGGGACTCGGCCTCCATATGGT	23	A	0.0427	0.0418

NC_005101.4-11873134	AGCGGCGCTACCCACCCTCCTTCGCAG	23	C	0.0435	0.059
NC_005101.4-11873134	AGCGGCGCTACCCACCCTCCTTCGCAG	27	G	0	0.4682
NC_005101.4-11879761	TTTTGCAAAACAACAGCTCCACCCCTC	25	C	0	0.4728
NC_005101.4-11892077	CACCTATTTACATCCTCTCCAGGAGTA	27	A	0	0.48
NC_005101.4-11910113	CCAATGAAAACCCCAGCTCCATTAAAT	12	C	0.0948	0.1058
NC_005101.4-12006944	ACGTACGGGACCTGTTCTCCTGTGGGA	12	C	0.0965	0.2836
NC_005101.4-12006944	ACGTACGGGACCTGTTCTCCTGTGGGA	14	G	0.0708	0.0846
NC_005101.4-12009645	CTGAGCTATACCCCTTCTCCACGCCCC	6	C	0.0413	0.0562
NC_005101.4-12009645	CTGAGCTATACCCCTTCTCCACGCCCC	23	G	0	0.48
NC_005101.4-12203254	GTTAAGCCCACCAGCTCTCCTATGCCC	3	T	0.05	0.4911
NC_005101.4-12203254	GTTAAGCCCACCAGCTCTCCTATGCCC	24	G	0.05	0.4911
NC_005101.4-12215218	GCCACTGATACTAAGTCTCCACAGCAA	1	G	0.2414	0.3179
NC_005101.4-12215218	GCCACTGATACTAAGTCTCCACAGCAA	7	G	0.0431	0.0585
NC_005101.4-12249609	CTTCTGAAGACCCTTTCTCCCAAATTA	1	C	0.0901	0.4967
NC_005101.4-12266537	CTGTGTCAAACTCTTGCTCCCTCTGCC	16	G	0.0244	0.0241
NC_005101.4-12272630	CCATCCTCAACCCTGTCTCCCTTCCCC	12	C	0.0609	0.0911
NC_005101.4-12285034	TTTCTCCCCACCTCCCCTCCAGTCCAG	3	T	0	0.4724
NC_005101.4-12306105	TGTCACACTACTAAAGCTCCAAACGTC	3	T	0.0656	0.0935
NC_005101.4-12321212	TTTCCCACTACACGGTCTCCCTCCTCA	7	A	0.0325	0.032
NC_005101.4-12321212	TTTCCCACTACACGGTCTCCCTCCTCA	16	T	0.2846	0.4618
NC_005101.4-12339103	GCCACAGACACACAGGCTCCCTGATCT	3	C	0	0.4839
NC_005101.4-12339103	GCCACAGACACACAGGCTCCCTGATCT	25	T	0.0413	0.0405
NC_005101.4-12359587	CTGGTAACCACCAAGTCTCCAATCTTT	3	G	0.1356	0.1264
NC_005101.4-12359587	CTGGTAACCACCAAGTCTCCAATCTTT	22	A	0.0763	0.0889
NC_005101.4-12371881	CTTAGGAGCACTCTCACTCCTAAGACC	8	G	0.2846	0.4036
NC_005101.4-12395174	CTGCCTCCCACTTAGTCTCCAGTTTTA	4	C	0.1818	0.2975
NC_005101.4-12404391	AATGGGTTTACATAATCTCCTTAACAA	7	T	0.0083	0.4769
NC_005101.4-12404391	AATGGGTTTACATAATCTCCTTAACAA	25	C	0.0248	0.0245
NC_005101.4-12499397	TCAACTGTGACTCTAGCTCCAAGACAT	5	C	0	0.4698
NC_005101.4-12582517	GGCAGAGAAACAGCTCCTCCTCCTTCC	23	C	0	0.488
NC_005101.4-12645635	TCTTTCTCCACACCTTCTCCAGCATGT	15	T	0.3729	0.3034
NC_005101.4-12645635	TCTTTCTCCACACCTTCTCCAGCATGT	24	A	0.3729	0.3034
NC_005101.4-12663674	CTGACCTCGACCAGCTCTCCTGACTTA	9	G	0.041	0.0401
NC_005101.4-12774069	AGGATCACCACCTGGCCTCCATTATTT	5	T	0.1138	0.414
NC_005101.4-12813642	GGACAAATCACTTAATCTCCTGAGACT	24	G	0.0089	0.4885
NC_005101.4-12988437	ACTCCTTAAACTGTGACTCCAACTCAC	2	C	0	0.4793
NC_005101.4-13059135	GTGCTTATCACAGTTGCTCCATGTGCA	9	C	0	0.4807
NC_005101.4-13059135	GTGCTTATCACAGTTGCTCCATGTGCA	21	A	0.0325	0.0476
NC_005101.4-13116494	TACCAAGCAACCAAAACTCCTAGGGAC	24	G	0.2069	0.2122
NC_005101.4-13126967	CCTTGATCCACATCCTCTCCACCGTGT	12	A	0.1316	0.4969
NC_005101.4-13126967	CCTTGATCCACATCCTCTCCACCGTGT	25	T	0.1304	0.4976
NC_005101.4-13277996	CCCCTCCAAACCCCAACTCCATCCCAT	15	A	0.1475	0.387
NC_005101.4-13326177	CAAATGAAAACAAGCACTCCTTCCTCA	21	T	0.0427	0.1049
NC_005101.4-13326177	CAAATGAAAACAAGCACTCCTTCCTCA	27	A	0.0431	0.0746
NC_005101.4-13353906	ATCCTGCTTACTGGGGCTCCACCTTCA	22	C	0.5462	0.397
NC_005101.4-13385039	CACACTGCTACTCTACCTCCTCAGACA	24	G	0	0.4765
NC_005101.4-13425022	AATCTGCTTACTTAGCCTCCTTCAAAT	4	C	0.2185	0.4957
NC_005101.4-13675972	GGGGATCTGACACCCTCTCCCCACCTC	2	G	0.0431	0.0422
NC_005101.4-13675972	GGGGATCTGACACCCTCTCCCCACCTC	13	C	0.0603	0.0585
NC_005101.4-13682842	ATTCACTAGACTCAATCTCCACGAGCA	23	G	0.1913	0.4991
NC_005101.4-13769196	TACCTTTCTACTTTAGCTCCAATCCTC	2	A	0	0.477

NC_005101.4-13890940	AGACTCCGTACAGGGACTCCCCCTGTC	12	A	0.0256	0.1049
NC_005101.4-13890940	AGACTCCGTACAGGGACTCCCCCTGTC	16	A	0.0256	0.1049
NC_005101.4-13914562	TTTTAGAGAACCAGGTCTCCTCACTAA	7	A	0.0085	0.481
NC_005101.4-14046366	ATGTCCCACACAAATCCTCCTCCTCCT	21	T	0.2437	0.4117
NC_005101.4-14302773	GGACTGATTACCGGACCTCCTTTCCCT	2	G	0.2521	0.4463
NC_005101.4-14302773	GGACTGATTACCGGACCTCCTTTCCCT	13	G	0.2269	0.4186
NC_005101.4-14327332	CTTTGCAGGACAGGACCTCCAGCTTGC	21	A	0.0089	0.4709
NC_005101.4-14499739	GTACCATTTACCATTTCTCCCTCATTG	1	G	0.0583	0.1025
NC_005101.4-14499739	GTACCATTTACCATTTCTCCCTCATTG	26	T	0.1583	0.4991
NC_005101.4-14567934	TCTCCTCATACTTTCCCTCCATTCTCT	22	T	0.0684	0.0973
NC_005101.4-14567934	TCTCCTCATACTTTCCCTCCATTCTCT	27	T	0.0783	0.4903
NC_005101.4-14628000	TGGGAACAGACCCTGGCTCCTCTGTAA	9	G	0.1351	0.4994
NC_005101.4-14707017	CCTTCTCCCACAACGACTCCTCGATTC	1	C	0.0169	0.0333
NC_005101.4-14707017	CCTTCTCCCACAACGACTCCTCGATTC	16	A	0.1453	0.1492
NC_005101.4-15057385	AGCAGATTTACATGCCCTCCCATCCTT	9	T	0.0609	0.0911
NC_005101.4-15224234	CTGTTCTTCACAGTCCCTCCCTCTTAC	9	C	0.0244	0.4915
NC_005101.4-15224234	CTGTTCTTCACAGTCCCTCCCTCTTAC	27	C	0.0246	0.4924
NC_005101.4-15241867	TACAAGAAAACACTTCCTCCTCTAGAC	1	T	0.0088	0.4676
NC_005101.4-15241867	TACAAGAAAACACTTCCTCCTCTAGAC	22	C	0.087	0.4234
NC_005101.4-15269008	TCCTGGTTAACAACATCTCCCCCTTTC	8	T	0	0.482
NC_005101.4-15393997	ATAACAACAACACATGCTCCACCTTCT	26	C	0	0.4851
NC_005101.4-15463936	ATAGAGTTTACCTTCCCTCCACTCTTC	1	A	0.1429	0.232
NC_005101.4-15477045	ACCAGTCTTACACTTACTCCTTATTTA	3	C	0.0431	0.0746
NC_005101.4-15477045	ACCAGTCTTACACTTACTCCTTATTTA	22	T	0.1552	0.2854
NC_005101.4-15574443	AGATATCATACATTGTCTCCCTTACCT	12	A	0.114	0.3836
NC_005101.4-15574443	AGATATCATACATTGTCTCCCTTACCT	26	C	0.0439	0.0595
NC_005101.4-15860262	ACATCAAAGACACATGCTCCTTTATGT	13	C	0.0496	0.0484
NC_005101.4-15867519	TACCAAGGGACCTCTCCTCCTATTGAT	2	A	0.0678	0.0812
NC_005101.4-15943692	ATTGCAGCAACACAGGCTCCTTTGTCT	3	T	0	0.4793
NC_005101.4-15943692	ATTGCAGCAACACAGGCTCCTTTGTCT	15	G	0.2602	0.464
NC_005101.4-16072247	ATGTTATAGACCTAACCTCCAGGGTGA	22	G	0.0973	0.1084
NC_005101.4-16072247	ATGTTATAGACCTAACCTCCAGGGTGA	23	G	0.0088	0.4693
NC_005101.4-16224033	CTTTCTAATACTACCACTCCTTATGGG	4	T	0.0174	0.4724
NC_005101.4-16224033	CTTTCTAATACTACCACTCCTTATGGG	26	G	0.0435	0.0425
NC_005101.4-16236800	CTGCTCCAAACTTGGTCTCCATGTCTC	15	G	0.2167	0.1932
NC_005101.4-16236800	CTGCTCCAAACTTGGTCTCCATGTCTC	24	T	0.55	0.3987
NC_005101.4-16370172	ATTCTTCCCACCTCTCCTCCTTCCCGG	9	C	0.0407	0.0398
NC_005101.4-16370172	ATTCTTCCCACCTCTCCTCCTTCCCGG	26	G	0.187	0.2203
NC_005101.4-16458966	CTTATACTCACCACCACTCCATACATA	12	C	0.2018	0.2719
NC_005101.4-16473311	CACAGCATTACCCATACTCCAGTTAAT	6	C	0	0.4864
NC_005101.4-16473311	CACAGCATTACCCATACTCCAGTTAAT	22	G	0	0.4864
NC_005101.4-16473787	CACAACATCACTCCAGCTCCCAGCACC	24	C	0	0.4813
NC_005101.4-16476709	TCTACAACAACCCTGTCTCCTCTCGTT	25	G	0.094	0.1049
NC_005101.4-16594784	TGATCTTCTACTTTAGCTCCTGCCTTT	4	T	0.1071	0.1327
NC_005101.4-16683605	TAAGGTGTAACAACTCCTCCATTTTCA	15	T	0.069	0.0666
NC_005101.4-16710655	ATGTGCCCTACTAAACCTCCCAGCACT	5	G	0.2478	0.4953
NC_005101.4-16785026	CAATATGCCACACCTACTCCCCACCCT	7	G	0.1293	0.1504
NC_005101.4-16785026	CAATATGCCACACCTACTCCCCACCCT	23	A	0	0.4724
NC_005101.4-16787554	GTCTCTTCCACAACTCCTCCAATACCC	12	A	0.0265	0.4774
NC_005101.4-16891886	AGGAGATCCACCCTTTCTCCTTGTGAT	9	C	0.025	0.4875
NC_005101.4-16891886	AGGAGATCCACCCTTTCTCCTTGTGAT	23	G	0.0248	0.4863

NC_005101.4-16957297	GGCTGAGAAACCAGTTCTCCCCACTCC	23	A	0.0833	0.1665
NC_005101.4-17136486	TATTAAACAACCAGAGCTCCCTGGGAC	23	G	0.0619	0.0765
NC_005101.4-17141300	CTGTTACCAACAACCTCTCCTGCTTCT	7	C	0.2049	0.2796
NC_005101.4-17216482	CAAGTCACAACTGAGTCTCCAAAGAAA	6	C	0.5	0.4144
NC_005101.4-17231928	AGGTCAAGAACGTCTCCTCCAACAGGT	23	C	0.1518	0.212
NC_005101.4-17379629	AGATGTCAGACTCCAACTCCAGGTATA	24	T	0.0909	0.0868
NC_005101.4-17379629	AGATGTCAGACTCCAACTCCAGGTATA	26	T	0.1186	0.1116
NC_005101.4-17391344	GAAGGACCAACATCTTCTCCCATTGAT	8	C	0	0.4807
NC_005101.4-17418505	TAACTCAGGACAATCTCTCCAGAGATA	25	A	0.0083	0.4783
NC_005101.4-17472597	ATTATAAGGACACATGCTCCACTATGT	6	A	0.0333	0.0328
NC_005101.4-17619744	TGATACGGCACTGCTTCTCCAATGGTG	7	G	0	0.4787
NC_005101.4-17756669	CCCATGTTCACTTGTCCTCCAATTCTT	23	T	0.3393	0.3468
NC_005101.4-17758049	GTAGACTCTACAAGCTCTCCCTCTCCA	24	T	0.0855	0.2361
NC_005101.4-17844684	TCTCTCTTCACATTCTCTCCTTCCATT	2	C	0.0569	0.0553
NC_005101.4-17907960	TCTTTCATTACCTCAGCTCCTTGAACC	13	T	0	0.4778
NC_005101.4-17907960	TCTTTCATTACCTCAGCTCCTTGAACC	16	G	0	0.4778
NC_005101.4-17966499	TGACTAACAACCTTAGCTCCTAGTGAT	12	C	0.1901	0.313
NC_005101.4-18017431	ATGGTGTTCACATGATCTCCTGGCAAT	12	A	0	0.4751
NC_005101.4-18180790	TTTAAGATGACTGAGGCTCCATCAATG	6	G	0	0.4793
NC_005101.4-18259891	TGCTTGCTCACAGAGTCTCCTTAAATG	4	T	0.0082	0.4755
NC_005101.4-18259891	TGCTTGCTCACAGAGTCTCCTTAAATG	6	G	0.0082	0.4755
NC_005101.4-18328906	TTAATGTGCACTGCCTCTCCTTTAACC	9	C	0.2193	0.4969
NC_005101.4-18351820	CTCTAAGACACATACGCTCCACAAGGT	12	A	0.0976	0.1073
NC_005101.4-18351820	CTCTAAGACACATACGCTCCACAAGGT	15	C	0.0407	0.0398
NC_005101.4-18354087	AGACCCCTCACAGCATCTCCTGTGGAG	26	A	0	0.4773
NC_005101.4-18355268	GGAAAGCAAACCTAAACTCCTAGGGTT	7	C	0.0435	0.0425
NC_005101.4-18372233	CAAAGATCCACAACATCTCCTTATACC	13	A	0	0.4751
NC_005101.4-18372233	CAAAGATCCACAACATCTCCTTATACC	24	T	0.1967	0.2157
NC_005101.4-18383817	TCCTCCTCTACCCCATCTCCATGATGG	16	T	0.125	0.4991
NC_005101.4-18412427	CCCGGCCATACTTACCCTCCCTTTCCC	15	C	0.2269	0.4316
NC_005101.4-18506562	GTGGCAAACACTGCACCTCCGCCATTT	26	T	0.2645	0.2647
NC_005101.4-18511060	TTTGATACTACTCTACCTCCCAAGGTG	9	T	0	0.4693
NC_005101.4-18529791	GCTCAAAAGACTGTGTCTCCAAATTGG	12	T	0.2342	0.2837
NC_005101.4-18555944	CGAAGCAAAACTATTCCTCCTGACTAA	1	C	0	0.4813
NC_005101.4-18634555	TACCTGGGAACCCAGGCTCCTTTTTCT	14	A	0.2627	0.4396
NC_005101.4-18680157	CTGGGTGCTACCCCAACTCCTGCTGTC	14	C	0.1327	0.2237
NC_005101.4-18687727	TCTCAGGACACTTAGACTCCCAGGTTC	15	G	0.193	0.2155
NC_005101.4-18687727	TCTCAGGACACTTAGACTCCCAGGTTC	16	A	0	0.4778
NC_005101.4-18689213	GTAGCCGAAACTATGGCTCCTTCCGTG	6	C	0.2054	0.4709
NC_005101.4-18689213	GTAGCCGAAACTATGGCTCCTTCCGTG	24	C	0.1607	0.4998
NC_005101.4-18724840	GAGTCACAGACCATGCCTCCGCCCCAT	27	T	0.0263	0.026
NC_005101.4-18776585	CTTTTACCAACCATGGCTCCTCCCAGA	21	T	0.3509	0.396
NC_005101.4-18877576	CTGCACCTCACCCTTTCTCCCTTCTCC	15	T	0.0672	0.065
NC_005101.4-18877576	CTGCACCTCACCCTTTCTCCCTTCTCC	23	T	0.0168	0.4743
NC_005101.4-19000172	TATTACGGCACACCCACTCCTCAAGAT	13	C	0.0342	0.0818
NC_005101.4-19148825	TGCAATCTCACGTAGGCTCCATGATTG	8	T	0	0.4793
NC_005101.4-19154087	ATGTGAATCACTGTATCTCCTTCATCA	14	T	0	0.4844
NC_005101.4-19166472	TATTTAGAGACATGCTCTCCCCCATCA	13	T	0.2602	0.427
NC_005101.4-19166622	CATTGTCGCACACAAACTCCTCACTAG	7	C	0.1967	0.4891
NC_005101.4-19173591	CACTATGCCACTGAAGCTCCCTGCAGA	24	C	0.0244	0.0241
NC_005101.4-19222758	ATGTCTTAGACACTACCTCCCATGCTA	25	C	0.0721	0.4854

NC_005101.4-19373291	CATAAGAGCACCACGCCTCCGGCTGCG	13	A	0	0.4912
NC_005101.4-19373698	TCCCTTGAGACAGGCTCTCCCTCACTG	3	C	0.0708	0.1006
NC_005101.4-19373698	TCCCTTGAGACAGGCTCTCCCTCACTG	22	T	0.0708	0.1006
NC_005101.4-19440832	TACAAACCAACCAGAGCTCCCAGGGAA	14	G	0.0656	0.0634
NC_005101.4-19469268	TGAGGACCCACTACCTCTCCTGGGCAT	13	A	0.0678	0.2815
NC_005101.4-19469268	TGAGGACCCACTACCTCTCCTGGGCAT	23	G	0.1709	0.4997
NC_005101.4-19477847	AACCTCTGAACCATCTCTCCTTGCCTC	21	T	0.082	0.1505
NC_005101.4-19483461	CACATCTGCACATGCCCTCCTGCTTCA	6	C	0.0744	0.1306
NC_005101.4-19483461	CACATCTGCACATGCCCTCCTGCTTCA	22	G	0	0.4832
NC_005101.4-19529788	TCTAATAAGACCACACCTCCTAATCCT	8	A	0.1488	0.1377
NC_005101.4-19529788	TCTAATAAGACCACACCTCCTAATCCT	15	A	0.5124	0.4369
NC_005101.4-19584862	TCACTTAGTACATCTGCTCCACCTAGT	12	A	0.0776	0.1058
NC_005101.4-19588911	CTGTCTGTGACATGCCCTCCACCCACT	23	C	0.275	0.3622
NC_005101.4-19588911	CTGTCTGTGACATGCCCTCCACCCACT	24	C	0.281	0.4628
NC_005101.4-19646218	TCCAAAGCAACTGTATCTCCTCTTTGA	4	A	0.1429	0.1478
NC_005101.4-19661446	CCCAGATCCACATAGGCTCCAGAGTGA	8	C	0.2353	0.4615
NC_005101.4-19682751	CTAGAGAGAACTGCCTCTCCTCTTCTA	14	C	0.0804	0.1093
NC_005101.4-19682751	CTAGAGAGAACTGCCTCTCCTCTTCTA	24	T	0.027	0.0267
NC_005101.4-19744076	CTTGCCCTCACCCAATCTCCACCCTTA	15	A	0.2	0.4328
NC_005101.4-19780824	TTCCTGCCGACTTTCTCTCCCCTTCCC	9	G	0.0339	0.0496
NC_005101.4-19783975	TTCACCAACACGATGGCTCCTCCAAGC	2	T	0.1186	0.1409
NC_005101.4-19795683	AGTACCGTCACCCTAGCTCCAGGGATC	6	C	0.2033	0.484
NC_005101.4-19806959	TTTGCTTTCACTGACCCTCCTTCCCCC	5	C	0.1802	0.498
NC_005101.4-19857732	TCCCTTCTGACTTCCCCTCCCCTTTGT	7	C	0.1074	0.1017
NC_005101.4-19881391	CTGCCTCAAACTTTCTCTCCTTTCCTC	1	C	0.1964	0.2934
NC_005101.4-19954289	AATGGTGCCACCCACCCTCCAGGCCAG	26	A	0.0517	0.0504
NC_005101.4-20027886	CCAGTGATCACTCTGTCTCCTCCACTT	26	T	0	0.4807
NC_005101.4-20068135	CAAACACAAACCACTCCTCCTCTCATC	22	C	0.1417	0.1597
NC_005101.4-20106890	GACGTCAGAACTGCTTCTCCGTCAGTC	24	A	0.0407	0.0705
NC_005101.4-20106890	GACGTCAGAACTGCTTCTCCGTCAGTC	27	C	0.1789	0.496
NC_005101.4-20136557	CTCCAAAGAACTCGTCCTCCATGTTAA	14	G	0.0407	0.0398
NC_005101.4-20136557	CTCCAAAGAACTCGTCCTCCATGTTAA	24	T	0.0813	0.1073
NC_005101.4-20231010	CCAAACATCACCTTTCCTCCCATGGGT	2	C	0.124	0.1163
NC_005101.4-20249189	GCCACCCATACTGCCCCTCCTGAGGAG	12	T	0.0744	0.4557
NC_005101.4-20249189	GCCACCCATACTGCCCCTCCTGAGGAG	22	G	0.1803	0.1904
NC_005101.4-20267161	GTCATATTCACCCCTTCTCCCCAGATG	23	A	0.0796	0.0926
NC_005101.4-20279123	AGCCACTGAACTTAGACTCCAGGCTGT	1	A	0.3243	0.4026
NC_005101.4-20279123	AGCCACTGAACTTAGACTCCAGGCTGT	24	C	0.1043	0.0989
NC_005101.4-20330393	AGAAGCAGGACTCTCTCTCCCTTCCTT	21	C	0.0261	0.4849
NC_005101.4-20330393	AGAAGCAGGACTCTCTCTCCCTTCCTT	22	T	0	0.4786
NC_005101.4-20390855	ACAACTTTCACCCGACCTCCCTGAATG	16	C	0.0522	0.0508
NC_005101.4-20411098	ACTTTCTTAACGTAGGCTCCTGTAACC	12	G	0.0439	0.0595
NC_005101.4-20413972	GAACAAGACACCTGATCTCCAACCCTG	23	C	0.0088	0.026
NC_005101.4-20416209	CTTAACATAACCGTTCCTCCTCCTCCT	5	A	0	0.4773
NC_005101.4-20416858	GAGGCAGGCACAGTGCCTCCCGGGGAG	8	G	0.2377	0.4489
NC_005101.4-20546326	TGGCTGTGGACTCTGTCTCCATTCCCA	13	C	0.0796	0.1239
NC_005101.4-20646678	TATTATAGTACACAATCTCCGGCCTAG	13	C	0.0088	0.483
NC_005101.4-20662366	GATATCCGCACTTGCTCTCCAAAGCTC	8	G	0.2439	0.4595
NC_005101.4-20662366	GATATCCGCACTTGCTCTCCAAAGCTC	13	T	0.1382	0.1695
NC_005101.4-20862316	TGTCTGAAGACAGGTCCTCCTCTATCC	14	G	0.2162	0.4026
NC_005101.4-20893948	GCCGATCACACAATCTCTCCCCACACC	1	G	0	0.4807

NC_005101.4-20893948	GCCGATCACACAATCTCTCCCCACACC	8	A	0.0732	0.0705
NC_005101.4-20899593	TCCATTAGTACTTTATCTCCTAGACGT	21	T	0.2066	0.4732
NC_005101.4-20906014	GAAATGTCCACTCTGCCTCCGCCATCA	8	C	0.1429	0.3067
NC_005101.4-20917618	GAGAGGGTTACAGGGCCTCCTTGTGAA	9	T	0.114	0.1229
NC_005101.4-21421617	AATCTTGTGACCAGGGCTCCTTGATAT	26	A	0.2167	0.3911
NC_005101.4-21460597	AGAGAGCATACGTGATCTCCGATGGCG	21	G	0	0.462
NC_005101.4-21460597	AGAGAGCATACGTGATCTCCGATGGCG	26	C	0.0086	0.4595
NC_005101.4-21503529	CTAGTATGCACCAGATCTCCTGCAACT	8	G	0.2358	0.4741
NC_005101.4-21511937	CAGGTACCCACACACCCTCCGTGATAG	21	G	0.0084	0.4859
NC_005101.4-21514259	GTCTCTTTCACCAACTCTCCTTATAGC	24	T	0.25	0.3299
NC_005101.4-21521428	TATGATGTCACGATAGCTCCTGTAGAT	2	A	0.0246	0.4822
NC_005101.4-21521428	TATGATGTCACGATAGCTCCTGTAGAT	26	A	0.0246	0.0243
NC_005101.4-21523475	AGCGAGTTTACTTCAGCTCCTTCATCA	24	A	0.0579	0.4978
NC_005101.4-21534900	AGAGTGAGAACACGGGCTCCGAGTACA	5	T	0.0593	0.0734
NC_005101.4-21534900	AGAGTGAGAACACGGGCTCCGAGTACA	13	C	0.0508	0.0965
NC_005101.4-21548988	CCCCACACTACTGTATCTCCCACTCTG	9	T	0.1157	0.1235
NC_005101.4-21548988	CCCCACACTACTGTATCTCCCACTCTG	14	T	0.1157	0.1235
NC_005101.4-21559053	GCAGACTGCACAGTAGCTCCCCAGAGC	21	C	0.0261	0.0257
NC_005101.4-21605868	CATATTCTGACTCGGCCTCCACCATCG	5	T	0.0083	0.4803
NC_005101.4-21634919	CATGGTGCCACTAAAGCTCCAAGCAAC	9	C	0.2562	0.4146
NC_005101.4-21673242	CACAGAATCACCAGATCTCCCACCTGG	22	A	0.2276	0.4389
NC_005101.4-21785040	GGGTTCTACACAAGACCTCCTCAGTCA	22	C	0	0.4807
NC_005101.4-21800149	TGCCGTGACACGGTGGCTCCTCCTAAC	12	G	0.122	0.1287
NC_005101.4-21805160	CACGTACGCACGCGTGCTCCAAATAGA	5	T	0.2213	0.2457
NC_005101.4-21805160	CACGTACGCACGCGTGCTCCAAATAGA	15	T	0.4215	0.4146
NC_005101.4-21819094	CTCTAACAGACCCAGCCTCCATAGCAC	8	A	0.225	0.4972
NC_005101.4-21819094	CTCTAACAGACCCAGCCTCCATAGCAC	14	A	0.1083	0.4972
NC_005101.4-21851454	ATCACCCTGACTGTACCTCCACGAGAA	14	T	0.0973	0.4373
NC_005101.4-21851454	ATCACCCTGACTGTACCTCCACGAGAA	22	C	0.1081	0.1338
NC_005101.4-21900862	CACACACACACACACACTCCCCTCTCC	5	C	0.0339	0.0333
NC_005101.4-21905821	TTCGCAATTACCAGAACTCCAGTTCTA	1	T	0.0992	0.1377
NC_005101.4-21905821	TTCGCAATTACCAGAACTCCAGTTCTA	4	G	0.041	0.0401
NC_005101.4-21916256	GTGGAACCCACCTAGTCTCCAGTAAAA	2	T	0.1167	0.1528
NC_005101.4-21927467	TGAATGAATACGTCCGCTCCCAAGCCT	16	G	0.0082	0.4822
NC_005101.4-21969595	GAGGTGTGCACCACCGCTCCCACCTGG	5	T	0.2288	0.3086
NC_005101.4-21994295	ATCCATCTCACAGTGCCTCCCTCAGTC	2	T	0.1901	0.3687
NC_005101.4-21994295	ATCCATCTCACAGTGCCTCCCTCAGTC	6	T	0.2149	0.418
NC_005101.4-21997399	TCAGTCCTCACAATTCCTCCGTGAGTC	21	G	0.1696	0.4043
NC_005101.4-22046177	TTCAGAGAGACCGCTACTCCCACATAA	1	T	0.087	0.4936
NC_005101.4-22046177	TTCAGAGAGACCGCTACTCCCACATAA	2	T	0.0172	0.4851
NC_005101.4-22166850	CACACAAAAACACACACTCCACACCAC	22	C	0.0574	0.0557
NC_005101.4-22171889	GAAATCGAAACCCTTTCTCCGACCCCT	5	T	0	0.4773
NC_005101.4-22171889	GAAATCGAAACCCTTTCTCCGACCCCT	6	C	0.0894	0.1001
NC_005101.4-22201704	CTCTTCAACACCGTGACTCCTCTGACC	12	C	0.1765	0.2389
NC_005101.4-22201704	CTCTTCAACACCGTGACTCCTCTGACC	25	A	0.2857	0.4844
NC_005101.4-22212188	TCTGTTTAGACCTGTGCTCCTGGAATC	12	C	0.2787	0.4807
NC_005101.4-22244678	CGTGTGTCAACTGGATCTCCTATAATT	1	C	0.0086	0.4803
NC_005101.4-22254691	AGTGCTGGGACCTAGGCTCCAAGGTTG	2	G	0.1282	0.1347
NC_005101.4-22329228	CTAAACGCCACCTAGTCTCCGAGGCTG	7	G	0.0442	0.0433
NC_005101.4-22341076	CCAAACAGAACCTCTCCTCCTCTTAAT	24	T	0.2083	0.4847
NC_005101.4-22380134	GGCAGAAGCACTGGCTCTCCTGTTTTA	7	A	0.2203	0.4948

NC_005101.4-22407317	GCTGGTGACACACCTCCTCCAATGAAG	13	C	0.0702	0.4444
NC_005101.4-22472158	GCAATAATGACGGGGGCTCCGTTCCCC	12	G	0.0678	0.1409
NC_005101.4-22489620	AAAGCTGTCACCAAGCCTCCGAGCTGG	1	A	0.1379	0.4946
NC_005101.4-22489620	AAAGCTGTCACCAAGCCTCCGAGCTGG	27	G	0.0672	0.1254
NC_005101.4-22491458	ACGGCTAACACCTCTCCTCCAATGCTC	1	A	0.1983	0.4729
NC_005101.4-22544541	GCATTAGCCACAGTGTCTCCAACCTCA	24	C	0	0.4839
NC_005101.4-22629912	CAATTCCAAACACCAACTCCCTGAGTC	21	C	0.1858	0.4975
NC_005101.4-22633071	AAAACCTACACGAGTCCTCCTTTGGGA	2	A	0.125	0.4871
NC_005101.4-22647234	TACCCCCAAACTCTTACTCCTATTCTT	8	A	0.0083	0.4783
NC_005101.4-22667431	TACCAGATGACTAGTTCTCCATAGCTG	25	C	0.0331	0.0325
NC_005101.4-22680512	GAGGCGAACACGGGACCTCCGTGGGAA	5	C	0	0.4793
NC_005101.4-22680512	GAGGCGAACACGGGACCTCCGTGGGAA	12	G	0.3051	0.4425
NC_005101.4-22692041	AACATTCCTACCCGCTCTCCTCACAAG	13	C	0.0656	0.0786
NC_005101.4-22692041	AACATTCCTACCCGCTCTCCTCACAAG	14	G	0.0328	0.0322
NC_005101.4-22698853	ACAACAAGGACACATGCTCCATGTTCA	24	T	0.1017	0.2466
NC_005101.4-22710291	TCCTCGGTCACTAAGGCTCCCTTCTCT	6	G	0.042	0.4949
NC_005101.4-22752501	ACTCAACCAACTCTTGCTCCATATATG	14	T	0.2358	0.2441
NC_005101.4-22805170	TATGTGTTTACATGGGCTCCTAGGACA	1	T	0.0333	0.0328
NC_005101.4-22805170	TATGTGTTTACATGGGCTCCTAGGACA	16	G	0.1597	0.1469
NC_005101.4-22950865	CAACACCCAACATAGTCTCCAGCTCCA	13	T	0	0.4746
NC_005101.4-22965032	GATCAATGGACTCCTGCTCCAAAGGAA	24	G	0.1721	0.1708
NC_005101.4-22987615	CACATACACACACACACTCCACCACAC	1	C	0.193	0.1744
NC_005101.4-22987615	CACATACACACACACACTCCACCACAC	13	C	0.0431	0.0422
NC_005101.4-23093156	GAAACTGAAACCAGGACTCCAGATGTC	25	G	0.3008	0.3472
NC_005101.4-23096977	CAGCCGGAGACTCCTGCTCCGCGGCGG	15	T	0.0328	0.0322
NC_005101.4-23119368	CAGAAGAAAACAGGGACTCCACAGAAG	9	A	0	0.48
NC_005101.4-23156827	CCACTCCTAACTTTCTCTCCCATGCTC	23	T	0.0087	0.4744
NC_005101.4-23159501	ACCCAAAGGACTTGACCTCCTATGTCA	2	C	0.0631	0.4896
NC_005101.4-23186246	ACCTGTCTAACAAAGCCTCCATCCAGG	21	A	0	0.4807
NC_005101.4-23216973	AACCGCCACACCATCCCTCCATGTTGC	8	A	0.1463	0.4992
NC_005101.4-23216973	AACCGCCACACCATCCCTCCATGTTGC	24	T	0	0.4793
NC_005101.4-23218189	CTGCCTGTCACTTGGTCTCCCCACCCT	25	C	0	0.4654
NC_005101.4-23224881	ATTCTCTGCACGCCCACTCCTGCCCAC	3	T	0.0574	0.4903
NC_005101.4-23224881	ATTCTCTGCACGCCCACTCCTGCCCAC	7	T	0.0082	0.4822
NC_005101.4-23274885	CTGTGAGAAACATCGTCTCCGACAGAC	15	G	0.0909	0.1447
NC_005101.4-23322098	GAGGGGAACACCGAGTCTCCTCTGGTC	1	G	0	0.48
NC_005101.4-23345716	TTTAAAAACACCTGACCTCCCTTCCCC	23	T	0.1765	0.214
NC_005101.4-23358078	TCACCGGTAACTCCAGCTCCAGGGGAT	26	A	0.2368	0.4981
NC_005101.4-23364318	AGACTTTTCACGTTCGCTCCTTTAGTT	14	T	0.2314	0.3374
NC_005101.4-23364318	AGACTTTTCACGTTCGCTCCTTTAGTT	16	G	0.1322	0.1235
NC_005101.4-23432014	ATAGAAACTACAATGACTCCTTGGGTT	8	C	0.1504	0.3683
NC_005101.4-23470207	GGGCTTGTGACTTTCTCTCCATAGTGG	3	G	0.082	0.4952
NC_005101.4-23470207	GGGCTTGTGACTTTCTCTCCATAGTGG	25	T	0.0813	0.1073
NC_005101.4-23473819	ACACGAATCACTAGAGCTCCTCCTAGT	4	C	0.0261	0.0257
NC_005101.4-23473819	ACACGAATCACTAGAGCTCCTCCTAGT	24	T	0.287	0.4395
NC_005101.4-23474000	GGTCAGCACACTGGCTCTCCCTGATCC	5	A	0.0894	0.4967
NC_005101.4-23474000	GGTCAGCACACTGGCTCTCCCTGATCC	9	C	0.0738	0.1839
NC_005101.4-23486407	TCCAAACCTACTTTCTCTCCTACGTAG	13	T	0.0496	0.0639
NC_005101.4-23522530	TCCAGTGTCACTCCCTCTCCCTGCTGT	12	T	0.1382	0.2203
NC_005101.4-23565458	CCACCTGTCACTTCTGCTCCAAAGGAT	12	T	0.1017	0.0965
NC_005101.4-23584996	GTAATGACCACCCTCCCTCCTCCAACC	8	C	0.0789	0.0758

NC_005101.4-23589536	AAGGTCTTGACTCCTTCTCCATCACAG	13	C	0.2049	0.4984
NC_005101.4-23603237	GGACCATCCACCCTCTCTCCTCCACTG	9	C	0.1721	0.1573
NC_005101.4-23610445	CTGGCCAGGACAAGCCCTCCTGGGATG	16	C	0.0351	0.0345
NC_005101.4-23619742	GGTGCAGCGACCCAAACTCCAGTTCTC	26	T	0.2162	0.2594
NC_005101.4-23626542	AAAAATCAGACACCGCCTCCCGCAGAG	21	C	0.2397	0.4913
NC_005101.4-23626542	AAAAATCAGACACCGCCTCCCGCAGAG	27	G	0.0492	0.0634
NC_005101.4-23756305	TGTTTTGTCACCTCATCTCCCTGGACT	3	T	0.0598	0.058
NC_005101.4-23757057	TTCATTGTTACTGGTTCTCCTCCTTTT	1	T	0.1579	0.2021
NC_005101.4-23769932	TCTGCTTTAACACAGGCTCCTCCTTTG	16	G	0.0177	0.4827
NC_005101.4-23780844	TAAGAACTTACCTGCTCTCCTCCGATG	3	A	0.1053	0.1153
NC_005101.4-23780844	TAAGAACTTACCTGCTCTCCTCCGATG	5	A	0.1062	0.4968
NC_005101.4-23793638	AGCCACAGCACAGAGCCTCCTGGAAAA	25	A	0.0088	0.4599
NC_005101.4-23807367	ATGGCTGGTACTTCACCTCCTTATTTT	12	T	0.1795	0.1908
NC_005101.4-23812163	CAGCCCTACACTATGGCTCCTCTTCCT	23	T	0.1607	0.1771
NC_005101.4-23813579	GACGGCGCCACGGTTCCTCCAGGGCGG	26	G	0.0847	0.0812
NC_005101.4-23816947	CCACAGTTGACTGGACCTCCCTTACCC	23	T	0.0656	0.0634
NC_005101.4-23825807	TAGTGGAACACATGACCTCCTCCCTTA	3	G	0.1754	0.2021
NC_005101.4-23825807	TAGTGGAACACATGACCTCCTCCCTTA	5	G	0.0614	0.1075
NC_005101.4-23833870	AAGGGAAACACAGCACCTCCAGCCTCT	16	C	0	0.4787
NC_005101.4-23848921	CTGTGGGAGACTCCATCTCCCTGGGGC	5	G	0.0522	0.4764
NC_005101.4-23848921	CTGTGGGAGACTCCATCTCCCTGGGGC	25	G	0.0345	0.0339
NC_005101.4-23859998	CTACAGAGCACGCAGGCTCCTCTCTGC	27	C	0.0732	0.4618
NC_005101.4-23896026	AAAGACTTGACAAACCCTCCCTGTCAC	12	A	0.188	0.2361
NC_005101.4-23902555	GGACAGACCACCACCCCTCCGGACTCT	9	C	0.0917	0.1997
NC_005101.4-23902555	GGACAGACCACCACCCCTCCGGACTCT	16	C	0.0413	0.0716
NC_005101.4-23918667	ACATTTTCCACACACTCTCCTTTATTC	6	T	0.1043	0.1588
NC_005101.4-23918667	ACATTTTCCACACACTCTCCTTTATTC	25	T	0.2609	0.3772
NC_005101.4-23923043	ACCAAAACAACAGAGCCTCCACAGGGA	12	A	0.0413	0.4835
NC_005101.4-23923043	ACCAAAACAACAGAGCCTCCACAGGGA	23	A	0.0246	0.0243
NC_005101.4-23924618	GGCTCTGACACCACTGCTCCTCTAGAT	27	T	0.0083	0.4847
NC_005101.4-23924763	TGCCATGGTACACTTACTCCTAGATAC	12	A	0.325	0.4358
NC_005101.4-23932211	CTTCCACAGACACTGGCTCCTCCTTCA	7	C	0.027	0.0267
NC_005101.4-23932211	CTTCCACAGACACTGGCTCCTCCTTCA	24	T	0.0268	0.0264
NC_005101.4-23940332	CTGGGGATCACTTCCACTCCAAGAAGG	14	C	0.0708	0.0683
NC_005101.4-23968239	TGTCCTGCCACGGCTTCTCCCTTTCTC	12	G	0.4417	0.4166
NC_005101.4-23977885	ACCTTGCAGACAGTGTCTCCCTGAAGA	5	T	0.2295	0.387
NC_005101.4-23977885	ACCTTGCAGACAGTGTCTCCCTGAAGA	7	C	0.1557	0.2903
NC_005101.4-23992447	ATAGCACAAACTGTGTCTCCTCCAGTG	1	A	0.0957	0.1219
NC_005101.4-23992447	ATAGCACAAACTGTGTCTCCTCCAGTG	23	C	0.1913	0.4537
NC_005101.4-24022067	GTTGTGGTGACTGCCCCTCCCCCCATC	2	T	0.3478	0.3593
NC_005101.4-24022067	GTTGTGGTGACTGCCCCTCCCCCCATC	26	T	0.0427	0.0418
NC_005101.4-24071832	CAGGAAGACACCCAGCCTCCTCCTCTG	1	C	0.0089	0.4789
NC_005101.4-24071832	CAGGAAGACACCCAGCCTCCTCCTCTG	2	A	0.2719	0.4935
NC_005101.4-24091879	ACAAAGTGCACCTCAGCTCCAGGTCAG	15	A	0.1417	0.1316
NC_005101.4-24094284	GTTTGCTCTACATAAGCTCCAGGCCTT	4	T	0.0328	0.0322
NC_005101.4-24188899	TGTACTGAGACCCTGTCTCCTTGCTCC	5	C	0	0.4793
NC_005101.4-24202182	TCACCTTGGACACACACTCCTTCCATT	14	A	0.0248	0.4913
NC_005101.4-24202182	TCACCTTGGACACACACTCCTTCCATT	26	T	0.2397	0.4913
NC_005101.4-24208138	ACTCACAGGACGGAGCCTCCACTGCTG	12	G	0.0246	0.0243
NC_005101.4-24232116	TCTTTAGTCACGTGCCCTCCTCTGAGC	12	G	0.0496	0.0484
NC_005101.4-24273282	CATGTAGCCACATTTCCTCCGAGTTCT	16	C	0.025	0.0247

NC_005101.4-24280424	TAGGCTATCACCATTGCTCCTGGTTGG	23	G	0.1583	0.1733
NC_005101.4-24285431	CTACTCAGTACCCAGTCTCCCTGGTGT	8	G	0.0598	0.4626
NC_005101.4-24285431	CTACTCAGTACCCAGTCTCCCTGGTGT	16	T	0	0.0488
NC_005101.4-24315438	CCCAGTGATACCCCCTCTCCACCCCAC	22	C	0.1261	0.1327
NC_005101.4-24332997	CTTTTGGAGACTTGGCCTCCTTGTATT	22	T	0.2667	0.2994
NC_005101.4-24333470	TGGGTGTACACGGCTGCTCCATCCATC	4	G	0.2439	0.4389
NC_005101.4-24333470	TGGGTGTACACGGCTGCTCCATCCATC	12	G	0.0407	0.4935
NC_005101.4-24352925	TGTGATCTCACAGTGGCTCCACCTCTC	2	G	0.0804	0.1983
NC_005101.4-24352925	TGTGATCTCACAGTGGCTCCACCTCTC	5	A	0.1518	0.4751
NC_005101.4-24375553	AAACTCCTCACACTGCCTCCTTAAGGG	7	C	0.1138	0.2499
NC_005101.4-24375553	AAACTCCTCACACTGCCTCCTTAAGGG	13	C	0	0.4807
NC_005101.4-24393185	GGGCAGGTAACATAGCCTCCTAGCATA	6	G	0	0.482
NC_005101.4-24393185	GGGCAGGTAACATAGCCTCCTAGCATA	9	A	0	0.482
NC_005101.4-24419961	GTCCTGACAACTTGACCTCCCTCGGGG	1	G	0.2857	0.4347
NC_005101.4-24423734	ATGGGAATGACCAAACCTCCTCTGGTC	12	C	0.0328	0.0322
NC_005101.4-24424221	GTCGTTTACACGACAGCTCCTAACATC	3	C	0.0086	0.4688
NC_005101.4-24424221	GTCGTTTACACGACAGCTCCTAACATC	25	A	0.0431	0.4768
NC_005101.4-24485074	ATACCCTGGACCCTCCCTCCAATAACT	3	A	0.0086	0.0255
NC_005101.4-24485074	ATACCCTGGACCCTCCCTCCAATAACT	26	C	0.0086	0.0255
NC_005101.4-24518611	CTCCACACCACCTTTCCTCCTTTCCTG	6	C	0.0579	0.4693
NC_005101.4-24518611	CTCCACACCACCTTTCCTCCTTTCCTG	7	A	0.0579	0.4693
NC_005101.4-24538870	CTTTCCAACACTGTAACTCCAGAGAAG	16	A	0.2213	0.4924
NC_005101.4-24599987	CACGTTGTCACTAAGACTCCTTGCATG	4	G	0.1765	0.188
NC_005101.4-24660049	CTGCTGTTCACCGTTTCTCCTGCAGCC	2	T	0.0833	0.4978
NC_005101.4-24667072	GGAATGATCACATTAGCTCCCAGAATC	2	G	0.0625	0.0771
NC_005101.4-24714612	CTCCTGAGAACTTTTGCTCCTGTATTT	1	C	0.0325	0.032
NC_005101.4-24717425	CAGCTGACTACTTTCCCTCCGTGAGAA	2	A	0	0.4773
NC_005101.4-24720048	AGATATTAAACACTAGCTCCTTAACGT	24	A	0	0.4757
NC_005101.4-24723427	ACTCCTCCAACCCCGCCTCCCCTTCCG	27	G	0.0171	0.4868
NC_005101.4-24742016	CTCATAGCCACTTTCTCTCCTGCCATT	26	T	0.1429	0.491
NC_005101.4-24745617	TCTTTCTCTACCGTAGCTCCCTCAATG	13	G	0.1148	0.1505
NC_005101.4-24749618	CCTTTCAGCACATTCGCTCCTAGCCAG	15	C	0.0265	0.0262
NC_005101.4-24778579	GCCTGCCTCACCATTCCTCCTGACATG	13	A	0.1138	0.2017
NC_005101.4-24778579	GCCTGCCTCACCATTCCTCCTGACATG	26	T	0.2114	0.2832
NC_005101.4-24865789	CAAATATGAACGTCATCTCCTCTTTTA	15	A	0	0.4793
NC_005101.4-24878126	AAAATAAAAACAAGGTCTCCATGATTG	23	G	0.2252	0.3817
NC_005101.4-24896084	CAAGTACACACCACCACTCCTGTGCAA	27	A	0.0172	0.4749
NC_005101.4-24910152	TGGCTAAGAACACAAACTCCCATTCTG	14	A	0.0244	0.4741
NC_005101.4-24911707	ATCACACCAACTGGCTCTCCAGTACTA	15	C	0.0357	0.0351
NC_005101.4-24940721	TCATCATAGACCTTGGCTCCCCTGCCT	6	A	0.0088	0.4676
NC_005101.4-25025455	AGACCGTAAACCCTGCCTCCCTCTTCA	5	C	0.0488	0.0476
NC_005101.4-25025455	AGACCGTAAACCCTGCCTCCCTCTTCA	9	A	0.0492	0.4866
NC_005101.4-25049947	ATTGTTATAACCTCCACTCCCAATATG	3	T	0	0.4839
NC_005101.4-25049947	ATTGTTATAACCTCCACTCCCAATATG	21	C	0	0.4851
NC_005101.4-25051087	TGCAGTAGCACGACTGCTCCATGTGTA	3	C	0.0325	0.032
NC_005101.4-25061188	CCTGCTTACACAGTATCTCCTGCCTCT	3	T	0.1795	0.3596
NC_005101.4-25061213	CTGGTGTCCACCAAACCTCCACGATGT	26	G	0.0526	0.0677
NC_005101.4-25078247	AGACACAGGACTGAGCCTCCCTTACTG	12	T	0.0082	0.0243
NC_005101.4-25078247	AGACACAGGACTGAGCCTCCCTTACTG	15	G	0.0909	0.4951
NC_005101.4-25145603	AGCAGCTCCACAAAGTCTCCAGTTCCA	21	A	0.0325	0.032
NC_005101.4-25154695	CATACCATGACCTACCCTCCTCCCTCA	8	T	0.1707	0.4471

NC_005101.4-25166753	CAATGGGCTACACCCTCTCCACATCTC	8	C	0.0885	0.1316
NC_005101.4-25166753	CAATGGGCTACACCCTCTCCACATCTC	21	A	0.1593	0.2171
NC_005101.4-25204461	TCCAGTGGAACCTAAGCTCCACCCACC	2	C	0.2479	0.418
NC_005101.4-25204461	TCCAGTGGAACCTAAGCTCCACCCACC	24	C	0.0082	0.0243
NC_005101.4-25204746	AGAGACCACACACTTTCTCCAGTTGTA	2	G	0.2033	0.2556
NC_005101.4-25274500	TCCATTCTGACTTCAACTCCAAACATT	5	T	0.0325	0.032
NC_005101.4-25303238	TGTAAAGACACTTTCTCTCCCTGTGTG	3	T	0	0.48
NC_005101.4-25306856	ACCCAAGGGACAGGGCCTCCCAGGCCT	21	C	0.3482	0.4193
NC_005101.4-25317314	ATGAAGCATACAGTAACTCCAATGTCT	7	C	0	0.4715
NC_005101.4-25415736	ACCTGACTCACGCATCCTCCAGTGGTA	12	G	0	0.4649
NC_005101.4-25437853	ATCAATGTTACTCACTCTCCTTCACTC	1	A	0.1271	0.119
NC_005101.4-25448181	GACCAGCCTACTACAGCTCCAGGAAGT	3	C	0.0167	0.0488
NC_005101.4-25448181	GACCAGCCTACTACAGCTCCAGGAAGT	13	A	0.025	0.0247
NC_005101.4-25493170	TCCGTGATAACCAGTGCTCCTTCAAGA	9	A	0.2759	0.3835
NC_005101.4-25546875	GTGAGAACCACTGTACCTCCTTTGTGG	14	T	0.2195	0.2989
NC_005101.4-25573313	GGTAGTAGAACTGAGACTCCTGTATAA	5	G	0.0804	0.0934
NC_005101.4-25597074	AAGTGCTTCACCTTTCCTCCCTTTTGT	23	T	0.2377	0.4126
NC_005101.4-25597074	AAGTGCTTCACCTTTCCTCCCTTTTGT	27	T	0.0826	0.4369
NC_005101.4-25599353	GCAGTCTCCACCCAGACTCCGCCATGT	2	C	0.0086	0.4729
NC_005101.4-25607957	GGGTTTAAGACCCTTCCTCCTGACCTC	2	G	0.0976	0.0928
NC_005101.4-25659280	CTAAAGAAGACCATCTCTCCCTCTTTC	15	C	0.0413	0.0405
NC_005101.4-25664364	ATGACCATTACCGATTCTCCATCAATT	12	C	0.075	0.0722
NC_005101.4-25664364	ATGACCATTACCGATTCTCCATCAATT	15	T	0.15	0.2188
NC_005101.4-25668512	AGAGCCATTACATTGACTCCCTGGGAA	24	G	0	0.4751
NC_005101.4-25668512	AGAGCCATTACATTGACTCCCTGGGAA	25	G	0.0417	0.0408
NC_005101.4-25729139	CCATTAAACACCAGGACTCCTTACCAG	15	G	0.0678	0.0655
NC_005101.4-25729139	CCATTAAACACCAGGACTCCTTACCAG	24	C	0.2586	0.4851
NC_005101.4-25765074	CATCTTGGAACTCAGTCTCCAGAACTC	25	C	0.0088	0.026
NC_005101.4-25767056	ATTCCTTCCACGTCCCCTCCAAGGTCT	15	C	0.1736	0.4004
NC_005101.4-25767056	ATTCCTTCCACGTCCCCTCCAAGGTCT	24	G	0.1901	0.3027
NC_005101.4-25849489	CTGCAAGGAACTTCAGCTCCCCAGGAC	12	T	0.0252	0.0249
NC_005101.4-25861509	TCTCCAGTCACTGTGCCTCCTTAAAGT	9	C	0.0885	0.1613
NC_005101.4-25872674	GAATAGGTGACTACAACTCCACTTGTC	5	A	0.0089	0.4709
NC_005101.4-25872674	GAATAGGTGACTACAACTCCACTTGTC	6	G	0.0982	0.1093
NC_005101.4-25894687	AACCAGCCCACCCACCCTCCACAAACT	1	A	0.0909	0.4951
NC_005101.4-25894687	AACCAGCCCACCCACCCTCCACAAACT	8	C	0.2893	0.4507
NC_005101.4-25947344	TGAGTGCAAACACTGACTCCTTGGTAC	6	G	0.187	0.1826
NC_005101.4-25947344	TGAGTGCAAACACTGACTCCTTGGTAC	15	G	0.0902	0.0861
NC_005101.4-25956134	TCCTCATATACCTTTACTCCTCCTAGA	16	A	0.0574	0.0861
NC_005101.4-25956134	TCCTCATATACCTTTACTCCTCCTAGA	22	C	0	0.4773
NC_005101.4-26009381	CACATATGCACTGCCACTCCTGTGACA	7	T	0.2167	0.32
NC_005101.4-26011032	ACCCAGTCCACTTCCTCTCCTGCAGGG	6	G	0.0517	0.0504
NC_005101.4-26054364	AAACAGAAAACCAACACTCCTCATGCC	9	A	0	0.4902
NC_005101.4-26066115	AGGGGAGGGACTCAAGCTCCTGCAAGC	23	C	0.0265	0.0262
NC_005101.4-26072279	GACTAGGGCACTGGGTCTCCTGAACGG	26	G	0.0246	0.0401
NC_005101.4-26114722	CTGTGGATGACTGGACCTCCAAACAAG	13	G	0.2348	0.4395
NC_005101.4-26124262	GAAGTTGTGACTGAGCCTCCATAGCAA	2	A	0.1228	0.1454
NC_005101.4-26125240	CTCAAATTCACCCTCTCTCCCCAATAA	12	C	0.2051	0.3457
NC_005101.4-26125240	CTCAAATTCACCCTCTCTCCCCAATAA	24	A	0.2542	0.3874
NC_005101.4-26164091	CTAGAGCAAACAGACCCTCCAAGCCAC	6	G	0.0081	0.4777
NC_005101.4-26164091	CTAGAGCAAACAGACCCTCCAAGCCAC	23	G	0.1885	0.4943

NC_005101.4-26277695	ACAGGGATCACCCGGCCTCCAGCTAAG	16	C	0.0244	0.4741
NC_005101.4-26287617	CTTTCCTGAACCGTGTCTCCTGTGTAC	24	G	0.0261	0.0752
NC_005101.4-26287617	CTTTCCTGAACCGTGTCTCCTGTGTAC	26	A	0.0261	0.0257
NC_005101.4-26293651	TACAGTATGACACGGTCTCCAAAGGAG	8	T	0.0244	0.0241
NC_005101.4-26293651	TACAGTATGACACGGTCTCCAAAGGAG	13	C	0.1951	0.4
NC_005101.4-26303872	ATTTACAGAACTTGACCTCCTTTTCCT	2	T	0.0083	0.4769
NC_005101.4-26305464	GCCAAATAAACTGTCTCTCCTATGAAG	9	A	0.2069	0.2501
NC_005101.4-26344485	TTAAAGTCAACTATTTCTCCTTAAGTG	12	T	0.0508	0.0655
NC_005101.4-26376526	TCCTAGACCACACGGCCTCCATTCGGG	2	C	0.0813	0.078
NC_005101.4-26376526	TCCTAGACCACACGGCCTCCATTCGGG	24	C	0.2114	0.4904
NC_005101.4-26376735	TGCAGCTCCACCTAGGCTCCACCCCAC	22	C	0.193	0.1744
NC_005101.4-26406671	TATGGGGCAACAGGCACTCCCACTGCT	3	T	0.0732	0.0854
NC_005101.4-26406671	TATGGGGCAACAGGCACTCCCACTGCT	6	G	0.0325	0.032
NC_005101.4-26410286	AGGAACCAGACACTCCCTCCCTCCCAT	27	T	0.2917	0.3622
NC_005101.4-26423943	GCTTCCACCACACCTCCTCCACTTTCC	14	C	0.027	0.4726
NC_005101.4-26434504	CATCACTACACCCATTCTCCCACCTCT	23	C	0.0661	0.0639
NC_005101.4-26473619	AGCATGAAGACCAGCACTCCAGTCCCC	9	G	0.036	0.0354
NC_005101.4-26473619	AGCATGAAGACCAGCACTCCAGTCCCC	27	C	0.027	0.0611
NC_005101.4-26510026	CTAGATACCACCCAACCTCCTAGCTTT	1	C	0.025	0.0247
NC_005101.4-26510026	CTAGATACCACCCAACCTCCTAGCTTT	2	T	0.0248	0.0245
NC_005101.4-26576790	TGTCTTGAGACTGGCTCTCCCACCATG	15	C	0.0833	0.1244
NC_005101.4-26576790	TGTCTTGAGACTGGCTCTCCCACCATG	26	T	0.15	0.255
NC_005101.4-26623938	AAACATCAGACAAGGACTCCTCCATCT	16	A	0	0.4807
NC_005101.4-26623938	AAACATCAGACAAGGACTCCTCCATCT	26	C	0.1026	0.1563
NC_005101.4-26664380	CACAGGTGCACAGCTGCTCCCCGATCT	1	C	0	0.4703
NC_005101.4-26707421	CCTGTCCCCACAGCCTCTCCAGGTGAT	3	T	0.2119	0.2406
NC_005101.4-26707421	CCTGTCCCCACAGCCTCTCCAGGTGAT	9	C	0.2288	0.4919
NC_005101.4-26759261	ACCTCTCTCACATCTCCTCCAGGTGCT	3	C	0.1339	0.1403
NC_005101.4-26774824	TCCTGACCCACACCTGCTCCAGAGACT	21	A	0	0.48
NC_005101.4-26789378	GTGACAGACACATCTCCTCCAACAAGA	13	T	0.0081	0.4777
NC_005101.4-26805041	GGGAATCTGACTCCTCCTCCGGTATCA	8	T	0.0574	0.0557
NC_005101.4-26805041	GGGAATCTGACTCCTCCTCCGGTATCA	23	T	0.1148	0.1774
NC_005101.4-26841809	CAGTTGGCTACACTTGCTCCAATGTTT	3	G	0.2705	0.2796
NC_005101.4-26841809	CAGTTGGCTACACTTGCTCCAATGTTT	23	T	0.0656	0.0935
NC_005101.4-26908854	TTCCACTTCACACTCCCTCCAGTAGCG	27	G	0.0246	0.0243
NC_005101.4-26920745	AAGGATGGAACAATACCTCCATAAGTT	3	G	0.0496	0.0484
NC_005101.4-26920745	AAGGATGGAACAATACCTCCATAAGTT	15	A	0.0248	0.4693
NC_005101.4-26957389	TTCCTTTTGACCTCTCCTCCTTTGTGT	2	T	0.0574	0.0557
NC_005101.4-26957389	TTCCTTTTGACCTCTCCTCCTTTGTGT	25	T	0.0246	0.0243
NC_005101.4-26958082	ATTCTCTCAACCGAGGCTCCCTCCTCT	13	G	0.0268	0.0436
NC_005101.4-26958082	ATTCTCTCAACCGAGGCTCCCTCCTCT	15	G	0.1696	0.5
NC_005101.4-26959374	TTTTGTACTACCCCCACTCCTAAGAGG	8	C	0.1681	0.2237
NC_005101.4-26959374	TTTTGTACTACCCCCACTCCTAAGAGG	9	T	0.1667	0.222
NC_005101.4-27025276	AAATTCTACACTGGATCTCCCACCAAC	15	A	0.0082	0.4755
NC_005101.4-27072495	TGGGGAATTACTGAGGCTCCATATCAG	15	G	0.0265	0.0765
NC_005101.4-27141736	AATGCTTACACATACACTCCACCCTCA	5	C	0.0167	0.4688
NC_005101.4-27141736	AATGCTTACACATACACTCCACCCTCA	27	A	0.0167	0.4688
NC_005101.4-27142622	AGAGGCATCACCTTTTCTCCAACATTA	5	G	0.2114	0.2832
NC_005101.4-27180594	TCTTCACCTACGTAGGCTCCCTAGCCT	9	T	0.1818	0.4628
NC_005101.4-27275259	GAGCAGATCACTAGGCCTCCTGGCCCA	21	T	0.0917	0.1172
NC_005101.4-27281591	GATCCATCAACAAACACTCCTTTCTGC	14	A	0.1565	0.1869

NC_005101.4-27282473	ACAATGTGTACCTTGGCTCCCTAGGTC	12	C	0.1653	0.1918
NC_005101.4-27290866	TCTTTTCAAACAGCTTCTCCATAAGAT	23	A	0.1261	0.3389
NC_005101.4-27304515	TGCTTCTACACCATAGCTCCTGCAACT	4	T	0.2033	0.208
NC_005101.4-27304515	TGCTTCTACACCATAGCTCCTGCAACT	22	G	0.0976	0.1073
NC_005101.4-27315267	GCTGCCTCTACATCAACTCCCACCCCA	3	T	0	0.4501
NC_005101.4-27315267	GCTGCCTCTACATCAACTCCCACCCCA	21	C	0.2193	0.499
NC_005101.4-27354842	ACAGTGGTCACGGACTCTCCCTCTAAA	7	G	0.0088	0.4923
NC_005101.4-27449810	CATGCATGCACACATACTCCACACATA	8	G	0.0783	0.0752
NC_005101.4-27511098	GGGGACAGGACTTAGTCTCCAGAAAGA	26	G	0.125	0.2188
NC_005101.4-27516520	AACCACCCTACCAGACCTCCTGACTCC	9	T	0.5714	0.4285
NC_005101.4-27516520	AACCACCCTACCAGACCTCCTGACTCC	15	A	0.5763	0.4305
NC_005101.4-27727508	CCATATCCCACTCCCCCTCCCCCTGCT	6	T	0.1724	0.1576
NC_005101.4-27730590	TCGTGAGAGACTCTTTCTCCTTCCTTC	2	C	0.0261	0.0257
NC_005101.4-27730590	TCGTGAGAGACTCTTTCTCCTTCCTTC	27	C	0	0.4814
NC_005101.4-27748915	ATGATGGTGACTCAGGCTCCTGCTACC	6	G	0.193	0.1884
NC_005101.4-27748915	ATGATGGTGACTCAGGCTCCTGCTACC	15	G	0.2105	0.4998
NC_005101.4-27792095	CTGCTTTGAACATGGACTCCTCTGATG	12	A	0.094	0.1773
NC_005101.4-27820724	GTAGCTTCAACATCCTCTCCTTCCATC	14	C	0.2683	0.2989
NC_005101.4-27847158	ACGGATTGGACCAGATCTCCCTCTGAA	2	C	0.2846	0.4618
NC_005101.4-27861069	AAACGTTAGACAAGTGCTCCACCAATG	2	A	0.1463	0.1494
NC_005101.4-27901699	TGGTAAAATACTGTGCCTCCTTATCAT	22	T	0.0082	0.479
NC_005101.4-27928954	TCCAGATAGACGTCATCTCCATTATAG	12	G	0.2393	0.4938
NC_005101.4-27928954	TCCAGATAGACGTCATCTCCATTATAG	23	T	0	0.4703
NC_005101.4-27989606	GGCCTCGACACTGTAACTCCTGAGTCC	7	G	0.0325	0.0476
NC_005101.4-27989606	GGCCTCGACACTGTAACTCCTGAGTCC	16	A	0	0.4793
NC_005101.4-28035038	CTGTATGCTACCATACCTCCATACTCA	26	C	0.0855	0.4991
NC_005101.4-28075318	CATGACACTACCGAGTCTCCAAAGAAA	15	G	0.0847	0.0812
NC_005101.4-28192756	AGCCTTTGAACAAAGACTCCTTGAACA	22	T	0.025	0.0247
NC_005101.4-28245493	TAAACATCCACCAGGTCTCCCATGACA	12	C	0.1301	0.1629
NC_005101.4-28246903	TCCCTGAGGACCAGGCCTCCACCGATT	1	T	0	0.4751
NC_005101.4-28246903	TCCCTGAGGACCAGGCCTCCACCGATT	9	G	0.1322	0.1653
NC_005101.4-28316860	AAAGAGGGGACTCAGGCTCCCGAGGTT	14	A	0.1565	0.2005
NC_005101.4-28353329	AAGAAGCAAACTCAGGCTCCTCACAAG	24	C	0.2541	0.2686
NC_005101.4-28433180	GAGCTAACCACATCCCCTCCCCCAGCT	4	C	0.2131	0.2032
NC_005101.4-28433180	GAGCTAACCACATCCCCTCCCCCAGCT	21	C	0.0992	0.1377
NC_005101.4-28473838	TCATAATAAACAGCACCTCCTCCCCAC	24	C	0.0833	0.0799
NC_005101.4-28526279	TGTCTCCTCACGTGATCTCCTCCCAAC	16	T	0.2276	0.2382
NC_005101.4-28529762	CAGTGAACAACGTGAACTCCAGAGAGT	13	T	0	0.4724
NC_005101.4-28529762	CAGTGAACAACGTGAACTCCAGAGAGT	22	G	0.1983	0.1786
NC_005101.4-28538414	TACACACTCACACACACTCCCCCACTT	1	T	0.0083	0.0247
NC_005101.4-28538414	TACACACTCACACACACTCCCCCACTT	9	C	0.0924	0.0882
NC_005101.4-28538535	CATACACTTACACACACTCCTCCACTT	22	C	0.2846	0.4618
NC_005101.4-28538686	CACACACATACACATACTCCTCCACTT	21	T	0.2358	0.3646
NC_005101.4-28554985	AAAACCTCCACGGACTCTCCTGAGGCT	5	C	0	0.4793
NC_005101.4-28554985	AAAACCTCCACGGACTCTCCTGAGGCT	9	C	0	0.4807
NC_005101.4-28600307	ATTTGTGGGACCCCAGCTCCCCTGAGC	5	G	0	0.4793
NC_005101.4-28610452	CTATTAGTCACCATTACTCCCAAGGCT	26	C	0	0.4813
NC_005101.4-28613207	CAAAGCGACACAACGCCTCCAGGTTTC	4	A	0.094	0.12
NC_005101.4-28641365	CTTGGACCTACTTGTTCTCCACATTTG	7	C	0.0268	0.0264
NC_005101.4-28659115	GTGCCCAGTACTCCATCTCCGGGGGTG	21	G	0	0.4715
NC_005101.4-28672045	ATCTGTTTGACTCTGACTCCTGGGATT	24	G	0.3304	0.4043

NC_005101.4-28672045	ATCTGTTTGACTCTGACTCCTGGGATT	27	T	0.0357	0.0351
NC_005101.4-28687370	TGTAACGTGACATGCCCTCCTATTTCT	3	T	0	0.4832
NC_005101.4-28714682	CCTGACACAACTGAGTCTCCAACAAAA	15	G	0.1538	0.4982
NC_005101.4-28784883	AGGCTGGCAACCCGAGCTCCATCCCTG	13	C	0.1197	0.1274
NC_005101.4-28784883	AGGCTGGCAACCCGAGCTCCATCCCTG	14	G	0.094	0.1049
NC_005101.4-28952076	TATCTCCTTACAGCCCCTCCCCATTCA	12	A	0	0.477
NC_005101.4-28952076	TATCTCCTTACAGCCCCTCCCCATTCA	25	T	0	0.477
NC_005101.4-29081594	TGCTACATCACCGCCCCTCCCCCATTA	25	T	0	0.4793
NC_005101.4-29119275	CATCCCTACACCGATCCTCCTCAGACA	13	G	0	0.4793
NC_005101.4-29119275	CATCCCTACACCGATCCTCCTCAGACA	15	T	0	0.4793
NC_005101.4-29227641	ATTCTGATCACAGTTTCTCCTCTCTCA	24	C	0.0248	0.0245
NC_005101.4-29282502	CTGACAGTTACTCCGTCTCCTTTATAG	2	T	0.0254	0.0251
NC_005101.4-29282502	CTGACAGTTACTCCGTCTCCTTTATAG	27	G	0.0345	0.0339
NC_005101.4-29312602	CTTTGAGAGACATCTACTCCATAGCAG	9	G	0.027	0.0267
NC_005101.4-29312602	CTTTGAGAGACATCTACTCCATAGCAG	26	A	0.0714	0.4974
NC_005101.4-29313787	CTGCTTTTCACTGTGTCTCCTCAATTC	23	A	0.2195	0.4952
NC_005101.4-29313787	CTGCTTTTCACTGTGTCTCCTCAATTC	24	A	0.0894	0.1145
NC_005101.4-29343810	TGAAGAAGTACCATGACTCCCTATAAA	8	G	0.1301	0.1356
NC_005101.4-29382424	TAAGAACACACAAGAGCTCCAAGGCAA	9	C	0.0522	0.0508
NC_005101.4-29384280	ACGTATTTTACAATGCCTCCTTAGTCC	12	A	0.1121	0.1358
NC_005101.4-29384280	ACGTATTTTACAATGCCTCCTTAGTCC	24	G	0.1293	0.2562
NC_005101.4-29417497	CATGCCTATACAAAATCTCCAGCCTTC	7	T	0.1653	0.2869
NC_005101.4-29428401	CAAAGATGGACCTGGCCTCCAGGTCCT	23	G	0.0984	0.1225
NC_005101.4-29447651	CAGGGAGCAACTATTGCTCCCAGCACT	8	C	0.1518	0.4512
NC_005101.4-29462785	TTTAAAGGGACAACTTCTCCCCAGACT	5	A	0.0174	0.4588
NC_005101.4-29481793	TCTCTCAGAACAGCAGCTCCAGCACCC	13	G	0.0407	0.0398
NC_005101.4-29481793	TCTCTCAGAACAGCAGCTCCAGCACCC	26	C	0.0325	0.032
NC_005101.4-29539611	ATCTTTCCCACCATACCTCCTTTCCTT	3	C	0.0508	0.4826
NC_005101.4-29539611	ATCTTTCCCACCATACCTCCTTTCCTT	23	T	0.0336	0.033
NC_005101.4-29554774	GCCCTCCCCACCTTCACTCCACCAAGT	2	C	0.1681	0.2203
NC_005101.4-29554774	GCCCTCCCCACCTTCACTCCACCAAGT	4	C	0.05	0.0488
NC_005101.4-29590143	CCTGTGAACACAGGAACTCCATAGTTG	7	A	0.0175	0.4846
NC_005101.4-29590143	CCTGTGAACACAGGAACTCCATAGTTG	26	T	0.1739	0.2268
NC_005101.4-29690961	TGCGACTAAACCAGATCTCCTTTGGCA	3	C	0.265	0.3855
NC_005101.4-29690961	TGCGACTAAACCAGATCTCCTTTGGCA	25	G	0.0513	0.05
NC_005101.4-29699256	CGAGAAGCAACTCTATCTCCAAGGAGA	2	G	0.05	0.0488
NC_005101.4-29705140	CAGACTTTCACCCCTACTCCTCCATCA	6	T	0.0513	0.05
NC_005101.4-29706425	ATCCCTTTCACCCTTTCTCCTCAGCCA	22	C	0.3496	0.373
NC_005101.4-29728280	ACATTGTCCACCCTGCCTCCAAGGGCA	4	T	0	0.4807
NC_005101.4-29744648	AATTCATAAACGGCAACTCCTAGAGTC	8	A	0.2893	0.4693
NC_005101.4-29744648	AATTCATAAACGGCAACTCCTAGAGTC	27	C	0.2893	0.4693
NC_005101.4-29795905	GGAATGCAGACACCAGCTCCCCAAAGT	1	G	0.2203	0.5
NC_005101.4-29795905	GGAATGCAGACACCAGCTCCCCAAAGT	6	G	0.2034	0.1827
NC_005101.4-29819006	GTAGGAAACACTGGGACTCCATTGCAT	26	A	0.0696	0.4864
NC_005101.4-29855591	AGATGTTCGACTTTCTCTCCTCTACCA	22	C	0.1833	0.2311
NC_005101.4-29855591	AGATGTTCGACTTTCTCTCCTCTACCA	23	T	0	0.4765
NC_005101.4-29861529	AACTGATGGACCCATCCTCCTTGTCAT	5	G	0	0.4773
NC_005101.4-29862946	ATTAAGCCAACCTTGGCTCCCTGAACC	6	G	0.2105	0.2659
NC_005101.4-29899891	AGAGGGATCACCAGGGCTCCGAGACCA	21	G	0.0504	0.0491
NC_005101.4-29907803	AGGCTGCCCACACAGGCTCCCTGCCAC	15	G	0.2035	0.297
NC_005101.4-29907803	AGGCTGCCCACACAGGCTCCCTGCCAC	16	G	0.0439	0.0595

NC_005101.4-29910004	TGGCAATTAACTCACACTCCCTGAGAC	16	A	0.0254	0.481
NC_005101.4-29930082	GTATGCTGGACCCACTCTCCTGAATCC	27	C	0.2333	0.255
NC_005101.4-29933755	CACTTCTACACATGCTCTCCCACACAT	23	C	0.2479	0.2975
NC_005101.4-29965349	GCCCTCAACACTGCCCCTCCCCCACAT	1	G	0.7611	0.5
NC_005101.4-29965349	GCCCTCAACACTGCCCCTCCCCCACAT	3	C	0.5702	0.463
NC_005101.4-29976893	TGGGTTGACACATCTTCTCCTGAAAGA	16	T	0.1311	0.3709
NC_005101.4-29987550	GGAATAAGGACAGGCTCTCCAGCTTTA	12	A	0	0.4787
NC_005101.4-29996176	TGGAGATGGACAGAAGCTCCCCAACAG	14	A	0.2583	0.3347
NC_005101.4-30042350	CTCATCAATACAGTCGCTCCAAAGAGT	12	A	0.1345	0.5
NC_005101.4-30042350	CTCATCAATACAGTCGCTCCAAAGAGT	16	G	0.025	0.0247
NC_005101.4-30044137	CTTCCTGTCACCCCTCCTCCCTGGAAC	6	T	0	0.4813
NC_005101.4-30078038	AACTGCGGGACCATTTCTCCACCTATG	15	T	0.0756	0.4542
NC_005101.4-30150163	AGATGGGCCACTGTCCCTCCTTCCTCT	4	T	0.2683	0.4662
NC_005101.4-30150163	AGATGGGCCACTGTCCCTCCTTCCTCT	9	C	0.122	0.4174
NC_005101.4-30175485	ATCCGAGTCACCATCCCTCCATGCCAT	6	A	0.0345	0.0339
NC_005101.4-30184791	ATGGTGTGCACTTGTACTCCCAGTGCC	2	T	0.1736	0.4889
NC_005101.4-30192272	CTCCATGGCACTCACACTCCCTTAGCT	5	A	0.1066	0.1708
NC_005101.4-30197595	TCTCTCTCTACCAGGGCTCCAGGTCTT	6	C	0.1786	0.2188
NC_005101.4-30218002	TAACTTTTCACAGCAGCTCCTAGCAAA	6	T	0	0.4682
NC_005101.4-30218002	TAACTTTTCACAGCAGCTCCTAGCAAA	21	T	0.2957	0.32
NC_005101.4-30242726	ACATGACATACCATCTCTCCAATCCAC	21	A	0	0.4807
NC_005101.4-30276601	GACTGCAGTACTATGTCTCCCATTGTA	15	G	0	0.4832
NC_005101.4-30335357	GTAAAAAAAACATACACTCCACTACGT	1	G	0.1892	0.3012
NC_005101.4-30335357	GTAAAAAAAACATACACTCCACTACGT	8	A	0	0.4746
NC_005101.4-30349339	ATTGTCTCTACCATGTCTCCTAAAATG	6	C	0.1538	0.1704
NC_005101.4-30404688	ATGCCTGTTACTCCACCTCCAAAATAC	8	T	0.2213	0.2095
NC_005101.4-30404688	ATGCCTGTTACTCCACCTCCAAAATAC	26	A	0.0902	0.0861
NC_005101.4-30415692	CTCCAACATACTCCACCTCCTTTGTAA	1	C	0.2033	0.1826
NC_005101.4-30415692	CTCCAACATACTCCACCTCCTTTGTAA	24	G	0.5085	0.3953
NC_005101.4-30416207	CTCCCTCCAACCTCTCCTCCCTGTCCT	26	C	0	0.4787
NC_005101.4-30432302	AAGGGATTGACAATATCTCCTACAGAC	13	A	0	0.48
NC_005101.4-30432302	AAGGGATTGACAATATCTCCTACAGAC	23	C	0	0.48
NC_005101.4-30523787	TGACAAGGAACCAACACTCCTCCCAAG	3	A	0.0357	0.0351
NC_005101.4-30566658	GTAACGACAACCTCACCTCCTTCCATC	5	C	0.0488	0.0476
NC_005101.4-30583295	AACCACTGAACTGTCTCTCCACCCCTT	4	C	0.1709	0.4997
NC_005101.4-30628244	AACCACTGTACTATCTCTCCAGCCCGG	25	C	0.1083	0.1597
NC_005101.4-30628244	AACCACTGTACTATCTCTCCAGCCCGG	27	G	0.3167	0.2665
NC_005101.4-30657589	GCTAAAGTCACTAGCACTCCACTGGGT	26	G	0.0088	0.4735
NC_005101.4-30667884	ACACTAGGCACACCAACTCCACAAGCC	24	A	0.0813	0.078
NC_005101.4-30667884	ACACTAGGCACACCAACTCCACAAGCC	26	C	0.3902	0.3239
NC_005101.4-30672147	TCAAAGTGGACCAGGTCTCCAGCTTCC	24	T	0.2212	0.3294
NC_005101.4-30677080	GATTAGCTAACGCTGTCTCCTTGATCC	12	G	0.1405	0.1306
NC_005101.4-30707822	GTGGTGCACACCTTTACTCCCATCGCT	9	C	0.1933	0.3367
NC_005101.4-30723049	CTGATCTCTACCTCAGCTCCACACTGT	14	C	0.0351	0.0345
NC_005101.4-30723049	CTGATCTCTACCTCAGCTCCACACTGT	21	A	0.0439	0.5
NC_005101.4-30777496	TGTAAGGGGACTTTGGCTCCCAGCACT	27	T	0	0.4807
NC_005101.4-30781797	AGAATCCCTACCACCCCTCCATACAAC	14	C	0.0574	0.071
NC_005101.4-30781989	CCTGTCCCAACCCCAGCTCCTGGATCA	2	C	0.2602	0.3516
NC_005101.4-30781989	CCTGTCCCAACCCCAGCTCCTGGATCA	21	T	0.1301	0.1629
NC_005101.4-30878911	CCCATCCATACCTTGGCTCCCTTCCTT	24	C	0	0.4624
NC_005101.4-30878911	CCCATCCATACCTTGGCTCCCTTCCTT	27	T	0.193	0.3221

NC_005101.4-30906784	CTGCCTGCAACTCCAGCTCCAGCAGAT	6	T	0.3814	0.4207
NC_005101.4-30915018	CACAATCTAACACCAGCTCCCGGGGCT	16	G	0.0331	0.0325
NC_005101.4-30915018	CACAATCTAACACCAGCTCCCGGGGCT	21	C	0.1405	0.4999
NC_005101.4-30961905	CACTGTCTTACAGCATCTCCCTCCCTG	1	C	0.0325	0.032
NC_005101.4-30977348	ACCCACCGAACCCTCTCTCCGTCCACC	7	C	0.1327	0.4435
NC_005101.4-30989040	TCTCCTGGTACTTCAGCTCCAGGGAAT	9	T	0	0.4787
NC_005101.4-30995686	GGGTCGTCAACATTTACTCCTCCCATG	23	C	0.2456	0.4038
NC_005101.4-31063394	AACCACTGAACTCTCTCTCCAGTTCTT	2	A	0.0522	0.0508
NC_005101.4-31068013	AGGAAGTTTACCAAATCTCCTTGCTGT	2	G	0.2049	0.3492
NC_005101.4-31081495	ATATGTCTGACTCTGCCTCCCAGTTAC	13	C	0.0625	0.499
NC_005101.4-31144589	CTTATCAGAACATAAGCTCCTTAAGAC	13	T	0.1417	0.1316
NC_005101.4-31231625	AGAGACTCCACCTCCTCTCCTACCGTG	8	C	0	0.4891
NC_005101.4-31231625	AGAGACTCCACCTCCTCTCCTACCGTG	25	G	0	0.4891
NC_005101.4-31238578	CACCTAAGCACTGGCGCTCCCACGATG	23	C	0.2764	0.3239
NC_005101.4-31242357	CTTGAGAAGACCTGTCCTCCTGGCTGA	14	G	0.0583	0.0566
NC_005101.4-31242357	CTTGAGAAGACCTGTCCTCCTGGCTGA	22	G	0.0417	0.0408
NC_005101.4-31249239	GCATGTAAGACCAAAGCTCCAGGCAAG	1	G	0.0496	0.0639
NC_005101.4-31252232	CGTCCCCCCACCACTCCTCCATCCTTC	2	G	0	0.4728
NC_005101.4-31252232	CGTCCCCCCACCACTCCTCCATCCTTC	6	C	0.2149	0.5
NC_005101.4-31266254	GACATATTTACTATACCTCCTCTGACC	26	C	0.0087	0.0257
NC_005101.4-31282304	TGCTTGGCTACTTTGGCTCCCAGTGGT	16	G	0	0.4807
NC_005101.4-31282304	TGCTTGGCTACTTTGGCTCCCAGTGGT	24	T	0.0082	0.4879
NC_005101.4-31285029	TTCTGTGCCACATTTACTCCAGAACAT	8	C	0.2414	0.4987
NC_005101.4-31368837	CATAGATAGACTCTATCTCCAATTCTG	27	G	0.0248	0.0245
NC_005101.4-31530109	GTTGTGTCCACCTGTTCTCCCCAATTC	1	G	0	0.4751
NC_005101.4-31530109	GTTGTGTCCACCTGTTCTCCCCAATTC	4	G	0.1301	0.1761
NC_005101.4-31593573	GCTTTACCCACTCACTCTCCCACGATT	23	C	0.0598	0.0896
NC_005101.4-31605323	AACAAGCAAACATGGACTCCCATTTTG	4	A	0.1593	0.2678
NC_005101.4-31625312	ACAGACAGTACAACCTCTCCTGCGTAC	13	A	0	0.4787
NC_005101.4-31625312	ACAGACAGTACAACCTCTCCTGCGTAC	22	G	0	0.4787
NC_005101.4-31629622	TTCATAGTGACTCTATCTCCGTAGTCT	3	C	0.0256	0.0253
NC_005101.4-31635089	GAGCAGAACACAAATACTCCTCTCCCC	9	C	0.0085	0.4854
NC_005101.4-31652966	AATCTAACAACAGTTGCTCCCTGGGCT	1	A	0	0.4779
NC_005101.4-31652966	AATCTAACAACAGTTGCTCCCTGGGCT	14	T	0	0.4779
NC_005101.4-31655342	AACTTTGGCACCTTCACTCCCATGGAC	14	T	0.0579	0.0716
NC_005101.4-31655342	AACTTTGGCACCTTCACTCCCATGGAC	22	A	0.0331	0.0325
NC_005101.4-31662536	CACCAGTGGACCAGCGCTCCTGACTCT	16	G	0.0082	0.4755
NC_005101.4-31673947	ACATTCTGTACTTTGCCTCCCTAAGTT	4	T	0.065	0.0629
NC_005101.4-31696652	AATGCGAACACATGCTCTCCTAAGAAC	5	C	0.0244	0.0241
NC_005101.4-31754622	GGGAGACGAACGAATACTCCTTCTTAG	26	A	0	0.0354
NC_005101.4-31759013	CACCTGTCCACTGTGCCTCCATCCATA	9	C	0.0084	0.0249
NC_005101.4-31780342	TTAATCAACACCTTAGCTCCTAGTGGA	7	A	0.0265	0.0262
NC_005101.4-31790218	CCAACTTAAACAGCATCTCCCAGGGCA	24	G	0.0661	0.0943
NC_005101.4-31805770	AATCCTTGAACCTTGTCTCCTGAGCAC	25	C	0.0407	0.0398
NC_005101.4-31849737	TCCCTCACGACTGTAACTCCCAGCCAG	9	G	0.0083	0.0247
NC_005101.4-31863105	GGAAGCTCAACCTCATCTCCTCAAGGT	1	G	0.0244	0.0241
NC_005101.4-31867216	AAGCCTCTCACTCTACCTCCATTCTGG	23	T	0	0.4654
NC_005101.4-31869800	CTCAGTTGCACCTCTTCTCCCCCTGTT	16	T	0.0328	0.0322
NC_005101.4-31870564	GACGACACCACTGGCTCTCCAGCTGGG	3	C	0.3115	0.4612
NC_005101.4-31904287	GCCTCAATGACTCTTGCTCCATCCTTC	23	C	0.2295	0.4773
NC_005101.4-31953038	GTTCAAATCACCACATCTCCTCATAAG	24	T	0.2017	0.4957

NC_005101.4-31982267	GCTAAGCAAACCTCATCTCCAAGTTTA	6	G	0.0614	0.0595
NC_005101.4-32014873	CTTTGAGTCACATTTCCTCCCTGTGAT	13	T	0.1681	0.1813
NC_005101.4-32014873	CTTTGAGTCACATTTCCTCCCTGTGAT	27	T	0.0085	0.481
NC_005101.4-32022860	ACAGAACTTACTCCTTCTCCAACATAT	13	C	0.0088	0.4796
NC_005101.4-32042616	TGTGGGAAGACAGAGTCTCCTCCACAG	16	T	0.0598	0.058
NC_005101.4-32057740	ACACCTCTGACGCTATCTCCTCATCAG	3	A	0.0769	0.0896
NC_005101.4-32244867	ATGTCTGAGACATAGACTCCTAAGATT	3	G	0.25	0.2562
NC_005101.4-32293235	AAATCACTTACAGTGGCTCCTAGGAGG	4	T	0	0.4833
NC_005101.4-32293235	AAATCACTTACAGTGGCTCCTAGGAGG	25	A	0.0087	0.4878
NC_005101.4-32306877	TGTCAGAGCACTGGTCCTCCTGGGGAG	7	A	0	0.48
NC_005101.4-32334015	CACCAATACACCCTATCTCCTTTTTAT	3	C	0.0256	0.0253
NC_005101.4-32334015	CACCAATACACCCTATCTCCTTTTTAT	8	A	0.0171	0.0336
NC_005101.4-32545203	ACAGCTATGACCAGAACTCCGGTGTTT	6	T	0.0088	0.4774
NC_005101.4-32556221	ACAAATACCACCTGCTCTCCCTTCTAT	13	T	0.2645	0.4751
NC_005101.4-32560905	AGGTTCCCCACACACTCTCCCAATTAT	3	G	0	0.4773
NC_005101.4-32588504	ATTACCAATACTGTGTCTCCTACCCGG	25	C	0.3884	0.4213
NC_005101.4-32603335	AATAGCCATACAAACGCTCCTCAGTAT	5	G	0.2832	0.39
NC_005101.4-32666646	TTCAATCAAACATACACTCCCATCCAT	24	C	0.0259	0.4866
NC_005101.4-32678165	GTTACACAAACTGTTTCTCCCAGCGTG	2	T	0.0248	0.4803
NC_005101.4-32678165	GTTACACAAACTGTTTCTCCCAGCGTG	16	T	0.175	0.2125
NC_005101.4-32709883	AATGCTGAGACCTCTGCTCCTAGTGTT	3	T	0.0325	0.032
NC_005101.4-32709883	AATGCTGAGACCTCTGCTCCTAGTGTT	26	T	0	0.4793
NC_005101.4-32713015	CATAGACAAACCTTCTCTCCAAAAGAA	5	G	0.0086	0.4729
NC_005101.4-32769240	TGTGCGATCACTCCACCTCCTTGTTCC	16	C	0.1066	0.1009
NC_005101.4-32773532	CTCTCAGTGACATTCTCTCCTTTCTTA	7	G	0.1121	0.1504
NC_005101.4-32788607	ACCAGCGCAACACCTACTCCATGCGTT	7	G	0.2683	0.3472
NC_005101.4-32788607	ACCAGCGCAACACCTACTCCATGCGTT	12	A	0.2358	0.3091
NC_005101.4-32803803	GATTTTCTAACTTGGGCTCCAGACATT	2	A	0	0.4728
NC_005101.4-32821854	AGGATGGGGACATCACCTCCTCAGTGA	3	G	0.252	0.3472
NC_005101.4-32947300	GAGGAGGGCACTGGAGCTCCATAACAG	22	T	0.177	0.2171
NC_005101.4-32948143	TTACGTGCAACCAGACCTCCAGAATCT	4	C	0.0826	0.0943
NC_005101.4-33021750	CTACAGGATACCTGACCTCCTCCTCCC	14	G	0.1951	0.3334
NC_005101.4-33028184	CAAGTGTCTACTCTGTCTCCTATGATA	3	A	0	0.4773
NC_005101.4-33028184	CAAGTGTCTACTCTGTCTCCTATGATA	23	T	0.2049	0.4193
NC_005101.4-33051713	GACCATTGAACTATGTCTCCTTCCCAT	13	A	0.0569	0.0553
NC_005101.4-33072629	TGGGGTTGGACTCAGGCTCCTTGGAGT	4	G	0.0246	0.0243
NC_005101.4-33120365	AGCTGATTTACAGTTTCTCCATTGAGC	21	A	0.0973	0.1239
NC_005101.4-33177235	GTAGAGAAAACATGGCCTCCTCTACAT	27	T	0.2167	0.2311
NC_005101.4-33232639	TGCTCAGAAACACTTGCTCCAGCGACC	22	G	0.2632	0.4501
NC_005101.4-33232639	TGCTCAGAAACACTTGCTCCAGCGACC	23	C	0.1304	0.1938
NC_005101.4-33235669	CCCATAGGAACAAAGGCTCCCCCATTG	21	C	0.2807	0.4606
NC_005101.4-33241435	AGTGACATCACATGAACTCCTCGTCTG	12	A	0	0.4807
NC_005101.4-33241435	AGTGACATCACATGAACTCCTCGTCTG	21	T	0	0.4807
NC_005101.4-33254395	AGCACAACCACCGATTCTCCTAGTTTT	12	C	0	0.4718
NC_005101.4-33260774	ACTCAGGTCACTAGGCCTCCACAGAAA	21	A	0.1626	0.2142
NC_005101.4-33298383	TATTCACTCACAATTGCTCCCACGGAT	24	G	0	0.4786
NC_005101.4-33305904	TCATCTGCTACACTGCCTCCTGCCTCA	2	C	0.0769	0.1347
NC_005101.4-33305904	TCATCTGCTACACTGCCTCCTGCCTCA	21	T	0	0.4807
NC_005101.4-33306398	TGGACTCACACTTAAGCTCCCCCTGTG	26	T	0.2114	0.2612
NC_005101.4-33357872	AAATTTCAAACCATTCCTCCTGCCTTC	9	A	0.1	0.18
NC_005101.4-33357872	AAATTTCAAACCATTCCTCCTGCCTTC	21	T	0.0167	0.4832

NC_005101.4-33365779	GTCACTGTCACAGAGCCTCCAGACCTT	12	A	0.0804	0.0771
NC_005101.4-33367386	ACATACTGCACATCTGCTCCTGCGGAC	21	T	0	0.4773
NC_005101.4-33367386	ACATACTGCACATCTGCTCCTGCGGAC	23	C	0	0.4793
NC_005101.4-33392768	GTACAGGTAACTCACTCTCCTCTCGGT	3	A	0.2417	0.3533
NC_005101.4-33397709	GGATGACGCACATCTACTCCAACAAGG	7	C	0.065	0.078
NC_005101.4-33445332	GCAGGACGGACAGGGTCTCCGTATTAA	8	G	0.0435	0.0425
NC_005101.4-33445332	GCAGGACGGACAGGGTCTCCGTATTAA	14	G	0.0261	0.0257
NC_005101.4-33445418	AGCAGGGAAACGCTCCCTCCTCGCTCT	3	C	0.1951	0.496
NC_005101.4-33450814	GACCAAACCACCCACCCTCCAGCGACT	14	A	0.0348	0.0342
NC_005101.4-33450814	GACCAAACCACCCACCCTCCAGCGACT	23	C	0.0259	0.0422
NC_005101.4-33490944	TCCTCATGGACATAGGCTCCACTAGAT	1	T	0.1053	0.1305
NC_005101.4-33507025	CTGTCCCCAACCTCCCCTCCCCCAGTG	5	C	0.1	0.1099
NC_005101.4-33556080	TTCTCTGGCACCTACTCTCCCCAGACT	14	A	0.2627	0.329
NC_005101.4-33583279	ACACTTTGGACAAAGTCTCCCAGATTG	4	C	0.1709	0.2235
NC_005101.4-33583279	ACACTTTGGACAAAGTCTCCCAGATTG	5	T	0	0.4839
NC_005101.4-33621609	AGGCTGACAACAGTGACTCCAATTCCC	2	G	0.1	0.495
NC_005101.4-33621609	AGGCTGACAACAGTGACTCCAATTCCC	24	T	0.0083	0.4783
NC_005101.4-33690891	GTAACTCACACAGGCCCTCCTCCAGTA	13	G	0.0976	0.1073
NC_005101.4-33728258	ATTTCTGAGACACCCTCTCCAATCCAG	15	C	0.0259	0.0255
NC_005101.4-33735440	GTGGAAATCACACCAGCTCCGAGAAAC	14	C	0.1271	0.4982
NC_005101.4-33748796	CCATCAGCTACTATATCTCCCTCCACA	13	A	0	0.4819
NC_005101.4-33751961	ACTGTCTCAACCTCTTCTCCTTAGAAC	27	C	0.0164	0.0322
NC_005101.4-33798117	CATTGGGCTACACTTCCTCCTAACACT	3	T	0	0.4787
NC_005101.4-33810924	ATATAATTTACTGGTTCTCCAGAACAA	3	A	0.0087	0.4704
NC_005101.4-33810924	ATATAATTTACTGGTTCTCCAGAACAA	14	G	0.0087	0.4704
NC_005101.4-33844152	CCCTTTAATACGGTTCCTCCTGTTGTG	6	T	0.2609	0.2268
NC_005101.4-33844152	CCCTTTAATACGGTTCCTCCTGTTGTG	24	T	0.3063	0.2594
NC_005101.4-33860936	CCTCCCGCAACACCTTCTCCTTCAATA	22	T	0	0.4773
NC_005101.4-33869416	TGTTCTCTGACTTATTCTCCCGAGACA	13	T	0.2308	0.4227
NC_005101.4-33997379	TGAGCTCTGACAGCCTCTCCTCCCTTC	8	T	0.1102	0.4939
NC_005101.4-34086075	GAGCTTCCCACGGACCCTCCCTACATG	13	G	0.0656	0.0786
NC_005101.4-34113170	TGAGCTGTCACTGGCCCTCCTGGCACT	6	T	0.0083	0.4816
NC_005101.4-34134852	GTAGCCCTAACAGTCACTCCATGGCTG	3	A	0.0427	0.058
NC_005101.4-34134852	GTAGCCCTAACAGTCACTCCATGGCTG	9	A	0.1102	0.1337
NC_005101.4-34201716	CCAGACCCCACCATAACTCCCAGAGCC	16	A	0	0.4827
NC_005101.4-34408682	ATTGCCTTCACCCCCTCTCCTTTGCAT	26	A	0.0569	0.4893
NC_005101.4-34468947	CTGACACTAACCTGGCCTCCAACAACT	26	C	0	0.4807
NC_005101.4-34540838	CATCTTCCTACCCTGTCTCCTTCCTTC	26	T	0.0087	0.4563
NC_005101.4-34540838	CATCTTCCTACCCTGTCTCCTTCCTTC	27	C	0.0087	0.4563
NC_005101.4-34567682	CAATTCATAACTGTCCCTCCCATGGAT	25	G	0	0.4764
NC_005101.4-34598598	AGGAAAACCACACTGTCTCCGGCCAGG	2	G	0.0631	0.0611
NC_005101.4-34639505	TTCTTTGTGACAGAGTCTCCTTGTGTA	3	C	0	0.4749
NC_005101.4-34669992	TGACTCAGGACTGAGTCTCCAGGGTGA	23	G	0.1707	0.2441
NC_005101.4-34691377	TAATCAATAACCCTCACTCCAACTGCT	3	A	0.0163	0.032
NC_005101.4-34691377	TAATCAATAACCCTCACTCCAACTGCT	22	A	0.0244	0.0241
NC_005101.4-34728670	ACTACAGAAACACACCCTCCATTTACC	8	A	0	0.4833
NC_005101.4-34793655	CATCCTGTGACCGCAGCTCCCCTTCGT	26	G	0.0168	0.033
NC_005101.4-34867144	GAGACAGTGACTCTGACTCCTCGGTCT	23	G	0	0.4779
NC_005101.4-34930119	CTGTCTTCTACCCCGTCTCCCAACACA	15	G	0.1405	0.4966
NC_005101.4-34930119	CTGTCTTCTACCCCGTCTCCCAACACA	24	C	0.0909	0.1163
NC_005101.4-34974790	CAAAATCAAACACCTACTCCCATTCCA	8	A	0.2645	0.3645

NC_005101.4-35018510	TGATCTAGCACCATGCCTCCCTGAATG	8	G	0.1138	0.1073
NC_005101.4-35029997	TGTTTAAAGACCCTGGCTCCTGTCTTC	7	A	0.0088	0.4843
NC_005101.4-35050316	ACGGCTGAAACTTCCCCTCCACTCTGA	8	A	0.0082	0.4822
NC_005101.4-35067973	ACCAAACACACCACTTCTCCCAACATA	16	T	0	0.4819
NC_005101.4-35096404	CCTCAGAGAACTTTGGCTCCTCCTTCT	24	T	0.0088	0.481
NC_005101.4-35118401	ATCCATGAGACCCTTTCTCCCTCTTCC	14	T	0.1966	0.2041
NC_005101.4-35118401	ATCCATGAGACCCTTTCTCCCTCTTCC	21	C	0.1538	0.2107
NC_005101.4-35627811	AGGACCCACACAACGGCTCCAGAGGAC	27	C	0.1239	0.5
NC_005101.4-35709987	TGGCCTCTTACCAAGCCTCCATTCTTT	26	T	0.2389	0.4021
NC_005101.4-35709987	TGGCCTCTTACCAAGCCTCCATTCTTT	27	T	0.2124	0.4934
NC_005101.4-35749892	GTGATCCAGACTGCTGCTCCTGAGTCT	5	T	0.1789	0.3141
NC_005101.4-35755173	CTACAGTCCACTCGGTCTCCTGCTGGT	24	T	0.1789	0.2832
NC_005101.4-35768698	CTCAGGCTGACTTTCCCTCCTCTCTTT	4	A	0.0431	0.4916
NC_005101.4-35799650	TTGCATATCACCTGGGCTCCTTCCACA	23	C	0.0496	0.109
NC_005101.4-35845074	CTGCATTGGACTCTAGCTCCAGGGGAT	6	T	0.1475	0.1904
NC_005101.4-35896014	TTTACAGGCACGTATTCTCCTGGAGTC	12	G	0.0504	0.0491
NC_005101.4-36141979	ACTCATGAGACATTTCCTCCAAAGCCA	6	T	0.0488	0.078
NC_005101.4-36167167	TTTCTCATCACTGTTTCTCCTCTGCAG	1	T	0.0488	0.4881
NC_005101.4-36176474	CCACCCTCCACACATCCTCCTCTTTTC	22	C	0.0254	0.0251
NC_005101.4-36191007	TCCTCTGAAACTTCAGCTCCTCTTACT	8	A	0.2589	0.4566
NC_005101.4-36264735	TCCTATCCCACCCCCTCTCCTTCTTCT	6	T	0.2411	0.2512
NC_005101.4-36264735	TCCTATCCCACCCCCTCTCCTTCTTCT	22	T	0.2478	0.2556
NC_005101.4-36322597	CCTCTCCAAACACTCACTCCCACCTCA	4	C	0.0265	0.0262
NC_005101.4-36383908	AAAACACGTACCTTGACTCCTAGTCTT	8	G	0	0.4827
NC_005101.4-36417797	GGTCCTGTCACTGGACCTCCTTTACCA	26	C	0.1239	0.1757
NC_005101.4-36509923	GACCGTTGCACACGACCTCCCTTGATC	5	G	0.0579	0.0562
NC_005101.4-36509923	GACCGTTGCACACGACCTCCCTTGATC	27	C	0.225	0.3347
NC_005101.4-36519783	AGCAGAAAGACTGGAGCTCCACCAGGG	2	G	0.2787	0.4565
NC_005101.4-36519783	AGCAGAAAGACTGGAGCTCCACCAGGG	9	G	0.0244	0.0241
NC_005101.4-36775468	ATCCGATAAACCATAACTCCTAAGGTG	13	A	0.0708	0.0846
NC_005101.4-36775468	ATCCGATAAACCATAACTCCTAAGGTG	27	G	0.0708	0.4934
NC_005101.4-36780552	GGAATGAGTACTCCTCCTCCTTCCTGG	9	T	0	0.4786
NC_005101.4-36780552	GGAATGAGTACTCCTCCTCCTTCCTGG	23	C	0	0.4786
NC_005101.4-36881300	ACAATGTCAACCAGAGCTCCCAGAGAC	14	G	0.2869	0.4056
NC_005101.4-36955081	ACTCTCAGGACAGACACTCCATTTCTG	8	G	0.1053	0.1153
NC_005101.4-36981268	CATGATGGTACAATTTCTCCCACCAAG	5	A	0.2946	0.3316
NC_005101.4-37022623	ACCAACCCAACTGTGTCTCCTCCATTT	24	A	0.3577	0.414
NC_005101.4-37038940	CCTCCTGTCACACCCACTCCCACCTCA	1	C	0.0598	0.058
NC_005101.4-37038940	CCTCCTGTCACACCCACTCCCACCTCA	25	T	0.0593	0.0576
NC_005101.4-37049651	CCCATGTCTACAAGATCTCCACCCCAA	27	A	0.2342	0.3587
NC_005101.4-37071045	CTACCTTTCACGCCTCCTCCTTCCATC	12	G	0.3214	0.3565
NC_005101.4-37071045	CTACCTTTCACGCCTCCTCCTTCCATC	14	C	0.3214	0.4807
NC_005101.4-37130055	AACTATGTAACTAACACTCCTGGATTA	4	T	0.0089	0.4824
NC_005101.4-37130055	AACTATGTAACTAACACTCCTGGATTA	27	A	0.3036	0.3838
NC_005101.4-37208573	GAGCCTGTCACTGGTCCTCCTTTTCCT	9	C	0.3153	0.2896
NC_005101.4-37514693	CACAGTGACACACTCACTCCAATGGTG	23	T	0.0164	0.4773
NC_005101.4-37648507	ACTCCATAGACACTTGCTCCGCTGTGA	7	T	0	0.4793
NC_005101.4-37648507	ACTCCATAGACACTTGCTCCGCTGTGA	27	A	0.122	0.3646
NC_005101.4-37692197	ACAATGTCCACACAGTCTCCACTATAC	2	C	0.0348	0.4165
NC_005101.4-37692197	ACAATGTCCACACAGTCTCCACTATAC	23	T	0.2	0.4849
NC_005101.4-37732096	ACTGCTCATACACATCCTCCCGGTGAG	22	G	0.1066	0.2796

NC_005101.4-37732096	ACTGCTCATACACATCCTCCCGGTGAG	24	T	0.2131	0.416
NC_005101.4-37736517	GTTTGTATAACTTCAACTCCACATCAA	7	A	0.1579	0.1454
NC_005101.4-37736517	GTTTGTATAACTTCAACTCCACATCAA	26	A	0	0.4654
NC_005101.4-37740117	CAGGTTTGAACTAGGCCTCCTCACACA	12	T	0.1982	0.425
NC_005101.4-37770333	TTCTCTGCAACTGCATCTCCTATCAAG	6	T	0.0328	0.0322
NC_005101.4-37770333	TTCTCTGCAACTGCATCTCCTATCAAG	23	T	0.0738	0.4903
NC_005101.4-37816541	CAACTACCCACAAGATCTCCTTAGATG	12	A	0.1707	0.2203
NC_005101.4-37890444	GGGGGAGAGACACAGCCTCCCACGTAT	21	C	0.1724	0.2739
NC_005101.4-37929512	GTGCCCCCCACCCCACCTCCTCTGGTC	14	C	0.1405	0.1982
NC_005101.4-37929512	GTGCCCCCCACCCCACCTCCTCTGGTC	16	C	0.0333	0.0328
NC_005101.4-37979160	TGAATACATACAGCCACTCCTCCATTA	26	T	0.041	0.0401
NC_005101.4-38087529	CGGACATGTACTAAGGCTCCAGGGTAA	9	T	0.1707	0.5
NC_005101.4-38087529	CGGACATGTACTAAGGCTCCAGGGTAA	22	G	0	0.4773
NC_005101.4-38167093	TCCATGACCACTCATCCTCCCAAAGGA	6	G	0.0082	0.479
NC_005101.4-38167093	TCCATGACCACTCATCCTCCCAAAGGA	8	C	0.0082	0.479
NC_005101.4-38179039	TGGAGGGTGACATCATCTCCACTATCT	13	T	0.0603	0.0585
NC_005101.4-38331020	GAGACGGCCACTGCATCTCCTTTATCT	9	C	0.2541	0.375
NC_005101.4-38334706	GACCTTGCCACCGCAGCTCCCATGGCA	2	A	0	0.4826
NC_005101.4-38334706	GACCTTGCCACCGCAGCTCCCATGGCA	23	T	0.2119	0.4508
NC_005101.4-38335714	CCCTCTCACACCCAGGCTCCTCTTAAA	6	T	0	0.4793
NC_005101.4-38335714	CCCTCTCACACCCAGGCTCCTCTTAAA	14	A	0	0.4793
NC_005101.4-38348152	TCAATGAATACGATTACTCCGTAAGTT	4	A	0.1429	0.2934
NC_005101.4-38389454	GCCCTCACGACTGTGTCTCCATGTCCA	22	T	0.0328	0.4866
NC_005101.4-38389454	GCCCTCACGACTGTGTCTCCATGTCCA	23	G	0.0656	0.0786
NC_005101.4-38401403	TGGCACTTCACTGCTTCTCCAGAGTCA	12	T	0.0714	0.0853
NC_005101.4-38433349	TCTGTAGCCACGCATCCTCCAGACAGA	4	G	0.0702	0.0677
NC_005101.4-38433349	TCTGTAGCCACGCATCCTCCAGACAGA	16	C	0.0982	0.1093
NC_005101.4-38527221	GACTCCACTACTCACCCTCCCACTTGT	23	C	0.0248	0.0562
NC_005101.4-38536933	TTTCTCATCACACTTCCTCCTGGCTTG	6	C	0.0813	0.0928
NC_005101.4-38632442	AACTTAACAACAGCATCTCCACGGCCC	22	C	0.1967	0.4999
NC_005101.4-38634692	CAGTCATGGACTAACCCTCCAGATCCA	1	C	0.0902	0.1009
NC_005101.4-38634692	CAGTCATGGACTAACCCTCCAGATCCA	2	A	0	0.4819
NC_005101.4-38698749	ACACCTTCTACTCGCCCTCCCTTGAGT	6	T	0.2	0.2204
NC_005101.4-38698749	ACACCTTCTACTCGCCCTCCCTTGAGT	14	G	0.1304	0.4998
NC_005101.4-38765692	TCGAGAAATACTGCCACTCCCTAGACT	4	A	0.0259	0.0255
NC_005101.4-38791409	AAAACACTAACCCTGTCTCCACCGTCT	7	C	0.1488	0.1516
NC_005101.4-38802988	TGAAATGTAACACGCTCTCCCTTACAT	13	C	0.0081	0.0241
NC_005101.4-38824248	CTGATGGTTACCCCAGCTCCTAGAACT	14	C	0.0244	0.0398
NC_005101.4-38824248	CTGATGGTTACCCCAGCTCCTAGAACT	25	A	0.1967	0.4999
NC_005101.4-38839616	CGCAACTCCACACTCCCTCCCCTTGCA	3	C	0.0244	0.0241
NC_005101.4-38839616	CGCAACTCCACACTCCCTCCCCTTGCA	23	T	0.1301	0.1494
NC_005101.4-38848711	AGATAAGACACAAGGTCTCCCTTTGCT	27	T	0.0259	0.0255
NC_005101.4-38853878	GCACCTGTCACCCTAGCTCCTCACTTC	2	C	0.0526	0.4555
NC_005101.4-38858287	GGGAGAAACACCATCTCTCCTTGACTG	1	G	0.2241	0.2622
NC_005101.4-38858287	GGGAGAAACACCATCTCTCCTTGACTG	8	A	0.0603	0.0585
NC_005101.4-38902696	TAAGCCAAGACCTTTTCTCCTCTTACA	12	C	0.1538	0.2604
NC_005101.4-38902696	TAAGCCAAGACCTTTTCTCCTCTTACA	26	C	0.1579	0.4994
NC_005101.4-38906385	GAACAGAACACCATTTCTCCCTAACTG	25	C	0.1043	0.1588
NC_005101.4-38922697	GTTCTGTAGACCAGGCCTCCTTGAACT	26	C	0.1552	0.4963
NC_005101.4-39013339	CCCATTCAGACACTCTCTCCCTTCCTG	2	C	0.1901	0.313
NC_005101.4-39013339	CCCATTCAGACACTCTCTCCCTTCCTG	3	C	0.1901	0.313

NC_005101.4-39029234	GTTGTGGGAACAGAGTCTCCTTGTTGT	4	G	0.0246	0.0243
NC_005101.4-39029234	GTTGTGGGAACAGAGTCTCCTTGTTGT	16	T	0	0.4765
NC_005101.4-39056763	AGAAAGTTTACCAAGTCTCCTTAACAC	27	C	0	0.0325
NC_005101.4-39067087	AGCCTCTGCACAAAGTCTCCTCCCACC	26	C	0	0.4793
NC_005101.4-39071693	CCCAGGACAACAGTGTCTCCTGGAATG	4	A	0.1074	0.4978
NC_005101.4-39075572	CCACGTCCCACCTCCTCTCCTACTGCA	5	G	0.0732	0.0705
NC_005101.4-39079650	TGCTGGGGCACTGAGCCTCCACAGGAC	6	G	0.0342	0.0336
NC_005101.4-39096301	TGATGGATCACGTAAGCTCCGTAACAG	16	G	0.0244	0.0241
NC_005101.4-39096301	TGATGGATCACGTAAGCTCCGTAACAG	21	G	0.0244	0.0241
NC_005101.4-39142734	ACTTAATCTACCTGGCCTCCCCGCTCC	22	C	0	0.4765
NC_005101.4-39180114	CCAGGAAGCACTGTTCCTCCACGGCAT	22	C	0	0.4844
NC_005101.4-39194989	TATATGCCCACCCCCGCTCCCCATACG	15	C	0.0167	0.0644
NC_005101.4-39219010	ACCCACCTCACTGCCACTCCAATTGTA	21	A	0	0.48
NC_005101.4-39331772	CAAGTTTAAACACTAGCTCCATAATGC	6	T	0.0667	0.0799
NC_005101.4-39331772	CAAGTTTAAACACTAGCTCCATAATGC	26	G	0.0083	0.0245
NC_005101.4-39369816	CCATCAGCCACCTCTCCTCCCCTTCTC	21	C	0.0678	0.1264
NC_005101.4-39374639	ATCAGGTTCACACTGTCTCCATCTCCT	21	A	0	0.482
NC_005101.4-39374639	ATCAGGTTCACACTGTCTCCATCTCCT	22	T	0.0086	0.4836
NC_005101.4-39381519	CTGATGATGACTTAATCTCCTGCACAG	21	T	0	0.4743
NC_005101.4-39388413	ATCACACTGACCTCTGCTCCATGCTCA	23	G	0	0.4715
NC_005101.4-39412318	ATTCCTCAAACCCTTCCTCCCGCCCAT	21	C	0.0508	0.493
NC_005101.4-39474358	CCCCTAGTAACACTTCCTCCAACAAGA	7	G	0.1478	0.1516
NC_005101.4-39474358	CCCCTAGTAACACTTCCTCCAACAAGA	8	T	0.1478	0.1516
NC_005101.4-39491109	CATCCAAGAACTGCTTCTCCCTTCTCA	5	C	0.0435	0.1067
NC_005101.4-39597566	GGAGAATAAACCTGTACTCCTGCCTAG	9	A	0.1933	0.4253
NC_005101.4-39601687	CCAGTAGCCACCACAGCTCCTGTGAGA	5	T	0.1475	0.3791
NC_005101.4-39601687	CCAGTAGCCACCACAGCTCCTGTGAGA	8	C	0.1885	0.2573
NC_005101.4-39653744	AAATGCCCTACAGGCTCTCCTACAGCC	22	A	0.5714	0.4285
NC_005101.4-39676920	AGAAAGCCAACCAATACTCCTATCCAT	27	T	0.2069	0.2501
NC_005101.4-39742579	TGTGGAATAACATGCTCTCCAACCTCA	1	T	0.0678	0.4305
NC_005101.4-39742579	TGTGGAATAACATGCTCTCCAACCTCA	2	G	0	0.4649
NC_005101.4-39767860	ACTATACAAACTTTCTCTCCATCAGTA	4	A	0.042	0.4723
NC_005101.4-39767860	ACTATACAAACTTTCTCTCCATCAGTA	22	T	0	0.4813
NC_005101.4-39851632	ATTTTTTGTACCATGTCTCCCATGCAC	9	T	0	0.4671
NC_005101.4-39851632	ATTTTTTGTACCATGTCTCCCATGCAC	27	C	0.0893	0.1014
NC_005101.4-39911712	AGAGAACAGACCTCCACTCCAAACTCC	5	A	0.1416	0.4996
NC_005101.4-39922266	AGAAAAGGGACCATTCCTCCATTGTTG	3	A	0.4711	0.3601
NC_005101.4-39941908	CCATGGACCACTTTTACTCCAACAGTT	5	G	0.0598	0.058
NC_005101.4-39974417	TACTGACCCACCTATCCTCCCCTCTTG	25	T	0	0.4832
NC_005101.4-39999821	CAGAAGCTGACCAGGCCTCCTTATAGT	2	A	0.1885	0.4588
NC_005101.4-39999821	CAGAAGCTGACCAGGCCTCCTTATAGT	8	T	0.0246	0.0401
NC_005101.4-40067340	TTGTTGATGACAAACTCTCCAACAAGG	4	T	0.1074	0.3512
NC_005101.4-40067340	TTGTTGATGACAAACTCTCCAACAAGG	5	T	0.0656	0.0786
NC_005101.4-40184612	TAGCAGGTGACACAGTCTCCAGTTATA	25	A	0.0684	0.4982
NC_005101.4-40184612	TAGCAGGTGACACAGTCTCCAGTTATA	26	T	0.0684	0.0973
NC_005101.4-40225286	ACCAGACTAACCTTATCTCCCTACTAT	5	G	0.0885	0.1613
NC_005101.4-40511456	GCTGTGAGGACAGTGTCTCCCATGCAT	15	G	0.0804	0.0771
NC_005101.4-40514684	CTGTTCATGACAGGAGCTCCAAAAAAT	3	G	0.0087	0.4817
NC_005101.4-40527648	ATATAAAATACAGTTGCTCCACTAACA	22	C	0.2342	0.349
NC_005101.4-40527648	ATATAAAATACAGTTGCTCCACTAACA	25	A	0.036	0.1023
NC_005101.4-40585738	TTAGTATTTACCCAGGCTCCAGTTACA	1	T	0.1404	0.2155

NC_005101.4-40645704	AACTTGTGAACCATATCTCCAATCCTG	14	T	0.2541	0.3492
NC_005101.4-40647925	CCCTGGGTCACCAAGTCTCCACACGAC	24	C	0.0333	0.4728
NC_005101.4-40653755	ATAGTCAATACAGCTGCTCCAGGGATC	1	A	0.2846	0.4777
NC_005101.4-40653755	ATAGTCAATACAGCTGCTCCAGGGATC	5	T	0	0.4793
NC_005101.4-40725020	ACAGGATCTACCACCGCTCCCAGCATC	16	G	0.0083	0.4783
NC_005101.4-40746098	TCATATCATACATTTCCTCCCTGCAAT	22	T	0	0.4851
NC_005101.4-40758378	CAGTGTCTAACTCCAGCTCCACAGGGT	16	G	0.041	0.0401
NC_005101.4-40782392	TGGCACCTCACTTAGTCTCCAGTGCTA	23	T	0	0.4793
NC_005101.4-40784605	TGCTCTCCCACTCTGTCTCCTAAGCTG	22	A	0.0254	0.487
NC_005101.4-40817050	AATATACCCACTTTCCCTCCTTGATCT	1	A	0	0.4779
NC_005101.4-40840022	CAGTTCACCACAGCCCCTCCTAGCCAT	1	C	0.025	0.4847
NC_005101.4-40840022	CAGTTCACCACAGCCCCTCCTAGCCAT	12	A	0.025	0.4847
NC_005101.4-40879434	CCCATCTCCACCTCTACTCCTTCTGTG	13	T	0.0089	0.4933
NC_005101.4-40879434	CCCATCTCCACCTCTACTCCTTCTGTG	24	T	0	0.4807
NC_005101.4-40897017	CATACACACACATGTTCTCCCTCTCTC	26	T	0.0339	0.0333
NC_005101.4-41036971	ACTTCAGTCACCTTTTCTCCTCGTCCC	22	C	0.0082	0.479
NC_005101.4-41045900	ATGGTCTCTACAGATTCTCCCTCCTTT	22	T	0.0769	0.074
NC_005101.4-41045900	ATGGTCTCTACAGATTCTCCCTCCTTT	23	C	0.0769	0.074
NC_005101.4-41060439	CCACCTTCCACCTCCCCTCCCATCCAT	12	C	0.042	0.0411
NC_005101.4-41101049	TTAACTGCTACTCTATCTCCTAACATT	8	C	0.0259	0.0255
NC_005101.4-41101049	TTAACTGCTACTCTATCTCCTAACATT	22	A	0.3362	0.4034
NC_005101.4-41111220	GAAGAGAGCACTATCACTCCTAGTGGT	21	T	0.0826	0.109
NC_005101.4-41199693	ACCAAGGCCACAGTATCTCCATTATAG	13	G	0.0259	0.0255
NC_005101.4-41199693	ACCAAGGCCACAGTATCTCCATTATAG	26	A	0	0.482
NC_005101.4-41208054	TTCTCATCCACTTTGACTCCTAACTCT	8	C	0.2066	0.2234
NC_005101.4-41253396	TGGATTAGGACTCTATCTCCCTCAATG	12	T	0.2846	0.4036
NC_005101.4-41260102	GTCATATTCACCCTCTCTCCTCCAACT	13	C	0.1842	0.4529
NC_005101.4-41281975	CTAGGAGTCACCACGGCTCCTTCAGGG	8	T	0	0.4793
NC_005101.4-41299578	AAACACTGCACCAAATCTCCTTCTCAA	21	T	0.0714	0.0689
NC_005101.4-41314459	TATCTCACTACAGCTTCTCCCAGGTCC	1	T	0.0348	0.4936
NC_005101.4-41322329	AAGTAGTTGACATATCCTCCTACATGC	14	A	0.0085	0.4776
NC_005101.4-41359493	CTACTTTTCACCTTTCCTCCCTTCTTT	9	C	0.1513	0.1539
NC_005101.4-41359493	CTACTTTTCACCTTTCCTCCCTTCTTT	27	T	0.1849	0.2076
NC_005101.4-41375239	CCTCAGAGAACCGTAGCTCCTCGTTGC	23	G	0.0714	0.4986
NC_005101.4-41414029	GCACACATGACTGGCACTCCGTATGGA	24	T	0.0163	0.4759
NC_005101.4-41414029	GCACACATGACTGGCACTCCGTATGGA	26	G	0.3008	0.4618
NC_005101.4-41441926	TGAGCCGCAACCGTGCCTCCTGCCCTC	7	G	0.0579	0.0562
NC_005101.4-41441926	TGAGCCGCAACCGTGCCTCCTGCCCTC	13	G	0.0417	0.4847
NC_005101.4-41458395	ACATAGATGACTATGTCTCCAATGACT	8	T	0.1736	0.2355
NC_005101.4-41461054	TGATGTACAACGACTCCTCCGTCTTAG	24	T	0.1933	0.2389
NC_005101.4-41523253	GGACTTCTAACCATCTCTCCTATCACA	1	G	0.1583	0.1866
NC_005101.4-41523253	GGACTTCTAACCATCTCTCCTATCACA	13	A	0.0333	0.0488
NC_005101.4-41562882	ATACTTAATACTCCTGCTCCAGATGCG	2	T	0.1239	0.1466
NC_005101.4-41562882	ATACTTAATACTCCTGCTCCAGATGCG	27	G	0.1053	0.1305
NC_005101.4-41576153	CCCGCATCCACCATAACTCCCCAAACT	4	G	0	0.4807
NC_005101.4-41576153	CCCGCATCCACCATAACTCCCCAAACT	27	T	0.0085	0.0251
NC_005101.4-41645590	CTTACGTCCACTCCTACTCCTTCTGTA	5	C	0.1293	0.2315
NC_005101.4-41645590	CTTACGTCCACTCCTACTCCTTCTGTA	7	T	0.0948	0.2188
NC_005101.4-41653954	TTCAACAAGACCACACCTCCTAACCCT	1	T	0.582	0.4126
NC_005101.4-41653954	TTCAACAAGACCACACCTCCTAACCCT	3	C	0.5537	0.4004
NC_005101.4-41668243	AAAAAAACAACAGTCTCTCCCCTTATG	6	A	0.0248	0.0716

NC_005101.4-41668243	AAAAAAACAACAGTCTCTCCCCTTATG	24	T	0.0496	0.4923
NC_005101.4-41702304	GCTGAAGAAACATCTTCTCCCTTCCTT	22	T	0.1736	0.4803
NC_005101.4-41722529	CACACACACACACAGACTCCAGCACAG	1	C	0.0603	0.0585
NC_005101.4-41722529	CACACACACACACAGACTCCAGCACAG	15	G	0	0.488
NC_005101.4-41782015	CGGAATGTCACCACCTCTCCACAGTCA	2	G	0.0976	0.1073
NC_005101.4-41799721	CCTCACTCCACATGTGCTCCTTTTCTG	23	T	0.0932	0.1337
NC_005101.4-41805075	GCCATGAGCACTCTCCCTCCAAAGTCC	2	C	0.1057	0.1425
NC_005101.4-41842411	CTGTTTTGTACTGTGCCTCCTATGCCT	16	C	0.113	0.1659
NC_005101.4-41844720	TGTGTACTCACACCTGCTCCTAGGTAG	5	T	0.2667	0.4132
NC_005101.4-41863042	TTTTGAATTACCCTTACTCCCTGTGAA	16	A	0	0.4841
NC_005101.4-41863042	TTTTGAATTACCCTTACTCCCTGTGAA	22	T	0.0357	0.0351
NC_005101.4-41936722	TGGGAAAACACTCCTGCTCCTCTTTTC	15	T	0.0614	0.483
NC_005101.4-41936722	TGGGAAAACACTCCTGCTCCTCTTTTC	26	T	0	0.4698
NC_005101.4-41937270	CAGTAATCCACAGCAACTCCTGTTTCA	15	A	0.1667	0.1528
NC_005101.4-41953438	CACTGACAAACAAAGCCTCCTCGCTGC	8	A	0	0.4819
NC_005101.4-41953438	CACTGACAAACAAAGCCTCCTCGCTGC	15	G	0.0328	0.048
NC_005101.4-41956687	AATTAGCAAACAATCTCTCCTTGTCCT	3	T	0	0.4765
NC_005101.4-41956687	AATTAGCAAACAATCTCTCCTTGTCCT	13	A	0.124	0.2704
NC_005101.4-41962949	CCACCCTGCACTTCCTCTCCCACTGAG	5	C	0.0325	0.032
NC_005101.4-41962949	CCACCCTGCACTTCCTCTCCCACTGAG	6	C	0.0488	0.0629
NC_005101.4-41973484	TCAGTCTTCACCCACCCTCCATCAAAA	21	A	0.1074	0.4999
NC_005101.4-41973484	TCAGTCTTCACCCACCCTCCATCAAAA	27	A	0.075	0.1597
NC_005101.4-42002323	TGACCCTGGACACCACCTCCCACATTC	2	G	0.0902	0.0861
NC_005101.4-42025704	CTAAAAGGAACCTCGGCTCCTGGAAGA	15	G	0.193	0.474
NC_005101.4-42025704	CTAAAAGGAACCTCGGCTCCTGGAAGA	27	A	0	0.4898
NC_005101.4-42029766	TAGAGCCTCACAGAGGCTCCTGTCTGC	7	C	0.0811	0.1414
NC_005101.4-42029766	TAGAGCCTCACAGAGGCTCCTGTCTGC	16	G	0.0439	0.0595
NC_005101.4-42033409	GCATTGAACACTAATGCTCCATAGCAT	23	A	0	0.477
NC_005101.4-42033409	GCATTGAACACTAATGCTCCATAGCAT	24	G	0.0982	0.498
NC_005101.4-42083481	AGACATTAAACCGACTCTCCTCCGTGG	23	C	0.157	0.2922
NC_005101.4-42173794	GTGGTGAACACTTCAGCTCCCTTCCCT	5	T	0.1765	0.2012
NC_005101.4-42173794	GTGGTGAACACTTCAGCTCCCTTCCCT	22	T	0.0504	0.0491
NC_005101.4-42201452	TCAGACTGAACTCAGACTCCTGGCCTC	1	T	0.0088	0.4676
NC_005101.4-42201452	TCAGACTGAACTCAGACTCCTGGCCTC	2	C	0.113	0.1067
NC_005101.4-42429097	GTCTTGATCACTCTGACTCCTACATTT	23	C	0.1864	0.2219
NC_005101.4-42464753	GAATAACTTACATGGTCTCCAGGAAGC	22	G	0.1017	0.1264
NC_005101.4-42464753	GAATAACTTACATGGTCTCCAGGAAGC	26	G	0.1017	0.4987
NC_005101.4-42491306	ACCCTGCCTACTCCTCCTCCATACCTG	1	A	0.125	0.1597
NC_005101.4-42491306	ACCCTGCCTACTCCTCCTCCATACCTG	7	C	0	0.4832
NC_005101.4-42538339	TTCATAATCACCTCCTCTCCCCAGCCG	2	T	0.0087	0.4782
NC_005101.4-42569127	GAAACACGTACAGGCTCTCCCTTTCAC	5	C	0.0488	0.0928
NC_005101.4-42585554	GTGGCAGCAACCATCCCTCCTCATCGG	23	A	0.0244	0.0241
NC_005101.4-42629920	CTAACCAAGACAGCATCTCCAATAACA	26	C	0.0085	0.481
NC_005101.4-42630449	TTCCCCAGCACCGTGACTCCTTGTCTA	12	C	0.025	0.0247
NC_005101.4-42631705	TTATCTGAAACATGGCCTCCTCTAGAG	9	A	0.0085	0.4854
NC_005101.4-42631705	TTATCTGAAACATGGCCTCCTCTAGAG	21	T	0.0254	0.0251
NC_005101.4-42644526	GAACTCTCCACTGCCTCTCCTGCACTG	13	G	0.0531	0.0517
NC_005101.4-42644526	GAACTCTCCACTGCCTCTCCTGCACTG	26	T	0.0619	0.4975
NC_005101.4-42679351	AGGCCCTGGACACTTCCTCCTCATGGA	1	A	0	0.4787
NC_005101.4-42686134	GGAGAAAGTACCTGCTCTCCATAAAGA	1	G	0.0261	0.4878
NC_005101.4-42686134	GGAGAAAGTACCTGCTCTCCATAAAGA	2	G	0.0087	0.4849

NC_005101.4-42689086	ACATCTATAACACCATCTCCAACTCTC	15	A	0.0164	0.4807
NC_005101.4-42709617	TCTATTCTGACCAAACCTCCCTCCAAG	7	C	0.1789	0.2499
NC_005101.4-42735255	CTCCACATGACTTCTTCTCCAGCAACC	2	T	0.0435	0.059
NC_005101.4-42735255	CTCCACATGACTTCTTCTCCAGCAACC	5	A	0.0174	0.4682
NC_005101.4-42747126	ACAGTTGACACTGAAGCTCCAGGGCTT	13	G	0.0175	0.0677
NC_005101.4-42760439	TAGGTCACCACCTCCTCTCCAAACAGC	8	C	0.113	0.1067
NC_005101.4-42760439	TAGGTCACCACCTCCTCTCCAAACAGC	14	C	0.193	0.2659
NC_005101.4-42761240	AATTCAATAACACTGACTCCTTAATTT	3	T	0.1966	0.4928
NC_005101.4-42761240	AATTCAATAACACTGACTCCTTAATTT	15	G	0.2308	0.4882
NC_005101.4-42822418	AGAAAATAGACCTTCTCTCCTTCCTCT	23	C	0.1842	0.3273
NC_005101.4-42843067	TGAAGACACACCCAGACTCCTACTCTC	24	T	0	0.4773
NC_005101.4-42876737	GAGGCCAGGACCCTCTCTCCTTGCCAT	24	C	0.187	0.2989
NC_005101.4-42888036	AGAATGTCTACATCATCTCCACGTGTG	1	A	0.0246	0.0243
NC_005101.4-42911628	TGGCTTCCTACGAAAGCTCCAGACACC	16	G	0.0081	0.0241
NC_005101.4-42911628	TGGCTTCCTACGAAAGCTCCAGACACC	22	G	0.0081	0.0241
NC_005101.4-42942067	GCCCAAGGGACTCATCCTCCTACACTA	1	G	0.0714	0.0689
NC_005101.4-43017918	CTCACATCTACCTTTCCTCCCACTGCT	2	T	0	0.474
NC_005101.4-43017918	CTCACATCTACCTTTCCTCCCACTGCT	3	C	0	0.474
NC_005101.4-43032404	TTCCTTAGAACATTACCTCCATTAGGA	1	T	0.1217	0.1588
NC_005101.4-43036841	ACCTATTGGACATTTACTCCATCACTA	16	A	0.2281	0.3324
NC_005101.4-43093135	TGGATGGACACTTAAACTCCTTGGGTT	4	A	0	0.4832
NC_005101.4-43112663	AGTGCTTGCACCACCACTCCCTTACAG	24	A	0.0877	0.4998
NC_005101.4-43112663	AGTGCTTGCACCACCACTCCCTTACAG	25	C	0.0877	0.4998
NC_005101.4-43123546	TGCCTGTGTACTGTTGCTCCCTGACAT	5	T	0.1803	0.285
NC_005101.4-43135188	CCGTGCTGAACTAGTGCTCCACCACAG	23	C	0.1186	0.2926
NC_005101.4-43139750	CAGAATCCCACTGGTGCTCCTTTTGCC	7	C	0.1186	0.1409
NC_005101.4-43139750	CAGAATCCCACTGGTGCTCCTTTTGCC	16	G	0.0167	0.0328
NC_005101.4-43139828	TTTTGGAGAACTCTGTCTCCTGTATTA	14	T	0.114	0.3836
NC_005101.4-43139828	TTTTGGAGAACTCTGTCTCCTGTATTA	24	A	0.0263	0.4676
NC_005101.4-43152788	GCGTCAAGAACCGAACCTCCATCTTGC	26	G	0.1463	0.1356
NC_005101.4-43316779	CCTGGGAACACTGTTCCTCCTTCTCCC	22	T	0	0.4793
NC_005101.4-43316779	CCTGGGAACACTGTTCCTCCTTCTCCC	26	C	0.1345	0.4983
NC_005101.4-43323615	GCACGTGCTACCCCAGCTCCACTGAAC	4	C	0.0088	0.4796
NC_005101.4-43323615	GCACGTGCTACCCCAGCTCCACTGAAC	16	G	0.0088	0.4796
NC_005101.4-43330632	ACTATAGAGACCCCGTCTCCCTTCCCA	8	A	0	0.0345
NC_005101.4-43453405	CTGCCAGTCACAGTGGCTCCCTCAGGG	1	C	0.1301	0.1494
NC_005101.4-43463097	GCTCTTTAAACCAAGACTCCATCTAAC	25	A	0	0.4909
NC_005101.4-43484581	TCTTTCCTGACCTCACCTCCCATCCGT	4	T	0	0.4839
NC_005101.4-43484581	TCTTTCCTGACCTCACCTCCCATCCGT	27	T	0.2845	0.3876
NC_005101.4-43487467	GAAAGGGGCACTCCACCTCCATTAGTT	14	C	0.1217	0.32
NC_005101.4-43487467	GAAAGGGGCACTCCACCTCCATTAGTT	16	C	0.0789	0.1528
NC_005101.4-43543830	AGTTGGACCACAATGACTCCAAGTGTT	13	A	0.0833	0.4932
NC_005101.4-43596728	CTGTTGTCCACTGACCCTCCTGCTTCT	12	T	0.0328	0.048
NC_005101.4-43602029	ATCCCAAGAACCCATGCTCCCACCTAT	14	A	0.1138	0.3516
NC_005101.4-43602029	ATCCCAAGAACCCATGCTCCCACCTAT	27	T	0	0.4793
NC_005101.4-43636432	AGTGTGCAGACATTTCCTCCAATGAAG	6	G	0.065	0.0629
NC_005101.4-43638122	CTTTACAAAACTGTAGCTCCTTTCTTT	16	G	0.1161	0.4996
NC_005101.4-43641270	GGTCTTAAAACAATGTCTCCCTGGGTG	9	A	0	0.4778
NC_005101.4-43641761	TATAAGTGGACAGAGCCTCCAATTCAC	2	A	0.0254	0.0251
NC_005101.4-43650095	TCTATCTCTACTTCCTCTCCTCCAGTA	3	T	0.2232	0.4394
NC_005101.4-43650095	TCTATCTCTACTTCCTCTCCTCCAGTA	6	C	0.115	0.2237

NC_005101.4-43717895	GTGGTTCCCACATATGCTCCCAACCCA	9	C	0.265	0.2778
NC_005101.4-43717895	GTGGTTCCCACATATGCTCCCAACCCA	25	C	0.1795	0.2423
NC_005101.4-43725091	GCTCCGGAGACTCCCTCTCCAGCTCCT	9	G	0.1565	0.2395
NC_005101.4-43725091	GCTCCGGAGACTCCCTCTCCAGCTCCT	24	T	0.0256	0.058
NC_005101.4-43728330	TGTGGGTAAACCGAGGCTCCAAACAAA	13	G	0.2735	0.4734
NC_005101.4-43729626	CCAATCTACACAAATACTCCACAGCAT	16	A	0.0164	0.0322
NC_005101.4-43729626	CCAATCTACACAAATACTCCACAGCAT	24	G	0.157	0.2474
NC_005101.4-43794732	AAGAAACTCACTGTTGCTCCCAACATT	7	C	0.0435	0.0752
NC_005101.4-43794732	AAGAAACTCACTGTTGCTCCCAACATT	8	T	0.1404	0.1884
NC_005101.4-43816610	CCTCCTGCAACTCCAGCTCCAGGAGAT	12	T	0.6283	0.4435
NC_005101.4-43816610	CCTCCTGCAACTCCAGCTCCAGGAGAT	13	C	0.6339	0.4394
NC_005101.4-43838713	GGGCCAGGCACTGAATCTCCAGAGGGA	21	A	0.0531	0.0846
NC_005101.4-44102174	CTCAGACCAACCCTGGCTCCTCTTCCT	23	T	0.0833	0.1244
NC_005101.4-44102174	CTCAGACCAACCCTGGCTCCTCTTCCT	25	C	0.0667	0.1099
NC_005101.4-44102626	CACTGCAGGACCACATCTCCTTCCTTA	15	A	0	0.4819
NC_005101.4-44102626	CACTGCAGGACCACATCTCCTTCCTTA	25	T	0.0661	0.109
NC_005101.4-44104999	GGCATGGTGACAAACTCTCCATTTCAA	21	A	0.0089	0.4751
NC_005101.4-44166431	AAGCAGTAAACATTCTCTCCACGCACA	2	A	0.2	0.3639
NC_005101.4-44205087	CAGACATGGACCCACACTCCTTGTCTC	22	T	0.0342	0.4918
NC_005101.4-44205087	CAGACATGGACCCACACTCCTTGTCTC	23	G	0.2542	0.4481
NC_005101.4-44205615	TGCATGTGTACATTTACTCCTTCAACA	2	G	0.0992	0.1516
NC_005101.4-44229478	GAGCTTGTCACTAAAGCTCCTCAGAAG	3	G	0.2478	0.4342
NC_005101.4-44229478	GAGCTTGTCACTAAAGCTCCTCAGAAG	13	A	0.1826	0.2458
NC_005101.4-44271995	AAATAGGGCACTTGGCCTCCCAAATCT	21	C	0.1261	0.1746
NC_005101.4-44271995	AAATAGGGCACTTGGCCTCCCAAATCT	24	A	0.0085	0.4655
NC_005101.4-44274195	AGAGTAGAAACCGAGGCTCCAGCCATA	12	C	0.0932	0.1041
NC_005101.4-44275640	ATGTTATACACTTTCACTCCCTTGAGG	2	T	0.1316	0.499
NC_005101.4-44305471	CTCTGAACCACAGGCTCTCCGTATGGG	1	C	0.0804	0.1093
NC_005101.4-44305471	CTCTGAACCACAGGCTCTCCGTATGGG	21	G	0.0804	0.1093
NC_005101.4-44320785	TATCTGAACACTCTTGCTCCAAGCAAC	6	G	0.0085	0.4854
NC_005101.4-44365799	CGGCCAGTCACACCGGCTCCTTCTGGT	2	G	0	0.4807
NC_005101.4-44365799	CGGCCAGTCACACCGGCTCCTTCTGGT	15	G	0.3077	0.45
NC_005101.4-44404567	GGGACCTTCACATGCTCTCCTGGGCAG	2	G	0.1789	0.2937
NC_005101.4-44418086	TTCAATACGACACCGTCTCCAATAGAC	9	G	0.1074	0.1017
NC_005101.4-44424490	TTTCTGAGCACTGGGACTCCAGGTATA	3	T	0.5431	0.4108
NC_005101.4-44424490	TTTCTGAGCACTGGGACTCCAGGTATA	27	A	0.5431	0.4108
NC_005101.4-44544318	GTGTTTTCCACTATTTCTCCTCTGTTG	8	C	0.1491	0.3568
NC_005101.4-44562284	GGAGGTATCACAATAACTCCCACTTAA	5	G	0	0.4826
NC_005101.4-44569184	AGTGACTAAACCATGCCTCCATTGTTG	22	T	0.0175	0.4846
NC_005101.4-44571141	CAGGCAAGCACCGTAGCTCCGCCCCAG	12	C	0.0732	0.4915
NC_005101.4-44571141	CAGGCAAGCACCGTAGCTCCGCCCCAG	13	G	0.065	0.0629
NC_005101.4-44591566	AGCCAAATGACTCTGTCTCCTTTCGTT	23	T	0	0.4813
NC_005101.4-44607278	CAGTGTACTACTTTGTCTCCCAGATCT	22	A	0.2314	0.4959
NC_005101.4-44616779	CTGGTCTCTACACACCCTCCTACATAT	6	C	0.0244	0.0241
NC_005101.4-44616779	CTGGTCTCTACACACCCTCCTACATAT	9	T	0.1382	0.1695
NC_005101.4-44661087	CCAACCTTAACTGTCCCTCCTCAGTCA	6	C	0.1949	0.329
NC_005101.4-44705037	AAGAAGGCCACACCTCCTCCAGCAAGA	2	A	0.1066	0.1154
NC_005101.4-44760324	GTGCTGTGTACTTCTCCTCCTCTGAAG	1	G	0.0579	0.0716
NC_005101.4-44760324	GTGCTGTGTACTTCTCCTCCTCTGAAG	22	C	0.2167	0.2188
NC_005101.4-44797490	TTATGTAGCACCCCAGCTCCTACAGCT	1	T	0.0661	0.0639
NC_005101.4-44797490	TTATGTAGCACCCCAGCTCCTACAGCT	16	G	0	0.4819

NC_005101.4-44812522	TGGACAGCGACAAGTTCTCCACGTACT	8	C	0.0667	0.4988
NC_005101.4-44851157	CCAGTTCTCACTGCTTCTCCAAGCCTT	23	G	0.1901	0.2704
NC_005101.4-44856252	TAAGGGGTTACAGTCCCTCCTGAGGAT	22	G	0.0244	0.0241
NC_005101.4-44859389	TCACCACCTACACACCCTCCCTGCTAG	23	G	0.2114	0.2937
NC_005101.4-44871446	ACAATGAACACCGGCTCTCCCGCTCTC	13	G	0.0976	0.1629
NC_005101.4-44871446	ACAATGAACACCGGCTCTCCCGCTCTC	21	C	0.0488	0.078
NC_005101.4-44887831	ACATTATCAACAGAGTCTCCTCACGGT	1	A	0	0.4793
NC_005101.4-44973945	TGAGGAACTACTGTGTCTCCTCCAATG	12	T	0.122	0.1425
NC_005101.4-45057999	CCATGTGTAACTGTGGCTCCAGGAGGA	5	G	0.2212	0.4986
NC_005101.4-45057999	CCATGTGTAACTGTGGCTCCAGGAGGA	21	A	0.0261	0.0257
NC_005101.4-45118539	ATTTCATGCACCGGAGCTCCCGCGTGG	26	G	0.2066	0.4966
NC_005101.4-45150807	ATGGTACCAACCGCAGCTCCAACAAAC	13	G	0.0488	0.0476
NC_005101.4-45150807	ATGGTACCAACCGCAGCTCCAACAAAC	26	A	0.0081	0.0241
NC_005101.4-45175900	GTACTGGTCACTCAACCTCCCTTTCCT	14	A	0.0172	0.4749
NC_005101.4-45193544	TCTCCCAATACCAGCGCTCCCTGTGAA	3	T	0.0345	0.0339
NC_005101.4-45193544	TCTCCCAATACCAGCGCTCCCTGTGAA	25	G	0.2288	0.2406
NC_005101.4-45198800	TGGATGAAAACTGTCCCTCCATTATTC	14	T	0	0.48
NC_005101.4-45208788	GTCTCCATCACTTGCCCTCCTGATGGT	24	T	0	0.4773
NC_005101.4-45258525	CATAATTACACTTTCTCTCCTTACATC	27	C	0.2703	0.3389
NC_005101.4-45299559	GCTCTTTCCACTTCCTCTCCACGTGGA	1	G	0.0496	0.0639
NC_005101.4-45299559	GCTCTTTCCACTTCCTCTCCACGTGGA	27	A	0.0579	0.4966
NC_005101.4-45377394	AGGAGGCTGACATAGCCTCCAAATTAT	14	A	0.2155	0.4803
NC_005101.4-45388423	AATCAGAGGACAACCACTCCGTCCCAT	27	T	0.113	0.18
NC_005101.4-45441993	AGGAGACACACAGAACCTCCCACTGAG	12	A	0.084	0.0805
NC_005101.4-45453750	TCCCCGGGAACTCTGGCTCCTCGTGGG	9	A	0.1624	0.1634
NC_005101.4-45470873	CAGCTCAGCACCTGAGCTCCAAGACTG	2	A	0.2439	0.4992
NC_005101.4-45473737	ACCCAAGCCACCCAACCTCCCTTGTAA	5	A	0	0.4728
NC_005101.4-45473737	ACCCAAGCCACCCAACCTCCCTTGTAA	27	A	0	0.4728
NC_005101.4-45567094	CTAGTTTCCACTCTCCCTCCCTTTCAG	8	C	0.0813	0.078
NC_005101.4-45567094	CTAGTTTCCACTCTCCCTCCCTTTCAG	9	C	0	0.4793
NC_005101.4-45581992	TATCTATCAACATGACCTCCACTGAGT	2	A	0.161	0.2406
NC_005101.4-45623110	ATATGATACACGGTCTCTCCTTGCTGC	12	G	0	0.4793
NC_005101.4-45642330	TCAATTGCCACCTTAGCTCCAACTGAC	26	A	0.05	0.4888
NC_005101.4-45675841	GGCCACTAGACTGCTTCTCCTTGGGCC	8	A	0.0082	0.4822
NC_005101.4-45675841	GGCCACTAGACTGCTTCTCCTTGGGCC	26	C	0.0569	0.0854
NC_005101.4-45723092	CTGACAGAAACCTCTCCTCCTGTTGTG	3	G	0.0259	0.0255
NC_005101.4-45735250	AAGCCAAACACACCCTCTCCTTCCATT	24	C	0.2727	0.4146
NC_005101.4-45764686	TAAAGGGACACCGTGTCTCCCAACCTA	24	C	0.0081	0.4777
NC_005101.4-45764686	TAAAGGGACACCGTGTCTCCCAACCTA	27	A	0.0082	0.479
NC_005101.4-45769082	AGTCCAGGTACTGTAGCTCCTACAGTT	22	A	0	0.4751
NC_005101.4-45769082	AGTCCAGGTACTGTAGCTCCTACAGTT	23	C	0	0.4751
NC_005101.4-45778709	TGGAATTGAACTTGAACTCCCTATATG	4	A	0	0.4807
NC_005101.4-45796993	TCTCGATACACATGACCTCCTTCCTCT	5	G	0.1228	0.1454
NC_005101.4-45796993	TCTCGATACACATGACCTCCTTCCTCT	23	C	0.1293	0.1504
NC_005101.4-45874946	CTAGTAGTAACCCGCCCTCCAGCGGCT	5	T	0	0.4779
NC_005101.4-45874946	CTAGTAGTAACCCGCCCTCCAGCGGCT	25	G	0.042	0.4886
NC_005101.4-45895475	GGGAAAGACACTCGTGCTCCCACAGGT	6	A	0.0081	0.4777
NC_005101.4-45895475	GGGAAAGACACTCGTGCTCCCACAGGT	14	G	0.0569	0.0553
NC_005101.4-45923856	GTACCGTGAACTTAGACTCCAAACTTA	26	T	0	0.4709
NC_005101.4-45923856	GTACCGTGAACTTAGACTCCAAACTTA	27	A	0	0.4709
NC_005101.4-45925493	TAACCATTGACCATGTCTCCAGCCCAT	7	T	0.0252	0.4797

NC_005101.4-45968204	CACACCCTCACTGTAGCTCCCAGGCAG	27	G	0.0909	0.1017
NC_005101.4-45999266	ATCCTGGGCACTATACCTCCTTTTGTT	9	C	0.2566	0.3081
NC_005101.4-45999266	ATCCTGGGCACTATACCTCCTTTTGTT	22	T	0.2456	0.3221
NC_005101.4-46007847	GAGAAAGGCACCAAGACTCCACTTGTG	8	G	0.2203	0.3436
NC_005101.4-46007847	GAGAAAGGCACCAAGACTCCACTTGTG	24	T	0.0085	0.4776
NC_005101.4-46021827	AACAGAAAGACTGAGCCTCCTTGTGGT	9	G	0.225	0.3047
NC_005101.4-46023045	ACATTTATTACACTGGCTCCCTCCTTC	4	T	0.2069	0.3179
NC_005101.4-46050946	AGGATTCTGACTGAGACTCCACCCAGT	8	T	0.2821	0.3
NC_005101.4-46055501	ACCTGGCCTACTGATGCTCCCCGTGTC	16	G	0.1391	0.2005
NC_005101.4-46055501	ACCTGGCCTACTGATGCTCCCCGTGTC	23	G	0.1379	0.199
NC_005101.4-46272390	GAGCCTAGTACAGAGACTCCTCCTTCA	4	C	0.0248	0.0245
NC_005101.4-46272390	GAGCCTAGTACAGAGACTCCTCCTTCA	5	C	0.0661	0.0639
NC_005101.4-46277157	ACGACACTCACCTTCCCTCCCTTACCA	15	C	0.1513	0.4997
NC_005101.4-46329139	CGCTGCCTGACACCCCCTCCTCTGCAC	1	C	0.2583	0.3441
NC_005101.4-46329139	CGCTGCCTGACACCCCCTCCTCTGCAC	23	T	0.124	0.1163
NC_005101.4-46364223	GGGAGGGGCACTCCAGCTCCTAACTAT	24	C	0.0082	0.0243
NC_005101.4-46364461	CAGTACAGGACGAACACTCCCATTTTG	6	C	0.0175	0.0512
NC_005101.4-46415364	GCCACCATGACAGCTTCTCCACATGTA	7	A	0.0088	0.481
NC_005101.4-46415364	GCCACCATGACAGCTTCTCCACATGTA	8	T	0.0088	0.481
NC_005101.4-46420472	ATGGACTAGACCCTTCCTCCCTCTCTG	15	T	0.0086	0.4768
NC_005101.4-46485931	TCCGGTGGTACTTGGCCTCCTGCAGCT	3	C	0.0244	0.0553
NC_005101.4-46489369	CACCTACACACCCAATCTCCTGATGCT	8	A	0.0656	0.0634
NC_005101.4-46544116	CCCCTAACTACTGAGGCTCCAGTTCCC	23	T	0.0168	0.4703
NC_005101.4-46556939	CAAAACATCACATGCCCTCCTTCCAGG	6	C	0.1417	0.1866
NC_005101.4-46556939	CAAAACATCACATGCCCTCCTTCCAGG	13	T	0.1417	0.5
NC_005101.4-46584658	AGACTTCTCACTACTTCTCCTCTCTAA	21	T	0.0089	0.4856
NC_005101.4-46592171	ACCAGCACCACATCAACTCCAGACTAT	26	A	0.1478	0.2204
NC_005101.4-46674723	GGCCCAGAAACCTTTCCTCCTCTCTCT	23	T	0.2269	0.4797
NC_005101.4-46688822	CAGCCGCTTACAGTGACTCCCACGTAG	4	C	0.2623	0.3354
NC_005101.4-46688822	CAGCCGCTTACAGTGACTCCCACGTAG	23	C	0	0.4773
NC_005101.4-46718133	CTGTGGTTAACCATGACTCCTGTAGCC	2	T	0	0.4786
NC_005101.4-46718133	CTGTGGTTAACCATGACTCCTGTAGCC	4	T	0.2783	0.4234
NC_005101.4-46909812	CTGAGTGTTACTGTTTCTCCTTTGGCC	14	T	0.0085	0.4842
NC_005101.4-46917266	GACCTTTGCACACTTCCTCCAAAGGTT	27	T	0.2114	0.3688
NC_005101.4-46926757	CTACCACAAACTACAACTCCCCCATCC	24	A	0.1161	0.2991
NC_005101.4-46993803	AGAGTCTGAACCCCTCCTCCACCTTTA	4	G	0.0424	0.0415
NC_005101.4-47001962	CCCCTTCCAACCCTTCCTCCTCTCACT	13	C	0.0082	0.0557
NC_005101.4-47004052	GGCCTGAAGACTTAGCCTCCTGCATGC	16	C	0.0351	0.0997
NC_005101.4-47022083	GTTGTTTTTACCTTTGCTCCTATGTCT	1	G	0.1639	0.2032
NC_005101.4-47022083	GTTGTTTTTACCTTTGCTCCTATGTCT	15	T	0.1311	0.1225
NC_005101.4-47033403	GCACGTGCCACTATGCCTCCACTGTGC	27	C	0.3017	0.4836
NC_005101.4-47078963	TGCTCTAGGACGTCTGCTCCCACCCAT	9	G	0	0.4793
NC_005101.4-47078963	TGCTCTAGGACGTCTGCTCCCACCCAT	12	G	0	0.4793
NC_005101.4-47079085	TTTGCTCTCACGGCCCCTCCATTCTTC	3	T	0.1066	0.4959
NC_005101.4-47079085	TTTGCTCTCACGGCCCCTCCATTCTTC	16	C	0	0.4793
NC_005101.4-47163592	GGATCCATCACTGTTCCTCCCAGAGCA	26	C	0.0244	0.0398
NC_005101.4-47196428	CACTGGCCTACATTAACTCCTGTCCCC	26	C	0.2683	0.484
NC_005101.4-47213686	TATGTGGTCACACTGTCTCCATAACCC	7	G	0	0.4787
NC_005101.4-47213686	TATGTGGTCACACTGTCTCCATAACCC	24	A	0	0.4787
NC_005101.4-47264731	GCCCTGAGCACACCTGCTCCCCCACTC	27	C	0.0982	0.1093
NC_005101.4-47331880	AGGAACCATACAGCCACTCCTTTCTAA	6	C	0.1525	0.1551

NC_005101.4-47331880	AGGAACCATACAGCCACTCCTTTCTAA	14	C	0.2458	0.4842
NC_005101.4-47347711	AGCAGTCTGACTTCCCCTCCCAGTGAA	16	C	0.2377	0.479
NC_005101.4-47349321	CACACACAGACATGGCCTCCACACTGT	3	C	0.0984	0.1505
NC_005101.4-47349321	CACACACAGACATGGCCTCCACACTGT	6	A	0.0165	0.4819
NC_005101.4-47447993	CTATAGGGAACTCTAGCTCCAGCTGTG	8	G	0.0738	0.0861
NC_005101.4-47493266	ATGCTCTGAACTGAAGCTCCTCCCCCT	6	C	0.0342	0.0336
NC_005101.4-47493266	ATGCTCTGAACTGAAGCTCCTCCCCCT	22	C	0.0342	0.4839
NC_005101.4-47503967	AACAAAGCCACACCCACTCCATCAAGA	1	A	0.2542	0.3529
NC_005101.4-47663536	GAAATCATAACTTGTTCTCCACAAAGT	12	T	0.0902	0.2573
NC_005101.4-47670266	CACTTGAAGACTTATTCTCCTATACTT	14	A	0.1579	0.4975
NC_005101.4-47670266	CACTTGAAGACTTATTCTCCTATACTT	15	T	0.1579	0.4975
NC_005101.4-47751977	CCATGTCCTACTCCAACTCCTCCCATG	7	C	0.0246	0.4852
NC_005101.4-48002237	GTTTCTGACACTTTATCTCCCTAGCAG	23	A	0.0177	0.4755
NC_005101.4-48010829	GCTTCATGCACTGTTTCTCCTTCACAC	2	C	0.161	0.2156
NC_005101.4-48047346	TTCCCCAGCACAAGACCTCCAATAATT	4	C	0.0902	0.1009
NC_005101.4-48082195	TCCTCATTCACCGTCTCTCCTAGAACA	12	C	0.0446	0.0771
NC_005101.4-48082195	TCCTCATTCACCGTCTCTCCTAGAACA	13	G	0.0631	0.492
NC_005101.4-48094309	GTATTTCCCACTGCAGCTCCCCTCCCA	27	A	0.0536	0.1478
NC_005101.4-48125118	CCACCTGAAACTTCACCTCCACTGAAT	3	A	0.1311	0.4979
NC_005101.4-48125118	CCACCTGAAACTTCACCTCCACTGAAT	25	A	0.0082	0.4755
NC_005101.4-48163230	CCAAGATTAACACTGTCTCCAGCATTG	7	T	0.0862	0.1855
NC_005101.4-48163230	CCAAGATTAACACTGTCTCCAGCATTG	13	C	0.0948	0.0903
NC_005101.4-48235293	TAGATCTTCACAGGGACTCCTTTTGCC	3	G	0	0.0342
NC_005101.4-48235293	TAGATCTTCACAGGGACTCCTTTTGCC	7	T	0.0261	0.0257
NC_005101.4-48235468	ATTACCAGGACAAGCTCTCCAACACTG	5	C	0.0252	0.0411
NC_005101.4-48235468	ATTACCAGGACAAGCTCTCCAACACTG	22	A	0	0.4813
NC_005101.4-48415588	ACGCCTGCAACTCCAGCTCCGACAGAT	13	C	0	0.48
NC_005101.4-48415588	ACGCCTGCAACTCCAGCTCCGACAGAT	23	C	0	0.48
NC_005101.4-48458087	ACTCTCCATACTCTCCCTCCATCCCAT	2	C	0.0992	0.4991
NC_005101.4-48526383	CATGGTAGGACATTCACTCCTTTAATC	13	T	0	0.4709
NC_005101.4-48526383	CATGGTAGGACATTCACTCCTTTAATC	27	C	0.181	0.2057
NC_005101.4-48569556	CTTTGACTCACCCATGCTCCTGTAGGT	24	A	0.0744	0.0716
NC_005101.4-48630699	CACTGGATCACCCTACCTCCAAAGGAC	15	A	0.0163	0.4759
NC_005101.4-48678701	GCTCAAAGCACCCATGCTCCCTTTAAC	21	C	0.1404	0.16
NC_005101.4-48738228	ATATTTGGAACTCCAGCTCCAAGGGAT	25	G	0.0244	0.0398
NC_005101.4-48738228	ATATTTGGAACTCCAGCTCCAAGGGAT	27	T	0.124	0.4999
NC_005101.4-48759847	CAGAGACTGACCCCTGCTCCCAGTTAT	15	T	0.1933	0.4638
NC_005101.4-48759847	CAGAGACTGACCCCTGCTCCCAGTTAT	23	G	0.0504	0.0958
NC_005101.4-48781808	ACAGTGATGACTGAGACTCCATTCCTA	22	T	0.1092	0.4045
NC_005101.4-48856803	CATGATCCAACTCCTGCTCCTCAGTTT	8	C	0	0.4793
NC_005101.4-48856803	CATGATCCAACTCCTGCTCCTCAGTTT	9	A	0.1057	0.1001
NC_005101.4-48877578	CAACAAGTGACCTAACCTCCATATTCC	25	T	0.1491	0.1672
NC_005101.4-48902480	TTCTTCCACACTCAGACTCCGTGCATG	9	C	0.0083	0.4783
NC_005101.4-48902480	TTCTTCCACACTCAGACTCCGTGCATG	21	G	0.0083	0.4783
NC_005101.4-48905295	TAACAAAAGACCTTGTCTCCGTCTATA	21	G	0.2975	0.3079
NC_005101.4-48905295	TAACAAAAGACCTTGTCTCCGTCTATA	23	C	0	0.4807
NC_005101.4-48932790	ACAACCCACACATTCCCTCCTGCCTCC	13	T	0.4018	0.491
NC_005101.4-48932790	ACAACCCACACATTCCCTCCTGCCTCC	21	T	0.0435	0.0425
NC_005101.4-48935919	TAGTTTGTGACACTCTCTCCATTTTAT	2	A	0	0.4786
NC_005101.4-48941949	CAAAAATAAACGCGTCCTCCTTCATTC	3	A	0	0.4751
NC_005101.4-48958245	CAAGTTTGTACAATTTCTCCTGAAGCT	8	G	0.2119	0.4273

NC_005101.4-48971220	CGGGCTCTTACACTACCTCCTGTTCCT	1	C	0.0574	0.1009
NC_005101.4-48971220	CGGGCTCTTACACTACCTCCTGTTCCT	23	T	0.2049	0.4677
NC_005101.4-49009387	ACTTCCATGACCACCTCTCCAGCAGCT	9	G	0	0.4703
NC_005101.4-49053729	AATGAGCAAACAGTAACTCCCCCTTCA	14	T	0	0.4787
NC_005101.4-49062343	CACACACACACTGTGTCTCCCTTACTT	21	C	0.0248	0.0245
NC_005101.4-49062343	CACACACACACTGTGTCTCCCTTACTT	23	T	0.0248	0.0245
NC_005101.4-49150089	TTCTTCATCACCACACCTCCTCTTCCT	15	A	0.1545	0.1425
NC_005101.4-49193311	TCAAACAGCACGGAAGCTCCTTCACAA	6	C	0.0085	0.4854
NC_005101.4-49193311	TCAAACAGCACGGAAGCTCCTTCACAA	12	G	0.0085	0.4854
NC_005101.4-49224293	TCTCCTCTAACCCTGACTCCCGTGTTG	24	G	0.0085	0.481
NC_005101.4-49402477	GAAATCACAACAACAGCTCCAGGCTAT	4	A	0	0.4751
NC_005101.4-49416249	TGCACAATCACCGCATCTCCAATCATC	9	C	0.0086	0.4893
NC_005101.4-49416249	TGCACAATCACCGCATCTCCAATCATC	12	C	0.0603	0.4729
NC_005101.4-49423217	TGTTTAGTAACTGAACCTCCTCGCTGC	8	T	0.0087	0.0425
NC_005101.4-49423217	TGTTTAGTAACTGAACCTCCTCGCTGC	23	G	0.1565	0.4301
NC_005101.4-49542758	TGTTTCTTGACAGAGTCTCCTGTAGCC	3	T	0.2459	0.316
NC_005101.4-49619803	AGAACACCCACACTCCCTCCATCTGGT	6	A	0.1966	0.3311
NC_005101.4-49621938	TTTCTCAATACAAGTTCTCCACTAAAT	1	T	0.1709	0.2235
NC_005101.4-49621938	TTTCTCAATACAAGTTCTCCACTAAAT	22	C	0.1368	0.1274
NC_005101.4-49685987	CATCACCCTACCCTAGCTCCTTCTCTT	16	G	0	0.4793
NC_005101.4-49688267	CAACCCAAGACAGAGTCTCCTCTGCTT	27	T	0.1583	0.4972
NC_005101.4-49699568	CACTGTGATACTGCTTCTCCCTCCTCT	6	T	0.1463	0.4997
NC_005101.4-49699568	CACTGTGATACTGCTTCTCCCTCCTCT	12	T	0.0244	0.0241
NC_005101.4-49702579	TCTGCTGTAACAAAGTCTCCAACAAAA	15	G	0.0086	0.4836
NC_005101.4-49712627	TTTTCTCTCACGTCTTCTCCACTTACC	12	G	0.1062	0.1757
NC_005101.4-49712627	TTTTCTCTCACGTCTTCTCCACTTACC	14	C	0.2281	0.3324
NC_005101.4-49739824	GCATGTGAGACCGGAACTCCCGTCTTG	21	C	0.1557	0.1968
NC_005101.4-49855188	TCCCTCTGAACACACCCTCCTCAAACC	16	C	0.0259	0.0585
NC_005101.4-49998090	ACCTCTCTCACATCTCCTCCATTTCCG	26	C	0.2479	0.3729
NC_005101.4-50082528	AGACAAGAAACAATCTCTCCTCATGTG	24	T	0.0083	0.4732
NC_005101.4-50370833	CATAGAAGCACCGGAACTCCACACACG	12	C	0.1721	0.311
NC_005101.4-50415262	TGCATGACCACTCTAGCTCCCAGTTAG	9	C	0.0345	0.0339
NC_005101.4-50453499	GACTGTGGGACCTCTGCTCCAGAGGAC	25	G	0.042	0.0411
NC_005101.4-50470335	CATGATGAAACACCAGCTCCCGGCTGG	14	C	0.0513	0.45
NC_005101.4-50499032	CGCATGAGGACCCAATCTCCACCAGTC	15	A	0	0.4757
NC_005101.4-50688137	GTGCTACACACTTTGTCTCCAAATCTC	1	G	0.0504	0.0491
NC_005101.4-50833312	TGTGTCAATACTTGGTCTCCAATTACT	12	T	0	0.4773
NC_005101.4-50833312	TGTGTCAATACTTGGTCTCCAATTACT	14	G	0	0.0322
NC_005101.4-50941527	TCATACATCACCATACCTCCAAAGAAC	1	T	0.087	0.0989
NC_005101.4-50941527	TCATACATCACCATACCTCCAAAGAAC	6	C	0	0.474
NC_005101.4-51044414	TGTTATTCAACACCGTCTCCATAGGTA	24	G	0.0492	0.048
NC_005101.4-51058417	TTTTGTTTCACTCTTACTCCTTAGATG	4	T	0.0446	0.0436
NC_005101.4-51058417	TTTTGTTTCACTCTTACTCCTTAGATG	21	T	0.0446	0.0436
NC_005101.4-51138322	TCCTCCTAGACCTTCCCTCCTTCCTTT	8	A	0.2605	0.2266
NC_005101.4-51138322	TCCTCCTAGACCTTCCCTCCTTCCTTT	25	T	0.2605	0.2266
NC_005101.4-51247488	CCAGACACTACTCACACTCCCTGGGTA	8	C	0.0248	0.0405
NC_005101.4-51380542	CATAGGAATACTCAACCTCCATCGAAT	23	C	0.2602	0.4072
NC_005101.4-51396688	TGATGGAGAACACCCTCTCCAAGCTTT	16	T	0	0.4793
NC_005101.4-51447388	GTCTTTGGCACCCTTTCTCCCTACTGT	6	T	0.2727	0.3601
NC_005101.4-51447388	GTCTTTGGCACCCTTTCTCCCTACTGT	21	C	0.0248	0.0245
NC_005101.4-51574359	TACAGCCATACAGTTCCTCCTGTAGAG	21	T	0	0.4833

NC_005101.4-51574419	AACATTCCCACTAGTGCTCCAGTGGAA	26	A	0.0085	0.4854
NC_005101.4-51672109	CCCTTCTCGACTTTAGCTCCTATCAGT	14	T	0.2155	0.2797
NC_005101.4-51741185	ATGCTGGTTACCCTAACTCCCAAGGAT	12	C	0.0333	0.0328
NC_005101.4-51758567	CCTACCTGCACCACGCCTCCATCCTAT	16	C	0.0984	0.4952
NC_005101.4-51758567	CCTACCTGCACCACGCCTCCATCCTAT	21	A	0.0984	0.4952
NC_005101.4-51879109	AGATGCCACACTGTAACTCCATCACAT	13	G	0.2541	0.3008
NC_005101.4-51879109	AGATGCCACACTGTAACTCCATCACAT	27	T	0.0574	0.4903
NC_005101.4-52050778	TCACAATTAACTGCAACTCCACACTCC	6	A	0	0.4807
NC_005101.4-52083992	CAAGTTCCTACTTTGCCTCCGACATCT	21	G	0.065	0.4904
NC_005101.4-52099267	ATATCCACGACCCCCTCTCCTCTGGAG	8	C	0.0086	0.4729
NC_005101.4-52112453	ATCAAAGCGACATGACCTCCGAAGATA	13	T	0.1826	0.4817
NC_005101.4-52112453	ATCAAAGCGACATGACCTCCGAAGATA	21	G	0.1826	0.4946
NC_005101.4-52116977	ATTAAGGTCACACGAGCTCCCTTCTCT	16	G	0.065	0.1356
NC_005101.4-52187927	ACTCCCATAACTATGCCTCCCTTTTCC	3	T	0	0.4751
NC_005101.4-52198491	TCCGCGATGACATAAGCTCCACTCTTG	14	A	0.1379	0.4987
NC_005101.4-52199695	AACTCATGAACTAGAGCTCCTCCCAGA	23	C	0.0083	0.4708
NC_005101.4-52199695	AACTCATGAACTAGAGCTCCTCCCAGA	24	C	0.2066	0.4835
NC_005101.4-52222164	TCGGTGATGACCAAGTCTCCTATATGA	14	A	0.1404	0.1454
NC_005101.4-52265603	CCTCTGACCACCAGATCTCCTTGCCGG	26	G	0.2069	0.3478
NC_005101.4-52435906	AACCACTAAACCATTGCTCCAACCTTT	7	T	0.2269	0.3554
NC_005101.4-52443163	GATGTTATGACAGGCCCTCCTCACTAC	13	G	0	0.4864
NC_005101.4-52464084	GCTCTCCCCACTGTTCCTCCCTTAGGC	7	C	0.042	0.0411
NC_005101.4-52480028	CTGTTACACACTCACGCTCCTGCTGCC	16	G	0.0268	0.0264
NC_005101.4-52526724	CTGATTCAAACTGAGGCTCCTGAGGAG	1	C	0.1261	0.1327
NC_005101.4-52526724	CTGATTCAAACTGAGGCTCCTGAGGAG	23	A	0.0085	0.481
NC_005101.4-52539707	CTATCAAGCACTGTCACTCCAGTTCAG	1	C	0	0.4909
NC_005101.4-52539707	CTATCAAGCACTGTCACTCCAGTTCAG	21	A	0.0089	0.4856
NC_005101.4-52579000	CCCAATCCGACCTATCCTCCCAACCCT	1	C	0.1552	0.4976
NC_005101.4-52581157	CTTTAAAACACATCTTCTCCTGCATGC	13	T	0.193	0.3615
NC_005101.4-52813439	CTCCAGATCACACTTCCTCCTATGTTA	16	C	0.0732	0.0854
NC_005101.4-52943940	ACAGCAATGACTCAGACTCCAAGATCA	4	G	0.0086	0.4803
NC_005101.4-53020024	CTCTCTCCCACTGTCTCTCCAGCCATG	25	A	0.0413	0.0405
NC_005101.4-53020183	ACTTTGCTCACTGTAGCTCCCACATCC	15	A	0.0339	0.0333
NC_005101.4-53022500	CTTGCCTCTACAGAACCTCCGGTAGTT	1	C	0	0.4778
NC_005101.4-53022500	CTTGCCTCTACAGAACCTCCGGTAGTT	8	C	0.0789	0.138
NC_005101.4-53064466	GCTTCTATGACCGATTCTCCCATTTGT	15	T	0.314	0.3467
NC_005101.4-53065908	TATTCAGTAACCTGGGCTCCTTGAGGC	3	T	0.1316	0.1814
NC_005101.4-53081752	TGCCATTAAACCAAGTCTCCATTTCAC	24	T	0.0085	0.4776
NC_005101.4-53102776	GCTGCCCACACCTCTTCTCCAGCATCA	25	T	0.1282	0.4977
NC_005101.4-53105253	CAACCATGCACTGCTCCTCCCCCATTC	2	A	0.0813	0.078
NC_005101.4-53109168	CATGATGGCACCATGCCTCCACTGTCA	3	T	0.281	0.3278
NC_005101.4-53149606	AAATTTTTCACTGTCTCTCCTGCATGA	2	A	0	0.4746
NC_005101.4-53149606	AAATTTTTCACTGTCTCTCCTGCATGA	27	A	0.3125	0.4454
NC_005101.4-53201360	GTCAATACTACTGTTTCTCCTGCGACT	24	G	0.0696	0.0671
NC_005101.4-53201360	GTCAATACTACTGTTTCTCCTGCGACT	27	T	0.2281	0.3221
NC_005101.4-53247444	TTCAGCTTAACACATTCTCCTCTATCT	6	C	0.0763	0.1041
NC_005101.4-53247444	TTCAGCTTAACACATTCTCCTCTATCT	24	A	0	0.4757
NC_005101.4-53273434	TTATAGGGAACTGTTACTCCATCTTTT	3	A	0.1858	0.2104
NC_005101.4-53281465	ACAAAGTGGACACACCCTCCTTTTGGT	1	A	0.027	0.0267
NC_005101.4-53287786	TTCCTCCTCACACACACTCCTTTTAAT	1	T	0.0171	0.4839
NC_005101.4-53287786	TTCCTCCTCACACACACTCCTTTTAAT	16	A	0.0171	0.4839

NC_005101.4-53318028	TTCTTGTTCACAATTCCTCCCTTGGTT	9	C	0.0086	0.4729
NC_005101.4-53318028	TTCTTGTTCACAATTCCTCCCTTGGTT	13	A	0.0086	0.4729
NC_005101.4-53351577	ACCCTTCCAACCATATCTCCAACTCAG	3	C	0.0336	0.033
NC_005101.4-53351577	ACCCTTCCAACCATATCTCCAACTCAG	25	C	0.0932	0.1041
NC_005101.4-53431677	ATTATTTCCACCTCTGCTCCAAGTATT	2	T	0	0.4819
NC_005101.4-53614049	AATCACATCACTTTCCCTCCTCAATTT	7	A	0.0333	0.4499
NC_005101.4-53614361	GAAATCCCCACTCCACCTCCTTCTATG	12	T	0	0.4832
NC_005101.4-53712785	GGGTGGTCTACATTACCTCCAAGGAAT	26	A	0	0.4773
NC_005101.4-53772984	GTACCTCCTACTCACACTCCTCCTAGT	21	T	0.1453	0.5
NC_005101.4-53846919	GGCCCTGTTACAGTTCCTCCTTTCCCT	25	C	0.0325	0.032
NC_005101.4-53929987	TACCTTGAAACTGTTTCTCCTTTTTTG	8	A	0	0.4731
NC_005101.4-53932153	CATTATCTCACTCAATCTCCTAAGTCT	13	C	0	0.4773
NC_005101.4-53939465	AGATGATAAACGAGACCTCCTAGACAC	13	A	0.2583	0.4922
NC_005101.4-53939465	AGATGATAAACGAGACCTCCTAGACAC	21	T	0	0.4819
NC_005101.4-53941236	CAAGCATGGACTTTGGCTCCATACAAT	8	G	0.0083	0.4783
NC_005101.4-53952764	GATTCTCCCACTTCTTCTCCTCTTCTT	24	T	0	0.4751
NC_005101.4-54071805	GTGAAAACTACAAGGTCTCCTTGGTAG	15	G	0.0259	0.0422
NC_005101.4-54090623	TACCATATGACAGGATCTCCCTTCTCT	24	C	0	0.4793
NC_005101.4-54395380	CCTAAAGCAACTCTGTCTCCATTAGCT	24	A	0.0776	0.0903
NC_005101.4-54441699	ATTAGTGTGACCATTCCTCCCATCATA	9	G	0.0261	0.0257
NC_005101.4-54441699	ATTAGTGTGACCATTCCTCCCATCATA	14	T	0.2	0.1938
NC_005101.4-54443052	AAAGGAGTGACTGATCCTCCCTCAGGA	16	C	0.0256	0.0253
NC_005101.4-54443052	AAAGGAGTGACTGATCCTCCCTCAGGA	23	C	0.0769	0.12
NC_005101.4-54525142	ACAGTCTCCACATCCCCTCCCTGCTAC	13	T	0.2683	0.4868
NC_005101.4-54559748	ATCTGAACCACTGGAGCTCCTGAGCCA	12	T	0.1967	0.4966
NC_005101.4-54571403	GACACCACCACCACAGCTCCCAAGGAC	12	C	0.0261	0.0257
NC_005101.4-54571403	GACACCACCACCACAGCTCCCAAGGAC	23	A	0	0.4764
NC_005101.4-54629720	TCATTCCACACTGTTTCTCCACCTTTG	21	A	0.2295	0.3354
NC_005101.4-54633847	AGCAGTATTACTCTTCCTCCAGGTGAG	6	T	0.1186	0.4987
NC_005101.4-54711259	CTTACCATCACATTTGCTCCATATTGG	25	T	0	0.4773
NC_005101.4-54735362	CAGTTTCTGACTGTAGCTCCTGGGTCT	24	G	0.2368	0.375
NC_005101.4-54740377	CCCTTGAACACAGGGGCTCCAAGAGGA	22	A	0.0169	0.4793
NC_005101.4-54771253	TTAAGGACCACCCATTCTCCACAGCAT	22	C	0.0847	0.4964
NC_005101.4-54771253	TTAAGGACCACCCATTCTCCACAGCAT	23	A	0	0.4813
NC_005101.4-54776302	TGCTTGCCTACCTGAGCTCCAGGGAGA	3	C	0.2479	0.3
NC_005101.4-54776302	TGCTTGCCTACCTGAGCTCCAGGGAGA	14	G	0.2479	0.3
NC_005101.4-54799119	CAGTTCTAGACACACTCTCCAATCCAG	9	G	0.0714	0.4297
NC_005101.4-54799119	CAGTTCTAGACACACTCTCCAATCCAG	15	C	0.0714	0.4297
NC_005101.4-54817652	AAACGTTACACTTACACTCCGTAAATA	5	G	0.2203	0.4884
NC_005101.4-54874853	GTGTCTCTGACCTTTGCTCCTTTATCA	7	C	0.0082	0.0243
NC_005101.4-54881211	CCCTTAAGTACTTCCTCTCCCTCCTTG	1	C	0	0.4751
NC_005101.4-54899028	ATTCTCTCCACTCAGTCTCCACATGCT	15	G	0.2439	0.4793
NC_005101.4-54899028	ATTCTCTCCACTCAGTCTCCACATGCT	23	A	0	0.4793
NC_005101.4-54922894	CACACTGTCACGGGGACTCCATGGACC	3	C	0.0085	0.4842
NC_005101.4-54967132	GTGATTTTGACCATCTCTCCTGCCCTG	4	A	0	0.4666
NC_005101.4-54967132	GTGATTTTGACCATCTCTCCTGCCCTG	12	C	0.115	0.5
NC_005101.4-55002157	CTCCTCCGTACCAGGCCTCCCCCTTCC	15	G	0.0088	0.483
NC_005101.4-55196351	CTGTAAGCCACAGATTCTCCACTTAAA	5	A	0	0.4703
NC_005101.4-55196351	CTGTAAGCCACAGATTCTCCACTTAAA	8	C	0.1	0.095
NC_005101.4-55268586	CTACAAAACACTCCCCCTCCAAGGAGG	26	G	0.0082	0.4822
NC_005101.4-55302517	TTAGTGAGCACACCAGCTCCAATGAAT	16	G	0.1	0.1099

NC_005101.4-55321934	GGTCGGCCCACCAGTTCTCCTATGCTC	5	G	0	0.4778
NC_005101.4-55322310	AGGGATGGAACTAGCACTCCTGAGTTT	16	A	0.0087	0.4782
NC_005101.4-55454085	AAATGAATGACTGTTTCTCCTCCTTCA	25	T	0.2439	0.3688
NC_005101.4-55478561	CCTGAGATGACCCTTTCTCCTTCTGGT	24	T	0	0.4793
NC_005101.4-55478561	CCTGAGATGACCCTTTCTCCTTCTGGT	25	G	0.1301	0.1216
NC_005101.4-55535557	GCCTGGGCTACTCCTACTCCAAGGCTG	8	C	0.2124	0.3136
NC_005101.4-55535557	GCCTGGGCTACTCCTACTCCAAGGCTG	24	G	0.0877	0.0839
NC_005101.4-55629510	AAACTGAAGACCTTTGCTCCTAACCAC	27	C	0.1712	0.4869
NC_005101.4-55722674	GCTTGCTCCACATTGTCTCCGAACGGT	16	T	0	0.4856
NC_005101.4-55722674	GCTTGCTCCACATTGTCTCCGAACGGT	21	G	0.0085	0.487
NC_005101.4-55761430	CTCTTCAAAACCAGTGCTCCTGCCCCA	15	T	0.0081	0.481
NC_005101.4-55761430	CTCTTCAAAACCAGTGCTCCTGCCCCA	16	G	0.0325	0.032
NC_005101.4-55849492	ACTGCCTCCACCCTGTCTCCCTTCTCC	8	C	0.0083	0.4803
NC_005101.4-55849492	ACTGCCTCCACCCTGTCTCCCTTCTCC	24	C	0.1074	0.1447
NC_005101.4-55858949	GTAATTGAGACCCTGGCTCCCTCTATT	12	C	0.0625	0.125
NC_005101.4-55858949	GTAATTGAGACCCTGGCTCCCTCTATT	24	T	0.0442	0.06
NC_005101.4-55872821	TCCTTTTTCACACTTTCTCCACGCCAC	22	C	0	0.4839
NC_005101.4-55921219	ATTCTCTGTACTGCTCCTCCCTCTACA	25	A	0.1057	0.4952
NC_005101.4-55999281	ACCAACAGGACCACGCCTCCTAATTGT	1	A	0	0.4724
NC_005101.4-55999281	ACCAACAGGACCACGCCTCCTAATTGT	7	A	0.0261	0.4782
NC_005101.4-56023245	CATTTAATCACAATGTCTCCTGTGTTA	2	A	0.0678	0.4718
NC_005101.4-56095960	GGCCTTTGTACTCCATCTCCCCACCTC	7	T	0.1721	0.2457
NC_005101.4-56112790	TGTGAGTCAACAAACACTCCACAGTTC	8	C	0.25	0.4539
NC_005101.4-56118674	TTGGGTTGCACTGCAGCTCCTTGGATT	6	T	0.252	0.356
NC_005101.4-56139668	CCTCTGTGGACATCAGCTCCTGACTTC	23	A	0.0252	0.0249
NC_005101.4-56145333	AATGTCCAGACACCAGCTCCCTTCGTT	21	C	0.2846	0.3888
NC_005101.4-56145333	AATGTCCAGACACCAGCTCCCTTCGTT	24	C	0.3333	0.4301
NC_005101.4-56180197	CCTGTTGGCACCACATCTCCATATCTG	6	T	0	0.4887
NC_005101.4-56180197	CCTGTTGGCACCACATCTCCATATCTG	15	A	0.0357	0.1327
NC_005101.4-56181023	TTCCCTCATACCCCCTCTCCACCCTTG	15	C	0.1653	0.2869
NC_005101.4-56188252	GTCAGACAGACCTCTGCTCCTTTTCAC	2	T	0	0.4793
NC_005101.4-56200533	ACACCAGTAACTCCATCTCCAGAAGGT	2	C	0.1167	0.2888
NC_005101.4-56300956	ATGATGTGCACATGGACTCCAGAACTC	24	A	0.0083	0.4835
NC_005101.4-56329622	GACTTTCTCACTGTATCTCCCAGCCAA	2	A	0	0.4793
NC_005101.4-56329622	GACTTTCTCACTGTATCTCCCAGCCAA	24	C	0.0732	0.0705
NC_005101.4-56392429	CTGTTGCCAACACGCACTCCATCTTTA	13	C	0.1271	0.298
NC_005101.4-56392429	CTGTTGCCAACACGCACTCCATCTTTA	15	C	0	0.4826
NC_005101.4-56395802	GGTTCATCTACATCTGCTCCATTGTGT	7	T	0.0165	0.4819
NC_005101.4-56401812	GTCTGTAACACAGAGCCTCCCCTAGCT	7	A	0.1557	0.4992
NC_005101.4-56401812	GTCTGTAACACAGAGCCTCCCCTAGCT	22	C	0.1557	0.4992
NC_005101.4-56477178	GTCCCCCCCACCTCACCTCCATCCTCA	9	C	0	0.4807
NC_005101.4-56477178	GTCCCCCCCACCTCACCTCCATCCTCA	14	C	0.1885	0.4943
NC_005101.4-56500784	CTTCTTAGAACCCTTTCTCCTCAGATG	6	T	0.0085	0.4776
NC_005101.4-56549905	TCACATGTGACCATACCTCCTCAAAGG	4	C	0	0.4765
NC_005101.4-56549905	TCACATGTGACCATACCTCCTCAAAGG	13	A	0	0.4765
NC_005101.4-56595745	ACCCATGCTACCGCCCCTCCTTGCATT	9	T	0.0163	0.032
NC_005101.4-56595745	ACCCATGCTACCGCCCCTCCTTGCATT	12	C	0.0732	0.1287
NC_005101.4-56604694	GTAGATGCTACTGGTTCTCCATAATAT	8	C	0.1382	0.1562
NC_005101.4-56623967	CTACAGCCAACACTGGCTCCTGGCAAT	27	T	0.1304	0.4454
NC_005101.4-56666547	GGCATGCACACAGCCTCTCCAAAGAAG	2	G	0.1405	0.1447
NC_005101.4-56666547	GGCATGCACACAGCCTCTCCAAAGAAG	5	T	0.0165	0.4877

NC_005101.4-56686017	TGTCCTAGGACAGTAACTCCAGAGTCT	27	T	0.0407	0.0854
NC_005101.4-56699664	GGGCCCCTCACTTCCCCTCCTTGCGTC	23	G	0.0667	0.1099
NC_005101.4-56699664	GGGCCCCTCACTTCCCCTCCTTGCGTC	24	C	0.0083	0.4803
NC_005101.4-56741386	GCATCTGTAACAGTTTCTCCCTACCGC	22	T	0	0.4807
NC_005101.4-56742320	TCCTTGCCTACCTGACCTCCAAATACC	6	G	0.069	0.1134
NC_005101.4-56742320	TCCTTGCCTACCTGACCTCCAAATACC	27	C	0.0609	0.1067
NC_005101.4-56876665	TGAGTCCAGACTTCAACTCCTGATCCT	5	T	0.2479	0.4481
NC_005101.4-56893376	TTTGATACAACCTATCCTCCAGTCCTC	26	T	0.065	0.4944
NC_005101.4-56910214	ATGTATCTGACCCTGGCTCCAGAGACT	14	T	0.1074	0.4213
NC_005101.4-57003082	GGGTGGTACACATGCCCTCCTGACACT	6	G	0.0263	0.026
NC_005101.4-57048863	TACAATACAACTTCAACTCCTGAGACT	22	G	0.1092	0.491
NC_005101.4-57048863	TACAATACAACTTCAACTCCTGAGACT	23	A	0.0417	0.0408
NC_005101.4-57057666	TAGTGAGCAACCTTTCCTCCCAGACAC	22	A	0.1597	0.4797
NC_005101.4-57068979	GTGATGACCACACTAACTCCTCACTCT	24	C	0.2358	0.4301
NC_005101.4-57080081	AGCTGTGCAACAGCAGCTCCCAGTTCC	8	C	0.0331	0.0325
NC_005101.4-57129422	CCCAGTGACACAACACCTCCAAAGGCC	1	C	0.065	0.1073
NC_005101.4-57129422	CCCAGTGACACAACACCTCCAAAGGCC	14	C	0.0244	0.0553
NC_005101.4-57163764	TGAAGTGAGACTTGCCCTCCTACCCCT	9	G	0.0336	0.0805
NC_005101.4-57163764	TGAAGTGAGACTTGCCCTCCTACCCCT	24	C	0.1429	0.4797
NC_005101.4-57183813	AACCAACACACTGAAACTCCCAAAGCA	13	G	0	0.4851
NC_005101.4-57188714	GGTCTCCAGACTTCATCTCCCATCTTG	8	A	0	0.4813
NC_005101.4-57233833	CTTTCAGTTACCCGAGCTCCATTCAAT	14	G	0.0333	0.0328
NC_005101.4-57280512	GCGTGCTGGACAAGCCCTCCTATCCCT	15	C	0.2602	0.3334
NC_005101.4-57374358	ATGTGATAAACATATTCTCCATGCTTA	24	C	0.0885	0.1466
NC_005101.4-57409541	ATTAGAGCAACTGGAACTCCCATACAC	7	G	0.0252	0.0249
NC_005101.4-57430864	AAACTCTGAACCATCTCTCCAGCTGAT	27	T	0	0.4841
NC_005101.4-57468606	TAGTAATAAACTAGTTCTCCTCTTCCT	13	A	0	0.4757
NC_005101.4-57517593	CAGAGTGGCACTGAGCCTCCTCCTGCC	1	C	0.042	0.0411
NC_005101.4-57517593	CAGAGTGGCACTGAGCCTCCTCCTGCC	2	A	0.042	0.0411
NC_005101.4-57538418	CTTTAAAGCACATATACTCCCTACCTC	13	T	0.1186	0.4977
NC_005101.4-57538418	CTTTAAAGCACATATACTCCCTACCTC	15	T	0.1186	0.1691
NC_005101.4-57651249	CTAGCATCAACGCTGCCTCCTGAATTT	13	C	0	0.4819
NC_005101.4-57651249	CTAGCATCAACGCTGCCTCCTGAATTT	23	A	0.1	0.42
NC_005101.4-57691729	ACTGCCTCAACCTCGTCTCCATCCAGT	26	G	0.1379	0.2622
NC_005101.4-57705958	AATGGTATAACACGGACTCCTCGCTCA	13	C	0.2917	0.4358
NC_005101.4-57768787	TGATGGAGGACGGAGTCTCCTGGGTAC	6	G	0.0246	0.0243
NC_005101.4-57768787	TGATGGAGGACGGAGTCTCCTGGGTAC	12	G	0.1475	0.4966
NC_005101.4-57806633	CTTCATTCCACCGTTTCTCCCCATTCC	13	G	0.018	0.0695
NC_005101.4-57822905	AGCACTACCACGCCTTCTCCACCACGG	6	T	0.2195	0.2989
NC_005101.4-57822905	AGCACTACCACGCCTTCTCCACCACGG	12	G	0.0163	0.032
NC_005101.4-57897589	GGATTACAGACATGACCTCCACAGCGG	21	A	0.0081	0.4777
NC_005101.4-57937562	CCTCCCCCCACCTCTGCTCCTTACTAA	9	C	0.0625	0.0934
NC_005101.4-57940267	TTCTCCATCACCATGTCTCCTTGTGCA	16	T	0.0569	0.0854
NC_005101.4-57949519	CAGTTGGTTACACCCTCTCCCTCAATA	25	A	0	0.4682
NC_005101.4-58015746	TATTCTTACACATGGGCTCCTGTGGTT	23	T	0.0254	0.487
NC_005101.4-58015746	TATTCTTACACATGGGCTCCTGTGGTT	24	G	0.025	0.4875
NC_005101.4-58029187	GAGTGTGACACCTTGTCTCCTGCCTGC	25	T	0	0.4807
NC_005101.4-58086321	TGTGGGAAAACTTCCTCTCCTAATAAT	2	G	0.1417	0.2608
NC_005101.4-58086321	TGTGGGAAAACTTCCTCTCCTAATAAT	25	A	0.1583	0.2125
NC_005101.4-58170107	TGCAGTCGCACCGATACTCCTGCTGTT	12	C	0	0.4793
NC_005101.4-58239528	AGACACTTGACATTGGCTCCATACACA	13	T	0.2333	0.4765

NC_005101.4-58240341	CTGACTCGGACCTGTGCTCCTATGGAG	8	G	0.0431	0.0585
NC_005101.4-58244827	TATGAAAGGACAAGAGCTCCACAGGAA	2	A	0.041	0.0401
NC_005101.4-58244827	TATGAAAGGACAAGAGCTCCACAGGAA	16	G	0.157	0.4978
NC_005101.4-58372133	ACCCATAATACTTTAGCTCCAGGGAAT	4	C	0.0579	0.0562
NC_005101.4-58372133	ACCCATAATACTTTAGCTCCAGGGAAT	27	T	0	0.48
NC_005101.4-58432747	TTCTCAGAGACATGCGCTCCTTGGCCA	8	A	0.075	0.1025
NC_005101.4-59037408	ACAACTGCAACTGTAGCTCCAAAGGAT	5	C	0.0081	0.0241
NC_005101.4-59241310	TATAAATATACATATGCTCCATCAATG	24	A	0.0089	0.4751
NC_005101.4-59256378	ACCTCGCACACTCTAACTCCAACCACA	25	A	0.0531	0.0846
NC_005101.4-59349451	ATGAAAGAAACAAACTCTCCTTTAGTG	2	T	0.0085	0.4776
NC_005101.4-59349451	ATGAAAGAAACAAACTCTCCTTTAGTG	24	A	0.0917	0.0875
NC_005101.4-59451750	TCTCAACTAACTTGGACTCCAGAGATC	9	A	0.0177	0.4755
NC_005101.4-59451750	TCTCAACTAACTTGGACTCCAGAGATC	16	A	0.2035	0.359
NC_005101.4-59452122	TGCTGAATCACTGAAACTCCAGATCTG	7	A	0.0531	0.4859
NC_005101.4-59452122	TGCTGAATCACTGAAACTCCAGATCTG	8	T	0.0088	0.4774
NC_005101.4-59641387	CCAACCCAAACACTCACTCCCTCCACC	9	A	0.009	0.4766
NC_005101.4-59677369	GGAACCAACACTTCTCCTCCTAGGTAA	26	A	0.0574	0.071
NC_005101.4-59703024	ACCACACACACATCCTCTCCTGACTCC	23	A	0.0086	0.4803
NC_005101.4-59775676	CACTGCCAAACACCTACTCCCACCTTA	23	C	0.2018	0.4221
NC_005101.4-59816774	AACAAGCAGACACATTCTCCCACACAA	9	G	0.0081	0.4777
NC_005101.4-59816774	AACAAGCAGACACATTCTCCCACACAA	26	A	0.0407	0.0398
NC_005101.4-59817153	GTGTCTGTAACATTCTCTCCTTACAGT	13	T	0.2	0.4911
NC_005101.4-59817153	GTGTCTGTAACATTCTCTCCTTACAGT	15	C	0.0413	0.1017
NC_005101.4-59966515	ATCCACATGACTTGAGCTCCACAATTC	22	C	0.124	0.3687
NC_005101.4-59994816	TCCTCTCTAACCCTCCCTCCTCTTCTT	2	C	0.2069	0.2252
NC_005101.4-59994816	TCCTCTCTAACCCTCCCTCCTCTTCTT	12	C	0.2743	0.4843
NC_005101.4-60057410	CCCTCCTGCACCCCTTCTCCACCTCTG	15	T	0	0.4819
NC_005101.4-60057410	CCCTCCTGCACCCCTTCTCCACCTCTG	21	A	0	0.4807
NC_005101.4-60093711	GTTACCTGTACCCCTTCTCCAAACTAG	6	C	0.0261	0.0257
NC_005101.4-60093711	GTTACCTGTACCCCTTCTCCAAACTAG	24	C	0.0721	0.1182
NC_005101.4-60141560	TACCACCCAACCAAAGCTCCCAGGGAC	16	G	0.2155	0.4916
NC_005101.4-60141560	TACCACCCAACCAAAGCTCCCAGGGAC	24	G	0.1368	0.1274
NC_005101.4-60183155	TAACTTAAAACAAATGCTCCTCTGTAG	4	C	0.0496	0.0484
NC_005101.4-60183155	TAACTTAAAACAAATGCTCCTCTGTAG	25	T	0.0496	0.0484
NC_005101.4-60235320	TCCTTTCTCACTGTTCCTCCTTCAAAA	15	T	0.1417	0.1866
NC_005101.4-60282815	CCCTTGGCCACCTCACCTCCACCCAAC	4	T	0	0.4787
NC_005101.4-60286470	CCCGTGAAGACCAGCTCTCCCATTGAG	3	C	0.1282	0.5
NC_005101.4-60317741	AAACTTGAGACGATATCTCCTAAACCA	1	A	0	0.4772
NC_005101.4-60317741	AAACTTGAGACGATATCTCCTAAACCA	4	C	0	0.4772
NC_005101.4-60322997	AAGGGAAGAACGCTGACTCCTGAGAGT	12	G	0	0.4868
NC_005101.4-60368457	AACAATAGAACTTTCTCTCCCTACCTA	27	A	0.2377	0.2095
NC_005101.4-60381139	ACACCTGTCACTGGGCCTCCCAACACT	4	C	0	0.4807
NC_005101.4-60455969	TATCTCGGTACTCTTCCTCCTCCTGAG	15	T	0	0.4826
NC_005101.4-60500593	TTCATGTCCACTTTCTCTCCTTTCTCT	13	T	0	0.4654
NC_005101.4-60526612	CACTAAGGTACACAATCTCCTTTGCCC	7	G	0.0407	0.0398
NC_005101.4-60526612	CACTAAGGTACACAATCTCCTTTGCCC	14	A	0.0488	0.4595
NC_005101.4-60530145	CTAGAAAGCACGTTTTCTCCATGGTTG	12	G	0.2314	0.4877
NC_005101.4-60538340	TCTTGGCTAACCAGTCCTCCCCTTTCC	2	C	0.0268	0.0264
NC_005101.4-60548808	TACTTTAAAACACACACTCCCACACGC	25	C	0	0.4709
NC_005101.4-60577716	AAGCTGTCCACTTATCCTCCTCACAGC	5	T	0.0732	0.4572
NC_005101.4-60577716	AAGCTGTCCACTTATCCTCCTCACAGC	27	C	0	0.4793

NC_005101.4-60592449	GTGTGAGGCACCCTGGCTCCCTGTCCT	13	C	0.2149	0.4983
NC_005101.4-60597192	TACCAGGGAACCAGAGCTCCAGCCCCA	21	A	0.2627	0.329
NC_005101.4-60597778	AGTATCACAACCTGGGCTCCACGTTCC	14	G	0.0244	0.0398
NC_005101.4-60597778	AGTATCACAACCTGGGCTCCACGTTCC	23	G	0.0407	0.0398
NC_005101.4-60602989	GGCAATGCCACTCACGCTCCTGCAGTT	16	G	0.187	0.4988
NC_005101.4-60652303	ACTGTGATGACCTTCCCTCCTTCTGAG	4	G	0.1463	0.4206
NC_005101.4-60652303	ACTGTGATGACCTTCCCTCCTTCTGAG	16	C	0.1557	0.1839
NC_005101.4-60658551	CTCCCTTTTACAGGACCTCCCGACACT	23	A	0.1786	0.2188
NC_005101.4-60667140	AAACCTGCTACTCGTGCTCCGAGGAGC	12	T	0.0325	0.032
NC_005101.4-60682230	TTCATTCCCACCCGCGCTCCCTCGGCA	22	T	0	0.4827
NC_005101.4-60682230	TTCATTCCCACCCGCGCTCCCTCGGCA	23	C	0.1316	0.138
NC_005101.4-60721241	CCACAGAAGACATTTTCTCCCTTTCCT	2	C	0.1826	0.4998
NC_005101.4-60745499	ACACTAGAGACCAAACCTCCATCGCAT	24	G	0	0.4807
NC_005101.4-60767782	TCTATTGGCACATTCTCTCCCTCTCTC	13	T	0.1	0.1099
NC_005101.4-60767782	TCTATTGGCACATTCTCTCCCTCTCTC	14	T	0.0084	0.4682
NC_005101.4-60826100	TTCAAGGGAACTGACACTCCCTGAGCA	4	A	0.0763	0.1481
NC_005101.4-60826100	TTCAAGGGAACTGACACTCCCTGAGCA	27	A	0.0169	0.4757
NC_005101.4-60880561	TGGGCTATTACCCCATCTCCTTTATTG	6	T	0.0088	0.4735
NC_005101.4-60916124	CAAGACATAACTGGGACTCCATCTTTT	2	A	0	0.4832
NC_005101.4-60916124	CAAGACATAACTGGGACTCCATCTTTT	3	A	0.0413	0.0405
NC_005101.4-60948472	GCCACCTATACACAAGCTCCACCCATT	9	T	0.0088	0.4843
NC_005101.4-60948472	GCCACCTATACACAAGCTCCACCCATT	22	C	0	0.4807
NC_005101.4-60956117	AAAAGGCGAACCTCCCCTCCCCCGATG	1	A	0	0.488
NC_005101.4-60970286	CTGCCTCCCACTAGTTCTCCACATTCT	6	T	0.2269	0.4045
NC_005101.4-60981014	GATGATGCAACAGACCCTCCCTTACAG	5	A	0	0.4703
NC_005101.4-60981014	GATGATGCAACAGACCCTCCCTTACAG	23	T	0	0.4703
NC_005101.4-61075630	TAAGTGGCAACTGCTACTCCAGCGATT	23	C	0.0265	0.0262
NC_005101.4-61075630	TAAGTGGCAACTGCTACTCCAGCGATT	26	T	0.1947	0.4981
NC_005101.4-61110140	GTAAGAATCACATTCTCTCCACATAGG	2	T	0.0348	0.0342
NC_005101.4-61154206	CAGCAAAGCACACTTACTCCTTTTTGA	8	G	0.0259	0.0255
NC_005101.4-61154206	CAGCAAAGCACACTTACTCCTTTTTGA	25	T	0.2348	0.3451
NC_005101.4-61203715	TGAAAGAACACTAAAGCTCCCTCTCCC	8	A	0.025	0.0566
NC_005101.4-61203715	TGAAAGAACACTAAAGCTCCCTCTCCC	15	A	0.0084	0.4761
NC_005101.4-61247296	TTGGTCTCTACATCTGCTCCATCTGCT	7	T	0.2439	0.4973
NC_005101.4-61249072	ATATCATGCACCTCTTCTCCACTAAGC	7	T	0.0265	0.4693
NC_005101.4-61249072	ATATCATGCACCTCTTCTCCACTAAGC	8	G	0.0517	0.0504
NC_005101.4-61253415	TCTCCCTCCACCATATCTCCTTACTCC	9	C	0.0248	0.0562
NC_005101.4-61260103	TACCAATCAACCAGAGCTCCAGGGTCT	6	A	0.0763	0.0734
NC_005101.4-61343726	AAGTATGCTACCACACCTCCAGGCACC	22	G	0.0813	0.4904
NC_005101.4-61358131	CCCCCTTGTACTTGTCCTCCACCATTG	15	T	0	0.4793
NC_005101.4-61358131	CCCCCTTGTACTTGTCCTCCACCATTG	22	C	0.1301	0.1216
NC_005101.4-61358299	CACTATCATACTGGATCTCCTCACTGG	27	G	0.0763	0.0734
NC_005101.4-61406302	ATTCCAACCACCCGGGCTCCTCACAGA	26	G	0.0088	0.4599
NC_005101.4-61406302	ATTCCAACCACCCGGGCTCCTCACAGA	27	A	0.0089	0.4617
NC_005101.4-61452857	CATGCTGAGACCGTGTCTCCAAACAAG	27	G	0.0439	0.463
NC_005101.4-61453203	AAAATACTAACTTCATCTCCTGCAGTG	23	C	0.1967	0.2032
NC_005101.4-61459418	CCTCGCACAACCGAGGCTCCACAAACT	1	C	0.0085	0.4854
NC_005101.4-61459418	CCTCGCACAACCGAGGCTCCACAAACT	13	G	0.0085	0.4854
NC_005101.4-61463242	CTGTATGGTACAGTTCCTCCCCAGAAG	12	A	0.2066	0.2474
NC_005101.4-61463242	CTGTATGGTACAGTTCCTCCCCAGAAG	25	A	0.084	0.1678
NC_005101.4-61522455	AAACTATCAACCTTTGCTCCTTTAATT	26	T	0.1982	0.4909

NC_005101.4-61543144	TGCTTCTTAACCCTTTCTCCTGCCTTT	23	C	0	0.4864
NC_005101.4-61575800	AGTCATCTCACTGGCCCTCCACCATTT	2	G	0.0083	0.0405
NC_005101.4-61575800	AGTCATCTCACTGGCCCTCCACCATTT	7	C	0.2101	0.4542
NC_005101.4-61636113	AGCATGCGTACCCCAACTCCTCACTAT	24	C	0.25	0.3299
NC_005101.4-61661894	CATGGAATAACATCTGCTCCATCATTT	6	A	0.1148	0.4966
NC_005101.4-61661894	CATGGAATAACATCTGCTCCATCATTT	14	C	0	0.4773
NC_005101.4-61690986	ATCTTTATCACATCCACTCCATTACTC	14	C	0.018	0.4821
NC_005101.4-61715008	CACTCCACAACCACATCTCCCGTGTTA	9	A	0	0.4902
NC_005101.4-61715008	CACTCCACAACCACATCTCCCGTGTTA	26	T	0.0609	0.1659
NC_005101.4-61747164	GGCTCCCACACTGCTTCTCCTGATATC	23	A	0.2917	0.4358
NC_005101.4-61747164	GGCTCCCACACTGCTTCTCCTGATATC	24	T	0.2583	0.3047
NC_005101.4-61764754	CACAACCGCACTCACGCTCCTGAACCC	26	C	0.0083	0.0247
NC_005101.4-61806062	CCTTTCTCCACAACTGCTCCTGACTCT	1	C	0.3058	0.4454
NC_005101.4-61841882	CATGATTAGACAGTCCCTCCATCAGAG	7	T	0.2033	0.2441
NC_005101.4-61852151	ATGGGGTAAACTGCCCCTCCTTGTCCC	1	A	0.1239	0.1162
NC_005101.4-61852151	ATGGGGTAAACTGCCCCTCCTTGTCCC	2	T	0.1228	0.1153
NC_005101.4-61880669	CCCAAACCAACCTCTGCTCCACCAGAG	3	C	0.122	0.4999
NC_005101.4-61885237	CCGCTTCAAACTTCTTCTCCCTGTCAC	8	A	0.0672	0.4921
NC_005101.4-61885237	CCGCTTCAAACTTCTTCTCCCTGTCAC	9	A	0.0254	0.0415
NC_005101.4-61887319	TTGTGCTCCACAAGCTCTCCTCCTAGA	3	G	0.0165	0.0325
NC_005101.4-61887319	TTGTGCTCCACAAGCTCTCCTCCTAGA	14	G	0.0165	0.0325
NC_005101.4-61888305	AAAAAGAGAACCAGTCCTCCCTCACCA	3	A	0.0588	0.0571
NC_005101.4-61898490	CACAATGAAACTGATGCTCCCTCTGCA	27	A	0.3036	0.375
NC_005101.4-61907084	AGGGCTACCACACTCTCTCCAATGTCT	7	A	0.0328	0.0322
NC_005101.4-61907084	AGGGCTACCACACTCTCTCCAATGTCT	23	T	0.0246	0.0243
NC_005101.4-61981968	TTCCTTGGCACGGTCTCTCCTCCACAA	12	G	0.1017	0.4367
NC_005101.4-62004414	ATAAGAGGCACAGGGTCTCCTTCTTGC	7	G	0.0348	0.0832
NC_005101.4-62006805	AAATCCAGGACACTCGCTCCTCGTGAT	16	G	0.2623	0.3947
NC_005101.4-62006805	AAATCCAGGACACTCGCTCCTCGTGAT	23	G	0.1475	0.4999
NC_005101.4-62038380	CTCCTAGCAACTTAGACTCCCAGCTAT	23	G	0.188	0.2604
NC_005101.4-62051347	GAGAGACACACGATACCTCCCTCCCAG	14	T	0	0.4715
NC_005101.4-62066662	TCATGTCCAACCTCTACTCCTCCCCTC	14	C	0.2655	0.3346
NC_005101.4-62086903	ATCAACAAAACTCTATCTCCCTCTCAG	15	A	0.1463	0.1629
NC_005101.4-62093581	CACCTCCTCACCTAACCTCCATTCCAC	8	T	0.2696	0.3547
NC_005101.4-62107097	CTGGCTCCTACGATCCCTCCTCTCCCT	9	T	0.0667	0.0799
NC_005101.4-62107097	CTGGCTCCTACGATCCCTCCTCTCCCT	13	A	0.2941	0.4253
NC_005101.4-62107511	ACCACAGACACTACTGCTCCCCAGAAA	15	T	0.0179	0.4943
NC_005101.4-62107511	ACCACAGACACTACTGCTCCCCAGAAA	16	G	0.036	0.1338
NC_005101.4-62126417	ATAGGTCCCACAGCCCCTCCTGGAGTT	21	T	0.0259	0.4893
NC_005101.4-62126417	ATAGGTCCCACAGCCCCTCCTGGAGTT	22	G	0.2564	0.3974
NC_005101.4-62149538	TACTAATTGACTTTCCCTCCACCTGTC	1	T	0.0083	0.0245
NC_005101.4-62149538	TACTAATTGACTTTCCCTCCACCTGTC	9	G	0.042	0.0411
NC_005101.4-62164024	TTTCCATGTACCATCCCTCCTTGCATT	12	C	0.2167	0.1932
NC_005101.4-62193361	CCATTAGTAACTCTACCTCCAAGAGAT	1	C	0.2735	0.355
NC_005101.4-62200381	TTCTCTCTCACTGCAGCTCCCGATGCT	21	C	0.0085	0.4626
NC_005101.4-62219519	CTGGCTTCAACTTTCTCTCCCTCTTCT	6	T	0	0.4787
NC_005101.4-62232624	ATTGAGGCGACAAGATCTCCTATGCTC	8	C	0.0333	0.0328
NC_005101.4-62303101	ATAGCTGTAACCTTGCCTCCTAACCAG	27	G	0.0847	0.0812
NC_005101.4-62334782	ACCACCATAACTTCAGCTCCATGGGCA	23	G	0.0407	0.0705
NC_005101.4-62357622	GCTCTTGCAACATGCCCTCCCACCAAA	22	A	0.0172	0.4851
NC_005101.4-62369536	CTAGTCCCTACATTTCCTCCCTCATTT	2	T	0	0.4751

NC_005101.4-62404620	TGGTTACCAACTTGGGCTCCCTCCTGT	15	G	0.193	0.2893
NC_005101.4-62424027	TATCAACTAACTTGCACTCCGCGTGGT	6	A	0.3305	0.4776
NC_005101.4-62435496	GAGCTGGAGACACCTGCTCCTTGGGGG	3	G	0.3097	0.4373
NC_005101.4-62435496	GAGCTGGAGACACCTGCTCCTTGGGGG	13	C	0.287	0.3353
NC_005101.4-62449724	CACACTCACACACAAACTCCTACAGAC	15	A	0.0083	0.4783
NC_005101.4-62464593	AGCCCTGGGACACTCACTCCAGTAATT	3	C	0.0526	0.0512
NC_005101.4-62476967	CACACGGAGACAAGTTCTCCATTATTG	22	T	0.0732	0.1425
NC_005101.4-62485407	TGGCCTCACACAGATTCTCCTAGTTAG	27	G	0.0714	0.0689
NC_005101.4-62494585	CTAATTCAGACCTCGACTCCTGGATTT	15	G	0.1197	0.1274
NC_005101.4-62494585	CTAATTCAGACCTCGACTCCTGGATTT	21	T	0.0256	0.0418
NC_005101.4-62517695	TGCACAGAGACAGCATCTCCACTACCA	22	C	0.225	0.3347
NC_005101.4-62548076	TACATAGCAACAGTGTCTCCAAAAGGA	1	T	0.1322	0.4997
NC_005101.4-62567267	GGAGTTAGAACTGTGGCTCCAATGTAA	1	G	0.124	0.259
NC_005101.4-62585298	AAATCTGCAACTGTCTCTCCCTGACCC	22	T	0	0.4813
NC_005101.4-62585298	AAATCTGCAACTGTCTCTCCCTGACCC	26	C	0.1074	0.1585
NC_005101.4-62592694	GAAAAGGCTACTGCATCTCCACTCTTA	6	G	0.1789	0.3604
NC_005101.4-62602604	GAACCGCACACTCAAGCTCCTCTTGTG	5	C	0.0164	0.4773
NC_005101.4-62604921	TCTTCCCATACACATCCTCCTTTAACT	6	C	0.2033	0.481
NC_005101.4-62610264	AAACGTTAAACACACGCTCCTGAAGGT	14	A	0.2478	0.3728
NC_005101.4-62674389	CACACACACACACACGCTCCCCTTAAT	15	C	0.0413	0.0562
NC_005101.4-62680656	AAATTATAAACCCTTCCTCCCAGGGTG	14	T	0.0333	0.0328
NC_005101.4-62702521	CCTCTCACCACTGCTGCTCCTAGGGTC	7	A	0.2193	0.375
NC_005101.4-62702521	CCTCTCACCACTGCTGCTCCTAGGGTC	23	G	0.2281	0.4114
NC_005101.4-62727928	ATGGAATTCACAGCCACTCCGCGGCAC	22	C	0.1345	0.1539
NC_005101.4-62727928	ATGGAATTCACAGCCACTCCGCGGCAC	23	G	0.0336	0.033
NC_005101.4-62753281	AACAGATGTACTGTCACTCCTCCTGGG	3	C	0.0614	0.0595
NC_005101.4-62753281	AACAGATGTACTGTCACTCCTCCTGGG	9	T	0.1053	0.1305
NC_005101.4-62815891	GACCACCCCACACCAGCTCCTCCCTCT	26	C	0.0984	0.1225
NC_005101.4-62832282	TGTGGAGAAACCAGGTCTCCGGGATGG	25	T	0.0526	0.4986
NC_005101.4-62832282	TGTGGAGAAACCAGGTCTCCGGGATGG	26	G	0.0526	0.4986
NC_005101.4-62855649	TTTCTTGGGACAGGGACTCCTCTGTCC	16	A	0.2124	0.4404
NC_005101.4-62855649	TTTCTTGGGACAGGGACTCCTCTGTCC	24	G	0.1565	0.1869
NC_005101.4-62894651	CCAGACGGCACGCAGCCTCCGTGGTGG	14	A	0.0336	0.033
NC_005101.4-63005448	CTGCTTCTCACTGGTTCTCCTGGGATC	22	G	0.0082	0.479
NC_005101.4-63029948	CACTGAAGGACAACATCTCCTTGGGTG	14	C	0.3025	0.4615
NC_005101.4-63029948	CACTGAAGGACAACATCTCCTTGGGTG	15	A	0	0.4718
NC_005101.4-63110034	GTGTCTGTCACCATTTCTCCTTAGACA	5	C	0.0259	0.0585
NC_005101.4-63121807	CCTTTATCTACCTCTCCTCCATCACCT	12	C	0.2727	0.4339
NC_005101.4-63121807	CCTTTATCTACCTCTCCTCCATCACCT	25	C	0.0248	0.0245
NC_005101.4-63136572	TGGTCGTGAACTCACCCTCCGATACTT	27	T	0	0.4819
NC_005101.4-63146837	CTTACCAAGACTCTATCTCCTCACACG	26	C	0.2083	0.2491
NC_005101.4-63193624	GAATCCCCAACTGTGCCTCCTTTCTAG	24	C	0.0732	0.1287
NC_005101.4-63201895	GATGCAGAAACCAAGACTCCACAGTTC	6	A	0.1157	0.4997
NC_005101.4-63219525	GTATCTGCCACAGTATCTCCCTCCTTC	3	A	0	0.4704
NC_005101.4-63219525	GTATCTGCCACAGTATCTCCCTCCTTC	12	A	0.2883	0.3285
NC_005101.4-63240379	TACCAAGGCACACTGGCTCCTGCTTGT	3	C	0.0088	0.4735
NC_005101.4-63240379	TACCAAGGCACACTGGCTCCTGCTTGT	5	A	0.2035	0.2104
NC_005101.4-63278292	AGAATGTGGACGATTTCTCCTTCTGTG	12	G	0.0333	0.0328
NC_005101.4-63298515	CCTTATGTAACTTTTCCTCCATCTATT	14	T	0.1491	0.2088
NC_005101.4-63373782	AGCAAAGCTACTAAAGCTCCACACTAT	3	C	0.2655	0.3982
NC_005101.4-63491803	CAACCCTATACCTAGGCTCCACTCAGT	5	C	0.0625	0.0934

NC_005101.4-63491803	CAACCCTATACCTAGGCTCCACTCAGT	9	T	0.1429	0.2934
NC_005101.4-63507483	AGTCCTCCCACACTTTCTCCACAAACA	15	T	0.2393	0.497
NC_005101.4-63507483	AGTCCTCCCACACTTTCTCCACAAACA	23	A	0.1017	0.1409
NC_005101.4-63522133	TCCTCAACTACTCTTCCTCCGTTTCGA	27	A	0.1849	0.4921
NC_005101.4-63522371	GTCAGACTTACACAGGCTCCCTGATAG	12	A	0.2034	0.3953
NC_005101.4-63522900	CCTCCGAGAACACATCCTCCTTTGTTG	5	C	0.1626	0.2832
NC_005101.4-63522900	CCTCCGAGAACACATCCTCCTTTGTTG	16	C	0.065	0.0928
NC_005101.4-63536439	GGATCCTAAACTCTAACTCCATTCACT	22	T	0.1696	0.4933
NC_005101.4-63613278	GGGCCTTTCACTGGACCTCCTTTCCCT	5	C	0.287	0.2458
NC_005101.4-63743739	TGTTCTAGTACCATGCCTCCCTGCCTG	21	C	0.0407	0.0705
NC_005101.4-63753663	AATGCCTGAACTGTGTCTCCTTTATGA	2	A	0.0973	0.0926
NC_005101.4-63815304	TAGACTCTAACCTGTTCTCCGCGCACG	23	G	0.0164	0.0786
NC_005101.4-63875133	GCCTGTCACACTCTGCCTCCTGTTATA	8	A	0.1157	0.1235
NC_005101.4-63875133	GCCTGTCACACTCTGCCTCCTGTTATA	9	C	0.1157	0.1235
NC_005101.4-63898680	ATTTGTCTCACATTGGCTCCTGCTATC	16	G	0.05	0.0488
NC_005101.4-64043833	TGACACTTGACTCACCCTCCATTAATA	12	T	0.2321	0.4871
NC_005101.4-64387143	AGTAGGTAGACGAGGCCTCCAGTTGAG	27	G	0.0345	0.0339
NC_005101.4-64475674	AAGCAAGTCACTTAACCTCCCGGTGAC	22	G	0.0256	0.0253
NC_005101.4-64524177	AACCAACCCACTGATTCTCCTTGGTTT	14	A	0.087	0.2005
NC_005101.4-64532895	ACATTCTATACCATCACTCCTACATAA	2	C	0.045	0.044
NC_005101.4-64608809	AGGTCACCTACATTGACTCCTGGGAGA	23	G	0.041	0.0401
NC_005101.4-64672236	TTGCAAGGAACCCTGGCTCCTTATCAA	21	T	0	0.4793
NC_005101.4-64709945	AGACTAACAACTGTAGCTCCCTCTTCT	9	A	0.0348	0.0832
NC_005101.4-64713295	GTATCCCCAACTGGGGCTCCATTTGGA	1	G	0.1754	0.16
NC_005101.4-64814312	TCCATGATCACTGCTACTCCTACCATT	24	C	0.1736	0.4933
NC_005101.4-64929931	TACTTCTCCACTTAGTCTCCCAGCCTG	16	T	0	0.4807
NC_005101.4-64997031	GCCTCTTCTACATTTTCTCCTTTTGTA	5	C	0.0991	0.0942
NC_005101.4-65053407	CTAGCTGATACATATGCTCCTGACACA	15	T	0.0442	0.4975
NC_005101.4-65129412	CATGTGGTCACCTGACCTCCACTGCCT	22	C	0.0855	0.0818
NC_005101.4-65240429	CACAAGGACACATGCTCTCCACTATGT	23	T	0.0342	0.0336
NC_005101.4-65240429	CACAAGGACACATGCTCTCCACTATGT	27	T	0.2435	0.4969
NC_005101.4-65397104	CTGCATTCCACACCTTCTCCCACCCCA	25	C	0.0826	0.0943
NC_005101.4-65438360	ATCACCCTTACCCCATCTCCAAGAGGA	23	G	0.1083	0.1025
NC_005101.4-65438765	GGTCCTGTCACTGGAACTCCTTTCCCT	12	T	0.0708	0.1006
NC_005101.4-65456616	TGCCAACCAACCAGAGCTCCAAGTGAC	4	C	0.1826	0.2332
NC_005101.4-65515401	TTGTACGAAACCATAGCTCCAGCTGCA	6	C	0.3947	0.4289
NC_005101.4-65515401	TTGTACGAAACCATAGCTCCAGCTGCA	8	A	0.1062	0.1898
NC_005101.4-65679165	TACCTTTTCACTCCAACTCCATGCTCT	2	A	0	0.4841
NC_005101.4-65679165	TACCTTTTCACTCCAACTCCATGCTCT	23	G	0	0.4841
NC_005101.4-65684226	GGACCAAGAACCTCCCCTCCATTGATG	1	G	0.2946	0.2637
NC_005101.4-65684226	GGACCAAGAACCTCCCCTCCATTGATG	16	C	0.3009	0.2678
NC_005101.4-65695998	CCCACATGCACACCTACTCCTTACAAT	8	G	0.1368	0.4123
NC_005101.4-65695998	CCCACATGCACACCTACTCCTTACAAT	27	T	0.0427	0.0418
NC_005101.4-65865608	AGTGGGGCAACTGTACCTCCCTTGATT	7	G	0.1739	0.4969
NC_005101.4-65935972	TCCCCTCCCACCAGGCCTCCCTATTCC	6	T	0.0756	0.491
NC_005101.4-65935972	TCCCCTCCCACCAGGCCTCCCTATTCC	7	C	0.0763	0.4896
NC_005101.4-66024667	CAAAATGGTACTTTGTCTCCTACCAAT	1	C	0.1607	0.3565
NC_005101.4-66024667	CAAAATGGTACTTTGTCTCCTACCAAT	2	A	0.1607	0.3565
NC_005101.4-66086795	CATTCTATGACTTCCTCTCCTCCATAT	24	A	0.0246	0.4717
NC_005101.4-66437665	TATCAACCAACCAGAGCTCCTAGGGAG	4	C	0.2869	0.2457
NC_005101.4-66437665	TATCAACCAACCAGAGCTCCTAGGGAG	7	C	0.0574	0.071

NC_005101.4-66502253	ACCATGATCACTGCAACTCCTATGAAG	23	T	0.4298	0.4221
NC_005101.4-66696881	AGTCAACCCACCAACTCTCCTTCACCC	24	A	0.0714	0.2698
NC_005101.4-66761418	CTCCTATCCACTTAGGCTCCATAGATT	6	A	0.0085	0.4842
NC_005101.4-66761418	CTCCTATCCACTTAGGCTCCATAGATT	7	T	0.0085	0.4842
NC_005101.4-67077325	TCTAATTGGACCATTACTCCATGTCTC	3	T	0	0.4751
NC_005101.4-67077325	TCTAATTGGACCATTACTCCATGTCTC	13	A	0.0583	0.0566
NC_005101.4-67090893	ATTGCTTTAACTTTCCCTCCATTTAAT	6	T	0.0614	0.0918
NC_005101.4-67160583	ACAAGGCCTACATATTCTCCTTCATAT	22	T	0	0.4813
NC_005101.4-67240390	TCTAACAAGACTACATCTCCTAATAGT	4	A	0.0268	0.0436
NC_005101.4-67240390	TCTAACAAGACTACATCTCCTAATAGT	24	T	0.0982	0.1553
NC_005101.4-67279569	TGCCAACCAACCAGGGCTCCCAGTGAC	2	G	0.6134	0.4253
NC_005101.4-67344572	TCTTATCCTACCAGTTCTCCGCACTCC	21	G	0.0413	0.0405
NC_005101.4-67347988	GGTGTCAGCACAGTTTCTCCATTTTCA	1	G	0.0948	0.0903
NC_005101.4-67347988	GGTGTCAGCACAGTTTCTCCATTTTCA	3	T	0.0948	0.0903
NC_005101.4-67448091	TTGAGATCCACAGTGCCTCCAGTATTC	12	A	0.041	0.4879
NC_005101.4-67448091	TTGAGATCCACAGTGCCTCCAGTATTC	16	C	0.0492	0.048
NC_005101.4-67506261	TTGTGTTCTACATTTGCTCCATTTGTT	4	T	0.1034	0.4946
NC_005101.4-67506261	TTGTGTTCTACATTTGCTCCATTTGTT	5	G	0.1034	0.4946
NC_005101.4-67667371	CTCACCAACACATGCCCTCCAGCCCCA	16	C	0	0.4793
NC_005101.4-67751253	ACCAATCTTACCCTCACTCCATTTTAT	5	A	0.2101	0.2266
NC_005101.4-67751253	ACCAATCTTACCCTCACTCCATTTTAT	24	T	0.2564	0.4956
NC_005101.4-67811318	TACTGACACACTTTTCCTCCTTTGAGT	2	A	0.0168	0.4743
NC_005101.4-67811318	TACTGACACACTTTTCCTCCTTTGAGT	3	C	0.3	0.4728
NC_005101.4-67812480	AAAGCTTTCACCTGGTCTCCAAATAAA	4	G	0.0598	0.058
NC_005101.4-67859448	AGAAGTGTCACTCTTTCTCCACATCCT	7	G	0.4035	0.4114
NC_005101.4-67859448	AGAAGTGTCACTCTTTCTCCACATCCT	26	C	0.3652	0.2985
NC_005101.4-68007030	TCTAAATCGACTCTGTCTCCTCCCATA	9	G	0.0982	0.1093
NC_005101.4-68210651	TTTCATCCCACTGTCCCTCCCCTTCTG	1	T	0	0.4891
NC_005101.4-68301289	AATAAGAGCACTAGGCCTCCATGCACT	27	T	0.0508	0.1264
NC_005101.4-68318039	AAGCCCATGACATATTCTCCTACTGAC	15	T	0	0.4786
NC_005101.4-68449969	CTCTCCCTTACTCTCTCTCCTCCACTT	21	T	0.0086	0.4768
NC_005101.4-68586369	ACCTTCATAACCAAAGCTCCATGGTCT	7	A	0.0684	0.4956
NC_005101.4-68586369	ACCTTCATAACCAAAGCTCCATGGTCT	21	A	0.2845	0.4375
NC_005101.4-68589827	GTGAGTAAAACAAATCCTCCATGAATA	7	A	0.0982	0.125
NC_005101.4-68597322	CATCAACCTACCCACCCTCCTCTCCCA	13	C	0.0496	0.0792
NC_005101.4-68597322	CATCAACCTACCCACCCTCCTCTCCCA	23	T	0.0248	0.0245
NC_005101.4-68648201	CGAGAGGGTACCTCAACTCCTACCTCT	2	G	0.0085	0.0418
NC_005101.4-68648201	CGAGAGGGTACCTCAACTCCTACCTCT	27	T	0	0.4807
NC_005101.4-68685977	ATAATCACAACTTTTTCTCCTGCTTTC	15	T	0.0619	0.4873
NC_005101.4-68699778	CCAAGATGAACTCTCACTCCTAACTAT	4	A	0.0331	0.0325
NC_005101.4-68763488	GTTGAGTCTACCTGAACTCCCTCTTTA	22	T	0.0179	0.4898
NC_005101.4-68763488	GTTGAGTCTACCTGAACTCCCTCTTTA	23	C	0.0179	0.4898
NC_005101.4-69057996	CTGCTCCAAACTTTGACTCCTTATTTC	6	C	0.2149	0.2759
NC_005101.4-69099270	TTCAATAATACCCAACCTCCTTTGTAC	25	T	0	0.4764
NC_005101.4-69199384	AATGGGGAGACTCTCGCTCCAGTCTCA	27	A	0.0603	0.0746
NC_005101.4-69256851	TAATAGCCAACTCGTCCTCCATTGTGT	22	T	0.1858	0.3396
NC_005101.4-69264990	CCCACTCCTACCTCAACTCCATCACCA	6	T	0.0513	0.0818
NC_005101.4-69264990	CCCACTCCTACCTCAACTCCATCACCA	8	C	0.1864	0.1961
NC_005101.4-69277285	GGTTGTCTCACCTGTGCTCCCAGGCAT	5	G	0.0268	0.0605
NC_005101.4-69288046	CTGACTTATACCACAGCTCCAAGTACC	21	A	0.0789	0.1814
NC_005101.4-69301390	TCAAACATCACATGTCCTCCCACTGGT	1	T	0.359	0.3054

NC_005101.4-69301390	TCAAACATCACATGTCCTCCCACTGGT	13	T	0.359	0.3054
NC_005101.4-69390581	AACACCCAGACACCTTCTCCTTCAGAT	4	A	0.0776	0.4916
NC_005101.4-69452772	ATTTCAGGTACAGCATCTCCAAGCTAA	1	A	0.157	0.2234
NC_005101.4-69452772	ATTTCAGGTACAGCATCTCCAAGCTAA	8	G	0.0083	0.4803
NC_005101.4-69487325	CACAATGACACACTTTCTCCAAAGAGT	9	C	0.0833	0.095
NC_005101.4-69512914	TCTGTGAAAACCCTGACTCCCAACATT	3	T	0.2456	0.4555
NC_005101.4-69512914	TCTGTGAAAACCCTGACTCCCAACATT	21	C	0.2456	0.4555
NC_005101.4-69661728	AACTCCCCAACCATTTCTCCTCCCCAT	8	C	0.2295	0.4656
NC_005101.4-69661728	AACTCCCCAACCATTTCTCCTCCCCAT	25	C	0.1066	0.2095
NC_005101.4-69729572	AGACAAAGAACTGCAACTCCTCAAGCA	9	A	0.1626	0.2382
NC_005101.4-69730357	AGAACCACTACATCTCCTCCCGCAGAG	9	T	0	0.4859
NC_005101.4-69820288	CTATGGTAAACTGTAGCTCCAAGAGCA	1	C	0.2105	0.2893
NC_005101.4-69900395	TGTCCTCCCACCTGTCCTCCTCTCTGG	9	C	0.2	0.4861
NC_005101.4-69907663	TAAGTATTTACAGATGCTCCCATCAGG	23	T	0.1913	0.4682
NC_005101.4-69958754	CACAGTGGTACAGGTACTCCAACAAGA	26	G	0	0.0339
NC_005101.4-69997836	CCAGGCTGTACTTGCCCTCCACAAATC	27	C	0.1405	0.4999
NC_005101.4-70015617	AATTGGGGCACACCTTCTCCTGGAGAC	7	G	0.1062	0.1006
NC_005101.4-70050530	AGCTCAGTAACTCCTTCTCCAAGGATG	2	G	0.1913	0.3772
NC_005101.4-70050530	AGCTCAGTAACTCCTTCTCCAAGGATG	16	T	0	0.474
NC_005101.4-70142855	TATATTTCCACTTGTTCTCCTGGCATC	14	G	0.0885	0.0846
NC_005101.4-70142855	TATATTTCCACTTGTTCTCCTGGCATC	23	G	0.0885	0.0846
NC_005101.4-70245842	TCAGTTCCTACTAAGACTCCCTTACAG	14	A	0.0536	0.0521
NC_005101.4-70301009	AGGAAAGAAACCCTTCCTCCATACTGA	14	T	0.2018	0.2476
NC_005101.4-70301009	AGGAAAGAAACCCTTCCTCCATACTGA	21	A	0.0965	0.3661
NC_005101.4-70345703	TCCACTGCAACTGGGGCTCCAACCTTG	4	A	0.0171	0.4839
NC_005101.4-70345703	TCCACTGCAACTGGGGCTCCAACCTTG	5	C	0.0345	0.0504
NC_005101.4-70424726	CGAGACCCCACCAGAGCTCCGAGAGAC	14	G	0.0248	0.0245
NC_005101.4-70832137	ACAAAGTCTACCTCTTCTCCATCCTCC	22	T	0.0804	0.1093
NC_005101.4-70912032	ACCATAAATACATGTGCTCCACTATGT	13	T	0.2743	0.3772
NC_005101.4-70912032	ACCATAAATACATGTGCTCCACTATGT	22	C	0.2566	0.3941
NC_005101.4-71179498	CAAAATGTCACTGGACCTCCTTACATC	7	G	0.122	0.1425
NC_005101.4-71340905	GATATTTAAACAGGCTCTCCAGTGGTA	5	T	0	0.4844
NC_005101.4-71464173	GGTATTACCACCTGTGCTCCAAGCAAG	4	A	0.0088	0.4796
NC_005101.4-71464173	GGTATTACCACCTGTGCTCCAAGCAAG	12	C	0.3246	0.463
NC_005101.4-71488802	TCCCTGTTAACCTCAGCTCCTCTGTTC	8	T	0	0.4757
NC_005101.4-71488802	TCCCTGTTAACCTCAGCTCCTCTGTTC	22	C	0.0083	0.0247
NC_005101.4-71492108	AAGCCACCCACATAGACTCCTGGGATT	8	C	0.0083	0.0247
NC_005101.4-71492108	AAGCCACCCACATAGACTCCTGGGATT	14	A	0.0083	0.0247
NC_005101.4-71557435	AACCCTTCCACCTTTCCTCCCTCTTTG	12	C	0.0259	0.0585
NC_005101.4-71557435	AACCCTTCCACCTTTCCTCCCTCTTTG	13	T	0.1197	0.1274
NC_005101.4-71571445	TATGTCATGACTATATCTCCATGCCAA	2	A	0.0744	0.0868
NC_005101.4-71571445	TATGTCATGACTATATCTCCATGCCAA	27	A	0.0085	0.4655
NC_005101.4-71743751	AACAATAAGACCATGCCTCCCCAACCA	15	G	0.0435	0.0425
NC_005101.4-71806392	TGATTACCTACCGTGTCTCCCTCCTTG	8	C	0.065	0.1216
NC_005101.4-71806392	TGATTACCTACCGTGTCTCCCTCCTTG	22	T	0.1545	0.208
NC_005101.4-71819672	CTCCACATCACAGCTACTCCTCCATTT	4	C	0.2101	0.2626
NC_005101.4-71819672	CTCCACATCACAGCTACTCCTCCATTT	25	T	0.0504	0.0958
NC_005101.4-71852309	GACTGCAGGACATGATCTCCTAGGACT	15	A	0	0.4728
NC_005101.4-71883765	TGTCCCTCCACCCCTGCTCCTTCTAAG	12	C	0.2083	0.4472
NC_005101.4-72005930	AGGAGACCCACAGCCTCTCCGCATGCT	21	G	0.065	0.078
NC_005101.4-72307341	GTTCCTGCAACCTTAACTCCCACCCTC	21	C	0.0259	0.0422

NC_005101.4-72384511	ATAAGAATAACTGCTGCTCCGTGAGCA	21	G	0.124	0.2474
NC_005101.4-72384511	ATAAGAATAACTGCTGCTCCGTGAGCA	26	C	0	0.4765
NC_005101.4-72435881	ACATTCCATACCCCCTCTCCTATGTCT	3	A	0.0407	0.0705
NC_005101.4-72455045	GTCAGCTCAACTTCCTCTCCCACAAGA	13	T	0.1391	0.2395
NC_005101.4-72455045	GTCAGCTCAACTTCCTCTCCCACAAGA	14	C	0.1391	0.2395
NC_005101.4-72465894	CACACACACACACACACTCCTAGATAC	12	A	0.0261	0.0257
NC_005101.4-72575543	CCAATCTCCACTCACTCTCCAACCTAG	3	A	0	0.4807
NC_005101.4-72822926	CATACTGCTACTCTGACTCCATCAACT	5	C	0	0.4807
NC_005101.4-72822926	CATACTGCTACTCTGACTCCATCAACT	15	G	0.1475	0.306
NC_005101.4-72964054	GTGATCACCACACTCGCTCCTTGTTTC	5	T	0	0.4832
NC_005101.4-72964054	GTGATCACCACACTCGCTCCTTGTTTC	16	G	0	0.4832
NC_005101.4-72978980	TCCTCTGCCACTGCACCTCCCTAAAAC	24	A	0	0.4787
NC_005101.4-72988017	AATGCTAGAACTTGAACTCCTTTCTCA	26	C	0.0813	0.3688
NC_005101.4-72992094	GCTCTCTTCACCTTCACTCCTCATCAC	1	G	0.0084	0.4682
NC_005101.4-72992094	GCTCTCTTCACCTTCACTCCTCATCAC	6	C	0.2185	0.3599
NC_005101.4-72998403	TCTGTTAGCACTTCAACTCCTTGGGAA	24	G	0.0325	0.0476
NC_005101.4-73016922	TTCAAACTTACTATCTCTCCTGCTTTT	9	T	0	0.4841
NC_005101.4-73016922	TTCAAACTTACTATCTCTCCTGCTTTT	26	T	0.2321	0.4807
NC_005101.4-73222803	AACATGCACACGTCCCCTCCTATGGGA	14	C	0.0086	0.0255
NC_005101.4-73240417	TTGCAATCCACTTCAGCTCCTTCATTC	1	T	0.0263	0.026
NC_005101.4-73268414	AATAAAGCCACACCCACTCCAACAATC	4	A	0	0.4793
NC_005101.4-73328056	TATCAATTTACTTGAACTCCAAAGCAT	13	T	0.125	0.1327
NC_005101.4-73401963	TCACAATACACTGCACCTCCAGCTCCA	6	A	0.1261	0.4994
NC_005101.4-73405630	AGTAGTGTTACACTTGCTCCACAACTA	25	C	0.0769	0.12
NC_005101.4-73635139	CTTCTTCCTACCCACCCTCCCTGTGTG	23	G	0	0.4751
NC_005101.4-73635139	CTTCTTCCTACCCACCCTCCCTGTGTG	26	T	0.082	0.0935
NC_005101.4-73662705	CAGTTGCCTACCTTCTCTCCTTCTTCT	7	C	0.082	0.0786
NC_005101.4-73664510	TCACCTTACACAGTGCCTCCAAACCCT	12	A	0.0163	0.0476
NC_005101.4-73664510	TCACCTTACACAGTGCCTCCAAACCCT	13	G	0.1967	0.4773
NC_005101.4-73745309	GCTCTCTGCACGTGATCTCCCTGTCCT	1	G	0	0.4606
NC_005101.4-73745309	GCTCTCTGCACGTGATCTCCCTGTCCT	14	G	0.087	0.0989
NC_005101.4-73747145	TGAGCATTCACACCCACTCCTTTATGT	4	G	0.0893	0.0853
NC_005101.4-73747145	TGAGCATTCACACCCACTCCTTTATGT	13	C	0.0088	0.4693
NC_005101.4-73804122	TTTCACAGAACTGAGGCTCCAGAAAAT	22	G	0.2414	0.3179
NC_005101.4-73804122	TTTCACAGAACTGAGGCTCCAGAAAAT	23	A	0.2414	0.3179
NC_005101.4-74040990	CATATACATACTCAATCTCCCTCTCTT	7	C	0	0.4833
NC_005101.4-74040990	CATATACATACTCAATCTCCCTCTCTT	22	T	0	0.4814
NC_005101.4-74421259	ATCTGACTCACTGCTCCTCCTTTTCCG	1	A	0	0.4764
NC_005101.4-74421259	ATCTGACTCACTGCTCCTCCTTTTCCG	26	C	0	0.4764
NC_005101.4-75104828	TGAAGTTCAACCAGAACTCCCAGGGAC	5	G	0.2155	0.2315
NC_005101.4-75104828	TGAAGTTCAACCAGAACTCCCAGGGAC	14	G	0.1478	0.1938
NC_005101.4-75112028	CTGTCTCTAACTGAAGCTCCAGAGAAC	1	C	0.1721	0.4984
NC_005101.4-75149671	CTATGTTGAACTTCATCTCCAGGAACA	4	T	0.1901	0.2922
NC_005101.4-75149671	CTATGTTGAACTTCATCTCCAGGAACA	16	T	0.2417	0.2491
NC_005101.4-75319791	ATCCCCCACACCCCTACTCCTGCCTCA	22	G	0.1712	0.4959
NC_005101.4-75319791	ATCCCCCACACCCCTACTCCTGCCTCA	24	C	0.1712	0.4959
NC_005101.4-75364258	TATTAGATAACAGCTCCTCCTTGCATA	13	G	0.0177	0.0348
NC_005101.4-75364258	TATTAGATAACAGCTCCTCCTTGCATA	16	C	0.2054	0.3418
NC_005101.4-75371669	AGAGACAGAACTTCAGCTCCACTAGGT	4	G	0.0357	0.4943
NC_005101.4-75420748	AACATAAGCACAGTAACTCCTTCCAAG	26	A	0.1293	0.2797
NC_005101.4-75455304	ACAGCATGAACACAGTCTCCTGCTCTC	25	C	0.1518	0.3418

NC_005101.4-75608530	TATTTTGAGACAGTGCCTCCTCTTGAC	21	T	0.2241	0.4281
NC_005101.4-75639390	AGTTCATATACAGAACCTCCTTCATGG	9	T	0.1721	0.2686
NC_005101.4-75872441	CTAGAATGTACCTCACCTCCCTCTTCC	27	C	0.1478	0.2204
NC_005101.4-76038462	CCTCCAAACACGTATCCTCCACTGCTA	14	A	0	0.4871
NC_005101.4-76204902	CCTCCTTTAACTCCAACTCCATTGTGT	7	T	0.0427	0.058
NC_005101.4-76232627	GTGGGCGTCACTTGTTCTCCAATGATC	6	C	0.0163	0.4759
NC_005101.4-76232627	GTGGGCGTCACTTGTTCTCCAATGATC	25	A	0.0163	0.4759
NC_005101.4-76236943	GTGTTCCCAACACATCCTCCTCCATTA	12	A	0	0.4832
NC_005101.4-76261408	AAGCAAAGCACAAGATCTCCAGAGCTT	3	G	0	0.4734
NC_005101.4-76269221	CACCCTCACACAAGTTCTCCCCACTTC	1	C	0.0625	0.0605
NC_005101.4-76269221	CACCCTCACACAAGTTCTCCCCACTTC	23	A	0.0089	0.4856
NC_005101.4-76286963	AGATATAGAACTTTTACTCCTCCAGCA	24	A	0.0256	0.0253
NC_005101.4-76313498	TTCTGTCTCACTACCACTCCTGTCCTG	7	C	0.0957	0.1659
NC_005101.4-76313498	TTCTGTCTCACTACCACTCCTGTCCTG	16	A	0.0965	0.5
NC_005101.4-76331213	GAACAAAGGACCTCCCCTCCCATTGCT	12	C	0.0339	0.0333
NC_005101.4-76331213	GAACAAAGGACCTCCCCTCCCATTGCT	16	C	0.1111	0.4977
NC_005101.4-76379541	CAATGCGAAACCACCACTCCCCCTACA	7	G	0.2049	0.4943
NC_005101.4-76379541	CAATGCGAAACCACCACTCCCCCTACA	25	A	0.0246	0.4822
NC_005101.4-76392897	CGGTCAGCTACAGTGACTCCCACCCAC	2	G	0.0407	0.0705
NC_005101.4-76482218	CAGCCTGGAACTCCAGCTCCATGGGTC	26	T	0.0667	0.0644
NC_005101.4-76500521	AGAAGCATTACTATCACTCCCAACTTG	12	T	0.115	0.4998
NC_005101.4-76500521	AGAAGCATTACTATCACTCCCAACTTG	13	A	0.115	0.4998
NC_005101.4-77496628	ATCCTATCCACCTCTCCTCCTTAGCCC	23	A	0	0.4807
NC_005101.4-77496628	ATCCTATCCACCTCTCCTCCTTAGCCC	25	C	0.0407	0.0553
NC_005101.4-77499595	CAACTGGAAACAGTGACTCCCCCACAT	6	G	0.0924	0.1327
NC_005101.4-77499595	CAACTGGAAACAGTGACTCCCCCACAT	27	T	0	0.4813
NC_005101.4-77523296	ATAGGTCGGACTGGCACTCCCGACGCT	6	T	0	0.4827
NC_005101.4-77523296	ATAGGTCGGACTGGCACTCCCGACGCT	25	G	0.0696	0.0671
NC_005101.4-77553478	ATCCCCATGACAGGGCCTCCGGCTGAA	15	G	0.0252	0.4797
NC_005101.4-77553478	ATCCCCATGACAGGGCCTCCGGCTGAA	21	G	0.0588	0.4859
NC_005101.4-77727960	CGCATGTGGACAGCAGCTCCTAAACTC	1	C	0.2667	0.3487
NC_005101.4-77727960	CGCATGTGGACAGCAGCTCCTAAACTC	2	G	0.1917	0.49
NC_005101.4-77819255	GGCACTGTTACAAGGTCTCCATGTGTT	21	A	0.0569	0.0553
NC_005101.4-77822115	TTATGTCCCACGAATACTCCTGGTATG	16	A	0.15	0.1528
NC_005101.4-77822115	TTATGTCCCACGAATACTCCTGGTATG	22	G	0.0932	0.1621
NC_005101.4-77831921	GGATCCAGGACTCCATCTCCGAGTGTC	26	T	0.0357	0.1014
NC_005101.4-77852481	TCTTCTCCCACACGGGCTCCTTCCCTC	14	G	0.0248	0.0245
NC_005101.4-77962720	CCGAGCTCCACGGAGGCTCCATCATGT	2	C	0.1557	0.2796
NC_005101.4-78005709	ACTAGACCCACATGTTCTCCTCCTCCT	5	G	0.0087	0.4849
NC_005101.4-78005709	ACTAGACCCACATGTTCTCCTCCTCCT	16	T	0.0522	0.4991
NC_005101.4-78008327	GGACTGGGGACCATGTCTCCGGCCCTG	13	A	0.0083	0.4732
NC_005101.4-78029478	CCTGTACTCACAGCGGCTCCTGCTCAC	14	C	0.0826	0.2295
NC_005101.4-78029478	CCTGTACTCACAGCGGCTCCTGCTCAC	26	A	0	0.4787
NC_005101.4-78036488	GTGCTGAACACTGATTCTCCCACCCCA	8	A	0.0684	0.066
NC_005101.4-78036488	GTGCTGAACACTGATTCTCCCACCCCA	26	C	0.0684	0.066
NC_005101.4-78058874	GTGACTGACACCAAGGCTCCAATTGAC	27	C	0.0424	0.0415
NC_005101.4-78080947	CACTGAATTACGCTCACTCCCAACTGA	8	T	0.0085	0.4823
NC_005101.4-78091694	GGACCCAATACTGTCCCTCCATGTAGA	22	T	0.0167	0.48
NC_005101.4-78128440	GAAGCTTGCACCTAACCTCCTTGTTTC	12	C	0.0789	0.4953
NC_005101.4-78128440	GAAGCTTGCACCTAACCTCCTTGTTTC	24	T	0.1404	0.4501
NC_005101.4-78147665	GACATCTATACCCTCCCTCCAAGGCTC	9	T	0.0957	0.4994

NC_005101.4-78151623	CTGTTCAGTACATTGTCTCCTTTTTAT	13	T	0.0531	0.499
NC_005101.4-78165191	ACTAGAGTTACATCTGCTCCTTAACTG	6	A	0	0.4793
NC_005101.4-78538145	GACTCACTAACTCCTCCTCCTTGCATA	6	A	0.0083	0.4769
NC_005101.4-78538145	GACTCACTAACTCCTCCTCCTTGCATA	9	A	0.0083	0.4769
NC_005101.4-78605292	CTATAAACCACCATCACTCCCATTTAT	25	T	0.1518	0.4885
NC_005101.4-78661589	AGTAGCCAGACAGGGACTCCCAAGTGG	15	G	0	0.4887
NC_005101.4-78661589	AGTAGCCAGACAGGGACTCCCAAGTGG	22	A	0.0265	0.4923
NC_005101.4-78681953	CTTCTGCAAACTCCCTCTCCCATTACC	22	A	0	0.4871
NC_005101.4-78701340	ATGGAGGGAACCATGCCTCCAGCCACA	23	C	0.4	0.32
NC_005101.4-78763638	TTCCTACCCACCTCCCCTCCCATCTGG	8	C	0.25	0.232
NC_005101.4-78763638	TTCCTACCCACCTCCCCTCCCATCTGG	21	C	0.1062	0.1006
NC_005101.4-78768819	CCATGGCATACTCTACCTCCAACATGA	1	C	0.2991	0.2544
NC_005101.4-78800468	CACAGGAAGACCCTGTCTCCTGACCAC	6	G	0.1653	0.4983
NC_005101.4-78821302	AACAAGGCCACACCTACTCCACAAGCT	25	G	0.0424	0.0415
NC_005101.4-78858239	AGCAGGCACACAATGTCTCCAGTGCAT	5	G	0.1789	0.189
NC_005101.4-78858239	AGCAGGCACACAATGTCTCCAGTGCAT	14	T	0.122	0.2885
NC_005101.4-78886978	GAACCTGATACCAACACTCCATCAGAG	5	C	0	0.4793
NC_005101.4-78903626	TATGTGACCACTCTCTCTCCTACATCC	4	G	0.027	0.044
NC_005101.4-79080146	AGTTTATTCACAGGAACTCCAGAAGGA	2	G	0.2353	0.3118
NC_005101.4-79087864	ATGAAGCACACTTGCCCTCCTTGAGCA	2	T	0.0517	0.4976
NC_005101.4-79087864	ATGAAGCACACTTGCCCTCCTTGAGCA	9	C	0.1478	0.18
NC_005101.4-79097790	GTGATGCTCACATGAACTCCTCTTTTG	13	T	0	0.033
NC_005101.4-79140747	TTTAATGCCACGTCATCTCCTAGTGGT	12	G	0.0252	0.0249
NC_005101.4-79190828	CATTGTTTAACCCGATCTCCTTAGAAA	14	G	0	0.4827
NC_005101.4-79201397	TACCCTGACACACCCACTCCATGCTGT	21	A	0.1721	0.1968
NC_005101.4-79287415	TACTCCCTCACCTTCACTCCGTACAAT	1	T	0.1197	0.1563
NC_005101.4-79287415	TACTCCCTCACCTTCACTCCGTACAAT	22	T	0.1261	0.1469
NC_005101.4-79293994	CTAGACGTAACTCTACCTCCAGCTTCT	6	C	0.0813	0.1216
NC_005101.4-79301549	CACCTCTCCACTGTTTCTCCAGCTTCT	14	T	0.0082	0.4755
NC_005101.4-79304976	TCCCTGGGCACATCTCCTCCTTCCTTC	23	C	0.009	0.4896
NC_005101.4-79304976	TCCCTGGGCACATCTCCTCCTTCCTTC	27	C	0	0.4854
NC_005101.4-79343766	TGGCAGTGAACTGTGGCTCCCAAGATT	15	G	0.0164	0.048
NC_005101.4-79344703	CTTCTCTCAACTGATGCTCCCACTCTG	23	C	0	0.4773
NC_005101.4-79382948	CCCAGCATCACCAGGGCTCCCTGTCTC	15	G	0.0254	0.0251
NC_005101.4-79382948	CCCAGCATCACCAGGGCTCCCTGTCTC	21	C	0.0508	0.0496
NC_005101.4-79425673	ATCTTTATTACCCAAGCTCCCCCTTGT	15	A	0	0.48
NC_005101.4-79425673	ATCTTTATTACCCAAGCTCCCCCTTGT	22	C	0.0833	0.095
NC_005101.4-79442853	GACATCTAAACCATGCCTCCTTTCTGT	25	T	0	0.4922
NC_005101.4-79442853	GACATCTAAACCATGCCTCCTTTCTGT	27	T	0	0.4922
NC_005101.4-79454076	CCACATCCCACATCTCCTCCTCCCATT	14	C	0.1368	0.3641
NC_005101.4-79584831	ATGGTATCAACATGAACTCCTCAATAC	8	C	0.075	0.4972
NC_005101.4-79616337	CAGGCTCTAACTCTCTCTCCTTAATTT	22	T	0	0.4765
NC_005101.4-79617222	CAGCTCTATACCTTCTCTCCATGTGCC	9	T	0.027	0.0267
NC_005101.4-79644892	AACCTCTTTACTCTATCTCCACAATAG	15	A	0.2768	0.4454
NC_005101.4-79706761	GTCTGTGGGACACTAGCTCCTACTGAC	1	G	0.1111	0.12
NC_005101.4-79717900	CTTACAGGGACAGCTCCTCCCAAGATT	26	T	0.2066	0.3327
NC_005101.4-79718005	CATGCAGTAACCACTCCTCCATGTTTA	5	C	0.0331	0.0325
NC_005101.4-79718005	CATGCAGTAACCACTCCTCCATGTTTA	15	T	0.1074	0.1447
NC_005101.4-79846929	TCTCTCCTCACTTCCGCTCCATCCCGT	16	G	0	0.477
NC_005101.4-79947517	CTCGTTCAAACCAGGACTCCAAACATG	3	C	0.0254	0.0251
NC_005101.4-79951253	AATAGTCACACGTGATCTCCATTAGGG	12	G	0.1947	0.4934

NC_005101.4-79951253	AATAGTCACACGTGATCTCCATTAGGG	26	G	0.193	0.2155
NC_005101.4-79999150	GAGAAGGTAACTCCTGCTCCAACAATA	12	T	0.2479	0.3557
NC_005101.4-80084900	TCATGGTAGACTACACCTCCTCAAGCT	6	G	0.0413	0.0405
NC_005101.4-80084900	TCATGGTAGACTACACCTCCTCAAGCT	14	C	0.05	0.0644
NC_005101.4-80108456	ATCTTGACTACTGGTCCTCCAGAACAA	7	A	0.1639	0.1774
NC_005101.4-80181183	GAGTATTCGACCTTGACTCCAGTACCT	9	G	0	0.4864
NC_005101.4-80181183	GAGTATTCGACCTTGACTCCAGTACCT	21	A	0.1842	0.2836
NC_005101.4-80208640	ACCCCCAGCACGAGGCCTCCTTTACAC	25	C	0.161	0.1481
NC_005101.4-80211644	GACACTTATACAATCACTCCCTTGGGG	22	T	0.0263	0.0429
NC_005101.4-80211644	GACACTTATACAATCACTCCCTTGGGG	24	G	0.087	0.4017
NC_005101.4-80221911	AAAAACACAACCCTCCCTCCAAGGGAA	1	A	0.1721	0.1708
NC_005101.4-80221911	AAAAACACAACCCTCCCTCCAAGGGAA	23	G	0	0.4807
NC_005101.4-80222524	AGGCATGTGACCTGCTCTCCACCTCCA	3	G	0.2712	0.4585
NC_005101.4-80270943	AGAGGCTGTACTCCTCCTCCTCGTTTG	23	G	0.0325	0.0476
NC_005101.4-80305690	CTAGCTCCCACAATTCCTCCTCCCTCT	1	C	0.0246	0.0243
NC_005101.4-80321408	AAGAAATTAACTTCAACTCCTGTTCCT	25	C	0.0413	0.0405
NC_005101.4-80400880	CTTCAGTCCACCCTGGCTCCCTCTGTC	1	C	0	0.4819
NC_005101.4-80414239	TTCTGGGCTACAATGGCTCCTTGGATT	1	T	0	0.4887
NC_005101.4-80414239	TTCTGGGCTACAATGGCTCCTTGGATT	6	G	0.0702	0.1153
NC_005101.4-80422287	AACCTGCTGACGTCATCTCCACCCCAT	7	C	0.2066	0.2355
NC_005101.4-80428795	TCACTCCTCACTGCATCTCCTTCAGAG	6	C	0.0982	0.1403
NC_005101.4-80428795	TCACTCCTCACTGCATCTCCTTCAGAG	12	T	0.2143	0.2698
NC_005101.4-80454704	TCCCTTTCCACCTGGACTCCAGATGTT	24	T	0.2759	0.3916
NC_005101.4-80454704	TCCCTTTCCACCTGGACTCCAGATGTT	25	G	0.0339	0.0333
NC_005101.4-80484085	CTCTTGTGGACAGCCGCTCCGTCTCCT	6	G	0	0.4833
NC_005101.4-80484085	CTCTTGTGGACAGCCGCTCCGTCTCCT	21	G	0	0.4833
NC_005101.4-80520756	GTCACATGCACAGCATCTCCTTCATTG	4	A	0.1967	0.4952
NC_005101.4-80520756	GTCACATGCACAGCATCTCCTTCATTG	25	T	0	0.4807
NC_005101.4-80521872	TGGCCCTGAACCCAGGCTCCCAGTGAA	1	T	0.1239	0.1162
NC_005101.4-80521872	TGGCCCTGAACCCAGGCTCCCAGTGAA	4	C	0.0517	0.0504
NC_005101.4-80527774	GACGCTAGCACACATGCTCCCTTAATC	15	T	0.0531	0.4968
NC_005101.4-80542825	ACAAATCACACGTATCCTCCCTGTCCA	5	A	0	0.4588
NC_005101.4-80585368	CTCTCTTGTACTCCTGCTCCAGGCTCT	8	G	0.094	0.0896
NC_005101.4-80587159	CAGACTCCCACTCGAGCTCCCTGATTT	7	C	0	0.4793
NC_005101.4-80587159	CAGACTCCCACTCGAGCTCCCTGATTT	14	G	0.0732	0.0854
NC_005101.4-80588849	GTGGAATAAACCCCTCCTCCTCAGGTT	4	G	0.0862	0.0825
NC_005101.4-80599794	AGAGGCATCACGGCAGCTCCACATGTG	4	G	0.087	0.32
NC_005101.4-80601660	TCTCACAAAACCTTTTCTCCCTCACCT	8	A	0.0087	0.4817
NC_005101.4-80607296	TAGGTAGGAACGTAGACTCCTAAGGCA	14	A	0	0.4731
NC_005101.4-80626394	GGTTGGAAGACACTTTCTCCTCCTGAT	4	T	0.0085	0.4698
NC_005101.4-80626394	GGTTGGAAGACACTTTCTCCTCCTGAT	12	A	0.0855	0.4868
NC_005101.4-80646513	ATCTACAAGACCCTTGCTCCCTTCCCT	8	A	0	0.4793
NC_005101.4-80669175	CAGTAACTCACTACCGCTCCTGAGTAA	25	T	0.2951	0.435
NC_005101.4-80669175	CAGTAACTCACTACCGCTCCTGAGTAA	27	A	0.041	0.0861
NC_005101.4-80684192	TACTCATTTACAGTGACTCCATGAAAA	25	A	0.2389	0.4944
NC_005101.4-80696685	CAGCTTTCAACAGCTTCTCCATGGACT	24	G	0.188	0.3054
NC_005101.4-80707796	GAGGACTCCACTCCCTCTCCAGACTCC	7	T	0.1261	0.5
NC_005101.4-80707796	GAGGACTCCACTCCCTCTCCAGACTCC	9	C	0.0917	0.4958
NC_005101.4-80709669	AAATAAACAACACCAACTCCGACAGCT	6	A	0	0.4751
NC_005101.4-80709669	AAATAAACAACACCAACTCCGACAGCT	13	C	0.2397	0.4605
NC_005101.4-80788154	GTTCTCCAAACACTTTCTCCCTTACTA	4	C	0.0609	0.059

NC_005101.4-80790427	CGACAATGGACTAAGCCTCCCCACTGT	9	G	0.2541	0.4822
NC_005101.4-80796327	CTGGTTGCTACTTGTGCTCCAGGTTAA	22	G	0	0.4773
NC_005101.4-80796327	CTGGTTGCTACTTGTGCTCCAGGTTAA	23	G	0.2033	0.2556
NC_005101.4-80817614	TGACTGTGGACTGAACCTCCGAATCGG	2	G	0.075	0.1316
NC_005101.4-80817614	TGACTGTGGACTGAACCTCCGAATCGG	25	C	0.075	0.1316
NC_005101.4-80880844	CATGACCCAACTGAGTCTCCACCAAAA	13	G	0.1833	0.255
NC_005101.4-80889380	ATCCACGGGACCACATCTCCCCATCAC	6	C	0.178	0.4896
NC_005101.4-80889380	ATCCACGGGACCACATCTCCCCATCAC	7	G	0.0333	0.0644
NC_005101.4-80899159	ACCAACCAGACACACCCTCCACCCTCA	24	C	0.2393	0.2107
NC_005101.4-80899159	ACCAACCAGACACACCCTCCACCCTCA	25	T	0	0.4833
NC_005101.4-80976743	CTATCTGGGACTCCTGCTCCAGGGTAT	5	C	0.1947	0.2171
NC_005101.4-80976743	CTATCTGGGACTCCTGCTCCAGGGTAT	7	G	0.1947	0.2171
NC_005101.4-80986809	CACACACACACACTTCCTCCTGAAGCA	13	C	0.2602	0.4595
NC_005101.4-81317648	CTTGGAAGGACTCACTCTCCTTCTCTC	15	C	0.188	0.2361
NC_005101.4-81482200	GCATGGGACACTTTTCCTCCTACTGGT	23	C	0.1057	0.1287
NC_005101.4-81619801	TTGATTACAACCACCTCTCCTTCCATT	12	C	0.0086	0.0422
NC_005101.4-81619801	TTGATTACAACCACCTCTCCTTCCATT	26	T	0.0427	0.058
NC_005101.4-81654359	AGCTGTTGAACCATCTCTCCATATCCA	16	T	0.0268	0.0264
NC_005101.4-81654359	AGCTGTTGAACCATCTCTCCATATCCA	22	T	0	0.4807
NC_005101.4-81712930	CTGCCTATAACTTGAGCTCCAGGGGTT	25	G	0.0265	0.0262
NC_005101.4-81738780	AGGACCAGGACATCTGCTCCAAGAAAT	23	G	0.0488	0.0476
NC_005101.4-81759073	TTATCACCTACTTGCTCTCCGGCCTGA	12	T	0.2162	0.4558
NC_005101.4-81842858	GCCACAAGGACACTTGCTCCATGTTCA	1	G	0	0.48
NC_005101.4-81842858	GCCACAAGGACACTTGCTCCATGTTCA	23	G	0.0492	0.1225
NC_005101.4-81917483	CAAACTGTTACCTTCGCTCCATGCACT	4	A	0	0.4793
NC_005101.4-81917483	CAAACTGTTACCTTCGCTCCATGCACT	15	C	0.0984	0.1082
NC_005101.4-81977303	TACTAATCAACCAGAGCTCCCAAGACT	16	G	0.1071	0.1014
NC_005101.4-81977303	TACTAATCAACCAGAGCTCCCAAGACT	24	G	0.0536	0.1014
NC_005101.4-82006265	GATACCTTGACTGCTTCTCCTCTGAAC	14	C	0.1217	0.3403
NC_005101.4-82141372	TTTCCCTGCACTGGGGCTCCAACACTG	13	G	0.3471	0.4041
NC_005101.4-82177984	AAGGAGGGAACCATGGCTCCAGCTACA	15	G	0.1161	0.1699
NC_005101.4-82182407	GGAACCAGCACTGACCCTCCAAAGCAG	6	C	0.3171	0.3472
NC_005101.4-82182407	GGAACCAGCACTGACCCTCCAAAGCAG	9	C	0.2049	0.2095
NC_005101.4-82338119	CCCACATTGACACTTCCTCCACTATGT	4	A	0.287	0.3814
NC_005101.4-82338119	CCCACATTGACACTTCCTCCACTATGT	7	T	0	0.4807
NC_005101.4-82622105	AGCACACTCACTCAGTCTCCTCCCTAC	27	C	0.1789	0.2612
NC_005101.4-82814170	GAATTCAATACCTGGACTCCACAGGGT	3	A	0	0.4765
NC_005101.4-82814170	GAATTCAATACCTGGACTCCACAGGGT	13	T	0.0248	0.0245
NC_005101.4-82883957	TGATCCTGCACAAAACCTCCTGGGTTT	8	G	0	0.4793
NC_005101.4-82883957	TGATCCTGCACAAAACCTCCTGGGTTT	22	G	0.0531	0.0517
NC_005101.4-82885890	AAAAGGATTACTGTTGCTCCAAATTTC	27	C	0.036	0.0526
NC_005101.4-82899615	GGCCTGCCCACTGACACTCCATTTTAG	16	A	0.0331	0.0325
NC_005101.4-82899989	GTGGTTTACACAACAGCTCCCAGGCAT	14	C	0.2348	0.4613
NC_005101.4-82899989	GTGGTTTACACAACAGCTCCCAGGCAT	15	A	0	0.48
NC_005101.4-82970750	GCTATTGTGACCAGCACTCCCTTCAAA	7	G	0.2193	0.463
NC_005101.4-82971608	CCTCTCCCCACTCCTCCTCCCCACCAG	13	C	0.0885	0.1316
NC_005101.4-83034530	AGTTGTAGGACCAGTTCTCCCATGCTT	15	T	0	0.4819
NC_005101.4-83052564	GCGATATTCACTGGGTCTCCTTTATTT	21	T	0.1167	0.1387
NC_005101.4-83073221	CTCTCTTTCACTTTTTCTCCCTGTCTC	22	T	0.0439	0.0429
NC_005101.4-83086368	CACATGCTCACTTTAACTCCTGCGACT	3	C	0.175	0.2491
NC_005101.4-83086368	CACATGCTCACTTTAACTCCTGCGACT	8	T	0.1583	0.2941

NC_005101.4-83167710	ATAAGGCTAACTCTGGCTCCTCCGTAG	27	G	0.15	0.2188
NC_005101.4-83246940	GGTCTAAGAACTTTGCCTCCTTTAACA	25	A	0.0085	0.4854
NC_005101.4-83246940	GGTCTAAGAACTTTGCCTCCTTTAACA	27	A	0.0171	0.4868
NC_005101.4-83285150	CTACTAAGAACACTTTCTCCATATACA	4	C	0.0811	0.4985
NC_005101.4-83285150	CTACTAAGAACACTTTCTCCATATACA	24	T	0.0811	0.4985
NC_005101.4-83394408	CAGCCCCTGACACACCCTCCTCCTTAT	6	C	0.1885	0.4943
NC_005101.4-83443277	TTGGAAGAGACTTGGTCTCCTTTAAAG	16	T	0	0.4734
NC_005101.4-83481202	GACTTAAATACCCTGACTCCACAATTT	9	T	0	0.4724
NC_005101.4-83481202	GACTTAAATACCCTGACTCCACAATTT	12	C	0.115	0.2237
NC_005101.4-83503267	TGCAAAGCAACCGAGACTCCAGGGACA	2	G	0.1626	0.1494
NC_005101.4-83503267	TGCAAAGCAACCGAGACTCCAGGGACA	12	C	0.1626	0.1494
NC_005101.4-83505481	ACTCCTGGGACACTTCCTCCCGTCATC	1	A	0.2131	0.2515
NC_005101.4-83505481	ACTCCTGGGACACTTCCTCCCGTCATC	25	A	0	0.4793
NC_005101.4-83539593	TTCACGAATACTTGTTCTCCATGAATC	6	G	0.0174	0.4833
NC_005101.4-83600172	CATTACCAGACGACAGCTCCTACTCTG	2	A	0	0.4819
NC_005101.4-83600172	CATTACCAGACGACAGCTCCTACTCTG	3	T	0	0.4819
NC_005101.4-83600309	GACAGAGATACATAAGCTCCACCTACA	15	A	0.0331	0.0484
NC_005101.4-83635310	GTCAGTCTAACACTTCCTCCTGTATAC	27	C	0.1207	0.3478
NC_005101.4-83641228	ATTTGTCCCACATGTGCTCCCTTCCAG	9	C	0.0348	0.0508
NC_005101.4-83642355	TCACTACTTACAGTGTCTCCAGACATT	13	G	0.0083	0.0247
NC_005101.4-83642355	TCACTACTTACAGTGTCTCCAGACATT	25	A	0	0.4703
NC_005101.4-83656604	ACAGATTGCACAGTCCCTCCATGTGAA	5	A	0.2213	0.4677
NC_005101.4-83656604	ACAGATTGCACAGTCCCTCCATGTGAA	8	G	0.0579	0.0868
NC_005101.4-83717918	AGACACAGAACACAGACTCCATTATCA	27	A	0	0.4813
NC_005101.4-83791316	CTTGCCATGACTCTTTCTCCTATTCCA	8	T	0.1	0.3299
NC_005101.4-83791316	CTTGCCATGACTCTTTCTCCTATTCCA	22	A	0	0.4703
NC_005101.4-83918286	GATTGCCACACCTTCTCTCCTGCGGTC	23	C	0.1622	0.5
NC_005101.4-83932819	GATGAATTTACTCTTCCTCCTACAGAG	6	A	0.0165	0.4751
NC_005101.4-83967204	CACCAAACAACAGTGTCTCCAAGCTCG	9	A	0.1538	0.1975
NC_005101.4-83967204	CACCAAACAACAGTGTCTCCAAGCTCG	26	C	0.0086	0.4803
NC_005101.4-83967860	CTGCCTTGCACTCGAGCTCCAAAGGGT	1	C	0.0254	0.0576
NC_005101.4-83967860	CTGCCTTGCACTCGAGCTCCAAAGGGT	14	G	0.0086	0.4803
NC_005101.4-83969559	CTTCTCCCCACATCACCTCCCTAGATG	27	G	0.2562	0.4213
NC_005101.4-83980443	CAGCATGATACCCACACTCCACTCCTA	15	C	0.0084	0.4859
NC_005101.4-84006541	CATCAAGCCACTCTACCTCCTTCCCTG	13	C	0.0976	0.1073
NC_005101.4-84024204	GGGAGCTACACTGGGTCTCCATTTCTC	7	T	0	0.482
NC_005101.4-84088082	AGAATGAGAACCTGACCTCCCTCTAAA	13	T	0.065	0.0629
NC_005101.4-84088082	AGAATGAGAACCTGACCTCCCTCTAAA	26	A	0.2439	0.3334
NC_005101.4-84131471	GGGTAATGGACACATACTCCTAGGTCA	4	T	0.1795	0.2171
NC_005101.4-84269264	AGGGCCATTACCTTTACTCCAGATTCA	5	C	0	0.4743
NC_005101.4-84303262	TGGATGCAGACCCTTCCTCCCTATTGA	4	A	0.1583	0.2491
NC_005101.4-84303262	TGGATGCAGACCCTTCCTCCCTATTGA	15	T	0	0.4787
NC_005101.4-84341970	TGGACAGCTACCTATCCTCCTCTGATA	3	G	0	0.4785
NC_005101.4-84341970	TGGACAGCTACCTATCCTCCTCTGATA	26	T	0.0088	0.4873
NC_005101.4-84393675	AGGCTTGTGACTGTGGCTCCGGCCCAC	25	C	0.2787	0.3624
NC_005101.4-84433315	TCCATGCTCACATAGGCTCCACATACC	27	C	0.2605	0.4117
NC_005101.4-84441579	CTCTGCCCAACTGTGTCTCCTTCTTGT	1	C	0	0.4773
NC_005101.4-84441579	CTCTGCCCAACTGTGTCTCCTTCTTGT	25	T	0.2131	0.2399
NC_005101.4-84448769	CCCCAGATAACCTCAGCTCCTGTGGTA	9	A	0	0.4807
NC_005101.4-84448769	CCCCAGATAACCTCAGCTCCTGTGGTA	27	A	0	0.4807
NC_005101.4-84450471	GATTTCCCTACTCTCCCTCCCTTAGTG	9	T	0	0.482

NC_005101.4-84450471	GATTTCCCTACTCTCCCTCCCTTAGTG	23	T	0.2609	0.4234
NC_005101.4-84464704	TCTTCTCACACCCTCGCTCCACACGTC	25	G	0.1639	0.3354
NC_005101.4-84493518	AACAGGGCCACCCAGACTCCAGCTTCA	9	C	0.0268	0.0264
NC_005101.4-84493518	AACAGGGCCACCCAGACTCCAGCTTCA	24	T	0.1712	0.3124
NC_005101.4-84500001	TGGTTTTGAACACCTGCTCCTTTTGCA	7	T	0.0084	0.4682
NC_005101.4-84518844	CCACTCTGGACAACAGCTCCTGTCCCT	5	T	0.0696	0.4936
NC_005101.4-84518844	CCACTCTGGACAACAGCTCCTGTCCCT	6	C	0.2018	0.3374
NC_005101.4-84528129	AGAATGCCCACCTGTACTCCTAACACT	3	A	0.1724	0.4946
NC_005101.4-84545142	GGTAGTGGAACACTGCCTCCAAACCCT	24	C	0.0354	0.0348
NC_005101.4-84606359	GCCTCCTGAACCATCTCTCCAGTTCAC	6	C	0.0089	0.4789
NC_005101.4-84664813	ATTAAGAATACCTGGCCTCCACACTTA	2	T	0.0783	0.1219
NC_005101.4-84664813	ATTAAGAATACCTGGCCTCCACACTTA	3	T	0.0789	0.1229
NC_005101.4-84713011	TCAAGTTAGACTTGGACTCCCAGTCCT	9	G	0.0256	0.0253
NC_005101.4-84728061	CTTCAAATCACAGGGCCTCCTGGGAAG	14	G	0.2906	0.4386
NC_005101.4-84728061	CTTCAAATCACAGGGCCTCCTGGGAAG	23	G	0.1261	0.188
NC_005101.4-84899965	AGTGTTAGCACAAATACTCCTGAGGAT	21	T	0.1333	0.2432
NC_005101.4-84949002	TGAATCCTCACTGGATCTCCTTCTGAT	24	T	0	0.4864
NC_005101.4-84968648	GGCAAGATCACGCTCCCTCCAGACACT	8	T	0	0.48
NC_005101.4-84968648	GGCAAGATCACGCTCCCTCCAGACACT	24	C	0.0744	0.1163
NC_005101.4-84996196	GTACCCCTAACCCCTCCTCCCTTCTTT	1	G	0.0744	0.0868
NC_005101.4-84996196	GTACCCCTAACCCCTCCTCCCTTCTTT	27	T	0.2231	0.2704
NC_005101.4-85028588	CCAAACTGCACATTTGCTCCAAATGTG	15	T	0.0325	0.032
NC_005101.4-85028588	CCAAACTGCACATTTGCTCCAAATGTG	16	G	0.0328	0.0322
NC_005101.4-85065687	ATGTTTTAGACTGTGTCTCCTCCTGAC	1	A	0.0598	0.0896
NC_005101.4-85065687	ATGTTTTAGACTGTGTCTCCTCCTGAC	9	G	0.2672	0.302
NC_005101.4-85068149	ATTTGCAAAACATTGGCTCCACGATTG	22	C	0.2137	0.2662
NC_005101.4-85127932	TGCAGCCATACTTCGACTCCTTTGGAG	15	G	0.084	0.3686
NC_005101.4-85127932	TGCAGCCATACTTCGACTCCTTTGGAG	26	A	0.0085	0.4738
NC_005101.4-85151367	ATTTGTGGTACCTGGACTCCACTGCAG	21	A	0.041	0.0557
NC_005101.4-85177677	CTTATCTTTACCAGAGCTCCCACAGCT	15	A	0.1368	0.1704
NC_005101.4-85186000	TTCAACAAGACCACACCTCCAATCCTT	9	G	0.6066	0.4226
NC_005101.4-85186000	TTCAACAAGACCACACCTCCAATCCTT	22	A	0.4672	0.3581
NC_005101.4-85208872	TTCCGTGCCACCTCATCTCCAACCCTC	9	C	0.1545	0.1425
NC_005101.4-85224900	TGAGACAGAACCCTGACTCCTCAAGTG	6	C	0.1382	0.1562
NC_005101.4-85262238	GGACAGACCACATCCTCTCCTTTGGAC	13	T	0.0325	0.4722
NC_005101.4-85330448	TGTTTGGAAACGCTCACTCCGTCATCT	1	T	0.2213	0.321
NC_005101.4-85330448	TGTTTGGAAACGCTCACTCCGTCATCT	12	G	0	0.4773
NC_005101.4-85332472	GTGGGGCCAACAGTCTCTCCAGAACAG	4	G	0.0427	0.0418
NC_005101.4-85332472	GTGGGGCCAACAGTCTCTCCAGAACAG	8	C	0.2203	0.334
NC_005101.4-85343744	AAAGAGATTACTATCACTCCTCCTTGA	2	A	0	0.4778
NC_005101.4-85371430	TTTCCTGGTACCCTTCCTCCACAAGAT	27	T	0.2288	0.4842
NC_005101.4-85386154	GGCTGGAATACAGAGACTCCTTTAATT	2	G	0.0517	0.0504
NC_005101.4-85413083	CTTTCTCGTACTGATTCTCCCGCACAA	7	C	0.0833	0.1387
NC_005101.4-85413083	CTTTCTCGTACTGATTCTCCCGCACAA	26	A	0.2167	0.455
NC_005101.4-85421076	ATCTTTGGAACTCGGGCTCCTTTGCTG	13	C	0.2437	0.3812
NC_005101.4-85508819	GGGTGTGCTACAATACCTCCAAGTCAT	5	G	0	0.4807
NC_005101.4-85508819	GGGTGTGCTACAATACCTCCAAGTCAT	21	A	0.2231	0.3601
NC_005101.4-85565524	TGGCTAGCTACTGTGTCTCCTGGGACC	9	T	0.0085	0.4655
NC_005101.4-85595643	ACTACAGATACTCTCCCTCCTACAATG	15	C	0.0656	0.0634
NC_005101.4-85600693	TTTACGGTTACTTTGCCTCCATGTCTT	5	C	0.0407	0.0398
NC_005101.4-85627926	CCATATGTCACCCACCCTCCGTGTTAT	21	G	0.0325	0.4722

NC_005101.4-85644508	CTCGTGATTACCTTTTCTCCTCAGATA	3	C	0.0083	0.4769
NC_005101.4-85647670	ATTTAAGACACTCAGCCTCCAAATGTG	1	A	0	0.4807
NC_005101.4-85654432	ACACACTAGACTTTCTCTCCAGGGTCC	26	C	0.281	0.418
NC_005101.4-85662473	TCCATGCTCACCATCCCTCCTGCCCTC	4	A	0.0252	0.0249
NC_005101.4-85690983	CCCTGTCTCACAGCTTCTCCTTTCCTT	6	T	0	0.4787
NC_005101.4-85690983	CCCTGTCTCACAGCTTCTCCTTTCCTT	14	C	0	0.4787
NC_005101.4-85744077	TATTGAATCACTTCCTCTCCTACAGGA	22	A	0	0.4793
NC_005101.4-85746415	TGCTCTGTCACTATGACTCCGCCAACT	5	C	0.2951	0.416
NC_005101.4-85746415	TGCTCTGTCACTATGACTCCGCCAACT	21	G	0.1557	0.321
NC_005101.4-85749629	ACCACAGAAACGACATCTCCTATACTG	24	A	0	0.4764
NC_005101.4-85754221	TGATCTCGAACAACAACTCCTGTTTAG	7	C	0.1652	0.1938
NC_005101.4-85843070	CCCAAGAAAACCAGAGCTCCTCTCCAA	5	A	0.2281	0.4698
NC_005101.4-85851062	TGATTCTGGACCACAGCTCCCTGCACC	24	C	0.0085	0.0251
NC_005101.4-85861978	CAGTAATAAACACTGTCTCCTATTTCT	9	A	0.0261	0.0257
NC_005101.4-85914241	TGCCAGAACACACAGCCTCCTTGTGGG	13	C	0.0796	0.0926
NC_005101.4-85940120	CCAGGTAGGACAGTGACTCCGAGGAAC	2	C	0.0588	0.0571
NC_005101.4-85940120	CCAGGTAGGACAGTGACTCCGAGGAAC	21	G	0.0085	0.4842
NC_005101.4-85948790	AATGGAGAGACTAAGCCTCCTCAATAT	24	A	0.0333	0.0799
NC_005101.4-85955545	TCAGCCTATACCCTAGCTCCCCATTTT	9	T	0.1721	0.5
NC_005101.4-85964322	CACTGCTCCACAGGAACTCCCAAGCCT	1	C	0.0721	0.086
NC_005101.4-85988402	TGGAGTTTTACGGTAGCTCCTGAGAGA	14	T	0.0081	0.0553
NC_005101.4-85995023	GTGTCTAGGACTGTTCCTCCAGAGTCG	14	T	0.0089	0.4789
NC_005101.4-85995023	GTGTCTAGGACTGTTCCTCCAGAGTCG	24	G	0.0796	0.0926
NC_005101.4-86033432	ATTCTCGAAACTTCTGCTCCATTTGTT	3	T	0	0.4724
NC_005101.4-86095947	GCAACAGAAACCAAGACTCCAAGGCAT	16	A	0.2759	0.2501
NC_005101.4-86158385	GGTGGAGTCACCTACTCTCCAGAATCT	21	A	0.0885	0.1006
NC_005101.4-86245000	CCACGTATGACACCAGCTCCAGAAGAT	4	C	0.281	0.4983
NC_005101.4-86245000	CCACGTATGACACCAGCTCCAGAAGAT	23	A	0.1653	0.2533
NC_005101.4-86346883	AACTGAATCACTGTGTCTCCTTCTTAC	12	T	0.0259	0.0255
NC_005101.4-86566346	GCACACAGCACACAAACTCCTGGGTTT	1	G	0.2602	0.4548
NC_005101.4-86566346	GCACACAGCACACAAACTCCTGGGTTT	23	G	0.0325	0.1073
NC_005101.4-86687911	ATGTGGCTAACGCACTCTCCCCTCTAA	3	G	0.0089	0.4824
NC_005101.4-86829350	CAAGGATATACAGCTGCTCCTGAGCTC	16	G	0.1885	0.2339
NC_005101.4-86875610	ATCTCTCTCACTTTTCCTCCTCCTCTC	26	T	0.3246	0.3061
NC_005101.4-86875610	ATCTCTCTCACTTTTCCTCCTCCTCTC	27	C	0.3158	0.3006
NC_005101.4-86882777	TTCTCCTCCACAGCCCCTCCCCCTCTC	27	C	0.2602	0.2263
NC_005101.4-86907111	TTCTCCTCCACAGCCCCTCCCCAATCT	5	C	0.5124	0.3811
NC_005101.4-87011484	AGTAGGATCACCCAAGCTCCTCTAACA	26	C	0.1901	0.4693
NC_005101.4-87727672	GGTAAGTCCACTGATGCTCCAGCACTC	22	G	0.187	0.2323
NC_005101.4-87793581	GAGTGTTTTACTCAGGCTCCTTTCTCA	4	T	0.2143	0.4362
NC_005101.4-87793581	GAGTGTTTTACTCAGGCTCCTTTCTCA	7	T	0.1339	0.3794
NC_005101.4-87823190	CCAAGTCAGACATGGCCTCCATGTGCT	8	A	0.178	0.2026
NC_005101.4-87860719	AACGTATGAACCCTGACTCCTCTTTCA	24	T	0.2703	0.4383
NC_005101.4-87935255	CAAAATTAAACATTAGCTCCTACTGTG	2	A	0.1765	0.4987
NC_005101.4-87935255	CAAAATTAAACATTAGCTCCTACTGTG	12	A	0.0417	0.0566
NC_005101.4-88070248	GGTGCGTGCACACATGCTCCTGAGAAT	6	G	0.1518	0.1843
NC_005101.4-88103654	CTACTACATACACTCTCTCCCTGTCCC	1	C	0.0268	0.0436
NC_005101.4-88103654	CTACTACATACACTCTCTCCCTGTCCC	23	G	0.0973	0.1084
NC_005101.4-88117917	AAATGCACTACATATGCTCCAATGTAC	9	T	0.0833	0.0799
NC_005101.4-88117917	AAATGCACTACATATGCTCCAATGTAC	12	A	0.1	0.1244
NC_005101.4-88251248	ACAAACCATACCTTTCCTCCTTTCTTC	13	T	0.0171	0.4807

NC_005101.4-88296100	TGGTCAACTACTTTGTCTCCCTCCTTC	2	G	0.187	0.2989
NC_005101.4-88675817	AGATGTGTGACAACAACTCCTAGTTTG	16	A	0.3917	0.3347
NC_005101.4-88857776	CACCCTTGCACCCTTGCTCCCTCCGAA	7	T	0.0424	0.1894
NC_005101.4-88892258	CCTTTAGCCACTGCTGCTCCTTTTCGA	8	C	0.3115	0.3947
NC_005101.4-89012223	TCTATATTCACTAAACCTCCCACCCTT	21	C	0.1652	0.1938
NC_005101.4-89033765	CTCTCTTCCACCCTGTCTCCCTCAGCA	6	T	0.1148	0.4979
NC_005101.4-89078569	AGATAATCCACTTCAGCTCCATTACCT	22	T	0.5798	0.4117
NC_005101.4-89078569	AGATAATCCACTTCAGCTCCATTACCT	23	T	0.5833	0.4132
NC_005101.4-89251641	CATCAATATACCTACTCTCCTTACCCA	3	T	0.1217	0.4969
NC_005101.4-89388323	CTACAGTCCACCACAGCTCCCTGTAGA	16	G	0	0.4793
NC_005101.4-89529820	GATTCCCCTACCTCAACTCCACCCCCA	9	T	0.0504	0.065
NC_005101.4-89580201	TTTCAGCTAACCTGGTCTCCATCACCA	13	T	0.1463	0.3041
NC_005101.4-89648636	TTGCAAACCACTTCAGCTCCTTTAGTT	24	A	0.1186	0.1116
NC_005101.4-89750929	ATGTGCTCTACAGGTTCTCCCTCACCT	8	C	0.065	0.0629
NC_005101.4-89750929	ATGTGCTCTACAGGTTCTCCCTCACCT	26	C	0.065	0.0629
NC_005101.4-89813602	TTTCTCACCACGGGATCTCCTTTCACA	12	G	0.0082	0.479
NC_005101.4-89900314	TAGCAGAATACCCTCTCTCCTGTGTCT	13	C	0.0357	0.0351
NC_005101.4-89900314	TAGCAGAATACCCTCTCTCCTGTGTCT	26	C	0.027	0.0267
NC_005101.4-90005267	GTCGGTGATACATCTGCTCCCATTGTT	3	C	0.1565	0.173
NC_005101.4-90005267	GTCGGTGATACATCTGCTCCCATTGTT	4	G	0.0603	0.0585
NC_005101.4-90023283	TGCAAGCCAACAATGGCTCCCATCACT	4	A	0.0168	0.4813
NC_005101.4-90023283	TGCAAGCCAACAATGGCTCCCATCACT	7	C	0.1525	0.2219
NC_005101.4-90086118	ACCTCCAGCACATGCTCTCCTGTCTTT	8	G	0	0.4814
NC_005101.4-90115905	GAACTGACTACACCCTCTCCTTGTAAT	23	G	0.193	0.3221
NC_005101.4-90604085	TAAGCCTACACTGATGCTCCACCTCCG	27	G	0.0179	0.4898
NC_005101.4-90608640	AATAACTCTACCAGCACTCCTACAGCG	23	C	0.2195	0.2989
NC_005101.4-91128163	TTTAAAAGCACACCTGCTCCTTCTCTG	24	T	0.1271	0.4971
NC_005101.4-91156249	AGAAAATTGACTTTCCCTCCTCCAATA	24	A	0.1518	0.1983
NC_005101.4-91160561	AAAGCCTATACCTTACCTCCTAATTCT	8	A	0.0442	0.0433
NC_005101.4-91246864	CATTTCCCCACCATTCCTCCAGACCTT	3	T	0	0.4649
NC_005101.4-91280251	TACCCTCATACAGGCCCTCCAACTTTG	24	T	0	0.4641
NC_005101.4-91280251	TACCCTCATACAGGCCCTCCAACTTTG	25	T	0.1062	0.1613
NC_005101.4-91317597	GTTGGTCTTACCTCAGCTCCCAGGTGT	24	G	0.0261	0.0257
NC_005101.4-91417401	GTAAAATGTACCTCAGCTCCTAACATT	7	T	0	0.4764
NC_005101.4-91722448	TCTCTCTCTACATCTTCTCCAGGCTCT	4	C	0	0.4728
NC_005101.4-91761490	CCGTAATGAACATGCTCTCCCAATGTA	3	G	0.0088	0.483
NC_005101.4-91894492	GTACCACACACCTTCTCTCCCTTTCAA	14	T	0.0517	0.462
NC_005101.4-92092151	GGCACTCATACCTGTACTCCGAGCTGA	21	G	0.0339	0.0333
NC_005101.4-92171426	GCCTGTCTGACTCTACCTCCCAAGTGC	2	C	0.0261	0.0257
NC_005101.4-92207607	CCCACTCCCACCTACCCTCCCATCACA	15	C	0.0333	0.0488
NC_005101.4-92243138	TGGACCACTACCAAACCTCCATGTAGC	22	T	0.4107	0.3367
NC_005101.4-92243138	TGGACCACTACCAAACCTCCATGTAGC	25	A	0.4107	0.3367
NC_005101.4-92357911	TCCTCCAGCACCATGTCTCCTTATAAC	12	C	0.0833	0.1099
NC_005101.4-92357911	TCCTCCAGCACCATGTCTCCTTATAAC	26	A	0.1333	0.4988
NC_005101.4-92502966	TGAATGGAAACTGAAGCTCCGATCATT	5	T	0.0965	0.4076
NC_005101.4-92502966	TGAATGGAAACTGAAGCTCCGATCATT	6	G	0	0.462
NC_005101.4-92673997	TTCTCTAGAACAGGTCCTCCCCTATCT	23	T	0.1802	0.3389
NC_005101.4-92724941	ATCAATAACACACCTACTCCAATAGGA	7	A	0	0.48
NC_005101.4-92793550	CCAAACCCTACCAGAGCTCCCAGGAAC	1	C	0.0254	0.0251
NC_005101.4-92793550	CCAAACCCTACCAGAGCTCCCAGGAAC	7	C	0.3761	0.3054
NC_005101.4-92888334	TCACTCATTACCCTCCCTCCTCTCCTA	7	A	0.065	0.078

NC_005101.4-92963465	CGTCTGAGTACATTTCCTCCATGATGG	1	C	0.0833	0.095
NC_005101.4-92963465	CGTCTGAGTACATTTCCTCCATGATGG	2	G	0.1333	0.1244
NC_005101.4-93104488	GTGATTTTAACCTTGGCTCCGATTTGG	21	G	0	0.4844
NC_005101.4-93106746	AGATATTATACCTTCTCTCCTTTCTCT	12	C	0.0631	0.0611
NC_005101.4-93126936	TGTTTCTACACCAGATCTCCTTACACA	8	A	0.0261	0.0257
NC_005101.4-93126936	TGTTTCTACACCAGATCTCCTTACACA	16	T	0	0.4875
NC_005101.4-93144322	CGGCCCTGAACCTGTTCTCCTGTCATT	2	G	0.0508	0.0655
NC_005101.4-93194318	AACAGGCACACAGATTCTCCTGAAGTT	25	G	0.1652	0.1659
NC_005101.4-93198923	AGATGGTAAACTGAGACTCCATTACTA	3	A	0.1532	0.1713
NC_005101.4-93198923	AGATGGTAAACTGAGACTCCATTACTA	26	T	0.0714	0.1172
NC_005101.4-93250123	ATCTCTCTCACTGAGTCTCCTTCATTC	16	T	0	0.4793
NC_005101.4-93335513	AAAGTTGCAACATTCTCTCCCTCAGTT	2	A	0.0513	0.0973
NC_005101.4-93335513	AAAGTTGCAACATTCTCTCCCTCAGTT	3	A	0.094	0.1049
NC_005101.4-93358432	TATCTACAGACCTGTCCTCCAGTTCAT	16	C	0.0254	0.0415
NC_005101.4-93384420	TACCCTAGCACTACTGCTCCTTCCTAC	7	A	0	0.4793
NC_005101.4-93388185	TTATTTCACACCCCATCTCCAGGGACT	12	C	0.0168	0.4743
NC_005101.4-93419106	ATTTCAATCACTGCCCCTCCTGCTGGT	6	A	0.0265	0.0262
NC_005101.4-93530021	TCCGAATCTACCTAAGCTCCCTGTACC	2	C	0.0813	0.1356
NC_005101.4-93566495	GGATTAGTCACCGCAGCTCCCACCCCC	14	C	0.0244	0.0398
NC_005101.4-93566495	GGATTAGTCACCGCAGCTCCCACCCCC	27	C	0.0732	0.1001
NC_005101.4-93572518	TTTTTAAGTACATCGTCTCCTTAGCCC	9	T	0.0085	0.4823
NC_005101.4-93664607	CGTGCCTGTACCTTCTCTCCCAGCTCA	7	T	0.0167	0.4765
NC_005101.4-93695123	CTCCGTCACACCTGCTCTCCCTGGGAT	4	C	0.0569	0.1954
NC_005101.4-93698441	GGATGAGGTACTCACTCTCCCTCAAGT	3	A	0.0169	0.4793
NC_005101.4-93698441	GGATGAGGTACTCACTCTCCCTCAAGT	5	G	0.0169	0.4793
NC_005101.4-93714959	CGATAAAAGACCCAACCTCCATGGAAG	1	C	0.1066	0.2457
NC_005101.4-93714959	CGATAAAAGACCCAACCTCCATGGAAG	2	G	0.1066	0.2457
NC_005101.4-93716576	AACTTTGAGACAGTAGCTCCATGTAGC	24	T	0.0259	0.4836
NC_005101.4-93725010	CGGCAAACAACACAGACTCCCAAGTTA	2	G	0.1322	0.1653
NC_005101.4-93768730	TTTCCAGGAACCGTTGCTCCAATCCAC	12	C	0.0265	0.0262
NC_005101.4-93768730	TTTCCAGGAACCGTTGCTCCAATCCAC	13	G	0	0.4786
NC_005101.4-93832672	CCACAGCAAACATGGGCTCCAGCAGGA	12	A	0.1739	0.3094
NC_005101.4-93832672	CCACAGCAAACATGGGCTCCAGCAGGA	25	G	0.0259	0.0255
NC_005101.4-93847886	CGTTTGTCTACATGCCCTCCCTCATGT	2	G	0	0.4773
NC_005101.4-93847886	CGTTTGTCTACATGCCCTCCCTCATGT	15	C	0	0.4773
NC_005101.4-93893725	AGAATGGGCACAGCTTCTCCTGACAGC	15	T	0.0826	0.2415
NC_005101.4-93924960	AATAGAAGCACCTTGGCTCCCGGATTA	22	G	0.0684	0.066
NC_005101.4-93950247	ACTCCACAAACCTATCCTCCTCACACA	6	A	0.1488	0.2415
NC_005101.4-93950247	ACTCCACAAACCTATCCTCCTCACACA	23	A	0	0.4807
NC_005101.4-94007287	TAGAAGAGAACATGAGCTCCATTCTCA	12	A	0	0.4793
NC_005101.4-94104799	AGGTCTTACACAACTTCTCCATCACAA	3	G	0.0721	0.086
NC_005101.4-94115414	ATGCGCTCCACTCCTCCTCCCCACAGG	4	C	0.0325	0.032
NC_005101.4-94115414	ATGCGCTCCACTCCTCCTCCCCACAGG	13	C	0.0163	0.032
NC_005101.4-94151183	CACTATGTCACCTGTCCTCCTTCATAG	9	C	0	0.4773
NC_005101.4-94158105	AAAGGTAATACAATGCCTCCCCACCCC	26	C	0.2149	0.3374
NC_005101.4-94166289	TATTCCTGTACTAGCTCTCCCTGCCTC	8	G	0.1983	0.343
NC_005101.4-94204983	GCCTCCCTGACTGGGACTCCATGGTTC	16	A	0.1949	0.3483
NC_005101.4-94217294	ACATCTCTCACACTATCTCCCCTCTGT	14	T	0	0.4793
NC_005101.4-94217294	ACATCTCTCACACTATCTCCCCTCTGT	26	G	0.1721	0.2903
NC_005101.4-94222059	TGTGCCAGGACTTGCCCTCCATTTAAG	8	G	0.1739	0.2519
NC_005101.4-94223121	TAGTTTGTTACCTGTCCTCCCTAGGTT	4	T	0	0.4813

NC_005101.4-94223121	TAGTTTGTTACCTGTCCTCCCTAGGTT	27	T	0.1949	0.4738
NC_005101.4-94237750	AATGGATATACAGTTGCTCCCCCTGCT	13	G	0.1849	0.2568
NC_005101.4-94257121	CAGCTGAGGACATCATCTCCTGCATCC	9	G	0.0744	0.0868
NC_005101.4-94291929	TGTGGTGGGACCTTGGCTCCTTCAGAA	5	G	0.0569	0.0705
NC_005101.4-94292051	TCTCTTGAAACAGGGTCTCCTGTGTCT	27	T	0	0.4793
NC_005101.4-94306244	CTCTTAGAAACCTGGGCTCCCTGTGTG	3	C	0.0246	0.0243
NC_005101.4-94309647	CACTACGGAACTGGCACTCCCCAAGTG	6	C	0.0082	0.4755
NC_005101.4-94309647	CACTACGGAACTGGCACTCCCCAAGTG	21	C	0.0082	0.4755
NC_005101.4-94349393	CAGGCACTCACTGAGGCTCCTTTCTGC	4	G	0.0811	0.4351
NC_005101.4-94349393	CAGGCACTCACTGAGGCTCCTTTCTGC	15	G	0.0614	0.1075
NC_005101.4-94411961	TATTCCCAAACATCACCTCCATGTGTG	2	A	0.1066	0.4992
NC_005101.4-94418788	TATGTTCAAACCTAATCTCCATGGTGA	4	G	0.1171	0.1103
NC_005101.4-94429226	CAGGCAGGCACCATTTCTCCAGACTCC	24	C	0.0885	0.499
NC_005101.4-94439579	GAAGATAAGACCAGGCCTCCTCCAAAA	25	A	0	0.4773
NC_005101.4-94494144	CCTGTAACAACAACAGCTCCTCACCCC	25	C	0.3445	0.2852
NC_005101.4-94496168	GGCACATCGACTGCCTCTCCCATTATA	6	A	0	0.4807
NC_005101.4-94501846	TCCACCGTCACACATCCTCCATGTAGA	14	A	0	0.4592
NC_005101.4-94504940	AACAAATGAACAAGGTCTCCTGTGTGG	22	G	0.0254	0.0251
NC_005101.4-94571142	CCTTCAGTCACCCAACCTCCTAGCCTA	16	C	0.0536	0.0521
NC_005101.4-94601184	TGTCCATGCACCCCTTCTCCTGCTTCA	22	G	0.2679	0.496
NC_005101.4-94621954	TCTGCCTTTACTGCCTCTCCCCTGGAT	9	T	0.0531	0.1316
NC_005101.4-94642211	CTGGCCGTGACGTCATCTCCAATCCCG	23	T	0	0.4749
NC_005101.4-94658881	ACAAAAACAACTCAGCCTCCCCTGCTT	3	A	0	0.4682
NC_005101.4-94740728	TTTCTGGAGACGGGTTCTCCCTCTGTA	12	G	0.0085	0.479
NC_005101.4-94774369	GTAGCTCAAACAATTTCTCCTCTTCCT	22	C	0.1271	0.4991
NC_005101.4-94817968	CAAGGTCCCACTCATGCTCCATAGTAT	22	T	0.161	0.1894
NC_005101.4-94829299	TAAGGACATACTGCCCCTCCCCTACAC	27	C	0.2295	0.4515
NC_005101.4-94840456	CAAGGTGCTACAGCTTCTCCATCAGAG	12	A	0	0.4765
NC_005101.4-94840930	CTTTGTTACACTATGGCTCCGTAGTGT	15	G	0.1532	0.3338
NC_005101.4-94889602	GTGAGACCAACATCATCTCCTTCAGAA	1	G	0.1239	0.1757
NC_005101.4-94889602	GTGAGACCAACATCATCTCCTTCAGAA	3	G	0.1228	0.1744
NC_005101.4-94896481	GTGGTGCACACCTTTGCTCCCAGCACC	26	C	0.0769	0.074
NC_005101.4-94909173	ATGGGCAGAACAACAGCTCCCTACAAA	9	A	0.0087	0.4744
NC_005101.4-94917938	CACACACACACACACACTCCACTGACA	2	A	0.0894	0.0854
NC_005101.4-94922954	ATGAAGGTAACCGGCTCTCCACATGGT	24	T	0	0.4827
NC_005101.4-94922954	ATGAAGGTAACCGGCTCTCCACATGGT	25	G	0.0263	0.026
NC_005101.4-94931806	CCAGTCATCACAACAGCTCCGTGAAGT	21	G	0.2846	0.3888
NC_005101.4-94931806	CCAGTCATCACAACAGCTCCGTGAAGT	22	T	0.2846	0.3888
NC_005101.4-94932241	CCATTCCCCACCCTTTCTCCCTGGGTA	25	G	0.0357	0.0853
NC_005101.4-94944188	GTAAATAAAACCTTTCCTCCTTCAAGC	6	T	0.2397	0.4146
NC_005101.4-95057671	GTGGCTATTACTACTGCTCCTGTCAGC	15	T	0.2759	0.3916
NC_005101.4-95057671	GTGGCTATTACTACTGCTCCTGTCAGC	22	G	0	0.4728
NC_005101.4-95060966	TGAGTTGCTACCTAACCTCCTTCTCAC	4	G	0.0744	0.2814
NC_005101.4-95062850	TCTTCCCATACAGAGTCTCCTGTGTTC	13	G	0.265	0.3504
NC_005101.4-95074619	AGATGCAGAACTCTCACTCCTCCAGCA	14	T	0	0.4844
NC_005101.4-95074619	AGATGCAGAACTCTCACTCCTCCAGCA	16	A	0	0.4856
NC_005101.4-95120018	CAAGGTCACACTCGTCCTCCTGGGCCT	13	C	0	0.4833
NC_005101.4-95120018	CAAGGTCACACTCGTCCTCCTGGGCCT	14	G	0.0259	0.0585
NC_005101.4-95214458	CACATCGGCACCACAACTCCAAACCTT	6	C	0.2114	0.4722
NC_005101.4-95214458	CACATCGGCACCACAACTCCAAACCTT	26	T	0.1626	0.2499
NC_005101.4-95229385	GCTGAGCGTACTCCTTCTCCCTGCTTC	16	T	0	0.4698

NC_005101.4-95231680	ATACATACAACATTGCCTCCATTTTAT	9	A	0.0083	0.4732
NC_005101.4-95237083	AGAAGATTCACTGTGTCTCCTGCGATT	23	C	0	0.4793
NC_005101.4-95244347	TCCACAACCACAGAGCCTCCCCTTTGC	6	A	0	0.4693
NC_005101.4-95258175	CCCTCCCCCACCTTCGCTCCTCACTAA	9	C	0.1849	0.4152
NC_005101.4-95298565	TCACTTTCAACTCGGGCTCCTACACCT	21	T	0.0917	0.4991
NC_005101.4-95298565	TCACTTTCAACTCGGGCTCCTACACCT	23	C	0.1167	0.2665
NC_005101.4-95303043	TGCAAGTCGACTGAAACTCCAGGACAG	3	C	0.1475	0.2279
NC_005101.4-95369846	GCGTGGTAGACAGCTCCTCCTTCTAAA	3	G	0.0992	0.1235
NC_005101.4-95369976	AGAGAGGCTACCCGTTCTCCAGTTGAT	14	G	0	0.4819
NC_005101.4-95426967	CAAGTAGGAACAGCAACTCCGCCAGAC	21	G	0.2033	0.2989
NC_005101.4-95426967	CAAGTAGGAACAGCAACTCCGCCAGAC	23	C	0.2114	0.3141
NC_005101.4-95445438	TGCAACGGCACATATCCTCCATGGAAT	6	C	0.05	0.0644
NC_005101.4-95445438	TGCAACGGCACATATCCTCCATGGAAT	15	T	0	0.4793
NC_005101.4-95469365	ATCTGGTACACAGGCTCTCCTTAGCAA	12	A	0.025	0.0247
NC_005101.4-95470829	AATACGTTAACCCACTCTCCTCTCGTC	26	T	0	0.4787
NC_005101.4-95486557	CTCTTTGACACACTTGCTCCATTGCCA	13	C	0.0172	0.0339
NC_005101.4-95486557	CTCTTTGACACACTTGCTCCATTGCCA	15	T	0	0.488
NC_005101.4-95562194	TGCAGGTGAACTAGTCCTCCATAAATC	2	G	0.3103	0.3995
NC_005101.4-95562194	TGCAGGTGAACTAGTCCTCCATAAATC	25	A	0.2845	0.4375
NC_005101.4-95562260	TGGCTGAACACAGGGTCTCCAACATTA	4	C	0.1393	0.1573
NC_005101.4-95700206	GGACTACTCACCCAGGCTCCTTCATCA	21	T	0.2562	0.4507
NC_005101.4-95727298	TTCCTGGTGACTAGTCCTCCCTGTTGC	4	C	0.2583	0.4622
NC_005101.4-95810162	TCCACTACAACCTCAACTCCCTGGCAT	2	C	0.0087	0.0425
NC_005101.4-96261203	ATAACATAGACATTTGCTCCCTCATGT	2	T	0	0.4698
NC_005101.4-96261203	ATAACATAGACATTTGCTCCCTCATGT	13	T	0	0.4715
NC_005101.4-96312525	TGCCCCACCACACGTGCTCCACTATGT	14	G	0.0732	0.0705
NC_005101.4-96316265	CTCCTCAGCACCATCCCTCCTGACTTC	15	C	0.0265	0.0262
NC_005101.4-96403368	CAAAGCTCTACATGGTCTCCCATGTCA	4	A	0	0.4773
NC_005101.4-96403368	CAAAGCTCTACATGGTCTCCCATGTCA	16	T	0	0.4773
NC_005101.4-96423730	TAAAAGAACACTGTGGCTCCTGCACAG	23	C	0	0.4779
NC_005101.4-96470767	AGTCAGAAGACCACGCCTCCTTTGCAT	23	T	0.0328	0.0322
NC_005101.4-96484362	TCTCCTATGACAGCACCTCCTCTAATT	16	C	0.0254	0.0251
NC_005101.4-96660789	CAAGGGAACACCTGATCTCCTGAGTCT	12	C	0.0246	0.0243
NC_005101.4-96660789	CAAGGGAACACCTGATCTCCTGAGTCT	21	T	0.0082	0.4755
NC_005101.4-96663870	TCCTTTGAGACTTAATCTCCCCTTCTT	16	T	0.1875	0.3418
NC_005101.4-96669954	AGATACAGAACCTCTGCTCCTCTAGCA	6	C	0.1967	0.4289
NC_005101.4-96719177	TGAATAAGAACATAGTCTCCACAAGTA	9	A	0.1271	0.1894
NC_005101.4-96886112	AGGTCCCTTACCCAGTCTCCCTGATTT	27	T	0.0948	0.1058
NC_005101.4-96932453	TTTCTCCTCACAGTTCCTCCATCCCTC	13	G	0	0.4765
NC_005101.4-96987879	TTGAAGAAAACTGTATCTCCCCCCTCA	1	T	0	0.4677
NC_005101.4-96987879	TTGAAGAAAACTGTATCTCCCCCCTCA	2	T	0	0.4677
NC_005101.4-96993347	ATGTATCAAACCATCCCTCCATTCCTG	16	C	0.1351	0.2531
NC_005101.4-97084432	GACATGGCCACACCTACTCCAACAAGT	4	A	0.3009	0.4715
NC_005101.4-97084432	GACATGGCCACACCTACTCCAACAAGT	16	A	0.2301	0.2678
NC_005101.4-97125734	TTGTAGTACACTTCAGCTCCCTATCAG	22	T	0.2373	0.2926
NC_005101.4-97127176	TGCTATCCCACCTCCCCTCCCTCTGGT	14	C	0.036	0.0526
NC_005101.4-97262367	ATTACCATTACCCGTTCTCCTGAGAAA	2	T	0	0.4859
NC_005101.4-97262367	ATTACCATTACCCGTTCTCCTGAGAAA	14	G	0	0.4859
NC_005101.4-97307504	TGCTGACGCACAGTCACTCCATATGAT	7	C	0.0248	0.0245
NC_005101.4-97574519	CCAATAACTACCACATCTCCTCTCGGT	24	C	0.2869	0.3667
NC_005101.4-97574519	CCAATAACTACCACATCTCCTCTCGGT	25	G	0.1475	0.2515

NC_005101.4-97579234	TAAATATATACCATGTCTCCTTTCTGT	1	T	0.0175	0.4778
NC_005101.4-97594385	CTTCCAAATACTTCAGCTCCCTCTTCT	4	C	0.2583	0.3441
NC_005101.4-97594385	CTTCCAAATACTTCAGCTCCCTCTTCT	22	T	0.025	0.0247
NC_005101.4-97709233	CTGCAACAAACCTGTACTCCCAGGTTA	1	C	0.0179	0.4922
NC_005101.4-97867950	TTTAAAGAAACTGATTCTCCTTTCCAT	15	T	0	0.4793
NC_005101.4-98068533	ACATGTCTTACCTCTGCTCCTAAGTCC	3	A	0.0427	0.4987
NC_005101.4-98068533	ACATGTCTTACCTCTGCTCCTAAGTCC	5	G	0	0.4868
NC_005101.4-98099234	TTTAATTAGACCTACCCTCCATTATTG	25	T	0.2768	0.4394
NC_005101.4-98182319	AACATAAACACAGAGACTCCCACAGTT	1	A	0.0085	0.4714
NC_005101.4-98201312	CCCAATAACACACCTCCTCCAGCAAGA	14	C	0.292	0.4309
NC_005101.4-98229854	CTAGAGAGGACTGACACTCCTTTCTAA	5	A	0	0.4693
NC_005101.4-98229854	CTAGAGAGGACTGACACTCCTTTCTAA	12	T	0	0.4709
NC_005101.4-98245953	CCACAATACACTGGCCCTCCTCTGTCA	8	A	0.1034	0.1134
NC_005101.4-98265520	TGAAAGAGGACACATGCTCCAAGCAAA	7	A	0	0.4773
NC_005101.4-98265520	TGAAAGAGGACACATGCTCCAAGCAAA	9	G	0	0.4773
NC_005101.4-98310094	TAAGTCCCTACAACAACTCCTGGTCTG	22	G	0.0248	0.0562
NC_005101.4-98394767	TGCAGTTAAACAATGACTCCAGGTTTA	2	G	0.2035	0.4648
NC_005101.4-98394767	TGCAGTTAAACAATGACTCCAGGTTTA	12	A	0.1579	0.2155
NC_005101.4-98492291	ATCATGCATACTATTGCTCCTACTCCC	22	A	0	0.4749
NC_005101.4-98498301	CTGTGTCCCACAGCACCTCCTCCCAAA	25	A	0.1405	0.4004
NC_005101.4-98617549	GCACTTCAAACCTCTCCTCCAGACATT	14	C	0.0244	0.0241
NC_005101.4-98673484	CTGTATAGGACAGGATCTCCATGTTAC	6	T	0	0.4887
NC_005101.4-98673484	CTGTATAGGACAGGATCTCCATGTTAC	25	T	0.115	0.1968
NC_005101.4-98677775	CCCTTTTCCACATTTTCTCCTGGCCAC	24	C	0.1074	0.1447
NC_005101.4-98792935	TGTCGGGTCACCGTGCCTCCCTACCTG	5	G	0.2017	0.2328
NC_005101.4-98814666	CTCACTGTTACCTGTTCTCCTGGAGGA	12	C	0.0413	0.0405
NC_005101.4-99067947	TGCAGCCAGACAGGAACTCCAATGAAG	6	C	0	0.4851
NC_005101.4-99205462	AATAGACCCACAACAGCTCCCAGGACT	14	C	0.1391	0.4425
NC_005101.4-99236440	TCATCAGGCACCGTTACTCCTTCCACT	12	C	0	0.4793
NC_005101.4-99425874	CAAATACCAACATGGCCTCCATAAACA	3	A	0.2667	0.4444
NC_005101.4-99425874	CAAATACCAACATGGCCTCCATAAACA	22	T	0	0.4773
NC_005101.4-99574617	TGCTATATTACAGTGGCTCCCAGTTGA	9	T	0.1271	0.1759
NC_005101.4-99574617	TGCTATATTACAGTGGCTCCCAGTTGA	27	A	0.1186	0.2344
NC_005101.4-99739690	GGATAGCTGACCCTGCCTCCTCTGCCT	13	C	0.1681	0.4994
NC_005101.4-99739836	TACAATGCAACAGGATCTCCATGATAC	5	A	0.0244	0.0241
NC_005101.4-99739836	TACAATGCAACAGGATCTCCATGATAC	25	T	0.082	0.4966
NC_005101.4-99821747	CATTATATCACCTGACCTCCAATTTAT	7	A	0.0826	0.109
NC_005101.4-99854937	CCAAGGCGGACCAGGTCTCCTGTTTAT	13	A	0.2435	0.4891
NC_005101.4-99854937	CCAAGGCGGACCAGGTCTCCTGTTTAT	23	T	0	0.4833
NC_005101.4-99905690	CTCCTTACTACTCACCCTCCTATGATT	23	T	0.1404	0.16
NC_005101.4-99914792	TATGCAAGCACAAGACCTCCATCACTC	14	G	0.0702	0.0677
NC_005101.4-99957942	GAACCTATGACTCCAGCTCCATATGTA	1	G	0	0.4772
NC_005101.4-100036251	TGATCTTCAACCTCTGCTCCATTTACT	21	A	0	0.4779
NC_005101.4-100119063	CTAATGTGGACAATGGCTCCTTAACTT	24	A	0	0.48
NC_005101.4-100169897	CACTTCCCAACCCCAACTCCTACCTTC	4	T	0	0.4793
NC_005101.4-100269217	TCCACTCTCACCTCACCTCCCTAGCAT	4	A	0.0431	0.0746
NC_005101.4-100323010	TGCTTTCTGACATTATCTCCATGCTAA	4	T	0.0261	0.0257
NC_005101.4-100323010	TGCTTTCTGACATTATCTCCATGCTAA	25	T	0.1053	0.1454
NC_005101.4-100356444	CTCTCAGATACTTTGCCTCCAACCAGG	14	T	0	0.4624
NC_005101.4-100356444	CTCTCAGATACTTTGCCTCCAACCAGG	15	G	0.1947	0.2036
NC_005101.4-100446602	CTCTTATATACCTACTCTCCTTAAGTT	24	A	0	0.4833

NC_005101.4-100446602	CTCTTATATACCTACTCTCCTTAAGTT	26	T	0.1391	0.4891
NC_005101.4-100472162	CCTTCCCCTACCCTTCCTCCTCCCTTG	21	T	0.0168	0.4661
NC_005101.4-100472162	CCTTCCCCTACCCTTCCTCCTCCCTTG	22	C	0.0084	0.4682
NC_005101.4-100520158	TGCTCTACCACCCCCACTCCCTCCCTC	14	C	0.1453	0.2299
NC_005101.4-100520158	TGCTCTACCACCCCCACTCCCTCCCTC	23	C	0.2712	0.362
NC_005101.4-100539851	AACACTTGTACACTGTCTCCTAGGCTC	26	T	0.1121	0.1923
NC_005101.4-100610804	CTTTCACAGACACATACTCCTCCAGAG	9	G	0.0259	0.0255
NC_005101.4-100801445	CACACACACACAGGATCTCCAGGTGGA	25	G	0.2655	0.4671
NC_005101.4-100942767	CCTTCAATAACTTCATCTCCCCTGGAT	21	C	0.1833	0.4728
NC_005101.4-100942767	CCTTCAATAACTTCATCTCCCCTGGAT	22	C	0.1917	0.4708
NC_005101.4-100993007	CTGATGGAAACTATCACTCCTTCATAA	22	T	0.2035	0.481
NC_005101.4-100996412	AGTAAGCAAACATAACCTCCAGCCTCA	23	C	0.1983	0.4866
NC_005101.4-101155010	TGTGTAAAGACCTCATCTCCAGAGCAC	16	T	0.1565	0.2268
NC_005101.4-101155010	TGTGTAAAGACCTCATCTCCAGAGCAC	24	G	0.0261	0.0257
NC_005101.4-101249320	CACCCTATCACAATCCCTCCACCTTCT	14	T	0.0982	0.4952
NC_005101.4-101254379	ATAACCAGAACACTTGCTCCACTATGT	15	T	0.0855	0.0973
NC_005101.4-101280169	CACGTATATACACACACTCCCAAGCAC	23	A	0.0086	0.4836
NC_005101.4-101453312	CCCTTTTCTACTCACCCTCCCCTCTTC	1	C	0.0354	0.0348
NC_005101.4-101579822	GAAGTTACTACATCTTCTCCCACTGTG	4	G	0	0.4841
NC_005101.4-101726524	TTGTGAGCTACTCTGCCTCCACTTACA	1	T	0	0.4902
NC_005101.4-101741239	TGGGTGGGAACACCCCCTCCCAGGTCA	12	A	0.0088	0.4693
NC_005101.4-101741239	TGGGTGGGAACACCCCCTCCCAGGTCA	25	T	0.2478	0.3982
NC_005101.4-101862831	CATCGACAGACCTGCTCTCCAGTGCAT	8	A	0.1228	0.4998
NC_005101.4-101862831	CATCGACAGACCTGCTCTCCAGTGCAT	16	T	0	0.4778
NC_005101.4-101906482	TTCCTACCCACTAGTCCTCCTCATTCC	2	T	0	0.4793
NC_005101.4-101906482	TTCCTACCCACTAGTCCTCCTCATTCC	23	A	0.1026	0.2235
NC_005101.4-102029891	CAGGAGATGACCTTGCCTCCAAGAACA	25	A	0	0.4786
NC_005101.4-102068220	GGCATCGTCACTGTGCCTCCAGTGGTG	12	T	0	0.4841
NC_005101.4-102068220	GGCATCGTCACTGTGCCTCCAGTGGTG	14	T	0.2054	0.2758
NC_005101.4-102142856	CTGCAACCTACCCAGCCTCCTCCACCA	13	C	0.1983	0.2172
NC_005101.4-102142856	CTGCAACCTACCCAGCCTCCTCCACCA	15	G	0	0.4813
NC_005101.4-102320059	GAACTGATTACTGGCTCTCCAGATTGT	14	G	0	0.4773
NC_005101.4-102353770	CCCTTTCCAACTGTTCCTCCTGATGAG	8	C	0.0424	0.0415
NC_005101.4-102353770	CCCTTTCCAACTGTTCCTCCTGATGAG	9	A	0.6053	0.4473
NC_005101.4-102410556	GATCAGCCCACTGGATCTCCCTTATAC	24	A	0	0.4765
NC_005101.4-102410556	GATCAGCCCACTGGATCTCCCTTATAC	25	T	0	0.4765
NC_005101.4-102420991	TAAGTGGTTACCTCCTCTCCTTTCCTC	6	G	0	0.4715
NC_005101.4-102420991	TAAGTGGTTACCTCCTCTCCTTTCCTC	8	T	0	0.4715
NC_005101.4-102472526	TCTCCCATTACCCCTTCTCCTGCTTCC	13	C	0.0328	0.048
NC_005101.4-102536482	ATGATCTCCACACCACCTCCTGCTGCT	9	C	0.0248	0.0562
NC_005101.4-102674573	ACGTGGACTACAGGTTCTCCATTTCTC	26	T	0.2982	0.2893
NC_005101.4-102695168	TATAGCATAACCATTCCTCCCTCAAAT	14	T	0.0342	0.0336
NC_005101.4-102707724	AAGACAGCTACAAAGTCTCCTTTCCGC	26	G	0.2523	0.461
NC_005101.4-102815174	AAATCATCCACACAGACTCCTAACGTG	25	G	0.1901	0.4933
NC_005101.4-102867120	TGCACAGGCACATACACTCCATACCAC	8	G	0.0789	0.0918
NC_005101.4-102867120	TGCACAGGCACATACACTCCATACCAC	21	A	0.0696	0.0671
NC_005101.4-103029778	AAATAACCCACCTCTACTCCTGCTATA	6	A	0.1356	0.2219
NC_005101.4-103046398	CCCCATCCCACTTCCCCTCCTCCTGCT	4	C	0.0656	0.2956
NC_005101.4-103170539	CCAAACACCACCAGAGCTCCCAGGGAC	26	A	0.0588	0.0571
NC_005101.4-103271551	CTTGCCAGAACCTTTTCTCCTTTCCTT	12	C	0.0678	0.0655
NC_005101.4-103385118	CACGCTCACACAAATTCTCCCACATAC	21	C	0.0174	0.0508

NC_005101.4-103385118	CACGCTCACACAAATTCTCCCACATAC	25	T	0	0.4778
NC_005101.4-103484738	TTCTACTTAACCCTTGCTCCTGTGATA	6	C	0.2212	0.4923
NC_005101.4-103504480	GAAACAAGTACTGGACCTCCCACAAGA	8	G	0.0244	0.0241
NC_005101.4-103542757	ATCTACTCTACCCTAACTCCTCTTTGG	26	G	0.2034	0.4977
NC_005101.4-103551394	ACCAGAGGTACCGCATCTCCCCTCTCT	26	C	0.0702	0.0677
NC_005101.4-103566749	TCACCCAGAACAACCTCTCCATGCTTG	23	G	0.2589	0.4617
NC_005101.4-103577399	CACAATTCTACAGAGCCTCCTTCTCTT	5	A	0	0.4851
NC_005101.4-103595029	CCAAGTTATACACTTGCTCCCTTTGGT	13	C	0.177	0.1757
NC_005101.4-103595412	AGAAAATCAACAGCTCCTCCCTACTAT	8	C	0.2727	0.4693
NC_005101.4-103595412	AGAAAATCAACAGCTCCTCCCTACTAT	9	A	0.1917	0.5
NC_005101.4-103654574	CCACCTCCCACCAGGCCTCCCCACTTT	4	C	0.0588	0.1181
NC_005101.4-103730964	AGGCTTAGAACCCAGGCTCCTGGGTCC	7	A	0.2377	0.3492
NC_005101.4-103845909	CACATGCTAACATAGTCTCCATATTCT	16	T	0.0804	0.0771
NC_005101.4-103861209	GGACTGACAACCACAGCTCCATGTGGT	16	G	0.1638	0.5
NC_005101.4-103861209	GGACTGACAACCACAGCTCCATGTGGT	21	A	0.0672	0.0805
NC_005101.4-103890900	TTGTAAATTACTGAATCTCCTGGAAGG	3	G	0.1311	0.4999
NC_005101.4-103890900	TTGTAAATTACTGAATCTCCTGGAAGG	12	T	0	0.4807
NC_005101.4-103974134	GGAACACTGACCTGGGCTCCATCCTCA	2	G	0.0407	0.0553
NC_005101.4-104059243	ACGCTCTATACATCCACTCCTTATGGT	25	G	0.082	0.4952
NC_005101.4-104067795	GTCTTTCCCACAGGGACTCCCTGCTAC	15	G	0	0.4807
NC_005101.4-104119729	CACACACACACACTCACTCCTATGCAT	13	C	0.0526	0.0512
NC_005101.4-104119729	CACACACACACACTCACTCCTATGCAT	22	A	0.0526	0.0512
NC_005101.4-104123532	CCTATCAATACTGCCACTCCTCATGGG	23	A	0	0.4734
NC_005101.4-104160339	AGGAGACACACAGACACTCCATGAGAA	24	A	0.4741	0.3617
NC_005101.4-104209080	ACATACTGCACCTGTTCTCCATCTCTT	4	T	0	0.4773
NC_005101.4-104256023	ACTCTGTACACCTGACCTCCTGTTTCC	14	G	0.0333	0.0328
NC_005101.4-104256023	ACTCTGTACACCTGACCTCCTGTTTCC	16	C	0.275	0.4416
NC_005101.4-104295116	CTAACTAGTACAGCATCTCCCACTTTA	13	G	0.1557	0.4997
NC_005101.4-104295116	CTAACTAGTACAGCATCTCCCACTTTA	21	C	0.157	0.4995
NC_005101.4-104305497	TAGAGAGCCACTAAGCCTCCCTGGCAG	1	T	0	0.4814
NC_005101.4-104305497	TAGAGAGCCACTAAGCCTCCCTGGCAG	13	A	0	0.482
NC_005101.4-104343866	GTACTTCTTACTAGTTCTCCCCGCCAG	8	T	0.0085	0.4655
NC_005101.4-104343866	GTACTTCTTACTAGTTCTCCCCGCCAG	22	C	0.2627	0.5
NC_005101.4-104402805	CACAACCACACATATTCTCCCAACATA	14	A	0	0.482
NC_005101.4-104402805	CACAACCACACATATTCTCCCAACATA	22	A	0	0.482
NC_005101.4-104411355	ATGTAGTCAACTGGGACTCCTTGTATC	12	T	0.0089	0.4856
NC_005101.4-104416296	TTACCAACCACATGCTCTCCCCACATG	1	T	0	0.4887
NC_005101.4-104416296	TTACCAACCACATGCTCTCCCCACATG	5	C	0.0619	0.1084
NC_005101.4-104418571	CCACAACATACCGTGCCTCCACCAAAC	27	C	0.0932	0.119
NC_005101.4-104420081	AACAATATCACAGAGGCTCCACAGTGT	5	A	0.2114	0.2499
NC_005101.4-104430504	GGGTCGTTAACTATGACTCCTATGCTC	5	C	0.0357	0.0689
NC_005101.4-104450521	TTAATCCCCACATCAGCTCCACACAGA	7	C	0.0496	0.0484
NC_005101.4-104528754	TAGCTTGCCACTGCTACTCCTCCAGAC	4	C	0.0569	0.0553
NC_005101.4-104528754	TAGCTTGCCACTGCTACTCCTCCAGAC	9	C	0.1382	0.1695
NC_005101.4-104534092	ATTGGCCACACAGTTACTCCTATAGGC	14	T	0.1622	0.149
NC_005101.4-104621186	CTATTAACCACCCTGGCTCCCAGCCTC	24	C	0.0583	0.4958
NC_005101.4-104740261	TTTCTCTTTACCTCTACTCCTTTCTTT	16	A	0.0756	0.4965
NC_005101.4-104778703	AAGAGTTCTACAGTTGCTCCAACGTTA	3	G	0.2167	0.4688
NC_005101.4-104815847	GAGTCTTATACTCGAGCTCCACCCAGG	2	A	0.1304	0.2204
NC_005101.4-104815847	GAGTCTTATACTCGAGCTCCACCCAGG	9	T	0.0614	0.1953
NC_005101.4-104912144	TACTGTTTCACTGTGTCTCCAGTGTTT	12	T	0.087	0.4997

NC_005101.4-104912144	TACTGTTTCACTGTGTCTCCAGTGTTT	15	G	0.087	0.4997
NC_005101.4-104913737	GTCTGACTCACTAATCCTCCCTAACGT	26	G	0	0.4632
NC_005101.4-104956488	CAAACAACAACCCTGTCTCCAATTCCC	3	A	0.0085	0.4776
NC_005101.4-104984084	TGTGGCATCACAGCTCCTCCAAAAGCC	12	A	0.2566	0.3294
NC_005101.4-105078763	GAGGAATTGACAGCCTCTCCGGCCTCT	6	A	0	0.4819
NC_005101.4-105078763	GAGGAATTGACAGCCTCTCCGGCCTCT	21	G	0.0909	0.172
NC_005101.4-105131105	CTACATCAGACTTTTGCTCCCACTATC	2	T	0	0.4671
NC_005101.4-105131105	CTACATCAGACTTTTGCTCCCACTATC	26	T	0.1696	0.1699
NC_005101.4-105285397	GCATGGGCCACTATGACTCCCCAAGTC	2	C	0	0.4731
NC_005101.4-105673517	TAGCAGTGTACTCTCTCTCCTTCATGG	22	T	0.0248	0.0245
NC_005101.4-105754078	ACTGTGTGGACCATCTCTCCCTGTATG	3	T	0	0.4819
NC_005101.4-105814231	TAACCCCTCACTCATGCTCCAGTGAGT	12	T	0.1624	0.3685
NC_005101.4-106053144	GGATCAATCACCTTACCTCCTGCCTGC	4	T	0	0.48
NC_005101.4-106109002	GCTGCTATTACTGAGTCTCCACCTAAG	5	C	0.1849	0.2076
NC_005101.4-106111184	GATCCTAGCACACTTGCTCCTTTTCAT	27	T	0.0446	0.4885
NC_005101.4-106162144	AATTTCTTCACTGAGACTCCCTGCTCT	6	C	0.05	0.095
NC_005101.4-106353176	GGATCTTACACCCTCACTCCTCTCCCT	1	G	0.1062	0.1162
NC_005101.4-106353176	GGATCTTACACCCTCACTCCTCTCCCT	2	G	0.1239	0.4912
NC_005101.4-106464035	CCTTATCCCACCACCCCTCCCCTGATT	13	A	0	0.4755
NC_005101.4-106464035	CCTTATCCCACCACCCCTCCCCTGATT	16	C	0.0982	0.0934
NC_005101.4-106485544	ACTACTCACACTGTGCCTCCTTAACTC	14	T	0.275	0.4416
NC_005101.4-106512786	CTCAGCAAAACCTTGTCTCCTAGGACA	23	G	0.0902	0.4943
NC_005101.4-106579971	AAAGCAAATACATGAGCTCCAGGTTAC	4	G	0	0.4854
NC_005101.4-106723824	CATGGACTGACCACAACTCCCTCTAAA	9	G	0.1681	0.1678
NC_005101.4-106723824	CATGGACTGACCACAACTCCCTCTAAA	23	C	0.2991	0.4823
NC_005101.4-106724646	TTAACTTACACTGCTACTCCAAGGAGT	21	A	0.1092	0.4977
NC_005101.4-106736092	GGCTGTGAAACCCCTGCTCCCAATAAA	23	A	0.0081	0.481
NC_005101.4-106770051	TCATACTGGACCAACACTCCTACCCCT	5	A	0.2119	0.4997
NC_005101.4-106790553	AGGTCTTCCACTATTTCTCCTTTCCTT	21	T	0.0083	0.4835
NC_005101.4-106846280	CCAGGGGAGACAGCTTCTCCTTCTGTG	13	G	0.0442	0.0433
NC_005101.4-106846280	CCAGGGGAGACAGCTTCTCCTTCTGTG	15	T	0	0.4887
NC_005101.4-106859319	TCAGCCACAACTAATCCTCCCTGCTTT	12	T	0	0.4773
NC_005101.4-106951994	CAAAGATCAACCTTGCCTCCAGGTAAA	22	G	0	0.4864
NC_005101.4-106963901	CTGGATGACACCTGACCTCCGGCTCTG	21	G	0	0.4871
NC_005101.4-106997843	GTTTGAGACACAGCTTCTCCTTGACCT	1	G	0.2683	0.3964
NC_005101.4-106997843	GTTTGAGACACAGCTTCTCCTTGACCT	26	C	0.0732	0.0854
NC_005101.4-107233841	TAATCCGAAACTTGGGCTCCTGGAGAT	7	G	0	0.4841
NC_005101.4-107256207	ATTCATACTACTCATTCTCCCTCATTC	12	T	0	0.4785
NC_005101.4-107256207	ATTCATACTACTCATTCTCCCTCATTC	14	A	0.25	0.3468
NC_005101.4-107298876	GGCATGTTGACATCCTCTCCCTCTGTA	22	T	0.188	0.2107
NC_005101.4-107311873	GCCCCTTTGACTGCTTCTCCTTAGCTA	5	C	0.0496	0.0484
NC_005101.4-107445783	ATGCCACACACTACTCCTCCGCAGCCC	21	G	0	0.4807
NC_005101.4-107451302	GCTGCCTTGACTCCCTCTCCCCTACAG	26	A	0	0.48
NC_005101.4-107451302	GCTGCCTTGACTCCCTCTCCCCTACAG	27	G	0	0.48
NC_005101.4-107889078	CATAGAGAAACCACAACTCCCTTAACC	24	A	0.018	0.461
NC_005101.4-107889078	CATAGAGAAACCACAACTCCCTTAACC	25	A	0.018	0.461
NC_005101.4-107942074	TGCATCTGCACAACAGCTCCAAAACAT	5	T	0.0085	0.0251
NC_005101.4-107942074	TGCATCTGCACAACAGCTCCAAAACAT	25	C	0.1171	0.2656
NC_005101.4-108072909	GGAGTTCACACACCTCCTCCCCTTTAC	4	G	0.0081	0.0241
NC_005101.4-108230304	CTGCTCTACACATTGTCTCCGTATTTC	6	C	0	0.4654
NC_005101.4-108230304	CTGCTCTACACATTGTCTCCGTATTTC	15	G	0.4407	0.3436

NC_005101.4-108268314	GCCAGGAACACTCTGTCTCCTAAGACC	12	T	0.0569	0.0553
NC_005101.4-108268314	GCCAGGAACACTCTGTCTCCTAAGACC	15	G	0	0.4793
NC_005101.4-108383616	TCATGCATTACCATCTCTCCTCCATCC	15	C	0.0427	0.0418
NC_005101.4-108586156	AACAAGGCAACACCTACTCCAAGGCCA	5	A	0.1736	0.1585
NC_005101.4-108586156	AACAAGGCAACACCTACTCCAAGGCCA	27	A	0.1818	0.4997
NC_005101.4-108731993	TGGATAAGCACATTCTCTCCTACTGAG	1	T	0.2435	0.264
NC_005101.4-108731993	TGGATAAGCACATTCTCTCCTACTGAG	8	G	0.3421	0.4529
NC_005101.4-108738647	TATCACCTTACCCCCTCTCCCACACAT	25	C	0.1074	0.1017
NC_005101.4-108756034	ACACATGAAACTCTCTCTCCTGGTCAT	21	T	0.0526	0.0512
NC_005101.4-108898667	AGGTCACCTACATTGACTCCTGGGAGT	23	G	0.2087	0.5
NC_005101.4-108898667	AGGTCACCTACATTGACTCCTGGGAGT	26	G	0.1983	0.1923
NC_005101.4-108902910	CAGATATCGACAACATCTCCTGTATTA	15	A	0.0252	0.0249
NC_005101.4-108911972	CCATAAAGCACCATCGCTCCCTGCCTG	3	A	0	0.4793
NC_005101.4-109104736	GCACTAGGGACAGTACCTCCAGCCTTA	7	G	0.1849	0.4082
NC_005101.4-109133665	AGTTTAACCACAAAGACTCCATTCCTC	7	A	0	0.4773
NC_005101.4-109213746	GTACCTCACACAAATCCTCCGCCCATT	21	G	0.1138	0.2017
NC_005101.4-109363518	TGAGTCGGCACCGGGTCTCCTGTGTAT	7	G	0	0.4807
NC_005101.4-109363518	TGAGTCGGCACCGGGTCTCCTGTGTAT	13	G	0.1736	0.4999
NC_005101.4-109374215	GATCCCCTGACACAGCCTCCCAGTGAG	6	C	0.2389	0.4873
NC_005101.4-109374215	GATCCCCTGACACAGCCTCCCAGTGAG	24	T	0	0.4778
NC_005101.4-109380680	TACCCACGCACACTTACTCCCACATAA	8	G	0	0.4787
NC_005101.4-109425633	AGTAGACATACTCTCTCTCCTTGTCAC	6	A	0.0598	0.4854
NC_005101.4-109524484	CAAGTTCTGACATTGACTCCAGCATTT	8	T	0.0351	0.0345
NC_005101.4-109530666	ACCACAAGGACAAGCGCTCCACCATAT	6	A	0.0894	0.4967
NC_005101.4-109530666	ACCACAAGGACAAGCGCTCCACCATAT	15	C	0	0.4793
NC_005101.4-109622192	CATTCTAGCACTACCACTCCATGCTTA	14	C	0.009	0.0267
NC_005101.4-109662506	CTCTATCAAACCAAATCTCCTAAGGAC	22	A	0.2018	0.2599
NC_005101.4-109669223	CCAGCTCCCACAGGGACTCCTAGGCTC	1	C	0	0.4868
NC_005101.4-109669223	CCAGCTCCCACAGGGACTCCTAGGCTC	25	C	0	0.4868
NC_005101.4-109837203	GTAATCCCTACCTTCACTCCCCAGGAA	8	C	0.1681	0.2203
NC_005101.4-109838617	ACTCTACTAACGGCAGCTCCATTAGCC	2	C	0.0446	0.0605
NC_005101.4-109838617	ACTCTACTAACGGCAGCTCCATTAGCC	14	C	0	0.4883
NC_005101.4-109876926	ATCTCCCTCACAATCCCTCCTAGACTT	7	C	0.0894	0.1145
NC_005101.4-109928706	CACAAGCACACTTGAACTCCAACACAC	5	A	0.1557	0.2095
NC_005101.4-109938347	TGCCAACCAACCAGAGCTCCTATGGAC	22	A	0.1667	0.1528
NC_005101.4-109938347	TGCCAACCAACCAGAGCTCCTATGGAC	26	A	0.2373	0.2091
NC_005101.4-109954914	CCAATAAGCACTGTTTCTCCTTTGTCT	2	C	0.1092	0.2852
NC_005101.4-109954914	CCAATAAGCACTGTTTCTCCTTTGTCT	4	A	0.1083	0.3622
NC_005101.4-109998332	TGCATTTACACTGAATCTCCATCCTAG	3	C	0.0263	0.026
NC_005101.4-109998332	TGCATTTACACTGAATCTCCATCCTAG	13	G	0.0526	0.0677
NC_005101.4-110115357	CCTATGAAGACCATCTCTCCTTTAGTC	1	C	0	0.4779
NC_005101.4-110115357	CCTATGAAGACCATCTCTCCTTTAGTC	4	A	0	0.4765
NC_005101.4-110151798	CCACCAACCACTGAGTCTCCAGACCTG	25	C	0.0325	0.032
NC_005101.4-110208459	ACAGAATAAACTAGTCCTCCATGGTTC	1	A	0.0776	0.0746
NC_005101.4-110233342	AACTCCATGACTAAGCCTCCCTTGATG	6	C	0.0081	0.0241
NC_005101.4-110255773	GTACATAGCACTCTCCCTCCTCTTAAC	6	T	0	0.4773
NC_005101.4-110255773	GTACATAGCACTCTCCCTCCTCTTAAC	21	T	0	0.4773
NC_005101.4-110259341	AAGCAGGAAACAGTATCTCCTTTTCAT	6	G	0.0732	0.4935
NC_005101.4-110280414	ATCTGCATGACACCATCTCCCTTTCCA	5	G	0.082	0.1505
NC_005101.4-110308944	CTGTAACCAACTACTTCTCCTTCCTTC	3	G	0.0901	0.1182
NC_005101.4-110308944	CTGTAACCAACTACTTCTCCTTCCTTC	15	T	0.0901	0.1182

NC_005101.4-110355699	CATGCATGCACCTTCACTCCACATAAA	1	C	0.1304	0.4976
NC_005101.4-110355699	CATGCATGCACCTTCACTCCACATAAA	7	T	0.0522	0.0508
NC_005101.4-110436594	CAGGCTGCGACTGGATCTCCTTCCCTT	4	G	0.2542	0.4305
NC_005101.4-110444986	ACACCCCCAACATGCTCTCCTACTCTC	7	C	0.3009	0.4342
NC_005101.4-110450787	ACGGAAAGAACAATAACTCCTGCCTTT	1	A	0	0.4851
NC_005101.4-110513965	AGAATCAGTACTGCAGCTCCTCCATGG	25	T	0.0492	0.0634
NC_005101.4-110589796	GTCGCAATAACAGAAACTCCAGAAAGA	4	G	0	0.4503
NC_005101.4-110637770	CTCACCCACACATGCACTCCTTTCCAC	2	T	0.0261	0.0425
NC_005101.4-110640725	TCCAAAAAGACCATACCTCCACCATCA	15	A	0.1271	0.1894
NC_005101.4-110640725	TCCAAAAAGACCATACCTCCACCATCA	21	A	0.042	0.0571
NC_005101.4-110656565	AAGGACATGACAGCTACTCCTAACCTA	21	T	0.0244	0.0241
NC_005101.4-110697837	GTACACTGCACACATGCTCCATACTTG	23	A	0.0083	0.4803
NC_005101.4-110717629	CATCCAACCACCCCTTCTCCTTCAAGC	1	C	0.042	0.0728
NC_005101.4-110717629	CATCCAACCACCCCTTCTCCTTCAAGC	13	C	0.0932	0.3388
NC_005101.4-110718998	TAGTCATGTACATTTCCTCCTGTGGCT	3	G	0	0.4779
NC_005101.4-110835483	ATTCTGCTCACAGTTCCTCCTCACTGA	1	A	0.0932	0.4991
NC_005101.4-110835483	ATTCTGCTCACAGTTCCTCCTCACTGA	7	C	0.0847	0.1116
NC_005101.4-110845133	GCTACATCAACCTCATCTCCTCTTTCC	9	A	0.0164	0.4737
NC_005101.4-110886934	CTAGAACCCACCATAGCTCCCAGGGAC	9	C	0.1102	0.329
NC_005101.4-110886934	CTAGAACCCACCATAGCTCCCAGGGAC	16	G	0.1102	0.5
NC_005101.4-111003377	CCTGGTCCCACCTTTACTCCTTTGTCT	8	C	0.0656	0.0634
NC_005101.4-111014533	ACTACACCCACCTCCCCTCCCTGGCAT	15	C	0.0083	0.4803
NC_005101.4-111029688	ACTAAAGGGACAAAGACTCCAACACAT	23	C	0.0177	0.0348
NC_005101.4-111038212	CTGACCTTTACATTTTCTCCCCTTCTT	26	T	0	0.4749
NC_005101.4-111195063	AATATAAGGACACATGCTCCACTATGT	21	A	0.0342	0.0336
NC_005101.4-111211824	CCAGTGTATACTTTCCCTCCATGGAGT	16	C	0	0.0348
NC_005101.4-111244418	CCATGCTCAACTTTCCCTCCTCCTAAG	4	T	0	0.4778
NC_005101.4-111244418	CCATGCTCAACTTTCCCTCCTCCTAAG	14	T	0.0351	0.0345
NC_005101.4-111317275	AGAAGGCAGACACAACCTCCTCAAGAA	14	A	0.0894	0.1425
NC_005101.4-111317275	AGAAGGCAGACACAACCTCCTCAAGAA	16	C	0.2764	0.3926
NC_005101.4-111430639	GTGCTACCCACGAACACTCCAGCATTT	8	C	0.0496	0.0943
NC_005101.4-111430639	GTGCTACCCACGAACACTCCAGCATTT	13	A	0.124	0.4988
NC_005101.4-111535589	TGGCTATACACACCAGCTCCACCTATA	24	T	0.1176	0.2328
NC_005101.4-111589817	TCCTACACCACCAGGGCTCCTGGAATT	27	T	0.0776	0.1786
NC_005101.4-111646072	GTGTTAACCACAACTTCTCCAGGTCAA	25	C	0.2655	0.39
NC_005101.4-111648018	TCTTCTCCAACCTCCCCTCCATCCTAT	27	T	0.2114	0.3041
NC_005101.4-111666363	AAAAGTCGCACACATCCTCCTTAAAAT	7	C	0.094	0.1492
NC_005101.4-111711168	GGCAGGTAAACTGTTTCTCCACTGTTG	14	T	0.0442	0.0433
NC_005101.4-111711168	GGCAGGTAAACTGTTTCTCCACTGTTG	22	C	0.2193	0.3061
NC_005101.4-111741571	TTTATCACAACTAAGTCTCCTTTTCTT	13	A	0.0089	0.4789
NC_005101.4-111771299	TCAGCTTGTACTGGCTCTCCTGACCAG	21	T	0.1463	0.2612
NC_005101.4-111771299	TCAGCTTGTACTGGCTCTCCTGACCAG	26	A	0.0894	0.1001
NC_005101.4-111831668	GGGTTGGTGACACCGTCTCCATCTTTT	4	T	0.0957	0.4986
NC_005101.4-111831668	GGGTTGGTGACACCGTCTCCATCTTTT	16	T	0.0957	0.4986
NC_005101.4-111853541	CAGTGAAAGACTGTATCTCCTAGTAAC	6	A	0	0.4786
NC_005101.4-111853541	CAGTGAAAGACTGTATCTCCTAGTAAC	24	T	0.1345	0.1254
NC_005101.4-111929019	CCTTGCTTTACTACTCCTCCACAAGTC	8	T	0	0.4773
NC_005101.4-111929019	CCTTGCTTTACTACTCCTCCACAAGTC	13	A	0.2683	0.319
NC_005101.4-111940743	GGGACACAGACTAAAGCTCCTCTAGGA	12	T	0.275	0.4622
NC_005101.4-111940743	GGGACACAGACTAAAGCTCCTCTAGGA	25	G	0.0417	0.4747
NC_005101.4-111961675	ACAGGCTGGACCTACCCTCCTCCTGAC	12	C	0.2105	0.2659

NC_005101.4-111961675	ACAGGCTGGACCTACCCTCCTCCTGAC	26	A	0	0.4778
NC_005101.4-111969826	TGAAAGATTACCATTTCTCCTTTAGCT	9	T	0.1333	0.3299
NC_005101.4-111969826	TGAAAGATTACCATTTCTCCTTTAGCT	26	C	0.2083	0.4416
NC_005101.4-112100825	TCTGAAGCTACAGGGTCTCCATGACAC	7	G	0.175	0.4297
NC_005101.4-112138505	ACAACGTGCACCATTCCTCCAACTGGG	5	C	0	0.4786
NC_005101.4-112138505	ACAACGTGCACCATTCCTCCAACTGGG	7	T	0.0431	0.0422
NC_005101.4-112175490	AAGTTAAAGACCCTGCCTCCAGGGACT	26	C	0.0081	0.481
NC_005101.4-112255383	CCTCTTTTCACCATCCCTCCTCCCTTT	22	C	0.2328	0.4034
NC_005101.4-112281832	TTTATTCTAACTGCTACTCCTTAATAA	26	A	0.0085	0.4823
NC_005101.4-112299621	TAACTCCATACAACGACTCCATGCAAT	14	C	0.1545	0.1826
NC_005101.4-112299621	TAACTCCATACAACGACTCCATGCAAT	22	T	0.2764	0.427
NC_005101.4-112309658	CTGGGTGGGACAGGTGCTCCACAAATA	21	A	0.0976	0.1216
NC_005101.4-112309658	CTGGGTGGGACAGGTGCTCCACAAATA	26	T	0	0.4807
NC_005101.4-112387056	ATTCTCCCCACCCCTACTCCCTTGGTA	15	T	0	0.4813
NC_005101.4-112392146	AATTGGTAGACAATGACTCCAACAATG	2	A	0.2353	0.4008
NC_005101.4-112392146	AATTGGTAGACAATGACTCCAACAATG	27	G	0.2101	0.4638
NC_005101.4-112564350	GAAGGAAGCACTGAAGCTCCTAAGGAA	3	A	0	0.4793
NC_005101.4-112574673	TCAATGCCTACCTTACCTCCTTACTTA	24	C	0	0.477
NC_005101.4-112611054	TCTCTTCTCACTGCCCCTCCAAGAGTT	8	T	0.05	0.0488
NC_005101.4-112611054	TCTCTTCTCACTGCCCCTCCAAGAGTT	24	A	0.125	0.1172
NC_005101.4-112612702	ACACTAGAGACCAAGCCTCCAGCACAC	9	G	0.0248	0.0245
NC_005101.4-112716082	AATGTGACCACCCAGCCTCCTGGTGCC	23	G	0.1789	0.2723
NC_005101.4-112718602	ATGTAGCCAACTCTTTCTCCAAAGCTC	25	C	0.0268	0.0264
NC_005101.4-113088581	AAGAGGGTGACTGACTCTCCCACATTC	14	A	0	0.4764
NC_005101.4-113088581	AAGAGGGTGACTGACTCTCCCACATTC	25	T	0	0.4764
NC_005101.4-113124300	GGATGGGAAACGAAAACTCCAAGGGTT	2	G	0	0.4751
NC_005101.4-113124300	GGATGGGAAACGAAAACTCCAAGGGTT	24	G	0.1967	0.4837
NC_005101.4-113128355	GTGTTAAGGACTGCAGCTCCCACTGGG	6	A	0.0439	0.4953
NC_005101.4-113142530	ACAGTATGGACGGGCGCTCCAGAGGAA	15	C	0	0.4765
NC_005101.4-113159789	ATGTGTAGCACACGACCTCCTGGCTGT	2	T	0	0.4793
NC_005101.4-113175627	AAAGCTGGGACAGGGACTCCATCTAAT	1	A	0	0.4703
NC_005101.4-113177729	TGCAGTGCAACTGCTTCTCCATAGTCA	1	T	0.0082	0.479
NC_005101.4-113188400	CCATGAGTAACACCTGCTCCCCTCCTC	6	A	0	0.4649
NC_005101.4-113188731	CAGCCCACGACCAGGGCTCCTGAGTTC	9	G	0	0.462
NC_005101.4-113188731	CAGCCCACGACCAGGGCTCCTGAGTTC	23	A	0	0.462
NC_005101.4-113194194	GCACAATCAACCCTTCCTCCCTGTGTG	3	A	0.125	0.4922
NC_005101.4-113206349	AAGTAAACAACCCAAACTCCATCTTGA	27	A	0.0424	0.0415
NC_005101.4-113258201	AACAGAATCACATTGCCTCCTCTCCCT	13	T	0.0248	0.4835
NC_005101.4-113291550	TTGCTGGTCACTCAGTCTCCCACTGAG	12	T	0	0.4743
NC_005101.4-113291550	TTGCTGGTCACTCAGTCTCCCACTGAG	15	G	0	0.4743
NC_005101.4-113293201	AGGGCCTCCACATGGCCTCCTCCTCAT	1	A	0	0.4819
NC_005101.4-113303683	AAAGTTCCCACCCTGCCTCCCTCTCCC	16	C	0	0.4787
NC_005101.4-113324517	AGCTCTATTACACTACCTCCAGTGAAA	21	A	0	0.4844
NC_005101.4-113327618	TATCTTACCACACCTCCTCCACCTTAG	14	C	0.0265	0.0262
NC_005101.4-113345754	ATCTCTCAGACCCAGTCTCCAAGCATG	26	T	0	0.4751
NC_005101.4-113345754	ATCTCTCAGACCCAGTCTCCAAGCATG	27	G	0	0.4751
NC_005101.4-113371630	CTAGTGCAGACAATCACTCCACCTCAT	5	T	0	0.4757
NC_005101.4-113381720	GGAAGCAGCACACCAGCTCCCATGAAC	16	G	0	0.4787
NC_005101.4-113421645	CTCCTAATTACAGTATCTCCAACCTTG	12	A	0	0.4841
NC_005101.4-113532865	TGCTTGCCAACCTGGCCTCCTTCGGCT	23	C	0.2124	0.499
NC_005101.4-113555339	CTTCGCAGAACTCATCCTCCCCTATCC	5	G	0.087	0.0832

NC_005101.4-113555339	CTTCGCAGAACTCATCCTCCCCTATCC	15	T	0.2957	0.4637
NC_005101.4-113594988	ACAAACCAAACAAAACCTCCTCCTCTT	6	C	0.0085	0.4714
NC_005101.4-113594988	ACAAACCAAACAAAACCTCCTCCTCTT	25	C	0.0085	0.4714
NC_005101.4-113655918	TGAGGCAGAACTGGGCCTCCAGCCCAT	26	A	0.042	0.0571
NC_005101.4-113668802	TCCTGTAATACCAGCGCTCCTGAGGAG	16	G	0	0.4779
NC_005101.4-113737711	CCCTCCTCCACCCTGTCTCCGCATTTC	21	G	0.25	0.3987
NC_005101.4-113737711	CCCTCCTCCACCCTGTCTCCGCATTTC	22	C	0.2479	0.2869
NC_005101.4-113818119	CCACTTCCTACGCCCTCTCCCATGAGA	12	G	0.1186	0.493
NC_005101.4-113825700	TCCCCCCTCACCTTCTCTCCCCCTTCC	27	C	0	0.4555
NC_005101.4-113842304	GGCTTACCCACAGGTTCTCCATCTCGG	9	C	0.0593	0.0576
NC_005101.4-113854756	CATGTTTCCACTCAGACTCCCAGAGAA	16	A	0.0345	0.4709
NC_005101.4-113854756	CATGTTTCCACTCAGACTCCCAGAGAA	24	A	0.0678	0.1116
NC_005101.4-113894386	GACTGAAATACTCCTACTCCGTCATGT	24	A	0.0714	0.4974
NC_005101.4-113899414	GGGGTAGGCACTCTCCCTCCTGAACTC	1	G	0.0083	0.4803
NC_005101.4-113899414	GGGGTAGGCACTCTCCCTCCTGAACTC	23	A	0.2314	0.3467
NC_005101.4-113903923	TCACAGCCCACCCTCCCTCCTCATGGC	7	C	0.0252	0.0249
NC_005101.4-113912724	CTGCATGTAACTGCCGCTCCTGAGGAT	15	C	0.1557	0.2095
NC_005101.4-113942723	AGTGGCAACACCTTTCCTCCCGGTCTA	21	C	0	0.4765
NC_005101.4-114087227	TTTGTGCTGACCCGGTCTCCAGGGTTC	13	C	0.0088	0.472
NC_005101.4-114090034	AACTGCAGAACCATTGCTCCATTCATC	3	C	0.0894	0.1562
NC_005101.4-114090184	GTAAATACCACGCGTGCTCCTCAGTCA	12	G	0.0732	0.0705
NC_005101.4-114090184	GTAAATACCACGCGTGCTCCTCAGTCA	14	G	0.0976	0.1356
NC_005101.4-114103871	ACCAATGGCACCCATACTCCTGACATG	5	A	0.0513	0.05
NC_005101.4-114105217	ATGATGCTCACTGGCACTCCCTGTTCT	2	T	0.0325	0.4854
NC_005101.4-114105217	ATGATGCTCACTGGCACTCCCTGTTCT	3	G	0.2439	0.4682
NC_005101.4-114247007	CAGCAGCACACATGCTCTCCTGCACAT	8	A	0.0488	0.4881
NC_005101.4-114247007	CAGCAGCACACATGCTCTCCTGCACAT	12	A	0	0.4793
NC_005101.4-114254040	CCTTCACTCACTCCTGCTCCACATGCC	3	T	0	0.4734
NC_005101.4-114254040	CCTTCACTCACTCCTGCTCCACATGCC	25	G	0.3814	0.456
NC_005101.4-114265117	TGCTAAGATACACAATCTCCCATTGTA	13	C	0.2269	0.4723
NC_005101.4-114288937	AGCAAGAACACACATGCTCCATCCATA	9	C	0.0268	0.0771
NC_005101.4-114293560	AGACCCAGCACATCACCTCCCCAGTGT	15	A	0.2054	0.4885
NC_005101.4-114323636	GTGGAGGGCACACCATCTCCCTGCCTG	1	G	0	0.4807
NC_005101.4-114323636	GTGGAGGGCACACCATCTCCCTGCCTG	12	A	0.2131	0.2956
NC_005101.4-114349316	TGACTGATTACTCTTCCTCCAGCGAGG	26	G	0.0744	0.0716
NC_005101.4-114351630	TCACCTGTAACTCCAGCTCCCAAGATC	2	C	0.0339	0.4757
NC_005101.4-114351630	TCACCTGTAACTCCAGCTCCCAAGATC	21	C	0.0325	0.032
NC_005101.4-114355069	CTGGGGAATACTGACTCTCCCTCTCAG	24	T	0.0082	0.0243
NC_005101.4-114390221	TTCCAGGTCACAAGCCCTCCTTCCTCT	14	G	0.0165	0.0325
NC_005101.4-114435581	ATCTGCTGTACCAGCTCTCCTGCAGTG	1	A	0.0345	0.4927
NC_005101.4-114497995	TAACTTCTGACATCTACTCCCCTGTCC	9	G	0.1875	0.4885
NC_005101.4-114497995	TAACTTCTGACATCTACTCCCCTGTCC	23	T	0.1875	0.3705
NC_005101.4-115343775	ATGATATCCACTCCCTCTCCTCCAATA	27	A	0	0.4793
NC_005101.4-115374906	CTTCAGCTAACTCACCCTCCTTCCACT	24	C	0.1475	0.1904
NC_005101.4-115427490	GGAAAACTTACTCTACCTCCCTAGATG	9	T	0	0.482
NC_005101.4-115427490	GGAAAACTTACTCTACCTCCCTAGATG	27	G	0.1368	0.1975
NC_005101.4-115433463	TCTGTCCCTACTCTTTCTCCAGGTTCT	25	T	0.1391	0.1588
NC_005101.4-115494954	CCACTGTGTACCTGAGCTCCTTTGAAC	14	G	0.05	0.4888
NC_005101.4-115497103	GCGCTCTCCACGTGAGCTCCTCCCTGG	2	C	0.0496	0.0484
NC_005101.4-115497103	GCGCTCTCCACGTGAGCTCCTCCCTGG	3	G	0.2276	0.2723
NC_005101.4-115509206	AAGGAGATGACAGCAGCTCCAGCCAAA	2	A	0.2833	0.4265

NC_005101.4-115537560	CAGAAGAGTACAGTCACTCCATGTGAT	3	G	0.0244	0.0241
NC_005101.4-115599214	CCCCAAAGAACTACAACTCCCAGCATT	3	C	0.0442	0.0765
NC_005101.4-115624011	ATCCCCAACACAGTATCTCCAATGTCC	21	A	0.193	0.3324
NC_005101.4-115634849	CCCACCATCACCTGTTCTCCTGCTTTT	27	T	0.0082	0.4755
NC_005101.4-115639303	AGCCCCAAGACGACTGCTCCTAGCACA	3	C	0.0877	0.0997
NC_005101.4-115639303	AGCCCCAAGACGACTGCTCCTAGCACA	8	A	0.0885	0.0846
NC_005101.4-115664150	CTGCAGAGCACGGTTTCTCCTCATATA	12	G	0.1639	0.2399
NC_005101.4-115687215	AAATGCTAGACATGCTCTCCTCCACGA	8	A	0.0732	0.0854
NC_005101.4-115687215	AAATGCTAGACATGCTCTCCTCCACGA	13	T	0	0.4793
NC_005101.4-115735441	GTAGTAACTACAGTTACTCCCTGGACT	13	G	0.0175	0.474
NC_005101.4-115735441	GTAGTAACTACAGTTACTCCCTGGACT	21	C	0.1228	0.1153
NC_005101.4-115741453	CCTGGAATCACCCTTTCTCCTGCATAA	12	C	0.1639	0.1904
NC_005101.4-115741453	CCTGGAATCACCCTTTCTCCTGCATAA	13	C	0.1721	0.2339
NC_005101.4-115750907	ACTGTCACCACCTCAACTCCCTCTCTG	1	A	0.2174	0.4395
NC_005101.4-115797167	TTTAGTCTAACCAAACCTCCCTTTGTC	26	T	0.1167	0.2432
NC_005101.4-115820803	AGAGTGGTTACCTGACCTCCACAAGCC	2	G	0	0.4773
NC_005101.4-115833323	CAAGTGCCCACTGTACCTCCCTTTCCT	5	T	0.0833	0.0799
NC_005101.4-115833323	CAAGTGCCCACTGTACCTCCCTTTCCT	22	T	0.0833	0.0799
NC_005101.4-115836270	TTCACTAGGACTAACTCTCCTATGAGG	23	T	0	0.4793
NC_005101.4-115956456	TTTGGCGTAACTTCCTCTCCCTATAAC	7	G	0.0333	0.1244
NC_005101.4-116070232	CCGCCCCCCACTTTGTCTCCAGCAGCT	12	T	0.018	0.4659
NC_005101.4-116070232	CCGCCCCCCACTTTGTCTCCAGCAGCT	27	T	0.1161	0.125
NC_005101.4-116082548	CAGAAGGAAACAACTCCTCCTCTCTGT	27	T	0	0.4875
NC_005101.4-116098239	GTGACAGTGACAGTCACTCCCGCAGAA	21	C	0.0263	0.476
NC_005101.4-116151612	GTAAACCGCACAACTGCTCCACAGTAC	22	C	0.0598	0.4626
NC_005101.4-116151612	GTAAACCGCACAACTGCTCCACAGTAC	26	A	0.0171	0.4807
NC_005101.4-116195150	TGCAGCTACACTGGAGCTCCAGGTTCA	6	C	0.0439	0.0429
NC_005101.4-116242776	AGTTGGAAGACTGGGACTCCACATGGA	24	T	0.0248	0.0405
NC_005101.4-116260674	AAATATATCACTAACACTCCACTTTAA	26	A	0.0177	0.4793
NC_005101.4-116275998	ATGCAAATTACATCCTCTCCTCCTCCT	7	A	0.0164	0.4807
NC_005101.4-116275998	ATGCAAATTACATCCTCTCCTCCTCCT	27	T	0	0.4773
NC_005101.4-116286890	ATAAATCTGACTCTGTCTCCTTACCCC	14	T	0.0085	0.4842
NC_005101.4-116299492	CTAAGATCTACTCTACCTCCCCCCACA	3	A	0	0.4807
NC_005101.4-116299492	CTAAGATCTACTCTACCTCCCCCCACA	27	A	0	0.4807
NC_005101.4-116312211	TTTGCTTGCACTTTTCCTCCCAGCTGA	6	T	0.1083	0.1458
NC_005101.4-116319565	CTCTGCCCTACCAGCCCTCCCCAAAAT	7	C	0	0.482
NC_005101.4-116340927	GCAGGAGAAACTGGGACTCCAGTGGTG	12	T	0.2623	0.2956
NC_005101.4-116344535	CTGGCATGCACATGCCCTCCCAAGAAA	4	G	0.094	0.2041
NC_005101.4-116344535	CTGGCATGCACATGCCCTCCCAAGAAA	26	A	0.0417	0.49
NC_005101.4-116386988	GCTAACTGGACACGAGCTCCCAAGATC	27	C	0.0169	0.0333
NC_005101.4-116451323	ACTGACTGGACCATCGCTCCTTTGGTG	16	G	0.1818	0.4877
NC_005101.4-116453306	GTAATTTAAACCATAGCTCCCTTTTCC	2	T	0.2018	0.4889
NC_005101.4-116455284	CCTCTAACCACACTTCCTCCATCACGT	12	A	0	0.4637
NC_005101.4-116465512	AGAGCATGTACACGCTCTCCCATGCTT	4	G	0.2185	0.4921
NC_005101.4-116607272	CTATGTGTCACACTCCCTCCCAATTCC	1	C	0.2435	0.4165
NC_005101.4-116614826	TTCACCACCACACCTTCTCCAGGTAAG	13	C	0.0513	0.05
NC_005101.4-116616765	AGCTGCAATACTTCATCTCCATCTCCC	14	C	0.0957	0.1067
NC_005101.4-116616765	AGCTGCAATACTTCATCTCCATCTCCC	24	T	0.0435	0.1067
NC_005101.4-116753490	GTGAATCAGACTTTAACTCCTCTTTCC	27	C	0.0435	0.059
NC_005101.4-116855611	CTGGAGAAAACTGTTTCTCCCTCCTTC	21	C	0.0583	0.4847
NC_005101.4-116855611	CTGGAGAAAACTGTTTCTCCCTCCTTC	27	C	0	0.4728

NC_005101.4-116867763	GATGTTTGAACAGGTCCTCCAGGAAAG	15	T	0.1681	0.4944
NC_005101.4-116869637	ACCTCACTCACAAGACCTCCCTGTATG	16	C	0.2389	0.3683
NC_005101.4-116943691	GTTTTGTGCACACAATCTCCTTGTGCT	22	T	0.0593	0.0734
NC_005101.4-116976936	ATCCAAGAAACTGGAACTCCAAGACAG	3	C	0.1982	0.5
NC_005101.4-116976936	ATCCAAGAAACTGGAACTCCAAGACAG	8	A	0	0.4746
NC_005101.4-116989367	TTTCTGTCCACATGAGCTCCAGCCACT	9	C	0.0244	0.0241
NC_005101.4-117013264	CTTAGGTGTACTGAACCTCCTTGTGTT	1	C	0.0168	0.033
NC_005101.4-117013264	CTTAGGTGTACTGAACCTCCTTGTGTT	13	G	0.0085	0.481
NC_005101.4-117028543	GGTCAGCCTACCACCTCTCCAACAGGT	1	G	0.0407	0.0705
NC_005101.4-117028543	GGTCAGCCTACCACCTCTCCAACAGGT	14	C	0.0407	0.4915
NC_005101.4-117129602	AGGAGAGCTACTGTCTCTCCCTCAACA	8	C	0.1441	0.4982
NC_005101.4-117133525	CAACCGAGGACATGCTCTCCTCCAGCT	27	T	0	0.4751
NC_005101.4-117134518	GGTGGCTGGACTGCGACTCCGTAAGTA	21	G	0.0804	0.0934
NC_005101.4-117146930	GGTTGAGGCACTGTCTCTCCAGCCCAG	25	C	0.123	0.2218
NC_005101.4-117149732	AGTGAGGTAACCCAGACTCCAAAGGGA	26	G	0.4146	0.3287
NC_005101.4-117158316	CAGTGAAAGACCCTATCTCCAGAGCTA	9	G	0.0488	0.0629
NC_005101.4-117173875	CCGATTAAGACATGACCTCCTAAGGAA	5	T	0	0.4731
NC_005101.4-117173875	CCGATTAAGACATGACCTCCTAAGGAA	8	A	0	0.4731
NC_005101.4-117198946	TCCCTTGTCACTCCTGCTCCTTCACAA	9	C	0.0976	0.1073
NC_005101.4-117198946	TCCCTTGTCACTCCTGCTCCTTCACAA	16	G	0.0813	0.1073
NC_005101.4-117200464	CAGACACTTACATAGTCTCCCTAGACT	1	C	0.0345	0.0504
NC_005101.4-117208246	AACTTGTACACGTGAGCTCCATTCTAG	9	C	0.0513	0.497
NC_005101.4-117208246	AACTTGTACACGTGAGCTCCATTCTAG	16	G	0.0345	0.0339
NC_005101.4-117211227	AAGGAAAGCACTGCGACTCCTGCCTCC	15	G	0	0.4827
NC_005101.4-117241401	GAAGTTATTACGTCTGCTCCTTAGTGT	5	T	0	0.4765
NC_005101.4-117305878	CAAACAAGCACATATACTCCACAAATG	15	T	0.0488	0.0629
NC_005101.4-117320899	GGTATGCACACTACCTCTCCCACCACC	8	A	0	0.48
NC_005101.4-117372736	CTGGAGGTCACTTGGTCTCCCTTGTCT	9	C	0.0488	0.0476
NC_005101.4-117379719	TCTGAGGATACAATGTCTCCATCTCCT	12	A	0.2368	0.235
NC_005101.4-117379719	TCTGAGGATACAATGTCTCCATCTCCT	26	C	0.2389	0.2367
NC_005101.4-117405974	CATTGCTTTACCTCTCCTCCATGTTCC	1	C	0	0.4793
NC_005101.4-117405974	CATTGCTTTACCTCTCCTCCATGTTCC	3	T	0.0488	0.4595
NC_005101.4-117414157	AGTGTGCTTACGTCACCTCCAACAGGT	12	G	0	0.4813
NC_005101.4-117414157	AGTGTGCTTACGTCACCTCCAACAGGT	26	G	0	0.4826
NC_005101.4-117431840	GCATGGGACACTCGGCCTCCTTCTCTG	6	G	0	0.4757
NC_005101.4-117472910	TGACTAGAAACTCTTGCTCCCATTGGT	6	A	0	0.4793
NC_005101.4-117472910	TGACTAGAAACTCTTGCTCCCATTGGT	9	A	0	0.4793
NC_005101.4-117499434	GACTGCATTACCACATCTCCCTCCCAA	6	C	0.0526	0.4945
NC_005101.4-117499434	GACTGCATTACCACATCTCCCTCCCAA	7	A	0	0.4724
NC_005101.4-117537601	ACTATGAAAACTCAGTCTCCTTCTGCA	25	G	0.1681	0.2494
NC_005101.4-117660909	ACTTCCTGCACCTGACCTCCCGTGATC	21	C	0.0738	0.4959
NC_005101.4-117677485	TCCGGGTTTACATCCCCTCCTAGCATT	4	G	0	0.4765
NC_005101.4-117776248	GAACTTGTAACTGTGTCTCCAGTTACA	14	T	0.2276	0.4072
NC_005101.4-117796578	CCTGCTGCCACTTGCTCTCCTCATCCT	23	A	0	0.4807
NC_005101.4-117829627	AGACCCACTACCAGCTCTCCCGGGTCC	23	G	0.2743	0.4098
NC_005101.4-117864759	GTTCTGCAAACCAGCACTCCAGTCACA	27	A	0	0.4846
NC_005101.4-117912096	GGAGGCTTGACTAGCCCTCCCATACCA	15	C	0.2232	0.2637
NC_005101.4-117912494	GTTATTGCTACTTGTCCTCCACATCTC	13	T	0	0.4682
NC_005101.4-117917659	ACTGAATCAACAAGTACTCCTGGGATT	3	T	0.0082	0.4755
NC_005101.4-117924909	CAGCTATTTACTGGGTCTCCTCACTCA	6	A	0.2764	0.4497
NC_005101.4-117948804	AACATCTTCACTGTTTCTCCCTTGGCT	15	T	0	0.4793

NC_005101.4-117949820	GCTCAAGTCACCCAGGCTCCAGAGTGT	1	G	0.0603	0.0746
NC_005101.4-117993061	ATGGGACGTACTGCACCTCCCAGCTTT	1	A	0.0087	0.466
NC_005101.4-118009579	CTTGTTCCCACCTGTTCTCCTTTTGGA	8	C	0.1017	0.1827
NC_005101.4-118065294	ATGATAAAAACTTCAACTCCTTGAAGA	8	A	0.0531	0.0517
NC_005101.4-118065294	ATGATAAAAACTTCAACTCCTTGAAGA	14	C	0.0531	0.0517
NC_005101.4-118112185	CTGTTTAAGACTTCATCTCCATCAGCA	8	A	0.0082	0.0243
NC_005101.4-118147696	GATCCCATAACAGTGACTCCCAGCAGT	26	G	0.2479	0.4246
NC_005101.4-118170193	CAATCCCTCACTTAGACTCCCTTCCTA	12	T	0.177	0.2302
NC_005101.4-118170193	CAATCCCTCACTTAGACTCCCTTCCTA	21	C	0.1532	0.3232
NC_005101.4-118183922	CTGGTGATAACATACTCTCCAAGAGTC	4	G	0.2124	0.5
NC_005101.4-118190112	GATCTGTCAACTGCTACTCCACGTCAT	2	A	0	0.4841
NC_005101.4-118190112	GATCTGTCAACTGCTACTCCACGTCAT	6	G	0	0.4841
NC_005101.4-118204503	TGTTGGACTACCCACCCTCCAAAGCCT	8	C	0.2035	0.1968
NC_005101.4-118209270	AACCTGTTTACCCTCCCTCCATGGACT	5	T	0	0.4807
NC_005101.4-118209270	AACCTGTTTACCCTCCCTCCATGGACT	6	G	0.1864	0.1827
NC_005101.4-118226707	ACTTAAGACACAATTCCTCCTCACTGT	16	C	0.1858	0.2237
NC_005101.4-118259543	CCTCACCCCACACTGGCTCCTGGGTTG	4	C	0.1818	0.1787
NC_005101.4-118290850	ACTCCCAAAACCAAATCTCCAGCTATG	25	A	0.0088	0.4693
NC_005101.4-118301211	CAGACATCAACTCTCCCTCCCTTTAAC	22	T	0	0.4813
NC_005101.4-118301446	TTGTTGGACACATGGGCTCCTTCTTCC	13	T	0	0.48
NC_005101.4-118301446	TTGTTGGACACATGGGCTCCTTCTTCC	14	G	0	0.48
NC_005101.4-118390482	AAAGTGTAGACTGGCTCTCCAGGCCTG	14	G	0	0.4819
NC_005101.4-118390482	AAAGTGTAGACTGGCTCTCCAGGCCTG	24	C	0	0.4819
NC_005101.4-118419746	CCTACACTGACACCGACTCCTCCATTT	22	C	0.0574	0.0557
NC_005101.4-118433258	TTTACTGCCACGATTCCTCCTTTTGTT	12	G	0.1901	0.4004
NC_005101.4-118433701	AATTAGGGGACTTACTCTCCTCGTGGA	6	G	0.1368	0.2107
NC_005101.4-118448668	CACCAAGCAACTCTGGCTCCCTCACGT	27	T	0.1739	0.4936
NC_005101.4-118451456	ACCATAGCTACAAGCTCTCCCATCCGC	25	C	0.0992	0.1377
NC_005101.4-118496572	TCCACATTCACACTTACTCCATCCTCT	6	A	0	0.4819
NC_005101.4-118496572	TCCACATTCACACTTACTCCATCCTCT	24	C	0	0.4819
NC_005101.4-118500082	GTACATGAAACTGCAGCTCCTTGCAGA	25	A	0.027	0.0778
NC_005101.4-118531301	TGGCTACACACTCTTGCTCCTCCTGCC	3	G	0	0.4693
NC_005101.4-118531301	TGGCTACACACTCTTGCTCCTCCTGCC	9	C	0.2966	0.4207
NC_005101.4-118561369	TAATGTCGTACACTGACTCCTGGTCCT	4	T	0.1532	0.4998
NC_005101.4-118561369	TAATGTCGTACACTGACTCCTGGTCCT	21	T	0.2212	0.4435
NC_005101.4-118599509	GTACCTGAGACCCAGTCTCCTTGGTCT	15	G	0	0.4749
NC_005101.4-118615780	GTCTGCTGCACAACTCCTCCCGTTTGC	13	A	0.1026	0.336
NC_005101.4-118615780	GTCTGCTGCACAACTCCTCCCGTTTGC	22	G	0.0684	0.066
NC_005101.4-118664370	TGCTGTAAAACTGAAGCTCCCATTGAT	13	G	0.0088	0.4889
NC_005101.4-118664370	TGCTGTAAAACTGAAGCTCCCATTGAT	21	C	0.0088	0.4889
NC_005101.4-118693611	TGTCCCTTGACTAGAACTCCTTGAGGA	9	G	0.0569	0.0705
NC_005101.4-118726830	TATCCACCGACCATGTCTCCCTCACAG	8	C	0.0738	0.1009
NC_005101.4-118754801	CAAGCCCAGACTTGCCCTCCAATCAGT	25	A	0.0885	0.499
NC_005101.4-118794710	ATTCCTGTCACGTCGTCTCCCTGGGAT	15	G	0	0.4793
NC_005101.4-118803110	AGGTTGTGCACCTGCACTCCTGAGACT	26	C	0.0661	0.0792
NC_005101.4-118873713	CCCCTTCACACCTCTTCTCCCTCTCTC	3	C	0.2295	0.4407
NC_005101.4-118873713	CCCCTTCACACCTCTTCTCCCTCTCTC	5	T	0.2295	0.402
NC_005101.4-118884268	AAACACAGAACCATTTCTCCAGCCCCT	16	T	0.2295	0.4737
NC_005101.4-118886388	TTCCTTCTAACTAAAGCTCCTGGTGCA	23	G	0.2397	0.3327
NC_005101.4-118929208	ACCTACTGAACCGGCTCTCCAGCCCTT	1	A	0.1478	0.3547
NC_005101.4-118937890	CTTTCTTTAACATTGTCTCCTTCCCCT	13	T	0.0588	0.0571

NC_005101.4-118970507	GAACATGTGACTGCCACTCCCCACATA	2	A	0	0.482
NC_005101.4-118970507	GAACATGTGACTGCCACTCCCCACATA	24	C	0.0508	0.0496
NC_005101.4-118976419	AGAAACCCTACAGGCACTCCTGTCAGA	9	T	0	0.4819
NC_005101.4-118976419	AGAAACCCTACAGGCACTCCTGTCAGA	23	T	0.1653	0.4369
NC_005101.4-119017452	AGACTTTTCACTGTCCCTCCCTTATTT	14	T	0.1653	0.3467
NC_005101.4-119017452	AGACTTTTCACTGTCCCTCCCTTATTT	25	T	0.0246	0.0401
NC_005101.4-119028854	ATCTTCTCAACTGAAGCTCCCTCCTCT	4	T	0.0598	0.1347
NC_005101.4-119037820	GTGTTTCCAACTCAGCCTCCCACGCAG	12	T	0	0.4819
NC_005101.4-119037820	GTGTTTCCAACTCAGCCTCCCACGCAG	25	C	0	0.4793
NC_005101.4-119058416	AACAAGGCCACGCCCACTCCAAATAAT	12	G	0.0672	0.0805
NC_005101.4-119058416	AACAAGGCCACGCCCACTCCAAATAAT	27	T	0.3036	0.2575
NC_005101.4-119058581	CTACATCTGACCTGTCCTCCATTCTTC	5	A	0	0.488
NC_005101.4-119058581	CTACATCTGACCTGTCCTCCATTCTTC	24	C	0.0259	0.4916
NC_005101.4-119131275	GTAACAAGCACATATTCTCCTTGTCTT	14	A	0.069	0.4905
NC_005101.4-119141155	AATCCAGAAACATTCTCTCCAATCTCC	2	A	0	0.0336
NC_005101.4-119141155	AATCCAGAAACATTCTCTCCAATCTCC	23	T	0.2137	0.3855
NC_005101.4-119147574	TATTCAACCACCATATCTCCTTTCCCA	3	T	0	0.4751
NC_005101.4-119147574	TATTCAACCACCATATCTCCTTTCCCA	13	A	0.0579	0.4863
NC_005101.4-119152272	GGTGAGGAGACTCCACCTCCTGTGGAT	14	C	0.0263	0.0429
NC_005101.4-119152272	GGTGAGGAGACTCCACCTCCTGTGGAT	21	T	0.0526	0.0512
NC_005101.4-119181219	CCAGTAAGCACTACTCCTCCATGGCCT	24	G	0.0517	0.0504
NC_005101.4-119216709	GTGAGAAAAACGTGGCCTCCTCAGTAG	26	A	0	0.4856
NC_005101.4-119337954	TTGTTTGAGACAGGGTCTCCTTGTACT	25	A	0.3197	0.2686
NC_005101.4-119411013	TGTAGACACACTGGTTCTCCTTTCTAA	5	G	0.0085	0.0253
NC_005101.4-119480794	ATTATGTCCACAACTACTCCATAGAAG	21	A	0	0.4724
NC_005101.4-119481523	AGTTGGCTAACACTGTCTCCTTGCCTG	26	T	0.0672	0.065
NC_005101.4-119481523	AGTTGGCTAACACTGTCTCCTTGCCTG	27	G	0.0672	0.065
NC_005101.4-119496788	CAGTCAGGGACCATAGCTCCCACTAGT	15	A	0.0248	0.0405
NC_005101.4-119502098	ACCATAGCAACACACTCTCCATCTCTC	3	C	0	0.4864
NC_005101.4-119557670	CTGTAAAGTACTGGAGCTCCTATCTAT	12	T	0.0083	0.4816
NC_005101.4-119580657	TCAGAGATAACCATGCCTCCTTGTCCT	8	T	0.1579	0.3706
NC_005101.4-119609207	ATGTATCACACACACACTCCCACACAC	15	C	0.0855	0.0818
NC_005101.4-119609207	ATGTATCACACACACACTCCCACACAC	27	C	0.0513	0.066
NC_005101.4-119626569	CAGGGAACCACCCCTTCTCCATGCTCC	9	C	0.0574	0.0557
NC_005101.4-119626569	CAGGGAACCACCCCTTCTCCATGCTCC	26	C	0.1967	0.4092
NC_005101.4-119641812	ATCAGAATGACTGAGACTCCATCTTAG	8	T	0	0.4912
NC_005101.4-119641812	ATCAGAATGACTGAGACTCCATCTTAG	27	G	0	0.4912
NC_005101.4-119747970	TCCAGCAAGACCACAGCTCCTACACTT	24	A	0.3577	0.4444
NC_005101.4-119762423	GGTGTGTTTACCTCCACTCCCTCTTGC	4	G	0.1513	0.2203
NC_005101.4-119868746	CTGCTCCAAACTTTGCCTCCATATAAT	23	A	0.0254	0.0251
NC_005101.4-119921432	GACCAGAGAACATTGGCTCCATGGATG	15	G	0.2683	0.4174
NC_005101.4-119949706	TATTGCTACACAGGGGCTCCTGTCTAG	14	G	0.0488	0.0476
NC_005101.4-120075971	ATGAGTTCCACTCCCTCTCCAGATGTA	5	G	0.094	0.1492
NC_005101.4-120093262	TAGGATCTGACACTAACTCCAGATAGA	15	A	0.113	0.4946
NC_005101.4-120097664	TTTACCTGCACCTTGTCTCCTTATTTG	14	T	0.1695	0.4987
NC_005101.4-120300587	TGCCACTGCACCAGGCCTCCTATGACT	8	G	0.1111	0.4987
NC_005101.4-120361810	CTCTAACACACTGCAGCTCCTTCGACA	25	A	0	0.4819
NC_005101.4-120390084	CATGACTCCACCCCACCTCCAGCTTTT	13	C	0.0252	0.0249
NC_005101.4-120416441	TCTTCTCATACCCAACCTCCACCTTTG	2	C	0.15	0.4988
NC_005101.4-120416441	TCTTCTCATACCCAACCTCCACCTTTG	24	T	0.041	0.4879
NC_005101.4-120431165	GAAGAGGGAACCAGCTCTCCTGGAACT	21	T	0.2195	0.4935

NC_005101.4-120442248	TTACTAGCAACTTCTGCTCCCTACAAG	12	T	0.1356	0.1827
NC_005101.4-120481638	TTCGTTGTGACAAGGTCTCCTTTAAGC	6	T	0.0508	0.0812
NC_005101.4-120481638	TTCGTTGTGACAAGGTCTCCTTTAAGC	25	A	0.0763	0.1041
NC_005101.4-120515940	CAAGAAAACACAATGGCTCCTAAGAAC	22	A	0.0769	0.12
NC_005101.4-120595077	CCCTTCCCAACTCTTTCTCCCTTCCCA	6	C	0.0708	0.0683
NC_005101.4-120616920	CCTTCAGCAACCTCTCCTCCTTTCCCC	13	T	0.0179	0.4483
NC_005101.4-120616920	CCTTCAGCAACCTCTCCTCCTTTCCCC	14	C	0	0.0351
NC_005101.4-120658875	GGCAGATTTACTTTGCCTCCTTAGACC	16	C	0.0492	0.4866
NC_005101.4-120677230	CTTAGCAAGACTATGACTCCTAAGACA	7	A	0	0.4755
NC_005101.4-120692516	GATACAGGGACTATCTCTCCAACAGTG	4	A	0.0732	0.1145
NC_005101.4-120718727	AAATAAGAGACAATCACTCCTCCTTTG	27	G	0	0.4755
NC_005101.4-120724639	GCCGAAAGCACCTTAGCTCCTCAGCTT	4	G	0.2114	0.2723
NC_005101.4-120764712	TTAAGGAAGACTGAGCCTCCCTCTCTC	12	T	0.1622	0.4785
NC_005101.4-120844413	CACTTATCAACATGCTCTCCAGAAATG	15	C	0.0254	0.0415
NC_005101.4-120860554	CCTCATTGCACTAACCCTCCAAGCTGT	16	C	0.0265	0.4693
NC_005101.4-120860554	CCTCATTGCACTAACCCTCCAAGCTGT	22	A	0	0.4641
NC_005101.4-120868139	TGTCCTTGGACGACATCTCCCTTCCCC	9	G	0.0667	0.0644
NC_005101.4-120915209	CTGCCTAGAACTTCAGCTCCAGTGGAT	22	G	0.1557	0.1708
NC_005101.4-120952064	TGTACATGCACTTCAGCTCCTTTAAGC	15	A	0.123	0.4992
NC_005101.4-120952064	TGTACATGCACTTCAGCTCCTTTAAGC	25	A	0.1311	0.4995
NC_005101.4-120957807	ATAGAGAGGACCCTGCCTCCACCATAA	2	T	0	0.4718
NC_005101.4-120981309	TGCTTGCCCACCCCACCTCCCCATCCA	26	C	0.1709	0.4325
NC_005101.4-121074666	TGATCATGGACAGTGGCTCCTAGACAC	14	T	0	0.48
NC_005101.4-121074666	TGATCATGGACAGTGGCTCCTAGACAC	22	A	0	0.48
NC_005101.4-121078541	CTCCCTCCGACCTAACCTCCAGAGTGC	25	T	0.1754	0.2155
NC_005101.4-121114593	CAATTTTCGACAGTCCCTCCTGAAGAG	3	A	0.3033	0.375
NC_005101.4-121123331	CTGTGCACCACGGCGCCTCCTCTTCTG	15	G	0.1301	0.1761
NC_005101.4-121147435	ACACCTAGAACCGCAGCTCCTCAGGCA	12	C	0.3008	0.4572
NC_005101.4-121148221	AATCTCACTACTTCAGCTCCCCAGGTG	2	A	0.2623	0.3947
NC_005101.4-121173758	AGTCCTCATACTTCTACTCCTGGAGAT	1	A	0	0.4728
NC_005101.4-121173758	AGTCCTCATACTTCTACTCCTGGAGAT	15	T	0	0.4728
NC_005101.4-121198874	ACCTCATGAACTTTTCCTCCTGTAGTT	26	T	0	0.4833
NC_005101.4-121226752	TCTCCCAGTACCCACCCTCCACCTTTG	6	C	0.252	0.481
NC_005101.4-121313702	TGGTATAGGACTCATTCTCCACTGAAA	9	G	0.082	0.1225
NC_005101.4-121313702	TGGTATAGGACTCATTCTCCACTGAAA	12	T	0.1803	0.4966
NC_005101.4-121432509	GGCTGCAGTACTTCTGCTCCCAGTCTG	26	T	0.1092	0.4253
NC_005101.4-121445467	TGTCCCCCAACTTCTTCTCCCTCAGAC	26	A	0.0082	0.479
NC_005101.4-121467871	ACCAAGCACACAGATCCTCCATCATCT	22	T	0.1545	0.1954
NC_005101.4-121467871	ACCAAGCACACAGATCCTCCATCATCT	26	C	0.0163	0.032
NC_005101.4-121492876	ATCCTTCAGACCTTTGCTCCAGGCACA	9	G	0.0263	0.026
NC_005101.4-121494611	ACAAGAGTAACGGCAACTCCTTTCCTC	27	C	0.1681	0.4547
NC_005101.4-121522308	CACATTTGCACCCGTCCTCCACATAAA	1	C	0.1478	0.1369
NC_005101.4-121522308	CACATTTGCACCCGTCCTCCACATAAA	14	G	0.0086	0.0255
NC_005101.4-121543477	GATGGCCCCACTCTCTCTCCATTCTGA	2	A	0	0.4814
NC_005101.4-121547631	GAAATTTAAACACAAGCTCCTGACTTC	22	G	0.0991	0.2403
NC_005101.4-121620937	AGCCTCCAGACCAGCCCTCCCAGTCCC	5	T	0.3279	0.3447
NC_005101.4-121620937	AGCCTCCAGACCAGCCCTCCCAGTCCC	16	C	0.0246	0.0243
NC_005101.4-121638495	ATCTTAGGTACGGAAACTCCATTCCAC	12	G	0.0083	0.4769
NC_005101.4-121638495	ATCTTAGGTACGGAAACTCCATTCCAC	13	G	0.0083	0.4769
NC_005101.4-121643054	GTTCTTAACACAGAATCTCCCTATTAT	9	C	0.0351	0.0345
NC_005101.4-121669344	AGCAACAGCACTCTGGCTCCTGGAACA	22	G	0.0248	0.4803

NC_005101.4-121787308	TCTCTGCACACAGCCTCTCCATGACTT	16	T	0.0446	0.0436
NC_005101.4-121846676	GGAGTGTAGACTAGTCCTCCATGGGAT	25	G	0	0.4778
NC_005101.4-121864973	TTTAGATATACCAGAGCTCCTCAGTAC	14	G	0.1696	0.1553
NC_005101.4-121865026	CAGACAAACACACTAGCTCCTAGCACT	4	A	0.1639	0.4891
NC_005101.4-121865026	CAGACAAACACACTAGCTCCTAGCACT	16	G	0	0.4773
NC_005101.4-121881168	CAGAGTAAAACATGCACTCCATCGACT	15	C	0.1652	0.3547
NC_005101.4-121881168	CAGAGTAAAACATGCACTCCATCGACT	16	A	0.0086	0.4866
NC_005101.4-121936155	TCCTACTCCACTGACACTCCCTCAGCC	1	T	0.0488	0.0476
NC_005101.4-121977765	AAACTGCATACACTGCCTCCAAGTACA	4	C	0.05	0.0488
NC_005101.4-122023891	GGTTCCCACACCCCTTCTCCATTACAG	7	C	0.2143	0.3659
NC_005101.4-122023891	GGTTCCCACACCCCTTCTCCATTACAG	23	T	0.2035	0.3772
NC_005101.4-122031244	CTTGGAATCACCTGACCTCCTTGTATC	6	A	0.025	0.0247
NC_005101.4-122250411	CAACCCCTCACCTACGCTCCACTACAC	16	G	0.0259	0.0255
NC_005101.4-122269252	CTCTCGTGTACTTTTGCTCCTTACATA	6	G	0.1111	0.4928
NC_005101.4-122278239	AAACGGTTAACGAAATCTCCATTCTGA	7	T	0.0756	0.0728
NC_005101.4-122278239	AAACGGTTAACGAAATCTCCATTCTGA	12	G	0.2017	0.4517
NC_005101.4-122385098	TGGTAGAGTACACTCACTCCTGCATGG	8	G	0.0265	0.0262
NC_005101.4-122385098	TGGTAGAGTACACTCACTCCTGCATGG	24	A	0.2124	0.4624
NC_005101.4-122398144	AGTTGTTCAACACTGACTCCTAGCCAC	1	A	0	0.4859
NC_005101.4-122428521	GAAGACGGTACTGTATCTCCTGGAGTT	6	C	0.0087	0.0257
NC_005101.4-122429912	GTCATGTGTACGTCTGCTCCTGCATGT	9	T	0.0085	0.0253
NC_005101.4-122442400	CAGCTGGCCACGGTGCCTCCATAGTCA	12	G	0.0089	0.4789
NC_005101.4-122442400	CAGCTGGCCACGGTGCCTCCATAGTCA	26	C	0.0089	0.4789
NC_005101.4-122460196	GACAAACACACCATTGCTCCAATTAGT	9	C	0.0086	0.4729
NC_005101.4-122460196	GACAAACACACCATTGCTCCAATTAGT	22	A	0.0086	0.4729
NC_005101.4-122549806	CCGCTGGCAACTTTATCTCCCAATCAG	3	G	0.0342	0.0336
NC_005101.4-122671884	TCAGTGATTACAGTTCCTCCACAATAT	21	A	0.0087	0.4744
NC_005101.4-122671884	TCAGTGATTACAGTTCCTCCACAATAT	24	A	0.0086	0.4729
NC_005101.4-122729571	AGTGCGAAGACCTAGACTCCAGCTACA	5	C	0.0336	0.0805
NC_005101.4-122783890	TTCTGCTCCACATCTCCTCCTAGCTGT	3	C	0.15	0.2665
NC_005101.4-122783890	TTCTGCTCCACATCTCCTCCTAGCTGT	6	C	0.3	0.4387
NC_005101.4-122801788	GAACACTTAACTCTGTCTCCCCGTCCC	22	C	0	0.4592
NC_005101.4-122805126	TCTACTCTCACAGATGCTCCAGAGAAC	4	A	0.0536	0.0853
NC_005101.4-122805126	TCTACTCTCACAGATGCTCCAGAGAAC	15	T	0.1518	0.1699
NC_005101.4-122881230	CTGCTGCACACGCACACTCCCCTTGTG	5	T	0.0574	0.0861
NC_005101.4-122900745	CTACCTGGCACACAGGCTCCCGGGATT	21	C	0	0.474
NC_005101.4-122900745	CTACCTGGCACACAGGCTCCCGGGATT	27	T	0.1667	0.1814
NC_005101.4-122902797	TGGCCTGTCACTGGGCCTCCTTTCCCT	13	G	0.2377	0.4435
NC_005101.4-122915581	ACCACGTGGACACGTGCTCCACTTGTT	14	G	0	0.4813
NC_005101.4-122915581	ACCACGTGGACACGTGCTCCACTTGTT	24	T	0.0504	0.0805
NC_005101.4-123049580	TATTCTCCCACAGACTCTCCCATCAGA	8	C	0.2478	0.3728
NC_005101.4-123130795	CGGACATGCACATACACTCCCACACAC	24	A	0.1026	0.4997
NC_005101.4-123159437	ATGGTGTTTACCGTTCCTCCATCTTCT	12	C	0.05	0.0799
NC_005101.4-123206338	AGGAAGGAGACTTAGACTCCTGATAAT	27	T	0.0088	0.4843
NC_005101.4-123213779	TACTTCGTTACAGATGCTCCAACGCAT	7	G	0.1885	0.2903
NC_005101.4-123245761	ACTGTGCCCACAGAGGCTCCTAGCTGC	5	T	0.0574	0.1154
NC_005101.4-123245761	ACTGTGCCCACAGAGGCTCCTAGCTGC	24	C	0.2314	0.2869
NC_005101.4-123293994	CTCTCTCTCACTGCTCCTCCTGCTGCT	14	C	0.1301	0.496
NC_005101.4-123318746	TGCCCTAAAACATTGGCTCCACTCACC	24	C	0.2101	0.4859
NC_005101.4-123333583	GTCCTTTGCACGGCAGCTCCTGCTTTT	12	G	0.0569	0.0705
NC_005101.4-123354161	TGATTCTGTACTTTACCTCCAGGGAAC	5	T	0.2895	0.3568

NC_005101.4-123393432	AAACAAATAACCTTGTCTCCTGTTGTA	2	A	0.0763	0.0734
NC_005101.4-123452650	CTGCCAATCACATCAGCTCCTCCCTAC	24	C	0.275	0.4358
NC_005101.4-123471432	CTGCCAGTTACCTCTTCTCCTGGCTTC	15	T	0.0168	0.4813
NC_005101.4-123471432	CTGCCAGTTACCTCTTCTCCTGGCTTC	25	T	0	0.4765
NC_005101.4-123478445	TTCTTCTGAACAGCGTCTCCTCACTAG	1	T	0.1138	0.1216
NC_005101.4-123478445	TTCTTCTGAACAGCGTCTCCTCACTAG	15	G	0.1301	0.1494
NC_005101.4-123498831	GGTTCTATTACACGTCCTCCACCTGGA	15	T	0.2295	0.4999
NC_005101.4-123498831	GGTTCTATTACACGTCCTCCACCTGGA	22	C	0.0164	0.4773
NC_005101.4-123500623	TTAAGGATAACCTGGTCTCCATCCTTT	5	G	0.0598	0.0896
NC_005101.4-123500623	TTAAGGATAACCTGGTCTCCATCCTTT	26	T	0.1913	0.48
NC_005101.4-123531603	CCCAGTGAGACCCCACCTCCTAAAGGT	16	C	0.2143	0.2188
NC_005101.4-123531603	CCCAGTGAGACCCCACCTCCTAAAGGT	24	A	0	0.477
NC_005101.4-123532367	ACTAACAGGACAACTCCTCCAGTGGAC	21	A	0	0.4793
NC_005101.4-123662356	TCCTCTTCTACTGAGGCTCCACCAGGA	3	C	0.0261	0.0257
NC_005101.4-123662356	TCCTCTTCTACTGAGGCTCCACCAGGA	7	T	0	0.48
NC_005101.4-123720415	GAAGTTGATACTATGTCTCCCACCATC	21	C	0.2035	0.2237
NC_005101.4-123734816	TGCATGAGAACCCGAGCTCCCAAATTA	14	G	0	0.4755
NC_005101.4-123823997	GTGTTTATAACAGCATCTCCCTAACAG	1	G	0	0.4793
NC_005101.4-123855152	GACAGGCTAACATCAGCTCCCGCTGTT	21	C	0	0.4751
NC_005101.4-123861402	AACAACTAAACCATTTCTCCATCCACA	8	A	0.0325	0.032
NC_005101.4-123900069	TGCACTAGGACATAGACTCCTTTGAGT	15	G	0.2033	0.208
NC_005101.4-123908422	CCTTACCACACAGCCTCTCCACGAGTG	4	T	0	0.4787
NC_005101.4-123911321	ATCTGGCAGACATCACCTCCTGGGATA	5	G	0.0085	0.487
NC_005101.4-123982823	CACTCCTGCACAGCTTCTCCATTGCAC	24	G	0	0.4793
NC_005101.4-124000664	TGTCAGCCTACATCTCCTCCAAGATGA	2	G	0	0.4757
NC_005101.4-124021992	GTCTCAGGCACTGGGACTCCTTAAAGC	12	T	0	0.4765
NC_005101.4-124021992	GTCTCAGGCACTGGGACTCCTTAAAGC	13	G	0	0.4743
NC_005101.4-124057791	AGGCTAGGCACATCCTCTCCCAATGAG	27	G	0.4336	0.3396
NC_005101.4-124063048	TCCCCTTTAACCTTCTCTCCCTCTTAC	7	T	0.065	0.0629
NC_005101.4-124063048	TCCCCTTTAACCTTCTCTCCCTCTTAC	24	T	0.065	0.0629
NC_005101.4-124080148	CGACCCAGCACCGATGCTCCCCAATCC	1	C	0.0427	0.0418
NC_005101.4-124080148	CGACCCAGCACCGATGCTCCCCAATCC	2	G	0	0.4772
NC_005101.4-124090671	CAATCCTTCACTGAAACTCCCTTCTGT	3	A	0.1017	0.493
NC_005101.4-124095584	GTAGCTTGAACTTCTTCTCCACGGCTG	13	T	0.0085	0.4776
NC_005101.4-124109088	GGCAGCAGCACTTCCCCTCCGTGCTCC	15	C	0.292	0.3081
NC_005101.4-124109088	GGCAGCAGCACTTCCCCTCCGTGCTCC	21	G	0	0.4779
NC_005101.4-124123336	TACAGTCAAACTACAGCTCCAAGCAGA	16	G	0	0.4833
NC_005101.4-124150352	CGCTCGGGGACCTCGACTCCTCTCGCT	12	C	0.0583	0.0566
NC_005101.4-124150352	CGCTCGGGGACCTCGACTCCTCTCGCT	27	T	0	0.4844
NC_005101.4-124346101	TCAATTTAAACCACAACTCCATTTAAA	4	A	0.0088	0.4861
NC_005101.4-124354473	TTCTCTGTGACCAGGTCTCCTGGGCTA	26	T	0	0.48
NC_005101.4-124357195	CTGGGTGGCACATTTTCTCCTCCTCAG	12	A	0	0.4912
NC_005101.4-124385882	TTCTGTGAGACATATTCTCCTGGTGTC	4	T	0.6239	0.4293
NC_005101.4-124390737	TGCCCCACCACCCCAGCTCCAGGGGAC	9	C	0.1951	0.3141
NC_005101.4-124391423	CGGTTAGCTACGGGTTCTCCCAGTCCT	2	G	0.0088	0.476
NC_005101.4-124420290	AACTCTGTTACCGCTGCTCCCACACAA	13	G	0.3008	0.436
NC_005101.4-124446539	GCCCCATCCACCCCTTCTCCATCACCC	22	T	0.4576	0.3529
NC_005101.4-124452861	AACATTCTCACCCTGTCTCCCGAAGAT	12	C	0.0254	0.0251
NC_005101.4-124464170	CCAGTTTGGACTCCAACTCCATGAGGC	5	T	0	0.477
NC_005101.4-124464170	CCAGTTTGGACTCCAACTCCATGAGGC	27	C	0.0179	0.0521
NC_005101.4-124619929	CCAGCCCTTACTCTCCCTCCCCCTTTT	13	C	0.041	0.0401

NC_005101.4-124624906	GAAAGATTTACTTTGGCTCCTGCTTTC	3	A	0.6016	0.4206
NC_005101.4-124624906	GAAAGATTTACTTTGGCTCCTGCTTTC	25	T	0.6016	0.4206
NC_005101.4-124690784	AGGATCTGAACTCAAGCTCCCATGCGT	4	A	0	0.4606
NC_005101.4-124690784	AGGATCTGAACTCAAGCTCCCATGCGT	26	G	0.1579	0.4998
NC_005101.4-124711441	GTTCTTTGAACATGAGCTCCATGGGAT	15	A	0	0.4826
NC_005101.4-124711441	GTTCTTTGAACATGAGCTCCATGGGAT	24	G	0.0593	0.0576
NC_005101.4-124810161	TTGAAGACTACACACTCTCCGTGAACA	21	G	0	0.4793
NC_005101.4-124853543	GGTGTCTTCACTATATCTCCAAAGTCT	7	T	0	0.477
NC_005101.4-124853543	GGTGTCTTCACTATATCTCCAAAGTCT	13	A	0	0.4755
NC_005101.4-124877956	TTCCTTCCAACAACATCTCCTTGGGTA	12	A	0	0.4854
NC_005101.4-124877956	TTCCTTCCAACAACATCTCCTTGGGTA	24	G	0	0.4854
NC_005101.4-124878899	TAGACACTAACATGGCCTCCTGAGGTA	1	T	0	0.4666
NC_005101.4-124922225	TCTCTCATTACATTCTCTCCCTTCTCA	8	T	0.0268	0.0264
NC_005101.4-124952508	TCTGACTCCACTCCACCTCCCTTGTGC	7	T	0.2222	0.4982
NC_005101.4-124969997	TTTTCACTGACCCTTCCTCCCTCCCAC	9	G	0.0336	0.033
NC_005101.4-125008076	TTCAACTGTACTGCCTCTCCCCTTTCC	2	T	0.0083	0.4816
NC_005101.4-125008076	TTCAACTGTACTGCCTCTCCCCTTTCC	25	T	0.1667	0.2061
NC_005101.4-125013537	AGAAAGAGCACTGCTCCTCCCCAAGCA	6	G	0.0738	0.071
NC_005101.4-125016083	CATTGCTGCACACTGACTCCCACCGCT	8	G	0.0407	0.0398
NC_005101.4-125035171	TGCTGCGCCACTGCCTCTCCAACCTTG	8	C	0	0.4787
NC_005101.4-125035171	TGCTGCGCCACTGCCTCTCCAACCTTG	26	T	0	0.4787
NC_005101.4-125038775	TGTCAACTGACTAAATCTCCAGGCCAA	9	G	0.0083	0.4803
NC_005101.4-125040152	ATATTTCCCACTCAGCCTCCATTGATG	9	C	0	0.4793
NC_005101.4-125076824	TCCAAATGTACCAGGTCTCCAGAGAGG	22	G	0.2368	0.2836
NC_005101.4-125196287	ACCTATATTACCAACCCTCCAAAGAAT	24	G	0.0625	0.0605
NC_005101.4-125243566	TCTCATTCAACTCTTTCTCCCTCCTCT	21	C	0.0776	0.4955
NC_005101.4-125251042	AATTTTTACACACCCCCTCCCTCTCCC	14	C	0.0708	0.0683
NC_005101.4-125251130	TTGAATGTTACCAACTCTCCATTCTCT	9	T	0.1026	0.4991
NC_005101.4-125251130	TTGAATGTTACCAACTCTCCATTCTCT	21	A	0.0508	0.0496
NC_005101.4-125257942	CCAACTCTAACTATGGCTCCAGAGGAT	23	A	0.2478	0.4793
NC_005101.4-125279836	GTGGAAACAACCTTGGCTCCAATTGGC	14	T	0.0357	0.0351
NC_005101.4-125311864	CAGGAGCCCACAGCTTCTCCCGCTTCT	21	C	0	0.4718
NC_005101.4-125332073	AGCTTTGCTACCGTCCCTCCCAGTCCC	9	T	0	0.4709
NC_005101.4-125332073	AGCTTTGCTACCGTCCCTCCCAGTCCC	12	C	0.0342	0.0336
NC_005101.4-125332634	TAACAACCCACCACACCTCCAGTGCTT	15	A	0.1789	0.2723
NC_005101.4-125369398	CTCTCTGGTACCTGCACTCCCACACCT	8	G	0.1138	0.1761
NC_005101.4-125369398	CTCTCTGGTACCTGCACTCCCACACCT	15	C	0.0732	0.0854
NC_005101.4-125387858	AGGGAGTATACAGGACCTCCCCATGGC	24	T	0.1217	0.3499
NC_005101.4-125387858	AGGGAGTATACAGGACCTCCCCATGGC	25	G	0.0087	0.4817
NC_005101.4-125401485	CAGCAACCAACACATGCTCCTTCAATA	5	A	0	0.4709
NC_005101.4-125401485	CAGCAACCAACACATGCTCCTTCAATA	14	A	0.0427	0.074
NC_005101.4-125435803	CATCTTGCCACCAACTCTCCATTTATC	26	T	0.2213	0.4924
NC_005101.4-125506172	GTGAGATGAACAACCCCTCCCTTCCCT	24	C	0.0427	0.0418
NC_005101.4-125506172	GTGAGATGAACAACCCCTCCCTTCCCT	26	C	0.178	0.2156
NC_005101.4-125549268	AATTTTTACACAGTGCCTCCATTATCT	4	T	0.0593	0.4273
NC_005101.4-125578403	AAATGCACAACGAAGTCTCCACTTCTC	9	A	0.0084	0.4761
NC_005101.4-125578403	AAATGCACAACGAAGTCTCCACTTCTC	12	G	0.0084	0.4761
NC_005101.4-125586168	AGGTGATAGACCAGTTCTCCTGGTCTC	23	G	0	0.4749
NC_005101.4-125587710	CTCACTGAAACAGCCCCTCCCACTGCA	2	T	0.1736	0.2474
NC_005101.4-125587710	CTCACTGAAACAGCCCCTCCCACTGCA	15	C	0.1736	0.4951
NC_005101.4-125603276	AAATTCTGCACCATCCCTCCAGTCCCT	9	C	0.0424	0.0889

NC_005101.4-125615466	GTTCCTGCCACTGTAACTCCATTTCTT	27	T	0	0.4773
NC_005101.4-125633881	TTAGTGAGCACTCTGCCTCCGTGGTTC	8	G	0.1983	0.4628
NC_005101.4-125633881	TTAGTGAGCACTCTGCCTCCGTGGTTC	16	C	0.2314	0.3811
NC_005101.4-125770055	CTAGTGCCTACCTTTTCTCCTTCAGTC	7	C	0.0984	0.1367
NC_005101.4-125770055	CTAGTGCCTACCTTTTCTCCTTCAGTC	27	C	0.1057	0.2203
NC_005101.4-125798413	ACTGAGAGCACTAGGTCTCCAGCTTCT	3	T	0.0086	0.4768
NC_005101.4-125826650	TTCCTCTCCACTGTAACTCCTCTGCTC	21	T	0.1789	0.2263
NC_005101.4-125906197	CTTCCCTTGACCTTACCTCCCGCCTTC	27	C	0.0508	0.0496
NC_005101.4-125907860	CTCTTGCCAACCCTGGCTCCCCAGCAT	22	C	0.2195	0.208
NC_005101.4-125916029	GAAGAAACGACAGCCACTCCTCACCTC	14	C	0.3197	0.3307
NC_005101.4-125938016	CCACTCAACACCAACACTCCTTTTCTT	6	C	0.0603	0.0585
NC_005101.4-125988648	CTGAAACAAACATGGTCTCCATGATAG	12	A	0.1071	0.2449
NC_005101.4-125988648	CTGAAACAAACATGGTCTCCATGATAG	26	A	0.0702	0.0839
NC_005101.4-126010096	GTGAACCATACACTTACTCCTGAGGCA	6	C	0.2137	0.3685
NC_005101.4-126018283	CCAACTGTAACGCCAGCTCCAAGGAGT	25	A	0.0164	0.4837
NC_005101.4-126159113	TGTTCTCAAACTTGATCTCCTGTATTA	3	T	0.1982	0.3587
NC_005101.4-126170460	TCTTCTGTGACCCAGCCTCCCAGTGGC	4	T	0	0.4807
NC_005101.4-126244284	CCTGTTACAACTTATTCTCCATGTGAG	1	C	0.2783	0.3772
NC_005101.4-126244284	CCTGTTACAACTTATTCTCCATGTGAG	23	G	0.287	0.3978
NC_005101.4-126354772	CAAGGGCTGACCCTAGCTCCTCCACAC	1	C	0.0168	0.4779
NC_005101.4-126354772	CAAGGGCTGACCCTAGCTCCTCCACAC	2	A	0.0084	0.4761
NC_005101.4-126391984	GAGGGACTTACTTTCTCTCCTAACTTC	22	A	0	0.4793
NC_005101.4-126395105	CAATATCAGACTTGCACTCCAGATATC	1	C	0.0088	0.476
NC_005101.4-126395105	CAATATCAGACTTGCACTCCAGATATC	9	G	0.0088	0.476
NC_005101.4-126431116	CTTGCAGTCACTGCCTCTCCTGGGAAG	22	G	0.2087	0.3938
NC_005101.4-126433943	TCCTTTCAAACTTTAACTCCCTATGTC	7	C	0.2314	0.3645
NC_005101.4-126515355	CTGCTCCAAACTTAGCCTCCATAACTC	16	C	0.1157	0.1235
NC_005101.4-126531797	GTGTGCTTCACACATCCTCCTTTGTAT	25	T	0	0.4772
NC_005101.4-126577244	AGAGGACTGACTCTCCCTCCTCCAGAA	2	G	0.2743	0.3294
NC_005101.4-126634756	TGATCCTCTACTTTTGCTCCTCAAATG	1	T	0.2941	0.3812
NC_005101.4-126634756	TGATCCTCTACTTTTGCTCCTCAAATG	12	T	0.1864	0.3138
NC_005101.4-126641971	TAACTTTGAACTCCATCTCCTGAGAGC	16	T	0	0.4773
NC_005101.4-126641971	TAACTTTGAACTCCATCTCCTGAGAGC	25	A	0.2951	0.387
NC_005101.4-126643110	TCTCCCAGAACTGGGCCTCCAGGCTGT	7	A	0.025	0.0247
NC_005101.4-126659116	TTTAGATCCACTGAGGCTCCATGTCAA	2	T	0	0.4746
NC_005101.4-126662875	ACAGTGTTCACAGTCTCTCCAGCGTGT	14	T	0.0083	0.4732
NC_005101.4-126662875	ACAGTGTTCACAGTCTCTCCAGCGTGT	23	C	0.0165	0.4751
NC_005101.4-126679670	GGGATAGATACAGATGCTCCATCTACA	26	C	0	0.4887
NC_005101.4-126679670	GGGATAGATACAGATGCTCCATCTACA	27	A	0	0.4887
NC_005101.4-126724589	ATGTGCTAGACCACGGCTCCTTTAAAA	7	T	0.2869	0.375
NC_005101.4-126724589	ATGTGCTAGACCACGGCTCCTTTAAAA	15	G	0.2869	0.375
NC_005101.4-126755152	CTACTACACACCCAGTCTCCTAGATCA	4	C	0	0.4807
NC_005101.4-126755152	CTACTACACACCCAGTCTCCTAGATCA	15	G	0	0.4807
NC_005101.4-126769951	TCAGAGACAACCAATGCTCCTGCTACT	8	C	0.0256	0.0253
NC_005101.4-126769951	TCAGAGACAACCAATGCTCCTGCTACT	26	C	0	0.4772
NC_005101.4-126795680	TTCCCTAATACTATTGCTCCTTCAATG	9	T	0.2213	0.3667
NC_005101.4-126812395	ATCCTTTCCACAGGGCCTCCTCGTGCT	15	G	0	0.474
NC_005101.4-126812395	ATCCTTTCCACAGGGCCTCCTCGTGCT	23	G	0.1167	0.1528
NC_005101.4-126826623	TACTCAGTAACCATCTCTCCAGCCTCT	25	T	0.0081	0.4777
NC_005101.4-126853350	CTGCCTGTAACTCTGGCTCCACAGGAT	22	C	0.0976	0.1356
NC_005101.4-126935233	AAATACTCAACAGTATCTCCAGACATT	6	C	0.0744	0.0868

NC_005101.4-126947048	TGCCCGCTTACTCATTCTCCGGCTGGA	5	C	0.1754	0.3706
NC_005101.4-126947048	TGCCCGCTTACTCATTCTCCGGCTGGA	15	T	0.2281	0.4384
NC_005101.4-126992914	ATGCATTCTACCAACACTCCCTTTGCA	26	C	0.0496	0.4628
NC_005101.4-127034139	TTACAGAGCACAGACCCTCCCCCTGAT	7	A	0.1071	0.1327
NC_005101.4-127111688	ATCGTTACCACAATCCCTCCCTCCAAC	4	G	0.0536	0.0853
NC_005101.4-127145058	ATGTATAAAACTCTAGCTCCAAGGAAT	1	A	0	0.4813
NC_005101.4-127146300	ATCACTTGTACTACTTCTCCCTATCAA	2	T	0.018	0.4746
NC_005101.4-127151715	TACAGGGTAACCCCAACTCCTATCTCC	6	G	0.1626	0.2017
NC_005101.4-127165787	CCAGTTCTCACTGGCACTCCATAGCAA	1	C	0.041	0.071
NC_005101.4-127165787	CCAGTTCTCACTGGCACTCCATAGCAA	24	G	0.1057	0.1425
NC_005101.4-127189204	TACCAACCAACCAGCTCTCCCAGGGAC	1	T	0.0924	0.0882
NC_005101.4-127205007	ATGCAGCAGACCACCCCTCCCATAATA	7	C	0.1441	0.3772
NC_005101.4-127205007	ATGCAGCAGACCACCCCTCCCATAATA	23	T	0.0631	0.1414
NC_005101.4-127218181	GTGTTAGAGACTTGGGCTCCACATCAG	23	A	0.0924	0.1181
NC_005101.4-127395764	TTGCCTGTAACTCTAGCTCCAGAGAAT	27	T	0.0248	0.0245
NC_005101.4-127421941	AAATGTTTTACAAACACTCCAACAGTC	26	T	0.0083	0.0245
NC_005101.4-127436846	CCACCTGTAACTCCAGCTCCAGGGCAC	12	T	0.319	0.291
NC_005101.4-127587266	CAAGGCTCGACACCCTCTCCCCCCAGT	9	G	0.5492	0.3984
NC_005101.4-127587266	CAAGGCTCGACACCCTCTCCCCCCAGT	16	T	0.5492	0.3984
NC_005101.4-127665581	CTCTGGCATACCAAGCCTCCTTTGTAA	8	A	0	0.4749
NC_005101.4-127665581	CTCTGGCATACCAAGCCTCCTTTGTAA	14	A	0	0.4764
NC_005101.4-127723363	AGCTTTGTGACTAAGTCTCCAGGCTTC	26	T	0.0088	0.4843
NC_005101.4-127723363	AGCTTTGTGACTAAGTCTCCAGGCTTC	27	C	0.0088	0.4843
NC_005101.4-127789846	GAGTTGAATACAGGGTCTCCCTACTTG	2	A	0.0325	0.032
NC_005101.4-127815032	TGTAGCTGCACCAGCTCTCCCTACTGC	4	A	0	0.4807
NC_005101.4-127937044	GTGCTGCCTACGACCTCTCCGAGGCGG	26	G	0.2793	0.2403
NC_005101.4-127974706	TGAAGGCAAACCCTAGCTCCTGTAGGT	8	A	0.2521	0.322
NC_005101.4-127976768	CCACAGATTACCCAAGCTCCCAGTTAT	24	T	0	0.4807
NC_005101.4-127986396	ACACTGAGAACTCCTCCTCCACTCCAC	9	A	0	0.48
NC_005101.4-127986396	ACACTGAGAACTCCTCCTCCACTCCAC	25	C	0	0.48
NC_005101.4-128001299	TGGGGCGCCACTTTAGCTCCCTTGCTC	13	T	0.0732	0.0854
NC_005101.4-128016216	AGTCTGGTTACCTCCCCTCCTCTACTC	24	A	0.0261	0.0257
NC_005101.4-128022823	AATTTTACCACCGCAGCTCCAGGATAT	13	G	0	0.4844
NC_005101.4-128022823	AATTTTACCACCGCAGCTCCAGGATAT	24	A	0	0.4844
NC_005101.4-128046674	AGTCTTTCTACATGGCCTCCTCTCATC	12	A	0.1681	0.4957
NC_005101.4-128051680	CAAGTCCGGACTTTGACTCCCACAGTG	8	G	0	0.4773
NC_005101.4-128094208	TTTGGGTCCACAGCATCTCCATCCGAT	24	C	0.1624	0.3311
NC_005101.4-128114046	ATATCGATCACCCAGCCTCCATTTTCA	7	A	0	0.48
NC_005101.4-128124268	CTGGTCTCCACACTTGCTCCCTACGTG	2	T	0.0246	0.4852
NC_005101.4-128124268	CTGGTCTCCACACTTGCTCCCTACGTG	24	C	0.0492	0.0786
NC_005101.4-128166209	GACTCACGAACCCCTCCTCCATCCTGT	7	C	0.3033	0.375
NC_005101.4-128166209	GACTCACGAACCCCTCCTCCATCCTGT	23	C	0.065	0.0629
NC_005101.4-128190733	TCATCCACCACCACACCTCCTCACTCT	23	A	0	0.4793
NC_005101.4-128205554	TGCTGAACGACACATGCTCCCGTTCCC	7	A	0.1176	0.1539
NC_005101.4-128215649	CCCAGATTCACTCCCACTCCTTAGAAA	26	A	0.2435	0.32
NC_005101.4-128264407	CTCATAGGTACCATATCTCCAAGAGCT	13	A	0.2308	0.4527
NC_005101.4-128278536	CCCCTAGAAACCTTCTCTCCTCGCAAT	23	G	0	0.4779
NC_005101.4-128355624	CTGTGAAAGACAGCCCCTCCTGCTCCC	2	T	0.225	0.3622
NC_005101.4-128355624	CTGTGAAAGACAGCCCCTCCTGCTCCC	4	T	0.0579	0.0562
NC_005101.4-128356088	ATATACCACACACATACTCCATGGGCA	15	T	0.0526	0.0512
NC_005101.4-128376913	TTCCTTAGTACTTCTGCTCCGATGAAT	26	A	0.1071	0.1172

NC_005101.4-128411351	CCAAGCATAACATTAACTCCCACGGTT	24	G	0.0598	0.058
NC_005101.4-128411351	CCAAGCATAACATTAACTCCCACGGTT	25	G	0.2137	0.4012
NC_005101.4-128419750	TCCTGAGGCACACAGACTCCCTTTCCC	4	T	0.1739	0.1869
NC_005101.4-128419750	TCCTGAGGCACACAGACTCCCTTTCCC	24	T	0	0.4851
NC_005101.4-128430181	CATGGGGCCACCCACACTCCAGGTAGC	7	G	0.2321	0.3157
NC_005101.4-128457296	AAGCTACCTACTATCTCTCCTAGGTAT	15	C	0.2521	0.2683
NC_005101.4-128457296	AAGCTACCTACTATCTCTCCTAGGTAT	27	T	0	0.4844
NC_005101.4-128509275	TTTGGAGCTACTCAGGCTCCAAGTCAG	9	T	0.1833	0.3099
NC_005101.4-128509275	TTTGGAGCTACTCAGGCTCCAAGTCAG	13	C	0.0417	0.0408
NC_005101.4-128533277	GTTGGTTTAACAGTGACTCCATACAAG	3	T	0.2479	0.2544
NC_005101.4-128593011	AATCTACTCACTCTTACTCCTCTTGCT	5	T	0	0.4793
NC_005101.4-129050098	ACCCAAGTGACCTTGCCTCCACAGGTT	9	G	0.1121	0.2057
NC_005101.4-129050098	ACCCAAGTGACCTTGCCTCCACAGGTT	22	C	0.1897	0.2378
NC_005101.4-129081944	TGACTCTTAACACTCTCTCCACCTCCT	3	A	0.0085	0.481
NC_005101.4-129082783	ATAACCCCTACATTCCCTCCCTTAATT	23	T	0	0.4773
NC_005101.4-129149724	CTAATCCTCACTTAACCTCCTGAGAAC	9	C	0.3158	0.4814
NC_005101.4-129305011	AGCCTCTCCACCACTTCTCCTGCTATC	27	C	0	0.4821
NC_005101.4-129305476	ACTATTGTGACAGGAGCTCCTGGAGCC	13	G	0.3197	0.4717
NC_005101.4-129357514	AGAGATAGAACTCTAGCTCCATCTGTA	26	T	0.2195	0.2778
NC_005101.4-129370948	CTAGCTGGCACTGGCACTCCATCGTCC	24	G	0.1545	0.1826
NC_005101.4-129436063	TAACCAAGAACTGTTTCTCCTGTTCCC	23	T	0	0.4787
NC_005101.4-129436063	TAACCAAGAACTGTTTCTCCTGTTCCC	27	C	0	0.4787
NC_005101.4-129850986	TACCTACCCACTCCTGCTCCACTGCCC	12	T	0.2397	0.313
NC_005101.4-129850986	TACCTACCCACTCCTGCTCCACTGCCC	26	C	0.1157	0.1235
NC_005101.4-129868008	CTCATGTTCACAGAGCCTCCCCACATC	7	T	0.177	0.3815
NC_005101.4-130317013	TTGTTGCCTACCAATTCTCCTAAAAGT	2	T	0	0.4819
NC_005101.4-130317013	TTGTTGCCTACCAATTCTCCTAAAAGT	9	T	0	0.4807
NC_005101.4-130424281	GTGAATCCTACATCTGCTCCCACAGGG	4	A	0	0.474
NC_005101.4-130424281	GTGAATCCTACATCTGCTCCCACAGGG	8	C	0.1478	0.4998
NC_005101.4-130461123	CTGCATTACACTTGTGCTCCTTTTCCC	8	A	0.0261	0.0257
NC_005101.4-130461123	CTGCATTACACTTGTGCTCCTTTTCCC	22	T	0.0261	0.0425
NC_005101.4-130542677	TCTCCTTGTACTGTACCTCCCGAATTT	21	C	0.0179	0.4592
NC_005101.4-130542677	TCTCCTTGTACTGTACCTCCCGAATTT	26	T	0.0625	0.4751
NC_005101.4-130600525	CACACACACACCCTTGCTCCATTCTCC	2	A	0.2288	0.3575
NC_005101.4-130600525	CACACACACACCCTTGCTCCATTCTCC	7	C	0.0083	0.0247
NC_005101.4-130658291	TTTGTCCACACCAGCCCTCCCAAGCAA	12	C	0.0339	0.0333
NC_005101.4-130825571	ACTTTAATAACAGTCACTCCAACTCAG	5	T	0.1148	0.4979
NC_005101.4-130886607	GAGAAAAGTACCCTCTCTCCAAGGTGA	24	G	0.2712	0.4757
NC_005101.4-131001815	AGTGTCAGCACCCCACCTCCATGAGAT	21	A	0.0732	0.1001
NC_005101.4-131406252	AACACAAGAACTTTGACTCCAGAGAAT	4	A	0	0.4772
NC_005101.4-131489081	CATGGAGGGACCATGGCTCCAACCACA	23	C	0.1504	0.1391
NC_005101.4-131614351	TACCCACCAACCAGAGCTCCCAGACAC	21	C	0.1818	0.2533
NC_005101.4-131629190	TCACACCATACCATGCCTCCAGCAAAC	15	G	0.2696	0.4333
NC_005101.4-131629190	TCACACCATACCATGCCTCCAGCAAAC	27	C	0.1709	0.2834
NC_005101.4-131828204	GAATGGGAAACCTGCCCTCCATGCCTA	12	C	0.1949	0.4939
NC_005101.4-131845498	GGCCTCTCTACTATCTCTCCCCATACA	6	C	0.1322	0.4983
NC_005101.4-131845498	GGCCTCTCTACTATCTCTCCCCATACA	24	T	0.3613	0.2961
NC_005101.4-131886419	AACTCCAGGACTCATGCTCCACTATGT	6	C	0	0.4887
NC_005101.4-132101391	TGGAGATTCACTCGACCTCCTCAAATA	3	G	0.036	0.086
NC_005101.4-132101391	TGGAGATTCACTCGACCTCCTCAAATA	13	C	0.1892	0.2656
NC_005101.4-132217987	TTCTTTGAGACTGTGTCTCCTACAGAT	21	T	0	0.4807

NC_005101.4-132286024	AAGCCAATGACATTGTCTCCTAGAGTT	13	T	0.1739	0.3772
NC_005101.4-132320887	TGTGGTATAACTATTGCTCCTACAATT	14	T	0.375	0.3367
NC_005101.4-132990306	AAAAAGAGCACCTTTCCTCCCTTACAG	8	G	0.0263	0.026
NC_005101.4-132990306	AAAAAGAGCACCTTTCCTCCCTTACAG	24	A	0.2589	0.3211
NC_005101.4-133146081	TGAAGTGACACGCATGCTCCATTGTAA	12	G	0.0738	0.071
NC_005101.4-133280663	AATGTCTACACCCTTACTCCCACAATA	12	C	0	0.4793
NC_005101.4-133309674	CACAAACACACTTTGTCTCCTTCTCTC	1	C	0.027	0.0267
NC_005101.4-133406310	TTGGACTTCACTCTTACTCCTTTAAAT	2	T	0.0342	0.066
NC_005101.4-133406310	TTGGACTTCACTCTTACTCCTTTAAAT	14	T	0.2222	0.3729
NC_005101.4-133407518	TATATGGACACTTGCCCTCCCATGAAC	15	C	0.2308	0.3935
NC_005101.4-133755590	TATAAACACACAGGACCTCCTCTGTCT	14	G	0.1271	0.1481
NC_005101.4-133815375	ATTCCCCTAACCCCTCCTCCTCCCATT	16	C	0.2213	0.1968
NC_005101.4-133833794	CTGTTAAGAACACCACCTCCCTTCTAC	1	C	0	0.4757
NC_005101.4-133833794	CTGTTAAGAACACCACCTCCCTTCTAC	5	T	0.0336	0.065
NC_005101.4-133848291	TACCAACCAACCAGGGCTCCAGGGACT	23	G	0	0.0496
NC_005101.4-133875219	ACAACAAGCACCCAACCTCCTACACTG	2	C	0.1217	0.4969
NC_005101.4-133886334	TACGAGCCAACCAGAGCTCCCAGGGAG	3	C	0.0348	0.0508
NC_005101.4-133889307	TCTTATAGAACCATCCCTCCTTTTAAA	5	A	0.1261	0.1338
NC_005101.4-134251451	TACGTTAAAACATACTCTCCCAGAGAA	3	C	0	0.4772
NC_005101.4-134360562	AAAGTGTGAACACATACTCCGTACACT	8	G	0.0089	0.4789
NC_005101.4-134360562	AAAGTGTGAACACATACTCCGTACACT	13	C	0.0088	0.481
NC_005101.4-134400737	TTAGTGATGACTGTTACTCCCTGTGTA	22	T	0.0526	0.0512
NC_005101.4-134588791	GTGCAAGCAACCACAACTCCTGGGAAT	23	G	0.6083	0.4297
NC_005101.4-134588791	GTGCAAGCAACCACAACTCCTGGGAAT	24	G	0.6083	0.4297
NC_005101.4-134840978	AACACAGTCACTGATGCTCCCATAGAA	6	A	0.114	0.235
NC_005101.4-134840978	AACACAGTCACTGATGCTCCCATAGAA	16	G	0.292	0.3081
NC_005101.4-134866765	TTTTCTTTCACCACACCTCCTACAAAC	14	C	0.0603	0.1786
NC_005101.4-134886133	AACTGGGATACATTTCCTCCTTTTTAT	26	A	0.0179	0.0351
NC_005101.4-134902518	GGATGAGTCACATGAACTCCAGCATGG	4	T	0.0609	0.4926
NC_005101.4-134902518	GGATGAGTCACATGAACTCCAGCATGG	15	A	0.0522	0.0508
NC_005101.4-135001343	CTCAAGTCCACTTAGTCTCCTGCAAAA	7	T	0.1667	0.2476
NC_005101.4-135087044	TCCTCTAGTACCATGTCTCCCTGGATT	3	C	0	0.4807
NC_005101.4-135087044	TCCTCTAGTACCATGTCTCCCTGGATT	13	A	0.0976	0.1216
NC_005101.4-135322060	CCAGATAGCACATCTTCTCCATTGACT	16	T	0.0252	0.4797
NC_005101.4-135483066	GGGAAGTGGACACAAGCTCCTACCTCT	2	G	0.1545	0.1695
NC_005101.4-135802479	CCTAAGACTACTTGTCCTCCTACAGTG	1	C	0.1083	0.2833
NC_005101.4-135804628	TGAGCATGGACAATCTCTCCTTTTGTG	12	A	0.339	0.4102
NC_005101.4-135990219	TGTATGAAAACAGAAACTCCAATAGAG	4	A	0	0.4749
NC_005101.4-135990219	TGTATGAAAACAGAAACTCCAATAGAG	16	A	0	0.4772
NC_005101.4-136181294	TACCTAGGTACCAGGGCTCCTCAGGCC	3	C	0.0084	0.4829
NC_005101.4-136226445	GTTAGACACACTGGATCTCCACAGAGT	2	T	0.0084	0.4723
NC_005101.4-136291792	GAGAGAAGAACTTGATCTCCAATGTTG	24	G	0.1709	0.1975
NC_005101.4-136300107	TGGCAGCCAACCAGCTCTCCTGTACCA	22	G	0.1681	0.2203
NC_005101.4-136307429	TGTGTGATGACTATGTCTCCCTTTGTG	8	T	0	0.4773
NC_005101.4-136307429	TGTGTGATGACTATGTCTCCCTTTGTG	23	T	0	0.4773
NC_005101.4-136549971	ACAGTTGGGACTTTGTCTCCCTTATTA	13	T	0	0.4764
NC_005101.4-136565631	AATTTCATCACCAATTCTCCAGTGGTA	21	A	0.0696	0.0989
NC_005101.4-136565631	AATTTCATCACCAATTCTCCAGTGGTA	22	G	0.0259	0.0422
NC_005101.4-136577923	CTGTGGTTAACACTGTCTCCAGAGGCA	5	G	0	0.4813
NC_005101.4-136600050	TGAGGGACCACACCCACTCCAGTTCTG	23	T	0.2276	0.4072
NC_005101.4-136600050	TGAGGGACCACACCCACTCCAGTTCTG	25	C	0.1301	0.1761

NC_005101.4-137225958	CATCAATCTACAGCTTCTCCAGCCTTC	7	T	0.2564	0.4649
NC_005101.4-137225958	CATCAATCTACAGCTTCTCCAGCCTTC	24	C	0.2564	0.355
NC_005101.4-137227997	GCACACACCACATCATCTCCTTGTGAG	2	C	0	0.4751
NC_005101.4-137227997	GCACACACCACATCATCTCCTTGTGAG	13	T	0.2377	0.4193
NC_005101.4-137274335	GGCAAGGCCACTTCTCCTCCTAGTATT	21	T	0.122	0.1826
NC_005101.4-137274335	GGCAAGGCCACTTCTCCTCCTAGTATT	26	T	0.2033	0.4967
NC_005101.4-137436245	AGTCCTTATACACTGTCTCCAGTACTC	3	T	0.5652	0.4268
NC_005101.4-137436245	AGTCCTTATACACTGTCTCCAGTACTC	9	T	0.5565	0.4165
NC_005101.4-137717010	GTAGTCTCTACTTATTCTCCACGATGA	25	T	0	0.4826
NC_005101.4-137788079	TCTCTCCGCACATTGCCTCCCTTATCC	8	G	0.0089	0.4824
NC_005101.4-137901704	GAGAAATGCACTGGTACTCCACTTCAT	12	T	0.075	0.4622
NC_005101.4-137901704	GAGAAATGCACTGGTACTCCACTTCAT	15	T	0.0992	0.0943
NC_005101.4-137904045	AGGTGCTTAACTCTTTCTCCAGCCTTA	7	T	0.114	0.4913
NC_005101.4-137957503	CTTCCTCCAACTCACGCTCCACTAGTT	15	C	0.2269	0.4886
NC_005101.4-137957503	CTTCCTCCAACTCACGCTCCACTAGTT	24	A	0.0084	0.4761
NC_005101.4-138024979	ATGCGAGTCACCAGTCCTCCTGTGTGT	7	G	0	0.4807
NC_005101.4-138083645	GGCATCATAACACCTCCTCCCAGCATA	21	C	0.0164	0.4807
NC_005101.4-138125472	AACGCTTGTACGTGCCCTCCCACAGAG	3	C	0.2764	0.4682
NC_005101.4-138225008	TTTCACTGCACTGTCTCTCCACAGTTT	7	T	0.1239	0.2036
NC_005101.4-138488808	CTGCCTCATACTGCTGCTCCTTATTGT	9	T	0.0246	0.0243
NC_005101.4-138488808	CTGCCTCATACTGCTGCTCCTTATTGT	24	T	0	0.4773
NC_005101.4-138501592	TAACTTTCCACCCCCTCTCCCATCCTT	3	A	0.0965	0.138
NC_005101.4-138548725	ACTTCTCAGACAGCCACTCCGTCATAC	21	G	0.1789	0.3334
NC_005101.4-138548725	ACTTCTCAGACAGCCACTCCGTCATAC	27	C	0.1951	0.4926
NC_005101.4-138684781	CTCAAAGCTACTGGATCTCCACAACAC	23	A	0	0.4807
NC_005101.4-138684781	CTCAAAGCTACTGGATCTCCACAACAC	26	A	0.0259	0.1209
NC_005101.4-138782999	TTGCTCCTCACAGCAGCTCCTTGGTGT	15	A	0	0.4773
NC_005101.4-138835095	AAGTTTATCACTCTGGCTCCATTCTTG	13	C	0.0179	0.0351
NC_005101.4-138835354	AGTCCCAGTACAAACTCTCCTTCATTT	8	G	0.2222	0.3729
NC_005101.4-138958882	CACAATTCCACCTGCCCTCCTAAGCCA	14	G	0.0504	0.065
NC_005101.4-138958882	CACAATTCCACCTGCCCTCCTAAGCCA	16	C	0.025	0.0247
NC_005101.4-138968450	TCTTTGCTTACACTGTCTCCTTTGCTT	1	T	0.0088	0.4599
NC_005101.4-138979953	TTCCTCCATACTTTGTCTCCTTTATTT	1	T	0	0.4786
NC_005101.4-138993453	TGCAAATACACTCCTTCTCCTTCTGAA	4	A	0.1379	0.3381
NC_005101.4-139035701	ATTCTGAAGACATTCCCTCCTCCTCAT	22	C	0.1429	0.2852
NC_005101.4-139035701	ATTCTGAAGACATTCCCTCCTCCTCAT	26	A	0.1333	0.1387
NC_005101.4-139254122	GCCCCTCCCACATGCTCTCCCACATCC	4	C	0.0252	0.0249
NC_005101.4-139310389	ATCAATCCCACTACCACTCCTTCATGA	6	T	0.1261	0.1327
NC_005101.4-139316514	GCCCATAGTACACAATCTCCCTGGATG	3	C	0.0446	0.1403
NC_005101.4-139316514	GCCCATAGTACACAATCTCCCTGGATG	8	G	0.0714	0.4841
NC_005101.4-139335961	ATGTTGGGGACTTGCACTCCCTTGGTC	26	T	0	0.4846
NC_005101.4-139335961	ATGTTGGGGACTTGCACTCCCTTGGTC	27	C	0	0.4833
NC_005101.4-139343238	CTGGTTCTAACTCAAGCTCCATCTGAT	2	T	0	0.4773
NC_005101.4-139343238	CTGGTTCTAACTCAAGCTCCATCTGAT	4	G	0.0165	0.0325
NC_005101.4-139352045	CTGTAGAGCACGAAGTCTCCTGATAAA	8	G	0.1818	0.2295
NC_005101.4-139376569	AAGTCCCAGACCATTTCTCCTGAGGTT	22	G	0.1034	0.1284
NC_005101.4-139427474	TTAGAGCAGACCAGGCCTCCAGGCTTT	23	G	0.2185	0.4567
NC_005101.4-139465244	GTTTCTTCCACCACAGCTCCTTCCACA	6	T	0	0.4677
NC_005101.4-139465244	GTTTCTTCCACCACAGCTCCTTCCACA	24	C	0.1356	0.1827
NC_005101.4-139474887	CAGCATGGAACTCAGCCTCCTTCCCTG	13	C	0	0.4807
NC_005101.4-139505118	TGGCCCTACACTGTAACTCCCAACCCT	8	A	0.2957	0.48

NC_005101.4-139560709	AGGCATCCCACAGTTTCTCCTGGCTCA	4	C	0.0902	0.5
NC_005101.4-139634161	GTCATCAGTACTGTGGCTCCCTCCTGC	13	G	0.0732	0.1001
NC_005101.4-139644911	CTATACAACACACCTACTCCACGACCA	4	T	0.123	0.2796
NC_005101.4-139644911	CTATACAACACACCTACTCCACGACCA	22	C	0	0.4807
NC_005101.4-139696127	TGCCCACCCACCCCAGCTCCTCACTTC	7	C	0.1765	0.2626
NC_005101.4-139728858	GCTCTTGAGACATGCTCTCCCATAGGT	2	C	0.1593	0.2171
NC_005101.4-139728858	GCTCTTGAGACATGCTCTCCCATAGGT	24	A	0	0.4807
NC_005101.4-139741422	ATCTGAGTGACACCAGCTCCCAGCACC	25	A	0	0.477
NC_005101.4-139796827	TTGTAGGATACCACCTCTCCCTGGGTT	21	C	0.1293	0.3793
NC_005101.4-139818139	CTAAGGGAAACTCTGTCTCCCTACTAC	15	G	0.2281	0.4778
NC_005101.4-139848283	TCCCCATCTACTCCTCCTCCTCTCAGA	9	T	0.0909	0.4507
NC_005101.4-139870275	GTCCCAAATACATAAACTCCTTCTGCC	8	A	0.2432	0.3232
NC_005101.4-139870275	GTCCCAAATACATAAACTCCTTCTGCC	23	C	0.2072	0.344
NC_005101.4-139876647	TCTTTTGCCACAGGTACTCCAGCAAGA	22	G	0.123	0.4992
NC_005101.4-139888571	TGGTGAATCACCCTGCCTCCTTCTCTC	21	T	0.1789	0.385
NC_005101.4-139921358	CTTGTCAGTACATTCCCTCCACAAACA	23	A	0.1653	0.4991
NC_005101.4-139925947	CTGAAGGCAACAAGCACTCCATGACGG	25	C	0.2155	0.2681
NC_005101.4-139925947	CTGAAGGCAACAAGCACTCCATGACGG	26	G	0.2	0.2332
NC_005101.4-139999980	TATTTGTGCACACTTTCTCCTCAATAA	24	A	0	0.4734
NC_005101.4-140001835	TTCCCACCAACTTCCCCTCCTCTGCTC	3	C	0.0579	0.0716
NC_005101.4-140044979	TAAAGACATACATTAGCTCCCCAGTCC	25	T	0	0.4854
NC_005101.4-140073163	CTGACCTCCACACCTGCTCCTGCTTAT	23	C	0.0442	0.0765
NC_005101.4-140080067	TGAATTGAAACACCCTCTCCAGAAGCA	5	T	0	0.4868
NC_005101.4-140097785	CATCGAGTCACTGCACCTCCTTTGTTA	5	G	0.0082	0.479
NC_005101.4-140100154	ACTTAGGAAACATGAGCTCCTAGCCTC	3	T	0.0163	0.4825
NC_005101.4-140181923	CACTACCTCACAACAGCTCCAAGTCAG	24	T	0.1405	0.3327
NC_005101.4-140188139	CCCAGCAAGACTGCACCTCCTAAGAAA	4	A	0	0.4743
NC_005101.4-140188139	CCCAGCAAGACTGCACCTCCTAAGAAA	25	A	0.084	0.4997
NC_005101.4-140191896	ATGAAGGGCACTGTCCCTCCCATGTCA	1	A	0.0084	0.4859
NC_005101.4-140191896	ATGAAGGGCACTGTCCCTCCCATGTCA	13	G	0.1092	0.1609
NC_005101.4-140196841	CTTGATATCACATAATCTCCTGAGTCT	16	T	0.0087	0.0257
NC_005101.4-140221714	AGCCCCCACACAGAAACTCCAAATGCA	26	C	0	0.4724
NC_005101.4-140280756	CTCCTTCACACCCGGCCTCCTGTAGCT	15	G	0.0246	0.0243
NC_005101.4-140280756	CTCCTTCACACCCGGCCTCCTGTAGCT	25	G	0.1639	0.2032
NC_005101.4-140281192	TCCTGAAACACCAGGCCTCCTTTCCTC	8	A	0.2435	0.3094
NC_005101.4-140282447	AATGACACCACGGAAGCTCCGACTGGT	12	G	0.0917	0.225
NC_005101.4-140282447	AATGACACCACGGAAGCTCCGACTGGT	21	G	0.1583	0.325
NC_005101.4-140337920	TGGGGAGACACAAGGGCTCCTGTAACT	9	C	0	0.4793
NC_005101.4-140349842	AAACCAGAAACATGCACTCCAGGTGTG	16	A	0.1532	0.3903
NC_005101.4-140349842	AAACCAGAAACATGCACTCCAGGTGTG	22	G	0.1062	0.1006
NC_005101.4-140361187	CGCTACCCTACCCACCCTCCATACTAT	2	G	0.0756	0.2012
NC_005101.4-140364751	CTTGGAAGAACAAGAGCTCCTAATCTC	26	T	0.0172	0.4786
NC_005101.4-140410041	TGGGTATCAACTCTTGCTCCTTTCTCT	26	C	0.2	0.4387
NC_005101.4-140428854	GTGGAGCAGACACCCTCTCCACCCGCT	5	A	0.1593	0.2171
NC_005101.4-140428854	GTGGAGCAGACACCCTCTCCACCCGCT	25	G	0	0.4807
NC_005101.4-140442732	AACCACTGAACCATGTCTCCAGGCCAA	14	T	0.2	0.3353
NC_005101.4-140442732	AACCACTGAACCATGTCTCCAGGCCAA	21	A	0.0259	0.0255
NC_005101.4-140467103	TGAACGTATACCTGCACTCCATCAGAG	5	C	0.1475	0.2157
NC_005101.4-140492971	CACAGAGAAACCCTTTCTCCAAAATAA	3	C	0.084	0.0805
NC_005101.4-140547313	AAGAAAGGAACCAGAACTCCACTAAGT	1	A	0	0.477
NC_005101.4-140547313	AAGAAAGGAACCAGAACTCCACTAAGT	3	G	0	0.4755

NC_005101.4-140568903	CCTATTTCCACCGGGCCTCCCTCTTCA	13	G	0.1597	0.4977
NC_005101.4-140588024	AAAAAGAGGACGTTCTCTCCCTGCCTG	12	G	0	0.4875
NC_005101.4-140694267	GTCAGACCAACAGTATCTCCTCTCTAA	2	T	0.1333	0.1665
NC_005101.4-140696879	AGCCTTTTAACTGCTGCTCCTTCTGCT	7	T	0.1885	0.4489
NC_005101.4-140720755	GATGGGTTAACTGCAGCTCCCAGAGAA	22	A	0	0.4813
NC_005101.4-140734616	CAACTCGCCACTAGTCCTCCCGGGCAC	22	G	0.1947	0.4968
NC_005101.4-140734616	CAACTCGCCACTAGTCCTCCCGGGCAC	25	C	0.1947	0.4968
NC_005101.4-140828684	AAAGAGGTAACATTGTCTCCAGCGTGG	9	A	0	0.4813
NC_005101.4-140828684	AAAGAGGTAACATTGTCTCCAGCGTGG	24	G	0	0.4813
NC_005101.4-140841640	GGATTTATAACACAGGCTCCCTGTGTT	12	A	0	0.4832
NC_005101.4-140879313	GTGGAACGAACCTGCTCTCCATGGTGT	24	G	0.1102	0.4982
NC_005101.4-140879313	GTGGAACGAACCTGCTCTCCATGGTGT	26	G	0.1102	0.4982
NC_005101.4-140970275	GTAAATGCTACAGCGCCTCCTCAGCAA	6	T	0.0083	0.4732
NC_005101.4-140970275	GTAAATGCTACAGCGCCTCCTCAGCAA	15	G	0.0248	0.0405
NC_005101.4-140986568	TTGAGAGTGACCCCGTCTCCTTACGTC	4	A	0.0569	0.4893
NC_005101.4-140986568	TTGAGAGTGACCCCGTCTCCTTACGTC	14	C	0.0163	0.032
NC_005101.4-141000357	TAGCACGTGACGCCATCTCCTCTCCAG	1	T	0.0087	0.4613
NC_005101.4-141011397	CTCCCCTTCACGAACCCTCCCTTACCA	12	G	0.0588	0.0571
NC_005101.4-141028512	CCCCAGCCCACTCACACTCCACTGAAT	14	A	0	0.4772
NC_005101.4-141028512	CCCCAGCCCACTCACACTCCACTGAAT	25	A	0	0.4772
NC_005101.4-141035535	ACTGAATGCACCAGGACTCCATGAGAA	16	A	0.2414	0.375
NC_005101.4-141060753	CTGGGGAAGACTGTCTCTCCATGTTCA	5	G	0.1557	0.1708
NC_005101.4-141060753	CTGGGGAAGACTGTCTCTCCATGTTCA	26	C	0.1405	0.2474
NC_005101.4-141104738	GGAAATGGGACTCACTCTCCGTTGTCC	14	A	0	0.488
NC_005101.4-141104738	GGAAATGGGACTCACTCTCCGTTGTCC	21	G	0.0862	0.2854
NC_005101.4-141147789	TCTTAACATACCAATGCTCCTTTATGG	6	A	0.0431	0.0585
NC_005101.4-141151693	CACTGAGAGACCTTGTCTCCTGGCGCT	23	G	0.1583	0.4416
NC_005101.4-141151693	CACTGAGAGACCTTGTCTCCTGGCGCT	24	C	0.0417	0.0875
NC_005101.4-141159466	GAACTTCTCACAGGGTCTCCATGGTCT	2	A	0.0328	0.0322
NC_005101.4-141201670	TCCTCAACTACATTTCCTCCTACTCCT	8	C	0.2542	0.424
NC_005101.4-141210337	CAGCGAAAGACCCTACCTCCTGTTTTG	23	T	0.2931	0.4851
NC_005101.4-141220475	TTGGCGTTCACTGTTCCTCCTGTGGAC	6	G	0	0.48
NC_005101.4-141254608	TTTAACTAAACTGCCTCTCCCAGGCAG	6	C	0.0973	0.1391
NC_005101.4-141306892	ACCAGCAAGACTTTGTCTCCATCACCA	22	T	0.1339	0.2876
NC_005101.4-141323369	TGTAAATCAACACAAGCTCCATCATAA	2	G	0.0168	0.4779
NC_005101.4-141323369	TGTAAATCAACACAAGCTCCATCATAA	21	A	0.1765	0.2852
NC_005101.4-141336896	TCTCTCTGCACCCTGACTCCCAATAAT	6	C	0.1966	0.3107
NC_005101.4-141350050	CCAGCTGCAACTCCAGCTCCAAGGAAG	14	C	0.1157	0.5
NC_005101.4-141350050	CCAGCTGCAACTCCAGCTCCAAGGAAG	16	G	0.1157	0.5
NC_005101.4-141356500	TTGACGATTACCCATACTCCCAGGAGG	13	C	0.0991	0.126
NC_005101.4-141361070	CAGCTCCTGACACTGCCTCCTTTCCAC	7	C	0.1983	0.2295
NC_005101.4-141369227	TGATGACTCACATGGCCTCCCAGCTAC	1	T	0.0702	0.0997
NC_005101.4-141372734	TAGGTCTCCACAGCACCTCCACTTGGC	12	A	0.0085	0.4776
NC_005101.4-141372734	TAGGTCTCCACAGCACCTCCACTTGGC	23	T	0.0085	0.4776
NC_005101.4-141406701	TGGTACCTGACTAGAACTCCAGCATTT	12	T	0.2705	0.3008
NC_005101.4-141409274	GCGATGGAAACCTAATCTCCTTGATTG	2	C	0.2623	0.3709
NC_005101.4-141514860	CACGGACAAACCACATCTCCTGTAGCA	16	T	0.041	0.071
NC_005101.4-141538075	AAAGAGAGAACAGCACCTCCGTCACAT	12	A	0	0.4793
NC_005101.4-141569982	CCATGCTGGACTGCTTCTCCTTCTCCT	5	G	0.2119	0.2526
NC_005101.4-141572998	GGTAAGGGCACTGGATCTCCGGGCACT	6	G	0.0084	0.0249
NC_005101.4-141572998	GGTAAGGGCACTGGATCTCCGGGCACT	21	G	0.1176	0.1398

NC_005101.4-141585493	TGCACAAGCACCTCTACTCCAACCAGC	8	G	0.2679	0.4841
NC_005101.4-141585493	TGCACAAGCACCTCTACTCCAACCAGC	14	C	0.0265	0.0926
NC_005101.4-141593068	GACATTGTGACCAGCTCTCCTTGTGAG	4	A	0.0082	0.0243
NC_005101.4-141593068	GACATTGTGACCAGCTCTCCTTGTGAG	16	T	0.123	0.1154
NC_005101.4-141612104	ACTCTTTTAACTCTACCTCCCACCAGA	14	T	0.0087	0.4849
NC_005101.4-141620896	CATGAATACACCAGAACTCCAGTTTGA	5	A	0.0083	0.4803
NC_005101.4-141685104	AACACCTTCACACAGCCTCCTATTTAT	21	T	0.0087	0.4878
NC_005101.4-141761189	GATATTTGCACTCCATCTCCTGTTGAT	3	T	0.2241	0.2622
NC_005101.4-141784553	ATTGTTCTTACCTCTTCTCCCTAACCT	12	C	0.1327	0.4998
NC_005101.4-141784553	ATTGTTCTTACCTCTTCTCCCTAACCT	26	C	0.0796	0.154
NC_005101.4-141800487	TGACCTGTCACCATCACTCCTATAAGT	6	T	0.0082	0.479
NC_005101.4-141800487	TGACCTGTCACCATCACTCCTATAAGT	7	G	0.1789	0.2723
NC_005101.4-141801896	ACTACTGCCACCATTCCTCCTATTGGG	9	C	0.1736	0.2704
NC_005101.4-141801896	ACTACTGCCACCATTCCTCCTATTGGG	23	T	0	0.4787
NC_005101.4-141862652	GTCTCTTGTACATCCTCTCCATTCCTT	22	T	0	0.4819
NC_005101.4-141899795	AGGAGTGAGACCCAAACTCCAGTCCAG	2	G	0.0619	0.0765
NC_005101.4-141899795	AGGAGTGAGACCCAAACTCCAGTCCAG	25	C	0.0965	0.1953
NC_005101.4-141930897	TTCTGCAGCACCTGAGCTCCCAGTTCG	27	G	0.0244	0.0241
NC_005101.4-141944857	CCCCGTGGTACAATTCCTCCACAGTGC	4	C	0	0.4698
NC_005101.4-141999235	CACTATAAGACTCTCCCTCCCATTTCT	5	A	0	0.4724
NC_005101.4-142012743	ACACTTGCCACCCCCTCTCCTTCCCAA	8	C	0.252	0.3381
NC_005101.4-142012743	ACACTTGCCACCCCCTCTCCTTCCCAA	12	C	0	0.4793
NC_005101.4-142018391	CTATGTATAACTCCACCTCCCTGTTCA	7	A	0	0.4793
NC_005101.4-142018391	CTATGTATAACTCCACCTCCCTGTTCA	12	T	0	0.4793
NC_005101.4-142023974	AAATGTCACACCTTCCCTCCTATCCTA	21	T	0	0.4765
NC_005101.4-142023974	AAATGTCACACCTTCCCTCCTATCCTA	25	C	0.2213	0.4126
NC_005101.4-142072720	ACGAGCTAAACGTTACCTCCTGCTAGA	3	G	0.1176	0.5
NC_005101.4-142090644	CCATCTGTAACTCCGGCTCCAGGGAAG	6	T	0.4286	0.3367
NC_005101.4-142090644	CCATCTGTAACTCCGGCTCCAGGGAAG	21	A	0.4286	0.3367
NC_005101.4-142187841	TGCATCTGCACCACAGCTCCCGCATCA	2	G	0.4672	0.3581
NC_005101.4-142187841	TGCATCTGCACCACAGCTCCCGCATCA	9	C	0.5854	0.427
NC_005101.4-142204405	CCCACCAAGACAGAGTCTCCAGTAGCC	21	A	0.1333	0.4965
NC_005101.4-142223305	GCTGCAGAAACCAGCCCTCCACTGCCT	14	G	0.0763	0.119
NC_005101.4-142223305	GCTGCAGAAACCAGCCCTCCACTGCCT	23	T	0	0.4772
NC_005101.4-142227999	TCCATAGGCACTCCTGCTCCCTCACCA	27	A	0.0574	0.071
NC_005101.4-142228788	ACCTCAGTAACGGGGCCTCCTCTTGTA	12	G	0.0738	0.1009
NC_005101.4-142320358	TCTTTGAGAACATGCTCTCCTAGTAAC	15	C	0.0413	0.0562
NC_005101.4-142320358	TCTTTGAGAACATGCTCTCCTAGTAAC	21	T	0	0.4807
NC_005101.4-142326975	TGTGGCTTTACCTCTCCTCCCTTCCTA	4	G	0.0965	0.1528
NC_005101.4-142350017	CCTAGGGCCACCTGACCTCCAGGCTAG	22	G	0.0672	0.065
NC_005101.4-142364559	TGTTTTGGGACAAGGTCTCCCTTGGAG	22	T	0	0.4839
NC_005101.4-142370678	GTGCTACTGACTTAGTCTCCTAAGAGA	6	A	0	0.4773
NC_005101.4-142370678	GTGCTACTGACTTAGTCTCCTAAGAGA	14	A	0.0984	0.1505
NC_005101.4-142378857	TGGCCTTGAACTGAGTCTCCTCCAGTT	3	G	0	0.4793
NC_005101.4-142425469	GCCCTCCTCACTTCCACTCCTGACTAC	16	A	0	0.4875
NC_005101.4-142448949	TTTAAGACCACCCATCCTCCTGCCTAA	12	C	0.2185	0.3319
NC_005101.4-142448949	TTTAAGACCACCCATCCTCCTGCCTAA	13	C	0.05	0.0644
NC_005101.4-142457365	TGTTCATTCACCTGATCTCCTGGGTTG	15	A	0	0.4839
NC_005101.4-142458759	GAGCTGCCTACTAATCCTCCCTCCTAG	1	G	0.0924	0.1469
NC_005101.4-142460092	CTGGTTTGAACCCTGTCTCCAGTCCTG	16	T	0.3525	0.4193
NC_005101.4-142468453	ACCATCGTAACTCCAGCTCCAAGTGAT	3	C	0.1983	0.2057

NC_005101.4-142468453	ACCATCGTAACTCCAGCTCCAAGTGAT	6	C	0.2155	0.244
NC_005101.4-142521407	CGTCCTCTCACTCTTCCTCCCTTCTGT	2	G	0.0331	0.0484
NC_005101.4-142627362	CACTGGGTCACCCGGGCTCCTGAAGGA	6	G	0.1736	0.3512
NC_005101.4-142750806	AAAGAATACACTTGCACTCCAATGTAT	7	T	0	0.4787
NC_005101.4-142750806	AAAGAATACACTTGCACTCCAATGTAT	23	T	0.2479	0.3467
NC_005101.4-142765967	CGGGTTACCACAGATTCTCCTCTTTAT	1	C	0.1885	0.3492
NC_005101.4-142787061	TACAATTCCACTGTGTCTCCTGAGATG	8	C	0.1475	0.1641
NC_005101.4-142810668	AAAGAGTGAACGCACCCTCCCTGTATC	2	A	0	0.4793
NC_005101.4-142824903	CACACACACACAGTCCCTCCCTCCTTC	14	T	0	0.4807
NC_005101.4-142845736	CTGACACTGACCTCACCTCCTAGTCCC	5	C	0.0984	0.1082
NC_005101.4-142846282	AACCTTCCAACCCCCGCTCCTCAGATA	15	C	0.0331	0.4628
NC_005101.4-142852449	AGATATCCGACCTTAGCTCCTCATCTT	9	G	0.1983	0.302
NC_005101.4-142852449	AGATATCCGACCTTAGCTCCTCATCTT	24	T	0.1379	0.4976
NC_005101.4-142867974	TCTCCCTGTACAGTCTCTCCCCGGAAC	14	T	0.0351	0.0512
NC_005101.4-142867974	TCTCCCTGTACAGTCTCTCCCCGGAAC	23	G	0.0614	0.0595
NC_005101.4-142893659	CATAACCTAACTGAGTCTCCAAAGAAA	14	A	0.0894	0.1145
NC_005101.4-142924401	CTGGAGTTCACTTGAACTCCGTGGGTC	21	G	0.2	0.4888
NC_005101.4-142949708	TTGTGGACCACACAATCTCCACAGCTA	1	T	0.0583	0.4941
NC_005101.4-142949708	TTGTGGACCACACAATCTCCACAGCTA	23	A	0.1736	0.4933
NC_005101.4-142976998	CAGAAATCAACACAATCTCCCCTCACC	27	C	0.0088	0.0262
NC_005101.4-143049857	GGTATGAGCACACCCACTCCCAGTCTT	5	T	0.0569	0.0854
NC_005101.4-143049857	GGTATGAGCACACCCACTCCCAGTCTT	24	T	0.0569	0.4915
NC_005101.4-143082998	AGACATGACACACTGTCTCCAGATACT	12	A	0	0.4912
NC_005101.4-143101776	AGACAAGGGACCAAAGCTCCGTGTGAA	23	G	0.0342	0.066
NC_005101.4-143171899	TACTGCTGAACTCGCTCTCCAGAACCT	2	A	0.0672	0.065
NC_005101.4-143171899	TACTGCTGAACTCGCTCTCCAGAACCT	13	C	0.1933	0.2012
NC_005101.4-143174096	GGAGGCAAAACTTTACCTCCACTTCAA	16	C	0.0991	0.4942
NC_005101.4-143214032	GTGCAGGCTACATGATCTCCTACTAAA	9	T	0.0776	0.0746
NC_005101.4-143214032	GTGCAGGCTACATGATCTCCTACTAAA	12	A	0.0862	0.0981
NC_005101.4-143242039	AACCATGCTACCATCCCTCCATCCTCG	27	G	0.0165	0.0325
NC_005101.4-143272247	CTACCACAGACTGCAGCTCCTCGCTGC	22	C	0.25	0.4641
NC_005101.4-143277214	ACTCAGCACACAGCTGCTCCTGTTGCC	27	C	0.1074	0.1306
NC_005101.4-143444579	CAGAGTGTAACACTCACTCCTGTTTGT	5	G	0.0325	0.032
NC_005101.4-143444579	CAGAGTGTAACACTCACTCCTGTTTGT	12	A	0.0325	0.032
NC_005101.4-143504084	ATGAAATCCACATCCACTCCCGCTCTC	15	C	0.0574	0.0557
NC_005101.4-143664925	CACGATGAGACACTGCCTCCAGAAAAA	7	G	0.2212	0.4599
NC_005101.4-143664925	CACGATGAGACACTGCCTCCAGAAAAA	14	T	0.2212	0.4242
NC_005101.4-143693776	TTCCACTTGACCTTCTCTCCCACAAGA	7	T	0.0424	0.0415
NC_005101.4-143699362	CACACATGCACACATACTCCTACACAA	3	C	0	0.4787
NC_005101.4-143794802	ACGCTCACGACAAAGACTCCCGCTCTC	3	G	0.1789	0.4944
NC_005101.4-143819453	GACACAGGCACTGGCTCTCCAGCCCAG	5	C	0.0492	0.048
NC_005101.4-143819453	GACACAGGCACTGGCTCTCCAGCCCAG	24	C	0	0.4787
NC_005101.4-143842965	ACTGATAGCACATGATCTCCTGGGCAA	6	T	0.2231	0.313
NC_005101.4-143851711	GGCTACTGTACCATGCCTCCCCACCTT	22	C	0.1017	0.0965
NC_005101.4-143867687	GGTTGTGAAACATGGTCTCCTTGTGGG	12	A	0	0.474
NC_005101.4-143872020	TGTGGTATTACCACATCTCCTGTTCTG	6	T	0.1579	0.4986
NC_005101.4-143872020	TGTGGTATTACCACATCTCCTGTTCTG	9	T	0.1579	0.2538
NC_005101.4-143931793	TTTACAATTACTGGAGCTCCGTTTGTC	7	A	0	0.4807
NC_005101.4-143931793	TTTACAATTACTGGAGCTCCGTTTGTC	12	T	0.0714	0.1014
NC_005101.4-143938778	ACCTCTGACACCAGTTCTCCAAGCTAA	2	C	0	0.4864
NC_005101.4-143938778	ACCTCTGACACCAGTTCTCCAAGCTAA	5	C	0	0.4864

NC_005101.4-143953818	TTACATCTGACCTTCCCTCCCTCTCTA	4	C	0	0.4909
NC_005101.4-144039030	CTAAAACTCACCACCCCTCCTGAGTCA	6	A	0	0.4859
NC_005101.4-144039030	CTAAAACTCACCACCCCTCCTGAGTCA	16	C	0.0614	0.1075
NC_005101.4-144045511	CCCGGAAAGACCTGATCTCCCAGGAGT	7	A	0.0081	0.4777
NC_005101.4-144226297	CATACTCCTACGGAGTCTCCTCCTGTG	6	T	0.0992	0.4959
NC_005101.4-144226297	CATACTCCTACGGAGTCTCCTCCTGTG	9	T	0.2439	0.3427
NC_005101.4-144284751	CTTCGGAGGACCACAGCTCCCTTCCCG	26	C	0.125	0.1327
NC_005101.4-144325284	TGCATTTTCACCTCTCCTCCTCCTGAG	14	C	0.1901	0.2704
NC_005101.4-144419896	ATATGATGAACCCTGACTCCCAAAGAG	26	A	0.2261	0.32
NC_005101.4-144480093	CTGCAGATCACTTCTGCTCCTCCAGAT	6	G	0.0164	0.4837
NC_005101.4-144490524	TTATGGAGCACACGGCCTCCATGCCAC	13	C	0.1593	0.3543
NC_005101.4-144490524	TTATGGAGCACACGGCCTCCATGCCAC	22	T	0	0.4912
NC_005101.4-144493157	GGCCCCAGCACAGGACCTCCCTTTCGA	25	C	0	0.4751
NC_005101.4-144599451	ACTGTCATTACAATGACTCCCCTTTTC	16	A	0	0.4807
NC_005101.4-144620995	CCTGAATGCACCTGATCTCCTCTGATC	7	T	0.1983	0.4983
NC_005101.4-144684583	TTCTCCCCAACTCTCTCTCCCTTCTCT	9	A	0	0.4773
NC_005101.4-144701213	GTTCTCTAAACGTCAGCTCCAATGACA	25	A	0.0085	0.4753
NC_005101.4-144763057	AGGCTCTGAACCTTTCCTCCTACCCTT	1	A	0.2276	0.2382
NC_005101.4-144763057	AGGCTCTGAACCTTTCCTCCTACCCTT	2	G	0.2276	0.2382
NC_005101.4-144763679	CAGAGAAAAACAGTGGCTCCGTTACTT	21	G	0.0082	0.4755
NC_005101.4-144784565	TACTGTGTGACTTGTCCTCCCACATTC	26	T	0.0439	0.0429
NC_005101.4-144819967	TATTCAGCAACCCCGCCTCCCAGTCTT	24	T	0.0976	0.1356
NC_005101.4-144821670	GGCTTCTGTACTTCTGCTCCCTGTGAC	13	T	0.1626	0.4389
NC_005101.4-144821861	AATAAGAAAACATGCCCTCCTTGTTTC	16	C	0.0256	0.0253
NC_005101.4-144821861	AATAAGAAAACATGCCCTCCTTGTTTC	23	G	0.0847	0.1116
NC_005101.4-144876309	GATGGGGCAACACTAGCTCCTGAGATT	9	A	0.2705	0.4435
NC_005101.4-144876309	GATGGGGCAACACTAGCTCCTGAGATT	25	A	0.1148	0.1641
NC_005101.4-144885857	TGATCCAAAACGAGGTCTCCATCTTAA	8	A	0.018	0.4704
NC_005101.4-144891705	AGAAATACCACCTTTGCTCCAGTACAA	8	C	0.1849	0.4703
NC_005101.4-144896495	AAAACTTGCACAGTTGCTCCGTTAAGT	12	A	0.0813	0.4944
NC_005101.4-144896495	AAAACTTGCACAGTTGCTCCGTTAAGT	21	G	0	0.4793
NC_005101.4-144909279	GTCTCTCCGACCTGCCCTCCAAGAACT	9	G	0.0536	0.0689
NC_005101.4-144915421	AACATCAGCACCTCTCCTCCCTGTCAA	8	G	0.0088	0.4889
NC_005101.4-144915421	AACATCAGCACCTCTCCTCCCTGTCAA	15	T	0.0351	0.0345
NC_005101.4-144915580	TGCCTTGTGACCACACCTCCTTATCTT	12	C	0.0333	0.0328
NC_005101.4-144915580	TGCCTTGTGACCACACCTCCTTATCTT	14	C	0.1951	0.3516
NC_005101.4-144919417	TTATCACTGACTTGTTCTCCATGGTCA	25	T	0.0083	0.4803
NC_005101.4-144937398	CCCTTCTCAACTTCTCCTCCGCTCCCG	21	G	0.1463	0.1761
NC_005101.4-144937398	CCCTTCTCAACTTCTCCTCCGCTCCCG	26	C	0	0.4793
NC_005101.4-144954176	CAGAATCTAACGCCATCTCCAGGTAGA	27	A	0.2033	0.3381
NC_005101.4-145000122	TTTTCTCCAACACTCCCTCCTTCAGTG	7	C	0.0976	0.1629
NC_005101.4-145004156	GAGTATCCAACCATCCCTCCCAACCAT	3	G	0.1186	0.2219
NC_005101.4-145004156	GAGTATCCAACCATCCCTCCCAACCAT	24	C	0.1453	0.3107
NC_005101.4-145013300	CCACAGAGCACTTGTGCTCCCACTTGA	21	C	0.0084	0.0249
NC_005101.4-145027280	GTCCACTGAACAATTTCTCCAATGTAT	6	C	0.0893	0.4998
NC_005101.4-145039061	ATAACATTTACACAGGCTCCTTGGGGA	16	G	0.1453	0.2299
NC_005101.4-145049409	AATGTTGAGACATGTTCTCCAAGTGTT	1	A	0	0.4844
NC_005101.4-145049409	AATGTTGAGACATGTTCTCCAAGTGTT	12	A	0	0.4856
NC_005101.4-145075862	TAATACTCAACTTTAACTCCTAGTGCA	1	T	0	0.4814
NC_005101.4-145125767	CTGAATCTTACTGCGTCTCCTCGGAGG	23	G	0	0.4755
NC_005101.4-145127254	GGGTTTTGTACTTTTGCTCCCAGTTCT	2	G	0.0517	0.0666

NC_005101.4-145154723	GTCATACCAACCCTGGCTCCTTCATTA	5	T	0	0.4773
NC_005101.4-145154723	GTCATACCAACCCTGGCTCCTTCATTA	24	A	0.1463	0.2263
NC_005101.4-145155981	CTGTGTGAAACAGAAACTCCTTAATAT	15	A	0.0169	0.4793
NC_005101.4-145155981	CTGTGTGAAACAGAAACTCCTTAATAT	24	A	0.0169	0.4793
NC_005101.4-145174915	ACCCAGCACACTTTTCCTCCATCTGAT	3	C	0	0.4807
NC_005101.4-145184941	AAGGTGGGCACACTTTCTCCACTGAAC	12	A	0	0.4743
NC_005101.4-145187603	AAAACCACCACCAGCACTCCCAGTGAT	3	A	0.0619	0.06
NC_005101.4-145187846	GTCTAAGCCACTCTTGCTCCTCAGAAA	22	C	0.1071	0.2698
NC_005101.4-145248580	GGTGGTCGCACATTTTCTCCTCCTCTC	14	T	0.0174	0.4864
NC_005101.4-145255102	TGCAGGAGCACAACAGCTCCATTTACC	13	A	0	0.4709
NC_005101.4-145396704	TACAGAAGCACCCTCCCTCCCTGTCTT	9	C	0.0721	0.0695
NC_005101.4-145488579	CTACACAGAACCTCTGCTCCTCTACTT	23	T	0	0.4787
NC_005101.4-145519183	ACAATACCCACCCCACCTCCTCTATCA	23	T	0	0.4793
NC_005101.4-145552607	ATGTTAGACACCCCACCTCCATTCCCT	15	A	0	0.4728
NC_005101.4-145591309	CGGACTCCAACCGGGACTCCAATGGAT	1	C	0	0.48
NC_005101.4-145591309	CGGACTCCAACCGGGACTCCAATGGAT	3	G	0	0.48
NC_005101.4-145617980	TGGCCTTCAACTGCTGCTCCTTCTGCT	4	C	0.0407	0.0398
NC_005101.4-145636401	TCAAATCACACACACCCTCCCAGTACG	27	G	0	0.4778
NC_005101.4-145636521	GGACTAGACACATCCTCTCCACTGAAG	4	C	0.1416	0.4996
NC_005101.4-145682974	TCCAAATAAACCATCCCTCCCTTACAT	4	A	0.1983	0.3811
NC_005101.4-145752569	GTTGATTCTACATGTTCTCCTCAAGAA	16	T	0.15	0.1932
NC_005101.4-145759896	CTAGTAATTACTGCTTCTCCTTTCCAT	27	T	0.0609	0.059
NC_005101.4-145785995	TATTATATAACCAACTCTCCCCTTTCC	27	C	0.1293	0.291
NC_005101.4-145795386	CCCATGCCAACCTTCCCTCCCATCTAT	12	C	0.1327	0.2494
NC_005101.4-145798315	GATCGGGCCACCTATCCTCCTTTTACA	4	C	0.0084	0.4829
NC_005101.4-145798315	GATCGGGCCACCTATCCTCCTTTTACA	16	C	0.0504	0.0491
NC_005101.4-145861316	TCCTCAACCACTCTGGCTCCTACAATC	6	A	0.325	0.2722
NC_005101.4-145958107	TTCTGGGCCACCTCACCTCCAACCCCC	12	C	0.1066	0.1436
NC_005101.4-145958107	TTCTGGGCCACCTCACCTCCAACCCCC	14	C	0.0167	0.4861
NC_005101.4-146045187	AGATTCTAAACTAAGGCTCCTTGCGCC	25	G	0	0.4772
NC_005101.4-146079518	CTGTTATCAACAAGATCTCCTGGGACA	7	T	0.1121	0.2057
NC_005101.4-146144289	GAATCTGTCACCCAACCTCCTAGTCAT	8	T	0.0894	0.1287
NC_005101.4-146165298	ATGCAATTTACTTGAACTCCTTTATTT	9	T	0	0.4807
NC_005101.4-146165298	ATGCAATTTACTTGAACTCCTTTATTT	15	A	0	0.4807
NC_005101.4-146193121	CTCCTCTCTACCAAATCTCCTTCTCTC	4	C	0	0.4779
NC_005101.4-146196478	TTGTGTGCTACTTTTCCTCCTTCCTTT	4	T	0.0331	0.0325
NC_005101.4-146196478	TTGTGTGCTACTTTTCCTCCTTCCTTT	12	T	0.2562	0.4077
NC_005101.4-146226205	CCCAGTGAAACCCTGTCTCCAAAAGTA	7	G	0.0164	0.0322
NC_005101.4-146351063	ATGCCTAGCACTTGGACTCCCTGTATC	8	G	0.2397	0.4732
NC_005101.4-146351063	ATGCCTAGCACTTGGACTCCCTGTATC	21	C	0.0244	0.0241
NC_005101.4-146448136	AGCTATACAACTTCTTCTCCAGTACCA	9	A	0	0.4793
NC_005101.4-146448136	AGCTATACAACTTCTTCTCCAGTACCA	27	A	0	0.4793
NC_005101.4-146499649	GACACTATTACTGGTGCTCCAATGTAC	26	A	0.0085	0.4842
NC_005101.4-146501614	TAGTGGAAGACCATGGCTCCAGGACCC	3	G	0.1864	0.3034
NC_005101.4-146501614	TAGTGGAAGACCATGGCTCCAGGACCC	5	G	0.1949	0.3086
NC_005101.4-146558518	AGGCCTTAAACTCAGTCTCCTTTCTTG	2	G	0.0574	0.1296
NC_005101.4-146758894	AGCTTAAACACATTGCCTCCCATCCAA	4	T	0.0588	0.0571
NC_005101.4-146790963	GCATTGCAGACCTTTGCTCCAACATCT	2	C	0.0081	0.4777
NC_005101.4-146790963	GCATTGCAGACCTTTGCTCCAACATCT	13	T	0.1789	0.4997
NC_005101.4-146881693	TATGGCATAACAGTCTCTCCAAACTGA	8	T	0	0.4773
NC_005101.4-146985623	CCCATCCAAACCTCCTCTCCTCCTCAG	13	T	0.0171	0.4807

NC_005101.4-146985623	CCCATCCAAACCTCCTCTCCTCCTCAG	14	C	0.0672	0.0958
NC_005101.4-146989536	TACGTATTGACTTTTGCTCCTTACCTG	1	T	0.0246	0.0243
NC_005101.4-146989536	TACGTATTGACTTTTGCTCCTTACCTG	25	C	0.0164	0.4837
NC_005101.4-146991340	GCTGTATCTACCCCCACTCCTGTATTA	2	C	0.0248	0.0245
NC_005101.4-146991340	GCTGTATCTACCCCCACTCCTGTATTA	7	T	0.0083	0.4835
NC_005101.4-147153917	CCCAGTGACACTCTCTCTCCCTAATGA	24	A	0.2281	0.3324
NC_005101.4-147286030	AGATCTGCCACCAGCTCTCCTGCTTCA	23	C	0.0894	0.4935
NC_005101.4-147405720	AGACTCCCAACTCTCCCTCCACGATGT	22	C	0.0082	0.4822
NC_005101.4-147405720	AGACTCCCAACTCTCCCTCCACGATGT	26	G	0.041	0.0401
NC_005101.4-147523836	CTGGCCTCCACAGGAACTCCACACTTT	14	G	0.2797	0.3914
NC_005101.4-147540476	AATGGCGTGACGGCTTCTCCTTTCTGT	12	G	0	0.4787
NC_005101.4-147540476	AATGGCGTGACGGCTTCTCCTTTCTGT	15	T	0	0.4787
NC_005101.4-147575592	GACAGTGTCACAACCCCTCCCTCTCCT	13	A	0	0.4909
NC_005101.4-147575592	GACAGTGTCACAACCCCTCCCTCTCCT	25	C	0	0.4909
NC_005101.4-147579985	AAGTGCTGAACCATGTCTCCAGCACAA	26	A	0.045	0.044
NC_005101.4-147605408	CCTTGTAGGACTCTCTCTCCTGAGTAA	8	G	0.1491	0.483
NC_005101.4-147608166	CTCAGCTAAACAGACTCTCCAAAGACT	12	A	0.0826	0.0792
NC_005101.4-147608166	CTCAGCTAAACAGACTCTCCAAAGACT	15	C	0.0826	0.0792
NC_005101.4-147608549	GGCACAGCAACCAACCCTCCCATGTTT	3	C	0.0164	0.4837
NC_005101.4-147625934	CTATACAGTACTTTCTCTCCAGAAGAG	24	A	0	0.4751
NC_005101.4-147635181	AACTAAAGAACCGCCTCTCCAAGCCCT	15	C	0.2295	0.4866
NC_005101.4-147653441	TTGATCACTACAGGACCTCCTCAACGG	26	G	0.05	0.0644
NC_005101.4-147655805	TACATAATTACAGTTGCTCCAAGTCAG	4	A	0	0.4793
NC_005101.4-147655805	TACATAATTACAGTTGCTCCAAGTCAG	24	T	0	0.4793
NC_005101.4-147660112	AGCTCCTGAACACAGGCTCCATTCATT	16	G	0	0.4779
NC_005101.4-147703885	CTTGCAAGCACTCCTACTCCCCGCAAC	23	G	0.0424	0.0734
NC_005101.4-147742209	ATCTGAAAGACACATCCTCCAGCAGCT	7	A	0.0331	0.0325
NC_005101.4-147742209	ATCTGAAAGACACATCCTCCAGCAGCT	8	A	0.1322	0.1516
NC_005101.4-147776437	AGATGTACCACTGCCACTCCTGAACTG	15	C	0.065	0.078
NC_005101.4-147776437	AGATGTACCACTGCCACTCCTGAACTG	16	A	0.082	0.4966
NC_005101.4-147821946	TATGAGGATACTCCTCCTCCCACCCAC	25	C	0.0442	0.0433
NC_005101.4-147822532	CATGCACAAACACAAACTCCCACACGT	4	G	0.0254	0.0251
NC_005101.4-147822532	CATGCACAAACACAAACTCCCACACGT	25	C	0	0.4757
NC_005101.4-147824194	GAAGGTGTAACTATTTCTCCCACCTCT	7	G	0.0684	0.0973
NC_005101.4-147857839	GTTGTCTCCACACCTCCTCCACTTACT	1	G	0.0256	0.0253
NC_005101.4-148119606	GAATTCATTACTGTGTCTCCATGGGAC	4	T	0.0081	0.4777
NC_005101.4-148144252	GCCACTCACACATGCCCTCCAGGAGAG	14	G	0.0407	0.0854
NC_005101.4-148302145	CATTAATTCACCTCTGCTCCACATGCT	24	T	0.0603	0.0746
NC_005101.4-148302145	CATTAATTCACCTCTGCTCCACATGCT	27	T	0.0603	0.0746
NC_005101.4-148309098	TCCAAGCGAACAGCTTCTCCATCCACA	7	C	0.1071	0.1172
NC_005101.4-148309098	TCCAAGCGAACAGCTTCTCCATCCACA	26	C	0.0177	0.0348
NC_005101.4-148357730	CACAGTGACACATGTCCTCCAATACAG	2	A	0.2213	0.2339
NC_005101.4-148448162	GATTGCCTCACATCACCTCCTCTTCCT	2	A	0	0.4755
NC_005101.4-148495921	AACTCATTAACTTGCTCTCCCTACGGC	25	G	0.2957	0.4234
NC_005101.4-148545110	CATAGAAGAACCAGCTCTCCTGTCCCC	3	T	0.0435	0.0425
NC_005101.4-148545110	CATAGAAGAACCAGCTCTCCTGTCCCC	12	C	0.1404	0.2659
NC_005101.4-148568440	CTTGCACATACACACACTCCTTGCTAG	7	C	0.0263	0.4889
NC_005101.4-148593445	CACACACACACACACCCTCCCATTAAA	1	C	0.3333	0.4384
NC_005101.4-148597527	CTCTTTGCTACCAGACCTCCATCATGG	12	C	0.0254	0.0251
NC_005101.4-148665930	CATACCTTCACCTTGGCTCCTCCAGAT	12	C	0.157	0.4933
NC_005101.4-148665930	CATACCTTCACCTTGGCTCCTCCAGAT	15	G	0	0.4787

NC_005101.4-148679804	TATCAACCAACCAGAGCTCCCAGTGAG	22	A	0.5328	0.4056
NC_005101.4-148760592	CCTCCTACAACCCACTCTCCCCAACTA	5	C	0.2066	0.4966
NC_005101.4-148805672	CGGTGATGGACACCCACTCCTATCCCA	2	G	0	0.482
NC_005101.4-148806970	CTGACTATCACCATGTCTCCTGCAATA	9	C	0.2623	0.3791
NC_005101.4-148858397	CTGCCTTTAACTAATTCTCCTCCTTTG	4	C	0	0.4743
NC_005101.4-148881961	AAATTCCTCACCTCCGCTCCCAAGGCA	15	C	0.1167	0.1244
NC_005101.4-148922358	AGCTGGCTAACAAGCCCTCCACCTCCA	14	G	0	0.4827
NC_005101.4-149024506	ACCATGCAGACGATTGCTCCTTAGGCT	4	A	0	0.4779
NC_005101.4-149044398	CTCCTTTGCACCCTTTCTCCTCACACT	8	G	0.0417	0.0408
NC_005101.4-149044398	CTCCTTTGCACCCTTTCTCCTCACACT	22	C	0.0826	0.4942
NC_005101.4-149133734	GTTCTTCAAACATTTCCTCCATTCTTT	14	T	0	0.4786
NC_005101.4-149133734	GTTCTTCAAACATTTCCTCCATTCTTT	26	T	0.0522	0.0508
NC_005101.4-149140713	CCACTATTCACTAGCTCTCCTGCAGCT	23	C	0.0894	0.1425
NC_005101.4-149200745	ACCTACTACACTGTGACTCCTCCAGCC	22	C	0.0244	0.0398
NC_005101.4-149207800	TTTAAAGCCACCCACACTCCTGTCCAT	15	C	0.0424	0.0415
NC_005101.4-149402792	TGTTAGACAACCATGGCTCCCAAGGAG	3	T	0	0.4851
NC_005101.4-149437050	GTATTGCCCACTTACTCTCCTTCTGGA	1	G	0.0088	0.4774
NC_005101.4-149437050	GTATTGCCCACTTACTCTCCTTCTGGA	16	T	0.0789	0.1229
NC_005101.4-149463150	ATAGCAGTAACCGTATCTCCTGCTGCT	12	C	0.041	0.0557
NC_005101.4-149463150	ATAGCAGTAACCGTATCTCCTGCTGCT	13	G	0.0083	0.4769
NC_005101.4-149463580	ATGTCAGCCACAGAGGCTCCTCAGAGA	8	C	0.0732	0.1001
NC_005101.4-149491782	TCATTTACTACAAAGCCTCCAGTTGTA	7	A	0.0508	0.4908
NC_005101.4-149532630	TTGCTCAGTACTTTCGCTCCTTCAGGG	14	T	0	0.4649
NC_005101.4-149557880	GATACACAAACAATTCCTCCGTAAGAT	8	A	0.0089	0.4709
NC_005101.4-149571777	CCATGATGGACTGTGACTCCTGTGACA	3	A	0.125	0.2052
NC_005101.4-149571777	CCATGATGGACTGTGACTCCTGTGACA	22	G	0.1062	0.5
NC_005101.4-149581897	CCACTAAGCACAATTTCTCCATGTCCT	4	C	0	0.4868
NC_005101.4-149581897	CCACTAAGCACAATTTCTCCATGTCCT	15	T	0.1282	0.2041
NC_005101.4-149654490	AGGACTCTGACTCTTCCTCCTCCACCT	9	G	0.0164	0.4837
NC_005101.4-149782936	TTCCAATCAACTCCTCCTCCCTTTCTT	2	T	0.2	0.3353
NC_005101.4-149817109	AGAATTGCAACACATTCTCCAGTTCCT	7	G	0.1765	0.4859
NC_005101.4-149817109	AGAATTGCAACACATTCTCCAGTTCCT	23	T	0.1333	0.1932
NC_005101.4-149923879	GACCTTGATACACTTTCTCCTCCAGTG	3	C	0.1316	0.4981
NC_005101.4-150008733	TTAAAGAACACTGACCCTCCTCCCCCA	23	C	0.0082	0.4755
NC_005101.4-150026123	CTACTTTAGACACTGCCTCCCTTAGCC	21	C	0	0.4728
NC_005101.4-150078648	AATGTGGCCACCAAGTCTCCTTTCTGT	22	T	0.2768	0.3517
NC_005101.4-150157627	CCATGTTCCACCTGGGCTCCTCCTACT	26	C	0	0.4787
NC_005101.4-150194476	CAGTATCAAACCCAAACTCCACAAGAT	15	A	0	0.4718
NC_005101.4-150210196	GCTCCCACCACTGTGACTCCCATGCTA	23	T	0.1008	0.494
NC_005101.4-150213923	CTCCCTGTCACTCCTGCTCCATGGAAT	7	G	0.0488	0.0928
NC_005101.4-150229350	CATCAACCTACAAACCCTCCTTAGCGA	26	G	0.1951	0.3516
NC_005101.4-150229350	CATCAACCTACAAACCCTCCTTAGCGA	27	A	0	0.4793
NC_005101.4-150230141	GCCTGGGCCACTCCCACTCCAACCGGT	7	G	0.0696	0.4482
NC_005101.4-150251267	CTCTCTTTAACCATTCCTCCATGATCC	9	A	0.0339	0.0655
NC_005101.4-150284869	AAGGATTTAACTATCTCTCCCTCCACT	4	G	0.0619	0.4986
NC_005101.4-150284869	AAGGATTTAACTATCTCTCCCTCCACT	21	C	0.0619	0.4986
NC_005101.4-150291591	TCAGGAACTACTCGTACTCCTACATGA	13	C	0	0.4755
NC_005101.4-150314760	TGTACTTGTACCCTGACTCCTCTCATA	16	A	0	0.4779
NC_005101.4-150462698	TATTCCTCCACTGGGACTCCTGCCTGT	9	C	0.0259	0.0255
NC_005101.4-150462698	TATTCCTCCACTGGGACTCCTGCCTGT	14	G	0.2	0.4817
NC_005101.4-150558083	CTTGGGTGGACTGCCTCTCCTGTTGCC	1	C	0.123	0.1296

NC_005101.4-150558083	CTTGGGTGGACTGCCTCTCCTGTTGCC	14	C	0.065	0.1356
NC_005101.4-150559867	AAAGAAACCACCAGGCCTCCAATCGTG	7	A	0.0667	0.1387
NC_005101.4-150559867	AAAGAAACCACCAGGCCTCCAATCGTG	23	T	0.0667	0.1387
NC_005101.4-150566509	AAAATGGCCACAGAGACTCCAGCTTTT	16	A	0.0082	0.479
NC_005101.4-150668535	ATCAGGGGAACAACTGCTCCGCCCATC	13	A	0.0678	0.1551
NC_005101.4-150706107	GACTATCAGACTGCATCTCCCTATGTA	16	T	0	0.4909
NC_005101.4-150725759	CAGACTGTAACATCAACTCCCAGTATA	5	C	0.0325	0.032
NC_005101.4-150725759	CAGACTGTAACATCAACTCCCAGTATA	12	A	0.0164	0.4737
NC_005101.4-150863079	GTAGCGCGAACATGTTCTCCGTATGTG	6	G	0.094	0.4994
NC_005101.4-150863079	GTAGCGCGAACATGTTCTCCGTATGTG	7	C	0.0924	0.1327
NC_005101.4-151049908	CACCATGGCACCAGACCTCCCTACTCC	1	C	0.1293	0.3525
NC_005101.4-151049908	CACCATGGCACCAGACCTCCCTACTCC	24	C	0.0172	0.0504
NC_005101.4-151103974	TGAGCTTCAACCTCAACTCCCAACCAT	7	T	0	0.4718
NC_005101.4-151103974	TGAGCTTCAACCTCAACTCCCAACCAT	25	C	0	0.4718
NC_005101.4-151174047	TCTGCATGTACACCAGCTCCACCAGCA	9	T	0.0894	0.0854
NC_005101.4-151174047	TCTGCATGTACACCAGCTCCACCAGCA	22	C	0.0976	0.0928
NC_005101.4-151184495	GAACTGAGAACCCTGGCTCCTCTTAGG	23	T	0.1	0.1099
NC_005101.4-151184495	GAACTGAGAACCCTGGCTCCTCTTAGG	26	G	0.0917	0.0875
NC_005101.4-151207596	ATAAGAAGGACACATGCTCCTCTATAT	1	A	0.0948	0.1058
NC_005101.4-151245099	GCTGTTTGCACTCTTCCTCCTTGGTCT	9	C	0.0598	0.12
NC_005101.4-151325253	TTATTTATTACTTATTCTCCTTACATC	16	T	0	0.4814
NC_005101.4-151325253	TTATTTATTACTTATTCTCCTTACATC	25	A	0.0265	0.0433
NC_005101.4-151464181	CGAGTGAAAACTGTTTCTCCACAGGGC	2	G	0.0268	0.0771
NC_005101.4-151464181	CGAGTGAAAACTGTTTCTCCACAGGGC	14	T	0.0708	0.0683
NC_005101.4-151519259	CACCATGGCACCTCCACTCCCCCAACA	7	G	0.2	0.2432
NC_005101.4-151519259	CACCATGGCACCTCCACTCCCCCAACA	23	C	0.0744	0.1017
NC_005101.4-151563214	AAAAATAAGACAGTATCTCCATGGTAA	25	T	0.0536	0.0521
NC_005101.4-151568311	AGTATTAGTACTCATCCTCCTCTGCTT	16	C	0.0439	0.138
NC_005101.4-151651827	AGAGTGAGTACCAAGGCTCCAGAGGCT	16	G	0	0.4793
NC_005101.4-151735062	TGTCTTTTAACCTGGCCTCCTCTATTA	24	A	0.1488	0.4991
NC_005101.4-152089658	TGCCTTCCCACCGTATCTCCAGGCAAT	12	C	0	0.4793
NC_005101.4-152089658	TGCCTTCCCACCGTATCTCCAGGCAAT	15	A	0	0.4793
NC_005101.4-152096227	TCTTCTCTCACTCCAACTCCTGTTATT	15	A	0.1391	0.2873
NC_005101.4-152146146	ACCCATTTCACCACCACTCCTACCTCA	14	C	0.2522	0.3547
NC_005101.4-152146146	ACCCATTTCACCACCACTCCTACCTCA	22	A	0.2389	0.4242
NC_005101.4-152399831	CTAAGGCCTACAACCTCTCCCAAAGCC	6	G	0.0435	0.0425
NC_005101.4-152399831	CTAAGGCCTACAACCTCTCCCAAAGCC	8	C	0.1754	0.4114
NC_005101.4-152529197	TGACAATAAACATTGACTCCTGGTATT	6	A	0.0826	0.2172
NC_005101.4-152560698	GGTCATCCCACCAGCTCTCCCTTATCC	8	C	0.1707	0.2556
NC_005101.4-152560698	GGTCATCCCACCAGCTCTCCCTTATCC	9	C	0.0244	0.0398
NC_005101.4-152745707	ATGTTCCTCACACTAACTCCAAGCCCC	14	T	0.1951	0.4497
NC_005101.4-152745707	ATGTTCCTCACACTAACTCCAAGCCCC	25	C	0.0325	0.0476
NC_005101.4-152762526	TGTCCCCACACAGCTCCTCCACCATTT	3	T	0.0732	0.0705
NC_005101.4-152966706	CTAGGGTGTACTTTCCCTCCTTTTGTT	26	T	0.188	0.1704
NC_005101.4-153367208	ACATGCACCACCCAGGCTCCTAGTCCC	22	A	0	0.48
NC_005101.4-153390878	CTGTTCCACACCTTGTCTCCATATTTC	26	T	0.0894	0.0854
NC_005101.4-153402500	CCAACTTCAACTGTCCCTCCCTGTACT	16	C	0	0.4793
NC_005101.4-153412344	CATAGTGGCACACCTACTCCAACACAC	7	G	0.0252	0.0411
NC_005101.4-153412344	CATAGTGGCACACCTACTCCAACACAC	8	G	0	0.4693
NC_005101.4-153587465	TCTCCCTCCACTCTATCTCCCCATCTC	7	T	0.1826	0.2072
NC_005101.4-153737222	ATCTTCTTTACTGAACCTCCCAAGTGA	7	T	0.0084	0.4829

NC_005101.4-153836466	AATATTGCCACTGGTTCTCCTTTAAAT	2	A	0.1667	0.42
NC_005101.4-153836466	AATATTGCCACTGGTTCTCCTTTAAAT	3	T	0	0.4718
NC_005101.4-153906039	TGTTATCGAACTCACTCTCCAAGCCCC	7	C	0	0.4786
NC_005101.4-153906039	TGTTATCGAACTCACTCTCCAAGCCCC	24	C	0.1552	0.1855
NC_005101.4-153919632	TGGGGTCCAACTCTGTCTCCTTGGATA	4	G	0	0.4807
NC_005101.4-153919632	TGGGGTCCAACTCTGTCTCCTTGGATA	16	T	0.2377	0.375
NC_005101.4-153975119	GGTGTGTCGACTCCTGCTCCCAACTTC	8	C	0	0.4887
NC_005101.4-153975119	GGTGTGTCGACTCCTGCTCCCAACTTC	23	A	0.0609	0.2072
NC_005101.4-154005131	GATGATGTGACTTAAACTCCTAGCCAG	24	C	0.0738	0.0861
NC_005101.4-154034390	GACCTGATCACAGCTGCTCCTGACTGC	8	T	0	0.4773
NC_005101.4-154053404	CTCCTACGAACTGGGACTCCCGGCATG	21	C	0.0085	0.4738
NC_005101.4-154141066	CATCTGACAACCACTACTCCCTAGGGG	12	C	0.2353	0.3118
NC_005101.4-154141066	CATCTGACAACCACTACTCCCTAGGGG	14	C	0.1	0.1387
NC_005101.4-154186585	ATGTTGTCCACACCACCTCCTGCCACA	7	T	0.0714	0.4871
NC_005101.4-154186585	ATGTTGTCCACACCACCTCCTGCCACA	26	C	0.2232	0.4193
NC_005101.4-154216883	GAGGTTGTCACTATACCTCCTTTGTTC	2	A	0.1111	0.1634
NC_005101.4-154216883	GAGGTTGTCACTATACCTCCTTTGTTC	3	G	0.2414	0.3995
NC_005101.4-154233830	TAGACATCAACTTGTTCTCCAGAGGCT	13	T	0.0256	0.0253
NC_005101.4-154278591	CTGTAGAATACTGCTGCTCCTGGGCTT	21	T	0.1066	0.4973
NC_005101.4-154278591	CTGTAGAATACTGCTGCTCCTGGGCTT	22	G	0.041	0.0401
NC_005101.4-154296665	GAGAACTGGACACTGGCTCCCACCTCT	23	C	0.1053	0.1153
NC_005101.4-154335647	ACTATCTTAACTCCAGCTCCAAGGGCT	12	T	0.0244	0.0241
NC_005101.4-154335647	ACTATCTTAACTCCAGCTCCAAGGGCT	27	T	0	0.4793
NC_005101.4-154373370	ATTACATACACTGCACCTCCCTTCCAC	5	C	0.1228	0.1884
NC_005101.4-154373370	ATTACATACACTGCACCTCCCTTCCAC	6	A	0	0.4864
NC_005101.4-154423141	TGTGGCTGCACGTCTCCTCCAGGTCAC	12	G	0.2035	0.2367
NC_005101.4-154432872	ATTTGAAAGACCTCAGCTCCCCTATCT	23	T	0.2807	0.4321
NC_005101.4-154506864	GGTTTTGAGACAAGGTCTCCCTCAGTC	21	C	0.0536	0.0521
NC_005101.4-154542710	CCACCTGCCACTAGATCTCCGCTGATA	26	T	0	0.4839
NC_005101.4-154629374	GCTGTTTCTACTACCCCTCCTTAAGCT	3	T	0	0.4868
NC_005101.4-154674096	CTTTCAACCACTCTGACTCCTGGTCTG	9	C	0.0085	0.4714
NC_005101.4-154705495	TATGATCACACATAGCCTCCTGGGTCT	2	A	0.0442	0.1239
NC_005101.4-154776040	AACACACATACACGGCCTCCTGAGAAA	13	C	0	0.4743
NC_005101.4-155344920	TCTCACACTACCTCTTCTCCACCTCCA	9	T	0.1951	0.2832
NC_005101.4-155344920	TCTCACACTACCTCTTCTCCACCTCCA	21	A	0.2033	0.2885
NC_005101.4-155373932	AATGTGGTCACAGGCTCTCCACAGACC	23	A	0.0086	0.0255
NC_005101.4-155388090	GGGTGCCTCACATAATCTCCCTTACAT	3	G	0.0082	0.479
NC_005101.4-155396135	TAATCTTGTACGAAGTCTCCTGGTGAG	15	G	0.1525	0.1691
NC_005101.4-155396135	TAATCTTGTACGAAGTCTCCTGGTGAG	22	G	0	0.4718
NC_005101.4-155456979	GGAGACCCCACCAGAGCTCCCAGGTAC	7	C	0.0614	0.138
NC_005101.4-155456979	GGAGACCCCACCAGAGCTCCCAGGTAC	24	G	0.0614	0.138
NC_005101.4-155487376	TGGAACCTCACTGGGACTCCTCTTGAA	13	G	0.5625	0.4119
NC_005101.4-155678360	ATAAAGGAAACCATGGCTCCATTTATG	24	T	0	0.4875
NC_005101.4-155678360	ATAAAGGAAACCATGGCTCCATTTATG	27	G	0	0.4875
NC_005101.4-155684503	AACAAGGCTACACCTACTCCAACAGAA	15	T	0.0877	0.2538
NC_005101.4-155721031	TCCTCATTCACAACCACTCCAGCTAGG	2	C	0.0424	0.487
NC_005101.4-155721031	TCCTCATTCACAACCACTCCAGCTAGG	15	C	0.0339	0.0333
NC_005101.4-155735546	GATTCATTAACAAACACTCCCTGGGCA	14	A	0.1913	0.2873
NC_005101.4-155775164	GTCCAACAGACAACTCCTCCCTACATC	7	C	0	0.482
NC_005101.4-155844488	CAGCTGCTTACAATTCCTCCGGCAAGC	9	T	0.0085	0.481
NC_005101.4-155844488	CAGCTGCTTACAATTCCTCCGGCAAGC	26	G	0.042	0.0411

NC_005101.4-155906159	TGGTCTCTGACCTCACCTCCCATTCAT	1	T	0.041	0.4879
NC_005101.4-155932219	CACACACACACACACACTCCAAGATTT	16	A	0.4914	0.3707
NC_005101.4-155933830	TTACTTAATACTCTGTCTCCTTGAGCA	4	C	0.036	0.0354
NC_005101.4-155940618	ACCCAGCACACAGTTCCTCCTACTCTC	21	T	0.2195	0.484
NC_005101.4-155955920	AAGGTGCTAACTTCCTCTCCCCTCCCA	2	A	0.1504	0.1686
NC_005101.4-155955920	AAGGTGCTAACTTCCTCTCCCCTCCCA	16	T	0	0.4555
NC_005101.4-155983007	CACAGCTTCACAGAGCCTCCTTCTGGA	3	C	0.065	0.4944
NC_005101.4-155983007	CACAGCTTCACAGAGCCTCCTTCTGGA	5	G	0.065	0.0928
NC_005101.4-155983758	CACATCCACACCTAACCTCCTGTGGTG	12	C	0.0342	0.0336
NC_005101.4-155991598	AAGACCCTGACCTTGGCTCCTTCGATC	9	G	0.1933	0.4977
NC_005101.4-155991598	AAGACCCTGACCTTGGCTCCTTCGATC	21	T	0.1393	0.2796
NC_005101.4-156016115	CATGATGATACCCCAGCTCCCATGTTA	3	T	0	0.4793
NC_005101.4-156715560	GAACCGAGAACGGGACCTCCCCATTGA	6	G	0.3717	0.3026
NC_005101.4-156715560	GAACCGAGAACGGGACCTCCCCATTGA	25	T	0.1947	0.1757
NC_005101.4-157201512	CCCACAGCTACCTACCCTCCTTCAAAC	14	A	0.0517	0.0666
NC_005101.4-157201512	CCCACAGCTACCTACCCTCCTTCAAAC	16	C	0	0.4864
NC_005101.4-157253230	ATGGCCTCTACTTCGGCTCCTGCCTCT	2	T	0.0893	0.0853
NC_005101.4-157382941	CACCCACACACACACCCTCCTCCACCT	14	A	0	0.474
NC_005101.4-157382941	CACCCACACACACACCCTCCTCCACCT	22	C	0	0.474
NC_005101.4-157413996	AAGATAGGCACTGTCTCTCCCAGGGCT	6	A	0.0174	0.48
NC_005101.4-157413996	AAGATAGGCACTGTCTCTCCCAGGGCT	8	G	0.0435	0.059
NC_005101.4-157539217	ACTCATTACACATAGGCTCCTTCGTGC	9	C	0.1624	0.4823
NC_005101.4-157539217	ACTCATTACACATAGGCTCCTTCGTGC	23	C	0.0084	0.4859
NC_005101.4-157557490	CTGTGCCTCACCACACCTCCAAGGATA	6	C	0.0579	0.1017
NC_005101.4-157557490	CTGTGCCTCACCACACCTCCAAGGATA	23	G	0	0.4787
NC_005101.4-157561017	CCCGTGCTAACTTGATCTCCAAATGCA	16	T	0.1034	0.4518
NC_005101.4-157579003	ATGGACTCAACATGGTCTCCAGCAGCA	16	T	0.0163	0.4825
NC_005101.4-157579003	ATGGACTCAACATGGTCTCCAGCAGCA	27	A	0.1789	0.3141
NC_005101.4-157579077	CTGACGTCAACATGGCCTCCTGCCACA	5	C	0.0656	0.0786
NC_005101.4-157579077	CTGACGTCAACATGGCCTCCTGCCACA	7	T	0.0656	0.0786
NC_005101.4-157621953	GACCACTGCACTCGATCTCCTTGTTCT	13	C	0	0.4793
NC_005101.4-157629945	TCAACAAGTACCATTGCTCCTGGTTGT	27	T	0.0083	0.4803
NC_005101.4-157647616	ATCCCAAGAACACCCCCTCCTCAGACT	12	A	0.0179	0.4871
NC_005101.4-157689113	CTCCCCAACACCCACACTCCCACCTCC	27	C	0	0.4743
NC_005101.4-157697371	TACCTCACAACAAAGGCTCCATTGGTG	13	A	0.1518	0.3612
NC_005101.4-157730235	GAGTCTAGAACACAGTCTCCTCCGGTG	23	C	0	0.48
NC_005101.4-157796132	CGTGAGTCTACATAACCTCCCTGCCCT	7	T	0.0855	0.1125
NC_005101.4-157812503	ACTGATAGGACCAGCCCTCCTGCTTTC	16	C	0	0.4751
NC_005101.4-157815115	GCTCTCTCCACTGCTCCTCCTGTCTCT	2	C	0.1826	0.2816
NC_005101.4-157834958	ACACGCCCTACAGCATCTCCCCTCCCC	7	C	0.2373	0.2926
NC_005101.4-157851143	TCTACCATGACATCACCTCCTTTCTCC	1	T	0.2397	0.2814
NC_005101.4-157851143	TCTACCATGACATCACCTCCTTTCTCC	21	T	0	0.4787
NC_005101.4-157866505	ACCAGTGACACACCTTCTCCAACAAGA	6	T	0.0569	0.0553
NC_005101.4-157917078	ATCGTGGTCACACATGCTCCTGTAGAG	22	G	0.1983	0.3332
NC_005101.4-157917078	ATCGTGGTCACACATGCTCCTGTAGAG	26	A	0.1379	0.375
NC_005101.4-157930276	AAGCCTGCAACTCCAGCTCCAGTGGAT	6	T	0.0508	0.0812
NC_005101.4-158027011	TCATGAAGAACATACTCTCCCCTTTCT	13	T	0.0244	0.0241
NC_005101.4-158027011	TCATGAAGAACATACTCTCCCCTTTCT	27	T	0	0.4793
NC_005101.4-158090651	TTTGTTGAGACAGGATCTCCCTCAGGG	12	A	0.1818	0.2415
NC_005101.4-158108624	GGAGCACAGACAACCTCTCCGACATTG	4	G	0.0574	0.1296
NC_005101.4-158122607	GTATTCACAACCGAGTCTCCATCTGTG	1	G	0.0339	0.0333

NC_005101.4-158122607	GTATTCACAACCGAGTCTCCATCTGTG	23	C	0.0678	0.1116
NC_005101.4-158200966	TACAAATAGACCTTACCTCCCAACACC	12	C	0	0.4827
NC_005101.4-158200966	TACAAATAGACCTTACCTCCCAACACC	26	C	0	0.4827
NC_005101.4-158209723	TTCTTCTCCACCTCCTCTCCCGTTCAG	22	G	0.0081	0.481
NC_005101.4-158224091	TAGGGACCTACACATACTCCGTATGAA	5	G	0.2185	0.4898
NC_005101.4-158253582	TTTATACATACATGTCCTCCCTATGTG	12	A	0.0348	0.0508
NC_005101.4-158253582	TTTATACATACATGTCCTCCCTATGTG	26	T	0.1739	0.3593
NC_005101.4-158264822	CTCATGGGGACAGTGGCTCCAGTCTAT	3	C	0.018	0.0354
NC_005101.4-158271438	CCCTCTCCCACGTCCCCTCCCCCAGCT	12	G	0	0.4688
NC_005101.4-158271438	CCCTCTCCCACGTCCCCTCCCCCAGCT	25	G	0.009	0.4726
NC_005101.4-158324614	TTACCTGTTACTCTCCCTCCTCCTATG	4	C	0	0.4765
NC_005101.4-158324614	TTACCTGTTACTCTCCCTCCTCCTATG	5	C	0	0.4751
NC_005101.4-158411725	GGTGCTGTAACTGCAGCTCCAGAGGAT	25	G	0	0.4807
NC_005101.4-158428047	ATTTACACCACAATATCTCCATTGAAG	8	C	0.0082	0.479
NC_005101.4-158428047	ATTTACACCACAATATCTCCATTGAAG	14	T	0.0082	0.479
NC_005101.4-158513723	AACATAATGACTTCGACTCCCTTTTAG	5	T	0.1513	0.5
NC_005101.4-158520794	CTCTGACTGACATTATCTCCACTAAGA	6	A	0.0246	0.0243
NC_005101.4-158549722	CAGTGGTTGACATTGTCTCCTCCAGCT	13	T	0.1639	0.4988
NC_005101.4-158576995	ATTACTTAAACTCAATCTCCATGGCTG	26	T	0.0813	0.078
NC_005101.4-158576995	ATTACTTAAACTCAATCTCCATGGCTG	27	G	0.187	0.2556
NC_005101.4-158577357	ACACTGGGTACATGTCCTCCTTTTTGA	22	T	0.0769	0.1347
NC_005101.4-158594255	TGCCTGGCAACGGAATCTCCAAGAGCT	2	G	0.2623	0.4737
NC_005101.4-158594255	TGCCTGGCAACGGAATCTCCAAGAGCT	22	A	0.0738	0.0861
NC_005101.4-158637910	AGAGTCTGGACCAGTGCTCCTGAAATA	14	G	0.2368	0.3473
NC_005101.4-158639502	TGTCCTATAACAGAAGCTCCGTGGAGG	9	A	0.0083	0.4783
NC_005101.4-158639502	TGTCCTATAACAGAAGCTCCGTGGAGG	24	G	0.0331	0.0325
NC_005101.4-158639606	CAGAGCAGGACAGACCCTCCTCTGACC	6	C	0.0894	0.1145
NC_005101.4-158639606	CAGAGCAGGACAGACCCTCCTCTGACC	27	C	0.0325	0.032
NC_005101.4-158979512	TCCTTTGCAACTGGCTCTCCTCCATGC	26	G	0.0244	0.0241
NC_005101.4-158980169	ACATAATCAACTAGGTCTCCCTTTTGC	16	T	0.157	0.4999
NC_005101.4-158980169	ACATAATCAACTAGGTCTCCCTTTTGC	27	C	0.3223	0.3687
NC_005101.4-158996597	CCTATAGGTACTTGATCTCCAGCTCTA	4	A	0	0.4773
NC_005101.4-158996597	CCTATAGGTACTTGATCTCCAGCTCTA	8	G	0.0569	0.0553
NC_005101.4-164079550	AATCTTTCCACTAATTCTCCATTACAG	7	T	0.0339	0.0333
NC_005101.4-164079550	AATCTTTCCACTAATTCTCCATTACAG	22	T	0.0331	0.0325
NC_005101.4-164176693	CTCTCTCTCACCCTGACTCCCACCCCC	3	C	0.0756	0.1469
NC_005101.4-164176693	CTCTCTCTCACCCTGACTCCCACCCCC	21	C	0.0252	0.4829
NC_005101.4-164212830	CTTGGCTTTACCTGCTCTCCTTGTGTT	12	C	0	0.48
NC_005101.4-164212830	CTTGGCTTTACCTGCTCTCCTTGTGTT	22	T	0	0.48
NC_005101.4-164290603	TACCAACCAACCAGAACTCCTTGGGAC	1	T	0.3966	0.3662
NC_005101.4-164290603	TACCAACCAACCAGAACTCCTTGGGAC	15	A	0.3966	0.3662
NC_005101.4-164292908	ACTCAATGCACTGGTGCTCCTTGTTTA	7	T	0	0.4793
NC_005101.4-164302277	CCCTTCTCCACTCTTCCTCCATTGTTC	9	C	0.0082	0.479
NC_005101.4-164308785	ATGCATATGACAGTTGCTCCTTTTCTG	1	A	0.0826	0.109
NC_005101.4-164322072	TATGAAGTCACTCATCCTCCTCAGTAT	15	T	0	0.4698
NC_005101.4-164485952	TGGACTCTGACAGTCCCTCCACTTCAG	4	A	0.0789	0.1229
NC_005101.4-164528741	AGCCTGAGGACTGGGGCTCCATCCCCA	4	C	0	0.4787
NC_005101.4-164528741	AGCCTGAGGACTGGGGCTCCATCCCCA	16	G	0.0744	0.1163
NC_005101.4-164531445	TGTTCTGTCACAGTGACTCCTGTTATG	23	T	0.0732	0.1145
NC_005101.4-164531445	TGTTCTGTCACAGTGACTCCTGTTATG	24	T	0	0.4793
NC_005101.4-164541311	GCTACTTCTACAGCACCTCCTCTACCC	3	T	0.0083	0.4803

NC_005101.4-164541311	GCTACTTCTACAGCACCTCCTCTACCC	4	A	0	0.48
NC_005101.4-164567189	CAGTTCCCCACAGCCTCTCCGTCTATA	21	G	0.0684	0.0973
NC_005101.4-164567189	CAGTTCCCCACAGCCTCTCCGTCTATA	25	A	0.0085	0.0418
NC_005101.4-164614649	TTCCCTGGTACCAGCACTCCGGAGCAT	9	T	0.0085	0.481
NC_005101.4-164614649	TTCCCTGGTACCAGCACTCCGGAGCAT	21	G	0.1102	0.1894
NC_005101.4-164667264	ACGCTCAATACGCTATCTCCCTGTCAC	15	A	0.1552	0.1855
NC_005101.4-164685220	TGAGGGCACACCCCGACTCCCGCTCTG	12	C	0.0268	0.0264
NC_005101.4-164733253	CAGAGCAGGACCAGTTCTCCTGCTCTT	6	C	0.025	0.4747
NC_005101.4-164733253	CAGAGCAGGACCAGTTCTCCTGCTCTT	27	T	0.0167	0.4728
NC_005101.4-164747996	CCCCGTAGCACAGCATCTCCTCCATGG	2	C	0.0174	0.0508
NC_005101.4-164747996	CCCCGTAGCACAGCATCTCCTCCATGG	16	T	0	0.4902
NC_005101.4-164748327	CATTAGACTACCTTCCCTCCTGTGTTA	5	A	0	0.4832
NC_005101.4-164748327	CATTAGACTACCTTCCCTCCTGTGTTA	23	T	0.2667	0.4688
NC_005101.4-164766715	CTCATGTGCACTGCTGCTCCAATAAAA	12	T	0.157	0.4966
NC_005101.4-164810542	TACTGTCCCACATATCCTCCATTGGCT	1	T	0	0.4898
NC_005101.4-164810542	TACTGTCCCACATATCCTCCATTGGCT	15	T	0.0536	0.4998
NC_005101.4-164832017	CTACCTATAACTCCAACTCCTATTGTC	22	A	0.125	0.4958
NC_005101.4-164934965	ACAAAACCAACCAGAGCTCCCAGGGAC	27	C	0.0531	0.0517
NC_005101.4-164962304	CCACCTGCAACCTCAGCTCCAGGGGCT	1	C	0.2114	0.2499
NC_005101.4-164993764	TTCTCATCAACTTCTCCTCCAAATTCC	15	T	0	0.4715
NC_005101.4-165099145	TGATTCTGGACATGGCCTCCTGCCTTG	8	G	0.087	0.4425
NC_005101.4-165395184	AATGCCCTCACGAATCCTCCTGAACTC	1	A	0.1333	0.3665
NC_005101.4-165395184	AATGCCCTCACGAATCCTCCTGAACTC	25	C	0.1667	0.4965
NC_005101.4-165403727	CGCAACCCCACTCTTGCTCCAAGCCTG	3	C	0.065	0.0928
NC_005101.4-165423758	ACATGAGACACCCCCGCTCCCTAAGAG	16	G	0.0265	0.0262
NC_005101.4-165437236	CTCCTAGACACACAGCCTCCTCCAACA	3	C	0.0424	0.0415
NC_005101.4-165441078	CTCAAGATGACCTGGACTCCTAAACAA	6	G	0.2232	0.4193
NC_005101.4-165445033	TGGAGGGCTACAGTTCCTCCCTGAAAG	5	G	0	0.0351
NC_005101.4-165445033	TGGAGGGCTACAGTTCCTCCCTGAAAG	21	C	0.0982	0.125
NC_005101.4-165485524	CATCAACCCACTCCATCTCCATCAACC	1	C	0.0833	0.095
NC_005101.4-165490467	AACGATAGAACAAACCCTCCTTCCTTT	3	C	0.2203	0.3034
NC_005101.4-165490467	AACGATAGAACAAACCCTCCTTCCTTT	4	G	0.0504	0.0491
NC_005101.4-165533232	TGTGCCCACACTGCCACTCCTCCACTC	3	T	0	0.4793
NC_005101.4-165533232	TGTGCCCACACTGCCACTCCTCCACTC	26	T	0.2195	0.2885
NC_005101.4-165572526	CAATCAAACACTGCACCTCCCCTTTCT	1	C	0.225	0.2722
NC_005101.4-165572526	CAATCAAACACTGCACCTCCCCTTTCT	3	A	0.2231	0.2704
NC_005101.4-165573368	ACCAAATAAACCTTTCCTCCCCGCTTG	23	G	0.2564	0.405
NC_005101.4-165573368	ACCAAATAAACCTTTCCTCCCCGCTTG	25	T	0.2155	0.4916
NC_005101.4-165690899	TGCATTTACACCTATGCTCCTCGCATT	9	C	0.2459	0.4737
NC_005101.4-165727702	ATTATAAGAACAAGCTCTCCTATTATG	15	C	0.0174	0.48
NC_005101.4-165727702	ATTATAAGAACAAGCTCTCCTATTATG	21	T	0.0174	0.48
NC_005101.4-165743889	TTATCTTTAACTCCAGCTCCAGGAGGT	2	T	0	0.4793
NC_005101.4-165774257	AGGACCACCACCACCACTCCCTGAATT	22	T	0.1429	0.4723
NC_005101.4-165774257	AGGACCACCACCACCACTCCCTGAATT	23	G	0.15	0.2994
NC_005101.4-165848759	CAGGCCAACACAGGGGCTCCTCCCCGC	25	C	0	0.48
NC_005101.4-165860427	GAAGACACTACCACTTCTCCAGCTTCT	2	A	0.2377	0.432
NC_005101.4-165900595	ATGCTTCATACATAGTCTCCTGCCTTA	22	G	0.2544	0.3999
NC_005101.4-165900595	ATGCTTCATACATAGTCTCCTGCCTTA	23	C	0.0446	0.4856
NC_005101.4-165954135	ATTCTCCCCACTCAGCCTCCTGAGCAC	2	T	0.1795	0.3409
NC_005101.4-165976361	TGGAATCTTACACCACCTCCCTTGCCT	14	C	0.2137	0.321
NC_005101.4-165984120	CACTGGCCCACAAATACTCCCACTCAG	23	C	0	0.4813

NC_005101.4-165984120	CACTGGCCCACAAATACTCCCACTCAG	27	G	0.1083	0.395
NC_005101.4-165994533	GGAGAGGGTACTTTTCCTCCACATTCT	16	C	0.0354	0.0348
NC_005101.4-165994533	GGAGAGGGTACTTTTCCTCCACATTCT	25	T	0.0089	0.4789
NC_005101.4-166019489	GAGAGGTTTACTTTGGCTCCAGGTAGA	3	G	0.0982	0.212
NC_005101.4-166021109	CCTCCTGTGACTTTAGCTCCGTGAGAT	21	G	0.125	0.4991
NC_005101.4-166071779	TACCACTCAACTACATCTCCAGCCCGG	4	C	0	0.4779
NC_005101.4-166071779	TACCACTCAACTACATCTCCAGCCCGG	5	A	0	0.4779
NC_005101.4-166130895	GCAGTATGCACTAGTCCTCCTCCGTGT	12	T	0.2295	0.3259
NC_005101.4-166130895	GCAGTATGCACTAGTCCTCCTCCGTGT	23	C	0.2645	0.3557
NC_005101.4-166182271	GAGCACAACACAAGAGCTCCAGGAACT	6	C	0.0407	0.0854
NC_005101.4-166288531	CACACACACACACACACTCCTTTTATA	14	A	0.2973	0.2778
NC_005101.4-166290733	ACTGTTCTTACAGCAGCTCCAACAGTA	8	T	0.1368	0.2361
NC_005101.4-166292677	TCAGGTAGGACTGTTACTCCACAGTGG	13	G	0.0256	0.0253
NC_005101.4-166355425	GGGATATGAACTTATCCTCCCACGTGT	15	T	0.2252	0.344
NC_005101.4-166375961	CAGGTTGGGACCCGCTCTCCTGAGTAC	1	C	0.1504	0.2738
NC_005101.4-166375961	CAGGTTGGGACCCGCTCTCCTGAGTAC	14	G	0	0.4875
NC_005101.4-166400293	CATTCTTCAACTGATTCTCCCATGCCT	1	C	0.0522	0.4936
NC_005101.4-166400293	CATTCTTCAACTGATTCTCCCATGCCT	2	A	0.0522	0.4936
NC_005101.4-166444748	TTTCACAGAACTTTCTCTCCCCTCTCC	4	C	0.2035	0.4309
NC_005101.4-166444748	TTTCACAGAACTTTCTCTCCCCTCTCC	5	A	0.0783	0.1219
NC_005101.4-166468076	TGAACTAAAACCTTTTCTCCCAAGAGA	15	T	0.1724	0.3835
NC_005101.4-166468552	TATTGTTGAACTCCACCTCCTTTAAGT	13	C	0.0351	0.0345
NC_005101.4-166468552	TATTGTTGAACTCCACCTCCTTTAAGT	27	T	0	0.4841
NC_005101.4-166491332	TGTACTCTCACCAGCACTCCTACCACA	3	T	0.1157	0.1235
NC_005101.4-166491332	TGTACTCTCACCAGCACTCCTACCACA	15	C	0.2397	0.3327
NC_005101.4-166667091	AATGCGCACACCCTTTCTCCCCGTGTG	5	C	0	0.4793
NC_005101.4-166667091	AATGCGCACACCCTTTCTCCCCGTGTG	22	C	0.2195	0.4618
NC_005101.4-166704681	GAATTAGCCACTGTAGCTCCATGACTG	5	T	0.2185	0.322
NC_005101.4-166704681	GAATTAGCCACTGTAGCTCCATGACTG	15	A	0.2185	0.322
NC_005101.4-166709781	TCAGGTGAGACTTGGTCTCCCAGCATG	26	T	0.0252	0.4886
NC_005101.4-166709781	TCAGGTGAGACTTGGTCTCCCAGCATG	27	G	0.175	0.3047
NC_005101.4-166731432	TGGGTTGCAACCGTGCCTCCACTATAC	12	C	0.2119	0.3483
NC_005101.4-166731432	TGGGTTGCAACCGTGCCTCCACTATAC	14	T	0.1597	0.3554
NC_005101.4-166887598	GGATGATGAACTCAAGCTCCAAACCTT	14	A	0.1404	0.3115
NC_005101.4-166922207	GCTGCAAACACCGGCTCTCCTGCCTTG	2	C	0.2991	0.3409
NC_005101.4-166923215	AGAAGTCCTACCTCTGCTCCGATTTCT	16	G	0.0877	0.1884
NC_005101.4-166923215	AGAAGTCCTACCTCTGCTCCGATTTCT	27	T	0.0702	0.0997
NC_005101.4-166927833	CACAAGTCAACACAGACTCCTCTGCAG	7	T	0	0.4807
NC_005101.4-166927833	CACAAGTCAACACAGACTCCTCTGCAG	12	A	0.1949	0.2156
NC_005101.4-167285246	TTTTTTCAAACAGGGACTCCTCATATA	6	T	0.3158	0.4962
NC_005101.4-167285246	TTTTTTCAAACAGGGACTCCTCATATA	25	A	0.0086	0.4643
NC_005101.4-167314309	AGGTAAAGAACTCCGTCTCCTCAGATG	14	C	0.0574	0.071
NC_005101.4-167314309	AGGTAAAGAACTCCGTCTCCTCAGATG	15	G	0.041	0.0401
NC_005101.4-167327443	ATAAGCCAGACTAAATCTCCTACCGCT	5	G	0.0083	0.4803
NC_005101.4-167351227	TGCCAACCAACCATAGCTCCCATGGAC	15	A	0.2328	0.2315
NC_005101.4-167351227	TGCCAACCAACCATAGCTCCCATGGAC	27	C	0.2328	0.2315
NC_005101.4-167359078	TAGGTTTGAACACCACCTCCTACTTAT	14	C	0.1316	0.1528
NC_005101.4-167380653	CAACATATGACTCTGTCTCCTTTTCCT	4	C	0.0088	0.4873
NC_005101.4-167451237	AAGTTATGGACATGGCCTCCCTTCATC	8	G	0.1026	0.1704
NC_005101.4-167472898	GGACAATGTACCCACCCTCCATTCAAC	4	C	0.3363	0.4207
NC_005101.4-167490969	CAATACAATACAGTGTCTCCCCTGGGG	2	A	0.1379	0.1431

NC_005101.4-167490969	CAATACAATACAGTGTCTCCCCTGGGG	26	G	0.0431	0.0422
NC_005101.4-167596595	ATCTACGCCACAGGTTCTCCACTTTTC	7	G	0	0.4807
NC_005101.4-167596859	ACTTCCAATACTCCATCTCCATTATGA	23	T	0	0.48
NC_005101.4-167839032	CCAAAAGCTACCAGCACTCCTTACATA	1	C	0.2358	0.3287
NC_005101.4-167839032	CCAAAAGCTACCAGCACTCCTTACATA	24	C	0.187	0.2203
NC_005101.4-167900005	ACCACAAGGACTCTTGCTCCACTATGT	26	G	0	0.032
NC_005101.4-167979805	TGCCAACCAACCAGAGCTCCTAGGGGA	14	G	0.0813	0.1216
NC_005101.4-167979805	TGCCAACCAACCAGAGCTCCTAGGGGA	24	G	0.0081	0.481
NC_005101.4-168010560	CCTGTTGGCACTTCATCTCCTGTTGTT	15	A	0.1795	0.2171
NC_005101.4-168107320	GTAGGTGAAACATGTCCTCCTGGATGG	27	G	0.0756	0.1033
NC_005101.4-168258245	CTCTCTGTAACTCCAGCTCCAAGGCTT	21	A	0.2034	0.2466
NC_005101.4-168258245	CTCTCTGTAACTCCAGCTCCAAGGCTT	23	G	0.0086	0.4836
NC_005101.4-168283832	GGGCAAATAACTTAGTCTCCAGACTAA	2	G	0.0263	0.026
NC_005101.4-168576714	ACTTGATGTACTGTCCCTCCACTGGAG	2	C	0.122	0.1145
NC_005101.4-168636374	GTGGATGCTACTCTTCCTCCCCTCTCT	12	T	0	0.4813
NC_005101.4-168636374	GTGGATGCTACTCTTCCTCCCCTCTCT	25	T	0.2605	0.3812
NC_005101.4-168834596	GAGTTGAATACACTGGCTCCTCATGGT	3	G	0.0583	0.0566
NC_005101.4-168896845	CAATTCATCACTGTTTCTCCTTAGCTG	13	G	0.0268	0.0264
NC_005101.4-168984630	ATAACAAAGACGCATGCTCCACTATTT	8	A	0.3304	0.3102
NC_005101.4-169049206	ATTTTGGTCACATTAACTCCATTTCCC	21	A	0.0756	0.4886
NC_005101.4-169228699	ACCCAGAAAACCATGTCTCCAAATACT	9	A	0	0.4693
NC_005101.4-169228699	ACCCAGAAAACCATGTCTCCAAATACT	26	C	0.0417	0.0408
NC_005101.4-169294453	ATAACTCACACTCTTCCTCCAAGACTC	22	A	0.0089	0.0264
NC_005101.4-169433752	CAATGGGAAACCCATTCTCCATTCAAT	8	A	0	0.4555
NC_005101.4-169433752	CAATGGGAAACCCATTCTCCATTCAAT	21	A	0.5575	0.4599
NC_005101.4-169499886	GCCTCTCACACTCCTTCTCCATTTACT	8	A	0.0164	0.4837
NC_005101.4-169648971	TACCTTACCACCTACCCTCCAACATTA	15	C	0.0244	0.0241
NC_005101.4-169743940	CTGTTTTGAACATTGTCTCCATATCTC	13	T	0.0826	0.0943
NC_005101.4-169743940	CTGTTTTGAACATTGTCTCCATATCTC	14	T	0.2314	0.3278
NC_005101.4-169882341	GCTCCCCCAACCATCCCTCCATTACAA	2	C	0.2321	0.4424
NC_005101.4-170054303	TTTCATGGGACAGATCCTCCTATCATA	8	G	0.2414	0.4405
NC_005101.4-170056253	CCCACAGACACTGCTCCTCCCCCCTGC	2	C	0.094	0.1049
NC_005101.4-170074631	ATGTTTCCCACCCCTTCTCCCTCACCT	25	C	0.1739	0.2758
NC_005101.4-170333175	CTAAAATACACTACCTCTCCTACCTTA	24	C	0.0847	0.0965
NC_005101.4-170349225	ACAAAAAGTACTGTGACTCCTGAGAGC	6	A	0.0259	0.0255
NC_005101.4-170440226	CTGCTTGTTACTACATCTCCAGGATAT	8	T	0.115	0.4774
NC_005101.4-170440226	CTGCTTGTTACTACATCTCCAGGATAT	25	T	0	0.4846
NC_005101.4-170450111	CTGTAGTTTACAGTGACTCCATAAGTC	13	G	0.1947	0.2036
NC_005101.4-170450111	CTGTAGTTTACAGTGACTCCATAAGTC	25	G	0.1913	0.264
NC_005101.4-170481224	CCCCCCATTACCTCCTCTCCTTCTCAG	15	C	0.1933	0.2012
NC_005101.4-170535836	GTAGCACTTACTGACTCTCCACACATT	24	C	0.1488	0.2759
NC_005101.4-170535836	GTAGCACTTACTGACTCTCCACACATT	25	A	0.1488	0.2759
NC_005101.4-170547446	TATCCTGCTACTCCTCCTCCTCTTTCC	6	T	0.2975	0.4481
NC_005101.4-170547446	TATCCTGCTACTCCTCCTCCTCTTTCC	15	T	0.0083	0.4803
NC_005101.4-170592730	ATGGTCTCTACAGGTGCTCCCTTTCCT	6	C	0.15	0.1932
NC_005101.4-170814322	GCTTCATATACAAGCACTCCCTCAGCA	9	T	0	0.4839
NC_005101.4-170814322	GCTTCATATACAAGCACTCCCTCAGCA	23	C	0.1695	0.1827
NC_005101.4-170817929	AAGATTCAGACATGTGCTCCCTGCCTC	3	G	0.0531	0.0517
NC_005101.4-170839763	ACCTTGATCACATGATCTCCTTGGCTT	24	G	0	0.4555
NC_005101.4-170842377	ATTCATTCCACGTTGTCTCCGTGTGGT	12	G	0.2417	0.49
NC_005101.4-170842377	ATTCATTCCACGTTGTCTCCGTGTGGT	21	G	0	0.4751

NC_005101.4-170888226	AACCCTGACACCATTACTCCTGCTATG	3	C	0.2051	0.2361
NC_005101.4-170888226	AACCCTGACACCATTACTCCTGCTATG	23	C	0.2051	0.2361
NC_005101.4-170895595	TTCAACAGTACCTAATCTCCAACTAGG	14	A	0.0508	0.0655
NC_005101.4-170932124	TATTTACTCACCAAAGCTCCTCAGTAT	24	G	0.2414	0.4144
NC_005101.4-171258213	GGAAAACTGACTTATGCTCCTGCTGTT	5	A	0.1565	0.5
NC_005101.4-171337753	AATATCTCTACATTAACTCCAACATTG	3	T	0.0588	0.0882
NC_005101.4-171385972	ATGAAATAAACAACTCCTCCACCCTTC	5	A	0	0.4751
NC_005101.4-171508381	AAGATAGGCACAGGATCTCCAAACAAC	14	G	0.281	0.3645
NC_005101.4-171596322	ACAATGGGGACACATGCTCCACTATGT	9	G	0.5726	0.4087
NC_005101.4-171596322	ACAATGGGGACACATGCTCCACTATGT	13	C	0.4622	0.3643
NC_005101.4-171639137	GGCACACTCACCCGGTCTCCTGGAGGT	14	G	0.0342	0.05
NC_005101.4-171639137	GGCACACTCACCCGGTCTCCTGGAGGT	27	T	0	0.4851
NC_005101.4-171885168	ACAACAAAGACATATGCTCCATTATTT	15	T	0.4912	0.4501
NC_005101.4-171941015	GGTTCTGTTACTCTTTCTCCTCTCTCT	7	G	0.1356	0.1961
NC_005101.4-171967341	CAGATATCTACATGGTCTCCCTCATCA	14	G	0.042	0.0411
NC_005101.4-172109012	GTAGCAAAAACCTCAGCTCCTTCAGTC	1	G	0.1833	0.4644
NC_005101.4-172109012	GTAGCAAAAACCTCAGCTCCTTCAGTC	9	A	0	0.4728
NC_005101.4-172157418	CATTCTCCCACCCCCTCTCCACATCTG	8	C	0.0732	0.1287
NC_005101.4-172157418	CATTCTCCCACCCCCTCTCCACATCTG	15	C	0.0407	0.0705
NC_005101.4-172221243	TGACAGATGACTGCAGCTCCTGTAAAC	14	C	0	0.4779
NC_005101.4-172221243	TGACAGATGACTGCAGCTCCTGTAAAC	22	G	0.0508	0.4908
NC_005101.4-172297558	GGTGGAGACACTAGGCCTCCATGGAAT	22	T	0.0678	0.0812
NC_005101.4-172330658	TTGAGCTCCACTCAGCCTCCAAGGTGA	8	C	0.0351	0.0345
NC_005101.4-172330658	TTGAGCTCCACTCAGCCTCCAAGGTGA	15	G	0.2632	0.3793
NC_005101.4-172411818	GTCTCTGGGACCAGCTCTCCTTCCCAT	1	G	0.0488	0.1073
NC_005101.4-172429279	AGCTACAAAACCCTCACTCCGCGTCCC	7	A	0	0.4793
NC_005101.4-172458180	TGACTCACAACCTAGTCTCCATAAGTA	14	A	0	0.0328
NC_005101.4-172478923	AGAGTACATACACAGCCTCCCCTCTGT	9	T	0.1293	0.5
NC_005101.4-172499632	CTGGTGAAAACAGGAGCTCCATAAACA	12	A	0.0796	0.1084
NC_005101.4-172521482	ATGTGCCTCACCCCAACTCCCTGCCCC	26	C	0	0.4728
NC_005101.4-172537631	GACCTTGGAACACTCACTCCTAACCGG	13	C	0.1538	0.2107
NC_005101.4-172537631	GACCTTGGAACACTCACTCCTAACCGG	26	G	0.1053	0.1305
NC_005101.4-172572417	ATGAGCAGAACTGACACTCCTTGTCAG	1	A	0.2087	0.3593
NC_005101.4-172572417	ATGAGCAGAACTGACACTCCTTGTCAG	6	C	0.1593	0.2302
NC_005101.4-172622233	TTACAGATAACTGGGACTCCTATCAAG	27	G	0.1864	0.2219
NC_005101.4-172624547	TGCTGCCATACCTTTGCTCCCCCATTG	12	C	0.0431	0.0746
NC_005101.4-172624547	TGCTGCCATACCTTTGCTCCCCCATTG	13	T	0.1379	0.4786
NC_005101.4-172626842	AATGAGGCCACAAGAGCTCCAATGAGT	1	A	0.2377	0.3984
NC_005101.4-172648428	ATTTACATCACTCCTCCTCCTCCTGCT	22	C	0.157	0.4999
NC_005101.4-172672458	TTCTTCAGCACCATGACTCCTGTGTGC	5	T	0.2703	0.2337
NC_005101.4-172687048	CCCAGATCCACTGTTCCTCCTCCATTT	15	T	0.2439	0.385
NC_005101.4-172687048	CCCAGATCCACTGTTCCTCCTCCATTT	16	C	0.1057	0.1695
NC_005101.4-172687350	ATTCTGTGGACCAGTTCTCCTGGTGTC	14	G	0.339	0.2815
NC_005101.4-172704143	TTAAATGTCACTTGCTCTCCACAGTAT	1	T	0.0085	0.479
NC_005101.4-172737121	AGCTGACTGACAGGCTCTCCCTTGCTT	9	G	0.0252	0.0249
NC_005101.4-172737121	AGCTGACTGACAGGCTCTCCCTTGCTT	26	T	0.1803	0.4463
NC_005101.4-172778299	ACAATCCTGACCTTGCCTCCTTTCTTC	4	A	0.1803	0.2741
NC_005101.4-172784870	ATCAAATTTACCTTTTCTCCTCAATCA	25	T	0.1304	0.4946
NC_005101.4-172784870	ATCAAATTTACCTTTTCTCCTCAATCA	27	A	0.1304	0.4946
NC_005101.4-172838150	CTTGAGAACACAAACTCTCCCGATGAA	21	C	0	0.4839
NC_005101.4-172872190	AGTGGGGGAACCATGGCTCCAGATGCA	1	A	0.0339	0.0812

NC_005101.4-172872190	AGTGGGGGAACCATGGCTCCAGATGCA	22	G	0.2083	0.4847
NC_005101.4-172910066	CTGCCTATAACTGCAGCTCCAGGAAAT	2	T	0.2017	0.3118
NC_005101.4-172956007	CTGTTGAATACTTGGCCTCCAGCTGTC	26	T	0.1707	0.3091
NC_005101.4-172992465	AACATGTCCACAAGTTCTCCGGCTCTC	8	C	0	0.4709
NC_005101.4-173025702	CCTGTTCTGACTCATTCTCCATCATTC	2	C	0.2787	0.4515
NC_005101.4-173027433	ACGTTTCACACCTAGTCTCCCTTCTCC	2	C	0	0.4887
NC_005101.4-173146755	AATCCCTTTACCACCCCTCCCAACATT	2	A	0	0.4793
NC_005101.4-173209630	GAGTGATCCACACCATCTCCCCTTCCC	7	T	0.177	0.4404
NC_005101.4-173209630	GAGTGATCCACACCATCTCCCCTTCCC	14	C	0.2035	0.4098
NC_005101.4-173271268	CCATAAGCCACATCCTCTCCCCCTCCC	15	C	0	0.0348
NC_005101.4-173271268	CCATAAGCCACATCCTCTCCCCCTCCC	21	C	0.0354	0.1006
NC_005101.4-173336900	CCGTGTGACACACTTCCTCCACACTAC	2	C	0	0.4807
NC_005101.4-173349078	AGAGCAGTGACTACTTCTCCATAGGGT	7	G	0.0446	0.0605
NC_005101.4-173478656	AGCAACCATACTGAAGCTCCAAGCACA	22	A	0.1062	0.1898
NC_005101.4-173679196	CTGCCTAGCACACCATCTCCAAAACAT	7	A	0	0.4773
NC_005101.4-173679196	CTGCCTAGCACACCATCTCCAAAACAT	13	C	0.0246	0.0243
NC_005101.4-173687117	AATAATAATACCTCAACTCCTATTCCC	26	C	0.1696	0.1983
NC_005101.4-173697990	CAGTGTACTACTTATGCTCCCTGCTTT	22	T	0.0244	0.0241
NC_005101.4-173702699	GTCTCAGGAACCCACACTCCGACTGAG	2	T	0.1017	0.4425
NC_005101.4-173702699	GTCTCAGGAACCCACACTCCGACTGAG	6	A	0	0.4844
NC_005101.4-173765524	CAAGGAGCAACACGGACTCCATGTTTG	13	C	0.041	0.4588
NC_005101.4-173851318	TAATGAGTAACACATTCTCCCTCAATT	1	T	0.0708	0.1006
NC_005101.4-173863104	GGGCTCTTTACTCTGCCTCCTATCTCA	3	G	0	0.4793
NC_005101.4-173863104	GGGCTCTTTACTCTGCCTCCTATCTCA	4	C	0.0894	0.1145
NC_005101.4-173882281	CTTTATAACACCTTAACTCCTGTACAG	1	C	0.1138	0.1494
NC_005101.4-173885895	GAACACAGAACAGTGGCTCCCAAGCAT	13	G	0.0175	0.4875
NC_005101.4-173889024	CCAGACCCTACCAGAGCTCCCAGGGAG	1	C	0.0413	0.0562
NC_005101.4-173889024	CCAGACCCTACCAGAGCTCCCAGGGAG	21	C	0.1417	0.1316
NC_005101.4-173956395	AGTTAAAGCACAATATCTCCTAGTTCT	2	G	0.1161	0.4996
NC_005101.4-173972087	CCATTTACCACCACTGCTCCTTATTTA	1	C	0.0088	0.4693
NC_005101.4-173986039	CACTCTACAACAACAGCTCCATCTGCT	9	A	0.1138	0.5
NC_005101.4-174019891	GCTCCTTCTACTGCTTCTCCTCCTGTT	4	C	0.1754	0.474
NC_005101.4-174107682	TATGTCCATACCTAGCCTCCCAATGAG	2	A	0.1983	0.3956
NC_005101.4-174107682	TATGTCCATACCTAGCCTCCCAATGAG	15	G	0.1983	0.3956
NC_005101.4-174140431	TCATTGGGAACATTTTCTCCTTAGTAG	14	T	0	0.4755
NC_005101.4-174140431	TCATTGGGAACATTTTCTCCTTAGTAG	21	T	0.0265	0.06
NC_005101.4-174165479	AGCTTCCTTACTAATGCTCCAATGCCA	25	C	0	0.4891
NC_005101.4-174298258	AGTTTGAAGACACATGCTCCACTATGT	6	G	0.9328	0.4977
NC_005101.4-174298258	AGTTTGAAGACACATGCTCCACTATGT	9	G	0.9322	0.4977
NC_005101.4-174314446	GGTGCGGCTACATCACCTCCACACAGG	8	C	0.0087	0.0257
NC_005101.4-174314446	GGTGCGGCTACATCACCTCCACACAGG	24	C	0.0261	0.0257
NC_005101.4-174325485	TGAGACAGGACACTACCTCCCAGAGTA	26	T	0.2787	0.2956
NC_005101.4-174341574	TATTCAGATACCAGAGCTCCTTTAATA	1	T	0.0084	0.4761
NC_005101.4-174341574	TATTCAGATACCAGAGCTCCTTTAATA	3	T	0.2712	0.3529
NC_005101.4-174369244	GTAGGAGAAACAACATCTCCTTAGCTG	24	G	0.1638	0.1646
NC_005101.4-174407451	AACAGTGAGACTGGGACTCCTTACACT	14	G	0.2764	0.3239
NC_005101.4-174407451	AACAGTGAGACTGGGACTCCTTACACT	22	T	0.2764	0.4793
NC_005101.4-174434764	AGTCCTGTCACTCTCTCTCCTTGCCTG	4	C	0.2353	0.4898
NC_005101.4-174454789	TATCCTGAGACCACCACTCCCTTAATT	2	A	0.0714	0.0689
NC_005101.4-174454789	TATCCTGAGACCACCACTCCCTTAATT	3	T	0.0714	0.0689
NC_005101.4-174568964	ACTGTACCTACATGACCTCCAGCATCA	23	C	0.0082	0.479

NC_005101.4-174582221	GGCTTTGTCACTACATCTCCTTCATTT	12	T	0.2101	0.2266
NC_005101.4-174710511	TTTGTGTCGACCAGCACTCCTGAGCAT	9	G	0.2727	0.3687
NC_005101.4-174887650	TAATGTGGCACTTTTGCTCCATTGCAA	9	C	0.2479	0.4308
NC_005101.4-174887650	TAATGTGGCACTTTTGCTCCATTGCAA	24	G	0.0246	0.0557
NC_005101.4-174981929	TCTTCCATTACCCTTCCTCCCTCCTTC	6	C	0.0252	0.0249
NC_005101.4-174981929	TCTTCCATTACCCTTCCTCCCTCCTTC	24	C	0.042	0.0882
NC_005101.4-175232531	AGTGATGTGACAGGCACTCCTTACGTC	9	G	0.0261	0.0257
NC_005101.4-175232531	AGTGATGTGACAGGCACTCCTTACGTC	24	C	0.0085	0.4823
NC_005101.4-175433696	AATCCAGAGACTGCCCCTCCAGTGTAT	22	G	0.2261	0.4864
NC_005101.4-175603020	ATGGTCTCTACTTTAGCTCCTTTCTCT	22	T	0	0.4827
NC_005101.4-175873886	AAACATTGAACAATCTCTCCACCCCTT	13	A	0.3214	0.4871
NC_005101.4-176038935	AGGGTTGTGACTAACTCTCCTGCATTA	3	G	0.0086	0.4768
NC_005101.4-176165873	TTTCCCCTGACCTGATCTCCCTTACAA	13	T	0.0442	0.0433
NC_005101.4-176184177	GTGTCACACACACACACTCCCTTCTCA	7	C	0.0531	0.0683
NC_005101.4-176184177	GTGTCACACACACACACTCCCTTCTCA	8	A	0.0265	0.4693
NC_005101.4-176520064	TATCAACAAACCAGACCTCCACTACTC	7	C	0.0574	0.0557
NC_005101.4-176602085	TCACTCTTCACTCAATCTCCTTCTAAT	14	A	0.188	0.2235
NC_005101.4-176805448	GCCCTTCCCACTCTCCCTCCTCTTCCT	15	C	0.2411	0.4952
NC_005101.4-176859964	TCCAATCTCACTCATCCTCCATACCCT	1	T	0.2203	0.4173
NC_005101.4-176859964	TCCAATCTCACTCATCCTCCATACCCT	2	C	0.1966	0.3311
NC_005101.4-176970456	GATCCATTCACTTTCTCTCCATCCATG	12	T	0.0088	0.0433
NC_005101.4-177029336	TTCCCCATTACTCCTGCTCCATTCTTC	3	C	0.0684	0.4938
NC_005101.4-177029336	TTCCCCATTACTCCTGCTCCATTCTTC	9	T	0.0339	0.0333
NC_005101.4-177032555	CACTCTTACACACCAACTCCAACCTTA	21	A	0.0417	0.0875
NC_005101.4-177032555	CACTCTTACACACCAACTCCAACCTTA	24	C	0.0417	0.0875
NC_005101.4-177217592	AATCAAATCACCCTTTCTCCTCTTCTC	24	T	0.1597	0.4949
NC_005101.4-177418375	GTGCCCTTCACCTTGACTCCTTCTGTA	9	C	0.188	0.2945
NC_005101.4-177437014	CTCATCCATACCAACTCTCCAAACCCT	5	T	0.3333	0.4644
NC_005101.4-177437014	CTCATCCATACCAACTCTCCAAACCCT	21	A	0.1583	0.1733
NC_005101.4-177468188	CAGGTACTGACAGAACCTCCCAAGGAA	9	G	0.1552	0.4666
NC_005101.4-177512119	GTGCAAAGGACAGCTACTCCTGAACAC	27	C	0.1339	0.125
NC_005101.4-177529331	GCCGTGATGACCTAAGCTCCAACTTAA	4	G	0.065	0.0928
NC_005101.4-177616531	AGTAGAGTCACACCCACTCCAGTAAAA	25	A	0	0.4709
NC_005101.4-177616531	AGTAGAGTCACACCCACTCCAGTAAAA	27	A	0.2435	0.3403
NC_005101.4-177629797	CAGGGCTCAACAGTTACTCCTACTAAT	7	T	0.2195	0.2885
NC_005101.4-177629797	CAGGGCTCAACAGTTACTCCTACTAAT	9	A	0.122	0.1562
NC_005101.4-177698930	TCTTTGGCTACACATTCTCCACCACTG	1	T	0.082	0.1082
NC_005101.4-177698930	TCTTTGGCTACACATTCTCCACCACTG	13	C	0.0246	0.0243
NC_005101.4-177745306	GCCAGTTGTACACTTTCTCCACAGAGC	1	G	0	0.4844
NC_005101.4-177763095	AGCTACTAGACCCACTCTCCCAAGCAC	3	C	0.0678	0.1116
NC_005101.4-177763095	AGCTACTAGACCCACTCTCCCAAGCAC	7	T	0.2222	0.2235
NC_005101.4-177783565	CTTTGTGCCACCCATGCTCCCGTAAGT	23	T	0.1161	0.1699
NC_005101.4-177862047	GCAAGACCAACAAGTGCTCCTAACTGT	4	A	0.0813	0.2017
NC_005101.4-177862047	GCAAGACCAACAAGTGCTCCTAACTGT	7	C	0.5738	0.416
NC_005101.4-177872849	CATCTGGATACGTCACCTCCACCGAAC	23	C	0.2308	0.4906
NC_005101.4-177924904	TACTTACAGACTGCTTCTCCTGCTGGC	9	G	0.0265	0.0262
NC_005101.4-177978925	GGTGGTGGTACTGTCTCTCCTGTTGAC	2	G	0.2458	0.4454
NC_005101.4-177978925	GGTGGTGGTACTGTCTCTCCTGTTGAC	9	T	0	0.4757
NC_005101.4-177989559	TTCCTGGGCACTAGACCTCCTGATGCC	22	G	0.1138	0.1356
NC_005101.4-178005593	AAACTCTACACTCAATCTCCCAAATGC	14	A	0.0084	0.4829
NC_005101.4-178005593	AAACTCTACACTCAATCTCCCAAATGC	16	T	0.0085	0.4842

NC_005101.4-178029678	CCTGTTCCCACACTTCCTCCCTCCATC	5	T	0.0924	0.1033
NC_005101.4-178048122	ATGCTGTAAACTTCTCCTCCATCTTTA	8	A	0.0163	0.032
NC_005101.4-178156631	TGAGGCTCCACTCCTCCTCCCTGAAGA	12	T	0.0085	0.487
NC_005101.4-178156631	TGAGGCTCCACTCCTCCTCCCTGAAGA	14	C	0.0085	0.487
NC_005101.4-178162274	CCAGATGTTACCGGAGCTCCTAGAGGG	12	C	0.0588	0.4886
NC_005101.4-178233827	ACCAGCTGTACCCAGGCTCCTGCTGCA	27	A	0	0.4819
NC_005101.4-178234936	ACAACTAATACTACGCCTCCTAGAACT	14	C	0.0982	0.0934
NC_005101.4-178237651	GAAAAGTACACTGAGTCTCCTTTAGGT	12	T	0.3051	0.4367
NC_005101.4-178267989	GCCTGAGCTACAGAGCCTCCCTACAAC	2	C	0	0.48
NC_005101.4-178267989	GCCTGAGCTACAGAGCCTCCCTACAAC	3	C	0.125	0.4991
NC_005101.4-178270753	ACCAGCATCACAGCTGCTCCCAGGGTT	4	A	0	0.4718
NC_005101.4-178316442	TGAGTTCAAACACTTCCTCCTGGAGAC	22	G	0.0833	0.1528
NC_005101.4-178317798	GTACCTGAGACCAGATCTCCAGGACCC	12	C	0.0083	0.0247
NC_005101.4-178317798	GTACCTGAGACCAGATCTCCAGGACCC	24	A	0	0.4765
NC_005101.4-178338309	GATAATTGAACATTTCCTCCTTTTAGA	7	T	0.018	0.4821
NC_005101.4-178339789	TGCTCTCAAACTCCAGCTCCTCTTGGT	24	T	0	0.4827
NC_005101.4-178347351	AGGAGATCTACTTGTTCTCCTAGTCCA	22	A	0	0.4772
NC_005101.4-178347948	GTTCAGAGGACCCAAGCTCCAGATGTT	1	G	0.0254	0.0251
NC_005101.4-178347948	GTTCAGAGGACCCAAGCTCCAGATGTT	15	A	0.1864	0.2219
NC_005101.4-178353285	CCACCTATGACAGCAGCTCCAGGGGAA	25	G	0.0894	0.1562
NC_005101.4-178393585	TAAATTCCTACCTTGCCTCCACTTCCA	7	C	0.0254	0.0415
NC_005101.4-178393585	TAAATTCCTACCTTGCCTCCACTTCCA	27	A	0.1271	0.1337
NC_005101.4-178396434	GTCACATATACCCGGCCTCCCTGCCTC	7	T	0.2131	0.4515
NC_005101.4-178396434	GTCACATATACCCGGCCTCCCTGCCTC	8	A	0.2131	0.3791
NC_005101.4-178402725	GTGCTCAGCACTTTACCTCCATGCAGT	1	G	0.25	0.4728
NC_005101.4-178402725	GTGCTCAGCACTTTACCTCCATGCAGT	3	G	0.2583	0.325
NC_005101.4-178404427	CATACACAAACAAAATCTCCAAACATT	8	A	0.2	0.2332
NC_005101.4-178404427	CATACACAAACAAAATCTCCAAACATT	25	A	0.0431	0.4893
NC_005101.4-178466466	AGCAGGGTTACTCAGTCTCCCCAGCCT	2	G	0	0.4743
NC_005101.4-178466466	AGCAGGGTTACTCAGTCTCCCCAGCCT	22	C	0.2627	0.2871
NC_005101.4-178622909	ACCCCCTCCACTCAGGCTCCATTCTCT	25	T	0	0.4765
NC_005101.4-178623685	TGTGCAGAGACACGTACTCCTTCCTCG	13	C	0.0085	0.4671
NC_005101.4-178623685	TGTGCAGAGACACGTACTCCTTCCTCG	27	G	0.0513	0.05
NC_005101.4-178658334	GTGTGTGTGACACTGTCTCCATCTCAG	9	G	0.0488	0.0476
NC_005101.4-178682357	AACCAGTGAACTCGGCCTCCTTAATAA	1	A	0.0508	0.0496
NC_005101.4-178682357	AACCAGTGAACTCGGCCTCCTTAATAA	13	C	0.1271	0.1337
NC_005101.4-178705573	CTTTAACCCACACATTCTCCGTAAGCT	25	G	0.1327	0.1968
NC_005101.4-178707036	TCTTCAGTAACACTCCCTCCTGTGTAC	12	A	0.1261	0.3554
NC_005101.4-178717269	TGCCTCAAAACCTATGCTCCATGTTAA	22	T	0.2195	0.3964
NC_005101.4-178747460	TTTATACACACCCCTACTCCCTCCTCT	5	T	0.0171	0.4772
NC_005101.4-178787844	GGCAGCCACACTACAGCTCCACTCCAG	12	T	0	0.4773
NC_005101.4-178800982	GCATCCATAACTCCAGCTCCAGAGGAT	1	G	0	0.4728
NC_005101.4-178804703	ATCACATCTACACTGACTCCTAATGCA	3	C	0.0427	0.0418
NC_005101.4-178854203	GCTGTTGAGACAGTTTCTCCAGGTGTC	23	G	0	0.4749
NC_005101.4-178867417	CTCGTTACCACTCTGCCTCCTGACTGC	3	C	0.1983	0.4426
NC_005101.4-178921266	CTGCTGTCCACTCCCACTCCCATGCTC	8	C	0.2389	0.4599
NC_005101.4-178988473	TAATTTGAAACATAATCTCCAATGTGA	1	T	0.2966	0.4655
NC_005101.4-179029864	GTTCACTGAACCCTGTCTCCTTCTCTG	2	T	0	0.4793
NC_005101.4-179031233	GAAATGGCAACCTTGACTCCTTTGGTA	25	G	0	0.48
NC_005101.4-179090936	CCCATCACAACACATGCTCCTCTGTAT	25	T	0.0413	0.0405
NC_005101.4-179104458	AATTATTTGACATTCTCTCCTTCCACA	22	T	0.009	0.044

NC_005101.4-179115448	GCAGCCATGACTGATGCTCCTGCCTAC	2	C	0.2083	0.2722
NC_005101.4-179121845	GTGTTGTCTACAGTCGCTCCATATGTT	4	T	0.0619	0.4493
NC_005101.4-179123588	ATGGTGGACACTGTGGCTCCTCAGCTT	16	G	0.0081	0.4777
NC_005101.4-179131041	GGGTAGGGGACTTGATCTCCTTTATCA	3	G	0.1429	0.1746
NC_005101.4-179308715	AAGTAAATCACTTGTGCTCCAATATTC	12	T	0.2459	0.2279
NC_005101.4-179308715	AAGTAAATCACTTGTGCTCCAATATTC	26	T	0	0.4751
NC_005101.4-179406818	TTCCTTTTTACACAGTCTCCTTTCCTT	24	C	0.1176	0.4991
NC_005101.4-179551959	ATTGTATAAACTCCAGCTCCAGAGGAT	2	T	0.1339	0.498
NC_005101.4-179559586	AATCTCCCAACATTAGCTCCTTTCCAG	24	C	0.1404	0.1454
NC_005101.4-179608334	ACCCCGTAGACAAGCTCTCCAACCATA	6	G	0	0.4813
NC_005101.4-179608334	ACCCCGTAGACAAGCTCTCCAACCATA	15	C	0.1949	0.3833
NC_005101.4-179663854	GCCTTTCTGACTCAGCCTCCTGAGCGT	16	C	0.0661	0.0639
NC_005101.4-179680713	CAACTCTGCACTGTTTCTCCACAAGAC	3	A	0.2627	0.4655
NC_005101.4-179680713	CAACTCTGCACTGTTTCTCCACAAGAC	14	T	0	0.4718
NC_005101.4-179754822	TCACATCCTACTACATCTCCTTAGGAG	25	G	0.0172	0.488
NC_005101.4-179782094	CCTCTCCCCACCTGTACTCCACAGCTC	1	C	0.0672	0.065
NC_005101.4-179811243	CAGACCGATACAGGAGCTCCCCTTTTC	7	G	0.15	0.1387
NC_005101.4-179819514	CCAAAGCAGACGTATCCTCCTGACCTA	27	A	0.009	0.4584
NC_005101.4-179833883	TATACAGAAACCTAGGCTCCAAGCACC	1	T	0.0256	0.0253
NC_005101.4-179833883	TATACAGAAACCTAGGCTCCAAGCACC	13	T	0.2456	0.396
NC_005101.4-179847308	GTCACTTCCACATACTCTCCTGACACA	7	T	0.0333	0.48
NC_005101.4-179893980	ACACACTTGACTTTCTCTCCTGAGAAA	24	G	0.0354	0.1006
NC_005101.4-179893980	ACACACTTGACTTTCTCTCCTGAGAAA	27	A	0.0354	0.1006
NC_005101.4-179935791	GCGTCTGTGACCCAACCTCCTAAAGGA	8	T	0.0796	0.0765
NC_005101.4-179976183	AGACATTGAACGTGTTCTCCTCCACAC	2	G	0	0.4826
NC_005101.4-179976183	AGACATTGAACGTGTTCTCCTCCACAC	12	G	0.0847	0.1551
NC_005101.4-180057677	ATTCCATTCACACAAGCTCCTTAAACA	25	A	0.0574	0.071
NC_005101.4-180057677	ATTCCATTCACACAAGCTCCTTAAACA	26	C	0.2459	0.4737
NC_005101.4-180095843	ATCAAATCCACTTCCACTCCATTCCTC	1	A	0.2083	0.315
NC_005101.4-180095843	ATCAAATCCACTTCCACTCCATTCCTC	26	T	0.1624	0.3771
NC_005101.4-180243045	TTCCTCCCCACCTTCTCTCCCAGTCAT	12	C	0.042	0.0411
NC_005101.4-180271021	TTCTCTGAAACAGTAGCTCCCTCTAAA	8	A	0	0.4813
NC_005101.4-180301081	ATCATCGAAACATCCCCTCCTTAGGGA	7	G	0	0.4846
NC_005101.4-180301081	ATCATCGAAACATCCCCTCCTTAGGGA	25	G	0.0263	0.4889
NC_005101.4-180465036	CTAACTTTCACATGCACTCCATAGTGC	27	C	0.125	0.1866
NC_005101.4-180475349	ATTTTATGAACCATTGCTCCAAAGCCT	4	T	0.0248	0.0245
NC_005101.4-180724121	CCTGGCCTTACTGGGACTCCATCAAGT	5	G	0.0246	0.0401
NC_005101.4-180770896	TTCACATAAACCACTTCTCCTACCAAA	4	A	0.2353	0.2796
NC_005101.4-180817669	CCCCTTCACACAATCGCTCCTCCCATC	22	C	0.2232	0.3705
NC_005101.4-180825880	ATTAAGTGGACTTCTTCTCCCCAACCT	15	T	0.0973	0.1239
NC_005101.4-180840028	CACTCAAGGACTTGGTCTCCAGTTAGC	7	A	0.0089	0.4789
NC_005101.4-180843381	GACAAGTTGACCCCACCTCCACCTTGA	9	G	0.0081	0.0553
NC_005101.4-180858213	CTGTTCTTGACTTCATCTCCTGATGGA	6	C	0.0435	0.0425
NC_005101.4-180878849	AGGCTCTGGACTTGATCTCCGATACAG	12	T	0.2459	0.4698
NC_005101.4-180878849	AGGCTCTGGACTTGATCTCCGATACAG	21	G	0.0325	0.0476
NC_005101.4-180926824	TCTTCTCTAACACTATCTCCTACTTTC	4	T	0.0087	0.4817
NC_005101.4-181103539	TCTGGTCTCACTGAGGCTCCCCACATA	15	G	0.2212	0.4242
NC_005101.4-181109744	CCCTGGTCCACTCCTCCTCCATTTCCC	7	T	0.2333	0.32
NC_005101.4-181162935	GCCCTTGGTACTGTTCCTCCAGCAATG	1	G	0.1864	0.4977
NC_005101.4-181162935	GCCCTTGGTACTGTTCCTCCAGCAATG	23	C	0.0254	0.0251
NC_005101.4-181216980	CCCCTTCCAACTAACCCTCCGCCATTC	1	C	0	0.4703

NC_005101.4-181253973	TGACCTCACACATTAGCTCCACGGTTA	22	C	0.0667	0.0644
NC_005101.4-181263572	TCTCCGCACACCAGCTCTCCACCTGAG	5	C	0	0.4793
NC_005101.4-181263572	TCTCCGCACACCAGCTCTCCACCTGAG	7	C	0.2927	0.3516
NC_005101.4-181311254	ATCAATGGAACACGAACTCCGACTGTT	13	C	0	0.4755
NC_005101.4-181311254	ATCAATGGAACACGAACTCCGACTGTT	23	C	0	0.4755
NC_005101.4-181350038	GATGAGCTCACAAACCCTCCTTTGAAT	6	G	0.2479	0.3811
NC_005101.4-181474401	CACATAAATACATTAACTCCATCATAT	7	A	0	0.4749
NC_005101.4-181474401	CACATAAATACATTAACTCCATCATAT	14	T	0	0.4749
NC_005101.4-181478217	AACTACACCACATCCCCTCCTTCTTTT	13	T	0.0661	0.0639
NC_005101.4-181484661	TGTACACTTACTGCTGCTCCCAACAAT	4	A	0.0488	0.0476
NC_005101.4-181484661	TGTACACTTACTGCTGCTCCCAACAAT	9	T	0.0574	0.4879
NC_005101.4-181514351	CCCTCCTGTACCCTGCCTCCCATCCCC	8	G	0.1148	0.1082
NC_005101.4-181514351	CCCTCCTGTACCCTGCCTCCCATCCCC	16	C	0.1148	0.1082
NC_005101.4-181535542	CATTTCTACACATGGACTCCTTCTCTT	9	C	0	0.4779
NC_005101.4-181540382	CACGATGCTACATGTTCTCCACGTAGT	5	A	0	0.4859
NC_005101.4-181540382	CACGATGCTACATGTTCTCCACGTAGT	21	A	0.0088	0.06
NC_005101.4-181541018	CTTAAGTCCACACAGCCTCCATGTGTG	7	T	0.1545	0.4999
NC_005101.4-181541018	CTTAAGTCCACACAGCCTCCATGTGTG	22	T	0.1626	0.4997
NC_005101.4-181543906	ATTCTGGGAACAGTTGCTCCAGGGTCC	2	T	0.1416	0.4996
NC_005101.4-181543906	ATTCTGGGAACAGTTGCTCCAGGGTCC	22	G	0.1416	0.4996
NC_005101.4-181564325	TGTTAAGACACAGGATCTCCCTATGAT	1	T	0.0526	0.0512
NC_005101.4-181595574	CACCACCTAACCTCAGCTCCTATAAGT	15	A	0.0261	0.0257
NC_005101.4-181595574	CACCACCTAACCTCAGCTCCTATAAGT	24	A	0	0.4778
NC_005101.4-181662021	TTGTGGAAAACTTCTCCTCCAATGTGT	16	C	0.009	0.4838
NC_005101.4-181680511	TTACAAATGACCCGACCTCCCATTCCA	13	C	0.2735	0.3895
NC_005101.4-181680511	TTACAAATGACCCGACCTCCCATTCCA	14	G	0.0256	0.0253
NC_005101.4-181735247	TTCCCAATGACTCCAGCTCCTTCCTAG	7	A	0.0957	0.1938
NC_005101.4-181762954	GACAACCTCACTTGCTCTCCAGGGCCC	22	G	0	0.477
NC_005101.4-181775952	ATGTTATTCACTCTTCCTCCTTCTCTC	2	T	0	0.4659
NC_005101.4-181835057	TATCCCGAGACAGTGTCTCCTGTATCT	8	A	0	0.4793
NC_005101.4-181852434	CAGACAACCACCCGCCCTCCCTTTAAA	14	G	0.0174	0.0342
NC_005101.4-181859678	CCATGGTACACAGCACCTCCAGTCTAC	21	A	0	0.4793
NC_005101.4-181895782	CTCTTTAACACCCTCCCTCCCTGGCAT	16	C	0	0.4819
NC_005101.4-181905067	GAAGGAGGGACGCAGCCTCCAGCATTG	5	G	0.0246	0.4852
NC_005101.4-181920859	CACTGTGCAACTCTTTCTCCAGTGCCA	27	A	0	0.4728
NC_005101.4-181927137	ATTGACCTAACACACACTCCTTTCACC	7	C	0.0244	0.0241
NC_005101.4-181943517	ACGTTATGAACTTCGTCTCCTAACGAT	15	G	0.0333	0.0799
NC_005101.4-181967215	CTCCCTCCTACTTCTTCTCCATGTCTT	5	C	0.084	0.0805
NC_005101.4-182001695	CTTGTAGATACCAATTCTCCAAATTGA	8	A	0.0088	0.4676
NC_005101.4-182001695	CTTGTAGATACCAATTCTCCAAATTGA	23	A	0	0.4654
NC_005101.4-182002546	ATTTAACCCACAGCAGCTCCCTCCCCA	14	C	0.2906	0.3159
NC_005101.4-182011978	ACATCGAGAACCCTAGCTCCCATGTAC	5	C	0.0902	0.4489
NC_005101.4-182011978	ACATCGAGAACCCTAGCTCCCATGTAC	7	A	0.2314	0.4942
NC_005101.4-182051645	TGCCTTGGCACTGCATCTCCGGGCCGC	26	G	0.2397	0.4557
NC_005101.4-182077454	CTCACCCCTACCCCAGCTCCCCAGGGA	14	C	0.124	0.1447
NC_005101.4-182079758	AGATTCCATACATTCACTCCTCAGATT	2	G	0.3504	0.4994
NC_005101.4-182079758	AGATTCCATACATTCACTCCTCAGATT	27	T	0.2458	0.4396
NC_005101.4-182188214	TTTATTGAGACAGTGTCTCCCTAAGTT	23	A	0.1518	0.498
NC_005101.4-182205456	TCTCTCCTTACTCTTCCTCCCTTCCCC	23	T	0	0.4793
NC_005101.4-182238217	TAGACAGTCACATCTTCTCCACAGACA	5	C	0.0732	0.1145
NC_005101.4-182246471	GTTCACAAGACTGGGACTCCTGCGAAT	5	A	0.0252	0.0249

NC_005101.4-182273695	AACAGCCACACCCCTCCTCCTTTGTGC	3	C	0.0431	0.0422
NC_005101.4-182274031	CCAGTGAGAACTGCTGCTCCCCTGATC	14	C	0.0081	0.0241
NC_005101.4-182274031	CCAGTGAGAACTGCTGCTCCCCTGATC	24	G	0.2213	0.432
NC_005101.4-182285436	ATTTGTCTAACTTCTGCTCCCCAACAG	1	A	0.1538	0.4982
NC_005101.4-182317377	TCTTGGTTCACAGTTTCTCCATCAGTC	26	T	0.0588	0.4965
NC_005101.4-182432385	TTTGAATCAACAGAAGCTCCTTTGTTT	7	T	0.2564	0.4553
NC_005101.4-182455320	CCACCCTTCACCTCCCCTCCTGTCTCT	4	C	0.1736	0.3771
NC_005101.4-182455320	CCACCCTTCACCTCCCCTCCTGTCTCT	7	T	0.0992	0.1235
NC_005101.4-182535834	AATACTGTGACTGGCCCTCCCCACGTT	5	C	0	0.4698
NC_005101.4-182548290	CCATAGTAGACACATCCTCCTGACCTT	2	C	0.0082	0.4822
NC_005101.4-182649710	CTGGTTCTTACACTCACTCCACAGCCT	5	T	0.25	0.4483
NC_005101.4-182653171	ACAACCTGAACCTCCCCTCCTGCTTAG	7	T	0	0.48
NC_005101.4-182653171	ACAACCTGAACCTCCCCTCCTGCTTAG	9	A	0	0.4832
NC_005101.4-182666144	CAGTTATTGACTGACACTCCATAAGCA	13	G	0.0259	0.0255
NC_005101.4-182680075	GTGCTCAGCACTTTCTCTCCTCTTCAT	4	C	0.0488	0.0629
NC_005101.4-182691202	TTTATATGGACTGCATCTCCCATCAGT	22	A	0.3097	0.4309
NC_005101.4-182700023	AGGAACAACACAAATGCTCCACGTAAT	23	G	0.1161	0.1553
NC_005101.4-182747023	CCTCTCTCAACCCCTTCTCCCAGCTTC	4	C	0.0244	0.0398
NC_005101.4-182771192	CTTTCCCCAACTCTCCCTCCCTCCCTC	24	C	0.1167	0.1932
NC_005101.4-182812172	GTTCCACGTACACATTCTCCACACTAG	24	C	0	0.4773
NC_005101.4-182873367	GAGAGAAGGACTTGGTCTCCAATGCTG	1	G	0.1176	0.1946
NC_005101.4-182884419	CCATTGGCGACATGAGCTCCAGTTGCA	8	C	0.0976	0.4984
NC_005101.4-182911530	CTCTGTGCTACATACCCTCCTCTCTCC	6	T	0.0656	0.0634
NC_005101.4-182914481	CACAGAAAGACCTGCTCTCCAGGGCGT	15	C	0	0.4793
NC_005101.4-182914481	CACAGAAAGACCTGCTCTCCAGGGCGT	26	G	0	0.4793
NC_005101.4-182921486	TCTTCTGAGACATTCTCTCCACCTTGC	1	T	0.0083	0.4769
NC_005101.4-182926530	TTACCTGGCACTACTCCTCCAAGTGTG	26	T	0.1967	0.4995
NC_005101.4-182981824	AACTTCCAAACACTCTCTCCTTTCTAA	21	T	0	0.4779
NC_005101.4-182986419	CCCCTCGGAACGCAGTCTCCACCAGCG	27	G	0	0.4787
NC_005101.4-183007378	TTCCGGATAACTTCTCCTCCCAGAACA	5	G	0	0.4787
NC_005101.4-183055425	ATGGGAACAACTTTAGCTCCCACTGAG	4	G	0.1368	0.497
NC_005101.4-183058888	AGGCTCTGCACTCAACCTCCAGCATCA	16	C	0.0244	0.0241
NC_005101.4-183060020	ATGCCCCCAACTGCCACTCCAAGCCAC	12	T	0.2735	0.4602
NC_005101.4-183060020	ATGCCCCCAACTGCCACTCCAAGCCAC	26	A	0.1034	0.3282
NC_005101.4-183103615	CCTACATATACACAGCCTCCTCGCTAT	27	T	0.2066	0.259
NC_005101.4-183251537	TACATTCTCACAAACCCTCCTGAAGCT	21	T	0.1901	0.2474
NC_005101.4-183328975	AGCCGCTTTACTCAGTCTCCTGGTTTC	1	A	0.0085	0.0253
NC_005101.4-183328975	AGCCGCTTTACTCAGTCTCCTGGTTTC	4	C	0.094	0.1049
NC_005101.4-183337997	CTGAAGGGCACCTGCACTCCTTAGAAG	15	C	0.2114	0.2723
NC_005101.4-183367156	AAAATTAACACCAATGCTCCTCAGATT	7	A	0.0086	0.4768
NC_005101.4-183377461	ACATCCAGGACCTTAGCTCCACTACAC	23	T	0.1892	0.4998
NC_005101.4-183404447	TCCCTCTTCACCTCCTCTCCATCTCTC	8	T	0.1842	0.4289
NC_005101.4-183404447	TCCCTCTTCACCTCCTCTCCATCTCTC	23	C	0.0614	0.0758
NC_005101.4-183431135	AAGGCAAGAACTGTCTCTCCCATCCTC	14	T	0	0.4779
NC_005101.4-183432330	AACAACGTAACAGAGACTCCCAATGCT	21	C	0	0.4703
NC_005101.4-183446362	TACCCTGGTACTATGGCTCCTTGGAAG	2	A	0	0.482
NC_005101.4-183446362	TACCCTGGTACTATGGCTCCTTGGAAG	24	G	0	0.482
NC_005101.4-183482289	CCTGCACGCACGCTCCCTCCACTTTTG	12	G	0.2105	0.4255
NC_005101.4-183517722	GACGAACTTACTGTTACTCCCTACATG	4	G	0.0083	0.4816
NC_005101.4-183540570	TGAACATTAACTATGGCTCCTTAATTA	26	T	0	0.482
NC_005101.4-183551008	TCCTCCCTCACCATCTCTCCCTTGGTG	2	C	0.1186	0.2926

NC_005101.4-183587716	AAATGCAGGACCTTCACTCCTACAGCA	5	G	0.0569	0.0705
NC_005101.4-183587716	AAATGCAGGACCTTCACTCCTACAGCA	22	A	0.0244	0.0241
NC_005101.4-183621924	GCCTCCAGAACCTTCCCTCCCCCAACA	14	T	0.0256	0.058
NC_005101.4-183630453	GCAATGCTAACACGCTCTCCTACCATA	22	A	0.2213	0.2218
NC_005101.4-183630453	GCAATGCTAACACGCTCTCCTACCATA	26	T	0.041	0.0401
NC_005101.4-183685611	TAACAAAGCACCCCGCCTCCCTCACCC	15	G	0.1121	0.4893
NC_005101.4-183758339	TTGTCATTAACAGGGCCTCCAGACCAG	1	T	0	0.4787
NC_005101.4-183758339	TTGTCATTAACAGGGCCTCCAGACCAG	2	T	0.2213	0.2573
NC_005101.4-183783254	TTCCAGGTCACAGTCTCTCCTGCTAAG	8	T	0.2328	0.4866
NC_005101.4-183783254	TTCCAGGTCACAGTCTCTCCTGCTAAG	25	A	0.0085	0.4753
NC_005101.4-183784787	TATCGGTGCACATGTTCTCCCTTCAAG	14	G	0	0.4793
NC_005101.4-183894146	TATCCTTAGACACTCTCTCCCCTCAGC	14	T	0	0.4765
NC_005101.4-183933999	ACTGGCTCTACTAAGGCTCCTCAGTCA	24	G	0.3333	0.3381
NC_005101.4-183978966	ACAAAACACACAACGTCTCCTTCTGTG	1	A	0	0.48
NC_005101.4-183978966	ACAAAACACACAACGTCTCCTTCTGTG	9	C	0.1	0.095
NC_005101.4-183993667	AAGGACAGAACAAGCTCTCCTTCTCCA	5	A	0.0177	0.4859
NC_005101.4-184016274	CACACACACACACCTGCTCCTCTTTCA	13	C	0.0531	0.0517
NC_005101.4-184041859	CTGCAAGAAACGCTTTCTCCTTTCTAA	25	T	0.0085	0.481
NC_005101.4-184059445	CTAAACAACACCTTACCTCCCCAGAAT	27	T	0.0965	0.0918
NC_005101.4-184061423	ATCCTGAGAACCCTGACTCCCAAGTCC	26	C	0.1478	0.18
NC_005101.4-184069062	TGTGTGCACACATGTTCTCCGCCATGT	21	G	0.0345	0.0981
NC_005101.4-184073380	GACAATAACACTATCTCTCCTATCCCA	13	A	0.1917	0.325
NC_005101.4-184073380	GACAATAACACTATCTCTCCTATCCCA	26	C	0.0248	0.0245
NC_005101.4-184109639	AGGCTTGGCACACACCCTCCAAGACCA	24	A	0.0163	0.4759
NC_005101.4-184123068	GATGTGATGACGGTAACTCCGTCAACA	8	T	0.1933	0.4949
NC_005101.4-184123068	GATGTGATGACGGTAACTCCGTCAACA	21	G	0.2712	0.362
NC_005101.4-184165157	GGTGTGGGAACCCTGCCTCCTCAGCCT	7	G	0.2869	0.321
NC_005101.4-184165157	GGTGTGGGAACCCTGCCTCCTCAGCCT	24	G	0.2869	0.321
NC_005101.4-184213865	CTGCGCATGACCCCTCCTCCCATTTCT	1	C	0.1176	0.1539
NC_005101.4-184213865	CTGCGCATGACCCCTCCTCCCATTTCT	2	T	0.0254	0.0251
NC_005101.4-184227331	GACTGATTCACCACCTCTCCATTTTCC	22	T	0.1964	0.2188
NC_005101.4-184231480	GAGATCACCACCACTGCTCCTTGGTGA	12	C	0.0656	0.0786
NC_005101.4-184231480	GAGATCACCACCACTGCTCCTTGGTGA	16	G	0	0.4807
NC_005101.4-184252843	CTACTAAGAACCAGGGCTCCATACCTG	22	T	0	0.4772
NC_005101.4-184266115	TACAAGTGGACACTGGCTCCTGAAGTA	25	G	0.0342	0.0336
NC_005101.4-184290135	CTTCTTCCCACAACCCCTCCCACTGTG	22	A	0.0082	0.479
NC_005101.4-184303078	GAGCTGACGACCCAGTCTCCTCCCTCA	27	A	0	0.4773
NC_005101.4-184343867	TATAAGACGACTCTTTCTCCTCCTCAC	8	C	0.3083	0.4166
NC_005101.4-184347936	TTCTCTTCCACCCACACTCCACAGAGA	9	C	0.1074	0.1853
NC_005101.4-184355285	GGCACTCACACCATCCCTCCTCACCCT	4	A	0.3333	0.4606
NC_005101.4-184362443	TTCTCTCCCACGTTTTCTCCTTGATGG	12	G	0.1466	0.244
NC_005101.4-184371859	GCCTGGGAGACCAAAACTCCTGCCTGG	16	A	0.1327	0.3294
NC_005101.4-184387285	CTCACAGCAACTGGTACTCCAGCCACA	16	A	0	0.4773
NC_005101.4-184409373	CAATGGGCCACCCTGCCTCCACACCGT	24	C	0.0574	0.4903
NC_005101.4-184409373	CAATGGGCCACCCTGCCTCCACACCGT	25	C	0.0574	0.4903
NC_005101.4-184413113	ATACCTTGTACTCACTCTCCCGGCTTT	7	T	0.0259	0.0255
NC_005101.4-184413113	ATACCTTGTACTCACTCTCCCGGCTTT	22	G	0.2845	0.4595
NC_005101.4-184518296	TCTCTTGCAACTGTATCTCCCAGAGTC	21	C	0.0345	0.0339
NC_005101.4-184567010	ACAGTAAATACCATTTCTCCATGGCCT	1	A	0.1513	0.4703
NC_005101.4-184567010	ACAGTAAATACCATTTCTCCATGGCCT	8	A	0.1513	0.3319
NC_005101.4-184642814	TACCATGCTACTCTTTCTCCCATCTTC	21	C	0.0442	0.0433

NC_005101.4-184642814	TACCATGCTACTCTTTCTCCCATCTTC	22	A	0.0442	0.0433
NC_005101.4-184681014	CATCACCAGACAGTGGCTCCCAGCATT	22	A	0.2066	0.259
NC_005101.4-184682742	TCTCTTAAAACGCTTCCTCCTTTGACA	12	G	0.1786	0.3047
NC_005101.4-184692660	CAACATCTCACGCCAGCTCCTTCCTTG	23	C	0	0.1014
NC_005101.4-184761200	CTGCTCCAAACTCTGCCTCCATGATTC	22	T	0	0.4856
NC_005101.4-184761200	CTGCTCCAAACTCTGCCTCCATGATTC	26	T	0	0.4844
NC_005101.4-184939114	GTTCTTGCCACCACCTCTCCCAACTCC	25	T	0	0.4807
NC_005101.4-184941140	GCCTCTTACACAGATTCTCCCTGCCGC	25	C	0.0678	0.0655
NC_005101.4-185036586	TTCACGCACACATGGACTCCCTTAGTG	6	G	0.2881	0.4029
NC_005101.4-185036586	TTCACGCACACATGGACTCCCTTAGTG	24	A	0	0.4743
NC_005101.4-185077397	TGGCAGGACACTACAGCTCCCCACCCC	25	C	0.2034	0.2926
NC_005101.4-185197844	TGATTGATTACCAGCCCTCCACTGCTG	21	A	0.0909	0.1017
NC_005101.4-185204093	CTGGAAAAGACCTTTCCTCCTTTAGTT	13	T	0.1339	0.125
NC_005101.4-185207867	ACATGCTAAACAAGAACTCCTAAGGCT	22	A	0.115	0.4961
NC_005101.4-185232133	CTGATTTCCACCCACGCTCCCACAAAT	2	T	0.0085	0.4776
NC_005101.4-185232133	CTGATTTCCACCCACGCTCCCACAAAT	15	C	0.0084	0.0411
NC_005101.4-185324395	GACTTACACACTGATGCTCCTGGTTCT	22	G	0	0.4807
NC_005101.4-185355661	CCCCACATCACGTGTTCTCCACGGGCC	15	T	0.2975	0.4246
NC_005101.4-185361910	GATCTGCACACTTTTACTCCAAGCAAT	26	A	0.0088	0.4735
NC_005101.4-185362281	AGAACGCTCACACCCACTCCACTTCTG	6	G	0.1463	0.1494
NC_005101.4-185369891	GCTATCAGAACACCATCTCCTAATGTA	23	A	0	0.4826
NC_005101.4-185378566	TGAAGACCGACTGAAACTCCAGCTTCC	8	C	0.0083	0.0247
NC_005101.4-185402255	CAGTAAAATACCCTGCCTCCAAGGGGT	14	T	0	0.477
NC_005101.4-185402255	CAGTAAAATACCCTGCCTCCAAGGGGT	16	C	0.2946	0.4789
NC_005101.4-185411757	TGCCTTTTAACCGCTCCTCCTCCGACA	23	C	0.1393	0.3008
NC_005101.4-185471120	ATTGACCTGACTTAAGCTCCAACTTGC	22	A	0.1513	0.1678
NC_005101.4-185471120	ATTGACCTGACTTAAGCTCCAACTTGC	26	G	0.1513	0.1678
NC_005101.4-185485559	AAATACCACACCTAGCCTCCGAGAATT	21	G	0.0163	0.032
NC_005101.4-185486756	TGGATTCACACCCTAACTCCCCGAATA	21	C	0.3388	0.4398
NC_005101.4-185521833	ATGGTGGTAACAAGGGCTCCTAACTTC	12	A	0.0357	0.1014
NC_005101.4-185626423	CCCTTTCCCACAGCCCCTCCTCTACCC	1	C	0.0756	0.0882
NC_005101.4-185805761	TTGTCGTAAACCTCTGCTCCAATGCAC	6	G	0	0.4787
NC_005101.4-185837545	CTTAACAAAACATGTCCTCCTTTTGTT	14	G	0.0169	0.4793
NC_005101.4-185855115	CCCTTGTCCACTGTTTCTCCACTCTTC	4	T	0.0407	0.0398
NC_005101.4-185859648	GCAGACTCAACAGCTCCTCCAGCCCTC	2	C	0.0407	0.0398
NC_005101.4-185859648	GCAGACTCAACAGCTCCTCCAGCCCTC	3	A	0	0.4793
NC_005101.4-185925067	GAAAGAGCCACAAGAGCTCCCGTTCAG	7	G	0.0081	0.0241
NC_005101.4-185925067	GAAAGAGCCACAAGAGCTCCCGTTCAG	21	C	0.2195	0.4703
NC_005101.4-185986170	CCCAGGATAACCAGGGCTCCATAACAC	27	C	0.2602	0.496
NC_005101.4-186010598	TGTCACCACACCGCCTCTCCTAGGATC	8	A	0	0.4773
NC_005101.4-186068836	TTCCAGATCACAGCCCCTCCCCTACTA	23	T	0	0.4715
NC_005101.4-186156246	CTGGCTGGCACATGAGCTCCTGACATC	2	T	0	0.4718
NC_005101.4-186191174	GCTCCTCCTACATGTGCTCCCATCATG	1	G	0.1382	0.4979
NC_005101.4-186191174	GCTCCTCCTACATGTGCTCCCATCATG	4	C	0.4309	0.356
NC_005101.4-186201476	CACACATGCACTCAGCCTCCCTCTCTA	4	A	0.0354	0.0348
NC_005101.4-186212635	TCTTCCCTCACACCTCCTCCTCCACTG	5	C	0.252	0.381
NC_005101.4-186212635	TCTTCCCTCACACCTCCTCCTCCACTG	22	C	0	0.4807
NC_005101.4-186222074	CCCTCGGACACACCCCCTCCCAAAGCC	5	C	0.1622	0.2837
NC_005101.4-186222074	CCCTCGGACACACCCCCTCCCAAAGCC	14	C	0.1802	0.349
NC_005101.4-186227905	CTAAACACCACATGTCCTCCAAGGGTC	6	C	0.252	0.373
NC_005101.4-186240094	AACAAGTCCACACAGCCTCCTTTATAA	7	T	0.0084	0.4797

NC_005101.4-186240094	AACAAGTCCACACAGCCTCCTTTATAA	14	A	0.1	0.1099
NC_005101.4-186288702	CATTGCTGGACCTGCCCTCCACACCTT	2	A	0.2869	0.4717
NC_005101.4-186288702	CATTGCTGGACCTGCCCTCCACACCTT	14	G	0.2705	0.2903
NC_005101.4-186321750	CACTTCTCTACAGCAGCTCCAGTTTAA	16	G	0.0885	0.1162
NC_005101.4-186331758	GTTTGTTCCACTCTCACTCCATGTTTG	5	G	0.0744	0.0716
NC_005101.4-186331758	GTTTGTTCCACTCTCACTCCATGTTTG	12	T	0.1818	0.2869
NC_005101.4-186375156	TTTACTTCCACATCGTCTCCATCAGCC	5	C	0	0.4746
NC_005101.4-186375156	TTTACTTCCACATCGTCTCCATCAGCC	15	G	0.2432	0.2778
NC_005101.4-186461466	CAATGGCGGACTGCGGCTCCTGCAAAT	22	G	0.1557	0.2796
NC_005101.4-186461466	CAATGGCGGACTGCGGCTCCTGCAAAT	27	T	0.175	0.315
NC_005101.4-186472653	ACCTTGTCTACTCTCCCTCCGTAATCA	1	A	0.0417	0.0722
NC_005101.4-186472653	ACCTTGTCTACTCTCCCTCCGTAATCA	21	G	0.041	0.0401
NC_005101.4-186484422	TCTGCTCTCACCCACCCTCCTGCCTCT	2	C	0.1167	0.4978
NC_005101.4-186484422	TCTGCTCTCACCCACCCTCCTGCCTCT	6	T	0.05	0.0488
NC_005101.4-186494336	ATAGCCCCAACAATGCCTCCAGGCAGA	25	A	0.0588	0.0571
NC_005101.4-186582335	AGGGGCAGGACCAGCTCTCCTGCTGAT	27	T	0	0.4793
NC_005101.4-186590739	CCATCCCACACAATCCCTCCCTCTTCT	27	T	0	0.4832
NC_005101.4-186602904	CTTATAACTACTTGGTCTCCTTCATAA	13	T	0.0083	0.4803
NC_005101.4-186613255	TAGCTCTCCACGCCTCCTCCTCCTACC	12	G	0.069	0.1855
NC_005101.4-186613255	TAGCTCTCCACGCCTCCTCCTCCTACC	26	C	0.1478	0.1369
NC_005101.4-186613995	TGTATTCCTACATAAACTCCTCTGATG	3	T	0.0763	0.487
NC_005101.4-186614321	GTCTATATCACACCCCCTCCTTCTGAG	2	T	0.1593	0.1613
NC_005101.4-186614321	GTCTATATCACACCCCCTCCTTCTGAG	15	C	0.1316	0.1528
NC_005101.4-186621224	TGTGTTTGAACAGTTACTCCCCAGCTG	4	G	0.0167	0.4861
NC_005101.4-186665084	ACTCTCCCCACTCCCACTCCTCCACAA	12	T	0.0902	0.4258
NC_005101.4-186668155	GTGCTACTGACTAGCACTCCACTCAGC	9	G	0.1949	0.4896
NC_005101.4-186668155	GTGCTACTGACTAGCACTCCACTCAGC	12	T	0.2	0.2665
NC_005101.4-186688189	TTCTTTGACACTATCTCTCCATGCTCT	13	A	0.2821	0.4227
NC_005101.4-186750799	TTCCTCCTCACACAGGCTCCAGCCCTG	23	C	0.1967	0.4999
NC_005101.4-186789526	AGTCATCTCACACTGCCTCCTCATGCC	12	A	0.0345	0.4786
NC_005101.4-186789526	AGTCATCTCACACTGCCTCCTCATGCC	25	G	0.1197	0.2604
NC_005101.4-186790241	CAGTCCTCTACTTCCTCTCCCTGCTTC	7	T	0	0.4819
NC_005101.4-186790241	CAGTCCTCTACTTCCTCTCCCTGCTTC	13	T	0.0325	0.032
NC_005101.4-186852615	GCCGTTAACACAAACACTCCTGACTTC	4	G	0.4035	0.4998
NC_005101.4-186852615	GCCGTTAACACAAACACTCCTGACTTC	16	A	0.0087	0.4782
NC_005101.4-186865656	ACCCAAAGGACTCTTCCTCCCTTGCAC	23	T	0	0.4839
NC_005101.4-186876769	TATGAAACTACATGCTCTCCTTTATCA	8	C	0.0081	0.0241
NC_005101.4-186889582	CAGGACTCCACCCCACCTCCCATGGTC	23	T	0	0.4731
NC_005101.4-186895226	GGTGAGAGGACTGTCCCTCCAGTGTAC	5	A	0.0164	0.4773
NC_005101.4-186961651	TAACCCCTCACCCATGCTCCTGAGAGC	6	C	0.0087	0.4744
NC_005101.4-187048601	CCTATCTCTACTTCATCTCCTTTCTCT	7	T	0	0.4909
NC_005101.4-187048601	CCTATCTCTACTTCATCTCCTTTCTCT	27	T	0	0.4909
NC_005101.4-187154064	ACACACCACACAAATCCTCCTGCTCCC	13	A	0	0.4743
NC_005101.4-187189871	CTGCCACTCACCTGGCCTCCTCCTCGG	24	T	0.0407	0.0553
NC_005101.4-187189871	CTGCCACTCACCTGGCCTCCTCCTCGG	26	G	0.0569	0.0705
NC_005101.4-187211518	GCCTGATCTACAGAAACTCCAGGCCAG	5	G	0.1368	0.2834
NC_005101.4-187211518	GCCTGATCTACAGAAACTCCAGGCCAG	6	A	0.2328	0.302
NC_005101.4-187219958	GAGGAAGCCACCCTCCCTCCCCTGGCC	1	G	0.2131	0.402
NC_005101.4-187248349	GTGCCTGTAACACTCTCTCCTGGCCTG	1	G	0	0.4778
NC_005101.4-187258771	GAGTGTGAAACTTGGACTCCTTCCGCA	5	G	0.0254	0.0576
NC_005101.4-187285289	GAACAACTTACCCTCACTCCAGCCATG	6	A	0	0.4787

NC_005101.4-187328488	AAAGACATCACCGACTCTCCCCGTCTG	12	C	0.0167	0.0328
NC_005101.4-187328488	AAAGACATCACCGACTCTCCCCGTCTG	22	C	0.1167	0.1244
NC_005101.4-187332039	AGAACCCATACCACACCTCCCTGTCCC	6	C	0	0.4671
NC_005101.4-187336186	CCAGCAGTCACCCTTGCTCCAGTACCA	3	A	0.0427	0.4928
NC_005101.4-187365255	GAGGGAGCCACAGCCACTCCACCTCGG	24	T	0.009	0.4726
NC_005101.4-187365703	GCCACACCCACAGGTTCTCCTGCTCCT	15	T	0.1513	0.5
NC_005101.4-187433623	CCCTGTTCCACAGACCCTCCCGGCATT	22	G	0.0488	0.0476
NC_005101.4-187447800	CTCATTGAAACATTGCCTCCCAAATTA	9	A	0.3223	0.4339
NC_005101.4-187469440	ACTCTGCACACTCCCCCTCCCCTCCAT	26	A	0.0957	0.1659
NC_005101.4-187481892	GACTGACATACACAGACTCCCAGCCCC	27	C	0.0413	0.4889
NC_005101.4-187491498	TTTTTGAGAACCATCTCTCCAGCCCAC	6	G	0.0407	0.0553
NC_005101.4-187492168	TACTCTATAACACCACCTCCAGGATAA	2	A	0	0.48
NC_005101.4-187492168	TACTCTATAACACCACCTCCAGGATAA	16	C	0.1739	0.3303
NC_005101.4-187502446	TCCACCCAGACCATCACTCCACCGACA	13	A	0	0.4746
NC_005101.4-187568365	TATCCCCAGACCAAGCCTCCCGGCCAT	21	C	0.0087	0.4849
NC_005101.4-187583991	AATGTCATCACCCAGACTCCGGTGGTT	25	G	0.0084	0.4761
NC_005101.4-187597083	CCACCCTCTACCCTCCCTCCCAACACA	2	C	0.0244	0.0553
NC_005101.4-187597083	CCACCCTCTACCCTCCCTCCCAACACA	6	C	0.0082	0.4755
NC_005101.4-187651929	CTGACAGTCACAGCCTCTCCTGATTCA	26	C	0.0744	0.1017
NC_005101.4-187651929	CTGACAGTCACAGCCTCTCCTGATTCA	27	A	0.0744	0.1017
NC_005101.4-187657496	GAACCTAGGACTGCGACTCCTAGTACA	12	T	0.0333	0.0488
NC_005101.4-187750386	GCAACGTCAACCTCGGCTCCTTCATAA	14	C	0	0.4813
NC_005101.4-187783514	CACCACAGGACATTACCTCCTCCTCCC	14	T	0	0.4793
NC_005101.4-187802442	AAAGCATGTACCACCACTCCATGTTCT	24	T	0.0172	0.0504
NC_005101.4-187820695	CACACACACACACACACTCCATCAACC	3	C	0	0.0348
NC_005101.4-187823283	TTGCCTACTACCTCCCCTCCCCCATAC	6	T	0	0.4819
NC_005101.4-187846271	GATAGGGCGACAGACGCTCCACCAGAA	3	T	0.3115	0.3624
NC_005101.4-187908689	ATGGCTACCACTCTCCCTCCCCTCTTT	25	T	0.0826	0.1787
NC_005101.4-187909048	AATCAAAAGACCCTGTCTCCTCTGGAA	7	A	0.0085	0.4854
NC_005101.4-187909048	AATCAAAAGACCCTGTCTCCTCTGGAA	14	T	0.0252	0.0249
NC_005101.4-187956129	GAATGTGTCACTTGCTCTCCATTTCAT	5	G	0.2906	0.3974
NC_005101.4-187971469	AGCCTACTGACCTGAGCTCCCCCACGA	3	C	0	0.4746
NC_005101.4-187993021	TGAAAACCCACCCACACTCCACACTCC	25	T	0.1167	0.1099
NC_005101.4-188021032	GGAACCCTCACCTCGTCTCCCACGGTG	5	C	0.2439	0.4389
NC_005101.4-188097148	ACTCTCTGTACCCCAGCTCCCTGGGAC	3	T	0.114	0.138
NC_005101.4-188105426	GAGGCGAGCACCACCACTCCCGTGTAC	5	C	0	0.4764
NC_005101.4-188105426	GAGGCGAGCACCACCACTCCCGTGTAC	6	G	0	0.4764
NC_005101.4-188113055	CCTACCAGCACCCGCGCTCCGCAGACC	3	T	0	0.4793
NC_005101.4-188117077	CATTCTCGAACTTCTGCTCCTTCCTCC	1	C	0.0265	0.0262
NC_005101.4-188157897	TGCAGTGAAACTGTTACTCCATACAGA	7	G	0.0833	0.0799
NC_005101.4-188198197	TGGAGTTAGACAGGGACTCCCCACAGG	16	A	0.1282	0.1347
NC_005101.4-188280025	GTCTAGAGGACACCTCCTCCCTCTCCC	1	G	0.0619	0.4693
NC_005101.4-188335277	CTAGGCTTTACATCAACTCCATTGCAG	24	G	0.1565	0.3303
NC_005101.4-188337594	CATAGTTAAACACTGTCTCCAAACAGA	26	G	0.0089	0.4856
NC_005101.4-188342835	GTGGTACATACCTTTTCTCCCAATACT	2	T	0.0932	0.0889
NC_005101.4-188342835	GTGGTACATACCTTTTCTCCCAATACT	16	T	0.0924	0.0882
NC_005101.4-188350580	CTCCCTGTCACTCCAGCTCCACAGTAT	16	G	0.0976	0.1073
NC_005101.4-188368553	GCGCTTGCCACTCGCCCTCCTGAGAAC	24	G	0	0.4743
NC_005101.4-188395677	CGTAGTGAGACCCTGTCTCCCAAATAC	4	A	0	0.4833
NC_005101.4-188398646	ATAGCCTGGACCGACTCTCCTCCCATA	12	C	0.0086	0.4803
NC_005101.4-188465521	ATAATGGGAACACGCACTCCAGGAAAT	14	G	0	0.477

NC_005101.4-188487051	AGGCATCACACTACCTCTCCCTCCCCC	25	C	0.0169	0.4793
NC_005101.4-188515881	GCGAGACAGACAGCAGCTCCCTGCAGT	3	G	0.0083	0.0245
NC_005101.4-188515881	GCGAGACAGACAGCAGCTCCCTGCAGT	26	G	0.0085	0.4671
NC_005101.4-188518713	CTCAGTGCCACTGATTCTCCACAATTC	13	G	0.0082	0.0401
NC_005101.4-188518713	CTCAGTGCCACTGATTCTCCACAATTC	15	T	0	0.4773
NC_005101.4-188534514	CCCTTTTTAACTCCACCTCCCTACTCA	6	T	0	0.4773
NC_005101.4-188557137	TCATATTTTACCCTAGCTCCTTCATAG	26	A	0	0.4779
NC_005101.4-188603080	AATGCATGAACTTTGACTCCTTCCCAG	5	C	0.0496	0.0484
NC_005101.4-188603080	AATGCATGAACTTTGACTCCTTCCCAG	6	A	0	0.4751
NC_005101.4-188654472	ACTCAGCCCACAGCTCCTCCCACGGTT	24	G	0	0.4743
NC_005101.4-188665231	GGCCGATGCACACCTGCTCCATAGAGA	21	A	0.0696	0.48
NC_005101.4-188682524	CACACACACACAATTTCTCCTCTTTCA	13	A	0	0.4773
NC_005101.4-188689886	CAGCCCAGCACCCTGCCTCCCTAACTG	14	T	0.0172	0.488
NC_005101.4-188689886	CAGCCCAGCACCCTGCCTCCCTAACTG	16	C	0.2544	0.4796
NC_005101.4-188701244	GCAATCGTAACCCTGGCTCCGGGGGAA	3	A	0.0609	0.059
NC_005101.4-188712056	CTGGGCAGTACCAGCTCTCCTTAGGGC	22	T	0.1681	0.2076
NC_005101.4-188732707	ACCTAGAGAACTCAGACTCCTTTCCCA	22	T	0.0342	0.066
NC_005101.4-188778464	AGAGTGACCACAGGCGCTCCTCTGACT	21	T	0.1382	0.2668
NC_005101.4-188780066	ATGCCCCACACAGAACCTCCTGTGCAG	15	A	0.0171	0.4807
NC_005101.4-188780066	ATGCCCCACACAGAACCTCCTGTGCAG	27	G	0.0342	0.4918
NC_005101.4-188808453	GTTGGCCTCACCTGGACTCCAGATCTT	21	A	0	0.4751
NC_005101.4-188811905	TATTCCCCCACTCTGTCTCCTCTTCAC	1	T	0.2193	0.4221
NC_005101.4-188831064	CAGATAACCACCAAGCCTCCTTCAGGA	2	A	0.0603	0.4937
NC_005101.4-188837268	ACCCCGCAGACAACTGCTCCCATCTGG	5	C	0.0252	0.0249
NC_005101.4-188850836	AGATCACTTACATTCTCTCCGCATGTG	13	T	0.0174	0.4764
NC_005101.4-188850836	AGATCACTTACATTCTCTCCGCATGTG	22	C	0.1652	0.304
NC_005101.4-188851657	CACAGCTTAACTGCTGCTCCACACGGT	7	T	0	0.4698
NC_005101.4-188851657	CACAGCTTAACTGCTGCTCCACACGGT	8	T	0	0.4698
NC_005101.4-188868926	TAGGAAATTACCCAGGCTCCATGGGTT	3	G	0.1217	0.1295
NC_005101.4-188868926	TAGGAAATTACCCAGGCTCCATGGGTT	13	C	0	0.4814
NC_005101.4-188921295	TCTCCTCCTACCTCCTCTCCATAGGCT	5	C	0	0.4807
NC_005101.4-188939587	ATGGTGCAAACATCGGCTCCACCAAGC	5	T	0.0088	0.0262
NC_005101.4-188947449	ACCCATCTCACAAGGCCTCCCAGAGCA	1	A	0	0.4773
NC_005101.4-188947449	ACCCATCTCACAAGGCCTCCCAGAGCA	2	C	0	0.4773
NC_005101.4-188958425	CCCAGTAACACATCTTCTCCAATGAAA	13	T	0.0087	0.4744
NC_005101.4-188986202	CGTGATGCCACGCTTGCTCCTTTCCTT	24	C	0	0.4773
NC_005101.4-188986202	CGTGATGCCACGCTTGCTCCTTTCCTT	26	T	0	0.4773
NC_005101.4-188986527	ACAACTGTCACCCACACTCCATACTGG	6	T	0.1207	0.199
NC_005101.4-189015919	GTAGGGGATACTAGTACTCCAACATTT	7	G	0	0.4787
NC_005101.4-189028444	CAGGAGAAGACTGAGACTCCTATATGG	22	A	0	0.4785
NC_005101.4-189056262	TCCTCTCCCACTCCCTCTCCCACATAT	4	T	0.0083	0.0245
NC_005101.4-189098607	ACATCTGAGACAGCATCTCCAGGGGAT	6	T	0.2114	0.4759
NC_005101.4-189098607	ACATCTGAGACAGCATCTCCAGGGGAT	21	A	0.1545	0.4995
NC_005101.4-189099531	CTCCCTGTCACTATTCCTCCTCTTCAA	16	C	0.2162	0.3178
NC_005101.4-189118864	GAACATGACACTGTTGCTCCCAATATC	26	T	0.0325	0.032
NC_005101.4-189121257	TAAGTCCTCACAAGCCCTCCACAGACA	6	C	0.2114	0.3926
NC_005101.4-189139129	CACAGTGAGACACTGTCTCCAGTAACA	9	G	0.05	0.0488
NC_005101.4-189148856	TCTCTCTCCACCTACACTCCTTGGAGA	8	C	0.1518	0.3316
NC_005101.4-189156128	CAGGCAAGGACAGTCTCTCCAGGACGC	25	C	0.0163	0.0476
NC_005101.4-189156128	CAGGCAAGGACAGTCTCTCCAGGACGC	26	G	0.2764	0.4881
NC_005101.4-189156683	AGAGGGGCCACCAAGACTCCCTGGGGA	5	G	0.2149	0.2533

NC_005101.4-189221352	GATGACAAGACTGAGGCTCCAAGATGT	14	A	0.1638	0.2188
NC_005101.4-189249067	CATAGTGAGACTCTGTCTCCAAAGACA	6	T	0.0085	0.481
NC_005101.4-189258486	AAACAAGCCACTGTGACTCCAGCAAAG	2	A	0	0.4793
NC_005101.4-189272481	CTATGTGTAACTCTCTCTCCTCCCTCC	24	C	0	0.0512
NC_005101.4-189291935	CCGGTAATCACAACCCCTCCCTCCCTT	2	C	0	0.4819
NC_005101.4-189291935	CCGGTAATCACAACCCCTCCCTCCCTT	4	G	0	0.4819
NC_005101.4-189308164	CAGTCTCCCACAGCCTCTCCCAGTGGC	22	A	0.0085	0.4698
NC_005101.4-189394516	CATAGAGAAACCTTATCTCCACTAAAA	3	T	0	0.4765
NC_005101.4-189472735	TTACCTGCAACTCCAGCTCCAGGGGAT	9	A	0.0265	0.0262
NC_005101.4-189508753	TATTTTGTGACAGGGTCTCCTGTAATC	27	C	0.177	0.2036
NC_005101.4-189558700	CAGTCTGTAACTCCAGCTCCAGGGGTC	5	C	0.178	0.1894
NC_005101.4-189579921	ACCCTCTTCACCCTCTCTCCACGTGCT	22	C	0	0.48
NC_005101.4-189579921	ACCCTCTTCACCCTCTCTCCACGTGCT	25	G	0	0.48
NC_005101.4-189601732	ACCTCGGCTACAGCCTCTCCTCATCGC	26	G	0.0085	0.4626
NC_005101.4-189624811	CTGTGTTTGACCCCTCCTCCTGCCCCT	16	C	0	0.4793
NC_005101.4-189723795	TGTAGAGAGACGCCGTCTCCACCAAAA	14	C	0	0.4922
NC_005101.4-189738641	AACACACCCACCAGCTCTCCATTGAGT	8	C	0	0.4807
NC_005101.4-189741586	GAATTACAGACTCTTCCTCCAAACAAG	21	A	0	0.4793
NC_005101.4-189769620	GAAGGCTTCACCTACTCTCCTCCACAG	14	A	0	0.48
NC_005101.4-189782716	GAGCCCTTAACACTCCCTCCATACCCC	3	G	0.2672	0.343
NC_005101.4-189871313	AGTTAAAGAACTGTTACTCCATCTGGT	8	G	0.161	0.1894
NC_005101.4-189874416	AACAGTGCCACCCAATCTCCGGAAACT	13	C	0	0.4807
NC_005101.4-189931009	AAGATCTTCACAGGACCTCCTCATTCA	16	C	0.0325	0.032
NC_005101.4-189975286	TGGAAAATTACAGCTGCTCCTTGGGTT	21	T	0.1239	0.2036
NC_005101.4-189982880	ATGGCAGAGACTCTGACTCCAGTAGAC	4	G	0.2689	0.4406
NC_005101.4-189982880	ATGGCAGAGACTCTGACTCCAGTAGAC	13	C	0.2542	0.4367
NC_005101.4-190016221	CTTAGTAACACCTCTCCTCCCTCTTCA	2	T	0.0171	0.4839
NC_005101.4-190016221	CTTAGTAACACCTCTCCTCCCTCTTCA	7	A	0.0085	0.4823
NC_005101.4-190025903	GTGTGTTGGACTTAGACTCCTGCCTAG	16	A	0	0.4749
NC_005101.4-190048043	ATATAACCCACCCCCTCTCCTCTTCAG	7	C	0	0.4793
NC_005101.4-190048043	ATATAACCCACCCCCTCTCCTCTTCAG	26	A	0.3171	0.373
NC_005101.4-190088261	TGGTTTGAGACAACCCCTCCAATCATT	9	G	0.0796	0.0926
NC_005101.4-190088261	TGGTTTGAGACAACCCCTCCAATCATT	13	A	0.2672	0.4313
NC_005101.4-190106062	TCCCTTTATACAGTCCCTCCCTCCCTC	25	C	0	0.4779
NC_005101.4-190106062	TCCCTTTATACAGTCCCTCCCTCCCTC	26	T	0.0417	0.4875
NC_005101.4-190739296	AGCTAAAGTACCATCTCTCCTGCTGTT	15	C	0.082	0.0786
NC_005101.4-190884421	ACACCAAAGACCCATGCTCCACTATGT	22	C	0.0083	0.4783
NC_005101.4-191022219	ACAAGAAAGACACATACTCCTCTATGT	15	T	0.575	0.4166
NC_005101.4-191022219	ACAAGAAAGACACATACTCCTCTATGT	23	T	0.575	0.4166
NC_005101.4-191254282	CACTCTAAAACCTCTTCTCCTGGCCAT	22	G	0.0496	0.0484
NC_005101.4-191254282	CACTCTAAAACCTCTTCTCCTGGCCAT	23	G	0.0496	0.0484
NC_005101.4-191390766	AGAAGAAGGACACATCCTCCTCTATGT	2	G	0.2696	0.258
NC_005101.4-191390766	AGAAGAAGGACACATCCTCCTCTATGT	12	A	0.2696	0.258
NC_005101.4-191421274	ACACAAAAGACACAACCTCCTCTATGT	12	A	0.5462	0.449
NC_005101.4-191421274	ACACAAAAGACACAACCTCCTCTATGT	14	A	0.5462	0.449
NC_005101.4-191954900	TCCCTTTGCACACAAGCTCCTACAACT	2	C	0.0732	0.0705
NC_005101.4-191954900	TCCCTTTGCACACAAGCTCCTACAACT	9	C	0.1382	0.1562
NC_005101.4-191986955	CATTCCATGACTTATTCTCCTTACTCT	26	C	0.0424	0.0415
NC_005101.4-192005273	CTAACCCATACCACCTCTCCCTACTTC	16	T	0.0088	0.481
NC_005101.4-192022901	AAGACTGACACCTTGGCTCCTTAAACA	27	A	0.0424	0.0576
NC_005101.4-192049895	CTATGAGATACTCCAACTCCTACCCAC	21	T	0	0.4826

NC_005101.4-192156126	GCTTGCAAGACAAGCCCTCCACCCCTG	1	G	0.0087	0.4613
NC_005101.4-192156126	GCTTGCAAGACAAGCCCTCCACCCCTG	24	C	0.0087	0.4613
NC_005101.4-192471340	TCCCCTTTCACTGGTGCTCCTACTAGG	4	C	0.1583	0.1458
NC_005101.4-192471340	TCCCCTTTCACTGGTGCTCCTACTAGG	23	C	0.1583	0.1458
NC_005101.4-192492100	ATGAACAATACATTTTCTCCAAGTGTC	21	A	0.0248	0.0245
NC_005101.4-192492100	ATGAACAATACATTTTCTCCAAGTGTC	25	G	0.3583	0.4525
NC_005101.4-192521972	TGAATAACAACACTATCTCCTTTAGCA	25	G	0.2373	0.4884
NC_005101.4-192651710	CAGACTGGAACCAGCTCTCCTTATCAG	22	T	0.069	0.0825
NC_005101.4-192691402	CACAGGGACACTTACCCTCCATAGTCA	2	A	0.2759	0.3179
NC_005101.4-192691402	CACAGGGACACTTACCCTCCATAGTCA	27	A	0.0427	0.1049
NC_005101.4-192732712	CTACCCAGAACTTGTGCTCCTTGCCTT	12	T	0.0813	0.2017
NC_005101.4-192753099	GTCATTAGCACATGTTCTCCCTCGCTT	13	T	0.2437	0.274
NC_005101.4-192753099	GTCATTAGCACATGTTCTCCCTCGCTT	24	G	0	0.4779
NC_005101.4-192957459	AAAGTCTTCACCAATCCTCCATCCAAT	23	C	0.0976	0.1356
NC_005101.4-192962826	AACCACTGCACCATCTCTCCATCTCTC	2	A	0.0252	0.0249
NC_005101.4-192962826	AACCACTGCACCATCTCTCCATCTCTC	25	C	0.0252	0.0249
NC_005101.4-193072473	AGCACCAAGACATGTGCTCCATGATTT	8	A	0	0.4765
NC_005101.4-193101100	ACACCAGACACTGGGGCTCCCTACCCC	26	C	0.1327	0.1968
NC_005101.4-193121612	TTGGAAGAAACACCTGCTCCTGTTCTG	15	T	0.069	0.4976
NC_005101.4-193173047	GCTTTCCACACTGCCTCTCCTCAATCT	7	C	0.1239	0.1466
NC_005101.4-193173047	GCTTTCCACACTGCCTCTCCTCAATCT	24	A	0.1239	0.2036
NC_005101.4-193199999	GAATTAACAACTAGTCCTCCTGTAGTG	9	A	0.188	0.497
NC_005101.4-193203144	CCCTGCGATACCTCTGCTCCAACAATC	6	C	0.157	0.465
NC_005101.4-193203144	CCCTGCGATACCTCTGCTCCAACAATC	26	T	0.1148	0.1505
NC_005101.4-193237750	GATCCTCTAACAGGACCTCCCTCAGCT	21	C	0.1066	0.1009
NC_005101.4-193281821	CTATGGACCACTCAGTCTCCTTTTTAT	3	A	0.6016	0.4206
NC_005101.4-193281821	CTATGGACCACTCAGTCTCCTTTTTAT	24	T	0.5935	0.4239
NC_005101.4-193296641	ACCACAGATACCTGTGCTCCTTCCTTC	5	C	0	0.4773
NC_005101.4-193329219	TCTATGCTTACAAAGGCTCCAACTGTC	12	A	0.0656	0.0634
NC_005101.4-193339211	CCCTCACCAACCATCTCTCCATCATAG	25	T	0.0168	0.4743
NC_005101.4-193339211	CCCTCACCAACCATCTCTCCATCATAG	27	G	0.157	0.172
NC_005101.4-193343344	ATAATACTAACAGTGCCTCCTTGAAAG	9	A	0.3193	0.3508
NC_005101.4-193343344	ATAATACTAACAGTGCCTCCTTGAAAG	26	A	0.0917	0.1025
NC_005101.4-193352209	CTCTCAGGTACCCTACCTCCACCCCAT	7	G	0.05	0.0799
NC_005101.4-193352209	CTCTCAGGTACCCTACCTCCACCCCAT	9	T	0.025	0.0247
NC_005101.4-193374068	CTTACTGTGACAGAGACTCCAAATGTT	7	G	0	0.4807
NC_005101.4-193400224	TGTTATGTAACTCTTGCTCCTCATAAG	6	T	0.0336	0.033
NC_005101.4-193406561	CTGTACTTCACCTCTGCTCCAAGGCCA	3	G	0.122	0.1954
NC_005101.4-193412296	TCACAACAAACTGTCACTCCAGCTGGT	8	A	0.0174	0.48
NC_005101.4-193598750	TTAGGCCATACATTTACTCCGTAAGTA	6	C	0.0088	0.0429
NC_005101.4-193616737	GGATCTTGCACACACTCTCCTACTGAA	3	A	0	0.4779
NC_005101.4-193703635	AGGTTCTCGACTTGAGCTCCTTCCCTG	13	T	0.045	0.0611
NC_005101.4-193729653	ATCTCACTCACAACTACTCCTTCGATT	23	C	0	0.4787
NC_005101.4-193729653	ATCTCACTCACAACTACTCCTTCGATT	24	G	0.0248	0.0245
NC_005101.4-193731715	ACCTAAGCCACATCACCTCCCCTCAAT	2	C	0.1707	0.1562
NC_005101.4-193731715	ACCTAAGCCACATCACCTCCCCTCAAT	22	C	0	0.4793
NC_005101.4-193768479	GAGACATCTACTTTTCCTCCCAATGCT	23	A	0	0.4786
NC_005101.4-193769859	GAAACATAGACTTGCTCTCCTGTGTGA	9	G	0.0609	0.0911
NC_005101.4-193826946	CAATGAGACACCTTGTCTCCTGTTCTG	12	C	0.2373	0.4999
NC_005101.4-193826946	CAATGAGACACCTTGTCTCCTGTTCTG	13	T	0.2373	0.4999
NC_005101.4-193876743	CTGCTCTGCACCTAGGCTCCTAGTACT	12	C	0.0407	0.0398

NC_005101.4-193876743	CTGCTCTGCACCTAGGCTCCTAGTACT	23	G	0.0407	0.0398
NC_005101.4-193929033	TGCAGGTCAACTCTCTCTCCTCGAGTA	22	C	0.0948	0.1646
NC_005101.4-193932095	AGCTTTGTGACAAATCCTCCCAAGATA	21	C	0.0488	0.0476
NC_005101.4-193937910	CTGCTGAATACTCATTCTCCTCCTTCA	22	C	0.0603	0.0585
NC_005101.4-194025860	CCTTCTTCCACATCTTCTCCTTTACTT	2	C	0.3451	0.4648
NC_005101.4-194025860	CCTTCTTCCACATCTTCTCCTTTACTT	8	C	0.0708	0.0683
NC_005101.4-194395645	CCTTTAAATACCTCTTCTCCTAGCCTC	4	T	0.3607	0.2956
NC_005101.4-194395645	CCTTTAAATACCTCTTCTCCTAGCCTC	23	G	0.3607	0.2956
NC_005101.4-194405566	TCTTGGCATACATACTCTCCAATATCT	12	A	0.3277	0.3643
NC_005101.4-194412451	AGATCTCCAACCCTACCTCCTCCTACT	4	T	0.041	0.0401
NC_005101.4-194878054	GTTACTTAAACACTGCCTCCTTACATT	1	G	0.6068	0.4227
NC_005101.4-195091948	AGCTCCTGCACCATGCCTCCCTGCATA	9	C	0.1282	0.2299
NC_005101.4-195527191	AACTCTTCCACCTGCCCTCCATGATGT	21	A	0	0.4844
NC_005101.4-195577727	AGACCGTCTACATTGACTCCTAGAAGA	22	A	0	0.4807
NC_005101.4-195609116	TTCTGGGGGACTGAAGCTCCTATGAGT	16	G	0.0163	0.0476
NC_005101.4-195626614	GCGAACAGAACGCAGCCTCCTCAGACC	13	C	0.0357	0.0351
NC_005101.4-195644513	TCCCTCTCAACCTCCACTCCCCCCTTT	24	C	0	0.4846
NC_005101.4-195652964	CCCTGTGACACACTCCCTCCACAAACA	1	C	0	0.4814
NC_005101.4-195687908	TCTCCTCTGACTCCAACTCCTGTTGAC	2	C	0.2051	0.4918
NC_005101.4-195706762	AGCCAATGAACAGTGTCTCCCGAGCCT	1	A	0.2857	0.3932
NC_005101.4-195718093	AGTATACACACACCTCCTCCAGAGAGA	13	C	0	0.4833
NC_005101.4-195718093	AGTATACACACACCTCCTCCAGAGAGA	15	T	0.0087	0.4817
NC_005101.4-195721768	GTACTGGCCACCCATCCTCCTGAGAGG	21	T	0.0357	0.0521
NC_005101.4-195731472	AATCCATGCACCCCTTCTCCTTCCTTC	16	T	0	0.4807
NC_005101.4-195731472	AATCCATGCACCCCTTCTCCTTCCTTC	22	T	0.0407	0.0553
NC_005101.4-195746281	CACCACACTACACCATCTCCTCCTTTT	22	C	0.0603	0.0585
NC_005101.4-195747688	AATGCCCTGACCGGTCCTCCAACATCT	27	T	0	0.4827
NC_005101.4-195784811	AACGATGAGACCATATCTCCAAAAATA	5	A	0	0.4746
NC_005101.4-195813476	AGAATGTGTACATCTCCTCCAGTACGA	8	G	0.0738	0.0861
NC_005101.4-195813476	AGAATGTGTACATCTCCTCCAGTACGA	25	C	0.2131	0.2515
NC_005101.4-195849487	CAGACTGCCACACCCACTCCAGTGGAG	3	G	0.5086	0.4108
NC_005101.4-195849487	CAGACTGCCACACCCACTCCAGTGGAG	13	C	0.1102	0.1041
NC_005101.4-195861637	TTTCTGAATACCACAACTCCTGCACAC	3	T	0.0087	0.466
NC_005101.4-195861637	TTTCTGAATACCACAACTCCTGCACAC	15	A	0.0087	0.466
NC_005101.4-195869838	CTTAGAGCAACAAAACCTCCTGCCTTG	14	A	0.0738	0.1009
NC_005101.4-195891677	TAATGTGAGACTCTAGCTCCTGAGGAC	2	A	0	0.4793
NC_005101.4-195891677	TAATGTGAGACTCTAGCTCCTGAGGAC	27	C	0	0.4793
NC_005101.4-195934095	GTGACAGGAACTTTGTCTCCCTCCACC	15	G	0.1062	0.3136
NC_005101.4-195984557	AGCTACTGAACTGGCTCTCCAGCCCTT	14	G	0.0171	0.4602
NC_005101.4-195985761	AACCGTACCACATGGACTCCAGAGGTC	4	C	0.0163	0.4854
NC_005101.4-195985761	AACCGTACCACATGGACTCCAGAGGTC	15	G	0.0163	0.032
NC_005101.4-196004732	GTCTGCCTGACTCTGTCTCCTAAGTGC	13	C	0.1322	0.2046
NC_005101.4-196037886	GTATCTTCCACCTCCTCTCCTCCCTAA	1	G	0	0.4819
NC_005101.4-196047590	AGCAATGTCACCCCCTCTCCCCAATAG	26	A	0.0163	0.032
NC_005101.4-196123949	TCAGGATGGACTCCAACTCCTGGGTTC	4	G	0	0.4793
NC_005101.4-196245114	AACGGTGTAACTCAAGCTCCAAGAGAT	1	A	0	0.4728
NC_005101.4-196253241	TGACAGACAACACCCTCTCCCACAAAG	1	T	0.0087	0.4817
NC_005101.4-196253241	TGACAGACAACACCCTCTCCCACAAAG	3	A	0.0086	0.4803
NC_005101.4-196291431	GCAAGCCAAACGCAGTCTCCGTCACTG	3	A	0.0089	0.0264
NC_005101.4-196291431	GCAAGCCAAACGCAGTCTCCGTCACTG	12	G	0	0.4807
NC_005101.4-196308638	CCAGAAGATACTGAGACTCCATCCAAC	21	A	0.225	0.2372

NC_005101.4-196313243	CCACCTATCACTCCAGCTCCAGGAATT	22	G	0.2348	0.4782
NC_005101.4-196320819	GGAGCACTGACTGCTCCTCCCAGCACT	6	A	0.0517	0.4905
NC_005101.4-196355697	AACAACGGAACCAGCCCTCCCTAAAGA	15	C	0	0.4773
NC_005101.4-196364047	CTCATAATCACTGTCACTCCAGTTCCA	8	T	0	0.4773
NC_005101.4-196393094	CTATCACATACTGCACCTCCATGAAGG	22	T	0.0083	0.4769
NC_005101.4-196406384	GTTTTCAAGACTCCTTCTCCTTCCTAT	4	T	0	0.4751
NC_005101.4-196406384	GTTTTCAAGACTCCTTCTCCTTCCTAT	26	A	0.2727	0.3771
NC_005101.4-196419281	CACATCACTACCTCCCCTCCCTTCCCC	1	C	0.1696	0.4996
NC_005101.4-196419281	CACATCACTACCTCCCCTCCCTTCCCC	27	C	0	0.0342
NC_005101.4-196419712	AACCCAACAACTGAACCTCCAGCTAAG	12	T	0.0165	0.4819
NC_005101.4-196423704	CACAAATGCACAGGGACTCCTATATAC	14	G	0.0696	0.0989
NC_005101.4-196428976	CACTCCCCGACCCCGCCTCCGATGGTC	21	G	0.0256	0.0418
NC_005101.4-196428976	CACTCCCCGACCCCGCCTCCGATGGTC	23	T	0	0.4772
NC_005101.4-196437463	GACATACCTACTGTCACTCCACTGATG	5	T	0	0.4793
NC_005101.4-196457719	CGGAGAGGCACACGCGCTCCGTCCCTT	2	G	0.0087	0.4903
NC_005101.4-196536574	GATTCATCTACTCTACCTCCCTGGCAG	12	T	0	0.4832
NC_005101.4-196664517	TAGCAGCAAACTTCACCTCCAGCTTCC	8	A	0.0089	0.4885
NC_005101.4-196664517	TAGCAGCAAACTTCACCTCCAGCTTCC	15	A	0	0.4871
NC_005101.4-197747372	GAGCCTGTAACTTCAGCTCCAGAATAT	23	A	0	0.4787
NC_005101.4-197838153	AGACATTCGACGGTTACTCCCTCCCAT	12	G	0.0169	0.4856
NC_005101.4-197851101	GCACTGACGACAATGTCTCCTTGTAGG	8	C	0.1074	0.1982
NC_005101.4-197851504	ATGGTGTGCACCGTCACTCCCCTCTCT	2	T	0	0.4793
NC_005101.4-197851504	ATGGTGTGCACCGTCACTCCCCTCTCT	14	T	0	0.4793
NC_005101.4-197908727	ACAAACACAACAGGAGCTCCAGATAGA	1	A	0.2212	0.4309
NC_005101.4-197914175	TCTAGGGATACTCTAACTCCATGTTAA	1	T	0	0.4826
NC_005101.4-198053699	CAAACTGTGACCGCAACTCCCTCATTG	12	C	0.0082	0.0243
NC_005101.4-198073808	AGTAGCAGTACCTGGACTCCGGAAGTG	27	G	0.0574	0.0557
NC_005101.4-198109884	AGGCACCTCACATGCCCTCCACGGAAG	21	A	0.0333	0.0328
NC_005101.4-198147500	GAAGTAGACACTGTTGCTCCTCTTATG	3	A	0.0088	0.4873
NC_005101.4-198158808	CTCAGCGACACCTTTACTCCTGAGGTC	7	G	0	0.4793
NC_005101.4-198213056	TCATTAACAACATCCACTCCCAGCCTT	16	A	0.5932	0.424
NC_005101.4-198300715	AACATAATTACACTCTCTCCACCCTAC	27	C	0.0431	0.0746
NC_005101.4-198308878	AACCTCTGGACTCAAACTCCGTGTGAA	14	A	0.0328	0.048
NC_005101.4-198308878	AACCTCTGGACTCAAACTCCGTGTGAA	16	A	0.1721	0.4992
NC_005101.4-198313496	CAGGGCTCCACCTCTGCTCCAGCATGG	9	C	0.1062	0.1316
NC_005101.4-198317031	CATGGATACACCGGTCCTCCGAGACCC	21	G	0.313	0.264
NC_005101.4-198418132	GACATAAATACACGCCCTCCAGTGTGT	25	T	0.0342	0.0336
NC_005101.4-198422994	CCTCCCTTGACTGCCCCTCCCCCAAAC	16	C	0	0.4793
NC_005101.4-198436282	CCCAGACTGACTGAAACTCCCGAGTGT	12	T	0.1121	0.1646
NC_005101.4-198436282	CCCAGACTGACTGAAACTCCCGAGTGT	22	G	0.1207	0.1284
NC_005101.4-198499786	ACCAGAGTCACAGGAACTCCATGACTT	8	T	0	0.4765
NC_005101.4-198503641	CCAAAAGCAACATGGTCTCCTTTCATT	1	C	0.0354	0.0348
NC_005101.4-198548744	CCCAGCAGGACTGATGCTCCTATGACC	23	T	0.1311	0.1505
NC_005101.4-198548744	CCCAGCAGGACTGATGCTCCTATGACC	27	C	0.0246	0.0243
NC_005101.4-198548838	GGGAAGGGCACACAGGCTCCCACCCCT	6	G	0.3025	0.2568
NC_005101.4-198548838	GGGAAGGGCACACAGGCTCCCACCCCT	9	C	0.5299	0.3895
NC_005101.4-198560305	ATTCTTTAGACTGGACCTCCTTATGGG	2	T	0.0085	0.4671
NC_005101.4-198560305	ATTCTTTAGACTGGACCTCCTTATGGG	3	T	0.0086	0.4688
NC_005101.4-198571142	CACATTGTTACTAAGTCTCCAGACCTT	5	T	0.0325	0.032
NC_005101.4-198579883	ATAATGGTGACGTCTGCTCCAGTGTTG	7	G	0.0625	0.0771
NC_005101.4-198599371	AAAAGCTGTACCCTGGCTCCCGGTTTT	14	T	0.1074	0.1017

NC_005101.4-198599371	AAAAGCTGTACCCTGGCTCCCGGTTTT	25	T	0.0083	0.4769
NC_005101.4-198635997	CAGAGAGAGACCTTGTCTCCAAGGGGA	13	T	0.1545	0.4174
NC_005101.4-198676419	TTCTCCTTCACAGGTCCTCCAACCCAC	9	C	0	0.4757
NC_005101.4-198676419	TTCTCCTTCACAGGTCCTCCAACCCAC	27	C	0.0339	0.4908
NC_005101.4-198707546	CACACACAGACTCTAGCTCCAGGGGCT	2	A	0	0.4891
NC_005101.4-198707763	AAAGAGGGCACTGTTTCTCCCGAGACT	1	A	0	0.4859
NC_005101.4-198713745	CCTGCTGTTACATGGGCTCCATGGAAA	7	G	0	0.4819
NC_005101.4-198742216	AGTTAAGTGACAACATCTCCCACTGTC	22	A	0.0446	0.0436
NC_005101.4-198754034	TAGCCTGAAACTCCAGCTCCTGCGGAT	22	G	0.1983	0.2057
NC_005101.4-198754034	TAGCCTGAAACTCCAGCTCCTGCGGAT	24	G	0.0339	0.0812
NC_005101.4-198774845	CATCTAGTAACTCTGACTCCTTGGACA	5	T	0.0672	0.494
NC_005101.4-198798304	CTCCGTGACACATCCTCTCCCAGTCTC	12	A	0.0325	0.4548
NC_005101.4-198826856	TTCCTTCCCACTTTCACTCCCTTACAC	15	C	0.0177	0.4887
NC_005101.4-198826856	TTCCTTCCCACTTTCACTCCCTTACAC	25	C	0.0442	0.0433
NC_005101.4-198841022	CACACTGGCACTGGCTCTCCCCAGCTC	3	C	0.0084	0.4761
NC_005101.4-198841022	CACACTGGCACTGGCTCTCCCCAGCTC	21	C	0.1345	0.1678
NC_005101.4-198847114	TGCCCTCAAACTGCTTCTCCTCCTACA	24	T	0	0.4793
NC_005101.4-198913782	AATACAAAGACCTTGCCTCCAGGTTGT	15	G	0.187	0.2778
NC_005101.4-198934237	GGAGAAACCACCCTTGCTCCGACAGTG	1	G	0.1639	0.2956
NC_005101.4-198935816	TCTAGATGAACGTTGCCTCCACTTGCA	9	A	0	0.4772
NC_005101.4-198935816	TCTAGATGAACGTTGCCTCCACTTGCA	12	G	0	0.4772
NC_005101.4-198958781	TGATTAGAAACAAGGTCTCCCCTTAGT	6	A	0.0244	0.0241
NC_005101.4-198959606	TCCCTTAAAACCTGCACTCCGAAGTGT	21	G	0.0513	0.0973
NC_005101.4-198968215	CCTCTTCCCACACTATCTCCATGGTTC	26	T	0.0342	0.4772
NC_005101.4-198974531	TGGCCTCAGACTCATGCTCCCCCTCAT	3	G	0.1583	0.4097
NC_005101.4-198981429	TCCTTGTACACTTTACCTCCCCTTCTT	8	A	0	0.4819
NC_005101.4-198986775	AGAATTGTTACTTGCACTCCTTCAGGT	13	T	0	0.4786
NC_005101.4-198986775	AGAATTGTTACTTGCACTCCTTCAGGT	27	T	0.1538	0.426
NC_005101.4-199009967	GGGTGAAGCACACGGACTCCATTTTCT	13	C	0	0.4779
NC_005101.4-199016326	GACCAGCTTACACCCCCTCCCCTGACC	25	A	0	0.4793
NC_005101.4-199073342	CTGCTTAAAACGGTAGCTCCAGGGGAT	8	A	0	0.4807
NC_005101.4-199128960	CATGTGTGGACGTCTCCTCCTTTCTTG	12	G	0.0085	0.4753
NC_005101.4-199128960	CATGTGTGGACGTCTCCTCCTTTCTTG	13	T	0.0254	0.0576
NC_005101.4-199136784	ATTTGTCACACCCTGTCTCCTCTGTGA	12	C	0.2602	0.385
NC_005101.4-199147124	CATCCACGGACTCCATCTCCTTAGCAT	8	G	0.0427	0.0418
NC_005101.4-199182717	CAGGGCCTCACTCCTCCTCCTGACTTC	4	G	0.0259	0.4545
NC_005101.4-199193397	TGTGCTCTCACTCTCCCTCCCATACTG	14	T	0	0.4819
NC_005101.4-199195240	CCAGTTGAAACCTTTGCTCCTGGAGTT	5	T	0.0175	0.0345
NC_005101.4-199195240	CCAGTTGAAACCTTTGCTCCTGGAGTT	9	A	0.027	0.4838
NC_005101.4-199196119	CAGCTTCTCACCCGGACTCCTTAGTAG	13	C	0.0531	0.0517
NC_005101.4-199241808	CTACTCCTCACCTCCTCTCCCAGCTGC	24	C	0.0991	0.4985
NC_005101.4-199275604	CTAGACATCACGAGTCCTCCCACAGGT	1	C	0.0783	0.4994
NC_005101.4-199279836	TCCCCTTAAACCTTTTCTCCTGCCCAT	21	T	0.0696	0.1588
NC_005101.4-199301744	ACAGGGCCCACAGAGCCTCCTTCGAGA	24	G	0	0.4793
NC_005101.4-199302935	GTTCCCTCTACCTACCCTCCCAGCTTT	22	A	0.1639	0.2399
NC_005101.4-199309886	TTACTAAGAACATTCCCTCCCTCAGCC	1	T	0.0603	0.4803
NC_005101.4-199309886	TTACTAAGAACATTCCCTCCCTCAGCC	12	A	0	0.4666
NC_005101.4-199330766	ATGCCCTCAACCTGCACTCCAGCCACT	21	A	0.0738	0.0861
NC_005101.4-199402558	TCACTTCTAACTCCAGCTCCAGGAGAC	26	A	0.0744	0.1163
NC_005101.4-199413490	CAGTAAACCACCAAACCTCCACGGCGA	25	C	0.1391	0.173
NC_005101.4-199488541	AGCCCCCCAACTCATTCTCCTCACAAA	3	C	0	0.4757

NC_005101.4-199488541	AGCCCCCCAACTCATTCTCCTCACAAA	4	C	0.161	0.3086
NC_005101.4-199614643	GTTGCACGAACCAGATCTCCTGTCTTC	8	G	0.2759	0.3916
NC_005101.4-199655543	AGGAAACACACCTCCTCTCCTAGGCTA	2	G	0.0248	0.0405
NC_005101.4-199734815	CTAAAACAAACCTGCTCTCCTTTAAGT	15	C	0	0.4883
NC_005101.4-199811511	TCACAACAAACCATGTCTCCCATGCCC	8	A	0	0.4807
NC_005101.4-199811511	TCACAACAAACCATGTCTCCCATGCCC	23	T	0	0.4807
NC_005101.4-199838513	TGCCATTGTACTCCAGCTCCCAGCAGT	26	G	0.025	0.0408
NC_005101.4-199851633	AGCCAAGTTACCTTTGCTCCTAGGTAT	26	A	0	0.4827
NC_005101.4-199859177	ATAAAGAACACAGTCACTCCACCAATA	14	T	0	0.4703
NC_005101.4-199859294	ATTTCTGTGACTCTGACTCCTGAGGAG	14	T	0.0084	0.4723
NC_005101.4-199859294	ATTTCTGTGACTCTGACTCCTGAGGAG	15	G	0.042	0.0571
NC_005101.4-199893573	GCTCAGAGCACAAGCCCTCCACCAGCA	13	A	0.0244	0.0241
NC_005101.4-199903158	GACCCTAGAACATGGGCTCCTTGACTG	12	A	0.0661	0.0639
NC_005101.4-199919576	CCTAAGGTAACTTAGTCTCCAGGCCAC	24	C	0.1043	0.4969
NC_005101.4-199919576	CCTAAGGTAACTTAGTCTCCAGGCCAC	26	A	0.0435	0.0425
NC_005101.4-199967690	CCTGTTTTTACATAGGCTCCTGTCACA	8	T	0.1391	0.2985
NC_005101.4-199967952	TCAAGCTTCACCTGGTCTCCCGGTGCA	22	G	0.0684	0.066
NC_005101.4-200012703	TACTCACGGACTGTGGCTCCAAACTAG	8	G	0.1917	0.4991
NC_005101.4-200022390	GTTCTGGGCACCATCTCTCCATCTTCT	24	T	0	0.4793
NC_005101.4-200032912	TCACTTCGTACAGTCTCTCCATGAAAG	7	C	0.1858	0.2104
NC_005101.4-200032912	TCACTTCGTACAGTCTCTCCATGAAAG	8	G	0.0265	0.0262
NC_005101.4-200048507	TAGGGCAGAACAGCAGCTCCCATTACA	25	A	0	0.4773
NC_005101.4-200051107	AATTGCTCTACAATGTCTCCATACCTC	1	A	0.2124	0.4968
NC_005101.4-200051107	AATTGCTCTACAATGTCTCCATACCTC	2	A	0.2143	0.496
NC_005101.4-200054398	CACACACATACTGTGGCTCCTCCCACA	27	A	0.2034	0.4948
NC_005101.4-200085462	TGTGGGAGGACTGTACCTCCACAACAG	26	A	0	0.4807
NC_005101.4-200108089	TCATGACTAACAGGAACTCCCTGTTGA	21	C	0.0598	0.4882
NC_005101.4-200112946	GAAGACTCCACCCCATCTCCTCCTCCT	22	C	0.2193	0.222
NC_005101.4-200169452	AGCGAAACCACTCATCCTCCAGAACTC	15	T	0	0.4787
NC_005101.4-200277758	TGCCTTTGGACGGGGCCTCCATTTGCC	12	G	0.0813	0.0928
NC_005101.4-200288624	AGAAGCCTTACTCAGACTCCATGTCTA	5	G	0.2087	0.32
NC_005101.4-200291821	TGTCCTCTTACCCCTTCTCCCTCTCTT	2	G	0.0976	0.4973
NC_005101.4-200295972	AGAGTCTGGACCACCCCTCCTTAACTG	24	A	0.0625	0.0771
NC_005101.4-200306171	CACTAACACACTCGGTCTCCTGTTCTC	14	G	0.0261	0.0425
NC_005101.4-200306171	CACTAACACACTCGGTCTCCTGTTCTC	27	C	0.0088	0.4547
NC_005101.4-200319350	AACAAACTTACCGTTTCTCCGTCAGGG	21	G	0.0813	0.1073
NC_005101.4-200329836	AGGGCGTCAACTGCTCCTCCGGGCTCT	5	C	0.0171	0.0336
NC_005101.4-200330346	ATGTCAACGACGGTACCTCCTGCGGTC	27	C	0	0.4856
NC_005101.4-200341706	TACTTCAAAACCTGTCCTCCACAAAAT	27	T	0.0085	0.0251
NC_005101.4-200360148	CTGGCTGTAACTCTACCTCCAGGGGAT	25	G	0.05	0.0644
NC_005101.4-200385559	CACCTCACGACCTCCTCTCCTCCTCAC	7	A	0	0.4844
NC_005101.4-200385559	CACCTCACGACCTCCTCTCCTCCTCAC	24	T	0.0336	0.4898
NC_005101.4-200393988	GATCAGGAGACTGTGGCTCCAAGAGGT	12	T	0	0.4832
NC_005101.4-200402222	CCAATGGCTACGAGACCTCCTTAATTT	16	C	0.0345	0.0504
NC_005101.4-200402222	CCAATGGCTACGAGACCTCCTTAATTT	25	T	0.0769	0.4882
NC_005101.4-200426568	ACCGTCCCCACCATTCCTCCAGGGAGT	6	C	0.0085	0.058
NC_005101.4-200465041	AGGTTTTCTACAATCCCTCCTGCTCCC	3	G	0.0603	0.0585
NC_005101.4-200465231	TCCAGGAGGACATCAACTCCCTGTGCC	21	C	0.187	0.3091
NC_005101.4-200465595	TTTATTCATACAGTTGCTCCTATAACC	14	T	0.1453	0.3771
NC_005101.4-200503454	TTGTGCTGCACACCTTCTCCATGCTGG	25	T	0.0909	0.0868
NC_005101.4-200519772	CTATACCCCACTGCATCTCCTTTCCCA	24	C	0	0.4793

NC_005101.4-200519772	CTATACCCCACTGCATCTCCTTTCCCA	27	A	0	0.4793
NC_005101.4-200569434	GCCTTCTTAACCCTCCCTCCCCTTGTG	3	C	0.2252	0.2271
NC_005101.4-200762154	CTCTGGCCCACAAACCCTCCTGCTCCT	15	C	0.5691	0.4072
NC_005101.4-200804109	TATGTCTTTACACGTCCTCCATTATTA	13	C	0.0439	0.463
NC_005101.4-200831702	CTCTGGATGACTTTCCCTCCGTTTCCT	21	G	0.1681	0.4971
NC_005101.4-200936497	GGGAACCATACCTGCTCTCCACCTACC	2	G	0	0.4772
NC_005101.4-201009238	AGGCACAGAACACTTGCTCCACAGGCC	6	C	0.2683	0.3287
NC_005101.4-201047075	CAGCTGAAAACACACGCTCCACCATCG	2	A	0	0.488
NC_005101.4-201047075	CAGCTGAAAACACACGCTCCACCATCG	22	C	0.0696	0.0671
NC_005101.4-201129241	CTGGACCCCACCCTTGCTCCAGGACTG	3	G	0.2437	0.3554
NC_005101.4-201138510	CCCAAATGTACCCTGTCTCCAAGATGT	27	T	0.2583	0.4472
NC_005101.4-201154766	TGATGAGAAACTCATGCTCCGTTTCCA	21	G	0.113	0.1369
NC_005101.4-201267099	AGGACATGAACACAAGCTCCAATCGCT	25	G	0.0082	0.0401
NC_005101.4-201306989	CCTCTTCCCACAATCCCTCCCCCAATG	25	A	0.2679	0.3264
NC_005101.4-201338449	AATTCCCTCACACTTTCTCCCTGTGTT	6	C	0.2632	0.2413
NC_005101.4-201361916	ACATCCTCAACTATTTCTCCCAAGCCA	5	C	0.2051	0.3457
NC_005101.4-201410115	AACTCAGGAACTGTTGCTCCAAATGCT	9	A	0	0.4779
NC_005101.4-201410115	AACTCAGGAACTGTTGCTCCAAATGCT	24	T	0.1933	0.2961
NC_005101.4-201871377	GGAGTTCTTACTGTTACTCCCTCCCAG	8	T	0.2377	0.321
NC_005101.4-201871377	GGAGTTCTTACTGTTACTCCCTCCCAG	12	T	0.1967	0.2032
NC_005101.4-201876620	TTCTACCAAACGCAGCCTCCTTTAGTT	26	T	0	0.48
NC_005101.4-201931724	GATGCCTGCACTTCTGCTCCCCACGGT	4	G	0.0244	0.4618
NC_005101.4-201931724	GATGCCTGCACTTCTGCTCCCCACGGT	24	C	0.2114	0.4793
NC_005101.4-201940082	GTAATATCCACCTTGGCTCCTCTGGAC	27	C	0.0992	0.4972
NC_005101.4-201944809	CCAAGAATTACTCCTGCTCCTGAAGGG	26	G	0	0.4641
NC_005101.4-202006512	ATCCCCCTAACTCCTTCTCCTCCCGTT	24	C	0.0325	0.032
NC_005101.4-202016789	CATCTGCCAACAGCTCCTCCAACAGGG	22	A	0.0407	0.0553
NC_005101.4-202049354	AGAGGAGAAACACTCCCTCCTCTAGGG	13	C	0.1475	0.4866
NC_005101.4-202072476	TCTGTACTCACCCTCACTCCTTAGTCC	7	C	0.3036	0.4641
NC_005101.4-202072476	TCTGTACTCACCCTCACTCCTTAGTCC	15	C	0.0174	0.0342
NC_005101.4-202120352	ATCAAGATAACAGCAACTCCATTGGAC	23	T	0.1356	0.4102
NC_005101.4-202120352	ATCAAGATAACAGCAACTCCATTGGAC	25	G	0.1356	0.4102
NC_005101.4-202131229	AATCCCCAAACCTGTTCTCCTTCCTTT	6	C	0	0.4807
NC_005101.4-202131229	AATCCCCAAACCTGTTCTCCTTCCTTT	12	C	0.0579	0.0562
NC_005101.4-202169891	ATGGCTTCCACAAAAGCTCCAGGCGAT	4	G	0.0917	0.1458
NC_005101.4-202169891	ATGGCTTCCACAAAAGCTCCAGGCGAT	6	T	0.0339	0.4535
NC_005101.4-202297458	TCAATTGCCACTGGCTCTCCTGATGTT	4	A	0.0085	0.4823
NC_005101.4-202393226	GCTGAGAGGACTGACTCTCCCCTTGGT	5	A	0	0.4779
NC_005101.4-202426554	TGTGAAGCAACTTTCCCTCCTTGCCCA	21	T	0	0.4819
NC_005101.4-202477583	TCTGCAGACACATGAGCTCCGGAAGGT	21	G	0	0.4807
NC_005101.4-202486328	AAGTAGAGAACTAGATCTCCACTAATT	5	A	0.0328	0.4891
NC_005101.4-202486328	AAGTAGAGAACTAGATCTCCACTAATT	24	A	0.0082	0.4822
NC_005101.4-202526647	TGTTGTGCTACCAGCACTCCTAAGTAA	14	G	0.025	0.0247
NC_005101.4-202540983	AGTCTGTATACAAAGACTCCCATGATA	3	T	0.1316	0.2088
NC_005101.4-202540983	AGTCTGTATACAAAGACTCCCATGATA	14	A	0.3009	0.3637
NC_005101.4-202579862	GTATAGTTGACATTCTCTCCAGTGGTC	8	T	0.0248	0.0245
NC_005101.4-202589514	CCCTCTCCTACCTCCACTCCCCCTGCT	23	C	0.2941	0.4682
NC_005101.4-202609439	ACCTACTGAACTATCTCTCCAGTCTCT	1	A	0	0.4773
NC_005101.4-202621412	TCTCCTCCTACTCCTACTCCTCCTTCC	26	C	0.4492	0.4066
NC_005101.4-202690470	GCTGTGAGCACCTCTCCTCCTTATACC	27	C	0.0256	0.0418
NC_005101.4-202745538	TCTTACAGGACATAAGCTCCCTCTACA	13	T	0	0.4718

NC_005101.4-202751280	TGCCCTCACACTTGGACTCCATTTCTA	22	T	0.0086	0.4836
NC_005101.4-202751280	TGCCCTCACACTTGGACTCCATTTCTA	25	C	0.2586	0.488
NC_005101.4-202765522	CCAGTTGCTACATTAACTCCTGTGTTT	1	C	0.1271	0.1621
NC_005101.4-202765522	CCAGTTGCTACATTAACTCCTGTGTTT	22	G	0.1261	0.1609
NC_005101.4-202783910	CTGCAGCAAACATGATCTCCTAGTATG	7	C	0.2203	0.3707
NC_005101.4-202783910	CTGCAGCAAACATGATCTCCTAGTATG	23	G	0.0083	0.4816
NC_005101.4-202800656	CGTATACACACTACTGCTCCATTTGCT	1	C	0.0085	0.481
NC_005101.4-202807057	CTGACTCTGACCATGGCTCCTCACCCA	3	G	0.0083	0.4816
NC_005101.4-202842017	GGCCTGCAGACTGTCCCTCCTTGAGCT	3	C	0.1475	0.4966
NC_005101.4-202843788	CTTAGGTACACACACGCTCCTTCCCCT	16	G	0.0085	0.4882
NC_005101.4-202843788	CTTAGGTACACACACGCTCCTTCCCCT	25	C	0.0085	0.4882
NC_005101.4-202861313	GGACACTTCACGGTTCCTCCATGACAT	24	A	0	0.4772
NC_005101.4-202864828	CTAGCTGTCACCCGTGCTCCCCAATGT	13	C	0.0351	0.0839
NC_005101.4-202864828	CTAGCTGTCACCCGTGCTCCCCAATGT	14	G	0.0357	0.4898
NC_005101.4-202868177	AACTGTCATACCATCGCTCCATTACTG	14	T	0	0.4757
NC_005101.4-202868177	AACTGTCATACCATCGCTCCATTACTG	15	C	0	0.4757
NC_005101.4-202872053	TAGGCAAGCACTGAGGCTCCACCCTCC	16	G	0	0.4757
NC_005101.4-202883019	TTTTCTCCAACTATGGCTCCTGTATAC	21	T	0.1667	0.235
NC_005101.4-202893602	AAAACATGAACCTGCTCTCCTGAGTAT	2	A	0.042	0.4638
NC_005101.4-202893602	AAAACATGAACCTGCTCTCCTGAGTAT	14	G	0.1167	0.1244
NC_005101.4-202987898	AGGTGAGGTACATTCCCTCCTACACAA	9	T	0	0.4773
NC_005101.4-203016540	ATAACAGTTACAGTAGCTCCTGATTAT	5	C	0.3033	0.432
NC_005101.4-203016540	ATAACAGTTACAGTAGCTCCTGATTAT	27	T	0	0.4807
NC_005101.4-203039045	GGAGAAATCACAGGGGCTCCCCCAAGG	26	G	0	0.477
NC_005101.4-203102910	GAGAAAGCGACCTGAGCTCCTAGCAGG	9	G	0.3036	0.4297
NC_005101.4-203106325	TTGCCTCCCACTTTAACTCCACCAAAT	9	C	0.0083	0.4835
NC_005101.4-203106325	TTGCCTCCCACTTTAACTCCACCAAAT	25	A	0.2917	0.4747
NC_005101.4-203128772	GTAAATTCCACGACCACTCCAAGTTAT	5	A	0	0.48
NC_005101.4-203153315	CACTCTTCAACATGAGCTCCCACTGTG	15	A	0.2602	0.427
NC_005101.4-203153315	CACTCTTCAACATGAGCTCCCACTGTG	24	T	0	0.4793
NC_005101.4-203176031	AAAACCCCTACCCATTCTCCTGGGTTT	23	G	0.1593	0.1757
NC_005101.4-203187246	CTCCACCACACACTCCCTCCATCCTTC	15	C	0.1951	0.3688
NC_005101.4-203238405	CCCCGTGTTACAGCGCCTCCTCTTCCA	4	C	0.1826	0.4962
NC_005101.4-203311546	GTTTGCCCCACCCCACCTCCCTGAGGC	3	T	0	0.4807
NC_005101.4-203313502	GGCCAGCCCACCCGTCCTCCTCCCTCT	5	A	0.1301	0.1216
NC_005101.4-203313502	GGCCAGCCCACCCGTCCTCCTCCCTCT	14	G	0.0244	0.0241
NC_005101.4-203318892	AGCTTGGTGACAGGTCCTCCGGATCCT	21	G	0.1491	0.1528
NC_005101.4-203359222	CTGACTTCCACATGTACTCCAGGTTAA	4	A	0.0088	0.483
NC_005101.4-203359222	CTGACTTCCACATGTACTCCAGGTTAA	8	C	0.1478	0.1516
NC_005101.4-203376566	TCTGTCAGCACAGATGCTCCTTGGAAC	2	C	0.1736	0.259
NC_005101.4-203383631	TAGCCCCACACTCGTGCTCCCATCGCT	2	A	0.2314	0.2533
NC_005101.4-203383631	TAGCCCCACACTCGTGCTCCCATCGCT	6	C	0.0984	0.1505
NC_005101.4-203396683	AGAAGGGTGACTGTCTCTCCTTCCCAC	21	T	0.2114	0.2937
NC_005101.4-203419848	GAAAAGGTCACTGGATCTCCTGGAGCT	16	T	0.25	0.4911
NC_005101.4-203451409	CCCGGACACACACTCTCTCCAAAGCTA	3	C	0.065	0.0629
NC_005101.4-203452731	AGCACCCAAACCCATTCTCCTACTTCC	2	G	0.2051	0.2235
NC_005101.4-203452731	AGCACCCAAACCCATTCTCCTACTTCC	14	A	0.0855	0.0818
NC_005101.4-203462560	ATAACAGAAACCAGCACTCCTCTAGAA	1	A	0.2696	0.3252
NC_005101.4-203462560	ATAACAGAAACCAGCACTCCTCTAGAA	13	A	0.0087	0.4744
NC_005101.4-203480027	ATAATAGTCACAGCAGCTCCACCTCCC	7	G	0.187	0.1695
NC_005101.4-203486606	GTCTGTCCTACTTGACCTCCTTGGCCA	23	G	0.0263	0.026

NC_005101.4-203510043	CAAAAGCCTACCACTTCTCCCTGTACA	7	C	0.0417	0.0408
NC_005101.4-203510043	CAAAAGCCTACCACTTCTCCCTGTACA	21	C	0.2083	0.2491
NC_005101.4-203547923	TGTTCGCACACAGCCCCTCCCTGATCT	1	T	0.0407	0.0398
NC_005101.4-203558220	CACCAAGGAACGTGGGCTCCCAGCTAG	13	T	0.188	0.3261
NC_005101.4-203558220	CACCAAGGAACGTGGGCTCCCAGCTAG	25	T	0.2373	0.4481
NC_005101.4-203661858	ATTCGAACCACTGTCTCTCCCATTATT	3	T	0.1017	0.1116
NC_005101.4-203661858	ATTCGAACCACTGTCTCTCCCATTATT	23	T	0.1466	0.5
NC_005101.4-203674637	GATTATGGGACCCAAGCTCCCATCTAC	4	T	0	0.4755
NC_005101.4-203674637	GATTATGGGACCCAAGCTCCCATCTAC	24	C	0.0531	0.1162
NC_005101.4-203683751	AGTTCTGAGACCTCCTCTCCTGCCTTC	2	G	0.2602	0.3334
NC_005101.4-203688639	CAGCAGGGCACACATCCTCCTGCTTTC	6	G	0.0496	0.0484
NC_005101.4-203688639	CAGCAGGGCACACATCCTCCTGCTTTC	22	G	0.3058	0.3421
NC_005101.4-203710038	ACAGCCTGCACTCATGCTCCGTTAACC	12	T	0.0488	0.0476
NC_005101.4-203723851	CTGCAAAATACATGCTCTCCTCAAGGA	5	A	0.1008	0.1398
NC_005101.4-203802917	GATAAGCTAACCCAGCCTCCTTACTCT	2	A	0	0.4826
NC_005101.4-203813323	GGTGATTAGACTCAGTCTCCAGCCCCT	24	C	0.0431	0.4916
NC_005101.4-203831297	GGGATGAAAACAGAGCCTCCAACAGCC	25	G	0.0407	0.484
NC_005101.4-203832263	AGTGGGGAGACAAGTCCTCCACCTTCC	8	A	0	0.4827
NC_005101.4-203857318	AGACGTGGCACAGGGGCTCCTGTTCAC	4	C	0.0244	0.0241
NC_005101.4-203859704	TGCATCACCACAAAGCCTCCCCCGGCG	2	G	0.025	0.0408
NC_005101.4-203884143	TGGAGTGGCACTGCATCTCCTTTGAGA	6	T	0.2821	0.4714
NC_005101.4-203884143	TGGAGTGGCACTGCATCTCCTTTGAGA	22	T	0.2821	0.3311
NC_005101.4-203884752	AGCCTCTTGACTCAGCCTCCCCAGCGC	2	G	0.0696	0.0671
NC_005101.4-203884752	AGCCTCTTGACTCAGCCTCCCCAGCGC	25	C	0.1724	0.199
NC_005101.4-203886850	TCCGATGGGACAGCAGCTCCTGGGCCT	3	C	0.0082	0.4822
NC_005101.4-203886850	TCCGATGGGACAGCAGCTCCTGGGCCT	8	G	0.0732	0.1954
NC_005101.4-203977301	TGAGTACTTACCCTACCTCCCAAGTAC	5	T	0.042	0.0411
NC_005101.4-203977301	TGAGTACTTACCCTACCTCCCAAGTAC	6	A	0.075	0.1025
NC_005101.4-203998271	CCACAATAAACCCTCCCTCCATCCATC	21	A	0.1624	0.2778
NC_005101.4-204036474	CTACCCAAGACTTCTACTCCCTTCATC	21	C	0.1043	0.0989
NC_005101.4-204036474	CTACCCAAGACTTCTACTCCCTTCATC	22	T	0	0.4851
NC_005101.4-204105432	GAGGAAGGGACTGGTCCTCCTCAACTC	2	A	0.125	0.232
NC_005101.4-204124006	TTCAATGGGACCCAGACTCCTCATTCT	1	T	0.0885	0.4276
NC_005101.4-204124006	TTCAATGGGACCCAGACTCCTCATTCT	2	T	0.0885	0.4276
NC_005101.4-204139541	GGGTTGAACACTCAGTCTCCTTTTCTC	5	T	0.2479	0.4714
NC_005101.4-204139541	GGGTTGAACACTCAGTCTCCTTTTCTC	8	A	0	0.4833
NC_005101.4-204212762	CCGTCGCAAACAAGGCCTCCTTGTGAA	5	C	0.1917	0.1997
NC_005101.4-204213759	CAAGGAGAGACCCTGTCTCCAAAAATA	3	A	0.2927	0.414
NC_005101.4-204214566	CTTCCTCCCACTGTCCCTCCTCTATAA	1	C	0.0796	0.0765
NC_005101.4-204223989	AGCAAACAAACGCTAACTCCGTTGCAA	7	C	0.2105	0.2155
NC_005101.4-204236540	CAGGATCTGACGCCCTCTCCTGGCCTC	4	G	0.0917	0.0875
NC_005101.4-204236540	CAGGATCTGACGCCCTCTCCTGGCCTC	15	C	0.0496	0.1235
NC_005101.4-204283135	CATCATAGAACTCTTCCTCCTCTTCCT	23	T	0	0.4793
NC_005101.4-204302291	CAATGAGATACAGGGCCTCCTCCGCCT	8	A	0	0.4765
NC_005101.4-204317539	AAGAGTCCCACTTTCTCTCCATGTATA	27	A	0.2793	0.4682
NC_005101.4-204323772	TATCAATCAACTAGACCTCCCAGAGCT	1	T	0.5179	0.3838
NC_005101.4-204435627	CACCTAAGTACACCATCTCCCATTTTC	1	C	0.1008	0.494
NC_005101.4-204435627	CACCTAAGTACACCATCTCCCATTTTC	5	T	0.1	0.495
NC_005101.4-204474319	CATTCACCCACAGAGCCTCCGAAGCCA	13	G	0.0894	0.1145
NC_005101.4-204474319	CATTCACCCACAGAGCCTCCGAAGCCA	21	G	0.2195	0.2885
NC_005101.4-204494874	TTCCCAGGCACACGAACTCCCATGCAC	14	G	0.0973	0.1084

NC_005101.4-204524225	ATCTTGTGAACAGCTCCTCCTTGCACA	4	T	0.2276	0.4904
NC_005101.4-204524225	ATCTTGTGAACAGCTCCTCCTTGCACA	15	T	0.2602	0.4722
NC_005101.4-204531758	AGCAAAACGACAGACCCTCCGGATGGA	8	C	0.1933	0.274
NC_005101.4-204534454	AGGACGCTGACTGATGCTCCCTGGGAC	5	C	0.1161	0.4952
NC_005101.4-204577170	CCACCCCTTACCTTATCTCCTAAAGTA	6	C	0.0917	0.1025
NC_005101.4-204577170	CCACCCCTTACCTTATCTCCTAAAGTA	14	T	0	0.4765
NC_005101.4-204596954	GCTGCCCTGACTCTCTCTCCGTGACGG	13	C	0.0265	0.4944
NC_005101.4-204596954	GCTGCCCTGACTCTCTCTCCGTGACGG	21	G	0.0696	0.0989
NC_005101.4-204607903	AAAACAAGGACTGCTGCTCCTCCACCA	4	A	0.2602	0.3141
NC_005101.4-204646959	CTAGTAATAACCAGCTCTCCTCTTGGG	23	T	0.0167	0.4765
NC_005101.4-204650628	AGGAAGGCCACAGCTCCTCCCCCTACG	22	C	0.0179	0.0351
NC_005101.4-204718104	GAATCCGCTACTGAATCTCCGAGGGCA	6	C	0	0.4793
NC_005101.4-204737730	TGGATCCTCACTGAGACTCCTGGTTAT	21	T	0.042	0.0411
NC_005101.4-204737730	TGGATCCTCACTGAGACTCCTGGTTAT	27	T	0.563	0.4045
NC_005101.4-204756507	CCTGTTATCACTACTTCTCCACCATCA	2	C	0.0169	0.0333
NC_005101.4-204761898	TTTGCTACCACTTCACCTCCATTGTCA	5	C	0	0.4773
NC_005101.4-204770572	TTCCTGACCACTCAGCCTCCAGAACTG	1	T	0.0244	0.0241
NC_005101.4-204770572	TTCCTGACCACTCAGCCTCCAGAACTG	24	A	0.1057	0.1145
NC_005101.4-204796572	CTATGCAGAACTTCATCTCCCTCACTC	9	A	0.0357	0.1626
NC_005101.4-204881374	ATCACCACCACACGAGCTCCCGTTGAC	14	G	0.2727	0.4507
NC_005101.4-204883044	TTCTGAGCCACACTGCCTCCACAGGAC	4	T	0.2358	0.4417
NC_005101.4-204899400	CTGGCTCCTACTGTGTCTCCTTTTCTG	21	T	0.2787	0.4837
NC_005101.4-204905225	GGGCTTTGCACACTCCCTCCCTTCGTC	24	C	0.1818	0.2647
NC_005101.4-204907706	AGGGTCAAGACGTGAACTCCTTGGGGA	2	G	0.041	0.0401
NC_005101.4-204907706	AGGGTCAAGACGTGAACTCCTTGGGGA	21	T	0	0.4819
NC_005101.4-204918847	TCTTCTTTGACTGTTTCTCCTTCAGGA	2	C	0.094	0.0896
NC_005101.4-204938144	TTCAAGAATACCTAATCTCCAGAATCA	23	A	0.2281	0.3424
NC_005101.4-204957153	AAGGAGCACACCCAGACTCCTTCTCTG	6	G	0.1057	0.1145
NC_005101.4-204970290	CCTAGCTGCACTGGAGCTCCCAGCCCT	16	G	0.1186	0.1409
NC_005101.4-204970290	CCTAGCTGCACTGGAGCTCCCAGCCCT	27	T	0.0085	0.4842
NC_005101.4-205001352	AGGAAAGAGACCATGACTCCTTTGATT	13	A	0.3083	0.4816
NC_005101.4-205032769	AGCTTGTCTACTGATTCTCCTGTGCGA	25	C	0.0593	0.0734
NC_005101.4-205032769	AGCTTGTCTACTGATTCTCCTGTGCGA	26	G	0.2	0.2665
NC_005101.4-205048284	CTGTCCTTCACCTCCCCTCCTACTCCC	16	C	0.2295	0.2741
NC_005101.4-205115390	CTAGGAGCCACAGGATCTCCTCCACAG	22	C	0.122	0.1425
NC_005101.4-205124635	GTCATCTCAACCCACTCTCCCCACCCT	7	T	0.1301	0.1494
NC_005101.4-205124635	GTCATCTCAACCCACTCTCCCCACCCT	14	A	0.1707	0.208
NC_005101.4-205127741	CCTCCCATAACATCCTCTCCCTTTCCT	8	T	0.1453	0.479
NC_005101.4-205127741	CCTCCCATAACATCCTCTCCCTTTCCT	15	C	0	0.4868
NC_005101.4-205131796	TCCATAAAGACAACTTCTCCCAAGCAT	16	T	0.0833	0.1099
NC_005101.4-205134699	AAATAGTAGACTGTCTCTCCCCGCAGT	22	C	0.1102	0.4997
NC_005101.4-205141205	CCACTATAGACAAAAGCTCCAAAGAGA	8	A	0.0163	0.4825
NC_005101.4-205141205	CCACTATAGACAAAAGCTCCAAAGAGA	21	A	0.122	0.1425
NC_005101.4-205147522	ATAATGTACACACGTCCTCCCATGCAC	6	G	0	0.4787
NC_005101.4-205147522	ATAATGTACACACGTCCTCCCATGCAC	16	C	0.0407	0.0705
NC_005101.4-205155856	AGGGATCTGACAGTCTCTCCAGCCTCT	1	A	0.082	0.4656
NC_005101.4-205159207	CTATGCTGCACCTCCTCTCCTCTCCCT	26	C	0.225	0.4575
NC_005101.4-205165998	TGCAGGAGGACCCAAGCTCCAGTCCCC	24	C	0.0325	0.4854
NC_005101.4-205262485	GAATGGTCAACCAGAACTCCAAGTTGG	4	T	0.0086	0.4729
NC_005101.4-205498214	CCTAATGTGACAGAGCCTCCCTCTCTT	25	C	0	0.4826
NC_005101.4-205639770	CACTGTAGCACTAGGACTCCTAACCCT	9	C	0.122	0.1425

NC_005101.4-205646358	ACAAGACTAACAAGTGCTCCTAACCAC	27	C	0.0917	0.1025
NC_005101.4-205654714	CAGGCACAGACATTCACTCCTGTAGTC	7	C	0	0.4787
NC_005101.4-205689139	GCAGAAAGAACCAGACCTCCATCCTGG	24	C	0.1983	0.4849
NC_005101.4-205725879	TGAGCCATCACCTCAACTCCAAGATTG	4	G	0.0667	0.095
NC_005101.4-205756800	GACTGCAAAACTCTTCCTCCTATTCCC	2	A	0.042	0.0728
NC_005101.4-205756800	GACTGCAAAACTCTTCCTCCTATTCCC	21	T	0.05	0.0488
NC_005101.4-205790455	TGGTGCTACACCACAGCTCCAGTTTTG	4	T	0	0.4841
NC_005101.4-205844694	ACAGACATAACTTCAGCTCCTCAGGGT	1	A	0.0325	0.032
NC_005101.4-205844694	ACAGACATAACTTCAGCTCCTCAGGGT	13	T	0.1066	0.4489
NC_005101.4-205895336	TCTTGTGTTACTGACACTCCATGGTCT	22	T	0.0427	0.0418
NC_005101.4-205897505	CACACATAGACATGTCCTCCAGACGCT	7	T	0.1951	0.4926
NC_005101.4-205897505	CACACATAGACATGTCCTCCAGACGCT	24	C	0.2114	0.3041
NC_005101.4-205918771	AGAATTTAAACAAAGTCTCCTGACCAA	24	C	0	0.4827
NC_005101.4-205928770	TGGGCTCAGACATTGTCTCCCACTGCA	8	A	0.0609	0.1369
NC_005101.4-205953810	CTGCAAGAAACTGAAGCTCCCAGACAA	1	C	0.0354	0.0517
NC_005101.4-205970852	CACATATAAACAAGACCTCCACCCATC	22	C	0.1345	0.2683
NC_005101.4-206036207	GTCCCAGGCACAAGGGCTCCCCAGGAT	8	G	0.0446	0.0436
NC_005101.4-206036207	GTCCCAGGCACAAGGGCTCCCCAGGAT	15	G	0.1071	0.2052
NC_005101.4-206036315	AAACATGGCACTAGTTCTCCAACCAGA	23	C	0.124	0.1853
NC_005101.4-206044271	AAAGACACCACAATATCTCCCATCTTA	4	G	0	0.477
NC_005101.4-206044271	AAAGACACCACAATATCTCCCATCTTA	6	C	0.1239	0.1316
NC_005101.4-206045349	TCCCACACCACCCTCACTCCACCAAAA	9	C	0.1083	0.1997
NC_005101.4-206045404	TTTGGTATTACAGTGCCTCCTCTTCTC	2	T	0.188	0.2945
NC_005101.4-206045404	TTTGGTATTACAGTGCCTCCTCTTCTC	22	C	0	0.4807
NC_005101.4-206050837	ATCCTTCCAACCTTGCCTCCTCTCCCT	6	T	0.2069	0.3478
NC_005101.4-206052651	GTTCTGTGGACACTAGCTCCCTTCTCT	13	C	0.1066	0.4984
NC_005101.4-206052651	GTTCTGTGGACACTAGCTCCCTTCTCT	26	C	0.2541	0.3307
NC_005101.4-206090788	GTATGGAAAACAACCACTCCACAGGCC	12	A	0.2719	0.3273
NC_005101.4-206090788	GTATGGAAAACAACCACTCCACAGGCC	25	G	0.0702	0.0677
NC_005101.4-206127980	TTCACAAAGACAGCCACTCCCCTAGTC	7	A	0.0164	0.4807
NC_005101.4-206136524	CCCCAGCTCACCCACACTCCTACATAA	1	C	0.041	0.4879
NC_005101.4-206136524	CCCCAGCTCACCCACACTCCTACATAA	2	C	0.0902	0.1154
NC_005101.4-206185095	ATAGATGACACTGTTGCTCCAGGTAGT	16	G	0.1736	0.2234
NC_005101.4-206185315	GAGAAAGTAACTTGTCCTCCCTGAGCC	22	T	0.2389	0.4493
NC_005101.4-206209728	GAGCCGAGGACCCTTTCTCCCATCCTC	6	G	0.0656	0.0786
NC_005101.4-206209728	GAGCCGAGGACCCTTTCTCCCATCCTC	23	T	0.041	0.0401
NC_005101.4-206210043	GAGTAAGACACTGTCCCTCCATGCAAC	22	T	0	0.4751
NC_005101.4-206217165	GTCAAAAGTACAGTGTCTCCAGAGCTG	9	T	0.0174	0.4764
NC_005101.4-206318647	GGAAAATGGACATAGGCTCCCAACCCC	16	G	0.1696	0.2254
NC_005101.4-206367187	TGGCCTTGAACTGAAGCTCCTAAGCTG	3	G	0.1897	0.199
NC_005101.4-206433775	CTCTTCCTTACATTCTCTCCAGCATTT	27	T	0.181	0.2188
NC_005101.4-206443881	TCCTGTGACACACCTTCTCCAACAAGG	8	A	0.0328	0.0322
NC_005101.4-206517204	CCAGAACAAACCTCCACTCCCAGAGAA	23	G	0	0.4839
NC_005101.4-206519388	CCCAGTCCCACAGCAGCTCCTCCTGGG	12	A	0	0.4833
NC_005101.4-206519388	CCCAGTCCCACAGCAGCTCCTCCTGGG	13	G	0	0.4833
NC_005101.4-206523068	ATCCACATTACAGCACCTCCTCCCTTT	8	T	0.2276	0.4497
NC_005101.4-206568568	GCTACGTCTACTTCCACTCCTCCATTT	6	G	0.0168	0.4813
NC_005101.4-206664806	ATTAAAGACACAAGAGCTCCTACAATA	2	T	0.0442	0.0433
NC_005101.4-206664806	ATTAAAGACACAAGAGCTCCTACAATA	3	T	0.0442	0.0433
NC_005101.4-206667858	GGGAATCTGACACACTCTCCTACTCCC	5	A	0.0328	0.0322
NC_005101.4-206682380	ACAGGACAAACTCTTACTCCTGCACTC	3	A	0	0.4749

NC_005101.4-206702355	ATCCCTGTAACGACAGCTCCAGGAAAT	24	A	0.0088	0.4693
NC_005101.4-206705133	GCACTCAGAACAAGAGCTCCCATCTGT	3	A	0	0.4778
NC_005101.4-206719304	TATCTGAGCACCCCATCTCCTTTTTAT	24	T	0.1789	0.4854
NC_005101.4-206761652	TATGAAATCACACTTTCTCCTGATTAC	24	T	0.1552	0.4999
NC_005101.4-206761652	TATGAAATCACACTTTCTCCTGATTAC	26	A	0.1579	0.2413
NC_005101.4-206775638	TTCTCTTCTACCTCCACTCCCACTCTG	21	C	0.2054	0.4617
NC_005101.4-206824599	TAAGTGGGCACCCTCCCTCCCAGGCGG	26	G	0.0252	0.4638
NC_005101.4-206825079	CGCGTGCGTACTGTGGCTCCCCAAGAC	8	G	0.087	0.1143
NC_005101.4-206856798	TACCCGTCCACTGCTGCTCCGTTTGAT	12	T	0.2083	0.4708
NC_005101.4-206856798	TACCCGTCCACTGCTGCTCCGTTTGAT	13	G	0.1488	0.1918
NC_005101.4-206873627	CCTAACACGACCAATCCTCCTACCCTA	24	C	0.1453	0.3771
NC_005101.4-206878929	GGTAGAGGAACTCTACCTCCTCTAAAT	16	C	0.1552	0.2739
NC_005101.4-206880000	TGCGTGTGCACACACACTCCACTCAGG	1	T	0.2033	0.319
NC_005101.4-206880000	TGCGTGTGCACACACACTCCACTCAGG	3	C	0.2459	0.3709
NC_005101.4-206881591	TTACCCAGCACTCTCTCTCCTTTTTGT	22	T	0.2051	0.2604
NC_005101.4-206882601	CATCCAACAACTACTACTCCATGAGGA	3	T	0	0.4779
NC_005101.4-206913456	CCAGCCTTCACCCCGTCTCCTCTGCCC	13	C	0.0569	0.0553
NC_005101.4-206913456	CCAGCCTTCACCCCGTCTCCTCTGCCC	14	C	0.1545	0.208
NC_005101.4-206924949	ACCATATCTACATTCTCTCCTCACTAC	1	A	0	0.4793
NC_005101.4-206924949	ACCATATCTACATTCTCTCCTCACTAC	3	C	0.1057	0.1001
NC_005101.4-206927543	CCTGCCTCCACCCCACCTCCACAGAGC	21	A	0	0.4671
NC_005101.4-206966255	CCAGGCCCAACCCCAGCTCCAACTATC	25	A	0	0.48
NC_005101.4-206966255	CCAGGCCCAACCCCAGCTCCAACTATC	27	C	0	0.48
NC_005101.4-207015914	CCTCTGATGACTCTAGCTCCTGTCAAG	7	A	0.5678	0.4066
NC_005101.4-207048915	TCATGCGATACTGTCACTCCAGAACTA	6	C	0.2083	0.3622
NC_005101.4-207064473	AGTGATAAAACTCTTGCTCCATTCGTC	2	G	0.0769	0.0896
NC_005101.4-207117144	TCTCAGAACACAGGTCCTCCTGCCCCT	24	C	0.1148	0.1641
NC_005101.4-207145305	TTAAATTAGACATTCTCTCCTGCCGTC	25	G	0.0603	0.0746
NC_005101.4-207181432	AAGGTCCCAACTTACACTCCTGTAACA	15	C	0.1475	0.1904
NC_005101.4-207226744	AACTAACACACATCTCCTCCAGGGAAC	7	C	0.3305	0.4066
NC_005101.4-207251037	AGATGGGGCACCGAGACTCCAGTGCAA	15	G	0	0.4813
NC_005101.4-207279853	AGGTTTTCAACCTTGGCTCCACACATC	13	T	0	0.4793
NC_005101.4-207279853	AGGTTTTCAACCTTGGCTCCACACATC	16	G	0	0.4793
NC_005101.4-207332531	CTATATAGTACTGATTCTCCTAGAGTA	22	A	0.0083	0.4708
NC_005101.4-207401061	TCTACCTTTACATGGGCTCCAAGGATT	22	A	0.5366	0.3926
NC_005101.4-207444065	AAGCGTACAACTCATCCTCCCTTCAAA	9	A	0.0177	0.4793
NC_005101.4-207444065	AAGCGTACAACTCATCCTCCCTTCAAA	26	A	0.2035	0.4944
NC_005101.4-207447420	CATAGACAAACGCTGCCTCCCGACTGC	23	A	0.0083	0.4747
NC_005101.4-207483061	TCCTTTGAGACAGGGTCTCCCCCTGAT	5	T	0.0263	0.026
NC_005101.4-207485325	CAGAATGAGACTTAGTCTCCCAAACAA	12	T	0.0084	0.4797
NC_005101.4-207489908	TTCGCCATTACTGTATCTCCAGGGGAT	2	T	0.2542	0.4535
NC_005101.4-207489908	TTCGCCATTACTGTATCTCCAGGGGAT	6	C	0	0.4718
NC_005101.4-207491407	AGCACTCACACAGAAGCTCCCTCATCA	9	C	0	0.4793
NC_005101.4-207492018	AGGAGCTAAACCCAAACTCCTGGGACT	25	A	0.0424	0.0415
NC_005101.4-207492967	TCTTAGACTACTCTCCCTCCTCTTCTG	5	A	0.2377	0.2339
NC_005101.4-207492967	TCTTAGACTACTCTCCCTCCTCTTCTG	12	T	0	0.4807
NC_005101.4-207525340	GATGAGTTCACGTACTCTCCTTCACTT	8	T	0.275	0.4525
NC_005101.4-207525340	GATGAGTTCACGTACTCTCCTTCACTT	27	T	0.1818	0.2046
NC_005101.4-207530298	TGAATTAACACCCAAGCTCCTTGCACT	24	C	0.1803	0.2032
NC_005101.4-207533119	GGTTAACGCACCCTTCCTCCCATGGCT	9	C	0.0167	0.0328
NC_005101.4-207583490	TCAAGTCAAACTCTGACTCCCCCGCCC	8	A	0.0089	0.4665

NC_005101.4-207583490	TCAAGTCAAACTCTGACTCCCCCGCCC	21	C	0.0354	0.0348
NC_005101.4-207596519	TTCTGGCCCACCATGTCTCCTTCATCT	13	A	0	0.4755
NC_005101.4-207606069	ACATTGAAGACACCGACTCCATCTCTT	27	T	0	0.4779
NC_005101.4-207673845	AGTTGGGACACGTGGTCTCCCTGCCTG	24	C	0.3554	0.4557
NC_005101.4-207673845	AGTTGGGACACGTGGTCTCCCTGCCTG	27	G	0.0488	0.0476
NC_005101.4-207699108	CTGCTGTGCACTCTCACTCCTGAGGAC	1	C	0.0357	0.0521
NC_005101.4-207746523	AGGCACCATACTTAGTCTCCTAATTCG	27	G	0	0.4813
NC_005101.4-207748919	ACATGAGCAACTGCTTCTCCCAGATAG	8	C	0.0696	0.4915
NC_005101.4-207766870	GATTCTAAAACGTTGGCTCCTGACTGT	7	A	0	0.4751
NC_005101.4-207766870	GATTCTAAAACGTTGGCTCCTGACTGT	24	C	0.1765	0.4829
NC_005101.4-207808556	CATCTGTCTACCCTTCCTCCATTCCTC	5	T	0.1849	0.2449
NC_005101.4-207811338	CTAGTGGGCACCAGGGCTCCAGCCTTT	5	T	0.1597	0.1469
NC_005101.4-207811338	CTAGTGGGCACCAGGGCTCCAGCCTTT	15	G	0.1933	0.2509
NC_005101.4-207818635	TGCTGCCATACTACGACTCCATCAAAA	16	A	0.1982	0.4026
NC_005101.4-207818635	TGCTGCCATACTACGACTCCATCAAAA	27	A	0.0811	0.4351
NC_005101.4-207906662	AGACCTTCTACTCCTTCTCCATGTTTT	5	C	0.2602	0.385
NC_005101.4-207906662	AGACCTTCTACTCCTTCTCCATGTTTT	27	T	0.2764	0.4497
NC_005101.4-207945939	ACATACAGAACCTGTTCTCCTCATTTA	13	T	0.0172	0.482
NC_005101.4-207945939	ACATACAGAACCTGTTCTCCTCATTTA	14	G	0.1102	0.119
NC_005101.4-207956507	GCTTGACCCACACAGGCTCCAAAGATA	2	C	0	0.4826
NC_005101.4-207956507	GCTTGACCCACACAGGCTCCAAAGATA	21	A	0	0.4826
NC_005101.4-207969975	AGCTTCTATACCCGGGCTCCAGCCGGC	9	T	0	0.4793
NC_005101.4-207973068	CGCAGAGACACAGTGGCTCCATTAATG	26	T	0	0.4751
NC_005101.4-207973068	CGCAGAGACACAGTGGCTCCATTAATG	27	G	0	0.4751
NC_005101.4-208009099	ACGAAAGCTACCTTGGCTCCAGAGCTC	2	C	0.2941	0.3643
NC_005101.4-208009099	ACGAAAGCTACCTTGGCTCCAGAGCTC	13	T	0.0083	0.4816
NC_005101.4-208014779	TTTTGAAAGACGCCACCTCCCTTTCTG	3	T	0	0.4751
NC_005101.4-208029861	CTCCAAGGAACATGAGCTCCTAACACC	23	A	0	0.4846
NC_005101.4-208041306	TGACCTTGAACTCAAGCTCCTTCAACT	21	T	0.0732	0.0705
NC_005101.4-208058756	TGAGAGTTCACTTAATCTCCCCATGTC	1	T	0.2389	0.359
NC_005101.4-208063006	TCTCTCCTCACGCTCCCTCCCCACCTG	3	T	0.0351	0.0677
NC_005101.4-208063006	TCTCTCCTCACGCTCCCTCCCCACCTG	6	C	0.0351	0.0677
NC_005101.4-208096474	GTAGGAGCAACATGGACTCCATTGGCT	16	A	0.1626	0.1761
NC_005101.4-208156204	CGTAGCCAGACAAATGCTCCAGCCTTC	1	C	0	0.4773
NC_005101.4-208156204	CGTAGCCAGACAAATGCTCCAGCCTTC	9	G	0.1818	0.2172
NC_005101.4-208180113	ACGACGGGCACAACTACTCCATGGGTT	5	C	0.1345	0.5
NC_005101.4-208187862	CTTCCCAACACTGCATCTCCCTTCTTC	1	C	0.2193	0.4353
NC_005101.4-208187862	CTTCCCAACACTGCATCTCCCTTCTTC	6	C	0.0439	0.0429
NC_005101.4-208293138	AATGGACTAACACAATCTCCTAGGGAT	26	A	0.1949	0.4138
NC_005101.4-208312617	TTGGGGTGCACAGCTTCTCCTGGCACC	24	C	0.0089	0.4824
NC_005101.4-208591243	GTGGTTTCCACGAATCCTCCATTGCAT	1	G	0.0732	0.0705
NC_005101.4-208591939	CTGTGGTGAACACGTACTCCCTGCTAG	13	C	0.1261	0.1639
NC_005101.4-208633319	GTGGGAGCTACCGAAGCTCCTAGGGTC	12	C	0	0.48
NC_005101.4-208784255	CTTTCCCTCACCCTGTCTCCAAGAGGT	6	C	0.2417	0.4958
NC_005101.4-208888069	CTCCAGACTACCCTCCCTCCGACAATT	9	T	0.1967	0.1774
NC_005101.4-208888069	CTCCAGACTACCCTCCCTCCGACAATT	25	A	0	0.4793
NC_005101.4-208898915	GACATAAGTACACATCCTCCCTCTAAA	21	C	0.036	0.0354
NC_005101.4-209107523	GGGCCTCAGACTGGACCTCCTGAGCCA	2	G	0	0.4868
NC_005101.4-209167517	TTAGCTGTAACTCCAGCTCCAGGGAAT	22	G	0.1818	0.1787
NC_005101.4-209167517	TTAGCTGTAACTCCAGCTCCAGGGAAT	26	A	0.1818	0.1787
NC_005101.4-209182948	CTGATCACAACTTCCCCTCCATCATCT	2	T	0.2167	0.2432

NC_005101.4-209182948	CTGATCACAACTTCCCCTCCATCATCT	21	A	0.2167	0.2432
NC_005101.4-209186682	AACTATTGAACCATCTCTCCATACCCT	24	C	0	0.4851
NC_005101.4-209219866	CTCTGAGACACTGGATCTCCAATCAGG	1	C	0.2281	0.4255
NC_005101.4-209287678	CATGACACAACTGAGCCTCCAATGAAA	14	A	0.1261	0.4723
NC_005101.4-209326341	TCTCCTGCCACATCTCCTCCACCTACA	4	C	0.2033	0.2556
NC_005101.4-209358061	GAAGAAGCAACGGGTTCTCCAAACTGA	5	A	0.1161	0.1699
NC_005101.4-209529236	CCTTTCGTAACACTTTCTCCTTTTCTC	7	G	0.0776	0.1058
NC_005101.4-209529236	CCTTTCGTAACACTTTCTCCTTTTCTC	14	T	0.0598	0.0896
NC_005101.4-209538143	CACTTCCTGACCCCCTCTCCAAGAGTT	3	C	0.1304	0.1516
NC_005101.4-209538143	CACTTCCTGACCCCCTCTCCAAGAGTT	4	T	0.0256	0.0253
NC_005101.4-209585128	AGGTAGATGACCTGACCTCCCTTCCCT	3	G	0.1933	0.2012
NC_005101.4-209585128	AGGTAGATGACCTGACCTCCCTTCCCT	23	T	0	0.4844
NC_005101.4-209598358	ACCTCTCGTACCCCATCTCCCCACACC	7	C	0.2203	0.4305
NC_005101.4-209696869	TACATGGGCACATGAACTCCTGGCTTA	12	A	0	0.4787
NC_005101.4-209702585	CTTACCTGCACAAGATCTCCATATAAT	24	T	0.0083	0.4803
NC_005101.4-209702585	CTTACCTGCACAAGATCTCCATATAAT	26	A	0.0082	0.0243
NC_005101.4-209724835	TTACAGTTTACATACACTCCTTCACTT	3	A	0	0.48
NC_005101.4-209724835	TTACAGTTTACATACACTCCTTCACTT	26	T	0.0088	0.0429
NC_005101.4-209756202	TCTATCATTACATTATCTCCCAGACCT	12	A	0.0446	0.0605
NC_005101.4-209756202	TCTATCATTACATTATCTCCCAGACCT	24	A	0	0.4854
NC_005101.4-209875980	CTTTCCTGGACATGAGCTCCATAGGAG	27	G	0.2	0.2311
NC_005101.4-209897515	CTCGACTTCACTCATCCTCCTATATCT	15	T	0.1488	0.4959
NC_005101.4-209914780	AATCCAGGGACTCAGCCTCCACATCCG	26	C	0.2295	0.3537
NC_005101.4-209928084	ACTTACCAGACATCAACTCCATCGTTC	12	A	0.2301	0.4135
NC_005101.4-209930370	AGATCAGCCACCCCGCCTCCATCTTCT	7	G	0.2541	0.3581
NC_005101.4-209930370	AGATCAGCCACCCCGCCTCCATCTTCT	14	C	0	0.4807
NC_005101.4-209934624	CCACAGTACACAATTCCTCCATGTATG	5	A	0	0.4931
NC_005101.4-209934624	CCACAGTACACAATTCCTCCATGTATG	26	T	0	0.4922
NC_005101.4-209962512	AACCTGGAGACATCGTCTCCATGGGCT	3	C	0.2435	0.4017
NC_005101.4-209979487	CATCCTTACACTCCTCCTCCTCCTCTT	27	T	0.2562	0.4146
NC_005101.4-209987410	AGGGACTGGACTTCAGCTCCAAGAGTT	12	T	0.0164	0.0322
NC_005101.4-209987410	AGGGACTGGACTTCAGCTCCAAGAGTT	15	A	0	0.4807
NC_005101.4-210023718	CTATGTAGAACACAAGCTCCTTGAGGA	15	A	0.0574	0.071
NC_005101.4-210023927	GATAGGAGTACTCTCTCTCCTTTGCCT	1	G	0.0083	0.4732
NC_005101.4-210144876	CCCACCACCACCCAGTCTCCAATGCTG	15	G	0	0.4793
NC_005101.4-210151314	CTGGATCTGACCGGACCTCCATGTGCT	22	T	0	0.4875
NC_005101.4-210165342	ATGAAGAGTACAGAAGCTCCATGTGGG	6	G	0.0244	0.0241
NC_005101.4-210211269	GAGCCCTTCACGCCAGCTCCTGCTTTA	13	C	0.0488	0.0629
NC_005101.4-210214257	AGAAGACCAACTGGGGCTCCATTGGAC	23	T	0	0.4773
NC_005101.4-210225785	TGAAGTGGCACATGCACTCCTGGCTCA	4	A	0	0.4793
NC_005101.4-210226527	AGGCGCCAGACCGCTGCTCCTGTTGCT	12	C	0	0.482
NC_005101.4-210226527	AGGCGCCAGACCGCTGCTCCTGTTGCT	14	C	0.0862	0.0981
NC_005101.4-210256121	CAGCTCAAGACAACGGCTCCACTTAAT	14	C	0.0684	0.4553
NC_005101.4-210258331	CAGCTCATAACTATAACTCCAGTTACA	9	A	0.1607	0.5
NC_005101.4-210266929	TCTCTGAACACTTAGGCTCCAGAGCTG	25	C	0.1681	0.4986
NC_005101.4-210297889	TACTTAGCCACAGCTGCTCCATTAGAG	6	A	0.0976	0.1216
NC_005101.4-210314982	TCAAGCAGAACTGCCTCTCCTTGAGTC	22	T	0	0.4793
NC_005101.4-210322493	CACACATGGACACTAACTCCCTAACGC	23	A	0.1186	0.4102
NC_005101.4-210322493	CACACATGGACACTAACTCCCTAACGC	26	G	0	0.4666
NC_005101.4-210332080	GGATTGAGCACTCTTCCTCCATGTTCT	21	A	0	0.4793
NC_005101.4-210336908	CACACACACACACCCTCTCCACAGTCT	1	C	0.1681	0.2618

NC_005101.4-210336908	CACACACACACACCCTCTCCACAGTCT	2	A	0.2143	0.3367
NC_005101.4-210346300	GCCTCGGTCACTGCCCCTCCTGGGTGG	6	G	0.0427	0.0418
NC_005101.4-210373138	AAACAACCCACTTGGGCTCCCCAACCC	26	C	0.0331	0.0325
NC_005101.4-210385925	TGTCCCCATACTCCCACTCCCACGGAA	23	C	0.1638	0.2797
NC_005101.4-210388778	TGTGAGCTGACAGATCCTCCCACTGAC	6	G	0.0256	0.0253
NC_005101.4-210390947	TTTCACGGTACAGTCGCTCCTTCTCTT	6	C	0.1667	0.3568
NC_005101.4-210393115	ATACACACAACTAACTCTCCTCCTTGT	4	C	0.0087	0.0257
NC_005101.4-210393115	ATACACACAACTAACTCTCCTCCTTGT	23	C	0	0.4887
NC_005101.4-210410030	TGTCGGCATACTCAAACTCCAGGTCTG	5	G	0.0248	0.0245
NC_005101.4-210451021	CTGTCAGTCACCTGTACTCCATTCCTG	26	T	0.0089	0.4665
NC_005101.4-210481553	GGATCAGAGACTGTGCCTCCCACAGTC	3	A	0	0.482
NC_005101.4-210574038	CTTTGTTGGACTTTGACTCCCTTGGTT	4	T	0	0.48
NC_005101.4-210612274	AGCCTGGAGACCCAGACTCCCTCCCAC	16	A	0.2705	0.3831
NC_005101.4-210633316	GGACAATCCACGCAACCTCCTCCTCTC	25	C	0.1624	0.2423
NC_005101.4-210633746	TACCCTAGAACCCTCTCTCCACATTTG	5	C	0.0504	0.065
NC_005101.4-210633746	TACCCTAGAACCCTCTCTCCACATTTG	15	C	0	0.4772
NC_005101.4-210646986	TGCGTTGATACCTTTCCTCCAGTGGAA	4	G	0.2137	0.2041
NC_005101.4-210646986	TGCGTTGATACCTTTCCTCCAGTGGAA	12	C	0.2222	0.3054
NC_005101.4-210716867	AAGGGCGTCACCCACGCTCCTCTTCCT	2	A	0	0.4793
NC_005101.4-210716867	AAGGGCGTCACCCACGCTCCTCTTCCT	15	C	0	0.4793
NC_005101.4-210722753	ACAAATCCCACCACGCCTCCCTCTCTG	14	C	0	0.4793
NC_005101.4-210725240	CATCTCCAAACCTCCCCTCCCCTGCAC	2	A	0.4836	0.3667
NC_005101.4-210725240	CATCTCCAAACCTCCCCTCCCCTGCAC	5	T	0.4876	0.3687
NC_005101.4-210740211	TCCTGAGCTACTCTTCCTCCTCAGCTG	24	G	0.175	0.2608
NC_005101.4-210777099	AGATAATGCACTCAAACTCCACATTTT	3	A	0.0855	0.4868
NC_005101.4-210780572	TACCTTCAGACACAGGCTCCCTGGCAG	27	G	0.2069	0.2378
NC_005101.4-210835113	CTCTTGCATACAGAAACTCCATGTTCC	22	T	0.0083	0.4816
NC_005101.4-210836413	CTTATCAACACTTCATCTCCAAACCCA	1	C	0.1121	0.4982
NC_005101.4-210836413	CTTATCAACACTTCATCTCCAAACCCA	8	A	0.3025	0.4008
NC_005101.4-210850756	AATGGAACCACCACACCTCCTCTCTTT	13	A	0.0325	0.032
NC_005101.4-210964877	GGGATCCTCACACACACTCCTGACACA	9	C	0.1681	0.2738
NC_005101.4-210964877	GGGATCCTCACACACACTCCTGACACA	15	C	0.1681	0.2738
NC_005101.4-210973929	ACACCTGTAACTCCAGCTCCACAGGAT	24	G	0.2295	0.2741
NC_005101.4-210982469	GTGTCTAGGACTCTTCCTCCCACTGTG	16	C	0.0976	0.1073
NC_005101.4-210985858	ATCGCTAATACTGATCCTCCTTTTCTC	4	G	0.0088	0.49
NC_005101.4-211008255	CTGCTTGGAACCCTCCCTCCTTGCGAC	15	C	0.2645	0.4923
NC_005101.4-211012461	GCAATGGCTACACAGCCTCCACCTCTG	23	C	0	0.4765
NC_005101.4-211012461	GCAATGGCTACACAGCCTCCACCTCTG	25	C	0.2149	0.2759
NC_005101.4-211064044	AGAGCTGGGACTGGAGCTCCCTGGAAG	3	A	0	0.4765
NC_005101.4-211100508	TGAAGACAAACATGAACTCCAGTGTAC	26	A	0.0246	0.0243
NC_005101.4-211100508	TGAAGACAAACATGAACTCCAGTGTAC	27	C	0	0.4751
NC_005101.4-211135398	CATGGCGTGACACCTTCTCCTCACACT	15	T	0.2358	0.2989
NC_005101.4-211149245	TGAACAACCACACACACTCCACACTCG	26	C	0.0513	0.05
NC_005101.4-211151837	ACTCTGAAAACCTCGGCTCCATTCTCT	21	A	0.0855	0.4602
NC_005101.4-211165391	AAAAATCCCACACACCCTCCTGTGTTC	4	A	0.018	0.4704
NC_005101.4-211165391	AAAAATCCCACACACCCTCCTGTGTTC	6	T	0.027	0.0267
NC_005101.4-211201482	CTGCTGCTCACCTCTGCTCCTCCTGCT	6	G	0	0.4807
NC_005101.4-211202409	GTCCTCCTCACCCAGCCTCCTGAACTT	5	T	0	0.4856
NC_005101.4-211240989	CACACTTCAACATGGTCTCCTCTGGAG	4	A	0.075	0.4875
NC_005101.4-211241406	ACCATTCCCACGGCTGCTCCGTATGCC	12	G	0	0.48
NC_005101.4-211241406	ACCATTCCCACGGCTGCTCCGTATGCC	13	G	0.2114	0.3688

NC_005101.4-211254463	TACTGTGGCACACGTACTCCACTGTTC	13	C	0.2589	0.3794
NC_005101.4-211254463	TACTGTGGCACACGTACTCCACTGTTC	15	T	0.2589	0.4119
NC_005101.4-211293225	GCTTCAGCCACTCACTCTCCCCACTGG	14	A	0.0776	0.0746
NC_005101.4-211345200	GCCTCGCTTACAGAGCCTCCTGTTTGT	16	C	0.2941	0.4117
NC_005101.4-211358872	ACTGACTGTACCATCTCTCCCGCACAT	9	T	0	0.4875
NC_005101.4-211421941	AATATTCACACAAGTACTCCTCGTAAT	15	T	0	0.4718
NC_005101.4-211506099	TCGAGCAGGACACTCACTCCTTCTCAG	2	C	0.252	0.356
NC_005101.4-211506099	TCGAGCAGGACACTCACTCCTTCTCAG	26	A	0	0.4807
NC_005101.4-211509032	TGCCGATGAACGGGACCTCCACTTTGG	5	G	0.0089	0.4665
NC_005101.4-211509032	TGCCGATGAACGGGACCTCCACTTTGG	21	A	0.0268	0.4709
NC_005101.4-211608755	AAGACAAGGACTGCCACTCCAGCTAGG	9	G	0.0254	0.0251
NC_005101.4-211608755	AAGACAAGGACTGCCACTCCAGCTAGG	16	A	0.1897	0.2622
NC_005101.4-211611466	GATCCTTCCACTCAGGCTCCCAAACAC	7	T	0.1707	0.208
NC_005101.4-211620852	TACTTATCCACTGACCCTCCTTGTGAC	4	T	0	0.4743
NC_005101.4-211675996	CAATGTGACACTGTGTCTCCAAAAGTA	9	C	0	0.4807
NC_005101.4-211716849	TATTAGTGCACCTCTGCTCCTGTATAA	23	T	0	0.4773
NC_005101.4-211751601	AGGACAACTACCACTGCTCCTTTCTTC	16	G	0.3058	0.3771
NC_005101.4-211813642	CAGGTTTCTACCCCAGCTCCTCAGTGA	16	G	0	0.48
NC_005101.4-211880475	GGCTGGAACACTGGAACTCCTCCTTTG	22	C	0	0.4807
NC_005101.4-211971966	GTGTGGCTGACTGTTCCTCCTTCCTCT	8	T	0.0167	0.0328
NC_005101.4-212004340	CAGCTCCTTACTTAGGCTCCCTTCCTG	9	T	0	0.4728
NC_005101.4-212004340	CAGCTCCTTACTTAGGCTCCCTTCCTG	13	T	0.075	0.4416
NC_005101.4-212028459	CTGTTTATCACGTTATCTCCAGAACCT	2	T	0	0.4856
NC_005101.4-212157218	AACTGAGTCACGCAGACTCCAGTCTTT	1	A	0.2645	0.3645
NC_005101.4-212202446	TCACATTCGACAGGGTCTCCTAGGTAA	5	A	0	0.4743
NC_005101.4-212222589	TGAAATAACACTTGTGCTCCACGGCTG	7	A	0	0.4826
NC_005101.4-212222589	TGAAATAACACTTGTGCTCCACGGCTG	24	G	0	0.4826
NC_005101.4-212244769	ATCAGGGACACATCCCCTCCCATCCCA	4	A	0.2857	0.4406
NC_005101.4-212259199	CACTTTCCAACCCCTCCTCCTGACTCT	25	T	0	0.4787
NC_005101.4-212308294	CCTAAATAAACAAACTCTCCCTTTCCT	27	T	0.0086	0.4866
NC_005101.4-212417264	TCAGCGTCTACGGAGTCTCCATGCTAT	12	G	0.0446	0.0771
NC_005101.4-212465228	CACTTTGCCACTTTGCCTCCCCTGAAC	5	T	0.1786	0.2817
NC_005101.4-212487465	TCCCATCTCACCAACGCTCCTGTCTTG	3	C	0.0894	0.0854
NC_005101.4-212487465	TCCCATCTCACCAACGCTCCTGTCTTG	12	C	0.0894	0.1695
NC_005101.4-212502349	GATCCTGACACCCACACTCCCTTATTG	24	A	0.0081	0.4777
NC_005101.4-212507232	TTTCCTCCAACCCTCCCTCCTCCTACA	2	T	0.2049	0.2095
NC_005101.4-212522305	TATGGGAAGACCATTTCTCCTTCTTAC	1	T	0.0932	0.0889
NC_005101.4-212675200	ACATCGTAAACAAAGACTCCTTTGCCT	6	G	0	0.4887
NC_005101.4-213538537	ATATTCTCCACATAGTCTCCTAGGAAG	4	T	0	0.48
NC_005101.4-213538537	ATATTCTCCACATAGTCTCCTAGGAAG	13	T	0.0417	0.0566
NC_005101.4-213549316	AGATAACACACTGTTTCTCCTCAAGAT	3	A	0.175	0.2125
NC_005101.4-213555977	ATTTATTCTACCTCAGCTCCTTCTATC	24	T	0	0.4859
NC_005101.4-213584614	TGTCCTCAAACTCTCACTCCTTCTGAC	2	G	0.124	0.1306
NC_005101.4-213786842	GGAATACTAACAGGCCCTCCAACTCAA	16	C	0.084	0.1107
NC_005101.4-213831819	TTAGCTTATACACCCCCTCCTATGCTG	22	A	0.1739	0.2758
NC_005101.4-213881797	TACATCTTCACATCTTCTCCTATCTTT	2	A	0.0345	0.0339
NC_005101.4-213881797	TACATCTTCACATCTTCTCCTATCTTT	6	C	0.0348	0.4936
NC_005101.4-213901116	CCTAACCTAACTCTTTCTCCTCTCCGG	25	C	0	0.477
NC_005101.4-213923464	TAGGCACAGACACCAGCTCCCACATAC	1	T	0.009	0.4726
NC_005101.4-214026297	AAATGAGAAACTTGTCCTCCAGTGTTT	9	A	0.1721	0.2686
NC_005101.4-214026297	AAATGAGAAACTTGTCCTCCAGTGTTT	21	A	0.041	0.4677

NC_005101.4-214047137	CTTCTCCTGACTCAGTCTCCCTCAAGC	6	C	0.2212	0.3396
NC_005101.4-214242599	TTCTCTGACACACCTGCTCCACTGTAC	2	T	0.0407	0.0705
NC_005101.4-214242599	TTCTCTGACACACCTGCTCCACTGTAC	16	G	0.1463	0.2142
NC_005101.4-214347594	GTTGGTTACACAACCTCTCCGAACCCA	8	A	0	0.4778
NC_005101.4-214347594	GTTGGTTACACAACCTCTCCGAACCCA	25	C	0.0598	0.074
NC_005101.4-214637615	GGATTAGCCACAATCTCTCCTAATGAA	3	A	0.0407	0.0398
NC_005101.4-214637615	GGATTAGCCACAATCTCTCCTAATGAA	21	T	0.1066	0.5
NC_005101.4-214713280	TGGATATCCACACAATCTCCAGAACAT	4	A	0.0948	0.1209
NC_005101.4-214792101	TCAGGATGTACCTCACCTCCCCATCTC	25	C	0	0.474
NC_005101.4-214984368	CCTACTTCTACCTCACCTCCCTGTATT	9	T	0.2054	0.2512
NC_005101.4-214984368	CCTACTTCTACCTCACCTCCCTGTATT	26	T	0.2054	0.2512
NC_005101.4-215077296	AACCCCTCCACCCCAGCTCCCAAGTAC	21	C	0.1504	0.3189
NC_005101.4-215139804	GTGCTAGAAACAAAGACTCCCTCAGCG	26	C	0.087	0.3499
NC_005101.4-215159469	GGGGATGTTACCCTTGCTCCACATCCT	3	G	0.1333	0.4644
NC_005101.4-215165697	ATCAAACAGACCCCTTCTCCTCCCCAT	6	A	0	0.4819
NC_005101.4-215168039	ACCACAGATACATGTGCTCCATGATGT	8	A	0.1083	0.4983
NC_005101.4-215195111	TTATCTTGTACTTATACTCCACCCTTG	1	T	0.0744	0.4933
NC_005101.4-215195111	TTATCTTGTACTTATACTCCACCCTTG	22	C	0.0992	0.1235
NC_005101.4-215308254	CCCCACCCCACCAGATCTCCTCCCTCC	1	C	0.0345	0.4786
NC_005101.4-215341068	TAACCAATCACACAGGCTCCCATACTC	26	T	0.0263	0.476
NC_005101.4-215382906	TTCCTCTTCACCCTACCTCCCATCCAG	13	C	0.2105	0.4038
NC_005101.4-215409460	TTTACTTTGACCTGTTCTCCATTTCTT	4	A	0.265	0.4158
NC_005101.4-215480983	CTAAGAAGGACAAATGCTCCATTATGT	8	G	0.0965	0.138
NC_005101.4-215632119	AGTCAGCCCACTAGCTCTCCTGAGTCA	23	A	0.1739	0.4954
NC_005101.4-215632119	AGTCAGCCCACTAGCTCTCCTGAGTCA	25	T	0	0.482
NC_005101.4-215660197	TAAATCAGTACCCTTCCTCCACACAAA	12	C	0.6216	0.4284
NC_005101.4-215660197	TAAATCAGTACCCTTCCTCCACACAAA	13	C	0.1593	0.2036
NC_005101.4-216064562	GTTGCCGTGACTGCTCCTCCATGATAG	7	G	0.0083	0.4783
NC_005101.4-216064562	GTTGCCGTGACTGCTCCTCCATGATAG	16	C	0.0167	0.48
NC_005101.4-216136755	CATACACGCACCACCCCTCCATACTTA	26	T	0	0.4819
NC_005101.4-216344568	CATGGAATGACCATGGCTCCAGCCGCA	21	A	0.0776	0.1058
NC_005101.4-216424696	GACCCCACAACTCTGTCTCCTTTCCTT	5	C	0.2358	0.4935
NC_005101.4-216528576	TTTACCACAACACGTCCTCCCATGGCA	14	G	0.0351	0.0345
NC_005101.4-216699514	TTTCTACCTACCCATTCTCCCAACTTC	6	A	0.0171	0.0336
NC_005101.4-216728162	CTACCCAGTACCTTGCCTCCATCCATC	5	C	0	0.4743
NC_005101.4-216728162	CTACCCAGTACCTTGCCTCCATCCATC	13	T	0	0.4743
NC_005101.4-216795898	CTGAGAATGACTTTGACTCCAGACCCT	24	C	0	0.4765
NC_005101.4-217002687	GGAACATGGACTTTATCTCCAGTGTTT	4	A	0.1417	0.1866
NC_005101.4-217002687	GGAACATGGACTTTATCTCCAGTGTTT	7	T	0.1417	0.5
NC_005101.4-217142788	ACTTGCCATACCCTGTCTCCTCAAGCA	1	A	0.0702	0.1153
NC_005101.4-217142788	ACTTGCCATACCCTGTCTCCTCAAGCA	15	G	0.1228	0.16
NC_005101.4-217183149	CCTCTTTTTACACTCTCTCCCTTCATC	6	T	0.1681	0.481
NC_005101.4-217183149	CCTCTTTTTACACTCTCTCCCTTCATC	8	T	0.0174	0.48
NC_005101.4-217414363	ATCAAGCCTACCATGTCTCCATGATTC	12	C	0.025	0.0247
NC_005101.4-217414363	ATCAAGCCTACCATGTCTCCATGATTC	22	T	0.075	0.1172
NC_005101.4-217481157	GGCTGCCCTACCATGGCTCCCTGCCAC	1	G	0.1405	0.2355
NC_005101.4-217630045	ATGTACTGTACAGTTCCTCCTATAATA	16	C	0.027	0.0267
NC_005101.4-217630045	ATGTACTGTACAGTTCCTCCTATAATA	21	T	0.0354	0.4624
NC_005101.4-217640522	ACTATATCTACTCCCTCTCCTTTTATT	6	A	0.1217	0.4969
NC_005101.4-217640522	ACTATATCTACTCCCTCTCCTTTTATT	13	C	0.0085	0.479
NC_005101.4-217650977	TGCAAACCAACCAGAGCTCCCAAGGAC	12	C	0.113	0.18

NC_005101.4-217650977	TGCAAACCAACCAGAGCTCCCAAGGAC	21	C	0.3846	0.3935
NC_005101.4-217662257	AAGTAGCACACTGGGTCTCCTGCTGCA	14	G	0.05	0.0488
NC_005101.4-217662257	AAGTAGCACACTGGGTCTCCTGCTGCA	15	G	0.0417	0.0566
NC_005101.4-217749085	GGTCTCTATACATACTCTCCTTTTGCT	1	G	0	0.4743
NC_005101.4-217749085	GGTCTCTATACATACTCTCCTTTTGCT	8	A	0.0417	0.0408
NC_005101.4-217764995	AATGCTCCCACTCCTACTCCAACCTCA	6	T	0.0263	0.026
NC_005101.4-217764995	AATGCTCCCACTCCTACTCCAACCTCA	12	T	0.1491	0.1814
NC_005101.4-217781353	CAGTGGCTGACAAACCCTCCCGCCTGT	1	C	0.0088	0.4889
NC_005101.4-217781353	CAGTGGCTGACAAACCCTCCCGCCTGT	21	C	0.1652	0.258
NC_005101.4-217781573	AAATCATTTACTCTGCCTCCCCACTAT	24	C	0.0256	0.0253
NC_005101.4-217877176	CTACCTGTAACTCACGCTCCAGGGATA	5	C	0.0407	0.1145
NC_005101.4-218002311	ACGTGGTCTACAATGGCTCCCTCTACT	5	G	0	0.4728
NC_005101.4-218193342	CCTGAGCCCACAGGGACTCCCAGAGAC	6	G	0	0.4814
NC_005101.4-218193342	CCTGAGCCCACAGGGACTCCCAGAGAC	12	A	0.0439	0.0429
NC_005101.4-218209027	TTGTGGAAAACATCTTCTCCTTAAGCA	13	T	0.0583	0.0566
NC_005101.4-218225131	ATTTTCATGACCATCTCTCCATACCTG	4	T	0.1405	0.3601
NC_005101.4-218225131	ATTTTCATGACCATCTCTCCATACCTG	21	A	0.0328	0.0322
NC_005101.4-218433584	CACCCTGTCACCTCTTCTCCAATGTTG	12	C	0.177	0.3637
NC_005101.4-218433584	CACCCTGTCACCTCTTCTCCAATGTTG	15	T	0	0.4793
NC_005101.4-218459506	TGCACGGTCACTTCCTCTCCACACTTT	6	G	0	0.4743
NC_005101.4-218610701	GGCTGATAAACAGCGCCTCCTCTCTAG	8	A	0	0.4793
NC_005101.4-218610701	GGCTGATAAACAGCGCCTCCTCTCTAG	9	A	0.1695	0.2466
NC_005101.4-218629126	ATTGAACCCACAATTCCTCCTGTCTTG	13	A	0.2542	0.334
NC_005101.4-218629126	ATTGAACCCACAATTCCTCCTGTCTTG	16	C	0.0252	0.0249
NC_005101.4-218634406	TGAACTGATACCACAGCTCCCTGTACC	2	G	0.5664	0.406
NC_005101.4-218634406	TGAACTGATACCACAGCTCCCTGTACC	25	A	0.5714	0.4082
NC_005101.4-218663619	CAGAACCTTACACTGCCTCCAGAACCT	16	C	0.1345	0.1539
NC_005101.4-218668328	CTTGTTTTCACACGTACTCCAAGCTGA	14	G	0.0435	0.0911
NC_005101.4-218673387	TCTGACGTCACAAGGTCTCCTTAGTGA	7	G	0.1696	0.1983
NC_005101.4-218772992	CTGCCTGTTACTCCAGCTCCAGAGTCC	21	A	0.0089	0.0436
NC_005101.4-218881169	CTGTTCCACACTCTGTCTCCATATTCC	21	A	0.075	0.1025
NC_005101.4-218881169	CTGTTCCACACTCTGTCTCCATATTCC	24	T	0.0833	0.095
NC_005101.4-218887405	CCCATGTATACCTGCCCTCCTCAAAGA	8	A	0.0168	0.4844
NC_005101.4-218887405	CCCATGTATACCTGCCCTCCTCAAAGA	27	A	0.0263	0.0429
NC_005101.4-218902360	CTGATTACTACCTGACCTCCGGATGAT	23	A	0.0488	0.078
NC_005101.4-218929688	TTAGGGAAAACAGTTCCTCCCCAGCCC	1	T	0.2083	0.4358
NC_005101.4-218929688	TTAGGGAAAACAGTTCCTCCCCAGCCC	2	T	0.2167	0.3832
NC_005101.4-218941733	ACCCATTCCACAAAGGCTCCTTCCATA	6	T	0.1545	0.319
NC_005101.4-218970682	GAAACGGCGACCAGTACTCCATGGTGC	2	A	0.2034	0.4948
NC_005101.4-218970682	GAAACGGCGACCAGTACTCCATGGTGC	5	C	0.0932	0.1041
NC_005101.4-218998752	GGCGCTTTGACCTAAGCTCCACAATGA	12	C	0.0083	0.0247
NC_005101.4-218998752	GGCGCTTTGACCTAAGCTCCACAATGA	14	A	0.225	0.4416
NC_005101.4-219054524	TACAATTGTACAAATCCTCCTATCTAG	6	T	0	0.4826
NC_005101.4-219110119	GCTCTGGGCACACATGCTCCTCATTGA	7	G	0.2232	0.4952
NC_005101.4-219217479	CACTGGCCCACCCACCCTCCTCACACA	22	C	0.0177	0.0348
NC_005101.4-219293903	CCAACCTTCACCTCCCCTCCCCCCACA	9	C	0	0.4868
NC_005101.4-219341184	GTGGGGCTCACTTGCCCTCCCTCACCT	16	C	0.1552	0.2252
NC_005101.4-219360471	TTAAACATGACCACCACTCCATAGTGT	26	G	0	0.4715
NC_005101.4-219360471	TTAAACATGACCACCACTCCATAGTGT	27	T	0	0.4715
NC_005101.4-219361176	ACCGACCAGACGTCTTCTCCAACAGCA	12	G	0.2564	0.45
NC_005101.4-219387963	TCTTTACAAACACCACCTCCTCCTTCC	6	A	0	0.4787

NC_005101.4-219398564	ATCCGATGAACAGCATCTCCACTTGTG	4	C	0.0407	0.0553
NC_005101.4-219398564	ATCCGATGAACAGCATCTCCACTTGTG	16	T	0.0082	0.4822
NC_005101.4-219471840	TCTATCTTTACATGAACTCCAGGGATT	1	T	0.2231	0.3601
NC_005101.4-219508895	CAAACCAAGACTTTGGCTCCTGGTGAG	3	A	0.1417	0.2372
NC_005101.4-219545984	CTGCCTGGAACGCTGGCTCCATGAGAC	26	A	0.1667	0.4981
NC_005101.4-219711892	CACGTGCCAACACAGCCTCCAGACCTG	23	A	0.0167	0.0328
NC_005101.4-219786966	GAAGGGGGTACCCAGGCTCCATCAGCA	5	G	0.1626	0.1629
NC_005101.4-219786966	GAAGGGGGTACCCAGGCTCCATCAGCA	12	C	0.1626	0.1629
NC_005101.4-219816643	AGCTACTCAACCATCTCTCCAGACCAC	3	C	0	0.4787
NC_005101.4-219906672	CACACACACACACACACTCCCCATTCT	7	C	0.2333	0.2778
NC_005101.4-219907552	TTGAGACTAACACTGTCTCCTACCACA	9	A	0	0.4807
NC_005101.4-219936018	AATTGGACCACTAGAACTCCCAGGGAC	3	T	0	0.4764
NC_005101.4-219986333	AAGTGGAACACAGACACTCCTCCCTTT	8	A	0	0.4793
NC_005101.4-220130931	TGCACTATCACTATTTCTCCTCCCTCA	1	T	0.2105	0.2893
NC_005101.4-220151386	AATTGTTGAACAGATTCTCCTTCCACT	8	G	0	0.4859
NC_005101.4-220180989	CAGCAAGCCACCCACTCTCCGTCCACA	3	G	0.1043	0.2005
NC_005101.4-220180989	CAGCAAGCCACCCACTCTCCGTCCACA	21	G	0.2087	0.2985
NC_005101.4-220219595	CTAGCGTTTACTGAACCTCCATTTTCT	6	G	0.0087	0.4903
NC_005101.4-220219595	CTAGCGTTTACTGAACCTCCATTTTCT	14	A	0.0087	0.4903
NC_005101.4-220283719	TTGTCATCCACATCAACTCCTTCAATC	9	C	0.0261	0.0257
NC_005101.4-220283719	TTGTCATCCACATCAACTCCTTCAATC	25	A	0.2105	0.3424
NC_005101.4-220295278	CTCTTTTCCACTCCAGCTCCTCCTGTG	1	C	0.0744	0.0716
NC_005101.4-220295278	CTCTTTTCCACTCCAGCTCCTCCTGTG	25	G	0.0083	0.4783
NC_005101.4-220317954	GGAACTAAAACACTACCTCCTTCTTCT	22	T	0	0.48
NC_005101.4-220607776	GTGCTGTTCACATTGTCTCCCTTTCCC	22	T	0.0917	0.1025
NC_005101.4-220607776	GTGCTGTTCACATTGTCTCCCTTTCCC	23	T	0.2	0.495
NC_005101.4-220735137	GGGGTTAGCACTGCTTCTCCACTGTGA	12	T	0	0.4793
NC_005101.4-220743477	TGTGTCCTCACACTGCCTCCAGGCATT	25	A	0.0345	0.4927
NC_005101.4-220789672	AGGTTTTAGACAACATCTCCAGCTGTT	1	A	0.2414	0.4905
NC_005101.4-220789672	AGGTTTTAGACAACATCTCCAGCTGTT	24	T	0.0427	0.4714
NC_005101.4-220853690	TCTTCTACCACTTCACCTCCTCCACCA	12	T	0.2124	0.4464
NC_005101.4-220918767	ATGCACAGAACCTGGCCTCCAAAGAAT	6	C	0.1057	0.1287
NC_005101.4-220928655	TTAAACAAAACCAGGCCTCCTGCACAT	23	C	0.0164	0.4807
NC_005101.4-221082404	TCCTTTTAGACAGCATCTCCATTGTCT	25	T	0.2114	0.4682
NC_005101.4-221086285	TTTAATACCACAGAGCCTCCTCTGAGT	25	A	0.2719	0.3919
NC_005101.4-221222918	CAGGGTAATACATTCACTCCTGCTGGG	3	G	0.1597	0.4186
NC_005101.4-221222918	CAGGGTAATACATTCACTCCTGCTGGG	22	G	0.161	0.4207
NC_005101.4-221268474	ACCCTACCCACCCTCTCTCCCCATAGT	12	C	0.1864	0.2815
NC_005101.4-221303622	ATGTCTTTAACTGAGGCTCCTTTCTCT	2	T	0.0407	0.0553
NC_005101.4-221379684	ATCATTTCTACTCTATCTCCCTGGTCC	14	T	0	0.4839
NC_005101.4-221461805	CTATCCTATACAGGTACTCCATGGTAC	3	A	0.0169	0.4677
NC_005101.4-221462787	TTGTACATGACCACAGCTCCTGCTGTA	1	T	0	0.032
NC_005101.4-221462787	TTGTACATGACCACAGCTCCTGCTGTA	22	G	0.0902	0.1296
NC_005101.4-221600967	TGAACTACAACTTTAGCTCCTGCCAAG	24	C	0.0254	0.0415
NC_005101.4-221752867	GGACCAAGTACCTCCTCTCCCACTGAT	12	C	0.0672	0.065
NC_005101.4-221766651	TACTACAGAACAGGGTCTCCTTACTGG	24	C	0.2602	0.4331
NC_005101.4-221796701	GTCACAGGAACAACTACTCCACGCACT	16	A	0	0.4765
NC_005101.4-221810181	ATGTCATCTACTCCCTCTCCAGCCTGC	27	C	0.1639	0.1641
NC_005101.4-221834238	TTGCAGAGGACATCTCCTCCAAGTTCA	6	G	0.2114	0.3141
NC_005101.4-221889811	AACACTCATACAGAGACTCCCAATCCC	22	A	0.1111	0.12
NC_005101.4-221889811	AACACTCATACAGAGACTCCCAATCCC	24	T	0	0.4851

NC_005101.4-221898840	TTCCATGCCACACAGGCTCCTGTGGAT	12	A	0.1301	0.2017
NC_005101.4-221898840	TTCCATGCCACACAGGCTCCTGTGGAT	22	G	0.0732	0.0854
NC_005101.4-222065787	GGCCCTCTCACTATCCCTCCACACACC	3	C	0.0244	0.0241
NC_005101.4-222065787	GGCCCTCTCACTATCCCTCCACACACC	13	A	0.0325	0.032
NC_005101.4-222078623	GACTATACCACTTTGGCTCCTGGTGGT	6	T	0.0826	0.4991
NC_005101.4-222078623	GACTATACCACTTTGGCTCCTGGTGGT	16	G	0.0826	0.4991
NC_005101.4-222298934	TGTCCGTGAACTTTGGCTCCAATACTT	5	C	0.065	0.0928
NC_005101.4-222298934	TGTCCGTGAACTTTGGCTCCAATACTT	6	G	0.0081	0.4777
NC_005101.4-222554922	GGCATTTTGACTTGTCCTCCTTACGAG	24	C	0.1504	0.4994
NC_005101.4-222569821	TTCTGTCCTACTCTTTCTCCTCTTCTA	26	T	0.0804	0.0771
NC_005101.4-222585713	TCAGCTGGCACGGAGACTCCAAAAGCA	7	G	0.0427	0.0418
NC_005101.4-222605481	CCGTGTCCCACCCCTCCTCCTCTGTTC	3	G	0.0826	0.1235
NC_005101.4-222605481	CCGTGTCCCACCCCTCCTCCTCTGTTC	26	T	0.0667	0.1387
NC_005101.4-222628023	GAGTCTCAGACTTGAGCTCCACAGCAG	12	T	0.1721	0.5
NC_005101.4-222646125	TGGGAAGTAACCATTGCTCCTTAAGTC	9	A	0.2017	0.4921
NC_005101.4-222663736	GGCCCCACTACTTCTCCTCCTACAATG	7	A	0.069	0.4946
NC_005101.4-222715500	AACTCAACTACTATATCTCCATTTAAT	15	A	0.0177	0.4715
NC_005101.4-222739685	CCTCACCCAACTTCATCTCCTCTTCCT	22	C	0.0902	0.0861
NC_005101.4-222739685	CCTCACCCAACTTCATCTCCTCTTCCT	24	T	0.1736	0.2474
NC_005101.4-222749384	AAAGTTAAGACAGCCTCTCCCACCCAC	3	A	0.1864	0.4908
NC_005101.4-222836402	ATAACAAGGACACTTGCTCCACTATAT	6	A	0.1186	0.1116
NC_005101.4-222838292	CTGGATTCTACTTTGGCTCCAACTCCT	5	A	0	0.4765
NC_005101.4-222867961	TAATTCAGAACATGATCTCCATGGTCA	8	G	0.2295	0.3537
NC_005101.4-223126471	TGCTAATGTACGCTATCTCCTGGTACT	7	T	0.0089	0.4617
NC_005101.4-223126471	TGCTAATGTACGCTATCTCCTGGTACT	12	G	0.0776	0.0746
NC_005101.4-223167652	GGAATTCAAACTTTCACTCCCCATGGC	3	A	0.2348	0.451
NC_005101.4-223177647	CCACCTGTCACTACAGCTCCATCTTTT	6	T	0.2719	0.4353
NC_005101.4-223208065	AGCATTTCTACTCCAGCTCCAAGAAAT	8	C	0.0579	0.0716
NC_005101.4-223237953	AGATATACCACCATCACTCCCTTTCTA	8	C	0.0085	0.4738
NC_005101.4-223241929	TGTTTCGACACGTTCTCTCCTGTAGCT	7	G	0.094	0.5
NC_005101.4-223241929	TGTTTCGACACGTTCTCTCCTGTAGCT	12	G	0.094	0.5
NC_005101.4-223245340	GAACCCCCAACTCTTGCTCCAGGACTT	1	G	0.0082	0.0243
NC_005101.4-223245340	GAACCCCCAACTCTTGCTCCAGGACTT	3	A	0.0082	0.0243
NC_005101.4-223269824	TGTAAAGTCACTGTCTCTCCCTTTTAT	22	T	0.0902	0.4992
NC_005101.4-223377524	CTTGGAACTACTGATCCTCCTGCCTCT	7	A	0	0.4734
NC_005101.4-223386956	GCAATAGACACAAGGGCTCCTAAGTGA	15	G	0	0.4844
NC_005101.4-223400594	AAGTCAGTCACCTTACCTCCAACACCA	23	C	0.2705	0.4193
NC_005101.4-223476123	ATCAGGAAAACAATCCCTCCCTCAAAT	15	C	0.0973	0.0926
NC_005101.4-223484915	AGAGTCTGCACAGGTCCTCCCTACCCC	9	C	0	0.4813
NC_005101.4-223545964	CTTTCTTCTACTCGTCCTCCATGTCTC	9	T	0.1453	0.2171
NC_005101.4-223602067	AGACAGATTACAGCATCTCCGGATGCT	9	T	0.2105	0.4555
NC_005101.4-223602067	AGACAGATTACAGCATCTCCGGATGCT	22	G	0.0526	0.0512
NC_005101.4-223609785	TGTGTAAAGACAGGAGCTCCAGACTAG	2	G	0	0.482
NC_005101.4-223617570	CTGCCTCTTACTAAATCTCCATTTCCA	1	C	0.0804	0.0771
NC_005101.4-223702219	CCCGTCAGCACTCAGGCTCCCAACAGA	25	A	0.0081	0.481
NC_005101.4-223710070	GATAGGTATACACATTCTCCATCAGAG	5	G	0	0.4826
NC_005101.4-223710070	GATAGGTATACACATTCTCCATCAGAG	9	T	0.042	0.0411
NC_005101.4-223744342	CCAGAGAACACCATCACTCCACCACAG	8	A	0.0522	0.3938
NC_005101.4-223744342	CCAGAGAACACCATCACTCCACCACAG	27	G	0.0811	0.3635
NC_005101.4-223791555	CCTCAGTAAACCCCATCTCCAGGGACA	24	G	0.0813	0.078
NC_005101.4-223805260	CTCCCTTCTACACCCTCTCCATTCCAT	3	C	0.2185	0.3319

NC_005101.4-223805260	CTCCCTTCTACACCCTCTCCATTCCAT	13	C	0.075	0.1025
NC_005101.4-223844945	ACTAAGTTTACATCCCCTCCCTTCATC	8	T	0	0.4765
NC_005101.4-223844945	ACTAAGTTTACATCCCCTCCCTTCATC	27	C	0	0.4765
NC_005101.4-223922205	TCATCGAAAACGGTATCTCCAGAGTTG	5	C	0.2627	0.4454
NC_005101.4-223942523	TATGACCCCACACTCACTCCTCAGACT	12	A	0	0.4793
NC_005101.4-223942523	TATGACCCCACACTCACTCCTCAGACT	25	A	0	0.4793
NC_005101.4-223963246	ACTGCATTCACTGCATCTCCAGGGTAG	4	G	0.2333	0.4265
NC_005101.4-223963246	ACTGCATTCACTGCATCTCCAGGGTAG	5	C	0.1736	0.3929
NC_005101.4-223971509	CTCTCTGTCACCCTCTCTCCAGCCTAC	7	G	0.2542	0.4757
NC_005101.4-224018257	TCTAACAAGACCACGCCTCCTAACAGT	14	C	0.0354	0.0348
NC_005101.4-224018257	TCTAACAAGACCACGCCTCCTAACAGT	27	T	0.0175	0.0345
NC_005101.4-224036565	TCTCAGTGGACTCCAGCTCCCTCAATT	1	T	0.0339	0.0333
NC_005101.4-224036565	TCTCAGTGGACTCCAGCTCCCTCAATT	26	T	0.3304	0.3499
NC_005101.4-224048962	CTCAGATTCACTCCACCTCCACAGGTC	3	C	0	0.4693
NC_005101.4-224048962	CTCAGATTCACTCCACCTCCACAGGTC	7	T	0	0.4693
NC_005101.4-224063682	CCACCTGTAACGTCTGCTCCATGGATT	5	C	0	0.4779
NC_005101.4-224088052	CGATGTTCCACCTTTCCTCCCTGTTTG	7	T	0.1624	0.2041
NC_005101.4-224114861	GAGTACAGCACATAGGCTCCTGGGAGA	2	A	0.123	0.4959
NC_005101.4-224116722	TCTGCCTCTACTGTCTCTCCTGCCCAA	13	G	0.0783	0.0752
NC_005101.4-224149082	TCTCCTCTCACTCTGTCTCCTCTTTGA	14	T	0.0435	0.059
NC_005101.4-224149082	TCTCCTCTCACTCTGTCTCCTCTTTGA	23	T	0.0174	0.4724
NC_005101.4-224241239	CCGCAGCTCACTTTCTCTCCTCTCTAA	2	C	0.0957	0.1067
NC_005101.4-224241239	CCGCAGCTCACTTTCTCTCCTCTCTAA	15	C	0	0.4846
NC_005101.4-224329622	CCTTCTCTTACGTGTCCTCCTCCCACT	26	C	0.2358	0.4572
NC_005101.4-224394272	CCACTCTGCACTCACCCTCCTACACAT	22	A	0.1301	0.2612
NC_005101.4-224398335	TCTAAAGAAACCGAGCCTCCATCTGGT	12	C	0.0163	0.0476
NC_005101.4-224514461	TTGGCCCCAACAGTATCTCCCTTATTG	1	T	0.0661	0.0639
NC_005101.4-224636451	AAAGCCGTTACAGATGCTCCCTGTCTT	8	T	0.0496	0.0484
NC_005101.4-224641054	CTGGTCAGCACACCGACTCCATCACTT	15	G	0	0.4868
NC_005101.4-224641054	CTGGTCAGCACACCGACTCCATCACTT	27	T	0	0.4868
NC_005101.4-224672594	AGAGTTCACACAAATGCTCCTCAGACA	5	T	0.0813	0.0928
NC_005101.4-224774860	CACGCCATTACAGATGCTCCAACATGT	14	A	0.0976	0.1629
NC_005101.4-224774860	CACGCCATTACAGATGCTCCAACATGT	24	A	0.0894	0.1287
NC_005101.4-224778394	TAACTACGAACCATCTCTCCAGCCTCC	6	A	0.1545	0.4995
NC_005101.4-224786764	TCTCTACATACGCAGGCTCCTATCAGC	13	C	0.0261	0.0257
NC_005101.4-224786764	TCTCTACATACGCAGGCTCCTATCAGC	23	T	0.0261	0.0257
NC_005101.4-224834673	GCACTTCTGACCAACACTCCAGCTTTG	23	C	0.0252	0.0249
NC_005101.4-224838396	AAAAACAAAACCTCACCTCCAGCCAGG	4	A	0.2566	0.4547
NC_005101.4-224838396	AAAAACAAAACCTCACCTCCAGCCAGG	6	C	0.2768	0.4665
NC_005101.4-224857351	TTATTTGACACGGACCCTCCATTAGAC	9	C	0.2689	0.4957
NC_005101.4-224857351	TTATTTGACACGGACCCTCCATTAGAC	12	G	0	0.4813
NC_005101.4-224894657	AGGTTTAATACTTTGCCTCCTTTCCTG	2	G	0.2066	0.1982
NC_005101.4-224916644	CACCACAGGACCTGTCCTCCAAGCTCT	2	A	0	0.4793
NC_005101.4-224916644	CACCACAGGACCTGTCCTCCAAGCTCT	27	T	0	0.4793
NC_005101.4-224924262	AAGGTCTCAACTCTGGCTCCAATGTTG	22	A	0.2212	0.4994
NC_005101.4-224980274	CTGCCTATGACCTCACCTCCACCTTCT	8	T	0.0541	0.0695
NC_005101.4-224980274	CTGCCTATGACCTCACCTCCACCTTCT	21	A	0.0446	0.0771
NC_005101.4-224990569	CCACATCCCACTAGGCCTCCTTGTCTG	16	C	0.3089	0.377
NC_005101.4-225046035	GACCTCCCCACCCCGACTCCCATGGTT	1	G	0.1074	0.1447
NC_005101.4-225046035	GACCTCCCCACCCCGACTCCCATGGTT	14	C	0.0333	0.0328
NC_005101.4-225058311	CTCTGTATCACTCCACCTCCCTCCCTG	7	A	0.175	0.2372

NC_005101.4-225060283	TCCACACCCACTCCCACTCCTGCTGGA	26	G	0.1111	0.1049
NC_005101.4-225120082	TAACCTTGAACTCCTGCTCCCAAGTGC	21	C	0.15	0.1665
NC_005101.4-225120082	TAACCTTGAACTCCTGCTCCCAAGTGC	22	A	0.0417	0.4847
NC_005101.4-225122349	ACTTCTCCAACGAAGACTCCTGGACTT	2	C	0.2353	0.3319
NC_005101.4-225136088	GGGGCAGTCACAAGCTCTCCACGTGGG	12	A	0.0536	0.4841
NC_005101.4-225136088	GGGGCAGTCACAAGCTCTCCACGTGGG	22	C	0.1071	0.2188
NC_005101.4-225145594	TAGCAAGCAACTCTAGCTCCAGGGGAT	25	G	0.1518	0.1699
NC_005101.4-225153385	TAATAAGGGACCGATCCTCCGAGGCCT	21	G	0.1639	0.285
NC_005101.4-225153385	TAATAAGGGACCGATCCTCCGAGGCCT	27	T	0.3361	0.4435
NC_005101.4-225175011	GTCAGTTATACATCTCCTCCAACAAGG	4	A	0.2846	0.4301
NC_005101.4-225175011	GTCAGTTATACATCTCCTCCAACAAGG	26	G	0.0244	0.0241
NC_005101.4-225200031	GGTCTCTCTACCTTTCCTCCCTCTTTT	12	C	0.0948	0.0903
NC_005101.4-225202382	GAACCTTCCACGCAGCCTCCCTCTCTT	27	T	0	0.4773
NC_005101.4-225243633	AGACATGTTACCTCTGCTCCGTCTGTT	9	T	0	0.4773
NC_005101.4-225243633	AGACATGTTACCTCTGCTCCGTCTGTT	23	C	0	0.4773
NC_005101.4-225303434	CTCGGATCCACTCTGCCTCCAAGATAG	6	A	0.2564	0.45
NC_005101.4-225319027	GTATGCATAACACATCCTCCTTCTGCT	3	A	0	0.4688
NC_005101.4-225331697	TCCTTCCCCACTCCCCCTCCTCTTCTC	21	T	0	0.4868
NC_005101.4-225341559	ACATGGCATACAGACGCTCCGGGACAC	8	A	0.0083	0.4747
NC_005101.4-225341559	ACATGGCATACAGACGCTCCGGGACAC	9	T	0.0083	0.4747
NC_005101.4-225371780	TACTGACTAACTGCAGCTCCCAAGATG	5	G	0.2231	0.323
NC_005101.4-225456404	TTGGCCCTGACTCTGTCTCCTTCAAGA	24	A	0	0.48
NC_005101.4-225462476	ATGCATTAAACAGTAACTCCTCATTCT	13	G	0.0424	0.0415
NC_005101.4-225647707	AATATGCCCACTCCACCTCCCGCATCT	12	T	0.1368	0.5
NC_005101.4-225647707	AATATGCCCACTCCACCTCCCGCATCT	22	G	0.1441	0.1894
NC_005101.4-225663431	GAATTCATGACTCGTGCTCCATATTCA	13	C	0.1913	0.2138
NC_005101.4-225663431	GAATTCATGACTCGTGCTCCATATTCA	26	C	0.307	0.375
NC_005101.4-225709995	CATGGATCTACTCTGTCTCCATGACTG	2	A	0	0.4827
NC_005101.4-225710109	GGTTCCTGGACAGCTTCTCCATTATGT	6	C	0.1818	0.2172
NC_005101.4-225719872	CAGTCTACAACTCCAGCTCCATGGGAT	22	T	0.0603	0.4866
NC_005101.4-225722692	TGTCCCCCAACCCCTACTCCAATATCC	15	T	0	0.4765
NC_005101.4-225779083	GTAAAAGCCACTCAGTCTCCCCCACAT	1	G	0	0.4832
NC_005101.4-225816486	CCTCATCCCACAGATCCTCCTTCAGGT	4	C	0.0417	0.0408
NC_005101.4-225833593	AACCAACGAACCTTCCCTCCAGCTCCC	6	A	0.0252	0.0249
NC_005101.4-225927297	ACAGCGCTTACTGCAGCTCCCTTACAT	21	C	0.2114	0.385
NC_005101.4-225929255	CTTTGTTTAACCAATCCTCCCTTCCCC	22	T	0	0.4846
NC_005101.4-225929585	TGCTATGACACCCAATCTCCCCTTTCC	3	C	0	0.4765
NC_005101.4-225929585	TGCTATGACACCCAATCTCCCCTTTCC	22	C	0.15	0.1387
NC_005101.4-225946080	TGGGTTGCCACTCTCCCTCCACTTCTC	23	T	0	0.4807
NC_005101.4-225952427	TACAAGGACACACCTACTCCAACACAG	21	A	0.0847	0.0965
NC_005101.4-225955647	CCGAATACCACATGACCTCCTCAAAGG	8	C	0	0.4827
NC_005101.4-225983270	TAAAGATGTACTGGGTCTCCGTGTGCC	12	T	0.0442	0.49
NC_005101.4-225983270	TAAAGATGTACTGGGTCTCCGTGTGCC	27	C	0.1491	0.4981
NC_005101.4-225991671	CATTGGGCGACTCTGCCTCCCACTCTG	7	G	0.0776	0.1058
NC_005101.4-225991671	CATTGGGCGACTCTGCCTCCCACTCTG	15	G	0.1043	0.1443
NC_005101.4-225998188	CATACGCTAACACTTGCTCCATCACAG	25	C	0	0.4751
NC_005101.4-226123929	AGAACCAGTACAACAGCTCCCTGCACC	5	C	0.2893	0.465
NC_005101.4-226269284	TATTACAAAACGAAGACTCCCTCATTC	1	T	0.0769	0.12
NC_005101.4-226353807	CCATACAATACCGATGCTCCTTTGCTT	12	C	0	0.4826
NC_005101.4-226558019	ATTGCCATGACACCAACTCCACGGCAG	21	A	0.041	0.071
NC_005101.4-226558019	ATTGCCATGACACCAACTCCACGGCAG	22	C	0.0082	0.479

NC_005101.4-226710740	TCATATCTTACACAGTCTCCTCTGACA	22	C	0.0081	0.4777
NC_005101.4-226844505	GAATTCTCAACATTAGCTCCTGCAGTT	1	G	0.1864	0.4987
NC_005101.4-226857181	GGTCTTATTACAATGGCTCCTTTCTGT	14	T	0.0517	0.0504
NC_005101.4-226857181	GGTCTTATTACAATGGCTCCTTTCTGT	15	G	0.1667	0.4981
NC_005101.4-226868587	AGGTGTTCAACCTCGTCTCCTGGTTTC	5	G	0.1626	0.2142
NC_005101.4-226868587	AGGTGTTCAACCTCGTCTCCTGGTTTC	14	C	0.0574	0.454
NC_005101.4-226955845	TCATAGCCCACGGCATCTCCACTGCTC	3	A	0.3197	0.3909
NC_005101.4-226955845	TCATAGCCCACGGCATCTCCACTGCTC	4	T	0.041	0.4677
NC_005101.4-227041729	AGCCCATCCACGCAGTCTCCCTTCAGC	14	A	0.1504	0.1686
NC_005101.4-227086963	CAGATGCCAACAGCTCCTCCTGGAGAT	8	C	0.161	0.2871
NC_005101.4-227086963	CAGATGCCAACAGCTCCTCCTGGAGAT	14	C	0.161	0.2871
NC_005101.4-227105346	ACGAAGCTGACCTAAACTCCTATCTCA	2	C	0.0496	0.0484
NC_005101.4-227105346	ACGAAGCTGACCTAAACTCCTATCTCA	4	A	0.05	0.4599
NC_005101.4-227205751	TTTGGGCAGACTTATTCTCCCTTTATT	3	T	0	0.4827
NC_005101.4-227214631	CCTGTGTCTACCAGCCCTCCTACACCA	6	G	0.0325	0.0629
NC_005101.4-227214631	CCTGTGTCTACCAGCCCTCCTACACCA	16	C	0.2114	0.3334
NC_005101.4-227239371	GGGACTCTCACAGCCACTCCATCAGCC	22	T	0.1897	0.3478
NC_005101.4-227239371	GGGACTCTCACAGCCACTCCATCAGCC	26	C	0.0085	0.4842
NC_005101.4-227270958	TGAAGGAAAACAAACTCTCCCCAGGAT	21	C	0	0.474
NC_005101.4-227308738	GGACTGGCTACAAGGTCTCCTGTTTAT	3	A	0.0089	0.4856
NC_005101.4-227343011	CGGCTTCTCACTCTGTCTCCTCACCGG	1	C	0.2764	0.377
NC_005101.4-227343011	CGGCTTCTCACTCTGTCTCCTCACCGG	6	T	0	0.4773
NC_005101.4-227365160	TTGCCTATAACTCCTTCTCCAGGGGAT	5	C	0.1066	0.1154
NC_005101.4-227385846	GGAAAAAAGACAGGGCCTCCAGAGGCG	16	C	0	0.4807
NC_005101.4-227390276	GCTTTGAAAACCTTAGCTCCCTCAGGG	3	T	0	0.4793
NC_005101.4-227407285	TCTAGTTTTACACCCTCTCCCTTCCTG	14	C	0.0932	0.0889
NC_005101.4-227407285	TCTAGTTTTACACCCTCTCCCTTCCTG	27	G	0.2393	0.2834
NC_005101.4-227423378	ATGCTATTCACACTTTCTCCTTCCGGT	25	G	0	0.48
NC_005101.4-227423378	ATGCTATTCACACTTTCTCCTTCCGGT	26	G	0	0.48
NC_005101.4-227433657	CTCCTCCACACACCCACTCCTATGTCA	5	T	0.0261	0.4903
NC_005101.4-227433657	CTCCTCCACACACCCACTCCTATGTCA	6	C	0.2	0.3898
NC_005101.4-227466120	GCCTCTCACACCTGAGCTCCTTACCAG	4	T	0.0165	0.4751
NC_005101.4-227466120	GCCTCTCACACCTGAGCTCCTTACCAG	16	G	0.0328	0.0322
NC_005101.4-227484504	TAGACTTGAACATCTGCTCCATAGGGC	25	G	0.2054	0.1843
NC_005101.4-227492366	GATGAGGGAACATGTCCTCCTTGGAGG	15	T	0.0488	0.4548
NC_005101.4-227506364	CTGATGTTTACTTTCCCTCCTGAAGGT	27	T	0.0088	0.476
NC_005101.4-227541971	CAGCTCATTACACCCTCTCCTCTTCCA	26	C	0.0656	0.0786
NC_005101.4-227549145	GCCCCCATAACCCAGCCTCCTCTAATC	2	C	0.0252	0.0249
NC_005101.4-227595984	TGTAGGTGTACCGTGTCTCCACCTTAT	12	C	0.1626	0.189
NC_005101.4-227648125	AGACAATTCACCCAAACTCCTGTCGTC	25	G	0.1818	0.2172
NC_005101.4-227686703	TGGCTTGAGACCTTGTCTCCTAAATGT	27	T	0.0598	0.058
NC_005101.4-227718748	CCCTCACTTACACAACCTCCAGAATTA	9	T	0	0.4731
NC_005101.4-227718748	CCCTCACTTACACAACCTCCAGAATTA	25	T	0	0.4731
NC_005101.4-227726611	ACCACCTGAACTACATCTCCAGTCCCT	2	C	0.1983	0.3374
NC_005101.4-227726611	ACCACCTGAACTACATCTCCAGTCCCT	4	A	0.0246	0.0243
NC_005101.4-227730087	CCCCATCTTACTTGCACTCCAGTTCCT	13	T	0	0.4715
NC_005101.4-227730087	CCCCATCTTACTTGCACTCCAGTTCCT	22	G	0.0973	0.4923
NC_005101.4-227776717	TGACATCACACATTTTCTCCTCTCACC	15	T	0.2314	0.4877
NC_005101.4-227845598	CTCTACCACACACAGCCTCCATGACGT	26	G	0.0756	0.4987
NC_005101.4-227895333	GGAGAGAGCACACACTCTCCGTCTTTC	21	G	0.0583	0.4783
NC_005101.4-227923339	AATGGAAATACACCCTCTCCTTCTCCT	9	T	0.0656	0.1082

NC_005101.4-227928505	TATTTATTGACATTTCCTCCTTTGCTG	26	T	0.0263	0.026
NC_005101.4-227952803	CAAAATAGAACATGTTCTCCACACTTC	25	T	0.2389	0.297
NC_005101.4-227955256	CCATGAACTACAAAGCCTCCCTGCTCT	23	G	0.0574	0.0557
NC_005101.4-228009971	TCTCTGCTTACTGGTTCTCCCTTCCCC	7	C	0	0.4743
NC_005101.4-228055440	CACCTAACGACCTGTGCTCCTGAGTGT	8	C	0.1545	0.1826
NC_005101.4-228078809	CACAAGACAACCTGAGCTCCATCTTGG	14	G	0.0656	0.0786
NC_005101.4-228168751	TTGATTCAAACATGACCTCCACGTGCA	23	G	0.2342	0.4883
NC_005101.4-228168751	TTGATTCAAACATGACCTCCACGTGCA	26	C	0.1802	0.1928
NC_005101.4-228200955	GCGCTCTCAACTCTGTCTCCCTCACAA	15	G	0.1161	0.1403
NC_005101.4-228235423	TGCTCCCTGACCCTGGCTCCTCTCGAT	24	C	0.3033	0.3984
NC_005101.4-228235423	TGCTCCCTGACCCTGGCTCCTCTCGAT	26	A	0	0.4773
NC_005101.4-228292577	TTCCATAGAACATTCACTCCTGACCTC	15	C	0.025	0.0247
NC_005101.4-228296721	CCAACAAGGACACTTCCTCCACTCTGT	3	A	0.0333	0.4728
NC_005101.4-228296721	CCAACAAGGACACTTCCTCCACTCTGT	12	A	0.0333	0.0328
NC_005101.4-228393692	GATACAAAGACCCACTCTCCATTCTAG	5	C	0.1624	0.3935
NC_005101.4-228438392	TTCCTCGAGACAGTTTCTCCACGTAGC	23	G	0.2321	0.2934
NC_005101.4-228485395	ATATCAACCACCAGACCTCCAGAGCTC	9	C	0.0082	0.4822
NC_005101.4-228485395	ATATCAACCACCAGACCTCCAGAGCTC	22	G	0	0.4819
NC_005101.4-228592259	GATCATTTCACCTAGCCTCCACTTAAT	1	G	0.0174	0.4764
NC_005101.4-228595520	CTGTCTCACACTTTCTCTCCTACCGTT	22	A	0.0081	0.4777
NC_005101.4-228613203	CATAGAAATACTGCAGCTCCTGACCAA	14	C	0.0263	0.476
NC_005101.4-228662589	CATGTCCGAACTGACCCTCCACACACT	14	A	0.1261	0.2961
NC_005101.4-228672372	ATGACCCCCACCCCATCTCCCCCATCC	12	C	0.0244	0.0241
NC_005101.4-228672372	ATGACCCCCACCCCATCTCCCCCATCC	22	C	0.2705	0.4634
NC_005101.4-228725532	CAAAGACTAACAAAGACTCCAGTACTG	12	A	0.3009	0.4135
NC_005101.4-228823886	TTATGAGCTACTTAGGCTCCCTCCAAT	1	T	0.1463	0.4992
NC_005101.4-228823886	TTATGAGCTACTTAGGCTCCCTCCAAT	6	A	0.1545	0.4979
NC_005101.4-228937327	TGCTTTGGCACTGAGTCTCCCACTGAT	13	G	0.1626	0.1629
NC_005101.4-228937327	TGCTTTGGCACTGAGTCTCCCACTGAT	24	T	0	0.4793
NC_005101.4-229018357	ATCTTCCTCACTCCCCCTCCCATCCCT	16	C	0.0855	0.497
NC_005101.4-229144245	CAAACTTTAACTACACCTCCAGGGCAC	5	C	0.0625	0.0771
NC_005101.4-229153759	CCTGTGTGTACCTGCACTCCAGCACAG	25	C	0.082	0.1367
NC_005101.4-229191047	AATGGAGACACACTACCTCCTTGAACC	25	A	0	0.4764
NC_005101.4-229206454	CTCTGAGAAACTAACTCTCCCATTCTT	1	C	0.0588	0.0728
NC_005101.4-229285649	GGTGGGTGCACTTTCTCTCCTGCTTCA	4	G	0.0333	0.0488
NC_005101.4-229285649	GGTGGGTGCACTTTCTCTCCTGCTTCA	26	C	0	0.4813
NC_005101.4-229353101	CCTAGTGACACACTTTCTCCCACACTG	2	C	0.0708	0.0683
NC_005101.4-229353101	CCTAGTGACACACTTTCTCCCACACTG	9	C	0.1416	0.1316
NC_005101.4-229459865	AATGCATTCACTTGCTCTCCCGCCTTC	22	G	0.1695	0.2091
NC_005101.4-229459865	AATGCATTCACTTGCTCTCCCGCCTTC	27	C	0.0085	0.4738
NC_005101.4-229489067	TCTAAGATCACCATGACTCCTGTGACA	14	T	0.1301	0.4997
NC_005101.4-229489067	TCTAAGATCACCATGACTCCTGTGACA	25	A	0.1301	0.1629
NC_005101.4-229606239	TTTCCTCCCACAACTCCTCCTAGATCC	7	C	0.0684	0.066
NC_005101.4-229614623	CACACACACACACAAGCTCCCCTTTCA	5	C	0.1466	0.1923
NC_005101.4-229614623	CACACACACACACAAGCTCCCCTTTCA	6	A	0.2414	0.3282
NC_005101.4-229636968	TTTATCACCACACACTCTCCAGTCATA	26	T	0	0.4832
NC_005101.4-229636968	TTTATCACCACACACTCTCCAGTCATA	27	A	0.0833	0.1244
NC_005101.4-229736817	ATTACAGAGACATACCCTCCACCATAA	7	G	0.0087	0.4903
NC_005101.4-229736817	ATTACAGAGACATACCCTCCACCATAA	21	A	0.1043	0.0989
NC_005101.4-229931491	GTTCTGAGTACTCTTCCTCCTCTGAAC	2	T	0.1864	0.2219
NC_005101.4-229931491	GTTCTGAGTACTCTTCCTCCTCTGAAC	5	T	0.1913	0.4969

NC_005101.4-230041771	CCTGTCTGCACCTCTCCTCCCTGTCCT	2	C	0.0325	0.032
NC_005101.4-230048296	TGTGAGAGGACCACAACTCCCAGCTTC	8	G	0.1382	0.1425
NC_005101.4-230055964	ACAAATAACACATATGCTCCTATATTA	12	A	0.1897	0.5
NC_005101.4-230236052	CTGGACCCAACTCCTTCTCCCTCAAAG	5	A	0.0261	0.0257
NC_005101.4-230249662	CCATCCAAAACGCTTTCTCCTTTTCTC	12	G	0.1382	0.4239
NC_005101.4-230249662	CCATCCAAAACGCTTTCTCCTTTTCTC	25	C	0.0813	0.078
NC_005101.4-230307939	TGCCCTGTCACTTTGTCTCCAGAGATC	15	G	0	0.4793
NC_005101.4-230307939	TGCCCTGTCACTTTGTCTCCAGAGATC	21	A	0.122	0.1826
NC_005101.4-230333486	TGAATCAGAACCTTACCTCCCCCTGCA	16	C	0.0085	0.481
NC_005101.4-230380663	CTCGGTTGTACTACTGCTCCTGTGAGT	3	C	0.193	0.3878
NC_005101.4-230380663	CTCGGTTGTACTACTGCTCCTGTGAGT	4	G	0.1826	0.3451
NC_005101.4-230392366	CAAATATCCACTCTGCCTCCCTTATAA	4	A	0	0.4703
NC_005101.4-230402795	TTTTGTATAACTCCCACTCCCGTCCCT	7	A	0.1607	0.4994
NC_005101.4-230402795	TTTTGTATAACTCCCACTCCCGTCCCT	21	C	0.0268	0.4933
NC_005101.4-230460874	TGAAACAAAACTTACCCTCCTCCACAA	23	C	0.0244	0.0241
NC_005101.4-230460874	TGAAACAAAACTTACCCTCCTCCACAA	24	A	0.1667	0.4861
NC_005101.4-230501405	AGTTTCAAAACCTTTACTCCTTTCCCT	2	G	0.2	0.2888
NC_005101.4-230501405	AGTTTCAAAACCTTTACTCCTTTCCCT	23	T	0.2017	0.2907
NC_005101.4-230539282	TTATTCTTCACCGGCTCTCCATGCAGT	24	C	0.1102	0.1481
NC_005101.4-230540819	GTGCACGGTACCACAGCTCCAAGGGAA	7	G	0	0.4793
NC_005101.4-230543040	CCCCTTGGCACAGTGACTCCATCTGCA	4	C	0.0932	0.1337
NC_005101.4-230575688	CCGATTATCACTGGGCCTCCTTGTGGT	26	G	0.1789	0.3516
NC_005101.4-230606775	CCCATCACTACCAACACTCCTTAAACA	1	C	0	0.4793
NC_005101.4-230645394	CCAGACTCCACCAGAGCTCCCAGGAAC	3	A	0.5917	0.4166
NC_005101.4-230645394	CCAGACTCCACCAGAGCTCCCAGGAAC	21	C	0.3917	0.315
NC_005101.4-230675965	GGGATATACACATGTACTCCTGGCACA	5	T	0.178	0.2156
NC_005101.4-230675965	GGGATATACACATGTACTCCTGGCACA	16	A	0.1724	0.2378
NC_005101.4-230762428	ATATGAGCCACAGGAACTCCTCGCTTT	15	A	0	0.4772
NC_005101.4-230791021	CTACAAGCCACCATGCCTCCCACCATG	27	G	0.1917	0.225
NC_005101.4-230799027	AACTACGTGACCTCACCTCCCACATGT	25	T	0.2195	0.3472
NC_005101.4-230861792	GGTGTCTGCACATAAGCTCCTTAAGTC	12	A	0.1429	0.4987
NC_005101.4-230862763	GTGATCTCTACAAGTTCTCCTTCTCAT	26	A	0.0089	0.4824
NC_005101.4-231009399	CTTACTGAAACCAGTTCTCCCTCATTT	14	G	0.0088	0.4889
NC_005101.4-231014868	GGGAATAAAACAAGTGCTCCTCTTGAT	22	C	0.0169	0.4856
NC_005101.4-231083219	CATTGCGTGACGTCAGCTCCTTCCTGA	7	G	0.1148	0.4988
NC_005101.4-231104928	TTTGCCCTCACAGATTCTCCTCCGTAG	24	G	0.2435	0.4364
NC_005101.4-231112482	CTGGTCTCTACTGCTGCTCCTGACTCC	4	G	0.0083	0.4835
NC_005101.4-231112482	CTGGTCTCTACTGCTGCTCCTGACTCC	25	T	0.2541	0.3984
NC_005101.4-231158099	GTTACCGAGACATGCGCTCCTTCCTCA	6	C	0.0083	0.4769
NC_005101.4-231158099	GTTACCGAGACATGCGCTCCTTCCTCA	16	G	0.1393	0.2218
NC_005101.4-231339653	AATGATAGAACACGAACTCCATGGGCT	6	T	0.2149	0.4959
NC_005101.4-231339653	AATGATAGAACACGAACTCCATGGGCT	25	G	0.2149	0.2533
NC_005101.4-231351394	GTGAAGTCTACAGCAGCTCCCCGGTTT	9	T	0	0.48
NC_005101.4-231351394	GTGAAGTCTACAGCAGCTCCCCGGTTT	15	A	0.2149	0.3374
NC_005101.4-231395994	ATTTTATCCACTTGTTCTCCACCAAAA	4	T	0.1339	0.1699
NC_005101.4-231421641	TCATCAGAGACAACCCCTCCTGCAGTG	8	A	0.0917	0.1997
NC_005101.4-231430584	ACTCTCTCCACCCCCACTCCTCACTCA	15	C	0.2846	0.4106
NC_005101.4-231447848	TAACTTTCTACAGCTTCTCCTTCCTTT	8	C	0.0325	0.032
NC_005101.4-231447848	TAACTTTCTACAGCTTCTCCTTCCTTT	12	A	0.1626	0.4072
NC_005101.4-231471780	ACCGGGACAACACTCACTCCTCTGTCT	4	G	0.0331	0.4849
NC_005101.4-231502630	TCTGTCATGACCTTTTCTCCTTGTGTC	16	T	0	0.4833

NC_005101.4-231521717	TAACACCACACCGCTGCTCCACCCACT	12	C	0.042	0.0411
NC_005101.4-231552792	CAGGAGGTGACCCAGACTCCAGGCCCT	16	A	0.0336	0.0491
NC_005101.4-231552792	CAGGAGGTGACCCAGACTCCAGGCCCT	27	T	0	0.4779
NC_005101.4-231604835	CAATCCACTACATTAACTCCCTCGATT	24	G	0.1074	0.1306
NC_005101.4-231604835	CAATCCACTACATTAACTCCCTCGATT	25	A	0.0083	0.4783
NC_005101.4-231662460	ATGCCCGTAACCTCAGCTCCAAGGGAT	2	T	0.1951	0.4444
NC_005101.4-231676680	CGGGACTGCACGTATGCTCCACCACAC	1	C	0.3636	0.4672
NC_005101.4-231678046	CTAACCGTAACTTCAGCTCCTCTGTGG	6	C	0.2308	0.3
NC_005101.4-231699069	CCAGCACTCACACCATCTCCTGATCCA	22	G	0.2114	0.3427
NC_005101.4-231711128	TCCACACCAACACTTCCTCCCCGCTTC	22	C	0	0.4813
NC_005101.4-231728848	AAACTCTTTACGGAGTCTCCATTAGTC	12	G	0.0407	0.0553
NC_005101.4-231728848	AAACTCTTTACGGAGTCTCCATTAGTC	24	A	0.2358	0.3472
NC_005101.4-231747259	AACCTGCAAACTGCGGCTCCAGGGAAT	15	G	0.3	0.3665
NC_005101.4-231747259	AACCTGCAAACTGCGGCTCCAGGGAAT	25	A	0.1261	0.1181
NC_005101.4-231783323	AAACCGGAAACAGTAGCTCCTGCCTGG	5	C	0.2946	0.4043
NC_005101.4-231783323	AAACCGGAAACAGTAGCTCCTGCCTGG	14	T	0.0526	0.0512
NC_005101.4-231849598	TTGGGCATGACCGATTCTCCTCTAGCC	6	C	0.0574	0.071
NC_005101.4-231849598	TTGGGCATGACCGATTCTCCTCTAGCC	22	C	0.0246	0.0243
NC_005101.4-231851200	CTGAGCCACACTCCGGCTCCAGGATTT	16	G	0	0.4787
NC_005101.4-231855000	GACTACGGAACTCTCCCTCCAGCTCCT	7	G	0.1157	0.4426
NC_005101.4-231855000	GACTACGGAACTCTCCCTCCAGCTCCT	13	C	0.0579	0.0868
NC_005101.4-231930891	GGGAGGGGAACAGTTCCTCCTTCTGCT	7	G	0.0738	0.0861
NC_005101.4-231930891	GGGAGGGGAACAGTTCCTCCTTCTGCT	25	G	0.0732	0.0854
NC_005101.4-231943918	GTCCTTCACACTGGTGCTCCATAAGTC	12	T	0.0339	0.0333
NC_005101.4-231966582	CTCATCTCTACCAGTTCTCCAGCCCCA	16	T	0.0085	0.4776
NC_005101.4-231979309	GATTCTTACACTTGATCTCCTTAGCCA	7	T	0.1148	0.1225
NC_005101.4-232093622	CAAATGGACACACTTCCTCCGCAAGCT	4	A	0.2034	0.2344
NC_005101.4-232100760	AGGCAGGGGACTTGTCCTCCTTCTTGA	1	A	0.2137	0.2299
NC_005101.4-232100760	AGGCAGGGGACTTGTCCTCCTTCTTGA	25	T	0.2393	0.3261
NC_005101.4-232107436	ATGTGTAATACATGTTCTCCGGGTTTA	12	A	0.0085	0.481
NC_005101.4-232124536	CAACACCAAACTGTATCTCCGTGTCTG	14	T	0.2479	0.4923
NC_005101.4-232134887	AACCTCTTAACAACTGCTCCATCTCTC	24	T	0.0609	0.1067
NC_005101.4-232167333	AGATGACTGACAGTCCCTCCCCTCACT	1	A	0	0.4807
NC_005101.4-232248306	TGGAGGCTGACAGAGGCTCCTTAATTC	1	T	0	0.48
NC_005101.4-232248306	TGGAGGCTGACAGAGGCTCCTTAATTC	2	G	0.0333	0.4861
NC_005101.4-232274059	GGATCCTCAACTTGGCCTCCAGGGGAG	3	A	0.0087	0.4903
NC_005101.4-232281701	AGGAAATTCACCCTCTCTCCTTTCGAG	23	T	0.0244	0.484
NC_005101.4-232407708	AAAGACTGAACCCTGTCTCCTGGCGGT	27	T	0.2333	0.255
NC_005101.4-232411414	ATTACTTGCACTCTTCCTCCAAGCCTT	22	A	0.2348	0.4976
NC_005101.4-232529444	GTAGCCCTTACTTTATCTCCACTTCTG	26	T	0.0083	0.0566
NC_005101.4-232533368	CCCTAGCTCACAGCTGCTCCCTTTTCA	2	C	0.0833	0.4932
NC_005101.4-232533368	CCCTAGCTCACAGCTGCTCCCTTTTCA	4	T	0.0083	0.4783
NC_005101.4-232594049	CATTTCTCTACCCCATCTCCCCTCTCT	12	C	0.3097	0.3772
NC_005101.4-232594049	CATTTCTCTACCCCATCTCCCCTCTCT	21	C	0.2348	0.27
NC_005101.4-232605407	CCGTGTCATACCCCTCCTCCATTTCAG	1	C	0.0167	0.0488
NC_005101.4-232668975	TCAAACACCACCCACCCTCCAGTCATT	3	A	0.3333	0.474
NC_005101.4-232668975	TCAAACACCACCCACCCTCCAGTCATT	25	A	0	0.4814
NC_005101.4-232704147	GCCCACCAGACCTTGTCTCCTTAAGGG	14	T	0.1197	0.1704
NC_005101.4-232722324	AAAAGCTACACACTATCTCCATTCTTT	6	C	0.2018	0.4796
NC_005101.4-232722324	AAAAGCTACACACTATCTCCATTCTTT	16	T	0.2018	0.4796
NC_005101.4-232767071	CTCCCTCTCACTCTCTCTCCTGTGTAT	26	A	0	0.4731

NC_005101.4-232769012	GAAAGAAGGACTCTCCCTCCTCTGCAG	15	C	0	0.4832
NC_005101.4-232842081	GGGCTGGTCACTCTGCCTCCTTTCTCT	3	G	0.0678	0.0655
NC_005101.4-232842081	GGGCTGGTCACTCTGCCTCCTTTCTCT	15	G	0.0085	0.481
NC_005101.4-232942429	GTGAGAGTAACTGTGTCTCCTCAAGAT	16	T	0.1597	0.5
NC_005101.4-232959937	TGAAGGAGTACATGGACTCCAGAGGAT	27	T	0.2459	0.2956
NC_005101.4-232997330	TACTCATCAACCCTAACTCCTCCCCTA	14	T	0	0.4757
NC_005101.4-233050377	AAATCATCCACTCCCTCTCCTTTCTTC	2	A	0.0086	0.4836
NC_005101.4-233050377	AAATCATCCACTCCCTCTCCTTTCTTC	26	T	0.0087	0.4849
NC_005101.4-233097894	TCACTCTGCACTGTCTCTCCACGAGTG	15	C	0.0917	0.1316
NC_005101.4-233118404	TCATTCCATACCTCCTCTCCCATCCTG	5	T	0.114	0.4353
NC_005101.4-233192463	TTGTTGCAAACACTGTCTCCATGTTTT	15	G	0.025	0.0247
NC_005101.4-233210331	TATAGGTCAACCACCCCTCCATTAATT	15	C	0.1739	0.2758
NC_005101.4-233210559	CCCTCTGTAACTCTAGCTCCAGGAGAT	7	G	0.314	0.2759
NC_005101.4-233269736	CACTGCTCCACCATGACTCCTTCTCTG	3	C	0.0427	0.0418
NC_005101.4-233269736	CACTGCTCCACCATGACTCCTTCTCTG	6	C	0	0.488
NC_005101.4-233282058	AATTGGTTCACCCATCCTCCAAACATG	7	T	0.0084	0.4829
NC_005101.4-233374279	ATGAACACTACGGTTACTCCCATTTTA	14	T	0.25	0.4248
NC_005101.4-233388646	TTACAGCAAACCACATCTCCTCAAAGA	23	A	0.2162	0.4704
NC_005101.4-233417428	AAATATTTCACGCATCCTCCCTTACTC	12	G	0.0536	0.0853
NC_005101.4-233456535	CTCCAAGGTACTGCCCCTCCTGCTTGG	13	G	0.3136	0.4396
NC_005101.4-233456535	CTCCAAGGTACTGCCCCTCCTGCTTGG	14	C	0.0333	0.0328
NC_005101.4-233463971	TCCTGATGCACTAGCTCTCCCTTTCCC	7	T	0	0.4832
NC_005101.4-233463971	TCCTGATGCACTAGCTCTCCCTTTCCC	16	T	0.025	0.0247
NC_005101.4-233477621	CTGGGTCAAACACAGTCTCCCCCTCCT	22	C	0	0.4773
NC_005101.4-233493042	ATATCCACAACTTCTTCTCCTTAACTT	22	T	0.1901	0.2704
NC_005101.4-233524726	GCTTCAAAGACAGTGCCTCCTTCTGAT	25	G	0.0902	0.1436
NC_005101.4-233538197	TCCCAGCCAACCCACCCTCCAAATTCC	16	C	0.0333	0.0328
NC_005101.4-233561081	AGCCTCGGGACAGCAGCTCCTACACAT	13	G	0.2358	0.373
NC_005101.4-233561081	AGCCTCGGGACAGCAGCTCCTACACAT	21	T	0	0.4793
NC_005101.4-233630069	TGGGCAGGGACCCTTCCTCCACACGCA	8	G	0.0439	0.4415
NC_005101.4-233630069	TGGGCAGGGACCCTTCCTCCACACGCA	25	G	0	0.474
NC_005101.4-233642937	GACCTTGTCACAGGAACTCCTTTTCTA	2	A	0	0.032
NC_005101.4-233668040	ATTGTATTGACCGCATCTCCTGGTGTA	21	T	0.0261	0.0257
NC_005101.4-233728793	TTGAACTGGACCAGTCCTCCTGAGCCC	5	A	0.2333	0.3987
NC_005101.4-233728793	TTGAACTGGACCAGTCCTCCTGAGCCC	24	G	0.0909	0.1017
NC_005101.4-234660888	ATGTGACACACGGCAACTCCTGAGAGT	9	C	0.0087	0.4782
NC_005101.4-234662232	GATTTGCACACATGCTCTCCTGAATGG	15	C	0.025	0.0408
NC_005101.4-234706332	GGCCAGACCACCAGTGCTCCCAGGGAC	9	C	0.4107	0.4641
NC_005101.4-234706332	GGCCAGACCACCAGTGCTCCCAGGGAC	14	G	0.4107	0.4641
NC_005101.4-234709932	GAGCGTACAACACTGGCTCCCATCTCA	22	A	0.123	0.4984
NC_005101.4-234730764	CACCCCAGAACTCACCCTCCTGAGGCT	2	A	0.082	0.1082
NC_005101.4-234730764	CACCCCAGAACTCACCCTCCTGAGGCT	3	C	0.082	0.1082
NC_005101.4-234769832	TCATGTCCAACGCTGGCTCCTTCCTGG	13	C	0.0789	0.0758
NC_005101.4-234786756	GAGCTGGTGACAGGGGCTCCTGCAATG	24	A	0.1475	0.263
NC_005101.4-234806362	AACCTCCTTACTTCTCCTCCATCTCCT	7	C	0.1858	0.359
NC_005101.4-234824095	TACCCCAATACTATTTCTCCTATCACA	6	C	0.0088	0.4676
NC_005101.4-234824095	TACCCCAATACTATTTCTCCTATCACA	8	A	0.0088	0.4676
NC_005101.4-235230139	CAGTGGTAGACAATGTCTCCCTGCCAA	3	G	0.0598	0.12
NC_005101.4-235253042	GTGTGCCTAACTACCACTCCATCGGGT	6	C	0.2358	0.3381
NC_005101.4-235253042	GTGTGCCTAACTACCACTCCATCGGGT	24	G	0.2358	0.4417
NC_005101.4-235258145	ATAGATTACACCTCACCTCCTGCCTCC	4	G	0.1239	0.1316

NC_005101.4-235262656	ACTATACTTACCCTCCCTCCCAATGAT	4	A	0.1795	0.3855
NC_005101.4-235307045	TGGTTTCCTACCTCCTCTCCAACTGTC	2	G	0.0862	0.0825
NC_005101.4-235307045	TGGTTTCCTACCTCCTCTCCAACTGTC	7	C	0.0603	0.0585
NC_005101.4-235385518	ACCAGAGGGACATGTGCTCCATGATGT	1	A	0.0569	0.0553
NC_005101.4-235401595	TTTGTGCCCACCAGGTCTCCGTAGACT	9	C	0.042	0.4886
NC_005101.4-235401595	TTTGTGCCCACCAGGTCTCCGTAGACT	25	A	0	0.4813
NC_005101.4-235465248	TCATCTACCACAATATCTCCTGGAAGG	9	C	0.1327	0.2104
NC_005101.4-235465248	TCATCTACCACAATATCTCCTGGAAGG	14	T	0.0435	0.4878
NC_005101.4-235474550	ACTCCACACACATGTCCTCCTAACATT	24	C	0.082	0.1082
NC_005101.4-235496534	TTGGTTTTCACAACTTCTCCCTTCATT	24	C	0.1842	0.2476
NC_005101.4-235533418	TTCGCAGTTACTTAGCCTCCTTTCTGT	26	G	0.0254	0.0251
NC_005101.4-235542115	TCGTGGCTTACTTTCTCTCCCAGAATG	2	C	0.0492	0.0634
NC_005101.4-235561535	TCCTAACTTACCCCTCCTCCAGATGGC	16	C	0.0909	0.1585
NC_005101.4-235565217	GAAGAATAGACAGGCTCTCCACAATGA	14	G	0.1491	0.3919
NC_005101.4-235615997	TGAGAAAGGACTTAGGCTCCATTGTCT	9	G	0.0588	0.2266
NC_005101.4-235615997	TGAGAAAGGACTTAGGCTCCATTGTCT	24	G	0.0588	0.0571
NC_005101.4-235726365	TCCAAAGAAACGCTCTCTCCTTTAGTT	21	T	0.0252	0.0249
NC_005101.4-235735348	CTTGTGTGGACTATCTCTCCTTTTCAG	2	T	0.082	0.1225
NC_005101.4-235769650	ACAACACTAACTCACTCTCCGAGCATT	25	A	0.2679	0.3838
NC_005101.4-235770051	GGAACCATTACTCAGCCTCCCACAGTG	12	T	0.0579	0.0562
NC_005101.4-235786486	TAAGCTGGTACTTCAGCTCCAGCTAAC	8	G	0.0957	0.1067
NC_005101.4-235880339	CATCCTATAACAGAATCTCCCAAGCTG	27	G	0	0.4902
NC_005101.4-235927950	TTTTCTTTTACCCTATCTCCTATGCCT	27	T	0.1504	0.1828
NC_005101.4-235931652	CTGTCACGGACAAGTGCTCCTCACCCA	8	G	0.2869	0.4379
NC_005101.4-235945484	GGCCTGGAAACATGGCCTCCTTGTTCT	3	C	0.0088	0.0433
NC_005101.4-235963563	TGATCGCTGACCCCTTCTCCACTTGAA	6	G	0.0656	0.1082
NC_005101.4-235963563	TGATCGCTGACCCCTTCTCCACTTGAA	21	A	0.0656	0.4952
NC_005101.4-236031574	GTGAAAGTCACAGAAGCTCCTAAGTGT	2	T	0	0.4871
NC_005101.4-236034308	TTGAAGTCAACAAGCTCTCCTTTTCAT	4	A	0.3277	0.4117
NC_005101.4-236036640	TGTAGGAAAACTTGTCCTCCAGGCAAA	5	G	0.0357	0.0853
NC_005101.4-236038363	AATGAGATCACTATTGCTCCACTCTAG	26	A	0.0992	0.1235
NC_005101.4-236048521	ATGGCATAAACTTTATCTCCATCTTCA	3	G	0.265	0.3107
NC_005101.4-236077715	CAAAGCTCAACACTGACTCCTTTGTCG	26	C	0.1441	0.1759
NC_005101.4-236096427	ATCCTGGGGACAGCAGCTCCTTCGCTG	24	G	0.065	0.4944
NC_005101.4-236098326	CATAGCCTTACATTCACTCCGAATCTG	2	A	0.0084	0.4859
NC_005101.4-236098326	CATAGCCTTACATTCACTCCGAATCTG	23	A	0.0084	0.4859
NC_005101.4-236118245	TCGCACTGCACAGCCACTCCGTGCTCA	3	G	0.0667	0.095
NC_005101.4-236122141	GTCTGTGAAACAGTAGCTCCTAGATGA	22	A	0	0.4807
NC_005101.4-236150675	GGATGTAAGACGTCTGCTCCGGCAACC	4	T	0.2759	0.4144
NC_005101.4-236197337	GAAATCTAAACACCTCCTCCCTACACA	13	C	0.1597	0.274
NC_005101.4-236197337	GAAATCTAAACACCTCCTCCCTACACA	15	T	0.1597	0.274
NC_005101.4-236202736	CCTTCTCTAACTCCAGCTCCAGGACAT	14	C	0.0796	0.154
NC_005101.4-236205711	TCCTCTCATACACGTGCTCCTCTCATA	23	T	0.1552	0.3916
NC_005101.4-236225498	AGATTTGAAACAGTTACTCCGTTTCTA	21	G	0.1624	0.5
NC_005101.4-236392270	AAACTGTGAACAGAACCTCCATGGACT	21	A	0.0083	0.0247
NC_005101.4-236451025	TCCAATAAGACCACACCTCCTAAGCCT	9	G	0.5575	0.4373
NC_005101.4-236469372	ATTCCTCCAACTGTGACTCCCATCCTC	2	T	0.0492	0.4914
NC_005101.4-236469372	ATTCCTCCAACTGTGACTCCCATCCTC	24	C	0.1545	0.1562
NC_005101.4-236469755	ACGTCATAGACGCTATCTCCAACTGAT	3	G	0.0976	0.496
NC_005101.4-236471992	GTGCTGATAACTTGTTCTCCTGACCTG	12	T	0.1393	0.1573
NC_005101.4-236488974	CACTTTAATACAATTTCTCCTTCCTTC	26	T	0.0248	0.0405

NC_005101.4-236488974	CACTTTAATACAATTTCTCCTTCCTTC	27	C	0.175	0.5
NC_005101.4-236491225	TCATATACCACGGCATCTCCACGATGG	12	G	0.1148	0.1225
NC_005101.4-236491225	TCATATACCACGGCATCTCCACGATGG	23	G	0.041	0.0401
NC_005101.4-236492098	TATCCTGCAACAGCTTCTCCCTCAAAC	2	A	0.0244	0.0241
NC_005101.4-236492098	TATCCTGCAACAGCTTCTCCCTCAAAC	4	C	0.0244	0.0241
NC_005101.4-236495178	TACAACCTGACCAAAGCTCCTCTAAGT	27	T	0.0902	0.1009
NC_005101.4-236495367	GGTTGGGTCACTTTCCCTCCCCTAACT	3	T	0	0.4898
NC_005101.4-236495367	GGTTGGGTCACTTTCCCTCCCCTAACT	15	C	0	0.4898
NC_005101.4-236530344	TGCTGACACACCGAGTCTCCCTTCTTT	9	C	0.0167	0.4861
NC_005101.4-236530447	CAGCGAGTGACAGTCTCTCCTCCACCA	15	C	0.0333	0.0328
NC_005101.4-236544434	TGACTTCTCACTACCTCTCCTACATAC	7	C	0.1217	0.4981
NC_005101.4-236544434	TGACTTCTCACTACCTCTCCTACATAC	26	A	0	0.4833
NC_005101.4-236548998	TGAATTTCTACCCCACCTCCTCCCTAG	27	G	0.0625	0.0605
NC_005101.4-236556604	GTGATGGGCACTACAGCTCCCTCACAG	4	A	0	0.4793
NC_005101.4-236699618	CTTGTGAACACAGCATCTCCAACAGTC	13	G	0.0244	0.0705
NC_005101.4-236699618	CTTGTGAACACAGCATCTCCAACAGTC	16	T	0.0407	0.0398
NC_005101.4-236711585	GGCCAGCTCACCTTTTCTCCTGCCGCC	16	T	0.0088	0.4735
NC_005101.4-236711585	GGCCAGCTCACCTTTTCTCCTGCCGCC	24	C	0	0.0348
NC_005101.4-236763309	GCTTTTCTGACTTTGCCTCCCGTGCTG	3	T	0.0088	0.472
NC_005101.4-236882495	GGAAATAATACTTCAACTCCTTTCCAT	13	T	0.2	0.4061
NC_005101.4-236944260	GATCCTCCCACCTTCCCTCCCGTTCTA	14	T	0.0083	0.4732
NC_005101.4-236944260	GATCCTCCCACCTTCCCTCCCGTTCTA	22	G	0.0496	0.109
NC_005101.4-236953667	GCTGGCGACACACTCACTCCCAACACT	6	C	0	0.4898
NC_005101.4-237017401	CAATTAAGCACAATGTCTCCATGGTTC	3	A	0.0932	0.119
NC_005101.4-237017401	CAATTAAGCACAATGTCTCCATGGTTC	12	A	0.1538	0.4997
NC_005101.4-237021813	GAAAGAGGAACTGGGGCTCCCATGTTG	8	G	0.1667	0.2061
NC_005101.4-237021813	GAAAGAGGAACTGGGGCTCCCATGTTG	14	G	0.0917	0.1458
NC_005101.4-237038163	AAAGTGTGCACTCAAACTCCCAGAAGT	2	A	0.2308	0.4416
NC_005101.4-237038163	AAAGTGTGCACTCAAACTCCCAGAAGT	25	A	0.2051	0.4956
NC_005101.4-237061221	TCCAGAGACACCGTCACTCCCAGCTCA	12	C	0.0513	0.4772
NC_005101.4-237083548	TTTTCAAATACACAAGCTCCCATCCAT	14	A	0.0536	0.1014
NC_005101.4-237083548	TTTTCAAATACACAAGCTCCCATCCAT	25	C	0.0442	0.3858
NC_005101.4-237087300	ATCGTCTTGACTGGAGCTCCTTTATTA	26	T	0.2541	0.4489
NC_005101.4-237111226	TTAGGAGCTACAATCCCTCCTCCTATT	6	A	0.0088	0.483
NC_005101.4-237129015	GGAACGTCTACCCTCTCTCCTTACACT	6	G	0.2417	0.3708
NC_005101.4-237135223	ATTTCCACAACTGAATCTCCGATTGAT	27	T	0.0446	0.0436
NC_005101.4-237148919	CAAGAGAGCACTGGCCCTCCACGCAGC	15	C	0.069	0.0666
NC_005101.4-237148919	CAAGAGAGCACTGGCCCTCCACGCAGC	21	A	0.0263	0.4935
NC_005101.4-237299597	GTCATTCCTACGTCGCCTCCAGTTCTG	1	G	0.1417	0.1458
NC_005101.4-237299597	GTCATTCCTACGTCGCCTCCAGTTCTG	15	G	0.0917	0.1172
NC_005101.4-237318476	CCCTAAAGGACTCTGTCTCCAAGAAAT	5	A	0.1739	0.2268
NC_005101.4-237347946	TACATATTCACCACCTCTCCACTCCTA	3	C	0.2124	0.4404
NC_005101.4-237359248	AGTCTACCCACCTACCCTCCTCACCCA	7	C	0.1176	0.1539
NC_005101.4-237366408	ATTCTCTCCACCCCTCCTCCCACCATC	21	C	0.0579	0.1163
NC_005101.4-237413911	GACACTGTGACAAGCACTCCCTTCACC	25	A	0.0085	0.0415
NC_005101.4-237436558	CACCTATAAACTCCAGCTCCTGAGGTT	9	A	0.0081	0.4777
NC_005101.4-237447912	AACGTTGAAACTGCAGCTCCTCAGGAT	4	G	0	0.4819
NC_005101.4-237447912	AACGTTGAAACTGCAGCTCCTCAGGAT	14	C	0.1721	0.2796
NC_005101.4-237449637	TCCAATCACACACCACCTCCTCATACC	12	A	0.1057	0.356
NC_005101.4-237449637	TCCAATCACACACCACCTCCTCATACC	27	C	0.1463	0.189
NC_005101.4-237469815	AAACTGTAAACCCTACCTCCAAATACA	14	T	0.2143	0.232

NC_005101.4-237499057	TCGCTTTGGACAACCTCTCCTGTTTAT	2	C	0.2541	0.3831
NC_005101.4-237499057	TCGCTTTGGACAACCTCTCCTGTTTAT	3	G	0.2623	0.3791
NC_005101.4-237518449	AACAAGACCACATCTACTCCAATGCAG	27	G	0.1083	0.1733
NC_005101.4-237543056	TTATTAAGAACTTACCCTCCCCACCTC	15	C	0.0855	0.142
NC_005101.4-237543056	TTATTAAGAACTTACCCTCCCCACCTC	16	C	0.2759	0.4749
NC_005101.4-237599712	CTGACCGTAACTGGCCCTCCGAGTTTC	6	C	0	0.4773
NC_005101.4-237599712	CTGACCGTAACTGGCCCTCCGAGTTTC	21	G	0.1382	0.2668
NC_005101.4-237640787	CAAAGGTGGACACTTGCTCCAACATTA	9	G	0.0855	0.4997
NC_005101.4-237640787	CAAAGGTGGACACTTGCTCCAACATTA	14	T	0.0855	0.4997
NC_005101.4-237698138	TGGAAGAGGACAGCCACTCCGTATGTC	15	C	0.0488	0.0476
NC_005101.4-237698138	TGGAAGAGGACAGCCACTCCGTATGTC	21	G	0.3577	0.4072
NC_005101.4-237844116	GCTGTGGCGACTGCCGCTCCACCACTA	9	G	0.0259	0.0255
NC_005101.4-237844116	GCTGTGGCGACTGCCGCTCCACCACTA	15	C	0.0086	0.4595
NC_005101.4-237853038	GGTGCTTAGACTTGAACTCCAGTCGTC	12	T	0.0435	0.0425
NC_005101.4-237853038	GGTGCTTAGACTTGAACTCCAGTCGTC	27	C	0.0439	0.0429
NC_005101.4-237890934	CAGCTCTTTACTTCATCTCCAGAGACA	24	G	0.0744	0.1306
NC_005101.4-237943446	ATGAGGCAAACCATCGCTCCACAGGTG	15	C	0.1789	0.4
NC_005101.4-237946741	CCATAACAAACATTTACTCCCACTGCC	6	A	0.1217	0.1588
NC_005101.4-238035484	CTATGGACCACTTAACCTCCTAAATCA	7	A	0.0331	0.4713
NC_005101.4-238038182	TTTGGCCCGACGCACTCTCCGTGTTTG	3	T	0.0593	0.0576
NC_005101.4-238048649	TGGCTCCTAACTTTAGCTCCTAGTGAA	14	T	0.0417	0.0722
NC_005101.4-238114926	TGATAATGGACTTAATCTCCGAAACTG	24	A	0.0948	0.0903
NC_005101.4-238114926	TGATAATGGACTTAATCTCCGAAACTG	27	G	0.4138	0.3282
NC_005101.4-238115383	CTCCCCAAGACAGAGTCTCCCTAGTAG	4	C	0.1593	0.2171
NC_005101.4-238301819	CTTTGAGGGACTCCTCCTCCTCGTTCT	24	T	0.0536	0.4943
NC_005101.4-238444514	TGAGCGGCTACTGTATCTCCCAAAACA	12	T	0.018	0.0695
NC_005101.4-238463862	CATTCACTGACATGGTCTCCACAGTCT	13	T	0.1207	0.1134
NC_005101.4-238528505	TTCCCCAGAACCATAGCTCCCATTGCT	5	C	0	0.4724
NC_005101.4-238543805	AGGGCTGCTACTGTTACTCCTTGGTCA	9	T	0.1057	0.4995
NC_005101.4-238547921	CCACCACCGACCTAGTCTCCTTTCAGC	27	C	0.0508	0.4884
NC_005101.4-238562091	TTTCCTCTGACTGTGACTCCTCCTGGA	21	T	0.0826	0.5
NC_005101.4-238576525	GTAGCATTAACAATTGCTCCGTTGTCA	15	T	0	0.4773
NC_005101.4-238617340	AATGCTGGCACACTTTCTCCTCTGCTC	26	T	0	0.4807
NC_005101.4-238618188	TGCTTGTTGACCTTGTCTCCAGAAGAC	21	A	0.2645	0.3645
NC_005101.4-238724844	ACTCCATTCACCCTTCCTCCTACAATT	22	A	0	0.474
NC_005101.4-238776395	TTAGGCTCAACAGTGTCTCCTAAATGT	9	A	0.025	0.0247
NC_005101.4-238776395	TTAGGCTCAACAGTGTCTCCTAAATGT	12	A	0.0259	0.4803
NC_005101.4-238784142	GTACATGTCACCTGCACTCCCTGCCAT	16	A	0.009	0.4838
NC_005101.4-238816468	CAGCTAAACACTCTGGCTCCAAGAGTA	16	G	0.0167	0.48
NC_005101.4-238816468	CAGCTAAACACTCTGGCTCCAAGAGTA	27	A	0.0083	0.4816
NC_005101.4-238835049	GAAGACAAAACGGGGCCTCCTGGGACC	13	G	0.0177	0.452
NC_005101.4-238887893	ATTGGAGTTACCTACTCTCCTATTTCT	25	T	0.2522	0.4976
NC_005101.4-238898640	TGGAGAGAGACAACAGCTCCCATTAGA	5	G	0.1864	0.2466
NC_005101.4-238942433	ACAGTACACACTACAGCTCCTGGATTA	8	A	0.2241	0.4976
NC_005101.4-238942433	ACAGTACACACTACAGCTCCTGGATTA	27	A	0.1466	0.5
NC_005101.4-238986098	CTGGCAAAGACTGTTTCTCCTGCTCTC	27	C	0.1197	0.3729
NC_005101.4-239013534	CAGGCTAGGACTGTACCTCCAGCACTC	7	A	0	0.4902
NC_005101.4-239013534	CAGGCTAGGACTGTACCTCCAGCACTC	21	A	0.1404	0.4186
NC_005101.4-239031323	GAACCCCAAACACTAACTCCCTGTCCC	12	A	0	0.488
NC_005101.4-239031323	GAACCCCAAACACTAACTCCCTGTCCC	14	T	0	0.488
NC_005101.4-239062818	ATGGTTGCCACTCGGACTCCGCCAACT	14	G	0.0902	0.1154

NC_005101.4-239062818	ATGGTTGCCACTCGGACTCCGCCAACT	25	A	0.1967	0.3791
NC_005101.4-239064548	AGGATAGTGACTGAAACTCCGTTAGGA	12	T	0.1607	0.232
NC_005101.4-239105368	TCTAATGCCACTGGACCTCCATTACAT	23	T	0	0.4793
NC_005101.4-239105368	TCTAATGCCACTGGACCTCCATTACAT	24	A	0	0.4793
NC_005101.4-239154770	GGTATTTGCACATGGTCTCCCAAGTTA	12	A	0.2439	0.3141
NC_005101.4-239178407	GCTTCAGTCACATGCTCTCCCCACCCA	27	A	0.0783	0.0752
NC_005101.4-239179180	TCAGATTAGACAGTCTCTCCTTTGTAT	3	A	0.0531	0.4912
NC_005101.4-239188997	CATTATGCTACTGTGTCTCCCCACAGT	4	T	0.0088	0.4735
NC_005101.4-239219101	ATTTTTACTACTTAGACTCCACTGCTG	13	T	0	0.4682
NC_005101.4-239303603	GTTCAATGTACTAATGCTCCTGTTTAC	13	A	0	0.4787
NC_005101.4-239367393	TTAAAGAAAACGGACTCTCCCATCCTC	26	T	0.2857	0.3932
NC_005101.4-239399245	AGCATTGCCACCTTGTCTCCATTAATT	23	T	0.3305	0.3991
NC_005101.4-239424795	TAACTGAAAACCCTCGCTCCATTTCAG	21	A	0.009	0.4531
NC_005101.4-239430140	ATATTTAGTACGTGTTCTCCTTTAGAT	12	G	0.2087	0.3403
NC_005101.4-239655235	CAGTAACCAACACTCTCTCCACTAATA	8	C	0.2689	0.3118
NC_005101.4-239820932	CCTGGGGACACTGTTTCTCCTGCTGTC	12	T	0	0.4793
NC_005101.4-239823768	CTGCATTTGACTATGGCTCCCACTTCA	25	T	0.0252	0.0249
NC_005101.4-239823768	CTGCATTTGACTATGGCTCCCACTTCA	26	C	0.0252	0.0249
NC_005101.4-239824771	CCCGGACCGACCAGTGCTCCCAGGGAC	3	C	0.0965	0.3168
NC_005101.4-239959114	GGATAAAGCACTTCCCCTCCCATCATG	9	C	0.113	0.4613
NC_005101.4-239959114	GGATAAAGCACTTCCCCTCCCATCATG	15	C	0.113	0.4613
NC_005101.4-239998204	GTGACTTCAACGGCCCCTCCCCTCTTC	12	G	0.1967	0.1774
NC_005101.4-239998204	GTGACTTCAACGGCCCCTCCCCTCTTC	15	C	0.1967	0.1774
NC_005101.4-240001331	ATAATTAGCACAGTGGCTCCTAGACAG	8	G	0.0265	0.0262
NC_005101.4-240075099	GCTGTTCTGACTCAAACTCCCCTCCAT	4	G	0.075	0.0722
NC_005101.4-240075099	GCTGTTCTGACTCAAACTCCCCTCCAT	7	C	0.8783	0.4926
NC_005101.4-240220777	AGACCACTCACTACTCCTCCCCAGGTT	12	T	0	0.4666
NC_005101.4-240223666	ACGCAAGCTACACTTCCTCCGACGGTG	3	G	0.1885	0.2339
NC_005101.4-240223666	ACGCAAGCTACACTTCCTCCGACGGTG	24	G	0.1639	0.316
NC_005101.4-240238595	CACTGAACCACACTGCCTCCAGAAATT	1	C	0.1624	0.2423
NC_005101.4-240238595	CACTGAACCACACTGCCTCCAGAAATT	2	A	0.1638	0.2315
NC_005101.4-240352490	TATGAAAATACACTACCTCCTTCTAGC	23	C	0.115	0.1239
NC_005101.4-240363874	TGTACAAAGACACATGCTCCACTATGT	27	T	0.082	0.0786
NC_005101.4-240385191	TTGTCCCACACAAACACTCCAAACAAA	2	T	0.1121	0.4991
NC_005101.4-240408809	ATCATCTGCACAATCACTCCAGCGTCG	24	G	0	0.4807
NC_005101.4-240495703	CCTGGTGAGACCCTGCCTCCAAAAGAG	25	G	0.0804	0.125
NC_005101.4-240578403	TAAAAAACCACACTTCCTCCTTGCCTG	12	A	0	0.4839
NC_005101.4-240578800	AAAACCTAGACCGAAGCTCCCTCCTTC	12	C	0.1382	0.1695
NC_005101.4-240578800	AAAACCTAGACCGAAGCTCCCTCCTTC	13	G	0.0407	0.0553
NC_005101.4-240674216	AATCAATCCACAATACCTCCACAGAAT	24	G	0	0.477
NC_005101.4-240748705	GGAGCCTTGACTGGCCCTCCTCTTCCG	3	A	0	0.4682
NC_005101.4-240748705	GGAGCCTTGACTGGCCCTCCTCTTCCG	27	G	0	0.4654
NC_005101.4-240752444	CCCTGCCTCACTCCCACTCCTCATCCC	14	C	0	0.4641
NC_005101.4-240799198	CACCTGGTGACCTCCTCTCCATCCTCA	9	G	0.0488	0.0476
NC_005101.4-240801497	CATACATTCACAGAGCCTCCTTTTCTA	2	A	0	0.4826
NC_005101.4-240801497	CATACATTCACAGAGCCTCCTTTTCTA	4	A	0	0.4826
NC_005101.4-240810916	TGTGCGGGCACTGCTTCTCCACTGTGC	7	G	0.1057	0.1001
NC_005101.4-240836949	TTACTGAGAACATCAACTCCACTCTCT	23	T	0.1525	0.3436
NC_005101.4-240851725	ACCAAGGCTACTTCTTCTCCTAGCATC	2	C	0.0083	0.0245
NC_005101.4-240854339	AAGCCAGGCACCTGAGCTCCTCATATG	16	G	0.042	0.0411
NC_005101.4-240869119	TCATCAGTAACACCCTCTCCTAAGGTT	27	T	0	0.4757

NC_005101.4-240896577	CCCAGCGAGACCCTGACTCCATACAAA	1	C	0.2295	0.4463
NC_005101.4-240924137	TAGCTCATAACTCCCGCTCCAGTGCTG	16	G	0.0407	0.4868
NC_005101.4-240924137	TAGCTCATAACTCCCGCTCCAGTGCTG	23	T	0.0976	0.1761
NC_005101.4-240999588	TTGTTCATTACCTCGACTCCCTTAGAT	3	G	0.0826	0.0943
NC_005101.4-240999588	TTGTTCATTACCTCGACTCCCTTAGAT	14	C	0.0252	0.0249
NC_005101.4-241077135	CGCCATATTACTCGGCCTCCCCATTCC	1	C	0.1429	0.1469
NC_005101.4-241077135	CGCCATATTACTCGGCCTCCCCATTCC	2	G	0.025	0.0247
NC_005101.4-241138261	GTCTTAGCCACATGCTCTCCTGAAACC	12	A	0	0.0325
NC_005101.4-241138261	GTCTTAGCCACATGCTCTCCTGAAACC	21	T	0	0.4765
NC_005101.4-241228364	CTAAGGCTGACAGAACCTCCAGAGCAC	4	A	0.125	0.1997
NC_005101.4-241256220	TACTGCCCCACCCCCTCTCCCTCCTTA	12	C	0.1057	0.1145
NC_005101.4-241256220	TACTGCCCCACCCCCTCTCCCTCCTTA	14	C	0.0164	0.0634
NC_005101.4-241275058	TGTTTTATCACATGACCTCCCTGTGTC	9	C	0.0439	0.0429
NC_005101.4-241387036	AAGGTGAGAACCGTGGCTCCATGAGAA	4	G	0.0569	0.0553
NC_005101.4-241414582	GGCAGAAAAACCTTTGCTCCCTGCCCA	25	C	0.0351	0.0345
NC_005101.4-241424270	AAAACAATCACTGTGGCTCCTGTGGGT	15	G	0.0442	0.0433
NC_005101.4-241428855	GAGTCAAAAACAATCTCTCCTCCTAAG	15	C	0.325	0.3533
NC_005101.4-241428855	GAGTCAAAAACAATCTCTCCTCCTAAG	24	T	0.325	0.3533
NC_005101.4-241459369	CCCCTCCTGACCTGCTCTCCCAGCCAG	25	C	0.1933	0.4682
NC_005101.4-241533877	TTCTCATCTACTAAAGCTCCTTTCACT	7	T	0.2479	0.3855
NC_005101.4-241621319	TGCCAATCAACCAGTTCTCCAAGGGAC	7	T	0.0084	0.4761
NC_005101.4-241655426	GTCCCTCGAACACTTGCTCCAAGCTAA	15	T	0	0.4807
NC_005101.4-241693666	ATTAGAACAACTATACCTCCTAACTGT	13	A	0.3333	0.4606
NC_005101.4-241709280	CCAGCATGTACGGTTGCTCCATTGCAG	5	C	0.175	0.2491
NC_005101.4-241709280	CCAGCATGTACGGTTGCTCCATTGCAG	9	T	0.0083	0.4816
NC_005101.4-241746349	TGCCTTTTGACTCCAGCTCCAGGAGAT	15	A	0.0331	0.0325
NC_005101.4-241828994	AGCCCCTCCACTGTTCCTCCCCAATTA	13	G	0.0984	0.1505
NC_005101.4-241845532	AGATCCCAGACAGGTCCTCCTGGCCAG	3	A	0	0.4793
NC_005101.4-241845532	AGATCCCAGACAGGTCCTCCTGGCCAG	15	T	0	0.4793
NC_005101.4-241980073	CGAGCTGGCACCTTGACTCCCCAAATA	16	A	0.0531	0.4574
NC_005101.4-242010299	CATGTACACACTTGTACTCCATTACAC	26	A	0.0531	0.0517
NC_005101.4-242049530	GGATCCCACACAGCATCTCCTCAGACT	27	T	0	0.4854
NC_005101.4-242072430	TCTTGTAAAACAAACTCTCCACACAGC	13	A	0.0085	0.4823
NC_005101.4-242089237	CAAGCAGTCACATTATCTCCAGACTCC	13	T	0	0.4728
NC_005101.4-242089237	CAAGCAGTCACATTATCTCCAGACTCC	15	A	0	0.4728
NC_005101.4-242141640	GCCCTCTAGACTTCCGCTCCTGTATAA	2	C	0.2696	0.42
NC_005101.4-242141640	GCCCTCTAGACTTCCGCTCCTGTATAA	24	A	0.1304	0.1516
NC_005101.4-242146310	ACCTTACAAACATTATCTCCCATCAGT	22	A	0.3193	0.3508
NC_005101.4-242146310	ACCTTACAAACATTATCTCCCATCAGT	27	T	0.2712	0.3436
NC_005101.4-242165136	TGTTTAACAACCTTTCCTCCAGGGGAG	16	C	0.0992	0.0943
NC_005101.4-242184209	CCGACGCCAACCTTAACTCCATCAACA	2	C	0.0732	0.0854
NC_005101.4-242184209	CCGACGCCAACCTTAACTCCATCAACA	5	C	0.1301	0.189
NC_005101.4-242369726	GGGTTGAACACACTTTCTCCATCTTCA	24	T	0	0.4793
NC_005101.4-242396609	AATGCTGAGACAAGTCCTCCATTATCC	15	T	0.1667	0.2188
NC_005101.4-242396609	AATGCTGAGACAAGTCCTCCATTATCC	27	C	0.0085	0.4698
NC_005101.4-242489082	TTGTTCCTTACTTACTCTCCATTCATT	9	T	0	0.4846
NC_005101.4-242587985	TCCCGTGTCACTCCCTCTCCGTGCTTC	7	G	0.0513	0.066
NC_005101.4-242622397	TCATTCCTCACCAACTCTCCCACATTT	13	A	0.0407	0.0398
NC_005101.4-242622397	TCATTCCTCACCAACTCTCCCACATTT	21	C	0.0244	0.0241
NC_005101.4-242658912	CCTAAACACACACAGCCTCCCTCGCTT	9	C	0.0417	0.0408
NC_005101.4-242658912	CCTAAACACACACAGCCTCCCTCGCTT	23	C	0.1849	0.322

NC_005101.4-242665706	ATGGCTGAAACCACCCCTCCACAGATT	3	G	0.0598	0.074
NC_005101.4-242693980	AGAACTGTGACCTTCCCTCCTTCTCCA	3	A	0	0.4826
NC_005101.4-242726599	GGAGGATATACTTAGGCTCCGATCCAT	21	G	0.042	0.0411
NC_005101.4-242726599	GGAGGATATACTTAGGCTCCGATCCAT	22	A	0	0.4779
NC_005101.4-242733614	CCTACTCCAACCCTTTCTCCAGTGCAG	2	C	0	0.4844
NC_005101.4-242733614	CCTACTCCAACCCTTTCTCCAGTGCAG	9	A	0.2373	0.4826
NC_005101.4-242779525	AACCACTTCACTGAAGCTCCTAATCTC	6	C	0.0254	0.0576
NC_005101.4-242806521	GGCTAGTATACATTGACTCCAATCTTT	8	A	0.0088	0.4861
NC_005101.4-242841262	AAGAAATGTACCTGCGCTCCACAAGTG	15	C	0.1393	0.4984
NC_005101.4-242841262	AAGAAATGTACCTGCGCTCCACAAGTG	16	G	0.123	0.1296
NC_005101.4-242892603	GTGCTGCCTACACACCCTCCCACTTCC	1	G	0.2213	0.4924
NC_005101.4-242892603	GTGCTGCCTACACACCCTCCCACTTCC	22	A	0.1138	0.1629
NC_005101.4-242894430	AGAGAAGGGACCAAGCCTCCATCACTG	12	C	0.1057	0.1562
NC_005101.4-242913307	AGAAATGGTACGGTATCTCCTAGCATC	5	A	0.2193	0.4581
NC_005101.4-242913307	AGAAATGGTACGGTATCTCCTAGCATC	12	G	0.0855	0.0818
NC_005101.4-242926730	ACGAGATCCACCTACTCTCCTTGACAC	9	C	0.0083	0.4847
NC_005101.4-242926730	ACGAGATCCACCTACTCTCCTTGACAC	14	A	0	0.4832
NC_005101.4-242972199	TTAAAGGAAACACTTTCTCCACAGACA	13	C	0.0696	0.4997
NC_005101.4-242993489	ACACCCACAACCCACACTCCTCTTCTG	23	T	0.0583	0.4525
NC_005101.4-243025509	GATCACTTGACTTCAGCTCCTGTCCTG	2	A	0	0.4624
NC_005101.4-243028804	GACACAACCACAGCTGCTCCCAACAGC	21	C	0.0431	0.1058
NC_005101.4-243050254	CCTCCTGACACAGCCCCTCCTCACTTC	16	C	0.0574	0.0557
NC_005101.4-243050479	ACAATCCTGACTCTGCCTCCTGAGTGT	5	T	0.3058	0.259
NC_005101.4-243050637	CTTGATGAAACTTCAGCTCCTTCATAG	13	T	0.0901	0.4821
NC_005101.4-243057727	TGTACAATGACCCCTGCTCCTTCAGGG	6	A	0.0424	0.0415
NC_005101.4-243057727	TGTACAATGACCCCTGCTCCTTCAGGG	8	T	0.181	0.3617
NC_005101.4-243071844	AACCTCTTCACCCGCCCTCCAGCCCCT	13	C	0.1721	0.2095
NC_005101.4-243071844	AACCTCTTCACCCGCCCTCCAGCCCCT	14	G	0.1393	0.321
NC_005101.4-243099438	TTGTGTCAGACCCTTCCTCCCAGGAAA	1	T	0.1	0.1244
NC_005101.4-243121283	AAGGTGAGCACAGTTGCTCCTTATTGT	13	G	0.1304	0.2816
NC_005101.4-243127980	TGATCCTAAACTCCTTCTCCTCCTGTT	5	C	0.0526	0.0677
NC_005101.4-243127980	TGATCCTAAACTCCTTCTCCTCCTGTT	25	G	0.2368	0.4676
NC_005101.4-243138557	AACCACTGAACCGGCTCTCCAGCCTCA	4	C	0.2314	0.4041
NC_005101.4-243220912	GGCCCATCCACACCACCTCCTACCCTG	4	C	0.1626	0.2612
NC_005101.4-243220912	GGCCCATCCACACCACCTCCTACCCTG	13	C	0	0.4793
NC_005101.4-243347681	CTGAGTCACACGATCACTCCTCCTTCA	13	A	0	0.4856
NC_005101.4-243362514	TGGATCTTCACCACTTCTCCATTTCTT	9	C	0.1261	0.1338
NC_005101.4-243362514	TGGATCTTCACCACTTCTCCATTTCTT	21	A	0.018	0.0354
NC_005101.4-243377702	AGAGGGCACACTCATGCTCCTCCATGT	5	G	0.2276	0.2499
NC_005101.4-243377702	AGAGGGCACACTCATGCTCCTCCATGT	9	C	0.0244	0.0241
NC_005101.4-243384502	AAACGCTGGACTTGAACTCCTCTTGAG	26	A	0	0.461
NC_005101.4-243482301	TGGGCCAGCACAATGTCTCCCTCTGCA	6	C	0.0789	0.1229
NC_005101.4-243482301	TGGGCCAGCACAATGTCTCCCTCTGCA	13	A	0	0.474
NC_005101.4-243488047	GGGCTTCAAACATGCGCTCCTTGGATG	16	G	0.161	0.4982
NC_005101.4-243512937	CAATCCACCACAGACACTCCCTAGAGT	24	G	0.1639	0.4999
NC_005101.4-243517244	GGTCAGCGCACTGTCTCTCCCTTCTCT	8	G	0.1368	0.3641
NC_005101.4-243522857	GAATGAGGAACAGGCTCTCCCAACCAC	8	G	0.122	0.2203
NC_005101.4-243524903	CAGCCAGCGACTCCAGCTCCATGGGAT	8	C	0	0.4832
NC_005101.4-243524903	CAGCCAGCGACTCCAGCTCCATGGGAT	21	A	0.1667	0.2994
NC_005101.4-243531708	GGTTGAAGAACAGGAGCTCCCTGATGT	5	G	0	0.48
NC_005101.4-243531708	GGTTGAAGAACAGGAGCTCCCTGATGT	16	G	0	0.48

NC_005101.4-243627734	CTCCAGGAGACAAATGCTCCTTCTGTT	16	G	0	0.4859
NC_005101.4-243627734	CTCCAGGAGACAAATGCTCCTTCTGTT	21	T	0	0.4846
NC_005101.4-243634136	GGAATTACTACTCTGGCTCCCACCCGA	25	C	0	0.4793
NC_005101.4-243667713	TCACCCCTCACTCTTTCTCCCTGTGTA	9	C	0.2883	0.4854
NC_005101.4-243695248	GATGTCCCCACCAGACCTCCCCACACC	1	G	0.3	0.4888
NC_005101.4-243720723	TACATTCTGACAGCGGCTCCTTTTCCT	14	C	0.2174	0.4333
NC_005101.4-243720723	TACATTCTGACAGCGGCTCCTTTTCCT	16	G	0.0435	0.0425
NC_005101.4-243724236	GGCACGTGGACACAAGCTCCCACCCCT	6	G	0.0789	0.0758
NC_005101.4-243766074	AAAAATAACACTATGGCTCCGTGAGTT	9	C	0	0.4786
NC_005101.4-243767801	TATGGAGAGACCTTGTCTCCAAACATC	2	A	0	0.474
NC_005101.4-243785208	AATAATATGACACAAGCTCCTTTTCCA	4	A	0	0.0328
NC_005101.4-243785208	AATAATATGACACAAGCTCCTTTTCCA	8	T	0.0084	0.4797
NC_005101.4-243787777	CTGCCACGCACAGAAGCTCCAGCAGAG	8	G	0.0804	0.491
NC_005101.4-243789331	CATTGAGAGACACTGACTCCAGTGATA	22	G	0	0.4779
NC_005101.4-243789331	CATTGAGAGACACTGACTCCAGTGATA	23	T	0	0.4779
NC_005101.4-243806960	TCCTCCTCGACTAGACCTCCTTAGACA	8	C	0.0084	0.4797
NC_005101.4-243864210	TTCTAAGCCACAGCAGCTCCTCTACCT	8	C	0.0569	0.4893
NC_005101.4-243882240	CGTGCCTCCACACACACTCCCCTATCT	2	G	0	0.4772
NC_005101.4-243882240	CGTGCCTCCACACACACTCCCCTATCT	3	T	0.1186	0.1551
NC_005101.4-243900060	GTGCAGATAACAGCAGCTCCAGCTACA	9	A	0	0.4793
NC_005101.4-243903931	CACAGTGATACACCATCTCCAACAAGG	6	T	0.0992	0.2172
NC_005101.4-243986374	ATCTCTTTAACATTCTCTCCAAGAGAT	24	A	0.2083	0.4941
NC_005101.4-243996668	CAAAGGGAAACCTGTCCTCCAAGTGTC	16	C	0.1597	0.1746
NC_005101.4-244009065	CTGCCCCAAACACCAGCTCCTGTGCCT	8	A	0	0.4751
NC_005101.4-244009065	CTGCCCCAAACACCAGCTCCTGTGCCT	22	G	0.0661	0.0792
NC_005101.4-244036048	TATTGGAGCACACAGGCTCCAGTGCTG	14	A	0.0413	0.4835
NC_005101.4-244052511	AGAAATGGCACCTTTTCTCCAAGCGTC	15	T	0	0.4728
NC_005101.4-244097386	GGTCTGACTACATAGTCTCCGTGTGCC	2	G	0	0.4807
NC_005101.4-244139946	CTGCTTCTAACTCTGTCTCCACTGGAA	14	T	0.045	0.044
NC_005101.4-244184643	TCAAAGACGACACTCTCTCCCACTCAG	25	C	0.2632	0.4945
NC_005101.4-244184643	TCAAAGACGACACTCTCTCCCACTCAG	26	A	0.2632	0.4945
NC_005101.4-244192179	GCTAATTTCACCTCACCTCCTTGACGT	8	T	0.2564	0.2945
NC_005101.4-244192179	GCTAATTTCACCTCACCTCCTTGACGT	26	G	0.2308	0.2778
NC_005101.4-244193758	CACTTGCTGACATGGCCTCCCATTTCC	24	T	0.1795	0.3855
NC_005101.4-244193758	CACTTGCTGACATGGCCTCCCATTTCC	27	C	0.0254	0.0251
NC_005101.4-244212057	AACAAAGTCACCGTGCCTCCAACAACC	6	A	0	0.4793
NC_005101.4-244212057	AACAAAGTCACCGTGCCTCCAACAACC	13	G	0.0894	0.2203
NC_005101.4-244238727	GGCCATCACACCGCAGCTCCATGGGTG	13	G	0.2667	0.3487
NC_005101.4-244261826	ACAACATTCACAGCAACTCCCACTGTG	7	T	0	0.4807
NC_005101.4-244291203	AGACTTTCTACGACTGCTCCTGACACA	1	A	0.1626	0.2142
NC_005101.4-244291203	AGACTTTCTACGACTGCTCCTGACACA	14	C	0.0813	0.078
NC_005101.4-244334716	CACGCAGAAACACAGGCTCCCCTGTGA	4	G	0.0086	0.4768
NC_005101.4-244373126	TAAACTCCTACACAGTCTCCCACCACT	1	T	0.2377	0.3984
NC_005101.4-244373126	TAAACTCCTACACAGTCTCCCACCACT	13	C	0.2459	0.435
NC_005101.4-244384953	ACATACAACACACACACTCCACACACA	15	C	0.1696	0.2385
NC_005101.4-244384953	ACATACAACACACACACTCCACACACA	24	C	0.1053	0.0997
NC_005101.4-244391881	GGACCCGACACTAGCACTCCTCCTGAA	6	C	0.0909	0.1017
NC_005101.4-244404874	CCAGCTTTCACATTTTCTCCCTATGTG	3	A	0.0656	0.0786
NC_005101.4-244404874	CCAGCTTTCACATTTTCTCCCTATGTG	14	T	0	0.4807
NC_005101.4-244440178	ATTCGTTTGACTCCCTCTCCCATCTAT	1	A	0.1083	0.1025
NC_005101.4-244440178	ATTCGTTTGACTCCCTCTCCCATCTAT	21	C	0.1667	0.4999

NC_005101.4-244619263	ATCCTTCAAACTTTGGCTCCCGACTTT	2	T	0.0084	0.0411
NC_005101.4-244619263	ATCCTTCAAACTTTGGCTCCCGACTTT	21	C	0	0.4677
NC_005101.4-244629966	ATAAGATAGACAAACCCTCCAGACTAT	27	T	0	0.48
NC_005101.4-244632365	ATATCAAGGACCCATGCTCCGTATGTT	8	G	0.2479	0.479
NC_005101.4-244758325	CCTTGATAGACCCTTGCTCCATGCTTA	3	T	0.1441	0.1481
NC_005101.4-244761099	GTCAAAGTCACTCACTCTCCGCACATT	1	G	0.5984	0.4193
NC_005101.4-244761099	GTCAAAGTCACTCACTCTCCGCACATT	15	C	0.5902	0.416
NC_005101.4-244822056	TCCTCTCCTACACTTCCTCCCCAACCG	23	A	0.0877	0.0997
NC_005101.4-244822056	TCCTCTCCTACACTTCCTCCCCAACCG	26	C	0	0.474
NC_005101.4-244878494	GACACAAACACAAACACTCCCACTGAC	27	C	0.0083	0.0405
NC_005101.4-245044326	TGAGCTGTTACACCAGCTCCTTGTTTG	9	T	0.0833	0.1099
NC_005101.4-245057563	CTCAGAAGGACCACCTCTCCTGTGCAG	13	A	0.2833	0.4888
NC_005101.4-245062183	CACATGCATACGGAAGCTCCCGTAGCC	21	C	0.05	0.0488
NC_005101.4-245062183	CACATGCATACGGAAGCTCCCGTAGCC	24	A	0.05	0.0488
NC_005101.4-245072185	GACTCTCCCACTCACCCTCCCAAGGTC	5	C	0.2605	0.4859
NC_005101.4-245280908	TGCAGAAACACGCTCTCTCCTTCCACC	13	C	0.0531	0.4912
NC_005101.4-245280908	TGCAGAAACACGCTCTCTCCTTCCACC	27	C	0.0439	0.4913
NC_005101.4-245461808	GCGATTTCCACATGGCCTCCCATGTTT	24	G	0.1475	0.1505
NC_005101.4-245522542	AGGACAGTCACCATGACTCCAGACCTC	2	G	0.2712	0.3034
NC_005101.4-245522542	AGGACAGTCACCATGACTCCAGACCTC	25	C	0.1849	0.2076
NC_005101.4-245697176	AACCTGGGTACTTAGACTCCGTGCTCT	2	A	0	0.4778
NC_005101.4-245697176	AACCTGGGTACTTAGACTCCGTGCTCT	21	G	0.0348	0.0342
NC_005101.4-245796780	TCTTTCTCCACATCCTCTCCTTGAACT	9	C	0.3058	0.4146
NC_005101.4-245796780	TCTTTCTCCACATCCTCTCCTTGAACT	26	C	0.0244	0.0241
NC_005101.4-245803531	CACAGTGACACACTTCCTCCAAAGGCA	9	C	0.0254	0.4842
NC_005101.4-245940772	ATTGTCTACACTGTGTCTCCATGCTAT	13	G	0.2276	0.2937
NC_005101.4-246012727	CTCTCTCCAACCTCCTCTCCTTCCCAT	5	C	0.2213	0.4588
NC_005101.4-246033716	AACTCATAGACTCTACCTCCAGCAGAA	4	T	0.027	0.126
NC_005101.4-246033716	AACTCATAGACTCTACCTCCAGCAGAA	6	A	0.027	0.126
NC_005101.4-246076855	AGAGGCAATACAATTTCTCCATGTAAT	23	G	0.175	0.4958
NC_005101.4-246116750	GACTCACCTACCGCAGCTCCAGGCTTC	5	C	0.275	0.4575
NC_005101.4-246273814	TGTTAGATGACTTGCTCTCCTAAGTTA	15	C	0.1316	0.4997
NC_005101.4-246344198	CATGCTACTACCATTTCTCCTTTACAT	12	C	0.2479	0.3645
NC_005101.4-246650784	AACGAAGACACACGTGCTCCACTTTGT	3	C	0.1293	0.1504
NC_005101.4-246650784	AACGAAGACACACGTGCTCCACTTTGT	14	G	0.0086	0.4836
NC_005101.4-246692859	CACTGTCACACTATCTCTCCTGTCACA	15	C	0.0744	0.5
NC_005101.4-246692859	CACTGTCACACTATCTCTCCTGTCACA	22	G	0.1901	0.2704
NC_005101.4-246737416	GCGAGAGCCACACCAGCTCCCAGGGGT	3	G	0.0086	0.4768
NC_005101.4-246737416	GCGAGAGCCACACCAGCTCCCAGGGGT	5	G	0.0086	0.4768
NC_005101.4-246765207	CTCTCACACACTCCTCCTCCTTCCTGT	1	C	0.0789	0.1075
NC_005101.4-246945280	GGATCCATCACATTGACTCCTCAGTCA	5	C	0	0.4778
NC_005101.4-246946268	TCTCCTACAACATAATCTCCAGGGAGA	12	A	0.0163	0.032
NC_005101.4-247022829	CTGCCGAGGACCTCTTCTCCCACAGCC	5	C	0	0.4773
NC_005101.4-247030033	CCTTTCAATACGCAAACTCCACCTGAT	12	G	0.2261	0.3403
NC_005101.4-247030033	CCTTTCAATACGCAAACTCCACCTGAT	25	G	0.0348	0.0508
NC_005101.4-247048375	CCAATTTACACTAGAGCTCCCACATAT	1	C	0.0084	0.4829
NC_005101.4-247128220	CTCCTGAACACTGCTTCTCCTGAGAGC	24	G	0.0435	0.059
NC_005101.4-247153423	GGATACTTTACTTTTGCTCCTACTGAT	5	A	0	0.4743
NC_005101.4-247153423	GGATACTTTACTTTTGCTCCTACTGAT	24	T	0.0588	0.0882
NC_005101.4-247419220	AAGGTCAGTACTAAATCTCCTTCATCA	25	T	0.0672	0.0805
NC_005101.4-247424881	AATGGTTTCACACTAACTCCTATCTGC	1	A	0.1207	0.1576

NC_005101.4-247459006	TTGGGGCAGACAGTGTCTCCAGTGTAG	4	G	0.2101	0.2389
NC_005101.4-247473401	AGACAAAACACCAAGGCTCCGAGGCAA	26	A	0.0513	0.05
NC_005101.4-247486396	AAAGAATGAACAAGCTCTCCTGATTCA	4	G	0.0331	0.0325
NC_005101.4-247502785	CTTAGAATCACCATCTCTCCTTCAACC	3	T	0	0.4734
NC_005101.4-247502785	CTTAGAATCACCATCTCTCCTTCAACC	25	A	0.0504	0.0491
NC_005101.4-247548335	GAAACAGGTACACCTGCTCCAACACCG	27	G	0.252	0.3964
NC_005101.4-247585979	AAGGTCATCACCTTAGCTCCTAGACAC	2	A	0.1333	0.1528
NC_005101.4-247585979	AAGGTCATCACCTTAGCTCCTAGACAC	27	C	0.025	0.0247
NC_005101.4-247591312	GTGAGATCAACATGTACTCCGAGGCTA	21	G	0.027	0.4682
NC_005101.4-247637392	AGATGAGGTACAATTTCTCCAACTGAA	15	T	0.1339	0.1553
NC_005101.4-247732783	AAAGTAAAGACTTCGCCTCCGTGAAGA	1	A	0.2203	0.334
NC_005101.4-247854249	CTCATTCCGACCCGCTCTCCCAGACAA	13	C	0.188	0.2945
NC_005101.4-247857477	TTTCATGAGACAAGACCTCCCACTGGT	4	C	0.1261	0.1327
NC_005101.4-247858952	AACAAAGAGACAGGAACTCCTGCCTTC	16	A	0	0.48
NC_005101.4-247936313	AGAAGGGACACTGTTGCTCCATTATAC	1	A	0.1545	0.1425
NC_005101.4-248098012	GGCAAGAGTACTTCCTCTCCTGTGTAT	1	G	0.2101	0.2852
NC_005101.4-248131774	TGTTCTTAGACTGCCTCTCCCTTTGCT	26	C	0.1092	0.1469
NC_005101.4-248131774	TGTTCTTAGACTGCCTCTCCCTTTGCT	27	T	0.1092	0.1469
NC_005101.4-248288995	ATAGCCAGTACCTTCCCTCCTCAGGGA	9	T	0.2035	0.1828
NC_005101.4-248288995	ATAGCCAGTACCTTCCCTCCTCAGGGA	16	C	0.3304	0.3418
NC_005101.4-248346578	TTTTGGAATACTTTTCCTCCTCTTGCG	5	G	0.0796	0.0926
NC_005101.4-248346578	TTTTGGAATACTTTTCCTCCTCTTGCG	26	C	0.0088	0.481
NC_005101.4-248454017	GTAACATGCACACATACTCCTTTCAAA	25	A	0.2295	0.2741
NC_005101.4-248457735	CCAATAAGAACTTGGGCTCCTTTATGA	3	A	0.0417	0.0408
NC_005101.4-248459002	TGACCTGTAACTCCTGCTCCTTCTGCT	4	C	0	0.0351
NC_005101.4-248492703	GATAAGAGTACCACAACTCCTACTCTA	25	C	0.0631	0.126
NC_005101.4-248525936	AGTTCCTCTACAGAATCTCCATCAGTG	3	T	0.4786	0.3641
NC_005101.4-248525936	AGTTCCTCTACAGAATCTCCATCAGTG	14	A	0.547	0.45
NC_005101.4-248725812	CAGCTATTTACATGGCCTCCCAGGCTT	8	T	0.0619	0.06
NC_005101.4-248725812	CAGCTATTTACATGGCCTCCCAGGCTT	26	T	0.0619	0.06
NC_005101.4-248764907	TGGAATTCTACTAGTTCTCCTTCCCAT	5	A	0.0085	0.4753
NC_005101.4-248764907	TGGAATTCTACTAGTTCTCCTTCCCAT	27	T	0.0085	0.4753
NC_005101.4-248839898	GATCACGCTACAGGCTCTCCGTCACCT	9	T	0.0678	0.0655
NC_005101.4-248883949	TGCGTGCACACTGCATCTCCTGGAGTG	1	T	0.1057	0.1425
NC_005101.4-248906319	TGCAACTTCACTGGAACTCCTGTTGGA	15	A	0.0702	0.4654
NC_005101.4-248930924	GCTTCAGACACTTTTACTCCCCCAAGA	26	G	0.1795	0.4578
NC_005101.4-248936795	CTGGTCGGTACACTCGCTCCTATTGGT	1	C	0.2456	0.4114
NC_005101.4-248936795	CTGGTCGGTACACTCGCTCCTATTGGT	5	T	0.2193	0.4953
NC_005101.4-249055817	TACTTACAGACCTTGCCTCCCTCTTCT	8	A	0	0.4793
NC_005101.4-249145205	TTCTAAGGAACAGTTTCTCCAGGTCAG	7	G	0.0263	0.0595
NC_005101.4-249188087	AGGGATTTGACACCCTCTCCTGGCCCG	27	G	0.0948	0.1058
NC_005101.4-249206809	CTGTCAGTCACTTTGCCTCCTTGGTTG	5	C	0	0.4819
NC_005101.4-249206809	CTGTCAGTCACTTTGCCTCCTTGGTTG	8	T	0.0661	0.4923
NC_005101.4-249298842	TTCATGTGAACTCATCCTCCCTCATCC	4	A	0	0.4779
NC_005101.4-249298842	TTCATGTGAACTCATCCTCCCTCATCC	12	T	0	0.4779
NC_005101.4-249376722	TGCTCTTGAACAGACCCTCCTGCTCTG	2	G	0.082	0.1225
NC_005101.4-249396865	GACTTCTTAACCTGTTCTCCATTTCAA	2	A	0.1271	0.1481
NC_005101.4-249396865	GACTTCTTAACCTGTTCTCCATTTCAA	4	T	0.1186	0.1409
NC_005101.4-249494380	TTACCTTGGACCGTGTCTCCATGCCTG	13	G	0.2586	0.2965
NC_005101.4-249619363	ATGAGAGGAACTCTAACTCCTAGATTC	22	A	0.0625	0.0605
NC_005101.4-249619363	ATGAGAGGAACTCTAACTCCTAGATTC	23	G	0.2768	0.4617

NC_005101.4-249651788	ACATGTAGTACATGCTCTCCAGTCTCT	3	A	0	0.4787
NC_005101.4-249669749	GTAATGGTTACAATTGCTCCATATAAG	2	T	0.0631	0.0611
NC_005101.4-249701062	TGGTCCAGCACTGGGACTCCTTGCTAT	3	G	0.1552	0.1576
NC_005101.4-249774518	AGTGGTGCTACAGGCTCTCCTACAAAT	2	G	0.0813	0.078
NC_005101.4-249806846	AGACACTAGACAAGTGCTCCATCTCCT	5	A	0	0.4833
NC_005101.4-249820280	AGCTCTGCCACTTACACTCCGCATGAA	5	C	0.0163	0.4825
NC_005101.4-249820280	AGCTCTGCCACTTACACTCCGCATGAA	21	G	0.1545	0.1562
NC_005101.4-249851978	GATTTGGTTACAGGTCCTCCTTAGAAA	2	A	0.0439	0.0429
NC_005101.4-249851978	GATTTGGTTACAGGTCCTCCTTAGAAA	15	T	0.0263	0.026
NC_005101.4-249898402	GCATATGCAACCTGCACTCCACTCATC	14	G	0.0424	0.0415
NC_005101.4-249898402	GCATATGCAACCTGCACTCCACTCATC	26	T	0.0508	0.4856
NC_005101.4-249906967	TGAGCTGAAACAAACCCTCCTTTAAGT	13	A	0.1008	0.1107
NC_005101.4-249921168	GATGCCATTACATGTCCTCCAACAGCT	5	C	0.0254	0.0251
NC_005101.4-249980633	CATCTCCTTACTTAGACTCCACTCAAG	3	T	0.1695	0.4987
NC_005101.4-249983220	TGCATTGCCACTGTGGCTCCCTTTGGG	12	T	0.0417	0.49
NC_005101.4-249983220	TGCATTGCCACTGTGGCTCCCTTTGGG	24	T	0	0.4832
NC_005101.4-250017371	AGGATCCTCACCTCTTCTCCATAGGCA	27	A	0.2167	0.2311
NC_005101.4-250018353	AAGAAAGATACTCAAGCTCCCACCCTG	12	T	0.1074	0.4213
NC_005101.4-250018353	AAGAAAGATACTCAAGCTCCCACCCTG	24	C	0.0246	0.0243
NC_005101.4-250043568	CTAGCTGCCACTCAGCCTCCGCCTTGT	4	G	0.0244	0.0241
NC_005101.4-250043568	CTAGCTGCCACTCAGCCTCCGCCTTGT	14	A	0	0.4793
NC_005101.4-250053699	GTCATTATTACTGCTCCTCCACAGGGT	24	G	0.1849	0.4991
NC_005101.4-250058070	CTGTGGTAGACTATACCTCCCCAAACA	12	T	0.0732	0.0854
NC_005101.4-250058070	CTGTGGTAGACTATACCTCCCCAAACA	13	A	0.075	0.4941
NC_005101.4-250172835	TGTTGGTGGACTGTGTCTCCTCAAACT	1	T	0	0.4813
NC_005101.4-250180724	GGTCCTGGGACCAATCCTCCATGGGTA	21	A	0.0087	0.4817
NC_005101.4-250252739	CAATCACTTACTGCTGCTCCACAGGAT	13	G	0.2712	0.3707
NC_005101.4-250257718	AGAGATGAAACAAATTCTCCATATTGG	16	T	0.2544	0.3661
NC_005101.4-250279527	CATCACCAAACTAGGCCTCCCTGCATA	2	A	0	0.4839
NC_005101.4-250279527	CATCACCAAACTAGGCCTCCCTGCATA	3	T	0.1186	0.4948
NC_005101.4-250283770	CAAGAATACACGTATTCTCCCCACACT	15	T	0.1017	0.1264
NC_005101.4-250285114	CCAGGAACCACGTTTGCTCCTTGTATT	12	G	0.0081	0.4777
NC_005101.4-250285114	CCAGGAACCACGTTTGCTCCTTGTATT	25	A	0.1885	0.2903
NC_005101.4-250289860	TGACCCCGAACCCGTGCTCCTCCTGCC	13	C	0.2712	0.3953
NC_005101.4-250289860	TGACCCCGAACCCGTGCTCCTCCTGCC	23	C	0.0417	0.0566
NC_005101.4-250297725	AAGAGTATTACCCAATCTCCTGATCTT	15	A	0.0492	0.4656
NC_005101.4-250343703	TCCTGCAGCACAGGAGCTCCGCCCGAA	4	T	0.0164	0.4807
NC_005101.4-250353467	CTTTGAGTCACATCAACTCCTTTCCCT	9	C	0.0081	0.0241
NC_005101.4-250353467	CTTTGAGTCACATCAACTCCTTTCCCT	23	T	0.0813	0.078
NC_005101.4-250362936	CATGAAACCACTGCACCTCCATCAACT	13	G	0.0732	0.0705
NC_005101.4-250362936	CATGAAACCACTGCACCTCCATCAACT	21	A	0.0732	0.0705
NC_005101.4-250410631	CCGTGTCCCACAACAGCTCCAGTAGAC	22	G	0.2541	0.3581
NC_005101.4-250433068	GAATGGTTGACCAGATCTCCCACCGTG	12	C	0.1504	0.1828
NC_005101.4-250433068	GAATGGTTGACCAGATCTCCCACCGTG	23	C	0.0531	0.0846
NC_005101.4-250470311	ACTCATAGAACTCATTCTCCATTTTCC	14	A	0.0789	0.0758
NC_005101.4-250470311	ACTCATAGAACTCATTCTCCATTTTCC	23	T	0.0789	0.0758
NC_005101.4-250495109	CCTGTCTCCACCTTCCCTCCGTCCAGT	25	A	0.2328	0.4991
NC_005101.4-250517367	ATATTTCCAACCTTGCCTCCTCCAGAA	1	A	0.0084	0.4859
NC_005101.4-250517367	ATATTTCCAACCTTGCCTCCTCCAGAA	13	T	0.0168	0.4873
NC_005101.4-250530316	ACTTGGGTAACACCCACTCCTTCCTCA	1	A	0.2414	0.457
NC_005101.4-250531966	GTAGCCCCCACTCTCACTCCCAGCCTG	1	G	0	0.48

NC_005101.4-250545802	ATGAGACTGACTGTGACTCCTGATAAG	2	T	0	0.4765
NC_005101.4-250559102	ATTCAGGGGACGGAGCCTCCTTCCCTG	27	G	0	0.4894
NC_005101.4-250589348	TCAAGACACACACTAACTCCTCCGCCA	24	G	0	0.4746
NC_005101.4-250673563	CAGTCTTCTACTTCCTCTCCCTGCCCT	15	C	0.0976	0.0928
NC_005101.4-250723752	AGACCACCAACTCTCCCTCCAGAATGA	1	A	0.0541	0.4558
NC_005101.4-250726128	GGGAATCCAACCTCAGCTCCTCATGCT	8	C	0.431	0.488
NC_005101.4-250726128	GGGAATCCAACCTCAGCTCCTCATGCT	23	A	0.2458	0.2156
NC_005101.4-250746958	TCCTCCAGGACCTCATCTCCCTCACTG	21	C	0.2645	0.4481
NC_005101.4-250754662	TCTCTGTCTACAGCATCTCCATGAGGA	16	T	0.178	0.2026
NC_005101.4-250808134	CACACACCCACAGGTGCTCCAACACCC	6	A	0.1818	0.5
NC_005101.4-250821609	AGAGATGGGACCTTTGCTCCTATGCAA	8	G	0.4667	0.3578
NC_005101.4-250829481	GTTCTCTCCACAACTTCTCCTCACATC	12	A	0.1913	0.4092
NC_005101.4-250829481	GTTCTCTCCACAACTTCTCCTCACATC	25	A	0.4035	0.4925
NC_005101.4-250831597	ACCCTTTCAACCTTTTCTCCTTCAGAG	3	C	0.1964	0.3157
NC_005101.4-250831597	ACCCTTTCAACCTTTTCTCCTTCAGAG	13	T	0.0354	0.4671
NC_005101.4-250833436	TGTCTTCTCACTTCTTCTCCAGAGCCT	12	T	0.5935	0.4174
NC_005101.4-250854648	ACCAGGCACACATCCCCTCCTTGTGAG	12	A	0.5935	0.4952
NC_005101.4-250862991	CATCCCGTCACCCTGCCTCCCTACAAG	7	G	0	0.4839
NC_005101.4-250862991	CATCCCGTCACCCTGCCTCCCTACAAG	14	T	0.1356	0.3953
NC_005101.4-250889143	CACTCTTGGACTCCGTCTCCTGAGTTA	14	C	0.25	0.4832
NC_005101.4-250906270	CCAAGGATGACCTTTACTCCAGATCCT	2	C	0.2458	0.298
NC_005101.4-250907311	CGGTGGCCCACGTGTCCTCCTCCAAGT	1	C	0.2906	0.4553
NC_005101.4-250907311	CGGTGGCCCACGTGTCCTCCTCCAAGT	25	A	0.0603	0.4435
NC_005101.4-250933403	CTGACAGTGACAGAGGCTCCTGTGATA	6	A	0	0.48
NC_005101.4-250933403	CTGACAGTGACAGAGGCTCCTGTGATA	27	A	0.0328	0.0322
NC_005101.4-250974425	GGGAGTACCACGCTCTCTCCCTCTCTG	23	C	0.3008	0.4741
NC_005101.4-251026111	CTCAGCCGAACAGAGACTCCAGGAGTG	9	A	0.0244	0.0241
NC_005101.4-251035057	AGTCAAGCCACTCAGCCTCCTCACGCA	1	A	0	0.4709
NC_005101.4-251035057	AGTCAAGCCACTCAGCCTCCTCACGCA	25	G	0.2807	0.3221
NC_005101.4-251157280	AACCATGGGACAAGGCCTCCAAATCTG	5	A	0.1639	0.1774
NC_005101.4-251204818	TGCCTTTGTACACTGCCTCCTCCTCTG	26	T	0	0.4787
NC_005101.4-251207643	GTTTGCCCAACCTGCCCTCCCCTCTGG	6	C	0.2276	0.4854
NC_005101.4-251254554	CAGGGACCAACTGGGCCTCCAGGACCA	15	G	0.0089	0.4789
NC_005101.4-251256060	CAAGTCACGACTTTCCCTCCTATCTTA	8	C	0.0661	0.0639
NC_005101.4-251256060	CAAGTCACGACTTTCCCTCCTATCTTA	22	A	0.1488	0.1787
NC_005101.4-251260686	GGGTGCTGGACTCAAACTCCTGTCTTC	5	G	0.1157	0.1653
NC_005101.4-251316336	TCAGAGACTACATCGTCTCCCAACAAA	1	T	0.0619	0.0765
NC_005101.4-251316336	TCAGAGACTACATCGTCTCCCAACAAA	24	C	0.0619	0.0765
NC_005101.4-251328657	GAGTAGCAAACCACGTCTCCTTAAGCA	13	A	0.0732	0.0705
NC_005101.4-251328657	GAGTAGCAAACCACGTCTCCTTAAGCA	22	T	0.187	0.2778
NC_005101.4-251333326	CAAGGGCTTACAAGGACTCCCCGGCAG	22	C	0.25	0.4803
NC_005101.4-251367510	CTGCTTCCCACACTCCCTCCTCTACCG	27	G	0.0569	0.0553
NC_005101.4-251437505	AGGGAGGGCACTGCAGCTCCACACCAT	21	A	0	0.4779
NC_005101.4-251445251	ATCCAGCCCACTCTGTCTCCTCAGTGC	7	C	0.1951	0.4881
NC_005101.4-251445251	ATCCAGCCCACTCTGTCTCCTCAGTGC	24	G	0.0894	0.1001
NC_005101.4-251454971	TAGACACACACAAAATCTCCACGAATT	5	C	0.1947	0.4859
NC_005101.4-251468883	GAGCAGGTGACTGCTGCTCCTGTGTTG	16	G	0.0813	0.0928
NC_005101.4-251468883	GAGCAGGTGACTGCTGCTCCTGTGTTG	25	T	0.0488	0.0476
NC_005101.4-251490661	GGCAGGGCTACCCATGCTCCTGGGAAA	2	G	0.0087	0.4782
NC_005101.4-251490661	GGCAGGGCTACCCATGCTCCTGGGAAA	26	A	0.2137	0.3504
NC_005101.4-251530896	TTCTTGTTAACTCCACCTCCGAGGCAT	5	T	0	0.4793

NC_005101.4-251530896	TTCTTGTTAACTCCACCTCCGAGGCAT	26	A	0.1721	0.2339
NC_005101.4-251557922	TCCCAGTCCACTCACCCTCCTGTGGGA	8	C	0	0.4728
NC_005101.4-251565605	GAGCCCACTACAACAGCTCCCAGCAGT	3	G	0.0336	0.033
NC_005101.4-251565605	GAGCCCACTACAACAGCTCCCAGCAGT	27	T	0.0522	0.173
NC_005101.4-251568550	GTGAGGAGAACTGTAACTCCTATCTGG	22	A	0.1947	0.4953
NC_005101.4-251568550	GTGAGGAGAACTGTAACTCCTATCTGG	23	T	0	0.4731
NC_005101.4-251600215	AGTGAAGCCACCTCCTCTCCTTATGAC	22	T	0.1316	0.3374
NC_005101.4-251681654	GGGAAGTGTACTGAGTCTCCACTGAGC	5	A	0.2857	0.322
NC_005101.4-251711582	TGTTACAAGACAAAGACTCCCTTCCCC	4	T	0.1239	0.2171
NC_005101.4-251711946	ATCTTTAAAACAAATCCTCCATTCTTG	27	G	0.161	0.1894
NC_005101.4-251723981	ATCCATACTACATACCCTCCTGTATCC	22	G	0.0179	0.4898
NC_005101.4-251729650	TACCCATTCACACCTTCTCCCAGCTCC	7	T	0.1652	0.3898
NC_005101.4-251729650	TACCCATTCACACCTTCTCCCAGCTCC	12	A	0	0.48
NC_005101.4-251754810	CAGAGCTGTACCTTGCCTCCTGTGCCT	14	T	0.2846	0.4301
NC_005101.4-251786792	TGATACTGTACTAGGCCTCCATTGTCT	8	G	0.1875	0.2385
NC_005101.4-251786792	TGATACTGTACTAGGCCTCCATTGTCT	13	A	0.1261	0.1182
NC_005101.4-251811105	AACTTTAGAACGTTGCCTCCTACCAGT	6	T	0	0.4787
NC_005101.4-251811105	AACTTTAGAACGTTGCCTCCTACCAGT	9	A	0.0252	0.0249
NC_005101.4-251830288	TAATGAGACACCTCTCCTCCCTCCTAC	8	A	0.0083	0.4803
NC_005101.4-251830288	TAATGAGACACCTCTCCTCCCTCCTAC	25	T	0.225	0.4847
NC_005101.4-251839565	TTTGTTGAGACATTGTCTCCTCTATCC	26	C	0.0574	0.071
NC_005101.4-251905538	TGCAGACCCACACAGGCTCCGTGATTC	22	T	0.0631	0.1103
NC_005101.4-251905538	TGCAGACCCACACAGGCTCCGTGATTC	27	C	0	0.4931
NC_005101.4-251914131	TCACCAGACACAGAAACTCCACACAAT	27	T	0	0.4807
NC_005101.4-251919402	GGTTTGAATACCGGTCCTCCACAGGCT	12	C	0.0172	0.4749
NC_005101.4-251928447	TTAGCAGGAACCCTCCCTCCTCCTTTG	26	T	0.0588	0.4859
NC_005101.4-251942355	CCAACAAGAACTATTCCTCCGTGGTTT	6	A	0.0089	0.4824
NC_005101.4-251968720	TTTCTGTGAACTTAACCTCCGTCACAT	21	G	0	0.4807
NC_005101.4-251982290	AGAGTAGCTACACGTGCTCCAAAGGGT	25	G	0.0342	0.0336
NC_005101.4-251982375	CAGTTGAGAACCTGCTCTCCCGCCAGT	23	C	0.0246	0.0243
NC_005101.4-252017396	ACTTTTCCGACAGACTCTCCGTACCCA	8	C	0.275	0.3708
NC_005101.4-252017396	ACTTTTCCGACAGACTCTCCGTACCCA	21	G	0.2353	0.322
NC_005101.4-252055040	CTGCGTCTCACGTTTTCTCCTTCCTCT	22	T	0.124	0.3851
NC_005101.4-252079225	GATAGGCAAACCACCCCTCCCAATCCT	24	T	0	0.4773
NC_005101.4-252121673	CGGTTCATGACCTTTCCTCCCGTATCA	21	C	0.0333	0.0328
NC_005101.4-252121673	CGGTTCATGACCTTTCCTCCCGTATCA	22	G	0.1583	0.3347
NC_005101.4-252122454	TAAAGCAGGACTTCCCCTCCAGGCCCA	8	G	0.1721	0.2573
NC_005101.4-252150594	TGAAGCTTAACCAACACTCCCTCACTC	24	A	0.2583	0.4416
NC_005101.4-252150594	TGAAGCTTAACCAACACTCCCTCACTC	26	T	0.2583	0.4416
NC_005101.4-252251752	CACAAACGGACAGCAGCTCCAAATGTT	5	A	0.0328	0.0634
NC_005101.4-252251752	CACAAACGGACAGCAGCTCCAAATGTT	8	G	0	0.4807
NC_005101.4-252287627	GAGGTGAGAACGGAGGCTCCATGAGGT	1	G	0.2261	0.2005
NC_005101.4-252287627	GAGGTGAGAACGGAGGCTCCATGAGGT	21	A	0	0.4856
NC_005101.4-252294589	AGTGCGCTGACTGTGACTCCTGAAGAA	24	A	0.0086	0.4729
NC_005101.4-252323485	ATCTAGTGTACAAAAGCTCCTACTGGA	14	A	0	0.4751
NC_005101.4-252404922	CATGCCTCCACACCACCTCCATCTGTC	7	T	0.1161	0.1553
NC_005101.4-252409541	CGATTAACTACCTCTGCTCCAGTCTTC	15	T	0.0246	0.0243
NC_005101.4-252410890	GTACGTGAGACGCTCGCTCCTCCAGTC	4	C	0.1121	0.2797
NC_005101.4-252438029	TTATGTAAGACAAGCGCTCCTGCCTGG	13	A	0.0588	0.0728
NC_005101.4-252438029	TTATGTAAGACAAGCGCTCCTGCCTGG	22	G	0.1261	0.1609
NC_005101.4-252480121	ATTGTGTTTACCCCTTCTCCAACCCCT	5	T	0.2583	0.3872

NC_005101.4-252480121	ATTGTGTTTACCCCTTCTCCAACCCCT	12	C	0.2672	0.4491
NC_005101.4-252480699	CTGTCTGACACAGGACCTCCCATCATT	22	A	0	0.4773
NC_005101.4-252485974	CCAGGAGTCACCCTCCCTCCAGTCCCT	22	G	0.0446	0.0436
NC_005101.4-252561110	TGTTGATATACTTGGCCTCCTTCTTGC	26	G	0.0732	0.0854
NC_005101.4-252613448	AGAAATGTGACCATCACTCCCACTGCC	4	A	0.0263	0.026
NC_005101.4-252613448	AGAAATGTGACCATCACTCCCACTGCC	22	A	0.2807	0.4321
NC_005101.4-252616686	CAACATCTCACCTCTTCTCCTAGTGTT	1	C	0	0.4819
NC_005101.4-252616686	CAACATCTCACCTCTTCTCCTAGTGTT	24	T	0.2231	0.2922
NC_005101.4-252660515	GTTACCTTGACTGGCTCTCCTGATAGA	4	A	0.1695	0.3138
NC_005101.4-252660515	GTTACCTTGACTGGCTCTCCTGATAGA	7	T	0.2119	0.4273
NC_005101.4-252693990	GTCAGTGACACAGTCCCTCCTTGGCGA	26	G	0.1083	0.1025
NC_005101.4-252720799	TTTCTGACCACAGCACCTCCATTAATG	12	A	0.0268	0.4885
NC_005101.4-252720799	TTTCTGACCACAGCACCTCCATTAATG	26	T	0	0.4841
NC_005101.4-252764556	GGTGATTTTACACCTCCTCCATTTTCC	16	C	0.1261	0.1327
NC_005101.4-252829191	ATTTCCTTCACCTTGACTCCATTGCTG	8	T	0.2846	0.4662
NC_005101.4-252829191	ATTTCCTTCACCTTGACTCCATTGCTG	23	T	0	0.4793
NC_005101.4-252846092	AGTCAGCAAACGGCAGCTCCTGTTGGC	12	G	0.0248	0.0245
NC_005101.4-252878993	AATTGTTGAACCGTCTCTCCAGTCCTT	23	T	0.4262	0.416
NC_005101.4-252884266	ACCATCCCCACTGCTCCTCCCTCTGCT	6	C	0	0.4793
NC_005101.4-252884266	ACCATCCCCACTGCTCCTCCCTCTGCT	12	T	0.0163	0.4825
NC_005101.4-252885174	AAGTACTTAACATGAACTCCAAGTGAT	22	A	0.0885	0.1757
NC_005101.4-252926759	AGCTAGGCCACTGCGTCTCCATTTAAT	14	C	0.3197	0.4258
NC_005101.4-252936961	ACTCTTTTAACTACCTCTCCCTTGGAC	1	A	0	0.4871
NC_005101.4-252936961	ACTCTTTTAACTACCTCTCCCTTGGAC	24	G	0	0.4871
NC_005101.4-252970780	CCAAATGCTACCCTTGCTCCGTGCGCC	24	C	0.1626	0.3239
NC_005101.4-252988880	ATGCCGTCTACCTCTCCTCCCCACCCT	5	C	0.0957	0.1219
NC_005101.4-252997501	GGTTTGAGTACTTGGACTCCTTTGCTT	9	T	0	0.4773
NC_005101.4-253050354	ATAAACGACACCTTAACTCCCATCTTT	16	A	0.0167	0.0328
NC_005101.4-253107670	AACTGACTAACTCCAGCTCCAGGCAAT	8	T	0.0407	0.0705
NC_005101.4-253115811	TTGAGGTTAACCCCAGCTCCTCCCCAT	27	T	0.2689	0.4008
NC_005101.4-253117450	CCAGCCTTGACTTTCCCTCCATGGTGG	4	G	0.0261	0.4817
NC_005101.4-253117450	CCAGCCTTGACTTTCCCTCCATGGTGG	25	T	0.0696	0.4954
NC_005101.4-253156341	GGTCGCAGAACTTGCTCTCCTTTTATC	5	G	0	0.4793
NC_005101.4-253212978	ATGGAAATTACATTGGCTCCATGAACA	26	C	0.0333	0.0328
NC_005101.4-253222000	CACACCGGGACTCTGTCTCCCTAAATG	6	C	0.1261	0.1609
NC_005101.4-253258181	GCCAGGAATACATAAGCTCCTGTCTCA	7	A	0.2389	0.2738
NC_005101.4-253327527	GGCATCGTTACTTAGCCTCCTTGTCCA	7	G	0.1301	0.1356
NC_005101.4-253327527	GGCATCGTTACTTAGCCTCCTTGTCCA	9	T	0.2276	0.3141
NC_005101.4-253346573	ACTCGCCACACCTGATCTCCACCCACA	5	G	0.1721	0.2339
NC_005101.4-253346573	ACTCGCCACACCTGATCTCCACCCACA	9	C	0.065	0.1494
NC_005101.4-253351968	GAATGCTGTACCACTTCTCCTGTTAGA	14	C	0	0.4832
NC_005101.4-253351968	GAATGCTGTACCACTTCTCCTGTTAGA	15	T	0.2149	0.2869
NC_005101.4-253389468	TTAACTTGTACATGTGCTCCATGAAAT	26	A	0.1607	0.4994
NC_005101.4-253461707	AGTTAGAACACACTATCTCCTTCTGTT	8	A	0.2276	0.3604
NC_005101.4-253461707	AGTTAGAACACACTATCTCCTTCTGTT	14	T	0.1789	0.2017
NC_005101.4-253466856	AAAATTAGCACATCACCTCCCAATATG	3	A	0.1368	0.2235
NC_005101.4-253474692	TCTTTTCCCACCACAACTCCCACATGT	12	C	0.0164	0.4773
NC_005101.4-253475327	AATAAATATACACAGCCTCCATTTGAT	7	T	0.0085	0.4626
NC_005101.4-253475327	AATAAATATACACAGCCTCCATTTGAT	23	T	0.2696	0.3898
NC_005101.4-253534897	CCTCCCTTCACTCCTACTCCAGGACCC	16	A	0.0442	0.4873
NC_005101.4-253577127	GTCCGACTTACACTCTCTCCAGACATC	4	C	0.0174	0.0342

NC_005101.4-253577127	GTCCGACTTACACTCTCTCCAGACATC	13	C	0	0.4883
NC_005101.4-253583056	TTGTACCACACATATCCTCCAATACAT	14	A	0.15	0.4978
NC_005101.4-253623964	CCTTTCTTCACAGCCCCTCCAGCATTC	15	C	0.1316	0.3168
NC_005101.4-253629187	TAAGAGGCTACAGTAGCTCCCTGACAT	25	C	0.1102	0.1481
NC_005101.4-253629187	TAAGAGGCTACAGTAGCTCCCTGACAT	27	T	0.1102	0.1894
NC_005101.4-253689147	GCAGCAGCCACTCATGCTCCCTACCAC	8	C	0.1171	0.2531
NC_005101.4-253797630	TACCAAACAACTCTTGCTCCCAGGGAG	12	T	0	0.4718
NC_005101.4-253824341	AGGATGCTGACAGCAACTCCGGCAGGA	2	G	0	0.0354
NC_005101.4-253824341	AGGATGCTGACAGCAACTCCGGCAGGA	15	A	0	0.4841
NC_005101.4-253916481	GAAGGACAGACTGAGTCTCCACTTGGG	22	C	0.0244	0.4741
NC_005101.4-253926283	ATGCATGAGACGCTCACTCCTTTCTTT	3	G	0.0957	0.1369
NC_005101.4-253957080	GGAGGTTATACCATCCCTCCTTTCTAT	22	T	0.0331	0.0484
NC_005101.4-253957080	GGAGGTTATACCATCCCTCCTTTCTAT	23	T	0	0.4728
NC_005101.4-253977325	TACTCCAACACCACGCCTCCCTGTTTG	2	A	0.0488	0.0476
NC_005101.4-253977325	TACTCCAACACCACGCCTCCCTGTTTG	16	C	0.1463	0.1629
NC_005101.4-254172688	TAGAAATAAACAAGATCTCCGAATGCC	21	G	0.2222	0.272
NC_005101.4-254177962	ATCCACAGCACTCTTTCTCCTGCACTC	4	C	0.0446	0.0934
NC_005101.4-254217579	TGCATACATACACATGCTCCCACACAT	7	C	0.2377	0.2573
NC_005101.4-254262978	ATCTGCAGCACCACACCTCCACAGTCC	2	T	0	0.4807
NC_005101.4-254324964	CCGTGAGAGACTTGGCCTCCATCGTAG	13	T	0.177	0.2556
NC_005101.4-254372344	TAATTGTGGACCACGCCTCCAGCAGTG	14	C	0.0439	0.0429
NC_005101.4-254395364	AGACTCCGGACTGGGACTCCCTGAGCT	4	C	0.2712	0.424
NC_005101.4-254395364	AGACTCCGGACTGGGACTCCCTGAGCT	16	A	0.2627	0.4207
NC_005101.4-254404580	ATTCTCTCTACAGAGGCTCCCTTCTTT	8	C	0.1316	0.1672
NC_005101.4-254444072	CTGTACAAAACATGGCCTCCAAACATT	5	A	0.2764	0.3334
NC_005101.4-254506620	AACCTCTTTACCCTCCCTCCCAACTCT	5	T	0.027	0.0267
NC_005101.4-254517022	CCTCCTCCCACTCTTTCTCCACCTCCT	1	C	0.193	0.3221
NC_005101.4-254517022	CCTCCTCCCACTCTTTCTCCACCTCCT	22	C	0.0259	0.0422
NC_005101.4-254572592	ATTTAGACAACCTCCTCTCCTTCATCA	14	C	0.1368	0.1704
NC_005101.4-254633314	ATCCTCTGGACAACCTCTCCCTATTAG	13	A	0.2542	0.4718
NC_005101.4-254639477	TATTTCATAACAGGTCCTCCCAGACCT	13	G	0.0574	0.0557
NC_005101.4-254680003	TAACGCAGGACTATGGCTCCTGTGTGT	7	A	0.1441	0.4991
NC_005101.4-254680003	TAACGCAGGACTATGGCTCCTGTGTGT	8	G	0.0504	0.065
NC_005101.4-254752979	TGAGAGAAAACTGAATCTCCATCAGGC	8	A	0.0776	0.1504
NC_005101.4-254801527	TTCCCTCTCACGTTGCCTCCTTTCCTA	12	G	0.1404	0.1454
NC_005101.4-254863177	AAAGTTAGAACGGGAACTCCACGTTTA	23	G	0.1053	0.1884
NC_005101.4-254869065	CCACAGAAAACTTCTGCTCCAAGCTAT	24	C	0.0492	0.0634
NC_005101.4-254906460	AGGATCAAGACATTTCCTCCTGGAGAA	15	T	0.0084	0.4723
NC_005101.4-254916810	GACTGGCAAACAATTCCTCCAGTGAGT	1	G	0.0351	0.0345
NC_005101.4-254916810	GACTGGCAAACAATTCCTCCAGTGAGT	6	G	0	0.4814
NC_005101.4-254973275	ATACCAACCACCAGAGCTCCCAGGGAT	25	G	0.0164	0.4837
NC_005101.4-255059464	TGACCAGCTACATACTCTCCATTACAA	27	A	0	0.4793
NC_005101.4-255120851	CTAGTCTGCACTCTTGCTCCTCATAGT	23	A	0.0084	0.4829
NC_005101.4-255170348	ATATTCTTAACCTGTCCTCCTCTGTTC	14	G	0.0583	0.1025
NC_005101.4-255171274	TTTCTAATAACACATTCTCCTGGTGAC	23	G	0.025	0.0408
NC_005101.4-255180566	GCATTCATAACTTTATCTCCTTGATAG	21	T	0.0435	0.059
NC_005101.4-255180566	GCATTCATAACTTTATCTCCTTGATAG	27	G	0.2696	0.4055
NC_005101.4-255195333	GCCTAGGTAACTAGCTCTCCAAATGCC	16	T	0.0804	0.0934
NC_005101.4-255201854	GGATCTGACACTCTGCCTCCTTCGGCA	16	C	0.0259	0.0255
NC_005101.4-255241867	CATCCACTTACCTGTTCTCCTCTCCTG	8	T	0.0088	0.4599
NC_005101.4-255241867	CATCCACTTACCTGTTCTCCTCTCCTG	15	T	0.0088	0.4599

NC_005101.4-255311214	AACCATCTCACCTTCTCTCCGCGTTGT	4	C	0.0569	0.1562
NC_005101.4-255311214	AACCATCTCACCTTCTCTCCGCGTTGT	21	G	0.1545	0.1425
NC_005101.4-255471983	TCTATAACAACCCAAACTCCATTTCTA	4	A	0.2185	0.4983
NC_005101.4-255583461	ATCTCAGTCACCACTGCTCCCTGACCA	1	A	0.2149	0.3729
NC_005101.4-255619212	CATATGTGCACATGAGCTCCTTCCCAC	4	A	0	0.4703
NC_005101.4-255619212	CATATGTGCACATGAGCTCCTTCCCAC	21	T	0	0.4703
NC_005101.4-255704904	TTCACACAAACGGAACCTCCTAAGTTC	12	G	0.1913	0.4092
NC_005101.4-255704904	TTCACACAAACGGAACCTCCTAAGTTC	16	C	0.0342	0.0973
NC_005101.4-255775083	CTGGGCCATACCCTAACTCCTTGCTAT	23	G	0.009	0.4838
NC_005101.4-255951406	GTATCCTCAACTGGTCCTCCCTTCAGT	21	C	0.1333	0.1387
NC_005101.4-255974132	ATTTGTGACACTCTTGCTCCAGCAGTT	3	T	0.1491	0.235
NC_005101.4-256060076	TACTCAAGAACCATCCCTCCGACCTCC	9	A	0.0598	0.074
NC_005101.4-256060076	TACTCAAGAACCATCCCTCCGACCTCC	21	G	0	0.488
NC_005101.4-256165700	CACCCAGTCACCCCGCCTCCTGTAATT	21	T	0	0.4819
NC_005101.4-256179033	TTCTGTGCCACAATTGCTCCACCACAT	12	A	0.0089	0.4856
NC_005101.4-256235162	CCCACTGATACCCACTCTCCAATGTGT	6	T	0.2397	0.4004
NC_005101.4-256276908	CACAGTTAGACACAGACTCCATGATAA	13	C	0	0.4773
NC_005101.4-256348198	GGGATGCAGACTTCCTCTCCACGCCTT	22	C	0.0833	0.1244
NC_005101.4-256393618	GACAGAGGGACTTCCTCTCCTCTGACT	8	G	0.1622	0.2068
NC_005101.4-256431570	AAGTCTTTAACTCCAGCTCCAACTGAT	21	A	0.1833	0.2888
NC_005101.4-256459761	GGAGTGGAGACAAAGACTCCTGTAGTG	27	G	0.0488	0.0476
NC_005101.4-256462153	CCTGAGGTGACCACAGCTCCTCTGTGG	2	C	0.2212	0.4547
NC_005101.4-256462153	CCTGAGGTGACCACAGCTCCTCTGTGG	6	G	0.0522	0.0508
NC_005101.4-256486738	TCGAGGTCCACATTTTCTCCTTCGTCT	16	T	0.1951	0.2382
NC_005101.4-256531253	TCCTTTAAAACACTAACTCCTTGCCAG	15	A	0	0.48
NC_005101.4-256531253	TCCTTTAAAACACTAACTCCTTGCCAG	27	G	0.3058	0.4077
NC_005101.4-256639540	GGGGAGAAAACAGAGTCTCCGATGATA	12	A	0.0593	0.0734
NC_005101.4-256714223	ATCCCGCATACTTTAACTCCTTTTACA	2	T	0	0.4868
NC_005101.4-256765184	CACTACCTTACAGAAGCTCCGTGGACT	21	G	0.0348	0.4891
NC_005101.4-256775404	CCAAAGCAGACCTGGCCTCCAGATCCT	16	C	0.1304	0.1516
NC_005101.4-256833871	TGTTGCAGAACACTGTCTCCAGGCCAT	1	T	0.0593	0.0734
NC_005101.4-256833871	TGTTGCAGAACACTGTCTCCAGGCCAT	7	A	0	0.4718
NC_005101.4-256855437	ATCTTCCTCACCAAGTCTCCAGACAAG	9	C	0	0.4793
NC_005101.4-256855437	ATCTTCCTCACCAAGTCTCCAGACAAG	15	G	0.065	0.4944
NC_005101.4-256883495	ACTGCTCTGACCCTGTCTCCAAGCTCT	1	A	0.0984	0.1225
NC_005101.4-257031557	GGGAACAACACTGTGACTCCCATATTC	2	G	0.0984	0.1225
NC_005101.4-257037509	CTTTGTGAGACATTCACTCCCAAGTAG	3	T	0	0.4688
NC_005101.4-257067613	AACAGCACAACTCTAACTCCTACGTAC	24	G	0.0569	0.0553
NC_005101.4-257070085	TCATGAGTCACACCCACTCCCTTTTCT	14	C	0	0.4793
NC_005101.4-257095235	TGGTAACAGACTGATACTCCACAAGCA	7	C	0.1261	0.2337
NC_005101.4-257125405	AGGCTGGTCACTGGACCTCCTTTCCCT	21	T	0.4261	0.3728
NC_005101.4-257125405	AGGCTGGTCACTGGACCTCCTTTCCCT	23	T	0.0948	0.0903
NC_005101.4-257174437	TTAATGGAAACAGAAACTCCATGTTTA	7	G	0.1017	0.1551
NC_005101.4-257215120	GAAAAAGGCACTGACCCTCCTGAGAAA	16	C	0	0.4709
NC_005101.4-257245490	CCCAACTCCACAGCTGCTCCCTCTGTA	4	A	0.0508	0.0496
NC_005101.4-257334724	CATGTAGACACAGGGACTCCAAGGGTT	7	G	0.0598	0.058
NC_005101.4-257355153	GCTCCCACCACAGTTTCTCCCCACCCT	1	G	0.0614	0.0595
NC_005101.4-257355153	GCTCCCACCACAGTTTCTCCCCACCCT	8	C	0.0442	0.0433
NC_005101.4-257364037	TAAGATCTGACTCACTCTCCGGGCCTG	21	G	0.1239	0.1162
NC_005101.4-257367345	AGAGCAGTCACTCCCCCTCCACACAAT	1	A	0.0783	0.4946
NC_005101.4-257390692	AGGCCATACACCTGTTCTCCCAGCATA	4	C	0.0536	0.4898

NC_005101.4-257390692	AGGCCATACACCTGTTCTCCCAGCATA	5	C	0.0625	0.0605
NC_005101.4-257401356	ATCCTGTACACTGCAACTCCCAACAAA	2	T	0	0.4772
NC_005101.4-257401356	ATCCTGTACACTGCAACTCCCAACAAA	23	A	0	0.4757
NC_005101.4-257538806	GAGCCAACCACACACGCTCCCTCAACA	12	A	0	0.4757
NC_005101.4-257538806	GAGCCAACCACACACGCTCCCTCAACA	16	G	0.1525	0.4999
NC_005101.4-257540303	AGCTTTGCCACTGAGCCTCCCCATCAC	22	C	0.0244	0.0398
NC_005101.4-257540303	AGCTTTGCCACTGAGCCTCCCCATCAC	26	A	0	0.4773
NC_005101.4-257541116	CTACTGCTCACACGGCCTCCAGTAAAA	21	A	0	0.4715
NC_005101.4-257546774	GATCTGCTGACTGTCTCTCCGGACATG	21	G	0.0246	0.0401
NC_005101.4-257565095	CACGCAGGGACTGGCTCTCCGTGACTA	3	C	0.0246	0.0243
NC_005101.4-257573736	ATGGAAGGCACTGGCACTCCACGCCAA	23	G	0	0.4787
NC_005101.4-257599754	GCAATTGTAACCCCAGCTCCAGGAGGA	12	C	0.025	0.0247
NC_005101.4-257619665	GTAACCTAGACCTAAACTCCTTATCAT	14	A	0	0.4779
NC_005101.4-257650804	CTCACCTGCACTCACCCTCCTCCACCA	6	C	0	0.0351
NC_005101.4-257728112	AACAGGAGAACCAGCACTCCTTTCTCC	15	C	0.025	0.0247
NC_005101.4-257738221	TGGGCAGACACATTTCCTCCAGTTGTG	6	A	0.0165	0.4877
NC_005101.4-257742319	AACGCAAGTACTCTGTCTCCTCTTTCT	3	C	0.0813	0.1216
NC_005101.4-257748511	GACAATCAGACTCAATCTCCTTTTTGT	21	T	0	0.474
NC_005101.4-257755923	GGCTAATGAACTCTGCCTCCTCCCTCC	6	A	0.2033	0.4915
NC_005101.4-257797480	CCCCTTCACACAGCCTCTCCCCTCCCC	22	C	0.2035	0.2494
NC_005101.4-257815284	TCCAGATACACACTTTCTCCACTTTCA	25	T	0.0588	0.4829
NC_005101.4-257815949	TGTGCACACACACCATCTCCACAGGTA	2	G	0.0957	0.1219
NC_005101.4-257815949	TGTGCACACACACCATCTCCACAGGTA	27	A	0.0261	0.0257
NC_005101.4-257829224	CTCATTAACACCAGTTCTCCTCGCTTC	9	C	0.0488	0.0928
NC_005101.4-257829601	CAACTCCCCACCCCATCTCCCGGCCTT	21	C	0.1339	0.1553
NC_005101.4-257840717	TTCAGTGCAACCTCTTCTCCAGCAACC	5	G	0.0174	0.0342
NC_005101.4-257840717	TTCAGTGCAACCTCTTCTCCAGCAACC	14	C	0.0348	0.0342
NC_005101.4-257858321	ATTCTCATGACCCTGTCTCCCTTGTGA	16	T	0	0.4773
NC_005101.4-257898605	CTGACCCCTACTTGACCTCCAGCCCTG	25	C	0	0.4637
NC_005101.4-257916908	ATGTTGATCACTTTTACTCCCAGAGTT	4	T	0.2167	0.3578
NC_005101.4-257953063	CCCAGCACAACTAGAACTCCCACACAG	13	A	0	0.4751
NC_005101.4-258013500	TCCAACCACACTGCTCCTCCTCAGCTA	7	C	0.1176	0.1398
NC_005101.4-258013500	TCCAACCACACTGCTCCTCCTCAGCTA	9	C	0.1765	0.2389
NC_005101.4-258158661	CTCAGATGGACAGTTACTCCCTGATGA	21	C	0.0261	0.0257
NC_005101.4-258178008	TGAACTGGGACATCAGCTCCATCCTTT	2	G	0.025	0.0247
NC_005101.4-258193842	AGGAAATGGACCTTTGCTCCTAAGAAC	5	A	0	0.4793
NC_005101.4-258193842	AGGAAATGGACCTTTGCTCCTAAGAAC	27	C	0.1463	0.4997
NC_005101.4-258226858	GACCTATGAACTCTGTCTCCTTGGGAC	24	G	0.3058	0.465
NC_005101.4-258226858	GACCTATGAACTCTGTCTCCTTGGGAC	27	C	0.3058	0.465
NC_005101.4-258226921	GATGAGGAAACACTGTCTCCACAGCTC	15	G	0.1897	0.4851
NC_005101.4-258282038	TGTTGGGAGACTGTTACTCCATACCTG	7	G	0.0177	0.4793
NC_005101.4-258282038	TGTTGGGAGACTGTTACTCCATACCTG	25	C	0.0354	0.0348
NC_005101.4-258293645	CATCTCCTTACAACACCTCCCACTTCT	25	T	0.1967	0.3354
NC_005101.4-258299615	CATGACTGTACACTGACTCCTCTTCCC	7	T	0.25	0.4911
NC_005101.4-258326032	AACTAGTACACTCTCCCTCCCCAATTC	23	A	0.0431	0.0422
NC_005101.4-258365875	CCATGGTCAACACATCCTCCTTTTACT	7	T	0.1102	0.119
NC_005101.4-258376833	TAAGGCTGGACCTGGTCTCCTAAACCC	23	A	0.1081	0.3944
NC_005101.4-258383714	CAACTCCACACTCCATCTCCACCATCT	3	A	0	0.4807
NC_005101.4-258383714	CAACTCCACACTCCATCTCCACCATCT	5	T	0	0.4807
NC_005101.4-258443312	TTATCTCCCACCAGACCTCCCCCCTCC	24	C	0.2456	0.4384
NC_005101.4-258443312	TTATCTCCCACCAGACCTCCCCCCTCC	27	C	0.1429	0.2698

NC_005101.4-258497485	ATTCAGTCTACAAGCTCTCCTGCACGA	6	G	0.0783	0.1219
NC_005101.4-258508497	CCAGGCCCCACACCTCCTCCACCGTGA	24	G	0.1624	0.3
NC_005101.4-258525033	TTAAACCACACTACCCCTCCCTCCACG	12	T	0.2797	0.2871
NC_005101.4-258536005	CTGGAGAAGACCAACTCTCCCTCACAC	14	A	0.0087	0.4817
NC_005101.4-258573547	TGCTAGACAACTCTTCCTCCCTTTCAT	4	T	0.0656	0.4914
NC_005101.4-258590375	GGACAGGGAACATTTCCTCCGGGAAAT	6	G	0.1696	0.3705
NC_005101.4-258642757	CCATGACAAACTCTATCTCCAATTCAG	12	T	0	0.4718
NC_005101.4-258654518	AAAGGAGACACTTGGACTCCCGCCCTT	4	G	0.2583	0.4416
NC_005101.4-258687681	TTATGGCCCACCAAGACTCCTCAGAGA	4	T	0.0256	0.4823
NC_005101.4-258687681	TTATGGCCCACCAAGACTCCTCAGAGA	8	C	0.0167	0.0328
NC_005101.4-258710384	ACATATATCACACCTCCTCCATAGGCA	5	A	0	0.4793
NC_005101.4-258710384	ACATATATCACACCTCCTCCATAGGCA	6	T	0.122	0.4999
NC_005101.4-258711051	CTCGGAGGCACACACCCTCCATATGCA	3	C	0	0.4807
NC_005101.4-258711051	CTCGGAGGCACACACCCTCCATATGCA	21	A	0	0.4807
NC_005101.4-258740015	GTCAGAGGCACCCCCACTCCCACTGTT	3	C	0.5702	0.4077
NC_005101.4-258827318	AAATAGTTAACTTCCCCTCCCCAACAG	27	G	0.1261	0.1639
NC_005101.4-258840239	AGTCCTAACACTACTCCTCCGTTAGGA	23	T	0	0.4819
NC_005101.4-258854469	TTTTCTTGGACTCCATCTCCAAGTGTC	6	T	0	0.4807
NC_005101.4-258856082	ATTTGAAAGACCCTTGCTCCTCAGATA	13	C	0.2623	0.4463
NC_005101.4-258888350	GTGAAGCACACGCAGTCTCCTCAGGAC	14	A	0.2893	0.3851
NC_005101.4-258890566	TTTCAAAGGACCTTAGCTCCCTCTGTG	8	G	0	0.4757
NC_005101.4-258890566	TTTCAAAGGACCTTAGCTCCCTCTGTG	15	A	0.1933	0.397
NC_005101.4-258915489	CACACTCTCACATACACTCCATCTCAC	27	C	0.0325	0.078
NC_005101.4-258915705	AGTACTCACACACACTCTCCCACATAC	25	T	0.2114	0.3141
NC_005101.4-259020075	CGTCACATGACCAAGCCTCCTCACAGG	1	C	0.009	0.4474
NC_005101.4-259037776	ATTGCCTGTACTCTAGCTCCAGGGGCT	2	T	0.0246	0.0243
NC_005101.4-259037776	ATTGCCTGTACTCTAGCTCCAGGGGCT	8	G	0.0984	0.1225
NC_005101.4-259041763	CACGTGAGAACACCGCCTCCCCCCTGT	3	C	0.0246	0.0243
NC_005101.4-259127992	AATGCACATACATATACTCCTGCACCC	26	C	0.1949	0.4939
NC_005101.4-259136053	ACAATCCTCACCCACTCTCCTGTGTCC	16	T	0.0084	0.4859
NC_005101.4-259148309	TTCTGGAGAACATGAGCTCCCCCCTTG	22	C	0.1167	0.1932
NC_005101.4-259149246	ACCAGGACCACTGGGGCTCCGGATCCA	21	G	0.2276	0.3688
NC_005101.4-259149246	ACCAGGACCACTGGGGCTCCGGATCCA	26	C	0.2927	0.4072
NC_005101.4-259149380	CCAGAACTAACAGCTGCTCCCGCATCT	26	C	0.2017	0.4743
NC_005101.4-259153622	GAGCTTTGCACAGGGCCTCCAAGAGCA	13	G	0.1864	0.2466
NC_005101.4-259153622	GAGCTTTGCACAGGGCCTCCAAGAGCA	16	C	0.2034	0.2585
NC_005101.4-259163971	ACCCACTCAACCACCTCTCCAGCCCTT	22	G	0.2759	0.2378
NC_005101.4-259178447	CTGGAGACCACGGTGTCTCCTCCTCTC	27	C	0.0976	0.0928
NC_005101.4-259179367	TAGTAACCCACCTACTCTCCCTACCTG	4	T	0.0083	0.4783
NC_005101.4-259237828	ATAAAGTGTACGTAAGCTCCTCAGTAC	13	T	0.1311	0.1904
NC_005101.4-259240760	TTTACTGTCACAGGGCCTCCGCTTTGT	21	G	0.042	0.4723
NC_005101.4-259250535	CTAAATATAACAGGACCTCCAGCTGTG	3	A	0.2018	0.235
NC_005101.4-259304431	TCTAAACCAACCTTCTCTCCATCAAGC	6	A	0.1081	0.2594
NC_005101.4-259310093	CAGCCAGGCACCTGCACTCCTCCCCAC	24	C	0.0917	0.1733
NC_005101.4-259316731	CAGAGGCACACCCCATCTCCCTCCACT	14	C	0.0427	0.0896
NC_005101.4-259388695	GCCCAGAGCACTCTCCCTCCTAAGCCC	9	C	0	0.0345
NC_005101.4-259388695	GCCCAGAGCACTCTCCCTCCTAAGCCC	13	C	0.1053	0.2021
NC_005101.4-259397818	ACTGACAGGACCAGAGCTCCACTTCAT	12	C	0.0492	0.0786
NC_005101.4-259403347	GTTCTGCTGACCCCAACTCCGTTCACA	21	G	0.124	0.1585
NC_005101.4-259415090	TTCCCTGATACAAGAGCTCCAAAACCC	25	C	0.0256	0.0253
NC_005101.4-259427025	TGAGAACAGACGCAGGCTCCTGCCTAT	12	G	0.1583	0.395

NC_005101.4-259431744	GCCATGCGAACAGCACCTCCAGCCCAC	14	C	0.1842	0.222
NC_005101.4-259466676	ATGCAGACGACTAAGCCTCCTCAGCTC	8	C	0	0.4793
NC_005101.4-259493383	TGCTGGCTTACTTGTCCTCCATCTTGA	13	T	0	0.4751
NC_005101.4-259494624	AATGTGCTTACTGGCCCTCCTACCTGA	7	C	0.2017	0.4844
NC_005101.4-259494624	AATGTGCTTACTGGCCCTCCTACCTGA	16	C	0.2051	0.2945
NC_005101.4-259520548	GTAGTGGCTACAGGTTCTCCTCCTCTC	22	C	0.1803	0.2956
NC_005101.4-259522419	GTGACACCAACAGAACCTCCTTCACCC	8	C	0.1066	0.1436
NC_005101.4-259532236	ATGGCGCCTACAAGATCTCCATTTGAA	4	G	0.1532	0.1713
NC_005101.4-259537715	GTCGGTCTCACCCCTCCTCCTCCTGTT	25	G	0.1724	0.4463
NC_005101.4-259537715	GTCGGTCTCACCCCTCCTCCTCCTGTT	26	T	0.1724	0.4463
NC_005101.4-259540457	TTGCTCAGCACTGTGACTCCATCACAG	14	T	0	0.4743
NC_005101.4-259567192	CAAAAAGACACCATTCCTCCAGCTCAG	6	A	0.2033	0.4523
NC_005101.4-259590163	CTTGCAAACACCAGGTCTCCAGTGCCC	21	A	0.2348	0.3728
NC_005101.4-259618846	TGTAGCTATACGAAAGCTCCCGGTGTG	6	C	0.0976	0.1073
NC_005101.4-259618846	TGTAGCTATACGAAAGCTCCCGGTGTG	13	A	0.0081	0.4777
NC_005101.4-259639949	TGCCTCTCCACCACATCTCCTTAGTCT	22	T	0.0164	0.4773
NC_005101.4-259639949	TGCCTCTCCACCACATCTCCTTAGTCT	26	C	0.122	0.208
NC_005101.4-259641109	CTCAGACCCACGGACACTCCCGCCATG	22	G	0.0625	0.0934
NC_005101.4-259652002	AGCTGGAGGACTCGCACTCCAGGATTT	14	G	0.041	0.0401
NC_005101.4-259652002	AGCTGGAGGACTCGCACTCCAGGATTT	15	C	0.041	0.0401
NC_005101.4-259691584	TGGCTCGCAACTCCAGCTCCCCTTCTG	6	C	0.1583	0.49
NC_005101.4-259733032	CCTCTTCTTACCATAACTCCTTTTCAG	6	T	0.0259	0.0255
NC_005101.4-259738196	CTGGGTGTTACCACATCTCCTACAAAA	13	A	0.0083	0.4847
NC_005101.4-259738196	CTGGGTGTTACCACATCTCCTACAAAA	15	A	0	0.4819
NC_005101.4-259758033	CCCTGGGCAACCTGTGCTCCCATGCAG	14	G	0.2683	0.4036
NC_005101.4-259761539	AGGAACTGTACCTGGGCTCCAGGCATG	23	G	0.1453	0.4999
NC_005101.4-259783332	TCCAGTCTCACAGCAGCTCCGGCAGGG	5	G	0.2845	0.4688
NC_005101.4-259783332	TCCAGTCTCACAGCAGCTCCGGCAGGG	24	A	0.0342	0.0336
NC_005101.4-259786780	TGCAGCACCACCACCGCTCCATTCTTG	16	G	0	0.4749
NC_005101.4-259821143	GCACTGACTACACCATCTCCTAGCCCT	21	T	0.1083	0.1025
NC_005101.4-259861057	CAAGATCCCACAGCCCCTCCTATATGT	14	C	0.2735	0.4553
NC_005101.4-259862664	CCACCCATTACAGACACTCCCTAGTTT	22	T	0.0804	0.0771
NC_005101.4-259871446	CACTATACCACACACCCTCCACAATCA	9	C	0.1167	0.1244
NC_005101.4-259871446	CACTATACCACACACCCTCCACAATCA	13	C	0.1157	0.1235
NC_005101.4-259900065	CTGAAAAAAACAATGACTCCATAAGAT	9	A	0.1681	0.5
NC_005101.4-259900065	CTGAAAAAAACAATGACTCCATAAGAT	12	A	0.0833	0.095
NC_005101.4-259914403	CTCACTGACACAGCAGCTCCTAGGGTC	4	A	0.0085	0.4842
NC_005101.4-259915445	CAACCCTACACCAGCCCTCCCTCTCCC	4	C	0.0508	0.0655
NC_005101.4-259922616	CCAAACGTCACGTGTCCTCCATGTGAC	12	G	0.2066	0.1853
NC_005101.4-259922616	CCAAACGTCACGTGTCCTCCATGTGAC	27	C	0.2066	0.1853
NC_005101.4-259929107	TGTGAGGCAACTGAGTCTCCAACAGAA	5	A	0.0574	0.071
NC_005101.4-259929107	TGTGAGGCAACTGAGTCTCCAACAGAA	15	G	0.1557	0.1839
NC_005101.4-259945970	GTCCAGCAAACGGTCACTCCCTCACCA	12	G	0.1176	0.2449
NC_005101.4-259981894	GTGAGATACACTACCTCTCCCTGGATG	4	A	0.1667	0.375
NC_005101.4-260136246	TTGGTTCCCACAACTCCTCCTTATGCC	24	T	0.0082	0.0401
NC_005101.4-260141222	GATTTGAGGACAGATCCTCCTAGGCTC	23	G	0.042	0.0411
NC_005101.4-260159351	TGGCCGCTGACCATCTCTCCAGCTGTC	6	G	0.1083	0.3533
NC_005101.4-260164770	TGCTGTGAGACTCTGTCTCCTCGTAAT	12	T	0	0.4765
NC_005101.4-260174496	CATAGTGGGACCCTGTCTCCAAAGCAA	12	C	0.0833	0.4932
NC_005101.4-260174496	CATAGTGGGACCCTGTCTCCAAAGCAA	13	C	0.0833	0.4932
NC_005101.4-260176788	GAAACTGCCACTGGGCCTCCTAACCAG	13	G	0	0.4819

NC_005101.4-260176788	GAAACTGCCACTGGGCCTCCTAACCAG	23	A	0.0413	0.0405
NC_005101.4-260188922	ACACAGTGAACACAGCCTCCAGCAAAC	3	A	0.4701	0.3596
NC_005101.4-260200498	CTGCTTATAACTCCAACTCCATTAGAT	14	C	0.0325	0.0629
NC_005101.4-260208716	AGTACCTGAACCACCTCTCCAGCTCCA	3	T	0.1197	0.1125
NC_005101.4-260208716	AGTACCTGAACCACCTCTCCAGCTCCA	14	C	0.3276	0.4144
NC_005101.4-260218311	TTACATAAGACCCCATCTCCATAAGAA	5	A	0.0246	0.0243
NC_005101.4-260292959	GGAAATAAAACCTCCCCTCCTGCCTTG	5	A	0	0.4832
NC_005101.4-260292959	GGAAATAAAACCTCCCCTCCTGCCTTG	26	T	0.0417	0.4708
NC_005101.4-260320112	AGTTATCATACTCTAGCTCCTCTTTGC	6	T	0.0169	0.0496
NC_005101.4-260320112	AGTTATCATACTCTAGCTCCTCTTTGC	8	A	0.1453	0.479
NC_005101.4-260344037	ACACCTATCACTCCAGCTCCAGGGCAT	5	C	0.084	0.1813
NC_005101.4-260361850	ACCTGCTTCACTTGCCCTCCATGATGA	2	C	0.187	0.3472
NC_005101.4-260361850	ACCTGCTTCACTTGCCCTCCATGATGA	23	G	0.2683	0.4106
NC_005101.4-260379592	CTGCCTATAACTCCAGCTCCAGGGAGA	14	C	0.193	0.4778
NC_005101.4-260404525	GAGACACACACACACCCTCCACCATTG	14	A	0.05	0.4888
NC_005101.4-260404525	GAGACACACACACACCCTCCACCATTG	21	A	0.0083	0.4816
NC_005101.4-260409045	GACAACCCGACTTCCCCTCCACTCTCT	9	G	0.0924	0.4994
NC_005101.4-260435946	TGGATACAGACTAGTTCTCCACACGCT	25	G	0.0901	0.1338
NC_005101.4-260506137	CCCTCTCCTACCTTCCCTCCATCCACC	13	T	0	0.4833
NC_005101.4-260555341	CCTGTGGCTACATGCCCTCCTGTGTTG	13	T	0.2478	0.4953
NC_005101.4-260556631	CACAGTCCCACTCTCCCTCCAACAATT	2	A	0.0488	0.0476
NC_005101.4-260556631	CACAGTCCCACTCTCCCTCCAACAATT	21	A	0.2541	0.432
NC_005101.4-260568516	TGCTCAGCGACCCTAACTCCCACGTTC	23	C	0.2459	0.4837
NC_005101.4-260587618	GATCCGGAAACTCTTCCTCCTATGAGA	13	C	0	0.4902
NC_005101.4-260622607	AACCACCCAACCATCTCTCCAGGCCTT	27	T	0.2105	0.3706
NC_005101.4-260661060	TAAGTGTCCACCCAAACTCCCTCCTTC	6	G	0.2222	0.4553
NC_005101.4-260661060	TAAGTGTCCACCCAAACTCCCTCCTTC	24	C	0.2222	0.4553
NC_005101.4-260681168	ACATTCTGTACGTTGTCTCCTTTCTCT	12	G	0.2212	0.3941
NC_005101.4-260681168	ACATTCTGTACGTTGTCTCCTTTCTCT	27	T	0.0088	0.4889
NC_005101.4-260726078	TGGTTCCTCACTGGGACTCCTCTTGGA	16	A	0.0598	0.058
NC_005101.4-260726245	CATCAGTCTACCAACTCTCCCGCACCC	4	C	0.0492	0.048
NC_005101.4-260726245	CATCAGTCTACCAACTCTCCCGCACCC	22	G	0.0656	0.0935
NC_005101.4-260755531	CACACACGCACACATACTCCCCCATAC	5	C	0.0256	0.4823
NC_005101.4-260755531	CACACACGCACACATACTCCCCCATAC	22	C	0	0.4868
NC_005101.4-260765759	TGAGTTCCAACACCACCTCCAGCTTCC	7	C	0.0167	0.0328
NC_005101.4-260765759	TGAGTTCCAACACCACCTCCAGCTTCC	22	G	0.1513	0.2796
NC_005101.4-260772525	AGATAGCTGACTCTGGCTCCTTTAAAA	15	G	0.0957	0.1516
NC_005101.4-260809578	TACAGGTCAACTTCGGCTCCACACTAG	15	G	0	0.4483
NC_005101.4-260809578	TACAGGTCAACTTCGGCTCCACACTAG	23	A	0	0.4483
NC_005101.4-260839597	CTTCCTGTAACTCTAGCTCCTGGGGAT	4	C	0.5043	0.3771
NC_005101.4-260839597	CTTCCTGTAACTCTAGCTCCTGGGGAT	23	G	0.5128	0.4444
NC_005101.4-260857605	AGTTGAAACACCCAGCCTCCTGCAGCG	3	T	0	0.4779
NC_005101.4-260882769	GTTCTCAGTACTGCCACTCCTAAATGA	9	T	0.2583	0.3347
NC_005101.4-260920978	ATCTGCCAGACTTCCCCTCCAGCTCCC	22	G	0.0357	0.0351
NC_005101.4-260947542	TTGCTTCCTACCAACGCTCCTTCCCCT	2	T	0.2167	0.255
NC_005101.4-260988307	CAGGTTGGCACGGGGCCTCCCGTTCAC	3	G	0.0082	0.0243
NC_005101.4-260988307	CAGGTTGGCACGGGGCCTCCCGTTCAC	22	G	0.0331	0.0325
NC_005101.4-261104576	TAGCTTATAACTCCAGCTCCATAGTTC	22	T	0.0083	0.4803
NC_005101.4-261122646	GTGTGGCCCACCACGCCTCCCATCCTC	14	C	0.1545	0.1954
NC_005101.4-261128600	CAGATTTGAACTCTGCCTCCCTCTGAC	13	C	0.0085	0.4738
NC_005101.4-261139986	CTGCTGAAGACCTCATCTCCTGGGGTC	27	C	0.0331	0.0325

NC_005101.4-261176045	TGAAGATGAACATAAACTCCTGATCCT	9	A	0.0088	0.026
NC_005101.4-261178607	CCAACACTAACTGAATCTCCATAGGCT	22	T	0	0.4793
NC_005101.4-261193688	AGACTGCATACTTTTCCTCCTATGTCT	5	T	0.009	0.4766
NC_005101.4-261193688	AGACTGCATACTTTTCCTCCTATGTCT	7	C	0.027	0.0267
NC_005101.4-261195223	CCTACAAGGACACATGCTCCACTATGC	14	A	0.0667	0.0644
NC_005101.4-261241734	ATAAAGCTTACAGATCCTCCGTGTGGA	4	A	0.1008	0.4921
NC_005101.4-261247111	CAACCACACACCTTCTCTCCAGCAGGC	14	T	0	0.4773
NC_005101.4-261251644	AGAGTTTGAACAAATGCTCCACCTTTC	1	A	0.0603	0.4937
NC_005101.4-261251644	AGAGTTTGAACAAATGCTCCACCTTTC	2	G	0.0603	0.4937
NC_005101.4-261269999	TGCTGTTATACATCACCTCCTGGAGGT	25	G	0.1081	0.4931
NC_005101.4-261332438	TCCAGTGACACATCTCCTCCAACAAGG	21	A	0.0531	0.0517
NC_005101.4-261344314	CAACCAGGGACAATCTCTCCAGGTACT	23	G	0.075	0.0875
NC_005101.4-261344314	CAACCAGGGACAATCTCTCCAGGTACT	25	A	0.125	0.1458
NC_005101.4-261382104	CCACGTCCCACACCTCCTCCTGTCTCC	4	C	0.0163	0.032
NC_005101.4-261382104	CCACGTCCCACACCTCCTCCTGTCTCC	14	C	0.1301	0.1356
NC_005101.4-261401966	TTCTTGCTGACTGGGCCTCCCACGTAG	23	C	0	0.4819
NC_005101.4-261429072	ATCAGTGTGACATGGGCTCCTTGCTTA	9	G	0.0496	0.0484
NC_005101.4-261485263	CATTTGCCCACCCTTCCTCCCAACAGC	7	C	0.0259	0.0255
NC_005101.4-261485263	CATTTGCCCACCCTTCCTCCCAACAGC	12	C	0.0531	0.4912
NC_005101.4-261507493	GGAAAGGGCACTTCCACTCCCTGCATG	27	G	0.2051	0.2484
NC_005101.4-261655274	CCTTCTGAGACTTCAGCTCCGTGTTTT	24	T	0	0.4724
NC_005101.4-261677834	CCAAGCATCACATGTTCTCCCATGGGT	3	A	0.1933	0.3554
NC_005101.4-261677834	CCAAGCATCACATGTTCTCCCATGGGT	12	A	0.05	0.4932
NC_005101.4-261716676	ATCCTTCACACCATGGCTCCTAAAGAC	24	A	0.0526	0.0512
NC_005101.4-261733272	CCTCCCTCTACTCCTCCTCCTCTCCCT	9	T	0.4955	0.4682
NC_005101.4-261733272	CCTCCCTCTACTCCTCCTCCTCTCCCT	14	C	0.4505	0.3587
NC_005101.4-261878067	CCACTAATAACTCCAGCTCCAGGAACT	26	C	0.2035	0.3495
NC_005101.4-261879456	AGGTTAGTCACCCTTCCTCCTGAGGAA	14	T	0	0.4827
NC_005101.4-261930823	ACCGTGCACACCTAAACTCCCTAGGAA	2	C	0	0.4793
NC_005101.4-261930823	ACCGTGCACACCTAAACTCCCTAGGAA	4	G	0	0.4793
NC_005101.4-262042606	TCTCACTACACCTGGGCTCCATGGTGC	6	C	0.0522	0.0989
NC_005101.4-262114777	CATCAACTAACTTGAACTCCTGGGGCT	6	A	0	0.4693
NC_005101.4-262114777	CATCAACTAACTTGAACTCCTGGGGCT	12	T	0.0085	0.4671
NC_005101.4-262119364	CCTTAAAGAACTCTGCCTCCACACTAT	7	A	0	0.4718
NC_005101.4-262128831	AGACTACAAACATCTTCTCCAAACATA	26	T	0.094	0.4947
NC_005101.4-262154411	TGCTGTGAGACTGTGTCTCCTGGACTG	23	G	0.2541	0.4755
NC_005101.4-262214100	CCACCACACACAGAAACTCCATGAAGA	22	T	0.0087	0.466
NC_005101.4-262214100	CCACCACACACAGAAACTCCATGAAGA	23	G	0.1017	0.1409
NC_005101.4-262269448	CCTCCCTCAACTCTACCTCCTCAGTCC	1	C	0.5546	0.4285
NC_005101.4-262269448	CCTCCCTCAACTCTACCTCCTCAGTCC	5	C	0.55	0.4328
NC_005101.4-262298388	GATCACAGCACAGGACCTCCACAGTAT	22	C	0	0.4787
NC_005101.4-262321412	TTTGCCCCCACTGCTTCTCCAATGATA	26	T	0.0345	0.0339
NC_005101.4-262445269	CCTGAATCTACCACCACTCCTGTTACT	22	G	0.0089	0.4789
NC_005101.4-262458136	GTACACAGGACTATTCCTCCTGCTGAT	4	C	0.1271	0.2406
NC_005101.4-262458136	GTACACAGGACTATTCCTCCTGCTGAT	6	C	0.1282	0.2423
NC_005101.4-262465503	ACCATATGGACATCTGCTCCACTTCAT	2	C	0.1833	0.4988
NC_005101.4-262465503	ACCATATGGACATCTGCTCCACTTCAT	6	A	0.15	0.1932
NC_005101.4-262516451	GCAGCCTTCACTGCAGCTCCTTTCCAC	6	C	0.0171	0.4734
NC_005101.4-262516451	GCAGCCTTCACTGCAGCTCCTTTCCAC	14	C	0.1795	0.2778
NC_005101.4-262598344	TTCCCTCATACCCTATCTCCAGTCTTT	15	A	0.1696	0.2758
NC_005101.4-262629960	ATAGTACCTACACATACTCCAGCAAAG	13	C	0.0513	0.0818

NC_005101.4-262629960	ATAGTACCTACACATACTCCAGCAAAG	26	A	0	0.4734
NC_005101.4-262655727	AATCACTAGACCAAGTCTCCGGTCCCC	21	G	0.1626	0.2723
NC_005101.4-262668478	TCAGTCATCACCCTCTCTCCACATCCT	15	C	0	0.4682
NC_005101.4-262668478	TCAGTCATCACCCTCTCTCCACATCCT	23	A	0	0.4682
NC_005101.4-262695381	CACCTGAACACCAGAGCTCCTGTGTGC	14	G	0.0492	0.048
NC_005101.4-262695381	CACCTGAACACCAGAGCTCCTGTGTGC	27	C	0.0574	0.4852
NC_005101.4-262698398	AAATTCTTAACCCATGCTCCTGTCTAT	26	A	0	0.0354
NC_005101.4-262703515	TTAAAGGGTACTTAAGCTCCCCAAACC	27	C	0.0992	0.0943
NC_005101.4-262716982	CCAGGAAAGACTATTTCTCCTGCTCTC	12	T	0.0265	0.0262
NC_005101.4-262757190	AGAAGTGTCACTATTGCTCCCCTAAGA	5	G	0.0513	0.4868
NC_005101.4-262757190	AGAAGTGTCACTATTGCTCCCCTAAGA	13	A	0.0513	0.4868
NC_005101.4-262764200	TCCGCCACTACCACTACTCCTTTATAT	4	G	0.0345	0.482
NC_005101.4-262764200	TCCGCCACTACCACTACTCCTTTATAT	15	T	0	0.4749
NC_005101.4-262835827	TTCTGAGAAACCATTGCTCCCTGATCG	27	G	0.0342	0.4553
NC_005101.4-262836441	TCCATCATTACTTACGCTCCTATCACT	15	C	0.1026	0.1274
NC_005101.4-262836441	TCCATCATTACTTACGCTCCTATCACT	16	G	0.1724	0.2854
NC_005101.4-262879366	TAACGCAACACAATCACTCCTCTATTA	5	G	0.1917	0.4525
NC_005101.4-262879366	TAACGCAACACAATCACTCCTCTATTA	12	A	0.1917	0.3708
NC_005101.4-262964199	CACAGTTTGACCCACCCTCCTGTGACT	7	T	0	0.4779
NC_005101.4-263124485	GCCTTCCTCACAGCTTCTCCTCAGCAT	26	A	0.036	0.0354
NC_005101.4-263149713	ATAGCAGGTACTCAAGCTCCGGGATAA	7	G	0.1311	0.4999
NC_005101.4-263168505	GGGCAAGTTACTTAACCTCCATAGCCC	15	A	0.812	0.4994
NC_005101.4-263267826	CACACACACACACAAGCTCCATGGCAG	6	A	0.0254	0.0415
NC_005101.4-263281412	CATCTTCAAACCATCCCTCCTATGGGA	26	G	0.0598	0.1347
NC_005101.4-263299919	TAAGCAATAACTTCATCTCCTGCTGAT	1	T	0.2609	0.3593
NC_005101.4-263328358	GAAGCTTTGACACCCCCTCCTCATCTT	13	C	0.0086	0.0255
NC_005101.4-263396565	CTGATCTCTACTGAATCTCCCAGGGTT	2	T	0.2083	0.2833
NC_005101.4-263396565	CTGATCTCTACTGAATCTCCCAGGGTT	4	A	0	0.4734
NC_005101.4-263408086	CCTCTGTGCACACAAGCTCCTCTGTGA	27	A	0.0244	0.0241
NC_005101.4-263485675	AGCGTATACACAGAACCTCCCTTCCAC	4	G	0.2927	0.4759
NC_005101.4-263539841	GTAGATGAAACACTGCCTCCCCTTTCC	15	G	0	0.0484
NC_005101.4-263539841	GTAGATGAAACACTGCCTCCCCTTTCC	16	C	0.0738	0.071
NC_005101.4-263547946	AGCCAAGACACAGCATCTCCTGTCTCT	26	C	0.084	0.4873
NC_005101.4-263574107	CATGGTATCACAGGCACTCCAGCCTCT	9	C	0	0.4807
NC_005101.4-263575894	GCAGAATGCACATCTGCTCCACAATGA	23	A	0.0948	0.0903
NC_005101.4-263609088	AGCTGCCTAACACTAGCTCCCCATGTA	3	C	0.042	0.4542
NC_005101.4-263612156	TCTCATCGTACTCCTTCTCCTTCTTGT	7	C	0.1681	0.1813
NC_005101.4-263653419	AGTGTGAGAACTTGAACTCCAGGGCTC	1	A	0.2583	0.4922
NC_005101.4-263653419	AGTGTGAGAACTTGAACTCCAGGGCTC	2	G	0.2583	0.4922
NC_005101.4-263683019	TGAAGCCCCACGAATCCTCCTTCCTTT	5	G	0.0089	0.4789
NC_005101.4-263725286	CCCCTCAGTACTTTCTCTCCCCATTTC	4	C	0.2389	0.4021
NC_005101.4-263725758	CATGATGTGACATACCCTCCCTCGTGG	23	C	0.0339	0.0333
NC_005101.4-263725758	CATGATGTGACATACCCTCCCTCGTGG	24	G	0	0.4757
NC_005101.4-263790605	TGAATTTCAACCCTTTCTCCTCCCACT	13	C	0	0.4786
NC_005101.4-263856070	TTGTCCTGAACCTGGTCTCCTTCATCT	27	T	0	0.4832
NC_005101.4-263862755	AGAGGATCCACACCTTCTCCTGGCCTC	3	A	0.2787	0.4463
NC_005101.4-263897624	CTTCTTTTTACCCACTCTCCCTGATTA	6	T	0	0.4793
NC_005101.4-263903929	TAGACAGCTACACCTACTCCAGTAACC	1	T	0.2586	0.3916
NC_005101.4-264007892	TGGGAGTTCACATCTCCTCCTGCTCCT	6	G	0.0446	0.4751
NC_005101.4-264028175	AATAAACACACTTATCCTCCACAATTG	1	A	0.1933	0.397
NC_005101.4-264028175	AATAAACACACTTATCCTCCACAATTG	24	A	0.0084	0.4723

NC_005101.4-264036497	AGGCAATGCACAGTATCTCCTTTAGTG	7	T	0.2101	0.491
NC_005101.4-264070410	CTTGAGAACACACCTACTCCAGAGCTC	16	A	0.025	0.4816
NC_005101.4-264149865	TTGTCCTTGACCTCCCCTCCCCAAGCA	15	C	0.1607	0.3838
NC_005101.4-264151552	TTTAGCCACACCCTTGCTCCTGTAGAG	3	T	0.2	0.4395
NC_005101.4-264154430	CTCACGGGAACTCTCTCTCCATGTTCT	6	G	0.1316	0.375
NC_005101.4-264158075	TCTGACAGTACAAAGCCTCCTGGCTTC	6	C	0.0348	0.4915
NC_005101.4-264158075	TCTGACAGTACAAAGCCTCCTGGCTTC	16	C	0.0348	0.0342
NC_005101.4-264164526	ACCACAAAAACCAGCTCTCCTATGCTG	8	A	0.2479	0.3
NC_005101.4-264231382	AGACAGAAGACCAAGGCTCCTTAAGGG	6	G	0.2589	0.4394
NC_005101.4-264232012	CTCCAGATTACTATACCTCCAATCCAG	1	C	0.2051	0.4772
NC_005101.4-264241634	ATCTCTTGCACTCTTCCTCCTGCTTTG	1	A	0.1557	0.432
NC_005101.4-264241928	AACACCTACACCTTGTCTCCTCTCCAG	23	T	0.0167	0.48
NC_005101.4-264241928	AACACCTACACCTTGTCTCCTCTCCAG	26	A	0.1176	0.4997
NC_005101.4-264451263	TGCCAACCAACCAGATCTCCTCAGGAC	6	A	0	0.4807
NC_005101.4-264473940	TATGAGGATACTCCCCCTCCTACTCAC	27	C	0.25	0.375
NC_005101.4-264509145	GCAGGAACAACTGCTTCTCCTCCCACT	13	G	0.178	0.1621
NC_005101.4-264525839	GTGTGTTTTACCTACACTCCATCCAAT	26	A	0.0265	0.0262
NC_005101.4-264547703	TGTGCCATGACATATGCTCCTGTTCCA	1	T	0.0522	0.0832
NC_005101.4-264554156	GCACTCTCCACATCTTCTCCAGCATCT	23	C	0.2033	0.3287
NC_005101.4-264560678	ACCTCTCTGACTCCATCTCCATACAAT	24	C	0.025	0.0408
NC_005101.4-264634217	TAGCATAGCACCCTTTCTCCTGAGTCT	12	C	0.0431	0.0422
NC_005101.4-264634217	TAGCATAGCACCCTTTCTCCTGAGTCT	14	T	0.0424	0.0415
NC_005101.4-264809580	CATGCGTCCACCCTTTCTCCATTCATG	6	G	0.0085	0.481
NC_005101.4-264925956	TCTTCTGTGACCATTTCTCCTTCCCAC	13	A	0.1017	0.1409
NC_005101.4-264947654	TGCCTGCCCACCCACTCTCCTGGGCTA	9	C	0.0252	0.0249
NC_005101.4-264949414	CACACATGCACTCCTCCTCCCACTACA	27	A	0.0609	0.059
NC_005101.4-265038237	GGGCGATGAACTCTCTCTCCAATGGGG	6	A	0.0877	0.4945
NC_005101.4-265038237	GGGCGATGAACTCTCTCTCCAATGGGG	23	T	0	0.4814
NC_005101.4-265069109	AGACCATGGACAGATGCTCCAGTCAGA	14	A	0.087	0.0832
NC_005101.4-265222876	CTCACTAGCACGGTGTCTCCTTTTGTT	21	T	0.0917	0.1172
NC_005101.4-265229792	TATCAATCAACCAGAGCTCCCACGGAC	16	G	0.0496	0.0484
NC_005101.4-265326912	ATGCTTGCAACACGTTCTCCAACTCTG	14	G	0.0492	0.0935
NC_005101.4-265394800	TCATGAACTACACTGTCTCCAAGTAAC	14	T	0	0.4641
NC_005101.4-265533473	ATAACAAGGACACAAGCTCCACTCTCA	4	A	0.0609	0.059
NC_005101.4-265533473	ATAACAAGGACACAAGCTCCACTCTCA	13	C	0.0174	0.0508
NC_005101.4-265538802	CTCCAAGGCACACCTTCTCCACAAGGT	22	C	0.0656	0.0935
NC_005101.4-265804949	ATCATTTTTACCTCTGCTCCAGGCACT	4	A	0.2269	0.3367
NC_005101.4-265826750	ATGAGCTAGACCCTGTCTCCTGTGTTG	6	C	0.0526	0.4925
NC_005101.4-265826750	ATGAGCTAGACCCTGTCTCCTGTGTTG	12	C	0.0526	0.4925
NC_005101.4-265853331	CTCTTTCACACTTTCCCTCCCTTCCTA	24	C	0.1983	0.3467
NC_005101.4-265866703	GGACCAAGCACCTCCCCTCCCACTGTT	4	C	0.0574	0.071
NC_005101.4-265891441	TACCAGCCAACCAGAGCTCCAGGGACT	25	A	0.4298	0.3557
NC_005101.4-265891917	ATTCATCTAACCTGAACTCCTCTAGTC	2	T	0.0593	0.4919
NC_005101.4-265903099	TCCAGGAATACTCTCACTCCTAAGCCC	13	C	0.2114	0.3516
NC_005101.4-265967487	GACTTTAATACTTCACCTCCAGTTGTT	1	G	0.0957	0.1219
NC_005101.4-265967487	GACTTTAATACTTCACCTCCAGTTGTT	15	A	0.0957	0.4986
NC_005101.4-265982142	CTCATCATGACTAAGTCTCCCATGTAT	26	A	0	0.477
NC_005101.4-266008741	CATACCAGCACCTAGTCTCCCTAAGCT	3	T	0.1833	0.2665
NC_005101.4-266140514	CATGAATGAACAGAACCTCCAATGAGT	2	A	0.1121	0.4982
NC_005101.4-266140514	CATGAATGAACAGAACCTCCAATGAGT	12	A	0.1121	0.4982
NC_005101.4-266293534	ATATCACTAACTCAGCCTCCCTCTCCC	1	A	0	0.4807

NC_005101.4-266293534	ATATCACTAACTCAGCCTCCCTCTCCC	25	C	0	0.4807
NC_005102.4-67224	CCTCTGGGTACCAAACCTCCTCAGCCT	3	T	0.0167	0.4688
NC_005102.4-126105	ATGGACTGAACCTGGACTCCAACCTCA	22	A	0.0488	0.0476
NC_005102.4-368249	TTTCAAGCTACAAGCTCTCCCCAAGTG	27	G	0.1597	0.4987
NC_005102.4-550892	ACTTCCATTACCCAGGCTCCATTTGAG	3	T	0.8348	0.4891
NC_005102.4-550892	ACTTCCATTACCCAGGCTCCATTTGAG	15	G	0.8348	0.4891
NC_005102.4-745251	CTTCATTCTACTCAACCTCCTGCGCAC	21	T	0.0407	0.4893
NC_005102.4-745251	CTTCATTCTACTCAACCTCCTGCGCAC	24	G	0	0.4793
NC_005102.4-818659	TCTCCTACGACTTGTCCTCCACCTTCA	9	G	0	0.4773
NC_005102.4-818921	CAAGGTGGAACTAGGCCTCCCTGCACA	5	G	0	0.477
NC_005102.4-903252	GCAACACAGACCTGGCCTCCAGGTTCT	15	G	0.0804	0.0771
NC_005102.4-1217205	TCATAAAGAACCTCAACTCCAATATTC	1	T	0	0.4846
NC_005102.4-1217205	TCATAAAGAACCTCAACTCCAATATTC	4	T	0	0.4846
NC_005102.4-1247579	ACCACTTGCACCTTGTCTCCATGCTCT	2	C	0.1579	0.4975
NC_005102.4-1265222	TAAGCCTAGACTCTATCTCCCAGGCAG	15	A	0.082	0.1505
NC_005102.4-1270374	TGGCTGAGAACAGGGTCTCCAATGGAG	27	G	0.0171	0.4868
NC_005102.4-1273607	CTGCTCTAAACTTCACCTCCATATCTC	22	T	0.1795	0.1634
NC_005102.4-1434619	CTTTAGCCCACACTGCCTCCCACCTGG	22	A	0.178	0.3914
NC_005102.4-1435021	TGGCAGAAAACCATTTCTCCTCTATGT	4	C	0.1696	0.4119
NC_005102.4-1435021	TGGCAGAAAACCATTTCTCCTCTATGT	7	A	0.0702	0.0677
NC_005102.4-1505655	TGCTTCTCAACTTAGCCTCCCTGTTCT	3	C	0	0.4826
NC_005102.4-1505655	TGCTTCTCAACTTAGCCTCCCTGTTCT	27	T	0	0.4826
NC_005102.4-1517713	TCTTATTCAACTGTGGCTCCCCTATTC	9	A	0	0.4793
NC_005102.4-1535692	GTTCTTACCACCCCACCTCCTGAGGCT	15	A	0	0.4807
NC_005102.4-1541495	CCAAGCCAAACCTTGACTCCTTCAGAC	24	A	0.124	0.1585
NC_005102.4-1543437	GAGGAAGCCACCCTTTCTCCTGTGAAG	12	C	0.0165	0.0639
NC_005102.4-1549712	AACCCCTAAACATGAGCTCCTTCCTTC	16	G	0.0083	0.4803
NC_005102.4-1560201	TAAATTATGACCTTCACTCCCCTATGT	7	A	0	0.4779
NC_005102.4-1618144	GGGACATCAACATAGACTCCAGTTGCC	3	G	0.0348	0.0342
NC_005102.4-1619682	TGAATTCTCACAGGGACTCCTCTTAGA	27	A	0.0439	0.0758
NC_005102.4-1641129	CACCACTTTACCCTGCCTCCCTAGTTG	14	T	0.0179	0.4898
NC_005102.4-1641129	CACCACTTTACCCTGCCTCCCTAGTTG	23	A	0	0.4841
NC_005102.4-1643003	AACCCTGATACAGGATCTCCCTGCTTC	8	A	0.0569	0.1145
NC_005102.4-1643003	AACCCTGATACAGGATCTCCCTGCTTC	9	T	0.0569	0.1145
NC_005102.4-1671569	CTGATATCAACCAAGGCTCCCTACTTT	4	A	0.123	0.4973
NC_005102.4-1681992	TTCCCCGAAACAGTCCCTCCCTGGTCA	7	G	0.0541	0.4909
NC_005102.4-1681992	TTCCCCGAAACAGTCCCTCCCTGGTCA	25	T	0.1532	0.3338
NC_005102.4-1778236	CCCACAGGGACAACTGCTCCTTGTTTA	21	T	0.1053	0.4994
NC_005102.4-1778236	CCCACAGGGACAACTGCTCCTTGTTTA	23	G	0.0263	0.026
NC_005102.4-1850852	CGTCCATCTACCAAGTCTCCAGCCTCT	1	C	0.1966	0.2423
NC_005102.4-1852809	ACTATCTCTACCTTCACTCCGAAATGT	2	C	0.2035	0.2618
NC_005102.4-1903340	CATCCACCTACACTGGCTCCTTGGGAT	21	T	0.0488	0.0476
NC_005102.4-1903340	CATCCACCTACACTGGCTCCTTGGGAT	26	A	0.1545	0.3964
NC_005102.4-1915637	TCTCTGTACACAAGTGCTCCCCTAGAT	1	T	0	0.4746
NC_005102.4-1918873	CCCACCTACACCAGGCCTCCATGGAAG	15	G	0.0593	0.0889
NC_005102.4-1918873	CCCACCTACACCAGGCCTCCATGGAAG	22	T	0.0593	0.0734
NC_005102.4-1919102	GTTCCCAGCACAGCAGCTCCAGAGTCC	15	A	0.1138	0.1494
NC_005102.4-1997286	CCACATTCTACACTGTCTCCTTTAGCA	2	C	0	0.4751
NC_005102.4-1997286	CCACATTCTACACTGTCTCCTTTAGCA	13	C	0.1736	0.4803
NC_005102.4-2027627	CCATTGACGACAAAAACTCCAAACAGA	6	G	0.0431	0.0585
NC_005102.4-2055352	AGGTGACCAACTGGTGCTCCTTAGGT	27	T	0.0082	0.479

NC_005102.4-2101295	TGTGGCTAGACTGGAACTCCCTATATA	1	T	0.5405	0.461
NC_005102.4-2101295	TGTGGCTAGACTGGAACTCCCTATATA	2	G	0.5405	0.461
NC_005102.4-2129892	TTTATTTATACAGTCTCTCCAGGCCCC	25	C	0.0789	0.1075
NC_005102.4-2175452	AGCACACAGACTGTTTCTCCACGCTTA	23	G	0.1864	0.4826
NC_005102.4-2177711	CCTTCCATGACATCTCCTCCATGCTCT	1	C	0.0088	0.472
NC_005102.4-2233753	CCCGATCTCACTTTCCCTCCTCTCTCC	6	T	0	0.4793
NC_005102.4-2260906	ACAGCCCTGACCAGCCCTCCATGCATG	4	G	0	0.4807
NC_005102.4-2260906	ACAGCCCTGACCAGCCCTCCATGCATG	26	T	0.0598	0.0896
NC_005102.4-2304670	GTGGTCCTCACACCAGCTCCCCAGTTA	27	A	0.1333	0.2188
NC_005102.4-2381686	AGAGTTAGGACCTGGCCTCCTCCAGGC	3	A	0	0.4703
NC_005102.4-2381686	AGAGTTAGGACCTGGCCTCCTCCAGGC	9	G	0.0924	0.214
NC_005102.4-2406983	GGTGATGTAACTCTCACTCCACTTCCC	1	G	0	0.4793
NC_005102.4-2438844	CAAAGCTAGACGATCTCTCCGTAGGAA	25	G	0.0488	0.078
NC_005102.4-2478482	AGGCTCTGGACCCAATCTCCGACACTA	15	A	0	0.474
NC_005102.4-2516826	ATCCACCCAACTCTGCCTCCTGAATAC	12	T	0.087	0.1869
NC_005102.4-2527890	GTGTTAATAACCAGCCCTCCTTCCCAG	3	G	0.0488	0.0476
NC_005102.4-2562670	TGGAATTAAACTCAGGCTCCCGGCATT	22	G	0.0813	0.0928
NC_005102.4-2562670	TGGAATTAAACTCAGGCTCCCGGCATT	23	G	0	0.4793
NC_005102.4-2562804	TTCTTTCATACTTGCACTCCCTTTATC	9	T	0.0424	0.0415
NC_005102.4-2581524	GTGGGGAGAACTCCTCCTCCCGACACA	26	C	0.0833	0.4932
NC_005102.4-2584857	CCAGACCTAACTTAGCCTCCTGTCTGT	13	T	0	0.4698
NC_005102.4-2613497	ATTGTCCAGACAACATCTCCAAGCCCA	2	T	0.178	0.487
NC_005102.4-2613497	ATTGTCCAGACAACATCTCCAAGCCCA	16	T	0.0085	0.481
NC_005102.4-2617160	CTGTGTGTAACTTGTCCTCCTCTTCTG	4	T	0.1475	0.2032
NC_005102.4-2617160	CTGTGTGTAACTTGTCCTCCTCTTCTG	24	T	0.1557	0.4959
NC_005102.4-2636579	GTAAGCCTAACCGTATCTCCCCACCAG	12	C	0.1545	0.2323
NC_005102.4-2636579	GTAAGCCTAACCGTATCTCCCCACCAG	21	C	0	0.4773
NC_005102.4-2664897	CCATCATGTACCCGGCCTCCTTCTGGT	14	G	0.0738	0.1009
NC_005102.4-2671362	GCCCGCACTACTGCCCCTCCCTTTACT	4	C	0.1696	0.2512
NC_005102.4-2679308	CTCCCCTGGACACCGTCTCCTGACCCC	25	C	0.0496	0.0484
NC_005102.4-2687717	CCTTCCTGAACACTAACTCCAAACCAT	27	T	0	0.4814
NC_005102.4-2692711	CTTCGGGGGACCTAGGCTCCCATCCTC	7	G	0.0603	0.0585
NC_005102.4-2702879	GCCCTATCCACAGGAGCTCCATTGAGT	14	G	0.0656	0.1225
NC_005102.4-2702879	GCCCTATCCACAGGAGCTCCATTGAGT	23	T	0.0656	0.1505
NC_005102.4-2733003	TGTTGCTATACGAGCCCTCCTGTGGTT	2	G	0	0.4793
NC_005102.4-2733003	TGTTGCTATACGAGCCCTCCTGTGGTT	7	T	0	0.4793
NC_005102.4-2749613	CTGCAAGGGACCTGGGCTCCTGATGCT	15	G	0.0081	0.0241
NC_005102.4-2764986	CCAGCCTACACTTGGTCTCCACAGTAA	22	C	0.2602	0.4072
NC_005102.4-2775408	CCCATCTGCACCAGTTCTCCTCACACT	2	C	0	0.4793
NC_005102.4-2806208	CCTGCGTACACAGCAGCTCCCTAGCAT	4	G	0	0.4793
NC_005102.4-2806208	CCTGCGTACACAGCAGCTCCCTAGCAT	22	T	0.2033	0.3381
NC_005102.4-2842434	CAGCTGCGCACCTTACCTCCCTAATGT	7	C	0.0431	0.0422
NC_005102.4-2842434	CAGCTGCGCACCTTACCTCCCTAATGT	24	A	0	0.4851
NC_005102.4-2864781	CTGATACTGACTGTAGCTCCTTTGCAG	12	T	0	0.4875
NC_005102.4-2888484	GTCGTGGTGACTCTTACTCCAGATAAC	3	C	0.1639	0.1774
NC_005102.4-2888754	ATCGGTTGCACTTCCTCTCCTCACTGG	8	G	0	0.482
NC_005102.4-2908861	GGCCGGGCCACGCCAGCTCCTCCGCAC	4	C	0.1311	0.1367
NC_005102.4-2943802	CCTCCACAGACTGTTTCTCCAGCTTGC	24	T	0.2478	0.2797
NC_005102.4-3183736	CCAAAGCAGACCTGCACTCCCAGCATC	3	A	0.0261	0.0257
NC_005102.4-3249215	AGAAACTCTACGCCCCCTCCCCCAACT	12	G	0.0744	0.1017
NC_005102.4-3251001	AGGTTAGCAACAGAACCTCCCCTACTA	26	T	0.1271	0.298

NC_005102.4-3256328	AGTCGCTCCACTGCCTCTCCTGGCTTA	26	T	0	0.4773
NC_005102.4-3256328	AGTCGCTCCACTGCCTCTCCTGGCTTA	27	A	0.0738	0.4943
NC_005102.4-3272800	CCATGCCCCACAGGACCTCCACGCACT	13	G	0	0.4819
NC_005102.4-3274013	GAGTACAAGACAGCAGCTCCGGGGTGT	21	G	0	0.477
NC_005102.4-3278923	CCATTCATCACCTGTCCTCCATAAGCC	3	A	0.3782	0.3067
NC_005102.4-3295187	TGCCTACCCACAGGAGCTCCGCATCCA	25	C	0.0984	0.1505
NC_005102.4-3303206	ACCTTTTAAACCATCTCTCCTGTGCCT	21	T	0	0.4832
NC_005102.4-3303206	ACCTTTTAAACCATCTCTCCTGTGCCT	22	G	0.0917	0.1172
NC_005102.4-3314237	CCCTCCAGAACGGCCCCTCCAAGTTTC	21	A	0.0325	0.4926
NC_005102.4-3328674	AAGGCCCCCACCTCCCCTCCCGGCTTT	22	G	0.0678	0.0812
NC_005102.4-3367574	GATAGCAGCACTATCTCTCCATCTTGA	13	A	0	0.4793
NC_005102.4-3373715	CACCAGGCCACATGGCCTCCACAGACT	13	T	0.0179	0.4898
NC_005102.4-3373715	CACCAGGCCACATGGCCTCCACAGACT	25	A	0	0.4871
NC_005102.4-3384990	TCATGTCCAACCACCTCTCCATCCTCA	27	A	0	0.4807
NC_005102.4-3394775	AGTCAGCCCACGAGGCCTCCTGACTAG	27	G	0.0508	0.4977
NC_005102.4-3400366	CACCAGCCCACATTTTCTCCAGAAGGA	23	A	0.0661	0.4983
NC_005102.4-3400366	CACCAGCCCACATTTTCTCCAGAAGGA	26	G	0.0661	0.4983
NC_005102.4-3471592	GTCCCACACACAATCCCTCCACACTTT	8	A	0.0684	0.5
NC_005102.4-3473769	TTCCTCCACACTGCAGCTCCATCATAC	16	G	0.2083	0.325
NC_005102.4-3496893	ATACCTGTGACCCAGTCTCCACACCCA	23	A	0	0.4902
NC_005102.4-3528393	CCCTCTGGAACTGTAACTCCAAACAAA	6	T	0.2602	0.377
NC_005102.4-3528393	CCCTCTGGAACTGTAACTCCAAACAAA	7	G	0.1951	0.3239
NC_005102.4-3549264	CATCTTCCCACTTGCTCTCCTATCCCA	13	T	0	0.4793
NC_005102.4-3549264	CATCTTCCCACTTGCTCTCCTATCCCA	22	A	0.0254	0.4896
NC_005102.4-3556212	CCATATTAAACTCTGCCTCCAATAATT	3	A	0.1667	0.4999
NC_005102.4-3556212	CCATATTAAACTCTGCCTCCAATAATT	26	T	0.0984	0.0935
NC_005102.4-3596728	ATCCGTAACACAGTTACTCCTTTACCT	4	C	0	0.48
NC_005102.4-3609480	CACACACACACACACACTCCTACATTT	9	C	0.0259	0.0255
NC_005102.4-3617066	TTGTGGCCTACCTGTACTCCTTTACAT	25	C	0	0.4839
NC_005102.4-3619131	CTCTTGCACACCTTGGCTCCCAACTCC	12	C	0.0246	0.0243
NC_005102.4-3637279	TGCCACCCAACAGTCTCTCCATGGGAC	5	A	0.2358	0.2885
NC_005102.4-3667205	ACAAATTGAACTAGATCTCCAGTCCCT	9	A	0.2566	0.2618
NC_005102.4-3667205	ACAAATTGAACTAGATCTCCAGTCCCT	22	G	0	0.4807
NC_005102.4-3685978	GAACAGAGCACAAGTCCTCCAGGGACA	12	A	0.1	0.1387
NC_005102.4-3685978	GAACAGAGCACAAGTCCTCCAGGGACA	25	A	0	0.4844
NC_005102.4-3689499	CCCCTAAGTACCCTCTCTCCTGTTAGA	24	T	0.1017	0.1409
NC_005102.4-3697653	CCAAGCGAAACGCCACCTCCTACAACC	12	G	0.0089	0.491
NC_005102.4-3716033	GTGCTGGGAACTAAACCTCCCTCCTCT	13	A	0.0336	0.1254
NC_005102.4-3727402	TGGGCTTGCACAGCCTCTCCTCTTCTA	12	A	0.1966	0.4293
NC_005102.4-3728363	ACCTGATGCACCCAGACTCCTGGCTGT	25	T	0.0328	0.048
NC_005102.4-3734877	AGAGAGGATACCCACCCTCCAGAGCAA	27	A	0	0.4793
NC_005102.4-3752998	AACACAGACACCCCTACTCCAGGGGCA	9	C	0.2314	0.4751
NC_005102.4-3775279	CTTTCGGTGACTCTTTCTCCCTGACAT	6	G	0.1736	0.2355
NC_005102.4-3775522	CTGTGTCCTACAGGGCCTCCCTCTCGC	1	C	0.0672	0.0958
NC_005102.4-3775522	CTGTGTCCTACAGGGCCTCCCTCTCGC	6	T	0.0667	0.095
NC_005102.4-3781453	CTCATCCTAACTGAACCTCCAAGCTAA	3	C	0.0084	0.4829
NC_005102.4-3781453	CTCATCCTAACTGAACCTCCAAGCTAA	22	A	0.161	0.3914
NC_005102.4-3785465	CACCATGTCACTGCTACTCCAGCCTGG	6	T	0	0.4825
NC_005102.4-3795683	TGCAACTGCACCCGGCCTCCCACGGCC	6	C	0	0.4793
NC_005102.4-3796709	ACCCGGCGTACCTCCTCTCCTCTGGCT	3	C	0.0826	0.1235
NC_005102.4-3796709	ACCCGGCGTACCTCCTCTCCTCTGGCT	12	C	0.0254	0.0251

NC_005102.4-3800006	CCTCTCACCACCCACCCTCCGCTGCCC	6	C	0.1083	0.1866
NC_005102.4-3806547	CCTGTAGGTACTGCTGCTCCAGGGTCC	26	C	0	0.482
NC_005102.4-3810789	CACACAAGCACCTTGGCTCCCCGTCAG	14	T	0.1333	0.4061
NC_005102.4-3810789	CACACAAGCACCTTGGCTCCCCGTCAG	23	G	0	0.48
NC_005102.4-3819012	GTCACCAGGACCTCCCCTCCCCAACTT	8	G	0.1261	0.2012
NC_005102.4-3821825	ACCAACCACACTCACCCTCCAGGGTAC	5	A	0.2358	0.3472
NC_005102.4-3830204	ACCCCTTGGACCTGGGCTCCCTCTGCT	1	A	0.2167	0.48
NC_005102.4-3833035	CTTCCTCCGACCTTTCCTCCAAGTACC	8	C	0	0.4793
NC_005102.4-3842309	CTCCAACCCACAGACCCTCCTGCCTCT	16	C	0.0246	0.0243
NC_005102.4-3851791	ATCACTGTAACCCTAGCTCCAGAGGAT	4	A	0	0.4793
NC_005102.4-3867233	ATCCCTGTAACTGCAGCTCCAGGGACT	1	A	0	0.4787
NC_005102.4-3869793	GAGGTCTGGACCTCATCTCCTGGAGGG	6	C	0	0.4773
NC_005102.4-3884643	TCCCCCACCACAGCATCTCCCCAGCAC	6	C	0.0164	0.4807
NC_005102.4-3924584	GTTGACATTACACATGCTCCCTGTGGG	25	G	0.1983	0.3793
NC_005102.4-3924637	CACCCAGAGACCAACCCTCCTGGAGCT	13	A	0	0.4772
NC_005102.4-3926274	TGACTGCTCACATGGGCTCCCTAGAGG	12	A	0.1707	0.2989
NC_005102.4-3926672	CTCGGGAGCACCGCAGCTCCTGTTTAT	6	G	0.1504	0.2494
NC_005102.4-3991473	ATCCACAGGACGCAAGCTCCCCTGCCT	25	C	0.025	0.0247
NC_005102.4-4013166	CTGAGAGCAACATCAGCTCCGGAGGCA	15	A	0	0.4764
NC_005102.4-4013166	CTGAGAGCAACATCAGCTCCGGAGGCA	21	G	0	0.4764
NC_005102.4-4027589	AGCAGGGCCACCGCTGCTCCGGAAAAC	12	C	0.1626	0.3141
NC_005102.4-4027589	AGCAGGGCCACCGCTGCTCCGGAAAAC	21	G	0.0081	0.4777
NC_005102.4-4028076	CAGGTCAGAACTGTCACTCCCACCAGG	2	A	0.1624	0.479
NC_005102.4-4051920	CCATCTATGACCTGGCCTCCATGTTTC	7	A	0.0171	0.4693
NC_005102.4-4051920	CCATCTATGACCTGGCCTCCATGTTTC	27	C	0.0171	0.4693
NC_005102.4-4052810	CCACCCAACACTCACCCTCCACAGATG	16	C	0.0342	0.0336
NC_005102.4-4055773	TAGCCGGGCACCGCAGCTCCTCAAGGG	6	G	0	0.4764
NC_005102.4-4064855	GACTTCAGCACTCCCCCTCCCCCATTC	1	G	0.1382	0.2203
NC_005102.4-4068707	GTGATACACACCTTTACTCCTAGCACT	7	C	0.1597	0.2509
NC_005102.4-4139326	CACCTCTAAACCATGACTCCATCCCAG	21	A	0.1429	0.3565
NC_005102.4-4236765	TTTGGTTCCACTGACTCTCCTTCTTTC	6	T	0.122	0.5
NC_005102.4-4244679	GTCAAGCTGACTCCATCTCCAAAGCAG	21	A	0	0.4703
NC_005102.4-4260365	GGACAGGGCACCCTCTCTCCTATCTTA	13	C	0.0492	0.0634
NC_005102.4-4260365	GGACAGGGCACCCTCTCTCCTATCTTA	27	A	0.2397	0.4732
NC_005102.4-4320728	CTGGGGAACACATCAGCTCCATGTGTC	5	G	0	0.4773
NC_005102.4-4320728	CTGGGGAACACATCAGCTCCATGTGTC	21	A	0	0.4773
NC_005102.4-4355701	TGTTTGGAGACAGGGTCTCCCAATTTA	15	G	0.027	0.0267
NC_005102.4-4356071	AGCTTCACTACAGGGTCTCCTCTTTTC	14	G	0.2973	0.4985
NC_005102.4-4846651	TGGAGTACTACTCAGTCTCCATCTCTT	24	T	0.0496	0.109
NC_005102.4-4846651	TGGAGTACTACTCAGTCTCCATCTCTT	25	C	0.0496	0.109
NC_005102.4-5092123	CCTAGTGAAACTTTGTCTCCCAAAATA	13	T	0.188	0.4602
NC_005102.4-5092123	CCTAGTGAAACTTTGTCTCCCAAAATA	23	A	0.193	0.2413
NC_005102.4-5167904	CTTCAGCACACAGAGCCTCCTGGTCCA	26	C	0.2417	0.4922
NC_005102.4-5240683	CACAGTGAGACACTTACTCCATCATGG	2	A	0.0488	0.0476
NC_005102.4-5331360	ATATGACAAACTACAGCTCCAAATGCA	13	A	0	0.48
NC_005102.4-5405869	AGTGGGTACACTCGAGCTCCTTACTAC	13	C	0.4865	0.3772
NC_005102.4-5416685	ATCCACAGAACCTTGTCTCCACCCATT	15	G	0.2439	0.2142
NC_005102.4-5416685	ATCCACAGAACCTTGTCTCCACCCATT	23	C	0.3008	0.2556
NC_005102.4-5521526	GTTTATGAAACAACCACTCCAAGGCTT	8	A	0	0.4793
NC_005102.4-5521526	GTTTATGAAACAACCACTCCAAGGCTT	24	G	0.2276	0.2937
NC_005102.4-5522743	GTCTTACAGACTCCACCTCCCTGTGGT	26	G	0.1967	0.4656

NC_005102.4-5539894	GGCTGTACCACCGCATCTCCCTTTACA	2	G	0	0.4832
NC_005102.4-5539894	GGCTGTACCACCGCATCTCCCTTTACA	24	T	0	0.4832
NC_005102.4-5557599	CACTCACCAACAAGTGCTCCTGGCACT	8	C	0.1736	0.2474
NC_005102.4-5557599	CACTCACCAACAAGTGCTCCTGGCACT	25	A	0	0.4807
NC_005102.4-5619045	CCTAAGTCAACCTCGGCTCCCCACGAG	1	C	0	0.4854
NC_005102.4-5619045	CCTAAGTCAACCTCGGCTCCCCACGAG	15	G	0.0261	0.0257
NC_005102.4-5620077	GGTCCTGACACTTCCTCTCCTTTGTCC	26	C	0.0268	0.0264
NC_005102.4-5633131	TGACCTGTGACTGAAGCTCCACCTGGG	7	G	0.0169	0.0333
NC_005102.4-5637519	GTGGATCTCACTCACTCTCCCCTCCCC	6	T	0.0174	0.48
NC_005102.4-5643407	TGGCCTAGGACCCAACCTCCCTCCTTT	2	G	0.0435	0.059
NC_005102.4-5643407	TGGCCTAGGACCCAACCTCCCTCCTTT	7	A	0.0531	0.4827
NC_005102.4-5649255	AAGAACTGCACGGGACCTCCCTGTGAT	15	A	0	0.4787
NC_005102.4-5658275	AGCTCGCGGACGGCAGCTCCACATCTT	2	G	0.0261	0.0257
NC_005102.4-5658275	AGCTCGCGGACGGCAGCTCCACATCTT	6	G	0.1652	0.1659
NC_005102.4-5664212	CTTCAGGACACAGACTCTCCTTCACAG	2	T	0	0.4846
NC_005102.4-5664212	CTTCAGGACACAGACTCTCCTTCACAG	24	A	0	0.4846
NC_005102.4-5690128	TATGCATTCACAACTACTCCAACCCAC	13	A	0.0089	0.0436
NC_005102.4-5715515	TTGGGGGTCACGAAAGCTCCTTGCATC	8	T	0.0083	0.4803
NC_005102.4-5731743	TTCAACTCCACAGGACCTCCCTTGCCT	6	C	0.1043	0.2873
NC_005102.4-5764939	TAAGCTGGCACCGGAACTCCTGATCCT	23	A	0	0.4755
NC_005102.4-5805020	GGTGTGAGAACTGTACCTCCTAGAGGA	14	T	0.1008	0.2076
NC_005102.4-5827115	CACGCATAGACCCAGCCTCCTATCTAT	2	A	0	0.4793
NC_005102.4-5827115	CACGCATAGACCCAGCCTCCTATCTAT	3	C	0.0325	0.032
NC_005102.4-5838095	GCAGCTTGAACTTGCCCTCCTGGACCT	14	G	0.0254	0.0251
NC_005102.4-5838095	GCAGCTTGAACTTGCCCTCCTGGACCT	23	G	0.0252	0.0249
NC_005102.4-5858337	CCCAAGCTAACCTGCACTCCTCCTCAC	9	A	0.018	0.4785
NC_005102.4-5858337	CCCAAGCTAACCTGCACTCCTCCTCAC	27	C	0.2162	0.4854
NC_005102.4-5869482	ATGGCCTCCACCGCAGCTCCCCCGCCC	1	A	0.0446	0.0605
NC_005102.4-5869482	ATGGCCTCCACCGCAGCTCCCCCGCCC	2	T	0.0088	0.4843
NC_005102.4-5875372	ACACTTAAGACAAATCCTCCAGAGACC	23	A	0.1186	0.4977
NC_005102.4-5875372	ACACTTAAGACAAATCCTCCAGAGACC	27	C	0.075	0.0875
NC_005102.4-5881130	CCGGCCAGGACAAAGACTCCTCCTACT	2	C	0.0744	0.0716
NC_005102.4-5881130	CCGGCCAGGACAAAGACTCCTCCTACT	24	T	0.175	0.3047
NC_005102.4-5900398	TTAAGGCCCACAGGCTCTCCCTGGGAC	14	G	0.0268	0.0264
NC_005102.4-5904439	TCTCCCTGTACACAGGCTCCAGTCACC	22	G	0.2655	0.406
NC_005102.4-5919150	CAGGACCTCACATCGCCTCCCTGGAGC	15	G	0.1186	0.1827
NC_005102.4-5919200	CCACTTCCTACGCCTTCTCCCTGGGGG	2	C	0.025	0.0247
NC_005102.4-5919200	CCACTTCCTACGCCTTCTCCCTGGGGG	26	G	0.125	0.1172
NC_005102.4-5948297	CCTCCAAGCACCCCTTCTCCTGTGCGA	25	C	0.0442	0.0433
NC_005102.4-5951055	AGGATTCAGACTCCAGCTCCAAGATAG	1	A	0	0.4819
NC_005102.4-5951055	AGGATTCAGACTCCAGCTCCAAGATAG	23	G	0.1765	0.4761
NC_005102.4-5989897	ACATGGGCTACCTGGGCTCCGCATGGA	12	C	0.0252	0.0249
NC_005102.4-6001986	TGCCCGAGAACCTGTCCTCCAGTGTCC	5	C	0.0976	0.0928
NC_005102.4-6016741	CCACTGAGCACCTAAGCTCCCTGGCGT	2	C	0	0.4793
NC_005102.4-6016741	CCACTGAGCACCTAAGCTCCCTGGCGT	23	G	0.2033	0.2668
NC_005102.4-6018769	AATCCCTTCACTTGGGCTCCAGGACAG	7	T	0.0169	0.4826
NC_005102.4-6032106	TGCCACTGCACTAAATCTCCAAGCCCA	4	C	0.0496	0.0484
NC_005102.4-6032106	TGCCACTGCACTAAATCTCCAAGCCCA	26	C	0.0496	0.0484
NC_005102.4-6100249	AGTTGCTGAACTCTCTCTCCAGCAGTC	2	G	0	0.4764
NC_005102.4-6110185	ATTATTCCCACCCTAACTCCCCTTACT	22	C	0.0526	0.0677
NC_005102.4-6114612	CAGGCATCCACTAGCCCTCCACCAGCT	6	A	0.1626	0.2142

NC_005102.4-6114612	CAGGCATCCACTAGCCCTCCACCAGCT	12	T	0.2927	0.4759
NC_005102.4-6147868	GCCTCTTGTACTCTAACTCCGCAGTGT	26	G	0.1463	0.1629
NC_005102.4-6283826	ACAGTCACCACCCCTGCTCCTGATCAG	7	A	0.1475	0.263
NC_005102.4-6306806	TCCTAGAGCACTAGTCCTCCAGCTTGG	5	A	0.2321	0.4641
NC_005102.4-6336048	ATATCTGTGACTACTTCTCCTTTCCCC	6	T	0.122	0.2441
NC_005102.4-6336962	TGCGCCATCACCCCAGCTCCTCTGTGA	15	A	0.1197	0.2107
NC_005102.4-6336962	TGCGCCATCACCCCAGCTCCTCTGTGA	23	T	0.0085	0.4842
NC_005102.4-6379923	GTTCAGACTACCTCGCCTCCCTGCAGC	12	C	0.1667	0.2719
NC_005102.4-6386255	ACTGAAAGTACTCTGCCTCCGGCCGCC	21	G	0.1239	0.1162
NC_005102.4-6388986	GCAGTAAGGACTGTTACTCCAAGCAGC	16	A	0	0.4718
NC_005102.4-6533889	ATACTCCAGACCCCTCCTCCATTCTCT	2	T	0	0.4734
NC_005102.4-6580951	CCTCAGTCCACTTTAGCTCCAATATTT	13	T	0.0351	0.0512
NC_005102.4-6605464	TGAATGTGCACAGGCTCTCCTGGGATG	4	A	0.1301	0.2263
NC_005102.4-6625491	GTAGGAAGTACGACCCCTCCTGCTCCC	3	A	0.2705	0.3492
NC_005102.4-6625491	GTAGGAAGTACGACCCCTCCTGCTCCC	12	G	0.0583	0.395
NC_005102.4-6629118	ATATTCAGGACTGACGCTCCTACTGGG	9	G	0.0261	0.0257
NC_005102.4-6631404	GACCCCGGCACCCAGGCTCCCTACGCC	25	G	0.0244	0.0398
NC_005102.4-6648466	TGAAAGGGCACAGGTCCTCCTGCTCCA	8	G	0	0.4826
NC_005102.4-6648466	TGAAAGGGCACAGGTCCTCCTGCTCCA	14	G	0.1525	0.1551
NC_005102.4-6732400	GAGAAGCAAACCGTAACTCCAGAAAAG	26	A	0.0169	0.0496
NC_005102.4-6732400	GAGAAGCAAACCGTAACTCCAGAAAAG	27	G	0.0169	0.0496
NC_005102.4-6788075	GGAAGGCGAACTGCTGCTCCAGAGTGG	8	G	0.0488	0.0629
NC_005102.4-6796583	TCCTTGCAGACAGAAGCTCCCCAGTGT	8	A	0.0083	0.4783
NC_005102.4-6799798	TTCCCAGGCACTGCTGCTCCAGAGACC	8	G	0	0.4793
NC_005102.4-6805662	ATGGTCTTTACCCTCCCTCCTCACACT	25	A	0.2787	0.4656
NC_005102.4-6805662	ATGGTCTTTACCCTCCCTCCTCACACT	26	C	0.0492	0.048
NC_005102.4-6828379	CTCAACCAGACACTCTCTCCTGTGTGT	1	C	0	0.033
NC_005102.4-6828379	CTCAACCAGACACTCTCTCCTGTGTGT	24	G	0.042	0.0728
NC_005102.4-6847355	GATATAACAACTTTTACTCCTTAGTGT	22	T	0	0.4813
NC_005102.4-6870449	GGAATCACGACGTTGGCTCCTTGGATG	2	G	0.0163	0.032
NC_005102.4-6870449	GGAATCACGACGTTGGCTCCTTGGATG	12	G	0.0163	0.4759
NC_005102.4-6874704	GCAAAATTCACCTGAGCTCCCCTAGCA	12	C	0.0917	0.1316
NC_005102.4-6907188	AGACGGAACACCAGGGCTCCAAGCCAA	4	C	0	0.48
NC_005102.4-6911335	GGGAGCGGCACAGATGCTCCTGGTGGT	6	C	0.2195	0.3287
NC_005102.4-6926613	GTCAGCTAAACCCCTCCTCCTTTAGCT	3	C	0	0.4833
NC_005102.4-6926613	GTCAGCTAAACCCCTCCTCCTTTAGCT	14	C	0.1121	0.1209
NC_005102.4-6929897	TGTGTGTCCACATGACCTCCCAGTTTA	25	T	0	0.4851
NC_005102.4-6929897	TGTGTGTCCACATGACCTCCCAGTTTA	27	A	0	0.4851
NC_005102.4-6932629	TTGAAGATAACTGAGCCTCCTGAGGAT	27	T	0.0813	0.1073
NC_005102.4-6941561	GCATTGTGTACTTGAGCTCCTTTTGTC	8	G	0.0631	0.0611
NC_005102.4-6945135	CCCCAGACAACTCCCCCTCCTTCCAAT	1	C	0.2212	0.3294
NC_005102.4-6945135	CCCCAGACAACTCCCCCTCCTTCCAAT	23	C	0.0522	0.0832
NC_005102.4-6951059	AGACCATTTACCTCTTCTCCTTGTCCC	7	T	0.0086	0.4768
NC_005102.4-6987461	GCCTGAAGCACCTGACCTCCTTTCACT	1	G	0.2	0.2311
NC_005102.4-6987461	GCCTGAAGCACCTGACCTCCTTTCACT	16	C	0.325	0.4416
NC_005102.4-6998404	CTGTGTAGCACTTACCCTCCCCGGCTC	23	G	0.0756	0.0882
NC_005102.4-7011160	GGAGCTCCCACCTTCCCTCCCCGCCCT	14	T	0	0.4851
NC_005102.4-7011160	GGAGCTCCCACCTTCCCTCCCCGCCCT	22	C	0	0.4851
NC_005102.4-7012687	GATATGGTGACTTGGTCTCCATATACT	14	G	0.1681	0.4997
NC_005102.4-7024210	CTCGACCTCACTCTTGCTCCCTCGTGG	7	C	0.2705	0.4379
NC_005102.4-7024210	CTCGACCTCACTCTTGCTCCCTCGTGG	23	C	0	0.4793

NC_005102.4-7036966	GGCTAAAACACCAGATCTCCCTCTGGG	4	T	0.0336	0.0958
NC_005102.4-7070444	TTGCCCCAAACTCTCCCTCCTGAGTGC	15	C	0	0.4826
NC_005102.4-7070444	TTGCCCCAAACTCTCCCTCCTGAGTGC	27	C	0.1765	0.3067
NC_005102.4-7152485	CAGTCAACTACCCCTGCTCCTGGGCTA	4	T	0.0165	0.4787
NC_005102.4-7160810	CAACCCCTTACATCAGCTCCCGCCCTG	6	C	0	0.4793
NC_005102.4-7172607	AATTTAAAGACCTGAGCTCCATCCCTG	1	A	0.0348	0.1295
NC_005102.4-7178393	CCTGCCCACACCATCCCTCCGGCGTGT	13	A	0.0088	0.4648
NC_005102.4-7210303	GAGGGGGAAACTGAGGCTCCGTGAGAA	21	G	0.1261	0.1609
NC_005102.4-7225356	TGAGCACTTACATCTGCTCCATAAACA	12	A	0	0.4779
NC_005102.4-7239140	TGAGCCGCAACAGCTCCTCCATAGGTT	4	G	0.0254	0.0251
NC_005102.4-7239140	TGAGCCGCAACAGCTCCTCCATAGGTT	22	T	0.0847	0.493
NC_005102.4-7261605	CGCTCTGACACTGCTCCTCCATCAAGT	14	C	0.0496	0.0484
NC_005102.4-7334817	TAGATGTGCACTGTTACTCCACTTTTA	1	T	0	0.4785
NC_005102.4-7369099	GCCCTTCTCACAGGCTCTCCCCACCGC	16	T	0.2034	0.4425
NC_005102.4-7399756	GAAGGGGTTACTGGAGCTCCAAGAAGT	3	A	0	0.4807
NC_005102.4-7399756	GAAGGGGTTACTGGAGCTCCAAGAAGT	8	T	0	0.4807
NC_005102.4-7439502	TGCATCTGCACAGAGACTCCTCGCATT	24	C	0.0244	0.0241
NC_005102.4-7450844	CATGCTGATACCAGTCCTCCCTTGTGT	8	A	0	0.4787
NC_005102.4-7517278	GCCAAGTACACCATCACTCCTCATTTA	2	C	0	0.474
NC_005102.4-7517278	GCCAAGTACACCATCACTCCTCATTTA	27	A	0.114	0.4935
NC_005102.4-7519400	GTGGAGCACACAGCCCCTCCCATGCTT	3	G	0	0.4555
NC_005102.4-7519400	GTGGAGCACACAGCCCCTCCCATGCTT	5	A	0	0.4555
NC_005102.4-7573178	CTCCTGGCCACCCTGCCTCCCCAGCCT	23	A	0.0619	0.4986
NC_005102.4-7649014	CCAGCGTCCACTGCACCTCCTTCACCT	3	A	0.0082	0.4822
NC_005102.4-7716289	TTCTCCAGCACTGTCTCTCCTGAGATG	25	A	0	0.4793
NC_005102.4-7756094	GTGGATTCCACCTGACCTCCACAAGCT	15	A	0	0.4793
NC_005102.4-7768683	CTGGTTCTGACTCATACTCCTTCAGGG	13	C	0.2033	0.4952
NC_005102.4-7768683	CTGGTTCTGACTCATACTCCTTCAGGG	14	A	0.0081	0.481
NC_005102.4-7777206	ACTCGTCTCACGCCTACTCCTGAATGG	5	G	0.1652	0.4782
NC_005102.4-7777206	ACTCGTCTCACGCCTACTCCTGAATGG	9	C	0.0342	0.0336
NC_005102.4-7778061	TCGTAGGCTACCTGGCCTCCTGGTAAT	1	T	0.123	0.4992
NC_005102.4-7831226	CTGTCACCTACCCCATCTCCCCGGACT	22	C	0.0902	0.1009
NC_005102.4-7835352	TGCAGAGGGACCTTTTCTCCCTCAGGA	12	C	0.0348	0.0342
NC_005102.4-7835352	TGCAGAGGGACCTTTTCTCCCTCAGGA	15	T	0.1339	0.1843
NC_005102.4-7879836	CTTGTGGAAACTGGTTCTCCTTTTCTA	1	C	0.0331	0.0484
NC_005102.4-7893372	ATTCTGCTGACCGCTCCTCCACCACTG	13	G	0.0407	0.0398
NC_005102.4-7896522	GGCCTTAACACAGCTGCTCCAGCAATC	1	G	0.0417	0.0408
NC_005102.4-7896522	GGCCTTAACACAGCTGCTCCAGCAATC	13	G	0.25	0.4644
NC_005102.4-7953901	GGTCTAGACACTCAGCCTCCCAGGGGC	2	G	0.0769	0.074
NC_005102.4-7959257	GAATCTCCCACTTTGCCTCCCGAGAGC	12	T	0.2373	0.324
NC_005102.4-7979222	GGCCAAATAACTGTGTCTCCTTTCTTC	3	C	0.1271	0.2871
NC_005102.4-7991857	AGCCCGTACACTTTTCCTCCTTCAGTA	6	G	0	0.4709
NC_005102.4-8015201	GCATTACCCACCTCTCCTCCTCTGAAA	1	G	0.027	0.0267
NC_005102.4-8042480	GCCTACTCTACTTTGCCTCCTGCCCCC	12	T	0.0085	0.0251
NC_005102.4-8042480	GCCTACTCTACTTTGCCTCCTGCCCCC	13	T	0	0.4793
NC_005102.4-8044516	CATCACCCCACTGTCGCTCCGGCCCAG	21	G	0.0407	0.4893
NC_005102.4-8045050	CCATGTTTTACCCACCCTCCCTGGGAA	15	C	0.0081	0.0241
NC_005102.4-8055671	CTGGCCTAGACTGGCCCTCCCAATTGC	26	G	0.0088	0.0262
NC_005102.4-8057624	TGAGAGGGAACGAATGCTCCTCACGGG	14	A	0.2	0.3639
NC_005102.4-8057624	TGAGAGGGAACGAATGCTCCTCACGGG	16	G	0.0088	0.4889
NC_005102.4-8063868	CATGAATGAACTGTTTCTCCTGGGATC	2	A	0	0.4883

NC_005102.4-8067842	CTTAGCCCCACAGCCTCTCCTAGCACT	12	A	0.0325	0.0629
NC_005102.4-8073210	GGGACCATGACGTCACCTCCCTTTAAA	21	C	0	0.4813
NC_005102.4-8077303	CCAGAGCAAACATACACTCCTGACCTG	25	C	0.0902	0.1296
NC_005102.4-8078556	TATGCTGACACCCACACTCCTTAAATT	16	A	0.175	0.3047
NC_005102.4-8107047	CAGCCCCTCACTGTGGCTCCATGGAGT	26	G	0.1148	0.1225
NC_005102.4-8158137	CACTCTGAAACCCAGTCTCCTACCTCC	6	T	0	0.4772
NC_005102.4-8184379	AGCAATACCACTGTCCCTCCTGACAGC	12	T	0.0087	0.4849
NC_005102.4-8200869	CTGACAGCTACCAGGACTCCCTCTGTG	6	A	0.2321	0.3838
NC_005102.4-8217455	CTCTTTGGCACTGCCCCTCCTCTAGTG	12	T	0	0.4718
NC_005102.4-8248698	TCAAGCTTCACAAGTTCTCCCCATGTC	7	T	0	0.4875
NC_005102.4-8296629	CGCTAGCCCACTCTGTCTCCCATGACA	2	G	0.0252	0.0571
NC_005102.4-8298158	CACAGGACCACAGGTCCTCCATCACAG	25	C	0.0833	0.095
NC_005102.4-8298158	CACAGGACCACAGGTCCTCCATCACAG	27	G	0.0763	0.0734
NC_005102.4-8331628	GAAGAGGGCACTAGATCTCCTGGAGCA	22	G	0.525	0.4233
NC_005102.4-8331628	GAAGAGGGCACTAGATCTCCTGGAGCA	23	G	0.525	0.4233
NC_005102.4-8341105	ATGCCTGTAACCACAGCTCCTGAGAGC	22	G	0.1475	0.2399
NC_005102.4-8341832	GGAGGAGCAACTATGCCTCCAGGCTCC	25	T	0	0.4793
NC_005102.4-8388420	TCCTTTATCACTCTCTCTCCATAGGAC	9	C	0	0.4772
NC_005102.4-8388420	TCCTTTATCACTCTCTCTCCATAGGAC	14	T	0.2119	0.4336
NC_005102.4-8396658	ACAGACTCCACCCCTGCTCCAGTCGTG	21	A	0.2295	0.4934
NC_005102.4-8398493	GTGAGCCCCACACCTTCTCCTTTCTGT	9	C	0.1626	0.5
NC_005102.4-8445595	GCAGACCAAACCAATGCTCCCTCTCCC	26	C	0.1652	0.1938
NC_005102.4-8478541	AAGCTCTGGACTGTGTCTCCTGAACTC	13	G	0	0.4765
NC_005102.4-8478541	AAGCTCTGGACTGTGTCTCCTGAACTC	15	G	0.0331	0.0484
NC_005102.4-8478619	TGGTGGCACACCTTTACTCCCAGCACT	9	C	0.0407	0.0553
NC_005102.4-8539439	TTCCTACGCACCATGTCTCCATGTTCC	13	A	0	0.4757
NC_005102.4-8580537	GCACATGTAACTCCAGCTCCCAGCGAT	25	G	0.0492	0.0786
NC_005102.4-8601280	TGTTTTCTCACCCTCCCTCCAGTACAG	4	T	0.2705	0.4755
NC_005102.4-8688997	TTGCCACCCACTCTGCCTCCTCCATTC	9	C	0.05	0.0644
NC_005102.4-8717688	TGTTTTGCTACCCTCTCTCCCCACACA	8	C	0.2602	0.377
NC_005102.4-8741035	AACCGCTGAACCATTTCTCCAGCGCCC	23	C	0.0263	0.026
NC_005102.4-8755080	CAGCTCTGGACTCTACCTCCAGCCTAT	6	C	0.0325	0.032
NC_005102.4-8755080	CAGCTCTGGACTCTACCTCCAGCCTAT	26	A	0	0.4793
NC_005102.4-8756833	GCATAACACACGAATGCTCCGCCATCA	5	A	0	0.4773
NC_005102.4-8756833	GCATAACACACGAATGCTCCGCCATCA	16	G	0.2195	0.4036
NC_005102.4-8758409	CTTCCCCTAACTAGCTCTCCATTTGTT	13	A	0.4425	0.3543
NC_005102.4-8795351	TGGCTCCTAACTCCGGCTCCTGTGCAT	7	C	0	0.488
NC_005102.4-8795351	TGGCTCCTAACTCCGGCTCCTGTGCAT	13	C	0.0603	0.1058
NC_005102.4-8808752	GCCCTCCCCACCTTCACTCCATTTCCT	13	T	0.0984	0.1774
NC_005102.4-8809064	GGCTTGGCAACCCATGCTCCATCCACT	24	C	0	0.4859
NC_005102.4-8809064	GGCTTGGCAACCCATGCTCCATCCACT	25	A	0	0.4859
NC_005102.4-8838543	CTGCTGGGGACCAAGCCTCCAGTACAT	26	A	0.0672	0.0958
NC_005102.4-8865087	CTCTGGAGCACTGGCTCTCCTGGAACT	9	C	0.0833	0.1387
NC_005102.4-8891960	CTGGGAAAGACTAGTTCTCCTGCTGTC	4	G	0.2328	0.3127
NC_005102.4-8902863	AGAGGGCAGACACAGGCTCCTTAACCT	23	A	0	0.4773
NC_005102.4-8943582	ATGCATCCAACCGATACTCCCTTTGAG	26	A	0	0.4787
NC_005102.4-8945582	AGCGCAGCCACCTTCCCTCCCCAGCTT	4	G	0.0086	0.4803
NC_005102.4-8945582	AGCGCAGCCACCTTCCCTCCCCAGCTT	12	C	0.0357	0.0351
NC_005102.4-8978148	GACCTCATGACTCTGTCTCCTTGTGCC	14	T	0.0488	0.0928
NC_005102.4-8984080	TGACTTTGTACCGGTCCTCCTATCTCC	13	G	0.0351	0.0345
NC_005102.4-8984080	TGACTTTGTACCGGTCCTCCTATCTCC	22	A	0.0089	0.4709

NC_005102.4-8988730	GCGTTTGTGACTCTAGCTCCAGGGGCT	3	G	0.0248	0.0245
NC_005102.4-8988730	GCGTTTGTGACTCTAGCTCCAGGGGCT	7	G	0.1066	0.1154
NC_005102.4-9004799	GTGGATTACACGTGGTCTCCTGCTCAT	26	A	0	0.4844
NC_005102.4-9022291	TCCAAGCTTACTTCACCTCCTGAGGTC	21	T	0	0.4868
NC_005102.4-9101965	AGGTGCTGAACCATCTCTCCCCATTGA	13	A	0.125	0.2052
NC_005102.4-9114563	CACCTCTCAACCCTCCCTCCCATACCC	9	A	0.122	0.4979
NC_005102.4-9165730	CACAGCGGGACTCTTTCTCCACTTTAT	7	G	0.0508	0.0496
NC_005102.4-9205965	TGCCTTGAGACAGCGTCTCCACTGCAC	14	C	0.0259	0.0255
NC_005102.4-9214006	GACACTTGCACTCACTCTCCCCTCAGC	16	T	0.0504	0.0491
NC_005102.4-9238610	CATAGCTAAACAATCTCTCCTGAACTG	12	A	0	0.4779
NC_005102.4-9270529	CATCAGGTGACCAGACCTCCTCCACCA	3	T	0.0431	0.4955
NC_005102.4-9276334	CTCTTGGTTACAGAAGCTCCTTTTCAT	14	A	0.065	0.0629
NC_005102.4-9278725	CTACTAAGGACAATGTCTCCTCCCTCC	27	C	0.1842	0.4473
NC_005102.4-9281684	ATGCTGAGCACAGACTCTCCCCAGGAG	13	G	0	0.48
NC_005102.4-9480731	GACACCACAACTTTGCCTCCTGGCGCG	25	G	0.0424	0.0415
NC_005102.4-9490880	TGGGCGCTGACGGAAGCTCCAAACCAA	5	C	0.2174	0.4962
NC_005102.4-9490880	TGGGCGCTGACGGAAGCTCCAAACCAA	23	A	0	0.474
NC_005102.4-9491065	TGCCTTCTCACTTCCTCTCCAGCCAGT	1	T	0.2	0.455
NC_005102.4-9501221	AATGGGACTACAAACTCTCCTGTGTCA	9	T	0	0.4793
NC_005102.4-9501221	AATGGGACTACAAACTCTCCTGTGTCA	22	G	0.1382	0.1562
NC_005102.4-9523371	GAGACAAGTACAGAGCCTCCCCTGGGA	25	G	0.0804	0.1093
NC_005102.4-9531132	GAACAACAAACCCCTCCTCCCCGTCAT	22	C	0.2373	0.3874
NC_005102.4-9574046	ACCTGTCTGACTCTGTCTCCCAAGTAT	9	G	0.1667	0.295
NC_005102.4-9580738	GAAACATCCACAAAGCCTCCAGCGATG	22	G	0.0431	0.0903
NC_005102.4-9588574	GAATGACTGACAGCCCCTCCTTCACAT	5	G	0.2066	0.4077
NC_005102.4-9661872	GCTGCGTTGACATCTGCTCCAAGATCC	6	G	0.1197	0.1975
NC_005102.4-9666855	CAGCCCGTCACCCAGGCTCCTGTCTTT	7	G	0.0325	0.032
NC_005102.4-9667000	TTCCTCACCACTACTGCTCCCTTTCTG	13	A	0	0.4728
NC_005102.4-9689356	TGTCCTTACACACATCCTCCTGCATCC	7	T	0.1382	0.3381
NC_005102.4-9689356	TGTCCTTACACACATCCTCCTGCATCC	24	A	0.2195	0.3646
NC_005102.4-9703497	GACACGCACACTTAGCCTCCTTCAACC	6	G	0.1333	0.1528
NC_005102.4-9703893	AAGCTTAGAACACTCACTCCTGGTCTC	6	T	0	0.4826
NC_005102.4-9729999	TCTCTACACACCATGCCTCCCTCTCAG	6	A	0	0.4773
NC_005102.4-9733595	CCCCACCCCACCTCCTCTCCATCCAGT	6	C	0.0796	0.1828
NC_005102.4-9733595	CCCCACCCCACCTCCTCTCCATCCAGT	9	C	0.1327	0.154
NC_005102.4-9755651	CAGAATTAGACCAGGCCTCCAGACCAA	5	A	0	0.4703
NC_005102.4-9792127	CCAAGTGTAACACCCACTCCTCCAGAT	25	G	0.2439	0.3688
NC_005102.4-9792127	CCAAGTGTAACACCCACTCCTCCAGAT	27	T	0	0.4807
NC_005102.4-9792155	GGGACCACAACCCTAGCTCCACGTTTT	13	C	0.0348	0.0342
NC_005102.4-9794932	CTTGTTGGTACTCGGCCTCCTGGTGGA	13	C	0.0244	0.0241
NC_005102.4-9794932	CTTGTTGGTACTCGGCCTCCTGGTGGA	14	G	0.0569	0.0705
NC_005102.4-9804978	GGTATTTCCACCCACCCTCCTCCTCCT	26	C	0.1897	0.4463
NC_005102.4-9804978	GGTATTTCCACCCACCCTCCTCCTCCT	27	T	0.1966	0.4473
NC_005102.4-9818288	ATCTTCCTAACTTAGTCTCCTTAGTGT	1	A	0.0744	0.4339
NC_005102.4-9818288	ATCTTCCTAACTTAGTCTCCTTAGTGT	3	C	0.0083	0.0247
NC_005102.4-9833370	ACACTGTCCACAGGCCCTCCAATGGGA	22	A	0.0089	0.4885
NC_005102.4-9839989	TGACTCAGGACCAGCTCTCCCCTCTTC	7	A	0	0.4688
NC_005102.4-9857624	GGAACTGGCACTGGCCCTCCTGTGTCC	1	G	0	0.4731
NC_005102.4-9879396	CAAAGGGCCACAGCAGCTCCGTTACTG	1	C	0	0.4793
NC_005102.4-9879396	CAAAGGGCCACAGCAGCTCCGTTACTG	3	A	0	0.4793
NC_005102.4-10135971	ACAAAGTAAACTGTCACTCCACTTGTA	26	T	0.0254	0.0415

NC_005102.4-10135971	ACAAAGTAAACTGTCACTCCACTTGTA	27	A	0.0342	0.0336
NC_005102.4-10164593	CAGGGAAAGACATTGACTCCTCCCCGC	21	T	0.009	0.4942
NC_005102.4-10164593	CAGGGAAAGACATTGACTCCTCCCCGC	25	C	0.1964	0.4228
NC_005102.4-10175625	TGGGGGCGAACTCGATCTCCTTGGGCA	7	C	0.041	0.0401
NC_005102.4-10175625	TGGGGGCGAACTCGATCTCCTTGGGCA	8	G	0.041	0.4879
NC_005102.4-10181024	GTCAGCGGAACAACCCCTCCAGCCATT	24	C	0.0579	0.1017
NC_005102.4-10221998	GGAAGGTGGACAGCATCTCCGTATCAC	15	A	0	0.4734
NC_005102.4-10230072	TAAAAAAGGACAGATGCTCCCCCAGGT	6	A	0	0.482
NC_005102.4-10230650	GGAGCTCACACCTTCTCTCCAACGGTC	3	A	0	0.4793
NC_005102.4-10291997	GCCGTTAGAACGCCTCCTCCTTTTGTA	3	C	0	0.4819
NC_005102.4-10291997	GCCGTTAGAACGCCTCCTCCTTTTGTA	15	T	0	0.4819
NC_005102.4-10294503	ATCCTTTCCACCATCACTCCGTGTAAA	1	A	0.0417	0.0722
NC_005102.4-10294503	ATCCTTTCCACCATCACTCCGTGTAAA	9	C	0.0331	0.0325
NC_005102.4-10298586	CATACACGGACCTGGTCTCCAAGGAAC	7	C	0.1803	0.4737
NC_005102.4-10332355	TCCACATCCACTGTAACTCCTAAATGT	25	T	0.0714	0.1478
NC_005102.4-10332355	TCCACATCCACTGTAACTCCTAAATGT	26	G	0.0696	0.1143
NC_005102.4-10345968	CCCAGTGGTACGGGAGCTCCAGAGGTT	1	C	0.1478	0.2204
NC_005102.4-10388777	AATCCTTTTACACAAGCTCCTCGTGTG	23	G	0	0.032
NC_005102.4-10399538	ATTGTGGGCACAGGCTCTCCAGTGGCC	9	C	0.1463	0.2263
NC_005102.4-10399538	ATTGTGGGCACAGGCTCTCCAGTGGCC	15	C	0.1545	0.1954
NC_005102.4-10402842	CAGAGGACAACGTTCTCTCCCACCACA	6	G	0.1382	0.208
NC_005102.4-10427020	CCACAAGGTACTGGGGCTCCCTGCCTC	9	T	0.0609	0.1067
NC_005102.4-10427020	CCACAAGGTACTGGGGCTCCCTGCCTC	12	T	0.0354	0.0348
NC_005102.4-10430461	TGGGCTTCTACTGTTGCTCCACTGAAT	23	T	0.2705	0.432
NC_005102.4-10437988	ATGGTGTGCACCAGCCCTCCTGGCTTT	5	T	0.1333	0.2778
NC_005102.4-10438183	CTCCTTAATACAGTCCCTCCTATGTGG	25	T	0.0085	0.4738
NC_005102.4-10449272	CTGCCATTTACACAGACTCCGGGGATT	4	C	0.0328	0.0322
NC_005102.4-10449272	CTGCCATTTACACAGACTCCGGGGATT	21	G	0.123	0.5
NC_005102.4-10463512	GTGTTCCTGACTTCCCCTCCCCCGCTC	12	T	0.3089	0.4206
NC_005102.4-10463512	GTGTTCCTGACTTCCCCTCCCCCGCTC	21	C	0.3306	0.4041
NC_005102.4-10534618	CCTGACAGAACCCCGCCTCCTCACACA	26	C	0.0991	0.126
NC_005102.4-10545619	CTACCTCCCACCATCCCTCCCCTTCCT	14	T	0.1207	0.1717
NC_005102.4-10546688	CACAGAGAAACCCTGTCTCCAAGAACC	2	A	0.0619	0.06
NC_005102.4-10548265	AGCACATTTACCGGGCCTCCTCACATC	4	A	0.2049	0.4588
NC_005102.4-10548265	AGCACATTTACCGGGCCTCCTCACATC	25	A	0	0.4807
NC_005102.4-10555985	AGGCCAACCACCAGGCCTCCCAGCCCT	16	C	0.0357	0.0351
NC_005102.4-10568955	CTTCTGAGGACCATTCCTCCCACGATG	23	C	0	0.4749
NC_005102.4-10568955	CTTCTGAGGACCATTCCTCCCACGATG	24	G	0.0256	0.0253
NC_005102.4-10574210	TCTCTCCACACCTGCACTCCCTTGCCA	14	G	0.0088	0.4547
NC_005102.4-10616026	CTGAGTCCCACATCACCTCCTTCAGAC	13	T	0	0.4844
NC_005102.4-10633706	AGCATTAACACCTTGTCTCCTCGACAC	4	A	0.1186	0.2091
NC_005102.4-10643944	AGGCAAATCACCTACCCTCCTGGTCTG	14	A	0.1062	0.1466
NC_005102.4-10652599	AGTCTGTCAACTCGAGCTCCAGGGGAT	14	G	0.065	0.1216
NC_005102.4-10686525	CCCACCTTTACAAGAGCTCCAGGGATT	26	T	0.2033	0.356
NC_005102.4-11003564	GCAGAGGTGACAGCTGCTCCCTGGTTA	13	G	0	0.4785
NC_005102.4-11003998	CTCTCAAAGACAAGATCTCCATGTCTT	14	G	0.0909	0.1306
NC_005102.4-11040567	GGAGGTGACACGCCAGCTCCCCTTGGC	23	T	0.0776	0.0746
NC_005102.4-11044732	AGTGATCCTACATTAACTCCGGATCAG	22	G	0	0.4709
NC_005102.4-11066722	TGCAGGATGACAGGCACTCCTTAGTTC	8	T	0.0244	0.0398
NC_005102.4-11069879	GATGAGCTGACTGTGCCTCCGTCTCCT	24	T	0	0.48
NC_005102.4-11086883	TACCCTCCCACGCTGTCTCCAAGGTTC	25	T	0	0.48

NC_005102.4-11096243	CTCCAGTGCACCCCTCCTCCAGACCCT	7	T	0	0.4813
NC_005102.4-11096243	CTCCAGTGCACCCCTCCTCCAGACCCT	27	T	0	0.4813
NC_005102.4-11100597	ACTCTTCAAACATAAGCTCCAACTCTC	12	A	0.225	0.3347
NC_005102.4-11100597	ACTCTTCAAACATAAGCTCCAACTCTC	13	T	0	0.48
NC_005102.4-11104494	GCACACCCCACCACAGCTCCTTAACTC	5	A	0.0738	0.4634
NC_005102.4-11105999	GGCCCCAGCACAGCATCTCCCTGGTGA	5	C	0	0.4751
NC_005102.4-11105999	GGCCCCAGCACAGCATCTCCCTGGTGA	23	G	0.2295	0.3947
NC_005102.4-11115814	GTCACTGCCACTAGCTCTCCAGGATTG	24	A	0	0.4773
NC_005102.4-11115814	GTCACTGCCACTAGCTCTCCAGGATTG	26	T	0.0656	0.4565
NC_005102.4-11143446	GCCGTGGGAACCAGGCCTCCAGCGTCC	4	G	0	0.4773
NC_005102.4-11147474	GACTTCCAGACAGGAACTCCTTGTATC	5	T	0.1417	0.4997
NC_005102.4-11158133	CCCTACATGACCGGAACTCCTGGGTTG	12	C	0	0.48
NC_005102.4-11188107	CTGCCCCACACCCGCGCTCCTTTCATT	13	C	0.0083	0.0245
NC_005102.4-11188107	CTGCCCCACACCCGCGCTCCTTTCATT	22	T	0	0.4787
NC_005102.4-11213823	TGACACATGACAGTGTCTCCTAAGCAA	7	A	0.1333	0.4988
NC_005102.4-11239687	ACGGGGTCAACTACAGCTCCTTTGGTG	5	G	0.6083	0.4233
NC_005102.4-11264462	CACCCCATCACTTGGCCTCCTTGTTAG	23	G	0.1475	0.2515
NC_005102.4-11305762	TCCGTGTGTACTCCATCTCCTGCTGAT	4	G	0.0083	0.4769
NC_005102.4-11305762	TCCGTGTGTACTCCATCTCCTGCTGAT	15	A	0.0492	0.1082
NC_005102.4-11319604	TACCCATGCACCCTGTCTCCTAGTATA	5	C	0	0.4773
NC_005102.4-11319604	TACCCATGCACCCTGTCTCCTAGTATA	25	A	0.0244	0.0241
NC_005102.4-11323585	ACAAGAGTTACGCGGCCTCCAGGATGT	15	G	0.0248	0.0245
NC_005102.4-11330808	AGCAAGAGCACTGGCTCTCCATCAGAA	26	A	0.0171	0.0336
NC_005102.4-11373699	GCATCCTTGACTTGACCTCCGGTCAGA	13	T	0	0.4787
NC_005102.4-11378684	GCCCCTCCCACTCCCTCTCCTGTCTAT	9	C	0.0732	0.0854
NC_005102.4-11393865	GTCACCCTTACCTGGCCTCCAAGGGCA	23	G	0.0333	0.0488
NC_005102.4-11416452	GGGCTCCTGACCCCTCCTCCCCAGGGC	9	G	0.124	0.2814
NC_005102.4-11431743	CAGTGTGAGACCCTGTCTCCAAAAGAC	21	A	0.0603	0.0746
NC_005102.4-11445509	TGGGTACAAACACTGGCTCCAGCCTCA	2	G	0.0083	0.0245
NC_005102.4-11559178	TCCTCCTTGACTGAGACTCCCTTTTGA	12	T	0.115	0.4994
NC_005102.4-11608329	GAGCTGTGAACCCCGTCTCCTGGACAG	14	C	0	0.4807
NC_005102.4-11683360	CTAGTCAGGACAGTGGCTCCCTGGGAC	1	C	0.0085	0.4854
NC_005102.4-11699684	GGTGGCATGACATCATCTCCCTGTCTG	16	T	0.1897	0.4946
NC_005102.4-11754840	GTGTCTTGGACTCCATCTCCCACAGAC	6	T	0.0164	0.4837
NC_005102.4-11754840	GTGTCTTGGACTCCATCTCCCACAGAC	13	C	0.0813	0.078
NC_005102.4-11756063	ATTATTGCCACTCCATCTCCTGGGTAC	8	C	0.0407	0.0398
NC_005102.4-11761204	TGTACCCTCACTGTCACTCCATTTCTG	24	T	0	0.4807
NC_005102.4-11793296	CACACGTGCACCAAGTCTCCCTTCCAA	6	G	0.1803	0.3624
NC_005102.4-11808689	TACAGCAGCACAGAGTCTCCAGACAGA	3	C	0.0417	0.1025
NC_005102.4-11847515	TCCCGCAGTACCTGCCCTCCACCAGAA	15	C	0.1795	0.2662
NC_005102.4-11898951	GTCCTCCCCACCCCTCCTCCCTATCTA	24	T	0.0085	0.4823
NC_005102.4-11923830	AACCTGCTTACCTTTTCTCCAAACACT	1	A	0.1311	0.1641
NC_005102.4-11945635	GTTCTTTGCACTGAGGCTCCTTGTGTG	8	G	0.1057	0.1695
NC_005102.4-11952311	ATTGAGGGCACCGGGCCTCCGCAGGCA	3	T	0.0244	0.484
NC_005102.4-11952311	ATTGAGGGCACCGGGCCTCCGCAGGCA	12	C	0.0325	0.032
NC_005102.4-11978179	CCAGCGGCTACAGGACCTCCAGGACTT	5	C	0.0407	0.4868
NC_005102.4-11978179	CCAGCGGCTACAGGACCTCCAGGACTT	6	G	0.2033	0.2989
NC_005102.4-11991372	CCAGGCCTGACCTACTCTCCCTGAGGA	3	A	0	0.4751
NC_005102.4-12027936	GTCACCAGGACCTCCCCTCCCAGGAGT	14	C	0.0574	0.0557
NC_005102.4-12032785	ATGCCAGTAACTCCATCTCCAGGGGCT	8	T	0.0407	0.0398
NC_005102.4-12039020	AGGCCAAAGACTGATTCTCCAAAGTTT	4	C	0.1062	0.1466

NC_005102.4-12047219	CTTATACCTACTGTGGCTCCATAAACA	3	T	0.0244	0.0241
NC_005102.4-12047219	CTTATACCTACTGTGGCTCCATAAACA	26	C	0.1301	0.2142
NC_005102.4-12060438	GAACATGGGACGATAACTCCGGCACTG	12	G	0.0248	0.0245
NC_005102.4-12060438	GAACATGGGACGATAACTCCGGCACTG	21	G	0.0248	0.0245
NC_005102.4-12080481	ACATGTTAGACAAGCACTCCATCCTTG	13	A	0.0244	0.0241
NC_005102.4-12080481	ACATGTTAGACAAGCACTCCATCCTTG	22	T	0	0.4793
NC_005102.4-12083355	GATGTCTTCACCTGCTCTCCAGCTGAT	2	A	0	0.48
NC_005102.4-12098589	GTTGGTCTAACCGTCTCTCCCCACCAC	8	T	0	0.48
NC_005102.4-12098589	GTTGGTCTAACCGTCTCTCCCCACCAC	13	G	0	0.4813
NC_005102.4-12109045	CCAGAGCACACATTTCCTCCTGAGCCA	6	G	0.0351	0.0677
NC_005102.4-12109045	CCAGAGCACACATTTCCTCCTGAGCCA	12	A	0.0177	0.0348
NC_005102.4-12109615	GAGCACCTCACGGTGTCTCCTCTTCGT	14	T	0.0738	0.1436
NC_005102.4-12109615	GAGCACCTCACGGTGTCTCCTCTTCGT	26	G	0.041	0.0401
NC_005102.4-12126808	CTGAGAAGCACATGCTCTCCACAGTAC	2	T	0.1786	0.3838
NC_005102.4-12129033	CATTATGCAACTGCATCTCCATGTTCA	16	T	0.0593	0.487
NC_005102.4-12129033	CATTATGCAACTGCATCTCCATGTTCA	27	A	0.0084	0.4797
NC_005102.4-12160394	AGGCTGAGAACGTGCTCTCCAAGTCCA	16	T	0.0084	0.4797
NC_005102.4-12170755	CCAGTAAGCACCATTCCTCCGTGGTCT	1	C	0.0661	0.0639
NC_005102.4-12170755	CCAGTAAGCACCATTCCTCCGTGGTCT	8	G	0.5785	0.418
NC_005102.4-12179724	CTCATGGCAACTTACTCTCCCAACATC	6	G	0.0522	0.4537
NC_005102.4-12179724	CTCATGGCAACTTACTCTCCCAACATC	27	C	0.0087	0.466
NC_005102.4-12188371	AGAGAGAGAACAGAGGCTCCAGCCCGG	7	A	0.1983	0.2797
NC_005102.4-12190399	CTGTATACCACCATTTCTCCCACTGCG	16	T	0	0.4703
NC_005102.4-12191981	ACTTGTCCCACTAAGTCTCCAGCTAGG	8	C	0	0.4757
NC_005102.4-12191981	ACTTGTCCCACTAAGTCTCCAGCTAGG	22	G	0	0.4757
NC_005102.4-12207395	TGAGGGGTTACCCGGCCTCCTGTGGAC	27	C	0.0536	0.0521
NC_005102.4-12210198	CCAACTAAGACACAAGCTCCGGGAGGC	21	G	0.0982	0.1403
NC_005102.4-12211873	CGGGTCACAACTCATCCTCCTTCTGAT	14	A	0.0492	0.0786
NC_005102.4-12236246	AGCCAGCCAACTATGGCTCCAGGGGTC	12	T	0	0.4637
NC_005102.4-12272376	GTGTATGACACTTGTACTCCTACATAT	14	G	0.0756	0.0728
NC_005102.4-12279990	GTGAAAGCCACCCGGTCTCCTGCCTCT	13	C	0	0.4793
NC_005102.4-12306990	AAAGGCAGGACGATGACTCCACAGTGT	12	G	0.1667	0.4965
NC_005102.4-12308845	TGTTTAAAAACACCAGCTCCATACCTC	13	C	0	0.48
NC_005102.4-12332636	AGGCTAAGTACTCATTCTCCTGAATGA	1	A	0.1475	0.306
NC_005102.4-12368451	GAGGCAGGGACGAGGACTCCTGGCCAG	13	A	0.1724	0.3179
NC_005102.4-12368451	GAGGCAGGGACGAGGACTCCTGGCCAG	16	A	0	0.4654
NC_005102.4-12377000	TTATCCATCACGTGCTCTCCTACTGTG	5	C	0.0089	0.4751
NC_005102.4-12377000	TTATCCATCACGTGCTCTCCTACTGTG	12	G	0.1696	0.499
NC_005102.4-12402907	AACCCCCTGACCTGGCCTCCAACTTAA	22	A	0	0.4654
NC_005102.4-12466180	CCTGGAAACACTGCTGCTCCCTGAGAT	23	G	0.1057	0.4106
NC_005102.4-12489499	ACCCCACCCACTTTGGCTCCTGACCCC	25	C	0	0.48
NC_005102.4-12493703	CAATCTGGAACTCCTCCTCCGCTCCCG	5	C	0.0165	0.4787
NC_005102.4-12511408	GAAAGCTACACTCTGTCTCCAGACTTC	14	T	0	0.4793
NC_005102.4-12526276	AACTTGGGAACGAGGCCTCCTGATTCC	9	A	0.1789	0.3516
NC_005102.4-12526276	AACTTGGGAACGAGGCCTCCTGATTCC	13	A	0	0.4825
NC_005102.4-12542404	TTCTTTGGCACTTACACTCCCGGGGTG	5	T	0.018	0.4951
NC_005102.4-12542404	TTCTTTGGCACTTACACTCCCGGGGTG	13	T	0	0.4931
NC_005102.4-12558019	GAGAAAACGACTCCCTCTCCTACAGCA	27	A	0.0932	0.4982
NC_005102.4-12562446	CCAGCCTCTACTGCTACTCCTGCCCAC	3	A	0.1111	0.1347
NC_005102.4-12572265	ACTGGAATGACGGCTCCTCCTAAGCTG	12	G	0.0259	0.0422
NC_005102.4-12579585	CCGGCTTAAACAAACACTCCACAGATT	3	G	0.1261	0.4977

NC_005102.4-12615061	CAAACAGCCACAGTGTCTCCAGGGCAC	3	A	0.0083	0.4732
NC_005102.4-12619323	CTGAGGATAACATCTACTCCACATGTG	12	A	0	0.4779
NC_005102.4-12637027	CCCCTCTCCACTGTCACTCCAAGACTC	16	A	0	0.4728
NC_005102.4-12647856	AAGGAACCCACCCAGGCTCCCGCAGCT	9	C	0	0.4813
NC_005102.4-12657143	CATTCATCCACCCAGCCTCCCACCCCG	12	C	0.0732	0.1001
NC_005102.4-12657143	CATTCATCCACCCAGCCTCCCACCCCG	15	G	0.2623	0.3447
NC_005102.4-12670792	ATTTTGCTAACTTCATCTCCACCATGA	25	T	0	0.4833
NC_005102.4-12710135	GTGTGACTCACTGTTACTCCCAGGGAA	8	T	0.1311	0.2279
NC_005102.4-12775431	AACTGCTGAACCATCTCTCCAGTCTCC	3	C	0.0325	0.032
NC_005102.4-12775431	AACTGCTGAACCATCTCTCCAGTCTCC	5	G	0.1707	0.1695
NC_005102.4-12809730	GGAGCTCAAACGTGAGCTCCCTCCTTG	12	G	0.0513	0.142
NC_005102.4-12833328	GTGTGGTCAACACCTACTCCTTAGGGT	22	T	0	0.4785
NC_005102.4-12857834	TGCCCACACACCGTCTCTCCTTGCTTC	12	C	0.123	0.4822
NC_005102.4-12858054	CATTCCAGGACTCTAGCTCCGAGCCAG	14	T	0.1057	0.2668
NC_005102.4-12858054	CATTCCAGGACTCTAGCTCCGAGCCAG	21	G	0.0325	0.0629
NC_005102.4-12859579	CTCCCCCACACCCTGGCTCCTGGAACA	7	C	0.065	0.0629
NC_005102.4-12859579	CTCCCCCACACCCTGGCTCCTGGAACA	8	A	0.1382	0.4952
NC_005102.4-12870751	GCTGTTTCTACCACTGCTCCTTTACTG	7	T	0.075	0.1172
NC_005102.4-12880914	ATTCATGTTACCTTAACTCCATCACTC	1	A	0.0089	0.4709
NC_005102.4-12880914	ATTCATGTTACCTTAACTCCATCACTC	13	T	0.1171	0.4985
NC_005102.4-12941137	CCACCTGAAACTTCAGCTCCAGGGCTT	27	T	0.1239	0.4996
NC_005102.4-12979648	TCCTGGAGGACTTGACCTCCTTCCACT	12	T	0	0.4883
NC_005102.4-12997634	CCACATATAACTCCATCTCCAGAGAAT	2	C	0.1391	0.2758
NC_005102.4-13004256	GGAGTGAAAACCTGCTCTCCATCCCAT	9	A	0.1765	0.2961
NC_005102.4-13028140	CACGGACTCACACTAGCTCCATGACAC	3	C	0.0714	0.0853
NC_005102.4-13028140	CACGGACTCACACTAGCTCCATGACAC	26	A	0.0714	0.0853
NC_005102.4-13033140	AGCCCTTGCACCCTCACTCCCACTGCG	5	C	0.082	0.1082
NC_005102.4-13033140	AGCCCTTGCACCCTCACTCCCACTGCG	12	C	0.1393	0.3492
NC_005102.4-13034523	TGATAATAGACTAAGCCTCCGACTGTA	22	A	0.0088	0.4913
NC_005102.4-13046307	ACACCAATCACTCCATCTCCAGTGCTT	25	C	0.0744	0.0716
NC_005102.4-13046307	ACACCAATCACTCCATCTCCAGTGCTT	26	T	0.2314	0.4787
NC_005102.4-13056377	TGTTAACTCACGCTGTCTCCGTGTGTG	1	T	0.1008	0.4615
NC_005102.4-13056377	TGTTAACTCACGCTGTCTCCGTGTGTG	21	G	0.1429	0.4377
NC_005102.4-13116389	GACGTCTTTACAAGACCTCCACACTCC	13	A	0	0.4558
NC_005102.4-13116772	CTTGGCTAAACTAGACCTCCCTCTCTC	6	C	0.0609	0.1516
NC_005102.4-13121531	ACTGGTCATACAGCTTCTCCCATGGCC	5	G	0.1057	0.1826
NC_005102.4-13121531	ACTGGTCATACAGCTTCTCCCATGGCC	15	T	0.1083	0.2125
NC_005102.4-13137984	CTGACTGAAACTTGAACTCCAGAAGGT	1	C	0.0496	0.1377
NC_005102.4-13137984	CTGACTGAAACTTGAACTCCAGAAGGT	24	A	0.0492	0.1367
NC_005102.4-13170185	AACCAGCACACCTGCCCTCCTACAATC	25	A	0.0976	0.1216
NC_005102.4-13189302	TGGAGCATCACGGAAGCTCCCTAAGGT	3	G	0.0256	0.4906
NC_005102.4-13201173	TGGAACCGCACCTGCTCTCCTGGTCTC	15	C	0.0598	0.0896
NC_005102.4-13229633	AGCAACGGCACTCTTCCTCCTCATTGC	6	C	0	0.4793
NC_005102.4-13229633	AGCAACGGCACTCTTCCTCCTCATTGC	25	T	0.065	0.4944
NC_005102.4-13291396	CCAGTCACAACTTCCACTCCTCTGGGC	12	T	0	0.4666
NC_005102.4-13335243	TCCTTCTCAACACTGTCTCCCCACCTG	22	C	0.0244	0.0553
NC_005102.4-13337104	TGATTTTCGACTTCTGCTCCTATCATT	9	G	0	0.4814
NC_005102.4-13384716	ACCTCTGGAACCTGCTCTCCTGCTCCT	16	T	0	0.4793
NC_005102.4-13481866	AGAGAAGGAACAAATTCTCCTAGAGGC	26	G	0.0609	0.059
NC_005102.4-13517643	TTCTTAGCTACTATTTCTCCCCCAATC	21	C	0.0625	0.491
NC_005102.4-13667396	CACGAGGTAACTGATCCTCCATCTTCC	23	C	0.0168	0.4873

NC_005102.4-13667704	ACTCCTTTCACAGAGCCTCCCTTGGGA	24	G	0	0.4757
NC_005102.4-13676331	CCGTTTCTCACCCCTCCTCCCTGGGTG	2	C	0.0574	0.1009
NC_005102.4-13678379	ATGTCGGTAACCCCTGCTCCAGGCAGG	3	G	0	0.4793
NC_005102.4-13678379	ATGTCGGTAACCCCTGCTCCAGGCAGG	4	T	0.0732	0.4967
NC_005102.4-13695014	GGTGTCCACACAAGGCCTCCTAGGGGC	5	T	0.0268	0.0264
NC_005102.4-13704105	CCGCACCCCACCCAGCCTCCTCGAGGT	2	C	0.0909	0.1017
NC_005102.4-13708885	TTAAGTTGAACCCTGACTCCGAATGGA	2	T	0	0.4787
NC_005102.4-13708885	TTAAGTTGAACCCTGACTCCGAATGGA	21	G	0.0328	0.048
NC_005102.4-13752935	CCTTAGGAGACTGTATCTCCGAGACCC	12	T	0	0.4773
NC_005102.4-13768910	CTGCTGTGGACAATTACTCCTCAGACA	13	A	0.0167	0.4765
NC_005102.4-13768910	CTGCTGTGGACAATTACTCCTCAGACA	14	T	0.0252	0.0249
NC_005102.4-13812263	ACGTGCTGCACCAACACTCCCAGCCCT	2	C	0.0174	0.4588
NC_005102.4-13954414	AGGGCTTCAACACAAGCTCCTGGGTTG	6	T	0.0256	0.0418
NC_005102.4-13972226	TTGCTGGGTACTCTCTCTCCCACTGAA	5	T	0.0252	0.0249
NC_005102.4-13995419	GGGCCTGTCACAAATTCTCCATCCCAT	26	A	0.2114	0.3926
NC_005102.4-13998777	TCACCTACCACCAGTACTCCCCATCTG	2	C	0.0084	0.0249
NC_005102.4-14047144	GCTTTTCAGACTCTGCCTCCTGCTGAT	9	G	0.1583	0.4941
NC_005102.4-14047144	GCTTTTCAGACTCTGCCTCCTGCTGAT	27	T	0.1765	0.491
NC_005102.4-14073351	ACATAGGCAACATTCGCTCCCATTTGG	15	C	0.1557	0.4992
NC_005102.4-14102965	CTATGGGAAACAGCCACTCCCAGACAT	15	C	0.0574	0.1009
NC_005102.4-14103944	TCCATGTCCACAGACTCTCCCGTATCA	22	G	0.1966	0.2778
NC_005102.4-14106947	ATAAGGGGAACCCAGTCTCCATGGATA	21	A	0.1626	0.1494
NC_005102.4-14294725	GCTGGGCAAACCTGGCCTCCACCCACT	4	G	0.1724	0.4345
NC_005102.4-14400397	GAAGCTCCCACCTCCTCTCCACTGTTT	25	T	0.009	0.4869
NC_005102.4-14458360	GAGACAGTCACACAGGCTCCCTTGTCT	2	A	0	0.4793
NC_005102.4-14471812	CAGTCTTCAACTGAAGCTCCCTGAGCC	22	T	0.0569	0.0553
NC_005102.4-14472269	AGGTGAGGAACTGAGGCTCCTTATACA	23	A	0.0427	0.0418
NC_005102.4-14473545	ACAATAGGCACATGGGCTCCTAGTGCA	5	T	0.2114	0.2937
NC_005102.4-14477847	GATAAGGTAACGGAGGCTCCGAGAGCT	21	G	0.0439	0.483
NC_005102.4-14540591	CCACCAGTAACATCAACTCCACAGATC	4	C	0.1043	0.1143
NC_005102.4-14553377	ATTTCAGTCACCCATGCTCCTTTCACT	4	T	0.0427	0.4947
NC_005102.4-14553377	ATTTCAGTCACCCATGCTCCTTTCACT	27	T	0.0427	0.4947
NC_005102.4-14562341	ACCATGCCTACGCTTGCTCCTCTAGGC	27	C	0.1316	0.5
NC_005102.4-14612210	TGAGGTCCCACTGCATCTCCAAGCGGG	7	C	0.0252	0.0571
NC_005102.4-14613339	AGATGTAGGACTCTAGCTCCTCTAGTT	2	G	0.1789	0.3041
NC_005102.4-14613339	AGATGTAGGACTCTAGCTCCTCTAGTT	26	T	0.0976	0.1356
NC_005102.4-14677557	GACAGGTAAACTGAGGCTCCAGGAAAG	6	G	0.0826	0.0792
NC_005102.4-14677557	GACAGGTAAACTGAGGCTCCAGGAAAG	24	A	0.0826	0.0792
NC_005102.4-14677620	TCTTGCCCTACAGCCGCTCCATCAACC	15	C	0.2054	0.2991
NC_005102.4-14680248	ATGGTGGGGACCAGGTCTCCAGTATCC	24	A	0.2857	0.4921
NC_005102.4-14695423	CTCCAAAGGACTCTGGCTCCTCAGATA	5	A	0.0593	0.5
NC_005102.4-14819065	CGTGATAAAACGACTGCTCCCTACCTG	15	T	0.0164	0.048
NC_005102.4-14898019	CTCAGGGCCACACGCTCTCCCTTTCTG	8	C	0.2348	0.3639
NC_005102.4-14898019	CTCAGGGCCACACGCTCTCCCTTTCTG	12	A	0.2348	0.3639
NC_005102.4-14913753	GACTCTGGTACCTTGCCTCCAAGCCTG	3	C	0.1057	0.4952
NC_005102.4-14946263	GTGCATGTGACGCCTCCTCCCCACAGT	12	G	0	0.4826
NC_005102.4-14966183	TGTTCCTACACTCTCCCTCCCTGCTTG	1	T	0.122	0.4988
NC_005102.4-14971000	CACACACACACCCAGACTCCTTGTGGG	12	C	0.036	0.0526
NC_005102.4-14971000	CACACACACACCCAGACTCCTTGTGGG	16	A	0	0.4909
NC_005102.4-14980993	CCTGGGCAGACATTCCCTCCCCGACTT	12	A	0.0333	0.0328
NC_005102.4-14980993	CCTGGGCAGACATTCCCTCCCCGACTT	22	C	0.2881	0.4481

NC_005102.4-15035263	GGAGATGTAACCTCTGCTCCAGGTTTT	21	A	0	0.4755
NC_005102.4-15036901	CCCAGGTCGACACAGACTCCTCGACAG	5	G	0.5678	0.4207
NC_005102.4-15036901	CCCAGGTCGACACAGACTCCTCGACAG	23	G	0	0.4734
NC_005102.4-15040346	CCAGGAGGCACAGCATCTCCAGTAGTG	16	T	0.1789	0.2937
NC_005102.4-15093075	TTCCTAACAACACCCCCTCCCATAGTC	4	C	0.177	0.3728
NC_005102.4-15097945	CCAAACATTACCCGTCCTCCACAAGTC	9	T	0	0.4793
NC_005102.4-15121337	AGCCAGCTCACTCTCACTCCTGCAACC	1	A	0.0085	0.487
NC_005102.4-15121337	AGCCAGCTCACTCTCACTCCTGCAACC	26	C	0.15	0.3299
NC_005102.4-15158996	GCTCTCCTTACTTGTTCTCCATCTTGA	22	T	0.2203	0.324
NC_005102.4-15159889	TCCTTGCCCACCGCAGCTCCCTTGCTG	15	A	0.1441	0.3682
NC_005102.4-15167805	CCAATAAAGACCCTACCTCCAAATAAG	3	A	0.2035	0.2237
NC_005102.4-15185819	CAATCCGGCACTGAGTCTCCCCACTCA	4	T	0.125	0.1626
NC_005102.4-15208224	GGCTTTTGCACGTCACCTCCCTCTGGG	24	T	0.2768	0.388
NC_005102.4-15228060	GAGGTTCAGACTTGAACTCCACAGACC	9	G	0.025	0.0247
NC_005102.4-15232913	CTGTGCAAGACTAGAACTCCTCCCCAT	22	C	0.1466	0.4179
NC_005102.4-15232913	CTGTGCAAGACTAGAACTCCTCCCCAT	24	C	0.1466	0.4937
NC_005102.4-15249558	ATTCACCCAACATTTCCTCCATTCAGT	12	A	0.175	0.4991
NC_005102.4-15260771	TAAACTGTGACCATAGCTCCCTTGTAG	21	C	0.0085	0.4842
NC_005102.4-15262817	CCTTTTAAGACAGTCTCTCCCAGAGAG	12	A	0.1849	0.2203
NC_005102.4-15262817	CCTTTTAAGACAGTCTCTCCCAGAGAG	14	T	0.1765	0.214
NC_005102.4-15308017	CCTTCGTGGACTTCAGCTCCCCCATTT	5	C	0.0678	0.0812
NC_005102.4-15309319	CATGATCTGACCCTGGCTCCCTCCTCT	8	T	0	0.4787
NC_005102.4-15309319	CATGATCTGACCCTGGCTCCCTCCTCT	23	C	0.0331	0.4672
NC_005102.4-15312550	CGTGATGCAACCATCTCTCCCGTTCAT	26	A	0.0328	0.048
NC_005102.4-15312719	TTCAAGGGCACCAGGCCTCCTCTGTAG	14	G	0.2033	0.3646
NC_005102.4-15312719	TTCAAGGGCACCAGGCCTCCTCTGTAG	22	C	0	0.4793
NC_005102.4-15323574	CATAGGCTAACGTAGGCTCCATTTCAA	4	A	0.0588	0.4886
NC_005102.4-15396956	GGCTCCCACACAGCCCCTCCCACCAGC	24	C	0.0177	0.0517
NC_005102.4-15414455	CTAAGAGCGACAGCACCTCCTGCTTCT	2	T	0.0424	0.4919
NC_005102.4-15414455	CTAAGAGCGACAGCACCTCCTGCTTCT	8	C	0.2542	0.3707
NC_005102.4-15424067	CTTCCCTGGACATGTCCTCCCACCGTC	13	T	0.0667	0.095
NC_005102.4-15424067	CTTCCCTGGACATGTCCTCCCACCGTC	25	G	0	0.4832
NC_005102.4-15430712	CGCGCTGGCACTGGGGCTCCCTTCTGT	3	C	0	0.4793
NC_005102.4-15443337	GTCTGAGGAACTGTCACTCCTCCCGCC	1	G	0.0268	0.0264
NC_005102.4-15443337	GTCTGAGGAACTGTCACTCCTCCCGCC	25	G	0.0357	0.0351
NC_005102.4-15443397	AGTATTCCCACCAACCCTCCACCCCCA	8	C	0.3621	0.2965
NC_005102.4-15485585	TTTGCACAGACCAGTCCTCCTCCCTTG	15	T	0	0.4749
NC_005102.4-15518014	GAGGCTGACACTGCTGCTCCTCCGTCA	15	T	0.4359	0.3409
NC_005102.4-15518792	TAATGAGCTACTATTTCTCCTGATAGA	13	A	0.0085	0.4698
NC_005102.4-15539753	CAGGCAATAACCTGGCCTCCCGGATTA	23	G	0.1157	0.1235
NC_005102.4-15544212	GTAAAGATGACCATGCCTCCTTCAGGG	13	A	0	0.4793
NC_005102.4-15560217	AGAATGTAAACTACAGCTCCTCATGTC	6	G	0.0244	0.0241
NC_005102.4-15560217	AGAATGTAAACTACAGCTCCTCATGTC	24	T	0.3171	0.4417
NC_005102.4-15564140	GCCAGTGGTACCTGGTCTCCTCTCTGC	9	T	0.0246	0.4717
NC_005102.4-15564140	GCCAGTGGTACCTGGTCTCCTCTCTGC	13	T	0	0.4807
NC_005102.4-15571267	TTCGCAACTACTGTCACTCCAGTTCTA	4	G	0.2232	0.2991
NC_005102.4-15584195	GTCATACACACTCTCTCTCCAGAGCCT	15	C	0.339	0.4173
NC_005102.4-15587965	TCTATGTCCACGTCCTCTCCTACTTTG	5	T	0	0.4868
NC_005102.4-15787315	CCTTTTAAAACCCACTCTCCTATCTTC	3	T	0.0357	0.0521
NC_005102.4-15787315	CCTTTTAAAACCCACTCTCCTATCTTC	7	A	0	0.4641
NC_005102.4-15825655	TTGCCCCTTACCACTCCTCCTTCCCAC	16	C	0.0261	0.0257

NC_005102.4-16075016	AGTCCCTGAACCCTTCCTCCAACTCTC	13	C	0.2393	0.2604
NC_005102.4-16309299	TCTATAATCACAGTCCCTCCAATTCAT	15	C	0.2821	0.3685
NC_005102.4-16431087	TAACCTAAAACATTGTCTCCATTTATG	4	C	0.9915	0.5
NC_005102.4-16470586	TCATAGACCACATCTTCTCCTCTCCTG	27	G	0.2114	0.3688
NC_005102.4-16477322	TCCTAAGTAACTTGAGCTCCTTACAGA	23	A	0.0088	0.49
NC_005102.4-16482917	TGCTAGTGTACAGGAACTCCAGGGAGG	2	G	0.1301	0.2142
NC_005102.4-16482917	TGCTAGTGTACAGGAACTCCAGGGAGG	7	T	0.2377	0.375
NC_005102.4-16564141	TATGTAAGAACCCAGGCTCCTTTAAGG	3	T	0.1593	0.4968
NC_005102.4-16583168	GGACAATGAACTCTCTCTCCCTTTGGG	5	A	0.1983	0.3278
NC_005102.4-17073049	CAGGCTACAACATTTACTCCATTTTTC	8	C	0.0331	0.0325
NC_005102.4-17073049	CAGGCTACAACATTTACTCCATTTTTC	12	A	0.3417	0.3347
NC_005102.4-17089439	CTGCTCCCAACTTTGTCTCCATATCCT	4	C	0.3913	0.451
NC_005102.4-17108127	ATTGTGATGACCCAGACTCCATCCTCC	27	C	0.0517	0.0504
NC_005102.4-17118906	GGTTGCAGGACTAGGTCTCCTGCTCCT	1	G	0.1167	0.1099
NC_005102.4-17118906	GGTTGCAGGACTAGGTCTCCTGCTCCT	23	C	0.1176	0.1107
NC_005102.4-17315802	AGTCATTCCACTTGTTCTCCTCTTCTT	25	C	0	0.4814
NC_005102.4-17315802	AGTCATTCCACTTGTTCTCCTCTTCTT	26	T	0	0.4807
NC_005102.4-17325587	TGACCTTGAACATTTCCTCCTCTATCT	15	T	0.0345	0.0666
NC_005102.4-17607282	TAGCCCTCAACGAATTCTCCAGCATCA	26	C	0.1416	0.2302
NC_005102.4-18649785	CATAAGCCTACATCCCCTCCATCCCCC	15	C	0.0442	0.4961
NC_005102.4-18662543	CAGTTCTTCACTCTTCCTCCTTTCCAA	9	C	0.0893	0.232
NC_005102.4-19772086	ATCCAGATGACCCAGACTCCATCCTCC	9	G	0.0268	0.0264
NC_005102.4-20038822	GTGAGGGTGACTCTGTCTCCCGAAATG	7	G	0.0259	0.0255
NC_005102.4-20092436	CTTTGGAGGACCACCCCTCCTGCTCCT	14	C	0.1391	0.173
NC_005102.4-20092436	CTTTGGAGGACCACCCCTCCTGCTCCT	15	C	0.1652	0.2072
NC_005102.4-20197885	ATCCAGGTTACCCAGTCTCCATCCTCC	15	G	0.2377	0.321
NC_005102.4-20212461	TTCCTAGATACCTATGCTCCAAACACA	24	C	0	0.4807
NC_005102.4-20318342	ATTGCCACCACACCTGCTCCCCACACA	9	C	0.3217	0.3252
NC_005102.4-20689791	ATCAAGATGACACAGTCTCCTGCCTCT	24	C	0.0684	0.0818
NC_005102.4-20882640	AGCATGAGCACTGTACCTCCTAAGGCA	2	G	0.25	0.4688
NC_005102.4-20979746	TTGTGTTACACTCCTTCTCCACAAGGC	7	T	0.0268	0.0264
NC_005102.4-21139632	GCCATGAGCACCAACACTCCTGAGGGC	24	G	0.0167	0.0328
NC_005102.4-21139632	GCCATGAGCACCAACACTCCTGAGGGC	26	G	0.0084	0.4761
NC_005102.4-21148261	GCATGAGAAACCCACCCTCCAAGAATA	1	G	0.2522	0.4926
NC_005102.4-21210814	TGGGACCCCACTTTCTCTCCACTGAAT	6	C	0.4123	0.3273
NC_005102.4-21313592	GGCCTTCTCACTCACACTCCAAATGGC	26	G	0.0488	0.0476
NC_005102.4-21386549	GAATGACCTACTGGTCCTCCAGGTTGG	8	C	0	0.4637
NC_005102.4-21386549	GAATGACCTACTGGTCCTCCAGGTTGG	15	T	0	0.4637
NC_005102.4-21422326	TTTGGGAAGACTGAGGCTCCAAATCAT	15	G	0.1111	0.1492
NC_005102.4-21669641	TAGACACCTACCTGGGCTCCAACTAAC	8	C	0	0.48
NC_005102.4-21685821	CTAAGGTTCACCCCTGCTCCATATAAT	6	G	0	0.4813
NC_005102.4-21693543	CACAACATTACAGTGACTCCTTGATAT	6	C	0.2333	0.4688
NC_005102.4-21744318	GGCAATGCAACAGGGACTCCTGACCTT	23	A	0.0082	0.4822
NC_005102.4-21783979	ATCTTTCATACATTGCCTCCAACCATT	9	T	0	0.4734
NC_005102.4-21822697	AAGCACCTCACCTGCACTCCTGTAGCA	16	A	0	0.4728
NC_005102.4-21840863	GGGAACAATACTAGTCCTCCTTGTCTA	15	T	0	0.4786
NC_005102.4-21840863	GGGAACAATACTAGTCCTCCTTGTCTA	27	A	0	0.4786
NC_005102.4-21858459	AGGGTATACACTACAGCTCCTTTGCAC	13	A	0	0.4764
NC_005102.4-21910546	AAACTTTTGACCCATCCTCCATCAGTA	14	A	0	0.4751
NC_005102.4-22027307	CCTGTGCACACAACCCCTCCTGAGTGT	21	T	0	0.4786
NC_005102.4-22028762	CCCTACACAACTCTGCCTCCACAATCG	4	T	0	0.4757

NC_005102.4-22086294	TTCCTTAGAACAGACTCTCCTTCCACG	4	C	0	0.4832
NC_005102.4-22175238	AACTATTGAACCATCTCTCCAGCCCCT	14	T	0.2232	0.1983
NC_005102.4-22204360	AAGAAAGAAACAGGAACTCCCTACTAG	4	A	0.0847	0.1264
NC_005102.4-22217697	CCCTGAACCACACTGTCTCCCAGCCCT	24	C	0	0.477
NC_005102.4-22228953	ACCGAGGCCACCTCACCTCCTGAGGAA	2	C	0.2683	0.3888
NC_005102.4-22277094	CTCCCTTGCACTGTCCCTCCTAGATAT	25	T	0	0.4807
NC_005102.4-22277094	CTCCCTTGCACTGTCCCTCCTAGATAT	27	T	0.2917	0.3441
NC_005102.4-22376457	AGTTGAGCAACATCATCTCCAACACTA	13	T	0.3008	0.4618
NC_005102.4-22376457	AGTTGAGCAACATCATCTCCAACACTA	26	T	0	0.4793
NC_005102.4-22405252	AGCCTGCCAACCTATGCTCCATCTACA	2	G	0.2927	0.427
NC_005102.4-22485676	GATTTTAACACTTGAACTCCTGGTCCA	16	A	0.009	0.4635
NC_005102.4-22538542	CACAGACGGACCTTCTCTCCAAGAAGA	3	C	0	0.4793
NC_005102.4-22538566	AGAGGAACTACATCTTCTCCTCCCACA	2	G	0.0357	0.0351
NC_005102.4-22539637	TAAACATCTACCTTCTCTCCCCAAGAA	5	C	0	0.48
NC_005102.4-22606846	GACAGCAGCACTATTTCTCCTCCTCCT	1	G	0.2564	0.272
NC_005102.4-22611063	CTCAGTGGGACCGAGTCTCCCCACTGT	4	A	0.1417	0.1597
NC_005102.4-22611063	CTCAGTGGGACCGAGTCTCCCCACTGT	26	G	0.1849	0.3319
NC_005102.4-22634506	GGTTCTTCTACTTCCTCTCCCATCACC	27	C	0.1864	0.4977
NC_005102.4-22652292	CCCACTCCCACTCCCACTCCCACTTTG	4	A	0.5913	0.4234
NC_005102.4-22652292	CCCACTCCCACTCCCACTCCCACTTTG	5	C	0.0847	0.0812
NC_005102.4-22675702	CTCCAGGGCACTTCTGCTCCCTTTCCT	26	C	0.2377	0.479
NC_005102.4-22675702	CTCCAGGGCACTTCTGCTCCCTTTCCT	27	T	0.1967	0.3354
NC_005102.4-22685535	TGAAAAGTCACTTTGCCTCCCTGAGCC	22	T	0.2321	0.4228
NC_005102.4-22695290	CCCGTCCCGACTTCTCCTCCTTCATCC	22	T	0	0.48
NC_005102.4-22699631	TGTGGATCTACTGCCTCTCCTCCCTAG	23	C	0.1304	0.1369
NC_005102.4-22711615	GCAAGCTAGACAAGCTCTCCACCCACT	27	T	0.0336	0.033
NC_005102.4-22725300	AAACCACCCACTGCCTCTCCCGCTTTG	22	G	0	0.482
NC_005102.4-22829300	CCCACCCTCACCGAGTCTCCTTCCTCT	8	T	0.009	0.492
NC_005102.4-22829300	CCCACCCTCACCGAGTCTCCTTCCTCT	13	G	0.009	0.492
NC_005102.4-22841557	ACACCCGACACATAATCTCCTGCCCAT	6	C	0.0769	0.1049
NC_005102.4-22843356	CTCAGCCACACTGTGCCTCCAGCTGTT	27	T	0	0.4841
NC_005102.4-22843703	TAGTGAGCCACAAACCCTCCATCACAC	15	C	0.0342	0.4868
NC_005102.4-22843703	TAGTGAGCCACAAACCCTCCATCACAC	24	A	0.0517	0.0504
NC_005102.4-22848955	CACATGTGTACACAGACTCCACACCAG	4	A	0	0.4793
NC_005102.4-22848955	CACATGTGTACACAGACTCCACACCAG	25	C	0	0.4793
NC_005102.4-22855623	TTAGGGAACACATAGCCTCCTGGGATA	1	T	0.0492	0.0634
NC_005102.4-22892954	CTGTCTTAAACACCACCTCCACCTCCC	2	T	0.1057	0.1001
NC_005102.4-22955695	TGAGCAAAAACAGAGACTCCCTTCCTG	7	A	0	0.4807
NC_005102.4-22981848	GCGTATAGCACTGCCTCTCCTCTTCCT	3	G	0.1966	0.4714
NC_005102.4-22981848	GCGTATAGCACTGCCTCTCCTCTTCCT	14	C	0.0254	0.4842
NC_005102.4-23005882	CCATTGGCTACGTTGCCTCCAGTCCCT	14	T	0.1901	0.323
NC_005102.4-23075030	ATGGCCTGAACTGGTTCTCCTGCCTTT	4	G	0	0.4793
NC_005102.4-23075030	ATGGCCTGAACTGGTTCTCCTGCCTTT	12	T	0	0.4793
NC_005102.4-23087644	CACTGCCTCACACACTCTCCTGACAAC	14	A	0	0.4786
NC_005102.4-23087644	CACTGCCTCACACACTCTCCTGACAAC	24	C	0	0.4786
NC_005102.4-23147926	CCACTTGTAACTATAGCTCCTCACATG	14	T	0	0.4891
NC_005102.4-23285455	ACGAGTCTCACTAGGTCTCCCTGGCTG	3	G	0.1597	0.1746
NC_005102.4-23294676	AACTAAGCCACCAGATCTCCAAGGCAG	26	A	0.0496	0.0484
NC_005102.4-23296540	CCATGGGTAACCCAACCTCCAAGTCTT	27	T	0	0.4807
NC_005102.4-23328868	CAGTTCACAACTCTAACTCCAAGATAT	3	G	0.2105	0.2893
NC_005102.4-23506463	TGATCTATAACTCCAGCTCCATGGGAT	7	A	0.439	0.3604

NC_005102.4-23514194	GAATTCCAGACCATGCCTCCCCTTCAG	15	G	0	0.4773
NC_005102.4-23645527	AATGTGGGCACAAGAACTCCAGGGCTT	13	A	0.0088	0.4693
NC_005102.4-23671532	CTAGGTTACACACAATCTCCACACTAT	2	T	0	0.4883
NC_005102.4-23690666	AAGGCATGCACACCCTCTCCCAGCCCA	9	C	0.1271	0.1621
NC_005102.4-23749399	ACTTATCACACAGCTCCTCCTTGTATC	3	T	0.0259	0.4836
NC_005102.4-23749399	ACTTATCACACAGCTCCTCCTTGTATC	12	A	0.1111	0.1347
NC_005102.4-23776628	CCTGCATCCACTCCTCCTCCATCTCCC	21	A	0.2458	0.481
NC_005102.4-23831419	GGAATGTCTACTCCTGCTCCAGACCAA	26	A	0.0609	0.059
NC_005102.4-24153702	GAAGAGGATACTCCCACTCCTGTTTTT	14	C	0.0354	0.0348
NC_005102.4-24292092	CTGTTTCAAACATGTGCTCCTTACGTA	13	T	0.0246	0.0243
NC_005102.4-24292092	CTGTTTCAAACATGTGCTCCTTACGTA	25	G	0.1393	0.1968
NC_005102.4-24364654	GGCAGACAGACATCCTCTCCTTCTGGT	22	T	0	0.4827
NC_005102.4-24409372	CACACTGCTACTGAGTCTCCTGGATTC	21	T	0.1626	0.1761
NC_005102.4-24469921	AGAACAATCACTCTCACTCCTCAGTAT	9	C	0.2101	0.2626
NC_005102.4-24523164	TAGTCAAACACTGTGCCTCCAACATAG	13	G	0.2353	0.4921
NC_005102.4-24574591	CATTTTGTCACCCCCACTCCTGCACTG	8	T	0	0.4779
NC_005102.4-24619759	TTTTGGAATACATTTCCTCCCACTGGG	8	A	0.1026	0.0973
NC_005102.4-24882431	AGAATAAACACCAGTGCTCCAGGTTGC	22	G	0.0957	0.1067
NC_005102.4-24882431	AGAATAAACACCAGTGCTCCAGGTTGC	25	T	0	0.4786
NC_005102.4-24911691	AATCCATCCACCAAGCCTCCAGTGAAT	4	C	0.1488	0.4901
NC_005102.4-24976574	AAGAATATCACTGAAGCTCCGAAGATT	12	T	0.2437	0.4186
NC_005102.4-24976574	AAGAATATCACTGAAGCTCCGAAGATT	21	G	0.225	0.3708
NC_005102.4-25141723	CACTCCCGCACCCCATCTCCACAAGGA	5	C	0.4333	0.3394
NC_005102.4-25141723	CACTCCCGCACCCCATCTCCACAAGGA	7	C	0.0579	0.1017
NC_005102.4-25360552	CACACACACACCTGGCCTCCCTACTCC	13	T	0	0.4833
NC_005102.4-25536033	TAATGTATGACTCATGCTCCATGTGTG	13	C	0	0.4751
NC_005102.4-25578937	CCCTACTGCACTATCGCTCCAGTCTTA	4	T	0.157	0.1585
NC_005102.4-25600328	AGTTACAGGACTGGATCTCCATGACAC	5	A	0.2049	0.3307
NC_005102.4-25751521	CACACACACACCAGGACTCCTAGGGAA	14	G	0.0259	0.0585
NC_005102.4-25751521	CACACACACACCAGGACTCCTAGGGAA	24	G	0.0431	0.0422
NC_005102.4-25803285	CCAATGTGAACATCAGCTCCTGAAATT	8	G	0.0256	0.0418
NC_005102.4-25871062	TATGAATACACTATTCCTCCTCAGTAG	6	A	0	0.4765
NC_005102.4-25871062	TATGAATACACTATTCCTCCTCAGTAG	14	T	0.1	0.1932
NC_005102.4-25897053	CATGAAAACACATAAGCTCCACATGCA	3	T	0.1557	0.1968
NC_005102.4-26121339	TTGCGTCCTACAGCTTCTCCTTGCATG	1	T	0	0.4724
NC_005102.4-26123577	AACTGTGACACTTTAGCTCCTTTTCAT	15	A	0.1638	0.343
NC_005102.4-26123577	AACTGTGACACTTTAGCTCCTTTTCAT	16	G	0.2105	0.396
NC_005102.4-26138119	ACCTATGTCACTTGCTCTCCATTCTGC	8	T	0.0357	0.0521
NC_005102.4-26168680	TGTTCTGATACTTTTTCTCCTGGCAGT	8	A	0.1681	0.2856
NC_005102.4-26172690	AGTCTCTGCACTTTGTCTCCTTATTTC	7	T	0.4103	0.3729
NC_005102.4-26172690	AGTCTCTGCACTTTGTCTCCTTATTTC	21	T	0.4188	0.3771
NC_005102.4-26230906	CTGAAGAAAACTGTGTCTCCCTTCCTC	9	A	0.036	0.0354
NC_005102.4-26255416	TATCAAGATACTCCAGCTCCCACCCAT	9	T	0.0175	0.4555
NC_005102.4-26255416	TATCAAGATACTCCAGCTCCCACCCAT	16	G	0	0.4654
NC_005102.4-26364306	GACACCACAACTTCAGCTCCTAACTTG	3	C	0.0976	0.1073
NC_005102.4-26364306	GACACCACAACTTCAGCTCCTAACTTG	13	T	0.1951	0.2612
NC_005102.4-26403478	AATATTGTTACGCTTTCTCCATTGCTG	27	G	0.1356	0.1961
NC_005102.4-26420585	TTGACCCTAACTGAGACTCCTACCAGT	3	G	0.2727	0.3327
NC_005102.4-26420585	TTGACCCTAACTGAGACTCCTACCAGT	12	T	0.0413	0.0562
NC_005102.4-26521207	AGGCAAAAGACTAACACTCCTTCTTAA	3	G	0	0.0342
NC_005102.4-26521207	AGGCAAAAGACTAACACTCCTTCTTAA	15	C	0.2018	0.2836

NC_005102.4-26521670	GAGATTAAAACTTAACCTCCTTGCTTC	23	G	0.0847	0.4367
NC_005102.4-26521670	GAGATTAAAACTTAACCTCCTTGCTTC	24	C	0.3162	0.4473
NC_005102.4-26542728	TCTGCCACCACGCCTTCTCCTCCATGA	2	C	0.2941	0.3812
NC_005102.4-26592251	CTCTGATTAACATTCCCTCCTTATGTG	24	T	0.2975	0.3967
NC_005102.4-26675902	ACTCAGTGCACACATACTCCTTGGAGA	25	A	0.0714	0.0853
NC_005102.4-26678025	CTCAAGTTCACCTTTCCTCCCAGGGTC	5	A	0.0531	0.4671
NC_005102.4-26678025	CTCAAGTTCACCTTTCCTCCCAGGGTC	7	T	0.1053	0.1153
NC_005102.4-26680099	TATGAGGATACTTGCACTCCCACCTAC	27	C	0.0348	0.0342
NC_005102.4-26777757	TCAATATTAACTGGAACTCCTCCCTCT	22	C	0.1138	0.2263
NC_005102.4-26777978	CATGCCAGCACAAGCTCTCCGACTCTG	9	C	0	0.4931
NC_005102.4-26826992	TCTGATCTTACTGGCTCTCCACCTCCC	27	C	0.1026	0.3813
NC_005102.4-26927630	CACAGTGACACAATTTCTCCAATAAGA	9	C	0.1304	0.1659
NC_005102.4-26932840	GTATTAGGCACCTGCTCTCCACTTCCT	1	G	0.1102	0.1621
NC_005102.4-26932840	GTATTAGGCACCTGCTCTCCACTTCCT	12	C	0.0261	0.0257
NC_005102.4-26939695	ACTATGAACACAAGCTCTCCCACCCTT	13	A	0.1405	0.2474
NC_005102.4-27015736	TCTCGCTGCACTACTCCTCCTACAAAT	5	G	0	0.4734
NC_005102.4-27015736	TCTCGCTGCACTACTCCTCCTACAAAT	13	A	0.1441	0.1759
NC_005102.4-27041801	TATGTAGGTACAATAGCTCCTTTACAT	2	A	0.1026	0.1274
NC_005102.4-27041801	TATGTAGGTACAATAGCTCCTTTACAT	7	G	0.0336	0.033
NC_005102.4-27102088	AACCAATTTACATCTGCTCCCATAGAT	2	A	0.0175	0.0512
NC_005102.4-27102088	AACCAATTTACATCTGCTCCCATAGAT	6	A	0.0263	0.0429
NC_005102.4-27164185	AAGCTTATGACAGAGACTCCCAGAGCA	6	T	0.0089	0.4789
NC_005102.4-27202088	AAATCCCCAACCTATCCTCCCTCTACT	5	C	0.1271	0.4991
NC_005102.4-27202088	AAATCCCCAACCTATCCTCCCTCTACT	15	T	0.0756	0.1033
NC_005102.4-27217381	CGTAACTTTACTCAACCTCCTCATTAA	15	A	0.0614	0.1075
NC_005102.4-27217381	CGTAACTTTACTCAACCTCCTCATTAA	21	T	0.125	0.4974
NC_005102.4-27281040	ACCACATGGACGTGTGCTCCACGATAT	12	G	0.0598	0.074
NC_005102.4-27317998	GAAAATCTGACCGGCTCTCCCAACACA	13	G	0.041	0.0401
NC_005102.4-27317998	GAAAATCTGACCGGCTCTCCCAACACA	15	C	0.0246	0.0243
NC_005102.4-27320666	TGTTAGAAGACTGGTCCTCCACAGAGA	16	C	0.0261	0.0257
NC_005102.4-27321190	GTTGGCCAAACATTGTCTCCGAACTTC	16	T	0.1053	0.4975
NC_005102.4-27322160	AGAATTAATACAGTTACTCCGGAGAGC	21	G	0.025	0.0247
NC_005102.4-27322160	AGAATTAATACAGTTACTCCGGAGAGC	23	A	0.0424	0.4698
NC_005102.4-27343247	ATTTAATAAACAGTGTCTCCAATGTTG	6	A	0.2735	0.4444
NC_005102.4-27343247	ATTTAATAAACAGTGTCTCCAATGTTG	24	G	0.2627	0.3991
NC_005102.4-27368020	CACTAGCTCACTGTACCTCCGAATCTG	7	C	0.123	0.1436
NC_005102.4-27427513	CTGAGTAGTACTAGTACTCCATTTTTA	1	C	0	0.4846
NC_005102.4-27427513	CTGAGTAGTACTAGTACTCCATTTTTA	4	A	0.2544	0.3999
NC_005102.4-27535788	TACCATCATACAAGTACTCCCTCCATT	1	T	0.1667	0.2888
NC_005102.4-27535788	TACCATCATACAAGTACTCCCTCCATT	16	A	0	0.4751
NC_005102.4-27622253	TGCTATGAAACAGTCTCTCCCCAAGAA	2	G	0.3276	0.4345
NC_005102.4-27638609	GATATTTCAACATGGCCTCCCTTAGAT	3	T	0.3186	0.4207
NC_005102.4-27638609	GATATTTCAACATGGCCTCCCTTAGAT	9	A	0.0177	0.0348
NC_005102.4-27720242	AAAAGTCAAACCCAAACTCCTCACAGA	25	A	0	0.4704
NC_005102.4-27765662	CATGCTGTCACCCTGCCTCCCACACTG	2	A	0.2295	0.4934
NC_005102.4-27765662	CATGCTGTCACCCTGCCTCCCACACTG	27	G	0.0574	0.0557
NC_005102.4-27851789	TCCTCTCTAACACATCCTCCAAAATCA	5	C	0.0088	0.4873
NC_005102.4-27851789	TCCTCTCTAACACATCCTCCAAAATCA	21	A	0.0088	0.4861
NC_005102.4-27909702	TTTGTACCAACACAGCCTCCTGAGGAA	12	A	0.3008	0.4572
NC_005102.4-27909702	TTTGTACCAACACAGCCTCCTGAGGAA	13	C	0	0.4793
NC_005102.4-27910948	TGGAACAAGACAAACACTCCTCCATTG	1	T	0.6404	0.4415

NC_005102.4-27910948	TGGAACAAGACAAACACTCCTCCATTG	8	A	0.6404	0.4415
NC_005102.4-27961552	AAGGTGTGTACCACGACTCCTGGATTT	14	C	0.0973	0.4944
NC_005102.4-27961552	AAGGTGTGTACCACGACTCCTGGATTT	24	A	0.0442	0.0433
NC_005102.4-28078244	CTGTGGCACACCAGTTCTCCAGCTCTA	13	A	0.1565	0.2268
NC_005102.4-28137140	ACCAATATTACTGAATCTCCAATCAAT	7	A	0.0531	0.1757
NC_005102.4-28156006	GCGTGCCAAACAGCCCCTCCCATCCAG	3	G	0.1057	0.1001
NC_005102.4-28228266	CTTGTTATTACCACCACTCCCAGATAA	25	T	0.0614	0.0595
NC_005102.4-28290917	AACCCTAAGACCTAACCTCCCTCATAA	4	C	0	0.4844
NC_005102.4-28290917	AACCCTAAGACCTAACCTCCCTCATAA	15	A	0.1597	0.1746
NC_005102.4-28294377	GTCTTTTTGACACCACCTCCCTCATTA	13	C	0.0331	0.0325
NC_005102.4-28294377	GTCTTTTTGACACCACCTCCCTCATTA	24	A	0.0331	0.0325
NC_005102.4-28305983	GTCATGTAAACACAACCTCCTTTCCTG	23	T	0.0917	0.1025
NC_005102.4-28419001	AGGTAAGTAACCCTAACTCCTCAGTAT	16	A	0.0826	0.0792
NC_005102.4-28419674	GCAGTGTTAACAAATGCTCCTGTAAGG	3	A	0.2212	0.2738
NC_005102.4-28430154	TACAGGCTAACTTTACCTCCAGTGTTT	1	T	0.025	0.0247
NC_005102.4-28488899	CAGTGAGCAACACCGTCTCCTCAAAAT	13	C	0.0252	0.0249
NC_005102.4-28490164	TGACCTACAACAGAAGCTCCTATCTCT	5	C	0.0085	0.481
NC_005102.4-28531114	CAGGTTCCTACCCCAACTCCTTTTGAT	5	T	0.0333	0.0328
NC_005102.4-28531114	CAGGTTCCTACCCCAACTCCTTTTGAT	21	T	0	0.48
NC_005102.4-28573407	CATCCTGTCACTGACACTCCTCAGTTA	27	A	0.187	0.2885
NC_005102.4-28656651	GTTTCTCAGACTTACCCTCCTGACTAT	16	C	0	0.4765
NC_005102.4-28660181	GTGTAAGCTACCTAAGCTCCAATGACT	8	C	0.1111	0.12
NC_005102.4-28660181	GTGTAAGCTACCTAAGCTCCAATGACT	13	T	0.0171	0.4649
NC_005102.4-28687696	CCCAGACTGACCTGCTCTCCTTGTTCC	5	G	0.2231	0.2922
NC_005102.4-28704858	CTTCGAGGTACATGTTCTCCATGAGTG	24	A	0.0088	0.4889
NC_005102.4-28758574	CAACAATGGACTAAAACTCCGAAACTC	7	T	0.0488	0.0476
NC_005102.4-28804039	TTTAAAAACACACTCGCTCCTTGCCTG	16	G	0.0345	0.0339
NC_005102.4-28860653	GTGGTAAATACATAGACTCCTAATACT	13	T	0.1786	0.2934
NC_005102.4-29020419	TTCATAACTACATTGACTCCTCACACA	1	T	0.25	0.4688
NC_005102.4-29020419	TTCATAACTACATTGACTCCTCACACA	22	C	0.2333	0.3299
NC_005102.4-29031578	TTGAGGATAACAATGTCTCCTTAGATA	27	A	0.275	0.3622
NC_005102.4-29039752	CCATGTGATACAGCGTCTCCGCACTGC	14	C	0.0656	0.0935
NC_005102.4-29149088	CCCATAGGAACTCAAACTCCATTATGC	26	G	0.0526	0.0997
NC_005102.4-29197954	TTCTCCATTACTGTACCTCCATTAGGA	14	T	0.1176	0.1254
NC_005102.4-29253451	TTGTTCTCCACCTCATCTCCTTTTCCT	24	T	0	0.4765
NC_005102.4-29289176	GGTGCCTACACTTAATCTCCTTCCTTC	16	T	0	0.4813
NC_005102.4-29290998	CCTCCTCATACCGCAGCTCCAGCTGGG	1	C	0.0083	0.4783
NC_005102.4-29290998	CCTCCTCATACCGCAGCTCCAGCTGGG	13	G	0.2857	0.3771
NC_005102.4-29328178	GGCACAGTGACTCATGCTCCAGTATAG	25	T	0	0.477
NC_005102.4-29328459	TTCTAGGGTACTGATTCTCCACGTAAG	8	G	0.0351	0.4846
NC_005102.4-29328459	TTCTAGGGTACTGATTCTCCACGTAAG	12	T	0.0348	0.4864
NC_005102.4-29339238	AATGACCCTACCTAAACTCCTAGTAAT	4	G	0.0088	0.4861
NC_005102.4-29339238	AATGACCCTACCTAAACTCCTAGTAAT	7	C	0.0088	0.4861
NC_005102.4-29383021	GAACACCACACGCGGCCTCCTCCACAG	12	G	0	0.4772
NC_005102.4-29396381	CAGTTCCCCACAGTGCCTCCAGTGTGT	21	A	0	0.4807
NC_005102.4-29407964	CCCTTTTGTACCAGAACTCCAAAGGAA	9	T	0	0.4779
NC_005102.4-29425917	TACAGAGACACTCCTCCTCCTGCAGTT	14	C	0	0.4779
NC_005102.4-29425917	TACAGAGACACTCCTCCTCCTGCAGTT	16	C	0	0.033
NC_005102.4-29521477	CACACACACACCCTTCCTCCTTGTCTT	6	A	0.1504	0.4994
NC_005102.4-29521477	CACACACACACCCTTCCTCCTTGTCTT	12	C	0.0439	0.0429
NC_005102.4-29521700	CACTCAGCAACCCCCTCTCCTCCTGGA	5	C	0.2564	0.3813

NC_005102.4-29551477	ATTATCCCAACTTCTTCTCCAGAGGGT	26	G	0.0177	0.0517
NC_005102.4-29555699	ACTCCATCGACCAGCTCTCCAATGAAT	9	G	0.1525	0.2219
NC_005102.4-29558134	AAGTCCATGACACCATCTCCCCTTCCT	8	T	0	0.4773
NC_005102.4-29573188	GTCATGGCCACTGGAGCTCCTCTCTTC	1	G	0.2833	0.4444
NC_005102.4-29598537	CCTTCAGCGACTGATGCTCCTCTTGCC	8	C	0	0.4813
NC_005102.4-29620800	GCGCTCCCTACACTTTCTCCTCCTGCT	3	G	0.0084	0.4859
NC_005102.4-29661590	TTCCTGTTCACTAACTCTCCTGTTAAC	7	T	0.0738	0.1154
NC_005102.4-29800205	AATGCACTAACCTGGGCTCCCAGGGAC	5	C	0.0813	0.1216
NC_005102.4-29800205	AATGCACTAACCTGGGCTCCCAGGGAC	6	A	0.0732	0.0705
NC_005102.4-29889382	CATGTGTGCACTCCTACTCCTGTGTAC	15	T	0.0163	0.4759
NC_005102.4-29907013	GACATCAAAACTTCATCTCCCTGCCTA	5	T	0.0348	0.0671
NC_005102.4-29907013	GACATCAAAACTTCATCTCCCTGCCTA	12	T	0.0614	0.0595
NC_005102.4-29915996	CATCTCCTGACCATGGCTCCTACTTAT	1	C	0	0.4841
NC_005102.4-29915996	CATCTCCTGACCATGGCTCCTACTTAT	2	A	0	0.4841
NC_005102.4-29944057	GTTGTGTAAACCACAGCTCCATGGACC	23	G	0.025	0.0247
NC_005102.4-29944231	TAGCTCAGCACACCTACTCCCTTCTAA	4	C	0.0085	0.4738
NC_005102.4-30073441	GAGCTTGTCACTCGACCTCCCTGAATC	6	T	0.3	0.32
NC_005102.4-30150842	GTGTAAATAACTGGGTCTCCAATAGCT	6	A	0.1333	0.2061
NC_005102.4-30184434	ATGGGCTTGACCTAGGCTCCCTGCATA	1	A	0.1345	0.1254
NC_005102.4-30184434	ATGGGCTTGACCTAGGCTCCCTGCATA	27	A	0.7311	0.4638
NC_005102.4-30192662	TGGGCACCTACCTGGGCTCCATCTTGA	8	C	0.1368	0.2235
NC_005102.4-30192662	TGGGCACCTACCTGGGCTCCATCTTGA	12	C	0.1724	0.2501
NC_005102.4-30275890	CAAGACTCCACCTCCCCTCCCCACACT	3	A	0.1709	0.4982
NC_005102.4-30308592	TGTGTCGATACATGGTCTCCTAAAGAT	2	G	0.2632	0.3878
NC_005102.4-30308592	TGTGTCGATACATGGTCTCCTAAAGAT	6	C	0.1754	0.2413
NC_005102.4-30402204	GAAGCTGGAACTTTTCCTCCAAATAAG	9	A	0.009	0.4766
NC_005102.4-30482629	CTGCTTCTTACAATTCCTCCCCCTCTT	23	C	0.0424	0.0734
NC_005102.4-30548134	AATACTTCAACCCTATCTCCTAATAAC	14	T	0.3043	0.4782
NC_005102.4-30563010	CAACCTATAACTCCATCTCCAGGGAAT	8	T	0.114	0.1075
NC_005102.4-30582773	ACATGAAACACCTCATCTCCATGCAGA	14	C	0.2034	0.2926
NC_005102.4-30688916	CTCCCTCCCACATCTCCTCCCATTGAT	8	C	0.1724	0.199
NC_005102.4-30688916	CTCCCTCCCACATCTCCTCCCATTGAT	14	C	0.0259	0.0255
NC_005102.4-30875015	CCTGGTGGCACCAAGCCTCCAGGGATG	4	G	0.0661	0.0792
NC_005102.4-30875015	CCTGGTGGCACCAAGCCTCCAGGGATG	14	A	0.0744	0.1017
NC_005102.4-30880548	TGGAAAATCACTGTAACTCCCAATAGC	3	G	0.0603	0.0585
NC_005102.4-30880548	TGGAAAATCACTGTAACTCCCAATAGC	14	T	0.0783	0.4055
NC_005102.4-30944920	ATATCTCTCACAGTACCTCCTACCATA	3	A	0	0.48
NC_005102.4-30944920	ATATCTCTCACAGTACCTCCTACCATA	14	T	0.2542	0.3707
NC_005102.4-30970871	CAATCCCCTACATGACCTCCATAGTAG	6	C	0.2393	0.4938
NC_005102.4-30988684	ATTAAAAGGACACATGCTCCACTATCT	7	A	0.1271	0.1621
NC_005102.4-30988684	ATTAAAAGGACACATGCTCCACTATCT	15	T	0.1271	0.1621
NC_005102.4-31004788	GTTGGTCTTACCTCTGCTCCCTGGTGT	9	T	0.0862	0.0825
NC_005102.4-31004788	GTTGGTCTTACCTCTGCTCCCTGGTGT	23	G	0.0172	0.0339
NC_005102.4-31055227	TTTAGATAAACTTATCCTCCTGCTGTG	1	T	0.0345	0.0504
NC_005102.4-31055227	TTTAGATAAACTTATCCTCCTGCTGTG	3	T	0.1466	0.5
NC_005102.4-31093272	TCTGCCTCTACAGTGACTCCTTCTTCA	5	C	0.2377	0.3984
NC_005102.4-31093272	TCTGCCTCTACAGTGACTCCTTCTTCA	23	C	0.123	0.2095
NC_005102.4-31325325	CCTTTTGAAACATGCTCTCCCTACATG	24	C	0.0661	0.4923
NC_005102.4-31400069	GATTTAACCACAGCTGCTCCACGGGGA	23	G	0.0333	0.0644
NC_005102.4-31615967	CCTCTGGATACGGTCTCTCCATCTTCC	2	C	0.0593	0.0734
NC_005102.4-31615967	CCTCTGGATACGGTCTCTCCATCTTCC	6	G	0.1864	0.334

NC_005102.4-31621509	TTACCGCACACCTACCCTCCAGACTCA	6	G	0.2083	0.2941
NC_005102.4-31621509	TTACCGCACACCTACCCTCCAGACTCA	9	C	0.1833	0.2061
NC_005102.4-31730139	ATCCCCTTCACCACTACTCCATTATCT	8	T	0.2397	0.465
NC_005102.4-31730139	ATCCCCTTCACCACTACTCCATTATCT	14	C	0.2417	0.3441
NC_005102.4-31830002	TGAAAGGTCACATAGTCTCCCAAGGAA	2	G	0.0513	0.066
NC_005102.4-31830002	TGAAAGGTCACATAGTCTCCCAAGGAA	6	G	0.0342	0.0336
NC_005102.4-31868823	CATGGGAGGACCCTGGCTCCAGCTGGA	1	C	0.5739	0.4936
NC_005102.4-31868823	CATGGGAGGACCCTGGCTCCAGCTGGA	6	G	0.5739	0.4936
NC_005102.4-31870857	AACACACAAACAGTCTCTCCTGATTAT	1	A	0.1707	0.1954
NC_005102.4-31870857	AACACACAAACAGTCTCTCCTGATTAT	24	T	0	0.4807
NC_005102.4-31898035	TCAGGATCCACTAGAGCTCCAAGCCAA	1	T	0.0598	0.1049
NC_005102.4-31898035	TCAGGATCCACTAGAGCTCCAAGCCAA	15	A	0.084	0.1107
NC_005102.4-31903754	ACATTGCATACCTGAGCTCCATTAGTT	16	G	0.1057	0.1287
NC_005102.4-31903754	ACATTGCATACCTGAGCTCCATTAGTT	23	T	0.122	0.4988
NC_005102.4-32001264	TCTTGCCACACTAGCACTCCCCTTCTC	9	C	0.0517	0.1284
NC_005102.4-32041260	TCCTCTCCCACCTCTCCTCCTGTACCC	7	C	0.0248	0.0245
NC_005102.4-32041260	TCCTCTCCCACCTCTCCTCCTGTACCC	24	A	0	0.4703
NC_005102.4-32073671	TTACTAGTAACGATTCCTCCAGTTGGT	4	C	0	0.4839
NC_005102.4-32075825	ACTGCAAACACCCCATCTCCTGGTGTT	12	C	0	0.4773
NC_005102.4-32242627	TACCTTTTCACATTGGCTCCAGGCTCC	2	A	0.25	0.4888
NC_005102.4-32255792	GAATCTATTACAGTATCTCCTGGGCCC	12	A	0.0417	0.0408
NC_005102.4-32255792	GAATCTATTACAGTATCTCCTGGGCCC	24	G	0.1949	0.2406
NC_005102.4-32447429	GTGGCCACCACCATCTCTCCTGTTGAC	8	C	0.0083	0.0245
NC_005102.4-32542516	AATAAGTAGACAGTGCCTCCAAGCTAT	3	T	0	0.4765
NC_005102.4-32542516	AATAAGTAGACAGTGCCTCCAAGCTAT	6	G	0.025	0.0408
NC_005102.4-32544962	TTTGTTCAAACTCCTCCTCCACCAGGT	21	A	0	0.4807
NC_005102.4-32546670	TAAACAATAACAAAGGCTCCCACTGTG	22	A	0.1803	0.4773
NC_005102.4-32573671	CCTAAGTGCACGTTCCCTCCTCCCATC	12	G	0.025	0.0408
NC_005102.4-32585942	GGATAGAATACAGCTCCTCCTGCTTAC	14	C	0.0431	0.4688
NC_005102.4-32644639	AAACTACAAACCAGATCTCCTTGTACA	8	A	0.0991	0.4985
NC_005102.4-32644639	AAACTACAAACCAGATCTCCTTGTACA	14	G	0.0268	0.0264
NC_005102.4-32676506	TCGGAAAGCACTATCCCTCCCTTCCTT	15	C	0	0.4814
NC_005102.4-32701713	GCCAGACATACTTGTGCTCCCCAAACT	13	T	0.2544	0.3836
NC_005102.4-32728010	CTACCTGAAACTCCAACTCCTGGGGGT	8	A	0.1652	0.3639
NC_005102.4-32774750	GCATTGCCTACAACACCTCCATGGCAG	23	G	0.0407	0.4868
NC_005102.4-32817212	CAAGTGGGTACAGTATCTCCCACATAG	23	C	0.041	0.4879
NC_005102.4-32840598	GTCTCACCAACATGGCCTCCCAATGTG	5	C	0.0588	0.4859
NC_005102.4-32901600	AGAAAAGTCACAGTGACTCCACAATTA	7	G	0.1653	0.3967
NC_005102.4-32918948	CTTTATTTGACTTTATCTCCTCCTGCT	5	A	0.1453	0.2299
NC_005102.4-33102963	AATTGCAACACAGTAACTCCTGTGCTG	9	C	0	0.4773
NC_005102.4-33143699	CCCTCTCCTACTTGAACTCCTCCATAC	9	T	0.0088	0.476
NC_005102.4-33143699	CCCTCTCCTACTTGAACTCCTCCATAC	13	T	0.0087	0.4744
NC_005102.4-33163583	GATCTCCTGACACCATCTCCTTCCTTG	3	T	0	0.4773
NC_005102.4-33260924	AGTAGGATGACTAAGACTCCAGTAGTT	1	A	0.0619	0.06
NC_005102.4-33297267	AGTAGATGCACCCCACCTCCTGGACTG	9	C	0.1638	0.2681
NC_005102.4-33297267	AGTAGATGCACCCCACCTCCTGGACTG	24	A	0	0.4839
NC_005102.4-33299450	GAAGACTCGACCACCTCTCCAGAACCA	9	G	0.0087	0.4849
NC_005102.4-33476934	AGTATGTTCACTTAAACTCCACCATCA	22	C	0.1026	0.1125
NC_005102.4-33554868	AGACCTGGGACCCTATCTCCACTCCCA	15	A	0.2212	0.3189
NC_005102.4-33558937	GCAGGACACACCCCTTCTCCTACCTGG	24	C	0.0684	0.0973
NC_005102.4-33718569	AAACCAACCACTTTATCTCCCTTCGCC	25	G	0	0.4851

NC_005102.4-33744357	CGCTGGCCAACACATCCTCCACTATAC	3	C	0	0.48
NC_005102.4-33744357	CGCTGGCCAACACATCCTCCACTATAC	9	A	0	0.48
NC_005102.4-33752761	CACGCCAACACTTGTGCTCCCTCAAGA	4	G	0	0.4807
NC_005102.4-33752761	CACGCCAACACTTGTGCTCCCTCAAGA	7	A	0	0.4807
NC_005102.4-33761123	GGTGCCCACACCTTGACTCCTACAGGT	9	C	0.2049	0.2686
NC_005102.4-33761123	GGTGCCCACACCTTGACTCCTACAGGT	23	C	0.065	0.078
NC_005102.4-33785133	ACTGAAGCAACCCCTGCTCCACTTGTT	26	T	0	0.4751
NC_005102.4-33876390	AGAGTGATCACCTAAGCTCCAGTAATA	2	G	0.0348	0.0671
NC_005102.4-33876390	AGAGTGATCACCTAAGCTCCAGTAATA	5	T	0.0783	0.0752
NC_005102.4-33884225	AAACTCCTCACTCTGGCTCCCCGGGAC	14	T	0.1356	0.4987
NC_005102.4-33987858	CCATTCAAGACGCTTACTCCTTTCCTG	2	C	0.3197	0.3667
NC_005102.4-33987858	CCATTCAAGACGCTTACTCCTTTCCTG	3	A	0.3197	0.4588
NC_005102.4-33998792	TAGCACACAACCTATTCTCCTATTTTT	7	A	0.2241	0.2122
NC_005102.4-33998792	TAGCACACAACCTATTCTCCTATTTTT	27	T	0.1197	0.3261
NC_005102.4-34050858	TTCCATTCAACCTCCTCTCCCAGCATT	23	G	0.1513	0.1398
NC_005102.4-34050858	TTCCATTCAACCTCCTCTCCCAGCATT	24	C	0.0504	0.0805
NC_005102.4-34206679	TGAGATGCCACCTTACCTCCACCCTCA	14	T	0	0.4813
NC_005102.4-34206679	TGAGATGCCACCTTACCTCCACCCTCA	15	A	0	0.4813
NC_005102.4-34265699	CTCTTGTGTACTTATGCTCCAGGTGAT	8	G	0.05	0.0488
NC_005102.4-34321991	GTCTAGTTGACAGATCCTCCAGACGAT	27	T	0.0496	0.0943
NC_005102.4-34408186	GCATCTGGAACCATGGCTCCCTCCATG	13	A	0.1786	0.5
NC_005102.4-34408186	GCATCTGGAACCATGGCTCCCTCCATG	16	G	0.0517	0.0504
NC_005102.4-34429594	GCATGGTCAACATTTCCTCCCCCAGTA	21	C	0.0413	0.0405
NC_005102.4-34429594	GCATGGTCAACATTTCCTCCCCCAGTA	27	A	0.1583	0.4622
NC_005102.4-34440249	GATGGCTGCACCATTGCTCCGTCATTC	21	G	0.1624	0.4578
NC_005102.4-34473820	TGCCCTGCCACGGCTTCTCCAGAAATA	3	C	0.125	0.2449
NC_005102.4-34473820	TGCCCTGCCACGGCTTCTCCAGAAATA	7	G	0.0796	0.154
NC_005102.4-34526842	ACATGGATCACACTGGCTCCACTGCCA	1	A	0.0763	0.4957
NC_005102.4-34604180	AGTAGGAACACAGCCCCTCCCGTGATT	22	G	0	0.4773
NC_005102.4-34622681	AACCTCCAAACAATCTCTCCAGTTAAC	27	C	0	0.4751
NC_005102.4-34774422	AAGCTGCCAACGTGGACTCCCTGAATG	22	T	0.0086	0.4866
NC_005102.4-34926264	TACATTCCTACTGGAACTCCACAGCCT	4	A	0.0982	0.0934
NC_005102.4-34960443	ATAACAAGGACATGTTCTCCACTATGC	4	A	0.0088	0.481
NC_005102.4-35018679	CCTGACTCAACTCTCCCTCCAGCCCCT	25	C	0.0175	0.4606
NC_005102.4-35110914	CAATTAAGAACAGATTCTCCTTTTGCA	15	T	0.0351	0.4846
NC_005102.4-35119694	TGTGGAAATACAGCTACTCCTTAAGGA	1	T	0.0901	0.3178
NC_005102.4-35119694	TGTGGAAATACAGCTACTCCTTAAGGA	2	G	0.0901	0.1023
NC_005102.4-35127415	AATCCACGCACTCGAGCTCCTTATGTA	14	G	0.0593	0.1041
NC_005102.4-35145843	TTCAGCTGGACTGTAGCTCCTGCAGCA	14	T	0	0.4807
NC_005102.4-35221823	ATTTCACCCACGGCCCCTCCCCGCCTC	12	G	0	0.48
NC_005102.4-35221823	ATTTCACCCACGGCCCCTCCCCGCCTC	23	G	0.0738	0.1009
NC_005102.4-35225684	ACCATACCCACTGTCTCTCCCCACTTG	3	C	0.0333	0.0644
NC_005102.4-35225684	ACCATACCCACTGTCTCTCCCCACTTG	26	T	0.1417	0.2125
NC_005102.4-35288820	GCACACATCACTGGAGCTCCTTGGCCA	4	C	0.1368	0.1975
NC_005102.4-35288820	GCACACATCACTGGAGCTCCTTGGCCA	5	A	0.0086	0.4866
NC_005102.4-35290563	GGGCTTAATACTGCTGCTCCTTGTAGA	23	G	0	0.4772
NC_005102.4-35387420	GGGGCATCAACTGTTACTCCAGTTTAT	6	A	0.0354	0.4715
NC_005102.4-35412548	CTTGGGGCTACCTCTTCTCCCTACACT	16	T	0	0.4772
NC_005102.4-35505750	AAAGCCTCTACAAAGACTCCCTCTCTT	21	C	0.0083	0.4803
NC_005102.4-35510033	TTCCCTCTTACCCATGCTCCTTGCTAC	9	T	0.065	0.0928
NC_005102.4-35510033	TTCCCTCTTACCCATGCTCCTTGCTAC	27	C	0	0.4751

NC_005102.4-35511212	TGTTCGTTAACCCTTCCTCCCCATTCA	12	C	0.0603	0.0585
NC_005102.4-35611242	ACCCAACATACACTCTCTCCTCTGTGG	4	C	0.0088	0.026
NC_005102.4-35656327	TGGTTGGAGACATTATCTCCAAGGAAA	2	G	0.281	0.3278
NC_005102.4-35657059	CCAGTCCCCACCCTTCCTCCGTCACCT	7	C	0.0569	0.0854
NC_005102.4-35657059	CCAGTCCCCACCCTTCCTCCGTCACCT	8	C	0.0488	0.0476
NC_005102.4-35678238	CCTGAATGCACGTTTGCTCCAGGAAGC	7	T	0.0614	0.0758
NC_005102.4-35735394	CAATGTTCCACACCATCTCCTTGCCCA	12	A	0	0.4902
NC_005102.4-35735394	CAATGTTCCACACCATCTCCTTGCCCA	14	C	0.0088	0.026
NC_005102.4-35768109	AAGGGTCCCACCCCTTCTCCCTTTGCT	6	T	0.0976	0.4944
NC_005102.4-35823089	ATGCCATTCACTGCCCCTCCCAGCATC	26	T	0	0.4827
NC_005102.4-35890336	ATGTGTGGAACAACTTCTCCATGACTG	23	G	0.2066	0.2474
NC_005102.4-35890826	GGAAATTGCACTGCTGCTCCAATCTCT	21	A	0	0.4807
NC_005102.4-35890826	GGAAATTGCACTGCTGCTCCAATCTCT	24	C	0.0244	0.0241
NC_005102.4-35916523	TGCACAATCACATAAACTCCTCAACCA	12	A	0.0246	0.0401
NC_005102.4-35932352	CACCTCTTCACCCTAGCTCCAGATCCA	23	A	0.2583	0.3441
NC_005102.4-35979768	CCCAGGAGTACTGTCCCTCCTAAGTTT	16	C	0	0.4912
NC_005102.4-35979768	CCCAGGAGTACTGTCCCTCCTAAGTTT	25	T	0	0.4912
NC_005102.4-36033461	CATAACATAACATACACTCCAGACATA	24	C	0.0342	0.0336
NC_005102.4-36172899	AAGTGAAATACGTACACTCCCAGAGTA	12	G	0.0261	0.059
NC_005102.4-36172899	AAGTGAAATACGTACACTCCCAGAGTA	21	C	0.2212	0.4435
NC_005102.4-36184934	TGTGGTATCACAAGAGCTCCGTTCAAA	3	T	0.0631	0.4635
NC_005102.4-36184934	TGTGGTATCACAAGAGCTCCGTTCAAA	4	G	0.2432	0.2531
NC_005102.4-36208534	ATGTCATCTACTGTGTCTCCATTCCTG	1	A	0.1967	0.4565
NC_005102.4-36208534	ATGTCATCTACTGTGTCTCCATTCCTG	7	T	0.1901	0.4507
NC_005102.4-36276966	CATGTGTGCACGCTGTCTCCATGTTTT	14	T	0.0325	0.032
NC_005102.4-36286633	GACTCCACAACCAAACCTCCTTTGCCT	5	C	0.0163	0.4825
NC_005102.4-36303188	TAATGTATCACCTGCCCTCCCTGCACC	12	C	0.1066	0.1296
NC_005102.4-36320842	AGGCTAGACACTGCCTCTCCCTTTTCC	2	G	0.2212	0.481
NC_005102.4-36320842	AGGCTAGACACTGCCTCTCCCTTTTCC	26	C	0	0.4807
NC_005102.4-36328200	TACTGTTCAACAGAGGCTCCCAGTGTC	25	G	0	0.4773
NC_005102.4-36330607	AGCTAGAAAACATTGCCTCCTTGGGAG	6	G	0.084	0.1107
NC_005102.4-36457216	TGAGTTCCTACTTCGACTCCTTCAATG	15	G	0.1368	0.4991
NC_005102.4-36478437	CCCTAACAAACCTTATCTCCAGTGGAT	4	T	0.1	0.3299
NC_005102.4-36509627	GTAACTCAAACCCTGCCTCCTTCAGTT	5	C	0.1429	0.4949
NC_005102.4-36583746	TATAACATGACAGAGCCTCCCTCTGCC	23	C	0.0446	0.0436
NC_005102.4-36601597	GGTCGGGAGACAGGTTCTCCTCAAGTG	7	G	0.2119	0.2526
NC_005102.4-36628107	GCTTAATCAACTTAATCTCCAACACAC	4	T	0	0.4793
NC_005102.4-36637607	CACTGGCTGACTCTGACTCCTCCTTCA	25	T	0	0.4778
NC_005102.4-36657472	ATACCAGGAACCCCATCTCCTTAGCAA	3	A	0	0.4786
NC_005102.4-36657472	ATACCAGGAACCCCATCTCCTTAGCAA	7	G	0	0.4786
NC_005102.4-36734816	GGTATGAGCACAGCATCTCCAAACTGA	5	T	0.2389	0.2856
NC_005102.4-36734816	GGTATGAGCACAGCATCTCCAAACTGA	27	A	0	0.4715
NC_005102.4-36784549	TGTTTCTCAACTCTGCCTCCTAAGGGA	4	T	0	0.4793
NC_005102.4-36790833	CACACACACACACACACTCCTGAGCCC	13	C	0.065	0.0629
NC_005102.4-36805276	GGTTGAAACACCCTGTCTCCTATGGAT	9	C	0.0413	0.0562
NC_005102.4-36832378	AAAGGGAAAACTGAGACTCCTCAGATG	5	G	0.036	0.0354
NC_005102.4-36844108	GCCTGGGCAACTCTTACTCCAATTGAC	27	C	0	0.033
NC_005102.4-36863542	ATCATACTTACTCCTTCTCCAGTTCCT	15	T	0.114	0.138
NC_005102.4-36863542	ATCATACTTACTCCTTCTCCAGTTCCT	16	T	0.0179	0.4641
NC_005102.4-36896316	ATGCTGTGCACTACTACTCCCAGAGAA	12	T	0	0.4864
NC_005102.4-36961173	AGAAGAATAACCAAGACTCCTAATCAC	24	T	0	0.4793

NC_005102.4-36965852	CAAACATAAACTTAACCTCCTCTTCAT	21	T	0.1545	0.2556
NC_005102.4-36965852	CAAACATAAACTTAACCTCCTCTTCAT	27	T	0.1626	0.2612
NC_005102.4-37002740	CCAACAGGAACCTTTGCTCCTTAGATC	8	G	0.0569	0.0705
NC_005102.4-37118589	ATATCGCTCACCCTTGCTCCTGGAGTT	21	T	0	0.4731
NC_005102.4-37122541	CAGCGGCTGACAATGGCTCCAACACCC	5	G	0.2807	0.3424
NC_005102.4-37135976	TGCTTGTTCACACACTCTCCACTCCTG	22	C	0.2203	0.2466
NC_005102.4-37188479	TATTTTGAGACAAGGGCTCCATGTGGC	16	G	0.1765	0.4186
NC_005102.4-37188479	TATTTTGAGACAAGGGCTCCATGTGGC	23	G	0.1933	0.3729
NC_005102.4-37199822	ATCCATTTCACCTCTGCTCCTCGAATG	23	G	0.1316	0.1953
NC_005102.4-37199878	TACATCATAACTAGAGCTCCCAAGCTT	1	T	0	0.4698
NC_005102.4-37237791	GCCCCCAGTACCATAGCTCCCACACGC	13	A	0.2054	0.3211
NC_005102.4-37237791	GCCCCCAGTACCATAGCTCCCACACGC	26	G	0	0.4922
NC_005102.4-37244674	CAATCTTTCACTGTATCTCCATGGATA	2	A	0.0082	0.0243
NC_005102.4-37255180	TTGCCCACCACGTTAGCTCCTACAGTC	1	T	0.1311	0.2399
NC_005102.4-37257224	TCCCATCAGACCCTCTCTCCCCACATA	25	A	0.1917	0.2125
NC_005102.4-37468627	GGGCAGATAACTACATCTCCATCAAGT	23	C	0.1626	0.2499
NC_005102.4-37501004	CTGCTCCAAACGTTGTCTCCATCTCTC	8	A	0.3814	0.4508
NC_005102.4-37503974	GGCTCGGCGACAGTGGCTCCAGCCGAG	25	G	0.0574	0.0557
NC_005102.4-37513540	ATAATTGTGACCACAGCTCCCCCCTGG	5	T	0.0331	0.4751
NC_005102.4-37529341	CCCAGTACTACCAAGCCTCCTGATCTC	1	C	0.0351	0.0839
NC_005102.4-37529341	CCCAGTACTACCAAGCCTCCTGATCTC	2	C	0.0351	0.0839
NC_005102.4-37616171	TAGCGTGAAACAAGAGCTCCATCTGCC	27	C	0	0.4703
NC_005102.4-37663788	TTGTTTGAGACAAAGTCTCCATATAGC	22	T	0.0087	0.4704
NC_005102.4-37663788	TTGTTTGAGACAAAGTCTCCATATAGC	23	A	0.0087	0.4704
NC_005102.4-37713844	TCACTTTAAACATTGTCTCCTTGACTC	3	A	0.1681	0.2494
NC_005102.4-37713844	TCACTTTAAACATTGTCTCCTTGACTC	27	C	0.1947	0.2797
NC_005102.4-37767985	CTTAGATTCACAAGATCTCCACAGGCT	21	A	0.1652	0.1938
NC_005102.4-37855817	CACCGGAGTACGGGCGCTCCCAACTTA	5	G	0.2162	0.2467
NC_005102.4-37929344	CTGATTTAGACATTGGCTCCATGAAAT	8	A	0.1538	0.1841
NC_005102.4-37929344	CTGATTTAGACATTGGCTCCATGAAAT	21	A	0	0.482
NC_005102.4-37937868	GCCATACATACAATTCCTCCAATCACA	7	C	0.1983	0.343
NC_005102.4-37937868	GCCATACATACAATTCCTCCAATCACA	8	A	0	0.48
NC_005102.4-37971258	GGGATGACGACAGTGGCTCCTGGACAG	8	C	0.1488	0.2415
NC_005102.4-37996028	CCGTTGCCCACCACAGCTCCTGTGCCC	3	G	0.252	0.356
NC_005102.4-37996028	CCGTTGCCCACCACAGCTCCTGTGCCC	24	G	0	0.4793
NC_005102.4-38013111	GTGAAAGTTACCTGCCCTCCAGGTAAC	8	T	0.0992	0.0943
NC_005102.4-38050889	GTGAGAACTACTTTGCCTCCTTGCATC	13	T	0	0.4671
NC_005102.4-38052113	CCGGCCGCCACCCTAACTCCTGAAGGA	2	C	0.1057	0.1001
NC_005102.4-38052113	CCGGCCGCCACCCTAACTCCTGAAGGA	6	C	0.3333	0.3381
NC_005102.4-38076436	GGTTGACCTACGTAGCCTCCAGTACTT	12	G	0.1092	0.2626
NC_005102.4-38076436	GGTTGACCTACGTAGCCTCCAGTACTT	15	G	0.1092	0.2626
NC_005102.4-38096240	ATGTGGTCCACACCCCCTCCCACCTAC	1	A	0	0.4832
NC_005102.4-38106747	CACACACCAACCACTTCTCCCTGGCCT	22	T	0.2521	0.3118
NC_005102.4-38128183	CAACATGAGACCTTTACTCCTGGCACA	5	A	0.2417	0.4166
NC_005102.4-38177453	CGGAAAGCTACCTAAACTCCACAGGAG	21	A	0.2566	0.4435
NC_005102.4-38188930	CACATGCAAACTGTGGCTCCCTCCCCT	8	A	0.0508	0.0655
NC_005102.4-38195518	CACTTCATCACCAAAACTCCTCTTTCC	6	C	0.0082	0.479
NC_005102.4-38220442	GGGTTCAACACTTGGTCTCCAGCTGTC	6	C	0.0932	0.1337
NC_005102.4-38234092	AGGATGACTACTCCACCTCCCCCACTG	13	C	0.1138	0.1356
NC_005102.4-38234092	AGGATGACTACTCCACCTCCCCCACTG	24	A	0.2358	0.4662
NC_005102.4-38247878	CAAATGTAAACTGGTTCTCCGTGGCCT	21	G	0.0081	0.4777

NC_005102.4-38260450	CTACCCTACACACTAACTCCAGGCCAT	13	C	0.0085	0.4753
NC_005102.4-38260450	CTACCCTACACACTAACTCCAGGCCAT	21	A	0.1949	0.2282
NC_005102.4-38330638	GACCAAGGTACTGTCACTCCAAGGACG	1	G	0.1017	0.1264
NC_005102.4-38330638	GACCAAGGTACTGTCACTCCAAGGACG	7	G	0.1017	0.4987
NC_005102.4-38385981	TAACGTGACACCTCAACTCCTGCAGCG	15	A	0.0756	0.0882
NC_005102.4-38385981	TAACGTGACACCTCAACTCCTGCAGCG	27	G	0.0678	0.0965
NC_005102.4-38469364	ATTTGGACCACATGAACTCCCTGTTGT	21	C	0.0569	0.0553
NC_005102.4-38479572	CAGATCAAAACCAGCTCTCCCTGGAAA	22	T	0.036	0.0526
NC_005102.4-38479572	CAGATCAAAACCAGCTCTCCCTGGAAA	23	G	0.036	0.0526
NC_005102.4-38544459	AATGGTAGAACAGAGGCTCCATGTTGT	2	A	0.123	0.1708
NC_005102.4-38544459	AATGGTAGAACAGAGGCTCCATGTTGT	8	G	0.1639	0.4988
NC_005102.4-38556206	CCTCAGTACACGTTTGCTCCCTCACTG	12	G	0.1053	0.0997
NC_005102.4-38556206	CCTCAGTACACGTTTGCTCCCTCACTG	26	T	0.2105	0.4846
NC_005102.4-38628010	CATTGTTCCACTATCACTCCTCAGTAA	1	C	0	0.4793
NC_005102.4-38628010	CATTGTTCCACTATCACTCCTCAGTAA	16	A	0.0244	0.0241
NC_005102.4-38778589	AGCATTAACACAGGCCCTCCTCCTGGG	2	G	0	0.4772
NC_005102.4-38778589	AGCATTAACACAGGCCCTCCTCCTGGG	23	C	0	0.4772
NC_005102.4-38907751	GTTAACTGCACTTATCCTCCATGCAGA	21	A	0.1391	0.1588
NC_005102.4-38950756	GATAGTGGTACCAATGCTCCTGTAACA	9	T	0	0.4833
NC_005102.4-39056921	TCCCTAGCTACTCAGTCTCCTACCAGC	21	T	0.025	0.0247
NC_005102.4-39061637	GCATTGCCCACATCCCCTCCTGGCCTT	9	C	0.3217	0.4395
NC_005102.4-39088592	CCCCTTTACACAGCCTCTCCCTTCCAT	22	T	0.1488	0.2046
NC_005102.4-39135917	TAGAAAGCAACATTTTCTCCAATGTTG	22	A	0.2906	0.4649
NC_005102.4-39167297	TATTCTTTCACTCTCTCTCCTTCATCA	1	T	0	0.4731
NC_005102.4-39185579	CTGGATAGTACAGAGTCTCCCAAGAAA	2	T	0.0526	0.0512
NC_005102.4-39378402	ATACAAGCCACAATTGCTCCTGGTGGA	13	A	0.0179	0.4688
NC_005102.4-39378402	ATACAAGCCACAATTGCTCCTGGTGGA	16	G	0.0179	0.4688
NC_005102.4-39476115	ATCACGTCCACCTTGCCTCCCTGTCCA	6	G	0.0574	0.071
NC_005102.4-39510895	TCCTTCTTAACCTAAACTCCTAAGTAT	8	T	0.1842	0.4981
NC_005102.4-39510895	TCCTTCTTAACCTAAACTCCTAAGTAT	26	A	0.1842	0.4981
NC_005102.4-39582268	CACTGGCAAACCCCAGCTCCACCAAAA	5	G	0.1765	0.274
NC_005102.4-39694763	CATTATGACACCTGATCTCCTTCCTAG	14	G	0.1417	0.2722
NC_005102.4-39694763	CATTATGACACCTGATCTCCTTCCTAG	24	C	0.0417	0.0722
NC_005102.4-39782854	ACCATACCAACCAGAGCTCCCAGGGCC	16	G	0.0263	0.026
NC_005102.4-39785625	TGAACCAGCACATAACCTCCTCCTCTG	8	G	0.0894	0.319
NC_005102.4-39872098	ACCCTGCTGACCAGTGCTCCTGTTTTT	14	G	0.0678	0.1264
NC_005102.4-39915486	AGATTCAGGACAGCCACTCCTGTATAC	4	T	0	0.4807
NC_005102.4-39966698	TTTTTCCTTACCCCAGCTCCCAAGATT	25	A	0.181	0.4982
NC_005102.4-39979873	GCTGTTATTACAGATGCTCCTCTTCCA	23	T	0.0702	0.1305
NC_005102.4-40096378	AGTGAATAGACATGTACTCCATTGAGA	5	A	0	0.4793
NC_005102.4-40096378	AGTGAATAGACATGTACTCCATTGAGA	15	T	0	0.4793
NC_005102.4-40126106	TATAGCTAGACATGGCCTCCATAAACT	13	T	0.1167	0.1665
NC_005102.4-40199569	CTCACAACTACTTCAACTCCCCTCCCT	7	A	0	0.4772
NC_005102.4-40299341	AATCCACTTACTATGTCTCCCACAAGA	5	C	0.0424	0.0889
NC_005102.4-40312344	GTATCACTGACATGACCTCCCGAATGT	22	G	0.069	0.0981
NC_005102.4-40322742	CTTCATAGTACAAATACTCCATGCTTT	1	C	0.2321	0.3922
NC_005102.4-40326573	AGTAAATCAACTCTGCCTCCCTCAGGG	21	C	0.1504	0.2237
NC_005102.4-40326573	AGTAAATCAACTCTGCCTCCCTCAGGG	24	A	0.2193	0.4676
NC_005102.4-40371987	AGAAGGAAAACGATGACTCCTCAAAGT	12	G	0	0.4693
NC_005102.4-40371987	AGAAGGAAAACGATGACTCCTCAAAGT	14	T	0.0598	0.1049
NC_005102.4-40591815	CTAATGTCCACCCATACTCCCATAGTT	22	A	0.1513	0.1678

NC_005102.4-40634035	TTCCAATTTACTTCATCTCCTCATACT	14	C	0.0248	0.0562
NC_005102.4-40634035	TTCCAATTTACTTCATCTCCTCATACT	24	T	0.1074	0.4951
NC_005102.4-40798955	GTAGGACCCACCTAGTCTCCCTCAGTG	14	A	0.1951	0.4072
NC_005102.4-40798955	GTAGGACCCACCTAGTCTCCCTCAGTG	16	T	0.1951	0.4072
NC_005102.4-40879518	ACATACCATACCTCATCTCCTCCCCGC	9	T	0.2288	0.4336
NC_005102.4-40879518	ACATACCATACCTCATCTCCTCCCCGC	25	C	0.2203	0.4367
NC_005102.4-40906841	TCACCAAGGACCCTCCCTCCTTACCAT	14	T	0.0259	0.0255
NC_005102.4-40976826	ATAACCAGGACAGAGGCTCCAGTATGT	24	A	0.0811	0.4959
NC_005102.4-40979308	AAGACCCAGACAATAGCTCCAGGATCC	24	A	0.1724	0.2854
NC_005102.4-41035505	GTTCTCTTCACTGCTTCTCCCAGGAGA	2	T	0.2917	0.4472
NC_005102.4-41082583	TCCTGCCCCACCTATTCTCCTCCCTAT	26	A	0.2119	0.4609
NC_005102.4-41135594	TCCCTCTGCACACCTGCTCCTGCTCCT	13	C	0.0175	0.0677
NC_005102.4-41157920	GATAAGACCACTTCAACTCCTTCAGTC	4	A	0.065	0.0629
NC_005102.4-41238989	ATGGCCTCTACATCAGCTCCTGCTTTC	15	A	0.3193	0.4983
NC_005102.4-41238989	ATGGCCTCTACATCAGCTCCTGCTTTC	22	G	0.3025	0.4997
NC_005102.4-41285902	GCCACTGTAACTGAAACTCCAGTCCCT	23	T	0	0.4841
NC_005102.4-41329988	TTTTCTCTCACCTCACCTCCACCCATT	1	T	0	0.4693
NC_005102.4-41329988	TTTTCTCTCACCTCACCTCCACCCATT	22	C	0	0.4693
NC_005102.4-41335709	TCTAGATCCACCAGCTCTCCTTTGTTG	16	T	0	0.4851
NC_005102.4-41480089	TAAATCATCACCCTAGCTCCCTTGCGT	4	A	0	0.4765
NC_005102.4-41480089	TAAATCATCACCCTAGCTCCCTTGCGT	25	C	0	0.4765
NC_005102.4-41491000	CTCTCTCCTACCCTCCCTCCTCCTTCT	25	T	0	0.4793
NC_005102.4-41502971	TGCCATCAGACCATTTCTCCACTGAGA	6	T	0.2232	0.2637
NC_005102.4-41524126	TGAGATCCTACTCATACTCCCACCTCA	2	G	0	0.0476
NC_005102.4-41524126	TGAGATCCTACTCATACTCCCACCTCA	12	T	0.0407	0.4893
NC_005102.4-41595777	ACTAACTTCACTGTTCCTCCTTTCGTC	24	C	0.2308	0.4977
NC_005102.4-41751113	TTAAACATCACTCATCCTCCACAGGTC	12	T	0.25	0.3578
NC_005102.4-42012690	GAACAACCAACAAACCCTCCCAAACTC	6	A	0.0593	0.4609
NC_005102.4-42012690	GAACAACCAACAAACCCTCCCAAACTC	21	C	0	0.4844
NC_005102.4-42025886	TTGTCCAATACAAACTCTCCTGGAGTC	8	A	0	0.4751
NC_005102.4-42025886	TTGTCCAATACAAACTCTCCTGGAGTC	9	T	0	0.4751
NC_005102.4-42168821	TACATTGTCACCTTGCCTCCCCTCTTC	5	T	0.0614	0.0918
NC_005102.4-42188977	CAGACATCAACCTGACCTCCTGGGGGA	16	C	0.2314	0.2869
NC_005102.4-42188977	CAGACATCAACCTGACCTCCTGGGGGA	24	G	0	0.4751
NC_005102.4-42345121	TGAAGAGGTACCAGTACTCCGTTTTAC	8	G	0.0442	0.0433
NC_005102.4-42345121	TGAAGAGGTACCAGTACTCCGTTTTAC	21	G	0.1786	0.2934
NC_005102.4-42381825	GCGACTTCAACAGCAACTCCTACTTTT	27	T	0.2417	0.4922
NC_005102.4-42400894	AGGATTGGAACCTGTCCTCCAGGGGAT	23	G	0.252	0.2441
NC_005102.4-42467486	ATATCCAGTACTATTGCTCCCCACCAT	6	C	0.2743	0.4171
NC_005102.4-42602776	ACTTATTCTACTTTTTCTCCTCTTCAG	1	A	0.1842	0.2476
NC_005102.4-42602776	ACTTATTCTACTTTTTCTCCTCTTCAG	2	C	0.1316	0.138
NC_005102.4-42614918	TGAACTCTCACTGAGACTCCTCCAGAT	26	A	0.1803	0.2157
NC_005102.4-42664794	AAACTGAAGACACAACCTCCAGTAGAC	9	G	0.0569	0.0553
NC_005102.4-43070293	TACTCTGTAACCAGGTCTCCTGATTCA	23	A	0	0.4856
NC_005102.4-43119822	TTAAAACCCACTCTTACTCCCCGCTTC	15	T	0.1327	0.4998
NC_005102.4-43212275	CACACGCTTACACTACCTCCTACTCCC	1	C	0.084	0.0805
NC_005102.4-43212275	CACACGCTTACACTACCTCCTACTCCC	13	C	0.0336	0.033
NC_005102.4-43248826	TCTCCCTAGACACGAGCTCCACTACTG	13	C	0.115	0.1968
NC_005102.4-43269690	ATGGGAATGACCGCCCCTCCCCCAATC	25	A	0.0431	0.0585
NC_005102.4-43272081	CTTGTTACCACATTGACTCCTGTTTTC	21	T	0	0.4859
NC_005102.4-43333630	TAGAAAAAGACTGGTCCTCCTAGTAGT	5	A	0	0.474

NC_005102.4-43496646	GACTCAGGAACGATGTCTCCATTTCTC	4	T	0.1552	0.4987
NC_005102.4-43496646	GACTCAGGAACGATGTCTCCATTTCTC	15	G	0.1552	0.2378
NC_005102.4-43532819	GGCTATCACACACTTCCTCCAGTACCA	24	A	0	0.4793
NC_005102.4-43605551	AGGCATCTGACATCCTCTCCCATGTAT	27	T	0.0431	0.0422
NC_005102.4-43810341	CCCAGAAGCACTCTCACTCCTAAGATG	22	A	0.1885	0.4943
NC_005102.4-43887960	TCTAACGTAACTATTCCTCCTCTTGGA	7	G	0.0088	0.481
NC_005102.4-44008601	CACAATCTTACAACACCTCCCTTTTGT	14	C	0.0331	0.0325
NC_005102.4-44013579	CTCTGCTCCACACTGTCTCCTTATTTC	13	C	0.0259	0.0255
NC_005102.4-44045504	TTTCTATCCACCTCTCCTCCTACCCCT	6	A	0.0541	0.4909
NC_005102.4-44056716	TGTTCATAAACTCCATCTCCAAACAAA	14	C	0.0333	0.4861
NC_005102.4-44064600	CACCCCAGAACAGGAGCTCCGTATCGA	16	G	0.0331	0.0325
NC_005102.4-44064600	CACCCCAGAACAGGAGCTCCGTATCGA	23	A	0.1917	0.4972
NC_005102.4-44066484	GTACAAATCACCCCTTCTCCCAGATTG	24	A	0.1864	0.4977
NC_005102.4-44081914	ACATTTACAACACAACCTCCTTTGACT	5	T	0	0.4846
NC_005102.4-44088541	CAATTATGTACCACAACTCCATCTGGC	4	T	0	0.4773
NC_005102.4-44100107	CATTTGTGCACACAGACTCCTTAGTAC	6	G	0.0672	0.0805
NC_005102.4-44143106	TTGCAAATGACTTCAGCTCCTTCAGTC	7	A	0.075	0.0722
NC_005102.4-44281894	GCCACCAAAACAAGACCTCCGGGTGTG	23	G	0.0574	0.0557
NC_005102.4-44296080	AACCCACACACCACTCCTCCATGCGCT	22	T	0	0.4773
NC_005102.4-44360021	ACCAGTGTGACTGCTTCTCCTGCTCTG	9	G	0.1667	0.3299
NC_005102.4-44364147	CCTCATTACACCCCGGCTCCCACTCTA	14	C	0.0678	0.1116
NC_005102.4-44393003	TTAGATTTGACTCCTACTCCACAGGAA	1	T	0.087	0.4936
NC_005102.4-44393003	TTAGATTTGACTCCTACTCCACAGGAA	2	T	0.0763	0.1041
NC_005102.4-44411952	GTTTCAGGGACTCCAACTCCTTCCTCT	3	T	0.0261	0.4782
NC_005102.4-44428183	AACAGACTGACCTGGCCTCCCAAGCCA	7	C	0.0526	0.4945
NC_005102.4-44428183	AACAGACTGACCTGGCCTCCCAAGCCA	12	C	0.0439	0.0429
NC_005102.4-44439766	CTTCCTCCAACCTCGCCTCCACATTCC	15	G	0.0357	0.0521
NC_005102.4-44452029	AACAAGAATACCTGCTCTCCAAGGCTG	15	C	0.193	0.2285
NC_005102.4-44454572	CCTGGATGTACTTAAGCTCCTCTGGCC	4	G	0.0435	0.0911
NC_005102.4-44458409	GCGCTTGCCACATGAACTCCTGATATC	16	A	0.2562	0.4889
NC_005102.4-44458409	GCGCTTGCCACATGAACTCCTGATATC	24	T	0.2562	0.4889
NC_005102.4-44475705	TCTCAAATAACCGCAACTCCCTGGTGA	12	C	0	0.4868
NC_005102.4-44475705	TCTCAAATAACCGCAACTCCCTGGTGA	15	A	0	0.4868
NC_005102.4-44484407	ATCTCTTACACCGCATCTCCTGCAAAA	13	G	0.2719	0.3661
NC_005102.4-44494484	TAAGCAACAACCTATTCTCCATCTCCG	14	A	0.3103	0.4666
NC_005102.4-44526184	CCAAACGACACTTGAACTCCTGATCCT	13	T	0	0.482
NC_005102.4-44565098	TGCAGTGAGACTCTGCCTCCCTCATTT	23	C	0.1463	0.2612
NC_005102.4-44569496	GCGGACACAACCAGTCCTCCCATGCCT	2	C	0.1008	0.4898
NC_005102.4-44569496	GCGGACACAACCAGTCCTCCCATGCCT	26	C	0	0.4703
NC_005102.4-44578005	CCACCCTTCACCGCACCTCCATAGAAC	5	C	0.0894	0.4935
NC_005102.4-44742328	GAGAGAAAGACTCATCCTCCCTAAGTA	27	A	0.0088	0.0429
NC_005102.4-44790455	ACCTTGATCACAAAGGCTCCAACCTAA	12	A	0.2295	0.3791
NC_005102.4-44813111	CCCAGCTGGACTGGGACTCCACAGTAC	6	C	0.0248	0.0245
NC_005102.4-44813111	CCCAGCTGGACTGGGACTCCACAGTAC	9	G	0.0254	0.0251
NC_005102.4-44895253	TTCACCACCACTTCCACTCCTCTCCTC	26	T	0	0.4793
NC_005102.4-44979189	TTCACTAATACAGTCGCTCCAAAAGCT	15	C	0.0085	0.0253
NC_005102.4-44979189	TTCACTAATACAGTCGCTCCAAAAGCT	16	G	0.0342	0.05
NC_005102.4-44983902	TTGTCACTTACCTCAGCTCCACGGTAC	22	C	0	0.4787
NC_005102.4-45015772	CCACCTTTAACTCCAGCTCCAGGATAT	5	C	0.05	0.0488
NC_005102.4-45085710	GCAGCAGACACCCACACTCCAGCCTTC	5	C	0.0738	0.1839
NC_005102.4-45085710	GCAGCAGACACCCACACTCCAGCCTTC	16	A	0	0.4773

NC_005102.4-45189206	AGGCTGAGAACTGCTGCTCCACATGTT	16	G	0.1607	0.2052
NC_005102.4-45189306	ATCGTAATAACTCTGCCTCCTTGTTTA	14	T	0.2069	0.4976
NC_005102.4-45220880	ACAGTGATTACAGAGGCTCCTGCTACA	5	T	0	0.4757
NC_005102.4-45221482	TGCTTTCAAACTGGGGCTCCAGCCCTG	12	T	0.0957	0.1938
NC_005102.4-45240480	CTGAGCTACACCTATCCTCCATTGTTG	3	G	0.0407	0.0854
NC_005102.4-45259919	TACCACAGAACTGGGACTCCCATCGCT	12	T	0	0.4574
NC_005102.4-45259919	TACCACAGAACTGGGACTCCCATCGCT	23	T	0	0.4606
NC_005102.4-45313985	CAGTGCTGGACCAGTGCTCCCCACCAA	22	C	0.0088	0.0595
NC_005102.4-45331104	AAGTGCATCACACACCCTCCTGAGCAT	12	A	0.1404	0.2021
NC_005102.4-45404801	TTTCCCACAACTCTGGCTCCTATGAAA	12	T	0.0263	0.026
NC_005102.4-45425507	TCATGGCTAACCCAGCCTCCTTTGTAA	16	C	0.1138	0.1629
NC_005102.4-45461074	TCCAACAAAACTTCATCTCCACGAGCA	23	G	0.1356	0.2219
NC_005102.4-45474203	TCAACGCGCACCCTCTCTCCGCGTCAT	7	C	0.0894	0.2556
NC_005102.4-45474203	TCAACGCGCACCCTCTCTCCGCGTCAT	21	G	0.1382	0.319
NC_005102.4-45488276	TCACAGATGACTCCTGCTCCAGTGTTT	27	T	0.1053	0.0997
NC_005102.4-45503693	ACTGATCGTACTTGATCTCCCGGCAGG	8	G	0.2308	0.4823
NC_005102.4-45503693	ACTGATCGTACTTGATCTCCCGGCAGG	22	G	0.1083	0.1997
NC_005102.4-45520102	CCCAAGGTCACTTTTACTCCAATGGTC	22	A	0	0.48
NC_005102.4-45520102	CCCAAGGTCACTTTTACTCCAATGGTC	24	G	0	0.48
NC_005102.4-45540772	ACATAGCACACCATGACTCCATCAAGC	13	A	0.0351	0.4778
NC_005102.4-45543134	GATTTAGGCACAGTGGCTCCTTGGAAA	24	G	0.157	0.2355
NC_005102.4-45544720	ACTGATTCCACAGCTCCTCCTGGGACT	5	A	0.1066	0.1009
NC_005102.4-45544720	ACTGATTCCACAGCTCCTCCTGGGACT	23	G	0.0902	0.4943
NC_005102.4-45551363	ATGATTGCCACCTCCTCTCCAAGCCCT	1	A	0	0.4826
NC_005102.4-45570959	AGAACTGTCACTCAGTCTCCATCCCAG	7	G	0.1382	0.2203
NC_005102.4-45591205	GTTTCTGAGACAGTGTCTCCACTGGTA	4	T	0	0.4743
NC_005102.4-45609707	CCAGGATGTACTAGAGCTCCAGTATAT	8	G	0.0661	0.0639
NC_005102.4-45683237	TCCTCCAGGACTCCTCCTCCAGACAAG	21	A	0	0.4793
NC_005102.4-45729309	CTGTTGCTCACTGCATCTCCTCATTGT	2	T	0	0.4807
NC_005102.4-45729309	CTGTTGCTCACTGCATCTCCTCATTGT	16	T	0.2213	0.4879
NC_005102.4-45732951	ATCCTTGAAACAGCAGCTCCTCACCGA	1	A	0.1207	0.4946
NC_005102.4-45732951	ATCCTTGAAACAGCAGCTCCTCACCGA	27	A	0.1207	0.2252
NC_005102.4-45765161	TGTAGGGTCACACCAGCTCCTTTATGT	16	G	0.1667	0.2778
NC_005102.4-45794448	TGGCCTTCTACTGCTCCTCCTCCCTTG	14	C	0.2034	0.3874
NC_005102.4-45802855	ACTGGTCCGACCTCTACTCCTGAATTA	8	C	0	0.4871
NC_005102.4-45804317	AGGATCCTCACTGAGACTCCTCTTGGG	5	T	0.1786	0.1626
NC_005102.4-45825023	ACATTCCCTACAGTGACTCCGCCCCCC	27	C	0.0265	0.0262
NC_005102.4-45835317	GAAGGTTCGACCTCCTCTCCTCCCATG	6	T	0.0407	0.0553
NC_005102.4-45835317	GAAGGTTCGACCTCCTCTCCTCCCATG	8	C	0.0976	0.4944
NC_005102.4-45843660	CGAGGGTGCACGAAAGCTCCAGACAAG	16	G	0.1964	0.3659
NC_005102.4-45844979	ACAAGCAGCACCGCCTCTCCCAGCACC	27	C	0.0089	0.0436
NC_005102.4-45849517	CTAGCACGCACTTCCCCTCCAGTAATC	7	C	0.0583	0.0566
NC_005102.4-45857783	TCCAGCTCTACCTAGCCTCCTTCAAAG	16	C	0.0088	0.4861
NC_005102.4-45884413	ATCCTGAGCACACACCCTCCAAGAGTC	2	T	0	0.482
NC_005102.4-45884413	ATCCTGAGCACACACCCTCCAAGAGTC	14	A	0	0.482
NC_005102.4-45884455	CATAGGGCCACATGCACTCCTTCACCA	13	T	0.2667	0.4599
NC_005102.4-45891312	GGCACAGATACTTCATCTCCCCAGGGG	15	A	0.0769	0.074
NC_005102.4-45894299	CAGAACCTGACCTTTCCTCCAAGAGTG	21	A	0.25	0.3578
NC_005102.4-45920541	TAACTACCAACCACGTCTCCAGCCTCT	6	A	0	0.4773
NC_005102.4-45920541	TAACTACCAACCACGTCTCCAGCCTCT	22	G	0.1463	0.3427
NC_005102.4-45938093	ACACCTGTTACCTTAACTCCTTGTTCA	7	G	0.2231	0.259

NC_005102.4-45978849	TGCATTAGGACCAGATCTCCTCCTTTA	22	C	0.0336	0.0491
NC_005102.4-45988350	AACAGCTCAACGTAGTCTCCAACTCTC	7	T	0.0085	0.4738
NC_005102.4-46011030	GAGACAAGGACAGCGACTCCAAGCATG	15	G	0.181	0.343
NC_005102.4-46019866	ACTTGGGTGACATTAACTCCAGTACCA	12	A	0	0.4778
NC_005102.4-46019866	ACTTGGGTGACATTAACTCCAGTACCA	25	C	0	0.0342
NC_005102.4-46027178	AACAGTAACACAGCAGCTCCTCTCCTA	2	A	0.0924	0.4949
NC_005102.4-46027178	AACAGTAACACAGCAGCTCCTCTCCTA	8	A	0.0924	0.4949
NC_005102.4-46038716	TGAGGGGTTACTCACGCTCCAATCGGT	1	T	0.0763	0.0889
NC_005102.4-46038716	TGAGGGGTTACTCACGCTCCAATCGGT	15	C	0.0504	0.0491
NC_005102.4-46039271	CTGCCACAGACACGGCCTCCAACGGGG	5	C	0.0696	0.1143
NC_005102.4-46214042	CAAAGGTCCACCTTCCCTCCTAATGAA	21	T	0	0.4765
NC_005102.4-46239273	AGGCCACTGACTGTGGCTCCTCAAGTT	15	G	0	0.4787
NC_005102.4-46239273	AGGCCACTGACTGTGGCTCCTCAAGTT	26	T	0	0.4787
NC_005102.4-46266560	CACATTAGTACTTTCTCTCCAATTAAT	4	A	0.2966	0.4971
NC_005102.4-46266560	CACATTAGTACTTTCTCTCCAATTAAT	7	A	0.225	0.4708
NC_005102.4-46269143	CATGCCTATACAAAAGCTCCTTCACTA	26	T	0.2321	0.4539
NC_005102.4-46288128	ACACCATCCACCTCCCCTCCCCCATGC	15	C	0.0089	0.4512
NC_005102.4-46389463	TGCACATGGACTGCACCTCCTCCGGTG	1	T	0.2049	0.375
NC_005102.4-46389463	TGCACATGGACTGCACCTCCTCCGGTG	26	T	0.2049	0.375
NC_005102.4-46398222	ACTGTGTACACAGTTCCTCCATTAGCA	3	T	0.2232	0.2876
NC_005102.4-46421046	CATGTATTCACATACCCTCCAGCCATG	26	T	0.0407	0.0398
NC_005102.4-46423429	AAGAGGGGCACCTCCTCTCCCACCAGA	5	G	0.2358	0.356
NC_005102.4-46423429	AAGAGGGGCACCTCCTCTCCCACCAGA	7	G	0.2358	0.356
NC_005102.4-46450798	ACCATCAGAACTGCCCCTCCTCCCTCA	4	A	0.1026	0.1125
NC_005102.4-46459297	GGCTACCCCACACTCCCTCCTTATTTC	3	C	0	0.4807
NC_005102.4-46470146	TGTTGTGAGACTGTGTCTCCTAGTAAT	13	G	0.4957	0.3729
NC_005102.4-46493501	ACCACGGTGACACCCTCTCCCCCAAGT	5	C	0.0492	0.0634
NC_005102.4-46493501	ACCACGGTGACACCCTCTCCCCCAAGT	26	G	0	0.4787
NC_005102.4-46576411	GCGGCCGGTACCTTGGCTCCAGTTCAG	6	C	0.1301	0.1356
NC_005102.4-46666683	CTAGATTACACTGCTGCTCCGTGTAGA	26	G	0.1	0.1528
NC_005102.4-46689434	AGCAGTCCCACAGTCGCTCCAGGGATG	12	A	0	0.4851
NC_005102.4-46695634	ATCTGACACACTATGCCTCCATATAAG	1	A	0.1217	0.2138
NC_005102.4-46707603	CCGGTGCTAACCTTACCTCCATGAGTG	2	C	0.2066	0.3027
NC_005102.4-46726099	ACAGGACTAACCCTTCCTCCTCGAGCA	21	T	0.0504	0.0491
NC_005102.4-46726099	ACAGGACTAACCCTTCCTCCTCGAGCA	23	G	0	0.4813
NC_005102.4-46770331	TTCTTCCAGACTACAGCTCCCTTGAAG	21	C	0.0588	0.0571
NC_005102.4-46770331	TTCTTCCAGACTACAGCTCCCTTGAAG	27	G	0.2542	0.4029
NC_005102.4-46915570	ACTAAGCATACTCTCCCTCCACCCAGT	9	T	0.0504	0.1107
NC_005102.4-46915570	ACTAAGCATACTCTCCCTCCACCCAGT	14	T	0.2353	0.4082
NC_005102.4-46930853	AGACTTTAGACTCCCCCTCCCACAAAC	27	C	0.0598	0.4527
NC_005102.4-46955684	GTTCACTGGACACATGCTCCACTAATG	21	A	0.2232	0.3102
NC_005102.4-46972970	CTGCTCACCACACACTCTCCTCATTCT	25	T	0	0.4751
NC_005102.4-46973850	AATGCACGAACATCAGCTCCACTTAAA	8	G	0.0244	0.0705
NC_005102.4-47008756	CCATTTGCAACTCTGGCTCCAGATGAC	7	G	0.0531	0.0517
NC_005102.4-47053566	CTGGAAAGCACCTTTACTCCCTGAGTC	1	C	0.2881	0.4757
NC_005102.4-47053566	CTGGAAAGCACCTTTACTCCCTGAGTC	25	G	0.2797	0.319
NC_005102.4-47142752	GCCTATTGAACCATAGCTCCTGTTGCC	22	G	0.0877	0.1305
NC_005102.4-47248981	CCAACCTAAACAATACCTCCTGGAGAT	14	T	0.0885	0.0846
NC_005102.4-47310402	TTAAGGAAAACAAGCCCTCCCATCTGC	15	C	0.1513	0.2076
NC_005102.4-47310402	TTAAGGAAAACAAGCCCTCCCATCTGC	23	T	0.025	0.0247
NC_005102.4-47368596	CATCTGAACACAGCTTCTCCTCCCTCC	24	C	0	0.4734

NC_005102.4-47391588	AGCAGTCACACTGCTCCTCCAGAGAGA	15	T	0	0.4793
NC_005102.4-47398363	TCTACTATGACCAGATCTCCTCTGAGC	12	C	0.0603	0.1209
NC_005102.4-47398363	TCTACTATGACCAGATCTCCTCTGAGC	16	T	0.0087	0.4817
NC_005102.4-47489770	ATCCCTCACACTACCCCTCCCTTCAAA	3	C	0.0333	0.48
NC_005102.4-47489770	ATCCCTCACACTACCCCTCCCTTCAAA	25	A	0.157	0.172
NC_005102.4-47491573	AGTACTAGAACCAGAACTCCAGCCCTC	6	T	0.0325	0.032
NC_005102.4-47510694	GACCCTGACACCACCACTCCTTCCCGT	13	A	0.1707	0.1826
NC_005102.4-47510694	GACCCTGACACCACCACTCCTTCCCGT	25	C	0.0325	0.032
NC_005102.4-47533387	GTATGTATAACATCATCTCCCATAATG	24	A	0.1709	0.1841
NC_005102.4-47561580	CAAACCAGTACTGGAGCTCCCACTGTA	13	G	0.0254	0.0251
NC_005102.4-47605600	CTGCATCTTACTGGACCTCCCTGAATT	1	C	0.0085	0.481
NC_005102.4-47623017	CAAGTAAGTACCAGTCCTCCTGCCTCA	5	T	0.0924	0.1469
NC_005102.4-47896420	CCTTATTCAACTCTCCCTCCTCTGCTT	9	A	0	0.4875
NC_005102.4-47997152	AGATGCTGAACGTTAGCTCCTAGCCTT	4	T	0.2033	0.4952
NC_005102.4-48079266	TAAAGAAAGACCTGGTCTCCTTCCAAT	6	A	0.2439	0.3141
NC_005102.4-48083826	AAAGTGTTCACATTGCCTCCAGTATTG	15	G	0	0.0336
NC_005102.4-48103754	TGTTGGCCAACTAGTACTCCATTGTAT	23	T	0	0.4734
NC_005102.4-48104735	CTAGACTTGACATTCCCTCCTTTTTCA	12	A	0.1368	0.3895
NC_005102.4-48104735	CTAGACTTGACATTCCCTCCTTTTTCA	25	T	0.1368	0.3895
NC_005102.4-48195400	GATAATAGTACTGTGCCTCCATGTCTT	15	G	0	0.4807
NC_005102.4-48348740	GCCTGTCCCACCTCTGCTCCTCATCCT	4	T	0	0.4819
NC_005102.4-48348740	GCCTGTCCCACCTCTGCTCCTCATCCT	13	T	0	0.0325
NC_005102.4-48356444	GTAGCCTCCACACCCACTCCGAACAAT	15	C	0.0569	0.0705
NC_005102.4-48413275	ACATTTACCACAAGCTCTCCTTGGAAG	13	A	0.0826	0.109
NC_005102.4-48444485	GTTACCCGTACTAGTTCTCCCTCATTT	23	C	0.0328	0.048
NC_005102.4-48597500	CCCCCTCCCACTTCCTCTCCATCTCAT	22	T	0	0.4728
NC_005102.4-48608211	ATCCTAACTACAGTCCCTCCTTCCCCT	16	C	0.0261	0.0257
NC_005102.4-48984444	CATTCATGTACCGAGGCTCCATGTGAC	13	G	0.1695	0.2585
NC_005102.4-49007744	CGCTGAATGACTTTAACTCCTTTGTCA	1	C	0.1833	0.3832
NC_005102.4-49017965	ATACACACAACCAAGTCTCCCGGCAAT	21	C	0.2368	0.2836
NC_005102.4-49228780	CTCCTACATACAGCCTCTCCACGGCTC	23	G	0	0.4751
NC_005102.4-49271232	ACCCTAACCACAGCCCCTCCTCTTTTC	2	C	0.5656	0.4126
NC_005102.4-49271232	ACCCTAACCACAGCCCCTCCTCTTTTC	25	T	0.5656	0.4126
NC_005102.4-49302992	AGAGCAAAGACTGCTCCTCCAGAGGTC	23	A	0.0256	0.0253
NC_005102.4-49302992	AGAGCAAAGACTGCTCCTCCAGAGGTC	25	G	0.2328	0.302
NC_005102.4-49486457	CTGCAGTTGACAACTCCTCCTGGATCA	24	A	0	0.488
NC_005102.4-49494440	GATGCACTTACAGCACCTCCTGTAATT	12	A	0.0976	0.1216
NC_005102.4-49522250	CAAACGTCAACTTCTGCTCCTCCACAT	13	T	0.0082	0.479
NC_005102.4-49522250	CAAACGTCAACTTCTGCTCCTCCACAT	27	T	0.1148	0.1082
NC_005102.4-49531553	GAATTAATCACTATGCCTCCTACAATG	14	T	0.0702	0.1454
NC_005102.4-49585811	TGGTAATTAACCTGTACTCCCACATAT	25	T	0.0088	0.4861
NC_005102.4-49585811	TGGTAATTAACCTGTACTCCCACATAT	26	A	0.0965	0.1229
NC_005102.4-49594107	AACCCTAATACTGTAGCTCCTCTAAAA	12	T	0	0.4693
NC_005102.4-49594107	AACCCTAATACTGTAGCTCCTCTAAAA	26	A	0.161	0.4991
NC_005102.4-49632356	GTACCTATCACTGGACCTCCTTTCACT	4	C	0.0252	0.0249
NC_005102.4-49632356	GTACCTATCACTGGACCTCCTTTCACT	25	A	0.0588	0.0571
NC_005102.4-49705029	CTGGAGGAGACTGCATCTCCACAGGAA	12	T	0	0.4773
NC_005102.4-49738607	AGCTAGCTCACACACCCTCCCCATAGT	12	A	0.1695	0.493
NC_005102.4-49738607	AGCTAGCTCACACACCCTCCCCATAGT	21	C	0.0169	0.4856
NC_005102.4-49853012	ATAGCAGTTACTCTCTCTCCACTTCAT	8	T	0.0598	0.074
NC_005102.4-49858102	TGAAAACAAACTGCTACTCCCAAGCCT	24	G	0.027	0.044

NC_005102.4-49858723	GTTCAGACCACAGAGACTCCTTAATTC	16	A	0	0.4728
NC_005102.4-49882531	CTCAGAGACACACCCTCTCCTGAAATA	13	C	0.1228	0.4986
NC_005102.4-49900075	GTGGGCACCACTGATTCTCCAGCTCTG	14	A	0.0984	0.1082
NC_005102.4-49952312	TGAGAAATGACTCAGTCTCCTCCATTG	2	G	0.0603	0.4643
NC_005102.4-49952312	TGAGAAATGACTCAGTCTCCTCCATTG	25	T	0.0431	0.0746
NC_005102.4-49987013	ATTCGAAGCACCATGACTCCATGTGTA	27	A	0	0.4703
NC_005102.4-50060500	GATCCTCCAACCTCAGCTCCTGAGCCC	22	G	0.2222	0.2484
NC_005102.4-50091535	TTTCCCCACACTTGTCCTCCTTTTCCC	14	G	0.0328	0.0322
NC_005102.4-50091535	TTTCCCCACACTTGTCCTCCTTTTCCC	23	T	0.0583	0.4525
NC_005102.4-50099170	GAAATAATCACAGAGCCTCCTTCATCC	21	T	0	0.4778
NC_005102.4-50101486	CACAGGAAAACATACCCTCCCCCCTCA	21	C	0	0.4724
NC_005102.4-50129983	TTTCACATGACGGCAGCTCCTAATCCC	26	C	0.0088	0.4676
NC_005102.4-50145353	GACAGCAGTACATCTCCTCCAGCTTTC	6	C	0	0.4839
NC_005102.4-50268254	TTCAATGGAACTTGATCTCCTTGATGG	4	A	0.1607	0.1771
NC_005102.4-50268254	TTCAATGGAACTTGATCTCCTTGATGG	5	A	0.1875	0.2254
NC_005102.4-50305418	CCTTTTCCAACCTCAACTCCTCCCCAG	12	C	0.0331	0.0484
NC_005102.4-50324044	CAAAGAGTAACCAGGACTCCAAGGATC	1	C	0.0261	0.0257
NC_005102.4-50353284	TTATGCTTCACAGGCTCTCCTGCACTG	13	G	0.0348	0.0342
NC_005102.4-50400118	CAATGAGAGACCAAAGCTCCTGACCAT	7	G	0.1504	0.1968
NC_005102.4-50400118	CAATGAGAGACCAAAGCTCCTGACCAT	16	G	0.1593	0.5
NC_005102.4-50428293	GAGCAGAGGACAGCACCTCCTAGACCA	9	G	0.1074	0.1447
NC_005102.4-50428293	GAGCAGAGGACAGCACCTCCTAGACCA	15	A	0	0.4751
NC_005102.4-50528703	CTATCTCACACGAATTCTCCCATTCAC	4	T	0.2672	0.4893
NC_005102.4-50531155	TTCTTCCCCACCATCTCTCCTCTCCTA	3	C	0.2544	0.2719
NC_005102.4-50531155	TTCTTCCCCACCATCTCTCCTCTCCTA	13	A	0.0088	0.4529
NC_005102.4-50575174	GGGAAAACTACTCTGCCTCCTCCCTAG	2	G	0.0965	0.1229
NC_005102.4-50576429	CAAAGCAGAACTATCTCTCCTAAAATC	13	A	0.0089	0.4665
NC_005102.4-50609129	TTCCTCTCCACCTCATCTCCCCTCCAG	9	C	0.0744	0.1163
NC_005102.4-50609129	TTCCTCTCCACCTCATCTCCCCTCCAG	21	C	0.1901	0.4966
NC_005102.4-50738188	CTGAGCTGTACGGAATCTCCATCCAGG	15	A	0.0579	0.0868
NC_005102.4-50738188	CTGAGCTGTACGGAATCTCCATCCAGG	21	A	0.1333	0.2061
NC_005102.4-50805064	AACCGAAGGACAGATACTCCCCCTACG	5	G	0.1316	0.2599
NC_005102.4-50805064	AACCGAAGGACAGATACTCCCCCTACG	26	C	0.0531	0.1006
NC_005102.4-50826404	CAAGAGCCAACAGAAGCTCCATCAAAC	7	C	0.1138	0.1216
NC_005102.4-50826404	CAAGAGCCAACAGAAGCTCCATCAAAC	14	A	0.2033	0.4417
NC_005102.4-50827718	CTCAATCCCACTCCTCCTCCCCCATGT	21	C	0.2459	0.3791
NC_005102.4-50836101	TGAATTCAGACCTGTTCTCCATTACAC	5	T	0	0.4793
NC_005102.4-50836101	TGAATTCAGACCTGTTCTCCATTACAC	22	T	0.0488	0.0476
NC_005102.4-50846161	ATAATGACAACACACTCTCCATTCCAG	4	A	0.0087	0.4849
NC_005102.4-50846161	ATAATGACAACACACTCTCCATTCCAG	22	T	0.2261	0.48
NC_005102.4-50903546	TCCCTAGCTACCTCTACTCCCACCTTC	7	G	0	0.4793
NC_005102.4-50909083	GGCTATAAAACCTGCTCTCCTTAATAA	7	A	0.0177	0.0846
NC_005102.4-50909083	GGCTATAAAACCTGCTCTCCTTAATAA	27	A	0.0179	0.0853
NC_005102.4-50923731	AGCCTCCTAACGCCCCCTCCCACCGTC	3	C	0.1176	0.4983
NC_005102.4-51032180	AATTCAGAAACCATGCCTCCCCTCCTC	15	G	0.0413	0.0405
NC_005102.4-51048141	ATGGGTCTCACCATTACTCCCAGCACT	26	C	0.2479	0.4753
NC_005102.4-51053721	TCCTAATGTACGACAACTCCAAAGGCA	1	T	0.0427	0.0418
NC_005102.4-51137030	TACACGTTTACACGTTCTCCACACCAG	6	G	0.0522	0.0508
NC_005102.4-51137030	TACACGTTTACACGTTCTCCACACCAG	14	G	0	0.474
NC_005102.4-51207986	CTGCCTGTTACCCATGCTCCAGGGGAT	9	T	0	0.4793
NC_005102.4-51253707	GCATTCAACACAAAAGCTCCATCTTTG	5	T	0	0.4827

NC_005102.4-51283579	CTACCTGCAACTCTAGCTCCAGGAGAC	1	C	0.2906	0.3895
NC_005102.4-51283579	CTACCTGCAACTCTAGCTCCAGGAGAC	21	A	0.042	0.0411
NC_005102.4-51289431	CTCAAGTTCACTAAGGCTCCAGACACT	7	T	0	0.4819
NC_005102.4-51289431	CTCAAGTTCACTAAGGCTCCAGACACT	21	A	0.0164	0.048
NC_005102.4-51316713	GGCCGACTCACAGGTCCTCCCTCCACC	25	A	0	0.4793
NC_005102.4-51316713	GGCCGACTCACAGGTCCTCCCTCCACC	26	C	0	0.4793
NC_005102.4-51319385	ACACCTTTGACCTTTGCTCCATGTTCC	4	C	0.0446	0.0436
NC_005102.4-51319385	ACACCTTTGACCTTTGCTCCATGTTCC	21	A	0	0.4807
NC_005102.4-51354179	CAAGGAACCACCCTTGCTCCACAGTTC	1	C	0.1639	0.1774
NC_005102.4-51354179	CAAGGAACCACCCTTGCTCCACAGTTC	8	C	0.0574	0.454
NC_005102.4-51399736	AGGACAGCAACAGAAGCTCCTGATGGG	22	G	0.0579	0.0716
NC_005102.4-51399736	AGGACAGCAACAGAAGCTCCTGATGGG	26	G	0.0084	0.4723
NC_005102.4-51404381	TATTTTGAGACCAAGTCTCCTGTGTCT	23	T	0.1949	0.1759
NC_005102.4-51429237	AGCAAGGCAACCAGTGCTCCAGCTCCC	14	G	0	0.4813
NC_005102.4-51435075	CTCCCTCAAACACACCCTCCACACTTG	25	T	0.1875	0.3102
NC_005102.4-51490362	AACTCAGTAACCATCACTCCAACACTC	7	G	0	0.4751
NC_005102.4-51521191	CATAGTGCTACCTGACCTCCTGGGCAT	1	C	0.1624	0.2171
NC_005102.4-51521191	CATAGTGCTACCTGACCTCCTGGGCAT	14	G	0.2222	0.2945
NC_005102.4-51585127	AACTAAAGGACCATTTCTCCTTGCCTC	22	T	0	0.4773
NC_005102.4-51625115	AATTTTAAGACAGGGTCTCCTCTAACC	1	A	0	0.4666
NC_005102.4-51696360	AGAAAACTGACCTAGGCTCCCACACTA	4	A	0.1818	0.1787
NC_005102.4-51742806	GGATCGGAAACCAAGACTCCAATCCTT	6	G	0.0789	0.4076
NC_005102.4-51746191	GCACAAAACACGGTTGCTCCTGAGGCA	12	G	0.0085	0.4714
NC_005102.4-51791554	CCGATAAGCACAGCTGCTCCTAGGTTG	1	C	0	0.4688
NC_005102.4-51804534	TAAAGGACCACAGGACCTCCTGGCTTC	15	A	0.0085	0.4776
NC_005102.4-51806162	AATGATTGCACCTTGTCTCCATGGTAT	1	A	0.1818	0.2415
NC_005102.4-51807986	CTGCTCCAAACTTTGTCTCCTTCCTTC	22	T	0.2439	0.2142
NC_005102.4-51807986	CTGCTCCAAACTTTGTCTCCTTCCTTC	25	T	0.2439	0.2142
NC_005102.4-51819557	ACACCTTCCACAACTTCTCCACCTTCC	12	A	0.0083	0.4769
NC_005102.4-51900812	AAAATGTTCACAGGTTCTCCTTTGTAA	2	A	0	0.4785
NC_005102.4-51900812	AAAATGTTCACAGGTTCTCCTTTGTAA	6	G	0.0721	0.4909
NC_005102.4-51962350	ATGGCATCCACCAAGACTCCAGGAAAC	12	C	0.1393	0.2095
NC_005102.4-52144233	CTTCAGGACACTGGAACTCCCGTGTCA	21	C	0.2049	0.2573
NC_005102.4-52144233	CTTCAGGACACTGGAACTCCCGTGTCA	22	G	0.2049	0.4959
NC_005102.4-52173987	AGTAAGATCACTTCCTCTCCTCGGACG	23	G	0	0.4731
NC_005102.4-52238433	ATATATGGTACAGTTTCTCCTACAGCC	1	A	0.0179	0.4807
NC_005102.4-52285986	TGCCAGGGCACCTGCCCTCCACCCACC	15	C	0.0254	0.0251
NC_005102.4-52285986	TGCCAGGGCACCTGCCCTCCACCCACC	27	C	0.2222	0.5
NC_005102.4-52349726	ACTTCCCACACTCATCCTCCTGACATG	1	A	0.1864	0.3138
NC_005102.4-52356439	ATGATCAGCACGTACCCTCCATTAGTG	3	G	0.175	0.2372
NC_005102.4-52397718	CTCTATCCTACAGTTCCTCCTGCCACA	5	A	0.2195	0.208
NC_005102.4-52411099	TCCTCTATGACAGTCACTCCATGATAA	24	A	0.0168	0.033
NC_005102.4-52527805	GCGCTGGAAACCAGAGCTCCTTTACTG	3	G	0.1724	0.4999
NC_005102.4-52527805	GCGCTGGAAACCAGAGCTCCTTTACTG	6	G	0.1826	0.2072
NC_005102.4-52611367	AGGTTTTAGACACATGCTCCCTCAGCC	9	G	0	0.4787
NC_005102.4-52684686	CAGTGAAAAACGATAGCTCCTGTGTGA	2	A	0.2458	0.4066
NC_005102.4-52684686	CAGTGAAAAACGATAGCTCCTGTGTGA	22	G	0.1453	0.289
NC_005102.4-52696710	CACCCAATAACTCCCTCTCCCATCTTC	13	C	0.2083	0.225
NC_005102.4-52751886	ATCTATCTGACTCTGCCTCCCAACTAT	13	C	0.0081	0.0241
NC_005102.4-52751886	ATCTATCTGACTCTGCCTCCCAACTAT	24	C	0.082	0.4407
NC_005102.4-52779639	TCCCTGTTTACAGCGTCTCCCTTAATG	14	C	0.0763	0.1041

NC_005102.4-52779639	TCCCTGTTTACAGCGTCTCCCTTAATG	15	G	0.2119	0.2644
NC_005102.4-52808899	CAAGGAGTGACATGAGCTCCTCACTCA	24	C	0.225	0.2833
NC_005102.4-52847622	TCCAGGCAGACTCTGTCTCCAAACATG	9	G	0.0732	0.1001
NC_005102.4-52936688	CCATCTGTTACCTCAGCTCCCAGCAAT	26	A	0.0336	0.033
NC_005102.4-53008448	GACACTGCTACAAGCCCTCCTTTGCAT	16	C	0.2797	0.4698
NC_005102.4-53049394	ATTCTAAGGACTCTGCCTCCTATGAGT	8	G	0.2069	0.2122
NC_005102.4-53049394	ATTCTAAGGACTCTGCCTCCTATGAGT	14	T	0.2328	0.291
NC_005102.4-53178079	CATGTTCTCACAGTGGCTCCTGCACCC	12	A	0.3153	0.4682
NC_005102.4-53204325	TGTCTCCCTACCCAGCCTCCACACCCT	1	T	0.036	0.0695
NC_005102.4-53212796	CAACCAAAGACCCATCCTCCTGAATAA	26	A	0.3652	0.2985
NC_005102.4-53212796	CAACCAAAGACCCATCCTCCTGAATAA	27	A	0.3652	0.2985
NC_005102.4-53254676	ATCTTTCTGACTCAGCCTCCCTAGTCT	6	T	0.5289	0.4481
NC_005102.4-53254676	ATCTTTCTGACTCAGCCTCCCTAGTCT	8	T	0.5289	0.4481
NC_005102.4-53256693	ATTCCTCATACACTAACTCCATTCGGA	8	A	0.1983	0.1918
NC_005102.4-53256693	ATTCCTCATACACTAACTCCATTCGGA	25	G	0.1583	0.1733
NC_005102.4-53353288	CTGGAACCCACCTTCCCTCCTACACAG	13	T	0.0976	0.1073
NC_005102.4-53353288	CTGGAACCCACCTTCCCTCCTACACAG	16	C	0.2846	0.4106
NC_005102.4-53360212	TGTTCTTACACAGCACCTCCACACCTC	8	A	0.1639	0.306
NC_005102.4-53369844	AATGAAAGGACAAGGTCTCCATAGAGT	2	A	0.25	0.2797
NC_005102.4-53540631	CACACACACACACACACTCCTAAATGG	5	C	0.2679	0.3367
NC_005102.4-53560311	GTGTTTAATACCGCACCTCCCTAGTAC	12	C	0.2589	0.3316
NC_005102.4-53628086	TTTAAAGGGACTTACTCTCCTGCCATC	4	A	0.0413	0.0405
NC_005102.4-53713023	TTCTCCCGTACTCCCTCTCCCAACTTC	22	A	0	0.4641
NC_005102.4-53833370	ATCGTAAGGACAGCATCTCCATTTTCA	4	G	0.0252	0.4638
NC_005102.4-53833370	ATCGTAAGGACAGCATCTCCATTTTCA	21	A	0.0084	0.4723
NC_005102.4-53862709	TTTAAAGATACTGTTTCTCCTCAAACA	8	A	0.2261	0.2985
NC_005102.4-53900936	AGTTGAGGAACTGGTTCTCCTGGTGTC	1	A	0.1316	0.3168
NC_005102.4-54081998	TCCCATGGAACCCTGCCTCCTCCCCAG	7	G	0.1138	0.1216
NC_005102.4-54081998	TCCCATGGAACCCTGCCTCCTCCCCAG	12	C	0.1138	0.1216
NC_005102.4-54097786	CCTGCTTCCACTTATTCTCCAGCACAA	5	C	0.3391	0.3898
NC_005102.4-54097786	CCTGCTTCCACTTATTCTCCAGCACAA	15	T	0.3362	0.4435
NC_005102.4-54206109	GGGTCTTTCACTTTCTCTCCCATTGAT	4	T	0.0492	0.048
NC_005102.4-54239141	CAGTCAAGCACATGCACTCCACATAAA	2	A	0.292	0.297
NC_005102.4-54289713	CAAGTCGCAACTTCAGCTCCTCTCTGG	1	C	0.0488	0.0476
NC_005102.4-54335407	TTCCCCACAACCACCTCTCCTTCGGAA	23	C	0.1951	0.2263
NC_005102.4-54399772	TACCAACCCACAAACGCTCCAGGAATC	4	C	0.1316	0.1814
NC_005102.4-54399772	TACCAACCCACAAACGCTCCAGGAATC	8	C	0.1071	0.4986
NC_005102.4-54447703	AGCAAGTGCACTACAGCTCCGTACCAC	1	A	0	0.4755
NC_005102.4-54448277	CTTTGCATTACCAAATCTCCATTAACA	25	A	0.027	0.492
NC_005102.4-54481207	ACTTTCTCCACACCCTCTCCACACTTA	5	T	0	0.4728
NC_005102.4-54481207	ACTTTCTCCACACCCTCTCCACACTTA	26	T	0	0.4728
NC_005102.4-54493388	TTGTTCTGCACCTCAGCTCCCCTCACC	1	T	0.1475	0.1904
NC_005102.4-54493388	TTGTTCTGCACCTCAGCTCCCCTCACC	21	C	0.0244	0.0241
NC_005102.4-54495640	AACACCATCACGTGCTCTCCACGAGCA	4	A	0.1639	0.4999
NC_005102.4-54495640	AACACCATCACGTGCTCTCCACGAGCA	23	G	0.3115	0.4463
NC_005102.4-54538788	ATGAAGGGAACATCCTCTCCCTCCCAG	6	G	0.0427	0.4928
NC_005102.4-54568252	CAACTTCAGACTGCGACTCCCGCTAAT	15	G	0.0248	0.0405
NC_005102.4-54629151	TGGGTGAGCACAGCCACTCCAGCCGCC	14	C	0.1197	0.4868
NC_005102.4-54629151	TGGGTGAGCACAGCCACTCCAGCCGCC	25	G	0.1197	0.4868
NC_005102.4-54638568	CCTGCTGCCACATCTGCTCCACTAGGA	12	A	0.1681	0.4435
NC_005102.4-54692075	TGGGAAATGACAGTAGCTCCTCAACTA	26	T	0.0348	0.4954

NC_005102.4-54715832	TTGGCAGTTACTCAGTCTCCTCACAGA	23	A	0.0517	0.0504
NC_005102.4-54746218	TTGCTAATAACCCCTGCTCCAGAGAAT	12	C	0.0085	0.4776
NC_005102.4-54842268	TTCCTAAGTACTACTCCTCCAACTCTT	27	T	0.2231	0.2109
NC_005102.4-54864537	ACTGAATTGACACTGGCTCCAGGGTCA	9	G	0.0252	0.0249
NC_005102.4-54864537	ACTGAATTGACACTGGCTCCAGGGTCA	23	G	0.2437	0.2266
NC_005102.4-54869717	CACAGCTGAACTAGGTCTCCAGCCACT	24	C	0.0082	0.0557
NC_005102.4-54887960	TCCCTGGATACAGGGTCTCCTGCTGAC	14	G	0	0.4793
NC_005102.4-54907197	TAACCTACCACTCTTTCTCCACCAATT	16	T	0	0.4793
NC_005102.4-54907197	TAACCTACCACTCTTTCTCCACCAATT	21	A	0.124	0.1447
NC_005102.4-54998990	TTTAAGGGCACTTATGCTCCAGAGCCT	13	T	0.0804	0.0934
NC_005102.4-55000935	ATGGTCAGAACCATGGCTCCAGCTGCA	12	C	0.0339	0.0333
NC_005102.4-55036174	ACATTTAAAACTGTTCCTCCTGGAATG	16	C	0	0.0328
NC_005102.4-55036174	ACATTTAAAACTGTTCCTCCTGGAATG	27	G	0	0.4844
NC_005102.4-55042873	CAGCCTCCAACTCATGCTCCATTTGCT	9	A	0.0492	0.4914
NC_005102.4-55074184	AATAAATAAACCTTTTCTCCCTGTAAG	12	C	0.1429	0.2575
NC_005102.4-55156980	ATCAAACACACACACTCTCCATGAGGA	16	T	0	0.4851
NC_005102.4-55237917	GTGTGGGTTACTCTACCTCCAACCCTT	9	T	0.2645	0.4426
NC_005102.4-55237917	GTGTGGGTTACTCTACCTCCAACCCTT	24	C	0.0082	0.0557
NC_005102.4-55240801	CCCACAGACACCTTCGCTCCTCAGTCT	15	C	0.0948	0.1209
NC_005102.4-55272368	ATACACTTCACCCACGCTCCTGTCTCC	4	C	0.0684	0.066
NC_005102.4-55272368	ATACACTTCACCCACGCTCCTGTCTCC	16	G	0.0517	0.0504
NC_005102.4-55291565	CAGTGTTGAACCCATTCTCCATGGTCA	22	T	0.2131	0.2956
NC_005102.4-55291565	CAGTGTTGAACCCATTCTCCATGGTCA	26	C	0.1322	0.1377
NC_005102.4-55308997	CGGTGATTCACCTAAGCTCCCTGACTC	1	C	0.124	0.1585
NC_005102.4-55316992	TCCTCTCTCACTTTCTCTCCATCCCTC	1	T	0.1739	0.1588
NC_005102.4-55316992	TCCTCTCTCACTTTCTCTCCATCCCTC	25	C	0.1739	0.1588
NC_005102.4-55317742	AGAATGGTCACATCAGCTCCCCCACCT	23	C	0.1951	0.3334
NC_005102.4-55335810	TTGCCTCTAACTGTTACTCCATCATCT	2	T	0.0588	0.0571
NC_005102.4-55390397	GAACTCAGTACTCTCTCTCCTCTGTGA	13	C	0.0082	0.479
NC_005102.4-55402144	CTCCCTGAGACAGCCCCTCCCATCCCA	21	C	0.2017	0.4463
NC_005102.4-55418821	CCTCCCATGACCTTTCCTCCTTCCTCT	7	A	0.3707	0.4435
NC_005102.4-55418821	CCTCCCATGACCTTTCCTCCTTCCTCT	25	T	0.2241	0.4987
NC_005102.4-55447240	ACACACATTACTATATCTCCTCACTGA	4	C	0.1518	0.5
NC_005102.4-55452468	ACCAAAGTAACCCATGCTCCCTGAAGA	21	C	0.2459	0.4914
NC_005102.4-55466204	ATGTCAGGCACTGTGCCTCCATCTTCT	14	T	0.0656	0.4612
NC_005102.4-55466204	ATGTCAGGCACTGTGCCTCCATCTTCT	15	G	0.0902	0.4973
NC_005102.4-55532562	CCTCTTTCCACAGCTACTCCATGCTCA	3	T	0	0.4698
NC_005102.4-55579598	AGTGAGCTAACACTTCCTCCATACTAC	2	G	0.2764	0.2937
NC_005102.4-55588255	CTACTTCTTACCTTCTCTCCTCTATTT	7	C	0.1557	0.1839
NC_005102.4-55590390	ATGTGACTGACCAATGCTCCTTATGTG	21	T	0.1463	0.3239
NC_005102.4-55634022	CACACACACACACTGCCTCCCCCAATA	22	C	0.2586	0.3662
NC_005102.4-55646079	CTGTTGGCTACACCTGCTCCTCATTAC	13	C	0	0.4793
NC_005102.4-55658738	AGTCCTTCAACAATAACTCCCAGCATG	9	A	0	0.4807
NC_005102.4-55714705	CTGGATACCACACCGTCTCCATGTGTC	15	G	0.0246	0.0401
NC_005102.4-55724144	GTGAAGCTCACTGAGTCTCCCTCTTTG	2	T	0.2301	0.3728
NC_005102.4-55776025	ATAAGCTGTACCTATCCTCCCACACAG	5	G	0.1271	0.1621
NC_005102.4-55776025	ATAAGCTGTACCTATCCTCCCACACAG	27	G	0.1271	0.1621
NC_005102.4-55812086	ATCCATGAGACATTGTCTCCAAAGGCA	13	T	0.0579	0.0562
NC_005102.4-55812086	ATCCATGAGACATTGTCTCCAAAGGCA	27	A	0.0992	0.3557
NC_005102.4-55813179	CATCAAGTCACATCTTCTCCCACTGAG	5	A	0.0536	0.0521
NC_005102.4-55848192	TGGTATTACACAGGTTCTCCTAGGGTA	8	A	0	0.4718

NC_005102.4-55848192	TGGTATTACACAGGTTCTCCTAGGGTA	23	G	0.0084	0.4723
NC_005102.4-55857167	AGAAAAGAGACAAAGGCTCCTTCTTAG	27	G	0.0084	0.4723
NC_005102.4-55859990	ATCTTTGTAACTTTTCCTCCTACTCCT	1	A	0.1271	0.4997
NC_005102.4-55859990	ATCTTTGTAACTTTTCCTCCTACTCCT	25	C	0.0588	0.1033
NC_005102.4-55874444	TGCCTCAGTACGGTCCCTCCCTGGCCT	14	T	0.2195	0.4741
NC_005102.4-55887269	TTATTGTAAACAGTGTCTCCTTAAATG	27	G	0.1071	0.1014
NC_005102.4-55903350	AACTGAGGGACTTGAGCTCCATGGGGA	1	A	0.0081	0.4777
NC_005102.4-55926046	GACACAGACACAGGCTCTCCAAAGACA	3	C	0.0163	0.0476
NC_005102.4-55927543	AACCCTGGAACTCCAACTCCTGAGGGA	16	A	0.0708	0.1466
NC_005102.4-55947889	CACACGTGCACATATCCTCCTACCCAA	6	G	0.0721	0.4931
NC_005102.4-55993942	CCAGGGATTACAACAACTCCAAGGGTA	4	G	0.1393	0.3008
NC_005102.4-56151409	GTGGGATGTACAGACCCTCCCTGAGCT	22	T	0.181	0.2188
NC_005102.4-56151409	GTGGGATGTACAGACCCTCCCTGAGCT	23	G	0.181	0.2188
NC_005102.4-56264863	TGCATCTTCACACCTTCTCCTGTTTCA	22	G	0.2131	0.2279
NC_005102.4-56275122	TATTGCTAAACCCCCTCTCCAGCCTCA	3	T	0.0357	0.0351
NC_005102.4-56277667	CCTTCTGTTACCTGTGCTCCCTTCACT	8	T	0.0894	0.1287
NC_005102.4-56311868	TGTATGCACACCTTCCCTCCCGGGATT	12	C	0.1092	0.2626
NC_005102.4-56311868	TGTATGCACACCTTCCCTCCCGGGATT	22	G	0	0.4844
NC_005102.4-56314335	CTAAGGAAAACCGATTCTCCCTTCCCC	7	A	0.1416	0.3136
NC_005102.4-56321588	GAGCAATGCACAACCTCTCCAATTGCC	15	C	0.0357	0.0521
NC_005102.4-56328653	GGGAACTGTACATCTTCTCCTGCCACT	5	A	0.2066	0.4398
NC_005102.4-56328653	GGGAACTGTACATCTTCTCCTGCCACT	14	C	0.0164	0.0322
NC_005102.4-56353625	GCAACTAGCACCTGGCCTCCACCCCTG	1	G	0.1565	0.1869
NC_005102.4-56353625	GCAACTAGCACCTGGCCTCCACCCCTG	4	A	0	0.4483
NC_005102.4-56366279	TTTTGGCCTACTCAGTCTCCTGTATGC	24	A	0	0.48
NC_005102.4-56373013	TCCCTCATAACACTGTCTCCACGGTTC	22	C	0.0625	0.0605
NC_005102.4-56414467	ACTAGAACCACCGTGCCTCCAGCCCTT	1	A	0.2174	0.3728
NC_005102.4-56414467	ACTAGAACCACCGTGCCTCCAGCCCTT	7	A	0	0.48
NC_005102.4-56423835	TCTTTTATCACATCGTCTCCATATCTC	1	T	0.0086	0.4893
NC_005102.4-56423835	TCTTTTATCACATCGTCTCCATATCTC	8	T	0.0431	0.0422
NC_005102.4-56465410	TGTCTGTCCACAGATTCTCCTCAGCCT	23	A	0.0504	0.065
NC_005102.4-56490203	CATTTCTCAACTGAGGCTCCCTATTCT	24	T	0	0.4743
NC_005102.4-56562466	TGCGTGGGTACTGAGACTCCCTGGAAG	4	G	0.2276	0.3427
NC_005102.4-56577267	GGGTCTCGCACATAGGCTCCGCCCAGC	7	C	0.2893	0.465
NC_005102.4-56577267	GGGTCTCGCACATAGGCTCCGCCCAGC	24	C	0.0413	0.0405
NC_005102.4-56625611	AACTGGCCCACATGAGCTCCAATACAC	12	A	0.3008	0.3472
NC_005102.4-56635233	TACAAAGGTACTGGATCTCCCGGAACT	2	A	0.1736	0.1982
NC_005102.4-56647323	CTTCCCCACACCTTTTCTCCTTCCCCT	15	T	0.1721	0.5
NC_005102.4-56647323	CTTCCCCACACCTTTTCTCCTTCCCCT	22	T	0.122	0.1425
NC_005102.4-56658842	GCTCTTTACACCGTTTCTCCTCAGGCT	13	G	0.3223	0.3327
NC_005102.4-56658842	GCTCTTTACACCGTTTCTCCTCAGGCT	15	T	0.0328	0.0322
NC_005102.4-56775370	GGTGTTGTCACGGCCTCTCCTGCCTCT	2	G	0.0574	0.071
NC_005102.4-56775370	GGTGTTGTCACGGCCTCTCCTGCCTCT	5	T	0.1393	0.4984
NC_005102.4-56863502	TGGGATGAGACCTGACCTCCTCCTCTC	8	A	0.1327	0.1391
NC_005102.4-56868889	GGTGTCCCTACTCATGCTCCAGAGGCT	8	C	0.0744	0.0716
NC_005102.4-56868889	GGTGTCCCTACTCATGCTCCAGAGGCT	24	G	0.05	0.0488
NC_005102.4-56870200	ATTACAGAAACGGTGGCTCCAAATGGC	14	T	0.1626	0.189
NC_005102.4-56883732	TAACTGAACACATGTTCTCCAAGGGTC	21	A	0.2149	0.4923
NC_005102.4-56884061	TAAATGATGACGATTCCTCCTGTGTCA	15	T	0.0083	0.4803
NC_005102.4-56938549	CACCTTAACACAAAAGCTCCAGACTAT	25	T	0.1803	0.2399
NC_005102.4-57125226	CTGCCCATAACTCCAGCTCCAGGGGAC	2	T	0.3514	0.3012

NC_005102.4-57125226	CTGCCCATAACTCCAGCTCCAGGGGAC	25	G	0.3514	0.3012
NC_005102.4-57168861	ATGAGAGAGACCTCCCCTCCATGCTCA	23	G	0.0325	0.032
NC_005102.4-57186669	AACAAACAAACATAGCCTCCAAGGTGG	15	G	0.0893	0.0853
NC_005102.4-57186669	AACAAACAAACATAGCCTCCAAGGTGG	21	A	0.0901	0.086
NC_005102.4-57188111	TAAATAAACACACTGTCTCCAAGCCAC	5	T	0.0089	0.4751
NC_005102.4-57213516	TTACAGACTACGATATCTCCCCGGACT	13	A	0	0.4786
NC_005102.4-57227713	CTCTGATCCACAATGACTCCACTTTCC	4	T	0	0.4841
NC_005102.4-57227713	CTCTGATCCACAATGACTCCACTTTCC	27	C	0.1429	0.3157
NC_005102.4-57399726	AGAATAGAAACCCTTCCTCCATACAAG	13	C	0.1405	0.1585
NC_005102.4-57576626	CAGAGAAACACTGATACTCCTGCCCAT	2	A	0.0084	0.0249
NC_005102.4-57576626	CAGAGAAACACTGATACTCCTGCCCAT	26	A	0.042	0.0411
NC_005102.4-57583423	CAGGTGTGCACAAGAGCTCCTGTTTCT	22	G	0	0.4677
NC_005102.4-57619826	CTGCAACTTACACAGACTCCTAGTATG	15	G	0.0168	0.033
NC_005102.4-57619826	CTGCAACTTACACAGACTCCTAGTATG	25	A	0	0.4793
NC_005102.4-57653930	GAAATGAGCACTTCTGCTCCTCTTTCA	12	T	0	0.4786
NC_005102.4-57703342	TTATTTGGGACAACATCTCCACTGCTC	4	T	0	0.4875
NC_005102.4-57703342	TTATTTGGGACAACATCTCCACTGCTC	9	G	0.0351	0.0345
NC_005102.4-57727180	AGTTATTTCACCAGCTCTCCTTTTTAA	12	C	0.0417	0.0408
NC_005102.4-57731313	ATCTGAGGGACCCTTTCTCCATTCTCA	8	G	0.1304	0.4454
NC_005102.4-57733447	CACACACACACCAACTCTCCTTCCAGC	14	A	0	0.488
NC_005102.4-57783425	CATCATGTAACCAGTCCTCCTGCTTTC	15	T	0.0082	0.4755
NC_005102.4-57783425	CATCATGTAACCAGTCCTCCTGCTTTC	21	T	0.1639	0.1774
NC_005102.4-57808103	TTGATGAAAACAGTTCCTCCCAGCCTC	13	G	0.2049	0.321
NC_005102.4-57840355	GTACCTGTCACCGAGCCTCCACAGTGC	12	C	0.0902	0.0861
NC_005102.4-57888985	ATGTGTGTCACATCTTCTCCATTCAGT	12	A	0.3171	0.4174
NC_005102.4-57888985	ATGTGTGTCACATCTTCTCCATTCAGT	26	G	0.3171	0.4239
NC_005102.4-57912924	CCGTTTCCCACTTTCTCTCCCCATTTC	6	T	0.2193	0.4473
NC_005102.4-57912924	CCGTTTCCCACTTTCTCTCCCCATTTC	25	T	0.2035	0.3772
NC_005102.4-57920459	GCTGGCCCCACAACTCCTCCGTGTATT	25	A	0	0.4891
NC_005102.4-57950638	CACTCAGCAACACGTTCTCCTTGGAAC	14	G	0	0.4807
NC_005102.4-57950638	CACTCAGCAACACGTTCTCCTTGGAAC	26	A	0.0574	0.4903
NC_005102.4-57952904	CATTACGAAACTAGAACTCCAAAGTTT	7	G	0.2564	0.4938
NC_005102.4-57958312	ATCTGCCTAACTCTGTCTCCCAAGCTC	16	T	0.0086	0.4893
NC_005102.4-57961065	TTCAGAGAAACCCTGACTCCAATAAAT	13	C	0.0656	0.0634
NC_005102.4-58015250	GTGAACAAAACACGTTCTCCTTTATTG	1	G	0.0517	0.0504
NC_005102.4-58025980	AGTACACTGACCCTGCCTCCCACAAAA	6	A	0.0085	0.4823
NC_005102.4-58088165	AGAGCCCCGACTGCCCCTCCAGAGGCC	6	C	0	0.4779
NC_005102.4-58098538	ATATAAGGCACCACTCCTCCCTGTCAT	2	T	0.2195	0.4036
NC_005102.4-58187259	AGGAGAAACACAAAATCTCCATGGCTA	5	G	0.0085	0.4714
NC_005102.4-58187259	AGGAGAAACACAAAATCTCCATGGCTA	16	T	0.0086	0.4729
NC_005102.4-58245487	TGGGTCTTCACAGCAGCTCCTCGAAGA	4	G	0.0085	0.487
NC_005102.4-58245487	TGGGTCTTCACAGCAGCTCCTCGAAGA	24	A	0.1538	0.4918
NC_005102.4-58255632	TATCCAAACACTGCGCCTCCATCCTGC	26	G	0.0894	0.1562
NC_005102.4-58272739	CACTCTTGAACAGCCTCTCCCAAAGCT	3	C	0	0.4856
NC_005102.4-58272739	CACTCTTGAACAGCCTCTCCCAAAGCT	7	T	0.2222	0.355
NC_005102.4-58275139	ACAATAAATACGGTCTCTCCTGAAGTC	3	A	0	0.4898
NC_005102.4-58275139	ACAATAAATACGGTCTCTCCTGAAGTC	25	G	0.0089	0.491
NC_005102.4-58298061	CCCTAACACACACTTCCTCCTACGTGC	8	A	0.2368	0.4415
NC_005102.4-58298061	CCCTAACACACACTTCCTCCTACGTGC	24	G	0.0088	0.4796
NC_005102.4-58304683	AGGGCGGGCACTAGGCCTCCACACTCT	5	C	0.1239	0.3026
NC_005102.4-58304683	AGGGCGGGCACTAGGCCTCCACACTCT	9	C	0	0.4841

NC_005102.4-58314691	CCACTGGCCACCCCATCTCCTTCATGT	14	C	0.2231	0.2474
NC_005102.4-58314691	CCACTGGCCACCCCATCTCCTTCATGT	23	C	0	0.48
NC_005102.4-58418878	GCCCTCAGGACCTGAGCTCCATCCTCA	6	C	0.265	0.4753
NC_005102.4-58473485	TCAGTGATAACTCTCCCTCCAAGTCCT	12	T	0.1491	0.235
NC_005102.4-58473485	TCAGTGATAACTCTCCCTCCAAGTCCT	24	T	0.0085	0.0251
NC_005102.4-58492302	AAAACCATCACAGTGACTCCTAGCAGA	7	A	0	0.4833
NC_005102.4-58503796	CTAGAACAAACCGTAGCTCCTAACCAT	22	A	0.0738	0.071
NC_005102.4-58521978	AATAAACAAACTCTTGCTCCCTTTGGG	6	A	0.1441	0.1894
NC_005102.4-58522587	AGTATGAGTACACTAGCTCCGTGTCTT	16	G	0	0.4539
NC_005102.4-58522587	AGTATGAGTACACTAGCTCCGTGTCTT	21	G	0	0.4558
NC_005102.4-58562428	ACCAGCACTACCCAGTCTCCATGACAC	25	C	0.1026	0.1563
NC_005102.4-58623400	CTTCTTCCAACCCCTTCTCCATTCCCG	3	T	0	0.4773
NC_005102.4-58629854	GCCATCATAACTCTAGCTCCAGGGGAT	3	C	0.3017	0.4545
NC_005102.4-58629854	GCCATCATAACTCTAGCTCCAGGGGAT	24	G	0.2845	0.4248
NC_005102.4-58642220	TGCATTGGCACAGCGCCTCCGAGCTAC	14	C	0.0763	0.0734
NC_005102.4-58642220	TGCATTGGCACAGCGCCTCCGAGCTAC	21	G	0.0593	0.0734
NC_005102.4-58665660	GTTAGACCCACAGTCCCTCCCTCCACT	1	G	0.1157	0.1787
NC_005102.4-58678887	ACAATTTCTACCCACTCTCCCCGACTA	24	A	0.1043	0.0989
NC_005102.4-58696532	GTTGTTCTGACGGGCACTCCTGGTGTC	6	T	0.0776	0.1786
NC_005102.4-58717930	GGAAGACCTACCAGCTCTCCTCCCACC	1	G	0.0081	0.0241
NC_005102.4-58763964	AACACAGCCACACCTACTCCAACACAG	9	C	0.1736	0.4999
NC_005102.4-58763964	AACACAGCCACACCTACTCCAACACAG	16	A	0.1322	0.1235
NC_005102.4-58775241	TTCTAACGCACGCTCTCTCCAACTGAG	7	C	0.1138	0.1216
NC_005102.4-58779492	GGCATGCTGACTTCGTCTCCTTTAGTT	4	A	0.1709	0.4991
NC_005102.4-58779492	GGCATGCTGACTTCGTCTCCTTTAGTT	24	A	0.1282	0.1347
NC_005102.4-58802300	AACCTCTTCACTCCATCTCCACAAATG	8	T	0	0.4728
NC_005102.4-58808982	TTGTCCACCACCCGTTCTCCCGCTCTA	15	T	0.025	0.4783
NC_005102.4-58808982	TTGTCCACCACCCGTTCTCCCGCTCTA	22	G	0.2131	0.4698
NC_005102.4-58862462	TGCCACTCTACAAACGCTCCTAAGCGC	15	C	0.0259	0.0255
NC_005102.4-58875648	AAACAGACCACTCTCTCTCCCTCCGGA	24	C	0.1607	0.3047
NC_005102.4-58963515	TGTGTAAGAACCGAATCTCCCTTTCCC	24	T	0.0082	0.0401
NC_005102.4-58987802	TGGCAGGGAACTGGTTCTCCATTCTAG	2	G	0.065	0.1356
NC_005102.4-58987802	TGGCAGGGAACTGGTTCTCCATTCTAG	9	A	0.2276	0.4
NC_005102.4-59038778	TAGCTACTGACTGTGACTCCTAAGTCT	3	G	0.225	0.3791
NC_005102.4-59038778	TAGCTACTGACTGTGACTCCTAAGTCT	12	T	0.0083	0.4769
NC_005102.4-59047172	GCTTTATTCACTCTCACTCCCAACCAT	6	A	0.0088	0.476
NC_005102.4-59062799	ATCCACAACACAGACCCTCCCTTAGTT	6	C	0.1157	0.1653
NC_005102.4-59131931	TCAGTTCATACTTGGGCTCCACATGAG	5	T	0.0171	0.4734
NC_005102.4-59143030	TTTACTTTTACAACCTCTCCGGGAGTC	21	G	0	0.4807
NC_005102.4-59183352	CATCTTAACACTCCCTCTCCCCAAATA	23	A	0.0684	0.4982
NC_005102.4-59270664	TCACAGATCACCAGAACTCCCCGCTTA	21	C	0	0.474
NC_005102.4-59270664	TCACAGATCACCAGAACTCCCCGCTTA	22	C	0	0.474
NC_005102.4-59292415	GAATGCACAACTTTTCCTCCTTGACCC	2	A	0.0083	0.4747
NC_005102.4-59310551	AGCTAAGGCACACCCTCTCCCCTGGTT	3	C	0.0081	0.0398
NC_005102.4-59310951	CCCCTCCCCACACCCTCTCCTGAGCTG	16	T	0	0.4875
NC_005102.4-59341870	GGTACATGCACTCCCCCTCCCCCACAT	25	C	0.0672	0.065
NC_005102.4-59347385	GATGACAGAACTGACACTCCGGTACAG	21	G	0.0261	0.059
NC_005102.4-59347385	GATGACAGAACTGACACTCCGGTACAG	25	C	0.0261	0.059
NC_005102.4-59374961	GCATCTGTAACCTGCACTCCTATGGGG	1	G	0.1538	0.4868
NC_005102.4-59374961	GCATCTGTAACCTGCACTCCTATGGGG	7	G	0	0.4749
NC_005102.4-59390963	AATAACAGGACCATCCCTCCTGAACTC	1	A	0.1066	0.4984

NC_005102.4-59397125	AGTAGTTACACGGTTACTCCATGGTAG	16	A	0.0169	0.0496
NC_005102.4-59397125	AGTAGTTACACGGTTACTCCATGGTAG	22	T	0.1017	0.1827
NC_005102.4-59415360	CAAATTGAAACACTCCCTCCAAGAGGG	22	A	0.025	0.0247
NC_005102.4-59439453	CCACTGAGGACCCCAACTCCACTTCCC	4	C	0.0339	0.0333
NC_005102.4-59441094	CTTCATGCCACCGAATCTCCAGTAGTA	7	G	0.0894	0.1001
NC_005102.4-59444978	CTATCTGTAACTCTAGCTCCAGTGGAT	24	G	0.0083	0.4835
NC_005102.4-59446812	GCAAGGAAAACAGCAGCTCCCCGTAAG	22	C	0.1405	0.1585
NC_005102.4-59453513	CAACACTCTACCAGAGCTCCCTTTTTT	21	C	0.115	0.1391
NC_005102.4-59453513	CAACACTCTACCAGAGCTCCCTTTTTT	22	T	0.2054	0.2385
NC_005102.4-59461951	AGCATCATTACACGGTCTCCATGAGCT	5	T	0	0.4793
NC_005102.4-59470791	CATGCCATCACTATGGCTCCATCAGCA	12	T	0	0.4912
NC_005102.4-59501512	TGCGTTGTTACTCCCCCTCCCTTCTCC	3	C	0.1382	0.1425
NC_005102.4-59524208	GTAGTACCCACTGCTCCTCCCACAGCA	5	T	0.0427	0.074
NC_005102.4-59524208	GTAGTACCCACTGCTCCTCCCACAGCA	26	C	0.1111	0.1049
NC_005102.4-59621457	GGCCAGCAAACTGGGACTCCTCCTGCT	2	G	0.041	0.0557
NC_005102.4-59624257	GCGGAGTCAACGGTTCCTCCAAGCAGC	3	G	0	0.0351
NC_005102.4-59624257	GCGGAGTCAACGGTTCCTCCAAGCAGC	24	C	0.0536	0.0521
NC_005102.4-59665850	TGAGACATCACTCCAGCTCCTCACAAT	21	T	0.0583	0.0566
NC_005102.4-59693182	TCCCTTTGGACACCGACTCCAGCAACG	14	C	0.1583	0.1733
NC_005102.4-59693182	TCCCTTTGGACACCGACTCCAGCAACG	16	A	0	0.4751
NC_005102.4-59736729	GGTATGTGCACTTTTACTCCGAAGACC	7	T	0	0.4832
NC_005102.4-59736729	GGTATGTGCACTTTTACTCCGAAGACC	8	G	0.1695	0.1827
NC_005102.4-59743119	GTCACCGAGACTGCTGCTCCTAAGGAA	2	T	0.2231	0.4966
NC_005102.4-59743119	GTCACCGAGACTGCTGCTCCTAAGGAA	7	G	0.1736	0.2234
NC_005102.4-59769954	CTGGACTCCACAGGCACTCCAGTCAAA	2	T	0.2966	0.4066
NC_005102.4-59769954	CTGGACTCCACAGGCACTCCAGTCAAA	4	G	0.2034	0.2585
NC_005102.4-59776031	TGTCTTGGCACAGGAGCTCCAGGTTAG	4	C	0.05	0.0488
NC_005102.4-59776031	TGTCTTGGCACAGGAGCTCCAGGTTAG	6	T	0.2083	0.4622
NC_005102.4-59793031	CTTCTTACCACCACCCCTCCCCCTGCC	3	T	0.0756	0.0882
NC_005102.4-59793031	CTTCTTACCACCACCCCTCCCCCTGCC	6	T	0	0.4703
NC_005102.4-59803316	ACTCTTGTGACCCTGGCTCCAACCACT	15	G	0.0496	0.4877
NC_005102.4-59911094	AGGCCTGTCACAAGATCTCCCAGAACT	7	G	0	0.4734
NC_005102.4-59912847	AGGATAGGGACAGTGCCTCCCACCATG	4	A	0	0.4703
NC_005102.4-59983169	CCAGCTGCAACCTCGGCTCCAGCCTCT	14	C	0.0328	0.0634
NC_005102.4-59989498	TCCTAGTCCACTACCCCTCCCCCATCC	25	T	0.1901	0.259
NC_005102.4-60030720	CGTCTCACCACAGAAGCTCCGAGACTG	1	C	0.025	0.0247
NC_005102.4-60030720	CGTCTCACCACAGAAGCTCCGAGACTG	4	C	0.2167	0.2888
NC_005102.4-60033876	CCCATTGCCACATCTCCTCCAACAAGG	7	G	0.0877	0.0839
NC_005102.4-60033876	CCCATTGCCACATCTCCTCCAACAAGG	27	G	0.0877	0.0839
NC_005102.4-60098244	AACCCTGTAACCCTGTCTCCTTACTTG	24	C	0	0.4764
NC_005102.4-60098244	AACCCTGTAACCCTGTCTCCTTACTTG	27	G	0.0522	0.0508
NC_005102.4-60131402	AGTCTACACACGTGTGCTCCTATGGGA	12	G	0.2017	0.422
NC_005102.4-60131402	AGTCTACACACGTGTGCTCCTATGGGA	26	G	0.0667	0.095
NC_005102.4-60134350	TTAACGGCCACCTCCTCTCCATCCCCA	5	C	0.0744	0.4951
NC_005102.4-60134350	TTAACGGCCACCTCCTCTCCATCCCCA	15	C	0.157	0.3327
NC_005102.4-60188322	ACTCTGTTCACAGAATCTCCTACTCAT	7	T	0.1382	0.208
NC_005102.4-60188322	ACTCTGTTCACAGAATCTCCTACTCAT	13	G	0.1545	0.1695
NC_005102.4-60194035	GGGATGCTTACTCTCCCTCCTTCCCTC	13	C	0.2195	0.3381
NC_005102.4-60200610	AAGACTCACACGGCACCTCCCTCCGAT	25	G	0.0165	0.0325
NC_005102.4-60203788	CCCCTGGGCACGCCCTCTCCAGCATTT	27	T	0.1532	0.4141
NC_005102.4-60235885	ACGTTTAATACACTAACTCCACACAAG	2	C	0	0.4772

NC_005102.4-60292611	CAAGCCACGACAGATGCTCCCAAGATG	7	A	0	0.4807
NC_005102.4-60292830	GTGTGATAGACGGCTTCTCCACTTTGG	6	A	0	0.4749
NC_005102.4-60292830	GTGTGATAGACGGCTTCTCCACTTTGG	22	C	0.0085	0.0253
NC_005102.4-60327424	GGCTTTTGCACGCCAGCTCCAGCCACT	2	G	0.0246	0.0243
NC_005102.4-60344497	CAACTTTTGACCTTAGCTCCATTAGAA	12	C	0.0862	0.4963
NC_005102.4-60366740	CACAAAGAAACCACAACTCCCATCCGG	26	G	0.0086	0.4729
NC_005102.4-60374790	GTAGCCCACACCCATCCTCCGGAGTGT	9	C	0.2	0.4849
NC_005102.4-60376107	GCAAGAGAAACTCATTCTCCCACCCAC	25	C	0.1327	0.5
NC_005102.4-60388569	AGATGCAGTACAAAGACTCCTGGAGAA	2	G	0.0424	0.0415
NC_005102.4-60389535	GTGAGGCAAACTTCATCTCCTACCACA	2	T	0	0.4832
NC_005102.4-60430732	GGGCAAACCACTTCATCTCCTGCTGTT	23	C	0.2358	0.4741
NC_005102.4-60443557	ATTCATAGGACCTGAACTCCAAACATG	23	A	0	0.4773
NC_005102.4-60455759	CATGTGGTGACCCCAACTCCTAATTTT	3	T	0.084	0.0805
NC_005102.4-60455759	CATGTGGTGACCCCAACTCCTAATTTT	12	C	0.0256	0.0253
NC_005102.4-60470114	ACATGGCCCACCTGCCCTCCGGGTAAA	25	A	0.0603	0.0585
NC_005102.4-60470453	GGCTTCAGGACATGGTCTCCTGTAGCC	22	G	0.094	0.0896
NC_005102.4-60521542	TATCATGCAACATGTGCTCCTTCACCT	25	C	0.1488	0.1377
NC_005102.4-60526338	CACCTCTGGACTCGATCTCCCTGATGC	23	G	0.0769	0.4947
NC_005102.4-60538563	CGACAATGGACTGTGCCTCCTCTATCG	25	T	0	0.4851
NC_005102.4-60538563	CGACAATGGACTGTGCCTCCTCTATCG	26	C	0	0.4851
NC_005102.4-60565090	TGTGGCAGGACACCTGCTCCCTGTATA	23	G	0.0252	0.4886
NC_005102.4-60599573	CAAGGCAATACTCGCTCTCCCTCTTTC	25	T	0	0.4724
NC_005102.4-60626883	GCCATCACAACGTAGTCTCCAGTTCTA	12	G	0.2195	0.2556
NC_005102.4-60631381	AGAAAATCTACCCAGACTCCCATCATT	26	T	0.0672	0.494
NC_005102.4-60669213	ATTAAGAGCACTCTGCCTCCAGGGTAC	24	G	0	0.4819
NC_005102.4-60822704	ATGATTTCTACAATTACTCCAGGATAT	1	A	0.0083	0.4732
NC_005102.4-60822704	ATGATTTCTACAATTACTCCAGGATAT	12	A	0.1176	0.2328
NC_005102.4-60844524	CCAACTAGCACTGCATCTCCCTGTACT	9	C	0.0902	0.1296
NC_005102.4-60844524	CCAACTAGCACTGCATCTCCCTGTACT	12	T	0.2417	0.3533
NC_005102.4-60853223	AGTGGCTGGACCAACTCTCCTTTGCTC	7	T	0.0088	0.4861
NC_005102.4-60863260	GTCTTCGCCACTCTCTCTCCCAACTCC	2	T	0.3	0.4328
NC_005102.4-60863260	GTCTTCGCCACTCTCTCTCCCAACTCC	22	A	0.3109	0.4435
NC_005102.4-60864904	AGCTTTGAAACCCCAGCTCCTTCTATC	24	T	0.1057	0.1145
NC_005102.4-60884765	GTCTCCCCCACCCTACCTCCTTTCCAT	16	C	0.0351	0.0345
NC_005102.4-60911852	TTCCAACTGACTCTTCCTCCCGGGCTG	22	G	0.0783	0.1067
NC_005102.4-60911852	TTCCAACTGACTCTTCCTCCCGGGCTG	25	C	0.0776	0.1058
NC_005102.4-60912961	TTTAGAATAACCATCTCTCCCAGACCT	21	C	0.0427	0.058
NC_005102.4-60912961	TTTAGAATAACCATCTCTCCCAGACCT	22	A	0.0085	0.481
NC_005102.4-60935846	CCATCGCGCACTATTCCTCCCTTCGGT	8	G	0.1557	0.2573
NC_005102.4-60949460	TTACGGGAGACCAGACCTCCAGACGGT	4	C	0.2231	0.3851
NC_005102.4-60949460	TTACGGGAGACCAGACCTCCAGACGGT	5	G	0.041	0.0557
NC_005102.4-60955321	ACAGTGGGCACATTATCTCCATGTAAA	13	T	0.2609	0.4092
NC_005102.4-60968851	ATAAGAACTACTTCAGCTCCAGGGACT	1	A	0.025	0.0247
NC_005102.4-61063606	AAGGTGTTAACATGTCCTCCTTCCAAC	2	A	0.0672	0.4921
NC_005102.4-61065326	CACCTGTCAACTCTAGCTCCAGGTGAT	7	T	0.0769	0.4987
NC_005102.4-61069186	ATTTCCCCCACTGCAGCTCCTTCCTCT	15	A	0	0.4832
NC_005102.4-61144413	TGAACAAAAACCAGATCTCCAGACTTG	15	A	0.0351	0.0345
NC_005102.4-61168260	GGGTAGGTAACAGCCTCTCCAGTGAAA	1	G	0.2119	0.319
NC_005102.4-61168260	GGGTAGGTAACAGCCTCTCCAGTGAAA	27	A	0.2479	0.3685
NC_005102.4-61198430	CCTGTTAGTACAGCTCCTCCTGCTGCT	16	C	0	0.4844
NC_005102.4-61206713	TTTGTATGCACCTATTCTCCACAATGA	4	G	0.0965	0.138

NC_005102.4-61276848	ACAAGTATTACCCACTCTCCAAAGATT	24	G	0.0252	0.0411
NC_005102.4-61308258	TAACATAAAACGTATGCTCCTTTGGAC	12	G	0.1892	0.4974
NC_005102.4-61351490	CCTTGAAAGACAGATGCTCCAGACGTC	25	G	0.2759	0.482
NC_005102.4-61387221	GTGCTGGAAACTGAAACTCCTAACAGT	1	G	0.1475	0.4988
NC_005102.4-61387221	GTGCTGGAAACTGAAACTCCTAACAGT	27	T	0.0574	0.4879
NC_005102.4-61408273	TCTTTTAAAACATTTGCTCCCTTTTCA	26	C	0.0702	0.2285
NC_005102.4-61451322	CTGTCTGTAACCCCAACTCCACAGGCA	8	T	0.065	0.0629
NC_005102.4-61486689	GAAGAAGGCACTGAATCTCCTGAAGTT	5	A	0.0325	0.0476
NC_005102.4-61486689	GAAGAAGGCACTGAATCTCCTGAAGTT	7	G	0	0.4793
NC_005102.4-61506990	ATGACAACAACCGAGTCTCCAAAGAAA	12	C	0.6016	0.4206
NC_005102.4-61517714	CTATTTGCTACCTGACCTCCATGAGGT	15	A	0	0.4793
NC_005102.4-61560200	AGCAAAGTTACAAACCCTCCTTTGAAT	26	A	0.0088	0.4774
NC_005102.4-61566568	ACCAGCACCACTCAAGCTCCTGGGTCA	5	G	0	0.4819
NC_005102.4-61599313	CATGCTGTAACCACGACTCCCCTTTTG	6	T	0.2018	0.4935
NC_005102.4-61601035	TCAAAGACTACGGTGTCTCCTTCCCTA	14	T	0.0413	0.0405
NC_005102.4-61628685	GTGGACGCCACCGTTTCTCCCCTCTTC	14	T	0.1653	0.4481
NC_005102.4-61646020	TTTCACCCCACCTCTTCTCCTCAGCTG	22	C	0	0.4728
NC_005102.4-61681976	AATCTGTTCACTGTCCCTCCTACAGAA	2	A	0	0.4793
NC_005102.4-61689421	TAGTCTGCCACCCGTTCTCCGAGAAAG	14	G	0.1964	0.3565
NC_005102.4-61690364	AGGCAAGTTACATCAGCTCCGAAGTTC	21	G	0.0598	0.074
NC_005102.4-61695990	CACAGTGAGACCTTGTCTCCCCATCCT	2	A	0	0.4793
NC_005102.4-61695990	CACAGTGAGACCTTGTCTCCCCATCCT	27	T	0	0.4793
NC_005102.4-61748153	TGTTCTCTGACTCCGTCTCCTGAGTTC	14	C	0.1818	0.4942
NC_005102.4-61752978	TCCCACATTACACATTCTCCTCAGCCT	5	A	0.0088	0.481
NC_005102.4-61861270	CTGTAACTTACGTTCCCTCCACGGAGG	22	C	0.1429	0.1327
NC_005102.4-61866717	TGGGTTCTCACAGCGCCTCCTCGCTCT	15	G	0.0446	0.0436
NC_005102.4-61866948	TTACCTGCAACTTCAGCTCCCGGGGAG	13	T	0.0082	0.479
NC_005102.4-61891638	TCTCTCCACACACATGCTCCTGAGCCT	5	T	0	0.4868
NC_005102.4-61921022	TCTTATCAGACATGGCCTCCTTGTCTC	25	C	0.1345	0.2328
NC_005102.4-61927447	GGGGAGGGGACATTGCCTCCTACCTAC	26	A	0.1653	0.2759
NC_005102.4-61937243	AATTTGCTAACTGTAACTCCAAAATCT	3	T	0.0603	0.0746
NC_005102.4-61965645	AACACACGGACACACTCTCCTCAGCGT	7	C	0.1966	0.4578
NC_005102.4-61965645	AACACACGGACACACTCTCCTCAGCGT	14	A	0	0.4826
NC_005102.4-62019962	AATGCTATGACCATGCCTCCATTAAGA	5	C	0.0083	0.4747
NC_005102.4-62031228	AACTCTGTAACTCCATCTCCAAGGGGT	9	A	0.161	0.2282
NC_005102.4-62111998	GGGAGTCCTACCTGACCTCCTGGCTTT	9	T	0.2397	0.4835
NC_005102.4-62111998	GGGAGTCCTACCTGACCTCCTGGCTTT	22	G	0	0.4793
NC_005102.4-62245122	AAGTCTTCAACTGTAACTCCAGCCAAG	13	G	0.2951	0.3259
NC_005102.4-62274712	CCGGTAAGCACTGTGCCTCCGTGGCTT	26	T	0.0862	0.4927
NC_005102.4-62283027	AAGGGAGCAACCTTAGCTCCTCCTTTG	9	A	0.1322	0.4959
NC_005102.4-62296554	AGCCCTAGCACTTGGTCTCCTCCTAAT	3	C	0.0756	0.0882
NC_005102.4-62320581	AGGCAGTGCACAAAAGCTCCTCCTTGG	16	G	0.0504	0.0491
NC_005102.4-62390759	GTAGCCCTCACCTGACCTCCACCAAGG	15	A	0.1913	0.4724
NC_005102.4-62456186	GAGGCAAATACCTTTCCTCCCTCTCCC	16	C	0.0339	0.0496
NC_005102.4-62458923	GGTGCAGGCACCCAGACTCCCACTGCA	2	G	0.1967	0.2032
NC_005102.4-62458923	GGTGCAGGCACCCAGACTCCCACTGCA	7	G	0	0.4773
NC_005102.4-62460840	CAACATTGCACACAAGCTCCAAAGGTC	6	T	0.2333	0.4132
NC_005102.4-62470817	AACAGCAGAACCATGGCTCCCAAGCAG	15	G	0.0488	0.0629
NC_005102.4-62470817	AACAGCAGAACCATGGCTCCCAAGCAG	27	G	0	0.4793
NC_005102.4-62502918	TGAGCTGAAACAGTGGCTCCCCCAGCC	23	C	0.1917	0.2372
NC_005102.4-62502918	TGAGCTGAAACAGTGGCTCCCCCAGCC	24	A	0.1917	0.2372

NC_005102.4-62536828	CTAAAATCCACTCCTGCTCCGGATGTG	12	T	0	0.4826
NC_005102.4-62536828	CTAAAATCCACTCCTGCTCCGGATGTG	21	G	0.042	0.0411
NC_005102.4-62540367	ATCTGTTACACAGTGGCTCCTCGACAC	22	C	0.2632	0.3706
NC_005102.4-62557993	CTTCCTAGCACAGTCCCTCCTCATCTC	25	C	0.193	0.2893
NC_005102.4-62581004	CAGACTCTGACCCTCGCTCCAAGCAAC	12	C	0	0.4807
NC_005102.4-62601963	GGCAATGTCACAAATTCTCCATGCATT	1	G	0	0.4728
NC_005102.4-62670911	TGACCTCACACATGTACTCCCTACCAT	13	T	0.025	0.0247
NC_005102.4-62670911	TGACCTCACACATGTACTCCCTACCAT	16	A	0.0083	0.4747
NC_005102.4-62737439	TTACATCTGACCCTTCCTCCCTTCCCT	1	T	0	0.4807
NC_005102.4-62771946	CAAAACATCACATGCCCTCCCGTGTGT	22	G	0.1017	0.1551
NC_005102.4-62850752	CTCGTGAACACAGATGCTCCACGGTCA	3	C	0	0.4793
NC_005102.4-62850752	CTCGTGAACACAGATGCTCCACGGTCA	22	C	0.2645	0.3729
NC_005102.4-62916168	TCACAACTTACACTCCCTCCTAGCACA	21	T	0.0357	0.0521
NC_005102.4-62917752	CCATCTGCCACTGAGTCTCCTTTGCTG	25	C	0.114	0.138
NC_005102.4-62923618	AAGGTGAAGACAGTGGCTCCAAGGTCC	24	G	0	0.4703
NC_005102.4-62923804	CCCGCACACACGGCAGCTCCCTTTCTT	25	C	0.1416	0.4887
NC_005102.4-62944328	CGGAAGTAAACTCTGTCTCCAACTGTG	2	G	0.0826	0.0943
NC_005102.4-62948676	AAATCTGCCACATTTCCTCCGCACCCC	16	C	0.1754	0.4444
NC_005102.4-62948676	AAATCTGCCACATTTCCTCCGCACCCC	24	C	0.1391	0.2268
NC_005102.4-62992997	GACTGCCAAACAGTGACTCCCAAGTCG	3	C	0.3058	0.4077
NC_005102.4-62992997	GACTGCCAAACAGTGACTCCCAAGTCG	26	C	0.0331	0.0325
NC_005102.4-63021014	GACCAGCCAACTCGTGCTCCTGTTGCC	25	G	0.0082	0.479
NC_005102.4-63092142	GACAGCCCCACCCCCACTCCCACCACC	15	C	0.2583	0.3708
NC_005102.4-63098614	AAGAAGGACACCTAAACTCCTGAGTAT	3	G	0	0.4864
NC_005102.4-63098614	AAGAAGGACACCTAAACTCCTGAGTAT	26	A	0.1441	0.2717
NC_005102.4-63143227	CCAAGACAGACCCTTCCTCCTGATATA	22	G	0.0744	0.4557
NC_005102.4-63240328	CTAAAAGCTACCTCAGCTCCATCATTA	8	C	0.1311	0.316
NC_005102.4-63252504	GGGCTTGGGACCAGTCCTCCTTTGCAG	1	G	0	0.4819
NC_005102.4-63252504	GGGCTTGGGACCAGTCCTCCTTTGCAG	16	C	0.0579	0.0716
NC_005102.4-63274086	TCTGCACACACGCCATCTCCAACTCGC	7	C	0.0331	0.0943
NC_005102.4-63274086	TCTGCACACACGCCATCTCCAACTCGC	8	A	0	0.4734
NC_005102.4-63344096	ACACCCCGGACTGGCTCTCCTTTCTAG	23	T	0.0579	0.4978
NC_005102.4-63344096	ACACCCCGGACTGGCTCTCCTTTCTAG	24	C	0.0579	0.4978
NC_005102.4-63363902	CCCATGAATACTTGGTCTCCAATTGAT	22	A	0.0086	0.4729
NC_005102.4-63368298	AGAAGACCAACTGAGACTCCTAGCCTA	23	G	0.3471	0.4582
NC_005102.4-63368298	AGAAGACCAACTGAGACTCCTAGCCTA	24	C	0.3471	0.4112
NC_005102.4-63379221	GCTCATAAAACATCCACTCCGACCCAC	21	G	0.025	0.0247
NC_005102.4-63379221	GCTCATAAAACATCCACTCCGACCCAC	27	C	0.0083	0.4847
NC_005102.4-63380922	GCCCGCTGCACCTGCGCTCCAGGCTTT	6	C	0.0163	0.4825
NC_005102.4-63458579	AAAATGACAACCATGTCTCCTCAAAGC	14	T	0	0.4659
NC_005102.4-63491843	CACCTGTCTACATTCGCTCCTCAGGTT	8	C	0.1405	0.1447
NC_005102.4-63491843	CACCTGTCTACATTCGCTCCTCAGGTT	15	C	0.0164	0.4807
NC_005102.4-63548393	GGGGCTAGCACATGCTCTCCTCTGGTA	25	G	0.1261	0.1928
NC_005102.4-63597069	TAATTTTTCACTGGTGCTCCGCCATCA	6	T	0	0.4787
NC_005102.4-63599838	TTTGCAACGACTGGCACTCCAAGCTGT	8	C	0.0244	0.0241
NC_005102.4-63624093	GCTAGGAGGACCAGATCTCCATGCAGG	25	A	0.0667	0.0644
NC_005102.4-63695284	ATCAAGTTCACATCAACTCCTGAGTTT	25	T	0.125	0.2491
NC_005102.4-63721002	CTGTGACAGACACCTCCTCCTCGGTGT	23	G	0.0407	0.0398
NC_005102.4-63769905	GTAAGGTGGACGGTATCTCCTTTCTCA	23	T	0	0.4765
NC_005102.4-63776444	GTTCAATGCACATGTACTCCTTATACC	2	T	0	0.4807
NC_005102.4-63784822	ATGAATGAAACTGATTCTCCCAATTGG	9	A	0.0089	0.4751

NC_005102.4-63784822	ATGAATGAAACTGATTCTCCCAATTGG	22	A	0.0177	0.4793
NC_005102.4-63868802	TAAAAGCTCACCGTCTCTCCTTAGTTT	16	T	0	0.4807
NC_005102.4-63913639	TGCAACACCACAGCTGCTCCCGTGCTT	2	G	0.2124	0.3982
NC_005102.4-63941760	CTGTGAGCTACGCGGCCTCCATTAGAA	21	A	0.1333	0.18
NC_005102.4-63958317	CAGATTGGGACTACTGCTCCATAGGTA	1	C	0.0348	0.0989
NC_005102.4-63958317	CAGATTGGGACTACTGCTCCATAGGTA	22	T	0.0087	0.4782
NC_005102.4-64034476	AACGATCCCACTGTCCCTCCATTTTCT	3	C	0.2051	0.4991
NC_005102.4-64034476	AACGATCCCACTGTCCCTCCATTTTCT	8	C	0.0508	0.4632
NC_005102.4-64071669	AACTTCCTGACTCTCCCTCCTAGGCAC	5	T	0	0.4807
NC_005102.4-64119174	ACTGTGAGAACCTGGTCTCCAACCAAC	3	T	0	0.4819
NC_005102.4-64120286	GTTCCTGCCACTTAGTCTCCTGGCACA	13	T	0	0.4807
NC_005102.4-64120286	GTTCCTGCCACTTAGTCTCCTGGCACA	26	C	0.0909	0.1163
NC_005102.4-64220188	ATCAGAGCTACACCTGCTCCCACTGTT	22	A	0.178	0.4454
NC_005102.4-64235657	TTATGGATGACAAGTTCTCCTACAGAT	21	T	0.1667	0.3578
NC_005102.4-64254802	TACAACAAAACACATGCTCCACTTTGT	16	G	0.0087	0.4849
NC_005102.4-64258819	TCGAGAGACACCCTTGCTCCGAGAGTT	2	C	0.0252	0.0249
NC_005102.4-64331358	ATAGTAAGCACTGCGGCTCCTGGATCG	15	G	0.2602	0.427
NC_005102.4-64331358	ATAGTAAGCACTGCGGCTCCTGGATCG	27	G	0.0407	0.0553
NC_005102.4-64336293	AGTGAGCAAACAAATTCTCCTCATAAT	23	A	0.0261	0.0257
NC_005102.4-64336293	AGTGAGCAAACAAATTCTCCTCATAAT	26	A	0.0087	0.4817
NC_005102.4-64354717	CTGGACATGACAGGACCTCCGTGCTCA	21	G	0.2033	0.2441
NC_005102.4-64354717	CTGGACATGACAGGACCTCCGTGCTCA	23	G	0.1626	0.2382
NC_005102.4-64357665	CACGTTCACACATCTGCTCCTATATGC	5	T	0	0.4912
NC_005102.4-64384958	GGAATGGCCACACTTCCTCCAGTCTTT	15	T	0.2167	0.255
NC_005102.4-64427551	AGAAAGCCAACATTCTCTCCTCCTAGC	12	A	0	0.4868
NC_005102.4-64456172	CATTGCAACACCCAGCCTCCCCAAAGA	3	T	0.2295	0.3259
NC_005102.4-64457233	CCATGACACACCGAGACTCCCTGAGTC	7	C	0.2195	0.319
NC_005102.4-64492053	AGCGAGGGGACCGCAGCTCCATTCTAA	3	C	0.2541	0.2796
NC_005102.4-64492053	AGCGAGGGGACCGCAGCTCCATTCTAA	21	A	0.0738	0.0861
NC_005102.4-64535565	TACTTAAATACACAGACTCCAGTAGTT	25	G	0.0536	0.0853
NC_005102.4-64553781	GACATCCCTACCTCGTCTCCTCTAATG	26	T	0.1786	0.232
NC_005102.4-64564746	TGAGATATGACTTTCTCTCCTCCTCTC	1	T	0	0.4666
NC_005102.4-64564746	TGAGATATGACTTTCTCTCCTCCTCTC	13	T	0.0172	0.4709
NC_005102.4-64604015	GTAACTAAAACGCCAACTCCAAGGTTA	12	G	0.252	0.2989
NC_005102.4-64640374	ATGTGTTAAACTGGTTCTCCGCCTGTG	21	G	0.2589	0.4617
NC_005102.4-64641266	TGCGTGGCTACACATTCTCCAGAGGCC	4	G	0.0702	0.0839
NC_005102.4-64641266	TGCGTGGCTACACATTCTCCAGAGGCC	5	T	0.2	0.293
NC_005102.4-64672907	TATCACAGAACCTATCCTCCGGTAGAA	24	A	0	0.4773
NC_005102.4-64722732	CTTTCTGTGACCCAGACTCCTCTCTTC	4	T	0.1176	0.5
NC_005102.4-64727527	GCTCTAGAGACTCTTACTCCTCCAAAT	6	A	0.0086	0.4836
NC_005102.4-64741112	CGGAAGATCACAGGATCTCCTTCCCAC	3	G	0.0087	0.4704
NC_005102.4-64741625	GACCCAATTACTTACACTCCACGGAAG	3	C	0.0085	0.4823
NC_005102.4-64772000	GCACTTGCTACCTTTCCTCCCCCAGCA	3	A	0.0407	0.0398
NC_005102.4-64788602	CAACAACCCACACTGCCTCCCTTGGCC	8	C	0.0246	0.0243
NC_005102.4-64807329	AGCCATCCAACCACAACTCCAGTTCCA	4	C	0.2195	0.484
NC_005102.4-64809657	GCAACTTCCACAGCAACTCCCACTCTT	2	C	0.2689	0.3319
NC_005102.4-64832928	ACTTTTTAAACTGAAGCTCCTATAAGT	7	T	0.0583	0.0566
NC_005102.4-64867227	TCTTTACTCACATGGCCTCCATAGGAG	13	T	0	0.4787
NC_005102.4-64867227	TCTTTACTCACATGGCCTCCATAGGAG	22	T	0	0.4787
NC_005102.4-64867266	CCTGCTTGGACATCTTCTCCTTCTGTG	26	T	0.1842	0.295
NC_005102.4-65045994	CTGTATCATACCTGCCCTCCTTTAGAC	15	C	0.2797	0.3664

NC_005102.4-65045994	CTGTATCATACCTGCCCTCCTTTAGAC	21	T	0.2564	0.2945
NC_005102.4-65058409	CAGAGCTGCACTGGGACTCCAACCTCC	6	C	0	0.4833
NC_005102.4-65248001	TCAATCAAGACAAACTCTCCTAGACAA	26	A	0.084	0.0958
NC_005102.4-65249725	AAGTCTTCCACCCCACCTCCTCTCTCT	1	A	0	0.4807
NC_005102.4-65249725	AAGTCTTCCACCCCACCTCCTCTCTCT	6	T	0.1789	0.2142
NC_005102.4-65315484	CACAGTGACACCCTTCCTCCAATAGTG	22	A	0.0261	0.0425
NC_005102.4-65396538	GAGTGCTCCACTGGCCCTCCTCTCCCT	6	C	0.0244	0.0241
NC_005102.4-65411978	GTGTCACATACATTCTCTCCCTTGTTT	1	G	0.2869	0.4193
NC_005102.4-65546680	CAACCTATGACATTATCTCCTAAGTGA	4	C	0.0088	0.483
NC_005102.4-65569586	TGATGGTCCACACTGCCTCCAAGAGTC	21	A	0	0.4902
NC_005102.4-65584751	AGGGACCACACTCCTCCTCCCTCAGCC	23	C	0.0244	0.0241
NC_005102.4-65680861	AAAGGTTTTACCAATCCTCCAACTGAT	9	T	0.124	0.5
NC_005102.4-65680861	AAAGGTTTTACCAATCCTCCAACTGAT	14	A	0.1818	0.2759
NC_005102.4-65737508	AGAACTGCAACATGAGCTCCATACATA	14	G	0	0.4875
NC_005102.4-65826286	CACACACACACGCACGCTCCAACTGTT	12	G	0.0084	0.0728
NC_005102.4-65826286	CACACACACACGCACGCTCCAACTGTT	14	A	0.0417	0.1025
NC_005102.4-65883812	GATACTTTGACATCAACTCCTCACCCT	26	C	0.0263	0.026
NC_005102.4-65901833	CATCTCCTCACATTATCTCCTCGTAAT	9	C	0.0732	0.1287
NC_005102.4-65920291	TTTGGGGTTACCCATGCTCCTGTGAGT	23	T	0	0.4887
NC_005102.4-66019753	CTCTCGGGTACTCTGCCTCCACCTTTC	6	G	0	0.4814
NC_005102.4-66019753	CTCTCGGGTACTCTGCCTCCACCTTTC	8	G	0.1053	0.1454
NC_005102.4-66043704	TTGAATAGGACGAAGTCTCCTTTTCCT	15	G	0.0328	0.0322
NC_005102.4-66076940	GCTACAAGGACACATCCTCCACTACGT	6	A	0	0.4555
NC_005102.4-66088369	GCTTGAACAACAAAGGCTCCTACCTGC	8	C	0.1228	0.4945
NC_005102.4-66094861	TACTACAATACCTACCCTCCAGTATAA	1	T	0.0702	0.0677
NC_005102.4-66094861	TACTACAATACCTACCCTCCAGTATAA	12	C	0.0088	0.4889
NC_005102.4-66157297	TTTCACATCACTTTGCCTCCTCATCCA	23	A	0	0.4786
NC_005102.4-66176011	GTGCCTAAAACCTGTTCTCCTCCAGAC	13	T	0.1057	0.1695
NC_005102.4-66176011	GTGCCTAAAACCTGTTCTCCTCCAGAC	23	C	0.0894	0.0854
NC_005102.4-66178074	CGTTAAGTCACAAAGTCTCCTTGGTTA	1	C	0	0.4751
NC_005102.4-66178074	CGTTAAGTCACAAAGTCTCCTTGGTTA	23	G	0	0.4751
NC_005102.4-66247496	GGCTGGATTACTTTCTCTCCTTCTACA	5	G	0.1057	0.1001
NC_005102.4-66300641	ATGCTACCTACTTCAACTCCTCCCTAC	2	T	0.1429	0.4994
NC_005102.4-66335242	TGACAAGTTACAAGATCTCCTCAGGAT	7	G	0	0.4854
NC_005102.4-66378041	GCTGTGAGGACCTCTCCTCCGTCCTTC	7	A	0.1017	0.4718
NC_005102.4-66378041	GCTGTGAGGACCTCTCCTCCGTCCTTC	21	G	0.0932	0.4698
NC_005102.4-66401791	CCGGAGGAAACTAAGTCTCCCCCTGTC	3	G	0.0254	0.0251
NC_005102.4-66452107	CATAAAGGTACACAAACTCCCCCTTAG	27	G	0	0.4851
NC_005102.4-66454949	CAGTGTTCTACAGTACCTCCTTGGAGT	24	G	0.0417	0.0566
NC_005102.4-66461745	AGCATGCACACAGTAGCTCCTATGTGA	9	C	0.0088	0.481
NC_005102.4-66461745	AGCATGCACACAGTAGCTCCTATGTGA	21	T	0.0088	0.481
NC_005102.4-66581234	TCCACTCTCACCAAGTCTCCATTCTTG	27	G	0	0.4807
NC_005102.4-66699075	TGGCTTATGACTCCGTCTCCGTGACAG	14	C	0.2276	0.2612
NC_005102.4-66728268	GAGCACAGCACAGCAACTCCTTCAGTA	12	A	0	0.4821
NC_005102.4-66731392	ACTTTCCTCACATTTCCTCCCTTGGCT	23	T	0.2566	0.3683
NC_005102.4-66733911	CAGGCACATACCACTACTCCCAGCTTT	4	G	0	0.4807
NC_005102.4-66733911	CAGGCACATACCACTACTCCCAGCTTT	15	T	0	0.4807
NC_005102.4-66781660	GAGGAACACACCACCACTCCTTGCTTA	4	G	0	0.4765
NC_005102.4-67391909	CATTGTGAAACACCATCTCCAGAGTAC	2	A	0.1071	0.1913
NC_005102.4-67418627	GCCTGGGACACAAAGCCTCCAAGACTA	6	G	0.2439	0.3926
NC_005102.4-67472460	AACATCCCAACTGGGTCTCCAATGAAA	22	A	0.0351	0.4846

NC_005102.4-67496842	GAGAATGATACTGACTCTCCTAAAATG	27	G	0	0.4731
NC_005102.4-67531620	AGCCCTTAGACCGTGTCTCCTTCAAAC	1	A	0.0171	0.4868
NC_005102.4-67531620	AGCCCTTAGACCGTGTCTCCTTCAAAC	13	G	0.0431	0.4836
NC_005102.4-67581060	CATTGAAGAACACGGCCTCCTGCCTCA	14	G	0	0.477
NC_005102.4-67581060	CATTGAAGAACACGGCCTCCTGCCTCA	25	T	0.1964	0.3264
NC_005102.4-67600859	CTGTGTGTAACTTCAGCTCCATGGGAT	5	G	0.187	0.356
NC_005102.4-67605086	TATGAGAAAACTGATTCTCCACGTCCT	1	T	0.1724	0.3916
NC_005102.4-67605086	TATGAGAAAACTGATTCTCCACGTCCT	15	T	0	0.4839
NC_005102.4-67625192	ATCTAACCCACCGTTTCTCCCTGCCCA	13	G	0.0976	0.0928
NC_005102.4-67708031	TGACTTGTAACAAAGACTCCACTGAGA	16	A	0	0.4793
NC_005102.4-67806798	GAGCTTCCGACTTGATCTCCAAATAGG	9	G	0.1301	0.1494
NC_005102.4-67875530	GATCAGCCAACTCAGCCTCCTGGGTAG	1	G	0.0167	0.0328
NC_005102.4-67875530	GATCAGCCAACTCAGCCTCCTGGGTAG	15	G	0.025	0.0247
NC_005102.4-68037754	CCCCCACCCACTCAGCCTCCCACTCAT	1	C	0.0246	0.0243
NC_005102.4-68037883	CCCCTACCCACTCAGTCTCCTATCCAT	8	C	0.1391	0.173
NC_005102.4-68094701	TTATGGACCACATTGTCTCCTTTCTAT	1	T	0	0.0943
NC_005102.4-68123808	TGTCTAATTACACTTCCTCCTCTTCAG	1	T	0.1102	0.1621
NC_005102.4-68123808	TGTCTAATTACACTTCCTCCTCTTCAG	7	A	0	0.4772
NC_005102.4-68151353	GTTTCTGACACAGGGCCTCCTGTGGTC	15	G	0.2149	0.389
NC_005102.4-68151607	TGGAAAGAAACCTGCACTCCATAATAT	25	T	0.0168	0.4779
NC_005102.4-68252366	CTTTGGCAAACAGAGACTCCTATTATA	21	T	0.2389	0.3189
NC_005102.4-68252366	CTTTGGCAAACAGAGACTCCTATTATA	24	T	0.2389	0.3189
NC_005102.4-68275593	AATGACACAACCGAGTCTCCAAAGAAA	13	G	0.0574	0.0557
NC_005102.4-68275593	AATGACACAACCGAGTCTCCAAAGAAA	21	A	0.0246	0.0243
NC_005102.4-68296858	CTGCTTGTTACTTAGCCTCCTTGCGTC	24	C	0.1453	0.1634
NC_005102.4-68452889	AGTAGACAGACATGACCTCCAGTGGAA	13	T	0.2017	0.3771
NC_005102.4-68462165	CTGTTAGTAACTGCTGCTCCCTGTCAA	25	C	0.0672	0.0805
NC_005102.4-68470437	CACAGCTGAACTTTGGCTCCAATACCT	5	G	0	0.48
NC_005102.4-68470437	CACAGCTGAACTTTGGCTCCAATACCT	9	A	0.1111	0.2662
NC_005102.4-68501587	TATGAATCTACAGGCCCTCCTTGCTTC	27	C	0.1652	0.4926
NC_005102.4-68546849	AACCTAAGGACATCTACTCCCTTGTCT	4	C	0	0.477
NC_005102.4-68546849	AACCTAAGGACATCTACTCCCTTGTCT	26	C	0.0885	0.1466
NC_005102.4-68552849	CTCAGAATGACTGATTCTCCCTAGCAA	15	T	0	0.4743
NC_005102.4-68552849	CTCAGAATGACTGATTCTCCCTAGCAA	26	A	0.1917	0.225
NC_005102.4-68666511	CCTACGTCTACTCAACCTCCGTGAGTG	4	A	0.0088	0.483
NC_005102.4-68697360	CTAATCACAACTGTATCTCCCAAGCAG	15	A	0	0.4773
NC_005102.4-68711491	ATAAGGTAAACCAATTCTCCGTCTCTC	21	G	0.0085	0.479
NC_005102.4-68775117	AGTGTTTCCACAAACTCTCCCAGCTTA	23	G	0	0.48
NC_005102.4-68871100	AGAGAGCATACTGCTTCTCCAAGCAGT	1	A	0.0087	0.4817
NC_005102.4-68893313	AGCACAGTCACTCTTGCTCCACTGTTT	1	A	0	0.4846
NC_005102.4-68893313	AGCACAGTCACTCTTGCTCCACTGTTT	7	G	0	0.4846
NC_005102.4-68991080	AAACACTGCACTGACCCTCCTGCTGGC	22	G	0.0265	0.0262
NC_005102.4-69060802	AGAGTAATAACATTGTCTCCAGGGATA	6	A	0	0.4606
NC_005102.4-69111177	CTCCTAACCACTCTGCCTCCCCTTCTT	14	T	0.2321	0.4297
NC_005102.4-69238328	ACCCTTGGCACCGCTTCTCCAAGTTGA	8	G	0.0522	0.0508
NC_005102.4-69421605	CTAGAAAAGACTATTCCTCCCATTCTC	12	T	0.1681	0.297
NC_005102.4-69450467	ACCCCTCCCACAATCTCTCCTACTTCT	3	C	0.1102	0.1041
NC_005102.4-69450467	ACCCCTCCCACAATCTCTCCTACTTCT	21	T	0.0085	0.481
NC_005102.4-69521492	AATCAAGCAACCAACCCTCCTGTTATT	15	C	0	0.4859
NC_005102.4-69545362	TATCAACCTACCAGACCTCCCCTTCAG	14	G	0	0.4912
NC_005102.4-69856456	CATGTAAATACACATACTCCTCATTCC	15	T	0.0174	0.0671

NC_005102.4-69863788	TCTCCCAATACACGGCCTCCTTCTTCA	14	G	0.0165	0.0792
NC_005102.4-69882947	CCATGGCAGACACCAACTCCCAACACT	5	G	0.0082	0.479
NC_005102.4-69882947	CCATGGCAGACACCAACTCCCAACACT	6	G	0.1057	0.1695
NC_005102.4-69924190	CAGTTACTTACTCATGCTCCTGTAAGT	6	A	0.252	0.3888
NC_005102.4-69966158	TAAAGCTTGACAGCATCTCCTCCTGTC	6	C	0.124	0.2109
NC_005102.4-70013115	ATAAACCTCACTGTCACTCCCTGTCAC	2	T	0.1518	0.1553
NC_005102.4-70013115	ATAAACCTCACTGTCACTCCCTGTCAC	9	C	0.1681	0.2494
NC_005102.4-70041967	CACTCTGCAACTCCAGCTCCAGGAGAT	5	C	0.2645	0.4481
NC_005102.4-70041967	CACTCTGCAACTCCAGCTCCAGGAGAT	22	G	0.2667	0.4444
NC_005102.4-70202020	CCTTGGTGGACTGCATCTCCTGAGTCT	25	T	0	0.4793
NC_005102.4-70204495	ATTCTTAAAACAACACCTCCTAATCCC	4	C	0.0179	0.4641
NC_005102.4-70339821	TCCAGTATGACAGACCCTCCTAAGTCC	26	C	0.0339	0.0496
NC_005102.4-70357909	AAAAGCATCACTTTATCTCCCAATGAT	7	A	0.0431	0.0422
NC_005102.4-70357909	AAAAGCATCACTTTATCTCCCAATGAT	8	T	0.0431	0.0422
NC_005102.4-70451255	CCTCCTCAAACGTACTCTCCTTCTGTC	21	T	0	0.4773
NC_005102.4-70454271	GTGGTCCTTACAGTCACTCCTTTCACA	13	G	0.1339	0.1403
NC_005102.4-70454271	GTGGTCCTTACAGTCACTCCTTTCACA	14	T	0.2883	0.3178
NC_005102.4-70601515	TCTCTTCCCACACTATCTCCCTCCACA	13	C	0.2459	0.402
NC_005102.4-70643714	CCTCATTCCACTCTTTCTCCCCTGTCT	26	C	0.113	0.2204
NC_005102.4-70732032	ACAAAATCTACAGAAGCTCCACCAATA	5	A	0.0085	0.4714
NC_005102.4-70769170	TACAGCAACACCAGACCTCCCCACTCC	27	C	0.1652	0.304
NC_005102.4-70792080	TTCCAGAAAACCCCAGCTCCGTTTCCA	21	G	0.0089	0.4856
NC_005102.4-70801530	TACCCGCTGACACACACTCCTTCCATA	6	G	0.1167	0.4265
NC_005102.4-70801530	TACCCGCTGACACACACTCCTTCCATA	12	A	0.084	0.494
NC_005102.4-70822394	AGATGATTAACGAGCCCTCCCTGTGAA	12	G	0	0.4851
NC_005102.4-70836330	CACATTCCCACTCTCCCTCCAAGAGGA	7	C	0.0855	0.0973
NC_005102.4-70839924	AATTGAATGACCTTTCCTCCCAATATG	9	G	0.0331	0.0325
NC_005102.4-70875206	TGACAGTTTACCCTCCCTCCTCTCCTT	6	G	0.1393	0.2903
NC_005102.4-70875206	TGACAGTTTACCCTCCCTCCTCTCCTT	15	C	0.0083	0.0245
NC_005102.4-70997062	CAAAGAGTAACTGGCACTCCCAAGGGT	12	T	0.0088	0.4693
NC_005102.4-71082550	CTGCTGAACACTTTCTCTCCGAGACCT	5	T	0.0407	0.0398
NC_005102.4-71129667	TGCCCTGAAACATTCCCTCCTGGGAGT	15	C	0	0.4793
NC_005102.4-71129667	TGCCCTGAAACATTCCCTCCTGGGAGT	24	G	0.1301	0.1629
NC_005102.4-71253014	AGAGTTGACACACACACTCCCATTTCC	15	C	0.0435	0.059
NC_005102.4-71269539	CACACACACACACAGCCTCCCTTCTTG	1	C	0.041	0.0401
NC_005102.4-71276677	GGTGCACAAACAGTAGCTCCAATACTC	22	A	0.0082	0.4822
NC_005102.4-71276677	GGTGCACAAACAGTAGCTCCAATACTC	26	T	0.0082	0.4822
NC_005102.4-71307171	GAGTGTAATACTCTTGCTCCTTAGAAG	7	A	0.0667	0.4888
NC_005102.4-71348117	AACGTTGACACCTTTTCTCCAGCCTAG	4	G	0.0171	0.4868
NC_005102.4-71348117	AACGTTGACACCTTTTCTCCAGCCTAG	23	C	0.0932	0.0889
NC_005102.4-71488908	TTACAAGCCACTTCAGCTCCTTCAATC	12	T	0.6066	0.4226
NC_005102.4-71577910	TAATGTCCTACTAAACCTCCTAAGACA	21	T	0.0179	0.477
NC_005102.4-71614247	ATCTGAAGCACATTCTCTCCTATCCAC	15	C	0.1301	0.2142
NC_005102.4-71713396	GATACTCCCACACTTCCTCCCAGCAGA	23	G	0.292	0.4873
NC_005102.4-71742196	TTACTGTGGACAGACTCTCCAAACAGG	4	C	0.1368	0.1274
NC_005102.4-71778984	GGTCAACCTACCAGCTCTCCCAGTCAC	9	T	0	0.4827
NC_005102.4-71779200	TGAGCTTAGACCAATTCTCCCACCTGC	26	G	0.0339	0.0333
NC_005102.4-71807650	ACAGTCCTCACGGGACCTCCACACTCT	9	C	0	0.4844
NC_005102.4-71807650	ACAGTCCTCACGGGACCTCCACACTCT	12	G	0.0336	0.033
NC_005102.4-71834286	TCAACTTCTACACAGTCTCCAAGCTTG	16	T	0.1271	0.1337
NC_005102.4-71903386	ACTGAACGAACCCTAACTCCGAAGCAG	24	G	0.0168	0.033

NC_005102.4-71908477	CATCTCCACACAAATGCTCCCTTCACT	26	C	0.2167	0.255
NC_005102.4-71948839	ATTTTGAACACACTCACTCCCCTCCCG	26	C	0.0256	0.0253
NC_005102.4-71948839	ATTTTGAACACACTCACTCCCCTCCCG	27	G	0.0256	0.0253
NC_005102.4-71988961	TGCAGAAGCACAAGGCCTCCTCCTTGG	22	C	0	0.4883
NC_005102.4-71997410	ACTGGGATGACATACACTCCTCCTCTA	8	T	0	0.4772
NC_005102.4-71997410	ACTGGGATGACATACACTCCTCCTCTA	16	A	0	0.4772
NC_005102.4-72008406	AAGATAATCACAGTGGCTCCCTGTGGC	5	T	0	0.48
NC_005102.4-72008406	AAGATAATCACAGTGGCTCCCTGTGGC	7	A	0	0.48
NC_005102.4-72048369	CACACCCCAACACAATCTCCAGCAAAA	4	A	0.0263	0.026
NC_005102.4-72048369	CACACCCCAACACAATCTCCAGCAAAA	6	C	0.0088	0.4873
NC_005102.4-72050943	CAGCAACAGACCGACCCTCCAAAGACA	6	A	0.1393	0.5
NC_005102.4-72132910	CCAGGCTGGACTGCAACTCCTTGTGTA	21	T	0	0.4787
NC_005102.4-72164396	AAGGCATGCACAACCACTCCTCACCTG	12	A	0	0.4793
NC_005102.4-72168042	ACATAGGATACTCATGCTCCTATGATA	15	T	0	0.4883
NC_005102.4-72180853	GCATGCACCACCATGCCTCCTGAATTA	3	A	0.0708	0.0846
NC_005102.4-72252502	TGTTTCTTGACACCCACTCCAGCGGAT	16	A	0.0089	0.4856
NC_005102.4-72252502	TGTTTCTTGACACCCACTCCAGCGGAT	24	G	0.1947	0.2913
NC_005102.4-72254878	TGCCAGATTACCCCACCTCCCTGTGGC	5	A	0.1875	0.2385
NC_005102.4-72275991	CAGGGACCAACGTCAGCTCCACACCGC	26	G	0	0.4793
NC_005102.4-72383603	AGGTCTGAAACAAGAACTCCCACGGAT	16	A	0.1552	0.3916
NC_005102.4-72394787	CATGTGGATACTCAGGCTCCCTTCTCC	8	A	0.0763	0.1041
NC_005102.4-72394787	CATGTGGATACTCAGGCTCCCTTCTCC	27	C	0	0.4856
NC_005102.4-72398729	ACCGAGGCAACCTTGCCTCCATCCACA	4	G	0.069	0.0825
NC_005102.4-72404422	AGGACTCTTACCCCTTCTCCCCCTGGG	8	T	0	0.4793
NC_005102.4-72406649	CTGTAACCCACGTTATCTCCAGGCAAG	1	C	0.0598	0.4854
NC_005102.4-72406649	CTGTAACCCACGTTATCTCCAGGCAAG	2	T	0.0593	0.4842
NC_005102.4-72408891	TGCTACGGTACTGCCACTCCCCTGGAG	6	C	0	0.4864
NC_005102.4-72408891	TGCTACGGTACTGCCACTCCCCTGGAG	25	G	0.0431	0.0422
NC_005102.4-72432379	GAACCATGCACACCATCTCCATAGCCC	16	T	0	0.4819
NC_005102.4-72452051	AATCCTTTTACAGAACCTCCGTGTTTA	3	T	0	0.4704
NC_005102.4-72452051	AATCCTTTTACAGAACCTCCGTGTTTA	21	G	0.018	0.0354
NC_005102.4-72487253	TCTGTTCTCACCTAAGCTCCTCCTCTG	2	C	0.041	0.0401
NC_005102.4-72487253	TCTGTTCTCACCTAAGCTCCTCCTCTG	14	A	0.0407	0.0398
NC_005102.4-72589620	CTCACCCCAACTCGTTCTCCTCTTCTG	6	C	0.3193	0.4406
NC_005102.4-72622064	AGGACTGCCACCACAGCTCCTGAGTTT	4	A	0	0.4793
NC_005102.4-72650801	ATAGTTGGTACCCTGACTCCATACATG	24	C	0.0325	0.032
NC_005102.4-72726210	AGAAGGCGGACACAGGCTCCCATTCCT	7	C	0	0.4864
NC_005102.4-72726210	AGAAGGCGGACACAGGCTCCCATTCCT	15	G	0	0.4864
NC_005102.4-72767067	ATCCCCCTAACACCTCCTCCTCCCATT	16	C	0.025	0.0247
NC_005102.4-72767067	ATCCCCCTAACACCTCCTCCTCCCATT	27	T	0.1405	0.172
NC_005102.4-72787024	AGTTGCGCTACACACCCTCCAAATGAT	7	G	0.1008	0.4152
NC_005102.4-72884556	TGCAACAGCACAGACCCTCCCCACAGA	15	C	0.1071	0.1327
NC_005102.4-72892598	TCAGAGTAAACATTTCCTCCTTGATAG	21	T	0.2072	0.3903
NC_005102.4-73206245	GTCCTGGTTACTGTATCTCCTTTCTCT	9	T	0.1186	0.4994
NC_005102.4-73206245	GTCCTGGTTACTGTATCTCCTTTCTCT	14	T	0.1356	0.4305
NC_005102.4-73309392	CAGTTTGTAACCTCTCCTCCATTCCTT	2	A	0.2087	0.4682
NC_005102.4-73309392	CAGTTTGTAACCTCTCCTCCATTCCTT	4	T	0.2087	0.3303
NC_005102.4-73324849	CTTACCTTCACCAATCCTCCAATCCTG	5	C	0.0086	0.4768
NC_005102.4-73393010	GCCAGAACTACTATTCCTCCACACATG	1	G	0.3063	0.4785
NC_005102.4-73434546	CTGTTCTAAACTTTGCCTCCTACACAC	15	G	0.2342	0.3285
NC_005102.4-73542368	AGGCTTTGGACCGCCCCTCCCTGGAGT	24	G	0.0254	0.0251

NC_005102.4-73885344	GACTAAGGCACACCACCTCCCACTGGC	3	C	0.2314	0.389
NC_005102.4-73885344	GACTAAGGCACACCACCTCCCACTGGC	13	C	0.0083	0.4769
NC_005102.4-74061726	GTTGGTAACACCACCTCTCCTTTTGAT	27	T	0.1885	0.2095
NC_005102.4-74075283	CATCAACCAACCAAACCTCCTTAGTAC	14	A	0.0087	0.466
NC_005102.4-74482393	AACCATTGAACCATCCCTCCATTCCTG	25	C	0.0246	0.0557
NC_005102.4-74482393	AACCATTGAACCATCCCTCCATTCCTG	26	T	0.124	0.4995
NC_005102.4-74585111	ACAAGCAATACAATGACTCCATCAGAT	6	C	0.1217	0.1443
NC_005102.4-74585111	ACAAGCAATACAATGACTCCATCAGAT	26	A	0.2544	0.415
NC_005102.4-74632365	AGCTGGAATACGAACACTCCCTTAGTA	6	G	0.2521	0.4921
NC_005102.4-75090801	CATATACACACTAAACCTCCTCTCTCT	4	A	0	0.4787
NC_005102.4-75090801	CATATACACACTAAACCTCCTCTCTCT	27	T	0	0.4751
NC_005102.4-75504413	GGACAAAGAACAAGGCCTCCACAATTA	9	A	0.0088	0.483
NC_005102.4-75504413	GGACAAAGAACAAGGCCTCCACAATTA	24	A	0.0088	0.483
NC_005102.4-75689783	TACTACCCTACATCTCCTCCCACACCT	5	A	0	0.4751
NC_005102.4-75689783	TACTACCCTACATCTCCTCCCACACCT	8	C	0.1557	0.4997
NC_005102.4-75741462	GATCCATTCACCATCCCTCCACAAAAC	3	T	0.2174	0.2332
NC_005102.4-75743731	ACCTAAAACACTAGTACTCCTTCACCT	6	A	0	0.4743
NC_005102.4-75743731	ACCTAAAACACTAGTACTCCTTCACCT	7	A	0	0.4743
NC_005102.4-75751846	CAATATGAGACCAGGACTCCTGAGATG	6	T	0.2667	0.4132
NC_005102.4-75809984	TTTACCACCACTATACCTCCTTTCCTG	13	A	0.2679	0.232
NC_005102.4-76082390	GCACTCCTAACCTCAACTCCTGCACAT	5	T	0.0661	0.0639
NC_005102.4-76085847	TTCGCTATGACTGTCACTCCATAAATG	26	T	0.065	0.0629
NC_005102.4-76157323	TATCTTATCACACCCCCTCCCAGCCCA	1	T	0.1795	0.3409
NC_005102.4-76157323	TATCTTATCACACCCCCTCCCAGCCCA	4	C	0.0427	0.1049
NC_005102.4-76219132	GAATACACAACCAGTCCTCCATACCCT	12	C	0.0708	0.0846
NC_005102.4-76225030	ATTGTGGATACTCTTGCTCCAGTCTCG	15	T	0.157	0.2234
NC_005102.4-76238992	ATTTAGACTACTGGTCCTCCTACAGTT	4	T	0.0413	0.0405
NC_005102.4-76238992	ATTTAGACTACTGGTCCTCCTACAGTT	13	G	0.3058	0.4398
NC_005102.4-76450927	GGATTAGCCACATACTCTCCCAGTGAG	27	G	0.1917	0.3347
NC_005102.4-76614003	CTGAGGGAAACTCCATCTCCAACTGCC	1	C	0.1062	0.1006
NC_005102.4-76747539	TTCCCCAGAACTGAGGCTCCAACATTG	2	T	0.1441	0.2717
NC_005102.4-76810869	TTTCCTTCAACCCCTTCTCCCTTCCAT	13	C	0	0.4922
NC_005102.4-77147617	TGTGATTTGACTGTGTCTCCCAAGAGA	5	A	0	0.4693
NC_005102.4-77147617	TGTGATTTGACTGTGTCTCCCAAGAGA	25	A	0.0171	0.0336
NC_005102.4-77159080	CCCAGCTCAACCCTCTCTCCATAGCTC	3	C	0.0252	0.491
NC_005102.4-77159080	CCCAGCTCAACCCTCTCTCCATAGCTC	24	G	0.1167	0.5
NC_005102.4-77438715	AACTGAGGAACATCAGCTCCTAAAGAA	7	G	0.0504	0.0491
NC_005102.4-77462925	AGCCAATCAACAGGTTCTCCAGGGCAG	8	C	0.0902	0.1154
NC_005102.4-77462925	AGCCAATCAACAGGTTCTCCAGGGCAG	23	G	0.2869	0.4588
NC_005102.4-77507083	CAGTCAATGACAAGTTCTCCAAAGCAA	27	A	0.1917	0.4847
NC_005102.4-77525090	CAGGGTACCACAGAGCCTCCAATTCAT	6	T	0.1138	0.1494
NC_005102.4-77525090	CAGGGTACCACAGAGCCTCCAATTCAT	13	G	0.2602	0.385
NC_005102.4-77526994	CATCAACCAACCAGACCTCCCAGTGCT	8	C	0.0504	0.0491
NC_005102.4-77526994	CATCAACCAACCAGACCTCCCAGTGCT	13	A	0.2712	0.3792
NC_005102.4-77568006	ACCAGCCACACACCTACTCCAGCCTCC	22	G	0.2295	0.3447
NC_005102.4-77603559	GTTTCAGGAACCTTTCCTCCTACGCAT	24	G	0.05	0.4644
NC_005102.4-77618090	CAGACTGCCACAGAGCCTCCGATTCAT	5	C	0	0.4659
NC_005102.4-77698558	CTACAGGAAACATCTTCTCCCCCTTCA	2	T	0.9569	0.4991
NC_005102.4-77698558	CTACAGGAAACATCTTCTCCCCCTTCA	3	A	0.4237	0.334
NC_005102.4-77874578	TAAACAAGAACACATGCTCCACTCTGT	14	A	0.2437	0.2961
NC_005102.4-77889061	TTGAATAAAACATAAACTCCTCCCTTA	7	A	0.1157	0.3079

NC_005102.4-77931151	AATGGCACGACTCAAGCTCCTTTCCTA	9	G	0.0496	0.0484
NC_005102.4-77953315	GGATTCATGACTTCCTCTCCATACACA	1	G	0.0165	0.4901
NC_005102.4-77953315	GGATTCATGACTTCCTCTCCATACACA	27	A	0	0.4773
NC_005102.4-77961564	TAGGATGACACAGAGCCTCCGTGTCAT	1	T	0.2213	0.4435
NC_005102.4-78009711	ACTTCTTCCACTGTCACTCCCAAAATG	9	C	0.6053	0.4415
NC_005102.4-78009711	ACTTCTTCCACTGTCACTCCCAAAATG	24	A	0.6053	0.4415
NC_005102.4-78011765	GATTTCCCCACCTAACCTCCTTGCCAT	25	C	0.0172	0.0504
NC_005102.4-78024691	AACAAAGAAACCACAGCTCCCTCCTTT	24	C	0.2735	0.4123
NC_005102.4-78088521	AGTACCAAGACTCAGACTCCACCACAT	27	T	0.1652	0.1516
NC_005102.4-78281076	CTACTTAAGACCATGACTCCTTTTCTC	4	C	0.8983	0.4948
NC_005102.4-78461722	ATGTTAGGAACCAAACCTCCATACACT	1	A	0.0081	0.481
NC_005102.4-78461722	ATGTTAGGAACCAAACCTCCATACACT	2	T	0.2764	0.3041
NC_005102.4-78635280	AAGACCAACACTTCTGCTCCTAAGGAA	21	T	0.1795	0.2544
NC_005102.4-78679432	TATAACAAGACACATGCTCCATTATGT	13	C	0.0708	0.0683
NC_005102.4-78724352	ATGAAACAGACAGTGCCTCCACTTTCA	21	A	0.2586	0.4927
NC_005102.4-78772613	TTATCATCTACATCATCTCCATGGTAG	21	A	0.0732	0.1145
NC_005102.4-78846535	CTGTTCCAAACAGAGTCTCCATATCCC	27	C	0.0442	0.06
NC_005102.4-78870350	AACCTGCACACTGCTTCTCCTGCATGT	4	C	0	0.4813
NC_005102.4-78870350	AACCTGCACACTGCTTCTCCTGCATGT	6	G	0	0.4813
NC_005102.4-78895360	TTAAAGAGAACTGATTCTCCCCCTTCC	23	C	0.0261	0.0257
NC_005102.4-78897293	CTTCCAAACACTGTAACTCCAGCTACA	12	T	0.0268	0.1093
NC_005102.4-79054527	TAACTTAAAACTGACACTCCATCACAT	23	C	0.025	0.4783
NC_005102.4-79166840	AAGAATCTCACTTTGCCTCCAAGAAGA	2	A	0.036	0.0695
NC_005102.4-79166840	AAGAATCTCACTTTGCCTCCAAGAAGA	23	G	0.0901	0.149
NC_005102.4-79257628	GCGCTGAGCACCTAACCTCCAATACCT	2	C	0.1532	0.2403
NC_005102.4-79314666	GAGCCAAGCACTCCCTCTCCATTCTCC	12	T	0.1121	0.1058
NC_005102.4-79314666	GAGCCAAGCACTCCCTCTCCATTCTCC	23	T	0.0088	0.4796
NC_005102.4-79355951	ATTCTCTACACTTGGGCTCCTACTACT	12	T	0	0.4755
NC_005102.4-79355951	ATTCTCTACACTTGGGCTCCTACTACT	22	A	0	0.4755
NC_005102.4-79356210	AAGGACAGCACGCTCACTCCTACTGGC	12	G	0.0526	0.0512
NC_005102.4-79361372	AGAGGGGGAACAGTCTCTCCAATGCTA	26	T	0.0684	0.0973
NC_005102.4-79361372	AGAGGGGGAACAGTCTCTCCAATGCTA	27	A	0.069	0.0981
NC_005102.4-79409899	GTTTCCAAAACATGTCCTCCTGCTGGT	12	A	0.0804	0.2512
NC_005102.4-79417019	TTCAGAAGCACAAGCTCTCCCTTGGAG	1	T	0.0265	0.4923
NC_005102.4-79417019	TTCAGAAGCACAAGCTCTCCCTTGGAG	5	G	0.0261	0.059
NC_005102.4-79419915	CCATTCCAGACCTGGTCTCCTGGAGTG	22	G	0.1754	0.2021
NC_005102.4-79437871	AGCTCTACCACATCACCTCCACATTTT	24	T	0	0.4718
NC_005102.4-79445972	TTTACAAGCACCAACTCTCCTACCACC	9	C	0.123	0.1296
NC_005102.4-79551355	AACTGCTGGACCATCTCTCCTGCCCCA	6	C	0.0588	0.0728
NC_005102.4-79551355	AACTGCTGGACCATCTCTCCTGCCCCA	23	C	0.0085	0.0251
NC_005102.4-79637203	TTAATCAGCACATCCCCTCCGCTTGTG	5	T	0	0.4793
NC_005102.4-79637203	TTAATCAGCACATCCCCTCCGCTTGTG	14	C	0.0569	0.0705
NC_005102.4-79637237	AGATGGAACACCAGGCCTCCTCACTCA	7	A	0.0082	0.479
NC_005102.4-79665992	GAGTTGATCACCGCTCCTCCTCCAGTG	12	C	0.0085	0.4738
NC_005102.4-79687693	GAGGTGGTTACTGGAACTCCTTTCTCA	26	C	0.0244	0.0241
NC_005102.4-79705169	GCATATGAAACAAACACTCCTTCACTG	6	T	0	0.4764
NC_005102.4-79758577	GAAGGACCGACCACTTCTCCAGCAAGA	8	C	0.1897	0.2122
NC_005102.4-79824662	CTGTATCACACTATGCCTCCCATCTCT	3	G	0	0.4773
NC_005102.4-79827916	AACAAGAGCACAATTTCTCCAAATCTT	22	A	0.2035	0.3081
NC_005102.4-79886227	CATAGGAACACCCAGCCTCCTGTCTAC	15	G	0.0763	0.1481
NC_005102.4-79925109	CTTACCCACACCCATCCTCCATTCAAT	22	T	0	0.4793

NC_005102.4-79935847	CCCTCACTGACCTCCACTCCACTTTCA	15	C	0.0536	0.1014
NC_005102.4-79936139	CTCTACAGCACTCGGTCTCCAAGTCAG	21	A	0	0.482
NC_005102.4-79944126	GGACTCTGGACTTTAGCTCCCTGCTGA	21	C	0.161	0.2156
NC_005102.4-79956334	CACAGAGCGACCACGGCTCCAACTGCC	22	A	0	0.4773
NC_005102.4-79960688	ATCAGCCACACTGAGACTCCTGCCCCT	5	G	0.2276	0.4444
NC_005102.4-79960688	ATCAGCCACACTGAGACTCCTGCCCCT	12	T	0.065	0.078
NC_005102.4-79981377	TTCCCAGGGACTGCCCCTCCCCACCAC	7	G	0	0.4793
NC_005102.4-79981377	TTCCCAGGGACTGCCCCTCCCCACCAC	8	G	0	0.4793
NC_005102.4-79987062	ACCGATAGGACAGGGCCTCCTCCAGCT	4	G	0.2195	0.4174
NC_005102.4-79996904	TGACAGGGAACATGTACTCCAGGGGGT	5	A	0.0263	0.026
NC_005102.4-79997492	CCTGCCATCACATTACCTCCTAGAAAT	24	A	0	0.4778
NC_005102.4-80017678	CTTGGATTTACGTTTGCTCCTGCCAAC	4	G	0.0342	0.0336
NC_005102.4-80017678	CTTGGATTTACGTTTGCTCCTGCCAAC	12	G	0.0598	0.058
NC_005102.4-80030682	GCCACCAGCACCCCTACTCCCACACTG	6	C	0.0081	0.0398
NC_005102.4-80122158	GTCTACCATACCCCAGCTCCTTGTTAG	4	T	0	0.474
NC_005102.4-80239948	ACAACCACTACCACATCTCCATGACTG	26	T	0.0492	0.0634
NC_005102.4-80428842	CTCAACTGAACCACCTCTCCAGACCTA	23	A	0	0.4764
NC_005102.4-80469736	AAAGAGAGCACCGTAACTCCTGGAACT	12	C	0.036	0.4746
NC_005102.4-80470901	CTTCTCTGCACTCTAACTCCTCTTCTG	13	C	0.1393	0.2339
NC_005102.4-80536520	CAAAGCACCACGCGCCCTCCTCATCCC	27	C	0.0776	0.0903
NC_005102.4-80548144	TGCCTATACACTGAGTCTCCAGGATGC	12	T	0	0.4779
NC_005102.4-80548144	TGCCTATACACTGAGTCTCCAGGATGC	13	G	0.0909	0.1017
NC_005102.4-80554714	TCCCCAAGTACACCTTCTCCACCTCCT	9	T	0	0.4793
NC_005102.4-80570760	TAAAACAAAACAGAAACTCCAAACCTA	12	A	0.0172	0.4749
NC_005102.4-80599077	AAGGTCCCCACCCTCCCTCCAACCCGA	23	C	0.0504	0.065
NC_005102.4-80630612	TCAGTAACTACAGCAGCTCCAGGGGAT	3	A	0	0.4743
NC_005102.4-80646824	CACTCATGAACAACCCCTCCGACCCCA	22	A	0.0089	0.4824
NC_005102.4-80721262	AACTGCTGAACCATATCTCCAACTCTC	3	C	0.0089	0.0264
NC_005102.4-80748185	ATCTCCCCCACCTGCTCTCCTGCCCTC	16	T	0.2377	0.2095
NC_005102.4-80748185	ATCTCCCCCACCTGCTCTCCTGCCCTC	22	G	0.2377	0.2095
NC_005102.4-80866770	TGTCTGACAACTTGGACTCCGGGGGCT	13	T	0.0085	0.4776
NC_005102.4-80866770	TGTCTGACAACTTGGACTCCGGGGGCT	16	A	0.0085	0.4776
NC_005102.4-80886735	ATTGTGGGAACATGGTCTCCAGGACTA	24	A	0	0.4875
NC_005102.4-80888345	ACTGCTGAGACAGCCCCTCCCTTCTTT	26	T	0	0.4793
NC_005102.4-80889460	GAACGTGAAACTCTTGCTCCACTGCTG	4	C	0	0.4851
NC_005102.4-80889460	GAACGTGAAACTCTTGCTCCACTGCTG	25	C	0.2261	0.3499
NC_005102.4-80909822	AACGACAAGACTGAGACTCCCTTCCTT	23	T	0.0085	0.4842
NC_005102.4-80923154	TACCTTCCCACCCCTGCTCCTCCCATT	3	C	0	0.4832
NC_005102.4-80923154	TACCTTCCCACCCCTGCTCCTCCCATT	16	G	0	0.4832
NC_005102.4-80938666	GCAATGCTAACTTGATCTCCCTCTCTT	5	T	0.082	0.1082
NC_005102.4-80939355	CACCTCTGTACACATGCTCCTGCCAGC	8	G	0.0569	0.0553
NC_005102.4-80939355	CACCTCTGTACACATGCTCCTGCCAGC	16	G	0.3089	0.4331
NC_005102.4-80994031	GTGGAGAACACACGGGCTCCGTACTAA	21	G	0.2893	0.323
NC_005102.4-81015066	ATTTTGACCACAGATACTCCAACTAGT	12	A	0	0.4772
NC_005102.4-81015066	ATTTTGACCACAGATACTCCAACTAGT	15	T	0.1724	0.4946
NC_005102.4-81019215	AGAACCCAAACCTCATCTCCAGTCCAT	1	A	0	0.4773
NC_005102.4-81040136	TAGGGTTCAACCACCCCTCCAGCCCAC	9	A	0.1161	0.1403
NC_005102.4-81040136	TAGGGTTCAACCACCCCTCCAGCCCAC	15	C	0.125	0.1478
NC_005102.4-81076556	AACTGAGCTACACCCACTCCCCCTTAC	5	G	0.0087	0.0257
NC_005102.4-81116367	TCTTTGCACACTGCCCCTCCACAGCCA	8	A	0	0.4793
NC_005102.4-81166230	ATCAAGTGAACAAGGACTCCCAGTTTC	5	A	0	0.4749

NC_005102.4-81189536	AAGCACTGAACAGCATCTCCTAGGGAC	8	G	0.0082	0.4755
NC_005102.4-81262088	TGCCTTCTCACACTGCCTCCTCCACCC	16	C	0	0.4755
NC_005102.4-81274900	ACTCTCACCACCAGCCCTCCTTCCCCC	22	T	0.0407	0.0705
NC_005102.4-81274900	ACTCTCACCACCAGCCCTCCTTCCCCC	23	C	0.0407	0.0705
NC_005102.4-81282666	TTGAAGGCTACCTCCCCTCCTGCACGA	25	C	0.0167	0.0328
NC_005102.4-81310093	CAACTTCCAACAGACGCTCCCATCTTT	24	C	0.2314	0.4112
NC_005102.4-81368174	CCAGAAACCACGTCACCTCCTCCAGAT	1	C	0.0082	0.479
NC_005102.4-81368174	CCAGAAACCACGTCACCTCCTCCAGAT	7	A	0.0082	0.479
NC_005102.4-81398335	TGGTCCCCAACCTTAGCTCCTAGCCAC	16	G	0.0407	0.0398
NC_005102.4-81449853	ATGGTTGTGACATAGCCTCCATGACAG	5	T	0	0.4807
NC_005102.4-81453457	CTGCCGTGCACACCTGCTCCTCACTTC	5	C	0	0.4715
NC_005102.4-81456818	TACTCTCGGACAGCTGCTCCTTGCATG	7	C	0.1301	0.3427
NC_005102.4-81481932	GGTGGAATGACTTGAGCTCCACAAAGC	5	G	0	0.4734
NC_005102.4-81484487	TGGGTGCCCACACTCTCTCCTTGCACA	3	G	0.0081	0.4777
NC_005102.4-81484487	TGGGTGCCCACACTCTCTCCTTGCACA	16	T	0.0081	0.4777
NC_005102.4-81506459	CTGTTCAACACTCCTTCTCCAGCCTGG	13	C	0	0.4793
NC_005102.4-81509929	TTTTCCCATACCCTTCCTCCCCCTCCC	8	A	0.0783	0.0752
NC_005102.4-81509929	TTTTCCCATACCCTTCCTCCCCCTCCC	13	C	0.0783	0.0752
NC_005102.4-81515624	ATTGCAAGCACACACCCTCCTGCCTGG	16	C	0.0424	0.0415
NC_005102.4-81516359	GTTTGACCTACACTCCCTCCCCACCTC	6	A	0.0336	0.033
NC_005102.4-81516359	GTTTGACCTACACTCCCTCCCCACCTC	14	T	0.0261	0.0257
NC_005102.4-81516596	TTTGGATGAACAAAGCCTCCTGCTTTT	16	C	0	0.4793
NC_005102.4-81528758	GTTCTGGCAACTCTTCCTCCCCAGTGT	1	G	0.0783	0.4986
NC_005102.4-81528758	GTTCTGGCAACTCTTCCTCCCCAGTGT	6	G	0.0783	0.4986
NC_005102.4-81555784	GTCCCCTGCACCTTATCTCCCGTAGCT	7	T	0.2439	0.3688
NC_005102.4-81555784	GTCCCCTGCACCTTATCTCCCGTAGCT	22	G	0.0081	0.4777
NC_005102.4-81606199	TGAGTATGCACTACCACTCCTGGCTTG	5	T	0.0086	0.4768
NC_005102.4-81606199	TGAGTATGCACTACCACTCCTGGCTTG	12	T	0	0.4764
NC_005102.4-81720906	AGCCTCCCGACACATGCTCCTCCTCCC	8	C	0.2689	0.4285
NC_005102.4-81720906	AGCCTCCCGACACATGCTCCTCCTCCC	26	C	0.1933	0.2012
NC_005102.4-81763346	GTAAGAGTGACTGTTTCTCCCCAAGGT	2	T	0.1228	0.1454
NC_005102.4-81763346	GTAAGAGTGACTGTTTCTCCCCAAGGT	22	C	0.2832	0.39
NC_005102.4-81779169	CCCTGGGGAACAGTTCCTCCTACCTCA	6	G	0.0248	0.0245
NC_005102.4-81798282	AGCGGACCAACCTCATCTCCACTTCTT	4	G	0	0.4793
NC_005102.4-81798282	AGCGGACCAACCTCATCTCCACTTCTT	12	C	0.0976	0.1216
NC_005102.4-81813025	CATAAGCATACATATTCTCCACGTTGT	22	C	0.1322	0.1653
NC_005102.4-81814829	AGGGAGGCGACACCTCCTCCCAGCTGG	22	A	0.1441	0.4991
NC_005102.4-81822701	CACTGATGCACCAGCTCTCCTTCCACA	6	A	0	0.4722
NC_005102.4-81822701	CACTGATGCACCAGCTCTCCTTCCACA	16	T	0.1463	0.1494
NC_005102.4-81836308	CCCCTTTCAACAGTGCCTCCCTCCTCC	23	C	0.0082	0.0557
NC_005102.4-81845074	GGACCTGTGACCGCTTCTCCAGACATA	21	A	0.0579	0.4913
NC_005102.4-81855335	TATTTGCCCACTTCTGCTCCCTGAGTG	5	T	0.1917	0.4983
NC_005102.4-81855335	TATTTGCCCACTTCTGCTCCCTGAGTG	7	C	0.084	0.0805
NC_005102.4-81878782	ACATCTCTCACCCAGTCTCCTTCAGAT	13	C	0.0982	0.5
NC_005102.4-81908351	CAATGGCCAACTTCTTCTCCATCCCTC	2	A	0	0.4851
NC_005102.4-81908351	CAATGGCCAACTTCTTCTCCATCCCTC	16	T	0.0254	0.0734
NC_005102.4-81916615	GTGTGGTAGACACCACCTCCAATCCCA	8	A	0	0.4786
NC_005102.4-81916615	GTGTGGTAGACACCACCTCCAATCCCA	21	A	0	0.4786
NC_005102.4-81988824	TACTGAGCTACCCCCTCTCCCTTCCTT	23	T	0.0413	0.0405
NC_005102.4-81988824	TACTGAGCTACCCCCTCTCCCTTCCTT	24	C	0	0.4751
NC_005102.4-82001839	TAGAGCTAAACAGAGTCTCCCAAGGAT	15	G	0.2066	0.2474

NC_005102.4-82008430	CTGCTTCCCACCAAGCCTCCGGTTGGA	21	G	0.0924	0.4186
NC_005102.4-82011407	TGTTTTGGGACGACAGCTCCAGGACAG	27	G	0	0.4856
NC_005102.4-82017172	AATAAGAGTACCTCCACTCCACACCTC	9	T	0.1739	0.1869
NC_005102.4-82017172	AATAAGAGTACCTCCACTCCACACCTC	22	C	0.1316	0.1528
NC_005102.4-82069423	CAGTAAGTGACCTGAGCTCCCTGGTGT	22	T	0.0417	0.0408
NC_005102.4-82083613	AGAGAGAGGACAGAGGCTCCTGGCTAG	12	A	0.0488	0.0629
NC_005102.4-82111837	CGAAGCCCCACAGATACTCCTGGGAGA	2	G	0.2308	0.2544
NC_005102.4-82111837	CGAAGCCCCACAGATACTCCTGGGAGA	8	C	0.0424	0.0415
NC_005102.4-82139634	TGTGGCAGCACAGACCCTCCACAGGTG	22	C	0.2276	0.4548
NC_005102.4-82151007	ATAACTGCTACCATGCCTCCCTCTTGA	6	T	0.1513	0.4983
NC_005102.4-82205054	AGCTGAGACACTAGTTCTCCTTCCTTC	25	T	0.0672	0.0805
NC_005102.4-82216427	AGAACAGATACGTCTCCTCCCCATTCA	12	G	0.0738	0.0861
NC_005102.4-82217354	CTCATGCACACTGCGGCTCCCATCATC	15	G	0.0407	0.0398
NC_005102.4-82218910	TCCAGGCAAACTGCACCTCCACTCTTT	22	C	0.045	0.044
NC_005102.4-82265808	TGCTGCTTGACTCCAGCTCCAGAGGTT	7	T	0	0.4746
NC_005102.4-82270661	GACCATGATACCCATCCTCCTCCAAGA	1	G	0.0901	0.086
NC_005102.4-82313832	AGCTCTAAGACTGTAACTCCATCTGTG	21	A	0.2149	0.1918
NC_005102.4-82320650	GGTACAGGCACAGGTCCTCCGTGGCAT	12	A	0.188	0.4123
NC_005102.4-82320650	GGTACAGGCACAGGTCCTCCGTGGCAT	26	A	0.2137	0.4227
NC_005102.4-82341536	AACGAATAAACGCTGTCTCCATTCGCT	4	G	0.2185	0.4703
NC_005102.4-82341536	AACGAATAAACGCTGTCTCCATTCGCT	23	T	0	0.4813
NC_005102.4-82366320	ACATCCCTGACTCAAGCTCCTTCCCAG	25	C	0.1951	0.3688
NC_005102.4-82366615	CTGCTGCTCACTGGTTCTCCAGAGTAT	15	T	0.2353	0.4152
NC_005102.4-82385172	ACCCCATTGACAATTCCTCCTTTCTCC	3	C	0.0244	0.484
NC_005102.4-82415050	CCTGCCTTGACAGTTTCTCCACCGAAC	24	G	0.0085	0.0251
NC_005102.4-82424210	ACCCTTTAAACCCCTTCTCCTGAGTAC	1	A	0	0.4793
NC_005102.4-82425837	AATGTTCAGACTATAGCTCCTGTGAAG	22	G	0.0087	0.4849
NC_005102.4-82425837	AATGTTCAGACTATAGCTCCTGTGAAG	27	G	0.087	0.4954
NC_005102.4-82428802	GGGGGTTGAACCCAGGCTCCGCTGTCG	21	G	0.1351	0.4994
NC_005102.4-82428802	GGGGGTTGAACCCAGGCTCCGCTGTCG	26	C	0.0351	0.0512
NC_005102.4-82429218	ATCCCTGTGACTTCACCTCCCAGAATT	12	T	0.0909	0.4951
NC_005102.4-82429218	ATCCCTGTGACTTCACCTCCCAGAATT	27	T	0.1057	0.1425
NC_005102.4-82431744	ATGAGAGTCACATCACCTCCATGTGCC	1	A	0	0.4793
NC_005102.4-82431744	ATGAGAGTCACATCACCTCCATGTGCC	15	A	0.1951	0.2937
NC_005102.4-82451807	CCGACCAGCACACCCACTCCTGCTGCA	3	G	0.0252	0.0249
NC_005102.4-82451807	CCGACCAGCACACCCACTCCTGCTGCA	15	C	0	0.4844
NC_005102.4-82457488	GAGAATGGCACCCCACCTCCTCCCACT	22	C	0.0084	0.4797
NC_005102.4-82465933	GGCCCTTCCACAGTGGCTCCAAACCCT	23	A	0.0179	0.0521
NC_005102.4-82493089	TGGCTTAAAACACTACCTCCATCACTT	14	T	0	0.4734
NC_005102.4-82493089	TGGCTTAAAACACTACCTCCATCACTT	24	A	0	0.4734
NC_005102.4-82494089	GACCATTGCACCAAAGCTCCTCGGAAC	16	G	0.0244	0.0241
NC_005102.4-82494089	GACCATTGCACCAAAGCTCCTCGGAAC	22	C	0.1707	0.4999
NC_005102.4-82511124	CCAGTCAGAACAAGCGCTCCAGAACAT	7	A	0.1967	0.3709
NC_005102.4-82511124	CCAGTCAGAACAAGCGCTCCAGAACAT	15	C	0	0.4807
NC_005102.4-82512623	TCTCACTGAACTGAAGCTCCAGAGATT	14	A	0	0.4743
NC_005102.4-82512623	TCTCACTGAACTGAAGCTCCAGAGATT	23	A	0	0.4743
NC_005102.4-82520056	ATTGGCTTAACCTGGGCTCCAAACCCC	2	T	0.0328	0.0322
NC_005102.4-82520056	ATTGGCTTAACCTGGGCTCCAAACCCC	22	A	0	0.4787
NC_005102.4-82553896	AAGGTAGGTACTGGTACTCCTGAGTCC	14	G	0.0083	0.4769
NC_005102.4-82569629	TGAGGACACACATGTGCTCCCTGTGTT	22	T	0.0667	0.495
NC_005102.4-82580683	TACACCTCAACCTCCTCTCCACCACAG	23	C	0	0.4787

NC_005102.4-82585375	GAGGTCATGACCCCTGCTCCCTGCCAA	3	G	0.0165	0.0325
NC_005102.4-82585375	GAGGTCATGACCCCTGCTCCCTGCCAA	25	C	0.0417	0.0566
NC_005102.4-82623511	AATGTCCAAACCTTAACTCCAGTCAGT	4	G	0.1983	0.2869
NC_005102.4-82623511	AATGTCCAAACCTTAACTCCAGTCAGT	8	A	0.0246	0.4852
NC_005102.4-82640094	TGGAGAATGACTGGGTCTCCATGGAAC	21	A	0.0417	0.4922
NC_005102.4-82646421	TCTGTGGAAACGGAATCTCCAGACAGG	9	A	0.2435	0.3938
NC_005102.4-82652956	CACATGATCACATGGCCTCCCTTGCCA	5	T	0.0345	0.482
NC_005102.4-82652956	CACATGATCACATGGCCTCCCTTGCCA	7	A	0.1466	0.1786
NC_005102.4-82670550	TACTATGTCACCTCGTCTCCAAGAAAT	27	T	0	0.4859
NC_005102.4-82688680	TACCACACCACAAACGCTCCTTGCTTT	6	C	0.0083	0.4835
NC_005102.4-82699087	TTATTTGATACGAGGTCTCCCTTACCT	12	G	0	0.4743
NC_005102.4-82704331	ACCATATTCACCAAGTCTCCTTATGAG	1	A	0.2276	0.2017
NC_005102.4-82704331	ACCATATTCACCAAGTCTCCTTATGAG	25	G	0.2276	0.2017
NC_005102.4-82751483	CTCAGCTCTACTCTACCTCCCTGGAGG	21	C	0.0252	0.0411
NC_005102.4-82777214	CCAATGTCTACTCATCCTCCATGTTGG	2	C	0.0348	0.0342
NC_005102.4-82799642	AGGTCAGTCACTCAGGCTCCAAGGCCA	12	T	0.2353	0.3415
NC_005102.4-82819055	CCAGAGTGCACTGGACCTCCATGAAGG	7	T	0.2035	0.3941
NC_005102.4-82823654	CATGCTGCCACCAAGTCTCCATATCAC	24	T	0	0.4751
NC_005102.4-82823654	CATGCTGCCACCAAGTCTCCATATCAC	25	C	0.1066	0.2339
NC_005102.4-82830177	GGTGCGCTAACTGCCCCTCCCTCCCAT	5	C	0.027	0.044
NC_005102.4-82848781	CCAACACATACCACTGCTCCTGACAGA	4	A	0.0345	0.482
NC_005102.4-82848781	CCAACACATACCACTGCTCCTGACAGA	5	C	0	0.4709
NC_005102.4-82875817	GTTGTGGTGACCCTCCCTCCCAACCAT	27	T	0.1239	0.1466
NC_005102.4-82877746	CAGGCATACACACACACTCCAATATGC	22	A	0.0177	0.0517
NC_005102.4-82908693	AATAAAACAACATACACTCCCAAAATA	3	T	0.1667	0.483
NC_005102.4-82913526	AAGAAAGGCACTCCCACTCCCTGCCCA	16	A	0.0783	0.0752
NC_005102.4-82990226	GTCTCCCTTACCCAGCCTCCTACTCTG	2	T	0	0.4773
NC_005102.4-83021441	CTCCCCTCCACATGCCCTCCTAGAATG	1	C	0	0.4793
NC_005102.4-83021441	CTCCCCTCCACATGCCCTCCTAGAATG	2	T	0	0.4793
NC_005102.4-83025010	GCTTGCCCTACCTTCTCTCCCTGAGAA	4	T	0	0.4793
NC_005102.4-83080431	AACAAGCTTACTGCTTCTCCCGCCTCT	21	C	0.0087	0.0425
NC_005102.4-83130694	GAGCATCTGACTCCTCCTCCCTCCCAA	3	G	0	0.4734
NC_005102.4-83222456	ACTTAAGTTACATCTCCTCCCTCCTTG	21	C	0.3167	0.455
NC_005102.4-83230437	CATGAGGAAACATCTCCTCCATACCAC	1	C	0.0672	0.065
NC_005102.4-83265411	AGCAACTTAACTGGGTCTCCATTACAG	1	A	0	0.4698
NC_005102.4-83265411	AGCAACTTAACTGGGTCTCCATTACAG	22	T	0	0.4698
NC_005102.4-83461446	TCCAATAAAACCACAACTCCCAATCCT	26	C	0.2321	0.3468
NC_005102.4-83491575	TCCCCTCCCACAGCTCCTCCCTCCATT	21	C	0	0.4787
NC_005102.4-83626450	AAGCTCGCCACTGCCACTCCATGACAT	7	G	0.2083	0.3441
NC_005102.4-83638032	ACAGAAAGTACTGTTTCTCCATGTAGA	27	A	0.0256	0.074
NC_005102.4-83703049	AAAATAGACACAGCATCTCCTGCCTCC	23	C	0.1949	0.4207
NC_005102.4-83725577	TTCACTTGAACCATCTCTCCAGCTTCA	23	C	0.2203	0.362
NC_005102.4-83841626	CACTCACAAACACTTACTCCAAGAGCT	7	C	0.0084	0.0411
NC_005102.4-83911407	TGACCCTCAACCCCTACTCCGTGCTGC	8	C	0	0.4819
NC_005102.4-83911407	TGACCCTCAACCCCTACTCCGTGCTGC	21	G	0	0.4819
NC_005102.4-83950700	CTGCTCCAAACTTTGACTCCGTAGTTC	5	T	0.1897	0.2965
NC_005102.4-83966035	GAGTTTGTGACCTCCTCTCCTACTCAT	12	C	0.2333	0.4988
NC_005102.4-83996007	TAACCACTGACACATCCTCCCAGCACA	8	T	0	0.4793
NC_005102.4-84002831	GCCATTGCAACCCCTTCTCCCTACCTG	2	C	0.2655	0.4135
NC_005102.4-84012719	GCTGAGCAGACTGTAGCTCCATTTCGT	6	G	0.2083	0.2941
NC_005102.4-84012719	GCTGAGCAGACTGTAGCTCCATTTCGT	26	G	0.2773	0.327

NC_005102.4-84055562	GCTGCTCTGACTGGCTCTCCAATAATC	15	C	0.0427	0.0896
NC_005102.4-84075704	CCTATCAGCACTCACACTCCCCGTCTT	15	C	0.1885	0.2686
NC_005102.4-84075704	CCTATCAGCACTCACACTCCCCGTCTT	24	T	0.2114	0.3516
NC_005102.4-84107308	GGCGAATTAACAACAGCTCCTTGACTG	3	C	0.1176	0.1398
NC_005102.4-84113174	TCAGCTCTGACCCTGTCTCCTAAGTCT	8	T	0.0248	0.0245
NC_005102.4-84113174	TCAGCTCTGACCCTGTCTCCTAAGTCT	21	T	0.0579	0.0716
NC_005102.4-84186483	GTAAGCAGGACATGGCCTCCTCTGGAA	12	A	0.1017	0.1116
NC_005102.4-84190083	TCTGCTGATACATTGACTCCATCATAT	13	T	0.0174	0.0989
NC_005102.4-84434653	AATGCTGTAACCTCCTCTCCCCTCACA	3	T	0.2143	0.4362
NC_005102.4-84464980	CAAGGGTGCACACTCTCTCCTATTAAA	9	C	0.0439	0.0595
NC_005102.4-84485021	TCTAAAAAGACTGACCCTCCATGTTTG	13	G	0.0083	0.0408
NC_005102.4-84499967	TTCATTGGAACTGAGACTCCTGAACCA	16	A	0.0087	0.466
NC_005102.4-84603035	ATAATAAGGACATGTACTCCACTCTGT	1	A	0.0531	0.4912
NC_005102.4-84603035	ATAATAAGGACATGTACTCCACTCTGT	21	A	0.0796	0.2104
NC_005102.4-84617338	CCACATCCTACACTTCCTCCCCTAACC	3	A	0.1901	0.3027
NC_005102.4-84962853	GTCTGTCGGACACCCTCTCCCCTCTTG	13	C	0.124	0.2234
NC_005102.4-85145372	AAACAAGTCACTTAGACTCCAGAGGAT	14	A	0.1102	0.1621
NC_005102.4-85288383	TACTCTTAAACCTCCTCTCCAATAATT	15	C	0.1538	0.4807
NC_005102.4-85370415	TTAATAAAGACCTTCCCTCCTCGACAG	1	T	0.0424	0.0415
NC_005102.4-85370415	TTAATAAAGACCTTCCCTCCTCGACAG	25	C	0.2542	0.4884
NC_005102.4-85410692	GTTCAGACTACATATCCTCCATTCTAA	1	G	0.25	0.4034
NC_005102.4-85410692	GTTCAGACTACATATCCTCCATTCTAA	27	A	0.0086	0.4595
NC_005102.4-85837807	GTGCTGCCTACGACCTCTCCGCGGTGG	1	G	0.0431	0.0422
NC_005102.4-85837807	GTGCTGCCTACGACCTCTCCGCGGTGG	15	C	0.0254	0.0251
NC_005102.4-85931084	AGTGAGTTTACTTACTCTCCAGGACTC	25	C	0.0089	0.4751
NC_005102.4-85940806	AGGCCAACAACCCAAGCTCCTTGGACA	4	C	0.2783	0.4864
NC_005102.4-85986810	AGGAGTCACACAGAAACTCCGAGTTGG	8	A	0	0.488
NC_005102.4-85986810	AGGAGTCACACAGAAACTCCGAGTTGG	23	G	0.0517	0.0504
NC_005102.4-85986955	TTGCCTCTAACACTGTCTCCTTCTGGA	15	G	0.069	0.0981
NC_005102.4-86027590	TGCCTGTGAACTCTGCCTCCAAGGGAT	24	G	0.0248	0.0405
NC_005102.4-86084157	ACATTATTAACATGGCCTCCAGGGCAT	15	G	0.1148	0.1641
NC_005102.4-86117249	GAGAAAGGAACCTTCTCTCCTCTTCCT	7	G	0.0813	0.4973
NC_005102.4-86183265	AGAGTTGGAACTCTTGCTCCTCCAGCT	12	T	0.065	0.0629
NC_005102.4-86219446	TGTGTCCTTACTAACGCTCCAACAGTA	4	G	0.041	0.0401
NC_005102.4-86223668	AGAATGTCTACATTTCCTCCTGGGATG	6	G	0	0.4724
NC_005102.4-86223668	AGAATGTCTACATTTCCTCCTGGGATG	23	G	0.0776	0.0903
NC_005102.4-86275464	TCCTGAGTCACACTGTCTCCCTCTCCT	7	G	0.0504	0.0491
NC_005102.4-86333448	CTAAGATCCACCCCTCCTCCATTTCCA	8	C	0.1466	0.1646
NC_005102.4-86337844	ACATATTCTACTCACCCTCCAAGGCCT	22	A	0.1518	0.2991
NC_005102.4-86465367	AGAGAAGTTACTGCAGCTCCTTCAGTA	1	A	0.0083	0.4835
NC_005102.4-86465367	AGAGAAGTTACTGCAGCTCCTTCAGTA	15	A	0.0083	0.4835
NC_005102.4-86505863	TTCTTAGCCACATATTCTCCTGTCCAT	9	C	0.2951	0.4515
NC_005102.4-86525099	CTCTCCCATACCCCTCCTCCCACTGCT	4	T	0.0325	0.0476
NC_005102.4-86525099	CTCTCCCATACCCCTCCTCCCACTGCT	7	C	0.0325	0.0476
NC_005102.4-86568705	AAAAACTTGACTCAGTCTCCAATGTAG	15	G	0.2458	0.3991
NC_005102.4-86568705	AAAAACTTGACTCAGTCTCCAATGTAG	22	A	0	0.4793
NC_005102.4-86574712	AAAACAAGCACACATGCTCCAATATGT	12	A	0.0167	0.48
NC_005102.4-86612539	GTGTGGCACACAGTTCCTCCTTTGTTG	1	G	0.2773	0.4542
NC_005102.4-86647620	CCACCTATCACACCCTCTCCAAGCACA	5	C	0.0328	0.0935
NC_005102.4-86682252	CTTCTTCTCACTTATCCTCCTTCTCTT	3	T	0.0252	0.4859
NC_005102.4-86682252	CTTCTTCTCACTTATCCTCCTTCTCTT	21	T	0.0252	0.4859

NC_005102.4-86689409	TCTTGCTTCACATCCTCTCCTGTATGA	25	T	0.1404	0.1744
NC_005102.4-86693636	CACAGATTCACCCTATCTCCAAATACT	23	A	0.15	0.5
NC_005102.4-86770960	AGTTGGAACACAAGGCCTCCCTTTTAC	6	G	0.2174	0.2204
NC_005102.4-86770960	AGTTGGAACACAAGGCCTCCCTTTTAC	27	C	0.0261	0.0257
NC_005102.4-86846908	TATCAACCAACCCTAGCTCCTAGGGAC	23	G	0	0.4807
NC_005102.4-86884154	CCCAGGGGCACTCTCACTCCTAAGTTC	3	C	0.2459	0.4866
NC_005102.4-86968992	CACCTTCAGACAGATCCTCCCTTACCC	5	T	0.225	0.4747
NC_005102.4-86968992	CACCTTCAGACAGATCCTCCCTTACCC	9	G	0.0992	0.1918
NC_005102.4-87019626	CTGGTGAAAACTGATTCTCCCGTATTA	4	G	0.1261	0.2389
NC_005102.4-87019626	CTGGTGAAAACTGATTCTCCCGTATTA	21	C	0	0.4813
NC_005102.4-87031544	TACCAAGCCACAATACCTCCTACAAGT	9	C	0.2564	0.4123
NC_005102.4-87031544	TACCAAGCCACAATACCTCCTACAAGT	13	A	0.0085	0.4626
NC_005102.4-87075914	TACAATGGCACAATTCCTCCAACACTG	5	A	0.1967	0.2157
NC_005102.4-87154100	AGAAGTATGACACACTCTCCTCCTTCA	4	A	0.0089	0.4885
NC_005102.4-87154100	AGAAGTATGACACACTCTCCTCCTTCA	5	G	0.0089	0.4885
NC_005102.4-87421186	GAATATGTGACCTACCCTCCTTAGCTG	4	T	0.125	0.232
NC_005102.4-87446781	ATAAAGGACACAAGCCCTCCTACATAA	1	A	0.0087	0.4849
NC_005102.4-87446781	ATAAAGGACACAAGCCCTCCTACATAA	2	T	0.0087	0.4849
NC_005102.4-87449996	ACTTCAGCAACCCACACTCCTCAATGA	9	A	0.2101	0.4999
NC_005102.4-87462527	TATGCCTCTACCTTTTCTCCATTTAAA	6	C	0.0619	0.0765
NC_005102.4-87463511	TTGAAAACCACTTCATCTCCTTCAGTC	1	T	0.3063	0.498
NC_005102.4-87463511	TTGAAAACCACTTCATCTCCTTCAGTC	12	T	0.0439	0.0429
NC_005102.4-87600172	TCCAGAGACACAGGAACTCCCACACAG	9	C	0.0248	0.0245
NC_005102.4-87600172	TCCAGAGACACAGGAACTCCCACACAG	24	A	0.0248	0.0245
NC_005102.4-87847922	GAGATTATCACATATTCTCCTTCTTAT	14	A	0.2101	0.2626
NC_005102.4-87937634	GGACCCAAAACTTCCCCTCCCATTGAT	21	C	0.0991	0.1103
NC_005102.4-88232668	GGTGAGCTGACCCCAACTCCTGTTTTA	1	G	0.0991	0.2778
NC_005102.4-88368573	ATAACATGGACACATGCTCCTCTATGT	12	A	0.2174	0.3547
NC_005102.4-88656866	TGAACCAGTACCACAGCTCCCTGCACA	12	C	0.0508	0.0496
NC_005102.4-88656866	TGAACCAGTACCACAGCTCCCTGCACA	25	A	0.0593	0.0576
NC_005102.4-88737069	CTAATGAAAACTGCAACTCCTATCTTC	2	T	0	0.4772
NC_005102.4-88737069	CTAATGAAAACTGCAACTCCTATCTTC	22	A	0	0.4772
NC_005102.4-88755726	ACTGCAATTACCCTCCCTCCCCTTTGC	22	C	0.0089	0.0264
NC_005102.4-88770503	ATTATGATTACAGTTTCTCCTTCAACT	13	G	0.0862	0.1134
NC_005102.4-88816930	AGGCTGAAGACAGATTCTCCAAAGGTA	7	A	0	0.4859
NC_005102.4-89128016	TAACATGGAACCTGATCTCCTCATTTC	16	T	0	0.4793
NC_005102.4-89206615	GAAGCAGGCACACTGACTCCAACAGAA	1	G	0.0417	0.0566
NC_005102.4-89206615	GAAGCAGGCACACTGACTCCAACAGAA	2	A	0.0417	0.4875
NC_005102.4-89328303	AAAACCTTTACACTTTCTCCCAGTATC	6	C	0.1316	0.1229
NC_005102.4-89328303	AAAACCTTTACACTTTCTCCCAGTATC	24	T	0.2719	0.4289
NC_005102.4-89446639	TTCCATTCCACTTACTCTCCCGTATAC	25	T	0.0357	0.0521
NC_005102.4-89497735	ATCTGGAATACATTTCCTCCTGCTTGC	12	A	0	0.4851
NC_005102.4-89579121	GCCAGGGGCACTCTCCCTCCTAAGAAC	26	A	0	0.4868
NC_005102.4-89694953	AACTATCTAACATTCTCTCCATAGTAG	5	A	0.0263	0.4861
NC_005102.4-89721546	GCTCCTAAGACTTTCTCTCCCTGAAAC	2	C	0.0177	0.452
NC_005102.4-89742576	GAACTGAGAACTGGACCTCCGTTGGAG	5	T	0.075	0.0722
NC_005102.4-89969897	GCCTGCAGGACCTTTCCTCCAGTCAAG	23	T	0.0087	0.4849
NC_005102.4-90140884	ACAGCACTTACTCTTCCTCCAGAGGAC	2	C	0.5897	0.4158
NC_005102.4-90140884	ACAGCACTTACTCTTCCTCCAGAGGAC	13	C	0.084	0.0805
NC_005102.4-90158628	TGGATCATCACCAGGACTCCTCTCCTC	9	C	0.0439	0.0429
NC_005102.4-90175952	GTGGCTTCTACACTTCCTCCATGTATG	25	A	0.252	0.2989

NC_005102.4-90193065	TACCAAACAACTAAAGCTCCCAGTGCC	7	A	0	0.4764
NC_005102.4-90272586	TTATCATACACATTTGCTCCTACATCA	1	T	0.0957	0.3728
NC_005102.4-90283588	GGACTCCTTACATCCTCTCCTACTGAG	3	A	0	0.4728
NC_005102.4-90283588	GGACTCCTTACATCCTCTCCTACTGAG	8	T	0	0.4728
NC_005102.4-90334335	GGCATTGGAACAAAGTCTCCCTTTACC	8	G	0.0569	0.0553
NC_005102.4-90413343	CCATACAGGACAGTCACTCCTATACGG	27	G	0.0089	0.4824
NC_005102.4-90420952	AGAGTCCTAACAAAGACTCCATTGAGT	16	A	0.0522	0.0508
NC_005102.4-90483915	CCCACTCTCACCTCAACTCCCTGGCAT	3	C	0.0508	0.0496
NC_005102.4-90483915	CCCACTCTCACCTCAACTCCCTGGCAT	14	C	0.0508	0.0496
NC_005102.4-90489768	CCCACTGGGACCCACCCTCCAACTGTT	5	C	0.175	0.4472
NC_005102.4-90489848	CCCCACCCCACCAGACCTCCTCACACC	1	C	0.1966	0.4416
NC_005102.4-90502288	ATTATATAAACCCATTCTCCATGACAA	7	T	0.1339	0.125
NC_005102.4-90529595	ATGTGGCACACATTATCTCCATATGTT	16	T	0.0488	0.0629
NC_005102.4-90594334	TACCAGCCAACCAGAGCTCCCAGAACT	4	C	0	0.4757
NC_005102.4-90632279	CCATGCTGAACCATCTCTCCAGCTTTG	4	T	0.0244	0.0705
NC_005102.4-90684714	TTTCTCTGTACTCCCTCTCCCCAACAC	7	T	0.0089	0.4751
NC_005102.4-90746195	TTGAATAAGACCACACCTCCTAACTGT	14	C	0.2072	0.4726
NC_005102.4-90786163	AAGATGCTTACTGCTGCTCCAAGCACT	14	C	0.0164	0.4807
NC_005102.4-90787897	TAGGAGAAAACATAAGCTCCATCGCTT	4	G	0.1171	0.4959
NC_005102.4-90787897	TAGGAGAAAACATAAGCTCCATCGCTT	14	A	0.1171	0.4959
NC_005102.4-90810338	AGAAGAGAAACGGCCTCTCCAAAGCAT	12	G	0.2295	0.4737
NC_005102.4-90815051	ATCCTTATGACCTTTTCTCCCCCTGAG	4	C	0.2301	0.4624
NC_005102.4-90931447	CAACCAAACACCAAGTCTCCACTGCAG	15	G	0.0244	0.0241
NC_005102.4-90931447	CAACCAAACACCAAGTCTCCACTGCAG	21	A	0.0407	0.0398
NC_005102.4-90979663	ACTTTTATGACATCATCTCCTTTCCTT	21	T	0.1057	0.1425
NC_005102.4-90982195	CGAATGTTTACTGTATCTCCCTAATCA	22	T	0.0261	0.0257
NC_005102.4-91051390	CAAGTTACCACTGGGTCTCCAAGGTAT	27	T	0.0783	0.0911
NC_005102.4-91121660	TCCAAGGAAACCTCTTCTCCCAACAAG	4	A	0.1081	0.499
NC_005102.4-91121660	TCCAAGGAAACCTCTTCTCCCAACAAG	27	G	0.1062	0.4996
NC_005102.4-91136652	TACATTCAAACTGGCACTCCTCGGTAA	22	C	0	0.477
NC_005102.4-91181944	TGGCAACCAACCATGGCTCCCAGGGAT	26	A	0	0.4844
NC_005102.4-91189163	AGTCCACAAACTGGTGCTCCTCCTCTG	21	T	0.1475	0.1641
NC_005102.4-91324895	GACCTTAGAACGGTGACTCCCAACCTT	1	G	0.2261	0.4588
NC_005102.4-91341038	ATGCTGTCAACGTGGTCTCCGCTCTTG	4	C	0.2927	0.385
NC_005102.4-91341038	ATGCTGTCAACGTGGTCTCCGCTCTTG	22	C	0	0.4793
NC_005102.4-91346792	ACCACCACTACCCTGCCTCCTGCACCC	9	T	0.1967	0.3537
NC_005102.4-91346792	ACCACCACTACCCTGCCTCCTGCACCC	27	C	0.0081	0.0241
NC_005102.4-91373430	ATGTTGAAAACAGAGACTCCCAGCCTG	6	G	0.1008	0.4406
NC_005102.4-91387575	ATTCCACGCACTCTTTCTCCTCACCCT	23	A	0	0.4807
NC_005102.4-91401911	CAACCTAAAACCAAGTCTCCATCCACT	2	A	0.2314	0.2759
NC_005102.4-91401911	CAACCTAAAACCAAGTCTCCATCCACT	24	C	0.1983	0.2046
NC_005102.4-91411521	CTACCTGCAACTTTACCTCCAGGGGTT	8	C	0.0325	0.032
NC_005102.4-91411521	CTACCTGCAACTTTACCTCCAGGGGTT	27	T	0.0325	0.032
NC_005102.4-91412574	CTGATAGGCACATAGGCTCCACGGCTT	5	T	0.1186	0.4977
NC_005102.4-91440423	CAACGGGACACACCACCTCCTCATATC	5	G	0.0496	0.0484
NC_005102.4-91452177	CACAGAACCACACGTGCTCCTTGAATG	5	G	0.2149	0.4923
NC_005102.4-91452177	CACAGAACCACACGTGCTCCTTGAATG	14	G	0.0744	0.1017
NC_005102.4-91482707	ACACACCCCACCCCCACTCCGTGCTTC	6	C	0.0083	0.4783
NC_005102.4-91482707	ACACACCCCACCCCCACTCCGTGCTTC	9	C	0.0167	0.4832
NC_005102.4-91486098	TGAATCAGAACAAAGCCTCCCTCCCAT	4	A	0	0.4793
NC_005102.4-91507807	CTGCCCCACACGAGATCTCCAGAGACT	21	A	0.0081	0.481

NC_005102.4-91664348	CCCTCCCCCACAGTGACTCCATGCCCT	21	A	0.0174	0.4833
NC_005102.4-91664348	CCCTCCCCCACAGTGACTCCATGCCCT	27	T	0	0.48
NC_005102.4-91669428	GAGCATGTGACCACGACTCCCTATTAA	22	T	0	0.4846
NC_005102.4-91688601	AGTGTAGCCACAGCGGCTCCTAACCAG	23	A	0.1653	0.2046
NC_005102.4-91690418	ATACTGAGGACAGCGCCTCCTCTCTCC	15	G	0.114	0.472
NC_005102.4-91705985	GCACAACTCACAGAGCCTCCAGTCCTC	26	T	0	0.4793
NC_005102.4-91724121	AGCCAAATGACGGAGCCTCCAGGACTG	12	G	0	0.4826
NC_005102.4-91731349	TCCCCACCCACTCTGTCTCCTTTCATT	7	C	0	0.4793
NC_005102.4-91731349	TCCCCACCCACTCTGTCTCCTTTCATT	25	A	0.3089	0.3604
NC_005102.4-91751465	CATACTGGCACTACAGCTCCTAAGGCT	3	T	0	0.4793
NC_005102.4-91751465	CATACTGGCACTACAGCTCCTAAGGCT	25	G	0.2439	0.3141
NC_005102.4-91754554	GCAGCAGTGACTTTCTCTCCTGTACCC	1	G	0.0259	0.0255
NC_005102.4-91754554	GCAGCAGTGACTTTCTCTCCTGTACCC	24	A	0.2069	0.2965
NC_005102.4-91757031	GGCCTGGCCACACTTCCTCCAGACGCA	16	C	0.1917	0.49
NC_005102.4-91764321	GTGCAGAAGACTGGCTCTCCAGGAGCA	15	C	0.2213	0.2457
NC_005102.4-91779931	GGCGAATGCACACCATCTCCTTATCCC	7	T	0	0.4807
NC_005102.4-91790583	GAGTGGAAGACATCCTCTCCTCTCCTG	21	T	0.0714	0.1771
NC_005102.4-91791329	CCCTACCCTACTGTTGCTCCTACTTGT	8	C	0.0248	0.0245
NC_005102.4-91793483	GAATTGTTAACTTTCTCTCCAGGGATT	22	G	0.0089	0.4789
NC_005102.4-91793483	GAATTGTTAACTTTCTCTCCAGGGATT	26	T	0	0.477
NC_005102.4-91797840	GTCTGGGGAACACTAACTCCTCCCATC	25	A	0.0081	0.481
NC_005102.4-91806758	CACACAGGAACACTCTCTCCACTTCAC	8	G	0.1074	0.1163
NC_005102.4-91869540	CTCACTACCACTCTAGCTCCAGGGGAT	26	A	0.2764	0.4682
NC_005102.4-91901405	CACATACACACCCAGGCTCCACAAGAT	21	A	0.2523	0.3772
NC_005102.4-91911392	ATTCGGCACACCCCTGCTCCCCGAGTG	4	C	0.2212	0.4873
NC_005102.4-91911392	ATTCGGCACACCCCTGCTCCCCGAGTG	15	T	0.0877	0.0839
NC_005102.4-91974091	AAGGACCCAACATCACCTCCACCATCA	7	C	0.1071	0.1327
NC_005102.4-91974091	AAGGACCCAACATCACCTCCACCATCA	21	A	0.009	0.4726
NC_005102.4-91993387	TGCTCTTTAACTGAAGCTCCACATCCA	23	A	0	0.4751
NC_005102.4-91994754	CTTCAAATCACCTTGCCTCCTTGTGTC	24	T	0.082	0.1082
NC_005102.4-92071867	AACCAAGCAACCCCGCCTCCACCCCAT	15	G	0.1008	0.2796
NC_005102.4-92071867	AACCAAGCAACCCCGCCTCCACCCCAT	16	C	0.0085	0.4655
NC_005102.4-92075242	CTTTAGGGCACCCTACCTCCCAAGCTG	3	T	0	0.4793
NC_005102.4-92091487	CACATCCAGACTAATACTCCACAGAGT	8	A	0.2119	0.4396
NC_005102.4-92091487	CACATCCAGACTAATACTCCACAGAGT	26	G	0.0085	0.4776
NC_005102.4-92107403	TCCTTCACCACCTGCTCTCCTGACCAT	16	T	0.0732	0.4471
NC_005102.4-92175573	TAACAACCAACCATATCTCCCAGGGGA	1	T	0.0263	0.026
NC_005102.4-92175573	TAACAACCAACCATATCTCCCAGGGGA	8	C	0.0263	0.026
NC_005102.4-92256253	TTTCTCCCCACCCCAACTCCACTATTG	3	T	0.0625	0.0771
NC_005102.4-92256253	TTTCTCCCCACCCCAACTCCACTATTG	5	T	0.0708	0.0846
NC_005102.4-92305179	CTTCTTGCCACCAGCCCTCCTTCTAGG	8	C	0.065	0.078
NC_005102.4-92338922	TCTGATGTGACCCTCTCTCCTCACTCA	6	T	0.2276	0.4206
NC_005102.4-92461694	CTGACCCAGACACTAACTCCTGACCTG	16	A	0.1161	0.1093
NC_005102.4-92469713	TAAAAGAAAACAGGAGCTCCCCAGAGT	26	G	0.2281	0.3706
NC_005102.4-92476031	TTGGGAATCACGGACCCTCCCTCATTT	7	A	0	0.4868
NC_005102.4-92476031	TTGGGAATCACGGACCCTCCCTCATTT	23	C	0.0427	0.058
NC_005102.4-92487035	GTGAACTCTACAAGTTCTCCCTCTCCA	2	T	0.0261	0.0257
NC_005102.4-92487035	GTGAACTCTACAAGTTCTCCCTCTCCA	25	C	0.2018	0.4676
NC_005102.4-92516934	TTATTGAGAACGACCCCTCCAGCTTTG	26	T	0.0246	0.4852
NC_005102.4-92529273	TGCGTTTTCACCTCTGCTCCACTGGTG	3	C	0.1789	0.3604
NC_005102.4-92538166	AGGTAATTCACATCACCTCCGAGATAG	2	G	0.1102	0.1481

NC_005102.4-92646979	TTATATCTCACCCCAACTCCCAGAGTC	12	C	0.1597	0.4045
NC_005102.4-92648520	ATGTGGGTCACACTGCCTCCGCTATAG	22	C	0.0984	0.1225
NC_005102.4-92654028	TTGTTGCTTACATTCTCTCCCAGCTCC	27	C	0.0259	0.4643
NC_005102.4-92707302	CCTCCCTGAACGTCCACTCCCTCTTTG	25	T	0.2222	0.3729
NC_005102.4-92727861	CACCCCCAAACCCCTTCTCCAAAGGTT	3	C	0.0261	0.0257
NC_005102.4-92727861	CACCCCCAAACCCCTTCTCCAAAGGTT	9	A	0.1062	0.1316
NC_005102.4-92789523	GCAGATAAAACTCCAACTCCTTGCCTT	21	T	0.0246	0.0243
NC_005102.4-92805583	CTAATGGCTACCTTCCCTCCAGGTATA	12	C	0.1802	0.2068
NC_005102.4-92846664	GTCCATAGCACACCCCCTCCCACACAT	26	A	0.2358	0.4239
NC_005102.4-92907904	AGACCATGTACCCACGCTCCTTCAAAC	27	C	0	0.4793
NC_005102.4-92912772	CACTGAGCAACCAAGCCTCCCATGCCT	4	T	0.1057	0.1145
NC_005102.4-92926076	TGACTCTGAACGGTACCTCCAAAACAG	7	T	0	0.4751
NC_005102.4-92934931	CCCCACATGACTTAACCTCCTCTGCAT	5	A	0	0.4787
NC_005102.4-92971922	CCCCTTGCCACCTCCCCTCCCTAACAT	1	C	0.1092	0.1033
NC_005102.4-92971922	CCCCTTGCCACCTCCCCTCCCTAACAT	7	G	0.0583	0.0566
NC_005102.4-92988398	CCTGCCCTTACTGTGGCTCCAGAGGGG	7	C	0.0354	0.0348
NC_005102.4-92997149	ACCCCTTGGACCACATCTCCTACAGAC	2	C	0.0756	0.1746
NC_005102.4-92997149	ACCCCTTGGACCACATCTCCTACAGAC	15	A	0.0168	0.4873
NC_005102.4-93037962	TGAGGTAGTACTGTCCCTCCAATTCTG	4	G	0.2437	0.4682
NC_005102.4-93057284	TGCTTCCGCACTGTATCTCCACCATGG	7	C	0.042	0.0411
NC_005102.4-93091533	TTAAGAGCTACCACTACTCCATGTTCA	21	A	0.1311	0.1505
NC_005102.4-93122981	TCCTCAATTACCCCATCTCCTTCTCTT	5	C	0.0084	0.4859
NC_005102.4-93122981	TCCTCAATTACCCCATCTCCTTCTCTT	26	T	0.0084	0.4859
NC_005102.4-93194365	TGAATGTTCACAACTACTCCGATTTCC	21	G	0.2541	0.454
NC_005102.4-93194365	TGAATGTTCACAACTACTCCGATTTCC	22	A	0.1557	0.4997
NC_005102.4-93209102	AGGCAGGAAACCCAGGCTCCTCCCTAG	4	C	0	0.4751
NC_005102.4-93209102	AGGCAGGAAACCCAGGCTCCTCCCTAG	22	C	0.3306	0.4246
NC_005102.4-93254946	GAGTCAGAAACACAGGCTCCCACTGTT	14	A	0.0175	0.474
NC_005102.4-93254946	GAGTCAGAAACACAGGCTCCCACTGTT	15	G	0.3596	0.3999
NC_005102.4-93267075	TGCTCCATGACAAAGACTCCCATGAAC	3	C	0.1062	0.1162
NC_005102.4-93297182	CATACATAAACTGTGTCTCCCCACTCC	24	C	0.082	0.0935
NC_005102.4-93362213	CTGAGTGCCACACCAACTCCAACAATG	26	T	0.0413	0.0405
NC_005102.4-93362213	CTGAGTGCCACACCAACTCCAACAATG	27	G	0.0413	0.0405
NC_005102.4-93385019	CCAGCCCACACACAGCCTCCCACAGCA	23	C	0.0351	0.0345
NC_005102.4-93385019	CCAGCCCACACACAGCCTCCCACAGCA	25	G	0.087	0.1143
NC_005102.4-93390282	CCTTCCCCCACAATCACTCCGAATCAC	5	C	0.2261	0.32
NC_005102.4-93390282	CCTTCCCCCACAATCACTCCGAATCAC	21	G	0.0345	0.4851
NC_005102.4-93409921	TAAAGGAACACTGGGACTCCAAGGCAC	16	A	0.2479	0.4959
NC_005102.4-93420783	ACTTCTCAAACATGCGCTCCTGCTATC	15	C	0	0.4844
NC_005102.4-93420783	ACTTCTCAAACATGCGCTCCTGCTATC	23	C	0	0.4844
NC_005102.4-93608940	ACCACCCCAACATCAGCTCCTGGTACT	12	A	0.1818	0.4997
NC_005102.4-93624215	CAAGGAGTCACTTGAACTCCGAGGTTC	7	G	0.307	0.4473
NC_005102.4-93665707	CCTGCTGCAACCAAGTCTCCAGCAGTC	5	C	0.0569	0.0553
NC_005102.4-93666096	CCAGTAGCCACCTGCCCTCCTGCTTTG	15	C	0	0.4819
NC_005102.4-93688608	CAGTGTGGAACCGTAACTCCCTGAAAG	4	T	0	0.4793
NC_005102.4-93688608	CAGTGTGGAACCGTAACTCCCTGAAAG	13	G	0	0.4793
NC_005102.4-93715002	CTGCCCCTAACCCACACTCCTTATCCA	15	C	0.0164	0.4807
NC_005102.4-93724825	AAATTGAAAACAGTTCCTCCACAAGAG	6	G	0.1148	0.1505
NC_005102.4-93724825	AAATTGAAAACAGTTCCTCCACAAGAG	21	A	0.0656	0.4891
NC_005102.4-93750208	CCATGAGTCACTCACTCTCCAGCCATT	16	T	0.0894	0.1145
NC_005102.4-93777828	CCACTCCTAACTCAAGCTCCTCTTCTA	15	A	0.2613	0.4531

NC_005102.4-93777828	CCACTCCTAACTCAAGCTCCTCTTCTA	27	A	0.2768	0.4043
NC_005102.4-93784689	ATTAATCCGACCTAATCTCCCAAGCAG	9	G	0	0.4868
NC_005102.4-93796519	AACCATGGCACCGCTTCTCCACTAGTC	13	G	0.1626	0.2612
NC_005102.4-93803246	GCCATTGCGACCGAATCTCCTTTACCT	8	C	0.0569	0.0705
NC_005102.4-93803246	GCCATTGCGACCGAATCTCCTTTACCT	12	C	0.065	0.0629
NC_005102.4-93827735	CTTTATGCCACTGTGGCTCCCACATTG	4	T	0.1	0.495
NC_005102.4-93829013	CTGTCTGTGACTTCAGCTCCAGAGAAT	7	G	0.1789	0.1629
NC_005102.4-93839558	AGTTGCCTCACCCCATCTCCTGTGGAG	3	T	0	0.4778
NC_005102.4-93839921	GGCTTTCAAACACTACCTCCCTGCTTC	4	T	0.275	0.4816
NC_005102.4-93854397	GTACTCTTGACCCTTCCTCCGTCCTTT	13	C	0.0163	0.032
NC_005102.4-93866502	TGACCTCTGACTCACTCTCCAAAGGAA	15	C	0	0.0322
NC_005102.4-93917256	GTCTTTCTCACCTCAGCTCCTCCCACA	5	T	0.0086	0.4643
NC_005102.4-93929552	GCTGTCTTTACCTCTCCTCCATCCTCT	4	G	0.0894	0.0854
NC_005102.4-93929552	GCTGTCTTTACCTCTCCTCCATCCTCT	7	T	0.2683	0.4417
NC_005102.4-93931165	TCAGTGGAGACGCTGGCTCCGCGGTCT	21	G	0.065	0.078
NC_005102.4-93931165	TCAGTGGAGACGCTGGCTCCGCGGTCT	22	C	0.0163	0.0476
NC_005102.4-93939541	TACCGACTTACCCTTGCTCCTAATGCT	4	C	0.0667	0.1099
NC_005102.4-93967146	CCCACATCAACCCTAGCTCCCAGCTTT	6	A	0.2195	0.4741
NC_005102.4-93989543	CTAAGTGGGACTGTGTCTCCTCAGTGT	24	G	0.2066	0.4339
NC_005102.4-94157422	GATAGGAAAACAGCCCCTCCTAACGAG	25	G	0.3008	0.3964
NC_005102.4-94160690	GTGTGTGAGACCTGACCTCCAGTACAT	23	T	0.2683	0.3964
NC_005102.4-94197308	CACACTCTGACCCCAGCTCCCCGGGCT	23	G	0.1709	0.4982
NC_005102.4-94206721	TCCTACACCACGAGATCTCCAAGGTTT	9	C	0.0431	0.0422
NC_005102.4-94215323	GCAGCTGGAACTCATTCTCCACTCTTG	15	T	0.0893	0.4898
NC_005102.4-94220947	AAATCTCACACCCATACTCCAAAACCT	4	T	0	0.4751
NC_005102.4-94220947	AAATCTCACACCCATACTCCAAAACCT	5	C	0.0246	0.0243
NC_005102.4-94237446	CTGGCTGTTACCTCATCTCCCGAAGAC	21	C	0.0252	0.0249
NC_005102.4-94256670	TGTATATAAACCGATCCTCCACATGAG	2	G	0.0354	0.0348
NC_005102.4-94256670	TGTATATAAACCGATCCTCCACATGAG	25	G	0.0177	0.4671
NC_005102.4-94272177	CCTGTGAAAACGTCATCTCCTCCTTCT	3	T	0.0702	0.0997
NC_005102.4-94272177	CCTGTGAAAACGTCATCTCCTCCTTCT	12	G	0.2456	0.3615
NC_005102.4-94284607	AGTGATGGGACACCATCTCCCTCGCTG	13	C	0.157	0.4557
NC_005102.4-94284607	AGTGATGGGACACCATCTCCCTCGCTG	24	G	0.157	0.4557
NC_005102.4-94308819	CTGGTCTGAACTGAGTCTCCACCTCAA	22	C	0.0826	0.109
NC_005102.4-94312289	GCCTGTAATACCAGCACTCCAGAGGCA	2	C	0.0172	0.457
NC_005102.4-94451779	CCTCCCAAGACCACACCTCCTTATCTT	9	G	0.2627	0.4776
NC_005102.4-94459752	TCCACCCCTACCAACCCTCCAACTGCC	2	C	0.0244	0.0241
NC_005102.4-94467346	GGTGTTCTCACTCAGCCTCCTGAGCAC	14	A	0.1138	0.4973
NC_005102.4-94482114	GTGCTAACCACCCCTGCTCCCACTGTA	8	C	0.123	0.4193
NC_005102.4-94519500	AGTAGGGAAACAACACCTCCCCCTGCA	6	G	0.0252	0.0249
NC_005102.4-94519500	AGTAGGGAAACAACACCTCCCCCTGCA	14	C	0.0084	0.0249
NC_005102.4-94622242	CACAGGCAAACCTCTTCTCCCAAACTA	15	T	0.0488	0.0629
NC_005102.4-94724136	CATGAAAACACAAGCACTCCAAGAGCA	25	G	0.0625	0.0605
NC_005102.4-94756593	CACACACACACACACACTCCCCTATAC	1	C	0.0339	0.0333
NC_005102.4-94756593	CACACACACACACACACTCCCCTATAC	9	C	0.2542	0.2466
NC_005102.4-94760818	AACTTGTTAACTCCAGCTCCAAGGGTT	23	G	0.2797	0.3483
NC_005102.4-94919355	CTGTCTGGAACCGTGGCTCCTTTGATT	12	C	0.125	0.1172
NC_005102.4-94959647	GCCTCACTGACGATGCCTCCCTCCACT	15	G	0.1092	0.1327
NC_005102.4-94959647	GCCTCACTGACGATGCCTCCCTCCACT	23	C	0.2353	0.2683
NC_005102.4-95020908	ATATTACACACCTAGCCTCCAAAGCGC	15	G	0.1083	0.2125
NC_005102.4-95080498	CCTTCGCCCACTTAGGCTCCTGCTCTC	6	G	0	0.4827

NC_005102.4-95080498	CCTTCGCCCACTTAGGCTCCTGCTCTC	27	C	0.1964	0.232
NC_005102.4-95082475	GTGCTCCCCACATTGGCTCCTGAGTCT	13	T	0	0.4793
NC_005102.4-95082475	GTGCTCCCCACATTGGCTCCTGAGTCT	14	T	0.252	0.381
NC_005102.4-95093150	CTACACAGCACTAGCTCTCCCTGACCC	1	C	0	0.4864
NC_005102.4-95093150	CTACACAGCACTAGCTCTCCCTGACCC	27	C	0.0439	0.0595
NC_005102.4-95095924	CCTGTCAGCACTGTGGCTCCAAACACC	6	C	0.0756	0.1609
NC_005102.4-95095924	CCTGTCAGCACTGTGGCTCCAAACACC	27	C	0.0417	0.0408
NC_005102.4-95129374	CGTCAAAACACTGTGGCTCCAGGCACC	14	T	0	0.4715
NC_005102.4-95129374	CGTCAAAACACTGTGGCTCCAGGCACC	16	G	0.0263	0.0758
NC_005102.4-95135677	TACAATCCGACCACACCTCCCTGGATC	12	C	0	0.4793
NC_005102.4-95160070	CTGGAGAAGACTGATTCTCCCTCTCTC	1	C	0.4911	0.3705
NC_005102.4-95160070	CTGGAGAAGACTGATTCTCCCTCTCTC	8	A	0.0345	0.0339
NC_005102.4-95189780	CCTTGCACTACTGTGTCTCCATCTCCA	5	G	0.082	0.0935
NC_005102.4-95219962	TCATTTAGCACAAGAACTCCAGAGGTG	24	G	0.0345	0.0504
NC_005102.4-95248735	TCCCATGGTACCGTTCCTCCCCCTTAT	12	C	0.0244	0.0241
NC_005102.4-95303419	TTGGCCAACACTGTTTCTCCGTTCCTA	21	G	0.069	0.0825
NC_005102.4-95303833	AATGATCGCACTTAACCTCCAAGCTTT	7	C	0.1626	0.4997
NC_005102.4-95324009	GCAGAAGTCACACGGCCTCCTCTAAGT	13	C	0.0407	0.0553
NC_005102.4-95350827	CTCCCTAAGACCCTCTCTCCCATCCTT	3	C	0	0.4807
NC_005102.4-95350827	CTCCCTAAGACCCTCTCTCCCATCCTT	6	T	0	0.4807
NC_005102.4-95363045	GCTAGATGAACCTTAACTCCTGAGCTA	21	T	0.0579	0.4951
NC_005102.4-95373265	TGACAGGCTACTGAACCTCCTGGCAGC	3	A	0.0333	0.0328
NC_005102.4-95373265	TGACAGGCTACTGAACCTCCTGGCAGC	27	C	0.0168	0.4743
NC_005102.4-95527330	TTCAGTGTGACACCCTCTCCTCTGCCT	5	G	0.1818	0.318
NC_005102.4-95527330	TTCAGTGTGACACCCTCTCCTCTGCCT	25	C	0	0.4703
NC_005102.4-95581931	CCTGCTCTAACCTACCCTCCCAAATTA	2	C	0.0268	0.0264
NC_005102.4-95581931	CCTGCTCTAACCTACCCTCCCAAATTA	27	A	0	0.4807
NC_005102.4-95582466	GTCTGAAGCACCGGTTCTCCCACCACT	13	G	0.0351	0.0345
NC_005102.4-95596939	ATTATGGGAACACTTGCTCCCTCCAAA	5	T	0	0.4772
NC_005102.4-95654047	CACACACATACAAGTACTCCTAGAATA	16	A	0.0084	0.4829
NC_005102.4-95686576	CAGTTTATTACTTTCCCTCCTCAAACA	1	C	0.0738	0.071
NC_005102.4-95686576	CAGTTTATTACTTTCCCTCCTCAAACA	3	G	0.041	0.071
NC_005102.4-95699018	TTCCACACAACCAGATCTCCAGGCGTG	13	A	0.1885	0.4126
NC_005102.4-95701548	CCCACTCCCACTCGTGCTCCTGGCGCC	23	G	0.1538	0.1975
NC_005102.4-95717762	ACCACCACTACCCCGCCTCCAATGTGC	7	A	0.2155	0.4108
NC_005102.4-95723131	AGGGAGAAAACACCAACTCCATCAGCT	8	A	0.0088	0.4547
NC_005102.4-95734571	TTCCCACAGACGCTTGCTCCTGTGTGT	12	G	0.0732	0.0854
NC_005102.4-95734571	TTCCCACAGACGCTTGCTCCTGTGTGT	21	T	0.1545	0.4967
NC_005102.4-95749022	CGTGCATGAACAAGCTCTCCTCTACTG	2	G	0	0.4773
NC_005102.4-95751838	GGACCTAGTACTGCGTCTCCCTTACTT	15	G	0.1405	0.5
NC_005102.4-95780871	GGCCCCAGCACCAGAGCTCCCTGGATG	24	G	0.1057	0.1695
NC_005102.4-95819718	CAACTTGGCACCTCCCCTCCATTTCAA	8	G	0.0738	0.071
NC_005102.4-95819718	CAACTTGGCACCTCCCCTCCATTTCAA	27	A	0	0.4751
NC_005102.4-95822356	AGTACTGCCACATCCACTCCTTGCCTG	2	G	0.1538	0.1563
NC_005102.4-95822356	AGTACTGCCACATCCACTCCTTGCCTG	22	T	0.2155	0.4643
NC_005102.4-95853416	TCTTTCCCTACTCTGACTCCATGTCAG	4	T	0.0085	0.4823
NC_005102.4-95994676	CAGTTCCTCACAAGGCCTCCTTACTTC	6	C	0.0244	0.0241
NC_005102.4-95998188	GGAAGCAAGACACGGTCTCCGTGCTCC	1	G	0	0.4807
NC_005102.4-95998188	GGAAGCAAGACACGGTCTCCGTGCTCC	21	G	0.123	0.1839
NC_005102.4-96012154	CACACACACACACAGACTCCTGGCACT	13	C	0.0431	0.0422
NC_005102.4-96051715	TGTACAGACACCAACCCTCCCAGTGCT	3	T	0.025	0.0247

NC_005102.4-96075360	CAACATCAAACCTAAGCTCCAGACTCT	26	C	0.0248	0.0245
NC_005102.4-96075360	CAACATCAAACCTAAGCTCCAGACTCT	27	T	0.0413	0.0405
NC_005102.4-96101352	TCTGCCTCCACTGATTCTCCAGATATA	26	T	0.1624	0.2299
NC_005102.4-96163284	ATAGTCACTACCTGTTCTCCATCTGGT	3	A	0.0982	0.1093
NC_005102.4-96163284	ATAGTCACTACCTGTTCTCCATCTGGT	5	T	0.0982	0.1093
NC_005102.4-96182754	ATCATAAGGACATGTCCTCCACTGTGT	2	T	0.125	0.2941
NC_005102.4-97185237	CAAGGCTCAACTCTGTCTCCTAAAACC	7	T	0.0086	0.4688
NC_005102.4-97185237	CAAGGCTCAACTCTGTCTCCTAAAACC	12	T	0.2414	0.4463
NC_005102.4-97187334	TTAAATCAAACCTCCCCTCCAAAGCCC	25	C	0.0246	0.0243
NC_005102.4-97215113	TCTCCAGCAACCTTGCCTCCCTAGGTT	3	T	0	0.48
NC_005102.4-97215113	TCTCCAGCAACCTTGCCTCCCTAGGTT	7	G	0	0.48
NC_005102.4-97248723	TAGGAGAAAACCCAGTCTCCTGTCCTG	3	G	0.0331	0.0325
NC_005102.4-97289373	CTTATCATGACAATGACTCCTTTGGCA	26	C	0.0965	0.4981
NC_005102.4-97319593	ATGTGTGGCACACTGCCTCCAATACTC	1	A	0.0536	0.4922
NC_005102.4-97324315	GAAATGTTTACCCATTCTCCTTACAGG	14	A	0	0.4731
NC_005102.4-97324315	GAAATGTTTACCCATTCTCCTTACAGG	22	T	0	0.4731
NC_005102.4-97345304	CCAGTCATGACTGTCCCTCCCATTGGT	2	C	0.0336	0.033
NC_005102.4-97345304	CCAGTCATGACTGTCCCTCCCATTGGT	24	T	0.0756	0.0728
NC_005102.4-97353253	TTCAACAAGACCATACCTCCCTATCTC	2	T	0.0991	0.4959
NC_005102.4-97401208	TACTCATCTACATCATCTCCCTCAGAT	1	T	0.0087	0.4704
NC_005102.4-97401208	TACTCATCTACATCATCTCCCTCAGAT	2	A	0.0776	0.1058
NC_005102.4-97452547	GACTGGCTGACTGATGCTCCTGCCTTT	2	A	0.0492	0.4952
NC_005102.4-97489012	AATAACCCTACTCTTTCTCCATTAAAG	12	T	0.0917	0.1025
NC_005102.4-97507801	TGATGACAAACATTACCTCCTCCTTAT	25	T	0.0328	0.0322
NC_005102.4-97521202	AGAAGAGTCACCAGTGCTCCTTTTGCT	2	G	0.0083	0.4835
NC_005102.4-97521202	AGAAGAGTCACCAGTGCTCCTTTTGCT	9	C	0.1513	0.3771
NC_005102.4-97545127	TGCCATTGGACTACATCTCCAGCCTCA	23	C	0.0656	0.0786
NC_005102.4-97545338	TGTGGTTCTACCTCCCCTCCTCTCACA	6	T	0.0254	0.4842
NC_005102.4-97545338	TGTGGTTCTACCTCCCCTCCTCTCACA	27	A	0.0336	0.033
NC_005102.4-97660594	TTTCCATAAACTTTTCCTCCTTCCCAG	3	T	0.0256	0.0253
NC_005102.4-97665269	AAGTTTCTGACCCCTGCTCCAAAGGAA	25	G	0.2479	0.2759
NC_005102.4-97670079	CCCTACCATACTGTGCCTCCTTTGAAT	6	C	0	0.4807
NC_005102.4-97670079	CCCTACCATACTGTGCCTCCTTTGAAT	9	T	0	0.4807
NC_005102.4-97682449	GATTCACAAACCACCACTCCCGTGCCT	6	A	0.0259	0.0422
NC_005102.4-97682449	GATTCACAAACCACCACTCCCGTGCCT	21	C	0.0259	0.0422
NC_005102.4-97685999	AACCCTGTCACATCAGCTCCATGGTTT	12	A	0.115	0.4986
NC_005102.4-97688624	GCACTCCTCACCCCCTCTCCCACACAG	3	A	0	0.4688
NC_005102.4-97765167	CCCCACACTACTCTTACTCCCTGGATA	12	T	0.0902	0.4943
NC_005102.4-97774620	GCATCTCAGACACACACTCCAGAACAG	14	A	0.0407	0.0398
NC_005102.4-97783819	CACACACACACCCATCCTCCTGTGTCT	12	C	0.0417	0.0566
NC_005102.4-97838179	ACCACTTGAACCATCTCTCCAGCTCAT	13	A	0.1488	0.4972
NC_005102.4-97863785	GGATCTTACACACTTGCTCCTCAGACC	9	C	0.1161	0.1843
NC_005102.4-97863785	GGATCTTACACACTTGCTCCTCAGACC	24	G	0.0796	0.1686
NC_005102.4-97873004	GAACTGTGAACATTACCTCCCTTGACA	25	A	0.1597	0.2852
NC_005102.4-97941157	GGCTATCTTACTATCTCTCCATCTTCT	12	T	0.0336	0.4898
NC_005102.4-98075051	AACAAGATCACCATTACTCCACCCTGG	12	C	0.0984	0.0935
NC_005102.4-98096209	CTCTGTGGTACATCTCCTCCTTTGATT	24	G	0.2328	0.4916
NC_005102.4-98139748	GATCCACCCACCTTTCCTCCACATCTC	14	T	0	0.4755
NC_005102.4-98159099	TCTATTAGAACGAGAGCTCCATGGAAT	6	T	0.0598	0.058
NC_005102.4-98211346	ATGGGCTGGACCTAGCCTCCCGACCCC	22	G	0.0446	0.0436
NC_005102.4-98211346	ATGGGCTGGACCTAGCCTCCCGACCCC	25	C	0.2054	0.4665

NC_005102.4-98254730	AGAGCTGATACAGGTCCTCCTCATATG	24	T	0.0246	0.0243
NC_005102.4-98262560	CAGTGATAAACATCATCTCCACTTTTA	3	G	0	0.4755
NC_005102.4-98289086	CCATAAGGAACTGGCGCTCCTTGCAGT	15	C	0	0.4734
NC_005102.4-98294801	CACTTTCCCACCATGCCTCCCTGCTTC	27	C	0.0579	0.4835
NC_005102.4-98314111	GGAAGCCCTACCTCTTCTCCCTAGCCA	6	C	0	0.477
NC_005102.4-98414933	ATCATAGACACAGCTGCTCCCACTGTT	21	C	0.115	0.1239
NC_005102.4-98433036	TTCTTTGTTACTCAATCTCCAAGGCCT	5	T	0	0.4793
NC_005102.4-98433036	TTCTTTGTTACTCAATCTCCAAGGCCT	26	C	0.0588	0.491
NC_005102.4-98434003	ATACAAGTAACAAACTCTCCTTTTAGT	6	A	0	0.4772
NC_005102.4-98481277	ATTACAGATACATCTGCTCCTGTATCC	16	G	0.0609	0.4878
NC_005102.4-98490170	CACATTCCAACTTAATCTCCTACAACT	2	A	0	0.4833
NC_005102.4-98511403	ATTGGAACTACTAGCCCTCCCTCCCTT	12	T	0	0.4743
NC_005102.4-98532859	ACTTCTGGCACTAGCTCTCCTGAGCAC	15	C	0	0.48
NC_005102.4-98532859	ACTTCTGGCACTAGCTCTCCTGAGCAC	16	T	0	0.4786
NC_005102.4-98707935	TAACCTGTCACTGGACCTCCATATTCT	2	A	0.0976	0.1216
NC_005102.4-98707935	TAACCTGTCACTGGACCTCCATATTCT	3	A	0.0976	0.1216
NC_005102.4-98710587	CTTACAGTCACCTTGGCTCCTACCACA	7	G	0.0776	0.1058
NC_005102.4-98764729	TGAAGAGATACTTATCCTCCATGTTCA	1	T	0.0588	0.4886
NC_005102.4-98764729	TGAAGAGATACTTATCCTCCATGTTCA	2	G	0.1933	0.4987
NC_005102.4-98811266	AACAACTGAACATGGCCTCCCCTTCTT	6	C	0	0.4819
NC_005102.4-98811266	AACAACTGAACATGGCCTCCCCTTCTT	23	T	0.1488	0.4426
NC_005102.4-98839123	CTTGCCTCGACCCAAGCTCCAGTCCAC	8	C	0.2358	0.481
NC_005102.4-98839123	CTTGCCTCGACCCAAGCTCCAGTCCAC	25	C	0.252	0.4703
NC_005102.4-98875175	CTATGGTCTACAGCTCCTCCAGAGTGA	1	C	0.025	0.0408
NC_005102.4-98875175	CTATGGTCTACAGCTCCTCCAGAGTGA	16	C	0	0.4779
NC_005102.4-98941620	ATGGGCTGGACCCAGGCTCCATACATA	15	G	0.0351	0.0345
NC_005102.4-98941620	ATGGGCTGGACCCAGGCTCCATACATA	22	T	0	0.4755
NC_005102.4-99226361	ATTACAGAGACACATGCTCCACTATGA	4	A	0.0339	0.0496
NC_005102.4-99282292	TGAGACCTCACCAGAGCTCCCAGGGAC	7	C	0.4262	0.3447
NC_005102.4-99412905	CAGCATCTCACCCTAACTCCCTCTTAG	6	T	0.177	0.3242
NC_005102.4-99412905	CAGCATCTCACCCTAACTCCCTCTTAG	14	T	0	0.4793
NC_005102.4-99428799	TAAGAAAGGACTCATTCTCCTGTGTGA	5	A	0.0177	0.4859
NC_005102.4-99484607	ACTTGAAGTACAGTCTCTCCCTGTGAA	1	A	0.0172	0.4786
NC_005102.4-99487685	ATCCACTGAACCATGTCTCCAGGAGCC	6	C	0.0982	0.3211
NC_005102.4-99487685	ATCCACTGAACCATGTCTCCAGGAGCC	25	G	0.0268	0.0264
NC_005102.4-99537617	TATGGTTAAACCACGTCTCCCACCTAA	5	G	0.0087	0.4817
NC_005102.4-99598557	CCCAGGAGTACTCTCACTCCTGAGCCC	16	A	0.2377	0.375
NC_005102.4-99631076	ACCAACTGCACTATTGCTCCAAGTCTC	23	G	0	0.4682
NC_005102.4-99676775	TAACATTCTACCATGACTCCGTTTGGT	12	C	0.0164	0.0322
NC_005102.4-99745644	ATTTATTACACAGTCTCTCCTAAGAAA	9	C	0.0442	0.0765
NC_005102.4-99767846	TGAGGGACAACTTCACCTCCATGAATA	27	A	0.0089	0.4512
NC_005102.4-99783645	ATATTAAATACCTCCTCTCCCATGTGT	4	T	0	0.4827
NC_005102.4-99783645	ATATTAAATACCTCCTCTCCCATGTGT	22	A	0	0.4827
NC_005102.4-99787233	TTCTCTTCTACCAAAGCTCCAAGGATT	12	C	0.0435	0.059
NC_005102.4-99807043	AGAAATGGTACTTGTCCTCCAGGGATA	7	G	0.082	0.0935
NC_005102.4-99807043	AGAAATGGTACTTGTCCTCCAGGGATA	9	T	0.0492	0.0634
NC_005102.4-99842933	TTCCAAACCACTGTTGCTCCTTCAAAT	8	C	0	0.4807
NC_005102.4-99898627	CACAACCTCACTGCAGCTCCTAGGAAT	2	A	0.0085	0.4714
NC_005102.4-99898627	CACAACCTCACTGCAGCTCCTAGGAAT	21	T	0.0085	0.4714
NC_005102.4-99908420	GTTGTATGCACTTCTCCTCCTTCATGC	7	T	0.0084	0.4829
NC_005102.4-99908420	GTTGTATGCACTTCTCCTCCTTCATGC	8	G	0.0084	0.4829

NC_005102.4-99959703	GGACCTAGGACTGTGACTCCTGTATTA	21	T	0	0.4793
NC_005102.4-100056484	GAAAGCGGGACAAGGACTCCTTATATC	12	A	0.0168	0.4703
NC_005102.4-100056484	GAAAGCGGGACAAGGACTCCTTATATC	27	C	0.0168	0.0491
NC_005102.4-100082267	ATTTTAAAGACAGTCCCTCCTTAATCT	5	T	0	0.4743
NC_005102.4-100082267	ATTTTAAAGACAGTCCCTCCTTAATCT	25	T	0	0.4743
NC_005102.4-100122251	CCTATTATTACCTACTCTCCTCACTTT	2	C	0.1667	0.4997
NC_005102.4-100122251	CCTATTATTACCTACTCTCCTCACTTT	27	T	0.0087	0.4782
NC_005102.4-100232631	ATACCCTACACGCCAACTCCTGTATTA	23	T	0.0325	0.0476
NC_005102.4-100237874	TGCATGACGACCCCAGCTCCAATGGAG	8	C	0.1008	0.1107
NC_005102.4-100257479	CACACACACACACACACTCCTAAGTTA	7	C	0.0902	0.0861
NC_005102.4-100307494	AAGTGGAGTACTCATCCTCCCTCAAGG	25	A	0.0796	0.4843
NC_005102.4-100317582	TCATTTTGCACACATTCTCCCTATAGG	14	A	0.0783	0.0911
NC_005102.4-100317582	TCATTTTGCACACATTCTCCCTATAGG	24	T	0.1379	0.1431
NC_005102.4-100318936	CAAGCTGCTACCTAATCTCCTACCAAT	4	G	0.1593	0.1613
NC_005102.4-100318936	CAAGCTGCTACCTAATCTCCTACCAAT	5	C	0.292	0.3294
NC_005102.4-100345770	ATCTTAACGACATTAACTCCAGTGGGA	4	T	0.3033	0.3909
NC_005102.4-100345770	ATCTTAACGACATTAACTCCAGTGGGA	9	G	0.0413	0.0405
NC_005102.4-100437334	CATGGGTACACATGCCCTCCTCACAGA	7	T	0.252	0.3091
NC_005102.4-100528739	TGTAACAGTACACATGCTCCACTATGT	14	A	0.0248	0.0245
NC_005102.4-100544003	ACTTTCCCCACCCCCACTCCGAGTCTG	5	T	0.0893	0.1626
NC_005102.4-100551160	TACTGCAATACTGCATCTCCAGTAGAC	13	G	0.2857	0.4082
NC_005102.4-100551955	TGCTCCTCCACTCACTCTCCCACTACT	15	C	0.281	0.4041
NC_005102.4-100584460	ATTCATGGCACTCTGACTCCTCTCTGA	2	T	0	0.4839
NC_005102.4-100639797	GGCCCATCAACACTAGCTCCCACATTC	1	G	0.2417	0.4025
NC_005102.4-100639797	GGCCCATCAACACTAGCTCCCACATTC	16	G	0.0082	0.4822
NC_005102.4-100640492	TATCAACTAACCTGACCTCCCAGAGCA	14	G	0.2054	0.1843
NC_005102.4-100645452	AGCCTCCAGACAATACCTCCTTTGAAT	15	A	0.0259	0.4688
NC_005102.4-100675160	ACCAGAAGCACAGCTTCTCCTCGGGGT	23	G	0.0333	0.4861
NC_005102.4-100675160	ACCAGAAGCACAGCTTCTCCTCGGGGT	24	G	0.0333	0.4861
NC_005102.4-100721031	CTCTGGTGCACCAGCACTCCCATAGTC	9	C	0	0.48
NC_005102.4-100721031	CTCTGGTGCACCAGCACTCCCATAGTC	21	C	0.1826	0.3353
NC_005102.4-100757500	CAAAGAAAAACTACCTCTCCTGTGTGT	6	A	0.0088	0.483
NC_005102.4-100766103	ACCAAGAGAACAGATTCTCCTCAGGTC	27	C	0.0932	0.0889
NC_005102.4-100779160	GCAGTCCTCACCACATCTCCCCACAAT	1	G	0.2397	0.4557
NC_005102.4-100796966	CTCTTCCTCACTGTTACTCCAACATGT	7	C	0.0088	0.4529
NC_005102.4-100796966	CTCTTCCTCACTGTTACTCCAACATGT	15	T	0.2261	0.4537
NC_005102.4-100804124	AGAGTTAGCACACATTCTCCCTTAGGG	15	T	0.1789	0.464
NC_005102.4-100822727	CTTTCCAAGACTCCAGCTCCCCTACCT	16	G	0.0756	0.0882
NC_005102.4-100874547	CCCCCTCCCACCAAGTCTCCAAACCTA	23	A	0.0756	0.1181
NC_005102.4-101029851	ACTGGATGAACTGGGGCTCCTCTGATG	16	G	0.2083	0.225
NC_005102.4-101032744	CCAGCCCCAACCATTCCTCCCCACCTA	14	T	0.2034	0.2219
NC_005102.4-101043968	CCACATTCCACTAGTTCTCCCCATCCC	25	C	0	0.4773
NC_005102.4-101064169	TGAGTTTGTACAAATCCTCCAGCCTCA	13	A	0.0089	0.4751
NC_005102.4-101104271	TTCTGTGAGACATTTCCTCCCTGCAAT	4	T	0	0.4764
NC_005102.4-101104271	TTCTGTGAGACATTTCCTCCCTGCAAT	5	G	0	0.4846
NC_005102.4-101119749	ACCACCACCACCACCACTCCTCTGGAT	27	T	0.0339	0.0333
NC_005102.4-101121324	AATAGCCGAACCATTTCTCCAGCTCCT	7	C	0	0.474
NC_005102.4-101187350	TGGAGGCTCACTCTTCCTCCTGCAAGT	9	C	0.0336	0.033
NC_005102.4-101299373	CTCTCTCCAACTCCAACTCCAGCAGCT	3	C	0.2295	0.4979
NC_005102.4-101459693	CAAGTTTTCACACAGTCTCCTCTATTT	16	T	0.0086	0.4768
NC_005102.4-101589515	AGTATTTTCACGGTGTCTCCCTCTAAG	12	G	0.0088	0.483

NC_005102.4-101589515	AGTATTTTCACGGTGTCTCCCTCTAAG	23	C	0.1404	0.3221
NC_005102.4-101606880	ATATGAGACACATAAACTCCAATATTC	1	A	0.0087	0.4744
NC_005102.4-101796032	CCAATTGCAACCACACCTCCTTCTGTA	5	T	0	0.4793
NC_005102.4-101810021	CGAGTTCTTACCTTAACTCCTGAGTTA	1	C	0.2114	0.2382
NC_005102.4-101810021	CGAGTTCTTACCTTAACTCCTGAGTTA	16	A	0.0163	0.0476
NC_005102.4-101852042	AAATAAAATACTCTATCTCCTGCTTGG	15	A	0.281	0.4481
NC_005102.4-101857237	ATCTACACGACTATAACTCCTCTGTTC	6	C	0.0522	0.0508
NC_005102.4-101857237	ATCTACACGACTATAACTCCTCTGTTC	8	C	0.0087	0.4744
NC_005102.4-101891611	AGTAACGGAACCACGCCTCCAAATATG	9	A	0.0086	0.4836
NC_005102.4-101897540	GACGATGCTACCACCCCTCCCCCCTCA	3	C	0	0.4793
NC_005102.4-101897540	GACGATGCTACCACCCCTCCCCCCTCA	7	G	0.0488	0.0629
NC_005102.4-101905818	TCCAATTCTACACATTCTCCATTGTCG	12	A	0.2035	0.1968
NC_005102.4-102076665	CATTTAGGCACTTGCTCTCCACAGTAC	15	C	0.3051	0.424
NC_005102.4-102076665	CATTTAGGCACTTGCTCTCCACAGTAC	16	T	0	0.4765
NC_005102.4-102101958	GTTCAAGGTACAATTTCTCCTGGCATA	13	A	0	0.4734
NC_005102.4-102106856	TTGAAGTAAACTGGCTCTCCCTCACCT	15	C	0.075	0.0722
NC_005102.4-102165447	AACACTAACACCGAGCCTCCAGGACAT	12	C	0.0268	0.0264
NC_005102.4-102309220	ACTGGTCTTACAGGAGCTCCTAAGCCC	25	C	0.2213	0.311
NC_005102.4-102309220	ACTGGTCTTACAGGAGCTCCTAAGCCC	27	C	0.0902	0.0861
NC_005102.4-102364913	TGCCCACCAACCAGAGCTCCAAGGCAT	27	T	0.0813	0.4926
NC_005102.4-102396866	CCTAGTGACACAAGTCCTCCAACAAGG	4	A	0.0783	0.0752
NC_005102.4-102647785	GTCTACCGTACAGTGGCTCCAGATGTT	2	T	0.0084	0.4797
NC_005102.4-102773455	ACCAAAAGTACCATCTCTCCTCCAGCA	9	T	0.0082	0.4755
NC_005102.4-102870183	ATTCCAATAACTTAGGCTCCAAGATCA	16	G	0.2241	0.3572
NC_005102.4-102871803	TGCAGATCTACTCTCCCTCCATTTCCT	2	G	0.2377	0.2573
NC_005102.4-102994875	CCAAACATCACATGTCCTCCTTCGGTC	24	G	0.2	0.375
NC_005102.4-103062728	TCTTTAAATACCCTTTCTCCCAGCCTC	16	T	0.0261	0.0257
NC_005102.4-103062728	TCTTTAAATACCCTTTCTCCCAGCCTC	24	C	0.2162	0.1928
NC_005102.4-103106874	CAGTAAGGGACAGGACCTCCCTGAGAT	27	T	0	0.4912
NC_005102.4-103600855	CCAGAGGACACGGGACCTCCTGTCATG	9	C	0.0574	0.1009
NC_005102.4-103678526	ACCCCACCCACCAGGTCTCCCCACTCT	16	T	0.55	0.42
NC_005102.4-103706114	TCTGGACTCACACACTCTCCTTCCGTC	25	G	0.0427	0.058
NC_005102.4-103850894	TCAACTGTGACACCACCTCCACGGCCC	3	A	0	0.4688
NC_005102.4-103850894	TCAACTGTGACACCACCTCCACGGCCC	4	A	0.1964	0.4943
NC_005102.4-103851092	ATTTGAGGCACTTGACCTCCATGCCCA	12	T	0	0.4819
NC_005102.4-103859633	ATTAGCCTGACTCTATCTCCTAGGATT	23	G	0	0.4807
NC_005102.4-103957213	TCTGAAATTACTTTGTCTCCTGGAGGA	4	G	0.0256	0.4823
NC_005102.4-103974837	CCTCCTCTCACCCTCCCTCCCCACTTG	4	C	0.3846	0.3504
NC_005102.4-103974837	CCTCCTCTCACCCTCCCTCCCCACTTG	13	C	0.3697	0.3014
NC_005102.4-104102393	TAAGGATGCACTTCTCCTCCTGGCCGT	25	C	0.1301	0.1216
NC_005102.4-104114201	GCTATTGACACAGAATCTCCAGGTGTC	25	G	0.1491	0.138
NC_005102.4-104175957	TGCAAGTTCACTTTCCCTCCTATAGGT	24	A	0.0248	0.0245
NC_005102.4-104189688	TGAAGGTAGACTGGGTCTCCTCAGACC	14	G	0.124	0.4951
NC_005102.4-104222934	GGCAGACCCACACAGGCTCCCTGATTG	1	G	0.0244	0.0241
NC_005102.4-104222934	GGCAGACCCACACAGGCTCCCTGATTG	16	G	0.0246	0.0243
NC_005102.4-104255676	AGCTATTGCACAATCACTCCGGATTTA	21	G	0.2613	0.3012
NC_005102.4-104343974	ATGCACCCGACCACATCTCCTGGCTGC	9	G	0.0261	0.0257
NC_005102.4-104350699	TGCCTTTGTACATGGCCTCCAACCCTC	13	T	0.0325	0.032
NC_005102.4-104350699	TGCCTTTGTACATGGCCTCCAACCCTC	21	A	0.0163	0.4854
NC_005102.4-104434223	TTCTGCCCCACACTCCCTCCAATGATA	26	T	0.0684	0.0818
NC_005102.4-104471564	CCCAGTAAGACAGGCACTCCTGAGGCA	7	A	0.0085	0.0251

NC_005102.4-104471564	CCCAGTAAGACAGGCACTCCTGAGGCA	26	C	0.2982	0.4321
NC_005102.4-104512360	TTGCACCATACCAAGGCTCCTTCTTTA	27	A	0	0.4778
NC_005102.4-104515206	ACCTTCCTAACTCCATCTCCTAACTCA	2	C	0.0252	0.0249
NC_005102.4-104519121	TTCTTCTCTACCAGTTCTCCATTTTTC	14	G	0.0085	0.4655
NC_005102.4-104519121	TTCTTCTCTACCAGTTCTCCATTTTTC	23	T	0	0.4677
NC_005102.4-104519862	CACCAGGCTACCTCTCCTCCCAGCAGT	24	C	0.0339	0.4856
NC_005102.4-104580673	CATGTCCATACCAGCTCTCCCAGCCAC	27	C	0.1121	0.4997
NC_005102.4-104658440	TCCAGTGCAACTCCTACTCCTTTGGGA	23	T	0.1217	0.2873
NC_005102.4-104671694	GACAGCAGTACTGTTTCTCCATGTGTG	25	G	0.0417	0.0408
NC_005102.4-104784059	GAGGCCACAACCAAATCTCCTTTTAAC	16	T	0.1721	0.2903
NC_005102.4-104794271	TAAAGTAACACAGTATCTCCCTAAGTG	8	A	0	0.4821
NC_005102.4-104809272	GTGCCTCCCACCACTTCTCCTTGCCCT	15	T	0.252	0.3646
NC_005102.4-105001084	CCACCTCCAACTGTGGCTCCAGACGAT	16	G	0.1391	0.5
NC_005102.4-105124896	ATCACCTGTACTAACACTCCACCTGTG	5	C	0.1607	0.4362
NC_005102.4-105129931	TCAGACTTCACTGCTCCTCCCAACACA	6	C	0.036	0.4785
NC_005102.4-105129970	AATAAAACTACAGTGGCTCCTGGGTGA	12	A	0.0336	0.4844
NC_005102.4-105156225	AACTCCAAGACAGTTTCTCCATCTATT	27	T	0	0.4891
NC_005102.4-105160316	GTAAACTTGACACAGCCTCCTTCATTT	4	A	0.0081	0.4777
NC_005102.4-105168259	GCTCTCCTCACCTTTCCTCCCTGCACT	24	C	0.1008	0.1254
NC_005102.4-105216751	CCACGGTCCACTTCATCTCCCCGTCAT	22	C	0.0081	0.4777
NC_005102.4-105229493	CAAGGTGTAACACCCTCTCCAAAACAA	8	T	0	0.4703
NC_005102.4-105270176	AGCCCTGTGACCCTAACTCCTGTCACT	6	T	0.3008	0.3888
NC_005102.4-105272343	TATCTCTGGACCGGTACTCCTGGGTTC	8	G	0	0.4826
NC_005102.4-105272343	TATCTCTGGACCGGTACTCCTGGGTTC	21	T	0.1695	0.2585
NC_005102.4-105353819	CTGAGAGCTACTTCCTCTCCCATTGTT	27	T	0.2833	0.375
NC_005102.4-105372396	GTGACATTTACTGGGCCTCCAGTGTTC	23	T	0	0.4677
NC_005102.4-105487824	TATCCACGCACTGATGCTCCCAGTGTC	2	A	0.1639	0.5
NC_005102.4-105487824	TATCCACGCACTGATGCTCCCAGTGTC	7	C	0.1721	0.4997
NC_005102.4-105496447	GCATCATTGACCAAGGCTCCATTCACC	16	G	0.2456	0.3706
NC_005102.4-105505765	ATCCCCCTAACCCTTCCTCCTCTCCTT	1	A	0.0776	0.1058
NC_005102.4-105511015	ATGTTATATACTTCATCTCCGCAGCAG	25	C	0.0169	0.4826
NC_005102.4-105548919	ATGTTTGATACTTGGTCTCCAGTTAGT	1	A	0.124	0.2355
NC_005102.4-105548919	ATGTTTGATACTTGGTCTCCAGTTAGT	7	G	0.2295	0.435
NC_005102.4-105560993	AAGATAACAACCAATCCTCCAGAATTC	15	T	0	0.4731
NC_005102.4-105580743	ATACTCCTCACGTCTGCTCCACCATCA	8	T	0.1667	0.2088
NC_005102.4-105628511	ATCTTAAACACTATCACTCCACTTCAT	13	A	0.0169	0.0496
NC_005102.4-105717807	CATTTAATAACACAATCTCCCAACAGA	2	A	0.0893	0.1172
NC_005102.4-105717807	CATTTAATAACACAATCTCCCAACAGA	15	A	0.0804	0.1093
NC_005102.4-105760742	CTGCTTTCCACAGATTCTCCTCCAGAC	15	T	0.0089	0.4789
NC_005102.4-105760742	CTGCTTTCCACAGATTCTCCTCCAGAC	22	C	0.0088	0.4774
NC_005102.4-105932470	ATCTTGGCCACTGCCACTCCTTTTCCT	16	A	0.1102	0.1481
NC_005102.4-105935226	TGCACTTTCACAGTATCTCCTGTTGGT	3	C	0.0708	0.0683
NC_005102.4-105935226	TGCACTTTCACAGTATCTCCTGTTGGT	13	G	0.0885	0.0846
NC_005102.4-105950540	CCAGCTCACACGTGTTCTCCAACGTTT	9	C	0.0259	0.0255
NC_005102.4-105950540	CCAGCTCACACGTGTTCTCCAACGTTT	24	G	0.2544	0.4796
NC_005102.4-106075936	AGCCCCTACACTCTATCTCCCTCCCCT	2	G	0	0.4814
NC_005102.4-106090643	GTTAAGAAAACAGTATCTCCCTGCTTC	27	C	0.0833	0.1528
NC_005102.4-106340683	TGCTTAAAGACCTCTCCTCCACTATAG	26	A	0	0.4757
NC_005102.4-106358165	TGAGGATCTACTTAGTCTCCTTGCCCA	9	T	0	0.4746
NC_005102.4-106359975	GGAAGGGTTACTCGAACTCCTTTCAAG	2	G	0.041	0.0557
NC_005102.4-106361448	TCTTCCATCACCCTCCCTCCTCCTCTG	4	T	0.2308	0.4473

NC_005102.4-106361448	TCTTCCATCACCCTCCCTCCTCCTCTG	16	C	0.084	0.0958
NC_005102.4-106397694	AACTGCAACACTCCACCTCCCACTTAA	6	C	0	0.4787
NC_005102.4-106403627	CTTGTTGATACATTGTCTCCTTCATGT	9	T	0.0164	0.0322
NC_005102.4-106413562	CAAGCTAGCACTGTTTCTCCTGCTCAG	4	G	0.0088	0.4843
NC_005102.4-106658308	AAAATGTCTACTCTCCCTCCCTTCCTA	5	T	0	0.4839
NC_005102.4-106671182	CCCAAGCCCACTCATACTCCTGCCCAT	16	A	0.1593	0.1613
NC_005102.4-106671182	CCCAAGCCCACTCATACTCCTGCCCAT	26	A	0.1607	0.1626
NC_005102.4-106701404	CCCACTCCCACCTTACCTCCCTAGCAT	1	C	0.0517	0.0504
NC_005102.4-106701404	CCCACTCCCACCTTACCTCCCTAGCAT	13	T	0.1379	0.1284
NC_005102.4-106862026	AACATGAGCACCATCCCTCCAACACAG	21	A	0.2314	0.3467
NC_005102.4-106870985	TCTACGGTCACCTTATCTCCAAAACAA	5	C	0.0439	0.0429
NC_005102.4-106874490	CTACAGAGAACGGGAGCTCCCTGTCAT	12	G	0	0.4793
NC_005102.4-106953353	AAGCAAATCACTCAGACTCCAGACCCG	27	G	0	0.4718
NC_005102.4-107007498	TCCAGGGCTACATTGTCTCCTAACTAC	24	C	0.0265	0.0262
NC_005102.4-107008845	GTGGAGAAAACTGATGCTCCCTCTCTT	12	T	0.0084	0.4797
NC_005102.4-107130833	CTGTCTTTGACTATATCTCCCCTGATC	22	C	0.2269	0.4117
NC_005102.4-107158478	CAGCCTGAGACCCAAGCTCCTGCTGAG	15	A	0	0.4793
NC_005102.4-107201252	CAAATAAACACCCAAGCTCCCATGTTT	3	A	0.0089	0.4617
NC_005102.4-107201252	CAAATAAACACCCAAGCTCCCATGTTT	4	A	0.0088	0.4648
NC_005102.4-107224468	CCATACAACACTGGAACTCCAATGAAT	14	G	0.0492	0.1505
NC_005102.4-107224468	CCATACAACACTGGAACTCCAATGAAT	15	A	0	0.4751
NC_005102.4-107228959	AACAGTCTTACCTATACTCCTCACCTG	2	A	0.2437	0.4592
NC_005102.4-107280776	TCCTTTTCTACCCATGCTCCATGTGTT	25	G	0.065	0.1356
NC_005102.4-107282092	ATTGCTCTGACCACGCCTCCCTCCATT	7	C	0.1074	0.1163
NC_005102.4-107306040	GAAATGGGGACATGATCTCCTAGGTAC	26	A	0.0769	0.4906
NC_005102.4-107341087	TCCCTGAGAACTCCAGCTCCACCTTCA	24	T	0	0.4715
NC_005102.4-107346494	CGGCCCATCACGGCTTCTCCTTTACTT	3	G	0.1724	0.4214
NC_005102.4-107346494	CGGCCCATCACGGCTTCTCCTTTACTT	26	T	0	0.4864
NC_005102.4-107491033	CTGATTGCCACCACACCTCCCTGTGAT	14	C	0.2439	0.3427
NC_005102.4-107586033	GCCCGGGCCACGCCTCCTCCAGCTGAT	15	T	0	0.4773
NC_005102.4-107595060	TGCGTGCGTACACACACTCCACATATT	12	A	0.0732	0.1425
NC_005102.4-107614849	ATGTTTTACACACTATCTCCATAAATA	2	T	0.0862	0.0825
NC_005102.4-107614849	ATGTTTTACACACTATCTCCATAAATA	21	A	0.0598	0.4714
NC_005102.4-107669811	TTCTTTACCACGTAGCCTCCTTCCCCA	3	C	0.041	0.0401
NC_005102.4-107705486	AGATGCCAAACCTTTACTCCTCCCTGT	26	G	0.2982	0.4186
NC_005102.4-107715754	TGCTACAGCACTGCCACTCCAGCCTTG	1	T	0.2195	0.4979
NC_005102.4-107715754	TGCTACAGCACTGCCACTCCAGCCTTG	6	C	0.2439	0.3688
NC_005102.4-107736130	TCACATACTACCTGTTCTCCTGGTCAG	2	C	0.2119	0.2871
NC_005102.4-107736130	TCACATACTACCTGTTCTCCTGGTCAG	23	G	0.0085	0.479
NC_005102.4-107768579	CTCTCTCCCACCCTCTCTCCTCCATAC	24	A	0.2941	0.4638
NC_005102.4-107768579	CTCTCTCCCACCCTCTCTCCTCCATAC	26	A	0	0.4819
NC_005102.4-107773745	ACGACAGAAACTTCAACTCCTTAGGTT	3	G	0.0087	0.4782
NC_005102.4-107773745	ACGACAGAAACTTCAACTCCTTAGGTT	12	T	0.0088	0.4774
NC_005102.4-107775047	ATGAGGCAAACCTCATCTCCATCTCAT	16	T	0	0.4787
NC_005102.4-107775047	ATGAGGCAAACCTCATCTCCATCTCAT	27	T	0	0.4773
NC_005102.4-107830519	TCATGAGGCACCTCTCCTCCTGTGCTG	4	T	0	0.4793
NC_005102.4-107840684	TTGTCTGTAACTAAGGCTCCAAAGGAC	14	A	0.0259	0.0255
NC_005102.4-107840684	TTGTCTGTAACTAAGGCTCCAAAGGAC	25	G	0	0.4637
NC_005102.4-107859113	TGCTAGACAACATAGCCTCCTTTTGAG	13	T	0.1765	0.4994
NC_005102.4-107877860	TCCTCCCATACCTCCTCTCCCATATCC	3	C	0.0263	0.026
NC_005102.4-107877860	TCCTCCCATACCTCCTCTCCCATATCC	21	C	0.0089	0.4933

NC_005102.4-107925789	TCCTCCCTCACACTTCCTCCTGTTAAT	3	C	0.0417	0.0408
NC_005102.4-108032937	TGCTCCAAAACCCGGGCTCCCACTCCA	3	C	0	0.4773
NC_005102.4-108034126	ACTAGTATCACATGGGCTCCGGTAGGA	5	G	0	0.4715
NC_005102.4-108034126	ACTAGTATCACATGGGCTCCGGTAGGA	22	G	0	0.4715
NC_005102.4-108044109	AAGTGAGAGACGCTGCCTCCAGATGTA	25	G	0.0328	0.4866
NC_005102.4-108089721	CCCCCATCCACTCTCTCTCCCCCATAG	12	T	0.2333	0.48
NC_005102.4-108317433	CAGCAACAAACTCTTGCTCCACCGAGC	27	C	0.0407	0.0398
NC_005102.4-108392436	CACCATTCCACAATTACTCCTAGTCAT	13	A	0.1765	0.3729
NC_005102.4-108415807	ATCTGCGTAACAGCCTCTCCTGTTCAT	2	T	0.1681	0.4923
NC_005102.4-108415807	ATCTGCGTAACAGCCTCTCCTGTTCAT	7	G	0.1053	0.1153
NC_005102.4-108476381	GACCATGCTACGCAGACTCCGAATAGC	5	A	0	0.4764
NC_005102.4-108476381	GACCATGCTACGCAGACTCCGAATAGC	14	A	0.0696	0.0671
NC_005102.4-108497758	TCCATCAGAACAGCAGCTCCTCTTGAC	8	G	0	0.4749
NC_005102.4-108529667	TGTTACGTAACTGCTTCTCCTACACCT	7	G	0.0609	0.4563
NC_005102.4-108529667	TGTTACGTAACTGCTTCTCCTACACCT	8	T	0.113	0.4976
NC_005102.4-108536121	TATGAGGACACTCCCACTCCCACCTCA	23	C	0.5862	0.4144
NC_005102.4-108572787	CTGCCACTGACCACCACTCCCTCAACA	4	C	0	0.48
NC_005102.4-108634877	GGTAGACTGACCAGCTCTCCTGCACCC	23	C	0.0435	0.0425
NC_005102.4-108642744	ACGTGTTAGACACCCCCTCCCCAACAC	6	T	0.0165	0.0943
NC_005102.4-108684599	CTGGAGAAGACTTGTTCTCCCTTTCTG	23	T	0.0083	0.4847
NC_005102.4-108693823	GCTGTCCCAACAGTCTCTCCAGCCATA	15	C	0.175	0.4233
NC_005102.4-108693823	GCTGTCCCAACAGTCTCTCCAGCCATA	26	T	0	0.4779
NC_005102.4-108697021	CATGATACAACTAAGTCTCCATCACAA	2	A	0.2131	0.2515
NC_005102.4-108697021	CATGATACAACTAAGTCTCCATCACAA	24	A	0.0246	0.0243
NC_005102.4-108709087	CATCTCCTCACTGCACCTCCGCCAATC	16	C	0.0087	0.4849
NC_005102.4-108709087	CATCTCCTCACTGCACCTCCGCCAATC	24	A	0.1217	0.4017
NC_005102.4-108769745	CATGTTGAAACTTAACCTCCAATGAAA	16	C	0	0.4807
NC_005102.4-108830504	ACCCCAGTTACGTTGTCTCCAGCAATG	27	G	0.0083	0.4769
NC_005102.4-108890869	TTCCATCCTACTGTTCCTCCTTGATGG	6	T	0	0.4827
NC_005102.4-108890869	TTCCATCCTACTGTTCCTCCTTGATGG	27	G	0.0696	0.0671
NC_005102.4-108947220	GTTAGATGCACAGAGGCTCCCAGCCAG	3	T	0.0513	0.05
NC_005102.4-108987732	CTGTGGTCCACAACCTCTCCATTCTGG	14	C	0.0331	0.0325
NC_005102.4-109020883	TGCTCTTGAACATAGTCTCCAGAGCAC	27	C	0	0.48
NC_005102.4-109050049	TGCTGGGGGACACGCCCTCCCCTCACC	2	G	0	0.4793
NC_005102.4-109050049	TGCTGGGGGACACGCCCTCCCCTCACC	8	G	0.0244	0.0241
NC_005102.4-109066531	CTGAAAATCACCTGTACTCCTTTAAAT	7	A	0.2632	0.4444
NC_005102.4-109083371	CTTACCCCTACCCTTCCTCCTTTCCCC	9	T	0	0.4841
NC_005102.4-109164758	CCCCTAAAAACACTTTCTCCATGCTTA	8	A	0.1417	0.1597
NC_005102.4-109224215	ACTCTGTTCACTTCCCCTCCATAACAG	2	C	0	0.4859
NC_005102.4-109224215	ACTCTGTTCACTTCCCCTCCATAACAG	3	T	0	0.4859
NC_005102.4-109226529	TACATCCAGACCCCAGCTCCTGCTAGC	7	C	0.314	0.3811
NC_005102.4-109250318	TTTATCCATACCCGTGCTCCAGGGTGT	14	G	0	0.4832
NC_005102.4-109277556	ACATGCTGTACCTTCCCTCCAGGGTCC	16	C	0	0.4793
NC_005102.4-109337415	ATGATGCAAACACTCTCTCCATTGGGT	4	A	0.0174	0.4864
NC_005102.4-109433887	ATTGGACCAACTGTCCCTCCATCTAGC	26	G	0.0902	0.4903
NC_005102.4-109455171	CTGCTGAGCACAGAGGCTCCTTCTTTC	22	T	0.1707	0.2989
NC_005102.4-109519177	ACAAGTGTGACCAGCTCTCCCGCCCTG	16	T	0.2832	0.406
NC_005102.4-109540249	ACATAACTAACCAGAACTCCTAACACT	7	C	0.2787	0.306
NC_005102.4-109540249	ACATAACTAACCAGAACTCCTAACACT	23	A	0	0.4773
NC_005102.4-109552544	ATGTCATATACATCTACTCCAACAATG	22	A	0.0348	0.4764
NC_005102.4-109621077	CAGGAAGCAACATTTTCTCCTCAGCCT	25	C	0.0089	0.0264

NC_005102.4-109639743	CCACACCTTACTGCATCTCCATCAAAC	13	G	0.0263	0.026
NC_005102.4-109669010	GCAGAACCCACAGAGCCTCCCCTGCCA	21	C	0	0.4746
NC_005102.4-109720065	CAAGAAACTACCCTGCCTCCTTATATC	21	T	0	0.4731
NC_005102.4-109789133	GCCTGAACTACTTGTTCTCCTTCCTCT	26	C	0.0569	0.0553
NC_005102.4-109794299	TTCTTTAAGACAAAGTCTCCTAGTGAA	1	T	0.0708	0.1006
NC_005102.4-109849794	CCACCTCCTACCTGGACTCCCACTTAC	5	C	0	0.4786
NC_005102.4-109849794	CCACCTCCTACCTGGACTCCCACTTAC	9	T	0	0.4786
NC_005102.4-109852831	CTCTCTGACACAAGTTCTCCATGGGTC	23	G	0.0336	0.033
NC_005102.4-109863449	GGCTACCCCACCCCCACTCCCAGCATT	2	G	0.1538	0.5
NC_005102.4-109863449	GGCTACCCCACCCCCACTCCCAGCATT	5	A	0	0.4693
NC_005102.4-110051453	TAACCTCGCACCTTTCCTCCTCTGGTT	8	G	0.1008	0.1107
NC_005102.4-110051453	TAACCTCGCACCTTTCCTCCTCTGGTT	16	C	0.025	0.0247
NC_005102.4-110084278	AAGTCACCCACAACCACTCCCAAGCAT	9	C	0.2276	0.4
NC_005102.4-110091100	AAAAATGAAACAGGAGCTCCAGATAAT	8	A	0.1478	0.1938
NC_005102.4-110098348	TCCACAGTCACAGCTCCTCCTGCAGGT	27	T	0.05	0.0799
NC_005102.4-110108639	GTTGTGCCCACAAAGCCTCCTGTCTAG	5	T	0.0339	0.4977
NC_005102.4-110108639	GTTGTGCCCACAAAGCCTCCTGTCTAG	16	C	0.0593	0.0889
NC_005102.4-110129179	AGCACAGTCACTCTGACTCCCTTTCTC	15	G	0.1624	0.289
NC_005102.4-110129179	AGCACAGTCACTCTGACTCCCTTTCTC	16	A	0.0085	0.481
NC_005102.4-110160424	GGGGCAATGACAGTTTCTCCCTATCTT	7	A	0.2759	0.4518
NC_005102.4-110168948	CTCCTACTCACTGTCCCTCCTTTTTAT	3	C	0	0.477
NC_005102.4-110273012	ATGTATTGCACTGCATCTCCAGCCTTG	14	C	0.1803	0.1904
NC_005102.4-110273012	ATGTATTGCACTGCATCTCCAGCCTTG	16	T	0.0246	0.0243
NC_005102.4-110280740	TGCTCTTTCACAGAATCTCCGCCTGAC	3	C	0.042	0.4761
NC_005102.4-110280740	TGCTCTTTCACAGAATCTCCGCCTGAC	21	G	0.178	0.4066
NC_005102.4-110320573	TGAGCCTTTACCCTCCCTCCACAGGAC	1	T	0	0.4807
NC_005102.4-110388826	TGTGCCTCCACTCTGGCTCCTTTTTAA	22	T	0	0.4841
NC_005102.4-110400706	GGTTCATCTACAGAGCCTCCTCACCAG	8	C	0.0084	0.4859
NC_005102.4-110400706	GGTTCATCTACAGAGCCTCCTCACCAG	9	T	0.0084	0.4859
NC_005102.4-110456558	CTACATGGCACCCGGACTCCCAGAAGC	4	C	0.0678	0.0812
NC_005102.4-110465776	CAGGTGGCCACTGCTCCTCCAAGTCAG	24	T	0	0.48
NC_005102.4-110509999	GCACACCGCACGTGAGCTCCAGGGTCT	1	G	0	0.4773
NC_005102.4-110525503	CAAGCCTGTACATAACCTCCATCTAGG	25	A	0.0619	0.4873
NC_005102.4-110584379	CTGAGCTTCACCCCCACTCCTGTGTGC	15	C	0.0593	0.0576
NC_005102.4-110588462	CATGCTGCCACTACAGCTCCCTGTCTC	3	T	0.1966	0.2171
NC_005102.4-110616201	AGGACTTGGACACATGCTCCTGACCCA	8	G	0	0.4793
NC_005102.4-110634681	GGTCTCACTACATAGTCTCCCCCGTCC	23	C	0.0579	0.4913
NC_005102.4-110638865	ATGCCTGTGACAAGGGCTCCCAGGATT	1	A	0	0.4856
NC_005102.4-110644778	AGTGACCTTACAGGACCTCCTCACAGG	15	A	0	0.4807
NC_005102.4-110707767	CTTTAGAAAACCTGTCCTCCTCCTTCC	5	A	0	0.4773
NC_005102.4-110771684	GAATTTATCACACAGGCTCCCTGGAAT	14	A	0.1017	0.3707
NC_005102.4-110796514	CCATTCATAACACCGGCTCCAGACTCA	13	C	0.0083	0.4835
NC_005102.4-110922661	ATCCTCCTGACCGTTCCTCCCTAGATT	12	C	0	0.4841
NC_005102.4-110988127	CACCCTGGGACTCATCCTCCTTCTTGC	16	C	0.0446	0.0436
NC_005102.4-110991635	ACAGAAGAGACATGGGCTCCAGCCAAG	13	T	0.0603	0.0585
NC_005102.4-110991635	ACAGAAGAGACATGGGCTCCAGCCAAG	24	C	0.0431	0.1209
NC_005102.4-110992023	GGGCTTGGGACTCACTCTCCTCTCTGC	5	T	0	0.4793
NC_005102.4-111038817	GTGACAGAAACAGCCTCTCCCTTACTG	6	A	0	0.4793
NC_005102.4-111038817	GTGACAGAAACAGCCTCTCCCTTACTG	23	T	0	0.4793
NC_005102.4-111101157	CACTCGAGTACTAGAGCTCCAGATGTG	5	C	0.2439	0.4682
NC_005102.4-111180957	GCCACCTCAACCCTGCCTCCTGTGTGG	9	A	0	0.4793

NC_005102.4-111254344	AACCACTAAACCATCTCTCCAGTCCAA	13	A	0.0783	0.0752
NC_005102.4-111254463	ATGTTAACCACCACTCCTCCACCAATG	15	T	0.0254	0.0251
NC_005102.4-111265164	TACAGCGCAACCTTATCTCCCTACGAT	15	A	0	0.4787
NC_005102.4-111338033	TGGCGACACACATTAGCTCCTTCAGCA	24	A	0	0.4793
NC_005102.4-111340685	TGACCGTATACGTCACCTCCTCTTCCT	8	A	0	0.4793
NC_005102.4-111340685	TGACCGTATACGTCACCTCCTCTTCCT	26	C	0.3171	0.319
NC_005102.4-111483074	TTCCTGAGTACCTCTGCTCCCAAGTGT	21	C	0.041	0.0401
NC_005102.4-111483108	CAGGTGTGCACCACATCTCCAGGTGCT	5	T	0	0.4807
NC_005102.4-111483108	CAGGTGTGCACCACATCTCCAGGTGCT	9	C	0.1393	0.2457
NC_005102.4-111511533	CATTGAAGAACCATTTCTCCCATCCCT	7	A	0	0.4793
NC_005102.4-111511533	CATTGAAGAACCATTTCTCCCATCCCT	9	A	0	0.4793
NC_005102.4-111517699	TTTTTGGAGACAGTGCCTCCTATAGCT	13	G	0.2456	0.3878
NC_005102.4-111557015	GGAGAAAGAACGTCATCTCCAGGGGGA	12	G	0	0.0488
NC_005102.4-111557015	GGAGAAAGAACGTCATCTCCAGGGGGA	16	T	0.1	0.4994
NC_005102.4-111576573	CCTGGCTCTACCATGCCTCCAAAACAC	14	T	0.0732	0.1001
NC_005102.4-111610345	GCATGGAGTACCTGAGCTCCCGGAACT	23	G	0.0965	0.1075
NC_005102.4-111612317	GTATGATAAACAAGTGCTCCACCGCTG	6	A	0.1071	0.1327
NC_005102.4-111612317	GTATGATAAACAAGTGCTCCACCGCTG	24	G	0.009	0.4838
NC_005102.4-111706390	AAAGTGAATACTGGTGCTCCTCTGACT	3	A	0	0.4677
NC_005102.4-111706390	AAAGTGAATACTGGTGCTCCTCTGACT	4	G	0.1176	0.1254
NC_005102.4-111721312	TACTATAGAACTTGTTCTCCTTGAATT	1	T	0	0.4793
NC_005102.4-111731399	CACACACACACACACCCTCCCTCTTCT	15	C	0.1062	0.1006
NC_005102.4-111731399	CACACACACACACACCCTCCCTCTTCT	21	C	0.0973	0.0926
NC_005102.4-111757931	GCCATTGCAACTCCCCCTCCCTTTCTA	3	C	0.0254	0.0251
NC_005102.4-111795674	TGTTGGACCACCTTTACTCCATGTTTG	8	C	0.2231	0.4995
NC_005102.4-111804100	CCCTTATTTACCTGAGCTCCTCTGCCC	6	A	0.1157	0.4983
NC_005102.4-111804100	CCCTTATTTACCTGAGCTCCTCTGCCC	12	C	0.124	0.1982
NC_005102.4-111824555	GATCTCCCCACCCTGACTCCTTTGTCA	8	C	0	0.4715
NC_005102.4-111829937	CCAATAACCACTGAAGCTCCCCTCTGG	8	C	0	0.4814
NC_005102.4-111849627	ACTCCTTCAACCCCTACTCCACGCCAG	23	G	0	0.4703
NC_005102.4-111858042	GGTGCAATGACCACAGCTCCAGCCTCC	7	A	0	0.4814
NC_005102.4-111895260	ACTGACCTGACCGGTTCTCCATCTGTG	13	G	0	0.4807
NC_005102.4-111905297	ATGATTCATACCACGACTCCTTTCCCC	9	T	0.009	0.4896
NC_005102.4-111905297	ATGATTCATACCACGACTCCTTTCCCC	15	G	0.0991	0.1103
NC_005102.4-111908457	TGGCTATTTACCCATGCTCCAGCACGC	26	G	0.025	0.4747
NC_005102.4-111964456	AACCACTGGACCATCTCTCCAGCCTGC	23	C	0.087	0.1143
NC_005102.4-111964456	AACCACTGGACCATCTCTCCAGCCTGC	25	T	0.0348	0.0342
NC_005102.4-111970100	CACACACACACACACGCTCCCTTCCCA	12	A	0.1491	0.138
NC_005102.4-111992794	GCTTGGGTTACTCCACCTCCCTGATCC	6	G	0	0.4724
NC_005102.4-112003619	CCCTCTGATACAGTTCCTCCTGCTTAG	7	G	0.2051	0.5
NC_005102.4-112013632	CCGTTAGCTACCCTCCCTCCATCTTCC	2	C	0.2049	0.2218
NC_005102.4-112013632	CCGTTAGCTACCCTCCCTCCATCTTCC	16	C	0.2358	0.4893
NC_005102.4-112016418	TTCCCTCAAACTCTCTCTCCATTGCAT	21	A	0.2017	0.2449
NC_005102.4-112045405	ATGTAGTCTACCATATCTCCTCCCATT	23	C	0.2479	0.3557
NC_005102.4-112051235	CCTGTCTGCACTTCCCCTCCAGCTGAG	6	C	0.2377	0.3492
NC_005102.4-112064667	GAGGTAATGACTCCATCTCCAGCTTCA	8	T	0.1382	0.1562
NC_005102.4-112069362	GTCCGAAGCACTGTTCCTCCAGCCCTA	5	G	0	0.4724
NC_005102.4-112069362	GTCCGAAGCACTGTTCCTCCAGCCCTA	14	T	0.115	0.3294
NC_005102.4-112075107	ACATAGATCACTTTTGCTCCGTGCTCT	7	A	0.2203	0.2702
NC_005102.4-112075107	ACATAGATCACTTTTGCTCCGTGCTCT	21	G	0.1356	0.1551
NC_005102.4-112089390	TGGTGCTCCACGTCTCCTCCAGCCCCT	9	C	0	0.4793

NC_005102.4-112091352	TATAATCAGACCCTTTCTCCTAGATTG	16	T	0	0.4715
NC_005102.4-112199150	CTAAGCCCTACCTTGTCTCCCTGCCTG	3	A	0.0325	0.4854
NC_005102.4-112232163	TGGGCAGGCACATGGGCTCCCTGCTGC	4	G	0.1121	0.1209
NC_005102.4-112232163	TGGGCAGGCACATGGGCTCCCTGCTGC	6	A	0	0.4786
NC_005102.4-112232231	CCAGCTGTCACCTTCACTCCTTCACCT	23	C	0.0894	0.0854
NC_005102.4-112232644	AGCCACATTACCTAGACTCCACCGCTC	23	C	0	0.4773
NC_005102.4-112267984	CCGTAATGTACGTTTTCTCCCTCCCAC	16	T	0.2213	0.2796
NC_005102.4-112279420	TAAGCCCCAACAATCCCTCCAGCCCCA	3	A	0	0.4793
NC_005102.4-112289280	CTACTTATGACCTCCCCTCCCCTGATC	9	G	0.0325	0.032
NC_005102.4-112462291	AGTCTCTGAACCATCTCTCCAGCTGTC	15	C	0.2437	0.4829
NC_005102.4-112686579	GCCCATACCACACTCGCTCCCCTAACT	16	G	0	0.4666
NC_005102.4-112686579	GCCCATACCACACTCGCTCCCCTAACT	23	T	0	0.4693
NC_005102.4-112725396	GGAGAGGGAACTCCACCTCCAGCAGAA	6	G	0	0.4793
NC_005102.4-112725396	GGAGAGGGAACTCCACCTCCAGCAGAA	22	G	0	0.4793
NC_005102.4-112768806	CCCCTCACCACCAGGCCTCCCCCATCC	7	A	0.0333	0.0328
NC_005102.4-112803996	CTGCCTGTAACTACAACTCCAAAGGAT	2	T	0.0948	0.1209
NC_005102.4-112805954	GCAGTTGATACTCCAGCTCCTTTTCCT	27	T	0	0.4772
NC_005102.4-112808204	AGCTCCCGGACACCAGCTCCTTTTCCT	7	C	0.0661	0.0792
NC_005102.4-112862265	CAGAGACTAACACTTTCTCCATTACAT	26	A	0.0164	0.4737
NC_005102.4-112862265	CAGAGACTAACACTTTCTCCATTACAT	27	T	0.0164	0.4737
NC_005102.4-112869923	AAGAGCTGAACTATTTCTCCAACCCCC	2	A	0.2411	0.2758
NC_005102.4-112950241	AGAGCATGTACTTTACCTCCAGAAGAC	16	C	0.0522	0.0508
NC_005102.4-112971639	ATGTAAAACACTCAGGCTCCTGAGAAT	4	T	0.0081	0.4777
NC_005102.4-112977460	ATGGGAAATACCAAGCCTCCTTCATGG	14	A	0.0263	0.0429
NC_005102.4-112977801	TCGTGTCCCACCTCTTCTCCCTTTGCT	3	G	0	0.4785
NC_005102.4-113004987	TGTCATGAGACCTCACCTCCCTAAGCT	14	C	0.1967	0.4226
NC_005102.4-113095132	AAATCCCCAACCCTGTCTCCACCTTTC	25	T	0.2797	0.2759
NC_005102.4-113095132	AAATCCCCAACCCTGTCTCCACCTTTC	27	C	0.2797	0.2759
NC_005102.4-113225605	TGACGCTCCACACACACTCCCATCTTC	2	G	0.0263	0.026
NC_005102.4-113225605	TGACGCTCCACACACACTCCCATCTTC	4	C	0.0088	0.4796
NC_005102.4-113227743	AAGGATCTAACACCTTCTCCTGGCCTC	7	C	0.1301	0.1216
NC_005102.4-113227743	AAGGATCTAACACCTTCTCCTGGCCTC	15	T	0.1301	0.1216
NC_005102.4-113258743	TGCAGAGACACCTGCACTCCTGCCCAC	16	A	0.0082	0.4755
NC_005102.4-113262369	CTGTCCCCTACCTGACCTCCATCACTG	8	C	0.0325	0.032
NC_005102.4-113269086	CAGCCAGCGACTCAGGCTCCTCACAGT	23	A	0	0.4793
NC_005102.4-113270299	TTCTGAATGACCCATGCTCCCTTCCCC	15	T	0	0.48
NC_005102.4-113273654	TGAACATAAACAGCTCCTCCTTAGGCG	26	C	0.2308	0.4671
NC_005102.4-113305670	GAAGAGGGGACTGGACCTCCATTGCAG	22	T	0	0.4793
NC_005102.4-113305670	GAAGAGGGGACTGGACCTCCATTGCAG	24	G	0.0325	0.032
NC_005102.4-113306213	TGTTTTAAAACAGGATCTCCTGGGCTG	27	G	0.2185	0.2796
NC_005102.4-113324318	CCACGGCAAACATTAACTCCCCGTTCC	25	T	0.0331	0.0325
NC_005102.4-113325720	CCCTACAGCACCACCACTCCAAACCTT	2	C	0.0672	0.1107
NC_005102.4-113335576	GGGCTTAAGACAGTTCCTCCAGATGAA	16	C	0.0252	0.0249
NC_005102.4-113362914	CAGCCTTATACTGAACCTCCAGGTGAG	26	A	0	0.4773
NC_005102.4-113367287	TCTGAATCGACCCTATCTCCACTTCCT	8	C	0.0714	0.1014
NC_005102.4-113381026	AACTGCGGAACCATCTCTCCAACTCTC	8	G	0	0.4864
NC_005102.4-113418106	CTTTCCCTGACCCTATCTCCAGCTCAG	15	A	0	0.4793
NC_005102.4-113471536	TTAAGGACCACTGCCTCTCCCGTAGGT	22	G	0	0.4773
NC_005102.4-113578332	GGGCAAGTCACCTGGCCTCCTGGGTCT	23	G	0.1789	0.4926
NC_005102.4-113587991	CACTCATAGACATGAGCTCCAAAGGAG	13	T	0.0893	0.4841
NC_005102.4-113635016	AAGCGCAGAACGGGAGCTCCAACGTGC	5	G	0	0.4773

NC_005102.4-113683943	GCCATTCTCACGATGTCTCCACACTCA	8	T	0	0.4773
NC_005102.4-113697941	CACAGAGAAACCCTGTCTCCAGAAGAA	21	A	0.5455	0.4041
NC_005102.4-113697941	CACAGAGAAACCCTGTCTCCAGAAGAA	24	A	0.5455	0.4041
NC_005102.4-113706827	GGAGTGACAACACACCCTCCACTTGTG	5	T	0.1417	0.4972
NC_005102.4-113706827	GGAGTGACAACACACCCTCCACTTGTG	14	A	0	0.4765
NC_005102.4-113723496	GCTCACTGAACCATCTCTCCAGTCACT	4	C	0.2605	0.4682
NC_005102.4-113723496	GCTCACTGAACCATCTCTCCAGTCACT	5	A	0.0169	0.4718
NC_005102.4-113726224	GAGGGCACGACTTTCTCTCCTCCCACC	9	G	0.2627	0.298
NC_005102.4-113730355	GTCTCATCCACCCGGCCTCCATTCATC	2	T	0.0171	0.4693
NC_005102.4-113730355	GTCTCATCCACCCGGCCTCCATTCATC	16	C	0	0.4772
NC_005102.4-113737537	CAGCCTCTAACTCCAGCTCCAGGATAT	5	C	0.2764	0.377
NC_005102.4-113844309	CTCCTTGTTACTGTCCCTCCCCAGATG	12	T	0	0.4757
NC_005102.4-113844309	CTCCTTGTTACTGTCCCTCCCCAGATG	13	G	0.2458	0.4454
NC_005102.4-113873726	CTGCCTCTAACTCTGGCTCCAGAGGAT	16	G	0.0261	0.4817
NC_005102.4-113956235	CCTCAGGGTACCGAGCCTCCAATGAAT	12	C	0.2667	0.4328
NC_005102.4-114009246	AGCAGGAGGACCAGGGCTCCACCATCA	4	A	0.2276	0.2937
NC_005102.4-114015942	AAAGGAAGCACACCTGCTCCCTCATCC	12	A	0	0.4793
NC_005102.4-114176052	ACACAGAACACCCGCGCTCCACCTCAG	6	G	0	0.4772
NC_005102.4-114267501	TGCTGGTTTACATGGTCTCCTCATGCC	24	T	0	0.4793
NC_005102.4-114267501	TGCTGGTTTACATGGTCTCCTCATGCC	25	G	0	0.4793
NC_005102.4-114290254	ACAGTGAGCACACAGGCTCCGAGGAAA	21	G	0.0165	0.4751
NC_005102.4-114336850	CTAGGATGAACTCAAACTCCTGGACCC	16	A	0.1593	0.2036
NC_005102.4-114347771	GTGCATCTGACACAACCTCCCATCTTA	4	C	0.0984	0.0935
NC_005102.4-114381479	CGTCACCATACACTGTCTCCTGCCACC	15	G	0.2276	0.2142
NC_005102.4-114381479	CGTCACCATACACTGTCTCCTGCCACC	24	C	0.0732	0.0705
NC_005102.4-114704354	AAACCTTTTACCCCAGCTCCAGGACTC	2	A	0	0.48
NC_005102.4-114704354	AAACCTTTTACCCCAGCTCCAGGACTC	7	T	0	0.48
NC_005102.4-114707196	GTGCAAGCAACTCACACTCCACATGTA	5	A	0.225	0.4025
NC_005102.4-114717340	TGTAGTCGGACACAAACTCCATGCTTT	8	G	0.0089	0.0264
NC_005102.4-114731870	TTGACAAGAACAATTCCTCCAACTTTT	1	T	0.161	0.5
NC_005102.4-114795486	TGGCCAATAACGGAGACTCCTGACATG	9	A	0	0.48
NC_005102.4-114815780	ACAACAGTCACTGTGTCTCCTCACCCT	4	A	0.1261	0.1639
NC_005102.4-114876828	AGCACAGCCACACTGACTCCCTCACAC	6	A	0.1111	0.4087
NC_005102.4-114876828	AGCACAGCCACACTGACTCCCTCACAC	26	A	0	0.4793
NC_005102.4-115069246	GAAATGCTTACATAGTCTCCCCCCTAA	26	A	0.2895	0.3836
NC_005102.4-115082253	GAGGGCCCCACTGATACTCCCCATGAT	6	C	0.0684	0.0818
NC_005102.4-115103437	CTTAGCCATACCAGCTCTCCTCAAAGT	14	G	0.2764	0.3239
NC_005102.4-115209736	ATGTTTGAGACAAAGCCTCCCTCACTG	2	T	0.1724	0.199
NC_005102.4-115230151	GCTGAGGACACTGGATCTCCACCAAAT	7	G	0.2881	0.424
NC_005102.4-115230151	GCTGAGGACACTGGATCTCCACCAAAT	8	A	0.2881	0.424
NC_005102.4-115342804	AACAAAGACACACCTACTCCAGTAAGT	22	G	0	0.4868
NC_005102.4-115345033	TTACAAACCACTTCATCTCCTTCAATC	24	A	0.2833	0.2888
NC_005102.4-115390552	CCTGTCAGTACAGCTGCTCCACGTTCT	7	A	0.0172	0.4905
NC_005102.4-115390552	CCTGTCAGTACAGCTGCTCCACGTTCT	14	C	0.0172	0.4905
NC_005102.4-115397525	TTACTCCTAACACCATCTCCTCTTCCC	6	C	0.0579	0.0868
NC_005102.4-115398047	AGTTTCCCCACTAGTTCTCCCTGGACT	6	C	0.0517	0.0825
NC_005102.4-115402074	TTGAGGCACACAGGAACTCCTTAGAAG	1	T	0.1379	0.1855
NC_005102.4-115493161	TGTCTCATTACCCAACCTCCACATTCC	26	C	0.041	0.0861
NC_005102.4-115494553	CTGCCTTGAACAGATGCTCCATAATAT	24	A	0.0086	0.4643
NC_005102.4-115664765	TGATGCAAAACAGAGCCTCCTGAATCA	9	A	0	0.488
NC_005102.4-115730483	GTGTTTAATACCAAAGCTCCTTCATAT	5	T	0.1982	0.4909

NC_005102.4-115730483	GTGTTTAATACCAAAGCTCCTTCATAT	13	A	0.2054	0.2637
NC_005102.4-115739604	CGCTTCCCAACTTTAGCTCCCTCTTCT	2	G	0	0.4743
NC_005102.4-115755301	TGTAAGGGAACATGCCCTCCTTTGTTT	13	T	0.0168	0.4813
NC_005102.4-115831243	CAAAGAATAACCAAGGCTCCAGCTGCA	26	C	0.2655	0.3242
NC_005102.4-116445033	CCTCCAGCTACTATCACTCCATCATCC	14	T	0.0667	0.1099
NC_005102.4-116452008	AACTCAAAGACATCCACTCCTGATAGC	6	A	0.0089	0.4789
NC_005102.4-116452008	AACTCAAAGACATCCACTCCTGATAGC	24	T	0.0268	0.4856
NC_005102.4-116526054	CAATGTGCTACTGGAGCTCCTTTAGCA	23	T	0	0.4773
NC_005102.4-116555447	CACACACACACACACGCTCCTCAACAC	14	A	0.2195	0.4777
NC_005102.4-116556902	TGAGGCTTAACACACCCTCCTTTCAGT	3	A	0	0.4807
NC_005102.4-116556902	TGAGGCTTAACACACCCTCCTTTCAGT	24	C	0.2193	0.415
NC_005102.4-116572883	ATGCCCTGGACATAATCTCCAGCACCA	22	G	0.1293	0.5
NC_005102.4-116657684	AGGGACCTGACTGCCTCTCCCCCCTCA	22	C	0.2479	0.3467
NC_005102.4-116657684	AGGGACCTGACTGCCTCTCCCCCCTCA	23	C	0.2541	0.4755
NC_005102.4-116658747	CGCTTAGACACCTGAGCTCCAATCATT	2	G	0.1429	0.214
NC_005102.4-116678246	CCAGCTGTCACAGTAGCTCCACCATCA	4	G	0.2033	0.4868
NC_005102.4-116706437	TACTGACCCACACTATCTCCCCAGTTG	27	G	0.0667	0.0644
NC_005102.4-116717339	AGTGCTTCCACATTACCTCCCAAACGA	15	A	0	0.4778
NC_005102.4-116717339	AGTGCTTCCACATTACCTCCCAAACGA	25	C	0	0.4778
NC_005102.4-116742850	GGGTTGGTCACATCACCTCCCAAAGTC	12	A	0.275	0.4358
NC_005102.4-116750442	TTGAGTCTCACTGGTTCTCCTCTCATT	27	T	0.0932	0.1041
NC_005102.4-116765995	GGAGGTTGCACTCCTCCTCCTCAGGTT	5	G	0.2521	0.3686
NC_005102.4-116765995	GGAGGTTGCACTCCTCCTCCTCAGGTT	23	A	0.2437	0.491
NC_005102.4-116785688	CCAGCTGTAACTGCCCCTCCTCTCGCC	12	T	0.1949	0.2156
NC_005102.4-116785688	CCAGCTGTAACTGCCCCTCCTCTCGCC	27	C	0.1949	0.4982
NC_005102.4-116816374	GGAAGGGTAACCTCTCCTCCCTGACGG	3	A	0.1667	0.1953
NC_005102.4-116816374	GGAAGGGTAACCTCTCCTCCCTGACGG	25	C	0.0435	0.0425
NC_005102.4-116906606	CTCCCTAACACTGTGACTCCCCGCTAC	22	C	0.0244	0.0241
NC_005102.4-116906606	CTCCCTAACACTGTGACTCCCCGCTAC	23	G	0.2276	0.3141
NC_005102.4-116924199	ACCTAAACTACCGTTTCTCCTGCCACC	1	A	0.1043	0.1443
NC_005102.4-116924199	ACCTAAACTACCGTTTCTCCTGCCACC	13	G	0	0.4778
NC_005102.4-116930178	TGCGGATTTACCTAGACTCCAGTTCTG	3	C	0.1417	0.1866
NC_005102.4-116959884	AGACAGAACACCCATGCTCCTGTTCCA	1	A	0	0.4832
NC_005102.4-116959884	AGACAGAACACCCATGCTCCTGTTCCA	21	T	0.0661	0.0792
NC_005102.4-117003892	AGTCCTCCCACAGCAGCTCCTGTCGGG	2	G	0	0.4751
NC_005102.4-117020280	TGCTCGCTCACTCTTCCTCCCTTTGAA	26	A	0.0246	0.4677
NC_005102.4-117029405	ATCTCACACACCTGATCTCCTGACTTG	27	G	0	0.4734
NC_005102.4-117045788	CAAGGGGACACAGTCTCTCCCCCACTT	6	G	0.1417	0.315
NC_005102.4-117045788	CAAGGGGACACAGTCTCTCCCCCACTT	14	T	0.0417	0.0722
NC_005102.4-117055500	TTACTGTCGACAGAGCCTCCTGACCTC	8	C	0.0348	0.0508
NC_005102.4-117124234	GTAAAGATGACATCCCCTCCACAAAAG	1	G	0.1525	0.2466
NC_005102.4-117141093	CACCTCCAGACTCACTCTCCCTTGTTT	1	C	0.0082	0.479
NC_005102.4-117148475	TTATCAAGCACACTCACTCCTGACTTT	8	G	0.161	0.2644
NC_005102.4-117158274	AGTTCAGTGACATGCCCTCCCACACTG	6	A	0.0083	0.4783
NC_005102.4-117158274	AGTTCAGTGACATGCCCTCCCACACTG	13	T	0.0083	0.4783
NC_005102.4-117167703	TTCATTCTGACATTAGCTCCTCTTCCA	15	A	0	0.4793
NC_005102.4-117174575	TGGCTCACCACTAAACCTCCAAGAAAC	4	C	0.1626	0.1629
NC_005102.4-117177292	CTCATGTGCACAAATCCTCCATGTGTG	4	A	0.2195	0.2989
NC_005102.4-117177292	CTCATGTGCACAAATCCTCCATGTGTG	24	T	0.1707	0.1954
NC_005102.4-117216915	GTCACAACCACTTTCTCTCCTCATCCC	14	T	0.187	0.4995
NC_005102.4-117220164	ATGTAGTTCACTCCCCCTCCCTGCCCC	7	T	0.1102	0.4982

NC_005102.4-117230179	TCTCATCTCACCCTCTCTCCTGCATGG	5	A	0.2276	0.4854
NC_005102.4-117245940	ATGCTTAAAACATCCACTCCACCCTTG	13	T	0.15	0.1665
NC_005102.4-117287400	GAAAGCACCACACCATCTCCATTCGAC	15	A	0.0089	0.4789
NC_005102.4-117332078	GTTTGGAGGACAGTAACTCCCTTTGGA	13	G	0.2017	0.2683
NC_005102.4-117338363	CTGATGCAGACCCTTACTCCGGCGGTC	22	G	0.0252	0.0249
NC_005102.4-117338777	GGCACCAGCACTAGCCCTCCCTCCTCT	1	G	0.193	0.2021
NC_005102.4-117338777	GGCACCAGCACTAGCCCTCCCTCCTCT	6	C	0.2018	0.5
NC_005102.4-117346299	GGTGTCTGCACCAGCCCTCCCTTCTGT	13	A	0	0.4793
NC_005102.4-117346299	GGTGTCTGCACCAGCCCTCCCTTCTGT	14	G	0.0488	0.4881
NC_005102.4-117437824	GCAAAAGCAACAAGGGCTCCTAATTGT	5	A	0	0.4826
NC_005102.4-117443628	AGCCTTCTCACACTCTCTCCTTTCATT	4	C	0.0168	0.4844
NC_005102.4-117512972	AACAGCTCAACCATCTCTCCAGGCCCA	21	A	0.0244	0.0241
NC_005102.4-117559183	AAAACCTGTACTCAATCTCCAGTACTG	9	T	0	0.4827
NC_005102.4-117565386	CAGACTCTCACAAAGGCTCCAAGCCTT	1	C	0.2213	0.3667
NC_005102.4-117565386	CAGACTCTCACAAAGGCTCCAAGCCTT	24	C	0	0.4819
NC_005102.4-117566814	GTAGCACAGACTTAGGCTCCATCTAGG	26	G	0.1066	0.1154
NC_005102.4-117571995	ATCTGTCAAACGGCCACTCCCAAGCGG	25	C	0	0.4819
NC_005102.4-117613239	AAAAGTTCCACGTGAGCTCCTCTGAAT	12	G	0.1875	0.4789
NC_005102.4-117621667	AGAAAAGTGACCCTCTCTCCAACTGAC	26	A	0.0789	0.0758
NC_005102.4-117659446	GGCCTCAGTACAAGGACTCCAACCAAT	4	C	0.0976	0.0928
NC_005102.4-117662531	ACCGAGTAAACTACATCTCCGTGCAGT	21	G	0.2373	0.3874
NC_005102.4-117662531	ACCGAGTAAACTACATCTCCGTGCAGT	27	T	0.2458	0.3833
NC_005102.4-117686599	CACTTAACTACCCTTTCTCCCTGGCTG	14	T	0.1148	0.1367
NC_005102.4-117736376	AAAAAGACCACACTCGCTCCTTACCAG	8	C	0	0.4922
NC_005102.4-117736376	AAAAAGACCACACTCGCTCCTTACCAG	22	T	0	0.4922
NC_005102.4-117769799	AAATTAAAAACACACCCTCCCTGGGTA	21	C	0.1356	0.1551
NC_005102.4-117781167	GATCTCCGGACTCTGTCTCCCAGGTGC	7	C	0.0583	0.0722
NC_005102.4-117781167	GATCTCCGGACTCTGTCTCCCAGGTGC	8	G	0	0.48
NC_005102.4-117793066	GTGGCTCTCACTTTTTCTCCCTGTGGA	1	G	0.0588	0.0571
NC_005102.4-117793066	GTGGCTCTCACTTTTTCTCCCTGTGGA	16	T	0.1345	0.1254
NC_005102.4-117819636	TGACTGGACACATGGGCTCCCTGCTTG	3	A	0	0.4787
NC_005102.4-117819636	TGACTGGACACATGGGCTCCCTGCTTG	7	G	0.1557	0.1708
NC_005102.4-117834959	TGATTGGTTACTTAGCCTCCATAGGCT	7	G	0.041	0.0401
NC_005102.4-117834959	TGATTGGTTACTTAGCCTCCATAGGCT	21	A	0.0833	0.1099
NC_005102.4-117886124	CATGTGTGCACCACCACTCCTGGTTCT	12	C	0.0248	0.0245
NC_005102.4-117918695	CCTTAACACACATTTTCTCCAGCAACG	12	A	0.0336	0.033
NC_005102.4-117940833	AGAGCAGCTACAAACACTCCCTTGAGT	15	C	0	0.4787
NC_005102.4-117942124	GAGACTGTCACCTTATCTCCAGCTTTG	7	G	0.0517	0.0504
NC_005102.4-117945549	TCAAGGGAGACTGAGCCTCCACAACCA	3	A	0.0085	0.481
NC_005102.4-117982249	CCTTATAGCACATGATCTCCGAAGTGT	6	T	0.1626	0.4997
NC_005102.4-117995607	CTGCGGTGAACCATCGCTCCGGCCTTT	4	C	0.2632	0.4038
NC_005102.4-117995607	CTGCGGTGAACCATCGCTCCGGCCTTT	21	G	0	0.4724
NC_005102.4-118010511	CGGCATGGGACAGGAGCTCCCATTCTC	2	G	0.009	0.4414
NC_005102.4-118011093	CACCCTCAGACCCTCACTCCCATCATA	7	C	0.2689	0.4008
NC_005102.4-118132597	ACCCTCACAACTCACCCTCCAGACCAG	21	A	0.0089	0.4824
NC_005102.4-118315933	ATTGGTGATACAATGGCTCCATTCTAA	2	T	0.2478	0.452
NC_005102.4-118327497	CAACCACACACACTAGCTCCAGGGGGA	5	C	0.0847	0.0812
NC_005102.4-118327497	CAACCACACACACTAGCTCCAGGGGGA	7	C	0.0254	0.0251
NC_005102.4-118342360	TGAAAGGACACATTTGCTCCTACATCA	2	G	0	0.4749
NC_005102.4-118342360	TGAAAGGACACATTTGCTCCTACATCA	22	A	0	0.4734
NC_005102.4-118355480	CTCAATGTCACACATTCTCCAGTGAAG	21	A	0.2054	0.2758

NC_005102.4-118363175	GTCTTTAGAACCCATGCTCCATCCATT	15	T	0	0.4793
NC_005102.4-118393514	CTTAAGACAACTAGTTCTCCCAACTCA	13	A	0	0.4703
NC_005102.4-118458834	CGCACTGGGACTTTCGCTCCATGTTTT	9	G	0.0244	0.0241
NC_005102.4-118464454	AGCAAACAGACCCAAACTCCACTGGAG	25	G	0.0174	0.48
NC_005102.4-118528552	CAGAAAAGTACATTGTCTCCAAGTGTA	3	G	0.0084	0.4829
NC_005102.4-118528552	CAGAAAAGTACATTGTCTCCAAGTGTA	9	T	0.0504	0.0805
NC_005102.4-118577605	TTAATTGTAACATCTACTCCTGATAGA	16	A	0.1171	0.1565
NC_005102.4-118577605	TTAATTGTAACATCTACTCCTGATAGA	27	A	0.1171	0.1565
NC_005102.4-118663677	GAAGGTCTCACCAGGCCTCCCTGTTCT	22	T	0.1339	0.4394
NC_005102.4-118739841	GATACCCACACTGATACTCCTACACCC	6	C	0.0086	0.0422
NC_005102.4-118785727	AGGACACCTACATCAGCTCCAATGTCT	1	A	0	0.4671
NC_005102.4-118792167	ATGAAGATGACATATACTCCAATTTCA	9	G	0.0268	0.0264
NC_005102.4-118792167	ATGAAGATGACATATACTCCAATTTCA	22	A	0	0.4755
NC_005102.4-118795457	ACCTCTGTTACCTTTGCTCCTTCATCT	14	T	0.1463	0.2142
NC_005102.4-118795457	ACCTCTGTTACCTTTGCTCCTTCATCT	25	T	0.1545	0.3091
NC_005102.4-118820244	TCAACTGTAACTCTAGCTCCAGGGGAT	24	G	0.479	0.4186
NC_005102.4-118820244	TCAACTGTAACTCTAGCTCCAGGGGAT	26	A	0.479	0.4186
NC_005102.4-118854974	AGCTATCAAACTGTTTCTCCACCTAAG	12	T	0.2054	0.3211
NC_005102.4-118870179	GCAAAAGGTACTGTGTCTCCTTCATCC	13	G	0.2203	0.4632
NC_005102.4-118877404	ACTGTCCCTACTCTACCTCCCTGGAAA	13	C	0.0789	0.138
NC_005102.4-118877482	ATCTCTGACACTTTCTCTCCTCGCTCA	23	G	0.094	0.1049
NC_005102.4-118877482	ATCTCTGACACTTTCTCTCCTCGCTCA	24	C	0.0342	0.066
NC_005102.4-118979584	ATCCCACCAACCATTCCTCCTAGCTGT	9	A	0.2523	0.2467
NC_005102.4-118979584	ATCCCACCAACCATTCCTCCTAGCTGT	25	T	0	0.4785
NC_005102.4-119056243	GTGCGTTTAACAAGGCCTCCCACCACT	5	G	0.1066	0.1708
NC_005102.4-119056243	GTGCGTTTAACAAGGCCTCCCACCACT	25	A	0	0.4807
NC_005102.4-119072873	AACTCAATCACCAGCCCTCCCTGGTAA	3	C	0.0789	0.0918
NC_005102.4-119072873	AACTCAATCACCAGCCCTCCCTGGTAA	8	T	0.1842	0.2719
NC_005102.4-119094784	ACTCTTGTTACTCCAGCTCCAACGGGA	22	A	0	0.4922
NC_005102.4-119195193	AAGTATGAAACACGAACTCCCTTAACT	14	G	0.2689	0.322
NC_005102.4-119229854	ACCAAAGCGACAGCACCTCCTGTGCAC	24	G	0.3033	0.4489
NC_005102.4-119230013	GCTCCCACTACCACAGCTCCATGGCAG	8	C	0.0252	0.0249
NC_005102.4-119346410	CGCTGCAACACACCGACTCCCTCCAGT	1	C	0.0593	0.0889
NC_005102.4-119369463	GAAAGTTCTACACCACCTCCATTAATT	21	A	0.1034	0.1284
NC_005102.4-119371657	GTTTCTGATACCTTGACTCCCTGCATG	8	A	0.0167	0.4765
NC_005102.4-119374195	AAACTCAACACTAGAGCTCCTGAATCT	21	T	0	0.4779
NC_005102.4-119379148	GGGCTATGCACAAGATCTCCTACAGTG	23	C	0.2541	0.321
NC_005102.4-119385125	TCCGGGTCAACCCTGCCTCCTTTCTTT	3	C	0	0.4832
NC_005102.4-119385125	TCCGGGTCAACCCTGCCTCCTTTCTTT	23	T	0	0.4832
NC_005102.4-119385612	TGAGTCCAAACAGCACCTCCAGCCTCT	8	A	0	0.4709
NC_005102.4-119387656	ATCAATGGCACACTCTCTCCCTTCTTC	8	G	0.009	0.4726
NC_005102.4-119458067	AGAGAAGAGACAGTGCCTCCACCTTAG	2	G	0.0574	0.071
NC_005102.4-119481412	AGGGGTGTAACCTTTCCTCCAGGGCTT	12	C	0.0345	0.0339
NC_005102.4-119487958	AACTGCTGAACTGTCTCTCCAGTCACT	1	A	0.3223	0.313
NC_005102.4-119487958	AACTGCTGAACTGTCTCTCCAGTCACT	25	A	0.3223	0.313
NC_005102.4-119590496	CCCTCCCCAACTCTCTCTCCCTGCTAC	8	C	0.0517	0.0504
NC_005102.4-119594752	TGCCTCACCACCTGCTCTCCTGTGATC	27	C	0.027	0.0267
NC_005102.4-119649343	ATATAGAACACCTTTTCTCCCTGCCTT	3	A	0.0171	0.4839
NC_005102.4-119652967	GTCTCTCCCACAACTGCTCCTAGGGGT	2	T	0.1026	0.3159
NC_005102.4-119652967	GTCTCTCCCACAACTGCTCCTAGGGGT	13	A	0	0.4749
NC_005102.4-119680511	TGGGCAAATACAGACCCTCCGTCCCAG	13	G	0.0901	0.1023

NC_005102.4-119702863	AAAGCAGAGACTGTCTCTCCTTCATAC	9	G	0.0256	0.4823
NC_005102.4-119707499	CTATCAACAACCAACCCTCCTTCCCCA	2	T	0.1138	0.1494
NC_005102.4-119904924	CTCTGAGCCACCTCACCTCCAACCCCA	13	T	0.1102	0.1041
NC_005102.4-119904924	CTCTGAGCCACCTCACCTCCAACCCCA	22	A	0.1102	0.1041
NC_005102.4-119924764	TTTGGAGTTACTGAGACTCCACCCAGG	8	T	0.1167	0.1528
NC_005102.4-119937227	ACCCGGGGAACTCAGTCTCCAACAGAG	2	C	0.0744	0.1447
NC_005102.4-119937227	ACCCGGGGAACTCAGTCTCCAACAGAG	4	C	0.157	0.4803
NC_005102.4-119946933	AGGCTGAACACTGCCCCTCCTCCAGTG	9	C	0.0248	0.0562
NC_005102.4-119964282	CCAGAGCTCACATGCGCTCCATCGTCA	3	A	0.3043	0.3353
NC_005102.4-119964282	CCAGAGCTCACATGCGCTCCATCGTCA	23	C	0	0.0339
NC_005102.4-119978139	CCCTTTGTCACAGTCACTCCTGAGACA	5	T	0	0.4728
NC_005102.4-119980288	CCATCTCCCACCCTCCCTCCTAGGTGT	4	T	0	0.4779
NC_005102.4-119987341	CACTAGGACACTATCTCTCCTAGGATT	24	G	0.2377	0.3401
NC_005102.4-119989249	AGTCCTGGGACAGTCTCTCCTGAGTTG	12	A	0.0084	0.4761
NC_005102.4-119999218	ACTGTCCCTACAGTCACTCCAAGGTCT	21	A	0.3033	0.4879
NC_005102.4-120014910	ATGCCTGTAACTCTAGCTCCAGGATAT	7	G	0.0246	0.0243
NC_005102.4-120015174	CTTTGGGCCACTGCTCCTCCTTCCCCA	2	T	0	0.4832
NC_005102.4-120037759	TACAAACACACCATTTCTCCTTAAAGT	13	A	0.0248	0.0405
NC_005102.4-120041185	TCCCTTTTAACTTAGCCTCCGGCAGAT	21	G	0.1917	0.4875
NC_005102.4-120044897	TACTCCTTGACCATCTCTCCTCACAGC	6	C	0.2683	0.4239
NC_005102.4-120044897	TACTCCTTGACCATCTCTCCTCACAGC	16	T	0.0163	0.032
NC_005102.4-120052946	TACACACACACACCACCTCCTGGACTC	1	T	0.0901	0.4444
NC_005102.4-120073253	GAGAGTACAACCTTGGCTCCATGAATG	1	G	0.2054	0.2637
NC_005102.4-120083328	CTTGCCAGGACATGAGCTCCGCTCCCC	26	C	0.1652	0.2072
NC_005102.4-120098888	TCTCTGGTCACCATCTCTCCCTATCTT	14	T	0.1138	0.2142
NC_005102.4-120098888	TCTCTGGTCACCATCTCTCCCTATCTT	21	C	0.1951	0.2263
NC_005102.4-120100546	CTTGGTGCCACTGTTACTCCAACCCAA	25	C	0	0.4743
NC_005102.4-120191276	AAGGTGTGCACCAGCACTCCTGCACCT	9	C	0.1933	0.2509
NC_005102.4-120218641	AACCCACCCACCCCATCTCCCAGCCAA	8	C	0.1593	0.3026
NC_005102.4-120218641	AACCCACCCACCCCATCTCCCAGCCAA	9	C	0.1579	0.3115
NC_005102.4-120227506	TATGATAGCACCCAGCCTCCAGGACAA	13	C	0.1513	0.2076
NC_005102.4-120229191	TCTCGGGAAACTAAGCCTCCCACCTTC	6	G	0.1709	0.1841
NC_005102.4-120332967	GTAGGGCCCACCTCGTCTCCTCATGCC	3	A	0.0165	0.4787
NC_005102.4-120332967	GTAGGGCCCACCTCGTCTCCTCATGCC	14	C	0.0165	0.4787
NC_005102.4-120348570	CTCTGTTTCACTTGGGCTCCGTCTGCA	21	G	0.0164	0.4737
NC_005102.4-120353061	CCACAGTCGACAATACCTCCATTACTT	22	T	0.2195	0.1954
NC_005102.4-120358255	AACAAACAAACCCAAACTCCAAGGCTG	22	A	0.069	0.0666
NC_005102.4-120366686	AGGTCCCACACAAGCTCTCCCAGTACT	23	G	0.0175	0.4925
NC_005102.4-120387179	AGGCATGGTACATTAGCTCCTCACTGT	9	T	0	0.4793
NC_005102.4-120387179	AGGCATGGTACATTAGCTCCTCACTGT	13	T	0	0.4793
NC_005102.4-120505949	CATCCTGCAACAGGAGCTCCTGAGAGC	15	A	0.05	0.0644
NC_005102.4-120505949	CATCCTGCAACAGGAGCTCCTGAGAGC	27	C	0.0085	0.4655
NC_005102.4-120526954	AGTTCATTTACTCAGCCTCCCCAGAAC	4	T	0.0732	0.1562
NC_005102.4-120549755	GCCTATGCTACATGAGCTCCTGAATCA	16	G	0.0427	0.058
NC_005102.4-120557975	ACTGCTCCTACCCGTACTCCATTGTCA	14	G	0.0917	0.1025
NC_005102.4-120557975	ACTGCTCCTACCCGTACTCCATTGTCA	24	G	0.1167	0.1244
NC_005102.4-120559232	AACAGTGACACACTTCCTCCTGCAATA	15	T	0.0244	0.484
NC_005102.4-120571348	TTACTTGAAACAAAGCCTCCACTGGAC	22	C	0.0085	0.4753
NC_005102.4-120571348	TTACTTGAAACAAAGCCTCCACTGGAC	24	G	0.0684	0.4868
NC_005102.4-120607968	CAAACAAGCACATAACCTCCAATAGTA	1	C	0.2735	0.4193
NC_005102.4-120607968	CAAACAAGCACATAACCTCCAATAGTA	16	C	0.0088	0.472

NC_005102.4-120642723	TGGATTGCCACTGTGCCTCCAACCCTG	5	T	0.065	0.4926
NC_005102.4-120642723	TGGATTGCCACTGTGCCTCCAACCCTG	16	C	0.2683	0.3646
NC_005102.4-120678737	TAGGGTCTTACTGCAGCTCCTGGAGTG	1	T	0.0083	0.4769
NC_005102.4-120689793	TAATTGAGAACCACTTCTCCGAGGTAG	5	T	0	0.4891
NC_005102.4-120689793	TAATTGAGAACCACTTCTCCGAGGTAG	16	T	0	0.4891
NC_005102.4-120693590	TATCTGGATACATTTCCTCCTTTCTGG	1	T	0.2793	0.4635
NC_005102.4-120693590	TATCTGGATACATTTCCTCCTTTCTGG	6	G	0.1504	0.2237
NC_005102.4-120702168	TGCTCTGTAACTCTAGCTCCAGAGGAA	14	T	0.0982	0.1699
NC_005102.4-120702168	TGCTCTGTAACTCTAGCTCCAGAGGAA	26	A	0.0265	0.0262
NC_005102.4-120736843	GACAAAGTGACACTGGCTCCTCCTGTT	14	T	0.2439	0.4926
NC_005102.4-120736843	GACAAAGTGACACTGGCTCCTCCTGTT	15	G	0.122	0.1145
NC_005102.4-120776618	TGAGGGAATACACTTGCTCCTGTGACT	25	A	0.0265	0.4873
NC_005102.4-120776915	CAGCCAGGGACAATGGCTCCCTGTCGG	6	A	0.2437	0.4829
NC_005102.4-120810440	ACTTACCACACCCCATCTCCTTGGCTC	21	T	0.0083	0.4732
NC_005102.4-120810440	ACTTACCACACCCCATCTCCTTGGCTC	25	C	0.157	0.2814
NC_005102.4-120821726	CTCTTTCTTACTCATCCTCCTCCTTCC	24	T	0.042	0.0411
NC_005102.4-120827671	TGCACATACACCAGGCCTCCTTCAGAA	22	T	0.1071	0.4424
NC_005102.4-120832214	ACTAAAGTCACACTGCCTCCTTCAGGA	2	C	0.0333	0.0328
NC_005102.4-120832214	ACTAAAGTCACACTGCCTCCTTCAGGA	4	A	0	0.4793
NC_005102.4-120833660	TTGACTGAAACTTCACCTCCATCATTT	3	G	0.0085	0.4655
NC_005102.4-120833660	TTGACTGAAACTTCACCTCCATCATTT	27	T	0.0085	0.4655
NC_005102.4-120846206	TGCAATCCGACAGTTACTCCTTAGCAC	9	G	0	0.4807
NC_005102.4-120856771	CACAGTTTCACAGTGGCTCCTAAAAGT	5	G	0	0.4787
NC_005102.4-120857389	GATGTCCCTACGATGGCTCCACATCAT	12	G	0.0982	0.4043
NC_005102.4-120916039	TGCTTTGCTACCATGCCTCCTGGAGCC	21	T	0.1803	0.2032
NC_005102.4-120958650	ACCAAGGCTACTCTGACTCCAGGCCTA	23	G	0.2927	0.4444
NC_005102.4-120959805	GAAAAGGTAACCCATGCTCCATACGGA	25	G	0.123	0.1708
NC_005102.4-120962904	GCCTTTCATACCTTGTCTCCAGGGTTC	12	C	0.0609	0.059
NC_005102.4-120982224	AGATGGGAAACTGAGGCTCCAAGCTAC	3	A	0	0.4813
NC_005102.4-120993637	GACAATACCACAGGTCCTCCACTTTGG	12	A	0	0.4765
NC_005102.4-120997375	CCAGAGGCCACAGGCCCTCCCTATCTC	26	T	0.1034	0.1431
NC_005102.4-121002193	AAGCGATGCACCATCTCTCCAGGCCCA	14	T	0.065	0.4904
NC_005102.4-121004627	CGCTTGCACACTCAATCTCCAACATGT	6	G	0.0917	0.1316
NC_005102.4-121004627	CGCTTGCACACTCAATCTCCAACATGT	7	C	0.0917	0.1316
NC_005102.4-121005922	ACTGGGTCCACCCCACCTCCATTCGTC	25	G	0	0.4819
NC_005102.4-121013464	GGGCATCCCACGTCAGCTCCTTTCTTG	3	G	0.2114	0.2263
NC_005102.4-121013464	GGGCATCCCACGTCAGCTCCTTTCTTG	12	G	0.2705	0.432
NC_005102.4-121083522	ACATATAACACAGCCTCTCCTCTCCCC	22	C	0.0084	0.4859
NC_005102.4-121083522	ACATATAACACAGCCTCTCCTCTCCCC	23	T	0	0.4832
NC_005102.4-121086990	AGGTAGGACACACCATCTCCCTCTGTC	8	A	0.0732	0.0705
NC_005102.4-121086990	AGGTAGGACACACCATCTCCCTCTGTC	22	T	0.0732	0.0705
NC_005102.4-121089872	TGAGTTGGGACAGGGACTCCTCAGCAG	4	G	0.3167	0.2665
NC_005102.4-121091359	TATGCATTGACTCTCACTCCTCAGGAT	22	C	0.0342	0.0818
NC_005102.4-121151857	ATCTTGCAAACTCTGGCTCCCTCGGTC	1	A	0.0522	0.1443
NC_005102.4-121250184	TGCCATGGAACTCTAACTCCAAGTAAT	15	A	0	0.4728
NC_005102.4-121251550	TTCAGGCTCACTCTTCCTCCACCATTG	6	G	0.114	0.1953
NC_005102.4-121251550	TTCAGGCTCACTCTTCCTCCACCATTG	8	T	0.0087	0.4903
NC_005102.4-121265856	CCAGCACACACAGCCACTCCCCAGATG	25	A	0.0678	0.493
NC_005102.4-121272544	CAAGAGGTTACCAGCCCTCCATTTTCA	13	A	0.0427	0.4882
NC_005102.4-121280880	CCTCTAGTCACAATCCCTCCTTACCTG	6	A	0	0.4819
NC_005102.4-121280880	CCTCTAGTCACAATCCCTCCTTACCTG	27	G	0.0667	0.0644

NC_005102.4-121296963	AGACTTCTTACCACATCTCCAGTGCAT	26	A	0	0.4773
NC_005102.4-121330836	CTCTTCTCAACCATAGCTCCTTCTCCC	1	C	0.1488	0.1377
NC_005102.4-121330836	CTCTTCTCAACCATAGCTCCTTCTCCC	9	A	0	0.4787
NC_005102.4-121335456	TCATTTGTGACTTACACTCCCTTTGGT	22	T	0.0424	0.0415
NC_005102.4-121335456	TCATTTGTGACTTACACTCCCTTTGGT	26	G	0.0424	0.0415
NC_005102.4-121353434	ATCTCTCTTACCCTCCCTCCCCATTGT	6	T	0	0.4787
NC_005102.4-121353434	ATCTCTCTTACCCTCCCTCCCCATTGT	16	C	0.0248	0.0405
NC_005102.4-121411708	GAGTCAAGCACCCATCCTCCTTTGGCT	9	C	0.0893	0.1014
NC_005102.4-121473491	TTACAAAGCACTTCTACTCCTTGGTTC	5	A	0.1102	0.1337
NC_005102.4-121577908	TTACATCAAACAAACCCTCCCCACCCC	27	C	0.1478	0.4962
NC_005102.4-121582150	TAAGCCCAGACCCTCTCTCCCTGTGGG	26	G	0.0738	0.071
NC_005102.4-121588191	CTCTCCCTCACCTTCCCTCCTCTATCT	2	T	0.0259	0.0255
NC_005102.4-121589734	TTCCAGACTACGGGATCTCCCAGCATC	12	G	0.0263	0.0758
NC_005102.4-121590311	CACCCCCACACTTGCCCTCCAATCTTG	15	C	0	0.4826
NC_005102.4-121590311	CACCCCCACACTTGCCCTCCAATCTTG	22	A	0	0.4826
NC_005102.4-121599536	GCTACTGACACTGTCACTCCGCACCTG	4	A	0.1066	0.1154
NC_005102.4-121623625	GTTCTGGGAACAGCTCCTCCTCCCTGG	15	T	0.0667	0.0799
NC_005102.4-121685348	CTTTGCATGACTCTACCTCCAGTTATA	6	C	0	0.4764
NC_005102.4-121685348	CTTTGCATGACTCTACCTCCAGTTATA	14	T	0.0948	0.1504
NC_005102.4-121687962	TCTAAGGTCACCTATACTCCACCTGTG	4	A	0.0439	0.0429
NC_005102.4-121746255	TGAAATGAAACCCAGGCTCCTCATTTG	1	T	0	0.48
NC_005102.4-121755648	TAGATCAAAACTCTGCCTCCTCCCCCT	26	C	0.1148	0.1082
NC_005102.4-121763561	GCCAGCCACACTGTTACTCCATTTATG	8	A	0.0847	0.0965
NC_005102.4-121820899	GAGGCTAATACACCTACTCCAAGCAAA	6	T	0.0894	0.0854
NC_005102.4-121823627	TTACCTGTAACTCTAGCTCCATAGGGT	14	T	0	0.4898
NC_005102.4-121856196	TAGTCCCATACTGCAGCTCCTAAGGAG	9	T	0.2308	0.2778
NC_005102.4-121856196	TAGTCCCATACTGCAGCTCCTAAGGAG	27	G	0.0254	0.0415
NC_005102.4-121858140	GAGGTCCTAACCTATGCTCCTACCTTT	12	C	0.0085	0.479
NC_005102.4-121862509	GAAAGGAACACCTTGGCTCCAGGACAT	13	T	0	0.4787
NC_005102.4-121862509	GAAAGGAACACCTTGGCTCCAGGACAT	21	A	0	0.4787
NC_005102.4-121983066	TATGATGACACCTTTGCTCCCATGATA	3	T	0.0088	0.49
NC_005102.4-121983066	TATGATGACACCTTTGCTCCCATGATA	26	T	0.0088	0.49
NC_005102.4-122030965	GGGATGACCACCTGCCCTCCTCCATAT	7	A	0	0.4807
NC_005102.4-122036800	TCTCTTTTCACATTAGCTCCTATGCGT	26	G	0.0902	0.1154
NC_005102.4-122074000	AAAGTCCCTACCCCCACTCCCTCCCAT	1	A	0.2414	0.3572
NC_005102.4-122107153	GCACTGACTACAAGCCCTCCAGGTGAC	5	T	0.0169	0.0333
NC_005102.4-122107910	GTAGCAGGAACCGATACTCCAGCTGTA	25	G	0	0.4846
NC_005102.4-122118912	TATCTCTCTACTTTTGCTCCTGCTGTT	26	T	0.041	0.0401
NC_005102.4-122143778	GTTCTCATGACCACATCTCCTGAACAC	1	G	0.041	0.0557
NC_005102.4-122148016	TCAGCACTTACGGTCCCTCCTCCGCTT	12	G	0.1008	0.0958
NC_005102.4-122148016	TCAGCACTTACGGTCCCTCCTCCGCTT	27	T	0.0168	0.4743
NC_005102.4-122181049	GGCATCTGGACCTGCCCTCCACTGTCT	22	C	0.1545	0.1562
NC_005102.4-122199267	TGACAACTGACATTCACTCCCGCCTCA	2	G	0.1488	0.389
NC_005102.4-122199267	TGACAACTGACATTCACTCCCGCCTCA	21	C	0.0992	0.4877
NC_005102.4-122199430	ACGGTGTCCACCTTCCCTCCAGGTTCC	2	C	0.1026	0.4997
NC_005102.4-122199430	ACGGTGTCCACCTTCCCTCCAGGTTCC	24	T	0.1026	0.4997
NC_005102.4-122257177	GGCTGCAGAACACAGCCTCCCTGCCCC	1	G	0.0496	0.0484
NC_005102.4-122257177	GGCTGCAGAACACAGCCTCCCTGCCCC	2	G	0.0413	0.0562
NC_005102.4-122278068	GAGACGCAGACATAGGCTCCCACTGTC	5	C	0.2017	0.2328
NC_005102.4-122278068	GAGACGCAGACATAGGCTCCCACTGTC	22	A	0.025	0.4847
NC_005102.4-122283952	CTTGTGTCCACACATGCTCCTGCGGGT	14	A	0	0.4773

NC_005102.4-122283952	CTTGTGTCCACACATGCTCCTGCGGGT	22	G	0.0407	0.0553
NC_005102.4-122284387	CTTATGCTCACAGCCTCTCCACGCCAT	4	A	0.1057	0.1287
NC_005102.4-122284387	CTTATGCTCACAGCCTCTCCACGCCAT	22	C	0.1707	0.1954
NC_005102.4-122285964	CCAATCATCACAAACTCTCCAAGTGGA	1	C	0	0.4875
NC_005102.4-122285964	CCAATCATCACAAACTCTCCAAGTGGA	2	C	0	0.4875
NC_005102.4-122309818	CAATTCGGGACCGTAGCTCCTTCTTAA	12	C	0	0.48
NC_005102.4-122362572	AACTCCATTACTTTGGCTCCATGTCTG	8	T	0.1736	0.5
NC_005102.4-122362572	AACTCCATTACTTTGGCTCCATGTCTG	13	T	0	0.4819
NC_005102.4-122369166	CCGAGAGAAACCCAGTCTCCTGAAAGA	2	C	0.087	0.1443
NC_005102.4-122369528	CCCGAGGATACCTCAGCTCCTTCCACT	9	T	0	0.4773
NC_005102.4-122372306	TGTGAAACAACTGTAGCTCCTATTTTA	6	A	0	0.4743
NC_005102.4-122393497	TGATGGACCACGGCCCCTCCCTGCTCG	3	A	0.1475	0.2741
NC_005102.4-122393497	TGATGGACCACGGCCCCTCCCTGCTCG	26	C	0.1148	0.3624
NC_005102.4-122438336	GTTTCTGGCACCTTTGCTCCTGCCGGG	24	C	0.042	0.0411
NC_005102.4-122438336	GTTTCTGGCACCTTTGCTCCTGCCGGG	26	G	0.1017	0.4977
NC_005102.4-122439347	AAGGTTCACACTTTTACTCCCAACACT	9	C	0.1405	0.2704
NC_005102.4-122444259	GCTAGTAACACAGTTTCTCCATGTCAC	21	A	0.0171	0.0336
NC_005102.4-122450921	GTGTCTTTCACCTCACCTCCGTCCGGG	15	A	0.0732	0.4952
NC_005102.4-122474303	AATCTCTGCACGTCCCCTCCACTCATT	3	T	0.1463	0.2142
NC_005102.4-122474303	AATCTCTGCACGTCCCCTCCACTCATT	12	G	0.0246	0.0243
NC_005102.4-122527591	GTTTGTTGAACCACCTCTCCATCCCCT	14	C	0.0991	0.1103
NC_005102.4-122527591	GTTTGTTGAACCACCTCTCCATCCCCT	25	C	0.0721	0.1023
NC_005102.4-122555409	TTGTGCCGTACTGTGACTCCTACGTGG	6	C	0.0081	0.4777
NC_005102.4-122555409	TTGTGCCGTACTGTGACTCCTACGTGG	24	G	0.0163	0.4881
NC_005102.4-122555938	CCCCTTAATACAGTTTCTCCTGTTGTG	15	T	0.0496	0.0484
NC_005102.4-122555938	CCCCTTAATACAGTTTCTCCTGTTGTG	24	T	0.05	0.0488
NC_005102.4-122565140	CAGCTGAGCACAGCTTCTCCCCAGGCC	15	T	0.1721	0.4973
NC_005102.4-122591601	CCCAACAACACACCTCCTCCAAAAGGC	16	C	0.125	0.2188
NC_005102.4-122645617	GGAGGCCACACCTCTTCTCCGGAGCTC	21	G	0.1393	0.4997
NC_005102.4-122668405	GCTAGTGACACCACCACTCCTAATCTC	3	T	0.1311	0.1641
NC_005102.4-122668405	GCTAGTGACACCACCACTCCTAATCTC	16	A	0.124	0.4999
NC_005102.4-122680128	CATCTTGTAACTCCAGCTCCAGGGCTC	5	T	0.1833	0.1665
NC_005102.4-122680128	CATCTTGTAACTCCAGCTCCAGGGCTC	13	C	0.1833	0.1665
NC_005102.4-122685617	CACACACCCACCATCCCTCCACATGTC	25	G	0.0172	0.0339
NC_005102.4-122698410	TGTACCAACACCATTCCTCCTCCGCCA	26	C	0.0083	0.4732
NC_005102.4-122698410	TGTACCAACACCATTCCTCCTCCGCCA	27	A	0.0083	0.4732
NC_005102.4-122702941	AGATTGCTAACCCTCCCTCCTAGGGGG	21	T	0.0083	0.4816
NC_005102.4-122706581	CTCTCCTTAACCTCATCTCCTTCAACA	6	C	0.0265	0.0262
NC_005102.4-122821934	TAGGCTCAGACCAAGCCTCCTCTGTCT	1	T	0.0082	0.4822
NC_005102.4-122830574	GGATTTGAAACCCATTCTCCACCACCT	24	A	0.0164	0.4837
NC_005102.4-122833840	TTGTTTGAGACAGGGTCTCCCTTTAGC	27	C	0.5478	0.4055
NC_005102.4-122838902	TCCTATTTCACAACACCTCCAGTTCCC	3	C	0.1239	0.5
NC_005102.4-122840008	GAGCTACAGACCCCAACTCCTTTATCA	13	C	0.041	0.0401
NC_005102.4-122907344	TAATTCTGTACAGGAACTCCATCCCTC	2	A	0	0.4677
NC_005102.4-122907344	TAATTCTGTACAGGAACTCCATCCCTC	21	A	0	0.4677
NC_005102.4-122918248	CCAGACCTGACGCTGGCTCCAGATCCA	1	C	0.0789	0.0918
NC_005102.4-122918248	CCAGACCTGACGCTGGCTCCAGATCCA	12	G	0.2124	0.2302
NC_005102.4-122929025	TCTCCTAGCACGAGGACTCCCTAATAA	9	C	0.0256	0.0253
NC_005102.4-123041923	TCTCTTCGTACTGAAGCTCCTACCTTG	2	C	0.2033	0.2778
NC_005102.4-123041923	TCTCTTCGTACTGAAGCTCCTACCTTG	7	C	0.2276	0.2937
NC_005102.4-123046679	AGCTAAGATACATAACCTCCCGCTCTT	22	G	0.188	0.2604

NC_005102.4-123054073	CTTCCAAGGACACTTTCTCCAACAAGA	3	T	0.0085	0.4655
NC_005102.4-123054073	CTTCCAAGGACACTTTCTCCAACAAGA	22	A	0.0085	0.4655
NC_005102.4-123076397	ACAATTGTAACAATCTCTCCAGTATCT	5	T	0.0177	0.4827
NC_005102.4-123121323	AGAGACCCTACACGGCCTCCATTTCCC	12	A	0.0246	0.0243
NC_005102.4-123129873	CTAATTCTGACAGAGCCTCCTCTCCAG	24	C	0.125	0.4297
NC_005102.4-123153201	CTTACATGCACATAATCTCCCTATGTG	23	A	0.0248	0.0405
NC_005102.4-123174392	CACCCTCCAACTTTACCTCCCATACTT	3	C	0.0833	0.095
NC_005102.4-123182893	ACTCCCTCTACAGGGGCTCCTTCTCAG	14	G	0.065	0.0629
NC_005102.4-123195371	CTTAGCTCTACTTAGACTCCTGGGTCT	13	T	0	0.477
NC_005102.4-123231366	GTGTGGAGGACGGTTTCTCCAGGGACA	12	G	0.0569	0.0553
NC_005102.4-123232374	GACGTGGGTACCCATGCTCCGTCTCCC	4	G	0.0656	0.0634
NC_005102.4-123232374	GACGTGGGTACCCATGCTCCGTCTCCC	27	C	0.0082	0.4822
NC_005102.4-123277567	CTGTTAGAAACAACATCTCCTATAGCC	1	C	0.1405	0.3327
NC_005102.4-123277567	CTGTTAGAAACAACATCTCCTATAGCC	13	A	0	0.4787
NC_005102.4-123375833	GTCCCTGAAACAGGCGCTCCCTAAGGG	12	A	0	0.48
NC_005102.4-123396109	TGGATGTGCACTCCCACTCCCAGCATT	7	T	0	0.4728
NC_005102.4-123427093	TAGATGTGAACTTAGTCTCCAGTTGTT	1	T	0	0.4751
NC_005102.4-123428960	ATGTAAAAAACCTGTTCTCCACCCAGA	6	A	0.0085	0.4698
NC_005102.4-123487085	CAAGTCATGACCTCAGCTCCTCGTTCC	4	G	0.0522	0.0508
NC_005102.4-123493698	ATATACAGCACCATTCCTCCCATCCAG	8	G	0.0248	0.4693
NC_005102.4-123574708	TTCATTCTCACACACACTCCTCATACA	27	A	0.0783	0.0752
NC_005102.4-123589449	CTAAAAAGCACCATTCCTCCTGCTAGG	4	A	0.0084	0.4682
NC_005102.4-123597863	CAGCTGTAGACCTCAGCTCCACACCTC	1	C	0.181	0.2681
NC_005102.4-123609715	GACCTGTTCACTGGCCCTCCAAAGCTC	6	G	0.2321	0.2934
NC_005102.4-123614472	AGGCTGTGCACACATACTCCCCTTGAT	5	T	0	0.4751
NC_005102.4-123684787	AGTGTTCCCACCTCCCCTCCCCCTCAT	5	T	0	0.4807
NC_005102.4-123684787	AGTGTTCCCACCTCCCCTCCCCCTCAT	26	A	0.0246	0.0243
NC_005102.4-123693917	CACCAGGAAACCACATCTCCTTGTTTC	22	T	0.265	0.3504
NC_005102.4-123695932	CACACACACACACACCCTCCCTCAGCC	4	A	0.0328	0.4866
NC_005102.4-123695932	CACACACACACACACCCTCCCTCAGCC	14	A	0.0976	0.0928
NC_005102.4-123701967	CAAAGCAATACCCTATCTCCACATCCC	15	A	0.0328	0.0322
NC_005102.4-123722154	TCTTCTCCTACCTCCTCTCCTTCCCCT	3	T	0.0082	0.479
NC_005102.4-123741945	TAATCATGCACCACTGCTCCTAGCTGG	24	C	0.1368	0.1563
NC_005102.4-123754205	TGTTTAAACACTGTATCTCCTCAGCCT	2	G	0.0593	0.0576
NC_005102.4-123775762	CACACGAAGACTCTCTCTCCTTCAGTA	3	C	0.0948	0.0903
NC_005102.4-123775762	CACACGAAGACTCTCTCTCCTTCAGTA	12	T	0.0087	0.4817
NC_005102.4-123827804	ATTATGTGGACTCTTCCTCCATGGTTA	4	A	0.0084	0.4682
NC_005102.4-123837789	CCCGCATTGACATAGGCTCCAAGCTCT	6	A	0.0082	0.4822
NC_005102.4-123957062	CTCTCTGGAACCTTAGCTCCCTACTCT	12	C	0.0261	0.0425
NC_005102.4-123957062	CTCTCTGGAACCTTAGCTCCCTACTCT	24	C	0.1304	0.1938
NC_005102.4-123960230	CAGATATCAACATGGCCTCCTGCAGTA	1	C	0.0488	0.4881
NC_005102.4-123960230	CAGATATCAACATGGCCTCCTGCAGTA	22	G	0.3171	0.4703
NC_005102.4-123965574	CTAGCATGCACCAGATCTCCTTTTCCT	15	A	0	0.4787
NC_005102.4-123970424	AACCACCGAACCCCATCTCCAGTAGCA	22	G	0	0.4826
NC_005102.4-123970424	AACCACCGAACCCCATCTCCAGTAGCA	23	T	0.2689	0.4779
NC_005102.4-124083274	GATGCAGATACACTGTCTCCCACAGCT	2	A	0	0.4793
NC_005102.4-124094666	GTGGCTGTGACTTCTTCTCCTTCCTGT	14	C	0	0.4851
NC_005102.4-124102627	CCTTTGGTCACACCACCTCCCTTCTCT	12	A	0.0081	0.481
NC_005102.4-124106365	ACTCCTCTTACACATTCTCCATCTAGT	6	T	0.2705	0.454
NC_005102.4-124137653	AAGGTGGGCACAGGAACTCCAACACTG	15	A	0.124	0.323
NC_005102.4-124192608	ATGGAAGCTACTGTGACTCCATGGAAG	8	C	0.2295	0.3447

NC_005102.4-124256367	GGATGATGGACCGAACCTCCAGAAGTG	1	G	0.2735	0.4444
NC_005102.4-124260830	AACTCCCTCACCAATGCTCCCGCACAT	5	C	0.2705	0.454
NC_005102.4-124260830	AACTCCCTCACCAATGCTCCCGCACAT	6	C	0.2951	0.416
NC_005102.4-124269959	CTCATGACCACCGAGTCTCCTGGGGAA	12	C	0.2623	0.3447
NC_005102.4-124286266	CTCCTAGTCACTCCTACTCCCTCTCAA	15	T	0.25	0.3264
NC_005102.4-124297835	CTGATCTCAACCGAACCTCCTTGTGTG	12	C	0.0407	0.0398
NC_005102.4-124334443	GCTTCTGCTACTGTAACTCCCCTGCTG	4	T	0.0357	0.4641
NC_005102.4-124375661	ATTGTAAGCACACCTCCTCCTCTTGTT	8	G	0	0.4793
NC_005102.4-124375661	ATTGTAAGCACACCTCCTCCTCTTGTT	15	T	0.0407	0.0398
NC_005102.4-124403382	ATCTGGACTACGTTCTCTCCCTTCCAC	12	G	0.0847	0.1827
NC_005102.4-124403382	ATCTGGACTACGTTCTCTCCCTTCCAC	27	C	0.0855	0.4956
NC_005102.4-124404557	CAAGAGCACACAACTTCTCCACATTCT	14	C	0.0083	0.4769
NC_005102.4-124404557	CAAGAGCACACAACTTCTCCACATTCT	16	T	0.0492	0.048
NC_005102.4-124410243	GTCATATGCACCTATTCTCCACAATGA	8	G	0.0855	0.1125
NC_005102.4-124430459	GAGACATTTACAGTGACTCCATATTCC	2	A	0.1545	0.2203
NC_005102.4-124444095	ATTCCATTCACCGCCTCTCCAGATGCA	12	C	0.0442	0.0926
NC_005102.4-124498716	TTGAAATAAACCTGGTCTCCAGCTCCT	5	A	0.125	0.4994
NC_005102.4-124498716	TTGAAATAAACCTGGTCTCCAGCTCCT	22	G	0.0714	0.1014
NC_005102.4-124534459	CCACCCTCGACAGTCACTCCTTACCTT	8	C	0	0.4819
NC_005102.4-124534459	CCACCCTCGACAGTCACTCCTTACCTT	22	T	0.1057	0.1425
NC_005102.4-124551254	CTGTCTGTAACTCCAGCTCCAGGACAA	4	T	0.0268	0.0264
NC_005102.4-124585670	CCAGGCGGCACCCCGTCTCCACGGTCC	12	C	0.069	0.0666
NC_005102.4-124596734	AGGTGCAGGACCGAAACTCCCTCTGGT	4	T	0.0583	0.4941
NC_005102.4-124596734	AGGTGCAGGACCGAAACTCCCTCTGGT	5	G	0.2066	0.3421
NC_005102.4-124599021	AATGCTGGCACCCTTACTCCCTGAGGT	6	T	0.0744	0.4913
NC_005102.4-124615601	TCAGTGGCCACAGTGACTCCCCCAAGG	24	A	0.0442	0.4873
NC_005102.4-124631339	TAAGTGCTGACAAGAACTCCATACTAG	1	T	0.0588	0.1609
NC_005102.4-124631339	TAAGTGCTGACAAGAACTCCATACTAG	3	A	0.0588	0.1609
NC_005102.4-124638369	ATAAAAGCCACACAGCCTCCGACGAAC	24	G	0.0086	0.0422
NC_005102.4-124661866	GGTTTCTTCACTGGATCTCCATCTTTC	8	T	0	0.4757
NC_005102.4-124661866	GGTTTCTTCACTGGATCTCCATCTTTC	26	T	0.0342	0.4602
NC_005102.4-124666425	TACCATAGCACCCACTCTCCTAGACAG	8	G	0.2049	0.4879
NC_005102.4-124678153	ACACGATGGACGTTCACTCCTTGGACT	5	G	0	0.4875
NC_005102.4-124697827	CTCATGCACACATACCCTCCCCTACCA	4	A	0.1391	0.2758
NC_005102.4-124697827	CTCATGCACACATACCCTCCCCTACCA	8	A	0.0696	0.4954
NC_005102.4-124793027	AGTCTCTACACACAGGCTCCTTGCTCA	9	C	0	0.48
NC_005102.4-124793027	AGTCTCTACACACAGGCTCCTTGCTCA	23	G	0.2137	0.3685
NC_005102.4-124841631	TCCCAGTAAACTTTCCCTCCAACCCAT	1	T	0.1043	0.4092
NC_005102.4-124841631	TCCCAGTAAACTTTCCCTCCAACCCAT	21	A	0.0336	0.033
NC_005102.4-124882843	GAGATCACCACAGCATCTCCAATATGG	27	G	0.0569	0.4988
NC_005102.4-124894946	GTGCTGAGAACCAAACCTCCATCCACT	23	C	0.0407	0.0398
NC_005102.4-124904954	TCTCCTCACACTGAGCCTCCTCTAGCT	23	T	0.1545	0.1425
NC_005102.4-124978844	TGAACATATACCACCCCTCCCACTCCT	15	C	0.0174	0.4864
NC_005102.4-124980387	ATGATGCACACCGATGCTCCTCACTTC	12	C	0.1607	0.496
NC_005102.4-124980387	ATGATGCACACCGATGCTCCTCACTTC	13	G	0.1579	0.2413
NC_005102.4-125012212	AGATCAGCAACCATAGCTCCTTGAGTT	16	G	0.0325	0.032
NC_005102.4-125060762	ACAGGGTTTACAGGTTCTCCCTAGTAT	22	T	0.2288	0.3388
NC_005102.4-125060931	TGACCCCTCACCTCTACTCCTGTGACC	24	G	0.2541	0.4056
NC_005102.4-125062351	GCTGATGTTACTCTTACTCCTTCAGCC	24	A	0.025	0.0875
NC_005102.4-125096038	AGGGAGATCACCACACCTCCCAGCTCC	6	G	0.0732	0.1287
NC_005102.4-125096038	AGGGAGATCACCACACCTCCCAGCTCC	27	C	0.1475	0.285

NC_005102.4-125271835	TCCCACCTCACCCACACTCCCATCCCA	15	C	0.0085	0.4823
NC_005102.4-125283879	CATAGGTGAACTTCTACTCCTGTTCCC	2	A	0.0085	0.0253
NC_005102.4-125283879	CATAGGTGAACTTCTACTCCTGTTCCC	4	A	0.0086	0.4729
NC_005102.4-125291006	ATCCCCCTGACTCTACCTCCTGTGTGC	9	G	0.0261	0.0257
NC_005102.4-125305269	TGTAAGATTACCTGTGCTCCTGAGCAT	2	G	0	0.4807
NC_005102.4-125305269	TGTAAGATTACCTGTGCTCCTGAGCAT	25	C	0.041	0.0861
NC_005102.4-125385134	GAGCTTTAAACAGCTGCTCCCATGTGT	27	T	0.1917	0.4972
NC_005102.4-125388523	TTCCTTCTCACAAGGGCTCCTTACTGT	26	G	0.0619	0.4735
NC_005102.4-125432240	GATTCACTCACTCACACTCCTGCCTCT	1	G	0.2049	0.321
NC_005102.4-125439249	AGAAACACAACGCCTTCTCCAACAAAA	12	G	0	0.4787
NC_005102.4-125492774	GAGAGGCTCACAGTAACTCCAGCGACT	24	G	0.3689	0.4126
NC_005102.4-125505216	GTTAGACCAACTGGTGCTCCTCCTTTT	15	T	0.1161	0.212
NC_005102.4-125588487	TCCTTTCTCACCTTTCCTCCTTCCCCT	12	C	0.1382	0.1954
NC_005102.4-125588487	TCCTTTCTCACCTTTCCTCCTTCCCCT	16	C	0.0325	0.032
NC_005102.4-125643554	ATCCCTCTAACCCTTCCTCCTCCCCTT	12	C	0.2368	0.2719
NC_005102.4-125643554	ATCCCTCTAACCCTTCCTCCTCCCCTT	21	T	0.3363	0.4464
NC_005102.4-125658915	TGAAGAAAGACTATTGCTCCCACTCTC	13	A	0.1721	0.2339
NC_005102.4-125677818	ATTCACTCCACTTGACCTCCTCAGGTC	23	A	0.0169	0.0333
NC_005102.4-125706688	GTGAAATTCACACATCCTCCTTTGGAC	21	T	0.2232	0.499
NC_005102.4-125722386	GCTGGCTCTACAGATGCTCCCTGGCTT	2	C	0	0.4724
NC_005102.4-125769244	CTTCTCTCCACTGTGTCTCCTCAACAG	3	T	0.0089	0.4512
NC_005102.4-125855678	TTTCCTCTCACACTTGCTCCTCTGCCT	21	T	0.0252	0.0411
NC_005102.4-126182903	ATCTGGAACACCCAGCCTCCTGCCCAT	22	G	0.1382	0.1425
NC_005102.4-126236952	ACTGGGAGGACAGTAGCTCCAGGACAC	4	G	0.0427	0.0418
NC_005102.4-126290932	TATATCAGCACTACTACTCCCATGCTT	13	A	0.2241	0.2501
NC_005102.4-126351117	TTTCCAAGCACCGAGGCTCCAGATGCT	5	C	0	0.4902
NC_005102.4-126351117	TTTCCAAGCACCGAGGCTCCAGATGCT	12	C	0.1404	0.2021
NC_005102.4-126461283	AGGTCATTCACAGTGCCTCCTTTAATA	7	T	0.1639	0.3354
NC_005102.4-126471662	GGTAACCCCACAAGGTCTCCTTGTGTC	2	G	0.0909	0.1017
NC_005102.4-126471662	GGTAACCCCACAAGGTCTCCTTGTGTC	24	T	0.0992	0.109
NC_005102.4-126499135	AGGTGGATTACTAAGGCTCCTTTCTAG	6	G	0.0744	0.4913
NC_005102.4-126499135	AGGTGGATTACTAAGGCTCCTTTCTAG	8	T	0.0579	0.0562
NC_005102.4-126507754	CAAAATGAGACGTTATCTCCTGATGCT	7	G	0.115	0.1239
NC_005102.4-126526641	GAAAGGTAAACATGGGCTCCACTCCCT	24	C	0.0769	0.4947
NC_005102.4-126633065	AGAAGTGGGACATAACCTCCTTATTTT	24	T	0.0932	0.119
NC_005102.4-126655002	TGTCACAGAACACTGCCTCCCGTAGCA	22	G	0.0083	0.4732
NC_005102.4-126655002	TGTCACAGAACACTGCCTCCCGTAGCA	24	A	0.0984	0.1774
NC_005102.4-126688234	CCCTGTAGCACTCCTTCTCCTCACCCC	2	C	0.0339	0.4585
NC_005102.4-126796299	AGCATTCTTACAGCTGCTCCCTACAGT	4	A	0.0667	0.0799
NC_005102.4-126796299	AGCATTCTTACAGCTGCTCCCTACAGT	16	G	0.0932	0.119
NC_005102.4-126878789	GTGCAGGGGACGGAACCTCCCAGCCAT	6	G	0.042	0.0411
NC_005102.4-126994401	ACCAACCAAACCAAAGCTCCATCAGCA	25	G	0	0.4728
NC_005102.4-127051764	TGCTCTTGTACTTTATCTCCAAAAGAA	3	C	0.0263	0.4889
NC_005102.4-127133126	ATTCAATCCACAGCTGCTCCTCTAGTG	8	C	0	0.4773
NC_005102.4-127173206	AAACCACTCACAGGTGCTCCTGTAGGT	16	G	0.0446	0.0605
NC_005102.4-127208512	CACCTTCTTACCCCTGCTCCTAAGGGT	3	C	0.0917	0.0875
NC_005102.4-127208512	CACCTTCTTACCCCTGCTCCTAAGGGT	21	T	0.0917	0.0875
NC_005102.4-127230513	ACCCTTTGTACACATGCTCCAATGTAT	8	G	0.252	0.3964
NC_005102.4-127230513	ACCCTTTGTACACATGCTCCAATGTAT	26	A	0	0.4793
NC_005102.4-127259354	TTCCAAGACACTCAGACTCCAGGTAAG	24	T	0.0263	0.026
NC_005102.4-127279368	CACTCACTCACTTTCTCTCCTCCCTCC	4	T	0.0354	0.4887

NC_005102.4-127279368	CACTCACTCACTTTCTCTCCTCCCTCC	23	C	0.0973	0.0926
NC_005102.4-127280747	AGTCTTGGAACCATTTCTCCGTCAACA	26	C	0.1707	0.2203
NC_005102.4-127284265	GTGAGATTCACAAATCCTCCCATGGTA	12	A	0.2564	0.3974
NC_005102.4-127301325	CTCTCTAAAACCGCACCTCCTCAGGAC	7	A	0	0.4807
NC_005102.4-127301325	CTCTCTAAAACCGCACCTCCTCAGGAC	26	A	0	0.4807
NC_005102.4-127313618	AAGAAATGTACCTGGCCTCCTCAGTAG	12	C	0.1754	0.3324
NC_005102.4-127356956	CATTGGTATACAGGAGCTCCAACTTCA	5	G	0	0.4724
NC_005102.4-127356956	CATTGGTATACAGGAGCTCCAACTTCA	14	G	0	0.4724
NC_005102.4-127391021	TGGGCAAATACCCCTTCTCCCTCTCCC	3	G	0.2069	0.2378
NC_005102.4-127391021	TGGGCAAATACCCCTTCTCCCTCTCCC	21	C	0.0086	0.4803
NC_005102.4-127429359	TGAAGATGTACAAAAACTCCTTAGCCA	9	T	0.0847	0.1116
NC_005102.4-127528064	AAGGGATAAACCAAATCTCCACTCCAC	22	C	0	0.48
NC_005102.4-127616363	AATCACTGAACCATTGCTCCAGCTCCC	27	C	0	0.4826
NC_005102.4-127624588	TTGACTCCTACCGGCTCTCCTCTGACC	12	C	0	0.4703
NC_005102.4-127624588	TTGACTCCTACCGGCTCTCCTCTGACC	27	C	0	0.4703
NC_005102.4-127755370	TGCAGACTGACCGACACTCCTGTGGCC	12	C	0.2314	0.389
NC_005102.4-127755370	TGCAGACTGACCGACACTCCTGTGGCC	22	G	0.2066	0.2704
NC_005102.4-127769205	TCCTATCACACCCTAACTCCAACACCT	5	A	0	0.4813
NC_005102.4-127769205	TCCTATCACACCCTAACTCCAACACCT	26	C	0.2185	0.422
NC_005102.4-127780623	CAGTGGAGAACGGTGGCTCCATGTGCT	1	C	0.0826	0.0792
NC_005102.4-127780623	CAGTGGAGAACGGTGGCTCCATGTGCT	25	G	0.124	0.4146
NC_005102.4-127849763	ATCACCTCCACGTTACCTCCACAGACT	1	A	0.065	0.0629
NC_005102.4-127910945	GTAAGTCAGACAATGGCTCCATCACTG	16	G	0.125	0.1626
NC_005102.4-128044488	ACATATCATACCCAGGCTCCAGAAGTG	21	A	0.3083	0.3708
NC_005102.4-128063653	TATTGACCAACTTATTCTCCAGGCACA	25	A	0.2353	0.4873
NC_005102.4-128076741	TCCAGCTGTACCCTACCTCCTGTCTTT	2	C	0	0.4765
NC_005102.4-128090769	CCTGCCTCCACTTCTACTCCCTGCTTC	2	C	0.0656	0.4988
NC_005102.4-128090769	CCTGCCTCCACTTCTACTCCCTGCTTC	15	T	0.0656	0.4988
NC_005102.4-128112393	GGACACTCTACAGAACCTCCAGCCTTG	6	C	0.3304	0.4263
NC_005102.4-128113709	CTTTCTCGGACCAGGGCTCCTGAGTAT	1	C	0.3077	0.4444
NC_005102.4-128113709	CTTTCTCGGACCAGGGCTCCTGAGTAT	8	G	0	0.4786
NC_005102.4-128135394	GGGTGAGATACCTAGCCTCCTTCTGTG	3	G	0.1083	0.1866
NC_005102.4-128135394	GGGTGAGATACCTAGCCTCCTTCTGTG	7	G	0.1	0.1099
NC_005102.4-128182418	CCTAGTTTTACACTCGCTCCTGAACCT	15	C	0	0.4709
NC_005102.4-128182418	CCTAGTTTTACACTCGCTCCTGAACCT	24	A	0	0.4724
NC_005102.4-128204679	AATTAAGAAACTGTGACTCCACGTTAT	23	G	0	0.4807
NC_005102.4-128211457	ATGATAGACACCAAAGCTCCAGGTCTA	8	A	0	0.4787
NC_005102.4-128255714	CTCCGTTTGACCTTTGCTCCAGTTTTC	5	G	0.0325	0.032
NC_005102.4-128255714	CTCCGTTTGACCTTTGCTCCAGTTTTC	26	T	0.3197	0.4193
NC_005102.4-128270158	CAAAGCAAAACTCCAGCTCCATGTAGT	3	A	0.0738	0.0861
NC_005102.4-128317164	CTAAATACAACACAGCCTCCTCTCCTG	9	A	0	0.4793
NC_005102.4-128317752	AACGCTAAGACCCAGACTCCTTAGACT	3	C	0.0167	0.1099
NC_005102.4-128342303	TTATATAAGACACTGTCTCCACCAAAA	5	A	0.0684	0.497
NC_005102.4-128413608	GAAGCCAAGACGACAGCTCCAGCTTCT	23	C	0	0.4649
NC_005102.4-128517746	AATGGTCTAACTGGGTCTCCAGATAGT	14	G	0	0.0935
NC_005102.4-128534092	TTAAGGGAAACCCTTGCTCCAATGGCA	22	A	0.0902	0.4984
NC_005102.4-128561957	ATCTTACCCACTCCTGCTCCAACTGCC	8	C	0.0244	0.0241
NC_005102.4-128652337	CTAACCAGAACCATATCTCCAACCTCC	7	A	0.2	0.32
NC_005102.4-128657840	CACACACACACACACACTCCTTTAGTT	8	A	0.1111	0.1049
NC_005102.4-128665991	TGGAACCTTACTCCGTCTCCTTTACAG	7	C	0.1622	0.149
NC_005102.4-128685577	TAGAATTGCACTCGGGCTCCGTGCCCT	6	T	0.2459	0.4773

NC_005102.4-128698783	GGGTGCTAGACTGTGTCTCCTTGCATG	9	G	0.0085	0.4776
NC_005102.4-128698783	GGGTGCTAGACTGTGTCTCCTTGCATG	24	C	0.042	0.0411
NC_005102.4-128713327	TCATCCAAGACTCCTGCTCCTTTCTCC	8	A	0.2432	0.4766
NC_005102.4-128713327	TCATCCAAGACTCCTGCTCCTTTCTCC	23	T	0	0.48
NC_005102.4-128795787	ATGAGCTCTACTTTGTCTCCCACTCAT	5	G	0.0089	0.4824
NC_005102.4-128795787	ATGAGCTCTACTTTGTCTCCCACTCAT	12	T	0.0619	0.154
NC_005102.4-128817418	GTCTAGCTCACTGCAACTCCATCCTCC	6	G	0.2541	0.4717
NC_005102.4-128897766	ATATTTGACACTTTCACTCCTCGGTAA	22	C	0	0.4821
NC_005102.4-128912619	TCCCAGCACACCCTTTCTCCCCACACT	15	T	0	0.4755
NC_005102.4-128919141	GTCATGGGAACCTTCTCTCCTAAGGTC	22	A	0.2602	0.4
NC_005102.4-128940112	AGTATTAGCACTGCGACTCCTACAGGT	26	G	0	0.4807
NC_005102.4-128940112	AGTATTAGCACTGCGACTCCTACAGGT	27	T	0	0.4807
NC_005102.4-129004057	CTAGCAACAACTAACTCTCCAGACGTG	23	A	0.1053	0.16
NC_005102.4-129043646	TCCCCCTTAACTCTTCCTCCACCACAT	4	C	0.0488	0.0476
NC_005102.4-129043646	TCCCCCTTAACTCTTCCTCCACCACAT	5	C	0	0.4793
NC_005102.4-129052102	CAGCAAACTACCCCATCTCCTTAATGC	1	C	0.2478	0.39
NC_005102.4-129102784	GTCATGGAAACTGATCCTCCTCTGCCT	26	C	0.2411	0.4512
NC_005102.4-129111588	TGTGTAGAAACTCAAACTCCGAGGTTA	21	G	0.2719	0.4889
NC_005102.4-129166928	GGAATTCTCACCTGGACTCCCTCTTCC	16	A	0	0.4751
NC_005102.4-129181237	AACACACCTACTTTCCCTCCCACTTGG	5	C	0.125	0.232
NC_005102.4-129235467	CTGGTACAGACTCAGGCTCCTTCTTGT	6	A	0.1864	0.2815
NC_005102.4-129320918	TTCTGTGTGACTCAGGCTCCTGGAACA	15	G	0	0.4864
NC_005102.4-129366117	TGAAGGACCACTGTGCCTCCAGAACAC	27	C	0.2162	0.4659
NC_005102.4-129409308	CTAGCAAAAACACTTCCTCCATGGATC	5	C	0.0847	0.0965
NC_005102.4-129509243	CTCCCCGCAACCCTCACTCCCTTCATC	7	G	0.042	0.0882
NC_005102.4-129515654	CATCATCCTACATGAACTCCACTGATC	6	T	0	0.4839
NC_005102.4-129643715	GACCTTGGCACGATTTCTCCTTCATGT	9	C	0.2213	0.3008
NC_005102.4-129643715	GACCTTGGCACGATTTCTCCTTCATGT	16	T	0	0.4728
NC_005102.4-129691554	GAGTGGGCTACCATTTCTCCTTCCCTC	3	G	0.094	0.4994
NC_005102.4-129766927	AACCTAAGTACTAGGACTCCAACATCC	14	G	0.1518	0.1699
NC_005102.4-129798851	ATACTGATTACTGAGGCTCCTGTGACT	4	C	0.0263	0.026
NC_005102.4-129798851	ATACTGATTACTGAGGCTCCTGTGACT	21	T	0.0263	0.026
NC_005102.4-129855133	TCCTTTATTACGGTATCTCCATTTATA	12	G	0.1316	0.4076
NC_005102.4-129855133	TCCTTTATTACGGTATCTCCATTTATA	21	A	0.0088	0.4796
NC_005102.4-129896854	GCCTCCTGTACCTGATCTCCAGGCAGT	6	C	0	0.488
NC_005102.4-129917880	CATAGACTTACTCTGCCTCCATTACTG	9	T	0.0982	0.4952
NC_005102.4-129917880	CATAGACTTACTCTGCCTCCATTACTG	26	T	0.0179	0.4841
NC_005102.4-129967671	TGCTAACGTACAGAAGCTCCTCTGTCT	7	C	0.0813	0.0928
NC_005102.4-129967671	TGCTAACGTACAGAAGCTCCTCTGTCT	8	G	0.0407	0.0398
NC_005102.4-130042752	TCTCCCCCAACTCCTTCTCCAGCTGAA	7	C	0.0588	0.0571
NC_005102.4-130072030	ATGTCTCACACCCTTTCTCCACGTGCC	12	C	0.0517	0.0504
NC_005102.4-130072030	ATGTCTCACACCCTTTCTCCACGTGCC	23	G	0.0167	0.4861
NC_005102.4-130097791	TTTGACCCCACCCTCACTCCTCAGCAC	21	T	0.0083	0.4847
NC_005102.4-130104558	CACACCTTCACTGCCTCTCCAACCTCC	25	T	0	0.4787
NC_005102.4-130109969	CTGCAGGCCACCCTACCTCCATGTTCC	27	C	0.0354	0.0348
NC_005102.4-130130447	AGTCCTTTTACTTCTCCTCCATCTTCT	1	A	0	0.4765
NC_005102.4-130204446	CTGTAGGAAACATGCTCTCCAGTCCTG	23	T	0.1034	0.1431
NC_005102.4-130204446	CTGTAGGAAACATGCTCTCCAGTCCTG	25	C	0	0.4786
NC_005102.4-130269878	TCAGGACCGACCTAGGCTCCCGACGTG	8	C	0.0488	0.0476
NC_005102.4-130269878	TCAGGACCGACCTAGGCTCCCGACGTG	25	G	0.1707	0.2323
NC_005102.4-130278830	TCCTGCCTGACTCAGACTCCAGGGGAA	16	A	0	0.4778

NC_005102.4-130312468	ATAGAGAAGACAAGCTCTCCACTGGGA	26	G	0.0088	0.4796
NC_005102.4-130336451	CTGCTGGGTACGCAGCCTCCCTTAAGA	12	G	0.0246	0.0243
NC_005102.4-130341526	CCCTGCCCTACTTCTCCTCCTTGTGCT	12	T	0.0672	0.065
NC_005102.4-130345122	CTAAACATGACTCCTTCTCCAGTATGA	6	C	0.1947	0.3728
NC_005102.4-130345122	CTAAACATGACTCCTTCTCCAGTATGA	24	A	0.1947	0.3728
NC_005102.4-130456402	GACTAATTGACAAATCCTCCGGACACA	21	G	0.0656	0.0634
NC_005102.4-130502547	CACACTCAAACACCCACTCCTACATAA	23	C	0.1382	0.4979
NC_005102.4-130507682	TACATGCTCACACCTTCTCCCATACAT	26	A	0.2881	0.4585
NC_005102.4-130592976	TCCTTCACAACATAAGCTCCGTTCGTG	1	T	0	0.4773
NC_005102.4-130592976	TCCTTCACAACATAAGCTCCGTTCGTG	21	G	0.2705	0.4056
NC_005102.4-130605873	ATGTACCTCACTCTCTCTCCCCAGAAA	7	C	0.2241	0.2739
NC_005102.4-130605873	ATGTACCTCACTCTCTCTCCCCAGAAA	26	A	0.0087	0.4849
NC_005102.4-130632648	CTAAGGAATACATATTCTCCTCTGTAG	24	G	0	0.4807
NC_005102.4-130654119	AAACTGCACACCACATCTCCACAGTAA	15	A	0.2655	0.4793
NC_005102.4-130774528	GGTGTAATCACACTGACTCCTAAAGCT	5	T	0.1034	0.1855
NC_005102.4-130774528	GGTGTAATCACACTGACTCCTAAAGCT	25	G	0	0.482
NC_005102.4-130784748	GTAATGTTGACTCTGCCTCCTCTTCTC	12	T	0	0.4813
NC_005102.4-130784748	GTAATGTTGACTCTGCCTCCTCTTCTC	15	G	0.2333	0.4328
NC_005102.4-130806103	TATCTACACACAAATGCTCCCCCGTTT	21	C	0.1624	0.4947
NC_005102.4-130848035	AGCTGACTGACCACTGCTCCTTAGGCA	9	G	0.0351	0.4875
NC_005102.4-130848035	AGCTGACTGACCACTGCTCCTTAGGCA	12	C	0.0351	0.4875
NC_005102.4-130850552	CAGCACAAGACGCCTCCTCCTTATTTC	12	G	0	0.4841
NC_005102.4-130859562	AGCTAAGAAACTGAGTCTCCTGGAAGA	1	A	0.0089	0.0264
NC_005102.4-130919112	CTGTGTAAAACATGTCCTCCATCATGA	21	A	0.2807	0.3615
NC_005102.4-130919112	CTGTGTAAAACATGTCCTCCATCATGA	25	T	0.0179	0.4592
NC_005102.4-131027402	TTTCTGATCACTTGGACTCCTTGGACT	22	T	0.1316	0.3273
NC_005102.4-131081612	GGACTTGTGACTCCAGCTCCATGTGTA	8	T	0.0261	0.4946
NC_005102.4-131096176	TACCATCTCACACAGCCTCCTCCATAC	2	A	0	0.4793
NC_005102.4-131102688	TTACCTTAAACCCTGCCTCCTGGTTTA	13	C	0.0261	0.0257
NC_005102.4-131144395	TTAATCAGCACACTCACTCCTCTTGGG	16	A	0.2203	0.3529
NC_005102.4-131144395	TTAATCAGCACACTCACTCCTCTTGGG	25	G	0.0085	0.4776
NC_005102.4-131209267	ACTGATCCTACATGCACTCCATGATTA	14	G	0.0261	0.0752
NC_005102.4-131261875	CTTACCTCTACTCTTCCTCCCTGACTG	6	C	0.1488	0.1377
NC_005102.4-131306261	TGCACATATACACACACTCCAAGAATT	16	A	0.2679	0.2934
NC_005102.4-131313930	TGGGGAAGGACACCGTCTCCAGCTACA	9	G	0.0089	0.4709
NC_005102.4-131313930	TGGGGAAGGACACCGTCTCCAGCTACA	15	G	0.0179	0.4731
NC_005102.4-131341881	CCCCGTGCCACCTTATCTCCTCTGGCA	4	C	0.0917	0.4416
NC_005102.4-131341881	CCCCGTGCCACCTTATCTCCTCTGGCA	7	G	0.0417	0.0566
NC_005102.4-131368038	GAGGATAGAACCATGACTCCCATTCGG	3	G	0.0081	0.4777
NC_005102.4-131374985	AACTGCTGGACCATATCTCCATCATCA	3	C	0.1565	0.1443
NC_005102.4-131408500	TCACACAGAACTTCTTCTCCAACCTTC	6	C	0.2069	0.3074
NC_005102.4-131441365	AGGAGTGCCACTGTTACTCCAGTGGAT	15	T	0.0333	0.0488
NC_005102.4-131461995	TGACTGGAGACTGGTCCTCCTATATTC	4	C	0.0756	0.0728
NC_005102.4-131461995	TGACTGGAGACTGGTCCTCCTATATTC	25	T	0.0756	0.0728
NC_005102.4-131524480	TTCTTTCTTACTGCCTCTCCAAAAGGA	22	A	0.0088	0.4581
NC_005102.4-131615099	CCACTCAGGACCCTCTCTCCCTCTGCC	12	C	0.1304	0.1219
NC_005102.4-131645561	TAATCACAAACTCCTCCTCCTGCTCCA	22	G	0.2017	0.3118
NC_005102.4-131652194	TGAGCACGAACCATCACTCCTTACTGT	7	C	0.0957	0.1067
NC_005102.4-131655168	CCTGGGGGCACCAAGTCTCCATAGGAT	8	G	0.3089	0.4
NC_005102.4-131729234	CTTCTTACCACTCTCCCTCCTTTTGTC	21	T	0	0.4764
NC_005102.4-131743267	CCCTCTTGCACCCCTTCTCCCCACAGT	1	C	0.0531	0.0517

NC_005102.4-131744703	GCCCTTTATACCCACTCTCCACCATTG	13	C	0.1707	0.4777
NC_005102.4-131777397	ACATGGAGGACCCTCCCTCCTTTTGAG	5	G	0.2049	0.479
NC_005102.4-131777397	ACATGGAGGACCCTCCCTCCTTTTGAG	27	G	0.0331	0.0325
NC_005102.4-131785750	CCACCACTCACTGCAGCTCCCACAGAG	26	A	0	0.4787
NC_005102.4-131822679	TATCCCAAGACTCTTGCTCCTTAAGTC	7	A	0.0417	0.0408
NC_005102.4-132063638	TTGTTAATCACTATGCCTCCATGTCTG	5	T	0.0082	0.0243
NC_005102.4-132188524	GAAAGGCTTACCCATTCTCCTGACTCT	7	C	0.175	0.2941
NC_005102.4-132189292	TTACCTTAGACACATGCTCCTAGCATG	23	G	0.2609	0.2758
NC_005102.4-132190658	TTCCTCACCACCCACACTCCTTCAAAT	13	C	0.1593	0.3346
NC_005102.4-132330992	CAATAGTGCACGCATTCTCCACTGCCT	14	A	0.069	0.1717
NC_005102.4-132366946	CTCAGAGACACCAGTGCTCCTTTGCCT	2	T	0.0696	0.3938
NC_005102.4-132439737	CTTATAGTCACTTACACTCCTTATAAT	22	T	0.0893	0.0853
NC_005102.4-132439737	CTTATAGTCACTTACACTCCTTATAAT	23	A	0.0877	0.1305
NC_005102.4-132512443	CGTACAGAGACTGCACCTCCTGAGATT	4	A	0.2414	0.3282
NC_005102.4-132602486	GAAGTTGAAACATTGTCTCCATGTACA	2	A	0.0661	0.0639
NC_005102.4-132613690	AAACCTAAGACTAATTCTCCTGGATAT	4	C	0	0.4827
NC_005102.4-132613690	AAACCTAAGACTAATTCTCCTGGATAT	14	A	0.2252	0.4869
NC_005102.4-132658972	CAGTGGCCCACAGGCTCTCCAGACTTC	8	C	0.0087	0.1067
NC_005102.4-132660630	AGCAGTCTCACCATCCCTCCAGGGGCT	9	C	0.2051	0.2945
NC_005102.4-132676363	AAGGGTTTCACAAGGTCTCCCCTTTAC	6	T	0	0.4833
NC_005102.4-132679137	TAACACGAGACTGTCCCTCCAACACAT	6	C	0.157	0.4988
NC_005102.4-132679137	TAACACGAGACTGTCCCTCCAACACAT	23	C	0	0.4813
NC_005102.4-132710446	CTCCCCCAAACATACTCTCCCTCGCAT	13	T	0	0.4793
NC_005102.4-132740033	AAGTCCCTCACAGTGACTCCCAATGAT	26	A	0.113	0.4976
NC_005102.4-132745204	CACCACACTACCCATGCTCCTGTCCCC	6	C	0.0248	0.0405
NC_005102.4-132788761	AGCCCTCTGACTCACTCTCCTGACAGT	1	A	0	0.4793
NC_005102.4-132790587	TTTCGAGTGACTTAAGCTCCATGTTAG	14	A	0.0667	0.1099
NC_005102.4-132790587	TTTCGAGTGACTTAAGCTCCATGTTAG	23	G	0.05	0.0488
NC_005102.4-132803900	TCCCTGTACACTAGACCTCCTTTTTCT	5	T	0	0.4839
NC_005102.4-132803900	TCCCTGTACACTAGACCTCCTTTTTCT	6	G	0.2672	0.4179
NC_005102.4-132805851	CTACCTGATACTCTAGCTCCCGAGGAT	2	T	0.1826	0.4986
NC_005102.4-132805851	CTACCTGATACTCTAGCTCCCGAGGAT	22	G	0.2435	0.2519
NC_005102.4-132812087	CCCTCTCTAACTCCCTCTCCCTCTCTT	8	T	0.0083	0.4769
NC_005102.4-132830937	TCCTAGGGAACACAAACTCCCACTGGG	12	A	0.1008	0.4983
NC_005102.4-132830937	TCCTAGGGAACACAAACTCCCACTGGG	27	G	0.084	0.0805
NC_005102.4-132845624	AGGAACTCTACATAAGCTCCCACATAA	2	G	0.0678	0.0655
NC_005102.4-132886234	AGACCCTCAACCTAGCCTCCTGGAAGC	5	C	0.1826	0.2332
NC_005102.4-132886234	AGACCCTCAACCTAGCCTCCTGGAAGC	14	A	0.1826	0.4962
NC_005102.4-132893760	AAACAATCAACTCCAACTCCTCAAACT	14	C	0.2521	0.4347
NC_005102.4-132904983	TGATTCTCCACAACCTCTCCTGCCTTT	14	C	0.0081	0.0241
NC_005102.4-132950068	GGGTAAGGGACTAAACCTCCGTACTGT	26	G	0.187	0.208
NC_005102.4-132979628	CCCCTCCCCACTCCCCCTCCTCCTCTC	12	T	0.0439	0.0429
NC_005102.4-133024031	CAGGCCTGGACACAAGCTCCTCTATAT	24	A	0	0.4671
NC_005102.4-133024031	CAGGCCTGGACACAAGCTCCTCTATAT	25	T	0.2252	0.3817
NC_005102.4-133027653	TGCTTTTGCACACCATCTCCAGGAGGC	13	C	0.0984	0.1367
NC_005102.4-133027653	TGCTTTTGCACACCATCTCCAGGAGGC	22	G	0	0.4787
NC_005102.4-133043991	TAAGCCTTAACTCTGTCTCCAGATATA	13	C	0.0517	0.4666
NC_005102.4-133102680	CATGAAGGCACACCACCTCCTATATCC	22	A	0.1293	0.5
NC_005102.4-133104408	TAAAGGAAGACCTTATCTCCTCCCATC	6	G	0.0783	0.0911
NC_005102.4-133126674	TAATCTCCTACTTACACTCCTTGACTG	1	T	0.2541	0.4973
NC_005102.4-133169652	AGAGCACTGACTACTCCTCCACTGGAT	1	A	0	0.48

NC_005102.4-133172150	CGTCAACCCACCAGCTCTCCCACACTC	15	C	0.0088	0.4889
NC_005102.4-133222141	ATGATCTCCACTTCAACTCCAGAGTCC	27	C	0.0088	0.483
NC_005102.4-133250615	GCCTGTACAACCCCGTCTCCAATCCCC	5	G	0	0.4864
NC_005102.4-133260309	TAGGCAAAGACTGTACCTCCTAGCTAT	2	A	0.2417	0.4416
NC_005102.4-133262325	ACATGCTACACCTTTCCTCCTTCTGGT	1	A	0.0084	0.4859
NC_005102.4-133269062	GTCTGGGGCACCAGGCCTCCATCCCTG	9	C	0.065	0.4926
NC_005102.4-133269062	GTCTGGGGCACCAGGCCTCCATCCCTG	21	A	0.0569	0.0705
NC_005102.4-133273414	AGAAAGCTGACTGAGTCTCCTCTCTGT	6	G	0.1803	0.4999
NC_005102.4-133273414	AGAAAGCTGACTGAGTCTCCTCTCTGT	13	G	0	0.4807
NC_005102.4-134128623	CACACACAAACACACACTCCAACACAC	1	C	0.0088	0.4923
NC_005102.4-134276069	CTTGGGACCACTTGTGCTCCTGTAAGT	21	T	0	0.4807
NC_005102.4-134295403	CCCTCCCTCACCTCTTCTCCTCCACTA	3	C	0	0.4793
NC_005102.4-134295403	CCCTCCCTCACCTCTTCTCCTCCACTA	16	T	0	0.4793
NC_005102.4-134303042	TAGGGCACTACCTTTTCTCCCTGCTCT	3	G	0	0.4704
NC_005102.4-134303042	TAGGGCACTACCTTTTCTCCCTGCTCT	15	T	0.036	0.425
NC_005102.4-134307176	GTCCTTGGAACTCTGCCTCCATGGTAT	8	G	0.0504	0.0491
NC_005102.4-134307176	GTCCTTGGAACTCTGCCTCCATGGTAT	16	C	0.0672	0.494
NC_005102.4-134389027	AAGATTGAAACAATCCCTCCAGGAGAC	5	T	0.0439	0.0918
NC_005102.4-134389199	CCTTTGAAGACTTCAACTCCATTTCTG	12	T	0.2033	0.4979
NC_005102.4-134482000	AGAGCGTGAACTATGCCTCCATGGTCA	25	T	0.1062	0.4887
NC_005102.4-134570894	TAGCTTTTCACAGTAACTCCCTTACTA	5	T	0.0082	0.4755
NC_005102.4-134583863	TTAAAGAGAACATTATCTCCGTTAGCT	16	T	0.0847	0.0965
NC_005102.4-134646712	GTTACTGTTACTGTGTCTCCTTAACTG	1	G	0.0417	0.0566
NC_005102.4-134646712	GTTACTGTTACTGTGTCTCCTTAACTG	7	G	0.0417	0.0566
NC_005102.4-134674844	AGTTGCTTTACTGCCCCTCCTGTCCCA	14	C	0.1429	0.1609
NC_005102.4-134779443	TTTCATGCTACTCGCCCTCCATGTCTT	14	G	0.0826	0.4972
NC_005102.4-134779443	TTTCATGCTACTCGCCCTCCATGTCTT	24	T	0.0328	0.0634
NC_005102.4-134786211	GAGTGAAAGACATCATCTCCCACAGGA	6	A	0.0804	0.0934
NC_005102.4-134790290	CTCTCACACACCTATGCTCCTTTTAGA	14	A	0.2301	0.4207
NC_005102.4-134820023	GGACTAGGGACTAGGTCTCCTGTTATT	9	G	0.2893	0.3421
NC_005102.4-134841730	CTAAATCCTACATCTACTCCTACTCAT	25	C	0.1271	0.2026
NC_005102.4-134921325	ACCCAGTCTACATAGGCTCCAGCTCTA	12	A	0.1754	0.1884
NC_005102.4-134935680	TAACTACAGACCAAATCTCCATTCCTT	22	T	0	0.4833
NC_005102.4-134935680	TAACTACAGACCAAATCTCCATTCCTT	23	T	0.2696	0.3147
NC_005102.4-135069609	TGGTCATGAACTCACTCTCCGAGTATA	7	T	0.025	0.4847
NC_005102.4-135078199	CACACATCCACTCTGACTCCATGCACT	4	A	0.0244	0.0553
NC_005102.4-135117633	CACTCAGCAACAGAAACTCCAAACTCT	4	T	0.178	0.3388
NC_005102.4-135118934	ACAACACAGACAGTTACTCCAAAGAAA	16	A	0	0.4779
NC_005102.4-135260813	GCCTGGTTGACTGTTCCTCCCACAGCA	26	C	0	0.4793
NC_005102.4-135260813	GCCTGGTTGACTGTTCCTCCCACAGCA	27	A	0	0.4793
NC_005102.4-135278435	GTCAAGCATACCCCACCTCCTTTCCCA	26	C	0.0427	0.074
NC_005102.4-135304891	CAATGCCAAACACTTTCTCCAAGTCAG	12	A	0.1429	0.1327
NC_005102.4-135304891	CAATGCCAAACACTTTCTCCAAGTCAG	27	G	0.1333	0.1244
NC_005102.4-135308556	CATTGAATCACCACCACTCCCTACCCT	3	T	0.0522	0.4364
NC_005102.4-135308556	CATTGAATCACCACCACTCCCTACCCT	5	G	0	0.4724
NC_005102.4-135326316	GACTGTTGAACACTGTCTCCATCTTTT	14	T	0.0339	0.0655
NC_005102.4-135332001	ATTATATGTACTTTTACTCCTTGAAGT	8	G	0.1681	0.2367
NC_005102.4-135342051	GTAACAAGTACATTAACTCCATGATTG	5	C	0.0702	0.0997
NC_005102.4-135344528	GTAGATATCACTCTGTCTCCATGTTGG	14	T	0.1417	0.3708
NC_005102.4-135344528	GTAGATATCACTCTGTCTCCATGTTGG	24	T	0.1417	0.3708
NC_005102.4-135349622	CCTGCTGCCACTCCGCCTCCTCTACTT	14	C	0.0976	0.1356

NC_005102.4-135362420	CACCATTTAACTATAACTCCCATTCTT	25	C	0.0256	0.0253
NC_005102.4-135363182	TATAACCCAACCGAGTCTCCAAAGAAA	3	T	0.0984	0.1641
NC_005102.4-135396984	CCACTCCCCACCCCCACTCCATACACA	1	C	0.025	0.0247
NC_005102.4-135396984	CCACTCCCCACCCCCACTCCATACACA	5	T	0.0331	0.0325
NC_005102.4-135443477	CATGGAAACACTCTTCCTCCCTCTTAT	26	A	0	0.4793
NC_005102.4-135444402	CTATGCCTGACAAATCCTCCGCCGATA	23	C	0.2931	0.4709
NC_005102.4-135545047	AGGAATGTGACTGGTGCTCCCATTAGC	27	C	0	0.4555
NC_005102.4-135548066	GAATGTAGCACAGTCACTCCTCAAATG	22	C	0	0.032
NC_005102.4-135667814	AGGATGAAAACTTGATCTCCTGAATAC	6	G	0.2893	0.3601
NC_005102.4-135740791	TCCCACCAAACAGAGCCTCCTAAGCAG	9	A	0.2049	0.4973
NC_005102.4-135776362	CTGAAGACTACTGATTCTCCCTTCCCT	27	T	0.2149	0.389
NC_005102.4-135781163	AGGCAAGAAACGTTATCTCCAAGTAAT	12	G	0.0252	0.0249
NC_005102.4-135792837	CCAGTTTTCACTTTGGCTCCTTGAGGA	2	C	0	0.4819
NC_005102.4-135792837	CCAGTTTTCACTTTGGCTCCTTGAGGA	3	A	0	0.4819
NC_005102.4-135814948	CCAAGCTCGACATGGTCTCCTTGGGCT	9	G	0	0.4807
NC_005102.4-135902667	GAAGGTATCACGCATGCTCCAGGGTTG	12	G	0.157	0.1982
NC_005102.4-135960186	ACAGCATCAACATCATCTCCATCAAAT	9	A	0.2991	0.4947
NC_005102.4-136048428	TGACTGTGGACTCTTTCTCCTCATGCC	25	G	0.2358	0.4523
NC_005102.4-136090028	TAAGCAACAACACCTGCTCCTTGATAC	26	A	0.2712	0.334
NC_005102.4-136149105	TGAAGTTCCACAACTTCTCCAACTGCC	2	G	0.2458	0.4066
NC_005102.4-136174589	AGTATGTTGACTTAGTCTCCTTTGGAA	4	A	0.0948	0.1504
NC_005102.4-136262665	GCCCCTCCCACCCCCTCTCCATTTCTT	8	C	0.0763	0.4939
NC_005102.4-136262665	GCCCCTCCCACCCCCTCTCCATTTCTT	12	C	0.0763	0.4939
NC_005102.4-136317154	ATTCTATGGACACTAGCTCCAGAGAGA	12	A	0.2435	0.4864
NC_005102.4-136345910	CATTTAGCCACATTGCCTCCTCCTAAA	5	T	0	0.4751
NC_005102.4-136375432	TTCAGAGAAACACGAGCTCCTTCACGT	14	G	0.2137	0.3
NC_005102.4-136375432	TTCAGAGAAACACGAGCTCCTTCACGT	16	G	0.1897	0.2739
NC_005102.4-136381220	CAACAATAGACCTTTCCTCCAAGCTCA	1	C	0.0246	0.0243
NC_005102.4-136381220	CAACAATAGACCTTTCCTCCAAGCTCA	27	A	0.0656	0.0935
NC_005102.4-136436028	TTTTTCTCAACATCATCTCCATGCCAA	3	T	0.0513	0.4325
NC_005102.4-136436028	TTTTTCTCAACATCATCTCCATGCCAA	22	T	0	0.4757
NC_005102.4-136441378	CCATCTATAACTGTAGCTCCGTGAATT	21	G	0	0.4819
NC_005102.4-136467266	CTTTTGAATACTGGGTCTCCACTTCAG	7	A	0.0424	0.0576
NC_005102.4-136467266	CTTTTGAATACTGGGTCTCCACTTCAG	21	A	0.2522	0.466
NC_005102.4-136545102	CAGTCAAGCACCTACTCTCCTATCTGA	27	A	0	0.4765
NC_005102.4-136606960	GCCCCTGTCACCAGGCCTCCACCCACA	8	T	0	0.4787
NC_005102.4-136606960	GCCCCTGTCACCAGGCCTCCACCCACA	15	G	0.325	0.3533
NC_005102.4-136609693	TTAGGATTGACATCAACTCCAGCATTT	8	T	0.0261	0.0257
NC_005102.4-136622979	AAACTGTTTACCCTGCCTCCACCATGT	8	T	0	0.4819
NC_005102.4-136633118	TGATGCGAGACCTACCCTCCAGGGGAA	16	C	0.0252	0.0249
NC_005102.4-136644513	GTCAGCAAGACCAAGCCTCCAACAGGT	8	A	0	0.4912
NC_005102.4-136644513	GTCAGCAAGACCAAGCCTCCAACAGGT	12	C	0.0973	0.1084
NC_005102.4-136696892	CCAGTGAAAACAGTATCTCCACTTTAG	3	A	0	0.4813
NC_005102.4-136755824	CTCTCCTCAACAGCTCCTCCAAAGTGT	2	T	0.0082	0.4755
NC_005102.4-136756286	AGATGGCTCACTTGATCTCCAGGACTT	2	G	0.042	0.449
NC_005102.4-136761096	TTGCCCTTCACAGTGACTCCTTCCAGT	4	C	0.2941	0.2961
NC_005102.4-136761096	TTGCCCTTCACAGTGACTCCTTCCAGT	16	A	0.2941	0.2961
NC_005102.4-136799988	TCTCCATGCACAGTCCCTCCTACTCCC	1	T	0.0826	0.2415
NC_005102.4-136799988	TCTCCATGCACAGTCCCTCCTACTCCC	25	C	0.2213	0.3307
NC_005102.4-136806057	TCTGGCCTCACACTACCTCCTCTACAG	2	C	0.225	0.3441
NC_005102.4-136875563	GGGAAAGGCACTCTATCTCCACGCTCC	22	C	0.0083	0.4783

NC_005102.4-136885186	TATGTGCATACCCAGTCTCCCTCAACA	15	G	0.0522	0.4537
NC_005102.4-136911980	TCGCATCTGACTCAGCCTCCTCTCCTG	5	A	0.0086	0.4836
NC_005102.4-136927519	TCCAACCAGACCTAATCTCCAATGCAG	14	A	0.1901	0.172
NC_005102.4-136927519	TCCAACCAGACCTAATCTCCAATGCAG	27	G	0.0909	0.172
NC_005102.4-137124761	ACAGGTCATACAGCGTCTCCTTTATAA	5	G	0.0902	0.0861
NC_005102.4-137124761	ACAGGTCATACAGCGTCTCCTTTATAA	7	C	0.0574	0.071
NC_005102.4-137151222	AGAACTCTTACAGCTTCTCCAATGGCT	12	A	0.0167	0.4728
NC_005102.4-137151222	AGAACTCTTACAGCTTCTCCAATGGCT	13	G	0.0583	0.1172
NC_005102.4-137175894	ATCAGCACCACGAGGGCTCCTGGCTGG	23	G	0.0348	0.0832
NC_005102.4-137177154	AGATCAATGACTGAGCCTCCTGACTTT	27	T	0.0088	0.4648
NC_005102.4-137210857	ACTGTGGAAACAGCAGCTCCAGTAGCC	4	G	0.1818	0.4997
NC_005102.4-137226652	TAGCTTTAAACATCTTCTCCACCAGAA	2	A	0	0.4724
NC_005102.4-137260119	CTGGCACTCACAGTTCCTCCAAGAAAC	14	T	0	0.4793
NC_005102.4-137325749	GAATTTCTGACCCTGTCTCCTTCATTT	12	C	0.0625	0.0771
NC_005102.4-137328435	TGCTTCTCTACTCCCTCTCCTGGTTCT	1	T	0	0.4787
NC_005102.4-137328435	TGCTTCTCTACTCCCTCTCCTGGTTCT	3	C	0.0165	0.0639
NC_005102.4-137415428	CACAGATCCACACAGGCTCCACGGTTG	26	T	0.0163	0.032
NC_005102.4-137484443	CTCAGAACAACCAAGACTCCAACCCAT	1	C	0.0351	0.0677
NC_005102.4-137570520	CTCTTTACCACAGCCACTCCTCAGATT	5	T	0.3115	0.4737
NC_005102.4-137570520	CTCTTTACCACAGCCACTCCTCAGATT	26	T	0	0.4773
NC_005102.4-137616237	AGCACCCACACAGTAACTCCAGAGGGT	12	A	0.0086	0.4643
NC_005102.4-137617400	GTTTCTAAAACCCTTGCTCCCACTCTG	21	C	0.114	0.3661
NC_005102.4-137692591	CTGGGAAGGACCCTTTCTCCAGCTGTC	15	T	0.225	0.325
NC_005102.4-137701771	CGTATGATGACTTAGACTCCATTACTC	1	C	0.2931	0.4071
NC_005102.4-137707879	ACTCAACACACTTTGTCTCCCTGAGTC	2	C	0.2314	0.3374
NC_005102.4-137707879	ACTCAACACACTTTGTCTCCCTGAGTC	3	T	0.2314	0.3374
NC_005102.4-137717968	ACCTCCCCAACCCCATCTCCCCAGCAT	3	C	0.0407	0.0705
NC_005102.4-137739160	TGAAGTTCTACACGGGCTCCATGGGAG	13	C	0	0.4793
NC_005102.4-137739160	TGAAGTTCTACACGGGCTCCATGGGAG	16	G	0	0.4793
NC_005102.4-137740867	GGTTTTGAGACACCATCTCCCTATGTA	7	G	0.0598	0.074
NC_005102.4-137740867	GGTTTTGAGACACCATCTCCCTATGTA	15	A	0.3017	0.4375
NC_005102.4-137748443	CCACACGAAACAGCAACTCCACATGAA	13	G	0.2832	0.4404
NC_005102.4-137749894	TGCTAATTGACACTTCCTCCCTTGCTG	21	C	0	0.474
NC_005102.4-137798338	ATTCACTAGACATCTTCTCCCTCAAAC	1	A	0.1148	0.4966
NC_005102.4-137798338	ATTCACTAGACATCTTCTCCCTCAAAC	8	A	0.0081	0.481
NC_005102.4-137881656	AGGTGAGTGACTATCACTCCATCATTG	4	T	0.1789	0.189
NC_005102.4-137908977	TCCTTTGTAACCCCTTCTCCAGCCAGA	6	T	0.2203	0.4632
NC_005102.4-137922707	CAAGTTGGTACTGACCCTCCAAACAAA	26	A	0.2368	0.4529
NC_005102.4-137932873	CCCACCCTGACCTCTTCTCCTTCCATT	3	C	0.2143	0.3838
NC_005102.4-137943079	GTCAGGCACACCAGCTCTCCTCACAGA	5	G	0.0894	0.0854
NC_005102.4-137943079	GTCAGGCACACCAGCTCTCCTCACAGA	12	C	0.2602	0.4331
NC_005102.4-137961580	CGTTCACTAACTCCAACTCCCTCTTTT	3	T	0	0.4751
NC_005102.4-137961580	CGTTCACTAACTCCAACTCCCTCTTTT	26	T	0.2705	0.4193
NC_005102.4-137962104	GCGACAACCACACCAACTCCCTATGTT	3	G	0.0957	0.0911
NC_005102.4-137974602	GTAACCCTCACACCTCCTCCAACTCCT	22	A	0.0783	0.0752
NC_005102.4-137986527	TTCCAATGGACCAACCCTCCAGAGACG	3	C	0	0.4731
NC_005102.4-137986527	TTCCAATGGACCAACCCTCCAGAGACG	26	C	0.0796	0.1084
NC_005102.4-138012085	GAATCCAACACGCAGCCTCCGTTGGAG	2	A	0.0331	0.0325
NC_005102.4-138012085	GAATCCAACACGCAGCCTCCGTTGGAG	8	A	0.0331	0.0639
NC_005102.4-138062107	TCTGCTGAGACCATCTCTCCAAGCCCC	9	G	0	0.4787
NC_005102.4-138062107	TCTGCTGAGACCATCTCTCCAAGCCCC	24	C	0.0328	0.0322

NC_005102.4-138072512	AGCCTGGCCACTGTTCCTCCTTCTAGC	9	C	0.0259	0.0255
NC_005102.4-138072512	AGCCTGGCCACTGTTCCTCCTTCTAGC	22	T	0.0259	0.0255
NC_005102.4-138093100	TAAACACTCACCCCCGCTCCTGTATAA	6	A	0	0.474
NC_005102.4-138131437	CTTCTTTCCACCTGAACTCCCAGTCCT	24	T	0	0.4832
NC_005102.4-138152359	CCAGCTGGCACCAGGCCTCCTTTCCAG	4	G	0.05	0.0488
NC_005102.4-138152359	CCAGCTGGCACCAGGCCTCCTTTCCAG	25	C	0.1667	0.1665
NC_005102.4-138166289	CAGGGACACACCTATGCTCCCATATTC	13	T	0	0.4833
NC_005102.4-138166289	CAGGGACACACCTATGCTCCCATATTC	15	T	0	0.4833
NC_005102.4-138168720	AGTAGAACAACACTATCTCCTTTTGCA	15	A	0.1724	0.1576
NC_005102.4-138168720	AGTAGAACAACACTATCTCCTTTTGCA	24	T	0.0609	0.0911
NC_005102.4-138185720	CGGTATGGTACTGCCTCTCCACAGCCA	2	G	0	0.482
NC_005102.4-138203398	AATGCTACCACGGAGCCTCCAACCTCA	27	A	0.1795	0.4227
NC_005102.4-138241397	GCGATGAACACCCAGTCTCCCCTGCCA	16	T	0.1875	0.3211
NC_005102.4-138241397	GCGATGAACACCCAGTCTCCCCTGCCA	27	A	0	0.4731
NC_005102.4-138257146	TCATTTCTTACAGTGACTCCAAGAATC	25	A	0.1818	0.4959
NC_005102.4-138259119	TATGGACACACTCTGGCTCCTGTTCAC	3	T	0.0796	0.0765
NC_005102.4-138259119	TATGGACACACTCTGGCTCCTGTTCAC	24	T	0	0.461
NC_005102.4-138276087	TCTGAAACTACAAGCTCTCCTAGTGCC	21	T	0.1552	0.375
NC_005102.4-138322287	GCACTGGAAACCTTGGCTCCAGAGACA	5	T	0.1322	0.4991
NC_005102.4-138322923	GAAGCTATCACTCCCTCTCCCTCTCCC	2	A	0.2314	0.3557
NC_005102.4-138322923	GAAGCTATCACTCCCTCTCCCTCTCCC	15	C	0.0492	0.048
NC_005102.4-138323840	GTTGGGAAGACCGTTTCTCCATTAGGT	2	T	0.0328	0.0322
NC_005102.4-138330094	TACCTGAGAACCATTCCTCCCCAGACA	6	G	0.157	0.1982
NC_005102.4-138330094	TACCTGAGAACCATTCCTCCCCAGACA	21	C	0.2314	0.4751
NC_005102.4-138330522	GAACTCTCGACATAGGCTCCCTGATGT	9	G	0.1545	0.1826
NC_005102.4-138330650	AAGTTTACAACCCGTTCTCCAACACTG	3	G	0.1842	0.463
NC_005102.4-138346558	AAATACACAACAAATCCTCCTGCCTCT	6	C	0.1167	0.1387
NC_005102.4-138346558	AAATACACAACAAATCCTCCTGCCTCT	22	G	0.0917	0.1172
NC_005102.4-138351876	GATGTGGCAACTTATGCTCCATGACCC	8	C	0.1271	0.2282
NC_005102.4-138351876	GATGTGGCAACTTATGCTCCATGACCC	9	A	0.0593	0.4738
NC_005102.4-138355162	TGAGTGGACACTGAGGCTCCTCCTAAA	1	T	0.1983	0.2057
NC_005102.4-138390765	TGTTACATTACATACACTCCTAAATAC	6	C	0.0684	0.066
NC_005102.4-138390765	TGTTACATTACATACACTCCTAAATAC	23	A	0.0684	0.066
NC_005102.4-138474017	TTAAGGAGCACAGCCCCTCCCCATTGT	13	G	0	0.4793
NC_005102.4-138474017	TTAAGGAGCACAGCCCCTCCCCATTGT	26	G	0.0894	0.1145
NC_005102.4-138504050	TTCTGTTGGACGCAGCCTCCAAACCGG	25	C	0.05	0.0799
NC_005102.4-138506820	CATATGCATACGAGCACTCCTGCATAT	14	G	0.1475	0.4966
NC_005102.4-138548146	CAGGGTAGAACCACCCCTCCAGAGTAC	25	T	0.1488	0.2172
NC_005102.4-138564010	GCAAGAACAACAGGTGCTCCCAACTAT	26	A	0.1667	0.4796
NC_005102.4-138592831	GTGTACACAACTTACCCTCCAGCACTG	13	T	0.0492	0.0935
NC_005102.4-138608574	GAACTGGAGACTGTTGCTCCGTGGTTA	15	T	0.1917	0.4747
NC_005102.4-138663084	CACTAACTAACCAGAGCTCCCAGGGAC	16	G	0.3833	0.32
NC_005102.4-138663084	CACTAACTAACCAGAGCTCCCAGGGAC	27	C	0.3833	0.32
NC_005102.4-138679065	GGAAATTGCACAATCACTCCTAACACA	21	T	0.1864	0.4908
NC_005102.4-138684380	CGCCTCACGACTGCTCCTCCGCAGCAG	12	T	0.0721	0.086
NC_005102.4-138698185	TTCCACATCACTGATCCTCCATACCAA	25	C	0	0.48
NC_005102.4-138718845	TACCTCCATACCGACCCTCCCCAGTGA	12	C	0.1345	0.1539
NC_005102.4-138735990	GAATGTGCTACTGCTCCTCCTCCTCCT	14	C	0.0083	0.4803
NC_005102.4-138735990	GAATGTGCTACTGCTCCTCCTCCTCCT	16	C	0.0744	0.1163
NC_005102.4-138741048	CTGCAGCCGACCGTCACTCCATTGCCC	13	G	0.2	0.2072
NC_005102.4-138784934	TGGATCTGCACCAGGACTCCTCCTGGA	14	G	0.115	0.1391

NC_005102.4-138820451	GAAGAGGGGACCGTGGCTCCAAGGGGG	16	G	0.0667	0.0799
NC_005102.4-138890347	TCTTCAGATACCCCAACTCCACCAACC	6	A	0.0246	0.0401
NC_005102.4-138929065	TACCCTTGGACTTGGCCTCCTCCAGAA	15	G	0.0783	0.1067
NC_005102.4-138932565	CGTAGATAAACTGCCACTCCAGTTGAT	2	G	0	0.4751
NC_005102.4-138941126	AGTCAGAGGACTAGATCTCCTGCTGTG	2	G	0.0661	0.4923
NC_005102.4-138941126	AGTCAGAGGACTAGATCTCCTGCTGTG	3	T	0.0661	0.4923
NC_005102.4-138980201	GGGGCTTTCACAAGCTCTCCTTCCTCT	1	G	0.3	0.4688
NC_005102.4-138980201	GGGGCTTTCACAAGCTCTCCTTCCTCT	13	A	0	0.4819
NC_005102.4-138982282	GTTCTTCCAACTCAGTCTCCTTTGTCT	23	T	0.1795	0.1908
NC_005102.4-139006622	ATCTTTCTCACATCTCCTCCAGATCCT	3	C	0.1008	0.1107
NC_005102.4-139039925	TCCCTTTATACCTTCTCTCCCCTCCCC	23	T	0.0522	0.0508
NC_005102.4-139039925	TCCCTTTATACCTTCTCTCCCCTCCCC	24	C	0.0085	0.0253
NC_005102.4-139292667	TGCTGCTCAACCATATCTCCAGTGCGC	26	G	0.252	0.2989
NC_005102.4-139346905	TTGGACTTGACAGAGCCTCCCTTTTCA	1	T	0.1488	0.1918
NC_005102.4-139346905	TTGGACTTGACAGAGCCTCCCTTTTCA	23	T	0.2049	0.4879
NC_005102.4-139347366	TCACACACCACACTGTCTCCTTTGATT	9	C	0.0424	0.0576
NC_005102.4-139378599	CCTCCAGCCACCAAGTCTCCACACTCC	7	G	0.0083	0.4835
NC_005102.4-139440439	TAGTTTTCTACAAGAGCTCCTACTGCA	2	A	0	0.4786
NC_005102.4-139440439	TAGTTTTCTACAAGAGCTCCTACTGCA	14	G	0.3077	0.3895
NC_005102.4-139525735	CCACTATTTACCCACACTCCACTCCTG	4	C	0.1754	0.3706
NC_005102.4-139525971	AGAACACAGACACAGTCTCCCTCTTCA	14	A	0.0256	0.0253
NC_005102.4-139525971	AGAACACAGACACAGTCTCCCTCTTCA	26	C	0.1624	0.1492
NC_005102.4-139546654	ATGCCCCACACATGGACTCCAACTTGT	27	T	0.0833	0.1665
NC_005102.4-139555743	AACTGTTCCACTGTTTCTCCCCTGCTC	14	T	0.0656	0.0634
NC_005102.4-139607598	CCTCCAAGGACCACAGCTCCTTTCTGA	13	A	0.042	0.4886
NC_005102.4-139608270	CTTGTCTTAACCTGTTCTCCAAATACA	27	A	0	0.4786
NC_005102.4-139634295	TCTTGGATTACTCTACCTCCCTGTCTC	14	T	0.1488	0.4983
NC_005102.4-139668364	ACTGTACTCACCTCACCTCCAGGTGGA	26	G	0.2276	0.464
NC_005102.4-139692406	TCTATCCGCACAATCTCTCCTGACAGC	8	G	0	0.4807
NC_005102.4-139695652	TCGGGCTCTACTCCCCCTCCTTCTTTT	3	G	0.1316	0.1229
NC_005102.4-139695652	TCGGGCTCTACTCCCCCTCCTTCTTTT	24	T	0.0088	0.4735
NC_005102.4-139739808	GGTGGTCTTACTGCGCCTCCTGAGGAT	27	T	0.1681	0.4743
NC_005102.4-139749465	ACCACCATCACAACACCTCCTGCCACT	14	C	0.0708	0.0846
NC_005102.4-139749465	ACCACCATCACAACACCTCCTGCCACT	25	A	0.0442	0.0433
NC_005102.4-139750778	TATTTCAAGACCTTCTCTCCAAATACC	12	C	0.0526	0.0512
NC_005102.4-139750778	TATTTCAAGACCTTCTCTCCAAATACC	27	C	0.0088	0.472
NC_005102.4-139759431	ATGGGCATCACCTGTTCTCCCTACCCT	22	T	0	0.4793
NC_005102.4-139793928	CCATCCCCTACAATTCCTCCATCATAG	27	G	0.0902	0.5
NC_005102.4-139882379	TGGCTGCTAACCAGCTCTCCAGGGCAG	6	G	0.0171	0.0336
NC_005102.4-139882379	TGGCTGCTAACCAGCTCTCCAGGGCAG	22	G	0	0.4772
NC_005102.4-139900493	CTACTATACACACAACCTCCTCTGATG	24	G	0.0325	0.0629
NC_005102.4-139900493	CTACTATACACACAACCTCCTCTGATG	25	A	0	0.4793
NC_005102.4-139927415	AGTCCCCACACCCCTGCTCCCTAACCT	21	C	0.1754	0.3324
NC_005102.4-139927415	AGTCCCCACACCCCTGCTCCCTAACCT	22	T	0.0609	0.059
NC_005102.4-140034812	CAGCCAGAGACAACTTCTCCAAGACCT	9	G	0.0826	0.4923
NC_005102.4-140036947	CTGCATCCTACAATGGCTCCAGGAAAA	7	C	0	0.48
NC_005102.4-140080322	GGGTGTTGAACTGCAGCTCCTCTTCCT	22	C	0.0976	0.496
NC_005102.4-140090003	AGACCTTGAACTCCTGCTCCTGAGTGT	9	A	0.0738	0.0861
NC_005102.4-140119190	AGCAGCACCACTGCTGCTCCTCTGCTG	3	C	0.2562	0.3421
NC_005102.4-140119190	AGCAGCACCACTGCTGCTCCTCTGCTG	12	T	0.0083	0.4783
NC_005102.4-140164009	CACAGTGAGACCCTGTCTCCAAAAGCC	3	C	0.2583	0.225

NC_005102.4-140189522	TCAAGCTGGACTCCAACTCCCGGACAT	21	C	0.0569	0.0854
NC_005102.4-140201993	TAAGGCTCCACTTTCTCTCCCTAATTC	13	T	0.094	0.4854
NC_005102.4-140224083	TTGCCTCCCACAGTCTCTCCTTCAGGA	1	T	0.2605	0.4377
NC_005102.4-140236773	CCAGTAAAAACAGTTGCTCCCACCCAC	21	C	0.1983	0.4916
NC_005102.4-140236773	CCAGTAAAAACAGTTGCTCCCACCCAC	24	C	0.1966	0.2778
NC_005102.4-140242951	TATACACCCACTCCCACTCCCCACTCC	3	T	0.1593	0.499
NC_005102.4-140278590	GAGACCACCACCATTACTCCATTCAAC	7	A	0.0909	0.2355
NC_005102.4-140278590	GAGACCACCACCATTACTCCATTCAAC	13	A	0	0.4751
NC_005102.4-140279229	TCCGCCATCACATGTTCTCCATACGGA	12	A	0.0598	0.12
NC_005102.4-140283593	TGACTTGCCACACAAGCTCCACTGAAC	27	C	0.1709	0.45
NC_005102.4-140372639	ATAGTCTAAACCCCACCTCCTTATATA	15	A	0.2143	0.4994
NC_005102.4-140380683	AGCGAGGCTACTGTTTCTCCTTAGGTT	16	T	0.0088	0.4796
NC_005102.4-140396559	AGGCGCTCCACTCCGCCTCCTAGGATT	21	T	0.1404	0.1884
NC_005102.4-140418281	TAACCCTGGACCATCTCTCCATCTCCC	15	C	0.0732	0.0705
NC_005102.4-140418281	TAACCCTGGACCATCTCTCCATCTCCC	16	T	0.1707	0.208
NC_005102.4-140440273	CACAGAGAAACATTGTCTCCGAGGAAA	14	T	0	0.4793
NC_005102.4-140440273	CACAGAGAAACATTGTCTCCGAGGAAA	16	T	0.1538	0.2107
NC_005102.4-140514134	GAACTGTACACAAGAACTCCTTTGATA	7	T	0.0085	0.479
NC_005102.4-140532828	ACCAATACCACAGTCTCTCCTTCTCAT	1	A	0.0085	0.4906
NC_005102.4-140532828	ACCAATACCACAGTCTCTCCTTCTCAT	3	C	0.0085	0.4906
NC_005102.4-140572598	GTGTAGTAGACCCATGCTCCAGGACAG	13	C	0.3197	0.4379
NC_005102.4-140572598	GTGTAGTAGACCCATGCTCCAGGACAG	14	A	0	0.4773
NC_005102.4-140620054	AGAAATCTTACTGTTACTCCTTCGTGT	24	G	0	0.4765
NC_005102.4-140637854	CCTCTACAAACAGTAGCTCCTCCAGAT	1	C	0	0.4832
NC_005102.4-140637854	CCTCTACAAACAGTAGCTCCTCCAGAT	22	C	0.1333	0.1528
NC_005102.4-140640627	TGATACTGGACTGGGTCTCCATCACAC	25	C	0.2479	0.4819
NC_005102.4-140717042	CCATTCCTTACTCCTCCTCCTCACAGT	3	A	0.0609	0.0752
NC_005102.4-140808805	CCACTATGGACTGTCCCTCCCCTATCA	14	T	0	0.4793
NC_005102.4-140809849	CACACTCACACACATACTCCACATATA	23	A	0.4188	0.4823
NC_005102.4-140818575	AGGACAAGGACCAAGCCTCCATCCTGT	22	T	0.3025	0.3771
NC_005102.4-140836675	CAGACTAAGACCCTGTCTCCAAACACA	9	G	0.0085	0.4776
NC_005102.4-140851419	CCAACTCTAACTTCAGCTCCTGGGTAT	1	C	0.0248	0.0245
NC_005102.4-140851419	CCAACTCTAACTTCAGCTCCTGGGTAT	12	T	0.0579	0.0562
NC_005102.4-140874983	TATCTTCCCACCCTTTCTCCTTACTCC	3	T	0	0.4755
NC_005102.4-140913216	TCTGCTCCCACACACCCTCCCCATTCT	14	A	0	0.4671
NC_005102.4-140995031	ATTTTGAAAACTATCTCTCCTCTCATA	13	A	0.2241	0.4666
NC_005102.4-141039810	GGAGCATGAACACTCACTCCAAGTCAT	27	T	0	0.4793
NC_005102.4-141045326	AGCTGTCCCACCTAGGCTCCTAGGCCT	3	C	0.1488	0.1918
NC_005102.4-141045326	AGCTGTCCCACCTAGGCTCCTAGGCCT	27	T	0.0084	0.4829
NC_005102.4-141076100	TTCTAATGGACACCTTCTCCACTAGAT	9	G	0.1327	0.1686
NC_005102.4-141149985	CGGGTTCCGACACTACCTCCCCAAACA	2	G	0.1228	0.1153
NC_005102.4-141194873	TTGTCCTCTACAGCCGCTCCAAGTTAC	6	C	0.2213	0.2573
NC_005102.4-141217911	CCCTCCTCCACGAGCCCTCCATCCCAT	12	G	0	0.4703
NC_005102.4-141288582	TGAAGAAGAACTTCCTCTCCTTTTTGA	2	G	0.1858	0.2618
NC_005102.4-141381245	CCTCATCCTACTCTTTCTCCTCAGTTT	16	T	0.3	0.42
NC_005102.4-141381734	GGACAGGTCACTGGACCTCCTTTCCCT	7	G	0.0342	0.0336
NC_005102.4-141384490	GTTGCTCTTACCCACACTCCTATTCAG	23	T	0.1858	0.3941
NC_005102.4-141397328	TGATAATGGACTGAACCTCCGAACTAT	1	T	0.3689	0.3008
NC_005102.4-141397328	TGATAATGGACTGAACCTCCGAACTAT	6	A	0.5738	0.4092
NC_005102.4-141407860	CCCAAAGTCACTTTCCCTCCCCCTGTT	2	C	0.1026	0.0973
NC_005102.4-141444934	TAACAACACACGAATTCTCCTTTAGAT	1	T	0.0342	0.0336

NC_005102.4-141445397	ATCCAGTCCACTCCTCCTCCAACACAG	5	A	0.2735	0.336
NC_005102.4-141485748	CTAGTCAGCACTTGGACTCCAAGCTTG	4	G	0.3333	0.4301
NC_005102.4-141551193	ACCGTGTGGACTTTGACTCCTCGAGAT	3	C	0.2417	0.2722
NC_005102.4-141551193	ACCGTGTGGACTTTGACTCCTCGAGAT	23	G	0.1885	0.2686
NC_005102.4-141565702	TTTCTAGTCACCCCTGCTCCTTCCTGT	26	G	0.1261	0.2467
NC_005102.4-141565702	TTTCTAGTCACCCCTGCTCCTTCCTGT	27	T	0.1261	0.2467
NC_005102.4-141570617	TTCTTCGGCACACTGGCTCCTTAGCCT	6	C	0.0328	0.048
NC_005102.4-141632541	ATGTAAACCACCACAGCTCCTGTCCAT	13	A	0.1557	0.4997
NC_005102.4-141657303	TCTCAGAGCACCATGGCTCCCCCTTTT	2	C	0.0492	0.0634
NC_005102.4-141657303	TCTCAGAGCACCATGGCTCCCCCTTTT	12	C	0.0492	0.0634
NC_005102.4-141664485	TGCTTAGTCACCTTGACTCCACTCCCG	4	T	0.2881	0.4305
NC_005102.4-141664485	TGCTTAGTCACCTTGACTCCACTCCCG	14	T	0.0593	0.1041
NC_005102.4-141684476	TGTAGAAATACAGTGCCTCCAAAATCT	2	G	0.0619	0.06
NC_005102.4-141684476	TGTAGAAATACAGTGCCTCCAAAATCT	26	C	0.2679	0.4297
NC_005102.4-141748838	CTGGCTAGGACATTTGCTCCTGTGCTG	23	T	0.0328	0.0322
NC_005102.4-141758888	ATACTCTTCACTACCACTCCCTGTCAC	1	A	0.0085	0.0418
NC_005102.4-141829128	GAAGGAGATACTGAGGCTCCGGCATGG	21	G	0.2124	0.3543
NC_005102.4-141829128	GAAGGAGATACTGAGGCTCCGGCATGG	26	G	0.0354	0.0517
NC_005102.4-141846165	CACATAAGCACTCCATCTCCTGCTATG	22	G	0.2295	0.285
NC_005102.4-141865510	TGTTACTGAACACTGCCTCCCACATGC	2	G	0.1043	0.48
NC_005102.4-141865510	TGTTACTGAACACTGCCTCCCACATGC	23	C	0.1207	0.1576
NC_005102.4-141885320	GGCTTGTCGACAATCTCTCCAGCCCCA	9	G	0.2941	0.4186
NC_005102.4-141941939	CACCCCCTGACTGGCTCTCCTTTGAAC	1	C	0.1681	0.2237
NC_005102.4-141960420	AGGGTTAAGACCTGGCCTCCTCAGATA	6	T	0	0.4819
NC_005102.4-141961260	CACACACACACACAATCTCCTCCTTTA	14	A	0.4336	0.3683
NC_005102.4-141976197	ATTGGCCACACTGTCTCTCCCACTCAG	2	T	0.161	0.2156
NC_005102.4-142090983	TTTCAGTGGACCCTGGCTCCTTTGCGT	6	G	0	0.4793
NC_005102.4-142090983	TTTCAGTGGACCCTGGCTCCTTTGCGT	26	G	0	0.4793
NC_005102.4-142137512	TCATCATCCACCAGGCCTCCCCACTCC	7	T	0	0.4807
NC_005102.4-142137512	TCATCATCCACCAGGCCTCCCCACTCC	27	C	0.1138	0.1356
NC_005102.4-142154059	AGCCTCACCACACTGCCTCCAAAGCTT	23	A	0.0083	0.4769
NC_005102.4-142168637	CTAACATGGACACCTCCTCCCCAAAGA	6	A	0.2435	0.4682
NC_005102.4-142191353	CCCAGCCCCACCACCTCTCCCATGTTT	22	A	0.1066	0.1009
NC_005102.4-142222282	GGCTTGCCCACTCATTCTCCATACATC	7	C	0.0446	0.0436
NC_005102.4-142228032	TATGCAATAACAACATCTCCCTCTTAA	12	A	0	0.4779
NC_005102.4-142246428	CCCGGCAGCACTTTGTCTCCATGGACT	3	C	0	0.482
NC_005102.4-142287210	GGTTCCACCACCAAGTCTCCACAACAC	6	C	0.1148	0.3709
NC_005102.4-142300436	AGTGGGTTGACTCCTCCTCCTCCAGTT	3	T	0.065	0.4926
NC_005102.4-142313420	GCCTGGATGACATAGCCTCCTGTCTCA	13	T	0.2456	0.4654
NC_005102.4-142313420	GCCTGGATGACATAGCCTCCTGTCTCA	25	T	0.1842	0.2599
NC_005102.4-142346541	ATATGGTTTACCAAGGCTCCTACCTCT	1	A	0	0.4715
NC_005102.4-142346541	ATATGGTTTACCAAGGCTCCTACCTCT	6	G	0.0531	0.0517
NC_005102.4-142366603	GACAGGGCCACCCCACCTCCTTGCAAA	8	C	0	0.4793
NC_005102.4-142366603	GACAGGGCCACCCCACCTCCTTGCAAA	12	C	0.0407	0.0398
NC_005102.4-142374431	CTTGCAAGTACATCGACTCCTTGACAG	14	C	0	0.48
NC_005102.4-142411603	TGTGACCATACAAACCCTCCTCTGCTG	14	A	0.1966	0.321
NC_005102.4-142411603	TGTGACCATACAAACCCTCCTCTGCTG	27	G	0.0598	0.058
NC_005102.4-142563056	TATGTGCATACTTAACCTCCCGATTAT	21	C	0.0342	0.066
NC_005102.4-142565016	GGGCTACTCACTCTGCCTCCTTAATAA	2	G	0.0531	0.1006
NC_005102.4-142565016	GGGCTACTCACTCTGCCTCCTTAATAA	3	G	0.0531	0.1006
NC_005102.4-142608421	ATGGTAATAACATAGCCTCCAGCAATT	16	C	0.2241	0.3995

NC_005102.4-142609338	TTCACACTAACCATATCTCCAAGTTCA	4	A	0.1382	0.4995
NC_005102.4-142632534	CAAAGCTTCACCCAAGCTCCTCCCACT	2	A	0.0081	0.0241
NC_005102.4-142641473	CCGTAACATACCACAGCTCCTTCCACA	8	A	0.0957	0.1219
NC_005102.4-142676952	CCAAGAGCCACCCCCTCTCCAACAGCT	5	G	0.0081	0.0398
NC_005102.4-142676952	CCAAGAGCCACCCCCTCTCCAACAGCT	12	C	0.2033	0.2885
NC_005102.4-142722881	CCTTTCTCAACACCTCCTCCTTACTCC	27	C	0.3077	0.3895
NC_005102.4-142736975	ATATTCCTGACTCTACCTCCAAGTGTG	23	G	0.2342	0.3389
NC_005102.4-142801915	CTGTGGCCTACCTCCTCTCCTTGCTGA	24	C	0.1043	0.4301
NC_005102.4-142808835	ACAATGAACACATGGGCTCCGGCCGTA	24	C	0.2632	0.3793
NC_005102.4-142808835	ACAATGAACACATGGGCTCCGGCCGTA	25	G	0.1897	0.2252
NC_005102.4-142825882	CTCTGCAGAACCCCTTCTCCATTTCTC	13	C	0.0252	0.0249
NC_005102.4-142825882	CTCTGCAGAACCCCTTCTCCATTTCTC	21	A	0.2759	0.3381
NC_005102.4-142826856	TGGAAAGTTACGTTTTCTCCTGCTTCC	12	G	0.0088	0.4774
NC_005102.4-142912351	AAGAATCCAACACTCTCTCCTGGACTC	8	C	0	0.4773
NC_005102.4-142959250	GAGAATGCCACCCTTTCTCCATGGTTA	7	G	0.0569	0.4952
NC_005102.4-143019197	GGAAGAGTAACCATCTCTCCAGCCTCC	2	G	0.0427	0.0418
NC_005102.4-143019197	GGAAGAGTAACCATCTCTCCAGCCTCC	8	T	0.0427	0.0418
NC_005102.4-143021868	TACAGTGCCACCATGTCTCCAGTGTTT	3	C	0.0574	0.0557
NC_005102.4-143035992	AAGATTACTACCTACCCTCCCTTGTCC	1	A	0.2017	0.3415
NC_005102.4-143074509	CTTTGCTTCACCTGTGCTCCACTTGTT	24	T	0.1681	0.1828
NC_005102.4-143095145	GAGTTCTTCACCCCGTCTCCCCTCTGA	14	C	0.0982	0.0934
NC_005102.4-143102046	AAAATTCTCACACAGCCTCCAATCCTG	4	A	0	0.4778
NC_005102.4-143122739	CAGGCACCCACGTGGTCTCCATCGGTG	24	G	0	0.4773
NC_005102.4-143146400	AGTGCCACCACCTTCCCTCCCCTGGCT	14	T	0.2857	0.3014
NC_005102.4-143167959	AGCTATAACACAGTATCTCCATCTGAC	13	G	0	0.4779
NC_005102.4-143208974	CAGGCCTAGACAATGGCTCCCTTCTCC	3	G	0.0417	0.0408
NC_005102.4-143246992	GGAGCTAATACCAACACTCCTCAAACT	16	A	0.0614	0.0595
NC_005102.4-143263180	CTCCGAAACACAGGTCCTCCAAGGCGA	4	C	0.2203	0.2466
NC_005102.4-143263297	GTGAAGTCCACTAAGTCTCCTGGTAAG	23	G	0.0917	0.325
NC_005102.4-143910967	GGGAGGGAGACCTCACCTCCTAGACAG	2	G	0.0625	0.0605
NC_005102.4-143910967	GGGAGGGAGACCTCACCTCCTAGACAG	3	G	0.0625	0.0605
NC_005102.4-143990780	ATCCCCCTAACCCTTTCTCCTCCCCTT	8	T	0.6121	0.4435
NC_005102.4-144197239	AGGATAAGGACAGCATCTCCCCCAAAA	13	G	0	0.4749
NC_005102.4-144197239	AGGATAAGGACAGCATCTCCCCCAAAA	24	A	0	0.4749
NC_005102.4-144317309	CTCAAGTGCACAGTATCTCCAATGTAC	6	G	0.0417	0.4622
NC_005102.4-144317309	CTCAAGTGCACAGTATCTCCAATGTAC	12	A	0.0083	0.4708
NC_005102.4-144321672	TCTTAGTTTACATTGGCTCCTTTCCAG	6	G	0.3898	0.3707
NC_005102.4-144424949	GATATTAGCACAAAAGCTCCCATTACC	21	C	0.1102	0.1041
NC_005102.4-144457088	TCAACACATACATTATCTCCTGGACAC	2	C	0.0877	0.4444
NC_005102.4-144457088	TCAACACATACATTATCTCCTGGACAC	4	A	0.2035	0.2367
NC_005102.4-144463077	CTAAGATGAACACTCACTCCTTCAATC	25	A	0.3025	0.4844
NC_005102.4-144476123	ATAACAAAGACACATTCTCCACTATAT	12	A	0.1736	0.1585
NC_005102.4-144485529	TTCCTCTTTACCAAACCTCCAACTTCT	9	T	0	0.4813
NC_005102.4-144485529	TTCCTCTTTACCAAACCTCCAACTTCT	22	A	0	0.4813
NC_005102.4-144503312	AACACAGGGACTATGTCTCCCTCTTCT	8	G	0.0172	0.4666
NC_005102.4-144515360	AGGTATGTGACCAAGGCTCCAATTCTC	15	G	0.1062	0.1162
NC_005102.4-144582701	AAGAATAGAACAGCAGCTCCTGAACTC	16	G	0.2432	0.4141
NC_005102.4-144623967	GTCTTTTTGACTATTCCTCCCTAAAGT	23	A	0.178	0.2026
NC_005102.4-144659814	AGGAGTTGCACTTTGTCTCCTTTTATC	6	T	0	0.4778
NC_005102.4-144659814	AGGAGTTGCACTTTGTCTCCTTTTATC	16	T	0.0789	0.0758
NC_005102.4-144745499	GTACCCAACACTCTACCTCCCCAAATT	4	C	0.0089	0.4856

NC_005102.4-144745499	GTACCCAACACTCTACCTCCCCAAATT	5	C	0.0357	0.0521
NC_005102.4-144952802	CAGCGATTTACCAAAGCTCCCCAGCTC	12	C	0.2845	0.343
NC_005102.4-145077684	CCATTCCTCACATGCTCTCCAACTCCA	7	C	0	0.4813
NC_005102.4-145121951	TCCCTCATTACCTCATCTCCTATTGCT	4	C	0.2743	0.481
NC_005102.4-145167359	TGAGGCCATACACAAACTCCTACTGCC	22	A	0	0.4793
NC_005102.4-145256603	TACCAACCAACTGGAGCTCCCAGGGAA	8	C	0	0.48
NC_005102.4-145256603	TACCAACCAACTGGAGCTCCCAGGGAA	23	G	0.0086	0.4803
NC_005102.4-145266797	CCCACATCAACAATTCCTCCCTTGTCT	2	C	0.0087	0.4878
NC_005102.4-145339692	AGATCAACAACCAACCCTCCTTAAAAT	14	A	0.0345	0.482
NC_005102.4-145339692	AGATCAACAACCAACCCTCCTTAAAAT	26	A	0.1026	0.0973
NC_005102.4-145365626	GTAACGAAGACACATGCTCCACTGTGT	5	C	0.439	0.3604
NC_005102.4-145923891	CAATCCTTGACCTTCTCTCCTAGCAAT	4	T	0	0.4793
NC_005102.4-145923891	CAATCCTTGACCTTCTCTCCTAGCAAT	21	T	0	0.4793
NC_005102.4-145944170	ACAGTAAACACGCATGCTCCACTATGA	4	G	0.2033	0.1954
NC_005102.4-145944170	ACAGTAAACACGCATGCTCCACTATGA	12	G	0.0488	0.0476
NC_005102.4-146036519	GGCCACTGGACTCAAGCTCCCTTGACT	22	T	0	0.4819
NC_005102.4-146037615	CACAACGAAACCCAGCCTCCAAAGAAC	16	C	0.6116	0.4246
NC_005102.4-146043105	AATTGTGTCACCCAGTCTCCACGTATC	5	G	0	0.4786
NC_005102.4-146043105	AATTGTGTCACCCAGTCTCCACGTATC	12	C	0.181	0.4937
NC_005102.4-146137685	ACCAAACCAACCAGAGCTCCCAAGGAC	15	A	0.0248	0.0245
NC_005102.4-146141979	CAGGGTACCACCATCCCTCCATTGTGA	5	G	0.0085	0.0253
NC_005102.4-146203096	CTCCTGAAAACAGACCCTCCGGACCAT	21	G	0.0813	0.4926
NC_005102.4-146380305	CTGGCTAGTACGGACCCTCCCAGTCTA	12	G	0.0531	0.0683
NC_005102.4-146413939	TGTTTTGAAACACCTTCTCCTTGGATG	23	G	0	0.4807
NC_005102.4-146413939	TGTTTTGAAACACCTTCTCCTTGGATG	27	G	0.2562	0.4004
NC_005102.4-146419140	AACCCTGCCACGAGGTCTCCCTTGAGC	16	T	0.181	0.4435
NC_005102.4-146473459	CGCATCAAGACCTTGCCTCCAAAAGAA	1	C	0.0435	0.4903
NC_005102.4-146507569	AGCTAAGCAACCCTGGCTCCTTGAAAT	6	A	0.2114	0.4444
NC_005102.4-146507569	AGCTAAGCAACCCTGGCTCCTTGAAAT	15	G	0	0.4807
NC_005102.4-146521349	GCCATCTCCACAGTCCCTCCTTTATCG	26	C	0.2417	0.4575
NC_005102.4-146562892	GGTCTCTACACAAAGTCTCCTTGTCTG	8	A	0.0656	0.4914
NC_005102.4-146569719	CTTTTTCACACCGCACCTCCCACTCCT	6	T	0.1138	0.1761
NC_005102.4-146569719	CTTTTTCACACCGCACCTCCCACTCCT	15	A	0	0.4807
NC_005102.4-146652032	ATTCATGCCACCCATCCTCCTTTGCCT	9	C	0.2602	0.4206
NC_005102.4-146655733	CTCCGGTACACACTCTCTCCCAATCAG	8	A	0.025	0.4816
NC_005102.4-146660689	CATCAGGTCACTCTTACTCCTTTGTAT	14	T	0	0.4793
NC_005102.4-146689969	CCTGGGACTACCTGCCCTCCAGGTGAG	1	C	0.0256	0.0253
NC_005102.4-146716532	AGCGTCCCCACCGGACCTCCCCCTCCT	3	C	0.025	0.0247
NC_005102.4-146716532	AGCGTCCCCACCGGACCTCCCCCTCCT	23	C	0.1833	0.2188
NC_005102.4-146722374	GCACTAGCCACAGTGTCTCCACACAAC	9	C	0.0325	0.032
NC_005102.4-146750145	TTCTCCAACACTTACTCTCCATCCTAG	23	C	0.0254	0.0251
NC_005102.4-146750145	TTCTCCAACACTTACTCTCCATCCTAG	27	G	0.0254	0.0251
NC_005102.4-146774952	TTGATCTCTACACCGGCTCCTGCTATT	5	T	0.0086	0.4893
NC_005102.4-146789615	CCAGAGAGAACAGACCCTCCCCAAGAG	15	C	0.2185	0.2796
NC_005102.4-146795082	CAGACGCCCACAATCTCTCCTTAGGAG	5	C	0	0.4844
NC_005102.4-146907806	AAAATACCAACCTAGGCTCCTACATAG	15	G	0.0932	0.1041
NC_005102.4-146907806	AAAATACCAACCTAGGCTCCTACATAG	27	G	0	0.4807
NC_005102.4-146962704	CCTAGCTGCACACTGCCTCCATGACAG	7	T	0.0976	0.0928
NC_005102.4-146962704	CCTAGCTGCACACTGCCTCCATGACAG	22	T	0.0976	0.0928
NC_005102.4-146991185	CACTTGGCTACTTGTTCTCCTGTGTAG	24	G	0.0348	0.0342
NC_005102.4-146992453	CACACACACACACATTCTCCATGCAAT	5	C	0.2124	0.3026

NC_005102.4-147011013	TCTCCCTACACCTGATCTCCACCCACC	6	C	0.2087	0.4833
NC_005102.4-147011013	TCTCCCTACACCTGATCTCCACCCACC	16	T	0.1624	0.2423
NC_005102.4-147030930	CACTCAGTCACGGCCTCTCCCTTCCTA	6	A	0.188	0.4982
NC_005102.4-147030930	CACTCAGTCACGGCCTCTCCCTTCCTA	13	G	0.1849	0.2568
NC_005102.4-147032666	GTCATGCAGACATGACCTCCCCGAGAT	23	G	0.2417	0.4622
NC_005102.4-147049388	TATTGTTGCACAGCTCCTCCCATTGTG	6	T	0.1933	0.2852
NC_005102.4-147049388	TATTGTTGCACAGCTCCTCCCATTGTG	16	C	0.0885	0.0846
NC_005102.4-147067575	GTCTCATTCACCGTGGCTCCATCTTCA	12	C	0.2162	0.2337
NC_005102.4-147068147	AAGGAGCACACATGATCTCCATTTACC	6	G	0.2342	0.4026
NC_005102.4-147069049	CCATCGGACACACTGCCTCCGCTTGAT	6	G	0.0083	0.4816
NC_005102.4-147069049	CCATCGGACACACTGCCTCCGCTTGAT	21	G	0.1933	0.214
NC_005102.4-147070450	ATCTGCTACACCGAGTCTCCCAGGTTG	27	G	0.187	0.2778
NC_005102.4-147072389	ACCACACGCACACACACTCCTACAGAT	7	C	0.1667	0.2476
NC_005102.4-147091225	GGTTGGAACACTCAAACTCCTCTCAAC	2	G	0	0.4787
NC_005102.4-147109286	GCCTGTGCCACCAGCCCTCCTGCATGG	24	A	0	0.4793
NC_005102.4-147132495	ATCTAACAAACACAGGCTCCAACCACT	12	A	0.0492	0.4914
NC_005102.4-147132495	ATCTAACAAACACAGGCTCCAACCACT	23	C	0.0082	0.0243
NC_005102.4-147139544	ATGTTTGCCACCATACCTCCTAGACAT	4	T	0	0.4728
NC_005102.4-147165233	CGGTGTGACACACAGTCTCCTTTGTTG	1	C	0	0.4772
NC_005102.4-147165233	CGGTGTGACACACAGTCTCCTTTGTTG	24	G	0.0345	0.0504
NC_005102.4-147183519	AGTAGTCTCACCAAAGCTCCCCGTTTG	22	C	0	0.4807
NC_005102.4-147193458	AAGATGACGACTTTACCTCCCAGACAC	9	G	0	0.4793
NC_005102.4-147206505	GATGTTCCTACACGGTCTCCTCCCTCT	15	G	0.1545	0.4239
NC_005102.4-147248537	TGATCCGAGACCGAAGCTCCTCACTAT	6	C	0.5	0.375
NC_005102.4-147297090	CAAGTGTCCACCACCTCTCCAGATGAT	9	C	0.1707	0.2556
NC_005102.4-147316349	GCAAATGCAACAGCTTCTCCATGGGAC	6	T	0	0.4654
NC_005102.4-147323086	TTGCTACCAACTCGCCCTCCTGCCTCT	13	C	0.2705	0.3307
NC_005102.4-147323086	TTGCTACCAACTCGCCCTCCTGCCTCT	26	C	0	0.4787
NC_005102.4-147336704	AGTTAGCCTACCATGCCTCCGAGGCGA	12	C	0.0263	0.026
NC_005102.4-147361455	TTCTCATGGACCTGGGCTCCTCTGACT	26	C	0.0813	0.1356
NC_005102.4-147399978	ATTCCACATACATGCACTCCTGCTGCC	3	T	0.0259	0.4643
NC_005102.4-147399978	ATTCCACATACATGCACTCCTGCTGCC	13	T	0.1121	0.4179
NC_005102.4-147426021	AGCCATGAAACTCTGTCTCCTCTTCAG	26	A	0.178	0.2759
NC_005102.4-147426021	AGCCATGAAACTCTGTCTCCTCTTCAG	27	G	0.1017	0.1827
NC_005102.4-147428969	GTGGCCCTAACCCAGCCTCCACTGGTC	5	C	0.0081	0.0241
NC_005102.4-147449385	CAGCCGCAGACACCGCCTCCCTCTTCA	14	C	0.2101	0.4542
NC_005102.4-147468156	CACCTGCTCACTGAGACTCCCCGTGTT	23	G	0.0083	0.4708
NC_005102.4-147482092	CCTCTGAGCACTGCTGCTCCCTCGGGT	23	C	0.1301	0.189
NC_005102.4-147527160	CCCCTGGCCACCACCACTCCACTCGCT	9	C	0.0738	0.1009
NC_005102.4-147573160	ACCAGACCTACTTACTCTCCTGGTCCC	21	T	0	0.4749
NC_005102.4-147591883	ATTTGCAAGACTTCCTCTCCCACCCTA	23	C	0.0431	0.0585
NC_005102.4-147594337	TGATCTCTGACACTCACTCCAATGTAC	21	A	0.1901	0.2814
NC_005102.4-147594337	TGATCTCTGACACTCACTCCAATGTAC	22	A	0.2066	0.3027
NC_005102.4-147602469	CACCCAAGGACAGCTCCTCCAGCTGTG	3	C	0.0427	0.4714
NC_005102.4-147648876	TGCATACACACACTTCCTCCCCAAGTG	4	A	0.0171	0.4839
NC_005102.4-147653630	GTAGGAGAGACAGCGGCTCCCACGCTG	2	T	0	0.4764
NC_005102.4-147685195	TCCCTTCCTACAGCTTCTCCATTTCCT	23	T	0.0789	0.1075
NC_005102.4-147785101	TGCAGATTCACCTCCTCTCCTTAGGCA	15	C	0.1316	0.1814
NC_005102.4-147785101	TGCAGATTCACCTCCTCTCCTTAGGCA	22	T	0	0.4698
NC_005102.4-147862676	AGCTACGAGACTAGTTCTCCAAAGACC	7	G	0.6066	0.4226
NC_005102.4-147888116	CGAGGACCGACTGAACCTCCTAGTTTG	22	A	0.0579	0.0868

NC_005102.4-147900929	AACACAATAACCTTAGCTCCAAGTGAT	23	G	0.0082	0.479
NC_005102.4-147929601	CCTCACTGAACTACATCTCCAGCCCTA	1	C	0.5526	0.4353
NC_005102.4-147929601	CCTCACTGAACTACATCTCCAGCCCTA	22	G	0.6	0.4765
NC_005102.4-147959264	ACGCTGGAAACCGCAGCTCCAAGGAAT	2	C	0.0081	0.481
NC_005102.4-147965468	TAATCAGGAACCAGGGCTCCAACATAG	25	T	0.0085	0.487
NC_005102.4-147965468	TAATCAGGAACCAGGGCTCCAACATAG	26	A	0.0085	0.487
NC_005102.4-148026822	CACACCGTAACAGGAGCTCCTCAAATA	7	G	0.0504	0.4898
NC_005102.4-148026822	CACACCGTAACAGGAGCTCCTCAAATA	9	A	0.0917	0.1733
NC_005102.4-148028695	AGGAGGGTAACCTCAGCTCCTCCAGTA	7	G	0.0167	0.0328
NC_005102.4-148028695	AGGAGGGTAACCTCAGCTCCTCCAGTA	26	T	0	0.482
NC_005102.4-148073367	ACTGCCCAGACAGTGTCTCCTACACAT	6	C	0	0.4773
NC_005102.4-148088505	TATTAGGAAACCGAGGCTCCCGGCTAC	27	C	0.178	0.456
NC_005102.4-148101364	GTCGCTGTGACAAATACTCCCACGTAG	24	G	0.2049	0.2095
NC_005102.4-148101531	CTTATGTGCACGTCTCCTCCTAGTTTT	12	G	0.0259	0.0255
NC_005102.4-148101531	CTTATGTGCACGTCTCCTCCTAGTTTT	24	T	0.2456	0.4945
NC_005102.4-148103509	GTTCCTCCAACCATCACTCCCACATCT	8	C	0.0085	0.0415
NC_005102.4-148115476	TTCAGCCTGACTGCCCCTCCTCCAGTG	26	T	0.0783	0.0752
NC_005102.4-148141527	ACGGTGAGCACCGCAGCTCCAACACTC	2	C	0	0.4793
NC_005102.4-148141527	ACGGTGAGCACCGCAGCTCCAACACTC	14	C	0.0813	0.078
NC_005102.4-148152356	CAGTTCCCCACTGCCCCTCCCCCACCA	2	A	0	0.4682
NC_005102.4-148159812	CAACAAGCCACATCCTCTCCCGTAGTT	6	A	0.114	0.138
NC_005102.4-148159812	CAACAAGCCACATCCTCTCCCGTAGTT	26	T	0.0789	0.0918
NC_005102.4-148180910	CACCCTGTCACTGTGTCTCCCAAATGC	23	A	0	0.4718
NC_005102.4-148186275	TATGGACAGACGGCTCCTCCCTGGCTG	12	G	0	0.4773
NC_005102.4-148186275	TATGGACAGACGGCTCCTCCCTGGCTG	14	C	0	0.4773
NC_005102.4-148191374	TCCCTGTGCACAGTTTCTCCTGCAATG	2	C	0	0.4693
NC_005102.4-148191374	TCCCTGTGCACAGTTTCTCCTGCAATG	9	C	0.0769	0.074
NC_005102.4-148213905	TTCTCTGAGACCCGAACTCCTAGCTCA	24	C	0	0.4807
NC_005102.4-148237170	CTCTGCCTTACTGGACCTCCTCTCAGG	1	C	0.0702	0.4846
NC_005102.4-148239842	TAGAAATCTACCTTCCCTCCTCATTTA	23	A	0	0.4743
NC_005102.4-148242014	CTCCTCAGAACCTCTGCTCCATCATAC	25	T	0	0.4826
NC_005102.4-148244066	TGCGCTCACACGGGTACTCCACTGTGC	8	A	0.2066	0.2704
NC_005102.4-148244066	TGCGCTCACACGGGTACTCCACTGTGC	13	G	0.2066	0.2704
NC_005102.4-148251234	GGAAGACACACTCTCGCTCCAGTGAGC	1	G	0	0.4765
NC_005102.4-148302462	TGTACTGCAACACTAACTCCTCCAGGT	16	A	0.1463	0.2612
NC_005102.4-148306000	CGATCCCACACTCAGCCTCCTATTCCT	3	A	0	0.4813
NC_005102.4-148400357	CAGCAGTGCACTGGACCTCCTGTGTTA	3	G	0.0738	0.4924
NC_005102.4-148404744	CTCTAACTGACCGTGTCTCCCCAGCAT	23	A	0.0672	0.4873
NC_005102.4-148414086	TTATGCGCAACTGTGACTCCAGTGACC	5	G	0.2627	0.4066
NC_005102.4-148414086	TTATGCGCAACTGTGACTCCAGTGACC	25	A	0.075	0.0722
NC_005102.4-148452640	TCTAAGAATACAGCTACTCCAATGTGT	26	G	0.2479	0.3596
NC_005102.4-148472149	AATGGCCTCACTGGTGCTCCGTGAGGG	21	G	0.2927	0.4497
NC_005102.4-148472421	TCCTTGGCCACTCAGCCTCCAGCTTTG	6	G	0.018	0.0354
NC_005102.4-148472421	TCCTTGGCCACTCAGCCTCCAGCTTTG	13	C	0.3063	0.4444
NC_005102.4-148484604	CTGTGTGTGACTCTGGCTCCACACCTA	4	T	0	0.4814
NC_005102.4-148484604	CTGTGTGTGACTCTGGCTCCACACCTA	27	A	0	0.4814
NC_005102.4-148486258	GCCTGAGCCACCCTGCCTCCTTCCTCT	12	C	0.0976	0.1073
NC_005102.4-148486258	GCCTGAGCCACCCTGCCTCCTTCCTCT	23	C	0.2314	0.4582
NC_005102.4-148512350	TTCTTAGGCACACCTCCTCCTCCTTTC	6	A	0	0.4773
NC_005102.4-148512350	TTCTTAGGCACACCTCCTCCTCCTTTC	12	A	0	0.4773
NC_005102.4-148518038	CACCAGGGAACCTACACTCCTTTATCC	24	A	0.0089	0.4856

NC_005102.4-148522626	CTCTACAAAACCTCCCCTCCTCCCCTG	15	C	0.0342	0.4772
NC_005102.4-148522626	CTCTACAAAACCTCCCCTCCTCCCCTG	23	C	0.0333	0.0488
NC_005102.4-148528096	AAAGAAAGCACTTGATCTCCTGAAAAT	26	A	0.0089	0.4709
NC_005102.4-148542337	CCAAGCTACACCTGCCCTCCCCATCCT	24	T	0.0088	0.49
NC_005102.4-148545576	ATGGACACCACCGCCACTCCCATGGCT	2	T	0.124	0.1306
NC_005102.4-148545576	ATGGACACCACCGCCACTCCCATGGCT	8	C	0.0083	0.4769
NC_005102.4-148550996	AGCACAGGAACAGGCACTCCACAGATG	12	A	0	0.4787
NC_005102.4-148562699	CCTCTCCACACCCCTCCTCCATCCCCA	23	C	0.6134	0.4253
NC_005102.4-148578863	GCGGGTCAGACTTCCTCTCCTTCAACT	5	G	0.0357	0.4943
NC_005102.4-148578863	GCGGGTCAGACTTCCTCTCCTTCAACT	27	T	0.0531	0.0683
NC_005102.4-148600070	ACCTTTTCCACATAGCCTCCTCACACT	25	A	0.0508	0.4994
NC_005102.4-148601138	AGGATCTCCACAAAGCCTCCCTAAGGG	23	A	0	0.4793
NC_005102.4-148618258	GACACGGAGACCCTGACTCCTGAAAGG	6	G	0.2358	0.3472
NC_005102.4-148669883	CTGAGGGTTACTTTGGCTCCCTCTATG	1	C	0	0.4832
NC_005102.4-148669883	CTGAGGGTTACTTTGGCTCCCTCTATG	16	G	0	0.4813
NC_005102.4-148684860	TTCATAAACACTTAGGCTCCTGAGGAG	4	A	0.0813	0.4944
NC_005102.4-148733634	ATCCACCTTACTCTGCCTCCCAAGCAG	25	C	0.0574	0.0557
NC_005102.4-148778682	TGGCCTTGAACTAGCTCTCCTGCATGC	26	G	0	0.0322
NC_005102.4-148869006	AGTTTTGCAACAAGGTCTCCATGTATC	9	A	0	0.4765
NC_005102.4-148933685	CAGGAAGGAACCCCACCTCCTTTCAAG	15	A	0.1538	0.1704
NC_005102.4-148985638	CACTCACACACAAACACTCCTGATACA	8	A	0.082	0.0935
NC_005102.4-148985638	CACTCACACACAAACACTCCTGATACA	15	C	0.0833	0.095
NC_005102.4-148994762	GACAATAGAACTTTCCCTCCAAGAGAA	12	T	0.1739	0.2138
NC_005102.4-148994762	GACAATAGAACTTTCCCTCCAAGAGAA	13	T	0	0.4654
NC_005102.4-149002547	CCTGGACCTACTCTCGCTCCCACTGCT	13	C	0.1695	0.3792
NC_005102.4-149002547	CCTGGACCTACTCTCGCTCCCACTGCT	16	G	0.1949	0.3664
NC_005102.4-149009040	TTCATGCCCACAGCACCTCCTCAGTGT	4	A	0.123	0.4992
NC_005102.4-149009040	TTCATGCCCACAGCACCTCCTCAGTGT	7	C	0.1311	0.1367
NC_005102.4-149021273	AAACATATGACCCCTCCTCCTTCCACT	9	G	0.0488	0.1073
NC_005102.4-149022902	CCCATTGGCACCTATACTCCTAGCTCT	6	T	0.0488	0.4444
NC_005102.4-149113285	CCAAAGCTTACCTGACCTCCAGGTTCT	21	A	0	0.4749
NC_005102.4-149116702	CATTTGCCAACCATTGCTCCCAAGATC	13	A	0	0.4793
NC_005102.4-149120062	ATACAGCAAACCCTGTCTCCAACAACA	2	T	0.1667	0.2188
NC_005102.4-149120062	ATACAGCAAACCCTGTCTCCAACAACA	6	G	0.1653	0.2172
NC_005102.4-149195841	AGAACCACTACTACGGCTCCAATGTCT	14	C	0	0.4807
NC_005102.4-149195841	AGAACCACTACTACGGCTCCAATGTCT	22	A	0.1453	0.1634
NC_005102.4-149236668	TGTGTTCCTACCTTCACTCCCAGAGTG	2	G	0.1062	0.1162
NC_005102.4-149256296	AAAAAAACAACCATGGCTCCTGGCTTG	6	A	0.0252	0.4435
NC_005102.4-149269572	AATATCATCACCTTCCCTCCCTCTGTG	9	C	0.2623	0.4952
NC_005102.4-149277295	ACAAAGCTTACCTGGTCTCCGAACTGC	21	G	0.0244	0.0241
NC_005102.4-149292874	ATCCTGGTGACTCTTGCTCCAGGACAT	14	T	0.0263	0.026
NC_005102.4-149319037	GGGTTCTTCACTTGCTCTCCACCTCAT	12	T	0.0776	0.0903
NC_005102.4-149365750	CTCAGGCTAACAGCAACTCCCGCCTTC	22	G	0.0268	0.0771
NC_005102.4-149402629	GGACCCAAGACACAGTCTCCTCCTTCC	13	C	0.1786	0.2449
NC_005102.4-149407231	ACTGTACCCACACGATCTCCAGACAGG	13	C	0.0826	0.0943
NC_005102.4-149409979	GAGCACATCACTAATCCTCCATACTGC	7	A	0.2373	0.3138
NC_005102.4-149411443	GCTGTTGTCACAGCAACTCCAGCAGAC	6	T	0	0.4715
NC_005102.4-149435163	ATGGTCTACACAGTGGCTCCTCTGGTG	5	T	0.1721	0.2095
NC_005102.4-149435163	ATGGTCTACACAGTGGCTCCTCTGGTG	7	T	0.0826	0.1235
NC_005102.4-149484896	CTTGGACTCACGTTTCCTCCTACCTTC	8	T	0.0508	0.4535
NC_005102.4-149484896	CTTGGACTCACGTTTCCTCCTACCTTC	23	C	0.0508	0.4535

NC_005102.4-149523016	CAGACTGTAACTTGACCTCCTGAAACA	16	C	0.0672	0.4661
NC_005102.4-149523016	CAGACTGTAACTTGACCTCCTGAAACA	27	A	0	0.4844
NC_005102.4-149531718	CTCCCTCTCACTCTTCCTCCCACTTTC	2	T	0.0696	0.0671
NC_005102.4-149531718	CTCCCTCTCACTCTTCCTCCCACTTTC	22	A	0.069	0.0666
NC_005102.4-149582978	GTTGGATTGACACTGTCTCCACTGTAC	15	G	0.0168	0.033
NC_005102.4-149582978	GTTGGATTGACACTGTCTCCACTGTAC	22	C	0	0.4743
NC_005102.4-149661613	AACCAGGCCACTGCCTCTCCTTCATTT	12	T	0	0.4724
NC_005102.4-149671684	CCTAAAAGCACCCAGCCTCCATCCTCC	1	C	0	0.4787
NC_005102.4-149671684	CCTAAAAGCACCCAGCCTCCATCCTCC	22	T	0.2479	0.4582
NC_005102.4-149722940	TTCTCAAGGACCCAAGCTCCACTCCCA	6	A	0	0.462
NC_005102.4-149776793	CCAAAGTAGACCTGGCCTCCAGGCTCT	21	A	0.0087	0.4817
NC_005102.4-149803378	CAACTCTCCACATCATCTCCTCCCCAA	4	C	0.3661	0.3316
NC_005102.4-149804537	TGAGGCTCCACCCTCACTCCCTGCCCT	12	C	0.3583	0.4958
NC_005102.4-149867930	ACAGAGATTACAGTATCTCCATATGTA	6	G	0.0427	0.0418
NC_005102.4-149899819	CATCTTTCCACCTCAACTCCTTTTGTT	9	C	0.1802	0.4104
NC_005102.4-149899819	CATCTTTCCACCTCAACTCCTTTTGTT	13	T	0	0.4909
NC_005102.4-149901711	TGTTCCAGTACTTACCCTCCAACACAA	3	T	0	0.4682
NC_005102.4-149903481	CACAGCCAGACACAGTCTCCAAAAGAC	27	C	0.1917	0.1997
NC_005102.4-149911961	ACGTTTCTGACAGATCCTCCTCTGTAC	14	A	0.0488	0.0476
NC_005102.4-149911961	ACGTTTCTGACAGATCCTCCTCTGTAC	27	C	0.0738	0.4924
NC_005102.4-149927197	CACTTTGCGACTCTGCCTCCAGCGATA	24	G	0	0.4832
NC_005102.4-149927197	CACTTTGCGACTCTGCCTCCAGCGATA	27	A	0	0.4832
NC_005102.4-149967878	GGTGCTTGGACTGGTCCTCCCTAGCAC	23	A	0	0.4793
NC_005102.4-150037560	GAGCTACAGACTCCACCTCCCCTGGTG	25	G	0.0082	0.4822
NC_005102.4-150041268	GATGAGGCTACAACGGCTCCATCCCTA	12	A	0	0.4746
NC_005102.4-150060491	GGCTCGAATACTCTGCCTCCTACATAC	7	A	0.0082	0.4822
NC_005102.4-150062976	AGGTAACACACATAATCTCCTACCCAT	27	T	0.2033	0.208
NC_005102.4-150080602	ACAATATGTACTGCTCCTCCGGAAGAT	4	A	0.2589	0.3102
NC_005102.4-150092909	TGCTTGGAGACAGGGTCTCCCTATTAC	3	C	0	0.4912
NC_005102.4-150098904	CCAACTGCAACTGCTGCTCCTTTTGAG	16	G	0	0.4773
NC_005102.4-150161012	AGGTCAGTCACGATGACTCCATCTGTT	4	T	0	0.4832
NC_005102.4-150161012	AGGTCAGTCACGATGACTCCATCTGTT	27	T	0	0.4832
NC_005102.4-150272468	GAAGACAACACTGGATCTCCTAGAACT	2	A	0.1983	0.3557
NC_005102.4-150315788	TGGGATCTGACAGCATCTCCTGGGGTC	3	G	0	0.4819
NC_005102.4-150326401	ACCTCAATGACAAGCTCTCCTTTTCTG	25	C	0.0087	0.4849
NC_005102.4-150341441	TATCAGGGTACACACACTCCCATCATG	13	C	0.0492	0.048
NC_005102.4-150354015	TTCTGTGCCACTCCCTCTCCCTCTACT	9	C	0.1597	0.4797
NC_005102.4-150485044	ATGGGAGGCACCCAGCCTCCCGGGCCT	22	G	0.125	0.4958
NC_005102.4-150485044	ATGGGAGGCACCCAGCCTCCCGGGCCT	24	G	0.125	0.4958
NC_005102.4-150499698	TTTCCTGTAACTCCAGCTCCACAGGAC	24	G	0.094	0.1347
NC_005102.4-150514060	TCCCAAACCACACTGTCTCCACCTAAC	21	A	0.2368	0.483
NC_005102.4-150522068	CATGGAATCACCAGAGCTCCAAGGAGA	9	C	0	0.4832
NC_005102.4-150522068	CATGGAATCACCAGAGCTCCAAGGAGA	24	G	0.1293	0.1923
NC_005102.4-150597899	TGCTTTGAGACAGGATCTCCCATTGCA	2	G	0.0492	0.048
NC_005102.4-150597899	TGCTTTGAGACAGGATCTCCCATTGCA	3	C	0.0492	0.048
NC_005102.4-150601393	TTCAATGGGACTTCCACTCCACAGGGA	4	A	0.0086	0.4866
NC_005102.4-150601393	TTCAATGGGACTTCCACTCCACAGGGA	15	C	0.0603	0.0585
NC_005102.4-150684672	AGGTTGTCTACCACAGCTCCTAATCTT	22	A	0.252	0.3381
NC_005102.4-150685408	CCCCCATCCACCCCTTCTCCTCTGATG	4	C	0	0.4793
NC_005102.4-150685408	CCCCCATCCACCCCTTCTCCTCTGATG	13	C	0.1951	0.2937
NC_005102.4-150686953	GGCCTTACAACCCCAACTCCAGAGGAC	4	C	0.0256	0.0418

NC_005102.4-150686953	GGCCTTACAACCCCAACTCCAGAGGAC	6	T	0.114	0.4289
NC_005102.4-150702439	CATGCTGGGACTTGAACTCCTCACGTA	3	T	0.0446	0.0605
NC_005102.4-150736518	AAGGCTGGCACCGCCACTCCTGGAATG	5	C	0.0354	0.0517
NC_005102.4-150736518	AAGGCTGGCACCGCCACTCCTGGAATG	13	G	0.0177	0.4464
NC_005102.4-150748325	CCACCACCTACTCTAGCTCCAGGGACC	26	C	0.0086	0.4803
NC_005102.4-150754920	CCAGTCTACACCCTTTCTCCTTTCTTC	8	A	0	0.4807
NC_005102.4-150811114	AGCCGCCACACCCAGCCTCCCTTCTCA	5	G	0.0165	0.4849
NC_005102.4-150811114	AGCCGCCACACCCAGCCTCCCTTCTCA	25	T	0.0165	0.4849
NC_005102.4-150818355	ATGAAGGAGACACTTACTCCGAAGCGG	25	C	0.2759	0.3478
NC_005102.4-150837289	TGCCCACACACCTGCCCTCCGCTCACA	24	C	0	0.4793
NC_005102.4-150890310	AAATGCTGAACCATCTCTCCAATCCCA	21	A	0.1947	0.2171
NC_005102.4-150941405	CTTCACCAAACACGTTCTCCGAGTTTC	15	T	0.0175	0.4606
NC_005102.4-150941511	GTGGTGCACACTTTTACTCCTAGCACT	4	G	0	0.4813
NC_005102.4-150996649	CTTGGGCAGACCCAAGCTCCTTGTACA	15	A	0.2478	0.4464
NC_005102.4-150998829	GGGAAGTGGACACTGGCTCCTACCCCT	5	A	0.0496	0.0792
NC_005102.4-151009088	TTATGGGCAACTCTGTCTCCTTACCAT	4	T	0.0082	0.479
NC_005102.4-151015399	AGTTGACCAACTCACTCTCCAGACACA	13	C	0.187	0.1954
NC_005102.4-151015399	AGTTGACCAACTCACTCTCCAGACACA	27	A	0	0.4793
NC_005102.4-151015606	GAATCATAGACAGGGTCTCCCAGTCTG	15	G	0.2066	0.2234
NC_005102.4-151019018	ATCATCTCTACCCATGCTCCTCAAGTC	24	A	0	0.4793
NC_005102.4-151030289	TAATCTCAAACTATTCCTCCGACCCCT	2	A	0	0.4743
NC_005102.4-151030289	TAATCTCAAACTATTCCTCCGACCCCT	4	T	0.2689	0.4844
NC_005102.4-151172346	TGTTTTAAAACACATTCTCCACACATA	15	T	0.2114	0.2382
NC_005102.4-151172346	TGTTTTAAAACACATTCTCCACACATA	27	A	0.3171	0.436
NC_005102.4-151187448	TCCACAGACACAGGCGCTCCAACAGCT	5	C	0.1786	0.1913
NC_005102.4-151202591	GTTTTCTCTACTTGATCTCCCCTGCCC	9	T	0.041	0.0401
NC_005102.4-151231557	CACTCCTTGACTTGGCCTCCTGAAAAT	13	T	0.0417	0.49
NC_005102.4-151235620	CCGTGGCGAACCCGCACTCCACCTTGC	2	C	0.1	0.1528
NC_005102.4-151235620	CCGTGGCGAACCCGCACTCCACCTTGC	14	G	0.1818	0.2295
NC_005102.4-151275006	CTTGCTGGCACACCCACTCCTTTCCTG	6	T	0.2213	0.2095
NC_005102.4-151275006	CTTGCTGGCACACCCACTCCTTTCCTG	16	A	0.0574	0.0557
NC_005102.4-151361969	CACCTATTAACCCACACTCCCCATCTG	13	C	0.0435	0.0425
NC_005102.4-151361969	CACCTATTAACCCACACTCCCCATCTG	27	G	0.1391	0.1869
NC_005102.4-151401382	CACTGCAACACAGGGCCTCCCGCGGCA	7	A	0.1404	0.2659
NC_005102.4-151401382	CACTGCAACACAGGGCCTCCCGCGGCA	21	C	0.1842	0.295
NC_005102.4-151427908	GGTAGCAACACATTAGCTCCACTGTAC	5	G	0.1947	0.4574
NC_005102.4-151427908	GGTAGCAACACATTAGCTCCACTGTAC	27	C	0.1316	0.2836
NC_005102.4-151442454	CATGACCACACTCCTGCTCCAATACAC	6	C	0.0082	0.4755
NC_005102.4-151454130	AGGACCTCCACACATTCTCCACTGCAT	2	G	0	0.4793
NC_005102.4-151454130	AGGACCTCCACACATTCTCCACTGCAT	14	A	0	0.4793
NC_005102.4-151492764	GTACCCATAACTGCAGCTCCAGAGGAT	6	C	0.0164	0.0322
NC_005102.4-151511771	GGCCCTCAAACCCGCGCTCCAGTCTTG	13	C	0.0163	0.032
NC_005102.4-151546249	TGGATATAGACTAGAGCTCCCAGGCCT	3	G	0.0163	0.032
NC_005102.4-151561692	TGGCTTGGTACAACCACTCCTTCCATT	9	T	0.2893	0.3851
NC_005102.4-151562129	CTGCTTCCCACACTGCCTCCAGACATC	24	C	0.2377	0.2796
NC_005102.4-151563806	CCAGCACAGACCACAGCTCCTCACCAC	24	C	0.2645	0.4877
NC_005102.4-151565865	GCTGCCGGAACTCAGCCTCCTCCAGGG	14	A	0.0089	0.4789
NC_005102.4-151576329	TCCAGCCACACAGCCACTCCTTTTTCA	26	C	0.2195	0.2203
NC_005102.4-151760288	CCTCAGTCCACCTTTGCTCCCTCTTTA	21	C	0	0.4773
NC_005102.4-151866512	ACTCATATGACTCAAACTCCCAAGTAT	23	A	0.2768	0.4119
NC_005102.4-151897255	ACCACTCCTACCCTAGCTCCATGAGCC	14	T	0.2231	0.2234

NC_005102.4-152011322	AACAATCAAACACTCTCTCCAGTATTG	3	C	0.0263	0.0429
NC_005102.4-152275915	TTCCTCCCTACACATCCTCCTTCCCTT	2	T	0.2605	0.3462
NC_005102.4-152275915	TTCCTCCCTACACATCCTCCTTCCCTT	27	T	0.1186	0.2702
NC_005102.4-152290736	ACACAGGGCACATCCTCTCCTGAGGCC	2	C	0.122	0.2556
NC_005102.4-152334241	CATGATGAGACCCTGTCTCCAAAACAA	25	C	0.5583	0.4025
NC_005102.4-152366402	TTTCCTTTCACCCACACTCCAGGTCAG	1	T	0.1875	0.1843
NC_005102.4-152366868	GAATGCTCCACGAATTCTCCTGTCATT	5	G	0.042	0.0728
NC_005102.4-152366868	GAATGCTCCACGAATTCTCCTGTCATT	27	T	0.1795	0.1908
NC_005102.4-152385903	GATGGTGAAACCCCGTCTCCACTGTAA	15	G	0.075	0.4941
NC_005102.4-152396380	TCCAGTCCCACTGGAACTCCTGCAGAT	14	G	0.0083	0.0247
NC_005102.4-152398950	CTGAGACATACCCAAGCTCCTGTGTTG	13	C	0.2232	0.491
NC_005102.4-152403313	TCCACGTGAACCGAAACTCCAGCAGTG	5	C	0	0.4819
NC_005102.4-152435694	TCCAGCCCCACACCTCCTCCTCACCTT	6	C	0.0082	0.479
NC_005102.4-152461279	AGTAGTACTACAGTCTCTCCCTCTCCC	26	C	0.1818	0.2046
NC_005102.4-152491434	GAGGTCCACACCAGGCCTCCCCTTCTG	6	C	0.0924	0.1033
NC_005102.4-152506783	GGAGATACAACTGAAGCTCCATGTGCA	8	C	0.2333	0.4765
NC_005102.4-152506783	GGAGATACAACTGAAGCTCCATGTGCA	21	A	0	0.4743
NC_005102.4-152509474	GACATTTAAACCAACACTCCCTTTCCT	15	C	0.0261	0.0257
NC_005102.4-152536527	TCTTGAAAAACTGAAACTCCATTCATT	26	T	0.113	0.1067
NC_005102.4-152568515	CACACAGTGACAGGTCCTCCTGTTTCA	16	C	0	0.4793
NC_005102.4-152630179	GGAATGAAGACTATTGCTCCTTAAATC	24	A	0.1417	0.225
NC_005102.4-152646336	AGGACAGATACTATGACTCCTCTCTAG	5	C	0.1356	0.4908
NC_005102.4-152646336	AGGACAGATACTATGACTCCTCTCTAG	25	T	0	0.48
NC_005102.4-152665975	TGCTGTCTAACAGGGTCTCCCATTGGT	15	G	0.0265	0.0262
NC_005102.4-152665975	TGCTGTCTAACAGGGTCTCCCATTGGT	27	T	0.0177	0.4934
NC_005102.4-152699049	CCTGCCTAGACTTCAGCTCCTTAACCT	16	G	0.124	0.4988
NC_005102.4-152722455	TGGCATGTCACCTGTGCTCCTTTCTTT	13	T	0.0744	0.0868
NC_005102.4-152752788	TGTTCTCACACAGTCTCTCCCCACCCC	12	A	0.1557	0.4984
NC_005102.4-152752788	TGTTCTCACACAGTCTCTCCCCACCCC	15	C	0.1557	0.4984
NC_005102.4-152766067	CCTCGTAATACCTAAGCTCCACTGAGA	23	T	0.1696	0.4789
NC_005102.4-152766342	AGGTCCAAGACAGCTGCTCCAAGCCAC	4	T	0.1026	0.1274
NC_005102.4-152796006	GTCGCTCACACACTTTCTCCATCCCAG	1	G	0.1653	0.2172
NC_005102.4-152796006	GTCGCTCACACACTTTCTCCATCCCAG	3	C	0.1653	0.2172
NC_005102.4-152811187	GTCGCTGTCACTGTGTCTCCTTGTTCT	3	C	0.0246	0.0243
NC_005102.4-152811187	GTCGCTGTCACTGTGTCTCCTTGTTCT	15	G	0.5798	0.4377
NC_005102.4-152903163	CATGCACAAACCCACACTCCAACACAA	1	C	0.1057	0.1145
NC_005102.4-152944848	CACTGACAAACTCAGGCTCCTCATCCC	26	C	0.0083	0.4816
NC_005102.4-152972415	TCTGCTGACACTTAGGCTCCACTTATT	25	A	0.0256	0.0253
NC_005102.4-153035704	CTGTGTGAGACTCCCGCTCCCACTTCT	12	T	0.1765	0.1609
NC_005102.4-153035704	CTGTGTGAGACTCCCGCTCCCACTTCT	24	T	0.3077	0.2604
NC_005102.4-153037956	TCATCAACTACACCATCTCCAAATTTA	24	T	0.1345	0.1254
NC_005102.4-153072038	TCAAAGCACACTCCATCTCCTCATTCC	27	C	0.0811	0.4896
NC_005102.4-153088396	ATGTTCACTACATCAGCTCCACAGAGG	5	T	0.0083	0.4835
NC_005102.4-153091535	CCACTGAATACTTGGTCTCCATTGATG	4	C	0	0.4773
NC_005102.4-153091535	CCACTGAATACTTGGTCTCCATTGATG	5	T	0.1983	0.5
NC_005102.4-153204726	ACAGCGACTACCCTGACTCCTTTAAGT	6	G	0.0965	0.1075
NC_005102.4-153204726	ACAGCGACTACCCTGACTCCTTTAAGT	7	A	0.113	0.1369
NC_005102.4-153208639	ACCCTTCCCACGCTGCCTCCTGCTGGA	12	G	0.065	0.0928
NC_005102.4-153215321	TGCATGATGACCAGTTCTCCCTCCCTC	8	T	0.0083	0.4783
NC_005102.4-153215321	TGCATGATGACCAGTTCTCCCTCCCTC	25	C	0.0167	0.0328
NC_005102.4-153225064	GAAACAAAAACAATCCCTCCCTCTGTG	6	A	0	0.4846

NC_005102.4-153288594	CAGTGAACCACCTATACTCCAGTTCCA	27	A	0.1565	0.4991
NC_005102.4-153368501	TTGCACCACACCTTTTCTCCTTCCCAT	1	T	0.0086	0.0255
NC_005102.4-153368501	TTGCACCACACCTTTTCTCCTTCCCAT	24	C	0.0259	0.0422
NC_005102.4-153388481	GCATGGCTGACCCTCCCTCCTCTTCTC	12	C	0.0169	0.4884
NC_005102.4-153450780	AGGGAGATTACCTCCTCTCCTGGTGAG	6	G	0.0672	0.065
NC_005102.4-153565618	GCATACATAACAGCTTCTCCATACATG	12	A	0.1833	0.1932
NC_005102.4-153570813	CCTCCTGTGACTTTAGCTCCAGGTGAT	5	C	0.0609	0.0752
NC_005102.4-153579575	ACCTCTCAAACTTTCTCTCCCTTTCAT	6	T	0	0.48
NC_005102.4-153579575	ACCTCTCAAACTTTCTCTCCCTTTCAT	9	A	0.2193	0.1953
NC_005102.4-153697417	CCTCAGTCAACTTCTCCTCCTGGAAGT	3	T	0.1327	0.1828
NC_005102.4-153709308	ATGCTATTCACTTATTCTCCATGTCTC	26	T	0	0.4682
NC_005102.4-153709308	ATGCTATTCACTTATTCTCCATGTCTC	27	C	0	0.4654
NC_005102.4-153751645	CCACTTCCCACCTTATCTCCTTGTTAG	4	C	0	0.4807
NC_005102.4-153753812	TCTGATAAGACACAGGCTCCTGGAATT	22	G	0.1404	0.1744
NC_005102.4-153756343	TGCATTTGCACCCATTCTCCATGCCTT	8	G	0.0256	0.0253
NC_005102.4-153756343	TGCATTTGCACCCATTCTCCATGCCTT	9	C	0.2373	0.493
NC_005102.4-153767865	AAAGCCTCCACATTTTCTCCCTCTCTC	9	C	0.027	0.0778
NC_005102.4-153770409	GGTTCATGAACTAAGTCTCCAGTGATC	8	G	0.1453	0.1908
NC_005102.4-153823942	AACTCTCTTACAAGGACTCCTATGATG	22	A	0.2564	0.4602
NC_005102.4-153833527	GCCTTTCTAACTGAGTCTCCACTTCTG	9	A	0	0.4793
NC_005102.4-154018523	CACCTCGGTACCCCATCTCCACAGAGG	6	C	0	0.4898
NC_005102.4-154039015	AAGCCTCGTACAAGGTCTCCGTGTTTG	21	G	0.1301	0.189
NC_005102.4-154072151	CTTCCCACCACAGCGTCTCCATTACAT	15	G	0.0569	0.0705
NC_005102.4-154072151	CTTCCCACCACAGCGTCTCCATTACAT	27	T	0.0081	0.481
NC_005102.4-154082251	TTGGACTCCACCCTTCCTCCTTTTGCC	2	T	0.0893	0.1771
NC_005102.4-154091396	GGCTGCAGGACTCCCACTCCTAGTCCA	16	A	0.0541	0.461
NC_005102.4-154091396	GGCTGCAGGACTCCCACTCCTAGTCCA	21	T	0.036	0.4659
NC_005102.4-154099722	TGATGTCGTACACTTACTCCCAATCAT	6	T	0.0593	0.4997
NC_005102.4-154135212	AGCCAAATGACTTTCACTCCGTCCCTT	4	C	0.0656	0.1082
NC_005102.4-154147666	AGCAAAGCCACCTCAGCTCCCATGAGA	8	C	0.0417	0.0408
NC_005102.4-154147666	AGCAAAGCCACCTCAGCTCCCATGAGA	12	C	0.1597	0.1746
NC_005102.4-154151178	GGCGACCTCACCCCCTCTCCTTCCTCT	2	G	0.15	0.2778
NC_005102.4-154151178	GGCGACCTCACCCCCTCTCCTTCCTCT	15	C	0.1356	0.3034
NC_005102.4-154197691	GTAGTTACCACCTCAGCTCCACTCTAC	12	C	0.0413	0.0405
NC_005102.4-154204495	CATCCTTACACTTTCCCTCCTTTCACG	7	T	0.2213	0.2686
NC_005102.4-154204495	CATCCTTACACTTTCCCTCCTTTCACG	16	C	0.1653	0.2533
NC_005102.4-154273600	CCAGGCTTGACCTTGTCTCCAGTTCTC	3	A	0	0.4765
NC_005102.4-154281792	CTTCCAGTCACGGCCTCTCCTTTCTCT	14	C	0	0.4807
NC_005102.4-154285946	TAGGAGATTACTTTCCCTCCCACTCCT	21	C	0.0407	0.0398
NC_005102.4-154285946	TAGGAGATTACTTTCCCTCCCACTCCT	22	A	0.0165	0.4787
NC_005102.4-154325318	AGCCTTCCAACCTCCCCTCCTGGTCAG	2	G	0.1622	0.2467
NC_005102.4-154325414	TGAAGGAGGACTCAGACTCCTGCCCTT	24	C	0	0.4793
NC_005102.4-154341504	CTTGATTTGACAGGGCCTCCTGCTACT	14	G	0.05	0.0488
NC_005102.4-154394575	AGAGCTTAAACCAGCTCTCCTAGGTCC	12	C	0.2432	0.2778
NC_005102.4-154407935	ACTTTACACACCTCTCCTCCAGTGCAG	1	A	0.0569	0.0854
NC_005102.4-154432287	CCCTGTGAAACGCTCGCTCCATCACTG	6	T	0.0089	0.4824
NC_005102.4-154433297	GTTGGGTCCACCTCCTCTCCCTGAAAC	26	A	0.0086	0.4836
NC_005102.4-154446396	CCTGTTATTACAGTATCTCCCCTGACT	1	C	0.1951	0.189
NC_005102.4-154446396	CCTGTTATTACAGTATCTCCCCTGACT	6	T	0.123	0.3307
NC_005102.4-154474602	AGGTGAGGGACACAAGCTCCACTAGTC	12	A	0.1901	0.2234
NC_005102.4-154486338	CTCTCTACAACATTTTCTCCAAACACT	7	A	0.0088	0.481

NC_005102.4-154493378	CTTCAACTGACAACGTCTCCCTTTCCT	15	G	0.1826	0.2072
NC_005102.4-154505048	CCATGTGACACGGTCCCTCCATACTCT	2	C	0.2667	0.4265
NC_005102.4-154512267	GATCTTTCCACCCTTTCTCCTGATCCC	1	G	0	0.4793
NC_005102.4-154556017	GAGCCTTAAACAAGGTCTCCCTGTGCT	12	A	0	0.4807
NC_005102.4-154566936	TGCCGCTGGACGTGAGCTCCATCCCCA	5	G	0.2478	0.3026
NC_005102.4-154576009	AGGTGTCACACCTCAGCTCCAATCCAC	1	A	0	0.4807
NC_005102.4-154576676	GACGTGATGACCAGGCCTCCAAAGAAT	3	C	0	0.4764
NC_005102.4-154593485	TGTGTCCACACATGCTCTCCTCTCTCA	5	T	0.2602	0.427
NC_005102.4-154600171	TTGGCTTTCACCAAGCCTCCAAACATG	4	G	0.3171	0.4174
NC_005102.4-154603517	GAAGGTCTCACTGGCTCTCCAGTACTG	8	T	0	0.4826
NC_005102.4-154608864	ACCCTCCCAACCCCAGCTCCCTGCTCC	24	C	0.319	0.4643
NC_005102.4-154624343	ACCCTGGTGACACTTTCTCCCCTCTAG	27	G	0.0684	0.066
NC_005102.4-154732305	GTAATCAAGACAACCCCTCCACTACAC	5	T	0.0088	0.4796
NC_005102.4-154736898	CTCCTGTGCACACTGCCTCCTGATGCC	3	C	0	0.4765
NC_005102.4-154736898	CTCCTGTGCACACTGCCTCCTGATGCC	26	C	0.15	0.1528
NC_005102.4-154737624	GGAATGCACACCCTAACTCCAACCCCG	26	C	0.0167	0.0328
NC_005102.4-154751868	GGTGCTCCAACCCAATCTCCATCTCCA	9	A	0.2869	0.4258
NC_005102.4-154755397	GAACACAGAACAAGGACTCCGAGTCCA	3	A	0	0.4787
NC_005102.4-154770003	CCACTCCTCACCCTCTCTCCCATCATT	21	C	0.0175	0.0345
NC_005102.4-154770003	CCACTCCTCACCCTCTCTCCCATCATT	22	A	0.0087	0.4782
NC_005102.4-154842489	AGAGGGAGGACTTTGCCTCCCCTGCAG	6	G	0.2459	0.4515
NC_005102.4-154842489	AGAGGGAGGACTTTGCCTCCCCTGCAG	21	C	0.122	0.1695
NC_005102.4-154860963	CCAAATATCACACACCCTCCACGGGTC	13	C	0.0598	0.058
NC_005102.4-154860963	CCAAATATCACACACCCTCCACGGGTC	23	G	0.0684	0.4938
NC_005102.4-154920731	ACCACTTAGACATACTCTCCTCCTTTA	12	A	0	0.4773
NC_005102.4-154981525	CTGCTCCACACACCATCTCCCCAGCAA	27	A	0.3652	0.3772
NC_005102.4-155013117	GGGACTGGGACCCTTACTCCCACCCCT	7	G	0.069	0.0825
NC_005102.4-155013117	GGGACTGGGACCCTTACTCCCACCCCT	27	T	0.265	0.3311
NC_005102.4-155042642	AGTTTACAAACTCCATCTCCAGTGTCT	8	A	0.1311	0.4966
NC_005102.4-155061356	CTGTTGTTGACGCCCTCTCCCCAGGAA	12	G	0.1933	0.317
NC_005102.4-155080451	AACAAAGCCACCCTTTCTCCTCACATC	8	C	0.252	0.319
NC_005102.4-155080451	AACAAAGCCACCCTTTCTCCTCACATC	27	C	0.3333	0.4036
NC_005102.4-155095016	CACCCGGTGACCACGGCTCCCTGTGCT	15	G	0.0885	0.1466
NC_005102.4-155135898	GATTCTTGCACATAGCCTCCAGAAAGC	14	A	0.0163	0.4682
NC_005102.4-155180278	ACAGTCCTAACAGGCGCTCCTTGTTTA	14	G	0	0.4793
NC_005102.4-155194492	GTACTGTGAACATTTGCTCCTTTGTTT	5	T	0	0.4751
NC_005102.4-155260965	GTATAACCTACACATCCTCCTATTACT	1	G	0.0877	0.1454
NC_005102.4-155260965	GTATAACCTACACATCCTCCTATTACT	16	C	0.114	0.1075
NC_005102.4-155286443	GTGCATCTGACTATAGCTCCCTGGACC	25	A	0.1217	0.3403
NC_005102.4-155323120	GACCGGAGAACAGCGCCTCCCTCAGTT	24	A	0	0.4793
NC_005102.4-155369940	CCTCAGGATACCTCAGCTCCCCAGGCT	5	A	0.3482	0.4789
NC_005102.4-155414184	GGACTAACTACAACACCTCCTTTATGG	16	C	0.2069	0.2739
NC_005102.4-155426412	CCCCTTCCCACCCTTTCTCCTTCTCTT	7	C	0.0246	0.0243
NC_005102.4-155426412	CCCCTTCCCACCCTTTCTCCTTCTCTT	25	C	0.1066	0.1154
NC_005102.4-155436924	CCGTGAATCACGACTGCTCCGTGTCCT	2	C	0.0569	0.0553
NC_005102.4-155436924	CCGTGAATCACGACTGCTCCGTGTCCT	13	A	0	0.4793
NC_005102.4-155440257	ATCCCCAGGACCTCTTCTCCTAGATGG	9	G	0.0261	0.0257
NC_005102.4-155457213	TGTCACACTACACCCTCTCCACATGCA	6	C	0.2232	0.212
NC_005102.4-155495740	CCCAACATCACATGTTCTCCATGGGTC	14	G	0.0167	0.0328
NC_005102.4-155501453	AATTAGCTCACTGCTTCTCCCTTCTAT	26	A	0.1351	0.3338
NC_005102.4-155517495	AGGGAGGTCACTGGGCCTCCTGGAGGG	15	G	0.252	0.381

NC_005102.4-155524631	CAAGTTTTCACTCAGCCTCCTAGGATA	23	G	0.1333	0.1387
NC_005102.4-155538850	TTTGCAGGAACTGATTCTCCCCACTAC	26	A	0.042	0.0411
NC_005102.4-155609316	CTACTTACAACAGTGACTCCTTCTTTT	25	T	0.018	0.4383
NC_005102.4-155609520	GATGTTTACACCCCTACTCCCATATAC	5	T	0.0804	0.0771
NC_005102.4-155614496	CTAGATATCACATGCCCTCCATGGGTC	13	T	0.1478	0.304
NC_005102.4-155614496	CTAGATATCACATGCCCTCCATGGGTC	24	G	0.0086	0.4866
NC_005102.4-155629391	ACGAACAGCACAGAGACTCCTCCTGCT	2	C	0.1111	0.1773
NC_005102.4-155683786	CAGGCAGAAACACAGCCTCCCAGATTC	24	A	0.161	0.2026
NC_005102.4-155697074	ACTAGCATCACATAGCCTCCACTAAAG	5	G	0.2605	0.4186
NC_005102.4-155757252	ACCTGGTTCACATGGCCTCCTGTGAAT	16	C	0.1736	0.4605
NC_005102.4-155762038	CCCCATCTCACTCCCACTCCCTCATCT	2	C	0.125	0.225
NC_005102.4-155770373	CCACTGGGGACTCCCCCTCCTGGCACA	6	G	0.0246	0.0243
NC_005102.4-155819757	TGATCCTAAACCACAGCTCCTCATTCT	16	G	0.0089	0.4751
NC_005102.4-155840720	ATCCTTCAGACTCCCCCTCCCAAGGGT	3	C	0.0256	0.0418
NC_005102.4-155840720	ATCCTTCAGACTCCCCCTCCCAAGGGT	24	G	0.1638	0.4836
NC_005102.4-155853904	TCCTCCGTGACACTTCCTCCTTCTACA	7	G	0.0256	0.0253
NC_005102.4-155863020	GTCTGTGTCACACTTCCTCCAGACACT	3	C	0.1639	0.2399
NC_005102.4-155863020	GTCTGTGTCACACTTCCTCCAGACACT	5	G	0.2951	0.4092
NC_005102.4-155885644	GGCACGGGTACACTTTCTCCTTGATCT	5	C	0.3	0.3665
NC_005102.4-155885644	GGCACGGGTACACTTTCTCCTTGATCT	8	G	0.0336	0.033
NC_005102.4-155904106	ACTGAACTTACCTTCACTCCCAGCATA	27	A	0.2119	0.4997
NC_005102.4-155919188	CTGTCAAAGACATCTTCTCCATCTATT	22	T	0.025	0.0247
NC_005102.4-155925975	AACTTAGGAACTCTCTCTCCTAAACTC	12	T	0.0259	0.0585
NC_005102.4-155928764	CTTCTGCCCACCAGCTCTCCCTTCTTT	14	G	0.0246	0.0243
NC_005102.4-155942661	GTATTCATCACGTTCTCTCCTTCACAC	7	A	0.0413	0.0716
NC_005102.4-155949348	TTCTTTCAAACCTGTTCTCCCTCGGTT	6	T	0.2358	0.2989
NC_005102.4-155949348	TTCTTTCAAACCTGTTCTCCCTCGGTT	24	G	0.0894	0.1425
NC_005102.4-155987404	CCCTCAGAAACGCTTCCTCCCAGTAAG	6	A	0	0.477
NC_005102.4-156012366	AACTCCAGGACAGCCCCTCCAGTGAAA	14	C	0.2857	0.4228
NC_005102.4-156043781	TCTCCATTCACTCTGACTCCTGTAATC	4	C	0	0.4787
NC_005102.4-156053583	ATACTCAATACCTTCTCTCCTCGCTCC	25	T	0.2683	0.3646
NC_005102.4-156068281	TCAGGGCACACTCCTTCTCCCTCCTAT	24	C	0	0.4807
NC_005102.4-156068281	TCAGGGCACACTCCTTCTCCCTCCTAT	27	T	0	0.4807
NC_005102.4-156071474	GTCCCAAAGACAGAAACTCCTAACTCT	22	A	0	0.4779
NC_005102.4-156071474	GTCCCAAAGACAGAAACTCCTAACTCT	23	A	0	0.4779
NC_005102.4-156092490	CACACGGGCACTTTATCTCCATGTCTT	14	T	0.1271	0.1759
NC_005102.4-156138447	ATAAATGCTACTGCCCCTCCCCTACTC	22	C	0.2417	0.4097
NC_005102.4-156161873	CTAGAATGAACACCTTCTCCCTTAGAT	14	C	0.0431	0.0422
NC_005102.4-156171457	CCATTCTTCACCCCATCTCCGCTCACT	22	C	0.2083	0.1997
NC_005102.4-156185647	AGTACAATCACTCGGGCTCCCGAGAAA	23	A	0.0684	0.066
NC_005102.4-156206455	CTTCTTCACACCAACTCTCCAACACTG	24	A	0	0.4787
NC_005102.4-156223372	AGGATCTTCACAGAACCTCCATCCTCT	16	C	0.0756	0.4931
NC_005102.4-156223372	AGGATCTTCACAGAACCTCCATCCTCT	24	C	0.1345	0.494
NC_005102.4-156230143	CAACCTCACACCACCTCTCCCTTCGCT	22	T	0.1709	0.4444
NC_005102.4-156230143	CAACCTCACACCACCTCTCCCTTCGCT	25	G	0.1513	0.3118
NC_005102.4-156249774	GGCAATAATACCCTTACTCCTTGATAT	24	A	0.1967	0.263
NC_005102.4-156262502	GGTTTCTGAACTTTACCTCCAGGCTCA	9	A	0	0.4718
NC_005102.4-156286734	CCCACAAAGACCTAACCTCCAGGATCT	7	A	0.2131	0.2399
NC_005102.4-156301002	CGTCCTCTGACCACCTCTCCCAGGCCC	24	G	0.0756	0.214
NC_005102.4-156316420	TTCCTCCTCACCTCTCCTCCCATCAGT	8	T	0.0083	0.4803
NC_005102.4-156316420	TTCCTCCTCACCTCTCCTCCCATCAGT	24	C	0.0331	0.0484

NC_005102.4-156318022	GGGTATGAAACACCAGCTCCCAGAAAA	12	A	0	0.477
NC_005102.4-156331387	CAAATCAGGACCATTCCTCCTTGCCCT	22	T	0	0.4649
NC_005102.4-156331387	CAAATCAGGACCATTCCTCCTTGCCCT	23	G	0.181	0.1923
NC_005102.4-156338853	ATCTCCGCAACTGCCTCTCCCTTAGCT	7	G	0.2893	0.313
NC_005102.4-156338853	ATCTCCGCAACTGCCTCTCCCTTAGCT	14	C	0	0.4751
NC_005102.4-156373921	CAGGATACAACAACACCTCCCTCATCT	9	A	0.0088	0.4861
NC_005102.4-156375459	GCTGGGAGAACAGGAGCTCCCCCACGG	16	G	0.3306	0.4481
NC_005102.4-156403558	ATTCTGAACACTGGGTCTCCAGATGGT	3	T	0	0.4715
NC_005102.4-156403558	ATTCTGAACACTGGGTCTCCAGATGGT	15	G	0.177	0.2171
NC_005102.4-156420025	AAAATTAGAACACTTCCTCCAGATCCA	12	A	0.2743	0.3396
NC_005102.4-156420025	AAAATTAGAACACTTCCTCCAGATCCA	22	G	0.1842	0.1953
NC_005102.4-156499006	GCCACCTCAACCCCAACTCCATTCCCT	7	T	0.0088	0.49
NC_005102.4-156503317	TCTCGGGGCACCCAAGCTCCAAGCTGT	12	C	0.0885	0.0846
NC_005102.4-156531658	CTGGTGTGCACAGTAACTCCCCTACCA	1	C	0.0252	0.0571
NC_005102.4-156531658	CTGGTGTGCACAGTAACTCCCCTACCA	6	G	0.1765	0.2389
NC_005102.4-156533981	CCTCTTGACACACATGCTCCTGACTCA	5	T	0	0.4751
NC_005102.4-156533981	CCTCTTGACACACATGCTCCTGACTCA	15	T	0.0325	0.032
NC_005102.4-156575667	GGACGTATCACAGGCACTCCCTTCTAC	3	A	0	0.4807
NC_005102.4-156575667	GGACGTATCACAGGCACTCCCTTCTAC	8	T	0.2213	0.3401
NC_005102.4-156580243	CCTCTGAGGACCATTTCTCCTGTAAGA	15	T	0.1538	0.2484
NC_005102.4-156740528	TTTCCTGTGACAGCAGCTCCTGGGAAT	12	A	0.0081	0.481
NC_005102.4-156788522	AGATCAGACACACAGACTCCACTCCAT	22	C	0	0.48
NC_005102.4-156807570	GAGACAGAGACTTCCTCTCCTTACACA	9	G	0.2222	0.4444
NC_005102.4-156840239	ATAGTCTTTACAACGCCTCCCTCCAAA	15	G	0	0.4832
NC_005102.4-156840239	ATAGTCTTTACAACGCCTCCCTCCAAA	16	C	0	0.4832
NC_005102.4-156893429	CTCAGAGGTACAGTAGCTCCAATACCT	25	C	0.0424	0.0734
NC_005102.4-156893429	CTCAGAGGTACAGTAGCTCCAATACCT	26	C	0.1966	0.3504
NC_005102.4-156918055	CCACTGGCCACCCGTTCTCCCTTTGTC	3	A	0.1913	0.2138
NC_005102.4-156953725	CCTACCTGCACTACTTCTCCCACATCT	6	C	0.1901	0.2355
NC_005102.4-156954567	CTCATCCTCACACTTCCTCCTGGACCT	16	C	0.1901	0.2474
NC_005102.4-156957143	GAAGGGTCCACATTAGCTCCCCGACTC	22	C	0.1951	0.2263
NC_005102.4-156967394	AATGTTCTGACCTTGACTCCATCAGTG	21	A	0	0.4773
NC_005102.4-156986408	TGGCCTACCACAACATCTCCCCCATCT	26	C	0.1429	0.188
NC_005102.4-157000901	TTAAGCAAAACAGTAACTCCACACTGT	24	C	0	0.4779
NC_005102.4-157036948	AAGCTGATCACTGCATCTCCGTCTTTG	6	G	0	0.4819
NC_005102.4-157036948	AAGCTGATCACTGCATCTCCGTCTTTG	21	G	0.0331	0.4877
NC_005102.4-157069664	GAAAACAACACCCACACTCCTTCTGGT	25	G	0.0254	0.0251
NC_005102.4-157088262	ACTTCTAACACTTCTTCTCCCTCCCGG	9	C	0.2397	0.2922
NC_005102.4-157143713	GCTCTTGGTACTTCGTCTCCAAATAGA	15	G	0.042	0.0411
NC_005102.4-157150706	TCTGTACAAACCTCAGCTCCAGATGGA	25	G	0.1721	0.3401
NC_005102.4-157174519	TGGGCATTCACTTTGTCTCCTAAGTGT	3	G	0.05	0.0488
NC_005102.4-157188100	AGAATCTCCACCAAGGCTCCTGGCCTC	14	A	0.3393	0.4362
NC_005102.4-157188100	AGAATCTCCACCAAGGCTCCTGGCCTC	27	C	0.3451	0.3396
NC_005102.4-157193290	CAAGATCCTACTCTACCTCCCACGACC	4	G	0.2241	0.3572
NC_005102.4-157193290	CAAGATCCTACTCTACCTCCCACGACC	23	C	0.287	0.3451
NC_005102.4-157249218	TATGCATACACTCATACTCCATAAGAA	15	T	0.2051	0.4693
NC_005102.4-157282990	CGTCCCTAAACCCTAGCTCCACACATG	2	G	0	0.4793
NC_005102.4-157282990	CGTCCCTAAACCCTAGCTCCACACATG	7	T	0.1463	0.4984
NC_005102.4-157324048	CAAAGGGCCACTCTGACTCCTTAATGA	3	A	0.1148	0.4407
NC_005102.4-157324048	CAAAGGGCCACTCTGACTCCTTAATGA	16	A	0.0328	0.0634
NC_005102.4-157335363	GCTCGTCGTACATTTTCTCCTGTGAGA	6	T	0	0.4724

NC_005102.4-157354414	TACCCCGCCACCTTTTCTCCTGGGTTC	1	T	0.0855	0.4444
NC_005102.4-157354414	TACCCCGCCACCTTTTCTCCTGGGTTC	27	C	0.0254	0.0734
NC_005102.4-157361176	GATAGCCTCACTCCACCTCCTGTCCCT	3	T	0.0569	0.0553
NC_005102.4-157410810	ACCTGGCAAACTCCCCCTCCCCACTTA	24	C	0	0.4807
NC_005102.4-157523325	AACACGTGGACTTGCCCTCCTGCTGAA	6	G	0	0.48
NC_005102.4-157546676	AACAAACAAACTACCCCTCCCTCATAG	12	T	0	0.4807
NC_005102.4-157557642	GATCGAGACACTATTTCTCCAACTCAA	4	C	0	0.4793
NC_005102.4-157561544	CTCTGTATGACCAGATCTCCCCTGCTC	12	C	0.0522	0.0671
NC_005102.4-157561544	CTCTGTATGACCAGATCTCCCCTGCTC	14	G	0.0263	0.026
NC_005102.4-157562444	CCCGGTCCCACAGCCCCTCCTACCTGA	3	C	0.1818	0.4426
NC_005102.4-157562444	CCCGGTCCCACAGCCCCTCCTACCTGA	15	C	0.1333	0.1528
NC_005102.4-157574971	TGCCTGGCTACTTATACTCCACGGATA	22	C	0.0603	0.0746
NC_005102.4-157683327	TCTTCTGCAACCCAAACTCCTGGTGAT	6	T	0.1008	0.494
NC_005102.4-157740197	CACTCAGTCACTGATTCTCCCCTTCTA	7	G	0.2602	0.377
NC_005102.4-157768011	CCATATAACACCACCTCTCCTCAGGCA	26	C	0	0.4779
NC_005102.4-157795276	CATTGCCAAACCGTAGCTCCTACCCTG	23	C	0.1121	0.1504
NC_005102.4-157834152	GAAGAAAGAACTTGACCTCCAGCCAAC	25	A	0.1983	0.3332
NC_005102.4-157837177	ATTAAATACACCAGGGCTCCAGGTGTA	21	A	0.2435	0.3593
NC_005102.4-157917467	TTATAGGCCACCATTCCTCCTCTTACT	14	T	0.1639	0.4979
NC_005102.4-157951198	GCAACTCCTACGTCAGCTCCTCAGTCA	5	C	0.187	0.1954
NC_005102.4-157951198	GCAACTCCTACGTCAGCTCCTCAGTCA	12	G	0	0.4793
NC_005102.4-157977416	GGCATGTCGACTTCCTCTCCGACTCCT	2	G	0.1795	0.1908
NC_005102.4-157977416	GGCATGTCGACTTCCTCTCCGACTCCT	12	T	0.1538	0.1841
NC_005102.4-157979321	AACCCTTTCACTAGTGCTCCTTCCTGA	14	G	0.0252	0.4829
NC_005102.4-157979321	AACCCTTTCACTAGTGCTCCTTCCTGA	26	G	0.0252	0.4829
NC_005102.4-158009387	CATGCAGACACATGAACTCCCACATAC	7	G	0.075	0.1458
NC_005102.4-158025507	TTAATGTTAACCTCCTCTCCAGGTTGT	8	T	0.0174	0.4588
NC_005102.4-158025507	TTAATGTTAACCTCCTCTCCAGGTTGT	22	G	0.0174	0.4588
NC_005102.4-158047317	CCTGTCTCGACCCTCCCTCCTGAGTTC	13	C	0.1057	0.1001
NC_005102.4-158047317	CCTGTCTCGACCCTCCCTCCTGAGTTC	15	C	0.0488	0.078
NC_005102.4-158055730	CTCTGCTCTACCCGGTCTCCAACACAC	16	T	0.0672	0.065
NC_005102.4-158055730	CTCTGCTCTACCCGGTCTCCAACACAC	26	A	0.1368	0.4997
NC_005102.4-158060983	TAAGCTAACACCTCATCTCCAACAATT	22	A	0	0.4807
NC_005102.4-158060983	TAAGCTAACACCTCATCTCCAACAATT	24	A	0	0.4807
NC_005102.4-158094636	TGCGCACATACCCACCCTCCATCTCTC	4	G	0	0.4786
NC_005102.4-158094636	TGCGCACATACCCACCCTCCATCTCTC	7	C	0.0517	0.4905
NC_005102.4-158098545	CTAATCACGACCAGCTCTCCAGACTCA	8	C	0.1538	0.4997
NC_005102.4-158098545	CTAATCACGACCAGCTCTCCAGACTCA	24	C	0.2051	0.272
NC_005102.4-158113751	GGTTTCAGGACTCTCCCTCCCCCTTTC	26	T	0.0357	0.0351
NC_005102.4-158207221	CTCCTAATCACCTCAGCTCCTTGCTCA	26	C	0.1913	0.4981
NC_005102.4-158258755	GCGTCTCACACCCTAGCTCCTGGTACA	2	C	0.0248	0.0245
NC_005102.4-158263136	CACAAAGGGACTGTAGCTCCACCAGGA	8	G	0.0084	0.4797
NC_005102.4-158263136	CACAAAGGGACTGTAGCTCCACCAGGA	22	C	0.0333	0.0328
NC_005102.4-158288859	AGTGGGGTTACCATAGCTCCTTCCTAT	4	G	0.2213	0.4852
NC_005102.4-158289877	TCATCAGGAACCTGGGCTCCCCTCTTG	16	G	0.0333	0.0488
NC_005102.4-158289877	TCATCAGGAACCTGGGCTCCCCTCTTG	23	T	0.042	0.4829
NC_005102.4-158312663	CCCTCGAGTACTTGGTCTCCACACACT	7	A	0	0.4793
NC_005102.4-158328527	AGGGAAGGAACAAACTCTCCTTTTTCT	13	A	0.2832	0.4887
NC_005102.4-158328527	AGGGAAGGAACAAACTCTCCTTTTTCT	22	T	0	0.4755
NC_005102.4-158354528	CCTACTTTAACAGTTTCTCCTTCCTAC	15	T	0	0.4833
NC_005102.4-158354528	CCTACTTTAACAGTTTCTCCTTCCTAC	23	C	0.1026	0.1274

NC_005102.4-158368310	ATGAAACAAACCCAAACTCCTATCTCC	21	T	0.2281	0.4114
NC_005102.4-158378873	GCTACTGGAACAGCCTCTCCTGATCCA	15	C	0.0407	0.0398
NC_005102.4-158414373	AAGGAAAAGACATCTGCTCCCATGCTT	8	A	0.1066	0.4984
NC_005102.4-158414373	AAGGAAAAGACATCTGCTCCCATGCTT	25	C	0.0984	0.1225
NC_005102.4-158437649	ATACCTTGTACATTCTCTCCCTCCTTC	3	A	0.042	0.4797
NC_005102.4-158437649	ATACCTTGTACATTCTCTCCCTCCTTC	23	C	0.0417	0.0408
NC_005102.4-158458291	CTGTGCTCCACCCCAGCTCCAGATGCT	27	T	0.3186	0.3136
NC_005102.4-158482919	CACTGTGAGACACTGACTCCTTATCTC	5	G	0.2787	0.4914
NC_005102.4-158482919	CACTGTGAGACACTGACTCCTTATCTC	13	C	0.2869	0.4879
NC_005102.4-158501982	CCTAAAGCCACCTCCACTCCATTCCTT	9	C	0.0435	0.0425
NC_005102.4-158590416	CTTCCACATACTCAACCTCCCATTTAA	12	T	0	0.477
NC_005102.4-158590416	CTTCCACATACTCAACCTCCCATTTAA	14	A	0	0.477
NC_005102.4-158592494	TTAGTTTCCACAGGATCTCCTGCCTCA	23	C	0.1525	0.2219
NC_005102.4-158602412	CCCCTTCCTACTCTGCCTCCAGTCCCC	26	C	0	0.4807
NC_005102.4-158610898	GGGTCAGCAACAGCCTCTCCCTGTCTC	27	C	0.1626	0.4973
NC_005102.4-158629919	ATGATTTCTACACAGTCTCCCTTTCTT	15	G	0.0333	0.0328
NC_005102.4-158682949	CTCTCACCCACCTTCCCTCCCCTCTCT	3	C	0.5536	0.4592
NC_005102.4-158732182	GAGCAACATACCTCTTCTCCAAACATC	4	C	0.1148	0.2157
NC_005102.4-158757449	ACCCTTTGCACCTGGCCTCCCTTCTCT	15	G	0.1463	0.189
NC_005102.4-158760651	CTCTCCTGCACGTTCTCTCCTTTGGAC	6	C	0.0598	0.074
NC_005102.4-158769780	TTGTTCCTCACCCTGTCTCCTTACCAG	1	T	0.0248	0.0245
NC_005102.4-158769780	TTGTTCCTCACCCTGTCTCCTTACCAG	4	T	0.0083	0.4803
NC_005102.4-159154690	GCACTGCAAACTGGATCTCCATGTTGC	1	G	0.2105	0.4186
NC_005102.4-159154690	GCACTGCAAACTGGATCTCCATGTTGC	7	C	0.2087	0.4165
NC_005102.4-159220827	GGCAGAAGCACCCTGTCTCCTTCAGGG	24	A	0.1593	0.2171
NC_005102.4-159274198	CCACCTGTCACCCCAGCTCCAAGGGCC	15	A	0.3083	0.3533
NC_005102.4-159293384	ATGATCCCGACTCAGCCTCCTAAGAGT	22	A	0.1148	0.2399
NC_005102.4-159320241	CCAGCAGTTACCAAACCTCCCTGAGGT	16	C	0.1626	0.3688
NC_005102.4-159348072	TAGCTATGTACCCTTGCTCCCCATTCA	6	A	0	0.4793
NC_005102.4-159379797	TGTGGCCGGACATGCCCTCCCTAAGGA	1	T	0.0348	0.0342
NC_005102.4-159379797	TGTGGCCGGACATGCCCTCCCTAAGGA	8	G	0.1316	0.1814
NC_005102.4-159405843	ATTCCTAGGACAGGCTCTCCCTCTGGT	15	C	0.0244	0.484
NC_005102.4-159410020	GGCTGAAAGACTGCATCTCCCCTAGGT	2	G	0.0504	0.0491
NC_005102.4-159414971	GAAGAGGGCACCAGGTCTCCTGAAACT	6	G	0.0894	0.0854
NC_005102.4-159414971	GAAGAGGGCACCAGGTCTCCTGAAACT	25	A	0.0894	0.0854
NC_005102.4-159429776	CCGTACCATACAGCTTCTCCAGGGACT	2	C	0.2333	0.42
NC_005102.4-159429776	CCGTACCATACAGCTTCTCCAGGGACT	9	T	0.0667	0.0799
NC_005102.4-159469183	GCTTTTCACACGCCTCCTCCTGTGACC	9	C	0.1102	0.2644
NC_005102.4-159472248	GGAGATGGCACACACCCTCCTTAGGGT	13	C	0.0085	0.4776
NC_005102.4-159472248	GGAGATGGCACACACCCTCCTTAGGGT	14	A	0	0.4757
NC_005102.4-159533514	TGTCCTACCACCTTCTCTCCAGTCCTG	7	A	0.0325	0.032
NC_005102.4-159534008	ACATCGGAGACATTTCCTCCACTCTGA	5	C	0.0446	0.0436
NC_005102.4-159562013	TGCACCCAGACCTGGCCTCCTGGCTAA	22	G	0.1789	0.4984
NC_005102.4-159572409	TTGGTAAGCACAGAAGCTCCTGGCTTC	15	A	0	0.4793
NC_005102.4-159572409	TTGGTAAGCACAGAAGCTCCTGGCTTC	16	G	0	0.4793
NC_005102.4-159605745	ATAGCCAAGACTACCTCTCCTCATGAG	3	A	0	0.4793
NC_005102.4-159605745	ATAGCCAAGACTACCTCTCCTCATGAG	13	A	0	0.4793
NC_005102.4-159609493	GACCCACTTACCAACCCTCCACCAATC	26	T	0.1765	0.4999
NC_005102.4-159643716	TGTGGCCCCACCCCGCCTCCATTCCCA	15	G	0	0.4841
NC_005102.4-159643716	TGTGGCCCCACCCCGCCTCCATTCCCA	16	C	0.0526	0.0512
NC_005102.4-159658946	GGCCTCCGCACTGGCTCTCCACCATAT	8	G	0.2966	0.4919

NC_005102.4-159658946	GGCCTCCGCACTGGCTCTCCACCATAT	14	G	0.0826	0.0792
NC_005102.4-159669819	CTCAATGTCACCCTATCTCCCTGACAG	13	C	0.1739	0.264
NC_005102.4-159669819	CTCAATGTCACCCTATCTCCCTGACAG	15	A	0.0087	0.4817
NC_005102.4-159673878	TCAGAAATCACCTCCACTCCGTTGCCT	21	G	0.1453	0.4987
NC_005102.4-159696681	CACTCCTTAACCCAGGCTCCCTAGTAG	12	C	0.0684	0.0973
NC_005102.4-159696681	CACTCCTTAACCCAGGCTCCCTAGTAG	22	T	0	0.4807
NC_005102.4-159708670	CCCATCCCCACTGACCCTCCTGAGGTA	1	C	0.1379	0.1717
NC_005102.4-159708670	CCCATCCCCACTGACCCTCCTGAGGTA	8	C	0.1897	0.2501
NC_005102.4-159738593	AGCTTCTACACAGCGTCTCCTCTACCT	2	G	0.2213	0.3401
NC_005102.4-159739005	GGAACCCTCACCTCACCTCCACCTCCT	25	C	0.1885	0.4879
NC_005102.4-159746746	GCCCTGCAAACTTCGACTCCCAGCATG	14	C	0.0901	0.349
NC_005102.4-159764720	CAGAAACTTACCAGCCCTCCCTTCACA	8	T	0.0976	0.4331
NC_005102.4-159764720	CAGAAACTTACCAGCCCTCCCTTCACA	15	C	0	0.4793
NC_005102.4-159772320	AAAGAATCCACAAGGTCTCCAGTGTCC	23	T	0	0.4764
NC_005102.4-159818268	CAGGCCAGGACAGCACCTCCCAACCAG	6	C	0	0.4793
NC_005102.4-159851300	GCAGTCCAGACTGATGCTCCACCATCA	4	G	0.1167	0.1244
NC_005102.4-159851300	GCAGTCCAGACTGATGCTCCACCATCA	14	A	0.1333	0.1665
NC_005102.4-159863887	GTCCCCTGCACTTTGCCTCCCGAGTAC	15	G	0.0756	0.0882
NC_005102.4-159928701	TTACATGTAACGTTTGCTCCACTAAGA	8	T	0	0.4764
NC_005102.4-159928701	TTACATGTAACGTTTGCTCCACTAAGA	22	C	0.0614	0.0595
NC_005102.4-159935279	CAGTAATTCACCTGGCCTCCTAGTCAC	21	T	0.2479	0.4671
NC_005102.4-159943747	GAATCAAGAACCTGGGCTCCAGCTCAT	3	A	0	0.4807
NC_005102.4-159964201	TCCTTGGCCACTCTCACTCCAGTGCCC	25	C	0.042	0.0411
NC_005102.4-159989493	GAAGTGGAGACAGCGGCTCCACGTGGC	25	G	0.2018	0.4969
NC_005102.4-159999215	CCCCCAAGCACCGGAGCTCCAGCCTCA	13	G	0.1901	0.2234
NC_005102.4-160020468	CTCTCAGTAACTGCCACTCCATAGGTC	23	A	0	0.4743
NC_005102.4-160021744	CTAGCCTGGACATGAGCTCCTGCAACA	22	G	0	0.4807
NC_005102.4-160038502	CAGTCCACCACTTTCTCTCCCTGTCCT	27	T	0.1933	0.4949
NC_005102.4-160043288	CCCAGTGAAACACCTCCTCCACCGTGT	23	C	0.1597	0.214
NC_005102.4-160053466	TGTAGCTAGACGTGTGCTCCATGATAT	3	T	0.0246	0.071
NC_005102.4-160085902	CAGATGGTTACTATGACTCCCCTGCCT	1	C	0.0087	0.4744
NC_005102.4-160085902	CAGATGGTTACTATGACTCCCCTGCCT	13	A	0.0087	0.4744
NC_005102.4-160111753	ATCAGAGGCACCCCTTCTCCCTGTCTC	2	T	0.157	0.313
NC_005102.4-160143113	GTCTTAACCACTCACTCTCCTCCATTA	2	T	0.0083	0.4769
NC_005102.4-160143113	GTCTTAACCACTCACTCTCCTCCATTA	12	T	0.0083	0.4747
NC_005102.4-160190392	AATGACCCAACTCCTCCTCCTCGTCTT	23	G	0.2017	0.2076
NC_005102.4-160190392	AATGACCCAACTCCTCCTCCTCGTCTT	26	T	0.2017	0.2076
NC_005102.4-160196008	ACCAAAGAGACCTACGCTCCATTCCTT	16	G	0.2034	0.3034
NC_005102.4-160211567	TGACCTCCTACCTACGCTCCAGGGTTA	13	T	0	0.4819
NC_005102.4-160211567	TGACCTCCTACCTACGCTCCAGGGTTA	21	A	0	0.4819
NC_005102.4-160233621	GAAGTGGATACCATGGCTCCTGCCCTC	8	A	0.1885	0.1968
NC_005102.4-160234860	ATGAATCTAACCAGGCCTCCTCTATTG	16	C	0.1639	0.1641
NC_005102.4-160247871	ACTAAGTGCACAGACGCTCCAAGTCCT	15	C	0.1892	0.4985
NC_005102.4-160264011	TCAGGTCCCACACTGCCTCCCCTCTCC	5	G	0.0261	0.4849
NC_005102.4-160264011	TCAGGTCCCACACTGCCTCCCCTCTCC	14	T	0.113	0.4976
NC_005102.4-160267271	CACACTCATACACAAACTCCTATACAC	23	T	0	0.482
NC_005102.4-160275763	CTCTGCAGGACAGCACCTCCATGTGTC	26	T	0	0.4743
NC_005102.4-160297860	CAGATTTGAACCCTAACTCCCTCACTT	14	T	0.0783	0.4903
NC_005102.4-160331300	AGCTGTGACACATGGTCTCCCAGTGCT	25	G	0.0579	0.0562
NC_005102.4-160331300	AGCTGTGACACATGGTCTCCCAGTGCT	27	T	0.0579	0.0562
NC_005102.4-160337190	ACATGTGTAACCCCAGCTCCAGAGGAG	4	T	0.0167	0.4832

NC_005102.4-160337190	ACATGTGTAACCCCAGCTCCAGAGGAG	21	A	0.1	0.4965
NC_005102.4-160369913	CCTCTGACCACTACCCCTCCCCACCTC	23	A	0	0.4902
NC_005102.4-160369913	CCTCTGACCACTACCCCTCCCCACCTC	25	C	0.0348	0.0342
NC_005102.4-160399333	CTGTGACAGACCCTCCCTCCAGTGGAC	2	T	0.0082	0.0243
NC_005102.4-160406237	ATGGAGATTACTATCCCTCCTTCCTTG	2	T	0.3	0.3394
NC_005102.4-160406237	ATGGAGATTACTATCCCTCCTTCCTTG	9	T	0	0.4787
NC_005102.4-160432242	CTGTAAGCTACCTGCCCTCCAGCTGCC	3	G	0.2857	0.4615
NC_005102.4-160454771	TTTCCCAGCACATGGTCTCCTGCTGTG	13	T	0.0583	0.4358
NC_005102.4-160454771	TTTCCCAGCACATGGTCTCCTGCTGTG	15	G	0	0.4832
NC_005102.4-160457381	ACCACAGCTACATACACTCCTGTGTCC	3	C	0.0084	0.4829
NC_005102.4-160457381	ACCACAGCTACATACACTCCTGTGTCC	4	A	0.0084	0.4829
NC_005102.4-160482760	CTGTCTGTAACTCTAGCTCCAGTAAGT	26	G	0.1393	0.5
NC_005102.4-160529386	CTAATCTTAACTGCCTCTCCTCCTGGC	6	C	0.1652	0.18
NC_005102.4-160567891	CTTTGTGGCACCAAACCTCCTCTGCCC	4	T	0.2308	0.4963
NC_005102.4-160569704	CACACTCGCACGGATTCTCCACCTCGG	26	G	0.0427	0.0896
NC_005102.4-160601429	GTCCTTAGCACCTTGCCTCCTCTGCTT	1	G	0.225	0.4816
NC_005102.4-160832157	TCATCCGATACGGAGCCTCCACATCAG	6	C	0.2377	0.375
NC_005102.4-160833686	CCATGAGAAACATGTCCTCCTTGGAAT	2	C	0.1951	0.4944
NC_005102.4-160855140	CTGCTGGGCACGAAGCCTCCAGCTGGA	2	T	0.0244	0.0241
NC_005102.4-160855140	CTGCTGGGCACGAAGCCTCCAGCTGGA	12	G	0.1721	0.4984
NC_005102.4-160856479	GCACTGGCTACTCCTCCTCCAGAGGAC	6	G	0.6083	0.4233
NC_005102.4-160860189	ACGGGCATCACACTTCCTCCTATGGTA	3	G	0.3043	0.4962
NC_005102.4-160865910	CTCACAACTACTCTGCCTCCCACAGCC	23	C	0	0.4912
NC_005102.4-160865910	CTCACAACTACTCTGCCTCCCACAGCC	26	C	0.0531	0.1006
NC_005102.4-160870429	TGTTTAAGCACAATGTCTCCATAGAAG	1	T	0.0614	0.1229
NC_005102.4-160924548	AGGGGTTCCACCAGCCCTCCGGTTCCC	7	T	0.1967	0.263
NC_005102.4-160928101	CCTAGGGAGACTGTTCCTCCCACTGTC	16	C	0.0619	0.06
NC_005102.4-160932627	CTGGCTGTGACTTACCCTCCGCACTCC	22	C	0.0082	0.0401
NC_005102.4-160932821	GCCCTTTGCACACACCCTCCCATTTCC	7	T	0	0.4724
NC_005102.4-160972874	CTGAGTGCTACGACTACTCCGATTTCC	7	G	0.0902	0.4997
NC_005102.4-161061749	TTTCCAGTCACAGCAACTCCGTTAGGT	15	A	0	0.48
NC_005102.4-161088997	TCCAAAACTACAGGGTCTCCACGACAC	1	T	0.0172	0.0339
NC_005102.4-161094037	GTTCTACCGACCCATCCTCCCTGCCTC	9	G	0.1488	0.4997
NC_005102.4-161130798	GAATTCATTACCTCGGCTCCCAAAGGC	14	C	0.0708	0.4953
NC_005102.4-161149880	GTCTGGCAAACCTGACCTCCCTCAGCC	7	C	0	0.4832
NC_005102.4-161151987	TGATGAGACACGACTACTCCTCTCACC	6	A	0.0088	0.483
NC_005102.4-161151987	TGATGAGACACGACTACTCCTCTCACC	25	A	0.0088	0.483
NC_005102.4-161170963	TCTGAGAGCACCTCTCCTCCGGTGTGC	21	G	0.2683	0.436
NC_005102.4-161197152	AGCTGAGGCACCGCACCTCCCTCTGCT	13	G	0.0593	0.4919
NC_005102.4-161277030	AGCTCTTATACACTGGCTCCCTAGTTA	4	T	0	0.4765
NC_005102.4-161312104	AGACCTCCCACGTACCCTCCACCTACC	12	G	0.0086	0.0255
NC_005102.4-161312104	AGACCTCCCACGTACCCTCCACCTACC	13	T	0.2478	0.4574
NC_005102.4-161343800	AAGCACTGAACCATCTCTCCAGCCCAC	2	A	0.3814	0.3086
NC_005102.4-161353638	AATAGCCCCACAAGAGCTCCACCTGCC	15	A	0.0164	0.048
NC_005102.4-161356406	AGGCCAAGGACCGGGACTCCGGCCGGG	26	G	0.0261	0.0257
NC_005102.4-161361345	TAGATGTCAACTCTGCCTCCACGTCCT	22	C	0.0328	0.0322
NC_005102.4-161366551	GGTTCCTCAACACTGCCTCCATTTCCC	3	T	0	0.4807
NC_005102.4-161374420	TATAGGGCCACACCCTCTCCCTACTCA	3	T	0	0.4793
NC_005102.4-161378298	CACCTGGACACGAGGGCTCCTACGATC	25	A	0.082	0.1082
NC_005102.4-161404602	CATGGCTGCACCCAGACTCCTAGACCC	22	A	0	0.4793
NC_005102.4-161411131	GCAGCACAAACCTGACCTCCCTTTGAC	2	C	0.0885	0.1316

NC_005102.4-161420607	TGCCCTTGAACTAAGGCTCCTCTTTTG	25	T	0.0763	0.1481
NC_005102.4-161435867	TCCCCCATCACCCAGACTCCTGGCTGT	26	G	0	0.48
NC_005102.4-161452906	ACCTCAGTGACCCCTTCTCCCTGCTCC	14	C	0.1901	0.259
NC_005102.4-161479698	GACACCATCACCTGCACTCCCAACAAT	25	A	0	0.4751
NC_005102.4-161501625	CATAGGAACACTTGATCTCCAGATGGT	27	T	0.2203	0.3138
NC_005102.4-161528720	GTGGTGATGACCCTGTCTCCTCATAGA	12	C	0.1034	0.0981
NC_005102.4-161530213	GTTCCTACCACCTACACTCCTGGCTAT	9	C	0.1933	0.2266
NC_005102.4-161530730	AATGGGTTGACAGCACCTCCTTCCTTC	23	C	0.2114	0.2612
NC_005102.4-161530730	AATGGGTTGACAGCACCTCCTTCCTTC	26	T	0.0407	0.0398
NC_005102.4-161533255	TTAATCTTGACTCGACCTCCCCATGTC	7	T	0	0.4728
NC_005102.4-161533255	TTAATCTTGACTCGACCTCCCCATGTC	21	C	0	0.4728
NC_005102.4-161546599	AATTAAGGCACTTCTACTCCCTTCTCC	2	A	0.2966	0.4508
NC_005102.4-161566931	TATGTCTAGACTGCAGCTCCCAATCCC	2	A	0.1405	0.2814
NC_005102.4-161765002	TCATGTGTTACAGCGCCTCCCTCCCTC	3	A	0.2623	0.3259
NC_005102.4-161765002	TCATGTGTTACAGCGCCTCCCTCCCTC	8	T	0.2787	0.3791
NC_005102.4-161775041	CTGGCGCCCACTGCTCCTCCCCAGGAA	22	C	0.0407	0.0398
NC_005102.4-161816814	GCTGGCCACACCTCCTCTCCAACTGAT	12	C	0	0.4922
NC_005102.4-161891336	AATTGCTGCACCATCTCTCCGGTGCCT	2	A	0.0579	0.0868
NC_005102.4-161935015	ATCAGAAGCACTGCAGCTCCTGTCGGG	4	A	0	0.4807
NC_005102.4-161949574	CCTGACCACACCTAGTCTCCAGCTTTC	4	G	0.1513	0.5
NC_005102.4-161949574	CCTGACCACACCTAGTCTCCAGCTTTC	16	T	0.2167	0.4765
NC_005102.4-161970536	GACACAGGGACCGCGACTCCAGGGCCG	13	G	0.0909	0.1306
NC_005102.4-162062914	TGCCAGGCCACAGGCTCTCCTCACGAA	24	C	0.025	0.4666
NC_005102.4-162078472	AGATGTCCCACCGTTGCTCCACCGTAT	15	T	0	0.4819
NC_005102.4-162078472	AGATGTCCCACCGTTGCTCCACCGTAT	16	G	0.1818	0.4628
NC_005102.4-162083063	GTGGGTCTCACCAAGTCTCCACAACTG	24	A	0.0082	0.479
NC_005102.4-162085988	GGCCAGGTGACACTCACTCCCTCCACT	4	C	0.1228	0.1454
NC_005102.4-162085988	GGCCAGGTGACACTCACTCCCTCCACT	16	A	0.009	0.4474
NC_005102.4-162099754	TCTTTCACAACCCCCACTCCTGCCTGG	1	T	0.0684	0.0818
NC_005102.4-162099754	TCTTTCACAACCCCCACTCCTGCCTGG	8	C	0.0769	0.0896
NC_005102.4-162100378	GTTTTGAATACCCATTCTCCACGGAAA	5	T	0.0932	0.1337
NC_005102.4-162100378	GTTTTGAATACCCATTCTCCACGGAAA	23	G	0.2627	0.4273
NC_005102.4-162116731	GTTTCTGAGACAGTCTCTCCCTAAACC	6	T	0.1197	0.4982
NC_005102.4-162149161	CTTCGTCCCACGCTTTCTCCTGTCTTT	4	C	0.2773	0.4682
NC_005102.4-162149161	CTTCGTCCCACGCTTTCTCCTGTCTTT	12	G	0.0583	0.0722
NC_005102.4-162162395	CTGATGGTCACACCAGCTCCTGTGGCT	5	T	0.009	0.4838
NC_005102.4-162191914	GGGTGGACTACTGGCTCTCCATCTAGG	23	C	0.1967	0.4914
NC_005102.4-162191914	GGGTGGACTACTGGCTCTCCATCTAGG	27	G	0.1736	0.2704
NC_005102.4-162206201	AGGTCTGAGACACCCACTCCTTTTTCT	14	C	0.027	0.4838
NC_005102.4-162210247	TACTGAGTCACACCCACTCCCGTGAGA	12	A	0.0336	0.0491
NC_005102.4-162210247	TACTGAGTCACACCCACTCCCGTGAGA	13	C	0.0336	0.0491
NC_005102.4-162212204	CAGTGAGACACCTTGTCTCCAAAGATA	9	C	0.2773	0.2961
NC_005102.4-162219364	GAAGCTGACACTCATACTCCTGCCTCC	21	T	0.2295	0.416
NC_005102.4-162231286	TGATAATGGACTAAACCTCCGAACCTG	16	C	0.5948	0.4179
NC_005102.4-162270090	CTTGCAGCCACGGCATCTCCCAAGGCC	3	T	0.1951	0.4722
NC_005102.4-162270090	CTTGCAGCCACGGCATCTCCCAAGGCC	16	T	0.1951	0.4722
NC_005102.4-162276621	AGAGAGATGACAGATGCTCCTCCACGG	25	C	0.1327	0.4242
NC_005102.4-162319494	TCTCTTGACACGGAAGCTCCTGGAACT	1	T	0.1538	0.2361
NC_005102.4-162327684	AGGGTTTAGACAAGGTCTCCCACAGTG	1	A	0.0088	0.4796
NC_005102.4-162327684	AGGGTTTAGACAAGGTCTCCCACAGTG	5	T	0.0348	0.0508
NC_005102.4-162331994	CTCCCTGCTACTTACACTCCTCCCCCC	15	C	0.0336	0.033

NC_005102.4-162331994	CTCCCTGCTACTTACACTCCTCCCCCC	23	C	0.2101	0.2012
NC_005102.4-162332700	ACAGTATAAACTGCATCTCCCCGGCTA	3	A	0.2213	0.2095
NC_005102.4-162332700	ACAGTATAAACTGCATCTCCCCGGCTA	23	G	0.0082	0.0401
NC_005102.4-162339336	TCCCCCAGCACCGAGACTCCAAGTACA	12	C	0.0261	0.4903
NC_005102.4-162339422	CCTCAACCGACCCCCTCTCCCTGCTCC	1	C	0.0336	0.4898
NC_005102.4-162339422	CCTCAACCGACCCCCTCTCCCTGCTCC	9	G	0.0339	0.0496
NC_005102.4-162340735	CATCACCAGACACATTCTCCTGACGAG	25	G	0.1695	0.334
NC_005102.4-162345939	GAAGATTCTACTAGCTCTCCAAGCTCA	4	G	0.0328	0.0322
NC_005102.4-162357728	CCTCCCACCACCCTTTCTCCCAACAGT	15	T	0	0.461
NC_005102.4-162357728	CCTCCCACCACCCTTTCTCCCAACAGT	22	A	0.0531	0.0517
NC_005102.4-162412900	TCCAAGCCGACCCTCCCTCCCACTGTG	8	C	0.0248	0.0245
NC_005102.4-162414066	ACACACACAACGGACCCTCCACGCCAG	23	G	0.0244	0.0241
NC_005102.4-162419044	GTCAGTAAAACATCAGCTCCGTCATCT	1	G	0.2114	0.4444
NC_005102.4-162434631	CTCCAGCTCACCACAGCTCCAAGCCCT	21	A	0	0.4819
NC_005102.4-162450459	CTTGGCAGCACTCTTCCTCCGTGCATG	21	G	0.2377	0.4489
NC_005102.4-162461836	CCAGCACATACAACTTCTCCACTGATG	23	T	0.2114	0.4497
NC_005102.4-162476356	AGCTAGGCCACTTCACCTCCCAGCTGT	7	G	0.1917	0.4358
NC_005102.4-162482348	CTGGGAGCCACCTTCCCTCCCTCCAGC	25	A	0	0.4844
NC_005102.4-162502919	CTATCCTGGACCACTACTCCTTGGCAT	22	T	0.1681	0.3014
NC_005102.4-162508187	CTCAGGATGACCCTCACTCCTGGACTC	4	A	0.25	0.4499
NC_005102.4-162508187	CTCAGGATGACCCTCACTCCTGGACTC	23	G	0.042	0.0411
NC_005102.4-162525833	TGTGGTACGACTTTATCTCCTATGCCT	3	T	0.2114	0.4926
NC_005102.4-162534948	CACACACACACCATGCCTCCCCTGTAT	21	C	0.0407	0.0854
NC_005102.4-162540784	ATTTACACAACACAAGCTCCCTGTCAG	24	T	0.1043	0.1143
NC_005102.4-162545348	CTTCAGTCAACCCTTGCTCCCTAGAGT	15	T	0	0.4764
NC_005102.4-162545348	CTTCAGTCAACCCTTGCTCCCTAGAGT	22	T	0.0847	0.0965
NC_005102.4-162557381	ATGTTCTGTACCCAATCTCCTCCACAG	15	A	0.0656	0.0786
NC_005102.4-162557381	ATGTTCTGTACCCAATCTCCTCCACAG	21	T	0	0.4793
NC_005102.4-162568603	GGGGGAACAACATCATCTCCACTGTAC	12	A	0.1849	0.2328
NC_005102.4-162570417	TGTCGTGAGACCCGGTCTCCAAAGGGA	5	G	0.0862	0.1431
NC_005102.4-162577319	ATGAAAGCCACTTCATCTCCCGAAGGA	2	T	0	0.4787
NC_005102.4-162587454	GAGAGAAGCACACTGGCTCCTTTAGGC	14	T	0.0877	0.396
NC_005102.4-162659281	CAAAGGGAGACTCAAGCTCCACTATCA	16	G	0.123	0.4992
NC_005102.4-162659281	CAAAGGGAGACTCAAGCTCCACTATCA	23	T	0.2314	0.3811
NC_005102.4-162702063	AGATATTATACCTTCCCTCCCATGAAC	16	C	0.1316	0.1229
NC_005102.4-162720382	ATATTAGACACTGATCCTCCTTCTGTC	12	T	0.0431	0.0422
NC_005102.4-162782163	CACAGAAAGACACCATCTCCAAACAAA	26	A	0	0.4793
NC_005102.4-162798311	AAGGACCAGACTGCTCCTCCTGCCATT	6	C	0.025	0.0247
NC_005102.4-162808144	CCATGCAGAACTCTGCCTCCTCTTCAC	1	C	0.1148	0.1225
NC_005102.4-162816903	TCCCAACACACATTTGCTCCTCAGCCT	3	C	0.2162	0.4967
NC_005102.4-162816903	TCCCAACACACATTTGCTCCTCAGCCT	21	T	0.2523	0.4444
NC_005102.4-162844219	CCGGTAAGCACAGTTCCTCCATGACCT	2	C	0.0625	0.0605
NC_005102.4-162852611	TCTCCATTTACCTCTTCTCCTTTTCCC	3	T	0.025	0.0247
NC_005102.4-162853293	CCCACCTGCACAGTAGCTCCCCATCTG	7	T	0	0.4718
NC_005102.4-162853293	CCCACCTGCACAGTAGCTCCCCATCTG	22	C	0.2308	0.479
NC_005102.4-162864514	CCAGGGCACACATTGACTCCCAGGCCT	12	A	0	0.48
NC_005102.4-162864514	CCAGGGCACACATTGACTCCCAGGCCT	13	T	0	0.4813
NC_005102.4-162898740	ACAGAGGAAACCATGACTCCTTCTCAC	15	G	0.0769	0.0896
NC_005102.4-162914344	TGCTCCAGGACTTCTGCTCCTTCCTCT	14	C	0	0.4902
NC_005102.4-162935236	TGTAAGCAGACACTCCCTCCCCAACTA	14	T	0.0431	0.0903
NC_005102.4-162937579	GCGGAGGACACAGTGGCTCCAATATCT	15	G	0.1653	0.2046

NC_005102.4-162947288	GCTAAAGCCACTGCTCCTCCACAGGAA	14	C	0.0435	0.0425
NC_005102.4-162948637	GATGCTAAAACATCCCCTCCATGGCCC	3	T	0	0.4819
NC_005102.4-162948637	GATGCTAAAACATCCCCTCCATGGCCC	21	A	0	0.4819
NC_005102.4-162968435	CAGCTGAGCACCCCATCTCCAATATGA	12	C	0.1525	0.4481
NC_005102.4-162974068	AGAGTAACCACACTCACTCCAGCCAAG	21	A	0.1368	0.272
NC_005102.4-162976659	TTTTGGGTAACAGCGACTCCTGAACAC	14	C	0	0.4826
NC_005102.4-162976659	TTTTGGGTAACAGCGACTCCTGAACAC	24	A	0.178	0.4609
NC_005102.4-163075385	TGTAGCCTGACCCCTACTCCACACACA	22	C	0.1	0.1244
NC_005102.4-163075385	TGTAGCCTGACCCCTACTCCACACACA	24	C	0.1176	0.4997
NC_005102.4-163111561	CTCACCTTGACCAATCCTCCTGGGGTC	13	A	0	0.48
NC_005102.4-163111561	CTCACCTTGACCAATCCTCCTGGGGTC	24	G	0.0417	0.0566
NC_005102.4-163113026	ATTTCCTAGACAGGAGCTCCCTTACTT	26	T	0.0081	0.4777
NC_005102.4-163127712	CTCTGTCCCACGGGTGCTCCTGGGATG	8	C	0.0081	0.0553
NC_005102.4-163166586	GTGGAGAGCACTCAGGCTCCCACCCAG	2	T	0.0086	0.4893
NC_005102.4-163166586	GTGGAGAGCACTCAGGCTCCCACCCAG	21	C	0.1552	0.4963
NC_005102.4-163178034	AAGAATTCAACTCTCACTCCCTACGCC	24	C	0.1667	0.3374
NC_005102.4-163186233	ATGGCAAGCACCCCAGCTCCCTTTGCA	2	T	0	0.4821
NC_005102.4-163202269	TGCCGGTCAACTGTGCCTCCTGTGTTA	4	C	0	0.4875
NC_005102.4-163202674	CACCCTTGCACACTAGCTCCACTCTCT	14	T	0	0.4793
NC_005102.4-163204117	ATTTATGAAACAAGGCCTCCACTTGAC	21	A	0	0.4751
NC_005102.4-163208142	GCCGCCTCCACAGCCCCTCCAAAGTGG	4	G	0.1034	0.4987
NC_005102.4-163208142	GCCGCCTCCACAGCCCCTCCAAAGTGG	8	C	0.094	0.1492
NC_005102.4-163220992	TCATTCTCCACAGCCTCTCCAGTTGTC	23	T	0	0.4755
NC_005102.4-163246941	TGGAAATAGACTTGACCTCCACAGCTC	12	T	0	0.4751
NC_005102.4-163272787	CAAACGAGCACGCCTGCTCCCGCCCCT	14	C	0.0588	0.0571
NC_005102.4-163277342	AGCAGCTTCACCTGGGCTCCAGGTCTC	4	A	0.25	0.4592
NC_005102.4-163284368	GATCCCAGAACACAAGCTCCATAGATA	7	A	0.0684	0.0818
NC_005102.4-163284368	GATCCCAGAACACAAGCTCCATAGATA	16	G	0.0342	0.4894
NC_005102.4-163305231	TGATTTGATACAGAGCCTCCAACACTC	4	T	0.3248	0.405
NC_005102.4-163305231	TGATTTGATACAGAGCCTCCAACACTC	24	A	0.0085	0.479
NC_005102.4-163335132	TAATCAATCACCTCTCCTCCCTGAGCT	12	C	0.0325	0.032
NC_005102.4-163369943	TATTTTGACACAAGGTCTCCATTGCTA	27	A	0	0.4891
NC_005102.4-163378797	GGCTTTCAGACCCAGGCTCCAAGGGTC	2	G	0.0325	0.0476
NC_005102.4-163378797	GGCTTTCAGACCCAGGCTCCAAGGGTC	26	T	0.0325	0.0476
NC_005102.4-163388522	TTCCAAGTGACTCCAGCTCCCAGGAAT	5	A	0	0.474
NC_005102.4-163396230	ACCTTCCTTACCCTCCCTCCAGTTCAC	27	C	0.0328	0.048
NC_005102.4-163411904	CCCACGGGAACCTCAGCTCCAGATACC	3	C	0.122	0.4174
NC_005102.4-163411904	CCCACGGGAACCTCAGCTCCAGATACC	6	G	0.2439	0.4548
NC_005102.4-163435368	AGGGAGAAGACCCTGTCTCCAAAACAT	6	G	0.2521	0.3599
NC_005102.4-163435368	AGGGAGAAGACCCTGTCTCCAAAACAT	9	G	0	0.4793
NC_005102.4-163441876	TGGGTGGTAACCGTGGCTCCATGGTAG	23	G	0.0163	0.0629
NC_005102.4-163442941	AAAGGCCACACCAGGCCTCCATCCCAC	13	A	0	0.4787
NC_005102.4-163448316	CCTAGGCCAACTGCATCTCCCACCTCA	15	A	0	0.4793
NC_005102.4-163448450	CCATCCTTCACGGATGCTCCTCCCCCC	15	T	0.0164	0.0634
NC_005102.4-163448450	CCATCCTTCACGGATGCTCCTCCCCCC	26	C	0.0165	0.0639
NC_005102.4-163451180	TTAATGTCCACTGTCTCTCCTCTAGAT	3	A	0	0.461
NC_005102.4-163488729	GCCGTCTCGACTTCCGCTCCGTGGGGA	4	G	0.0168	0.0491
NC_005102.4-163488729	GCCGTCTCGACTTCCGCTCCGTGGGGA	15	C	0.1261	0.3893
NC_005102.4-163495766	CATAGTGAGACCCATCCTCCAGCACAG	1	C	0.187	0.1954
NC_005102.4-163520916	CTGCTCTACACCTTGACTCCCTCTCTG	8	A	0.0084	0.4797
NC_005102.4-163530318	GAGTTGTTAACAGGTCCTCCCAGTTTG	9	A	0.0085	0.4776

NC_005102.4-163566344	GACCATTGAACCGTCTCTCCATCCTCG	12	C	0.1488	0.2172
NC_005102.4-163566344	GACCATTGAACCGTCTCTCCATCCTCG	22	T	0	0.4751
NC_005102.4-163603711	CAGGAATGCACTGTTCCTCCTGCAGTT	12	T	0	0.4793
NC_005102.4-163616766	CTGTCTGTAACTCCATCTCCTGGGGAT	7	G	0.0667	0.4911
NC_005102.4-163629796	ATTTAAAATACTGCATCTCCAGTCCTT	23	T	0.0625	0.4952
NC_005102.4-163633828	TTCTTCCTCACTTGCTCTCCTTACTTT	13	T	0.0672	0.0805
NC_005102.4-163634735	CACACAGAAACTGAAACTCCCACTGTG	16	A	0	0.4793
NC_005102.4-163704789	TAACATTTAACCACAGCTCCGTGGATA	21	G	0	0.4793
NC_005102.4-163715561	CCAAGGTTCACCAGTCCTCCTGCCAGG	5	G	0.2087	0.4724
NC_005102.4-163730557	CTCTCAGAGACACAAACTCCCAAAGAA	23	A	0	0.4751
NC_005102.4-163770849	CAGCTGGGCACTCTCACTCCCACTTCT	8	G	0	0.4751
NC_005102.4-163775941	GTATCTGTAACTGCAGCTCCAGGGGAT	2	T	0	0.4832
NC_005102.4-163809000	ACTACTCTGACTTCAGCTCCATGGGGA	7	C	0.2689	0.4008
NC_005102.4-163824803	GACTCAAGCACTCTGGCTCCGCCCCAC	8	G	0.2787	0.435
NC_005102.4-163824803	GACTCAAGCACTCTGGCTCCGCCCCAC	27	C	0.2787	0.435
NC_005102.4-163853409	ATGCGATGCACACCGCCTCCACGTGCG	2	T	0.0083	0.4769
NC_005102.4-163868940	GTTTGTGTCACTAGTCCTCCCGTCACA	7	G	0.2566	0.4242
NC_005102.4-163870932	CAATAGTGCACCAGATCTCCCTTCCAT	4	T	0.0732	0.3888
NC_005102.4-163870932	CAATAGTGCACCAGATCTCCCTTCCAT	26	A	0.0569	0.208
NC_005102.4-163881185	ACTGAGCTCACTGCTACTCCGGAGGCT	2	C	0.1382	0.2323
NC_005102.4-163881185	ACTGAGCTCACTGCTACTCCGGAGGCT	25	G	0.0081	0.481
NC_005102.4-163887509	TGTCATGACACGTGTGCTCCCTCACAA	16	G	0.1593	0.2302
NC_005102.4-163891879	CTGGCTACCACAAATCCTCCAGCAGCT	3	G	0.0574	0.0557
NC_005102.4-163891879	CTGGCTACCACAAATCCTCCAGCAGCT	26	C	0	0.4773
NC_005102.4-163903688	TCCCCACAGACTCTACCTCCTGCCCTG	8	A	0	0.4778
NC_005102.4-163903688	TCCCCACAGACTCTACCTCCTGCCCTG	15	A	0.0348	0.0508
NC_005102.4-163907420	TGATCCTGGACCTGATCTCCCTCCCTG	1	T	0	0.4846
NC_005102.4-163907420	TGATCCTGGACCTGATCTCCCTCCCTG	2	G	0	0.4846
NC_005102.4-163940840	CCTAAGTGCACCGAGACTCCACTGCCT	1	C	0	0.4793
NC_005102.4-163940840	CCTAAGTGCACCGAGACTCCACTGCCT	21	A	0.1463	0.4992
NC_005102.4-163945843	GATCCATCCACGCCACCTCCTCTGACC	15	A	0	0.4773
NC_005102.4-163969120	TGTTTTTTAACCGTCTCTCCTGGACAG	13	G	0.1579	0.2155
NC_005102.4-163969120	TGTTTTTTAACCGTCTCTCCTGGACAG	14	T	0.1667	0.463
NC_005102.4-163969942	GAGCTAGGGACTGCCACTCCCCCATCT	4	C	0.0708	0.0683
NC_005102.4-163969942	GAGCTAGGGACTGCCACTCCCCCATCT	27	T	0.0708	0.0683
NC_005102.4-164038443	GTTCTCAGCACTCCTGCTCCAACACTC	9	C	0.1967	0.4995
NC_005102.4-164040500	ACACACACCACTGTCCCTCCAAGGACA	22	A	0	0.4793
NC_005102.4-164062431	GAGCCTGGCACTTCAGCTCCAAGGTGG	13	T	0	0.4827
NC_005102.4-164066540	ACGCCTGTAACTCCAGCTCCACAGATT	14	C	0.2683	0.484
NC_005102.4-164080628	GAGAGCCCCACCTTCACTCCCCTTTCA	7	C	0.1102	0.1337
NC_005102.4-164119283	GGCACATGCACAGTCCCTCCTCTCGAT	12	A	0.0081	0.481
NC_005102.4-164121961	CAAGTACGCACACACACTCCTGTGCAC	6	A	0.0177	0.4912
NC_005102.4-164121961	CAAGTACGCACACACACTCCTGTGCAC	7	C	0.0177	0.4912
NC_005102.4-164127879	CCAAGCTCCACCCCAGCTCCTAGTCTT	26	T	0	0.4793
NC_005102.4-164137346	AACGCCACGACTAAAGCTCCTTGGAGA	8	C	0.0246	0.0243
NC_005102.4-164176927	CCCTTTATAACTCTTTCTCCATAGTCC	1	C	0.0082	0.479
NC_005102.4-164177140	ATATCTCCAACAGGTTCTCCAACACGA	1	A	0.1393	0.2095
NC_005102.4-164190910	CTTGAAAGAACACCCTCTCCGGCTGGA	14	C	0.0331	0.0325
NC_005102.4-164212351	GCCAATTAAACCGTCTCTCCCCACAGT	13	G	0.0244	0.0241
NC_005102.4-164212351	GCCAATTAAACCGTCTCTCCCCACAGT	26	G	0.1074	0.4999
NC_005102.4-164223276	TTTCTCAGAACTCAGGCTCCGTCTTTG	21	G	0	0.4793

NC_005102.4-164226530	CTGAGACCAACACGGTCTCCATGGCAG	26	A	0.0089	0.4885
NC_005102.4-164259328	AAGAGTTCAACTGCTTCTCCCACACAA	7	T	0	0.4813
NC_005102.4-164266945	CTCCACCACACCCCGCCTCCAGAGGGT	15	G	0.0256	0.0253
NC_005102.4-164266945	CTCCACCACACCCCGCCTCCAGAGGGT	27	T	0.0948	0.1358
NC_005102.4-164324984	GAGAGAGGAACATCAGCTCCATCCCCC	8	G	0.0246	0.0401
NC_005102.4-164339959	CCCACATACACCTTATCTCCTGAAGCA	5	C	0.0086	0.4803
NC_005102.4-164384718	CAACAGCATACTCTCTCTCCGAGTCCA	4	C	0.0732	0.2668
NC_005102.4-164467555	ACTGTGACTACCATGGCTCCGAGAAGT	13	A	0.2683	0.3888
NC_005102.4-164493239	GTCCCACATACCCCGCCTCCCACCGCA	5	C	0.1351	0.2137
NC_005102.4-164552367	TTCTCTGCTACGCAGGCTCCCACGGCT	1	T	0.0256	0.0253
NC_005102.4-164552367	TTCTCTGCTACGCAGGCTCCCACGGCT	8	C	0.1624	0.1908
NC_005102.4-164557152	CGGGTAGAGACCCAGACTCCACAACAT	22	C	0.025	0.0247
NC_005102.4-164557948	CCCCAGTTCACGTCTTCTCCTCTCCGG	24	C	0.1429	0.1626
NC_005102.4-164573040	CGCCCCTCTACCCATGCTCCAAGGCCA	1	C	0.0252	0.0249
NC_005102.4-164573040	CGCCCCTCTACCCATGCTCCAAGGCCA	8	C	0.2373	0.3529
NC_005102.4-164578222	CCGCTTGAGACCCTGTCTCCAAAACAA	3	G	0.3475	0.298
NC_005102.4-164578222	CCGCTTGAGACCCTGTCTCCAAAACAA	8	A	0.3475	0.298
NC_005102.4-164622454	CCAGCTGTGACTCCAGCTCCTGGAGAT	9	G	0	0.48
NC_005102.4-164631521	ACCACACATACACACACTCCTCACCAC	15	C	0.1074	0.1163
NC_005102.4-164678123	ACTGGCCTCACTGCTCCTCCTTATCCC	22	T	0.0488	0.4497
NC_005102.4-164705343	GAGGTGAAAACGCCCACTCCCTCTTCT	21	C	0.0667	0.4911
NC_005102.4-164713186	TTAGAAAACACACAGGCTCCTGTCCTG	1	T	0.2719	0.483
NC_005102.4-164713186	TTAGAAAACACACAGGCTCCTGTCCTG	9	C	0.2719	0.483
NC_005102.4-164720325	TAGACCATGACCGAAACTCCTTCTCTT	7	A	0	0.4813
NC_005102.4-164732974	ATCTGCTCAACCGGGCCTCCCCTTCCC	16	C	0	0.4832
NC_005102.4-164740709	GTCTTCCTCACCCCCACTCCCCATCAC	12	C	0.0631	0.0778
NC_005102.4-164748866	CAGGAATTCACCGCAGCTCCCCGAGAG	22	C	0.2232	0.4566
NC_005102.4-164765519	GAAGCCTGGACTCCATCTCCCGCATTG	21	C	0.0593	0.0734
NC_005102.4-164771158	TCTTGGGGCACACTGTCTCCGGCTTCT	21	G	0.0325	0.078
NC_005102.4-164775782	GAACTGAGTACCCACACTCCTCTCTCT	12	C	0.0833	0.1244
NC_005102.4-164783846	GTGCTGCCAACGCCCCCTCCTCCACCA	12	G	0.1951	0.2832
NC_005102.4-164783846	GTGCTGCCAACGCCCCCTCCTCCACCA	27	A	0.0325	0.032
NC_005102.4-164797211	GCCCTTCTGACGCTCCCTCCCCCACGA	2	C	0.0351	0.0345
NC_005102.4-164797211	GCCCTTCTGACGCTCCCTCCCCCACGA	26	G	0.0351	0.0345
NC_005102.4-164803223	GGGGAGGGCACAGGCACTCCCGCAACA	3	G	0.2193	0.2719
NC_005102.4-164803223	GGGGAGGGCACAGGCACTCCCGCAACA	22	G	0.2105	0.4698
NC_005102.4-164835713	GTTTCCTACACGCTGACTCCGCCCCAG	3	T	0	0.4773
NC_005102.4-164835713	GTTTCCTACACGCTGACTCCGCCCCAG	14	T	0.123	0.1573
NC_005102.4-164862294	TTTGGGGTGACGCCCCCTCCAGCCAGT	2	T	0.1327	0.4373
NC_005102.4-164899750	TTCGTACACACACATACTCCATGCATG	27	G	0	0.4773
NC_005102.4-164918492	GCATCACCTACCAGCCCTCCGTTTCTA	4	T	0	0.4743
NC_005102.4-164944924	GACCTTCCGACCCTGCCTCCACCTCCC	8	C	0	0.4807
NC_005102.4-164951240	CTGTCTCTGACTGCAGCTCCAGGGGAT	14	C	0.3083	0.3708
NC_005102.4-164978972	TGCTGCTCCACAGCACCTCCTTTACCC	3	C	0	0.48
NC_005102.4-165002477	GGCCAGAAGACACTCGCTCCTAACGTG	13	C	0.1545	0.1562
NC_005102.4-165027234	TGCCCCAGTACAGCAGCTCCTCCTGGA	8	G	0.1083	0.1025
NC_005102.4-165043918	TTGGACATAACTGACTCTCCAAGCCAG	23	G	0	0.4718
NC_005102.4-165053560	AGTTCTCTGACTTAAGCTCCACCCACT	2	G	0.1057	0.1001
NC_005102.4-165053560	AGTTCTCTGACTTAAGCTCCACCCACT	8	T	0	0.4773
NC_005102.4-165062348	ACGTCCCACACCCTACCTCCCCAAGCA	8	A	0.1	0.1528
NC_005102.4-165062348	ACGTCCCACACCCTACCTCCCCAAGCA	9	C	0.1983	0.2869

NC_005102.4-165065847	CTTTAAGCCACCTATGCTCCTGCCTGT	24	C	0.2143	0.4157
NC_005102.4-165069345	ACCTGTGCTACAACACCTCCCCCACCC	1	A	0.0769	0.4416
NC_005102.4-165069345	ACCTGTGCTACAACACCTCCCCCACCC	23	C	0.1667	0.2088
NC_005102.4-165083085	GCTCCTGAAACCAGGCCTCCAGCTGGG	2	C	0.2276	0.3926
NC_005102.4-165097169	ACCACTGACACCTCCACTCCAGGAACA	5	C	0.082	0.1774
NC_005102.4-165097169	ACCACTGACACCTCCACTCCAGGAACA	9	C	0.082	0.1774
NC_005102.4-165127703	CCGTGATGGACAGTATCTCCTGAACCA	22	G	0.0656	0.1082
NC_005102.4-165127703	CCGTGATGGACAGTATCTCCTGAACCA	27	A	0.1092	0.4999
NC_005102.4-165172458	TCCTAACTCACCGAGTCTCCAAGAATA	12	C	0.0087	0.4817
NC_005102.4-165172458	TCCTAACTCACCGAGTCTCCAAGAATA	14	A	0.0086	0.4803
NC_005102.4-165185256	TATCAAACTACCACATCTCCTTTTGTG	16	T	0.178	0.4982
NC_005102.4-165185256	TATCAAACTACCACATCTCCTTTTGTG	27	G	0.0833	0.1665
NC_005102.4-165188764	ATTCTTCTGACTCAGCCTCCTGGGTCA	2	T	0.0246	0.0401
NC_005102.4-165188764	ATTCTTCTGACTCAGCCTCCTGGGTCA	23	G	0.2101	0.4592
NC_005102.4-165243401	TTCTCTTCTACAGAGTCTCCTCCCCTC	7	T	0	0.4813
NC_005102.4-165243401	TTCTCTTCTACAGAGTCTCCTCCCCTC	22	C	0.1695	0.2815
NC_005102.4-165248596	AGGCTAAAAACCTGGGCTCCAAGTTCT	14	G	0.0089	0.0436
NC_005102.4-165265543	TCCATCCACACAGTGTCTCCTGTTCAT	24	T	0.0833	0.4888
NC_005102.4-165269706	TCCTGTTTGACTGTCACTCCACTGGGC	6	T	0	0.4757
NC_005102.4-165287855	CGTCTTCCCACTGCTTCTCCTGTGCAG	5	T	0	0.4826
NC_005102.4-165294197	GGATCCAGAACATAGACTCCTAACCAC	6	C	0.1391	0.1295
NC_005102.4-165341035	TCTGCTCTCACTATAGCTCCATAGACT	12	T	0	0.4772
NC_005102.4-165356109	CAATTACCCACACTGACTCCTGTAGAG	1	C	0.0085	0.481
NC_005102.4-165356109	CAATTACCCACACTGACTCCTGTAGAG	3	A	0.0085	0.481
NC_005102.4-165424573	CCGTCAGGCACAGCAGCTCCATCTCCA	2	C	0.1597	0.1746
NC_005102.4-165428638	ATACGCCAGACATTATCTCCTCGGTGT	22	C	0	0.4844
NC_005102.4-165452086	CCCAGCGGAACCACCGCTCCCTTCAAC	1	C	0.0083	0.0405
NC_005102.4-165452086	CCCAGCGGAACCACCGCTCCCTTCAAC	6	C	0	0.4787
NC_005102.4-165521496	CCGAGGGAGACTAAGGCTCCTTCCTCA	26	C	0.1043	0.4915
NC_005102.4-165521496	CCGAGGGAGACTAAGGCTCCTTCCTCA	27	A	0.0175	0.4698
NC_005102.4-165534270	CCCAGATAAACCAGTTCTCCTGTCTTC	27	C	0.0171	0.4602
NC_005102.4-165540569	GCTGTGACCACCGAGGCTCCTATTCCC	12	C	0	0.4807
NC_005102.4-165543694	AACCTCTGTACCATCCCTCCAGACTCT	8	G	0.0407	0.0398
NC_005102.4-165577035	GCAGCGAACACTCTGGCTCCAGGAGCA	5	C	0.1626	0.4992
NC_005102.4-165577035	GCAGCGAACACTCTGGCTCCAGGAGCA	6	G	0.0407	0.0398
NC_005102.4-165583496	TACAGAGAGACCCTGTCTCCTCACAAA	24	C	0.0894	0.1287
NC_005102.4-165619236	ACTGACGCCACGGGCTCTCCCAGCCCT	1	A	0.025	0.0247
NC_005102.4-165626524	GTGGGAGAAACGACCCCTCCAGCGGAG	22	G	0.0085	0.487
NC_005102.4-165626524	GTGGGAGAAACGACCCCTCCAGCGGAG	23	C	0.265	0.3409
NC_005102.4-165651373	GAGCCAATCACAGATCCTCCTGAAACC	3	G	0.1638	0.1923
NC_005102.4-165651373	GAGCCAATCACAGATCCTCCTGAAACC	16	C	0.1638	0.1923
NC_005102.4-165654204	CGCCAATGAACCACACCTCCCAGCCTG	1	C	0.0513	0.4938
NC_005102.4-165654204	CGCCAATGAACCACACCTCCCAGCCTG	6	A	0.0862	0.4999
NC_005102.4-165657628	ATCAGAGGCACGGGGTCTCCTCGGCCT	15	G	0.0325	0.0629
NC_005102.4-165657628	ATCAGAGGCACGGGGTCTCCTCGGCCT	22	C	0.0813	0.078
NC_005102.4-165674718	GCCACGTGCACATGGACTCCCAGATAC	13	T	0	0.48
NC_005102.4-165684707	TACCTCAACACTCCACCTCCTCGAGTG	23	G	0.2602	0.4497
NC_005102.4-165731415	AGTGAGCAAACCCAGCCTCCACCATCC	24	A	0	0.4793
NC_005102.4-165765025	ACCAATGAGACACCACCTCCGGAAGTT	23	A	0.0254	0.0251
NC_005102.4-165787368	GGAAGGAGAACGGCAGCTCCCACACAT	8	G	0.0246	0.0243
NC_005102.4-165787368	GGAAGGAGAACGGCAGCTCCCACACAT	12	G	0.0082	0.0401

NC_005102.4-165797516	TTAAAGATCACACAACCTCCATACAGA	14	A	0.0328	0.4837
NC_005102.4-165810239	TGGCTCCAAACATGGCCTCCTGCCTCC	7	C	0.0265	0.0262
NC_005102.4-165832786	TTGAAGGTGACATGTACTCCACGGGCT	21	A	0.0357	0.4841
NC_005102.4-165833785	CTCCACATAACCCAGCCTCCAGACTAA	2	T	0.0084	0.4859
NC_005102.4-165833785	CTCCACATAACCCAGCCTCCAGACTAA	8	T	0.0333	0.0328
NC_005102.4-165878845	TTTCCTCCTACCTGGCCTCCACTCCTG	7	C	0.0407	0.0398
NC_005102.4-165878845	TTTCCTCCTACCTGGCCTCCACTCCTG	16	C	0.0081	0.0241
NC_005102.4-165929972	ACATTTTCAACCTACACTCCTACACTT	8	C	0.027	0.044
NC_005102.4-165936998	CTGGCCTCCACCTGGCCTCCTGTTGTT	6	C	0.0769	0.1347
NC_005102.4-165936998	CTGGCCTCCACCTGGCCTCCTGTTGTT	8	C	0.1327	0.4986
NC_005102.4-165982367	TGCTTCCTTACCCAGCCTCCCTTTGTT	13	C	0.0684	0.066
NC_005102.4-165982367	TGCTTCCTTACCCAGCCTCCCTTTGTT	14	A	0.2522	0.3147
NC_005102.4-166004339	AGGTTGAGAACCACTGCTCCTGGGGAA	21	T	0	0.4787
NC_005102.4-166014696	ATGATGGTGACGCTGCCTCCCTGAGTA	12	G	0.1441	0.4982
NC_005102.4-166069875	CTGAAGGAGACTGGCTCTCCCTCCTGT	21	C	0.0796	0.1391
NC_005102.4-166116594	TACTTTGTTACAGCTGCTCCCACCTGC	27	C	0.1885	0.5
NC_005102.4-166139898	GTTTCCCTAACTGCATCTCCAACAAGC	13	G	0.0084	0.4829
NC_005102.4-166139898	GTTTCCCTAACTGCATCTCCAACAAGC	22	A	0.0084	0.4829
NC_005102.4-166148252	GGAGTGGGAACTTACTCTCCTGTGACT	1	G	0.036	0.0354
NC_005102.4-166148252	GGAGTGGGAACTTACTCTCCTGTGACT	2	G	0	0.4785
NC_005102.4-166157867	GTTACTGACACTCACTCTCCGTTCCTA	21	G	0.0973	0.1968
NC_005102.4-166164945	TACAATGACACATGCGCTCCTCCCCAG	5	A	0	0.4786
NC_005102.4-166166832	GCCCCATTCACAGCATCTCCAACACAG	22	A	0.0168	0.033
NC_005102.4-166193304	CACTTAAGAACAATGCCTCCATCAGCA	25	G	0.0804	0.4933
NC_005102.4-166224211	CCTTCAGGGACTCCTGCTCCTCTGGAA	7	G	0.1017	0.1409
NC_005102.4-166224211	CCTTCAGGGACTCCTGCTCCTCTGGAA	27	A	0.1897	0.4999
NC_005102.4-166227430	TGGGTGTGAACTATTTCTCCTATTATG	9	A	0.1709	0.4991
NC_005102.4-166227430	TGGGTGTGAACTATTTCTCCTATTATG	24	T	0.0085	0.4714
NC_005102.4-166246272	GTTCTTTGTACATTATCTCCTAGCTAG	8	G	0.0248	0.0245
NC_005102.4-166275450	AGATCAAATACTGAAACTCCCCATCTG	3	A	0.2054	0.2876
NC_005102.4-166320636	GTCAGCCCTACATCGCCTCCATGGTAG	14	C	0.1382	0.1287
NC_005102.4-166342761	ATTGCTATGACCGCTCCTCCCATCATA	7	A	0.2645	0.2647
NC_005102.4-166342761	ATTGCTATGACCGCTCCTCCCATCATA	13	G	0.2667	0.2311
NC_005102.4-166357154	GACATCCCCACTGTGCCTCCATGCCTC	5	T	0.125	0.1172
NC_005102.4-166377620	TGAGGTCATACATAAGCTCCACCCAGT	12	A	0.0336	0.033
NC_005102.4-166377620	TGAGGTCATACATAAGCTCCACCCAGT	21	A	0	0.4677
NC_005102.4-166379265	TTTGTAACCACTGAGCCTCCTCTCCAG	24	C	0.2333	0.2888
NC_005102.4-166415374	CACTCCTGAACAAATCCTCCTCCCTCT	7	T	0	0.4807
NC_005102.4-166415374	CACTCCTGAACAAATCCTCCTCCCTCT	14	A	0	0.4807
NC_005102.4-166417713	TGTGTTGGAACACCTCCTCCTCGTGAT	6	T	0.2927	0.427
NC_005102.4-166419591	ATGCCTCTAACACGCCCTCCTCTGACT	14	G	0.0328	0.048
NC_005102.4-166441836	TGAGCAGGAACTTGACCTCCGCTGCAG	23	T	0.125	0.1866
NC_005102.4-166443312	CTGACAAGCACATAGCCTCCGTCTCAG	1	C	0.2261	0.4425
NC_005102.4-166443312	CTGACAAGCACATAGCCTCCGTCTCAG	2	T	0.2241	0.3662
NC_005102.4-166452633	CCTTATAGCACTATGACTCCCACAAAG	22	A	0	0.4793
NC_005102.4-166465173	AACCAATACACTTTGCCTCCACCTGAA	22	C	0	0.4773
NC_005102.4-166482336	CTGGTAAGGACGCTGGCTCCTGTGGAG	13	C	0.0813	0.0928
NC_005102.4-166498483	GATCCTTGTACATCCTCTCCTGTCCCC	24	C	0.0083	0.4708
NC_005102.4-166580074	AGCCCTCCCACACACGCTCCTCAGGCT	4	C	0.2033	0.4703
NC_005102.4-166600195	ACTCCTCAGACACTGCCTCCCCTCTGC	1	A	0.0083	0.4783
NC_005102.4-166600195	ACTCCTCAGACACTGCCTCCCCTCTGC	21	C	0.0083	0.4783

NC_005102.4-166609392	CAGTCATGTACCTGACCTCCAGCCTTG	2	A	0.2276	0.4973
NC_005102.4-166609392	CAGTCATGTACCTGACCTCCAGCCTTG	15	A	0	0.4793
NC_005102.4-166665776	CATAAAGGAACTTCAGCTCCTGGAGCT	21	T	0.0813	0.078
NC_005102.4-166694296	CCAGGAGTCACCTCCTCTCCAGCCTAC	6	A	0	0.4724
NC_005102.4-166755441	GTTCCGATCACAGCCCCTCCCCACCTC	1	G	0.0508	0.5
NC_005102.4-166778224	GTTGAGATGACAGTAGCTCCGCAGACA	14	T	0.0088	0.4693
NC_005102.4-166832680	TCAATTCACACGTACTCTCCACGTGCC	12	G	0	0.48
NC_005102.4-166832680	TCAATTCACACGTACTCTCCACGTGCC	27	C	0.2167	0.2311
NC_005102.4-166856431	ACAAAGGATACTGTGCCTCCTCCTCCG	26	C	0.0413	0.0562
NC_005102.4-166856431	ACAAAGGATACTGTGCCTCCTCCTCCG	27	G	0	0.4819
NC_005102.4-166881439	TGTTTTAAGACTGGGCCTCCTACTGTT	26	T	0	0.4793
NC_005102.4-166882581	GGCACATGCACGTACCCTCCTACAGAC	16	C	0.0246	0.0243
NC_005102.4-166883547	ACAGCAAGCACTTTACCTCCTCAAAAC	2	C	0.0619	0.4994
NC_005102.4-166902444	GCCGTGCACACCAGGCCTCCTGTCCTG	5	T	0.0084	0.0249
NC_005102.4-166931516	TCCAATGCAACTTCTCCTCCGAACCAT	5	A	0.0667	0.0799
NC_005102.4-166936139	AATGTGGGGACTAGGGCTCCTTTGGCT	3	T	0	0.4787
NC_005102.4-166974958	GCACTCACTACTTGCTCTCCCGGTGCA	22	G	0.0894	0.4106
NC_005102.4-166975467	TTTAAGGTCACAGACACTCCTGCTGCT	12	A	0.0179	0.4871
NC_005102.4-166987091	CGCTGGGCGACTCCACCTCCCCAGGGC	2	G	0	0.4503
NC_005102.4-166993646	GGCTTAAATACTCGGCCTCCACACGCA	8	A	0	0.4704
NC_005102.4-166993646	GGCTTAAATACTCGGCCTCCACACGCA	14	G	0.036	0.0695
NC_005102.4-167023120	ACAGAAAGGACCACCGCTCCTTGCCAG	22	T	0	0.4832
NC_005102.4-167080821	CCATTGATAACGTCATCTCCCAGATTT	12	G	0.0357	0.0521
NC_005102.4-167105738	GTGCTGATAACCTGGCCTCCAGTCCTC	16	C	0.2301	0.3026
NC_005102.4-167115205	CCGGTTCACACCTATGCTCCACTCAGT	2	C	0.1707	0.208
NC_005102.4-167115205	CCGGTTCACACCTATGCTCCACTCAGT	26	G	0.1951	0.2832
NC_005102.4-167595217	CCACATGGCACATCTGCTCCCCATCTC	8	G	0.3214	0.2698
NC_005102.4-167596950	CTGTACTACACTGTAGCTCCTTAATGA	7	T	0	0.4751
NC_005102.4-167874237	CCATCATCCACCCTTCCTCCCTCTGTT	12	C	0.2353	0.3599
NC_005102.4-167874237	CCATCATCCACCCTTCCTCCCTCTGTT	14	T	0	0.4779
NC_005102.4-167905543	TTACCCAGTACTGAGGCTCCTTGAATC	16	G	0.2034	0.2344
NC_005102.4-167925404	TATCCTCCCACTTGGTCTCCTGGGTCC	14	G	0.1583	0.1597
NC_005102.4-167939059	AACCAGGTCACTTGGTCTCCTCAGGTT	5	A	0.0265	0.0262
NC_005102.4-167939059	AACCAGGTCACTTGGTCTCCTCAGGTT	26	T	0.0796	0.0926
NC_005102.4-168022793	CCTTCTACCACGTGGCCTCCAGGTGTT	16	C	0.1475	0.285
NC_005102.4-168030178	GTAATGACTACGTCACCTCCAGAGTCC	8	C	0	0.4793
NC_005102.4-168030178	GTAATGACTACGTCACCTCCAGAGTCC	12	G	0	0.4793
NC_005102.4-168034399	AATGCAAGCACTTTGTCTCCTGAACCA	2	A	0	0.4807
NC_005102.4-168042948	CTTAAGGCCACAGCACCTCCAATGGCT	22	A	0.2276	0.4854
NC_005102.4-168042948	CTTAAGGCCACAGCACCTCCAATGGCT	24	G	0.2276	0.4854
NC_005102.4-168043949	CAGAGGCCAACCTTAGCTCCTGTTCCT	14	T	0	0.4787
NC_005102.4-168061565	CATCCACAGACCTGCCCTCCAGCAGGC	2	A	0.0082	0.4822
NC_005102.4-168061565	CATCCACAGACCTGCCCTCCAGCAGGC	3	T	0.0082	0.4822
NC_005102.4-168085789	TTTGAAGGCACGAACCCTCCGTCTCAG	21	G	0.1228	0.1884
NC_005102.4-168085940	CGGAAGTCTACCTGAGCTCCAGTTTTC	2	G	0.084	0.4406
NC_005102.4-168085940	CGGAAGTCTACCTGAGCTCCAGTTTTC	9	T	0	0.4813
NC_005102.4-168109636	TCTACAGGAACCGCCCCTCCCTCGGCT	22	T	0.0427	0.4947
NC_005102.4-168116070	TGGGTTGAAACAGTGTCTCCACTCTTT	12	A	0.0339	0.4718
NC_005102.4-168116070	TGGGTTGAAACAGTGTCTCCACTCTTT	14	T	0.188	0.5
NC_005102.4-168157918	CCTCTTGTAACTCCAGCTCCAGGAGGT	22	G	0.2411	0.5
NC_005102.4-168188365	AATATCTGAACCCCTTCTCCCAAGAGT	13	C	0.2667	0.3832

NC_005102.4-168188365	AATATCTGAACCCCTTCTCCCAAGAGT	14	C	0	0.4813
NC_005102.4-168276162	TAACCCTGCACCTCCCCTCCTGTGGCT	25	G	0.0407	0.0398
NC_005102.4-168296348	GCAGTGGGCACCTCCCCTCCTGTACCG	12	C	0.025	0.0247
NC_005102.4-168296348	GCAGTGGGCACCTCCCCTCCTGTACCG	26	C	0.0417	0.0566
NC_005102.4-168301201	CTGCCAACAACTCCCTCTCCACTCCCC	24	C	0.1532	0.1713
NC_005102.4-168349502	TCTTATTCAACGCCATCTCCTGAACCA	12	G	0	0.4793
NC_005102.4-168354401	GCTATTTTTACATTCACTCCAACAGTG	12	A	0.0177	0.4912
NC_005102.4-168408867	TTGCAGCTCACACATCCTCCGTTCATA	3	G	0.0328	0.0322
NC_005102.4-168408867	TTGCAGCTCACACATCCTCCGTTCATA	21	G	0.157	0.3771
NC_005102.4-168414142	GTACTTCCCACAGACCCTCCTGACACC	1	G	0.0708	0.4912
NC_005102.4-168414142	GTACTTCCCACAGACCCTCCTGACACC	4	C	0.1339	0.3418
NC_005102.4-168417162	TATGTGTAGACGTTCTCTCCAGCTGTA	6	G	0.0354	0.0683
NC_005102.4-168417162	TATGTGTAGACGTTCTCTCCAGCTGTA	22	G	0.1754	0.3706
NC_005102.4-168482901	GGTACAAAGACTCAGGCTCCGTTGCCA	21	G	0.0325	0.032
NC_005102.4-168504181	GGCAGACATACAAGCACTCCATCATCT	4	A	0.2301	0.2171
NC_005102.4-168504181	GGCAGACATACAAGCACTCCATCATCT	8	A	0.0263	0.026
NC_005102.4-168521728	AGAACACTCACTGCTCCTCCAGAGCAA	22	G	0.281	0.4041
NC_005102.4-168528914	CACACACACACGGTGGCTCCTCACCAT	25	C	0.0082	0.479
NC_005102.4-168558740	TGTTTAGTAACAGTTCCTCCTCCATGT	15	T	0.0179	0.4922
NC_005102.4-168558740	TGTTTAGTAACAGTTCCTCCTCCATGT	22	C	0.1081	0.2068
NC_005102.4-168569806	GTTGAGGTCACCCAGACTCCTACAAGT	1	G	0.1803	0.4914
NC_005102.4-168603724	GATCATTTCACCCTCACTCCAGTGGGA	1	G	0.1933	0.4829
NC_005102.4-168603724	GATCATTTCACCCTCACTCCAGTGGGA	13	C	0.2605	0.4592
NC_005102.4-168626890	TTAAGATCTACCTGTACTCCCTGATCC	7	T	0.1667	0.3168
NC_005102.4-168626890	TTAAGATCTACCTGTACTCCCTGATCC	26	C	0.0085	0.0253
NC_005102.4-168627467	GACTGAACCACTGACACTCCTCATAGA	2	A	0.1579	0.2538
NC_005102.4-168627467	GACTGAACCACTGACACTCCTCATAGA	23	A	0.0354	0.4953
NC_005102.4-168650825	TGCTTCCACACTCAAGCTCCTGCCGAG	4	T	0.1864	0.2219
NC_005102.4-168679567	CGCCCTTGCACTCTTCCTCCCTTTGTA	1	C	0.2683	0.481
NC_005102.4-168679567	CGCCCTTGCACTCTTCCTCCCTTTGTA	2	G	0.2602	0.3334
NC_005102.4-168683095	GAGTCCATCACAGTGTCTCCATCACCC	7	A	0	0.4765
NC_005102.4-168696418	CGTCATGTCACATGAGCTCCCTTTACT	3	T	0.1833	0.4832
NC_005102.4-168696418	CGTCATGTCACATGAGCTCCCTTTACT	5	A	0.1833	0.4832
NC_005102.4-168705745	GCATCATAAACTTGGCCTCCGTCTTTA	2	C	0.252	0.3287
NC_005102.4-168705745	GCATCATAAACTTGGCCTCCGTCTTTA	23	C	0.0488	0.0476
NC_005102.4-168730194	CAATGGGAAACCCTTTCTCCAGAAATA	7	G	0.1239	0.1316
NC_005102.4-168736638	GAGTGTGGAACAATACCTCCCGTGACT	12	A	0.113	0.1938
NC_005102.4-168736638	GAGTGTGGAACAATACCTCCCGTGACT	21	C	0.2807	0.3221
NC_005102.4-168751849	GGATTACAAACCCTCACTCCTGGCTCT	13	C	0	0.482
NC_005102.4-168817742	ATAGAGCCGACCCCTCCTCCCCCCACC	27	C	0.0087	0.466
NC_005102.4-168913961	TGTCTGCAGACCCTCACTCCTACCCCA	3	T	0.1074	0.1306
NC_005102.4-168919425	CATACACACACTCTTCCTCCTCCTGTC	23	C	0.065	0.4904
NC_005102.4-168963915	GTGACTGAGACCGCAGCTCCTGTCCCA	13	G	0.1545	0.2556
NC_005102.4-168974829	CTTGTTCCTACTTGAACTCCTAACATT	1	C	0.2033	0.3287
NC_005102.4-168990443	ACATACTACACACACACTCCACACACC	15	C	0.1333	0.1665
NC_005102.4-168990443	ACATACTACACACACACTCCACACACC	21	A	0.1333	0.1665
NC_005102.4-168991225	GCTTTGCACACACTCTCTCCTATTCCT	15	C	0.1453	0.4999
NC_005102.4-168993111	GTCTCTGAGACAATGGCTCCTCCAAGT	4	T	0	0.4751
NC_005102.4-168993111	GTCTCTGAGACAATGGCTCCTCCAAGT	27	T	0	0.4728
NC_005102.4-169001098	CGAGAGAATACCAAGTCTCCAGGAGCT	1	C	0.0086	0.4729
NC_005102.4-169001098	CGAGAGAATACCAAGTCTCCAGGAGCT	2	G	0.0086	0.4729

NC_005102.4-169045195	CAAGTCTCCACTGAGTCTCCTCTCGCT	12	T	0.1983	0.2415
NC_005102.4-169045195	CAAGTCTCCACTGAGTCTCCTCTCGCT	13	G	0.1983	0.2415
NC_005102.4-169474687	AATAATTACACACAGACTCCCTTTGCA	9	C	0.0342	0.0336
NC_005102.4-169484060	ACTGGCCTCACTGGGGCTCCATGCAGG	5	G	0.0738	0.071
NC_005102.4-169484060	ACTGGCCTCACTGGGGCTCCATGCAGG	6	C	0.1488	0.1653
NC_005102.4-169484731	TTCTAAGCAACACTGTCTCCTATGCTT	2	T	0.1983	0.4942
NC_005102.4-169484731	TTCTAAGCAACACTGTCTCCTATGCTT	13	C	0.3689	0.3909
NC_005102.4-169539117	ACCTTGAAGACATCAACTCCTTGATGG	12	A	0.1842	0.4889
NC_005102.4-169539117	ACCTTGAAGACATCAACTCCTTGATGG	13	T	0.1842	0.4889
NC_005102.4-169541640	TTCTGTAGCACATCATCTCCCTTATAT	26	A	0.0976	0.1629
NC_005102.4-169569171	AGGGAAGTGACCTGATCTCCTCATTAA	22	C	0.1626	0.2142
NC_005102.4-169597963	TCACTACCCACCCCCACTCCTCTTCTT	12	C	0	0.4922
NC_005102.4-169597963	TCACTACCCACCCCCACTCCTCTTCTT	26	T	0.1071	0.4943
NC_005102.4-169628158	CCAGTCAATACGTACTCTCCTACTGTG	25	G	0.4833	0.3665
NC_005102.4-169629963	CCTCCCAGCACAGTACCTCCTGATAGA	15	A	0.125	0.5
NC_005102.4-169629963	CCTCCCAGCACAGTACCTCCTGATAGA	23	A	0.0083	0.4816
NC_005102.4-169649390	TCGGTTCAGACCGTGGCTCCATTCCCA	24	C	0.0244	0.0241
NC_005102.4-169650072	TCTAACAAGACCACACCTCCACCATTA	2	C	0.1966	0.3107
NC_005102.4-169650072	TCTAACAAGACCACACCTCCACCATTA	15	A	0.188	0.2945
NC_005102.4-169665015	CCAGAGAGCACAGCACCTCCAGAAGGC	21	A	0.1901	0.2355
NC_005102.4-169670877	ATCTAAAGTACCCATCCTCCCCATATT	12	C	0.0345	0.0339
NC_005102.4-169673923	ACCAGCTCCACGTTGGCTCCCACACAG	12	G	0.1951	0.2612
NC_005102.4-169673923	ACCAGCTCCACGTTGGCTCCCACACAG	25	C	0.1057	0.1001
NC_005102.4-169682056	ATTCTGAATACATAGACTCCATTCCTA	5	T	0.281	0.4819
NC_005102.4-169682056	ATTCTGAATACATAGACTCCATTCCTA	26	T	0.0165	0.0325
NC_005102.4-169690149	GAACGATGGACTGTACCTCCAACCTTG	5	G	0.0244	0.0398
NC_005102.4-169690149	GAACGATGGACTGTACCTCCAACCTTG	13	G	0.0732	0.0854
NC_005102.4-169706216	AAGGGGGAAACCGTGCCTCCATTTAAA	13	G	0.0631	0.0778
NC_005102.4-169792495	ATTACATATACCCAGACTCCATGAAAG	22	T	0	0.4765
NC_005102.4-169792495	ATTACATATACCCAGACTCCATGAAAG	24	A	0	0.4765
NC_005102.4-169855638	GTGAAGAAAACTGACGCTCCTTACCTC	14	A	0	0.4728
NC_005102.4-169855897	TTTGACCATACTCCCCCTCCCCTTTCT	16	C	0.0536	0.0521
NC_005102.4-169886539	GCATCTCCTACCTCGTCTCCTCTCTGA	15	G	0.2764	0.2937
NC_005102.4-169901698	TCTCTAGACACCAGCTCTCCTCCGCAT	24	G	0.252	0.4967
NC_005102.4-169908715	CTCAACTTCACTACCTCTCCATTGTAT	3	C	0.1786	0.3922
NC_005102.4-169923599	ATGCAATAAACTTGTCCTCCCGTCACA	22	G	0.0862	0.4905
NC_005102.4-169942800	CGTGACACCACAGTCCCTCCAATCCCT	1	C	0.2389	0.3189
NC_005102.4-169942800	CGTGACACCACAGTCCCTCCAATCCCT	6	C	0.193	0.1884
NC_005102.4-170000998	GTAAATCCCACAGCTCCTCCATGAGCA	12	A	0.042	0.4682
NC_005102.4-170005556	TGATACCATACACTTGCTCCCAACTTG	22	A	0.1339	0.1843
NC_005102.4-170011978	TTCATCTCTACTCTGACTCCTCCAGTA	22	C	0.0259	0.0255
NC_005102.4-170055701	AGAAAAAGCACAGCAGCTCCTTCTGCC	7	A	0.2195	0.4106
NC_005102.4-170103757	CAGGTCTCTACTTGAGCTCCTACCCTG	7	T	0	0.4826
NC_005102.4-170133031	GACCCACCTACCCTAACTCCCCCAAAC	24	A	0	0.488
NC_005102.4-170159030	CTGCCCCGCACACTTGCTCCTGCCTCC	5	C	0.1901	0.4213
NC_005102.4-170159030	CTGCCCCGCACACTTGCTCCTGCCTCC	7	C	0.0083	0.4847
NC_005102.4-170184075	TGCCCACAGACTCCAACTCCAAAGGAT	6	A	0.042	0.4886
NC_005102.4-170184075	TGCCCACAGACTCCAACTCCAAAGGAT	16	A	0.2458	0.3086
NC_005102.4-170228901	GAGACTCTCACTGAGGCTCCCTCTCCT	6	T	0.122	0.356
NC_005102.4-170244060	TGCATTGTCACAGAGACTCCTGACATC	5	T	0.1545	0.4999
NC_005102.4-170244060	TGCATTGTCACAGAGACTCCTGACATC	23	A	0	0.4793

NC_005102.4-170253193	GGAACGGCAACCTGCACTCCCCCATGT	8	C	0.1951	0.4984
NC_005102.4-170253295	TGGTTATCAACAGCGACTCCCTGACCT	14	C	0.1138	0.1356
NC_005102.4-170301705	CTGTGTCTGACCCATGCTCCACAGAGG	15	T	0	0.4814
NC_005102.4-170306215	GGTAACAGGACAAAGGCTCCTTCCTGG	13	A	0.0167	0.4728
NC_005102.4-170312191	CTCTCCCCAACTCTTTCTCCCTGCCAT	15	T	0	0.48
NC_005102.4-170315029	GGGACACACACCTGTGCTCCCAGCACC	16	G	0.1681	0.2494
NC_005102.4-170320523	GAATCTGTGACCTGTTCTCCTGGTCAG	2	A	0.252	0.2203
NC_005102.4-170320523	GAATCTGTGACCTGTTCTCCTGGTCAG	26	A	0.5528	0.4331
NC_005102.4-170323858	TTTTGTTTGACAATCCCTCCAGTACTC	16	C	0.1557	0.2339
NC_005102.4-170323858	TTTTGTTTGACAATCCCTCCAGTACTC	23	T	0	0.4793
NC_005102.4-170335336	CACTGGGCCACATTGCCTCCTCCCTCC	13	T	0	0.4779
NC_005102.4-170343146	TAAATAAATACGCTCACTCCCAGTCAT	2	A	0.0244	0.0241
NC_005102.4-170343146	TAAATAAATACGCTCACTCCCAGTCAT	5	T	0.0244	0.0241
NC_005102.4-170355967	CAGCATCCCACTCCATCTCCAACCTGG	16	T	0.0085	0.4753
NC_005102.4-170356791	TGTTGTGAAACACCGCCTCCTGTATCC	4	T	0.0244	0.0241
NC_005102.4-170360140	CAGCTACCAACCTGACCTCCCCTTCAT	6	A	0.0579	0.0716
NC_005102.4-170360140	CAGCTACCAACCTGACCTCCCCTTCAT	26	A	0.1983	0.4942
NC_005102.4-170385649	TTCCCACCCACTGCTGCTCCAGACAGC	6	A	0.2333	0.2432
NC_005102.4-170385649	TTCCCACCCACTGCTGCTCCAGACAGC	12	T	0.2333	0.2432
NC_005102.4-170390896	TCAAATCAAACGAAGGCTCCTGGCCTC	12	G	0.0248	0.0245
NC_005102.4-170390896	TCAAATCAAACGAAGGCTCCTGGCCTC	27	C	0.0417	0.0408
NC_005102.4-170392010	ATCACACAGACTCATTCTCCTCACAGA	8	A	0.0244	0.0241
NC_005102.4-170439140	TTGCTTGCAACCACGGCTCCAGGAGAG	25	G	0.2672	0.4893
NC_005102.4-170461339	TATATTGTCACATGAGCTCCATACTAC	23	A	0.0342	0.4807
NC_005102.4-170480816	AGAGATGTTACCAAGGCTCCAACAGTC	9	T	0.0696	0.4969
NC_005102.4-170493941	AATAGATGAACGGGTTCTCCCAGGCCA	25	C	0.0328	0.4837
NC_005102.4-170511856	CAGCTGAGAACAGATCCTCCTTGTGGG	1	C	0.2328	0.4545
NC_005102.4-170539202	GCATCTGTAACTCCAGCTCCTCACCTT	26	T	0	0.4709
NC_005102.4-170539969	ACCAGCTTCACCTTTCCTCCTCTGGCT	1	A	0	0.4827
NC_005102.4-170575695	GTGGAGTGGACAGGTCCTCCAGGGCCG	23	G	0.0342	0.4839
NC_005102.4-170575695	GTGGAGTGGACAGGTCCTCCAGGGCCG	26	C	0.2174	0.27
NC_005102.4-170597054	AGCTTTGCCACTCATCCTCCTCTTCCA	14	A	0	0.4793
NC_005102.4-170640865	TGGGCCTCCACACCCCCTCCCAGGACA	12	A	0.0732	0.0705
NC_005102.4-170640865	TGGGCCTCCACACCCCCTCCCAGGACA	21	C	0.1463	0.189
NC_005102.4-170646388	CAATCATTCACGGGGTCTCCCATGCAC	26	A	0.065	0.0629
NC_005102.4-170666615	TTGATTCCAACTGGGCCTCCGTCTGGA	21	G	0.0351	0.0512
NC_005102.4-170683971	GGAGACTGGACTCCGCCTCCACCTCTG	1	G	0.0625	0.4885
NC_005102.4-170683971	GGAGACTGGACTCCGCCTCCACCTCTG	23	C	0.0357	0.0351
NC_005102.4-170743329	GTTCCAAGTACTGCCACTCCCTTCCTG	9	T	0	0.4807
NC_005102.4-170800748	CCTGATGCTACTTCCTCTCCACTGTGG	4	G	0.0593	0.1041
NC_005102.4-170800748	CCTGATGCTACTTCCTCTCCACTGTGG	7	G	0.0513	0.0973
NC_005102.4-170814097	ATGTGGTTCACAGAATCTCCTGCACTC	8	T	0.0407	0.0398
NC_005102.4-170814097	ATGTGGTTCACAGAATCTCCTGCACTC	14	A	0	0.4793
NC_005102.4-170823750	GCCGTGAGCACCAACGCTCCCAGGCTA	3	C	0.1736	0.2474
NC_005102.4-170823750	GCCGTGAGCACCAACGCTCCCAGGCTA	16	G	0.1557	0.3581
NC_005102.4-170841654	GGGAGGGACACACAGACTCCATGTGAC	1	G	0.1858	0.481
NC_005102.4-170850651	CATACTCACACACACACTCCTCACACA	2	A	0.3109	0.397
NC_005102.4-170850651	CATACTCACACACACACTCCTCACACA	4	A	0.175	0.1597
NC_005102.4-170850923	CACATACTCACACACACTCCTCATGTA	3	C	0.0609	0.42
NC_005102.4-170850923	CACATACTCACACACACTCCTCATGTA	26	T	0.1043	0.2138
NC_005102.4-170861929	GTGTCCCCCACCCTACCTCCCTGGCAC	26	A	0.0086	0.4729

NC_005102.4-170902103	CACATGTACACATTCACTCCACAGAGA	25	A	0.125	0.2449
NC_005102.4-170921946	CCGTGGCGCACAACACCTCCACACAAG	7	C	0.0168	0.4743
NC_005102.4-170927803	TGTGTGGGGACAAGTCCTCCCTGGTTG	12	A	0.0354	0.4624
NC_005102.4-170927803	TGTGTGGGGACAAGTCCTCCCTGGTTG	26	T	0.2124	0.4276
NC_005102.4-170936218	AACGCCATCACACACTCTCCACATGCA	4	G	0.2705	0.4717
NC_005102.4-170936218	AACGCCATCACACACTCTCCACATGCA	25	G	0.2623	0.3447
NC_005102.4-170942318	TGCTCGGGCACACCACCTCCATCCAGT	6	G	0.2846	0.3287
NC_005102.4-170942318	TGCTCGGGCACACCACCTCCATCCAGT	23	C	0.1463	0.2263
NC_005102.4-170951968	CAGTCACATACACCCTCTCCAAGCCGG	24	C	0.0407	0.0398
NC_005102.4-170959679	CACACAGAGACACACACTCCTGTGTGC	23	T	0.0256	0.0253
NC_005102.4-170964499	CACCTCTGAACTTTTCCTCCCTTTTCC	13	T	0	0.4734
NC_005102.4-170995904	CCTTGTGAGACCTTGTCTCCAAAAGCG	27	G	0	0.4773
NC_005102.4-171053111	TCCGAAGCAACATGAGCTCCAGCCTCC	23	C	0.0407	0.0398
NC_005102.4-171073384	GGTGGGGAAACTGAGGCTCCAGAGTTG	7	G	0.1951	0.2382
NC_005102.4-171075168	TGTTTCCATACAAAGTCTCCTGGGTAA	12	A	0	0.4787
NC_005102.4-171078003	GGCCTCAGCACTGTGACTCCATAGGAT	3	C	0.0252	0.0249
NC_005102.4-171081143	CACAGAGAGACTTGGTCTCCATTGCAA	13	T	0.1552	0.375
NC_005102.4-171145875	ACGCTGGCCACCATGTCTCCCTGATTC	13	A	0	0.4793
NC_005102.4-171202604	ACAAAGGAAACATTATCTCCAAGCTGT	1	A	0.0672	0.1254
NC_005102.4-171212209	ATCATGGAGACCCCTTCTCCTCCTCCA	1	A	0	0.4807
NC_005102.4-171212209	ATCATGGAGACCCCTTCTCCTCCTCCA	14	C	0.2759	0.3662
NC_005102.4-171266562	AGCAAGGGGACCACTCCTCCATGCCTG	5	A	0.2377	0.4973
NC_005102.4-171266562	AGCAAGGGGACCACTCCTCCATGCCTG	9	G	0.025	0.0247
NC_005102.4-171280332	CGAGGCCGGACAAGTCCTCCACAGCCT	7	C	0	0.4773
NC_005102.4-171280332	CGAGGCCGGACAAGTCCTCCACAGCCT	22	C	0.0246	0.0401
NC_005102.4-171298517	GACTTCTTCACAGTCTCTCCCTCTGCA	9	C	0.2213	0.454
NC_005102.4-171326549	CCACTGCGGACTCAAGCTCCTCCCCCT	25	C	0.5929	0.4309
NC_005102.4-171357450	GGGAAGGAGACAGTGGCTCCCACGTAG	9	G	0.2114	0.3334
NC_005102.4-171357450	GGGAAGGAGACAGTGGCTCCCACGTAG	22	A	0.0081	0.481
NC_005102.4-171361415	AAGTGAGACACCACAACTCCCAGTTGC	16	A	0	0.4724
NC_005102.4-171361415	AAGTGAGACACCACAACTCCCAGTTGC	27	C	0.1786	0.3157
NC_005102.4-171367793	GGAAGTTCCACAGTGTCTCCCAACCAG	1	G	0.1525	0.1961
NC_005102.4-171367793	GGAAGTTCCACAGTGTCTCCCAACCAG	14	T	0.1453	0.4626
NC_005102.4-171430161	TCATGGATGACAGAATCTCCAGCTATG	15	A	0	0.4743
NC_005102.4-171434968	TGCTCACCCACCCAACCTCCTTCTCTC	6	A	0.0732	0.1287
NC_005102.4-171444998	GGCGATACCACCCTGTCTCCTGGCGCC	3	C	0.1681	0.2237
NC_005102.4-171449001	GGTAAAGGCACCGGTGCTCCCTGAGGA	13	G	0.1833	0.4728
NC_005102.4-171449001	GGTAAAGGCACCGGTGCTCCCTGAGGA	15	T	0.0083	0.0245
NC_005102.4-171483450	ACAGAAGGCACAGGGTCTCCCTAGGAA	3	A	0.2101	0.2852
NC_005102.4-171483450	ACAGAAGGCACAGGGTCTCCCTAGGAA	8	G	0.2185	0.4285
NC_005102.4-171502628	GGACATGTGACTACTGCTCCTGGACAT	26	A	0.1026	0.1274
NC_005102.4-171515790	AAGCCCAAGACCACCCCTCCTTTCATC	7	A	0	0.4813
NC_005102.4-171531093	TGATAACTCACACAAACTCCCTCCCAA	1	T	0.0088	0.483
NC_005102.4-171544295	TTCTCCATGACAGTTCCTCCAGCCTGA	13	G	0.2479	0.4227
NC_005102.4-171560884	AAGAGGGTAACCCTAGCTCCTAGCAAC	26	A	0.1795	0.4087
NC_005102.4-171568030	GAGTTCAGTACACTGACTCCACATTGC	1	G	0.2544	0.4473
NC_005102.4-171576876	CCGAAGATCACGTGTCCTCCGTGGGTC	3	G	0.0086	0.4836
NC_005102.4-171584498	TTGAGTGACACTGGCTCTCCCGTAGGC	21	C	0.1901	0.3601
NC_005102.4-171598859	TACCTTGTCACTTGAGCTCCCTAGTAA	1	T	0.2276	0.4881
NC_005102.4-171598859	TACCTTGTCACTTGAGCTCCCTAGTAA	12	T	0.1707	0.4979
NC_005102.4-171611708	GAACTTAGCACAGAAACTCCAGGCAGG	3	A	0.0252	0.0249

NC_005102.4-171629600	ATACTAGGTACACACACTCCTAGGTGT	25	T	0.0625	0.4968
NC_005102.4-171633351	GGCTTTGAGACCCTTCCTCCTAGCTTC	27	C	0.0924	0.491
NC_005102.4-171638548	TCATGTCCAACTTGGACTCCACCCACA	16	A	0	0.4793
NC_005102.4-171638548	TCATGTCCAACTTGGACTCCACCCACA	23	C	0	0.4793
NC_005102.4-171655041	TTCAACTGAACACCGGCTCCAGGGAAT	14	C	0.0246	0.0401
NC_005102.4-171655041	TTCAACTGAACACCGGCTCCAGGGAAT	16	G	0	0.4773
NC_005102.4-171675194	CTCAGGTCCACCGTGGCTCCCCTCTAG	13	G	0	0.477
NC_005102.4-171689352	ATGAAAGTCACACCTACTCCTGCTCTT	23	C	0.025	0.0247
NC_005102.4-171698866	GGAATTGGGACTTTATCTCCCAGCCCG	14	T	0	0.4793
NC_005102.4-171735521	TGCAGGGCTACTCATACTCCTTTCCTG	8	C	0	0.4807
NC_005102.4-171750729	GATGCCAGGACTGTGTCTCCCACTGCA	8	G	0	0.4793
NC_005102.4-171750729	GATGCCAGGACTGTGTCTCCCACTGCA	12	T	0	0.4793
NC_005102.4-171781942	CCATGTGTAACTCAAGCTCCAGGGGCT	15	A	0	0.4773
NC_005102.4-171789222	TGAAGCAGGACTTGTGCTCCCCTCCTT	27	T	0	0.4724
NC_005102.4-171797838	CTCTCTGGTACCATTGCTCCTGTGCTG	14	T	0.0331	0.4849
NC_005102.4-171799301	GTGTCTGTCACGGTCACTCCCCGTGCC	12	G	0.0348	0.0342
NC_005102.4-171799301	GTGTCTGTCACGGTCACTCCCCGTGCC	24	T	0.0351	0.0345
NC_005102.4-171838355	CCATCCAGCACAGTCACTCCTTCAGGT	3	A	0	0.4807
NC_005102.4-171873648	CATCAACTCACCAGCTCTCCCTGTAAC	16	T	0	0.4773
NC_005102.4-171880559	TTTTAACAAACAAGGCCTCCGTTGCTT	21	G	0.0085	0.0253
NC_005102.4-171976273	ATCCTCCCTACCTTTTCTCCTACAGAA	8	C	0.2174	0.2332
NC_005102.4-171976273	ATCCTCCCTACCTTTTCTCCTACAGAA	22	A	0.0957	0.18
NC_005102.4-171986939	CTAGTAGGGACACTGTCTCCCTTGTTA	13	C	0.0256	0.4928
NC_005102.4-172063922	CCCTTGCAGACCCTTCCTCCTTTACTC	27	C	0.0085	0.479
NC_005102.4-172074047	CCAGTAGGCACCACTGCTCCATGGTCT	16	G	0.0339	0.0333
NC_005102.4-172137141	GTGCGCATCACTTGTTCTCCTAATGAC	13	T	0.0082	0.479
NC_005102.4-172142413	CTCCGGTGCACACTCCCTCCCATGTTG	5	G	0	0.4887
NC_005102.4-172155040	GGAGAGTTAACCCAAACTCCCAAGTTA	13	C	0	0.4826
NC_005102.4-172163993	GCTTCCATGACTTAGTCTCCTCAGCCC	1	G	0	0.4819
NC_005102.4-172302967	TGCCCTTGAACATATTCTCCTACGGAG	13	T	0	0.4734
NC_005102.4-172324368	TTGTGCCTCACTCCCCCTCCCTCTCAT	15	C	0.0165	0.4787
NC_005102.4-172331725	CCTAATCCAACAGCTACTCCTCTAGTC	23	T	0.0089	0.4454
NC_005102.4-172331725	CCTAATCCAACAGCTACTCCTCTAGTC	26	T	0.0089	0.4454
NC_005102.4-172385187	AACGTCCCTACGAGGCCTCCGCTCATC	23	T	0	0.4718
NC_005102.4-172432637	GTACCCCAAACAACATCTCCTTGTGCC	15	A	0.0579	0.1017
NC_005102.4-172432637	GTACCCCAAACAACATCTCCTTGTGCC	22	T	0	0.4787
NC_005102.4-172457441	AAAATGAGCACCTAACCTCCCAGTTAC	15	A	0	0.4807
NC_005102.4-172457579	GTCAAGTGAACAAACTCTCCTAAAGTG	2	T	0.0259	0.0585
NC_005102.4-172457579	GTCAAGTGAACAAACTCTCCTAAAGTG	15	C	0.0259	0.4955
NC_005102.4-172461963	CTCTTGTGGACCGGGCCTCCAGTTCAA	12	C	0	0.4773
NC_005102.4-172465182	CTGTGAGACACACCACCTCCAAGCAGG	1	C	0.0174	0.4891
NC_005102.4-172465182	CTGTGAGACACACCACCTCCAAGCAGG	2	T	0.0174	0.4891
NC_005102.4-172534061	CACAACTGCACTCTGCCTCCATGGCCT	4	A	0.1653	0.4877
NC_005102.4-172614482	AACCTGTGGACTCAGGCTCCTGAGCCT	16	G	0	0.4807
NC_005102.4-172651804	GCAGATGTAACTCTGCCTCCTGAGCAC	1	G	0	0.4779
NC_005102.4-172651804	GCAGATGTAACTCTGCCTCCTGAGCAC	3	A	0	0.4779
NC_005102.4-172746549	GACACCTGAACAAGATCTCCTTGCTTT	7	T	0.0171	0.4807
NC_005102.4-172800331	CATTCCGGAACTCTCCCTCCACCCTCC	26	C	0.0579	0.0562
NC_005102.4-172809337	TTGTCCATAACTCCAGCTCCAGGGCAG	6	C	0.1293	0.5
NC_005102.4-172809337	TTGTCCATAACTCCAGCTCCAGGGCAG	21	A	0.1217	0.173
NC_005102.4-172829890	CACACACACACATGTCCTCCCTTTATG	13	T	0	0.4891

NC_005102.4-172833848	GATCTGGGGACCAAGCCTCCAGCCCAT	24	C	0.2034	0.4677
NC_005102.4-172848186	TCAGTCACCACCTCCTCTCCTCCTTCA	2	C	0.1239	0.1613
NC_005102.4-172848186	TCAGTCACCACCTCCTCTCCTCCTTCA	13	T	0.0885	0.4953
NC_005102.4-172898033	TCTGTCTGGACACCATCTCCTCCCCAC	1	T	0.0086	0.4836
NC_005102.4-172913283	GGCTGTGCTACCCTGTCTCCTAGTGAC	3	C	0	0.4793
NC_005102.4-173001890	GAATGCTTGACTTCACCTCCTACAGGA	6	C	0	0.4793
NC_005102.4-173001890	GAATGCTTGACTTCACCTCCTACAGGA	22	A	0.1864	0.2926
NC_005102.4-173016562	TTGTGTGACACAGTGGCTCCTTAGACC	2	T	0.1345	0.3415
NC_005102.4-173016766	TATAGTAAGACCCTGTCTCCACATTAA	3	T	0.2	0.2994
NC_005102.4-173023664	ATGACCCCTACCCTTTCTCCTGCAATT	15	T	0.2114	0.2723
NC_005102.4-173029345	AGAAGTGGAACATCCCCTCCTGGAGGG	4	A	0.2174	0.3451
NC_005102.4-173353335	GGTGTGTGAACCCACACTCCAGGTCTG	2	G	0.0702	0.4925
NC_005102.4-173353335	GGTGTGTGAACCCACACTCCAGGTCTG	12	C	0	0.48
NC_005102.4-173440851	GTTGGTCTTACCTGTGCTCCTGCGGCT	25	G	0.0583	0.0566
NC_005102.4-173656699	GTGTCATGGACATGTTCTCCTCAATCC	25	T	0	0.4728
NC_005102.4-173675650	ATAGGAGCCACCTCAGCTCCAGGTCAT	12	C	0.0446	0.0436
NC_005102.4-173685245	TTAAAAAAGACGCCCTCTCCAGCCCCC	2	T	0.0917	0.1025
NC_005102.4-173685518	GGCATGGGGACCACGTCTCCATGCGCC	26	C	0.0083	0.0405
NC_005102.4-173690003	CCGGTCTTGACGCCTTCTCCCACAGCA	2	C	0.0246	0.0243
NC_005102.4-173713933	TCTTTGTGTACACTCCCTCCCCCTCCC	8	G	0	0.4832
NC_005102.4-173713933	TCTTTGTGTACACTCCCTCCCCCTCCC	14	T	0.0339	0.4908
NC_005102.4-173714746	GGTTTCACTACGGTGTCTCCATACATG	15	G	0.0413	0.0562
NC_005102.4-173717400	ATAGTTTCAACCCTATCTCCCTGTTTC	9	A	0.0984	0.4966
NC_005102.4-173717400	ATAGTTTCAACCCTATCTCCCTGTTTC	12	C	0.0407	0.0553
NC_005102.4-173761073	CCTTACTGCACAGCTTCTCCCACCTCT	9	C	0.0083	0.4835
NC_005102.4-173761073	CCTTACTGCACAGCTTCTCCCACCTCT	27	T	0.2017	0.2449
NC_005102.4-173766776	TATCAAATGACAATTGCTCCTCAGCTG	9	G	0.0261	0.0257
NC_005102.4-173790209	TAGGTGAGGACTCTGGCTCCTGTCATC	2	A	0.2542	0.2926
NC_005102.4-173792974	TGTCGACGCACGCATGCTCCTAAGTAT	4	C	0	0.4793
NC_005102.4-173804848	GGCATGCACACATGTGCTCCCACTGTG	24	T	0.2602	0.2832
NC_005102.4-173871191	CATTCAACCACAGCAACTCCCTCCTTG	7	A	0.0082	0.479
NC_005102.4-173871191	CATTCAACCACAGCAACTCCCTCCTTG	25	T	0.1721	0.2218
NC_005102.4-173876195	ATTTGTCCCACCTCCCCTCCCATCCTG	12	C	0.1356	0.1264
NC_005102.4-173876195	ATTTGTCCCACCTCCCCTCCCATCCTG	22	A	0.2137	0.4947
NC_005102.4-173936411	CCAATCATGACAACATCTCCAGAGAGA	15	A	0.0169	0.4826
NC_005102.4-173955534	CTGCCTTCAACCCAAGCTCCAGGGGAT	5	C	0.1393	0.1296
NC_005102.4-173965081	ATTCTCTGTACCCCTTCTCCCTGAGGT	2	T	0	0.4846
NC_005102.4-173980883	TAAGGACATACTTGACCTCCTGTGGAC	24	G	0.122	0.1145
NC_005102.4-174028124	TGCCACAGGACAGTGTCTCCGGAAGGC	2	G	0.2034	0.334
NC_005102.4-174028124	TGCCACAGGACAGTGTCTCCGGAAGGC	27	C	0.1466	0.4955
NC_005102.4-174070087	AGAAAGTCAACTGGGACTCCAGGTTCC	3	A	0.0325	0.032
NC_005102.4-174101841	CTGGAGGAAACACTGTCTCCCAGTGTT	24	T	0.1901	0.2474
NC_005102.4-174120939	TTCCTAATAACAGCCCCTCCCGTTTCC	21	C	0.1339	0.125
NC_005102.4-174144181	GAGACGCAGACCTGGGCTCCTGCTGCT	5	C	0.1721	0.2218
NC_005102.4-174144181	GAGACGCAGACCTGGGCTCCTGCTGCT	23	C	0.2295	0.3791
NC_005102.4-174166749	TGCGTTGTAACCTTGCCTCCCGCCCCC	23	C	0.1724	0.2739
NC_005102.4-174169271	GTTGTGTGGACAGTGTCTCCCTAAGAT	2	T	0.2034	0.4793
NC_005102.4-174186768	TTAACCCCAACTCGGGCTCCTTCCACA	26	C	0.0261	0.0425
NC_005102.4-174213584	CGTTCACACACCCAGGCTCCCATCTGG	2	G	0.0413	0.0405
NC_005102.4-174245796	GCCCAATCTACCTCCTCTCCTTATAGT	23	A	0	0.48
NC_005102.4-174245796	GCCCAATCTACCTCCTCTCCTTATAGT	27	T	0.2185	0.2796

NC_005102.4-174266554	CTCTCACCCACCACATCTCCTCAGCTA	27	A	0.0081	0.4777
NC_005102.4-174274632	CTCACTCTCACTTGCTCTCCTGTGTGC	9	C	0.084	0.1398
NC_005102.4-174276308	TCCTCTCTCACCTCAACTCCCAGCATG	16	A	0.2195	0.4952
NC_005102.4-174303590	GATGCCCCCACAGAGTCTCCTCAAAGA	16	T	0.0738	0.0861
NC_005102.4-174303590	GATGCCCCCACAGAGTCTCCTCAAAGA	26	G	0.0082	0.0243
NC_005102.4-174305783	TGAGATTCTACTTCACCTCCTCTTATG	23	T	0.0252	0.0249
NC_005102.4-174310341	CTCTCCATGACTCTTTCTCCTCCATGG	16	T	0	0.48
NC_005102.4-174317568	CTGTCAGCAACTCCAGCTCCAGGGCAT	8	C	0.0325	0.0476
NC_005102.4-174342243	GGAAGAGTCACTGTGGCTCCCCACCTA	12	T	0	0.4751
NC_005102.4-174352234	TATGATGAAACACGCGCTCCCAGTGTG	13	C	0.041	0.0401
NC_005102.4-174357652	CTATGTCACACAGGACCTCCACCTCAG	2	T	0.0413	0.0405
NC_005102.4-174494347	GAGCAGTTAACGCGGGCTCCTTCAGCT	12	G	0.0708	0.0846
NC_005102.4-174494347	GAGCAGTTAACGCGGGCTCCTTCAGCT	13	C	0.0354	0.0348
NC_005102.4-174512104	TGTGTGGATACGTATGCTCCATCCATT	12	G	0.05	0.4911
NC_005102.4-174550339	GTACTCTGCACCCTTACTCCATCATCA	13	C	0.065	0.0629
NC_005102.4-174550339	GTACTCTGCACCCTTACTCCATCATCA	25	T	0.2114	0.2723
NC_005102.4-174552844	GGGTGCTGGACTGGGTCTCCAAGACAA	21	A	0.0357	0.0351
NC_005102.4-174581593	TCCAGCACAACAGGAACTCCAGCCTCA	12	A	0.252	0.4106
NC_005102.4-174581990	CTGCCACTGACAAATGCTCCACGACAT	23	G	0	0.4813
NC_005102.4-174582550	TCACTTTTTACCTGGACTCCTCATCAG	13	T	0.281	0.418
NC_005102.4-174584194	GAGTAATGGACCAGAACTCCAATGCTC	7	T	0.1875	0.4856
NC_005102.4-174585435	ACAGCTTGGACATGAACTCCTTTGAAG	26	A	0.0407	0.0854
NC_005102.4-174621899	GCCCACGTCACAGAGCCTCCCAGGTCC	7	G	0.1964	0.3157
NC_005102.4-174623084	GGGACATAAACAACCCCTCCAACTCAG	13	A	0.1917	0.4875
NC_005102.4-174632359	CAGTGAGAGACCTTATCTCCAAAGCAA	9	G	0.2	0.3252
NC_005102.4-174702742	CCAGTTGTAACTCTACCTCCAGGGAAT	5	T	0	0.4793
NC_005102.4-174751438	GAAACAGTTACATTGGCTCCTGGTTTC	5	C	0	0.4819
NC_005102.4-174753975	ACACATGGGACCTTCTCTCCTACACCA	27	A	0	0.4807
NC_005102.4-174759758	CCTCCACGCACTCCTCCTCCCGCTTTT	21	C	0.2846	0.4741
NC_005102.4-174759758	CCTCCACGCACTCCTCCTCCCGCTTTT	24	T	0.1092	0.4977
NC_005102.4-174766169	GATGGGGACACACTGGCTCCGGTGCCC	12	A	0	0.4813
NC_005102.4-174766169	GATGGGGACACACTGGCTCCGGTGCCC	14	T	0.0763	0.1481
NC_005102.4-174767218	CCTCTGAGCACTGTCTCTCCTTCTTCA	4	C	0.0244	0.0398
NC_005102.4-174772538	CAAGTCAGGACAAACCCTCCTGTTTTT	8	G	0.0254	0.4655
NC_005102.4-174772538	CAAGTCAGGACAAACCCTCCTGTTTTT	24	T	0.0254	0.4655
NC_005102.4-174818762	TCTATGCACACAGTTACTCCACCACCC	24	A	0	0.4887
NC_005102.4-174818908	ACAGCAGAGACTCAGGCTCCTTCAGCT	14	A	0	0.4793
NC_005102.4-174821896	TCTGACTCTACCCAAGCTCCCTGCAGG	3	T	0.0244	0.0241
NC_005102.4-174828469	GGTAGAAACACAGAAACTCCAGTAGAT	7	A	0.2203	0.2344
NC_005102.4-174828469	GGTAGAAACACAGAAACTCCAGTAGAT	23	T	0.1441	0.4957
NC_005102.4-174843502	TCACTAATGACACTTCCTCCAGAGTGT	14	T	0	0.482
NC_005102.4-174871321	AACGTCAACACATGCCCTCCAGCAATG	8	A	0.1453	0.1773
NC_005102.4-174912048	TTGTTGGTAACCCTCACTCCCCAAGGT	7	G	0.0744	0.1017
NC_005102.4-174945384	TATCCTTCCACAGTAGCTCCATTAAAG	16	G	0.0756	0.1327
NC_005102.4-174945384	TATCCTTCCACAGTAGCTCCATTAAAG	21	A	0.0756	0.4435
NC_005102.4-174956201	CGTCTGGTGACAATTCCTCCCCCACCT	13	A	0	0.4654
NC_005102.4-174981852	AACTTCAGAACCCGTTCTCCCTGTGAG	6	C	0.1983	0.4787
NC_005102.4-174991292	ACTGTCCTAACATGTGCTCCCCCATCA	3	T	0	0.4793
NC_005102.4-175018015	TGCCTGGGCACTGTGTCTCCCCTCCAT	1	T	0.2602	0.4722
NC_005102.4-175018015	TGCCTGGGCACTGTGTCTCCCCTCCAT	15	G	0.2602	0.3427
NC_005102.4-175030033	CTAGGTCTCACCTTCCCTCCTCCATGC	15	C	0.1849	0.5

NC_005102.4-175043211	CTGCTTGTAACTTGGTCTCCAACATGG	6	T	0	0.4751
NC_005102.4-175043211	CTGCTTGTAACTTGGTCTCCAACATGG	8	T	0	0.4751
NC_005102.4-175048110	TTTATTGACACAAAGTCTCCTGCTGCC	16	T	0.2807	0.396
NC_005102.4-175081983	TCTGTACTCACGCTGCCTCCCTTTCTC	6	A	0.0089	0.4789
NC_005102.4-175085064	TCCAGGCTCACAGCACCTCCTCCAGGC	7	C	0	0.4851
NC_005102.4-175091029	AAAAGAAGCACTCCAACTCCTCCTTAC	1	A	0	0.4765
NC_005102.4-175099236	GCCAGGATCACGAGTCCTCCAATGGGC	22	A	0.1849	0.494
NC_005102.4-175100732	GGATGCTGAACAGGCTCTCCAGTCCCT	3	A	0.0244	0.0241
NC_005102.4-175100732	GGATGCTGAACAGGCTCTCCAGTCCCT	21	A	0.0244	0.0241
NC_005102.4-175107874	ACCTCGGCTACCCACACTCCCGCCACT	6	G	0.0855	0.1704
NC_005102.4-175123392	CTTGTGCCGACAGCTTCTCCTGCCCGT	26	G	0.1593	0.2171
NC_005102.4-175152115	GACATAATTACTAGCACTCCAGAACCA	12	T	0	0.4637
NC_005102.4-175189059	AAGGCCTTCACAGATGCTCCCACAGGT	5	C	0.0756	0.1033
NC_005102.4-175191381	ATCCTTACAACATGCCCTCCTCCAGCC	1	A	0.1885	0.2339
NC_005102.4-175193289	ATGTCACATACTAACCCTCCTGCTTGC	1	A	0	0.4807
NC_005102.4-175231817	TATCCACCAACCCCAGCTCCTGCAGTG	22	G	0.2683	0.3381
NC_005102.4-175249909	TGTGTGTGTACATTGGCTCCGTCATGA	9	T	0.2033	0.2668
NC_005102.4-175249909	TGTGTGTGTACATTGGCTCCGTCATGA	12	A	0.2114	0.4389
NC_005102.4-175264537	CTAGAGCAGACACCCTCTCCCCTACCC	4	G	0.0089	0.4856
NC_005102.4-175269343	AAGCCCCCTACTTCCCCTCCAACCATA	26	T	0.2162	0.4854
NC_005102.4-175302847	GGCATCCTCACTATGCCTCCACAGTCA	2	G	0	0.4793
NC_005102.4-175305790	ACAACAGTGACAACAGCTCCCAGTGAT	2	C	0.1217	0.4991
NC_005102.4-175305790	ACAACAGTGACAACAGCTCCCAGTGAT	3	A	0	0.4807
NC_005102.4-175310348	CATTCAGCTACCACTCCTCCTACTGCA	5	C	0.2787	0.435
NC_005102.4-175311444	AATGTTCAGACACTGACTCCTTCATCT	14	T	0.1901	0.2922
NC_005102.4-175314601	CTTTAGAGGACCCATGCTCCGCTCCCA	1	C	0.0261	0.0257
NC_005102.4-175314647	ACACCCGCAACTATGGCTCCAGGGATT	6	C	0.2066	0.2922
NC_005102.4-175332374	CTCACAGAAACCTCTGCTCCCTACAAT	23	A	0.0522	0.0508
NC_005102.4-175355171	GCTGCCCAAACACACACTCCTCCACCT	9	A	0.0244	0.4777
NC_005102.4-175387048	ATTTGACTTACAGCCACTCCATAAGAT	23	A	0.3008	0.4174
NC_005102.4-175439406	GGGGTCAGTACTGTCTCTCCATGTGGG	4	G	0.0439	0.0429
NC_005102.4-175468975	TCACCTCCCACTAGGCCTCCTGGTGAA	27	A	0	0.4641
NC_005102.4-175545767	CATGAGAACACATTGGCTCCAAGTCCC	9	C	0	0.4807
NC_005102.4-175558440	CCCCTCTGCACAGCAGCTCCACACTTG	8	G	0.1176	0.1107
NC_005102.4-175565175	ACCAGAGCCACCTTCTCTCCACCCTGA	12	C	0.0738	0.0861
NC_005102.4-175588293	GAGAGGGGAACCCTTACTCCAGGCTGT	13	C	0.0442	0.4923
NC_005102.4-175589676	AGGGACAGCACCATCCCTCCTCTCGGT	2	G	0.2869	0.2796
NC_005102.4-175618923	AACCGCTGAACCCTCTCTCCACCCCCC	24	C	0	0.4765
NC_005102.4-175625939	CTGCTTGCAACTCCAGCTCCAGGGGAG	14	C	0.3033	0.2573
NC_005102.4-175631220	CAAGTTAATACCTTTTCTCCATCATGA	22	T	0.0351	0.0512
NC_005102.4-175635644	CCCTGATATACCACATCTCCTTCTGCG	26	C	0.287	0.3451
NC_005102.4-175635644	CCCTGATATACCACATCTCCTTCTGCG	27	G	0.2478	0.4464
NC_005102.4-175644516	CTCACCACCACCCGTGCTCCGGGCTGC	13	C	0	0.4826
NC_005102.4-175682954	CTGTGGGTCACTTGTTCTCCAAAGGGA	26	G	0	0.4688
NC_005102.4-175741604	ATCAAATACACTGTCCCTCCCTCCTCT	8	A	0.0976	0.4944
NC_005102.4-175783953	ATGCTGGTAACAGTCACTCCTGCTTCT	1	A	0.1301	0.4331
NC_005102.4-175783953	ATGCTGGTAACAGTCACTCCTGCTTCT	14	T	0	0.4793
NC_005102.4-175784178	GCATATCCTACACATTCTCCCATGCAC	25	C	0.125	0.1997
NC_005102.4-175793434	ATGAAGGTCACACTAGCTCCCAGTCAT	1	A	0.1933	0.327
NC_005102.4-175793434	ATGAAGGTCACACTAGCTCCCAGTCAT	24	T	0.0248	0.0245
NC_005102.4-175825758	CAGAAACAGACTTTAGCTCCCCACGCT	1	C	0	0.4819

NC_005102.4-175831531	CAGGTTAAAACAAGCTCTCCAACACAG	24	A	0.1849	0.4844
NC_005102.4-175854463	GCAGAGACAACTCCAGCTCCTGCAAGT	16	G	0.041	0.0557
NC_005102.4-175857187	TGAGAGCTAACATCTCCTCCTGTCCCT	2	G	0.2049	0.4852
NC_005102.4-175857187	TGAGAGCTAACATCTCCTCCTGTCCCT	5	A	0	0.4807
NC_005102.4-175878421	GTGCCCAGCACTAATGCTCCTACACAA	13	A	0.2358	0.4417
NC_005102.4-175878421	GTGCCCAGCACTAATGCTCCTACACAA	23	C	0.2195	0.2989
NC_005102.4-176101564	CTGTAAAGGACCCATACTCCATTCTCA	6	A	0	0.4826
NC_005102.4-176101564	CTGTAAAGGACCCATACTCCATTCTCA	25	T	0.084	0.0805
NC_005102.4-176106279	AGAGTTATGACTTAGGCTCCAAAGGAA	13	T	0.1008	0.1254
NC_005102.4-176107084	CCTCTGCCCACCAACCCTCCTGTCTTG	25	T	0	0.48
NC_005102.4-176113782	ATTGCATACACAGGAACTCCCTCCTTC	21	C	0	0.4832
NC_005102.4-176129361	GTCACAGTCACTCACGCTCCACAGCCA	3	C	0	0.4654
NC_005102.4-176129361	GTCACAGTCACTCACGCTCCACAGCCA	5	C	0.094	0.1347
NC_005102.4-176134628	CGAACTCACACCTCCACTCCCAGAAAG	1	C	0	0.4728
NC_005102.4-176138926	ACCACATCCACAGTGGCTCCCAACCAT	13	G	0.3089	0.4331
NC_005102.4-176138926	ACCACATCCACAGTGGCTCCCAACCAT	16	G	0.1057	0.1145
NC_005102.4-176148747	CAACAGTGGACTAAACCTCCCAAACTG	4	C	0.431	0.3478
NC_005102.4-176148747	CAACAGTGGACTAAACCTCCCAAACTG	16	C	0.431	0.3478
NC_005102.4-176204122	GAAAAGAAAACAGGAACTCCTAACAGC	12	A	0.0089	0.4933
NC_005102.4-176218423	GTTACCTTGACTGTCCCTCCCCTACCC	2	T	0	0.4841
NC_005102.4-176230635	CACCCTGCCACCTTCACTCCTGGCATG	3	C	0	0.48
NC_005102.4-176230635	CACCCTGCCACCTTCACTCCTGGCATG	7	G	0.0609	0.4903
NC_005102.4-176263131	TTTGAGCTCACAGATTCTCCCAAGATC	2	T	0	0.4755
NC_005102.4-176263131	TTTGAGCTCACAGATTCTCCCAAGATC	3	T	0	0.4755
NC_005102.4-176298284	GGAATGTAGACCATCACTCCCCTGCAG	6	G	0.0248	0.0245
NC_005102.4-176298284	GGAATGTAGACCATCACTCCCCTGCAG	14	T	0.2479	0.2975
NC_005102.4-176304442	TCTGTCATGACAATGGCTCCTACACAT	2	C	0.2149	0.3278
NC_005102.4-176304442	TCTGTCATGACAATGGCTCCTACACAT	5	T	0.2149	0.3278
NC_005102.4-176340553	AGTCATCTCACATGAGCTCCTGCCACC	27	C	0.1057	0.1562
NC_005102.4-176357949	TGTCAGAACACTCGGCCTCCCCTAAAG	13	C	0.1579	0.3706
NC_005102.4-176366502	AAATGGAGGACCCTTCCTCCCAAACAA	5	G	0.2583	0.4816
NC_005102.4-176379399	AGAGGATGAACACTGACTCCAGAGGAC	27	C	0.3162	0.3596
NC_005102.4-176414692	TAGTAGTCTACCACATCTCCTGACTCT	1	T	0.0082	0.4755
NC_005102.4-176414692	TAGTAGTCTACCACATCTCCTGACTCT	7	T	0	0.4751
NC_005102.4-176429274	CTCTCCTAGACCGTAACTCCAACCCTC	9	G	0.0083	0.4769
NC_005102.4-176429274	CTCTCCTAGACCGTAACTCCAACCCTC	13	G	0.025	0.0247
NC_005102.4-176448533	AAGTCTGATACTTTCTCTCCTACCATC	2	A	0.0354	0.0348
NC_005102.4-176448533	AAGTCTGATACTTTCTCTCCTACCATC	24	C	0.009	0.4766
NC_005102.4-176504471	CACGTGGGGACCTAACCTCCCAGGATG	13	T	0.0084	0.4859
NC_005102.4-176504471	CACGTGGGGACCTAACCTCCCAGGATG	27	G	0.2083	0.2941
NC_005102.4-176504494	GATGGTAGTACCATGTCTCCCAAGTCT	4	G	0.2155	0.302
NC_005102.4-176504494	GATGGTAGTACCATGTCTCCCAAGTCT	6	T	0	0.4734
NC_005102.4-176518995	ACAATGTGCACCTGCTCTCCTACTCTC	22	A	0	0.48
NC_005102.4-176549998	TGATGTGACACCTCTGCTCCTTATTAG	21	T	0	0.48
NC_005102.4-176616147	GAGCTTCTCACCTCAGCTCCGAAGTTC	21	G	0	0.4814
NC_005102.4-176627879	GAAGTCAAGACCATTTCTCCCCCAACA	6	C	0.124	0.4978
NC_005102.4-176703607	CCTGCCCGCACTGTTCCTCCAGATCCT	12	T	0.0169	0.4826
NC_005102.4-176704093	CCGATCTCTACCCCAGCTCCACACCCA	25	C	0.0909	0.4966
NC_005102.4-176737956	CAGTAAGAAACGGGCCCTCCTACCAAT	12	G	0.0244	0.0241
NC_005102.4-176737956	CAGTAAGAAACGGGCCCTCCTACCAAT	22	A	0.1639	0.2515
NC_005102.4-176748737	GGTCTCTACACCTAGACTCCCAGGATA	26	T	0	0.4793

NC_005102.4-176810059	AGAAGTGAGACCCTTTCTCCTTGCTCC	27	C	0.0081	0.4777
NC_005102.4-176824468	ATATGCCTCACCGCTACTCCCTTTCCT	14	C	0.0179	0.0351
NC_005102.4-176849348	ACCAGTGGTACACTGGCTCCGCTGTCT	21	G	0.0496	0.4628
NC_005102.4-176861300	TTGATTCTGACAGCCTCTCCTCATCAG	9	G	0.1789	0.1761
NC_005102.4-176876231	AAGGTATATACTGCCACTCCCTGGCCT	9	T	0.0089	0.4789
NC_005102.4-176915263	GGGGCTACCACAGAATCTCCTCCTCTC	23	C	0	0.4764
NC_005102.4-176926153	ATTGGCAGGACTTGTTCTCCGAGCCAA	7	A	0	0.4814
NC_005102.4-176949639	CAATGATAAACAGAAACTCCAGGCATG	5	G	0.0248	0.0716
NC_005102.4-176949639	CAATGATAAACAGAAACTCCAGGCATG	23	G	0	0.4819
NC_005102.4-176962352	TTCCCAACAACCAGCCCTCCACCAACA	7	A	0.0179	0.4592
NC_005102.4-176978906	TGTCTTTTAACCTGCCCTCCAACACAC	15	C	0.0082	0.479
NC_005102.4-176978906	TGTCTTTTAACCTGCCCTCCAACACAC	25	C	0.0325	0.032
NC_005102.4-176985450	TCCTAGTCTACCACATCTCCTTCCTGC	7	T	0	0.4868
NC_005102.4-177080414	CTGGCTGAGACCCAACCTCCTACTATC	14	A	0	0.4793
NC_005102.4-177080414	CTGGCTGAGACCCAACCTCCTACTATC	25	A	0	0.4793
NC_005102.4-177087173	AGTGGCTGCACAATATCTCCGAACCCC	21	G	0.0082	0.4822
NC_005102.4-177087173	AGTGGCTGCACAATATCTCCGAACCCC	23	A	0.0082	0.4822
NC_005102.4-177127160	TGTGAGTACACTGTTGCTCCCTTCAGA	9	C	0.036	0.0354
NC_005102.4-177200930	TCTCTGGGGACAGAAGCTCCCTGGTGA	22	T	0.0488	0.0476
NC_005102.4-177248444	CTAGGTTTTACACAACCTCCTCTAGCA	2	T	0.0517	0.0504
NC_005102.4-177248444	CTAGGTTTTACACAACCTCCTCTAGCA	13	C	0.1293	0.5
NC_005102.4-177276371	TACAGTCCTACCTCTACTCCAGACTCA	25	T	0.05	0.0488
NW_007906147.1-12821	TTGTTGACTACCCATGCTCCTTCACAG	13	C	0.0492	0.048
NW_007906147.1-12821	TTGTTGACTACCCATGCTCCTTCACAG	15	T	0.0328	0.0322
NC_005103.4-258551	CTGGTTGGGACTCCGTCTCCTCACTAT	5	T	0	0.4891
NC_005103.4-258551	CTGGTTGGGACTCCGTCTCCTCACTAT	6	T	0.0957	0.466
NC_005103.4-273043	CACACCAACACGCTGCCTCCATCATCC	14	T	0.2295	0.3947
NC_005103.4-273043	CACACCAACACGCTGCCTCCATCATCC	21	A	0.1557	0.1573
NC_005103.4-284648	CCCAGCCACACAGCCCCTCCTTTTCCC	14	C	0.0696	0.2268
NC_005103.4-284648	CCCAGCCACACAGCCCCTCCTTTTCCC	24	T	0.1217	0.1869
NC_005103.4-288398	GTAACAGTCACAAAGGCTCCCTGGTAG	27	G	0.1092	0.317
NC_005103.4-299984	AACGTCTTGACTCTACCTCCAAGGTGC	4	G	0	0.4793
NC_005103.4-316055	GTATGGGAGACCCGAACTCCTGCCCTT	21	T	0	0.4826
NC_005103.4-316055	GTATGGGAGACCCGAACTCCTGCCCTT	26	T	0	0.4807
NC_005103.4-320369	TTTGTAGGTACATACACTCCAGCACAC	2	T	0	0.4751
NC_005103.4-324777	CCCATCACTACCTAATCTCCTATCTCC	1	C	0.2137	0.289
NC_005103.4-334719	CCTGGCCACACCCTGCCTCCTAATCAA	22	A	0	0.4751
NC_005103.4-334719	CCTGGCCACACCCTGCCTCCTAATCAA	26	A	0.2645	0.4481
NC_005103.4-381306	CTACCTGCAACTCCACCTCCATGAGAC	21	A	0	0.4793
NC_005103.4-381306	CTACCTGCAACTCCACCTCCATGAGAC	22	T	0.0081	0.0241
NC_005103.4-389354	AAGGAAATGACCTACACTCCATCCCTG	24	C	0.0244	0.0241
NC_005103.4-392032	TCTGGTCTCACCCTACCTCCTAGAAGA	1	T	0	0.4833
NC_005103.4-407103	CAGAAGTTAACTCGGGCTCCGCTTTCC	14	G	0.2893	0.4004
NC_005103.4-419127	AGCTCCTACACAGAGCCTCCGTGAGGC	21	G	0.0776	0.2315
NC_005103.4-427439	AAGCCTGTCACCTCATCTCCTTGGAAA	4	C	0.084	0.4661
NC_005103.4-427439	AAGCCTGTCACCTCATCTCCTTGGAAA	15	A	0.0672	0.4957
NC_005103.4-485389	TCCTGTTTTACCCGGACTCCTATAGCA	9	T	0.2137	0.4671
NC_005103.4-485389	TCCTGTTTTACCCGGACTCCTATAGCA	13	C	0	0.4743
NC_005103.4-534891	CCTGGCCTCACCTTGTCTCCTACACGG	22	A	0	0.482
NC_005103.4-534891	CCTGGCCTCACCTTGTCTCCTACACGG	25	C	0	0.482
NC_005103.4-712600	CTAGATTACACCTTTCCTCCTTGCTTG	12	C	0.0917	0.1025

NC_005103.4-712600	CTAGATTACACCTTTCCTCCTTGCTTG	13	T	0.1901	0.3851
NC_005103.4-714897	TTCTTTAGGACTGGATCTCCTGCTGGA	15	A	0	0.4666
NC_005103.4-722897	ACTCTCCTTACCCTGCCTCCTCCCTTG	8	T	0	0.48
NC_005103.4-722897	ACTCTCCTTACCCTGCCTCCTCCCTTG	12	C	0	0.48
NC_005103.4-729493	GGATCCTGCACCAGCTCTCCTCGGCAT	23	G	0.1917	0.3872
NC_005103.4-756498	CATTCCAGAACACAGTCTCCAACTTTG	5	C	0.1186	0.1691
NC_005103.4-783142	CTTCCCACCACCGGATCTCCCTCTTCC	5	C	0.0252	0.0249
NC_005103.4-783142	CTTCCCACCACCGGATCTCCCTCTTCC	13	G	0.1186	0.334
NC_005103.4-815144	AGGGGTGAGACTAGCCCTCCTCCACTC	8	A	0	0.4793
NC_005103.4-828386	GAGCATGTGACAGAGTCTCCTGACATC	3	G	0	0.4807
NC_005103.4-919121	GGGGCTTTCACCTTTCCTCCCAGTGAA	9	C	0.1316	0.4529
NC_005103.4-919121	GGGGCTTTCACCTTTCCTCCCAGTGAA	27	A	0	0.4902
NC_005103.4-941731	CCCTTCATAACCTGTTCTCCAGCTCAC	15	T	0	0.48
NC_005103.4-977812	TGACTAGGTACCTGTCCTCCACTGTCA	22	C	0	0.4654
NC_005103.4-977812	TGACTAGGTACCTGTCCTCCACTGTCA	23	T	0.0259	0.0255
NC_005103.4-982346	CACAGTACAACTCCATCTCCAGGTAGT	26	G	0.0083	0.4783
NC_005103.4-993450	TTGTTCAACACAGTTCCTCCCGCGTTC	26	T	0.0084	0.4761
NC_005103.4-1061708	AGGTAAGTAACCCACACTCCTGTAAGT	8	T	0.025	0.0247
NC_005103.4-1061708	AGGTAAGTAACCCACACTCCTGTAAGT	22	G	0.025	0.0247
NC_005103.4-1075030	CTTCTCGTCACTTTCTCTCCTGGTGTT	6	C	0.1111	0.3596
NC_005103.4-1079672	AGTATCGTAACCTAACCTCCATAATTG	7	G	0	0.4856
NC_005103.4-1088750	TATGTCTGGACACAGTCTCCTGAGTAG	27	G	0.0488	0.0476
NC_005103.4-1100195	AATATGGGGACCTGAACTCCAATCCTT	24	C	0.1721	0.375
NC_005103.4-1108064	ACCCCACTCACCCTGTCTCCTGCTTTA	4	C	0.1488	0.4532
NC_005103.4-1108064	ACCCCACTCACCCTGTCTCCTGCTTTA	5	C	0.1148	0.3447
NC_005103.4-1114424	ACACACTATACATGTACTCCACATATA	22	C	0.1833	0.4265
NC_005103.4-1116964	TGAGGTAGGACAAGTACTCCATACCCA	27	A	0.0684	0.066
NC_005103.4-1140266	GACTCACACACTCAAACTCCTTTCCAC	27	C	0.1949	0.4655
NC_005103.4-1140399	ACTGCCCCCACACACCCTCCTTCTCAA	27	A	0	0.4813
NC_005103.4-1226665	TGTGGCTAGACTTTATCTCCAACGTAC	8	A	0	0.4793
NC_005103.4-1226665	TGTGGCTAGACTTTATCTCCAACGTAC	23	C	0	0.4793
NC_005103.4-1230102	TGCTATGGCACACACCCTCCCATCCCA	3	C	0	0.4743
NC_005103.4-1261467	AGTTCCCCAACAGTCTCTCCCAGATCC	8	C	0.2051	0.4868
NC_005103.4-1272578	CTGCCTCAGACCTGAACTCCTACCTCT	9	G	0.0488	0.0476
NC_005103.4-1287738	GTCCTCAGCACAAAGTCTCCACATATA	26	T	0	0.4793
NC_005103.4-1292715	TATGCTGCGACAATGGCTCCTGCCATC	12	A	0.0085	0.4823
NC_005103.4-1443859	TATGTGAAAACCCAACCTCCATAGGTT	16	C	0.1333	0.1665
NC_005103.4-1448080	CAGAACACAACACCATCTCCTTCACAG	26	A	0.2137	0.1908
NC_005103.4-1558269	GCATCTTCCACATTAACTCCAATGAGG	14	T	0.1008	0.3014
NC_005103.4-1576278	AGATGTCACACAATGGCTCCTTGGTAA	5	G	0.0325	0.0476
NC_005103.4-1842886	ACCAAGACAACATGATCTCCCTTTTCT	14	G	0.6016	0.4206
NC_005103.4-1842886	ACCAAGACAACATGATCTCCCTTTTCT	15	A	0.6016	0.4206
NC_005103.4-1859822	ATCCCCCTAACTCTTCCTCCCCCTTCC	16	C	0.0446	0.0436
NC_005103.4-1938523	ACATAGGTTACTCTCACTCCCTATTGA	25	T	0.0336	0.4779
NC_005103.4-1939129	AGCTGCTAAACTACATCTCCCTAGTGT	22	T	0	0.4814
NC_005103.4-1950464	CATATGGCTACTCATCCTCCTATCAAG	16	C	0.0678	0.0965
NC_005103.4-2094723	ATATCAAGTACCTGGACTCCTGGTTAT	8	G	0.0357	0.0689
NC_005103.4-2168239	CCAGTTGCTACTGTAGCTCCCAGTGTT	21	C	0.0082	0.479
NC_005103.4-2178707	TGTCCAGCTACTCAGACTCCCTGGACC	16	A	0	0.4793
NC_005103.4-2199881	CTGTCCTTGACCATCTCTCCTCTTCTT	3	G	0.0744	0.0868
NC_005103.4-2358491	GAACAGCACACTCTCTCTCCATTCTCC	7	C	0.0089	0.4751

NC_005103.4-2358491	GAACAGCACACTCTCTCCATTCTCC	8	A	0	0.477
NC_005103.4-2362235	TGCTCACTCACCATTGCTCCACCTCAG	21	A	0.1186	0.3529
NC_005103.4-2387777	GGACAAAGGACAGGAGCTCCAAATGGG	23	A	0	0.4778
NC_005103.4-2430490	CAGTAAGAGACCTTGTCTCCAAACATA	13	T	0	0.4751
NC_005103.4-2495915	TGCCTGACAACCTGAGCTCCATCCATG	1	T	0.5984	0.4193
NC_005103.4-2495915	TGCCTGACAACCTGAGCTCCATCCATG	26	T	0.2397	0.2109
NC_005103.4-2602367	CTCAACTCAACTGCCTCTCCCTACACT	14	C	0.5772	0.4106
NC_005103.4-2624938	TGTCCTGCCACGGCTTCTCCACTATAT	3	T	0.0833	0.1244
NC_005103.4-2624938	TGTCCTGCCACGGCTTCTCCACTATAT	4	C	0.0833	0.1244
NC_005103.4-2641901	ACCAACTGAACCATAGCTCCAGCCCCC	24	C	0.045	0.0778
NC_005103.4-2708191	TCCTCCAGTACCCTCCCTCCCATTAGT	5	C	0.0261	0.0752
NC_005103.4-2734986	TTTGTCAGTACTCCCACTCCAAATTGT	22	A	0.1228	0.4501
NC_005103.4-2750035	TACATTTAAACCCCTGCTCCACCATTT	4	A	0.0085	0.479
NC_005103.4-2751790	AAGGTGTACACCATCACTCCTAGCATC	3	G	0	0.4778
NC_005103.4-2786564	CCAAACATCACGTGCTCTCCATGGGTC	8	T	0.2705	0.3909
NC_005103.4-2786564	CCAAACATCACGTGCTCTCCATGGGTC	21	A	0	0.4807
NC_005103.4-2812591	TATGTGAGTACACTGTCTCCTCTCTTC	2	A	0.0246	0.0243
NC_005103.4-2818812	GCTGTCAGGACTATTGCTCCTATGTTA	1	G	0.0171	0.4772
NC_005103.4-2841467	ACAGGTGCCACAGCCTCTCCCACAGCA	9	C	0.1393	0.2218
NC_005103.4-2864707	TGGATCCGCACTGGGACTCCTCTCACT	24	C	0	0.4778
NC_005103.4-3286208	TCTTGTGTAACCAACTCTCCCCAATTA	2	C	0	0.4778
NC_005103.4-3286208	TCTTGTGTAACCAACTCTCCCCAATTA	3	T	0	0.4778
NC_005103.4-3316521	GAACTGAGGACAGGACCTCCGTTGAAG	4	C	0.0246	0.0243
NC_005103.4-3316521	GAACTGAGGACAGGACCTCCGTTGAAG	13	G	0.0246	0.0243
NC_005103.4-3367995	ATGTTAGCCACTTTCCCTCCTCTCCCA	7	G	0.3913	0.3252
NC_005103.4-3367995	ATGTTAGCCACTTTCCCTCCTCTCCCA	9	C	0.0603	0.4937
NC_005103.4-3640194	CACCACTGTACAGTCGCTCCTATAATC	16	G	0.1066	0.1154
NC_005103.4-3873655	TGGTGTCTGACTGACCCTCCTATCCTG	21	T	0.0354	0.0517
NC_005103.4-3968915	AGAGATGCCACCACCACTCCTGACAAA	27	A	0	0.4793
NC_005103.4-3980880	ATCTCAGGTACATGCGCTCCTCCTGTG	4	T	0	0.4755
NC_005103.4-4022574	AATGCCGGCACCTCCACTCCTACTGCC	7	G	0.2712	0.4908
NC_005103.4-4032307	CACTTGACCACCACTGCTCCATGGCTG	24	G	0.0244	0.0241
NC_005103.4-4032307	CACTTGACCACCACTGCTCCATGGCTG	27	G	0.1333	0.4861
NC_005103.4-4066632	CTTACTGCCACTCTCCCTCCCCCTTCT	2	T	0.0244	0.0398
NC_005103.4-4174361	GCGGGAACCACACATGCTCCCCCTATG	3	G	0.0175	0.1153
NC_005103.4-4174361	GCGGGAACCACACATGCTCCCCCTATG	25	A	0	0.4778
NC_005103.4-4187459	ACCACTGTCACAAGAGCTCCCACACTG	6	T	0.0081	0.0553
NC_005103.4-4187953	AGCTCCCACACCATCACTCCATTTCAC	9	C	0.1765	0.4186
NC_005103.4-4191592	ACAGGAGCTACCTCACCTCCTTATTTT	2	C	0.0603	0.1058
NC_005103.4-4191592	ACAGGAGCTACCTCACCTCCTTATTTT	3	A	0.1652	0.2458
NC_005103.4-4234289	CTACTTCCCACCCTCCCTCCTCAGTGC	25	T	0	0.4677
NC_005103.4-4243139	AACTAGGATACACTGTCTCCGACTTTG	16	T	0.0089	0.0264
NC_005103.4-4254293	CCAATCTCTACAAGGCCTCCAAAAGAG	12	A	0.0088	0.4843
NC_005103.4-4254293	CCAATCTCTACAAGGCCTCCAAAAGAG	13	A	0.0088	0.4843
NC_005103.4-4255190	AGACCTGGCACCCAGCCTCCAGCCTTG	26	T	0	0.4819
NC_005103.4-4255934	TCCCTTCACACTGGCTCTCCTGCCCTG	9	C	0.1475	0.1774
NC_005103.4-4268920	CCTGCTATCACATTTTCTCCATTGGGG	12	A	0.1475	0.3447
NC_005103.4-4268920	CCTGCTATCACATTTTCTCCATTGGGG	14	T	0	0.4787
NC_005103.4-4270190	GATATTCCCACGCCTGCTCCTAAAGGC	26	G	0.3063	0.4104
NC_005103.4-4270834	TCTCCTCTCACTGCCTCTCCTCTCCTC	1	T	0.1008	0.0958
NC_005103.4-4277390	CTATCCCAGACTGCTCCTCCTATGTCT	7	C	0.122	0.1425

NC_005103.4-4298529	CTGCCTGGGACAACTTCTCCTAGACTG	13	A	0.1441	0.319
NC_005103.4-4298529	CTGCCTGGGACAACTTCTCCTAGACTG	22	A	0.2051	0.4123
NC_005103.4-4354298	TCTCTAGCTACGACTTCTCCCAAGCTT	12	G	0.161	0.319
NC_005103.4-4401117	GTTAGCTCCACTTCCTCTCCTCACAGT	27	T	0.1441	0.5
NC_005103.4-4446214	AGTGGAGAAACTTCAGCTCCCAAATAT	23	A	0.0246	0.0243
NC_005103.4-4501423	TGAAATGTAACCCCAGCTCCAGAAGGG	6	T	0.0085	0.4776
NC_005103.4-4506497	TTTCAATACACACAGCCTCCTTGGGCT	26	C	0.0579	0.0716
NC_005103.4-4523822	CCAGCATGAACAAAGTCTCCAGCCCTG	7	T	0.0082	0.4755
NC_005103.4-4549774	CGCTGTTGTACCTTCTCTCCCAACCAC	2	G	0.1322	0.4972
NC_005103.4-4549774	CGCTGTTGTACCTTCTCTCCCAACCAC	12	C	0.042	0.0411
NC_005103.4-4583667	TATTTATCAACCATGGCTCCTTCTGCC	21	T	0.1034	0.4405
NC_005103.4-4586849	TACATCCTGACTGCAGCTCCCATCCCT	2	A	0.0579	0.1017
NC_005103.4-4591439	CTAACTTCCACCTGGCCTCCATCTGGT	8	C	0.2203	0.493
NC_005103.4-4591578	AACACCCACACACTGGCTCCAGCAACT	7	C	0.1545	0.1425
NC_005103.4-4591578	AACACCCACACACTGGCTCCAGCAACT	22	G	0.1626	0.4992
NC_005103.4-4636388	TGGAGAGGCACTGTGGCTCCATGGATC	4	A	0	0.4787
NC_005103.4-4637667	AGAGAGCTAACACTCCCTCCCTTGGTC	26	T	0	0.4793
NC_005103.4-4641770	AGCATGGCCACACCTACTCCAACACTG	16	A	0.2281	0.3324
NC_005103.4-4654245	CTGTCTCAGACAGCATCTCCAAAGCTT	12	A	0.2439	0.3427
NC_005103.4-4655993	AAAGCCACAACTTCTTCTCCTTCACAC	8	C	0.0244	0.0241
NC_005103.4-4655993	AAAGCCACAACTTCTTCTCCTTCACAC	26	A	0	0.4793
NC_005103.4-4665255	TGTGTGTGCACACATCCTCCAGCCCCA	24	C	0.0965	0.0918
NC_005103.4-4673770	GTCTTTTAAACTCAGGCTCCCATTCTC	4	T	0.2083	0.3347
NC_005103.4-4673770	GTCTTTTAAACTCAGGCTCCCATTCTC	14	A	0	0.4765
NC_005103.4-4674044	GCTGATTGTACACACACTCCTGATGCT	22	G	0.2143	0.3565
NC_005103.4-4700501	CCGACACTTACTAGCACTCCAGACTTT	2	C	0.2328	0.3525
NC_005103.4-4700501	CCGACACTTACTAGCACTCCAGACTTT	22	G	0.2155	0.343
NC_005103.4-4713636	CAAGAGGTCACAAAAGCTCCAGTTCCC	21	A	0.1525	0.2815
NC_005103.4-4715143	GAGAGGCAGACACCAGCTCCTTCCTGT	25	T	0.2439	0.3041
NC_005103.4-4717643	CTCTCACTGACTGTCACTCCAGGCTAT	3	C	0.1864	0.2926
NC_005103.4-4717643	CTCTCACTGACTGTCACTCCAGGCTAT	21	A	0.0924	0.1033
NC_005103.4-4814293	GTACTCCCTACTTCCTCTCCCTGACAT	5	T	0.2131	0.4515
NC_005103.4-4814293	GTACTCCCTACTTCCTCTCCCTGACAT	14	C	0.2066	0.3601
NC_005103.4-4835522	ATTGGACAGACTGGTTCTCCCAGCATA	25	A	0.0085	0.4738
NC_005103.4-4836403	TGAACACTAACCAGGTCTCCTACTTCC	12	C	0.1429	0.4682
NC_005103.4-4885780	GTAACACGGACACAGGCTCCCTGAGGA	8	G	0	0.4819
NC_005103.4-4910827	ACCACTGCCACCCCACCTCCTGCATAG	1	A	0.1707	0.2778
NC_005103.4-4910827	ACCACTGCCACCCCACCTCCTGCATAG	15	A	0.1382	0.2989
NC_005103.4-4937669	CCTAGTGGCACACATCCTCCAACAAGA	22	A	0.065	0.0928
NC_005103.4-4938300	ATCTTTCCTACCTCTGCTCCTGCTGAA	6	T	0.2397	0.3771
NC_005103.4-4938300	ATCTTTCCTACCTCTGCTCCTGCTGAA	16	G	0.1949	0.2526
NC_005103.4-4985379	AGAACGGAGACCCTTCCTCCTCCAGTA	1	A	0.177	0.3026
NC_005103.4-4985379	AGAACGGAGACCCTTCCTCCTCCAGTA	15	T	0	0.4755
NC_005103.4-5048052	TCACATGTAACTCTGTCTCCAAGGGAT	25	G	0.2353	0.4898
NC_005103.4-5048052	TCACATGTAACTCTGTCTCCAAGGGAT	26	A	0.2353	0.4898
NC_005103.4-5084248	CAGCACACAACTCTGCCTCCAGTGCCT	27	T	0.0982	0.1093
NC_005103.4-5137352	CGTATAATCACCTTCACTCCTTCAATC	2	G	0.0085	0.4714
NC_005103.4-5137352	CGTATAATCACCTTCACTCCTTCAATC	25	A	0.0085	0.4714
NC_005103.4-5157395	GACAGAAGAACTTAGTCTCCAATGTTG	15	G	0.1083	0.1172
NC_005103.4-5173140	GGTAATGAAACAAGTACTCCATGAGGA	16	A	0	0.4793
NC_005103.4-5173140	GGTAATGAAACAAGTACTCCATGAGGA	26	G	0.2458	0.4609

NC_005103.4-5175723	GACAAGAAAACAAAGTCTCCTTAGCAC	4	A	0.0179	0.477
NC_005103.4-5221372	TGAATCTTCACATCTCCTCCCAGGATC	26	T	0	0.4787
NC_005103.4-5261557	GAAAGGAAGACTTGATCTCCACCAAGC	1	G	0.1545	0.2203
NC_005103.4-5303430	CCAGAGACTACTGGCTCTCCATGGGTC	23	G	0.0268	0.0436
NC_005103.4-5331099	TTTAATGACACCAGTCCTCCATGAGGA	16	C	0	0.4793
NC_005103.4-5354459	AATGACCTGACATTGTCTCCAACAATA	12	A	0.0413	0.0562
NC_005103.4-5354459	AATGACCTGACATTGTCTCCAACAATA	27	A	0.0084	0.4859
NC_005103.4-5359633	CTCTTCATGACCCCACCTCCAGCAGCT	5	T	0	0.4787
NC_005103.4-5372234	TTTCTTCCCACAGTTCCTCCTCCATTT	13	G	0.123	0.1436
NC_005103.4-5457726	CAAGGGCTTACTAATACTCCACGGTGT	23	G	0.2759	0.4463
NC_005103.4-5460174	GAGGCAATCACAAACCCTCCCTGCTGT	6	A	0	0.4787
NC_005103.4-5463207	CCCTTATAAACTGGCCCTCCTCCTACC	7	T	0.0087	0.4782
NC_005103.4-5610376	ACATCTATCACATCATCTCCAAGTACC	24	T	0	0.477
NC_005103.4-5649547	TGAGGTTCAACGTGCTCTCCTACACAG	12	G	0.1639	0.316
NC_005103.4-5649547	TGAGGTTCAACGTGCTCTCCTACACAG	21	T	0.2377	0.2686
NC_005103.4-5687156	ATCCTCTCAACAGGAACTCCACAGCTG	27	G	0.0442	0.0433
NC_005103.4-5691059	TGTGGCTTCACCCAACCTCCTGTGGAT	12	C	0.2213	0.4435
NC_005103.4-5698186	GAGATATATACTGCTGCTCCTAAAGCA	25	G	0.0672	0.4661
NC_005103.4-5702646	CTTATGCAGACATTTTCTCCATCTGCA	27	A	0.042	0.0411
NC_005103.4-5706005	TCATTCCTAACAGCCGCTCCTACTTAG	16	G	0.0661	0.0639
NC_005103.4-5720401	ATGCCTGTAACCCTAACTCCAGGGGAT	3	G	0.1583	0.325
NC_005103.4-5724861	ATGTCTTCTACTACATCTCCACAGACA	1	A	0.2281	0.4846
NC_005103.4-5724861	ATGTCTTCTACTACATCTCCACAGACA	23	A	0.0174	0.4764
NC_005103.4-5739237	CCCATCAGGACCAGCTCTCCTGCTCTT	24	T	0.027	0.0267
NC_005103.4-5746669	GAGGCCGGAACAGAAGCTCCAGGGAAG	21	A	0.1593	0.1757
NC_005103.4-5770368	GGCTGCATAACAGGCCCTCCCTCACTC	8	T	0.0708	0.0683
NC_005103.4-5801265	GTCTGAGCCACAGGATCTCCAGGTCAG	7	G	0.0569	0.0553
NC_005103.4-5801265	GTCTGAGCCACAGGATCTCCAGGTCAG	12	A	0.1138	0.1629
NC_005103.4-5808603	CTGCTCCAAACTTGGTCTCCATATATA	22	T	0.0661	0.0943
NC_005103.4-5848790	GGCCCCCATACTTGGACTCCTACTTTA	8	A	0.1416	0.4342
NC_005103.4-5853773	TGTGTGCTCACTGAAACTCCGCTTATT	26	T	0	0.4765
NC_005103.4-5954946	AATAATAGCACATATGCTCCTTCCTCA	1	A	0	0.4826
NC_005103.4-5954946	AATAATAGCACATATGCTCCTTCCTCA	2	A	0	0.4826
NC_005103.4-5960993	CTTCCCCAAACAGTGCCTCCCGCCAAG	27	G	0.0083	0.4783
NC_005103.4-5963508	ACCTCTGTAACTCTAGCTCCAGGGAAT	16	G	0.1271	0.4991
NC_005103.4-5994220	GCTCCTCCAACAACACCTCCTAACAGT	1	G	0.339	0.2926
NC_005103.4-5994220	GCTCCTCCAACAACACCTCCTAACAGT	2	C	0.339	0.2926
NC_005103.4-6004724	GAGCCGATGACTAGGTCTCCACCCTGT	5	C	0	0.4854
NC_005103.4-6004724	GAGCCGATGACTAGGTCTCCACCCTGT	13	A	0.1351	0.2403
NC_005103.4-6005157	GGCCCTGTGACACATGCTCCAGTCATG	14	A	0.2773	0.3067
NC_005103.4-6005381	CTCTATGTGACACATGCTCCAGTCATG	5	A	0.6102	0.424
NC_005103.4-6011302	AGTGGTAGTACACACCCTCCCCAGCAC	5	G	0	0.4773
NC_005103.4-6011302	AGTGGTAGTACACACCCTCCCCAGCAC	24	G	0.1967	0.2399
NC_005103.4-6027404	ACAGCTGCAACATGGTCTCCCTCTGGG	5	C	0.0732	0.0705
NC_005103.4-6030821	CTGGAATTAACTCCAGCTCCACATACA	24	T	0.0164	0.0322
NC_005103.4-6096056	CTCACTCCAACATCTACTCCACCTATG	25	A	0.0083	0.4783
NC_005103.4-6137932	GGAACATCAACTCTCCCTCCCACCATT	1	G	0.0442	0.0433
NC_005103.4-6204648	CTGGCTGTAACAGCCTCTCCACTTACC	26	C	0.0177	0.4793
NC_005103.4-6302537	GGAGCCTTCACAGGACCTCCCGTGGTG	14	G	0.0252	0.0249
NC_005103.4-6349795	GCACCTGTAACTCAGGCTCCAGGGGAT	25	G	0.0085	0.4714
NC_005103.4-6491290	ACACTGGGCACTCACTCTCCTGACTGG	26	G	0.2167	0.1932

NC_005103.4-6491290	ACACTGGGCACTCACTCTCCTGACTGG	27	G	0.2167	0.1932
NC_005103.4-6498486	ATCCTAAAGACTCTCCCTCCATGTTGA	9	G	0.0265	0.0262
NC_005103.4-6498486	ATCCTAAAGACTCTCCCTCCATGTTGA	14	T	0.0169	0.4718
NC_005103.4-6535147	AGACCACAGACAGTGCCTCCATGGTCA	12	A	0	0.4724
NC_005103.4-6535643	TGCCTAGTAACTCCAGCTCCCGGGCAT	7	G	0	0.4793
NC_005103.4-6535643	TGCCTAGTAACTCCAGCTCCCGGGCAT	9	A	0.0263	0.026
NC_005103.4-6538526	CAGTTCCTCACAGGATCTCCTCAGCAA	6	C	0.3008	0.4301
NC_005103.4-6538526	CAGTTCCTCACAGGATCTCCTCAGCAA	25	C	0.2439	0.3141
NC_005103.4-6563197	CCCAGCAATACATCTTCTCCATAAAGT	9	T	0.5702	0.4353
NC_005103.4-6578610	AGAAGCCCAACGAAGCCTCCCTACAGG	7	C	0.0413	0.0405
NC_005103.4-6579612	CTGATCCCTACGCCTGCTCCGAGCTGG	27	G	0.193	0.2893
NC_005103.4-6583349	GCCACCCTCACTTGCTCTCCATCATTG	9	C	0.2667	0.455
NC_005103.4-6584891	AAGAGCCATACCTACCCTCCCACTGCA	6	C	0.1652	0.3353
NC_005103.4-6584891	AAGAGCCATACCTACCCTCCCACTGCA	14	A	0.1565	0.3593
NC_005103.4-6603358	AACCCATAGACCATTCCTCCTGCCTTA	21	T	0.1008	0.4971
NC_005103.4-6603358	AACCCATAGACCATTCCTCCTGCCTTA	22	G	0.1008	0.0958
NC_005103.4-6605965	CATCATGGCACAGATGCTCCAATCACC	7	G	0.2358	0.481
NC_005103.4-6605965	CATCATGGCACAGATGCTCCAATCACC	16	G	0.0163	0.4825
NC_005103.4-6639658	GTAACTAACACGCCATCTCCATTGCGG	5	C	0.0826	0.109
NC_005103.4-6639658	GTAACTAACACGCCATCTCCATTGCGG	26	G	0.0588	0.0728
NC_005103.4-6643682	AGTCTTAGGACTTGCTCTCCACGGGGC	23	G	0.0244	0.0241
NC_005103.4-6644174	AGGCGTGCCACCTCACCTCCTAGTGTT	5	G	0.252	0.4703
NC_005103.4-6644174	AGGCGTGCCACCTCACCTCCTAGTGTT	12	C	0.0248	0.0245
NC_005103.4-6687727	GATATTATGACAGGTCCTCCTCTGATA	24	G	0	0.4793
NC_005103.4-6697950	CCCAGAGACACACCCCCTCCTGTTATG	4	A	0.0261	0.0257
NC_005103.4-6697950	CCCAGAGACACACCCCCTCCTGTTATG	14	C	0.0175	0.0345
NC_005103.4-6732132	TTAACTGCTACATCACCTCCCGAGTCT	13	T	0.1491	0.1672
NC_005103.4-6732132	TTAACTGCTACATCACCTCCCGAGTCT	16	C	0.0526	0.4902
NC_005103.4-6763757	CTCCCCCCTACTCTGTCTCCCACTCAA	7	C	0	0.4909
NC_005103.4-6763757	CTCCCCCCTACTCTGTCTCCCACTCAA	13	C	0	0.4909
NC_005103.4-6776239	CACACCTCTACCTCCTCTCCTGATATG	9	T	0	0.4793
NC_005103.4-6777154	ACGGCCTTCACTTGTGCTCCAATGGGT	2	C	0	0.4793
NC_005103.4-6842376	TGCAAGGTGACTGTGGCTCCAGAAGCA	5	A	0.025	0.0408
NC_005103.4-6905065	AAAGCTGGGACGTGAGCTCCCTAAGTG	4	G	0.2586	0.3282
NC_005103.4-6905065	AAAGCTGGGACGTGAGCTCCCTAAGTG	12	G	0.1293	0.1786
NC_005103.4-6905222	AGTAATAGAACCCTTTCTCCCATCACA	3	T	0.2564	0.3813
NC_005103.4-6906832	TACCCTCTTACCTGACCTCCCAACCCC	14	G	0.0354	0.0517
NC_005103.4-6906832	TACCCTCTTACCTGACCTCCCAACCCC	24	C	0.0354	0.0517
NC_005103.4-6945137	GACCTGGACACTCAGGCTCCTAGAATC	9	C	0	0.4819
NC_005103.4-6962967	TCCGTCAGCACCGGGGCTCCTGCTACA	13	G	0.0325	0.032
NC_005103.4-6975478	CTGGCAGCCACCCTTCCTCCAAAACTG	24	A	0	0.4765
NC_005103.4-6975738	GACAGCTGGACACAACCTCCCTTTCAG	15	A	0.0085	0.4738
NC_005103.4-7017549	CTTGATTGGACCCAGTCTCCAGGCAGG	26	G	0.2035	0.1828
NC_005103.4-7080882	CCCATTTGAACCGTTTCTCCAGACTAA	12	C	0.0169	0.0333
NC_005103.4-7087264	TGAAGGGGTACACAGCCTCCTGCCCCT	7	G	0.1913	0.2005
NC_005103.4-7087264	TGAAGGGGTACACAGCCTCCTGCCCCT	15	G	0.181	0.2188
NC_005103.4-7097600	AGCTCGGGGACTCCTCCTCCTGAAGGT	6	G	0.2566	0.359
NC_005103.4-7097600	AGCTCGGGGACTCCTCCTCCTGAAGGT	8	G	0.1579	0.1744
NC_005103.4-7107352	GAGGCACACACAGCCCCTCCCTGCTAT	1	G	0.1736	0.2109
NC_005103.4-7107352	GAGGCACACACAGCCCCTCCCTGCTAT	2	A	0.1695	0.2344
NC_005103.4-7161887	AGCCGTTGTACAACTGCTCCTTTCCTG	22	T	0	0.4793

NC_005103.4-7173052	AGTAGGATCACTCGCGCTCCTAAACTG	14	G	0.0342	0.0336
NC_005103.4-7175929	GACGAGCCCACTTCGGCTCCACAGTTC	24	G	0	0.4765
NC_005103.4-7230086	CAGTCCCTGACCTCAGCTCCAGAGGCA	12	C	0.0342	0.0336
NC_005103.4-7240903	GGATCAGCAACAGCTGCTCCAGGTCAC	12	A	0.0082	0.479
NC_005103.4-7246135	GATCCCAGAACAGTGGCTCCTCTACAG	12	A	0	0.4764
NC_005103.4-7289851	GACTCAGGTACAGTGCCTCCAGCCTTG	8	G	0.0246	0.0557
NC_005103.4-7301054	TACTCAAAGACTCGGGCTCCTGCACTC	3	C	0.0086	0.4729
NC_005103.4-7304924	CCAGGACTTACTCCTCCTCCCTCTTCT	25	T	0	0.482
NC_005103.4-7314881	GGACCACCAACCTTTGCTCCATGTACC	13	T	0	0.4793
NC_005103.4-7314881	GGACCACCAACCTTTGCTCCATGTACC	26	C	0.0081	0.0241
NC_005103.4-7336482	CACACTTGCACACACACTCCTTTGGGA	12	A	0.0252	0.0249
NC_005103.4-7346530	GCATCAGACACCATGACTCCTGTAAGG	12	C	0	0.4755
NC_005103.4-7346530	GCATCAGACACCATGACTCCTGTAAGG	16	A	0	0.4755
NC_005103.4-7352722	ACTCTAAATACAGCCTCTCCCTTCCCT	4	C	0	0.4807
NC_005103.4-7352722	ACTCTAAATACAGCCTCTCCCTTCCCT	23	T	0.0248	0.4863
NC_005103.4-7378221	CCCCTCCTCACTGGCTCTCCTGCATTA	8	T	0	0.48
NC_005103.4-7407817	TGACAGCAGACAAGCTCTCCCATGCTC	2	G	0.0357	0.4986
NC_005103.4-7408482	CTCCTGCTTACCCTCACTCCTGCTGGC	26	G	0.0339	0.0496
NC_005103.4-7431929	CAGACCGCCACAAGTGCTCCTCCAGAT	7	G	0.1351	0.3635
NC_005103.4-7436923	AAGGACTGGACCACTCCTCCAGCCACA	13	A	0.1789	0.2499
NC_005103.4-7436923	AAGGACTGGACCACTCCTCCAGCCACA	15	T	0.1789	0.2499
NC_005103.4-7440869	CTGTCTCTCACTTACTCTCCTTCCTTC	2	T	0	0.4883
NC_005103.4-7467799	ACTTATTGAACTGCTCCTCCTACAGAC	6	T	0.0244	0.0398
NC_005103.4-7494522	TTTCTGCCCACCTTTACTCCAAACAAT	2	T	0	0.4773
NC_005103.4-7494522	TTTCTGCCCACCTTTACTCCAAACAAT	23	A	0	0.4773
NC_005103.4-7546046	CTCATATCAACCCTTACTCCCACTCAG	23	C	0.0417	0.0408
NC_005103.4-7567513	ACCCTTGACACACCTCCTCCTACATAG	14	C	0.2119	0.4336
NC_005103.4-7625624	TAAATACGAACACCCCCTCCCATAGGT	8	G	0.1966	0.3935
NC_005103.4-7625624	TAAATACGAACACCCCCTCCCATAGGT	24	A	0.0763	0.4896
NC_005103.4-7651098	TAAGAACAAACCCAAGCTCCAAGACAC	3	A	0.0268	0.4751
NC_005103.4-7709344	CTGAGTGTGACTGTCTCTCCTTCACTG	1	C	0.0976	0.0928
NC_005103.4-7739717	GGATAAGAAACCAATTCTCCCTGCTCC	8	A	0.2155	0.302
NC_005103.4-7739717	GGATAAGAAACCAATTCTCCCTGCTCC	23	G	0.0254	0.0415
NC_005103.4-7745235	CAGTACGTCACTAGGACTCCAGGCCCT	6	C	0.0569	0.1425
NC_005103.4-7760366	GCGTCTCAGACAGCAGCTCCTTACTCT	2	C	0	0.4751
NC_005103.4-7760366	GCGTCTCAGACAGCAGCTCCTTACTCT	3	G	0.0167	0.095
NC_005103.4-7783919	AATGTCTCAACTAGCTCTCCATTAGTT	21	A	0.0165	0.0325
NC_005103.4-7788146	TAAACAGAAACTGACCCTCCGCAGGAA	21	G	0.0738	0.1708
NC_005103.4-7842040	GTGAGAGCCACTCTTCCTCCATTGTGT	27	T	0.2705	0.3307
NC_005103.4-7869284	AGGCAACCAACCATTTCTCCAATACTG	27	G	0.2193	0.3473
NC_005103.4-7874075	TGCCACCGTACCTCAGCTCCTCGATCT	7	C	0.1301	0.496
NC_005103.4-7933995	TGAAGAGACACTGAAACTCCAGTCCTC	27	C	0.0268	0.0436
NC_005103.4-7942273	CCGGTCTCGACCTGCTCTCCTTTACCA	3	G	0.0248	0.0405
NC_005103.4-7996965	CATTCGGTAACTCCAGCTCCAAAGCAT	23	A	0	0.4902
NC_005103.4-7996965	CATTCGGTAACTCCAGCTCCAAAGCAT	27	T	0.0614	0.1075
NC_005103.4-8012566	CATGCTGACACTTATGCTCCCCCGGTG	23	C	0.0265	0.0262
NC_005103.4-8019190	AACTGTGTGACCCGCCCTCCTCCCCTC	15	C	0.0435	0.0425
NC_005103.4-8019190	AACTGTGTGACCCGCCCTCCTCCCCTC	27	C	0.1207	0.3662
NC_005103.4-8033075	ATTTATAGCACTTCCTCTCCTGTCTTC	8	G	0	0.4778
NC_005103.4-8061692	GGCAGTGACACAGTGGCTCCAGCCCGG	25	C	0.0678	0.0965
NC_005103.4-8061747	AGTGAGACCACATGGACTCCCTATCAT	22	T	0.0089	0.4751

NC_005103.4-8082775	AGTGATGTTACTTGGTCTCCCAGGGCT	1	A	0.1351	0.3817
NC_005103.4-8082775	AGTGATGTTACTTGGTCTCCCAGGGCT	26	C	0	0.4931
NC_005103.4-8100607	CAGTCATCCACATGGCCTCCTGTGTCA	6	A	0.0588	0.1033
NC_005103.4-8101276	TGGAGAGCCACATGGACTCCATTGGCA	1	T	0.15	0.4132
NC_005103.4-8101645	CAGACATCCACATGGTCTCCGGTGGCA	13	T	0.1695	0.1551
NC_005103.4-8101645	CAGACATCCACATGGTCTCCGGTGGCA	16	T	0.3419	0.3261
NC_005103.4-8143536	CCCAATCCCACTCATCCTCCATTTCCT	3	C	0.065	0.0629
NC_005103.4-8148043	GCACGTGCCACACGTTCTCCATTAGTT	14	G	0	0.4778
NC_005103.4-8148043	GCACGTGCCACACGTTCTCCATTAGTT	23	T	0	0.4778
NC_005103.4-8163171	TTCACGTGCACAGCGTCTCCTGCGGCC	23	C	0.1622	0.386
NC_005103.4-8171640	TTATGGCCCACTTCAACTCCGGAGAAC	21	G	0.0973	0.4994
NC_005103.4-8235417	TCATGTCTAACTCCTGCTCCAAAGCTT	15	T	0.0168	0.4779
NC_005103.4-8235417	TCATGTCTAACTCCTGCTCCAAAGCTT	26	T	0.0168	0.4779
NC_005103.4-8267380	GAAATAAGTACACCCTCTCCACGCAGT	23	G	0	0.488
NC_005103.4-8297003	TAGCCAAGCACCGCCTCTCCAGCTCAT	13	G	0.0252	0.0249
NC_005103.4-8330437	TTCTGTGCCACCTTTCCTCCAACCCCC	12	C	0.0082	0.4822
NC_005103.4-8429665	ACTGTGCCCACTAACACTCCCTCATGG	5	T	0.0175	0.4902
NC_005103.4-8429665	ACTGTGCCCACTAACACTCCCTCATGG	6	G	0.1207	0.2622
NC_005103.4-8435968	AGTTAGAAAACAATTCCTCCAACAAGA	23	C	0.0246	0.0243
NC_005103.4-8439714	GGCTGCAGCACTTGCTCTCCCCTCTGC	26	G	0.1842	0.235
NC_005103.4-8521791	TACGGTATCACATGGTCTCCAATCTCC	3	C	0.1441	0.2156
NC_005103.4-8521791	TACGGTATCACATGGTCTCCAATCTCC	12	A	0.1261	0.4994
NC_005103.4-8554684	CTTAAGACAACAGAGGCTCCTTGAAGT	27	T	0.0738	0.1009
NC_005103.4-8576052	TTGTGCATGACCTGATCTCCCACAGGT	21	C	0.2982	0.3615
NC_005103.4-8576052	TTGTGCATGACCTGATCTCCCACAGGT	23	C	0	0.4814
NC_005103.4-8622242	ATGACTCTGACAGGTTCTCCTTCTCCC	1	A	0	0.4793
NC_005103.4-8622242	ATGACTCTGACAGGTTCTCCTTCTCCC	4	A	0	0.4793
NC_005103.4-8818871	TTGTAACGTACATCATCTCCTTGACAA	8	G	0.0244	0.484
NC_005103.4-8892866	GTGTTCCAAACTGCCACTCCCAATGTT	9	A	0.0336	0.033
NC_005103.4-8967152	GATGGAGAAACCTCAGCTCCCTGGAGA	23	G	0.113	0.5
NC_005103.4-9021925	GGATCTTTCACTATCTCTCCATCTCTC	1	G	0.1368	0.1274
NC_005103.4-9026672	TCCAACAGCACAGACCCTCCCCCAATA	26	T	0	0.48
NC_005103.4-9027531	TCCAGGAACACATGTCCTCCAACGTTG	3	C	0.0435	0.0425
NC_005103.4-9027531	TCCAGGAACACATGTCCTCCAACGTTG	23	C	0.0435	0.0425
NC_005103.4-9075234	ATCCAAACCACATAGCCTCCCCCCAAA	15	G	0.0354	0.0348
NC_005103.4-9101551	GGCACGTAGACAGCACCTCCTGCCAAA	5	C	0.0087	0.4817
NC_005103.4-9101551	GGCACGTAGACAGCACCTCCTGCCAAA	6	G	0.0256	0.0253
NC_005103.4-9140565	AAATTCATGACATCTCCTCCTTGTGTG	6	C	0.2314	0.3467
NC_005103.4-9186584	TTCGTCTGCACGGCTGCTCCCAGATTA	4	G	0	0.4779
NC_005103.4-9187323	GTTTAATGAACTTGGCCTCCATAGGCT	12	T	0.0252	0.0249
NC_005103.4-9211437	CAGTGTGGTACATGAACTCCTATTGTT	26	T	0	0.4813
NC_005103.4-9224720	AGTGTCCACACCATTGCTCCTCAGCAC	26	A	0.0333	0.48
NC_005103.4-9317051	TCCTTCCACACCTCCCCTCCATCCAGG	27	G	0.1322	0.1377
NC_005103.4-9339931	CGAGATGATACTGTGTCTCCATCATGA	16	T	0	0.4704
NC_005103.4-9367795	TTTTGCAATACATTGTCTCCTGTATCT	23	T	0.0862	0.0825
NC_005103.4-9413399	TGTCCTGAGACCCCTCCTCCATTTATT	6	T	0	0.4755
NC_005103.4-9414899	TCTTTCTTCACCTGCACTCCCACCAGA	3	T	0.1786	0.3367
NC_005103.4-9438348	GACCAAGTGACTGTAACTCCCTGATAC	13	G	0.0325	0.032
NC_005103.4-9473942	TGACGTCTCACTGTACCTCCTCCCTAA	24	C	0.122	0.1826
NC_005103.4-9494780	TAAAATACTACAGACCCTCCCAAAGCT	8	C	0.094	0.1773
NC_005103.4-9494780	TAAAATACTACAGACCCTCCCAAAGCT	22	A	0	0.4772

NC_005103.4-9521086	GATCCTGTGACCCTTGCTCCCTAGAGT	13	C	0	0.4793
NC_005103.4-9694191	CAACTGACCACACTGCCTCCACAGCTG	3	A	0.0625	0.5
NC_005103.4-9704299	AGATACAGGACTTCAACTCCCATAAAG	25	A	0.0179	0.4641
NC_005103.4-9716783	ATTGCTTTCACCCAGACTCCATGACTT	5	C	0	0.4912
NC_005103.4-9716783	ATTGCTTTCACCCAGACTCCATGACTT	9	C	0.0088	0.4923
NC_005103.4-9726353	ACAGTCTTCACATGGTCTCCTACTGAT	4	G	0.2241	0.462
NC_005103.4-9758810	ACCTGCCTGACCTGCTCTCCTTTTGCA	4	T	0.025	0.0247
NC_005103.4-9758810	ACCTGCCTGACCTGCTCTCCTTTTGCA	14	G	0.1	0.1387
NC_005103.4-9773344	ACCAAGTGTACCCCACCTCCCCACACT	12	C	0.0244	0.0241
NC_005103.4-9802643	CTACTAATGACAGGATCTCCTTCCTTT	5	T	0.2295	0.3259
NC_005103.4-9802643	CTACTAATGACAGGATCTCCTTCCTTT	23	C	0.0569	0.0705
NC_005103.4-9819619	GCTTTAAGCACAACCCCTCCTTGGATG	2	C	0.1008	0.2076
NC_005103.4-9842813	GAGTGTGAGACAGTGACTCCTCACAAT	4	T	0.0339	0.493
NC_005103.4-9842813	GAGTGTGAGACAGTGACTCCTCACAAT	5	G	0	0.0333
NC_005103.4-9868673	CTGCCTGCAACTCCAGCTCCGTGGCTT	5	C	0.0244	0.484
NC_005103.4-9868673	CTGCCTGCAACTCCAGCTCCGTGGCTT	6	T	0.0244	0.484
NC_005103.4-9909399	CACACAGACACTGCAGCTCCCCGCAGC	21	C	0.1967	0.2157
NC_005103.4-9909399	CACACAGACACTGCAGCTCCCCGCAGC	23	G	0.1557	0.1573
NC_005103.4-9922139	GTTAAACAGACACCTCCTCCATTTGTC	25	G	0.2193	0.222
NC_005103.4-9929890	AGGTGCAGGACAGGCCCTCCTGAGTAC	5	G	0.1803	0.263
NC_005103.4-10023322	CTATGCTGCACAATTCCTCCCCCTTTC	13	A	0.157	0.2474
NC_005103.4-10023322	CTATGCTGCACAATTCCTCCCCCTTTC	23	C	0.0496	0.0484
NC_005103.4-10039770	GGGAAATTTACCACCCCTCCCCCAACA	7	T	0	0.4654
NC_005103.4-10039770	GGGAAATTTACCACCCCTCCCCCAACA	9	T	0.0175	0.4606
NC_005103.4-10048659	GAAGAGGGCACAGAATCTCCTGGAACT	9	C	0.3417	0.2833
NC_005103.4-10048659	GAAGAGGGCACAGAATCTCCTGGAACT	13	G	0.3417	0.2833
NC_005103.4-10093959	TGCGTTCTAACACTCTCTCCCCTACTT	8	T	0.157	0.1853
NC_005103.4-10093959	TGCGTTCTAACACTCTCTCCCCTACTT	22	C	0.1488	0.1787
NC_005103.4-10118507	CACTGGTCTACCGTGACTCCTGTAGGC	6	G	0.2069	0.4927
NC_005103.4-10118507	CACTGGTCTACCGTGACTCCTGTAGGC	12	C	0.1897	0.3282
NC_005103.4-10137442	GGGAGACAGACATTTTCTCCCTACTTC	3	G	0	0.477
NC_005103.4-10137442	GGGAGACAGACATTTTCTCCCTACTTC	7	C	0	0.477
NC_005103.4-10173326	TCCAGAGGCACTGACCCTCCTGTCTTC	2	C	0.1707	0.2203
NC_005103.4-10185019	CTTCTTTCAACCTTCTCTCCCGCCCAT	15	C	0.5294	0.3893
NC_005103.4-10185019	CTTCTTTCAACCTTCTCTCCCGCCCAT	22	G	0.55	0.4444
NC_005103.4-10233747	AACCACTGAACCATCTCTCCTGACCCA	16	T	0	0.4793
NC_005103.4-10241607	GCAGCTTCTACAATGGCTCCCACTATT	25	A	0	0.4746
NC_005103.4-10281248	CAACCGTCCACCATCTCTCCTTTCCTC	6	G	0.0579	0.0716
NC_005103.4-10375233	TGATTAAGAACACCAGCTCCTCTTCAG	26	A	0.1789	0.3239
NC_005103.4-10436307	GAAAAATCCACCCCTGCTCCCCAAAAG	16	G	0.2269	0.3812
NC_005103.4-10475666	ATCCCAACAACCCTCTCTCCATGTTCT	26	C	0.0431	0.0422
NC_005103.4-10476588	GCACTTAAAACACGGACTCCTCTCATA	1	G	0.1593	0.4968
NC_005103.4-10476588	GCACTTAAAACACGGACTCCTCTCATA	15	G	0.1478	0.18
NC_005103.4-10485285	TGGGCGACTACACATGCTCCCCCATAT	1	T	0.2222	0.4386
NC_005103.4-10505381	TGTGGTCTGACGATGTCTCCCAAGTTC	26	T	0.0862	0.5
NC_005103.4-10546704	ATTATGTAGACTCGGTCTCCATCGTTC	24	G	0.1148	0.1225
NC_005103.4-10585406	TGCCGTGATACTCCCTCTCCATGCTGT	5	G	0.0492	0.4656
NC_005103.4-10657870	GACCATAGCACTAGTCCTCCTTGGAAT	4	C	0.1186	0.2466
NC_005103.4-10657870	GACCATAGCACTAGTCCTCCTTGGAAT	14	G	0	0.4839
NC_005103.4-10680089	GGGATTAAGACACCCTCTCCTGTGCAT	27	T	0.009	0.4869
NC_005103.4-10696061	GGTAAGTACACAGAACCTCCCCAACAC	25	C	0	0.4813

NC_005103.4-10756334	CACTGATAGACCTGCGCTCCTGGTGTC	9	G	0.1518	0.1403
NC_005103.4-10756334	CACTGATAGACCTGCGCTCCTGGTGTC	15	C	0.1327	0.2856
NC_005103.4-10772067	GCATTGGTGACTGACGCTCCCTCTGTG	7	G	0	0.4864
NC_005103.4-10808319	ACACGGAGAACCAAAGCTCCTCAGACC	2	C	0.0973	0.1239
NC_005103.4-10808319	ACACGGAGAACCAAAGCTCCTCAGACC	27	C	0.018	0.4704
NC_005103.4-10871920	GCCCTCAGTACAGCGTCTCCCTTCCCA	26	C	0	0.4793
NC_005103.4-10871920	GCCCTCAGTACAGCGTCTCCCTTCCCA	27	A	0	0.4793
NC_005103.4-10909162	CCATCCGTAACTCTAGCTCCCGGGGAT	21	C	0.2783	0.4301
NC_005103.4-10987640	GTCTCAAGGACAGCATCTCCTGCTTCT	13	G	0.0254	0.0415
NC_005103.4-11118914	TAAAACGACACTGTCTCTCCTCCACAT	13	G	0	0.4819
NC_005103.4-11118914	TAAAACGACACTGTCTCTCCTCCACAT	25	C	0	0.4819
NC_005103.4-11132189	TCTTTCTCAACCTCCACTCCATTTTAT	4	T	0	0.4859
NC_005103.4-11132189	TCTTTCTCAACCTCCACTCCATTTTAT	7	T	0	0.4859
NC_005103.4-11152601	ACATATCTAACACAGACTCCTTAGCAT	6	T	0.1818	0.2647
NC_005103.4-11152601	ACATATCTAACACAGACTCCTTAGCAT	9	A	0.2605	0.397
NC_005103.4-11303310	CCCATGCTTACAAGTACTCCTACACAT	6	G	0.1583	0.1597
NC_005103.4-11303310	CCCATGCTTACAAGTACTCCTACACAT	27	T	0.0083	0.4747
NC_005103.4-11332206	CTCCCATGAACAATAGCTCCTGGGTCT	1	C	0.2314	0.2869
NC_005103.4-11332206	CTCCCATGAACAATAGCTCCTGGGTCT	16	G	0.2869	0.4634
NC_005103.4-11351858	CTCCCAACCACAAGTGCTCCATTTCAT	4	C	0.0598	0.058
NC_005103.4-11367340	CAGTCAACAACAGACCCTCCATCTTCA	3	G	0.0171	0.4693
NC_005103.4-11373236	AATTATGGCACCAACACTCCCTGTATA	3	T	0	0.4827
NC_005103.4-11585937	TACGTTACCACAGAGCCTCCTTAGCCC	4	G	0.2054	0.2512
NC_005103.4-11610304	CAGCAGATCACCCGTTCTCCTGATACT	8	T	0.0252	0.4829
NC_005103.4-11610304	CAGCAGATCACCCGTTCTCCTGATACT	24	T	0.075	0.0875
NC_005103.4-11652504	GCCACAAGGACACCTGCTCCACTATGT	12	A	0.0783	0.0911
NC_005103.4-11756201	ACTCATAGCACTCCAACTCCAGAAATA	9	C	0.1639	0.4988
NC_005103.4-11760260	TATTTACACACTCTTCCTCCCATCAAC	22	A	0.0984	0.1641
NC_005103.4-11760260	TATTTACACACTCTTCCTCCCATCAAC	27	C	0.0164	0.048
NC_005103.4-11766857	CTGAAAAGTACACAGTCTCCTCCATAA	14	A	0.0325	0.032
NC_005103.4-11854329	TTAAGTGCTACATCCTCTCCATTTGGG	5	G	0.0756	0.1327
NC_005103.4-11854329	TTAAGTGCTACATCCTCTCCATTTGGG	22	T	0.0924	0.1181
NC_005103.4-11858392	TTCCTACTGACTAGTGCTCCCGATACA	26	C	0.0331	0.0325
NC_005103.4-11919008	GTAAGCCCGACTCTCACTCCGTGTTTC	3	A	0	0.4891
NC_005103.4-11919008	GTAAGCCCGACTCTCACTCCGTGTTTC	21	G	0.1293	0.1646
NC_005103.4-11947356	GCAAGGGAAACACCCCCTCCACTGCTG	13	C	0.0901	0.1182
NC_005103.4-11966779	TCCCCAAGTACCTTGTCTCCAGCTAAG	25	A	0.0328	0.0634
NC_005103.4-12052807	ATACTCCACACTGATACTCCATTGATA	2	T	0	0.4765
NC_005103.4-12052807	ATACTCCACACTGATACTCCATTGATA	13	G	0.0744	0.0716
NC_005103.4-12088976	CACTGTTTCACACTTACTCCCACACTT	2	A	0.1885	0.4903
NC_005103.4-12092952	AAAGGGTTTACAGAATCTCCCTCCATG	24	C	0.0513	0.4807
NC_005103.4-12092952	AAAGGGTTTACAGAATCTCCCTCCATG	25	A	0.0513	0.4807
NC_005103.4-12104042	CTTCTTAAGACTCTGTCTCCTTTCCCA	1	C	0.0179	0.477
NC_005103.4-12104042	CTTCTTAAGACTCTGTCTCCTTTCCCA	22	T	0.1964	0.2575
NC_005103.4-12158899	GAGACTTTGACACTCTCTCCTGCAGCA	21	T	0.1057	0.1145
NC_005103.4-12158899	GAGACTTTGACACTCTCTCCTGCAGCA	23	C	0	0.4773
NC_005103.4-12227047	AGTACATCTACTCATTCTCCTTTATAG	5	C	0.2119	0.2644
NC_005103.4-12227047	AGTACATCTACTCATTCTCCTTTATAG	13	C	0.2051	0.2604
NC_005103.4-12234451	TCTGCCCTTACACCATCTCCTGTTTTG	14	C	0.0976	0.2017
NC_005103.4-12261179	CTGCAACTTACTGCCTCTCCTAAACAT	4	C	0.0744	0.1017
NC_005103.4-12261179	CTGCAACTTACTGCCTCTCCTAAACAT	24	A	0.1074	0.1017

NC_005103.4-12300151	TCACTAACAACTGAAACTCCCACTGCT	5	T	0.1043	0.1443
NC_005103.4-12300151	TCACTAACAACTGAAACTCCCACTGCT	7	A	0.1391	0.1588
NC_005103.4-12309694	ATCCCCCTTACTGCCTCTCCATAGTCC	21	A	0.0439	0.0595
NC_005103.4-12349693	ACAATCCCTACTTTTGCTCCTCGAATG	15	T	0.0083	0.4816
NC_005103.4-12349693	ACAATCCCTACTTTTGCTCCTCGAATG	23	G	0.0084	0.4829
NC_005103.4-12359779	TCTTAGCTAACAATAACTCCCAAACAC	27	C	0.0089	0.4856
NC_005103.4-12384145	ACTAAGGTTACAGTTTCTCCTGGGGCT	16	T	0	0.4856
NC_005103.4-12384145	ACTAAGGTTACAGTTTCTCCTGGGGCT	25	G	0.1176	0.1539
NC_005103.4-12439256	AGCCAGACAACAGTGTCTCCAGTGAAA	23	T	0.0932	0.1337
NC_005103.4-12471252	ACACTACAAACTAGCTCTCCCAGCTCC	3	A	0.2397	0.2922
NC_005103.4-12471828	CCCCGTCCCACTATTGCTCCGAACACA	16	G	0.0256	0.0253
NC_005103.4-12609863	ACAATACCAACCAGAGCTCCCAGAGAC	9	A	0.0427	0.0418
NC_005103.4-12615811	GTGAAATATACAGTCTCTCCCATAGAT	7	T	0.0708	0.4624
NC_005103.4-12626255	ACCCTGCACACCTCACCTCCATGTTCT	12	C	0.0252	0.0249
NC_005103.4-12626255	ACCCTGCACACCTCACCTCCATGTTCT	21	A	0.1322	0.2647
NC_005103.4-12724582	CTCAACCCAACCAGGCCTCCATACTCC	24	C	0	0.4912
NC_005103.4-12724582	CTCAACCCAACCAGGCCTCCATACTCC	26	C	0.1404	0.3793
NC_005103.4-12789757	CCATCTCAAACATTCACTCCTACCTCT	5	C	0.0417	0.0408
NC_005103.4-12888035	GCCAAGGACACCCTCTCTCCACTTTCT	9	C	0.2069	0.3572
NC_005103.4-12894788	CCCCTGCCCACCACCCCTCCACACACA	12	C	0.1026	0.4982
NC_005103.4-12894788	CCCCTGCCCACCACCCCTCCACACACA	26	C	0.1074	0.1447
NC_005103.4-12897862	CTGATGTCTACAAGTCCTCCTGATTCC	7	T	0.0721	0.4854
NC_005103.4-12897862	CTGATGTCTACAAGTCCTCCTGATTCC	15	T	0.0721	0.4854
NC_005103.4-13176826	TTGACTTCCACCCTGGCTCCTCCTTCC	9	C	0.2735	0.4193
NC_005103.4-13189145	TTCCCTCTCACCCTCTCTCCCTATACC	1	T	0	0.4779
NC_005103.4-13240401	TGTTTGAACACACAAACTCCAGAGGAT	24	G	0.0261	0.0257
NC_005103.4-13290913	TTAGCACATACTAACACTCCCAATGAT	21	C	0	0.4765
NC_005103.4-13393394	AATGCTTCTACTACCACTCCACCAACA	7	T	0	0.4773
NC_005103.4-13435972	CAAAGCCACACACCTACTCCAACTAGG	7	C	0.0263	0.026
NC_005103.4-13442760	CCCCGCTTAACGTCTCCTCCCTCATCA	5	G	0	0.4793
NC_005103.4-13442760	CCCCGCTTAACGTCTCCTCCCTCATCA	12	G	0.2602	0.427
NC_005103.4-13443512	AGAATTTGTACAATCTCTCCCATTCTA	4	A	0.1518	0.2876
NC_005103.4-13445069	ACGTGGTACACACGGCCTCCTCCTGTC	23	C	0.1982	0.3944
NC_005103.4-13445069	ACGTGGTACACACGGCCTCCTCCTGTC	24	T	0.1982	0.3944
NC_005103.4-13447102	CATCTAGAGACCCAGGCTCCTGCCACC	5	T	0.2881	0.4585
NC_005103.4-13473292	CAACTTCCAACTGAATCTCCCTGCCCT	21	C	0.0342	0.0336
NC_005103.4-13477916	TGCTGGGTGACAGAGCCTCCTTAAGGT	2	G	0.045	0.044
NC_005103.4-13480150	GCAACAGCGACGTCTCCTCCATGTGTT	4	A	0.2195	0.319
NC_005103.4-13480150	GCAACAGCGACGTCTCCTCCATGTGTT	9	G	0.1138	0.1073
NC_005103.4-13510640	TGGCTTCTCACCCTCTCTCCTGTCTCT	21	T	0.2735	0.2361
NC_005103.4-13552817	TCCTTGTCTACTCTGACTCCTTCATTG	27	G	0.1475	0.4914
NC_005103.4-13794453	GTTGAAGGCACCCAGGCTCCAGTGTTT	15	G	0.2049	0.4755
NC_005103.4-13794453	GTTGAAGGCACCCAGGCTCCAGTGTTT	23	T	0.1803	0.2741
NC_005103.4-13801933	CAAACCAGAACCCCTCCTCCCCCTCTG	4	A	0	0.4854
NC_005103.4-13804453	TATGCAGTTACAGCTGCTCCTCTGTCC	4	G	0.0804	0.1403
NC_005103.4-13804453	TATGCAGTTACAGCTGCTCCTCTGTCC	22	C	0.0265	0.0262
NC_005103.4-13811863	CTACTTCTTACTGACTCTCCTCTGACT	1	C	0.1102	0.1481
NC_005103.4-13811863	CTACTTCTTACTGACTCTCCTCTGACT	23	T	0	0.4786
NC_005103.4-13922219	CTATAGAAGACTAATTCTCCCTCTCTC	5	A	0.5862	0.4345
NC_005103.4-14003229	GATAGCAAAACAACTTCTCCTGGGAAG	14	C	0.0087	0.466
NC_005103.4-14421384	GTGCTTAGCACTGCAACTCCTGGTCTG	14	C	0.1707	0.2668

NC_005103.4-14427542	AGAAGACCCACCAGAGCTCCCAGGGAC	5	G	0.0244	0.0241
NC_005103.4-14427542	AGAAGACCCACCAGAGCTCCCAGGGAC	24	G	0.2195	0.1954
NC_005103.4-14434207	TCAGAATAAACCCTTGCTCCTTTTGTT	7	T	0.2174	0.4704
NC_005103.4-14434207	TCAGAATAAACCCTTGCTCCTTTTGTT	22	T	0.0259	0.4729
NC_005103.4-14515020	ATAGCTGCAACCAAGGCTCCTCGGGAG	22	C	0.0244	0.484
NC_005103.4-14515020	ATAGCTGCAACCAAGGCTCCTCGGGAG	24	G	0.0244	0.0241
NC_005103.4-14620111	CAATTGAAAACAATGACTCCTCTTCCC	4	T	0.3036	0.4871
NC_005103.4-14620111	CAATTGAAAACAATGACTCCTCTTCCC	27	C	0.3036	0.3468
NC_005103.4-14627080	TCTCTCCCTACTGGAACTCCAACATCT	27	T	0	0.4887
NC_005103.4-14712697	CCGTAGAATACCAGGACTCCAAATGCT	12	C	0.0256	0.0253
NC_005103.4-14786486	CCTGATCACACCTACACTCCTTTATGC	25	T	0.0513	0.0973
NC_005103.4-14874058	TGAGGCACCACTTTCCCTCCAGTAACT	22	G	0	0.4807
NC_005103.4-14994317	GTGCCAGGCACTTGCTCTCCATACCCG	27	G	0.2276	0.4331
NC_005103.4-14997718	ACTCATTTTACATTTCCTCCTGTGATT	9	T	0.0172	0.4666
NC_005103.4-15110376	AGTGTAGCTACTTCTGCTCCTTTAAAT	12	T	0	0.4807
NC_005103.4-15110376	AGTGTAGCTACTTCTGCTCCTTTAAAT	25	A	0	0.4793
NC_005103.4-15156355	TTCTGCACCACCTCACCTCCAACTCCT	5	G	0.0082	0.479
NC_005103.4-15157285	ATATTGAAGACATACACTCCTCCCCTC	3	A	0.009	0.4766
NC_005103.4-15396467	AGGATTCCCACAAGAACTCCATACTAT	3	G	0.0504	0.0491
NC_005103.4-15450007	AACCAATAAACCATCTCTCCTGCCCTC	6	A	0	0.4856
NC_005103.4-15468970	ATCAACAGGACTTGTGCTCCTTGGACC	1	A	0	0.4813
NC_005103.4-15495222	AGGATGAGTACCCAGGCTCCAGACACA	12	C	0.124	0.4889
NC_005103.4-15573660	ACAGTAAGCACAGGATCTCCTACCTCC	4	G	0.1789	0.2937
NC_005103.4-15573660	ACAGTAAGCACAGGATCTCCTACCTCC	27	C	0.0407	0.0398
NC_005103.4-15604734	GTCTTTCTAACACTGACTCCACACCAC	2	T	0.2195	0.4572
NC_005103.4-15655163	TCCCCATCCACTGCCTCTCCTCTTACA	1	T	0.0526	0.0677
NC_005103.4-15743965	TCGAATGCTACAGTGTCTCCCTATAAG	1	T	0	0.4751
NC_005103.4-15743965	TCGAATGCTACAGTGTCTCCCTATAAG	3	G	0.0328	0.0322
NC_005103.4-15744717	AGTGAGGATACCATGGCTCCTTCAGAG	16	G	0.0325	0.032
NC_005103.4-15744717	AGTGAGGATACCATGGCTCCTTCAGAG	26	A	0.1721	0.2573
NC_005103.4-15792317	TGATTAAACACTTAGTCTCCAGCATTT	25	T	0	0.4765
NC_005103.4-15808781	TCCGCTCACACTTCCACTCCTAGAACA	4	G	0.0339	0.0496
NC_005103.4-15808781	TCCGCTCACACTTCCACTCCTAGAACA	23	G	0.2069	0.3179
NC_005103.4-15809909	GCAGGGACAACCACTGCTCCTCGTTAT	2	C	0.0252	0.0249
NC_005103.4-15874971	TGAACCACCACGTTGGCTCCCCAGACA	12	G	0.0593	0.4508
NC_005103.4-15874971	TGAACCACCACGTTGGCTCCCCAGACA	13	T	0.2373	0.4535
NC_005103.4-15907895	AAAGTATTCACTGTGTCTCCCGCTTAA	21	C	0.2276	0.3516
NC_005103.4-15984287	CAAGTCCTAACATGGGCTCCAGCTTTT	27	T	0	0.4682
NC_005103.4-16081871	GCTATTCTGACAGCTTCTCCTTCACTT	12	A	0.1875	0.2254
NC_005103.4-16121692	GTAACAGCCACTCGTCCTCCATAAGGA	13	C	0.2397	0.2922
NC_005103.4-16121692	GTAACAGCCACTCGTCCTCCATAAGGA	14	G	0.1765	0.2389
NC_005103.4-16131573	ACTAGAGGGACCAGTACTCCTTGCAGG	5	G	0.1638	0.302
NC_005103.4-16145573	GTCACAAGAACATGAGCTCCTTACTAG	13	T	0.0513	0.1125
NC_005103.4-16145573	GTCACAAGAACATGAGCTCCTTACTAG	25	T	0	0.4839
NC_005103.4-16196237	TGCATGTACACCTGGGCTCCCATGTGC	26	G	0.0678	0.0655
NC_005103.4-16196237	TGCATGTACACCTGGGCTCCCATGTGC	27	C	0.2564	0.4807
NC_005103.4-16248164	GCTACATAAACATTCTCTCCTTTGATC	12	A	0.0088	0.476
NC_005103.4-16248164	GCTACATAAACATTCTCTCCTTTGATC	27	C	0.0086	0.0255
NC_005103.4-16258274	CAGCCTTTAACACTGTCTCCTGAGTAT	24	G	0.1339	0.491
NC_005103.4-16299907	CTGGAGCACACTACTTCTCCTTCTCTC	13	A	0.0085	0.481
NC_005103.4-16396221	GCCTTCTGAACATCATCTCCCCCACCC	26	C	0.025	0.0247

NC_005103.4-16396221	GCCTTCTGAACATCATCTCCCCCACCC	27	C	0.0248	0.0245
NC_005103.4-16425847	AATGTGTATACTTCCACTCCTTCTTAA	24	T	0.069	0.1284
NC_005103.4-16575305	TTGCTTTCAACCCTAACTCCTCATTTT	26	T	0	0.4765
NC_005103.4-16601064	TTCAGCATCACTTCATCTCCTCCAGGA	5	G	0	0.4922
NC_005103.4-16627556	AACACATTCACCTTAACTCCCAGGTAA	8	T	0.0932	0.2156
NC_005103.4-16627556	AACACATTCACCTTAACTCCCAGGTAA	23	G	0	0.4734
NC_005103.4-16671753	CTGAAACCTACAACAGCTCCCTGCCTG	26	T	0.0813	0.1216
NC_005103.4-16993712	TATCAACCAACCAGCCCTCCAAATGCT	14	G	0.1842	0.2476
NC_005103.4-17049494	GTAGAAGGTACCAGACCTCCTCAAATG	5	A	0.1356	0.1691
NC_005103.4-17116396	ATTGCTATGACAAATGCTCCCTTCAGA	7	A	0.1316	0.2476
NC_005103.4-17133639	CTGATCCAAACTAAGCCTCCATATCCT	7	C	0.1083	0.1172
NC_005103.4-17624003	CCAATATAAACTCTGACTCCCACAAGT	16	A	0.1754	0.3324
NC_005103.4-17716724	CTGCCTGTTACCATGCCTCCTCCATCA	13	A	0.0087	0.0257
NC_005103.4-17717923	ATCTGAAATACCCAGTCTCCTTCAGTT	12	C	0.0413	0.1017
NC_005103.4-17717923	ATCTGAAATACCCAGTCTCCTTCAGTT	14	A	0.2727	0.3512
NC_005103.4-17740913	GTTTCCAAGACAACGTCTCCTGCAGTG	15	G	0	0.4813
NC_005103.4-17843988	CTTCTTGTGACTGTGCCTCCGGGATCG	27	G	0.0177	0.452
NC_005103.4-17964796	AGCCCCCTTACCCCTCCTCCTCCCCTT	3	C	0.1207	0.2252
NC_005103.4-17980854	TCTCCTTCCACTGCCTCTCCCTATTCC	9	C	0.009	0.4942
NC_005103.4-18231146	CTTGGATTAACATTGTCTCCCTGTGCA	24	T	0.1695	0.3034
NC_005103.4-18307246	TCATATAAAACAAATTCTCCTTCATAA	1	T	0.0526	0.4846
NC_005103.4-18307246	TCATATAAAACAAATTCTCCTTCATAA	23	C	0.0435	0.059
NC_005103.4-18315499	TCTGTCAAAACAACTGCTCCATGCATG	22	T	0.0776	0.5
NC_005103.4-18365022	GAAAGATTAACCATCACTCCAATTTCT	2	A	0	0.4709
NC_005103.4-18399000	TGTGTACCCACTCAAACTCCCTCCTCC	1	T	0.0661	0.0792
NC_005103.4-18399000	TGTGTACCCACTCAAACTCCCTCCTCC	26	C	0.25	0.4599
NC_005103.4-18419579	CTTAGTTGAACAATGTCTCCTTTAGGA	2	T	0	0.4814
NC_005103.4-18444282	CAGAGACTCACTCATTCTCCAACAGAA	3	G	0.1739	0.2873
NC_005103.4-18511844	CAACATTGGACCGGTGCTCCTTTTACA	4	C	0.0488	0.4881
NC_005103.4-18511844	CAACATTGGACCGGTGCTCCTTTTACA	22	T	0.0407	0.0398
NC_005103.4-18516855	CTGCCTCATACAATCCCTCCCTCCCCC	27	C	0.0085	0.4698
NC_005103.4-18587424	TTCCTGATTACCAAGTCTCCTGAATCA	24	A	0.2632	0.4321
NC_005103.4-18659540	AAGTGCATCACTTGGCCTCCTGATGTG	3	G	0.0082	0.0243
NC_005103.4-18667965	GAAGTGACCACAAAATCTCCCTCTTCA	5	T	0.0847	0.2466
NC_005103.4-18795676	TCCAGTACAACACTTTCTCCCTAGATA	5	G	0.0492	0.4837
NC_005103.4-18854682	TACACTAGAACTTGTGCTCCTTGTGTG	22	T	0.0354	0.0348
NC_005103.4-19019733	AAGACCAAAACTTCTGCTCCAAGCAAT	8	A	0	0.4764
NC_005103.4-19019733	AAGACCAAAACTTCTGCTCCAAGCAAT	22	A	0.0086	0.4768
NC_005103.4-19073842	TCCTCCATTACCCTCTCTCCTTCTTTG	2	C	0.2035	0.3495
NC_005103.4-19073842	TCCTCCATTACCCTCTCTCCTTCTTTG	25	T	0	0.4814
NC_005103.4-19164677	ATATTATAAACACAGCCTCCATTGCTT	6	A	0.2072	0.3727
NC_005103.4-19171674	CTGCCTTTAACTGCAACTCCAGAAGTT	4	C	0.225	0.3533
NC_005103.4-19171674	CTGCCTTTAACTGCAACTCCAGAAGTT	7	T	0.225	0.3533
NC_005103.4-19173578	CAAATACCAACCTAACCTCCAAGCCAC	3	A	0.1885	0.2686
NC_005103.4-19367350	GGTTCTCCAACATGCACTCCTTGTTCC	1	G	0.082	0.2032
NC_005103.4-19543899	AACACCTTTACCAGTCCTCCATTCGAT	24	C	0.1	0.1528
NC_005103.4-19707733	ATCAGAGTAACCACATCTCCAGTTGCA	24	T	0.1789	0.3688
NC_005103.4-19719803	TGTGCTGTAACCCTTCCTCCTGTTTGG	13	C	0.0957	0.18
NC_005103.4-19747707	CTAACCCACACGCTCTCTCCAACCACT	6	C	0.0085	0.0251
NC_005103.4-19747707	CTAACCCACACGCTCTCTCCAACCACT	15	C	0.0085	0.0251
NC_005103.4-19964026	AACACAGGGACTCTCTCTCCTGCGAAT	8	G	0.1148	0.1225

NC_005103.4-20013812	TATGCTCCAACCCCCACTCCCTCCCAC	25	C	0.0088	0.49
NC_005103.4-20212923	AGCTTTGTCACATTTCCTCCTTTCCTT	7	G	0.0427	0.0418
NC_005103.4-20313011	CCAGTCTTCACAGCTTCTCCTATTGAT	5	T	0	0.4793
NC_005103.4-20313011	CCAGTCTTCACAGCTTCTCCTATTGAT	14	C	0.0619	0.0765
NC_005103.4-20343953	AGCATGCTAACCCTTTCTCCTTAAATG	15	T	0.1379	0.2739
NC_005103.4-20347138	CTGCTCTAAACTTTGTCTCCATATATC	14	T	0.0252	0.0249
NC_005103.4-20347138	CTGCTCTAAACTTTGTCTCCATATATC	24	T	0.0336	0.033
NC_005103.4-20348626	TACCAACAAACCACAGCTCCCTGGGAC	16	G	0.0672	0.1107
NC_005103.4-20466704	CACACAATCACAGGCCCTCCCTTATCT	1	C	0	0.4793
NC_005103.4-20466704	CACACAATCACAGGCCCTCCCTTATCT	2	A	0	0.4793
NC_005103.4-20514796	TCAGATATCACCAGAACTCCCAGGGAC	7	A	0.0893	0.1014
NC_005103.4-20514796	TCAGATATCACCAGAACTCCCAGGGAC	24	G	0.1071	0.4483
NC_005103.4-20566488	GGGCCTACCACCAGGTCTCCATCCTGT	8	C	0.0488	0.0629
NC_005103.4-20591429	CTCAGACAGACATGCTCTCCTCTACAT	7	C	0	0.4813
NC_005103.4-20591429	CTCAGACAGACATGCTCTCCTCTACAT	22	C	0.1949	0.4997
NC_005103.4-20633628	AATCAATGCACAATGTCTCCTAACAAC	21	T	0.0256	0.0253
NC_005103.4-20646574	AATAGTTTCACATGGCCTCCCTTTATC	21	C	0.2883	0.2467
NC_005103.4-20652008	CCCAGCAGTACCCTCCCTCCCGGTCAC	9	T	0	0.4703
NC_005103.4-20652008	CCCAGCAGTACCCTCCCTCCCGGTCAC	21	C	0	0.4718
NC_005103.4-20731345	ATGTGTTATACTCTCCCTCCAATGTAA	9	T	0	0.4755
NC_005103.4-20731345	ATGTGTTATACTCTCCCTCCAATGTAA	12	T	0.1478	0.1516
NC_005103.4-20805621	AACAACAATACCAGTGCTCCCAGAGAC	3	C	0.0965	0.4953
NC_005103.4-20881718	TGTATACGCACATGGACTCCAAATTAG	4	A	0.1667	0.3578
NC_005103.4-20890288	AGTAGGCTTACACATTCTCCTTGTAGT	6	G	0.1148	0.4966
NC_005103.4-20890288	AGTAGGCTTACACATTCTCCTTGTAGT	23	G	0.1148	0.1225
NC_005103.4-20928560	CACATGAAGACTTGAGCTCCCCAGTAC	14	G	0.0413	0.4863
NC_005103.4-21023369	AATGTATATACCTCTGCTCCCGCACAT	21	C	0.065	0.0629
NC_005103.4-21023369	AATGTATATACCTCTGCTCCCGCACAT	22	G	0	0.4793
NC_005103.4-21039181	ATGTCCTCCACAAGACCTCCAAGGATT	6	C	0.2321	0.2575
NC_005103.4-21039181	ATGTCCTCCACAAGACCTCCAAGGATT	23	G	0.2321	0.2575
NC_005103.4-21162775	TCTCTCCCCACACTCTCTCCTTCCATG	27	G	0.0536	0.1913
NC_005103.4-21165856	CCCTTCCTTACTTCACCTCCCTGTAAT	4	T	0.2589	0.4665
NC_005103.4-21165856	CCCTTCCTTACTTCACCTCCCTGTAAT	14	C	0	0.4793
NC_005103.4-21207464	GCAGTTCACACAATCCCTCCCTCAAGC	14	T	0.2377	0.4755
NC_005103.4-21208399	ATCACAAGGACACATGCTCCACTAAGT	6	A	0.0667	0.0644
NC_005103.4-21228855	TAAACACTTACTAAGTCTCCTGGGTAT	4	A	0.2222	0.4734
NC_005103.4-21228855	TAAACACTTACTAAGTCTCCTGGGTAT	23	G	0.0862	0.4999
NC_005103.4-21234278	ATCTACAATACAACTCCTCCTGCACCT	22	G	0.475	0.3708
NC_005103.4-21234278	ATCTACAATACAACTCCTCCTGCACCT	23	C	0.475	0.3708
NC_005103.4-21327768	ATATACAGCACTCTCCCTCCCCACGTT	2	T	0.1441	0.4931
NC_005103.4-21327768	ATATACAGCACTCTCCCTCCCCACGTT	14	T	0	0.4871
NC_005103.4-21340983	ATGCTCCAAACTTTGCCTCCATAATCT	15	G	0.1034	0.4976
NC_005103.4-21367910	CGCAGCTGGACGTAATCTCCCCCTTTG	12	G	0.0088	0.476
NC_005103.4-21369944	ACTGTCAGCACTCAGACTCCTAAACAA	15	G	0	0.4757
NC_005103.4-21375705	CCCTGCATCACTCCTTCTCCAGACTCA	14	C	0.0082	0.4755
NC_005103.4-21375705	CCCTGCATCACTCCTTCTCCAGACTCA	16	T	0.0082	0.4755
NC_005103.4-21472821	AGTAATAGGACTCAGACTCCTTCTCAA	12	T	0.1901	0.3687
NC_005103.4-21472821	AGTAATAGGACTCAGACTCCTTCTCAA	27	A	0.0826	0.0943
NC_005103.4-21483082	CAGCGTGATACGTGAGCTCCTGCAGAA	13	T	0.2276	0.4944
NC_005103.4-21530254	ATCACTCAGACACTGTCTCCTCTGGTC	16	T	0.0081	0.481
NC_005103.4-21590915	TTATGGCTGACTCGAACTCCATTGAGA	13	C	0.0877	0.0839

NC_005103.4-21590915	TTATGGCTGACTCGAACTCCATTGAGA	27	A	0	0.474
NC_005103.4-21618399	CAGAATGGCACAGAGACTCCAGGTCAC	24	T	0.1356	0.2219
NC_005103.4-21673416	ATGATCTCTACAGGTTCTCCCTCCTCT	1	A	0.2689	0.2328
NC_005103.4-21677984	GGCTCAAGTACCTCAACTCCAGCTCAG	1	G	0.0164	0.0322
NC_005103.4-21802148	CAACCAGGGACTTCTACTCCGTCTTTG	22	T	0	0.4718
NC_005103.4-21833416	TATGCAACCACTGACCCTCCCCAAGTG	6	A	0	0.4778
NC_005103.4-21836285	CCCCATGCCACATTAGCTCCAAGCATT	12	A	0.5041	0.377
NC_005103.4-21836285	CCCCATGCCACATTAGCTCCAAGCATT	13	T	0.3577	0.4854
NC_005103.4-21895841	AGACGTTTCACTTCAGCTCCAAGTGTA	5	G	0	0.4854
NC_005103.4-21922740	CAGCATGCCACCATAGCTCCCAACATG	8	C	0.0667	0.495
NC_005103.4-21928846	CAATGTCTAACCCCGCCTCCTGATGTC	15	G	0.0083	0.4847
NC_005103.4-21971744	GACCCGAAAACCCAACCTCCTTGTCTT	6	G	0.0492	0.4891
NC_005103.4-22086401	GTGCCTCCCACCTCATCTCCTTCCCAC	2	T	0.0804	0.4952
NC_005103.4-22086401	GTGCCTCCCACCTCATCTCCTTCCCAC	3	G	0.0619	0.06
NC_005103.4-22161389	ATACATTGGACTCCTTCTCCAACCACT	4	C	0	0.4868
NC_005103.4-22161389	ATACATTGGACTCCTTCTCCAACCACT	14	C	0.1597	0.1469
NC_005103.4-22165885	TTCCAACGCACAAGGGCTCCTCACTAG	8	G	0.1624	0.1492
NC_005103.4-22182537	TGGGTGAGAACCTCTCCTCCGTGTCTC	26	T	0.0084	0.4829
NC_005103.4-22182654	AGAGCCTGGACCCAACCTCCTCAGATG	7	T	0.0081	0.0241
NC_005103.4-22213043	GTGGACAATACCAAGGCTCCATCAGAT	16	G	0.1382	0.1826
NC_005103.4-22241216	CAGAGGACAACTCTTACTCCCTAGCCA	7	A	0.0541	0.4951
NC_005103.4-22281484	ATCTTCAGAACATGCTCTCCAAATCCT	12	A	0.0583	0.0722
NC_005103.4-22337578	CTTGAGAAGACTGAGTCTCCCTCTCAG	25	C	0.1557	0.1708
NC_005103.4-22337578	CTTGAGAAGACTGAGTCTCCCTCTCAG	27	G	0.1557	0.1708
NC_005103.4-22352761	GTTTAAGTCACAGACGCTCCTGCCTCC	23	C	0.0526	0.4606
NC_005103.4-22379049	ACACTCTGTACACCCTCTCCTTGAGAA	2	C	0.2522	0.4782
NC_005103.4-22379049	ACACTCTGTACACCCTCTCCTTGAGAA	13	C	0.2193	0.2599
NC_005103.4-22443525	CTACTCCAAACTTTGTCTCCATCTTTC	1	C	0.1849	0.2328
NC_005103.4-22484370	TGTGTAGGGACCACTTCTCCTGATATG	9	G	0	0.4787
NC_005103.4-22484370	TGTGTAGGGACCACTTCTCCTGATATG	14	C	0	0.4787
NC_005103.4-22513056	AGCCTCAGCACACAGTCTCCTGTGCCT	2	G	0.1967	0.3624
NC_005103.4-22513056	AGCCTCAGCACACAGTCTCCTGTGCCT	6	C	0.2459	0.306
NC_005103.4-22556661	ACAACATTCACATGGGCTCCAGCATTA	21	A	0	0.4793
NC_005103.4-22556661	ACAACATTCACATGGGCTCCAGCATTA	27	A	0.3089	0.3604
NC_005103.4-22556698	TGGACATCAACATGGTCTCCAGTAGCA	8	C	0.4492	0.4454
NC_005103.4-22628899	GGTTTATAAACAGCTTCTCCATCTAAC	26	A	0	0.4778
NC_005103.4-22628899	GGTTTATAAACAGCTTCTCCATCTAAC	27	C	0	0.4778
NC_005103.4-22692268	CACGGAGACACAGAGGCTCCCTCGCCT	2	A	0.2328	0.4688
NC_005103.4-22692611	ACAGAGGCAACCCTTTCTCCCCACGTT	21	C	0.075	0.1025
NC_005103.4-22725460	GGACATTTGACATTTGCTCCTTGTTTT	6	T	0.0174	0.4915
NC_005103.4-22856504	GTACTGAGAACGCATGCTCCTCCTGCA	2	T	0.0424	0.487
NC_005103.4-22856504	GTACTGAGAACGCATGCTCCTCCTGCA	12	G	0.2222	0.2834
NC_005103.4-22982847	TTACAAACTACACCCTCTCCAAATACC	9	T	0.0804	0.0934
NC_005103.4-22983568	TCTACCTAAACTTGCTCTCCATCTGAA	14	G	0.0168	0.033
NC_005103.4-22983568	TCTACCTAAACTTGCTCTCCATCTGAA	22	T	0	0.4743
NC_005103.4-22999926	GCTCTTTTCACATCTCCTCCTGGCTCC	24	C	0	0.4871
NC_005103.4-22999926	GCTCTTTTCACATCTCCTCCTGGCTCC	25	T	0.0536	0.1014
NC_005103.4-23003079	ATTGGTAGGACAGTTTCTCCAATGAGG	2	T	0.2185	0.4703
NC_005103.4-23012388	CACTAGCTAACACACTCTCCACCCATT	14	A	0.1092	0.188
NC_005103.4-23012388	CACTAGCTAACACACTCTCCACCCATT	16	T	0.175	0.4233
NC_005103.4-23015930	AGCACCCTAACTTGTCCTCCGTGTGGG	21	G	0	0.033

NC_005103.4-23040419	AGCTCCCACACTGCAACTCCGAGACCT	21	G	0.1197	0.4991
NC_005103.4-23040419	AGCTCCCACACTGCAACTCCGAGACCT	27	T	0.1197	0.1704
NC_005103.4-23067384	GGAGAAGGAACAAATTCTCCTAGAGGC	23	G	0.1864	0.2466
NC_005103.4-23091366	GAGACATTCACACATCCTCCCTTGGGA	12	A	0.0087	0.0752
NC_005103.4-23096278	CCCAGGGACACTATTCCTCCCCTGGGT	25	G	0.094	0.1634
NC_005103.4-23313028	AATCTAAACACCCCAGCTCCCATCTAA	13	C	0.0268	0.0605
NC_005103.4-23329440	GACTCAGAAACTGAATCTCCATTGCTT	27	T	0.0256	0.4714
NC_005103.4-23493753	CCAAAGTCAACTGAAGCTCCATCAGTT	21	A	0	0.4851
NC_005103.4-23596898	TGTGTGATAACCAGCTCTCCGTCACTG	21	G	0.0252	0.4797
NC_005103.4-23596898	TGTGTGATAACCAGCTCTCCGTCACTG	26	T	0.2185	0.2449
NC_005103.4-23687520	ACAGAAAAGACGTCAACTCCATTGTAA	9	G	0.0619	0.06
NC_005103.4-23747137	AAAACACCTACCTCACCTCCCTGTGCT	2	A	0.0708	0.1162
NC_005103.4-23747137	AAAACACCTACCTCACCTCCCTGTGCT	7	C	0.0088	0.0262
NC_005103.4-23747174	ATAGTTTTAACCAACTCTCCTTCTATC	21	T	0.2456	0.3521
NC_005103.4-23747174	ATAGTTTTAACCAACTCTCCTTCTATC	27	C	0.0354	0.0348
NC_005103.4-23785770	CTGTCTTTGACTTAGGCTCCAGCATCT	8	T	0.2583	0.4233
NC_005103.4-23785770	CTGTCTTTGACTTAGGCTCCAGCATCT	21	A	0.0333	0.0328
NC_005103.4-23845031	AGCATTCCTACCAAGACTCCAAGTTCA	26	C	0.0522	0.0508
NC_005103.4-23959471	TTCTTCCACACTGTGCCTCCTGGATAT	24	A	0.2479	0.4087
NC_005103.4-24032870	TAAAGAGTCACATGAGCTCCTCCTGTG	16	G	0.1583	0.1866
NC_005103.4-24044365	TTTACTCAAACCGTCCCTCCCAGGAAG	4	A	0.0082	0.479
NC_005103.4-24098966	TCACAAACAACCAGATCTCCACACCAC	9	A	0.1786	0.2575
NC_005103.4-24116034	TGAATATGCACCTGTTCTCCACTGCAC	7	T	0.0661	0.4628
NC_005103.4-24116034	TGAATATGCACCTGTTCTCCACTGCAC	8	G	0.0082	0.0401
NC_005103.4-24137530	TGGGTCTCTACATCTACTCCCATATAT	2	G	0.225	0.4097
NC_005103.4-24137530	TGGGTCTCTACATCTACTCCCATATAT	13	T	0.0083	0.4732
NC_005103.4-24146304	AGACAAGTTACTGTTTCTCCTTCTGCT	2	G	0.2521	0.4567
NC_005103.4-24302621	AGGGACCTGACAGATGCTCCAAACAGT	16	G	0.0268	0.0264
NC_005103.4-24302621	AGGGACCTGACAGATGCTCCAAACAGT	26	G	0.193	0.2778
NC_005103.4-24310326	CCTTGCTCCACATCCTCTCCACCCAGT	13	T	0.1416	0.4981
NC_005103.4-24310326	CCTTGCTCCACATCCTCTCCACCCAGT	15	C	0.0439	0.0429
NC_005103.4-24366246	TGCACTCCCACCACTGCTCCAGCATTC	1	T	0	0.4793
NC_005103.4-24366246	TGCACTCCCACCACTGCTCCAGCATTC	2	G	0	0.4793
NC_005103.4-24366836	CCGCTGCCTACATGGCCTCCAAGTACC	16	C	0.2288	0.4138
NC_005103.4-24366836	CCGCTGCCTACATGGCCTCCAAGTACC	23	G	0.0336	0.0491
NC_005103.4-24437690	CTCCAGTGCACACTTCCTCCAACAAGG	1	C	0.0413	0.0405
NC_005103.4-24437690	CTCCAGTGCACACTTCCTCCAACAAGG	27	G	0.2645	0.3645
NC_005103.4-24474662	AGGCTCAAGACAGAATCTCCATTATAA	25	T	0.0259	0.0255
NC_005103.4-24510772	GTCCACTCCACCAGACCTCCCCACTCC	12	C	0.3559	0.424
NC_005103.4-24608123	GTATTTTAAACACTTGCTCCCTAGTTA	5	T	0.1228	0.1884
NC_005103.4-24608123	GTATTTTAAACACTTGCTCCCTAGTTA	24	G	0.0435	0.0425
NC_005103.4-24651721	TCTTTTCCTACTTTCCCTCCTCGCTTC	14	T	0.0569	0.0705
[truncated: 495,876 more chars]
