# Supplementary material for: Comparative phylogeography of two commensal rat species (Rattus tanezumi and Rattus norvegicus) in China: Insights from mitochondrial DNA, microsatellite, and 2b‐RAD data
Source: Ecol Evol. 2022 Oct 13;12(10):e9409. doi: 10.1002/ece3.9409 (PMC9557235; doi:10.1002/ece3.9409)
Supplement: Supplementary file 17 — Table S11 [file ECE3-12-e9409-s005.pdf]

A1	A2	B	O	Z-scores	p value
CS15	JO7	CS10	*R.nitidus*	0.029493	0.071125
CS3	JO7	CS10	*R.nitidus*	-0.00991	0.070247
GD34	JO7	CS10	*R.nitidus*	0.014085	0.069962
GD44	JO7	CS10	*R.nitidus*	0.013711	0.072045
CS23	JO7	CS10	*R.nitidus*	-0.061355	0.071262
GD49	JO7	CS10	*R.nitidus*	0.089207	0.074161
CS12	JO7	CS10	*R.nitidus*	0.009643	0.073468
GD25	JO7	CS10	*R.nitidus*	0.054307	0.072901
SC24	JO7	CS10	*R.nitidus*	0.015311	0.07291
CS28	JO7	CS10	*R.nitidus*	-0.036217	0.07325
CS24	JO7	CS10	*R.nitidus*	-0.051625	0.073313
GD32	JO7	CS10	*R.nitidus*	0.025667	0.074205
CS18	JO7	CS10	*R.nitidus*	-0.039216	0.072869
CS30	JO7	CS10	*R.nitidus*	-0.054795	0.071606
SC5	JO7	CS10	*R.nitidus*	0.016605	0.076094
SC14	JO7	CS10	*R.nitidus*	-0.000907	0.072528
SC18	JO7	CS10	*R.nitidus*	0.024952	0.070824
CS20	JO7	CS10	*R.nitidus*	-0.024194	0.070337
GD33	JO7	CS10	*R.nitidus*	0.032345	0.072783
GD42	JO7	CS10	*R.nitidus*	0.041346	0.072447
GD21	JO7	CS10	*R.nitidus*	0.113861	0.06929
GD41	JO7	CS10	*R.nitidus*	-0.005484	0.070865
GD35	JO7	CS10	*R.nitidus*	0.03663	0.074113
CS17	JO7	CS10	*R.nitidus*	-0.011055	0.077047
CS25	JO7	CS10	*R.nitidus*	-0.0403	0.072998
CS22	JO7	CS10	*R.nitidus*	0.00188	0.071669
GD40	JO7	CS10	*R.nitidus*	0.067841	0.069836
SC26	JO7	CS10	*R.nitidus*	-0.024324	0.072672
SC6	JO7	CS10	*R.nitidus*	0.014006	0.070873
CS21	JO7	CS10	*R.nitidus*	-0.036036	0.072514
CS11	JO7	CS10	*R.nitidus*	0.001817	0.073034
GD46	JO7	CS10	*R.nitidus*	-0.02302	0.071531
CS4	JO7	CS10	*R.nitidus*	-0.037102	0.069988
GD22	JO7	CS10	*R.nitidus*	0.102389	0.070554
GD30	JO7	CS10	*R.nitidus*	0.155604	0.073521
GD50	JO7	CS10	*R.nitidus*	0.061267	0.075123
SC15	JO7	CS10	*R.nitidus*	-0.027264	0.074477
JO8	JO7	CS10	*R.nitidus*	0.006944	0.074329
SC12	JO7	CS10	*R.nitidus*	-0.021719	0.069821
GD31	JO7	CS10	*R.nitidus*	0.075543	0.072343
GD26	JO7	CS10	*R.nitidus*	0.088314	0.071785
SC10	JO7	CS10	*R.nitidus*	0.102786	0.073386
CS14	JO7	CS10	*R.nitidus*	0.001899	0.073375
GD48	JO7	CS10	*R.nitidus*	0.029493	0.072391
SC27	JO7	CS10	*R.nitidus*	0.006387	0.071995
CS13	JO7	CS10	*R.nitidus*	-0.04023	0.073097
CS16	JO7	CS10	*R.nitidus*	0.015224	0.07361
CS31	JO7	CS10	*R.nitidus*	-0.030899	0.072834
CS19	JO7	CS10	*R.nitidus*	-0.030841	0.074033
GD45	JO7	CS10	*R.nitidus*	0.107103	0.074918
JO5	JO7	CS10	*R.nitidus*	0.056968	0.068908
SC32	JO7	CS10	*R.nitidus*	0.05	0.071385
GD21	JO8	CS10	*R.nitidus*	0.07401	0.069686
SC42	JO7	CS10	*R.nitidus*	0.1736	0.06991
CS6	JO7	CS10	*R.nitidus*	-0.069982	0.069206

MY6	JO7	CS10	*R.nitidus*	-0.010397	0.072448
SC45	JO7	CS10	*R.nitidus*	0.045155	0.071317
JO3	JO7	CS10	*R.nitidus*	0.016304	0.074608
SC31	JO7	CS10	*R.nitidus*	-0.022183	0.071981
JW6	JO7	CS10	*R.nitidus*	-0.027907	0.067965
JO1	JO7	CS10	*R.nitidus*	0.021277	0.068654
GD33	JO8	CS10	*R.nitidus*	-0.018365	0.072426
MY4	JO7	CS10	*R.nitidus*	-0.047081	0.074441
GD22	JO8	CS10	*R.nitidus*	0.056637	0.07082
CS12	JO8	CS10	*R.nitidus*	-0.008902	0.073129
GD32	JO8	CS10	*R.nitidus*	-0.034225	0.076568
CS11	JO8	CS10	*R.nitidus*	-0.024281	0.072751
GD25	JO8	CS10	*R.nitidus*	0.029053	0.069833
JW7	JO7	CS10	*R.nitidus*	0.054435	0.072896
SC28	JO7	CS10	*R.nitidus*	-0.002768	0.071571
GD35	JO8	CS10	*R.nitidus*	0.01385	0.073391
SC44	JO7	CS10	*R.nitidus*	-0.012218	0.07377
GD29	JO8	CS10	*R.nitidus*	0.112875	0.067557
GD44	JO8	CS10	*R.nitidus*	-0.006591	0.072356
SC34	JO7	CS10	*R.nitidus*	0.006157	0.069992
SC35	JO7	CS10	*R.nitidus*	0.011515	0.069926
GD26	JO8	CS10	*R.nitidus*	0.040952	0.072565
JW4	JO7	CS10	*R.nitidus*	0.205993	0.069627
GD34	JO8	CS10	*R.nitidus*	-0.049587	0.069072
JO7	JO8	CS10	*R.nitidus*	-0.006944	0.074329
GD31	JO8	CS10	*R.nitidus*	0.024272	0.071807
GD41	JO8	CS10	*R.nitidus*	-0.032015	0.071135
CS15	JO8	CS10	*R.nitidus*	-0.008287	0.071507
GD40	JO8	CS10	*R.nitidus*	0.048713	0.070693
GD42	JO8	CS10	*R.nitidus*	-0.002876	0.072429
GD30	JO8	CS10	*R.nitidus*	0.089535	0.075832
GD46	JO8	CS10	*R.nitidus*	-0.08978	0.06869
GD49	JO8	CS10	*R.nitidus*	0.049605	0.074018
CS16	JO8	CS10	*R.nitidus*	-0.02975	0.072926
GD45	JO8	CS10	*R.nitidus*	0.04157	0.076579
GD50	JO8	CS10	*R.nitidus*	0.024811	0.075318
CS3	JO8	CS10	*R.nitidus*	-0.028958	0.072608
CS13	JO8	CS10	*R.nitidus*	-0.046602	0.075253
CS20	JO8	CS10	*R.nitidus*	-0.04698	0.071429
CS19	JO8	CS10	*R.nitidus*	-0.052052	0.073515
GD48	JO8	CS10	*R.nitidus*	0.0125	0.070723
CS31	JO8	CS10	*R.nitidus*	-0.076546	0.07262
CS14	JO8	CS10	*R.nitidus*	-0.00754	0.074941
CS17	JO8	CS10	*R.nitidus*	-0.011317	0.075825
CS18	JO8	CS10	*R.nitidus*	-0.053465	0.073015
SC34	JO8	CS10	*R.nitidus*	-0.033028	0.071107
CS22	JO8	CS10	*R.nitidus*	-0.051411	0.071057
CS24	JO8	CS10	*R.nitidus*	-0.055046	0.070789
CS25	JO8	CS10	*R.nitidus*	-0.04572	0.073437
SC27	JO8	CS10	*R.nitidus*	-0.023474	0.069135
SC24	JO8	CS10	*R.nitidus*	-0.008876	0.069856
CS28	JO8	CS10	*R.nitidus*	-0.059856	0.073712
CS4	JO8	CS10	*R.nitidus*	-0.038925	0.070121
SC12	JO8	CS10	*R.nitidus*	-0.05773	0.069871
SC14	JO8	CS10	*R.nitidus*	-0.018519	0.070647
SC31	JO8	CS10	*R.nitidus*	-0.011289	0.072924

SC35	JO8	CS10	*R.nitidus*	-0.004817	0.070453
CS21	JO8	CS10	*R.nitidus*	-0.055028	0.070897
SC5	JO8	CS10	*R.nitidus*	-0.016008	0.075282
SC6	JO8	CS10	*R.nitidus*	-0.017958	0.07256
SC18	JO8	CS10	*R.nitidus*	-0.006224	0.070065
SC28	JO8	CS10	*R.nitidus*	-0.045726	0.071793
SC26	JO8	CS10	*R.nitidus*	-0.08373	0.070936
CS30	JO8	CS10	*R.nitidus*	-0.106299	0.0694
CS6	JO8	CS10	*R.nitidus*	-0.080818	0.071365
CS23	JO8	CS10	*R.nitidus*	-0.075382	0.072919
MY6	JO8	CS10	*R.nitidus*	-0.031098	0.07303
JO1	JO8	CS10	*R.nitidus*	-0.024691	0.070046
SC32	JO8	CS10	*R.nitidus*	-0.003842	0.071836
SC42	JO8	CS10	*R.nitidus*	0.134217	0.070926
SC10	JO8	CS10	*R.nitidus*	0.062181	0.073978
MY4	JO8	CS10	*R.nitidus*	-0.075259	0.072375
JO3	JO8	CS10	*R.nitidus*	0.001109	0.073217
SC15	JO8	CS10	*R.nitidus*	-0.041206	0.072673
JW7	JO8	CS10	*R.nitidus*	0.030457	0.073758
SC45	JO8	CS10	*R.nitidus*	0.020774	0.071976
JW6	JO8	CS10	*R.nitidus*	-0.005384	0.070951
SC44	JO8	CS10	*R.nitidus*	-0.020468	0.071099
JW4	JO8	CS10	*R.nitidus*	0.183477	0.069267
JO5	JO8	CS10	*R.nitidus*	0.045367	0.069187
GD21	CS11	CS10	*R.nitidus*	0.120482	0.065965
JO8	CS11	CS10	*R.nitidus*	0.024281	0.072751
GD25	CS11	CS10	*R.nitidus*	0.065505	0.067727
JO7	CS11	CS10	*R.nitidus*	-0.001817	0.073034
GD26	CS11	CS10	*R.nitidus*	0.053465	0.068212
GD29	CS11	CS10	*R.nitidus*	0.128769	0.0666
GD30	CS11	CS10	*R.nitidus*	0.125	0.070597
GD22	CS11	CS10	*R.nitidus*	0.070068	0.067464
GD41	CS11	CS10	*R.nitidus*	0.019815	0.066283
CS12	CS11	CS10	*R.nitidus*	0.005035	0.070237
GD40	CS11	CS10	*R.nitidus*	0.072266	0.065526
CS17	CS11	CS10	*R.nitidus*	0.010753	0.069247
GD45	CS11	CS10	*R.nitidus*	0.07086	0.070617
GD31	CS11	CS10	*R.nitidus*	0.066713	0.067859
GD33	CS11	CS10	*R.nitidus*	0.017442	0.067012
CS13	CS11	CS10	*R.nitidus*	-0.042857	0.072233
GD35	CS11	CS10	*R.nitidus*	0.035475	0.067568
GD50	CS11	CS10	*R.nitidus*	0.018548	0.071386
GD42	CS11	CS10	*R.nitidus*	0.030426	0.067808
CS21	CS11	CS10	*R.nitidus*	-0.025263	0.065317
CS15	CS11	CS10	*R.nitidus*	0.04	0.068345
CS22	CS11	CS10	*R.nitidus*	0.003592	0.067752
GD34	CS11	CS10	*R.nitidus*	0.010821	0.066218
GD49	CS11	CS10	*R.nitidus*	0.082506	0.070097
GD32	CS11	CS10	*R.nitidus*	0.031746	0.070429
GD46	CS11	CS10	*R.nitidus*	0.000668	0.067183
CS28	CS11	CS10	*R.nitidus*	-0.035714	0.066472
GD48	CS11	CS10	*R.nitidus*	0.04669	0.068449
GD44	CS11	CS10	*R.nitidus*	0.021277	0.068204
SC27	CS11	CS10	*R.nitidus*	-0.001419	0.065896
SC5	CS11	CS10	*R.nitidus*	-0.001386	0.070936
CS14	CS11	CS10	*R.nitidus*	0.001153	0.070575

CS30	CS11	CS10	*R.nitidus*	-0.057307	0.067129
CS3	CS11	CS10	*R.nitidus*	-0.026012	0.067543
SC12	CS11	CS10	*R.nitidus*	-0.002807	0.067593
SC26	CS11	CS10	*R.nitidus*	-0.020139	0.065339
CS16	CS11	CS10	*R.nitidus*	0.014199	0.068534
CS25	CS11	CS10	*R.nitidus*	-0.016825	0.069525
CS19	CS11	CS10	*R.nitidus*	-0.023669	0.068122
CS18	CS11	CS10	*R.nitidus*	-0.03331	0.066465
SC14	CS11	CS10	*R.nitidus*	0.015829	0.067739
CS24	CS11	CS10	*R.nitidus*	-0.043704	0.067624
CS4	CS11	CS10	*R.nitidus*	-0.014104	0.067109
SC35	CS11	CS10	*R.nitidus*	0.046322	0.06506
SC34	CS11	CS10	*R.nitidus*	0.025956	0.067081
CS23	CS11	CS10	*R.nitidus*	-0.061324	0.065355
SC28	CS11	CS10	*R.nitidus*	-0.026389	0.065789
SC15	CS11	CS10	*R.nitidus*	-0.00145	0.068143
SC18	CS11	CS10	*R.nitidus*	0.046544	0.065773
CS20	CS11	CS10	*R.nitidus*	-0.027379	0.067388
CS6	CS11	CS10	*R.nitidus*	-0.042316	0.067911
MY4	CS11	CS10	*R.nitidus*	-0.034317	0.067848
SC42	CS11	CS10	*R.nitidus*	0.228571	0.062542
JW4	CS11	CS10	*R.nitidus*	0.21328	0.065411
SC24	CS11	CS10	*R.nitidus*	0.009091	0.067993
SC10	CS11	CS10	*R.nitidus*	0.073291	0.067319
CS31	CS11	CS10	*R.nitidus*	-0.021244	0.070443
SC44	CS11	CS10	*R.nitidus*	0.00616	0.066135
JW6	CS11	CS10	*R.nitidus*	0.011566	0.073303
SC6	CS11	CS10	*R.nitidus*	0.00211	0.067977
MY6	CS11	CS10	*R.nitidus*	-0.004707	0.067537
JO5	CS11	CS10	*R.nitidus*	0.026441	0.066725
GD30	GD21	CS10	*R.nitidus*	0.032197	0.065913
JO7	GD21	CS10	*R.nitidus*	-0.113861	0.06929
SC31	CS11	CS10	*R.nitidus*	-0.010253	0.065153
JO8	GD21	CS10	*R.nitidus*	-0.07401	0.069686
CS11	GD21	CS10	*R.nitidus*	-0.120482	0.065965
GD29	GD21	CS10	*R.nitidus*	0.002235	0.064647
CS12	GD21	CS10	*R.nitidus*	-0.135246	0.066918
SC32	CS11	CS10	*R.nitidus*	0.059075	0.067909
SC45	CS11	CS10	*R.nitidus*	0.040718	0.067851
JO1	CS11	CS10	*R.nitidus*	0.007618	0.066523
GD34	GD21	CS10	*R.nitidus*	-0.146517	0.060253
JW7	CS11	CS10	*R.nitidus*	0.042553	0.070399
JO3	CS11	CS10	*R.nitidus*	0.039021	0.07118
GD44	GD21	CS10	*R.nitidus*	-0.132898	0.064097
GD45	GD21	CS10	*R.nitidus*	-0.048458	0.068926
CS14	GD21	CS10	*R.nitidus*	-0.131492	0.065826
CS13	GD21	CS10	*R.nitidus*	-0.144703	0.06587
CS21	GD21	CS10	*R.nitidus*	-0.147468	0.063787
GD26	GD21	CS10	*R.nitidus*	-0.07971	0.062794
GD32	GD21	CS10	*R.nitidus*	-0.089974	0.064799
GD25	GD21	CS10	*R.nitidus*	-0.045936	0.064441
GD41	GD21	CS10	*R.nitidus*	-0.112554	0.063375
SC5	GD21	CS10	*R.nitidus*	-0.113072	0.06704
GD22	GD21	CS10	*R.nitidus*	-0.056723	0.061523
GD50	GD21	CS10	*R.nitidus*	-0.129318	0.068611
GD48	GD21	CS10	*R.nitidus*	-0.116505	0.067025

CS30	GD21	CS10	*R.nitidus*	-0.18516	0.064281
GD40	GD21	CS10	*R.nitidus*	-0.066802	0.064422
CS4	GD21	CS10	*R.nitidus*	-0.135034	0.062289
GD31	GD21	CS10	*R.nitidus*	-0.080092	0.063451
SC12	GD21	CS10	*R.nitidus*	-0.126019	0.063784
GD49	GD21	CS10	*R.nitidus*	-0.022241	0.068048
SC6	GD21	CS10	*R.nitidus*	-0.120613	0.064739
CS3	GD21	CS10	*R.nitidus*	-0.137217	0.063202
GD33	GD21	CS10	*R.nitidus*	-0.134723	0.063058
CS25	GD21	CS10	*R.nitidus*	-0.146455	0.065192
CS18	GD21	CS10	*R.nitidus*	-0.161899	0.065036
SC10	GD21	CS10	*R.nitidus*	-0.059016	0.064503
GD35	GD21	CS10	*R.nitidus*	-0.081119	0.062779
GD42	GD21	CS10	*R.nitidus*	-0.107762	0.063629
CS16	GD21	CS10	*R.nitidus*	-0.112777	0.062883
CS15	GD21	CS10	*R.nitidus*	-0.104275	0.065022
CS19	GD21	CS10	*R.nitidus*	-0.137374	0.065281
CS20	GD21	CS10	*R.nitidus*	-0.154145	0.064071
CS17	GD21	CS10	*R.nitidus*	-0.108635	0.066706
SC27	GD21	CS10	*R.nitidus*	-0.115756	0.063717
GD46	GD21	CS10	*R.nitidus*	-0.167328	0.062634
CS28	GD21	CS10	*R.nitidus*	-0.15304	0.064643
SC14	GD21	CS10	*R.nitidus*	-0.100577	0.064123
CS22	GD21	CS10	*R.nitidus*	-0.120498	0.062743
SC15	GD21	CS10	*R.nitidus*	-0.125415	0.065403
CS24	GD21	CS10	*R.nitidus*	-0.165003	0.063335
SC44	GD21	CS10	*R.nitidus*	-0.111825	0.064593
SC28	GD21	CS10	*R.nitidus*	-0.139185	0.063525
CS23	GD21	CS10	*R.nitidus*	-0.17224	0.063552
CS6	GD21	CS10	*R.nitidus*	-0.171906	0.063698
SC18	GD21	CS10	*R.nitidus*	-0.101266	0.064167
SC31	GD21	CS10	*R.nitidus*	-0.136588	0.064395
JW6	GD21	CS10	*R.nitidus*	-0.106732	0.068836
CS31	GD21	CS10	*R.nitidus*	-0.15322	0.063427
SC45	GD21	CS10	*R.nitidus*	-0.067445	0.062241
SC42	GD21	CS10	*R.nitidus*	0.143245	0.061579
SC24	GD21	CS10	*R.nitidus*	-0.122265	0.063998
JW7	GD21	CS10	*R.nitidus*	-0.089666	0.067143
SC26	GD21	CS10	*R.nitidus*	-0.148472	0.062455
SC34	GD21	CS10	*R.nitidus*	-0.096936	0.065075
SC32	GD21	CS10	*R.nitidus*	-0.077073	0.063148
MY4	GD21	CS10	*R.nitidus*	-0.172526	0.06327
GD45	GD22	CS10	*R.nitidus*	-0.023121	0.068837
MY6	GD21	CS10	*R.nitidus*	-0.134271	0.064059
CS11	GD22	CS10	*R.nitidus*	-0.070068	0.067464
SC35	GD21	CS10	*R.nitidus*	-0.077996	0.06263
JO8	GD22	CS10	*R.nitidus*	-0.056637	0.07082
CS13	GD22	CS10	*R.nitidus*	-0.103938	0.066746
GD35	GD22	CS10	*R.nitidus*	-0.019471	0.064041
GD44	GD22	CS10	*R.nitidus*	-0.051433	0.065443
CS14	GD22	CS10	*R.nitidus*	-0.066917	0.066792
JO5	GD21	CS10	*R.nitidus*	-0.069592	0.064012
GD33	GD22	CS10	*R.nitidus*	-0.05864	0.062705
GD31	GD22	CS10	*R.nitidus*	-0.020316	0.066297
JW4	GD21	CS10	*R.nitidus*	0.109554	0.066857
GD32	GD22	CS10	*R.nitidus*	-0.04932	0.065598

GD21	GD22	CS10	*R.nitidus*	0.056723	0.061523
JO1	GD21	CS10	*R.nitidus*	-0.130297	0.064271
GD41	GD22	CS10	*R.nitidus*	-0.067466	0.064239
CS19	GD22	CS10	*R.nitidus*	-0.088293	0.06605
JO3	GD21	CS10	*R.nitidus*	-0.084477	0.067334
JO7	GD22	CS10	*R.nitidus*	-0.102389	0.070554
GD25	GD22	CS10	*R.nitidus*	-0.012793	0.06389
GD49	GD22	CS10	*R.nitidus*	0.058651	0.069182
GD46	GD22	CS10	*R.nitidus*	-0.099709	0.062408
CS30	GD22	CS10	*R.nitidus*	-0.132498	0.063348
GD30	GD22	CS10	*R.nitidus*	0.089707	0.065564
GD42	GD22	CS10	*R.nitidus*	-0.067181	0.062948
GD50	GD22	CS10	*R.nitidus*	-0.023445	0.067455
GD40	GD22	CS10	*R.nitidus*	0.003984	0.062304
CS18	GD22	CS10	*R.nitidus*	-0.111184	0.064712
GD26	GD22	CS10	*R.nitidus*	-0.017075	0.064137
GD29	GD22	CS10	*R.nitidus*	0.076112	0.063054
CS16	GD22	CS10	*R.nitidus*	-0.063591	0.063002
CS20	GD22	CS10	*R.nitidus*	-0.105398	0.064895
GD34	GD22	CS10	*R.nitidus*	-0.08427	0.061402
CS12	GD22	CS10	*R.nitidus*	-0.060106	0.066789
CS22	GD22	CS10	*R.nitidus*	-0.061849	0.064593
GD48	GD22	CS10	*R.nitidus*	-0.042373	0.065006
SC15	GD22	CS10	*R.nitidus*	-0.068439	0.064133
CS23	GD22	CS10	*R.nitidus*	-0.118135	0.062825
SC14	GD22	CS10	*R.nitidus*	-0.056005	0.063071
CS17	GD22	CS10	*R.nitidus*	-0.053405	0.066144
CS15	GD22	CS10	*R.nitidus*	-0.048348	0.065846
CS25	GD22	CS10	*R.nitidus*	-0.102326	0.065749
SC5	GD22	CS10	*R.nitidus*	-0.079448	0.068299
CS21	GD22	CS10	*R.nitidus*	-0.095298	0.06419
CS28	GD22	CS10	*R.nitidus*	-0.104195	0.066101
SC18	GD22	CS10	*R.nitidus*	-0.032323	0.064035
SC31	GD22	CS10	*R.nitidus*	-0.088624	0.06547
JW6	GD22	CS10	*R.nitidus*	-0.053719	0.070141
CS24	GD22	CS10	*R.nitidus*	-0.107285	0.064236
CS3	GD22	CS10	*R.nitidus*	-0.095641	0.064194
SC35	GD22	CS10	*R.nitidus*	-0.019363	0.06195
MY4	GD22	CS10	*R.nitidus*	-0.109244	0.065028
CS31	GD22	CS10	*R.nitidus*	-0.094924	0.065456
SC26	GD22	CS10	*R.nitidus*	-0.084165	0.063458
JO1	GD22	CS10	*R.nitidus*	-0.074847	0.064572
SC34	GD22	CS10	*R.nitidus*	-0.042105	0.062726
MY6	GD22	CS10	*R.nitidus*	-0.077947	0.0645
JW4	GD22	CS10	*R.nitidus*	0.163121	0.064466
SC45	GD22	CS10	*R.nitidus*	-0.017341	0.064081
SC6	GD22	CS10	*R.nitidus*	-0.061097	0.066623
SC12	GD22	CS10	*R.nitidus*	-0.077264	0.063171
SC24	GD22	CS10	*R.nitidus*	-0.067017	0.064532
JO7	GD25	CS10	*R.nitidus*	-0.054307	0.072901
SC32	GD22	CS10	*R.nitidus*	-0.023602	0.063092
SC28	GD22	CS10	*R.nitidus*	-0.098186	0.063393
JO5	GD22	CS10	*R.nitidus*	-0.019783	0.063392
SC10	GD22	CS10	*R.nitidus*	0.006716	0.066033
CS4	GD22	CS10	*R.nitidus*	-0.087445	0.061663
SC42	GD22	CS10	*R.nitidus*	0.169321	0.062099

GD31	GD25	CS10	*R.nitidus*	0.005482	0.064374
JW7	GD22	CS10	*R.nitidus*	-0.032811	0.067364
GD22	GD25	CS10	*R.nitidus*	0.012793	0.06389
GD26	GD25	CS10	*R.nitidus*	0.005275	0.065019
SC27	GD22	CS10	*R.nitidus*	-0.059627	0.062323
SC44	GD22	CS10	*R.nitidus*	-0.072559	0.062944
CS11	GD25	CS10	*R.nitidus*	-0.065505	0.067727
GD30	GD25	CS10	*R.nitidus*	0.098478	0.067586
GD33	GD25	CS10	*R.nitidus*	-0.055273	0.065954
GD32	GD25	CS10	*R.nitidus*	-0.029022	0.068145
CS6	GD22	CS10	*R.nitidus*	-0.11908	0.064531
GD29	GD25	CS10	*R.nitidus*	0.066572	0.065145
JO8	GD25	CS10	*R.nitidus*	-0.029053	0.069833
JO3	GD22	CS10	*R.nitidus*	-0.033133	0.067063
GD40	GD25	CS10	*R.nitidus*	-0.009922	0.062492
GD21	GD25	CS10	*R.nitidus*	0.045936	0.064441
GD41	GD25	CS10	*R.nitidus*	-0.038743	0.064722
GD50	GD25	CS10	*R.nitidus*	0.007266	0.069079
GD45	GD25	CS10	*R.nitidus*	0.036269	0.070284
CS17	GD25	CS10	*R.nitidus*	-0.055849	0.068234
GD49	GD25	CS10	*R.nitidus*	0.039758	0.068574
GD48	GD25	CS10	*R.nitidus*	-0.014575	0.065876
CS21	GD25	CS10	*R.nitidus*	-0.09375	0.06593
GD46	GD25	CS10	*R.nitidus*	-0.079529	0.064218
CS25	GD25	CS10	*R.nitidus*	-0.092974	0.066146
CS12	GD25	CS10	*R.nitidus*	-0.07446	0.066775
CS18	GD25	CS10	*R.nitidus*	-0.100942	0.06615
GD35	GD25	CS10	*R.nitidus*	-0.029635	0.06397
SC18	GD25	CS10	*R.nitidus*	-0.03819	0.065743
CS19	GD25	CS10	*R.nitidus*	-0.073388	0.065784
CS4	GD25	CS10	*R.nitidus*	-0.068602	0.064665
CS16	GD25	CS10	*R.nitidus*	-0.064336	0.065946
GD34	GD25	CS10	*R.nitidus*	-0.056976	0.065068
SC6	GD25	CS10	*R.nitidus*	-0.063536	0.065783
GD42	GD25	CS10	*R.nitidus*	-0.040801	0.065105
CS31	GD25	CS10	*R.nitidus*	-0.096195	0.065548
SC31	GD25	CS10	*R.nitidus*	-0.080297	0.063784
GD44	GD25	CS10	*R.nitidus*	-0.050343	0.065579
CS3	GD25	CS10	*R.nitidus*	-0.083217	0.065958
CS13	GD25	CS10	*R.nitidus*	-0.093662	0.067505
CS22	GD25	CS10	*R.nitidus*	-0.052126	0.064612
CS15	GD25	CS10	*R.nitidus*	-0.051476	0.065235
CS24	GD25	CS10	*R.nitidus*	-0.084652	0.064976
SC35	GD25	CS10	*R.nitidus*	-0.025517	0.063945
CS30	GD25	CS10	*R.nitidus*	-0.116984	0.06578
SC14	GD25	CS10	*R.nitidus*	-0.054399	0.064836
CS14	GD25	CS10	*R.nitidus*	-0.071478	0.067394
SC24	GD25	CS10	*R.nitidus*	-0.057891	0.066027
SC42	GD25	CS10	*R.nitidus*	0.166181	0.062351
CS20	GD25	CS10	*R.nitidus*	-0.096115	0.066107
CS28	GD25	CS10	*R.nitidus*	-0.095455	0.066014
SC34	GD25	CS10	*R.nitidus*	-0.039281	0.063449
SC15	GD25	CS10	*R.nitidus*	-0.070341	0.066881
JW7	GD25	CS10	*R.nitidus*	-0.027273	0.068333
CS23	GD25	CS10	*R.nitidus*	-0.114458	0.064417
SC12	GD25	CS10	*R.nitidus*	-0.070839	0.064333

SC5	GD25	CS10	*R.nitidus*	-0.069493	0.068214
GD21	GD26	CS10	*R.nitidus*	0.07971	0.062794
CS6	GD25	CS10	*R.nitidus*	-0.105941	0.065236
SC26	GD25	CS10	*R.nitidus*	-0.084239	0.065163
SC10	GD25	CS10	*R.nitidus*	0.000719	0.064214
JO8	GD26	CS10	*R.nitidus*	-0.040952	0.072565
MY6	GD25	CS10	*R.nitidus*	-0.072364	0.064428
JW6	GD25	CS10	*R.nitidus*	-0.043001	0.070995
JO7	GD26	CS10	*R.nitidus*	-0.088314	0.071785
SC28	GD25	CS10	*R.nitidus*	-0.092517	0.064503
SC27	GD25	CS10	*R.nitidus*	-0.07186	0.063838
JW4	GD25	CS10	*R.nitidus*	0.165483	0.064648
GD30	GD26	CS10	*R.nitidus*	0.10796	0.066339
SC44	GD25	CS10	*R.nitidus*	-0.064677	0.063802
CS11	GD26	CS10	*R.nitidus*	-0.053465	0.068212
SC45	GD25	CS10	*R.nitidus*	-0.027273	0.065293
JO3	GD25	CS10	*R.nitidus*	-0.017256	0.068423
MY4	GD25	CS10	*R.nitidus*	-0.105802	0.065002
SC32	GD25	CS10	*R.nitidus*	-0.019359	0.063306
GD22	GD26	CS10	*R.nitidus*	0.017075	0.064137
GD41	GD26	CS10	*R.nitidus*	-0.049512	0.062875
CS12	GD26	CS10	*R.nitidus*	-0.048319	0.066768
GD31	GD26	CS10	*R.nitidus*	0.032925	0.066038
GD29	GD26	CS10	*R.nitidus*	0.073171	0.062776
JO5	GD25	CS10	*R.nitidus*	-0.033793	0.063831
GD32	GD26	CS10	*R.nitidus*	-0.015038	0.062223
GD35	GD26	CS10	*R.nitidus*	-0.015695	0.065018
GD42	GD26	CS10	*R.nitidus*	-0.020233	0.065702
GD49	GD26	CS10	*R.nitidus*	0.037267	0.066666
JO1	GD25	CS10	*R.nitidus*	-0.085357	0.064306
GD25	GD26	CS10	*R.nitidus*	-0.005275	0.065019
GD33	GD26	CS10	*R.nitidus*	-0.053165	0.063567
GD44	GD26	CS10	*R.nitidus*	-0.038401	0.065934
CS30	GD26	CS10	*R.nitidus*	-0.112336	0.064944
CS17	GD26	CS10	*R.nitidus*	-0.038596	0.068994
CS22	GD26	CS10	*R.nitidus*	-0.060751	0.064806
CS28	GD26	CS10	*R.nitidus*	-0.090778	0.066072
GD40	GD26	CS10	*R.nitidus*	0.01614	0.062219
GD46	GD26	CS10	*R.nitidus*	-0.07377	0.062614
GD45	GD26	CS10	*R.nitidus*	0.03255	0.068857
GD50	GD26	CS10	*R.nitidus*	-0.019355	0.067313
CS16	GD26	CS10	*R.nitidus*	-0.054125	0.065226
GD34	GD26	CS10	*R.nitidus*	-0.063232	0.062622
CS14	GD26	CS10	*R.nitidus*	-0.059419	0.068257
CS19	GD26	CS10	*R.nitidus*	-0.066265	0.067001
CS15	GD26	CS10	*R.nitidus*	-0.036113	0.066925
CS21	GD26	CS10	*R.nitidus*	-0.076375	0.066469
SC6	GD26	CS10	*R.nitidus*	-0.067705	0.066107
CS13	GD26	CS10	*R.nitidus*	-0.080656	0.068001
SC10	GD26	CS10	*R.nitidus*	0.010331	0.066379
GD48	GD26	CS10	*R.nitidus*	-0.030008	0.066239
CS3	GD26	CS10	*R.nitidus*	-0.078405	0.066731
SC14	GD26	CS10	*R.nitidus*	-0.04867	0.065191
SC26	GD26	CS10	*R.nitidus*	-0.082219	0.065679
SC28	GD26	CS10	*R.nitidus*	-0.081605	0.064925
SC12	GD26	CS10	*R.nitidus*	-0.071572	0.063814

CS18	GD26	CS10	*R.nitidus*	-0.095593	0.065839
SC15	GD26	CS10	*R.nitidus*	-0.053705	0.064945
SC18	GD26	CS10	*R.nitidus*	-0.027197	0.064174
CS24	GD26	CS10	*R.nitidus*	-0.089583	0.06487
CS20	GD26	CS10	*R.nitidus*	-0.084204	0.066676
JW7	GD26	CS10	*R.nitidus*	-0.009509	0.069711
CS25	GD26	CS10	*R.nitidus*	-0.072629	0.066477
CS23	GD26	CS10	*R.nitidus*	-0.101421	0.063134
SC5	GD26	CS10	*R.nitidus*	-0.060924	0.067117
SC42	GD26	CS10	*R.nitidus*	0.173913	0.062356
JO3	GD26	CS10	*R.nitidus*	-0.020801	0.069662
CS31	GD26	CS10	*R.nitidus*	-0.086231	0.065391
CS4	GD26	CS10	*R.nitidus*	-0.076923	0.06328
SC35	GD26	CS10	*R.nitidus*	-0.025574	0.063855
CS11	GD29	CS10	*R.nitidus*	-0.128769	0.0666
SC34	GD26	CS10	*R.nitidus*	-0.032744	0.064053
CS6	GD26	CS10	*R.nitidus*	-0.098563	0.065438
JW6	GD26	CS10	*R.nitidus*	-0.059701	0.071234
JW4	GD26	CS10	*R.nitidus*	0.185933	0.067106
SC24	GD26	CS10	*R.nitidus*	-0.056164	0.064483
GD42	GD29	CS10	*R.nitidus*	-0.11853	0.063804
SC27	GD26	CS10	*R.nitidus*	-0.056099	0.064854
GD35	GD29	CS10	*R.nitidus*	-0.071972	0.064519
GD22	GD29	CS10	*R.nitidus*	-0.076112	0.063054
JO8	GD29	CS10	*R.nitidus*	-0.112875	0.067557
SC32	GD26	CS10	*R.nitidus*	-0.012667	0.0649
MY6	GD26	CS10	*R.nitidus*	-0.061883	0.065676
JO7	GD29	CS10	*R.nitidus*	-0.156514	0.067585
MY4	GD26	CS10	*R.nitidus*	-0.08502	0.0653
SC31	GD26	CS10	*R.nitidus*	-0.066883	0.066055
SC45	GD26	CS10	*R.nitidus*	-0.012024	0.06454
SC44	GD26	CS10	*R.nitidus*	-0.052239	0.064749
GD30	GD29	CS10	*R.nitidus*	0.031579	0.065387
GD21	GD29	CS10	*R.nitidus*	-0.002235	0.064647
GD26	GD29	CS10	*R.nitidus*	-0.073171	0.062776
JO5	GD26	CS10	*R.nitidus*	-0.003989	0.064685
GD31	GD29	CS10	*R.nitidus*	-0.048276	0.063587
GD48	GD29	CS10	*R.nitidus*	-0.092874	0.063973
GD33	GD29	CS10	*R.nitidus*	-0.130469	0.06316
JO1	GD26	CS10	*R.nitidus*	-0.067775	0.063911
CS14	GD29	CS10	*R.nitidus*	-0.133838	0.066993
GD41	GD29	CS10	*R.nitidus*	-0.114138	0.063486
CS12	GD29	CS10	*R.nitidus*	-0.116342	0.065331
GD45	GD29	CS10	*R.nitidus*	-0.042857	0.068594
GD46	GD29	CS10	*R.nitidus*	-0.164341	0.064697
CS13	GD29	CS10	*R.nitidus*	-0.155654	0.066217
GD25	GD29	CS10	*R.nitidus*	-0.066572	0.065145
GD50	GD29	CS10	*R.nitidus*	-0.090009	0.066481
CS19	GD29	CS10	*R.nitidus*	-0.136667	0.064704
GD32	GD29	CS10	*R.nitidus*	-0.109495	0.064563
CS25	GD29	CS10	*R.nitidus*	-0.145778	0.063889
CS20	GD29	CS10	*R.nitidus*	-0.151803	0.064092
GD34	GD29	CS10	*R.nitidus*	-0.159967	0.061509
CS15	GD29	CS10	*R.nitidus*	-0.099564	0.06563
GD40	GD29	CS10	*R.nitidus*	-0.063492	0.063329
CS21	GD29	CS10	*R.nitidus*	-0.145833	0.063972

SC15	GD29	CS10	*R.nitidus*	-0.13058	0.064412
GD44	GD29	CS10	*R.nitidus*	-0.131635	0.065408
CS3	GD29	CS10	*R.nitidus*	-0.153946	0.063267
SC10	GD29	CS10	*R.nitidus*	-0.052069	0.066206
CS28	GD29	CS10	*R.nitidus*	-0.150331	0.065291
CS17	GD29	CS10	*R.nitidus*	-0.111713	0.06746
GD49	GD29	CS10	*R.nitidus*	-0.033159	0.067222
SC14	GD29	CS10	*R.nitidus*	-0.10971	0.0627
CS30	GD29	CS10	*R.nitidus*	-0.17942	0.061769
CS16	GD29	CS10	*R.nitidus*	-0.1279	0.063912
SC31	GD29	CS10	*R.nitidus*	-0.127306	0.063252
CS18	GD29	CS10	*R.nitidus*	-0.150685	0.064103
CS4	GD29	CS10	*R.nitidus*	-0.13852	0.06149
SC18	GD29	CS10	*R.nitidus*	-0.086436	0.064503
CS23	GD29	CS10	*R.nitidus*	-0.16441	0.061875
SC35	GD29	CS10	*R.nitidus*	-0.076087	0.063371
SC42	GD29	CS10	*R.nitidus*	0.107744	0.062002
CS6	GD29	CS10	*R.nitidus*	-0.154864	0.063444
CS22	GD29	CS10	*R.nitidus*	-0.122909	0.063494
JW7	GD29	CS10	*R.nitidus*	-0.087343	0.066512
JO8	GD30	CS10	*R.nitidus*	-0.089535	0.075832
CS24	GD29	CS10	*R.nitidus*	-0.155836	0.06336
JO3	GD29	CS10	*R.nitidus*	-0.093145	0.064798
SC27	GD29	CS10	*R.nitidus*	-0.120436	0.063
SC32	GD29	CS10	*R.nitidus*	-0.095208	0.064338
SC5	GD29	CS10	*R.nitidus*	-0.130976	0.068713
SC12	GD29	CS10	*R.nitidus*	-0.13038	0.064645
SC28	GD29	CS10	*R.nitidus*	-0.15	0.063438
JO1	GD29	CS10	*R.nitidus*	-0.1284	0.064565
JO7	GD30	CS10	*R.nitidus*	-0.155604	0.073521
CS31	GD29	CS10	*R.nitidus*	-0.157413	0.065137
SC26	GD29	CS10	*R.nitidus*	-0.147151	0.064711
SC6	GD29	CS10	*R.nitidus*	-0.129929	0.064094
CS11	GD30	CS10	*R.nitidus*	-0.125	0.070597
GD25	GD30	CS10	*R.nitidus*	-0.098478	0.067586
MY6	GD29	CS10	*R.nitidus*	-0.127646	0.063119
JW6	GD29	CS10	*R.nitidus*	-0.12628	0.068021
SC24	GD29	CS10	*R.nitidus*	-0.117722	0.063788
SC34	GD29	CS10	*R.nitidus*	-0.084353	0.063444
GD21	GD30	CS10	*R.nitidus*	-0.032197	0.065913
JO5	GD29	CS10	*R.nitidus*	-0.087516	0.063962
JW4	GD29	CS10	*R.nitidus*	0.10903	0.066418
SC44	GD29	CS10	*R.nitidus*	-0.11541	0.065096
GD46	GD30	CS10	*R.nitidus*	-0.171429	0.064747
MY4	GD29	CS10	*R.nitidus*	-0.159669	0.064077
GD34	GD30	CS10	*R.nitidus*	-0.160406	0.062515
SC45	GD29	CS10	*R.nitidus*	-0.083907	0.064425
GD26	GD30	CS10	*R.nitidus*	-0.10796	0.066339
GD31	GD30	CS10	*R.nitidus*	-0.105121	0.066338
GD50	GD30	CS10	*R.nitidus*	-0.10124	0.071251
GD41	GD30	CS10	*R.nitidus*	-0.105769	0.064174
GD35	GD30	CS10	*R.nitidus*	-0.106419	0.067094
CS28	GD30	CS10	*R.nitidus*	-0.164948	0.068344
GD49	GD30	CS10	*R.nitidus*	-0.066132	0.070792
CS12	GD30	CS10	*R.nitidus*	-0.122483	0.070729
SC5	GD30	CS10	*R.nitidus*	-0.130709	0.070061

GD22	GD30	CS10	*R.nitidus*	-0.089707	0.065564
CS30	GD30	CS10	*R.nitidus*	-0.172749	0.067059
CS17	GD30	CS10	*R.nitidus*	-0.114428	0.070635
GD29	GD30	CS10	*R.nitidus*	-0.031579	0.065387
GD45	GD30	CS10	*R.nitidus*	-0.056405	0.068999
GD32	GD30	CS10	*R.nitidus*	-0.134634	0.070388
CS16	GD30	CS10	*R.nitidus*	-0.115323	0.067402
CS24	GD30	CS10	*R.nitidus*	-0.155573	0.065605
CS3	GD30	CS10	*R.nitidus*	-0.1407	0.066737
GD48	GD30	CS10	*R.nitidus*	-0.066863	0.068003
CS22	GD30	CS10	*R.nitidus*	-0.12609	0.067308
GD33	GD30	CS10	*R.nitidus*	-0.112731	0.067177
CS15	GD30	CS10	*R.nitidus*	-0.10306	0.069057
SC10	GD30	CS10	*R.nitidus*	-0.078448	0.069903
GD44	GD30	CS10	*R.nitidus*	-0.119565	0.068315
CS13	GD30	CS10	*R.nitidus*	-0.146382	0.069513
CS14	GD30	CS10	*R.nitidus*	-0.120355	0.071371
CS21	GD30	CS10	*R.nitidus*	-0.161562	0.06737
GD40	GD30	CS10	*R.nitidus*	-0.066327	0.066458
GD42	GD30	CS10	*R.nitidus*	-0.125815	0.066977
SC26	GD30	CS10	*R.nitidus*	-0.140123	0.067162
SC6	GD30	CS10	*R.nitidus*	-0.135891	0.067221
SC24	GD30	CS10	*R.nitidus*	-0.128627	0.067928
SC18	GD30	CS10	*R.nitidus*	-0.081699	0.067458
SC27	GD30	CS10	*R.nitidus*	-0.135474	0.066652
CS20	GD30	CS10	*R.nitidus*	-0.152767	0.068381
SC12	GD30	CS10	*R.nitidus*	-0.128083	0.066527
CS19	GD30	CS10	*R.nitidus*	-0.12966	0.068776
CS18	GD30	CS10	*R.nitidus*	-0.150394	0.067593
SC14	GD30	CS10	*R.nitidus*	-0.10456	0.067189
CS23	GD30	CS10	*R.nitidus*	-0.154383	0.066929
JW4	GD30	CS10	*R.nitidus*	0.114618	0.069554
SC28	GD30	CS10	*R.nitidus*	-0.140856	0.065647
CS31	GD30	CS10	*R.nitidus*	-0.136139	0.068605
SC15	GD30	CS10	*R.nitidus*	-0.123077	0.069234
CS6	GD30	CS10	*R.nitidus*	-0.162602	0.066968
CS25	GD30	CS10	*R.nitidus*	-0.141196	0.070731
SC45	GD30	CS10	*R.nitidus*	-0.087853	0.066302
CS4	GD30	CS10	*R.nitidus*	-0.135812	0.064163
JO1	GD30	CS10	*R.nitidus*	-0.149644	0.06453
GD26	GD31	CS10	*R.nitidus*	-0.032925	0.066038
JW7	GD30	CS10	*R.nitidus*	-0.092803	0.070951
JO3	GD30	CS10	*R.nitidus*	-0.121154	0.070373
SC35	GD30	CS10	*R.nitidus*	-0.07192	0.066661
SC42	GD30	CS10	*R.nitidus*	0.120498	0.064347
SC34	GD30	CS10	*R.nitidus*	-0.084261	0.067276
JO7	GD31	CS10	*R.nitidus*	-0.075543	0.072343
CS11	GD31	CS10	*R.nitidus*	-0.066713	0.067859
MY4	GD30	CS10	*R.nitidus*	-0.162569	0.067215
SC31	GD30	CS10	*R.nitidus*	-0.136914	0.067122
GD30	GD31	CS10	*R.nitidus*	0.105121	0.066338
JO8	GD31	CS10	*R.nitidus*	-0.024272	0.071807
GD32	GD31	CS10	*R.nitidus*	-0.033304	0.069249
GD25	GD31	CS10	*R.nitidus*	-0.005482	0.064374
SC32	GD30	CS10	*R.nitidus*	-0.086687	0.067648
SC44	GD30	CS10	*R.nitidus*	-0.105691	0.065214

JW6	GD30	CS10	*R.nitidus*	-0.129202	0.072783
MY6	GD30	CS10	*R.nitidus*	-0.133122	0.066087
CS12	GD31	CS10	*R.nitidus*	-0.057143	0.06654
GD41	GD31	CS10	*R.nitidus*	-0.045872	0.066367
JO5	GD30	CS10	*R.nitidus*	-0.079937	0.067482
CS16	GD31	CS10	*R.nitidus*	-0.051049	0.066959
GD21	GD31	CS10	*R.nitidus*	0.080092	0.063451
CS13	GD31	CS10	*R.nitidus*	-0.096891	0.067892
GD45	GD31	CS10	*R.nitidus*	0	0.069519
GD42	GD31	CS10	*R.nitidus*	-0.029056	0.065747
GD34	GD31	CS10	*R.nitidus*	-0.055901	0.064564
GD48	GD31	CS10	*R.nitidus*	-0.023057	0.068067
GD29	GD31	CS10	*R.nitidus*	0.048276	0.063587
GD22	GD31	CS10	*R.nitidus*	0.020316	0.066297
GD35	GD31	CS10	*R.nitidus*	-0.022438	0.064932
GD44	GD31	CS10	*R.nitidus*	-0.051571	0.067601
GD49	GD31	CS10	*R.nitidus*	0.049778	0.071574
CS22	GD31	CS10	*R.nitidus*	-0.066239	0.067126
GD40	GD31	CS10	*R.nitidus*	0.01766	0.064677
GD33	GD31	CS10	*R.nitidus*	-0.049254	0.065056
SC6	GD31	CS10	*R.nitidus*	-0.056485	0.065502
GD50	GD31	CS10	*R.nitidus*	-0.025093	0.069425
CS24	GD31	CS10	*R.nitidus*	-0.103175	0.066616
CS14	GD31	CS10	*R.nitidus*	-0.054765	0.069645
CS28	GD31	CS10	*R.nitidus*	-0.087121	0.065813
CS31	GD31	CS10	*R.nitidus*	-0.085629	0.067671
CS19	GD31	CS10	*R.nitidus*	-0.101732	0.068532
CS21	GD31	CS10	*R.nitidus*	-0.087324	0.067464
SC5	GD31	CS10	*R.nitidus*	-0.068017	0.069191
GD46	GD31	CS10	*R.nitidus*	-0.068755	0.06404
SC12	GD31	CS10	*R.nitidus*	-0.06344	0.064474
CS15	GD31	CS10	*R.nitidus*	-0.047983	0.066382
CS20	GD31	CS10	*R.nitidus*	-0.098947	0.066491
CS6	GD31	CS10	*R.nitidus*	-0.103348	0.068355
CS17	GD31	CS10	*R.nitidus*	-0.055348	0.069841
SC26	GD31	CS10	*R.nitidus*	-0.0811	0.06484
SC14	GD31	CS10	*R.nitidus*	-0.050859	0.065153
CS18	GD31	CS10	*R.nitidus*	-0.100858	0.067676
SC35	GD31	CS10	*R.nitidus*	-0.02814	0.063738
CS23	GD31	CS10	*R.nitidus*	-0.114685	0.065946
SC31	GD31	CS10	*R.nitidus*	-0.071478	0.06589
SC32	GD31	CS10	*R.nitidus*	-0.008731	0.064579
SC18	GD31	CS10	*R.nitidus*	-0.021914	0.066193
SC24	GD31	CS10	*R.nitidus*	-0.054945	0.066508
JO3	GD31	CS10	*R.nitidus*	-0.028007	0.071116
CS3	GD31	CS10	*R.nitidus*	-0.089425	0.065978
CS4	GD31	CS10	*R.nitidus*	-0.07079	0.065408
CS25	GD31	CS10	*R.nitidus*	-0.092974	0.066576
CS30	GD31	CS10	*R.nitidus*	-0.126386	0.066875
SC10	GD31	CS10	*R.nitidus*	0.005054	0.066404
SC15	GD31	CS10	*R.nitidus*	-0.062902	0.066866
JW7	GD31	CS10	*R.nitidus*	-0.050202	0.069221
SC28	GD31	CS10	*R.nitidus*	-0.09193	0.064898
MY6	GD31	CS10	*R.nitidus*	-0.071127	0.065551
SC45	GD31	CS10	*R.nitidus*	-0.034955	0.065387
SC27	GD31	CS10	*R.nitidus*	-0.049931	0.065387

GD31	GD32	CS10	*R.nitidus*	0.033304	0.069249
CS12	GD32	CS10	*R.nitidus*	-0.01664	0.068759
SC42	GD31	CS10	*R.nitidus*	0.162099	0.06377
SC34	GD31	CS10	*R.nitidus*	-0.030261	0.065787
JO8	GD32	CS10	*R.nitidus*	0.034225	0.076568
JO5	GD31	CS10	*R.nitidus*	-0.035664	0.066509
JW6	GD31	CS10	*R.nitidus*	-0.06345	0.071392
JO1	GD31	CS10	*R.nitidus*	-0.07476	0.065336
GD40	GD32	CS10	*R.nitidus*	0.042742	0.065765
GD34	GD32	CS10	*R.nitidus*	-0.018182	0.0639
GD21	GD32	CS10	*R.nitidus*	0.089974	0.064799
JW4	GD31	CS10	*R.nitidus*	0.152443	0.067089
SC44	GD31	CS10	*R.nitidus*	-0.060172	0.065343
JO7	GD32	CS10	*R.nitidus*	-0.025667	0.074205
GD48	GD32	CS10	*R.nitidus*	0.010387	0.066125
GD22	GD32	CS10	*R.nitidus*	0.04932	0.065598
MY4	GD31	CS10	*R.nitidus*	-0.099067	0.067058
GD25	GD32	CS10	*R.nitidus*	0.029022	0.068145
GD33	GD32	CS10	*R.nitidus*	-0.025243	0.065554
CS18	GD32	CS10	*R.nitidus*	-0.064241	0.069364
GD42	GD32	CS10	*R.nitidus*	-0.017715	0.068953
GD49	GD32	CS10	*R.nitidus*	0.085239	0.071134
GD35	GD32	CS10	*R.nitidus*	0.012605	0.068373
GD26	GD32	CS10	*R.nitidus*	0.015038	0.062223
CS11	GD32	CS10	*R.nitidus*	-0.031746	0.070429
GD29	GD32	CS10	*R.nitidus*	0.109495	0.064563
CS14	GD32	CS10	*R.nitidus*	-0.025424	0.069449
GD30	GD32	CS10	*R.nitidus*	0.134634	0.070388
CS13	GD32	CS10	*R.nitidus*	-0.046189	0.068986
GD46	GD32	CS10	*R.nitidus*	-0.054264	0.066386
CS16	GD32	CS10	*R.nitidus*	-0.031226	0.068034
GD45	GD32	CS10	*R.nitidus*	0.033708	0.072371
CS28	GD32	CS10	*R.nitidus*	-0.057899	0.069466
CS17	GD32	CS10	*R.nitidus*	-0.008864	0.072341
CS4	GD32	CS10	*R.nitidus*	-0.045101	0.065562
SC6	GD32	CS10	*R.nitidus*	-0.020882	0.069498
SC12	GD32	CS10	*R.nitidus*	-0.03211	0.067054
SC10	GD32	CS10	*R.nitidus*	0.055686	0.068228
CS19	GD32	CS10	*R.nitidus*	-0.034351	0.06859
GD44	GD32	CS10	*R.nitidus*	0.003562	0.06968
GD41	GD32	CS10	*R.nitidus*	-0.027778	0.068456
CS22	GD32	CS10	*R.nitidus*	-0.02381	0.067525
CS25	GD32	CS10	*R.nitidus*	-0.034056	0.070049
CS30	GD32	CS10	*R.nitidus*	-0.069767	0.069605
GD50	GD32	CS10	*R.nitidus*	0.017635	0.071209
CS31	GD32	CS10	*R.nitidus*	-0.050654	0.069458
CS15	GD32	CS10	*R.nitidus*	-0.000763	0.068375
CS20	GD32	CS10	*R.nitidus*	-0.046923	0.068765
CS3	GD32	CS10	*R.nitidus*	-0.051322	0.068796
CS21	GD32	CS10	*R.nitidus*	-0.034612	0.069998
CS23	GD32	CS10	*R.nitidus*	-0.080364	0.067607
SC44	GD32	CS10	*R.nitidus*	0.010552	0.06775
JW6	GD32	CS10	*R.nitidus*	0.01277	0.07289
CS24	GD32	CS10	*R.nitidus*	-0.059761	0.067887
SC5	GD32	CS10	*R.nitidus*	0.002427	0.072026
SC15	GD32	CS10	*R.nitidus*	-0.002439	0.068269

SC34	GD32	CS10	*R.nitidus*	0.001522	0.066607
CS6	GD32	CS10	*R.nitidus*	-0.07172	0.069126
SC32	GD32	CS10	*R.nitidus*	0.039246	0.068038
SC18	GD32	CS10	*R.nitidus*	0.041599	0.067915
SC14	GD32	CS10	*R.nitidus*	0.005473	0.068086
JW4	GD32	CS10	*R.nitidus*	0.216718	0.067485
JO7	CS12	CS10	*R.nitidus*	-0.009643	0.073468
JO1	GD32	CS10	*R.nitidus*	-0.025354	0.067679
SC26	GD32	CS10	*R.nitidus*	-0.025542	0.067431
JW7	GD32	CS10	*R.nitidus*	0.038188	0.069515
JO5	GD32	CS10	*R.nitidus*	0.051482	0.067971
MY4	GD32	CS10	*R.nitidus*	-0.062156	0.070985
SC28	GD32	CS10	*R.nitidus*	-0.015711	0.065962
GD33	CS12	CS10	*R.nitidus*	0.004814	0.066648
SC42	GD32	CS10	*R.nitidus*	0.206238	0.064314
JO8	CS12	CS10	*R.nitidus*	0.008902	0.073129
SC35	GD32	CS10	*R.nitidus*	0.045697	0.066122
GD32	CS12	CS10	*R.nitidus*	0.01664	0.068759
SC24	GD32	CS10	*R.nitidus*	-0.012618	0.066761
SC31	GD32	CS10	*R.nitidus*	-0.024133	0.068695
SC27	GD32	CS10	*R.nitidus*	-0.003108	0.067844
GD29	CS12	CS10	*R.nitidus*	0.116342	0.065331
GD21	CS12	CS10	*R.nitidus*	0.135246	0.066918
GD50	CS12	CS10	*R.nitidus*	0.029167	0.070938
JO3	GD32	CS10	*R.nitidus*	0.00543	0.070626
CS11	CS12	CS10	*R.nitidus*	-0.005035	0.070237
GD30	CS12	CS10	*R.nitidus*	0.122483	0.070729
SC45	GD32	CS10	*R.nitidus*	0.031746	0.06647
MY6	GD32	CS10	*R.nitidus*	-0.033988	0.069288
GD25	CS12	CS10	*R.nitidus*	0.07446	0.066775
GD45	CS12	CS10	*R.nitidus*	0.059446	0.070564
CS14	CS12	CS10	*R.nitidus*	0	0.071393
GD49	CS12	CS10	*R.nitidus*	0.094694	0.069339
GD22	CS12	CS10	*R.nitidus*	0.060106	0.066789
GD41	CS12	CS10	*R.nitidus*	0.015089	0.06606
CS13	CS12	CS10	*R.nitidus*	-0.056818	0.071436
GD46	CS12	CS10	*R.nitidus*	-0.007948	0.067847
GD34	CS12	CS10	*R.nitidus*	0.002052	0.065619
GD42	CS12	CS10	*R.nitidus*	0.019844	0.066631
GD26	CS12	CS10	*R.nitidus*	0.048319	0.066768
CS19	CS12	CS10	*R.nitidus*	-0.012403	0.068729
GD31	CS12	CS10	*R.nitidus*	0.057143	0.06654
CS18	CS12	CS10	*R.nitidus*	-0.030735	0.066945
CS3	CS12	CS10	*R.nitidus*	-0.008252	0.066668
GD35	CS12	CS10	*R.nitidus*	0.04878	0.067725
GD40	CS12	CS10	*R.nitidus*	0.070827	0.066135
SC26	CS12	CS10	*R.nitidus*	-0.013235	0.066818
CS24	CS12	CS10	*R.nitidus*	-0.046384	0.066799
CS21	CS12	CS10	*R.nitidus*	-0.022288	0.065905
CS30	CS12	CS10	*R.nitidus*	-0.059004	0.066828
CS25	CS12	CS10	*R.nitidus*	-0.011485	0.067974
CS22	CS12	CS10	*R.nitidus*	-0.003779	0.066869
SC12	CS12	CS10	*R.nitidus*	-0.001462	0.066851
GD48	CS12	CS10	*R.nitidus*	0.042074	0.068055
SC15	CS12	CS10	*R.nitidus*	-0.005385	0.066906
CS28	CS12	CS10	*R.nitidus*	-0.022801	0.065981

CS17	CS12	CS10	*R.nitidus*	0.00883	0.070835
CS31	CS12	CS10	*R.nitidus*	-0.037736	0.067301
SC18	CS12	CS10	*R.nitidus*	0.060427	0.06753
CS4	CS12	CS10	*R.nitidus*	-0.027007	0.064364
CS16	CS12	CS10	*R.nitidus*	0.007982	0.064796
GD44	CS12	CS10	*R.nitidus*	0.026647	0.067566
CS15	CS12	CS10	*R.nitidus*	0.033684	0.066348
CS20	CS12	CS10	*R.nitidus*	-0.018195	0.067815
JW4	CS12	CS10	*R.nitidus*	0.213256	0.06614
SC44	CS12	CS10	*R.nitidus*	0.016094	0.065915
SC31	CS12	CS10	*R.nitidus*	-0.009413	0.068049
SC34	CS12	CS10	*R.nitidus*	0.026588	0.064715
SC32	CS12	CS10	*R.nitidus*	0.059043	0.068141
SC5	CS12	CS10	*R.nitidus*	0.014524	0.068404
SC35	CS12	CS10	*R.nitidus*	0.050355	0.064598
CS23	CS12	CS10	*R.nitidus*	-0.045388	0.065684
SC10	CS12	CS10	*R.nitidus*	0.096125	0.067995
SC14	CS12	CS10	*R.nitidus*	0.017921	0.067387
SC6	CS12	CS10	*R.nitidus*	-0.007582	0.069321
CS11	GD33	CS10	*R.nitidus*	-0.017442	0.067012
MY6	CS12	CS10	*R.nitidus*	-0.012023	0.067695
JO7	GD33	CS10	*R.nitidus*	-0.032345	0.072783
SC24	CS12	CS10	*R.nitidus*	0.020378	0.066753
SC28	CS12	CS10	*R.nitidus*	-0.02169	0.065314
SC27	CS12	CS10	*R.nitidus*	0.016591	0.065484
JO8	GD33	CS10	*R.nitidus*	0.018365	0.072426
CS12	GD33	CS10	*R.nitidus*	-0.004814	0.066648
JO3	CS12	CS10	*R.nitidus*	0.019124	0.069914
SC42	CS12	CS10	*R.nitidus*	0.242259	0.063423
JW7	CS12	CS10	*R.nitidus*	0.034884	0.071018
JW6	CS12	CS10	*R.nitidus*	0.00188	0.07275
CS6	CS12	CS10	*R.nitidus*	-0.052632	0.065765
GD34	GD33	CS10	*R.nitidus*	-0.028855	0.063784
SC45	CS12	CS10	*R.nitidus*	0.06355	0.067352
GD21	GD33	CS10	*R.nitidus*	0.134723	0.063058
JO1	CS12	CS10	*R.nitidus*	0.000747	0.066365
GD42	GD33	CS10	*R.nitidus*	0	0.066532
MY4	CS12	CS10	*R.nitidus*	-0.047238	0.069151
CS13	GD33	CS10	*R.nitidus*	-0.042904	0.066966
GD30	GD33	CS10	*R.nitidus*	0.112731	0.067177
GD46	GD33	CS10	*R.nitidus*	-0.033077	0.06441
GD25	GD33	CS10	*R.nitidus*	0.055273	0.065954
GD22	GD33	CS10	*R.nitidus*	0.05864	0.062705
GD31	GD33	CS10	*R.nitidus*	0.049254	0.065056
JO5	CS12	CS10	*R.nitidus*	0.045488	0.065394
GD29	GD33	CS10	*R.nitidus*	0.130469	0.06316
CS22	GD33	CS10	*R.nitidus*	-0.008672	0.065165
GD49	GD33	CS10	*R.nitidus*	0.074692	0.070274
CS14	GD33	CS10	*R.nitidus*	-0.012574	0.067104
GD32	GD33	CS10	*R.nitidus*	0.025243	0.065554
GD44	GD33	CS10	*R.nitidus*	-0.005279	0.065053
GD45	GD33	CS10	*R.nitidus*	0.058252	0.068826
GD35	GD33	CS10	*R.nitidus*	0.035714	0.06337
GD41	GD33	CS10	*R.nitidus*	0.004552	0.064742
GD26	GD33	CS10	*R.nitidus*	0.053165	0.063567
GD48	GD33	CS10	*R.nitidus*	0.021533	0.067973

CS17	GD33	CS10	*R.nitidus*	0.001411	0.067736
CS31	GD33	CS10	*R.nitidus*	-0.036577	0.065462
CS20	GD33	CS10	*R.nitidus*	-0.043738	0.066786
CS25	GD33	CS10	*R.nitidus*	-0.032867	0.067131
GD40	GD33	CS10	*R.nitidus*	0.053546	0.064405
GD50	GD33	CS10	*R.nitidus*	0.012903	0.07047
CS16	GD33	CS10	*R.nitidus*	-0.007138	0.064044
CS21	GD33	CS10	*R.nitidus*	-0.038714	0.065953
CS30	GD33	CS10	*R.nitidus*	-0.078029	0.065546
CS28	GD33	CS10	*R.nitidus*	-0.057329	0.06541
CS23	GD33	CS10	*R.nitidus*	-0.06658	0.06397
CS15	GD33	CS10	*R.nitidus*	0.003911	0.066429
CS3	GD33	CS10	*R.nitidus*	-0.043272	0.06587
CS19	GD33	CS10	*R.nitidus*	-0.027349	0.067457
SC31	GD33	CS10	*R.nitidus*	-0.034437	0.066363
JW4	GD33	CS10	*R.nitidus*	0.220946	0.066532
CS24	GD33	CS10	*R.nitidus*	-0.047423	0.066748
CS18	GD33	CS10	*R.nitidus*	-0.05325	0.066006
SC6	GD33	CS10	*R.nitidus*	-0.014151	0.065899
SC28	GD33	CS10	*R.nitidus*	-0.028724	0.064518
SC10	GD33	CS10	*R.nitidus*	0.044551	0.067754
SC5	GD33	CS10	*R.nitidus*	-0.007037	0.069222
SC44	GD33	CS10	*R.nitidus*	-0.009402	0.065256
SC14	GD33	CS10	*R.nitidus*	0.011075	0.06509
CS6	GD33	CS10	*R.nitidus*	-0.068587	0.064885
SC26	GD33	CS10	*R.nitidus*	-0.038102	0.064311
JW6	GD33	CS10	*R.nitidus*	0.017683	0.071738
SC35	GD33	CS10	*R.nitidus*	0.019949	0.065855
SC24	GD33	CS10	*R.nitidus*	-0.011273	0.063754
MY4	GD33	CS10	*R.nitidus*	-0.049399	0.064863
CS4	GD33	CS10	*R.nitidus*	-0.034806	0.064115
GD26	GD34	CS10	*R.nitidus*	0.063232	0.062622
SC27	GD33	CS10	*R.nitidus*	-0.005886	0.063896
SC12	GD33	CS10	*R.nitidus*	-0.023984	0.066449
SC18	GD33	CS10	*R.nitidus*	0.01546	0.065131
GD25	GD34	CS10	*R.nitidus*	0.056976	0.065068
SC42	GD33	CS10	*R.nitidus*	0.210954	0.063696
GD29	GD34	CS10	*R.nitidus*	0.159967	0.061509
SC45	GD33	CS10	*R.nitidus*	0.042735	0.064975
SC15	GD33	CS10	*R.nitidus*	-0.015162	0.065705
JW7	GD33	CS10	*R.nitidus*	0.035938	0.069444
SC34	GD33	CS10	*R.nitidus*	0.000657	0.064196
SC32	GD33	CS10	*R.nitidus*	0.037849	0.064794
JO5	GD33	CS10	*R.nitidus*	0.035363	0.064948
CS11	GD34	CS10	*R.nitidus*	-0.010821	0.066218
GD21	GD34	CS10	*R.nitidus*	0.146517	0.060253
JO3	GD33	CS10	*R.nitidus*	0.021672	0.068756
JO7	GD34	CS10	*R.nitidus*	-0.014085	0.069962
MY6	GD33	CS10	*R.nitidus*	-0.020039	0.06467
GD31	GD34	CS10	*R.nitidus*	0.055901	0.064564
GD48	GD34	CS10	*R.nitidus*	0.047619	0.065008
GD30	GD34	CS10	*R.nitidus*	0.160406	0.062515
JO8	GD34	CS10	*R.nitidus*	0.049587	0.069072
GD42	GD34	CS10	*R.nitidus*	0.015115	0.063871
CS12	GD34	CS10	*R.nitidus*	-0.002052	0.065619
GD35	GD34	CS10	*R.nitidus*	0.041574	0.064737

GD46	GD34	CS10	*R.nitidus*	-0.017829	0.062288
GD45	GD34	CS10	*R.nitidus*	0.075916	0.067703
CS14	GD34	CS10	*R.nitidus*	-0.012953	0.066118
GD32	GD34	CS10	*R.nitidus*	0.018182	0.0639
GD22	GD34	CS10	*R.nitidus*	0.08427	0.061402
GD49	GD34	CS10	*R.nitidus*	0.08863	0.067084
GD44	GD34	CS10	*R.nitidus*	0.006051	0.066163
CS20	GD34	CS10	*R.nitidus*	-0.035387	0.064811
CS31	GD34	CS10	*R.nitidus*	-0.028031	0.064745
CS15	GD34	CS10	*R.nitidus*	0.017187	0.065273
GD33	GD34	CS10	*R.nitidus*	0.028855	0.063784
GD41	GD34	CS10	*R.nitidus*	0.012461	0.062911
CS13	GD34	CS10	*R.nitidus*	-0.038362	0.067524
GD40	GD34	CS10	*R.nitidus*	0.069881	0.063062
GD50	GD34	CS10	*R.nitidus*	0.024545	0.066862
SC12	GD34	CS10	*R.nitidus*	-0.022786	0.064797
CS19	GD34	CS10	*R.nitidus*	-0.019986	0.065175
SC10	GD34	CS10	*R.nitidus*	0.048044	0.066231
SC5	GD34	CS10	*R.nitidus*	-0.004775	0.068521
CS18	GD34	CS10	*R.nitidus*	-0.03467	0.065156
CS25	GD34	CS10	*R.nitidus*	-0.023569	0.065712
CS28	GD34	CS10	*R.nitidus*	-0.050073	0.065795
JO1	GD33	CS10	*R.nitidus*	-0.021122	0.066071
CS16	GD34	CS10	*R.nitidus*	-0.008974	0.063312
CS21	GD34	CS10	*R.nitidus*	-0.026281	0.064791
SC26	GD34	CS10	*R.nitidus*	-0.025132	0.062657
CS17	GD34	CS10	*R.nitidus*	-0.000693	0.067439
CS4	GD34	CS10	*R.nitidus*	-0.023591	0.062113
SC27	GD34	CS10	*R.nitidus*	-0.001982	0.063605
SC42	GD34	CS10	*R.nitidus*	0.230176	0.062139
CS24	GD34	CS10	*R.nitidus*	-0.045299	0.064738
MY6	GD34	CS10	*R.nitidus*	-0.020156	0.063223
SC18	GD34	CS10	*R.nitidus*	0.022054	0.064206
CS22	GD34	CS10	*R.nitidus*	-0.011952	0.06449
CS3	GD34	CS10	*R.nitidus*	-0.032732	0.065343
CS6	GD34	CS10	*R.nitidus*	-0.051984	0.064746
SC24	GD34	CS10	*R.nitidus*	-0.005431	0.064665
CS23	GD34	CS10	*R.nitidus*	-0.062252	0.062428
SC14	GD34	CS10	*R.nitidus*	0.012467	0.064381
MY4	GD34	CS10	*R.nitidus*	-0.046605	0.063668
CS30	GD34	CS10	*R.nitidus*	-0.06768	0.063379
JO3	GD34	CS10	*R.nitidus*	0.030023	0.068004
SC6	GD34	CS10	*R.nitidus*	-0.007984	0.065039
SC31	GD34	CS10	*R.nitidus*	-0.020516	0.064812
SC15	GD34	CS10	*R.nitidus*	-0.017747	0.065539
SC35	GD34	CS10	*R.nitidus*	0.031936	0.063556
GD22	GD35	CS10	*R.nitidus*	0.019471	0.064041
SC44	GD34	CS10	*R.nitidus*	-0.001372	0.063753
GD21	GD35	CS10	*R.nitidus*	0.081119	0.062779
JO1	GD34	CS10	*R.nitidus*	-0.024437	0.063456
JO8	GD35	CS10	*R.nitidus*	-0.01385	0.073391
SC32	GD34	CS10	*R.nitidus*	0.035123	0.063341
JW4	GD34	CS10	*R.nitidus*	0.218443	0.064479
SC28	GD34	CS10	*R.nitidus*	-0.035286	0.062575
GD26	GD35	CS10	*R.nitidus*	0.015695	0.065018
SC45	GD34	CS10	*R.nitidus*	0.037013	0.063222

SC34	GD34	CS10	*R.nitidus*	0.018905	0.064646
JW6	GD34	CS10	*R.nitidus*	0.022165	0.068351
JO5	GD34	CS10	*R.nitidus*	0.036891	0.063126
JO7	GD35	CS10	*R.nitidus*	-0.03663	0.074113
GD32	GD35	CS10	*R.nitidus*	-0.012605	0.068373
GD33	GD35	CS10	*R.nitidus*	-0.035714	0.06337
GD34	GD35	CS10	*R.nitidus*	-0.041574	0.064737
CS11	GD35	CS10	*R.nitidus*	-0.035475	0.067568
GD31	GD35	CS10	*R.nitidus*	0.022438	0.064932
JW7	GD34	CS10	*R.nitidus*	0.053417	0.069035
GD45	GD35	CS10	*R.nitidus*	0.046713	0.068627
GD40	GD35	CS10	*R.nitidus*	0.026335	0.063118
GD48	GD35	CS10	*R.nitidus*	-0.010492	0.067192
GD30	GD35	CS10	*R.nitidus*	0.106419	0.067094
CS12	GD35	CS10	*R.nitidus*	-0.04878	0.067725
CS14	GD35	CS10	*R.nitidus*	-0.03603	0.068697
GD41	GD35	CS10	*R.nitidus*	-0.02855	0.064161
GD25	GD35	CS10	*R.nitidus*	0.029635	0.06397
CS13	GD35	CS10	*R.nitidus*	-0.064962	0.067708
CS16	GD35	CS10	*R.nitidus*	-0.035928	0.065172
GD29	GD35	CS10	*R.nitidus*	0.071972	0.064519
CS19	GD35	CS10	*R.nitidus*	-0.047755	0.066768
GD42	GD35	CS10	*R.nitidus*	-0.029163	0.06417
CS4	GD35	CS10	*R.nitidus*	-0.049598	0.063943
SC6	GD35	CS10	*R.nitidus*	-0.043003	0.064384
CS3	GD35	CS10	*R.nitidus*	-0.045796	0.064849
CS15	GD35	CS10	*R.nitidus*	-0.016556	0.067126
CS18	GD35	CS10	*R.nitidus*	-0.070827	0.066327
CS23	GD35	CS10	*R.nitidus*	-0.089382	0.06524
SC26	GD35	CS10	*R.nitidus*	-0.052283	0.063923
GD46	GD35	CS10	*R.nitidus*	-0.065137	0.06368
GD49	GD35	CS10	*R.nitidus*	0.059603	0.069037
GD44	GD35	CS10	*R.nitidus*	-0.026667	0.066793
GD50	GD35	CS10	*R.nitidus*	-0.017724	0.067505
CS21	GD35	CS10	*R.nitidus*	-0.058317	0.066436
CS30	GD35	CS10	*R.nitidus*	-0.089655	0.066559
CS24	GD35	CS10	*R.nitidus*	-0.075833	0.066028
SC35	GD35	CS10	*R.nitidus*	-0.004107	0.065016
CS28	GD35	CS10	*R.nitidus*	-0.080586	0.066802
SC5	GD35	CS10	*R.nitidus*	-0.035562	0.070938
CS17	GD35	CS10	*R.nitidus*	-0.033503	0.068763
SC10	GD35	CS10	*R.nitidus*	0.024983	0.065869
SC32	GD35	CS10	*R.nitidus*	0.013423	0.064559
CS20	GD35	CS10	*R.nitidus*	-0.066166	0.066061
JO1	GD35	CS10	*R.nitidus*	-0.042412	0.064538
SC34	GD35	CS10	*R.nitidus*	-0.020311	0.065284
SC12	GD35	CS10	*R.nitidus*	-0.055034	0.065787
SC31	GD35	CS10	*R.nitidus*	-0.052117	0.065248
CS22	GD35	CS10	*R.nitidus*	-0.040218	0.066612
SC14	GD35	CS10	*R.nitidus*	-0.014295	0.065866
SC15	GD35	CS10	*R.nitidus*	-0.043123	0.068105
CS25	GD35	CS10	*R.nitidus*	-0.04742	0.066239
SC28	GD35	CS10	*R.nitidus*	-0.060109	0.065178
JO7	GD40	CS10	*R.nitidus*	-0.067841	0.069836
SC18	GD35	CS10	*R.nitidus*	-0.015162	0.065935
CS31	GD35	CS10	*R.nitidus*	-0.068149	0.067581

JO8	GD40	CS10	*R.nitidus*	-0.048713	0.070693
SC44	GD35	CS10	*R.nitidus*	-0.038678	0.065588
JO3	GD35	CS10	*R.nitidus*	-0.010526	0.069648
SC24	GD35	CS10	*R.nitidus*	-0.040759	0.064812
JW7	GD35	CS10	*R.nitidus*	0.006405	0.069792
CS6	GD35	CS10	*R.nitidus*	-0.092711	0.065898
CS12	GD40	CS10	*R.nitidus*	-0.070827	0.066135
GD26	GD40	CS10	*R.nitidus*	-0.01614	0.062219
GD30	GD40	CS10	*R.nitidus*	0.066327	0.066458
MY6	GD35	CS10	*R.nitidus*	-0.054878	0.065497
SC42	GD35	CS10	*R.nitidus*	0.206061	0.063093
GD25	GD40	CS10	*R.nitidus*	0.009922	0.062492
GD34	GD40	CS10	*R.nitidus*	-0.069881	0.063062
GD45	GD40	CS10	*R.nitidus*	-0.001646	0.066464
JW4	GD35	CS10	*R.nitidus*	0.201503	0.063911
JW6	GD35	CS10	*R.nitidus*	-0.033929	0.072293
GD22	GD40	CS10	*R.nitidus*	-0.003984	0.062304
GD21	GD40	CS10	*R.nitidus*	0.066802	0.064422
MY4	GD35	CS10	*R.nitidus*	-0.088633	0.066305
GD41	GD40	CS10	*R.nitidus*	-0.055516	0.062947
CS11	GD40	CS10	*R.nitidus*	-0.072266	0.065526
SC45	GD35	CS10	*R.nitidus*	0.013316	0.064529
GD32	GD40	CS10	*R.nitidus*	-0.042742	0.065765
GD29	GD40	CS10	*R.nitidus*	0.063492	0.063329
GD49	GD40	CS10	*R.nitidus*	0.026961	0.068338
SC27	GD35	CS10	*R.nitidus*	-0.03391	0.064195
JO5	GD35	CS10	*R.nitidus*	-0.007534	0.066279
GD33	GD40	CS10	*R.nitidus*	-0.053546	0.064405
GD31	GD40	CS10	*R.nitidus*	-0.01766	0.064677
GD46	GD40	CS10	*R.nitidus*	-0.079829	0.062736
GD35	GD40	CS10	*R.nitidus*	-0.026335	0.063118
CS21	GD40	CS10	*R.nitidus*	-0.099342	0.063762
CS28	GD40	CS10	*R.nitidus*	-0.098731	0.063608
GD50	GD40	CS10	*R.nitidus*	-0.037815	0.067766
GD44	GD40	CS10	*R.nitidus*	-0.06213	0.06353
CS23	GD40	CS10	*R.nitidus*	-0.12045	0.063046
CS13	GD40	CS10	*R.nitidus*	-0.101932	0.066108
CS14	GD40	CS10	*R.nitidus*	-0.0625	0.06642
GD48	GD40	CS10	*R.nitidus*	-0.029321	0.066537
SC26	GD40	CS10	*R.nitidus*	-0.088599	0.062417
CS17	GD40	CS10	*R.nitidus*	-0.06528	0.066893
CS16	GD40	CS10	*R.nitidus*	-0.073007	0.064381
GD42	GD40	CS10	*R.nitidus*	-0.052402	0.064202
CS15	GD40	CS10	*R.nitidus*	-0.048232	0.065233
CS18	GD40	CS10	*R.nitidus*	-0.111111	0.063417
CS4	GD40	CS10	*R.nitidus*	-0.097386	0.062297
CS24	GD40	CS10	*R.nitidus*	-0.118213	0.061694
CS19	GD40	CS10	*R.nitidus*	-0.094715	0.064409
SC5	GD40	CS10	*R.nitidus*	-0.075233	0.065451
SC44	GD40	CS10	*R.nitidus*	-0.07438	0.062598
SC14	GD40	CS10	*R.nitidus*	-0.058177	0.063826
SC10	GD40	CS10	*R.nitidus*	-0.006671	0.06524
SC12	GD40	CS10	*R.nitidus*	-0.088616	0.063901
CS30	GD40	CS10	*R.nitidus*	-0.123005	0.06239
CS22	GD40	CS10	*R.nitidus*	-0.076568	0.062668
JW4	GD40	CS10	*R.nitidus*	0.151102	0.06489

CS3	GD40	CS10	*R.nitidus*	-0.089009	0.063634
CS20	GD40	CS10	*R.nitidus*	-0.098658	0.06365
MY6	GD40	CS10	*R.nitidus*	-0.08183	0.062983
SC18	GD40	CS10	*R.nitidus*	-0.038594	0.06247
CS25	GD40	CS10	*R.nitidus*	-0.08684	0.063771
JW6	GD40	CS10	*R.nitidus*	-0.056131	0.069914
JO7	GD41	CS10	*R.nitidus*	0.005484	0.070865
SC27	GD40	CS10	*R.nitidus*	-0.073424	0.061212
CS31	GD40	CS10	*R.nitidus*	-0.099933	0.064686
SC34	GD40	CS10	*R.nitidus*	-0.047433	0.062624
SC28	GD40	CS10	*R.nitidus*	-0.097513	0.062852
JO8	GD41	CS10	*R.nitidus*	0.032015	0.071135
JW7	GD40	CS10	*R.nitidus*	-0.03718	0.067222
SC6	GD40	CS10	*R.nitidus*	-0.077181	0.061631
SC15	GD40	CS10	*R.nitidus*	-0.088255	0.063346
SC45	GD40	CS10	*R.nitidus*	-0.028086	0.061542
SC42	GD40	CS10	*R.nitidus*	0.159757	0.061685
JO3	GD40	CS10	*R.nitidus*	-0.020179	0.06771
SC32	GD40	CS10	*R.nitidus*	-0.026762	0.062004
SC24	GD40	CS10	*R.nitidus*	-0.060095	0.063046
GD25	GD41	CS10	*R.nitidus*	0.038743	0.064722
CS6	GD40	CS10	*R.nitidus*	-0.123967	0.063515
MY4	GD40	CS10	*R.nitidus*	-0.118922	0.063198
SC31	GD40	CS10	*R.nitidus*	-0.088444	0.063754
SC35	GD40	CS10	*R.nitidus*	-0.024731	0.061127
GD30	GD41	CS10	*R.nitidus*	0.105769	0.064174
JO5	GD40	CS10	*R.nitidus*	-0.029202	0.063728
JO1	GD40	CS10	*R.nitidus*	-0.07732	0.064283
GD35	GD41	CS10	*R.nitidus*	0.02855	0.064161
GD50	GD41	CS10	*R.nitidus*	0.029557	0.067878
CS19	GD41	CS10	*R.nitidus*	-0.024341	0.06695
GD21	GD41	CS10	*R.nitidus*	0.112554	0.063375
CS18	GD41	CS10	*R.nitidus*	-0.045333	0.064987
GD40	GD41	CS10	*R.nitidus*	0.055516	0.062947
GD22	GD41	CS10	*R.nitidus*	0.067466	0.064239
GD44	GD41	CS10	*R.nitidus*	0.005591	0.067954
GD33	GD41	CS10	*R.nitidus*	-0.004552	0.064742
GD45	GD41	CS10	*R.nitidus*	0.035683	0.070057
GD31	GD41	CS10	*R.nitidus*	0.045872	0.066367
GD32	GD41	CS10	*R.nitidus*	0.027778	0.068456
GD46	GD41	CS10	*R.nitidus*	-0.045416	0.062873
GD42	GD41	CS10	*R.nitidus*	0.007913	0.063123
CS13	GD41	CS10	*R.nitidus*	-0.044564	0.066841
CS30	GD41	CS10	*R.nitidus*	-0.084099	0.064596
GD34	GD41	CS10	*R.nitidus*	-0.012461	0.062911
CS12	GD41	CS10	*R.nitidus*	-0.015089	0.06606
GD26	GD41	CS10	*R.nitidus*	0.049512	0.062875
CS24	GD41	CS10	*R.nitidus*	-0.055437	0.065287
SC5	GD41	CS10	*R.nitidus*	-0.011748	0.06842
CS31	GD41	CS10	*R.nitidus*	-0.044003	0.065803
GD29	GD41	CS10	*R.nitidus*	0.114138	0.063486
CS17	GD41	CS10	*R.nitidus*	-0.009194	0.068489
GD48	GD41	CS10	*R.nitidus*	0.047939	0.06707
CS21	GD41	CS10	*R.nitidus*	-0.038785	0.063614
CS22	GD41	CS10	*R.nitidus*	-0.023553	0.064564
GD49	GD41	CS10	*R.nitidus*	0.096104	0.067799

CS11	GD41	CS10	*R.nitidus*	-0.019815	0.066283
CS25	GD41	CS10	*R.nitidus*	-0.023778	0.066604
CS3	GD41	CS10	*R.nitidus*	-0.046296	0.063907
CS16	GD41	CS10	*R.nitidus*	-0.007338	0.063661
CS20	GD41	CS10	*R.nitidus*	-0.045543	0.065603
CS6	GD41	CS10	*R.nitidus*	-0.06237	0.065128
SC6	GD41	CS10	*R.nitidus*	-0.017568	0.066838
SC34	GD41	CS10	*R.nitidus*	0.015636	0.063942
CS14	GD41	CS10	*R.nitidus*	-0.010589	0.066565
SC27	GD41	CS10	*R.nitidus*	-0.019836	0.063732
CS4	GD41	CS10	*R.nitidus*	-0.031375	0.063077
CS15	GD41	CS10	*R.nitidus*	0.008453	0.064893
CS23	GD41	CS10	*R.nitidus*	-0.066841	0.064097
CS28	GD41	CS10	*R.nitidus*	-0.046921	0.06691
SC42	GD41	CS10	*R.nitidus*	0.22351	0.061876
SC14	GD41	CS10	*R.nitidus*	-0.002749	0.06446
JW4	GD41	CS10	*R.nitidus*	0.216892	0.062819
SC32	GD41	CS10	*R.nitidus*	0.030976	0.063609
SC12	GD41	CS10	*R.nitidus*	-0.01945	0.064385
SC10	GD41	CS10	*R.nitidus*	0.05	0.065972
SC18	GD41	CS10	*R.nitidus*	0.013542	0.064281
SC15	GD41	CS10	*R.nitidus*	-0.014337	0.065258
MY6	GD41	CS10	*R.nitidus*	-0.017207	0.062766
MY4	GD41	CS10	*R.nitidus*	-0.050414	0.063649
SC35	GD41	CS10	*R.nitidus*	0.027815	0.062993
GD22	GD42	CS10	*R.nitidus*	0.067181	0.062948
SC31	GD41	CS10	*R.nitidus*	-0.026017	0.063967
JW6	GD41	CS10	*R.nitidus*	-0.000917	0.069865
SC26	GD41	CS10	*R.nitidus*	-0.035053	0.067385
SC24	GD41	CS10	*R.nitidus*	-0.016598	0.064824
SC44	GD41	CS10	*R.nitidus*	-0.008433	0.063824
GD31	GD42	CS10	*R.nitidus*	0.029056	0.065747
SC28	GD41	CS10	*R.nitidus*	-0.041001	0.064469
GD30	GD42	CS10	*R.nitidus*	0.125815	0.066977
SC45	GD41	CS10	*R.nitidus*	0.028667	0.064531
GD26	GD42	CS10	*R.nitidus*	0.020233	0.065702
GD21	GD42	CS10	*R.nitidus*	0.107762	0.063629
CS11	GD42	CS10	*R.nitidus*	-0.030426	0.067808
JO7	GD42	CS10	*R.nitidus*	-0.041346	0.072447
GD34	GD42	CS10	*R.nitidus*	-0.015115	0.063871
GD25	GD42	CS10	*R.nitidus*	0.040801	0.065105
GD44	GD42	CS10	*R.nitidus*	0.004847	0.066238
CS12	GD42	CS10	*R.nitidus*	-0.019844	0.066631
GD32	GD42	CS10	*R.nitidus*	0.017715	0.068953
JO3	GD41	CS10	*R.nitidus*	0.029898	0.068413
JW7	GD41	CS10	*R.nitidus*	0.036585	0.069154
JO8	GD42	CS10	*R.nitidus*	0.002876	0.072429
GD33	GD42	CS10	*R.nitidus*	0	0.066532
CS13	GD42	CS10	*R.nitidus*	-0.053596	0.068036
JO1	GD41	CS10	*R.nitidus*	-0.016	0.065082
GD40	GD42	CS10	*R.nitidus*	0.052402	0.064202
CS15	GD42	CS10	*R.nitidus*	-0.008048	0.066256
JO5	GD41	CS10	*R.nitidus*	0.037798	0.065074
GD48	GD42	CS10	*R.nitidus*	0.023377	0.066456
GD41	GD42	CS10	*R.nitidus*	-0.007913	0.063123
GD29	GD42	CS10	*R.nitidus*	0.11853	0.063804

CS19	GD42	CS10	*R.nitidus*	-0.032011	0.066807
GD46	GD42	CS10	*R.nitidus*	-0.038884	0.063921
GD50	GD42	CS10	*R.nitidus*	0.00499	0.06867
CS21	GD42	CS10	*R.nitidus*	-0.051587	0.065993
CS17	GD42	CS10	*R.nitidus*	-0.012319	0.068888
CS28	GD42	CS10	*R.nitidus*	-0.062361	0.067718
SC6	GD42	CS10	*R.nitidus*	-0.023842	0.066701
GD45	GD42	CS10	*R.nitidus*	0.048957	0.068894
CS14	GD42	CS10	*R.nitidus*	-0.033198	0.067171
SC26	GD42	CS10	*R.nitidus*	-0.04123	0.066701
GD35	GD42	CS10	*R.nitidus*	0.029163	0.06417
CS20	GD42	CS10	*R.nitidus*	-0.045872	0.066453
CS25	GD42	CS10	*R.nitidus*	-0.033723	0.067254
SC32	GD42	CS10	*R.nitidus*	0.026513	0.063836
CS30	GD42	CS10	*R.nitidus*	-0.090116	0.064941
SC27	GD42	CS10	*R.nitidus*	-0.014384	0.065072
CS16	GD42	CS10	*R.nitidus*	-0.025798	0.06396
GD49	GD42	CS10	*R.nitidus*	0.085944	0.068206
CS31	GD42	CS10	*R.nitidus*	-0.039589	0.06599
SC31	GD42	CS10	*R.nitidus*	-0.044283	0.066307
SC15	GD42	CS10	*R.nitidus*	-0.035125	0.067487
CS18	GD42	CS10	*R.nitidus*	-0.058155	0.067138
SC14	GD42	CS10	*R.nitidus*	0.007468	0.065075
SC35	GD42	CS10	*R.nitidus*	0.016426	0.063504
CS3	GD42	CS10	*R.nitidus*	-0.043566	0.064177
JO3	GD42	CS10	*R.nitidus*	0.018745	0.069504
SC28	GD42	CS10	*R.nitidus*	-0.047358	0.064452
SC18	GD42	CS10	*R.nitidus*	0	0.065663
SC10	GD42	CS10	*R.nitidus*	0.043232	0.06688
CS22	GD42	CS10	*R.nitidus*	-0.03106	0.064429
JO1	GD42	CS10	*R.nitidus*	-0.028705	0.065247
CS24	GD42	CS10	*R.nitidus*	-0.055712	0.065508
SC34	GD42	CS10	*R.nitidus*	0.01019	0.065669
SC45	GD42	CS10	*R.nitidus*	0.029034	0.064213
CS6	GD42	CS10	*R.nitidus*	-0.070175	0.064879
CS23	GD42	CS10	*R.nitidus*	-0.074567	0.065491
MY4	GD42	CS10	*R.nitidus*	-0.055479	0.065704
SC24	GD42	CS10	*R.nitidus*	-0.01257	0.064947
JO7	GD44	CS10	*R.nitidus*	-0.013711	0.072045
JW7	GD42	CS10	*R.nitidus*	0.003284	0.068227
SC5	GD42	CS10	*R.nitidus*	-0.019622	0.068982
SC44	GD42	CS10	*R.nitidus*	-0.015217	0.065503
SC12	GD42	CS10	*R.nitidus*	-0.035568	0.064768
GD30	GD44	CS10	*R.nitidus*	0.119565	0.068315
JO8	GD44	CS10	*R.nitidus*	0.006591	0.072356
SC42	GD42	CS10	*R.nitidus*	0.232386	0.061203
CS4	GD42	CS10	*R.nitidus*	-0.029139	0.062597
JW6	GD42	CS10	*R.nitidus*	-0.019774	0.070366
JW4	GD42	CS10	*R.nitidus*	0.200947	0.063637
CS11	GD44	CS10	*R.nitidus*	-0.021277	0.068204
MY6	GD42	CS10	*R.nitidus*	-0.023066	0.064073
JO5	GD42	CS10	*R.nitidus*	0.024155	0.065604
GD22	GD44	CS10	*R.nitidus*	0.051433	0.065443
GD32	GD44	CS10	*R.nitidus*	-0.003562	0.06968
GD29	GD44	CS10	*R.nitidus*	0.131635	0.065408
GD31	GD44	CS10	*R.nitidus*	0.051571	0.067601

CS14	GD44	CS10	*R.nitidus*	-0.016609	0.067693
GD25	GD44	CS10	*R.nitidus*	0.050343	0.065579
CS12	GD44	CS10	*R.nitidus*	-0.026647	0.067566
GD34	GD44	CS10	*R.nitidus*	-0.006051	0.066163
GD46	GD44	CS10	*R.nitidus*	-0.032308	0.066449
GD26	GD44	CS10	*R.nitidus*	0.038401	0.065934
GD21	GD44	CS10	*R.nitidus*	0.132898	0.064097
GD41	GD44	CS10	*R.nitidus*	-0.005591	0.067954
GD48	GD44	CS10	*R.nitidus*	0.039834	0.067082
CS17	GD44	CS10	*R.nitidus*	0	0.070882
CS18	GD44	CS10	*R.nitidus*	-0.062187	0.066611
CS20	GD44	CS10	*R.nitidus*	-0.053259	0.067384
GD40	GD44	CS10	*R.nitidus*	0.06213	0.06353
CS31	GD44	CS10	*R.nitidus*	-0.049556	0.065851
GD33	GD44	CS10	*R.nitidus*	0.005279	0.065053
GD35	GD44	CS10	*R.nitidus*	0.026667	0.066793
CS22	GD44	CS10	*R.nitidus*	-0.040264	0.06553
SC10	GD44	CS10	*R.nitidus*	0.035197	0.065959
CS23	GD44	CS10	*R.nitidus*	-0.072931	0.063868
SC27	GD44	CS10	*R.nitidus*	-0.021368	0.066505
CS28	GD44	CS10	*R.nitidus*	-0.053992	0.067714
SC5	GD44	CS10	*R.nitidus*	-0.020059	0.069885
GD45	GD44	CS10	*R.nitidus*	0.045245	0.070797
GD49	GD44	CS10	*R.nitidus*	0.086312	0.069411
GD42	GD44	CS10	*R.nitidus*	-0.004847	0.066238
SC15	GD44	CS10	*R.nitidus*	-0.011722	0.067131
GD50	GD44	CS10	*R.nitidus*	0.022727	0.068922
CS24	GD44	CS10	*R.nitidus*	-0.063657	0.067038
CS16	GD44	CS10	*R.nitidus*	-0.006259	0.064968
CS13	GD44	CS10	*R.nitidus*	-0.067675	0.068798
CS3	GD44	CS10	*R.nitidus*	-0.037842	0.065249
CS19	GD44	CS10	*R.nitidus*	-0.040175	0.066426
SC18	GD44	CS10	*R.nitidus*	-0.008975	0.064784
CS4	GD44	CS10	*R.nitidus*	-0.054922	0.064319
CS21	GD44	CS10	*R.nitidus*	-0.040222	0.065164
SC34	GD44	CS10	*R.nitidus*	0.011042	0.065022
SC35	GD44	CS10	*R.nitidus*	0.028986	0.064894
CS15	GD44	CS10	*R.nitidus*	0.002037	0.065596
CS25	GD44	CS10	*R.nitidus*	-0.048798	0.068478
SC24	GD44	CS10	*R.nitidus*	-0.026163	0.066848
SC31	GD44	CS10	*R.nitidus*	-0.028112	0.066949
JO3	GD44	CS10	*R.nitidus*	0.007924	0.069865
CS30	GD44	CS10	*R.nitidus*	-0.068052	0.067141
SC28	GD44	CS10	*R.nitidus*	-0.052405	0.066269
SC14	GD44	CS10	*R.nitidus*	-0.011445	0.066631
SC12	GD44	CS10	*R.nitidus*	-0.040541	0.064567
JW6	GD44	CS10	*R.nitidus*	-0.013369	0.071138
SC42	GD44	CS10	*R.nitidus*	0.212031	0.06298
SC44	GD44	CS10	*R.nitidus*	-0.014609	0.066389
CS6	GD44	CS10	*R.nitidus*	-0.069434	0.066784
MY4	GD44	CS10	*R.nitidus*	-0.06392	0.067751
GD31	GD45	CS10	*R.nitidus*	0	0.069519
SC26	GD44	CS10	*R.nitidus*	-0.040356	0.066038
SC45	GD44	CS10	*R.nitidus*	0.025641	0.064905
JO7	GD45	CS10	*R.nitidus*	-0.107103	0.074918
SC6	GD44	CS10	*R.nitidus*	-0.016595	0.066503

MY6	GD44	CS10	*R.nitidus*	-0.030007	0.066926
GD25	GD45	CS10	*R.nitidus*	-0.036269	0.070284
JW4	GD44	CS10	*R.nitidus*	0.179846	0.067101
JW7	GD44	CS10	*R.nitidus*	0.014178	0.068851
SC32	GD44	CS10	*R.nitidus*	0.036678	0.066861
GD34	GD45	CS10	*R.nitidus*	-0.075916	0.067703
GD33	GD45	CS10	*R.nitidus*	-0.058252	0.068826
CS11	GD45	CS10	*R.nitidus*	-0.07086	0.070617
GD26	GD45	CS10	*R.nitidus*	-0.03255	0.068857
CS12	GD45	CS10	*R.nitidus*	-0.059446	0.070564
JO8	GD45	CS10	*R.nitidus*	-0.04157	0.076579
GD32	GD45	CS10	*R.nitidus*	-0.033708	0.072371
JO5	GD44	CS10	*R.nitidus*	0.027407	0.065375
GD49	GD45	CS10	*R.nitidus*	0.036926	0.072607
GD48	GD45	CS10	*R.nitidus*	-0.007797	0.071035
GD29	GD45	CS10	*R.nitidus*	0.042857	0.068594
JO1	GD44	CS10	*R.nitidus*	-0.021588	0.064665
GD46	GD45	CS10	*R.nitidus*	-0.097628	0.071298
GD42	GD45	CS10	*R.nitidus*	-0.048957	0.068894
GD30	GD45	CS10	*R.nitidus*	0.056405	0.068999
GD21	GD45	CS10	*R.nitidus*	0.048458	0.068926
SC6	GD45	CS10	*R.nitidus*	-0.057526	0.07152
CS15	GD45	CS10	*R.nitidus*	-0.040519	0.071109
GD35	GD45	CS10	*R.nitidus*	-0.046713	0.068627
GD22	GD45	CS10	*R.nitidus*	0.023121	0.068837
CS16	GD45	CS10	*R.nitidus*	-0.065916	0.071931
CS28	GD45	CS10	*R.nitidus*	-0.093178	0.068707
CS20	GD45	CS10	*R.nitidus*	-0.07188	0.071371
SC10	GD45	CS10	*R.nitidus*	0.004808	0.070176
GD40	GD45	CS10	*R.nitidus*	0.001646	0.066464
CS13	GD45	CS10	*R.nitidus*	-0.078176	0.070815
GD41	GD45	CS10	*R.nitidus*	-0.035683	0.070057
GD44	GD45	CS10	*R.nitidus*	-0.045245	0.070797
CS30	GD45	CS10	*R.nitidus*	-0.108424	0.0689
GD50	GD45	CS10	*R.nitidus*	-0.030801	0.072814
CS31	GD45	CS10	*R.nitidus*	-0.1025	0.069978
CS18	GD45	CS10	*R.nitidus*	-0.093227	0.071406
SC24	GD45	CS10	*R.nitidus*	-0.031422	0.069621
CS14	GD45	CS10	*R.nitidus*	-0.05198	0.072723
CS6	GD45	CS10	*R.nitidus*	-0.066322	0.070578
SC18	GD45	CS10	*R.nitidus*	0.002473	0.070941
SC28	GD45	CS10	*R.nitidus*	-0.072612	0.068001
CS23	GD45	CS10	*R.nitidus*	-0.079475	0.072547
CS19	GD45	CS10	*R.nitidus*	-0.067425	0.072124
CS17	GD45	CS10	*R.nitidus*	-0.06867	0.071615
SC15	GD45	CS10	*R.nitidus*	-0.041567	0.069488
CS22	GD45	CS10	*R.nitidus*	-0.059822	0.070079
SC31	GD45	CS10	*R.nitidus*	-0.046317	0.070488
CS25	GD45	CS10	*R.nitidus*	-0.076547	0.073555
SC26	GD45	CS10	*R.nitidus*	-0.064906	0.069264
SC5	GD45	CS10	*R.nitidus*	-0.06089	0.072988
CS3	GD45	CS10	*R.nitidus*	-0.055024	0.070412
SC27	GD45	CS10	*R.nitidus*	-0.042768	0.070376
CS24	GD45	CS10	*R.nitidus*	-0.079119	0.069732
SC34	GD45	CS10	*R.nitidus*	-0.012579	0.068573
JW6	GD45	CS10	*R.nitidus*	-0.093652	0.074987

SC44	GD45	CS10	*R.nitidus*	-0.034208	0.066397
SC14	GD45	CS10	*R.nitidus*	-0.025424	0.07082
CS21	GD45	CS10	*R.nitidus*	-0.07425	0.069648
CS4	GD45	CS10	*R.nitidus*	-0.069369	0.067218
JW4	GD45	CS10	*R.nitidus*	0.164251	0.069018
SC12	GD45	CS10	*R.nitidus*	-0.065201	0.067853
SC45	GD45	CS10	*R.nitidus*	0.001584	0.068151
JW7	GD45	CS10	*R.nitidus*	-0.038863	0.071175
MY6	GD45	CS10	*R.nitidus*	-0.047994	0.068252
SC35	GD45	CS10	*R.nitidus*	-0.006907	0.069128
SC32	GD45	CS10	*R.nitidus*	0.003123	0.067408
GD22	CS13	CS10	*R.nitidus*	0.103938	0.066746
JO1	GD45	CS10	*R.nitidus*	-0.054878	0.068758
GD33	CS13	CS10	*R.nitidus*	0.042904	0.066966
GD26	CS13	CS10	*R.nitidus*	0.080656	0.068001
SC42	GD45	CS10	*R.nitidus*	0.19727	0.065894
GD40	CS13	CS10	*R.nitidus*	0.101932	0.066108
CS11	CS13	CS10	*R.nitidus*	0.042857	0.072233
MY4	GD45	CS10	*R.nitidus*	-0.07903	0.067887
GD21	CS13	CS10	*R.nitidus*	0.144703	0.06587
JO7	CS13	CS10	*R.nitidus*	0.04023	0.073097
JO3	GD45	CS10	*R.nitidus*	-0.036832	0.073567
GD29	CS13	CS10	*R.nitidus*	0.155654	0.066217
GD34	CS13	CS10	*R.nitidus*	0.038362	0.067524
JO8	CS13	CS10	*R.nitidus*	0.046602	0.075253
GD32	CS13	CS10	*R.nitidus*	0.046189	0.068986
JO5	GD45	CS10	*R.nitidus*	-0.014458	0.069786
GD35	CS13	CS10	*R.nitidus*	0.064962	0.067708
CS22	CS13	CS10	*R.nitidus*	0.041086	0.068037
GD30	CS13	CS10	*R.nitidus*	0.146382	0.069513
GD49	CS13	CS10	*R.nitidus*	0.112006	0.070694
GD45	CS13	CS10	*R.nitidus*	0.078176	0.070815
GD25	CS13	CS10	*R.nitidus*	0.093662	0.067505
GD42	CS13	CS10	*R.nitidus*	0.053596	0.068036
CS16	CS13	CS10	*R.nitidus*	0.046602	0.069398
GD31	CS13	CS10	*R.nitidus*	0.096891	0.067892
CS17	CS13	CS10	*R.nitidus*	0.048966	0.072071
GD48	CS13	CS10	*R.nitidus*	0.078871	0.069727
CS15	CS13	CS10	*R.nitidus*	0.078764	0.069669
CS31	CS13	CS10	*R.nitidus*	0.013804	0.069691
CS21	CS13	CS10	*R.nitidus*	0.00721	0.067303
CS12	CS13	CS10	*R.nitidus*	0.056818	0.071436
CS20	CS13	CS10	*R.nitidus*	-0.002791	0.067981
CS3	CS13	CS10	*R.nitidus*	0.008708	0.067344
CS18	CS13	CS10	*R.nitidus*	-0.006056	0.068414
CS19	CS13	CS10	*R.nitidus*	0.016229	0.070361
CS23	CS13	CS10	*R.nitidus*	-0.02687	0.065548
GD50	CS13	CS10	*R.nitidus*	0.063812	0.072246
GD44	CS13	CS10	*R.nitidus*	0.067675	0.068798
CS4	CS13	CS10	*R.nitidus*	0.017341	0.067083
GD41	CS13	CS10	*R.nitidus*	0.044564	0.066841
CS30	CS13	CS10	*R.nitidus*	-0.034892	0.068065
CS28	CS13	CS10	*R.nitidus*	0.009623	0.070331
CS6	CS13	CS10	*R.nitidus*	-0.016629	0.068868
CS14	CS13	CS10	*R.nitidus*	0.046192	0.071654
GD46	CS13	CS10	*R.nitidus*	0.035446	0.068317

SC18	CS13	CS10	*R.nitidus*	0.069065	0.067964
SC26	CS13	CS10	*R.nitidus*	0.008608	0.066608
SC12	CS13	CS10	*R.nitidus*	0.031915	0.068255
SC31	CS13	CS10	*R.nitidus*	0.015548	0.068441
SC27	CS13	CS10	*R.nitidus*	0.041667	0.067366
CS25	CS13	CS10	*R.nitidus*	0.016442	0.070688
SC32	CS13	CS10	*R.nitidus*	0.085457	0.069026
SC10	CS13	CS10	*R.nitidus*	0.096012	0.068479
JW7	CS13	CS10	*R.nitidus*	0.077513	0.070968
JO8	GD46	CS10	*R.nitidus*	0.08978	0.06869
SC5	CS13	CS10	*R.nitidus*	0.029518	0.071084
SC28	CS13	CS10	*R.nitidus*	0.001435	0.065923
SC34	CS13	CS10	*R.nitidus*	0.050678	0.067687
SC6	CS13	CS10	*R.nitidus*	0.029187	0.068725
SC15	CS13	CS10	*R.nitidus*	0.028529	0.067624
SC45	CS13	CS10	*R.nitidus*	0.066235	0.069923
CS24	CS13	CS10	*R.nitidus*	-0.01251	0.06807
MY4	CS13	CS10	*R.nitidus*	-0.009503	0.068629
GD25	GD46	CS10	*R.nitidus*	0.079529	0.064218
SC14	CS13	CS10	*R.nitidus*	0.044693	0.066416
GD31	GD46	CS10	*R.nitidus*	0.068755	0.06404
SC44	CS13	CS10	*R.nitidus*	0.028674	0.068515
SC24	CS13	CS10	*R.nitidus*	0.046643	0.068856
JO3	CS13	CS10	*R.nitidus*	0.067194	0.071939
GD21	GD46	CS10	*R.nitidus*	0.167328	0.062634
GD22	GD46	CS10	*R.nitidus*	0.099709	0.062408
SC42	CS13	CS10	*R.nitidus*	0.259953	0.062885
SC35	CS13	CS10	*R.nitidus*	0.074689	0.065928
GD33	GD46	CS10	*R.nitidus*	0.033077	0.06441
JW4	CS13	CS10	*R.nitidus*	0.253156	0.065941
JW6	CS13	CS10	*R.nitidus*	0.030928	0.075084
MY6	CS13	CS10	*R.nitidus*	0.031361	0.06776
GD48	GD46	CS10	*R.nitidus*	0.08319	0.066538
JO7	GD46	CS10	*R.nitidus*	0.02302	0.071531
CS12	GD46	CS10	*R.nitidus*	0.007948	0.067847
GD34	GD46	CS10	*R.nitidus*	0.017829	0.062288
GD50	GD46	CS10	*R.nitidus*	0.054731	0.067139
CS13	GD46	CS10	*R.nitidus*	-0.035446	0.068317
GD26	GD46	CS10	*R.nitidus*	0.07377	0.062614
GD29	GD46	CS10	*R.nitidus*	0.164341	0.064697
GD40	GD46	CS10	*R.nitidus*	0.079829	0.062736
GD30	GD46	CS10	*R.nitidus*	0.171429	0.064747
GD49	GD46	CS10	*R.nitidus*	0.112	0.067922
GD41	GD46	CS10	*R.nitidus*	0.045416	0.062873
JO5	CS13	CS10	*R.nitidus*	0.073654	0.067636
JO1	CS13	CS10	*R.nitidus*	0.036403	0.067104
GD44	GD46	CS10	*R.nitidus*	0.032308	0.066449
GD32	GD46	CS10	*R.nitidus*	0.054264	0.066386
CS11	GD46	CS10	*R.nitidus*	-0.000668	0.067183
GD45	GD46	CS10	*R.nitidus*	0.097628	0.071298
CS18	GD46	CS10	*R.nitidus*	-0.037812	0.064956
GD35	GD46	CS10	*R.nitidus*	0.065137	0.06368
CS15	GD46	CS10	*R.nitidus*	0.020325	0.066931
GD42	GD46	CS10	*R.nitidus*	0.038884	0.063921
CS4	GD46	CS10	*R.nitidus*	-0.013021	0.063636
CS16	GD46	CS10	*R.nitidus*	0.00692	0.067255

SC18	GD46	CS10	*R.nitidus*	0.04954	0.063383
CS23	GD46	CS10	*R.nitidus*	-0.042949	0.063046
CS22	GD46	CS10	*R.nitidus*	-0.002045	0.063803
CS19	GD46	CS10	*R.nitidus*	-0.017735	0.066793
SC5	GD46	CS10	*R.nitidus*	0.001361	0.067063
SC6	GD46	CS10	*R.nitidus*	0.004808	0.064203
SC27	GD46	CS10	*R.nitidus*	0.008796	0.063682
CS20	GD46	CS10	*R.nitidus*	-0.02912	0.065529
CS6	GD46	CS10	*R.nitidus*	-0.039528	0.065602
CS21	GD46	CS10	*R.nitidus*	-0.025624	0.065272
CS14	GD46	CS10	*R.nitidus*	-0.001394	0.069305
CS30	GD46	CS10	*R.nitidus*	-0.051554	0.065717
SC10	GD46	CS10	*R.nitidus*	0.062753	0.065475
CS17	GD46	CS10	*R.nitidus*	0.015328	0.068919
JW7	GD46	CS10	*R.nitidus*	0.050889	0.066639
JO3	GD46	CS10	*R.nitidus*	0.0556	0.067402
CS31	GD46	CS10	*R.nitidus*	-0.02248	0.065516
CS24	GD46	CS10	*R.nitidus*	-0.030986	0.065309
SC12	GD46	CS10	*R.nitidus*	-0.00944	0.064927
CS25	GD46	CS10	*R.nitidus*	-0.009421	0.067751
CS3	GD46	CS10	*R.nitidus*	-0.008696	0.065286
SC26	GD46	CS10	*R.nitidus*	-0.011858	0.064022
SC24	GD46	CS10	*R.nitidus*	0.016506	0.064731
SC42	GD46	CS10	*R.nitidus*	0.227545	0.062137
CS28	GD46	CS10	*R.nitidus*	-0.032727	0.067676
SC31	GD46	CS10	*R.nitidus*	-0.013342	0.06449
JO8	GD48	CS10	*R.nitidus*	-0.0125	0.070723
SC14	GD46	CS10	*R.nitidus*	0.028859	0.064503
SC35	GD46	CS10	*R.nitidus*	0.049702	0.062672
SC28	GD46	CS10	*R.nitidus*	-0.023396	0.063445
SC15	GD46	CS10	*R.nitidus*	0.007097	0.066078
MY6	GD46	CS10	*R.nitidus*	0.002681	0.065224
SC44	GD46	CS10	*R.nitidus*	0.00827	0.065225
MY4	GD46	CS10	*R.nitidus*	-0.031528	0.065792
JW6	GD46	CS10	*R.nitidus*	0.038043	0.070983
GD31	GD48	CS10	*R.nitidus*	0.023057	0.068067
JW4	GD46	CS10	*R.nitidus*	0.239351	0.065489
SC32	GD46	CS10	*R.nitidus*	0.051713	0.065268
GD40	GD48	CS10	*R.nitidus*	0.029321	0.066537
JO7	GD48	CS10	*R.nitidus*	-0.029493	0.072391
SC34	GD46	CS10	*R.nitidus*	0.031767	0.063732
CS11	GD48	CS10	*R.nitidus*	-0.04669	0.068449
GD35	GD48	CS10	*R.nitidus*	0.010492	0.067192
GD26	GD48	CS10	*R.nitidus*	0.030008	0.066239
GD22	GD48	CS10	*R.nitidus*	0.042373	0.065006
SC45	GD46	CS10	*R.nitidus*	0.048716	0.064243
GD46	GD48	CS10	*R.nitidus*	-0.08319	0.066538
JO5	GD46	CS10	*R.nitidus*	0.044341	0.064509
JO1	GD46	CS10	*R.nitidus*	-0.006676	0.065564
GD25	GD48	CS10	*R.nitidus*	0.014575	0.065876
GD49	GD48	CS10	*R.nitidus*	0.057277	0.071718
GD21	GD48	CS10	*R.nitidus*	0.116505	0.067025
GD41	GD48	CS10	*R.nitidus*	-0.047939	0.06707
GD30	GD48	CS10	*R.nitidus*	0.066863	0.068003
GD50	GD48	CS10	*R.nitidus*	-0.032946	0.071726
GD33	GD48	CS10	*R.nitidus*	-0.021533	0.067973

CS13	GD48	CS10	*R.nitidus*	-0.078871	0.069727
GD45	GD48	CS10	*R.nitidus*	0.007797	0.071035
CS21	GD48	CS10	*R.nitidus*	-0.074363	0.067001
CS14	GD48	CS10	*R.nitidus*	-0.042143	0.070116
GD32	GD48	CS10	*R.nitidus*	-0.010387	0.066125
GD34	GD48	CS10	*R.nitidus*	-0.047619	0.065008
GD29	GD48	CS10	*R.nitidus*	0.092874	0.063973
GD44	GD48	CS10	*R.nitidus*	-0.039834	0.067082
CS23	GD48	CS10	*R.nitidus*	-0.097262	0.066847
CS17	GD48	CS10	*R.nitidus*	-0.048536	0.07036
CS12	GD48	CS10	*R.nitidus*	-0.042074	0.068055
CS16	GD48	CS10	*R.nitidus*	-0.050177	0.067046
CS20	GD48	CS10	*R.nitidus*	-0.065217	0.066757
CS24	GD48	CS10	*R.nitidus*	-0.072605	0.067184
CS18	GD48	CS10	*R.nitidus*	-0.076197	0.069641
GD42	GD48	CS10	*R.nitidus*	-0.023377	0.066456
CS28	GD48	CS10	*R.nitidus*	-0.086207	0.068484
CS3	GD48	CS10	*R.nitidus*	-0.057565	0.066392
CS4	GD48	CS10	*R.nitidus*	-0.046495	0.066116
SC6	GD48	CS10	*R.nitidus*	-0.046749	0.066578
SC12	GD48	CS10	*R.nitidus*	-0.052632	0.067634
CS22	GD48	CS10	*R.nitidus*	-0.048458	0.067124
CS15	GD48	CS10	*R.nitidus*	-0.02807	0.068117
CS19	GD48	CS10	*R.nitidus*	-0.059309	0.067535
SC31	GD48	CS10	*R.nitidus*	-0.05841	0.067686
MY6	GD48	CS10	*R.nitidus*	-0.039501	0.066577
SC15	GD48	CS10	*R.nitidus*	-0.051515	0.067249
CS30	GD48	CS10	*R.nitidus*	-0.093023	0.068174
SC5	GD48	CS10	*R.nitidus*	-0.012696	0.070132
SC35	GD48	CS10	*R.nitidus*	0.010784	0.064721
SC10	GD48	CS10	*R.nitidus*	0.026831	0.067428
SC14	GD48	CS10	*R.nitidus*	-0.035562	0.067297
CS25	GD48	CS10	*R.nitidus*	-0.053064	0.068384
JW6	GD48	CS10	*R.nitidus*	-0.021336	0.07112
SC26	GD48	CS10	*R.nitidus*	-0.068214	0.066411
GD26	GD49	CS10	*R.nitidus*	-0.037267	0.066666
SC34	GD48	CS10	*R.nitidus*	-0.022857	0.065272
JO3	GD48	CS10	*R.nitidus*	-0.021541	0.069392
SC42	GD48	CS10	*R.nitidus*	0.168395	0.064813
MY4	GD48	CS10	*R.nitidus*	-0.074236	0.067413
SC32	GD48	CS10	*R.nitidus*	-0.000727	0.067262
CS31	GD48	CS10	*R.nitidus*	-0.066517	0.066871
SC28	GD48	CS10	*R.nitidus*	-0.059371	0.065414
SC27	GD48	CS10	*R.nitidus*	-0.037223	0.065388
CS6	GD48	CS10	*R.nitidus*	-0.09368	0.066514
SC18	GD48	CS10	*R.nitidus*	0.004518	0.067793
JW7	GD48	CS10	*R.nitidus*	0.006606	0.068491
SC24	GD48	CS10	*R.nitidus*	-0.019868	0.067142
GD25	GD49	CS10	*R.nitidus*	-0.039758	0.068574
GD29	GD49	CS10	*R.nitidus*	0.033159	0.067222
CS12	GD49	CS10	*R.nitidus*	-0.094694	0.069339
JO1	GD48	CS10	*R.nitidus*	-0.040627	0.067448
CS11	GD49	CS10	*R.nitidus*	-0.082506	0.070097
GD31	GD49	CS10	*R.nitidus*	-0.049778	0.071574
GD30	GD49	CS10	*R.nitidus*	0.066132	0.070792
JO5	GD48	CS10	*R.nitidus*	0.013728	0.066311

JO7	GD49	CS10	*R.nitidus*	-0.089207	0.074161
JW4	GD48	CS10	*R.nitidus*	0.177609	0.06624
GD33	GD49	CS10	*R.nitidus*	-0.074692	0.070274
SC44	GD48	CS10	*R.nitidus*	-0.03386	0.06575
GD44	GD49	CS10	*R.nitidus*	-0.086312	0.069411
JO8	GD49	CS10	*R.nitidus*	-0.049605	0.074018
SC45	GD48	CS10	*R.nitidus*	-0.002131	0.065645
CS14	GD49	CS10	*R.nitidus*	-0.069207	0.071444
GD34	GD49	CS10	*R.nitidus*	-0.08863	0.067084
GD21	GD49	CS10	*R.nitidus*	0.022241	0.068048
GD46	GD49	CS10	*R.nitidus*	-0.112	0.067922
GD32	GD49	CS10	*R.nitidus*	-0.085239	0.071134
GD22	GD49	CS10	*R.nitidus*	-0.058651	0.069182
GD50	GD49	CS10	*R.nitidus*	-0.066946	0.074821
CS15	GD49	CS10	*R.nitidus*	-0.057813	0.068739
GD35	GD49	CS10	*R.nitidus*	-0.059603	0.069037
GD45	GD49	CS10	*R.nitidus*	-0.036926	0.072607
GD42	GD49	CS10	*R.nitidus*	-0.085944	0.068206
CS16	GD49	CS10	*R.nitidus*	-0.076804	0.068663
GD41	GD49	CS10	*R.nitidus*	-0.096104	0.067799
GD40	GD49	CS10	*R.nitidus*	-0.026961	0.068338
CS23	GD49	CS10	*R.nitidus*	-0.120189	0.066928
CS18	GD49	CS10	*R.nitidus*	-0.119497	0.069679
CS13	GD49	CS10	*R.nitidus*	-0.112006	0.070694
CS30	GD49	CS10	*R.nitidus*	-0.142857	0.068027
SC32	GD49	CS10	*R.nitidus*	-0.046584	0.067764
CS20	GD49	CS10	*R.nitidus*	-0.133077	0.068605
CS28	GD49	CS10	*R.nitidus*	-0.105651	0.070563
CS3	GD49	CS10	*R.nitidus*	-0.116904	0.067941
CS17	GD49	CS10	*R.nitidus*	-0.064437	0.071947
CS4	GD49	CS10	*R.nitidus*	-0.09084	0.067312
CS21	GD49	CS10	*R.nitidus*	-0.120127	0.068096
GD48	GD49	CS10	*R.nitidus*	-0.057277	0.071718
CS24	GD49	CS10	*R.nitidus*	-0.116242	0.069199
SC15	GD49	CS10	*R.nitidus*	-0.088697	0.068323
SC42	GD49	CS10	*R.nitidus*	0.1655	0.065288
SC12	GD49	CS10	*R.nitidus*	-0.087363	0.067365
SC28	GD49	CS10	*R.nitidus*	-0.102229	0.065814
SC27	GD49	CS10	*R.nitidus*	-0.076511	0.068435
SC31	GD49	CS10	*R.nitidus*	-0.112471	0.068668
SC35	GD49	CS10	*R.nitidus*	-0.028073	0.0681
CS19	GD49	CS10	*R.nitidus*	-0.082181	0.069231
SC24	GD49	CS10	*R.nitidus*	-0.091053	0.067123
CS31	GD49	CS10	*R.nitidus*	-0.112006	0.070285
SC14	GD49	CS10	*R.nitidus*	-0.06155	0.068298
JW7	GD49	CS10	*R.nitidus*	-0.014019	0.072765
SC5	GD49	CS10	*R.nitidus*	-0.092874	0.07159
JO3	GD49	CS10	*R.nitidus*	-0.019755	0.073075
SC10	GD49	CS10	*R.nitidus*	-0.026046	0.069858
SC6	GD49	CS10	*R.nitidus*	-0.089774	0.069436
JW4	GD49	CS10	*R.nitidus*	0.159073	0.06931
SC45	GD49	CS10	*R.nitidus*	-0.048145	0.068178
MY6	GD49	CS10	*R.nitidus*	-0.103528	0.068707
SC18	GD49	CS10	*R.nitidus*	-0.047297	0.06707
JO7	GD50	CS10	*R.nitidus*	-0.061267	0.075123
SC26	GD49	CS10	*R.nitidus*	-0.11026	0.069954

JW6	GD49	CS10	*R.nitidus*	-0.075078	0.072155
MY4	GD49	CS10	*R.nitidus*	-0.109642	0.069857
GD32	GD50	CS10	*R.nitidus*	-0.017635	0.071209
GD25	GD50	CS10	*R.nitidus*	-0.007266	0.069079
CS6	GD49	CS10	*R.nitidus*	-0.121262	0.068458
SC34	GD49	CS10	*R.nitidus*	-0.044174	0.065563
SC44	GD49	CS10	*R.nitidus*	-0.0744	0.067232
GD31	GD50	CS10	*R.nitidus*	0.025093	0.069425
JO1	GD49	CS10	*R.nitidus*	-0.079485	0.068372
CS11	GD50	CS10	*R.nitidus*	-0.018548	0.071386
GD21	GD50	CS10	*R.nitidus*	0.129318	0.068611
CS17	GD50	CS10	*R.nitidus*	-0.003419	0.072454
JO8	GD50	CS10	*R.nitidus*	-0.024811	0.075318
GD26	GD50	CS10	*R.nitidus*	0.019355	0.067313
JO5	GD49	CS10	*R.nitidus*	-0.060032	0.067319
GD29	GD50	CS10	*R.nitidus*	0.090009	0.066481
CS12	GD50	CS10	*R.nitidus*	-0.029167	0.070938
GD33	GD50	CS10	*R.nitidus*	-0.012903	0.07047
CS13	GD50	CS10	*R.nitidus*	-0.063812	0.072246
GD45	GD50	CS10	*R.nitidus*	0.030801	0.072814
GD41	GD50	CS10	*R.nitidus*	-0.029557	0.067878
GD48	GD50	CS10	*R.nitidus*	0.032946	0.071726
GD34	GD50	CS10	*R.nitidus*	-0.024545	0.066862
GD30	GD50	CS10	*R.nitidus*	0.10124	0.071251
CS19	GD50	CS10	*R.nitidus*	-0.055738	0.070628
GD22	GD50	CS10	*R.nitidus*	0.023445	0.067455
GD40	GD50	CS10	*R.nitidus*	0.037815	0.067766
GD49	GD50	CS10	*R.nitidus*	0.066946	0.074821
CS16	GD50	CS10	*R.nitidus*	-0.031981	0.06863
GD46	GD50	CS10	*R.nitidus*	-0.054731	0.067139
CS14	GD50	CS10	*R.nitidus*	-0.008958	0.071091
GD42	GD50	CS10	*R.nitidus*	-0.00499	0.06867
CS18	GD50	CS10	*R.nitidus*	-0.066285	0.067236
GD35	GD50	CS10	*R.nitidus*	0.017724	0.067505
SC24	GD50	CS10	*R.nitidus*	-0.060065	0.067883
CS3	GD50	CS10	*R.nitidus*	-0.066882	0.067653
CS28	GD50	CS10	*R.nitidus*	-0.097778	0.069263
SC18	GD50	CS10	*R.nitidus*	-0.01269	0.069507
SC10	GD50	CS10	*R.nitidus*	0.017529	0.070176
CS15	GD50	CS10	*R.nitidus*	-0.011094	0.070078
CS21	GD50	CS10	*R.nitidus*	-0.061224	0.068015
CS23	GD50	CS10	*R.nitidus*	-0.080841	0.06814
SC5	GD50	CS10	*R.nitidus*	-0.054841	0.073161
SC15	GD50	CS10	*R.nitidus*	-0.030431	0.068811
SC14	GD50	CS10	*R.nitidus*	-0.023256	0.068676
SC12	GD50	CS10	*R.nitidus*	-0.083199	0.069972
MY6	GD50	CS10	*R.nitidus*	-0.040221	0.068167
CS6	GD50	CS10	*R.nitidus*	-0.074875	0.068358
CS22	GD50	CS10	*R.nitidus*	-0.042834	0.06819
CS20	GD50	CS10	*R.nitidus*	-0.067742	0.067765
SC44	GD50	CS10	*R.nitidus*	-0.038851	0.068977
SC31	GD50	CS10	*R.nitidus*	-0.042254	0.068439
SC45	GD50	CS10	*R.nitidus*	0.004819	0.06819
CS24	GD50	CS10	*R.nitidus*	-0.053037	0.068468
CS31	GD50	CS10	*R.nitidus*	-0.065687	0.069018
SC34	GD50	CS10	*R.nitidus*	0.001591	0.065196

SC6	GD50	CS10	*R.nitidus*	-0.041085	0.068111
CS25	GD50	CS10	*R.nitidus*	-0.057661	0.069055
CS30	GD50	CS10	*R.nitidus*	-0.09205	0.06834
GD44	GD50	CS10	*R.nitidus*	-0.022727	0.068922
SC35	GD50	CS10	*R.nitidus*	-0.006457	0.066244
JW7	GD50	CS10	*R.nitidus*	-0.022265	0.073113
CS4	GD50	CS10	*R.nitidus*	-0.061807	0.065446
JW4	GD50	CS10	*R.nitidus*	0.149837	0.068434
SC27	GD50	CS10	*R.nitidus*	-0.056697	0.065612
SC26	GD50	CS10	*R.nitidus*	-0.0552	0.069954
JW6	GD50	CS10	*R.nitidus*	-0.05274	0.074563
SC28	GD50	CS10	*R.nitidus*	-0.071659	0.066511
JO1	GD50	CS10	*R.nitidus*	-0.056406	0.068498
SC32	GD50	CS10	*R.nitidus*	0	0.069045
JO3	GD50	CS10	*R.nitidus*	-0.041554	0.072922
CS11	CS14	CS10	*R.nitidus*	-0.001153	0.070575
GD29	CS14	CS10	*R.nitidus*	0.133838	0.066993
GD26	CS14	CS10	*R.nitidus*	0.059419	0.068257
SC42	GD50	CS10	*R.nitidus*	0.195155	0.065517
GD31	CS14	CS10	*R.nitidus*	0.054765	0.069645
GD30	CS14	CS10	*R.nitidus*	0.120355	0.071371
GD25	CS14	CS10	*R.nitidus*	0.071478	0.067394
MY4	GD50	CS10	*R.nitidus*	-0.082998	0.069546
JO7	CS14	CS10	*R.nitidus*	-0.001899	0.073375
GD22	CS14	CS10	*R.nitidus*	0.066917	0.066792
JO8	CS14	CS10	*R.nitidus*	0.00754	0.074941
GD33	CS14	CS10	*R.nitidus*	0.012574	0.067104
CS12	CS14	CS10	*R.nitidus*	0	0.071393
GD34	CS14	CS10	*R.nitidus*	0.012953	0.066118
GD40	CS14	CS10	*R.nitidus*	0.0625	0.06642
GD35	CS14	CS10	*R.nitidus*	0.03603	0.068697
JO5	GD50	CS10	*R.nitidus*	-0.004055	0.067664
CS16	CS14	CS10	*R.nitidus*	0.020021	0.068492
GD21	CS14	CS10	*R.nitidus*	0.131492	0.065826
GD48	CS14	CS10	*R.nitidus*	0.042143	0.070116
GD45	CS14	CS10	*R.nitidus*	0.05198	0.072723
CS20	CS14	CS10	*R.nitidus*	-0.030075	0.067662
CS13	CS14	CS10	*R.nitidus*	-0.046192	0.071654
CS18	CS14	CS10	*R.nitidus*	-0.045586	0.067196
CS3	CS14	CS10	*R.nitidus*	-0.025162	0.067814
CS28	CS14	CS10	*R.nitidus*	-0.033385	0.067375
GD50	CS14	CS10	*R.nitidus*	0.008958	0.071091
CS21	CS14	CS10	*R.nitidus*	-0.034532	0.066658
GD32	CS14	CS10	*R.nitidus*	0.025424	0.069449
GD46	CS14	CS10	*R.nitidus*	0.001394	0.069305
CS22	CS14	CS10	*R.nitidus*	-0.007942	0.068257
CS30	CS14	CS10	*R.nitidus*	-0.060517	0.067363
CS24	CS14	CS10	*R.nitidus*	-0.046423	0.068484
GD44	CS14	CS10	*R.nitidus*	0.016609	0.067693
GD49	CS14	CS10	*R.nitidus*	0.069207	0.071444
GD41	CS14	CS10	*R.nitidus*	0.010589	0.066565
GD42	CS14	CS10	*R.nitidus*	0.033198	0.067171
CS31	CS14	CS10	*R.nitidus*	-0.026276	0.068876
CS15	CS14	CS10	*R.nitidus*	0.044395	0.070129
SC12	CS14	CS10	*R.nitidus*	-0.009682	0.068391
CS17	CS14	CS10	*R.nitidus*	0.014201	0.073689

CS23	CS14	CS10	*R.nitidus*	-0.06377	0.066365
CS19	CS14	CS10	*R.nitidus*	-0.019505	0.069353
SC34	CS14	CS10	*R.nitidus*	0.025535	0.06651
MY4	CS14	CS10	*R.nitidus*	-0.041696	0.069358
SC42	CS14	CS10	*R.nitidus*	0.229003	0.06305
SC44	CS14	CS10	*R.nitidus*	0.002082	0.067499
SC6	CS14	CS10	*R.nitidus*	0.004283	0.068798
CS25	CS14	CS10	*R.nitidus*	-0.019665	0.067893
SC28	CS14	CS10	*R.nitidus*	-0.02591	0.066504
SC35	CS14	CS10	*R.nitidus*	0.040956	0.065231
SC27	CS14	CS10	*R.nitidus*	-0.004909	0.065201
JW6	CS14	CS10	*R.nitidus*	-0.00455	0.075371
JW4	CS14	CS10	*R.nitidus*	0.21616	0.067882
SC18	CS14	CS10	*R.nitidus*	0.024735	0.067637
SC31	CS14	CS10	*R.nitidus*	-0.015007	0.0664
JO1	CS14	CS10	*R.nitidus*	0.002101	0.068085
GD22	CS15	CS10	*R.nitidus*	0.048348	0.065846
SC14	CS14	CS10	*R.nitidus*	0.010135	0.065923
CS4	CS14	CS10	*R.nitidus*	-0.021648	0.067092
SC10	CS14	CS10	*R.nitidus*	0.064562	0.066713
GD26	CS15	CS10	*R.nitidus*	0.036113	0.066925
SC5	CS14	CS10	*R.nitidus*	0.006993	0.070112
SC15	CS14	CS10	*R.nitidus*	-0.004376	0.068116
GD30	CS15	CS10	*R.nitidus*	0.10306	0.069057
JO7	CS15	CS10	*R.nitidus*	-0.029493	0.071125
SC26	CS14	CS10	*R.nitidus*	-0.028374	0.067024
SC32	CS14	CS10	*R.nitidus*	0.052136	0.0688
JW7	CS14	CS10	*R.nitidus*	0.033727	0.070596
SC24	CS14	CS10	*R.nitidus*	0.010352	0.067659
CS6	CS14	CS10	*R.nitidus*	-0.061391	0.067369
JO3	CS14	CS10	*R.nitidus*	0.028302	0.071095
MY6	CS14	CS10	*R.nitidus*	-0.014493	0.068612
GD21	CS15	CS10	*R.nitidus*	0.104275	0.065022
JO5	CS14	CS10	*R.nitidus*	0.033311	0.067092
JO8	CS15	CS10	*R.nitidus*	0.008287	0.071507
CS11	CS15	CS10	*R.nitidus*	-0.04	0.068345
SC45	CS14	CS10	*R.nitidus*	0.042553	0.069681
GD25	CS15	CS10	*R.nitidus*	0.051476	0.065235
GD29	CS15	CS10	*R.nitidus*	0.099564	0.06563
GD32	CS15	CS10	*R.nitidus*	0.000763	0.068375
GD48	CS15	CS10	*R.nitidus*	0.02807	0.068117
GD42	CS15	CS10	*R.nitidus*	0.008048	0.066256
GD33	CS15	CS10	*R.nitidus*	-0.003911	0.066429
CS14	CS15	CS10	*R.nitidus*	-0.044395	0.070129
CS12	CS15	CS10	*R.nitidus*	-0.033684	0.066348
GD45	CS15	CS10	*R.nitidus*	0.040519	0.071109
GD49	CS15	CS10	*R.nitidus*	0.057813	0.068739
GD44	CS15	CS10	*R.nitidus*	-0.002037	0.065596
GD31	CS15	CS10	*R.nitidus*	0.047983	0.066382
CS13	CS15	CS10	*R.nitidus*	-0.078764	0.069669
GD50	CS15	CS10	*R.nitidus*	0.011094	0.070078
GD34	CS15	CS10	*R.nitidus*	-0.017187	0.065273
SC6	CS15	CS10	*R.nitidus*	-0.025714	0.065901
GD35	CS15	CS10	*R.nitidus*	0.016556	0.067126
CS30	CS15	CS10	*R.nitidus*	-0.085507	0.065642
CS17	CS15	CS10	*R.nitidus*	-0.044565	0.066676

CS23	CS15	CS10	*R.nitidus*	-0.085774	0.064729
CS24	CS15	CS10	*R.nitidus*	-0.071111	0.065962
GD41	CS15	CS10	*R.nitidus*	-0.008453	0.064893
CS28	CS15	CS10	*R.nitidus*	-0.04736	0.066065
GD40	CS15	CS10	*R.nitidus*	0.048232	0.065233
CS20	CS15	CS10	*R.nitidus*	-0.054348	0.066626
CS25	CS15	CS10	*R.nitidus*	-0.038267	0.068933
CS19	CS15	CS10	*R.nitidus*	-0.045944	0.066937
SC15	CS15	CS10	*R.nitidus*	-0.02641	0.06612
SC10	CS15	CS10	*R.nitidus*	0.044818	0.066962
CS22	CS15	CS10	*R.nitidus*	-0.023588	0.065812
SC28	CS15	CS10	*R.nitidus*	-0.054747	0.063978
SC34	CS15	CS10	*R.nitidus*	0.008125	0.06554
SC18	CS15	CS10	*R.nitidus*	0.021216	0.065358
CS16	CS15	CS10	*R.nitidus*	-0.030754	0.065114
CS21	CS15	CS10	*R.nitidus*	-0.054054	0.065096
GD46	CS15	CS10	*R.nitidus*	-0.020325	0.066931
SC12	CS15	CS10	*R.nitidus*	-0.033677	0.066976
SC35	CS15	CS10	*R.nitidus*	0.019828	0.064256
CS18	CS15	CS10	*R.nitidus*	-0.064609	0.066784
SC14	CS15	CS10	*R.nitidus*	-0.014483	0.066601
SC5	CS15	CS10	*R.nitidus*	-0.018367	0.070174
CS4	CS15	CS10	*R.nitidus*	-0.037263	0.063922
MY4	CS15	CS10	*R.nitidus*	-0.064472	0.067352
CS31	CS15	CS10	*R.nitidus*	-0.046131	0.067152
CS3	CS15	CS10	*R.nitidus*	-0.041076	0.066602
SC45	CS15	CS10	*R.nitidus*	0.018219	0.067046
SC26	CS15	CS10	*R.nitidus*	-0.047883	0.064668
SC32	CS15	CS10	*R.nitidus*	0.028787	0.06585
JO5	CS15	CS10	*R.nitidus*	0.023114	0.066406
JW6	CS15	CS10	*R.nitidus*	-0.029517	0.070908
GD31	CS16	CS10	*R.nitidus*	0.051049	0.066959
SC31	CS15	CS10	*R.nitidus*	-0.039947	0.065363
SC27	CS15	CS10	*R.nitidus*	-0.027992	0.064009
JO1	CS15	CS10	*R.nitidus*	-0.021618	0.063458
SC42	CS15	CS10	*R.nitidus*	0.205171	0.061541
SC44	CS15	CS10	*R.nitidus*	-0.017409	0.067346
JW7	CS15	CS10	*R.nitidus*	0	0.067632
CS6	CS15	CS10	*R.nitidus*	-0.073401	0.067427
JW4	CS15	CS10	*R.nitidus*	0.184053	0.06638
SC24	CS15	CS10	*R.nitidus*	-0.013624	0.065157
GD30	CS16	CS10	*R.nitidus*	0.115323	0.067402
JO8	CS16	CS10	*R.nitidus*	0.02975	0.072926
CS13	CS16	CS10	*R.nitidus*	-0.046602	0.069398
JO3	CS15	CS10	*R.nitidus*	0.010654	0.067992
JO7	CS16	CS10	*R.nitidus*	-0.015224	0.07361
MY6	CS15	CS10	*R.nitidus*	-0.028455	0.06708
GD45	CS16	CS10	*R.nitidus*	0.065916	0.071931
GD40	CS16	CS10	*R.nitidus*	0.073007	0.064381
GD21	CS16	CS10	*R.nitidus*	0.112777	0.062883
GD29	CS16	CS10	*R.nitidus*	0.1279	0.063912
GD49	CS16	CS10	*R.nitidus*	0.076804	0.068663
CS11	CS16	CS10	*R.nitidus*	-0.014199	0.068534
CS15	CS16	CS10	*R.nitidus*	0.030754	0.065114
GD22	CS16	CS10	*R.nitidus*	0.063591	0.063002
CS12	CS16	CS10	*R.nitidus*	-0.007982	0.064796

GD41	CS16	CS10	*R.nitidus*	0.007338	0.063661
GD25	CS16	CS10	*R.nitidus*	0.064336	0.065946
CS14	CS16	CS10	*R.nitidus*	-0.020021	0.068492
GD32	CS16	CS10	*R.nitidus*	0.031226	0.068034
GD26	CS16	CS10	*R.nitidus*	0.054125	0.065226
GD46	CS16	CS10	*R.nitidus*	-0.00692	0.067255
GD35	CS16	CS10	*R.nitidus*	0.035928	0.065172
GD34	CS16	CS10	*R.nitidus*	0.008974	0.063312
GD48	CS16	CS10	*R.nitidus*	0.050177	0.067046
GD33	CS16	CS10	*R.nitidus*	0.007138	0.064044
CS22	CS16	CS10	*R.nitidus*	-0.005069	0.066354
CS18	CS16	CS10	*R.nitidus*	-0.031825	0.066451
GD42	CS16	CS10	*R.nitidus*	0.025798	0.06396
SC5	CS16	CS10	*R.nitidus*	-0.000695	0.069209
CS17	CS16	CS10	*R.nitidus*	-0.004391	0.069165
GD44	CS16	CS10	*R.nitidus*	0.006259	0.064968
GD50	CS16	CS10	*R.nitidus*	0.031981	0.06863
CS31	CS16	CS10	*R.nitidus*	-0.035769	0.066577
CS30	CS16	CS10	*R.nitidus*	-0.071325	0.065593
CS3	CS16	CS10	*R.nitidus*	-0.025162	0.065028
SC18	CS16	CS10	*R.nitidus*	0.03307	0.065286
CS21	CS16	CS10	*R.nitidus*	-0.03644	0.0659
CS24	CS16	CS10	*R.nitidus*	-0.050528	0.065441
CS28	CS16	CS10	*R.nitidus*	-0.034776	0.065178
CS19	CS16	CS10	*R.nitidus*	-0.01551	0.06598
SC10	CS16	CS10	*R.nitidus*	0.075069	0.066962
SC12	CS16	CS10	*R.nitidus*	-0.018815	0.066555
SC14	CS16	CS10	*R.nitidus*	0.006207	0.064614
CS20	CS16	CS10	*R.nitidus*	-0.029372	0.066243
SC24	CS16	CS10	*R.nitidus*	0.006925	0.064364
SC6	CS16	CS10	*R.nitidus*	-0.015839	0.065546
SC27	CS16	CS10	*R.nitidus*	-0.00212	0.063795
SC35	CS16	CS10	*R.nitidus*	0.040431	0.063489
CS25	CS16	CS10	*R.nitidus*	-0.021352	0.067026
CS23	CS16	CS10	*R.nitidus*	-0.052929	0.063424
JW4	CS16	CS10	*R.nitidus*	0.224828	0.06504
SC42	CS16	CS10	*R.nitidus*	0.235227	0.060856
SC45	CS16	CS10	*R.nitidus*	0.037396	0.06579
SC32	CS16	CS10	*R.nitidus*	0.045678	0.065327
SC15	CS16	CS10	*R.nitidus*	-0.011038	0.06555
JO5	CS16	CS10	*R.nitidus*	0.036161	0.064015
SC34	CS16	CS10	*R.nitidus*	0.013094	0.065279
JW6	CS16	CS10	*R.nitidus*	0.001818	0.072928
JO3	CS16	CS10	*R.nitidus*	0.027069	0.069297
SC26	CS16	CS10	*R.nitidus*	-0.028101	0.063849
CS6	CS16	CS10	*R.nitidus*	-0.056759	0.065149
CS4	CS16	CS10	*R.nitidus*	-0.022965	0.064438
MY6	CS16	CS10	*R.nitidus*	-0.015775	0.065048
SC44	CS16	CS10	*R.nitidus*	0	0.064918
SC28	CS16	CS10	*R.nitidus*	-0.044097	0.065264
JO7	CS17	CS10	*R.nitidus*	0.011055	0.077047
SC31	CS16	CS10	*R.nitidus*	-0.035495	0.066294
GD22	CS17	CS10	*R.nitidus*	0.053405	0.066144
CS11	CS17	CS10	*R.nitidus*	-0.010753	0.069247
GD42	CS17	CS10	*R.nitidus*	0.012319	0.068888
GD29	CS17	CS10	*R.nitidus*	0.111713	0.06746

JO1	CS16	CS10	*R.nitidus*	0	0.065452
MY4	CS16	CS10	*R.nitidus*	-0.04502	0.066822
CS14	CS17	CS10	*R.nitidus*	-0.014201	0.073689
GD21	CS17	CS10	*R.nitidus*	0.108635	0.066706
GD25	CS17	CS10	*R.nitidus*	0.055849	0.068234
JO8	CS17	CS10	*R.nitidus*	0.011317	0.075825
GD40	CS17	CS10	*R.nitidus*	0.06528	0.066893
JW7	CS16	CS10	*R.nitidus*	0.037069	0.070216
GD30	CS17	CS10	*R.nitidus*	0.114428	0.070635
GD26	CS17	CS10	*R.nitidus*	0.038596	0.068994
GD35	CS17	CS10	*R.nitidus*	0.033503	0.068763
CS12	CS17	CS10	*R.nitidus*	-0.00883	0.070835
GD41	CS17	CS10	*R.nitidus*	0.009194	0.068489
GD32	CS17	CS10	*R.nitidus*	0.008864	0.072341
CS20	CS17	CS10	*R.nitidus*	-0.036585	0.068324
GD44	CS17	CS10	*R.nitidus*	0	0.070882
GD31	CS17	CS10	*R.nitidus*	0.055348	0.069841
CS31	CS17	CS10	*R.nitidus*	-0.032046	0.070227
CS13	CS17	CS10	*R.nitidus*	-0.048966	0.072071
GD48	CS17	CS10	*R.nitidus*	0.048536	0.07036
CS16	CS17	CS10	*R.nitidus*	0.004391	0.069165
GD46	CS17	CS10	*R.nitidus*	-0.015328	0.068919
GD49	CS17	CS10	*R.nitidus*	0.064437	0.071947
CS30	CS17	CS10	*R.nitidus*	-0.080309	0.068293
SC5	CS17	CS10	*R.nitidus*	-0.009811	0.071343
GD45	CS17	CS10	*R.nitidus*	0.06867	0.071615
GD33	CS17	CS10	*R.nitidus*	-0.001411	0.067736
CS18	CS17	CS10	*R.nitidus*	-0.046457	0.068099
CS28	CS17	CS10	*R.nitidus*	-0.04754	0.069376
CS22	CS17	CS10	*R.nitidus*	-0.018634	0.070544
GD34	CS17	CS10	*R.nitidus*	0.000693	0.067439
GD50	CS17	CS10	*R.nitidus*	0.003419	0.072454
CS15	CS17	CS10	*R.nitidus*	0.044565	0.066676
SC28	CS17	CS10	*R.nitidus*	-0.046792	0.066144
SC6	CS17	CS10	*R.nitidus*	-0.01597	0.068963
CS25	CS17	CS10	*R.nitidus*	-0.037721	0.072169
SC14	CS17	CS10	*R.nitidus*	0.006163	0.070051
CS23	CS17	CS10	*R.nitidus*	-0.067887	0.066339
CS6	CS17	CS10	*R.nitidus*	-0.036125	0.069623
CS19	CS17	CS10	*R.nitidus*	-0.02707	0.069204
SC26	CS17	CS10	*R.nitidus*	-0.025468	0.067297
CS21	CS17	CS10	*R.nitidus*	-0.037066	0.068288
SC27	CS17	CS10	*R.nitidus*	-0.024219	0.066203
CS4	CS17	CS10	*R.nitidus*	-0.026475	0.067522
CS3	CS17	CS10	*R.nitidus*	-0.024257	0.069239
JO8	CS18	CS10	*R.nitidus*	0.053465	0.073015
CS24	CS17	CS10	*R.nitidus*	-0.062806	0.06845
SC10	CS17	CS10	*R.nitidus*	0.06003	0.06985
SC12	CS17	CS10	*R.nitidus*	-0.02071	0.068087
SC15	CS17	CS10	*R.nitidus*	-0.016013	0.068925
SC32	CS17	CS10	*R.nitidus*	0.051163	0.069789
SC18	CS17	CS10	*R.nitidus*	0.027068	0.069266
SC42	CS17	CS10	*R.nitidus*	0.21781	0.063856
SC34	CS17	CS10	*R.nitidus*	0.016296	0.069219
JO3	CS17	CS10	*R.nitidus*	0.023729	0.071377
SC31	CS17	CS10	*R.nitidus*	-0.040847	0.068301

GD22	CS18	CS10	*R.nitidus*	0.111184	0.064712
JW6	CS17	CS10	*R.nitidus*	-0.00206	0.075703
SC24	CS17	CS10	*R.nitidus*	-0.004539	0.067967
JW4	CS17	CS10	*R.nitidus*	0.208151	0.067534
MY4	CS17	CS10	*R.nitidus*	-0.049814	0.069257
SC44	CS17	CS10	*R.nitidus*	-0.010582	0.06871
JO1	CS17	CS10	*R.nitidus*	-0.024775	0.066337
MY6	CS17	CS10	*R.nitidus*	-0.014577	0.068161
JO5	CS17	CS10	*R.nitidus*	0.011236	0.068257
SC35	CS17	CS10	*R.nitidus*	0.026929	0.065767
SC45	CS17	CS10	*R.nitidus*	0.022661	0.068114
JO7	CS18	CS10	*R.nitidus*	0.039216	0.072869
JW7	CS17	CS10	*R.nitidus*	0.023853	0.071334
GD31	CS18	CS10	*R.nitidus*	0.100858	0.067676
GD25	CS18	CS10	*R.nitidus*	0.100942	0.06615
CS11	CS18	CS10	*R.nitidus*	0.03331	0.066465
GD41	CS18	CS10	*R.nitidus*	0.045333	0.064987
GD32	CS18	CS10	*R.nitidus*	0.064241	0.069364
GD29	CS18	CS10	*R.nitidus*	0.150685	0.064103
GD33	CS18	CS10	*R.nitidus*	0.05325	0.066006
GD26	CS18	CS10	*R.nitidus*	0.095593	0.065839
GD50	CS18	CS10	*R.nitidus*	0.066285	0.067236
CS12	CS18	CS10	*R.nitidus*	0.030735	0.066945
GD40	CS18	CS10	*R.nitidus*	0.111111	0.063417
GD30	CS18	CS10	*R.nitidus*	0.150394	0.067593
GD34	CS18	CS10	*R.nitidus*	0.03467	0.065156
GD21	CS18	CS10	*R.nitidus*	0.161899	0.065036
GD35	CS18	CS10	*R.nitidus*	0.070827	0.066327
CS13	CS18	CS10	*R.nitidus*	0.006056	0.068414
GD44	CS18	CS10	*R.nitidus*	0.062187	0.066611
GD45	CS18	CS10	*R.nitidus*	0.093227	0.071406
GD42	CS18	CS10	*R.nitidus*	0.058155	0.067138
GD48	CS18	CS10	*R.nitidus*	0.076197	0.069641
GD46	CS18	CS10	*R.nitidus*	0.037812	0.064956
GD49	CS18	CS10	*R.nitidus*	0.119497	0.069679
CS15	CS18	CS10	*R.nitidus*	0.064609	0.066784
CS19	CS18	CS10	*R.nitidus*	0.014058	0.066784
CS17	CS18	CS10	*R.nitidus*	0.046457	0.068099
CS14	CS18	CS10	*R.nitidus*	0.045586	0.067196
CS21	CS18	CS10	*R.nitidus*	0.018744	0.064309
CS23	CS18	CS10	*R.nitidus*	-0.020193	0.066079
CS20	CS18	CS10	*R.nitidus*	0.005268	0.065719
CS24	CS18	CS10	*R.nitidus*	-0.013962	0.064046
CS30	CS18	CS10	*R.nitidus*	-0.026799	0.064562
CS28	CS18	CS10	*R.nitidus*	0.004666	0.066482
CS16	CS18	CS10	*R.nitidus*	0.031825	0.066451
SC6	CS18	CS10	*R.nitidus*	0.032928	0.066211
CS3	CS18	CS10	*R.nitidus*	0.010744	0.066505
CS31	CS18	CS10	*R.nitidus*	0.006691	0.065679
SC12	CS18	CS10	*R.nitidus*	0.021924	0.065057
CS22	CS18	CS10	*R.nitidus*	0.035889	0.065078
CS4	CS18	CS10	*R.nitidus*	0.011015	0.063479
SC15	CS18	CS10	*R.nitidus*	0.026114	0.065208
SC5	CS18	CS10	*R.nitidus*	0.037723	0.068962
SC18	CS18	CS10	*R.nitidus*	0.075485	0.064047
SC24	CS18	CS10	*R.nitidus*	0.042583	0.063931

CS25	CS18	CS10	*R.nitidus*	0.024348	0.068206
SC14	CS18	CS10	*R.nitidus*	0.040194	0.065211
SC28	CS18	CS10	*R.nitidus*	0.008374	0.064704
SC10	CS18	CS10	*R.nitidus*	0.108584	0.06574
SC32	CS18	CS10	*R.nitidus*	0.08606	0.063957
SC31	CS18	CS10	*R.nitidus*	0.012057	0.064548
SC27	CS18	CS10	*R.nitidus*	0.044012	0.06423
SC34	CS18	CS10	*R.nitidus*	0.064246	0.062439
CS6	CS18	CS10	*R.nitidus*	-0.016964	0.064159
JW4	CS18	CS10	*R.nitidus*	0.2582	0.065032
SC44	CS18	CS10	*R.nitidus*	0.034386	0.06459
JO3	CS18	CS10	*R.nitidus*	0.071197	0.067402
SC35	CS18	CS10	*R.nitidus*	0.07703	0.062368
JW7	CS18	CS10	*R.nitidus*	0.062657	0.068652
SC26	CS18	CS10	*R.nitidus*	0.013277	0.062527
JO7	CS19	CS10	*R.nitidus*	0.030841	0.074033
JO8	CS19	CS10	*R.nitidus*	0.052052	0.073515
JW6	CS18	CS10	*R.nitidus*	0.033363	0.071818
MY4	CS18	CS10	*R.nitidus*	-0.003608	0.066969
CS11	CS19	CS10	*R.nitidus*	0.023669	0.068122
JO5	CS18	CS10	*R.nitidus*	0.093278	0.064739
MY6	CS18	CS10	*R.nitidus*	0.026761	0.065571
GD22	CS19	CS10	*R.nitidus*	0.088293	0.06605
GD30	CS19	CS10	*R.nitidus*	0.12966	0.068776
JO1	CS18	CS10	*R.nitidus*	0.034028	0.064956
GD26	CS19	CS10	*R.nitidus*	0.066265	0.067001
GD21	CS19	CS10	*R.nitidus*	0.137374	0.065281
SC45	CS18	CS10	*R.nitidus*	0.084615	0.064018
GD32	CS19	CS10	*R.nitidus*	0.034351	0.06859
SC42	CS18	CS10	*R.nitidus*	0.260794	0.061383
GD29	CS19	CS10	*R.nitidus*	0.136667	0.064704
GD25	CS19	CS10	*R.nitidus*	0.073388	0.065784
GD33	CS19	CS10	*R.nitidus*	0.027349	0.067457
CS12	CS19	CS10	*R.nitidus*	0.012403	0.068729
GD31	CS19	CS10	*R.nitidus*	0.101732	0.068532
GD42	CS19	CS10	*R.nitidus*	0.032011	0.066807
GD40	CS19	CS10	*R.nitidus*	0.094715	0.064409
GD41	CS19	CS10	*R.nitidus*	0.024341	0.06695
GD45	CS19	CS10	*R.nitidus*	0.067425	0.072124
CS13	CS19	CS10	*R.nitidus*	-0.016229	0.070361
GD34	CS19	CS10	*R.nitidus*	0.019986	0.065175
GD49	CS19	CS10	*R.nitidus*	0.082181	0.069231
GD50	CS19	CS10	*R.nitidus*	0.055738	0.070628
GD35	CS19	CS10	*R.nitidus*	0.047755	0.066768
GD46	CS19	CS10	*R.nitidus*	0.017735	0.066793
CS14	CS19	CS10	*R.nitidus*	0.019505	0.069353
CS18	CS19	CS10	*R.nitidus*	-0.014058	0.066784
GD44	CS19	CS10	*R.nitidus*	0.040175	0.066426
CS17	CS19	CS10	*R.nitidus*	0.02707	0.069204
CS20	CS19	CS10	*R.nitidus*	-0.01479	0.068862
GD48	CS19	CS10	*R.nitidus*	0.059309	0.067535
CS21	CS19	CS10	*R.nitidus*	-0.007725	0.066264
CS30	CS19	CS10	*R.nitidus*	-0.035796	0.067299
CS28	CS19	CS10	*R.nitidus*	-0.025217	0.067783
CS3	CS19	CS10	*R.nitidus*	-0.003367	0.067228
CS31	CS19	CS10	*R.nitidus*	-0.00077	0.066981

CS25	CS19	CS10	*R.nitidus*	0.014056	0.070853
CS16	CS19	CS10	*R.nitidus*	0.01551	0.06598
SC5	CS19	CS10	*R.nitidus*	0.016667	0.071073
CS4	CS19	CS10	*R.nitidus*	0.001536	0.066806
CS24	CS19	CS10	*R.nitidus*	-0.011609	0.068384
SC10	CS19	CS10	*R.nitidus*	0.079587	0.066187
CS15	CS19	CS10	*R.nitidus*	0.045944	0.066937
SC15	CS19	CS10	*R.nitidus*	0.024006	0.067849
SC6	CS19	CS10	*R.nitidus*	0.017033	0.067485
SC14	CS19	CS10	*R.nitidus*	0.03274	0.06838
CS22	CS19	CS10	*R.nitidus*	0.011774	0.066883
CS23	CS19	CS10	*R.nitidus*	-0.038752	0.066265
SC18	CS19	CS10	*R.nitidus*	0.065599	0.066109
SC12	CS19	CS10	*R.nitidus*	0.011989	0.066782
SC31	CS19	CS10	*R.nitidus*	-0.000696	0.066811
SC26	CS19	CS10	*R.nitidus*	-0.001405	0.064928
SC27	CS19	CS10	*R.nitidus*	0.030281	0.068273
SC28	CS19	CS10	*R.nitidus*	-0.000697	0.065465
SC34	CS19	CS10	*R.nitidus*	0.048151	0.065809
SC44	CS19	CS10	*R.nitidus*	0.020863	0.067005
SC42	CS19	CS10	*R.nitidus*	0.252137	0.062631
SC24	CS19	CS10	*R.nitidus*	0.033309	0.066767
JW4	CS19	CS10	*R.nitidus*	0.246295	0.066758
SC35	CS19	CS10	*R.nitidus*	0.072514	0.064478
SC32	CS19	CS10	*R.nitidus*	0.071585	0.066661
MY6	CS19	CS10	*R.nitidus*	0.016243	0.06846
JO7	CS20	CS10	*R.nitidus*	0.024194	0.070337
JO8	CS20	CS10	*R.nitidus*	0.04698	0.071429
JW6	CS19	CS10	*R.nitidus*	0.055295	0.073677
CS11	CS20	CS10	*R.nitidus*	0.027379	0.067388
CS6	CS19	CS10	*R.nitidus*	-0.038596	0.066142
JO5	CS19	CS10	*R.nitidus*	0.064888	0.065723
JO1	CS19	CS10	*R.nitidus*	0.014716	0.067097
JW7	CS19	CS10	*R.nitidus*	0.057741	0.069349
JO3	CS19	CS10	*R.nitidus*	0.063167	0.069679
MY4	CS19	CS10	*R.nitidus*	-0.023704	0.07022
GD26	CS20	CS10	*R.nitidus*	0.084204	0.066676
SC45	CS19	CS10	*R.nitidus*	0.075905	0.067358
GD25	CS20	CS10	*R.nitidus*	0.096115	0.066107
GD22	CS20	CS10	*R.nitidus*	0.105398	0.064895
GD31	CS20	CS10	*R.nitidus*	0.098947	0.066491
GD32	CS20	CS10	*R.nitidus*	0.046923	0.068765
GD30	CS20	CS10	*R.nitidus*	0.152767	0.068381
GD21	CS20	CS10	*R.nitidus*	0.154145	0.064071
GD29	CS20	CS10	*R.nitidus*	0.151803	0.064092
GD34	CS20	CS10	*R.nitidus*	0.035387	0.064811
GD40	CS20	CS10	*R.nitidus*	0.098658	0.06365
GD49	CS20	CS10	*R.nitidus*	0.133077	0.068605
GD50	CS20	CS10	*R.nitidus*	0.067742	0.067765
GD48	CS20	CS10	*R.nitidus*	0.065217	0.066757
CS12	CS20	CS10	*R.nitidus*	0.018195	0.067815
GD41	CS20	CS10	*R.nitidus*	0.045543	0.065603
GD35	CS20	CS10	*R.nitidus*	0.066166	0.066061
GD46	CS20	CS10	*R.nitidus*	0.02912	0.065529
GD45	CS20	CS10	*R.nitidus*	0.07188	0.071371
CS15	CS20	CS10	*R.nitidus*	0.054348	0.066626

CS13	CS20	CS10	*R.nitidus*	0.002791	0.067981
GD42	CS20	CS10	*R.nitidus*	0.045872	0.066453
GD33	CS20	CS10	*R.nitidus*	0.043738	0.066786
GD44	CS20	CS10	*R.nitidus*	0.053259	0.067384
CS24	CS20	CS10	*R.nitidus*	-0.002259	0.066248
CS25	CS20	CS10	*R.nitidus*	0.011513	0.067916
CS14	CS20	CS10	*R.nitidus*	0.030075	0.067662
CS19	CS20	CS10	*R.nitidus*	0.01479	0.068862
CS21	CS20	CS10	*R.nitidus*	0.003653	0.062599
CS22	CS20	CS10	*R.nitidus*	0.034884	0.065715
CS28	CS20	CS10	*R.nitidus*	-0.020833	0.06693
CS30	CS20	CS10	*R.nitidus*	-0.032328	0.065942
CS16	CS20	CS10	*R.nitidus*	0.029372	0.066243
CS18	CS20	CS10	*R.nitidus*	-0.005268	0.065719
CS3	CS20	CS10	*R.nitidus*	0.007843	0.061219
SC10	CS20	CS10	*R.nitidus*	0.089943	0.065759
CS4	CS20	CS10	*R.nitidus*	0.015373	0.064187
CS31	CS20	CS10	*R.nitidus*	-0.005011	0.067357
CS17	CS20	CS10	*R.nitidus*	0.036585	0.068324
SC15	CS20	CS10	*R.nitidus*	0.019579	0.066616
CS23	CS20	CS10	*R.nitidus*	-0.038494	0.065375
SC14	CS20	CS10	*R.nitidus*	0.040513	0.065007
SC26	CS20	CS10	*R.nitidus*	0.002815	0.063839
SC5	CS20	CS10	*R.nitidus*	0.034317	0.068623
SC18	CS20	CS10	*R.nitidus*	0.071481	0.063739
SC24	CS20	CS10	*R.nitidus*	0.042674	0.063347
SC34	CS20	CS10	*R.nitidus*	0.055443	0.063654
SC35	CS20	CS10	*R.nitidus*	0.071809	0.063128
SC27	CS20	CS10	*R.nitidus*	0.0337	0.064456
SC6	CS20	CS10	*R.nitidus*	0.022989	0.064291
SC31	CS20	CS10	*R.nitidus*	0.006303	0.063669
SC28	CS20	CS10	*R.nitidus*	0.008868	0.06529
MY4	CS20	CS10	*R.nitidus*	-0.01238	0.066597
SC12	CS20	CS10	*R.nitidus*	0.019324	0.065147
CS6	CS20	CS10	*R.nitidus*	-0.035611	0.061687
SC32	CS20	CS10	*R.nitidus*	0.080877	0.063925
JW4	CS20	CS10	*R.nitidus*	0.23816	0.064474
SC45	CS20	CS10	*R.nitidus*	0.07747	0.065333
SC44	CS20	CS10	*R.nitidus*	0.032436	0.065794
JO7	CS21	CS10	*R.nitidus*	0.036036	0.072514
MY6	CS20	CS10	*R.nitidus*	0.015678	0.065643
JO8	CS21	CS10	*R.nitidus*	0.055028	0.070897
JW6	CS20	CS10	*R.nitidus*	0.007752	0.071688
SC42	CS20	CS10	*R.nitidus*	0.245644	0.062618
JW7	CS20	CS10	*R.nitidus*	0.047193	0.068319
JO3	CS20	CS10	*R.nitidus*	0.063796	0.067931
GD25	CS21	CS10	*R.nitidus*	0.09375	0.06593
JO1	CS20	CS10	*R.nitidus*	0.01932	0.065488
GD30	CS21	CS10	*R.nitidus*	0.161562	0.06737
JO5	CS20	CS10	*R.nitidus*	0.084836	0.063844
GD26	CS21	CS10	*R.nitidus*	0.076375	0.066469
GD21	CS21	CS10	*R.nitidus*	0.147468	0.063787
GD29	CS21	CS10	*R.nitidus*	0.145833	0.063972
GD22	CS21	CS10	*R.nitidus*	0.095298	0.06419
GD31	CS21	CS10	*R.nitidus*	0.087324	0.067464
CS12	CS21	CS10	*R.nitidus*	0.022288	0.065905

GD33	CS21	CS10	*R.nitidus*	0.038714	0.065953
GD41	CS21	CS10	*R.nitidus*	0.038785	0.063614
GD40	CS21	CS10	*R.nitidus*	0.099342	0.063762
GD34	CS21	CS10	*R.nitidus*	0.026281	0.064791
GD32	CS21	CS10	*R.nitidus*	0.034612	0.069998
GD45	CS21	CS10	*R.nitidus*	0.07425	0.069648
GD44	CS21	CS10	*R.nitidus*	0.040222	0.065164
GD35	CS21	CS10	*R.nitidus*	0.058317	0.066436
GD42	CS21	CS10	*R.nitidus*	0.051587	0.065993
GD46	CS21	CS10	*R.nitidus*	0.025624	0.065272
GD49	CS21	CS10	*R.nitidus*	0.120127	0.068096
CS11	CS21	CS10	*R.nitidus*	0.025263	0.065317
GD48	CS21	CS10	*R.nitidus*	0.074363	0.067001
CS16	CS21	CS10	*R.nitidus*	0.03644	0.0659
GD50	CS21	CS10	*R.nitidus*	0.061224	0.068015
CS18	CS21	CS10	*R.nitidus*	-0.018744	0.064309
CS15	CS21	CS10	*R.nitidus*	0.054054	0.065096
CS14	CS21	CS10	*R.nitidus*	0.034532	0.066658
CS13	CS21	CS10	*R.nitidus*	-0.00721	0.067303
CS19	CS21	CS10	*R.nitidus*	0.007725	0.066264
CS24	CS21	CS10	*R.nitidus*	-0.013027	0.06515
CS20	CS21	CS10	*R.nitidus*	-0.003653	0.062599
CS23	CS21	CS10	*R.nitidus*	-0.038817	0.06406
CS17	CS21	CS10	*R.nitidus*	0.037066	0.068288
SC5	CS21	CS10	*R.nitidus*	0.023729	0.067463
CS25	CS21	CS10	*R.nitidus*	0.003359	0.068191
CS31	CS21	CS10	*R.nitidus*	-0.009407	0.066002
SC15	CS21	CS10	*R.nitidus*	0.012528	0.065023
SC18	CS21	CS10	*R.nitidus*	0.06282	0.063844
CS28	CS21	CS10	*R.nitidus*	-0.027864	0.066001
SC12	CS21	CS10	*R.nitidus*	0.013333	0.065511
CS22	CS21	CS10	*R.nitidus*	0.02207	0.06449
CS30	CS21	CS10	*R.nitidus*	-0.037806	0.063623
SC31	CS21	CS10	*R.nitidus*	0.01142	0.064748
SC26	CS21	CS10	*R.nitidus*	-0.003514	0.063105
SC10	CS21	CS10	*R.nitidus*	0.087872	0.065448
SC27	CS21	CS10	*R.nitidus*	0.025892	0.064928
SC14	CS21	CS10	*R.nitidus*	0.042759	0.066117
CS4	CS21	CS10	*R.nitidus*	-0.00916	0.062908
SC6	CS21	CS10	*R.nitidus*	0.021429	0.06514
SC24	CS21	CS10	*R.nitidus*	0.03307	0.064606
SC34	CS21	CS10	*R.nitidus*	0.044352	0.063835
JW6	CS21	CS10	*R.nitidus*	0.023111	0.070605
SC32	CS21	CS10	*R.nitidus*	0.081387	0.064468
SC45	CS21	CS10	*R.nitidus*	0.071031	0.06497
JW4	CS21	CS10	*R.nitidus*	0.246228	0.065463
SC35	CS21	CS10	*R.nitidus*	0.076976	0.063905
MY6	CS21	CS10	*R.nitidus*	0.018698	0.065435
SC28	CS21	CS10	*R.nitidus*	0.003463	0.064752
JW7	CS21	CS10	*R.nitidus*	0.06486	0.067717
MY4	CS21	CS10	*R.nitidus*	-0.017908	0.067226
CS3	CS21	CS10	*R.nitidus*	-0.003493	0.065014
SC44	CS21	CS10	*R.nitidus*	0.031315	0.064135
JO7	CS22	CS10	*R.nitidus*	-0.00188	0.071669
JO3	CS21	CS10	*R.nitidus*	0.07086	0.069039
JO5	CS21	CS10	*R.nitidus*	0.075589	0.063429

SC42	CS21	CS10	*R.nitidus*	0.24971	0.060976
GD21	CS22	CS10	*R.nitidus*	0.120498	0.062743
JO8	CS22	CS10	*R.nitidus*	0.051411	0.071057
CS6	CS21	CS10	*R.nitidus*	-0.041404	0.063691
GD30	CS22	CS10	*R.nitidus*	0.12609	0.067308
GD26	CS22	CS10	*R.nitidus*	0.060751	0.064806
CS11	CS22	CS10	*R.nitidus*	-0.003592	0.067752
JO1	CS21	CS10	*R.nitidus*	0.025903	0.065499
GD22	CS22	CS10	*R.nitidus*	0.061849	0.064593
GD29	CS22	CS10	*R.nitidus*	0.122909	0.063494
CS12	CS22	CS10	*R.nitidus*	0.003779	0.066869
GD25	CS22	CS10	*R.nitidus*	0.052126	0.064612
GD32	CS22	CS10	*R.nitidus*	0.02381	0.067525
GD35	CS22	CS10	*R.nitidus*	0.040218	0.066612
GD33	CS22	CS10	*R.nitidus*	0.008672	0.065165
GD31	CS22	CS10	*R.nitidus*	0.066239	0.067126
GD34	CS22	CS10	*R.nitidus*	0.011952	0.06449
GD44	CS22	CS10	*R.nitidus*	0.040264	0.06553
CS13	CS22	CS10	*R.nitidus*	-0.041086	0.068037
GD49	CS22	CS10	*R.nitidus*	0.089132	0.068053
GD41	CS22	CS10	*R.nitidus*	0.023553	0.064564
GD45	CS22	CS10	*R.nitidus*	0.059822	0.070079
GD42	CS22	CS10	*R.nitidus*	0.03106	0.064429
GD50	CS22	CS10	*R.nitidus*	0.042834	0.06819
GD40	CS22	CS10	*R.nitidus*	0.076568	0.062668
CS15	CS22	CS10	*R.nitidus*	0.023588	0.065812
CS16	CS22	CS10	*R.nitidus*	0.005069	0.066354
GD48	CS22	CS10	*R.nitidus*	0.048458	0.067124
CS21	CS22	CS10	*R.nitidus*	-0.02207	0.06449
CS30	CS22	CS10	*R.nitidus*	-0.061597	0.066813
CS19	CS22	CS10	*R.nitidus*	-0.011774	0.066883
CS24	CS22	CS10	*R.nitidus*	-0.04507	0.063859
CS25	CS22	CS10	*R.nitidus*	-0.018039	0.067681
GD46	CS22	CS10	*R.nitidus*	0.002045	0.063803
CS31	CS22	CS10	*R.nitidus*	-0.032186	0.065429
CS28	CS22	CS10	*R.nitidus*	-0.039154	0.065689
SC6	CS22	CS10	*R.nitidus*	-0.001448	0.065732
CS18	CS22	CS10	*R.nitidus*	-0.035889	0.065078
CS14	CS22	CS10	*R.nitidus*	0.007942	0.068257
SC15	CS22	CS10	*R.nitidus*	0.002252	0.065037
CS20	CS22	CS10	*R.nitidus*	-0.034884	0.065715
CS4	CS22	CS10	*R.nitidus*	-0.020657	0.062438
SC10	CS22	CS10	*R.nitidus*	0.084328	0.064826
CS17	CS22	CS10	*R.nitidus*	0.018634	0.070544
CS23	CS22	CS10	*R.nitidus*	-0.061335	0.063707
SC27	CS22	CS10	*R.nitidus*	0.016094	0.064299
CS3	CS22	CS10	*R.nitidus*	-0.01719	0.066482
SC26	CS22	CS10	*R.nitidus*	-0.023239	0.06437
SC24	CS22	CS10	*R.nitidus*	0.010669	0.064193
SC5	CS22	CS10	*R.nitidus*	0.018813	0.069467
SC12	CS22	CS10	*R.nitidus*	-0.006555	0.065655
SC14	CS22	CS10	*R.nitidus*	0.020626	0.065403
SC18	CS22	CS10	*R.nitidus*	0.046338	0.065395
SC35	CS22	CS10	*R.nitidus*	0.052746	0.063285
SC28	CS22	CS10	*R.nitidus*	-0.014472	0.064651
SC42	CS22	CS10	*R.nitidus*	0.244656	0.061327

CS6	CS22	CS10	*R.nitidus*	-0.047534	0.063986
SC32	CS22	CS10	*R.nitidus*	0.063282	0.064637
SC31	CS22	CS10	*R.nitidus*	-0.01188	0.0656
JW7	CS22	CS10	*R.nitidus*	0.053587	0.068909
SC44	CS22	CS10	*R.nitidus*	0.004252	0.06592
JW4	CS22	CS10	*R.nitidus*	0.244569	0.066284
JO1	CS22	CS10	*R.nitidus*	0.009957	0.067225
SC45	CS22	CS10	*R.nitidus*	0.044317	0.064933
JO3	CS22	CS10	*R.nitidus*	0.047281	0.070021
JO7	CS23	CS10	*R.nitidus*	0.061355	0.071262
JW6	CS22	CS10	*R.nitidus*	0.037906	0.072124
MY6	CS22	CS10	*R.nitidus*	-0.002892	0.064829
SC34	CS22	CS10	*R.nitidus*	0.028846	0.064096
MY4	CS22	CS10	*R.nitidus*	-0.038942	0.067498
JO8	CS23	CS10	*R.nitidus*	0.075382	0.072919
JO5	CS22	CS10	*R.nitidus*	0.04954	0.065507
GD25	CS23	CS10	*R.nitidus*	0.114458	0.064417
GD26	CS23	CS10	*R.nitidus*	0.101421	0.063134
GD21	CS23	CS10	*R.nitidus*	0.17224	0.063552
CS11	CS23	CS10	*R.nitidus*	0.061324	0.065355
GD29	CS23	CS10	*R.nitidus*	0.16441	0.061875
GD22	CS23	CS10	*R.nitidus*	0.118135	0.062825
GD45	CS23	CS10	*R.nitidus*	0.079475	0.072547
GD31	CS23	CS10	*R.nitidus*	0.114685	0.065946
GD40	CS23	CS10	*R.nitidus*	0.12045	0.063046
GD41	CS23	CS10	*R.nitidus*	0.066841	0.064097
GD35	CS23	CS10	*R.nitidus*	0.089382	0.06524
GD30	CS23	CS10	*R.nitidus*	0.154383	0.066929
GD34	CS23	CS10	*R.nitidus*	0.062252	0.062428
GD50	CS23	CS10	*R.nitidus*	0.080841	0.06814
CS12	CS23	CS10	*R.nitidus*	0.045388	0.065684
GD44	CS23	CS10	*R.nitidus*	0.072931	0.063868
GD48	CS23	CS10	*R.nitidus*	0.097262	0.066847
GD46	CS23	CS10	*R.nitidus*	0.042949	0.063046
GD32	CS23	CS10	*R.nitidus*	0.080364	0.067607
CS13	CS23	CS10	*R.nitidus*	0.02687	0.065548
GD42	CS23	CS10	*R.nitidus*	0.074567	0.065491
CS17	CS23	CS10	*R.nitidus*	0.067887	0.066339
CS24	CS23	CS10	*R.nitidus*	0.018053	0.065773
CS18	CS23	CS10	*R.nitidus*	0.020193	0.066079
GD33	CS23	CS10	*R.nitidus*	0.06658	0.06397
CS21	CS23	CS10	*R.nitidus*	0.038817	0.06406
CS25	CS23	CS10	*R.nitidus*	0.03202	0.068286
CS22	CS23	CS10	*R.nitidus*	0.061335	0.063707
CS16	CS23	CS10	*R.nitidus*	0.052929	0.063424
CS20	CS23	CS10	*R.nitidus*	0.038494	0.065375
GD49	CS23	CS10	*R.nitidus*	0.120189	0.066928
CS14	CS23	CS10	*R.nitidus*	0.06377	0.066365
CS31	CS23	CS10	*R.nitidus*	0.030303	0.06634
SC10	CS23	CS10	*R.nitidus*	0.118911	0.066922
SC6	CS23	CS10	*R.nitidus*	0.056645	0.065195
CS19	CS23	CS10	*R.nitidus*	0.038752	0.066265
CS15	CS23	CS10	*R.nitidus*	0.085774	0.064729
CS30	CS23	CS10	*R.nitidus*	-0.006711	0.065024
CS28	CS23	CS10	*R.nitidus*	0.01542	0.065374
SC12	CS23	CS10	*R.nitidus*	0.032684	0.064259

SC18	CS23	CS10	*R.nitidus*	0.097109	0.063554
CS3	CS23	CS10	*R.nitidus*	0.03829	0.063512
SC14	CS23	CS10	*R.nitidus*	0.06467	0.065879
SC5	CS23	CS10	*R.nitidus*	0.050847	0.068763
SC24	CS23	CS10	*R.nitidus*	0.064286	0.064542
CS4	CS23	CS10	*R.nitidus*	0.03908	0.062512
SC15	CS23	CS10	*R.nitidus*	0.058999	0.064785
SC31	CS23	CS10	*R.nitidus*	0.036313	0.064409
SC34	CS23	CS10	*R.nitidus*	0.075172	0.06302
SC32	CS23	CS10	*R.nitidus*	0.103257	0.065107
SC27	CS23	CS10	*R.nitidus*	0.055322	0.064913
SC26	CS23	CS10	*R.nitidus*	0.027454	0.064132
SC35	CS23	CS10	*R.nitidus*	0.101509	0.062739
SC45	CS23	CS10	*R.nitidus*	0.098886	0.064218
JO3	CS23	CS10	*R.nitidus*	0.088976	0.066488
CS6	CS23	CS10	*R.nitidus*	0.002586	0.064084
MY4	CS23	CS10	*R.nitidus*	0.013552	0.065936
SC28	CS23	CS10	*R.nitidus*	0.012354	0.064365
SC42	CS23	CS10	*R.nitidus*	0.284897	0.060122
JW6	CS23	CS10	*R.nitidus*	0.038793	0.070619
JO5	CS23	CS10	*R.nitidus*	0.098384	0.062755
JW7	CS23	CS10	*R.nitidus*	0.08313	0.066187
JW4	CS23	CS10	*R.nitidus*	0.266124	0.063835
JO8	CS24	CS10	*R.nitidus*	0.055046	0.070789
JO7	CS24	CS10	*R.nitidus*	0.051625	0.073313
GD21	CS24	CS10	*R.nitidus*	0.165003	0.063335
JO1	CS23	CS10	*R.nitidus*	0.053997	0.064834
GD29	CS24	CS10	*R.nitidus*	0.155836	0.06336
GD26	CS24	CS10	*R.nitidus*	0.089583	0.06487
MY6	CS23	CS10	*R.nitidus*	0.044944	0.063717
GD25	CS24	CS10	*R.nitidus*	0.084652	0.064976
GD32	CS24	CS10	*R.nitidus*	0.059761	0.067887
GD31	CS24	CS10	*R.nitidus*	0.103175	0.066616
CS11	CS24	CS10	*R.nitidus*	0.043704	0.067624
GD33	CS24	CS10	*R.nitidus*	0.047423	0.066748
GD22	CS24	CS10	*R.nitidus*	0.107285	0.064236
SC44	CS23	CS10	*R.nitidus*	0.047091	0.065038
GD40	CS24	CS10	*R.nitidus*	0.118213	0.061694
GD35	CS24	CS10	*R.nitidus*	0.075833	0.066028
GD41	CS24	CS10	*R.nitidus*	0.055437	0.065287
GD34	CS24	CS10	*R.nitidus*	0.045299	0.064738
CS12	CS24	CS10	*R.nitidus*	0.046384	0.066799
GD44	CS24	CS10	*R.nitidus*	0.063657	0.067038
GD30	CS24	CS10	*R.nitidus*	0.155573	0.065605
GD49	CS24	CS10	*R.nitidus*	0.116242	0.069199
GD42	CS24	CS10	*R.nitidus*	0.055712	0.065508
GD46	CS24	CS10	*R.nitidus*	0.030986	0.065309
GD45	CS24	CS10	*R.nitidus*	0.079119	0.069732
CS13	CS24	CS10	*R.nitidus*	0.01251	0.06807
GD48	CS24	CS10	*R.nitidus*	0.072605	0.067184
CS19	CS24	CS10	*R.nitidus*	0.011609	0.068384
CS16	CS24	CS10	*R.nitidus*	0.050528	0.065441
GD50	CS24	CS10	*R.nitidus*	0.053037	0.068468
CS17	CS24	CS10	*R.nitidus*	0.062806	0.06845
CS15	CS24	CS10	*R.nitidus*	0.071111	0.065962
CS14	CS24	CS10	*R.nitidus*	0.046423	0.068484

CS21	CS24	CS10	*R.nitidus*	0.013027	0.06515
CS23	CS24	CS10	*R.nitidus*	-0.018053	0.065773
CS20	CS24	CS10	*R.nitidus*	0.002259	0.066248
CS25	CS24	CS10	*R.nitidus*	0.029272	0.068621
CS18	CS24	CS10	*R.nitidus*	0.013962	0.064046
CS30	CS24	CS10	*R.nitidus*	-0.027888	0.066115
CS22	CS24	CS10	*R.nitidus*	0.04507	0.063859
SC10	CS24	CS10	*R.nitidus*	0.13463	0.063629
SC5	CS24	CS10	*R.nitidus*	0.05086	0.068225
SC6	CS24	CS10	*R.nitidus*	0.040404	0.065461
CS4	CS24	CS10	*R.nitidus*	0.026886	0.061967
SC14	CS24	CS10	*R.nitidus*	0.05081	0.063627
SC15	CS24	CS10	*R.nitidus*	0.036462	0.064173
CS28	CS24	CS10	*R.nitidus*	0.009693	0.065767
CS31	CS24	CS10	*R.nitidus*	0.013783	0.065946
SC27	CS24	CS10	*R.nitidus*	0.042296	0.063095
SC28	CS24	CS10	*R.nitidus*	0.018665	0.063667
SC24	CS24	CS10	*R.nitidus*	0.046086	0.064231
SC18	CS24	CS10	*R.nitidus*	0.071958	0.065567
SC26	CS24	CS10	*R.nitidus*	0.017143	0.063601
JW4	CS24	CS10	*R.nitidus*	0.280308	0.063425
JO3	CS24	CS10	*R.nitidus*	0.070105	0.066621
SC32	CS24	CS10	*R.nitidus*	0.101935	0.064657
SC44	CS24	CS10	*R.nitidus*	0.051508	0.065369
JW7	CS24	CS10	*R.nitidus*	0.080142	0.066081
JO8	CS25	CS10	*R.nitidus*	0.04572	0.073437
SC12	CS24	CS10	*R.nitidus*	0.030724	0.065467
SC34	CS24	CS10	*R.nitidus*	0.075145	0.06339
SC45	CS24	CS10	*R.nitidus*	0.081818	0.065662
CS6	CS24	CS10	*R.nitidus*	-0.007488	0.066181
SC42	CS24	CS10	*R.nitidus*	0.284355	0.061175
CS3	CS24	CS10	*R.nitidus*	0.005405	0.066738
JW6	CS24	CS10	*R.nitidus*	0.06968	0.07273
SC35	CS24	CS10	*R.nitidus*	0.086894	0.062521
JO7	CS25	CS10	*R.nitidus*	0.0403	0.072998
JO1	CS24	CS10	*R.nitidus*	0.041785	0.065681
SC31	CS24	CS10	*R.nitidus*	0.02983	0.064618
MY4	CS24	CS10	*R.nitidus*	-0.000724	0.067675
GD21	CS25	CS10	*R.nitidus*	0.146455	0.065192
CS11	CS25	CS10	*R.nitidus*	0.016825	0.069525
JO5	CS24	CS10	*R.nitidus*	0.084709	0.064869
GD25	CS25	CS10	*R.nitidus*	0.092974	0.066146
MY6	CS24	CS10	*R.nitidus*	0.032328	0.065612
GD22	CS25	CS10	*R.nitidus*	0.102326	0.065749
GD30	CS25	CS10	*R.nitidus*	0.141196	0.070731
GD26	CS25	CS10	*R.nitidus*	0.072629	0.066477
GD31	CS25	CS10	*R.nitidus*	0.092974	0.066576
GD34	CS25	CS10	*R.nitidus*	0.023569	0.065712
GD32	CS25	CS10	*R.nitidus*	0.034056	0.070049
GD45	CS25	CS10	*R.nitidus*	0.076547	0.073555
GD29	CS25	CS10	*R.nitidus*	0.145778	0.063889
CS13	CS25	CS10	*R.nitidus*	-0.016442	0.070688
GD48	CS25	CS10	*R.nitidus*	0.053064	0.068384
GD50	CS25	CS10	*R.nitidus*	0.057661	0.069055
GD40	CS25	CS10	*R.nitidus*	0.08684	0.063771
GD49	CS25	CS10	*R.nitidus*	0.108239	0.070801

GD41	CS25	CS10	*R.nitidus*	0.023778	0.066604
GD46	CS25	CS10	*R.nitidus*	0.009421	0.067751
GD44	CS25	CS10	*R.nitidus*	0.048798	0.068478
GD33	CS25	CS10	*R.nitidus*	0.032867	0.067131
CS12	CS25	CS10	*R.nitidus*	0.011485	0.067974
GD35	CS25	CS10	*R.nitidus*	0.04742	0.066239
CS15	CS25	CS10	*R.nitidus*	0.038267	0.068933
GD42	CS25	CS10	*R.nitidus*	0.033723	0.067254
CS16	CS25	CS10	*R.nitidus*	0.021352	0.067026
CS14	CS25	CS10	*R.nitidus*	0.019665	0.067893
CS18	CS25	CS10	*R.nitidus*	-0.024348	0.068206
CS20	CS25	CS10	*R.nitidus*	-0.011513	0.067916
CS28	CS25	CS10	*R.nitidus*	-0.034783	0.070545
CS22	CS25	CS10	*R.nitidus*	0.018039	0.067681
CS21	CS25	CS10	*R.nitidus*	-0.003359	0.068191
CS24	CS25	CS10	*R.nitidus*	-0.029272	0.068621
CS19	CS25	CS10	*R.nitidus*	-0.014056	0.070853
SC5	CS25	CS10	*R.nitidus*	0.016231	0.070539
CS17	CS25	CS10	*R.nitidus*	0.037721	0.072169
SC6	CS25	CS10	*R.nitidus*	0.020408	0.066821
CS23	CS25	CS10	*R.nitidus*	-0.03202	0.068286
CS4	CS25	CS10	*R.nitidus*	0.008246	0.066389
SC10	CS25	CS10	*R.nitidus*	0.061255	0.068006
CS3	CS25	CS10	*R.nitidus*	-0.014377	0.066569
SC24	CS25	CS10	*R.nitidus*	0.033577	0.065397
SC26	CS25	CS10	*R.nitidus*	-0.001405	0.066216
SC31	CS25	CS10	*R.nitidus*	0.004875	0.065944
SC18	CS25	CS10	*R.nitidus*	0.056422	0.064856
SC12	CS25	CS10	*R.nitidus*	-0.001433	0.067189
CS31	CS25	CS10	*R.nitidus*	-0.015556	0.069894
SC14	CS25	CS10	*R.nitidus*	0.0247	0.067813
SC15	CS25	CS10	*R.nitidus*	0.015742	0.067293
CS6	CS25	CS10	*R.nitidus*	-0.042785	0.065828
CS30	CS25	CS10	*R.nitidus*	-0.043414	0.067135
SC42	CS25	CS10	*R.nitidus*	0.250739	0.063312
SC28	CS25	CS10	*R.nitidus*	-0.012023	0.067167
SC34	CS25	CS10	*R.nitidus*	0.041152	0.065188
SC35	CS25	CS10	*R.nitidus*	0.061379	0.063385
SC44	CS25	CS10	*R.nitidus*	0.00504	0.067164
SC27	CS25	CS10	*R.nitidus*	0.022825	0.066273
MY4	CS25	CS10	*R.nitidus*	-0.021583	0.067445
JW6	CS25	CS10	*R.nitidus*	0.041483	0.072341
SC45	CS25	CS10	*R.nitidus*	0.070413	0.064704
JW4	CS25	CS10	*R.nitidus*	0.232082	0.066282
JW7	CS25	CS10	*R.nitidus*	0.052941	0.070537
JO8	CS28	CS10	*R.nitidus*	0.059856	0.073712
SC32	CS25	CS10	*R.nitidus*	0.070866	0.067643
JO3	CS25	CS10	*R.nitidus*	0.069786	0.068673
JO7	CS28	CS10	*R.nitidus*	0.036217	0.07325
CS11	CS28	CS10	*R.nitidus*	0.035714	0.066472
MY6	CS25	CS10	*R.nitidus*	0.013428	0.067348
JO1	CS25	CS10	*R.nitidus*	0.004811	0.065964
GD21	CS28	CS10	*R.nitidus*	0.15304	0.064643
GD25	CS28	CS10	*R.nitidus*	0.095455	0.066014
GD26	CS28	CS10	*R.nitidus*	0.090778	0.066072
GD30	CS28	CS10	*R.nitidus*	0.164948	0.068344

GD22	CS28	CS10	*R.nitidus*	0.104195	0.066101
JO5	CS25	CS10	*R.nitidus*	0.072046	0.066941
CS12	CS28	CS10	*R.nitidus*	0.022801	0.065981
GD31	CS28	CS10	*R.nitidus*	0.087121	0.065813
GD40	CS28	CS10	*R.nitidus*	0.098731	0.063608
GD42	CS28	CS10	*R.nitidus*	0.062361	0.067718
GD45	CS28	CS10	*R.nitidus*	0.093178	0.068707
GD33	CS28	CS10	*R.nitidus*	0.057329	0.06541
GD41	CS28	CS10	*R.nitidus*	0.046921	0.06691
CS13	CS28	CS10	*R.nitidus*	-0.009623	0.070331
GD35	CS28	CS10	*R.nitidus*	0.080586	0.066802
GD44	CS28	CS10	*R.nitidus*	0.053992	0.067714
GD48	CS28	CS10	*R.nitidus*	0.086207	0.068484
GD46	CS28	CS10	*R.nitidus*	0.032727	0.067676
GD29	CS28	CS10	*R.nitidus*	0.150331	0.065291
GD49	CS28	CS10	*R.nitidus*	0.105651	0.070563
CS17	CS28	CS10	*R.nitidus*	0.04754	0.069376
GD34	CS28	CS10	*R.nitidus*	0.050073	0.065795
GD32	CS28	CS10	*R.nitidus*	0.057899	0.069466
CS16	CS28	CS10	*R.nitidus*	0.034776	0.065178
CS22	CS28	CS10	*R.nitidus*	0.039154	0.065689
CS18	CS28	CS10	*R.nitidus*	-0.004666	0.066482
GD50	CS28	CS10	*R.nitidus*	0.097778	0.069263
CS23	CS28	CS10	*R.nitidus*	-0.01542	0.065374
CS21	CS28	CS10	*R.nitidus*	0.027864	0.066001
CS20	CS28	CS10	*R.nitidus*	0.020833	0.06693
CS15	CS28	CS10	*R.nitidus*	0.04736	0.066065
CS31	CS28	CS10	*R.nitidus*	-0.00885	0.068733
CS14	CS28	CS10	*R.nitidus*	0.033385	0.067375
CS24	CS28	CS10	*R.nitidus*	-0.009693	0.065767
SC6	CS28	CS10	*R.nitidus*	0.045701	0.067548
SC5	CS28	CS10	*R.nitidus*	0.047473	0.068053
CS19	CS28	CS10	*R.nitidus*	0.025217	0.067783
CS3	CS28	CS10	*R.nitidus*	0.016342	0.066094
SC10	CS28	CS10	*R.nitidus*	0.111025	0.066929
CS4	CS28	CS10	*R.nitidus*	0.015921	0.064923
CS30	CS28	CS10	*R.nitidus*	-0.033135	0.066664
SC14	CS28	CS10	*R.nitidus*	0.048611	0.067177
SC28	CS28	CS10	*R.nitidus*	0.017679	0.064792
SC12	CS28	CS10	*R.nitidus*	0.030116	0.065874
SC24	CS28	CS10	*R.nitidus*	0.053948	0.065643
SC18	CS28	CS10	*R.nitidus*	0.084243	0.068232
SC27	CS28	CS10	*R.nitidus*	0.042089	0.064852
SC26	CS28	CS10	*R.nitidus*	0.008289	0.065297
SC15	CS28	CS10	*R.nitidus*	0.027575	0.06795
SC31	CS28	CS10	*R.nitidus*	0.021995	0.064894
SC35	CS28	CS10	*R.nitidus*	0.065071	0.065091
JW4	CS28	CS10	*R.nitidus*	0.257862	0.067941
SC34	CS28	CS10	*R.nitidus*	0.075655	0.065285
CS25	CS28	CS10	*R.nitidus*	0.034783	0.070545
SC44	CS28	CS10	*R.nitidus*	0.046317	0.065934
JO8	CS30	CS10	*R.nitidus*	0.106299	0.0694
MY6	CS28	CS10	*R.nitidus*	0.032641	0.066712
JW6	CS28	CS10	*R.nitidus*	0.061165	0.071982
SC32	CS28	CS10	*R.nitidus*	0.104037	0.06572
CS6	CS28	CS10	*R.nitidus*	-0.006467	0.066971

JW7	CS28	CS10	*R.nitidus*	0.083871	0.069804
MY4	CS28	CS10	*R.nitidus*	0.003058	0.0676
SC42	CS28	CS10	*R.nitidus*	0.267214	0.062341
SC45	CS28	CS10	*R.nitidus*	0.074101	0.066046
JO1	CS28	CS10	*R.nitidus*	0.040062	0.065377
JO3	CS28	CS10	*R.nitidus*	0.072667	0.071505
GD22	CS30	CS10	*R.nitidus*	0.132498	0.063348
GD26	CS30	CS10	*R.nitidus*	0.112336	0.064944
GD21	CS30	CS10	*R.nitidus*	0.18516	0.064281
JO7	CS30	CS10	*R.nitidus*	0.054795	0.071606
JO5	CS28	CS10	*R.nitidus*	0.083587	0.065174
GD32	CS30	CS10	*R.nitidus*	0.069767	0.069605
GD29	CS30	CS10	*R.nitidus*	0.17942	0.061769
GD31	CS30	CS10	*R.nitidus*	0.126386	0.066875
GD34	CS30	CS10	*R.nitidus*	0.06768	0.063379
CS11	CS30	CS10	*R.nitidus*	0.057307	0.067129
GD30	CS30	CS10	*R.nitidus*	0.172749	0.067059
GD33	CS30	CS10	*R.nitidus*	0.078029	0.065546
GD25	CS30	CS10	*R.nitidus*	0.116984	0.06578
GD42	CS30	CS10	*R.nitidus*	0.090116	0.064941
GD40	CS30	CS10	*R.nitidus*	0.123005	0.06239
GD46	CS30	CS10	*R.nitidus*	0.051554	0.065717
CS12	CS30	CS10	*R.nitidus*	0.059004	0.066828
GD45	CS30	CS10	*R.nitidus*	0.108424	0.0689
GD49	CS30	CS10	*R.nitidus*	0.142857	0.068027
GD50	CS30	CS10	*R.nitidus*	0.09205	0.06834
CS13	CS30	CS10	*R.nitidus*	0.034892	0.068065
GD35	CS30	CS10	*R.nitidus*	0.089655	0.066559
CS14	CS30	CS10	*R.nitidus*	0.060517	0.067363
CS18	CS30	CS10	*R.nitidus*	0.026799	0.064562
CS20	CS30	CS10	*R.nitidus*	0.032328	0.065942
CS21	CS30	CS10	*R.nitidus*	0.037806	0.063623
CS19	CS30	CS10	*R.nitidus*	0.035796	0.067299
CS15	CS30	CS10	*R.nitidus*	0.085507	0.065642
GD44	CS30	CS10	*R.nitidus*	0.068052	0.067141
GD41	CS30	CS10	*R.nitidus*	0.084099	0.064596
CS23	CS30	CS10	*R.nitidus*	0.006711	0.065024
CS17	CS30	CS10	*R.nitidus*	0.080309	0.068293
CS24	CS30	CS10	*R.nitidus*	0.027888	0.066115
GD48	CS30	CS10	*R.nitidus*	0.093023	0.068174
CS22	CS30	CS10	*R.nitidus*	0.061597	0.066813
CS28	CS30	CS10	*R.nitidus*	0.033135	0.066664
SC5	CS30	CS10	*R.nitidus*	0.05644	0.068716
CS25	CS30	CS10	*R.nitidus*	0.043414	0.067135
SC10	CS30	CS10	*R.nitidus*	0.13275	0.064434
SC6	CS30	CS10	*R.nitidus*	0.065734	0.066612
SC12	CS30	CS10	*R.nitidus*	0.043165	0.065675
CS16	CS30	CS10	*R.nitidus*	0.071325	0.065593
CS31	CS30	CS10	*R.nitidus*	0.048203	0.065119
SC24	CS30	CS10	*R.nitidus*	0.07148	0.064653
SC27	CS30	CS10	*R.nitidus*	0.068635	0.063407
CS4	CS30	CS10	*R.nitidus*	0.045488	0.063437
SC26	CS30	CS10	*R.nitidus*	0.034266	0.062589
SC35	CS30	CS10	*R.nitidus*	0.10372	0.062851
SC15	CS30	CS10	*R.nitidus*	0.057034	0.066024
SC14	CS30	CS10	*R.nitidus*	0.070313	0.065496

SC28	CS30	CS10	*R.nitidus*	0.03458	0.063832
SC18	CS30	CS10	*R.nitidus*	0.097938	0.06613
SC31	CS30	CS10	*R.nitidus*	0.047484	0.06551
JW4	CS30	CS10	*R.nitidus*	0.270692	0.064681
CS3	CS30	CS10	*R.nitidus*	0.038777	0.065161
SC32	CS30	CS10	*R.nitidus*	0.113703	0.06558
SC34	CS30	CS10	*R.nitidus*	0.08642	0.063339
SC42	CS30	CS10	*R.nitidus*	0.291154	0.060069
SC44	CS30	CS10	*R.nitidus*	0.067202	0.062714
JO3	CS30	CS10	*R.nitidus*	0.102668	0.067746
CS6	CS30	CS10	*R.nitidus*	0.012997	0.066571
SC45	CS30	CS10	*R.nitidus*	0.104423	0.064999
MY4	CS30	CS10	*R.nitidus*	0.017806	0.065993
JO8	CS31	CS10	*R.nitidus*	0.076546	0.07262
GD30	CS31	CS10	*R.nitidus*	0.136139	0.068605
GD22	CS31	CS10	*R.nitidus*	0.094924	0.065456
JW6	CS30	CS10	*R.nitidus*	0.069877	0.070326
CS11	CS31	CS10	*R.nitidus*	0.021244	0.070443
JW7	CS30	CS10	*R.nitidus*	0.095674	0.066292
GD35	CS31	CS10	*R.nitidus*	0.068149	0.067581
JO1	CS30	CS10	*R.nitidus*	0.054814	0.064573
JO7	CS31	CS10	*R.nitidus*	0.030899	0.072834
GD33	CS31	CS10	*R.nitidus*	0.036577	0.065462
GD34	CS31	CS10	*R.nitidus*	0.028031	0.064745
JO5	CS30	CS10	*R.nitidus*	0.104017	0.0616
GD26	CS31	CS10	*R.nitidus*	0.086231	0.065391
GD25	CS31	CS10	*R.nitidus*	0.096195	0.065548
CS21	CS31	CS10	*R.nitidus*	0.009407	0.066002
MY6	CS30	CS10	*R.nitidus*	0.054379	0.06539
GD21	CS31	CS10	*R.nitidus*	0.15322	0.063427
CS19	CS31	CS10	*R.nitidus*	0.00077	0.066981
GD48	CS31	CS10	*R.nitidus*	0.066517	0.066871
GD41	CS31	CS10	*R.nitidus*	0.044003	0.065803
CS15	CS31	CS10	*R.nitidus*	0.046131	0.067152
GD29	CS31	CS10	*R.nitidus*	0.157413	0.065137
GD45	CS31	CS10	*R.nitidus*	0.1025	0.069978
CS25	CS31	CS10	*R.nitidus*	0.015556	0.069894
GD49	CS31	CS10	*R.nitidus*	0.112006	0.070285
GD31	CS31	CS10	*R.nitidus*	0.085629	0.067671
GD40	CS31	CS10	*R.nitidus*	0.099933	0.064686
GD44	CS31	CS10	*R.nitidus*	0.049556	0.065851
CS14	CS31	CS10	*R.nitidus*	0.026276	0.068876
CS28	CS31	CS10	*R.nitidus*	0.00885	0.068733
CS12	CS31	CS10	*R.nitidus*	0.037736	0.067301
GD32	CS31	CS10	*R.nitidus*	0.050654	0.069458
GD50	CS31	CS10	*R.nitidus*	0.065687	0.069018
GD42	CS31	CS10	*R.nitidus*	0.039589	0.06599
SC18	CS31	CS10	*R.nitidus*	0.072848	0.065752
GD46	CS31	CS10	*R.nitidus*	0.02248	0.065516
CS17	CS31	CS10	*R.nitidus*	0.032046	0.070227
CS16	CS31	CS10	*R.nitidus*	0.035769	0.066577
SC10	CS31	CS10	*R.nitidus*	0.10643	0.066692
CS4	CS31	CS10	*R.nitidus*	0.010007	0.063239
CS13	CS31	CS10	*R.nitidus*	-0.013804	0.069691
CS20	CS31	CS10	*R.nitidus*	0.005011	0.067357
CS3	CS31	CS10	*R.nitidus*	0.01194	0.066331

CS18	CS31	CS10	*R.nitidus*	-0.006691	0.065679
SC32	CS31	CS10	*R.nitidus*	0.089005	0.067043
CS24	CS31	CS10	*R.nitidus*	-0.013783	0.065946
CS23	CS31	CS10	*R.nitidus*	-0.030303	0.06634
JW7	CS31	CS10	*R.nitidus*	0.091316	0.068801
CS22	CS31	CS10	*R.nitidus*	0.032186	0.065429
SC14	CS31	CS10	*R.nitidus*	0.039611	0.067365
SC6	CS31	CS10	*R.nitidus*	0.033886	0.067485
SC44	CS31	CS10	*R.nitidus*	0.03639	0.06629
SC34	CS31	CS10	*R.nitidus*	0.054843	0.064209
JO7	CS3	CS10	*R.nitidus*	0.00991	0.070247
SC5	CS31	CS10	*R.nitidus*	0.024691	0.069189
CS30	CS31	CS10	*R.nitidus*	-0.048203	0.065119
JO1	CS31	CS10	*R.nitidus*	0.019355	0.06604
SC15	CS31	CS10	*R.nitidus*	0.018462	0.068476
SC12	CS31	CS10	*R.nitidus*	0.017204	0.065555
JW6	CS31	CS10	*R.nitidus*	0.064366	0.07191
JW4	CS31	CS10	*R.nitidus*	0.269755	0.063214
CS6	CS31	CS10	*R.nitidus*	-0.015221	0.06745
SC31	CS31	CS10	*R.nitidus*	0.020364	0.067088
GD29	CS3	CS10	*R.nitidus*	0.153946	0.063267
SC42	CS31	CS10	*R.nitidus*	0.271267	0.061146
JO8	CS3	CS10	*R.nitidus*	0.028958	0.072608
SC24	CS31	CS10	*R.nitidus*	0.032377	0.065248
SC35	CS31	CS10	*R.nitidus*	0.070815	0.064161
SC26	CS31	CS10	*R.nitidus*	0.005678	0.065362
GD25	CS3	CS10	*R.nitidus*	0.083217	0.065958
SC28	CS31	CS10	*R.nitidus*	0.0036	0.064545
SC27	CS31	CS10	*R.nitidus*	0.030973	0.065351
GD22	CS3	CS10	*R.nitidus*	0.095641	0.064194
GD34	CS3	CS10	*R.nitidus*	0.032732	0.065343
SC45	CS31	CS10	*R.nitidus*	0.070721	0.065061
MY4	CS31	CS10	*R.nitidus*	-0.016801	0.067432
JO3	CS31	CS10	*R.nitidus*	0.059016	0.07002
GD30	CS3	CS10	*R.nitidus*	0.1407	0.066737
MY6	CS31	CS10	*R.nitidus*	0.015086	0.068375
JO5	CS31	CS10	*R.nitidus*	0.081237	0.063972
GD46	CS3	CS10	*R.nitidus*	0.008696	0.065286
CS12	CS3	CS10	*R.nitidus*	0.008252	0.066668
CS14	CS3	CS10	*R.nitidus*	0.025162	0.067814
GD26	CS3	CS10	*R.nitidus*	0.078405	0.066731
GD33	CS3	CS10	*R.nitidus*	0.043272	0.06587
GD21	CS3	CS10	*R.nitidus*	0.137217	0.063202
GD35	CS3	CS10	*R.nitidus*	0.045796	0.064849
CS11	CS3	CS10	*R.nitidus*	0.026012	0.067543
GD40	CS3	CS10	*R.nitidus*	0.089009	0.063634
GD41	CS3	CS10	*R.nitidus*	0.046296	0.063907
GD45	CS3	CS10	*R.nitidus*	0.055024	0.070412
GD31	CS3	CS10	*R.nitidus*	0.089425	0.065978
CS17	CS3	CS10	*R.nitidus*	0.024257	0.069239
GD32	CS3	CS10	*R.nitidus*	0.051322	0.068796
GD44	CS3	CS10	*R.nitidus*	0.037842	0.065249
GD48	CS3	CS10	*R.nitidus*	0.057565	0.066392
CS22	CS3	CS10	*R.nitidus*	0.01719	0.066482
CS31	CS3	CS10	*R.nitidus*	-0.01194	0.066331
CS18	CS3	CS10	*R.nitidus*	-0.010744	0.066505

GD49	CS3	CS10	*R.nitidus*	0.116904	0.067941
CS13	CS3	CS10	*R.nitidus*	-0.008708	0.067344
CS19	CS3	CS10	*R.nitidus*	0.003367	0.067228
CS15	CS3	CS10	*R.nitidus*	0.041076	0.066602
GD42	CS3	CS10	*R.nitidus*	0.043566	0.064177
CS25	CS3	CS10	*R.nitidus*	0.014377	0.066569
CS24	CS3	CS10	*R.nitidus*	-0.005405	0.066738
CS23	CS3	CS10	*R.nitidus*	-0.03829	0.063512
GD50	CS3	CS10	*R.nitidus*	0.066882	0.067653
CS16	CS3	CS10	*R.nitidus*	0.025162	0.065028
CS4	CS3	CS10	*R.nitidus*	0.015825	0.063971
SC31	CS3	CS10	*R.nitidus*	-0.007057	0.064848
SC10	CS3	CS10	*R.nitidus*	0.079678	0.06488
CS28	CS3	CS10	*R.nitidus*	-0.016342	0.066094
CS20	CS3	CS10	*R.nitidus*	-0.007843	0.061219
CS6	CS3	CS10	*R.nitidus*	-0.04568	0.061594
CS21	CS3	CS10	*R.nitidus*	0.003493	0.065014
SC35	CS3	CS10	*R.nitidus*	0.056357	0.062547
SC5	CS3	CS10	*R.nitidus*	0.009682	0.068749
SC6	CS3	CS10	*R.nitidus*	0.0188	0.064639
SC27	CS3	CS10	*R.nitidus*	0.016949	0.064432
SC26	CS3	CS10	*R.nitidus*	-0.000711	0.064658
JO7	SC5	CS10	*R.nitidus*	-0.016605	0.076094
SC28	CS3	CS10	*R.nitidus*	-0.009213	0.064252
CS30	CS3	CS10	*R.nitidus*	-0.038777	0.065161
SC24	CS3	CS10	*R.nitidus*	0.037147	0.065697
SC45	CS3	CS10	*R.nitidus*	0.063202	0.065787
JW4	CS3	CS10	*R.nitidus*	0.234807	0.06464
JW6	CS3	CS10	*R.nitidus*	-0.00625	0.070454
SC18	CS3	CS10	*R.nitidus*	0.07083	0.064449
SC12	CS3	CS10	*R.nitidus*	0.002144	0.063885
SC15	CS3	CS10	*R.nitidus*	0.023581	0.066394
SC14	CS3	CS10	*R.nitidus*	0.038596	0.06537
SC34	CS3	CS10	*R.nitidus*	0.045612	0.064551
GD21	SC5	CS10	*R.nitidus*	0.113072	0.06704
SC42	CS3	CS10	*R.nitidus*	0.241194	0.059941
MY6	CS3	CS10	*R.nitidus*	0.008457	0.065008
SC32	CS3	CS10	*R.nitidus*	0.071529	0.064323
JO5	CS3	CS10	*R.nitidus*	0.065505	0.063659
JO8	SC5	CS10	*R.nitidus*	0.016008	0.075282
GD26	SC5	CS10	*R.nitidus*	0.060924	0.067117
GD30	SC5	CS10	*R.nitidus*	0.130709	0.070061
JO3	CS3	CS10	*R.nitidus*	0.035656	0.068908
SC44	CS3	CS10	*R.nitidus*	0.016727	0.065197
JW7	CS3	CS10	*R.nitidus*	0.058966	0.067109
GD22	SC5	CS10	*R.nitidus*	0.079448	0.068299
GD25	SC5	CS10	*R.nitidus*	0.069493	0.068214
JO1	CS3	CS10	*R.nitidus*	0.012023	0.064976
MY4	CS3	CS10	*R.nitidus*	-0.017806	0.065744
CS13	SC5	CS10	*R.nitidus*	-0.029518	0.071084
GD29	SC5	CS10	*R.nitidus*	0.130976	0.068713
CS11	SC5	CS10	*R.nitidus*	0.001386	0.070936
GD32	SC5	CS10	*R.nitidus*	-0.002427	0.072026
GD31	SC5	CS10	*R.nitidus*	0.068017	0.069191
GD35	SC5	CS10	*R.nitidus*	0.035562	0.070938
CS21	SC5	CS10	*R.nitidus*	-0.023729	0.067463

CS28	SC5	CS10	*R.nitidus*	-0.047473	0.068053
CS19	SC5	CS10	*R.nitidus*	-0.016667	0.071073
CS12	SC5	CS10	*R.nitidus*	-0.014524	0.068404
GD42	SC5	CS10	*R.nitidus*	0.019622	0.068982
GD41	SC5	CS10	*R.nitidus*	0.011748	0.06842
GD34	SC5	CS10	*R.nitidus*	0.004775	0.068521
GD40	SC5	CS10	*R.nitidus*	0.075233	0.065451
GD45	SC5	CS10	*R.nitidus*	0.06089	0.072988
GD48	SC5	CS10	*R.nitidus*	0.012696	0.070132
GD49	SC5	CS10	*R.nitidus*	0.092874	0.07159
CS24	SC5	CS10	*R.nitidus*	-0.05086	0.068225
SC26	SC5	CS10	*R.nitidus*	-0.030169	0.068513
CS30	SC5	CS10	*R.nitidus*	-0.05644	0.068716
GD44	SC5	CS10	*R.nitidus*	0.020059	0.069885
CS20	SC5	CS10	*R.nitidus*	-0.034317	0.068623
GD46	SC5	CS10	*R.nitidus*	-0.001361	0.067063
GD33	SC5	CS10	*R.nitidus*	0.007037	0.069222
CS4	SC5	CS10	*R.nitidus*	-0.017009	0.066527
GD50	SC5	CS10	*R.nitidus*	0.054841	0.073161
CS14	SC5	CS10	*R.nitidus*	-0.006993	0.070112
SC15	SC5	CS10	*R.nitidus*	0.001524	0.069361
CS16	SC5	CS10	*R.nitidus*	0.000695	0.069209
CS15	SC5	CS10	*R.nitidus*	0.018367	0.070174
CS25	SC5	CS10	*R.nitidus*	-0.016231	0.070539
CS23	SC5	CS10	*R.nitidus*	-0.050847	0.068763
SC18	SC5	CS10	*R.nitidus*	0.047162	0.068559
SC24	SC5	CS10	*R.nitidus*	0.02331	0.068799
CS3	SC5	CS10	*R.nitidus*	-0.009682	0.068749
CS18	SC5	CS10	*R.nitidus*	-0.037723	0.068962
JW4	SC5	CS10	*R.nitidus*	0.213986	0.068659
SC44	SC5	CS10	*R.nitidus*	0.012669	0.068224
SC14	SC5	CS10	*R.nitidus*	0.017899	0.069706
SC28	SC5	CS10	*R.nitidus*	-0.014754	0.066587
SC10	SC5	CS10	*R.nitidus*	0.086486	0.07012
CS22	SC5	CS10	*R.nitidus*	-0.018813	0.069467
SC35	SC5	CS10	*R.nitidus*	0.043415	0.066693
CS31	SC5	CS10	*R.nitidus*	-0.024691	0.069189
SC6	SC5	CS10	*R.nitidus*	0.002269	0.070193
SC27	SC5	CS10	*R.nitidus*	0.005267	0.068956
SC12	SC5	CS10	*R.nitidus*	-0.014949	0.069406
JO8	SC6	CS10	*R.nitidus*	0.017958	0.07256
CS17	SC5	CS10	*R.nitidus*	0.009811	0.071343
JW6	SC5	CS10	*R.nitidus*	0.009928	0.076524
GD30	SC6	CS10	*R.nitidus*	0.135891	0.067221
JO5	SC5	CS10	*R.nitidus*	0.071603	0.0684
SC45	SC5	CS10	*R.nitidus*	0.053441	0.06728
MY6	SC5	CS10	*R.nitidus*	0	0.068596
GD26	SC6	CS10	*R.nitidus*	0.067705	0.066107
CS6	SC5	CS10	*R.nitidus*	-0.050287	0.067741
SC42	SC5	CS10	*R.nitidus*	0.252012	0.065079
SC31	SC5	CS10	*R.nitidus*	-0.013274	0.068537
SC34	SC5	CS10	*R.nitidus*	0.030432	0.067882
JO3	SC5	CS10	*R.nitidus*	0.0211	0.074334
SC32	SC5	CS10	*R.nitidus*	0.054929	0.0674
MY4	SC5	CS10	*R.nitidus*	-0.03396	0.069049
JO7	SC6	CS10	*R.nitidus*	-0.014006	0.070873

JO1	SC5	CS10	*R.nitidus*	0.003631	0.069669
CS11	SC6	CS10	*R.nitidus*	-0.00211	0.067977
GD50	SC6	CS10	*R.nitidus*	0.041085	0.068111
JW7	SC5	CS10	*R.nitidus*	0.050091	0.076631
GD29	SC6	CS10	*R.nitidus*	0.129929	0.064094
GD32	SC6	CS10	*R.nitidus*	0.020882	0.069498
GD33	SC6	CS10	*R.nitidus*	0.014151	0.065899
GD34	SC6	CS10	*R.nitidus*	0.007984	0.065039
GD49	SC6	CS10	*R.nitidus*	0.089774	0.069436
GD31	SC6	CS10	*R.nitidus*	0.056485	0.065502
GD22	SC6	CS10	*R.nitidus*	0.061097	0.066623
GD46	SC6	CS10	*R.nitidus*	-0.004808	0.064203
GD42	SC6	CS10	*R.nitidus*	0.023842	0.066701
GD48	SC6	CS10	*R.nitidus*	0.046749	0.066578
GD21	SC6	CS10	*R.nitidus*	0.120613	0.064739
CS15	SC6	CS10	*R.nitidus*	0.025714	0.065901
CS28	SC6	CS10	*R.nitidus*	-0.045701	0.067548
GD25	SC6	CS10	*R.nitidus*	0.063536	0.065783
CS14	SC6	CS10	*R.nitidus*	-0.004283	0.068798
CS17	SC6	CS10	*R.nitidus*	0.01597	0.068963
SC5	SC6	CS10	*R.nitidus*	-0.002269	0.070193
GD45	SC6	CS10	*R.nitidus*	0.057526	0.07152
CS16	SC6	CS10	*R.nitidus*	0.015839	0.065546
GD41	SC6	CS10	*R.nitidus*	0.017568	0.066838
GD35	SC6	CS10	*R.nitidus*	0.043003	0.064384
CS3	SC6	CS10	*R.nitidus*	-0.0188	0.064639
GD44	SC6	CS10	*R.nitidus*	0.016595	0.066503
CS13	SC6	CS10	*R.nitidus*	-0.029187	0.068725
GD40	SC6	CS10	*R.nitidus*	0.077181	0.061631
CS18	SC6	CS10	*R.nitidus*	-0.032928	0.066211
CS12	SC6	CS10	*R.nitidus*	0.007582	0.069321
CS25	SC6	CS10	*R.nitidus*	-0.020408	0.066821
CS20	SC6	CS10	*R.nitidus*	-0.022989	0.064291
SC28	SC6	CS10	*R.nitidus*	-0.040724	0.065941
SC10	SC6	CS10	*R.nitidus*	0.089231	0.065575
CS21	SC6	CS10	*R.nitidus*	-0.021429	0.06514
SC32	SC6	CS10	*R.nitidus*	0.05287	0.064913
CS31	SC6	CS10	*R.nitidus*	-0.033886	0.067485
SC27	SC6	CS10	*R.nitidus*	0.00075	0.06593
CS24	SC6	CS10	*R.nitidus*	-0.040404	0.065461
SC12	SC6	CS10	*R.nitidus*	-0.014925	0.06558
CS19	SC6	CS10	*R.nitidus*	-0.017033	0.067485
CS30	SC6	CS10	*R.nitidus*	-0.065734	0.066612
CS22	SC6	CS10	*R.nitidus*	0.001448	0.065732
SC15	SC6	CS10	*R.nitidus*	-0.012365	0.065699
CS23	SC6	CS10	*R.nitidus*	-0.056645	0.065195
SC34	SC6	CS10	*R.nitidus*	0.027818	0.065269
CS4	SC6	CS10	*R.nitidus*	-0.011057	0.064183
JW6	SC6	CS10	*R.nitidus*	-0.013405	0.073453
JW4	SC6	CS10	*R.nitidus*	0.217885	0.064784
JO7	SC10	CS10	*R.nitidus*	-0.102786	0.073386
SC18	SC6	CS10	*R.nitidus*	0.035938	0.066067
SC14	SC6	CS10	*R.nitidus*	0.010487	0.068374
JO1	SC6	CS10	*R.nitidus*	-0.005208	0.067611
SC35	SC6	CS10	*R.nitidus*	0.043801	0.0639
SC44	SC6	CS10	*R.nitidus*	0.009687	0.065767

JW7	SC6	CS10	*R.nitidus*	0.031818	0.06978
JO8	SC10	CS10	*R.nitidus*	-0.062181	0.073978
SC26	SC6	CS10	*R.nitidus*	-0.036594	0.066213
SC24	SC6	CS10	*R.nitidus*	0.011512	0.064381
CS6	SC6	CS10	*R.nitidus*	-0.052434	0.06507
JO5	SC6	CS10	*R.nitidus*	0.056245	0.067114
SC42	SC6	CS10	*R.nitidus*	0.26801	0.061198
GD21	SC10	CS10	*R.nitidus*	0.059016	0.064503
SC45	SC6	CS10	*R.nitidus*	0.040693	0.065875
SC31	SC6	CS10	*R.nitidus*	-0.004458	0.066752
JO3	SC6	CS10	*R.nitidus*	0.030977	0.070167
GD46	SC10	CS10	*R.nitidus*	-0.062753	0.065475
GD25	SC10	CS10	*R.nitidus*	-0.000719	0.064214
GD31	SC10	CS10	*R.nitidus*	-0.005054	0.066404
CS12	SC10	CS10	*R.nitidus*	-0.096125	0.067995
MY6	SC6	CS10	*R.nitidus*	-0.000726	0.065367
GD32	SC10	CS10	*R.nitidus*	-0.055686	0.068228
GD26	SC10	CS10	*R.nitidus*	-0.010331	0.066379
CS13	SC10	CS10	*R.nitidus*	-0.096012	0.068479
GD50	SC10	CS10	*R.nitidus*	-0.017529	0.070176
GD35	SC10	CS10	*R.nitidus*	-0.024983	0.065869
MY4	SC6	CS10	*R.nitidus*	-0.03655	0.065781
GD45	SC10	CS10	*R.nitidus*	-0.004808	0.070176
CS18	SC10	CS10	*R.nitidus*	-0.108584	0.06574
GD48	SC10	CS10	*R.nitidus*	-0.026831	0.067428
CS11	SC10	CS10	*R.nitidus*	-0.073291	0.067319
CS21	SC10	CS10	*R.nitidus*	-0.087872	0.065448
GD49	SC10	CS10	*R.nitidus*	0.026046	0.069858
GD34	SC10	CS10	*R.nitidus*	-0.048044	0.066231
GD44	SC10	CS10	*R.nitidus*	-0.035197	0.065959
CS23	SC10	CS10	*R.nitidus*	-0.118911	0.066922
CS25	SC10	CS10	*R.nitidus*	-0.061255	0.068006
GD42	SC10	CS10	*R.nitidus*	-0.043232	0.06688
GD22	SC10	CS10	*R.nitidus*	-0.006716	0.066033
GD30	SC10	CS10	*R.nitidus*	0.078448	0.069903
CS14	SC10	CS10	*R.nitidus*	-0.064562	0.066713
GD29	SC10	CS10	*R.nitidus*	0.052069	0.066206
CS22	SC10	CS10	*R.nitidus*	-0.084328	0.064826
CS28	SC10	CS10	*R.nitidus*	-0.111025	0.066929
CS17	SC10	CS10	*R.nitidus*	-0.06003	0.06985
GD33	SC10	CS10	*R.nitidus*	-0.044551	0.067754
GD41	SC10	CS10	*R.nitidus*	-0.05	0.065972
CS16	SC10	CS10	*R.nitidus*	-0.075069	0.066962
GD40	SC10	CS10	*R.nitidus*	0.006671	0.06524
SC6	SC10	CS10	*R.nitidus*	-0.089231	0.065575
SC14	SC10	CS10	*R.nitidus*	-0.053077	0.067053
SC5	SC10	CS10	*R.nitidus*	-0.086486	0.07012
JW6	SC10	CS10	*R.nitidus*	-0.082791	0.074307
CS19	SC10	CS10	*R.nitidus*	-0.079587	0.066187
SC31	SC10	CS10	*R.nitidus*	-0.098537	0.065274
CS20	SC10	CS10	*R.nitidus*	-0.089943	0.065759
SC34	SC10	CS10	*R.nitidus*	-0.035184	0.063665
SC27	SC10	CS10	*R.nitidus*	-0.075061	0.063113
CS24	SC10	CS10	*R.nitidus*	-0.13463	0.063629
SC24	SC10	CS10	*R.nitidus*	-0.078928	0.065397
CS31	SC10	CS10	*R.nitidus*	-0.10643	0.066692

CS3	SC10	CS10	*R.nitidus*	-0.079678	0.06488
JO7	CS4	CS10	*R.nitidus*	0.037102	0.069988
SC18	SC10	CS10	*R.nitidus*	-0.043408	0.067174
SC32	SC10	CS10	*R.nitidus*	-0.030008	0.06417
SC45	SC10	CS10	*R.nitidus*	-0.032508	0.065173
SC26	SC10	CS10	*R.nitidus*	-0.10916	0.063804
CS15	SC10	CS10	*R.nitidus*	-0.044818	0.066962
CS30	SC10	CS10	*R.nitidus*	-0.13275	0.064434
GD25	CS4	CS10	*R.nitidus*	0.068602	0.064665
SC15	SC10	CS10	*R.nitidus*	-0.08165	0.066919
SC12	SC10	CS10	*R.nitidus*	-0.099114	0.062761
SC42	SC10	CS10	*R.nitidus*	0.192105	0.061574
CS4	SC10	CS10	*R.nitidus*	-0.090204	0.063958
GD31	CS4	CS10	*R.nitidus*	0.07079	0.065408
SC28	SC10	CS10	*R.nitidus*	-0.119507	0.063512
JO8	CS4	CS10	*R.nitidus*	0.038925	0.070121
JW7	SC10	CS10	*R.nitidus*	-0.007605	0.068932
SC44	SC10	CS10	*R.nitidus*	-0.075643	0.063828
SC35	SC10	CS10	*R.nitidus*	-0.033001	0.064489
JO3	SC10	CS10	*R.nitidus*	-0.045416	0.069383
CS12	CS4	CS10	*R.nitidus*	0.027007	0.064364
JO5	SC10	CS10	*R.nitidus*	-0.028679	0.06585
CS11	CS4	CS10	*R.nitidus*	0.014104	0.067109
GD21	CS4	CS10	*R.nitidus*	0.135034	0.062289
JW4	SC10	CS10	*R.nitidus*	0.176385	0.066814
MY4	SC10	CS10	*R.nitidus*	-0.130301	0.067264
MY6	SC10	CS10	*R.nitidus*	-0.096313	0.066548
CS6	SC10	CS10	*R.nitidus*	-0.113433	0.065807
GD29	CS4	CS10	*R.nitidus*	0.13852	0.06149
GD32	CS4	CS10	*R.nitidus*	0.045101	0.065562
JO1	SC10	CS10	*R.nitidus*	-0.087023	0.064738
GD45	CS4	CS10	*R.nitidus*	0.069369	0.067218
GD30	CS4	CS10	*R.nitidus*	0.135812	0.064163
CS16	CS4	CS10	*R.nitidus*	0.022965	0.064438
GD42	CS4	CS10	*R.nitidus*	0.029139	0.062597
GD26	CS4	CS10	*R.nitidus*	0.076923	0.06328
GD22	CS4	CS10	*R.nitidus*	0.087445	0.061663
GD33	CS4	CS10	*R.nitidus*	0.034806	0.064115
CS22	CS4	CS10	*R.nitidus*	0.020657	0.062438
GD50	CS4	CS10	*R.nitidus*	0.061807	0.065446
CS25	CS4	CS10	*R.nitidus*	-0.008246	0.066389
GD41	CS4	CS10	*R.nitidus*	0.031375	0.063077
GD34	CS4	CS10	*R.nitidus*	0.023591	0.062113
CS20	CS4	CS10	*R.nitidus*	-0.015373	0.064187
GD48	CS4	CS10	*R.nitidus*	0.046495	0.066116
GD35	CS4	CS10	*R.nitidus*	0.049598	0.063943
GD46	CS4	CS10	*R.nitidus*	0.013021	0.063636
GD44	CS4	CS10	*R.nitidus*	0.054922	0.064319
GD40	CS4	CS10	*R.nitidus*	0.097386	0.062297
CS14	CS4	CS10	*R.nitidus*	0.021648	0.067092
CS17	CS4	CS10	*R.nitidus*	0.026475	0.067522
CS18	CS4	CS10	*R.nitidus*	-0.011015	0.063479
CS21	CS4	CS10	*R.nitidus*	0.00916	0.062908
CS30	CS4	CS10	*R.nitidus*	-0.045488	0.063437
SC6	CS4	CS10	*R.nitidus*	0.011057	0.064183
SC31	CS4	CS10	*R.nitidus*	-0.004854	0.063425

GD49	CS4	CS10	*R.nitidus*	0.09084	0.067312
CS13	CS4	CS10	*R.nitidus*	-0.017341	0.067083
CS15	CS4	CS10	*R.nitidus*	0.037263	0.063922
SC14	CS4	CS10	*R.nitidus*	0.02332	0.063635
SC35	CS4	CS10	*R.nitidus*	0.049214	0.062939
CS19	CS4	CS10	*R.nitidus*	-0.001536	0.066806
SC12	CS4	CS10	*R.nitidus*	0.000697	0.064071
SC5	CS4	CS10	*R.nitidus*	0.017009	0.066527
CS24	CS4	CS10	*R.nitidus*	-0.026886	0.061967
SC28	CS4	CS10	*R.nitidus*	-0.005491	0.062773
CS23	CS4	CS10	*R.nitidus*	-0.03908	0.062512
SC32	CS4	CS10	*R.nitidus*	0.062987	0.0636
SC44	CS4	CS10	*R.nitidus*	0.015862	0.062022
CS6	CS4	CS10	*R.nitidus*	-0.027654	0.062662
CS28	CS4	CS10	*R.nitidus*	-0.015921	0.064923
SC42	CS4	CS10	*R.nitidus*	0.261765	0.060681
JW4	CS4	CS10	*R.nitidus*	0.243132	0.063395
SC27	CS4	CS10	*R.nitidus*	0.015732	0.062525
CS31	CS4	CS10	*R.nitidus*	-0.010007	0.063239
CS3	CS4	CS10	*R.nitidus*	-0.015825	0.063971
SC15	CS4	CS10	*R.nitidus*	0.01362	0.064111
SC45	CS4	CS10	*R.nitidus*	0.055102	0.064769
MY4	CS4	CS10	*R.nitidus*	-0.025234	0.06642
SC10	CS4	CS10	*R.nitidus*	0.090204	0.063958
GD30	SC12	CS10	*R.nitidus*	0.128083	0.066527
SC18	CS4	CS10	*R.nitidus*	0.048201	0.063485
JW7	CS4	CS10	*R.nitidus*	0.045714	0.064519
SC34	CS4	CS10	*R.nitidus*	0.044295	0.062399
JW6	CS4	CS10	*R.nitidus*	0.017241	0.070112
JO1	CS4	CS10	*R.nitidus*	0.009763	0.064489
GD22	SC12	CS10	*R.nitidus*	0.077264	0.063171
SC26	CS4	CS10	*R.nitidus*	-0.008678	0.061745
MY6	CS4	CS10	*R.nitidus*	0.00977	0.065491
GD21	SC12	CS10	*R.nitidus*	0.126019	0.063784
GD33	SC12	CS10	*R.nitidus*	0.023984	0.066449
GD32	SC12	CS10	*R.nitidus*	0.03211	0.067054
JO3	CS4	CS10	*R.nitidus*	0.058462	0.066673
SC24	CS4	CS10	*R.nitidus*	0.021023	0.063358
JO8	SC12	CS10	*R.nitidus*	0.05773	0.069871
GD34	SC12	CS10	*R.nitidus*	0.022786	0.064797
GD26	SC12	CS10	*R.nitidus*	0.071572	0.063814
GD25	SC12	CS10	*R.nitidus*	0.070839	0.064333
JO7	SC12	CS10	*R.nitidus*	0.021719	0.069821
GD41	SC12	CS10	*R.nitidus*	0.01945	0.064385
JO5	CS4	CS10	*R.nitidus*	0.061324	0.062954
GD29	SC12	CS10	*R.nitidus*	0.13038	0.064645
CS11	SC12	CS10	*R.nitidus*	0.002807	0.067593
GD35	SC12	CS10	*R.nitidus*	0.055034	0.065787
GD42	SC12	CS10	*R.nitidus*	0.035568	0.064768
GD45	SC12	CS10	*R.nitidus*	0.065201	0.067853
CS14	SC12	CS10	*R.nitidus*	0.009682	0.068391
GD31	SC12	CS10	*R.nitidus*	0.06344	0.064474
CS28	SC12	CS10	*R.nitidus*	-0.030116	0.065874
GD40	SC12	CS10	*R.nitidus*	0.088616	0.063901
CS15	SC12	CS10	*R.nitidus*	0.033677	0.066976
GD48	SC12	CS10	*R.nitidus*	0.052632	0.067634

CS12	SC12	CS10	*R.nitidus*	0.001462	0.066851
CS21	SC12	CS10	*R.nitidus*	-0.013333	0.065511
CS25	SC12	CS10	*R.nitidus*	0.001433	0.067189
GD46	SC12	CS10	*R.nitidus*	0.00944	0.064927
GD44	SC12	CS10	*R.nitidus*	0.040541	0.064567
CS31	SC12	CS10	*R.nitidus*	-0.017204	0.065555
CS20	SC12	CS10	*R.nitidus*	-0.019324	0.065147
CS18	SC12	CS10	*R.nitidus*	-0.021924	0.065057
CS13	SC12	CS10	*R.nitidus*	-0.031915	0.068255
GD50	SC12	CS10	*R.nitidus*	0.083199	0.069972
SC5	SC12	CS10	*R.nitidus*	0.014949	0.069406
CS19	SC12	CS10	*R.nitidus*	-0.011989	0.066782
GD49	SC12	CS10	*R.nitidus*	0.087363	0.067365
CS16	SC12	CS10	*R.nitidus*	0.018815	0.066555
SC10	SC12	CS10	*R.nitidus*	0.099114	0.062761
CS6	SC12	CS10	*R.nitidus*	-0.047548	0.065525
SC32	SC12	CS10	*R.nitidus*	0.073408	0.06576
SC18	SC12	CS10	*R.nitidus*	0.076265	0.064991
CS17	SC12	CS10	*R.nitidus*	0.02071	0.068087
CS4	SC12	CS10	*R.nitidus*	-0.000697	0.064071
CS3	SC12	CS10	*R.nitidus*	-0.002144	0.063885
SC15	SC12	CS10	*R.nitidus*	0.010938	0.064906
SC14	SC12	CS10	*R.nitidus*	0.018561	0.066395
CS24	SC12	CS10	*R.nitidus*	-0.030724	0.065467
CS23	SC12	CS10	*R.nitidus*	-0.032684	0.064259
SC26	SC12	CS10	*R.nitidus*	-0.016999	0.062707
SC44	SC12	CS10	*R.nitidus*	0.01664	0.063526
CS22	SC12	CS10	*R.nitidus*	0.006555	0.065655
SC45	SC12	CS10	*R.nitidus*	0.064024	0.064373
JO3	SC12	CS10	*R.nitidus*	0.071038	0.066852
CS30	SC12	CS10	*R.nitidus*	-0.043165	0.065675
SC34	SC12	CS10	*R.nitidus*	0.047655	0.064524
SC24	SC12	CS10	*R.nitidus*	0.026886	0.06386
CS11	SC14	CS10	*R.nitidus*	-0.015829	0.067739
SC31	SC12	CS10	*R.nitidus*	-0.002859	0.065519
SC6	SC12	CS10	*R.nitidus*	0.014925	0.06558
JO5	SC12	CS10	*R.nitidus*	0.072646	0.062869
JO7	SC14	CS10	*R.nitidus*	0.000907	0.072528
JW6	SC12	CS10	*R.nitidus*	0.04562	0.07047
SC28	SC12	CS10	*R.nitidus*	-0.006154	0.062584
SC27	SC12	CS10	*R.nitidus*	0.022047	0.064513
SC42	SC12	CS10	*R.nitidus*	0.27082	0.059907
JO8	SC14	CS10	*R.nitidus*	0.018519	0.070647
GD22	SC14	CS10	*R.nitidus*	0.056005	0.063071
GD21	SC14	CS10	*R.nitidus*	0.100577	0.064123
SC35	SC12	CS10	*R.nitidus*	0.071852	0.061294
JW4	SC12	CS10	*R.nitidus*	0.250174	0.062859
GD32	SC14	CS10	*R.nitidus*	-0.005473	0.068086
GD25	SC14	CS10	*R.nitidus*	0.054399	0.064836
JO1	SC12	CS10	*R.nitidus*	0.006677	0.065112
MY4	SC12	CS10	*R.nitidus*	-0.029689	0.065564
JW7	SC12	CS10	*R.nitidus*	0.077132	0.067163
MY6	SC12	CS10	*R.nitidus*	0.005054	0.065784
GD29	SC14	CS10	*R.nitidus*	0.10971	0.0627
GD31	SC14	CS10	*R.nitidus*	0.050859	0.065153
GD33	SC14	CS10	*R.nitidus*	-0.011075	0.06509

GD30	SC14	CS10	*R.nitidus*	0.10456	0.067189
CS13	SC14	CS10	*R.nitidus*	-0.044693	0.066416
GD34	SC14	CS10	*R.nitidus*	-0.012467	0.064381
GD26	SC14	CS10	*R.nitidus*	0.04867	0.065191
GD49	SC14	CS10	*R.nitidus*	0.06155	0.068298
GD48	SC14	CS10	*R.nitidus*	0.035562	0.067297
CS22	SC14	CS10	*R.nitidus*	-0.020626	0.065403
GD44	SC14	CS10	*R.nitidus*	0.011445	0.066631
CS15	SC14	CS10	*R.nitidus*	0.014483	0.066601
CS14	SC14	CS10	*R.nitidus*	-0.010135	0.065923
CS18	SC14	CS10	*R.nitidus*	-0.040194	0.065211
GD35	SC14	CS10	*R.nitidus*	0.014295	0.065866
SC15	SC14	CS10	*R.nitidus*	-0.018972	0.06551
GD40	SC14	CS10	*R.nitidus*	0.058177	0.063826
SC12	SC14	CS10	*R.nitidus*	-0.018561	0.066395
CS17	SC14	CS10	*R.nitidus*	-0.006163	0.070051
CS24	SC14	CS10	*R.nitidus*	-0.05081	0.063627
CS20	SC14	CS10	*R.nitidus*	-0.040513	0.065007
CS12	SC14	CS10	*R.nitidus*	-0.017921	0.067387
GD50	SC14	CS10	*R.nitidus*	0.023256	0.068676
GD45	SC14	CS10	*R.nitidus*	0.025424	0.07082
SC24	SC14	CS10	*R.nitidus*	-0.004471	0.067373
GD42	SC14	CS10	*R.nitidus*	-0.007468	0.065075
CS31	SC14	CS10	*R.nitidus*	-0.039611	0.067365
SC10	SC14	CS10	*R.nitidus*	0.053077	0.067053
SC28	SC14	CS10	*R.nitidus*	-0.035445	0.063715
GD46	SC14	CS10	*R.nitidus*	-0.028859	0.064503
SC5	SC14	CS10	*R.nitidus*	-0.017899	0.069706
CS23	SC14	CS10	*R.nitidus*	-0.06467	0.065879
GD41	SC14	CS10	*R.nitidus*	0.002749	0.06446
CS3	SC14	CS10	*R.nitidus*	-0.038596	0.06537
CS19	SC14	CS10	*R.nitidus*	-0.03274	0.06838
CS16	SC14	CS10	*R.nitidus*	-0.006207	0.064614
SC32	SC14	CS10	*R.nitidus*	0.041601	0.066788
CS25	SC14	CS10	*R.nitidus*	-0.0247	0.067813
CS28	SC14	CS10	*R.nitidus*	-0.048611	0.067177
JW6	SC14	CS10	*R.nitidus*	0.013514	0.069781
CS21	SC14	CS10	*R.nitidus*	-0.042759	0.066117
CS30	SC14	CS10	*R.nitidus*	-0.070313	0.065496
JO7	SC15	CS10	*R.nitidus*	0.027264	0.074477
SC18	SC14	CS10	*R.nitidus*	0.02795	0.066659
SC34	SC14	CS10	*R.nitidus*	0.018437	0.065224
JO3	SC14	CS10	*R.nitidus*	0.032012	0.067731
MY6	SC14	CS10	*R.nitidus*	-0.019959	0.065346
SC35	SC14	CS10	*R.nitidus*	0.03543	0.063118
CS4	SC14	CS10	*R.nitidus*	-0.02332	0.063635
JW4	SC14	CS10	*R.nitidus*	0.213904	0.063628
MY4	SC14	CS10	*R.nitidus*	-0.059201	0.066349
SC31	SC14	CS10	*R.nitidus*	-0.031084	0.067339
CS6	SC14	CS10	*R.nitidus*	-0.062684	0.065098
JW7	SC14	CS10	*R.nitidus*	0.043437	0.067533
SC26	SC14	CS10	*R.nitidus*	-0.040089	0.064921
SC27	SC14	CS10	*R.nitidus*	-0.016211	0.062086
SC44	SC14	CS10	*R.nitidus*	-0.010638	0.065064
GD21	SC15	CS10	*R.nitidus*	0.125415	0.065403
CS11	SC15	CS10	*R.nitidus*	0.00145	0.068143

JO5	SC14	CS10	*R.nitidus*	0.043182	0.064672
SC42	SC14	CS10	*R.nitidus*	0.244094	0.062136
JO1	SC14	CS10	*R.nitidus*	-0.002182	0.064358
GD31	SC15	CS10	*R.nitidus*	0.062902	0.066866
GD40	SC15	CS10	*R.nitidus*	0.088255	0.063346
GD26	SC15	CS10	*R.nitidus*	0.053705	0.064945
SC6	SC14	CS10	*R.nitidus*	-0.010487	0.068374
SC45	SC14	CS10	*R.nitidus*	0.035915	0.066673
GD44	SC15	CS10	*R.nitidus*	0.011722	0.067131
GD45	SC15	CS10	*R.nitidus*	0.041567	0.069488
JO8	SC15	CS10	*R.nitidus*	0.041206	0.072673
GD34	SC15	CS10	*R.nitidus*	0.017747	0.065539
CS12	SC15	CS10	*R.nitidus*	0.005385	0.066906
GD22	SC15	CS10	*R.nitidus*	0.068439	0.064133
CS17	SC15	CS10	*R.nitidus*	0.016013	0.068925
GD30	SC15	CS10	*R.nitidus*	0.123077	0.069234
GD32	SC15	CS10	*R.nitidus*	0.002439	0.068269
GD29	SC15	CS10	*R.nitidus*	0.13058	0.064412
GD49	SC15	CS10	*R.nitidus*	0.088697	0.068323
GD41	SC15	CS10	*R.nitidus*	0.014337	0.065258
CS14	SC15	CS10	*R.nitidus*	0.004376	0.068116
GD25	SC15	CS10	*R.nitidus*	0.070341	0.066881
GD35	SC15	CS10	*R.nitidus*	0.043123	0.068105
CS19	SC15	CS10	*R.nitidus*	-0.024006	0.067849
CS15	SC15	CS10	*R.nitidus*	0.02641	0.06612
GD33	SC15	CS10	*R.nitidus*	0.015162	0.065705
CS23	SC15	CS10	*R.nitidus*	-0.058999	0.064785
GD46	SC15	CS10	*R.nitidus*	-0.007097	0.066078
GD42	SC15	CS10	*R.nitidus*	0.035125	0.067487
CS18	SC15	CS10	*R.nitidus*	-0.026114	0.065208
GD48	SC15	CS10	*R.nitidus*	0.051515	0.067249
GD50	SC15	CS10	*R.nitidus*	0.030431	0.068811
CS13	SC15	CS10	*R.nitidus*	-0.028529	0.067624
CS30	SC15	CS10	*R.nitidus*	-0.057034	0.066024
SC24	SC15	CS10	*R.nitidus*	0.011041	0.066001
CS25	SC15	CS10	*R.nitidus*	-0.015742	0.067293
CS3	SC15	CS10	*R.nitidus*	-0.023581	0.066394
CS28	SC15	CS10	*R.nitidus*	-0.027575	0.06795
CS16	SC15	CS10	*R.nitidus*	0.011038	0.06555
SC42	SC15	CS10	*R.nitidus*	0.26319	0.062129
SC44	SC15	CS10	*R.nitidus*	0.010679	0.065555
SC18	SC15	CS10	*R.nitidus*	0.048544	0.066663
SC12	SC15	CS10	*R.nitidus*	-0.010938	0.064906
CS22	SC15	CS10	*R.nitidus*	-0.002252	0.065037
MY4	SC15	CS10	*R.nitidus*	-0.040531	0.067128
SC5	SC15	CS10	*R.nitidus*	-0.001524	0.069361
CS20	SC15	CS10	*R.nitidus*	-0.019579	0.066616
SC6	SC15	CS10	*R.nitidus*	0.012365	0.065699
SC31	SC15	CS10	*R.nitidus*	-0.007395	0.06643
CS21	SC15	CS10	*R.nitidus*	-0.012528	0.065023
CS24	SC15	CS10	*R.nitidus*	-0.036462	0.064173
CS6	SC15	CS10	*R.nitidus*	-0.050989	0.065827
SC28	SC15	CS10	*R.nitidus*	-0.015825	0.065968
MY6	SC15	CS10	*R.nitidus*	-0.006608	0.06537
CS31	SC15	CS10	*R.nitidus*	-0.018462	0.068476
JW7	SC15	CS10	*R.nitidus*	0.066355	0.068879

SC26	SC15	CS10	*R.nitidus*	-0.024024	0.062871
SC27	SC15	CS10	*R.nitidus*	0.008842	0.063192
JO3	SC15	CS10	*R.nitidus*	0.054772	0.068609
JO7	SC18	CS10	*R.nitidus*	-0.024952	0.070824
JW6	SC15	CS10	*R.nitidus*	0.038573	0.073208
SC45	SC15	CS10	*R.nitidus*	0.056012	0.066057
SC10	SC15	CS10	*R.nitidus*	0.08165	0.066919
JW4	SC15	CS10	*R.nitidus*	0.237099	0.064899
CS4	SC15	CS10	*R.nitidus*	-0.01362	0.064111
SC14	SC15	CS10	*R.nitidus*	0.018972	0.06551
JO8	SC18	CS10	*R.nitidus*	0.006224	0.070065
SC32	SC15	CS10	*R.nitidus*	0.066824	0.066758
SC34	SC15	CS10	*R.nitidus*	0.037262	0.065457
JO1	SC15	CS10	*R.nitidus*	-0.002271	0.064877
GD35	SC18	CS10	*R.nitidus*	0.015162	0.065935
SC35	SC15	CS10	*R.nitidus*	0.050267	0.064121
GD22	SC18	CS10	*R.nitidus*	0.032323	0.064035
GD40	SC18	CS10	*R.nitidus*	0.038594	0.06247
GD26	SC18	CS10	*R.nitidus*	0.027197	0.064174
GD41	SC18	CS10	*R.nitidus*	-0.013542	0.064281
GD34	SC18	CS10	*R.nitidus*	-0.022054	0.064206
CS19	SC18	CS10	*R.nitidus*	-0.065599	0.066109
GD29	SC18	CS10	*R.nitidus*	0.086436	0.064503
GD30	SC18	CS10	*R.nitidus*	0.081699	0.067458
GD31	SC18	CS10	*R.nitidus*	0.021914	0.066193
GD49	SC18	CS10	*R.nitidus*	0.047297	0.06707
GD21	SC18	CS10	*R.nitidus*	0.101266	0.064167
GD50	SC18	CS10	*R.nitidus*	0.01269	0.069507
GD46	SC18	CS10	*R.nitidus*	-0.04954	0.063383
CS11	SC18	CS10	*R.nitidus*	-0.046544	0.065773
GD44	SC18	CS10	*R.nitidus*	0.008975	0.064784
GD42	SC18	CS10	*R.nitidus*	0	0.065663
GD25	SC18	CS10	*R.nitidus*	0.03819	0.065743
JO5	SC15	CS10	*R.nitidus*	0.06391	0.065082
CS20	SC18	CS10	*R.nitidus*	-0.071481	0.063739
CS30	SC18	CS10	*R.nitidus*	-0.097938	0.06613
GD48	SC18	CS10	*R.nitidus*	-0.004518	0.067793
GD32	SC18	CS10	*R.nitidus*	-0.041599	0.067915
CS13	SC18	CS10	*R.nitidus*	-0.069065	0.067964
CS14	SC18	CS10	*R.nitidus*	-0.024735	0.067637
CS12	SC18	CS10	*R.nitidus*	-0.060427	0.06753
GD33	SC18	CS10	*R.nitidus*	-0.01546	0.065131
GD45	SC18	CS10	*R.nitidus*	-0.002473	0.070941
CS25	SC18	CS10	*R.nitidus*	-0.056422	0.064856
CS21	SC18	CS10	*R.nitidus*	-0.06282	0.063844
SC15	SC18	CS10	*R.nitidus*	-0.048544	0.066663
CS22	SC18	CS10	*R.nitidus*	-0.046338	0.065395
SC26	SC18	CS10	*R.nitidus*	-0.080558	0.064475
CS31	SC18	CS10	*R.nitidus*	-0.072848	0.065752
SC10	SC18	CS10	*R.nitidus*	0.043408	0.067174
SC6	SC18	CS10	*R.nitidus*	-0.035938	0.066067
CS3	SC18	CS10	*R.nitidus*	-0.07083	0.064449
CS16	SC18	CS10	*R.nitidus*	-0.03307	0.065286
CS17	SC18	CS10	*R.nitidus*	-0.027068	0.069266
CS24	SC18	CS10	*R.nitidus*	-0.071958	0.065567
CS15	SC18	CS10	*R.nitidus*	-0.021216	0.065358

SC34	SC18	CS10	*R.nitidus*	-0.016591	0.064736
CS6	SC18	CS10	*R.nitidus*	-0.101852	0.063546
CS18	SC18	CS10	*R.nitidus*	-0.075485	0.064047
SC12	SC18	CS10	*R.nitidus*	-0.076265	0.064991
SC5	SC18	CS10	*R.nitidus*	-0.047162	0.068559
SC14	SC18	CS10	*R.nitidus*	-0.02795	0.066659
SC45	SC18	CS10	*R.nitidus*	-0.00541	0.065825
CS4	SC18	CS10	*R.nitidus*	-0.048201	0.063485
CS28	SC18	CS10	*R.nitidus*	-0.084243	0.068232
CS23	SC18	CS10	*R.nitidus*	-0.097109	0.063554
SC32	SC18	CS10	*R.nitidus*	0.011914	0.062012
MY4	SC18	CS10	*R.nitidus*	-0.097826	0.063884
JO7	SC24	CS10	*R.nitidus*	-0.015311	0.07291
SC42	SC18	CS10	*R.nitidus*	0.208993	0.062298
SC28	SC18	CS10	*R.nitidus*	-0.083135	0.061972
SC24	SC18	CS10	*R.nitidus*	-0.057518	0.061896
SC31	SC18	CS10	*R.nitidus*	-0.062016	0.065171
MY6	SC18	CS10	*R.nitidus*	-0.060213	0.063317
SC35	SC18	CS10	*R.nitidus*	-0.000759	0.062531
SC27	SC18	CS10	*R.nitidus*	-0.037383	0.065208
JO1	SC18	CS10	*R.nitidus*	-0.045455	0.065643
SC44	SC18	CS10	*R.nitidus*	-0.053557	0.062806
GD31	SC24	CS10	*R.nitidus*	0.054945	0.066508
JW6	SC18	CS10	*R.nitidus*	0.004739	0.069833
GD32	SC24	CS10	*R.nitidus*	0.012618	0.066761
JW7	SC18	CS10	*R.nitidus*	0.037418	0.067559
JW4	SC18	CS10	*R.nitidus*	0.201595	0.064935
JO8	SC24	CS10	*R.nitidus*	0.008876	0.069856
CS11	SC24	CS10	*R.nitidus*	-0.009091	0.067993
GD29	SC24	CS10	*R.nitidus*	0.117722	0.063788
GD42	SC24	CS10	*R.nitidus*	0.01257	0.064947
CS14	SC24	CS10	*R.nitidus*	-0.010352	0.067659
GD21	SC24	CS10	*R.nitidus*	0.122265	0.063998
GD35	SC24	CS10	*R.nitidus*	0.040759	0.064812
JO3	SC18	CS10	*R.nitidus*	0.013212	0.067627
CS12	SC24	CS10	*R.nitidus*	-0.020378	0.066753
GD22	SC24	CS10	*R.nitidus*	0.067017	0.064532
GD34	SC24	CS10	*R.nitidus*	0.005431	0.064665
GD50	SC24	CS10	*R.nitidus*	0.060065	0.067883
GD30	SC24	CS10	*R.nitidus*	0.128627	0.067928
GD25	SC24	CS10	*R.nitidus*	0.057891	0.066027
JO5	SC18	CS10	*R.nitidus*	0.013997	0.064935
GD26	SC24	CS10	*R.nitidus*	0.056164	0.064483
GD46	SC24	CS10	*R.nitidus*	-0.016506	0.064731
GD40	SC24	CS10	*R.nitidus*	0.060095	0.063046
CS17	SC24	CS10	*R.nitidus*	0.004539	0.067967
GD41	SC24	CS10	*R.nitidus*	0.016598	0.064824
GD33	SC24	CS10	*R.nitidus*	0.011273	0.063754
GD48	SC24	CS10	*R.nitidus*	0.019868	0.067142
CS19	SC24	CS10	*R.nitidus*	-0.033309	0.066767
SC5	SC24	CS10	*R.nitidus*	-0.02331	0.068799
CS13	SC24	CS10	*R.nitidus*	-0.046643	0.068856
GD45	SC24	CS10	*R.nitidus*	0.031422	0.069621
CS24	SC24	CS10	*R.nitidus*	-0.046086	0.064231
CS4	SC24	CS10	*R.nitidus*	-0.021023	0.063358
GD49	SC24	CS10	*R.nitidus*	0.091053	0.067123

CS21	SC24	CS10	*R.nitidus*	-0.03307	0.064606
SC27	SC24	CS10	*R.nitidus*	0.002974	0.064618
CS20	SC24	CS10	*R.nitidus*	-0.042674	0.063347
CS22	SC24	CS10	*R.nitidus*	-0.010669	0.064193
GD44	SC24	CS10	*R.nitidus*	0.026163	0.066848
CS15	SC24	CS10	*R.nitidus*	0.013624	0.065157
CS18	SC24	CS10	*R.nitidus*	-0.042583	0.063931
SC26	SC24	CS10	*R.nitidus*	-0.039098	0.063079
CS16	SC24	CS10	*R.nitidus*	-0.006925	0.064364
JW4	SC24	CS10	*R.nitidus*	0.220028	0.065139
CS28	SC24	CS10	*R.nitidus*	-0.053948	0.065643
SC6	SC24	CS10	*R.nitidus*	-0.011512	0.064381
MY4	SC24	CS10	*R.nitidus*	-0.057778	0.064935
SC45	SC24	CS10	*R.nitidus*	0.043769	0.063381
CS23	SC24	CS10	*R.nitidus*	-0.064286	0.064542
SC44	SC24	CS10	*R.nitidus*	-0.009901	0.064266
CS31	SC24	CS10	*R.nitidus*	-0.032377	0.065248
SC35	SC24	CS10	*R.nitidus*	0.033724	0.063249
SC32	SC24	CS10	*R.nitidus*	0.050633	0.063816
SC10	SC24	CS10	*R.nitidus*	0.078928	0.065397
JO5	SC24	CS10	*R.nitidus*	0.045315	0.064195
SC31	SC24	CS10	*R.nitidus*	-0.028932	0.064905
CS6	SC24	CS10	*R.nitidus*	-0.075358	0.06432
SC18	SC24	CS10	*R.nitidus*	0.057518	0.061896
CS25	SC24	CS10	*R.nitidus*	-0.033577	0.065397
CS3	SC24	CS10	*R.nitidus*	-0.037147	0.065697
CS30	SC24	CS10	*R.nitidus*	-0.07148	0.064653
JO7	SC26	CS10	*R.nitidus*	0.024324	0.072672
JO8	SC26	CS10	*R.nitidus*	0.08373	0.070936
SC28	SC24	CS10	*R.nitidus*	-0.042191	0.06382
CS11	SC26	CS10	*R.nitidus*	0.020139	0.065339
JO1	SC24	CS10	*R.nitidus*	-0.008136	0.065766
SC34	SC24	CS10	*R.nitidus*	0.024481	0.06322
SC14	SC24	CS10	*R.nitidus*	0.004471	0.067373
SC12	SC24	CS10	*R.nitidus*	-0.026886	0.06386
SC15	SC24	CS10	*R.nitidus*	-0.011041	0.066001
GD21	SC26	CS10	*R.nitidus*	0.148472	0.062455
SC42	SC24	CS10	*R.nitidus*	0.244005	0.061794
JW6	SC24	CS10	*R.nitidus*	-0.000939	0.071821
GD26	SC26	CS10	*R.nitidus*	0.082219	0.065679
GD50	SC26	CS10	*R.nitidus*	0.0552	0.069954
GD22	SC26	CS10	*R.nitidus*	0.084165	0.063458
MY6	SC24	CS10	*R.nitidus*	-0.017725	0.064539
GD25	SC26	CS10	*R.nitidus*	0.084239	0.065163
GD45	SC26	CS10	*R.nitidus*	0.064906	0.069264
GD31	SC26	CS10	*R.nitidus*	0.0811	0.06484
JO3	SC24	CS10	*R.nitidus*	0.043689	0.067424
GD42	SC26	CS10	*R.nitidus*	0.04123	0.066701
JW7	SC24	CS10	*R.nitidus*	0.052536	0.067983
GD34	SC26	CS10	*R.nitidus*	0.025132	0.062657
GD41	SC26	CS10	*R.nitidus*	0.035053	0.067385
CS19	SC26	CS10	*R.nitidus*	0.001405	0.064928
CS12	SC26	CS10	*R.nitidus*	0.013235	0.066818
GD44	SC26	CS10	*R.nitidus*	0.040356	0.066038
GD48	SC26	CS10	*R.nitidus*	0.068214	0.066411
GD29	SC26	CS10	*R.nitidus*	0.147151	0.064711

SC6	SC26	CS10	*R.nitidus*	0.036594	0.066213
CS14	SC26	CS10	*R.nitidus*	0.028374	0.067024
CS13	SC26	CS10	*R.nitidus*	-0.008608	0.066608
GD30	SC26	CS10	*R.nitidus*	0.140123	0.067162
CS22	SC26	CS10	*R.nitidus*	0.023239	0.06437
GD40	SC26	CS10	*R.nitidus*	0.088599	0.062417
GD32	SC26	CS10	*R.nitidus*	0.025542	0.067431
CS23	SC26	CS10	*R.nitidus*	-0.027454	0.064132
GD33	SC26	CS10	*R.nitidus*	0.038102	0.064311
CS17	SC26	CS10	*R.nitidus*	0.025468	0.067297
GD35	SC26	CS10	*R.nitidus*	0.052283	0.063923
CS18	SC26	CS10	*R.nitidus*	-0.013277	0.062527
GD46	SC26	CS10	*R.nitidus*	0.011858	0.064022
GD49	SC26	CS10	*R.nitidus*	0.11026	0.069954
SC5	SC26	CS10	*R.nitidus*	0.030169	0.068513
CS20	SC26	CS10	*R.nitidus*	-0.002815	0.063839
CS16	SC26	CS10	*R.nitidus*	0.028101	0.063849
SC14	SC26	CS10	*R.nitidus*	0.040089	0.064921
CS30	SC26	CS10	*R.nitidus*	-0.034266	0.062589
CS25	SC26	CS10	*R.nitidus*	0.001405	0.066216
CS4	SC26	CS10	*R.nitidus*	0.008678	0.061745
CS15	SC26	CS10	*R.nitidus*	0.047883	0.064668
CS31	SC26	CS10	*R.nitidus*	-0.005678	0.065362
SC12	SC26	CS10	*R.nitidus*	0.016999	0.062707
SC32	SC26	CS10	*R.nitidus*	0.077554	0.064036
SC10	SC26	CS10	*R.nitidus*	0.10916	0.063804
CS21	SC26	CS10	*R.nitidus*	0.003514	0.063105
CS28	SC26	CS10	*R.nitidus*	-0.008289	0.065297
CS24	SC26	CS10	*R.nitidus*	-0.017143	0.063601
JW4	SC26	CS10	*R.nitidus*	0.251701	0.065452
CS3	SC26	CS10	*R.nitidus*	0.000711	0.064658
SC24	SC26	CS10	*R.nitidus*	0.039098	0.063079
SC15	SC26	CS10	*R.nitidus*	0.024024	0.062871
SC42	SC26	CS10	*R.nitidus*	0.259861	0.060206
SC28	SC26	CS10	*R.nitidus*	0.010997	0.062782
SC27	SC26	CS10	*R.nitidus*	0.030628	0.062548
SC35	SC26	CS10	*R.nitidus*	0.080866	0.061232
JW7	SC26	CS10	*R.nitidus*	0.094086	0.06718
SC34	SC26	CS10	*R.nitidus*	0.062147	0.062144
SC44	SC26	CS10	*R.nitidus*	0.029126	0.065033
CS6	SC26	CS10	*R.nitidus*	-0.025993	0.064424
JW6	SC26	CS10	*R.nitidus*	0.039341	0.071207
JO1	SC26	CS10	*R.nitidus*	0.018896	0.064444
SC18	SC26	CS10	*R.nitidus*	0.080558	0.064475
SC31	SC26	CS10	*R.nitidus*	0.017319	0.063578
GD25	SC27	CS10	*R.nitidus*	0.07186	0.063838
SC45	SC26	CS10	*R.nitidus*	0.083639	0.064295
MY6	SC26	CS10	*R.nitidus*	0.017147	0.064754
GD32	SC27	CS10	*R.nitidus*	0.003108	0.067844
JO5	SC26	CS10	*R.nitidus*	0.080172	0.063996
JO8	SC27	CS10	*R.nitidus*	0.023474	0.069135
GD26	SC27	CS10	*R.nitidus*	0.056099	0.064854
MY4	SC26	CS10	*R.nitidus*	-0.00988	0.064468
GD21	SC27	CS10	*R.nitidus*	0.115756	0.063717
JO3	SC26	CS10	*R.nitidus*	0.072987	0.066964
GD45	SC27	CS10	*R.nitidus*	0.042768	0.070376

CS11	SC27	CS10	*R.nitidus*	0.001419	0.065896
JO7	SC27	CS10	*R.nitidus*	-0.006387	0.071995
GD29	SC27	CS10	*R.nitidus*	0.120436	0.063
GD41	SC27	CS10	*R.nitidus*	0.019836	0.063732
GD49	SC27	CS10	*R.nitidus*	0.076511	0.068435
CS15	SC27	CS10	*R.nitidus*	0.027992	0.064009
GD40	SC27	CS10	*R.nitidus*	0.073424	0.061212
GD35	SC27	CS10	*R.nitidus*	0.03391	0.064195
GD46	SC27	CS10	*R.nitidus*	-0.008796	0.063682
GD22	SC27	CS10	*R.nitidus*	0.059627	0.062323
GD33	SC27	CS10	*R.nitidus*	0.005886	0.063896
GD30	SC27	CS10	*R.nitidus*	0.135474	0.066652
GD42	SC27	CS10	*R.nitidus*	0.014384	0.065072
CS23	SC27	CS10	*R.nitidus*	-0.055322	0.064913
GD44	SC27	CS10	*R.nitidus*	0.021368	0.066505
CS14	SC27	CS10	*R.nitidus*	0.004909	0.065201
CS31	SC27	CS10	*R.nitidus*	-0.030973	0.065351
CS12	SC27	CS10	*R.nitidus*	-0.016591	0.065484
CS13	SC27	CS10	*R.nitidus*	-0.041667	0.067366
CS19	SC27	CS10	*R.nitidus*	-0.030281	0.068273
GD31	SC27	CS10	*R.nitidus*	0.049931	0.065387
CS30	SC27	CS10	*R.nitidus*	-0.068635	0.063407
GD50	SC27	CS10	*R.nitidus*	0.056697	0.065612
GD34	SC27	CS10	*R.nitidus*	0.001982	0.063605
CS18	SC27	CS10	*R.nitidus*	-0.044012	0.06423
CS24	SC27	CS10	*R.nitidus*	-0.042296	0.063095
SC24	SC27	CS10	*R.nitidus*	-0.002974	0.064618
SC10	SC27	CS10	*R.nitidus*	0.075061	0.063113
GD48	SC27	CS10	*R.nitidus*	0.037223	0.065388
CS3	SC27	CS10	*R.nitidus*	-0.016949	0.064432
SC6	SC27	CS10	*R.nitidus*	-0.00075	0.06593
SC32	SC27	CS10	*R.nitidus*	0.050889	0.063683
CS20	SC27	CS10	*R.nitidus*	-0.0337	0.064456
SC14	SC27	CS10	*R.nitidus*	0.016211	0.062086
CS25	SC27	CS10	*R.nitidus*	-0.022825	0.066273
CS17	SC27	CS10	*R.nitidus*	0.024219	0.066203
CS16	SC27	CS10	*R.nitidus*	0.00212	0.063795
SC28	SC27	CS10	*R.nitidus*	-0.032258	0.063911
CS28	SC27	CS10	*R.nitidus*	-0.042089	0.064852
CS22	SC27	CS10	*R.nitidus*	-0.016094	0.064299
CS21	SC27	CS10	*R.nitidus*	-0.025892	0.064928
CS4	SC27	CS10	*R.nitidus*	-0.015732	0.062525
SC35	SC27	CS10	*R.nitidus*	0.048961	0.062608
JW6	SC27	CS10	*R.nitidus*	0.021043	0.070539
SC26	SC27	CS10	*R.nitidus*	-0.030628	0.062548
SC5	SC27	CS10	*R.nitidus*	-0.005267	0.068956
MY6	SC27	CS10	*R.nitidus*	-0.013112	0.063943
GD30	SC28	CS10	*R.nitidus*	0.140856	0.065647
JO7	SC28	CS10	*R.nitidus*	0.002768	0.071571
SC31	SC27	CS10	*R.nitidus*	-0.01949	0.064771
JW7	SC27	CS10	*R.nitidus*	0.047393	0.067634
SC15	SC27	CS10	*R.nitidus*	-0.008842	0.063192
SC18	SC27	CS10	*R.nitidus*	0.037383	0.065208
CS6	SC27	CS10	*R.nitidus*	-0.060606	0.063741
SC42	SC27	CS10	*R.nitidus*	0.255971	0.0618
SC12	SC27	CS10	*R.nitidus*	-0.022047	0.064513

MY4	SC27	CS10	*R.nitidus*	-0.051028	0.065254
SC45	SC27	CS10	*R.nitidus*	0.034261	0.064735
GD34	SC28	CS10	*R.nitidus*	0.035286	0.062575
GD25	SC28	CS10	*R.nitidus*	0.092517	0.064503
GD21	SC28	CS10	*R.nitidus*	0.139185	0.063525
GD22	SC28	CS10	*R.nitidus*	0.098186	0.063393
SC34	SC27	CS10	*R.nitidus*	0.03221	0.062319
SC44	SC27	CS10	*R.nitidus*	-0.004539	0.063588
GD41	SC28	CS10	*R.nitidus*	0.041001	0.064469
GD42	SC28	CS10	*R.nitidus*	0.047358	0.064452
JW4	SC27	CS10	*R.nitidus*	0.208638	0.063293
GD35	SC28	CS10	*R.nitidus*	0.060109	0.065178
GD31	SC28	CS10	*R.nitidus*	0.09193	0.064898
GD26	SC28	CS10	*R.nitidus*	0.081605	0.064925
CS11	SC28	CS10	*R.nitidus*	0.026389	0.065789
JO8	SC28	CS10	*R.nitidus*	0.045726	0.071793
JO3	SC27	CS10	*R.nitidus*	0.027195	0.067101
GD46	SC28	CS10	*R.nitidus*	0.023396	0.063445
GD29	SC28	CS10	*R.nitidus*	0.15	0.063438
GD32	SC28	CS10	*R.nitidus*	0.015711	0.065962
GD45	SC28	CS10	*R.nitidus*	0.072612	0.068001
GD40	SC28	CS10	*R.nitidus*	0.097513	0.062852
JO1	SC27	CS10	*R.nitidus*	0.000728	0.063628
JO5	SC27	CS10	*R.nitidus*	0.045073	0.064943
GD48	SC28	CS10	*R.nitidus*	0.059371	0.065414
CS21	SC28	CS10	*R.nitidus*	-0.003463	0.064752
CS3	SC28	CS10	*R.nitidus*	0.009213	0.064252
CS15	SC28	CS10	*R.nitidus*	0.054747	0.063978
CS16	SC28	CS10	*R.nitidus*	0.044097	0.065264
CS12	SC28	CS10	*R.nitidus*	0.02169	0.065314
CS19	SC28	CS10	*R.nitidus*	0.000697	0.065465
GD33	SC28	CS10	*R.nitidus*	0.028724	0.064518
CS13	SC28	CS10	*R.nitidus*	-0.001435	0.065923
SC10	SC28	CS10	*R.nitidus*	0.119507	0.063512
CS23	SC28	CS10	*R.nitidus*	-0.012354	0.064365
CS22	SC28	CS10	*R.nitidus*	0.014472	0.064651
CS31	SC28	CS10	*R.nitidus*	-0.0036	0.064545
GD49	SC28	CS10	*R.nitidus*	0.102229	0.065814
GD50	SC28	CS10	*R.nitidus*	0.071659	0.066511
CS30	SC28	CS10	*R.nitidus*	-0.03458	0.063832
SC31	SC28	CS10	*R.nitidus*	0.008791	0.063736
SC27	SC28	CS10	*R.nitidus*	0.032258	0.063911
CS14	SC28	CS10	*R.nitidus*	0.02591	0.066504
GD44	SC28	CS10	*R.nitidus*	0.052405	0.066269
CS17	SC28	CS10	*R.nitidus*	0.046792	0.066144
CS25	SC28	CS10	*R.nitidus*	0.012023	0.067167
SC6	SC28	CS10	*R.nitidus*	0.040724	0.065941
SC18	SC28	CS10	*R.nitidus*	0.083135	0.061972
CS6	SC28	CS10	*R.nitidus*	-0.019751	0.062968
SC14	SC28	CS10	*R.nitidus*	0.035445	0.063715
CS18	SC28	CS10	*R.nitidus*	-0.008374	0.064704
CS24	SC28	CS10	*R.nitidus*	-0.018665	0.063667
CS28	SC28	CS10	*R.nitidus*	-0.017679	0.064792
CS20	SC28	CS10	*R.nitidus*	-0.008868	0.06529
JW7	SC28	CS10	*R.nitidus*	0.074749	0.068173
SC26	SC28	CS10	*R.nitidus*	-0.010997	0.062782

SC42	SC28	CS10	*R.nitidus*	0.272125	0.06112
SC44	SC28	CS10	*R.nitidus*	0.046859	0.058325
SC5	SC28	CS10	*R.nitidus*	0.014754	0.066587
JO3	SC28	CS10	*R.nitidus*	0.052754	0.067513
MY4	SC28	CS10	*R.nitidus*	-0.016973	0.063403
GD21	SC31	CS10	*R.nitidus*	0.136588	0.064395
GD22	SC31	CS10	*R.nitidus*	0.088624	0.06547
SC45	SC28	CS10	*R.nitidus*	0.062362	0.062608
SC15	SC28	CS10	*R.nitidus*	0.015825	0.065968
CS4	SC28	CS10	*R.nitidus*	0.005491	0.062773
JO8	SC31	CS10	*R.nitidus*	0.011289	0.072924
JO7	SC31	CS10	*R.nitidus*	0.022183	0.071981
JO1	SC28	CS10	*R.nitidus*	0.006047	0.064748
GD45	SC31	CS10	*R.nitidus*	0.046317	0.070488
SC32	SC28	CS10	*R.nitidus*	0.088476	0.062322
GD32	SC31	CS10	*R.nitidus*	0.024133	0.068695
SC34	SC28	CS10	*R.nitidus*	0.055675	0.062733
JW4	SC28	CS10	*R.nitidus*	0.247956	0.064194
JW6	SC28	CS10	*R.nitidus*	0.02468	0.072523
SC35	SC28	CS10	*R.nitidus*	0.073381	0.062545
SC12	SC28	CS10	*R.nitidus*	0.006154	0.062584
GD46	SC31	CS10	*R.nitidus*	0.013342	0.06449
CS12	SC31	CS10	*R.nitidus*	0.009413	0.068049
SC24	SC28	CS10	*R.nitidus*	0.042191	0.06382
GD29	SC31	CS10	*R.nitidus*	0.127306	0.063252
JO5	SC28	CS10	*R.nitidus*	0.091954	0.061502
CS11	SC31	CS10	*R.nitidus*	0.010253	0.065153
GD41	SC31	CS10	*R.nitidus*	0.026017	0.063967
MY6	SC28	CS10	*R.nitidus*	0.01198	0.061982
GD42	SC31	CS10	*R.nitidus*	0.044283	0.066307
GD35	SC31	CS10	*R.nitidus*	0.052117	0.065248
GD33	SC31	CS10	*R.nitidus*	0.034437	0.066363
GD25	SC31	CS10	*R.nitidus*	0.080297	0.063784
GD44	SC31	CS10	*R.nitidus*	0.028112	0.066949
CS15	SC31	CS10	*R.nitidus*	0.039947	0.065363
GD49	SC31	CS10	*R.nitidus*	0.112471	0.068668
CS18	SC31	CS10	*R.nitidus*	-0.012057	0.064548
GD30	SC31	CS10	*R.nitidus*	0.136914	0.067122
GD50	SC31	CS10	*R.nitidus*	0.042254	0.068439
GD26	SC31	CS10	*R.nitidus*	0.066883	0.066055
GD31	SC31	CS10	*R.nitidus*	0.071478	0.06589
CS21	SC31	CS10	*R.nitidus*	-0.01142	0.064748
CS3	SC31	CS10	*R.nitidus*	0.007057	0.064848
CS16	SC31	CS10	*R.nitidus*	0.035495	0.066294
GD40	SC31	CS10	*R.nitidus*	0.088444	0.063754
CS20	SC31	CS10	*R.nitidus*	-0.006303	0.063669
GD34	SC31	CS10	*R.nitidus*	0.020516	0.064812
CS13	SC31	CS10	*R.nitidus*	-0.015548	0.068441
CS25	SC31	CS10	*R.nitidus*	-0.004875	0.065944
CS23	SC31	CS10	*R.nitidus*	-0.036313	0.064409
CS14	SC31	CS10	*R.nitidus*	0.015007	0.0664
GD48	SC31	CS10	*R.nitidus*	0.05841	0.067686
CS4	SC31	CS10	*R.nitidus*	0.004854	0.063425
CS30	SC31	CS10	*R.nitidus*	-0.047484	0.06551
CS28	SC31	CS10	*R.nitidus*	-0.021995	0.064894
JW6	SC31	CS10	*R.nitidus*	0.046263	0.072562

SC14	SC31	CS10	*R.nitidus*	0.031084	0.067339
CS17	SC31	CS10	*R.nitidus*	0.040847	0.068301
SC5	SC31	CS10	*R.nitidus*	0.013274	0.068537
CS31	SC31	CS10	*R.nitidus*	-0.020364	0.067088
CS19	SC31	CS10	*R.nitidus*	0.000696	0.066811
CS22	SC31	CS10	*R.nitidus*	0.01188	0.0656
JW4	SC31	CS10	*R.nitidus*	0.248804	0.065346
SC24	SC31	CS10	*R.nitidus*	0.028932	0.064905
SC35	SC31	CS10	*R.nitidus*	0.058737	0.063964
JO1	SC31	CS10	*R.nitidus*	0.015173	0.064736
SC27	SC31	CS10	*R.nitidus*	0.01949	0.064771
SC32	SC31	CS10	*R.nitidus*	0.071376	0.064305
JW7	SC31	CS10	*R.nitidus*	0.093044	0.066044
SC12	SC31	CS10	*R.nitidus*	0.002859	0.065519
CS24	SC31	CS10	*R.nitidus*	-0.02983	0.064618
SC44	SC31	CS10	*R.nitidus*	0.020483	0.065243
SC18	SC31	CS10	*R.nitidus*	0.062016	0.065171
SC45	SC31	CS10	*R.nitidus*	0.060606	0.064139
SC10	SC31	CS10	*R.nitidus*	0.098537	0.065274
JO3	SC31	CS10	*R.nitidus*	0.058552	0.068465
SC6	SC31	CS10	*R.nitidus*	0.004458	0.066752
SC15	SC31	CS10	*R.nitidus*	0.007395	0.06643
MY4	SC31	CS10	*R.nitidus*	-0.022695	0.065941
SC26	SC31	CS10	*R.nitidus*	-0.017319	0.063578
JO5	SC31	CS10	*R.nitidus*	0.061314	0.064955
GD30	SC32	CS10	*R.nitidus*	0.086687	0.067648
SC34	SC31	CS10	*R.nitidus*	0.042343	0.066022
SC28	SC31	CS10	*R.nitidus*	-0.008791	0.063736
GD26	SC32	CS10	*R.nitidus*	0.012667	0.0649
JO7	SC32	CS10	*R.nitidus*	-0.05	0.071385
GD34	SC32	CS10	*R.nitidus*	-0.035123	0.063341
CS6	SC31	CS10	*R.nitidus*	-0.026638	0.067251
CS11	SC32	CS10	*R.nitidus*	-0.059075	0.067909
JO8	SC32	CS10	*R.nitidus*	0.003842	0.071836
MY6	SC31	CS10	*R.nitidus*	0.015449	0.064601
SC42	SC31	CS10	*R.nitidus*	0.245261	0.062323
CS13	SC32	CS10	*R.nitidus*	-0.085457	0.069026
GD32	SC32	CS10	*R.nitidus*	-0.039246	0.068038
CS17	SC32	CS10	*R.nitidus*	-0.051163	0.069789
GD41	SC32	CS10	*R.nitidus*	-0.030976	0.063609
GD45	SC32	CS10	*R.nitidus*	-0.003123	0.067408
GD35	SC32	CS10	*R.nitidus*	-0.013423	0.064559
GD50	SC32	CS10	*R.nitidus*	0	0.069045
GD21	SC32	CS10	*R.nitidus*	0.077073	0.063148
GD42	SC32	CS10	*R.nitidus*	-0.026513	0.063836
GD25	SC32	CS10	*R.nitidus*	0.019359	0.063306
GD22	SC32	CS10	*R.nitidus*	0.023602	0.063092
GD31	SC32	CS10	*R.nitidus*	0.008731	0.064579
GD44	SC32	CS10	*R.nitidus*	-0.036678	0.066861
CS15	SC32	CS10	*R.nitidus*	-0.028787	0.06585
CS20	SC32	CS10	*R.nitidus*	-0.080877	0.063925
CS22	SC32	CS10	*R.nitidus*	-0.063282	0.064637
GD29	SC32	CS10	*R.nitidus*	0.095208	0.064338
CS12	SC32	CS10	*R.nitidus*	-0.059043	0.068141
CS18	SC32	CS10	*R.nitidus*	-0.08606	0.063957
GD48	SC32	CS10	*R.nitidus*	0.000727	0.067262

GD40	SC32	CS10	*R.nitidus*	0.026762	0.062004
GD46	SC32	CS10	*R.nitidus*	-0.051713	0.065268
GD49	SC32	CS10	*R.nitidus*	0.046584	0.067764
SC6	SC32	CS10	*R.nitidus*	-0.05287	0.064913
GD33	SC32	CS10	*R.nitidus*	-0.037849	0.064794
SC27	SC32	CS10	*R.nitidus*	-0.050889	0.063683
CS14	SC32	CS10	*R.nitidus*	-0.052136	0.0688
CS25	SC32	CS10	*R.nitidus*	-0.070866	0.067643
CS21	SC32	CS10	*R.nitidus*	-0.081387	0.064468
CS28	SC32	CS10	*R.nitidus*	-0.104037	0.06572
CS16	SC32	CS10	*R.nitidus*	-0.045678	0.065327
SC18	SC32	CS10	*R.nitidus*	-0.011914	0.062012
CS4	SC32	CS10	*R.nitidus*	-0.062987	0.0636
CS23	SC32	CS10	*R.nitidus*	-0.103257	0.065107
SC14	SC32	CS10	*R.nitidus*	-0.041601	0.066788
SC24	SC32	CS10	*R.nitidus*	-0.050633	0.063816
CS19	SC32	CS10	*R.nitidus*	-0.071585	0.066661
SC34	SC32	CS10	*R.nitidus*	-0.0131	0.06381
SC12	SC32	CS10	*R.nitidus*	-0.073408	0.06576
SC28	SC32	CS10	*R.nitidus*	-0.088476	0.062322
CS24	SC32	CS10	*R.nitidus*	-0.101935	0.064657
SC15	SC32	CS10	*R.nitidus*	-0.066824	0.066758
JW6	SC32	CS10	*R.nitidus*	-0.036517	0.072247
SC5	SC32	CS10	*R.nitidus*	-0.054929	0.0674
SC44	SC32	CS10	*R.nitidus*	-0.047404	0.062782
SC31	SC32	CS10	*R.nitidus*	-0.071376	0.064305
CS30	SC32	CS10	*R.nitidus*	-0.113703	0.06558
MY4	SC32	CS10	*R.nitidus*	-0.095729	0.065222
CS3	SC32	CS10	*R.nitidus*	-0.071529	0.064323
JO8	SC34	CS10	*R.nitidus*	0.033028	0.071107
SC45	SC32	CS10	*R.nitidus*	-0.009545	0.064961
CS31	SC32	CS10	*R.nitidus*	-0.089005	0.067043
SC10	SC32	CS10	*R.nitidus*	0.030008	0.06417
JO1	SC32	CS10	*R.nitidus*	-0.050873	0.066017
CS6	SC32	CS10	*R.nitidus*	-0.110704	0.063727
JO5	SC32	CS10	*R.nitidus*	0.001415	0.064145
SC26	SC32	CS10	*R.nitidus*	-0.077554	0.064036
JW4	SC32	CS10	*R.nitidus*	0.192039	0.065165
GD33	SC34	CS10	*R.nitidus*	-0.000657	0.064196
GD29	SC34	CS10	*R.nitidus*	0.084353	0.063444
SC35	SC32	CS10	*R.nitidus*	-0.014903	0.061926
GD32	SC34	CS10	*R.nitidus*	-0.001522	0.066607
GD30	SC34	CS10	*R.nitidus*	0.084261	0.067276
JW7	SC32	CS10	*R.nitidus*	0.017958	0.068402
GD34	SC34	CS10	*R.nitidus*	-0.018905	0.064646
JO7	SC34	CS10	*R.nitidus*	-0.006157	0.069992
GD22	SC34	CS10	*R.nitidus*	0.042105	0.062726
GD42	SC34	CS10	*R.nitidus*	-0.01019	0.065669
CS11	SC34	CS10	*R.nitidus*	-0.025956	0.067081
JO3	SC32	CS10	*R.nitidus*	-0.014844	0.066914
MY6	SC32	CS10	*R.nitidus*	-0.054533	0.063371
GD31	SC34	CS10	*R.nitidus*	0.030261	0.065787
GD48	SC34	CS10	*R.nitidus*	0.022857	0.065272
GD40	SC34	CS10	*R.nitidus*	0.047433	0.062624
SC42	SC32	CS10	*R.nitidus*	0.231001	0.061981
GD44	SC34	CS10	*R.nitidus*	-0.011042	0.065022

GD41	SC34	CS10	*R.nitidus*	-0.015636	0.063942
GD21	SC34	CS10	*R.nitidus*	0.096936	0.065075
CS12	SC34	CS10	*R.nitidus*	-0.026588	0.064715
GD25	SC34	CS10	*R.nitidus*	0.039281	0.063449
GD46	SC34	CS10	*R.nitidus*	-0.031767	0.063732
GD49	SC34	CS10	*R.nitidus*	0.044174	0.065563
GD26	SC34	CS10	*R.nitidus*	0.032744	0.064053
CS13	SC34	CS10	*R.nitidus*	-0.050678	0.067687
GD45	SC34	CS10	*R.nitidus*	0.012579	0.068573
GD35	SC34	CS10	*R.nitidus*	0.020311	0.065284
CS17	SC34	CS10	*R.nitidus*	-0.016296	0.069219
CS4	SC34	CS10	*R.nitidus*	-0.044295	0.062399
CS23	SC34	CS10	*R.nitidus*	-0.075172	0.06302
CS20	SC34	CS10	*R.nitidus*	-0.055443	0.063654
CS18	SC34	CS10	*R.nitidus*	-0.064246	0.062439
CS22	SC34	CS10	*R.nitidus*	-0.028846	0.064096
CS30	SC34	CS10	*R.nitidus*	-0.08642	0.063339
SC14	SC34	CS10	*R.nitidus*	-0.018437	0.065224
CS14	SC34	CS10	*R.nitidus*	-0.025535	0.06651
CS19	SC34	CS10	*R.nitidus*	-0.048151	0.065809
CS15	SC34	CS10	*R.nitidus*	-0.008125	0.06554
CS24	SC34	CS10	*R.nitidus*	-0.075145	0.06339
CS31	SC34	CS10	*R.nitidus*	-0.054843	0.064209
CS21	SC34	CS10	*R.nitidus*	-0.044352	0.063835
CS3	SC34	CS10	*R.nitidus*	-0.045612	0.064551
GD50	SC34	CS10	*R.nitidus*	-0.001591	0.065196
CS25	SC34	CS10	*R.nitidus*	-0.041152	0.065188
SC45	SC34	CS10	*R.nitidus*	0.017206	0.065286
CS16	SC34	CS10	*R.nitidus*	-0.013094	0.065279
CS28	SC34	CS10	*R.nitidus*	-0.075655	0.065285
SC26	SC34	CS10	*R.nitidus*	-0.062147	0.062144
CS6	SC34	CS10	*R.nitidus*	-0.064964	0.063207
SC18	SC34	CS10	*R.nitidus*	0.016591	0.064736
SC42	SC34	CS10	*R.nitidus*	0.199637	0.059773
SC24	SC34	CS10	*R.nitidus*	-0.024481	0.06322
CS11	SC35	CS10	*R.nitidus*	-0.046322	0.06506
SC10	SC34	CS10	*R.nitidus*	0.035184	0.063665
SC6	SC34	CS10	*R.nitidus*	-0.027818	0.065269
SC27	SC34	CS10	*R.nitidus*	-0.03221	0.062319
SC31	SC34	CS10	*R.nitidus*	-0.042343	0.066022
JW4	SC34	CS10	*R.nitidus*	0.213277	0.062937
MY4	SC34	CS10	*R.nitidus*	-0.076923	0.064422
SC5	SC34	CS10	*R.nitidus*	-0.030432	0.067882
SC12	SC34	CS10	*R.nitidus*	-0.047655	0.064524
GD29	SC35	CS10	*R.nitidus*	0.076087	0.063371
GD30	SC35	CS10	*R.nitidus*	0.07192	0.066661
SC15	SC34	CS10	*R.nitidus*	-0.037262	0.065457
JO8	SC35	CS10	*R.nitidus*	0.004817	0.070453
GD26	SC35	CS10	*R.nitidus*	0.025574	0.063855
JW7	SC34	CS10	*R.nitidus*	0.008651	0.067717
JO1	SC34	CS10	*R.nitidus*	-0.027445	0.064937
GD21	SC35	CS10	*R.nitidus*	0.077996	0.06263
GD25	SC35	CS10	*R.nitidus*	0.025517	0.063945
MY6	SC34	CS10	*R.nitidus*	-0.033099	0.063716
SC28	SC34	CS10	*R.nitidus*	-0.055675	0.062733
GD48	SC35	CS10	*R.nitidus*	-0.010784	0.064721

SC35	SC34	CS10	*R.nitidus*	0.0188	0.062073
SC44	SC34	CS10	*R.nitidus*	-0.033479	0.062349
GD33	SC35	CS10	*R.nitidus*	-0.019949	0.065855
SC32	SC34	CS10	*R.nitidus*	0.0131	0.06381
GD45	SC35	CS10	*R.nitidus*	0.006907	0.069128
JW6	SC34	CS10	*R.nitidus*	0.003571	0.069199
JO7	SC35	CS10	*R.nitidus*	-0.011515	0.069926
GD40	SC35	CS10	*R.nitidus*	0.024731	0.061127
JO5	SC34	CS10	*R.nitidus*	0.025271	0.065277
JO3	SC34	CS10	*R.nitidus*	0.045666	0.06702
GD42	SC35	CS10	*R.nitidus*	-0.016426	0.063504
GD44	SC35	CS10	*R.nitidus*	-0.028986	0.064894
GD41	SC35	CS10	*R.nitidus*	-0.027815	0.062993
GD46	SC35	CS10	*R.nitidus*	-0.049702	0.062672
GD32	SC35	CS10	*R.nitidus*	-0.045697	0.066122
GD22	SC35	CS10	*R.nitidus*	0.019363	0.06195
GD31	SC35	CS10	*R.nitidus*	0.02814	0.063738
GD34	SC35	CS10	*R.nitidus*	-0.031936	0.063556
CS25	SC35	CS10	*R.nitidus*	-0.061379	0.063385
CS12	SC35	CS10	*R.nitidus*	-0.050355	0.064598
CS17	SC35	CS10	*R.nitidus*	-0.026929	0.065767
GD35	SC35	CS10	*R.nitidus*	0.004107	0.065016
CS19	SC35	CS10	*R.nitidus*	-0.072514	0.064478
SC6	SC35	CS10	*R.nitidus*	-0.043801	0.0639
CS28	SC35	CS10	*R.nitidus*	-0.065071	0.065091
CS22	SC35	CS10	*R.nitidus*	-0.052746	0.063285
CS13	SC35	CS10	*R.nitidus*	-0.074689	0.065928
CS16	SC35	CS10	*R.nitidus*	-0.040431	0.063489
CS21	SC35	CS10	*R.nitidus*	-0.076976	0.063905
CS23	SC35	CS10	*R.nitidus*	-0.101509	0.062739
GD50	SC35	CS10	*R.nitidus*	0.006457	0.066244
CS24	SC35	CS10	*R.nitidus*	-0.086894	0.062521
CS3	SC35	CS10	*R.nitidus*	-0.056357	0.062547
SC34	SC35	CS10	*R.nitidus*	-0.0188	0.062073
SC12	SC35	CS10	*R.nitidus*	-0.071852	0.061294
CS4	SC35	CS10	*R.nitidus*	-0.049214	0.062939
CS15	SC35	CS10	*R.nitidus*	-0.019828	0.064256
SC15	SC35	CS10	*R.nitidus*	-0.050267	0.064121
SC26	SC35	CS10	*R.nitidus*	-0.080866	0.061232
SC18	SC35	CS10	*R.nitidus*	0.000759	0.062531
SC44	SC35	CS10	*R.nitidus*	-0.050549	0.061597
SC14	SC35	CS10	*R.nitidus*	-0.03543	0.063118
CS18	SC35	CS10	*R.nitidus*	-0.07703	0.062368
JO5	SC35	CS10	*R.nitidus*	0.003539	0.063938
JO1	SC35	CS10	*R.nitidus*	-0.055676	0.064248
CS31	SC35	CS10	*R.nitidus*	-0.070815	0.064161
SC5	SC35	CS10	*R.nitidus*	-0.043415	0.066693
SC24	SC35	CS10	*R.nitidus*	-0.033724	0.063249
CS20	SC35	CS10	*R.nitidus*	-0.071809	0.063128
SC27	SC35	CS10	*R.nitidus*	-0.048961	0.062608
SC32	SC35	CS10	*R.nitidus*	0.014903	0.061926
SC42	SC35	CS10	*R.nitidus*	0.212575	0.062338
CS30	SC35	CS10	*R.nitidus*	-0.10372	0.062851
JW7	SC35	CS10	*R.nitidus*	0.020481	0.064994
SC10	SC35	CS10	*R.nitidus*	0.033001	0.064489
CS11	CS6	CS10	*R.nitidus*	0.042316	0.067911

JW6	SC35	CS10	*R.nitidus*	-0.007124	0.070243
SC28	SC35	CS10	*R.nitidus*	-0.073381	0.062545
CS14	SC35	CS10	*R.nitidus*	-0.040956	0.065231
JO8	CS6	CS10	*R.nitidus*	0.080818	0.071365
MY6	SC35	CS10	*R.nitidus*	-0.05434	0.064031
GD30	CS6	CS10	*R.nitidus*	0.162602	0.066968
CS6	SC35	CS10	*R.nitidus*	-0.098408	0.061949
SC31	SC35	CS10	*R.nitidus*	-0.058737	0.063964
JW4	SC35	CS10	*R.nitidus*	0.212163	0.06449
GD32	CS6	CS10	*R.nitidus*	0.07172	0.069126
GD21	CS6	CS10	*R.nitidus*	0.171906	0.063698
JO3	SC35	CS10	*R.nitidus*	0.013396	0.067902
SC45	SC35	CS10	*R.nitidus*	0.002926	0.063357
MY4	SC35	CS10	*R.nitidus*	-0.09596	0.063862
GD25	CS6	CS10	*R.nitidus*	0.105941	0.065236
JO7	CS6	CS10	*R.nitidus*	0.069982	0.069206
GD29	CS6	CS10	*R.nitidus*	0.154864	0.063444
GD49	CS6	CS10	*R.nitidus*	0.121262	0.068458
GD26	CS6	CS10	*R.nitidus*	0.098563	0.065438
CS17	CS6	CS10	*R.nitidus*	0.036125	0.069623
GD45	CS6	CS10	*R.nitidus*	0.066322	0.070578
CS12	CS6	CS10	*R.nitidus*	0.052632	0.065765
CS20	CS6	CS10	*R.nitidus*	0.035611	0.061687
GD44	CS6	CS10	*R.nitidus*	0.069434	0.066784
CS19	CS6	CS10	*R.nitidus*	0.038596	0.066142
GD34	CS6	CS10	*R.nitidus*	0.051984	0.064746
CS18	CS6	CS10	*R.nitidus*	0.016964	0.064159
CS14	CS6	CS10	*R.nitidus*	0.061391	0.067369
CS23	CS6	CS10	*R.nitidus*	-0.002586	0.064084
GD42	CS6	CS10	*R.nitidus*	0.070175	0.064879
GD40	CS6	CS10	*R.nitidus*	0.123967	0.063515
GD35	CS6	CS10	*R.nitidus*	0.092711	0.065898
GD22	CS6	CS10	*R.nitidus*	0.11908	0.064531
CS13	CS6	CS10	*R.nitidus*	0.016629	0.068868
GD31	CS6	CS10	*R.nitidus*	0.103348	0.068355
GD33	CS6	CS10	*R.nitidus*	0.068587	0.064885
SC6	CS6	CS10	*R.nitidus*	0.052434	0.06507
GD48	CS6	CS10	*R.nitidus*	0.09368	0.066514
GD41	CS6	CS10	*R.nitidus*	0.06237	0.065128
CS21	CS6	CS10	*R.nitidus*	0.041404	0.063691
CS16	CS6	CS10	*R.nitidus*	0.056759	0.065149
GD46	CS6	CS10	*R.nitidus*	0.039528	0.065602
CS31	CS6	CS10	*R.nitidus*	0.015221	0.06745
SC31	CS6	CS10	*R.nitidus*	0.026638	0.067251
GD50	CS6	CS10	*R.nitidus*	0.074875	0.068358
CS22	CS6	CS10	*R.nitidus*	0.047534	0.063986
SC26	CS6	CS10	*R.nitidus*	0.025993	0.064424
CS15	CS6	CS10	*R.nitidus*	0.073401	0.067427
SC45	CS6	CS10	*R.nitidus*	0.094752	0.065955
CS4	CS6	CS10	*R.nitidus*	0.027654	0.062662
CS24	CS6	CS10	*R.nitidus*	0.007488	0.066181
SC18	CS6	CS10	*R.nitidus*	0.101852	0.063546
SC15	CS6	CS10	*R.nitidus*	0.050989	0.065827
SC12	CS6	CS10	*R.nitidus*	0.047548	0.065525
CS28	CS6	CS10	*R.nitidus*	0.006467	0.066971
CS30	CS6	CS10	*R.nitidus*	-0.012997	0.066571

SC5	CS6	CS10	*R.nitidus*	0.050287	0.067741
CS25	CS6	CS10	*R.nitidus*	0.042785	0.065828
SC28	CS6	CS10	*R.nitidus*	0.019751	0.062968
JO5	CS6	CS10	*R.nitidus*	0.102695	0.06436
JW6	CS6	CS10	*R.nitidus*	0.06036	0.070173
CS3	CS6	CS10	*R.nitidus*	0.04568	0.061594
GD21	SC42	CS10	*R.nitidus*	-0.143245	0.061579
GD22	SC42	CS10	*R.nitidus*	-0.169321	0.062099
JW4	CS6	CS10	*R.nitidus*	0.253868	0.064066
SC14	CS6	CS10	*R.nitidus*	0.062684	0.065098
SC10	CS6	CS10	*R.nitidus*	0.113433	0.065807
MY4	CS6	CS10	*R.nitidus*	0.005185	0.067377
CS11	SC42	CS10	*R.nitidus*	-0.228571	0.062542
JO7	SC42	CS10	*R.nitidus*	-0.1736	0.06991
JO3	CS6	CS10	*R.nitidus*	0.10859	0.068748
MY6	CS6	CS10	*R.nitidus*	0.044299	0.065764
SC42	CS6	CS10	*R.nitidus*	0.26189	0.06172
GD25	SC42	CS10	*R.nitidus*	-0.166181	0.062351
SC24	CS6	CS10	*R.nitidus*	0.075358	0.06432
SC44	CS6	CS10	*R.nitidus*	0.055597	0.063762
SC27	CS6	CS10	*R.nitidus*	0.060606	0.063741
GD34	SC42	CS10	*R.nitidus*	-0.230176	0.062139
SC34	CS6	CS10	*R.nitidus*	0.064964	0.063207
JO8	SC42	CS10	*R.nitidus*	-0.134217	0.070926
SC35	CS6	CS10	*R.nitidus*	0.098408	0.061949
CS13	SC42	CS10	*R.nitidus*	-0.259953	0.062885
GD30	SC42	CS10	*R.nitidus*	-0.120498	0.064347
GD31	SC42	CS10	*R.nitidus*	-0.162099	0.06377
GD29	SC42	CS10	*R.nitidus*	-0.107744	0.062002
GD32	SC42	CS10	*R.nitidus*	-0.206238	0.064314
GD45	SC42	CS10	*R.nitidus*	-0.19727	0.065894
SC32	CS6	CS10	*R.nitidus*	0.110704	0.063727
JW7	CS6	CS10	*R.nitidus*	0.094835	0.0676
JO1	CS6	CS10	*R.nitidus*	0.039187	0.067697
GD40	SC42	CS10	*R.nitidus*	-0.159757	0.061685
GD46	SC42	CS10	*R.nitidus*	-0.227545	0.062137
GD26	SC42	CS10	*R.nitidus*	-0.173913	0.062356
CS14	SC42	CS10	*R.nitidus*	-0.229003	0.06305
GD44	SC42	CS10	*R.nitidus*	-0.212031	0.06298
GD49	SC42	CS10	*R.nitidus*	-0.1655	0.065288
CS28	SC42	CS10	*R.nitidus*	-0.267214	0.062341
GD41	SC42	CS10	*R.nitidus*	-0.22351	0.061876
CS12	SC42	CS10	*R.nitidus*	-0.242259	0.063423
GD35	SC42	CS10	*R.nitidus*	-0.206061	0.063093
GD48	SC42	CS10	*R.nitidus*	-0.168395	0.064813
CS15	SC42	CS10	*R.nitidus*	-0.205171	0.061541
CS30	SC42	CS10	*R.nitidus*	-0.291154	0.060069
CS16	SC42	CS10	*R.nitidus*	-0.235227	0.060856
GD42	SC42	CS10	*R.nitidus*	-0.232386	0.061203
GD33	SC42	CS10	*R.nitidus*	-0.210954	0.063696
SC12	SC42	CS10	*R.nitidus*	-0.27082	0.059907
CS19	SC42	CS10	*R.nitidus*	-0.252137	0.062631
CS18	SC42	CS10	*R.nitidus*	-0.260794	0.061383
CS25	SC42	CS10	*R.nitidus*	-0.250739	0.063312
SC6	SC42	CS10	*R.nitidus*	-0.26801	0.061198
CS21	SC42	CS10	*R.nitidus*	-0.24971	0.060976

CS24	SC42	CS10	*R.nitidus*	-0.284355	0.061175
CS4	SC42	CS10	*R.nitidus*	-0.261765	0.060681
CS22	SC42	CS10	*R.nitidus*	-0.244656	0.061327
CS3	SC42	CS10	*R.nitidus*	-0.241194	0.059941
MY4	SC42	CS10	*R.nitidus*	-0.278138	0.063537
CS31	SC42	CS10	*R.nitidus*	-0.271267	0.061146
CS17	SC42	CS10	*R.nitidus*	-0.21781	0.063856
SC27	SC42	CS10	*R.nitidus*	-0.255971	0.0618
SC15	SC42	CS10	*R.nitidus*	-0.26319	0.062129
GD50	SC42	CS10	*R.nitidus*	-0.195155	0.065517
SC28	SC42	CS10	*R.nitidus*	-0.272125	0.06112
SC10	SC42	CS10	*R.nitidus*	-0.192105	0.061574
SC35	SC42	CS10	*R.nitidus*	-0.212575	0.062338
CS23	SC42	CS10	*R.nitidus*	-0.284897	0.060122
SC14	SC42	CS10	*R.nitidus*	-0.244094	0.062136
JW4	SC42	CS10	*R.nitidus*	0.014189	0.06418
SC18	SC42	CS10	*R.nitidus*	-0.208993	0.062298
SC44	SC42	CS10	*R.nitidus*	-0.246951	0.060935
SC45	SC42	CS10	*R.nitidus*	-0.215034	0.062692
CS20	SC42	CS10	*R.nitidus*	-0.245644	0.062618
CS6	SC42	CS10	*R.nitidus*	-0.26189	0.06172
SC24	SC42	CS10	*R.nitidus*	-0.244005	0.061794
SC26	SC42	CS10	*R.nitidus*	-0.259861	0.060206
SC32	SC42	CS10	*R.nitidus*	-0.231001	0.061981
SC5	SC42	CS10	*R.nitidus*	-0.252012	0.065079
SC34	SC42	CS10	*R.nitidus*	-0.199637	0.059773
MY6	SC42	CS10	*R.nitidus*	-0.255168	0.062645
SC31	SC42	CS10	*R.nitidus*	-0.245261	0.062323
GD26	SC44	CS10	*R.nitidus*	0.052239	0.064749
CS11	SC44	CS10	*R.nitidus*	-0.00616	0.066135
JO3	SC42	CS10	*R.nitidus*	-0.150035	0.066408
JW6	SC42	CS10	*R.nitidus*	-0.159341	0.068602
GD32	SC44	CS10	*R.nitidus*	-0.010552	0.06775
JO8	SC44	CS10	*R.nitidus*	0.020468	0.071099
JO7	SC44	CS10	*R.nitidus*	0.012218	0.07377
JO1	SC42	CS10	*R.nitidus*	-0.254321	0.061858
GD34	SC44	CS10	*R.nitidus*	0.001372	0.063753
GD31	SC44	CS10	*R.nitidus*	0.060172	0.065343
JW7	SC42	CS10	*R.nitidus*	-0.172775	0.066691
GD29	SC44	CS10	*R.nitidus*	0.11541	0.065096
GD30	SC44	CS10	*R.nitidus*	0.105691	0.065214
GD25	SC44	CS10	*R.nitidus*	0.064677	0.063802
JO5	SC42	CS10	*R.nitidus*	-0.198534	0.063484
CS14	SC44	CS10	*R.nitidus*	-0.002082	0.067499
GD33	SC44	CS10	*R.nitidus*	0.009402	0.065256
CS18	SC44	CS10	*R.nitidus*	-0.034386	0.06459
GD41	SC44	CS10	*R.nitidus*	0.008433	0.063824
GD21	SC44	CS10	*R.nitidus*	0.111825	0.064593
CS25	SC44	CS10	*R.nitidus*	-0.00504	0.067164
CS12	SC44	CS10	*R.nitidus*	-0.016094	0.065915
CS13	SC44	CS10	*R.nitidus*	-0.028674	0.068515
GD22	SC44	CS10	*R.nitidus*	0.072559	0.062944
GD40	SC44	CS10	*R.nitidus*	0.07438	0.062598
CS22	SC44	CS10	*R.nitidus*	-0.004252	0.06592
CS21	SC44	CS10	*R.nitidus*	-0.031315	0.064135
GD48	SC44	CS10	*R.nitidus*	0.03386	0.06575

CS19	SC44	CS10	*R.nitidus*	-0.020863	0.067005
GD44	SC44	CS10	*R.nitidus*	0.014609	0.066389
CS15	SC44	CS10	*R.nitidus*	0.017409	0.067346
GD49	SC44	CS10	*R.nitidus*	0.0744	0.067232
CS3	SC44	CS10	*R.nitidus*	-0.016727	0.065197
CS20	SC44	CS10	*R.nitidus*	-0.032436	0.065794
CS28	SC44	CS10	*R.nitidus*	-0.046317	0.065934
GD35	SC44	CS10	*R.nitidus*	0.038678	0.065588
GD45	SC44	CS10	*R.nitidus*	0.034208	0.066397
GD50	SC44	CS10	*R.nitidus*	0.038851	0.068977
CS17	SC44	CS10	*R.nitidus*	0.010582	0.06871
CS30	SC44	CS10	*R.nitidus*	-0.067202	0.062714
CS6	SC44	CS10	*R.nitidus*	-0.055597	0.063762
GD42	SC44	CS10	*R.nitidus*	0.015217	0.065503
GD46	SC44	CS10	*R.nitidus*	-0.00827	0.065225
CS16	SC44	CS10	*R.nitidus*	0	0.064918
SC32	SC44	CS10	*R.nitidus*	0.047404	0.062782
SC10	SC44	CS10	*R.nitidus*	0.075643	0.063828
CS31	SC44	CS10	*R.nitidus*	-0.03639	0.06629
SC18	SC44	CS10	*R.nitidus*	0.053557	0.062806
SC15	SC44	CS10	*R.nitidus*	-0.010679	0.065555
SC5	SC44	CS10	*R.nitidus*	-0.012669	0.068224
CS4	SC44	CS10	*R.nitidus*	-0.015862	0.062022
CS24	SC44	CS10	*R.nitidus*	-0.051508	0.065369
CS23	SC44	CS10	*R.nitidus*	-0.047091	0.065038
MY6	SC44	CS10	*R.nitidus*	-0.018625	0.0633
SC34	SC44	CS10	*R.nitidus*	0.033479	0.062349
SC27	SC44	CS10	*R.nitidus*	0.004539	0.063588
SC24	SC44	CS10	*R.nitidus*	0.009901	0.064266
SC42	SC44	CS10	*R.nitidus*	0.246951	0.060935
MY4	SC44	CS10	*R.nitidus*	-0.050218	0.064148
JW4	SC44	CS10	*R.nitidus*	0.219697	0.064668
SC26	SC44	CS10	*R.nitidus*	-0.029126	0.065033
SC6	SC44	CS10	*R.nitidus*	-0.009687	0.065767
SC12	SC44	CS10	*R.nitidus*	-0.01664	0.063526
SC45	SC44	CS10	*R.nitidus*	0.035451	0.064242
SC14	SC44	CS10	*R.nitidus*	0.010638	0.065064
SC35	SC44	CS10	*R.nitidus*	0.050549	0.061597
SC31	SC44	CS10	*R.nitidus*	-0.020483	0.065243
GD25	SC45	CS10	*R.nitidus*	0.027273	0.065293
JO7	SC45	CS10	*R.nitidus*	-0.045155	0.071317
JO5	SC44	CS10	*R.nitidus*	0.059676	0.063184
JW6	SC44	CS10	*R.nitidus*	0.022079	0.072478
JW7	SC44	CS10	*R.nitidus*	0.042934	0.06867
GD22	SC45	CS10	*R.nitidus*	0.017341	0.064081
SC28	SC44	CS10	*R.nitidus*	-0.046859	0.058325
GD21	SC45	CS10	*R.nitidus*	0.067445	0.062241
CS13	SC45	CS10	*R.nitidus*	-0.066235	0.069923
CS11	SC45	CS10	*R.nitidus*	-0.040718	0.067851
GD29	SC45	CS10	*R.nitidus*	0.083907	0.064425
JO1	SC44	CS10	*R.nitidus*	-0.014717	0.065586
JO8	SC45	CS10	*R.nitidus*	-0.020774	0.071976
JO3	SC44	CS10	*R.nitidus*	0.031771	0.068263
GD35	SC45	CS10	*R.nitidus*	-0.013316	0.064529
GD31	SC45	CS10	*R.nitidus*	0.034955	0.065387
GD49	SC45	CS10	*R.nitidus*	0.048145	0.068178

GD41	SC45	CS10	*R.nitidus*	-0.028667	0.064531
GD32	SC45	CS10	*R.nitidus*	-0.031746	0.06647
CS15	SC45	CS10	*R.nitidus*	-0.018219	0.067046
GD48	SC45	CS10	*R.nitidus*	0.002131	0.065645
GD30	SC45	CS10	*R.nitidus*	0.087853	0.066302
CS12	SC45	CS10	*R.nitidus*	-0.06355	0.067352
GD50	SC45	CS10	*R.nitidus*	-0.004819	0.06819
CS17	SC45	CS10	*R.nitidus*	-0.022661	0.068114
GD26	SC45	CS10	*R.nitidus*	0.012024	0.06454
GD40	SC45	CS10	*R.nitidus*	0.028086	0.061542
GD45	SC45	CS10	*R.nitidus*	-0.001584	0.068151
CS19	SC45	CS10	*R.nitidus*	-0.075905	0.067358
GD33	SC45	CS10	*R.nitidus*	-0.042735	0.064975
GD46	SC45	CS10	*R.nitidus*	-0.048716	0.064243
GD42	SC45	CS10	*R.nitidus*	-0.029034	0.064213
GD34	SC45	CS10	*R.nitidus*	-0.037013	0.063222
CS21	SC45	CS10	*R.nitidus*	-0.071031	0.06497
GD44	SC45	CS10	*R.nitidus*	-0.025641	0.064905
SC24	SC45	CS10	*R.nitidus*	-0.043769	0.063381
CS28	SC45	CS10	*R.nitidus*	-0.074101	0.066046
CS14	SC45	CS10	*R.nitidus*	-0.042553	0.069681
CS3	SC45	CS10	*R.nitidus*	-0.063202	0.065787
CS22	SC45	CS10	*R.nitidus*	-0.044317	0.064933
SC28	SC45	CS10	*R.nitidus*	-0.062362	0.062608
CS20	SC45	CS10	*R.nitidus*	-0.07747	0.065333
CS30	SC45	CS10	*R.nitidus*	-0.104423	0.064999
CS23	SC45	CS10	*R.nitidus*	-0.098886	0.064218
SC34	SC45	CS10	*R.nitidus*	-0.017206	0.065286
SC10	SC45	CS10	*R.nitidus*	0.032508	0.065173
CS4	SC45	CS10	*R.nitidus*	-0.055102	0.064769
SC18	SC45	CS10	*R.nitidus*	0.00541	0.065825
SC26	SC45	CS10	*R.nitidus*	-0.083639	0.064295
CS18	SC45	CS10	*R.nitidus*	-0.084615	0.064018
CS31	SC45	CS10	*R.nitidus*	-0.070721	0.065061
SC27	SC45	CS10	*R.nitidus*	-0.034261	0.064735
SC14	SC45	CS10	*R.nitidus*	-0.035915	0.066673
SC12	SC45	CS10	*R.nitidus*	-0.064024	0.064373
MY6	SC45	CS10	*R.nitidus*	-0.055949	0.066262
SC35	SC45	CS10	*R.nitidus*	-0.002926	0.063357
CS25	SC45	CS10	*R.nitidus*	-0.070413	0.064704
CS24	SC45	CS10	*R.nitidus*	-0.081818	0.065662
JW4	SC45	CS10	*R.nitidus*	0.204369	0.063411
JW7	SC45	CS10	*R.nitidus*	0.003425	0.068818
SC31	SC45	CS10	*R.nitidus*	-0.060606	0.064139
SC5	SC45	CS10	*R.nitidus*	-0.053441	0.06728
SC6	SC45	CS10	*R.nitidus*	-0.040693	0.065875
SC32	SC45	CS10	*R.nitidus*	0.009545	0.064961
CS16	SC45	CS10	*R.nitidus*	-0.037396	0.06579
MY4	SC45	CS10	*R.nitidus*	-0.086219	0.065931
CS6	SC45	CS10	*R.nitidus*	-0.094752	0.065955
SC44	SC45	CS10	*R.nitidus*	-0.035451	0.064242
JO1	SC45	CS10	*R.nitidus*	-0.048708	0.066294
JO3	SC45	CS10	*R.nitidus*	0.005439	0.068865
SC15	SC45	CS10	*R.nitidus*	-0.056012	0.066057
SC42	SC45	CS10	*R.nitidus*	0.215034	0.062692
GD21	MY4	CS10	*R.nitidus*	0.172526	0.06327

GD29	MY4	CS10	*R.nitidus*	0.159669	0.064077
GD35	MY4	CS10	*R.nitidus*	0.088633	0.066305
JW6	SC45	CS10	*R.nitidus*	-0.030384	0.072069
CS11	MY4	CS10	*R.nitidus*	0.034317	0.067848
JO8	MY4	CS10	*R.nitidus*	0.075259	0.072375
JO7	MY4	CS10	*R.nitidus*	0.047081	0.074441
GD41	MY4	CS10	*R.nitidus*	0.050414	0.063649
JO5	SC45	CS10	*R.nitidus*	0.000729	0.065014
GD25	MY4	CS10	*R.nitidus*	0.105802	0.065002
CS12	MY4	CS10	*R.nitidus*	0.047238	0.069151
GD32	MY4	CS10	*R.nitidus*	0.062156	0.070985
GD30	MY4	CS10	*R.nitidus*	0.162569	0.067215
GD22	MY4	CS10	*R.nitidus*	0.109244	0.065028
CS13	MY4	CS10	*R.nitidus*	0.009503	0.068629
CS18	MY4	CS10	*R.nitidus*	0.003608	0.066969
CS24	MY4	CS10	*R.nitidus*	0.000724	0.067675
GD50	MY4	CS10	*R.nitidus*	0.082998	0.069546
GD46	MY4	CS10	*R.nitidus*	0.031528	0.065792
GD48	MY4	CS10	*R.nitidus*	0.074236	0.067413
GD40	MY4	CS10	*R.nitidus*	0.118922	0.063198
SC5	MY4	CS10	*R.nitidus*	0.03396	0.069049
GD26	MY4	CS10	*R.nitidus*	0.08502	0.0653
GD31	MY4	CS10	*R.nitidus*	0.099067	0.067058
GD49	MY4	CS10	*R.nitidus*	0.109642	0.069857
GD33	MY4	CS10	*R.nitidus*	0.049399	0.064863
CS15	MY4	CS10	*R.nitidus*	0.064472	0.067352
GD45	MY4	CS10	*R.nitidus*	0.07903	0.067887
CS20	MY4	CS10	*R.nitidus*	0.01238	0.066597
GD34	MY4	CS10	*R.nitidus*	0.046605	0.063668
GD44	MY4	CS10	*R.nitidus*	0.06392	0.067751
CS19	MY4	CS10	*R.nitidus*	0.023704	0.07022
CS25	MY4	CS10	*R.nitidus*	0.021583	0.067445
CS17	MY4	CS10	*R.nitidus*	0.049814	0.069257
CS16	MY4	CS10	*R.nitidus*	0.04502	0.066822
CS23	MY4	CS10	*R.nitidus*	-0.013552	0.065936
SC15	MY4	CS10	*R.nitidus*	0.040531	0.067128
GD42	MY4	CS10	*R.nitidus*	0.055479	0.065704
CS3	MY4	CS10	*R.nitidus*	0.017806	0.065744
CS4	MY4	CS10	*R.nitidus*	0.025234	0.06642
CS14	MY4	CS10	*R.nitidus*	0.041696	0.069358
SC24	MY4	CS10	*R.nitidus*	0.057778	0.064935
SC6	MY4	CS10	*R.nitidus*	0.03655	0.065781
CS31	MY4	CS10	*R.nitidus*	0.016801	0.067432
CS21	MY4	CS10	*R.nitidus*	0.017908	0.067226
SC31	MY4	CS10	*R.nitidus*	0.022695	0.065941
SC32	MY4	CS10	*R.nitidus*	0.095729	0.065222
CS30	MY4	CS10	*R.nitidus*	-0.017806	0.065993
CS28	MY4	CS10	*R.nitidus*	-0.003058	0.0676
CS22	MY4	CS10	*R.nitidus*	0.038942	0.067498
SC27	MY4	CS10	*R.nitidus*	0.051028	0.065254
SC14	MY4	CS10	*R.nitidus*	0.059201	0.066349
SC10	MY4	CS10	*R.nitidus*	0.130301	0.067264
CS11	MY6	CS10	*R.nitidus*	0.004707	0.067537
SC12	MY4	CS10	*R.nitidus*	0.029689	0.065564
JW7	MY4	CS10	*R.nitidus*	0.094618	0.069801
SC28	MY4	CS10	*R.nitidus*	0.016973	0.063403

JO3	MY4	CS10	*R.nitidus*	0.088618	0.069531
SC45	MY4	CS10	*R.nitidus*	0.086219	0.065931
SC35	MY4	CS10	*R.nitidus*	0.09596	0.063862
JO8	MY6	CS10	*R.nitidus*	0.031098	0.07303
JW6	MY4	CS10	*R.nitidus*	0.078067	0.07141
JO1	MY4	CS10	*R.nitidus*	0.038295	0.065955
SC26	MY4	CS10	*R.nitidus*	0.00988	0.064468
JO5	MY4	CS10	*R.nitidus*	0.094059	0.065772
SC44	MY4	CS10	*R.nitidus*	0.050218	0.064148
SC18	MY4	CS10	*R.nitidus*	0.097826	0.063884
JO7	MY6	CS10	*R.nitidus*	0.010397	0.072448
SC34	MY4	CS10	*R.nitidus*	0.076923	0.064422
MY6	MY4	CS10	*R.nitidus*	0.046759	0.057735
CS6	MY4	CS10	*R.nitidus*	-0.005185	0.067377
GD34	MY6	CS10	*R.nitidus*	0.020156	0.063223
CS12	MY6	CS10	*R.nitidus*	0.012023	0.067695
JW4	MY4	CS10	*R.nitidus*	0.273118	0.065632
GD21	MY6	CS10	*R.nitidus*	0.134271	0.064059
SC42	MY4	CS10	*R.nitidus*	0.278138	0.063537
GD35	MY6	CS10	*R.nitidus*	0.054878	0.065497
GD41	MY6	CS10	*R.nitidus*	0.017207	0.062766
GD42	MY6	CS10	*R.nitidus*	0.023066	0.064073
GD29	MY6	CS10	*R.nitidus*	0.127646	0.063119
GD32	MY6	CS10	*R.nitidus*	0.033988	0.069288
GD46	MY6	CS10	*R.nitidus*	-0.002681	0.065224
CS15	MY6	CS10	*R.nitidus*	0.028455	0.06708
GD30	MY6	CS10	*R.nitidus*	0.133122	0.066087
GD22	MY6	CS10	*R.nitidus*	0.077947	0.0645
GD26	MY6	CS10	*R.nitidus*	0.061883	0.065676
GD44	MY6	CS10	*R.nitidus*	0.030007	0.066926
GD45	MY6	CS10	*R.nitidus*	0.047994	0.068252
CS19	MY6	CS10	*R.nitidus*	-0.016243	0.06846
GD25	MY6	CS10	*R.nitidus*	0.072364	0.064428
GD40	MY6	CS10	*R.nitidus*	0.08183	0.062983
GD31	MY6	CS10	*R.nitidus*	0.071127	0.065551
CS13	MY6	CS10	*R.nitidus*	-0.031361	0.06776
GD33	MY6	CS10	*R.nitidus*	0.020039	0.06467
CS30	MY6	CS10	*R.nitidus*	-0.054379	0.06539
GD48	MY6	CS10	*R.nitidus*	0.039501	0.066577
CS22	MY6	CS10	*R.nitidus*	0.002892	0.064829
CS28	MY6	CS10	*R.nitidus*	-0.032641	0.066712
GD49	MY6	CS10	*R.nitidus*	0.103528	0.068707
CS17	MY6	CS10	*R.nitidus*	0.014577	0.068161
SC12	MY6	CS10	*R.nitidus*	-0.005054	0.065784
SC5	MY6	CS10	*R.nitidus*	0	0.068596
CS25	MY6	CS10	*R.nitidus*	-0.013428	0.067348
CS24	MY6	CS10	*R.nitidus*	-0.032328	0.065612
SC6	MY6	CS10	*R.nitidus*	0.000726	0.065367
GD50	MY6	CS10	*R.nitidus*	0.040221	0.068167
CS18	MY6	CS10	*R.nitidus*	-0.026761	0.065571
CS14	MY6	CS10	*R.nitidus*	0.014493	0.068612
CS31	MY6	CS10	*R.nitidus*	-0.015086	0.068375
SC14	MY6	CS10	*R.nitidus*	0.019959	0.065346
CS4	MY6	CS10	*R.nitidus*	-0.00977	0.065491
SC18	MY6	CS10	*R.nitidus*	0.060213	0.063317
SC10	MY6	CS10	*R.nitidus*	0.096313	0.066548

CS16	MY6	CS10	*R.nitidus*	0.015775	0.065048
CS21	MY6	CS10	*R.nitidus*	-0.018698	0.065435
SC34	MY6	CS10	*R.nitidus*	0.033099	0.063716
SC28	MY6	CS10	*R.nitidus*	-0.01198	0.061982
SC24	MY6	CS10	*R.nitidus*	0.017725	0.064539
SC44	MY6	CS10	*R.nitidus*	0.018625	0.0633
SC15	MY6	CS10	*R.nitidus*	0.006608	0.06537
SC26	MY6	CS10	*R.nitidus*	-0.017147	0.064754
CS23	MY6	CS10	*R.nitidus*	-0.044944	0.063717
CS3	MY6	CS10	*R.nitidus*	-0.008457	0.065008
SC35	MY6	CS10	*R.nitidus*	0.05434	0.064031
SC27	MY6	CS10	*R.nitidus*	0.013112	0.063943
GD30	JW4	CS10	*R.nitidus*	-0.114618	0.069554
GD29	JW4	CS10	*R.nitidus*	-0.10903	0.066418
MY4	MY6	CS10	*R.nitidus*	-0.046759	0.057735
GD25	JW4	CS10	*R.nitidus*	-0.165483	0.064648
GD21	JW4	CS10	*R.nitidus*	-0.109554	0.066857
SC45	MY6	CS10	*R.nitidus*	0.055949	0.066262
GD34	JW4	CS10	*R.nitidus*	-0.218443	0.064479
GD22	JW4	CS10	*R.nitidus*	-0.163121	0.064466
SC31	MY6	CS10	*R.nitidus*	-0.015449	0.064601
JO5	MY6	CS10	*R.nitidus*	0.061239	0.065292
CS6	MY6	CS10	*R.nitidus*	-0.044299	0.065764
SC32	MY6	CS10	*R.nitidus*	0.054533	0.063371
JO8	JW4	CS10	*R.nitidus*	-0.183477	0.069267
GD41	JW4	CS10	*R.nitidus*	-0.216892	0.062819
GD31	JW4	CS10	*R.nitidus*	-0.152443	0.067089
JW4	MY6	CS10	*R.nitidus*	0.241947	0.064462
GD26	JW4	CS10	*R.nitidus*	-0.185933	0.067106
JW6	MY6	CS10	*R.nitidus*	0.042254	0.070325
GD40	JW4	CS10	*R.nitidus*	-0.151102	0.06489
JO1	MY6	CS10	*R.nitidus*	-0.002108	0.064897
JO7	JW4	CS10	*R.nitidus*	-0.205993	0.069627
CS12	JW4	CS10	*R.nitidus*	-0.213256	0.06614
GD35	JW4	CS10	*R.nitidus*	-0.201503	0.063911
JW7	MY6	CS10	*R.nitidus*	0.054878	0.068598
JO3	MY6	CS10	*R.nitidus*	0.053061	0.068554
GD44	JW4	CS10	*R.nitidus*	-0.179846	0.067101
SC42	MY6	CS10	*R.nitidus*	0.255168	0.062645
CS11	JW4	CS10	*R.nitidus*	-0.21328	0.065411
GD32	JW4	CS10	*R.nitidus*	-0.216718	0.067485
GD42	JW4	CS10	*R.nitidus*	-0.200947	0.063637
GD33	JW4	CS10	*R.nitidus*	-0.220946	0.066532
CS18	JW4	CS10	*R.nitidus*	-0.2582	0.065032
CS28	JW4	CS10	*R.nitidus*	-0.257862	0.067941
GD50	JW4	CS10	*R.nitidus*	-0.149837	0.068434
GD46	JW4	CS10	*R.nitidus*	-0.239351	0.065489
GD49	JW4	CS10	*R.nitidus*	-0.159073	0.06931
GD45	JW4	CS10	*R.nitidus*	-0.164251	0.069018
CS20	JW4	CS10	*R.nitidus*	-0.23816	0.064474
GD48	JW4	CS10	*R.nitidus*	-0.177609	0.06624
CS13	JW4	CS10	*R.nitidus*	-0.253156	0.065941
CS4	JW4	CS10	*R.nitidus*	-0.243132	0.063395
CS16	JW4	CS10	*R.nitidus*	-0.224828	0.06504
CS14	JW4	CS10	*R.nitidus*	-0.21616	0.067882
CS23	JW4	CS10	*R.nitidus*	-0.266124	0.063835

CS3	JW4	CS10	*R.nitidus*	-0.234807	0.06464
CS17	JW4	CS10	*R.nitidus*	-0.208151	0.067534
CS31	JW4	CS10	*R.nitidus*	-0.269755	0.063214
CS22	JW4	CS10	*R.nitidus*	-0.244569	0.066284
CS15	JW4	CS10	*R.nitidus*	-0.184053	0.06638
CS19	JW4	CS10	*R.nitidus*	-0.246295	0.066758
CS21	JW4	CS10	*R.nitidus*	-0.246228	0.065463
SC5	JW4	CS10	*R.nitidus*	-0.213986	0.068659
SC44	JW4	CS10	*R.nitidus*	-0.219697	0.064668
SC10	JW4	CS10	*R.nitidus*	-0.176385	0.066814
SC31	JW4	CS10	*R.nitidus*	-0.248804	0.065346
SC12	JW4	CS10	*R.nitidus*	-0.250174	0.062859
CS25	JW4	CS10	*R.nitidus*	-0.232082	0.066282
CS30	JW4	CS10	*R.nitidus*	-0.270692	0.064681
SC32	JW4	CS10	*R.nitidus*	-0.192039	0.065165
SC15	JW4	CS10	*R.nitidus*	-0.237099	0.064899
CS6	JW4	CS10	*R.nitidus*	-0.253868	0.064066
GD26	JW6	CS10	*R.nitidus*	0.059701	0.071234
CS24	JW4	CS10	*R.nitidus*	-0.280308	0.063425
SC35	JW4	CS10	*R.nitidus*	-0.212163	0.06449
SC6	JW4	CS10	*R.nitidus*	-0.217885	0.064784
SC27	JW4	CS10	*R.nitidus*	-0.208638	0.063293
SC28	JW4	CS10	*R.nitidus*	-0.247956	0.064194
GD30	JW6	CS10	*R.nitidus*	0.129202	0.072783
JW6	JW4	CS10	*R.nitidus*	-0.183527	0.068536
SC18	JW4	CS10	*R.nitidus*	-0.201595	0.064935
SC42	JW4	CS10	*R.nitidus*	-0.014189	0.06418
CS12	JW6	CS10	*R.nitidus*	-0.00188	0.07275
SC14	JW4	CS10	*R.nitidus*	-0.213904	0.063628
SC45	JW4	CS10	*R.nitidus*	-0.204369	0.063411
GD32	JW6	CS10	*R.nitidus*	-0.01277	0.07289
MY4	JW4	CS10	*R.nitidus*	-0.273118	0.065632
SC24	JW4	CS10	*R.nitidus*	-0.220028	0.065139
JO3	JW4	CS10	*R.nitidus*	-0.157765	0.067867
SC26	JW4	CS10	*R.nitidus*	-0.251701	0.065452
GD31	JW6	CS10	*R.nitidus*	0.06345	0.071392
CS11	JW6	CS10	*R.nitidus*	-0.011566	0.073303
SC34	JW4	CS10	*R.nitidus*	-0.213277	0.062937
JO1	JW4	CS10	*R.nitidus*	-0.216216	0.06518
GD22	JW6	CS10	*R.nitidus*	0.053719	0.070141
JO5	JW4	CS10	*R.nitidus*	-0.167913	0.065102
GD25	JW6	CS10	*R.nitidus*	0.043001	0.070995
GD29	JW6	CS10	*R.nitidus*	0.12628	0.068021
MY6	JW4	CS10	*R.nitidus*	-0.241947	0.064462
JW7	JW4	CS10	*R.nitidus*	-0.141994	0.066971
JO7	JW6	CS10	*R.nitidus*	0.027907	0.067965
JO8	JW6	CS10	*R.nitidus*	0.005384	0.070951
GD21	JW6	CS10	*R.nitidus*	0.106732	0.068836
GD40	JW6	CS10	*R.nitidus*	0.056131	0.069914
GD33	JW6	CS10	*R.nitidus*	-0.017683	0.071738
GD49	JW6	CS10	*R.nitidus*	0.075078	0.072155
CS16	JW6	CS10	*R.nitidus*	-0.001818	0.072928
CS20	JW6	CS10	*R.nitidus*	-0.007752	0.071688
GD46	JW6	CS10	*R.nitidus*	-0.038043	0.070983
GD41	JW6	CS10	*R.nitidus*	0.000917	0.069865
CS14	JW6	CS10	*R.nitidus*	0.00455	0.075371

GD42	JW6	CS10	*R.nitidus*	0.019774	0.070366
CS23	JW6	CS10	*R.nitidus*	-0.038793	0.070619
CS21	JW6	CS10	*R.nitidus*	-0.023111	0.070605
GD35	JW6	CS10	*R.nitidus*	0.033929	0.072293
SC6	JW6	CS10	*R.nitidus*	0.013405	0.073453
GD50	JW6	CS10	*R.nitidus*	0.05274	0.074563
CS31	JW6	CS10	*R.nitidus*	-0.064366	0.07191
GD34	JW6	CS10	*R.nitidus*	-0.022165	0.068351
GD48	JW6	CS10	*R.nitidus*	0.021336	0.07112
CS18	JW6	CS10	*R.nitidus*	-0.033363	0.071818
SC32	JW6	CS10	*R.nitidus*	0.036517	0.072247
GD45	JW6	CS10	*R.nitidus*	0.093652	0.074987
SC28	JW6	CS10	*R.nitidus*	-0.02468	0.072523
SC12	JW6	CS10	*R.nitidus*	-0.04562	0.07047
CS22	JW6	CS10	*R.nitidus*	-0.037906	0.072124
SC5	JW6	CS10	*R.nitidus*	-0.009928	0.076524
CS19	JW6	CS10	*R.nitidus*	-0.055295	0.073677
GD44	JW6	CS10	*R.nitidus*	0.013369	0.071138
CS25	JW6	CS10	*R.nitidus*	-0.041483	0.072341
CS13	JW6	CS10	*R.nitidus*	-0.030928	0.075084
CS15	JW6	CS10	*R.nitidus*	0.029517	0.070908
CS30	JW6	CS10	*R.nitidus*	-0.069877	0.070326
SC27	JW6	CS10	*R.nitidus*	-0.021043	0.070539
SC10	JW6	CS10	*R.nitidus*	0.082791	0.074307
CS17	JW6	CS10	*R.nitidus*	0.00206	0.075703
SC31	JW6	CS10	*R.nitidus*	-0.046263	0.072562
CS24	JW6	CS10	*R.nitidus*	-0.06968	0.07273
SC18	JW6	CS10	*R.nitidus*	-0.004739	0.069833
SC26	JW6	CS10	*R.nitidus*	-0.039341	0.071207
CS28	JW6	CS10	*R.nitidus*	-0.061165	0.071982
CS6	JW6	CS10	*R.nitidus*	-0.06036	0.070173
SC45	JW6	CS10	*R.nitidus*	0.030384	0.072069
CS3	JW6	CS10	*R.nitidus*	0.00625	0.070454
SC44	JW6	CS10	*R.nitidus*	-0.022079	0.072478
CS4	JW6	CS10	*R.nitidus*	-0.017241	0.070112
SC15	JW6	CS10	*R.nitidus*	-0.038573	0.073208
SC14	JW6	CS10	*R.nitidus*	-0.013514	0.069781
SC24	JW6	CS10	*R.nitidus*	0.000939	0.071821
SC34	JW6	CS10	*R.nitidus*	-0.003571	0.069199
JO7	JW7	CS10	*R.nitidus*	-0.054435	0.072896
GD45	JW7	CS10	*R.nitidus*	0.038863	0.071175
SC35	JW6	CS10	*R.nitidus*	0.007124	0.070243
MY6	JW6	CS10	*R.nitidus*	-0.042254	0.070325
GD29	JW7	CS10	*R.nitidus*	0.087343	0.066512
JO3	JW6	CS10	*R.nitidus*	0.020496	0.073172
JO1	JW6	CS10	*R.nitidus*	-0.020702	0.070828
MY4	JW6	CS10	*R.nitidus*	-0.078067	0.07141
GD50	JW7	CS10	*R.nitidus*	0.022265	0.073113
JW4	JW6	CS10	*R.nitidus*	0.183527	0.068536
JO5	JW6	CS10	*R.nitidus*	0.045699	0.069876
SC42	JW6	CS10	*R.nitidus*	0.159341	0.068602
CS14	JW7	CS10	*R.nitidus*	-0.033727	0.070596
JW7	JW6	CS10	*R.nitidus*	0.02821	0.073365
GD44	JW7	CS10	*R.nitidus*	-0.014178	0.068851
GD30	JW7	CS10	*R.nitidus*	0.092803	0.070951
CS12	JW7	CS10	*R.nitidus*	-0.034884	0.071018

GD41	JW7	CS10	*R.nitidus*	-0.036585	0.069154
GD49	JW7	CS10	*R.nitidus*	0.014019	0.072765
GD46	JW7	CS10	*R.nitidus*	-0.050889	0.066639
GD32	JW7	CS10	*R.nitidus*	-0.038188	0.069515
GD26	JW7	CS10	*R.nitidus*	0.009509	0.069711
GD40	JW7	CS10	*R.nitidus*	0.03718	0.067222
JO8	JW7	CS10	*R.nitidus*	-0.030457	0.073758
CS11	JW7	CS10	*R.nitidus*	-0.042553	0.070399
GD31	JW7	CS10	*R.nitidus*	0.050202	0.069221
CS13	JW7	CS10	*R.nitidus*	-0.077513	0.070968
GD22	JW7	CS10	*R.nitidus*	0.032811	0.067364
GD25	JW7	CS10	*R.nitidus*	0.027273	0.068333
GD21	JW7	CS10	*R.nitidus*	0.089666	0.067143
GD42	JW7	CS10	*R.nitidus*	-0.003284	0.068227
CS16	JW7	CS10	*R.nitidus*	-0.037069	0.070216
CS17	JW7	CS10	*R.nitidus*	-0.023853	0.071334
GD48	JW7	CS10	*R.nitidus*	-0.006606	0.068491
GD33	JW7	CS10	*R.nitidus*	-0.035938	0.069444
GD34	JW7	CS10	*R.nitidus*	-0.053417	0.069035
GD35	JW7	CS10	*R.nitidus*	-0.006405	0.069792
CS22	JW7	CS10	*R.nitidus*	-0.053587	0.068909
SC24	JW7	CS10	*R.nitidus*	-0.052536	0.067983
SC6	JW7	CS10	*R.nitidus*	-0.031818	0.06978
CS21	JW7	CS10	*R.nitidus*	-0.06486	0.067717
CS19	JW7	CS10	*R.nitidus*	-0.057741	0.069349
CS20	JW7	CS10	*R.nitidus*	-0.047193	0.068319
CS15	JW7	CS10	*R.nitidus*	0	0.067632
SC27	JW7	CS10	*R.nitidus*	-0.047393	0.067634
CS18	JW7	CS10	*R.nitidus*	-0.062657	0.068652
SC14	JW7	CS10	*R.nitidus*	-0.043437	0.067533
CS30	JW7	CS10	*R.nitidus*	-0.095674	0.066292
SC45	JW7	CS10	*R.nitidus*	-0.003425	0.068818
CS25	JW7	CS10	*R.nitidus*	-0.052941	0.070537
CS3	JW7	CS10	*R.nitidus*	-0.058966	0.067109
JO8	JO1	CS10	*R.nitidus*	0.024691	0.070046
SC34	JW7	CS10	*R.nitidus*	-0.008651	0.067717
CS24	JW7	CS10	*R.nitidus*	-0.080142	0.066081
SC5	JW7	CS10	*R.nitidus*	-0.050091	0.076631
MY6	JW7	CS10	*R.nitidus*	-0.054878	0.068598
SC26	JW7	CS10	*R.nitidus*	-0.094086	0.06718
JO3	JW7	CS10	*R.nitidus*	-0.016034	0.069494
CS28	JW7	CS10	*R.nitidus*	-0.083871	0.069804
SC35	JW7	CS10	*R.nitidus*	-0.020481	0.064994
SC31	JW7	CS10	*R.nitidus*	-0.093044	0.066044
CS4	JW7	CS10	*R.nitidus*	-0.045714	0.064519
CS31	JW7	CS10	*R.nitidus*	-0.091316	0.068801
SC12	JW7	CS10	*R.nitidus*	-0.077132	0.067163
CS23	JW7	CS10	*R.nitidus*	-0.08313	0.066187
JO1	JW7	CS10	*R.nitidus*	-0.041927	0.067522
SC10	JW7	CS10	*R.nitidus*	0.007605	0.068932
SC28	JW7	CS10	*R.nitidus*	-0.074749	0.068173
JW4	JW7	CS10	*R.nitidus*	0.141994	0.066971
CS6	JW7	CS10	*R.nitidus*	-0.094835	0.0676
SC18	JW7	CS10	*R.nitidus*	-0.037418	0.067559
SC42	JW7	CS10	*R.nitidus*	0.172775	0.066691
SC15	JW7	CS10	*R.nitidus*	-0.066355	0.068879

JO7	JO1	CS10	*R.nitidus*	-0.021277	0.068654
GD21	JO1	CS10	*R.nitidus*	0.130297	0.064271
SC32	JW7	CS10	*R.nitidus*	-0.017958	0.068402
SC44	JW7	CS10	*R.nitidus*	-0.042934	0.06867
CS11	JO1	CS10	*R.nitidus*	-0.007618	0.066523
JO5	JW7	CS10	*R.nitidus*	0.010354	0.067724
JW6	JW7	CS10	*R.nitidus*	-0.02821	0.073365
GD22	JO1	CS10	*R.nitidus*	0.074847	0.064572
MY4	JW7	CS10	*R.nitidus*	-0.094618	0.069801
GD30	JO1	CS10	*R.nitidus*	0.149644	0.06453
CS12	JO1	CS10	*R.nitidus*	-0.000747	0.066365
GD42	JO1	CS10	*R.nitidus*	0.028705	0.065247
GD35	JO1	CS10	*R.nitidus*	0.042412	0.064538
GD25	JO1	CS10	*R.nitidus*	0.085357	0.064306
GD26	JO1	CS10	*R.nitidus*	0.067775	0.063911
GD34	JO1	CS10	*R.nitidus*	0.024437	0.063456
CS19	JO1	CS10	*R.nitidus*	-0.014716	0.067097
GD40	JO1	CS10	*R.nitidus*	0.07732	0.064283
GD41	JO1	CS10	*R.nitidus*	0.016	0.065082
GD32	JO1	CS10	*R.nitidus*	0.025354	0.067679
CS15	JO1	CS10	*R.nitidus*	0.021618	0.063458
GD48	JO1	CS10	*R.nitidus*	0.040627	0.067448
GD33	JO1	CS10	*R.nitidus*	0.021122	0.066071
GD50	JO1	CS10	*R.nitidus*	0.056406	0.068498
GD31	JO1	CS10	*R.nitidus*	0.07476	0.065336
CS14	JO1	CS10	*R.nitidus*	-0.002101	0.068085
CS4	JO1	CS10	*R.nitidus*	-0.009763	0.064489
CS31	JO1	CS10	*R.nitidus*	-0.019355	0.06604
CS13	JO1	CS10	*R.nitidus*	-0.036403	0.067104
CS24	JO1	CS10	*R.nitidus*	-0.041785	0.065681
CS28	JO1	CS10	*R.nitidus*	-0.040062	0.065377
GD45	JO1	CS10	*R.nitidus*	0.054878	0.068758
CS25	JO1	CS10	*R.nitidus*	-0.004811	0.065964
CS23	JO1	CS10	*R.nitidus*	-0.053997	0.064834
GD44	JO1	CS10	*R.nitidus*	0.021588	0.064665
GD46	JO1	CS10	*R.nitidus*	0.006676	0.065564
CS20	JO1	CS10	*R.nitidus*	-0.01932	0.065488
SC5	JO1	CS10	*R.nitidus*	-0.003631	0.069669
CS18	JO1	CS10	*R.nitidus*	-0.034028	0.064956
GD49	JO1	CS10	*R.nitidus*	0.079485	0.068372
SC14	JO1	CS10	*R.nitidus*	0.002182	0.064358
SC15	JO1	CS10	*R.nitidus*	0.002271	0.064877
GD29	JO1	CS10	*R.nitidus*	0.1284	0.064565
SC12	JO1	CS10	*R.nitidus*	-0.006677	0.065112
CS16	JO1	CS10	*R.nitidus*	0	0.065452
CS3	JO1	CS10	*R.nitidus*	-0.012023	0.064976
CS17	JO1	CS10	*R.nitidus*	0.024775	0.066337
CS21	JO1	CS10	*R.nitidus*	-0.025903	0.065499
SC27	JO1	CS10	*R.nitidus*	-0.000728	0.063628
JW7	JO1	CS10	*R.nitidus*	0.041927	0.067522
SC28	JO1	CS10	*R.nitidus*	-0.006047	0.064748
CS30	JO1	CS10	*R.nitidus*	-0.054814	0.064573
JO7	JO3	CS10	*R.nitidus*	-0.016304	0.074608
CS6	JO1	CS10	*R.nitidus*	-0.039187	0.067697
SC10	JO1	CS10	*R.nitidus*	0.087023	0.064738
JW4	JO1	CS10	*R.nitidus*	0.216216	0.06518

SC24	JO1	CS10	*R.nitidus*	0.008136	0.065766
MY6	JO1	CS10	*R.nitidus*	0.002108	0.064897
SC6	JO1	CS10	*R.nitidus*	0.005208	0.067611
SC26	JO1	CS10	*R.nitidus*	-0.018896	0.064444
CS22	JO1	CS10	*R.nitidus*	-0.009957	0.067225
SC35	JO1	CS10	*R.nitidus*	0.055676	0.064248
GD22	JO3	CS10	*R.nitidus*	0.033133	0.067063
CS11	JO3	CS10	*R.nitidus*	-0.039021	0.07118
SC34	JO1	CS10	*R.nitidus*	0.027445	0.064937
SC44	JO1	CS10	*R.nitidus*	0.014717	0.065586
GD42	JO3	CS10	*R.nitidus*	-0.018745	0.069504
GD21	JO3	CS10	*R.nitidus*	0.084477	0.067334
SC31	JO1	CS10	*R.nitidus*	-0.015173	0.064736
SC32	JO1	CS10	*R.nitidus*	0.050873	0.066017
SC42	JO1	CS10	*R.nitidus*	0.254321	0.061858
GD26	JO3	CS10	*R.nitidus*	0.020801	0.069662
JO8	JO3	CS10	*R.nitidus*	-0.001109	0.073217
GD25	JO3	CS10	*R.nitidus*	0.017256	0.068423
MY4	JO1	CS10	*R.nitidus*	-0.038295	0.065955
JO5	JO1	CS10	*R.nitidus*	0.057791	0.065086
GD30	JO3	CS10	*R.nitidus*	0.121154	0.070373
GD29	JO3	CS10	*R.nitidus*	0.093145	0.064798
GD40	JO3	CS10	*R.nitidus*	0.020179	0.06771
GD33	JO3	CS10	*R.nitidus*	-0.021672	0.068756
SC45	JO1	CS10	*R.nitidus*	0.048708	0.066294
CS12	JO3	CS10	*R.nitidus*	-0.019124	0.069914
JW6	JO1	CS10	*R.nitidus*	0.020702	0.070828
GD41	JO3	CS10	*R.nitidus*	-0.029898	0.068413
JO3	JO1	CS10	*R.nitidus*	0.045562	0.068137
GD46	JO3	CS10	*R.nitidus*	-0.0556	0.067402
GD35	JO3	CS10	*R.nitidus*	0.010526	0.069648
GD34	JO3	CS10	*R.nitidus*	-0.030023	0.068004
SC18	JO1	CS10	*R.nitidus*	0.045455	0.065643
GD31	JO3	CS10	*R.nitidus*	0.028007	0.071116
CS15	JO3	CS10	*R.nitidus*	-0.010654	0.067992
GD32	JO3	CS10	*R.nitidus*	-0.00543	0.070626
CS16	JO3	CS10	*R.nitidus*	-0.027069	0.069297
GD50	JO3	CS10	*R.nitidus*	0.041554	0.072922
GD45	JO3	CS10	*R.nitidus*	0.036832	0.073567
CS20	JO3	CS10	*R.nitidus*	-0.063796	0.067931
CS19	JO3	CS10	*R.nitidus*	-0.063167	0.069679
GD44	JO3	CS10	*R.nitidus*	-0.007924	0.069865
GD49	JO3	CS10	*R.nitidus*	0.019755	0.073075
CS31	JO3	CS10	*R.nitidus*	-0.059016	0.07002
CS24	JO3	CS10	*R.nitidus*	-0.070105	0.066621
CS14	JO3	CS10	*R.nitidus*	-0.028302	0.071095
GD48	JO3	CS10	*R.nitidus*	0.021541	0.069392
CS13	JO3	CS10	*R.nitidus*	-0.067194	0.071939
SC5	JO3	CS10	*R.nitidus*	-0.0211	0.074334
SC6	JO3	CS10	*R.nitidus*	-0.030977	0.070167
CS21	JO3	CS10	*R.nitidus*	-0.07086	0.069039
CS30	JO3	CS10	*R.nitidus*	-0.102668	0.067746
CS22	JO3	CS10	*R.nitidus*	-0.047281	0.070021
CS23	JO3	CS10	*R.nitidus*	-0.088976	0.066488
SC34	JO3	CS10	*R.nitidus*	-0.045666	0.06702
CS17	JO3	CS10	*R.nitidus*	-0.023729	0.071377

CS25	JO3	CS10	*R.nitidus*	-0.069786	0.068673
SC27	JO3	CS10	*R.nitidus*	-0.027195	0.067101
SC18	JO3	CS10	*R.nitidus*	-0.013212	0.067627
JW6	JO3	CS10	*R.nitidus*	-0.020496	0.073172
CS18	JO3	CS10	*R.nitidus*	-0.071197	0.067402
SC10	JO3	CS10	*R.nitidus*	0.045416	0.069383
CS28	JO3	CS10	*R.nitidus*	-0.072667	0.071505
JO5	JO3	CS10	*R.nitidus*	0.023865	0.067445
SC12	JO3	CS10	*R.nitidus*	-0.071038	0.066852
SC32	JO3	CS10	*R.nitidus*	0.014844	0.066914
SC26	JO3	CS10	*R.nitidus*	-0.072987	0.066964
SC31	JO3	CS10	*R.nitidus*	-0.058552	0.068465
MY4	JO3	CS10	*R.nitidus*	-0.088618	0.069531
SC44	JO3	CS10	*R.nitidus*	-0.031771	0.068263
CS4	JO3	CS10	*R.nitidus*	-0.058462	0.066673
JO1	JO3	CS10	*R.nitidus*	-0.045562	0.068137
JW4	JO3	CS10	*R.nitidus*	0.157765	0.067867
CS6	JO3	CS10	*R.nitidus*	-0.10859	0.068748
SC45	JO3	CS10	*R.nitidus*	-0.005439	0.068865
SC15	JO3	CS10	*R.nitidus*	-0.054772	0.068609
SC24	JO3	CS10	*R.nitidus*	-0.043689	0.067424
SC14	JO3	CS10	*R.nitidus*	-0.032012	0.067731
SC28	JO3	CS10	*R.nitidus*	-0.052754	0.067513
JW7	JO3	CS10	*R.nitidus*	0.016034	0.069494
SC42	JO3	CS10	*R.nitidus*	0.150035	0.066408
GD22	JO5	CS10	*R.nitidus*	0.019783	0.063392
GD21	JO5	CS10	*R.nitidus*	0.069592	0.064012
SC35	JO3	CS10	*R.nitidus*	-0.013396	0.067902
MY6	JO3	CS10	*R.nitidus*	-0.053061	0.068554
CS3	JO3	CS10	*R.nitidus*	-0.035656	0.068908
GD44	JO5	CS10	*R.nitidus*	-0.027407	0.065375
GD32	JO5	CS10	*R.nitidus*	-0.051482	0.067971
GD29	JO5	CS10	*R.nitidus*	0.087516	0.063962
GD35	JO5	CS10	*R.nitidus*	0.007534	0.066279
GD30	JO5	CS10	*R.nitidus*	0.079937	0.067482
GD31	JO5	CS10	*R.nitidus*	0.035664	0.066509
JO8	JO5	CS10	*R.nitidus*	-0.045367	0.069187
GD45	JO5	CS10	*R.nitidus*	0.014458	0.069786
GD41	JO5	CS10	*R.nitidus*	-0.037798	0.065074
CS13	JO5	CS10	*R.nitidus*	-0.073654	0.067636
JO7	JO5	CS10	*R.nitidus*	-0.056968	0.068908
GD50	JO5	CS10	*R.nitidus*	0.004055	0.067664
GD26	JO5	CS10	*R.nitidus*	0.003989	0.064685
GD25	JO5	CS10	*R.nitidus*	0.033793	0.063831
CS11	JO5	CS10	*R.nitidus*	-0.026441	0.066725
GD33	JO5	CS10	*R.nitidus*	-0.035363	0.064948
GD40	JO5	CS10	*R.nitidus*	0.029202	0.063728
GD46	JO5	CS10	*R.nitidus*	-0.044341	0.064509
GD48	JO5	CS10	*R.nitidus*	-0.013728	0.066311
CS18	JO5	CS10	*R.nitidus*	-0.093278	0.064739
CS12	JO5	CS10	*R.nitidus*	-0.045488	0.065394
GD42	JO5	CS10	*R.nitidus*	-0.024155	0.065604
CS15	JO5	CS10	*R.nitidus*	-0.023114	0.066406
GD49	JO5	CS10	*R.nitidus*	0.060032	0.067319
CS28	JO5	CS10	*R.nitidus*	-0.083587	0.065174
GD34	JO5	CS10	*R.nitidus*	-0.036891	0.063126

CS30	JO5	CS10	*R.nitidus*	-0.104017	0.0616
CS20	JO5	CS10	*R.nitidus*	-0.084836	0.063844
SC6	JO5	CS10	*R.nitidus*	-0.056245	0.067114
CS17	JO5	CS10	*R.nitidus*	-0.011236	0.068257
CS16	JO5	CS10	*R.nitidus*	-0.036161	0.064015
CS4	JO5	CS10	*R.nitidus*	-0.061324	0.062954
CS24	JO5	CS10	*R.nitidus*	-0.084709	0.064869
SC5	JO5	CS10	*R.nitidus*	-0.071603	0.0684
CS25	JO5	CS10	*R.nitidus*	-0.072046	0.066941
CS14	JO5	CS10	*R.nitidus*	-0.033311	0.067092
CS19	JO5	CS10	*R.nitidus*	-0.064888	0.065723
CS22	JO5	CS10	*R.nitidus*	-0.04954	0.065507
CS3	JO5	CS10	*R.nitidus*	-0.065505	0.063659
CS31	JO5	CS10	*R.nitidus*	-0.081237	0.063972
SC12	JO5	CS10	*R.nitidus*	-0.072646	0.062869
SC31	JO5	CS10	*R.nitidus*	-0.061314	0.064955
CS23	JO5	CS10	*R.nitidus*	-0.098384	0.062755
SC27	JO5	CS10	*R.nitidus*	-0.045073	0.064943
SC42	JO5	CS10	*R.nitidus*	0.198534	0.063484
JW6	JO5	CS10	*R.nitidus*	-0.045699	0.069876
SC24	JO5	CS10	*R.nitidus*	-0.045315	0.064195
JO3	JO5	CS10	*R.nitidus*	-0.023865	0.067445
CS21	JO5	CS10	*R.nitidus*	-0.075589	0.063429
SC14	JO5	CS10	*R.nitidus*	-0.043182	0.064672
JW7	JO5	CS10	*R.nitidus*	-0.010354	0.067724
MY4	JO5	CS10	*R.nitidus*	-0.094059	0.065772
SC15	JO5	CS10	*R.nitidus*	-0.06391	0.065082
SC10	JO5	CS10	*R.nitidus*	0.028679	0.06585
GD25	JO7	JO10	*R.nitidus*	0.071061	0.06913
GD30	JO7	JO10	*R.nitidus*	0.183882	0.072322
JW4	JO5	CS10	*R.nitidus*	0.167913	0.065102
MY6	JO5	CS10	*R.nitidus*	-0.061239	0.065292
SC35	JO5	CS10	*R.nitidus*	-0.003539	0.063938
GD32	JO7	JO10	*R.nitidus*	0.059638	0.073634
SC28	JO5	CS10	*R.nitidus*	-0.091954	0.061502
GD22	JO7	JO10	*R.nitidus*	0.100646	0.070077
SC18	JO5	CS10	*R.nitidus*	-0.013997	0.064935
SC32	JO5	CS10	*R.nitidus*	-0.001415	0.064145
JO8	JO7	JO10	*R.nitidus*	-0.0181	0.074141
SC26	JO5	CS10	*R.nitidus*	-0.080172	0.063996
CS11	JO7	JO10	*R.nitidus*	0.006648	0.071719
GD21	JO7	JO10	*R.nitidus*	0.161092	0.068607
CS6	JO5	CS10	*R.nitidus*	-0.102695	0.06436
GD34	JO7	JO10	*R.nitidus*	0.020992	0.070526
SC34	JO5	CS10	*R.nitidus*	-0.025271	0.065277
GD40	JO7	JO10	*R.nitidus*	0.058107	0.071832
GD45	JO7	JO10	*R.nitidus*	0.101904	0.0744
GD29	JO7	JO10	*R.nitidus*	0.127799	0.06938
GD41	JO7	JO10	*R.nitidus*	-0.006604	0.070415
SC44	JO5	CS10	*R.nitidus*	-0.059676	0.063184
SC45	JO5	CS10	*R.nitidus*	-0.000729	0.065014
GD31	JO7	JO10	*R.nitidus*	0.089069	0.071761
GD26	JO7	JO10	*R.nitidus*	0.100098	0.070777
GD35	JO7	JO10	*R.nitidus*	0.02737	0.068793
CS18	JO7	JO10	*R.nitidus*	-0.03229	0.071177
JO1	JO5	CS10	*R.nitidus*	-0.057791	0.065086

GD44	JO7	JO10	*R.nitidus*	0.010859	0.068685
GD42	JO7	JO10	*R.nitidus*	0.052023	0.070022
CS12	JO7	JO10	*R.nitidus*	0.018219	0.071519
GD33	JO7	JO10	*R.nitidus*	0.014286	0.070124
GD49	JO7	JO10	*R.nitidus*	0.106667	0.071637
CS13	JO7	JO10	*R.nitidus*	-0.063913	0.073001
GD46	JO7	JO10	*R.nitidus*	0.01256	0.072524
GD50	JO7	JO10	*R.nitidus*	0.057051	0.073988
CS25	JO7	JO10	*R.nitidus*	0.01592	0.070116
CS21	JO7	JO10	*R.nitidus*	-0.041466	0.071951
CS22	JO7	JO10	*R.nitidus*	0.029732	0.070005
CS16	JO7	JO10	*R.nitidus*	-0.000996	0.071949
SC10	JO7	JO10	*R.nitidus*	0.104628	0.070393
CS20	JO7	JO10	*R.nitidus*	-0.05103	0.06995
SC34	JO7	JO10	*R.nitidus*	0.013295	0.068309
CS4	JO7	JO10	*R.nitidus*	-0.027128	0.067074
CS14	JO7	JO10	*R.nitidus*	0.025616	0.073276
GD48	JO7	JO10	*R.nitidus*	0.052838	0.071411
CS17	JO7	JO10	*R.nitidus*	0.020492	0.074459
SC18	JO7	JO10	*R.nitidus*	0.064257	0.072265
CS3	JO7	JO10	*R.nitidus*	-0.043216	0.069335
SC6	JO7	JO10	*R.nitidus*	0.018682	0.068301
SC14	JO7	JO10	*R.nitidus*	0.053097	0.07068
CS28	JO7	JO10	*R.nitidus*	0.010363	0.071113
CS30	JO7	JO10	*R.nitidus*	-0.044284	0.071152
SC28	JO7	JO10	*R.nitidus*	0.013752	0.071406
CS24	JO7	JO10	*R.nitidus*	-0.033133	0.068851
CS15	JO7	JO10	*R.nitidus*	0.012708	0.072359
SC5	JO7	JO10	*R.nitidus*	0.035608	0.074742
SC26	JO7	JO10	*R.nitidus*	-0.003799	0.068871
JW6	JO7	JO10	*R.nitidus*	0.005042	0.068834
CS19	JO7	JO10	*R.nitidus*	0.005976	0.070648
MY6	JO7	JO10	*R.nitidus*	-0.005941	0.06992
CS23	JO7	JO10	*R.nitidus*	-0.052734	0.070053
SC31	JO7	JO10	*R.nitidus*	0.028116	0.071313
SC27	JO7	JO10	*R.nitidus*	0.031527	0.070812
MY4	JO7	JO10	*R.nitidus*	-0.042829	0.070774
JO3	JO7	JO10	*R.nitidus*	-0.002389	0.072358
SC12	JO7	JO10	*R.nitidus*	0.007021	0.069846
SC15	JO7	JO10	*R.nitidus*	0.013972	0.068154
JW7	JO7	JO10	*R.nitidus*	0.027655	0.073087
SC42	JO7	JO10	*R.nitidus*	0.137631	0.06921
GD21	JO8	JO10	*R.nitidus*	0.151601	0.071018
SC24	JO7	JO10	*R.nitidus*	0.044177	0.071204
SC44	JO7	JO10	*R.nitidus*	-0.014925	0.070814
CS6	JO7	JO10	*R.nitidus*	-0.033797	0.068529
JO1	JO7	JO10	*R.nitidus*	0.025093	0.068401
SC32	JO7	JO10	*R.nitidus*	0.051657	0.070291
SC35	JO7	JO10	*R.nitidus*	0.021113	0.069149
JW4	JO7	JO10	*R.nitidus*	0.126025	0.070168
SC45	JO7	JO10	*R.nitidus*	0.022044	0.068864
GD22	JO8	JO10	*R.nitidus*	0.099415	0.071971
GD48	JO8	JO10	*R.nitidus*	0.056818	0.073253
CS31	JO7	JO10	*R.nitidus*	0.026104	0.070802
GD26	JO8	JO10	*R.nitidus*	0.061412	0.070207
CS11	JO8	JO10	*R.nitidus*	0	0.071349

GD31	JO8	JO10	*R.nitidus*	0.060254	0.072309
GD32	JO8	JO10	*R.nitidus*	0.022002	0.076208
CS12	JO8	JO10	*R.nitidus*	0.026681	0.075397
GD29	JO8	JO10	*R.nitidus*	0.114286	0.070838
GD49	JO8	JO10	*R.nitidus*	0.112281	0.074585
JO7	JO8	JO10	*R.nitidus*	0.0181	0.074141
CS20	JO8	JO10	*R.nitidus*	-0.032393	0.072823
JO5	JO7	JO10	*R.nitidus*	0.055713	0.070822
GD33	JO8	JO10	*R.nitidus*	-0.01494	0.071971
CS13	JO8	JO10	*R.nitidus*	-0.050157	0.077397
GD40	JO8	JO10	*R.nitidus*	0.056158	0.070171
CS23	JO8	JO10	*R.nitidus*	-0.041195	0.072033
GD45	JO8	JO10	*R.nitidus*	0.090369	0.073169
GD25	JO8	JO10	*R.nitidus*	0.055497	0.071466
CS17	JO8	JO10	*R.nitidus*	0.039387	0.076742
GD46	JO8	JO10	*R.nitidus*	-0.012257	0.071867
GD42	JO8	JO10	*R.nitidus*	0.048338	0.072449
GD35	JO8	JO10	*R.nitidus*	0.044838	0.072361
GD41	JO8	JO10	*R.nitidus*	-0.008929	0.073002
CS15	JO8	JO10	*R.nitidus*	0.007172	0.071566
GD30	JO8	JO10	*R.nitidus*	0.170673	0.076805
CS21	JO8	JO10	*R.nitidus*	-0.019	0.072408
GD34	JO8	JO10	*R.nitidus*	-0.018367	0.072058
GD44	JO8	JO10	*R.nitidus*	0.024768	0.070609
CS19	JO8	JO10	*R.nitidus*	0.001049	0.071937
CS18	JO8	JO10	*R.nitidus*	-0.014568	0.071051
SC5	JO8	JO10	*R.nitidus*	0.029352	0.076073
GD50	JO8	JO10	*R.nitidus*	0.056779	0.075358
SC18	JO8	JO10	*R.nitidus*	0.069119	0.070432
CS22	JO8	JO10	*R.nitidus*	0.032362	0.069964
SC6	JO8	JO10	*R.nitidus*	0.018311	0.072313
SC12	JO8	JO10	*R.nitidus*	0.005274	0.070614
CS14	JO8	JO10	*R.nitidus*	0.033673	0.075591
CS16	JO8	JO10	*R.nitidus*	-0.026151	0.073024
SC10	JO8	JO10	*R.nitidus*	0.113147	0.069081
CS24	JO8	JO10	*R.nitidus*	-0.015504	0.069465
SC32	JO8	JO10	*R.nitidus*	0.030457	0.070123
CS25	JO8	JO10	*R.nitidus*	0.029598	0.074409
SC34	JO8	JO10	*R.nitidus*	0.009054	0.070255
CS31	JO8	JO10	*R.nitidus*	0.001075	0.072433
JW6	JO8	JO10	*R.nitidus*	0.043348	0.072231
SC15	JO8	JO10	*R.nitidus*	0.022581	0.073765
CS28	JO8	JO10	*R.nitidus*	0.017058	0.072282
CS30	JO8	JO10	*R.nitidus*	-0.05131	0.070471
JO5	JO8	JO10	*R.nitidus*	0.061203	0.071547
CS3	JO8	JO10	*R.nitidus*	-0.036194	0.072954
SC42	JO8	JO10	*R.nitidus*	0.139963	0.069743
CS4	JO8	JO10	*R.nitidus*	-0.000995	0.07008
SC14	JO8	JO10	*R.nitidus*	0.046512	0.071044
SC26	JO8	JO10	*R.nitidus*	-0.025773	0.069058
MY6	JO8	JO10	*R.nitidus*	0.009356	0.072344
CS6	JO8	JO10	*R.nitidus*	-0.026178	0.071953
SC28	JO8	JO10	*R.nitidus*	-0.002119	0.071774
SC24	JO8	JO10	*R.nitidus*	0.047059	0.071056
JW7	JO8	JO10	*R.nitidus*	0.046171	0.073941
SC44	JO8	JO10	*R.nitidus*	0.005258	0.072961

SC45	JO8	JO10	*R.nitidus*	0.024845	0.071471
SC35	JO8	JO10	*R.nitidus*	0.045311	0.071855
JW4	JO8	JO10	*R.nitidus*	0.157279	0.070206
SC31	JO8	JO10	*R.nitidus*	0.043651	0.072672
JO3	JO8	JO10	*R.nitidus*	0.014815	0.075337
SC27	JO8	JO10	*R.nitidus*	0.024742	0.070549
MY4	JO8	JO10	*R.nitidus*	-0.029805	0.072691
JO1	JO8	JO10	*R.nitidus*	0.021277	0.070945
JO7	CS11	JO10	*R.nitidus*	-0.006648	0.071719
JO8	CS11	JO10	*R.nitidus*	0	0.071349
GD21	CS11	JO10	*R.nitidus*	0.156783	0.065018
GD25	CS11	JO10	*R.nitidus*	0.046598	0.066406
GD26	CS11	JO10	*R.nitidus*	0.053547	0.066443
GD30	CS11	JO10	*R.nitidus*	0.124409	0.069554
GD29	CS11	JO10	*R.nitidus*	0.083911	0.064932
GD31	CS11	JO10	*R.nitidus*	0.043573	0.068192
GD22	CS11	JO10	*R.nitidus*	0.063492	0.06535
GD40	CS11	JO10	*R.nitidus*	0.05181	0.065306
GD32	CS11	JO10	*R.nitidus*	0.031939	0.069266
GD45	CS11	JO10	*R.nitidus*	0.052125	0.069674
GD34	CS11	JO10	*R.nitidus*	0.026567	0.066081
GD33	CS11	JO10	*R.nitidus*	-0.005495	0.066031
GD42	CS11	JO10	*R.nitidus*	0.01787	0.066823
GD49	CS11	JO10	*R.nitidus*	0.087869	0.06835
GD35	CS11	JO10	*R.nitidus*	0.030238	0.067254
GD44	CS11	JO10	*R.nitidus*	0.011923	0.067727
GD50	CS11	JO10	*R.nitidus*	0.022613	0.071602
CS12	CS11	JO10	*R.nitidus*	-0.004274	0.069097
GD46	CS11	JO10	*R.nitidus*	0.010776	0.067122
GD41	CS11	JO10	*R.nitidus*	-0.020423	0.066332
GD48	CS11	JO10	*R.nitidus*	0.036162	0.067451
CS20	CS11	JO10	*R.nitidus*	-0.049849	0.065223
CS15	CS11	JO10	*R.nitidus*	-0.006623	0.070044
CS14	CS11	JO10	*R.nitidus*	-0.002421	0.071259
CS17	CS11	JO10	*R.nitidus*	0.038943	0.066364
CS21	CS11	JO10	*R.nitidus*	-0.013383	0.066282
CS18	CS11	JO10	*R.nitidus*	-0.026475	0.06593
CS23	CS11	JO10	*R.nitidus*	-0.052399	0.065246
CS31	CS11	JO10	*R.nitidus*	0.008621	0.06803
CS25	CS11	JO10	*R.nitidus*	0.015825	0.066603
CS13	CS11	JO10	*R.nitidus*	-0.083333	0.071938
CS30	CS11	JO10	*R.nitidus*	-0.055178	0.067023
CS22	CS11	JO10	*R.nitidus*	0.022693	0.06592
SC5	CS11	JO10	*R.nitidus*	0.014881	0.070126
CS16	CS11	JO10	*R.nitidus*	-0.013304	0.066684
CS3	CS11	JO10	*R.nitidus*	-0.065676	0.065696
CS28	CS11	JO10	*R.nitidus*	-0.017586	0.06502
CS19	CS11	JO10	*R.nitidus*	0.006173	0.066636
CS24	CS11	JO10	*R.nitidus*	-0.028016	0.066179
SC24	CS11	JO10	*R.nitidus*	0.009552	0.066207
SC14	CS11	JO10	*R.nitidus*	0.035897	0.068327
CS4	CS11	JO10	*R.nitidus*	-0.011095	0.064941
SC15	CS11	JO10	*R.nitidus*	0.011219	0.065867
SC26	CS11	JO10	*R.nitidus*	-0.003023	0.06619
SC18	CS11	JO10	*R.nitidus*	0.046945	0.066499
SC10	CS11	JO10	*R.nitidus*	0.082222	0.066656

SC6	CS11	JO10	*R.nitidus*	0.015106	0.064943
SC28	CS11	JO10	*R.nitidus*	-0.038226	0.065448
SC31	CS11	JO10	*R.nitidus*	0.007407	0.065008
SC12	CS11	JO10	*R.nitidus*	0.004435	0.064012
SC27	CS11	JO10	*R.nitidus*	0.010023	0.066617
SC34	CS11	JO10	*R.nitidus*	0.017857	0.066317
MY4	CS11	JO10	*R.nitidus*	-0.016643	0.066411
SC35	CS11	JO10	*R.nitidus*	0.042785	0.065581
MY6	CS11	JO10	*R.nitidus*	0.015793	0.065871
JO3	CS11	JO10	*R.nitidus*	0.02418	0.071428
JW6	CS11	JO10	*R.nitidus*	0.051081	0.072193
JO1	CS11	JO10	*R.nitidus*	0.009608	0.065169
SC32	CS11	JO10	*R.nitidus*	0.054937	0.066104
JW4	CS11	JO10	*R.nitidus*	0.145125	0.06525
JO8	GD21	JO10	*R.nitidus*	-0.151601	0.071018
SC42	CS11	JO10	*R.nitidus*	0.166561	0.063555
JO7	GD21	JO10	*R.nitidus*	-0.161092	0.068607
SC44	CS11	JO10	*R.nitidus*	-0.00148	0.066598
JO5	CS11	JO10	*R.nitidus*	0.034282	0.066914
JW7	CS11	JO10	*R.nitidus*	0.03128	0.067915
CS11	GD21	JO10	*R.nitidus*	-0.156783	0.065018
CS6	CS11	JO10	*R.nitidus*	-0.022117	0.066851
GD31	GD21	JO10	*R.nitidus*	-0.124298	0.063971
GD32	GD21	JO10	*R.nitidus*	-0.14026	0.064625
SC45	CS11	JO10	*R.nitidus*	0.025564	0.065652
GD22	GD21	JO10	*R.nitidus*	-0.099772	0.061192
GD30	GD21	JO10	*R.nitidus*	-0.031853	0.068013
GD35	GD21	JO10	*R.nitidus*	-0.131365	0.063246
GD34	GD21	JO10	*R.nitidus*	-0.17757	0.061132
GD41	GD21	JO10	*R.nitidus*	-0.208967	0.059898
GD45	GD21	JO10	*R.nitidus*	-0.112847	0.06525
GD29	GD21	JO10	*R.nitidus*	-0.093344	0.064328
GD26	GD21	JO10	*R.nitidus*	-0.131783	0.060998
GD44	GD21	JO10	*R.nitidus*	-0.176917	0.062527
GD42	GD21	JO10	*R.nitidus*	-0.164634	0.060616
CS12	GD21	JO10	*R.nitidus*	-0.172632	0.065277
GD33	GD21	JO10	*R.nitidus*	-0.211735	0.063987
CS13	GD21	JO10	*R.nitidus*	-0.214334	0.063844
GD25	GD21	JO10	*R.nitidus*	-0.111027	0.064345
GD46	GD21	JO10	*R.nitidus*	-0.205615	0.061361
GD49	GD21	JO10	*R.nitidus*	-0.086146	0.065932
CS14	GD21	JO10	*R.nitidus*	-0.176307	0.066016
GD48	GD21	JO10	*R.nitidus*	-0.156514	0.063874
CS15	GD21	JO10	*R.nitidus*	-0.176674	0.063902
CS20	GD21	JO10	*R.nitidus*	-0.210856	0.063255
CS19	GD21	JO10	*R.nitidus*	-0.159801	0.063903
GD40	GD21	JO10	*R.nitidus*	-0.125984	0.063045
CS17	GD21	JO10	*R.nitidus*	-0.141935	0.066465
GD50	GD21	JO10	*R.nitidus*	-0.16321	0.066034
CS22	GD21	JO10	*R.nitidus*	-0.140777	0.063401
CS18	GD21	JO10	*R.nitidus*	-0.187542	0.064489
CS24	GD21	JO10	*R.nitidus*	-0.188366	0.062492
CS21	GD21	JO10	*R.nitidus*	-0.173709	0.063396
CS23	GD21	JO10	*R.nitidus*	-0.201072	0.062946
CS31	GD21	JO10	*R.nitidus*	-0.166311	0.064646
CS3	GD21	JO10	*R.nitidus*	-0.198479	0.064444

CS30	GD21	JO10	*R.nitidus*	-0.216833	0.062954
CS16	GD21	JO10	*R.nitidus*	-0.167231	0.063525
CS4	GD21	JO10	*R.nitidus*	-0.177539	0.062623
SC6	GD21	JO10	*R.nitidus*	-0.148575	0.063002
SC14	GD21	JO10	*R.nitidus*	-0.129183	0.063
CS25	GD21	JO10	*R.nitidus*	-0.159379	0.065363
CS28	GD21	JO10	*R.nitidus*	-0.177778	0.063364
SC5	GD21	JO10	*R.nitidus*	-0.136364	0.067988
SC12	GD21	JO10	*R.nitidus*	-0.158625	0.061614
SC24	GD21	JO10	*R.nitidus*	-0.157426	0.061829
SC28	GD21	JO10	*R.nitidus*	-0.184123	0.063574
SC27	GD21	JO10	*R.nitidus*	-0.143836	0.06439
SC26	GD21	JO10	*R.nitidus*	-0.174902	0.061952
SC35	GD21	JO10	*R.nitidus*	-0.123912	0.063682
SC15	GD21	JO10	*R.nitidus*	-0.161202	0.063121
SC32	GD21	JO10	*R.nitidus*	-0.113881	0.063162
SC31	GD21	JO10	*R.nitidus*	-0.147815	0.064199
SC10	GD21	JO10	*R.nitidus*	-0.08209	0.064462
JW6	GD21	JO10	*R.nitidus*	-0.139576	0.068865
JW7	GD21	JO10	*R.nitidus*	-0.136326	0.066784
SC45	GD21	JO10	*R.nitidus*	-0.126789	0.063058
MY6	GD21	JO10	*R.nitidus*	-0.145527	0.063476
SC18	GD21	JO10	*R.nitidus*	-0.129563	0.064001
JW4	GD21	JO10	*R.nitidus*	0.005575	0.065856
SC44	GD21	JO10	*R.nitidus*	-0.162618	0.063733
SC34	GD21	JO10	*R.nitidus*	-0.144289	0.064124
SC42	GD21	JO10	*R.nitidus*	0.039521	0.063005
JO7	GD22	JO10	*R.nitidus*	-0.100646	0.070077
MY4	GD21	JO10	*R.nitidus*	-0.182063	0.063369
JO1	GD21	JO10	*R.nitidus*	-0.162929	0.062673
CS11	GD22	JO10	*R.nitidus*	-0.063492	0.06535
JO3	GD21	JO10	*R.nitidus*	-0.135852	0.067532
GD30	GD22	JO10	*R.nitidus*	0.073267	0.066085
JO8	GD22	JO10	*R.nitidus*	-0.099415	0.071971
GD21	GD22	JO10	*R.nitidus*	0.099772	0.061192
GD31	GD22	JO10	*R.nitidus*	-0.026442	0.064093
JO5	GD21	JO10	*R.nitidus*	-0.128904	0.063641
CS6	GD21	JO10	*R.nitidus*	-0.196557	0.063372
GD33	GD22	JO10	*R.nitidus*	-0.095403	0.062632
GD25	GD22	JO10	*R.nitidus*	-0.038401	0.063361
GD29	GD22	JO10	*R.nitidus*	0.017588	0.062414
GD26	GD22	JO10	*R.nitidus*	-0.034903	0.062092
GD34	GD22	JO10	*R.nitidus*	-0.079646	0.060772
GD35	GD22	JO10	*R.nitidus*	-0.030053	0.06354
CS12	GD22	JO10	*R.nitidus*	-0.070977	0.06446
GD40	GD22	JO10	*R.nitidus*	-0.021676	0.061844
GD32	GD22	JO10	*R.nitidus*	-0.040529	0.065754
GD45	GD22	JO10	*R.nitidus*	-0.037351	0.066906
GD41	GD22	JO10	*R.nitidus*	-0.110759	0.062856
GD46	GD22	JO10	*R.nitidus*	-0.094627	0.063522
GD50	GD22	JO10	*R.nitidus*	-0.035647	0.065131
GD42	GD22	JO10	*R.nitidus*	-0.066774	0.064148
GD44	GD22	JO10	*R.nitidus*	-0.078906	0.064441
CS14	GD22	JO10	*R.nitidus*	-0.074301	0.06563
GD48	GD22	JO10	*R.nitidus*	-0.044554	0.064615
CS16	GD22	JO10	*R.nitidus*	-0.075305	0.063797

GD49	GD22	JO10	*R.nitidus*	0.019689	0.064247
CS18	GD22	JO10	*R.nitidus*	-0.101389	0.063457
CS20	GD22	JO10	*R.nitidus*	-0.121668	0.064587
CS24	GD22	JO10	*R.nitidus*	-0.096978	0.060143
CS23	GD22	JO10	*R.nitidus*	-0.117166	0.063001
CS13	GD22	JO10	*R.nitidus*	-0.125428	0.063841
CS25	GD22	JO10	*R.nitidus*	-0.065965	0.064301
CS22	GD22	JO10	*R.nitidus*	-0.043206	0.063804
CS15	GD22	JO10	*R.nitidus*	-0.08	0.063756
CS17	GD22	JO10	*R.nitidus*	-0.037683	0.064691
CS28	GD22	JO10	*R.nitidus*	-0.086676	0.06702
CS19	GD22	JO10	*R.nitidus*	-0.06831	0.065469
CS31	GD22	JO10	*R.nitidus*	-0.073205	0.065237
CS4	GD22	JO10	*R.nitidus*	-0.092008	0.062331
CS3	GD22	JO10	*R.nitidus*	-0.125864	0.06322
SC10	GD22	JO10	*R.nitidus*	0.009838	0.064209
CS21	GD22	JO10	*R.nitidus*	-0.08745	0.061953
SC12	GD22	JO10	*R.nitidus*	-0.072395	0.060444
SC15	GD22	JO10	*R.nitidus*	-0.060732	0.063079
SC24	GD22	JO10	*R.nitidus*	-0.066804	0.062246
CS30	GD22	JO10	*R.nitidus*	-0.130022	0.062209
SC28	GD22	JO10	*R.nitidus*	-0.103729	0.061733
SC32	GD22	JO10	*R.nitidus*	-0.020288	0.062084
SC26	GD22	JO10	*R.nitidus*	-0.084218	0.063077
SC34	GD22	JO10	*R.nitidus*	-0.051215	0.062459
SC27	GD22	JO10	*R.nitidus*	-0.052381	0.063299
SC35	GD22	JO10	*R.nitidus*	-0.020988	0.062028
SC14	GD22	JO10	*R.nitidus*	-0.049225	0.062302
SC5	GD22	JO10	*R.nitidus*	-0.061972	0.06434
SC18	GD22	JO10	*R.nitidus*	-0.029016	0.063296
SC6	GD22	JO10	*R.nitidus*	-0.05087	0.062962
SC31	GD22	JO10	*R.nitidus*	-0.067244	0.064872
JW4	GD22	JO10	*R.nitidus*	0.081871	0.066178
SC45	GD22	JO10	*R.nitidus*	-0.039229	0.0627
MY6	GD22	JO10	*R.nitidus*	-0.057162	0.062335
JW6	GD22	JO10	*R.nitidus*	-0.061131	0.070504
SC42	GD22	JO10	*R.nitidus*	0.10006	0.062061
JW7	GD22	JO10	*R.nitidus*	-0.057481	0.069057
CS6	GD22	JO10	*R.nitidus*	-0.10846	0.064719
JO7	GD25	JO10	*R.nitidus*	-0.071061	0.06913
MY4	GD22	JO10	*R.nitidus*	-0.1	0.064387
JO8	GD25	JO10	*R.nitidus*	-0.055497	0.071466
SC44	GD22	JO10	*R.nitidus*	-0.084926	0.061598
JO1	GD22	JO10	*R.nitidus*	-0.066007	0.061678
JO5	GD22	JO10	*R.nitidus*	-0.035446	0.062165
GD21	GD25	JO10	*R.nitidus*	0.111027	0.064345
JO3	GD22	JO10	*R.nitidus*	-0.058774	0.070869
GD26	GD25	JO10	*R.nitidus*	0.013765	0.065322
GD33	GD25	JO10	*R.nitidus*	-0.05887	0.065406
GD31	GD25	JO10	*R.nitidus*	0.033497	0.063944
CS11	GD25	JO10	*R.nitidus*	-0.046598	0.066406
GD30	GD25	JO10	*R.nitidus*	0.125926	0.067712
GD32	GD25	JO10	*R.nitidus*	-0.001675	0.066475
GD46	GD25	JO10	*R.nitidus*	-0.052797	0.064243
GD29	GD25	JO10	*R.nitidus*	0.04053	0.064398
GD34	GD25	JO10	*R.nitidus*	-0.018225	0.065941

GD42	GD25	JO10	*R.nitidus*	-0.042345	0.064964
GD35	GD25	JO10	*R.nitidus*	-0.010569	0.063227
CS13	GD25	JO10	*R.nitidus*	-0.099924	0.066172
GD41	GD25	JO10	*R.nitidus*	-0.066461	0.063483
CS14	GD25	JO10	*R.nitidus*	-0.052395	0.066519
GD49	GD25	JO10	*R.nitidus*	0.058664	0.066275
GD45	GD25	JO10	*R.nitidus*	0.039439	0.066987
CS16	GD25	JO10	*R.nitidus*	-0.060178	0.064273
CS15	GD25	JO10	*R.nitidus*	-0.063422	0.065429
GD22	GD25	JO10	*R.nitidus*	0.038401	0.063361
GD50	GD25	JO10	*R.nitidus*	0.011505	0.066789
CS12	GD25	JO10	*R.nitidus*	-0.059701	0.065344
CS19	GD25	JO10	*R.nitidus*	-0.042584	0.064485
GD44	GD25	JO10	*R.nitidus*	-0.0489	0.066003
GD48	GD25	JO10	*R.nitidus*	0	0.064409
CS21	GD25	JO10	*R.nitidus*	-0.061372	0.064693
CS18	GD25	JO10	*R.nitidus*	-0.075431	0.064212
CS25	GD25	JO10	*R.nitidus*	-0.034431	0.064813
GD40	GD25	JO10	*R.nitidus*	-0.017557	0.061521
CS22	GD25	JO10	*R.nitidus*	-0.014771	0.06545
CS24	GD25	JO10	*R.nitidus*	-0.055224	0.061389
CS20	GD25	JO10	*R.nitidus*	-0.092163	0.065336
CS23	GD25	JO10	*R.nitidus*	-0.088842	0.062606
CS28	GD25	JO10	*R.nitidus*	-0.073038	0.065529
CS31	GD25	JO10	*R.nitidus*	-0.039969	0.066348
SC10	GD25	JO10	*R.nitidus*	0.031818	0.062834
CS17	GD25	JO10	*R.nitidus*	-0.014672	0.065795
CS30	GD25	JO10	*R.nitidus*	-0.094325	0.06358
SC5	GD25	JO10	*R.nitidus*	-0.034822	0.065599
CS4	GD25	JO10	*R.nitidus*	-0.057951	0.06292
SC24	GD25	JO10	*R.nitidus*	-0.035819	0.062771
CS3	GD25	JO10	*R.nitidus*	-0.102179	0.065385
SC6	GD25	JO10	*R.nitidus*	-0.034934	0.062195
SC15	GD25	JO10	*R.nitidus*	-0.042456	0.064047
SC12	GD25	JO10	*R.nitidus*	-0.050558	0.062866
SC18	GD25	JO10	*R.nitidus*	-0.007513	0.063672
SC14	GD25	JO10	*R.nitidus*	-0.021583	0.061709
SC27	GD25	JO10	*R.nitidus*	-0.032823	0.062745
SC26	GD25	JO10	*R.nitidus*	-0.064888	0.062828
SC28	GD25	JO10	*R.nitidus*	-0.089167	0.063071
SC32	GD25	JO10	*R.nitidus*	0	0.063776
SC34	GD25	JO10	*R.nitidus*	-0.027027	0.06185
SC31	GD25	JO10	*R.nitidus*	-0.045161	0.063796
JO8	GD26	JO10	*R.nitidus*	-0.061412	0.070207
JW4	GD25	JO10	*R.nitidus*	0.0938	0.064997
JO3	GD25	JO10	*R.nitidus*	-0.043876	0.067417
CS6	GD25	JO10	*R.nitidus*	-0.080486	0.065031
MY4	GD25	JO10	*R.nitidus*	-0.086294	0.064103
SC45	GD25	JO10	*R.nitidus*	-0.025954	0.062169
SC44	GD25	JO10	*R.nitidus*	-0.0571	0.062177
JO5	GD25	JO10	*R.nitidus*	-0.034686	0.061748
JO7	GD26	JO10	*R.nitidus*	-0.100098	0.070777
SC35	GD25	JO10	*R.nitidus*	-0.003655	0.063623
JW7	GD25	JO10	*R.nitidus*	-0.025594	0.068046
GD30	GD26	JO10	*R.nitidus*	0.109756	0.064137
JW6	GD25	JO10	*R.nitidus*	-0.038618	0.069898

GD25	GD26	JO10	*R.nitidus*	-0.013765	0.065322
GD21	GD26	JO10	*R.nitidus*	0.131783	0.060998
GD22	GD26	JO10	*R.nitidus*	0.034903	0.062092
MY6	GD25	JO10	*R.nitidus*	-0.039028	0.063665
SC42	GD25	JO10	*R.nitidus*	0.112418	0.064226
GD34	GD26	JO10	*R.nitidus*	-0.034025	0.062401
GD33	GD26	JO10	*R.nitidus*	-0.074041	0.06251
JO1	GD25	JO10	*R.nitidus*	-0.066323	0.064371
GD31	GD26	JO10	*R.nitidus*	0.024213	0.065497
GD40	GD26	JO10	*R.nitidus*	-0.009125	0.064579
GD35	GD26	JO10	*R.nitidus*	-0.02057	0.064289
GD44	GD26	JO10	*R.nitidus*	-0.05033	0.065954
GD32	GD26	JO10	*R.nitidus*	-0.003337	0.065327
CS12	GD26	JO10	*R.nitidus*	-0.040787	0.065861
CS13	GD26	JO10	*R.nitidus*	-0.114103	0.06547
GD48	GD26	JO10	*R.nitidus*	-0.014419	0.063451
GD42	GD26	JO10	*R.nitidus*	-0.021987	0.064387
CS15	GD26	JO10	*R.nitidus*	-0.06676	0.065957
GD50	GD26	JO10	*R.nitidus*	-0.0254	0.064607
CS14	GD26	JO10	*R.nitidus*	-0.063604	0.066593
CS16	GD26	JO10	*R.nitidus*	-0.061239	0.064395
GD41	GD26	JO10	*R.nitidus*	-0.076923	0.063268
GD46	GD26	JO10	*R.nitidus*	-0.053217	0.062119
GD49	GD26	JO10	*R.nitidus*	0.046296	0.063732
GD45	GD26	JO10	*R.nitidus*	0.017415	0.067155
GD29	GD26	JO10	*R.nitidus*	0.039298	0.062912
CS17	GD26	JO10	*R.nitidus*	-0.017921	0.064492
CS18	GD26	JO10	*R.nitidus*	-0.082923	0.0648
CS21	GD26	JO10	*R.nitidus*	-0.058464	0.063216
CS22	GD26	JO10	*R.nitidus*	-0.035868	0.06499
CS19	GD26	JO10	*R.nitidus*	-0.03244	0.064743
CS20	GD26	JO10	*R.nitidus*	-0.090719	0.063472
CS25	GD26	JO10	*R.nitidus*	-0.042569	0.063446
CS28	GD26	JO10	*R.nitidus*	-0.063016	0.06552
CS23	GD26	JO10	*R.nitidus*	-0.088737	0.063726
CS31	GD26	JO10	*R.nitidus*	-0.052283	0.063712
SC10	GD26	JO10	*R.nitidus*	0.036417	0.064776
SC5	GD26	JO10	*R.nitidus*	-0.031433	0.065451
SC14	GD26	JO10	*R.nitidus*	-0.027178	0.063727
CS4	GD26	JO10	*R.nitidus*	-0.074202	0.062782
SC12	GD26	JO10	*R.nitidus*	-0.054054	0.06257
SC6	GD26	JO10	*R.nitidus*	-0.041518	0.064181
CS24	GD26	JO10	*R.nitidus*	-0.08153	0.062522
CS30	GD26	JO10	*R.nitidus*	-0.101845	0.061938
SC35	GD26	JO10	*R.nitidus*	-0.019068	0.063049
CS3	GD26	JO10	*R.nitidus*	-0.109705	0.065396
SC18	GD26	JO10	*R.nitidus*	-0.008203	0.064666
SC24	GD26	JO10	*R.nitidus*	-0.043571	0.064751
SC32	GD26	JO10	*R.nitidus*	0.002113	0.061888
SC34	GD26	JO10	*R.nitidus*	-0.034004	0.063465
JW4	GD26	JO10	*R.nitidus*	0.104244	0.067307
SC28	GD26	JO10	*R.nitidus*	-0.076379	0.064048
JW6	GD26	JO10	*R.nitidus*	-0.04	0.071906
SC27	GD26	JO10	*R.nitidus*	-0.034941	0.063791
MY6	GD26	JO10	*R.nitidus*	-0.034795	0.064857
SC15	GD26	JO10	*R.nitidus*	-0.044286	0.06309

JW7	GD26	JO10	*R.nitidus*	-0.03649	0.06899
SC45	GD26	JO10	*R.nitidus*	-0.016606	0.063387
CS6	GD26	JO10	*R.nitidus*	-0.079853	0.064852
MY4	GD26	JO10	*R.nitidus*	-0.073394	0.0641
SC31	GD26	JO10	*R.nitidus*	-0.047131	0.064671
JO7	GD29	JO10	*R.nitidus*	-0.127799	0.06938
JO1	GD26	JO10	*R.nitidus*	-0.055973	0.062327
JO8	GD29	JO10	*R.nitidus*	-0.114286	0.070838
SC26	GD26	JO10	*R.nitidus*	-0.070323	0.063672
JO3	GD26	JO10	*R.nitidus*	-0.038793	0.071668
CS11	GD29	JO10	*R.nitidus*	-0.083911	0.064932
SC42	GD26	JO10	*R.nitidus*	0.119552	0.064646
SC44	GD26	JO10	*R.nitidus*	-0.056671	0.063437
GD22	GD29	JO10	*R.nitidus*	-0.017588	0.062414
JO5	GD26	JO10	*R.nitidus*	-0.023093	0.064182
GD30	GD29	JO10	*R.nitidus*	0.076419	0.066037
CS12	GD29	JO10	*R.nitidus*	-0.08702	0.065549
GD31	GD29	JO10	*R.nitidus*	-0.01495	0.064576
GD33	GD29	JO10	*R.nitidus*	-0.111888	0.066034
GD34	GD29	JO10	*R.nitidus*	-0.086556	0.063492
GD26	GD29	JO10	*R.nitidus*	-0.039298	0.062912
GD32	GD29	JO10	*R.nitidus*	-0.040835	0.065167
GD25	GD29	JO10	*R.nitidus*	-0.04053	0.064398
GD21	GD29	JO10	*R.nitidus*	0.093344	0.064328
GD41	GD29	JO10	*R.nitidus*	-0.106635	0.064503
GD48	GD29	JO10	*R.nitidus*	-0.058469	0.06392
CS13	GD29	JO10	*R.nitidus*	-0.139045	0.065468
GD46	GD29	JO10	*R.nitidus*	-0.100597	0.064269
GD35	GD29	JO10	*R.nitidus*	-0.040121	0.063146
GD45	GD29	JO10	*R.nitidus*	-0.026042	0.066594
GD42	GD29	JO10	*R.nitidus*	-0.06737	0.063723
GD40	GD29	JO10	*R.nitidus*	-0.044174	0.06318
GD50	GD29	JO10	*R.nitidus*	-0.059259	0.06628
GD44	GD29	JO10	*R.nitidus*	-0.101218	0.065284
CS14	GD29	JO10	*R.nitidus*	-0.104225	0.067769
GD49	GD29	JO10	*R.nitidus*	-0.002791	0.065308
CS16	GD29	JO10	*R.nitidus*	-0.09952	0.066131
CS20	GD29	JO10	*R.nitidus*	-0.119916	0.064669
CS19	GD29	JO10	*R.nitidus*	-0.071326	0.064587
CS18	GD29	JO10	*R.nitidus*	-0.102941	0.063832
CS15	GD29	JO10	*R.nitidus*	-0.100975	0.06566
CS17	GD29	JO10	*R.nitidus*	-0.048692	0.066198
CS21	GD29	JO10	*R.nitidus*	-0.091343	0.063112
SC5	GD29	JO10	*R.nitidus*	-0.07148	0.066053
CS3	GD29	JO10	*R.nitidus*	-0.131579	0.063686
CS28	GD29	JO10	*R.nitidus*	-0.085631	0.065295
CS22	GD29	JO10	*R.nitidus*	-0.065911	0.06498
SC10	GD29	JO10	*R.nitidus*	0.010094	0.06402
CS31	GD29	JO10	*R.nitidus*	-0.084487	0.065811
CS24	GD29	JO10	*R.nitidus*	-0.109687	0.062564
SC12	GD29	JO10	*R.nitidus*	-0.077931	0.062688
CS30	GD29	JO10	*R.nitidus*	-0.124265	0.06301
CS25	GD29	JO10	*R.nitidus*	-0.068891	0.065661
SC6	GD29	JO10	*R.nitidus*	-0.068166	0.062853
SC15	GD29	JO10	*R.nitidus*	-0.075391	0.062955
CS4	GD29	JO10	*R.nitidus*	-0.107143	0.062799

SC24	GD29	JO10	*R.nitidus*	-0.062457	0.062256
CS23	GD29	JO10	*R.nitidus*	-0.122105	0.063158
SC26	GD29	JO10	*R.nitidus*	-0.089009	0.062461
SC18	GD29	JO10	*R.nitidus*	-0.032	0.064834
SC14	GD29	JO10	*R.nitidus*	-0.054258	0.062953
SC32	GD29	JO10	*R.nitidus*	-0.042553	0.064118
CS6	GD29	JO10	*R.nitidus*	-0.099853	0.063242
SC42	GD29	JO10	*R.nitidus*	0.084082	0.062975
SC31	GD29	JO10	*R.nitidus*	-0.062041	0.064477
SC44	GD29	JO10	*R.nitidus*	-0.075833	0.063361
JO7	GD30	JO10	*R.nitidus*	-0.183882	0.072322
JW6	GD29	JO10	*R.nitidus*	-0.089972	0.069111
SC27	GD29	JO10	*R.nitidus*	-0.056631	0.064178
JO3	GD29	JO10	*R.nitidus*	-0.080577	0.065571
SC28	GD29	JO10	*R.nitidus*	-0.106472	0.06331
SC34	GD29	JO10	*R.nitidus*	-0.05164	0.063662
MY4	GD29	JO10	*R.nitidus*	-0.103714	0.063025
SC35	GD29	JO10	*R.nitidus*	-0.033357	0.062955
JW4	GD29	JO10	*R.nitidus*	0.06578	0.065884
JO1	GD29	JO10	*R.nitidus*	-0.072297	0.06233
SC45	GD29	JO10	*R.nitidus*	-0.056669	0.063986
GD26	GD30	JO10	*R.nitidus*	-0.109756	0.064137
JO5	GD29	JO10	*R.nitidus*	-0.055361	0.061378
CS11	GD30	JO10	*R.nitidus*	-0.124409	0.069554
GD21	GD30	JO10	*R.nitidus*	0.031853	0.068013
GD25	GD30	JO10	*R.nitidus*	-0.125926	0.067712
JO8	GD30	JO10	*R.nitidus*	-0.170673	0.076805
GD22	GD30	JO10	*R.nitidus*	-0.073267	0.066085
JW7	GD29	JO10	*R.nitidus*	-0.072943	0.068712
GD31	GD30	JO10	*R.nitidus*	-0.096102	0.06816
MY6	GD29	JO10	*R.nitidus*	-0.063492	0.062564
GD34	GD30	JO10	*R.nitidus*	-0.1303	0.066493
GD35	GD30	JO10	*R.nitidus*	-0.100862	0.066956
CS12	GD30	JO10	*R.nitidus*	-0.12636	0.069918
GD45	GD30	JO10	*R.nitidus*	-0.081362	0.068367
GD33	GD30	JO10	*R.nitidus*	-0.147964	0.066098
GD44	GD30	JO10	*R.nitidus*	-0.143756	0.065564
GD49	GD30	JO10	*R.nitidus*	-0.068966	0.0676
GD42	GD30	JO10	*R.nitidus*	-0.145387	0.065924
GD29	GD30	JO10	*R.nitidus*	-0.076419	0.066037
GD41	GD30	JO10	*R.nitidus*	-0.154052	0.065903
GD50	GD30	JO10	*R.nitidus*	-0.105263	0.068086
GD40	GD30	JO10	*R.nitidus*	-0.110044	0.066244
CS13	GD30	JO10	*R.nitidus*	-0.178689	0.068278
CS15	GD30	JO10	*R.nitidus*	-0.133279	0.069304
GD48	GD30	JO10	*R.nitidus*	-0.076255	0.067675
GD32	GD30	JO10	*R.nitidus*	-0.110895	0.068556
CS17	GD30	JO10	*R.nitidus*	-0.090312	0.069979
CS18	GD30	JO10	*R.nitidus*	-0.147656	0.065266
GD46	GD30	JO10	*R.nitidus*	-0.162325	0.066583
CS19	GD30	JO10	*R.nitidus*	-0.126656	0.067612
CS16	GD30	JO10	*R.nitidus*	-0.126299	0.067097
CS22	GD30	JO10	*R.nitidus*	-0.096463	0.068126
CS14	GD30	JO10	*R.nitidus*	-0.129006	0.07016
CS23	GD30	JO10	*R.nitidus*	-0.147311	0.065955
CS20	GD30	JO10	*R.nitidus*	-0.156101	0.06682

CS21	GD30	JO10	*R.nitidus*	-0.137984	0.066823
CS28	GD30	JO10	*R.nitidus*	-0.148116	0.067259
CS24	GD30	JO10	*R.nitidus*	-0.13285	0.065716
CS30	GD30	JO10	*R.nitidus*	-0.16196	0.06507
CS25	GD30	JO10	*R.nitidus*	-0.1225	0.067002
SC6	GD30	JO10	*R.nitidus*	-0.105645	0.065252
SC14	GD30	JO10	*R.nitidus*	-0.094821	0.065403
SC10	GD30	JO10	*R.nitidus*	-0.051452	0.067198
CS3	GD30	JO10	*R.nitidus*	-0.177723	0.067114
CS31	GD30	JO10	*R.nitidus*	-0.10522	0.068926
SC26	GD30	JO10	*R.nitidus*	-0.139464	0.065724
SC15	GD30	JO10	*R.nitidus*	-0.128	0.066567
SC28	GD30	JO10	*R.nitidus*	-0.152516	0.064792
SC5	GD30	JO10	*R.nitidus*	-0.102871	0.067694
SC24	GD30	JO10	*R.nitidus*	-0.118974	0.064323
CS4	GD30	JO10	*R.nitidus*	-0.130568	0.065112
SC31	GD30	JO10	*R.nitidus*	-0.125286	0.067067
SC18	GD30	JO10	*R.nitidus*	-0.075348	0.065566
SC35	GD30	JO10	*R.nitidus*	-0.075841	0.065594
SC34	GD30	JO10	*R.nitidus*	-0.109943	0.066975
SC42	GD30	JO10	*R.nitidus*	0.043785	0.066447
SC45	GD30	JO10	*R.nitidus*	-0.095238	0.065785
SC32	GD30	JO10	*R.nitidus*	-0.068666	0.068451
MY6	GD30	JO10	*R.nitidus*	-0.106913	0.066303
SC44	GD30	JO10	*R.nitidus*	-0.119415	0.065007
CS6	GD30	JO10	*R.nitidus*	-0.151993	0.066429
JW6	GD30	JO10	*R.nitidus*	-0.157663	0.073886
SC12	GD30	JO10	*R.nitidus*	-0.130366	0.065125
JW4	GD30	JO10	*R.nitidus*	0.037326	0.069555
SC27	GD30	JO10	*R.nitidus*	-0.106858	0.065687
MY4	GD30	JO10	*R.nitidus*	-0.151858	0.065876
GD21	GD31	JO10	*R.nitidus*	0.124298	0.063971
JO7	GD31	JO10	*R.nitidus*	-0.089069	0.071761
CS11	GD31	JO10	*R.nitidus*	-0.043573	0.068192
JW7	GD30	JO10	*R.nitidus*	-0.106533	0.073737
JO3	GD30	JO10	*R.nitidus*	-0.128179	0.070955
JO5	GD30	JO10	*R.nitidus*	-0.098491	0.064874
GD22	GD31	JO10	*R.nitidus*	0.026442	0.064093
GD25	GD31	JO10	*R.nitidus*	-0.033497	0.063944
JO1	GD30	JO10	*R.nitidus*	-0.143414	0.065586
GD30	GD31	JO10	*R.nitidus*	0.096102	0.06816
GD29	GD31	JO10	*R.nitidus*	0.01495	0.064576
JO8	GD31	JO10	*R.nitidus*	-0.060254	0.072309
GD33	GD31	JO10	*R.nitidus*	-0.065047	0.065725
GD26	GD31	JO10	*R.nitidus*	-0.024213	0.065497
CS12	GD31	JO10	*R.nitidus*	-0.042537	0.065171
GD34	GD31	JO10	*R.nitidus*	-0.028226	0.066042
GD40	GD31	JO10	*R.nitidus*	-0.004769	0.061772
GD45	GD31	JO10	*R.nitidus*	-0.006312	0.067426
GD32	GD31	JO10	*R.nitidus*	-0.009717	0.070148
GD35	GD31	JO10	*R.nitidus*	-0.01248	0.063884
GD44	GD31	JO10	*R.nitidus*	-0.054899	0.066227
GD41	GD31	JO10	*R.nitidus*	-0.078678	0.065433
GD42	GD31	JO10	*R.nitidus*	-0.030424	0.064402
GD48	GD31	JO10	*R.nitidus*	-0.01913	0.067895
GD49	GD31	JO10	*R.nitidus*	0.050134	0.066686

CS16	GD31	JO10	*R.nitidus*	-0.049666	0.066637
CS13	GD31	JO10	*R.nitidus*	-0.098054	0.066375
CS14	GD31	JO10	*R.nitidus*	-0.043576	0.067585
GD46	GD31	JO10	*R.nitidus*	-0.045381	0.064727
CS22	GD31	JO10	*R.nitidus*	-0.032738	0.066256
CS15	GD31	JO10	*R.nitidus*	-0.058267	0.067014
CS17	GD31	JO10	*R.nitidus*	-0.017652	0.068491
CS18	GD31	JO10	*R.nitidus*	-0.087772	0.064806
CS21	GD31	JO10	*R.nitidus*	-0.068483	0.06533
CS24	GD31	JO10	*R.nitidus*	-0.077508	0.064105
CS25	GD31	JO10	*R.nitidus*	-0.045555	0.066573
CS20	GD31	JO10	*R.nitidus*	-0.098592	0.066365
CS19	GD31	JO10	*R.nitidus*	-0.055891	0.065133
CS30	GD31	JO10	*R.nitidus*	-0.105019	0.065662
CS28	GD31	JO10	*R.nitidus*	-0.055857	0.065886
CS23	GD31	JO10	*R.nitidus*	-0.098361	0.063033
SC6	GD31	JO10	*R.nitidus*	-0.023188	0.063566
GD50	GD31	JO10	*R.nitidus*	-0.024299	0.069623
CS4	GD31	JO10	*R.nitidus*	-0.072359	0.065326
SC18	GD31	JO10	*R.nitidus*	-0.002315	0.06416
SC10	GD31	JO10	*R.nitidus*	0.029148	0.065656
CS3	GD31	JO10	*R.nitidus*	-0.104012	0.066376
SC5	GD31	JO10	*R.nitidus*	-0.04236	0.068275
SC26	GD31	JO10	*R.nitidus*	-0.068406	0.062873
SC24	GD31	JO10	*R.nitidus*	-0.033716	0.062869
SC28	GD31	JO10	*R.nitidus*	-0.089102	0.064615
SC12	GD31	JO10	*R.nitidus*	-0.061495	0.064373
SC14	GD31	JO10	*R.nitidus*	-0.026948	0.063848
SC27	GD31	JO10	*R.nitidus*	-0.026846	0.063128
SC15	GD31	JO10	*R.nitidus*	-0.042773	0.064582
JW6	GD31	JO10	*R.nitidus*	-0.051556	0.071981
SC42	GD31	JO10	*R.nitidus*	0.11536	0.064352
CS31	GD31	JO10	*R.nitidus*	-0.043914	0.066658
SC31	GD31	JO10	*R.nitidus*	-0.04507	0.064941
SC32	GD31	JO10	*R.nitidus*	0.007067	0.065539
JW4	GD31	JO10	*R.nitidus*	0.077465	0.067502
CS6	GD31	JO10	*R.nitidus*	-0.087223	0.066953
MY4	GD31	JO10	*R.nitidus*	-0.084187	0.066179
SC45	GD31	JO10	*R.nitidus*	-0.041547	0.064303
SC35	GD31	JO10	*R.nitidus*	-0.017009	0.064474
JO3	GD31	JO10	*R.nitidus*	-0.028054	0.07093
JO8	GD32	JO10	*R.nitidus*	-0.022002	0.076208
SC34	GD31	JO10	*R.nitidus*	-0.034458	0.063792
JO7	GD32	JO10	*R.nitidus*	-0.059638	0.073634
JO1	GD31	JO10	*R.nitidus*	-0.064888	0.064421
JW7	GD31	JO10	*R.nitidus*	-0.053524	0.068635
JO5	GD31	JO10	*R.nitidus*	-0.025326	0.064816
MY6	GD31	JO10	*R.nitidus*	-0.05	0.064757
CS11	GD32	JO10	*R.nitidus*	-0.031939	0.069266
GD30	GD32	JO10	*R.nitidus*	0.110895	0.068556
GD22	GD32	JO10	*R.nitidus*	0.040529	0.065754
SC44	GD31	JO10	*R.nitidus*	-0.059823	0.065583
GD26	GD32	JO10	*R.nitidus*	0.003337	0.065327
GD29	GD32	JO10	*R.nitidus*	0.040835	0.065167
GD25	GD32	JO10	*R.nitidus*	0.001675	0.066475
GD40	GD32	JO10	*R.nitidus*	-0.004143	0.06676

GD31	GD32	JO10	*R.nitidus*	0.009717	0.070148
GD21	GD32	JO10	*R.nitidus*	0.14026	0.064625
GD35	GD32	JO10	*R.nitidus*	-0.001729	0.066326
GD41	GD32	JO10	*R.nitidus*	-0.074459	0.066287
GD33	GD32	JO10	*R.nitidus*	-0.067662	0.066692
GD44	GD32	JO10	*R.nitidus*	-0.028259	0.068526
GD34	GD32	JO10	*R.nitidus*	-0.012987	0.06631
CS12	GD32	JO10	*R.nitidus*	-0.032132	0.069358
GD48	GD32	JO10	*R.nitidus*	-0.00277	0.067321
CS13	GD32	JO10	*R.nitidus*	-0.092535	0.068145
CS16	GD32	JO10	*R.nitidus*	-0.051442	0.067252
GD49	GD32	JO10	*R.nitidus*	0.064786	0.06799
GD42	GD32	JO10	*R.nitidus*	-0.038294	0.065595
GD46	GD32	JO10	*R.nitidus*	-0.046713	0.064426
GD45	GD32	JO10	*R.nitidus*	-0.003006	0.069372
CS19	GD32	JO10	*R.nitidus*	-0.032308	0.067066
GD50	GD32	JO10	*R.nitidus*	-0.002081	0.067113
CS14	GD32	JO10	*R.nitidus*	-0.044084	0.069992
CS15	GD32	JO10	*R.nitidus*	-0.046368	0.068479
CS23	GD32	JO10	*R.nitidus*	-0.09084	0.064934
CS30	GD32	JO10	*R.nitidus*	-0.089141	0.0671
CS20	GD32	JO10	*R.nitidus*	-0.083975	0.064289
CS31	GD32	JO10	*R.nitidus*	-0.039967	0.068308
SC6	GD32	JO10	*R.nitidus*	-0.01139	0.066677
CS3	GD32	JO10	*R.nitidus*	-0.098669	0.066399
SC5	GD32	JO10	*R.nitidus*	0	0.070457
CS17	GD32	JO10	*R.nitidus*	-0.008751	0.069745
CS21	GD32	JO10	*R.nitidus*	-0.052749	0.065743
CS4	GD32	JO10	*R.nitidus*	-0.072374	0.065051
SC18	GD32	JO10	*R.nitidus*	0.027778	0.066771
SC14	GD32	JO10	*R.nitidus*	-0.001506	0.06619
SC10	GD32	JO10	*R.nitidus*	0.04653	0.067141
CS18	GD32	JO10	*R.nitidus*	-0.076389	0.065616
CS25	GD32	JO10	*R.nitidus*	-0.028169	0.066585
SC12	GD32	JO10	*R.nitidus*	-0.030023	0.066316
SC24	GD32	JO10	*R.nitidus*	-0.016936	0.065655
CS24	GD32	JO10	*R.nitidus*	-0.069675	0.063789
CS28	GD32	JO10	*R.nitidus*	-0.054927	0.067907
CS22	GD32	JO10	*R.nitidus*	-0.019936	0.065277
SC15	GD32	JO10	*R.nitidus*	-0.035658	0.066306
SC27	GD32	JO10	*R.nitidus*	-0.00854	0.066427
SC31	GD32	JO10	*R.nitidus*	-0.023186	0.066569
SC28	GD32	JO10	*R.nitidus*	-0.05106	0.067216
SC32	GD32	JO10	*R.nitidus*	0.021012	0.065221
SC26	GD32	JO10	*R.nitidus*	-0.035115	0.066328
SC42	GD32	JO10	*R.nitidus*	0.119403	0.064401
SC34	GD32	JO10	*R.nitidus*	-0.037123	0.066281
CS6	GD32	JO10	*R.nitidus*	-0.07775	0.067335
JW6	GD32	JO10	*R.nitidus*	0.004154	0.072308
SC44	GD32	JO10	*R.nitidus*	-0.03632	0.066247
JW7	GD32	JO10	*R.nitidus*	-0.009242	0.069907
MY4	GD32	JO10	*R.nitidus*	-0.064566	0.066338
SC35	GD32	JO10	*R.nitidus*	0.01935	0.067443
MY6	GD32	JO10	*R.nitidus*	-0.029434	0.066314
JW4	GD32	JO10	*R.nitidus*	0.129191	0.068083
JO1	GD32	JO10	*R.nitidus*	-0.039143	0.06625

SC45	GD32	JO10	*R.nitidus*	-0.005405	0.066193
JO5	GD32	JO10	*R.nitidus*	-0.005376	0.067291
JO7	CS12	JO10	*R.nitidus*	-0.018219	0.071519
JO8	CS12	JO10	*R.nitidus*	-0.026681	0.075397
JO3	GD32	JO10	*R.nitidus*	-0.021195	0.071683
CS11	CS12	JO10	*R.nitidus*	0.004274	0.069097
GD21	CS12	JO10	*R.nitidus*	0.172632	0.065277
GD30	CS12	JO10	*R.nitidus*	0.12636	0.069918
GD31	CS12	JO10	*R.nitidus*	0.042537	0.065171
GD25	CS12	JO10	*R.nitidus*	0.059701	0.065344
GD26	CS12	JO10	*R.nitidus*	0.040787	0.065861
GD29	CS12	JO10	*R.nitidus*	0.08702	0.065549
GD35	CS12	JO10	*R.nitidus*	0.043834	0.066122
GD44	CS12	JO10	*R.nitidus*	0.017336	0.06648
GD33	CS12	JO10	*R.nitidus*	-0.008621	0.067411
GD42	CS12	JO10	*R.nitidus*	0.016236	0.065167
GD46	CS12	JO10	*R.nitidus*	0.013393	0.066385
GD34	CS12	JO10	*R.nitidus*	0.022939	0.065854
GD45	CS12	JO10	*R.nitidus*	0.047695	0.066753
GD49	CS12	JO10	*R.nitidus*	0.089125	0.065822
GD48	CS12	JO10	*R.nitidus*	0.039566	0.066604
CS13	CS12	JO10	*R.nitidus*	-0.084691	0.068782
GD32	CS12	JO10	*R.nitidus*	0.032132	0.069358
GD41	CS12	JO10	*R.nitidus*	-0.016358	0.065593
GD40	CS12	JO10	*R.nitidus*	0.056122	0.065233
CS15	CS12	JO10	*R.nitidus*	-0.002278	0.06752
CS16	CS12	JO10	*R.nitidus*	-0.018382	0.065873
GD22	CS12	JO10	*R.nitidus*	0.070977	0.06446
CS20	CS12	JO10	*R.nitidus*	-0.024487	0.066609
CS17	CS12	JO10	*R.nitidus*	0.039414	0.070724
CS18	CS12	JO10	*R.nitidus*	-0.014107	0.066633
CS14	CS12	JO10	*R.nitidus*	-0.00117	0.0697
CS23	CS12	JO10	*R.nitidus*	-0.029794	0.066631
CS21	CS12	JO10	*R.nitidus*	0.000765	0.067185
CS25	CS12	JO10	*R.nitidus*	0.041765	0.067624
CS24	CS12	JO10	*R.nitidus*	-0.020375	0.064513
GD50	CS12	JO10	*R.nitidus*	0.04584	0.069277
CS19	CS12	JO10	*R.nitidus*	0.022418	0.067318
CS3	CS12	JO10	*R.nitidus*	-0.038308	0.066436
CS31	CS12	JO10	*R.nitidus*	0.004105	0.067463
CS28	CS12	JO10	*R.nitidus*	-0.001645	0.066362
SC5	CS12	JO10	*R.nitidus*	0.031179	0.068505
CS22	CS12	JO10	*R.nitidus*	0.032488	0.067056
CS30	CS12	JO10	*R.nitidus*	-0.046289	0.066843
SC10	CS12	JO10	*R.nitidus*	0.10717	0.066137
CS4	CS12	JO10	*R.nitidus*	-0.012928	0.065168
SC12	CS12	JO10	*R.nitidus*	0.014243	0.065947
SC15	CS12	JO10	*R.nitidus*	0.01691	0.066384
SC18	CS12	JO10	*R.nitidus*	0.061503	0.066066
SC14	CS12	JO10	*R.nitidus*	0.036732	0.068122
SC34	CS12	JO10	*R.nitidus*	0.024106	0.065792
SC32	CS12	JO10	*R.nitidus*	0.059316	0.067049
SC28	CS12	JO10	*R.nitidus*	-0.030757	0.065505
SC31	CS12	JO10	*R.nitidus*	0.015897	0.065828
SC6	CS12	JO10	*R.nitidus*	0.028418	0.06723
SC42	CS12	JO10	*R.nitidus*	0.17357	0.064399

JW6	CS12	JO10	*R.nitidus*	0.014881	0.073522
JW4	CS12	JO10	*R.nitidus*	0.151017	0.066991
SC24	CS12	JO10	*R.nitidus*	0.022388	0.065353
CS6	CS12	JO10	*R.nitidus*	-0.014388	0.067636
SC27	CS12	JO10	*R.nitidus*	0.031571	0.067106
SC26	CS12	JO10	*R.nitidus*	0.003785	0.065579
JO3	CS12	JO10	*R.nitidus*	-0.002616	0.070006
SC44	CS12	JO10	*R.nitidus*	0.004669	0.066284
MY4	CS12	JO10	*R.nitidus*	-0.019787	0.067223
SC45	CS12	JO10	*R.nitidus*	0.044892	0.065673
JW7	CS12	JO10	*R.nitidus*	0.024762	0.070586
MY6	CS12	JO10	*R.nitidus*	0.017582	0.065963
GD21	GD33	JO10	*R.nitidus*	0.211735	0.063987
SC35	CS12	JO10	*R.nitidus*	0.054154	0.064871
JO5	CS12	JO10	*R.nitidus*	0.037267	0.065516
GD25	GD33	JO10	*R.nitidus*	0.05887	0.065406
JO7	GD33	JO10	*R.nitidus*	-0.014286	0.070124
GD30	GD33	JO10	*R.nitidus*	0.147964	0.066098
GD26	GD33	JO10	*R.nitidus*	0.074041	0.06251
JO8	GD33	JO10	*R.nitidus*	0.01494	0.071971
CS11	GD33	JO10	*R.nitidus*	0.005495	0.066031
GD22	GD33	JO10	*R.nitidus*	0.095403	0.062632
GD29	GD33	JO10	*R.nitidus*	0.111888	0.066034
GD31	GD33	JO10	*R.nitidus*	0.065047	0.065725
GD32	GD33	JO10	*R.nitidus*	0.067662	0.066692
JO1	CS12	JO10	*R.nitidus*	0.013867	0.065061
CS12	GD33	JO10	*R.nitidus*	0.008621	0.067411
GD35	GD33	JO10	*R.nitidus*	0.054475	0.064301
GD40	GD33	JO10	*R.nitidus*	0.053272	0.065136
GD42	GD33	JO10	*R.nitidus*	0.020194	0.064546
GD34	GD33	JO10	*R.nitidus*	0.032598	0.063028
GD50	GD33	JO10	*R.nitidus*	0.039548	0.066764
GD48	GD33	JO10	*R.nitidus*	0.047359	0.06592
GD46	GD33	JO10	*R.nitidus*	0.004065	0.0635
GD41	GD33	JO10	*R.nitidus*	-0.004023	0.065752
CS13	GD33	JO10	*R.nitidus*	-0.048057	0.0654
CS14	GD33	JO10	*R.nitidus*	0.003536	0.067638
CS15	GD33	JO10	*R.nitidus*	-0.004965	0.065601
GD45	GD33	JO10	*R.nitidus*	0.053097	0.067292
CS16	GD33	JO10	*R.nitidus*	0.002048	0.065159
CS19	GD33	JO10	*R.nitidus*	0.02024	0.065676
GD49	GD33	JO10	*R.nitidus*	0.087687	0.066978
CS17	GD33	JO10	*R.nitidus*	0.031159	0.065195
GD44	GD33	JO10	*R.nitidus*	0.012559	0.065422
CS31	GD33	JO10	*R.nitidus*	0.02622	0.06647
CS28	GD33	JO10	*R.nitidus*	-0.007491	0.067567
CS20	GD33	JO10	*R.nitidus*	-0.033263	0.064945
SC6	GD33	JO10	*R.nitidus*	0.032694	0.063529
SC5	GD33	JO10	*R.nitidus*	0.031994	0.066002
SC10	GD33	JO10	*R.nitidus*	0.083095	0.065941
CS24	GD33	JO10	*R.nitidus*	-0.009993	0.064149
CS4	GD33	JO10	*R.nitidus*	-0.009246	0.064134
CS3	GD33	JO10	*R.nitidus*	-0.048341	0.064379
CS21	GD33	JO10	*R.nitidus*	0.006122	0.061831
CS25	GD33	JO10	*R.nitidus*	0.023256	0.064981
CS22	GD33	JO10	*R.nitidus*	0.035186	0.065809

SC12	GD33	JO10	*R.nitidus*	0.013112	0.062279
SC28	GD33	JO10	*R.nitidus*	-0.010653	0.064171
CS18	GD33	JO10	*R.nitidus*	-0.008487	0.062899
SC26	GD33	JO10	*R.nitidus*	0.003413	0.064273
MY6	GD33	JO10	*R.nitidus*	0.028886	0.063609
CS30	GD33	JO10	*R.nitidus*	-0.041274	0.064651
SC24	GD33	JO10	*R.nitidus*	0.018455	0.063081
JO8	GD34	JO10	*R.nitidus*	0.018367	0.072058
JW4	GD33	JO10	*R.nitidus*	0.173423	0.067024
SC35	GD33	JO10	*R.nitidus*	0.047358	0.064292
SC27	GD33	JO10	*R.nitidus*	0.038194	0.064363
SC15	GD33	JO10	*R.nitidus*	0.023305	0.063643
CS6	GD33	JO10	*R.nitidus*	-0.021308	0.063226
SC14	GD33	JO10	*R.nitidus*	0.05	0.063146
SC18	GD33	JO10	*R.nitidus*	0.050735	0.065007
SC31	GD33	JO10	*R.nitidus*	0.017687	0.065657
CS23	GD33	JO10	*R.nitidus*	-0.030007	0.065003
SC34	GD33	JO10	*R.nitidus*	0.026153	0.062745
GD22	GD34	JO10	*R.nitidus*	0.079646	0.060772
SC42	GD33	JO10	*R.nitidus*	0.171171	0.064303
JO7	GD34	JO10	*R.nitidus*	-0.020992	0.070526
SC32	GD33	JO10	*R.nitidus*	0.067035	0.062918
JO3	GD33	JO10	*R.nitidus*	0.026886	0.070935
MY4	GD33	JO10	*R.nitidus*	-0.00559	0.065366
SC45	GD33	JO10	*R.nitidus*	0.044912	0.063552
GD29	GD34	JO10	*R.nitidus*	0.086556	0.063492
SC44	GD33	JO10	*R.nitidus*	0.006383	0.063503
JW6	GD33	JO10	*R.nitidus*	0.055556	0.068985
GD26	GD34	JO10	*R.nitidus*	0.034025	0.062401
CS11	GD34	JO10	*R.nitidus*	-0.026567	0.066081
JW7	GD33	JO10	*R.nitidus*	0.046968	0.069465
GD33	GD34	JO10	*R.nitidus*	-0.032598	0.063028
GD50	GD34	JO10	*R.nitidus*	0.00365	0.066063
JO1	GD33	JO10	*R.nitidus*	0.014344	0.064864
GD40	GD34	JO10	*R.nitidus*	0.033886	0.063291
GD31	GD34	JO10	*R.nitidus*	0.028226	0.066042
GD21	GD34	JO10	*R.nitidus*	0.17757	0.061132
GD42	GD34	JO10	*R.nitidus*	-0.010569	0.063602
CS12	GD34	JO10	*R.nitidus*	-0.022939	0.065854
JO5	GD33	JO10	*R.nitidus*	0.043964	0.063497
GD46	GD34	JO10	*R.nitidus*	-0.028455	0.062748
GD49	GD34	JO10	*R.nitidus*	0.054717	0.066441
GD25	GD34	JO10	*R.nitidus*	0.018225	0.065941
GD41	GD34	JO10	*R.nitidus*	-0.048741	0.061721
GD30	GD34	JO10	*R.nitidus*	0.1303	0.066493
GD45	GD34	JO10	*R.nitidus*	0.040672	0.066257
SC6	GD34	JO10	*R.nitidus*	-0.004184	0.064299
CS20	GD34	JO10	*R.nitidus*	-0.070877	0.065068
CS25	GD34	JO10	*R.nitidus*	-0.007184	0.065468
CS16	GD34	JO10	*R.nitidus*	-0.03762	0.064113
GD32	GD34	JO10	*R.nitidus*	0.012987	0.06631
GD48	GD34	JO10	*R.nitidus*	0.023985	0.063735
GD44	GD34	JO10	*R.nitidus*	-0.023034	0.065487
CS13	GD34	JO10	*R.nitidus*	-0.081229	0.06531
GD35	GD34	JO10	*R.nitidus*	0.016368	0.065012
CS14	GD34	JO10	*R.nitidus*	-0.033957	0.065939

CS19	GD34	JO10	*R.nitidus*	-0.011679	0.064381
CS23	GD34	JO10	*R.nitidus*	-0.076265	0.064361
CS22	GD34	JO10	*R.nitidus*	-0.003511	0.065188
CS17	GD34	JO10	*R.nitidus*	-0.002901	0.066834
SC26	GD34	JO10	*R.nitidus*	-0.035957	0.062599
SC12	GD34	JO10	*R.nitidus*	-0.027046	0.062812
CS15	GD34	JO10	*R.nitidus*	-0.035641	0.064825
CS4	GD34	JO10	*R.nitidus*	-0.038408	0.064415
CS6	GD34	JO10	*R.nitidus*	-0.051546	0.065775
SC10	GD34	JO10	*R.nitidus*	0.053381	0.065217
CS18	GD34	JO10	*R.nitidus*	-0.044554	0.063828
MY4	GD34	JO10	*R.nitidus*	-0.049203	0.063734
CS24	GD34	JO10	*R.nitidus*	-0.046796	0.063146
CS21	GD34	JO10	*R.nitidus*	-0.034722	0.063718
SC24	GD34	JO10	*R.nitidus*	-0.01198	0.061541
CS28	GD34	JO10	*R.nitidus*	-0.039216	0.06698
CS30	GD34	JO10	*R.nitidus*	-0.080562	0.063532
SC18	GD34	JO10	*R.nitidus*	0.016643	0.064622
SC5	GD34	JO10	*R.nitidus*	0.000733	0.066951
JW4	GD34	JO10	*R.nitidus*	0.141818	0.065885
SC14	GD34	JO10	*R.nitidus*	0.006887	0.06357
SC32	GD34	JO10	*R.nitidus*	0.024793	0.064303
JO5	GD34	JO10	*R.nitidus*	0.004079	0.062813
SC15	GD34	JO10	*R.nitidus*	-0.021739	0.063857
MY6	GD34	JO10	*R.nitidus*	-0.010359	0.063725
SC27	GD34	JO10	*R.nitidus*	-0.008511	0.064222
GD22	GD35	JO10	*R.nitidus*	0.030053	0.06354
SC44	GD34	JO10	*R.nitidus*	-0.025018	0.062464
JO3	GD34	JO10	*R.nitidus*	0.004299	0.069345
CS3	GD34	JO10	*R.nitidus*	-0.078091	0.065008
SC34	GD34	JO10	*R.nitidus*	-0.000693	0.062857
CS31	GD34	JO10	*R.nitidus*	-0.00968	0.065899
JW6	GD34	JO10	*R.nitidus*	0.027027	0.068286
SC28	GD34	JO10	*R.nitidus*	-0.053296	0.062891
SC31	GD34	JO10	*R.nitidus*	-0.016393	0.065431
SC45	GD34	JO10	*R.nitidus*	0.000682	0.063921
JW7	GD34	JO10	*R.nitidus*	0.020548	0.069635
JO7	GD35	JO10	*R.nitidus*	-0.02737	0.068793
SC42	GD34	JO10	*R.nitidus*	0.15003	0.063679
SC35	GD34	JO10	*R.nitidus*	0.011268	0.063668
GD25	GD35	JO10	*R.nitidus*	0.010569	0.063227
GD45	GD35	JO10	*R.nitidus*	0.025483	0.067093
CS12	GD35	JO10	*R.nitidus*	-0.043834	0.066122
GD33	GD35	JO10	*R.nitidus*	-0.054475	0.064301
GD32	GD35	JO10	*R.nitidus*	0.001729	0.066326
GD29	GD35	JO10	*R.nitidus*	0.040121	0.063146
GD42	GD35	JO10	*R.nitidus*	-0.022508	0.064404
GD31	GD35	JO10	*R.nitidus*	0.01248	0.063884
JO8	GD35	JO10	*R.nitidus*	-0.044838	0.072361
GD34	GD35	JO10	*R.nitidus*	-0.016368	0.065012
GD26	GD35	JO10	*R.nitidus*	0.02057	0.064289
JO1	GD34	JO10	*R.nitidus*	-0.024308	0.064148
GD30	GD35	JO10	*R.nitidus*	0.100862	0.066956
GD21	GD35	JO10	*R.nitidus*	0.131365	0.063246
CS28	GD35	JO10	*R.nitidus*	-0.053464	0.06597
CS11	GD35	JO10	*R.nitidus*	-0.030238	0.067254

GD44	GD35	JO10	*R.nitidus*	-0.050167	0.064499
CS24	GD35	JO10	*R.nitidus*	-0.065217	0.06205
CS14	GD35	JO10	*R.nitidus*	-0.03647	0.06752
GD41	GD35	JO10	*R.nitidus*	-0.062844	0.062585
SC6	GD35	JO10	*R.nitidus*	-0.016405	0.062206
GD40	GD35	JO10	*R.nitidus*	0.007949	0.061576
CS4	GD35	JO10	*R.nitidus*	-0.051883	0.061546
CS20	GD35	JO10	*R.nitidus*	-0.081545	0.063965
CS13	GD35	JO10	*R.nitidus*	-0.093177	0.067346
GD48	GD35	JO10	*R.nitidus*	-0.008496	0.067615
GD46	GD35	JO10	*R.nitidus*	-0.046912	0.063132
CS17	GD35	JO10	*R.nitidus*	-0.009848	0.065912
CS18	GD35	JO10	*R.nitidus*	-0.055919	0.064101
GD49	GD35	JO10	*R.nitidus*	0.039049	0.06638
GD50	GD35	JO10	*R.nitidus*	-0.032738	0.067387
SC24	GD35	JO10	*R.nitidus*	-0.031341	0.063686
CS16	GD35	JO10	*R.nitidus*	-0.04911	0.064518
SC10	GD35	JO10	*R.nitidus*	0.050109	0.06369
SC34	GD35	JO10	*R.nitidus*	-0.011527	0.06264
SC14	GD35	JO10	*R.nitidus*	0.001435	0.064659
CS15	GD35	JO10	*R.nitidus*	-0.046528	0.065644
SC31	GD35	JO10	*R.nitidus*	-0.035221	0.063079
CS30	GD35	JO10	*R.nitidus*	-0.083458	0.06415
SC18	GD35	JO10	*R.nitidus*	-0.00387	0.064283
SC12	GD35	JO10	*R.nitidus*	-0.034657	0.062313
CS25	GD35	JO10	*R.nitidus*	-0.016188	0.064311
CS19	GD35	JO10	*R.nitidus*	-0.019389	0.066612
SC26	GD35	JO10	*R.nitidus*	-0.042194	0.06273
SC5	GD35	JO10	*R.nitidus*	-0.014948	0.066865
CS22	GD35	JO10	*R.nitidus*	-0.011486	0.062498
CS21	GD35	JO10	*R.nitidus*	-0.053696	0.063481
CS23	GD35	JO10	*R.nitidus*	-0.084588	0.062908
JW6	GD35	JO10	*R.nitidus*	-0.008729	0.070096
CS6	GD35	JO10	*R.nitidus*	-0.074581	0.065957
MY6	GD35	JO10	*R.nitidus*	-0.021277	0.064156
CS3	GD35	JO10	*R.nitidus*	-0.07421	0.06503
JO8	GD40	JO10	*R.nitidus*	-0.056158	0.070171
CS31	GD35	JO10	*R.nitidus*	-0.027946	0.064779
SC15	GD35	JO10	*R.nitidus*	-0.026946	0.06365
SC42	GD35	JO10	*R.nitidus*	0.141538	0.062932
JO3	GD35	JO10	*R.nitidus*	-0.042266	0.069762
CS11	GD40	JO10	*R.nitidus*	-0.05181	0.065306
SC27	GD35	JO10	*R.nitidus*	-0.015681	0.06223
SC28	GD35	JO10	*R.nitidus*	-0.060206	0.063327
JW4	GD35	JO10	*R.nitidus*	0.122807	0.065104
GD21	GD40	JO10	*R.nitidus*	0.125984	0.063045
JO7	GD40	JO10	*R.nitidus*	-0.058107	0.071832
GD22	GD40	JO10	*R.nitidus*	0.021676	0.061844
JW7	GD35	JO10	*R.nitidus*	-0.021258	0.067633
SC35	GD35	JO10	*R.nitidus*	0.006541	0.06339
JO1	GD35	JO10	*R.nitidus*	-0.037683	0.06187
SC32	GD35	JO10	*R.nitidus*	0.016039	0.064305
GD30	GD40	JO10	*R.nitidus*	0.110044	0.066244
GD26	GD40	JO10	*R.nitidus*	0.009125	0.064579
SC45	GD35	JO10	*R.nitidus*	-0.00072	0.061229
SC44	GD35	JO10	*R.nitidus*	-0.042254	0.064301

GD25	GD40	JO10	*R.nitidus*	0.017557	0.061521
GD32	GD40	JO10	*R.nitidus*	0.004143	0.06676
MY4	GD35	JO10	*R.nitidus*	-0.061254	0.065256
GD35	GD40	JO10	*R.nitidus*	-0.007949	0.061576
GD29	GD40	JO10	*R.nitidus*	0.044174	0.06318
GD34	GD40	JO10	*R.nitidus*	-0.033886	0.063291
JO5	GD35	JO10	*R.nitidus*	-0.018545	0.062573
GD49	GD40	JO10	*R.nitidus*	0.056459	0.065555
GD48	GD40	JO10	*R.nitidus*	0.001604	0.064467
GD31	GD40	JO10	*R.nitidus*	0.004769	0.061772
CS12	GD40	JO10	*R.nitidus*	-0.056122	0.065233
GD45	GD40	JO10	*R.nitidus*	0.014407	0.06549
CS25	GD40	JO10	*R.nitidus*	-0.03505	0.064003
GD41	GD40	JO10	*R.nitidus*	-0.061785	0.063976
CS21	GD40	JO10	*R.nitidus*	-0.071983	0.061948
CS22	GD40	JO10	*R.nitidus*	-0.029703	0.064846
CS31	GD40	JO10	*R.nitidus*	-0.042766	0.06492
CS3	GD40	JO10	*R.nitidus*	-0.103654	0.062591
CS13	GD40	JO10	*R.nitidus*	-0.111752	0.066627
CS28	GD40	JO10	*R.nitidus*	-0.052437	0.06587
SC14	GD40	JO10	*R.nitidus*	-0.027454	0.062551
GD33	GD40	JO10	*R.nitidus*	-0.053272	0.065136
SC27	GD40	JO10	*R.nitidus*	-0.040194	0.062826
CS4	GD40	JO10	*R.nitidus*	-0.069571	0.062659
CS19	GD40	JO10	*R.nitidus*	-0.045219	0.065176
GD44	GD40	JO10	*R.nitidus*	-0.043895	0.062644
CS17	GD40	JO10	*R.nitidus*	-0.020725	0.064917
CS18	GD40	JO10	*R.nitidus*	-0.078947	0.062543
CS23	GD40	JO10	*R.nitidus*	-0.094722	0.060078
GD50	GD40	JO10	*R.nitidus*	-0.000878	0.066426
CS15	GD40	JO10	*R.nitidus*	-0.060649	0.065964
SC15	GD40	JO10	*R.nitidus*	-0.044523	0.063237
GD42	GD40	JO10	*R.nitidus*	-0.029817	0.062932
CS24	GD40	JO10	*R.nitidus*	-0.077377	0.060354
CS20	GD40	JO10	*R.nitidus*	-0.091912	0.06306
GD46	GD40	JO10	*R.nitidus*	-0.05251	0.064496
CS14	GD40	JO10	*R.nitidus*	-0.048626	0.067222
CS16	GD40	JO10	*R.nitidus*	-0.073329	0.064537
CS6	GD40	JO10	*R.nitidus*	-0.080597	0.064943
SC6	GD40	JO10	*R.nitidus*	-0.032028	0.060563
SC12	GD40	JO10	*R.nitidus*	-0.059474	0.061553
SC10	GD40	JO10	*R.nitidus*	0.036749	0.063009
SC34	GD40	JO10	*R.nitidus*	-0.035261	0.061274
SC5	GD40	JO10	*R.nitidus*	-0.04034	0.066037
SC24	GD40	JO10	*R.nitidus*	-0.039046	0.061541
MY6	GD40	JO10	*R.nitidus*	-0.037037	0.062801
JW4	GD40	JO10	*R.nitidus*	0.091508	0.065788
CS30	GD40	JO10	*R.nitidus*	-0.10167	0.062458
SC28	GD40	JO10	*R.nitidus*	-0.079944	0.062302
SC26	GD40	JO10	*R.nitidus*	-0.057559	0.0621
JO8	GD41	JO10	*R.nitidus*	0.008929	0.073002
SC18	GD40	JO10	*R.nitidus*	-0.014126	0.062882
JW7	GD40	JO10	*R.nitidus*	-0.042795	0.066277
JW6	GD40	JO10	*R.nitidus*	-0.038826	0.072349
JO5	GD40	JO10	*R.nitidus*	-0.020819	0.063155
SC32	GD40	JO10	*R.nitidus*	-0.004144	0.062444

SC45	GD40	JO10	*R.nitidus*	-0.025965	0.061324
SC44	GD40	JO10	*R.nitidus*	-0.052632	0.063117
SC31	GD40	JO10	*R.nitidus*	-0.045423	0.062297
SC35	GD40	JO10	*R.nitidus*	-0.004101	0.060974
CS11	GD41	JO10	*R.nitidus*	0.020423	0.066332
SC42	GD40	JO10	*R.nitidus*	0.122665	0.062232
GD22	GD41	JO10	*R.nitidus*	0.110759	0.062856
MY4	GD40	JO10	*R.nitidus*	-0.079498	0.063337
GD29	GD41	JO10	*R.nitidus*	0.106635	0.064503
GD46	GD41	JO10	*R.nitidus*	0.018067	0.06283
JO7	GD41	JO10	*R.nitidus*	0.006604	0.070415
JO1	GD40	JO10	*R.nitidus*	-0.048143	0.062608
GD35	GD41	JO10	*R.nitidus*	0.062844	0.062585
GD34	GD41	JO10	*R.nitidus*	0.048741	0.061721
JO3	GD40	JO10	*R.nitidus*	-0.02616	0.06849
CS13	GD41	JO10	*R.nitidus*	-0.037722	0.066337
GD32	GD41	JO10	*R.nitidus*	0.074459	0.066287
GD25	GD41	JO10	*R.nitidus*	0.066461	0.063483
GD45	GD41	JO10	*R.nitidus*	0.061419	0.067014
GD48	GD41	JO10	*R.nitidus*	0.067234	0.062959
GD26	GD41	JO10	*R.nitidus*	0.076923	0.063268
GD42	GD41	JO10	*R.nitidus*	0.05083	0.060378
GD40	GD41	JO10	*R.nitidus*	0.061785	0.063976
GD21	GD41	JO10	*R.nitidus*	0.208967	0.059898
CS22	GD41	JO10	*R.nitidus*	0.033922	0.063769
CS19	GD41	JO10	*R.nitidus*	0.03026	0.066042
CS17	GD41	JO10	*R.nitidus*	0.048375	0.066557
CS3	GD41	JO10	*R.nitidus*	-0.046099	0.064182
GD31	GD41	JO10	*R.nitidus*	0.078678	0.065433
GD30	GD41	JO10	*R.nitidus*	0.154052	0.065903
GD49	GD41	JO10	*R.nitidus*	0.121186	0.067342
GD33	GD41	JO10	*R.nitidus*	0.004023	0.065752
CS16	GD41	JO10	*R.nitidus*	0.011283	0.064087
CS12	GD41	JO10	*R.nitidus*	0.016358	0.065593
CS20	GD41	JO10	*R.nitidus*	-0.025105	0.065455
CS18	GD41	JO10	*R.nitidus*	0	0.064683
CS21	GD41	JO10	*R.nitidus*	0.008874	0.063053
GD44	GD41	JO10	*R.nitidus*	0.021559	0.063399
CS15	GD41	JO10	*R.nitidus*	0.010563	0.066003
CS30	GD41	JO10	*R.nitidus*	-0.033962	0.063072
CS25	GD41	JO10	*R.nitidus*	0.033356	0.064926
SC5	GD41	JO10	*R.nitidus*	0.046852	0.066077
GD50	GD41	JO10	*R.nitidus*	0.049098	0.067503
CS14	GD41	JO10	*R.nitidus*	0.017033	0.067567
SC10	GD41	JO10	*R.nitidus*	0.097983	0.06354
SC42	GD41	JO10	*R.nitidus*	0.17955	0.06274
SC12	GD41	JO10	*R.nitidus*	0.030812	0.064976
JO1	GD41	JO10	*R.nitidus*	0.023578	0.063481
CS28	GD41	JO10	*R.nitidus*	0.015683	0.065677
JW7	GD41	JO10	*R.nitidus*	0.027706	0.068154
JO3	GD41	JO10	*R.nitidus*	0.021151	0.068902
CS6	GD41	JO10	*R.nitidus*	-0.013981	0.064228
CS24	GD41	JO10	*R.nitidus*	0	0.062513
CS23	GD41	JO10	*R.nitidus*	-0.027952	0.064503
SC28	GD41	JO10	*R.nitidus*	-0.001453	0.06416
SC26	GD41	JO10	*R.nitidus*	0.013122	0.064362

SC27	GD41	JO10	*R.nitidus*	0.037091	0.063165
CS31	GD41	JO10	*R.nitidus*	0.026886	0.064656
SC35	GD41	JO10	*R.nitidus*	0.070671	0.06392
CS4	GD41	JO10	*R.nitidus*	0.00071	0.063391
SC32	GD41	JO10	*R.nitidus*	0.074773	0.062811
SC6	GD41	JO10	*R.nitidus*	0.034826	0.065338
GD25	GD42	JO10	*R.nitidus*	0.042345	0.064964
JO8	GD42	JO10	*R.nitidus*	-0.048338	0.072449
JW4	GD41	JO10	*R.nitidus*	0.154532	0.065387
SC14	GD41	JO10	*R.nitidus*	0.054506	0.06275
SC18	GD41	JO10	*R.nitidus*	0.057143	0.065314
SC24	GD41	JO10	*R.nitidus*	0.018665	0.062737
JO5	GD41	JO10	*R.nitidus*	0.053672	0.062641
GD33	GD42	JO10	*R.nitidus*	-0.020194	0.064546
SC31	GD41	JO10	*R.nitidus*	0.028767	0.065196
MY4	GD41	JO10	*R.nitidus*	0.00072	0.063413
JW6	GD41	JO10	*R.nitidus*	0.020115	0.072545
SC34	GD41	JO10	*R.nitidus*	0.039858	0.064101
SC15	GD41	JO10	*R.nitidus*	0.030568	0.063272
GD30	GD42	JO10	*R.nitidus*	0.145387	0.065924
SC45	GD41	JO10	*R.nitidus*	0.048729	0.06263
GD21	GD42	JO10	*R.nitidus*	0.164634	0.060616
SC44	GD41	JO10	*R.nitidus*	0.018025	0.063481
MY6	GD41	JO10	*R.nitidus*	0.031834	0.06361
GD26	GD42	JO10	*R.nitidus*	0.021987	0.064387
JO7	GD42	JO10	*R.nitidus*	-0.052023	0.070022
GD44	GD42	JO10	*R.nitidus*	-0.023654	0.065385
CS15	GD42	JO10	*R.nitidus*	-0.02432	0.065793
CS16	GD42	JO10	*R.nitidus*	-0.027758	0.064544
GD41	GD42	JO10	*R.nitidus*	-0.05083	0.060378
CS18	GD42	JO10	*R.nitidus*	-0.036301	0.064475
GD29	GD42	JO10	*R.nitidus*	0.06737	0.063723
CS11	GD42	JO10	*R.nitidus*	-0.01787	0.066823
GD40	GD42	JO10	*R.nitidus*	0.029817	0.062932
GD46	GD42	JO10	*R.nitidus*	-0.014706	0.06255
GD49	GD42	JO10	*R.nitidus*	0.081938	0.067472
GD22	GD42	JO10	*R.nitidus*	0.066774	0.064148
CS12	GD42	JO10	*R.nitidus*	-0.016236	0.065167
GD31	GD42	JO10	*R.nitidus*	0.030424	0.064402
GD35	GD42	JO10	*R.nitidus*	0.022508	0.064404
GD32	GD42	JO10	*R.nitidus*	0.038294	0.065595
CS21	GD42	JO10	*R.nitidus*	-0.032809	0.064352
CS30	GD42	JO10	*R.nitidus*	-0.072948	0.061797
GD50	GD42	JO10	*R.nitidus*	-0.007216	0.069052
CS14	GD42	JO10	*R.nitidus*	-0.026969	0.066158
GD48	GD42	JO10	*R.nitidus*	0.024086	0.064637
CS17	GD42	JO10	*R.nitidus*	0.013971	0.06707
GD34	GD42	JO10	*R.nitidus*	0.010569	0.063602
CS31	GD42	JO10	*R.nitidus*	0.001493	0.064275
CS25	GD42	JO10	*R.nitidus*	-0.007174	0.065411
SC12	GD42	JO10	*R.nitidus*	-0.012667	0.064197
CS13	GD42	JO10	*R.nitidus*	-0.069236	0.066299
GD45	GD42	JO10	*R.nitidus*	0.043286	0.06587
CS23	GD42	JO10	*R.nitidus*	-0.067455	0.065401
CS4	GD42	JO10	*R.nitidus*	-0.02439	0.06372
CS28	GD42	JO10	*R.nitidus*	-0.031555	0.066261

CS20	GD42	JO10	*R.nitidus*	-0.060501	0.065402
CS22	GD42	JO10	*R.nitidus*	0.00838	0.065198
CS19	GD42	JO10	*R.nitidus*	-0.002157	0.065705
SC15	GD42	JO10	*R.nitidus*	-0.014337	0.065332
CS24	GD42	JO10	*R.nitidus*	-0.038814	0.062887
CS3	GD42	JO10	*R.nitidus*	-0.072187	0.064325
SC26	GD42	JO10	*R.nitidus*	-0.018355	0.064359
SC14	GD42	JO10	*R.nitidus*	0.021368	0.062781
CS6	GD42	JO10	*R.nitidus*	-0.048565	0.065381
JW7	GD42	JO10	*R.nitidus*	-0.027027	0.067966
SC27	GD42	JO10	*R.nitidus*	0.005087	0.06357
MY6	GD42	JO10	*R.nitidus*	0.004811	0.064732
SC5	GD42	JO10	*R.nitidus*	0.005069	0.065594
SC32	GD42	JO10	*R.nitidus*	0.032526	0.064316
SC35	GD42	JO10	*R.nitidus*	0.025052	0.06398
JO3	GD42	JO10	*R.nitidus*	-0.020761	0.068722
JW4	GD42	JO10	*R.nitidus*	0.116569	0.064771
JW6	GD42	JO10	*R.nitidus*	-0.030039	0.070932
SC10	GD42	JO10	*R.nitidus*	0.058824	0.065926
JO7	GD44	JO10	*R.nitidus*	-0.010859	0.068685
SC6	GD42	JO10	*R.nitidus*	-0.00495	0.063335
SC24	GD42	JO10	*R.nitidus*	-0.009306	0.063451
GD25	GD44	JO10	*R.nitidus*	0.0489	0.066003
JO8	GD44	JO10	*R.nitidus*	-0.024768	0.070609
SC18	GD42	JO10	*R.nitidus*	0.016862	0.064476
GD30	GD44	JO10	*R.nitidus*	0.143756	0.065564
MY4	GD42	JO10	*R.nitidus*	-0.035562	0.064237
SC34	GD42	JO10	*R.nitidus*	0.008421	0.062885
CS11	GD44	JO10	*R.nitidus*	-0.011923	0.067727
SC31	GD42	JO10	*R.nitidus*	-0.012422	0.066738
SC45	GD42	JO10	*R.nitidus*	0	0.063266
SC28	GD42	JO10	*R.nitidus*	-0.04796	0.064863
SC44	GD42	JO10	*R.nitidus*	-0.023547	0.06403
GD26	GD44	JO10	*R.nitidus*	0.05033	0.065954
GD31	GD44	JO10	*R.nitidus*	0.054899	0.066227
GD29	GD44	JO10	*R.nitidus*	0.101218	0.065284
GD49	GD44	JO10	*R.nitidus*	0.103327	0.067374
SC42	GD42	JO10	*R.nitidus*	0.166253	0.063059
GD32	GD44	JO10	*R.nitidus*	0.028259	0.068526
GD45	GD44	JO10	*R.nitidus*	0.056147	0.067953
GD42	GD44	JO10	*R.nitidus*	0.023654	0.065385
GD46	GD44	JO10	*R.nitidus*	0.00326	0.064872
JO1	GD42	JO10	*R.nitidus*	-0.01654	0.063282
GD41	GD44	JO10	*R.nitidus*	-0.021559	0.063399
GD33	GD44	JO10	*R.nitidus*	-0.012559	0.065422
GD34	GD44	JO10	*R.nitidus*	0.023034	0.065487
JO5	GD42	JO10	*R.nitidus*	0.010815	0.064075
CS15	GD44	JO10	*R.nitidus*	-0.026276	0.06459
GD22	GD44	JO10	*R.nitidus*	0.078906	0.064441
GD21	GD44	JO10	*R.nitidus*	0.176917	0.062527
CS13	GD44	JO10	*R.nitidus*	-0.084627	0.067587
GD50	GD44	JO10	*R.nitidus*	0.033708	0.066664
CS30	GD44	JO10	*R.nitidus*	-0.055887	0.063398
CS17	GD44	JO10	*R.nitidus*	0.010078	0.068466
CS12	GD44	JO10	*R.nitidus*	-0.017336	0.06648
SC10	GD44	JO10	*R.nitidus*	0.064945	0.064733

CS14	GD44	JO10	*R.nitidus*	-0.014296	0.067979
CS16	GD44	JO10	*R.nitidus*	-0.019787	0.065277
CS19	GD44	JO10	*R.nitidus*	0.000774	0.066455
GD40	GD44	JO10	*R.nitidus*	0.043895	0.062644
GD35	GD44	JO10	*R.nitidus*	0.050167	0.064499
CS28	GD44	JO10	*R.nitidus*	-0.022961	0.066262
CS23	GD44	JO10	*R.nitidus*	-0.057795	0.064172
CS31	GD44	JO10	*R.nitidus*	-0.011655	0.067253
GD48	GD44	JO10	*R.nitidus*	0.046696	0.06487
CS22	GD44	JO10	*R.nitidus*	-0.0039	0.065959
CS20	GD44	JO10	*R.nitidus*	-0.056948	0.065415
SC42	GD44	JO10	*R.nitidus*	0.15703	0.062742
CS21	GD44	JO10	*R.nitidus*	-0.022861	0.064294
SC24	GD44	JO10	*R.nitidus*	0.006971	0.064441
SC27	GD44	JO10	*R.nitidus*	0.006002	0.065884
CS18	GD44	JO10	*R.nitidus*	-0.038754	0.065367
SC35	GD44	JO10	*R.nitidus*	0.044412	0.064435
CS3	GD44	JO10	*R.nitidus*	-0.062121	0.064589
SC15	GD44	JO10	*R.nitidus*	0.009946	0.06565
SC34	GD44	JO10	*R.nitidus*	0.009587	0.064727
SC12	GD44	JO10	*R.nitidus*	-0.014738	0.064067
CS24	GD44	JO10	*R.nitidus*	-0.034456	0.064922
JW6	GD44	JO10	*R.nitidus*	0.00691	0.068932
SC32	GD44	JO10	*R.nitidus*	0.04821	0.064908
CS25	GD44	JO10	*R.nitidus*	-0.000765	0.063783
JO8	GD45	JO10	*R.nitidus*	-0.090369	0.073169
SC5	GD44	JO10	*R.nitidus*	0.006966	0.068487
CS11	GD45	JO10	*R.nitidus*	-0.052125	0.069674
SC18	GD44	JO10	*R.nitidus*	0.012638	0.06474
SC6	GD44	JO10	*R.nitidus*	0.015198	0.064269
CS4	GD44	JO10	*R.nitidus*	-0.040152	0.062356
JW7	GD44	JO10	*R.nitidus*	-0.002809	0.069511
JW4	GD44	JO10	*R.nitidus*	0.11838	0.069652
GD26	GD45	JO10	*R.nitidus*	-0.017415	0.067155
SC14	GD44	JO10	*R.nitidus*	0.015094	0.064354
SC45	GD44	JO10	*R.nitidus*	0.018882	0.065919
SC26	GD44	JO10	*R.nitidus*	-0.013788	0.06384
SC28	GD44	JO10	*R.nitidus*	-0.038314	0.064505
GD31	GD45	JO10	*R.nitidus*	0.006312	0.067426
MY6	GD44	JO10	*R.nitidus*	0.00295	0.063542
SC31	GD44	JO10	*R.nitidus*	0.002137	0.065557
JO3	GD44	JO10	*R.nitidus*	-0.013889	0.068155
GD30	GD45	JO10	*R.nitidus*	0.081362	0.068367
GD32	GD45	JO10	*R.nitidus*	0.003006	0.069372
GD22	GD45	JO10	*R.nitidus*	0.037351	0.066906
GD29	GD45	JO10	*R.nitidus*	0.026042	0.066594
CS6	GD44	JO10	*R.nitidus*	-0.045677	0.064932
MY4	GD44	JO10	*R.nitidus*	-0.035392	0.064801
JO7	GD45	JO10	*R.nitidus*	-0.101904	0.0744
JO1	GD44	JO10	*R.nitidus*	-0.00147	0.063187
GD21	GD45	JO10	*R.nitidus*	0.112847	0.06525
SC44	GD44	JO10	*R.nitidus*	-0.01414	0.063242
GD25	GD45	JO10	*R.nitidus*	-0.039439	0.066987
JO5	GD44	JO10	*R.nitidus*	0.019751	0.062619
GD35	GD45	JO10	*R.nitidus*	-0.025483	0.067093
GD40	GD45	JO10	*R.nitidus*	-0.014407	0.06549

GD44	GD45	JO10	*R.nitidus*	-0.056147	0.067953
GD41	GD45	JO10	*R.nitidus*	-0.061419	0.067014
CS14	GD45	JO10	*R.nitidus*	-0.046167	0.069835
GD42	GD45	JO10	*R.nitidus*	-0.043286	0.06587
CS13	GD45	JO10	*R.nitidus*	-0.097697	0.068941
GD49	GD45	JO10	*R.nitidus*	0.055233	0.067119
GD48	GD45	JO10	*R.nitidus*	-0.001921	0.069613
CS22	GD45	JO10	*R.nitidus*	-0.032721	0.067331
CS19	GD45	JO10	*R.nitidus*	-0.033681	0.068981
CS12	GD45	JO10	*R.nitidus*	-0.047695	0.066753
CS18	GD45	JO10	*R.nitidus*	-0.079154	0.067331
GD33	GD45	JO10	*R.nitidus*	-0.053097	0.067292
SC15	GD45	JO10	*R.nitidus*	-0.036335	0.066411
CS21	GD45	JO10	*R.nitidus*	-0.059655	0.067396
CS17	GD45	JO10	*R.nitidus*	-0.013344	0.068864
GD50	GD45	JO10	*R.nitidus*	-0.033473	0.070078
GD34	GD45	JO10	*R.nitidus*	-0.040672	0.066257
CS15	GD45	JO10	*R.nitidus*	-0.057074	0.067541
CS24	GD45	JO10	*R.nitidus*	-0.071828	0.066204
SC26	GD45	JO10	*R.nitidus*	-0.046687	0.064744
CS31	GD45	JO10	*R.nitidus*	-0.050539	0.06821
SC32	GD45	JO10	*R.nitidus*	0.010164	0.064528
SC6	GD45	JO10	*R.nitidus*	-0.013535	0.066272
CS30	GD45	JO10	*R.nitidus*	-0.09699	0.065405
SC28	GD45	JO10	*R.nitidus*	-0.066236	0.065092
CS16	GD45	JO10	*R.nitidus*	-0.062097	0.067342
CS20	GD45	JO10	*R.nitidus*	-0.080696	0.06699
CS4	GD45	JO10	*R.nitidus*	-0.071261	0.064723
JO3	GD45	JO10	*R.nitidus*	-0.040196	0.071394
SC31	GD45	JO10	*R.nitidus*	-0.02568	0.068228
CS23	GD45	JO10	*R.nitidus*	-0.068059	0.06728
JO7	CS13	JO10	*R.nitidus*	0.063913	0.073001
SC27	GD45	JO10	*R.nitidus*	-0.017028	0.06602
SC12	GD45	JO10	*R.nitidus*	-0.047692	0.064514
CS28	GD45	JO10	*R.nitidus*	-0.069136	0.067128
CS25	GD45	JO10	*R.nitidus*	-0.02762	0.067817
JO1	GD45	JO10	*R.nitidus*	-0.0478	0.065431
SC42	GD45	JO10	*R.nitidus*	0.137701	0.064517
CS3	GD45	JO10	*R.nitidus*	-0.0939	0.069079
SC18	GD45	JO10	*R.nitidus*	0.007463	0.066667
JO5	GD45	JO10	*R.nitidus*	-0.028022	0.064462
JW6	GD45	JO10	*R.nitidus*	-0.082177	0.072308
CS6	GD45	JO10	*R.nitidus*	-0.06518	0.068299
SC10	GD45	JO10	*R.nitidus*	0.039106	0.067487
SC45	GD45	JO10	*R.nitidus*	-0.008793	0.064933
SC14	GD45	JO10	*R.nitidus*	0	0.066223
SC44	GD45	JO10	*R.nitidus*	-0.038804	0.064319
GD22	CS13	JO10	*R.nitidus*	0.125428	0.063841
JW7	GD45	JO10	*R.nitidus*	-0.056604	0.068646
SC5	GD45	JO10	*R.nitidus*	-0.01454	0.068082
SC34	GD45	JO10	*R.nitidus*	-0.023772	0.06609
SC24	GD45	JO10	*R.nitidus*	-0.030977	0.065572
SC35	GD45	JO10	*R.nitidus*	-0.003155	0.065215
GD34	CS13	JO10	*R.nitidus*	0.081229	0.06531
CS11	CS13	JO10	*R.nitidus*	0.083333	0.071938
MY6	GD45	JO10	*R.nitidus*	-0.021143	0.067081

MY4	GD45	JO10	*R.nitidus*	-0.065066	0.066921
GD29	CS13	JO10	*R.nitidus*	0.139045	0.065468
GD32	CS13	JO10	*R.nitidus*	0.092535	0.068145
GD50	CS13	JO10	*R.nitidus*	0.091837	0.071882
GD25	CS13	JO10	*R.nitidus*	0.099924	0.066172
GD30	CS13	JO10	*R.nitidus*	0.178689	0.068278
JO8	CS13	JO10	*R.nitidus*	0.050157	0.077397
GD40	CS13	JO10	*R.nitidus*	0.111752	0.066627
JW4	GD45	JO10	*R.nitidus*	0.088336	0.068589
GD45	CS13	JO10	*R.nitidus*	0.097697	0.068941
GD35	CS13	JO10	*R.nitidus*	0.093177	0.067346
GD44	CS13	JO10	*R.nitidus*	0.084627	0.067587
GD31	CS13	JO10	*R.nitidus*	0.098054	0.066375
GD41	CS13	JO10	*R.nitidus*	0.037722	0.066337
GD21	CS13	JO10	*R.nitidus*	0.214334	0.063844
GD33	CS13	JO10	*R.nitidus*	0.048057	0.0654
CS12	CS13	JO10	*R.nitidus*	0.084691	0.068782
CS31	CS13	JO10	*R.nitidus*	0.072757	0.067557
SC6	CS13	JO10	*R.nitidus*	0.078067	0.064978
GD26	CS13	JO10	*R.nitidus*	0.114103	0.06547
CS16	CS13	JO10	*R.nitidus*	0.065126	0.069605
GD42	CS13	JO10	*R.nitidus*	0.069236	0.066299
CS23	CS13	JO10	*R.nitidus*	0.01044	0.064139
CS15	CS13	JO10	*R.nitidus*	0.077851	0.06943
CS17	CS13	JO10	*R.nitidus*	0.116667	0.073409
CS28	CS13	JO10	*R.nitidus*	0.051387	0.067961
GD46	CS13	JO10	*R.nitidus*	0.072316	0.067277
CS24	CS13	JO10	*R.nitidus*	0.045008	0.066125
CS25	CS13	JO10	*R.nitidus*	0.088872	0.06865
CS18	CS13	JO10	*R.nitidus*	0.038522	0.067286
CS21	CS13	JO10	*R.nitidus*	0.045697	0.066221
GD48	CS13	JO10	*R.nitidus*	0.104478	0.068528
SC5	CS13	JO10	*R.nitidus*	0.077042	0.06865
GD49	CS13	JO10	*R.nitidus*	0.142283	0.067972
CS30	CS13	JO10	*R.nitidus*	0.00317	0.066535
SC15	CS13	JO10	*R.nitidus*	0.070229	0.067016
CS22	CS13	JO10	*R.nitidus*	0.088993	0.068443
CS14	CS13	JO10	*R.nitidus*	0.084993	0.071175
SC18	CS13	JO10	*R.nitidus*	0.102896	0.067875
CS20	CS13	JO10	*R.nitidus*	0.016055	0.066876
CS19	CS13	JO10	*R.nitidus*	0.077409	0.067976
SC24	CS13	JO10	*R.nitidus*	0.07624	0.066431
SC35	CS13	JO10	*R.nitidus*	0.108808	0.066199
SC32	CS13	JO10	*R.nitidus*	0.119908	0.067825
SC44	CS13	JO10	*R.nitidus*	0.047328	0.06593
SC31	CS13	JO10	*R.nitidus*	0.072079	0.066184
SC28	CS13	JO10	*R.nitidus*	0.02322	0.06618
CS3	CS13	JO10	*R.nitidus*	0.001577	0.065588
JO1	CS13	JO10	*R.nitidus*	0.065623	0.06495
MY6	CS13	JO10	*R.nitidus*	0.075569	0.065635
SC42	CS13	JO10	*R.nitidus*	0.223314	0.064143
CS4	CS13	JO10	*R.nitidus*	0.047292	0.06657
SC12	CS13	JO10	*R.nitidus*	0.06155	0.06408
SC10	CS13	JO10	*R.nitidus*	0.141445	0.064722
JO5	CS13	JO10	*R.nitidus*	0.102952	0.065087
SC27	CS13	JO10	*R.nitidus*	0.08699	0.066527

JW7	CS13	JO10	*R.nitidus*	0.108716	0.071108
SC45	CS13	JO10	*R.nitidus*	0.077891	0.065492
SC26	CS13	JO10	*R.nitidus*	0.054364	0.066782
MY4	CS13	JO10	*R.nitidus*	0.03914	0.067236
JO3	CS13	JO10	*R.nitidus*	0.077324	0.071537
CS6	CS13	JO10	*R.nitidus*	0.030744	0.067393
GD31	GD46	JO10	*R.nitidus*	0.045381	0.064727
JW4	CS13	JO10	*R.nitidus*	0.210117	0.067229
JO8	GD46	JO10	*R.nitidus*	0.012257	0.071867
SC34	CS13	JO10	*R.nitidus*	0.072948	0.065249
JW6	CS13	JO10	*R.nitidus*	0.091549	0.07345
CS11	GD46	JO10	*R.nitidus*	-0.010776	0.067122
CS12	GD46	JO10	*R.nitidus*	-0.013393	0.066385
GD32	GD46	JO10	*R.nitidus*	0.046713	0.064426
GD29	GD46	JO10	*R.nitidus*	0.100597	0.064269
GD35	GD46	JO10	*R.nitidus*	0.046912	0.063132
GD22	GD46	JO10	*R.nitidus*	0.094627	0.063522
GD25	GD46	JO10	*R.nitidus*	0.052797	0.064243
GD34	GD46	JO10	*R.nitidus*	0.028455	0.062748
GD41	GD46	JO10	*R.nitidus*	-0.018067	0.06283
JO7	GD46	JO10	*R.nitidus*	-0.01256	0.072524
GD30	GD46	JO10	*R.nitidus*	0.162325	0.066583
GD45	GD46	JO10	*R.nitidus*	0.057587	0.067367
GD42	GD46	JO10	*R.nitidus*	0.014706	0.06255
GD21	GD46	JO10	*R.nitidus*	0.205615	0.061361
CS18	GD46	JO10	*R.nitidus*	-0.035137	0.066397
CS28	GD46	JO10	*R.nitidus*	-0.014012	0.066224
CS20	GD46	JO10	*R.nitidus*	-0.053997	0.066247
GD26	GD46	JO10	*R.nitidus*	0.053217	0.062119
CS30	GD46	JO10	*R.nitidus*	-0.055887	0.064647
CS25	GD46	JO10	*R.nitidus*	0.012894	0.066915
CS15	GD46	JO10	*R.nitidus*	-0.030523	0.067243
GD48	GD46	JO10	*R.nitidus*	0.054529	0.064176
GD33	GD46	JO10	*R.nitidus*	-0.004065	0.0635
GD50	GD46	JO10	*R.nitidus*	0.013527	0.065071
GD40	GD46	JO10	*R.nitidus*	0.05251	0.064496
CS31	GD46	JO10	*R.nitidus*	0.000754	0.066292
GD44	GD46	JO10	*R.nitidus*	-0.00326	0.064872
GD49	GD46	JO10	*R.nitidus*	0.095941	0.067651
CS22	GD46	JO10	*R.nitidus*	0.014306	0.065246
SC34	GD46	JO10	*R.nitidus*	0.013986	0.063095
CS19	GD46	JO10	*R.nitidus*	-0.002165	0.06707
SC15	GD46	JO10	*R.nitidus*	0.000713	0.064187
CS23	GD46	JO10	*R.nitidus*	-0.047619	0.064412
CS13	GD46	JO10	*R.nitidus*	-0.072316	0.067277
SC35	GD46	JO10	*R.nitidus*	0.037921	0.063673
CS16	GD46	JO10	*R.nitidus*	-0.021137	0.065104
SC6	GD46	JO10	*R.nitidus*	0.010101	0.063612
CS24	GD46	JO10	*R.nitidus*	-0.028571	0.064024
SC10	GD46	JO10	*R.nitidus*	0.067773	0.06448
SC28	GD46	JO10	*R.nitidus*	-0.039576	0.063892
CS14	GD46	JO10	*R.nitidus*	-0.019288	0.069344
CS17	GD46	JO10	*R.nitidus*	0.018477	0.067847
CS21	GD46	JO10	*R.nitidus*	-0.026823	0.064545
JW4	GD46	JO10	*R.nitidus*	0.150038	0.066065
MY6	GD46	JO10	*R.nitidus*	0.011134	0.06485

SC45	GD46	JO10	*R.nitidus*	0.022346	0.063758
SC31	GD46	JO10	*R.nitidus*	-0.001337	0.064451
CS4	GD46	JO10	*R.nitidus*	-0.023677	0.064088
SC5	GD46	JO10	*R.nitidus*	0.002183	0.066045
CS3	GD46	JO10	*R.nitidus*	-0.056712	0.067007
SC44	GD46	JO10	*R.nitidus*	-0.010079	0.06273
MY4	GD46	JO10	*R.nitidus*	-0.028955	0.064819
CS6	GD46	JO10	*R.nitidus*	-0.03037	0.066161
SC27	GD46	JO10	*R.nitidus*	0.003602	0.064261
GD21	GD48	JO10	*R.nitidus*	0.156514	0.063874
SC18	GD46	JO10	*R.nitidus*	0.041698	0.064591
SC14	GD46	JO10	*R.nitidus*	0.020379	0.062824
SC42	GD46	JO10	*R.nitidus*	0.162567	0.06209
SC12	GD46	JO10	*R.nitidus*	-0.016819	0.062942
SC26	GD46	JO10	*R.nitidus*	-0.021663	0.062848
GD30	GD48	JO10	*R.nitidus*	0.076255	0.067675
SC24	GD46	JO10	*R.nitidus*	0.008553	0.063538
GD22	GD48	JO10	*R.nitidus*	0.044554	0.064615
SC32	GD46	JO10	*R.nitidus*	0.036749	0.063171
GD40	GD48	JO10	*R.nitidus*	-0.001604	0.064467
GD45	GD48	JO10	*R.nitidus*	0.001921	0.069613
JW6	GD46	JO10	*R.nitidus*	0.014409	0.070692
GD31	GD48	JO10	*R.nitidus*	0.01913	0.067895
GD50	GD48	JO10	*R.nitidus*	-0.044732	0.070207
CS11	GD48	JO10	*R.nitidus*	-0.036162	0.067451
GD41	GD48	JO10	*R.nitidus*	-0.067234	0.062959
JO3	GD46	JO10	*R.nitidus*	0.00531	0.0698
GD25	GD48	JO10	*R.nitidus*	0	0.064409
JO7	GD48	JO10	*R.nitidus*	-0.052838	0.071411
GD46	GD48	JO10	*R.nitidus*	-0.054529	0.064176
GD35	GD48	JO10	*R.nitidus*	0.008496	0.067615
GD29	GD48	JO10	*R.nitidus*	0.058469	0.06392
JO1	GD46	JO10	*R.nitidus*	-0.001383	0.063252
JW7	GD46	JO10	*R.nitidus*	0.005329	0.067606
JO8	GD48	JO10	*R.nitidus*	-0.056818	0.073253
GD32	GD48	JO10	*R.nitidus*	0.00277	0.067321
GD49	GD48	JO10	*R.nitidus*	0.050524	0.066821
CS13	GD48	JO10	*R.nitidus*	-0.104478	0.068528
GD34	GD48	JO10	*R.nitidus*	-0.023985	0.063735
JO5	GD46	JO10	*R.nitidus*	0.01339	0.062326
GD26	GD48	JO10	*R.nitidus*	0.014419	0.063451
CS15	GD48	JO10	*R.nitidus*	-0.0546	0.067792
CS12	GD48	JO10	*R.nitidus*	-0.039566	0.066604
CS25	GD48	JO10	*R.nitidus*	-0.031466	0.065303
GD33	GD48	JO10	*R.nitidus*	-0.047359	0.06592
CS17	GD48	JO10	*R.nitidus*	-0.01403	0.067214
CS23	GD48	JO10	*R.nitidus*	-0.093303	0.065939
CS18	GD48	JO10	*R.nitidus*	-0.071219	0.066775
GD44	GD48	JO10	*R.nitidus*	-0.046696	0.06487
GD42	GD48	JO10	*R.nitidus*	-0.024086	0.064637
SC14	GD48	JO10	*R.nitidus*	-0.014179	0.06567
CS30	GD48	JO10	*R.nitidus*	-0.091861	0.066271
CS20	GD48	JO10	*R.nitidus*	-0.0757	0.065746
CS3	GD48	JO10	*R.nitidus*	-0.086353	0.065636
CS21	GD48	JO10	*R.nitidus*	-0.062409	0.065627
CS14	GD48	JO10	*R.nitidus*	-0.040785	0.067816

SC26	GD48	JO10	*R.nitidus*	-0.055394	0.064268
CS28	GD48	JO10	*R.nitidus*	-0.052756	0.067017
CS19	GD48	JO10	*R.nitidus*	-0.038371	0.067831
SC32	GD48	JO10	*R.nitidus*	0.009608	0.066465
SC18	GD48	JO10	*R.nitidus*	0.009479	0.065501
SC12	GD48	JO10	*R.nitidus*	-0.045718	0.063232
SC6	GD48	JO10	*R.nitidus*	-0.034252	0.064597
SC35	GD48	JO10	*R.nitidus*	0.005147	0.065141
JW6	GD48	JO10	*R.nitidus*	-0.018887	0.070238
CS4	GD48	JO10	*R.nitidus*	-0.054693	0.064323
JW4	GD48	JO10	*R.nitidus*	0.095382	0.067103
CS22	GD48	JO10	*R.nitidus*	-0.031963	0.06728
CS16	GD48	JO10	*R.nitidus*	-0.056171	0.066473
SC28	GD48	JO10	*R.nitidus*	-0.052515	0.065076
CS6	GD48	JO10	*R.nitidus*	-0.079537	0.065308
SC34	GD48	JO10	*R.nitidus*	-0.034918	0.0646
SC42	GD48	JO10	*R.nitidus*	0.117647	0.065996
SC44	GD48	JO10	*R.nitidus*	-0.031808	0.065136
CS31	GD48	JO10	*R.nitidus*	-0.026066	0.066172
SC5	GD48	JO10	*R.nitidus*	-0.001601	0.069044
CS24	GD48	JO10	*R.nitidus*	-0.070336	0.063932
SC31	GD48	JO10	*R.nitidus*	-0.041973	0.066266
SC10	GD48	JO10	*R.nitidus*	0.03973	0.065433
JO5	GD48	JO10	*R.nitidus*	-0.012801	0.066273
SC45	GD48	JO10	*R.nitidus*	-0.016357	0.063709
SC24	GD48	JO10	*R.nitidus*	-0.015361	0.064274
JW7	GD48	JO10	*R.nitidus*	-0.022686	0.069744
SC15	GD48	JO10	*R.nitidus*	-0.037291	0.066456
JO1	GD48	JO10	*R.nitidus*	-0.043382	0.066481
MY6	GD48	JO10	*R.nitidus*	-0.025344	0.064044
JO7	GD49	JO10	*R.nitidus*	-0.106667	0.071637
JO8	GD49	JO10	*R.nitidus*	-0.112281	0.074585
SC27	GD48	JO10	*R.nitidus*	-0.02197	0.06553
GD30	GD49	JO10	*R.nitidus*	0.068966	0.0676
GD26	GD49	JO10	*R.nitidus*	-0.046296	0.063732
MY4	GD48	JO10	*R.nitidus*	-0.064829	0.065441
JO3	GD48	JO10	*R.nitidus*	-0.047745	0.070825
GD31	GD49	JO10	*R.nitidus*	-0.050134	0.066686
GD25	GD49	JO10	*R.nitidus*	-0.058664	0.066275
GD22	GD49	JO10	*R.nitidus*	-0.019689	0.064247
GD44	GD49	JO10	*R.nitidus*	-0.103327	0.067374
CS14	GD49	JO10	*R.nitidus*	-0.084	0.06826
GD34	GD49	JO10	*R.nitidus*	-0.054717	0.066441
GD33	GD49	JO10	*R.nitidus*	-0.087687	0.066978
CS11	GD49	JO10	*R.nitidus*	-0.087869	0.06835
GD32	GD49	JO10	*R.nitidus*	-0.064786	0.06799
CS20	GD49	JO10	*R.nitidus*	-0.128389	0.066284
CS13	GD49	JO10	*R.nitidus*	-0.142283	0.067972
GD48	GD49	JO10	*R.nitidus*	-0.050524	0.066821
GD46	GD49	JO10	*R.nitidus*	-0.095941	0.067651
GD29	GD49	JO10	*R.nitidus*	0.002791	0.065308
GD21	GD49	JO10	*R.nitidus*	0.086146	0.065932
CS18	GD49	JO10	*R.nitidus*	-0.097134	0.06534
CS23	GD49	JO10	*R.nitidus*	-0.108974	0.065718
CS17	GD49	JO10	*R.nitidus*	-0.033981	0.067835
CS19	GD49	JO10	*R.nitidus*	-0.060855	0.067226

CS12	GD49	JO10	*R.nitidus*	-0.089125	0.065822
GD35	GD49	JO10	*R.nitidus*	-0.039049	0.06638
CS16	GD49	JO10	*R.nitidus*	-0.099448	0.067003
GD41	GD49	JO10	*R.nitidus*	-0.121186	0.067342
CS21	GD49	JO10	*R.nitidus*	-0.099279	0.064457
CS22	GD49	JO10	*R.nitidus*	-0.067098	0.065362
GD45	GD49	JO10	*R.nitidus*	-0.055233	0.067119
GD40	GD49	JO10	*R.nitidus*	-0.056459	0.065555
GD50	GD49	JO10	*R.nitidus*	-0.079793	0.069537
SC6	GD49	JO10	*R.nitidus*	-0.070024	0.067603
GD42	GD49	JO10	*R.nitidus*	-0.081938	0.067472
SC5	GD49	JO10	*R.nitidus*	-0.077239	0.070389
CS28	GD49	JO10	*R.nitidus*	-0.08843	0.067213
SC45	GD49	JO10	*R.nitidus*	-0.052504	0.064083
CS15	GD49	JO10	*R.nitidus*	-0.088188	0.068393
SC10	GD49	JO10	*R.nitidus*	-0.009592	0.066486
SC18	GD49	JO10	*R.nitidus*	-0.039864	0.066396
CS4	GD49	JO10	*R.nitidus*	-0.100311	0.064789
CS24	GD49	JO10	*R.nitidus*	-0.106612	0.063691
CS3	GD49	JO10	*R.nitidus*	-0.135891	0.065762
SC31	GD49	JO10	*R.nitidus*	-0.087024	0.066512
CS25	GD49	JO10	*R.nitidus*	-0.068328	0.067225
MY4	GD49	JO10	*R.nitidus*	-0.083662	0.065074
SC32	GD49	JO10	*R.nitidus*	-0.026214	0.064778
SC14	GD49	JO10	*R.nitidus*	-0.045807	0.064737
SC27	GD49	JO10	*R.nitidus*	-0.053906	0.065355
CS6	GD49	JO10	*R.nitidus*	-0.108333	0.066514
CS31	GD49	JO10	*R.nitidus*	-0.08826	0.068681
CS30	GD49	JO10	*R.nitidus*	-0.127642	0.065605
SC24	GD49	JO10	*R.nitidus*	-0.083399	0.0639
JW4	GD49	JO10	*R.nitidus*	0.080335	0.068593
SC12	GD49	JO10	*R.nitidus*	-0.077708	0.064044
JW6	GD49	JO10	*R.nitidus*	-0.090011	0.072606
SC15	GD49	JO10	*R.nitidus*	-0.074803	0.065859
SC26	GD49	JO10	*R.nitidus*	-0.086381	0.066941
JO7	GD50	JO10	*R.nitidus*	-0.057051	0.073988
GD21	GD50	JO10	*R.nitidus*	0.16321	0.066034
MY6	GD49	JO10	*R.nitidus*	-0.061383	0.064334
SC42	GD49	JO10	*R.nitidus*	0.096528	0.064493
JO8	GD50	JO10	*R.nitidus*	-0.056779	0.075358
JW7	GD49	JO10	*R.nitidus*	-0.050949	0.070837
SC28	GD49	JO10	*R.nitidus*	-0.111285	0.06489
SC35	GD49	JO10	*R.nitidus*	-0.027712	0.064688
JO1	GD49	JO10	*R.nitidus*	-0.077971	0.064987
GD22	GD50	JO10	*R.nitidus*	0.035647	0.065131
SC34	GD49	JO10	*R.nitidus*	-0.042254	0.063677
SC44	GD49	JO10	*R.nitidus*	-0.08867	0.065111
GD45	GD50	JO10	*R.nitidus*	0.033473	0.070078
JO5	GD49	JO10	*R.nitidus*	-0.063745	0.064855
JO3	GD49	JO10	*R.nitidus*	-0.054381	0.073983
GD29	GD50	JO10	*R.nitidus*	0.059259	0.06628
GD32	GD50	JO10	*R.nitidus*	0.002081	0.067113
GD25	GD50	JO10	*R.nitidus*	-0.011505	0.066789
CS12	GD50	JO10	*R.nitidus*	-0.04584	0.069277
CS13	GD50	JO10	*R.nitidus*	-0.091837	0.071882
CS11	GD50	JO10	*R.nitidus*	-0.022613	0.071602

CS18	GD50	JO10	*R.nitidus*	-0.062292	0.067805
GD31	GD50	JO10	*R.nitidus*	0.024299	0.069623
GD35	GD50	JO10	*R.nitidus*	0.032738	0.067387
GD34	GD50	JO10	*R.nitidus*	-0.00365	0.066063
CS30	GD50	JO10	*R.nitidus*	-0.074561	0.068132
GD41	GD50	JO10	*R.nitidus*	-0.049098	0.067503
GD30	GD50	JO10	*R.nitidus*	0.105263	0.068086
GD46	GD50	JO10	*R.nitidus*	-0.013527	0.065071
SC10	GD50	JO10	*R.nitidus*	0.044877	0.068183
GD40	GD50	JO10	*R.nitidus*	0.000878	0.066426
CS14	GD50	JO10	*R.nitidus*	-0.020202	0.073431
GD26	GD50	JO10	*R.nitidus*	0.0254	0.064607
SC6	GD50	JO10	*R.nitidus*	-0.009275	0.067389
GD33	GD50	JO10	*R.nitidus*	-0.039548	0.066764
GD49	GD50	JO10	*R.nitidus*	0.079793	0.069537
GD44	GD50	JO10	*R.nitidus*	-0.033708	0.066664
GD48	GD50	JO10	*R.nitidus*	0.044732	0.070207
GD42	GD50	JO10	*R.nitidus*	0.007216	0.069052
CS16	GD50	JO10	*R.nitidus*	-0.039773	0.06969
CS20	GD50	JO10	*R.nitidus*	-0.072925	0.068242
CS24	GD50	JO10	*R.nitidus*	-0.044712	0.066317
CS23	GD50	JO10	*R.nitidus*	-0.072148	0.068159
CS22	GD50	JO10	*R.nitidus*	-0.008432	0.069382
CS4	GD50	JO10	*R.nitidus*	-0.059016	0.066176
CS17	GD50	JO10	*R.nitidus*	-0.002586	0.071438
SC5	GD50	JO10	*R.nitidus*	-0.029255	0.071517
CS15	GD50	JO10	*R.nitidus*	-0.048801	0.070259
SC14	GD50	JO10	*R.nitidus*	-0.003215	0.066286
MY6	GD50	JO10	*R.nitidus*	-0.010604	0.066423
CS21	GD50	JO10	*R.nitidus*	-0.049313	0.067108
MY4	GD50	JO10	*R.nitidus*	-0.066942	0.06777
CS25	GD50	JO10	*R.nitidus*	-0.02294	0.068879
SC18	GD50	JO10	*R.nitidus*	0.019931	0.067851
CS19	GD50	JO10	*R.nitidus*	-0.041771	0.070077
SC24	GD50	JO10	*R.nitidus*	-0.036424	0.067148
CS28	GD50	JO10	*R.nitidus*	-0.064431	0.070216
SC12	GD50	JO10	*R.nitidus*	-0.039037	0.066097
SC34	GD50	JO10	*R.nitidus*	-0.012325	0.064702
CS3	GD50	JO10	*R.nitidus*	-0.095718	0.067156
CS31	GD50	JO10	*R.nitidus*	-0.027074	0.068239
JW7	GD50	JO10	*R.nitidus*	-0.032686	0.071837
SC26	GD50	JO10	*R.nitidus*	-0.035304	0.068776
JW4	GD50	JO10	*R.nitidus*	0.093162	0.071015
SC35	GD50	JO10	*R.nitidus*	0.016461	0.065033
SC27	GD50	JO10	*R.nitidus*	-0.020713	0.066379
SC15	GD50	JO10	*R.nitidus*	-0.011765	0.069626
SC44	GD50	JO10	*R.nitidus*	-0.049615	0.06637
GD21	CS14	JO10	*R.nitidus*	0.176307	0.066016
SC45	GD50	JO10	*R.nitidus*	0.009426	0.066418
JO3	GD50	JO10	*R.nitidus*	-0.053743	0.072515
JO7	CS14	JO10	*R.nitidus*	-0.025616	0.073276
JW6	GD50	JO10	*R.nitidus*	-0.044276	0.07332
CS6	GD50	JO10	*R.nitidus*	-0.062446	0.068269
SC31	GD50	JO10	*R.nitidus*	-0.012048	0.068805
SC32	GD50	JO10	*R.nitidus*	0.021399	0.068331
JO8	CS14	JO10	*R.nitidus*	-0.033673	0.075591

SC42	GD50	JO10	*R.nitidus*	0.146501	0.067831
GD25	CS14	JO10	*R.nitidus*	0.052395	0.066519
GD29	CS14	JO10	*R.nitidus*	0.104225	0.067769
GD26	CS14	JO10	*R.nitidus*	0.063604	0.066593
GD35	CS14	JO10	*R.nitidus*	0.03647	0.06752
SC28	GD50	JO10	*R.nitidus*	-0.05985	0.066434
GD46	CS14	JO10	*R.nitidus*	0.019288	0.069344
GD41	CS14	JO10	*R.nitidus*	-0.017033	0.067567
GD30	CS14	JO10	*R.nitidus*	0.129006	0.07016
GD50	CS14	JO10	*R.nitidus*	0.020202	0.073431
JO5	GD50	JO10	*R.nitidus*	-0.013502	0.065641
GD44	CS14	JO10	*R.nitidus*	0.014296	0.067979
JO1	GD50	JO10	*R.nitidus*	-0.034119	0.068244
CS16	CS14	JO10	*R.nitidus*	-0.01171	0.069597
CS11	CS14	JO10	*R.nitidus*	0.002421	0.071259
GD42	CS14	JO10	*R.nitidus*	0.026969	0.066158
CS19	CS14	JO10	*R.nitidus*	0.018139	0.068301
GD31	CS14	JO10	*R.nitidus*	0.043576	0.067585
CS15	CS14	JO10	*R.nitidus*	0.002427	0.069036
GD45	CS14	JO10	*R.nitidus*	0.046167	0.069835
CS23	CS14	JO10	*R.nitidus*	-0.050595	0.065718
GD22	CS14	JO10	*R.nitidus*	0.074301	0.06563
GD48	CS14	JO10	*R.nitidus*	0.040785	0.067816
CS13	CS14	JO10	*R.nitidus*	-0.084993	0.071175
SC15	CS14	JO10	*R.nitidus*	0.009709	0.067677
SC10	CS14	JO10	*R.nitidus*	0.087953	0.066724
GD32	CS14	JO10	*R.nitidus*	0.044084	0.069992
CS17	CS14	JO10	*R.nitidus*	0.04284	0.072794
GD49	CS14	JO10	*R.nitidus*	0.084	0.06826
GD34	CS14	JO10	*R.nitidus*	0.033957	0.065939
CS12	CS14	JO10	*R.nitidus*	0.00117	0.0697
CS31	CS14	JO10	*R.nitidus*	0.009615	0.068592
CS24	CS14	JO10	*R.nitidus*	-0.015987	0.067751
SC26	CS14	JO10	*R.nitidus*	-0.004435	0.067297
GD33	CS14	JO10	*R.nitidus*	-0.003536	0.067638
GD40	CS14	JO10	*R.nitidus*	0.048626	0.067222
SC14	CS14	JO10	*R.nitidus*	0.037445	0.06798
SC6	CS14	JO10	*R.nitidus*	0.029213	0.066305
CS3	CS14	JO10	*R.nitidus*	-0.052795	0.066904
CS25	CS14	JO10	*R.nitidus*	0.021196	0.068421
SC18	CS14	JO10	*R.nitidus*	0.040449	0.06713
CS4	CS14	JO10	*R.nitidus*	-0.01107	0.066131
CS28	CS14	JO10	*R.nitidus*	-0.012914	0.067984
SC44	CS14	JO10	*R.nitidus*	0.004438	0.067514
CS21	CS14	JO10	*R.nitidus*	-0.012821	0.066655
CS20	CS14	JO10	*R.nitidus*	-0.03852	0.06585
CS18	CS14	JO10	*R.nitidus*	-0.024615	0.068062
CS22	CS14	JO10	*R.nitidus*	0.019862	0.069224
CS30	CS14	JO10	*R.nitidus*	-0.050433	0.069212
JW4	CS14	JO10	*R.nitidus*	0.152799	0.066005
MY6	CS14	JO10	*R.nitidus*	0.017291	0.067447
SC28	CS14	JO10	*R.nitidus*	-0.03028	0.067685
SC5	CS14	JO10	*R.nitidus*	0.027468	0.070972
GD22	CS15	JO10	*R.nitidus*	0.08	0.063756
SC24	CS14	JO10	*R.nitidus*	0.022711	0.067643
SC27	CS14	JO10	*R.nitidus*	0.016807	0.066592

JW7	CS14	JO10	*R.nitidus*	0.025352	0.071486
SC31	CS14	JO10	*R.nitidus*	0.016949	0.066477
SC34	CS14	JO10	*R.nitidus*	0.020408	0.066358
SC12	CS14	JO10	*R.nitidus*	0.007965	0.066075
GD30	CS15	JO10	*R.nitidus*	0.133279	0.069304
JO8	CS15	JO10	*R.nitidus*	-0.007172	0.071566
JO1	CS14	JO10	*R.nitidus*	0.010309	0.067549
JO5	CS14	JO10	*R.nitidus*	0.037599	0.066467
GD26	CS15	JO10	*R.nitidus*	0.06676	0.065957
SC32	CS14	JO10	*R.nitidus*	0.060105	0.067824
JO3	CS14	JO10	*R.nitidus*	0.013135	0.071761
GD49	CS15	JO10	*R.nitidus*	0.088188	0.068393
MY4	CS14	JO10	*R.nitidus*	-0.016248	0.068683
SC42	CS14	JO10	*R.nitidus*	0.17064	0.065207
JW6	CS14	JO10	*R.nitidus*	0.031311	0.072891
GD46	CS15	JO10	*R.nitidus*	0.030523	0.067243
GD21	CS15	JO10	*R.nitidus*	0.176674	0.063902
SC45	CS14	JO10	*R.nitidus*	0.034691	0.067601
GD50	CS15	JO10	*R.nitidus*	0.048801	0.070259
SC35	CS14	JO10	*R.nitidus*	0.056122	0.065744
JO7	CS15	JO10	*R.nitidus*	-0.012708	0.072359
CS11	CS15	JO10	*R.nitidus*	0.006623	0.070044
GD40	CS15	JO10	*R.nitidus*	0.060649	0.065964
CS22	CS15	JO10	*R.nitidus*	0.026475	0.067827
GD25	CS15	JO10	*R.nitidus*	0.063422	0.065429
GD29	CS15	JO10	*R.nitidus*	0.100975	0.06566
CS31	CS15	JO10	*R.nitidus*	0.021429	0.068325
GD34	CS15	JO10	*R.nitidus*	0.035641	0.064825
GD33	CS15	JO10	*R.nitidus*	0.004965	0.065601
GD31	CS15	JO10	*R.nitidus*	0.058267	0.067014
GD32	CS15	JO10	*R.nitidus*	0.046368	0.068479
GD44	CS15	JO10	*R.nitidus*	0.026276	0.06459
CS23	CS15	JO10	*R.nitidus*	-0.045079	0.066281
GD35	CS15	JO10	*R.nitidus*	0.046528	0.065644
GD45	CS15	JO10	*R.nitidus*	0.057074	0.067541
CS12	CS15	JO10	*R.nitidus*	0.002278	0.06752
CS20	CS15	JO10	*R.nitidus*	-0.03177	0.066092
CS13	CS15	JO10	*R.nitidus*	-0.077851	0.06943
GD48	CS15	JO10	*R.nitidus*	0.0546	0.067792
SC12	CS15	JO10	*R.nitidus*	0.003639	0.066191
GD41	CS15	JO10	*R.nitidus*	-0.010563	0.066003
CS21	CS15	JO10	*R.nitidus*	-0.004481	0.066905
GD42	CS15	JO10	*R.nitidus*	0.02432	0.065793
SC10	CS15	JO10	*R.nitidus*	0.087085	0.064874
SC6	CS15	JO10	*R.nitidus*	0.029501	0.066569
SC27	CS15	JO10	*R.nitidus*	0.020753	0.066773
CS18	CS15	JO10	*R.nitidus*	-0.018307	0.067515
CS30	CS15	JO10	*R.nitidus*	-0.045073	0.067396
SC42	CS15	JO10	*R.nitidus*	0.175192	0.065355
CS17	CS15	JO10	*R.nitidus*	0.023175	0.067723
CS16	CS15	JO10	*R.nitidus*	-0.011161	0.066972
CS19	CS15	JO10	*R.nitidus*	0.015528	0.067528
CS4	CS15	JO10	*R.nitidus*	-0.001462	0.066606
SC14	CS15	JO10	*R.nitidus*	0.045655	0.067188
CS14	CS15	JO10	*R.nitidus*	-0.002427	0.069036
CS28	CS15	JO10	*R.nitidus*	0	0.067274

SC28	CS15	JO10	*R.nitidus*	-0.025449	0.0663
CS24	CS15	JO10	*R.nitidus*	-0.013878	0.065425
CS25	CS15	JO10	*R.nitidus*	0.026054	0.067515
SC18	CS15	JO10	*R.nitidus*	0.059542	0.066607
CS3	CS15	JO10	*R.nitidus*	-0.044393	0.067066
SC35	CS15	JO10	*R.nitidus*	0.060932	0.066333
SC5	CS15	JO10	*R.nitidus*	0.03532	0.068147
SC45	CS15	JO10	*R.nitidus*	0.033582	0.064738
JW7	CS15	JO10	*R.nitidus*	0.022915	0.069071
SC15	CS15	JO10	*R.nitidus*	0.022026	0.067396
GD21	CS16	JO10	*R.nitidus*	0.167231	0.063525
JO8	CS16	JO10	*R.nitidus*	0.026151	0.073024
SC32	CS15	JO10	*R.nitidus*	0.065886	0.066503
SC44	CS15	JO10	*R.nitidus*	0.006716	0.067876
SC31	CS15	JO10	*R.nitidus*	0.022399	0.065492
JO3	CS15	JO10	*R.nitidus*	0.022589	0.06915
JO1	CS15	JO10	*R.nitidus*	0.019217	0.065245
CS6	CS15	JO10	*R.nitidus*	-0.015103	0.067947
JW6	CS15	JO10	*R.nitidus*	0.025819	0.0718
SC34	CS15	JO10	*R.nitidus*	0.02788	0.065414
SC26	CS15	JO10	*R.nitidus*	0.002983	0.065975
MY4	CS15	JO10	*R.nitidus*	-0.008811	0.067746
GD30	CS16	JO10	*R.nitidus*	0.126299	0.067097
SC24	CS15	JO10	*R.nitidus*	0.021339	0.066012
CS11	CS16	JO10	*R.nitidus*	0.013304	0.066684
JO5	CS15	JO10	*R.nitidus*	0.05313	0.065452
GD22	CS16	JO10	*R.nitidus*	0.075305	0.063797
GD29	CS16	JO10	*R.nitidus*	0.09952	0.066131
JO7	CS16	JO10	*R.nitidus*	0.000996	0.071949
GD26	CS16	JO10	*R.nitidus*	0.061239	0.064395
JW4	CS15	JO10	*R.nitidus*	0.150901	0.065496
CS12	CS16	JO10	*R.nitidus*	0.018382	0.065873
MY6	CS15	JO10	*R.nitidus*	0.029539	0.066212
GD50	CS16	JO10	*R.nitidus*	0.039773	0.06969
GD34	CS16	JO10	*R.nitidus*	0.03762	0.064113
GD31	CS16	JO10	*R.nitidus*	0.049666	0.066637
GD46	CS16	JO10	*R.nitidus*	0.021137	0.065104
CS20	CS16	JO10	*R.nitidus*	-0.02755	0.063982
GD25	CS16	JO10	*R.nitidus*	0.060178	0.064273
CS22	CS16	JO10	*R.nitidus*	0.035195	0.066221
CS25	CS16	JO10	*R.nitidus*	0.03826	0.064866
GD33	CS16	JO10	*R.nitidus*	-0.002048	0.065159
CS15	CS16	JO10	*R.nitidus*	0.011161	0.066972
CS31	CS16	JO10	*R.nitidus*	0.017018	0.066222
GD35	CS16	JO10	*R.nitidus*	0.04911	0.064518
GD41	CS16	JO10	*R.nitidus*	-0.011283	0.064087
GD42	CS16	JO10	*R.nitidus*	0.027758	0.064544
SC6	CS16	JO10	*R.nitidus*	0.029456	0.064362
GD32	CS16	JO10	*R.nitidus*	0.051442	0.067252
GD49	CS16	JO10	*R.nitidus*	0.099448	0.067003
CS13	CS16	JO10	*R.nitidus*	-0.065126	0.069605
GD44	CS16	JO10	*R.nitidus*	0.019787	0.065277
CS3	CS16	JO10	*R.nitidus*	-0.039124	0.065239
GD45	CS16	JO10	*R.nitidus*	0.062097	0.067342
CS17	CS16	JO10	*R.nitidus*	0.052144	0.067367
GD40	CS16	JO10	*R.nitidus*	0.073329	0.064537

SC10	CS16	JO10	*R.nitidus*	0.100364	0.064345
SC5	CS16	JO10	*R.nitidus*	0.033432	0.067027
GD48	CS16	JO10	*R.nitidus*	0.056171	0.066473
CS24	CS16	JO10	*R.nitidus*	-0.014343	0.062631
CS23	CS16	JO10	*R.nitidus*	-0.030258	0.063252
CS19	CS16	JO10	*R.nitidus*	0.0265	0.064531
CS14	CS16	JO10	*R.nitidus*	0.01171	0.069597
SC28	CS16	JO10	*R.nitidus*	-0.028919	0.064046
CS21	CS16	JO10	*R.nitidus*	-0.008902	0.066045
SC15	CS16	JO10	*R.nitidus*	0.020564	0.066695
CS18	CS16	JO10	*R.nitidus*	-0.007582	0.065163
JW6	CS16	JO10	*R.nitidus*	0.051456	0.072102
SC27	CS16	JO10	*R.nitidus*	0.024335	0.065229
SC14	CS16	JO10	*R.nitidus*	0.038519	0.065512
SC45	CS16	JO10	*R.nitidus*	0.036898	0.064032
CS28	CS16	JO10	*R.nitidus*	0.002392	0.066571
SC12	CS16	JO10	*R.nitidus*	0.005882	0.063688
MY6	CS16	JO10	*R.nitidus*	0.023639	0.065424
SC26	CS16	JO10	*R.nitidus*	0	0.06331
SC31	CS16	JO10	*R.nitidus*	0.006662	0.06384
JO8	CS17	JO10	*R.nitidus*	-0.039387	0.076742
CS30	CS16	JO10	*R.nitidus*	-0.052877	0.064983
SC42	CS16	JO10	*R.nitidus*	0.182686	0.063375
SC24	CS16	JO10	*R.nitidus*	0.025335	0.063695
SC18	CS16	JO10	*R.nitidus*	0.054448	0.064328
JO5	CS16	JO10	*R.nitidus*	0.04216	0.063856
SC32	CS16	JO10	*R.nitidus*	0.059515	0.064621
SC35	CS16	JO10	*R.nitidus*	0.05758	0.06502
CS4	CS16	JO10	*R.nitidus*	-0.002242	0.064604
JW7	CS16	JO10	*R.nitidus*	0.02521	0.068694
MY4	CS16	JO10	*R.nitidus*	-0.010941	0.065729
SC34	CS16	JO10	*R.nitidus*	0.023134	0.06306
SC44	CS16	JO10	*R.nitidus*	0.006818	0.064625
JW4	CS16	JO10	*R.nitidus*	0.170472	0.065162
CS6	CS16	JO10	*R.nitidus*	-0.013688	0.066942
GD31	CS17	JO10	*R.nitidus*	0.017652	0.068491
GD22	CS17	JO10	*R.nitidus*	0.037683	0.064691
JO3	CS16	JO10	*R.nitidus*	0.025217	0.071242
JO7	CS17	JO10	*R.nitidus*	-0.020492	0.074459
CS11	CS17	JO10	*R.nitidus*	-0.038943	0.066364
GD29	CS17	JO10	*R.nitidus*	0.048692	0.066198
GD42	CS17	JO10	*R.nitidus*	-0.013971	0.06707
GD21	CS17	JO10	*R.nitidus*	0.141935	0.066465
GD32	CS17	JO10	*R.nitidus*	0.008751	0.069745
GD30	CS17	JO10	*R.nitidus*	0.090312	0.069979
GD26	CS17	JO10	*R.nitidus*	0.017921	0.064492
JO1	CS16	JO10	*R.nitidus*	0.022206	0.063496
GD49	CS17	JO10	*R.nitidus*	0.033981	0.067835
CS13	CS17	JO10	*R.nitidus*	-0.116667	0.073409
GD34	CS17	JO10	*R.nitidus*	0.002901	0.066834
GD25	CS17	JO10	*R.nitidus*	0.014672	0.065795
CS12	CS17	JO10	*R.nitidus*	-0.039414	0.070724
GD48	CS17	JO10	*R.nitidus*	0.01403	0.067214
SC6	CS17	JO10	*R.nitidus*	-0.01414	0.06491
GD35	CS17	JO10	*R.nitidus*	0.009848	0.065912
CS28	CS17	JO10	*R.nitidus*	-0.04802	0.066978

GD45	CS17	JO10	*R.nitidus*	0.013344	0.068864
CS21	CS17	JO10	*R.nitidus*	-0.03769	0.066403
CS15	CS17	JO10	*R.nitidus*	-0.023175	0.067723
CS18	CS17	JO10	*R.nitidus*	-0.057096	0.067093
CS16	CS17	JO10	*R.nitidus*	-0.052144	0.067367
CS24	CS17	JO10	*R.nitidus*	-0.063452	0.065831
CS31	CS17	JO10	*R.nitidus*	-0.016225	0.067512
SC12	CS17	JO10	*R.nitidus*	-0.03295	0.065495
GD33	CS17	JO10	*R.nitidus*	-0.031159	0.065195
GD40	CS17	JO10	*R.nitidus*	0.020725	0.064917
GD41	CS17	JO10	*R.nitidus*	-0.048375	0.066557
GD46	CS17	JO10	*R.nitidus*	-0.018477	0.067847
CS20	CS17	JO10	*R.nitidus*	-0.07649	0.066762
CS3	CS17	JO10	*R.nitidus*	-0.084718	0.067239
SC32	CS17	JO10	*R.nitidus*	0.030469	0.067949
SC5	CS17	JO10	*R.nitidus*	-0.014286	0.070386
SC24	CS17	JO10	*R.nitidus*	-0.020392	0.066402
CS30	CS17	JO10	*R.nitidus*	-0.08793	0.066207
GD44	CS17	JO10	*R.nitidus*	-0.010078	0.068466
SC10	CS17	JO10	*R.nitidus*	0.046166	0.066808
SC14	CS17	JO10	*R.nitidus*	0.009501	0.068348
SC28	CS17	JO10	*R.nitidus*	-0.07177	0.065687
CS14	CS17	JO10	*R.nitidus*	-0.04284	0.072794
SC15	CS17	JO10	*R.nitidus*	-0.0288	0.067646
SC26	CS17	JO10	*R.nitidus*	-0.029595	0.067649
CS23	CS17	JO10	*R.nitidus*	-0.070922	0.065428
CS19	CS17	JO10	*R.nitidus*	-0.012998	0.067411
GD50	CS17	JO10	*R.nitidus*	0.002586	0.071438
CS22	CS17	JO10	*R.nitidus*	-0.009456	0.067252
CS25	CS17	JO10	*R.nitidus*	-0.020227	0.067193
MY6	CS17	JO10	*R.nitidus*	-0.008271	0.065283
SC18	CS17	JO10	*R.nitidus*	0.015079	0.066812
SC35	CS17	JO10	*R.nitidus*	0.012112	0.064841
CS4	CS17	JO10	*R.nitidus*	-0.050433	0.06688
CS6	CS17	JO10	*R.nitidus*	-0.042445	0.068794
JW7	CS17	JO10	*R.nitidus*	0.019417	0.068943
SC44	CS17	JO10	*R.nitidus*	-0.04127	0.066695
JW6	CS17	JO10	*R.nitidus*	-0.003191	0.073982
SC42	CS17	JO10	*R.nitidus*	0.139828	0.064764
GD29	CS18	JO10	*R.nitidus*	0.102941	0.063832
GD31	CS18	JO10	*R.nitidus*	0.087772	0.064806
SC27	CS17	JO10	*R.nitidus*	-0.022932	0.066498
JW4	CS17	JO10	*R.nitidus*	0.126817	0.067335
SC31	CS17	JO10	*R.nitidus*	-0.028235	0.065375
GD22	CS18	JO10	*R.nitidus*	0.101389	0.063457
SC34	CS17	JO10	*R.nitidus*	-0.012739	0.065524
JO3	CS17	JO10	*R.nitidus*	-0.01487	0.071875
GD32	CS18	JO10	*R.nitidus*	0.076389	0.065616
GD30	CS18	JO10	*R.nitidus*	0.147656	0.065266
JO8	CS18	JO10	*R.nitidus*	0.014568	0.071051
GD35	CS18	JO10	*R.nitidus*	0.055919	0.064101
JO5	CS17	JO10	*R.nitidus*	-0.010109	0.067682
GD25	CS18	JO10	*R.nitidus*	0.075431	0.064212
SC45	CS17	JO10	*R.nitidus*	-0.007758	0.066332
GD44	CS18	JO10	*R.nitidus*	0.038754	0.065367
GD21	CS18	JO10	*R.nitidus*	0.187542	0.064489

GD33	CS18	JO10	*R.nitidus*	0.008487	0.062899
JO7	CS18	JO10	*R.nitidus*	0.03229	0.071177
GD49	CS18	JO10	*R.nitidus*	0.097134	0.06534
MY4	CS17	JO10	*R.nitidus*	-0.04835	0.067563
GD48	CS18	JO10	*R.nitidus*	0.071219	0.066775
JO1	CS17	JO10	*R.nitidus*	-0.030469	0.065621
GD46	CS18	JO10	*R.nitidus*	0.035137	0.066397
GD26	CS18	JO10	*R.nitidus*	0.082923	0.0648
CS12	CS18	JO10	*R.nitidus*	0.014107	0.066633
GD50	CS18	JO10	*R.nitidus*	0.062292	0.067805
CS14	CS18	JO10	*R.nitidus*	0.024615	0.068062
GD45	CS18	JO10	*R.nitidus*	0.079154	0.067331
CS23	CS18	JO10	*R.nitidus*	-0.028414	0.062023
GD42	CS18	JO10	*R.nitidus*	0.036301	0.064475
GD34	CS18	JO10	*R.nitidus*	0.044554	0.063828
GD41	CS18	JO10	*R.nitidus*	0	0.064683
SC6	CS18	JO10	*R.nitidus*	0.041667	0.063439
CS13	CS18	JO10	*R.nitidus*	-0.038522	0.067286
CS16	CS18	JO10	*R.nitidus*	0.007582	0.065163
GD40	CS18	JO10	*R.nitidus*	0.078947	0.062543
CS15	CS18	JO10	*R.nitidus*	0.018307	0.067515
SC15	CS18	JO10	*R.nitidus*	0.025522	0.063803
SC10	CS18	JO10	*R.nitidus*	0.115758	0.064793
CS24	CS18	JO10	*R.nitidus*	-0.01168	0.062855
SC32	CS18	JO10	*R.nitidus*	0.075332	0.063274
CS21	CS18	JO10	*R.nitidus*	0.010742	0.063399
SC5	CS18	JO10	*R.nitidus*	0.041727	0.066764
CS17	CS18	JO10	*R.nitidus*	0.057096	0.067093
CS19	CS18	JO10	*R.nitidus*	0.035294	0.064287
CS20	CS18	JO10	*R.nitidus*	-0.030075	0.06371
CS31	CS18	JO10	*R.nitidus*	0.02439	0.064288
CS11	CS18	JO10	*R.nitidus*	0.026475	0.06593
CS28	CS18	JO10	*R.nitidus*	0.029459	0.065269
SC14	CS18	JO10	*R.nitidus*	0.044798	0.064624
CS3	CS18	JO10	*R.nitidus*	-0.04021	0.062433
CS22	CS18	JO10	*R.nitidus*	0.047463	0.065213
SC42	CS18	JO10	*R.nitidus*	0.190141	0.061517
SC28	CS18	JO10	*R.nitidus*	-0.011177	0.063703
SC26	CS18	JO10	*R.nitidus*	0.015284	0.064161
SC34	CS18	JO10	*R.nitidus*	0.04428	0.062137
SC12	CS18	JO10	*R.nitidus*	0.024187	0.063738
MY6	CS18	JO10	*R.nitidus*	0.039114	0.063832
SC24	CS18	JO10	*R.nitidus*	0.032885	0.062755
CS25	CS18	JO10	*R.nitidus*	0.050274	0.066347
SC27	CS18	JO10	*R.nitidus*	0.042232	0.064386
MY4	CS18	JO10	*R.nitidus*	0.001495	0.065325
CS30	CS18	JO10	*R.nitidus*	-0.036604	0.063755
JW7	CS18	JO10	*R.nitidus*	0.034767	0.067962
SC18	CS18	JO10	*R.nitidus*	0.077454	0.062704
JO3	CS18	JO10	*R.nitidus*	0.040386	0.066886
CS6	CS18	JO10	*R.nitidus*	-0.0046	0.063093
JW6	CS18	JO10	*R.nitidus*	0.044677	0.069442
CS4	CS18	JO10	*R.nitidus*	-0.000805	0.063168
SC44	CS18	JO10	*R.nitidus*	0.016862	0.062489
SC31	CS18	JO10	*R.nitidus*	0.030236	0.062934
SC45	CS18	JO10	*R.nitidus*	0.050872	0.063061

JO8	CS19	JO10	*R.nitidus*	-0.001049	0.071937
GD21	CS19	JO10	*R.nitidus*	0.159801	0.063903
GD22	CS19	JO10	*R.nitidus*	0.06831	0.065469
JW4	CS18	JO10	*R.nitidus*	0.167957	0.064791
CS11	CS19	JO10	*R.nitidus*	-0.006173	0.066636
GD40	CS19	JO10	*R.nitidus*	0.045219	0.065176
SC35	CS18	JO10	*R.nitidus*	0.065609	0.060547
JO7	CS19	JO10	*R.nitidus*	-0.005976	0.070648
GD45	CS19	JO10	*R.nitidus*	0.033681	0.068981
GD33	CS19	JO10	*R.nitidus*	-0.02024	0.065676
JO1	CS18	JO10	*R.nitidus*	0.02788	0.063171
GD46	CS19	JO10	*R.nitidus*	0.002165	0.06707
CS12	CS19	JO10	*R.nitidus*	-0.022418	0.067318
JO5	CS18	JO10	*R.nitidus*	0.065751	0.064198
CS14	CS19	JO10	*R.nitidus*	-0.018139	0.068301
GD31	CS19	JO10	*R.nitidus*	0.055891	0.065133
GD29	CS19	JO10	*R.nitidus*	0.071326	0.064587
GD41	CS19	JO10	*R.nitidus*	-0.03026	0.066042
GD30	CS19	JO10	*R.nitidus*	0.126656	0.067612
GD35	CS19	JO10	*R.nitidus*	0.019389	0.066612
GD49	CS19	JO10	*R.nitidus*	0.060855	0.067226
CS18	CS19	JO10	*R.nitidus*	-0.035294	0.064287
CS16	CS19	JO10	*R.nitidus*	-0.0265	0.064531
GD25	CS19	JO10	*R.nitidus*	0.042584	0.064485
GD34	CS19	JO10	*R.nitidus*	0.011679	0.064381
GD42	CS19	JO10	*R.nitidus*	0.002157	0.065705
CS15	CS19	JO10	*R.nitidus*	-0.015528	0.067528
CS17	CS19	JO10	*R.nitidus*	0.012998	0.067411
GD26	CS19	JO10	*R.nitidus*	0.03244	0.064743
CS30	CS19	JO10	*R.nitidus*	-0.063091	0.064
GD32	CS19	JO10	*R.nitidus*	0.032308	0.067066
GD50	CS19	JO10	*R.nitidus*	0.041771	0.070077
CS20	CS19	JO10	*R.nitidus*	-0.073778	0.063978
CS21	CS19	JO10	*R.nitidus*	-0.021486	0.064022
CS23	CS19	JO10	*R.nitidus*	-0.066929	0.063872
GD44	CS19	JO10	*R.nitidus*	-0.000774	0.066455
CS13	CS19	JO10	*R.nitidus*	-0.077409	0.067976
CS25	CS19	JO10	*R.nitidus*	0.028332	0.067012
GD48	CS19	JO10	*R.nitidus*	0.038371	0.067831
CS24	CS19	JO10	*R.nitidus*	-0.030822	0.062871
SC28	CS19	JO10	*R.nitidus*	-0.031939	0.064308
CS28	CS19	JO10	*R.nitidus*	-0.016194	0.06542
SC10	CS19	JO10	*R.nitidus*	0.076923	0.067339
SC24	CS19	JO10	*R.nitidus*	0.001538	0.064387
SC45	CS19	JO10	*R.nitidus*	0.024372	0.064032
SC12	CS19	JO10	*R.nitidus*	-0.003698	0.063508
CS3	CS19	JO10	*R.nitidus*	-0.070018	0.064504
JO3	CS19	JO10	*R.nitidus*	0.008094	0.069137
SC6	CS19	JO10	*R.nitidus*	0.003751	0.064412
CS31	CS19	JO10	*R.nitidus*	-0.000803	0.067155
JW6	CS19	JO10	*R.nitidus*	0.038922	0.070874
SC14	CS19	JO10	*R.nitidus*	0.020528	0.065109
SC5	CS19	JO10	*R.nitidus*	0.01514	0.067812
CS4	CS19	JO10	*R.nitidus*	-0.021845	0.062844
SC32	CS19	JO10	*R.nitidus*	0.043478	0.064028
JO5	CS19	JO10	*R.nitidus*	0.026515	0.064228

GD21	CS20	JO10	*R.nitidus*	0.210856	0.063255
SC18	CS19	JO10	*R.nitidus*	0.043028	0.065286
JW7	CS19	JO10	*R.nitidus*	0.004613	0.068605
SC42	CS19	JO10	*R.nitidus*	0.170227	0.063234
SC15	CS19	JO10	*R.nitidus*	-0.000765	0.064553
CS6	CS19	JO10	*R.nitidus*	-0.037348	0.066345
CS11	CS20	JO10	*R.nitidus*	0.049849	0.065223
JW4	CS19	JO10	*R.nitidus*	0.14441	0.06697
SC26	CS19	JO10	*R.nitidus*	-0.014022	0.06532
MY4	CS19	JO10	*R.nitidus*	-0.037066	0.06547
SC27	CS19	JO10	*R.nitidus*	0.01434	0.064702
GD32	CS20	JO10	*R.nitidus*	0.083975	0.064289
SC34	CS19	JO10	*R.nitidus*	0.015228	0.062993
JO8	CS20	JO10	*R.nitidus*	0.032393	0.072823
SC31	CS19	JO10	*R.nitidus*	0.004357	0.06508
GD26	CS20	JO10	*R.nitidus*	0.090719	0.063472
SC35	CS19	JO10	*R.nitidus*	0.041877	0.06309
JO7	CS20	JO10	*R.nitidus*	0.05103	0.06995
GD40	CS20	JO10	*R.nitidus*	0.091912	0.06306
SC44	CS19	JO10	*R.nitidus*	-0.012103	0.064765
JO1	CS19	JO10	*R.nitidus*	-0.003668	0.063955
GD29	CS20	JO10	*R.nitidus*	0.119916	0.064669
GD45	CS20	JO10	*R.nitidus*	0.080696	0.06699
MY6	CS19	JO10	*R.nitidus*	0.013393	0.063482
GD25	CS20	JO10	*R.nitidus*	0.092163	0.065336
GD31	CS20	JO10	*R.nitidus*	0.098592	0.066365
GD41	CS20	JO10	*R.nitidus*	0.025105	0.065455
GD30	CS20	JO10	*R.nitidus*	0.156101	0.06682
GD48	CS20	JO10	*R.nitidus*	0.0757	0.065746
GD44	CS20	JO10	*R.nitidus*	0.056948	0.065415
GD50	CS20	JO10	*R.nitidus*	0.072925	0.068242
CS22	CS19	JO10	*R.nitidus*	0.008914	0.065509
CS13	CS20	JO10	*R.nitidus*	-0.016055	0.066876
GD22	CS20	JO10	*R.nitidus*	0.121668	0.064587
GD35	CS20	JO10	*R.nitidus*	0.081545	0.063965
CS15	CS20	JO10	*R.nitidus*	0.03177	0.066092
GD42	CS20	JO10	*R.nitidus*	0.060501	0.065402
CS12	CS20	JO10	*R.nitidus*	0.024487	0.066609
CS21	CS20	JO10	*R.nitidus*	0.048515	0.063755
CS16	CS20	JO10	*R.nitidus*	0.02755	0.063982
GD46	CS20	JO10	*R.nitidus*	0.053997	0.066247
GD34	CS20	JO10	*R.nitidus*	0.070877	0.065068
GD33	CS20	JO10	*R.nitidus*	0.033263	0.064945
GD49	CS20	JO10	*R.nitidus*	0.128389	0.066284
CS23	CS20	JO10	*R.nitidus*	-0.015152	0.063328
SC15	CS20	JO10	*R.nitidus*	0.054846	0.064659
SC5	CS20	JO10	*R.nitidus*	0.072368	0.065777
CS14	CS20	JO10	*R.nitidus*	0.03852	0.06585
CS28	CS20	JO10	*R.nitidus*	0.034029	0.064919
CS17	CS20	JO10	*R.nitidus*	0.07649	0.066762
CS22	CS20	JO10	*R.nitidus*	0.080232	0.066435
SC31	CS20	JO10	*R.nitidus*	0.051584	0.061973
CS24	CS20	JO10	*R.nitidus*	0.028823	0.06491
SC14	CS20	JO10	*R.nitidus*	0.075	0.063341
SC34	CS20	JO10	*R.nitidus*	0.068791	0.063755
CS4	CS20	JO10	*R.nitidus*	0.038017	0.062964

SC18	CS20	JO10	*R.nitidus*	0.094384	0.06438
MY4	CS20	JO10	*R.nitidus*	0.023138	0.064209
CS30	CS20	JO10	*R.nitidus*	-0.011468	0.064151
CS19	CS20	JO10	*R.nitidus*	0.073778	0.063978
SC26	CS20	JO10	*R.nitidus*	0.037445	0.064
JW6	CS20	JO10	*R.nitidus*	0.05619	0.07151
SC42	CS20	JO10	*R.nitidus*	0.211735	0.06161
SC32	CS20	JO10	*R.nitidus*	0.097122	0.063904
SC12	CS20	JO10	*R.nitidus*	0.050998	0.064215
CS25	CS20	JO10	*R.nitidus*	0.075949	0.065589
CS3	CS20	JO10	*R.nitidus*	-0.020942	0.06209
CS18	CS20	JO10	*R.nitidus*	0.030075	0.06371
MY6	CS20	JO10	*R.nitidus*	0.057102	0.064049
SC45	CS20	JO10	*R.nitidus*	0.072823	0.063525
SC35	CS20	JO10	*R.nitidus*	0.083333	0.063087
CS31	CS20	JO10	*R.nitidus*	0.048239	0.066104
SC10	CS20	JO10	*R.nitidus*	0.127924	0.065066
SC6	CS20	JO10	*R.nitidus*	0.062791	0.063381
JO3	CS20	JO10	*R.nitidus*	0.06295	0.068658
JW4	CS20	JO10	*R.nitidus*	0.175019	0.066196
JO1	CS20	JO10	*R.nitidus*	0.046875	0.064267
SC24	CS20	JO10	*R.nitidus*	0.059132	0.062975
GD26	CS21	JO10	*R.nitidus*	0.058464	0.063216
GD29	CS21	JO10	*R.nitidus*	0.091343	0.063112
JO8	CS21	JO10	*R.nitidus*	0.019	0.072408
SC27	CS20	JO10	*R.nitidus*	0.063181	0.063594
JW7	CS20	JO10	*R.nitidus*	0.052055	0.069122
SC28	CS20	JO10	*R.nitidus*	0.01551	0.065267
CS6	CS20	JO10	*R.nitidus*	0.025641	0.064087
GD30	CS21	JO10	*R.nitidus*	0.137984	0.066823
SC44	CS20	JO10	*R.nitidus*	0.046886	0.062922
GD22	CS21	JO10	*R.nitidus*	0.08745	0.061953
GD33	CS21	JO10	*R.nitidus*	-0.006122	0.061831
CS12	CS21	JO10	*R.nitidus*	-0.000765	0.067185
CS11	CS21	JO10	*R.nitidus*	0.013383	0.066282
JO5	CS20	JO10	*R.nitidus*	0.090973	0.063708
GD32	CS21	JO10	*R.nitidus*	0.052749	0.065743
JO7	CS21	JO10	*R.nitidus*	0.041466	0.071951
GD40	CS21	JO10	*R.nitidus*	0.071983	0.061948
GD50	CS21	JO10	*R.nitidus*	0.049313	0.067108
GD35	CS21	JO10	*R.nitidus*	0.053696	0.063481
GD48	CS21	JO10	*R.nitidus*	0.062409	0.065627
GD25	CS21	JO10	*R.nitidus*	0.061372	0.064693
GD21	CS21	JO10	*R.nitidus*	0.173709	0.063396
GD41	CS21	JO10	*R.nitidus*	-0.008874	0.063053
GD31	CS21	JO10	*R.nitidus*	0.068483	0.06533
GD45	CS21	JO10	*R.nitidus*	0.059655	0.067396
GD34	CS21	JO10	*R.nitidus*	0.034722	0.063718
GD46	CS21	JO10	*R.nitidus*	0.026823	0.064545
GD42	CS21	JO10	*R.nitidus*	0.032809	0.064352
GD49	CS21	JO10	*R.nitidus*	0.099279	0.064457
CS14	CS21	JO10	*R.nitidus*	0.012821	0.066655
SC10	CS21	JO10	*R.nitidus*	0.097923	0.064869
CS13	CS21	JO10	*R.nitidus*	-0.045697	0.066221
CS16	CS21	JO10	*R.nitidus*	0.008902	0.066045
CS18	CS21	JO10	*R.nitidus*	-0.010742	0.063399

CS17	CS21	JO10	*R.nitidus*	0.03769	0.066403
CS22	CS21	JO10	*R.nitidus*	0.045777	0.065419
GD44	CS21	JO10	*R.nitidus*	0.022861	0.064294
SC12	CS21	JO10	*R.nitidus*	0.013503	0.065204
SC28	CS21	JO10	*R.nitidus*	-0.013255	0.064254
SC15	CS21	JO10	*R.nitidus*	0.020347	0.063983
SC27	CS21	JO10	*R.nitidus*	0.025604	0.063621
SC45	CS21	JO10	*R.nitidus*	0.034557	0.064546
SC32	CS21	JO10	*R.nitidus*	0.06735	0.06444
CS28	CS21	JO10	*R.nitidus*	-0.001536	0.065926
SC42	CS21	JO10	*R.nitidus*	0.181173	0.061724
CS4	CS21	JO10	*R.nitidus*	-0.014129	0.062452
CS25	CS21	JO10	*R.nitidus*	0.037456	0.065568
SC34	CS21	JO10	*R.nitidus*	0.040471	0.062639
SC31	CS21	JO10	*R.nitidus*	0.030551	0.063022
CS23	CS21	JO10	*R.nitidus*	-0.046693	0.06213
CS15	CS21	JO10	*R.nitidus*	0.004481	0.066905
SC6	CS21	JO10	*R.nitidus*	0.032258	0.06465
CS30	CS21	JO10	*R.nitidus*	-0.049767	0.063815
SC14	CS21	JO10	*R.nitidus*	0.046329	0.063273
CS6	CS21	JO10	*R.nitidus*	-0.011299	0.063039
CS20	CS21	JO10	*R.nitidus*	-0.048515	0.063755
CS19	CS21	JO10	*R.nitidus*	0.021486	0.064022
JW7	CS21	JO10	*R.nitidus*	0.033575	0.068554
CS3	CS21	JO10	*R.nitidus*	-0.049954	0.063318
SC5	CS21	JO10	*R.nitidus*	0.033022	0.065422
CS31	CS21	JO10	*R.nitidus*	0.01283	0.064837
SC18	CS21	JO10	*R.nitidus*	0.055976	0.065691
JO7	CS22	JO10	*R.nitidus*	-0.029732	0.070005
SC24	CS21	JO10	*R.nitidus*	0.022761	0.063527
SC44	CS21	JO10	*R.nitidus*	0.013899	0.063079
GD22	CS22	JO10	*R.nitidus*	0.043206	0.063804
JO8	CS22	JO10	*R.nitidus*	-0.032362	0.069964
JW6	CS21	JO10	*R.nitidus*	0.038132	0.070736
SC35	CS21	JO10	*R.nitidus*	0.067647	0.06215
SC26	CS21	JO10	*R.nitidus*	0.001458	0.06453
JO1	CS21	JO10	*R.nitidus*	0.017279	0.064523
MY4	CS21	JO10	*R.nitidus*	-0.008094	0.063602
GD26	CS22	JO10	*R.nitidus*	0.035868	0.06499
GD29	CS22	JO10	*R.nitidus*	0.065911	0.06498
JW4	CS21	JO10	*R.nitidus*	0.160093	0.065011
GD32	CS22	JO10	*R.nitidus*	0.019936	0.065277
GD31	CS22	JO10	*R.nitidus*	0.032738	0.066256
CS24	CS21	JO10	*R.nitidus*	-0.01112	0.062897
CS11	CS22	JO10	*R.nitidus*	-0.022693	0.06592
GD21	CS22	JO10	*R.nitidus*	0.140777	0.063401
MY6	CS21	JO10	*R.nitidus*	0.031025	0.064421
JO3	CS21	JO10	*R.nitidus*	0.042478	0.06925
GD35	CS22	JO10	*R.nitidus*	0.011486	0.062498
CS12	CS22	JO10	*R.nitidus*	-0.032488	0.067056
CS17	CS22	JO10	*R.nitidus*	0.009456	0.067252
GD34	CS22	JO10	*R.nitidus*	0.003511	0.065188
JO5	CS21	JO10	*R.nitidus*	0.057496	0.064734
CS16	CS22	JO10	*R.nitidus*	-0.035195	0.066221
GD41	CS22	JO10	*R.nitidus*	-0.033922	0.063769
GD25	CS22	JO10	*R.nitidus*	0.014771	0.06545

GD30	CS22	JO10	*R.nitidus*	0.096463	0.068126
CS20	CS22	JO10	*R.nitidus*	-0.080232	0.066435
GD33	CS22	JO10	*R.nitidus*	-0.035186	0.065809
CS15	CS22	JO10	*R.nitidus*	-0.026475	0.067827
CS14	CS22	JO10	*R.nitidus*	-0.019862	0.069224
CS28	CS22	JO10	*R.nitidus*	-0.042742	0.065149
GD42	CS22	JO10	*R.nitidus*	-0.00838	0.065198
CS3	CS22	JO10	*R.nitidus*	-0.084261	0.065659
GD45	CS22	JO10	*R.nitidus*	0.032721	0.067331
CS18	CS22	JO10	*R.nitidus*	-0.047463	0.065213
CS13	CS22	JO10	*R.nitidus*	-0.088993	0.068443
GD44	CS22	JO10	*R.nitidus*	0.0039	0.065959
GD40	CS22	JO10	*R.nitidus*	0.029703	0.064846
CS4	CS22	JO10	*R.nitidus*	-0.047337	0.062348
GD50	CS22	JO10	*R.nitidus*	0.008432	0.069382
GD46	CS22	JO10	*R.nitidus*	-0.014306	0.065246
CS30	CS22	JO10	*R.nitidus*	-0.083333	0.066576
SC6	CS22	JO10	*R.nitidus*	-0.008271	0.063921
SC15	CS22	JO10	*R.nitidus*	-0.007593	0.065379
SC26	CS22	JO10	*R.nitidus*	-0.030303	0.065303
GD49	CS22	JO10	*R.nitidus*	0.067098	0.065362
SC27	CS22	JO10	*R.nitidus*	-0.002259	0.064013
SC31	CS22	JO10	*R.nitidus*	-0.009427	0.065123
GD48	CS22	JO10	*R.nitidus*	0.031963	0.06728
SC10	CS22	JO10	*R.nitidus*	0.070498	0.065253
CS19	CS22	JO10	*R.nitidus*	-0.008914	0.065509
SC28	CS22	JO10	*R.nitidus*	-0.049962	0.066
SC5	CS22	JO10	*R.nitidus*	0.002269	0.068217
SC14	CS22	JO10	*R.nitidus*	0.011161	0.064291
CS21	CS22	JO10	*R.nitidus*	-0.045777	0.065419
SC24	CS22	JO10	*R.nitidus*	-0.011808	0.064923
CS23	CS22	JO10	*R.nitidus*	-0.078176	0.064516
CS24	CS22	JO10	*R.nitidus*	-0.062076	0.062472
SC12	CS22	JO10	*R.nitidus*	-0.023006	0.063857
SC45	CS22	JO10	*R.nitidus*	0.001497	0.064033
JW7	CS22	JO10	*R.nitidus*	0.011152	0.068717
CS25	CS22	JO10	*R.nitidus*	-0.009677	0.067795
SC34	CS22	JO10	*R.nitidus*	-0.004428	0.064467
CS31	CS22	JO10	*R.nitidus*	-0.019048	0.064718
JO8	CS23	JO10	*R.nitidus*	0.041195	0.072033
SC42	CS22	JO10	*R.nitidus*	0.146149	0.062809
JO3	CS22	JO10	*R.nitidus*	-0.003466	0.068591
SC18	CS22	JO10	*R.nitidus*	0.0125	0.065833
CS6	CS22	JO10	*R.nitidus*	-0.057748	0.064923
SC44	CS22	JO10	*R.nitidus*	-0.024738	0.066314
SC35	CS22	JO10	*R.nitidus*	0.02099	0.063522
SC32	CS22	JO10	*R.nitidus*	0.031579	0.06352
JO7	CS23	JO10	*R.nitidus*	0.052734	0.070053
JO1	CS22	JO10	*R.nitidus*	-0.008915	0.065835
GD46	CS23	JO10	*R.nitidus*	0.047619	0.064412
GD35	CS23	JO10	*R.nitidus*	0.084588	0.062908
JW4	CS22	JO10	*R.nitidus*	0.133333	0.06591
JW6	CS22	JO10	*R.nitidus*	0.028487	0.069522
GD32	CS23	JO10	*R.nitidus*	0.09084	0.064934
GD40	CS23	JO10	*R.nitidus*	0.094722	0.060078
JO5	CS22	JO10	*R.nitidus*	0.015083	0.065156

CS11	CS23	JO10	*R.nitidus*	0.052399	0.065246
GD45	CS23	JO10	*R.nitidus*	0.068059	0.06728
MY6	CS22	JO10	*R.nitidus*	-0.007564	0.064359
GD41	CS23	JO10	*R.nitidus*	0.027952	0.064503
GD29	CS23	JO10	*R.nitidus*	0.122105	0.063158
CS13	CS23	JO10	*R.nitidus*	-0.01044	0.064139
MY4	CS22	JO10	*R.nitidus*	-0.051223	0.065071
GD22	CS23	JO10	*R.nitidus*	0.117166	0.063001
GD30	CS23	JO10	*R.nitidus*	0.147311	0.065955
GD49	CS23	JO10	*R.nitidus*	0.108974	0.065718
GD21	CS23	JO10	*R.nitidus*	0.201072	0.062946
GD50	CS23	JO10	*R.nitidus*	0.072148	0.068159
GD34	CS23	JO10	*R.nitidus*	0.076265	0.064361
CS12	CS23	JO10	*R.nitidus*	0.029794	0.066631
CS19	CS23	JO10	*R.nitidus*	0.066929	0.063872
GD25	CS23	JO10	*R.nitidus*	0.088842	0.062606
GD44	CS23	JO10	*R.nitidus*	0.057795	0.064172
CS3	CS23	JO10	*R.nitidus*	-0.004873	0.062855
GD26	CS23	JO10	*R.nitidus*	0.088737	0.063726
GD48	CS23	JO10	*R.nitidus*	0.093303	0.065939
GD31	CS23	JO10	*R.nitidus*	0.098361	0.063033
GD33	CS23	JO10	*R.nitidus*	0.030007	0.065003
SC6	CS23	JO10	*R.nitidus*	0.06692	0.0639
GD42	CS23	JO10	*R.nitidus*	0.067455	0.065401
CS28	CS23	JO10	*R.nitidus*	0.04	0.065325
CS21	CS23	JO10	*R.nitidus*	0.046693	0.06213
SC18	CS23	JO10	*R.nitidus*	0.102219	0.063697
CS31	CS23	JO10	*R.nitidus*	0.051672	0.064594
CS15	CS23	JO10	*R.nitidus*	0.045079	0.066281
CS22	CS23	JO10	*R.nitidus*	0.078176	0.064516
SC26	CS23	JO10	*R.nitidus*	0.037763	0.064438
CS24	CS23	JO10	*R.nitidus*	0.025224	0.063353
SC44	CS23	JO10	*R.nitidus*	0.038806	0.064057
CS30	CS23	JO10	*R.nitidus*	-0.009494	0.064082
SC5	CS23	JO10	*R.nitidus*	0.063309	0.065647
CS14	CS23	JO10	*R.nitidus*	0.050595	0.065718
CS16	CS23	JO10	*R.nitidus*	0.030258	0.063252
CS17	CS23	JO10	*R.nitidus*	0.070922	0.065428
SC28	CS23	JO10	*R.nitidus*	-0.000742	0.064727
CS18	CS23	JO10	*R.nitidus*	0.028414	0.062023
CS20	CS23	JO10	*R.nitidus*	0.015152	0.063328
SC32	CS23	JO10	*R.nitidus*	0.092461	0.06373
SC27	CS23	JO10	*R.nitidus*	0.060876	0.063353
JW6	CS23	JO10	*R.nitidus*	0.058491	0.069297
SC14	CS23	JO10	*R.nitidus*	0.072714	0.063916
SC15	CS23	JO10	*R.nitidus*	0.058069	0.064113
JO3	CS23	JO10	*R.nitidus*	0.071993	0.069427
JO5	CS23	JO10	*R.nitidus*	0.080963	0.062787
MY4	CS23	JO10	*R.nitidus*	0.024078	0.064075
JW7	CS23	JO10	*R.nitidus*	0.04946	0.068476
SC24	CS23	JO10	*R.nitidus*	0.061086	0.062194
MY6	CS23	JO10	*R.nitidus*	0.065868	0.062582
GD25	CS24	JO10	*R.nitidus*	0.055224	0.061389
SC45	CS23	JO10	*R.nitidus*	0.069904	0.063991
SC31	CS23	JO10	*R.nitidus*	0.05641	0.06236
SC12	CS23	JO10	*R.nitidus*	0.037093	0.062981

SC10	CS23	JO10	*R.nitidus*	0.133975	0.065258
JO8	CS24	JO10	*R.nitidus*	0.015504	0.069465
CS4	CS23	JO10	*R.nitidus*	0.033573	0.063551
CS11	CS24	JO10	*R.nitidus*	0.028016	0.066179
SC42	CS23	JO10	*R.nitidus*	0.214971	0.062718
JO1	CS23	JO10	*R.nitidus*	0.045918	0.063954
CS6	CS23	JO10	*R.nitidus*	0.033613	0.0645
SC34	CS23	JO10	*R.nitidus*	0.069343	0.06241
SC35	CS23	JO10	*R.nitidus*	0.091308	0.062081
GD22	CS24	JO10	*R.nitidus*	0.096978	0.060143
CS12	CS24	JO10	*R.nitidus*	0.020375	0.064513
JW4	CS23	JO10	*R.nitidus*	0.187595	0.064779
JO7	CS24	JO10	*R.nitidus*	0.033133	0.068851
GD35	CS24	JO10	*R.nitidus*	0.065217	0.06205
GD33	CS24	JO10	*R.nitidus*	0.009993	0.064149
GD34	CS24	JO10	*R.nitidus*	0.046796	0.063146
GD21	CS24	JO10	*R.nitidus*	0.188366	0.062492
GD31	CS24	JO10	*R.nitidus*	0.077508	0.064105
GD45	CS24	JO10	*R.nitidus*	0.071828	0.066204
GD46	CS24	JO10	*R.nitidus*	0.028571	0.064024
GD41	CS24	JO10	*R.nitidus*	0	0.062513
CS18	CS24	JO10	*R.nitidus*	0.01168	0.062855
GD30	CS24	JO10	*R.nitidus*	0.13285	0.065716
GD29	CS24	JO10	*R.nitidus*	0.109687	0.062564
GD42	CS24	JO10	*R.nitidus*	0.038814	0.062887
CS13	CS24	JO10	*R.nitidus*	-0.045008	0.066125
GD48	CS24	JO10	*R.nitidus*	0.070336	0.063932
GD26	CS24	JO10	*R.nitidus*	0.08153	0.062522
GD40	CS24	JO10	*R.nitidus*	0.077377	0.060354
GD49	CS24	JO10	*R.nitidus*	0.106612	0.063691
GD32	CS24	JO10	*R.nitidus*	0.069675	0.063789
CS30	CS24	JO10	*R.nitidus*	-0.037007	0.063457
GD44	CS24	JO10	*R.nitidus*	0.034456	0.064922
SC6	CS24	JO10	*R.nitidus*	0.045181	0.06171
CS22	CS24	JO10	*R.nitidus*	0.062076	0.062472
CS21	CS24	JO10	*R.nitidus*	0.01112	0.062897
CS16	CS24	JO10	*R.nitidus*	0.014343	0.062631
CS14	CS24	JO10	*R.nitidus*	0.015987	0.067751
SC5	CS24	JO10	*R.nitidus*	0.049373	0.067345
CS17	CS24	JO10	*R.nitidus*	0.063452	0.065831
CS25	CS24	JO10	*R.nitidus*	0.04864	0.065043
CS28	CS24	JO10	*R.nitidus*	0.02459	0.065026
GD50	CS24	JO10	*R.nitidus*	0.044712	0.066317
CS31	CS24	JO10	*R.nitidus*	0.033762	0.064721
CS19	CS24	JO10	*R.nitidus*	0.030822	0.062871
CS15	CS24	JO10	*R.nitidus*	0.013878	0.065425
SC12	CS24	JO10	*R.nitidus*	0.026275	0.060994
SC10	CS24	JO10	*R.nitidus*	0.125286	0.062667
CS20	CS24	JO10	*R.nitidus*	-0.028823	0.06491
JW4	CS24	JO10	*R.nitidus*	0.196386	0.06414
SC27	CS24	JO10	*R.nitidus*	0.03605	0.062052
CS23	CS24	JO10	*R.nitidus*	-0.025224	0.063353
CS3	CS24	JO10	*R.nitidus*	-0.040258	0.0627
SC18	CS24	JO10	*R.nitidus*	0.058366	0.062957
SC26	CS24	JO10	*R.nitidus*	0.019302	0.062856
CS4	CS24	JO10	*R.nitidus*	0.009857	0.062089

SC28	CS24	JO10	*R.nitidus*	-0.005385	0.062861
JO5	CS24	JO10	*R.nitidus*	0.054116	0.062242
SC14	CS24	JO10	*R.nitidus*	0.052793	0.06302
SC15	CS24	JO10	*R.nitidus*	0.034893	0.063213
SC35	CS24	JO10	*R.nitidus*	0.070776	0.061487
JW6	CS24	JO10	*R.nitidus*	0.080285	0.068374
JO8	CS25	JO10	*R.nitidus*	-0.029598	0.074409
SC44	CS24	JO10	*R.nitidus*	0.022781	0.06326
SC32	CS24	JO10	*R.nitidus*	0.079848	0.063202
CS6	CS24	JO10	*R.nitidus*	0.003493	0.062251
JO7	CS25	JO10	*R.nitidus*	-0.01592	0.070116
GD30	CS25	JO10	*R.nitidus*	0.1225	0.067002
CS11	CS25	JO10	*R.nitidus*	-0.015825	0.066603
SC24	CS24	JO10	*R.nitidus*	0.032689	0.061698
GD21	CS25	JO10	*R.nitidus*	0.159379	0.065363
GD35	CS25	JO10	*R.nitidus*	0.016188	0.064311
JO1	CS24	JO10	*R.nitidus*	0.032042	0.062289
SC31	CS24	JO10	*R.nitidus*	0.035661	0.061659
CS12	CS25	JO10	*R.nitidus*	-0.041765	0.067624
GD31	CS25	JO10	*R.nitidus*	0.045555	0.066573
SC45	CS24	JO10	*R.nitidus*	0.048835	0.062318
MY4	CS24	JO10	*R.nitidus*	-0.00228	0.063754
GD33	CS25	JO10	*R.nitidus*	-0.023256	0.064981
SC34	CS24	JO10	*R.nitidus*	0.05287	0.062321
JO3	CS24	JO10	*R.nitidus*	0.024801	0.06681
GD34	CS25	JO10	*R.nitidus*	0.007184	0.065468
GD29	CS25	JO10	*R.nitidus*	0.068891	0.065661
GD22	CS25	JO10	*R.nitidus*	0.065965	0.064301
SC42	CS24	JO10	*R.nitidus*	0.202581	0.061434
JW7	CS24	JO10	*R.nitidus*	0.050193	0.066005
MY6	CS24	JO10	*R.nitidus*	0.041478	0.063845
GD40	CS25	JO10	*R.nitidus*	0.03505	0.064003
CS13	CS25	JO10	*R.nitidus*	-0.088872	0.06865
GD42	CS25	JO10	*R.nitidus*	0.007174	0.065411
GD32	CS25	JO10	*R.nitidus*	0.028169	0.066585
CS16	CS25	JO10	*R.nitidus*	-0.03826	0.064866
GD25	CS25	JO10	*R.nitidus*	0.034431	0.064813
GD26	CS25	JO10	*R.nitidus*	0.042569	0.063446
CS20	CS25	JO10	*R.nitidus*	-0.075949	0.065589
GD46	CS25	JO10	*R.nitidus*	-0.012894	0.066915
GD50	CS25	JO10	*R.nitidus*	0.02294	0.068879
GD48	CS25	JO10	*R.nitidus*	0.031466	0.065303
GD45	CS25	JO10	*R.nitidus*	0.02762	0.067817
CS14	CS25	JO10	*R.nitidus*	-0.021196	0.068421
CS19	CS25	JO10	*R.nitidus*	-0.028332	0.067012
CS17	CS25	JO10	*R.nitidus*	0.020227	0.067193
GD44	CS25	JO10	*R.nitidus*	0.000765	0.063783
CS18	CS25	JO10	*R.nitidus*	-0.050274	0.066347
GD41	CS25	JO10	*R.nitidus*	-0.033356	0.064926
CS3	CS25	JO10	*R.nitidus*	-0.09253	0.064914
CS23	CS25	JO10	*R.nitidus*	-0.065104	0.064812
SC18	CS25	JO10	*R.nitidus*	0.031758	0.067193
SC12	CS25	JO10	*R.nitidus*	-0.017611	0.065002
GD49	CS25	JO10	*R.nitidus*	0.068328	0.067225
SC6	CS25	JO10	*R.nitidus*	-0.003852	0.06409
SC15	CS25	JO10	*R.nitidus*	-0.003915	0.065248

SC27	CS25	JO10	*R.nitidus*	0	0.065244
CS15	CS25	JO10	*R.nitidus*	-0.026054	0.067515
CS21	CS25	JO10	*R.nitidus*	-0.037456	0.065568
CS30	CS25	JO10	*R.nitidus*	-0.075722	0.065562
JW4	CS25	JO10	*R.nitidus*	0.127217	0.068121
SC24	CS25	JO10	*R.nitidus*	-0.006051	0.066998
SC10	CS25	JO10	*R.nitidus*	0.056967	0.067989
CS4	CS25	JO10	*R.nitidus*	-0.02692	0.065794
JO3	CS25	JO10	*R.nitidus*	0.008787	0.069815
CS22	CS25	JO10	*R.nitidus*	0.009677	0.067795
SC45	CS25	JO10	*R.nitidus*	0.013793	0.063301
SC42	CS25	JO10	*R.nitidus*	0.163696	0.063731
CS28	CS25	JO10	*R.nitidus*	-0.039075	0.067082
SC35	CS25	JO10	*R.nitidus*	0.02687	0.064195
SC5	CS25	JO10	*R.nitidus*	-0.000747	0.067015
JW6	CS25	JO10	*R.nitidus*	0.0286	0.070827
CS24	CS25	JO10	*R.nitidus*	-0.04864	0.065043
SC14	CS25	JO10	*R.nitidus*	0.016703	0.064718
SC26	CS25	JO10	*R.nitidus*	-0.018827	0.066274
MY6	CS25	JO10	*R.nitidus*	0.000749	0.063517
SC28	CS25	JO10	*R.nitidus*	-0.051476	0.06588
MY4	CS25	JO10	*R.nitidus*	-0.040847	0.065293
CS31	CS25	JO10	*R.nitidus*	-0.02126	0.067086
GD31	CS28	JO10	*R.nitidus*	0.055857	0.065886
SC31	CS25	JO10	*R.nitidus*	-0.00883	0.063261
JO1	CS25	JO10	*R.nitidus*	-0.015206	0.064503
SC32	CS25	JO10	*R.nitidus*	0.03062	0.066885
JW7	CS25	JO10	*R.nitidus*	-0.01011	0.069097
GD32	CS28	JO10	*R.nitidus*	0.054927	0.067907
CS6	CS25	JO10	*R.nitidus*	-0.053524	0.064987
SC44	CS25	JO10	*R.nitidus*	-0.034014	0.064335
CS12	CS28	JO10	*R.nitidus*	0.001645	0.066362
GD30	CS28	JO10	*R.nitidus*	0.148116	0.067259
GD29	CS28	JO10	*R.nitidus*	0.085631	0.065295
JO7	CS28	JO10	*R.nitidus*	-0.010363	0.071113
SC34	CS25	JO10	*R.nitidus*	0.007283	0.064616
GD40	CS28	JO10	*R.nitidus*	0.052437	0.06587
JO8	CS28	JO10	*R.nitidus*	-0.017058	0.072282
CS13	CS28	JO10	*R.nitidus*	-0.051387	0.067961
GD44	CS28	JO10	*R.nitidus*	0.022961	0.066262
GD35	CS28	JO10	*R.nitidus*	0.053464	0.06597
GD49	CS28	JO10	*R.nitidus*	0.08843	0.067213
GD33	CS28	JO10	*R.nitidus*	0.007491	0.067567
GD22	CS28	JO10	*R.nitidus*	0.086676	0.06702
GD34	CS28	JO10	*R.nitidus*	0.039216	0.06698
CS11	CS28	JO10	*R.nitidus*	0.017586	0.06502
GD41	CS28	JO10	*R.nitidus*	-0.015683	0.065677
JO5	CS25	JO10	*R.nitidus*	0.022104	0.064568
GD25	CS28	JO10	*R.nitidus*	0.073038	0.065529
GD21	CS28	JO10	*R.nitidus*	0.177778	0.063364
CS14	CS28	JO10	*R.nitidus*	0.012914	0.067984
GD26	CS28	JO10	*R.nitidus*	0.063016	0.06552
GD45	CS28	JO10	*R.nitidus*	0.069136	0.067128
GD46	CS28	JO10	*R.nitidus*	0.014012	0.066224
CS3	CS28	JO10	*R.nitidus*	-0.049719	0.065365
CS16	CS28	JO10	*R.nitidus*	-0.002392	0.066571

CS30	CS28	JO10	*R.nitidus*	-0.050909	0.068035
GD48	CS28	JO10	*R.nitidus*	0.052756	0.067017
CS19	CS28	JO10	*R.nitidus*	0.016194	0.06542
CS18	CS28	JO10	*R.nitidus*	-0.029459	0.065269
GD50	CS28	JO10	*R.nitidus*	0.064431	0.070216
CS25	CS28	JO10	*R.nitidus*	0.039075	0.067082
CS20	CS28	JO10	*R.nitidus*	-0.034029	0.064919
CS17	CS28	JO10	*R.nitidus*	0.04802	0.066978
GD42	CS28	JO10	*R.nitidus*	0.031555	0.066261
CS23	CS28	JO10	*R.nitidus*	-0.04	0.065325
SC5	CS28	JO10	*R.nitidus*	0.028081	0.068399
SC12	CS28	JO10	*R.nitidus*	0.016627	0.065429
CS6	CS28	JO10	*R.nitidus*	-0.020903	0.066543
SC32	CS28	JO10	*R.nitidus*	0.066294	0.064191
CS15	CS28	JO10	*R.nitidus*	0	0.067274
SC26	CS28	JO10	*R.nitidus*	-0.010861	0.065156
SC28	CS28	JO10	*R.nitidus*	-0.017433	0.064479
SC14	CS28	JO10	*R.nitidus*	0.031201	0.065981
SC6	CS28	JO10	*R.nitidus*	0.030159	0.063422
CS21	CS28	JO10	*R.nitidus*	0.001536	0.065926
CS22	CS28	JO10	*R.nitidus*	0.042742	0.065149
CS24	CS28	JO10	*R.nitidus*	-0.02459	0.065026
JW4	CS28	JO10	*R.nitidus*	0.172227	0.067485
SC18	CS28	JO10	*R.nitidus*	0.052083	0.065584
SC24	CS28	JO10	*R.nitidus*	0.016509	0.06334
CS4	CS28	JO10	*R.nitidus*	-0.00463	0.062529
CS31	CS28	JO10	*R.nitidus*	0.010373	0.065783
SC34	CS28	JO10	*R.nitidus*	0.039627	0.064042
SC31	CS28	JO10	*R.nitidus*	0.011024	0.064769
SC35	CS28	JO10	*R.nitidus*	0.036474	0.0658
GD22	CS30	JO10	*R.nitidus*	0.130022	0.062209
JW7	CS28	JO10	*R.nitidus*	0.028884	0.069306
SC45	CS28	JO10	*R.nitidus*	0.037606	0.06561
SC10	CS28	JO10	*R.nitidus*	0.105263	0.066807
SC27	CS28	JO10	*R.nitidus*	0.028135	0.064904
GD41	CS30	JO10	*R.nitidus*	0.033962	0.063072
MY4	CS28	JO10	*R.nitidus*	-0.014984	0.065843
GD33	CS30	JO10	*R.nitidus*	0.041274	0.064651
MY6	CS28	JO10	*R.nitidus*	0.027885	0.065419
GD30	CS30	JO10	*R.nitidus*	0.16196	0.06507
JO1	CS28	JO10	*R.nitidus*	0.007855	0.066054
JW6	CS28	JO10	*R.nitidus*	0.034205	0.069982
SC42	CS28	JO10	*R.nitidus*	0.181946	0.062967
SC15	CS28	JO10	*R.nitidus*	0.009868	0.066225
CS11	CS30	JO10	*R.nitidus*	0.055178	0.067023
GD31	CS30	JO10	*R.nitidus*	0.105019	0.065662
SC44	CS28	JO10	*R.nitidus*	0.003155	0.065953
GD25	CS30	JO10	*R.nitidus*	0.094325	0.06358
JO7	CS30	JO10	*R.nitidus*	0.044284	0.071152
JO8	CS30	JO10	*R.nitidus*	0.05131	0.070471
GD35	CS30	JO10	*R.nitidus*	0.083458	0.06415
CS14	CS30	JO10	*R.nitidus*	0.050433	0.069212
JO5	CS28	JO10	*R.nitidus*	0.039322	0.064986
GD34	CS30	JO10	*R.nitidus*	0.080562	0.063532
GD40	CS30	JO10	*R.nitidus*	0.10167	0.062458
GD26	CS30	JO10	*R.nitidus*	0.101845	0.061938

GD21	CS30	JO10	*R.nitidus*	0.216833	0.062954
GD42	CS30	JO10	*R.nitidus*	0.072948	0.061797
CS16	CS30	JO10	*R.nitidus*	0.052877	0.064983
GD29	CS30	JO10	*R.nitidus*	0.124265	0.06301
CS19	CS30	JO10	*R.nitidus*	0.063091	0.064
GD46	CS30	JO10	*R.nitidus*	0.055887	0.064647
GD45	CS30	JO10	*R.nitidus*	0.09699	0.065405
GD50	CS30	JO10	*R.nitidus*	0.074561	0.068132
CS15	CS30	JO10	*R.nitidus*	0.045073	0.067396
CS24	CS30	JO10	*R.nitidus*	0.037007	0.063457
GD49	CS30	JO10	*R.nitidus*	0.127642	0.065605
CS3	CS30	JO10	*R.nitidus*	-0.0032	0.065044
CS25	CS30	JO10	*R.nitidus*	0.075722	0.065562
CS21	CS30	JO10	*R.nitidus*	0.049767	0.063815
GD32	CS30	JO10	*R.nitidus*	0.089141	0.0671
CS13	CS30	JO10	*R.nitidus*	-0.00317	0.066535
JO3	CS28	JO10	*R.nitidus*	0.025686	0.071819
CS17	CS30	JO10	*R.nitidus*	0.08793	0.066207
CS12	CS30	JO10	*R.nitidus*	0.046289	0.066843
CS20	CS30	JO10	*R.nitidus*	0.011468	0.064151
GD44	CS30	JO10	*R.nitidus*	0.055887	0.063398
GD48	CS30	JO10	*R.nitidus*	0.091861	0.066271
CS23	CS30	JO10	*R.nitidus*	0.009494	0.064082
CS28	CS30	JO10	*R.nitidus*	0.050909	0.068035
SC31	CS30	JO10	*R.nitidus*	0.074887	0.063659
CS4	CS30	JO10	*R.nitidus*	0.043044	0.062851
SC6	CS30	JO10	*R.nitidus*	0.079554	0.062626
SC12	CS30	JO10	*R.nitidus*	0.054517	0.064016
CS18	CS30	JO10	*R.nitidus*	0.036604	0.063755
CS22	CS30	JO10	*R.nitidus*	0.083333	0.066576
SC10	CS30	JO10	*R.nitidus*	0.146677	0.062862
SC32	CS30	JO10	*R.nitidus*	0.105991	0.062594
CS31	CS30	JO10	*R.nitidus*	0.077807	0.066032
JO3	CS30	JO10	*R.nitidus*	0.067446	0.070459
SC26	CS30	JO10	*R.nitidus*	0.04236	0.063289
SC14	CS30	JO10	*R.nitidus*	0.084034	0.062933
SC15	CS30	JO10	*R.nitidus*	0.065481	0.065889
SC35	CS30	JO10	*R.nitidus*	0.099148	0.063444
SC34	CS30	JO10	*R.nitidus*	0.077795	0.06302
JW7	CS30	JO10	*R.nitidus*	0.078505	0.067342
SC44	CS30	JO10	*R.nitidus*	0.054011	0.064227
MY4	CS30	JO10	*R.nitidus*	0.035687	0.065366
MY6	CS30	JO10	*R.nitidus*	0.080092	0.065944
SC5	CS30	JO10	*R.nitidus*	0.065943	0.066465
SC45	CS30	JO10	*R.nitidus*	0.089683	0.063824
GD21	CS31	JO10	*R.nitidus*	0.166311	0.064646
SC24	CS30	JO10	*R.nitidus*	0.06537	0.064044
GD25	CS31	JO10	*R.nitidus*	0.039969	0.066348
SC27	CS30	JO10	*R.nitidus*	0.080192	0.062729
CS11	CS31	JO10	*R.nitidus*	-0.008621	0.06803
GD29	CS31	JO10	*R.nitidus*	0.084487	0.065811
JW4	CS30	JO10	*R.nitidus*	0.199237	0.063864
SC18	CS30	JO10	*R.nitidus*	0.093079	0.064527
JO1	CS30	JO10	*R.nitidus*	0.056445	0.063367
GD30	CS31	JO10	*R.nitidus*	0.10522	0.068926
CS12	CS31	JO10	*R.nitidus*	-0.004105	0.067463

GD34	CS31	JO10	*R.nitidus*	0.00968	0.065899
CS6	CS30	JO10	*R.nitidus*	0.025431	0.064233
JO5	CS30	JO10	*R.nitidus*	0.097276	0.062457
SC42	CS30	JO10	*R.nitidus*	0.228477	0.063411
GD22	CS31	JO10	*R.nitidus*	0.073205	0.065237
SC28	CS30	JO10	*R.nitidus*	0.026174	0.062415
GD32	CS31	JO10	*R.nitidus*	0.039967	0.068308
JW6	CS30	JO10	*R.nitidus*	0.076132	0.0692
JO8	CS31	JO10	*R.nitidus*	-0.001075	0.072433
JO7	CS31	JO10	*R.nitidus*	-0.026104	0.070802
GD49	CS31	JO10	*R.nitidus*	0.08826	0.068681
GD40	CS31	JO10	*R.nitidus*	0.042766	0.06492
CS13	CS31	JO10	*R.nitidus*	-0.072757	0.067557
GD45	CS31	JO10	*R.nitidus*	0.050539	0.06821
GD31	CS31	JO10	*R.nitidus*	0.043914	0.066658
GD26	CS31	JO10	*R.nitidus*	0.052283	0.063712
CS16	CS31	JO10	*R.nitidus*	-0.017018	0.066222
CS17	CS31	JO10	*R.nitidus*	0.016225	0.067512
GD46	CS31	JO10	*R.nitidus*	-0.000754	0.066292
CS22	CS31	JO10	*R.nitidus*	0.019048	0.064718
GD44	CS31	JO10	*R.nitidus*	0.011655	0.067253
GD33	CS31	JO10	*R.nitidus*	-0.02622	0.06647
GD35	CS31	JO10	*R.nitidus*	0.027946	0.064779
GD41	CS31	JO10	*R.nitidus*	-0.026886	0.064656
CS23	CS31	JO10	*R.nitidus*	-0.051672	0.064594
CS28	CS31	JO10	*R.nitidus*	-0.010373	0.065783
GD50	CS31	JO10	*R.nitidus*	0.027074	0.068239
CS18	CS31	JO10	*R.nitidus*	-0.02439	0.064288
SC5	CS31	JO10	*R.nitidus*	0.010125	0.06786
CS15	CS31	JO10	*R.nitidus*	-0.021429	0.068325
GD42	CS31	JO10	*R.nitidus*	-0.001493	0.064275
CS14	CS31	JO10	*R.nitidus*	-0.009615	0.068592
GD48	CS31	JO10	*R.nitidus*	0.026066	0.066172
CS20	CS31	JO10	*R.nitidus*	-0.048239	0.066104
CS21	CS31	JO10	*R.nitidus*	-0.01283	0.064837
SC24	CS31	JO10	*R.nitidus*	-0.001524	0.063702
CS19	CS31	JO10	*R.nitidus*	0.000803	0.067155
SC27	CS31	JO10	*R.nitidus*	0.012719	0.065831
SC12	CS31	JO10	*R.nitidus*	-0.003067	0.065184
CS24	CS31	JO10	*R.nitidus*	-0.033762	0.064721
SC28	CS31	JO10	*R.nitidus*	-0.029412	0.064478
SC35	CS31	JO10	*R.nitidus*	0.036009	0.065168
SC14	CS31	JO10	*R.nitidus*	0.027516	0.066818
SC15	CS31	JO10	*R.nitidus*	0.003218	0.068408
SC10	CS31	JO10	*R.nitidus*	0.091193	0.066815
CS30	CS31	JO10	*R.nitidus*	-0.077807	0.066032
CS25	CS31	JO10	*R.nitidus*	0.02126	0.067086
SC32	CS31	JO10	*R.nitidus*	0.044988	0.066036
CS11	CS3	JO10	*R.nitidus*	0.065676	0.065696
CS3	CS31	JO10	*R.nitidus*	-0.059618	0.064459
SC26	CS31	JO10	*R.nitidus*	-0.017319	0.065208
SC6	CS31	JO10	*R.nitidus*	0.016936	0.065076
SC42	CS31	JO10	*R.nitidus*	0.183299	0.062693
JO8	CS3	JO10	*R.nitidus*	0.036194	0.072954
JW4	CS31	JO10	*R.nitidus*	0.166156	0.065613
JO7	CS3	JO10	*R.nitidus*	0.043216	0.069335

CS4	CS31	JO10	*R.nitidus*	-0.024691	0.063577
JO1	CS31	JO10	*R.nitidus*	0.000755	0.064428
SC18	CS31	JO10	*R.nitidus*	0.037588	0.064788
JO5	CS31	JO10	*R.nitidus*	0.032774	0.06438
MY6	CS31	JO10	*R.nitidus*	0.008507	0.066926
MY4	CS31	JO10	*R.nitidus*	-0.03885	0.065148
CS6	CS31	JO10	*R.nitidus*	-0.033031	0.065964
SC31	CS31	JO10	*R.nitidus*	0.009834	0.067026
SC34	CS31	JO10	*R.nitidus*	0.018519	0.064026
JW7	CS31	JO10	*R.nitidus*	0.035122	0.069818
GD26	CS3	JO10	*R.nitidus*	0.109705	0.065396
GD30	CS3	JO10	*R.nitidus*	0.177723	0.067114
JW6	CS31	JO10	*R.nitidus*	0.039354	0.07138
SC44	CS31	JO10	*R.nitidus*	-0.002318	0.066221
SC45	CS31	JO10	*R.nitidus*	0.019985	0.065122
GD48	CS3	JO10	*R.nitidus*	0.086353	0.065636
GD29	CS3	JO10	*R.nitidus*	0.131579	0.063686
GD45	CS3	JO10	*R.nitidus*	0.0939	0.069079
GD35	CS3	JO10	*R.nitidus*	0.07421	0.06503
CS12	CS3	JO10	*R.nitidus*	0.038308	0.066436
CS15	CS3	JO10	*R.nitidus*	0.044393	0.067066
CS14	CS3	JO10	*R.nitidus*	0.052795	0.066904
CS13	CS3	JO10	*R.nitidus*	-0.001577	0.065588
GD21	CS3	JO10	*R.nitidus*	0.198479	0.064444
GD41	CS3	JO10	*R.nitidus*	0.046099	0.064182
GD33	CS3	JO10	*R.nitidus*	0.048341	0.064379
JO3	CS31	JO10	*R.nitidus*	-0.000892	0.070264
GD31	CS3	JO10	*R.nitidus*	0.104012	0.066376
GD42	CS3	JO10	*R.nitidus*	0.072187	0.064325
GD49	CS3	JO10	*R.nitidus*	0.135891	0.065762
GD22	CS3	JO10	*R.nitidus*	0.125864	0.06322
GD44	CS3	JO10	*R.nitidus*	0.062121	0.064589
GD50	CS3	JO10	*R.nitidus*	0.095718	0.067156
GD40	CS3	JO10	*R.nitidus*	0.103654	0.062591
GD32	CS3	JO10	*R.nitidus*	0.098669	0.066399
GD46	CS3	JO10	*R.nitidus*	0.056712	0.067007
CS20	CS3	JO10	*R.nitidus*	0.020942	0.06209
GD34	CS3	JO10	*R.nitidus*	0.078091	0.065008
CS23	CS3	JO10	*R.nitidus*	0.004873	0.062855
CS17	CS3	JO10	*R.nitidus*	0.084718	0.067239
SC18	CS3	JO10	*R.nitidus*	0.098865	0.064123
CS16	CS3	JO10	*R.nitidus*	0.039124	0.065239
GD25	CS3	JO10	*R.nitidus*	0.102179	0.065385
CS28	CS3	JO10	*R.nitidus*	0.049719	0.065365
CS6	CS3	JO10	*R.nitidus*	0.044133	0.062767
CS31	CS3	JO10	*R.nitidus*	0.059618	0.064459
SC12	CS3	JO10	*R.nitidus*	0.045419	0.063032
CS30	CS3	JO10	*R.nitidus*	0.0032	0.065044
CS4	CS3	JO10	*R.nitidus*	0.056574	0.063422
SC26	CS3	JO10	*R.nitidus*	0.040299	0.06414
SC15	CS3	JO10	*R.nitidus*	0.064063	0.065228
CS19	CS3	JO10	*R.nitidus*	0.070018	0.064504
CS18	CS3	JO10	*R.nitidus*	0.04021	0.062433
JW7	CS3	JO10	*R.nitidus*	0.075298	0.06686
SC6	CS3	JO10	*R.nitidus*	0.065676	0.062609
JO3	CS3	JO10	*R.nitidus*	0.039106	0.068585

SC28	CS3	JO10	*R.nitidus*	0.014063	0.064192
CS22	CS3	JO10	*R.nitidus*	0.084261	0.065659
CS21	CS3	JO10	*R.nitidus*	0.049954	0.063318
SC27	CS3	JO10	*R.nitidus*	0.062452	0.063277
JO8	SC5	JO10	*R.nitidus*	-0.029352	0.076073
SC31	CS3	JO10	*R.nitidus*	0.059361	0.061412
CS24	CS3	JO10	*R.nitidus*	0.040258	0.0627
CS25	CS3	JO10	*R.nitidus*	0.09253	0.064914
GD29	SC5	JO10	*R.nitidus*	0.07148	0.066053
JW6	CS3	JO10	*R.nitidus*	0.05746	0.071053
SC5	CS3	JO10	*R.nitidus*	0.064054	0.067016
JO5	CS3	JO10	*R.nitidus*	0.089286	0.063972
SC10	CS3	JO10	*R.nitidus*	0.122433	0.062038
GD22	SC5	JO10	*R.nitidus*	0.061972	0.06434
JW4	CS3	JO10	*R.nitidus*	0.194856	0.064944
JO7	SC5	JO10	*R.nitidus*	-0.035608	0.074742
SC42	CS3	JO10	*R.nitidus*	0.212737	0.062259
SC24	CS3	JO10	*R.nitidus*	0.071146	0.065585
MY4	CS3	JO10	*R.nitidus*	0.032307	0.065514
SC34	CS3	JO10	*R.nitidus*	0.070744	0.063468
SC32	CS3	JO10	*R.nitidus*	0.099702	0.06467
SC14	CS3	JO10	*R.nitidus*	0.088102	0.063579
SC35	CS3	JO10	*R.nitidus*	0.096629	0.06247
CS12	SC5	JO10	*R.nitidus*	-0.031179	0.068505
SC45	CS3	JO10	*R.nitidus*	0.071051	0.062467
SC44	CS3	JO10	*R.nitidus*	0.04262	0.062862
GD21	SC5	JO10	*R.nitidus*	0.136364	0.067988
MY6	CS3	JO10	*R.nitidus*	0.068576	0.063722
JO1	CS3	JO10	*R.nitidus*	0.047945	0.063708
GD40	SC5	JO10	*R.nitidus*	0.04034	0.066037
GD34	SC5	JO10	*R.nitidus*	-0.000733	0.066951
GD31	SC5	JO10	*R.nitidus*	0.04236	0.068275
CS11	SC5	JO10	*R.nitidus*	-0.014881	0.070126
GD26	SC5	JO10	*R.nitidus*	0.031433	0.065451
CS18	SC5	JO10	*R.nitidus*	-0.041727	0.066764
GD30	SC5	JO10	*R.nitidus*	0.102871	0.067694
GD32	SC5	JO10	*R.nitidus*	0	0.070457
GD33	SC5	JO10	*R.nitidus*	-0.031994	0.066002
GD25	SC5	JO10	*R.nitidus*	0.034822	0.065599
CS20	SC5	JO10	*R.nitidus*	-0.072368	0.065777
CS23	SC5	JO10	*R.nitidus*	-0.063309	0.065647
GD42	SC5	JO10	*R.nitidus*	-0.005069	0.065594
GD48	SC5	JO10	*R.nitidus*	0.001601	0.069044
GD49	SC5	JO10	*R.nitidus*	0.077239	0.070389
CS13	SC5	JO10	*R.nitidus*	-0.077042	0.06865
GD46	SC5	JO10	*R.nitidus*	-0.002183	0.066045
CS4	SC5	JO10	*R.nitidus*	-0.031627	0.067293
GD45	SC5	JO10	*R.nitidus*	0.01454	0.068082
CS16	SC5	JO10	*R.nitidus*	-0.033432	0.067027
SC12	SC5	JO10	*R.nitidus*	-0.008439	0.066572
CS14	SC5	JO10	*R.nitidus*	-0.027468	0.070972
GD35	SC5	JO10	*R.nitidus*	0.014948	0.066865
CS19	SC5	JO10	*R.nitidus*	-0.01514	0.067812
CS28	SC5	JO10	*R.nitidus*	-0.028081	0.068399
CS30	SC5	JO10	*R.nitidus*	-0.065943	0.066465
SC15	SC5	JO10	*R.nitidus*	-0.011401	0.066479

GD44	SC5	JO10	*R.nitidus*	-0.006966	0.068487
SC14	SC5	JO10	*R.nitidus*	0.016878	0.068609
CS17	SC5	JO10	*R.nitidus*	0.014286	0.070386
CS31	SC5	JO10	*R.nitidus*	-0.010125	0.06786
CS24	SC5	JO10	*R.nitidus*	-0.049373	0.067345
SC6	SC5	JO10	*R.nitidus*	0.002368	0.066444
GD41	SC5	JO10	*R.nitidus*	-0.046852	0.066077
SC28	SC5	JO10	*R.nitidus*	-0.043478	0.067644
CS25	SC5	JO10	*R.nitidus*	0.000747	0.067015
CS15	SC5	JO10	*R.nitidus*	-0.03532	0.068147
MY4	SC5	JO10	*R.nitidus*	-0.041316	0.067847
SC32	SC5	JO10	*R.nitidus*	0.027581	0.067973
CS21	SC5	JO10	*R.nitidus*	-0.033022	0.065422
CS22	SC5	JO10	*R.nitidus*	-0.002269	0.068217
JO7	SC6	JO10	*R.nitidus*	-0.018682	0.068301
GD50	SC5	JO10	*R.nitidus*	0.029255	0.071517
SC24	SC5	JO10	*R.nitidus*	0.001634	0.068501
SC45	SC5	JO10	*R.nitidus*	0.016813	0.066739
JO8	SC6	JO10	*R.nitidus*	-0.018311	0.072313
CS3	SC5	JO10	*R.nitidus*	-0.064054	0.067016
JO5	SC5	JO10	*R.nitidus*	0.028947	0.066775
SC10	SC5	JO10	*R.nitidus*	0.0819	0.068559
SC34	SC5	JO10	*R.nitidus*	-0.000776	0.066075
SC42	SC5	JO10	*R.nitidus*	0.166088	0.065748
SC26	SC5	JO10	*R.nitidus*	-0.037361	0.067587
MY6	SC5	JO10	*R.nitidus*	-0.00374	0.067152
SC35	SC5	JO10	*R.nitidus*	0.02864	0.067326
JO3	SC5	JO10	*R.nitidus*	-0.028061	0.073541
SC18	SC5	JO10	*R.nitidus*	0.033107	0.068045
JO1	SC5	JO10	*R.nitidus*	-0.012559	0.066916
CS11	SC6	JO10	*R.nitidus*	-0.015106	0.064943
GD22	SC6	JO10	*R.nitidus*	0.05087	0.062962
SC27	SC5	JO10	*R.nitidus*	0.005766	0.066645
SC31	SC5	JO10	*R.nitidus*	-0.004688	0.068228
GD29	SC6	JO10	*R.nitidus*	0.068166	0.062853
CS6	SC5	JO10	*R.nitidus*	-0.03972	0.066742
GD30	SC6	JO10	*R.nitidus*	0.105645	0.065252
SC44	SC5	JO10	*R.nitidus*	-0.018868	0.0667
GD41	SC6	JO10	*R.nitidus*	-0.034826	0.065338
JW4	SC5	JO10	*R.nitidus*	0.124902	0.068475
GD31	SC6	JO10	*R.nitidus*	0.023188	0.063566
JW7	SC5	JO10	*R.nitidus*	-0.008016	0.072352
GD26	SC6	JO10	*R.nitidus*	0.041518	0.064181
GD33	SC6	JO10	*R.nitidus*	-0.032694	0.063529
GD45	SC6	JO10	*R.nitidus*	0.013535	0.066272
CS16	SC6	JO10	*R.nitidus*	-0.029456	0.064362
CS15	SC6	JO10	*R.nitidus*	-0.029501	0.066569
GD32	SC6	JO10	*R.nitidus*	0.01139	0.066677
JW6	SC5	JO10	*R.nitidus*	0.005982	0.074644
GD40	SC6	JO10	*R.nitidus*	0.032028	0.060563
CS25	SC6	JO10	*R.nitidus*	0.003852	0.06409
GD21	SC6	JO10	*R.nitidus*	0.148575	0.063002
GD35	SC6	JO10	*R.nitidus*	0.016405	0.062206
GD25	SC6	JO10	*R.nitidus*	0.034934	0.062195
GD42	SC6	JO10	*R.nitidus*	0.00495	0.063335
CS12	SC6	JO10	*R.nitidus*	-0.028418	0.06723

CS28	SC6	JO10	*R.nitidus*	-0.030159	0.063422
GD44	SC6	JO10	*R.nitidus*	-0.015198	0.064269
GD46	SC6	JO10	*R.nitidus*	-0.010101	0.063612
CS21	SC6	JO10	*R.nitidus*	-0.032258	0.06465
CS13	SC6	JO10	*R.nitidus*	-0.078067	0.064978
SC5	SC6	JO10	*R.nitidus*	-0.002368	0.066444
GD34	SC6	JO10	*R.nitidus*	0.004184	0.064299
CS14	SC6	JO10	*R.nitidus*	-0.029213	0.066305
SC24	SC6	JO10	*R.nitidus*	-0.008032	0.061962
CS3	SC6	JO10	*R.nitidus*	-0.065676	0.062609
CS31	SC6	JO10	*R.nitidus*	-0.016936	0.065076
GD49	SC6	JO10	*R.nitidus*	0.070024	0.067603
CS24	SC6	JO10	*R.nitidus*	-0.045181	0.06171
SC15	SC6	JO10	*R.nitidus*	-0.013855	0.064842
GD50	SC6	JO10	*R.nitidus*	0.009275	0.067389
CS4	SC6	JO10	*R.nitidus*	-0.029112	0.062571
CS19	SC6	JO10	*R.nitidus*	-0.003751	0.064412
GD48	SC6	JO10	*R.nitidus*	0.034252	0.064597
SC12	SC6	JO10	*R.nitidus*	-0.017378	0.063925
SC31	SC6	JO10	*R.nitidus*	-0.003218	0.065128
JW4	SC6	JO10	*R.nitidus*	0.149381	0.066744
CS23	SC6	JO10	*R.nitidus*	-0.06692	0.0639
CS30	SC6	JO10	*R.nitidus*	-0.079554	0.062626
CS20	SC6	JO10	*R.nitidus*	-0.062791	0.063381
CS17	SC6	JO10	*R.nitidus*	0.01414	0.06491
SC32	SC6	JO10	*R.nitidus*	0.028191	0.063836
CS18	SC6	JO10	*R.nitidus*	-0.041667	0.063439
CS22	SC6	JO10	*R.nitidus*	0.008271	0.063921
SC26	SC6	JO10	*R.nitidus*	-0.039401	0.063959
MY4	SC6	JO10	*R.nitidus*	-0.039261	0.063052
SC18	SC6	JO10	*R.nitidus*	0.019295	0.064518
SC10	SC6	JO10	*R.nitidus*	0.072785	0.064464
SC14	SC6	JO10	*R.nitidus*	0.014263	0.063768
SC45	SC6	JO10	*R.nitidus*	0.00625	0.062304
JO8	SC10	JO10	*R.nitidus*	-0.113147	0.069081
GD25	SC10	JO10	*R.nitidus*	-0.031818	0.062834
SC34	SC6	JO10	*R.nitidus*	0.006923	0.063856
JO3	SC6	JO10	*R.nitidus*	0.002636	0.067306
SC28	SC6	JO10	*R.nitidus*	-0.069992	0.063792
SC27	SC6	JO10	*R.nitidus*	-0.009531	0.064341
GD30	SC10	JO10	*R.nitidus*	0.051452	0.067198
CS6	SC6	JO10	*R.nitidus*	-0.037213	0.061968
JO7	SC10	JO10	*R.nitidus*	-0.104628	0.070393
GD40	SC10	JO10	*R.nitidus*	-0.036749	0.063009
SC42	SC6	JO10	*R.nitidus*	0.178962	0.061562
JW7	SC6	JO10	*R.nitidus*	-0.003976	0.067627
GD31	SC10	JO10	*R.nitidus*	-0.029148	0.065656
SC35	SC6	JO10	*R.nitidus*	0.01992	0.06263
GD26	SC10	JO10	*R.nitidus*	-0.036417	0.064776
GD22	SC10	JO10	*R.nitidus*	-0.009838	0.064209
SC44	SC6	JO10	*R.nitidus*	-0.029641	0.064849
JO5	SC6	JO10	*R.nitidus*	0.023166	0.064931
GD33	SC10	JO10	*R.nitidus*	-0.083095	0.065941
GD21	SC10	JO10	*R.nitidus*	0.08209	0.064462
CS11	SC10	JO10	*R.nitidus*	-0.082222	0.066656
JW6	SC6	JO10	*R.nitidus*	-0.009756	0.070117

GD44	SC10	JO10	*R.nitidus*	-0.064945	0.064733
GD49	SC10	JO10	*R.nitidus*	0.009592	0.066486
MY6	SC6	JO10	*R.nitidus*	0.001528	0.062216
GD45	SC10	JO10	*R.nitidus*	-0.039106	0.067487
GD32	SC10	JO10	*R.nitidus*	-0.04653	0.067141
CS12	SC10	JO10	*R.nitidus*	-0.10717	0.066137
GD50	SC10	JO10	*R.nitidus*	-0.044877	0.068183
GD46	SC10	JO10	*R.nitidus*	-0.067773	0.06448
GD48	SC10	JO10	*R.nitidus*	-0.03973	0.065433
JO1	SC6	JO10	*R.nitidus*	-0.01474	0.063299
GD42	SC10	JO10	*R.nitidus*	-0.058824	0.065926
CS14	SC10	JO10	*R.nitidus*	-0.087953	0.066724
CS13	SC10	JO10	*R.nitidus*	-0.141445	0.064722
GD41	SC10	JO10	*R.nitidus*	-0.097983	0.06354
CS16	SC10	JO10	*R.nitidus*	-0.100364	0.064345
GD29	SC10	JO10	*R.nitidus*	-0.010094	0.06402
CS25	SC10	JO10	*R.nitidus*	-0.056967	0.067989
GD35	SC10	JO10	*R.nitidus*	-0.050109	0.06369
CS28	SC10	JO10	*R.nitidus*	-0.105263	0.066807
GD34	SC10	JO10	*R.nitidus*	-0.053381	0.065217
CS30	SC10	JO10	*R.nitidus*	-0.146677	0.062862
CS19	SC10	JO10	*R.nitidus*	-0.076923	0.067339
CS15	SC10	JO10	*R.nitidus*	-0.087085	0.064874
SC12	SC10	JO10	*R.nitidus*	-0.105174	0.062736
CS21	SC10	JO10	*R.nitidus*	-0.097923	0.064869
CS17	SC10	JO10	*R.nitidus*	-0.046166	0.066808
CS24	SC10	JO10	*R.nitidus*	-0.125286	0.062667
CS18	SC10	JO10	*R.nitidus*	-0.115758	0.064793
JW7	SC10	JO10	*R.nitidus*	-0.034895	0.067577
SC15	SC10	JO10	*R.nitidus*	-0.093043	0.065494
CS20	SC10	JO10	*R.nitidus*	-0.127924	0.065066
JO3	SC10	JO10	*R.nitidus*	-0.065814	0.057735
CS23	SC10	JO10	*R.nitidus*	-0.133975	0.065258
CS22	SC10	JO10	*R.nitidus*	-0.070498	0.065253
SC32	SC10	JO10	*R.nitidus*	-0.045455	0.064329
SC6	SC10	JO10	*R.nitidus*	-0.072785	0.064464
SC26	SC10	JO10	*R.nitidus*	-0.113497	0.063856
SC28	SC10	JO10	*R.nitidus*	-0.141694	0.064038
SC27	SC10	JO10	*R.nitidus*	-0.077253	0.063685
CS3	SC10	JO10	*R.nitidus*	-0.122433	0.062038
SC5	SC10	JO10	*R.nitidus*	-0.0819	0.068559
SC14	SC10	JO10	*R.nitidus*	-0.058584	0.065635
JW6	SC10	JO10	*R.nitidus*	-0.058291	0.070536
SC44	SC10	JO10	*R.nitidus*	-0.105098	0.063779
GD30	CS4	JO10	*R.nitidus*	0.130568	0.065112
SC31	SC10	JO10	*R.nitidus*	-0.081746	0.064674
CS31	SC10	JO10	*R.nitidus*	-0.091193	0.066815
CS4	SC10	JO10	*R.nitidus*	-0.109145	0.06382
JO5	SC10	JO10	*R.nitidus*	-0.064388	0.065762
CS6	SC10	JO10	*R.nitidus*	-0.106646	0.064531
MY4	SC10	JO10	*R.nitidus*	-0.115325	0.065302
SC18	SC10	JO10	*R.nitidus*	-0.061655	0.065326
JO8	CS4	JO10	*R.nitidus*	0.000995	0.07008
JO1	SC10	JO10	*R.nitidus*	-0.089518	0.062929
SC35	SC10	JO10	*R.nitidus*	-0.049489	0.063117
GD31	CS4	JO10	*R.nitidus*	0.072359	0.065326

GD22	CS4	JO10	*R.nitidus*	0.092008	0.062331
GD26	CS4	JO10	*R.nitidus*	0.074202	0.062782
SC24	SC10	JO10	*R.nitidus*	-0.093133	0.064356
SC34	SC10	JO10	*R.nitidus*	-0.068404	0.063566
GD21	CS4	JO10	*R.nitidus*	0.177539	0.062623
GD33	CS4	JO10	*R.nitidus*	0.009246	0.064134
CS11	CS4	JO10	*R.nitidus*	0.011095	0.064941
SC42	SC10	JO10	*R.nitidus*	0.100642	0.064785
GD25	CS4	JO10	*R.nitidus*	0.057951	0.06292
MY6	SC10	JO10	*R.nitidus*	-0.077619	0.063947
JO7	CS4	JO10	*R.nitidus*	0.027128	0.067074
SC45	SC10	JO10	*R.nitidus*	-0.069106	0.065172
GD40	CS4	JO10	*R.nitidus*	0.069571	0.062659
GD50	CS4	JO10	*R.nitidus*	0.059016	0.066176
JW4	SC10	JO10	*R.nitidus*	0.080848	0.065602
GD45	CS4	JO10	*R.nitidus*	0.071261	0.064723
GD35	CS4	JO10	*R.nitidus*	0.051883	0.061546
GD32	CS4	JO10	*R.nitidus*	0.072374	0.065051
CS13	CS4	JO10	*R.nitidus*	-0.047292	0.06657
GD34	CS4	JO10	*R.nitidus*	0.038408	0.064415
GD29	CS4	JO10	*R.nitidus*	0.107143	0.062799
GD48	CS4	JO10	*R.nitidus*	0.054693	0.064323
CS12	CS4	JO10	*R.nitidus*	0.012928	0.065168
CS28	CS4	JO10	*R.nitidus*	0.00463	0.062529
GD42	CS4	JO10	*R.nitidus*	0.02439	0.06372
CS19	CS4	JO10	*R.nitidus*	0.021845	0.062844
GD49	CS4	JO10	*R.nitidus*	0.100311	0.064789
CS22	CS4	JO10	*R.nitidus*	0.047337	0.062348
CS15	CS4	JO10	*R.nitidus*	0.001462	0.066606
CS16	CS4	JO10	*R.nitidus*	0.002242	0.064604
CS17	CS4	JO10	*R.nitidus*	0.050433	0.06688
GD44	CS4	JO10	*R.nitidus*	0.040152	0.062356
GD46	CS4	JO10	*R.nitidus*	0.023677	0.064088
SC18	CS4	JO10	*R.nitidus*	0.047619	0.064896
SC5	CS4	JO10	*R.nitidus*	0.031627	0.067293
GD41	CS4	JO10	*R.nitidus*	-0.00071	0.063391
SC45	CS4	JO10	*R.nitidus*	0.043066	0.065064
CS20	CS4	JO10	*R.nitidus*	-0.038017	0.062964
SC12	CS4	JO10	*R.nitidus*	0.020896	0.063028
SC31	CS4	JO10	*R.nitidus*	0.020393	0.062427
CS14	CS4	JO10	*R.nitidus*	0.01107	0.066131
SC42	CS4	JO10	*R.nitidus*	0.199609	0.0604
CS18	CS4	JO10	*R.nitidus*	0.000805	0.063168
SC44	CS4	JO10	*R.nitidus*	0.011914	0.063364
SC26	CS4	JO10	*R.nitidus*	0.009366	0.062741
SC15	CS4	JO10	*R.nitidus*	0.022539	0.063222
JW7	CS4	JO10	*R.nitidus*	0.041182	0.065806
CS25	CS4	JO10	*R.nitidus*	0.02692	0.065794
CS23	CS4	JO10	*R.nitidus*	-0.033573	0.063551
JO1	CS4	JO10	*R.nitidus*	0.012482	0.062741
JW6	CS4	JO10	*R.nitidus*	0.033898	0.069187
CS6	CS4	JO10	*R.nitidus*	-0.006481	0.062535
MY6	CS4	JO10	*R.nitidus*	0.030191	0.062832
CS21	CS4	JO10	*R.nitidus*	0.014129	0.062452
CS3	CS4	JO10	*R.nitidus*	-0.056574	0.063422
CS24	CS4	JO10	*R.nitidus*	-0.009857	0.062089

JW4	CS4	JO10	*R.nitidus*	0.156369	0.063361
SC28	CS4	JO10	*R.nitidus*	-0.017216	0.063471
JO7	SC12	JO10	*R.nitidus*	-0.007021	0.069846
SC10	CS4	JO10	*R.nitidus*	0.109145	0.06382
CS30	CS4	JO10	*R.nitidus*	-0.043044	0.062851
CS31	CS4	JO10	*R.nitidus*	0.024691	0.063577
SC14	CS4	JO10	*R.nitidus*	0.041007	0.061834
SC6	CS4	JO10	*R.nitidus*	0.029112	0.062571
JO3	CS4	JO10	*R.nitidus*	0.024829	0.067362
SC24	CS4	JO10	*R.nitidus*	0.025849	0.063465
JO8	SC12	JO10	*R.nitidus*	-0.005274	0.070614
SC27	CS4	JO10	*R.nitidus*	0.026412	0.062679
MY4	CS4	JO10	*R.nitidus*	-0.010355	0.063499
GD30	SC12	JO10	*R.nitidus*	0.130366	0.065125
SC32	CS4	JO10	*R.nitidus*	0.062777	0.063111
SC35	CS4	JO10	*R.nitidus*	0.05132	0.062323
GD32	SC12	JO10	*R.nitidus*	0.030023	0.066316
SC34	CS4	JO10	*R.nitidus*	0.037249	0.062012
GD21	SC12	JO10	*R.nitidus*	0.158625	0.061614
GD29	SC12	JO10	*R.nitidus*	0.077931	0.062688
GD25	SC12	JO10	*R.nitidus*	0.050558	0.062866
GD49	SC12	JO10	*R.nitidus*	0.077708	0.064044
GD40	SC12	JO10	*R.nitidus*	0.059474	0.061553
GD26	SC12	JO10	*R.nitidus*	0.054054	0.06257
GD44	SC12	JO10	*R.nitidus*	0.014738	0.064067
GD22	SC12	JO10	*R.nitidus*	0.072395	0.060444
JO5	CS4	JO10	*R.nitidus*	0.048148	0.063565
GD46	SC12	JO10	*R.nitidus*	0.016819	0.062942
GD34	SC12	JO10	*R.nitidus*	0.027046	0.062812
GD48	SC12	JO10	*R.nitidus*	0.045718	0.063232
GD33	SC12	JO10	*R.nitidus*	-0.013112	0.062279
CS11	SC12	JO10	*R.nitidus*	-0.004435	0.064012
GD31	SC12	JO10	*R.nitidus*	0.061495	0.064373
CS15	SC12	JO10	*R.nitidus*	-0.003639	0.066191
GD50	SC12	JO10	*R.nitidus*	0.039037	0.066097
GD42	SC12	JO10	*R.nitidus*	0.012667	0.064197
CS23	SC12	JO10	*R.nitidus*	-0.037093	0.062981
CS12	SC12	JO10	*R.nitidus*	-0.014243	0.065947
CS18	SC12	JO10	*R.nitidus*	-0.024187	0.063738
CS30	SC12	JO10	*R.nitidus*	-0.054517	0.064016
CS14	SC12	JO10	*R.nitidus*	-0.007965	0.066075
CS20	SC12	JO10	*R.nitidus*	-0.050998	0.064215
SC34	SC12	JO10	*R.nitidus*	0.023847	0.064259
GD45	SC12	JO10	*R.nitidus*	0.047692	0.064514
GD35	SC12	JO10	*R.nitidus*	0.034657	0.062313
CS21	SC12	JO10	*R.nitidus*	-0.013503	0.065204
CS13	SC12	JO10	*R.nitidus*	-0.06155	0.06408
SC10	SC12	JO10	*R.nitidus*	0.105174	0.062736
SC15	SC12	JO10	*R.nitidus*	0.006623	0.064821
SC26	SC12	JO10	*R.nitidus*	-0.016575	0.062435
GD41	SC12	JO10	*R.nitidus*	-0.030812	0.064976
CS3	SC12	JO10	*R.nitidus*	-0.045419	0.063032
SC31	SC12	JO10	*R.nitidus*	0.009412	0.064152
CS28	SC12	JO10	*R.nitidus*	-0.016627	0.065429
CS25	SC12	JO10	*R.nitidus*	0.017611	0.065002
CS24	SC12	JO10	*R.nitidus*	-0.026275	0.060994

SC32	SC12	JO10	*R.nitidus*	0.061699	0.064898
CS17	SC12	JO10	*R.nitidus*	0.03295	0.065495
CS22	SC12	JO10	*R.nitidus*	0.023006	0.063857
SC14	SC12	JO10	*R.nitidus*	0.025812	0.062732
CS19	SC12	JO10	*R.nitidus*	0.003698	0.063508
SC24	SC12	JO10	*R.nitidus*	0.015924	0.06223
CS31	SC12	JO10	*R.nitidus*	0.003067	0.065184
CS16	SC12	JO10	*R.nitidus*	-0.005882	0.063688
CS6	SC12	JO10	*R.nitidus*	-0.02562	0.064933
CS4	SC12	JO10	*R.nitidus*	-0.020896	0.063028
JW6	SC12	JO10	*R.nitidus*	0.027218	0.0692
SC6	SC12	JO10	*R.nitidus*	0.017378	0.063925
SC5	SC12	JO10	*R.nitidus*	0.008439	0.066572
SC18	SC12	JO10	*R.nitidus*	0.059216	0.06316
SC42	SC12	JO10	*R.nitidus*	0.197055	0.061571
JO1	SC12	JO10	*R.nitidus*	0.004739	0.063393
JW4	SC12	JO10	*R.nitidus*	0.154567	0.064228
SC27	SC12	JO10	*R.nitidus*	0.027562	0.062764
JO3	SC12	JO10	*R.nitidus*	0.018119	0.067174
SC28	SC12	JO10	*R.nitidus*	-0.028878	0.062351
JW7	SC12	JO10	*R.nitidus*	0.019527	0.065802
SC35	SC12	JO10	*R.nitidus*	0.061389	0.061647
JO8	SC14	JO10	*R.nitidus*	-0.046512	0.071044
CS11	SC14	JO10	*R.nitidus*	-0.035897	0.068327
GD25	SC14	JO10	*R.nitidus*	0.021583	0.061709
GD21	SC14	JO10	*R.nitidus*	0.129183	0.063
JO7	SC14	JO10	*R.nitidus*	-0.053097	0.07068
SC45	SC12	JO10	*R.nitidus*	0.031605	0.062053
JO5	SC12	JO10	*R.nitidus*	0.04739	0.063518
GD32	SC14	JO10	*R.nitidus*	0.001506	0.06619
GD44	SC14	JO10	*R.nitidus*	-0.015094	0.064354
GD22	SC14	JO10	*R.nitidus*	0.049225	0.062302
GD29	SC14	JO10	*R.nitidus*	0.054258	0.062953
GD30	SC14	JO10	*R.nitidus*	0.094821	0.065403
MY4	SC12	JO10	*R.nitidus*	-0.030023	0.063988
CS14	SC14	JO10	*R.nitidus*	-0.037445	0.06798
GD31	SC14	JO10	*R.nitidus*	0.026948	0.063848
GD42	SC14	JO10	*R.nitidus*	-0.021368	0.062781
MY6	SC12	JO10	*R.nitidus*	0.013067	0.064036
GD26	SC14	JO10	*R.nitidus*	0.027178	0.063727
GD40	SC14	JO10	*R.nitidus*	0.027454	0.062551
GD50	SC14	JO10	*R.nitidus*	0.003215	0.066286
CS30	SC14	JO10	*R.nitidus*	-0.084034	0.062933
GD33	SC14	JO10	*R.nitidus*	-0.05	0.063146
GD41	SC14	JO10	*R.nitidus*	-0.054506	0.06275
CS13	SC14	JO10	*R.nitidus*	-0.094382	0.064619
GD35	SC14	JO10	*R.nitidus*	-0.001435	0.064659
CS21	SC14	JO10	*R.nitidus*	-0.046329	0.063273
CS12	SC14	JO10	*R.nitidus*	-0.036732	0.068122
GD45	SC14	JO10	*R.nitidus*	0	0.066223
CS31	SC14	JO10	*R.nitidus*	-0.027516	0.066818
SC18	SC14	JO10	*R.nitidus*	0.012417	0.065013
GD49	SC14	JO10	*R.nitidus*	0.045807	0.064737
CS28	SC14	JO10	*R.nitidus*	-0.031201	0.065981
GD34	SC14	JO10	*R.nitidus*	-0.006887	0.06357
SC26	SC14	JO10	*R.nitidus*	-0.038522	0.062982

GD46	SC14	JO10	*R.nitidus*	-0.020379	0.062824
CS20	SC14	JO10	*R.nitidus*	-0.075	0.063341
CS17	SC14	JO10	*R.nitidus*	-0.009501	0.068348
SC32	SC14	JO10	*R.nitidus*	0.024116	0.063297
GD48	SC14	JO10	*R.nitidus*	0.014179	0.06567
SC5	SC14	JO10	*R.nitidus*	-0.016878	0.068609
SC6	SC14	JO10	*R.nitidus*	-0.014263	0.063768
CS15	SC14	JO10	*R.nitidus*	-0.045655	0.067188
CS24	SC14	JO10	*R.nitidus*	-0.052793	0.06302
CS16	SC14	JO10	*R.nitidus*	-0.038519	0.065512
SC24	SC14	JO10	*R.nitidus*	-0.016575	0.064989
CS18	SC14	JO10	*R.nitidus*	-0.044798	0.064624
CS19	SC14	JO10	*R.nitidus*	-0.020528	0.065109
SC31	SC14	JO10	*R.nitidus*	-0.021061	0.064879
SC15	SC14	JO10	*R.nitidus*	-0.017171	0.064018
SC35	SC14	JO10	*R.nitidus*	0.019908	0.062347
SC28	SC14	JO10	*R.nitidus*	-0.061006	0.063174
CS23	SC14	JO10	*R.nitidus*	-0.072714	0.063916
SC12	SC14	JO10	*R.nitidus*	-0.025812	0.062732
JW4	SC14	JO10	*R.nitidus*	0.11161	0.063467
CS25	SC14	JO10	*R.nitidus*	-0.016703	0.064718
CS4	SC14	JO10	*R.nitidus*	-0.041007	0.061834
SC27	SC14	JO10	*R.nitidus*	-0.018438	0.059753
JO3	SC14	JO10	*R.nitidus*	-0.03196	0.068275
JO7	SC15	JO10	*R.nitidus*	-0.013972	0.068154
CS22	SC14	JO10	*R.nitidus*	-0.011161	0.064291
CS3	SC14	JO10	*R.nitidus*	-0.088102	0.063579
SC10	SC14	JO10	*R.nitidus*	0.058584	0.065635
SC44	SC14	JO10	*R.nitidus*	-0.03268	0.063341
JW6	SC14	JO10	*R.nitidus*	-0.00796	0.070533
SC34	SC14	JO10	*R.nitidus*	-0.007819	0.063024
MY4	SC14	JO10	*R.nitidus*	-0.058383	0.064571
GD21	SC15	JO10	*R.nitidus*	0.161202	0.063121
GD32	SC15	JO10	*R.nitidus*	0.035658	0.066306
CS11	SC15	JO10	*R.nitidus*	-0.011219	0.065867
GD26	SC15	JO10	*R.nitidus*	0.044286	0.06309
SC45	SC14	JO10	*R.nitidus*	0.004532	0.064874
MY6	SC14	JO10	*R.nitidus*	-0.01462	0.065056
SC42	SC14	JO10	*R.nitidus*	0.162181	0.062423
CS6	SC14	JO10	*R.nitidus*	-0.056809	0.063106
JO8	SC15	JO10	*R.nitidus*	-0.022581	0.073765
GD30	SC15	JO10	*R.nitidus*	0.128	0.066567
CS12	SC15	JO10	*R.nitidus*	-0.01691	0.066384
JW7	SC14	JO10	*R.nitidus*	-0.01574	0.067895
GD41	SC15	JO10	*R.nitidus*	-0.030568	0.063272
GD22	SC15	JO10	*R.nitidus*	0.060732	0.063079
GD40	SC15	JO10	*R.nitidus*	0.044523	0.063237
GD29	SC15	JO10	*R.nitidus*	0.075391	0.062955
JO5	SC14	JO10	*R.nitidus*	0.009685	0.064124
CS17	SC15	JO10	*R.nitidus*	0.0288	0.067646
JO1	SC14	JO10	*R.nitidus*	-0.013503	0.062287
GD31	SC15	JO10	*R.nitidus*	0.042773	0.064582
CS21	SC15	JO10	*R.nitidus*	-0.020347	0.063983
GD25	SC15	JO10	*R.nitidus*	0.042456	0.064047
CS13	SC15	JO10	*R.nitidus*	-0.070229	0.067016
GD33	SC15	JO10	*R.nitidus*	-0.023305	0.063643

CS22	SC15	JO10	*R.nitidus*	0.007593	0.065379
GD45	SC15	JO10	*R.nitidus*	0.036335	0.066411
CS25	SC15	JO10	*R.nitidus*	0.003915	0.065248
CS19	SC15	JO10	*R.nitidus*	0.000765	0.064553
GD34	SC15	JO10	*R.nitidus*	0.021739	0.063857
CS4	SC15	JO10	*R.nitidus*	-0.022539	0.063222
CS20	SC15	JO10	*R.nitidus*	-0.054846	0.064659
CS15	SC15	JO10	*R.nitidus*	-0.022026	0.067396
SC10	SC15	JO10	*R.nitidus*	0.093043	0.065494
GD42	SC15	JO10	*R.nitidus*	0.014337	0.065332
CS30	SC15	JO10	*R.nitidus*	-0.065481	0.065889
GD49	SC15	JO10	*R.nitidus*	0.074803	0.065859
CS16	SC15	JO10	*R.nitidus*	-0.020564	0.066695
CS24	SC15	JO10	*R.nitidus*	-0.034893	0.063213
GD35	SC15	JO10	*R.nitidus*	0.026946	0.06365
GD50	SC15	JO10	*R.nitidus*	0.011765	0.069626
SC18	SC15	JO10	*R.nitidus*	0.031014	0.065321
GD44	SC15	JO10	*R.nitidus*	-0.009946	0.06565
SC14	SC15	JO10	*R.nitidus*	0.017171	0.064018
GD48	SC15	JO10	*R.nitidus*	0.037291	0.066456
SC24	SC15	JO10	*R.nitidus*	-0.002447	0.063937
SC5	SC15	JO10	*R.nitidus*	0.011401	0.066479
CS3	SC15	JO10	*R.nitidus*	-0.064063	0.065228
CS14	SC15	JO10	*R.nitidus*	-0.009709	0.067677
CS28	SC15	JO10	*R.nitidus*	-0.009868	0.066225
SC6	SC15	JO10	*R.nitidus*	0.013855	0.064842
SC44	SC15	JO10	*R.nitidus*	-0.017087	0.064243
CS18	SC15	JO10	*R.nitidus*	-0.025522	0.063803
SC12	SC15	JO10	*R.nitidus*	-0.006623	0.064821
SC42	SC15	JO10	*R.nitidus*	0.179219	0.062539
SC31	SC15	JO10	*R.nitidus*	-0.003448	0.064024
CS23	SC15	JO10	*R.nitidus*	-0.058069	0.064113
SC28	SC15	JO10	*R.nitidus*	-0.043867	0.064504
SC34	SC15	JO10	*R.nitidus*	0.017959	0.063957
SC32	SC15	JO10	*R.nitidus*	0.047771	0.064459
MY4	SC15	JO10	*R.nitidus*	-0.034875	0.063227
CS31	SC15	JO10	*R.nitidus*	-0.003218	0.068408
JW7	SC15	JO10	*R.nitidus*	0.013725	0.068604
JW4	SC15	JO10	*R.nitidus*	0.123007	0.065828
SC27	SC15	JO10	*R.nitidus*	0.003376	0.065086
GD30	SC18	JO10	*R.nitidus*	0.075348	0.065566
GD46	SC15	JO10	*R.nitidus*	-0.000713	0.064187
GD25	SC18	JO10	*R.nitidus*	0.007513	0.063672
SC26	SC15	JO10	*R.nitidus*	-0.015748	0.062269
SC45	SC15	JO10	*R.nitidus*	0.021002	0.064686
CS6	SC15	JO10	*R.nitidus*	-0.031002	0.063658
JO1	SC15	JO10	*R.nitidus*	-0.002379	0.062497
JO8	SC18	JO10	*R.nitidus*	-0.069119	0.070432
SC35	SC15	JO10	*R.nitidus*	0.038929	0.063759
GD46	SC18	JO10	*R.nitidus*	-0.041698	0.064591
MY6	SC15	JO10	*R.nitidus*	0.006798	0.062693
CS11	SC18	JO10	*R.nitidus*	-0.046945	0.066499
GD48	SC18	JO10	*R.nitidus*	-0.009479	0.065501
GD31	SC18	JO10	*R.nitidus*	0.002315	0.06416
GD41	SC18	JO10	*R.nitidus*	-0.057143	0.065314
JO5	SC15	JO10	*R.nitidus*	0.033844	0.063489

CS12	SC18	JO10	*R.nitidus*	-0.061503	0.066066
GD29	SC18	JO10	*R.nitidus*	0.032	0.064834
JW6	SC15	JO10	*R.nitidus*	0.024291	0.071615
JO7	SC18	JO10	*R.nitidus*	-0.064257	0.072265
CS17	SC18	JO10	*R.nitidus*	-0.015079	0.066812
GD32	SC18	JO10	*R.nitidus*	-0.027778	0.066771
GD22	SC18	JO10	*R.nitidus*	0.029016	0.063296
JO3	SC15	JO10	*R.nitidus*	0	0.068647
GD42	SC18	JO10	*R.nitidus*	-0.016862	0.064476
GD26	SC18	JO10	*R.nitidus*	0.008203	0.064666
CS21	SC18	JO10	*R.nitidus*	-0.055976	0.065691
GD35	SC18	JO10	*R.nitidus*	0.00387	0.064283
CS14	SC18	JO10	*R.nitidus*	-0.040449	0.06713
CS31	SC18	JO10	*R.nitidus*	-0.037588	0.064788
GD21	SC18	JO10	*R.nitidus*	0.129563	0.064001
CS24	SC18	JO10	*R.nitidus*	-0.058366	0.062957
CS28	SC18	JO10	*R.nitidus*	-0.052083	0.065584
GD34	SC18	JO10	*R.nitidus*	-0.016643	0.064622
CS4	SC18	JO10	*R.nitidus*	-0.047619	0.064896
CS13	SC18	JO10	*R.nitidus*	-0.102896	0.067875
GD40	SC18	JO10	*R.nitidus*	0.014126	0.062882
GD45	SC18	JO10	*R.nitidus*	-0.007463	0.066667
CS25	SC18	JO10	*R.nitidus*	-0.031758	0.067193
GD44	SC18	JO10	*R.nitidus*	-0.012638	0.06474
GD33	SC18	JO10	*R.nitidus*	-0.050735	0.065007
GD49	SC18	JO10	*R.nitidus*	0.039864	0.066396
CS20	SC18	JO10	*R.nitidus*	-0.094384	0.06438
GD50	SC18	JO10	*R.nitidus*	-0.019931	0.067851
SC10	SC18	JO10	*R.nitidus*	0.061655	0.065326
CS18	SC18	JO10	*R.nitidus*	-0.077454	0.062704
CS15	SC18	JO10	*R.nitidus*	-0.059542	0.066607
CS22	SC18	JO10	*R.nitidus*	-0.0125	0.065833
CS3	SC18	JO10	*R.nitidus*	-0.098865	0.064123
CS16	SC18	JO10	*R.nitidus*	-0.054448	0.064328
SC26	SC18	JO10	*R.nitidus*	-0.076539	0.063917
CS23	SC18	JO10	*R.nitidus*	-0.102219	0.063697
SC6	SC18	JO10	*R.nitidus*	-0.019295	0.064518
CS6	SC18	JO10	*R.nitidus*	-0.084298	0.064368
CS19	SC18	JO10	*R.nitidus*	-0.043028	0.065286
SC42	SC18	JO10	*R.nitidus*	0.149243	0.063599
SC32	SC18	JO10	*R.nitidus*	0.013147	0.063903
CS30	SC18	JO10	*R.nitidus*	-0.093079	0.064527
SC12	SC18	JO10	*R.nitidus*	-0.059216	0.06316
SC35	SC18	JO10	*R.nitidus*	0	0.062659
JO8	SC24	JO10	*R.nitidus*	-0.047059	0.071056
SC34	SC18	JO10	*R.nitidus*	-0.029221	0.064074
JO5	SC18	JO10	*R.nitidus*	-0.003228	0.063675
JO7	SC24	JO10	*R.nitidus*	-0.044177	0.071204
JW4	SC18	JO10	*R.nitidus*	0.101613	0.066954
SC15	SC18	JO10	*R.nitidus*	-0.031014	0.065321
SC24	SC18	JO10	*R.nitidus*	-0.05	0.06242
SC28	SC18	JO10	*R.nitidus*	-0.086505	0.062681
JO1	SC18	JO10	*R.nitidus*	-0.045714	0.064955
SC5	SC18	JO10	*R.nitidus*	-0.033107	0.068045
MY4	SC18	JO10	*R.nitidus*	-0.074281	0.063409
JW7	SC18	JO10	*R.nitidus*	-0.010309	0.068328

SC27	SC18	JO10	*R.nitidus*	-0.017888	0.06535
GD25	SC24	JO10	*R.nitidus*	0.035819	0.062771
SC14	SC18	JO10	*R.nitidus*	-0.012417	0.065013
MY6	SC18	JO10	*R.nitidus*	-0.039777	0.063491
GD26	SC24	JO10	*R.nitidus*	0.043571	0.064751
JW6	SC18	JO10	*R.nitidus*	-0.005252	0.07146
SC44	SC18	JO10	*R.nitidus*	-0.051607	0.064746
JO3	SC18	JO10	*R.nitidus*	-0.044264	0.070257
CS12	SC24	JO10	*R.nitidus*	-0.022388	0.065353
GD35	SC24	JO10	*R.nitidus*	0.031341	0.063686
SC31	SC18	JO10	*R.nitidus*	-0.027642	0.065359
SC45	SC18	JO10	*R.nitidus*	-0.023829	0.064787
GD48	SC24	JO10	*R.nitidus*	0.015361	0.064274
GD29	SC24	JO10	*R.nitidus*	0.062457	0.062256
GD22	SC24	JO10	*R.nitidus*	0.066804	0.062246
CS11	SC24	JO10	*R.nitidus*	-0.009552	0.066207
GD41	SC24	JO10	*R.nitidus*	-0.018665	0.062737
CS14	SC24	JO10	*R.nitidus*	-0.022711	0.067643
CS15	SC24	JO10	*R.nitidus*	-0.021339	0.066012
GD33	SC24	JO10	*R.nitidus*	-0.018455	0.063081
GD45	SC24	JO10	*R.nitidus*	0.030977	0.065572
CS19	SC24	JO10	*R.nitidus*	-0.001538	0.064387
GD46	SC24	JO10	*R.nitidus*	-0.008553	0.063538
GD44	SC24	JO10	*R.nitidus*	-0.006971	0.064441
GD30	SC24	JO10	*R.nitidus*	0.118974	0.064323
GD21	SC24	JO10	*R.nitidus*	0.157426	0.061829
CS17	SC24	JO10	*R.nitidus*	0.020392	0.066402
CS16	SC24	JO10	*R.nitidus*	-0.025335	0.063695
CS18	SC24	JO10	*R.nitidus*	-0.032885	0.062755
GD31	SC24	JO10	*R.nitidus*	0.033716	0.062869
CS30	SC24	JO10	*R.nitidus*	-0.06537	0.064044
GD50	SC24	JO10	*R.nitidus*	0.036424	0.067148
CS4	SC24	JO10	*R.nitidus*	-0.025849	0.063465
CS21	SC24	JO10	*R.nitidus*	-0.022761	0.063527
SC18	SC24	JO10	*R.nitidus*	0.05	0.06242
GD34	SC24	JO10	*R.nitidus*	0.01198	0.061541
GD40	SC24	JO10	*R.nitidus*	0.039046	0.061541
CS3	SC24	JO10	*R.nitidus*	-0.071146	0.065585
GD49	SC24	JO10	*R.nitidus*	0.083399	0.0639
GD32	SC24	JO10	*R.nitidus*	0.016936	0.065655
GD42	SC24	JO10	*R.nitidus*	0.009306	0.063451
CS24	SC24	JO10	*R.nitidus*	-0.032689	0.061698
SC5	SC24	JO10	*R.nitidus*	-0.001634	0.068501
CS13	SC24	JO10	*R.nitidus*	-0.07624	0.066431
SC31	SC24	JO10	*R.nitidus*	-0.001574	0.064456
SC6	SC24	JO10	*R.nitidus*	0.008032	0.061962
CS23	SC24	JO10	*R.nitidus*	-0.061086	0.062194
SC15	SC24	JO10	*R.nitidus*	0.002447	0.063937
CS22	SC24	JO10	*R.nitidus*	0.011808	0.064923
SC35	SC24	JO10	*R.nitidus*	0.029992	0.061727
CS31	SC24	JO10	*R.nitidus*	0.001524	0.063702
SC34	SC24	JO10	*R.nitidus*	0.007955	0.062949
CS6	SC24	JO10	*R.nitidus*	-0.044035	0.062414
CS20	SC24	JO10	*R.nitidus*	-0.059132	0.062975
SC10	SC24	JO10	*R.nitidus*	0.093133	0.064356
CS28	SC24	JO10	*R.nitidus*	-0.016509	0.06334

SC27	SC24	JO10	*R.nitidus*	0.012831	0.063118
SC32	SC24	JO10	*R.nitidus*	0.045732	0.063863
SC26	SC24	JO10	*R.nitidus*	-0.024845	0.064044
SC14	SC24	JO10	*R.nitidus*	0.016575	0.064989
JW4	SC24	JO10	*R.nitidus*	0.119691	0.067831
SC12	SC24	JO10	*R.nitidus*	-0.015924	0.06223
CS25	SC24	JO10	*R.nitidus*	0.006051	0.066998
GD31	SC26	JO10	*R.nitidus*	0.068406	0.062873
JO5	SC24	JO10	*R.nitidus*	0.027331	0.062963
JO8	SC26	JO10	*R.nitidus*	0.025773	0.069058
CS12	SC26	JO10	*R.nitidus*	-0.003785	0.065579
MY6	SC24	JO10	*R.nitidus*	-0.001537	0.063058
JW6	SC24	JO10	*R.nitidus*	-0.027835	0.071844
JW7	SC24	JO10	*R.nitidus*	-0.003027	0.068211
SC44	SC24	JO10	*R.nitidus*	-0.023274	0.062942
JO7	SC26	JO10	*R.nitidus*	0.003799	0.068871
JO3	SC24	JO10	*R.nitidus*	-0.008643	0.068466
SC28	SC24	JO10	*R.nitidus*	-0.053945	0.061684
SC42	SC24	JO10	*R.nitidus*	0.177564	0.060237
MY4	SC24	JO10	*R.nitidus*	-0.043143	0.063139
GD35	SC26	JO10	*R.nitidus*	0.042194	0.06273
GD29	SC26	JO10	*R.nitidus*	0.089009	0.062461
GD30	SC26	JO10	*R.nitidus*	0.139464	0.065724
CS11	SC26	JO10	*R.nitidus*	0.003023	0.06619
SC45	SC24	JO10	*R.nitidus*	0.018972	0.06211
GD25	SC26	JO10	*R.nitidus*	0.064888	0.062828
GD49	SC26	JO10	*R.nitidus*	0.086381	0.066941
GD32	SC26	JO10	*R.nitidus*	0.035115	0.066328
GD21	SC26	JO10	*R.nitidus*	0.174902	0.061952
JO1	SC24	JO10	*R.nitidus*	-0.016923	0.062533
CS16	SC26	JO10	*R.nitidus*	0	0.06331
GD45	SC26	JO10	*R.nitidus*	0.046687	0.064744
GD41	SC26	JO10	*R.nitidus*	-0.013122	0.064362
CS13	SC26	JO10	*R.nitidus*	-0.054364	0.066782
GD34	SC26	JO10	*R.nitidus*	0.035957	0.062599
GD48	SC26	JO10	*R.nitidus*	0.055394	0.064268
GD50	SC26	JO10	*R.nitidus*	0.035304	0.068776
GD26	SC26	JO10	*R.nitidus*	0.070323	0.063672
GD44	SC26	JO10	*R.nitidus*	0.013788	0.06384
GD40	SC26	JO10	*R.nitidus*	0.057559	0.0621
CS19	SC26	JO10	*R.nitidus*	0.014022	0.06532
GD33	SC26	JO10	*R.nitidus*	-0.003413	0.064273
GD22	SC26	JO10	*R.nitidus*	0.084218	0.063077
GD42	SC26	JO10	*R.nitidus*	0.018355	0.064359
CS21	SC26	JO10	*R.nitidus*	-0.001458	0.06453
CS22	SC26	JO10	*R.nitidus*	0.030303	0.065303
CS18	SC26	JO10	*R.nitidus*	-0.015284	0.064161
CS23	SC26	JO10	*R.nitidus*	-0.037763	0.064438
GD46	SC26	JO10	*R.nitidus*	0.021663	0.062848
CS15	SC26	JO10	*R.nitidus*	-0.002983	0.065975
SC6	SC26	JO10	*R.nitidus*	0.039401	0.063959
SC44	SC26	JO10	*R.nitidus*	0.001564	0.063722
CS14	SC26	JO10	*R.nitidus*	0.004435	0.067297
CS24	SC26	JO10	*R.nitidus*	-0.019302	0.062856
SC24	SC26	JO10	*R.nitidus*	0.024845	0.064044
CS17	SC26	JO10	*R.nitidus*	0.029595	0.067649

SC31	SC26	JO10	*R.nitidus*	0.024333	0.065
SC10	SC26	JO10	*R.nitidus*	0.113497	0.063856
SC12	SC26	JO10	*R.nitidus*	0.016575	0.062435
CS3	SC26	JO10	*R.nitidus*	-0.040299	0.06414
CS6	SC26	JO10	*R.nitidus*	-0.014604	0.064894
SC5	SC26	JO10	*R.nitidus*	0.037361	0.067587
CS4	SC26	JO10	*R.nitidus*	-0.009366	0.062741
CS20	SC26	JO10	*R.nitidus*	-0.037445	0.064
CS28	SC26	JO10	*R.nitidus*	0.010861	0.065156
SC28	SC26	JO10	*R.nitidus*	-0.010204	0.062978
SC42	SC26	JO10	*R.nitidus*	0.190445	0.06205
SC18	SC26	JO10	*R.nitidus*	0.076539	0.063917
CS25	SC26	JO10	*R.nitidus*	0.018827	0.066274
SC34	SC26	JO10	*R.nitidus*	0.042825	0.0635
SC35	SC26	JO10	*R.nitidus*	0.064492	0.061944
CS30	SC26	JO10	*R.nitidus*	-0.04236	0.063289
CS31	SC26	JO10	*R.nitidus*	0.017319	0.065208
JW4	SC26	JO10	*R.nitidus*	0.149963	0.064568
MY6	SC26	JO10	*R.nitidus*	0.028322	0.063525
JO5	SC26	JO10	*R.nitidus*	0.050193	0.063577
SC32	SC26	JO10	*R.nitidus*	0.064715	0.064124
SC14	SC26	JO10	*R.nitidus*	0.038522	0.062982
MY4	SC26	JO10	*R.nitidus*	-0.010256	0.06398
SC15	SC26	JO10	*R.nitidus*	0.015748	0.062269
JO7	SC27	JO10	*R.nitidus*	-0.031527	0.070812
JO8	SC27	JO10	*R.nitidus*	-0.024742	0.070549
JW7	SC26	JO10	*R.nitidus*	0.040394	0.069371
SC27	SC26	JO10	*R.nitidus*	0.026613	0.061012
GD22	SC27	JO10	*R.nitidus*	0.052381	0.063299
GD34	SC27	JO10	*R.nitidus*	0.008511	0.064222
JW6	SC26	JO10	*R.nitidus*	0.054348	0.069124
SC45	SC26	JO10	*R.nitidus*	0.048611	0.063914
CS11	SC27	JO10	*R.nitidus*	-0.010023	0.066617
JO1	SC26	JO10	*R.nitidus*	0.015286	0.062733
GD42	SC27	JO10	*R.nitidus*	-0.005087	0.06357
GD32	SC27	JO10	*R.nitidus*	0.00854	0.066427
GD29	SC27	JO10	*R.nitidus*	0.056631	0.064178
JO3	SC26	JO10	*R.nitidus*	0.02803	0.065435
GD25	SC27	JO10	*R.nitidus*	0.032823	0.062745
GD31	SC27	JO10	*R.nitidus*	0.026846	0.063128
GD48	SC27	JO10	*R.nitidus*	0.02197	0.06553
GD46	SC27	JO10	*R.nitidus*	-0.003602	0.064261
GD40	SC27	JO10	*R.nitidus*	0.040194	0.062826
CS12	SC27	JO10	*R.nitidus*	-0.031571	0.067106
GD21	SC27	JO10	*R.nitidus*	0.143836	0.06439
CS13	SC27	JO10	*R.nitidus*	-0.08699	0.066527
GD33	SC27	JO10	*R.nitidus*	-0.038194	0.064363
GD44	SC27	JO10	*R.nitidus*	-0.006002	0.065884
GD26	SC27	JO10	*R.nitidus*	0.034941	0.063791
GD30	SC27	JO10	*R.nitidus*	0.106858	0.065687
GD41	SC27	JO10	*R.nitidus*	-0.037091	0.063165
CS18	SC27	JO10	*R.nitidus*	-0.042232	0.064386
CS16	SC27	JO10	*R.nitidus*	-0.024335	0.065229
CS19	SC27	JO10	*R.nitidus*	-0.01434	0.064702
SC10	SC27	JO10	*R.nitidus*	0.077253	0.063685
GD45	SC27	JO10	*R.nitidus*	0.017028	0.06602

GD35	SC27	JO10	*R.nitidus*	0.015681	0.06223
CS22	SC27	JO10	*R.nitidus*	0.002259	0.064013
SC18	SC27	JO10	*R.nitidus*	0.017888	0.06535
CS20	SC27	JO10	*R.nitidus*	-0.063181	0.063594
CS14	SC27	JO10	*R.nitidus*	-0.016807	0.066592
CS28	SC27	JO10	*R.nitidus*	-0.028135	0.064904
GD49	SC27	JO10	*R.nitidus*	0.053906	0.065355
GD50	SC27	JO10	*R.nitidus*	0.020713	0.066379
CS21	SC27	JO10	*R.nitidus*	-0.025604	0.063621
CS31	SC27	JO10	*R.nitidus*	-0.012719	0.065831
CS23	SC27	JO10	*R.nitidus*	-0.060876	0.063353
SC6	SC27	JO10	*R.nitidus*	0.009531	0.064341
SC26	SC27	JO10	*R.nitidus*	-0.026613	0.061012
CS30	SC27	JO10	*R.nitidus*	-0.080192	0.062729
SC32	SC27	JO10	*R.nitidus*	0.034746	0.061767
SC34	SC27	JO10	*R.nitidus*	0.011281	0.062315
CS3	SC27	JO10	*R.nitidus*	-0.062452	0.063277
CS15	SC27	JO10	*R.nitidus*	-0.020753	0.066773
SC15	SC27	JO10	*R.nitidus*	-0.003376	0.065086
SC35	SC27	JO10	*R.nitidus*	0.037154	0.063056
CS17	SC27	JO10	*R.nitidus*	0.022932	0.066498
JW4	SC27	JO10	*R.nitidus*	0.12012	0.0654
SC14	SC27	JO10	*R.nitidus*	0.018438	0.059753
CS25	SC27	JO10	*R.nitidus*	0	0.065244
SC5	SC27	JO10	*R.nitidus*	-0.005766	0.066645
CS24	SC27	JO10	*R.nitidus*	-0.03605	0.062052
MY6	SC27	JO10	*R.nitidus*	-0.001481	0.064574
CS11	SC28	JO10	*R.nitidus*	0.038226	0.065448
CS4	SC27	JO10	*R.nitidus*	-0.026412	0.062679
SC28	SC27	JO10	*R.nitidus*	-0.056924	0.063014
CS6	SC27	JO10	*R.nitidus*	-0.044636	0.062554
SC12	SC27	JO10	*R.nitidus*	-0.027562	0.062764
SC42	SC27	JO10	*R.nitidus*	0.171528	0.062013
SC24	SC27	JO10	*R.nitidus*	-0.012831	0.063118
SC45	SC27	JO10	*R.nitidus*	0.00536	0.063774
MY4	SC27	JO10	*R.nitidus*	-0.046722	0.065369
JW7	SC27	JO10	*R.nitidus*	0.005269	0.065114
SC31	SC27	JO10	*R.nitidus*	-0.007949	0.064344
GD21	SC28	JO10	*R.nitidus*	0.184123	0.063574
GD29	SC28	JO10	*R.nitidus*	0.106472	0.06331
GD46	SC28	JO10	*R.nitidus*	0.039576	0.063892
JO5	SC27	JO10	*R.nitidus*	0.023987	0.064302
GD32	SC28	JO10	*R.nitidus*	0.05106	0.067216
JO7	SC28	JO10	*R.nitidus*	-0.013752	0.071406
JO1	SC27	JO10	*R.nitidus*	-0.009302	0.063677
SC44	SC27	JO10	*R.nitidus*	-0.023987	0.061449
GD33	SC28	JO10	*R.nitidus*	0.010653	0.064171
JW6	SC27	JO10	*R.nitidus*	0.003996	0.068902
GD35	SC28	JO10	*R.nitidus*	0.060206	0.063327
GD25	SC28	JO10	*R.nitidus*	0.089167	0.063071
JO3	SC27	JO10	*R.nitidus*	-0.012185	0.068154
JO8	SC28	JO10	*R.nitidus*	0.002119	0.071774
GD48	SC28	JO10	*R.nitidus*	0.052515	0.065076
CS12	SC28	JO10	*R.nitidus*	0.030757	0.065505
GD44	SC28	JO10	*R.nitidus*	0.038314	0.064505
GD30	SC28	JO10	*R.nitidus*	0.152516	0.064792

GD26	SC28	JO10	*R.nitidus*	0.076379	0.064048
GD42	SC28	JO10	*R.nitidus*	0.04796	0.064863
GD49	SC28	JO10	*R.nitidus*	0.111285	0.06489
CS17	SC28	JO10	*R.nitidus*	0.07177	0.065687
CS13	SC28	JO10	*R.nitidus*	-0.02322	0.06618
GD40	SC28	JO10	*R.nitidus*	0.079944	0.062302
GD31	SC28	JO10	*R.nitidus*	0.089102	0.064615
GD22	SC28	JO10	*R.nitidus*	0.103729	0.061733
CS15	SC28	JO10	*R.nitidus*	0.025449	0.0663
GD45	SC28	JO10	*R.nitidus*	0.066236	0.065092
CS18	SC28	JO10	*R.nitidus*	0.011177	0.063703
GD34	SC28	JO10	*R.nitidus*	0.053296	0.062891
CS31	SC28	JO10	*R.nitidus*	0.029412	0.064478
CS28	SC28	JO10	*R.nitidus*	0.017433	0.064479
GD41	SC28	JO10	*R.nitidus*	0.001453	0.06416
CS14	SC28	JO10	*R.nitidus*	0.03028	0.067685
CS22	SC28	JO10	*R.nitidus*	0.049962	0.066
CS19	SC28	JO10	*R.nitidus*	0.031939	0.064308
GD50	SC28	JO10	*R.nitidus*	0.05985	0.066434
CS4	SC28	JO10	*R.nitidus*	0.017216	0.063471
CS3	SC28	JO10	*R.nitidus*	-0.014063	0.064192
JW6	SC28	JO10	*R.nitidus*	0.017154	0.071156
SC5	SC28	JO10	*R.nitidus*	0.043478	0.067644
CS21	SC28	JO10	*R.nitidus*	0.013255	0.064254
CS16	SC28	JO10	*R.nitidus*	0.028919	0.064046
SC31	SC28	JO10	*R.nitidus*	0.044622	0.063162
CS6	SC28	JO10	*R.nitidus*	0.012136	0.065628
CS20	SC28	JO10	*R.nitidus*	-0.01551	0.065267
CS30	SC28	JO10	*R.nitidus*	-0.026174	0.062415
SC18	SC28	JO10	*R.nitidus*	0.086505	0.062681
CS23	SC28	JO10	*R.nitidus*	0.000742	0.064727
JO3	SC28	JO10	*R.nitidus*	0.013675	0.067826
CS24	SC28	JO10	*R.nitidus*	0.005385	0.062861
SC34	SC28	JO10	*R.nitidus*	0.057409	0.063531
JO1	SC28	JO10	*R.nitidus*	0.01531	0.062051
SC35	SC28	JO10	*R.nitidus*	0.081853	0.061861
JW4	SC28	JO10	*R.nitidus*	0.148628	0.064364
CS25	SC28	JO10	*R.nitidus*	0.051476	0.06588
SC27	SC28	JO10	*R.nitidus*	0.056924	0.063014
JO5	SC28	JO10	*R.nitidus*	0.081818	0.060186
SC26	SC28	JO10	*R.nitidus*	0.010204	0.062978
SC6	SC28	JO10	*R.nitidus*	0.069992	0.063792
SC42	SC28	JO10	*R.nitidus*	0.220752	0.061905
JO8	SC31	JO10	*R.nitidus*	-0.043651	0.072672
SC24	SC28	JO10	*R.nitidus*	0.053945	0.061684
SC10	SC28	JO10	*R.nitidus*	0.141694	0.064038
GD21	SC31	JO10	*R.nitidus*	0.147815	0.064199
SC15	SC28	JO10	*R.nitidus*	0.043867	0.064504
SC12	SC28	JO10	*R.nitidus*	0.028878	0.062351
SC14	SC28	JO10	*R.nitidus*	0.061006	0.063174
MY6	SC28	JO10	*R.nitidus*	0.035525	0.064259
SC32	SC28	JO10	*R.nitidus*	0.087621	0.062647
GD35	SC31	JO10	*R.nitidus*	0.035221	0.063079
GD26	SC31	JO10	*R.nitidus*	0.047131	0.064671
GD41	SC31	JO10	*R.nitidus*	-0.028767	0.065196
GD31	SC31	JO10	*R.nitidus*	0.04507	0.064941

MY4	SC28	JO10	*R.nitidus*	-0.001511	0.06422
JW7	SC28	JO10	*R.nitidus*	0.02505	0.065163
GD25	SC31	JO10	*R.nitidus*	0.045161	0.063796
SC44	SC28	JO10	*R.nitidus*	0.034152	0.059247
SC45	SC28	JO10	*R.nitidus*	0.044601	0.062839
GD30	SC31	JO10	*R.nitidus*	0.125286	0.067067
JO7	SC31	JO10	*R.nitidus*	-0.028116	0.071313
GD22	SC31	JO10	*R.nitidus*	0.067244	0.064872
CS12	SC31	JO10	*R.nitidus*	-0.015897	0.065828
GD44	SC31	JO10	*R.nitidus*	-0.002137	0.065557
GD33	SC31	JO10	*R.nitidus*	-0.017687	0.065657
GD29	SC31	JO10	*R.nitidus*	0.062041	0.064477
CS11	SC31	JO10	*R.nitidus*	-0.007407	0.065008
GD42	SC31	JO10	*R.nitidus*	0.012422	0.066738
GD34	SC31	JO10	*R.nitidus*	0.016393	0.065431
GD49	SC31	JO10	*R.nitidus*	0.087024	0.066512
CS14	SC31	JO10	*R.nitidus*	-0.016949	0.066477
GD45	SC31	JO10	*R.nitidus*	0.02568	0.068228
CS13	SC31	JO10	*R.nitidus*	-0.072079	0.066184
GD40	SC31	JO10	*R.nitidus*	0.045423	0.062297
GD50	SC31	JO10	*R.nitidus*	0.012048	0.068805
CS17	SC31	JO10	*R.nitidus*	0.028235	0.065375
CS30	SC31	JO10	*R.nitidus*	-0.074887	0.063659
GD32	SC31	JO10	*R.nitidus*	0.023186	0.066569
CS24	SC31	JO10	*R.nitidus*	-0.035661	0.061659
CS20	SC31	JO10	*R.nitidus*	-0.051584	0.061973
CS21	SC31	JO10	*R.nitidus*	-0.030551	0.063022
CS22	SC31	JO10	*R.nitidus*	0.009427	0.065123
CS19	SC31	JO10	*R.nitidus*	-0.004357	0.06508
CS18	SC31	JO10	*R.nitidus*	-0.030236	0.062934
SC15	SC31	JO10	*R.nitidus*	0.003448	0.064024
GD46	SC31	JO10	*R.nitidus*	0.001337	0.064451
GD48	SC31	JO10	*R.nitidus*	0.041973	0.066266
SC14	SC31	JO10	*R.nitidus*	0.021061	0.064879
SC18	SC31	JO10	*R.nitidus*	0.027642	0.065359
SC10	SC31	JO10	*R.nitidus*	0.081746	0.064674
CS15	SC31	JO10	*R.nitidus*	-0.022399	0.065492
CS28	SC31	JO10	*R.nitidus*	-0.011024	0.064769
CS16	SC31	JO10	*R.nitidus*	-0.006662	0.06384
SC6	SC31	JO10	*R.nitidus*	0.003218	0.065128
CS4	SC31	JO10	*R.nitidus*	-0.020393	0.062427
MY4	SC31	JO10	*R.nitidus*	-0.033577	0.063904
JW7	SC31	JO10	*R.nitidus*	0.039526	0.066299
SC44	SC31	JO10	*R.nitidus*	-0.009554	0.063713
JW4	SC31	JO10	*R.nitidus*	0.146545	0.063745
JO7	SC32	JO10	*R.nitidus*	-0.051657	0.070291
SC34	SC31	JO10	*R.nitidus*	0.013168	0.063973
CS25	SC31	JO10	*R.nitidus*	0.00883	0.063261
JW6	SC31	JO10	*R.nitidus*	0.027725	0.071751
JO5	SC31	JO10	*R.nitidus*	0.02381	0.06497
CS23	SC31	JO10	*R.nitidus*	-0.05641	0.06236
SC32	SC31	JO10	*R.nitidus*	0.048099	0.065085
SC12	SC31	JO10	*R.nitidus*	-0.009412	0.064152
SC27	SC31	JO10	*R.nitidus*	0.007949	0.064344
CS31	SC31	JO10	*R.nitidus*	-0.009834	0.067026
SC5	SC31	JO10	*R.nitidus*	0.004688	0.068228

CS3	SC31	JO10	*R.nitidus*	-0.059361	0.061412
GD22	SC32	JO10	*R.nitidus*	0.020288	0.062084
JO3	SC31	JO10	*R.nitidus*	-0.000853	0.069512
SC24	SC31	JO10	*R.nitidus*	0.001574	0.064456
SC26	SC31	JO10	*R.nitidus*	-0.024333	0.065
SC35	SC31	JO10	*R.nitidus*	0.032086	0.064336
GD26	SC32	JO10	*R.nitidus*	-0.002113	0.061888
SC28	SC31	JO10	*R.nitidus*	-0.044622	0.063162
JO8	SC32	JO10	*R.nitidus*	-0.030457	0.070123
SC42	SC31	JO10	*R.nitidus*	0.166667	0.061697
CS6	SC31	JO10	*R.nitidus*	-0.033639	0.063406
SC45	SC31	JO10	*R.nitidus*	0.010598	0.063852
MY6	SC31	JO10	*R.nitidus*	0.006632	0.063092
GD25	SC32	JO10	*R.nitidus*	0	0.063776
CS11	SC32	JO10	*R.nitidus*	-0.054937	0.066104
GD21	SC32	JO10	*R.nitidus*	0.113881	0.063162
CS13	SC32	JO10	*R.nitidus*	-0.119908	0.067825
GD32	SC32	JO10	*R.nitidus*	-0.021012	0.065221
JO1	SC31	JO10	*R.nitidus*	-0.010703	0.063767
GD30	SC32	JO10	*R.nitidus*	0.068666	0.068451
CS19	SC32	JO10	*R.nitidus*	-0.043478	0.064028
GD34	SC32	JO10	*R.nitidus*	-0.024793	0.064303
CS12	SC32	JO10	*R.nitidus*	-0.059316	0.067049
CS15	SC32	JO10	*R.nitidus*	-0.065886	0.066503
GD45	SC32	JO10	*R.nitidus*	-0.010164	0.064528
GD31	SC32	JO10	*R.nitidus*	-0.007067	0.065539
GD46	SC32	JO10	*R.nitidus*	-0.036749	0.063171
CS16	SC32	JO10	*R.nitidus*	-0.059515	0.064621
CS22	SC32	JO10	*R.nitidus*	-0.031579	0.06352
CS17	SC32	JO10	*R.nitidus*	-0.030469	0.067949
CS14	SC32	JO10	*R.nitidus*	-0.060105	0.067824
GD29	SC32	JO10	*R.nitidus*	0.042553	0.064118
GD44	SC32	JO10	*R.nitidus*	-0.04821	0.064908
SC5	SC32	JO10	*R.nitidus*	-0.027581	0.067973
CS31	SC32	JO10	*R.nitidus*	-0.044988	0.066036
CS18	SC32	JO10	*R.nitidus*	-0.075332	0.063274
CS20	SC32	JO10	*R.nitidus*	-0.097122	0.063904
GD49	SC32	JO10	*R.nitidus*	0.026214	0.064778
GD35	SC32	JO10	*R.nitidus*	-0.016039	0.064305
GD41	SC32	JO10	*R.nitidus*	-0.074773	0.062811
GD33	SC32	JO10	*R.nitidus*	-0.067035	0.062918
SC18	SC32	JO10	*R.nitidus*	-0.013147	0.063903
SC6	SC32	JO10	*R.nitidus*	-0.028191	0.063836
GD48	SC32	JO10	*R.nitidus*	-0.009608	0.066465
GD40	SC32	JO10	*R.nitidus*	0.004144	0.062444
CS3	SC32	JO10	*R.nitidus*	-0.099702	0.06467
GD42	SC32	JO10	*R.nitidus*	-0.032526	0.064316
GD50	SC32	JO10	*R.nitidus*	-0.021399	0.068331
CS21	SC32	JO10	*R.nitidus*	-0.06735	0.06444
CS25	SC32	JO10	*R.nitidus*	-0.03062	0.066885
CS28	SC32	JO10	*R.nitidus*	-0.066294	0.064191
CS23	SC32	JO10	*R.nitidus*	-0.092461	0.06373
SC35	SC32	JO10	*R.nitidus*	-0.017474	0.06164
SC14	SC32	JO10	*R.nitidus*	-0.024116	0.063297
SC15	SC32	JO10	*R.nitidus*	-0.047771	0.064459
CS24	SC32	JO10	*R.nitidus*	-0.079848	0.063202

SC31	SC32	JO10	*R.nitidus*	-0.048099	0.065085
CS30	SC32	JO10	*R.nitidus*	-0.105991	0.062594
JW4	SC32	JO10	*R.nitidus*	0.105543	0.065297
SC28	SC32	JO10	*R.nitidus*	-0.087621	0.062647
SC42	SC32	JO10	*R.nitidus*	0.151045	0.063206
SC26	SC32	JO10	*R.nitidus*	-0.064715	0.064124
JO1	SC32	JO10	*R.nitidus*	-0.052467	0.064236
GD21	SC34	JO10	*R.nitidus*	0.144289	0.064124
SC10	SC32	JO10	*R.nitidus*	0.045455	0.064329
MY6	SC32	JO10	*R.nitidus*	-0.036029	0.06319
CS4	SC32	JO10	*R.nitidus*	-0.062777	0.063111
JO5	SC32	JO10	*R.nitidus*	-0.011211	0.063684
SC12	SC32	JO10	*R.nitidus*	-0.061699	0.064898
CS11	SC34	JO10	*R.nitidus*	-0.017857	0.066317
SC24	SC32	JO10	*R.nitidus*	-0.045732	0.063863
JO7	SC34	JO10	*R.nitidus*	-0.013295	0.068309
CS6	SC32	JO10	*R.nitidus*	-0.080406	0.065056
JW7	SC32	JO10	*R.nitidus*	-0.003083	0.067628
SC45	SC32	JO10	*R.nitidus*	-0.029658	0.064029
JW6	SC32	JO10	*R.nitidus*	-0.012859	0.068806
SC27	SC32	JO10	*R.nitidus*	-0.034746	0.061767
JO8	SC34	JO10	*R.nitidus*	-0.009054	0.070255
GD29	SC34	JO10	*R.nitidus*	0.05164	0.063662
CS12	SC34	JO10	*R.nitidus*	-0.024106	0.065792
SC34	SC32	JO10	*R.nitidus*	-0.032209	0.063493
SC44	SC32	JO10	*R.nitidus*	-0.060241	0.063893
CS13	SC34	JO10	*R.nitidus*	-0.072948	0.065249
GD32	SC34	JO10	*R.nitidus*	0.037123	0.066281
GD34	SC34	JO10	*R.nitidus*	0.000693	0.062857
GD26	SC34	JO10	*R.nitidus*	0.034004	0.063465
GD25	SC34	JO10	*R.nitidus*	0.027027	0.06185
MY4	SC32	JO10	*R.nitidus*	-0.076982	0.064444
GD44	SC34	JO10	*R.nitidus*	-0.009587	0.064727
GD33	SC34	JO10	*R.nitidus*	-0.026153	0.062745
GD46	SC34	JO10	*R.nitidus*	-0.013986	0.063095
JO3	SC32	JO10	*R.nitidus*	-0.048183	0.067637
GD30	SC34	JO10	*R.nitidus*	0.109943	0.066975
GD35	SC34	JO10	*R.nitidus*	0.011527	0.06264
GD31	SC34	JO10	*R.nitidus*	0.034458	0.063792
GD41	SC34	JO10	*R.nitidus*	-0.039858	0.064101
CS15	SC34	JO10	*R.nitidus*	-0.02788	0.065414
GD22	SC34	JO10	*R.nitidus*	0.051215	0.062459
CS14	SC34	JO10	*R.nitidus*	-0.020408	0.066358
GD42	SC34	JO10	*R.nitidus*	-0.008421	0.062885
CS3	SC34	JO10	*R.nitidus*	-0.070744	0.063468
CS22	SC34	JO10	*R.nitidus*	0.004428	0.064467
CS17	SC34	JO10	*R.nitidus*	0.012739	0.065524
CS16	SC34	JO10	*R.nitidus*	-0.023134	0.06306
GD50	SC34	JO10	*R.nitidus*	0.012325	0.064702
GD45	SC34	JO10	*R.nitidus*	0.023772	0.06609
GD40	SC34	JO10	*R.nitidus*	0.035261	0.061274
SC6	SC34	JO10	*R.nitidus*	-0.006923	0.063856
GD49	SC34	JO10	*R.nitidus*	0.042254	0.063677
GD48	SC34	JO10	*R.nitidus*	0.034918	0.0646
CS21	SC34	JO10	*R.nitidus*	-0.040471	0.062639
SC10	SC34	JO10	*R.nitidus*	0.068404	0.063566

SC35	SC34	JO10	*R.nitidus*	0.02864	0.062108
CS6	SC34	JO10	*R.nitidus*	-0.04331	0.063312
CS24	SC34	JO10	*R.nitidus*	-0.05287	0.062321
CS31	SC34	JO10	*R.nitidus*	-0.018519	0.064026
SC12	SC34	JO10	*R.nitidus*	-0.023847	0.064259
CS18	SC34	JO10	*R.nitidus*	-0.04428	0.062137
JW4	SC34	JO10	*R.nitidus*	0.134156	0.06261
CS19	SC34	JO10	*R.nitidus*	-0.015228	0.062993
SC24	SC34	JO10	*R.nitidus*	-0.007955	0.062949
SC27	SC34	JO10	*R.nitidus*	-0.011281	0.062315
CS25	SC34	JO10	*R.nitidus*	-0.007283	0.064616
CS20	SC34	JO10	*R.nitidus*	-0.068791	0.063755
CS23	SC34	JO10	*R.nitidus*	-0.069343	0.06241
SC18	SC34	JO10	*R.nitidus*	0.029221	0.064074
SC14	SC34	JO10	*R.nitidus*	0.007819	0.063024
SC15	SC34	JO10	*R.nitidus*	-0.017959	0.063957
SC42	SC34	JO10	*R.nitidus*	0.147418	0.062647
CS28	SC34	JO10	*R.nitidus*	-0.039627	0.064042
JW7	SC34	JO10	*R.nitidus*	-0.004883	0.067513
SC44	SC34	JO10	*R.nitidus*	-0.036462	0.063203
SC28	SC34	JO10	*R.nitidus*	-0.057409	0.063531
CS30	SC34	JO10	*R.nitidus*	-0.077795	0.06302
SC5	SC34	JO10	*R.nitidus*	0.000776	0.066075
JO7	SC35	JO10	*R.nitidus*	-0.021113	0.069149
CS4	SC34	JO10	*R.nitidus*	-0.037249	0.062012
JW6	SC34	JO10	*R.nitidus*	0.01763	0.070679
SC26	SC34	JO10	*R.nitidus*	-0.042825	0.0635
GD21	SC35	JO10	*R.nitidus*	0.123912	0.063682
GD22	SC35	JO10	*R.nitidus*	0.020988	0.062028
SC31	SC34	JO10	*R.nitidus*	-0.013168	0.063973
JO5	SC34	JO10	*R.nitidus*	0.008475	0.064531
MY4	SC34	JO10	*R.nitidus*	-0.045488	0.062509
SC32	SC34	JO10	*R.nitidus*	0.032209	0.063493
GD31	SC35	JO10	*R.nitidus*	0.017009	0.064474
JO8	SC35	JO10	*R.nitidus*	-0.045311	0.071855
GD30	SC35	JO10	*R.nitidus*	0.075841	0.065594
MY6	SC34	JO10	*R.nitidus*	-0.002999	0.063439
GD44	SC35	JO10	*R.nitidus*	-0.044412	0.064435
GD41	SC35	JO10	*R.nitidus*	-0.070671	0.06392
JO3	SC34	JO10	*R.nitidus*	0.012195	0.068567
GD29	SC35	JO10	*R.nitidus*	0.033357	0.062955
SC45	SC34	JO10	*R.nitidus*	0.005263	0.063966
JO1	SC34	JO10	*R.nitidus*	-0.024006	0.061694
CS18	SC35	JO10	*R.nitidus*	-0.065609	0.060547
GD32	SC35	JO10	*R.nitidus*	-0.01935	0.067443
CS13	SC35	JO10	*R.nitidus*	-0.108808	0.066199
CS20	SC35	JO10	*R.nitidus*	-0.083333	0.063087
GD34	SC35	JO10	*R.nitidus*	-0.011268	0.063668
CS21	SC35	JO10	*R.nitidus*	-0.067647	0.06215
GD42	SC35	JO10	*R.nitidus*	-0.025052	0.06398
CS22	SC35	JO10	*R.nitidus*	-0.02099	0.063522
CS11	SC35	JO10	*R.nitidus*	-0.042785	0.065581
GD49	SC35	JO10	*R.nitidus*	0.027712	0.064688
GD26	SC35	JO10	*R.nitidus*	0.019068	0.063049
GD45	SC35	JO10	*R.nitidus*	0.003155	0.065215
GD50	SC35	JO10	*R.nitidus*	-0.016461	0.065033

CS15	SC35	JO10	*R.nitidus*	-0.060932	0.066333
CS30	SC35	JO10	*R.nitidus*	-0.099148	0.063444
CS12	SC35	JO10	*R.nitidus*	-0.054154	0.064871
GD46	SC35	JO10	*R.nitidus*	-0.037921	0.063673
CS23	SC35	JO10	*R.nitidus*	-0.091308	0.062081
CS28	SC35	JO10	*R.nitidus*	-0.036474	0.0658
CS19	SC35	JO10	*R.nitidus*	-0.041877	0.06309
CS31	SC35	JO10	*R.nitidus*	-0.036009	0.065168
GD33	SC35	JO10	*R.nitidus*	-0.047358	0.064292
GD35	SC35	JO10	*R.nitidus*	-0.006541	0.06339
GD25	SC35	JO10	*R.nitidus*	0.003655	0.063623
CS17	SC35	JO10	*R.nitidus*	-0.012112	0.064841
GD40	SC35	JO10	*R.nitidus*	0.004101	0.060974
CS4	SC35	JO10	*R.nitidus*	-0.05132	0.062323
GD48	SC35	JO10	*R.nitidus*	-0.005147	0.065141
CS16	SC35	JO10	*R.nitidus*	-0.05758	0.06502
SC12	SC35	JO10	*R.nitidus*	-0.061389	0.061647
CS14	SC35	JO10	*R.nitidus*	-0.056122	0.065744
SC31	SC35	JO10	*R.nitidus*	-0.032086	0.064336
CS3	SC35	JO10	*R.nitidus*	-0.096629	0.06247
CS25	SC35	JO10	*R.nitidus*	-0.02687	0.064195
SC18	SC35	JO10	*R.nitidus*	0	0.062659
MY6	SC35	JO10	*R.nitidus*	-0.034483	0.063412
CS6	SC35	JO10	*R.nitidus*	-0.075252	0.063253
CS24	SC35	JO10	*R.nitidus*	-0.070776	0.061487
SC15	SC35	JO10	*R.nitidus*	-0.038929	0.063759
SC26	SC35	JO10	*R.nitidus*	-0.064492	0.061944
JO1	SC35	JO10	*R.nitidus*	-0.044323	0.064141
SC6	SC35	JO10	*R.nitidus*	-0.01992	0.06263
SC5	SC35	JO10	*R.nitidus*	-0.02864	0.067326
JO8	CS6	JO10	*R.nitidus*	0.026178	0.071953
SC14	SC35	JO10	*R.nitidus*	-0.019908	0.062347
JW6	SC35	JO10	*R.nitidus*	0.000982	0.070169
SC10	SC35	JO10	*R.nitidus*	0.049489	0.063117
JO5	SC35	JO10	*R.nitidus*	-0.009132	0.06338
JW7	SC35	JO10	*R.nitidus*	-0.01003	0.064639
JO7	CS6	JO10	*R.nitidus*	0.033797	0.068529
SC24	SC35	JO10	*R.nitidus*	-0.029992	0.061727
SC42	SC35	JO10	*R.nitidus*	0.141503	0.064037
SC27	SC35	JO10	*R.nitidus*	-0.037154	0.063056
MY4	SC35	JO10	*R.nitidus*	-0.08009	0.063355
SC28	SC35	JO10	*R.nitidus*	-0.081853	0.061861
JO3	SC35	JO10	*R.nitidus*	-0.030357	0.069012
GD30	CS6	JO10	*R.nitidus*	0.151993	0.066429
GD26	CS6	JO10	*R.nitidus*	0.079853	0.064852
SC34	SC35	JO10	*R.nitidus*	-0.02864	0.062108
GD21	CS6	JO10	*R.nitidus*	0.196557	0.063372
SC44	SC35	JO10	*R.nitidus*	-0.05748	0.063039
JW4	SC35	JO10	*R.nitidus*	0.116692	0.064253
GD25	CS6	JO10	*R.nitidus*	0.080486	0.065031
SC45	SC35	JO10	*R.nitidus*	-0.01581	0.064104
SC32	SC35	JO10	*R.nitidus*	0.017474	0.06164
CS11	CS6	JO10	*R.nitidus*	0.022117	0.066851
GD32	CS6	JO10	*R.nitidus*	0.07775	0.067335
GD22	CS6	JO10	*R.nitidus*	0.10846	0.064719
GD41	CS6	JO10	*R.nitidus*	0.013981	0.064228

GD40	CS6	JO10	*R.nitidus*	0.080597	0.064943
GD48	CS6	JO10	*R.nitidus*	0.079537	0.065308
GD31	CS6	JO10	*R.nitidus*	0.087223	0.066953
GD50	CS6	JO10	*R.nitidus*	0.062446	0.068269
CS12	CS6	JO10	*R.nitidus*	0.014388	0.067636
CS15	CS6	JO10	*R.nitidus*	0.015103	0.067947
GD29	CS6	JO10	*R.nitidus*	0.099853	0.063242
CS28	CS6	JO10	*R.nitidus*	0.020903	0.066543
CS4	CS6	JO10	*R.nitidus*	0.006481	0.062535
GD35	CS6	JO10	*R.nitidus*	0.074581	0.065957
CS18	CS6	JO10	*R.nitidus*	0.0046	0.063093
CS17	CS6	JO10	*R.nitidus*	0.042445	0.068794
GD33	CS6	JO10	*R.nitidus*	0.021308	0.063226
SC10	CS6	JO10	*R.nitidus*	0.106646	0.064531
SC18	CS6	JO10	*R.nitidus*	0.084298	0.064368
GD34	CS6	JO10	*R.nitidus*	0.051546	0.065775
GD44	CS6	JO10	*R.nitidus*	0.045677	0.064932
CS13	CS6	JO10	*R.nitidus*	-0.030744	0.067393
SC14	CS6	JO10	*R.nitidus*	0.056809	0.063106
CS31	CS6	JO10	*R.nitidus*	0.033031	0.065964
CS3	CS6	JO10	*R.nitidus*	-0.044133	0.062767
CS24	CS6	JO10	*R.nitidus*	-0.003493	0.062251
SC5	CS6	JO10	*R.nitidus*	0.03972	0.066742
SC26	CS6	JO10	*R.nitidus*	0.014604	0.064894
GD45	CS6	JO10	*R.nitidus*	0.06518	0.068299
GD42	CS6	JO10	*R.nitidus*	0.048565	0.065381
GD49	CS6	JO10	*R.nitidus*	0.108333	0.066514
GD46	CS6	JO10	*R.nitidus*	0.03037	0.066161
CS16	CS6	JO10	*R.nitidus*	0.013688	0.066942
CS14	CS6	JO10	*R.nitidus*	0.033439	0.067553
CS25	CS6	JO10	*R.nitidus*	0.053524	0.064987
CS30	CS6	JO10	*R.nitidus*	-0.025431	0.064233
SC15	CS6	JO10	*R.nitidus*	0.031002	0.063658
SC6	CS6	JO10	*R.nitidus*	0.037213	0.061968
CS21	CS6	JO10	*R.nitidus*	0.011299	0.063039
SC42	CS6	JO10	*R.nitidus*	0.185	0.061775
CS19	CS6	JO10	*R.nitidus*	0.037348	0.066345
CS22	CS6	JO10	*R.nitidus*	0.057748	0.064923
CS23	CS6	JO10	*R.nitidus*	-0.033613	0.0645
SC44	CS6	JO10	*R.nitidus*	0.029791	0.06326
CS20	CS6	JO10	*R.nitidus*	-0.025641	0.064087
JO8	SC42	JO10	*R.nitidus*	-0.139963	0.069743
SC12	CS6	JO10	*R.nitidus*	0.02562	0.064933
SC24	CS6	JO10	*R.nitidus*	0.044035	0.062414
MY4	CS6	JO10	*R.nitidus*	-0.00155	0.065736
JW4	CS6	JO10	*R.nitidus*	0.16561	0.064886
JO7	SC42	JO10	*R.nitidus*	-0.137631	0.06921
MY6	CS6	JO10	*R.nitidus*	0.033794	0.063967
SC31	CS6	JO10	*R.nitidus*	0.033639	0.063406
JO3	CS6	JO10	*R.nitidus*	0.059037	0.068455
SC45	CS6	JO10	*R.nitidus*	0.048099	0.06421
CS11	SC42	JO10	*R.nitidus*	-0.166561	0.063555
SC34	CS6	JO10	*R.nitidus*	0.04331	0.063312
SC32	CS6	JO10	*R.nitidus*	0.080406	0.065056
SC27	CS6	JO10	*R.nitidus*	0.044636	0.062554
JO1	CS6	JO10	*R.nitidus*	0.017871	0.065254

JW6	CS6	JO10	*R.nitidus*	0.055172	0.067566
GD21	SC42	JO10	*R.nitidus*	-0.039521	0.063005
GD25	SC42	JO10	*R.nitidus*	-0.112418	0.064226
SC28	CS6	JO10	*R.nitidus*	-0.012136	0.065628
GD29	SC42	JO10	*R.nitidus*	-0.084082	0.062975
GD40	SC42	JO10	*R.nitidus*	-0.122665	0.062232
SC35	CS6	JO10	*R.nitidus*	0.075252	0.063253
CS12	SC42	JO10	*R.nitidus*	-0.17357	0.064399
GD34	SC42	JO10	*R.nitidus*	-0.15003	0.063679
GD22	SC42	JO10	*R.nitidus*	-0.10006	0.062061
GD32	SC42	JO10	*R.nitidus*	-0.119403	0.064401
JW7	CS6	JO10	*R.nitidus*	0.053232	0.067305
GD41	SC42	JO10	*R.nitidus*	-0.17955	0.06274
CS18	SC42	JO10	*R.nitidus*	-0.190141	0.061517
GD33	SC42	JO10	*R.nitidus*	-0.171171	0.064303
GD48	SC42	JO10	*R.nitidus*	-0.117647	0.065996
GD31	SC42	JO10	*R.nitidus*	-0.11536	0.064352
JO5	CS6	JO10	*R.nitidus*	0.072037	0.063847
GD30	SC42	JO10	*R.nitidus*	-0.043785	0.066447
CS21	SC42	JO10	*R.nitidus*	-0.181173	0.061724
GD35	SC42	JO10	*R.nitidus*	-0.141538	0.062932
GD26	SC42	JO10	*R.nitidus*	-0.119552	0.064646
GD49	SC42	JO10	*R.nitidus*	-0.096528	0.064493
GD46	SC42	JO10	*R.nitidus*	-0.162567	0.06209
CS17	SC42	JO10	*R.nitidus*	-0.139828	0.064764
CS13	SC42	JO10	*R.nitidus*	-0.223314	0.064143
GD45	SC42	JO10	*R.nitidus*	-0.137701	0.064517
GD42	SC42	JO10	*R.nitidus*	-0.166253	0.063059
CS15	SC42	JO10	*R.nitidus*	-0.175192	0.065355
CS3	SC42	JO10	*R.nitidus*	-0.212737	0.062259
GD44	SC42	JO10	*R.nitidus*	-0.15703	0.062742
CS23	SC42	JO10	*R.nitidus*	-0.214971	0.062718
SC6	SC42	JO10	*R.nitidus*	-0.178962	0.061562
CS31	SC42	JO10	*R.nitidus*	-0.183299	0.062693
GD50	SC42	JO10	*R.nitidus*	-0.146501	0.067831
CS14	SC42	JO10	*R.nitidus*	-0.17064	0.065207
CS22	SC42	JO10	*R.nitidus*	-0.146149	0.062809
CS25	SC42	JO10	*R.nitidus*	-0.163696	0.063731
SC12	SC42	JO10	*R.nitidus*	-0.197055	0.061571
CS16	SC42	JO10	*R.nitidus*	-0.182686	0.063375
SC5	SC42	JO10	*R.nitidus*	-0.166088	0.065748
SC10	SC42	JO10	*R.nitidus*	-0.100642	0.064785
CS19	SC42	JO10	*R.nitidus*	-0.170227	0.063234
CS20	SC42	JO10	*R.nitidus*	-0.211735	0.06161
CS28	SC42	JO10	*R.nitidus*	-0.181946	0.062967
SC44	SC42	JO10	*R.nitidus*	-0.183995	0.061624
SC32	SC42	JO10	*R.nitidus*	-0.151045	0.063206
SC28	SC42	JO10	*R.nitidus*	-0.220752	0.061905
SC14	SC42	JO10	*R.nitidus*	-0.162181	0.062423
JW4	SC42	JO10	*R.nitidus*	0.006964	0.066025
SC35	SC42	JO10	*R.nitidus*	-0.141503	0.064037
CS24	SC42	JO10	*R.nitidus*	-0.202581	0.061434
CS30	SC42	JO10	*R.nitidus*	-0.228477	0.063411
JO8	SC44	JO10	*R.nitidus*	-0.005258	0.072961
SC15	SC42	JO10	*R.nitidus*	-0.179219	0.062539
SC34	SC42	JO10	*R.nitidus*	-0.147418	0.062647

SC31	SC42	JO10	*R.nitidus*	-0.166667	0.061697
CS6	SC42	JO10	*R.nitidus*	-0.185	0.061775
JW7	SC42	JO10	*R.nitidus*	-0.12305	0.066996
CS4	SC42	JO10	*R.nitidus*	-0.199609	0.0604
GD25	SC44	JO10	*R.nitidus*	0.0571	0.062177
JW6	SC42	JO10	*R.nitidus*	-0.100534	0.06932
SC18	SC42	JO10	*R.nitidus*	-0.149243	0.063599
SC27	SC42	JO10	*R.nitidus*	-0.171528	0.062013
SC24	SC42	JO10	*R.nitidus*	-0.177564	0.060237
SC26	SC42	JO10	*R.nitidus*	-0.190445	0.06205
JO7	SC44	JO10	*R.nitidus*	0.014925	0.070814
GD34	SC44	JO10	*R.nitidus*	0.025018	0.062464
CS11	SC44	JO10	*R.nitidus*	0.00148	0.066598
GD30	SC44	JO10	*R.nitidus*	0.119415	0.065007
GD31	SC44	JO10	*R.nitidus*	0.059823	0.065583
SC45	SC42	JO10	*R.nitidus*	-0.153209	0.063125
MY4	SC42	JO10	*R.nitidus*	-0.196182	0.063042
JO5	SC42	JO10	*R.nitidus*	-0.156463	0.06395
GD44	SC44	JO10	*R.nitidus*	0.01414	0.063242
JO3	SC42	JO10	*R.nitidus*	-0.111468	0.067137
CS12	SC44	JO10	*R.nitidus*	-0.004669	0.066284
GD22	SC44	JO10	*R.nitidus*	0.084926	0.061598
MY6	SC42	JO10	*R.nitidus*	-0.16833	0.062669
GD45	SC44	JO10	*R.nitidus*	0.038804	0.064319
GD46	SC44	JO10	*R.nitidus*	0.010079	0.06273
JO1	SC42	JO10	*R.nitidus*	-0.194617	0.06219
GD29	SC44	JO10	*R.nitidus*	0.075833	0.063361
GD32	SC44	JO10	*R.nitidus*	0.03632	0.066247
GD50	SC44	JO10	*R.nitidus*	0.049615	0.06637
CS17	SC44	JO10	*R.nitidus*	0.04127	0.066695
CS28	SC44	JO10	*R.nitidus*	-0.003155	0.065953
CS30	SC44	JO10	*R.nitidus*	-0.054011	0.064227
CS16	SC44	JO10	*R.nitidus*	-0.006818	0.064625
GD26	SC44	JO10	*R.nitidus*	0.056671	0.063437
GD21	SC44	JO10	*R.nitidus*	0.162618	0.063733
GD41	SC44	JO10	*R.nitidus*	-0.018025	0.063481
GD33	SC44	JO10	*R.nitidus*	-0.006383	0.063503
CS22	SC44	JO10	*R.nitidus*	0.024738	0.066314
GD48	SC44	JO10	*R.nitidus*	0.031808	0.065136
CS18	SC44	JO10	*R.nitidus*	-0.016862	0.062489
CS23	SC44	JO10	*R.nitidus*	-0.038806	0.064057
SC5	SC44	JO10	*R.nitidus*	0.018868	0.0667
GD40	SC44	JO10	*R.nitidus*	0.052632	0.063117
GD42	SC44	JO10	*R.nitidus*	0.023547	0.06403
CS20	SC44	JO10	*R.nitidus*	-0.046886	0.062922
GD35	SC44	JO10	*R.nitidus*	0.042254	0.064301
CS15	SC44	JO10	*R.nitidus*	-0.006716	0.067876
CS14	SC44	JO10	*R.nitidus*	-0.004438	0.067514
CS4	SC44	JO10	*R.nitidus*	-0.011914	0.063364
CS13	SC44	JO10	*R.nitidus*	-0.047328	0.06593
GD49	SC44	JO10	*R.nitidus*	0.08867	0.065111
SC26	SC44	JO10	*R.nitidus*	-0.001564	0.063722
SC14	SC44	JO10	*R.nitidus*	0.03268	0.063341
CS3	SC44	JO10	*R.nitidus*	-0.04262	0.062862
SC6	SC44	JO10	*R.nitidus*	0.029641	0.064849
CS21	SC44	JO10	*R.nitidus*	-0.013899	0.063079

CS19	SC44	JO10	*R.nitidus*	0.012103	0.064765
CS25	SC44	JO10	*R.nitidus*	0.034014	0.064335
SC18	SC44	JO10	*R.nitidus*	0.051607	0.064746
CS31	SC44	JO10	*R.nitidus*	0.002318	0.066221
SC31	SC44	JO10	*R.nitidus*	0.009554	0.063713
CS24	SC44	JO10	*R.nitidus*	-0.022781	0.06326
SC27	SC44	JO10	*R.nitidus*	0.023987	0.061449
JW4	SC44	JO10	*R.nitidus*	0.138931	0.064949
SC34	SC44	JO10	*R.nitidus*	0.036462	0.063203
SC24	SC44	JO10	*R.nitidus*	0.023274	0.062942
JO5	SC44	JO10	*R.nitidus*	0.052535	0.060751
JW7	SC44	JO10	*R.nitidus*	0.027641	0.068206
MY6	SC44	JO10	*R.nitidus*	0.014264	0.064494
SC12	SC44	JO10	*R.nitidus*	0.01	0.062688
SC45	SC44	JO10	*R.nitidus*	0.026005	0.062691
SC10	SC44	JO10	*R.nitidus*	0.105098	0.063779
JO8	SC45	JO10	*R.nitidus*	-0.024845	0.071471
JW6	SC44	JO10	*R.nitidus*	0.026694	0.070561
SC15	SC44	JO10	*R.nitidus*	0.017087	0.064243
GD21	SC45	JO10	*R.nitidus*	0.126789	0.063058
JO1	SC44	JO10	*R.nitidus*	-0.003165	0.063644
JO7	SC45	JO10	*R.nitidus*	-0.022044	0.068864
SC28	SC44	JO10	*R.nitidus*	-0.034152	0.059247
SC32	SC44	JO10	*R.nitidus*	0.060241	0.063893
JO3	SC44	JO10	*R.nitidus*	0.003521	0.067408
MY4	SC44	JO10	*R.nitidus*	-0.029748	0.063797
SC35	SC44	JO10	*R.nitidus*	0.05748	0.063039
CS6	SC44	JO10	*R.nitidus*	-0.029791	0.06326
SC42	SC44	JO10	*R.nitidus*	0.183995	0.061624
GD50	SC45	JO10	*R.nitidus*	-0.009426	0.066418
GD45	SC45	JO10	*R.nitidus*	0.008793	0.064933
CS15	SC45	JO10	*R.nitidus*	-0.033582	0.064738
GD31	SC45	JO10	*R.nitidus*	0.041547	0.064303
CS14	SC45	JO10	*R.nitidus*	-0.034691	0.067601
GD40	SC45	JO10	*R.nitidus*	0.025965	0.061324
GD32	SC45	JO10	*R.nitidus*	0.005405	0.066193
GD49	SC45	JO10	*R.nitidus*	0.052504	0.064083
CS11	SC45	JO10	*R.nitidus*	-0.025564	0.065652
GD41	SC45	JO10	*R.nitidus*	-0.048729	0.06263
CS23	SC45	JO10	*R.nitidus*	-0.069904	0.063991
GD25	SC45	JO10	*R.nitidus*	0.025954	0.062169
GD35	SC45	JO10	*R.nitidus*	0.00072	0.061229
GD42	SC45	JO10	*R.nitidus*	0	0.063266
CS25	SC45	JO10	*R.nitidus*	-0.013793	0.063301
CS18	SC45	JO10	*R.nitidus*	-0.050872	0.063061
GD26	SC45	JO10	*R.nitidus*	0.016606	0.063387
GD33	SC45	JO10	*R.nitidus*	-0.044912	0.063552
GD29	SC45	JO10	*R.nitidus*	0.056669	0.063986
CS30	SC45	JO10	*R.nitidus*	-0.089683	0.063824
CS16	SC45	JO10	*R.nitidus*	-0.036898	0.064032
GD48	SC45	JO10	*R.nitidus*	0.016357	0.063709
GD22	SC45	JO10	*R.nitidus*	0.039229	0.0627
GD30	SC45	JO10	*R.nitidus*	0.095238	0.065785
CS12	SC45	JO10	*R.nitidus*	-0.044892	0.065673
CS17	SC45	JO10	*R.nitidus*	0.007758	0.066332
CS13	SC45	JO10	*R.nitidus*	-0.077891	0.065492

GD34	SC45	JO10	*R.nitidus*	-0.000682	0.063921
SC6	SC45	JO10	*R.nitidus*	-0.00625	0.062304
GD44	SC45	JO10	*R.nitidus*	-0.018882	0.065919
CS24	SC45	JO10	*R.nitidus*	-0.048835	0.062318
CS3	SC45	JO10	*R.nitidus*	-0.071051	0.062467
SC14	SC45	JO10	*R.nitidus*	-0.004532	0.064874
GD46	SC45	JO10	*R.nitidus*	-0.022346	0.063758
CS22	SC45	JO10	*R.nitidus*	-0.001497	0.064033
CS20	SC45	JO10	*R.nitidus*	-0.072823	0.063525
CS21	SC45	JO10	*R.nitidus*	-0.034557	0.064546
SC32	SC45	JO10	*R.nitidus*	0.029658	0.064029
CS19	SC45	JO10	*R.nitidus*	-0.024372	0.064032
CS28	SC45	JO10	*R.nitidus*	-0.037606	0.06561
SC24	SC45	JO10	*R.nitidus*	-0.018972	0.06211
CS4	SC45	JO10	*R.nitidus*	-0.043066	0.065064
SC42	SC45	JO10	*R.nitidus*	0.153209	0.063125
SC10	SC45	JO10	*R.nitidus*	0.069106	0.065172
SC44	SC45	JO10	*R.nitidus*	-0.026005	0.062691
CS31	SC45	JO10	*R.nitidus*	-0.019985	0.065122
SC34	SC45	JO10	*R.nitidus*	-0.005263	0.063966
SC26	SC45	JO10	*R.nitidus*	-0.048611	0.063914
SC5	SC45	JO10	*R.nitidus*	-0.016813	0.066739
SC12	SC45	JO10	*R.nitidus*	-0.031605	0.062053
MY4	SC45	JO10	*R.nitidus*	-0.05467	0.064538
JO7	MY4	JO10	*R.nitidus*	0.042829	0.070774
SC35	SC45	JO10	*R.nitidus*	0.01581	0.064104
GD26	MY4	JO10	*R.nitidus*	0.073394	0.0641
GD21	MY4	JO10	*R.nitidus*	0.182063	0.063369
SC15	SC45	JO10	*R.nitidus*	-0.021002	0.064686
GD25	MY4	JO10	*R.nitidus*	0.086294	0.064103
CS6	SC45	JO10	*R.nitidus*	-0.048099	0.06421
SC18	SC45	JO10	*R.nitidus*	0.023829	0.064787
JO5	SC45	JO10	*R.nitidus*	0.006886	0.063588
JW7	SC45	JO10	*R.nitidus*	0.001887	0.067933
SC28	SC45	JO10	*R.nitidus*	-0.044601	0.062839
SC27	SC45	JO10	*R.nitidus*	-0.00536	0.063774
JO3	SC45	JO10	*R.nitidus*	-0.010336	0.066855
MY6	SC45	JO10	*R.nitidus*	-0.018335	0.064267
JW4	SC45	JO10	*R.nitidus*	0.139241	0.065831
JW6	SC45	JO10	*R.nitidus*	0.009852	0.070643
GD32	MY4	JO10	*R.nitidus*	0.064566	0.066338
SC31	SC45	JO10	*R.nitidus*	-0.010598	0.063852
GD45	MY4	JO10	*R.nitidus*	0.065066	0.066921
GD46	MY4	JO10	*R.nitidus*	0.028955	0.064819
GD31	MY4	JO10	*R.nitidus*	0.084187	0.066179
GD44	MY4	JO10	*R.nitidus*	0.035392	0.064801
GD42	MY4	JO10	*R.nitidus*	0.035562	0.064237
JO8	MY4	JO10	*R.nitidus*	0.029805	0.072691
CS12	MY4	JO10	*R.nitidus*	0.019787	0.067223
GD41	MY4	JO10	*R.nitidus*	-0.00072	0.063413
GD29	MY4	JO10	*R.nitidus*	0.103714	0.063025
CS15	MY4	JO10	*R.nitidus*	0.008811	0.067746
JO1	SC45	JO10	*R.nitidus*	-0.032826	0.064588
GD48	MY4	JO10	*R.nitidus*	0.064829	0.065441
GD34	MY4	JO10	*R.nitidus*	0.049203	0.063734
GD22	MY4	JO10	*R.nitidus*	0.1	0.064387

CS22	MY4	JO10	*R.nitidus*	0.051223	0.065071
CS13	MY4	JO10	*R.nitidus*	-0.03914	0.067236
GD30	MY4	JO10	*R.nitidus*	0.151858	0.065876
CS18	MY4	JO10	*R.nitidus*	-0.001495	0.065325
CS23	MY4	JO10	*R.nitidus*	-0.024078	0.064075
CS14	MY4	JO10	*R.nitidus*	0.016248	0.068683
GD33	MY4	JO10	*R.nitidus*	0.00559	0.065366
GD35	MY4	JO10	*R.nitidus*	0.061254	0.065256
GD40	MY4	JO10	*R.nitidus*	0.079498	0.063337
GD50	MY4	JO10	*R.nitidus*	0.066942	0.06777
CS21	MY4	JO10	*R.nitidus*	0.008094	0.063602
CS17	MY4	JO10	*R.nitidus*	0.04835	0.067563
CS16	MY4	JO10	*R.nitidus*	0.010941	0.065729
CS20	MY4	JO10	*R.nitidus*	-0.023138	0.064209
CS28	MY4	JO10	*R.nitidus*	0.014984	0.065843
CS4	MY4	JO10	*R.nitidus*	0.010355	0.063499
GD49	MY4	JO10	*R.nitidus*	0.083662	0.065074
CS30	MY4	JO10	*R.nitidus*	-0.035687	0.065366
CS31	MY4	JO10	*R.nitidus*	0.03885	0.065148
CS19	MY4	JO10	*R.nitidus*	0.037066	0.06547
CS11	MY4	JO10	*R.nitidus*	0.016643	0.066411
SC27	MY4	JO10	*R.nitidus*	0.046722	0.065369
MY6	MY4	JO10	*R.nitidus*	0.061538	0.058673
SC28	MY4	JO10	*R.nitidus*	0.001511	0.06422
CS25	MY4	JO10	*R.nitidus*	0.040847	0.065293
SC12	MY4	JO10	*R.nitidus*	0.030023	0.063988
CS24	MY4	JO10	*R.nitidus*	0.00228	0.063754
JW6	MY4	JO10	*R.nitidus*	0.073812	0.071701
SC44	MY4	JO10	*R.nitidus*	0.029748	0.063797
SC32	MY4	JO10	*R.nitidus*	0.076982	0.064444
SC14	MY4	JO10	*R.nitidus*	0.058383	0.064571
CS3	MY4	JO10	*R.nitidus*	-0.032307	0.065514
GD21	MY6	JO10	*R.nitidus*	0.145527	0.063476
SC34	MY4	JO10	*R.nitidus*	0.045488	0.062509
JO3	MY4	JO10	*R.nitidus*	0.055802	0.070733
SC6	MY4	JO10	*R.nitidus*	0.039261	0.063052
SC5	MY4	JO10	*R.nitidus*	0.041316	0.067847
SC10	MY4	JO10	*R.nitidus*	0.115325	0.065302
CS6	MY4	JO10	*R.nitidus*	0.00155	0.065736
JO8	MY6	JO10	*R.nitidus*	-0.009356	0.072344
SC18	MY4	JO10	*R.nitidus*	0.074281	0.063409
SC15	MY4	JO10	*R.nitidus*	0.034875	0.063227
SC42	MY4	JO10	*R.nitidus*	0.196182	0.063042
SC31	MY4	JO10	*R.nitidus*	0.033577	0.063904
SC24	MY4	JO10	*R.nitidus*	0.043143	0.063139
SC35	MY4	JO10	*R.nitidus*	0.08009	0.063355
SC26	MY4	JO10	*R.nitidus*	0.010256	0.06398
JW4	MY4	JO10	*R.nitidus*	0.169123	0.065696
JO5	MY4	JO10	*R.nitidus*	0.065611	0.063522
GD33	MY6	JO10	*R.nitidus*	-0.028886	0.063609
SC45	MY4	JO10	*R.nitidus*	0.05467	0.064538
GD31	MY6	JO10	*R.nitidus*	0.05	0.064757
JO7	MY6	JO10	*R.nitidus*	0.005941	0.06992
JO1	MY4	JO10	*R.nitidus*	0.020974	0.062254
GD25	MY6	JO10	*R.nitidus*	0.039028	0.063665
GD34	MY6	JO10	*R.nitidus*	0.010359	0.063725

GD42	MY6	JO10	*R.nitidus*	-0.004811	0.064732
GD32	MY6	JO10	*R.nitidus*	0.029434	0.066314
GD49	MY6	JO10	*R.nitidus*	0.061383	0.064334
GD44	MY6	JO10	*R.nitidus*	-0.00295	0.063542
GD35	MY6	JO10	*R.nitidus*	0.021277	0.064156
JW7	MY4	JO10	*R.nitidus*	0.064246	0.069355
CS11	MY6	JO10	*R.nitidus*	-0.015793	0.065871
GD22	MY6	JO10	*R.nitidus*	0.057162	0.062335
GD26	MY6	JO10	*R.nitidus*	0.034795	0.064857
CS13	MY6	JO10	*R.nitidus*	-0.075569	0.065635
GD45	MY6	JO10	*R.nitidus*	0.021143	0.067081
GD29	MY6	JO10	*R.nitidus*	0.063492	0.062564
GD30	MY6	JO10	*R.nitidus*	0.106913	0.066303
GD50	MY6	JO10	*R.nitidus*	0.010604	0.066423
GD41	MY6	JO10	*R.nitidus*	-0.031834	0.06361
CS21	MY6	JO10	*R.nitidus*	-0.031025	0.064421
CS16	MY6	JO10	*R.nitidus*	-0.023639	0.065424
CS12	MY6	JO10	*R.nitidus*	-0.017582	0.065963
GD48	MY6	JO10	*R.nitidus*	0.025344	0.064044
GD40	MY6	JO10	*R.nitidus*	0.037037	0.062801
GD46	MY6	JO10	*R.nitidus*	-0.011134	0.06485
CS31	MY6	JO10	*R.nitidus*	-0.008507	0.066926
CS22	MY6	JO10	*R.nitidus*	0.007564	0.064359
SC10	MY6	JO10	*R.nitidus*	0.077619	0.063947
CS4	MY6	JO10	*R.nitidus*	-0.030191	0.062832
CS28	MY6	JO10	*R.nitidus*	-0.027885	0.065419
SC18	MY6	JO10	*R.nitidus*	0.039777	0.063491
CS18	MY6	JO10	*R.nitidus*	-0.039114	0.063832
SC28	MY6	JO10	*R.nitidus*	-0.035525	0.064259
SC5	MY6	JO10	*R.nitidus*	0.00374	0.067152
CS23	MY6	JO10	*R.nitidus*	-0.065868	0.062582
SC26	MY6	JO10	*R.nitidus*	-0.028322	0.063525
CS17	MY6	JO10	*R.nitidus*	0.008271	0.065283
SC42	MY6	JO10	*R.nitidus*	0.16833	0.062669
CS15	MY6	JO10	*R.nitidus*	-0.029539	0.066212
CS3	MY6	JO10	*R.nitidus*	-0.068576	0.063722
SC44	MY6	JO10	*R.nitidus*	-0.014264	0.064494
CS14	MY6	JO10	*R.nitidus*	-0.017291	0.067447
MY4	MY6	JO10	*R.nitidus*	-0.061538	0.058673
SC12	MY6	JO10	*R.nitidus*	-0.013067	0.064036
CS25	MY6	JO10	*R.nitidus*	-0.000749	0.063517
SC35	MY6	JO10	*R.nitidus*	0.034483	0.063412
SC45	MY6	JO10	*R.nitidus*	0.018335	0.064267
SC6	MY6	JO10	*R.nitidus*	-0.001528	0.062216
CS20	MY6	JO10	*R.nitidus*	-0.057102	0.064049
SC14	MY6	JO10	*R.nitidus*	0.01462	0.065056
SC32	MY6	JO10	*R.nitidus*	0.036029	0.06319
CS24	MY6	JO10	*R.nitidus*	-0.041478	0.063845
SC31	MY6	JO10	*R.nitidus*	-0.006632	0.063092
CS19	MY6	JO10	*R.nitidus*	-0.013393	0.063482
CS30	MY6	JO10	*R.nitidus*	-0.080092	0.065944
SC15	MY6	JO10	*R.nitidus*	-0.006798	0.062693
JW7	MY6	JO10	*R.nitidus*	0.018674	0.068788
SC27	MY6	JO10	*R.nitidus*	0.001481	0.064574
SC24	MY6	JO10	*R.nitidus*	0.001537	0.063058
JW6	MY6	JO10	*R.nitidus*	0.047379	0.071123

JW4	MY6	JO10	*R.nitidus*	0.148754	0.065608
JO5	MY6	JO10	*R.nitidus*	0.027798	0.06266
JO3	MY6	JO10	*R.nitidus*	0.012216	0.066607
SC34	MY6	JO10	*R.nitidus*	0.002999	0.063439
GD25	JW4	JO10	*R.nitidus*	-0.0938	0.064997
CS6	MY6	JO10	*R.nitidus*	-0.033794	0.063967
JO7	JW4	JO10	*R.nitidus*	-0.126025	0.070168
CS13	JW4	JO10	*R.nitidus*	-0.210117	0.067229
JO1	MY6	JO10	*R.nitidus*	-0.021513	0.06211
CS11	JW4	JO10	*R.nitidus*	-0.145125	0.06525
GD30	JW4	JO10	*R.nitidus*	-0.037326	0.069555
GD41	JW4	JO10	*R.nitidus*	-0.154532	0.065387
GD44	JW4	JO10	*R.nitidus*	-0.11838	0.069652
JO8	JW4	JO10	*R.nitidus*	-0.157279	0.070206
GD50	JW4	JO10	*R.nitidus*	-0.093162	0.071015
GD29	JW4	JO10	*R.nitidus*	-0.06578	0.065884
GD35	JW4	JO10	*R.nitidus*	-0.122807	0.065104
GD42	JW4	JO10	*R.nitidus*	-0.116569	0.064771
CS28	JW4	JO10	*R.nitidus*	-0.172227	0.067485
GD32	JW4	JO10	*R.nitidus*	-0.129191	0.068083
GD22	JW4	JO10	*R.nitidus*	-0.081871	0.066178
GD45	JW4	JO10	*R.nitidus*	-0.088336	0.068589
GD31	JW4	JO10	*R.nitidus*	-0.077465	0.067502
GD46	JW4	JO10	*R.nitidus*	-0.150038	0.066065
GD33	JW4	JO10	*R.nitidus*	-0.173423	0.067024
CS12	JW4	JO10	*R.nitidus*	-0.151017	0.066991
CS17	JW4	JO10	*R.nitidus*	-0.126817	0.067335
CS16	JW4	JO10	*R.nitidus*	-0.170472	0.065162
GD34	JW4	JO10	*R.nitidus*	-0.141818	0.065885
GD26	JW4	JO10	*R.nitidus*	-0.104244	0.067307
GD40	JW4	JO10	*R.nitidus*	-0.091508	0.065788
CS14	JW4	JO10	*R.nitidus*	-0.152799	0.066005
CS19	JW4	JO10	*R.nitidus*	-0.14441	0.06697
CS21	JW4	JO10	*R.nitidus*	-0.160093	0.065011
GD21	JW4	JO10	*R.nitidus*	-0.005575	0.065856
CS15	JW4	JO10	*R.nitidus*	-0.150901	0.065496
SC15	JW4	JO10	*R.nitidus*	-0.123007	0.065828
GD49	JW4	JO10	*R.nitidus*	-0.080335	0.068593
GD48	JW4	JO10	*R.nitidus*	-0.095382	0.067103
CS20	JW4	JO10	*R.nitidus*	-0.175019	0.066196
CS25	JW4	JO10	*R.nitidus*	-0.127217	0.068121
CS31	JW4	JO10	*R.nitidus*	-0.166156	0.065613
CS23	JW4	JO10	*R.nitidus*	-0.187595	0.064779
SC28	JW4	JO10	*R.nitidus*	-0.148628	0.064364
SC18	JW4	JO10	*R.nitidus*	-0.101613	0.066954
CS22	JW4	JO10	*R.nitidus*	-0.133333	0.06591
CS18	JW4	JO10	*R.nitidus*	-0.167957	0.064791
CS30	JW4	JO10	*R.nitidus*	-0.199237	0.063864
SC10	JW4	JO10	*R.nitidus*	-0.080848	0.065602
CS24	JW4	JO10	*R.nitidus*	-0.196386	0.06414
SC14	JW4	JO10	*R.nitidus*	-0.11161	0.063467
CS6	JW4	JO10	*R.nitidus*	-0.16561	0.064886
SC12	JW4	JO10	*R.nitidus*	-0.154567	0.064228
SC35	JW4	JO10	*R.nitidus*	-0.116692	0.064253
SC5	JW4	JO10	*R.nitidus*	-0.124902	0.068475
SC42	JW4	JO10	*R.nitidus*	-0.006964	0.066025

CS3	JW4	JO10	*R.nitidus*	-0.194856	0.064944
SC27	JW4	JO10	*R.nitidus*	-0.12012	0.0654
CS4	JW4	JO10	*R.nitidus*	-0.156369	0.063361
SC32	JW4	JO10	*R.nitidus*	-0.105543	0.065297
SC44	JW4	JO10	*R.nitidus*	-0.138931	0.064949
GD22	JW6	JO10	*R.nitidus*	0.061131	0.070504
SC6	JW4	JO10	*R.nitidus*	-0.149381	0.066744
SC45	JW4	JO10	*R.nitidus*	-0.139241	0.065831
JO7	JW6	JO10	*R.nitidus*	-0.005042	0.068834
SC34	JW4	JO10	*R.nitidus*	-0.134156	0.06261
GD21	JW6	JO10	*R.nitidus*	0.139576	0.068865
SC24	JW4	JO10	*R.nitidus*	-0.119691	0.067831
GD25	JW6	JO10	*R.nitidus*	0.038618	0.069898
MY6	JW4	JO10	*R.nitidus*	-0.148754	0.065608
SC26	JW4	JO10	*R.nitidus*	-0.149963	0.064568
JO8	JW6	JO10	*R.nitidus*	-0.043348	0.072231
SC31	JW4	JO10	*R.nitidus*	-0.146545	0.063745
CS11	JW6	JO10	*R.nitidus*	-0.051081	0.072193
GD26	JW6	JO10	*R.nitidus*	0.04	0.071906
GD33	JW6	JO10	*R.nitidus*	-0.055556	0.068985
GD29	JW6	JO10	*R.nitidus*	0.089972	0.069111
JW6	JW4	JO10	*R.nitidus*	-0.087901	0.070972
MY4	JW4	JO10	*R.nitidus*	-0.169123	0.065696
GD32	JW6	JO10	*R.nitidus*	-0.004154	0.072308
GD30	JW6	JO10	*R.nitidus*	0.157663	0.073886
CS13	JW6	JO10	*R.nitidus*	-0.091549	0.07345
GD34	JW6	JO10	*R.nitidus*	-0.027027	0.068286
GD40	JW6	JO10	*R.nitidus*	0.038826	0.072349
JO3	JW4	JO10	*R.nitidus*	-0.115169	0.068057
JO5	JW4	JO10	*R.nitidus*	-0.107388	0.066136
JO1	JW4	JO10	*R.nitidus*	-0.142429	0.064561
GD48	JW6	JO10	*R.nitidus*	0.018887	0.070238
GD45	JW6	JO10	*R.nitidus*	0.082177	0.072308
GD31	JW6	JO10	*R.nitidus*	0.051556	0.071981
JW7	JW4	JO10	*R.nitidus*	-0.082759	0.067977
GD44	JW6	JO10	*R.nitidus*	-0.00691	0.068932
CS30	JW6	JO10	*R.nitidus*	-0.076132	0.0692
CS15	JW6	JO10	*R.nitidus*	-0.025819	0.0718
CS23	JW6	JO10	*R.nitidus*	-0.058491	0.069297
GD49	JW6	JO10	*R.nitidus*	0.090011	0.072606
GD50	JW6	JO10	*R.nitidus*	0.044276	0.07332
CS12	JW6	JO10	*R.nitidus*	-0.014881	0.073522
CS20	JW6	JO10	*R.nitidus*	-0.05619	0.07151
GD35	JW6	JO10	*R.nitidus*	0.008729	0.070096
CS16	JW6	JO10	*R.nitidus*	-0.051456	0.072102
CS21	JW6	JO10	*R.nitidus*	-0.038132	0.070736
CS17	JW6	JO10	*R.nitidus*	0.003191	0.073982
GD42	JW6	JO10	*R.nitidus*	0.030039	0.070932
GD41	JW6	JO10	*R.nitidus*	-0.020115	0.072545
SC18	JW6	JO10	*R.nitidus*	0.005252	0.07146
CS22	JW6	JO10	*R.nitidus*	-0.028487	0.069522
SC6	JW6	JO10	*R.nitidus*	0.009756	0.070117
GD46	JW6	JO10	*R.nitidus*	-0.014409	0.070692
CS31	JW6	JO10	*R.nitidus*	-0.039354	0.07138
CS28	JW6	JO10	*R.nitidus*	-0.034205	0.069982
SC26	JW6	JO10	*R.nitidus*	-0.054348	0.069124

CS25	JW6	JO10	*R.nitidus*	-0.0286	0.070827
SC14	JW6	JO10	*R.nitidus*	0.00796	0.070533
CS3	JW6	JO10	*R.nitidus*	-0.05746	0.071053
CS19	JW6	JO10	*R.nitidus*	-0.038922	0.070874
CS18	JW6	JO10	*R.nitidus*	-0.044677	0.069442
SC32	JW6	JO10	*R.nitidus*	0.012859	0.068806
CS14	JW6	JO10	*R.nitidus*	-0.031311	0.072891
SC12	JW6	JO10	*R.nitidus*	-0.027218	0.0692
SC27	JW6	JO10	*R.nitidus*	-0.003996	0.068902
SC42	JW6	JO10	*R.nitidus*	0.100534	0.06932
SC31	JW6	JO10	*R.nitidus*	-0.027725	0.071751
CS24	JW6	JO10	*R.nitidus*	-0.080285	0.068374
SC45	JW6	JO10	*R.nitidus*	-0.009852	0.070643
SC5	JW6	JO10	*R.nitidus*	-0.005982	0.074644
SC10	JW6	JO10	*R.nitidus*	0.058291	0.070536
JO8	JW7	JO10	*R.nitidus*	-0.046171	0.073941
MY6	JW6	JO10	*R.nitidus*	-0.047379	0.071123
CS4	JW6	JO10	*R.nitidus*	-0.033898	0.069187
CS6	JW6	JO10	*R.nitidus*	-0.055172	0.067566
SC15	JW6	JO10	*R.nitidus*	-0.024291	0.071615
SC44	JW6	JO10	*R.nitidus*	-0.026694	0.070561
SC24	JW6	JO10	*R.nitidus*	0.027835	0.071844
JO5	JW6	JO10	*R.nitidus*	0.02505	0.069852
GD40	JW7	JO10	*R.nitidus*	0.042795	0.066277
SC28	JW6	JO10	*R.nitidus*	-0.017154	0.071156
JO1	JW6	JO10	*R.nitidus*	-0.027158	0.06899
CS12	JW7	JO10	*R.nitidus*	-0.024762	0.070586
SC34	JW6	JO10	*R.nitidus*	-0.01763	0.070679
SC35	JW6	JO10	*R.nitidus*	-0.000982	0.070169
GD42	JW7	JO10	*R.nitidus*	0.027027	0.067966
GD34	JW7	JO10	*R.nitidus*	-0.020548	0.069635
JW4	JW6	JO10	*R.nitidus*	0.087901	0.070972
GD31	JW7	JO10	*R.nitidus*	0.053524	0.068635
GD26	JW7	JO10	*R.nitidus*	0.03649	0.06899
MY4	JW6	JO10	*R.nitidus*	-0.073812	0.071701
GD21	JW7	JO10	*R.nitidus*	0.136326	0.066784
CS14	JW7	JO10	*R.nitidus*	-0.025352	0.071486
GD41	JW7	JO10	*R.nitidus*	-0.027706	0.068154
JW7	JW6	JO10	*R.nitidus*	-0.005482	0.071083
GD25	JW7	JO10	*R.nitidus*	0.025594	0.068046
GD44	JW7	JO10	*R.nitidus*	0.002809	0.069511
GD30	JW7	JO10	*R.nitidus*	0.106533	0.073737
JO7	JW7	JO10	*R.nitidus*	-0.027655	0.073087
GD32	JW7	JO10	*R.nitidus*	0.009242	0.069907
CS11	JW7	JO10	*R.nitidus*	-0.03128	0.067915
GD22	JW7	JO10	*R.nitidus*	0.057481	0.069057
CS16	JW7	JO10	*R.nitidus*	-0.02521	0.068694
GD33	JW7	JO10	*R.nitidus*	-0.046968	0.069465
JO3	JW6	JO10	*R.nitidus*	-0.017585	0.07246
CS19	JW7	JO10	*R.nitidus*	-0.004613	0.068605
CS21	JW7	JO10	*R.nitidus*	-0.033575	0.068554
GD49	JW7	JO10	*R.nitidus*	0.050949	0.070837
GD29	JW7	JO10	*R.nitidus*	0.072943	0.068712
CS15	JW7	JO10	*R.nitidus*	-0.022915	0.069071
CS17	JW7	JO10	*R.nitidus*	-0.019417	0.068943
CS18	JW7	JO10	*R.nitidus*	-0.034767	0.067962

GD48	JW7	JO10	*R.nitidus*	0.022686	0.069744
GD35	JW7	JO10	*R.nitidus*	0.021258	0.067633
GD45	JW7	JO10	*R.nitidus*	0.056604	0.068646
CS30	JW7	JO10	*R.nitidus*	-0.078505	0.067342
CS25	JW7	JO10	*R.nitidus*	0.01011	0.069097
CS20	JW7	JO10	*R.nitidus*	-0.052055	0.069122
SC10	JW7	JO10	*R.nitidus*	0.034895	0.067577
GD50	JW7	JO10	*R.nitidus*	0.032686	0.071837
CS13	JW7	JO10	*R.nitidus*	-0.108716	0.071108
SC6	JW7	JO10	*R.nitidus*	0.003976	0.067627
GD46	JW7	JO10	*R.nitidus*	-0.005329	0.067606
CS4	JW7	JO10	*R.nitidus*	-0.041182	0.065806
CS24	JW7	JO10	*R.nitidus*	-0.050193	0.066005
CS31	JW7	JO10	*R.nitidus*	-0.035122	0.069818
SC27	JW7	JO10	*R.nitidus*	-0.005269	0.065114
SC42	JW7	JO10	*R.nitidus*	0.12305	0.066996
MY4	JW7	JO10	*R.nitidus*	-0.064246	0.069355
JW6	JW7	JO10	*R.nitidus*	0.005482	0.071083
SC31	JW7	JO10	*R.nitidus*	-0.039526	0.066299
SC14	JW7	JO10	*R.nitidus*	0.01574	0.067895
MY6	JW7	JO10	*R.nitidus*	-0.018674	0.068788
SC15	JW7	JO10	*R.nitidus*	-0.013725	0.068604
SC26	JW7	JO10	*R.nitidus*	-0.040394	0.069371
CS23	JW7	JO10	*R.nitidus*	-0.04946	0.068476
SC32	JW7	JO10	*R.nitidus*	0.003083	0.067628
CS22	JW7	JO10	*R.nitidus*	-0.011152	0.068717
JO3	JW7	JO10	*R.nitidus*	-0.032914	0.068654
CS3	JW7	JO10	*R.nitidus*	-0.075298	0.06686
CS28	JW7	JO10	*R.nitidus*	-0.028884	0.069306
JO7	JO1	JO10	*R.nitidus*	-0.025093	0.068401
SC28	JW7	JO10	*R.nitidus*	-0.02505	0.065163
SC24	JW7	JO10	*R.nitidus*	0.003027	0.068211
SC5	JW7	JO10	*R.nitidus*	0.008016	0.072352
SC45	JW7	JO10	*R.nitidus*	-0.001887	0.067933
SC35	JW7	JO10	*R.nitidus*	0.01003	0.064639
JO1	JW7	JO10	*R.nitidus*	-0.028958	0.068293
CS6	JW7	JO10	*R.nitidus*	-0.053232	0.067305
SC18	JW7	JO10	*R.nitidus*	0.010309	0.068328
GD25	JO1	JO10	*R.nitidus*	0.066323	0.064371
SC34	JW7	JO10	*R.nitidus*	0.004883	0.067513
GD35	JO1	JO10	*R.nitidus*	0.037683	0.06187
GD31	JO1	JO10	*R.nitidus*	0.064888	0.064421
GD29	JO1	JO10	*R.nitidus*	0.072297	0.06233
GD21	JO1	JO10	*R.nitidus*	0.162929	0.062673
SC44	JW7	JO10	*R.nitidus*	-0.027641	0.068206
GD30	JO1	JO10	*R.nitidus*	0.143414	0.065586
JW4	JW7	JO10	*R.nitidus*	0.082759	0.067977
CS12	JO1	JO10	*R.nitidus*	-0.013867	0.065061
JO8	JO1	JO10	*R.nitidus*	-0.021277	0.070945
GD26	JO1	JO10	*R.nitidus*	0.055973	0.062327
JO5	JW7	JO10	*R.nitidus*	0.01813	0.06662
GD44	JO1	JO10	*R.nitidus*	0.00147	0.063187
GD41	JO1	JO10	*R.nitidus*	-0.023578	0.063481
SC12	JW7	JO10	*R.nitidus*	-0.019527	0.065802
CS11	JO1	JO10	*R.nitidus*	-0.009608	0.065169
GD33	JO1	JO10	*R.nitidus*	-0.014344	0.064864

GD32	JO1	JO10	*R.nitidus*	0.039143	0.06625
CS15	JO1	JO10	*R.nitidus*	-0.019217	0.065245
GD22	JO1	JO10	*R.nitidus*	0.066007	0.061678
GD40	JO1	JO10	*R.nitidus*	0.048143	0.062608
CS16	JO1	JO10	*R.nitidus*	-0.022206	0.063496
GD50	JO1	JO10	*R.nitidus*	0.034119	0.068244
GD45	JO1	JO10	*R.nitidus*	0.0478	0.065431
CS18	JO1	JO10	*R.nitidus*	-0.02788	0.063171
CS17	JO1	JO10	*R.nitidus*	0.030469	0.065621
CS25	JO1	JO10	*R.nitidus*	0.015206	0.064503
CS13	JO1	JO10	*R.nitidus*	-0.065623	0.06495
GD34	JO1	JO10	*R.nitidus*	0.024308	0.064148
CS3	JO1	JO10	*R.nitidus*	-0.047945	0.063708
CS22	JO1	JO10	*R.nitidus*	0.008915	0.065835
CS19	JO1	JO10	*R.nitidus*	0.003668	0.063955
GD49	JO1	JO10	*R.nitidus*	0.077971	0.064987
SC6	JO1	JO10	*R.nitidus*	0.01474	0.063299
CS30	JO1	JO10	*R.nitidus*	-0.056445	0.063367
CS31	JO1	JO10	*R.nitidus*	-0.000755	0.064428
CS14	JO1	JO10	*R.nitidus*	-0.010309	0.067549
GD42	JO1	JO10	*R.nitidus*	0.01654	0.063282
CS21	JO1	JO10	*R.nitidus*	-0.017279	0.064523
SC18	JO1	JO10	*R.nitidus*	0.045714	0.064955
GD48	JO1	JO10	*R.nitidus*	0.043382	0.066481
GD46	JO1	JO10	*R.nitidus*	0.001383	0.063252
CS24	JO1	JO10	*R.nitidus*	-0.032042	0.062289
SC27	JO1	JO10	*R.nitidus*	0.009302	0.063677
SC32	JO1	JO10	*R.nitidus*	0.052467	0.064236
CS4	JO1	JO10	*R.nitidus*	-0.012482	0.062741
SC14	JO1	JO10	*R.nitidus*	0.013503	0.062287
SC24	JO1	JO10	*R.nitidus*	0.016923	0.062533
CS20	JO1	JO10	*R.nitidus*	-0.046875	0.064267
SC34	JO1	JO10	*R.nitidus*	0.024006	0.061694
SC15	JO1	JO10	*R.nitidus*	0.002379	0.062497
SC45	JO1	JO10	*R.nitidus*	0.032826	0.064588
SC28	JO1	JO10	*R.nitidus*	-0.01531	0.062051
CS23	JO1	JO10	*R.nitidus*	-0.045918	0.063954
CS28	JO1	JO10	*R.nitidus*	-0.007855	0.066054
SC31	JO1	JO10	*R.nitidus*	0.010703	0.063767
JW4	JO1	JO10	*R.nitidus*	0.142429	0.064561
SC10	JO1	JO10	*R.nitidus*	0.089518	0.062929
CS6	JO1	JO10	*R.nitidus*	-0.017871	0.065254
SC12	JO1	JO10	*R.nitidus*	-0.004739	0.063393
SC35	JO1	JO10	*R.nitidus*	0.044323	0.064141
SC5	JO1	JO10	*R.nitidus*	0.012559	0.066916
SC42	JO1	JO10	*R.nitidus*	0.194617	0.06219
GD32	JO3	JO10	*R.nitidus*	0.021195	0.071683
CS12	JO3	JO10	*R.nitidus*	0.002616	0.070006
SC26	JO1	JO10	*R.nitidus*	-0.015286	0.062733
GD21	JO3	JO10	*R.nitidus*	0.135852	0.067532
GD34	JO3	JO10	*R.nitidus*	-0.004299	0.069345
GD30	JO3	JO10	*R.nitidus*	0.128179	0.070955
JO3	JO1	JO10	*R.nitidus*	0.004348	0.06778
JO8	JO3	JO10	*R.nitidus*	-0.014815	0.075337
GD31	JO3	JO10	*R.nitidus*	0.028054	0.07093
GD33	JO3	JO10	*R.nitidus*	-0.026886	0.070935

SC44	JO1	JO10	*R.nitidus*	0.003165	0.063644
GD29	JO3	JO10	*R.nitidus*	0.080577	0.065571
CS11	JO3	JO10	*R.nitidus*	-0.02418	0.071428
JW6	JO1	JO10	*R.nitidus*	0.027158	0.06899
GD45	JO3	JO10	*R.nitidus*	0.040196	0.071394
GD22	JO3	JO10	*R.nitidus*	0.058774	0.070869
MY6	JO1	JO10	*R.nitidus*	0.021513	0.06211
MY4	JO1	JO10	*R.nitidus*	-0.020974	0.062254
CS15	JO3	JO10	*R.nitidus*	-0.022589	0.06915
JO5	JO1	JO10	*R.nitidus*	0.033646	0.064011
JW7	JO1	JO10	*R.nitidus*	0.028958	0.068293
GD26	JO3	JO10	*R.nitidus*	0.038793	0.071668
JO7	JO3	JO10	*R.nitidus*	0.002389	0.072358
GD44	JO3	JO10	*R.nitidus*	0.013889	0.068155
CS19	JO3	JO10	*R.nitidus*	-0.008094	0.069137
GD25	JO3	JO10	*R.nitidus*	0.043876	0.067417
CS17	JO3	JO10	*R.nitidus*	0.01487	0.071875
GD48	JO3	JO10	*R.nitidus*	0.047745	0.070825
CS16	JO3	JO10	*R.nitidus*	-0.025217	0.071242
GD46	JO3	JO10	*R.nitidus*	-0.00531	0.0698
GD50	JO3	JO10	*R.nitidus*	0.053743	0.072515
GD35	JO3	JO10	*R.nitidus*	0.042266	0.069762
GD49	JO3	JO10	*R.nitidus*	0.054381	0.073983
CS13	JO3	JO10	*R.nitidus*	-0.077324	0.071537
GD42	JO3	JO10	*R.nitidus*	0.020761	0.068722
CS3	JO3	JO10	*R.nitidus*	-0.039106	0.068585
SC10	JO3	JO10	*R.nitidus*	0.065814	0.068221
GD40	JO3	JO10	*R.nitidus*	0.02616	0.06849
CS24	JO3	JO10	*R.nitidus*	-0.024801	0.06681
GD41	JO3	JO10	*R.nitidus*	-0.021151	0.068902
CS25	JO3	JO10	*R.nitidus*	-0.008787	0.069815
CS14	JO3	JO10	*R.nitidus*	-0.013135	0.071761
SC5	JO3	JO10	*R.nitidus*	0.028061	0.073541
CS20	JO3	JO10	*R.nitidus*	-0.06295	0.068658
SC12	JO3	JO10	*R.nitidus*	-0.018119	0.067174
CS18	JO3	JO10	*R.nitidus*	-0.040386	0.066886
SC32	JO3	JO10	*R.nitidus*	0.048183	0.067637
CS23	JO3	JO10	*R.nitidus*	-0.071993	0.069427
CS21	JO3	JO10	*R.nitidus*	-0.042478	0.06925
CS31	JO3	JO10	*R.nitidus*	0.000892	0.070264
CS22	JO3	JO10	*R.nitidus*	0.003466	0.068591
SC31	JO3	JO10	*R.nitidus*	0.000853	0.069512
CS28	JO3	JO10	*R.nitidus*	-0.025686	0.071819
JO5	JO3	JO10	*R.nitidus*	0.031409	0.066673
SC34	JO3	JO10	*R.nitidus*	-0.012195	0.068567
SC26	JO3	JO10	*R.nitidus*	-0.02803	0.065435
SC14	JO3	JO10	*R.nitidus*	0.03196	0.068275
CS6	JO3	JO10	*R.nitidus*	-0.059037	0.068455
CS30	JO3	JO10	*R.nitidus*	-0.067446	0.070459
SC18	JO3	JO10	*R.nitidus*	0.044264	0.070257
SC24	JO3	JO10	*R.nitidus*	0.008643	0.068466
JW6	JO3	JO10	*R.nitidus*	0.017585	0.07246
SC6	JO3	JO10	*R.nitidus*	-0.002636	0.067306
SC44	JO3	JO10	*R.nitidus*	-0.003521	0.067408
SC35	JO3	JO10	*R.nitidus*	0.030357	0.069012
GD21	JO5	JO10	*R.nitidus*	0.128904	0.063641

SC42	JO3	JO10	*R.nitidus*	0.111468	0.067137
SC15	JO3	JO10	*R.nitidus*	0	0.068647
CS4	JO3	JO10	*R.nitidus*	-0.024829	0.067362
JW4	JO3	JO10	*R.nitidus*	0.115169	0.068057
CS11	JO5	JO10	*R.nitidus*	-0.034282	0.066914
GD30	JO5	JO10	*R.nitidus*	0.098491	0.064874
GD22	JO5	JO10	*R.nitidus*	0.035446	0.062165
SC27	JO3	JO10	*R.nitidus*	0.012185	0.068154
GD25	JO5	JO10	*R.nitidus*	0.034686	0.061748
JO8	JO5	JO10	*R.nitidus*	-0.061203	0.071547
MY4	JO3	JO10	*R.nitidus*	-0.055802	0.070733
JW7	JO3	JO10	*R.nitidus*	0.032914	0.068654
MY6	JO3	JO10	*R.nitidus*	-0.012216	0.066607
JO1	JO3	JO10	*R.nitidus*	-0.004348	0.06778
GD26	JO5	JO10	*R.nitidus*	0.023093	0.064182
SC28	JO3	JO10	*R.nitidus*	-0.013675	0.067826
CS12	JO5	JO10	*R.nitidus*	-0.037267	0.065516
JO7	JO5	JO10	*R.nitidus*	-0.055713	0.070822
GD48	JO5	JO10	*R.nitidus*	0.012801	0.066273
GD34	JO5	JO10	*R.nitidus*	-0.004079	0.062813
GD32	JO5	JO10	*R.nitidus*	0.005376	0.067291
CS19	JO5	JO10	*R.nitidus*	-0.026515	0.064228
GD33	JO5	JO10	*R.nitidus*	-0.043964	0.063497
GD35	JO5	JO10	*R.nitidus*	0.018545	0.062573
CS23	JO5	JO10	*R.nitidus*	-0.080963	0.062787
GD29	JO5	JO10	*R.nitidus*	0.055361	0.061378
SC45	JO3	JO10	*R.nitidus*	0.010336	0.066855
CS13	JO5	JO10	*R.nitidus*	-0.102952	0.065087
GD31	JO5	JO10	*R.nitidus*	0.025326	0.064816
GD41	JO5	JO10	*R.nitidus*	-0.053672	0.062641
GD46	JO5	JO10	*R.nitidus*	-0.01339	0.062326
CS17	JO5	JO10	*R.nitidus*	0.010109	0.067682
CS18	JO5	JO10	*R.nitidus*	-0.065751	0.064198
CS15	JO5	JO10	*R.nitidus*	-0.05313	0.065452
CS25	JO5	JO10	*R.nitidus*	-0.022104	0.064568
CS24	JO5	JO10	*R.nitidus*	-0.054116	0.062242
CS30	JO5	JO10	*R.nitidus*	-0.097276	0.062457
GD40	JO5	JO10	*R.nitidus*	0.020819	0.063155
GD42	JO5	JO10	*R.nitidus*	-0.010815	0.064075
CS16	JO5	JO10	*R.nitidus*	-0.04216	0.063856
GD44	JO5	JO10	*R.nitidus*	-0.019751	0.062619
CS31	JO5	JO10	*R.nitidus*	-0.032774	0.06438
GD45	JO5	JO10	*R.nitidus*	0.028022	0.064462
GD49	JO5	JO10	*R.nitidus*	0.063745	0.064855
CS4	JO5	JO10	*R.nitidus*	-0.048148	0.063565
CS21	JO5	JO10	*R.nitidus*	-0.057496	0.064734
SC10	JO5	JO10	*R.nitidus*	0.064388	0.065762
SC18	JO5	JO10	*R.nitidus*	0.003228	0.063675
GD50	JO5	JO10	*R.nitidus*	0.013502	0.065641
SC15	JO5	JO10	*R.nitidus*	-0.033844	0.063489
SC35	JO5	JO10	*R.nitidus*	0.009132	0.06338
CS14	JO5	JO10	*R.nitidus*	-0.037599	0.066467
CS28	JO5	JO10	*R.nitidus*	-0.039322	0.064986
SC14	JO5	JO10	*R.nitidus*	-0.009685	0.064124
SC26	JO5	JO10	*R.nitidus*	-0.050193	0.063577
SC34	JO5	JO10	*R.nitidus*	-0.008475	0.064531

CS20	JO5	JO10	*R.nitidus*	-0.090973	0.063708
SC44	JO5	JO10	*R.nitidus*	-0.052535	0.060751
SC6	JO5	JO10	*R.nitidus*	-0.023166	0.064931
CS6	JO5	JO10	*R.nitidus*	-0.072037	0.063847
CS22	JO5	JO10	*R.nitidus*	-0.015083	0.065156
SC31	JO5	JO10	*R.nitidus*	-0.02381	0.06497
CS3	JO5	JO10	*R.nitidus*	-0.089286	0.063972
SC5	JO5	JO10	*R.nitidus*	-0.028947	0.066775
SC12	JO5	JO10	*R.nitidus*	-0.04739	0.063518
SC45	JO5	JO10	*R.nitidus*	-0.006886	0.063588
JO1	JO5	JO10	*R.nitidus*	-0.033646	0.064011
JW6	JO5	JO10	*R.nitidus*	-0.02505	0.069852
GD21	JO7	GD24	*R.nitidus*	0.122921	0.072057
JW4	JO5	JO10	*R.nitidus*	0.107388	0.066136
SC24	JO5	JO10	*R.nitidus*	-0.027331	0.062963
GD30	JO7	GD24	*R.nitidus*	0.164162	0.07389
GD26	JO7	GD24	*R.nitidus*	0.10668	0.074998
GD40	JO7	GD24	*R.nitidus*	0.103518	0.073362
SC27	JO5	JO10	*R.nitidus*	-0.023987	0.064302
MY6	JO5	JO10	*R.nitidus*	-0.027798	0.06266
SC28	JO5	JO10	*R.nitidus*	-0.081818	0.060186
GD22	JO7	GD24	*R.nitidus*	0.128008	0.073613
SC32	JO5	JO10	*R.nitidus*	0.011211	0.063684
GD46	JO7	GD24	*R.nitidus*	0.002043	0.07461
MY4	JO5	JO10	*R.nitidus*	-0.065611	0.063522
SC42	JO5	JO10	*R.nitidus*	0.156463	0.06395
JO8	JO7	GD24	*R.nitidus*	0.030744	0.077144
JO3	JO5	JO10	*R.nitidus*	-0.031409	0.066673
GD41	JO7	GD24	*R.nitidus*	0.039674	0.074453
GD42	JO7	GD24	*R.nitidus*	0.094241	0.074903
CS11	JO7	GD24	*R.nitidus*	0.036649	0.076624
CS13	JO7	GD24	*R.nitidus*	-0.011853	0.076617
JW7	JO5	JO10	*R.nitidus*	-0.01813	0.06662
GD45	JO7	GD24	*R.nitidus*	0.134293	0.077181
GD31	JO7	GD24	*R.nitidus*	0.105152	0.074193
GD32	JO7	GD24	*R.nitidus*	0.047018	0.076515
CS12	JO7	GD24	*R.nitidus*	0.023887	0.075046
GD29	JO7	GD24	*R.nitidus*	0.135109	0.070775
CS19	JO7	GD24	*R.nitidus*	-0.009626	0.077665
GD50	JO7	GD24	*R.nitidus*	0.07931	0.076586
GD34	JO7	GD24	*R.nitidus*	0.035821	0.074477
GD25	JO7	GD24	*R.nitidus*	0.07717	0.074969
CS14	JO7	GD24	*R.nitidus*	0.028446	0.076838
GD44	JO7	GD24	*R.nitidus*	0.046218	0.072628
GD48	JO7	GD24	*R.nitidus*	0.030303	0.074128
CS25	JO7	GD24	*R.nitidus*	0	0.075215
CS3	JO7	GD24	*R.nitidus*	0.018519	0.074654
GD33	JO7	GD24	*R.nitidus*	0.04065	0.077009
GD35	JO7	GD24	*R.nitidus*	0.058402	0.075919
GD49	JO7	GD24	*R.nitidus*	0.139089	0.07424
CS17	JO7	GD24	*R.nitidus*	0.041341	0.050103
SC6	JO7	GD24	*R.nitidus*	0.026427	0.07577
CS15	JO7	GD24	*R.nitidus*	0.062171	0.074426
CS4	JO7	GD24	*R.nitidus*	0.005045	0.072191
CS16	JO7	GD24	*R.nitidus*	0.032397	0.077705
CS31	JO7	GD24	*R.nitidus*	0.008556	0.075406

SC27	JO7	GD24	*R.nitidus*	0.051363	0.073086
SC35	JO7	GD24	*R.nitidus*	0.048485	0.070965
SC24	JO7	GD24	*R.nitidus*	0.062227	0.072511
CS18	JO7	GD24	*R.nitidus*	-0.030177	0.076875
CS24	JO7	GD24	*R.nitidus*	-0.020474	0.074379
SC12	JO7	GD24	*R.nitidus*	0.003195	0.073217
CS21	JO7	GD24	*R.nitidus*	-0.009063	0.07523
SC32	JO7	GD24	*R.nitidus*	0.065539	0.074141
SC34	JO7	GD24	*R.nitidus*	0.044444	0.071028
CS20	JO7	GD24	*R.nitidus*	0.001001	0.073563
SC5	JO7	GD24	*R.nitidus*	0.062305	0.07733
SC15	JO7	GD24	*R.nitidus*	0.012959	0.076918
CS22	JO7	GD24	*R.nitidus*	0.048884	0.073868
JO1	JO7	GD24	*R.nitidus*	0.045226	0.073547
CS28	JO7	GD24	*R.nitidus*	0.019101	0.07526
SC31	JO7	GD24	*R.nitidus*	0.015795	0.073139
CS30	JO7	GD24	*R.nitidus*	-0.017897	0.074752
CS23	JO7	GD24	*R.nitidus*	-0.02588	0.075408
SC18	JO7	GD24	*R.nitidus*	0.075027	0.073055
SC42	JO7	GD24	*R.nitidus*	0.2329	0.070414
SC28	JO7	GD24	*R.nitidus*	0.046316	0.074236
GD29	JO8	GD24	*R.nitidus*	0.089704	0.072339
SC10	JO7	GD24	*R.nitidus*	0.122713	0.073844
MY6	JO7	GD24	*R.nitidus*	0.008439	0.07237
SC44	JO7	GD24	*R.nitidus*	0.032597	0.075159
CS6	JO7	GD24	*R.nitidus*	-0.021299	0.073103
SC14	JO7	GD24	*R.nitidus*	0.039256	0.073493
GD22	JO8	GD24	*R.nitidus*	0.077079	0.074674
SC26	JO7	GD24	*R.nitidus*	-0.005133	0.073526
MY4	JO7	GD24	*R.nitidus*	-0.017932	0.072755
JW7	JO7	GD24	*R.nitidus*	0.088202	0.07712
JO5	JO7	GD24	*R.nitidus*	0.096517	0.071684
JW6	JO7	GD24	*R.nitidus*	-0.015679	0.069872
GD25	JO8	GD24	*R.nitidus*	0.033369	0.071563
JW4	JO7	GD24	*R.nitidus*	0.22412	0.073044
JO7	JO8	GD24	*R.nitidus*	-0.030744	0.077144
GD33	JO8	GD24	*R.nitidus*	-0.038176	0.075849
CS11	JO8	GD24	*R.nitidus*	-0.003148	0.07493
GD35	JO8	GD24	*R.nitidus*	0.035417	0.07519
JO3	JO7	GD24	*R.nitidus*	0.023457	0.077267
GD31	JO8	GD24	*R.nitidus*	0.047983	0.073573
SC45	JO7	GD24	*R.nitidus*	0.070883	0.073029
GD44	JO8	GD24	*R.nitidus*	0.016234	0.075211
GD34	JO8	GD24	*R.nitidus*	-0.040598	0.074372
GD50	JO8	GD24	*R.nitidus*	0.02657	0.076631
CS16	JO8	GD24	*R.nitidus*	-0.026432	0.076648
CS13	JO8	GD24	*R.nitidus*	-0.021421	0.076716
CS12	JO8	GD24	*R.nitidus*	0.00111	0.07435
GD21	JO8	GD24	*R.nitidus*	0.075269	0.072826
GD26	JO8	GD24	*R.nitidus*	0.032715	0.072959
GD30	JO8	GD24	*R.nitidus*	0.104348	0.078109
GD42	JO8	GD24	*R.nitidus*	0.042463	0.074728
CS14	JO8	GD24	*R.nitidus*	0.003341	0.078878
CS18	JO8	GD24	*R.nitidus*	-0.061135	0.07446
CS17	JO8	GD24	*R.nitidus*	0.007973	0.077502
GD41	JO8	GD24	*R.nitidus*	0.004251	0.075254

GD45	JO8	GD24	*R.nitidus*	0.067248	0.07849
CS31	JO8	GD24	*R.nitidus*	-0.050401	0.075519
GD46	JO8	GD24	*R.nitidus*	-0.068777	0.072502
CS20	JO8	GD24	*R.nitidus*	-0.027056	0.075938
GD48	JO8	GD24	*R.nitidus*	-0.00663	0.07138
SC24	JO8	GD24	*R.nitidus*	0.02381	0.074868
SC26	JO8	GD24	*R.nitidus*	-0.074398	0.074537
GD40	JO8	GD24	*R.nitidus*	0.066386	0.072901
GD32	JO8	GD24	*R.nitidus*	-0.041413	0.077875
SC35	JO8	GD24	*R.nitidus*	0.028825	0.073449
CS21	JO8	GD24	*R.nitidus*	-0.043022	0.073312
CS15	JO8	GD24	*R.nitidus*	0.015086	0.075364
GD49	JO8	GD24	*R.nitidus*	0.092683	0.074404
SC28	JO8	GD24	*R.nitidus*	-0.003432	0.074303
CS6	JO8	GD24	*R.nitidus*	-0.047085	0.074009
CS30	JO8	GD24	*R.nitidus*	-0.079955	0.072667
SC44	JO8	GD24	*R.nitidus*	0.022346	0.074477
CS22	JO8	GD24	*R.nitidus*	-0.01566	0.075091
CS25	JO8	GD24	*R.nitidus*	-0.018702	0.077248
SC10	JO8	GD24	*R.nitidus*	0.070615	0.075957
CS28	JO8	GD24	*R.nitidus*	-0.016092	0.073661
SC18	JO8	GD24	*R.nitidus*	0.053381	0.074303
SC45	JO8	GD24	*R.nitidus*	0.040526	0.072775
CS19	JO8	GD24	*R.nitidus*	-0.041002	0.075443
CS23	JO8	GD24	*R.nitidus*	-0.046187	0.07535
SC27	JO8	GD24	*R.nitidus*	0.009879	0.074551
CS24	JO8	GD24	*R.nitidus*	-0.036782	0.073344
SC31	JO8	GD24	*R.nitidus*	0.011518	0.074117
SC5	JO8	GD24	*R.nitidus*	0.021459	0.079281
CS3	JO8	GD24	*R.nitidus*	-0.009879	0.075821
MY4	JO8	GD24	*R.nitidus*	-0.051668	0.075017
JW6	JO8	GD24	*R.nitidus*	-0.019817	0.075202
CS4	JO8	GD24	*R.nitidus*	-0.008574	0.073169
SC12	JO8	GD24	*R.nitidus*	-0.040678	0.073291
SC6	JO8	GD24	*R.nitidus*	-0.02694	0.075409
SC15	JO8	GD24	*R.nitidus*	-0.009009	0.077495
SC14	JO8	GD24	*R.nitidus*	0.02139	0.075185
SC34	JO8	GD24	*R.nitidus*	-0.009534	0.072863
JO5	JO8	GD24	*R.nitidus*	0.083149	0.072858
JW7	JO8	GD24	*R.nitidus*	0.053613	0.076493
SC32	JO8	GD24	*R.nitidus*	0.003212	0.074122
SC42	JO8	GD24	*R.nitidus*	0.184044	0.071486
MY6	JO8	GD24	*R.nitidus*	-0.01559	0.075609
JO1	JO8	GD24	*R.nitidus*	-0.006472	0.073227
JO3	JO8	GD24	*R.nitidus*	-0.008883	0.076106
JW4	JO8	GD24	*R.nitidus*	0.191111	0.072687
JO7	CS11	GD24	*R.nitidus*	-0.036649	0.076624
GD30	CS11	GD24	*R.nitidus*	0.124474	0.072218
GD32	CS11	GD24	*R.nitidus*	0.023387	0.071322
GD21	CS11	GD24	*R.nitidus*	0.105563	0.068095
GD22	CS11	GD24	*R.nitidus*	0.066667	0.070284
GD25	CS11	GD24	*R.nitidus*	0.060583	0.070185
GD31	CS11	GD24	*R.nitidus*	0.066412	0.069968
GD42	CS11	GD24	*R.nitidus*	0.046477	0.068513
GD26	CS11	GD24	*R.nitidus*	0.035011	0.069449
GD29	CS11	GD24	*R.nitidus*	0.089636	0.068808

GD35	CS11	GD24	*R.nitidus*	0.034763	0.069757
JO8	CS11	GD24	*R.nitidus*	0.003148	0.07493
GD45	CS11	GD24	*R.nitidus*	0.081444	0.072734
CS21	CS11	GD24	*R.nitidus*	-0.026398	0.067172
CS14	CS11	GD24	*R.nitidus*	-0.013004	0.073761
CS17	CS11	GD24	*R.nitidus*	0.036075	0.071591
GD49	CS11	GD24	*R.nitidus*	0.109836	0.071928
CS20	CS11	GD24	*R.nitidus*	-0.029389	0.069406
GD40	CS11	GD24	*R.nitidus*	0.070803	0.068061
CS16	CS11	GD24	*R.nitidus*	-0.00905	0.071678
GD44	CS11	GD24	*R.nitidus*	0.018141	0.071545
CS12	CS11	GD24	*R.nitidus*	-0.001099	0.07157
GD33	CS11	GD24	*R.nitidus*	-0.005084	0.068779
CS28	CS11	GD24	*R.nitidus*	-0.023018	0.068584
SC12	CS11	GD24	*R.nitidus*	-0.023585	0.069169
GD46	CS11	GD24	*R.nitidus*	-0.007496	0.069281
GD34	CS11	GD24	*R.nitidus*	-0.003554	0.067836
CS23	CS11	GD24	*R.nitidus*	-0.065637	0.068281
CS13	CS11	GD24	*R.nitidus*	-0.050171	0.071516
CS25	CS11	GD24	*R.nitidus*	-0.020542	0.071663
CS19	CS11	GD24	*R.nitidus*	-0.03431	0.071346
GD41	CS11	GD24	*R.nitidus*	0.014043	0.067445
GD50	CS11	GD24	*R.nitidus*	0.017889	0.073281
CS3	CS11	GD24	*R.nitidus*	-0.029221	0.068472
CS6	CS11	GD24	*R.nitidus*	-0.035029	0.068826
GD48	CS11	GD24	*R.nitidus*	0.00793	0.071038
SC5	CS11	GD24	*R.nitidus*	0	0.073106
SC6	CS11	GD24	*R.nitidus*	-0.021944	0.070915
CS30	CS11	GD24	*R.nitidus*	-0.058039	0.069862
CS31	CS11	GD24	*R.nitidus*	-0.02437	0.072493
CS18	CS11	GD24	*R.nitidus*	-0.058214	0.069811
CS4	CS11	GD24	*R.nitidus*	-0.010989	0.068249
CS15	CS11	GD24	*R.nitidus*	0.047564	0.073648
SC26	CS11	GD24	*R.nitidus*	-0.040435	0.067495
SC18	CS11	GD24	*R.nitidus*	0.053864	0.068631
SC15	CS11	GD24	*R.nitidus*	-0.01876	0.070291
SC31	CS11	GD24	*R.nitidus*	-0.007813	0.069233
SC28	CS11	GD24	*R.nitidus*	-0.022621	0.069304
JO8	GD21	GD24	*R.nitidus*	-0.075269	0.072826
SC14	CS11	GD24	*R.nitidus*	0.019847	0.069973
SC10	CS11	GD24	*R.nitidus*	0.068075	0.070589
JW6	CS11	GD24	*R.nitidus*	-0.008188	0.074063
JW4	CS11	GD24	*R.nitidus*	0.200303	0.068608
CS22	CS11	GD24	*R.nitidus*	0.013557	0.070902
JW7	CS11	GD24	*R.nitidus*	0.026419	0.074165
CS24	CS11	GD24	*R.nitidus*	-0.056067	0.069837
SC44	CS11	GD24	*R.nitidus*	0.018447	0.068352
SC24	CS11	GD24	*R.nitidus*	0.000775	0.070341
SC27	CS11	GD24	*R.nitidus*	0.000799	0.068463
SC32	CS11	GD24	*R.nitidus*	0.047731	0.069194
MY6	CS11	GD24	*R.nitidus*	-0.016554	0.069523
SC42	CS11	GD24	*R.nitidus*	0.25509	0.064045
MY4	CS11	GD24	*R.nitidus*	-0.040182	0.069688
SC45	CS11	GD24	*R.nitidus*	0.024257	0.071117
JO5	CS11	GD24	*R.nitidus*	0.028832	0.068805
GD22	GD21	GD24	*R.nitidus*	-0.045207	0.065728

GD31	GD21	GD24	*R.nitidus*	-0.064869	0.067937
SC34	CS11	GD24	*R.nitidus*	0.028483	0.069944
JO1	CS11	GD24	*R.nitidus*	-0.003912	0.066929
SC35	CS11	GD24	*R.nitidus*	0.043445	0.067002
JO7	GD21	GD24	*R.nitidus*	-0.122921	0.072057
GD33	GD21	GD24	*R.nitidus*	-0.142857	0.064603
GD26	GD21	GD24	*R.nitidus*	-0.080888	0.063895
GD46	GD21	GD24	*R.nitidus*	-0.163462	0.064302
CS13	GD21	GD24	*R.nitidus*	-0.129935	0.067511
GD45	GD21	GD24	*R.nitidus*	-0.052632	0.069876
CS11	GD21	GD24	*R.nitidus*	-0.105563	0.068095
GD42	GD21	GD24	*R.nitidus*	-0.088163	0.066704
JO3	CS11	GD24	*R.nitidus*	0.014744	0.074634
GD35	GD21	GD24	*R.nitidus*	-0.071926	0.065449
GD49	GD21	GD24	*R.nitidus*	-0.012797	0.068221
GD32	GD21	GD24	*R.nitidus*	-0.093432	0.066515
GD30	GD21	GD24	*R.nitidus*	0.015	0.06914
GD34	GD21	GD24	*R.nitidus*	-0.134683	0.063785
GD25	GD21	GD24	*R.nitidus*	-0.043239	0.064482
CS24	GD21	GD24	*R.nitidus*	-0.152838	0.065992
CS15	GD21	GD24	*R.nitidus*	-0.086411	0.067782
GD44	GD21	GD24	*R.nitidus*	-0.118699	0.065803
CS19	GD21	GD24	*R.nitidus*	-0.125647	0.068353
GD29	GD21	GD24	*R.nitidus*	-0.018669	0.066152
CS21	GD21	GD24	*R.nitidus*	-0.129771	0.066013
CS17	GD21	GD24	*R.nitidus*	-0.090977	0.069215
GD48	GD21	GD24	*R.nitidus*	-0.120107	0.067719
SC14	GD21	GD24	*R.nitidus*	-0.090975	0.066873
CS18	GD21	GD24	*R.nitidus*	-0.159317	0.066849
CS16	GD21	GD24	*R.nitidus*	-0.109262	0.066535
GD41	GD21	GD24	*R.nitidus*	-0.112431	0.065372
GD40	GD21	GD24	*R.nitidus*	-0.045079	0.066315
CS12	GD21	GD24	*R.nitidus*	-0.114693	0.068245
CS22	GD21	GD24	*R.nitidus*	-0.099856	0.065049
CS30	GD21	GD24	*R.nitidus*	-0.164918	0.065568
GD50	GD21	GD24	*R.nitidus*	-0.101825	0.070938
CS31	GD21	GD24	*R.nitidus*	-0.138973	0.066575
SC5	GD21	GD24	*R.nitidus*	-0.102545	0.068803
SC26	GD21	GD24	*R.nitidus*	-0.150523	0.064457
SC10	GD21	GD24	*R.nitidus*	-0.049533	0.067491
CS14	GD21	GD24	*R.nitidus*	-0.123258	0.067656
SC24	GD21	GD24	*R.nitidus*	-0.113997	0.066477
SC15	GD21	GD24	*R.nitidus*	-0.127407	0.067149
CS28	GD21	GD24	*R.nitidus*	-0.130236	0.06596
CS3	GD21	GD24	*R.nitidus*	-0.12894	0.066223
CS20	GD21	GD24	*R.nitidus*	-0.137241	0.067309
SC18	GD21	GD24	*R.nitidus*	-0.069248	0.067153
SC34	GD21	GD24	*R.nitidus*	-0.082985	0.0658
JW6	GD21	GD24	*R.nitidus*	-0.103791	0.070733
CS4	GD21	GD24	*R.nitidus*	-0.118056	0.064238
CS6	GD21	GD24	*R.nitidus*	-0.151223	0.065092
CS23	GD21	GD24	*R.nitidus*	-0.156444	0.065804
SC12	GD21	GD24	*R.nitidus*	-0.123682	0.066273
CS25	GD21	GD24	*R.nitidus*	-0.131255	0.067158
SC27	GD21	GD24	*R.nitidus*	-0.103676	0.06747
SC6	GD21	GD24	*R.nitidus*	-0.132479	0.06914

SC28	GD21	GD24	*R.nitidus*	-0.124826	0.065691
SC35	GD21	GD24	*R.nitidus*	-0.062678	0.066226
SC45	GD21	GD24	*R.nitidus*	-0.063446	0.064399
JO8	GD22	GD24	*R.nitidus*	-0.077079	0.074674
SC42	GD21	GD24	*R.nitidus*	0.171577	0.062302
JO3	GD21	GD24	*R.nitidus*	-0.079545	0.071544
SC31	GD21	GD24	*R.nitidus*	-0.111186	0.065773
GD31	GD22	GD24	*R.nitidus*	-0.008389	0.068429
GD26	GD22	GD24	*R.nitidus*	-0.03431	0.066478
SC32	GD21	GD24	*R.nitidus*	-0.078571	0.06644
GD21	GD22	GD24	*R.nitidus*	0.045207	0.065728
CS11	GD22	GD24	*R.nitidus*	-0.066667	0.070284
SC44	GD21	GD24	*R.nitidus*	-0.085694	0.066307
JW7	GD21	GD24	*R.nitidus*	-0.082687	0.071008
MY4	GD21	GD24	*R.nitidus*	-0.14708	0.065747
GD25	GD22	GD24	*R.nitidus*	-0.0248	0.067164
GD34	GD22	GD24	*R.nitidus*	-0.094718	0.06341
GD46	GD22	GD24	*R.nitidus*	-0.099839	0.063363
JW4	GD21	GD24	*R.nitidus*	0.114449	0.066626
JO7	GD22	GD24	*R.nitidus*	-0.128008	0.073613
MY6	GD21	GD24	*R.nitidus*	-0.120397	0.066108
GD32	GD22	GD24	*R.nitidus*	-0.04364	0.068572
CS12	GD22	GD24	*R.nitidus*	-0.06054	0.069219
CS21	GD22	GD24	*R.nitidus*	-0.093471	0.066456
CS13	GD22	GD24	*R.nitidus*	-0.09849	0.069632
GD44	GD22	GD24	*R.nitidus*	-0.044935	0.067414
GD33	GD22	GD24	*R.nitidus*	-0.092709	0.066563
JO5	GD21	GD24	*R.nitidus*	-0.057534	0.065878
GD35	GD22	GD24	*R.nitidus*	-0.02313	0.067479
GD30	GD22	GD24	*R.nitidus*	0.066667	0.070714
CS28	GD22	GD24	*R.nitidus*	-0.103886	0.068501
GD49	GD22	GD24	*R.nitidus*	0.064278	0.071796
JO1	GD21	GD24	*R.nitidus*	-0.123326	0.065174
CS18	GD22	GD24	*R.nitidus*	-0.120776	0.067372
GD45	GD22	GD24	*R.nitidus*	-0.013146	0.070044
GD29	GD22	GD24	*R.nitidus*	0.035294	0.065918
CS20	GD22	GD24	*R.nitidus*	-0.10452	0.068528
GD42	GD22	GD24	*R.nitidus*	-0.057596	0.065724
CS19	GD22	GD24	*R.nitidus*	-0.093636	0.070239
GD41	GD22	GD24	*R.nitidus*	-0.073191	0.065085
GD40	GD22	GD24	*R.nitidus*	0.012687	0.065196
CS14	GD22	GD24	*R.nitidus*	-0.077088	0.070108
CS23	GD22	GD24	*R.nitidus*	-0.119718	0.066233
CS17	GD22	GD24	*R.nitidus*	-0.048834	0.069509
CS30	GD22	GD24	*R.nitidus*	-0.128243	0.065757
SC5	GD22	GD24	*R.nitidus*	-0.07169	0.069678
CS24	GD22	GD24	*R.nitidus*	-0.110207	0.066376
GD48	GD22	GD24	*R.nitidus*	-0.077324	0.066803
CS4	GD22	GD24	*R.nitidus*	-0.082627	0.066385
CS15	GD22	GD24	*R.nitidus*	-0.04682	0.068577
CS3	GD22	GD24	*R.nitidus*	-0.096126	0.066876
CS31	GD22	GD24	*R.nitidus*	-0.093493	0.068095
SC6	GD22	GD24	*R.nitidus*	-0.080056	0.069384
SC10	GD22	GD24	*R.nitidus*	-0.002221	0.068796
SC18	GD22	GD24	*R.nitidus*	-0.018018	0.06774
CS16	GD22	GD24	*R.nitidus*	-0.07288	0.06546

GD50	GD22	GD24	*R.nitidus*	-0.018812	0.071757
SC12	GD22	GD24	*R.nitidus*	-0.09744	0.065007
CS22	GD22	GD24	*R.nitidus*	-0.055556	0.068229
SC27	GD22	GD24	*R.nitidus*	-0.052031	0.066437
SC34	GD22	GD24	*R.nitidus*	-0.041144	0.066459
JW4	GD22	GD24	*R.nitidus*	0.164706	0.068216
SC26	GD22	GD24	*R.nitidus*	-0.09937	0.066145
JO7	GD25	GD24	*R.nitidus*	-0.07717	0.074969
SC24	GD22	GD24	*R.nitidus*	-0.071221	0.068081
SC42	GD22	GD24	*R.nitidus*	0.187783	0.063651
CS25	GD22	GD24	*R.nitidus*	-0.10162	0.068412
MY6	GD22	GD24	*R.nitidus*	-0.086771	0.066673
SC45	GD22	GD24	*R.nitidus*	-0.024691	0.067198
MY4	GD22	GD24	*R.nitidus*	-0.114119	0.067989
SC14	GD22	GD24	*R.nitidus*	-0.048985	0.065701
JW7	GD22	GD24	*R.nitidus*	-0.043919	0.071777
SC44	GD22	GD24	*R.nitidus*	-0.05878	0.066193
GD22	GD25	GD24	*R.nitidus*	0.0248	0.067164
SC15	GD22	GD24	*R.nitidus*	-0.082845	0.067478
GD40	GD25	GD24	*R.nitidus*	0.011015	0.065189
SC28	GD22	GD24	*R.nitidus*	-0.095871	0.066509
SC32	GD22	GD24	*R.nitidus*	-0.027046	0.066059
JW6	GD22	GD24	*R.nitidus*	-0.071161	0.071605
GD30	GD25	GD24	*R.nitidus*	0.078846	0.071634
CS6	GD22	GD24	*R.nitidus*	-0.110945	0.066966
SC31	GD22	GD24	*R.nitidus*	-0.081119	0.066997
JO3	GD22	GD24	*R.nitidus*	-0.05137	0.072204
GD42	GD25	GD24	*R.nitidus*	-0.022184	0.066488
JO1	GD22	GD24	*R.nitidus*	-0.0819	0.066375
SC35	GD22	GD24	*R.nitidus*	-0.017556	0.066455
JO8	GD25	GD24	*R.nitidus*	-0.033369	0.071563
CS15	GD25	GD24	*R.nitidus*	-0.047068	0.069518
JO5	GD22	GD24	*R.nitidus*	-0.015625	0.065131
GD35	GD25	GD24	*R.nitidus*	-0.022481	0.06731
GD45	GD25	GD24	*R.nitidus*	0.042266	0.071674
GD33	GD25	GD24	*R.nitidus*	-0.071487	0.068505
GD31	GD25	GD24	*R.nitidus*	0.00954	0.06711
GD49	GD25	GD24	*R.nitidus*	0.047798	0.069843
CS12	GD25	GD24	*R.nitidus*	-0.068438	0.069451
CS13	GD25	GD24	*R.nitidus*	-0.093385	0.069316
GD44	GD25	GD24	*R.nitidus*	-0.034599	0.067921
GD46	GD25	GD24	*R.nitidus*	-0.078818	0.066893
GD21	GD25	GD24	*R.nitidus*	0.043239	0.064482
GD34	GD25	GD24	*R.nitidus*	-0.052202	0.06768
GD26	GD25	GD24	*R.nitidus*	-0.000839	0.068018
GD29	GD25	GD24	*R.nitidus*	0.038492	0.065402
CS11	GD25	GD24	*R.nitidus*	-0.060583	0.070185
CS23	GD25	GD24	*R.nitidus*	-0.110542	0.067135
GD48	GD25	GD24	*R.nitidus*	-0.034451	0.067599
GD32	GD25	GD24	*R.nitidus*	-0.010762	0.071015
CS28	GD25	GD24	*R.nitidus*	-0.083402	0.068309
SC6	GD25	GD24	*R.nitidus*	-0.081493	0.068882
CS21	GD25	GD24	*R.nitidus*	-0.091181	0.07022
CS17	GD25	GD24	*R.nitidus*	-0.043408	0.070741
GD41	GD25	GD24	*R.nitidus*	-0.041497	0.06635
CS19	GD25	GD24	*R.nitidus*	-0.075457	0.069032

CS30	GD25	GD24	*R.nitidus*	-0.10878	0.066781
CS14	GD25	GD24	*R.nitidus*	-0.076744	0.070014
CS25	GD25	GD24	*R.nitidus*	-0.081664	0.068539
CS18	GD25	GD24	*R.nitidus*	-0.105341	0.068331
GD50	GD25	GD24	*R.nitidus*	0.017034	0.069628
CS20	GD25	GD24	*R.nitidus*	-0.087706	0.069135
CS22	GD25	GD24	*R.nitidus*	-0.034769	0.066331
SC5	GD25	GD24	*R.nitidus*	-0.06365	0.069776
SC15	GD25	GD24	*R.nitidus*	-0.078025	0.067855
CS24	GD25	GD24	*R.nitidus*	-0.083782	0.067429
CS16	GD25	GD24	*R.nitidus*	-0.068589	0.069488
CS4	GD25	GD24	*R.nitidus*	-0.06054	0.066107
CS6	GD25	GD24	*R.nitidus*	-0.087706	0.066744
SC10	GD25	GD24	*R.nitidus*	0.004673	0.06606
JW4	GD25	GD24	*R.nitidus*	0.173878	0.067918
SC26	GD25	GD24	*R.nitidus*	-0.09139	0.0678
SC14	GD25	GD24	*R.nitidus*	-0.043866	0.067363
SC45	GD25	GD24	*R.nitidus*	-0.02673	0.066637
SC12	GD25	GD24	*R.nitidus*	-0.082506	0.06749
SC24	GD25	GD24	*R.nitidus*	-0.053354	0.06879
SC18	GD25	GD24	*R.nitidus*	-0.021012	0.068415
SC27	GD25	GD24	*R.nitidus*	-0.059713	0.065627
CS31	GD25	GD24	*R.nitidus*	-0.084261	0.068052
CS3	GD25	GD24	*R.nitidus*	-0.087461	0.067613
JO7	GD26	GD24	*R.nitidus*	-0.10668	0.074998
SC44	GD25	GD24	*R.nitidus*	-0.039504	0.067582
SC28	GD25	GD24	*R.nitidus*	-0.079699	0.067005
GD21	GD26	GD24	*R.nitidus*	0.080888	0.063895
SC32	GD25	GD24	*R.nitidus*	-0.025868	0.066931
JO1	GD25	GD24	*R.nitidus*	-0.088598	0.066391
SC34	GD25	GD24	*R.nitidus*	-0.025698	0.065502
SC31	GD25	GD24	*R.nitidus*	-0.065089	0.066342
JO3	GD25	GD24	*R.nitidus*	-0.028519	0.070266
GD45	GD26	GD24	*R.nitidus*	0.041188	0.070483
GD22	GD26	GD24	*R.nitidus*	0.03431	0.066478
GD29	GD26	GD24	*R.nitidus*	0.055372	0.0644
GD31	GD26	GD24	*R.nitidus*	0.039967	0.066675
SC42	GD25	GD24	*R.nitidus*	0.196772	0.063868
GD32	GD26	GD24	*R.nitidus*	0.001156	0.066445
SC35	GD25	GD24	*R.nitidus*	-0.020579	0.067639
JW7	GD25	GD24	*R.nitidus*	-0.025328	0.073701
JO8	GD26	GD24	*R.nitidus*	-0.032715	0.072959
MY4	GD25	GD24	*R.nitidus*	-0.100457	0.067061
GD42	GD26	GD24	*R.nitidus*	0.011102	0.067583
JW6	GD25	GD24	*R.nitidus*	-0.062628	0.073106
MY6	GD25	GD24	*R.nitidus*	-0.06936	0.067089
GD25	GD26	GD24	*R.nitidus*	0.000839	0.068018
GD33	GD26	GD24	*R.nitidus*	-0.063492	0.065766
GD30	GD26	GD24	*R.nitidus*	0.096929	0.069117
CS13	GD26	GD24	*R.nitidus*	-0.069121	0.069148
GD40	GD26	GD24	*R.nitidus*	0.039937	0.064406
JO5	GD25	GD24	*R.nitidus*	-0.020301	0.067488
GD35	GD26	GD24	*R.nitidus*	-0.007469	0.068193
CS11	GD26	GD24	*R.nitidus*	-0.035011	0.069449
CS17	GD26	GD24	*R.nitidus*	-0.006757	0.070686
CS19	GD26	GD24	*R.nitidus*	-0.054985	0.070155

GD41	GD26	GD24	*R.nitidus*	-0.038111	0.065461
GD50	GD26	GD24	*R.nitidus*	0.009036	0.069906
GD46	GD26	GD24	*R.nitidus*	-0.067048	0.064182
CS12	GD26	GD24	*R.nitidus*	-0.02818	0.0683
CS14	GD26	GD24	*R.nitidus*	-0.05015	0.068651
CS31	GD26	GD24	*R.nitidus*	-0.070988	0.067756
CS16	GD26	GD24	*R.nitidus*	-0.04271	0.066939
CS21	GD26	GD24	*R.nitidus*	-0.059159	0.068589
GD34	GD26	GD24	*R.nitidus*	-0.053773	0.064976
GD44	GD26	GD24	*R.nitidus*	-0.013228	0.0672
SC10	GD26	GD24	*R.nitidus*	0.015849	0.068893
GD49	GD26	GD24	*R.nitidus*	0.068008	0.06924
CS22	GD26	GD24	*R.nitidus*	-0.041385	0.06702
CS28	GD26	GD24	*R.nitidus*	-0.075531	0.069362
CS4	GD26	GD24	*R.nitidus*	-0.054452	0.065344
GD48	GD26	GD24	*R.nitidus*	-0.04265	0.067671
CS3	GD26	GD24	*R.nitidus*	-0.07029	0.067678
CS20	GD26	GD24	*R.nitidus*	-0.070402	0.069206
SC42	GD26	GD24	*R.nitidus*	0.207991	0.063303
CS18	GD26	GD24	*R.nitidus*	-0.087693	0.068224
CS23	GD26	GD24	*R.nitidus*	-0.095068	0.067263
CS15	GD26	GD24	*R.nitidus*	-0.013314	0.069658
SC18	GD26	GD24	*R.nitidus*	0.003873	0.065755
SC12	GD26	GD24	*R.nitidus*	-0.076694	0.065865
SC45	GD26	GD24	*R.nitidus*	-0.010432	0.066047
MY6	GD26	GD24	*R.nitidus*	-0.055716	0.067762
CS25	GD26	GD24	*R.nitidus*	-0.065975	0.06975
SC27	GD26	GD24	*R.nitidus*	-0.035242	0.066753
SC15	GD26	GD24	*R.nitidus*	-0.055765	0.065793
CS30	GD26	GD24	*R.nitidus*	-0.105747	0.066862
SC24	GD26	GD24	*R.nitidus*	-0.047547	0.068716
CS24	GD26	GD24	*R.nitidus*	-0.078686	0.066023
CS6	GD26	GD24	*R.nitidus*	-0.068441	0.066889
SC6	GD26	GD24	*R.nitidus*	-0.069663	0.069805
JO8	GD29	GD24	*R.nitidus*	-0.089704	0.072339
JO1	GD26	GD24	*R.nitidus*	-0.058115	0.065815
SC32	GD26	GD24	*R.nitidus*	-0.009468	0.066446
JW6	GD26	GD24	*R.nitidus*	-0.057282	0.072492
MY4	GD26	GD24	*R.nitidus*	-0.06927	0.067399
SC5	GD26	GD24	*R.nitidus*	-0.037262	0.069648
SC28	GD26	GD24	*R.nitidus*	-0.059779	0.067402
CS11	GD29	GD24	*R.nitidus*	-0.089636	0.068808
JO7	GD29	GD24	*R.nitidus*	-0.135109	0.070775
SC35	GD26	GD24	*R.nitidus*	-0.008811	0.065821
SC34	GD26	GD24	*R.nitidus*	-0.017417	0.066364
JW4	GD26	GD24	*R.nitidus*	0.195755	0.067538
SC26	GD26	GD24	*R.nitidus*	-0.085312	0.06717
JO5	GD26	GD24	*R.nitidus*	0.006623	0.067945
SC14	GD26	GD24	*R.nitidus*	-0.028986	0.066767
SC31	GD26	GD24	*R.nitidus*	-0.046132	0.066887
GD25	GD29	GD24	*R.nitidus*	-0.038492	0.065402
JO3	GD26	GD24	*R.nitidus*	-0.022433	0.071866
JW7	GD26	GD24	*R.nitidus*	0.000894	0.071713
GD40	GD29	GD24	*R.nitidus*	-0.023364	0.063662
GD32	GD29	GD24	*R.nitidus*	-0.083799	0.06635
GD26	GD29	GD24	*R.nitidus*	-0.055372	0.0644

CS12	GD29	GD24	*R.nitidus*	-0.092047	0.065762
GD21	GD29	GD24	*R.nitidus*	0.018669	0.066152
SC44	GD26	GD24	*R.nitidus*	-0.022239	0.066957
GD30	GD29	GD24	*R.nitidus*	0.027594	0.066738
GD31	GD29	GD24	*R.nitidus*	-0.009458	0.066068
GD48	GD29	GD24	*R.nitidus*	-0.090345	0.065008
GD22	GD29	GD24	*R.nitidus*	-0.035294	0.065918
GD42	GD29	GD24	*R.nitidus*	-0.073469	0.06544
CS15	GD29	GD24	*R.nitidus*	-0.062412	0.068917
GD45	GD29	GD24	*R.nitidus*	-0.011722	0.068864
GD34	GD29	GD24	*R.nitidus*	-0.120799	0.064776
GD35	GD29	GD24	*R.nitidus*	-0.042879	0.065385
CS21	GD29	GD24	*R.nitidus*	-0.113116	0.064678
GD49	GD29	GD24	*R.nitidus*	0.005671	0.069311
CS30	GD29	GD24	*R.nitidus*	-0.140613	0.065568
GD46	GD29	GD24	*R.nitidus*	-0.127768	0.066952
GD33	GD29	GD24	*R.nitidus*	-0.119111	0.06703
CS24	GD29	GD24	*R.nitidus*	-0.124088	0.064831
SC5	GD29	GD24	*R.nitidus*	-0.085921	0.069906
CS19	GD29	GD24	*R.nitidus*	-0.115585	0.066476
CS14	GD29	GD24	*R.nitidus*	-0.10661	0.069318
GD44	GD29	GD24	*R.nitidus*	-0.092622	0.06785
CS25	GD29	GD24	*R.nitidus*	-0.112426	0.066668
CS17	GD29	GD24	*R.nitidus*	-0.063798	0.069045
CS13	GD29	GD24	*R.nitidus*	-0.121969	0.067612
CS28	GD29	GD24	*R.nitidus*	-0.113971	0.067248
GD50	GD29	GD24	*R.nitidus*	-0.055233	0.069015
GD41	GD29	GD24	*R.nitidus*	-0.086151	0.065613
CS18	GD29	GD24	*R.nitidus*	-0.128826	0.065588
CS22	GD29	GD24	*R.nitidus*	-0.079684	0.066167
SC10	GD29	GD24	*R.nitidus*	-0.018505	0.066119
CS16	GD29	GD24	*R.nitidus*	-0.108146	0.066262
CS23	GD29	GD24	*R.nitidus*	-0.139087	0.06488
SC28	GD29	GD24	*R.nitidus*	-0.111898	0.06643
CS20	GD29	GD24	*R.nitidus*	-0.120084	0.065596
SC14	GD29	GD24	*R.nitidus*	-0.074616	0.062987
SC12	GD29	GD24	*R.nitidus*	-0.11213	0.066554
CS31	GD29	GD24	*R.nitidus*	-0.124077	0.066523
SC26	GD29	GD24	*R.nitidus*	-0.13132	0.066332
SC35	GD29	GD24	*R.nitidus*	-0.041847	0.065354
SC44	GD29	GD24	*R.nitidus*	-0.066762	0.066212
SC6	GD29	GD24	*R.nitidus*	-0.120552	0.065054
MY4	GD29	GD24	*R.nitidus*	-0.127874	0.065273
JW7	GD29	GD24	*R.nitidus*	-0.060657	0.069713
SC31	GD29	GD24	*R.nitidus*	-0.09501	0.064756
CS6	GD29	GD24	*R.nitidus*	-0.114223	0.064182
JW4	GD29	GD24	*R.nitidus*	0.133484	0.067693
CS3	GD29	GD24	*R.nitidus*	-0.127338	0.066353
JO7	GD30	GD24	*R.nitidus*	-0.164162	0.07389
SC15	GD29	GD24	*R.nitidus*	-0.116679	0.066162
SC18	GD29	GD24	*R.nitidus*	-0.03982	0.065988
JO5	GD29	GD24	*R.nitidus*	-0.057775	0.065056
SC45	GD29	GD24	*R.nitidus*	-0.059617	0.065242
CS4	GD29	GD24	*R.nitidus*	-0.104925	0.063314
SC24	GD29	GD24	*R.nitidus*	-0.094084	0.065824
SC34	GD29	GD24	*R.nitidus*	-0.053207	0.064089

JO3	GD29	GD24	*R.nitidus*	-0.070707	0.068964
SC27	GD29	GD24	*R.nitidus*	-0.08	0.063922
SC32	GD29	GD24	*R.nitidus*	-0.062849	0.066243
GD22	GD30	GD24	*R.nitidus*	-0.066667	0.070714
SC42	GD29	GD24	*R.nitidus*	0.154126	0.063434
GD21	GD30	GD24	*R.nitidus*	-0.015	0.06914
JO1	GD29	GD24	*R.nitidus*	-0.103013	0.065428
GD34	GD30	GD24	*R.nitidus*	-0.133404	0.066375
GD26	GD30	GD24	*R.nitidus*	-0.096929	0.069117
JW6	GD29	GD24	*R.nitidus*	-0.091856	0.071292
JO8	GD30	GD24	*R.nitidus*	-0.104348	0.078109
MY6	GD29	GD24	*R.nitidus*	-0.104822	0.06496
GD33	GD30	GD24	*R.nitidus*	-0.123829	0.067582
CS14	GD30	GD24	*R.nitidus*	-0.121503	0.073161
GD25	GD30	GD24	*R.nitidus*	-0.078846	0.071634
CS12	GD30	GD24	*R.nitidus*	-0.104185	0.072858
GD31	GD30	GD24	*R.nitidus*	-0.081238	0.069783
GD48	GD30	GD24	*R.nitidus*	-0.079002	0.069344
CS11	GD30	GD24	*R.nitidus*	-0.124474	0.072218
GD32	GD30	GD24	*R.nitidus*	-0.122917	0.070912
CS24	GD30	GD24	*R.nitidus*	-0.146383	0.067374
GD41	GD30	GD24	*R.nitidus*	-0.101382	0.068414
GD29	GD30	GD24	*R.nitidus*	-0.027594	0.066738
CS16	GD30	GD24	*R.nitidus*	-0.11714	0.069789
CS19	GD30	GD24	*R.nitidus*	-0.125214	0.070854
GD35	GD30	GD24	*R.nitidus*	-0.080142	0.069353
CS25	GD30	GD24	*R.nitidus*	-0.125442	0.071719
CS21	GD30	GD24	*R.nitidus*	-0.151491	0.068717
GD50	GD30	GD24	*R.nitidus*	-0.077088	0.072338
CS15	GD30	GD24	*R.nitidus*	-0.098074	0.071444
GD42	GD30	GD24	*R.nitidus*	-0.102041	0.068067
CS23	GD30	GD24	*R.nitidus*	-0.146242	0.069278
SC10	GD30	GD24	*R.nitidus*	-0.065331	0.070945
GD46	GD30	GD24	*R.nitidus*	-0.159541	0.067093
GD40	GD30	GD24	*R.nitidus*	-0.049225	0.067606
CS30	GD30	GD24	*R.nitidus*	-0.153779	0.069752
GD49	GD30	GD24	*R.nitidus*	-0.039195	0.072831
CS13	GD30	GD24	*R.nitidus*	-0.140474	0.070734
CS17	GD30	GD24	*R.nitidus*	-0.100701	0.072868
CS20	GD30	GD24	*R.nitidus*	-0.140879	0.069636
GD45	GD30	GD24	*R.nitidus*	-0.031873	0.070229
CS31	GD30	GD24	*R.nitidus*	-0.130511	0.070092
CS3	GD30	GD24	*R.nitidus*	-0.135225	0.069237
CS18	GD30	GD24	*R.nitidus*	-0.143914	0.069664
SC6	GD30	GD24	*R.nitidus*	-0.143946	0.070328
CS22	GD30	GD24	*R.nitidus*	-0.100933	0.068982
SC15	GD30	GD24	*R.nitidus*	-0.129004	0.071634
GD44	GD30	GD24	*R.nitidus*	-0.098953	0.069825
SC28	GD30	GD24	*R.nitidus*	-0.129139	0.067386
SC18	GD30	GD24	*R.nitidus*	-0.066265	0.069067
SC14	GD30	GD24	*R.nitidus*	-0.093698	0.070617
SC44	GD30	GD24	*R.nitidus*	-0.087254	0.0666
SC32	GD30	GD24	*R.nitidus*	-0.085597	0.070677
SC42	GD30	GD24	*R.nitidus*	0.14276	0.065772
CS28	GD30	GD24	*R.nitidus*	-0.155172	0.071144
SC5	GD30	GD24	*R.nitidus*	-0.120432	0.071885

SC34	GD30	GD24	*R.nitidus*	-0.074387	0.069221
SC26	GD30	GD24	*R.nitidus*	-0.141803	0.0701
JW4	GD30	GD24	*R.nitidus*	0.11552	0.069684
SC35	GD30	GD24	*R.nitidus*	-0.051029	0.069266
MY6	GD30	GD24	*R.nitidus*	-0.114382	0.068413
JO8	GD31	GD24	*R.nitidus*	-0.047983	0.073573
SC12	GD30	GD24	*R.nitidus*	-0.124051	0.069353
SC24	GD30	GD24	*R.nitidus*	-0.124587	0.069267
GD45	GD31	GD24	*R.nitidus*	-0.005634	0.071211
SC27	GD30	GD24	*R.nitidus*	-0.108894	0.06979
SC31	GD30	GD24	*R.nitidus*	-0.127608	0.068611
JW7	GD30	GD24	*R.nitidus*	-0.092555	0.073972
GD30	GD31	GD24	*R.nitidus*	0.081238	0.069783
GD26	GD31	GD24	*R.nitidus*	-0.039967	0.066675
CS6	GD30	GD24	*R.nitidus*	-0.136364	0.067732
JO3	GD30	GD24	*R.nitidus*	-0.102513	0.073113
CS4	GD30	GD24	*R.nitidus*	-0.119935	0.06717
JO5	GD30	GD24	*R.nitidus*	-0.066557	0.067448
JW6	GD30	GD24	*R.nitidus*	-0.13892	0.074841
GD35	GD31	GD24	*R.nitidus*	-0.026101	0.067567
GD25	GD31	GD24	*R.nitidus*	-0.00954	0.06711
GD22	GD31	GD24	*R.nitidus*	0.008389	0.068429
GD33	GD31	GD24	*R.nitidus*	-0.077494	0.069046
SC45	GD30	GD24	*R.nitidus*	-0.086849	0.068568
JO7	GD31	GD24	*R.nitidus*	-0.105152	0.074193
GD46	GD31	GD24	*R.nitidus*	-0.084237	0.066277
CS11	GD31	GD24	*R.nitidus*	-0.066412	0.069968
GD21	GD31	GD24	*R.nitidus*	0.064869	0.067937
GD32	GD31	GD24	*R.nitidus*	-0.041985	0.070496
MY4	GD30	GD24	*R.nitidus*	-0.14322	0.068578
GD42	GD31	GD24	*R.nitidus*	-0.029668	0.067324
JO1	GD30	GD24	*R.nitidus*	-0.134615	0.066472
GD44	GD31	GD24	*R.nitidus*	-0.05427	0.069723
GD49	GD31	GD24	*R.nitidus*	0.047529	0.07105
CS12	GD31	GD24	*R.nitidus*	-0.06371	0.069036
GD29	GD31	GD24	*R.nitidus*	0.009458	0.066068
CS15	GD31	GD24	*R.nitidus*	-0.052059	0.069588
GD34	GD31	GD24	*R.nitidus*	-0.071613	0.067507
GD40	GD31	GD24	*R.nitidus*	0.020292	0.06718
CS17	GD31	GD24	*R.nitidus*	-0.063218	0.072444
GD50	GD31	GD24	*R.nitidus*	-0.029293	0.072326
CS18	GD31	GD24	*R.nitidus*	-0.129921	0.06895
GD41	GD31	GD24	*R.nitidus*	-0.066046	0.066822
CS28	GD31	GD24	*R.nitidus*	-0.089552	0.068685
CS25	GD31	GD24	*R.nitidus*	-0.101483	0.069779
CS13	GD31	GD24	*R.nitidus*	-0.103611	0.069208
CS3	GD31	GD24	*R.nitidus*	-0.110762	0.068417
GD48	GD31	GD24	*R.nitidus*	-0.060115	0.069076
CS20	GD31	GD24	*R.nitidus*	-0.10888	0.068242
CS23	GD31	GD24	*R.nitidus*	-0.13297	0.067016
CS30	GD31	GD24	*R.nitidus*	-0.133117	0.068698
CS14	GD31	GD24	*R.nitidus*	-0.076312	0.071493
SC28	GD31	GD24	*R.nitidus*	-0.092508	0.066757
SC10	GD31	GD24	*R.nitidus*	-0.007132	0.068933
CS16	GD31	GD24	*R.nitidus*	-0.071317	0.069772
SC34	GD31	GD24	*R.nitidus*	-0.038023	0.067258

SC5	GD31	GD24	*R.nitidus*	-0.076744	0.070129
CS19	GD31	GD24	*R.nitidus*	-0.121261	0.069234
SC32	GD31	GD24	*R.nitidus*	-0.034357	0.067318
CS21	GD31	GD24	*R.nitidus*	-0.097147	0.068458
MY6	GD31	GD24	*R.nitidus*	-0.089216	0.067081
JO7	GD32	GD24	*R.nitidus*	-0.047018	0.076515
CS22	GD31	GD24	*R.nitidus*	-0.059922	0.069581
SC15	GD31	GD24	*R.nitidus*	-0.087843	0.069399
JO8	GD32	GD24	*R.nitidus*	0.041413	0.077875
SC12	GD31	GD24	*R.nitidus*	-0.086045	0.068316
CS24	GD31	GD24	*R.nitidus*	-0.118431	0.068461
SC31	GD31	GD24	*R.nitidus*	-0.075019	0.066527
CS31	GD31	GD24	*R.nitidus*	-0.089182	0.069323
MY4	GD31	GD24	*R.nitidus*	-0.10935	0.068083
SC14	GD31	GD24	*R.nitidus*	-0.058378	0.067229
JO5	GD31	GD24	*R.nitidus*	-0.03976	0.068669
SC35	GD31	GD24	*R.nitidus*	-0.037405	0.067758
SC6	GD31	GD24	*R.nitidus*	-0.081679	0.068175
SC24	GD31	GD24	*R.nitidus*	-0.072686	0.069608
CS6	GD31	GD24	*R.nitidus*	-0.10209	0.069414
CS4	GD31	GD24	*R.nitidus*	-0.079027	0.067274
SC18	GD31	GD24	*R.nitidus*	-0.020884	0.068173
GD25	GD32	GD24	*R.nitidus*	0.010762	0.071015
JW6	GD31	GD24	*R.nitidus*	-0.088119	0.073306
SC26	GD31	GD24	*R.nitidus*	-0.103053	0.066968
SC27	GD31	GD24	*R.nitidus*	-0.050965	0.067755
GD26	GD32	GD24	*R.nitidus*	-0.001156	0.066445
GD31	GD32	GD24	*R.nitidus*	0.041985	0.070496
JW4	GD31	GD24	*R.nitidus*	0.145544	0.069895
GD30	GD32	GD24	*R.nitidus*	0.122917	0.070912
SC42	GD31	GD24	*R.nitidus*	0.183787	0.065181
JW7	GD31	GD24	*R.nitidus*	-0.059369	0.073634
SC44	GD31	GD24	*R.nitidus*	-0.051777	0.06842
SC45	GD31	GD24	*R.nitidus*	-0.05175	0.067824
JO1	GD31	GD24	*R.nitidus*	-0.089025	0.068264
GD35	GD32	GD24	*R.nitidus*	0.029091	0.070794
GD21	GD32	GD24	*R.nitidus*	0.093432	0.066515
JO3	GD31	GD24	*R.nitidus*	-0.032258	0.074895
GD33	GD32	GD24	*R.nitidus*	-0.04661	0.066445
CS11	GD32	GD24	*R.nitidus*	-0.023387	0.071322
CS13	GD32	GD24	*R.nitidus*	-0.041073	0.071198
GD22	GD32	GD24	*R.nitidus*	0.04364	0.068572
GD46	GD32	GD24	*R.nitidus*	-0.059798	0.065801
CS12	GD32	GD24	*R.nitidus*	-0.011054	0.069265
GD45	GD32	GD24	*R.nitidus*	0.05235	0.073621
GD29	GD32	GD24	*R.nitidus*	0.083799	0.06635
GD34	GD32	GD24	*R.nitidus*	-0.030723	0.067355
GD41	GD32	GD24	*R.nitidus*	-0.036347	0.068668
GD48	GD32	GD24	*R.nitidus*	-0.007099	0.066027
GD44	GD32	GD24	*R.nitidus*	0.006705	0.069803
GD50	GD32	GD24	*R.nitidus*	0.029933	0.073063
CS22	GD32	GD24	*R.nitidus*	-0.011226	0.069767
CS17	GD32	GD24	*R.nitidus*	0.00937	0.073766
CS19	GD32	GD24	*R.nitidus*	-0.043082	0.070369
SC24	GD32	GD24	*R.nitidus*	-0.012898	0.070168
GD40	GD32	GD24	*R.nitidus*	0.047535	0.066983

CS24	GD32	GD24	*R.nitidus*	-0.063993	0.069014
CS20	GD32	GD24	*R.nitidus*	-0.047855	0.068231
CS3	GD32	GD24	*R.nitidus*	-0.059216	0.069734
CS28	GD32	GD24	*R.nitidus*	-0.0518	0.070581
CS15	GD32	GD24	*R.nitidus*	0.011657	0.070404
GD49	GD32	GD24	*R.nitidus*	0.096559	0.072153
CS16	GD32	GD24	*R.nitidus*	-0.028668	0.069287
SC5	GD32	GD24	*R.nitidus*	0.006891	0.073108
SC31	GD32	GD24	*R.nitidus*	-0.012998	0.069885
GD42	GD32	GD24	*R.nitidus*	0	0.069533
SC12	GD32	GD24	*R.nitidus*	-0.048882	0.068871
CS14	GD32	GD24	*R.nitidus*	-0.023589	0.07242
CS18	GD32	GD24	*R.nitidus*	-0.080833	0.070724
SC6	GD32	GD24	*R.nitidus*	-0.030227	0.072045
CS23	GD32	GD24	*R.nitidus*	-0.084967	0.069923
SC27	GD32	GD24	*R.nitidus*	0.001667	0.068373
SC14	GD32	GD24	*R.nitidus*	-0.001643	0.067973
SC18	GD32	GD24	*R.nitidus*	0.053097	0.068831
CS31	GD32	GD24	*R.nitidus*	-0.053004	0.072327
CS21	GD32	GD24	*R.nitidus*	-0.042776	0.069982
CS25	GD32	GD24	*R.nitidus*	-0.041736	0.071506
SC28	GD32	GD24	*R.nitidus*	-0.019492	0.068598
JO7	CS12	GD24	*R.nitidus*	-0.023887	0.075046
MY6	GD32	GD24	*R.nitidus*	-0.028689	0.070067
SC10	GD32	GD24	*R.nitidus*	0.055038	0.069284
CS4	GD32	GD24	*R.nitidus*	-0.048101	0.068699
CS30	GD32	GD24	*R.nitidus*	-0.071861	0.071235
MY4	GD32	GD24	*R.nitidus*	-0.058081	0.069917
JW4	GD32	GD24	*R.nitidus*	0.216055	0.069182
JO3	GD32	GD24	*R.nitidus*	0.012871	0.072991
SC15	GD32	GD24	*R.nitidus*	-0.021645	0.069004
SC32	GD32	GD24	*R.nitidus*	0.024618	0.069076
SC45	GD32	GD24	*R.nitidus*	0.017413	0.068985
CS6	GD32	GD24	*R.nitidus*	-0.074271	0.068868
JW7	GD32	GD24	*R.nitidus*	0.036929	0.071837
SC44	GD32	GD24	*R.nitidus*	0.020017	0.06875
JO1	GD32	GD24	*R.nitidus*	-0.022599	0.068621
SC26	GD32	GD24	*R.nitidus*	-0.047337	0.069468
SC42	GD32	GD24	*R.nitidus*	0.218277	0.064642
JO8	CS12	GD24	*R.nitidus*	-0.00111	0.07435
JW6	GD32	GD24	*R.nitidus*	0.008658	0.073924
GD21	CS12	GD24	*R.nitidus*	0.114693	0.068245
SC34	GD32	GD24	*R.nitidus*	-0.008244	0.067395
SC35	GD32	GD24	*R.nitidus*	0.04698	0.066943
GD25	CS12	GD24	*R.nitidus*	0.068438	0.069451
GD41	CS12	GD24	*R.nitidus*	0.006767	0.067308
JO5	GD32	GD24	*R.nitidus*	0.042429	0.070019
GD30	CS12	GD24	*R.nitidus*	0.104185	0.072858
GD22	CS12	GD24	*R.nitidus*	0.06054	0.069219
GD34	CS12	GD24	*R.nitidus*	-0.007508	0.06703
GD33	CS12	GD24	*R.nitidus*	-0.012889	0.068518
CS21	CS12	GD24	*R.nitidus*	-0.023943	0.067503
CS11	CS12	GD24	*R.nitidus*	0.001099	0.07157
GD46	CS12	GD24	*R.nitidus*	-0.011924	0.067843
GD48	CS12	GD24	*R.nitidus*	0.009275	0.068946
GD35	CS12	GD24	*R.nitidus*	0.048818	0.069558

GD45	CS12	GD24	*R.nitidus*	0.066838	0.071908
GD42	CS12	GD24	*R.nitidus*	0.031783	0.068584
CS14	CS12	GD24	*R.nitidus*	-0.010883	0.071703
GD40	CS12	GD24	*R.nitidus*	0.068356	0.067495
GD31	CS12	GD24	*R.nitidus*	0.06371	0.069036
GD29	CS12	GD24	*R.nitidus*	0.092047	0.065762
GD50	CS12	GD24	*R.nitidus*	0.035651	0.072714
GD44	CS12	GD24	*R.nitidus*	0.026549	0.070253
GD32	CS12	GD24	*R.nitidus*	0.011054	0.069265
GD26	CS12	GD24	*R.nitidus*	0.02818	0.0683
SC6	CS12	GD24	*R.nitidus*	-0.035117	0.071491
CS13	CS12	GD24	*R.nitidus*	-0.059801	0.071244
CS19	CS12	GD24	*R.nitidus*	-0.02124	0.071575
CS16	CS12	GD24	*R.nitidus*	-0.003797	0.065498
CS28	CS12	GD24	*R.nitidus*	-0.018535	0.067394
CS31	CS12	GD24	*R.nitidus*	-0.032007	0.069529
CS20	CS12	GD24	*R.nitidus*	-0.015323	0.068715
CS30	CS12	GD24	*R.nitidus*	-0.055326	0.067896
CS4	CS12	GD24	*R.nitidus*	-0.018608	0.06862
SC24	CS12	GD24	*R.nitidus*	0.007987	0.069279
GD49	CS12	GD24	*R.nitidus*	0.10453	0.070261
SC5	CS12	GD24	*R.nitidus*	0.022709	0.070303
CS25	CS12	GD24	*R.nitidus*	-0.009885	0.068577
CS3	CS12	GD24	*R.nitidus*	-0.014901	0.068304
CS23	CS12	GD24	*R.nitidus*	-0.050766	0.069349
CS17	CS12	GD24	*R.nitidus*	0.029172	0.072563
CS15	CS12	GD24	*R.nitidus*	0.04662	0.067836
CS18	CS12	GD24	*R.nitidus*	-0.049877	0.067965
CS22	CS12	GD24	*R.nitidus*	0.014155	0.067999
SC26	CS12	GD24	*R.nitidus*	-0.03537	0.067333
JW6	CS12	GD24	*R.nitidus*	-0.005247	0.074903
SC32	CS12	GD24	*R.nitidus*	0.046154	0.07048
CS24	CS12	GD24	*R.nitidus*	-0.048696	0.068028
SC31	CS12	GD24	*R.nitidus*	-0.002342	0.069303
SC14	CS12	GD24	*R.nitidus*	0.013449	0.069058
SC12	CS12	GD24	*R.nitidus*	-0.025869	0.069353
SC45	CS12	GD24	*R.nitidus*	0.046079	0.069541
MY6	CS12	GD24	*R.nitidus*	-0.01248	0.069487
SC10	CS12	GD24	*R.nitidus*	0.085141	0.068849
JW7	CS12	GD24	*R.nitidus*	0.030242	0.072542
JO5	CS12	GD24	*R.nitidus*	0.04789	0.067855
SC15	CS12	GD24	*R.nitidus*	-0.01268	0.067952
JO3	CS12	GD24	*R.nitidus*	0.000884	0.073276
JO8	GD33	GD24	*R.nitidus*	0.038176	0.075849
JO7	GD33	GD24	*R.nitidus*	-0.04065	0.077009
JW4	CS12	GD24	*R.nitidus*	0.207865	0.068765
SC44	CS12	GD24	*R.nitidus*	0.02482	0.068184
GD22	GD33	GD24	*R.nitidus*	0.092709	0.066563
GD21	GD33	GD24	*R.nitidus*	0.142857	0.064603
SC27	CS12	GD24	*R.nitidus*	0.020084	0.067847
SC18	CS12	GD24	*R.nitidus*	0.066774	0.06848
SC28	CS12	GD24	*R.nitidus*	-0.027138	0.067467
MY4	CS12	GD24	*R.nitidus*	-0.039714	0.069705
JO1	CS12	GD24	*R.nitidus*	-0.001643	0.067378
CS6	CS12	GD24	*R.nitidus*	-0.042607	0.067765
GD42	GD33	GD24	*R.nitidus*	0.042517	0.069212

GD25	GD33	GD24	*R.nitidus*	0.071487	0.068505
SC34	CS12	GD24	*R.nitidus*	0.018489	0.066475
GD48	GD33	GD24	*R.nitidus*	0.015399	0.06767
SC42	CS12	GD24	*R.nitidus*	0.261529	0.063372
SC35	CS12	GD24	*R.nitidus*	0.053822	0.066037
GD29	GD33	GD24	*R.nitidus*	0.119111	0.06703
GD30	GD33	GD24	*R.nitidus*	0.123829	0.067582
GD41	GD33	GD24	*R.nitidus*	0.028333	0.06845
GD26	GD33	GD24	*R.nitidus*	0.063492	0.065766
CS12	GD33	GD24	*R.nitidus*	0.012889	0.068518
GD46	GD33	GD24	*R.nitidus*	-0.016779	0.066802
GD34	GD33	GD24	*R.nitidus*	0.009174	0.0672
GD45	GD33	GD24	*R.nitidus*	0.085661	0.070374
GD32	GD33	GD24	*R.nitidus*	0.04661	0.066445
GD44	GD33	GD24	*R.nitidus*	0.023392	0.068265
CS11	GD33	GD24	*R.nitidus*	0.005084	0.068779
GD31	GD33	GD24	*R.nitidus*	0.077494	0.069046
CS19	GD33	GD24	*R.nitidus*	-0.007746	0.069288
GD50	GD33	GD24	*R.nitidus*	0.043655	0.071534
CS14	GD33	GD24	*R.nitidus*	0.000741	0.070693
CS25	GD33	GD24	*R.nitidus*	-0.010878	0.0695
CS21	GD33	GD24	*R.nitidus*	-0.01295	0.068027
CS22	GD33	GD24	*R.nitidus*	0.022777	0.06863
GD35	GD33	GD24	*R.nitidus*	0.066076	0.066563
GD40	GD33	GD24	*R.nitidus*	0.080619	0.066325
CS18	GD33	GD24	*R.nitidus*	-0.036842	0.069304
CS15	GD33	GD24	*R.nitidus*	0.026647	0.069276
SC24	GD33	GD24	*R.nitidus*	0.009673	0.067883
CS13	GD33	GD24	*R.nitidus*	-0.028107	0.069204
SC15	GD33	GD24	*R.nitidus*	-0.006098	0.067938
CS31	GD33	GD24	*R.nitidus*	-0.009916	0.068907
GD49	GD33	GD24	*R.nitidus*	0.120272	0.071554
SC14	GD33	GD24	*R.nitidus*	0.038905	0.067894
CS28	GD33	GD24	*R.nitidus*	-0.018838	0.068426
SC28	GD33	GD24	*R.nitidus*	0.000745	0.066896
CS16	GD33	GD24	*R.nitidus*	0	0.067748
SC26	GD33	GD24	*R.nitidus*	-0.031828	0.066863
CS30	GD33	GD24	*R.nitidus*	-0.050038	0.068167
SC5	GD33	GD24	*R.nitidus*	0.020882	0.072233
CS20	GD33	GD24	*R.nitidus*	-0.015141	0.069363
CS17	GD33	GD24	*R.nitidus*	0.029074	0.070441
SC10	GD33	GD24	*R.nitidus*	0.066059	0.070449
SC42	GD33	GD24	*R.nitidus*	0.248292	0.065625
SC18	GD33	GD24	*R.nitidus*	0.061033	0.068219
CS6	GD33	GD24	*R.nitidus*	-0.028266	0.067344
SC12	GD33	GD24	*R.nitidus*	-0.015789	0.067942
CS23	GD33	GD24	*R.nitidus*	-0.037383	0.068432
CS3	GD33	GD24	*R.nitidus*	-0.024831	0.068816
CS24	GD33	GD24	*R.nitidus*	-0.028073	0.067986
JW4	GD33	GD24	*R.nitidus*	0.244804	0.069752
SC6	GD33	GD24	*R.nitidus*	-0.004521	0.068951
CS4	GD33	GD24	*R.nitidus*	0	0.067351
JW7	GD33	GD24	*R.nitidus*	0.060177	0.07399
JW6	GD33	GD24	*R.nitidus*	0.027695	0.075212
SC27	GD33	GD24	*R.nitidus*	0.028804	0.068884
JO7	GD34	GD24	*R.nitidus*	-0.035821	0.074477

SC45	GD33	GD24	*R.nitidus*	0.058692	0.069623
JO1	GD33	GD24	*R.nitidus*	-0.002214	0.067935
CS11	GD34	GD24	*R.nitidus*	0.003554	0.067836
SC34	GD33	GD24	*R.nitidus*	0.031069	0.066553
GD31	GD34	GD24	*R.nitidus*	0.071613	0.067507
JO5	GD33	GD24	*R.nitidus*	0.05981	0.068365
GD25	GD34	GD24	*R.nitidus*	0.052202	0.06768
SC31	GD33	GD24	*R.nitidus*	-0.00073	0.068071
SC44	GD33	GD24	*R.nitidus*	0.02891	0.066921
JO8	GD34	GD24	*R.nitidus*	0.040598	0.074372
MY4	GD33	GD24	*R.nitidus*	-0.025811	0.06652
SC35	GD33	GD24	*R.nitidus*	0.048516	0.068065
SC32	GD33	GD24	*R.nitidus*	0.050698	0.068147
GD40	GD34	GD24	*R.nitidus*	0.081633	0.065494
GD35	GD34	GD24	*R.nitidus*	0.049423	0.067613
JO3	GD33	GD24	*R.nitidus*	0.039301	0.072371
MY6	GD33	GD24	*R.nitidus*	-0.003571	0.066189
CS13	GD34	GD24	*R.nitidus*	-0.031723	0.068853
GD29	GD34	GD24	*R.nitidus*	0.120799	0.064776
GD46	GD34	GD24	*R.nitidus*	-0.024514	0.065131
GD22	GD34	GD24	*R.nitidus*	0.094718	0.06341
GD45	GD34	GD24	*R.nitidus*	0.076572	0.069712
GD32	GD34	GD24	*R.nitidus*	0.030723	0.067355
GD26	GD34	GD24	*R.nitidus*	0.053773	0.064976
GD30	GD34	GD24	*R.nitidus*	0.133404	0.066375
GD49	GD34	GD24	*R.nitidus*	0.118611	0.069713
GD21	GD34	GD24	*R.nitidus*	0.134683	0.063785
GD42	GD34	GD24	*R.nitidus*	0.026132	0.066473
GD50	GD34	GD24	*R.nitidus*	0.029297	0.071284
GD48	GD34	GD24	*R.nitidus*	0.011538	0.06632
CS31	GD34	GD24	*R.nitidus*	-0.02036	0.068027
GD44	GD34	GD24	*R.nitidus*	0.012626	0.069279
CS18	GD34	GD24	*R.nitidus*	-0.044328	0.067045
CS4	GD34	GD24	*R.nitidus*	-0.012491	0.065683
CS12	GD34	GD24	*R.nitidus*	0.007508	0.06703
GD33	GD34	GD24	*R.nitidus*	-0.009174	0.0672
SC6	GD34	GD24	*R.nitidus*	-0.023014	0.068977
CS15	GD34	GD24	*R.nitidus*	0.028281	0.067121
CS20	GD34	GD24	*R.nitidus*	-0.034432	0.067964
CS16	GD34	GD24	*R.nitidus*	-0.012848	0.065506
SC5	GD34	GD24	*R.nitidus*	0.008296	0.07218
SC26	GD34	GD24	*R.nitidus*	-0.036846	0.065075
SC28	GD34	GD24	*R.nitidus*	-0.020483	0.065783
CS30	GD34	GD24	*R.nitidus*	-0.06025	0.06643
CS3	GD34	GD24	*R.nitidus*	-0.030053	0.067691
CS19	GD34	GD24	*R.nitidus*	-0.02	0.069001
SC24	GD34	GD24	*R.nitidus*	-0.003023	0.067733
SC10	GD34	GD24	*R.nitidus*	0.053151	0.067485
GD41	GD34	GD24	*R.nitidus*	0.010283	0.066741
CS22	GD34	GD24	*R.nitidus*	0	0.066631
CS14	GD34	GD24	*R.nitidus*	-0.015306	0.068386
CS21	GD34	GD24	*R.nitidus*	-0.021214	0.067406
CS24	GD34	GD24	*R.nitidus*	-0.040335	0.067812
SC18	GD34	GD24	*R.nitidus*	0.045879	0.066823
JW4	GD34	GD24	*R.nitidus*	0.226048	0.067413
CS23	GD34	GD24	*R.nitidus*	-0.059911	0.065188

CS25	GD34	GD24	*R.nitidus*	-0.018983	0.067313
SC12	GD34	GD24	*R.nitidus*	-0.032895	0.066753
CS28	GD34	GD24	*R.nitidus*	-0.03742	0.067496
CS17	GD34	GD24	*R.nitidus*	0.023379	0.069763
JO8	GD35	GD24	*R.nitidus*	-0.035417	0.07519
SC15	GD34	GD24	*R.nitidus*	-0.02566	0.066657
JW6	GD34	GD24	*R.nitidus*	0.015123	0.072003
SC45	GD34	GD24	*R.nitidus*	0.033455	0.066349
JW7	GD34	GD24	*R.nitidus*	0.054659	0.073547
SC14	GD34	GD24	*R.nitidus*	0.02185	0.065943
SC34	GD34	GD24	*R.nitidus*	0.026753	0.066157
JO7	GD35	GD24	*R.nitidus*	-0.058402	0.075919
SC31	GD34	GD24	*R.nitidus*	-0.009475	0.065097
GD22	GD35	GD24	*R.nitidus*	0.02313	0.067479
MY4	GD34	GD24	*R.nitidus*	-0.03806	0.06593
SC27	GD34	GD24	*R.nitidus*	0.005913	0.067104
SC32	GD34	GD24	*R.nitidus*	0.031137	0.065803
GD21	GD35	GD24	*R.nitidus*	0.071926	0.065449
SC35	GD34	GD24	*R.nitidus*	0.039552	0.066011
SC44	GD34	GD24	*R.nitidus*	0.022539	0.065874
GD33	GD35	GD24	*R.nitidus*	-0.066076	0.066563
SC42	GD34	GD24	*R.nitidus*	0.255582	0.064245
JO3	GD34	GD24	*R.nitidus*	0.023789	0.072841
CS6	GD34	GD24	*R.nitidus*	-0.036237	0.065938
GD34	GD35	GD24	*R.nitidus*	-0.049423	0.067613
GD31	GD35	GD24	*R.nitidus*	0.026101	0.067567
GD45	GD35	GD24	*R.nitidus*	0.04479	0.069013
GD42	GD35	GD24	*R.nitidus*	-0.015	0.066511
MY6	GD34	GD24	*R.nitidus*	-0.019737	0.06603
CS12	GD35	GD24	*R.nitidus*	-0.048818	0.069558
GD48	GD35	GD24	*R.nitidus*	-0.049819	0.070573
GD32	GD35	GD24	*R.nitidus*	-0.029091	0.070794
GD30	GD35	GD24	*R.nitidus*	0.080142	0.069353
JO5	GD34	GD24	*R.nitidus*	0.046595	0.065766
JO1	GD34	GD24	*R.nitidus*	-0.017921	0.066683
GD46	GD35	GD24	*R.nitidus*	-0.074879	0.066348
GD26	GD35	GD24	*R.nitidus*	0.007469	0.068193
GD41	GD35	GD24	*R.nitidus*	-0.042219	0.066636
CS13	GD35	GD24	*R.nitidus*	-0.064199	0.069878
GD50	GD35	GD24	*R.nitidus*	-0.018127	0.069971
GD44	GD35	GD24	*R.nitidus*	-0.029412	0.068941
CS22	GD35	GD24	*R.nitidus*	-0.027166	0.069088
CS11	GD35	GD24	*R.nitidus*	-0.034763	0.069757
CS17	GD35	GD24	*R.nitidus*	-0.026688	0.071125
GD25	GD35	GD24	*R.nitidus*	0.022481	0.06731
GD49	GD35	GD24	*R.nitidus*	0.070671	0.070708
GD29	GD35	GD24	*R.nitidus*	0.042879	0.065385
GD40	GD35	GD24	*R.nitidus*	0.026895	0.065631
CS20	GD35	GD24	*R.nitidus*	-0.065379	0.068373
CS25	GD35	GD24	*R.nitidus*	-0.051869	0.069302
CS24	GD35	GD24	*R.nitidus*	-0.080997	0.067437
CS21	GD35	GD24	*R.nitidus*	-0.058696	0.068478
CS23	GD35	GD24	*R.nitidus*	-0.092661	0.067472
CS18	GD35	GD24	*R.nitidus*	-0.083143	0.068444
SC12	GD35	GD24	*R.nitidus*	-0.065201	0.068135
CS14	GD35	GD24	*R.nitidus*	-0.046247	0.071996

CS28	GD35	GD24	*R.nitidus*	-0.07613	0.068789
MY4	GD35	GD24	*R.nitidus*	-0.084695	0.067435
CS16	GD35	GD24	*R.nitidus*	-0.048006	0.067213
SC28	GD35	GD24	*R.nitidus*	-0.052006	0.067631
CS30	GD35	GD24	*R.nitidus*	-0.087481	0.068461
SC34	GD35	GD24	*R.nitidus*	-0.020818	0.065029
CS4	GD35	GD24	*R.nitidus*	-0.052244	0.067617
SC10	GD35	GD24	*R.nitidus*	0.029008	0.06856
CS15	GD35	GD24	*R.nitidus*	-0.009594	0.070448
SC24	GD35	GD24	*R.nitidus*	-0.049574	0.066984
SC27	GD35	GD24	*R.nitidus*	-0.036406	0.066598
CS19	GD35	GD24	*R.nitidus*	-0.054773	0.070437
SC15	GD35	GD24	*R.nitidus*	-0.051488	0.069562
JW7	GD35	GD24	*R.nitidus*	-0.001813	0.073325
JW6	GD35	GD24	*R.nitidus*	-0.053974	0.073249
SC26	GD35	GD24	*R.nitidus*	-0.069322	0.067772
CS31	GD35	GD24	*R.nitidus*	-0.064388	0.069326
SC45	GD35	GD24	*R.nitidus*	0.005228	0.067812
SC35	GD35	GD24	*R.nitidus*	0	0.06683
CS6	GD35	GD24	*R.nitidus*	-0.081269	0.06792
SC42	GD35	GD24	*R.nitidus*	0.227008	0.064746
JO8	GD40	GD24	*R.nitidus*	-0.066386	0.072901
SC31	GD35	GD24	*R.nitidus*	-0.040512	0.067545
SC44	GD35	GD24	*R.nitidus*	-0.023449	0.068062
CS3	GD35	GD24	*R.nitidus*	-0.051841	0.06681
MY6	GD35	GD24	*R.nitidus*	-0.054896	0.06696
JO7	GD40	GD24	*R.nitidus*	-0.103518	0.073362
SC5	GD35	GD24	*R.nitidus*	-0.02473	0.073093
SC18	GD35	GD24	*R.nitidus*	-0.003968	0.06714
JO5	GD35	GD24	*R.nitidus*	-0.007452	0.067179
CS11	GD40	GD24	*R.nitidus*	-0.070803	0.068061
GD29	GD40	GD24	*R.nitidus*	0.023364	0.063662
SC14	GD35	GD24	*R.nitidus*	-0.019564	0.067328
JO1	GD35	GD24	*R.nitidus*	-0.041636	0.066657
SC6	GD35	GD24	*R.nitidus*	-0.065169	0.067955
SC32	GD35	GD24	*R.nitidus*	0.000733	0.06665
JW4	GD35	GD24	*R.nitidus*	0.195122	0.066394
GD42	GD40	GD24	*R.nitidus*	-0.047393	0.066414
GD32	GD40	GD24	*R.nitidus*	-0.047535	0.066983
GD30	GD40	GD24	*R.nitidus*	0.049225	0.067606
GD50	GD40	GD24	*R.nitidus*	-0.043318	0.068909
GD22	GD40	GD24	*R.nitidus*	-0.012687	0.065196
GD35	GD40	GD24	*R.nitidus*	-0.026895	0.065631
JO3	GD35	GD24	*R.nitidus*	-0.02307	0.072804
GD25	GD40	GD24	*R.nitidus*	-0.011015	0.065189
CS24	GD40	GD24	*R.nitidus*	-0.127496	0.064497
GD21	GD40	GD24	*R.nitidus*	0.045079	0.066315
GD26	GD40	GD24	*R.nitidus*	-0.039937	0.064406
CS25	GD40	GD24	*R.nitidus*	-0.08817	0.066904
GD33	GD40	GD24	*R.nitidus*	-0.080619	0.066325
GD49	GD40	GD24	*R.nitidus*	0.035871	0.068969
GD46	GD40	GD24	*R.nitidus*	-0.096698	0.064943
CS13	GD40	GD24	*R.nitidus*	-0.107807	0.067931
SC18	GD40	GD24	*R.nitidus*	-0.028549	0.064186
GD31	GD40	GD24	*R.nitidus*	-0.020292	0.06718
CS23	GD40	GD24	*R.nitidus*	-0.126119	0.066299

CS18	GD40	GD24	*R.nitidus*	-0.133739	0.067044
GD45	GD40	GD24	*R.nitidus*	0.002591	0.068495
CS12	GD40	GD24	*R.nitidus*	-0.068356	0.067495
GD48	GD40	GD24	*R.nitidus*	-0.072727	0.067625
SC26	GD40	GD24	*R.nitidus*	-0.112491	0.065714
CS20	GD40	GD24	*R.nitidus*	-0.106095	0.066564
GD34	GD40	GD24	*R.nitidus*	-0.081633	0.065494
CS17	GD40	GD24	*R.nitidus*	-0.053417	0.069569
GD41	GD40	GD24	*R.nitidus*	-0.060109	0.064787
CS21	GD40	GD24	*R.nitidus*	-0.099636	0.067274
SC6	GD40	GD24	*R.nitidus*	-0.09692	0.067093
CS3	GD40	GD24	*R.nitidus*	-0.099693	0.066119
CS4	GD40	GD24	*R.nitidus*	-0.094923	0.065974
GD44	GD40	GD24	*R.nitidus*	-0.069594	0.065391
CS30	GD40	GD24	*R.nitidus*	-0.128399	0.065439
SC12	GD40	GD24	*R.nitidus*	-0.110549	0.065828
CS19	GD40	GD24	*R.nitidus*	-0.105828	0.068684
CS6	GD40	GD24	*R.nitidus*	-0.114996	0.066372
CS14	GD40	GD24	*R.nitidus*	-0.071481	0.07051
CS28	GD40	GD24	*R.nitidus*	-0.097276	0.066702
CS16	GD40	GD24	*R.nitidus*	-0.08768	0.067882
SC27	GD40	GD24	*R.nitidus*	-0.068966	0.06531
CS31	GD40	GD24	*R.nitidus*	-0.096386	0.068908
SC35	GD40	GD24	*R.nitidus*	-0.023538	0.064975
SC44	GD40	GD24	*R.nitidus*	-0.059091	0.065886
SC5	GD40	GD24	*R.nitidus*	-0.075166	0.068439
SC15	GD40	GD24	*R.nitidus*	-0.101208	0.066432
CS15	GD40	GD24	*R.nitidus*	-0.045157	0.068615
SC34	GD40	GD24	*R.nitidus*	-0.049012	0.064475
SC14	GD40	GD24	*R.nitidus*	-0.055637	0.065534
SC10	GD40	GD24	*R.nitidus*	-0.008949	0.068036
JO1	GD40	GD24	*R.nitidus*	-0.096536	0.067287
JW6	GD40	GD24	*R.nitidus*	-0.076172	0.072148
CS22	GD40	GD24	*R.nitidus*	-0.067301	0.065719
SC31	GD40	GD24	*R.nitidus*	-0.086402	0.066033
MY4	GD40	GD24	*R.nitidus*	-0.12675	0.065971
SC24	GD40	GD24	*R.nitidus*	-0.070015	0.066165
JW7	GD40	GD24	*R.nitidus*	-0.045534	0.070976
SC32	GD40	GD24	*R.nitidus*	-0.043353	0.066847
SC45	GD40	GD24	*R.nitidus*	-0.041298	0.064417
JO8	GD41	GD24	*R.nitidus*	-0.004251	0.075254
GD21	GD41	GD24	*R.nitidus*	0.112431	0.065372
JO7	GD41	GD24	*R.nitidus*	-0.039674	0.074453
CS11	GD41	GD24	*R.nitidus*	-0.014043	0.067445
SC28	GD40	GD24	*R.nitidus*	-0.093363	0.065083
JW4	GD40	GD24	*R.nitidus*	0.143279	0.06746
JO3	GD40	GD24	*R.nitidus*	-0.039655	0.070534
GD32	GD41	GD24	*R.nitidus*	0.036347	0.068668
GD35	GD41	GD24	*R.nitidus*	0.042219	0.066636
SC42	GD40	GD24	*R.nitidus*	0.189829	0.063867
GD30	GD41	GD24	*R.nitidus*	0.101382	0.068414
GD22	GD41	GD24	*R.nitidus*	0.073191	0.065085
MY6	GD40	GD24	*R.nitidus*	-0.093863	0.06519
JO5	GD40	GD24	*R.nitidus*	-0.029454	0.064704
CS13	GD41	GD24	*R.nitidus*	-0.04051	0.068815
CS17	GD41	GD24	*R.nitidus*	0.008416	0.070218

GD25	GD41	GD24	*R.nitidus*	0.041497	0.06635
GD26	GD41	GD24	*R.nitidus*	0.038111	0.065461
CS19	GD41	GD24	*R.nitidus*	-0.026647	0.069691
GD40	GD41	GD24	*R.nitidus*	0.060109	0.064787
CS12	GD41	GD24	*R.nitidus*	-0.006767	0.067308
GD50	GD41	GD24	*R.nitidus*	0.035332	0.070808
GD29	GD41	GD24	*R.nitidus*	0.086151	0.065613
GD44	GD41	GD24	*R.nitidus*	0.008795	0.06884
GD42	GD41	GD24	*R.nitidus*	0.034707	0.064625
GD49	GD41	GD24	*R.nitidus*	0.11803	0.069516
GD45	GD41	GD24	*R.nitidus*	0.058447	0.0702
GD31	GD41	GD24	*R.nitidus*	0.066046	0.066822
GD46	GD41	GD24	*R.nitidus*	-0.045167	0.065178
GD48	GD41	GD24	*R.nitidus*	0.015582	0.068511
CS15	GD41	GD24	*R.nitidus*	0.017088	0.067756
CS18	GD41	GD24	*R.nitidus*	-0.053623	0.067076
GD33	GD41	GD24	*R.nitidus*	-0.028333	0.06845
GD34	GD41	GD24	*R.nitidus*	-0.010283	0.066741
SC18	GD41	GD24	*R.nitidus*	0.0278	0.066475
CS20	GD41	GD24	*R.nitidus*	-0.044776	0.068891
SC12	GD41	GD24	*R.nitidus*	-0.032714	0.066737
CS4	GD41	GD24	*R.nitidus*	-0.025716	0.066034
CS22	GD41	GD24	*R.nitidus*	-0.008843	0.066863
SC5	GD41	GD24	*R.nitidus*	-0.008296	0.070015
CS21	GD41	GD24	*R.nitidus*	-0.036723	0.066579
CS30	GD41	GD24	*R.nitidus*	-0.078571	0.068372
SC35	GD41	GD24	*R.nitidus*	0.034125	0.067678
CS14	GD41	GD24	*R.nitidus*	-0.018532	0.070205
CS23	GD41	GD24	*R.nitidus*	-0.066187	0.066527
SC15	GD41	GD24	*R.nitidus*	-0.023622	0.066953
CS16	GD41	GD24	*R.nitidus*	-0.014782	0.065481
CS24	GD41	GD24	*R.nitidus*	-0.053241	0.066173
SC14	GD41	GD24	*R.nitidus*	0.002288	0.064869
SC10	GD41	GD24	*R.nitidus*	0.052672	0.067588
SC24	GD41	GD24	*R.nitidus*	-0.020737	0.065967
CS25	GD41	GD24	*R.nitidus*	-0.024	0.068273
SC34	GD41	GD24	*R.nitidus*	0.015707	0.065631
SC6	GD41	GD24	*R.nitidus*	-0.033208	0.069817
SC26	GD41	GD24	*R.nitidus*	-0.048673	0.067672
CS28	GD41	GD24	*R.nitidus*	-0.037037	0.067643
CS3	GD41	GD24	*R.nitidus*	-0.050983	0.067983
JO7	GD42	GD24	*R.nitidus*	-0.094241	0.074903
SC27	GD41	GD24	*R.nitidus*	-0.009245	0.065623
CS31	GD41	GD24	*R.nitidus*	-0.038217	0.066879
JO3	GD41	GD24	*R.nitidus*	0.011424	0.071458
MY4	GD41	GD24	*R.nitidus*	-0.05	0.064143
CS6	GD41	GD24	*R.nitidus*	-0.048611	0.067211
SC45	GD41	GD24	*R.nitidus*	0.021577	0.067602
SC31	GD41	GD24	*R.nitidus*	-0.017024	0.066271
SC44	GD41	GD24	*R.nitidus*	0.013772	0.065687
JO5	GD41	GD24	*R.nitidus*	0.04059	0.065745
GD32	GD42	GD24	*R.nitidus*	0	0.069533
JW7	GD41	GD24	*R.nitidus*	0.026802	0.071759
SC28	GD41	GD24	*R.nitidus*	-0.031418	0.067294
SC32	GD41	GD24	*R.nitidus*	0.025148	0.06567
GD30	GD42	GD24	*R.nitidus*	0.102041	0.068067

GD25	GD42	GD24	*R.nitidus*	0.022184	0.066488
SC42	GD41	GD24	*R.nitidus*	0.245921	0.063792
CS11	GD42	GD24	*R.nitidus*	-0.046477	0.068513
JO8	GD42	GD24	*R.nitidus*	-0.042463	0.074728
CS13	GD42	GD24	*R.nitidus*	-0.068524	0.068941
GD33	GD42	GD24	*R.nitidus*	-0.042517	0.069212
GD46	GD42	GD24	*R.nitidus*	-0.062784	0.064234
JO1	GD41	GD24	*R.nitidus*	-0.018797	0.065257
JW6	GD41	GD24	*R.nitidus*	-0.025126	0.072404
MY6	GD41	GD24	*R.nitidus*	-0.022075	0.064577
GD49	GD42	GD24	*R.nitidus*	0.097421	0.069333
GD45	GD42	GD24	*R.nitidus*	0.047125	0.06971
JW4	GD41	GD24	*R.nitidus*	0.214176	0.065438
GD22	GD42	GD24	*R.nitidus*	0.057596	0.065724
GD41	GD42	GD24	*R.nitidus*	-0.034707	0.064625
GD26	GD42	GD24	*R.nitidus*	-0.011102	0.067583
CS14	GD42	GD24	*R.nitidus*	-0.052513	0.069808
GD35	GD42	GD24	*R.nitidus*	0.015	0.066511
GD34	GD42	GD24	*R.nitidus*	-0.026132	0.066473
GD44	GD42	GD24	*R.nitidus*	-0.021016	0.067968
GD48	GD42	GD24	*R.nitidus*	-0.030132	0.067975
CS16	GD42	GD24	*R.nitidus*	-0.049327	0.066403
GD29	GD42	GD24	*R.nitidus*	0.073469	0.06544
GD31	GD42	GD24	*R.nitidus*	0.029668	0.067324
CS21	GD42	GD24	*R.nitidus*	-0.066287	0.066768
GD21	GD42	GD24	*R.nitidus*	0.088163	0.066704
CS25	GD42	GD24	*R.nitidus*	-0.051013	0.068412
GD40	GD42	GD24	*R.nitidus*	0.047393	0.066414
CS15	GD42	GD24	*R.nitidus*	-0.015129	0.069672
CS12	GD42	GD24	*R.nitidus*	-0.031783	0.068584
SC5	GD42	GD24	*R.nitidus*	-0.03099	0.069461
SC12	GD42	GD24	*R.nitidus*	-0.055762	0.065639
CS20	GD42	GD24	*R.nitidus*	-0.059701	0.067691
CS19	GD42	GD24	*R.nitidus*	-0.053707	0.068046
CS23	GD42	GD24	*R.nitidus*	-0.08966	0.066425
SC6	GD42	GD24	*R.nitidus*	-0.051685	0.069305
GD50	GD42	GD24	*R.nitidus*	-0.01741	0.070592
CS3	GD42	GD24	*R.nitidus*	-0.065393	0.067276
CS30	GD42	GD24	*R.nitidus*	-0.094519	0.065346
SC10	GD42	GD24	*R.nitidus*	0.021522	0.066341
SC24	GD42	GD24	*R.nitidus*	-0.038491	0.066595
CS24	GD42	GD24	*R.nitidus*	-0.079727	0.066324
SC45	GD42	GD24	*R.nitidus*	0.008929	0.066861
CS17	GD42	GD24	*R.nitidus*	-0.022568	0.069356
CS22	GD42	GD24	*R.nitidus*	-0.035871	0.066609
CS18	GD42	GD24	*R.nitidus*	-0.082793	0.066791
CS4	GD42	GD24	*R.nitidus*	-0.041999	0.065687
JW6	GD42	GD24	*R.nitidus*	-0.061866	0.072271
SC15	GD42	GD24	*R.nitidus*	-0.062745	0.068318
SC28	GD42	GD24	*R.nitidus*	-0.053933	0.066373
MY6	GD42	GD24	*R.nitidus*	-0.044886	0.067174
CS28	GD42	GD24	*R.nitidus*	-0.064464	0.068265
SC34	GD42	GD24	*R.nitidus*	-0.002959	0.065716
JO3	GD42	GD24	*R.nitidus*	-0.01982	0.071764
CS31	GD42	GD24	*R.nitidus*	-0.055466	0.066406
SC32	GD42	GD24	*R.nitidus*	0.006662	0.065382

SC18	GD42	GD24	*R.nitidus*	-0.000782	0.066755
JW4	GD42	GD24	*R.nitidus*	0.180924	0.066292
JW7	GD42	GD24	*R.nitidus*	-0.03352	0.070935
JO7	GD44	GD24	*R.nitidus*	-0.046218	0.072628
JO5	GD42	GD24	*R.nitidus*	0.014383	0.066953
SC26	GD42	GD24	*R.nitidus*	-0.068264	0.068192
SC35	GD42	GD24	*R.nitidus*	0.009482	0.06624
JO8	GD44	GD24	*R.nitidus*	-0.016234	0.075211
JO1	GD42	GD24	*R.nitidus*	-0.039493	0.066698
MY4	GD42	GD24	*R.nitidus*	-0.068807	0.066289
SC14	GD42	GD24	*R.nitidus*	-0.012782	0.065508
SC27	GD42	GD24	*R.nitidus*	-0.026094	0.066169
SC31	GD42	GD24	*R.nitidus*	-0.042661	0.066644
GD30	GD44	GD24	*R.nitidus*	0.098953	0.069825
GD21	GD44	GD24	*R.nitidus*	0.118699	0.065803
SC42	GD42	GD24	*R.nitidus*	0.238582	0.06297
CS6	GD42	GD24	*R.nitidus*	-0.073038	0.065908
GD25	GD44	GD24	*R.nitidus*	0.034599	0.067921
GD33	GD44	GD24	*R.nitidus*	-0.023392	0.068265
SC44	GD42	GD24	*R.nitidus*	-0.014096	0.067166
GD49	GD44	GD24	*R.nitidus*	0.096386	0.070703
GD31	GD44	GD24	*R.nitidus*	0.05427	0.069723
GD29	GD44	GD24	*R.nitidus*	0.092622	0.06785
GD50	GD44	GD24	*R.nitidus*	0.016016	0.068671
GD26	GD44	GD24	*R.nitidus*	0.013228	0.0672
GD32	GD44	GD24	*R.nitidus*	-0.006705	0.069803
CS11	GD44	GD24	*R.nitidus*	-0.018141	0.071545
GD35	GD44	GD24	*R.nitidus*	0.029412	0.068941
CS21	GD44	GD24	*R.nitidus*	-0.041889	0.067654
GD42	GD44	GD24	*R.nitidus*	0.021016	0.067968
GD22	GD44	GD24	*R.nitidus*	0.044935	0.067414
GD45	GD44	GD24	*R.nitidus*	0.05848	0.071277
CS14	GD44	GD24	*R.nitidus*	-0.026944	0.072055
CS13	GD44	GD24	*R.nitidus*	-0.070866	0.070688
CS18	GD44	GD24	*R.nitidus*	-0.07619	0.068162
CS12	GD44	GD24	*R.nitidus*	-0.026549	0.070253
CS19	GD44	GD24	*R.nitidus*	-0.053746	0.069146
CS28	GD44	GD24	*R.nitidus*	-0.052326	0.069636
GD41	GD44	GD24	*R.nitidus*	-0.008795	0.06884
CS24	GD44	GD24	*R.nitidus*	-0.067755	0.068495
GD34	GD44	GD24	*R.nitidus*	-0.012626	0.069279
CS23	GD44	GD24	*R.nitidus*	-0.076563	0.066698
GD40	GD44	GD24	*R.nitidus*	0.069594	0.065391
SC10	GD44	GD24	*R.nitidus*	0.035357	0.068743
SC14	GD44	GD24	*R.nitidus*	-0.011962	0.068939
CS30	GD44	GD24	*R.nitidus*	-0.06988	0.069678
CS22	GD44	GD24	*R.nitidus*	-0.028022	0.068695
GD48	GD44	GD24	*R.nitidus*	-0.005602	0.069441
GD46	GD44	GD24	*R.nitidus*	-0.040816	0.067201
CS31	GD44	GD24	*R.nitidus*	-0.052109	0.068904
CS20	GD44	GD24	*R.nitidus*	-0.050623	0.068872
SC5	GD44	GD24	*R.nitidus*	-0.010535	0.071394
SC27	GD44	GD24	*R.nitidus*	-0.01201	0.068899
CS17	GD44	GD24	*R.nitidus*	0.002408	0.073224
CS3	GD44	GD24	*R.nitidus*	-0.043991	0.067445
CS15	GD44	GD24	*R.nitidus*	0.003814	0.069578

CS4	GD44	GD24	*R.nitidus*	-0.047356	0.066868
SC15	GD44	GD24	*R.nitidus*	-0.022783	0.068774
SC24	GD44	GD24	*R.nitidus*	-0.032232	0.069177
SC6	GD44	GD24	*R.nitidus*	-0.030645	0.068857
CS16	GD44	GD24	*R.nitidus*	-0.02093	0.067577
JW7	GD44	GD24	*R.nitidus*	0.003854	0.07288
SC18	GD44	GD24	*R.nitidus*	0.006645	0.068056
SC26	GD44	GD24	*R.nitidus*	-0.057471	0.067855
JW4	GD44	GD24	*R.nitidus*	0.18328	0.069745
CS25	GD44	GD24	*R.nitidus*	-0.054927	0.070536
MY4	GD44	GD24	*R.nitidus*	-0.067141	0.068169
SC31	GD44	GD24	*R.nitidus*	-0.024176	0.067104
SC42	GD44	GD24	*R.nitidus*	0.233458	0.065789
SC35	GD44	GD24	*R.nitidus*	0.028832	0.067875
SC12	GD44	GD24	*R.nitidus*	-0.059181	0.068528
SC44	GD44	GD24	*R.nitidus*	-0.000801	0.068732
GD25	GD45	GD24	*R.nitidus*	-0.042266	0.071674
CS6	GD44	GD24	*R.nitidus*	-0.063025	0.068876
SC34	GD44	GD24	*R.nitidus*	0.013804	0.067915
JO7	GD45	GD24	*R.nitidus*	-0.134293	0.077181
JO1	GD44	GD24	*R.nitidus*	-0.028997	0.067737
SC28	GD44	GD24	*R.nitidus*	-0.049089	0.067957
GD31	GD45	GD24	*R.nitidus*	0.005634	0.071211
GD26	GD45	GD24	*R.nitidus*	-0.041188	0.070483
JW6	GD44	GD24	*R.nitidus*	-0.028986	0.073215
GD21	GD45	GD24	*R.nitidus*	0.052632	0.069876
GD50	GD45	GD24	*R.nitidus*	-0.030238	0.075349
CS11	GD45	GD24	*R.nitidus*	-0.081444	0.072734
GD34	GD45	GD24	*R.nitidus*	-0.076572	0.069712
GD29	GD45	GD24	*R.nitidus*	0.011722	0.068864
JO8	GD45	GD24	*R.nitidus*	-0.067248	0.07849
SC32	GD44	GD24	*R.nitidus*	0.025424	0.068248
JO3	GD44	GD24	*R.nitidus*	-0.01086	0.072864
CS16	GD45	GD24	*R.nitidus*	-0.081287	0.073105
SC45	GD44	GD24	*R.nitidus*	0.017309	0.06789
GD40	GD45	GD24	*R.nitidus*	-0.002591	0.068495
CS18	GD45	GD24	*R.nitidus*	-0.111392	0.070468
CS13	GD45	GD24	*R.nitidus*	-0.098207	0.072439
GD33	GD45	GD24	*R.nitidus*	-0.085661	0.070374
GD44	GD45	GD24	*R.nitidus*	-0.05848	0.071277
CS12	GD45	GD24	*R.nitidus*	-0.066838	0.071908
CS19	GD45	GD24	*R.nitidus*	-0.094128	0.073066
JO5	GD44	GD24	*R.nitidus*	0.028549	0.067486
GD42	GD45	GD24	*R.nitidus*	-0.047125	0.06971
MY6	GD44	GD24	*R.nitidus*	-0.041379	0.067875
GD30	GD45	GD24	*R.nitidus*	0.031873	0.070229
GD46	GD45	GD24	*R.nitidus*	-0.10942	0.069071
CS20	GD45	GD24	*R.nitidus*	-0.088481	0.072212
CS24	GD45	GD24	*R.nitidus*	-0.09792	0.070478
GD22	GD45	GD24	*R.nitidus*	0.013146	0.070044
CS14	GD45	GD24	*R.nitidus*	-0.067708	0.075567
GD32	GD45	GD24	*R.nitidus*	-0.05235	0.073621
CS15	GD45	GD24	*R.nitidus*	-0.049138	0.073489
SC10	GD45	GD24	*R.nitidus*	0.008518	0.072779
CS4	GD45	GD24	*R.nitidus*	-0.086921	0.068857
GD49	GD45	GD24	*R.nitidus*	0.050256	0.073866

CS23	GD45	GD24	*R.nitidus*	-0.106557	0.074004
GD35	GD45	GD24	*R.nitidus*	-0.04479	0.069013
GD41	GD45	GD24	*R.nitidus*	-0.058447	0.0702
SC18	GD45	GD24	*R.nitidus*	-0.007895	0.071252
SC24	GD45	GD24	*R.nitidus*	-0.048454	0.072389
GD48	GD45	GD24	*R.nitidus*	-0.058462	0.073044
CS21	GD45	GD24	*R.nitidus*	-0.094449	0.070505
CS17	GD45	GD24	*R.nitidus*	-0.056687	0.073587
CS30	GD45	GD24	*R.nitidus*	-0.112875	0.070702
SC6	GD45	GD24	*R.nitidus*	-0.087719	0.073136
SC5	GD45	GD24	*R.nitidus*	-0.066285	0.07388
SC44	GD45	GD24	*R.nitidus*	-0.028404	0.068821
SC14	GD45	GD24	*R.nitidus*	-0.037643	0.071966
CS22	GD45	GD24	*R.nitidus*	-0.061909	0.070939
SC26	GD45	GD24	*R.nitidus*	-0.086124	0.069029
CS25	GD45	GD24	*R.nitidus*	-0.087598	0.07383
SC45	GD45	GD24	*R.nitidus*	-0.026205	0.06889
CS28	GD45	GD24	*R.nitidus*	-0.099647	0.069171
SC32	GD45	GD24	*R.nitidus*	-0.00995	0.071103
CS31	GD45	GD24	*R.nitidus*	-0.115591	0.072202
SC34	GD45	GD24	*R.nitidus*	-0.023392	0.069385
SC28	GD45	GD24	*R.nitidus*	-0.086627	0.067883
JW4	GD45	GD24	*R.nitidus*	0.149435	0.071184
JW6	GD45	GD24	*R.nitidus*	-0.109756	0.076089
JW7	GD45	GD24	*R.nitidus*	-0.049345	0.073936
SC12	GD45	GD24	*R.nitidus*	-0.084202	0.071384
MY4	GD45	GD24	*R.nitidus*	-0.086634	0.071222
JO7	CS13	GD24	*R.nitidus*	0.011853	0.076617
SC42	GD45	GD24	*R.nitidus*	0.198337	0.067145
CS3	GD45	GD24	*R.nitidus*	-0.08557	0.070806
JO5	GD45	GD24	*R.nitidus*	-0.022034	0.068603
JO1	GD45	GD24	*R.nitidus*	-0.061439	0.070204
SC31	GD45	GD24	*R.nitidus*	-0.052464	0.071055
MY6	GD45	GD24	*R.nitidus*	-0.05848	0.069828
SC15	GD45	GD24	*R.nitidus*	-0.06955	0.071849
JO3	GD45	GD24	*R.nitidus*	-0.052052	0.075322
CS6	GD45	GD24	*R.nitidus*	-0.077413	0.071869
GD29	CS13	GD24	*R.nitidus*	0.121969	0.067612
SC35	GD45	GD24	*R.nitidus*	-0.019753	0.071746
GD25	CS13	GD24	*R.nitidus*	0.093385	0.069316
GD30	CS13	GD24	*R.nitidus*	0.140474	0.070734
GD32	CS13	GD24	*R.nitidus*	0.041073	0.071198
GD45	CS13	GD24	*R.nitidus*	0.098207	0.072439
GD40	CS13	GD24	*R.nitidus*	0.107807	0.067931
GD31	CS13	GD24	*R.nitidus*	0.103611	0.069208
SC27	GD45	GD24	*R.nitidus*	-0.043874	0.070143
JO8	CS13	GD24	*R.nitidus*	0.021421	0.076716
GD26	CS13	GD24	*R.nitidus*	0.069121	0.069148
GD50	CS13	GD24	*R.nitidus*	0.066298	0.074308
GD46	CS13	GD24	*R.nitidus*	0.028853	0.069929
GD48	CS13	GD24	*R.nitidus*	0.044971	0.072164
GD44	CS13	GD24	*R.nitidus*	0.070866	0.070688
GD22	CS13	GD24	*R.nitidus*	0.09849	0.069632
GD21	CS13	GD24	*R.nitidus*	0.129935	0.067511
GD42	CS13	GD24	*R.nitidus*	0.068524	0.068941
GD49	CS13	GD24	*R.nitidus*	0.133906	0.070897

CS11	CS13	GD24	*R.nitidus*	0.050171	0.071516
GD33	CS13	GD24	*R.nitidus*	0.028107	0.069204
GD41	CS13	GD24	*R.nitidus*	0.04051	0.068815
CS12	CS13	GD24	*R.nitidus*	0.059801	0.071244
GD35	CS13	GD24	*R.nitidus*	0.064199	0.069878
CS3	CS13	GD24	*R.nitidus*	0.010518	0.069463
GD34	CS13	GD24	*R.nitidus*	0.031723	0.068853
CS20	CS13	GD24	*R.nitidus*	0	0.07051
CS16	CS13	GD24	*R.nitidus*	0.044517	0.07087
CS28	CS13	GD24	*R.nitidus*	0.017467	0.07072
CS15	CS13	GD24	*R.nitidus*	0.091422	0.070806
SC6	CS13	GD24	*R.nitidus*	0.014008	0.07199
CS17	CS13	GD24	*R.nitidus*	0.071676	0.072519
CS24	CS13	GD24	*R.nitidus*	-0.014808	0.069251
SC14	CS13	GD24	*R.nitidus*	0.053125	0.067039
SC18	CS13	GD24	*R.nitidus*	0.079936	0.071168
CS23	CS13	GD24	*R.nitidus*	-0.02558	0.069208
CS4	CS13	GD24	*R.nitidus*	0.027244	0.068425
CS22	CS13	GD24	*R.nitidus*	0.057028	0.069869
SC34	CS13	GD24	*R.nitidus*	0.055732	0.069781
SC15	CS13	GD24	*R.nitidus*	0.016835	0.070372
CS30	CS13	GD24	*R.nitidus*	-0.03336	0.069559
CS31	CS13	GD24	*R.nitidus*	0.015544	0.071394
CS18	CS13	GD24	*R.nitidus*	-0.022388	0.071199
CS21	CS13	GD24	*R.nitidus*	0.008661	0.069143
SC5	CS13	GD24	*R.nitidus*	0.033858	0.071999
CS14	CS13	GD24	*R.nitidus*	0.044881	0.072333
SC35	CS13	GD24	*R.nitidus*	0.078522	0.068572
SC31	CS13	GD24	*R.nitidus*	0.026563	0.069179
SC44	CS13	GD24	*R.nitidus*	0.044953	0.068416
CS19	CS13	GD24	*R.nitidus*	0.011092	0.073383
CS25	CS13	GD24	*R.nitidus*	0.014718	0.071781
SC10	CS13	GD24	*R.nitidus*	0.088997	0.069617
SC32	CS13	GD24	*R.nitidus*	0.076675	0.069645
SC12	CS13	GD24	*R.nitidus*	0.012658	0.069479
JW6	CS13	GD24	*R.nitidus*	0.005258	0.075933
CS6	CS13	GD24	*R.nitidus*	-0.008467	0.06985
SC45	CS13	GD24	*R.nitidus*	0.052504	0.071861
SC26	CS13	GD24	*R.nitidus*	-0.010383	0.069022
JO3	CS13	GD24	*R.nitidus*	0.036738	0.075747
JW4	CS13	GD24	*R.nitidus*	0.244833	0.06748
JO7	GD46	GD24	*R.nitidus*	-0.002043	0.07461
SC27	CS13	GD24	*R.nitidus*	0.04964	0.068508
SC28	CS13	GD24	*R.nitidus*	0.0064	0.06802
GD40	GD46	GD24	*R.nitidus*	0.096698	0.064943
SC42	CS13	GD24	*R.nitidus*	0.282528	0.064822
JO8	GD46	GD24	*R.nitidus*	0.068777	0.072502
SC24	CS13	GD24	*R.nitidus*	0.037855	0.069948
JO5	CS13	GD24	*R.nitidus*	0.087094	0.068197
GD29	GD46	GD24	*R.nitidus*	0.127768	0.066952
GD30	GD46	GD24	*R.nitidus*	0.159541	0.067093
GD33	GD46	GD24	*R.nitidus*	0.016779	0.066802
JW7	CS13	GD24	*R.nitidus*	0.07483	0.07397
GD35	GD46	GD24	*R.nitidus*	0.074879	0.066348
JO1	CS13	GD24	*R.nitidus*	0.029435	0.068585
CS17	GD46	GD24	*R.nitidus*	0.038583	0.070417

MY4	CS13	GD24	*R.nitidus*	-0.010612	0.069571
GD42	GD46	GD24	*R.nitidus*	0.062784	0.064234
GD44	GD46	GD24	*R.nitidus*	0.040816	0.067201
CS12	GD46	GD24	*R.nitidus*	0.011924	0.067843
CS13	GD46	GD24	*R.nitidus*	-0.028853	0.069929
GD21	GD46	GD24	*R.nitidus*	0.163462	0.064302
GD22	GD46	GD24	*R.nitidus*	0.099839	0.063363
GD31	GD46	GD24	*R.nitidus*	0.084237	0.066277
GD34	GD46	GD24	*R.nitidus*	0.024514	0.065131
GD41	GD46	GD24	*R.nitidus*	0.045167	0.065178
CS25	GD46	GD24	*R.nitidus*	-0.001486	0.069935
GD50	GD46	GD24	*R.nitidus*	0.055276	0.070164
GD45	GD46	GD24	*R.nitidus*	0.10942	0.069071
CS18	GD46	GD24	*R.nitidus*	-0.040801	0.066915
CS11	GD46	GD24	*R.nitidus*	0.007496	0.069281
MY6	CS13	GD24	*R.nitidus*	0.018153	0.069587
GD25	GD46	GD24	*R.nitidus*	0.078818	0.066893
GD26	GD46	GD24	*R.nitidus*	0.067048	0.064182
CS23	GD46	GD24	*R.nitidus*	-0.034875	0.065988
GD32	GD46	GD24	*R.nitidus*	0.059798	0.065801
CS24	GD46	GD24	*R.nitidus*	-0.026738	0.066569
CS14	GD46	GD24	*R.nitidus*	0	0.072126
CS28	GD46	GD24	*R.nitidus*	-0.011924	0.067143
SC18	GD46	GD24	*R.nitidus*	0.070983	0.064627
GD49	GD46	GD24	*R.nitidus*	0.128694	0.06801
CS20	GD46	GD24	*R.nitidus*	-0.017429	0.067511
GD48	GD46	GD24	*R.nitidus*	0.05717	0.065971
SC10	GD46	GD24	*R.nitidus*	0.066814	0.065676
CS16	GD46	GD24	*R.nitidus*	0.005368	0.068961
CS30	GD46	GD24	*R.nitidus*	-0.039872	0.066856
CS22	GD46	GD24	*R.nitidus*	0.017306	0.066059
CS15	GD46	GD24	*R.nitidus*	0.031988	0.070512
SC28	GD46	GD24	*R.nitidus*	-0.010264	0.065743
CS21	GD46	GD24	*R.nitidus*	-0.013758	0.067348
CS19	GD46	GD24	*R.nitidus*	-0.016579	0.069118
CS6	GD46	GD24	*R.nitidus*	-0.018692	0.067246
CS3	GD46	GD24	*R.nitidus*	-0.007479	0.067745
SC35	GD46	GD24	*R.nitidus*	0.063846	0.065898
SC34	GD46	GD24	*R.nitidus*	0.040293	0.065038
SC12	GD46	GD24	*R.nitidus*	-0.019461	0.06671
SC26	GD46	GD24	*R.nitidus*	-0.018396	0.066252
JW4	GD46	GD24	*R.nitidus*	0.236576	0.066818
SC27	GD46	GD24	*R.nitidus*	0.02069	0.066269
SC42	GD46	GD24	*R.nitidus*	0.259088	0.062264
JO8	GD48	GD24	*R.nitidus*	0.00663	0.07138
CS31	GD46	GD24	*R.nitidus*	-0.009788	0.067749
SC6	GD46	GD24	*R.nitidus*	-0.003846	0.069349
SC44	GD46	GD24	*R.nitidus*	0.033758	0.066131
SC31	GD46	GD24	*R.nitidus*	0.008386	0.065613
JO5	GD46	GD24	*R.nitidus*	0.05267	0.065179
SC24	GD46	GD24	*R.nitidus*	0.015385	0.065793
CS11	GD48	GD24	*R.nitidus*	-0.00793	0.071038
JO3	GD46	GD24	*R.nitidus*	0.035746	0.070973
SC5	GD46	GD24	*R.nitidus*	0.017126	0.06956
SC45	GD46	GD24	*R.nitidus*	0.050847	0.067848
JO1	GD46	GD24	*R.nitidus*	-0.005935	0.06463

SC32	GD46	GD24	*R.nitidus*	0.051187	0.06729
CS4	GD46	GD24	*R.nitidus*	0.002175	0.065376
SC14	GD46	GD24	*R.nitidus*	0.037258	0.066798
JW6	GD46	GD24	*R.nitidus*	0.022	0.072498
MY6	GD46	GD24	*R.nitidus*	0.002193	0.066684
SC15	GD46	GD24	*R.nitidus*	-0.003145	0.068011
JO7	GD48	GD24	*R.nitidus*	-0.030303	0.074128
GD40	GD48	GD24	*R.nitidus*	0.072727	0.067625
GD32	GD48	GD24	*R.nitidus*	0.007099	0.066027
JW7	GD46	GD24	*R.nitidus*	0.051235	0.070628
GD25	GD48	GD24	*R.nitidus*	0.034451	0.067599
GD41	GD48	GD24	*R.nitidus*	-0.015582	0.068511
GD31	GD48	GD24	*R.nitidus*	0.060115	0.069076
GD26	GD48	GD24	*R.nitidus*	0.04265	0.067671
GD44	GD48	GD24	*R.nitidus*	0.005602	0.069441
GD35	GD48	GD24	*R.nitidus*	0.049819	0.070573
GD30	GD48	GD24	*R.nitidus*	0.079002	0.069344
MY4	GD46	GD24	*R.nitidus*	-0.023449	0.066778
GD22	GD48	GD24	*R.nitidus*	0.077324	0.066803
GD21	GD48	GD24	*R.nitidus*	0.120107	0.067719
CS17	GD48	GD24	*R.nitidus*	0.009362	0.072701
GD46	GD48	GD24	*R.nitidus*	-0.05717	0.065971
CS13	GD48	GD24	*R.nitidus*	-0.044971	0.072164
CS18	GD48	GD24	*R.nitidus*	-0.053989	0.069507
GD29	GD48	GD24	*R.nitidus*	0.090345	0.065008
CS31	GD48	GD24	*R.nitidus*	-0.030691	0.069994
CS20	GD48	GD24	*R.nitidus*	-0.030844	0.069109
CS12	GD48	GD24	*R.nitidus*	-0.009275	0.068946
CS22	GD48	GD24	*R.nitidus*	-0.000816	0.069135
GD34	GD48	GD24	*R.nitidus*	-0.011538	0.06632
GD33	GD48	GD24	*R.nitidus*	-0.015399	0.06767
GD42	GD48	GD24	*R.nitidus*	0.030132	0.067975
CS25	GD48	GD24	*R.nitidus*	-0.02294	0.071528
GD45	GD48	GD24	*R.nitidus*	0.058462	0.073044
CS23	GD48	GD24	*R.nitidus*	-0.065131	0.068682
CS19	GD48	GD24	*R.nitidus*	-0.033079	0.070703
CS21	GD48	GD24	*R.nitidus*	-0.040816	0.069757
SC5	GD48	GD24	*R.nitidus*	0.031561	0.071859
CS16	GD48	GD24	*R.nitidus*	-0.025703	0.068069
CS30	GD48	GD24	*R.nitidus*	-0.059177	0.069274
GD49	GD48	GD24	*R.nitidus*	0.100101	0.072211
GD50	GD48	GD24	*R.nitidus*	0.011677	0.073091
CS4	GD48	GD24	*R.nitidus*	-0.003987	0.067573
CS15	GD48	GD24	*R.nitidus*	0.012048	0.070962
CS3	GD48	GD24	*R.nitidus*	-0.034426	0.070038
CS28	GD48	GD24	*R.nitidus*	-0.048969	0.069784
CS14	GD48	GD24	*R.nitidus*	-0.016327	0.072253
SC28	GD48	GD24	*R.nitidus*	-0.019841	0.068153
SC14	GD48	GD24	*R.nitidus*	0.011914	0.069382
SC12	GD48	GD24	*R.nitidus*	-0.0375	0.069056
SC35	GD48	GD24	*R.nitidus*	0.047619	0.067642
SC15	GD48	GD24	*R.nitidus*	-0.028814	0.069853
SC44	GD48	GD24	*R.nitidus*	0.020576	0.067559
SC32	GD48	GD24	*R.nitidus*	0.018669	0.068167
CS24	GD48	GD24	*R.nitidus*	-0.033058	0.068716
MY4	GD48	GD24	*R.nitidus*	-0.046645	0.067751

SC10	GD48	GD24	*R.nitidus*	0.055961	0.069635
SC6	GD48	GD24	*R.nitidus*	-0.035275	0.069948
SC24	GD48	GD24	*R.nitidus*	0.011785	0.06956
JO3	GD48	GD24	*R.nitidus*	0.006629	0.072772
JW4	GD48	GD24	*R.nitidus*	0.205578	0.067712
SC26	GD48	GD24	*R.nitidus*	-0.05	0.068539
JW6	GD48	GD24	*R.nitidus*	0.005176	0.072204
CS11	GD49	GD24	*R.nitidus*	-0.109836	0.071928
CS6	GD48	GD24	*R.nitidus*	-0.048454	0.068998
SC18	GD48	GD24	*R.nitidus*	0.05675	0.070658
SC34	GD48	GD24	*R.nitidus*	0.012851	0.069144
GD30	GD49	GD24	*R.nitidus*	0.039195	0.072831
SC27	GD48	GD24	*R.nitidus*	0.009804	0.067853
MY6	GD48	GD24	*R.nitidus*	-0.017927	0.067985
JO8	GD49	GD24	*R.nitidus*	-0.092683	0.074404
SC42	GD48	GD24	*R.nitidus*	0.222868	0.065221
GD25	GD49	GD24	*R.nitidus*	-0.047798	0.069843
GD29	GD49	GD24	*R.nitidus*	-0.005671	0.069311
SC31	GD48	GD24	*R.nitidus*	-0.013312	0.069523
SC45	GD48	GD24	*R.nitidus*	0.018268	0.066672
JW7	GD48	GD24	*R.nitidus*	0.042654	0.072326
GD21	GD49	GD24	*R.nitidus*	0.012797	0.068221
JO7	GD49	GD24	*R.nitidus*	-0.139089	0.07424
GD34	GD49	GD24	*R.nitidus*	-0.118611	0.069713
GD33	GD49	GD24	*R.nitidus*	-0.120272	0.071554
JO1	GD48	GD24	*R.nitidus*	-0.008793	0.068716
CS12	GD49	GD24	*R.nitidus*	-0.10453	0.070261
GD32	GD49	GD24	*R.nitidus*	-0.096559	0.072153
GD22	GD49	GD24	*R.nitidus*	-0.064278	0.071796
GD35	GD49	GD24	*R.nitidus*	-0.070671	0.070708
JO5	GD48	GD24	*R.nitidus*	0.047773	0.069312
GD46	GD49	GD24	*R.nitidus*	-0.128694	0.06801
GD41	GD49	GD24	*R.nitidus*	-0.11803	0.069516
GD40	GD49	GD24	*R.nitidus*	-0.035871	0.068969
GD26	GD49	GD24	*R.nitidus*	-0.068008	0.06924
GD45	GD49	GD24	*R.nitidus*	-0.050256	0.073866
CS24	GD49	GD24	*R.nitidus*	-0.132253	0.070422
GD31	GD49	GD24	*R.nitidus*	-0.047529	0.07105
GD42	GD49	GD24	*R.nitidus*	-0.097421	0.069333
CS13	GD49	GD24	*R.nitidus*	-0.133906	0.070897
CS14	GD49	GD24	*R.nitidus*	-0.110831	0.073902
GD48	GD49	GD24	*R.nitidus*	-0.100101	0.072211
CS20	GD49	GD24	*R.nitidus*	-0.144381	0.069153
CS21	GD49	GD24	*R.nitidus*	-0.135792	0.068673
GD50	GD49	GD24	*R.nitidus*	-0.054084	0.076386
CS16	GD49	GD24	*R.nitidus*	-0.109258	0.068667
CS25	GD49	GD24	*R.nitidus*	-0.123288	0.072167
SC6	GD49	GD24	*R.nitidus*	-0.11863	0.070881
CS19	GD49	GD24	*R.nitidus*	-0.108202	0.070576
SC10	GD49	GD24	*R.nitidus*	-0.04876	0.070576
GD44	GD49	GD24	*R.nitidus*	-0.096386	0.070703
CS31	GD49	GD24	*R.nitidus*	-0.133741	0.070929
CS18	GD49	GD24	*R.nitidus*	-0.143093	0.070115
SC5	GD49	GD24	*R.nitidus*	-0.096639	0.071639
CS15	GD49	GD24	*R.nitidus*	-0.08	0.07155
CS28	GD49	GD24	*R.nitidus*	-0.130245	0.07038

CS3	GD49	GD24	*R.nitidus*	-0.133951	0.069022
CS23	GD49	GD24	*R.nitidus*	-0.149832	0.069412
SC27	GD49	GD24	*R.nitidus*	-0.087531	0.069301
CS4	GD49	GD24	*R.nitidus*	-0.111294	0.067959
CS30	GD49	GD24	*R.nitidus*	-0.149293	0.069692
JW4	GD49	GD24	*R.nitidus*	0.132275	0.07179
CS6	GD49	GD24	*R.nitidus*	-0.123793	0.068429
CS17	GD49	GD24	*R.nitidus*	-0.070009	0.071847
JO7	GD50	GD24	*R.nitidus*	-0.07931	0.076586
JO1	GD49	GD24	*R.nitidus*	-0.101307	0.068778
CS22	GD49	GD24	*R.nitidus*	-0.091213	0.068903
SC32	GD49	GD24	*R.nitidus*	-0.077551	0.067737
SC35	GD49	GD24	*R.nitidus*	-0.042079	0.06836
SC45	GD49	GD24	*R.nitidus*	-0.065756	0.069213
SC12	GD49	GD24	*R.nitidus*	-0.11474	0.068052
SC14	GD49	GD24	*R.nitidus*	-0.080882	0.069829
SC31	GD49	GD24	*R.nitidus*	-0.127153	0.069999
SC15	GD49	GD24	*R.nitidus*	-0.13198	0.067735
MY6	GD49	GD24	*R.nitidus*	-0.117162	0.0692
SC18	GD49	GD24	*R.nitidus*	-0.060198	0.070195
CS11	GD50	GD24	*R.nitidus*	-0.017889	0.073281
JW6	GD49	GD24	*R.nitidus*	-0.108597	0.072032
MY4	GD49	GD24	*R.nitidus*	-0.126149	0.069719
SC24	GD49	GD24	*R.nitidus*	-0.127011	0.070509
SC26	GD49	GD24	*R.nitidus*	-0.142145	0.070224
SC34	GD49	GD24	*R.nitidus*	-0.065789	0.067275
SC28	GD49	GD24	*R.nitidus*	-0.118699	0.067275
JW7	GD49	GD24	*R.nitidus*	-0.047619	0.073561
GD45	GD50	GD24	*R.nitidus*	0.030238	0.075349
GD26	GD50	GD24	*R.nitidus*	-0.009036	0.069906
JO5	GD49	GD24	*R.nitidus*	-0.071901	0.068881
SC42	GD49	GD24	*R.nitidus*	0.175694	0.065478
JO8	GD50	GD24	*R.nitidus*	-0.02657	0.076631
GD25	GD50	GD24	*R.nitidus*	-0.017034	0.069628
GD34	GD50	GD24	*R.nitidus*	-0.029297	0.071284
SC44	GD49	GD24	*R.nitidus*	-0.080617	0.068016
GD32	GD50	GD24	*R.nitidus*	-0.029933	0.073063
JO3	GD49	GD24	*R.nitidus*	-0.046344	0.075749
GD30	GD50	GD24	*R.nitidus*	0.077088	0.072338
GD44	GD50	GD24	*R.nitidus*	-0.016016	0.068671
GD22	GD50	GD24	*R.nitidus*	0.018812	0.071757
GD21	GD50	GD24	*R.nitidus*	0.101825	0.070938
GD41	GD50	GD24	*R.nitidus*	-0.035332	0.070808
GD46	GD50	GD24	*R.nitidus*	-0.055276	0.070164
GD35	GD50	GD24	*R.nitidus*	0.018127	0.069971
GD29	GD50	GD24	*R.nitidus*	0.055233	0.069015
CS13	GD50	GD24	*R.nitidus*	-0.066298	0.074308
CS15	GD50	GD24	*R.nitidus*	-0.00976	0.07499
GD40	GD50	GD24	*R.nitidus*	0.043318	0.068909
GD48	GD50	GD24	*R.nitidus*	-0.011677	0.073091
GD42	GD50	GD24	*R.nitidus*	0.01741	0.070592
CS17	GD50	GD24	*R.nitidus*	0.003633	0.073966
CS21	GD50	GD24	*R.nitidus*	-0.058722	0.070007
GD31	GD50	GD24	*R.nitidus*	0.029293	0.072326
CS12	GD50	GD24	*R.nitidus*	-0.035651	0.072714
GD33	GD50	GD24	*R.nitidus*	-0.043655	0.071534

CS19	GD50	GD24	*R.nitidus*	-0.071749	0.071342
CS22	GD50	GD24	*R.nitidus*	-0.023318	0.069558
CS18	GD50	GD24	*R.nitidus*	-0.065545	0.070796
CS16	GD50	GD24	*R.nitidus*	-0.036534	0.069482
CS23	GD50	GD24	*R.nitidus*	-0.081651	0.070427
GD49	GD50	GD24	*R.nitidus*	0.054084	0.076386
CS28	GD50	GD24	*R.nitidus*	-0.093985	0.070534
SC14	GD50	GD24	*R.nitidus*	-0.017513	0.068088
SC26	GD50	GD24	*R.nitidus*	-0.070859	0.070524
SC6	GD50	GD24	*R.nitidus*	-0.071103	0.070707
SC18	GD50	GD24	*R.nitidus*	0.000933	0.071765
SC27	GD50	GD24	*R.nitidus*	-0.042962	0.066684
CS25	GD50	GD24	*R.nitidus*	-0.052727	0.071345
CS24	GD50	GD24	*R.nitidus*	-0.039216	0.069608
SC5	GD50	GD24	*R.nitidus*	-0.04797	0.076249
SC24	GD50	GD24	*R.nitidus*	-0.073516	0.069955
SC31	GD50	GD24	*R.nitidus*	-0.037351	0.068271
SC10	GD50	GD24	*R.nitidus*	0.005455	0.071295
CS20	GD50	GD24	*R.nitidus*	-0.054243	0.071268
CS14	GD50	GD24	*R.nitidus*	-0.016484	0.075054
CS4	GD50	GD24	*R.nitidus*	-0.044866	0.06857
SC42	GD50	GD24	*R.nitidus*	0.214586	0.067476
SC45	GD50	GD24	*R.nitidus*	-0.009025	0.071394
JO8	CS14	GD24	*R.nitidus*	-0.003341	0.078878
JO1	GD50	GD24	*R.nitidus*	-0.071048	0.070156
MY4	GD50	GD24	*R.nitidus*	-0.08326	0.069502
JO3	GD50	GD24	*R.nitidus*	-0.038845	0.075117
SC34	GD50	GD24	*R.nitidus*	0.004378	0.066929
JW7	GD50	GD24	*R.nitidus*	-0.031729	0.07577
JO7	CS14	GD24	*R.nitidus*	-0.028446	0.076838
CS30	GD50	GD24	*R.nitidus*	-0.086477	0.070944
SC15	GD50	GD24	*R.nitidus*	-0.038211	0.069867
JO5	GD50	GD24	*R.nitidus*	-0.002662	0.068977
JW6	GD50	GD24	*R.nitidus*	-0.066441	0.074824
MY6	GD50	GD24	*R.nitidus*	-0.041415	0.069266
CS3	GD50	GD24	*R.nitidus*	-0.079156	0.069995
CS31	GD50	GD24	*R.nitidus*	-0.0625	0.070139
SC12	GD50	GD24	*R.nitidus*	-0.08684	0.070544
SC44	GD50	GD24	*R.nitidus*	-0.02634	0.071243
GD21	CS14	GD24	*R.nitidus*	0.123258	0.067656
SC32	GD50	GD24	*R.nitidus*	-0.002611	0.071993
SC28	GD50	GD24	*R.nitidus*	-0.057996	0.068398
CS11	CS14	GD24	*R.nitidus*	0.013004	0.073761
JW4	GD50	GD24	*R.nitidus*	0.166968	0.070461
SC35	GD50	GD24	*R.nitidus*	0.0018	0.069866
CS6	GD50	GD24	*R.nitidus*	-0.060965	0.072118
GD32	CS14	GD24	*R.nitidus*	0.023589	0.07242
GD31	CS14	GD24	*R.nitidus*	0.076312	0.071493
GD34	CS14	GD24	*R.nitidus*	0.015306	0.068386
GD45	CS14	GD24	*R.nitidus*	0.067708	0.075567
GD30	CS14	GD24	*R.nitidus*	0.121503	0.073161
CS19	CS14	GD24	*R.nitidus*	-0.019344	0.072854
GD33	CS14	GD24	*R.nitidus*	-0.000741	0.070693
CS21	CS14	GD24	*R.nitidus*	-0.025984	0.069202
GD22	CS14	GD24	*R.nitidus*	0.077088	0.070108
GD25	CS14	GD24	*R.nitidus*	0.076744	0.070014

GD42	CS14	GD24	*R.nitidus*	0.052513	0.069808
GD26	CS14	GD24	*R.nitidus*	0.05015	0.068651
CS18	CS14	GD24	*R.nitidus*	-0.054531	0.071556
GD29	CS14	GD24	*R.nitidus*	0.10661	0.069318
CS22	CS14	GD24	*R.nitidus*	0.014366	0.07077
GD49	CS14	GD24	*R.nitidus*	0.110831	0.073902
GD44	CS14	GD24	*R.nitidus*	0.026944	0.072055
CS4	CS14	GD24	*R.nitidus*	-0.009317	0.06863
GD48	CS14	GD24	*R.nitidus*	0.016327	0.072253
GD50	CS14	GD24	*R.nitidus*	0.016484	0.075054
CS20	CS14	GD24	*R.nitidus*	-0.023865	0.070929
CS16	CS14	GD24	*R.nitidus*	0.008413	0.071663
CS12	CS14	GD24	*R.nitidus*	0.010883	0.071703
SC10	CS14	GD24	*R.nitidus*	0.071203	0.071396
CS17	CS14	GD24	*R.nitidus*	0.05168	0.076059
CS13	CS14	GD24	*R.nitidus*	-0.044881	0.072333
GD41	CS14	GD24	*R.nitidus*	0.018532	0.070205
CS25	CS14	GD24	*R.nitidus*	-0.010612	0.070563
CS28	CS14	GD24	*R.nitidus*	-0.012669	0.069697
CS24	CS14	GD24	*R.nitidus*	-0.041809	0.069792
SC6	CS14	GD24	*R.nitidus*	-0.004831	0.072072
GD35	CS14	GD24	*R.nitidus*	0.046247	0.071996
SC18	CS14	GD24	*R.nitidus*	0.044917	0.071664
SC12	CS14	GD24	*R.nitidus*	-0.020882	0.070384
SC14	CS14	GD24	*R.nitidus*	0.022087	0.069419
GD46	CS14	GD24	*R.nitidus*	0	0.072126
GD40	CS14	GD24	*R.nitidus*	0.071481	0.07051
CS30	CS14	GD24	*R.nitidus*	-0.047428	0.071492
CS15	CS14	GD24	*R.nitidus*	0.067516	0.072447
CS23	CS14	GD24	*R.nitidus*	-0.057453	0.070259
CS3	CS14	GD24	*R.nitidus*	-0.019308	0.070322
CS31	CS14	GD24	*R.nitidus*	-0.024431	0.072088
SC15	CS14	GD24	*R.nitidus*	-0.01319	0.071078
SC5	CS14	GD24	*R.nitidus*	0.015337	0.072282
SC32	CS14	GD24	*R.nitidus*	0.043375	0.071908
JW6	CS14	GD24	*R.nitidus*	-0.017727	0.077115
JW4	CS14	GD24	*R.nitidus*	0.213669	0.070651
MY4	CS14	GD24	*R.nitidus*	-0.038431	0.07068
SC27	CS14	GD24	*R.nitidus*	0.011102	0.06864
SC24	CS14	GD24	*R.nitidus*	0.009317	0.071411
SC35	CS14	GD24	*R.nitidus*	0.052955	0.069167
SC34	CS14	GD24	*R.nitidus*	0.034375	0.069493
JO7	CS15	GD24	*R.nitidus*	-0.062171	0.074426
SC26	CS14	GD24	*R.nitidus*	-0.043011	0.068676
SC28	CS14	GD24	*R.nitidus*	-0.015164	0.070047
SC31	CS14	GD24	*R.nitidus*	0	0.069876
GD21	CS15	GD24	*R.nitidus*	0.086411	0.067782
GD31	CS15	GD24	*R.nitidus*	0.052059	0.069588
SC42	CS14	GD24	*R.nitidus*	0.252241	0.065675
JW7	CS14	GD24	*R.nitidus*	0.034123	0.074655
CS6	CS14	GD24	*R.nitidus*	-0.043369	0.070996
GD30	CS15	GD24	*R.nitidus*	0.098074	0.071444
JO3	CS14	GD24	*R.nitidus*	0.014719	0.077641
GD26	CS15	GD24	*R.nitidus*	0.013314	0.069658
GD32	CS15	GD24	*R.nitidus*	-0.011657	0.070404
MY6	CS14	GD24	*R.nitidus*	-0.017028	0.069966

SC45	CS14	GD24	*R.nitidus*	0.037867	0.072156
JO5	CS14	GD24	*R.nitidus*	0.046494	0.069953
CS11	CS15	GD24	*R.nitidus*	-0.047564	0.073648
CS14	CS15	GD24	*R.nitidus*	-0.067516	0.072447
GD22	CS15	GD24	*R.nitidus*	0.04682	0.068577
CS12	CS15	GD24	*R.nitidus*	-0.04662	0.067836
JO8	CS15	GD24	*R.nitidus*	-0.015086	0.075364
GD35	CS15	GD24	*R.nitidus*	0.009594	0.070448
SC44	CS14	GD24	*R.nitidus*	0.023346	0.07036
GD41	CS15	GD24	*R.nitidus*	-0.017088	0.067756
JO1	CS14	GD24	*R.nitidus*	-0.002362	0.070115
CS22	CS15	GD24	*R.nitidus*	-0.013492	0.070223
GD34	CS15	GD24	*R.nitidus*	-0.028281	0.067121
GD29	CS15	GD24	*R.nitidus*	0.062412	0.068917
GD49	CS15	GD24	*R.nitidus*	0.08	0.07155
GD25	CS15	GD24	*R.nitidus*	0.047068	0.069518
CS30	CS15	GD24	*R.nitidus*	-0.084194	0.069253
GD44	CS15	GD24	*R.nitidus*	-0.003814	0.069578
GD40	CS15	GD24	*R.nitidus*	0.045157	0.068615
CS17	CS15	GD24	*R.nitidus*	-0.023753	0.07219
GD33	CS15	GD24	*R.nitidus*	-0.026647	0.069276
CS24	CS15	GD24	*R.nitidus*	-0.07597	0.06828
CS13	CS15	GD24	*R.nitidus*	-0.091422	0.070806
CS20	CS15	GD24	*R.nitidus*	-0.056904	0.070029
GD50	CS15	GD24	*R.nitidus*	0.00976	0.07499
GD46	CS15	GD24	*R.nitidus*	-0.031988	0.070512
GD45	CS15	GD24	*R.nitidus*	0.049138	0.073489
CS3	CS15	GD24	*R.nitidus*	-0.044551	0.070437
GD42	CS15	GD24	*R.nitidus*	0.015129	0.069672
SC14	CS15	GD24	*R.nitidus*	-0.016129	0.069062
GD48	CS15	GD24	*R.nitidus*	-0.012048	0.070962
CS21	CS15	GD24	*R.nitidus*	-0.053118	0.067666
CS25	CS15	GD24	*R.nitidus*	-0.041063	0.072611
CS18	CS15	GD24	*R.nitidus*	-0.084328	0.07014
SC6	CS15	GD24	*R.nitidus*	-0.051012	0.070257
JW4	CS15	GD24	*R.nitidus*	0.182443	0.070015
CS19	CS15	GD24	*R.nitidus*	-0.051447	0.071987
CS23	CS15	GD24	*R.nitidus*	-0.089286	0.069405
SC44	CS15	GD24	*R.nitidus*	-0.006977	0.068092
SC5	CS15	GD24	*R.nitidus*	-0.015083	0.072082
SC28	CS15	GD24	*R.nitidus*	-0.056942	0.068297
SC31	CS15	GD24	*R.nitidus*	-0.034612	0.067918
SC27	CS15	GD24	*R.nitidus*	-0.026149	0.068071
CS16	CS15	GD24	*R.nitidus*	-0.059158	0.067041
CS28	CS15	GD24	*R.nitidus*	-0.043294	0.068972
CS31	CS15	GD24	*R.nitidus*	-0.050209	0.070984
SC26	CS15	GD24	*R.nitidus*	-0.070433	0.068182
CS6	CS15	GD24	*R.nitidus*	-0.068313	0.069528
SC12	CS15	GD24	*R.nitidus*	-0.057296	0.069817
JW7	CS15	GD24	*R.nitidus*	-0.009407	0.072784
SC24	CS15	GD24	*R.nitidus*	-0.025994	0.0684
SC10	CS15	GD24	*R.nitidus*	0.031323	0.069449
JO7	CS16	GD24	*R.nitidus*	-0.032397	0.077705
SC32	CS15	GD24	*R.nitidus*	0.012928	0.070497
JW6	CS15	GD24	*R.nitidus*	-0.057927	0.074268
SC45	CS15	GD24	*R.nitidus*	0.003873	0.070464

GD26	CS16	GD24	*R.nitidus*	0.04271	0.066939
SC42	CS15	GD24	*R.nitidus*	0.22994	0.06421
CS4	CS15	GD24	*R.nitidus*	-0.030395	0.068163
SC18	CS15	GD24	*R.nitidus*	0.026963	0.068957
SC35	CS15	GD24	*R.nitidus*	0.023134	0.067796
SC15	CS15	GD24	*R.nitidus*	-0.0456	0.070889
GD21	CS16	GD24	*R.nitidus*	0.109262	0.066535
SC34	CS15	GD24	*R.nitidus*	0.000763	0.06676
MY4	CS15	GD24	*R.nitidus*	-0.070348	0.068705
JO8	CS16	GD24	*R.nitidus*	0.026432	0.076648
JO3	CS15	GD24	*R.nitidus*	-0.013901	0.074411
GD32	CS16	GD24	*R.nitidus*	0.028668	0.069287
MY6	CS15	GD24	*R.nitidus*	-0.043774	0.06894
GD22	CS16	GD24	*R.nitidus*	0.07288	0.06546
CS11	CS16	GD24	*R.nitidus*	0.00905	0.071678
JO5	CS15	GD24	*R.nitidus*	0.027439	0.069784
JO1	CS15	GD24	*R.nitidus*	-0.03054	0.066424
GD41	CS16	GD24	*R.nitidus*	0.014782	0.065481
GD44	CS16	GD24	*R.nitidus*	0.02093	0.067577
GD35	CS16	GD24	*R.nitidus*	0.048006	0.067213
GD49	CS16	GD24	*R.nitidus*	0.109258	0.068667
GD45	CS16	GD24	*R.nitidus*	0.081287	0.073105
GD25	CS16	GD24	*R.nitidus*	0.068589	0.069488
GD50	CS16	GD24	*R.nitidus*	0.036534	0.069482
CS21	CS16	GD24	*R.nitidus*	-0.024578	0.067295
GD42	CS16	GD24	*R.nitidus*	0.049327	0.066403
GD31	CS16	GD24	*R.nitidus*	0.071317	0.069772
GD29	CS16	GD24	*R.nitidus*	0.108146	0.066262
CS12	CS16	GD24	*R.nitidus*	0.003797	0.065498
SC12	CS16	GD24	*R.nitidus*	-0.028103	0.070046
GD34	CS16	GD24	*R.nitidus*	0.012848	0.065506
GD40	CS16	GD24	*R.nitidus*	0.08768	0.067882
CS13	CS16	GD24	*R.nitidus*	-0.044517	0.07087
GD48	CS16	GD24	*R.nitidus*	0.025703	0.068069
SC18	CS16	GD24	*R.nitidus*	0.057325	0.067934
CS14	CS16	GD24	*R.nitidus*	-0.008413	0.071663
CS22	CS16	GD24	*R.nitidus*	0.018474	0.069031
CS19	CS16	GD24	*R.nitidus*	-0.009901	0.069047
CS17	CS16	GD24	*R.nitidus*	0.034238	0.072837
SC15	CS16	GD24	*R.nitidus*	-0.00982	0.06976
CS15	CS16	GD24	*R.nitidus*	0.059158	0.067041
SC6	CS16	GD24	*R.nitidus*	-0.023126	0.070249
CS4	CS16	GD24	*R.nitidus*	-0.003118	0.067898
GD46	CS16	GD24	*R.nitidus*	-0.005368	0.068961
CS28	CS16	GD24	*R.nitidus*	-0.009251	0.068972
CS25	CS16	GD24	*R.nitidus*	-0.008607	0.069238
CS20	CS16	GD24	*R.nitidus*	-0.017398	0.068654
CS18	CS16	GD24	*R.nitidus*	-0.037677	0.069708
CS24	CS16	GD24	*R.nitidus*	-0.04149	0.066352
CS31	CS16	GD24	*R.nitidus*	-0.022979	0.070051
CS30	CS16	GD24	*R.nitidus*	-0.0552	0.067698
SC44	CS16	GD24	*R.nitidus*	0.026688	0.067663
SC34	CS16	GD24	*R.nitidus*	0.024806	0.067348
CS23	CS16	GD24	*R.nitidus*	-0.042702	0.069247
SC10	CS16	GD24	*R.nitidus*	0.07917	0.067779
SC26	CS16	GD24	*R.nitidus*	-0.033742	0.066432

SC5	CS16	GD24	*R.nitidus*	0.012869	0.071417
CS3	CS16	GD24	*R.nitidus*	-0.01371	0.068517
JW4	CS16	GD24	*R.nitidus*	0.225352	0.067487
SC31	CS16	GD24	*R.nitidus*	-0.016591	0.068734
JO5	CS16	GD24	*R.nitidus*	0.054306	0.066682
CS6	CS16	GD24	*R.nitidus*	-0.033613	0.068278
SC14	CS16	GD24	*R.nitidus*	0.016807	0.066682
SC42	CS16	GD24	*R.nitidus*	0.268146	0.063359
SC24	CS16	GD24	*R.nitidus*	0.010023	0.067614
SC28	CS16	GD24	*R.nitidus*	-0.02707	0.068638
MY4	CS16	GD24	*R.nitidus*	-0.035285	0.068241
SC35	CS16	GD24	*R.nitidus*	0.054846	0.066319
SC27	CS16	GD24	*R.nitidus*	0.017433	0.066497
JO3	CS16	GD24	*R.nitidus*	0.021777	0.073736
JO1	CS16	GD24	*R.nitidus*	0.003147	0.067168
GD32	CS17	GD24	*R.nitidus*	-0.00937	0.073766
JW7	CS16	GD24	*R.nitidus*	0.041136	0.073737
SC45	CS16	GD24	*R.nitidus*	0.041149	0.069582
SC32	CS16	GD24	*R.nitidus*	0.043044	0.068882
GD25	CS17	GD24	*R.nitidus*	0.043408	0.070741
GD31	CS17	GD24	*R.nitidus*	0.063218	0.072444
JW6	CS16	GD24	*R.nitidus*	-0.006098	0.074684
GD33	CS17	GD24	*R.nitidus*	-0.029074	0.070441
GD49	CS17	GD24	*R.nitidus*	0.070009	0.071847
MY6	CS16	GD24	*R.nitidus*	-0.013688	0.06847
GD22	CS17	GD24	*R.nitidus*	0.048834	0.069509
CS12	CS17	GD24	*R.nitidus*	-0.029172	0.072563
JO8	CS17	GD24	*R.nitidus*	-0.007973	0.077502
GD45	CS17	GD24	*R.nitidus*	0.056687	0.073587
JO7	CS17	GD24	*R.nitidus*	-0.041341	0.050103
GD26	CS17	GD24	*R.nitidus*	0.006757	0.070686
CS16	CS17	GD24	*R.nitidus*	-0.034238	0.072837
GD34	CS17	GD24	*R.nitidus*	-0.023379	0.069763
GD44	CS17	GD24	*R.nitidus*	-0.002408	0.073224
CS14	CS17	GD24	*R.nitidus*	-0.05168	0.076059
GD48	CS17	GD24	*R.nitidus*	-0.009362	0.072701
GD21	CS17	GD24	*R.nitidus*	0.090977	0.069215
GD42	CS17	GD24	*R.nitidus*	0.022568	0.069356
GD29	CS17	GD24	*R.nitidus*	0.063798	0.069045
CS11	CS17	GD24	*R.nitidus*	-0.036075	0.071591
GD30	CS17	GD24	*R.nitidus*	0.100701	0.072868
GD40	CS17	GD24	*R.nitidus*	0.053417	0.069569
CS20	CS17	GD24	*R.nitidus*	-0.056884	0.071644
CS22	CS17	GD24	*R.nitidus*	-0.017515	0.073043
GD35	CS17	GD24	*R.nitidus*	0.026688	0.071125
CS25	CS17	GD24	*R.nitidus*	-0.049011	0.074473
CS13	CS17	GD24	*R.nitidus*	-0.071676	0.072519
GD50	CS17	GD24	*R.nitidus*	-0.003633	0.073966
CS15	CS17	GD24	*R.nitidus*	0.023753	0.07219
CS23	CS17	GD24	*R.nitidus*	-0.085316	0.070946
GD41	CS17	GD24	*R.nitidus*	-0.008416	0.070218
CS21	CS17	GD24	*R.nitidus*	-0.051217	0.070424
CS31	CS17	GD24	*R.nitidus*	-0.048171	0.072036
CS30	CS17	GD24	*R.nitidus*	-0.090312	0.070304
CS24	CS17	GD24	*R.nitidus*	-0.077601	0.070201
CS18	CS17	GD24	*R.nitidus*	-0.08	0.071166

GD46	CS17	GD24	*R.nitidus*	-0.038583	0.070417
SC26	CS17	GD24	*R.nitidus*	-0.064673	0.068844
SC14	CS17	GD24	*R.nitidus*	-0.009926	0.071057
CS19	CS17	GD24	*R.nitidus*	-0.044425	0.073695
SC10	CS17	GD24	*R.nitidus*	0.037705	0.072609
SC18	CS17	GD24	*R.nitidus*	0.015422	0.071171
CS4	CS17	GD24	*R.nitidus*	-0.042728	0.069276
MY6	CS17	GD24	*R.nitidus*	-0.025943	0.070629
SC12	CS17	GD24	*R.nitidus*	-0.053686	0.071411
JW4	CS17	GD24	*R.nitidus*	0.193264	0.070287
CS3	CS17	GD24	*R.nitidus*	-0.04403	0.071307
CS28	CS17	GD24	*R.nitidus*	-0.048415	0.0703
SC31	CS17	GD24	*R.nitidus*	-0.040496	0.070455
SC42	CS17	GD24	*R.nitidus*	0.221731	0.065477
SC34	CS17	GD24	*R.nitidus*	-0.002463	0.07026
SC6	CS17	GD24	*R.nitidus*	-0.047974	0.072487
SC44	CS17	GD24	*R.nitidus*	-0.017312	0.072183
JO1	CS17	GD24	*R.nitidus*	-0.039474	0.069032
SC32	CS17	GD24	*R.nitidus*	0.022259	0.07185
SC27	CS17	GD24	*R.nitidus*	-0.02906	0.068655
SC5	CS17	GD24	*R.nitidus*	-0.026464	0.071672
JW7	CS17	GD24	*R.nitidus*	0.005128	0.075089
SC28	CS17	GD24	*R.nitidus*	-0.065413	0.069475
GD25	CS18	GD24	*R.nitidus*	0.105341	0.068331
SC15	CS17	GD24	*R.nitidus*	-0.050994	0.071445
MY4	CS17	GD24	*R.nitidus*	-0.058121	0.071161
SC24	CS17	GD24	*R.nitidus*	-0.026251	0.070389
GD26	CS18	GD24	*R.nitidus*	0.087693	0.068224
JW6	CS17	GD24	*R.nitidus*	-0.04382	0.07814
GD30	CS18	GD24	*R.nitidus*	0.143914	0.069664
GD21	CS18	GD24	*R.nitidus*	0.159317	0.066849
GD29	CS18	GD24	*R.nitidus*	0.128826	0.065588
SC45	CS17	GD24	*R.nitidus*	-0.005627	0.072418
SC35	CS17	GD24	*R.nitidus*	0.018868	0.067892
JO8	CS18	GD24	*R.nitidus*	0.061135	0.07446
CS6	CS17	GD24	*R.nitidus*	-0.050938	0.07164
JO7	CS18	GD24	*R.nitidus*	0.030177	0.076875
CS12	CS18	GD24	*R.nitidus*	0.049877	0.067965
GD40	CS18	GD24	*R.nitidus*	0.133739	0.067044
JO3	CS17	GD24	*R.nitidus*	-0.005535	0.074666
GD34	CS18	GD24	*R.nitidus*	0.044328	0.067045
GD46	CS18	GD24	*R.nitidus*	0.040801	0.066915
GD49	CS18	GD24	*R.nitidus*	0.143093	0.070115
GD31	CS18	GD24	*R.nitidus*	0.129921	0.06895
CS14	CS18	GD24	*R.nitidus*	0.054531	0.071556
JO5	CS17	GD24	*R.nitidus*	0.002429	0.071047
GD42	CS18	GD24	*R.nitidus*	0.082793	0.066791
GD22	CS18	GD24	*R.nitidus*	0.120776	0.067372
GD33	CS18	GD24	*R.nitidus*	0.036842	0.069304
GD41	CS18	GD24	*R.nitidus*	0.053623	0.067076
CS11	CS18	GD24	*R.nitidus*	0.058214	0.069811
CS13	CS18	GD24	*R.nitidus*	0.022388	0.071199
GD32	CS18	GD24	*R.nitidus*	0.080833	0.070724
GD50	CS18	GD24	*R.nitidus*	0.065545	0.070796
CS30	CS18	GD24	*R.nitidus*	-0.00846	0.066475
CS15	CS18	GD24	*R.nitidus*	0.084328	0.07014

CS16	CS18	GD24	*R.nitidus*	0.037677	0.069708
CS20	CS18	GD24	*R.nitidus*	0.021339	0.067055
CS22	CS18	GD24	*R.nitidus*	0.070866	0.066915
GD48	CS18	GD24	*R.nitidus*	0.053989	0.069507
GD45	CS18	GD24	*R.nitidus*	0.111392	0.070468
GD35	CS18	GD24	*R.nitidus*	0.083143	0.068444
CS3	CS18	GD24	*R.nitidus*	0.025023	0.068447
GD44	CS18	GD24	*R.nitidus*	0.07619	0.068162
CS23	CS18	GD24	*R.nitidus*	-0.003891	0.067759
CS21	CS18	GD24	*R.nitidus*	0.04251	0.066221
CS25	CS18	GD24	*R.nitidus*	0.048341	0.070924
SC14	CS18	GD24	*R.nitidus*	0.057276	0.067576
SC10	CS18	GD24	*R.nitidus*	0.117131	0.068438
SC5	CS18	GD24	*R.nitidus*	0.054504	0.071304
CS28	CS18	GD24	*R.nitidus*	0.031489	0.069136
SC32	CS18	GD24	*R.nitidus*	0.087609	0.067324
CS24	CS18	GD24	*R.nitidus*	0.000959	0.064929
SC6	CS18	GD24	*R.nitidus*	0.0304	0.070493
CS17	CS18	GD24	*R.nitidus*	0.08	0.071166
SC12	CS18	GD24	*R.nitidus*	0.016381	0.068359
SC18	CS18	GD24	*R.nitidus*	0.109512	0.067644
CS31	CS18	GD24	*R.nitidus*	0.025941	0.067085
SC44	CS18	GD24	*R.nitidus*	0.064615	0.066355
SC27	CS18	GD24	*R.nitidus*	0.06874	0.068631
CS19	CS18	GD24	*R.nitidus*	0.019588	0.069189
SC42	CS18	GD24	*R.nitidus*	0.297937	0.062584
JO1	CS18	GD24	*R.nitidus*	0.040248	0.068577
JW7	CS18	GD24	*R.nitidus*	0.070755	0.072575
MY6	CS18	GD24	*R.nitidus*	0.029141	0.067324
JW6	CS18	GD24	*R.nitidus*	0.030693	0.074645
SC24	CS18	GD24	*R.nitidus*	0.048309	0.068787
SC45	CS18	GD24	*R.nitidus*	0.088006	0.067995
CS4	CS18	GD24	*R.nitidus*	0.034662	0.064987
GD22	CS19	GD24	*R.nitidus*	0.093636	0.070239
GD32	CS19	GD24	*R.nitidus*	0.043082	0.070369
JW4	CS18	GD24	*R.nitidus*	0.256672	0.06709
CS6	CS18	GD24	*R.nitidus*	0.010827	0.066827
SC15	CS18	GD24	*R.nitidus*	0.029362	0.067865
SC26	CS18	GD24	*R.nitidus*	0.011494	0.066395
SC31	CS18	GD24	*R.nitidus*	0.034776	0.067668
SC34	CS18	GD24	*R.nitidus*	0.081839	0.065194
MY4	CS18	GD24	*R.nitidus*	0.011006	0.068396
JO8	CS19	GD24	*R.nitidus*	0.041002	0.075443
SC35	CS18	GD24	*R.nitidus*	0.095532	0.065954
GD45	CS19	GD24	*R.nitidus*	0.094128	0.073066
GD26	CS19	GD24	*R.nitidus*	0.054985	0.070155
GD29	CS19	GD24	*R.nitidus*	0.115585	0.066476
JO7	CS19	GD24	*R.nitidus*	0.009626	0.077665
CS13	CS19	GD24	*R.nitidus*	-0.011092	0.073383
GD25	CS19	GD24	*R.nitidus*	0.075457	0.069032
GD44	CS19	GD24	*R.nitidus*	0.053746	0.069146
SC28	CS18	GD24	*R.nitidus*	0.02866	0.067548
CS16	CS19	GD24	*R.nitidus*	0.009901	0.069047
GD35	CS19	GD24	*R.nitidus*	0.054773	0.070437
CS17	CS19	GD24	*R.nitidus*	0.044425	0.073695
JO3	CS18	GD24	*R.nitidus*	0.062389	0.07112

GD30	CS19	GD24	*R.nitidus*	0.125214	0.070854
JO5	CS18	GD24	*R.nitidus*	0.111283	0.068915
CS12	CS19	GD24	*R.nitidus*	0.02124	0.071575
GD48	CS19	GD24	*R.nitidus*	0.033079	0.070703
GD31	CS19	GD24	*R.nitidus*	0.121261	0.069234
GD21	CS19	GD24	*R.nitidus*	0.125647	0.068353
GD41	CS19	GD24	*R.nitidus*	0.026647	0.069691
CS11	CS19	GD24	*R.nitidus*	0.03431	0.071346
GD42	CS19	GD24	*R.nitidus*	0.053707	0.068046
GD40	CS19	GD24	*R.nitidus*	0.105828	0.068684
CS25	CS19	GD24	*R.nitidus*	0.028571	0.071712
GD46	CS19	GD24	*R.nitidus*	0.016579	0.069118
GD49	CS19	GD24	*R.nitidus*	0.108202	0.070576
GD34	CS19	GD24	*R.nitidus*	0.02	0.069001
CS14	CS19	GD24	*R.nitidus*	0.019344	0.072854
GD33	CS19	GD24	*R.nitidus*	0.007746	0.069288
CS4	CS19	GD24	*R.nitidus*	0.02053	0.070442
CS23	CS19	GD24	*R.nitidus*	-0.026667	0.069526
CS15	CS19	GD24	*R.nitidus*	0.051447	0.071987
GD50	CS19	GD24	*R.nitidus*	0.071749	0.071342
SC27	CS19	GD24	*R.nitidus*	0.050736	0.072626
SC6	CS19	GD24	*R.nitidus*	0.007899	0.071598
SC5	CS19	GD24	*R.nitidus*	0.029482	0.073571
CS18	CS19	GD24	*R.nitidus*	-0.019588	0.069189
CS22	CS19	GD24	*R.nitidus*	0.036503	0.069358
CS20	CS19	GD24	*R.nitidus*	-0.00457	0.070205
SC10	CS19	GD24	*R.nitidus*	0.085969	0.072538
SC44	CS19	GD24	*R.nitidus*	0.043513	0.069582
CS3	CS19	GD24	*R.nitidus*	0.005571	0.069187
CS24	CS19	GD24	*R.nitidus*	-0.01184	0.068413
SC28	CS19	GD24	*R.nitidus*	0.014096	0.069522
CS6	CS19	GD24	*R.nitidus*	-0.007797	0.069487
CS21	CS19	GD24	*R.nitidus*	0.005571	0.069261
CS31	CS19	GD24	*R.nitidus*	0.008666	0.070497
SC18	CS19	GD24	*R.nitidus*	0.090986	0.069277
CS28	CS19	GD24	*R.nitidus*	-0.000853	0.07051
SC12	CS19	GD24	*R.nitidus*	0	0.070595
JO3	CS19	GD24	*R.nitidus*	0.052438	0.072271
JW4	CS19	GD24	*R.nitidus*	0.242231	0.070204
SC24	CS19	GD24	*R.nitidus*	0.038907	0.069445
GD25	CS20	GD24	*R.nitidus*	0.087706	0.069135
MY6	CS19	GD24	*R.nitidus*	0.017228	0.070019
JW6	CS19	GD24	*R.nitidus*	0.041489	0.075534
SC35	CS19	GD24	*R.nitidus*	0.090202	0.069967
CS30	CS19	GD24	*R.nitidus*	-0.022862	0.06855
MY4	CS19	GD24	*R.nitidus*	-0.013957	0.071024
GD30	CS20	GD24	*R.nitidus*	0.140879	0.069636
SC14	CS19	GD24	*R.nitidus*	0.049051	0.070279
SC45	CS19	GD24	*R.nitidus*	0.072713	0.070644
SC26	CS19	GD24	*R.nitidus*	-0.008648	0.068664
SC15	CS19	GD24	*R.nitidus*	0.016653	0.070033
SC32	CS19	GD24	*R.nitidus*	0.066667	0.071236
JO5	CS19	GD24	*R.nitidus*	0.075548	0.06893
SC31	CS19	GD24	*R.nitidus*	0.018419	0.068722
JO8	CS20	GD24	*R.nitidus*	0.027056	0.075938
JO7	CS20	GD24	*R.nitidus*	-0.001001	0.073563

SC34	CS19	GD24	*R.nitidus*	0.063035	0.069153
GD21	CS20	GD24	*R.nitidus*	0.137241	0.067309
CS13	CS20	GD24	*R.nitidus*	0	0.07051
JW7	CS19	GD24	*R.nitidus*	0.058325	0.073877
GD22	CS20	GD24	*R.nitidus*	0.10452	0.068528
GD26	CS20	GD24	*R.nitidus*	0.070402	0.069206
GD42	CS20	GD24	*R.nitidus*	0.059701	0.067691
SC42	CS19	GD24	*R.nitidus*	0.289063	0.064316
GD33	CS20	GD24	*R.nitidus*	0.015141	0.069363
GD44	CS20	GD24	*R.nitidus*	0.050623	0.068872
CS11	CS20	GD24	*R.nitidus*	0.029389	0.069406
GD34	CS20	GD24	*R.nitidus*	0.034432	0.067964
GD35	CS20	GD24	*R.nitidus*	0.065379	0.068373
JO1	CS19	GD24	*R.nitidus*	0.021622	0.070167
GD48	CS20	GD24	*R.nitidus*	0.030844	0.069109
GD41	CS20	GD24	*R.nitidus*	0.044776	0.068891
GD29	CS20	GD24	*R.nitidus*	0.120084	0.065596
GD31	CS20	GD24	*R.nitidus*	0.10888	0.068242
CS12	CS20	GD24	*R.nitidus*	0.015323	0.068715
CS14	CS20	GD24	*R.nitidus*	0.023865	0.070929
GD50	CS20	GD24	*R.nitidus*	0.054243	0.071268
GD49	CS20	GD24	*R.nitidus*	0.144381	0.069153
GD32	CS20	GD24	*R.nitidus*	0.047855	0.068231
CS21	CS20	GD24	*R.nitidus*	0.007092	0.067477
GD45	CS20	GD24	*R.nitidus*	0.088481	0.072212
SC24	CS20	GD24	*R.nitidus*	0.033832	0.067037
CS18	CS20	GD24	*R.nitidus*	-0.021339	0.067055
CS4	CS20	GD24	*R.nitidus*	0.01815	0.066446
CS28	CS20	GD24	*R.nitidus*	-0.011419	0.069457
SC14	CS20	GD24	*R.nitidus*	0.044731	0.066653
SC15	CS20	GD24	*R.nitidus*	0.000804	0.069018
GD46	CS20	GD24	*R.nitidus*	0.017429	0.067511
SC10	CS20	GD24	*R.nitidus*	0.082881	0.068228
CS30	CS20	GD24	*R.nitidus*	-0.033097	0.067956
CS17	CS20	GD24	*R.nitidus*	0.056884	0.071644
SC27	CS20	GD24	*R.nitidus*	0.039782	0.067823
GD40	CS20	GD24	*R.nitidus*	0.106095	0.066564
CS23	CS20	GD24	*R.nitidus*	-0.038059	0.067875
SC18	CS20	GD24	*R.nitidus*	0.092683	0.066668
CS22	CS20	GD24	*R.nitidus*	0.041773	0.069296
CS24	CS20	GD24	*R.nitidus*	-0.006683	0.068565
CS19	CS20	GD24	*R.nitidus*	0.00457	0.070205
SC5	CS20	GD24	*R.nitidus*	0.036063	0.071347
SC6	CS20	GD24	*R.nitidus*	0	0.068956
CS16	CS20	GD24	*R.nitidus*	0.017398	0.068654
CS6	CS20	GD24	*R.nitidus*	-0.01919	0.065835
CS3	CS20	GD24	*R.nitidus*	0.003282	0.063471
CS15	CS20	GD24	*R.nitidus*	0.056904	0.070029
SC31	CS20	GD24	*R.nitidus*	0.014879	0.067122
JW6	CS20	GD24	*R.nitidus*	-0.00956	0.074599
MY6	CS20	GD24	*R.nitidus*	0.007446	0.067581
SC28	CS20	GD24	*R.nitidus*	0.011424	0.068162
CS25	CS20	GD24	*R.nitidus*	0.010949	0.069969
CS31	CS20	GD24	*R.nitidus*	-0.003197	0.067598
SC45	CS20	GD24	*R.nitidus*	0.066876	0.068396
SC26	CS20	GD24	*R.nitidus*	-0.010196	0.067882

SC12	CS20	GD24	*R.nitidus*	0.005335	0.068888
GD22	CS21	GD24	*R.nitidus*	0.093471	0.066456
SC35	CS20	GD24	*R.nitidus*	0.078417	0.065878
MY4	CS20	GD24	*R.nitidus*	-0.011372	0.067879
JW4	CS20	GD24	*R.nitidus*	0.236719	0.068644
JW7	CS20	GD24	*R.nitidus*	0.04461	0.072621
SC34	CS20	GD24	*R.nitidus*	0.058868	0.066869
JO3	CS20	GD24	*R.nitidus*	0.041182	0.070758
JO5	CS20	GD24	*R.nitidus*	0.083459	0.068539
GD30	CS21	GD24	*R.nitidus*	0.151491	0.068717
SC32	CS20	GD24	*R.nitidus*	0.069856	0.066342
JO1	CS20	GD24	*R.nitidus*	0.014749	0.069461
JO7	CS21	GD24	*R.nitidus*	0.009063	0.07523
SC42	CS20	GD24	*R.nitidus*	0.272999	0.061856
GD31	CS21	GD24	*R.nitidus*	0.097147	0.068458
SC44	CS20	GD24	*R.nitidus*	0.048632	0.066777
JO8	CS21	GD24	*R.nitidus*	0.043022	0.073312
CS11	CS21	GD24	*R.nitidus*	0.026398	0.067172
GD40	CS21	GD24	*R.nitidus*	0.099636	0.067274
GD50	CS21	GD24	*R.nitidus*	0.058722	0.070007
GD46	CS21	GD24	*R.nitidus*	0.013758	0.067348
GD21	CS21	GD24	*R.nitidus*	0.129771	0.066013
CS14	CS21	GD24	*R.nitidus*	0.025984	0.069202
GD34	CS21	GD24	*R.nitidus*	0.021214	0.067406
GD26	CS21	GD24	*R.nitidus*	0.059159	0.068589
GD41	CS21	GD24	*R.nitidus*	0.036723	0.066579
GD45	CS21	GD24	*R.nitidus*	0.094449	0.070505
CS20	CS21	GD24	*R.nitidus*	-0.007092	0.067477
CS12	CS21	GD24	*R.nitidus*	0.023943	0.067503
CS17	CS21	GD24	*R.nitidus*	0.051217	0.070424
GD29	CS21	GD24	*R.nitidus*	0.113116	0.064678
CS30	CS21	GD24	*R.nitidus*	-0.040296	0.065126
GD42	CS21	GD24	*R.nitidus*	0.066287	0.066768
GD25	CS21	GD24	*R.nitidus*	0.091181	0.07022
CS15	CS21	GD24	*R.nitidus*	0.053118	0.067666
CS19	CS21	GD24	*R.nitidus*	-0.005571	0.069261
CS13	CS21	GD24	*R.nitidus*	-0.008661	0.069143
GD32	CS21	GD24	*R.nitidus*	0.042776	0.069982
GD48	CS21	GD24	*R.nitidus*	0.040816	0.069757
GD35	CS21	GD24	*R.nitidus*	0.058696	0.068478
CS28	CS21	GD24	*R.nitidus*	-0.013389	0.067804
SC6	CS21	GD24	*R.nitidus*	0.000784	0.069111
GD33	CS21	GD24	*R.nitidus*	0.01295	0.068027
CS3	CS21	GD24	*R.nitidus*	-0.009569	0.06721
GD49	CS21	GD24	*R.nitidus*	0.135792	0.068673
CS22	CS21	GD24	*R.nitidus*	0.038811	0.066133
CS25	CS21	GD24	*R.nitidus*	0.005445	0.06944
GD44	CS21	GD24	*R.nitidus*	0.041889	0.067654
SC12	CS21	GD24	*R.nitidus*	-0.007698	0.067671
SC10	CS21	GD24	*R.nitidus*	0.081377	0.068289
CS18	CS21	GD24	*R.nitidus*	-0.04251	0.066221
CS16	CS21	GD24	*R.nitidus*	0.024578	0.067295
CS31	CS21	GD24	*R.nitidus*	-0.008058	0.068321
SC31	CS21	GD24	*R.nitidus*	0.016484	0.066926
SC26	CS21	GD24	*R.nitidus*	-0.019335	0.066243
CS23	CS21	GD24	*R.nitidus*	-0.042871	0.066294

SC24	CS21	GD24	*R.nitidus*	0.023052	0.068503
SC14	CS21	GD24	*R.nitidus*	0.046743	0.067036
SC27	CS21	GD24	*R.nitidus*	0.032993	0.067515
SC15	CS21	GD24	*R.nitidus*	-0.00081	0.068275
CS24	CS21	GD24	*R.nitidus*	-0.018472	0.066799
JW6	CS21	GD24	*R.nitidus*	0	0.073391
SC28	CS21	GD24	*R.nitidus*	0.008365	0.066305
SC5	CS21	GD24	*R.nitidus*	0.026647	0.067773
JW4	CS21	GD24	*R.nitidus*	0.240842	0.067884
SC44	CS21	GD24	*R.nitidus*	0.043807	0.065437
MY6	CS21	GD24	*R.nitidus*	0.005279	0.067768
SC45	CS21	GD24	*R.nitidus*	0.056689	0.068011
JW7	CS21	GD24	*R.nitidus*	0.058011	0.072458
GD29	CS22	GD24	*R.nitidus*	0.079684	0.066167
SC18	CS21	GD24	*R.nitidus*	0.080735	0.066573
GD33	CS22	GD24	*R.nitidus*	-0.022777	0.06863
GD22	CS22	GD24	*R.nitidus*	0.055556	0.068229
SC42	CS21	GD24	*R.nitidus*	0.277337	0.061621
GD30	CS22	GD24	*R.nitidus*	0.100933	0.068982
GD26	CS22	GD24	*R.nitidus*	0.041385	0.06702
SC35	CS21	GD24	*R.nitidus*	0.08104	0.066545
JO3	CS21	GD24	*R.nitidus*	0.047957	0.072514
SC34	CS21	GD24	*R.nitidus*	0.048967	0.066952
SC32	CS21	GD24	*R.nitidus*	0.064615	0.066223
JO7	CS22	GD24	*R.nitidus*	-0.048884	0.073868
CS6	CS21	GD24	*R.nitidus*	-0.021804	0.066836
MY4	CS21	GD24	*R.nitidus*	-0.01947	0.068833
CS4	CS21	GD24	*R.nitidus*	-0.00339	0.065697
GD41	CS22	GD24	*R.nitidus*	0.008843	0.066863
CS11	CS22	GD24	*R.nitidus*	-0.013557	0.070902
JO8	CS22	GD24	*R.nitidus*	0.01566	0.075091
GD34	CS22	GD24	*R.nitidus*	0	0.066631
CS18	CS22	GD24	*R.nitidus*	-0.070866	0.066915
GD21	CS22	GD24	*R.nitidus*	0.099856	0.065049
JO5	CS21	GD24	*R.nitidus*	0.076746	0.06713
GD31	CS22	GD24	*R.nitidus*	0.059922	0.069581
GD50	CS22	GD24	*R.nitidus*	0.023318	0.069558
GD32	CS22	GD24	*R.nitidus*	0.011226	0.069767
GD40	CS22	GD24	*R.nitidus*	0.067301	0.065719
GD35	CS22	GD24	*R.nitidus*	0.027166	0.069088
GD25	CS22	GD24	*R.nitidus*	0.034769	0.066331
CS23	CS22	GD24	*R.nitidus*	-0.077246	0.066769
CS21	CS22	GD24	*R.nitidus*	-0.038811	0.066133
CS17	CS22	GD24	*R.nitidus*	0.017515	0.073043
GD42	CS22	GD24	*R.nitidus*	0.035871	0.066609
CS12	CS22	GD24	*R.nitidus*	-0.014155	0.067999
CS20	CS22	GD24	*R.nitidus*	-0.041773	0.069296
GD45	CS22	GD24	*R.nitidus*	0.061909	0.070939
CS16	CS22	GD24	*R.nitidus*	-0.018474	0.069031
CS31	CS22	GD24	*R.nitidus*	-0.044575	0.066446
GD48	CS22	GD24	*R.nitidus*	0.000816	0.069135
GD44	CS22	GD24	*R.nitidus*	0.028022	0.068695
CS28	CS22	GD24	*R.nitidus*	-0.048401	0.066275
CS15	CS22	GD24	*R.nitidus*	0.013492	0.070223
JO1	CS21	GD24	*R.nitidus*	0.018032	0.069035
GD46	CS22	GD24	*R.nitidus*	-0.017306	0.066059

GD49	CS22	GD24	*R.nitidus*	0.091213	0.068903
SC28	CS22	GD24	*R.nitidus*	-0.0224	0.067589
CS13	CS22	GD24	*R.nitidus*	-0.057028	0.069869
CS19	CS22	GD24	*R.nitidus*	-0.036503	0.069358
CS25	CS22	GD24	*R.nitidus*	-0.025575	0.070038
CS3	CS22	GD24	*R.nitidus*	-0.039344	0.067356
CS14	CS22	GD24	*R.nitidus*	-0.014366	0.07077
SC6	CS22	GD24	*R.nitidus*	-0.031422	0.069146
SC24	CS22	GD24	*R.nitidus*	-0.007813	0.067245
CS24	CS22	GD24	*R.nitidus*	-0.07297	0.064609
CS30	CS22	GD24	*R.nitidus*	-0.071429	0.068795
SC5	CS22	GD24	*R.nitidus*	0.004702	0.070024
CS4	CS22	GD24	*R.nitidus*	-0.037267	0.06478
SC18	CS22	GD24	*R.nitidus*	0.046053	0.069374
SC26	CS22	GD24	*R.nitidus*	-0.052836	0.065828
SC45	CS22	GD24	*R.nitidus*	0.027516	0.068
JO1	CS22	GD24	*R.nitidus*	-0.008607	0.068385
JW4	CS22	GD24	*R.nitidus*	0.22179	0.066761
JO7	CS23	GD24	*R.nitidus*	0.02588	0.075408
MY4	CS22	GD24	*R.nitidus*	-0.056439	0.068122
MY6	CS22	GD24	*R.nitidus*	-0.030398	0.065917
SC44	CS22	GD24	*R.nitidus*	0.006182	0.068207
SC10	CS22	GD24	*R.nitidus*	0.060827	0.066704
SC32	CS22	GD24	*R.nitidus*	0.03506	0.067055
SC31	CS22	GD24	*R.nitidus*	-0.018968	0.067758
SC35	CS22	GD24	*R.nitidus*	0.042789	0.065096
SC27	CS22	GD24	*R.nitidus*	0.004862	0.067641
JW7	CS22	GD24	*R.nitidus*	0.031823	0.072867
SC12	CS22	GD24	*R.nitidus*	-0.042164	0.068852
SC14	CS22	GD24	*R.nitidus*	0.011006	0.067725
CS11	CS23	GD24	*R.nitidus*	0.065637	0.068281
SC34	CS22	GD24	*R.nitidus*	0.015982	0.065319
JO8	CS23	GD24	*R.nitidus*	0.046187	0.07535
SC15	CS22	GD24	*R.nitidus*	-0.022895	0.066543
SC42	CS22	GD24	*R.nitidus*	0.253442	0.062398
CS12	CS23	GD24	*R.nitidus*	0.050766	0.069349
GD25	CS23	GD24	*R.nitidus*	0.110542	0.067135
GD30	CS23	GD24	*R.nitidus*	0.146242	0.069278
CS6	CS22	GD24	*R.nitidus*	-0.054187	0.065756
GD29	CS23	GD24	*R.nitidus*	0.139087	0.06488
JW6	CS22	GD24	*R.nitidus*	0.011	0.07422
GD32	CS23	GD24	*R.nitidus*	0.084967	0.069923
JO5	CS22	GD24	*R.nitidus*	0.041765	0.067889
GD22	CS23	GD24	*R.nitidus*	0.119718	0.066233
GD21	CS23	GD24	*R.nitidus*	0.156444	0.065804
JO3	CS22	GD24	*R.nitidus*	0.014912	0.073035
CS13	CS23	GD24	*R.nitidus*	0.02558	0.069208
GD50	CS23	GD24	*R.nitidus*	0.081651	0.070427
GD31	CS23	GD24	*R.nitidus*	0.13297	0.067016
GD34	CS23	GD24	*R.nitidus*	0.059911	0.065188
GD42	CS23	GD24	*R.nitidus*	0.08966	0.066425
CS17	CS23	GD24	*R.nitidus*	0.085316	0.070946
CS14	CS23	GD24	*R.nitidus*	0.057453	0.070259
GD45	CS23	GD24	*R.nitidus*	0.106557	0.074004
CS15	CS23	GD24	*R.nitidus*	0.089286	0.069405
GD46	CS23	GD24	*R.nitidus*	0.034875	0.065988

GD40	CS23	GD24	*R.nitidus*	0.126119	0.066299
GD49	CS23	GD24	*R.nitidus*	0.149832	0.069412
CS30	CS23	GD24	*R.nitidus*	-0.004136	0.065963
GD33	CS23	GD24	*R.nitidus*	0.037383	0.068432
GD44	CS23	GD24	*R.nitidus*	0.076563	0.066698
GD26	CS23	GD24	*R.nitidus*	0.095068	0.067263
GD48	CS23	GD24	*R.nitidus*	0.065131	0.068682
CS20	CS23	GD24	*R.nitidus*	0.038059	0.067875
CS19	CS23	GD24	*R.nitidus*	0.026667	0.069526
GD41	CS23	GD24	*R.nitidus*	0.066187	0.066527
GD35	CS23	GD24	*R.nitidus*	0.092661	0.067472
CS16	CS23	GD24	*R.nitidus*	0.042702	0.069247
JW4	CS23	GD24	*R.nitidus*	0.25076	0.06754
CS31	CS23	GD24	*R.nitidus*	0.029887	0.068851
CS6	CS23	GD24	*R.nitidus*	0.021053	0.06798
CS3	CS23	GD24	*R.nitidus*	0.032803	0.06858
CS21	CS23	GD24	*R.nitidus*	0.042871	0.066294
CS18	CS23	GD24	*R.nitidus*	0.003891	0.067759
SC45	CS23	GD24	*R.nitidus*	0.090768	0.067771
SC34	CS23	GD24	*R.nitidus*	0.080521	0.06623
JO3	CS23	GD24	*R.nitidus*	0.066071	0.070711
JW6	CS23	GD24	*R.nitidus*	0.012573	0.074464
SC32	CS23	GD24	*R.nitidus*	0.091116	0.067999
CS25	CS23	GD24	*R.nitidus*	0.029596	0.070789
SC28	CS23	GD24	*R.nitidus*	0.016756	0.066817
SC42	CS23	GD24	*R.nitidus*	0.308343	0.062223
CS22	CS23	GD24	*R.nitidus*	0.077246	0.066769
CS24	CS23	GD24	*R.nitidus*	0.015531	0.06655
CS28	CS23	GD24	*R.nitidus*	0.028428	0.069081
SC5	CS23	GD24	*R.nitidus*	0.055101	0.071033
JO8	CS24	GD24	*R.nitidus*	0.036782	0.073344
SC27	CS23	GD24	*R.nitidus*	0.066879	0.068312
SC35	CS23	GD24	*R.nitidus*	0.105223	0.068071
CS4	CS23	GD24	*R.nitidus*	0.050086	0.064835
JO5	CS23	GD24	*R.nitidus*	0.102964	0.066363
SC15	CS23	GD24	*R.nitidus*	0.042484	0.067788
MY4	CS23	GD24	*R.nitidus*	0.013997	0.068503
GD29	CS24	GD24	*R.nitidus*	0.124088	0.064831
SC6	CS23	GD24	*R.nitidus*	0.037721	0.069604
SC10	CS23	GD24	*R.nitidus*	0.12224	0.069773
SC44	CS23	GD24	*R.nitidus*	0.064171	0.066303
SC12	CS23	GD24	*R.nitidus*	0.014582	0.068934
SC14	CS23	GD24	*R.nitidus*	0.072617	0.067881
JW7	CS23	GD24	*R.nitidus*	0.064959	0.071768
SC24	CS23	GD24	*R.nitidus*	0.057325	0.068894
SC26	CS23	GD24	*R.nitidus*	0.015267	0.068535
JO1	CS23	GD24	*R.nitidus*	0.047104	0.068712
SC31	CS23	GD24	*R.nitidus*	0.046296	0.06704
JO7	CS24	GD24	*R.nitidus*	0.020474	0.074379
GD26	CS24	GD24	*R.nitidus*	0.078686	0.066023
SC18	CS23	GD24	*R.nitidus*	0.115196	0.067585
GD22	CS24	GD24	*R.nitidus*	0.110207	0.066376
GD32	CS24	GD24	*R.nitidus*	0.063993	0.069014
GD25	CS24	GD24	*R.nitidus*	0.083782	0.067429
GD40	CS24	GD24	*R.nitidus*	0.127496	0.064497
MY6	CS23	GD24	*R.nitidus*	0.035494	0.06741

GD30	CS24	GD24	*R.nitidus*	0.146383	0.067374
GD34	CS24	GD24	*R.nitidus*	0.040335	0.067812
GD31	CS24	GD24	*R.nitidus*	0.118431	0.068461
CS11	CS24	GD24	*R.nitidus*	0.056067	0.069837
GD21	CS24	GD24	*R.nitidus*	0.152838	0.065992
GD33	CS24	GD24	*R.nitidus*	0.028073	0.067986
CS12	CS24	GD24	*R.nitidus*	0.048696	0.068028
GD45	CS24	GD24	*R.nitidus*	0.09792	0.070478
GD42	CS24	GD24	*R.nitidus*	0.079727	0.066324
CS15	CS24	GD24	*R.nitidus*	0.07597	0.06828
GD50	CS24	GD24	*R.nitidus*	0.039216	0.069608
GD46	CS24	GD24	*R.nitidus*	0.026738	0.066569
GD41	CS24	GD24	*R.nitidus*	0.053241	0.066173
CS31	CS24	GD24	*R.nitidus*	0.018949	0.066409
CS14	CS24	GD24	*R.nitidus*	0.041809	0.069792
GD49	CS24	GD24	*R.nitidus*	0.132253	0.070422
CS28	CS24	GD24	*R.nitidus*	0.025847	0.068418
SC14	CS24	GD24	*R.nitidus*	0.058581	0.06535
GD44	CS24	GD24	*R.nitidus*	0.067755	0.068495
GD35	CS24	GD24	*R.nitidus*	0.080997	0.067437
CS30	CS24	GD24	*R.nitidus*	-0.019214	0.067922
CS25	CS24	GD24	*R.nitidus*	0.037489	0.068839
CS20	CS24	GD24	*R.nitidus*	0.006683	0.068565
CS18	CS24	GD24	*R.nitidus*	-0.000959	0.064929
CS13	CS24	GD24	*R.nitidus*	0.014808	0.069251
GD48	CS24	GD24	*R.nitidus*	0.033058	0.068716
CS22	CS24	GD24	*R.nitidus*	0.07297	0.064609
SC12	CS24	GD24	*R.nitidus*	0.014504	0.068691
SC10	CS24	GD24	*R.nitidus*	0.127642	0.0669
SC15	CS24	GD24	*R.nitidus*	0.02385	0.065897
CS19	CS24	GD24	*R.nitidus*	0.01184	0.068413
CS3	CS24	GD24	*R.nitidus*	0.007614	0.06724
JO7	CS25	GD24	*R.nitidus*	0	0.075215
CS16	CS24	GD24	*R.nitidus*	0.04149	0.066352
SC6	CS24	GD24	*R.nitidus*	0.02484	0.068922
SC42	CS24	GD24	*R.nitidus*	0.308953	0.062716
JO8	CS25	GD24	*R.nitidus*	0.018702	0.077248
CS21	CS24	GD24	*R.nitidus*	0.018472	0.066799
CS23	CS24	GD24	*R.nitidus*	-0.015531	0.06655
SC28	CS24	GD24	*R.nitidus*	0.032514	0.067694
SC27	CS24	GD24	*R.nitidus*	0.054514	0.065922
JW7	CS24	GD24	*R.nitidus*	0.081716	0.071164
JO5	CS24	GD24	*R.nitidus*	0.09589	0.066318
CS17	CS24	GD24	*R.nitidus*	0.077601	0.070201
SC26	CS24	GD24	*R.nitidus*	0.001601	0.06629
SC5	CS24	GD24	*R.nitidus*	0.059738	0.070793
SC31	CS24	GD24	*R.nitidus*	0.044129	0.066158
SC24	CS24	GD24	*R.nitidus*	0.04238	0.067063
SC34	CS24	GD24	*R.nitidus*	0.081848	0.065219
JO3	CS24	GD24	*R.nitidus*	0.050938	0.072198
JO1	CS24	GD24	*R.nitidus*	0.041732	0.068358
SC32	CS24	GD24	*R.nitidus*	0.092667	0.067105
CS4	CS24	GD24	*R.nitidus*	0.042816	0.064123
GD21	CS25	GD24	*R.nitidus*	0.131255	0.067158
GD22	CS25	GD24	*R.nitidus*	0.10162	0.068412
CS6	CS24	GD24	*R.nitidus*	0.01107	0.066243

SC35	CS24	GD24	*R.nitidus*	0.095779	0.063731
SC18	CS24	GD24	*R.nitidus*	0.088797	0.067591
JW6	CS24	GD24	*R.nitidus*	0.050725	0.074281
JW4	CS24	GD24	*R.nitidus*	0.2738	0.066713
GD26	CS25	GD24	*R.nitidus*	0.065975	0.06975
MY4	CS24	GD24	*R.nitidus*	-0.000794	0.067412
SC44	CS24	GD24	*R.nitidus*	0.069021	0.067443
SC45	CS24	GD24	*R.nitidus*	0.077882	0.067881
CS11	CS25	GD24	*R.nitidus*	0.020542	0.071663
GD25	CS25	GD24	*R.nitidus*	0.081664	0.068539
GD34	CS25	GD24	*R.nitidus*	0.018983	0.067313
GD33	CS25	GD24	*R.nitidus*	0.010878	0.0695
MY6	CS24	GD24	*R.nitidus*	0.02439	0.066843
GD41	CS25	GD24	*R.nitidus*	0.024	0.068273
GD32	CS25	GD24	*R.nitidus*	0.041736	0.071506
GD42	CS25	GD24	*R.nitidus*	0.051013	0.068412
GD44	CS25	GD24	*R.nitidus*	0.054927	0.070536
CS12	CS25	GD24	*R.nitidus*	0.009885	0.068577
CS20	CS25	GD24	*R.nitidus*	-0.010949	0.069969
GD45	CS25	GD24	*R.nitidus*	0.087598	0.07383
GD48	CS25	GD24	*R.nitidus*	0.02294	0.071528
GD49	CS25	GD24	*R.nitidus*	0.123288	0.072167
GD50	CS25	GD24	*R.nitidus*	0.052727	0.071345
GD46	CS25	GD24	*R.nitidus*	0.001486	0.069935
CS23	CS25	GD24	*R.nitidus*	-0.029596	0.070789
SC24	CS25	GD24	*R.nitidus*	0.0278	0.069274
GD30	CS25	GD24	*R.nitidus*	0.125442	0.071719
GD40	CS25	GD24	*R.nitidus*	0.08817	0.066904
CS17	CS25	GD24	*R.nitidus*	0.049011	0.074473
GD29	CS25	GD24	*R.nitidus*	0.112426	0.066668
GD31	CS25	GD24	*R.nitidus*	0.101483	0.069779
SC10	CS25	GD24	*R.nitidus*	0.065534	0.071249
SC12	CS25	GD24	*R.nitidus*	-0.018283	0.070387
CS4	CS25	GD24	*R.nitidus*	0.016143	0.068568
SC26	CS25	GD24	*R.nitidus*	-0.013057	0.067884
CS15	CS25	GD24	*R.nitidus*	0.041063	0.072611
GD35	CS25	GD24	*R.nitidus*	0.051869	0.069302
SC5	CS25	GD24	*R.nitidus*	0.019395	0.072078
CS30	CS25	GD24	*R.nitidus*	-0.038741	0.06772
CS3	CS25	GD24	*R.nitidus*	-0.015004	0.068792
CS24	CS25	GD24	*R.nitidus*	-0.037489	0.068839
SC18	CS25	GD24	*R.nitidus*	0.07225	0.069002
SC6	CS25	GD24	*R.nitidus*	-0.004847	0.070277
SC28	CS25	GD24	*R.nitidus*	-0.006265	0.068793
CS31	CS25	GD24	*R.nitidus*	-0.012356	0.069768
SC32	CS25	GD24	*R.nitidus*	0.058962	0.06943
CS18	CS25	GD24	*R.nitidus*	-0.048341	0.070924
CS19	CS25	GD24	*R.nitidus*	-0.028571	0.071712
SC45	CS25	GD24	*R.nitidus*	0.060918	0.068634
CS14	CS25	GD24	*R.nitidus*	0.010612	0.070563
CS13	CS25	GD24	*R.nitidus*	-0.014718	0.071781
CS16	CS25	GD24	*R.nitidus*	0.008607	0.069238
CS6	CS25	GD24	*R.nitidus*	-0.024954	0.067493
CS21	CS25	GD24	*R.nitidus*	-0.005445	0.06944
SC15	CS25	GD24	*R.nitidus*	0.001649	0.070503
CS28	CS25	GD24	*R.nitidus*	-0.019608	0.070843

SC31	CS25	GD24	*R.nitidus*	0.010679	0.066595
CS22	CS25	GD24	*R.nitidus*	0.025575	0.070038
SC42	CS25	GD24	*R.nitidus*	0.279319	0.064642
SC44	CS25	GD24	*R.nitidus*	0.026899	0.067968
JO8	CS28	GD24	*R.nitidus*	0.016092	0.073661
JO7	CS28	GD24	*R.nitidus*	-0.019101	0.07526
MY4	CS25	GD24	*R.nitidus*	-0.019841	0.068252
SC14	CS25	GD24	*R.nitidus*	0.035074	0.068118
JO3	CS25	GD24	*R.nitidus*	0.045654	0.072301
SC27	CS25	GD24	*R.nitidus*	0.029695	0.068874
JO1	CS25	GD24	*R.nitidus*	0.006061	0.069167
GD25	CS28	GD24	*R.nitidus*	0.083402	0.068309
SC34	CS25	GD24	*R.nitidus*	0.051459	0.067482
SC35	CS25	GD24	*R.nitidus*	0.065334	0.066809
GD42	CS28	GD24	*R.nitidus*	0.064464	0.068265
GD22	CS28	GD24	*R.nitidus*	0.103886	0.068501
JW6	CS25	GD24	*R.nitidus*	0.0199	0.076463
GD44	CS28	GD24	*R.nitidus*	0.052326	0.069636
JW4	CS25	GD24	*R.nitidus*	0.226357	0.070091
JW7	CS25	GD24	*R.nitidus*	0.048159	0.073442
CS15	CS28	GD24	*R.nitidus*	0.043294	0.068972
JO5	CS25	GD24	*R.nitidus*	0.082671	0.067957
GD32	CS28	GD24	*R.nitidus*	0.0518	0.070581
GD48	CS28	GD24	*R.nitidus*	0.048969	0.069784
GD21	CS28	GD24	*R.nitidus*	0.130236	0.06596
MY6	CS25	GD24	*R.nitidus*	0.008668	0.069765
CS11	CS28	GD24	*R.nitidus*	0.023018	0.068584
GD49	CS28	GD24	*R.nitidus*	0.130245	0.07038
GD34	CS28	GD24	*R.nitidus*	0.03742	0.067496
GD26	CS28	GD24	*R.nitidus*	0.075531	0.069362
CS14	CS28	GD24	*R.nitidus*	0.012669	0.069697
GD30	CS28	GD24	*R.nitidus*	0.155172	0.071144
GD40	CS28	GD24	*R.nitidus*	0.097276	0.066702
GD45	CS28	GD24	*R.nitidus*	0.099647	0.069171
CS23	CS28	GD24	*R.nitidus*	-0.028428	0.069081
GD46	CS28	GD24	*R.nitidus*	0.011924	0.067143
GD41	CS28	GD24	*R.nitidus*	0.037037	0.067643
GD33	CS28	GD24	*R.nitidus*	0.018838	0.068426
CS31	CS28	GD24	*R.nitidus*	-0.015284	0.071092
GD29	CS28	GD24	*R.nitidus*	0.113971	0.067248
CS16	CS28	GD24	*R.nitidus*	0.009251	0.068972
GD31	CS28	GD24	*R.nitidus*	0.089552	0.068685
GD35	CS28	GD24	*R.nitidus*	0.07613	0.068789
CS21	CS28	GD24	*R.nitidus*	0.013389	0.067804
CS30	CS28	GD24	*R.nitidus*	-0.039548	0.067918
SC10	CS28	GD24	*R.nitidus*	0.102564	0.067347
CS19	CS28	GD24	*R.nitidus*	0.000853	0.07051
CS22	CS28	GD24	*R.nitidus*	0.048401	0.066275
CS20	CS28	GD24	*R.nitidus*	0.011419	0.069457
CS24	CS28	GD24	*R.nitidus*	-0.025847	0.068418
CS13	CS28	GD24	*R.nitidus*	-0.017467	0.07072
CS12	CS28	GD24	*R.nitidus*	0.018535	0.067394
CS17	CS28	GD24	*R.nitidus*	0.048415	0.0703
SC15	CS28	GD24	*R.nitidus*	0	0.069612
SC14	CS28	GD24	*R.nitidus*	0.044839	0.069468
CS3	CS28	GD24	*R.nitidus*	-0.005106	0.069865

SC5	CS28	GD24	*R.nitidus*	0.036484	0.069883
SC24	CS28	GD24	*R.nitidus*	0.032618	0.068418
GD50	CS28	GD24	*R.nitidus*	0.093985	0.070534
SC6	CS28	GD24	*R.nitidus*	0.021222	0.069928
CS18	CS28	GD24	*R.nitidus*	-0.031489	0.069136
SC28	CS28	GD24	*R.nitidus*	0.012007	0.06675
SC27	CS28	GD24	*R.nitidus*	0.037587	0.068167
CS25	CS28	GD24	*R.nitidus*	0.019608	0.070843
JW4	CS28	GD24	*R.nitidus*	0.242925	0.071022
SC31	CS28	GD24	*R.nitidus*	0.021259	0.066804
JW6	CS28	GD24	*R.nitidus*	0.021622	0.074489
CS4	CS28	GD24	*R.nitidus*	0.009228	0.066845
CS6	CS28	GD24	*R.nitidus*	-0.00885	0.067405
SC12	CS28	GD24	*R.nitidus*	0.000854	0.06864
JO5	CS28	GD24	*R.nitidus*	0.069983	0.066909
GD21	CS30	GD24	*R.nitidus*	0.164918	0.065568
SC18	CS28	GD24	*R.nitidus*	0.079487	0.069645
SC42	CS28	GD24	*R.nitidus*	0.286193	0.063528
SC35	CS28	GD24	*R.nitidus*	0.061056	0.067592
JO3	CS28	GD24	*R.nitidus*	0.045579	0.074827
GD26	CS30	GD24	*R.nitidus*	0.105747	0.066862
SC26	CS28	GD24	*R.nitidus*	-0.01519	0.07035
SC32	CS28	GD24	*R.nitidus*	0.088983	0.068642
GD45	CS30	GD24	*R.nitidus*	0.112875	0.070702
SC44	CS28	GD24	*R.nitidus*	0.053333	0.067484
GD33	CS30	GD24	*R.nitidus*	0.050038	0.068167
SC34	CS28	GD24	*R.nitidus*	0.06425	0.068857
GD35	CS30	GD24	*R.nitidus*	0.087481	0.068461
JO1	CS28	GD24	*R.nitidus*	0.03322	0.06802
JW7	CS28	GD24	*R.nitidus*	0.064116	0.073815
SC45	CS28	GD24	*R.nitidus*	0.060435	0.069475
JO8	CS30	GD24	*R.nitidus*	0.079955	0.072667
GD44	CS30	GD24	*R.nitidus*	0.06988	0.069678
GD40	CS30	GD24	*R.nitidus*	0.128399	0.065439
GD46	CS30	GD24	*R.nitidus*	0.039872	0.066856
GD22	CS30	GD24	*R.nitidus*	0.128243	0.065757
GD49	CS30	GD24	*R.nitidus*	0.149293	0.069692
GD48	CS30	GD24	*R.nitidus*	0.059177	0.069274
GD41	CS30	GD24	*R.nitidus*	0.078571	0.068372
JO7	CS30	GD24	*R.nitidus*	0.017897	0.074752
GD25	CS30	GD24	*R.nitidus*	0.10878	0.066781
MY4	CS28	GD24	*R.nitidus*	-0.002479	0.068351
GD32	CS30	GD24	*R.nitidus*	0.071861	0.071235
MY6	CS28	GD24	*R.nitidus*	0.019449	0.069081
GD31	CS30	GD24	*R.nitidus*	0.133117	0.068698
GD29	CS30	GD24	*R.nitidus*	0.140613	0.065568
GD30	CS30	GD24	*R.nitidus*	0.153779	0.069752
CS13	CS30	GD24	*R.nitidus*	0.03336	0.069559
GD34	CS30	GD24	*R.nitidus*	0.06025	0.06643
CS11	CS30	GD24	*R.nitidus*	0.058039	0.069862
CS12	CS30	GD24	*R.nitidus*	0.055326	0.067896
GD50	CS30	GD24	*R.nitidus*	0.086477	0.070944
CS15	CS30	GD24	*R.nitidus*	0.084194	0.069253
GD42	CS30	GD24	*R.nitidus*	0.094519	0.065346
CS23	CS30	GD24	*R.nitidus*	0.004136	0.065963
CS16	CS30	GD24	*R.nitidus*	0.0552	0.067698

CS21	CS30	GD24	*R.nitidus*	0.040296	0.065126
CS24	CS30	GD24	*R.nitidus*	0.019214	0.067922
CS31	CS30	GD24	*R.nitidus*	0.045287	0.066431
CS18	CS30	GD24	*R.nitidus*	0.00846	0.066475
SC12	CS30	GD24	*R.nitidus*	0.020048	0.068507
CS22	CS30	GD24	*R.nitidus*	0.071429	0.068795
SC32	CS30	GD24	*R.nitidus*	0.095008	0.067648
SC27	CS30	GD24	*R.nitidus*	0.07233	0.066737
CS17	CS30	GD24	*R.nitidus*	0.090312	0.070304
CS4	CS30	GD24	*R.nitidus*	0.049755	0.066571
JW4	CS30	GD24	*R.nitidus*	0.269585	0.066381
CS25	CS30	GD24	*R.nitidus*	0.038741	0.06772
CS19	CS30	GD24	*R.nitidus*	0.022862	0.06855
CS3	CS30	GD24	*R.nitidus*	0.034625	0.06769
CS14	CS30	GD24	*R.nitidus*	0.047428	0.071492
MY4	CS30	GD24	*R.nitidus*	0.015504	0.06846
SC10	CS30	GD24	*R.nitidus*	0.1251	0.066554
SC15	CS30	GD24	*R.nitidus*	0.028814	0.067211
CS20	CS30	GD24	*R.nitidus*	0.033097	0.067956
SC45	CS30	GD24	*R.nitidus*	0.094108	0.066344
SC5	CS30	GD24	*R.nitidus*	0.055645	0.071023
CS28	CS30	GD24	*R.nitidus*	0.039548	0.067918
SC42	CS30	GD24	*R.nitidus*	0.308504	0.061976
SC14	CS30	GD24	*R.nitidus*	0.069268	0.067567
SC28	CS30	GD24	*R.nitidus*	0.038735	0.065759
SC26	CS30	GD24	*R.nitidus*	0.020553	0.066144
SC44	CS30	GD24	*R.nitidus*	0.082181	0.066023
SC31	CS30	GD24	*R.nitidus*	0.054545	0.066763
JW6	CS30	GD24	*R.nitidus*	0.048128	0.072329
SC34	CS30	GD24	*R.nitidus*	0.089992	0.065021
SC6	CS30	GD24	*R.nitidus*	0.036491	0.070268
SC18	CS30	GD24	*R.nitidus*	0.109578	0.068112
GD25	CS31	GD24	*R.nitidus*	0.084261	0.068052
JO1	CS30	GD24	*R.nitidus*	0.037647	0.067553
JO8	CS31	GD24	*R.nitidus*	0.050401	0.075519
MY6	CS30	GD24	*R.nitidus*	0.043143	0.066666
SC24	CS30	GD24	*R.nitidus*	0.057371	0.069087
CS6	CS30	GD24	*R.nitidus*	0.023709	0.068011
JO5	CS30	GD24	*R.nitidus*	0.105645	0.06482
JO3	CS30	GD24	*R.nitidus*	0.087993	0.071537
SC35	CS30	GD24	*R.nitidus*	0.104708	0.065142
JW7	CS30	GD24	*R.nitidus*	0.089962	0.071784
GD31	CS31	GD24	*R.nitidus*	0.089182	0.069323
CS13	CS31	GD24	*R.nitidus*	-0.015544	0.071394
GD33	CS31	GD24	*R.nitidus*	0.009916	0.068907
JO7	CS31	GD24	*R.nitidus*	-0.008556	0.075406
CS11	CS31	GD24	*R.nitidus*	0.02437	0.072493
GD41	CS31	GD24	*R.nitidus*	0.038217	0.066879
GD32	CS31	GD24	*R.nitidus*	0.053004	0.072327
GD29	CS31	GD24	*R.nitidus*	0.124077	0.066523
GD49	CS31	GD24	*R.nitidus*	0.133741	0.070929
CS14	CS31	GD24	*R.nitidus*	0.024431	0.072088
GD21	CS31	GD24	*R.nitidus*	0.138973	0.066575
GD22	CS31	GD24	*R.nitidus*	0.093493	0.068095
GD40	CS31	GD24	*R.nitidus*	0.096386	0.068908
GD45	CS31	GD24	*R.nitidus*	0.115591	0.072202

GD30	CS31	GD24	*R.nitidus*	0.130511	0.070092
CS15	CS31	GD24	*R.nitidus*	0.050209	0.070984
GD44	CS31	GD24	*R.nitidus*	0.052109	0.068904
CS12	CS31	GD24	*R.nitidus*	0.032007	0.069529
GD34	CS31	GD24	*R.nitidus*	0.02036	0.068027
CS25	CS31	GD24	*R.nitidus*	0.012356	0.069768
GD26	CS31	GD24	*R.nitidus*	0.070988	0.067756
GD35	CS31	GD24	*R.nitidus*	0.064388	0.069326
CS30	CS31	GD24	*R.nitidus*	-0.045287	0.066431
CS23	CS31	GD24	*R.nitidus*	-0.029887	0.068851
CS24	CS31	GD24	*R.nitidus*	-0.018949	0.066409
CS17	CS31	GD24	*R.nitidus*	0.048171	0.072036
CS16	CS31	GD24	*R.nitidus*	0.022979	0.070051
GD42	CS31	GD24	*R.nitidus*	0.055466	0.066406
GD50	CS31	GD24	*R.nitidus*	0.0625	0.070139
CS21	CS31	GD24	*R.nitidus*	0.008058	0.068321
CS22	CS31	GD24	*R.nitidus*	0.044575	0.066446
GD46	CS31	GD24	*R.nitidus*	0.009788	0.067749
CS18	CS31	GD24	*R.nitidus*	-0.025941	0.067085
GD48	CS31	GD24	*R.nitidus*	0.030691	0.069994
CS20	CS31	GD24	*R.nitidus*	0.003197	0.067598
SC5	CS31	GD24	*R.nitidus*	0.026634	0.072365
SC34	CS31	GD24	*R.nitidus*	0.053945	0.066499
CS28	CS31	GD24	*R.nitidus*	0.015284	0.071092
JW4	CS31	GD24	*R.nitidus*	0.268235	0.067999
MY4	CS31	GD24	*R.nitidus*	-0.021155	0.068569
CS19	CS31	GD24	*R.nitidus*	-0.008666	0.070497
SC10	CS31	GD24	*R.nitidus*	0.105263	0.067459
SC35	CS31	GD24	*R.nitidus*	0.073529	0.066963
SC27	CS31	GD24	*R.nitidus*	0.038884	0.06781
SC18	CS31	GD24	*R.nitidus*	0.084843	0.069308
SC12	CS31	GD24	*R.nitidus*	-0.00487	0.068181
CS3	CS31	GD24	*R.nitidus*	0.009106	0.068253
JW6	CS31	GD24	*R.nitidus*	0.038176	0.075911
JO3	CS31	GD24	*R.nitidus*	0.034906	0.073164
SC44	CS31	GD24	*R.nitidus*	0.050571	0.068311
MY6	CS31	GD24	*R.nitidus*	0.004023	0.068397
SC6	CS31	GD24	*R.nitidus*	0.008045	0.070165
JW7	CS31	GD24	*R.nitidus*	0.086294	0.071867
SC28	CS31	GD24	*R.nitidus*	0.009002	0.06783
GD30	CS3	GD24	*R.nitidus*	0.135225	0.069237
CS4	CS31	GD24	*R.nitidus*	0.015485	0.06547
SC14	CS31	GD24	*R.nitidus*	0.046374	0.069187
JO8	CS3	GD24	*R.nitidus*	0.009879	0.075821
SC24	CS31	GD24	*R.nitidus*	0.022951	0.06876
SC15	CS31	GD24	*R.nitidus*	0.00088	0.070962
SC45	CS31	GD24	*R.nitidus*	0.053386	0.067869
CS11	CS3	GD24	*R.nitidus*	0.029221	0.068472
SC26	CS31	GD24	*R.nitidus*	-0.011981	0.06876
SC32	CS31	GD24	*R.nitidus*	0.069594	0.069784
CS6	CS31	GD24	*R.nitidus*	-0.006867	0.067682
SC31	CS31	GD24	*R.nitidus*	0.025765	0.067169
JO1	CS31	GD24	*R.nitidus*	0.0089	0.067724
SC42	CS31	GD24	*R.nitidus*	0.30243	0.062734
GD26	CS3	GD24	*R.nitidus*	0.07029	0.067678
GD29	CS3	GD24	*R.nitidus*	0.127338	0.066353

GD22	CS3	GD24	*R.nitidus*	0.096126	0.066876
GD21	CS3	GD24	*R.nitidus*	0.12894	0.066223
GD33	CS3	GD24	*R.nitidus*	0.024831	0.068816
JO7	CS3	GD24	*R.nitidus*	-0.018519	0.074654
GD34	CS3	GD24	*R.nitidus*	0.030053	0.067691
GD35	CS3	GD24	*R.nitidus*	0.051841	0.06681
GD42	CS3	GD24	*R.nitidus*	0.065393	0.067276
GD44	CS3	GD24	*R.nitidus*	0.043991	0.067445
JO5	CS31	GD24	*R.nitidus*	0.078289	0.067941
GD46	CS3	GD24	*R.nitidus*	0.007479	0.067745
CS19	CS3	GD24	*R.nitidus*	-0.005571	0.069187
CS16	CS3	GD24	*R.nitidus*	0.01371	0.068517
GD25	CS3	GD24	*R.nitidus*	0.087461	0.067613
GD31	CS3	GD24	*R.nitidus*	0.110762	0.068417
GD45	CS3	GD24	*R.nitidus*	0.08557	0.070806
CS13	CS3	GD24	*R.nitidus*	-0.010518	0.069463
GD49	CS3	GD24	*R.nitidus*	0.133951	0.069022
GD32	CS3	GD24	*R.nitidus*	0.059216	0.069734
CS21	CS3	GD24	*R.nitidus*	0.009569	0.06721
CS18	CS3	GD24	*R.nitidus*	-0.025023	0.068447
CS15	CS3	GD24	*R.nitidus*	0.044551	0.070437
GD41	CS3	GD24	*R.nitidus*	0.050983	0.067983
GD48	CS3	GD24	*R.nitidus*	0.034426	0.070038
CS30	CS3	GD24	*R.nitidus*	-0.034625	0.06769
GD50	CS3	GD24	*R.nitidus*	0.079156	0.069995
CS12	CS3	GD24	*R.nitidus*	0.014901	0.068304
GD40	CS3	GD24	*R.nitidus*	0.099693	0.066119
CS20	CS3	GD24	*R.nitidus*	-0.003282	0.063471
CS14	CS3	GD24	*R.nitidus*	0.019308	0.070322
CS23	CS3	GD24	*R.nitidus*	-0.032803	0.06858
CS31	CS3	GD24	*R.nitidus*	-0.009106	0.068253
SC10	CS3	GD24	*R.nitidus*	0.076498	0.065864
CS24	CS3	GD24	*R.nitidus*	-0.007614	0.06724
SC5	CS3	GD24	*R.nitidus*	0.015468	0.071168
CS17	CS3	GD24	*R.nitidus*	0.04403	0.071307
SC32	CS3	GD24	*R.nitidus*	0.062548	0.0661
SC28	CS3	GD24	*R.nitidus*	-0.002412	0.06789
CS25	CS3	GD24	*R.nitidus*	0.015004	0.068792
CS4	CS3	GD24	*R.nitidus*	0.025855	0.066748
SC18	CS3	GD24	*R.nitidus*	0.095477	0.067908
MY6	CS3	GD24	*R.nitidus*	-0.003861	0.067007
SC12	CS3	GD24	*R.nitidus*	-0.0144	0.067947
JW6	CS3	GD24	*R.nitidus*	-0.019289	0.074545
SC14	CS3	GD24	*R.nitidus*	0.044917	0.066408
SC15	CS3	GD24	*R.nitidus*	0.005714	0.067249
JO3	CS3	GD24	*R.nitidus*	0.011894	0.071272
SC42	CS3	GD24	*R.nitidus*	0.27046	0.061543
CS22	CS3	GD24	*R.nitidus*	0.039344	0.067356
SC44	CS3	GD24	*R.nitidus*	0.032468	0.067615
SC6	CS3	GD24	*R.nitidus*	0.001612	0.067954
SC27	CS3	GD24	*R.nitidus*	0.02494	0.06605
CS28	CS3	GD24	*R.nitidus*	0.005106	0.069865
SC35	CS3	GD24	*R.nitidus*	0.065385	0.065932
JW4	CS3	GD24	*R.nitidus*	0.229624	0.0693
SC26	CS3	GD24	*R.nitidus*	-0.010317	0.066087
SC31	CS3	GD24	*R.nitidus*	0.003978	0.065622

JO8	SC5	GD24	*R.nitidus*	-0.021459	0.079281
CS6	CS3	GD24	*R.nitidus*	-0.022173	0.063985
SC24	CS3	GD24	*R.nitidus*	0.039571	0.068824
MY4	CS3	GD24	*R.nitidus*	-0.014752	0.066564
SC45	CS3	GD24	*R.nitidus*	0.060079	0.067691
GD32	SC5	GD24	*R.nitidus*	-0.006891	0.073108
SC34	CS3	GD24	*R.nitidus*	0.052429	0.067448
JO1	CS3	GD24	*R.nitidus*	0.009441	0.068843
GD31	SC5	GD24	*R.nitidus*	0.076744	0.070129
JW7	CS3	GD24	*R.nitidus*	0.051852	0.069706
JO7	SC5	GD24	*R.nitidus*	-0.062305	0.07733
JO5	CS3	GD24	*R.nitidus*	0.073841	0.067029
GD34	SC5	GD24	*R.nitidus*	-0.008296	0.07218
GD26	SC5	GD24	*R.nitidus*	0.037262	0.069648
GD29	SC5	GD24	*R.nitidus*	0.085921	0.069906
GD40	SC5	GD24	*R.nitidus*	0.075166	0.068439
CS12	SC5	GD24	*R.nitidus*	-0.022709	0.070303
CS11	SC5	GD24	*R.nitidus*	0	0.073106
GD49	SC5	GD24	*R.nitidus*	0.096639	0.071639
GD21	SC5	GD24	*R.nitidus*	0.102545	0.068803
GD44	SC5	GD24	*R.nitidus*	0.010535	0.071394
GD45	SC5	GD24	*R.nitidus*	0.066285	0.07388
GD42	SC5	GD24	*R.nitidus*	0.03099	0.069461
CS19	SC5	GD24	*R.nitidus*	-0.029482	0.073571
GD30	SC5	GD24	*R.nitidus*	0.120432	0.071885
GD22	SC5	GD24	*R.nitidus*	0.07169	0.069678
GD41	SC5	GD24	*R.nitidus*	0.008296	0.070015
GD25	SC5	GD24	*R.nitidus*	0.06365	0.069776
CS30	SC5	GD24	*R.nitidus*	-0.055645	0.071023
CS20	SC5	GD24	*R.nitidus*	-0.036063	0.071347
GD48	SC5	GD24	*R.nitidus*	-0.031561	0.071859
CS17	SC5	GD24	*R.nitidus*	0.026464	0.071672
CS15	SC5	GD24	*R.nitidus*	0.015083	0.072082
GD33	SC5	GD24	*R.nitidus*	-0.020882	0.072233
CS13	SC5	GD24	*R.nitidus*	-0.033858	0.071999
GD35	SC5	GD24	*R.nitidus*	0.02473	0.073093
GD50	SC5	GD24	*R.nitidus*	0.04797	0.076249
CS24	SC5	GD24	*R.nitidus*	-0.059738	0.070793
CS21	SC5	GD24	*R.nitidus*	-0.026647	0.067773
SC6	SC5	GD24	*R.nitidus*	-0.021382	0.073773
SC32	SC5	GD24	*R.nitidus*	0.03862	0.070773
CS16	SC5	GD24	*R.nitidus*	-0.012869	0.071417
SC26	SC5	GD24	*R.nitidus*	-0.051325	0.069821
GD46	SC5	GD24	*R.nitidus*	-0.017126	0.06956
CS18	SC5	GD24	*R.nitidus*	-0.054504	0.071304
SC42	SC5	GD24	*R.nitidus*	0.273206	0.066513
SC27	SC5	GD24	*R.nitidus*	0.015358	0.071188
CS31	SC5	GD24	*R.nitidus*	-0.026634	0.072365
CS14	SC5	GD24	*R.nitidus*	-0.015337	0.072282
CS28	SC5	GD24	*R.nitidus*	-0.036484	0.069883
CS22	SC5	GD24	*R.nitidus*	-0.004702	0.070024
SC14	SC5	GD24	*R.nitidus*	0.018182	0.070395
JW6	SC5	GD24	*R.nitidus*	-0.034205	0.077375
SC18	SC5	GD24	*R.nitidus*	0.059807	0.07073
CS25	SC5	GD24	*R.nitidus*	-0.019395	0.072078
JW4	SC5	GD24	*R.nitidus*	0.192459	0.071496

SC44	SC5	GD24	*R.nitidus*	0.031279	0.067861
SC15	SC5	GD24	*R.nitidus*	-0.021044	0.071462
SC28	SC5	GD24	*R.nitidus*	-0.015441	0.067264
CS23	SC5	GD24	*R.nitidus*	-0.055101	0.071033
CS6	SC5	GD24	*R.nitidus*	-0.043965	0.068737
SC34	SC5	GD24	*R.nitidus*	0.020701	0.071137
SC10	SC5	GD24	*R.nitidus*	0.060189	0.071579
SC24	SC5	GD24	*R.nitidus*	0.01463	0.072629
CS3	SC5	GD24	*R.nitidus*	-0.015468	0.071168
JO8	SC6	GD24	*R.nitidus*	0.02694	0.075409
JW7	SC5	GD24	*R.nitidus*	0.01749	0.079943
JO3	SC5	GD24	*R.nitidus*	-0.015177	0.075917
GD26	SC6	GD24	*R.nitidus*	0.069663	0.069805
SC12	SC5	GD24	*R.nitidus*	-0.03937	0.070797
CS4	SC5	GD24	*R.nitidus*	-0.016458	0.068692
SC45	SC5	GD24	*R.nitidus*	0.041459	0.069866
JO1	SC5	GD24	*R.nitidus*	-0.010569	0.069864
SC35	SC5	GD24	*R.nitidus*	0.039935	0.069345
SC31	SC5	GD24	*R.nitidus*	-0.004819	0.069113
CS11	SC6	GD24	*R.nitidus*	0.021944	0.070915
MY6	SC5	GD24	*R.nitidus*	-0.021358	0.070424
JO5	SC5	GD24	*R.nitidus*	0.059837	0.070117
GD31	SC6	GD24	*R.nitidus*	0.081679	0.068175
GD21	SC6	GD24	*R.nitidus*	0.132479	0.06914
MY4	SC5	GD24	*R.nitidus*	-0.042097	0.070216
JO7	SC6	GD24	*R.nitidus*	-0.026427	0.07577
GD22	SC6	GD24	*R.nitidus*	0.080056	0.069384
GD30	SC6	GD24	*R.nitidus*	0.143946	0.070328
GD29	SC6	GD24	*R.nitidus*	0.120552	0.065054
CS15	SC6	GD24	*R.nitidus*	0.051012	0.070257
GD34	SC6	GD24	*R.nitidus*	0.023014	0.068977
GD49	SC6	GD24	*R.nitidus*	0.11863	0.070881
GD25	SC6	GD24	*R.nitidus*	0.081493	0.068882
CS21	SC6	GD24	*R.nitidus*	-0.000784	0.069111
CS17	SC6	GD24	*R.nitidus*	0.047974	0.072487
GD32	SC6	GD24	*R.nitidus*	0.030227	0.072045
GD42	SC6	GD24	*R.nitidus*	0.051685	0.069305
CS23	SC6	GD24	*R.nitidus*	-0.037721	0.069604
GD45	SC6	GD24	*R.nitidus*	0.087719	0.073136
GD35	SC6	GD24	*R.nitidus*	0.065169	0.067955
CS14	SC6	GD24	*R.nitidus*	0.004831	0.072072
GD41	SC6	GD24	*R.nitidus*	0.033208	0.069817
GD33	SC6	GD24	*R.nitidus*	0.004521	0.068951
CS30	SC6	GD24	*R.nitidus*	-0.036491	0.070268
CS12	SC6	GD24	*R.nitidus*	0.035117	0.071491
CS16	SC6	GD24	*R.nitidus*	0.023126	0.070249
GD40	SC6	GD24	*R.nitidus*	0.09692	0.067093
CS22	SC6	GD24	*R.nitidus*	0.031422	0.069146
GD44	SC6	GD24	*R.nitidus*	0.030645	0.068857
SC12	SC6	GD24	*R.nitidus*	-0.010879	0.068024
GD48	SC6	GD24	*R.nitidus*	0.035275	0.069948
CS28	SC6	GD24	*R.nitidus*	-0.021222	0.069928
GD50	SC6	GD24	*R.nitidus*	0.071103	0.070707
SC32	SC6	GD24	*R.nitidus*	0.063847	0.0695
CS13	SC6	GD24	*R.nitidus*	-0.014008	0.07199
CS25	SC6	GD24	*R.nitidus*	0.004847	0.070277

GD46	SC6	GD24	*R.nitidus*	0.003846	0.069349
CS20	SC6	GD24	*R.nitidus*	0	0.068956
CS3	SC6	GD24	*R.nitidus*	-0.001612	0.067954
SC26	SC6	GD24	*R.nitidus*	-0.029711	0.069285
SC27	SC6	GD24	*R.nitidus*	0.030534	0.069858
SC18	SC6	GD24	*R.nitidus*	0.071986	0.069487
CS18	SC6	GD24	*R.nitidus*	-0.0304	0.070493
SC15	SC6	GD24	*R.nitidus*	-0.009466	0.069602
CS19	SC6	GD24	*R.nitidus*	-0.007899	0.071598
SC44	SC6	GD24	*R.nitidus*	0.038211	0.068159
SC42	SC6	GD24	*R.nitidus*	0.302597	0.06252
CS31	SC6	GD24	*R.nitidus*	-0.008045	0.070165
CS24	SC6	GD24	*R.nitidus*	-0.02484	0.068922
SC28	SC6	GD24	*R.nitidus*	-0.020661	0.068833
JO7	SC10	GD24	*R.nitidus*	-0.122713	0.073844
SC35	SC6	GD24	*R.nitidus*	0.068823	0.06722
SC24	SC6	GD24	*R.nitidus*	0.024914	0.06837
SC5	SC6	GD24	*R.nitidus*	0.021382	0.073773
CS4	SC6	GD24	*R.nitidus*	0.009195	0.067678
SC14	SC6	GD24	*R.nitidus*	0.04465	0.071213
SC34	SC6	GD24	*R.nitidus*	0.046964	0.06799
JW6	SC6	GD24	*R.nitidus*	-0.014099	0.077183
CS6	SC6	GD24	*R.nitidus*	-0.019119	0.068012
SC31	SC6	GD24	*R.nitidus*	0.023007	0.067411
GD22	SC10	GD24	*R.nitidus*	0.002221	0.068796
JO5	SC6	GD24	*R.nitidus*	0.078226	0.067494
MY4	SC6	GD24	*R.nitidus*	-0.022275	0.067997
JO3	SC6	GD24	*R.nitidus*	0.033333	0.07245
JW4	SC6	GD24	*R.nitidus*	0.239289	0.067425
SC10	SC6	GD24	*R.nitidus*	0.110351	0.069573
GD32	SC10	GD24	*R.nitidus*	-0.055038	0.069284
GD30	SC10	GD24	*R.nitidus*	0.065331	0.070945
GD25	SC10	GD24	*R.nitidus*	-0.004673	0.06606
GD42	SC10	GD24	*R.nitidus*	-0.021522	0.066341
JO8	SC10	GD24	*R.nitidus*	-0.070615	0.075957
MY6	SC6	GD24	*R.nitidus*	0.009479	0.068386
GD21	SC10	GD24	*R.nitidus*	0.049533	0.067491
SC45	SC6	GD24	*R.nitidus*	0.047342	0.069015
GD40	SC10	GD24	*R.nitidus*	0.008949	0.068036
GD45	SC10	GD24	*R.nitidus*	-0.008518	0.072779
JO1	SC6	GD24	*R.nitidus*	0.005063	0.068144
GD33	SC10	GD24	*R.nitidus*	-0.066059	0.070449
GD26	SC10	GD24	*R.nitidus*	-0.015849	0.068893
GD46	SC10	GD24	*R.nitidus*	-0.066814	0.065676
GD48	SC10	GD24	*R.nitidus*	-0.055961	0.069635
CS14	SC10	GD24	*R.nitidus*	-0.071203	0.071396
GD41	SC10	GD24	*R.nitidus*	-0.052672	0.067588
CS12	SC10	GD24	*R.nitidus*	-0.085141	0.068849
GD34	SC10	GD24	*R.nitidus*	-0.053151	0.067485
CS17	SC10	GD24	*R.nitidus*	-0.037705	0.072609
CS11	SC10	GD24	*R.nitidus*	-0.068075	0.070589
GD31	SC10	GD24	*R.nitidus*	0.007132	0.068933
GD35	SC10	GD24	*R.nitidus*	-0.029008	0.06856
GD29	SC10	GD24	*R.nitidus*	0.018505	0.066119
CS13	SC10	GD24	*R.nitidus*	-0.088997	0.069617
GD49	SC10	GD24	*R.nitidus*	0.04876	0.070576

CS22	SC10	GD24	*R.nitidus*	-0.060827	0.066704
CS16	SC10	GD24	*R.nitidus*	-0.07917	0.067779
GD44	SC10	GD24	*R.nitidus*	-0.035357	0.068743
SC5	SC10	GD24	*R.nitidus*	-0.060189	0.071579
CS23	SC10	GD24	*R.nitidus*	-0.12224	0.069773
CS25	SC10	GD24	*R.nitidus*	-0.065534	0.071249
CS21	SC10	GD24	*R.nitidus*	-0.081377	0.068289
SC28	SC10	GD24	*R.nitidus*	-0.103242	0.067017
SC31	SC10	GD24	*R.nitidus*	-0.078098	0.067093
CS15	SC10	GD24	*R.nitidus*	-0.031323	0.069449
GD50	SC10	GD24	*R.nitidus*	-0.005455	0.071295
CS30	SC10	GD24	*R.nitidus*	-0.1251	0.066554
CS28	SC10	GD24	*R.nitidus*	-0.102564	0.067347
CS19	SC10	GD24	*R.nitidus*	-0.085969	0.072538
SC34	SC10	GD24	*R.nitidus*	-0.030225	0.066032
CS31	SC10	GD24	*R.nitidus*	-0.105263	0.067459
CS20	SC10	GD24	*R.nitidus*	-0.082881	0.068228
SC42	SC10	GD24	*R.nitidus*	0.210345	0.063671
CS18	SC10	GD24	*R.nitidus*	-0.117131	0.068438
CS3	SC10	GD24	*R.nitidus*	-0.076498	0.065864
JW4	SC10	GD24	*R.nitidus*	0.17785	0.068055
CS6	SC10	GD24	*R.nitidus*	-0.092308	0.067272
SC15	SC10	GD24	*R.nitidus*	-0.089136	0.068876
SC12	SC10	GD24	*R.nitidus*	-0.106608	0.065779
CS4	SC10	GD24	*R.nitidus*	-0.084243	0.067091
SC35	SC10	GD24	*R.nitidus*	-0.020478	0.066931
CS24	SC10	GD24	*R.nitidus*	-0.127642	0.0669
SC27	SC10	GD24	*R.nitidus*	-0.060773	0.066394
JW7	SC10	GD24	*R.nitidus*	-0.022175	0.071276
JW6	SC10	GD24	*R.nitidus*	-0.079918	0.075574
SC14	SC10	GD24	*R.nitidus*	-0.04712	0.067782
JO8	CS4	GD24	*R.nitidus*	0.008574	0.073169
SC6	SC10	GD24	*R.nitidus*	-0.110351	0.069573
SC24	SC10	GD24	*R.nitidus*	-0.07371	0.069554
JO3	SC10	GD24	*R.nitidus*	-0.04977	0.072349
SC44	SC10	GD24	*R.nitidus*	-0.05198	0.066633
SC32	SC10	GD24	*R.nitidus*	-0.030973	0.066261
JO7	CS4	GD24	*R.nitidus*	-0.005045	0.072191
SC18	SC10	GD24	*R.nitidus*	-0.018584	0.068843
JO1	SC10	GD24	*R.nitidus*	-0.082203	0.067233
SC45	SC10	GD24	*R.nitidus*	-0.038494	0.068062
GD25	CS4	GD24	*R.nitidus*	0.06054	0.066107
SC26	SC10	GD24	*R.nitidus*	-0.12037	0.067118
GD29	CS4	GD24	*R.nitidus*	0.104925	0.063314
GD34	CS4	GD24	*R.nitidus*	0.012491	0.065683
GD40	CS4	GD24	*R.nitidus*	0.094923	0.065974
GD31	CS4	GD24	*R.nitidus*	0.079027	0.067274
MY4	SC10	GD24	*R.nitidus*	-0.113333	0.069502
GD30	CS4	GD24	*R.nitidus*	0.119935	0.06717
JO5	SC10	GD24	*R.nitidus*	-0.027523	0.068283
CS11	CS4	GD24	*R.nitidus*	0.010989	0.068249
MY6	SC10	GD24	*R.nitidus*	-0.092396	0.067555
GD21	CS4	GD24	*R.nitidus*	0.118056	0.064238
GD26	CS4	GD24	*R.nitidus*	0.054452	0.065344
GD46	CS4	GD24	*R.nitidus*	-0.002175	0.065376
GD42	CS4	GD24	*R.nitidus*	0.041999	0.065687

CS18	CS4	GD24	*R.nitidus*	-0.034662	0.064987
CS13	CS4	GD24	*R.nitidus*	-0.027244	0.068425
GD45	CS4	GD24	*R.nitidus*	0.086921	0.068857
CS12	CS4	GD24	*R.nitidus*	0.018608	0.06862
GD22	CS4	GD24	*R.nitidus*	0.082627	0.066385
GD50	CS4	GD24	*R.nitidus*	0.044866	0.06857
GD41	CS4	GD24	*R.nitidus*	0.025716	0.066034
GD32	CS4	GD24	*R.nitidus*	0.048101	0.068699
CS22	CS4	GD24	*R.nitidus*	0.037267	0.06478
SC5	CS4	GD24	*R.nitidus*	0.016458	0.068692
CS24	CS4	GD24	*R.nitidus*	-0.042816	0.064123
CS28	CS4	GD24	*R.nitidus*	-0.009228	0.066845
GD33	CS4	GD24	*R.nitidus*	0	0.067351
CS20	CS4	GD24	*R.nitidus*	-0.01815	0.066446
GD48	CS4	GD24	*R.nitidus*	0.003987	0.067573
GD44	CS4	GD24	*R.nitidus*	0.047356	0.066868
CS17	CS4	GD24	*R.nitidus*	0.042728	0.069276
CS25	CS4	GD24	*R.nitidus*	-0.016143	0.068568
CS15	CS4	GD24	*R.nitidus*	0.030395	0.068163
SC6	CS4	GD24	*R.nitidus*	-0.009195	0.067678
CS6	CS4	GD24	*R.nitidus*	-0.014822	0.065519
GD35	CS4	GD24	*R.nitidus*	0.052244	0.067617
SC14	CS4	GD24	*R.nitidus*	0.019186	0.065984
CS16	CS4	GD24	*R.nitidus*	0.003118	0.067898
CS19	CS4	GD24	*R.nitidus*	-0.02053	0.070442
CS14	CS4	GD24	*R.nitidus*	0.009317	0.06863
CS21	CS4	GD24	*R.nitidus*	0.00339	0.065697
SC28	CS4	GD24	*R.nitidus*	-0.005393	0.065495
SC32	CS4	GD24	*R.nitidus*	0.049961	0.065269
SC34	CS4	GD24	*R.nitidus*	0.036009	0.064729
SC35	CS4	GD24	*R.nitidus*	0.048499	0.067005
SC31	CS4	GD24	*R.nitidus*	0	0.066112
CS3	CS4	GD24	*R.nitidus*	-0.025855	0.066748
CS23	CS4	GD24	*R.nitidus*	-0.050086	0.064835
GD49	CS4	GD24	*R.nitidus*	0.111294	0.067959
SC24	CS4	GD24	*R.nitidus*	0.012719	0.066313
SC44	CS4	GD24	*R.nitidus*	0.030465	0.065811
SC12	CS4	GD24	*R.nitidus*	-0.02498	0.06665
SC15	CS4	GD24	*R.nitidus*	-0.004029	0.065926
SC10	CS4	GD24	*R.nitidus*	0.084243	0.067091
JW6	CS4	GD24	*R.nitidus*	-0.010721	0.071468
SC18	CS4	GD24	*R.nitidus*	0.063011	0.066742
CS31	CS4	GD24	*R.nitidus*	-0.015485	0.06547
CS30	CS4	GD24	*R.nitidus*	-0.049755	0.066571
MY4	CS4	GD24	*R.nitidus*	-0.035971	0.068341
JO8	SC12	GD24	*R.nitidus*	0.040678	0.073291
SC26	CS4	GD24	*R.nitidus*	-0.033233	0.065071
JW7	CS4	GD24	*R.nitidus*	0.02723	0.069564
MY6	CS4	GD24	*R.nitidus*	-0.007031	0.067247
SC27	CS4	GD24	*R.nitidus*	0.01932	0.066292
JO7	SC12	GD24	*R.nitidus*	-0.003195	0.073217
JO3	CS4	GD24	*R.nitidus*	0.030783	0.070823
GD34	SC12	GD24	*R.nitidus*	0.032895	0.066753
CS11	SC12	GD24	*R.nitidus*	0.023585	0.069169
JO1	CS4	GD24	*R.nitidus*	-0.000784	0.066614
JW4	CS4	GD24	*R.nitidus*	0.226033	0.066376

SC45	CS4	GD24	*R.nitidus*	0.04528	0.067433
GD31	SC12	GD24	*R.nitidus*	0.086045	0.068316
JO5	CS4	GD24	*R.nitidus*	0.062844	0.066196
SC42	CS4	GD24	*R.nitidus*	0.282324	0.062241
GD41	SC12	GD24	*R.nitidus*	0.032714	0.066737
GD35	SC12	GD24	*R.nitidus*	0.065201	0.068135
GD26	SC12	GD24	*R.nitidus*	0.076694	0.065865
GD32	SC12	GD24	*R.nitidus*	0.048882	0.068871
GD25	SC12	GD24	*R.nitidus*	0.082506	0.06749
CS14	SC12	GD24	*R.nitidus*	0.020882	0.070384
GD33	SC12	GD24	*R.nitidus*	0.015789	0.067942
GD21	SC12	GD24	*R.nitidus*	0.123682	0.066273
GD45	SC12	GD24	*R.nitidus*	0.084202	0.071384
GD48	SC12	GD24	*R.nitidus*	0.0375	0.069056
GD40	SC12	GD24	*R.nitidus*	0.110549	0.065828
GD30	SC12	GD24	*R.nitidus*	0.124051	0.069353
GD46	SC12	GD24	*R.nitidus*	0.019461	0.06671
CS13	SC12	GD24	*R.nitidus*	-0.012658	0.069479
SC10	SC12	GD24	*R.nitidus*	0.106608	0.065779
GD50	SC12	GD24	*R.nitidus*	0.08684	0.070544
GD29	SC12	GD24	*R.nitidus*	0.11213	0.066554
CS12	SC12	GD24	*R.nitidus*	0.025869	0.069353
CS23	SC12	GD24	*R.nitidus*	-0.014582	0.068934
CS28	SC12	GD24	*R.nitidus*	-0.000854	0.06864
GD22	SC12	GD24	*R.nitidus*	0.09744	0.065007
CS22	SC12	GD24	*R.nitidus*	0.042164	0.068852
CS15	SC12	GD24	*R.nitidus*	0.057296	0.069817
GD49	SC12	GD24	*R.nitidus*	0.11474	0.068052
CS17	SC12	GD24	*R.nitidus*	0.053686	0.071411
SC18	SC12	GD24	*R.nitidus*	0.113208	0.066927
CS20	SC12	GD24	*R.nitidus*	-0.005335	0.068888
SC28	SC12	GD24	*R.nitidus*	0.023952	0.066813
GD42	SC12	GD24	*R.nitidus*	0.055762	0.065639
CS19	SC12	GD24	*R.nitidus*	0	0.070595
SC27	SC12	GD24	*R.nitidus*	0.050757	0.066159
GD44	SC12	GD24	*R.nitidus*	0.059181	0.068528
CS4	SC12	GD24	*R.nitidus*	0.02498	0.06665
CS30	SC12	GD24	*R.nitidus*	-0.020048	0.068507
CS31	SC12	GD24	*R.nitidus*	0.00487	0.068181
CS24	SC12	GD24	*R.nitidus*	-0.014504	0.068691
SC5	SC12	GD24	*R.nitidus*	0.03937	0.070797
CS16	SC12	GD24	*R.nitidus*	0.028103	0.070046
CS18	SC12	GD24	*R.nitidus*	-0.016381	0.068359
SC34	SC12	GD24	*R.nitidus*	0.070578	0.066988
CS21	SC12	GD24	*R.nitidus*	0.007698	0.067671
SC31	SC12	GD24	*R.nitidus*	0.025357	0.068223
SC24	SC12	GD24	*R.nitidus*	0.037531	0.067305
CS3	SC12	GD24	*R.nitidus*	0.0144	0.067947
SC42	SC12	GD24	*R.nitidus*	0.306366	0.061595
SC26	SC12	GD24	*R.nitidus*	-0.010815	0.065325
JO5	SC12	GD24	*R.nitidus*	0.090528	0.067476
SC6	SC12	GD24	*R.nitidus*	0.010879	0.068024
CS25	SC12	GD24	*R.nitidus*	0.018283	0.070387
JO8	SC14	GD24	*R.nitidus*	-0.02139	0.075185
MY4	SC12	GD24	*R.nitidus*	-0.01129	0.06751
SC14	SC12	GD24	*R.nitidus*	0.047578	0.067599

SC15	SC12	GD24	*R.nitidus*	0.017918	0.067753
SC45	SC12	GD24	*R.nitidus*	0.075232	0.066784
MY6	SC12	GD24	*R.nitidus*	0.013557	0.069522
JO7	SC14	GD24	*R.nitidus*	-0.039256	0.073493
JW4	SC12	GD24	*R.nitidus*	0.253354	0.066936
GD32	SC14	GD24	*R.nitidus*	0.001643	0.067973
GD41	SC14	GD24	*R.nitidus*	-0.002288	0.064869
GD26	SC14	GD24	*R.nitidus*	0.028986	0.066767
SC35	SC12	GD24	*R.nitidus*	0.099749	0.063674
CS6	SC12	GD24	*R.nitidus*	-0.015728	0.067987
JW6	SC12	GD24	*R.nitidus*	0.040041	0.072896
GD40	SC14	GD24	*R.nitidus*	0.055637	0.065534
SC44	SC12	GD24	*R.nitidus*	0.052585	0.066583
JW7	SC12	GD24	*R.nitidus*	0.084536	0.072248
GD34	SC14	GD24	*R.nitidus*	-0.02185	0.065943
SC32	SC12	GD24	*R.nitidus*	0.080262	0.067978
GD22	SC14	GD24	*R.nitidus*	0.048985	0.065701
GD30	SC14	GD24	*R.nitidus*	0.093698	0.070617
JO3	SC12	GD24	*R.nitidus*	0.060793	0.07124
CS13	SC14	GD24	*R.nitidus*	-0.053125	0.067039
GD29	SC14	GD24	*R.nitidus*	0.074616	0.062987
GD35	SC14	GD24	*R.nitidus*	0.019564	0.067328
JO1	SC12	GD24	*R.nitidus*	0.014913	0.068144
GD31	SC14	GD24	*R.nitidus*	0.058378	0.067229
CS14	SC14	GD24	*R.nitidus*	-0.022087	0.069419
GD50	SC14	GD24	*R.nitidus*	0.017513	0.068088
GD33	SC14	GD24	*R.nitidus*	-0.038905	0.067894
CS11	SC14	GD24	*R.nitidus*	-0.019847	0.069973
CS12	SC14	GD24	*R.nitidus*	-0.013449	0.069058
GD21	SC14	GD24	*R.nitidus*	0.090975	0.066873
GD44	SC14	GD24	*R.nitidus*	0.011962	0.068939
CS19	SC14	GD24	*R.nitidus*	-0.049051	0.070279
GD25	SC14	GD24	*R.nitidus*	0.043866	0.067363
CS16	SC14	GD24	*R.nitidus*	-0.016807	0.066682
CS20	SC14	GD24	*R.nitidus*	-0.044731	0.066653
CS17	SC14	GD24	*R.nitidus*	0.009926	0.071057
GD49	SC14	GD24	*R.nitidus*	0.080882	0.069829
GD45	SC14	GD24	*R.nitidus*	0.037643	0.071966
GD42	SC14	GD24	*R.nitidus*	0.012782	0.065508
GD46	SC14	GD24	*R.nitidus*	-0.037258	0.066798
CS21	SC14	GD24	*R.nitidus*	-0.046743	0.067036
GD48	SC14	GD24	*R.nitidus*	-0.011914	0.069382
CS31	SC14	GD24	*R.nitidus*	-0.046374	0.069187
CS22	SC14	GD24	*R.nitidus*	-0.011006	0.067725
SC34	SC14	GD24	*R.nitidus*	0.019247	0.064948
CS23	SC14	GD24	*R.nitidus*	-0.072617	0.067881
SC15	SC14	GD24	*R.nitidus*	-0.04386	0.067884
CS3	SC14	GD24	*R.nitidus*	-0.044917	0.066408
CS25	SC14	GD24	*R.nitidus*	-0.035074	0.068118
SC12	SC14	GD24	*R.nitidus*	-0.047578	0.067599
CS15	SC14	GD24	*R.nitidus*	0.016129	0.069062
SC5	SC14	GD24	*R.nitidus*	-0.018182	0.070395
SC28	SC14	GD24	*R.nitidus*	-0.035354	0.06653
SC10	SC14	GD24	*R.nitidus*	0.04712	0.067782
SC26	SC14	GD24	*R.nitidus*	-0.062762	0.066961
CS6	SC14	GD24	*R.nitidus*	-0.056743	0.065718

CS18	SC14	GD24	*R.nitidus*	-0.057276	0.067576
CS30	SC14	GD24	*R.nitidus*	-0.069268	0.067567
CS28	SC14	GD24	*R.nitidus*	-0.044839	0.069468
SC24	SC14	GD24	*R.nitidus*	-0.01598	0.069092
SC6	SC14	GD24	*R.nitidus*	-0.04465	0.071213
SC42	SC14	GD24	*R.nitidus*	0.271053	0.06206
SC18	SC14	GD24	*R.nitidus*	0.039233	0.068294
CS4	SC14	GD24	*R.nitidus*	-0.019186	0.065984
CS24	SC14	GD24	*R.nitidus*	-0.058581	0.06535
SC44	SC14	GD24	*R.nitidus*	0.001698	0.065778
SC45	SC14	GD24	*R.nitidus*	0.02126	0.066812
JW7	SC14	GD24	*R.nitidus*	0.027174	0.071331
MY6	SC14	GD24	*R.nitidus*	-0.036293	0.065918
JO5	SC14	GD24	*R.nitidus*	0.043257	0.066173
SC32	SC14	GD24	*R.nitidus*	0.025618	0.066302
SC27	SC14	GD24	*R.nitidus*	-0.011442	0.062868
SC35	SC14	GD24	*R.nitidus*	0.028854	0.064738
JO8	SC15	GD24	*R.nitidus*	0.009009	0.077495
JW6	SC14	GD24	*R.nitidus*	-0.018367	0.071954
JW4	SC14	GD24	*R.nitidus*	0.20827	0.066734
GD26	SC15	GD24	*R.nitidus*	0.055765	0.065793
JO1	SC14	GD24	*R.nitidus*	-0.013083	0.065316
SC31	SC14	GD24	*R.nitidus*	-0.026138	0.066655
GD21	SC15	GD24	*R.nitidus*	0.127407	0.067149
JO7	SC15	GD24	*R.nitidus*	-0.012959	0.076918
GD33	SC15	GD24	*R.nitidus*	0.006098	0.067938
GD42	SC15	GD24	*R.nitidus*	0.062745	0.068318
JO3	SC14	GD24	*R.nitidus*	0.004374	0.071728
GD46	SC15	GD24	*R.nitidus*	0.003145	0.068011
MY4	SC14	GD24	*R.nitidus*	-0.065183	0.066998
GD48	SC15	GD24	*R.nitidus*	0.028814	0.069853
GD22	SC15	GD24	*R.nitidus*	0.082845	0.067478
GD25	SC15	GD24	*R.nitidus*	0.078025	0.067855
GD35	SC15	GD24	*R.nitidus*	0.051488	0.069562
GD40	SC15	GD24	*R.nitidus*	0.101208	0.066432
CS11	SC15	GD24	*R.nitidus*	0.01876	0.070291
GD41	SC15	GD24	*R.nitidus*	0.023622	0.066953
GD49	SC15	GD24	*R.nitidus*	0.13198	0.067735
CS18	SC15	GD24	*R.nitidus*	-0.029362	0.067865
GD32	SC15	GD24	*R.nitidus*	0.021645	0.069004
GD30	SC15	GD24	*R.nitidus*	0.129004	0.071634
CS15	SC15	GD24	*R.nitidus*	0.0456	0.070889
CS21	SC15	GD24	*R.nitidus*	0.00081	0.068275
CS20	SC15	GD24	*R.nitidus*	-0.000804	0.069018
GD31	SC15	GD24	*R.nitidus*	0.087843	0.069399
CS23	SC15	GD24	*R.nitidus*	-0.042484	0.067788
GD50	SC15	GD24	*R.nitidus*	0.038211	0.069867
GD29	SC15	GD24	*R.nitidus*	0.116679	0.066162
CS3	SC15	GD24	*R.nitidus*	-0.005714	0.067249
CS22	SC15	GD24	*R.nitidus*	0.022895	0.066543
GD45	SC15	GD24	*R.nitidus*	0.06955	0.071849
CS12	SC15	GD24	*R.nitidus*	0.01268	0.067952
CS30	SC15	GD24	*R.nitidus*	-0.028814	0.067211
GD34	SC15	GD24	*R.nitidus*	0.02566	0.066657
GD44	SC15	GD24	*R.nitidus*	0.022783	0.068774
SC18	SC15	GD24	*R.nitidus*	0.080176	0.069543

CS14	SC15	GD24	*R.nitidus*	0.01319	0.071078
SC12	SC15	GD24	*R.nitidus*	-0.017918	0.067753
CS25	SC15	GD24	*R.nitidus*	-0.001649	0.070503
SC27	SC15	GD24	*R.nitidus*	0.035846	0.067039
CS16	SC15	GD24	*R.nitidus*	0.00982	0.06976
CS17	SC15	GD24	*R.nitidus*	0.050994	0.071445
CS19	SC15	GD24	*R.nitidus*	-0.016653	0.070033
CS31	SC15	GD24	*R.nitidus*	-0.00088	0.070962
SC14	SC15	GD24	*R.nitidus*	0.04386	0.067884
CS13	SC15	GD24	*R.nitidus*	-0.016835	0.070372
CS28	SC15	GD24	*R.nitidus*	0	0.069612
SC34	SC15	GD24	*R.nitidus*	0.05503	0.066819
CS24	SC15	GD24	*R.nitidus*	-0.02385	0.065897
SC6	SC15	GD24	*R.nitidus*	0.009466	0.069602
CS4	SC15	GD24	*R.nitidus*	0.004029	0.065926
SC31	SC15	GD24	*R.nitidus*	0.015357	0.067538
SC5	SC15	GD24	*R.nitidus*	0.021044	0.071462
SC24	SC15	GD24	*R.nitidus*	0.015679	0.069407
JW6	SC15	GD24	*R.nitidus*	0.013757	0.075239
JO7	SC18	GD24	*R.nitidus*	-0.075027	0.073055
MY4	SC15	GD24	*R.nitidus*	-0.023829	0.068579
SC28	SC15	GD24	*R.nitidus*	0.005902	0.068408
SC35	SC15	GD24	*R.nitidus*	0.071973	0.06715
JW7	SC15	GD24	*R.nitidus*	0.063674	0.074069
SC42	SC15	GD24	*R.nitidus*	0.301379	0.064074
GD21	SC18	GD24	*R.nitidus*	0.069248	0.067153
SC10	SC15	GD24	*R.nitidus*	0.089136	0.068876
CS6	SC15	GD24	*R.nitidus*	-0.019312	0.06745
SC44	SC15	GD24	*R.nitidus*	0.041876	0.068009
SC26	SC15	GD24	*R.nitidus*	-0.027569	0.066474
JO1	SC15	GD24	*R.nitidus*	0.008432	0.069038
SC45	SC15	GD24	*R.nitidus*	0.05848	0.066722
JO3	SC15	GD24	*R.nitidus*	0.030359	0.070775
JW4	SC15	GD24	*R.nitidus*	0.233071	0.068708
SC32	SC15	GD24	*R.nitidus*	0.068481	0.069517
GD25	SC18	GD24	*R.nitidus*	0.021012	0.068415
GD22	SC18	GD24	*R.nitidus*	0.018018	0.06774
GD40	SC18	GD24	*R.nitidus*	0.028549	0.064186
JO8	SC18	GD24	*R.nitidus*	-0.053381	0.074303
GD26	SC18	GD24	*R.nitidus*	-0.003873	0.065755
GD29	SC18	GD24	*R.nitidus*	0.03982	0.065988
MY6	SC15	GD24	*R.nitidus*	0	0.065853
GD44	SC18	GD24	*R.nitidus*	-0.006645	0.068056
CS11	SC18	GD24	*R.nitidus*	-0.053864	0.068631
GD45	SC18	GD24	*R.nitidus*	0.007895	0.071252
CS12	SC18	GD24	*R.nitidus*	-0.066774	0.06848
GD32	SC18	GD24	*R.nitidus*	-0.053097	0.068831
JO5	SC15	GD24	*R.nitidus*	0.080617	0.065458
GD31	SC18	GD24	*R.nitidus*	0.020884	0.068173
GD50	SC18	GD24	*R.nitidus*	-0.000933	0.071765
GD41	SC18	GD24	*R.nitidus*	-0.0278	0.066475
CS19	SC18	GD24	*R.nitidus*	-0.090986	0.069277
CS24	SC18	GD24	*R.nitidus*	-0.088797	0.067591
GD42	SC18	GD24	*R.nitidus*	0.000782	0.066755
CS30	SC18	GD24	*R.nitidus*	-0.109578	0.068112
CS15	SC18	GD24	*R.nitidus*	-0.026963	0.068957

CS13	SC18	GD24	*R.nitidus*	-0.079936	0.071168
GD30	SC18	GD24	*R.nitidus*	0.066265	0.069067
CS14	SC18	GD24	*R.nitidus*	-0.044917	0.071664
GD33	SC18	GD24	*R.nitidus*	-0.061033	0.068219
GD34	SC18	GD24	*R.nitidus*	-0.045879	0.066823
GD46	SC18	GD24	*R.nitidus*	-0.070983	0.064627
GD35	SC18	GD24	*R.nitidus*	0.003968	0.06714
CS20	SC18	GD24	*R.nitidus*	-0.092683	0.066668
CS23	SC18	GD24	*R.nitidus*	-0.115196	0.067585
CS17	SC18	GD24	*R.nitidus*	-0.015422	0.071171
GD49	SC18	GD24	*R.nitidus*	0.060198	0.070195
GD48	SC18	GD24	*R.nitidus*	-0.05675	0.070658
SC12	SC18	GD24	*R.nitidus*	-0.113208	0.066927
CS25	SC18	GD24	*R.nitidus*	-0.07225	0.069002
CS22	SC18	GD24	*R.nitidus*	-0.046053	0.069374
CS21	SC18	GD24	*R.nitidus*	-0.080735	0.066573
CS16	SC18	GD24	*R.nitidus*	-0.057325	0.067934
CS6	SC18	GD24	*R.nitidus*	-0.107546	0.064658
CS3	SC18	GD24	*R.nitidus*	-0.095477	0.067908
CS28	SC18	GD24	*R.nitidus*	-0.079487	0.069645
CS31	SC18	GD24	*R.nitidus*	-0.084843	0.069308
JO7	SC24	GD24	*R.nitidus*	-0.062227	0.072511
CS18	SC18	GD24	*R.nitidus*	-0.109512	0.067644
MY6	SC18	GD24	*R.nitidus*	-0.090219	0.067001
SC27	SC18	GD24	*R.nitidus*	-0.041108	0.067959
SC26	SC18	GD24	*R.nitidus*	-0.11986	0.066477
SC24	SC18	GD24	*R.nitidus*	-0.084364	0.065571
SC31	SC18	GD24	*R.nitidus*	-0.070435	0.067353
SC28	SC18	GD24	*R.nitidus*	-0.099373	0.065432
CS4	SC18	GD24	*R.nitidus*	-0.063011	0.066742
SC35	SC18	GD24	*R.nitidus*	-0.014468	0.06667
SC5	SC18	GD24	*R.nitidus*	-0.059807	0.07073
JW6	SC18	GD24	*R.nitidus*	-0.044968	0.07409
SC10	SC18	GD24	*R.nitidus*	0.018584	0.068843
SC32	SC18	GD24	*R.nitidus*	-0.019248	0.066688
SC15	SC18	GD24	*R.nitidus*	-0.080176	0.069543
SC44	SC18	GD24	*R.nitidus*	-0.055456	0.067087
SC6	SC18	GD24	*R.nitidus*	-0.071986	0.069487
JO1	SC18	GD24	*R.nitidus*	-0.06563	0.068168
SC14	SC18	GD24	*R.nitidus*	-0.039233	0.068294
JO3	SC18	GD24	*R.nitidus*	-0.037684	0.073301
JW7	SC18	GD24	*R.nitidus*	-0.002125	0.070381
CS11	SC24	GD24	*R.nitidus*	-0.000775	0.070341
GD31	SC24	GD24	*R.nitidus*	0.072686	0.069608
SC45	SC18	GD24	*R.nitidus*	-0.029285	0.067532
JO8	SC24	GD24	*R.nitidus*	-0.02381	0.074868
JO5	SC18	GD24	*R.nitidus*	0	0.065558
SC34	SC18	GD24	*R.nitidus*	-0.035473	0.067651
JW4	SC18	GD24	*R.nitidus*	0.174267	0.06912
SC42	SC18	GD24	*R.nitidus*	0.22894	0.063986
GD26	SC24	GD24	*R.nitidus*	0.047547	0.068716
GD22	SC24	GD24	*R.nitidus*	0.071221	0.068081
GD49	SC24	GD24	*R.nitidus*	0.127011	0.070509
GD41	SC24	GD24	*R.nitidus*	0.020737	0.065967
MY4	SC18	GD24	*R.nitidus*	-0.116939	0.066207
GD42	SC24	GD24	*R.nitidus*	0.038491	0.066595

GD35	SC24	GD24	*R.nitidus*	0.049574	0.066984
GD25	SC24	GD24	*R.nitidus*	0.053354	0.06879
CS12	SC24	GD24	*R.nitidus*	-0.007987	0.069279
GD44	SC24	GD24	*R.nitidus*	0.032232	0.069177
GD32	SC24	GD24	*R.nitidus*	0.012898	0.070168
GD30	SC24	GD24	*R.nitidus*	0.124587	0.069267
GD21	SC24	GD24	*R.nitidus*	0.113997	0.066477
GD48	SC24	GD24	*R.nitidus*	-0.011785	0.06956
CS18	SC24	GD24	*R.nitidus*	-0.048309	0.068787
GD29	SC24	GD24	*R.nitidus*	0.094084	0.065824
SC5	SC24	GD24	*R.nitidus*	-0.01463	0.072629
CS13	SC24	GD24	*R.nitidus*	-0.037855	0.069948
SC10	SC24	GD24	*R.nitidus*	0.07371	0.069554
GD34	SC24	GD24	*R.nitidus*	0.003023	0.067733
GD46	SC24	GD24	*R.nitidus*	-0.015385	0.065793
GD33	SC24	GD24	*R.nitidus*	-0.009673	0.067883
GD40	SC24	GD24	*R.nitidus*	0.070015	0.066165
CS15	SC24	GD24	*R.nitidus*	0.025994	0.0684
CS14	SC24	GD24	*R.nitidus*	-0.009317	0.071411
SC27	SC24	GD24	*R.nitidus*	0.022184	0.069422
SC15	SC24	GD24	*R.nitidus*	-0.015679	0.069407
CS21	SC24	GD24	*R.nitidus*	-0.023052	0.068503
CS19	SC24	GD24	*R.nitidus*	-0.038907	0.069445
GD50	SC24	GD24	*R.nitidus*	0.073516	0.069955
CS22	SC24	GD24	*R.nitidus*	0.007813	0.067245
CS20	SC24	GD24	*R.nitidus*	-0.033832	0.067037
CS25	SC24	GD24	*R.nitidus*	-0.0278	0.069274
CS23	SC24	GD24	*R.nitidus*	-0.057325	0.068894
GD45	SC24	GD24	*R.nitidus*	0.048454	0.072389
CS30	SC24	GD24	*R.nitidus*	-0.057371	0.069087
CS3	SC24	GD24	*R.nitidus*	-0.039571	0.068824
MY4	SC24	GD24	*R.nitidus*	-0.049423	0.066965
CS17	SC24	GD24	*R.nitidus*	0.026251	0.070389
CS16	SC24	GD24	*R.nitidus*	-0.010023	0.067614
SC14	SC24	GD24	*R.nitidus*	0.01598	0.069092
SC28	SC24	GD24	*R.nitidus*	-0.031171	0.067726
CS24	SC24	GD24	*R.nitidus*	-0.04238	0.067063
SC6	SC24	GD24	*R.nitidus*	-0.024914	0.06837
SC35	SC24	GD24	*R.nitidus*	0.04553	0.066526
SC12	SC24	GD24	*R.nitidus*	-0.037531	0.067305
CS28	SC24	GD24	*R.nitidus*	-0.032618	0.068418
SC44	SC24	GD24	*R.nitidus*	0.01364	0.067324
CS4	SC24	GD24	*R.nitidus*	-0.012719	0.066313
JW4	SC24	GD24	*R.nitidus*	0.206731	0.067557
CS31	SC24	GD24	*R.nitidus*	-0.022951	0.06876
SC26	SC24	GD24	*R.nitidus*	-0.046571	0.067152
CS6	SC24	GD24	*R.nitidus*	-0.057823	0.066403
GD25	SC26	GD24	*R.nitidus*	0.09139	0.0678
SC42	SC24	GD24	*R.nitidus*	0.277598	0.063875
SC18	SC24	GD24	*R.nitidus*	0.084364	0.065571
JO8	SC26	GD24	*R.nitidus*	0.074398	0.074537
SC45	SC24	GD24	*R.nitidus*	0.041322	0.065839
SC31	SC24	GD24	*R.nitidus*	-0.008382	0.067557
JO3	SC24	GD24	*R.nitidus*	0.015426	0.072954
GD26	SC26	GD24	*R.nitidus*	0.085312	0.06717
JW6	SC24	GD24	*R.nitidus*	-0.030043	0.075236

GD22	SC26	GD24	*R.nitidus*	0.09937	0.066145
JW7	SC24	GD24	*R.nitidus*	0.038934	0.071788
GD35	SC26	GD24	*R.nitidus*	0.069322	0.067772
SC32	SC24	GD24	*R.nitidus*	0.046991	0.067811
SC34	SC24	GD24	*R.nitidus*	0.029437	0.065725
MY6	SC24	GD24	*R.nitidus*	-0.021224	0.067328
GD32	SC26	GD24	*R.nitidus*	0.047337	0.069468
JO5	SC24	GD24	*R.nitidus*	0.05464	0.066451
CS12	SC26	GD24	*R.nitidus*	0.03537	0.067333
GD42	SC26	GD24	*R.nitidus*	0.068264	0.068192
GD29	SC26	GD24	*R.nitidus*	0.13132	0.066332
GD30	SC26	GD24	*R.nitidus*	0.141803	0.0701
JO7	SC26	GD24	*R.nitidus*	0.005133	0.073526
GD34	SC26	GD24	*R.nitidus*	0.036846	0.065075
JO1	SC24	GD24	*R.nitidus*	-0.006536	0.067993
GD21	SC26	GD24	*R.nitidus*	0.150523	0.064457
GD48	SC26	GD24	*R.nitidus*	0.05	0.068539
GD49	SC26	GD24	*R.nitidus*	0.142145	0.070224
GD50	SC26	GD24	*R.nitidus*	0.070859	0.070524
CS13	SC26	GD24	*R.nitidus*	0.010383	0.069022
CS16	SC26	GD24	*R.nitidus*	0.033742	0.066432
GD40	SC26	GD24	*R.nitidus*	0.112491	0.065714
CS18	SC26	GD24	*R.nitidus*	-0.011494	0.066395
GD44	SC26	GD24	*R.nitidus*	0.057471	0.067855
GD31	SC26	GD24	*R.nitidus*	0.103053	0.066968
GD41	SC26	GD24	*R.nitidus*	0.048673	0.067672
CS11	SC26	GD24	*R.nitidus*	0.040435	0.067495
GD45	SC26	GD24	*R.nitidus*	0.086124	0.069029
GD33	SC26	GD24	*R.nitidus*	0.031828	0.066863
CS28	SC26	GD24	*R.nitidus*	0.01519	0.07035
GD46	SC26	GD24	*R.nitidus*	0.018396	0.066252
CS23	SC26	GD24	*R.nitidus*	-0.015267	0.068535
CS4	SC26	GD24	*R.nitidus*	0.033233	0.065071
CS21	SC26	GD24	*R.nitidus*	0.019335	0.066243
CS24	SC26	GD24	*R.nitidus*	-0.001601	0.06629
CS15	SC26	GD24	*R.nitidus*	0.070433	0.068182
CS19	SC26	GD24	*R.nitidus*	0.008648	0.068664
CS20	SC26	GD24	*R.nitidus*	0.010196	0.067882
CS17	SC26	GD24	*R.nitidus*	0.064673	0.068844
CS14	SC26	GD24	*R.nitidus*	0.043011	0.068676
SC27	SC26	GD24	*R.nitidus*	0.052174	0.065625
MY4	SC26	GD24	*R.nitidus*	0.003934	0.064689
SC44	SC26	GD24	*R.nitidus*	0.060855	0.067306
JW6	SC26	GD24	*R.nitidus*	0.024793	0.07385
JO3	SC26	GD24	*R.nitidus*	0.063194	0.070897
SC45	SC26	GD24	*R.nitidus*	0.09688	0.065761
SC35	SC26	GD24	*R.nitidus*	0.101133	0.064491
CS22	SC26	GD24	*R.nitidus*	0.052836	0.065828
CS25	SC26	GD24	*R.nitidus*	0.013057	0.067884
CS3	SC26	GD24	*R.nitidus*	0.010317	0.066087
SC32	SC26	GD24	*R.nitidus*	0.086235	0.065423
CS31	SC26	GD24	*R.nitidus*	0.011981	0.06876
JO8	SC27	GD24	*R.nitidus*	-0.009879	0.074551
SC24	SC26	GD24	*R.nitidus*	0.046571	0.067152
SC42	SC26	GD24	*R.nitidus*	0.297886	0.062003
CS6	SC26	GD24	*R.nitidus*	0	0.065152

SC12	SC26	GD24	*R.nitidus*	0.010815	0.065325
JO7	SC27	GD24	*R.nitidus*	-0.051363	0.073086
CS30	SC26	GD24	*R.nitidus*	-0.020553	0.066144
SC28	SC26	GD24	*R.nitidus*	0.034202	0.06621
SC15	SC26	GD24	*R.nitidus*	0.027569	0.066474
SC5	SC26	GD24	*R.nitidus*	0.051325	0.069821
JW7	SC26	GD24	*R.nitidus*	0.09751	0.071063
SC18	SC26	GD24	*R.nitidus*	0.11986	0.066477
SC34	SC26	GD24	*R.nitidus*	0.077165	0.064187
SC10	SC26	GD24	*R.nitidus*	0.12037	0.067118
SC6	SC26	GD24	*R.nitidus*	0.029711	0.069285
JO5	SC26	GD24	*R.nitidus*	0.095238	0.067474
GD26	SC27	GD24	*R.nitidus*	0.035242	0.066753
SC14	SC26	GD24	*R.nitidus*	0.062762	0.066961
SC31	SC26	GD24	*R.nitidus*	0.046473	0.065974
JW4	SC26	GD24	*R.nitidus*	0.260903	0.067191
MY6	SC26	GD24	*R.nitidus*	0.021538	0.065151
GD32	SC27	GD24	*R.nitidus*	-0.001667	0.068373
GD21	SC27	GD24	*R.nitidus*	0.103676	0.06747
GD31	SC27	GD24	*R.nitidus*	0.050965	0.067755
GD34	SC27	GD24	*R.nitidus*	-0.005913	0.067104
CS11	SC27	GD24	*R.nitidus*	-0.000799	0.068463
GD48	SC27	GD24	*R.nitidus*	-0.009804	0.067853
GD33	SC27	GD24	*R.nitidus*	-0.028804	0.068884
GD42	SC27	GD24	*R.nitidus*	0.026094	0.066169
GD41	SC27	GD24	*R.nitidus*	0.009245	0.065623
JO1	SC26	GD24	*R.nitidus*	0.032231	0.065408
CS12	SC27	GD24	*R.nitidus*	-0.020084	0.067847
GD22	SC27	GD24	*R.nitidus*	0.052031	0.066437
CS24	SC27	GD24	*R.nitidus*	-0.054514	0.065922
CS18	SC27	GD24	*R.nitidus*	-0.06874	0.068631
GD30	SC27	GD24	*R.nitidus*	0.108894	0.06979
GD25	SC27	GD24	*R.nitidus*	0.059713	0.065627
GD40	SC27	GD24	*R.nitidus*	0.068966	0.06531
CS21	SC27	GD24	*R.nitidus*	-0.032993	0.067515
GD45	SC27	GD24	*R.nitidus*	0.043874	0.070143
CS25	SC27	GD24	*R.nitidus*	-0.029695	0.068874
GD50	SC27	GD24	*R.nitidus*	0.042962	0.066684
CS23	SC27	GD24	*R.nitidus*	-0.066879	0.068312
GD46	SC27	GD24	*R.nitidus*	-0.02069	0.066269
GD49	SC27	GD24	*R.nitidus*	0.087531	0.069301
SC10	SC27	GD24	*R.nitidus*	0.060773	0.066394
CS14	SC27	GD24	*R.nitidus*	-0.011102	0.06864
CS19	SC27	GD24	*R.nitidus*	-0.050736	0.072626
CS22	SC27	GD24	*R.nitidus*	-0.004862	0.067641
CS17	SC27	GD24	*R.nitidus*	0.02906	0.068655
GD44	SC27	GD24	*R.nitidus*	0.01201	0.068899
CS28	SC27	GD24	*R.nitidus*	-0.037587	0.068167
CS13	SC27	GD24	*R.nitidus*	-0.04964	0.068508
GD29	SC27	GD24	*R.nitidus*	0.08	0.063922
SC5	SC27	GD24	*R.nitidus*	-0.015358	0.071188
SC6	SC27	GD24	*R.nitidus*	-0.030534	0.069858
CS15	SC27	GD24	*R.nitidus*	0.026149	0.068071
CS30	SC27	GD24	*R.nitidus*	-0.07233	0.066737
CS16	SC27	GD24	*R.nitidus*	-0.017433	0.066497
CS20	SC27	GD24	*R.nitidus*	-0.039782	0.067823

SC15	SC27	GD24	*R.nitidus*	-0.035846	0.067039
SC35	SC27	GD24	*R.nitidus*	0.04526	0.065373
SC26	SC27	GD24	*R.nitidus*	-0.052174	0.065625
SC14	SC27	GD24	*R.nitidus*	0.011442	0.062868
CS31	SC27	GD24	*R.nitidus*	-0.038884	0.06781
SC12	SC27	GD24	*R.nitidus*	-0.050757	0.066159
SC34	SC27	GD24	*R.nitidus*	0.025	0.064088
SC44	SC27	GD24	*R.nitidus*	0.006034	0.065755
CS6	SC27	GD24	*R.nitidus*	-0.057452	0.066032
GD35	SC27	GD24	*R.nitidus*	0.036406	0.066598
SC42	SC27	GD24	*R.nitidus*	0.277926	0.063242
CS3	SC27	GD24	*R.nitidus*	-0.02494	0.06605
JO8	SC28	GD24	*R.nitidus*	0.003432	0.074303
MY4	SC27	GD24	*R.nitidus*	-0.059289	0.066013
CS4	SC27	GD24	*R.nitidus*	-0.01932	0.066292
GD25	SC28	GD24	*R.nitidus*	0.079699	0.067005
SC24	SC27	GD24	*R.nitidus*	-0.022184	0.069422
JW6	SC27	GD24	*R.nitidus*	-0.010537	0.072695
SC18	SC27	GD24	*R.nitidus*	0.041108	0.067959
SC28	SC27	GD24	*R.nitidus*	-0.033972	0.067564
JO5	SC27	GD24	*R.nitidus*	0.045061	0.067007
SC31	SC27	GD24	*R.nitidus*	-0.018441	0.066994
JO1	SC27	GD24	*R.nitidus*	-0.010067	0.066156
SC45	SC27	GD24	*R.nitidus*	0.024155	0.066599
JO7	SC28	GD24	*R.nitidus*	-0.046316	0.074236
MY6	SC27	GD24	*R.nitidus*	-0.028974	0.065555
GD30	SC28	GD24	*R.nitidus*	0.129139	0.067386
GD33	SC28	GD24	*R.nitidus*	-0.000745	0.066896
JW4	SC27	GD24	*R.nitidus*	0.198635	0.067337
GD22	SC28	GD24	*R.nitidus*	0.095871	0.066509
GD29	SC28	GD24	*R.nitidus*	0.111898	0.06643
GD40	SC28	GD24	*R.nitidus*	0.093363	0.065083
CS12	SC28	GD24	*R.nitidus*	0.027138	0.067467
GD31	SC28	GD24	*R.nitidus*	0.092508	0.066757
GD26	SC28	GD24	*R.nitidus*	0.059779	0.067402
SC32	SC27	GD24	*R.nitidus*	0.028907	0.064655
JW7	SC27	GD24	*R.nitidus*	0.030837	0.072303
GD32	SC28	GD24	*R.nitidus*	0.019492	0.068598
GD21	SC28	GD24	*R.nitidus*	0.124826	0.065691
JO3	SC27	GD24	*R.nitidus*	-0.005386	0.071386
CS11	SC28	GD24	*R.nitidus*	0.022621	0.069304
GD46	SC28	GD24	*R.nitidus*	0.010264	0.065743
GD34	SC28	GD24	*R.nitidus*	0.020483	0.065783
CS25	SC28	GD24	*R.nitidus*	0.006265	0.068793
CS24	SC28	GD24	*R.nitidus*	-0.032514	0.067694
GD50	SC28	GD24	*R.nitidus*	0.057996	0.068398
GD48	SC28	GD24	*R.nitidus*	0.019841	0.068153
GD49	SC28	GD24	*R.nitidus*	0.118699	0.067275
CS13	SC28	GD24	*R.nitidus*	-0.0064	0.06802
CS30	SC28	GD24	*R.nitidus*	-0.038735	0.065759
CS31	SC28	GD24	*R.nitidus*	-0.009002	0.06783
CS20	SC28	GD24	*R.nitidus*	-0.011424	0.068162
GD41	SC28	GD24	*R.nitidus*	0.031418	0.067294
CS18	SC28	GD24	*R.nitidus*	-0.02866	0.067548
CS21	SC28	GD24	*R.nitidus*	-0.008365	0.066305
CS19	SC28	GD24	*R.nitidus*	-0.014096	0.069522

GD45	SC28	GD24	*R.nitidus*	0.086627	0.067883
GD42	SC28	GD24	*R.nitidus*	0.053933	0.066373
GD44	SC28	GD24	*R.nitidus*	0.049089	0.067957
CS23	SC28	GD24	*R.nitidus*	-0.016756	0.066817
CS3	SC28	GD24	*R.nitidus*	0.002412	0.06789
SC24	SC28	GD24	*R.nitidus*	0.031171	0.067726
CS17	SC28	GD24	*R.nitidus*	0.065413	0.069475
SC18	SC28	GD24	*R.nitidus*	0.099373	0.065432
CS15	SC28	GD24	*R.nitidus*	0.056942	0.068297
CS14	SC28	GD24	*R.nitidus*	0.015164	0.070047
GD35	SC28	GD24	*R.nitidus*	0.052006	0.067631
CS16	SC28	GD24	*R.nitidus*	0.02707	0.068638
SC14	SC28	GD24	*R.nitidus*	0.035354	0.06653
JW4	SC28	GD24	*R.nitidus*	0.227061	0.066432
SC10	SC28	GD24	*R.nitidus*	0.103242	0.067017
SC15	SC28	GD24	*R.nitidus*	-0.005902	0.068408
SC27	SC28	GD24	*R.nitidus*	0.033972	0.067564
CS22	SC28	GD24	*R.nitidus*	0.0224	0.067589
SC45	SC28	GD24	*R.nitidus*	0.045267	0.065664
CS28	SC28	GD24	*R.nitidus*	-0.012007	0.06675
MY4	SC28	GD24	*R.nitidus*	-0.026899	0.066567
CS6	SC28	GD24	*R.nitidus*	-0.011485	0.066774
SC34	SC28	GD24	*R.nitidus*	0.052251	0.064583
SC42	SC28	GD24	*R.nitidus*	0.3	0.062581
SC26	SC28	GD24	*R.nitidus*	-0.034202	0.06621
SC31	SC28	GD24	*R.nitidus*	0.013115	0.06611
SC32	SC28	GD24	*R.nitidus*	0.065359	0.065634
JW7	SC28	GD24	*R.nitidus*	0.045786	0.070336
SC44	SC28	GD24	*R.nitidus*	0.057332	0.060693
SC5	SC28	GD24	*R.nitidus*	0.015441	0.067264
SC6	SC28	GD24	*R.nitidus*	0.020661	0.068833
JO8	SC31	GD24	*R.nitidus*	-0.011518	0.074117
CS4	SC28	GD24	*R.nitidus*	0.005393	0.065495
MY6	SC28	GD24	*R.nitidus*	-0.004758	0.064896
SC12	SC28	GD24	*R.nitidus*	-0.023952	0.066813
GD21	SC31	GD24	*R.nitidus*	0.111186	0.065773
JO3	SC28	GD24	*R.nitidus*	0.008749	0.071902
JO7	SC31	GD24	*R.nitidus*	-0.015795	0.073139
JO5	SC28	GD24	*R.nitidus*	0.087912	0.062449
SC35	SC28	GD24	*R.nitidus*	0.072299	0.06611
GD29	SC31	GD24	*R.nitidus*	0.09501	0.064756
CS11	SC31	GD24	*R.nitidus*	0.007813	0.069233
GD30	SC31	GD24	*R.nitidus*	0.127608	0.068611
JW6	SC28	GD24	*R.nitidus*	-0.020513	0.075418
GD31	SC31	GD24	*R.nitidus*	0.075019	0.066527
GD35	SC31	GD24	*R.nitidus*	0.040512	0.067545
GD26	SC31	GD24	*R.nitidus*	0.046132	0.066887
JO1	SC28	GD24	*R.nitidus*	-0.009402	0.066144
CS14	SC31	GD24	*R.nitidus*	0	0.069876
GD22	SC31	GD24	*R.nitidus*	0.081119	0.066997
GD48	SC31	GD24	*R.nitidus*	0.013312	0.069523
GD34	SC31	GD24	*R.nitidus*	0.009475	0.065097
GD42	SC31	GD24	*R.nitidus*	0.042661	0.066644
CS21	SC31	GD24	*R.nitidus*	-0.016484	0.066926
GD32	SC31	GD24	*R.nitidus*	0.012998	0.069885
GD40	SC31	GD24	*R.nitidus*	0.086402	0.066033

CS22	SC31	GD24	*R.nitidus*	0.018968	0.067758
CS17	SC31	GD24	*R.nitidus*	0.040496	0.070455
GD41	SC31	GD24	*R.nitidus*	0.017024	0.066271
GD50	SC31	GD24	*R.nitidus*	0.037351	0.068271
CS23	SC31	GD24	*R.nitidus*	-0.046296	0.06704
CS16	SC31	GD24	*R.nitidus*	0.016591	0.068734
CS12	SC31	GD24	*R.nitidus*	0.002342	0.069303
GD25	SC31	GD24	*R.nitidus*	0.065089	0.066342
GD49	SC31	GD24	*R.nitidus*	0.127153	0.069999
CS15	SC31	GD24	*R.nitidus*	0.034612	0.067918
SC5	SC31	GD24	*R.nitidus*	0.004819	0.069113
CS19	SC31	GD24	*R.nitidus*	-0.018419	0.068722
GD33	SC31	GD24	*R.nitidus*	0.00073	0.068071
GD45	SC31	GD24	*R.nitidus*	0.052464	0.071055
CS13	SC31	GD24	*R.nitidus*	-0.026563	0.069179
CS28	SC31	GD24	*R.nitidus*	-0.021259	0.066804
CS18	SC31	GD24	*R.nitidus*	-0.034776	0.067668
SC24	SC31	GD24	*R.nitidus*	0.008382	0.067557
GD44	SC31	GD24	*R.nitidus*	0.024176	0.067104
SC10	SC31	GD24	*R.nitidus*	0.078098	0.067093
CS20	SC31	GD24	*R.nitidus*	-0.014879	0.067122
GD46	SC31	GD24	*R.nitidus*	-0.008386	0.065613
CS24	SC31	GD24	*R.nitidus*	-0.044129	0.066158
SC15	SC31	GD24	*R.nitidus*	-0.015357	0.067538
SC34	SC31	GD24	*R.nitidus*	0.044586	0.067338
SC18	SC31	GD24	*R.nitidus*	0.070435	0.067353
CS30	SC31	GD24	*R.nitidus*	-0.054545	0.066763
CS31	SC31	GD24	*R.nitidus*	-0.025765	0.067169
CS25	SC31	GD24	*R.nitidus*	-0.010679	0.066595
CS4	SC31	GD24	*R.nitidus*	0	0.066112
CS3	SC31	GD24	*R.nitidus*	-0.003978	0.065622
CS6	SC31	GD24	*R.nitidus*	-0.024741	0.06672
JO3	SC31	GD24	*R.nitidus*	0.030541	0.072298
SC28	SC31	GD24	*R.nitidus*	-0.013115	0.06611
MY6	SC31	GD24	*R.nitidus*	-0.003882	0.067191
JO7	SC32	GD24	*R.nitidus*	-0.065539	0.074141
SC6	SC31	GD24	*R.nitidus*	-0.023007	0.067411
JW6	SC31	GD24	*R.nitidus*	0.025666	0.075117
SC12	SC31	GD24	*R.nitidus*	-0.025357	0.068223
SC42	SC31	GD24	*R.nitidus*	0.261421	0.062612
JO8	SC32	GD24	*R.nitidus*	-0.003212	0.074122
SC14	SC31	GD24	*R.nitidus*	0.026138	0.066655
SC44	SC31	GD24	*R.nitidus*	0.03067	0.066141
CS11	SC32	GD24	*R.nitidus*	-0.047731	0.069194
GD25	SC32	GD24	*R.nitidus*	0.025868	0.066931
SC27	SC31	GD24	*R.nitidus*	0.018441	0.066994
SC35	SC31	GD24	*R.nitidus*	0.051387	0.065822
JO5	SC31	GD24	*R.nitidus*	0.058544	0.064714
SC26	SC31	GD24	*R.nitidus*	-0.046473	0.065974
GD30	SC32	GD24	*R.nitidus*	0.085597	0.070677
SC45	SC31	GD24	*R.nitidus*	0.043478	0.066053
JO1	SC31	GD24	*R.nitidus*	0.001626	0.065943
SC32	SC31	GD24	*R.nitidus*	0.051696	0.067933
GD40	SC32	GD24	*R.nitidus*	0.043353	0.066847
JW4	SC31	GD24	*R.nitidus*	0.234843	0.067554
GD22	SC32	GD24	*R.nitidus*	0.027046	0.066059

JW7	SC31	GD24	*R.nitidus*	0.069744	0.069144
GD42	SC32	GD24	*R.nitidus*	-0.006662	0.065382
GD48	SC32	GD24	*R.nitidus*	-0.018669	0.068167
GD31	SC32	GD24	*R.nitidus*	0.034357	0.067318
GD34	SC32	GD24	*R.nitidus*	-0.031137	0.065803
GD45	SC32	GD24	*R.nitidus*	0.00995	0.071103
GD21	SC32	GD24	*R.nitidus*	0.078571	0.06644
GD29	SC32	GD24	*R.nitidus*	0.062849	0.066243
GD46	SC32	GD24	*R.nitidus*	-0.051187	0.06729
MY4	SC31	GD24	*R.nitidus*	-0.035019	0.066049
GD41	SC32	GD24	*R.nitidus*	-0.025148	0.06567
GD32	SC32	GD24	*R.nitidus*	-0.024618	0.069076
GD26	SC32	GD24	*R.nitidus*	0.009468	0.066446
GD49	SC32	GD24	*R.nitidus*	0.077551	0.067737
CS16	SC32	GD24	*R.nitidus*	-0.043044	0.068882
CS24	SC32	GD24	*R.nitidus*	-0.092667	0.067105
GD33	SC32	GD24	*R.nitidus*	-0.050698	0.068147
CS14	SC32	GD24	*R.nitidus*	-0.043375	0.071908
CS17	SC32	GD24	*R.nitidus*	-0.022259	0.07185
CS20	SC32	GD24	*R.nitidus*	-0.069856	0.066342
CS21	SC32	GD24	*R.nitidus*	-0.064615	0.066223
GD50	SC32	GD24	*R.nitidus*	0.002611	0.071993
GD35	SC32	GD24	*R.nitidus*	-0.000733	0.06665
GD44	SC32	GD24	*R.nitidus*	-0.025424	0.068248
CS15	SC32	GD24	*R.nitidus*	-0.012928	0.070497
CS12	SC32	GD24	*R.nitidus*	-0.046154	0.07048
CS13	SC32	GD24	*R.nitidus*	-0.076675	0.069645
SC15	SC32	GD24	*R.nitidus*	-0.068481	0.069517
SC6	SC32	GD24	*R.nitidus*	-0.063847	0.0695
CS23	SC32	GD24	*R.nitidus*	-0.091116	0.067999
SC34	SC32	GD24	*R.nitidus*	-0.001627	0.065276
CS30	SC32	GD24	*R.nitidus*	-0.095008	0.067648
CS25	SC32	GD24	*R.nitidus*	-0.058962	0.06943
SC10	SC32	GD24	*R.nitidus*	0.030973	0.066261
SC14	SC32	GD24	*R.nitidus*	-0.025618	0.066302
SC5	SC32	GD24	*R.nitidus*	-0.03862	0.070773
SC27	SC32	GD24	*R.nitidus*	-0.028907	0.064655
CS18	SC32	GD24	*R.nitidus*	-0.087609	0.067324
CS4	SC32	GD24	*R.nitidus*	-0.049961	0.065269
CS28	SC32	GD24	*R.nitidus*	-0.088983	0.068642
SC24	SC32	GD24	*R.nitidus*	-0.046991	0.067811
CS19	SC32	GD24	*R.nitidus*	-0.066667	0.071236
CS22	SC32	GD24	*R.nitidus*	-0.03506	0.067055
SC28	SC32	GD24	*R.nitidus*	-0.065359	0.065634
SC26	SC32	GD24	*R.nitidus*	-0.086235	0.065423
SC45	SC32	GD24	*R.nitidus*	-0.00485	0.067379
CS3	SC32	GD24	*R.nitidus*	-0.062548	0.0661
SC12	SC32	GD24	*R.nitidus*	-0.080262	0.067978
CS6	SC32	GD24	*R.nitidus*	-0.08058	0.065843
SC44	SC32	GD24	*R.nitidus*	-0.024651	0.065045
SC18	SC32	GD24	*R.nitidus*	0.019248	0.066688
JW7	SC32	GD24	*R.nitidus*	0.022293	0.072284
SC31	SC32	GD24	*R.nitidus*	-0.051696	0.067933
JO7	SC34	GD24	*R.nitidus*	-0.044444	0.071028
GD21	SC34	GD24	*R.nitidus*	0.082985	0.0658
SC42	SC32	GD24	*R.nitidus*	0.253503	0.062883

GD25	SC34	GD24	*R.nitidus*	0.025698	0.065502
JO5	SC32	GD24	*R.nitidus*	0.01252	0.066419
JW4	SC32	GD24	*R.nitidus*	0.186191	0.067311
CS12	SC34	GD24	*R.nitidus*	-0.018489	0.066475
JO3	SC32	GD24	*R.nitidus*	-0.026817	0.072196
JW6	SC32	GD24	*R.nitidus*	-0.040082	0.073211
SC35	SC32	GD24	*R.nitidus*	0.002469	0.065875
CS11	SC34	GD24	*R.nitidus*	-0.028483	0.069944
GD22	SC34	GD24	*R.nitidus*	0.041144	0.066459
GD46	SC34	GD24	*R.nitidus*	-0.040293	0.065038
GD29	SC34	GD24	*R.nitidus*	0.053207	0.064089
MY6	SC32	GD24	*R.nitidus*	-0.050702	0.065783
MY4	SC32	GD24	*R.nitidus*	-0.082524	0.065505
GD50	SC34	GD24	*R.nitidus*	-0.004378	0.066929
GD44	SC34	GD24	*R.nitidus*	-0.013804	0.067915
JO1	SC32	GD24	*R.nitidus*	-0.044597	0.067314
JO8	SC34	GD24	*R.nitidus*	0.009534	0.072863
GD31	SC34	GD24	*R.nitidus*	0.038023	0.067258
CS31	SC32	GD24	*R.nitidus*	-0.069594	0.069784
CS19	SC34	GD24	*R.nitidus*	-0.063035	0.069153
GD30	SC34	GD24	*R.nitidus*	0.074387	0.069221
GD32	SC34	GD24	*R.nitidus*	0.008244	0.067395
GD26	SC34	GD24	*R.nitidus*	0.017417	0.066364
GD41	SC34	GD24	*R.nitidus*	-0.015707	0.065631
CS13	SC34	GD24	*R.nitidus*	-0.055732	0.069781
CS16	SC34	GD24	*R.nitidus*	-0.024806	0.067348
SC5	SC34	GD24	*R.nitidus*	-0.020701	0.071137
GD45	SC34	GD24	*R.nitidus*	0.023392	0.069385
CS14	SC34	GD24	*R.nitidus*	-0.034375	0.069493
GD35	SC34	GD24	*R.nitidus*	0.020818	0.065029
GD33	SC34	GD24	*R.nitidus*	-0.031069	0.066553
CS24	SC34	GD24	*R.nitidus*	-0.081848	0.065219
GD34	SC34	GD24	*R.nitidus*	-0.026753	0.066157
CS25	SC34	GD24	*R.nitidus*	-0.051459	0.067482
SC15	SC34	GD24	*R.nitidus*	-0.05503	0.066819
GD40	SC34	GD24	*R.nitidus*	0.049012	0.064475
GD48	SC34	GD24	*R.nitidus*	-0.012851	0.069144
CS22	SC34	GD24	*R.nitidus*	-0.015982	0.065319
GD49	SC34	GD24	*R.nitidus*	0.065789	0.067275
GD42	SC34	GD24	*R.nitidus*	0.002959	0.065716
CS21	SC34	GD24	*R.nitidus*	-0.048967	0.066952
SC14	SC34	GD24	*R.nitidus*	-0.019247	0.064948
CS20	SC34	GD24	*R.nitidus*	-0.058868	0.066869
CS15	SC34	GD24	*R.nitidus*	-0.000763	0.06676
CS3	SC34	GD24	*R.nitidus*	-0.052429	0.067448
CS17	SC34	GD24	*R.nitidus*	0.002463	0.07026
CS28	SC34	GD24	*R.nitidus*	-0.06425	0.068857
SC12	SC34	GD24	*R.nitidus*	-0.070578	0.066988
CS18	SC34	GD24	*R.nitidus*	-0.081839	0.065194
CS4	SC34	GD24	*R.nitidus*	-0.036009	0.064729
SC10	SC34	GD24	*R.nitidus*	0.030225	0.066032
SC26	SC34	GD24	*R.nitidus*	-0.077165	0.064187
CS30	SC34	GD24	*R.nitidus*	-0.089992	0.065021
SC44	SC34	GD24	*R.nitidus*	-0.019481	0.064032
JW4	SC34	GD24	*R.nitidus*	0.204214	0.065578
CS23	SC34	GD24	*R.nitidus*	-0.080521	0.06623

SC18	SC34	GD24	*R.nitidus*	0.035473	0.067651
CS6	SC34	GD24	*R.nitidus*	-0.056743	0.065595
CS31	SC34	GD24	*R.nitidus*	-0.053945	0.066499
SC35	SC34	GD24	*R.nitidus*	0.022314	0.063521
MY4	SC34	GD24	*R.nitidus*	-0.086538	0.066341
SC27	SC34	GD24	*R.nitidus*	-0.025	0.064088
SC6	SC34	GD24	*R.nitidus*	-0.046964	0.06799
SC32	SC34	GD24	*R.nitidus*	0.001627	0.065276
MY6	SC34	GD24	*R.nitidus*	-0.045959	0.065568
JO7	SC35	GD24	*R.nitidus*	-0.048485	0.070965
JO8	SC35	GD24	*R.nitidus*	-0.028825	0.073449
SC45	SC34	GD24	*R.nitidus*	0.010836	0.065853
GD21	SC35	GD24	*R.nitidus*	0.062678	0.066226
JO5	SC34	GD24	*R.nitidus*	0.024077	0.067725
SC24	SC34	GD24	*R.nitidus*	-0.029437	0.065725
JW6	SC34	GD24	*R.nitidus*	-0.026104	0.072883
SC28	SC34	GD24	*R.nitidus*	-0.052251	0.064583
SC42	SC34	GD24	*R.nitidus*	0.223881	0.060744
GD30	SC35	GD24	*R.nitidus*	0.051029	0.069266
CS11	SC35	GD24	*R.nitidus*	-0.043445	0.067002
SC31	SC34	GD24	*R.nitidus*	-0.044586	0.067338
GD41	SC35	GD24	*R.nitidus*	-0.034125	0.067678
GD33	SC35	GD24	*R.nitidus*	-0.048516	0.068065
JO1	SC34	GD24	*R.nitidus*	-0.038554	0.066972
JW7	SC34	GD24	*R.nitidus*	0.002944	0.069537
GD35	SC35	GD24	*R.nitidus*	0	0.06683
GD22	SC35	GD24	*R.nitidus*	0.017556	0.066455
CS15	SC35	GD24	*R.nitidus*	-0.023134	0.067796
CS14	SC35	GD24	*R.nitidus*	-0.052955	0.069167
GD44	SC35	GD24	*R.nitidus*	-0.028832	0.067875
GD32	SC35	GD24	*R.nitidus*	-0.04698	0.066943
GD49	SC35	GD24	*R.nitidus*	0.042079	0.06836
GD46	SC35	GD24	*R.nitidus*	-0.063846	0.065898
GD25	SC35	GD24	*R.nitidus*	0.020579	0.067639
GD42	SC35	GD24	*R.nitidus*	-0.009482	0.06624
GD29	SC35	GD24	*R.nitidus*	0.041847	0.065354
GD48	SC35	GD24	*R.nitidus*	-0.047619	0.067642
JO3	SC34	GD24	*R.nitidus*	0.015058	0.070525
GD26	SC35	GD24	*R.nitidus*	0.008811	0.065821
GD45	SC35	GD24	*R.nitidus*	0.019753	0.071746
CS31	SC35	GD24	*R.nitidus*	-0.073529	0.066963
GD31	SC35	GD24	*R.nitidus*	0.037405	0.067758
CS13	SC35	GD24	*R.nitidus*	-0.078522	0.068572
CS16	SC35	GD24	*R.nitidus*	-0.054846	0.066319
CS20	SC35	GD24	*R.nitidus*	-0.078417	0.065878
CS12	SC35	GD24	*R.nitidus*	-0.053822	0.066037
GD34	SC35	GD24	*R.nitidus*	-0.039552	0.066011
CS17	SC35	GD24	*R.nitidus*	-0.018868	0.067892
GD50	SC35	GD24	*R.nitidus*	-0.0018	0.069866
CS21	SC35	GD24	*R.nitidus*	-0.08104	0.066545
CS25	SC35	GD24	*R.nitidus*	-0.065334	0.066809
CS24	SC35	GD24	*R.nitidus*	-0.095779	0.063731
SC5	SC35	GD24	*R.nitidus*	-0.039935	0.069345
CS3	SC35	GD24	*R.nitidus*	-0.065385	0.065932
GD40	SC35	GD24	*R.nitidus*	0.023538	0.064975
CS18	SC35	GD24	*R.nitidus*	-0.095532	0.065954

SC10	SC35	GD24	*R.nitidus*	0.020478	0.066931
CS28	SC35	GD24	*R.nitidus*	-0.061056	0.067592
SC12	SC35	GD24	*R.nitidus*	-0.099749	0.063674
CS23	SC35	GD24	*R.nitidus*	-0.105223	0.068071
CS4	SC35	GD24	*R.nitidus*	-0.048499	0.067005
CS6	SC35	GD24	*R.nitidus*	-0.092147	0.064703
SC31	SC35	GD24	*R.nitidus*	-0.051387	0.065822
SC28	SC35	GD24	*R.nitidus*	-0.072299	0.06611
CS22	SC35	GD24	*R.nitidus*	-0.042789	0.065096
CS19	SC35	GD24	*R.nitidus*	-0.090202	0.069967
SC44	SC35	GD24	*R.nitidus*	-0.039377	0.064422
SC6	SC35	GD24	*R.nitidus*	-0.068823	0.06722
SC14	SC35	GD24	*R.nitidus*	-0.028854	0.064738
JO1	SC35	GD24	*R.nitidus*	-0.068852	0.066118
CS30	SC35	GD24	*R.nitidus*	-0.104708	0.065142
SC24	SC35	GD24	*R.nitidus*	-0.04553	0.066526
MY6	SC35	GD24	*R.nitidus*	-0.073634	0.066626
JW7	SC35	GD24	*R.nitidus*	0.008205	0.069084
JO3	SC35	GD24	*R.nitidus*	-0.017164	0.071551
JO8	CS6	GD24	*R.nitidus*	0.047085	0.074009
MY4	SC35	GD24	*R.nitidus*	-0.105901	0.065867
GD21	CS6	GD24	*R.nitidus*	0.151223	0.065092
SC34	SC35	GD24	*R.nitidus*	-0.022314	0.063521
SC18	SC35	GD24	*R.nitidus*	0.014468	0.06667
SC15	SC35	GD24	*R.nitidus*	-0.071973	0.06715
JW6	SC35	GD24	*R.nitidus*	-0.031536	0.073667
SC27	SC35	GD24	*R.nitidus*	-0.04526	0.065373
CS12	CS6	GD24	*R.nitidus*	0.042607	0.067765
SC26	SC35	GD24	*R.nitidus*	-0.101133	0.064491
GD34	CS6	GD24	*R.nitidus*	0.036237	0.065938
JO5	SC35	GD24	*R.nitidus*	0.004785	0.066624
SC42	SC35	GD24	*R.nitidus*	0.230769	0.063879
SC45	SC35	GD24	*R.nitidus*	-0.007414	0.065303
SC32	SC35	GD24	*R.nitidus*	-0.002469	0.065875
GD33	CS6	GD24	*R.nitidus*	0.028266	0.067344
JW4	SC35	GD24	*R.nitidus*	0.20266	0.066303
GD26	CS6	GD24	*R.nitidus*	0.068441	0.066889
CS13	CS6	GD24	*R.nitidus*	0.008467	0.06985
GD49	CS6	GD24	*R.nitidus*	0.123793	0.068429
GD30	CS6	GD24	*R.nitidus*	0.136364	0.067732
CS15	CS6	GD24	*R.nitidus*	0.068313	0.069528
JO7	CS6	GD24	*R.nitidus*	0.021299	0.073103
GD40	CS6	GD24	*R.nitidus*	0.114996	0.066372
CS18	CS6	GD24	*R.nitidus*	-0.010827	0.066827
GD46	CS6	GD24	*R.nitidus*	0.018692	0.067246
GD45	CS6	GD24	*R.nitidus*	0.077413	0.071869
GD50	CS6	GD24	*R.nitidus*	0.060965	0.072118
GD44	CS6	GD24	*R.nitidus*	0.063025	0.068876
GD41	CS6	GD24	*R.nitidus*	0.048611	0.067211
CS11	CS6	GD24	*R.nitidus*	0.035029	0.068826
GD25	CS6	GD24	*R.nitidus*	0.087706	0.066744
GD29	CS6	GD24	*R.nitidus*	0.114223	0.064182
GD22	CS6	GD24	*R.nitidus*	0.110945	0.066966
GD35	CS6	GD24	*R.nitidus*	0.081269	0.06792
CS22	CS6	GD24	*R.nitidus*	0.054187	0.065756
GD31	CS6	GD24	*R.nitidus*	0.10209	0.069414

GD48	CS6	GD24	*R.nitidus*	0.048454	0.068998
GD32	CS6	GD24	*R.nitidus*	0.074271	0.068868
GD42	CS6	GD24	*R.nitidus*	0.073038	0.065908
CS4	CS6	GD24	*R.nitidus*	0.014822	0.065519
CS14	CS6	GD24	*R.nitidus*	0.043369	0.070996
CS21	CS6	GD24	*R.nitidus*	0.021804	0.066836
CS16	CS6	GD24	*R.nitidus*	0.033613	0.068278
CS17	CS6	GD24	*R.nitidus*	0.050938	0.07164
CS25	CS6	GD24	*R.nitidus*	0.024954	0.067493
CS20	CS6	GD24	*R.nitidus*	0.01919	0.065835
SC12	CS6	GD24	*R.nitidus*	0.015728	0.067987
SC28	CS6	GD24	*R.nitidus*	0.011485	0.066774
CS3	CS6	GD24	*R.nitidus*	0.022173	0.063985
CS19	CS6	GD24	*R.nitidus*	0.007797	0.069487
CS30	CS6	GD24	*R.nitidus*	-0.023709	0.068011
CS24	CS6	GD24	*R.nitidus*	-0.01107	0.066243
SC42	CS6	GD24	*R.nitidus*	0.284553	0.062014
MY6	CS6	GD24	*R.nitidus*	0.0216	0.067515
JO3	CS6	GD24	*R.nitidus*	0.075	0.070077
SC10	CS6	GD24	*R.nitidus*	0.092308	0.067272
SC45	CS6	GD24	*R.nitidus*	0.07172	0.067032
SC5	CS6	GD24	*R.nitidus*	0.043965	0.068737
SC44	CS6	GD24	*R.nitidus*	0.059259	0.064692
SC31	CS6	GD24	*R.nitidus*	0.024741	0.06672
CS23	CS6	GD24	*R.nitidus*	-0.021053	0.06798
SC32	CS6	GD24	*R.nitidus*	0.08058	0.065843
JW7	CS6	GD24	*R.nitidus*	0.068467	0.072046
SC14	CS6	GD24	*R.nitidus*	0.056743	0.065718
CS31	CS6	GD24	*R.nitidus*	0.006867	0.067682
CS28	CS6	GD24	*R.nitidus*	0.00885	0.067405
MY4	CS6	GD24	*R.nitidus*	-0.005677	0.067792
JO1	CS6	GD24	*R.nitidus*	0.0208	0.069473
SC15	CS6	GD24	*R.nitidus*	0.019312	0.06745
SC6	CS6	GD24	*R.nitidus*	0.019119	0.068012
JO7	SC42	GD24	*R.nitidus*	-0.2329	0.070414
JO5	CS6	GD24	*R.nitidus*	0.092257	0.066294
GD25	SC42	GD24	*R.nitidus*	-0.196772	0.063868
JW6	CS6	GD24	*R.nitidus*	0.032888	0.072188
SC18	CS6	GD24	*R.nitidus*	0.107546	0.064658
JW4	CS6	GD24	*R.nitidus*	0.234916	0.067701
SC35	CS6	GD24	*R.nitidus*	0.092147	0.064703
GD22	SC42	GD24	*R.nitidus*	-0.187783	0.063651
SC27	CS6	GD24	*R.nitidus*	0.057452	0.066032
SC26	CS6	GD24	*R.nitidus*	0	0.065152
GD35	SC42	GD24	*R.nitidus*	-0.227008	0.064746
SC34	CS6	GD24	*R.nitidus*	0.056743	0.065595
GD31	SC42	GD24	*R.nitidus*	-0.183787	0.065181
SC24	CS6	GD24	*R.nitidus*	0.057823	0.066403
JO8	SC42	GD24	*R.nitidus*	-0.184044	0.071486
GD30	SC42	GD24	*R.nitidus*	-0.14276	0.065772
GD45	SC42	GD24	*R.nitidus*	-0.198337	0.067145
GD40	SC42	GD24	*R.nitidus*	-0.189829	0.063867
GD29	SC42	GD24	*R.nitidus*	-0.154126	0.063434
GD42	SC42	GD24	*R.nitidus*	-0.238582	0.06297
CS13	SC42	GD24	*R.nitidus*	-0.282528	0.064822
GD32	SC42	GD24	*R.nitidus*	-0.218277	0.064642

GD21	SC42	GD24	*R.nitidus*	-0.171577	0.062302
CS11	SC42	GD24	*R.nitidus*	-0.25509	0.064045
CS15	SC42	GD24	*R.nitidus*	-0.22994	0.06421
GD26	SC42	GD24	*R.nitidus*	-0.207991	0.063303
GD48	SC42	GD24	*R.nitidus*	-0.222868	0.065221
GD41	SC42	GD24	*R.nitidus*	-0.245921	0.063792
GD44	SC42	GD24	*R.nitidus*	-0.233458	0.065789
GD46	SC42	GD24	*R.nitidus*	-0.259088	0.062264
GD34	SC42	GD24	*R.nitidus*	-0.255582	0.064245
CS16	SC42	GD24	*R.nitidus*	-0.268146	0.063359
GD50	SC42	GD24	*R.nitidus*	-0.214586	0.067476
CS14	SC42	GD24	*R.nitidus*	-0.252241	0.065675
CS12	SC42	GD24	*R.nitidus*	-0.261529	0.063372
CS20	SC42	GD24	*R.nitidus*	-0.272999	0.061856
CS18	SC42	GD24	*R.nitidus*	-0.297937	0.062584
GD33	SC42	GD24	*R.nitidus*	-0.248292	0.065625
CS4	SC42	GD24	*R.nitidus*	-0.282324	0.062241
CS28	SC42	GD24	*R.nitidus*	-0.286193	0.063528
CS17	SC42	GD24	*R.nitidus*	-0.221731	0.065477
GD49	SC42	GD24	*R.nitidus*	-0.175694	0.065478
CS25	SC42	GD24	*R.nitidus*	-0.279319	0.064642
CS24	SC42	GD24	*R.nitidus*	-0.308953	0.062716
SC27	SC42	GD24	*R.nitidus*	-0.277926	0.063242
CS30	SC42	GD24	*R.nitidus*	-0.308504	0.061976
SC10	SC42	GD24	*R.nitidus*	-0.210345	0.063671
CS22	SC42	GD24	*R.nitidus*	-0.253442	0.062398
CS3	SC42	GD24	*R.nitidus*	-0.27046	0.061543
CS21	SC42	GD24	*R.nitidus*	-0.277337	0.061621
SC18	SC42	GD24	*R.nitidus*	-0.22894	0.063986
SC12	SC42	GD24	*R.nitidus*	-0.306366	0.061595
CS23	SC42	GD24	*R.nitidus*	-0.308343	0.062223
CS31	SC42	GD24	*R.nitidus*	-0.30243	0.062734
SC6	SC42	GD24	*R.nitidus*	-0.302597	0.06252
SC32	SC42	GD24	*R.nitidus*	-0.253503	0.062883
SC15	SC42	GD24	*R.nitidus*	-0.301379	0.064074
SC14	SC42	GD24	*R.nitidus*	-0.271053	0.06206
JW7	SC42	GD24	*R.nitidus*	-0.215221	0.067697
SC31	SC42	GD24	*R.nitidus*	-0.261421	0.062612
SC5	SC42	GD24	*R.nitidus*	-0.273206	0.066513
CS19	SC42	GD24	*R.nitidus*	-0.289063	0.064316
SC34	SC42	GD24	*R.nitidus*	-0.223881	0.060744
CS11	SC44	GD24	*R.nitidus*	-0.018447	0.068352
JW4	SC42	GD24	*R.nitidus*	-0.017426	0.066909
GD30	SC44	GD24	*R.nitidus*	0.087254	0.0666
GD22	SC44	GD24	*R.nitidus*	0.05878	0.066193
SC28	SC42	GD24	*R.nitidus*	-0.3	0.062581
SC24	SC42	GD24	*R.nitidus*	-0.277598	0.063875
SC26	SC42	GD24	*R.nitidus*	-0.297886	0.062003
SC45	SC42	GD24	*R.nitidus*	-0.23741	0.063728
MY4	SC42	GD24	*R.nitidus*	-0.299247	0.064318
GD40	SC44	GD24	*R.nitidus*	0.059091	0.065886
JO7	SC44	GD24	*R.nitidus*	-0.032597	0.075159
JO8	SC44	GD24	*R.nitidus*	-0.022346	0.074477
SC35	SC42	GD24	*R.nitidus*	-0.230769	0.063879
JW6	SC42	GD24	*R.nitidus*	-0.214953	0.068725
CS6	SC42	GD24	*R.nitidus*	-0.284553	0.062014

GD21	SC44	GD24	*R.nitidus*	0.085694	0.066307
JO3	SC42	GD24	*R.nitidus*	-0.195324	0.067337
GD42	SC44	GD24	*R.nitidus*	0.014096	0.067166
GD26	SC44	GD24	*R.nitidus*	0.022239	0.066957
GD31	SC44	GD24	*R.nitidus*	0.051777	0.06842
GD29	SC44	GD24	*R.nitidus*	0.066762	0.066212
MY6	SC42	GD24	*R.nitidus*	-0.282116	0.064865
CS12	SC44	GD24	*R.nitidus*	-0.02482	0.068184
GD33	SC44	GD24	*R.nitidus*	-0.02891	0.066921
JO1	SC42	GD24	*R.nitidus*	-0.27947	0.063012
JO5	SC42	GD24	*R.nitidus*	-0.229748	0.064233
GD48	SC44	GD24	*R.nitidus*	-0.020576	0.067559
SC44	SC42	GD24	*R.nitidus*	-0.260221	0.061714
GD25	SC44	GD24	*R.nitidus*	0.039504	0.067582
GD50	SC44	GD24	*R.nitidus*	0.02634	0.071243
CS25	SC44	GD24	*R.nitidus*	-0.026899	0.067968
GD45	SC44	GD24	*R.nitidus*	0.028404	0.068821
GD41	SC44	GD24	*R.nitidus*	-0.013772	0.065687
GD35	SC44	GD24	*R.nitidus*	0.023449	0.068062
GD34	SC44	GD24	*R.nitidus*	-0.022539	0.065874
CS13	SC44	GD24	*R.nitidus*	-0.044953	0.068416
CS17	SC44	GD24	*R.nitidus*	0.017312	0.072183
GD32	SC44	GD24	*R.nitidus*	-0.020017	0.06875
CS19	SC44	GD24	*R.nitidus*	-0.043513	0.069582
CS18	SC44	GD24	*R.nitidus*	-0.064615	0.066355
CS23	SC44	GD24	*R.nitidus*	-0.064171	0.066303
CS21	SC44	GD24	*R.nitidus*	-0.043807	0.065437
GD46	SC44	GD24	*R.nitidus*	-0.033758	0.066131
GD49	SC44	GD24	*R.nitidus*	0.080617	0.068016
CS22	SC44	GD24	*R.nitidus*	-0.006182	0.068207
GD44	SC44	GD24	*R.nitidus*	0.000801	0.068732
CS20	SC44	GD24	*R.nitidus*	-0.048632	0.066777
CS14	SC44	GD24	*R.nitidus*	-0.023346	0.07036
SC6	SC44	GD24	*R.nitidus*	-0.038211	0.068159
CS30	SC44	GD24	*R.nitidus*	-0.082181	0.066023
SC28	SC44	GD24	*R.nitidus*	-0.057332	0.060693
CS15	SC44	GD24	*R.nitidus*	0.006977	0.068092
SC32	SC44	GD24	*R.nitidus*	0.024651	0.065045
CS4	SC44	GD24	*R.nitidus*	-0.030465	0.065811
JW6	SC44	GD24	*R.nitidus*	-0.019211	0.073831
SC18	SC44	GD24	*R.nitidus*	0.055456	0.067087
CS24	SC44	GD24	*R.nitidus*	-0.069021	0.067443
CS16	SC44	GD24	*R.nitidus*	-0.026688	0.067663
CS3	SC44	GD24	*R.nitidus*	-0.032468	0.067615
CS28	SC44	GD24	*R.nitidus*	-0.053333	0.067484
SC26	SC44	GD24	*R.nitidus*	-0.060855	0.067306
SC24	SC44	GD24	*R.nitidus*	-0.01364	0.067324
SC10	SC44	GD24	*R.nitidus*	0.05198	0.066633
JW4	SC44	GD24	*R.nitidus*	0.200154	0.067065
CS31	SC44	GD24	*R.nitidus*	-0.050571	0.068311
SC5	SC44	GD24	*R.nitidus*	-0.031279	0.067861
SC14	SC44	GD24	*R.nitidus*	-0.001698	0.065778
SC12	SC44	GD24	*R.nitidus*	-0.052585	0.066583
GD30	SC45	GD24	*R.nitidus*	0.086849	0.068568
GD25	SC45	GD24	*R.nitidus*	0.02673	0.066637
JO5	SC44	GD24	*R.nitidus*	0.035349	0.064685

JW7	SC44	GD24	*R.nitidus*	0.008114	0.071351
SC31	SC44	GD24	*R.nitidus*	-0.03067	0.066141
GD29	SC45	GD24	*R.nitidus*	0.059617	0.065242
JO3	SC44	GD24	*R.nitidus*	-0.007036	0.071641
MY4	SC44	GD24	*R.nitidus*	-0.06699	0.066366
MY6	SC44	GD24	*R.nitidus*	-0.04372	0.066595
SC45	SC44	GD24	*R.nitidus*	0.008264	0.067255
SC27	SC44	GD24	*R.nitidus*	-0.006034	0.065755
SC15	SC44	GD24	*R.nitidus*	-0.041876	0.068009
JO7	SC45	GD24	*R.nitidus*	-0.070883	0.073029
SC35	SC44	GD24	*R.nitidus*	0.039377	0.064422
SC34	SC44	GD24	*R.nitidus*	0.019481	0.064032
SC42	SC44	GD24	*R.nitidus*	0.260221	0.061714
CS6	SC44	GD24	*R.nitidus*	-0.059259	0.064692
GD22	SC45	GD24	*R.nitidus*	0.024691	0.067198
GD26	SC45	GD24	*R.nitidus*	0.010432	0.066047
GD45	SC45	GD24	*R.nitidus*	0.026205	0.06889
GD35	SC45	GD24	*R.nitidus*	-0.005228	0.067812
GD48	SC45	GD24	*R.nitidus*	-0.018268	0.066672
CS12	SC45	GD24	*R.nitidus*	-0.046079	0.069541
JO8	SC45	GD24	*R.nitidus*	-0.040526	0.072775
JO1	SC44	GD24	*R.nitidus*	-0.036885	0.068311
CS17	SC45	GD24	*R.nitidus*	0.005627	0.072418
CS19	SC45	GD24	*R.nitidus*	-0.072713	0.070644
GD42	SC45	GD24	*R.nitidus*	-0.008929	0.066861
GD31	SC45	GD24	*R.nitidus*	0.05175	0.067824
CS14	SC45	GD24	*R.nitidus*	-0.037867	0.072156
GD21	SC45	GD24	*R.nitidus*	0.063446	0.064399
GD44	SC45	GD24	*R.nitidus*	-0.017309	0.06789
CS11	SC45	GD24	*R.nitidus*	-0.024257	0.071117
GD49	SC45	GD24	*R.nitidus*	0.065756	0.069213
GD46	SC45	GD24	*R.nitidus*	-0.050847	0.067848
GD41	SC45	GD24	*R.nitidus*	-0.021577	0.067602
GD32	SC45	GD24	*R.nitidus*	-0.017413	0.068985
CS13	SC45	GD24	*R.nitidus*	-0.052504	0.071861
CS22	SC45	GD24	*R.nitidus*	-0.027516	0.068
GD33	SC45	GD24	*R.nitidus*	-0.058692	0.069623
GD40	SC45	GD24	*R.nitidus*	0.041298	0.064417
GD34	SC45	GD24	*R.nitidus*	-0.033455	0.066349
CS25	SC45	GD24	*R.nitidus*	-0.060918	0.068634
CS15	SC45	GD24	*R.nitidus*	-0.003873	0.070464
GD50	SC45	GD24	*R.nitidus*	0.009025	0.071394
CS28	SC45	GD24	*R.nitidus*	-0.060435	0.069475
CS24	SC45	GD24	*R.nitidus*	-0.077882	0.067881
CS23	SC45	GD24	*R.nitidus*	-0.090768	0.067771
CS16	SC45	GD24	*R.nitidus*	-0.041149	0.069582
SC5	SC45	GD24	*R.nitidus*	-0.041459	0.069866
CS21	SC45	GD24	*R.nitidus*	-0.056689	0.068011
CS20	SC45	GD24	*R.nitidus*	-0.066876	0.068396
SC6	SC45	GD24	*R.nitidus*	-0.047342	0.069015
CS18	SC45	GD24	*R.nitidus*	-0.088006	0.067995
SC18	SC45	GD24	*R.nitidus*	0.029285	0.067532
CS30	SC45	GD24	*R.nitidus*	-0.094108	0.066344
SC34	SC45	GD24	*R.nitidus*	-0.010836	0.065853
MY4	SC45	GD24	*R.nitidus*	-0.079263	0.06743
SC24	SC45	GD24	*R.nitidus*	-0.041322	0.065839

SC12	SC45	GD24	*R.nitidus*	-0.075232	0.066784
CS4	SC45	GD24	*R.nitidus*	-0.04528	0.067433
SC10	SC45	GD24	*R.nitidus*	0.038494	0.068062
SC32	SC45	GD24	*R.nitidus*	0.00485	0.067379
SC27	SC45	GD24	*R.nitidus*	-0.024155	0.066599
SC14	SC45	GD24	*R.nitidus*	-0.02126	0.066812
CS6	SC45	GD24	*R.nitidus*	-0.07172	0.067032
JO7	MY4	GD24	*R.nitidus*	0.017932	0.072755
CS11	MY4	GD24	*R.nitidus*	0.040182	0.069688
CS3	SC45	GD24	*R.nitidus*	-0.060079	0.067691
JO1	SC45	GD24	*R.nitidus*	-0.049538	0.069238
SC26	SC45	GD24	*R.nitidus*	-0.09688	0.065761
JW6	SC45	GD24	*R.nitidus*	-0.051546	0.072682
CS31	SC45	GD24	*R.nitidus*	-0.053386	0.067869
JW4	SC45	GD24	*R.nitidus*	0.206758	0.067064
GD21	MY4	GD24	*R.nitidus*	0.14708	0.065747
JO3	SC45	GD24	*R.nitidus*	-0.011525	0.071272
SC42	SC45	GD24	*R.nitidus*	0.23741	0.063728
JO8	MY4	GD24	*R.nitidus*	0.051668	0.075017
SC35	SC45	GD24	*R.nitidus*	0.007414	0.065303
SC15	SC45	GD24	*R.nitidus*	-0.05848	0.066722
GD29	MY4	GD24	*R.nitidus*	0.127874	0.065273
SC31	SC45	GD24	*R.nitidus*	-0.043478	0.066053
GD25	MY4	GD24	*R.nitidus*	0.100457	0.067061
JW7	SC45	GD24	*R.nitidus*	0.000975	0.07211
SC28	SC45	GD24	*R.nitidus*	-0.045267	0.065664
SC44	SC45	GD24	*R.nitidus*	-0.008264	0.067255
GD44	MY4	GD24	*R.nitidus*	0.067141	0.068169
GD30	MY4	GD24	*R.nitidus*	0.14322	0.068578
GD32	MY4	GD24	*R.nitidus*	0.058081	0.069917
GD42	MY4	GD24	*R.nitidus*	0.068807	0.066289
JO5	SC45	GD24	*R.nitidus*	0.016234	0.067717
GD41	MY4	GD24	*R.nitidus*	0.05	0.064143
GD26	MY4	GD24	*R.nitidus*	0.06927	0.067399
GD50	MY4	GD24	*R.nitidus*	0.08326	0.069502
GD49	MY4	GD24	*R.nitidus*	0.126149	0.069719
MY6	SC45	GD24	*R.nitidus*	-0.056845	0.069103
GD40	MY4	GD24	*R.nitidus*	0.12675	0.065971
GD22	MY4	GD24	*R.nitidus*	0.114119	0.067989
GD33	MY4	GD24	*R.nitidus*	0.025811	0.06652
GD31	MY4	GD24	*R.nitidus*	0.10935	0.068083
CS17	MY4	GD24	*R.nitidus*	0.058121	0.071161
CS14	MY4	GD24	*R.nitidus*	0.038431	0.07068
GD34	MY4	GD24	*R.nitidus*	0.03806	0.06593
CS12	MY4	GD24	*R.nitidus*	0.039714	0.069705
CS31	MY4	GD24	*R.nitidus*	0.021155	0.068569
GD45	MY4	GD24	*R.nitidus*	0.086634	0.071222
CS16	MY4	GD24	*R.nitidus*	0.035285	0.068241
GD35	MY4	GD24	*R.nitidus*	0.084695	0.067435
CS22	MY4	GD24	*R.nitidus*	0.056439	0.068122
CS18	MY4	GD24	*R.nitidus*	-0.011006	0.068396
SC12	MY4	GD24	*R.nitidus*	0.01129	0.06751
CS24	MY4	GD24	*R.nitidus*	0.000794	0.067412
CS3	MY4	GD24	*R.nitidus*	0.014752	0.066564
CS13	MY4	GD24	*R.nitidus*	0.010612	0.069571
CS21	MY4	GD24	*R.nitidus*	0.01947	0.068833

GD46	MY4	GD24	*R.nitidus*	0.023449	0.066778
CS25	MY4	GD24	*R.nitidus*	0.019841	0.068252
SC5	MY4	GD24	*R.nitidus*	0.042097	0.070216
GD48	MY4	GD24	*R.nitidus*	0.046645	0.067751
CS19	MY4	GD24	*R.nitidus*	0.013957	0.071024
SC10	MY4	GD24	*R.nitidus*	0.113333	0.069502
CS20	MY4	GD24	*R.nitidus*	0.011372	0.067879
SC14	MY4	GD24	*R.nitidus*	0.065183	0.066998
CS28	MY4	GD24	*R.nitidus*	0.002479	0.068351
CS15	MY4	GD24	*R.nitidus*	0.070348	0.068705
JW6	MY4	GD24	*R.nitidus*	0.05285	0.075441
CS6	MY4	GD24	*R.nitidus*	0.005677	0.067792
CS4	MY4	GD24	*R.nitidus*	0.035971	0.068341
CS23	MY4	GD24	*R.nitidus*	-0.013997	0.068503
SC45	MY4	GD24	*R.nitidus*	0.079263	0.06743
SC6	MY4	GD24	*R.nitidus*	0.022275	0.067997
CS30	MY4	GD24	*R.nitidus*	-0.015504	0.06846
SC31	MY4	GD24	*R.nitidus*	0.035019	0.066049
SC27	MY4	GD24	*R.nitidus*	0.059289	0.066013
SC35	MY4	GD24	*R.nitidus*	0.105901	0.065867
JO3	MY4	GD24	*R.nitidus*	0.063406	0.071945
SC18	MY4	GD24	*R.nitidus*	0.116939	0.066207
SC15	MY4	GD24	*R.nitidus*	0.023829	0.068579
JO7	MY6	GD24	*R.nitidus*	-0.008439	0.07237
JW4	MY4	GD24	*R.nitidus*	0.267685	0.066224
JW7	MY4	GD24	*R.nitidus*	0.086873	0.073911
GD25	MY6	GD24	*R.nitidus*	0.06936	0.067089
SC28	MY4	GD24	*R.nitidus*	0.026899	0.066567
SC26	MY4	GD24	*R.nitidus*	-0.003934	0.064689
JO1	MY4	GD24	*R.nitidus*	0.03451	0.067864
SC24	MY4	GD24	*R.nitidus*	0.049423	0.066965
GD26	MY6	GD24	*R.nitidus*	0.055716	0.067762
SC32	MY4	GD24	*R.nitidus*	0.082524	0.065505
JO5	MY4	GD24	*R.nitidus*	0.095912	0.068277
SC34	MY4	GD24	*R.nitidus*	0.086538	0.066341
SC42	MY4	GD24	*R.nitidus*	0.299247	0.064318
GD45	MY6	GD24	*R.nitidus*	0.05848	0.069828
SC44	MY4	GD24	*R.nitidus*	0.06699	0.066366
JO8	MY6	GD24	*R.nitidus*	0.01559	0.075609
MY6	MY4	GD24	*R.nitidus*	0.035047	0.061224
GD21	MY6	GD24	*R.nitidus*	0.120397	0.066108
GD48	MY6	GD24	*R.nitidus*	0.017927	0.067985
CS12	MY6	GD24	*R.nitidus*	0.01248	0.069487
CS11	MY6	GD24	*R.nitidus*	0.016554	0.069523
GD33	MY6	GD24	*R.nitidus*	0.003571	0.066189
GD32	MY6	GD24	*R.nitidus*	0.028689	0.070067
GD44	MY6	GD24	*R.nitidus*	0.041379	0.067875
CS17	MY6	GD24	*R.nitidus*	0.025943	0.070629
GD34	MY6	GD24	*R.nitidus*	0.019737	0.06603
GD30	MY6	GD24	*R.nitidus*	0.114382	0.068413
GD42	MY6	GD24	*R.nitidus*	0.044886	0.067174
CS14	MY6	GD24	*R.nitidus*	0.017028	0.069966
GD50	MY6	GD24	*R.nitidus*	0.041415	0.069266
CS20	MY6	GD24	*R.nitidus*	-0.007446	0.067581
GD41	MY6	GD24	*R.nitidus*	0.022075	0.064577
GD49	MY6	GD24	*R.nitidus*	0.117162	0.0692

CS15	MY6	GD24	*R.nitidus*	0.043774	0.06894
GD29	MY6	GD24	*R.nitidus*	0.104822	0.06496
GD22	MY6	GD24	*R.nitidus*	0.086771	0.066673
GD31	MY6	GD24	*R.nitidus*	0.089216	0.067081
CS28	MY6	GD24	*R.nitidus*	-0.019449	0.069081
GD35	MY6	GD24	*R.nitidus*	0.054896	0.06696
GD40	MY6	GD24	*R.nitidus*	0.093863	0.06519
CS16	MY6	GD24	*R.nitidus*	0.013688	0.06847
SC5	MY6	GD24	*R.nitidus*	0.021358	0.070424
GD46	MY6	GD24	*R.nitidus*	-0.002193	0.066684
CS13	MY6	GD24	*R.nitidus*	-0.018153	0.069587
CS30	MY6	GD24	*R.nitidus*	-0.043143	0.066666
CS22	MY6	GD24	*R.nitidus*	0.030398	0.065917
CS4	MY6	GD24	*R.nitidus*	0.007031	0.067247
CS25	MY6	GD24	*R.nitidus*	-0.008668	0.069765
CS24	MY6	GD24	*R.nitidus*	-0.02439	0.066843
SC6	MY6	GD24	*R.nitidus*	-0.009479	0.068386
SC34	MY6	GD24	*R.nitidus*	0.045959	0.065568
CS6	MY6	GD24	*R.nitidus*	-0.0216	0.067515
CS21	MY6	GD24	*R.nitidus*	-0.005279	0.067768
JW7	MY6	GD24	*R.nitidus*	0.061303	0.072592
SC26	MY6	GD24	*R.nitidus*	-0.021538	0.065151
CS23	MY6	GD24	*R.nitidus*	-0.035494	0.06741
SC12	MY6	GD24	*R.nitidus*	-0.013557	0.069522
SC15	MY6	GD24	*R.nitidus*	0	0.065853
CS19	MY6	GD24	*R.nitidus*	-0.017228	0.070019
CS18	MY6	GD24	*R.nitidus*	-0.029141	0.067324
SC27	MY6	GD24	*R.nitidus*	0.028974	0.065555
SC45	MY6	GD24	*R.nitidus*	0.056845	0.069103
JO3	MY6	GD24	*R.nitidus*	0.039604	0.071337
SC42	MY6	GD24	*R.nitidus*	0.282116	0.064865
JW4	MY6	GD24	*R.nitidus*	0.246019	0.066457
SC44	MY6	GD24	*R.nitidus*	0.04372	0.066595
SC10	MY6	GD24	*R.nitidus*	0.092396	0.067555
MY4	MY6	GD24	*R.nitidus*	-0.035047	0.061224
SC28	MY6	GD24	*R.nitidus*	0.004758	0.064896
CS31	MY6	GD24	*R.nitidus*	-0.004023	0.068397
SC24	MY6	GD24	*R.nitidus*	0.021224	0.067328
JW6	MY6	GD24	*R.nitidus*	0.036017	0.073062
JO1	MY6	GD24	*R.nitidus*	-0.002347	0.065845
SC32	MY6	GD24	*R.nitidus*	0.050702	0.065783
SC18	MY6	GD24	*R.nitidus*	0.090219	0.067001
CS3	MY6	GD24	*R.nitidus*	0.003861	0.067007
SC14	MY6	GD24	*R.nitidus*	0.036293	0.065918
CS11	JW4	GD24	*R.nitidus*	-0.200303	0.068608
JO8	JW4	GD24	*R.nitidus*	-0.191111	0.072687
GD25	JW4	GD24	*R.nitidus*	-0.173878	0.067918
GD29	JW4	GD24	*R.nitidus*	-0.133484	0.067693
JO7	JW4	GD24	*R.nitidus*	-0.22412	0.073044
SC31	MY6	GD24	*R.nitidus*	0.003882	0.067191
SC35	MY6	GD24	*R.nitidus*	0.073634	0.066626
GD48	JW4	GD24	*R.nitidus*	-0.205578	0.067712
GD30	JW4	GD24	*R.nitidus*	-0.11552	0.069684
JO5	MY6	GD24	*R.nitidus*	0.06827	0.066078
GD42	JW4	GD24	*R.nitidus*	-0.180924	0.066292
GD40	JW4	GD24	*R.nitidus*	-0.143279	0.06746

GD46	JW4	GD24	*R.nitidus*	-0.236576	0.066818
CS12	JW4	GD24	*R.nitidus*	-0.207865	0.068765
GD45	JW4	GD24	*R.nitidus*	-0.149435	0.071184
GD32	JW4	GD24	*R.nitidus*	-0.216055	0.069182
GD35	JW4	GD24	*R.nitidus*	-0.195122	0.066394
GD49	JW4	GD24	*R.nitidus*	-0.132275	0.07179
GD21	JW4	GD24	*R.nitidus*	-0.114449	0.066626
GD22	JW4	GD24	*R.nitidus*	-0.164706	0.068216
GD31	JW4	GD24	*R.nitidus*	-0.145544	0.069895
CS21	JW4	GD24	*R.nitidus*	-0.240842	0.067884
GD50	JW4	GD24	*R.nitidus*	-0.166968	0.070461
GD26	JW4	GD24	*R.nitidus*	-0.195755	0.067538
CS16	JW4	GD24	*R.nitidus*	-0.225352	0.067487
CS14	JW4	GD24	*R.nitidus*	-0.213669	0.070651
GD34	JW4	GD24	*R.nitidus*	-0.226048	0.067413
CS20	JW4	GD24	*R.nitidus*	-0.236719	0.068644
CS30	JW4	GD24	*R.nitidus*	-0.269585	0.066381
CS23	JW4	GD24	*R.nitidus*	-0.25076	0.06754
GD33	JW4	GD24	*R.nitidus*	-0.244804	0.069752
GD41	JW4	GD24	*R.nitidus*	-0.214176	0.065438
CS19	JW4	GD24	*R.nitidus*	-0.242231	0.070204
CS13	JW4	GD24	*R.nitidus*	-0.244833	0.06748
GD44	JW4	GD24	*R.nitidus*	-0.18328	0.069745
CS15	JW4	GD24	*R.nitidus*	-0.182443	0.070015
CS28	JW4	GD24	*R.nitidus*	-0.242925	0.071022
CS17	JW4	GD24	*R.nitidus*	-0.193264	0.070287
CS22	JW4	GD24	*R.nitidus*	-0.22179	0.066761
SC24	JW4	GD24	*R.nitidus*	-0.206731	0.067557
SC10	JW4	GD24	*R.nitidus*	-0.17785	0.068055
CS18	JW4	GD24	*R.nitidus*	-0.256672	0.06709
SC12	JW4	GD24	*R.nitidus*	-0.253354	0.066936
CS4	JW4	GD24	*R.nitidus*	-0.226033	0.066376
CS24	JW4	GD24	*R.nitidus*	-0.2738	0.066713
SC5	JW4	GD24	*R.nitidus*	-0.192459	0.071496
SC14	JW4	GD24	*R.nitidus*	-0.20827	0.066734
SC28	JW4	GD24	*R.nitidus*	-0.227061	0.066432
SC35	JW4	GD24	*R.nitidus*	-0.20266	0.066303
CS25	JW4	GD24	*R.nitidus*	-0.226357	0.070091
CS3	JW4	GD24	*R.nitidus*	-0.229624	0.0693
SC6	JW4	GD24	*R.nitidus*	-0.239289	0.067425
CS31	JW4	GD24	*R.nitidus*	-0.268235	0.067999
SC44	JW4	GD24	*R.nitidus*	-0.200154	0.067065
SC32	JW4	GD24	*R.nitidus*	-0.186191	0.067311
SC27	JW4	GD24	*R.nitidus*	-0.198635	0.067337
SC18	JW4	GD24	*R.nitidus*	-0.174267	0.06912
SC31	JW4	GD24	*R.nitidus*	-0.234843	0.067554
JO8	JW6	GD24	*R.nitidus*	0.019817	0.075202
GD25	JW6	GD24	*R.nitidus*	0.062628	0.073106
JW6	JW4	GD24	*R.nitidus*	-0.187755	0.071156
JO5	JW4	GD24	*R.nitidus*	-0.158986	0.067717
GD26	JW6	GD24	*R.nitidus*	0.057282	0.072492
JO1	JW4	GD24	*R.nitidus*	-0.216654	0.06829
CS11	JW6	GD24	*R.nitidus*	0.008188	0.074063
SC26	JW4	GD24	*R.nitidus*	-0.260903	0.067191
SC15	JW4	GD24	*R.nitidus*	-0.233071	0.068708
CS12	JW6	GD24	*R.nitidus*	0.005247	0.074903

MY6	JW4	GD24	*R.nitidus*	-0.246019	0.066457
GD30	JW6	GD24	*R.nitidus*	0.13892	0.074841
JW7	JW4	GD24	*R.nitidus*	-0.131304	0.070577
SC42	JW4	GD24	*R.nitidus*	0.017426	0.066909
GD32	JW6	GD24	*R.nitidus*	-0.008658	0.073924
MY4	JW4	GD24	*R.nitidus*	-0.267685	0.066224
SC34	JW4	GD24	*R.nitidus*	-0.204214	0.065578
GD35	JW6	GD24	*R.nitidus*	0.053974	0.073249
GD22	JW6	GD24	*R.nitidus*	0.071161	0.071605
JO7	JW6	GD24	*R.nitidus*	0.015679	0.069872
SC45	JW4	GD24	*R.nitidus*	-0.206758	0.067064
GD31	JW6	GD24	*R.nitidus*	0.088119	0.073306
JO3	JW4	GD24	*R.nitidus*	-0.167614	0.069911
GD21	JW6	GD24	*R.nitidus*	0.103791	0.070733
GD33	JW6	GD24	*R.nitidus*	-0.027695	0.075212
CS13	JW6	GD24	*R.nitidus*	-0.005258	0.075933
GD41	JW6	GD24	*R.nitidus*	0.025126	0.072404
GD44	JW6	GD24	*R.nitidus*	0.028986	0.073215
GD50	JW6	GD24	*R.nitidus*	0.066441	0.074824
CS6	JW4	GD24	*R.nitidus*	-0.234916	0.067701
GD29	JW6	GD24	*R.nitidus*	0.091856	0.071292
GD46	JW6	GD24	*R.nitidus*	-0.022	0.072498
GD34	JW6	GD24	*R.nitidus*	-0.015123	0.072003
CS28	JW6	GD24	*R.nitidus*	-0.021622	0.074489
CS14	JW6	GD24	*R.nitidus*	0.017727	0.077115
CS22	JW6	GD24	*R.nitidus*	-0.011	0.07422
CS3	JW6	GD24	*R.nitidus*	0.019289	0.074545
SC6	JW6	GD24	*R.nitidus*	0.014099	0.077183
SC5	JW6	GD24	*R.nitidus*	0.034205	0.077375
CS17	JW6	GD24	*R.nitidus*	0.04382	0.07814
GD45	JW6	GD24	*R.nitidus*	0.109756	0.076089
GD40	JW6	GD24	*R.nitidus*	0.076172	0.072148
GD48	JW6	GD24	*R.nitidus*	-0.005176	0.072204
CS23	JW6	GD24	*R.nitidus*	-0.012573	0.074464
SC14	JW6	GD24	*R.nitidus*	0.018367	0.071954
GD49	JW6	GD24	*R.nitidus*	0.108597	0.072032
CS30	JW6	GD24	*R.nitidus*	-0.048128	0.072329
SC12	JW6	GD24	*R.nitidus*	-0.040041	0.072896
GD42	JW6	GD24	*R.nitidus*	0.061866	0.072271
CS25	JW6	GD24	*R.nitidus*	-0.0199	0.076463
CS31	JW6	GD24	*R.nitidus*	-0.038176	0.075911
CS16	JW6	GD24	*R.nitidus*	0.006098	0.074684
CS18	JW6	GD24	*R.nitidus*	-0.030693	0.074645
SC24	JW6	GD24	*R.nitidus*	0.030043	0.075236
CS15	JW6	GD24	*R.nitidus*	0.057927	0.074268
SC15	JW6	GD24	*R.nitidus*	-0.013757	0.075239
SC32	JW6	GD24	*R.nitidus*	0.040082	0.073211
SC42	JW6	GD24	*R.nitidus*	0.214953	0.068725
CS24	JW6	GD24	*R.nitidus*	-0.050725	0.074281
SC10	JW6	GD24	*R.nitidus*	0.079918	0.075574
CS4	JW6	GD24	*R.nitidus*	0.010721	0.071468
SC34	JW6	GD24	*R.nitidus*	0.026104	0.072883
CS19	JW6	GD24	*R.nitidus*	-0.041489	0.075534
CS20	JW6	GD24	*R.nitidus*	0.00956	0.074599
CS21	JW6	GD24	*R.nitidus*	0	0.073391
SC35	JW6	GD24	*R.nitidus*	0.031536	0.073667

SC27	JW6	GD24	*R.nitidus*	0.010537	0.072695
CS6	JW6	GD24	*R.nitidus*	-0.032888	0.072188
JO1	JW6	GD24	*R.nitidus*	-0.001011	0.073407
JO3	JW6	GD24	*R.nitidus*	0.014528	0.076806
JW7	JW6	GD24	*R.nitidus*	0.046275	0.074957
SC18	JW6	GD24	*R.nitidus*	0.044968	0.07409
SC26	JW6	GD24	*R.nitidus*	-0.024793	0.07385
SC45	JW6	GD24	*R.nitidus*	0.051546	0.072682
SC28	JW6	GD24	*R.nitidus*	0.020513	0.075418
SC31	JW6	GD24	*R.nitidus*	-0.025666	0.075117
GD30	JW7	GD24	*R.nitidus*	0.092555	0.073972
CS11	JW7	GD24	*R.nitidus*	-0.026419	0.074165
JO5	JW6	GD24	*R.nitidus*	0.082745	0.070909
GD45	JW7	GD24	*R.nitidus*	0.049345	0.073936
MY6	JW6	GD24	*R.nitidus*	-0.036017	0.073062
GD42	JW7	GD24	*R.nitidus*	0.03352	0.070935
GD46	JW7	GD24	*R.nitidus*	-0.051235	0.070628
SC44	JW6	GD24	*R.nitidus*	0.019211	0.073831
MY4	JW6	GD24	*R.nitidus*	-0.05285	0.075441
GD26	JW7	GD24	*R.nitidus*	-0.000894	0.071713
GD21	JW7	GD24	*R.nitidus*	0.082687	0.071008
GD25	JW7	GD24	*R.nitidus*	0.025328	0.073701
GD44	JW7	GD24	*R.nitidus*	-0.003854	0.07288
GD22	JW7	GD24	*R.nitidus*	0.043919	0.071777
GD29	JW7	GD24	*R.nitidus*	0.060657	0.069713
JW4	JW6	GD24	*R.nitidus*	0.187755	0.071156
JO7	JW7	GD24	*R.nitidus*	-0.088202	0.07712
GD40	JW7	GD24	*R.nitidus*	0.045534	0.070976
CS13	JW7	GD24	*R.nitidus*	-0.07483	0.07397
GD34	JW7	GD24	*R.nitidus*	-0.054659	0.073547
JO8	JW7	GD24	*R.nitidus*	-0.053613	0.076493
GD41	JW7	GD24	*R.nitidus*	-0.026802	0.071759
GD50	JW7	GD24	*R.nitidus*	0.031729	0.07577
GD33	JW7	GD24	*R.nitidus*	-0.060177	0.07399
CS25	JW7	GD24	*R.nitidus*	-0.048159	0.073442
GD31	JW7	GD24	*R.nitidus*	0.059369	0.073634
CS30	JW7	GD24	*R.nitidus*	-0.089962	0.071784
GD32	JW7	GD24	*R.nitidus*	-0.036929	0.071837
CS20	JW7	GD24	*R.nitidus*	-0.04461	0.072621
CS15	JW7	GD24	*R.nitidus*	0.009407	0.072784
CS12	JW7	GD24	*R.nitidus*	-0.030242	0.072542
CS24	JW7	GD24	*R.nitidus*	-0.081716	0.071164
CS28	JW7	GD24	*R.nitidus*	-0.064116	0.073815
CS17	JW7	GD24	*R.nitidus*	-0.005128	0.075089
GD35	JW7	GD24	*R.nitidus*	0.001813	0.073325
CS18	JW7	GD24	*R.nitidus*	-0.070755	0.072575
SC12	JW7	GD24	*R.nitidus*	-0.084536	0.072248
GD48	JW7	GD24	*R.nitidus*	-0.042654	0.072326
CS22	JW7	GD24	*R.nitidus*	-0.031823	0.072867
CS31	JW7	GD24	*R.nitidus*	-0.086294	0.071867
CS14	JW7	GD24	*R.nitidus*	-0.034123	0.074655
SC18	JW7	GD24	*R.nitidus*	0.002125	0.070381
SC24	JW7	GD24	*R.nitidus*	-0.038934	0.071788
SC26	JW7	GD24	*R.nitidus*	-0.09751	0.071063
CS19	JW7	GD24	*R.nitidus*	-0.058325	0.073877
GD49	JW7	GD24	*R.nitidus*	0.047619	0.073561

SC44	JW7	GD24	*R.nitidus*	-0.008114	0.071351
SC10	JW7	GD24	*R.nitidus*	0.022175	0.071276
CS16	JW7	GD24	*R.nitidus*	-0.041136	0.073737
SC14	JW7	GD24	*R.nitidus*	-0.027174	0.071331
CS21	JW7	GD24	*R.nitidus*	-0.058011	0.072458
MY6	JW7	GD24	*R.nitidus*	-0.061303	0.072592
SC45	JW7	GD24	*R.nitidus*	-0.000975	0.07211
SC15	JW7	GD24	*R.nitidus*	-0.063674	0.074069
SC31	JW7	GD24	*R.nitidus*	-0.069744	0.069144
SC42	JW7	GD24	*R.nitidus*	0.215221	0.067697
JO3	JW7	GD24	*R.nitidus*	-0.033784	0.072791
SC35	JW7	GD24	*R.nitidus*	-0.008205	0.069084
SC32	JW7	GD24	*R.nitidus*	-0.022293	0.072284
JW6	JW7	GD24	*R.nitidus*	-0.046275	0.074957
CS23	JW7	GD24	*R.nitidus*	-0.064959	0.071768
CS3	JW7	GD24	*R.nitidus*	-0.051852	0.069706
JO8	JO1	GD24	*R.nitidus*	0.006472	0.073227
SC5	JW7	GD24	*R.nitidus*	-0.01749	0.079943
SC34	JW7	GD24	*R.nitidus*	-0.002944	0.069537
CS4	JW7	GD24	*R.nitidus*	-0.02723	0.069564
SC6	JW7	GD24	*R.nitidus*	-0.052247	0.074915
JO5	JW7	GD24	*R.nitidus*	0.034551	0.071237
SC27	JW7	GD24	*R.nitidus*	-0.030837	0.072303
CS11	JO1	GD24	*R.nitidus*	0.003912	0.066929
SC28	JW7	GD24	*R.nitidus*	-0.045786	0.070336
CS12	JO1	GD24	*R.nitidus*	0.001643	0.067378
CS6	JW7	GD24	*R.nitidus*	-0.068467	0.072046
GD26	JO1	GD24	*R.nitidus*	0.058115	0.065815
GD30	JO1	GD24	*R.nitidus*	0.134615	0.066472
JW4	JW7	GD24	*R.nitidus*	0.131304	0.070577
GD49	JO1	GD24	*R.nitidus*	0.101307	0.068778
GD22	JO1	GD24	*R.nitidus*	0.0819	0.066375
MY4	JW7	GD24	*R.nitidus*	-0.086873	0.073911
JO1	JW7	GD24	*R.nitidus*	-0.042181	0.07132
GD35	JO1	GD24	*R.nitidus*	0.041636	0.066657
GD25	JO1	GD24	*R.nitidus*	0.088598	0.066391
GD32	JO1	GD24	*R.nitidus*	0.022599	0.068621
JO7	JO1	GD24	*R.nitidus*	-0.045226	0.073547
GD31	JO1	GD24	*R.nitidus*	0.089025	0.068264
GD44	JO1	GD24	*R.nitidus*	0.028997	0.067737
GD34	JO1	GD24	*R.nitidus*	0.017921	0.066683
CS14	JO1	GD24	*R.nitidus*	0.002362	0.070115
GD21	JO1	GD24	*R.nitidus*	0.123326	0.065174
GD50	JO1	GD24	*R.nitidus*	0.071048	0.070156
GD40	JO1	GD24	*R.nitidus*	0.096536	0.067287
CS13	JO1	GD24	*R.nitidus*	-0.029435	0.068585
GD48	JO1	GD24	*R.nitidus*	0.008793	0.068716
GD29	JO1	GD24	*R.nitidus*	0.103013	0.065428
GD33	JO1	GD24	*R.nitidus*	0.002214	0.067935
GD45	JO1	GD24	*R.nitidus*	0.061439	0.070204
CS23	JO1	GD24	*R.nitidus*	-0.047104	0.068712
CS18	JO1	GD24	*R.nitidus*	-0.040248	0.068577
CS19	JO1	GD24	*R.nitidus*	-0.021622	0.070167
CS30	JO1	GD24	*R.nitidus*	-0.037647	0.067553
CS17	JO1	GD24	*R.nitidus*	0.039474	0.069032
CS25	JO1	GD24	*R.nitidus*	-0.006061	0.069167

GD46	JO1	GD24	*R.nitidus*	0.005935	0.06463
CS22	JO1	GD24	*R.nitidus*	0.008607	0.068385
GD41	JO1	GD24	*R.nitidus*	0.018797	0.065257
SC12	JO1	GD24	*R.nitidus*	-0.014913	0.068144
CS3	JO1	GD24	*R.nitidus*	-0.009441	0.068843
CS15	JO1	GD24	*R.nitidus*	0.03054	0.066424
GD42	JO1	GD24	*R.nitidus*	0.039493	0.066698
CS31	JO1	GD24	*R.nitidus*	-0.0089	0.067724
CS4	JO1	GD24	*R.nitidus*	0.000784	0.066614
CS16	JO1	GD24	*R.nitidus*	-0.003147	0.067168
CS24	JO1	GD24	*R.nitidus*	-0.041732	0.068358
SC42	JO1	GD24	*R.nitidus*	0.27947	0.063012
SC28	JO1	GD24	*R.nitidus*	0.009402	0.066144
CS21	JO1	GD24	*R.nitidus*	-0.018032	0.069035
SC27	JO1	GD24	*R.nitidus*	0.010067	0.066156
CS20	JO1	GD24	*R.nitidus*	-0.014749	0.069461
SC5	JO1	GD24	*R.nitidus*	0.010569	0.069864
SC6	JO1	GD24	*R.nitidus*	-0.005063	0.068144
SC10	JO1	GD24	*R.nitidus*	0.082203	0.067233
SC32	JO1	GD24	*R.nitidus*	0.044597	0.067314
SC31	JO1	GD24	*R.nitidus*	-0.001626	0.065943
SC15	JO1	GD24	*R.nitidus*	-0.008432	0.069038
SC26	JO1	GD24	*R.nitidus*	-0.032231	0.065408
SC35	JO1	GD24	*R.nitidus*	0.068852	0.066118
SC44	JO1	GD24	*R.nitidus*	0.036885	0.068311
SC14	JO1	GD24	*R.nitidus*	0.013083	0.065316
CS6	JO1	GD24	*R.nitidus*	-0.0208	0.069473
SC18	JO1	GD24	*R.nitidus*	0.06563	0.068168
SC24	JO1	GD24	*R.nitidus*	0.006536	0.067993
SC34	JO1	GD24	*R.nitidus*	0.038554	0.066972
CS11	JO3	GD24	*R.nitidus*	-0.014744	0.074634
CS12	JO3	GD24	*R.nitidus*	-0.000884	0.073276
GD29	JO3	GD24	*R.nitidus*	0.070707	0.068964
JO5	JO1	GD24	*R.nitidus*	0.070319	0.06746
GD26	JO3	GD24	*R.nitidus*	0.022433	0.071866
GD30	JO3	GD24	*R.nitidus*	0.102513	0.073113
GD33	JO3	GD24	*R.nitidus*	-0.039301	0.072371
CS28	JO1	GD24	*R.nitidus*	-0.03322	0.06802
GD21	JO3	GD24	*R.nitidus*	0.079545	0.071544
GD25	JO3	GD24	*R.nitidus*	0.028519	0.070266
JO8	JO3	GD24	*R.nitidus*	0.008883	0.076106
GD31	JO3	GD24	*R.nitidus*	0.032258	0.074895
GD32	JO3	GD24	*R.nitidus*	-0.012871	0.072991
MY6	JO1	GD24	*R.nitidus*	0.002347	0.065845
JW6	JO1	GD24	*R.nitidus*	0.001011	0.073407
JW4	JO1	GD24	*R.nitidus*	0.216654	0.06829
GD40	JO3	GD24	*R.nitidus*	0.039655	0.070534
GD42	JO3	GD24	*R.nitidus*	0.01982	0.071764
GD44	JO3	GD24	*R.nitidus*	0.01086	0.072864
SC45	JO1	GD24	*R.nitidus*	0.049538	0.069238
JO3	JO1	GD24	*R.nitidus*	0.029956	0.072237
MY4	JO1	GD24	*R.nitidus*	-0.03451	0.067864
JW7	JO1	GD24	*R.nitidus*	0.042181	0.07132
GD48	JO3	GD24	*R.nitidus*	-0.006629	0.072772
GD41	JO3	GD24	*R.nitidus*	-0.011424	0.071458
GD50	JO3	GD24	*R.nitidus*	0.038845	0.075117

CS14	JO3	GD24	*R.nitidus*	-0.014719	0.077641
GD22	JO3	GD24	*R.nitidus*	0.05137	0.072204
CS17	JO3	GD24	*R.nitidus*	0.005535	0.074666
CS15	JO3	GD24	*R.nitidus*	0.013901	0.074411
CS23	JO3	GD24	*R.nitidus*	-0.066071	0.070711
GD46	JO3	GD24	*R.nitidus*	-0.035746	0.070973
GD35	JO3	GD24	*R.nitidus*	0.02307	0.072804
GD34	JO3	GD24	*R.nitidus*	-0.023789	0.072841
GD45	JO3	GD24	*R.nitidus*	0.052052	0.075322
CS28	JO3	GD24	*R.nitidus*	-0.045579	0.074827
GD49	JO3	GD24	*R.nitidus*	0.046344	0.075749
CS24	JO3	GD24	*R.nitidus*	-0.050938	0.072198
CS20	JO3	GD24	*R.nitidus*	-0.041182	0.070758
JO7	JO3	GD24	*R.nitidus*	-0.023457	0.077267
CS18	JO3	GD24	*R.nitidus*	-0.062389	0.07112
SC15	JO3	GD24	*R.nitidus*	-0.030359	0.070775
CS30	JO3	GD24	*R.nitidus*	-0.087993	0.071537
CS13	JO3	GD24	*R.nitidus*	-0.036738	0.075747
SC24	JO3	GD24	*R.nitidus*	-0.015426	0.072954
CS16	JO3	GD24	*R.nitidus*	-0.021777	0.073736
SC14	JO3	GD24	*R.nitidus*	-0.004374	0.071728
CS21	JO3	GD24	*R.nitidus*	-0.047957	0.072514
SC10	JO3	GD24	*R.nitidus*	0.04977	0.072349
CS22	JO3	GD24	*R.nitidus*	-0.014912	0.073035
CS19	JO3	GD24	*R.nitidus*	-0.052438	0.072271
SC12	JO3	GD24	*R.nitidus*	-0.060793	0.07124
SC31	JO3	GD24	*R.nitidus*	-0.030541	0.072298
SC27	JO3	GD24	*R.nitidus*	0.005386	0.071386
CS25	JO3	GD24	*R.nitidus*	-0.045654	0.072301
SC34	JO3	GD24	*R.nitidus*	-0.015058	0.070525
SC26	JO3	GD24	*R.nitidus*	-0.063194	0.070897
SC42	JO3	GD24	*R.nitidus*	0.195324	0.067337
SC18	JO3	GD24	*R.nitidus*	0.037684	0.073301
SC44	JO3	GD24	*R.nitidus*	0.007036	0.071641
SC32	JO3	GD24	*R.nitidus*	0.026817	0.072196
SC35	JO3	GD24	*R.nitidus*	0.017164	0.071551
SC45	JO3	GD24	*R.nitidus*	0.011525	0.071272
JW7	JO3	GD24	*R.nitidus*	0.033784	0.072791
MY4	JO3	GD24	*R.nitidus*	-0.063406	0.071945
CS3	JO3	GD24	*R.nitidus*	-0.011894	0.071272
CS31	JO3	GD24	*R.nitidus*	-0.034906	0.073164
SC5	JO3	GD24	*R.nitidus*	0.015177	0.075917
SC6	JO3	GD24	*R.nitidus*	-0.033333	0.07245
JO1	JO3	GD24	*R.nitidus*	-0.029956	0.072237
CS6	JO3	GD24	*R.nitidus*	-0.075	0.070077
JO8	JO5	GD24	*R.nitidus*	-0.083149	0.072858
CS4	JO3	GD24	*R.nitidus*	-0.030783	0.070823
SC28	JO3	GD24	*R.nitidus*	-0.008749	0.071902
GD21	JO5	GD24	*R.nitidus*	0.057534	0.065878
GD22	JO5	GD24	*R.nitidus*	0.015625	0.065131
JW4	JO3	GD24	*R.nitidus*	0.167614	0.069911
MY6	JO3	GD24	*R.nitidus*	-0.039604	0.071337
GD32	JO5	GD24	*R.nitidus*	-0.042429	0.070019
JO7	JO5	GD24	*R.nitidus*	-0.096517	0.071684
GD34	JO5	GD24	*R.nitidus*	-0.046595	0.065766
CS11	JO5	GD24	*R.nitidus*	-0.028832	0.068805

GD30	JO5	GD24	*R.nitidus*	0.066557	0.067448
JW6	JO3	GD24	*R.nitidus*	-0.014528	0.076806
GD42	JO5	GD24	*R.nitidus*	-0.014383	0.066953
JO5	JO3	GD24	*R.nitidus*	0.062827	0.072131
CS14	JO5	GD24	*R.nitidus*	-0.046494	0.069953
GD29	JO5	GD24	*R.nitidus*	0.057775	0.065056
GD41	JO5	GD24	*R.nitidus*	-0.04059	0.065745
GD40	JO5	GD24	*R.nitidus*	0.029454	0.064704
GD45	JO5	GD24	*R.nitidus*	0.022034	0.068603
GD25	JO5	GD24	*R.nitidus*	0.020301	0.067488
CS15	JO5	GD24	*R.nitidus*	-0.027439	0.069784
GD26	JO5	GD24	*R.nitidus*	-0.006623	0.067945
CS22	JO5	GD24	*R.nitidus*	-0.041765	0.067889
GD31	JO5	GD24	*R.nitidus*	0.03976	0.068669
GD33	JO5	GD24	*R.nitidus*	-0.05981	0.068365
SC6	JO5	GD24	*R.nitidus*	-0.078226	0.067494
CS25	JO5	GD24	*R.nitidus*	-0.082671	0.067957
CS16	JO5	GD24	*R.nitidus*	-0.054306	0.066682
SC12	JO5	GD24	*R.nitidus*	-0.090528	0.067476
CS20	JO5	GD24	*R.nitidus*	-0.083459	0.068539
CS24	JO5	GD24	*R.nitidus*	-0.09589	0.066318
CS28	JO5	GD24	*R.nitidus*	-0.069983	0.066909
CS18	JO5	GD24	*R.nitidus*	-0.111283	0.068915
CS12	JO5	GD24	*R.nitidus*	-0.04789	0.067855
CS21	JO5	GD24	*R.nitidus*	-0.076746	0.06713
CS23	JO5	GD24	*R.nitidus*	-0.102964	0.066363
CS19	JO5	GD24	*R.nitidus*	-0.075548	0.06893
GD35	JO5	GD24	*R.nitidus*	0.007452	0.067179
GD44	JO5	GD24	*R.nitidus*	-0.028549	0.067486
GD50	JO5	GD24	*R.nitidus*	0.002662	0.068977
GD46	JO5	GD24	*R.nitidus*	-0.05267	0.065179
GD49	JO5	GD24	*R.nitidus*	0.071901	0.068881
GD48	JO5	GD24	*R.nitidus*	-0.047773	0.069312
CS13	JO5	GD24	*R.nitidus*	-0.087094	0.068197
CS17	JO5	GD24	*R.nitidus*	-0.002429	0.071047
SC24	JO5	GD24	*R.nitidus*	-0.05464	0.066451
CS30	JO5	GD24	*R.nitidus*	-0.105645	0.06482
JW4	JO5	GD24	*R.nitidus*	0.158986	0.067717
MY4	JO5	GD24	*R.nitidus*	-0.095912	0.068277
CS3	JO5	GD24	*R.nitidus*	-0.073841	0.067029
SC18	JO5	GD24	*R.nitidus*	0	0.065558
SC28	JO5	GD24	*R.nitidus*	-0.087912	0.062449
SC44	JO5	GD24	*R.nitidus*	-0.035349	0.064685
JW6	JO5	GD24	*R.nitidus*	-0.082745	0.070909
SC31	JO5	GD24	*R.nitidus*	-0.058544	0.064714
SC15	JO5	GD24	*R.nitidus*	-0.080617	0.065458
CS4	JO5	GD24	*R.nitidus*	-0.062844	0.066196
CS31	JO5	GD24	*R.nitidus*	-0.078289	0.067941
SC5	JO5	GD24	*R.nitidus*	-0.059837	0.070117
SC10	JO5	GD24	*R.nitidus*	0.027523	0.068283
SC42	JO5	GD24	*R.nitidus*	0.229748	0.064233
GD25	JO7	GD28	*R.nitidus*	0.055966	0.071639
JO1	JO5	GD24	*R.nitidus*	-0.070319	0.06746
JO3	JO5	GD24	*R.nitidus*	-0.062827	0.072131
SC14	JO5	GD24	*R.nitidus*	-0.043257	0.066173
GD29	JO7	GD28	*R.nitidus*	0.104854	0.069824

SC32	JO5	GD24	*R.nitidus*	-0.01252	0.066419
SC27	JO5	GD24	*R.nitidus*	-0.045061	0.067007
CS6	JO5	GD24	*R.nitidus*	-0.092257	0.066294
SC26	JO5	GD24	*R.nitidus*	-0.095238	0.067474
GD21	JO7	GD28	*R.nitidus*	0.115128	0.071393
CS11	JO7	GD28	*R.nitidus*	-0.011213	0.075223
GD31	JO7	GD28	*R.nitidus*	0.041215	0.074269
SC35	JO5	GD24	*R.nitidus*	-0.004785	0.066624
SC45	JO5	GD24	*R.nitidus*	-0.016234	0.067717
GD32	JO7	GD28	*R.nitidus*	0.031567	0.076872
GD22	JO7	GD28	*R.nitidus*	0.084615	0.071189
SC34	JO5	GD24	*R.nitidus*	-0.024077	0.067725
JO8	JO7	GD28	*R.nitidus*	0.001587	0.077758
GD30	JO7	GD28	*R.nitidus*	0.128702	0.073995
GD26	JO7	GD28	*R.nitidus*	0.078629	0.074967
JW7	JO5	GD24	*R.nitidus*	-0.034551	0.071237
GD45	JO7	GD28	*R.nitidus*	0.071951	0.075727
MY6	JO5	GD24	*R.nitidus*	-0.06827	0.066078
CS18	JO7	GD28	*R.nitidus*	-0.044239	0.074489
GD46	JO7	GD28	*R.nitidus*	-0.011387	0.070842
GD33	JO7	GD28	*R.nitidus*	0.030422	0.073771
CS17	JO7	GD28	*R.nitidus*	-0.012155	0.076766
CS12	JO7	GD28	*R.nitidus*	-0.009698	0.076023
CS20	JO7	GD28	*R.nitidus*	-0.019685	0.072536
CS30	JO7	GD28	*R.nitidus*	-0.053215	0.072707
CS24	JO7	GD28	*R.nitidus*	-0.027197	0.074186
SC10	JO7	GD28	*R.nitidus*	0.095494	0.073824
GD35	JO7	GD28	*R.nitidus*	0.023398	0.074074
GD34	JO7	GD28	*R.nitidus*	0.023256	0.072831
CS23	JO7	GD28	*R.nitidus*	-0.033401	0.074364
GD41	JO7	GD28	*R.nitidus*	-0.018386	0.072927
GD40	JO7	GD28	*R.nitidus*	0.047094	0.072957
GD42	JO7	GD28	*R.nitidus*	0.029592	0.074983
GD50	JO7	GD28	*R.nitidus*	0.029377	0.076584
CS13	JO7	GD28	*R.nitidus*	-0.040469	0.075646
GD44	JO7	GD28	*R.nitidus*	-0.024691	0.074805
CS15	JO7	GD28	*R.nitidus*	0.012552	0.074226
SC26	JO7	GD28	*R.nitidus*	-0.04888	0.072659
GD48	JO7	GD28	*R.nitidus*	0.036458	0.071761
CS31	JO7	GD28	*R.nitidus*	-0.002153	0.071269
CS21	JO7	GD28	*R.nitidus*	-0.053307	0.073466
CS19	JO7	GD28	*R.nitidus*	-0.008316	0.075051
CS16	JO7	GD28	*R.nitidus*	-0.009605	0.074229
GD49	JO7	GD28	*R.nitidus*	0.09122	0.072553
CS4	JO7	GD28	*R.nitidus*	-0.040877	0.072661
SC24	JO7	GD28	*R.nitidus*	0.005336	0.073424
SC6	JO7	GD28	*R.nitidus*	-0.019048	0.069209
SC12	JO7	GD28	*R.nitidus*	-0.042508	0.070772
CS22	JO7	GD28	*R.nitidus*	0.019958	0.072227
SC27	JO7	GD28	*R.nitidus*	0.001037	0.071751
CS14	JO7	GD28	*R.nitidus*	-0.005336	0.076546
SC44	JO7	GD28	*R.nitidus*	-0.020063	0.074211
SC15	JO7	GD28	*R.nitidus*	-0.017486	0.074951
CS25	JO7	GD28	*R.nitidus*	-0.015512	0.072787
CS28	JO7	GD28	*R.nitidus*	-0.042316	0.072248
CS6	JO7	GD28	*R.nitidus*	-0.034836	0.070443

SC5	JO7	GD28	*R.nitidus*	-0.001055	0.073678
JW4	JO7	GD28	*R.nitidus*	0.149563	0.071367
SC34	JO7	GD28	*R.nitidus*	0.020121	0.070091
CS3	JO7	GD28	*R.nitidus*	-0.012948	0.07109
SC31	JO7	GD28	*R.nitidus*	-0.009775	0.073037
SC32	JO7	GD28	*R.nitidus*	0.035015	0.072334
SC28	JO7	GD28	*R.nitidus*	-0.029661	0.072156
SC14	JO7	GD28	*R.nitidus*	0.01119	0.072098
SC18	JO7	GD28	*R.nitidus*	0.013934	0.073042
MY4	JO7	GD28	*R.nitidus*	-0.023061	0.072149
JW7	JO7	GD28	*R.nitidus*	0.010539	0.073912
GD32	JO8	GD28	*R.nitidus*	-0.013953	0.078121
JO1	JO7	GD28	*R.nitidus*	-0.010194	0.071871
GD35	JO8	GD28	*R.nitidus*	0.006179	0.073798
MY6	JO7	GD28	*R.nitidus*	-0.02766	0.069379
SC35	JO7	GD28	*R.nitidus*	0.017734	0.070084
SC42	JO7	GD28	*R.nitidus*	0.145519	0.069467
JO3	JO7	GD28	*R.nitidus*	0.001235	0.075051
JO5	JO7	GD28	*R.nitidus*	0.030211	0.073171
GD42	JO8	GD28	*R.nitidus*	0.008155	0.074755
JW6	JO7	GD28	*R.nitidus*	0.001859	0.071329
CS12	JO8	GD28	*R.nitidus*	-0.0033	0.076325
SC45	JO7	GD28	*R.nitidus*	0.029258	0.070658
GD26	JO8	GD28	*R.nitidus*	0.036286	0.074397
GD41	JO8	GD28	*R.nitidus*	-0.02359	0.074116
JO7	JO8	GD28	*R.nitidus*	-0.001587	0.077758
GD21	JO8	GD28	*R.nitidus*	0.083095	0.072043
GD31	JO8	GD28	*R.nitidus*	-0.002172	0.074808
CS16	JO8	GD28	*R.nitidus*	-0.041037	0.075292
GD33	JO8	GD28	*R.nitidus*	-0.020141	0.074295
GD34	JO8	GD28	*R.nitidus*	-0.032129	0.073054
GD30	JO8	GD28	*R.nitidus*	0.086435	0.077653
CS15	JO8	GD28	*R.nitidus*	-0.007299	0.075279
CS17	JO8	GD28	*R.nitidus*	-0.007726	0.077617
GD29	JO8	GD28	*R.nitidus*	0.067202	0.071675
GD45	JO8	GD28	*R.nitidus*	0.007481	0.078371
GD40	JO8	GD28	*R.nitidus*	0.029536	0.071947
CS11	JO8	GD28	*R.nitidus*	-0.016966	0.076368
GD44	JO8	GD28	*R.nitidus*	-0.049009	0.074372
GD25	JO8	GD28	*R.nitidus*	0.023555	0.073829
GD22	JO8	GD28	*R.nitidus*	0.051793	0.072847
GD46	JO8	GD28	*R.nitidus*	-0.061376	0.070795
GD50	JO8	GD28	*R.nitidus*	-0.014337	0.075136
GD49	JO8	GD28	*R.nitidus*	0.04878	0.074312
CS13	JO8	GD28	*R.nitidus*	-0.026911	0.076435
SC5	JO8	GD28	*R.nitidus*	-0.015957	0.075462
CS30	JO8	GD28	*R.nitidus*	-0.090414	0.072731
CS14	JO8	GD28	*R.nitidus*	0.009544	0.078512
GD48	JO8	GD28	*R.nitidus*	0.02452	0.072715
SC15	JO8	GD28	*R.nitidus*	-0.011148	0.075084
CS22	JO8	GD28	*R.nitidus*	0.001116	0.073112
CS20	JO8	GD28	*R.nitidus*	-0.031612	0.072566
CS24	JO8	GD28	*R.nitidus*	-0.026077	0.072198
CS19	JO8	GD28	*R.nitidus*	-0.02063	0.0724
CS28	JO8	GD28	*R.nitidus*	-0.023516	0.07312
SC34	JO8	GD28	*R.nitidus*	-0.025667	0.071462

SC10	JO8	GD28	*R.nitidus*	0.068415	0.073427
JW4	JO8	GD28	*R.nitidus*	0.118436	0.074867
SC12	JO8	GD28	*R.nitidus*	-0.050605	0.071561
CS6	JO8	GD28	*R.nitidus*	-0.040948	0.071838
SC14	JO8	GD28	*R.nitidus*	0.007194	0.074613
CS18	JO8	GD28	*R.nitidus*	-0.057297	0.07308
SC42	JO8	GD28	*R.nitidus*	0.123944	0.070586
CS25	JO8	GD28	*R.nitidus*	-0.012672	0.07485
CS23	JO8	GD28	*R.nitidus*	-0.036881	0.072344
CS21	JO8	GD28	*R.nitidus*	-0.069792	0.071574
JW7	JO8	GD28	*R.nitidus*	-0.002356	0.075513
SC31	JO8	GD28	*R.nitidus*	0.001015	0.072318
SC27	JO8	GD28	*R.nitidus*	-0.022269	0.072764
SC45	JO8	GD28	*R.nitidus*	0.008325	0.072674
SC26	JO8	GD28	*R.nitidus*	-0.096501	0.071728
CS31	JO8	GD28	*R.nitidus*	-0.034973	0.073416
MY4	JO8	GD28	*R.nitidus*	-0.031646	0.073396
CS3	JO8	GD28	*R.nitidus*	-0.022293	0.071794
CS4	JO8	GD28	*R.nitidus*	-0.027311	0.071695
SC6	JO8	GD28	*R.nitidus*	-0.058698	0.075797
JO1	JO8	GD28	*R.nitidus*	-0.043663	0.071768
JO5	JO8	GD28	*R.nitidus*	0.032362	0.072684
SC44	JO8	GD28	*R.nitidus*	-0.017505	0.073195
SC18	JO8	GD28	*R.nitidus*	0.001147	0.072849
JW6	JO8	GD28	*R.nitidus*	0.023006	0.074143
SC28	JO8	GD28	*R.nitidus*	-0.055928	0.072178
SC35	JO8	GD28	*R.nitidus*	0.011944	0.075691
SC32	JO8	GD28	*R.nitidus*	0.002064	0.07236
SC24	JO8	GD28	*R.nitidus*	-0.001091	0.073276
JO3	JO8	GD28	*R.nitidus*	0.010063	0.074736
MY6	JO8	GD28	*R.nitidus*	-0.017699	0.072626
JO8	CS11	GD28	*R.nitidus*	0.016966	0.076368
GD22	CS11	GD28	*R.nitidus*	0.065262	0.067617
GD31	CS11	GD28	*R.nitidus*	0.04465	0.069503
JO7	CS11	GD28	*R.nitidus*	0.011213	0.075223
GD41	CS11	GD28	*R.nitidus*	0.007948	0.06799
GD30	CS11	GD28	*R.nitidus*	0.112642	0.071377
GD35	CS11	GD28	*R.nitidus*	0.022711	0.069074
GD32	CS11	GD28	*R.nitidus*	0.027287	0.071902
GD40	CS11	GD28	*R.nitidus*	0.050595	0.066512
GD25	CS11	GD28	*R.nitidus*	0.068726	0.068249
CS12	CS11	GD28	*R.nitidus*	0.015608	0.070893
GD45	CS11	GD28	*R.nitidus*	0.042465	0.071559
GD21	CS11	GD28	*R.nitidus*	0.132102	0.066394
CS13	CS11	GD28	*R.nitidus*	-0.043812	0.072066
GD50	CS11	GD28	*R.nitidus*	0.038328	0.073962
GD29	CS11	GD28	*R.nitidus*	0.101238	0.068549
CS15	CS11	GD28	*R.nitidus*	0.04023	0.069116
GD48	CS11	GD28	*R.nitidus*	0.04222	0.069341
GD34	CS11	GD28	*R.nitidus*	0.017495	0.067718
CS24	CS11	GD28	*R.nitidus*	-0.021739	0.067828
CS16	CS11	GD28	*R.nitidus*	-0.001149	0.070107
CS22	CS11	GD28	*R.nitidus*	0.021446	0.06801
GD44	CS11	GD28	*R.nitidus*	0.00228	0.068585
GD46	CS11	GD28	*R.nitidus*	0.002985	0.06795
GD26	CS11	GD28	*R.nitidus*	0.038095	0.068032

CS19	CS11	GD28	*R.nitidus*	0	0.06974
GD33	CS11	GD28	*R.nitidus*	0.007708	0.066761
SC12	CS11	GD28	*R.nitidus*	-0.005604	0.067565
GD49	CS11	GD28	*R.nitidus*	0.083065	0.071587
SC28	CS11	GD28	*R.nitidus*	-0.051322	0.069209
CS25	CS11	GD28	*R.nitidus*	0	0.069147
CS20	CS11	GD28	*R.nitidus*	-0.020015	0.06644
GD42	CS11	GD28	*R.nitidus*	0.024318	0.068525
CS21	CS11	GD28	*R.nitidus*	-0.031907	0.066159
CS23	CS11	GD28	*R.nitidus*	-0.044118	0.067995
CS14	CS11	GD28	*R.nitidus*	0.019108	0.071691
CS18	CS11	GD28	*R.nitidus*	-0.033594	0.066741
CS17	CS11	GD28	*R.nitidus*	0.030216	0.067398
SC15	CS11	GD28	*R.nitidus*	0.004777	0.068264
SC31	CS11	GD28	*R.nitidus*	0.005323	0.06775
CS4	CS11	GD28	*R.nitidus*	-0.01664	0.067084
SC24	CS11	GD28	*R.nitidus*	-0.00458	0.069973
CS31	CS11	GD28	*R.nitidus*	-0.007365	0.069306
SC26	CS11	GD28	*R.nitidus*	-0.039877	0.066342
SC10	CS11	GD28	*R.nitidus*	0.067901	0.068542
CS28	CS11	GD28	*R.nitidus*	-0.045918	0.068946
CS3	CS11	GD28	*R.nitidus*	-0.028135	0.066744
SC6	CS11	GD28	*R.nitidus*	-0.0347	0.068592
MY6	CS11	GD28	*R.nitidus*	-0.008378	0.068125
CS30	CS11	GD28	*R.nitidus*	-0.044427	0.068331
SC44	CS11	GD28	*R.nitidus*	-0.00152	0.069364
SC35	CS11	GD28	*R.nitidus*	0.054217	0.066014
SC27	CS11	GD28	*R.nitidus*	-0.011041	0.068198
JW4	CS11	GD28	*R.nitidus*	0.176607	0.067236
CS6	CS11	GD28	*R.nitidus*	-0.019151	0.066882
SC18	CS11	GD28	*R.nitidus*	0.031636	0.067782
SC5	CS11	GD28	*R.nitidus*	-0.005405	0.071843
JO3	CS11	GD28	*R.nitidus*	0.032759	0.071805
SC42	CS11	GD28	*R.nitidus*	0.21549	0.064464
GD26	GD21	GD28	*R.nitidus*	-0.113527	0.064794
SC14	CS11	GD28	*R.nitidus*	0.015152	0.069603
JO5	CS11	GD28	*R.nitidus*	0.014275	0.068326
JO1	CS11	GD28	*R.nitidus*	-0.014832	0.066427
JO7	GD21	GD28	*R.nitidus*	-0.115128	0.071393
JW7	CS11	GD28	*R.nitidus*	0.00789	0.07056
GD29	GD21	GD28	*R.nitidus*	-0.045416	0.065614
MY4	CS11	GD28	*R.nitidus*	-0.008327	0.068184
GD31	GD21	GD28	*R.nitidus*	-0.120346	0.066542
SC34	CS11	GD28	*R.nitidus*	0.026515	0.066923
SC32	CS11	GD28	*R.nitidus*	0.058366	0.068413
GD22	GD21	GD28	*R.nitidus*	-0.078064	0.063556
GD30	GD21	GD28	*R.nitidus*	-0.030602	0.06807
GD25	GD21	GD28	*R.nitidus*	-0.072384	0.064166
SC45	CS11	GD28	*R.nitidus*	0.030511	0.069817
JW6	CS11	GD28	*R.nitidus*	0.052247	0.073578
JO8	GD21	GD28	*R.nitidus*	-0.083095	0.072043
GD40	GD21	GD28	*R.nitidus*	-0.108712	0.064765
CS13	GD21	GD28	*R.nitidus*	-0.159652	0.067844
CS15	GD21	GD28	*R.nitidus*	-0.128093	0.066333
GD44	GD21	GD28	*R.nitidus*	-0.16843	0.066386
CS11	GD21	GD28	*R.nitidus*	-0.132102	0.066394

GD48	GD21	GD28	*R.nitidus*	-0.113514	0.066041
CS14	GD21	GD28	*R.nitidus*	-0.135095	0.067862
GD41	GD21	GD28	*R.nitidus*	-0.153785	0.06358
GD34	GD21	GD28	*R.nitidus*	-0.15625	0.061491
GD33	GD21	GD28	*R.nitidus*	-0.175313	0.063016
GD46	GD21	GD28	*R.nitidus*	-0.176883	0.064153
CS12	GD21	GD28	*R.nitidus*	-0.136364	0.067162
GD32	GD21	GD28	*R.nitidus*	-0.117041	0.066116
CS31	GD21	GD28	*R.nitidus*	-0.150489	0.06559
CS18	GD21	GD28	*R.nitidus*	-0.178521	0.066442
CS22	GD21	GD28	*R.nitidus*	-0.118805	0.064299
GD42	GD21	GD28	*R.nitidus*	-0.148988	0.063461
SC12	GD21	GD28	*R.nitidus*	-0.149566	0.066139
CS21	GD21	GD28	*R.nitidus*	-0.172366	0.065626
GD35	GD21	GD28	*R.nitidus*	-0.111548	0.064668
SC6	GD21	GD28	*R.nitidus*	-0.17482	0.06735
CS20	GD21	GD28	*R.nitidus*	-0.173631	0.066062
GD45	GD21	GD28	*R.nitidus*	-0.116214	0.069928
CS24	GD21	GD28	*R.nitidus*	-0.158892	0.066156
GD50	GD21	GD28	*R.nitidus*	-0.152765	0.069571
CS17	GD21	GD28	*R.nitidus*	-0.113063	0.06874
CS3	GD21	GD28	*R.nitidus*	-0.157246	0.063856
GD49	GD21	GD28	*R.nitidus*	-0.059946	0.068858
CS30	GD21	GD28	*R.nitidus*	-0.191154	0.06562
SC18	GD21	GD28	*R.nitidus*	-0.125843	0.066698
CS16	GD21	GD28	*R.nitidus*	-0.126087	0.064872
CS4	GD21	GD28	*R.nitidus*	-0.161609	0.064171
CS19	GD21	GD28	*R.nitidus*	-0.135994	0.067989
SC15	GD21	GD28	*R.nitidus*	-0.145695	0.06575
CS6	GD21	GD28	*R.nitidus*	-0.169881	0.065589
SC31	GD21	GD28	*R.nitidus*	-0.131793	0.066417
SC5	GD21	GD28	*R.nitidus*	-0.148537	0.068067
SC34	GD21	GD28	*R.nitidus*	-0.111728	0.066235
SC10	GD21	GD28	*R.nitidus*	-0.083758	0.06669
CS25	GD21	GD28	*R.nitidus*	-0.14771	0.066131
SC14	GD21	GD28	*R.nitidus*	-0.129149	0.066228
SC35	GD21	GD28	*R.nitidus*	-0.091292	0.06519
SC42	GD21	GD28	*R.nitidus*	0.116938	0.0624
CS23	GD21	GD28	*R.nitidus*	-0.16875	0.066613
JW6	GD21	GD28	*R.nitidus*	-0.101758	0.071059
SC44	GD21	GD28	*R.nitidus*	-0.134209	0.065483
JW4	GD21	GD28	*R.nitidus*	0.050835	0.068464
SC27	GD21	GD28	*R.nitidus*	-0.151982	0.065764
CS28	GD21	GD28	*R.nitidus*	-0.174118	0.066731
MY6	GD21	GD28	*R.nitidus*	-0.150822	0.065871
GD29	GD22	GD28	*R.nitidus*	0.041952	0.064887
SC24	GD21	GD28	*R.nitidus*	-0.146589	0.06647
SC45	GD21	GD28	*R.nitidus*	-0.101322	0.066613
SC26	GD21	GD28	*R.nitidus*	-0.186453	0.066009
MY4	GD21	GD28	*R.nitidus*	-0.156003	0.065833
SC28	GD21	GD28	*R.nitidus*	-0.18408	0.065554
SC32	GD21	GD28	*R.nitidus*	-0.102824	0.066734
GD34	GD22	GD28	*R.nitidus*	-0.066004	0.062215
JO7	GD22	GD28	*R.nitidus*	-0.084615	0.071189
GD31	GD22	GD28	*R.nitidus*	-0.033248	0.06659
JW7	GD21	GD28	*R.nitidus*	-0.143234	0.071301

GD26	GD22	GD28	*R.nitidus*	-0.025295	0.063736
JO5	GD21	GD28	*R.nitidus*	-0.117855	0.065783
JO8	GD22	GD28	*R.nitidus*	-0.051793	0.072847
CS11	GD22	GD28	*R.nitidus*	-0.065262	0.067617
GD32	GD22	GD28	*R.nitidus*	-0.04912	0.066303
GD25	GD22	GD28	*R.nitidus*	-0.016773	0.066608
GD30	GD22	GD28	*R.nitidus*	0.070577	0.066783
JO3	GD21	GD28	*R.nitidus*	-0.101473	0.068831
JO1	GD21	GD28	*R.nitidus*	-0.169757	0.063336
GD42	GD22	GD28	*R.nitidus*	-0.070248	0.064524
CS23	GD22	GD28	*R.nitidus*	-0.100282	0.064959
GD21	GD22	GD28	*R.nitidus*	0.078064	0.063556
GD44	GD22	GD28	*R.nitidus*	-0.072013	0.067746
GD35	GD22	GD28	*R.nitidus*	-0.025581	0.063684
CS13	GD22	GD28	*R.nitidus*	-0.094084	0.068553
GD48	GD22	GD28	*R.nitidus*	-0.025372	0.06531
CS14	GD22	GD28	*R.nitidus*	-0.056378	0.068148
GD40	GD22	GD28	*R.nitidus*	-0.010663	0.063412
GD50	GD22	GD28	*R.nitidus*	-0.046935	0.069989
CS19	GD22	GD28	*R.nitidus*	-0.071429	0.066689
CS22	GD22	GD28	*R.nitidus*	-0.04062	0.064674
GD45	GD22	GD28	*R.nitidus*	-0.04363	0.067922
GD33	GD22	GD28	*R.nitidus*	-0.062057	0.06474
CS17	GD22	GD28	*R.nitidus*	-0.039552	0.068391
CS12	GD22	GD28	*R.nitidus*	-0.049927	0.068528
GD46	GD22	GD28	*R.nitidus*	-0.08206	0.062886
GD49	GD22	GD28	*R.nitidus*	0.029652	0.066701
GD41	GD22	GD28	*R.nitidus*	-0.072428	0.063868
CS21	GD22	GD28	*R.nitidus*	-0.095568	0.064748
CS16	GD22	GD28	*R.nitidus*	-0.059155	0.064678
CS31	GD22	GD28	*R.nitidus*	-0.076183	0.065295
CS28	GD22	GD28	*R.nitidus*	-0.104863	0.065681
SC24	GD22	GD28	*R.nitidus*	-0.07261	0.066467
SC18	GD22	GD28	*R.nitidus*	-0.028295	0.066749
SC5	GD22	GD28	*R.nitidus*	-0.073225	0.067726
CS18	GD22	GD28	*R.nitidus*	-0.104181	0.066155
CS15	GD22	GD28	*R.nitidus*	-0.049689	0.067373
CS24	GD22	GD28	*R.nitidus*	-0.080921	0.066076
CS30	GD22	GD28	*R.nitidus*	-0.119581	0.064775
SC12	GD22	GD28	*R.nitidus*	-0.078757	0.064804
CS25	GD22	GD28	*R.nitidus*	-0.082902	0.067344
SC27	GD22	GD28	*R.nitidus*	-0.066762	0.064738
SC14	GD22	GD28	*R.nitidus*	-0.052482	0.066686
SC31	GD22	GD28	*R.nitidus*	-0.065142	0.066836
CS3	GD22	GD28	*R.nitidus*	-0.091904	0.064721
CS20	GD22	GD28	*R.nitidus*	-0.094101	0.064531
JW4	GD22	GD28	*R.nitidus*	0.12101	0.067688
JO8	GD25	GD28	*R.nitidus*	-0.023555	0.073829
SC42	GD22	GD28	*R.nitidus*	0.151211	0.062454
SC28	GD22	GD28	*R.nitidus*	-0.116129	0.065214
SC44	GD22	GD28	*R.nitidus*	-0.067014	0.066288
SC10	GD22	GD28	*R.nitidus*	0.008234	0.067409
MY6	GD22	GD28	*R.nitidus*	-0.075672	0.065981
CS4	GD22	GD28	*R.nitidus*	-0.086987	0.063562
SC26	GD22	GD28	*R.nitidus*	-0.099237	0.066334
SC6	GD22	GD28	*R.nitidus*	-0.089374	0.067912

SC35	GD22	GD28	*R.nitidus*	-0.00419	0.064861
JO3	GD22	GD28	*R.nitidus*	-0.039931	0.071431
MY4	GD22	GD28	*R.nitidus*	-0.080344	0.066204
SC15	GD22	GD28	*R.nitidus*	-0.068063	0.065687
GD22	GD25	GD28	*R.nitidus*	0.016773	0.066608
SC34	GD22	GD28	*R.nitidus*	-0.03391	0.063616
GD26	GD25	GD28	*R.nitidus*	-0.003407	0.066986
CS13	GD25	GD28	*R.nitidus*	-0.096875	0.068847
JW7	GD22	GD28	*R.nitidus*	-0.076522	0.070947
GD49	GD25	GD28	*R.nitidus*	0.043238	0.069432
GD44	GD25	GD28	*R.nitidus*	-0.060068	0.068301
GD30	GD25	GD28	*R.nitidus*	0.080705	0.069493
JW6	GD22	GD28	*R.nitidus*	-0.032381	0.070298
GD31	GD25	GD28	*R.nitidus*	-0.008711	0.066407
GD42	GD25	GD28	*R.nitidus*	-0.046784	0.066132
CS6	GD22	GD28	*R.nitidus*	-0.097834	0.065486
JO1	GD22	GD28	*R.nitidus*	-0.091608	0.064606
JO7	GD25	GD28	*R.nitidus*	-0.055966	0.071639
JO5	GD22	GD28	*R.nitidus*	-0.040831	0.065445
CS11	GD25	GD28	*R.nitidus*	-0.068726	0.068249
GD32	GD25	GD28	*R.nitidus*	-0.015302	0.067979
SC45	GD22	GD28	*R.nitidus*	-0.025974	0.066403
GD29	GD25	GD28	*R.nitidus*	0.047657	0.066225
CS19	GD25	GD28	*R.nitidus*	-0.048411	0.06406
GD34	GD25	GD28	*R.nitidus*	-0.049521	0.065895
SC32	GD22	GD28	*R.nitidus*	-0.020055	0.063997
CS15	GD25	GD28	*R.nitidus*	-0.051938	0.065267
CS21	GD25	GD28	*R.nitidus*	-0.09003	0.066854
GD41	GD25	GD28	*R.nitidus*	-0.052758	0.065285
GD40	GD25	GD28	*R.nitidus*	-0.019139	0.065123
GD21	GD25	GD28	*R.nitidus*	0.072384	0.064166
CS14	GD25	GD28	*R.nitidus*	-0.055686	0.069379
GD48	GD25	GD28	*R.nitidus*	0.008929	0.066801
GD50	GD25	GD28	*R.nitidus*	-0.004854	0.071111
CS20	GD25	GD28	*R.nitidus*	-0.08015	0.066435
CS17	GD25	GD28	*R.nitidus*	-0.033113	0.069049
GD33	GD25	GD28	*R.nitidus*	-0.050874	0.065479
CS12	GD25	GD28	*R.nitidus*	-0.057661	0.068549
CS25	GD25	GD28	*R.nitidus*	-0.069455	0.066159
GD35	GD25	GD28	*R.nitidus*	-0.040597	0.063701
CS30	GD25	GD28	*R.nitidus*	-0.102326	0.066393
CS3	GD25	GD28	*R.nitidus*	-0.084421	0.066152
GD45	GD25	GD28	*R.nitidus*	0.002712	0.068802
CS18	GD25	GD28	*R.nitidus*	-0.088128	0.06627
CS16	GD25	GD28	*R.nitidus*	-0.058544	0.068979
GD46	GD25	GD28	*R.nitidus*	-0.073684	0.065663
CS23	GD25	GD28	*R.nitidus*	-0.095518	0.06345
CS31	GD25	GD28	*R.nitidus*	-0.064618	0.06588
SC26	GD25	GD28	*R.nitidus*	-0.100752	0.064872
SC24	GD25	GD28	*R.nitidus*	-0.065034	0.067618
SC18	GD25	GD28	*R.nitidus*	-0.050157	0.066086
SC32	GD25	GD28	*R.nitidus*	-0.018059	0.065767
SC10	GD25	GD28	*R.nitidus*	0.002342	0.064089
JW6	GD25	GD28	*R.nitidus*	-0.018888	0.072192
SC28	GD25	GD28	*R.nitidus*	-0.105023	0.06453
CS24	GD25	GD28	*R.nitidus*	-0.054795	0.065367

CS28	GD25	GD28	*R.nitidus*	-0.094719	0.065242
SC44	GD25	GD28	*R.nitidus*	-0.0625	0.06589
CS22	GD25	GD28	*R.nitidus*	-0.027714	0.064056
SC12	GD25	GD28	*R.nitidus*	-0.070878	0.066657
SC15	GD25	GD28	*R.nitidus*	-0.06378	0.067311
JW4	GD25	GD28	*R.nitidus*	0.139888	0.069249
JW7	GD25	GD28	*R.nitidus*	-0.060345	0.071685
SC5	GD25	GD28	*R.nitidus*	-0.069074	0.068044
CS6	GD25	GD28	*R.nitidus*	-0.080343	0.065002
CS4	GD25	GD28	*R.nitidus*	-0.074019	0.064109
SC6	GD25	GD28	*R.nitidus*	-0.088304	0.066794
JO3	GD25	GD28	*R.nitidus*	-0.023084	0.071165
SC34	GD25	GD28	*R.nitidus*	-0.027656	0.064465
SC45	GD25	GD28	*R.nitidus*	-0.028682	0.066528
SC42	GD25	GD28	*R.nitidus*	0.169775	0.063786
SC14	GD25	GD28	*R.nitidus*	-0.039641	0.066806
GD21	GD26	GD28	*R.nitidus*	0.113527	0.064794
JO8	GD26	GD28	*R.nitidus*	-0.036286	0.074397
GD30	GD26	GD28	*R.nitidus*	0.087786	0.069357
GD32	GD26	GD28	*R.nitidus*	-0.004689	0.066353
SC27	GD25	GD28	*R.nitidus*	-0.065909	0.064055
JO1	GD25	GD28	*R.nitidus*	-0.096774	0.064314
GD29	GD26	GD28	*R.nitidus*	0.067625	0.062493
SC31	GD25	GD28	*R.nitidus*	-0.054625	0.065571
SC35	GD25	GD28	*R.nitidus*	-0.010574	0.065394
GD25	GD26	GD28	*R.nitidus*	0.003407	0.066986
GD33	GD26	GD28	*R.nitidus*	-0.045412	0.06285
MY4	GD25	GD28	*R.nitidus*	-0.083521	0.066031
JO7	GD26	GD28	*R.nitidus*	-0.078629	0.074967
MY6	GD25	GD28	*R.nitidus*	-0.067278	0.064725
GD42	GD26	GD28	*R.nitidus*	-0.013675	0.066661
GD49	GD26	GD28	*R.nitidus*	0.032864	0.06657
JO5	GD25	GD28	*R.nitidus*	-0.043678	0.065532
GD46	GD26	GD28	*R.nitidus*	-0.061932	0.062073
GD45	GD26	GD28	*R.nitidus*	0.011561	0.069653
GD22	GD26	GD28	*R.nitidus*	0.025295	0.063736
CS16	GD26	GD28	*R.nitidus*	-0.03901	0.064923
GD44	GD26	GD28	*R.nitidus*	-0.044619	0.065927
GD31	GD26	GD28	*R.nitidus*	0.014493	0.06496
GD48	GD26	GD28	*R.nitidus*	-0.0188	0.066917
GD41	GD26	GD28	*R.nitidus*	-0.052152	0.063997
CS11	GD26	GD28	*R.nitidus*	-0.038095	0.068032
CS19	GD26	GD28	*R.nitidus*	-0.04	0.066493
CS12	GD26	GD28	*R.nitidus*	-0.017899	0.067834
GD34	GD26	GD28	*R.nitidus*	-0.040171	0.063392
CS18	GD26	GD28	*R.nitidus*	-0.072006	0.0657
CS21	GD26	GD28	*R.nitidus*	-0.069435	0.066078
CS15	GD26	GD28	*R.nitidus*	-0.022272	0.066998
GD35	GD26	GD28	*R.nitidus*	-0.028286	0.066457
GD40	GD26	GD28	*R.nitidus*	0	0.06264
SC6	GD26	GD28	*R.nitidus*	-0.07079	0.067885
CS13	GD26	GD28	*R.nitidus*	-0.061209	0.068357
CS14	GD26	GD28	*R.nitidus*	-0.030075	0.068792
CS25	GD26	GD28	*R.nitidus*	-0.044313	0.067072
CS20	GD26	GD28	*R.nitidus*	-0.057816	0.066416
SC27	GD26	GD28	*R.nitidus*	-0.047584	0.066776

GD50	GD26	GD28	*R.nitidus*	-0.03493	0.066305
CS3	GD26	GD28	*R.nitidus*	-0.065185	0.065597
CS30	GD26	GD28	*R.nitidus*	-0.094457	0.065925
SC10	GD26	GD28	*R.nitidus*	0.020737	0.066236
SC14	GD26	GD28	*R.nitidus*	-0.032304	0.067376
SC18	GD26	GD28	*R.nitidus*	-0.020658	0.066486
CS31	GD26	GD28	*R.nitidus*	-0.046142	0.066622
MY6	GD26	GD28	*R.nitidus*	-0.058209	0.066644
CS17	GD26	GD28	*R.nitidus*	-0.002362	0.070406
SC34	GD26	GD28	*R.nitidus*	-0.016643	0.065534
SC24	GD26	GD28	*R.nitidus*	-0.052515	0.067358
SC12	GD26	GD28	*R.nitidus*	-0.059443	0.065041
SC5	GD26	GD28	*R.nitidus*	-0.040152	0.066322
SC26	GD26	GD28	*R.nitidus*	-0.090777	0.067355
CS22	GD26	GD28	*R.nitidus*	-0.029726	0.066714
JO3	GD26	GD28	*R.nitidus*	-0.025528	0.069193
CS28	GD26	GD28	*R.nitidus*	-0.079239	0.065965
CS24	GD26	GD28	*R.nitidus*	-0.052267	0.066154
CS23	GD26	GD28	*R.nitidus*	-0.073381	0.0636
JW6	GD26	GD28	*R.nitidus*	-0.032291	0.074052
JO8	GD29	GD28	*R.nitidus*	-0.067202	0.071675
SC45	GD26	GD28	*R.nitidus*	-0.00369	0.066024
SC32	GD26	GD28	*R.nitidus*	-0.005801	0.066723
SC31	GD26	GD28	*R.nitidus*	-0.033668	0.06693
JW4	GD26	GD28	*R.nitidus*	0.153724	0.067942
CS4	GD26	GD28	*R.nitidus*	-0.071953	0.064087
SC35	GD26	GD28	*R.nitidus*	0.003631	0.064768
MY4	GD26	GD28	*R.nitidus*	-0.056255	0.065509
JO1	GD26	GD28	*R.nitidus*	-0.064493	0.064545
CS6	GD26	GD28	*R.nitidus*	-0.058779	0.066417
SC44	GD26	GD28	*R.nitidus*	-0.03209	0.065334
GD32	GD29	GD28	*R.nitidus*	-0.089439	0.066823
SC15	GD26	GD28	*R.nitidus*	-0.046582	0.067585
GD26	GD29	GD28	*R.nitidus*	-0.067625	0.062493
JW7	GD26	GD28	*R.nitidus*	-0.043001	0.072112
SC28	GD26	GD28	*R.nitidus*	-0.088674	0.065586
JO5	GD26	GD28	*R.nitidus*	-0.019188	0.065894
SC42	GD26	GD28	*R.nitidus*	0.173833	0.063888
GD21	GD29	GD28	*R.nitidus*	0.045416	0.065614
CS14	GD29	GD28	*R.nitidus*	-0.095378	0.068246
GD50	GD29	GD28	*R.nitidus*	-0.088178	0.069543
JO7	GD29	GD28	*R.nitidus*	-0.104854	0.069824
GD22	GD29	GD28	*R.nitidus*	-0.041952	0.064887
GD46	GD29	GD28	*R.nitidus*	-0.129401	0.063116
CS18	GD29	GD28	*R.nitidus*	-0.128223	0.065623
GD25	GD29	GD28	*R.nitidus*	-0.047657	0.066225
GD34	GD29	GD28	*R.nitidus*	-0.106906	0.063797
GD40	GD29	GD28	*R.nitidus*	-0.052504	0.063461
GD44	GD29	GD28	*R.nitidus*	-0.121578	0.067068
CS11	GD29	GD28	*R.nitidus*	-0.101238	0.068549
GD30	GD29	GD28	*R.nitidus*	0.014477	0.067916
CS13	GD29	GD28	*R.nitidus*	-0.129317	0.069123
CS15	GD29	GD28	*R.nitidus*	-0.081884	0.068078
GD33	GD29	GD28	*R.nitidus*	-0.10348	0.063466
GD48	GD29	GD28	*R.nitidus*	-0.060329	0.065217
GD31	GD29	GD28	*R.nitidus*	-0.060345	0.066976

GD49	GD29	GD28	*R.nitidus*	-0.031365	0.068369
CS21	GD29	GD28	*R.nitidus*	-0.126145	0.064452
CS19	GD29	GD28	*R.nitidus*	-0.091523	0.067086
CS30	GD29	GD28	*R.nitidus*	-0.148969	0.06446
CS17	GD29	GD28	*R.nitidus*	-0.067757	0.068791
SC10	GD29	GD28	*R.nitidus*	-0.03286	0.067949
CS23	GD29	GD28	*R.nitidus*	-0.129643	0.064317
GD35	GD29	GD28	*R.nitidus*	-0.070488	0.065726
CS12	GD29	GD28	*R.nitidus*	-0.088838	0.067353
GD42	GD29	GD28	*R.nitidus*	-0.098347	0.064376
CS31	GD29	GD28	*R.nitidus*	-0.117293	0.066535
GD45	GD29	GD28	*R.nitidus*	-0.076498	0.068836
CS20	GD29	GD28	*R.nitidus*	-0.112766	0.065853
GD41	GD29	GD28	*R.nitidus*	-0.097621	0.066109
SC6	GD29	GD28	*R.nitidus*	-0.133138	0.065871
SC28	GD29	GD28	*R.nitidus*	-0.143597	0.065298
SC18	GD29	GD28	*R.nitidus*	-0.063685	0.066025
CS4	GD29	GD28	*R.nitidus*	-0.13231	0.063309
CS16	GD29	GD28	*R.nitidus*	-0.104258	0.065784
CS22	GD29	GD28	*R.nitidus*	-0.08584	0.065456
SC24	GD29	GD28	*R.nitidus*	-0.103152	0.065671
SC12	GD29	GD28	*R.nitidus*	-0.104499	0.066395
CS28	GD29	GD28	*R.nitidus*	-0.138009	0.065977
CS3	GD29	GD28	*R.nitidus*	-0.122041	0.06513
CS25	GD29	GD28	*R.nitidus*	-0.1	0.067106
SC26	GD29	GD28	*R.nitidus*	-0.136686	0.065275
SC14	GD29	GD28	*R.nitidus*	-0.092352	0.064413
SC5	GD29	GD28	*R.nitidus*	-0.10962	0.067718
SC45	GD29	GD28	*R.nitidus*	-0.069454	0.06748
JO8	GD30	GD28	*R.nitidus*	-0.086435	0.077653
CS24	GD29	GD28	*R.nitidus*	-0.113962	0.065004
SC27	GD29	GD28	*R.nitidus*	-0.114645	0.064267
SC42	GD29	GD28	*R.nitidus*	0.104468	0.063435
MY4	GD29	GD28	*R.nitidus*	-0.11372	0.065817
SC34	GD29	GD28	*R.nitidus*	-0.063457	0.064707
SC44	GD29	GD28	*R.nitidus*	-0.09186	0.065869
SC15	GD29	GD28	*R.nitidus*	-0.117378	0.06688
JW4	GD29	GD28	*R.nitidus*	0.108129	0.066943
JO7	GD30	GD28	*R.nitidus*	-0.128702	0.073995
SC35	GD29	GD28	*R.nitidus*	-0.039855	0.063754
GD22	GD30	GD28	*R.nitidus*	-0.070577	0.066783
GD34	GD30	GD28	*R.nitidus*	-0.116649	0.06467
SC31	GD29	GD28	*R.nitidus*	-0.087077	0.065048
JW6	GD29	GD28	*R.nitidus*	-0.067698	0.069707
GD35	GD30	GD28	*R.nitidus*	-0.081385	0.068495
GD25	GD30	GD28	*R.nitidus*	-0.080705	0.069493
GD31	GD30	GD28	*R.nitidus*	-0.087081	0.068752
CS11	GD30	GD28	*R.nitidus*	-0.112642	0.071377
JO3	GD29	GD28	*R.nitidus*	-0.056131	0.067759
SC32	GD29	GD28	*R.nitidus*	-0.068592	0.066595
GD49	GD30	GD28	*R.nitidus*	-0.04908	0.073228
JO5	GD29	GD28	*R.nitidus*	-0.079388	0.064205
GD41	GD30	GD28	*R.nitidus*	-0.108974	0.065754
CS6	GD29	GD28	*R.nitidus*	-0.112855	0.065596
GD21	GD30	GD28	*R.nitidus*	0.030602	0.06807
GD44	GD30	GD28	*R.nitidus*	-0.125234	0.069792

JW7	GD29	GD28	*R.nitidus*	-0.094323	0.072293
MY6	GD29	GD28	*R.nitidus*	-0.107659	0.064593
CS21	GD30	GD28	*R.nitidus*	-0.142631	0.067757
GD26	GD30	GD28	*R.nitidus*	-0.087786	0.069357
GD45	GD30	GD28	*R.nitidus*	-0.054294	0.073021
CS17	GD30	GD28	*R.nitidus*	-0.081315	0.071681
GD50	GD30	GD28	*R.nitidus*	-0.086189	0.071707
GD48	GD30	GD28	*R.nitidus*	-0.040201	0.067817
GD40	GD30	GD28	*R.nitidus*	-0.057762	0.065862
GD29	GD30	GD28	*R.nitidus*	-0.014477	0.067916
JO1	GD29	GD28	*R.nitidus*	-0.121898	0.06422
CS18	GD30	GD28	*R.nitidus*	-0.135593	0.068098
GD33	GD30	GD28	*R.nitidus*	-0.115424	0.066157
CS20	GD30	GD28	*R.nitidus*	-0.123408	0.068121
CS12	GD30	GD28	*R.nitidus*	-0.074074	0.073149
CS13	GD30	GD28	*R.nitidus*	-0.126388	0.071439
CS4	GD30	GD28	*R.nitidus*	-0.12253	0.067145
CS30	GD30	GD28	*R.nitidus*	-0.146959	0.069693
CS24	GD30	GD28	*R.nitidus*	-0.11755	0.067506
CS14	GD30	GD28	*R.nitidus*	-0.095359	0.072153
CS3	GD30	GD28	*R.nitidus*	-0.125729	0.066462
CS22	GD30	GD28	*R.nitidus*	-0.092715	0.06735
GD46	GD30	GD28	*R.nitidus*	-0.137255	0.065796
SC5	GD30	GD28	*R.nitidus*	-0.100408	0.070373
GD42	GD30	GD28	*R.nitidus*	-0.10942	0.066932
GD32	GD30	GD28	*R.nitidus*	-0.097336	0.073214
CS23	GD30	GD28	*R.nitidus*	-0.131537	0.068145
SC27	GD30	GD28	*R.nitidus*	-0.111837	0.069734
SC28	GD30	GD28	*R.nitidus*	-0.142974	0.067233
CS19	GD30	GD28	*R.nitidus*	-0.098415	0.069548
CS16	GD30	GD28	*R.nitidus*	-0.091135	0.069401
CS15	GD30	GD28	*R.nitidus*	-0.083195	0.071653
SC34	GD30	GD28	*R.nitidus*	-0.083196	0.068182
CS25	GD30	GD28	*R.nitidus*	-0.106438	0.072253
SC18	GD30	GD28	*R.nitidus*	-0.084175	0.068038
JW6	GD30	GD28	*R.nitidus*	-0.096774	0.073719
MY6	GD30	GD28	*R.nitidus*	-0.103563	0.067384
CS28	GD30	GD28	*R.nitidus*	-0.148673	0.068111
SC42	GD30	GD28	*R.nitidus*	0.126287	0.065991
SC10	GD30	GD28	*R.nitidus*	-0.052856	0.071133
CS31	GD30	GD28	*R.nitidus*	-0.104452	0.069571
SC26	GD30	GD28	*R.nitidus*	-0.144338	0.068773
CS6	GD30	GD28	*R.nitidus*	-0.122074	0.067145
SC15	GD30	GD28	*R.nitidus*	-0.113712	0.070484
SC24	GD30	GD28	*R.nitidus*	-0.116935	0.067901
SC6	GD30	GD28	*R.nitidus*	-0.130972	0.068452
JO5	GD30	GD28	*R.nitidus*	-0.069787	0.068447
SC31	GD30	GD28	*R.nitidus*	-0.103044	0.069482
JO8	GD31	GD28	*R.nitidus*	0.002172	0.074808
SC44	GD30	GD28	*R.nitidus*	-0.078925	0.067319
JW7	GD30	GD28	*R.nitidus*	-0.122302	0.074594
SC32	GD30	GD28	*R.nitidus*	-0.055511	0.070633
SC12	GD30	GD28	*R.nitidus*	-0.097808	0.069443
SC14	GD30	GD28	*R.nitidus*	-0.086249	0.069619
JO1	GD30	GD28	*R.nitidus*	-0.139002	0.066632
SC45	GD30	GD28	*R.nitidus*	-0.069544	0.069731

SC35	GD30	GD28	*R.nitidus*	-0.040848	0.068941
GD25	GD31	GD28	*R.nitidus*	0.008711	0.066407
JO3	GD30	GD28	*R.nitidus*	-0.105799	0.072385
JW4	GD30	GD28	*R.nitidus*	0.093162	0.070539
GD30	GD31	GD28	*R.nitidus*	0.087081	0.068752
MY4	GD30	GD28	*R.nitidus*	-0.116585	0.069232
GD29	GD31	GD28	*R.nitidus*	0.060345	0.066976
CS12	GD31	GD28	*R.nitidus*	-0.036036	0.068851
JO7	GD31	GD28	*R.nitidus*	-0.041215	0.074269
GD41	GD31	GD28	*R.nitidus*	-0.052239	0.066124
GD26	GD31	GD28	*R.nitidus*	-0.014493	0.06496
CS16	GD31	GD28	*R.nitidus*	-0.035405	0.068641
GD33	GD31	GD28	*R.nitidus*	-0.043551	0.067783
GD34	GD31	GD28	*R.nitidus*	-0.032118	0.066531
GD32	GD31	GD28	*R.nitidus*	-0.008629	0.070002
GD48	GD31	GD28	*R.nitidus*	0.01327	0.070387
GD44	GD31	GD28	*R.nitidus*	-0.054005	0.069424
GD45	GD31	GD28	*R.nitidus*	-0.015489	0.0696
GD42	GD31	GD28	*R.nitidus*	-0.03259	0.067761
CS22	GD31	GD28	*R.nitidus*	-0.022293	0.067412
CS14	GD31	GD28	*R.nitidus*	-0.023144	0.070606
CS28	GD31	GD28	*R.nitidus*	-0.083963	0.067938
CS15	GD31	GD28	*R.nitidus*	-0.020171	0.068076
GD22	GD31	GD28	*R.nitidus*	0.033248	0.06659
CS13	GD31	GD28	*R.nitidus*	-0.072337	0.069049
CS11	GD31	GD28	*R.nitidus*	-0.04465	0.069503
GD21	GD31	GD28	*R.nitidus*	0.120346	0.066542
CS25	GD31	GD28	*R.nitidus*	-0.0584	0.0679
CS21	GD31	GD28	*R.nitidus*	-0.071094	0.068744
SC5	GD31	GD28	*R.nitidus*	-0.045383	0.070178
GD49	GD31	GD28	*R.nitidus*	0.054493	0.071325
CS30	GD31	GD28	*R.nitidus*	-0.101161	0.06868
GD46	GD31	GD28	*R.nitidus*	-0.054668	0.065692
CS20	GD31	GD28	*R.nitidus*	-0.066246	0.068933
CS18	GD31	GD28	*R.nitidus*	-0.088468	0.069318
GD35	GD31	GD28	*R.nitidus*	-0.022222	0.066878
CS23	GD31	GD28	*R.nitidus*	-0.082409	0.066856
CS19	GD31	GD28	*R.nitidus*	-0.064777	0.069477
GD40	GD31	GD28	*R.nitidus*	0.009121	0.065117
GD50	GD31	GD28	*R.nitidus*	-0.00993	0.070334
CS24	GD31	GD28	*R.nitidus*	-0.064337	0.068086
SC24	GD31	GD28	*R.nitidus*	-0.04414	0.06826
CS3	GD31	GD28	*R.nitidus*	-0.072684	0.067619
SC6	GD31	GD28	*R.nitidus*	-0.059052	0.066604
SC15	GD31	GD28	*R.nitidus*	-0.045783	0.068664
SC12	GD31	GD28	*R.nitidus*	-0.048048	0.067567
CS31	GD31	GD28	*R.nitidus*	-0.048293	0.068309
SC32	GD31	GD28	*R.nitidus*	-0.001474	0.067507
SC26	GD31	GD28	*R.nitidus*	-0.086957	0.067193
SC18	GD31	GD28	*R.nitidus*	-0.016247	0.067898
JW4	GD31	GD28	*R.nitidus*	0.154098	0.069704
SC31	GD31	GD28	*R.nitidus*	-0.039574	0.067652
JO7	GD32	GD28	*R.nitidus*	-0.031567	0.076872
JO8	GD32	GD28	*R.nitidus*	0.013953	0.078121
CS17	GD31	GD28	*R.nitidus*	-0.017456	0.071153
JW6	GD31	GD28	*R.nitidus*	-0.014463	0.074674

JW7	GD31	GD28	*R.nitidus*	-0.055089	0.072981
CS4	GD31	GD28	*R.nitidus*	-0.068807	0.06679
SC28	GD31	GD28	*R.nitidus*	-0.093897	0.066908
SC10	GD31	GD28	*R.nitidus*	0.018211	0.067171
SC44	GD31	GD28	*R.nitidus*	-0.045814	0.067757
SC14	GD31	GD28	*R.nitidus*	-0.035687	0.067331
JO3	GD31	GD28	*R.nitidus*	-0.002806	0.073687
SC35	GD31	GD28	*R.nitidus*	0.004619	0.065101
MY6	GD31	GD28	*R.nitidus*	-0.058777	0.065987
JO5	GD31	GD28	*R.nitidus*	-0.033333	0.067426
SC42	GD31	GD28	*R.nitidus*	0.182228	0.063251
SC27	GD31	GD28	*R.nitidus*	-0.038766	0.068077
SC45	GD31	GD28	*R.nitidus*	-0.012355	0.069181
SC34	GD31	GD28	*R.nitidus*	-0.012879	0.067781
JO1	GD31	GD28	*R.nitidus*	-0.070988	0.066549
GD30	GD32	GD28	*R.nitidus*	0.097336	0.073214
CS6	GD31	GD28	*R.nitidus*	-0.068882	0.069563
CS12	GD32	GD28	*R.nitidus*	-0.005937	0.070603
CS11	GD32	GD28	*R.nitidus*	-0.027287	0.071902
GD31	GD32	GD28	*R.nitidus*	0.008629	0.070002
GD35	GD32	GD28	*R.nitidus*	-0.010064	0.069916
GD41	GD32	GD28	*R.nitidus*	-0.046555	0.070566
GD21	GD32	GD28	*R.nitidus*	0.117041	0.066116
GD26	GD32	GD28	*R.nitidus*	0.004689	0.066353
MY4	GD31	GD28	*R.nitidus*	-0.066667	0.068315
GD45	GD32	GD28	*R.nitidus*	0.004278	0.072608
GD50	GD32	GD28	*R.nitidus*	0.003367	0.07307
CS15	GD32	GD28	*R.nitidus*	0.002457	0.072311
CS13	GD32	GD28	*R.nitidus*	-0.055144	0.072156
CS21	GD32	GD28	*R.nitidus*	-0.050202	0.069901
GD25	GD32	GD28	*R.nitidus*	0.015302	0.067979
CS20	GD32	GD28	*R.nitidus*	-0.044571	0.067943
GD22	GD32	GD28	*R.nitidus*	0.04912	0.066303
GD42	GD32	GD28	*R.nitidus*	-0.027128	0.069308
GD29	GD32	GD28	*R.nitidus*	0.089439	0.066823
CS16	GD32	GD28	*R.nitidus*	-0.024411	0.071035
GD49	GD32	GD28	*R.nitidus*	0.077922	0.072758
GD34	GD32	GD28	*R.nitidus*	-0.020508	0.067247
GD48	GD32	GD28	*R.nitidus*	0.00998	0.066621
GD44	GD32	GD28	*R.nitidus*	-0.02277	0.071537
GD40	GD32	GD28	*R.nitidus*	0.00793	0.068054
CS30	GD32	GD28	*R.nitidus*	-0.074692	0.070806
CS17	GD32	GD28	*R.nitidus*	0.00519	0.074853
GD33	GD32	GD28	*R.nitidus*	-0.028481	0.066551
CS14	GD32	GD28	*R.nitidus*	-0.007475	0.07297
CS28	GD32	GD28	*R.nitidus*	-0.062665	0.070127
GD46	GD32	GD28	*R.nitidus*	-0.04912	0.068206
SC10	GD32	GD28	*R.nitidus*	0.049828	0.069128
CS24	GD32	GD28	*R.nitidus*	-0.043253	0.068754
SC14	GD32	GD28	*R.nitidus*	-0.007513	0.068869
SC12	GD32	GD28	*R.nitidus*	-0.042749	0.069078
CS22	GD32	GD28	*R.nitidus*	-0.004303	0.069029
CS23	GD32	GD28	*R.nitidus*	-0.066884	0.068681
CS4	GD32	GD28	*R.nitidus*	-0.05042	0.066987
JW4	GD32	GD28	*R.nitidus*	0.191327	0.069317
SC6	GD32	GD28	*R.nitidus*	-0.046473	0.070558

CS18	GD32	GD28	*R.nitidus*	-0.062346	0.068778
CS25	GD32	GD28	*R.nitidus*	-0.019442	0.070277
JW6	GD32	GD28	*R.nitidus*	0.029508	0.074612
SC42	GD32	GD28	*R.nitidus*	0.18637	0.065969
SC18	GD32	GD28	*R.nitidus*	0.017331	0.070933
CS19	GD32	GD28	*R.nitidus*	-0.015833	0.069289
SC5	GD32	GD28	*R.nitidus*	-0.006029	0.074856
SC34	GD32	GD28	*R.nitidus*	-0.00659	0.069165
SC32	GD32	GD28	*R.nitidus*	0.033501	0.069795
MY4	GD32	GD28	*R.nitidus*	-0.038851	0.070966
SC24	GD32	GD28	*R.nitidus*	-0.035383	0.069221
SC26	GD32	GD28	*R.nitidus*	-0.06357	0.069892
CS3	GD32	GD28	*R.nitidus*	-0.054122	0.069215
CS31	GD32	GD28	*R.nitidus*	-0.040672	0.069947
JO7	CS12	GD28	*R.nitidus*	0.009698	0.076023
JO3	GD32	GD28	*R.nitidus*	0.001992	0.071849
JO8	CS12	GD28	*R.nitidus*	0.0033	0.076325
JW7	GD32	GD28	*R.nitidus*	-0.017682	0.073787
SC28	GD32	GD28	*R.nitidus*	-0.057384	0.069038
GD22	CS12	GD28	*R.nitidus*	0.049927	0.068528
GD30	CS12	GD28	*R.nitidus*	0.074074	0.073149
GD26	CS12	GD28	*R.nitidus*	0.017899	0.067834
GD21	CS12	GD28	*R.nitidus*	0.136364	0.067162
GD31	CS12	GD28	*R.nitidus*	0.036036	0.068851
SC35	GD32	GD28	*R.nitidus*	0.053018	0.067277
SC15	GD32	GD28	*R.nitidus*	-0.019383	0.068191
SC44	GD32	GD28	*R.nitidus*	-0.004367	0.070725
SC27	GD32	GD28	*R.nitidus*	-0.028476	0.069204
CS6	GD32	GD28	*R.nitidus*	-0.0625	0.070083
GD41	CS12	GD28	*R.nitidus*	-0.015994	0.067738
SC31	GD32	GD28	*R.nitidus*	-0.008065	0.070305
SC45	GD32	GD28	*R.nitidus*	0.015523	0.070514
MY6	GD32	GD28	*R.nitidus*	-0.039037	0.070228
JO5	GD32	GD28	*R.nitidus*	0.001664	0.06881
JO1	GD32	GD28	*R.nitidus*	-0.044248	0.069353
GD45	CS12	GD28	*R.nitidus*	0.021222	0.072873
CS17	CS12	GD28	*R.nitidus*	-0.001229	0.072037
GD34	CS12	GD28	*R.nitidus*	0.002259	0.066585
GD42	CS12	GD28	*R.nitidus*	-0.006211	0.06912
GD32	CS12	GD28	*R.nitidus*	0.005937	0.070603
GD29	CS12	GD28	*R.nitidus*	0.088838	0.067353
GD40	CS12	GD28	*R.nitidus*	0.039463	0.06603
GD48	CS12	GD28	*R.nitidus*	0.040472	0.070137
GD44	CS12	GD28	*R.nitidus*	-0.004065	0.068437
CS11	CS12	GD28	*R.nitidus*	-0.015608	0.070893
CS14	CS12	GD28	*R.nitidus*	0.002475	0.073462
GD49	CS12	GD28	*R.nitidus*	0.059233	0.070379
GD25	CS12	GD28	*R.nitidus*	0.057661	0.068549
GD33	CS12	GD28	*R.nitidus*	-0.012232	0.067897
CS13	CS12	GD28	*R.nitidus*	-0.068771	0.071255
GD46	CS12	GD28	*R.nitidus*	-0.022382	0.068741
CS21	CS12	GD28	*R.nitidus*	-0.042484	0.067842
GD35	CS12	GD28	*R.nitidus*	0.020093	0.069388
SC10	CS12	GD28	*R.nitidus*	0.066176	0.06999
SC18	CS12	GD28	*R.nitidus*	0.0278	0.067867
CS18	CS12	GD28	*R.nitidus*	-0.038972	0.066698

CS3	CS12	GD28	*R.nitidus*	-0.028571	0.066106
CS22	CS12	GD28	*R.nitidus*	0.005917	0.065103
CS16	CS12	GD28	*R.nitidus*	-0.023841	0.067602
CS20	CS12	GD28	*R.nitidus*	-0.029056	0.067304
SC14	CS12	GD28	*R.nitidus*	0	0.070653
CS31	CS12	GD28	*R.nitidus*	-0.032007	0.068046
CS24	CS12	GD28	*R.nitidus*	-0.037768	0.06766
SC6	CS12	GD28	*R.nitidus*	-0.052632	0.069544
SC28	CS12	GD28	*R.nitidus*	-0.076728	0.068534
GD50	CS12	GD28	*R.nitidus*	0.030027	0.073447
CS23	CS12	GD28	*R.nitidus*	-0.045381	0.067228
CS15	CS12	GD28	*R.nitidus*	0.013142	0.068641
CS4	CS12	GD28	*R.nitidus*	-0.046243	0.067532
JO3	CS12	GD28	*R.nitidus*	0.010073	0.070195
SC5	CS12	GD28	*R.nitidus*	-0.008907	0.072042
SC24	CS12	GD28	*R.nitidus*	-0.015898	0.068196
JO8	GD33	GD28	*R.nitidus*	0.020141	0.074295
CS28	CS12	GD28	*R.nitidus*	-0.058876	0.066978
CS25	CS12	GD28	*R.nitidus*	-0.011986	0.068677
CS30	CS12	GD28	*R.nitidus*	-0.064892	0.066911
MY4	CS12	GD28	*R.nitidus*	-0.028685	0.069126
CS6	CS12	GD28	*R.nitidus*	-0.041246	0.066155
JW6	CS12	GD28	*R.nitidus*	0.025164	0.073895
JO5	CS12	GD28	*R.nitidus*	0.004918	0.06724
MY6	CS12	GD28	*R.nitidus*	-0.022943	0.067795
CS19	CS12	GD28	*R.nitidus*	-0.00605	0.068856
GD26	GD33	GD28	*R.nitidus*	0.045412	0.06285
SC12	CS12	GD28	*R.nitidus*	-0.023987	0.066894
JW4	CS12	GD28	*R.nitidus*	0.178037	0.068872
SC34	CS12	GD28	*R.nitidus*	0.014646	0.066527
SC15	CS12	GD28	*R.nitidus*	-0.023216	0.070154
CS11	GD33	GD28	*R.nitidus*	-0.007708	0.066761
JW7	CS12	GD28	*R.nitidus*	-0.005198	0.072202
SC35	CS12	GD28	*R.nitidus*	0.051522	0.067242
SC27	CS12	GD28	*R.nitidus*	-0.014274	0.068573
SC31	CS12	GD28	*R.nitidus*	-0.008607	0.07006
GD32	GD33	GD28	*R.nitidus*	0.028481	0.066551
GD31	GD33	GD28	*R.nitidus*	0.043551	0.067783
SC26	CS12	GD28	*R.nitidus*	-0.05	0.068687
GD22	GD33	GD28	*R.nitidus*	0.062057	0.06474
GD29	GD33	GD28	*R.nitidus*	0.10348	0.063466
JO7	GD33	GD28	*R.nitidus*	-0.030422	0.073771
SC32	CS12	GD28	*R.nitidus*	0.040902	0.068096
SC45	CS12	GD28	*R.nitidus*	0.0264	0.070106
SC42	CS12	GD28	*R.nitidus*	0.221109	0.064253
GD25	GD33	GD28	*R.nitidus*	0.050874	0.065479
GD42	GD33	GD28	*R.nitidus*	0	0.065461
SC44	CS12	GD28	*R.nitidus*	-0.00986	0.069489
GD49	GD33	GD28	*R.nitidus*	0.071429	0.0699
GD40	GD33	GD28	*R.nitidus*	0.029388	0.064767
GD30	GD33	GD28	*R.nitidus*	0.115424	0.066157
GD44	GD33	GD28	*R.nitidus*	-0.017327	0.067666
GD21	GD33	GD28	*R.nitidus*	0.175313	0.063016
JO1	CS12	GD28	*R.nitidus*	-0.032718	0.067204
GD46	GD33	GD28	*R.nitidus*	-0.021169	0.065029
GD34	GD33	GD28	*R.nitidus*	-0.002012	0.062787

CS15	GD33	GD28	*R.nitidus*	0.008658	0.067746
GD50	GD33	GD28	*R.nitidus*	0.00603	0.072777
GD35	GD33	GD28	*R.nitidus*	0.0256	0.065205
CS12	GD33	GD28	*R.nitidus*	0.012232	0.067897
CS14	GD33	GD28	*R.nitidus*	0.008689	0.067646
SC24	GD33	GD28	*R.nitidus*	-0.01444	0.067448
CS13	GD33	GD28	*R.nitidus*	-0.032	0.067658
CS24	GD33	GD28	*R.nitidus*	-0.019832	0.067032
GD41	GD33	GD28	*R.nitidus*	0	0.067134
GD45	GD33	GD28	*R.nitidus*	0.035647	0.06771
SC26	GD33	GD28	*R.nitidus*	-0.049822	0.066704
CS16	GD33	GD28	*R.nitidus*	0.003623	0.064847
SC5	GD33	GD28	*R.nitidus*	-0.004615	0.06802
GD48	GD33	GD28	*R.nitidus*	0.034247	0.066263
CS23	GD33	GD28	*R.nitidus*	-0.037116	0.066999
CS28	GD33	GD28	*R.nitidus*	-0.0472	0.067626
JW6	GD33	GD28	*R.nitidus*	0.026706	0.072532
SC42	GD33	GD28	*R.nitidus*	0.203359	0.065139
SC45	GD33	GD28	*R.nitidus*	0.029496	0.065998
CS4	GD33	GD28	*R.nitidus*	-0.035928	0.065899
CS3	GD33	GD28	*R.nitidus*	-0.034074	0.06694
CS30	GD33	GD28	*R.nitidus*	-0.054836	0.066005
SC12	GD33	GD28	*R.nitidus*	-0.016333	0.066833
CS21	GD33	GD28	*R.nitidus*	-0.04134	0.067317
CS17	GD33	GD28	*R.nitidus*	0.029771	0.069557
CS18	GD33	GD28	*R.nitidus*	-0.048529	0.067684
CS19	GD33	GD28	*R.nitidus*	0.006107	0.068682
SC27	GD33	GD28	*R.nitidus*	-0.005036	0.065977
SC15	GD33	GD28	*R.nitidus*	-0.003794	0.067276
CS20	GD33	GD28	*R.nitidus*	-0.030021	0.066863
MY6	GD33	GD28	*R.nitidus*	-0.015681	0.066339
JW4	GD33	GD28	*R.nitidus*	0.172628	0.069572
SC6	GD33	GD28	*R.nitidus*	-0.035529	0.068588
SC18	GD33	GD28	*R.nitidus*	0.016717	0.066488
CS25	GD33	GD28	*R.nitidus*	-0.008455	0.068222
CS22	GD33	GD28	*R.nitidus*	0.014837	0.067014
JO7	GD34	GD28	*R.nitidus*	-0.023256	0.072831
SC34	GD33	GD28	*R.nitidus*	0.020217	0.064424
SC10	GD33	GD28	*R.nitidus*	0.053869	0.068055
CS31	GD33	GD28	*R.nitidus*	-0.003776	0.066088
SC32	GD33	GD28	*R.nitidus*	0.033213	0.067076
SC31	GD33	GD28	*R.nitidus*	-0.005022	0.066759
SC35	GD33	GD28	*R.nitidus*	0.036646	0.065603
JW7	GD33	GD28	*R.nitidus*	0.008865	0.071614
SC28	GD33	GD28	*R.nitidus*	-0.0462	0.065033
CS6	GD33	GD28	*R.nitidus*	-0.040396	0.067417
SC44	GD33	GD28	*R.nitidus*	0	0.06564
JO5	GD33	GD28	*R.nitidus*	0.015407	0.065894
GD30	GD34	GD28	*R.nitidus*	0.116649	0.06467
SC14	GD33	GD28	*R.nitidus*	0.014174	0.066942
JO3	GD33	GD28	*R.nitidus*	0.022414	0.069129
MY4	GD33	GD28	*R.nitidus*	-0.016047	0.066042
GD22	GD34	GD28	*R.nitidus*	0.066004	0.062215
JO8	GD34	GD28	*R.nitidus*	0.032129	0.073054
GD42	GD34	GD28	*R.nitidus*	-0.016115	0.065298
GD41	GD34	GD28	*R.nitidus*	-0.010934	0.065245

GD25	GD34	GD28	*R.nitidus*	0.049521	0.065895
GD21	GD34	GD28	*R.nitidus*	0.15625	0.061491
GD31	GD34	GD28	*R.nitidus*	0.032118	0.066531
JO1	GD33	GD28	*R.nitidus*	-0.030258	0.065131
CS11	GD34	GD28	*R.nitidus*	-0.017495	0.067718
GD32	GD34	GD28	*R.nitidus*	0.020508	0.067247
GD46	GD34	GD28	*R.nitidus*	-0.021649	0.064269
CS12	GD34	GD28	*R.nitidus*	-0.002259	0.066585
CS14	GD34	GD28	*R.nitidus*	-0.012186	0.069257
GD26	GD34	GD28	*R.nitidus*	0.040171	0.063392
GD50	GD34	GD28	*R.nitidus*	0.000957	0.071502
GD48	GD34	GD28	*R.nitidus*	0.053622	0.066189
CS21	GD34	GD28	*R.nitidus*	-0.041847	0.065814
CS18	GD34	GD28	*R.nitidus*	-0.040757	0.066577
GD35	GD34	GD28	*R.nitidus*	0.012136	0.065295
CS15	GD34	GD28	*R.nitidus*	0.007846	0.067152
GD29	GD34	GD28	*R.nitidus*	0.106906	0.063797
GD40	GD34	GD28	*R.nitidus*	0.033673	0.06442
CS19	GD34	GD28	*R.nitidus*	-0.008922	0.066291
SC5	GD34	GD28	*R.nitidus*	-0.01949	0.068124
GD44	GD34	GD28	*R.nitidus*	-0.025898	0.069223
GD33	GD34	GD28	*R.nitidus*	0.002012	0.062787
GD45	GD34	GD28	*R.nitidus*	0.019039	0.06881
CS13	GD34	GD28	*R.nitidus*	-0.041311	0.069008
CS20	GD34	GD28	*R.nitidus*	-0.036429	0.065807
CS3	GD34	GD28	*R.nitidus*	-0.037834	0.065372
SC6	GD34	GD28	*R.nitidus*	-0.042274	0.066566
SC18	GD34	GD28	*R.nitidus*	0.005271	0.066889
CS22	GD34	GD28	*R.nitidus*	-0.002221	0.064826
GD49	GD34	GD28	*R.nitidus*	0.067405	0.068089
CS17	GD34	GD28	*R.nitidus*	0.007593	0.069652
CS24	GD34	GD28	*R.nitidus*	-0.031603	0.066827
JW4	GD34	GD28	*R.nitidus*	0.162041	0.068613
CS16	GD34	GD28	*R.nitidus*	-0.017143	0.064285
CS28	GD34	GD28	*R.nitidus*	-0.074016	0.065895
SC31	GD34	GD28	*R.nitidus*	-0.010029	0.066318
CS25	GD34	GD28	*R.nitidus*	-0.020104	0.067012
CS6	GD34	GD28	*R.nitidus*	-0.044294	0.06644
CS31	GD34	GD28	*R.nitidus*	-0.017625	0.066803
SC35	GD34	GD28	*R.nitidus*	0.034458	0.066232
JW7	GD34	GD28	*R.nitidus*	0.003623	0.071814
CS30	GD34	GD28	*R.nitidus*	-0.068807	0.065692
CS4	GD34	GD28	*R.nitidus*	-0.037956	0.064235
SC32	GD34	GD28	*R.nitidus*	0.024496	0.066821
CS23	GD34	GD28	*R.nitidus*	-0.052478	0.066343
SC42	GD34	GD28	*R.nitidus*	0.205648	0.063096
SC34	GD34	GD28	*R.nitidus*	0.010784	0.066445
SC10	GD34	GD28	*R.nitidus*	0.037707	0.067715
JO3	GD34	GD28	*R.nitidus*	0.008688	0.069037
SC27	GD34	GD28	*R.nitidus*	-0.022338	0.065928
MY6	GD34	GD28	*R.nitidus*	-0.030702	0.064773
GD25	GD35	GD28	*R.nitidus*	0.040597	0.063701
SC28	GD34	GD28	*R.nitidus*	-0.06478	0.065396
SC12	GD34	GD28	*R.nitidus*	-0.036792	0.066445
JO1	GD34	GD28	*R.nitidus*	-0.046931	0.065769
JO7	GD35	GD28	*R.nitidus*	-0.023398	0.074074

MY4	GD34	GD28	*R.nitidus*	-0.027778	0.066245
GD26	GD35	GD28	*R.nitidus*	0.028286	0.066457
JO8	GD35	GD28	*R.nitidus*	-0.006179	0.073798
SC14	GD34	GD28	*R.nitidus*	0.001451	0.066269
SC15	GD34	GD28	*R.nitidus*	-0.031923	0.067193
SC45	GD34	GD28	*R.nitidus*	0.018638	0.066508
SC24	GD34	GD28	*R.nitidus*	-0.027166	0.067
SC26	GD34	GD28	*R.nitidus*	-0.052632	0.066865
JW6	GD34	GD28	*R.nitidus*	0.02178	0.071086
CS11	GD35	GD28	*R.nitidus*	-0.022711	0.069074
GD30	GD35	GD28	*R.nitidus*	0.081385	0.068495
SC44	GD34	GD28	*R.nitidus*	-0.015186	0.065919
GD21	GD35	GD28	*R.nitidus*	0.111548	0.064668
JO5	GD34	GD28	*R.nitidus*	0.002882	0.065925
GD48	GD35	GD28	*R.nitidus*	0.015748	0.066861
GD42	GD35	GD28	*R.nitidus*	-0.016407	0.065262
GD32	GD35	GD28	*R.nitidus*	0.010064	0.069916
CS12	GD35	GD28	*R.nitidus*	-0.020093	0.069388
GD34	GD35	GD28	*R.nitidus*	-0.012136	0.065295
GD29	GD35	GD28	*R.nitidus*	0.070488	0.065726
GD22	GD35	GD28	*R.nitidus*	0.025581	0.063684
GD33	GD35	GD28	*R.nitidus*	-0.0256	0.065205
CS25	GD35	GD28	*R.nitidus*	-0.025522	0.067582
CS20	GD35	GD28	*R.nitidus*	-0.046805	0.065982
GD41	GD35	GD28	*R.nitidus*	-0.032099	0.064848
CS18	GD35	GD28	*R.nitidus*	-0.053788	0.067932
CS16	GD35	GD28	*R.nitidus*	-0.023845	0.067844
SC12	GD35	GD28	*R.nitidus*	-0.040801	0.067044
CS31	GD35	GD28	*R.nitidus*	-0.026563	0.066509
CS14	GD35	GD28	*R.nitidus*	-0.017267	0.071103
GD49	GD35	GD28	*R.nitidus*	0.071986	0.068303
CS17	GD35	GD28	*R.nitidus*	-0.006354	0.069389
SC14	GD35	GD28	*R.nitidus*	-0.003715	0.067686
CS28	GD35	GD28	*R.nitidus*	-0.063542	0.066497
GD31	GD35	GD28	*R.nitidus*	0.022222	0.066878
CS19	GD35	GD28	*R.nitidus*	-0.017625	0.068167
GD40	GD35	GD28	*R.nitidus*	0.016625	0.0643
GD46	GD35	GD28	*R.nitidus*	-0.042673	0.065488
CS13	GD35	GD28	*R.nitidus*	-0.04958	0.070466
GD45	GD35	GD28	*R.nitidus*	0.031306	0.0681
GD44	GD35	GD28	*R.nitidus*	-0.043739	0.06688
SC24	GD35	GD28	*R.nitidus*	-0.035115	0.068065
GD50	GD35	GD28	*R.nitidus*	-0.001004	0.068924
SC28	GD35	GD28	*R.nitidus*	-0.072878	0.066908
CS24	GD35	GD28	*R.nitidus*	-0.032875	0.065723
SC6	GD35	GD28	*R.nitidus*	-0.052161	0.067418
SC35	GD35	GD28	*R.nitidus*	0.02568	0.065144
SC18	GD35	GD28	*R.nitidus*	-0.01414	0.067931
CS4	GD35	GD28	*R.nitidus*	-0.047302	0.064758
CS15	GD35	GD28	*R.nitidus*	-0.003701	0.069205
CS3	GD35	GD28	*R.nitidus*	-0.040816	0.065038
JW7	GD35	GD28	*R.nitidus*	-0.021998	0.071124
SC45	GD35	GD28	*R.nitidus*	0.02071	0.066595
CS21	GD35	GD28	*R.nitidus*	-0.057455	0.066586
SC34	GD35	GD28	*R.nitidus*	-0.002224	0.066559
SC44	GD35	GD28	*R.nitidus*	-0.026758	0.067394

CS23	GD35	GD28	*R.nitidus*	-0.056576	0.066341
CS6	GD35	GD28	*R.nitidus*	-0.059459	0.068097
CS22	GD35	GD28	*R.nitidus*	-0.001479	0.066376
SC32	GD35	GD28	*R.nitidus*	0.021898	0.065385
SC10	GD35	GD28	*R.nitidus*	0.039352	0.06637
SC42	GD35	GD28	*R.nitidus*	0.198922	0.063758
SC5	GD35	GD28	*R.nitidus*	-0.023975	0.068865
MY6	GD35	GD28	*R.nitidus*	-0.037064	0.065481
CS30	GD35	GD28	*R.nitidus*	-0.058689	0.067262
SC15	GD35	GD28	*R.nitidus*	-0.02657	0.068425
SC26	GD35	GD28	*R.nitidus*	-0.060161	0.06628
SC27	GD35	GD28	*R.nitidus*	-0.03621	0.066335
SC31	GD35	GD28	*R.nitidus*	-0.018362	0.066165
MY4	GD35	GD28	*R.nitidus*	-0.047232	0.066248
JW4	GD35	GD28	*R.nitidus*	0.159574	0.067945
JO3	GD35	GD28	*R.nitidus*	-0.009919	0.072244
GD25	GD40	GD28	*R.nitidus*	0.019139	0.065123
CS11	GD40	GD28	*R.nitidus*	-0.050595	0.066512
GD33	GD40	GD28	*R.nitidus*	-0.029388	0.064767
JO8	GD40	GD28	*R.nitidus*	-0.029536	0.071947
GD30	GD40	GD28	*R.nitidus*	0.057762	0.065862
JW6	GD35	GD28	*R.nitidus*	-0.00402	0.071835
GD26	GD40	GD28	*R.nitidus*	0	0.06264
JO5	GD35	GD28	*R.nitidus*	-0.019593	0.066357
GD29	GD40	GD28	*R.nitidus*	0.052504	0.063461
JO7	GD40	GD28	*R.nitidus*	-0.047094	0.072957
GD21	GD40	GD28	*R.nitidus*	0.108712	0.064765
GD49	GD40	GD28	*R.nitidus*	0.026293	0.066022
GD31	GD40	GD28	*R.nitidus*	-0.009121	0.065117
GD48	GD40	GD28	*R.nitidus*	0.000868	0.065643
JO1	GD35	GD28	*R.nitidus*	-0.036003	0.065446
CS18	GD40	GD28	*R.nitidus*	-0.086391	0.065581
GD50	GD40	GD28	*R.nitidus*	-0.025352	0.069295
GD22	GD40	GD28	*R.nitidus*	0.010663	0.063412
GD32	GD40	GD28	*R.nitidus*	-0.00793	0.068054
CS13	GD40	GD28	*R.nitidus*	-0.083905	0.067798
GD35	GD40	GD28	*R.nitidus*	-0.016625	0.0643
GD34	GD40	GD28	*R.nitidus*	-0.033673	0.06442
CS17	GD40	GD28	*R.nitidus*	-0.024096	0.067499
CS12	GD40	GD28	*R.nitidus*	-0.039463	0.06603
CS30	GD40	GD28	*R.nitidus*	-0.091116	0.064608
GD41	GD40	GD28	*R.nitidus*	-0.044586	0.064188
GD44	GD40	GD28	*R.nitidus*	-0.059764	0.065399
CS25	GD40	GD28	*R.nitidus*	-0.054315	0.066316
GD42	GD40	GD28	*R.nitidus*	-0.026295	0.065794
CS19	GD40	GD28	*R.nitidus*	-0.052713	0.067959
CS22	GD40	GD28	*R.nitidus*	-0.034743	0.064104
CS20	GD40	GD28	*R.nitidus*	-0.07132	0.064326
CS14	GD40	GD28	*R.nitidus*	-0.031157	0.068276
GD45	GD40	GD28	*R.nitidus*	-0.001751	0.067015
CS21	GD40	GD28	*R.nitidus*	-0.082902	0.065543
SC35	GD40	GD28	*R.nitidus*	0.009468	0.061048
CS15	GD40	GD28	*R.nitidus*	-0.031594	0.067149
SC45	GD40	GD28	*R.nitidus*	-0.007429	0.066069
CS31	GD40	GD28	*R.nitidus*	-0.050152	0.065726
CS23	GD40	GD28	*R.nitidus*	-0.078947	0.064937

GD46	GD40	GD28	*R.nitidus*	-0.057212	0.062956
SC18	GD40	GD28	*R.nitidus*	-0.01811	0.064688
CS16	GD40	GD28	*R.nitidus*	-0.053991	0.066211
SC6	GD40	GD28	*R.nitidus*	-0.08238	0.065155
CS24	GD40	GD28	*R.nitidus*	-0.073958	0.064666
JO8	GD41	GD28	*R.nitidus*	0.02359	0.074116
SC24	GD40	GD28	*R.nitidus*	-0.045245	0.065419
SC27	GD40	GD28	*R.nitidus*	-0.058691	0.064671
SC44	GD40	GD28	*R.nitidus*	-0.048451	0.065399
CS6	GD40	GD28	*R.nitidus*	-0.084639	0.065456
SC42	GD40	GD28	*R.nitidus*	0.168719	0.06316
SC26	GD40	GD28	*R.nitidus*	-0.077592	0.065378
JW6	GD40	GD28	*R.nitidus*	-0.014085	0.070219
SC15	GD40	GD28	*R.nitidus*	-0.060351	0.066339
CS28	GD40	GD28	*R.nitidus*	-0.078696	0.065943
JO1	GD40	GD28	*R.nitidus*	-0.068914	0.062174
JW7	GD40	GD28	*R.nitidus*	-0.042125	0.070227
CS3	GD40	GD28	*R.nitidus*	-0.067757	0.064166
SC5	GD40	GD28	*R.nitidus*	-0.051948	0.066757
SC28	GD40	GD28	*R.nitidus*	-0.095165	0.064846
JO5	GD40	GD28	*R.nitidus*	-0.035338	0.065551
MY4	GD40	GD28	*R.nitidus*	-0.065886	0.064667
CS4	GD40	GD28	*R.nitidus*	-0.08	0.06411
SC10	GD40	GD28	*R.nitidus*	0.018237	0.067584
SC34	GD40	GD28	*R.nitidus*	-0.019076	0.063037
SC31	GD40	GD28	*R.nitidus*	-0.045649	0.066296
SC12	GD40	GD28	*R.nitidus*	-0.068787	0.065288
SC14	GD40	GD28	*R.nitidus*	-0.030015	0.065467
CS11	GD41	GD28	*R.nitidus*	-0.007948	0.06799
GD26	GD41	GD28	*R.nitidus*	0.052152	0.063997
JW4	GD40	GD28	*R.nitidus*	0.126803	0.068318
MY6	GD40	GD28	*R.nitidus*	-0.059341	0.063422
GD22	GD41	GD28	*R.nitidus*	0.072428	0.063868
JO3	GD40	GD28	*R.nitidus*	-0.011545	0.070502
SC32	GD40	GD28	*R.nitidus*	-0.005904	0.064768
GD32	GD41	GD28	*R.nitidus*	0.046555	0.070566
GD34	GD41	GD28	*R.nitidus*	0.010934	0.065245
GD25	GD41	GD28	*R.nitidus*	0.052758	0.065285
GD21	GD41	GD28	*R.nitidus*	0.153785	0.06358
GD30	GD41	GD28	*R.nitidus*	0.108974	0.065754
GD46	GD41	GD28	*R.nitidus*	-0.015399	0.063706
JO7	GD41	GD28	*R.nitidus*	0.018386	0.072927
GD45	GD41	GD28	*R.nitidus*	0.032022	0.069177
GD35	GD41	GD28	*R.nitidus*	0.032099	0.064848
CS21	GD41	GD28	*R.nitidus*	-0.033684	0.065693
GD33	GD41	GD28	*R.nitidus*	0	0.067134
GD31	GD41	GD28	*R.nitidus*	0.052239	0.066124
GD29	GD41	GD28	*R.nitidus*	0.097621	0.066109
CS12	GD41	GD28	*R.nitidus*	0.015994	0.067738
GD40	GD41	GD28	*R.nitidus*	0.044586	0.064188
CS19	GD41	GD28	*R.nitidus*	0.006623	0.066604
GD48	GD41	GD28	*R.nitidus*	0.067029	0.065587
CS13	GD41	GD28	*R.nitidus*	-0.031414	0.069626
CS3	GD41	GD28	*R.nitidus*	-0.037437	0.06563
CS20	GD41	GD28	*R.nitidus*	-0.026874	0.066137
CS22	GD41	GD28	*R.nitidus*	0.014232	0.064884

CS15	GD41	GD28	*R.nitidus*	0.022578	0.068132
CS14	GD41	GD28	*R.nitidus*	0.010909	0.069851
CS24	GD41	GD28	*R.nitidus*	-0.017571	0.065806
CS4	GD41	GD28	*R.nitidus*	-0.027286	0.064184
CS17	GD41	GD28	*R.nitidus*	0.025287	0.069387
CS31	GD41	GD28	*R.nitidus*	-0.00858	0.065625
GD42	GD41	GD28	*R.nitidus*	0.017223	0.063488
SC27	GD41	GD28	*R.nitidus*	-0.012223	0.065085
SC6	GD41	GD28	*R.nitidus*	-0.036226	0.068006
CS30	GD41	GD28	*R.nitidus*	-0.056294	0.067466
GD44	GD41	GD28	*R.nitidus*	-0.007772	0.067895
GD49	GD41	GD28	*R.nitidus*	0.107566	0.066421
CS18	GD41	GD28	*R.nitidus*	-0.028853	0.065615
GD50	GD41	GD28	*R.nitidus*	0.031351	0.069766
SC24	GD41	GD28	*R.nitidus*	-0.019374	0.065927
SC14	GD41	GD28	*R.nitidus*	0.009901	0.064497
CS16	GD41	GD28	*R.nitidus*	0.004444	0.066984
SC26	GD41	GD28	*R.nitidus*	-0.042661	0.067907
SC31	GD41	GD28	*R.nitidus*	0.005789	0.066841
SC32	GD41	GD28	*R.nitidus*	0.042305	0.065164
CS28	GD41	GD28	*R.nitidus*	-0.037307	0.066436
CS6	GD41	GD28	*R.nitidus*	-0.029908	0.066496
SC34	GD41	GD28	*R.nitidus*	0.030814	0.065206
SC18	GD41	GD28	*R.nitidus*	0.014638	0.066325
SC15	GD41	GD28	*R.nitidus*	-0.003968	0.066064
CS23	GD41	GD28	*R.nitidus*	-0.039007	0.06673
CS25	GD41	GD28	*R.nitidus*	-0.002182	0.065447
MY4	GD41	GD28	*R.nitidus*	-0.012696	0.064899
SC35	GD41	GD28	*R.nitidus*	0.047076	0.064118
SC5	GD41	GD28	*R.nitidus*	-0.002268	0.069563
JW4	GD41	GD28	*R.nitidus*	0.178681	0.066633
SC12	GD41	GD28	*R.nitidus*	-0.008209	0.065927
JW7	GD41	GD28	*R.nitidus*	-0.003724	0.071081
JO7	GD42	GD28	*R.nitidus*	-0.029592	0.074983
SC10	GD41	GD28	*R.nitidus*	0.058466	0.065475
SC28	GD41	GD28	*R.nitidus*	-0.047148	0.066125
GD30	GD42	GD28	*R.nitidus*	0.10942	0.066932
JO5	GD41	GD28	*R.nitidus*	0.019985	0.066114
SC42	GD41	GD28	*R.nitidus*	0.215244	0.062133
GD26	GD42	GD28	*R.nitidus*	0.013675	0.066661
SC44	GD41	GD28	*R.nitidus*	0.009985	0.065361
CS11	GD42	GD28	*R.nitidus*	-0.024318	0.068525
GD45	GD42	GD28	*R.nitidus*	0.030812	0.068835
JW6	GD41	GD28	*R.nitidus*	0.030928	0.070849
GD34	GD42	GD28	*R.nitidus*	0.016115	0.065298
GD35	GD42	GD28	*R.nitidus*	0.016407	0.065262
JO3	GD41	GD28	*R.nitidus*	0.016493	0.0694
SC45	GD41	GD28	*R.nitidus*	0.032258	0.066179
GD31	GD42	GD28	*R.nitidus*	0.03259	0.067761
GD40	GD42	GD28	*R.nitidus*	0.026295	0.065794
JO8	GD42	GD28	*R.nitidus*	-0.008155	0.074755
JO1	GD41	GD28	*R.nitidus*	-0.014706	0.064475
GD41	GD42	GD28	*R.nitidus*	-0.017223	0.063488
MY6	GD41	GD28	*R.nitidus*	-0.007225	0.065325
CS17	GD42	GD28	*R.nitidus*	0.012365	0.070449
GD29	GD42	GD28	*R.nitidus*	0.098347	0.064376

GD33	GD42	GD28	*R.nitidus*	0	0.065461
GD44	GD42	GD28	*R.nitidus*	-0.019983	0.067223
GD21	GD42	GD28	*R.nitidus*	0.148988	0.063461
GD25	GD42	GD28	*R.nitidus*	0.046784	0.066132
GD32	GD42	GD28	*R.nitidus*	0.027128	0.069308
CS24	GD42	GD28	*R.nitidus*	-0.030643	0.066871
CS19	GD42	GD28	*R.nitidus*	-0.004481	0.067745
CS12	GD42	GD28	*R.nitidus*	0.006211	0.06912
SC5	GD42	GD28	*R.nitidus*	-0.010558	0.0692
SC6	GD42	GD28	*R.nitidus*	-0.049587	0.067535
GD22	GD42	GD28	*R.nitidus*	0.070248	0.064524
GD48	GD42	GD28	*R.nitidus*	0.026654	0.064857
GD46	GD42	GD28	*R.nitidus*	-0.020591	0.064765
CS31	GD42	GD28	*R.nitidus*	-0.008682	0.065591
CS3	GD42	GD28	*R.nitidus*	-0.040058	0.06557
CS16	GD42	GD28	*R.nitidus*	-0.006747	0.066931
CS21	GD42	GD28	*R.nitidus*	-0.05278	0.06695
CS28	GD42	GD28	*R.nitidus*	-0.055285	0.06726
CS20	GD42	GD28	*R.nitidus*	-0.033732	0.067269
CS13	GD42	GD28	*R.nitidus*	-0.040801	0.06993
SC27	GD42	GD28	*R.nitidus*	-0.015026	0.066798
GD49	GD42	GD28	*R.nitidus*	0.089073	0.066719
GD50	GD42	GD28	*R.nitidus*	0.011853	0.070761
SC32	GD42	GD28	*R.nitidus*	0.032895	0.065519
SC14	GD42	GD28	*R.nitidus*	0.011038	0.06614
SC12	GD42	GD28	*R.nitidus*	-0.030574	0.065332
CS15	GD42	GD28	*R.nitidus*	0.002237	0.067912
CS14	GD42	GD28	*R.nitidus*	-0.011128	0.068869
CS22	GD42	GD28	*R.nitidus*	0.002187	0.066394
CS23	GD42	GD28	*R.nitidus*	-0.052857	0.068621
SC18	GD42	GD28	*R.nitidus*	0.007686	0.066014
CS18	GD42	GD28	*R.nitidus*	-0.045582	0.067732
CS25	GD42	GD28	*R.nitidus*	-0.011194	0.067599
CS30	GD42	GD28	*R.nitidus*	-0.059468	0.066589
JW4	GD42	GD28	*R.nitidus*	0.161339	0.066333
MY4	GD42	GD28	*R.nitidus*	-0.020104	0.066792
JO8	GD44	GD28	*R.nitidus*	0.049009	0.074372
JW6	GD42	GD28	*R.nitidus*	-0.006073	0.07212
CS4	GD42	GD28	*R.nitidus*	-0.036797	0.065368
JW7	GD42	GD28	*R.nitidus*	-0.034799	0.071898
JO3	GD42	GD28	*R.nitidus*	0.0097	0.07035
CS6	GD42	GD28	*R.nitidus*	-0.051223	0.066727
SC42	GD42	GD28	*R.nitidus*	0.216068	0.062635
SC10	GD42	GD28	*R.nitidus*	0.044799	0.065202
SC44	GD42	GD28	*R.nitidus*	-0.003846	0.067699
JO1	GD42	GD28	*R.nitidus*	-0.031594	0.065645
GD25	GD44	GD28	*R.nitidus*	0.060068	0.068301
GD29	GD44	GD28	*R.nitidus*	0.121578	0.067068
SC34	GD42	GD28	*R.nitidus*	0.019694	0.065314
SC35	GD42	GD28	*R.nitidus*	0.038298	0.064115
SC31	GD42	GD28	*R.nitidus*	-0.011404	0.068457
SC26	GD42	GD28	*R.nitidus*	-0.046709	0.067678
JO5	GD42	GD28	*R.nitidus*	0.009826	0.06777
SC15	GD42	GD28	*R.nitidus*	-0.028749	0.068388
SC24	GD42	GD28	*R.nitidus*	-0.018032	0.066655
GD22	GD44	GD28	*R.nitidus*	0.072013	0.067746

SC28	GD42	GD28	*R.nitidus*	-0.061119	0.067469
GD21	GD44	GD28	*R.nitidus*	0.16843	0.066386
SC45	GD42	GD28	*R.nitidus*	0.029155	0.065723
GD31	GD44	GD28	*R.nitidus*	0.054005	0.069424
GD34	GD44	GD28	*R.nitidus*	0.025898	0.069223
GD50	GD44	GD28	*R.nitidus*	0.024728	0.068401
CS11	GD44	GD28	*R.nitidus*	-0.00228	0.068585
GD48	GD44	GD28	*R.nitidus*	0.065574	0.067191
GD33	GD44	GD28	*R.nitidus*	0.017327	0.067666
JO7	GD44	GD28	*R.nitidus*	0.024691	0.074805
GD30	GD44	GD28	*R.nitidus*	0.125234	0.069792
GD42	GD44	GD28	*R.nitidus*	0.019983	0.067223
GD40	GD44	GD28	*R.nitidus*	0.059764	0.065399
GD45	GD44	GD28	*R.nitidus*	0.051961	0.070846
GD32	GD44	GD28	*R.nitidus*	0.02277	0.071537
MY6	GD42	GD28	*R.nitidus*	-0.016776	0.065845
GD35	GD44	GD28	*R.nitidus*	0.043739	0.06688
GD26	GD44	GD28	*R.nitidus*	0.044619	0.065927
CS14	GD44	GD28	*R.nitidus*	0.009132	0.068678
CS15	GD44	GD28	*R.nitidus*	0.017464	0.067319
CS20	GD44	GD28	*R.nitidus*	-0.021341	0.067661
SC14	GD44	GD28	*R.nitidus*	0.01249	0.068209
GD46	GD44	GD28	*R.nitidus*	0.0025	0.066082
CS12	GD44	GD28	*R.nitidus*	0.004065	0.068437
GD41	GD44	GD28	*R.nitidus*	0.007772	0.067895
GD49	GD44	GD28	*R.nitidus*	0.104545	0.068891
CS13	GD44	GD28	*R.nitidus*	-0.044847	0.068191
CS21	GD44	GD28	*R.nitidus*	-0.028137	0.066732
SC31	GD44	GD28	*R.nitidus*	0.008112	0.068638
CS22	GD44	GD28	*R.nitidus*	0.01536	0.066889
CS23	GD44	GD28	*R.nitidus*	-0.039816	0.066389
SC12	GD44	GD28	*R.nitidus*	-0.015861	0.068442
CS25	GD44	GD28	*R.nitidus*	-0.013503	0.067858
SC10	GD44	GD28	*R.nitidus*	0.058417	0.067912
SC15	GD44	GD28	*R.nitidus*	0.009717	0.068891
CS17	GD44	GD28	*R.nitidus*	0.03097	0.070099
CS16	GD44	GD28	*R.nitidus*	0.011655	0.066715
CS6	GD44	GD28	*R.nitidus*	-0.022314	0.068227
JW4	GD44	GD28	*R.nitidus*	0.170144	0.069926
JO1	GD44	GD28	*R.nitidus*	-0.006993	0.066616
CS28	GD44	GD28	*R.nitidus*	-0.04424	0.068177
CS19	GD44	GD28	*R.nitidus*	0.003984	0.067555
CS18	GD44	GD28	*R.nitidus*	-0.033463	0.068569
SC32	GD44	GD28	*R.nitidus*	0.056371	0.068305
SC6	GD44	GD28	*R.nitidus*	-0.02148	0.067637
CS24	GD44	GD28	*R.nitidus*	-0.021259	0.068097
JO8	GD45	GD28	*R.nitidus*	-0.007481	0.078371
CS30	GD44	GD28	*R.nitidus*	-0.034673	0.067251
CS4	GD44	GD28	*R.nitidus*	-0.032207	0.065527
SC34	GD44	GD28	*R.nitidus*	0.047655	0.06717
SC45	GD44	GD28	*R.nitidus*	0.043818	0.069293
SC27	GD44	GD28	*R.nitidus*	0.007026	0.068315
CS31	GD44	GD28	*R.nitidus*	-0.004058	0.068147
JO3	GD44	GD28	*R.nitidus*	0.032028	0.071836
MY6	GD44	GD28	*R.nitidus*	-0.007825	0.067223
CS3	GD44	GD28	*R.nitidus*	-0.019923	0.067898

SC5	GD44	GD28	*R.nitidus*	0.008842	0.069049
SC24	GD44	GD28	*R.nitidus*	-0.008744	0.068529
JO5	GD44	GD28	*R.nitidus*	0.018519	0.067975
SC44	GD44	GD28	*R.nitidus*	0.010359	0.069425
JO7	GD45	GD28	*R.nitidus*	-0.071951	0.075727
SC18	GD44	GD28	*R.nitidus*	0.011609	0.067564
GD26	GD45	GD28	*R.nitidus*	-0.011561	0.069653
JW7	GD44	GD28	*R.nitidus*	0.020154	0.070368
SC26	GD44	GD28	*R.nitidus*	-0.037936	0.067342
SC42	GD44	GD28	*R.nitidus*	0.214194	0.065162
SC35	GD44	GD28	*R.nitidus*	0.059939	0.065561
SC28	GD44	GD28	*R.nitidus*	-0.043582	0.068871
JW6	GD44	GD28	*R.nitidus*	0.054639	0.072449
CS11	GD45	GD28	*R.nitidus*	-0.042465	0.071559
GD29	GD45	GD28	*R.nitidus*	0.076498	0.068836
CS12	GD45	GD28	*R.nitidus*	-0.021222	0.072873
GD21	GD45	GD28	*R.nitidus*	0.116214	0.069928
GD34	GD45	GD28	*R.nitidus*	-0.019039	0.06881
GD22	GD45	GD28	*R.nitidus*	0.04363	0.067922
MY4	GD44	GD28	*R.nitidus*	-0.014151	0.067147
GD31	GD45	GD28	*R.nitidus*	0.015489	0.0696
GD40	GD45	GD28	*R.nitidus*	0.001751	0.067015
CS18	GD45	GD28	*R.nitidus*	-0.049622	0.071569
GD48	GD45	GD28	*R.nitidus*	0.025026	0.073371
GD25	GD45	GD28	*R.nitidus*	-0.002712	0.068802
GD35	GD45	GD28	*R.nitidus*	-0.031306	0.0681
CS13	GD45	GD28	*R.nitidus*	-0.057922	0.073177
GD46	GD45	GD28	*R.nitidus*	-0.065033	0.069256
GD41	GD45	GD28	*R.nitidus*	-0.032022	0.069177
CS19	GD45	GD28	*R.nitidus*	-0.024605	0.072154
CS16	GD45	GD28	*R.nitidus*	-0.025295	0.072021
CS20	GD45	GD28	*R.nitidus*	-0.041952	0.071829
GD30	GD45	GD28	*R.nitidus*	0.054294	0.073021
GD33	GD45	GD28	*R.nitidus*	-0.035647	0.06771
GD32	GD45	GD28	*R.nitidus*	-0.004278	0.072608
GD44	GD45	GD28	*R.nitidus*	-0.051961	0.070846
CS31	GD45	GD28	*R.nitidus*	-0.064947	0.073034
GD49	GD45	GD28	*R.nitidus*	0.059059	0.0728
CS17	GD45	GD28	*R.nitidus*	-0.00448	0.073951
CS25	GD45	GD28	*R.nitidus*	-0.037004	0.072493
CS28	GD45	GD28	*R.nitidus*	-0.067663	0.070761
CS23	GD45	GD28	*R.nitidus*	-0.047779	0.072378
CS3	GD45	GD28	*R.nitidus*	-0.038194	0.071486
CS30	GD45	GD28	*R.nitidus*	-0.071681	0.071884
CS15	GD45	GD28	*R.nitidus*	-0.010961	0.071617
SC28	GD45	GD28	*R.nitidus*	-0.078283	0.068955
CS21	GD45	GD28	*R.nitidus*	-0.048293	0.070712
GD50	GD45	GD28	*R.nitidus*	-0.008602	0.074418
GD42	GD45	GD28	*R.nitidus*	-0.030812	0.068835
SC18	GD45	GD28	*R.nitidus*	0.007036	0.072529
CS14	GD45	GD28	*R.nitidus*	-0.010453	0.074159
CS6	GD45	GD28	*R.nitidus*	-0.031905	0.074373
SC42	GD45	GD28	*R.nitidus*	0.193931	0.067491
SC32	GD45	GD28	*R.nitidus*	0.043261	0.070616
CS4	GD45	GD28	*R.nitidus*	-0.053112	0.069562
SC26	GD45	GD28	*R.nitidus*	-0.06245	0.070143

SC31	GD45	GD28	*R.nitidus*	-0.010475	0.071783
SC5	GD45	GD28	*R.nitidus*	-0.049003	0.071908
SC15	GD45	GD28	*R.nitidus*	-0.037229	0.072462
CS22	GD45	GD28	*R.nitidus*	-0.016536	0.070709
SC6	GD45	GD28	*R.nitidus*	-0.053691	0.071815
SC34	GD45	GD28	*R.nitidus*	0.004105	0.068973
CS24	GD45	GD28	*R.nitidus*	-0.042517	0.069689
JW4	GD45	GD28	*R.nitidus*	0.165194	0.071268
MY4	GD45	GD28	*R.nitidus*	-0.041975	0.069244
SC10	GD45	GD28	*R.nitidus*	0.025231	0.070079
JO1	GD45	GD28	*R.nitidus*	-0.042502	0.07062
SC14	GD45	GD28	*R.nitidus*	-0.011523	0.071847
JW6	GD45	GD28	*R.nitidus*	-0.044268	0.07767
JO7	CS13	GD28	*R.nitidus*	0.040469	0.075646
SC24	GD45	GD28	*R.nitidus*	-0.031826	0.070201
SC27	GD45	GD28	*R.nitidus*	-0.022333	0.070782
JO5	GD45	GD28	*R.nitidus*	-0.017722	0.071102
JW7	GD45	GD28	*R.nitidus*	-0.037267	0.074486
MY6	GD45	GD28	*R.nitidus*	-0.032527	0.069933
SC35	GD45	GD28	*R.nitidus*	0.031811	0.069417
SC12	GD45	GD28	*R.nitidus*	-0.044481	0.072302
JO8	CS13	GD28	*R.nitidus*	0.026911	0.076435
CS11	CS13	GD28	*R.nitidus*	0.043812	0.072066
CS12	CS13	GD28	*R.nitidus*	0.068771	0.071255
GD21	CS13	GD28	*R.nitidus*	0.159652	0.067844
SC44	GD45	GD28	*R.nitidus*	-0.010187	0.069397
GD32	CS13	GD28	*R.nitidus*	0.055144	0.072156
SC45	GD45	GD28	*R.nitidus*	0.017827	0.06902
GD29	CS13	GD28	*R.nitidus*	0.129317	0.069123
GD40	CS13	GD28	*R.nitidus*	0.083905	0.067798
GD41	CS13	GD28	*R.nitidus*	0.031414	0.069626
JO3	GD45	GD28	*R.nitidus*	-0.009165	0.073284
GD25	CS13	GD28	*R.nitidus*	0.096875	0.068847
GD35	CS13	GD28	*R.nitidus*	0.04958	0.070466
GD49	CS13	GD28	*R.nitidus*	0.106655	0.072169
GD26	CS13	GD28	*R.nitidus*	0.061209	0.068357
GD31	CS13	GD28	*R.nitidus*	0.072337	0.069049
GD46	CS13	GD28	*R.nitidus*	0.036446	0.069057
GD34	CS13	GD28	*R.nitidus*	0.041311	0.069008
CS18	CS13	GD28	*R.nitidus*	-0.007557	0.069162
CS21	CS13	GD28	*R.nitidus*	-0.000812	0.069403
GD42	CS13	GD28	*R.nitidus*	0.040801	0.06993
GD30	CS13	GD28	*R.nitidus*	0.126388	0.071439
GD50	CS13	GD28	*R.nitidus*	0.085299	0.074322
GD22	CS13	GD28	*R.nitidus*	0.094084	0.068553
CS17	CS13	GD28	*R.nitidus*	0.064815	0.071406
CS24	CS13	GD28	*R.nitidus*	0.011102	0.06927
CS22	CS13	GD28	*R.nitidus*	0.066335	0.067339
SC12	CS13	GD28	*R.nitidus*	0.025327	0.069977
GD48	CS13	GD28	*R.nitidus*	0.076105	0.070532
CS15	CS13	GD28	*R.nitidus*	0.074944	0.070259
SC24	CS13	GD28	*R.nitidus*	0.028324	0.071438
SC14	CS13	GD28	*R.nitidus*	0.045385	0.067671
GD33	CS13	GD28	*R.nitidus*	0.032	0.067658
GD44	CS13	GD28	*R.nitidus*	0.044847	0.068191
GD45	CS13	GD28	*R.nitidus*	0.057922	0.073177

CS19	CS13	GD28	*R.nitidus*	0.035654	0.070915
SC27	CS13	GD28	*R.nitidus*	0.031596	0.068355
CS25	CS13	GD28	*R.nitidus*	0.031915	0.071498
CS3	CS13	GD28	*R.nitidus*	-0.000813	0.068474
SC18	CS13	GD28	*R.nitidus*	0.049245	0.069069
CS16	CS13	GD28	*R.nitidus*	0.056955	0.070803
SC26	CS13	GD28	*R.nitidus*	-0.013535	0.068075
SC34	CS13	GD28	*R.nitidus*	0.051163	0.068423
CS28	CS13	GD28	*R.nitidus*	-0.000873	0.070591
CS20	CS13	GD28	*R.nitidus*	0.000791	0.068121
SC15	CS13	GD28	*R.nitidus*	0.027615	0.070375
CS14	CS13	GD28	*R.nitidus*	0.068493	0.073899
SC31	CS13	GD28	*R.nitidus*	0.034509	0.069598
CS30	CS13	GD28	*R.nitidus*	-0.025348	0.069894
CS23	CS13	GD28	*R.nitidus*	-0.013721	0.070228
SC32	CS13	GD28	*R.nitidus*	0.076292	0.068993
SC28	CS13	GD28	*R.nitidus*	-0.026807	0.068995
MY4	CS13	GD28	*R.nitidus*	0.019496	0.069914
JO8	GD46	GD28	*R.nitidus*	0.061376	0.070795
MY6	CS13	GD28	*R.nitidus*	0.026232	0.068006
CS6	CS13	GD28	*R.nitidus*	0.005068	0.068734
JW4	CS13	GD28	*R.nitidus*	0.205423	0.067705
JO7	GD46	GD28	*R.nitidus*	0.011387	0.070842
SC5	CS13	GD28	*R.nitidus*	0.023163	0.071647
SC10	CS13	GD28	*R.nitidus*	0.085209	0.069436
SC6	CS13	GD28	*R.nitidus*	-0.003091	0.070612
CS4	CS13	GD28	*R.nitidus*	0.01634	0.068216
SC42	CS13	GD28	*R.nitidus*	0.238496	0.065254
JW6	CS13	GD28	*R.nitidus*	0.056034	0.076375
JO5	CS13	GD28	*R.nitidus*	0.049245	0.068786
JW7	CS13	GD28	*R.nitidus*	0.039652	0.074978
CS31	CS13	GD28	*R.nitidus*	0.030431	0.068104
GD34	GD46	GD28	*R.nitidus*	0.021649	0.064269
GD22	GD46	GD28	*R.nitidus*	0.08206	0.062886
GD25	GD46	GD28	*R.nitidus*	0.073684	0.065663
SC35	CS13	GD28	*R.nitidus*	0.087812	0.067099
GD30	GD46	GD28	*R.nitidus*	0.137255	0.065796
GD31	GD46	GD28	*R.nitidus*	0.054668	0.065692
GD21	GD46	GD28	*R.nitidus*	0.176883	0.064153
SC45	CS13	GD28	*R.nitidus*	0.054805	0.070436
SC44	CS13	GD28	*R.nitidus*	0.019732	0.069694
CS13	GD46	GD28	*R.nitidus*	-0.036446	0.069057
JO3	CS13	GD28	*R.nitidus*	0.037838	0.07185
GD42	GD46	GD28	*R.nitidus*	0.020591	0.064765
CS12	GD46	GD28	*R.nitidus*	0.022382	0.068741
GD44	GD46	GD28	*R.nitidus*	-0.0025	0.066082
GD50	GD46	GD28	*R.nitidus*	0.044177	0.068361
GD29	GD46	GD28	*R.nitidus*	0.129401	0.063116
GD33	GD46	GD28	*R.nitidus*	0.021169	0.065029
GD35	GD46	GD28	*R.nitidus*	0.042673	0.065488
JO1	CS13	GD28	*R.nitidus*	0.011924	0.067348
CS14	GD46	GD28	*R.nitidus*	0.010078	0.069839
CS11	GD46	GD28	*R.nitidus*	-0.002985	0.06795
GD32	GD46	GD28	*R.nitidus*	0.04912	0.068206
GD26	GD46	GD28	*R.nitidus*	0.061932	0.062073
CS19	GD46	GD28	*R.nitidus*	-0.004542	0.069408

GD41	GD46	GD28	*R.nitidus*	0.015399	0.063706
CS30	GD46	GD28	*R.nitidus*	-0.044741	0.066909
CS17	GD46	GD28	*R.nitidus*	0.032334	0.069739
GD48	GD46	GD28	*R.nitidus*	0.081106	0.064691
CS31	GD46	GD28	*R.nitidus*	-0.00319	0.067096
CS18	GD46	GD28	*R.nitidus*	-0.037064	0.066949
CS28	GD46	GD28	*R.nitidus*	-0.0313	0.066035
CS21	GD46	GD28	*R.nitidus*	-0.032951	0.067708
CS15	GD46	GD28	*R.nitidus*	0.009848	0.068938
GD45	GD46	GD28	*R.nitidus*	0.065033	0.069256
SC6	GD46	GD28	*R.nitidus*	-0.025307	0.066126
SC15	GD46	GD28	*R.nitidus*	-0.001587	0.066058
CS23	GD46	GD28	*R.nitidus*	-0.030627	0.065752
GD49	GD46	GD28	*R.nitidus*	0.104421	0.068224
SC12	GD46	GD28	*R.nitidus*	-0.012289	0.064368
SC27	GD46	GD28	*R.nitidus*	-0.007553	0.065643
SC14	GD46	GD28	*R.nitidus*	0.019417	0.065825
CS6	GD46	GD28	*R.nitidus*	-0.022273	0.065764
CS24	GD46	GD28	*R.nitidus*	-0.014471	0.065636
CS16	GD46	GD28	*R.nitidus*	0.007698	0.069031
SC34	GD46	GD28	*R.nitidus*	0.030259	0.063806
CS20	GD46	GD28	*R.nitidus*	-0.023878	0.067174
GD40	GD46	GD28	*R.nitidus*	0.057212	0.062956
SC10	GD46	GD28	*R.nitidus*	0.058252	0.064543
CS22	GD46	GD28	*R.nitidus*	0.024981	0.064763
SC26	GD46	GD28	*R.nitidus*	-0.034207	0.065641
JW7	GD46	GD28	*R.nitidus*	0.007505	0.0708
SC31	GD46	GD28	*R.nitidus*	0.010359	0.064837
CS25	GD46	GD28	*R.nitidus*	0.008258	0.068728
JW6	GD46	GD28	*R.nitidus*	0.047813	0.069866
CS3	GD46	GD28	*R.nitidus*	-0.015613	0.067394
JO1	GD46	GD28	*R.nitidus*	-0.023792	0.063943
SC45	GD46	GD28	*R.nitidus*	0.038123	0.065798
SC32	GD46	GD28	*R.nitidus*	0.045522	0.066898
SC5	GD46	GD28	*R.nitidus*	-0.00686	0.066673
SC18	GD46	GD28	*R.nitidus*	0.038132	0.064409
JO3	GD46	GD28	*R.nitidus*	0.030411	0.069662
CS4	GD46	GD28	*R.nitidus*	-0.02407	0.064045
MY4	GD46	GD28	*R.nitidus*	-0.006798	0.065789
JO7	GD48	GD28	*R.nitidus*	-0.036458	0.071761
JW4	GD46	GD28	*R.nitidus*	0.192874	0.066853
GD30	GD48	GD28	*R.nitidus*	0.040201	0.067817
SC24	GD46	GD28	*R.nitidus*	0.00076	0.065716
MY6	GD46	GD28	*R.nitidus*	-0.000736	0.063663
SC44	GD46	GD28	*R.nitidus*	0.00761	0.064742
SC28	GD46	GD28	*R.nitidus*	-0.047584	0.063415
GD29	GD48	GD28	*R.nitidus*	0.060329	0.065217
GD26	GD48	GD28	*R.nitidus*	0.0188	0.066917
SC42	GD46	GD28	*R.nitidus*	0.217208	0.06281
GD32	GD48	GD28	*R.nitidus*	-0.00998	0.066621
GD21	GD48	GD28	*R.nitidus*	0.113514	0.066041
SC35	GD46	GD28	*R.nitidus*	0.050651	0.063027
GD31	GD48	GD28	*R.nitidus*	-0.01327	0.070387
GD40	GD48	GD28	*R.nitidus*	-0.000868	0.065643
JO8	GD48	GD28	*R.nitidus*	-0.02452	0.072715
GD25	GD48	GD28	*R.nitidus*	-0.008929	0.066801

GD45	GD48	GD28	*R.nitidus*	-0.025026	0.073371
CS12	GD48	GD28	*R.nitidus*	-0.040472	0.070137
GD34	GD48	GD28	*R.nitidus*	-0.053622	0.066189
GD46	GD48	GD28	*R.nitidus*	-0.081106	0.064691
GD22	GD48	GD28	*R.nitidus*	0.025372	0.06531
CS16	GD48	GD28	*R.nitidus*	-0.054805	0.068114
JO5	GD46	GD28	*R.nitidus*	0.015521	0.065449
CS13	GD48	GD28	*R.nitidus*	-0.076105	0.070532
GD33	GD48	GD28	*R.nitidus*	-0.034247	0.066263
GD44	GD48	GD28	*R.nitidus*	-0.065574	0.067191
CS11	GD48	GD28	*R.nitidus*	-0.04222	0.069341
GD42	GD48	GD28	*R.nitidus*	-0.026654	0.064857
CS24	GD48	GD28	*R.nitidus*	-0.060507	0.067125
SC5	GD48	GD28	*R.nitidus*	-0.020185	0.068682
CS25	GD48	GD28	*R.nitidus*	-0.048173	0.068196
GD35	GD48	GD28	*R.nitidus*	-0.015748	0.066861
CS14	GD48	GD28	*R.nitidus*	-0.043689	0.07105
GD49	GD48	GD28	*R.nitidus*	0.042532	0.069558
CS23	GD48	GD28	*R.nitidus*	-0.082278	0.068404
GD41	GD48	GD28	*R.nitidus*	-0.067029	0.065587
CS20	GD48	GD28	*R.nitidus*	-0.053945	0.066438
CS18	GD48	GD28	*R.nitidus*	-0.072157	0.06794
CS28	GD48	GD28	*R.nitidus*	-0.090439	0.06824
CS15	GD48	GD28	*R.nitidus*	-0.029851	0.070248
CS31	GD48	GD28	*R.nitidus*	-0.046167	0.066378
SC12	GD48	GD28	*R.nitidus*	-0.056394	0.067782
CS3	GD48	GD28	*R.nitidus*	-0.052803	0.066933
GD50	GD48	GD28	*R.nitidus*	-0.04371	0.073499
CS19	GD48	GD28	*R.nitidus*	-0.039396	0.06827
CS30	GD48	GD28	*R.nitidus*	-0.087427	0.067629
SC10	GD48	GD28	*R.nitidus*	0.028317	0.067023
CS4	GD48	GD28	*R.nitidus*	-0.058544	0.066035
SC14	GD48	GD28	*R.nitidus*	-0.022853	0.066349
CS21	GD48	GD28	*R.nitidus*	-0.073416	0.067228
CS17	GD48	GD28	*R.nitidus*	-0.021115	0.071519
SC27	GD48	GD28	*R.nitidus*	-0.042502	0.066119
CS22	GD48	GD28	*R.nitidus*	-0.032099	0.068731
SC18	GD48	GD28	*R.nitidus*	-0.012701	0.066903
SC44	GD48	GD28	*R.nitidus*	-0.038363	0.067883
SC24	GD48	GD28	*R.nitidus*	-0.042045	0.064431
MY6	GD48	GD28	*R.nitidus*	-0.048249	0.066089
SC26	GD48	GD28	*R.nitidus*	-0.086314	0.068089
JO1	GD48	GD28	*R.nitidus*	-0.061241	0.065813
SC35	GD48	GD28	*R.nitidus*	0.013428	0.065727
SC6	GD48	GD28	*R.nitidus*	-0.080422	0.066745
SC42	GD48	GD28	*R.nitidus*	0.152713	0.064802
CS6	GD48	GD28	*R.nitidus*	-0.071546	0.066227
SC45	GD48	GD28	*R.nitidus*	-0.01346	0.067538
JW4	GD48	GD28	*R.nitidus*	0.137318	0.069444
SC34	GD48	GD28	*R.nitidus*	-0.019794	0.06435
JO7	GD49	GD28	*R.nitidus*	-0.09122	0.072553
SC31	GD48	GD28	*R.nitidus*	-0.037867	0.068455
SC15	GD48	GD28	*R.nitidus*	-0.049278	0.068796
SC32	GD48	GD28	*R.nitidus*	-0.008972	0.066358
SC28	GD48	GD28	*R.nitidus*	-0.082324	0.066653
JW6	GD48	GD28	*R.nitidus*	0.004175	0.070552

CS11	GD49	GD28	*R.nitidus*	-0.083065	0.071587
JO8	GD49	GD28	*R.nitidus*	-0.04878	0.074312
JW7	GD48	GD28	*R.nitidus*	-0.017045	0.071602
GD40	GD49	GD28	*R.nitidus*	-0.026293	0.066022
MY4	GD48	GD28	*R.nitidus*	-0.059738	0.067846
GD26	GD49	GD28	*R.nitidus*	-0.032864	0.06657
GD44	GD49	GD28	*R.nitidus*	-0.104545	0.068891
JO3	GD48	GD28	*R.nitidus*	-0.025046	0.070361
CS12	GD49	GD28	*R.nitidus*	-0.059233	0.070379
CS17	GD49	GD28	*R.nitidus*	-0.05396	0.071918
GD25	GD49	GD28	*R.nitidus*	-0.043238	0.069432
GD46	GD49	GD28	*R.nitidus*	-0.104421	0.068224
GD22	GD49	GD28	*R.nitidus*	-0.029652	0.066701
CS18	GD49	GD28	*R.nitidus*	-0.115385	0.06992
GD33	GD49	GD28	*R.nitidus*	-0.071429	0.0699
GD34	GD49	GD28	*R.nitidus*	-0.067405	0.068089
GD21	GD49	GD28	*R.nitidus*	0.059946	0.068858
CS24	GD49	GD28	*R.nitidus*	-0.098007	0.06885
JO5	GD48	GD28	*R.nitidus*	-0.019656	0.066398
GD35	GD49	GD28	*R.nitidus*	-0.071986	0.068303
GD30	GD49	GD28	*R.nitidus*	0.04908	0.073228
GD48	GD49	GD28	*R.nitidus*	-0.042532	0.069558
GD31	GD49	GD28	*R.nitidus*	-0.054493	0.071325
GD29	GD49	GD28	*R.nitidus*	0.031365	0.068369
GD32	GD49	GD28	*R.nitidus*	-0.077922	0.072758
GD42	GD49	GD28	*R.nitidus*	-0.089073	0.066719
CS20	GD49	GD28	*R.nitidus*	-0.1136	0.070172
CS4	GD49	GD28	*R.nitidus*	-0.102646	0.06616
GD41	GD49	GD28	*R.nitidus*	-0.107566	0.066421
CS19	GD49	GD28	*R.nitidus*	-0.07479	0.070236
CS25	GD49	GD28	*R.nitidus*	-0.089076	0.069708
GD50	GD49	GD28	*R.nitidus*	-0.068627	0.074198
CS30	GD49	GD28	*R.nitidus*	-0.138233	0.069032
SC10	GD49	GD28	*R.nitidus*	-0.023102	0.070176
CS15	GD49	GD28	*R.nitidus*	-0.053156	0.070094
SC12	GD49	GD28	*R.nitidus*	-0.084495	0.068146
CS14	GD49	GD28	*R.nitidus*	-0.068823	0.072295
CS13	GD49	GD28	*R.nitidus*	-0.106655	0.072169
GD45	GD49	GD28	*R.nitidus*	-0.059059	0.0728
CS16	GD49	GD28	*R.nitidus*	-0.062657	0.069312
CS23	GD49	GD28	*R.nitidus*	-0.101958	0.067323
CS28	GD49	GD28	*R.nitidus*	-0.129362	0.070329
CS21	GD49	GD28	*R.nitidus*	-0.112943	0.067255
CS3	GD49	GD28	*R.nitidus*	-0.108766	0.069697
MY4	GD49	GD28	*R.nitidus*	-0.08	0.069688
SC5	GD49	GD28	*R.nitidus*	-0.075833	0.072077
SC6	GD49	GD28	*R.nitidus*	-0.112006	0.068787
SC15	GD49	GD28	*R.nitidus*	-0.097826	0.067771
CS22	GD49	GD28	*R.nitidus*	-0.067332	0.068082
SC45	GD49	GD28	*R.nitidus*	-0.042834	0.070077
SC24	GD49	GD28	*R.nitidus*	-0.111658	0.069262
SC26	GD49	GD28	*R.nitidus*	-0.119841	0.069583
CS31	GD49	GD28	*R.nitidus*	-0.108362	0.071133
SC28	GD49	GD28	*R.nitidus*	-0.127023	0.06787
SC35	GD49	GD28	*R.nitidus*	-0.022293	0.066164
SC27	GD49	GD28	*R.nitidus*	-0.083945	0.068261

SC42	GD49	GD28	*R.nitidus*	0.134577	0.065078
SC31	GD49	GD28	*R.nitidus*	-0.086056	0.071533
CS6	GD49	GD28	*R.nitidus*	-0.096499	0.068561
SC32	GD49	GD28	*R.nitidus*	-0.037097	0.069325
JO8	GD50	GD28	*R.nitidus*	0.014337	0.075136
SC34	GD49	GD28	*R.nitidus*	-0.040128	0.066197
SC14	GD49	GD28	*R.nitidus*	-0.062201	0.068495
JW6	GD49	GD28	*R.nitidus*	-0.078801	0.072256
JO3	GD49	GD28	*R.nitidus*	-0.044	0.072988
SC18	GD49	GD28	*R.nitidus*	-0.067532	0.069911
JO7	GD50	GD28	*R.nitidus*	-0.029377	0.076584
JO1	GD49	GD28	*R.nitidus*	-0.085782	0.067897
CS11	GD50	GD28	*R.nitidus*	-0.038328	0.073962
GD30	GD50	GD28	*R.nitidus*	0.086189	0.071707
GD22	GD50	GD28	*R.nitidus*	0.046935	0.069989
JO5	GD49	GD28	*R.nitidus*	-0.076292	0.068358
GD42	GD50	GD28	*R.nitidus*	-0.011853	0.070761
JW4	GD49	GD28	*R.nitidus*	0.119658	0.070172
SC44	GD49	GD28	*R.nitidus*	-0.081104	0.069108
JW7	GD49	GD28	*R.nitidus*	-0.064975	0.074315
MY6	GD49	GD28	*R.nitidus*	-0.078163	0.069571
CS15	GD50	GD28	*R.nitidus*	-0.026362	0.072399
CS12	GD50	GD28	*R.nitidus*	-0.030027	0.073447
GD26	GD50	GD28	*R.nitidus*	0.03493	0.066305
CS13	GD50	GD28	*R.nitidus*	-0.085299	0.074322
GD34	GD50	GD28	*R.nitidus*	-0.000957	0.071502
CS16	GD50	GD28	*R.nitidus*	-0.041812	0.070359
CS19	GD50	GD28	*R.nitidus*	-0.035242	0.072719
GD21	GD50	GD28	*R.nitidus*	0.152765	0.069571
CS24	GD50	GD28	*R.nitidus*	-0.025	0.070671
CS22	GD50	GD28	*R.nitidus*	-0.002708	0.070718
GD29	GD50	GD28	*R.nitidus*	0.088178	0.069543
GD32	GD50	GD28	*R.nitidus*	-0.003367	0.07307
GD35	GD50	GD28	*R.nitidus*	0.001004	0.068924
GD48	GD50	GD28	*R.nitidus*	0.04371	0.073499
GD45	GD50	GD28	*R.nitidus*	0.008602	0.074418
GD31	GD50	GD28	*R.nitidus*	0.00993	0.070334
GD46	GD50	GD28	*R.nitidus*	-0.044177	0.068361
CS14	GD50	GD28	*R.nitidus*	-0.030303	0.07449
GD44	GD50	GD28	*R.nitidus*	-0.024728	0.068401
CS17	GD50	GD28	*R.nitidus*	-0.01087	0.073614
GD25	GD50	GD28	*R.nitidus*	0.004854	0.071111
CS20	GD50	GD28	*R.nitidus*	-0.02954	0.072143
GD33	GD50	GD28	*R.nitidus*	-0.00603	0.072777
GD40	GD50	GD28	*R.nitidus*	0.025352	0.069295
CS21	GD50	GD28	*R.nitidus*	-0.055794	0.071994
GD41	GD50	GD28	*R.nitidus*	-0.031351	0.069766
CS31	GD50	GD28	*R.nitidus*	-0.031993	0.069715
GD49	GD50	GD28	*R.nitidus*	0.068627	0.074198
CS28	GD50	GD28	*R.nitidus*	-0.100659	0.070125
CS23	GD50	GD28	*R.nitidus*	-0.067869	0.070062
SC10	GD50	GD28	*R.nitidus*	0.017164	0.071612
SC6	GD50	GD28	*R.nitidus*	-0.059621	0.070206
CS3	GD50	GD28	*R.nitidus*	-0.056239	0.071455
CS30	GD50	GD28	*R.nitidus*	-0.074775	0.072777
CS18	GD50	GD28	*R.nitidus*	-0.064935	0.070544

SC28	GD50	GD28	*R.nitidus*	-0.081365	0.069642
SC35	GD50	GD28	*R.nitidus*	0.023458	0.06789
CS25	GD50	GD28	*R.nitidus*	-0.024658	0.071593
SC45	GD50	GD28	*R.nitidus*	0.007042	0.072071
SC42	GD50	GD28	*R.nitidus*	0.198407	0.067062
SC18	GD50	GD28	*R.nitidus*	-0.020128	0.0728
SC26	GD50	GD28	*R.nitidus*	-0.070191	0.070579
SC12	GD50	GD28	*R.nitidus*	-0.055013	0.072104
SC5	GD50	GD28	*R.nitidus*	-0.037443	0.073499
SC15	GD50	GD28	*R.nitidus*	-0.030937	0.071927
SC44	GD50	GD28	*R.nitidus*	-0.027027	0.072024
GD26	CS14	GD28	*R.nitidus*	0.030075	0.068792
CS6	GD50	GD28	*R.nitidus*	-0.044505	0.072123
SC14	GD50	GD28	*R.nitidus*	-0.010274	0.07148
JW4	GD50	GD28	*R.nitidus*	0.156776	0.071156
JW7	GD50	GD28	*R.nitidus*	-0.043663	0.076013
JO1	GD50	GD28	*R.nitidus*	-0.071115	0.069535
CS4	GD50	GD28	*R.nitidus*	-0.062715	0.069334
SC27	GD50	GD28	*R.nitidus*	-0.051373	0.068806
SC24	GD50	GD28	*R.nitidus*	-0.054054	0.071048
SC32	GD50	GD28	*R.nitidus*	0.004322	0.070416
MY4	GD50	GD28	*R.nitidus*	-0.062663	0.06922
SC31	GD50	GD28	*R.nitidus*	-0.015742	0.068885
GD25	CS14	GD28	*R.nitidus*	0.055686	0.069379
JO8	CS14	GD28	*R.nitidus*	-0.009544	0.078512
JO5	GD50	GD28	*R.nitidus*	-0.012357	0.069597
SC34	GD50	GD28	*R.nitidus*	0.020495	0.067249
JO7	CS14	GD28	*R.nitidus*	0.005336	0.076546
GD31	CS14	GD28	*R.nitidus*	0.023144	0.070606
GD32	CS14	GD28	*R.nitidus*	0.007475	0.07297
MY6	GD50	GD28	*R.nitidus*	-0.03856	0.068716
GD21	CS14	GD28	*R.nitidus*	0.135095	0.067862
JW6	GD50	GD28	*R.nitidus*	-0.013905	0.073139
GD22	CS14	GD28	*R.nitidus*	0.056378	0.068148
JO3	GD50	GD28	*R.nitidus*	-0.023952	0.072912
GD48	CS14	GD28	*R.nitidus*	0.043689	0.07105
GD42	CS14	GD28	*R.nitidus*	0.011128	0.068869
GD50	CS14	GD28	*R.nitidus*	0.030303	0.07449
CS12	CS14	GD28	*R.nitidus*	-0.002475	0.073462
GD46	CS14	GD28	*R.nitidus*	-0.010078	0.069839
GD30	CS14	GD28	*R.nitidus*	0.095359	0.072153
CS11	CS14	GD28	*R.nitidus*	-0.019108	0.071691
GD29	CS14	GD28	*R.nitidus*	0.095378	0.068246
CS16	CS14	GD28	*R.nitidus*	-0.014475	0.072576
GD40	CS14	GD28	*R.nitidus*	0.031157	0.068276
CS13	CS14	GD28	*R.nitidus*	-0.068493	0.073899
GD34	CS14	GD28	*R.nitidus*	0.012186	0.069257
GD45	CS14	GD28	*R.nitidus*	0.010453	0.074159
CS15	CS14	GD28	*R.nitidus*	0.021879	0.070448
CS23	CS14	GD28	*R.nitidus*	-0.061864	0.068601
GD44	CS14	GD28	*R.nitidus*	-0.009132	0.068678
GD49	CS14	GD28	*R.nitidus*	0.068823	0.072295
GD35	CS14	GD28	*R.nitidus*	0.017267	0.071103
GD33	CS14	GD28	*R.nitidus*	-0.008689	0.067646
CS17	CS14	GD28	*R.nitidus*	0.014493	0.071396
CS22	CS14	GD28	*R.nitidus*	-0.00495	0.067868

GD41	CS14	GD28	*R.nitidus*	-0.010909	0.069851
CS25	CS14	GD28	*R.nitidus*	-0.020425	0.070274
CS21	CS14	GD28	*R.nitidus*	-0.054618	0.068176
CS24	CS14	GD28	*R.nitidus*	-0.035473	0.070268
CS4	CS14	GD28	*R.nitidus*	-0.040258	0.068211
CS28	CS14	GD28	*R.nitidus*	-0.060052	0.069145
SC44	CS14	GD28	*R.nitidus*	-0.01548	0.070487
CS3	CS14	GD28	*R.nitidus*	-0.044534	0.068052
SC45	CS14	GD28	*R.nitidus*	0.021244	0.070031
CS19	CS14	GD28	*R.nitidus*	-0.010851	0.071233
SC18	CS14	GD28	*R.nitidus*	0.000785	0.069319
JW4	CS14	GD28	*R.nitidus*	0.162809	0.069642
CS18	CS14	GD28	*R.nitidus*	-0.057536	0.069361
CS31	CS14	GD28	*R.nitidus*	-0.027778	0.068763
CS20	CS14	GD28	*R.nitidus*	-0.04072	0.06758
CS30	CS14	GD28	*R.nitidus*	-0.056634	0.069128
CS6	CS14	GD28	*R.nitidus*	-0.053827	0.066584
SC15	CS14	GD28	*R.nitidus*	-0.017785	0.071739
SC31	CS14	GD28	*R.nitidus*	-0.008915	0.069576
SC28	CS14	GD28	*R.nitidus*	-0.066453	0.069384
SC10	CS14	GD28	*R.nitidus*	0.046751	0.069645
SC6	CS14	GD28	*R.nitidus*	-0.03962	0.070414
GD26	CS15	GD28	*R.nitidus*	0.022272	0.066998
SC12	CS14	GD28	*R.nitidus*	-0.027135	0.069725
JO5	CS14	GD28	*R.nitidus*	0.003808	0.069058
JO8	CS15	GD28	*R.nitidus*	0.007299	0.075279
MY4	CS14	GD28	*R.nitidus*	-0.023238	0.070031
SC24	CS14	GD28	*R.nitidus*	-0.015385	0.071584
SC27	CS14	GD28	*R.nitidus*	-0.026646	0.068863
GD30	CS15	GD28	*R.nitidus*	0.083195	0.071653
SC14	CS14	GD28	*R.nitidus*	-0.002271	0.069222
SC32	CS14	GD28	*R.nitidus*	0.032488	0.070287
SC26	CS14	GD28	*R.nitidus*	-0.060514	0.067825
SC35	CS14	GD28	*R.nitidus*	0.039641	0.066905
GD32	CS15	GD28	*R.nitidus*	-0.002457	0.072311
GD31	CS15	GD28	*R.nitidus*	0.020171	0.068076
MY6	CS14	GD28	*R.nitidus*	-0.023975	0.068767
CS12	CS15	GD28	*R.nitidus*	-0.013142	0.068641
JW6	CS14	GD28	*R.nitidus*	0.018162	0.076023
SC34	CS14	GD28	*R.nitidus*	0.013595	0.068114
SC5	CS14	GD28	*R.nitidus*	-0.012598	0.072063
GD21	CS15	GD28	*R.nitidus*	0.128093	0.066333
SC42	CS14	GD28	*R.nitidus*	0.194668	0.066405
JO7	CS15	GD28	*R.nitidus*	-0.012552	0.074226
JO3	CS14	GD28	*R.nitidus*	0.00457	0.072339
JW7	CS14	GD28	*R.nitidus*	-0.008729	0.074576
CS11	CS15	GD28	*R.nitidus*	-0.04023	0.069116
GD29	CS15	GD28	*R.nitidus*	0.081884	0.068078
GD44	CS15	GD28	*R.nitidus*	-0.017464	0.067319
JO1	CS14	GD28	*R.nitidus*	-0.03066	0.068192
GD33	CS15	GD28	*R.nitidus*	-0.008658	0.067746
GD25	CS15	GD28	*R.nitidus*	0.051938	0.065267
GD45	CS15	GD28	*R.nitidus*	0.010961	0.071617
GD42	CS15	GD28	*R.nitidus*	-0.002237	0.067912
GD22	CS15	GD28	*R.nitidus*	0.049689	0.067373
GD34	CS15	GD28	*R.nitidus*	-0.007846	0.067152

GD35	CS15	GD28	*R.nitidus*	0.003701	0.069205
CS14	CS15	GD28	*R.nitidus*	-0.021879	0.070448
GD41	CS15	GD28	*R.nitidus*	-0.022578	0.068132
CS16	CS15	GD28	*R.nitidus*	-0.032037	0.068129
CS24	CS15	GD28	*R.nitidus*	-0.044481	0.068922
GD46	CS15	GD28	*R.nitidus*	-0.009848	0.068938
GD49	CS15	GD28	*R.nitidus*	0.053156	0.070094
CS13	CS15	GD28	*R.nitidus*	-0.074944	0.070259
CS19	CS15	GD28	*R.nitidus*	-0.02439	0.06918
CS21	CS15	GD28	*R.nitidus*	-0.059052	0.066913
SC12	CS15	GD28	*R.nitidus*	-0.029319	0.067392
SC14	CS15	GD28	*R.nitidus*	-0.010679	0.068375
CS18	CS15	GD28	*R.nitidus*	-0.06506	0.068068
CS20	CS15	GD28	*R.nitidus*	-0.046458	0.067413
CS17	CS15	GD28	*R.nitidus*	-0.018094	0.0682
GD40	CS15	GD28	*R.nitidus*	0.031594	0.067149
GD48	CS15	GD28	*R.nitidus*	0.029851	0.070248
GD50	CS15	GD28	*R.nitidus*	0.026362	0.072399
CS31	CS15	GD28	*R.nitidus*	-0.024938	0.069021
CS22	CS15	GD28	*R.nitidus*	-0.004812	0.066671
SC26	CS15	GD28	*R.nitidus*	-0.060232	0.066404
SC44	CS15	GD28	*R.nitidus*	-0.016588	0.068856
CS28	CS15	GD28	*R.nitidus*	-0.051993	0.066052
SC24	CS15	GD28	*R.nitidus*	-0.026054	0.068719
SC27	CS15	GD28	*R.nitidus*	-0.03289	0.066351
JW6	CS15	GD28	*R.nitidus*	0.003191	0.073279
CS3	CS15	GD28	*R.nitidus*	-0.040444	0.067115
CS25	CS15	GD28	*R.nitidus*	-0.022222	0.07044
SC45	CS15	GD28	*R.nitidus*	0.006093	0.069535
CS6	CS15	GD28	*R.nitidus*	-0.045119	0.067078
SC28	CS15	GD28	*R.nitidus*	-0.078634	0.066535
JW4	CS15	GD28	*R.nitidus*	0.164263	0.06816
CS23	CS15	GD28	*R.nitidus*	-0.068536	0.067392
SC5	CS15	GD28	*R.nitidus*	-0.018692	0.071885
CS4	CS15	GD28	*R.nitidus*	-0.040253	0.066396
SC10	CS15	GD28	*R.nitidus*	0.042722	0.068574
CS30	CS15	GD28	*R.nitidus*	-0.071371	0.067429
MY6	CS15	GD28	*R.nitidus*	-0.027544	0.067212
JW7	CS15	GD28	*R.nitidus*	-0.030126	0.071915
SC35	CS15	GD28	*R.nitidus*	0.036819	0.065923
SC6	CS15	GD28	*R.nitidus*	-0.053485	0.068008
MY4	CS15	GD28	*R.nitidus*	-0.034091	0.069809
SC34	CS15	GD28	*R.nitidus*	0.013688	0.066432
GD25	CS16	GD28	*R.nitidus*	0.058544	0.068979
CS11	CS16	GD28	*R.nitidus*	0.001149	0.070107
SC31	CS15	GD28	*R.nitidus*	-0.018409	0.06646
JO1	CS15	GD28	*R.nitidus*	-0.033805	0.065017
SC15	CS15	GD28	*R.nitidus*	-0.015962	0.069924
SC32	CS15	GD28	*R.nitidus*	0.027842	0.068054
SC42	CS15	GD28	*R.nitidus*	0.200631	0.064023
JO8	CS16	GD28	*R.nitidus*	0.041037	0.075292
GD30	CS16	GD28	*R.nitidus*	0.091135	0.069401
GD32	CS16	GD28	*R.nitidus*	0.024411	0.071035
SC18	CS15	GD28	*R.nitidus*	0.01278	0.067646
GD21	CS16	GD28	*R.nitidus*	0.126087	0.064872
JO3	CS15	GD28	*R.nitidus*	0.004409	0.068678

CS12	CS16	GD28	*R.nitidus*	0.023841	0.067602
JO7	CS16	GD28	*R.nitidus*	0.009605	0.074229
GD34	CS16	GD28	*R.nitidus*	0.017143	0.064285
GD40	CS16	GD28	*R.nitidus*	0.053991	0.066211
JO5	CS15	GD28	*R.nitidus*	0.013783	0.068095
GD50	CS16	GD28	*R.nitidus*	0.041812	0.070359
GD22	CS16	GD28	*R.nitidus*	0.059155	0.064678
GD26	CS16	GD28	*R.nitidus*	0.03901	0.064923
GD48	CS16	GD28	*R.nitidus*	0.054805	0.068114
GD29	CS16	GD28	*R.nitidus*	0.104258	0.065784
CS18	CS16	GD28	*R.nitidus*	-0.033097	0.066403
GD45	CS16	GD28	*R.nitidus*	0.025295	0.072021
SC10	CS16	GD28	*R.nitidus*	0.070162	0.068679
GD31	CS16	GD28	*R.nitidus*	0.035405	0.068641
GD35	CS16	GD28	*R.nitidus*	0.023845	0.067844
CS15	CS16	GD28	*R.nitidus*	0.032037	0.068129
GD41	CS16	GD28	*R.nitidus*	-0.004444	0.066984
GD33	CS16	GD28	*R.nitidus*	-0.003623	0.064847
GD46	CS16	GD28	*R.nitidus*	-0.007698	0.069031
CS22	CS16	GD28	*R.nitidus*	0.014778	0.066635
GD44	CS16	GD28	*R.nitidus*	-0.011655	0.066715
GD49	CS16	GD28	*R.nitidus*	0.062657	0.069312
CS4	CS16	GD28	*R.nitidus*	-0.025806	0.06676
SC24	CS16	GD28	*R.nitidus*	-0.009353	0.067671
CS14	CS16	GD28	*R.nitidus*	0.014475	0.072576
CS23	CS16	GD28	*R.nitidus*	-0.032813	0.066737
CS25	CS16	GD28	*R.nitidus*	-0.007194	0.067289
GD42	CS16	GD28	*R.nitidus*	0.006747	0.066931
SC18	CS16	GD28	*R.nitidus*	0.021617	0.067544
SC14	CS16	GD28	*R.nitidus*	0.007004	0.067316
CS13	CS16	GD28	*R.nitidus*	-0.056955	0.070803
CS21	CS16	GD28	*R.nitidus*	-0.041763	0.066532
CS30	CS16	GD28	*R.nitidus*	-0.058635	0.065112
CS31	CS16	GD28	*R.nitidus*	-0.011844	0.067412
CS19	CS16	GD28	*R.nitidus*	0.008354	0.06722
CS24	CS16	GD28	*R.nitidus*	-0.024834	0.065321
CS17	CS16	GD28	*R.nitidus*	0.01699	0.071162
CS20	CS16	GD28	*R.nitidus*	-0.025385	0.06667
SC15	CS16	GD28	*R.nitidus*	-0.005795	0.068954
CS28	CS16	GD28	*R.nitidus*	-0.030848	0.066452
SC5	CS16	GD28	*R.nitidus*	-0.009577	0.070457
CS3	CS16	GD28	*R.nitidus*	-0.02492	0.064492
SC32	CS16	GD28	*R.nitidus*	0.038118	0.066953
SC27	CS16	GD28	*R.nitidus*	-0.016013	0.067043
SC6	CS16	GD28	*R.nitidus*	-0.048662	0.067578
CS6	CS16	GD28	*R.nitidus*	-0.03104	0.066393
SC12	CS16	GD28	*R.nitidus*	-0.024272	0.065864
SC26	CS16	GD28	*R.nitidus*	-0.050926	0.06461
JW4	CS16	GD28	*R.nitidus*	0.1979	0.06751
SC34	CS16	GD28	*R.nitidus*	0.015456	0.067009
JO7	CS17	GD28	*R.nitidus*	0.012155	0.076766
SC45	CS16	GD28	*R.nitidus*	0.025404	0.068191
GD30	CS17	GD28	*R.nitidus*	0.081315	0.071681
SC44	CS16	GD28	*R.nitidus*	-0.0088	0.068709
JO3	CS16	GD28	*R.nitidus*	0.030357	0.070176
SC31	CS16	GD28	*R.nitidus*	-0.015719	0.067687

MY4	CS16	GD28	*R.nitidus*	-0.019697	0.068563
JW6	CS16	GD28	*R.nitidus*	0.044351	0.073662
JO8	CS17	GD28	*R.nitidus*	0.007726	0.077617
SC35	CS16	GD28	*R.nitidus*	0.051013	0.06624
GD44	CS17	GD28	*R.nitidus*	-0.03097	0.070099
SC42	CS16	GD28	*R.nitidus*	0.222788	0.063236
SC28	CS16	GD28	*R.nitidus*	-0.068182	0.067059
CS12	CS17	GD28	*R.nitidus*	0.001229	0.072037
GD29	CS17	GD28	*R.nitidus*	0.067757	0.068791
GD33	CS17	GD28	*R.nitidus*	-0.029771	0.069557
CS11	CS17	GD28	*R.nitidus*	-0.030216	0.067398
GD41	CS17	GD28	*R.nitidus*	-0.025287	0.069387
GD25	CS17	GD28	*R.nitidus*	0.033113	0.069049
GD40	CS17	GD28	*R.nitidus*	0.024096	0.067499
GD42	CS17	GD28	*R.nitidus*	-0.012365	0.070449
JW7	CS16	GD28	*R.nitidus*	0.012195	0.072766
GD26	CS17	GD28	*R.nitidus*	0.002362	0.070406
JO1	CS16	GD28	*R.nitidus*	-0.019231	0.066091
GD32	CS17	GD28	*R.nitidus*	-0.00519	0.074853
GD22	CS17	GD28	*R.nitidus*	0.039552	0.068391
GD35	CS17	GD28	*R.nitidus*	0.006354	0.069389
JO5	CS16	GD28	*R.nitidus*	0.013281	0.066645
GD45	CS17	GD28	*R.nitidus*	0.00448	0.073951
MY6	CS16	GD28	*R.nitidus*	-0.015385	0.066621
GD31	CS17	GD28	*R.nitidus*	0.017456	0.071153
CS3	CS17	GD28	*R.nitidus*	-0.044194	0.068516
GD21	CS17	GD28	*R.nitidus*	0.113063	0.06874
CS16	CS17	GD28	*R.nitidus*	-0.01699	0.071162
GD49	CS17	GD28	*R.nitidus*	0.05396	0.071918
CS30	CS17	GD28	*R.nitidus*	-0.086192	0.068805
SC10	CS17	GD28	*R.nitidus*	0.035117	0.070569
GD46	CS17	GD28	*R.nitidus*	-0.032334	0.069739
SC6	CS17	GD28	*R.nitidus*	-0.066667	0.070448
SC5	CS17	GD28	*R.nitidus*	-0.030303	0.073462
GD50	CS17	GD28	*R.nitidus*	0.01087	0.073614
CS20	CS17	GD28	*R.nitidus*	-0.049615	0.069131
CS15	CS17	GD28	*R.nitidus*	0.018094	0.0682
GD34	CS17	GD28	*R.nitidus*	-0.007593	0.069652
CS13	CS17	GD28	*R.nitidus*	-0.064815	0.071406
SC24	CS17	GD28	*R.nitidus*	-0.037252	0.069932
CS14	CS17	GD28	*R.nitidus*	-0.014493	0.071396
GD48	CS17	GD28	*R.nitidus*	0.021115	0.071519
SC14	CS17	GD28	*R.nitidus*	-0.020117	0.070544
SC18	CS17	GD28	*R.nitidus*	-0.007432	0.069848
SC12	CS17	GD28	*R.nitidus*	-0.041701	0.068636
SC42	CS17	GD28	*R.nitidus*	0.184421	0.064588
CS25	CS17	GD28	*R.nitidus*	-0.028319	0.072804
CS4	CS17	GD28	*R.nitidus*	-0.051915	0.068269
CS21	CS17	GD28	*R.nitidus*	-0.056362	0.068321
CS22	CS17	GD28	*R.nitidus*	-0.009442	0.069932
SC27	CS17	GD28	*R.nitidus*	-0.049615	0.068248
CS18	CS17	GD28	*R.nitidus*	-0.060632	0.067667
CS19	CS17	GD28	*R.nitidus*	-0.01847	0.071442
SC35	CS17	GD28	*R.nitidus*	0.015176	0.066687
CS23	CS17	GD28	*R.nitidus*	-0.063652	0.068685
CS24	CS17	GD28	*R.nitidus*	-0.043706	0.070165

CS6	CS17	GD28	*R.nitidus*	-0.039234	0.069351
CS28	CS17	GD28	*R.nitidus*	-0.06853	0.069415
CS31	CS17	GD28	*R.nitidus*	-0.037872	0.069663
SC31	CS17	GD28	*R.nitidus*	-0.033524	0.068501
SC44	CS17	GD28	*R.nitidus*	-0.036013	0.07062
SC15	CS17	GD28	*R.nitidus*	-0.037262	0.070331
SC32	CS17	GD28	*R.nitidus*	0.030691	0.070104
SC34	CS17	GD28	*R.nitidus*	-0.003265	0.068269
JW4	CS17	GD28	*R.nitidus*	0.170976	0.069473
SC26	CS17	GD28	*R.nitidus*	-0.07009	0.069327
JO1	CS17	GD28	*R.nitidus*	-0.060708	0.067324
GD29	CS18	GD28	*R.nitidus*	0.128223	0.065623
MY4	CS17	GD28	*R.nitidus*	-0.031376	0.070865
JW6	CS17	GD28	*R.nitidus*	0.013857	0.074598
SC28	CS17	GD28	*R.nitidus*	-0.092671	0.068522
CS12	CS18	GD28	*R.nitidus*	0.038972	0.066698
GD31	CS18	GD28	*R.nitidus*	0.088468	0.069318
JO3	CS17	GD28	*R.nitidus*	0.018217	0.072417
JW7	CS17	GD28	*R.nitidus*	-0.019108	0.07432
GD21	CS18	GD28	*R.nitidus*	0.178521	0.066442
SC45	CS17	GD28	*R.nitidus*	-0.008821	0.070514
GD40	CS18	GD28	*R.nitidus*	0.086391	0.065581
GD26	CS18	GD28	*R.nitidus*	0.072006	0.0657
CS11	CS18	GD28	*R.nitidus*	0.033594	0.066741
JO8	CS18	GD28	*R.nitidus*	0.057297	0.07308
GD35	CS18	GD28	*R.nitidus*	0.053788	0.067932
GD48	CS18	GD28	*R.nitidus*	0.072157	0.06794
GD25	CS18	GD28	*R.nitidus*	0.088128	0.06627
GD41	CS18	GD28	*R.nitidus*	0.028853	0.065615
JO5	CS17	GD28	*R.nitidus*	-0.028783	0.069458
JO7	CS18	GD28	*R.nitidus*	0.044239	0.074489
MY6	CS17	GD28	*R.nitidus*	-0.027397	0.067834
GD45	CS18	GD28	*R.nitidus*	0.049622	0.071569
GD30	CS18	GD28	*R.nitidus*	0.135593	0.068098
GD46	CS18	GD28	*R.nitidus*	0.037064	0.066949
GD49	CS18	GD28	*R.nitidus*	0.115385	0.06992
GD32	CS18	GD28	*R.nitidus*	0.062346	0.068778
GD33	CS18	GD28	*R.nitidus*	0.048529	0.067684
GD22	CS18	GD28	*R.nitidus*	0.104181	0.066155
GD34	CS18	GD28	*R.nitidus*	0.040757	0.066577
CS13	CS18	GD28	*R.nitidus*	0.007557	0.069162
GD44	CS18	GD28	*R.nitidus*	0.033463	0.068569
CS14	CS18	GD28	*R.nitidus*	0.057536	0.069361
CS16	CS18	GD28	*R.nitidus*	0.033097	0.066403
CS23	CS18	GD28	*R.nitidus*	0.004859	0.066106
CS15	CS18	GD28	*R.nitidus*	0.06506	0.068068
GD42	CS18	GD28	*R.nitidus*	0.045582	0.067732
CS22	CS18	GD28	*R.nitidus*	0.06469	0.064741
CS20	CS18	GD28	*R.nitidus*	0.019361	0.065544
SC15	CS18	GD28	*R.nitidus*	0.030936	0.067189
GD50	CS18	GD28	*R.nitidus*	0.064935	0.070544
CS19	CS18	GD28	*R.nitidus*	0.037229	0.067535
SC34	CS18	GD28	*R.nitidus*	0.066009	0.063981
JW6	CS18	GD28	*R.nitidus*	0.049797	0.071922
CS3	CS18	GD28	*R.nitidus*	0.01756	0.065039
CS4	CS18	GD28	*R.nitidus*	0.008818	0.065187

CS24	CS18	GD28	*R.nitidus*	0.021277	0.065313
SC26	CS18	GD28	*R.nitidus*	-0.006897	0.064377
CS17	CS18	GD28	*R.nitidus*	0.060632	0.067667
CS25	CS18	GD28	*R.nitidus*	0.056895	0.069644
CS21	CS18	GD28	*R.nitidus*	0.014403	0.064646
SC31	CS18	GD28	*R.nitidus*	0.033898	0.065172
CS31	CS18	GD28	*R.nitidus*	0.026251	0.065558
JO3	CS18	GD28	*R.nitidus*	0.063234	0.069592
SC14	CS18	GD28	*R.nitidus*	0.042748	0.065917
SC12	CS18	GD28	*R.nitidus*	0.023659	0.066209
SC6	CS18	GD28	*R.nitidus*	0.008709	0.065773
SC10	CS18	GD28	*R.nitidus*	0.109501	0.065943
CS28	CS18	GD28	*R.nitidus*	0.005155	0.068665
SC24	CS18	GD28	*R.nitidus*	0.028777	0.066904
MY4	CS18	GD28	*R.nitidus*	0.029134	0.065954
CS30	CS18	GD28	*R.nitidus*	-0.013278	0.065725
JW7	CS18	GD28	*R.nitidus*	0.03016	0.072736
SC27	CS18	GD28	*R.nitidus*	0.031797	0.06642
SC42	CS18	GD28	*R.nitidus*	0.241401	0.062655
SC28	CS18	GD28	*R.nitidus*	-0.01171	0.065291
GD26	CS19	GD28	*R.nitidus*	0.04	0.066493
SC32	CS18	GD28	*R.nitidus*	0.078783	0.065179
SC44	CS18	GD28	*R.nitidus*	0.035549	0.066524
SC5	CS18	GD28	*R.nitidus*	0.033108	0.069192
SC18	CS18	GD28	*R.nitidus*	0.066347	0.066931
GD29	CS19	GD28	*R.nitidus*	0.091523	0.067086
CS6	CS18	GD28	*R.nitidus*	0.008876	0.06517
SC45	CS18	GD28	*R.nitidus*	0.070938	0.065293
JO7	CS19	GD28	*R.nitidus*	0.008316	0.075051
SC35	CS18	GD28	*R.nitidus*	0.09	0.064563
GD30	CS19	GD28	*R.nitidus*	0.098415	0.069548
GD35	CS19	GD28	*R.nitidus*	0.017625	0.068167
JO5	CS18	GD28	*R.nitidus*	0.066154	0.067553
JO8	CS19	GD28	*R.nitidus*	0.02063	0.0724
MY6	CS18	GD28	*R.nitidus*	0.027048	0.064934
CS13	CS19	GD28	*R.nitidus*	-0.035654	0.070915
GD31	CS19	GD28	*R.nitidus*	0.064777	0.069477
JW4	CS18	GD28	*R.nitidus*	0.213554	0.067217
GD32	CS19	GD28	*R.nitidus*	0.015833	0.069289
CS14	CS19	GD28	*R.nitidus*	0.010851	0.071233
CS11	CS19	GD28	*R.nitidus*	0	0.06974
CS12	CS19	GD28	*R.nitidus*	0.00605	0.068856
GD45	CS19	GD28	*R.nitidus*	0.024605	0.072154
GD50	CS19	GD28	*R.nitidus*	0.035242	0.072719
GD41	CS19	GD28	*R.nitidus*	-0.006623	0.066604
JO1	CS18	GD28	*R.nitidus*	0.017268	0.064567
GD21	CS19	GD28	*R.nitidus*	0.135994	0.067989
GD22	CS19	GD28	*R.nitidus*	0.071429	0.066689
CS15	CS19	GD28	*R.nitidus*	0.02439	0.06918
GD25	CS19	GD28	*R.nitidus*	0.048411	0.06406
GD40	CS19	GD28	*R.nitidus*	0.052713	0.067959
GD34	CS19	GD28	*R.nitidus*	0.008922	0.066291
CS22	CS19	GD28	*R.nitidus*	0.013865	0.068276
CS21	CS19	GD28	*R.nitidus*	-0.037175	0.067927
CS20	CS19	GD28	*R.nitidus*	-0.028336	0.067363
CS23	CS19	GD28	*R.nitidus*	-0.033881	0.063968

CS17	CS19	GD28	*R.nitidus*	0.01847	0.071442
GD33	CS19	GD28	*R.nitidus*	-0.006107	0.068682
GD42	CS19	GD28	*R.nitidus*	0.004481	0.067745
CS31	CS19	GD28	*R.nitidus*	0.003398	0.067999
GD49	CS19	GD28	*R.nitidus*	0.07479	0.070236
GD46	CS19	GD28	*R.nitidus*	0.004542	0.069408
CS16	CS19	GD28	*R.nitidus*	-0.008354	0.06722
GD44	CS19	GD28	*R.nitidus*	-0.003984	0.067555
GD48	CS19	GD28	*R.nitidus*	0.039396	0.06827
CS3	CS19	GD28	*R.nitidus*	-0.027985	0.067917
CS24	CS19	GD28	*R.nitidus*	-0.00816	0.067283
CS25	CS19	GD28	*R.nitidus*	0.01105	0.071672
SC28	CS19	GD28	*R.nitidus*	-0.040767	0.064321
SC31	CS19	GD28	*R.nitidus*	0.003737	0.066644
CS18	CS19	GD28	*R.nitidus*	-0.037229	0.067535
SC15	CS19	GD28	*R.nitidus*	0.004174	0.068109
CS30	CS19	GD28	*R.nitidus*	-0.036454	0.067085
CS6	CS19	GD28	*R.nitidus*	-0.033236	0.067323
JO7	CS20	GD28	*R.nitidus*	0.019685	0.072536
SC14	CS19	GD28	*R.nitidus*	0.018779	0.068675
SC6	CS19	GD28	*R.nitidus*	-0.026899	0.067192
SC5	CS19	GD28	*R.nitidus*	0	0.07113
JW7	CS19	GD28	*R.nitidus*	-0.003824	0.072583
SC24	CS19	GD28	*R.nitidus*	0.000818	0.068605
SC18	CS19	GD28	*R.nitidus*	0.035088	0.068407
CS28	CS19	GD28	*R.nitidus*	-0.045769	0.06797
JO3	CS19	GD28	*R.nitidus*	0.031905	0.070651
SC32	CS19	GD28	*R.nitidus*	0.0472	0.068374
SC10	CS19	GD28	*R.nitidus*	0.056634	0.068162
JW6	CS19	GD28	*R.nitidus*	0.052128	0.072626
CS4	CS19	GD28	*R.nitidus*	-0.020087	0.068235
SC26	CS19	GD28	*R.nitidus*	-0.036434	0.06779
SC35	CS19	GD28	*R.nitidus*	0.06862	0.067179
SC27	CS19	GD28	*R.nitidus*	0.008929	0.069162
JO8	CS20	GD28	*R.nitidus*	0.031612	0.072566
JO5	CS19	GD28	*R.nitidus*	0.02589	0.067967
SC12	CS19	GD28	*R.nitidus*	-0.010172	0.067283
SC44	CS19	GD28	*R.nitidus*	8.00E-04	0.067821
GD32	CS20	GD28	*R.nitidus*	0.044571	0.067943
SC42	CS19	GD28	*R.nitidus*	0.213997	0.064291
MY6	CS19	GD28	*R.nitidus*	-0.000767	0.068042
GD25	CS20	GD28	*R.nitidus*	0.08015	0.066435
GD21	CS20	GD28	*R.nitidus*	0.173631	0.066062
JO1	CS19	GD28	*R.nitidus*	-0.019531	0.067147
JW4	CS19	GD28	*R.nitidus*	0.181676	0.068122
SC34	CS19	GD28	*R.nitidus*	0.034981	0.066545
GD34	CS20	GD28	*R.nitidus*	0.036429	0.065807
GD29	CS20	GD28	*R.nitidus*	0.112766	0.065853
CS12	CS20	GD28	*R.nitidus*	0.029056	0.067304
SC45	CS19	GD28	*R.nitidus*	0.04422	0.067622
GD30	CS20	GD28	*R.nitidus*	0.123408	0.068121
MY4	CS19	GD28	*R.nitidus*	-0.009554	0.069618
GD41	CS20	GD28	*R.nitidus*	0.026874	0.066137
GD33	CS20	GD28	*R.nitidus*	0.030021	0.066863
GD40	CS20	GD28	*R.nitidus*	0.07132	0.064326
GD50	CS20	GD28	*R.nitidus*	0.02954	0.072143

GD22	CS20	GD28	*R.nitidus*	0.094101	0.064531
GD42	CS20	GD28	*R.nitidus*	0.033732	0.067269
GD31	CS20	GD28	*R.nitidus*	0.066246	0.068933
CS11	CS20	GD28	*R.nitidus*	0.020015	0.06644
GD46	CS20	GD28	*R.nitidus*	0.023878	0.067174
GD48	CS20	GD28	*R.nitidus*	0.053945	0.066438
CS21	CS20	GD28	*R.nitidus*	-0.011055	0.063957
CS3	CS20	GD28	*R.nitidus*	0.007543	0.062092
CS24	CS20	GD28	*R.nitidus*	0.014214	0.06699
CS30	CS20	GD28	*R.nitidus*	-0.025118	0.066332
GD35	CS20	GD28	*R.nitidus*	0.046805	0.065982
CS13	CS20	GD28	*R.nitidus*	-0.000791	0.068121
SC5	CS20	GD28	*R.nitidus*	0.022918	0.069859
CS15	CS20	GD28	*R.nitidus*	0.046458	0.067413
GD44	CS20	GD28	*R.nitidus*	0.021341	0.067661
GD26	CS20	GD28	*R.nitidus*	0.057816	0.066416
SC14	CS20	GD28	*R.nitidus*	0.039275	0.065171
GD45	CS20	GD28	*R.nitidus*	0.041952	0.071829
CS16	CS20	GD28	*R.nitidus*	0.025385	0.06667
GD49	CS20	GD28	*R.nitidus*	0.1136	0.070172
SC28	CS20	GD28	*R.nitidus*	-0.023438	0.06506
CS14	CS20	GD28	*R.nitidus*	0.04072	0.06758
CS18	CS20	GD28	*R.nitidus*	-0.019361	0.065544
SC32	CS20	GD28	*R.nitidus*	0.068887	0.06552
CS17	CS20	GD28	*R.nitidus*	0.049615	0.069131
CS19	CS20	GD28	*R.nitidus*	0.028336	0.067363
CS25	CS20	GD28	*R.nitidus*	0.030443	0.067141
CS22	CS20	GD28	*R.nitidus*	0.055993	0.067993
SC26	CS20	GD28	*R.nitidus*	-0.017067	0.06692
CS4	CS20	GD28	*R.nitidus*	0	0.065929
SC6	CS20	GD28	*R.nitidus*	-0.008828	0.065462
CS23	CS20	GD28	*R.nitidus*	-0.020677	0.064082
SC35	CS20	GD28	*R.nitidus*	0.077903	0.064391
CS31	CS20	GD28	*R.nitidus*	0.015032	0.066161
JW6	CS20	GD28	*R.nitidus*	0.019646	0.073033
CS28	CS20	GD28	*R.nitidus*	-0.01956	0.068191
SC18	CS20	GD28	*R.nitidus*	0.06068	0.066222
SC31	CS20	GD28	*R.nitidus*	0.020046	0.065285
SC15	CS20	GD28	*R.nitidus*	0.013754	0.068369
SC34	CS20	GD28	*R.nitidus*	0.053064	0.065606
SC12	CS20	GD28	*R.nitidus*	0.015528	0.066508
SC27	CS20	GD28	*R.nitidus*	0.015994	0.066074
GD21	CS21	GD28	*R.nitidus*	0.172366	0.065626
GD26	CS21	GD28	*R.nitidus*	0.069435	0.066078
SC10	CS20	GD28	*R.nitidus*	0.090267	0.065394
CS6	CS20	GD28	*R.nitidus*	-0.00641	0.06412
JW7	CS20	GD28	*R.nitidus*	0.007612	0.072726
JO1	CS20	GD28	*R.nitidus*	0.000762	0.065864
GD25	CS21	GD28	*R.nitidus*	0.09003	0.066854
GD31	CS21	GD28	*R.nitidus*	0.071094	0.068744
SC45	CS20	GD28	*R.nitidus*	0.057767	0.065323
SC42	CS20	GD28	*R.nitidus*	0.229856	0.064091
JO3	CS20	GD28	*R.nitidus*	0.050667	0.068645
JW4	CS20	GD28	*R.nitidus*	0.194167	0.066865
SC44	CS20	GD28	*R.nitidus*	0.026398	0.06696
JO7	CS21	GD28	*R.nitidus*	0.053307	0.073466

JO8	CS21	GD28	*R.nitidus*	0.069792	0.071574
SC24	CS20	GD28	*R.nitidus*	0.019078	0.066744
MY6	CS20	GD28	*R.nitidus*	0.011211	0.065201
GD44	CS21	GD28	*R.nitidus*	0.028137	0.066732
MY4	CS20	GD28	*R.nitidus*	0.011346	0.066621
GD29	CS21	GD28	*R.nitidus*	0.126145	0.064452
CS11	CS21	GD28	*R.nitidus*	0.031907	0.066159
GD40	CS21	GD28	*R.nitidus*	0.082902	0.065543
GD34	CS21	GD28	*R.nitidus*	0.041847	0.065814
JO5	CS20	GD28	*R.nitidus*	0.053585	0.066064
GD35	CS21	GD28	*R.nitidus*	0.057455	0.066586
GD22	CS21	GD28	*R.nitidus*	0.095568	0.064748
GD42	CS21	GD28	*R.nitidus*	0.05278	0.06695
GD50	CS21	GD28	*R.nitidus*	0.055794	0.071994
GD45	CS21	GD28	*R.nitidus*	0.048293	0.070712
GD41	CS21	GD28	*R.nitidus*	0.033684	0.065693
CS17	CS21	GD28	*R.nitidus*	0.056362	0.068321
CS15	CS21	GD28	*R.nitidus*	0.059052	0.066913
GD46	CS21	GD28	*R.nitidus*	0.032951	0.067708
CS20	CS21	GD28	*R.nitidus*	0.011055	0.063957
GD48	CS21	GD28	*R.nitidus*	0.073416	0.067228
GD30	CS21	GD28	*R.nitidus*	0.142631	0.067757
CS12	CS21	GD28	*R.nitidus*	0.042484	0.067842
GD33	CS21	GD28	*R.nitidus*	0.04134	0.067317
CS18	CS21	GD28	*R.nitidus*	-0.014403	0.064646
CS16	CS21	GD28	*R.nitidus*	0.041763	0.066532
SC26	CS21	GD28	*R.nitidus*	-0.010929	0.065682
CS13	CS21	GD28	*R.nitidus*	0.000812	0.069403
CS4	CS21	GD28	*R.nitidus*	-0.003436	0.065867
SC14	CS21	GD28	*R.nitidus*	0.058015	0.064792
GD32	CS21	GD28	*R.nitidus*	0.050202	0.069901
CS14	CS21	GD28	*R.nitidus*	0.054618	0.068176
CS30	CS21	GD28	*R.nitidus*	-0.023444	0.065297
CS22	CS21	GD28	*R.nitidus*	0.053663	0.066731
CS19	CS21	GD28	*R.nitidus*	0.037175	0.067927
GD49	CS21	GD28	*R.nitidus*	0.112943	0.067255
SC12	CS21	GD28	*R.nitidus*	0.018011	0.06618
CS3	CS21	GD28	*R.nitidus*	0.013462	0.065295
SC10	CS21	GD28	*R.nitidus*	0.092874	0.065162
SC6	CS21	GD28	*R.nitidus*	0.00399	0.065844
CS24	CS21	GD28	*R.nitidus*	0.016115	0.065047
SC15	CS21	GD28	*R.nitidus*	0.023007	0.064836
SC35	CS21	GD28	*R.nitidus*	0.092879	0.064662
CS31	CS21	GD28	*R.nitidus*	0.026379	0.065365
SC42	CS21	GD28	*R.nitidus*	0.24586	0.062913
SC27	CS21	GD28	*R.nitidus*	0.026255	0.064387
SC28	CS21	GD28	*R.nitidus*	-0.010133	0.065549
SC32	CS21	GD28	*R.nitidus*	0.080831	0.063646
SC34	CS21	GD28	*R.nitidus*	0.057604	0.065065
CS23	CS21	GD28	*R.nitidus*	-0.007056	0.064201
CS28	CS21	GD28	*R.nitidus*	-0.016892	0.067341
CS25	CS21	GD28	*R.nitidus*	0.04093	0.068428
SC5	CS21	GD28	*R.nitidus*	0.031988	0.067314
JO1	CS21	GD28	*R.nitidus*	0.013857	0.066064
SC24	CS21	GD28	*R.nitidus*	0.019826	0.065765
JO7	CS22	GD28	*R.nitidus*	-0.019958	0.072227

SC45	CS21	GD28	*R.nitidus*	0.065152	0.065497
SC18	CS21	GD28	*R.nitidus*	0.060729	0.066097
JW4	CS21	GD28	*R.nitidus*	0.220141	0.06732
SC31	CS21	GD28	*R.nitidus*	0.037441	0.064454
MY4	CS21	GD28	*R.nitidus*	0.024559	0.066035
JW7	CS21	GD28	*R.nitidus*	0.041627	0.069169
SC44	CS21	GD28	*R.nitidus*	0.033977	0.066135
CS6	CS21	GD28	*R.nitidus*	0.003	0.063841
GD40	CS22	GD28	*R.nitidus*	0.034743	0.064104
JW6	CS21	GD28	*R.nitidus*	0.054435	0.071998
GD49	CS22	GD28	*R.nitidus*	0.067332	0.068082
GD45	CS22	GD28	*R.nitidus*	0.016536	0.070709
MY6	CS21	GD28	*R.nitidus*	0.025622	0.065161
GD46	CS22	GD28	*R.nitidus*	-0.024981	0.064763
JO3	CS21	GD28	*R.nitidus*	0.07489	0.069863
CS11	CS22	GD28	*R.nitidus*	-0.021446	0.06801
JO8	CS22	GD28	*R.nitidus*	-0.001116	0.073112
JO5	CS21	GD28	*R.nitidus*	0.061162	0.065413
GD31	CS22	GD28	*R.nitidus*	0.022293	0.067412
CS17	CS22	GD28	*R.nitidus*	0.009442	0.069932
GD22	CS22	GD28	*R.nitidus*	0.04062	0.064674
CS13	CS22	GD28	*R.nitidus*	-0.066335	0.067339
GD25	CS22	GD28	*R.nitidus*	0.027714	0.064056
CS12	CS22	GD28	*R.nitidus*	-0.005917	0.065103
GD34	CS22	GD28	*R.nitidus*	0.002221	0.064826
GD30	CS22	GD28	*R.nitidus*	0.092715	0.06735
GD50	CS22	GD28	*R.nitidus*	0.002708	0.070718
GD48	CS22	GD28	*R.nitidus*	0.032099	0.068731
GD21	CS22	GD28	*R.nitidus*	0.118805	0.064299
CS20	CS22	GD28	*R.nitidus*	-0.055993	0.067993
GD32	CS22	GD28	*R.nitidus*	0.004303	0.069029
GD26	CS22	GD28	*R.nitidus*	0.029726	0.066714
CS30	CS22	GD28	*R.nitidus*	-0.071189	0.066324
GD42	CS22	GD28	*R.nitidus*	-0.002187	0.066394
GD33	CS22	GD28	*R.nitidus*	-0.014837	0.067014
GD29	CS22	GD28	*R.nitidus*	0.08584	0.065456
CS14	CS22	GD28	*R.nitidus*	0.00495	0.067868
GD44	CS22	GD28	*R.nitidus*	-0.01536	0.066889
CS19	CS22	GD28	*R.nitidus*	-0.013865	0.068276
GD35	CS22	GD28	*R.nitidus*	0.001479	0.066376
CS16	CS22	GD28	*R.nitidus*	-0.014778	0.066635
CS28	CS22	GD28	*R.nitidus*	-0.070191	0.066363
CS25	CS22	GD28	*R.nitidus*	-0.019983	0.068633
CS15	CS22	GD28	*R.nitidus*	0.004812	0.066671
CS23	CS22	GD28	*R.nitidus*	-0.061538	0.06594
CS18	CS22	GD28	*R.nitidus*	-0.06469	0.064741
SC31	CS22	GD28	*R.nitidus*	-0.017638	0.065573
GD41	CS22	GD28	*R.nitidus*	-0.014232	0.064884
JW4	CS22	GD28	*R.nitidus*	0.190204	0.066206
JO3	CS22	GD28	*R.nitidus*	0.018034	0.069487
SC10	CS22	GD28	*R.nitidus*	0.05679	0.065194
SC42	CS22	GD28	*R.nitidus*	0.207277	0.063448
SC6	CS22	GD28	*R.nitidus*	-0.04827	0.066648
CS24	CS22	GD28	*R.nitidus*	-0.049638	0.065923
CS21	CS22	GD28	*R.nitidus*	-0.053663	0.066731
SC35	CS22	GD28	*R.nitidus*	0.044444	0.066929

SC24	CS22	GD28	*R.nitidus*	-0.030159	0.066519
CS3	CS22	GD28	*R.nitidus*	-0.041425	0.066212
SC5	CS22	GD28	*R.nitidus*	-0.015485	0.069728
SC34	CS22	GD28	*R.nitidus*	0.009063	0.065094
GD21	CS23	GD28	*R.nitidus*	0.16875	0.066613
SC15	CS22	GD28	*R.nitidus*	-0.016639	0.067098
JW6	CS22	GD28	*R.nitidus*	0.038502	0.072585
SC27	CS22	GD28	*R.nitidus*	-0.015237	0.066072
MY4	CS22	GD28	*R.nitidus*	-0.035541	0.066917
SC12	CS22	GD28	*R.nitidus*	-0.030428	0.066914
GD26	CS23	GD28	*R.nitidus*	0.073381	0.0636
MY6	CS22	GD28	*R.nitidus*	-0.029459	0.065268
JO8	CS23	GD28	*R.nitidus*	0.036881	0.072344
CS31	CS22	GD28	*R.nitidus*	-0.03	0.065908
JO1	CS22	GD28	*R.nitidus*	-0.025974	0.06669
SC26	CS22	GD28	*R.nitidus*	-0.066406	0.065162
SC18	CS22	GD28	*R.nitidus*	0.00659	0.067795
CS4	CS22	GD28	*R.nitidus*	-0.063674	0.064997
GD32	CS23	GD28	*R.nitidus*	0.066884	0.068681
SC14	CS22	GD28	*R.nitidus*	0.001553	0.066899
CS6	CS22	GD28	*R.nitidus*	-0.054767	0.0659
SC45	CS22	GD28	*R.nitidus*	0.017516	0.066667
JO5	CS22	GD28	*R.nitidus*	0.006405	0.066833
SC28	CS22	GD28	*R.nitidus*	-0.062857	0.065612
SC32	CS22	GD28	*R.nitidus*	0.033279	0.065782
SC44	CS22	GD28	*R.nitidus*	-0.018125	0.067143
GD40	CS23	GD28	*R.nitidus*	0.078947	0.064937
GD33	CS23	GD28	*R.nitidus*	0.037116	0.066999
CS11	CS23	GD28	*R.nitidus*	0.044118	0.067995
GD35	CS23	GD28	*R.nitidus*	0.056576	0.066341
JO7	CS23	GD28	*R.nitidus*	0.033401	0.074364
GD44	CS23	GD28	*R.nitidus*	0.039816	0.066389
JW7	CS22	GD28	*R.nitidus*	-0.006972	0.071052
GD30	CS23	GD28	*R.nitidus*	0.131537	0.068145
GD49	CS23	GD28	*R.nitidus*	0.101958	0.067323
GD22	CS23	GD28	*R.nitidus*	0.100282	0.064959
GD41	CS23	GD28	*R.nitidus*	0.039007	0.06673
GD29	CS23	GD28	*R.nitidus*	0.129643	0.064317
GD25	CS23	GD28	*R.nitidus*	0.095518	0.06345
GD50	CS23	GD28	*R.nitidus*	0.067869	0.070062
CS13	CS23	GD28	*R.nitidus*	0.013721	0.070228
GD46	CS23	GD28	*R.nitidus*	0.030627	0.065752
GD31	CS23	GD28	*R.nitidus*	0.082409	0.066856
GD34	CS23	GD28	*R.nitidus*	0.052478	0.066343
GD42	CS23	GD28	*R.nitidus*	0.052857	0.068621
CS12	CS23	GD28	*R.nitidus*	0.045381	0.067228
CS28	CS23	GD28	*R.nitidus*	-0.007589	0.067867
CS3	CS23	GD28	*R.nitidus*	0.018304	0.063791
CS30	CS23	GD28	*R.nitidus*	-0.014038	0.064791
CS16	CS23	GD28	*R.nitidus*	0.032813	0.066737
SC5	CS23	GD28	*R.nitidus*	0.030511	0.069894
SC12	CS23	GD28	*R.nitidus*	0.010236	0.066429
CS25	CS23	GD28	*R.nitidus*	0.030881	0.069776
CS4	CS23	GD28	*R.nitidus*	0.015625	0.064955
CS24	CS23	GD28	*R.nitidus*	0.023097	0.067042
GD45	CS23	GD28	*R.nitidus*	0.047779	0.072378

CS15	CS23	GD28	*R.nitidus*	0.068536	0.067392
CS14	CS23	GD28	*R.nitidus*	0.061864	0.068601
CS18	CS23	GD28	*R.nitidus*	-0.004859	0.066106
SC31	CS23	GD28	*R.nitidus*	0.038695	0.06535
SC15	CS23	GD28	*R.nitidus*	0.040098	0.066145
SC44	CS23	GD28	*R.nitidus*	0.028059	0.066189
GD48	CS23	GD28	*R.nitidus*	0.082278	0.068404
CS21	CS23	GD28	*R.nitidus*	0.007056	0.064201
CS17	CS23	GD28	*R.nitidus*	0.063652	0.068685
SC27	CS23	GD28	*R.nitidus*	0.031546	0.065897
SC42	CS23	GD28	*R.nitidus*	0.247332	0.062433
SC45	CS23	GD28	*R.nitidus*	0.07027	0.065674
JW4	CS23	GD28	*R.nitidus*	0.208238	0.066015
SC35	CS23	GD28	*R.nitidus*	0.090008	0.065238
SC26	CS23	GD28	*R.nitidus*	-0.006088	0.066963
CS22	CS23	GD28	*R.nitidus*	0.061538	0.06594
MY6	CS23	GD28	*R.nitidus*	0.026965	0.063972
SC28	CS23	GD28	*R.nitidus*	-0.033071	0.06451
CS31	CS23	GD28	*R.nitidus*	0.030879	0.066173
SC10	CS23	GD28	*R.nitidus*	0.099602	0.067551
CS19	CS23	GD28	*R.nitidus*	0.033881	0.063968
JW7	CS23	GD28	*R.nitidus*	0.024276	0.070772
SC14	CS23	GD28	*R.nitidus*	0.049736	0.064945
SC18	CS23	GD28	*R.nitidus*	0.06759	0.066508
CS6	CS23	GD28	*R.nitidus*	0.01131	0.063819
SC6	CS23	GD28	*R.nitidus*	0.015212	0.067077
SC32	CS23	GD28	*R.nitidus*	0.075228	0.067148
JO7	CS24	GD28	*R.nitidus*	0.027197	0.074186
CS12	CS24	GD28	*R.nitidus*	0.037768	0.06766
MY4	CS23	GD28	*R.nitidus*	0.026965	0.066309
SC34	CS23	GD28	*R.nitidus*	0.061178	0.064578
SC24	CS23	GD28	*R.nitidus*	0.027451	0.066486
JW6	CS23	GD28	*R.nitidus*	0.032385	0.072949
GD25	CS24	GD28	*R.nitidus*	0.054795	0.065367
JO1	CS23	GD28	*R.nitidus*	0.014832	0.066867
JO5	CS23	GD28	*R.nitidus*	0.05325	0.066161
GD29	CS24	GD28	*R.nitidus*	0.113962	0.065004
GD35	CS24	GD28	*R.nitidus*	0.032875	0.065723
JO3	CS23	GD28	*R.nitidus*	0.062943	0.068738
JO8	CS24	GD28	*R.nitidus*	0.026077	0.072198
GD21	CS24	GD28	*R.nitidus*	0.158892	0.066156
GD22	CS24	GD28	*R.nitidus*	0.080921	0.066076
GD33	CS24	GD28	*R.nitidus*	0.019832	0.067032
GD46	CS24	GD28	*R.nitidus*	0.014471	0.065636
CS13	CS24	GD28	*R.nitidus*	-0.011102	0.06927
GD32	CS24	GD28	*R.nitidus*	0.043253	0.068754
GD44	CS24	GD28	*R.nitidus*	0.021259	0.068097
CS11	CS24	GD28	*R.nitidus*	0.021739	0.067828
GD48	CS24	GD28	*R.nitidus*	0.060507	0.067125
GD41	CS24	GD28	*R.nitidus*	0.017571	0.065806
GD42	CS24	GD28	*R.nitidus*	0.030643	0.066871
GD31	CS24	GD28	*R.nitidus*	0.064337	0.068086
CS18	CS24	GD28	*R.nitidus*	-0.021277	0.065313
GD34	CS24	GD28	*R.nitidus*	0.031603	0.066827
GD30	CS24	GD28	*R.nitidus*	0.11755	0.067506
CS19	CS24	GD28	*R.nitidus*	0.00816	0.067283

CS14	CS24	GD28	*R.nitidus*	0.035473	0.070268
CS23	CS24	GD28	*R.nitidus*	-0.023097	0.067042
GD26	CS24	GD28	*R.nitidus*	0.052267	0.066154
CS3	CS24	GD28	*R.nitidus*	-0.017782	0.066812
CS15	CS24	GD28	*R.nitidus*	0.044481	0.068922
CS17	CS24	GD28	*R.nitidus*	0.043706	0.070165
GD45	CS24	GD28	*R.nitidus*	0.042517	0.069689
GD50	CS24	GD28	*R.nitidus*	0.025	0.070671
GD40	CS24	GD28	*R.nitidus*	0.073958	0.064666
CS28	CS24	GD28	*R.nitidus*	-0.017071	0.067614
SC14	CS24	GD28	*R.nitidus*	0.030278	0.065119
GD49	CS24	GD28	*R.nitidus*	0.098007	0.06885
CS6	CS24	GD28	*R.nitidus*	-0.009285	0.065805
CS4	CS24	GD28	*R.nitidus*	-0.003817	0.064978
CS20	CS24	GD28	*R.nitidus*	-0.014214	0.06699
JW4	CS24	GD28	*R.nitidus*	0.220353	0.066508
SC44	CS24	GD28	*R.nitidus*	0.020525	0.066508
CS21	CS24	GD28	*R.nitidus*	-0.016115	0.065047
SC24	CS24	GD28	*R.nitidus*	0.004827	0.065961
CS30	CS24	GD28	*R.nitidus*	-0.042424	0.06583
JO8	CS25	GD28	*R.nitidus*	0.012672	0.07485
CS22	CS24	GD28	*R.nitidus*	0.049638	0.065923
SC28	CS24	GD28	*R.nitidus*	-0.026464	0.066263
MY6	CS24	GD28	*R.nitidus*	0.007837	0.067015
CS25	CS24	GD28	*R.nitidus*	0.023663	0.069558
SC32	CS24	GD28	*R.nitidus*	0.072065	0.066875
SC5	CS24	GD28	*R.nitidus*	0.026138	0.070245
CS16	CS24	GD28	*R.nitidus*	0.024834	0.065321
CS31	CS24	GD28	*R.nitidus*	0.015345	0.066092
SC15	CS24	GD28	*R.nitidus*	0.005093	0.06552
MY4	CS24	GD28	*R.nitidus*	0.007806	0.067515
SC6	CS24	GD28	*R.nitidus*	-0.001631	0.067885
SC10	CS24	GD28	*R.nitidus*	0.094262	0.066003
SC12	CS24	GD28	*R.nitidus*	0.004085	0.067204
SC34	CS24	GD28	*R.nitidus*	0.057279	0.063809
JO1	CS24	GD28	*R.nitidus*	0	0.066447
GD26	CS25	GD28	*R.nitidus*	0.044313	0.067072
SC31	CS24	GD28	*R.nitidus*	0.024409	0.066561
SC45	CS24	GD28	*R.nitidus*	0.049844	0.066187
JW6	CS24	GD28	*R.nitidus*	0.05491	0.071308
SC18	CS24	GD28	*R.nitidus*	0.03894	0.067
SC35	CS24	GD28	*R.nitidus*	0.078905	0.065179
GD21	CS25	GD28	*R.nitidus*	0.14771	0.066131
SC26	CS24	GD28	*R.nitidus*	-0.028436	0.066323
GD25	CS25	GD28	*R.nitidus*	0.069455	0.066159
JO3	CS24	GD28	*R.nitidus*	0.029412	0.069452
GD32	CS25	GD28	*R.nitidus*	0.019442	0.070277
SC27	CS24	GD28	*R.nitidus*	0.008446	0.064832
SC42	CS24	GD28	*R.nitidus*	0.241514	0.06295
GD34	CS25	GD28	*R.nitidus*	0.020104	0.067012
JW7	CS24	GD28	*R.nitidus*	0.01433	0.071346
JO7	CS25	GD28	*R.nitidus*	0.015512	0.072787
GD31	CS25	GD28	*R.nitidus*	0.0584	0.0679
JO5	CS24	GD28	*R.nitidus*	0.042345	0.065759
CS11	CS25	GD28	*R.nitidus*	0	0.069147
GD48	CS25	GD28	*R.nitidus*	0.048173	0.068196

GD22	CS25	GD28	*R.nitidus*	0.082902	0.067344
GD42	CS25	GD28	*R.nitidus*	0.011194	0.067599
GD41	CS25	GD28	*R.nitidus*	0.002182	0.065447
GD33	CS25	GD28	*R.nitidus*	0.008455	0.068222
GD30	CS25	GD28	*R.nitidus*	0.106438	0.072253
GD44	CS25	GD28	*R.nitidus*	0.013503	0.067858
GD46	CS25	GD28	*R.nitidus*	-0.008258	0.068728
GD29	CS25	GD28	*R.nitidus*	0.1	0.067106
CS12	CS25	GD28	*R.nitidus*	0.011986	0.068677
GD49	CS25	GD28	*R.nitidus*	0.089076	0.069708
GD45	CS25	GD28	*R.nitidus*	0.037004	0.072493
CS30	CS25	GD28	*R.nitidus*	-0.039169	0.066359
SC14	CS25	GD28	*R.nitidus*	0.014786	0.068776
CS3	CS25	GD28	*R.nitidus*	-0.040036	0.066146
CS14	CS25	GD28	*R.nitidus*	0.020425	0.070274
CS17	CS25	GD28	*R.nitidus*	0.028319	0.072804
SC12	CS25	GD28	*R.nitidus*	-0.020194	0.067901
GD35	CS25	GD28	*R.nitidus*	0.025522	0.067582
CS13	CS25	GD28	*R.nitidus*	-0.031915	0.071498
GD40	CS25	GD28	*R.nitidus*	0.054315	0.066316
CS19	CS25	GD28	*R.nitidus*	-0.01105	0.071672
CS16	CS25	GD28	*R.nitidus*	0.007194	0.067289
CS18	CS25	GD28	*R.nitidus*	-0.056895	0.069644
CS15	CS25	GD28	*R.nitidus*	0.022222	0.07044
SC5	CS25	GD28	*R.nitidus*	-0.00712	0.069454
CS20	CS25	GD28	*R.nitidus*	-0.030443	0.067141
GD50	CS25	GD28	*R.nitidus*	0.024658	0.071593
SC10	CS25	GD28	*R.nitidus*	0.049674	0.067247
SC26	CS25	GD28	*R.nitidus*	-0.037096	0.066543
CS4	CS25	GD28	*R.nitidus*	-0.015358	0.067873
SC18	CS25	GD28	*R.nitidus*	0.026622	0.0691
CS24	CS25	GD28	*R.nitidus*	-0.023663	0.069558
JW7	CS25	GD28	*R.nitidus*	-0.00779	0.072981
JW4	CS25	GD28	*R.nitidus*	0.180982	0.069224
SC6	CS25	GD28	*R.nitidus*	-0.026059	0.066155
SC34	CS25	GD28	*R.nitidus*	0.036364	0.067183
CS21	CS25	GD28	*R.nitidus*	-0.04093	0.068428
CS28	CS25	GD28	*R.nitidus*	-0.055993	0.069777
CS23	CS25	GD28	*R.nitidus*	-0.030881	0.069776
MY6	CS25	GD28	*R.nitidus*	-0.002383	0.06841
SC27	CS25	GD28	*R.nitidus*	0.000814	0.067847
CS22	CS25	GD28	*R.nitidus*	0.019983	0.068633
CS31	CS25	GD28	*R.nitidus*	-0.008264	0.067407
SC31	CS25	GD28	*R.nitidus*	0.005348	0.066895
SC28	CS25	GD28	*R.nitidus*	-0.052033	0.067504
JW6	CS25	GD28	*R.nitidus*	0.047862	0.072618
JO7	CS28	GD28	*R.nitidus*	0.042316	0.072248
CS6	CS25	GD28	*R.nitidus*	-0.039106	0.066618
SC15	CS25	GD28	*R.nitidus*	0.005013	0.069209
SC42	CS25	GD28	*R.nitidus*	0.222295	0.064526
SC24	CS25	GD28	*R.nitidus*	-0.000822	0.068386
CS11	CS28	GD28	*R.nitidus*	0.045918	0.068946
SC32	CS25	GD28	*R.nitidus*	0.050199	0.068957
SC45	CS25	GD28	*R.nitidus*	0.048896	0.066691
MY4	CS25	GD28	*R.nitidus*	-0.010277	0.066679
SC35	CS25	GD28	*R.nitidus*	0.061051	0.065195

JO5	CS25	GD28	*R.nitidus*	0.036496	0.067405
GD26	CS28	GD28	*R.nitidus*	0.079239	0.065965
SC44	CS25	GD28	*R.nitidus*	-0.006541	0.069238
JO3	CS25	GD28	*R.nitidus*	0.037104	0.072119
GD29	CS28	GD28	*R.nitidus*	0.138009	0.065977
JO1	CS25	GD28	*R.nitidus*	-0.025621	0.067288
GD31	CS28	GD28	*R.nitidus*	0.083963	0.067938
GD41	CS28	GD28	*R.nitidus*	0.037307	0.066436
GD32	CS28	GD28	*R.nitidus*	0.062665	0.070127
JO8	CS28	GD28	*R.nitidus*	0.023516	0.07312
GD30	CS28	GD28	*R.nitidus*	0.148673	0.068111
GD49	CS28	GD28	*R.nitidus*	0.129362	0.070329
GD44	CS28	GD28	*R.nitidus*	0.04424	0.068177
GD34	CS28	GD28	*R.nitidus*	0.074016	0.065895
CS12	CS28	GD28	*R.nitidus*	0.058876	0.066978
GD33	CS28	GD28	*R.nitidus*	0.0472	0.067626
GD22	CS28	GD28	*R.nitidus*	0.104863	0.065681
CS19	CS28	GD28	*R.nitidus*	0.045769	0.06797
GD48	CS28	GD28	*R.nitidus*	0.090439	0.06824
GD35	CS28	GD28	*R.nitidus*	0.063542	0.066497
CS16	CS28	GD28	*R.nitidus*	0.030848	0.066452
GD21	CS28	GD28	*R.nitidus*	0.174118	0.066731
GD42	CS28	GD28	*R.nitidus*	0.055285	0.06726
CS13	CS28	GD28	*R.nitidus*	0.000873	0.070591
CS23	CS28	GD28	*R.nitidus*	0.007589	0.067867
GD45	CS28	GD28	*R.nitidus*	0.067663	0.070761
CS22	CS28	GD28	*R.nitidus*	0.070191	0.066363
GD46	CS28	GD28	*R.nitidus*	0.0313	0.066035
CS25	CS28	GD28	*R.nitidus*	0.055993	0.069777
CS30	CS28	GD28	*R.nitidus*	-0.010526	0.067131
CS14	CS28	GD28	*R.nitidus*	0.060052	0.069145
GD40	CS28	GD28	*R.nitidus*	0.078696	0.065943
CS15	CS28	GD28	*R.nitidus*	0.051993	0.066052
CS24	CS28	GD28	*R.nitidus*	0.017071	0.067614
CS20	CS28	GD28	*R.nitidus*	0.01956	0.068191
CS31	CS28	GD28	*R.nitidus*	0.034934	0.066513
CS17	CS28	GD28	*R.nitidus*	0.06853	0.069415
GD50	CS28	GD28	*R.nitidus*	0.100659	0.070125
SC5	CS28	GD28	*R.nitidus*	0.03599	0.069668
SC18	CS28	GD28	*R.nitidus*	0.063812	0.068724
SC28	CS28	GD28	*R.nitidus*	-0.002609	0.0657
SC10	CS28	GD28	*R.nitidus*	0.118151	0.065912
SC26	CS28	GD28	*R.nitidus*	-0.000833	0.069016
CS4	CS28	GD28	*R.nitidus*	0.007712	0.066294
CS18	CS28	GD28	*R.nitidus*	-0.005155	0.068665
GD25	CS28	GD28	*R.nitidus*	0.094719	0.065242
CS3	CS28	GD28	*R.nitidus*	0.009322	0.067436
SC15	CS28	GD28	*R.nitidus*	0.032172	0.067532
SC27	CS28	GD28	*R.nitidus*	0.042808	0.065647
SC6	CS28	GD28	*R.nitidus*	0.021533	0.069066
SC12	CS28	GD28	*R.nitidus*	0.037037	0.067483
SC35	CS28	GD28	*R.nitidus*	0.08	0.067105
CS21	CS28	GD28	*R.nitidus*	0.016892	0.067341
SC31	CS28	GD28	*R.nitidus*	0.044963	0.067403
MY6	CS28	GD28	*R.nitidus*	0.036636	0.068251
SC32	CS28	GD28	*R.nitidus*	0.110638	0.068359

JO7	CS30	GD28	*R.nitidus*	0.053215	0.072707
SC14	CS28	GD28	*R.nitidus*	0.060935	0.066622
SC24	CS28	GD28	*R.nitidus*	0.031383	0.066961
GD26	CS30	GD28	*R.nitidus*	0.094457	0.065925
SC44	CS28	GD28	*R.nitidus*	0.051871	0.067223
CS6	CS28	GD28	*R.nitidus*	0.010695	0.067
JW7	CS28	GD28	*R.nitidus*	0.055907	0.071004
SC45	CS28	GD28	*R.nitidus*	0.074858	0.068788
CS11	CS30	GD28	*R.nitidus*	0.044427	0.068331
SC34	CS28	GD28	*R.nitidus*	0.092715	0.065172
JO8	CS30	GD28	*R.nitidus*	0.090414	0.072731
GD25	CS30	GD28	*R.nitidus*	0.102326	0.066393
GD31	CS30	GD28	*R.nitidus*	0.101161	0.06868
GD41	CS30	GD28	*R.nitidus*	0.056294	0.067466
GD33	CS30	GD28	*R.nitidus*	0.054836	0.066005
GD30	CS30	GD28	*R.nitidus*	0.146959	0.069693
CS12	CS30	GD28	*R.nitidus*	0.064892	0.066911
JW4	CS28	GD28	*R.nitidus*	0.223567	0.066774
JO3	CS28	GD28	*R.nitidus*	0.068392	0.072424
GD29	CS30	GD28	*R.nitidus*	0.148969	0.06446
SC42	CS28	GD28	*R.nitidus*	0.269068	0.061463
GD21	CS30	GD28	*R.nitidus*	0.191154	0.06562
GD44	CS30	GD28	*R.nitidus*	0.034673	0.067251
GD22	CS30	GD28	*R.nitidus*	0.119581	0.064775
MY4	CS28	GD28	*R.nitidus*	0.039967	0.0677
GD32	CS30	GD28	*R.nitidus*	0.074692	0.070806
GD48	CS30	GD28	*R.nitidus*	0.087427	0.067629
GD35	CS30	GD28	*R.nitidus*	0.058689	0.067262
JW6	CS28	GD28	*R.nitidus*	0.065934	0.071412
CS13	CS30	GD28	*R.nitidus*	0.025348	0.069894
JO5	CS28	GD28	*R.nitidus*	0.056572	0.066973
CS15	CS30	GD28	*R.nitidus*	0.071371	0.067429
JO1	CS28	GD28	*R.nitidus*	0.029636	0.066724
GD34	CS30	GD28	*R.nitidus*	0.068807	0.065692
CS21	CS30	GD28	*R.nitidus*	0.023444	0.065297
GD46	CS30	GD28	*R.nitidus*	0.044741	0.066909
GD45	CS30	GD28	*R.nitidus*	0.071681	0.071884
CS22	CS30	GD28	*R.nitidus*	0.071189	0.066324
SC5	CS30	GD28	*R.nitidus*	0.045902	0.071106
CS14	CS30	GD28	*R.nitidus*	0.056634	0.069128
CS25	CS30	GD28	*R.nitidus*	0.039169	0.066359
GD42	CS30	GD28	*R.nitidus*	0.059468	0.066589
SC10	CS30	GD28	*R.nitidus*	0.118548	0.065558
GD40	CS30	GD28	*R.nitidus*	0.091116	0.064608
CS28	CS30	GD28	*R.nitidus*	0.010526	0.067131
GD49	CS30	GD28	*R.nitidus*	0.138233	0.069032
GD50	CS30	GD28	*R.nitidus*	0.074775	0.072777
CS17	CS30	GD28	*R.nitidus*	0.086192	0.068805
CS18	CS30	GD28	*R.nitidus*	0.013278	0.065725
SC42	CS30	GD28	*R.nitidus*	0.26943	0.062051
CS4	CS30	GD28	*R.nitidus*	0.033003	0.063739
CS16	CS30	GD28	*R.nitidus*	0.058635	0.065112
CS19	CS30	GD28	*R.nitidus*	0.036454	0.067085
SC27	CS30	GD28	*R.nitidus*	0.049917	0.064777
CS3	CS30	GD28	*R.nitidus*	0.026337	0.066037
SC6	CS30	GD28	*R.nitidus*	0.023346	0.067151

CS31	CS30	GD28	*R.nitidus*	0.053442	0.063984
SC12	CS30	GD28	*R.nitidus*	0.029009	0.066369
CS6	CS30	GD28	*R.nitidus*	0.028668	0.065698
SC15	CS30	GD28	*R.nitidus*	0.050298	0.066734
CS20	CS30	GD28	*R.nitidus*	0.025118	0.066332
SC14	CS30	GD28	*R.nitidus*	0.0608	0.066428
SC28	CS30	GD28	*R.nitidus*	0.002392	0.065351
JW4	CS30	GD28	*R.nitidus*	0.235527	0.067439
SC24	CS30	GD28	*R.nitidus*	0.047087	0.066869
JO1	CS30	GD28	*R.nitidus*	0.024545	0.064721
SC34	CS30	GD28	*R.nitidus*	0.081867	0.062433
SC44	CS30	GD28	*R.nitidus*	0.05	0.066965
MY4	CS30	GD28	*R.nitidus*	0.036154	0.068998
CS23	CS30	GD28	*R.nitidus*	0.014038	0.064791
CS24	CS30	GD28	*R.nitidus*	0.042424	0.06583
JW7	CS30	GD28	*R.nitidus*	0.061787	0.070861
JW6	CS30	GD28	*R.nitidus*	0.074807	0.071635
JO7	CS31	GD28	*R.nitidus*	0.002153	0.071269
SC45	CS30	GD28	*R.nitidus*	0.084691	0.067309
SC18	CS30	GD28	*R.nitidus*	0.081169	0.067029
SC35	CS30	GD28	*R.nitidus*	0.107741	0.064722
SC32	CS30	GD28	*R.nitidus*	0.094003	0.067171
SC26	CS30	GD28	*R.nitidus*	0.011032	0.065553
GD21	CS31	GD28	*R.nitidus*	0.150489	0.06559
JO3	CS30	GD28	*R.nitidus*	0.092251	0.07095
GD31	CS31	GD28	*R.nitidus*	0.048293	0.068309
JO8	CS31	GD28	*R.nitidus*	0.034973	0.073416
GD30	CS31	GD28	*R.nitidus*	0.104452	0.069571
GD26	CS31	GD28	*R.nitidus*	0.046142	0.066622
SC31	CS30	GD28	*R.nitidus*	0.058824	0.06719
GD44	CS31	GD28	*R.nitidus*	0.004058	0.068147
GD32	CS31	GD28	*R.nitidus*	0.040672	0.069947
GD29	CS31	GD28	*R.nitidus*	0.117293	0.066535
MY6	CS30	GD28	*R.nitidus*	0.042835	0.067398
GD22	CS31	GD28	*R.nitidus*	0.076183	0.065295
CS12	CS31	GD28	*R.nitidus*	0.032007	0.068046
GD33	CS31	GD28	*R.nitidus*	0.003776	0.066088
GD49	CS31	GD28	*R.nitidus*	0.108362	0.071133
GD45	CS31	GD28	*R.nitidus*	0.064947	0.073034
JO5	CS30	GD28	*R.nitidus*	0.070344	0.065074
CS16	CS31	GD28	*R.nitidus*	0.011844	0.067412
CS11	CS31	GD28	*R.nitidus*	0.007365	0.069306
GD34	CS31	GD28	*R.nitidus*	0.017625	0.066803
GD25	CS31	GD28	*R.nitidus*	0.064618	0.06588
GD46	CS31	GD28	*R.nitidus*	0.00319	0.067096
CS25	CS31	GD28	*R.nitidus*	0.008264	0.067407
GD50	CS31	GD28	*R.nitidus*	0.031993	0.069715
GD48	CS31	GD28	*R.nitidus*	0.046167	0.066378
CS23	CS31	GD28	*R.nitidus*	-0.030879	0.066173
CS30	CS31	GD28	*R.nitidus*	-0.053442	0.063984
CS21	CS31	GD28	*R.nitidus*	-0.026379	0.065365
GD35	CS31	GD28	*R.nitidus*	0.026563	0.066509
CS14	CS31	GD28	*R.nitidus*	0.027778	0.068763
CS15	CS31	GD28	*R.nitidus*	0.024938	0.069021
CS18	CS31	GD28	*R.nitidus*	-0.026251	0.065558
GD42	CS31	GD28	*R.nitidus*	0.008682	0.065591

CS19	CS31	GD28	*R.nitidus*	-0.003398	0.067999
SC5	CS31	GD28	*R.nitidus*	-0.0057	0.070184
CS28	CS31	GD28	*R.nitidus*	-0.034934	0.066513
GD41	CS31	GD28	*R.nitidus*	0.00858	0.065625
CS13	CS31	GD28	*R.nitidus*	-0.030431	0.068104
GD40	CS31	GD28	*R.nitidus*	0.050152	0.065726
CS22	CS31	GD28	*R.nitidus*	0.03	0.065908
CS20	CS31	GD28	*R.nitidus*	-0.015032	0.066161
CS24	CS31	GD28	*R.nitidus*	-0.015345	0.066092
SC6	CS31	GD28	*R.nitidus*	-0.021173	0.068569
SC35	CS31	GD28	*R.nitidus*	0.058314	0.062892
CS17	CS31	GD28	*R.nitidus*	0.037872	0.069663
SC12	CS31	GD28	*R.nitidus*	-0.008842	0.066288
SC18	CS31	GD28	*R.nitidus*	0.033844	0.066641
SC32	CS31	GD28	*R.nitidus*	0.059113	0.067679
SC14	CS31	GD28	*R.nitidus*	0.023372	0.067915
SC34	CS31	GD28	*R.nitidus*	0.0384	0.064095
CS3	CS31	GD28	*R.nitidus*	-0.015612	0.066102
CS4	CS31	GD28	*R.nitidus*	-0.020442	0.065713
SC28	CS31	GD28	*R.nitidus*	-0.041803	0.066342
CS6	CS31	GD28	*R.nitidus*	-0.024829	0.06599
SC44	CS31	GD28	*R.nitidus*	0.013029	0.066483
SC31	CS31	GD28	*R.nitidus*	0.013158	0.067455
MY6	CS31	GD28	*R.nitidus*	-0.009615	0.069071
SC10	CS31	GD28	*R.nitidus*	0.089271	0.065878
JW4	CS31	GD28	*R.nitidus*	0.208991	0.066827
SC26	CS31	GD28	*R.nitidus*	-0.028616	0.067347
JO3	CS31	GD28	*R.nitidus*	0.02595	0.072476
SC15	CS31	GD28	*R.nitidus*	-0.001702	0.068209
GD29	CS3	GD28	*R.nitidus*	0.122041	0.06513
JO7	CS3	GD28	*R.nitidus*	0.012948	0.07109
JW7	CS31	GD28	*R.nitidus*	0.02863	0.071969
GD35	CS3	GD28	*R.nitidus*	0.040816	0.065038
SC45	CS31	GD28	*R.nitidus*	0.031671	0.066645
SC27	CS31	GD28	*R.nitidus*	-0.000826	0.066067
SC24	CS31	GD28	*R.nitidus*	-0.004039	0.065938
SC42	CS31	GD28	*R.nitidus*	0.244048	0.061959
JO1	CS31	GD28	*R.nitidus*	-0.022047	0.065775
GD32	CS3	GD28	*R.nitidus*	0.054122	0.069215
GD31	CS3	GD28	*R.nitidus*	0.072684	0.067619
GD21	CS3	GD28	*R.nitidus*	0.157246	0.063856
GD45	CS3	GD28	*R.nitidus*	0.038194	0.071486
JW6	CS31	GD28	*R.nitidus*	0.055556	0.072148
GD30	CS3	GD28	*R.nitidus*	0.125729	0.066462
GD41	CS3	GD28	*R.nitidus*	0.037437	0.06563
JO8	CS3	GD28	*R.nitidus*	0.022293	0.071794
MY4	CS31	GD28	*R.nitidus*	-0.012739	0.068284
CS12	CS3	GD28	*R.nitidus*	0.028571	0.066106
CS13	CS3	GD28	*R.nitidus*	0.000813	0.068474
GD22	CS3	GD28	*R.nitidus*	0.091904	0.064721
CS14	CS3	GD28	*R.nitidus*	0.044534	0.068052
GD25	CS3	GD28	*R.nitidus*	0.084421	0.066152
GD42	CS3	GD28	*R.nitidus*	0.040058	0.06557
CS17	CS3	GD28	*R.nitidus*	0.044194	0.068516
CS11	CS3	GD28	*R.nitidus*	0.028135	0.066744
CS22	CS3	GD28	*R.nitidus*	0.041425	0.066212

JO5	CS31	GD28	*R.nitidus*	0.032	0.065348
GD26	CS3	GD28	*R.nitidus*	0.065185	0.065597
CS16	CS3	GD28	*R.nitidus*	0.02492	0.064492
GD40	CS3	GD28	*R.nitidus*	0.067757	0.064166
GD44	CS3	GD28	*R.nitidus*	0.019923	0.067898
CS19	CS3	GD28	*R.nitidus*	0.027985	0.067917
GD46	CS3	GD28	*R.nitidus*	0.015613	0.067394
GD34	CS3	GD28	*R.nitidus*	0.037834	0.065372
GD48	CS3	GD28	*R.nitidus*	0.052803	0.066933
GD33	CS3	GD28	*R.nitidus*	0.034074	0.06694
CS20	CS3	GD28	*R.nitidus*	-0.007543	0.062092
CS18	CS3	GD28	*R.nitidus*	-0.01756	0.065039
CS24	CS3	GD28	*R.nitidus*	0.017782	0.066812
CS21	CS3	GD28	*R.nitidus*	-0.013462	0.065295
CS30	CS3	GD28	*R.nitidus*	-0.026337	0.066037
SC10	CS3	GD28	*R.nitidus*	0.076923	0.064143
GD49	CS3	GD28	*R.nitidus*	0.108766	0.069697
SC6	CS3	GD28	*R.nitidus*	-0.0048	0.063394
GD50	CS3	GD28	*R.nitidus*	0.056239	0.071455
SC12	CS3	GD28	*R.nitidus*	-0.000806	0.064911
CS15	CS3	GD28	*R.nitidus*	0.040444	0.067115
CS28	CS3	GD28	*R.nitidus*	-0.009322	0.067436
SC27	CS3	GD28	*R.nitidus*	0.012668	0.065066
SC24	CS3	GD28	*R.nitidus*	0.022259	0.067671
SC28	CS3	GD28	*R.nitidus*	-0.041599	0.064237
SC14	CS3	GD28	*R.nitidus*	0.049489	0.065082
CS4	CS3	GD28	*R.nitidus*	0.006774	0.065634
SC32	CS3	GD28	*R.nitidus*	0.062696	0.064634
JW4	CS3	GD28	*R.nitidus*	0.205696	0.065232
SC42	CS3	GD28	*R.nitidus*	0.237367	0.06221
CS6	CS3	GD28	*R.nitidus*	-0.015521	0.062391
SC26	CS3	GD28	*R.nitidus*	-0.018706	0.064717
SC5	CS3	GD28	*R.nitidus*	0.00936	0.067756
CS23	CS3	GD28	*R.nitidus*	-0.018304	0.063791
CS31	CS3	GD28	*R.nitidus*	0.015612	0.066102
SC35	CS3	GD28	*R.nitidus*	0.068147	0.063057
SC15	CS3	GD28	*R.nitidus*	0.023654	0.064801
SC44	CS3	GD28	*R.nitidus*	0.012275	0.066153
SC18	CS3	GD28	*R.nitidus*	0.065767	0.065965
GD25	SC5	GD28	*R.nitidus*	0.069074	0.068044
SC31	CS3	GD28	*R.nitidus*	0.018735	0.065449
MY6	CS3	GD28	*R.nitidus*	0.008475	0.062894
JO7	SC5	GD28	*R.nitidus*	0.001055	0.073678
JO3	CS3	GD28	*R.nitidus*	0.022769	0.070104
JW7	CS3	GD28	*R.nitidus*	0.02595	0.069364
SC34	CS3	GD28	*R.nitidus*	0.051145	0.065005
JO8	SC5	GD28	*R.nitidus*	0.015957	0.075462
MY4	CS3	GD28	*R.nitidus*	0.013057	0.065114
SC45	CS3	GD28	*R.nitidus*	0.04406	0.066287
JO1	CS3	GD28	*R.nitidus*	-0.009562	0.065536
GD30	SC5	GD28	*R.nitidus*	0.100408	0.070373
JW6	CS3	GD28	*R.nitidus*	0.021561	0.072107
GD40	SC5	GD28	*R.nitidus*	0.051948	0.066757
CS25	CS3	GD28	*R.nitidus*	0.040036	0.066146
GD33	SC5	GD28	*R.nitidus*	0.004615	0.06802
GD32	SC5	GD28	*R.nitidus*	0.006029	0.074856

GD45	SC5	GD28	*R.nitidus*	0.049003	0.071908
GD21	SC5	GD28	*R.nitidus*	0.148537	0.068067
CS16	SC5	GD28	*R.nitidus*	0.009577	0.070457
GD49	SC5	GD28	*R.nitidus*	0.075833	0.072077
JO5	CS3	GD28	*R.nitidus*	0.049606	0.065505
GD42	SC5	GD28	*R.nitidus*	0.010558	0.0692
CS12	SC5	GD28	*R.nitidus*	0.008907	0.072042
GD44	SC5	GD28	*R.nitidus*	-0.008842	0.069049
CS19	SC5	GD28	*R.nitidus*	0	0.07113
CS11	SC5	GD28	*R.nitidus*	0.005405	0.071843
GD22	SC5	GD28	*R.nitidus*	0.073225	0.067726
GD26	SC5	GD28	*R.nitidus*	0.040152	0.066322
GD35	SC5	GD28	*R.nitidus*	0.023975	0.068865
GD34	SC5	GD28	*R.nitidus*	0.01949	0.068124
GD48	SC5	GD28	*R.nitidus*	0.020185	0.068682
GD31	SC5	GD28	*R.nitidus*	0.045383	0.070178
CS17	SC5	GD28	*R.nitidus*	0.030303	0.073462
CS14	SC5	GD28	*R.nitidus*	0.012598	0.072063
GD50	SC5	GD28	*R.nitidus*	0.037443	0.073499
CS3	SC5	GD28	*R.nitidus*	-0.00936	0.067756
CS18	SC5	GD28	*R.nitidus*	-0.033108	0.069192
CS28	SC5	GD28	*R.nitidus*	-0.03599	0.069668
GD41	SC5	GD28	*R.nitidus*	0.002268	0.069563
SC14	SC5	GD28	*R.nitidus*	0.022183	0.069325
SC10	SC5	GD28	*R.nitidus*	0.07099	0.071977
CS15	SC5	GD28	*R.nitidus*	0.018692	0.071885
SC12	SC5	GD28	*R.nitidus*	-0.009937	0.07041
CS13	SC5	GD28	*R.nitidus*	-0.023163	0.071647
CS23	SC5	GD28	*R.nitidus*	-0.030511	0.069894
GD46	SC5	GD28	*R.nitidus*	0.00686	0.066673
CS24	SC5	GD28	*R.nitidus*	-0.026138	0.070245
SC18	SC5	GD28	*R.nitidus*	0.038774	0.071519
SC6	SC5	GD28	*R.nitidus*	-0.024411	0.071842
CS22	SC5	GD28	*R.nitidus*	0.015485	0.069728
CS25	SC5	GD28	*R.nitidus*	0.00712	0.069454
CS30	SC5	GD28	*R.nitidus*	-0.045902	0.071106
CS31	SC5	GD28	*R.nitidus*	0.0057	0.070184
SC26	SC5	GD28	*R.nitidus*	-0.042714	0.069557
JO3	SC5	GD28	*R.nitidus*	0.008681	0.073407
SC24	SC5	GD28	*R.nitidus*	0.006019	0.071164
SC35	SC5	GD28	*R.nitidus*	0.053366	0.068528
CS21	SC5	GD28	*R.nitidus*	-0.031988	0.067314
SC31	SC5	GD28	*R.nitidus*	0.005641	0.06945
SC34	SC5	GD28	*R.nitidus*	0.036615	0.069013
SC32	SC5	GD28	*R.nitidus*	0.058032	0.071073
CS20	SC5	GD28	*R.nitidus*	-0.022918	0.069859
GD30	SC6	GD28	*R.nitidus*	0.130972	0.068452
GD21	SC6	GD28	*R.nitidus*	0.17482	0.06735
JW4	SC5	GD28	*R.nitidus*	0.178049	0.069262
SC44	SC5	GD28	*R.nitidus*	0.012464	0.069561
CS4	SC5	GD28	*R.nitidus*	-0.022672	0.068626
JO1	SC5	GD28	*R.nitidus*	-0.013169	0.069451
MY6	SC5	GD28	*R.nitidus*	-0.004839	0.069687
JO8	SC6	GD28	*R.nitidus*	0.058698	0.075797
SC15	SC5	GD28	*R.nitidus*	0.00262	0.071651
JO7	SC6	GD28	*R.nitidus*	0.019048	0.069209

SC27	SC5	GD28	*R.nitidus*	-0.000868	0.070259
SC42	SC5	GD28	*R.nitidus*	0.242739	0.067232
GD25	SC6	GD28	*R.nitidus*	0.088304	0.066794
JO5	SC5	GD28	*R.nitidus*	0.041247	0.069689
JW7	SC5	GD28	*R.nitidus*	0.007535	0.075929
JW6	SC5	GD28	*R.nitidus*	0.01594	0.074171
MY4	SC5	GD28	*R.nitidus*	-0.00083	0.069744
SC45	SC5	GD28	*R.nitidus*	0.043838	0.069303
GD35	SC6	GD28	*R.nitidus*	0.052161	0.067418
GD26	SC6	GD28	*R.nitidus*	0.07079	0.067885
SC28	SC5	GD28	*R.nitidus*	-0.038716	0.07011
CS6	SC5	GD28	*R.nitidus*	-0.033773	0.06729
CS17	SC6	GD28	*R.nitidus*	0.066667	0.070448
GD31	SC6	GD28	*R.nitidus*	0.059052	0.066604
GD42	SC6	GD28	*R.nitidus*	0.049587	0.067535
GD40	SC6	GD28	*R.nitidus*	0.08238	0.065155
GD41	SC6	GD28	*R.nitidus*	0.036226	0.068006
GD32	SC6	GD28	*R.nitidus*	0.046473	0.070558
GD29	SC6	GD28	*R.nitidus*	0.133138	0.065871
CS11	SC6	GD28	*R.nitidus*	0.0347	0.068592
GD33	SC6	GD28	*R.nitidus*	0.035529	0.068588
GD45	SC6	GD28	*R.nitidus*	0.053691	0.071815
GD22	SC6	GD28	*R.nitidus*	0.089374	0.067912
GD34	SC6	GD28	*R.nitidus*	0.042274	0.066566
CS16	SC6	GD28	*R.nitidus*	0.048662	0.067578
CS18	SC6	GD28	*R.nitidus*	-0.008709	0.065773
CS15	SC6	GD28	*R.nitidus*	0.053485	0.068008
CS12	SC6	GD28	*R.nitidus*	0.052632	0.069544
CS22	SC6	GD28	*R.nitidus*	0.04827	0.066648
GD49	SC6	GD28	*R.nitidus*	0.112006	0.068787
CS13	SC6	GD28	*R.nitidus*	0.003091	0.070612
SC26	SC6	GD28	*R.nitidus*	-0.016863	0.066434
GD44	SC6	GD28	*R.nitidus*	0.02148	0.067637
CS20	SC6	GD28	*R.nitidus*	0.008828	0.065462
SC5	SC6	GD28	*R.nitidus*	0.024411	0.071842
CS21	SC6	GD28	*R.nitidus*	-0.00399	0.065844
CS25	SC6	GD28	*R.nitidus*	0.026059	0.066155
GD48	SC6	GD28	*R.nitidus*	0.080422	0.066745
CS24	SC6	GD28	*R.nitidus*	0.001631	0.067885
CS28	SC6	GD28	*R.nitidus*	-0.021533	0.069066
GD50	SC6	GD28	*R.nitidus*	0.059621	0.070206
CS30	SC6	GD28	*R.nitidus*	-0.023346	0.067151
GD46	SC6	GD28	*R.nitidus*	0.025307	0.066126
SC24	SC6	GD28	*R.nitidus*	0.024055	0.067615
CS3	SC6	GD28	*R.nitidus*	0.0048	0.063394
CS14	SC6	GD28	*R.nitidus*	0.03962	0.070414
CS23	SC6	GD28	*R.nitidus*	-0.015212	0.067077
JW7	SC6	GD28	*R.nitidus*	0.033084	0.07334
CS31	SC6	GD28	*R.nitidus*	0.021173	0.068569
MY6	SC6	GD28	*R.nitidus*	0.024311	0.06655
CS19	SC6	GD28	*R.nitidus*	0.026899	0.067192
SC27	SC6	GD28	*R.nitidus*	0.022594	0.068497
SC14	SC6	GD28	*R.nitidus*	0.039069	0.068385
CS6	SC6	GD28	*R.nitidus*	0.002538	0.062731
JO7	SC10	GD28	*R.nitidus*	-0.095494	0.073824
SC31	SC6	GD28	*R.nitidus*	0.044118	0.064969

SC18	SC6	GD28	*R.nitidus*	0.05815	0.06606
SC45	SC6	GD28	*R.nitidus*	0.060231	0.068383
SC10	SC6	GD28	*R.nitidus*	0.109672	0.066879
SC32	SC6	GD28	*R.nitidus*	0.074627	0.067023
SC35	SC6	GD28	*R.nitidus*	0.09	0.066946
CS4	SC6	GD28	*R.nitidus*	0.010912	0.066436
SC28	SC6	GD28	*R.nitidus*	-0.034599	0.067038
JW4	SC6	GD28	*R.nitidus*	0.20239	0.067825
SC12	SC6	GD28	*R.nitidus*	0.014382	0.06732
SC15	SC6	GD28	*R.nitidus*	0.023336	0.068678
JW6	SC6	GD28	*R.nitidus*	0.037344	0.072519
JO1	SC6	GD28	*R.nitidus*	0.007582	0.06747
SC34	SC6	GD28	*R.nitidus*	0.069421	0.065865
MY4	SC6	GD28	*R.nitidus*	0.017945	0.066805
JO8	SC10	GD28	*R.nitidus*	-0.068415	0.073427
SC42	SC6	GD28	*R.nitidus*	0.269257	0.060803
GD30	SC10	GD28	*R.nitidus*	0.052856	0.071133
SC44	SC6	GD28	*R.nitidus*	0.035508	0.068209
JO3	SC6	GD28	*R.nitidus*	0.046614	0.070658
GD22	SC10	GD28	*R.nitidus*	-0.008234	0.067409
GD33	SC10	GD28	*R.nitidus*	-0.053869	0.068055
GD46	SC10	GD28	*R.nitidus*	-0.058252	0.064543
GD45	SC10	GD28	*R.nitidus*	-0.025231	0.070079
GD29	SC10	GD28	*R.nitidus*	0.03286	0.067949
GD21	SC10	GD28	*R.nitidus*	0.083758	0.06669
CS11	SC10	GD28	*R.nitidus*	-0.067901	0.068542
GD35	SC10	GD28	*R.nitidus*	-0.039352	0.06637
CS17	SC10	GD28	*R.nitidus*	-0.035117	0.070569
GD34	SC10	GD28	*R.nitidus*	-0.037707	0.067715
JO5	SC6	GD28	*R.nitidus*	0.056511	0.068171
CS12	SC10	GD28	*R.nitidus*	-0.066176	0.06999
GD26	SC10	GD28	*R.nitidus*	-0.020737	0.066236
GD25	SC10	GD28	*R.nitidus*	-0.002342	0.064089
GD44	SC10	GD28	*R.nitidus*	-0.058417	0.067912
CS13	SC10	GD28	*R.nitidus*	-0.085209	0.069436
CS19	SC10	GD28	*R.nitidus*	-0.056634	0.068162
GD40	SC10	GD28	*R.nitidus*	-0.018237	0.067584
GD31	SC10	GD28	*R.nitidus*	-0.018211	0.067171
GD42	SC10	GD28	*R.nitidus*	-0.044799	0.065202
GD32	SC10	GD28	*R.nitidus*	-0.049828	0.069128
CS30	SC10	GD28	*R.nitidus*	-0.118548	0.065558
GD48	SC10	GD28	*R.nitidus*	-0.028317	0.067023
GD41	SC10	GD28	*R.nitidus*	-0.058466	0.065475
SC28	SC10	GD28	*R.nitidus*	-0.139719	0.065955
CS23	SC10	GD28	*R.nitidus*	-0.099602	0.067551
CS31	SC10	GD28	*R.nitidus*	-0.089271	0.065878
CS15	SC10	GD28	*R.nitidus*	-0.042722	0.068574
GD50	SC10	GD28	*R.nitidus*	-0.017164	0.071612
CS22	SC10	GD28	*R.nitidus*	-0.05679	0.065194
CS14	SC10	GD28	*R.nitidus*	-0.046751	0.069645
CS3	SC10	GD28	*R.nitidus*	-0.076923	0.064143
CS25	SC10	GD28	*R.nitidus*	-0.049674	0.067247
CS18	SC10	GD28	*R.nitidus*	-0.109501	0.065943
SC12	SC10	GD28	*R.nitidus*	-0.093382	0.065519
GD49	SC10	GD28	*R.nitidus*	0.023102	0.070176
CS24	SC10	GD28	*R.nitidus*	-0.094262	0.066003

CS16	SC10	GD28	*R.nitidus*	-0.070162	0.068679
SC42	SC10	GD28	*R.nitidus*	0.176812	0.062151
SC18	SC10	GD28	*R.nitidus*	-0.042179	0.066921
SC34	SC10	GD28	*R.nitidus*	-0.027241	0.064869
CS4	SC10	GD28	*R.nitidus*	-0.089683	0.06523
SC5	SC10	GD28	*R.nitidus*	-0.07099	0.071977
SC26	SC10	GD28	*R.nitidus*	-0.126263	0.06502
CS21	SC10	GD28	*R.nitidus*	-0.092874	0.065162
SC14	SC10	GD28	*R.nitidus*	-0.053219	0.066436
JW4	SC10	GD28	*R.nitidus*	0.147107	0.068926
SC31	SC10	GD28	*R.nitidus*	-0.06031	0.065813
CS6	SC10	GD28	*R.nitidus*	-0.088211	0.066166
SC15	SC10	GD28	*R.nitidus*	-0.07556	0.067028
SC27	SC10	GD28	*R.nitidus*	-0.077626	0.066851
SC32	SC10	GD28	*R.nitidus*	-0.018634	0.067337
CS28	SC10	GD28	*R.nitidus*	-0.118151	0.065912
SC24	SC10	GD28	*R.nitidus*	-0.080131	0.066117
JO1	SC10	GD28	*R.nitidus*	-0.094259	0.0653
CS20	SC10	GD28	*R.nitidus*	-0.090267	0.065394
SC6	SC10	GD28	*R.nitidus*	-0.109672	0.066879
SC44	SC10	GD28	*R.nitidus*	-0.067682	0.068076
MY4	SC10	GD28	*R.nitidus*	-0.09166	0.068155
SC45	SC10	GD28	*R.nitidus*	-0.036301	0.067149
GD21	CS4	GD28	*R.nitidus*	0.161609	0.064171
JO3	SC10	GD28	*R.nitidus*	-0.043396	0.072138
JO8	CS4	GD28	*R.nitidus*	0.027311	0.071695
JW7	SC10	GD28	*R.nitidus*	-0.048035	0.071106
MY6	SC10	GD28	*R.nitidus*	-0.085246	0.065552
JO7	CS4	GD28	*R.nitidus*	0.040877	0.072661
JW6	SC10	GD28	*R.nitidus*	-0.06424	0.07457
SC35	SC10	GD28	*R.nitidus*	-0.009394	0.065514
CS15	CS4	GD28	*R.nitidus*	0.040253	0.066396
GD30	CS4	GD28	*R.nitidus*	0.12253	0.067145
GD48	CS4	GD28	*R.nitidus*	0.058544	0.066035
GD40	CS4	GD28	*R.nitidus*	0.08	0.06411
JO5	SC10	GD28	*R.nitidus*	-0.052453	0.067519
CS12	CS4	GD28	*R.nitidus*	0.046243	0.067532
GD26	CS4	GD28	*R.nitidus*	0.071953	0.064087
CS21	CS4	GD28	*R.nitidus*	0.003436	0.065867
GD34	CS4	GD28	*R.nitidus*	0.037956	0.064235
GD45	CS4	GD28	*R.nitidus*	0.053112	0.069562
GD31	CS4	GD28	*R.nitidus*	0.068807	0.06679
CS11	CS4	GD28	*R.nitidus*	0.01664	0.067084
CS13	CS4	GD28	*R.nitidus*	-0.01634	0.068216
GD35	CS4	GD28	*R.nitidus*	0.047302	0.064758
CS20	CS4	GD28	*R.nitidus*	0	0.065929
CS3	CS4	GD28	*R.nitidus*	-0.006774	0.065634
CS25	CS4	GD28	*R.nitidus*	0.015358	0.067873
GD22	CS4	GD28	*R.nitidus*	0.086987	0.063562
GD25	CS4	GD28	*R.nitidus*	0.074019	0.064109
GD49	CS4	GD28	*R.nitidus*	0.102646	0.06616
CS17	CS4	GD28	*R.nitidus*	0.051915	0.068269
SC5	CS4	GD28	*R.nitidus*	0.022672	0.068626
CS18	CS4	GD28	*R.nitidus*	-0.008818	0.065187
GD42	CS4	GD28	*R.nitidus*	0.036797	0.065368
GD32	CS4	GD28	*R.nitidus*	0.05042	0.066987

GD29	CS4	GD28	*R.nitidus*	0.13231	0.063309
GD41	CS4	GD28	*R.nitidus*	0.027286	0.064184
CS16	CS4	GD28	*R.nitidus*	0.025806	0.06676
CS14	CS4	GD28	*R.nitidus*	0.040258	0.068211
CS19	CS4	GD28	*R.nitidus*	0.020087	0.068235
GD44	CS4	GD28	*R.nitidus*	0.032207	0.065527
GD46	CS4	GD28	*R.nitidus*	0.02407	0.064045
CS22	CS4	GD28	*R.nitidus*	0.063674	0.064997
GD50	CS4	GD28	*R.nitidus*	0.062715	0.069334
GD33	CS4	GD28	*R.nitidus*	0.035928	0.065899
CS28	CS4	GD28	*R.nitidus*	-0.007712	0.066294
CS24	CS4	GD28	*R.nitidus*	0.003817	0.064978
CS31	CS4	GD28	*R.nitidus*	0.020442	0.065713
SC15	CS4	GD28	*R.nitidus*	0.01893	0.06627
SC12	CS4	GD28	*R.nitidus*	0.004831	0.065594
JW6	CS4	GD28	*R.nitidus*	0.036108	0.07162
CS30	CS4	GD28	*R.nitidus*	-0.033003	0.063739
CS23	CS4	GD28	*R.nitidus*	-0.015625	0.064955
SC18	CS4	GD28	*R.nitidus*	0.041229	0.06624
SC10	CS4	GD28	*R.nitidus*	0.089683	0.06523
SC42	CS4	GD28	*R.nitidus*	0.243648	0.062408
SC35	CS4	GD28	*R.nitidus*	0.06827	0.0647
JO1	CS4	GD28	*R.nitidus*	0	0.065549
SC6	CS4	GD28	*R.nitidus*	-0.010912	0.066436
GD21	SC12	GD28	*R.nitidus*	0.149566	0.066139
SC32	CS4	GD28	*R.nitidus*	0.069841	0.065748
JO5	CS4	GD28	*R.nitidus*	0.047049	0.065139
SC14	CS4	GD28	*R.nitidus*	0.027778	0.064859
JO8	SC12	GD28	*R.nitidus*	0.050605	0.071561
SC27	CS4	GD28	*R.nitidus*	0.011719	0.064609
SC34	CS4	GD28	*R.nitidus*	0.054764	0.063501
SC45	CS4	GD28	*R.nitidus*	0.051262	0.06555
CS6	CS4	GD28	*R.nitidus*	-0.000995	0.064118
JW4	CS4	GD28	*R.nitidus*	0.211831	0.064523
MY6	CS4	GD28	*R.nitidus*	0.015079	0.066119
SC44	CS4	GD28	*R.nitidus*	0.021226	0.064533
MY4	CS4	GD28	*R.nitidus*	0.012851	0.066431
SC28	CS4	GD28	*R.nitidus*	-0.023108	0.063637
SC31	CS4	GD28	*R.nitidus*	0.017336	0.064771
SC24	CS4	GD28	*R.nitidus*	0.013386	0.065448
SC26	CS4	GD28	*R.nitidus*	-0.022693	0.06378
JO3	CS4	GD28	*R.nitidus*	0.06023	0.068903
CS12	SC12	GD28	*R.nitidus*	0.023987	0.066894
JO7	SC12	GD28	*R.nitidus*	0.042508	0.070772
GD44	SC12	GD28	*R.nitidus*	0.015861	0.068442
GD32	SC12	GD28	*R.nitidus*	0.042749	0.069078
GD33	SC12	GD28	*R.nitidus*	0.016333	0.066833
JW7	CS4	GD28	*R.nitidus*	0.013527	0.069624
GD42	SC12	GD28	*R.nitidus*	0.030574	0.065332
GD26	SC12	GD28	*R.nitidus*	0.059443	0.065041
CS11	SC12	GD28	*R.nitidus*	0.005604	0.067565
GD25	SC12	GD28	*R.nitidus*	0.070878	0.066657
GD45	SC12	GD28	*R.nitidus*	0.044481	0.072302
GD30	SC12	GD28	*R.nitidus*	0.097808	0.069443
CS17	SC12	GD28	*R.nitidus*	0.041701	0.068636
GD22	SC12	GD28	*R.nitidus*	0.078757	0.064804

GD31	SC12	GD28	*R.nitidus*	0.048048	0.067567
CS13	SC12	GD28	*R.nitidus*	-0.025327	0.069977
GD41	SC12	GD28	*R.nitidus*	0.008209	0.065927
GD29	SC12	GD28	*R.nitidus*	0.104499	0.066395
GD48	SC12	GD28	*R.nitidus*	0.056394	0.067782
GD46	SC12	GD28	*R.nitidus*	0.012289	0.064368
GD50	SC12	GD28	*R.nitidus*	0.055013	0.072104
CS22	SC12	GD28	*R.nitidus*	0.030428	0.066914
CS21	SC12	GD28	*R.nitidus*	-0.018011	0.06618
CS16	SC12	GD28	*R.nitidus*	0.024272	0.065864
CS15	SC12	GD28	*R.nitidus*	0.029319	0.067392
GD35	SC12	GD28	*R.nitidus*	0.040801	0.067044
CS24	SC12	GD28	*R.nitidus*	-0.004085	0.067204
SC5	SC12	GD28	*R.nitidus*	0.009937	0.07041
GD40	SC12	GD28	*R.nitidus*	0.068787	0.065288
CS20	SC12	GD28	*R.nitidus*	-0.015528	0.066508
CS23	SC12	GD28	*R.nitidus*	-0.010236	0.066429
GD49	SC12	GD28	*R.nitidus*	0.084495	0.068146
SC6	SC12	GD28	*R.nitidus*	-0.014382	0.06732
CS3	SC12	GD28	*R.nitidus*	0.000806	0.064911
GD34	SC12	GD28	*R.nitidus*	0.036792	0.066445
CS28	SC12	GD28	*R.nitidus*	-0.037037	0.067483
CS30	SC12	GD28	*R.nitidus*	-0.029009	0.066369
CS19	SC12	GD28	*R.nitidus*	0.010172	0.067283
CS14	SC12	GD28	*R.nitidus*	0.027135	0.069725
CS18	SC12	GD28	*R.nitidus*	-0.023659	0.066209
CS31	SC12	GD28	*R.nitidus*	0.008842	0.066288
CS25	SC12	GD28	*R.nitidus*	0.020194	0.067901
SC28	SC12	GD28	*R.nitidus*	-0.028144	0.065342
SC18	SC12	GD28	*R.nitidus*	0.068421	0.066863
SC32	SC12	GD28	*R.nitidus*	0.075797	0.067651
JO7	SC14	GD28	*R.nitidus*	-0.01119	0.072098
SC42	SC12	GD28	*R.nitidus*	0.261624	0.062063
SC35	SC12	GD28	*R.nitidus*	0.085833	0.064167
CS4	SC12	GD28	*R.nitidus*	-0.004831	0.065594
SC15	SC12	GD28	*R.nitidus*	0.020264	0.068009
JW6	SC12	GD28	*R.nitidus*	0.053608	0.071217
SC31	SC12	GD28	*R.nitidus*	0.012638	0.066621
SC24	SC12	GD28	*R.nitidus*	0.010135	0.066645
JW4	SC12	GD28	*R.nitidus*	0.210612	0.066522
SC34	SC12	GD28	*R.nitidus*	0.057823	0.065075
CS6	SC12	GD28	*R.nitidus*	-0.023891	0.065662
SC10	SC12	GD28	*R.nitidus*	0.093382	0.065519
GD33	SC14	GD28	*R.nitidus*	-0.014174	0.066942
JW7	SC12	GD28	*R.nitidus*	0.038043	0.070931
SC27	SC12	GD28	*R.nitidus*	0.0188	0.06557
JO8	SC14	GD28	*R.nitidus*	-0.007194	0.074613
MY6	SC12	GD28	*R.nitidus*	0.009129	0.06858
SC44	SC12	GD28	*R.nitidus*	0.015343	0.067606
SC14	SC12	GD28	*R.nitidus*	0.031223	0.06638
SC26	SC12	GD28	*R.nitidus*	-0.034541	0.0653
MY4	SC12	GD28	*R.nitidus*	0.002467	0.067344
GD31	SC14	GD28	*R.nitidus*	0.035687	0.067331
GD26	SC14	GD28	*R.nitidus*	0.032304	0.067376
CS11	SC14	GD28	*R.nitidus*	-0.015152	0.069603
JO5	SC12	GD28	*R.nitidus*	0.055178	0.065102

SC45	SC12	GD28	*R.nitidus*	0.057709	0.064977
GD45	SC14	GD28	*R.nitidus*	0.011523	0.071847
JO3	SC12	GD28	*R.nitidus*	0.057541	0.070055
GD42	SC14	GD28	*R.nitidus*	-0.011038	0.06614
GD30	SC14	GD28	*R.nitidus*	0.086249	0.069619
GD40	SC14	GD28	*R.nitidus*	0.030015	0.065467
GD22	SC14	GD28	*R.nitidus*	0.052482	0.066686
GD21	SC14	GD28	*R.nitidus*	0.129149	0.066228
GD44	SC14	GD28	*R.nitidus*	-0.01249	0.068209
GD32	SC14	GD28	*R.nitidus*	0.007513	0.068869
JO1	SC12	GD28	*R.nitidus*	-0.007759	0.065958
GD29	SC14	GD28	*R.nitidus*	0.092352	0.064413
GD34	SC14	GD28	*R.nitidus*	-0.001451	0.066269
CS14	SC14	GD28	*R.nitidus*	0.002271	0.069222
GD49	SC14	GD28	*R.nitidus*	0.062201	0.068495
GD25	SC14	GD28	*R.nitidus*	0.039641	0.066806
CS15	SC14	GD28	*R.nitidus*	0.010679	0.068375
GD46	SC14	GD28	*R.nitidus*	-0.019417	0.065825
GD35	SC14	GD28	*R.nitidus*	0.003715	0.067686
CS12	SC14	GD28	*R.nitidus*	0	0.070653
CS19	SC14	GD28	*R.nitidus*	-0.018779	0.068675
CS13	SC14	GD28	*R.nitidus*	-0.045385	0.067671
GD48	SC14	GD28	*R.nitidus*	0.022853	0.066349
GD50	SC14	GD28	*R.nitidus*	0.010274	0.07148
GD41	SC14	GD28	*R.nitidus*	-0.009901	0.064497
CS17	SC14	GD28	*R.nitidus*	0.020117	0.070544
CS16	SC14	GD28	*R.nitidus*	-0.007004	0.067316
SC6	SC14	GD28	*R.nitidus*	-0.039069	0.068385
SC28	SC14	GD28	*R.nitidus*	-0.063194	0.06624
SC24	SC14	GD28	*R.nitidus*	-0.020991	0.066901
CS18	SC14	GD28	*R.nitidus*	-0.042748	0.065917
CS31	SC14	GD28	*R.nitidus*	-0.023372	0.067915
CS22	SC14	GD28	*R.nitidus*	-0.001553	0.066899
CS25	SC14	GD28	*R.nitidus*	-0.014786	0.068776
SC27	SC14	GD28	*R.nitidus*	-0.028986	0.063851
SC5	SC14	GD28	*R.nitidus*	-0.022183	0.069325
CS28	SC14	GD28	*R.nitidus*	-0.060935	0.066622
CS20	SC14	GD28	*R.nitidus*	-0.039275	0.065171
SC18	SC14	GD28	*R.nitidus*	0.015477	0.066855
CS30	SC14	GD28	*R.nitidus*	-0.0608	0.066428
SC35	SC14	GD28	*R.nitidus*	0.036029	0.063816
CS23	SC14	GD28	*R.nitidus*	-0.049736	0.064945
JO8	SC15	GD28	*R.nitidus*	0.011148	0.075084
CS3	SC14	GD28	*R.nitidus*	-0.049489	0.065082
CS24	SC14	GD28	*R.nitidus*	-0.030278	0.065119
SC42	SC14	GD28	*R.nitidus*	0.226223	0.062492
SC12	SC14	GD28	*R.nitidus*	-0.031223	0.06638
CS21	SC14	GD28	*R.nitidus*	-0.058015	0.064792
JW7	SC14	GD28	*R.nitidus*	-0.011074	0.069379
SC26	SC14	GD28	*R.nitidus*	-0.064516	0.066311
CS4	SC14	GD28	*R.nitidus*	-0.027778	0.064859
MY6	SC14	GD28	*R.nitidus*	-0.021824	0.066462
SC10	SC14	GD28	*R.nitidus*	0.053219	0.066436
SC34	SC14	GD28	*R.nitidus*	0.021294	0.064527
JO1	SC14	GD28	*R.nitidus*	-0.03097	0.063629
JW6	SC14	GD28	*R.nitidus*	0.015448	0.070674

GD22	SC15	GD28	*R.nitidus*	0.068063	0.065687
GD29	SC15	GD28	*R.nitidus*	0.117378	0.06688
SC44	SC14	GD28	*R.nitidus*	-0.010435	0.066739
CS11	SC15	GD28	*R.nitidus*	-0.004777	0.068264
JO5	SC14	GD28	*R.nitidus*	0.019742	0.065732
GD21	SC15	GD28	*R.nitidus*	0.145695	0.06575
MY4	SC14	GD28	*R.nitidus*	-0.027712	0.067986
GD26	SC15	GD28	*R.nitidus*	0.046582	0.067585
JW4	SC14	GD28	*R.nitidus*	0.178824	0.067181
CS6	SC14	GD28	*R.nitidus*	-0.045605	0.063481
SC15	SC14	GD28	*R.nitidus*	-0.02109	0.067908
SC32	SC14	GD28	*R.nitidus*	0.039063	0.066356
JO7	SC15	GD28	*R.nitidus*	0.017486	0.074951
JO3	SC14	GD28	*R.nitidus*	0.011324	0.069969
SC45	SC14	GD28	*R.nitidus*	0.02502	0.068243
GD25	SC15	GD28	*R.nitidus*	0.06378	0.067311
GD32	SC15	GD28	*R.nitidus*	0.019383	0.068191
GD45	SC15	GD28	*R.nitidus*	0.037229	0.072462
GD31	SC15	GD28	*R.nitidus*	0.045783	0.068664
GD48	SC15	GD28	*R.nitidus*	0.049278	0.068796
GD30	SC15	GD28	*R.nitidus*	0.113712	0.070484
GD35	SC15	GD28	*R.nitidus*	0.02657	0.068425
SC31	SC14	GD28	*R.nitidus*	-0.01141	0.067802
CS13	SC15	GD28	*R.nitidus*	-0.027615	0.070375
GD40	SC15	GD28	*R.nitidus*	0.060351	0.066339
GD46	SC15	GD28	*R.nitidus*	0.001587	0.066058
CS12	SC15	GD28	*R.nitidus*	0.023216	0.070154
GD41	SC15	GD28	*R.nitidus*	0.003968	0.066064
GD33	SC15	GD28	*R.nitidus*	0.003794	0.067276
CS15	SC15	GD28	*R.nitidus*	0.015962	0.069924
GD42	SC15	GD28	*R.nitidus*	0.028749	0.068388
CS25	SC15	GD28	*R.nitidus*	-0.005013	0.069209
GD34	SC15	GD28	*R.nitidus*	0.031923	0.067193
CS31	SC15	GD28	*R.nitidus*	0.001702	0.068209
GD44	SC15	GD28	*R.nitidus*	-0.009717	0.068891
SC6	SC15	GD28	*R.nitidus*	-0.023336	0.068678
CS14	SC15	GD28	*R.nitidus*	0.017785	0.071739
CS23	SC15	GD28	*R.nitidus*	-0.040098	0.066145
CS28	SC15	GD28	*R.nitidus*	-0.032172	0.067532
SC5	SC15	GD28	*R.nitidus*	-0.00262	0.071651
GD50	SC15	GD28	*R.nitidus*	0.030937	0.071927
CS20	SC15	GD28	*R.nitidus*	-0.013754	0.068369
CS17	SC15	GD28	*R.nitidus*	0.037262	0.070331
CS21	SC15	GD28	*R.nitidus*	-0.023007	0.064836
CS4	SC15	GD28	*R.nitidus*	-0.01893	0.06627
CS16	SC15	GD28	*R.nitidus*	0.005795	0.068954
SC10	SC15	GD28	*R.nitidus*	0.07556	0.067028
GD49	SC15	GD28	*R.nitidus*	0.097826	0.067771
CS19	SC15	GD28	*R.nitidus*	-0.004174	0.068109
SC31	SC15	GD28	*R.nitidus*	0.013393	0.066862
SC12	SC15	GD28	*R.nitidus*	-0.020264	0.068009
CS6	SC15	GD28	*R.nitidus*	-0.02911	0.066422
CS18	SC15	GD28	*R.nitidus*	-0.030936	0.067189
SC24	SC15	GD28	*R.nitidus*	-0.002664	0.068329
SC35	SC15	GD28	*R.nitidus*	0.060201	0.067261
SC18	SC15	GD28	*R.nitidus*	0.031915	0.068333

CS22	SC15	GD28	*R.nitidus*	0.016639	0.067098
SC34	SC15	GD28	*R.nitidus*	0.037768	0.063681
SC28	SC15	GD28	*R.nitidus*	-0.047496	0.067761
CS24	SC15	GD28	*R.nitidus*	-0.005093	0.06552
CS30	SC15	GD28	*R.nitidus*	-0.050298	0.066734
MY4	SC15	GD28	*R.nitidus*	-0.013267	0.068742
CS3	SC15	GD28	*R.nitidus*	-0.023654	0.064801
JW7	SC15	GD28	*R.nitidus*	0.01921	0.073599
SC14	SC15	GD28	*R.nitidus*	0.02109	0.067908
SC26	SC15	GD28	*R.nitidus*	-0.043404	0.067438
JW6	SC15	GD28	*R.nitidus*	0.044907	0.074419
GD31	SC18	GD28	*R.nitidus*	0.016247	0.067898
JO7	SC18	GD28	*R.nitidus*	-0.013934	0.073042
GD32	SC18	GD28	*R.nitidus*	-0.017331	0.070933
JW4	SC15	GD28	*R.nitidus*	0.185096	0.068639
GD25	SC18	GD28	*R.nitidus*	0.050157	0.066086
CS12	SC18	GD28	*R.nitidus*	-0.0278	0.067867
SC27	SC15	GD28	*R.nitidus*	0.003643	0.066684
GD29	SC18	GD28	*R.nitidus*	0.063685	0.066025
SC42	SC15	GD28	*R.nitidus*	0.242984	0.063067
SC32	SC15	GD28	*R.nitidus*	0.058304	0.067949
GD35	SC18	GD28	*R.nitidus*	0.01414	0.067931
SC45	SC15	GD28	*R.nitidus*	0.039004	0.067116
GD21	SC18	GD28	*R.nitidus*	0.125843	0.066698
JO8	SC18	GD28	*R.nitidus*	-0.001147	0.072849
CS11	SC18	GD28	*R.nitidus*	-0.031636	0.067782
MY6	SC15	GD28	*R.nitidus*	-0.009796	0.067011
GD33	SC18	GD28	*R.nitidus*	-0.016717	0.066488
JO3	SC15	GD28	*R.nitidus*	0.02317	0.070161
SC44	SC15	GD28	*R.nitidus*	0.006034	0.067248
GD26	SC18	GD28	*R.nitidus*	0.020658	0.066486
GD34	SC18	GD28	*R.nitidus*	-0.005271	0.066889
GD22	SC18	GD28	*R.nitidus*	0.028295	0.066749
GD30	SC18	GD28	*R.nitidus*	0.084175	0.068038
GD42	SC18	GD28	*R.nitidus*	-0.007686	0.066014
GD45	SC18	GD28	*R.nitidus*	-0.007036	0.072529
CS16	SC18	GD28	*R.nitidus*	-0.021617	0.067544
JO5	SC15	GD28	*R.nitidus*	0.032506	0.066074
GD49	SC18	GD28	*R.nitidus*	0.067532	0.069911
GD41	SC18	GD28	*R.nitidus*	-0.014638	0.066325
JO1	SC15	GD28	*R.nitidus*	-0.023038	0.066219
GD44	SC18	GD28	*R.nitidus*	-0.011609	0.067564
CS17	SC18	GD28	*R.nitidus*	0.007432	0.069848
GD50	SC18	GD28	*R.nitidus*	0.020128	0.0728
GD48	SC18	GD28	*R.nitidus*	0.012701	0.066903
CS22	SC18	GD28	*R.nitidus*	-0.00659	0.067795
GD46	SC18	GD28	*R.nitidus*	-0.038132	0.064409
CS14	SC18	GD28	*R.nitidus*	-0.000785	0.069319
CS15	SC18	GD28	*R.nitidus*	-0.01278	0.067646
GD40	SC18	GD28	*R.nitidus*	0.01811	0.064688
CS3	SC18	GD28	*R.nitidus*	-0.065767	0.065965
SC14	SC18	GD28	*R.nitidus*	-0.015477	0.066855
SC5	SC18	GD28	*R.nitidus*	-0.038774	0.071519
SC10	SC18	GD28	*R.nitidus*	0.042179	0.066921
CS30	SC18	GD28	*R.nitidus*	-0.081169	0.067029
SC26	SC18	GD28	*R.nitidus*	-0.088488	0.067129

CS19	SC18	GD28	*R.nitidus*	-0.035088	0.068407
SC6	SC18	GD28	*R.nitidus*	-0.05815	0.06606
CS21	SC18	GD28	*R.nitidus*	-0.060729	0.066097
CS13	SC18	GD28	*R.nitidus*	-0.049245	0.069069
CS18	SC18	GD28	*R.nitidus*	-0.066347	0.066931
SC28	SC18	GD28	*R.nitidus*	-0.10064	0.065915
JW7	SC18	GD28	*R.nitidus*	0.001058	0.071286
CS6	SC18	GD28	*R.nitidus*	-0.069991	0.064753
JO7	SC24	GD28	*R.nitidus*	-0.005336	0.073424
JW4	SC18	GD28	*R.nitidus*	0.158155	0.068974
CS20	SC18	GD28	*R.nitidus*	-0.06068	0.066222
JO5	SC18	GD28	*R.nitidus*	-0.003534	0.066248
SC31	SC18	GD28	*R.nitidus*	-0.029412	0.066797
CS23	SC18	GD28	*R.nitidus*	-0.06759	0.066508
CS25	SC18	GD28	*R.nitidus*	-0.026622	0.0691
CS24	SC18	GD28	*R.nitidus*	-0.03894	0.067
CS28	SC18	GD28	*R.nitidus*	-0.063812	0.068724
SC44	SC18	GD28	*R.nitidus*	-0.040516	0.067893
JO1	SC18	GD28	*R.nitidus*	-0.058201	0.066028
JO3	SC18	GD28	*R.nitidus*	-0.007512	0.070489
SC15	SC18	GD28	*R.nitidus*	-0.031915	0.068333
SC12	SC18	GD28	*R.nitidus*	-0.068421	0.066863
SC34	SC18	GD28	*R.nitidus*	-0.005072	0.066245
CS31	SC18	GD28	*R.nitidus*	-0.033844	0.066641
SC32	SC18	GD28	*R.nitidus*	0.017606	0.06585
CS4	SC18	GD28	*R.nitidus*	-0.041229	0.06624
SC42	SC18	GD28	*R.nitidus*	0.206775	0.06354
SC27	SC18	GD28	*R.nitidus*	-0.035119	0.06779
SC45	SC18	GD28	*R.nitidus*	-0.004355	0.067251
SC24	SC18	GD28	*R.nitidus*	-0.065922	0.064157
JW6	SC18	GD28	*R.nitidus*	0.017429	0.07141
GD32	SC24	GD28	*R.nitidus*	0.035383	0.069221
SC35	SC18	GD28	*R.nitidus*	0.019475	0.064126
CS11	SC24	GD28	*R.nitidus*	0.00458	0.069973
GD22	SC24	GD28	*R.nitidus*	0.07261	0.066467
GD31	SC24	GD28	*R.nitidus*	0.04414	0.06826
GD25	SC24	GD28	*R.nitidus*	0.065034	0.067618
MY4	SC18	GD28	*R.nitidus*	-0.05807	0.066347
MY6	SC18	GD28	*R.nitidus*	-0.053782	0.065172
JO8	SC24	GD28	*R.nitidus*	0.001091	0.073276
GD29	SC24	GD28	*R.nitidus*	0.103152	0.065671
GD26	SC24	GD28	*R.nitidus*	0.052515	0.067358
GD40	SC24	GD28	*R.nitidus*	0.045245	0.065419
GD30	SC24	GD28	*R.nitidus*	0.116935	0.067901
GD33	SC24	GD28	*R.nitidus*	0.01444	0.067448
GD21	SC24	GD28	*R.nitidus*	0.146589	0.06647
GD48	SC24	GD28	*R.nitidus*	0.042045	0.064431
GD35	SC24	GD28	*R.nitidus*	0.035115	0.068065
GD34	SC24	GD28	*R.nitidus*	0.027166	0.067
CS30	SC24	GD28	*R.nitidus*	-0.047087	0.066869
GD41	SC24	GD28	*R.nitidus*	0.019374	0.065927
CS16	SC24	GD28	*R.nitidus*	0.009353	0.067671
GD50	SC24	GD28	*R.nitidus*	0.054054	0.071048
CS17	SC24	GD28	*R.nitidus*	0.037252	0.069932
CS12	SC24	GD28	*R.nitidus*	0.015898	0.068196
GD44	SC24	GD28	*R.nitidus*	0.008744	0.068529

SC12	SC24	GD28	*R.nitidus*	-0.010135	0.066645
CS4	SC24	GD28	*R.nitidus*	-0.013386	0.065448
GD42	SC24	GD28	*R.nitidus*	0.018032	0.066655
CS25	SC24	GD28	*R.nitidus*	0.000822	0.068386
GD49	SC24	GD28	*R.nitidus*	0.111658	0.069262
CS20	SC24	GD28	*R.nitidus*	-0.019078	0.066744
SC26	SC24	GD28	*R.nitidus*	-0.043261	0.067266
CS21	SC24	GD28	*R.nitidus*	-0.019826	0.065765
GD45	SC24	GD28	*R.nitidus*	0.031826	0.070201
CS6	SC24	GD28	*R.nitidus*	-0.037931	0.066706
CS3	SC24	GD28	*R.nitidus*	-0.022259	0.067671
CS13	SC24	GD28	*R.nitidus*	-0.028324	0.071438
CS22	SC24	GD28	*R.nitidus*	0.030159	0.066519
CS31	SC24	GD28	*R.nitidus*	0.004039	0.065938
CS15	SC24	GD28	*R.nitidus*	0.026054	0.068719
SC5	SC24	GD28	*R.nitidus*	-0.006019	0.071164
GD46	SC24	GD28	*R.nitidus*	-0.00076	0.065716
SC6	SC24	GD28	*R.nitidus*	-0.024055	0.067615
CS18	SC24	GD28	*R.nitidus*	-0.028777	0.066904
CS14	SC24	GD28	*R.nitidus*	0.015385	0.071584
CS23	SC24	GD28	*R.nitidus*	-0.027451	0.066486
CS19	SC24	GD28	*R.nitidus*	-0.000818	0.068605
SC14	SC24	GD28	*R.nitidus*	0.020991	0.066901
CS24	SC24	GD28	*R.nitidus*	-0.004827	0.065961
CS28	SC24	GD28	*R.nitidus*	-0.031383	0.066961
SC32	SC24	GD28	*R.nitidus*	0.056059	0.066339
SC35	SC24	GD28	*R.nitidus*	0.067268	0.063766
JO5	SC24	GD28	*R.nitidus*	0.03209	0.065974
JW4	SC24	GD28	*R.nitidus*	0.17502	0.067249
SC10	SC24	GD28	*R.nitidus*	0.080131	0.066117
SC34	SC24	GD28	*R.nitidus*	0.043154	0.062943
SC27	SC24	GD28	*R.nitidus*	0.009322	0.066259
JW7	SC24	GD28	*R.nitidus*	0.018711	0.072124
SC45	SC24	GD28	*R.nitidus*	0.04876	0.066806
MY4	SC24	GD28	*R.nitidus*	-0.004946	0.066938
GD21	SC26	GD28	*R.nitidus*	0.186453	0.066009
SC15	SC24	GD28	*R.nitidus*	0.002664	0.068329
SC42	SC24	GD28	*R.nitidus*	0.248331	0.063018
MY6	SC24	GD28	*R.nitidus*	0.001645	0.066027
SC18	SC24	GD28	*R.nitidus*	0.065922	0.064157
SC31	SC24	GD28	*R.nitidus*	0.013019	0.065804
GD25	SC26	GD28	*R.nitidus*	0.100752	0.064872
SC28	SC24	GD28	*R.nitidus*	-0.049828	0.067345
JO7	SC26	GD28	*R.nitidus*	0.04888	0.072659
CS11	SC26	GD28	*R.nitidus*	0.039877	0.066342
GD32	SC26	GD28	*R.nitidus*	0.06357	0.069892
SC44	SC24	GD28	*R.nitidus*	0.000864	0.068232
JW6	SC24	GD28	*R.nitidus*	0.01824	0.072415
JO8	SC26	GD28	*R.nitidus*	0.096501	0.071728
GD29	SC26	GD28	*R.nitidus*	0.136686	0.065275
GD34	SC26	GD28	*R.nitidus*	0.052632	0.066865
GD41	SC26	GD28	*R.nitidus*	0.042661	0.067907
GD33	SC26	GD28	*R.nitidus*	0.049822	0.066704
JO3	SC24	GD28	*R.nitidus*	0.030825	0.06867
CS12	SC26	GD28	*R.nitidus*	0.05	0.068687
GD22	SC26	GD28	*R.nitidus*	0.099237	0.066334

GD42	SC26	GD28	*R.nitidus*	0.046709	0.067678
GD26	SC26	GD28	*R.nitidus*	0.090777	0.067355
GD35	SC26	GD28	*R.nitidus*	0.060161	0.06628
JO1	SC24	GD28	*R.nitidus*	-0.005724	0.066343
GD46	SC26	GD28	*R.nitidus*	0.034207	0.065641
CS15	SC26	GD28	*R.nitidus*	0.060232	0.066404
GD31	SC26	GD28	*R.nitidus*	0.086957	0.067193
GD44	SC26	GD28	*R.nitidus*	0.037936	0.067342
GD30	SC26	GD28	*R.nitidus*	0.144338	0.068773
CS20	SC26	GD28	*R.nitidus*	0.017067	0.06692
CS30	SC26	GD28	*R.nitidus*	-0.011032	0.065553
CS21	SC26	GD28	*R.nitidus*	0.010929	0.065682
CS24	SC26	GD28	*R.nitidus*	0.028436	0.066323
GD49	SC26	GD28	*R.nitidus*	0.119841	0.069583
CS19	SC26	GD28	*R.nitidus*	0.036434	0.06779
CS22	SC26	GD28	*R.nitidus*	0.066406	0.065162
CS25	SC26	GD28	*R.nitidus*	0.037096	0.066543
CS14	SC26	GD28	*R.nitidus*	0.060514	0.067825
CS17	SC26	GD28	*R.nitidus*	0.07009	0.069327
GD45	SC26	GD28	*R.nitidus*	0.06245	0.070143
SC10	SC26	GD28	*R.nitidus*	0.126263	0.06502
CS28	SC26	GD28	*R.nitidus*	0.000833	0.069016
CS4	SC26	GD28	*R.nitidus*	0.022693	0.06378
CS13	SC26	GD28	*R.nitidus*	0.013535	0.068075
GD48	SC26	GD28	*R.nitidus*	0.086314	0.068089
GD40	SC26	GD28	*R.nitidus*	0.077592	0.065378
GD50	SC26	GD28	*R.nitidus*	0.070191	0.070579
SC27	SC26	GD28	*R.nitidus*	0.042918	0.066416
CS3	SC26	GD28	*R.nitidus*	0.018706	0.064717
CS16	SC26	GD28	*R.nitidus*	0.050926	0.06461
CS31	SC26	GD28	*R.nitidus*	0.028616	0.067347
SC24	SC26	GD28	*R.nitidus*	0.043261	0.067266
SC18	SC26	GD28	*R.nitidus*	0.088488	0.067129
SC44	SC26	GD28	*R.nitidus*	0.046257	0.066461
SC28	SC26	GD28	*R.nitidus*	0.004983	0.065637
CS18	SC26	GD28	*R.nitidus*	0.006897	0.064377
SC5	SC26	GD28	*R.nitidus*	0.042714	0.069557
JW6	SC26	GD28	*R.nitidus*	0.076433	0.071101
CS23	SC26	GD28	*R.nitidus*	0.006088	0.066963
SC12	SC26	GD28	*R.nitidus*	0.034541	0.0653
SC6	SC26	GD28	*R.nitidus*	0.016863	0.066434
SC34	SC26	GD28	*R.nitidus*	0.084518	0.06474
SC35	SC26	GD28	*R.nitidus*	0.108065	0.064553
SC42	SC26	GD28	*R.nitidus*	0.267275	0.062033
JW7	SC26	GD28	*R.nitidus*	0.067218	0.069879
SC45	SC26	GD28	*R.nitidus*	0.086109	0.06775
JW4	SC26	GD28	*R.nitidus*	0.229199	0.067098
SC15	SC26	GD28	*R.nitidus*	0.043404	0.067438
JO1	SC26	GD28	*R.nitidus*	0.020973	0.065273
CS6	SC26	GD28	*R.nitidus*	0.014423	0.064528
JO5	SC26	GD28	*R.nitidus*	0.070715	0.067299
SC32	SC26	GD28	*R.nitidus*	0.093157	0.065993
SC31	SC26	GD28	*R.nitidus*	0.057236	0.066807
SC14	SC26	GD28	*R.nitidus*	0.064516	0.066311
GD31	SC27	GD28	*R.nitidus*	0.038766	0.068077
JO3	SC26	GD28	*R.nitidus*	0.073356	0.068096

CS11	SC27	GD28	*R.nitidus*	0.011041	0.068198
GD22	SC27	GD28	*R.nitidus*	0.066762	0.064738
GD26	SC27	GD28	*R.nitidus*	0.047584	0.066776
GD41	SC27	GD28	*R.nitidus*	0.012223	0.065085
GD25	SC27	GD28	*R.nitidus*	0.065909	0.064055
GD30	SC27	GD28	*R.nitidus*	0.111837	0.069734
CS12	SC27	GD28	*R.nitidus*	0.014274	0.068573
MY4	SC26	GD28	*R.nitidus*	0.039352	0.063741
JO7	SC27	GD28	*R.nitidus*	-0.001037	0.071751
GD34	SC27	GD28	*R.nitidus*	0.022338	0.065928
MY6	SC26	GD28	*R.nitidus*	0.036753	0.064254
GD45	SC27	GD28	*R.nitidus*	0.022333	0.070782
GD35	SC27	GD28	*R.nitidus*	0.03621	0.066335
JO8	SC27	GD28	*R.nitidus*	0.022269	0.072764
CS19	SC27	GD28	*R.nitidus*	-0.008929	0.069162
GD40	SC27	GD28	*R.nitidus*	0.058691	0.064671
CS14	SC27	GD28	*R.nitidus*	0.026646	0.068863
GD44	SC27	GD28	*R.nitidus*	-0.007026	0.068315
GD29	SC27	GD28	*R.nitidus*	0.114645	0.064267
GD33	SC27	GD28	*R.nitidus*	0.005036	0.065977
CS15	SC27	GD28	*R.nitidus*	0.03289	0.066351
GD32	SC27	GD28	*R.nitidus*	0.028476	0.069204
GD21	SC27	GD28	*R.nitidus*	0.151982	0.065764
GD46	SC27	GD28	*R.nitidus*	0.007553	0.065643
CS16	SC27	GD28	*R.nitidus*	0.016013	0.067043
CS17	SC27	GD28	*R.nitidus*	0.049615	0.068248
CS22	SC27	GD28	*R.nitidus*	0.015237	0.066072
SC12	SC27	GD28	*R.nitidus*	-0.0188	0.06557
GD42	SC27	GD28	*R.nitidus*	0.015026	0.066798
SC34	SC27	GD28	*R.nitidus*	0.042678	0.065848
GD49	SC27	GD28	*R.nitidus*	0.083945	0.068261
GD50	SC27	GD28	*R.nitidus*	0.051373	0.068806
GD48	SC27	GD28	*R.nitidus*	0.042502	0.066119
CS23	SC27	GD28	*R.nitidus*	-0.031546	0.065897
CS20	SC27	GD28	*R.nitidus*	-0.015994	0.066074
CS28	SC27	GD28	*R.nitidus*	-0.042808	0.065647
CS13	SC27	GD28	*R.nitidus*	-0.031596	0.068355
CS30	SC27	GD28	*R.nitidus*	-0.049917	0.064777
SC42	SC27	GD28	*R.nitidus*	0.245166	0.062249
CS18	SC27	GD28	*R.nitidus*	-0.031797	0.06642
SC5	SC27	GD28	*R.nitidus*	0.000868	0.070259
CS21	SC27	GD28	*R.nitidus*	-0.026255	0.064387
SC35	SC27	GD28	*R.nitidus*	0.065381	0.064811
SC26	SC27	GD28	*R.nitidus*	-0.042918	0.066416
CS3	SC27	GD28	*R.nitidus*	-0.012668	0.065066
SC6	SC27	GD28	*R.nitidus*	-0.022594	0.068497
CS24	SC27	GD28	*R.nitidus*	-0.008446	0.064832
CS25	SC27	GD28	*R.nitidus*	-0.000814	0.067847
SC31	SC27	GD28	*R.nitidus*	0.008987	0.066572
SC10	SC27	GD28	*R.nitidus*	0.077626	0.066851
CS6	SC27	GD28	*R.nitidus*	-0.030833	0.065721
CS31	SC27	GD28	*R.nitidus*	0.000826	0.066067
SC15	SC27	GD28	*R.nitidus*	-0.003643	0.066684
SC32	SC27	GD28	*R.nitidus*	0.06328	0.065086
JW6	SC27	GD28	*R.nitidus*	0.037696	0.069662
SC28	SC27	GD28	*R.nitidus*	-0.049601	0.066727

JO5	SC27	GD28	*R.nitidus*	0.033275	0.064578
JO8	SC28	GD28	*R.nitidus*	0.055928	0.072178
SC14	SC27	GD28	*R.nitidus*	0.028986	0.063851
CS4	SC27	GD28	*R.nitidus*	-0.011719	0.064609
SC24	SC27	GD28	*R.nitidus*	-0.009322	0.066259
JO1	SC27	GD28	*R.nitidus*	-0.007426	0.063673
JO3	SC27	GD28	*R.nitidus*	0.021505	0.070052
SC44	SC27	GD28	*R.nitidus*	0.00354	0.066529
MY4	SC27	GD28	*R.nitidus*	-0.012618	0.066502
JW7	SC27	GD28	*R.nitidus*	0.019123	0.069812
SC18	SC27	GD28	*R.nitidus*	0.035119	0.06779
GD26	SC28	GD28	*R.nitidus*	0.088674	0.065586
JW4	SC27	GD28	*R.nitidus*	0.180769	0.06757
MY6	SC27	GD28	*R.nitidus*	-0.007059	0.065438
GD25	SC28	GD28	*R.nitidus*	0.105023	0.06453
JO7	SC28	GD28	*R.nitidus*	0.029661	0.072156
SC45	SC27	GD28	*R.nitidus*	0.039075	0.065689
CS11	SC28	GD28	*R.nitidus*	0.051322	0.069209
CS12	SC28	GD28	*R.nitidus*	0.076728	0.068534
GD49	SC28	GD28	*R.nitidus*	0.127023	0.06787
GD29	SC28	GD28	*R.nitidus*	0.143597	0.065298
GD22	SC28	GD28	*R.nitidus*	0.116129	0.065214
GD48	SC28	GD28	*R.nitidus*	0.082324	0.066653
GD40	SC28	GD28	*R.nitidus*	0.095165	0.064846
GD45	SC28	GD28	*R.nitidus*	0.078283	0.068955
GD30	SC28	GD28	*R.nitidus*	0.142974	0.067233
GD21	SC28	GD28	*R.nitidus*	0.18408	0.065554
GD46	SC28	GD28	*R.nitidus*	0.047584	0.063415
GD44	SC28	GD28	*R.nitidus*	0.043582	0.068871
CS14	SC28	GD28	*R.nitidus*	0.066453	0.069384
CS30	SC28	GD28	*R.nitidus*	-0.002392	0.065351
SC5	SC28	GD28	*R.nitidus*	0.038716	0.07011
CS21	SC28	GD28	*R.nitidus*	0.010133	0.065549
GD32	SC28	GD28	*R.nitidus*	0.057384	0.069038
CS17	SC28	GD28	*R.nitidus*	0.092671	0.068522
CS20	SC28	GD28	*R.nitidus*	0.023438	0.06506
CS28	SC28	GD28	*R.nitidus*	0.002609	0.0657
CS13	SC28	GD28	*R.nitidus*	0.026807	0.068995
CS22	SC28	GD28	*R.nitidus*	0.062857	0.065612
GD33	SC28	GD28	*R.nitidus*	0.0462	0.065033
GD35	SC28	GD28	*R.nitidus*	0.072878	0.066908
CS16	SC28	GD28	*R.nitidus*	0.068182	0.067059
GD50	SC28	GD28	*R.nitidus*	0.081365	0.069642
CS24	SC28	GD28	*R.nitidus*	0.026464	0.066263
GD34	SC28	GD28	*R.nitidus*	0.06478	0.065396
GD41	SC28	GD28	*R.nitidus*	0.047148	0.066125
SC10	SC28	GD28	*R.nitidus*	0.139719	0.065955
GD42	SC28	GD28	*R.nitidus*	0.061119	0.067469
CS23	SC28	GD28	*R.nitidus*	0.033071	0.06451
CS4	SC28	GD28	*R.nitidus*	0.023108	0.063637
SC14	SC28	GD28	*R.nitidus*	0.063194	0.06624
CS18	SC28	GD28	*R.nitidus*	0.01171	0.065291
SC6	SC28	GD28	*R.nitidus*	0.034599	0.067038
GD31	SC28	GD28	*R.nitidus*	0.093897	0.066908
CS31	SC28	GD28	*R.nitidus*	0.041803	0.066342
SC34	SC28	GD28	*R.nitidus*	0.086207	0.064437

CS15	SC28	GD28	*R.nitidus*	0.078634	0.066535
CS25	SC28	GD28	*R.nitidus*	0.052033	0.067504
CS19	SC28	GD28	*R.nitidus*	0.040767	0.064321
SC44	SC28	GD28	*R.nitidus*	0.075145	0.062804
SC15	SC28	GD28	*R.nitidus*	0.047496	0.067761
SC18	SC28	GD28	*R.nitidus*	0.10064	0.065915
SC12	SC28	GD28	*R.nitidus*	0.028144	0.065342
CS3	SC28	GD28	*R.nitidus*	0.041599	0.064237
MY6	SC28	GD28	*R.nitidus*	0.035801	0.065774
SC26	SC28	GD28	*R.nitidus*	-0.004983	0.065637
SC32	SC28	GD28	*R.nitidus*	0.10989	0.065219
SC24	SC28	GD28	*R.nitidus*	0.049828	0.067345
GD22	SC31	GD28	*R.nitidus*	0.065142	0.066836
SC45	SC28	GD28	*R.nitidus*	0.07577	0.065057
CS11	SC31	GD28	*R.nitidus*	-0.005323	0.06775
JO5	SC28	GD28	*R.nitidus*	0.094073	0.063916
GD30	SC31	GD28	*R.nitidus*	0.103044	0.069482
CS6	SC28	GD28	*R.nitidus*	0.027096	0.064513
SC27	SC28	GD28	*R.nitidus*	0.049601	0.066727
JO7	SC31	GD28	*R.nitidus*	0.009775	0.073037
GD29	SC31	GD28	*R.nitidus*	0.087077	0.065048
SC31	SC28	GD28	*R.nitidus*	0.05	0.065605
JW4	SC28	GD28	*R.nitidus*	0.228689	0.066608
GD44	SC31	GD28	*R.nitidus*	-0.008112	0.068638
GD32	SC31	GD28	*R.nitidus*	0.008065	0.070305
JW6	SC28	GD28	*R.nitidus*	0.054487	0.071795
GD33	SC31	GD28	*R.nitidus*	0.005022	0.066759
JW7	SC28	GD28	*R.nitidus*	0.050053	0.07155
JO8	SC31	GD28	*R.nitidus*	-0.001015	0.072318
JO3	SC28	GD28	*R.nitidus*	0.049729	0.069471
JO1	SC28	GD28	*R.nitidus*	0.011374	0.06454
SC42	SC28	GD28	*R.nitidus*	0.281122	0.062612
GD42	SC31	GD28	*R.nitidus*	0.011404	0.068457
MY4	SC28	GD28	*R.nitidus*	0.033898	0.066256
SC35	SC28	GD28	*R.nitidus*	0.104661	0.065637
GD40	SC31	GD28	*R.nitidus*	0.045649	0.066296
GD26	SC31	GD28	*R.nitidus*	0.033668	0.06693
GD34	SC31	GD28	*R.nitidus*	0.010029	0.066318
GD25	SC31	GD28	*R.nitidus*	0.054625	0.065571
CS19	SC31	GD28	*R.nitidus*	-0.003737	0.066644
GD31	SC31	GD28	*R.nitidus*	0.039574	0.067652
GD46	SC31	GD28	*R.nitidus*	-0.010359	0.064837
GD35	SC31	GD28	*R.nitidus*	0.018362	0.066165
GD21	SC31	GD28	*R.nitidus*	0.131793	0.066417
CS15	SC31	GD28	*R.nitidus*	0.018409	0.06646
CS12	SC31	GD28	*R.nitidus*	0.008607	0.07006
CS30	SC31	GD28	*R.nitidus*	-0.058824	0.06719
CS13	SC31	GD28	*R.nitidus*	-0.034509	0.069598
GD45	SC31	GD28	*R.nitidus*	0.010475	0.071783
GD48	SC31	GD28	*R.nitidus*	0.037867	0.068455
GD50	SC31	GD28	*R.nitidus*	0.015742	0.068885
GD41	SC31	GD28	*R.nitidus*	-0.005789	0.066841
CS20	SC31	GD28	*R.nitidus*	-0.020046	0.065285
GD49	SC31	GD28	*R.nitidus*	0.086056	0.071533
SC15	SC31	GD28	*R.nitidus*	-0.013393	0.066862
CS16	SC31	GD28	*R.nitidus*	0.015719	0.067687

SC12	SC31	GD28	*R.nitidus*	-0.012638	0.066621
CS17	SC31	GD28	*R.nitidus*	0.033524	0.068501
SC18	SC31	GD28	*R.nitidus*	0.029412	0.066797
CS31	SC31	GD28	*R.nitidus*	-0.013158	0.067455
SC27	SC31	GD28	*R.nitidus*	-0.008987	0.066572
SC5	SC31	GD28	*R.nitidus*	-0.005641	0.06945
CS24	SC31	GD28	*R.nitidus*	-0.024409	0.066561
CS14	SC31	GD28	*R.nitidus*	0.008915	0.069576
SC28	SC31	GD28	*R.nitidus*	-0.05	0.065605
CS22	SC31	GD28	*R.nitidus*	0.017638	0.065573
CS21	SC31	GD28	*R.nitidus*	-0.037441	0.064454
SC10	SC31	GD28	*R.nitidus*	0.06031	0.065813
CS18	SC31	GD28	*R.nitidus*	-0.033898	0.065172
CS28	SC31	GD28	*R.nitidus*	-0.044963	0.067403
CS4	SC31	GD28	*R.nitidus*	-0.017336	0.064771
SC24	SC31	GD28	*R.nitidus*	-0.013019	0.065804
CS23	SC31	GD28	*R.nitidus*	-0.038695	0.06535
SC35	SC31	GD28	*R.nitidus*	0.042468	0.065451
SC42	SC31	GD28	*R.nitidus*	0.211462	0.062336
SC14	SC31	GD28	*R.nitidus*	0.01141	0.067802
CS25	SC31	GD28	*R.nitidus*	-0.005348	0.066895
JO1	SC31	GD28	*R.nitidus*	-0.020129	0.066382
CS6	SC31	GD28	*R.nitidus*	-0.023181	0.066167
JW6	SC31	GD28	*R.nitidus*	0.04791	0.074628
SC26	SC31	GD28	*R.nitidus*	-0.057236	0.066807
JO8	SC32	GD28	*R.nitidus*	-0.002064	0.07236
CS3	SC31	GD28	*R.nitidus*	-0.018735	0.065449
JW7	SC31	GD28	*R.nitidus*	0.030992	0.069604
JO7	SC32	GD28	*R.nitidus*	-0.035015	0.072334
MY4	SC31	GD28	*R.nitidus*	-0.014582	0.066735
SC44	SC31	GD28	*R.nitidus*	0.00565	0.066128
SC6	SC31	GD28	*R.nitidus*	-0.044118	0.064969
SC45	SC31	GD28	*R.nitidus*	0.025875	0.064835
CS11	SC32	GD28	*R.nitidus*	-0.058366	0.068413
GD22	SC32	GD28	*R.nitidus*	0.020055	0.063997
JW4	SC31	GD28	*R.nitidus*	0.183077	0.068037
SC34	SC31	GD28	*R.nitidus*	0.033411	0.065403
GD25	SC32	GD28	*R.nitidus*	0.018059	0.065767
SC32	SC31	GD28	*R.nitidus*	0.047276	0.066389
GD21	SC32	GD28	*R.nitidus*	0.102824	0.066734
JO5	SC31	GD28	*R.nitidus*	0.018298	0.06589
GD31	SC32	GD28	*R.nitidus*	0.001474	0.067507
MY6	SC31	GD28	*R.nitidus*	-0.002318	0.06725
JO3	SC31	GD28	*R.nitidus*	0.031787	0.069513
GD32	SC32	GD28	*R.nitidus*	-0.033501	0.069795
GD29	SC32	GD28	*R.nitidus*	0.068592	0.066595
GD49	SC32	GD28	*R.nitidus*	0.037097	0.069325
GD44	SC32	GD28	*R.nitidus*	-0.056371	0.068305
CS15	SC32	GD28	*R.nitidus*	-0.027842	0.068054
GD46	SC32	GD28	*R.nitidus*	-0.045522	0.066898
GD50	SC32	GD28	*R.nitidus*	-0.004322	0.070416
GD40	SC32	GD28	*R.nitidus*	0.005904	0.064768
GD45	SC32	GD28	*R.nitidus*	-0.043261	0.070616
GD34	SC32	GD28	*R.nitidus*	-0.024496	0.066821
CS28	SC32	GD28	*R.nitidus*	-0.110638	0.068359
GD30	SC32	GD28	*R.nitidus*	0.055511	0.070633

CS12	SC32	GD28	*R.nitidus*	-0.040902	0.068096
GD26	SC32	GD28	*R.nitidus*	0.005801	0.066723
CS31	SC32	GD28	*R.nitidus*	-0.059113	0.067679
GD48	SC32	GD28	*R.nitidus*	0.008972	0.066358
CS18	SC32	GD28	*R.nitidus*	-0.078783	0.065179
CS22	SC32	GD28	*R.nitidus*	-0.033279	0.065782
CS17	SC32	GD28	*R.nitidus*	-0.030691	0.070104
CS13	SC32	GD28	*R.nitidus*	-0.076292	0.068993
CS20	SC32	GD28	*R.nitidus*	-0.068887	0.06552
GD33	SC32	GD28	*R.nitidus*	-0.033213	0.067076
GD41	SC32	GD28	*R.nitidus*	-0.042305	0.065164
GD35	SC32	GD28	*R.nitidus*	-0.021898	0.065385
CS30	SC32	GD28	*R.nitidus*	-0.094003	0.067171
CS16	SC32	GD28	*R.nitidus*	-0.038118	0.066953
CS25	SC32	GD28	*R.nitidus*	-0.050199	0.068957
SC27	SC32	GD28	*R.nitidus*	-0.06328	0.065086
CS14	SC32	GD28	*R.nitidus*	-0.032488	0.070287
CS19	SC32	GD28	*R.nitidus*	-0.0472	0.068374
GD42	SC32	GD28	*R.nitidus*	-0.032895	0.065519
CS4	SC32	GD28	*R.nitidus*	-0.069841	0.065748
CS24	SC32	GD28	*R.nitidus*	-0.072065	0.066875
SC28	SC32	GD28	*R.nitidus*	-0.10989	0.065219
CS21	SC32	GD28	*R.nitidus*	-0.080831	0.063646
SC35	SC32	GD28	*R.nitidus*	0.003273	0.066363
SC12	SC32	GD28	*R.nitidus*	-0.075797	0.067651
SC26	SC32	GD28	*R.nitidus*	-0.093157	0.065993
CS23	SC32	GD28	*R.nitidus*	-0.075228	0.067148
SC5	SC32	GD28	*R.nitidus*	-0.058032	0.071073
CS3	SC32	GD28	*R.nitidus*	-0.062696	0.064634
SC18	SC32	GD28	*R.nitidus*	-0.017606	0.06585
CS6	SC32	GD28	*R.nitidus*	-0.077366	0.064122
SC34	SC32	GD28	*R.nitidus*	-0.009788	0.064048
SC10	SC32	GD28	*R.nitidus*	0.018634	0.067337
SC15	SC32	GD28	*R.nitidus*	-0.058304	0.067949
SC6	SC32	GD28	*R.nitidus*	-0.074627	0.067023
JW4	SC32	GD28	*R.nitidus*	0.151869	0.06783
JW6	SC32	GD28	*R.nitidus*	-0.009326	0.070115
SC24	SC32	GD28	*R.nitidus*	-0.056059	0.066339
JO8	SC34	GD28	*R.nitidus*	0.025667	0.071462
SC42	SC32	GD28	*R.nitidus*	0.204027	0.06253
SC14	SC32	GD28	*R.nitidus*	-0.039063	0.066356
MY4	SC32	GD28	*R.nitidus*	-0.058489	0.065666
GD25	SC34	GD28	*R.nitidus*	0.027656	0.064465
GD29	SC34	GD28	*R.nitidus*	0.063457	0.064707
SC31	SC32	GD28	*R.nitidus*	-0.047276	0.066389
JO3	SC32	GD28	*R.nitidus*	-0.023216	0.069816
GD35	SC34	GD28	*R.nitidus*	0.002224	0.066559
JW7	SC32	GD28	*R.nitidus*	-0.026002	0.070686
GD40	SC34	GD28	*R.nitidus*	0.019076	0.063037
CS11	SC34	GD28	*R.nitidus*	-0.026515	0.066923
GD31	SC34	GD28	*R.nitidus*	0.012879	0.067781
GD21	SC34	GD28	*R.nitidus*	0.111728	0.066235
GD30	SC34	GD28	*R.nitidus*	0.083196	0.068182
GD50	SC34	GD28	*R.nitidus*	-0.020495	0.067249
JO5	SC32	GD28	*R.nitidus*	-0.016733	0.066169
SC45	SC32	GD28	*R.nitidus*	-0.021277	0.065467

GD22	SC34	GD28	*R.nitidus*	0.03391	0.063616
JO7	SC34	GD28	*R.nitidus*	-0.020121	0.070091
SC44	SC32	GD28	*R.nitidus*	-0.0497	0.066745
MY6	SC32	GD28	*R.nitidus*	-0.052092	0.06559
CS14	SC34	GD28	*R.nitidus*	-0.013595	0.068114
JO1	SC32	GD28	*R.nitidus*	-0.070873	0.066281
GD49	SC34	GD28	*R.nitidus*	0.040128	0.066197
GD41	SC34	GD28	*R.nitidus*	-0.030814	0.065206
GD44	SC34	GD28	*R.nitidus*	-0.047655	0.06717
GD26	SC34	GD28	*R.nitidus*	0.016643	0.065534
GD34	SC34	GD28	*R.nitidus*	-0.010784	0.066445
CS19	SC34	GD28	*R.nitidus*	-0.034981	0.066545
GD45	SC34	GD28	*R.nitidus*	-0.004105	0.068973
GD48	SC34	GD28	*R.nitidus*	0.019794	0.06435
GD42	SC34	GD28	*R.nitidus*	-0.019694	0.065314
GD32	SC34	GD28	*R.nitidus*	0.00659	0.069165
CS12	SC34	GD28	*R.nitidus*	-0.014646	0.066527
CS16	SC34	GD28	*R.nitidus*	-0.015456	0.067009
CS17	SC34	GD28	*R.nitidus*	0.003265	0.068269
CS31	SC34	GD28	*R.nitidus*	-0.0384	0.064095
CS24	SC34	GD28	*R.nitidus*	-0.057279	0.063809
CS23	SC34	GD28	*R.nitidus*	-0.061178	0.064578
CS22	SC34	GD28	*R.nitidus*	-0.009063	0.065094
GD33	SC34	GD28	*R.nitidus*	-0.020217	0.064424
SC18	SC34	GD28	*R.nitidus*	0.005072	0.066245
CS3	SC34	GD28	*R.nitidus*	-0.051145	0.065005
CS13	SC34	GD28	*R.nitidus*	-0.051163	0.068423
SC24	SC34	GD28	*R.nitidus*	-0.043154	0.062943
GD46	SC34	GD28	*R.nitidus*	-0.030259	0.063806
CS20	SC34	GD28	*R.nitidus*	-0.053064	0.065606
CS21	SC34	GD28	*R.nitidus*	-0.057604	0.065065
SC10	SC34	GD28	*R.nitidus*	0.027241	0.064869
CS18	SC34	GD28	*R.nitidus*	-0.066009	0.063981
SC31	SC34	GD28	*R.nitidus*	-0.033411	0.065403
SC26	SC34	GD28	*R.nitidus*	-0.084518	0.06474
CS30	SC34	GD28	*R.nitidus*	-0.081867	0.062433
SC27	SC34	GD28	*R.nitidus*	-0.042678	0.065848
CS15	SC34	GD28	*R.nitidus*	-0.013688	0.066432
JW7	SC34	GD28	*R.nitidus*	-0.016899	0.069016
SC28	SC34	GD28	*R.nitidus*	-0.086207	0.064437
SC6	SC34	GD28	*R.nitidus*	-0.069421	0.065865
SC12	SC34	GD28	*R.nitidus*	-0.057823	0.065075
CS4	SC34	GD28	*R.nitidus*	-0.054764	0.063501
SC5	SC34	GD28	*R.nitidus*	-0.036615	0.069013
CS28	SC34	GD28	*R.nitidus*	-0.092715	0.065172
CS25	SC34	GD28	*R.nitidus*	-0.036364	0.067183
SC42	SC34	GD28	*R.nitidus*	0.174411	0.060615
MY6	SC34	GD28	*R.nitidus*	-0.039782	0.064308
SC35	SC34	GD28	*R.nitidus*	0.023293	0.063642
GD26	SC35	GD28	*R.nitidus*	-0.003631	0.064768
CS6	SC34	GD28	*R.nitidus*	-0.053055	0.06293
SC14	SC34	GD28	*R.nitidus*	-0.021294	0.064527
JO3	SC34	GD28	*R.nitidus*	0.020628	0.069793
SC32	SC34	GD28	*R.nitidus*	0.009788	0.064048
SC15	SC34	GD28	*R.nitidus*	-0.037768	0.063681
CS11	SC35	GD28	*R.nitidus*	-0.054217	0.066014

JW6	SC34	GD28	*R.nitidus*	0.013388	0.070029
JO8	SC35	GD28	*R.nitidus*	-0.011944	0.075691
GD33	SC35	GD28	*R.nitidus*	-0.036646	0.065603
JO1	SC34	GD28	*R.nitidus*	-0.054357	0.064985
JO5	SC34	GD28	*R.nitidus*	-0.000813	0.065258
JW4	SC34	GD28	*R.nitidus*	0.170389	0.06395
GD30	SC35	GD28	*R.nitidus*	0.040848	0.068941
GD31	SC35	GD28	*R.nitidus*	-0.004619	0.065101
SC45	SC34	GD28	*R.nitidus*	0.000769	0.064343
CS13	SC35	GD28	*R.nitidus*	-0.087812	0.067099
MY4	SC34	GD28	*R.nitidus*	-0.052923	0.066458
GD29	SC35	GD28	*R.nitidus*	0.039855	0.063754
JO7	SC35	GD28	*R.nitidus*	-0.017734	0.070084
GD34	SC35	GD28	*R.nitidus*	-0.034458	0.066232
GD44	SC35	GD28	*R.nitidus*	-0.059939	0.065561
GD35	SC35	GD28	*R.nitidus*	-0.02568	0.065144
SC44	SC34	GD28	*R.nitidus*	-0.029851	0.065118
GD21	SC35	GD28	*R.nitidus*	0.091292	0.06519
GD40	SC35	GD28	*R.nitidus*	-0.009468	0.061048
GD41	SC35	GD28	*R.nitidus*	-0.047076	0.064118
GD25	SC35	GD28	*R.nitidus*	0.010574	0.065394
GD32	SC35	GD28	*R.nitidus*	-0.053018	0.067277
CS12	SC35	GD28	*R.nitidus*	-0.051522	0.067242
GD22	SC35	GD28	*R.nitidus*	0.00419	0.064861
GD50	SC35	GD28	*R.nitidus*	-0.023458	0.06789
CS25	SC35	GD28	*R.nitidus*	-0.061051	0.065195
CS21	SC35	GD28	*R.nitidus*	-0.092879	0.064662
CS20	SC35	GD28	*R.nitidus*	-0.077903	0.064391
GD49	SC35	GD28	*R.nitidus*	0.022293	0.066164
CS19	SC35	GD28	*R.nitidus*	-0.06862	0.067179
GD45	SC35	GD28	*R.nitidus*	-0.031811	0.069417
CS24	SC35	GD28	*R.nitidus*	-0.078905	0.065179
CS4	SC35	GD28	*R.nitidus*	-0.06827	0.0647
GD46	SC35	GD28	*R.nitidus*	-0.050651	0.063027
GD48	SC35	GD28	*R.nitidus*	-0.013428	0.065727
CS18	SC35	GD28	*R.nitidus*	-0.09	0.064563
CS16	SC35	GD28	*R.nitidus*	-0.051013	0.06624
GD42	SC35	GD28	*R.nitidus*	-0.038298	0.064115
CS17	SC35	GD28	*R.nitidus*	-0.015176	0.066687
SC10	SC35	GD28	*R.nitidus*	0.009394	0.065514
SC26	SC35	GD28	*R.nitidus*	-0.108065	0.064553
CS15	SC35	GD28	*R.nitidus*	-0.036819	0.065923
CS14	SC35	GD28	*R.nitidus*	-0.039641	0.066905
CS28	SC35	GD28	*R.nitidus*	-0.08	0.067105
SC6	SC35	GD28	*R.nitidus*	-0.09	0.066946
CS23	SC35	GD28	*R.nitidus*	-0.090008	0.065238
CS3	SC35	GD28	*R.nitidus*	-0.068147	0.063057
SC5	SC35	GD28	*R.nitidus*	-0.053366	0.068528
CS30	SC35	GD28	*R.nitidus*	-0.107741	0.064722
SC15	SC35	GD28	*R.nitidus*	-0.060201	0.067261
CS22	SC35	GD28	*R.nitidus*	-0.044444	0.066929
SC24	SC35	GD28	*R.nitidus*	-0.067268	0.063766
CS31	SC35	GD28	*R.nitidus*	-0.058314	0.062892
SC42	SC35	GD28	*R.nitidus*	0.180079	0.061748
SC31	SC35	GD28	*R.nitidus*	-0.042468	0.065451
SC28	SC35	GD28	*R.nitidus*	-0.104661	0.065637

SC18	SC35	GD28	*R.nitidus*	-0.019475	0.064126
SC44	SC35	GD28	*R.nitidus*	-0.060556	0.065795
CS6	SC35	GD28	*R.nitidus*	-0.085278	0.064143
SC45	SC35	GD28	*R.nitidus*	-0.013093	0.064616
GD22	CS6	GD28	*R.nitidus*	0.097834	0.065486
JO3	SC35	GD28	*R.nitidus*	-0.015315	0.069164
SC12	SC35	GD28	*R.nitidus*	-0.085833	0.064167
SC14	SC35	GD28	*R.nitidus*	-0.036029	0.063816
JW7	SC35	GD28	*R.nitidus*	-0.029622	0.06868
JW4	SC35	GD28	*R.nitidus*	0.153053	0.067352
SC27	SC35	GD28	*R.nitidus*	-0.065381	0.064811
GD30	CS6	GD28	*R.nitidus*	0.122074	0.067145
JO8	CS6	GD28	*R.nitidus*	0.040948	0.071838
JW6	SC35	GD28	*R.nitidus*	0	0.070559
JO5	SC35	GD28	*R.nitidus*	-0.025539	0.06565
SC34	SC35	GD28	*R.nitidus*	-0.023293	0.063642
SC32	SC35	GD28	*R.nitidus*	-0.003273	0.066363
GD31	CS6	GD28	*R.nitidus*	0.068882	0.069563
JO7	CS6	GD28	*R.nitidus*	0.034836	0.070443
GD21	CS6	GD28	*R.nitidus*	0.169881	0.065589
GD26	CS6	GD28	*R.nitidus*	0.058779	0.066417
MY4	SC35	GD28	*R.nitidus*	-0.079327	0.065962
JO1	SC35	GD28	*R.nitidus*	-0.088113	0.064742
GD49	CS6	GD28	*R.nitidus*	0.096499	0.068561
GD44	CS6	GD28	*R.nitidus*	0.022314	0.068227
CS12	CS6	GD28	*R.nitidus*	0.041246	0.066155
CS16	CS6	GD28	*R.nitidus*	0.03104	0.066393
GD41	CS6	GD28	*R.nitidus*	0.029908	0.066496
MY6	SC35	GD28	*R.nitidus*	-0.067353	0.065408
GD45	CS6	GD28	*R.nitidus*	0.031905	0.074373
GD33	CS6	GD28	*R.nitidus*	0.040396	0.067417
CS13	CS6	GD28	*R.nitidus*	-0.005068	0.068734
GD29	CS6	GD28	*R.nitidus*	0.112855	0.065596
CS19	CS6	GD28	*R.nitidus*	0.033236	0.067323
GD25	CS6	GD28	*R.nitidus*	0.080343	0.065002
CS11	CS6	GD28	*R.nitidus*	0.019151	0.066882
GD32	CS6	GD28	*R.nitidus*	0.0625	0.070083
GD50	CS6	GD28	*R.nitidus*	0.044505	0.072123
CS17	CS6	GD28	*R.nitidus*	0.039234	0.069351
GD42	CS6	GD28	*R.nitidus*	0.051223	0.066727
CS23	CS6	GD28	*R.nitidus*	-0.01131	0.063819
GD35	CS6	GD28	*R.nitidus*	0.059459	0.068097
GD40	CS6	GD28	*R.nitidus*	0.084639	0.065456
CS31	CS6	GD28	*R.nitidus*	0.024829	0.06599
GD46	CS6	GD28	*R.nitidus*	0.022273	0.065764
CS18	CS6	GD28	*R.nitidus*	-0.008876	0.06517
GD34	CS6	GD28	*R.nitidus*	0.044294	0.06644
CS14	CS6	GD28	*R.nitidus*	0.053827	0.066584
CS21	CS6	GD28	*R.nitidus*	-0.003	0.063841
GD48	CS6	GD28	*R.nitidus*	0.071546	0.066227
CS15	CS6	GD28	*R.nitidus*	0.045119	0.067078
CS3	CS6	GD28	*R.nitidus*	0.015521	0.062391
SC28	CS6	GD28	*R.nitidus*	-0.027096	0.064513
SC24	CS6	GD28	*R.nitidus*	0.037931	0.066706
SC5	CS6	GD28	*R.nitidus*	0.033773	0.06729
SC14	CS6	GD28	*R.nitidus*	0.045605	0.063481

CS24	CS6	GD28	*R.nitidus*	0.009285	0.065805
SC44	CS6	GD28	*R.nitidus*	0.039562	0.065087
CS20	CS6	GD28	*R.nitidus*	0.00641	0.06412
SC12	CS6	GD28	*R.nitidus*	0.023891	0.065662
CS30	CS6	GD28	*R.nitidus*	-0.028668	0.065698
JW7	CS6	GD28	*R.nitidus*	0.036453	0.07087
SC18	CS6	GD28	*R.nitidus*	0.069991	0.064753
CS22	CS6	GD28	*R.nitidus*	0.054767	0.0659
SC34	CS6	GD28	*R.nitidus*	0.053055	0.06293
SC26	CS6	GD28	*R.nitidus*	-0.014423	0.064528
CS28	CS6	GD28	*R.nitidus*	-0.010695	0.067
CS25	CS6	GD28	*R.nitidus*	0.039106	0.066618
SC32	CS6	GD28	*R.nitidus*	0.077366	0.064122
SC10	CS6	GD28	*R.nitidus*	0.088211	0.066166
CS4	CS6	GD28	*R.nitidus*	0.000995	0.064118
SC31	CS6	GD28	*R.nitidus*	0.023181	0.066167
SC6	CS6	GD28	*R.nitidus*	-0.002538	0.062731
JO5	CS6	GD28	*R.nitidus*	0.05363	0.065894
JW6	CS6	GD28	*R.nitidus*	0.050989	0.069944
SC27	CS6	GD28	*R.nitidus*	0.030833	0.065721
JW4	CS6	GD28	*R.nitidus*	0.199511	0.064839
GD30	SC42	GD28	*R.nitidus*	-0.126287	0.065991
SC15	CS6	GD28	*R.nitidus*	0.02911	0.066422
JO3	CS6	GD28	*R.nitidus*	0.080074	0.069077
GD31	SC42	GD28	*R.nitidus*	-0.182228	0.063251
GD42	SC42	GD28	*R.nitidus*	-0.216068	0.062635
SC35	CS6	GD28	*R.nitidus*	0.085278	0.064143
GD33	SC42	GD28	*R.nitidus*	-0.203359	0.065139
JO1	CS6	GD28	*R.nitidus*	0.001656	0.065876
GD45	SC42	GD28	*R.nitidus*	-0.193931	0.067491
SC42	CS6	GD28	*R.nitidus*	0.227786	0.062343
SC45	CS6	GD28	*R.nitidus*	0.054143	0.065359
GD25	SC42	GD28	*R.nitidus*	-0.169775	0.063786
MY6	CS6	GD28	*R.nitidus*	0.022293	0.064028
JO7	SC42	GD28	*R.nitidus*	-0.145519	0.069467
CS11	SC42	GD28	*R.nitidus*	-0.21549	0.064464
GD32	SC42	GD28	*R.nitidus*	-0.18637	0.065969
JO8	SC42	GD28	*R.nitidus*	-0.123944	0.070586
MY4	CS6	GD28	*R.nitidus*	0.012077	0.065409
GD21	SC42	GD28	*R.nitidus*	-0.116938	0.0624
GD29	SC42	GD28	*R.nitidus*	-0.104468	0.063435
GD22	SC42	GD28	*R.nitidus*	-0.151211	0.062454
GD40	SC42	GD28	*R.nitidus*	-0.168719	0.06316
GD49	SC42	GD28	*R.nitidus*	-0.134577	0.065078
GD34	SC42	GD28	*R.nitidus*	-0.205648	0.063096
GD35	SC42	GD28	*R.nitidus*	-0.198922	0.063758
CS13	SC42	GD28	*R.nitidus*	-0.238496	0.065254
GD48	SC42	GD28	*R.nitidus*	-0.152713	0.064802
CS19	SC42	GD28	*R.nitidus*	-0.213997	0.064291
CS21	SC42	GD28	*R.nitidus*	-0.24586	0.062913
GD41	SC42	GD28	*R.nitidus*	-0.215244	0.062133
CS14	SC42	GD28	*R.nitidus*	-0.194668	0.066405
CS23	SC42	GD28	*R.nitidus*	-0.247332	0.062433
GD26	SC42	GD28	*R.nitidus*	-0.173833	0.063888
GD44	SC42	GD28	*R.nitidus*	-0.214194	0.065162
CS12	SC42	GD28	*R.nitidus*	-0.221109	0.064253

CS30	SC42	GD28	*R.nitidus*	-0.26943	0.062051
CS18	SC42	GD28	*R.nitidus*	-0.241401	0.062655
CS15	SC42	GD28	*R.nitidus*	-0.200631	0.064023
CS22	SC42	GD28	*R.nitidus*	-0.207277	0.063448
GD46	SC42	GD28	*R.nitidus*	-0.217208	0.06281
GD50	SC42	GD28	*R.nitidus*	-0.198407	0.067062
CS24	SC42	GD28	*R.nitidus*	-0.241514	0.06295
CS28	SC42	GD28	*R.nitidus*	-0.269068	0.061463
CS17	SC42	GD28	*R.nitidus*	-0.184421	0.064588
SC6	SC42	GD28	*R.nitidus*	-0.269257	0.060803
CS25	SC42	GD28	*R.nitidus*	-0.222295	0.064526
SC26	SC42	GD28	*R.nitidus*	-0.267275	0.062033
CS16	SC42	GD28	*R.nitidus*	-0.222788	0.063236
CS20	SC42	GD28	*R.nitidus*	-0.229856	0.064091
SC15	SC42	GD28	*R.nitidus*	-0.242984	0.063067
CS4	SC42	GD28	*R.nitidus*	-0.243648	0.062408
SC10	SC42	GD28	*R.nitidus*	-0.176812	0.062151
SC44	SC42	GD28	*R.nitidus*	-0.229295	0.062633
SC27	SC42	GD28	*R.nitidus*	-0.245166	0.062249
CS3	SC42	GD28	*R.nitidus*	-0.237367	0.06221
SC5	SC42	GD28	*R.nitidus*	-0.242739	0.067232
JO8	SC44	GD28	*R.nitidus*	0.017505	0.073195
CS6	SC42	GD28	*R.nitidus*	-0.227786	0.062343
CS31	SC42	GD28	*R.nitidus*	-0.244048	0.061959
MY6	SC42	GD28	*R.nitidus*	-0.229465	0.064023
SC31	SC42	GD28	*R.nitidus*	-0.211462	0.062336
JW4	SC42	GD28	*R.nitidus*	0.001416	0.065063
SC34	SC42	GD28	*R.nitidus*	-0.174411	0.060615
JO7	SC44	GD28	*R.nitidus*	0.020063	0.074211
JW7	SC42	GD28	*R.nitidus*	-0.192813	0.06869
MY4	SC42	GD28	*R.nitidus*	-0.232166	0.064035
GD26	SC44	GD28	*R.nitidus*	0.03209	0.065334
JO1	SC42	GD28	*R.nitidus*	-0.256181	0.062254
GD31	SC44	GD28	*R.nitidus*	0.045814	0.067757
JW6	SC42	GD28	*R.nitidus*	-0.139892	0.068744
SC24	SC42	GD28	*R.nitidus*	-0.248331	0.063018
SC12	SC42	GD28	*R.nitidus*	-0.261624	0.062063
JO5	SC42	GD28	*R.nitidus*	-0.205903	0.064042
SC35	SC42	GD28	*R.nitidus*	-0.180079	0.061748
SC28	SC42	GD28	*R.nitidus*	-0.281122	0.062612
SC14	SC42	GD28	*R.nitidus*	-0.226223	0.062492
SC18	SC42	GD28	*R.nitidus*	-0.206775	0.06354
SC32	SC42	GD28	*R.nitidus*	-0.204027	0.06253
CS11	SC44	GD28	*R.nitidus*	0.00152	0.069364
SC45	SC42	GD28	*R.nitidus*	-0.207766	0.064905
GD25	SC44	GD28	*R.nitidus*	0.0625	0.06589
GD22	SC44	GD28	*R.nitidus*	0.067014	0.066288
GD42	SC44	GD28	*R.nitidus*	0.003846	0.067699
JO3	SC42	GD28	*R.nitidus*	-0.136578	0.067058
GD33	SC44	GD28	*R.nitidus*	0	0.06564
GD21	SC44	GD28	*R.nitidus*	0.134209	0.065483
GD30	SC44	GD28	*R.nitidus*	0.078925	0.067319
GD35	SC44	GD28	*R.nitidus*	0.026758	0.067394
CS18	SC44	GD28	*R.nitidus*	-0.035549	0.066524
GD41	SC44	GD28	*R.nitidus*	-0.009985	0.065361
GD44	SC44	GD28	*R.nitidus*	-0.010359	0.069425

CS12	SC44	GD28	*R.nitidus*	0.00986	0.069489
CS15	SC44	GD28	*R.nitidus*	0.016588	0.068856
GD40	SC44	GD28	*R.nitidus*	0.048451	0.065399
GD45	SC44	GD28	*R.nitidus*	0.010187	0.069397
GD29	SC44	GD28	*R.nitidus*	0.09186	0.065869
CS22	SC44	GD28	*R.nitidus*	0.018125	0.067143
GD32	SC44	GD28	*R.nitidus*	0.004367	0.070725
CS28	SC44	GD28	*R.nitidus*	-0.051871	0.067223
CS17	SC44	GD28	*R.nitidus*	0.036013	0.07062
CS23	SC44	GD28	*R.nitidus*	-0.028059	0.066189
CS19	SC44	GD28	*R.nitidus*	-8.00E-04	0.067821
GD48	SC44	GD28	*R.nitidus*	0.038363	0.067883
GD34	SC44	GD28	*R.nitidus*	0.015186	0.065919
CS21	SC44	GD28	*R.nitidus*	-0.033977	0.066135
GD49	SC44	GD28	*R.nitidus*	0.081104	0.069108
SC6	SC44	GD28	*R.nitidus*	-0.035508	0.068209
CS13	SC44	GD28	*R.nitidus*	-0.019732	0.069694
CS14	SC44	GD28	*R.nitidus*	0.01548	0.070487
CS16	SC44	GD28	*R.nitidus*	0.0088	0.068709
GD46	SC44	GD28	*R.nitidus*	-0.00761	0.064742
CS20	SC44	GD28	*R.nitidus*	-0.026398	0.06696
CS4	SC44	GD28	*R.nitidus*	-0.021226	0.064533
SC14	SC44	GD28	*R.nitidus*	0.010435	0.066739
SC32	SC44	GD28	*R.nitidus*	0.0497	0.066745
CS6	SC44	GD28	*R.nitidus*	-0.039562	0.065087
SC5	SC44	GD28	*R.nitidus*	-0.012464	0.069561
CS24	SC44	GD28	*R.nitidus*	-0.020525	0.066508
SC10	SC44	GD28	*R.nitidus*	0.067682	0.068076
GD50	SC44	GD28	*R.nitidus*	0.027027	0.072024
CS3	SC44	GD28	*R.nitidus*	-0.012275	0.066153
CS30	SC44	GD28	*R.nitidus*	-0.05	0.066965
SC15	SC44	GD28	*R.nitidus*	-0.006034	0.067248
JO1	SC44	GD28	*R.nitidus*	-0.031436	0.067468
JO7	SC45	GD28	*R.nitidus*	-0.029258	0.070658
SC28	SC44	GD28	*R.nitidus*	-0.075145	0.062804
CS25	SC44	GD28	*R.nitidus*	0.006541	0.069238
SC45	SC44	GD28	*R.nitidus*	0.02799	0.064865
JW7	SC44	GD28	*R.nitidus*	0.002086	0.071697
MY4	SC44	GD28	*R.nitidus*	-0.019324	0.065926
JW4	SC44	GD28	*R.nitidus*	0.189935	0.067325
JW6	SC44	GD28	*R.nitidus*	0.04837	0.073483
JO5	SC44	GD28	*R.nitidus*	0.030858	0.065008
CS31	SC44	GD28	*R.nitidus*	-0.013029	0.066483
SC27	SC44	GD28	*R.nitidus*	-0.00354	0.066529
SC35	SC44	GD28	*R.nitidus*	0.060556	0.065795
JO3	SC44	GD28	*R.nitidus*	0.023508	0.069392
MY6	SC44	GD28	*R.nitidus*	-0.01454	0.066825
SC18	SC44	GD28	*R.nitidus*	0.040516	0.067893
SC12	SC44	GD28	*R.nitidus*	-0.015343	0.067606
SC26	SC44	GD28	*R.nitidus*	-0.046257	0.066461
SC24	SC44	GD28	*R.nitidus*	-0.000864	0.068232
SC31	SC44	GD28	*R.nitidus*	-0.00565	0.066128
SC42	SC44	GD28	*R.nitidus*	0.229295	0.062633
SC34	SC44	GD28	*R.nitidus*	0.029851	0.065118
GD41	SC45	GD28	*R.nitidus*	-0.032258	0.066179
GD22	SC45	GD28	*R.nitidus*	0.025974	0.066403

GD30	SC45	GD28	*R.nitidus*	0.069544	0.069731
GD29	SC45	GD28	*R.nitidus*	0.069454	0.06748
GD33	SC45	GD28	*R.nitidus*	-0.029496	0.065998
JO8	SC45	GD28	*R.nitidus*	-0.008325	0.072674
CS11	SC45	GD28	*R.nitidus*	-0.030511	0.069817
GD25	SC45	GD28	*R.nitidus*	0.028682	0.066528
GD26	SC45	GD28	*R.nitidus*	0.00369	0.066024
CS15	SC45	GD28	*R.nitidus*	-0.006093	0.069535
GD34	SC45	GD28	*R.nitidus*	-0.018638	0.066508
GD21	SC45	GD28	*R.nitidus*	0.101322	0.066613
CS12	SC45	GD28	*R.nitidus*	-0.0264	0.070106
GD48	SC45	GD28	*R.nitidus*	0.01346	0.067538
CS19	SC45	GD28	*R.nitidus*	-0.04422	0.067622
GD35	SC45	GD28	*R.nitidus*	-0.02071	0.066595
GD46	SC45	GD28	*R.nitidus*	-0.038123	0.065798
GD45	SC45	GD28	*R.nitidus*	-0.017827	0.06902
CS22	SC45	GD28	*R.nitidus*	-0.017516	0.066667
GD42	SC45	GD28	*R.nitidus*	-0.029155	0.065723
GD31	SC45	GD28	*R.nitidus*	0.012355	0.069181
GD49	SC45	GD28	*R.nitidus*	0.042834	0.070077
CS13	SC45	GD28	*R.nitidus*	-0.054805	0.070436
GD32	SC45	GD28	*R.nitidus*	-0.015523	0.070514
CS16	SC45	GD28	*R.nitidus*	-0.025404	0.068191
GD44	SC45	GD28	*R.nitidus*	-0.043818	0.069293
GD50	SC45	GD28	*R.nitidus*	-0.007042	0.072071
SC10	SC45	GD28	*R.nitidus*	0.036301	0.067149
GD40	SC45	GD28	*R.nitidus*	0.007429	0.066069
CS17	SC45	GD28	*R.nitidus*	0.008821	0.070514
CS4	SC45	GD28	*R.nitidus*	-0.051262	0.06555
CS3	SC45	GD28	*R.nitidus*	-0.04406	0.066287
CS20	SC45	GD28	*R.nitidus*	-0.057767	0.065323
CS31	SC45	GD28	*R.nitidus*	-0.031671	0.066645
CS21	SC45	GD28	*R.nitidus*	-0.065152	0.065497
CS18	SC45	GD28	*R.nitidus*	-0.070938	0.065293
CS14	SC45	GD28	*R.nitidus*	-0.021244	0.070031
CS23	SC45	GD28	*R.nitidus*	-0.07027	0.065674
CS25	SC45	GD28	*R.nitidus*	-0.048896	0.066691
SC27	SC45	GD28	*R.nitidus*	-0.039075	0.065689
SC28	SC45	GD28	*R.nitidus*	-0.07577	0.065057
JO5	SC45	GD28	*R.nitidus*	-0.014851	0.066775
SC44	SC45	GD28	*R.nitidus*	-0.02799	0.064865
CS30	SC45	GD28	*R.nitidus*	-0.084691	0.067309
MY4	SC45	GD28	*R.nitidus*	-0.039557	0.067011
SC14	SC45	GD28	*R.nitidus*	-0.02502	0.068243
SC5	SC45	GD28	*R.nitidus*	-0.043838	0.069303
CS24	SC45	GD28	*R.nitidus*	-0.049844	0.066187
CS28	SC45	GD28	*R.nitidus*	-0.074858	0.068788
SC24	SC45	GD28	*R.nitidus*	-0.04876	0.066806
SC32	SC45	GD28	*R.nitidus*	0.021277	0.065467
CS6	SC45	GD28	*R.nitidus*	-0.054143	0.065359
JW6	SC45	GD28	*R.nitidus*	-0.01217	0.072358
SC6	SC45	GD28	*R.nitidus*	-0.060231	0.068383
SC18	SC45	GD28	*R.nitidus*	0.004355	0.067251
SC12	SC45	GD28	*R.nitidus*	-0.057709	0.064977
SC15	SC45	GD28	*R.nitidus*	-0.039004	0.067116
SC34	SC45	GD28	*R.nitidus*	-0.000769	0.064343

GD29	MY4	GD28	*R.nitidus*	0.11372	0.065817
JW7	SC45	GD28	*R.nitidus*	-0.021569	0.071481
SC26	SC45	GD28	*R.nitidus*	-0.086109	0.06775
MY6	SC45	GD28	*R.nitidus*	-0.04379	0.067582
GD26	MY4	GD28	*R.nitidus*	0.056255	0.065509
SC31	SC45	GD28	*R.nitidus*	-0.025875	0.064835
CS12	MY4	GD28	*R.nitidus*	0.028685	0.069126
GD21	MY4	GD28	*R.nitidus*	0.156003	0.065833
SC42	SC45	GD28	*R.nitidus*	0.207766	0.064905
JO3	SC45	GD28	*R.nitidus*	-0.002588	0.071314
SC35	SC45	GD28	*R.nitidus*	0.013093	0.064616
JO1	SC45	GD28	*R.nitidus*	-0.068936	0.066931
GD25	MY4	GD28	*R.nitidus*	0.083521	0.066031
JW4	SC45	GD28	*R.nitidus*	0.166263	0.067518
JO7	MY4	GD28	*R.nitidus*	0.023061	0.072149
JO8	MY4	GD28	*R.nitidus*	0.031646	0.073396
GD32	MY4	GD28	*R.nitidus*	0.038851	0.070966
GD35	MY4	GD28	*R.nitidus*	0.047232	0.066248
GD42	MY4	GD28	*R.nitidus*	0.020104	0.066792
GD31	MY4	GD28	*R.nitidus*	0.066667	0.068315
GD30	MY4	GD28	*R.nitidus*	0.116585	0.069232
CS11	MY4	GD28	*R.nitidus*	0.008327	0.068184
CS14	MY4	GD28	*R.nitidus*	0.023238	0.070031
GD22	MY4	GD28	*R.nitidus*	0.080344	0.066204
CS17	MY4	GD28	*R.nitidus*	0.031376	0.070865
CS23	MY4	GD28	*R.nitidus*	-0.026965	0.066309
GD44	MY4	GD28	*R.nitidus*	0.014151	0.067147
CS15	MY4	GD28	*R.nitidus*	0.034091	0.069809
GD40	MY4	GD28	*R.nitidus*	0.065886	0.064667
GD33	MY4	GD28	*R.nitidus*	0.016047	0.066042
GD46	MY4	GD28	*R.nitidus*	0.006798	0.065789
GD34	MY4	GD28	*R.nitidus*	0.027778	0.066245
CS16	MY4	GD28	*R.nitidus*	0.019697	0.068563
GD41	MY4	GD28	*R.nitidus*	0.012696	0.064899
SC10	MY4	GD28	*R.nitidus*	0.09166	0.068155
CS13	MY4	GD28	*R.nitidus*	-0.019496	0.069914
GD45	MY4	GD28	*R.nitidus*	0.041975	0.069244
GD50	MY4	GD28	*R.nitidus*	0.062663	0.06922
CS3	MY4	GD28	*R.nitidus*	-0.013057	0.065114
CS18	MY4	GD28	*R.nitidus*	-0.029134	0.065954
SC18	MY4	GD28	*R.nitidus*	0.05807	0.066347
SC5	MY4	GD28	*R.nitidus*	0.00083	0.069744
SC6	MY4	GD28	*R.nitidus*	-0.017945	0.066805
CS22	MY4	GD28	*R.nitidus*	0.035541	0.066917
SC14	MY4	GD28	*R.nitidus*	0.027712	0.067986
CS20	MY4	GD28	*R.nitidus*	-0.011346	0.066621
CS4	MY4	GD28	*R.nitidus*	-0.012851	0.066431
CS24	MY4	GD28	*R.nitidus*	-0.007806	0.067515
GD48	MY4	GD28	*R.nitidus*	0.059738	0.067846
CS25	MY4	GD28	*R.nitidus*	0.010277	0.066679
SC24	MY4	GD28	*R.nitidus*	0.004946	0.066938
CS19	MY4	GD28	*R.nitidus*	0.009554	0.069618
SC12	MY4	GD28	*R.nitidus*	-0.002467	0.067344
CS21	MY4	GD28	*R.nitidus*	-0.024559	0.066035
CS30	MY4	GD28	*R.nitidus*	-0.036154	0.068998
CS28	MY4	GD28	*R.nitidus*	-0.039967	0.0677

CS31	MY4	GD28	*R.nitidus*	0.012739	0.068284
SC31	MY4	GD28	*R.nitidus*	0.014582	0.066735
JW4	MY4	GD28	*R.nitidus*	0.200491	0.068165
SC28	MY4	GD28	*R.nitidus*	-0.033898	0.066256
SC34	MY4	GD28	*R.nitidus*	0.052923	0.066458
SC15	MY4	GD28	*R.nitidus*	0.013267	0.068742
SC42	MY4	GD28	*R.nitidus*	0.232166	0.064035
MY6	MY4	GD28	*R.nitidus*	0.004802	0.059254
GD26	MY6	GD28	*R.nitidus*	0.058209	0.066644
GD31	MY6	GD28	*R.nitidus*	0.058777	0.065987
JO7	MY6	GD28	*R.nitidus*	0.02766	0.069379
GD49	MY4	GD28	*R.nitidus*	0.08	0.069688
JO5	MY4	GD28	*R.nitidus*	0.044728	0.068217
SC26	MY4	GD28	*R.nitidus*	-0.039352	0.063741
SC32	MY4	GD28	*R.nitidus*	0.058489	0.065666
GD34	MY6	GD28	*R.nitidus*	0.030702	0.064773
JW7	MY4	GD28	*R.nitidus*	0.017839	0.072134
SC35	MY4	GD28	*R.nitidus*	0.079327	0.065962
SC27	MY4	GD28	*R.nitidus*	0.012618	0.066502
CS12	MY6	GD28	*R.nitidus*	0.022943	0.067795
GD22	MY6	GD28	*R.nitidus*	0.075672	0.065981
JW6	MY4	GD28	*R.nitidus*	0.059385	0.070948
JO1	MY4	GD28	*R.nitidus*	-0.012998	0.067159
SC45	MY4	GD28	*R.nitidus*	0.039557	0.067011
SC44	MY4	GD28	*R.nitidus*	0.019324	0.065926
CS6	MY4	GD28	*R.nitidus*	-0.012077	0.065409
GD25	MY6	GD28	*R.nitidus*	0.067278	0.064725
JO3	MY4	GD28	*R.nitidus*	0.037341	0.070698
GD29	MY6	GD28	*R.nitidus*	0.107659	0.064593
JO8	MY6	GD28	*R.nitidus*	0.017699	0.072626
CS11	MY6	GD28	*R.nitidus*	0.008378	0.068125
GD30	MY6	GD28	*R.nitidus*	0.103563	0.067384
GD42	MY6	GD28	*R.nitidus*	0.016776	0.065845
GD40	MY6	GD28	*R.nitidus*	0.059341	0.063422
GD41	MY6	GD28	*R.nitidus*	0.007225	0.065325
GD50	MY6	GD28	*R.nitidus*	0.03856	0.068716
GD45	MY6	GD28	*R.nitidus*	0.032527	0.069933
GD21	MY6	GD28	*R.nitidus*	0.150822	0.065871
GD32	MY6	GD28	*R.nitidus*	0.039037	0.070228
CS24	MY6	GD28	*R.nitidus*	-0.007837	0.067015
CS19	MY6	GD28	*R.nitidus*	0.000767	0.068042
CS15	MY6	GD28	*R.nitidus*	0.027544	0.067212
GD49	MY6	GD28	*R.nitidus*	0.078163	0.069571
CS13	MY6	GD28	*R.nitidus*	-0.026232	0.068006
GD33	MY6	GD28	*R.nitidus*	0.015681	0.066339
GD35	MY6	GD28	*R.nitidus*	0.037064	0.065481
CS20	MY6	GD28	*R.nitidus*	-0.011211	0.065201
CS22	MY6	GD28	*R.nitidus*	0.029459	0.065268
GD44	MY6	GD28	*R.nitidus*	0.007825	0.067223
CS4	MY6	GD28	*R.nitidus*	-0.015079	0.066119
GD46	MY6	GD28	*R.nitidus*	0.000736	0.063663
GD48	MY6	GD28	*R.nitidus*	0.048249	0.066089
CS28	MY6	GD28	*R.nitidus*	-0.036636	0.068251
CS16	MY6	GD28	*R.nitidus*	0.015385	0.066621
SC6	MY6	GD28	*R.nitidus*	-0.024311	0.06655
SC12	MY6	GD28	*R.nitidus*	-0.009129	0.06858

CS3	MY6	GD28	*R.nitidus*	-0.008475	0.062894
SC28	MY6	GD28	*R.nitidus*	-0.035801	0.065774
CS31	MY6	GD28	*R.nitidus*	0.009615	0.069071
CS14	MY6	GD28	*R.nitidus*	0.023975	0.068767
SC10	MY6	GD28	*R.nitidus*	0.085246	0.065552
SC27	MY6	GD28	*R.nitidus*	0.007059	0.065438
CS23	MY6	GD28	*R.nitidus*	-0.026965	0.063972
SC15	MY6	GD28	*R.nitidus*	0.009796	0.067011
CS18	MY6	GD28	*R.nitidus*	-0.027048	0.064934
CS17	MY6	GD28	*R.nitidus*	0.027397	0.067834
CS25	MY6	GD28	*R.nitidus*	0.002383	0.06841
SC18	MY6	GD28	*R.nitidus*	0.053782	0.065172
SC32	MY6	GD28	*R.nitidus*	0.052092	0.06559
CS30	MY6	GD28	*R.nitidus*	-0.042835	0.067398
CS21	MY6	GD28	*R.nitidus*	-0.025622	0.065161
SC44	MY6	GD28	*R.nitidus*	0.01454	0.066825
SC26	MY6	GD28	*R.nitidus*	-0.036753	0.064254
SC34	MY6	GD28	*R.nitidus*	0.039782	0.064308
JO1	MY6	GD28	*R.nitidus*	-0.019763	0.064473
SC31	MY6	GD28	*R.nitidus*	0.002318	0.06725
SC5	MY6	GD28	*R.nitidus*	0.004839	0.069687
SC45	MY6	GD28	*R.nitidus*	0.04379	0.067582
SC14	MY6	GD28	*R.nitidus*	0.021824	0.066462
CS6	MY6	GD28	*R.nitidus*	-0.022293	0.064028
JW4	MY6	GD28	*R.nitidus*	0.207267	0.068103
SC24	MY6	GD28	*R.nitidus*	-0.001645	0.066027
JW6	MY6	GD28	*R.nitidus*	0.066017	0.069903
GD21	JW4	GD28	*R.nitidus*	-0.050835	0.068464
GD25	JW4	GD28	*R.nitidus*	-0.139888	0.069249
MY4	MY6	GD28	*R.nitidus*	-0.004802	0.059254
GD30	JW4	GD28	*R.nitidus*	-0.093162	0.070539
JO5	MY6	GD28	*R.nitidus*	0.034154	0.066387
JO8	JW4	GD28	*R.nitidus*	-0.118436	0.074867
GD45	JW4	GD28	*R.nitidus*	-0.165194	0.071268
JO3	MY6	GD28	*R.nitidus*	0.034164	0.069667
SC42	MY6	GD28	*R.nitidus*	0.229465	0.064023
SC35	MY6	GD28	*R.nitidus*	0.067353	0.065408
GD32	JW4	GD28	*R.nitidus*	-0.191327	0.069317
GD42	JW4	GD28	*R.nitidus*	-0.161339	0.066333
JO7	JW4	GD28	*R.nitidus*	-0.149563	0.071367
CS11	JW4	GD28	*R.nitidus*	-0.176607	0.067236
JW7	MY6	GD28	*R.nitidus*	0.027245	0.071815
GD46	JW4	GD28	*R.nitidus*	-0.192874	0.066853
GD29	JW4	GD28	*R.nitidus*	-0.108129	0.066943
GD22	JW4	GD28	*R.nitidus*	-0.12101	0.067688
GD49	JW4	GD28	*R.nitidus*	-0.119658	0.070172
GD40	JW4	GD28	*R.nitidus*	-0.126803	0.068318
CS17	JW4	GD28	*R.nitidus*	-0.170976	0.069473
GD44	JW4	GD28	*R.nitidus*	-0.170144	0.069926
CS12	JW4	GD28	*R.nitidus*	-0.178037	0.068872
GD26	JW4	GD28	*R.nitidus*	-0.153724	0.067942
GD31	JW4	GD28	*R.nitidus*	-0.154098	0.069704
CS13	JW4	GD28	*R.nitidus*	-0.205423	0.067705
GD33	JW4	GD28	*R.nitidus*	-0.172628	0.069572
GD34	JW4	GD28	*R.nitidus*	-0.162041	0.068613
CS14	JW4	GD28	*R.nitidus*	-0.162809	0.069642

CS30	JW4	GD28	*R.nitidus*	-0.235527	0.067439
SC10	JW4	GD28	*R.nitidus*	-0.147107	0.068926
GD41	JW4	GD28	*R.nitidus*	-0.178681	0.066633
GD35	JW4	GD28	*R.nitidus*	-0.159574	0.067945
CS16	JW4	GD28	*R.nitidus*	-0.1979	0.06751
CS21	JW4	GD28	*R.nitidus*	-0.220141	0.06732
CS15	JW4	GD28	*R.nitidus*	-0.164263	0.06816
GD48	JW4	GD28	*R.nitidus*	-0.137318	0.069444
SC34	JW4	GD28	*R.nitidus*	-0.170389	0.06395
SC24	JW4	GD28	*R.nitidus*	-0.17502	0.067249
SC35	JW4	GD28	*R.nitidus*	-0.153053	0.067352
GD50	JW4	GD28	*R.nitidus*	-0.156776	0.071156
CS24	JW4	GD28	*R.nitidus*	-0.220353	0.066508
SC12	JW4	GD28	*R.nitidus*	-0.210612	0.066522
CS18	JW4	GD28	*R.nitidus*	-0.213554	0.067217
CS3	JW4	GD28	*R.nitidus*	-0.205696	0.065232
CS31	JW4	GD28	*R.nitidus*	-0.208991	0.066827
SC26	JW4	GD28	*R.nitidus*	-0.229199	0.067098
SC14	JW4	GD28	*R.nitidus*	-0.178824	0.067181
CS19	JW4	GD28	*R.nitidus*	-0.181676	0.068122
CS20	JW4	GD28	*R.nitidus*	-0.194167	0.066865
CS23	JW4	GD28	*R.nitidus*	-0.208238	0.066015
SC27	JW4	GD28	*R.nitidus*	-0.180769	0.06757
CS22	JW4	GD28	*R.nitidus*	-0.190204	0.066206
CS4	JW4	GD28	*R.nitidus*	-0.211831	0.064523
SC42	JW4	GD28	*R.nitidus*	-0.001416	0.065063
SC45	JW4	GD28	*R.nitidus*	-0.166263	0.067518
JO1	JW4	GD28	*R.nitidus*	-0.210912	0.066589
CS25	JW4	GD28	*R.nitidus*	-0.180982	0.069224
CS6	JW4	GD28	*R.nitidus*	-0.199511	0.064839
SC28	JW4	GD28	*R.nitidus*	-0.228689	0.066608
SC5	JW4	GD28	*R.nitidus*	-0.178049	0.069262
CS28	JW4	GD28	*R.nitidus*	-0.223567	0.066774
SC6	JW4	GD28	*R.nitidus*	-0.20239	0.067825
JO5	JW4	GD28	*R.nitidus*	-0.155502	0.067891
MY6	JW4	GD28	*R.nitidus*	-0.207267	0.068103
SC31	JW4	GD28	*R.nitidus*	-0.183077	0.068037
SC15	JW4	GD28	*R.nitidus*	-0.185096	0.068639
CS11	JW6	GD28	*R.nitidus*	-0.052247	0.073578
SC18	JW4	GD28	*R.nitidus*	-0.158155	0.068974
JO8	JW6	GD28	*R.nitidus*	-0.023006	0.074143
CS12	JW6	GD28	*R.nitidus*	-0.025164	0.073895
SC32	JW4	GD28	*R.nitidus*	-0.151869	0.06783
JW6	JW4	GD28	*R.nitidus*	-0.12095	0.072299
GD30	JW6	GD28	*R.nitidus*	0.096774	0.073719
JO3	JW4	GD28	*R.nitidus*	-0.106693	0.072185
GD26	JW6	GD28	*R.nitidus*	0.032291	0.074052
MY4	JW4	GD28	*R.nitidus*	-0.200491	0.068165
GD22	JW6	GD28	*R.nitidus*	0.032381	0.070298
GD32	JW6	GD28	*R.nitidus*	-0.029508	0.074612
GD31	JW6	GD28	*R.nitidus*	0.014463	0.074674
JW7	JW4	GD28	*R.nitidus*	-0.134892	0.071479
GD50	JW6	GD28	*R.nitidus*	0.013905	0.073139
GD25	JW6	GD28	*R.nitidus*	0.018888	0.072192
GD45	JW6	GD28	*R.nitidus*	0.044268	0.07767
CS14	JW6	GD28	*R.nitidus*	-0.018162	0.076023

CS18	JW6	GD28	*R.nitidus*	-0.049797	0.071922
GD21	JW6	GD28	*R.nitidus*	0.101758	0.071059
SC44	JW4	GD28	*R.nitidus*	-0.189935	0.067325
JO7	JW6	GD28	*R.nitidus*	-0.001859	0.071329
GD49	JW6	GD28	*R.nitidus*	0.078801	0.072256
GD35	JW6	GD28	*R.nitidus*	0.00402	0.071835
GD40	JW6	GD28	*R.nitidus*	0.014085	0.070219
GD29	JW6	GD28	*R.nitidus*	0.067698	0.069707
CS28	JW6	GD28	*R.nitidus*	-0.065934	0.071412
GD42	JW6	GD28	*R.nitidus*	0.006073	0.07212
CS3	JW6	GD28	*R.nitidus*	-0.021561	0.072107
CS31	JW6	GD28	*R.nitidus*	-0.055556	0.072148
CS23	JW6	GD28	*R.nitidus*	-0.032385	0.072949
CS21	JW6	GD28	*R.nitidus*	-0.054435	0.071998
GD41	JW6	GD28	*R.nitidus*	-0.030928	0.070849
GD44	JW6	GD28	*R.nitidus*	-0.054639	0.072449
CS4	JW6	GD28	*R.nitidus*	-0.036108	0.07162
GD33	JW6	GD28	*R.nitidus*	-0.026706	0.072532
CS17	JW6	GD28	*R.nitidus*	-0.013857	0.074598
CS24	JW6	GD28	*R.nitidus*	-0.05491	0.071308
CS22	JW6	GD28	*R.nitidus*	-0.038502	0.072585
SC28	JW6	GD28	*R.nitidus*	-0.054487	0.071795
SC6	JW6	GD28	*R.nitidus*	-0.037344	0.072519
GD46	JW6	GD28	*R.nitidus*	-0.047813	0.069866
CS20	JW6	GD28	*R.nitidus*	-0.019646	0.073033
GD48	JW6	GD28	*R.nitidus*	-0.004175	0.070552
CS13	JW6	GD28	*R.nitidus*	-0.056034	0.076375
GD34	JW6	GD28	*R.nitidus*	-0.02178	0.071086
SC15	JW6	GD28	*R.nitidus*	-0.044907	0.074419
CS15	JW6	GD28	*R.nitidus*	-0.003191	0.073279
SC12	JW6	GD28	*R.nitidus*	-0.053608	0.071217
SC14	JW6	GD28	*R.nitidus*	-0.015448	0.070674
SC32	JW6	GD28	*R.nitidus*	0.009326	0.070115
CS16	JW6	GD28	*R.nitidus*	-0.044351	0.073662
CS30	JW6	GD28	*R.nitidus*	-0.074807	0.071635
CS19	JW6	GD28	*R.nitidus*	-0.052128	0.072626
SC34	JW6	GD28	*R.nitidus*	-0.013388	0.070029
SC18	JW6	GD28	*R.nitidus*	-0.017429	0.07141
JW4	JW6	GD28	*R.nitidus*	0.12095	0.072299
MY6	JW6	GD28	*R.nitidus*	-0.066017	0.069903
SC26	JW6	GD28	*R.nitidus*	-0.076433	0.071101
CS25	JW6	GD28	*R.nitidus*	-0.047862	0.072618
SC5	JW6	GD28	*R.nitidus*	-0.01594	0.074171
SC24	JW6	GD28	*R.nitidus*	-0.01824	0.072415
SC35	JW6	GD28	*R.nitidus*	0	0.070559
CS6	JW6	GD28	*R.nitidus*	-0.050989	0.069944
JW7	JW6	GD28	*R.nitidus*	-0.017261	0.072513
SC10	JW6	GD28	*R.nitidus*	0.06424	0.07457
JO7	JW7	GD28	*R.nitidus*	-0.010539	0.073912
SC42	JW6	GD28	*R.nitidus*	0.139892	0.068744
SC45	JW6	GD28	*R.nitidus*	0.01217	0.072358
GD26	JW7	GD28	*R.nitidus*	0.043001	0.072112
GD33	JW7	GD28	*R.nitidus*	-0.008865	0.071614
SC27	JW6	GD28	*R.nitidus*	-0.037696	0.069662
SC31	JW6	GD28	*R.nitidus*	-0.04791	0.074628
GD30	JW7	GD28	*R.nitidus*	0.122302	0.074594

GD40	JW7	GD28	*R.nitidus*	0.042125	0.070227
GD31	JW7	GD28	*R.nitidus*	0.055089	0.072981
SC44	JW6	GD28	*R.nitidus*	-0.04837	0.073483
CS11	JW7	GD28	*R.nitidus*	-0.00789	0.07056
GD22	JW7	GD28	*R.nitidus*	0.076522	0.070947
GD25	JW7	GD28	*R.nitidus*	0.060345	0.071685
GD35	JW7	GD28	*R.nitidus*	0.021998	0.071124
GD21	JW7	GD28	*R.nitidus*	0.143234	0.071301
JO8	JW7	GD28	*R.nitidus*	0.002356	0.075513
MY4	JW6	GD28	*R.nitidus*	-0.059385	0.070948
JO3	JW6	GD28	*R.nitidus*	-0.013547	0.074814
JO5	JW6	GD28	*R.nitidus*	0.004215	0.069053
GD46	JW7	GD28	*R.nitidus*	-0.007505	0.0708
GD32	JW7	GD28	*R.nitidus*	0.017682	0.073787
JO1	JW6	GD28	*R.nitidus*	-0.055731	0.068138
GD48	JW7	GD28	*R.nitidus*	0.017045	0.071602
CS17	JW7	GD28	*R.nitidus*	0.019108	0.07432
GD29	JW7	GD28	*R.nitidus*	0.094323	0.072293
GD42	JW7	GD28	*R.nitidus*	0.034799	0.071898
GD49	JW7	GD28	*R.nitidus*	0.064975	0.074315
CS16	JW7	GD28	*R.nitidus*	-0.012195	0.072766
CS21	JW7	GD28	*R.nitidus*	-0.041627	0.069169
GD45	JW7	GD28	*R.nitidus*	0.037267	0.074486
CS24	JW7	GD28	*R.nitidus*	-0.01433	0.071346
CS19	JW7	GD28	*R.nitidus*	0.003824	0.072583
CS12	JW7	GD28	*R.nitidus*	0.005198	0.072202
SC6	JW7	GD28	*R.nitidus*	-0.033084	0.07334
GD34	JW7	GD28	*R.nitidus*	-0.003623	0.071814
GD44	JW7	GD28	*R.nitidus*	-0.020154	0.070368
CS23	JW7	GD28	*R.nitidus*	-0.024276	0.070772
CS3	JW7	GD28	*R.nitidus*	-0.02595	0.069364
CS30	JW7	GD28	*R.nitidus*	-0.061787	0.070861
GD41	JW7	GD28	*R.nitidus*	0.003724	0.071081
SC10	JW7	GD28	*R.nitidus*	0.048035	0.071106
SC28	JW7	GD28	*R.nitidus*	-0.050053	0.07155
CS28	JW7	GD28	*R.nitidus*	-0.055907	0.071004
CS14	JW7	GD28	*R.nitidus*	0.008729	0.074576
CS15	JW7	GD28	*R.nitidus*	0.030126	0.071915
CS18	JW7	GD28	*R.nitidus*	-0.03016	0.072736
CS20	JW7	GD28	*R.nitidus*	-0.007612	0.072726
GD50	JW7	GD28	*R.nitidus*	0.043663	0.076013
CS4	JW7	GD28	*R.nitidus*	-0.013527	0.069624
CS13	JW7	GD28	*R.nitidus*	-0.039652	0.074978
SC14	JW7	GD28	*R.nitidus*	0.011074	0.069379
CS25	JW7	GD28	*R.nitidus*	0.00779	0.072981
CS22	JW7	GD28	*R.nitidus*	0.006972	0.071052
SC31	JW7	GD28	*R.nitidus*	-0.030992	0.069604
SC12	JW7	GD28	*R.nitidus*	-0.038043	0.070931
CS6	JW7	GD28	*R.nitidus*	-0.036453	0.07087
SC26	JW7	GD28	*R.nitidus*	-0.067218	0.069879
SC35	JW7	GD28	*R.nitidus*	0.029622	0.06868
SC27	JW7	GD28	*R.nitidus*	-0.019123	0.069812
JW6	JW7	GD28	*R.nitidus*	0.017261	0.072513
CS31	JW7	GD28	*R.nitidus*	-0.02863	0.071969
SC34	JW7	GD28	*R.nitidus*	0.016899	0.069016
SC32	JW7	GD28	*R.nitidus*	0.026002	0.070686

SC42	JW7	GD28	*R.nitidus*	0.192813	0.06869
GD22	JO1	GD28	*R.nitidus*	0.091608	0.064606
SC15	JW7	GD28	*R.nitidus*	-0.01921	0.073599
JO7	JO1	GD28	*R.nitidus*	0.010194	0.071871
JO8	JO1	GD28	*R.nitidus*	0.043663	0.071768
SC5	JW7	GD28	*R.nitidus*	-0.007535	0.075929
SC18	JW7	GD28	*R.nitidus*	-0.001058	0.071286
JO5	JW7	GD28	*R.nitidus*	0.020305	0.069856
JW4	JW7	GD28	*R.nitidus*	0.134892	0.071479
JO3	JW7	GD28	*R.nitidus*	0.008858	0.07099
GD21	JO1	GD28	*R.nitidus*	0.169757	0.063336
MY6	JW7	GD28	*R.nitidus*	-0.027245	0.071815
SC24	JW7	GD28	*R.nitidus*	-0.018711	0.072124
JO1	JW7	GD28	*R.nitidus*	-0.032461	0.068164
MY4	JW7	GD28	*R.nitidus*	-0.017839	0.072134
SC44	JW7	GD28	*R.nitidus*	-0.002086	0.071697
SC45	JW7	GD28	*R.nitidus*	0.021569	0.071481
GD41	JO1	GD28	*R.nitidus*	0.014706	0.064475
CS12	JO1	GD28	*R.nitidus*	0.032718	0.067204
GD33	JO1	GD28	*R.nitidus*	0.030258	0.065131
GD29	JO1	GD28	*R.nitidus*	0.121898	0.06422
GD26	JO1	GD28	*R.nitidus*	0.064493	0.064545
GD35	JO1	GD28	*R.nitidus*	0.036003	0.065446
GD40	JO1	GD28	*R.nitidus*	0.068914	0.062174
GD30	JO1	GD28	*R.nitidus*	0.139002	0.066632
GD34	JO1	GD28	*R.nitidus*	0.046931	0.065769
GD31	JO1	GD28	*R.nitidus*	0.070988	0.066549
CS15	JO1	GD28	*R.nitidus*	0.033805	0.065017
CS11	JO1	GD28	*R.nitidus*	0.014832	0.066427
GD44	JO1	GD28	*R.nitidus*	0.006993	0.066616
GD32	JO1	GD28	*R.nitidus*	0.044248	0.069353
GD25	JO1	GD28	*R.nitidus*	0.096774	0.064314
CS24	JO1	GD28	*R.nitidus*	0	0.066447
GD49	JO1	GD28	*R.nitidus*	0.085782	0.067897
CS16	JO1	GD28	*R.nitidus*	0.019231	0.066091
CS20	JO1	GD28	*R.nitidus*	-0.000762	0.065864
SC5	JO1	GD28	*R.nitidus*	0.013169	0.069451
CS4	JO1	GD28	*R.nitidus*	0	0.065549
GD50	JO1	GD28	*R.nitidus*	0.071115	0.069535
GD45	JO1	GD28	*R.nitidus*	0.042502	0.07062
SC14	JO1	GD28	*R.nitidus*	0.03097	0.063629
CS21	JO1	GD28	*R.nitidus*	-0.013857	0.066064
CS13	JO1	GD28	*R.nitidus*	-0.011924	0.067348
GD42	JO1	GD28	*R.nitidus*	0.031594	0.065645
CS19	JO1	GD28	*R.nitidus*	0.019531	0.067147
GD48	JO1	GD28	*R.nitidus*	0.061241	0.065813
CS30	JO1	GD28	*R.nitidus*	-0.024545	0.064721
GD46	JO1	GD28	*R.nitidus*	0.023792	0.063943
CS31	JO1	GD28	*R.nitidus*	0.022047	0.065775
CS28	JO1	GD28	*R.nitidus*	-0.029636	0.066724
SC18	JO1	GD28	*R.nitidus*	0.058201	0.066028
CS23	JO1	GD28	*R.nitidus*	-0.014832	0.066867
CS14	JO1	GD28	*R.nitidus*	0.03066	0.068192
SC6	JO1	GD28	*R.nitidus*	-0.007582	0.06747
SC27	JO1	GD28	*R.nitidus*	0.007426	0.063673
CS18	JO1	GD28	*R.nitidus*	-0.017268	0.064567

CS25	JO1	GD28	*R.nitidus*	0.025621	0.067288
SC26	JO1	GD28	*R.nitidus*	-0.020973	0.065273
SC15	JO1	GD28	*R.nitidus*	0.023038	0.066219
CS3	JO1	GD28	*R.nitidus*	0.009562	0.065536
CS17	JO1	GD28	*R.nitidus*	0.060708	0.067324
CS22	JO1	GD28	*R.nitidus*	0.025974	0.06669
SC24	JO1	GD28	*R.nitidus*	0.005724	0.066343
CS6	JO1	GD28	*R.nitidus*	-0.001656	0.065876
JW6	JO1	GD28	*R.nitidus*	0.055731	0.068138
JO8	JO3	GD28	*R.nitidus*	-0.010063	0.074736
SC31	JO1	GD28	*R.nitidus*	0.020129	0.066382
SC10	JO1	GD28	*R.nitidus*	0.094259	0.0653
JW4	JO1	GD28	*R.nitidus*	0.210912	0.066589
JW7	JO1	GD28	*R.nitidus*	0.032461	0.068164
SC12	JO1	GD28	*R.nitidus*	0.007759	0.065958
SC34	JO1	GD28	*R.nitidus*	0.054357	0.064985
MY6	JO1	GD28	*R.nitidus*	0.019763	0.064473
SC28	JO1	GD28	*R.nitidus*	-0.011374	0.06454
GD21	JO3	GD28	*R.nitidus*	0.101473	0.068831
SC32	JO1	GD28	*R.nitidus*	0.070873	0.066281
MY4	JO1	GD28	*R.nitidus*	0.012998	0.067159
SC35	JO1	GD28	*R.nitidus*	0.088113	0.064742
GD29	JO3	GD28	*R.nitidus*	0.056131	0.067759
SC44	JO1	GD28	*R.nitidus*	0.031436	0.067468
GD31	JO3	GD28	*R.nitidus*	0.002806	0.073687
SC45	JO1	GD28	*R.nitidus*	0.068936	0.066931
SC42	JO1	GD28	*R.nitidus*	0.256181	0.062254
GD33	JO3	GD28	*R.nitidus*	-0.022414	0.069129
GD35	JO3	GD28	*R.nitidus*	0.009919	0.072244
GD45	JO3	GD28	*R.nitidus*	0.009165	0.073284
JO3	JO1	GD28	*R.nitidus*	0.059743	0.070432
CS13	JO3	GD28	*R.nitidus*	-0.037838	0.07185
GD26	JO3	GD28	*R.nitidus*	0.025528	0.069193
JO7	JO3	GD28	*R.nitidus*	-0.001235	0.075051
CS11	JO3	GD28	*R.nitidus*	-0.032759	0.071805
CS19	JO3	GD28	*R.nitidus*	-0.031905	0.070651
GD41	JO3	GD28	*R.nitidus*	-0.016493	0.0694
GD40	JO3	GD28	*R.nitidus*	0.011545	0.070502
CS14	JO3	GD28	*R.nitidus*	-0.00457	0.072339
GD48	JO3	GD28	*R.nitidus*	0.025046	0.070361
JO5	JO1	GD28	*R.nitidus*	0.062814	0.065885
GD44	JO3	GD28	*R.nitidus*	-0.032028	0.071836
GD25	JO3	GD28	*R.nitidus*	0.023084	0.071165
GD30	JO3	GD28	*R.nitidus*	0.105799	0.072385
CS23	JO3	GD28	*R.nitidus*	-0.062943	0.068738
CS17	JO3	GD28	*R.nitidus*	-0.018217	0.072417
GD46	JO3	GD28	*R.nitidus*	-0.030411	0.069662
GD22	JO3	GD28	*R.nitidus*	0.039931	0.071431
GD32	JO3	GD28	*R.nitidus*	-0.001992	0.071849
CS30	JO3	GD28	*R.nitidus*	-0.092251	0.07095
CS24	JO3	GD28	*R.nitidus*	-0.029412	0.069452
GD49	JO3	GD28	*R.nitidus*	0.044	0.072988
GD50	JO3	GD28	*R.nitidus*	0.023952	0.072912
GD34	JO3	GD28	*R.nitidus*	-0.008688	0.069037
GD42	JO3	GD28	*R.nitidus*	-0.0097	0.07035
CS18	JO3	GD28	*R.nitidus*	-0.063234	0.069592

SC5	JO3	GD28	*R.nitidus*	-0.008681	0.073407
SC14	JO3	GD28	*R.nitidus*	-0.011324	0.069969
CS3	JO3	GD28	*R.nitidus*	-0.022769	0.070104
CS15	JO3	GD28	*R.nitidus*	-0.004409	0.068678
CS20	JO3	GD28	*R.nitidus*	-0.050667	0.068645
SC12	JO3	GD28	*R.nitidus*	-0.057541	0.070055
CS4	JO3	GD28	*R.nitidus*	-0.06023	0.068903
CS16	JO3	GD28	*R.nitidus*	-0.030357	0.070176
CS12	JO3	GD28	*R.nitidus*	-0.010073	0.070195
CS22	JO3	GD28	*R.nitidus*	-0.018034	0.069487
CS28	JO3	GD28	*R.nitidus*	-0.068392	0.072424
SC34	JO3	GD28	*R.nitidus*	-0.020628	0.069793
SC6	JO3	GD28	*R.nitidus*	-0.046614	0.070658
CS21	JO3	GD28	*R.nitidus*	-0.07489	0.069863
SC27	JO3	GD28	*R.nitidus*	-0.021505	0.070052
SC45	JO3	GD28	*R.nitidus*	0.002588	0.071314
SC26	JO3	GD28	*R.nitidus*	-0.073356	0.068096
CS31	JO3	GD28	*R.nitidus*	-0.02595	0.072476
CS25	JO3	GD28	*R.nitidus*	-0.037104	0.072119
SC24	JO3	GD28	*R.nitidus*	-0.030825	0.06867
SC18	JO3	GD28	*R.nitidus*	0.007512	0.070489
SC44	JO3	GD28	*R.nitidus*	-0.023508	0.069392
SC32	JO3	GD28	*R.nitidus*	0.023216	0.069816
SC28	JO3	GD28	*R.nitidus*	-0.049729	0.069471
SC10	JO3	GD28	*R.nitidus*	0.043396	0.072138
SC42	JO3	GD28	*R.nitidus*	0.136578	0.067058
SC15	JO3	GD28	*R.nitidus*	-0.02317	0.070161
JO1	JO3	GD28	*R.nitidus*	-0.059743	0.070432
JW7	JO3	GD28	*R.nitidus*	-0.008858	0.07099
GD26	JO5	GD28	*R.nitidus*	0.019188	0.065894
JW4	JO3	GD28	*R.nitidus*	0.106693	0.072185
MY6	JO3	GD28	*R.nitidus*	-0.034164	0.069667
MY4	JO3	GD28	*R.nitidus*	-0.037341	0.070698
SC31	JO3	GD28	*R.nitidus*	-0.031787	0.069513
GD21	JO5	GD28	*R.nitidus*	0.117855	0.065783
SC35	JO3	GD28	*R.nitidus*	0.015315	0.069164
GD45	JO5	GD28	*R.nitidus*	0.017722	0.071102
CS6	JO3	GD28	*R.nitidus*	-0.080074	0.069077
JO8	JO5	GD28	*R.nitidus*	-0.032362	0.072684
GD30	JO5	GD28	*R.nitidus*	0.069787	0.068447
CS11	JO5	GD28	*R.nitidus*	-0.014275	0.068326
GD33	JO5	GD28	*R.nitidus*	-0.015407	0.065894
JW6	JO3	GD28	*R.nitidus*	0.013547	0.074814
GD29	JO5	GD28	*R.nitidus*	0.079388	0.064205
JO7	JO5	GD28	*R.nitidus*	-0.030211	0.073171
GD40	JO5	GD28	*R.nitidus*	0.035338	0.065551
CS12	JO5	GD28	*R.nitidus*	-0.004918	0.06724
CS18	JO5	GD28	*R.nitidus*	-0.066154	0.067553
CS16	JO5	GD28	*R.nitidus*	-0.013281	0.066645
GD46	JO5	GD28	*R.nitidus*	-0.015521	0.065449
GD41	JO5	GD28	*R.nitidus*	-0.019985	0.066114
GD50	JO5	GD28	*R.nitidus*	0.012357	0.069597
GD25	JO5	GD28	*R.nitidus*	0.043678	0.065532
GD22	JO5	GD28	*R.nitidus*	0.040831	0.065445
GD49	JO5	GD28	*R.nitidus*	0.076292	0.068358
GD31	JO5	GD28	*R.nitidus*	0.033333	0.067426

CS14	JO5	GD28	*R.nitidus*	-0.003808	0.069058
GD35	JO5	GD28	*R.nitidus*	0.019593	0.066357
GD48	JO5	GD28	*R.nitidus*	0.019656	0.066398
GD34	JO5	GD28	*R.nitidus*	-0.002882	0.065925
GD42	JO5	GD28	*R.nitidus*	-0.009826	0.06777
CS13	JO5	GD28	*R.nitidus*	-0.049245	0.068786
CS24	JO5	GD28	*R.nitidus*	-0.042345	0.065759
GD32	JO5	GD28	*R.nitidus*	-0.001664	0.06881
CS21	JO5	GD28	*R.nitidus*	-0.061162	0.065413
CS20	JO5	GD28	*R.nitidus*	-0.053585	0.066064
CS30	JO5	GD28	*R.nitidus*	-0.070344	0.065074
CS19	JO5	GD28	*R.nitidus*	-0.02589	0.067967
CS22	JO5	GD28	*R.nitidus*	-0.006405	0.066833
GD44	JO5	GD28	*R.nitidus*	-0.018519	0.067975
CS15	JO5	GD28	*R.nitidus*	-0.013783	0.068095
CS23	JO5	GD28	*R.nitidus*	-0.05325	0.066161
SC12	JO5	GD28	*R.nitidus*	-0.055178	0.065102
SC10	JO5	GD28	*R.nitidus*	0.052453	0.067519
CS28	JO5	GD28	*R.nitidus*	-0.056572	0.066973
CS17	JO5	GD28	*R.nitidus*	0.028783	0.069458
SC5	JO5	GD28	*R.nitidus*	-0.041247	0.069689
SC15	JO5	GD28	*R.nitidus*	-0.032506	0.066074
CS31	JO5	GD28	*R.nitidus*	-0.032	0.065348
CS6	JO5	GD28	*R.nitidus*	-0.05363	0.065894
CS3	JO5	GD28	*R.nitidus*	-0.049606	0.065505
CS25	JO5	GD28	*R.nitidus*	-0.036496	0.067405
SC35	JO5	GD28	*R.nitidus*	0.025539	0.06565
MY4	JO5	GD28	*R.nitidus*	-0.044728	0.068217
SC18	JO5	GD28	*R.nitidus*	0.003534	0.066248
SC14	JO5	GD28	*R.nitidus*	-0.019742	0.065732
JO8	JO7	HS3	*R.nitidus*	0.015131	0.073164
CS4	JO5	GD28	*R.nitidus*	-0.047049	0.065139
SC24	JO5	GD28	*R.nitidus*	-0.03209	0.065974
SC6	JO5	GD28	*R.nitidus*	-0.056511	0.068171
SC26	JO5	GD28	*R.nitidus*	-0.070715	0.067299
SC34	JO5	GD28	*R.nitidus*	0.000813	0.065258
SC42	JO5	GD28	*R.nitidus*	0.205903	0.064042
SC28	JO5	GD28	*R.nitidus*	-0.094073	0.063916
SC27	JO5	GD28	*R.nitidus*	-0.033275	0.064578
JW7	JO5	GD28	*R.nitidus*	-0.020305	0.069856
SC32	JO5	GD28	*R.nitidus*	0.016733	0.066169
GD30	JO7	HS3	*R.nitidus*	0.174102	0.073268
JW4	JO5	GD28	*R.nitidus*	0.155502	0.067891
SC45	JO5	GD28	*R.nitidus*	0.014851	0.066775
GD26	JO7	HS3	*R.nitidus*	0.108401	0.071224
SC31	JO5	GD28	*R.nitidus*	-0.018298	0.06589
JO1	JO5	GD28	*R.nitidus*	-0.062814	0.065885
GD34	JO7	HS3	*R.nitidus*	0.024043	0.072061
GD31	JO7	HS3	*R.nitidus*	0.10163	0.07269
GD22	JO7	HS3	*R.nitidus*	0.128535	0.070794
MY6	JO5	GD28	*R.nitidus*	-0.034154	0.066387
CS13	JO7	HS3	*R.nitidus*	-0.037645	0.073643
JO3	JO5	GD28	*R.nitidus*	-0.004472	0.070445
GD35	JO7	HS3	*R.nitidus*	0.053931	0.073966
JW6	JO5	GD28	*R.nitidus*	-0.004215	0.069053
GD40	JO7	HS3	*R.nitidus*	0.065237	0.070224

GD25	JO7	HS3	*R.nitidus*	0.054206	0.07185
GD33	JO7	HS3	*R.nitidus*	0.054645	0.073644
GD44	JO7	HS3	*R.nitidus*	0.034323	0.071618
GD32	JO7	HS3	*R.nitidus*	0.062959	0.0747
CS14	JO7	HS3	*R.nitidus*	0.004776	0.073068
GD29	JO7	HS3	*R.nitidus*	0.175573	0.068621
GD45	JO7	HS3	*R.nitidus*	0.13058	0.075073
GD42	JO7	HS3	*R.nitidus*	0.076181	0.074262
GD46	JO7	HS3	*R.nitidus*	-0.024231	0.071377
GD50	JO7	HS3	*R.nitidus*	0.066876	0.075077
GD48	JO7	HS3	*R.nitidus*	0.046773	0.072295
CS15	JO7	HS3	*R.nitidus*	0.045624	0.070965
CS16	JO7	HS3	*R.nitidus*	0.028409	0.072879
CS11	JO7	HS3	*R.nitidus*	0.016499	0.072082
GD21	JO7	HS3	*R.nitidus*	0.137417	0.069563
SC44	JO5	GD28	*R.nitidus*	-0.030858	0.065008
CS12	JO7	HS3	*R.nitidus*	0.020173	0.073326
CS22	JO7	HS3	*R.nitidus*	0.011204	0.0709
GD49	JO7	HS3	*R.nitidus*	0.109769	0.073855
GD41	JO7	HS3	*R.nitidus*	0.000925	0.071456
SC10	JO7	HS3	*R.nitidus*	0.102041	0.073012
SC27	JO7	HS3	*R.nitidus*	0.010223	0.071346
CS24	JO7	HS3	*R.nitidus*	-0.041785	0.071918
CS19	JO7	HS3	*R.nitidus*	-0.022578	0.073456
CS6	JO7	HS3	*R.nitidus*	-0.053309	0.069003
CS18	JO7	HS3	*R.nitidus*	-0.022451	0.073856
SC14	JO7	HS3	*R.nitidus*	0.012856	0.071341
JO3	JO7	HS3	*R.nitidus*	0.035242	0.073819
CS20	JO7	HS3	*R.nitidus*	-0.004476	0.070321
CS17	JO7	HS3	*R.nitidus*	0.02229	0.075886
CS31	JO7	HS3	*R.nitidus*	-0.016252	0.074136
SC32	JO7	HS3	*R.nitidus*	0.051011	0.070176
CS3	JO7	HS3	*R.nitidus*	-0.00542	0.071611
JO7	JO8	HS3	*R.nitidus*	-0.015131	0.073164
GD25	JO8	HS3	*R.nitidus*	0.02343	0.069705
CS30	JO7	HS3	*R.nitidus*	-0.046489	0.072787
SC6	JO7	HS3	*R.nitidus*	0.027103	0.070488
JO1	JO7	HS3	*R.nitidus*	0.022341	0.069064
CS21	JO7	HS3	*R.nitidus*	-0.030769	0.072498
SC24	JO7	HS3	*R.nitidus*	0.01165	0.07087
SC18	JO7	HS3	*R.nitidus*	0.036609	0.069717
CS4	JO7	HS3	*R.nitidus*	-0.020942	0.06971
SC15	JO7	HS3	*R.nitidus*	0.016457	0.072013
CS23	JO7	HS3	*R.nitidus*	-0.037615	0.071056
CS28	JO7	HS3	*R.nitidus*	-0.017068	0.072903
SC31	JO7	HS3	*R.nitidus*	0.00878	0.071507
SC34	JO7	HS3	*R.nitidus*	0.027753	0.068113
SC45	JO7	HS3	*R.nitidus*	0.026591	0.070635
CS25	JO7	HS3	*R.nitidus*	-0.01215	0.071823
SC5	JO7	HS3	*R.nitidus*	0.043112	0.073234
GD30	JO8	HS3	*R.nitidus*	0.103488	0.07625
JW7	JO7	HS3	*R.nitidus*	0.062626	0.072197
JW4	JO7	HS3	*R.nitidus*	0.192053	0.069265
SC35	JO7	HS3	*R.nitidus*	0.020833	0.069936
SC12	JO7	HS3	*R.nitidus*	-0.004798	0.069038
MY4	JO7	HS3	*R.nitidus*	-0.032381	0.071636

JW6	JO7	HS3	*R.nitidus*	-0.029457	0.067791
JO5	JO7	HS3	*R.nitidus*	0.076383	0.068556
SC28	JO7	HS3	*R.nitidus*	0.013133	0.070762
SC26	JO7	HS3	*R.nitidus*	-0.026364	0.071069
MY6	JO7	HS3	*R.nitidus*	0.010436	0.071736
SC42	JO7	HS3	*R.nitidus*	0.192216	0.066075
GD31	JO8	HS3	*R.nitidus*	0.03831	0.071551
SC44	JO7	HS3	*R.nitidus*	0.001896	0.071962
CS11	JO8	HS3	*R.nitidus*	-0.020966	0.072485
GD32	JO8	HS3	*R.nitidus*	-0.011957	0.076077
GD26	JO8	HS3	*R.nitidus*	0.047755	0.071103
CS12	JO8	HS3	*R.nitidus*	-0.010924	0.073213
GD50	JO8	HS3	*R.nitidus*	0.017112	0.073772
GD22	JO8	HS3	*R.nitidus*	0.076241	0.071511
GD21	JO8	HS3	*R.nitidus*	0.086473	0.069827
GD29	JO8	HS3	*R.nitidus*	0.125557	0.067412
CS17	JO8	HS3	*R.nitidus*	-0.001034	0.075211
GD34	JO8	HS3	*R.nitidus*	-0.045539	0.070687
GD44	JO8	HS3	*R.nitidus*	0.004794	0.072636
CS16	JO8	HS3	*R.nitidus*	-0.03164	0.072235
CS25	JO8	HS3	*R.nitidus*	-0.03001	0.073061
GD48	JO8	HS3	*R.nitidus*	0.018555	0.071456
GD33	JO8	HS3	*R.nitidus*	-0.005545	0.073147
CS14	JO8	HS3	*R.nitidus*	-0.021113	0.073524
CS20	JO8	HS3	*R.nitidus*	-0.039234	0.071973
SC5	JO8	HS3	*R.nitidus*	0	0.075212
SC32	JO8	HS3	*R.nitidus*	-0.009597	0.071602
GD49	JO8	HS3	*R.nitidus*	0.051111	0.073241
CS15	JO8	HS3	*R.nitidus*	-0.006536	0.072111
SC42	JO8	HS3	*R.nitidus*	0.146444	0.068894
GD35	JO8	HS3	*R.nitidus*	0.021257	0.072068
GD45	JO8	HS3	*R.nitidus*	0.052934	0.076543
GD42	JO8	HS3	*R.nitidus*	0.024085	0.071571
GD40	JO8	HS3	*R.nitidus*	0.038033	0.07032
CS13	JO8	HS3	*R.nitidus*	-0.061144	0.076038
GD41	JO8	HS3	*R.nitidus*	-0.028463	0.06969
SC27	JO8	HS3	*R.nitidus*	-0.021926	0.070437
SC6	JO8	HS3	*R.nitidus*	-0.018815	0.073166
CS28	JO8	HS3	*R.nitidus*	-0.043254	0.074528
CS19	JO8	HS3	*R.nitidus*	-0.058116	0.072053
CS18	JO8	HS3	*R.nitidus*	-0.054348	0.073363
CS21	JO8	HS3	*R.nitidus*	-0.067362	0.071459
SC35	JO8	HS3	*R.nitidus*	-0.007859	0.071451
CS4	JO8	HS3	*R.nitidus*	-0.032407	0.070006
SC12	JO8	HS3	*R.nitidus*	-0.046859	0.07022
SC14	JO8	HS3	*R.nitidus*	-0.015888	0.070564
CS30	JO8	HS3	*R.nitidus*	-0.098619	0.068889
CS3	JO8	HS3	*R.nitidus*	-0.031823	0.071918
CS22	JO8	HS3	*R.nitidus*	-0.04431	0.07124
CS6	JO8	HS3	*R.nitidus*	-0.079412	0.071715
GD46	JO8	HS3	*R.nitidus*	-0.096339	0.067963
SC24	JO8	HS3	*R.nitidus*	-0.023162	0.070544
SC10	JO8	HS3	*R.nitidus*	0.045643	0.07287
CS31	JO8	HS3	*R.nitidus*	-0.063872	0.073296
SC31	JO8	HS3	*R.nitidus*	0.001867	0.071999
CS23	JO8	HS3	*R.nitidus*	-0.065714	0.071769

CS24	JO8	HS3	*R.nitidus*	-0.055951	0.072076
SC18	JO8	HS3	*R.nitidus*	0.006322	0.07037
SC15	JO8	HS3	*R.nitidus*	-0.012133	0.072801
SC28	JO8	HS3	*R.nitidus*	-0.04467	0.071158
SC26	JO8	HS3	*R.nitidus*	-0.096805	0.07153
SC45	JO8	HS3	*R.nitidus*	-0.010536	0.071929
JW7	JO8	HS3	*R.nitidus*	0.028513	0.074176
SC34	JO8	HS3	*R.nitidus*	-0.023386	0.070326
SC44	JO8	HS3	*R.nitidus*	-0.019019	0.070221
JW6	JO8	HS3	*R.nitidus*	-0.012195	0.070485
MY6	JO8	HS3	*R.nitidus*	-0.029326	0.070024
JO3	JO8	HS3	*R.nitidus*	0.010011	0.073615
JW4	JO8	HS3	*R.nitidus*	0.164717	0.070783
JO5	JO8	HS3	*R.nitidus*	0.050098	0.070285
MY4	JO8	HS3	*R.nitidus*	-0.073473	0.06973
JO1	JO8	HS3	*R.nitidus*	-0.028571	0.070847
GD32	CS11	HS3	*R.nitidus*	0.05142	0.069336
GD25	CS11	HS3	*R.nitidus*	0.062981	0.067227
GD40	CS11	HS3	*R.nitidus*	0.062458	0.063969
JO8	CS11	HS3	*R.nitidus*	0.020966	0.072485
GD33	CS11	HS3	*R.nitidus*	0.030303	0.066824
JO7	CS11	HS3	*R.nitidus*	-0.016499	0.072082
GD22	CS11	HS3	*R.nitidus*	0.086629	0.066116
GD29	CS11	HS3	*R.nitidus*	0.134405	0.06609
CS18	CS11	HS3	*R.nitidus*	-0.042723	0.066988
GD21	CS11	HS3	*R.nitidus*	0.131274	0.065017
CS19	CS11	HS3	*R.nitidus*	-0.030943	0.067282
GD35	CS11	HS3	*R.nitidus*	0.041272	0.065688
GD30	CS11	HS3	*R.nitidus*	0.137579	0.069536
CS20	CS11	HS3	*R.nitidus*	-0.031403	0.066433
GD48	CS11	HS3	*R.nitidus*	0.045324	0.068003
GD44	CS11	HS3	*R.nitidus*	0.030856	0.068548
GD50	CS11	HS3	*R.nitidus*	0.018868	0.07204
GD42	CS11	HS3	*R.nitidus*	0.041181	0.067417
CS12	CS11	HS3	*R.nitidus*	0.019076	0.068764
GD26	CS11	HS3	*R.nitidus*	0.067513	0.067867
CS3	CS11	HS3	*R.nitidus*	-0.034533	0.065784
GD34	CS11	HS3	*R.nitidus*	0.008453	0.065343
GD49	CS11	HS3	*R.nitidus*	0.100231	0.07027
GD41	CS11	HS3	*R.nitidus*	0.017651	0.06488
CS13	CS11	HS3	*R.nitidus*	-0.049847	0.071542
SC10	CS11	HS3	*R.nitidus*	0.066762	0.066782
GD45	CS11	HS3	*R.nitidus*	0.083927	0.069695
GD46	CS11	HS3	*R.nitidus*	-0.012295	0.066678
CS25	CS11	HS3	*R.nitidus*	-0.011808	0.068906
CS15	CS11	HS3	*R.nitidus*	0.052795	0.067925
SC24	CS11	HS3	*R.nitidus*	-0.010722	0.066686
CS14	CS11	HS3	*R.nitidus*	-0.011507	0.069367
CS23	CS11	HS3	*R.nitidus*	-0.061658	0.065744
CS28	CS11	HS3	*R.nitidus*	-0.036078	0.066852
CS21	CS11	HS3	*R.nitidus*	-0.0349	0.064645
CS16	CS11	HS3	*R.nitidus*	0.007239	0.068236
JW4	CS11	HS3	*R.nitidus*	0.199313	0.065977
CS22	CS11	HS3	*R.nitidus*	-0.00438	0.068286
CS24	CS11	HS3	*R.nitidus*	-0.05291	0.066759
SC5	CS11	HS3	*R.nitidus*	0.004184	0.069492

SC44	CS11	HS3	*R.nitidus*	0.002094	0.065551
CS30	CS11	HS3	*R.nitidus*	-0.056328	0.067878
SC31	CS11	HS3	*R.nitidus*	0.006246	0.065456
SC14	CS11	HS3	*R.nitidus*	0.015246	0.068633
CS31	CS11	HS3	*R.nitidus*	-0.019084	0.069627
SC18	CS11	HS3	*R.nitidus*	0.037196	0.065554
JW7	CS11	HS3	*R.nitidus*	0.033015	0.070415
SC15	CS11	HS3	*R.nitidus*	0.017151	0.068066
SC28	CS11	HS3	*R.nitidus*	-0.031052	0.065285
MY6	CS11	HS3	*R.nitidus*	0.001366	0.066822
SC6	CS11	HS3	*R.nitidus*	0.007719	0.067225
CS17	CS11	HS3	*R.nitidus*	0.026846	0.069294
SC27	CS11	HS3	*R.nitidus*	-0.011603	0.066746
SC45	CS11	HS3	*R.nitidus*	0.026518	0.068451
SC35	CS11	HS3	*R.nitidus*	0.042936	0.064651
CS4	CS11	HS3	*R.nitidus*	-0.020729	0.066885
CS11	GD21	HS3	*R.nitidus*	-0.131274	0.065017
SC12	CS11	HS3	*R.nitidus*	-0.002867	0.066597
SC34	CS11	HS3	*R.nitidus*	0.03678	0.066556
GD30	GD21	HS3	*R.nitidus*	0.015023	0.066917
JO3	CS11	HS3	*R.nitidus*	0.043478	0.070974
SC42	CS11	HS3	*R.nitidus*	0.231973	0.061135
JO5	CS11	HS3	*R.nitidus*	0.030034	0.066371
GD33	GD21	HS3	*R.nitidus*	-0.1248	0.063539
SC26	CS11	HS3	*R.nitidus*	-0.032328	0.065441
MY4	CS11	HS3	*R.nitidus*	-0.033006	0.066317
CS6	CS11	HS3	*R.nitidus*	-0.03976	0.066183
JW6	CS11	HS3	*R.nitidus*	0.010909	0.07248
JO1	CS11	HS3	*R.nitidus*	-0.002849	0.066751
JO8	GD21	HS3	*R.nitidus*	-0.086473	0.069827
GD31	GD21	HS3	*R.nitidus*	-0.079148	0.063807
SC32	CS11	HS3	*R.nitidus*	0.054913	0.068564
GD26	GD21	HS3	*R.nitidus*	-0.079446	0.063377
GD42	GD21	HS3	*R.nitidus*	-0.107306	0.062229
GD29	GD21	HS3	*R.nitidus*	0	0.0643
GD34	GD21	HS3	*R.nitidus*	-0.147882	0.061979
CS13	GD21	HS3	*R.nitidus*	-0.161793	0.065493
JO7	GD21	HS3	*R.nitidus*	-0.137417	0.069563
GD35	GD21	HS3	*R.nitidus*	-0.089825	0.063714
GD50	GD21	HS3	*R.nitidus*	-0.143236	0.068524
CS14	GD21	HS3	*R.nitidus*	-0.150495	0.065908
GD45	GD21	HS3	*R.nitidus*	-0.047993	0.068195
GD46	GD21	HS3	*R.nitidus*	-0.188142	0.061489
GD25	GD21	HS3	*R.nitidus*	-0.062812	0.06312
GD32	GD21	HS3	*R.nitidus*	-0.080311	0.064162
GD22	GD21	HS3	*R.nitidus*	-0.048151	0.061298
GD41	GD21	HS3	*R.nitidus*	-0.127814	0.061209
GD49	GD21	HS3	*R.nitidus*	-0.023769	0.066759
CS12	GD21	HS3	*R.nitidus*	-0.136456	0.067771
SC6	GD21	HS3	*R.nitidus*	-0.129156	0.066717
GD44	GD21	HS3	*R.nitidus*	-0.127193	0.064531
GD40	GD21	HS3	*R.nitidus*	-0.077808	0.064257
CS22	GD21	HS3	*R.nitidus*	-0.134514	0.062982
CS3	GD21	HS3	*R.nitidus*	-0.151005	0.063209
CS18	GD21	HS3	*R.nitidus*	-0.171905	0.065091
CS20	GD21	HS3	*R.nitidus*	-0.165049	0.064833

SC15	GD21	HS3	*R.nitidus*	-0.121935	0.065806
CS17	GD21	HS3	*R.nitidus*	-0.109015	0.067386
CS28	GD21	HS3	*R.nitidus*	-0.156821	0.064666
GD48	GD21	HS3	*R.nitidus*	-0.122977	0.066629
CS21	GD21	HS3	*R.nitidus*	-0.165305	0.063459
SC18	GD21	HS3	*R.nitidus*	-0.117296	0.064129
CS15	GD21	HS3	*R.nitidus*	-0.110533	0.065161
SC10	GD21	HS3	*R.nitidus*	-0.078187	0.064906
CS19	GD21	HS3	*R.nitidus*	-0.153386	0.065051
CS25	GD21	HS3	*R.nitidus*	-0.151575	0.06498
CS23	GD21	HS3	*R.nitidus*	-0.178928	0.064435
CS16	GD21	HS3	*R.nitidus*	-0.124837	0.063958
SC12	GD21	HS3	*R.nitidus*	-0.13505	0.063985
JW7	GD21	HS3	*R.nitidus*	-0.10728	0.067245
CS6	GD21	HS3	*R.nitidus*	-0.182361	0.062959
CS31	GD21	HS3	*R.nitidus*	-0.153846	0.064519
JW4	GD21	HS3	*R.nitidus*	0.085271	0.067292
CS24	GD21	HS3	*R.nitidus*	-0.181275	0.063636
CS30	GD21	HS3	*R.nitidus*	-0.195696	0.064369
CS4	GD21	HS3	*R.nitidus*	-0.148194	0.061146
SC26	GD21	HS3	*R.nitidus*	-0.167404	0.063105
SC24	GD21	HS3	*R.nitidus*	-0.147362	0.064212
SC35	GD21	HS3	*R.nitidus*	-0.092308	0.064481
SC27	GD21	HS3	*R.nitidus*	-0.133721	0.063823
SC31	GD21	HS3	*R.nitidus*	-0.124538	0.064281
SC5	GD21	HS3	*R.nitidus*	-0.123432	0.066723
SC42	GD21	HS3	*R.nitidus*	0.134409	0.060997
SC14	GD21	HS3	*R.nitidus*	-0.117836	0.064316
JO1	GD21	HS3	*R.nitidus*	-0.153061	0.064124
JO8	GD22	HS3	*R.nitidus*	-0.076241	0.071511
MY6	GD21	HS3	*R.nitidus*	-0.136015	0.064548
JO3	GD21	HS3	*R.nitidus*	-0.092038	0.068283
JW6	GD21	HS3	*R.nitidus*	-0.129085	0.068391
SC32	GD21	HS3	*R.nitidus*	-0.090495	0.064917
GD26	GD22	HS3	*R.nitidus*	-0.023449	0.064093
SC34	GD21	HS3	*R.nitidus*	-0.101313	0.06475
GD32	GD22	HS3	*R.nitidus*	-0.053128	0.06518
SC44	GD21	HS3	*R.nitidus*	-0.124839	0.063867
SC28	GD21	HS3	*R.nitidus*	-0.15856	0.063453
JO5	GD21	HS3	*R.nitidus*	-0.08375	0.063353
SC45	GD21	HS3	*R.nitidus*	-0.092088	0.062324
JO7	GD22	HS3	*R.nitidus*	-0.128535	0.070794
GD25	GD22	HS3	*R.nitidus*	-0.038407	0.064522
MY4	GD21	HS3	*R.nitidus*	-0.17304	0.063684
GD40	GD22	HS3	*R.nitidus*	-0.017123	0.061879
GD21	GD22	HS3	*R.nitidus*	0.048151	0.061298
GD42	GD22	HS3	*R.nitidus*	-0.078234	0.064107
GD33	GD22	HS3	*R.nitidus*	-0.068855	0.063169
GD41	GD22	HS3	*R.nitidus*	-0.092494	0.063764
GD46	GD22	HS3	*R.nitidus*	-0.12936	0.060791
GD34	GD22	HS3	*R.nitidus*	-0.107908	0.06123
CS12	GD22	HS3	*R.nitidus*	-0.077079	0.067351
CS11	GD22	HS3	*R.nitidus*	-0.086629	0.066116
GD30	GD22	HS3	*R.nitidus*	0.065543	0.065722
GD44	GD22	HS3	*R.nitidus*	-0.056632	0.0652
GD29	GD22	HS3	*R.nitidus*	0.065561	0.062701

GD35	GD22	HS3	*R.nitidus*	-0.03273	0.06351
CS15	GD22	HS3	*R.nitidus*	-0.059412	0.066072
CS17	GD22	HS3	*R.nitidus*	-0.060298	0.065861
GD31	GD22	HS3	*R.nitidus*	-0.027376	0.06719
CS14	GD22	HS3	*R.nitidus*	-0.090967	0.065829
GD50	GD22	HS3	*R.nitidus*	-0.050771	0.068498
GD49	GD22	HS3	*R.nitidus*	0.041543	0.069821
SC6	GD22	HS3	*R.nitidus*	-0.076826	0.066831
GD45	GD22	HS3	*R.nitidus*	-0.028712	0.069535
CS19	GD22	HS3	*R.nitidus*	-0.113095	0.066794
CS21	GD22	HS3	*R.nitidus*	-0.121783	0.063963
SC12	GD22	HS3	*R.nitidus*	-0.096609	0.062541
CS22	GD22	HS3	*R.nitidus*	-0.083496	0.064329
CS23	GD22	HS3	*R.nitidus*	-0.135083	0.063332
CS13	GD22	HS3	*R.nitidus*	-0.130921	0.06522
SC18	GD22	HS3	*R.nitidus*	-0.05668	0.063868
SC10	GD22	HS3	*R.nitidus*	-0.017796	0.065901
CS3	GD22	HS3	*R.nitidus*	-0.120395	0.064186
CS16	GD22	HS3	*R.nitidus*	-0.082383	0.063816
CS24	GD22	HS3	*R.nitidus*	-0.131048	0.063
CS25	GD22	HS3	*R.nitidus*	-0.112299	0.067042
SC24	GD22	HS3	*R.nitidus*	-0.103106	0.064023
CS20	GD22	HS3	*R.nitidus*	-0.125402	0.065085
CS4	GD22	HS3	*R.nitidus*	-0.106717	0.062053
SC26	GD22	HS3	*R.nitidus*	-0.112658	0.06337
CS31	GD22	HS3	*R.nitidus*	-0.107287	0.065808
CS18	GD22	HS3	*R.nitidus*	-0.130719	0.064648
JW4	GD22	HS3	*R.nitidus*	0.131839	0.064967
JW7	GD22	HS3	*R.nitidus*	-0.050414	0.067148
JO7	GD25	HS3	*R.nitidus*	-0.054206	0.07185
SC28	GD22	HS3	*R.nitidus*	-0.123077	0.063095
JW6	GD22	HS3	*R.nitidus*	-0.075519	0.06924
CS6	GD22	HS3	*R.nitidus*	-0.137135	0.06473
CS30	GD22	HS3	*R.nitidus*	-0.151333	0.063531
MY4	GD22	HS3	*R.nitidus*	-0.126467	0.064852
JO1	GD22	HS3	*R.nitidus*	-0.099629	0.063676
SC5	GD22	HS3	*R.nitidus*	-0.096386	0.067039
SC31	GD22	HS3	*R.nitidus*	-0.08516	0.06445
SC15	GD22	HS3	*R.nitidus*	-0.074967	0.065083
CS28	GD22	HS3	*R.nitidus*	-0.119781	0.066362
SC27	GD22	HS3	*R.nitidus*	-0.090102	0.06204
SC45	GD22	HS3	*R.nitidus*	-0.04886	0.065739
SC14	GD22	HS3	*R.nitidus*	-0.081646	0.063672
SC42	GD22	HS3	*R.nitidus*	0.161428	0.06274
MY6	GD22	HS3	*R.nitidus*	-0.089571	0.06431
SC35	GD22	HS3	*R.nitidus*	-0.042513	0.062535
JO8	GD25	HS3	*R.nitidus*	-0.02343	0.069705
CS11	GD25	HS3	*R.nitidus*	-0.062981	0.067227
SC34	GD22	HS3	*R.nitidus*	-0.052566	0.063072
GD31	GD25	HS3	*R.nitidus*	0.02	0.065955
SC32	GD22	HS3	*R.nitidus*	-0.044739	0.063132
JO3	GD22	HS3	*R.nitidus*	-0.049475	0.067779
GD30	GD25	HS3	*R.nitidus*	0.098655	0.0691
SC44	GD22	HS3	*R.nitidus*	-0.095206	0.062667
GD33	GD25	HS3	*R.nitidus*	-0.039911	0.066072
CS12	GD25	HS3	*R.nitidus*	-0.069977	0.067158

GD34	GD25	HS3	*R.nitidus*	-0.053875	0.06451
GD45	GD25	HS3	*R.nitidus*	0.050213	0.070313
CS19	GD25	HS3	*R.nitidus*	-0.07703	0.065959
GD21	GD25	HS3	*R.nitidus*	0.062812	0.06312
CS20	GD25	HS3	*R.nitidus*	-0.094744	0.066882
JO5	GD22	HS3	*R.nitidus*	-0.039533	0.064195
GD44	GD25	HS3	*R.nitidus*	-0.032432	0.066178
GD41	GD25	HS3	*R.nitidus*	-0.041605	0.06474
GD42	GD25	HS3	*R.nitidus*	-0.027972	0.066167
CS16	GD25	HS3	*R.nitidus*	-0.062588	0.066147
GD32	GD25	HS3	*R.nitidus*	0	0.069282
GD26	GD25	HS3	*R.nitidus*	0.022137	0.065831
GD22	GD25	HS3	*R.nitidus*	0.038407	0.064522
CS25	GD25	HS3	*R.nitidus*	-0.082562	0.066338
CS22	GD25	HS3	*R.nitidus*	-0.049169	0.064063
GD29	GD25	HS3	*R.nitidus*	0.075071	0.064504
CS17	GD25	HS3	*R.nitidus*	-0.043379	0.067806
CS21	GD25	HS3	*R.nitidus*	-0.097527	0.065397
CS14	GD25	HS3	*R.nitidus*	-0.077568	0.067607
GD35	GD25	HS3	*R.nitidus*	-0.017557	0.063279
GD50	GD25	HS3	*R.nitidus*	0.00738	0.068753
GD46	GD25	HS3	*R.nitidus*	-0.086957	0.063785
CS24	GD25	HS3	*R.nitidus*	-0.086835	0.065295
GD40	GD25	HS3	*R.nitidus*	-0.011486	0.062423
CS13	GD25	HS3	*R.nitidus*	-0.096591	0.066198
GD49	GD25	HS3	*R.nitidus*	0.04565	0.068021
GD48	GD25	HS3	*R.nitidus*	-0.008326	0.064159
SC5	GD25	HS3	*R.nitidus*	-0.056407	0.06718
CS28	GD25	HS3	*R.nitidus*	-0.088146	0.065527
SC12	GD25	HS3	*R.nitidus*	-0.069832	0.06495
SC14	GD25	HS3	*R.nitidus*	-0.054841	0.064317
SC18	GD25	HS3	*R.nitidus*	-0.032601	0.065568
CS4	GD25	HS3	*R.nitidus*	-0.064624	0.063145
SC28	GD25	HS3	*R.nitidus*	-0.090226	0.063935
CS30	GD25	HS3	*R.nitidus*	-0.113781	0.0658
SC15	GD25	HS3	*R.nitidus*	-0.04821	0.065949
CS18	GD25	HS3	*R.nitidus*	-0.098439	0.064613
SC42	GD25	HS3	*R.nitidus*	0.166765	0.061552
SC6	GD25	HS3	*R.nitidus*	-0.057463	0.064976
SC10	GD25	HS3	*R.nitidus*	-0.003655	0.063317
SC27	GD25	HS3	*R.nitidus*	-0.076139	0.063005
CS3	GD25	HS3	*R.nitidus*	-0.08908	0.065209
CS31	GD25	HS3	*R.nitidus*	-0.08642	0.065411
SC32	GD25	HS3	*R.nitidus*	-0.02008	0.064165
SC34	GD25	HS3	*R.nitidus*	-0.025589	0.063295
CS23	GD25	HS3	*R.nitidus*	-0.109469	0.063944
JW7	GD25	HS3	*R.nitidus*	-0.030911	0.069331
SC24	GD25	HS3	*R.nitidus*	-0.067643	0.064897
CS15	GD25	HS3	*R.nitidus*	-0.04682	0.065951
SC26	GD25	HS3	*R.nitidus*	-0.093515	0.06499
CS6	GD25	HS3	*R.nitidus*	-0.100072	0.063991
MY4	GD25	HS3	*R.nitidus*	-0.09868	0.063679
JO5	GD25	HS3	*R.nitidus*	-0.029228	0.065429
GD30	GD26	HS3	*R.nitidus*	0.092981	0.067787
GD25	GD26	HS3	*R.nitidus*	-0.022137	0.065831
JO1	GD25	HS3	*R.nitidus*	-0.093575	0.06443

JO3	GD25	HS3	*R.nitidus*	-0.014155	0.067687
JW4	GD25	HS3	*R.nitidus*	0.15575	0.066897
JO7	GD26	HS3	*R.nitidus*	-0.108401	0.071224
JO8	GD26	HS3	*R.nitidus*	-0.047755	0.071103
JW6	GD25	HS3	*R.nitidus*	-0.039595	0.070183
SC35	GD25	HS3	*R.nitidus*	-0.026518	0.065148
SC31	GD25	HS3	*R.nitidus*	-0.057026	0.063923
SC45	GD25	HS3	*R.nitidus*	-0.03662	0.066474
GD29	GD26	HS3	*R.nitidus*	0.074476	0.062004
GD21	GD26	HS3	*R.nitidus*	0.079446	0.063377
SC44	GD25	HS3	*R.nitidus*	-0.056348	0.063139
MY6	GD25	HS3	*R.nitidus*	-0.056211	0.06386
GD41	GD26	HS3	*R.nitidus*	-0.071266	0.06373
GD31	GD26	HS3	*R.nitidus*	0.025681	0.066346
CS11	GD26	HS3	*R.nitidus*	-0.067513	0.067867
GD46	GD26	HS3	*R.nitidus*	-0.101657	0.061604
GD42	GD26	HS3	*R.nitidus*	-0.028369	0.066433
GD34	GD26	HS3	*R.nitidus*	-0.076616	0.063042
GD22	GD26	HS3	*R.nitidus*	0.023449	0.064093
GD40	GD26	HS3	*R.nitidus*	-0.001437	0.06218
GD32	GD26	HS3	*R.nitidus*	-0.006417	0.063658
GD49	GD26	HS3	*R.nitidus*	0.039251	0.067186
CS17	GD26	HS3	*R.nitidus*	-0.043262	0.069184
GD45	GD26	HS3	*R.nitidus*	0.03964	0.069348
CS13	GD26	HS3	*R.nitidus*	-0.105336	0.067632
CS20	GD26	HS3	*R.nitidus*	-0.094164	0.066827
GD50	GD26	HS3	*R.nitidus*	-0.034323	0.066592
GD44	GD26	HS3	*R.nitidus*	-0.033832	0.066583
CS25	GD26	HS3	*R.nitidus*	-0.077808	0.066115
CS28	GD26	HS3	*R.nitidus*	-0.101091	0.066818
CS14	GD26	HS3	*R.nitidus*	-0.080327	0.067561
CS12	GD26	HS3	*R.nitidus*	-0.062987	0.066768
GD33	GD26	HS3	*R.nitidus*	-0.049658	0.064981
CS16	GD26	HS3	*R.nitidus*	-0.066	0.065368
GD35	GD26	HS3	*R.nitidus*	-0.023134	0.065835
CS18	GD26	HS3	*R.nitidus*	-0.106412	0.066352
SC6	GD26	HS3	*R.nitidus*	-0.072011	0.066039
GD48	GD26	HS3	*R.nitidus*	-0.040296	0.067295
CS30	GD26	HS3	*R.nitidus*	-0.127899	0.065094
CS19	GD26	HS3	*R.nitidus*	-0.085193	0.066939
CS15	GD26	HS3	*R.nitidus*	-0.044206	0.067201
CS21	GD26	HS3	*R.nitidus*	-0.097577	0.066574
SC26	GD26	HS3	*R.nitidus*	-0.102931	0.066004
CS24	GD26	HS3	*R.nitidus*	-0.109091	0.064107
CS22	GD26	HS3	*R.nitidus*	-0.072514	0.065197
SC18	GD26	HS3	*R.nitidus*	-0.037842	0.063619
SC14	GD26	HS3	*R.nitidus*	-0.059172	0.066355
JW4	GD26	HS3	*R.nitidus*	0.1606	0.068012
CS3	GD26	HS3	*R.nitidus*	-0.097035	0.065687
SC44	GD26	HS3	*R.nitidus*	-0.064605	0.063464
MY4	GD26	HS3	*R.nitidus*	-0.09263	0.064548
SC24	GD26	HS3	*R.nitidus*	-0.086301	0.064445
JW6	GD26	HS3	*R.nitidus*	-0.076512	0.070235
SC32	GD26	HS3	*R.nitidus*	-0.026864	0.064474
SC5	GD26	HS3	*R.nitidus*	-0.068966	0.066707
CS23	GD26	HS3	*R.nitidus*	-0.112052	0.064322

SC28	GD26	HS3	*R.nitidus*	-0.098572	0.063754
SC10	GD26	HS3	*R.nitidus*	-0.005658	0.066124
CS31	GD26	HS3	*R.nitidus*	-0.093418	0.065355
SC42	GD26	HS3	*R.nitidus*	0.164071	0.062596
SC15	GD26	HS3	*R.nitidus*	-0.053425	0.065068
CS4	GD26	HS3	*R.nitidus*	-0.092359	0.063108
JO8	GD29	HS3	*R.nitidus*	-0.125557	0.067412
SC45	GD26	HS3	*R.nitidus*	-0.033175	0.065499
SC12	GD26	HS3	*R.nitidus*	-0.087636	0.062421
GD31	GD29	HS3	*R.nitidus*	-0.051898	0.06517
MY6	GD26	HS3	*R.nitidus*	-0.06471	0.065112
JW7	GD26	HS3	*R.nitidus*	-0.027265	0.070323
SC27	GD26	HS3	*R.nitidus*	-0.072464	0.064556
JO7	GD29	HS3	*R.nitidus*	-0.175573	0.068621
GD30	GD29	HS3	*R.nitidus*	0.024033	0.066488
CS6	GD26	HS3	*R.nitidus*	-0.106134	0.064547
SC31	GD26	HS3	*R.nitidus*	-0.056063	0.065062
GD26	GD29	HS3	*R.nitidus*	-0.074476	0.062004
GD32	GD29	HS3	*R.nitidus*	-0.100088	0.064943
SC34	GD26	HS3	*R.nitidus*	-0.033686	0.064098
SC35	GD26	HS3	*R.nitidus*	-0.031735	0.063835
JO1	GD26	HS3	*R.nitidus*	-0.089436	0.06497
JO3	GD26	HS3	*R.nitidus*	-0.031587	0.070047
GD25	GD29	HS3	*R.nitidus*	-0.075071	0.064504
JO5	GD26	HS3	*R.nitidus*	-0.017426	0.064154
GD40	GD29	HS3	*R.nitidus*	-0.081043	0.062395
GD50	GD29	HS3	*R.nitidus*	-0.109026	0.067762
GD21	GD29	HS3	*R.nitidus*	0	0.0643
CS15	GD29	HS3	*R.nitidus*	-0.100128	0.066317
GD44	GD29	HS3	*R.nitidus*	-0.127234	0.06536
GD22	GD29	HS3	*R.nitidus*	-0.065561	0.062701
GD33	GD29	HS3	*R.nitidus*	-0.123887	0.064706
CS11	GD29	HS3	*R.nitidus*	-0.134405	0.06609
CS17	GD29	HS3	*R.nitidus*	-0.109267	0.067135
GD45	GD29	HS3	*R.nitidus*	-0.036503	0.067862
GD34	GD29	HS3	*R.nitidus*	-0.167224	0.062924
GD41	GD29	HS3	*R.nitidus*	-0.126573	0.064218
GD49	GD29	HS3	*R.nitidus*	-0.032456	0.068712
CS12	GD29	HS3	*R.nitidus*	-0.119588	0.066013
CS18	GD29	HS3	*R.nitidus*	-0.163065	0.064088
CS14	GD29	HS3	*R.nitidus*	-0.149225	0.067089
CS23	GD29	HS3	*R.nitidus*	-0.175711	0.062553
CS13	GD29	HS3	*R.nitidus*	-0.17113	0.065477
CS3	GD29	HS3	*R.nitidus*	-0.167539	0.064276
GD35	GD29	HS3	*R.nitidus*	-0.075104	0.065367
CS20	GD29	HS3	*R.nitidus*	-0.163903	0.065344
GD42	GD29	HS3	*R.nitidus*	-0.118352	0.064272
SC6	GD29	HS3	*R.nitidus*	-0.139323	0.063678
GD48	GD29	HS3	*R.nitidus*	-0.103252	0.064021
SC18	GD29	HS3	*R.nitidus*	-0.09712	0.063527
GD46	GD29	HS3	*R.nitidus*	-0.179706	0.064208
CS30	GD29	HS3	*R.nitidus*	-0.190412	0.062467
CS22	GD29	HS3	*R.nitidus*	-0.137615	0.064074
SC27	GD29	HS3	*R.nitidus*	-0.137662	0.063051
SC10	GD29	HS3	*R.nitidus*	-0.066031	0.066149
CS24	GD29	HS3	*R.nitidus*	-0.173531	0.063567

SC14	GD29	HS3	*R.nitidus*	-0.124283	0.062495
CS16	GD29	HS3	*R.nitidus*	-0.134566	0.064709
SC34	GD29	HS3	*R.nitidus*	-0.079818	0.063481
CS19	GD29	HS3	*R.nitidus*	-0.158537	0.065666
CS28	GD29	HS3	*R.nitidus*	-0.162618	0.065843
SC12	GD29	HS3	*R.nitidus*	-0.136626	0.064619
CS21	GD29	HS3	*R.nitidus*	-0.165184	0.064836
SC28	GD29	HS3	*R.nitidus*	-0.162655	0.064262
JW4	GD29	HS3	*R.nitidus*	0.079398	0.065804
MY4	GD29	HS3	*R.nitidus*	-0.163226	0.063831
SC15	GD29	HS3	*R.nitidus*	-0.125337	0.065005
CS25	GD29	HS3	*R.nitidus*	-0.152815	0.065853
JO7	GD30	HS3	*R.nitidus*	-0.174102	0.073268
CS31	GD29	HS3	*R.nitidus*	-0.169697	0.064854
SC5	GD29	HS3	*R.nitidus*	-0.137977	0.067595
SC31	GD29	HS3	*R.nitidus*	-0.117978	0.06373
SC44	GD29	HS3	*R.nitidus*	-0.124005	0.064957
JO8	GD30	HS3	*R.nitidus*	-0.103488	0.07625
SC45	GD29	HS3	*R.nitidus*	-0.104193	0.06576
MY6	GD29	HS3	*R.nitidus*	-0.129747	0.063487
CS4	GD29	HS3	*R.nitidus*	-0.154293	0.061629
SC26	GD29	HS3	*R.nitidus*	-0.167519	0.063625
SC35	GD29	HS3	*R.nitidus*	-0.08447	0.063507
SC32	GD29	HS3	*R.nitidus*	-0.108892	0.064187
SC42	GD29	HS3	*R.nitidus*	0.102376	0.062218
GD32	GD30	HS3	*R.nitidus*	-0.117417	0.070182
GD25	GD30	HS3	*R.nitidus*	-0.098655	0.0691
CS6	GD29	HS3	*R.nitidus*	-0.164894	0.063296
JW6	GD29	HS3	*R.nitidus*	-0.142123	0.068136
GD33	GD30	HS3	*R.nitidus*	-0.101167	0.067681
GD35	GD30	HS3	*R.nitidus*	-0.094595	0.067345
GD22	GD30	HS3	*R.nitidus*	-0.065543	0.065722
GD26	GD30	HS3	*R.nitidus*	-0.092981	0.067787
SC24	GD29	HS3	*R.nitidus*	-0.143132	0.062678
CS11	GD30	HS3	*R.nitidus*	-0.137579	0.069536
JW7	GD29	HS3	*R.nitidus*	-0.108696	0.066393
JO3	GD29	HS3	*R.nitidus*	-0.103631	0.065808
JO1	GD29	HS3	*R.nitidus*	-0.144955	0.063931
CS12	GD30	HS3	*R.nitidus*	-0.121264	0.071272
GD21	GD30	HS3	*R.nitidus*	-0.015023	0.066917
CS16	GD30	HS3	*R.nitidus*	-0.12205	0.068704
GD44	GD30	HS3	*R.nitidus*	-0.099019	0.067776
GD42	GD30	HS3	*R.nitidus*	-0.108635	0.067552
JO5	GD29	HS3	*R.nitidus*	-0.098168	0.063876
GD50	GD30	HS3	*R.nitidus*	-0.103061	0.070959
GD46	GD30	HS3	*R.nitidus*	-0.17332	0.064281
CS19	GD30	HS3	*R.nitidus*	-0.138298	0.069807
GD29	GD30	HS3	*R.nitidus*	-0.024033	0.066488
GD40	GD30	HS3	*R.nitidus*	-0.070751	0.06529
GD31	GD30	HS3	*R.nitidus*	-0.092793	0.069281
GD45	GD30	HS3	*R.nitidus*	-0.03966	0.068446
GD41	GD30	HS3	*R.nitidus*	-0.108867	0.065792
GD49	GD30	HS3	*R.nitidus*	-0.057199	0.071127
CS13	GD30	HS3	*R.nitidus*	-0.155332	0.068312
CS18	GD30	HS3	*R.nitidus*	-0.159055	0.067388
GD48	GD30	HS3	*R.nitidus*	-0.065945	0.068395

CS23	GD30	HS3	*R.nitidus*	-0.160714	0.066978
CS31	GD30	HS3	*R.nitidus*	-0.133774	0.067989
CS14	GD30	HS3	*R.nitidus*	-0.126749	0.070747
CS17	GD30	HS3	*R.nitidus*	-0.114382	0.07003
CS22	GD30	HS3	*R.nitidus*	-0.128492	0.066793
CS21	GD30	HS3	*R.nitidus*	-0.174014	0.067337
CS20	GD30	HS3	*R.nitidus*	-0.16382	0.067781
GD34	GD30	HS3	*R.nitidus*	-0.151423	0.065087
CS4	GD30	HS3	*R.nitidus*	-0.132873	0.064663
CS15	GD30	HS3	*R.nitidus*	-0.105478	0.069226
CS24	GD30	HS3	*R.nitidus*	-0.156402	0.065127
SC28	GD30	HS3	*R.nitidus*	-0.159285	0.065668
JW6	GD30	HS3	*R.nitidus*	-0.140904	0.07304
CS30	GD30	HS3	*R.nitidus*	-0.171938	0.067226
SC10	GD30	HS3	*R.nitidus*	-0.074813	0.06908
JO7	GD31	HS3	*R.nitidus*	-0.10163	0.07269
CS3	GD30	HS3	*R.nitidus*	-0.149278	0.066454
SC27	GD30	HS3	*R.nitidus*	-0.133125	0.066408
SC12	GD30	HS3	*R.nitidus*	-0.130709	0.067574
CS25	GD30	HS3	*R.nitidus*	-0.129676	0.070928
CS28	GD30	HS3	*R.nitidus*	-0.154569	0.069242
SC26	GD30	HS3	*R.nitidus*	-0.158915	0.06815
SC14	GD30	HS3	*R.nitidus*	-0.105551	0.066947
SC32	GD30	HS3	*R.nitidus*	-0.086381	0.068131
JO8	GD31	HS3	*R.nitidus*	-0.03831	0.071551
SC45	GD30	HS3	*R.nitidus*	-0.097467	0.068812
SC6	GD30	HS3	*R.nitidus*	-0.138315	0.066901
JW4	GD30	HS3	*R.nitidus*	0.096801	0.070315
SC31	GD30	HS3	*R.nitidus*	-0.119301	0.067743
SC5	GD30	HS3	*R.nitidus*	-0.128931	0.070367
SC15	GD30	HS3	*R.nitidus*	-0.110395	0.070441
MY4	GD30	HS3	*R.nitidus*	-0.15655	0.066459
SC18	GD30	HS3	*R.nitidus*	-0.092084	0.067505
SC34	GD30	HS3	*R.nitidus*	-0.075818	0.067339
SC24	GD30	HS3	*R.nitidus*	-0.144984	0.06676
JW7	GD30	HS3	*R.nitidus*	-0.101711	0.071647
CS11	GD31	HS3	*R.nitidus*	-0.075284	0.066909
JO3	GD30	HS3	*R.nitidus*	-0.122841	0.071476
JO1	GD30	HS3	*R.nitidus*	-0.159684	0.065226
SC44	GD30	HS3	*R.nitidus*	-0.108749	0.064197
CS6	GD30	HS3	*R.nitidus*	-0.159514	0.066657
GD30	GD31	HS3	*R.nitidus*	0.092793	0.069281
JO5	GD30	HS3	*R.nitidus*	-0.083072	0.066567
MY6	GD30	HS3	*R.nitidus*	-0.121932	0.066252
SC42	GD30	HS3	*R.nitidus*	0.116917	0.06389
GD34	GD31	HS3	*R.nitidus*	-0.065303	0.065442
SC35	GD30	HS3	*R.nitidus*	-0.072699	0.066726
GD25	GD31	HS3	*R.nitidus*	-0.02	0.065955
GD49	GD31	HS3	*R.nitidus*	0.05174	0.070017
GD21	GD31	HS3	*R.nitidus*	0.079148	0.063807
GD50	GD31	HS3	*R.nitidus*	-0.034451	0.068543
GD29	GD31	HS3	*R.nitidus*	0.051898	0.06517
GD26	GD31	HS3	*R.nitidus*	-0.025681	0.066346
CS14	GD31	HS3	*R.nitidus*	-0.072622	0.069275
GD45	GD31	HS3	*R.nitidus*	0.000895	0.070462
GD40	GD31	HS3	*R.nitidus*	0.000753	0.064662

GD48	GD31	HS3	*R.nitidus*	-0.033884	0.067997
CS12	GD31	HS3	*R.nitidus*	-0.06006	0.067194
GD22	GD31	HS3	*R.nitidus*	0.027376	0.06719
GD46	GD31	HS3	*R.nitidus*	-0.093077	0.064483
GD32	GD31	HS3	*R.nitidus*	-0.02122	0.069702
CS17	GD31	HS3	*R.nitidus*	-0.055342	0.071396
GD33	GD31	HS3	*R.nitidus*	-0.041291	0.067077
CS28	GD31	HS3	*R.nitidus*	-0.089714	0.067673
CS24	GD31	HS3	*R.nitidus*	-0.119306	0.066025
GD41	GD31	HS3	*R.nitidus*	-0.059598	0.066236
GD44	GD31	HS3	*R.nitidus*	-0.04844	0.067728
GD35	GD31	HS3	*R.nitidus*	-0.028593	0.065969
CS31	GD31	HS3	*R.nitidus*	-0.085301	0.067209
CS23	GD31	HS3	*R.nitidus*	-0.131228	0.066326
CS30	GD31	HS3	*R.nitidus*	-0.136126	0.067889
CS22	GD31	HS3	*R.nitidus*	-0.077691	0.06684
SC12	GD31	HS3	*R.nitidus*	-0.072329	0.066509
SC6	GD31	HS3	*R.nitidus*	-0.0625	0.0654
GD42	GD31	HS3	*R.nitidus*	-0.031528	0.066511
SC15	GD31	HS3	*R.nitidus*	-0.05942	0.069223
CS13	GD31	HS3	*R.nitidus*	-0.110948	0.066919
CS15	GD31	HS3	*R.nitidus*	-0.050751	0.067553
SC35	GD31	HS3	*R.nitidus*	-0.035565	0.064653
SC34	GD31	HS3	*R.nitidus*	-0.031989	0.066101
SC27	GD31	HS3	*R.nitidus*	-0.061841	0.065257
SC31	GD31	HS3	*R.nitidus*	-0.062587	0.066447
SC28	GD31	HS3	*R.nitidus*	-0.101418	0.06526
CS16	GD31	HS3	*R.nitidus*	-0.064655	0.068205
CS3	GD31	HS3	*R.nitidus*	-0.110065	0.066039
CS18	GD31	HS3	*R.nitidus*	-0.122405	0.067336
CS20	GD31	HS3	*R.nitidus*	-0.113781	0.067006
SC10	GD31	HS3	*R.nitidus*	-0.010347	0.068255
MY4	GD31	HS3	*R.nitidus*	-0.105491	0.066766
SC18	GD31	HS3	*R.nitidus*	-0.032967	0.067003
CS21	GD31	HS3	*R.nitidus*	-0.11174	0.067427
CS19	GD31	HS3	*R.nitidus*	-0.121916	0.068335
SC14	GD31	HS3	*R.nitidus*	-0.060124	0.064539
JW4	GD31	HS3	*R.nitidus*	0.12988	0.067949
CS4	GD31	HS3	*R.nitidus*	-0.091346	0.064354
CS25	GD31	HS3	*R.nitidus*	-0.096154	0.06734
SC26	GD31	HS3	*R.nitidus*	-0.103641	0.064681
JW6	GD31	HS3	*R.nitidus*	-0.089826	0.071735
CS6	GD31	HS3	*R.nitidus*	-0.115187	0.067097
SC5	GD31	HS3	*R.nitidus*	-0.0707	0.068255
JO7	GD32	HS3	*R.nitidus*	-0.062959	0.0747
CS11	GD32	HS3	*R.nitidus*	-0.05142	0.069336
JW7	GD31	HS3	*R.nitidus*	-0.064198	0.070979
GD25	GD32	HS3	*R.nitidus*	0	0.069282
SC44	GD31	HS3	*R.nitidus*	-0.064167	0.065278
SC24	GD31	HS3	*R.nitidus*	-0.082116	0.066134
GD33	GD32	HS3	*R.nitidus*	-0.030392	0.066286
MY6	GD31	HS3	*R.nitidus*	-0.073205	0.066544
SC32	GD31	HS3	*R.nitidus*	-0.021636	0.065623
SC45	GD31	HS3	*R.nitidus*	-0.053147	0.067405
JO5	GD31	HS3	*R.nitidus*	-0.045007	0.066123
SC42	GD31	HS3	*R.nitidus*	0.159488	0.065265

JO8	GD32	HS3	*R.nitidus*	0.011957	0.076077
GD40	GD32	HS3	*R.nitidus*	0.014827	0.065418
JO1	GD31	HS3	*R.nitidus*	-0.094011	0.067177
GD22	GD32	HS3	*R.nitidus*	0.053128	0.06518
CS12	GD32	HS3	*R.nitidus*	-0.025848	0.068413
JO3	GD31	HS3	*R.nitidus*	-0.037563	0.071592
GD34	GD32	HS3	*R.nitidus*	-0.038104	0.064359
GD26	GD32	HS3	*R.nitidus*	0.006417	0.063658
GD41	GD32	HS3	*R.nitidus*	-0.052539	0.066864
GD29	GD32	HS3	*R.nitidus*	0.100088	0.064943
GD35	GD32	HS3	*R.nitidus*	-0.003344	0.066731
GD30	GD32	HS3	*R.nitidus*	0.117417	0.070182
CS18	GD32	HS3	*R.nitidus*	-0.089078	0.067917
GD21	GD32	HS3	*R.nitidus*	0.080311	0.064162
GD50	GD32	HS3	*R.nitidus*	-0.00418	0.069383
CS25	GD32	HS3	*R.nitidus*	-0.054306	0.069076
CS23	GD32	HS3	*R.nitidus*	-0.103422	0.065755
GD48	GD32	HS3	*R.nitidus*	-0.009643	0.066219
GD31	GD32	HS3	*R.nitidus*	0.02122	0.069702
CS14	GD32	HS3	*R.nitidus*	-0.050394	0.069357
CS22	GD32	HS3	*R.nitidus*	-0.062047	0.066864
CS31	GD32	HS3	*R.nitidus*	-0.070074	0.069221
GD49	GD32	HS3	*R.nitidus*	0.073499	0.069942
GD42	GD32	HS3	*R.nitidus*	-0.026549	0.068111
GD44	GD32	HS3	*R.nitidus*	0.000898	0.068117
GD45	GD32	HS3	*R.nitidus*	0.02994	0.072559
CS15	GD32	HS3	*R.nitidus*	-0.01947	0.068274
SC14	GD32	HS3	*R.nitidus*	-0.028324	0.067878
CS16	GD32	HS3	*R.nitidus*	-0.053883	0.068332
CS13	GD32	HS3	*R.nitidus*	-0.072998	0.068695
GD46	GD32	HS3	*R.nitidus*	-0.088133	0.064103
SC18	GD32	HS3	*R.nitidus*	0.015523	0.066102
CS21	GD32	HS3	*R.nitidus*	-0.072893	0.069931
CS19	GD32	HS3	*R.nitidus*	-0.065334	0.068086
SC10	GD32	HS3	*R.nitidus*	0.021531	0.066856
CS17	GD32	HS3	*R.nitidus*	-0.022857	0.07146
CS30	GD32	HS3	*R.nitidus*	-0.093042	0.069943
SC12	GD32	HS3	*R.nitidus*	-0.059006	0.067264
CS28	GD32	HS3	*R.nitidus*	-0.078254	0.069833
MY6	GD32	HS3	*R.nitidus*	-0.050343	0.066979
SC26	GD32	HS3	*R.nitidus*	-0.062549	0.066964
SC6	GD32	HS3	*R.nitidus*	-0.041569	0.068003
JW4	GD32	HS3	*R.nitidus*	0.184652	0.068081
CS24	GD32	HS3	*R.nitidus*	-0.097403	0.065655
CS4	GD32	HS3	*R.nitidus*	-0.077105	0.065446
CS20	GD32	HS3	*R.nitidus*	-0.075851	0.067603
SC34	GD32	HS3	*R.nitidus*	-0.015361	0.067242
CS3	GD32	HS3	*R.nitidus*	-0.082612	0.068039
SC5	GD32	HS3	*R.nitidus*	-0.017915	0.072105
SC42	GD32	HS3	*R.nitidus*	0.192455	0.063971
SC31	GD32	HS3	*R.nitidus*	-0.018911	0.068192
SC45	GD32	HS3	*R.nitidus*	-0.010008	0.066862
SC35	GD32	HS3	*R.nitidus*	0.022481	0.066062
SC28	GD32	HS3	*R.nitidus*	-0.056	0.06587
MY4	GD32	HS3	*R.nitidus*	-0.080032	0.067157
SC32	GD32	HS3	*R.nitidus*	0.005587	0.067517

GD22	CS12	HS3	*R.nitidus*	0.077079	0.067351
SC15	GD32	HS3	*R.nitidus*	-0.012235	0.068316
CS6	GD32	HS3	*R.nitidus*	-0.09736	0.067596
SC27	GD32	HS3	*R.nitidus*	-0.035101	0.067273
SC24	GD32	HS3	*R.nitidus*	-0.055468	0.066309
JW7	GD32	HS3	*R.nitidus*	0.00722	0.069438
JO5	GD32	HS3	*R.nitidus*	0.023585	0.068308
JO1	GD32	HS3	*R.nitidus*	-0.054157	0.067013
JO8	CS12	HS3	*R.nitidus*	0.010924	0.073213
GD34	CS12	HS3	*R.nitidus*	-0.000697	0.06593
SC44	GD32	HS3	*R.nitidus*	-0.017227	0.066145
JW6	GD32	HS3	*R.nitidus*	-0.01712	0.071389
GD29	CS12	HS3	*R.nitidus*	0.119588	0.066013
GD21	CS12	HS3	*R.nitidus*	0.136456	0.067771
JO7	CS12	HS3	*R.nitidus*	-0.020173	0.073326
GD26	CS12	HS3	*R.nitidus*	0.062987	0.066768
JO3	GD32	HS3	*R.nitidus*	-0.010018	0.070577
GD50	CS12	HS3	*R.nitidus*	0.028476	0.071433
GD25	CS12	HS3	*R.nitidus*	0.069977	0.067158
GD45	CS12	HS3	*R.nitidus*	0.066721	0.06944
CS11	CS12	HS3	*R.nitidus*	-0.019076	0.068764
GD48	CS12	HS3	*R.nitidus*	0.040467	0.069477
GD32	CS12	HS3	*R.nitidus*	0.025848	0.068413
GD46	CS12	HS3	*R.nitidus*	-0.024194	0.067389
GD33	CS12	HS3	*R.nitidus*	0.018182	0.067457
GD31	CS12	HS3	*R.nitidus*	0.06006	0.067194
GD30	CS12	HS3	*R.nitidus*	0.121264	0.071272
GD44	CS12	HS3	*R.nitidus*	0.044428	0.068412
GD49	CS12	HS3	*R.nitidus*	0.104132	0.070153
CS18	CS12	HS3	*R.nitidus*	-0.038404	0.068016
CS31	CS12	HS3	*R.nitidus*	-0.033228	0.068877
GD42	CS12	HS3	*R.nitidus*	0.024566	0.06851
CS19	CS12	HS3	*R.nitidus*	-0.024845	0.06901
SC10	CS12	HS3	*R.nitidus*	0.085736	0.067668
GD41	CS12	HS3	*R.nitidus*	0.007719	0.065279
CS13	CS12	HS3	*R.nitidus*	-0.07551	0.07027
GD40	CS12	HS3	*R.nitidus*	0.055914	0.066067
CS21	CS12	HS3	*R.nitidus*	-0.036955	0.065049
GD35	CS12	HS3	*R.nitidus*	0.053343	0.067544
CS17	CS12	HS3	*R.nitidus*	0.005513	0.070649
CS22	CS12	HS3	*R.nitidus*	-0.014515	0.065697
CS14	CS12	HS3	*R.nitidus*	-0.021598	0.070948
SC5	CS12	HS3	*R.nitidus*	0.014556	0.068086
CS15	CS12	HS3	*R.nitidus*	0.029567	0.066131
SC24	CS12	HS3	*R.nitidus*	-0.00593	0.066904
CS24	CS12	HS3	*R.nitidus*	-0.059809	0.066698
CS23	CS12	HS3	*R.nitidus*	-0.051395	0.066309
SC32	CS12	HS3	*R.nitidus*	0.049401	0.069324
CS20	CS12	HS3	*R.nitidus*	-0.020558	0.06728
CS4	CS12	HS3	*R.nitidus*	-0.034357	0.064474
CS16	CS12	HS3	*R.nitidus*	-0.014908	0.065368
SC15	CS12	HS3	*R.nitidus*	0.005439	0.066723
SC12	CS12	HS3	*R.nitidus*	-0.011128	0.067669
SC14	CS12	HS3	*R.nitidus*	0.010007	0.068559
CS30	CS12	HS3	*R.nitidus*	-0.062791	0.067713
SC35	CS12	HS3	*R.nitidus*	0.047109	0.06528

JW4	CS12	HS3	*R.nitidus*	0.204978	0.067213
SC31	CS12	HS3	*R.nitidus*	0.007267	0.068294
CS3	CS12	HS3	*R.nitidus*	-0.019742	0.065639
SC42	CS12	HS3	*R.nitidus*	0.238181	0.063664
CS25	CS12	HS3	*R.nitidus*	-0.006844	0.068512
CS28	CS12	HS3	*R.nitidus*	-0.028759	0.067885
MY4	CS12	HS3	*R.nitidus*	-0.044919	0.067819
JO5	CS12	HS3	*R.nitidus*	0.037834	0.065681
JW6	CS12	HS3	*R.nitidus*	-0.002822	0.072627
SC28	CS12	HS3	*R.nitidus*	-0.039245	0.065414
SC6	CS12	HS3	*R.nitidus*	-0.011202	0.068753
SC45	CS12	HS3	*R.nitidus*	0.046289	0.06779
SC34	CS12	HS3	*R.nitidus*	0.026198	0.064644
SC18	CS12	HS3	*R.nitidus*	0.045727	0.06754
SC26	CS12	HS3	*R.nitidus*	-0.030008	0.066799
SC27	CS12	HS3	*R.nitidus*	0.000757	0.066341
GD31	GD33	HS3	*R.nitidus*	0.041291	0.067077
GD25	GD33	HS3	*R.nitidus*	0.039911	0.066072
JO8	GD33	HS3	*R.nitidus*	0.005545	0.073147
SC44	CS12	HS3	*R.nitidus*	0.007424	0.066149
GD21	GD33	HS3	*R.nitidus*	0.1248	0.063539
CS12	GD33	HS3	*R.nitidus*	-0.018182	0.067457
GD41	GD33	HS3	*R.nitidus*	-0.011547	0.064535
JW7	CS12	HS3	*R.nitidus*	0.026126	0.071182
CS6	CS12	HS3	*R.nitidus*	-0.054033	0.065593
JO7	GD33	HS3	*R.nitidus*	-0.054645	0.073644
JO3	CS12	HS3	*R.nitidus*	0.021757	0.070188
GD26	GD33	HS3	*R.nitidus*	0.049658	0.064981
JO1	CS12	HS3	*R.nitidus*	-0.011512	0.066454
MY6	CS12	HS3	*R.nitidus*	-0.007829	0.067495
GD22	GD33	HS3	*R.nitidus*	0.068855	0.063169
GD32	GD33	HS3	*R.nitidus*	0.030392	0.066286
GD48	GD33	HS3	*R.nitidus*	0.0124	0.067712
GD34	GD33	HS3	*R.nitidus*	-0.043358	0.065642
GD49	GD33	HS3	*R.nitidus*	0.072631	0.069669
CS15	GD33	HS3	*R.nitidus*	-0.005337	0.066585
GD30	GD33	HS3	*R.nitidus*	0.101167	0.067681
GD42	GD33	HS3	*R.nitidus*	-0.004754	0.067049
CS11	GD33	HS3	*R.nitidus*	-0.030303	0.066824
CS18	GD33	HS3	*R.nitidus*	-0.064156	0.065228
CS17	GD33	HS3	*R.nitidus*	-0.007174	0.068306
GD29	GD33	HS3	*R.nitidus*	0.123887	0.064706
CS31	GD33	HS3	*R.nitidus*	-0.043964	0.065989
GD45	GD33	HS3	*R.nitidus*	0.058047	0.069658
CS28	GD33	HS3	*R.nitidus*	-0.066278	0.06678
GD46	GD33	HS3	*R.nitidus*	-0.0607	0.063242
CS19	GD33	HS3	*R.nitidus*	-0.046709	0.067587
GD40	GD33	HS3	*R.nitidus*	0.03118	0.062264
GD50	GD33	HS3	*R.nitidus*	0.000934	0.070334
CS16	GD33	HS3	*R.nitidus*	-0.023057	0.065425
GD35	GD33	HS3	*R.nitidus*	0.02687	0.064181
CS30	GD33	HS3	*R.nitidus*	-0.089583	0.066188
CS14	GD33	HS3	*R.nitidus*	-0.035135	0.067148
CS3	GD33	HS3	*R.nitidus*	-0.06383	0.065443
GD44	GD33	HS3	*R.nitidus*	0.00152	0.064902
SC15	GD33	HS3	*R.nitidus*	-0.013928	0.066016

CS24	GD33	HS3	*R.nitidus*	-0.069182	0.066827
SC18	GD33	HS3	*R.nitidus*	0	0.065764
CS20	GD33	HS3	*R.nitidus*	-0.05526	0.06617
SC10	GD33	HS3	*R.nitidus*	0.023109	0.068276
SC12	GD33	HS3	*R.nitidus*	-0.038384	0.066736
CS13	GD33	HS3	*R.nitidus*	-0.068336	0.066106
SC5	GD33	HS3	*R.nitidus*	-0.012784	0.069554
SC27	GD33	HS3	*R.nitidus*	-0.026473	0.064458
SC24	GD33	HS3	*R.nitidus*	-0.04186	0.064731
CS4	GD33	HS3	*R.nitidus*	-0.048649	0.064657
CS23	GD33	HS3	*R.nitidus*	-0.076115	0.065073
SC6	GD33	HS3	*R.nitidus*	-0.020436	0.066756
CS22	GD33	HS3	*R.nitidus*	-0.025624	0.065425
CS25	GD33	HS3	*R.nitidus*	-0.038678	0.067695
SC45	GD33	HS3	*R.nitidus*	0.019359	0.06578
CS21	GD33	HS3	*R.nitidus*	-0.060667	0.065521
SC28	GD33	HS3	*R.nitidus*	-0.05	0.064194
CS6	GD33	HS3	*R.nitidus*	-0.077135	0.064702
SC32	GD33	HS3	*R.nitidus*	0.022957	0.065721
JW4	GD33	HS3	*R.nitidus*	0.199861	0.069009
JO7	GD34	HS3	*R.nitidus*	-0.024043	0.072061
SC14	GD33	HS3	*R.nitidus*	-0.005937	0.065544
JO5	GD33	HS3	*R.nitidus*	0.02122	0.065749
MY4	GD33	HS3	*R.nitidus*	-0.057394	0.065094
JW6	GD33	HS3	*R.nitidus*	-0.009009	0.071592
SC31	GD33	HS3	*R.nitidus*	-0.027388	0.066623
SC26	GD33	HS3	*R.nitidus*	-0.062331	0.065097
JW7	GD33	HS3	*R.nitidus*	0.015823	0.070332
SC34	GD33	HS3	*R.nitidus*	0.000662	0.063729
MY6	GD33	HS3	*R.nitidus*	-0.02549	0.064618
SC35	GD33	HS3	*R.nitidus*	0.005913	0.065595
SC42	GD33	HS3	*R.nitidus*	0.199027	0.064585
GD50	GD34	HS3	*R.nitidus*	0.015654	0.068519
GD29	GD34	HS3	*R.nitidus*	0.167224	0.062924
SC44	GD33	HS3	*R.nitidus*	-0.028513	0.064709
GD30	GD34	HS3	*R.nitidus*	0.151423	0.065087
GD21	GD34	HS3	*R.nitidus*	0.147882	0.061979
GD25	GD34	HS3	*R.nitidus*	0.053875	0.06451
CS16	GD34	HS3	*R.nitidus*	-0.011711	0.064195
GD31	GD34	HS3	*R.nitidus*	0.065303	0.065442
GD42	GD34	HS3	*R.nitidus*	0.021669	0.06404
GD49	GD34	HS3	*R.nitidus*	0.098093	0.067701
JO1	GD33	HS3	*R.nitidus*	-0.041444	0.065589
JO8	GD34	HS3	*R.nitidus*	0.045539	0.070687
JO3	GD33	HS3	*R.nitidus*	0.011811	0.069135
CS11	GD34	HS3	*R.nitidus*	-0.008453	0.065343
GD22	GD34	HS3	*R.nitidus*	0.107908	0.06123
GD32	GD34	HS3	*R.nitidus*	0.038104	0.064359
GD45	GD34	HS3	*R.nitidus*	0.079825	0.068211
GD44	GD34	HS3	*R.nitidus*	0.018462	0.066214
GD41	GD34	HS3	*R.nitidus*	0.009456	0.063568
CS22	GD34	HS3	*R.nitidus*	-0.016903	0.06421
GD33	GD34	HS3	*R.nitidus*	0.043358	0.065642
GD35	GD34	HS3	*R.nitidus*	0.042665	0.065567
CS14	GD34	HS3	*R.nitidus*	-0.020395	0.065847
GD26	GD34	HS3	*R.nitidus*	0.076616	0.063042

CS12	GD34	HS3	*R.nitidus*	0.000697	0.06593
CS13	GD34	HS3	*R.nitidus*	-0.048312	0.06643
CS20	GD34	HS3	*R.nitidus*	-0.037433	0.066333
GD40	GD34	HS3	*R.nitidus*	0.065714	0.063408
CS28	GD34	HS3	*R.nitidus*	-0.051699	0.066755
GD48	GD34	HS3	*R.nitidus*	0.04833	0.066063
CS18	GD34	HS3	*R.nitidus*	-0.035837	0.064883
CS17	GD34	HS3	*R.nitidus*	0.007785	0.068107
CS15	GD34	HS3	*R.nitidus*	0.021583	0.066682
GD46	GD34	HS3	*R.nitidus*	-0.033673	0.06126
CS19	GD34	HS3	*R.nitidus*	-0.028209	0.066001
SC31	GD34	HS3	*R.nitidus*	-0.000668	0.064305
CS21	GD34	HS3	*R.nitidus*	-0.03469	0.066074
SC32	GD34	HS3	*R.nitidus*	0.030687	0.064605
CS3	GD34	HS3	*R.nitidus*	-0.036986	0.065995
SC27	GD34	HS3	*R.nitidus*	-0.011394	0.065055
JW7	GD34	HS3	*R.nitidus*	0.049206	0.069846
SC26	GD34	HS3	*R.nitidus*	-0.038514	0.063745
CS24	GD34	HS3	*R.nitidus*	-0.053446	0.064595
CS25	GD34	HS3	*R.nitidus*	-0.01717	0.065614
CS6	GD34	HS3	*R.nitidus*	-0.052047	0.065053
SC5	GD34	HS3	*R.nitidus*	0	0.069215
CS23	GD34	HS3	*R.nitidus*	-0.061745	0.064394
CS4	GD34	HS3	*R.nitidus*	-0.026157	0.061599
JO8	GD35	HS3	*R.nitidus*	-0.021257	0.072068
SC44	GD34	HS3	*R.nitidus*	-0.004196	0.064101
JW4	GD34	HS3	*R.nitidus*	0.210774	0.065893
SC6	GD34	HS3	*R.nitidus*	-0.004717	0.065218
SC34	GD34	HS3	*R.nitidus*	0.025049	0.064825
SC35	GD34	HS3	*R.nitidus*	0.028689	0.06488
JO3	GD34	HS3	*R.nitidus*	0.03289	0.068457
SC14	GD34	HS3	*R.nitidus*	0.005333	0.06562
JO1	GD34	HS3	*R.nitidus*	-0.03455	0.063778
CS30	GD34	HS3	*R.nitidus*	-0.069965	0.064322
SC24	GD34	HS3	*R.nitidus*	-0.026712	0.064446
CS31	GD34	HS3	*R.nitidus*	-0.022939	0.065653
JW6	GD34	HS3	*R.nitidus*	0.012853	0.069432
GD21	GD35	HS3	*R.nitidus*	0.089825	0.063714
SC12	GD34	HS3	*R.nitidus*	-0.025726	0.065635
SC10	GD34	HS3	*R.nitidus*	0.039409	0.066134
CS11	GD35	HS3	*R.nitidus*	-0.041272	0.065688
SC42	GD34	HS3	*R.nitidus*	0.228022	0.062787
GD30	GD35	HS3	*R.nitidus*	0.094595	0.067345
SC18	GD34	HS3	*R.nitidus*	0.014593	0.065577
JO7	GD35	HS3	*R.nitidus*	-0.053931	0.073966
GD25	GD35	HS3	*R.nitidus*	0.017557	0.063279
JO5	GD34	HS3	*R.nitidus*	0.034137	0.064411
SC28	GD34	HS3	*R.nitidus*	-0.04192	0.062802
SC15	GD34	HS3	*R.nitidus*	-0.005521	0.066704
GD26	GD35	HS3	*R.nitidus*	0.023134	0.065835
MY4	GD34	HS3	*R.nitidus*	-0.043243	0.064542
SC45	GD34	HS3	*R.nitidus*	0.023684	0.065327
GD31	GD35	HS3	*R.nitidus*	0.028593	0.065969
MY6	GD34	HS3	*R.nitidus*	-0.014657	0.06405
GD32	GD35	HS3	*R.nitidus*	0.003344	0.066731
CS13	GD35	HS3	*R.nitidus*	-0.078838	0.06687

GD41	GD35	HS3	*R.nitidus*	-0.038433	0.063145
GD49	GD35	HS3	*R.nitidus*	0.063584	0.068197
GD29	GD35	HS3	*R.nitidus*	0.075104	0.065367
GD44	GD35	HS3	*R.nitidus*	-0.014844	0.066651
GD45	GD35	HS3	*R.nitidus*	0.051724	0.067858
CS15	GD35	HS3	*R.nitidus*	-0.016892	0.066809
GD42	GD35	HS3	*R.nitidus*	-0.025994	0.06421
GD22	GD35	HS3	*R.nitidus*	0.03273	0.06351
GD34	GD35	HS3	*R.nitidus*	-0.042665	0.065567
GD33	GD35	HS3	*R.nitidus*	-0.02687	0.064181
CS25	GD35	HS3	*R.nitidus*	-0.044352	0.066707
GD48	GD35	HS3	*R.nitidus*	-0.01318	0.067763
GD46	GD35	HS3	*R.nitidus*	-0.079282	0.063201
CS12	GD35	HS3	*R.nitidus*	-0.053343	0.067544
SC5	GD35	HS3	*R.nitidus*	-0.038434	0.070527
CS17	GD35	HS3	*R.nitidus*	-0.035216	0.068039
GD40	GD35	HS3	*R.nitidus*	0.005869	0.062676
CS19	GD35	HS3	*R.nitidus*	-0.059328	0.067358
SC10	GD35	HS3	*R.nitidus*	0.016949	0.066244
CS16	GD35	HS3	*R.nitidus*	-0.046685	0.065291
CS24	GD35	HS3	*R.nitidus*	-0.088256	0.065637
CS23	GD35	HS3	*R.nitidus*	-0.094822	0.065391
SC6	GD35	HS3	*R.nitidus*	-0.044521	0.064178
CS18	GD35	HS3	*R.nitidus*	-0.077949	0.066245
GD50	GD35	HS3	*R.nitidus*	-0.034612	0.06809
SC32	GD35	HS3	*R.nitidus*	0.008021	0.065171
CS14	GD35	HS3	*R.nitidus*	-0.049451	0.067808
SC35	GD35	HS3	*R.nitidus*	-0.011757	0.065884
CS21	GD35	HS3	*R.nitidus*	-0.074123	0.06592
CS28	GD35	HS3	*R.nitidus*	-0.0837	0.067745
CS22	GD35	HS3	*R.nitidus*	-0.051055	0.06577
CS3	GD35	HS3	*R.nitidus*	-0.052342	0.064882
SC27	GD35	HS3	*R.nitidus*	-0.050104	0.064907
SC12	GD35	HS3	*R.nitidus*	-0.063758	0.065915
SC15	GD35	HS3	*R.nitidus*	-0.036792	0.06896
CS4	GD35	HS3	*R.nitidus*	-0.056478	0.064314
CS31	GD35	HS3	*R.nitidus*	-0.067148	0.066614
CS30	GD35	HS3	*R.nitidus*	-0.095007	0.066871
JO7	GD40	HS3	*R.nitidus*	-0.065237	0.070224
CS20	GD35	HS3	*R.nitidus*	-0.069911	0.06689
SC14	GD35	HS3	*R.nitidus*	-0.027229	0.066208
CS6	GD35	HS3	*R.nitidus*	-0.099436	0.065833
SC18	GD35	HS3	*R.nitidus*	-0.024478	0.065943
MY4	GD35	HS3	*R.nitidus*	-0.08728	0.065035
SC45	GD35	HS3	*R.nitidus*	-0.009453	0.06528
MY6	GD35	HS3	*R.nitidus*	-0.054091	0.065392
SC26	GD35	HS3	*R.nitidus*	-0.068687	0.064298
SC24	GD35	HS3	*R.nitidus*	-0.061841	0.064675
GD25	GD40	HS3	*R.nitidus*	0.011486	0.062423
SC28	GD35	HS3	*R.nitidus*	-0.077922	0.064927
SC34	GD35	HS3	*R.nitidus*	-0.020353	0.064418
SC31	GD35	HS3	*R.nitidus*	-0.037231	0.065041
JW6	GD35	HS3	*R.nitidus*	-0.044524	0.071488
JO5	GD35	HS3	*R.nitidus*	-0.021262	0.066015
SC44	GD35	HS3	*R.nitidus*	-0.047216	0.065822
JO1	GD35	HS3	*R.nitidus*	-0.059492	0.064388

SC42	GD35	HS3	*R.nitidus*	0.201865	0.063899
JO3	GD35	HS3	*R.nitidus*	-0.013799	0.070532
JO8	GD40	HS3	*R.nitidus*	-0.038033	0.07032
JW4	GD35	HS3	*R.nitidus*	0.176634	0.066057
CS13	GD40	HS3	*R.nitidus*	-0.104762	0.064891
GD35	GD40	HS3	*R.nitidus*	-0.005869	0.062676
GD32	GD40	HS3	*R.nitidus*	-0.014827	0.065418
GD50	GD40	HS3	*R.nitidus*	-0.03866	0.067925
JW7	GD35	HS3	*R.nitidus*	-0.006436	0.070756
GD26	GD40	HS3	*R.nitidus*	0.001437	0.06218
GD46	GD40	HS3	*R.nitidus*	-0.08642	0.062134
GD31	GD40	HS3	*R.nitidus*	-0.000753	0.064662
GD22	GD40	HS3	*R.nitidus*	0.017123	0.061879
GD21	GD40	HS3	*R.nitidus*	0.077808	0.064257
CS16	GD40	HS3	*R.nitidus*	-0.065529	0.064343
CS12	GD40	HS3	*R.nitidus*	-0.055914	0.066067
GD45	GD40	HS3	*R.nitidus*	0.010717	0.066681
CS23	GD40	HS3	*R.nitidus*	-0.113575	0.063597
CS17	GD40	HS3	*R.nitidus*	-0.048423	0.066506
CS11	GD40	HS3	*R.nitidus*	-0.062458	0.063969
CS19	GD40	HS3	*R.nitidus*	-0.093772	0.065545
GD33	GD40	HS3	*R.nitidus*	-0.03118	0.062264
GD44	GD40	HS3	*R.nitidus*	-0.041921	0.063435
CS14	GD40	HS3	*R.nitidus*	-0.060425	0.06652
GD29	GD40	HS3	*R.nitidus*	0.081043	0.062395
GD30	GD40	HS3	*R.nitidus*	0.070751	0.06529
CS24	GD40	HS3	*R.nitidus*	-0.114685	0.061431
CS3	GD40	HS3	*R.nitidus*	-0.086713	0.062278
CS18	GD40	HS3	*R.nitidus*	-0.109091	0.064188
CS30	GD40	HS3	*R.nitidus*	-0.117246	0.063843
GD48	GD40	HS3	*R.nitidus*	-0.021412	0.065539
CS15	GD40	HS3	*R.nitidus*	-0.032472	0.065432
GD49	GD40	HS3	*R.nitidus*	0.036484	0.068181
GD41	GD40	HS3	*R.nitidus*	-0.051986	0.061802
CS25	GD40	HS3	*R.nitidus*	-0.074024	0.065256
CS22	GD40	HS3	*R.nitidus*	-0.07014	0.062806
GD42	GD40	HS3	*R.nitidus*	-0.037199	0.063724
GD34	GD40	HS3	*R.nitidus*	-0.065714	0.063408
SC5	GD40	HS3	*R.nitidus*	-0.064164	0.064955
SC34	GD40	HS3	*R.nitidus*	-0.02988	0.062352
SC14	GD40	HS3	*R.nitidus*	-0.056728	0.063739
CS31	GD40	HS3	*R.nitidus*	-0.085992	0.064232
JW6	GD40	HS3	*R.nitidus*	-0.045253	0.06935
CS21	GD40	HS3	*R.nitidus*	-0.102341	0.062764
SC12	GD40	HS3	*R.nitidus*	-0.084712	0.064001
SC18	GD40	HS3	*R.nitidus*	-0.033803	0.062071
CS20	GD40	HS3	*R.nitidus*	-0.094976	0.063705
CS28	GD40	HS3	*R.nitidus*	-0.092806	0.064853
JO5	GD40	HS3	*R.nitidus*	-0.027027	0.063779
SC35	GD40	HS3	*R.nitidus*	-0.020039	0.062428
MY4	GD40	HS3	*R.nitidus*	-0.109838	0.06282
MY6	GD40	HS3	*R.nitidus*	-0.068602	0.062358
SC45	GD40	HS3	*R.nitidus*	-0.036569	0.063876
CS4	GD40	HS3	*R.nitidus*	-0.092359	0.061919
SC10	GD40	HS3	*R.nitidus*	-0.00622	0.064856
SC44	GD40	HS3	*R.nitidus*	-0.068772	0.062637

GD31	GD41	HS3	*R.nitidus*	0.059598	0.066236
SC24	GD40	HS3	*R.nitidus*	-0.072222	0.063086
GD21	GD41	HS3	*R.nitidus*	0.127814	0.061209
SC15	GD40	HS3	*R.nitidus*	-0.064428	0.063412
SC31	GD40	HS3	*R.nitidus*	-0.06369	0.06458
JO8	GD41	HS3	*R.nitidus*	0.028463	0.06969
JW4	GD40	HS3	*R.nitidus*	0.14698	0.065773
CS6	GD40	HS3	*R.nitidus*	-0.11354	0.062852
SC32	GD40	HS3	*R.nitidus*	-0.023825	0.063623
JO7	GD41	HS3	*R.nitidus*	-0.000925	0.071456
SC26	GD40	HS3	*R.nitidus*	-0.095111	0.06161
JW7	GD40	HS3	*R.nitidus*	-0.031671	0.067384
JO1	GD40	HS3	*R.nitidus*	-0.084106	0.065255
SC27	GD40	HS3	*R.nitidus*	-0.070199	0.061758
SC42	GD40	HS3	*R.nitidus*	0.170366	0.062278
JO3	GD40	HS3	*R.nitidus*	-0.003084	0.069004
GD26	GD41	HS3	*R.nitidus*	0.071266	0.06373
SC6	GD40	HS3	*R.nitidus*	-0.067669	0.062515
SC28	GD40	HS3	*R.nitidus*	-0.09825	0.06306
CS11	GD41	HS3	*R.nitidus*	-0.017651	0.06488
CS12	GD41	HS3	*R.nitidus*	-0.007719	0.065279
GD33	GD41	HS3	*R.nitidus*	0.011547	0.064535
GD35	GD41	HS3	*R.nitidus*	0.038433	0.063145
GD22	GD41	HS3	*R.nitidus*	0.092494	0.063764
GD30	GD41	HS3	*R.nitidus*	0.108867	0.065792
GD32	GD41	HS3	*R.nitidus*	0.052539	0.066864
CS17	GD41	HS3	*R.nitidus*	0.004338	0.068646
CS18	GD41	HS3	*R.nitidus*	-0.044548	0.063568
GD50	GD41	HS3	*R.nitidus*	0.029175	0.068636
GD48	GD41	HS3	*R.nitidus*	0.059483	0.066909
GD34	GD41	HS3	*R.nitidus*	-0.009456	0.063568
GD44	GD41	HS3	*R.nitidus*	0.027464	0.066134
GD29	GD41	HS3	*R.nitidus*	0.126573	0.064218
CS20	GD41	HS3	*R.nitidus*	-0.046588	0.064638
GD40	GD41	HS3	*R.nitidus*	0.051986	0.061802
GD25	GD41	HS3	*R.nitidus*	0.041605	0.06474
CS14	GD41	HS3	*R.nitidus*	-0.016194	0.066454
CS15	GD41	HS3	*R.nitidus*	0.016097	0.065272
GD42	GD41	HS3	*R.nitidus*	0.022977	0.062922
CS13	GD41	HS3	*R.nitidus*	-0.05073	0.065649
SC10	GD41	HS3	*R.nitidus*	0.043262	0.064287
GD45	GD41	HS3	*R.nitidus*	0.052174	0.068011
CS21	GD41	HS3	*R.nitidus*	-0.045514	0.063442
GD46	GD41	HS3	*R.nitidus*	-0.051607	0.061722
GD49	GD41	HS3	*R.nitidus*	0.112652	0.067599
SC44	GD41	HS3	*R.nitidus*	-0.007829	0.063736
SC15	GD41	HS3	*R.nitidus*	0.002905	0.06377
CS25	GD41	HS3	*R.nitidus*	-0.015272	0.066218
CS31	GD41	HS3	*R.nitidus*	-0.034532	0.065318
CS16	GD41	HS3	*R.nitidus*	-0.005446	0.063028
SC18	GD41	HS3	*R.nitidus*	0.011544	0.064119
CS19	GD41	HS3	*R.nitidus*	-0.028533	0.06577
JW7	GD41	HS3	*R.nitidus*	0.02963	0.068159
SC5	GD41	HS3	*R.nitidus*	-0.004213	0.067249
CS22	GD41	HS3	*R.nitidus*	-0.023874	0.063703
CS24	GD41	HS3	*R.nitidus*	-0.059786	0.064561

SC6	GD41	HS3	*R.nitidus*	-0.010989	0.065444
JO3	GD41	HS3	*R.nitidus*	0.024682	0.068526
SC45	GD41	HS3	*R.nitidus*	0.020932	0.065142
SC34	GD41	HS3	*R.nitidus*	0.024624	0.063935
CS23	GD41	HS3	*R.nitidus*	-0.061599	0.063828
JW4	GD41	HS3	*R.nitidus*	0.2	0.063143
CS28	GD41	HS3	*R.nitidus*	-0.043155	0.065826
SC35	GD41	HS3	*R.nitidus*	0.031123	0.063401
MY4	GD41	HS3	*R.nitidus*	-0.042538	0.062416
SC26	GD41	HS3	*R.nitidus*	-0.042424	0.065708
SC24	GD41	HS3	*R.nitidus*	-0.03244	0.063048
SC27	GD41	HS3	*R.nitidus*	-0.024458	0.061772
CS3	GD41	HS3	*R.nitidus*	-0.050734	0.063349
CS30	GD41	HS3	*R.nitidus*	-0.080542	0.06429
SC14	GD41	HS3	*R.nitidus*	-0.006285	0.062674
CS4	GD41	HS3	*R.nitidus*	-0.035762	0.062079
SC28	GD41	HS3	*R.nitidus*	-0.045839	0.064354
SC12	GD41	HS3	*R.nitidus*	-0.01828	0.06293
JO7	GD42	HS3	*R.nitidus*	-0.076181	0.074262
SC42	GD41	HS3	*R.nitidus*	0.227021	0.061892
GD22	GD42	HS3	*R.nitidus*	0.078234	0.064107
MY6	GD41	HS3	*R.nitidus*	-0.003318	0.061311
JO5	GD41	HS3	*R.nitidus*	0.035762	0.063381
SC31	GD41	HS3	*R.nitidus*	-0.002026	0.063691
CS6	GD41	HS3	*R.nitidus*	-0.058123	0.062694
JW6	GD41	HS3	*R.nitidus*	-0.008272	0.068515
JO1	GD41	HS3	*R.nitidus*	-0.025624	0.063604
GD34	GD42	HS3	*R.nitidus*	-0.021669	0.06404
GD25	GD42	HS3	*R.nitidus*	0.027972	0.066167
CS11	GD42	HS3	*R.nitidus*	-0.041181	0.067417
JO8	GD42	HS3	*R.nitidus*	-0.024085	0.071571
GD30	GD42	HS3	*R.nitidus*	0.108635	0.067552
GD49	GD42	HS3	*R.nitidus*	0.095111	0.067749
GD29	GD42	HS3	*R.nitidus*	0.118352	0.064272
GD32	GD42	HS3	*R.nitidus*	0.026549	0.068111
GD40	GD42	HS3	*R.nitidus*	0.037199	0.063724
GD46	GD42	HS3	*R.nitidus*	-0.062872	0.063423
CS16	GD42	HS3	*R.nitidus*	-0.037293	0.065679
GD41	GD42	HS3	*R.nitidus*	-0.022977	0.062922
GD35	GD42	HS3	*R.nitidus*	0.025994	0.06421
CS14	GD42	HS3	*R.nitidus*	-0.050859	0.067667
GD21	GD42	HS3	*R.nitidus*	0.107306	0.062229
SC32	GD41	HS3	*R.nitidus*	0.030096	0.062484
GD33	GD42	HS3	*R.nitidus*	0.004754	0.067049
GD26	GD42	HS3	*R.nitidus*	0.028369	0.066433
GD31	GD42	HS3	*R.nitidus*	0.031528	0.066511
GD44	GD42	HS3	*R.nitidus*	0.008137	0.066583
CS15	GD42	HS3	*R.nitidus*	-0.013032	0.068208
GD45	GD42	HS3	*R.nitidus*	0.060198	0.069367
CS21	GD42	HS3	*R.nitidus*	-0.07072	0.065744
CS24	GD42	HS3	*R.nitidus*	-0.073602	0.066382
CS13	GD42	HS3	*R.nitidus*	-0.071923	0.068379
CS22	GD42	HS3	*R.nitidus*	-0.0413	0.064211
CS12	GD42	HS3	*R.nitidus*	-0.024566	0.06851
CS20	GD42	HS3	*R.nitidus*	-0.053876	0.066154
GD48	GD42	HS3	*R.nitidus*	0.012411	0.06591

GD50	GD42	HS3	*R.nitidus*	-0.009165	0.068481
CS30	GD42	HS3	*R.nitidus*	-0.095481	0.065272
SC5	GD42	HS3	*R.nitidus*	-0.023994	0.068942
CS18	GD42	HS3	*R.nitidus*	-0.069907	0.066373
SC10	GD42	HS3	*R.nitidus*	0.024496	0.066583
CS19	GD42	HS3	*R.nitidus*	-0.04878	0.067378
CS23	GD42	HS3	*R.nitidus*	-0.083445	0.066185
CS28	GD42	HS3	*R.nitidus*	-0.068216	0.067175
CS25	GD42	HS3	*R.nitidus*	-0.037631	0.06868
SC26	GD42	HS3	*R.nitidus*	-0.05988	0.066374
SC15	GD42	HS3	*R.nitidus*	-0.034508	0.065652
CS6	GD42	HS3	*R.nitidus*	-0.082686	0.064424
SC6	GD42	HS3	*R.nitidus*	-0.033081	0.066975
SC24	GD42	HS3	*R.nitidus*	-0.041549	0.063755
SC12	GD42	HS3	*R.nitidus*	-0.042582	0.064428
SC28	GD42	HS3	*R.nitidus*	-0.066482	0.065435
SC45	GD42	HS3	*R.nitidus*	0.005464	0.065732
SC14	GD42	HS3	*R.nitidus*	-0.008978	0.065338
JO1	GD42	HS3	*R.nitidus*	-0.046748	0.065771
SC27	GD42	HS3	*R.nitidus*	-0.033403	0.063667
SC18	GD42	HS3	*R.nitidus*	-0.014399	0.066131
CS17	GD42	HS3	*R.nitidus*	-0.012427	0.068956
CS3	GD42	HS3	*R.nitidus*	-0.057666	0.065113
MY6	GD42	HS3	*R.nitidus*	-0.025	0.064518
CS4	GD42	HS3	*R.nitidus*	-0.040667	0.062398
SC31	GD42	HS3	*R.nitidus*	-0.03148	0.066201
JW4	GD42	HS3	*R.nitidus*	0.172295	0.06465
SC34	GD42	HS3	*R.nitidus*	0.004118	0.06537
SC44	GD42	HS3	*R.nitidus*	-0.02984	0.066019
JW6	GD42	HS3	*R.nitidus*	-0.048872	0.070732
JO5	GD42	HS3	*R.nitidus*	0.011173	0.065099
JW7	GD42	HS3	*R.nitidus*	-0.022575	0.067902
JO3	GD42	HS3	*R.nitidus*	0.001641	0.070629
SC42	GD42	HS3	*R.nitidus*	0.224758	0.062013
MY4	GD42	HS3	*R.nitidus*	-0.061495	0.064882
GD21	GD44	HS3	*R.nitidus*	0.127193	0.064531
CS31	GD42	HS3	*R.nitidus*	-0.041176	0.06672
SC32	GD42	HS3	*R.nitidus*	0.017906	0.063991
GD31	GD44	HS3	*R.nitidus*	0.04844	0.067728
SC35	GD42	HS3	*R.nitidus*	0.006028	0.06392
GD33	GD44	HS3	*R.nitidus*	-0.00152	0.064902
JO8	GD44	HS3	*R.nitidus*	-0.004794	0.072636
JO7	GD44	HS3	*R.nitidus*	-0.034323	0.071618
CS13	GD44	HS3	*R.nitidus*	-0.091304	0.068388
GD25	GD44	HS3	*R.nitidus*	0.032432	0.066178
GD32	GD44	HS3	*R.nitidus*	-0.000898	0.068117
CS12	GD44	HS3	*R.nitidus*	-0.044428	0.068412
GD22	GD44	HS3	*R.nitidus*	0.056632	0.0652
CS17	GD44	HS3	*R.nitidus*	-0.007651	0.071431
CS11	GD44	HS3	*R.nitidus*	-0.030856	0.068548
CS20	GD44	HS3	*R.nitidus*	-0.068474	0.06621
GD42	GD44	HS3	*R.nitidus*	-0.008137	0.066583
GD30	GD44	HS3	*R.nitidus*	0.099019	0.067776
GD35	GD44	HS3	*R.nitidus*	0.014844	0.066651
GD29	GD44	HS3	*R.nitidus*	0.127234	0.06536
CS15	GD44	HS3	*R.nitidus*	-0.005559	0.066264

GD26	GD44	HS3	*R.nitidus*	0.033832	0.066583
GD48	GD44	HS3	*R.nitidus*	0.019574	0.066688
GD50	GD44	HS3	*R.nitidus*	0.000928	0.069438
GD40	GD44	HS3	*R.nitidus*	0.041921	0.063435
CS30	GD44	HS3	*R.nitidus*	-0.079942	0.066524
GD34	GD44	HS3	*R.nitidus*	-0.018462	0.066214
CS21	GD44	HS3	*R.nitidus*	-0.065263	0.065418
CS22	GD44	HS3	*R.nitidus*	-0.061848	0.066226
CS3	GD44	HS3	*R.nitidus*	-0.059669	0.064206
CS18	GD44	HS3	*R.nitidus*	-0.080377	0.066014
CS23	GD44	HS3	*R.nitidus*	-0.085512	0.064767
CS19	GD44	HS3	*R.nitidus*	-0.06622	0.067637
SC12	GD44	HS3	*R.nitidus*	-0.055021	0.064412
SC14	GD44	HS3	*R.nitidus*	-0.034757	0.067034
SC5	GD44	HS3	*R.nitidus*	-0.028295	0.069326
GD41	GD44	HS3	*R.nitidus*	-0.027464	0.066134
GD49	GD44	HS3	*R.nitidus*	0.079615	0.069174
CS14	GD44	HS3	*R.nitidus*	-0.039548	0.06718
CS24	GD44	HS3	*R.nitidus*	-0.090566	0.066701
CS31	GD44	HS3	*R.nitidus*	-0.062453	0.066773
GD46	GD44	HS3	*R.nitidus*	-0.059375	0.065587
SC28	GD44	HS3	*R.nitidus*	-0.072674	0.065887
SC18	GD44	HS3	*R.nitidus*	-0.024115	0.064691
CS4	GD44	HS3	*R.nitidus*	-0.073487	0.065761
CS16	GD44	HS3	*R.nitidus*	-0.024338	0.066156
SC35	GD44	HS3	*R.nitidus*	0.013268	0.065005
SC44	GD44	HS3	*R.nitidus*	-0.029542	0.066074
GD45	GD44	HS3	*R.nitidus*	0.046961	0.069099
SC15	GD44	HS3	*R.nitidus*	-0.011111	0.067051
CS25	GD44	HS3	*R.nitidus*	-0.057607	0.067454
CS28	GD44	HS3	*R.nitidus*	-0.070933	0.067608
JW6	GD44	HS3	*R.nitidus*	-0.028362	0.070651
MY4	GD44	HS3	*R.nitidus*	-0.072307	0.066109
JW4	GD44	HS3	*R.nitidus*	0.160986	0.066916
SC31	GD44	HS3	*R.nitidus*	-0.019661	0.06535
SC6	GD44	HS3	*R.nitidus*	-0.023324	0.066917
SC10	GD44	HS3	*R.nitidus*	0.014894	0.0662
SC45	GD44	HS3	*R.nitidus*	-0.001422	0.066663
JW7	GD44	HS3	*R.nitidus*	-0.000851	0.069945
GD25	GD45	HS3	*R.nitidus*	-0.050213	0.070313
SC24	GD44	HS3	*R.nitidus*	-0.061947	0.065939
SC26	GD44	HS3	*R.nitidus*	-0.065597	0.06629
SC27	GD44	HS3	*R.nitidus*	-0.04717	0.066361
GD21	GD45	HS3	*R.nitidus*	0.047993	0.068195
GD32	GD45	HS3	*R.nitidus*	-0.02994	0.072559
JO5	GD44	HS3	*R.nitidus*	0.008493	0.065603
SC32	GD44	HS3	*R.nitidus*	0.016878	0.06708
MY6	GD44	HS3	*R.nitidus*	-0.034605	0.067081
GD33	GD45	HS3	*R.nitidus*	-0.058047	0.069658
SC34	GD44	HS3	*R.nitidus*	0.006263	0.066294
CS6	GD44	HS3	*R.nitidus*	-0.08659	0.065662
JO7	GD45	HS3	*R.nitidus*	-0.13058	0.075073
CS14	GD45	HS3	*R.nitidus*	-0.067977	0.073345
JO1	GD44	HS3	*R.nitidus*	-0.044554	0.066041
SC42	GD44	HS3	*R.nitidus*	0.203671	0.064122
GD31	GD45	HS3	*R.nitidus*	-0.000895	0.070462

JO3	GD44	HS3	*R.nitidus*	0.002421	0.071475
JO8	GD45	HS3	*R.nitidus*	-0.052934	0.076543
GD22	GD45	HS3	*R.nitidus*	0.028712	0.069535
CS11	GD45	HS3	*R.nitidus*	-0.083927	0.069695
GD42	GD45	HS3	*R.nitidus*	-0.060198	0.069367
GD26	GD45	HS3	*R.nitidus*	-0.03964	0.069348
GD30	GD45	HS3	*R.nitidus*	0.03966	0.068446
GD34	GD45	HS3	*R.nitidus*	-0.079825	0.068211
CS12	GD45	HS3	*R.nitidus*	-0.066721	0.06944
GD35	GD45	HS3	*R.nitidus*	-0.051724	0.067858
CS15	GD45	HS3	*R.nitidus*	-0.049313	0.070812
GD46	GD45	HS3	*R.nitidus*	-0.121683	0.069141
GD41	GD45	HS3	*R.nitidus*	-0.052174	0.068011
CS20	GD45	HS3	*R.nitidus*	-0.087302	0.071388
CS30	GD45	HS3	*R.nitidus*	-0.115702	0.068492
GD29	GD45	HS3	*R.nitidus*	0.036503	0.067862
GD49	GD45	HS3	*R.nitidus*	0.032258	0.072176
CS17	GD45	HS3	*R.nitidus*	-0.074576	0.071846
CS16	GD45	HS3	*R.nitidus*	-0.083534	0.071531
CS13	GD45	HS3	*R.nitidus*	-0.094279	0.071913
GD48	GD45	HS3	*R.nitidus*	-0.023645	0.071901
GD44	GD45	HS3	*R.nitidus*	-0.046961	0.069099
CS22	GD45	HS3	*R.nitidus*	-0.070204	0.06991
CS18	GD45	HS3	*R.nitidus*	-0.103721	0.071379
CS28	GD45	HS3	*R.nitidus*	-0.102415	0.069135
GD40	GD45	HS3	*R.nitidus*	-0.010717	0.066681
CS3	GD45	HS3	*R.nitidus*	-0.074819	0.070707
CS31	GD45	HS3	*R.nitidus*	-0.105705	0.070266
CS19	GD45	HS3	*R.nitidus*	-0.083333	0.071332
GD50	GD45	HS3	*R.nitidus*	-0.053971	0.072566
SC10	GD45	HS3	*R.nitidus*	-0.00161	0.070532
CS25	GD45	HS3	*R.nitidus*	-0.076613	0.072583
CS21	GD45	HS3	*R.nitidus*	-0.094531	0.070612
SC12	GD45	HS3	*R.nitidus*	-0.069413	0.068556
CS23	GD45	HS3	*R.nitidus*	-0.097692	0.071495
CS24	GD45	HS3	*R.nitidus*	-0.098441	0.069419
SC18	GD45	HS3	*R.nitidus*	-0.011494	0.068531
CS4	GD45	HS3	*R.nitidus*	-0.086889	0.067063
SC26	GD45	HS3	*R.nitidus*	-0.084848	0.068526
SC5	GD45	HS3	*R.nitidus*	-0.075428	0.072377
JW7	GD45	HS3	*R.nitidus*	-0.049856	0.072517
SC24	GD45	HS3	*R.nitidus*	-0.060222	0.068027
SC15	GD45	HS3	*R.nitidus*	-0.036058	0.069412
SC44	GD45	HS3	*R.nitidus*	-0.043165	0.067153
JO5	GD45	HS3	*R.nitidus*	-0.02809	0.068972
SC6	GD45	HS3	*R.nitidus*	-0.068775	0.070877
JO8	CS13	HS3	*R.nitidus*	0.061144	0.076038
JW6	GD45	HS3	*R.nitidus*	-0.115304	0.074228
SC27	GD45	HS3	*R.nitidus*	-0.05919	0.068904
SC42	GD45	HS3	*R.nitidus*	0.186725	0.064882
JO7	CS13	HS3	*R.nitidus*	0.037645	0.073643
MY6	GD45	HS3	*R.nitidus*	-0.046384	0.068757
SC45	GD45	HS3	*R.nitidus*	-0.011952	0.069216
SC31	GD45	HS3	*R.nitidus*	-0.04006	0.069621
CS6	GD45	HS3	*R.nitidus*	-0.074935	0.069642
JO1	GD45	HS3	*R.nitidus*	-0.072141	0.068026

SC14	GD45	HS3	*R.nitidus*	-0.040062	0.070881
GD21	CS13	HS3	*R.nitidus*	0.161793	0.065493
GD30	CS13	HS3	*R.nitidus*	0.155332	0.068312
SC28	GD45	HS3	*R.nitidus*	-0.090548	0.067275
GD26	CS13	HS3	*R.nitidus*	0.105336	0.067632
CS11	CS13	HS3	*R.nitidus*	0.049847	0.071542
SC32	GD45	HS3	*R.nitidus*	-0.003934	0.068447
SC34	GD45	HS3	*R.nitidus*	-0.014331	0.067464
GD31	CS13	HS3	*R.nitidus*	0.110948	0.066919
GD25	CS13	HS3	*R.nitidus*	0.096591	0.066198
GD34	CS13	HS3	*R.nitidus*	0.048312	0.06643
GD33	CS13	HS3	*R.nitidus*	0.068336	0.066106
SC35	GD45	HS3	*R.nitidus*	-0.010828	0.067837
GD42	CS13	HS3	*R.nitidus*	0.071923	0.068379
JO3	GD45	HS3	*R.nitidus*	-0.052288	0.073571
GD40	CS13	HS3	*R.nitidus*	0.104762	0.064891
JW4	GD45	HS3	*R.nitidus*	0.148329	0.070874
GD32	CS13	HS3	*R.nitidus*	0.072998	0.068695
MY4	GD45	HS3	*R.nitidus*	-0.083137	0.067346
CS12	CS13	HS3	*R.nitidus*	0.07551	0.07027
GD41	CS13	HS3	*R.nitidus*	0.05073	0.065649
GD50	CS13	HS3	*R.nitidus*	0.069528	0.072479
GD48	CS13	HS3	*R.nitidus*	0.091523	0.069499
GD22	CS13	HS3	*R.nitidus*	0.130921	0.06522
GD46	CS13	HS3	*R.nitidus*	0.033029	0.067912
CS3	CS13	HS3	*R.nitidus*	0.008076	0.065648
CS17	CS13	HS3	*R.nitidus*	0.068966	0.072251
GD29	CS13	HS3	*R.nitidus*	0.17113	0.065477
GD49	CS13	HS3	*R.nitidus*	0.128059	0.069462
CS28	CS13	HS3	*R.nitidus*	0.01684	0.06986
GD44	CS13	HS3	*R.nitidus*	0.091304	0.068388
CS14	CS13	HS3	*R.nitidus*	0.043902	0.071678
SC24	CS13	HS3	*R.nitidus*	0.030824	0.067488
GD35	CS13	HS3	*R.nitidus*	0.078838	0.06687
SC15	CS13	HS3	*R.nitidus*	0.051632	0.067901
GD45	CS13	HS3	*R.nitidus*	0.094279	0.071913
CS19	CS13	HS3	*R.nitidus*	0.014118	0.070397
SC14	CS13	HS3	*R.nitidus*	0.049088	0.067912
CS31	CS13	HS3	*R.nitidus*	0.027111	0.068942
CS22	CS13	HS3	*R.nitidus*	0.045018	0.066829
CS16	CS13	HS3	*R.nitidus*	0.054634	0.068312
CS18	CS13	HS3	*R.nitidus*	-0.003788	0.068204
SC35	CS13	HS3	*R.nitidus*	0.08042	0.066423
CS30	CS13	HS3	*R.nitidus*	-0.025354	0.068251
MY6	CS13	HS3	*R.nitidus*	0.046329	0.067212
CS25	CS13	HS3	*R.nitidus*	0.032114	0.070674
SC10	CS13	HS3	*R.nitidus*	0.099025	0.067709
CS24	CS13	HS3	*R.nitidus*	-0.009412	0.066795
CS21	CS13	HS3	*R.nitidus*	0.004367	0.067127
SC34	CS13	HS3	*R.nitidus*	0.06744	0.068152
SC28	CS13	HS3	*R.nitidus*	-0.000733	0.06568
CS20	CS13	HS3	*R.nitidus*	0.004222	0.067727
JW6	CS13	HS3	*R.nitidus*	0.037002	0.074089
CS23	CS13	HS3	*R.nitidus*	-0.020364	0.066013
JW7	CS13	HS3	*R.nitidus*	0.084265	0.0706
CS15	CS13	HS3	*R.nitidus*	0.094283	0.070361

SC44	CS13	HS3	*R.nitidus*	0.035062	0.067003
SC27	CS13	HS3	*R.nitidus*	0.03693	0.066827
JO8	GD46	HS3	*R.nitidus*	0.096339	0.067963
SC5	CS13	HS3	*R.nitidus*	0.039589	0.068771
CS6	CS13	HS3	*R.nitidus*	-0.009797	0.068003
SC42	CS13	HS3	*R.nitidus*	0.266124	0.062744
GD25	GD46	HS3	*R.nitidus*	0.086957	0.063785
SC6	CS13	HS3	*R.nitidus*	0.041783	0.068465
SC12	CS13	HS3	*R.nitidus*	0.034582	0.067207
CS4	CS13	HS3	*R.nitidus*	0.018012	0.066024
JO7	GD46	HS3	*R.nitidus*	0.024231	0.071377
SC32	CS13	HS3	*R.nitidus*	0.086207	0.069521
SC18	CS13	HS3	*R.nitidus*	0.066278	0.067148
SC31	CS13	HS3	*R.nitidus*	0.044881	0.067508
JW4	CS13	HS3	*R.nitidus*	0.255443	0.066172
GD21	GD46	HS3	*R.nitidus*	0.188142	0.061489
JO5	CS13	HS3	*R.nitidus*	0.080946	0.066023
GD22	GD46	HS3	*R.nitidus*	0.12936	0.060791
MY4	CS13	HS3	*R.nitidus*	0.005162	0.067617
JO3	CS13	HS3	*R.nitidus*	0.078759	0.071555
GD35	GD46	HS3	*R.nitidus*	0.079282	0.063201
SC26	CS13	HS3	*R.nitidus*	0.00369	0.066705
GD45	GD46	HS3	*R.nitidus*	0.121683	0.069141
GD29	GD46	HS3	*R.nitidus*	0.179706	0.064208
GD33	GD46	HS3	*R.nitidus*	0.0607	0.063242
JO1	CS13	HS3	*R.nitidus*	0.034107	0.066853
CS12	GD46	HS3	*R.nitidus*	0.024194	0.067389
GD41	GD46	HS3	*R.nitidus*	0.051607	0.061722
GD50	GD46	HS3	*R.nitidus*	0.064333	0.067582
CS11	GD46	HS3	*R.nitidus*	0.012295	0.066678
GD49	GD46	HS3	*R.nitidus*	0.127434	0.068258
GD42	GD46	HS3	*R.nitidus*	0.062872	0.063423
GD30	GD46	HS3	*R.nitidus*	0.17332	0.064281
CS18	GD46	HS3	*R.nitidus*	-0.029133	0.065867
GD26	GD46	HS3	*R.nitidus*	0.101657	0.061604
CS13	GD46	HS3	*R.nitidus*	-0.033029	0.067912
SC45	CS13	HS3	*R.nitidus*	0.060452	0.069428
GD40	GD46	HS3	*R.nitidus*	0.08642	0.062134
GD34	GD46	HS3	*R.nitidus*	0.033673	0.06126
CS20	GD46	HS3	*R.nitidus*	-0.019372	0.064913
GD32	GD46	HS3	*R.nitidus*	0.088133	0.064103
CS30	GD46	HS3	*R.nitidus*	-0.037857	0.06632
GD31	GD46	HS3	*R.nitidus*	0.093077	0.064483
GD48	GD46	HS3	*R.nitidus*	0.096407	0.064695
GD44	GD46	HS3	*R.nitidus*	0.059375	0.065587
CS16	GD46	HS3	*R.nitidus*	0.016231	0.067368
CS14	GD46	HS3	*R.nitidus*	-0.000713	0.06839
CS4	GD46	HS3	*R.nitidus*	-0.007246	0.06283
CS21	GD46	HS3	*R.nitidus*	-0.027796	0.065539
CS19	GD46	HS3	*R.nitidus*	-0.01584	0.068174
CS31	GD46	HS3	*R.nitidus*	-0.003698	0.064989
CS25	GD46	HS3	*R.nitidus*	0.00677	0.067858
CS28	GD46	HS3	*R.nitidus*	-0.016284	0.067267
SC26	GD46	HS3	*R.nitidus*	-0.008742	0.06264
SC6	GD46	HS3	*R.nitidus*	0.01669	0.064856
CS22	GD46	HS3	*R.nitidus*	0.006198	0.063817

CS17	GD46	HS3	*R.nitidus*	0.039347	0.069225
CS15	GD46	HS3	*R.nitidus*	0.033126	0.067431
CS3	GD46	HS3	*R.nitidus*	-0.005435	0.064225
CS6	GD46	HS3	*R.nitidus*	-0.027933	0.064323
SC45	GD46	HS3	*R.nitidus*	0.047011	0.064678
CS24	GD46	HS3	*R.nitidus*	-0.026148	0.065058
SC5	GD46	HS3	*R.nitidus*	0.018724	0.067672
SC31	GD46	HS3	*R.nitidus*	0.015464	0.06321
CS23	GD46	HS3	*R.nitidus*	-0.031511	0.063349
SC34	GD46	HS3	*R.nitidus*	0.049698	0.063343
SC35	GD46	HS3	*R.nitidus*	0.061486	0.062027
SC24	GD46	HS3	*R.nitidus*	0.008397	0.063685
SC10	GD46	HS3	*R.nitidus*	0.067447	0.063649
JW6	GD46	HS3	*R.nitidus*	0.041209	0.070103
MY4	GD46	HS3	*R.nitidus*	-0.015884	0.063938
SC15	GD46	HS3	*R.nitidus*	0.030891	0.065785
SC12	GD46	HS3	*R.nitidus*	0.003397	0.064349
JW7	GD46	HS3	*R.nitidus*	0.054591	0.067846
SC32	GD46	HS3	*R.nitidus*	0.058944	0.06438
SC28	GD46	HS3	*R.nitidus*	-0.020988	0.062458
SC14	GD46	HS3	*R.nitidus*	0.033243	0.064349
SC27	GD46	HS3	*R.nitidus*	0.00755	0.063566
SC18	GD46	HS3	*R.nitidus*	0.058023	0.062508
JO7	GD48	HS3	*R.nitidus*	-0.046773	0.072295
GD33	GD48	HS3	*R.nitidus*	-0.0124	0.067712
MY6	GD46	HS3	*R.nitidus*	0.020791	0.063432
SC44	GD46	HS3	*R.nitidus*	0.018156	0.064331
CS11	GD48	HS3	*R.nitidus*	-0.045324	0.068003
JW4	GD46	HS3	*R.nitidus*	0.226833	0.066509
GD31	GD48	HS3	*R.nitidus*	0.033884	0.067997
GD34	GD48	HS3	*R.nitidus*	-0.04833	0.066063
GD21	GD48	HS3	*R.nitidus*	0.122977	0.066629
GD35	GD48	HS3	*R.nitidus*	0.01318	0.067763
GD29	GD48	HS3	*R.nitidus*	0.103252	0.064021
JO3	GD46	HS3	*R.nitidus*	0.064071	0.067937
GD30	GD48	HS3	*R.nitidus*	0.065945	0.068395
JO8	GD48	HS3	*R.nitidus*	-0.018555	0.071456
GD32	GD48	HS3	*R.nitidus*	0.009643	0.066219
GD25	GD48	HS3	*R.nitidus*	0.008326	0.064159
JO1	GD46	HS3	*R.nitidus*	-0.004057	0.063772
GD41	GD48	HS3	*R.nitidus*	-0.059483	0.066909
CS12	GD48	HS3	*R.nitidus*	-0.040467	0.069477
GD42	GD48	HS3	*R.nitidus*	-0.012411	0.06591
SC42	GD46	HS3	*R.nitidus*	0.239083	0.061598
CS21	GD48	HS3	*R.nitidus*	-0.083393	0.067642
JO5	GD46	HS3	*R.nitidus*	0.047941	0.063745
GD26	GD48	HS3	*R.nitidus*	0.040296	0.067295
GD40	GD48	HS3	*R.nitidus*	0.021412	0.065539
GD22	GD48	HS3	*R.nitidus*	0.063729	0.065744
CS22	GD48	HS3	*R.nitidus*	-0.044394	0.067632
CS31	GD48	HS3	*R.nitidus*	-0.057515	0.068197
CS13	GD48	HS3	*R.nitidus*	-0.091523	0.069499
GD50	GD48	HS3	*R.nitidus*	-0.034585	0.072087
CS17	GD48	HS3	*R.nitidus*	-0.032437	0.070382
GD46	GD48	HS3	*R.nitidus*	-0.096407	0.064695
SC10	GD48	HS3	*R.nitidus*	0.019331	0.068568

CS16	GD48	HS3	*R.nitidus*	-0.051412	0.065876
GD49	GD48	HS3	*R.nitidus*	0.079772	0.07264
CS30	GD48	HS3	*R.nitidus*	-0.094874	0.068517
GD44	GD48	HS3	*R.nitidus*	-0.019574	0.066688
CS20	GD48	HS3	*R.nitidus*	-0.063142	0.067381
CS25	GD48	HS3	*R.nitidus*	-0.03914	0.069671
CS4	GD48	HS3	*R.nitidus*	-0.047342	0.064915
CS14	GD48	HS3	*R.nitidus*	-0.052863	0.068793
CS15	GD48	HS3	*R.nitidus*	-0.024673	0.068347
CS24	GD48	HS3	*R.nitidus*	-0.077922	0.067215
GD45	GD48	HS3	*R.nitidus*	0.023645	0.071901
SC24	GD48	HS3	*R.nitidus*	-0.037707	0.066554
CS28	GD48	HS3	*R.nitidus*	-0.080128	0.0698
SC35	GD48	HS3	*R.nitidus*	0.015407	0.066855
CS19	GD48	HS3	*R.nitidus*	-0.066154	0.067748
CS18	GD48	HS3	*R.nitidus*	-0.074265	0.068153
SC26	GD48	HS3	*R.nitidus*	-0.078871	0.067364
SC45	GD48	HS3	*R.nitidus*	-0.015173	0.067085
SC28	GD48	HS3	*R.nitidus*	-0.062044	0.067031
SC34	GD48	HS3	*R.nitidus*	-0.008708	0.066791
JW4	GD48	HS3	*R.nitidus*	0.163688	0.067544
CS23	GD48	HS3	*R.nitidus*	-0.09157	0.066742
SC31	GD48	HS3	*R.nitidus*	-0.033213	0.068152
SC27	GD48	HS3	*R.nitidus*	-0.037472	0.065971
CS3	GD48	HS3	*R.nitidus*	-0.06512	0.06696
SC14	GD48	HS3	*R.nitidus*	-0.034182	0.067125
SC42	GD48	HS3	*R.nitidus*	0.177518	0.065919
JW6	GD48	HS3	*R.nitidus*	-0.029384	0.071193
SC5	GD48	HS3	*R.nitidus*	-0.003065	0.070349
SC6	GD48	HS3	*R.nitidus*	-0.041075	0.067748
JO8	GD49	HS3	*R.nitidus*	-0.051111	0.073241
MY4	GD48	HS3	*R.nitidus*	-0.069559	0.067393
SC32	GD48	HS3	*R.nitidus*	-0.005216	0.067817
JW7	GD48	HS3	*R.nitidus*	0.00678	0.069791
SC15	GD48	HS3	*R.nitidus*	-0.033282	0.068785
JO3	GD48	HS3	*R.nitidus*	-0.017677	0.069485
SC18	GD48	HS3	*R.nitidus*	0.000762	0.067267
GD31	GD49	HS3	*R.nitidus*	-0.05174	0.070017
CS11	GD49	HS3	*R.nitidus*	-0.100231	0.07027
SC12	GD48	HS3	*R.nitidus*	-0.055124	0.068285
GD25	GD49	HS3	*R.nitidus*	-0.04565	0.068021
MY6	GD48	HS3	*R.nitidus*	-0.028149	0.066195
SC44	GD48	HS3	*R.nitidus*	-0.034483	0.066176
GD26	GD49	HS3	*R.nitidus*	-0.039251	0.067186
JO5	GD48	HS3	*R.nitidus*	0.013304	0.067431
JO1	GD48	HS3	*R.nitidus*	-0.049311	0.06831
JO7	GD49	HS3	*R.nitidus*	-0.109769	0.073855
GD33	GD49	HS3	*R.nitidus*	-0.072631	0.069669
CS13	GD49	HS3	*R.nitidus*	-0.128059	0.069462
GD22	GD49	HS3	*R.nitidus*	-0.041543	0.069821
GD45	GD49	HS3	*R.nitidus*	-0.032258	0.072176
GD46	GD49	HS3	*R.nitidus*	-0.127434	0.068258
CS6	GD48	HS3	*R.nitidus*	-0.085714	0.066467
GD44	GD49	HS3	*R.nitidus*	-0.079615	0.069174
GD21	GD49	HS3	*R.nitidus*	0.023769	0.066759
GD41	GD49	HS3	*R.nitidus*	-0.112652	0.067599

GD32	GD49	HS3	*R.nitidus*	-0.073499	0.069942
GD40	GD49	HS3	*R.nitidus*	-0.036484	0.068181
CS21	GD49	HS3	*R.nitidus*	-0.141487	0.067324
CS16	GD49	HS3	*R.nitidus*	-0.088441	0.068748
GD30	GD49	HS3	*R.nitidus*	0.057199	0.071127
CS18	GD49	HS3	*R.nitidus*	-0.13454	0.069332
GD29	GD49	HS3	*R.nitidus*	0.032456	0.068712
CS12	GD49	HS3	*R.nitidus*	-0.104132	0.070153
SC15	GD49	HS3	*R.nitidus*	-0.08744	0.069325
SC14	GD49	HS3	*R.nitidus*	-0.070444	0.067593
CS3	GD49	HS3	*R.nitidus*	-0.12988	0.06823
GD42	GD49	HS3	*R.nitidus*	-0.095111	0.067749
GD34	GD49	HS3	*R.nitidus*	-0.098093	0.067701
GD35	GD49	HS3	*R.nitidus*	-0.063584	0.068197
SC26	GD49	HS3	*R.nitidus*	-0.130233	0.069573
CS31	GD49	HS3	*R.nitidus*	-0.123052	0.069993
CS15	GD49	HS3	*R.nitidus*	-0.067247	0.068398
GD50	GD49	HS3	*R.nitidus*	-0.077167	0.074223
CS23	GD49	HS3	*R.nitidus*	-0.13948	0.06697
CS22	GD49	HS3	*R.nitidus*	-0.094118	0.067474
CS30	GD49	HS3	*R.nitidus*	-0.145663	0.067793
GD48	GD49	HS3	*R.nitidus*	-0.079772	0.07264
CS14	GD49	HS3	*R.nitidus*	-0.087363	0.071495
SC10	GD49	HS3	*R.nitidus*	-0.039872	0.069255
MY4	GD49	HS3	*R.nitidus*	-0.119403	0.069145
CS19	GD49	HS3	*R.nitidus*	-0.100164	0.069057
SC12	GD49	HS3	*R.nitidus*	-0.091193	0.066389
SC31	GD49	HS3	*R.nitidus*	-0.100154	0.06889
JW6	GD49	HS3	*R.nitidus*	-0.084034	0.072435
CS17	GD49	HS3	*R.nitidus*	-0.069959	0.072017
CS20	GD49	HS3	*R.nitidus*	-0.145286	0.068456
SC27	GD49	HS3	*R.nitidus*	-0.090208	0.066645
SC34	GD49	HS3	*R.nitidus*	-0.044479	0.065415
SC35	GD49	HS3	*R.nitidus*	-0.032357	0.067722
CS25	GD49	HS3	*R.nitidus*	-0.108766	0.071791
CS4	GD49	HS3	*R.nitidus*	-0.103448	0.066323
CS28	GD49	HS3	*R.nitidus*	-0.112572	0.070673
JO7	GD50	HS3	*R.nitidus*	-0.066876	0.075077
CS24	GD49	HS3	*R.nitidus*	-0.131558	0.067457
SC6	GD49	HS3	*R.nitidus*	-0.102424	0.07097
SC45	GD49	HS3	*R.nitidus*	-0.060461	0.068386
SC18	GD49	HS3	*R.nitidus*	-0.055927	0.068266
SC42	GD49	HS3	*R.nitidus*	0.15019	0.065822
JO8	GD50	HS3	*R.nitidus*	-0.017112	0.073772
SC44	GD49	HS3	*R.nitidus*	-0.082998	0.06577
SC5	GD49	HS3	*R.nitidus*	-0.102172	0.070713
SC24	GD49	HS3	*R.nitidus*	-0.121309	0.066348
GD25	GD50	HS3	*R.nitidus*	-0.00738	0.068753
SC32	GD49	HS3	*R.nitidus*	-0.058366	0.068381
MY6	GD49	HS3	*R.nitidus*	-0.094427	0.068244
SC28	GD49	HS3	*R.nitidus*	-0.120556	0.065156
JW7	GD49	HS3	*R.nitidus*	-0.021842	0.072547
JW4	GD49	HS3	*R.nitidus*	0.132029	0.070245
CS6	GD49	HS3	*R.nitidus*	-0.125828	0.067477
CS11	GD50	HS3	*R.nitidus*	-0.018868	0.07204
GD42	GD50	HS3	*R.nitidus*	0.009165	0.068481

GD40	GD50	HS3	*R.nitidus*	0.03866	0.067925
GD21	GD50	HS3	*R.nitidus*	0.143236	0.068524
JO3	GD49	HS3	*R.nitidus*	-0.025617	0.073534
CS12	GD50	HS3	*R.nitidus*	-0.028476	0.071433
GD33	GD50	HS3	*R.nitidus*	-0.000934	0.070334
GD41	GD50	HS3	*R.nitidus*	-0.029175	0.068636
CS16	GD50	HS3	*R.nitidus*	-0.029827	0.069325
GD32	GD50	HS3	*R.nitidus*	0.00418	0.069383
JO1	GD49	HS3	*R.nitidus*	-0.09589	0.068524
CS13	GD50	HS3	*R.nitidus*	-0.069528	0.072479
GD26	GD50	HS3	*R.nitidus*	0.034323	0.066592
JO5	GD49	HS3	*R.nitidus*	-0.071994	0.065983
GD31	GD50	HS3	*R.nitidus*	0.034451	0.068543
GD44	GD50	HS3	*R.nitidus*	-0.000928	0.069438
GD45	GD50	HS3	*R.nitidus*	0.053971	0.072566
CS19	GD50	HS3	*R.nitidus*	-0.063175	0.071294
GD30	GD50	HS3	*R.nitidus*	0.103061	0.070959
GD29	GD50	HS3	*R.nitidus*	0.109026	0.067762
CS18	GD50	HS3	*R.nitidus*	-0.060181	0.068686
CS17	GD50	HS3	*R.nitidus*	0.006908	0.072999
GD34	GD50	HS3	*R.nitidus*	-0.015654	0.068519
CS20	GD50	HS3	*R.nitidus*	-0.063518	0.070794
GD35	GD50	HS3	*R.nitidus*	0.034612	0.06809
GD48	GD50	HS3	*R.nitidus*	0.034585	0.072087
GD49	GD50	HS3	*R.nitidus*	0.077167	0.074223
CS21	GD50	HS3	*R.nitidus*	-0.065653	0.06958
CS24	GD50	HS3	*R.nitidus*	-0.048927	0.068307
SC10	GD50	HS3	*R.nitidus*	0.012853	0.07024
CS14	GD50	HS3	*R.nitidus*	-0.019802	0.072226
GD46	GD50	HS3	*R.nitidus*	-0.064333	0.067582
CS23	GD50	HS3	*R.nitidus*	-0.070796	0.069595
SC5	GD50	HS3	*R.nitidus*	-0.048696	0.072923
CS30	GD50	HS3	*R.nitidus*	-0.086337	0.069764
CS15	GD50	HS3	*R.nitidus*	-0.00974	0.07128
CS22	GD50	HS3	*R.nitidus*	-0.031199	0.068313
CS4	GD50	HS3	*R.nitidus*	-0.058964	0.065854
CS3	GD50	HS3	*R.nitidus*	-0.070319	0.06853
SC14	GD50	HS3	*R.nitidus*	-0.024213	0.067751
SC42	GD50	HS3	*R.nitidus*	0.199729	0.065535
SC24	GD50	HS3	*R.nitidus*	-0.074897	0.067848
SC15	GD50	HS3	*R.nitidus*	-0.007719	0.070233
CS25	GD50	HS3	*R.nitidus*	-0.045302	0.070052
CS28	GD50	HS3	*R.nitidus*	-0.095745	0.070783
CS31	GD50	HS3	*R.nitidus*	-0.059233	0.068142
SC28	GD50	HS3	*R.nitidus*	-0.071661	0.066704
CS6	GD50	HS3	*R.nitidus*	-0.065657	0.0691
SC6	GD50	HS3	*R.nitidus*	-0.03682	0.068245
JO1	GD50	HS3	*R.nitidus*	-0.06129	0.06928
SC45	GD50	HS3	*R.nitidus*	-0.002453	0.07073
SC12	GD50	HS3	*R.nitidus*	-0.070502	0.070396
SC27	GD50	HS3	*R.nitidus*	-0.05892	0.065127
SC26	GD50	HS3	*R.nitidus*	-0.063218	0.06884
MY4	GD50	HS3	*R.nitidus*	-0.066015	0.068488
JO8	CS14	HS3	*R.nitidus*	0.021113	0.073524
SC18	GD50	HS3	*R.nitidus*	-0.008547	0.069836
SC31	GD50	HS3	*R.nitidus*	-0.008648	0.069072

MY6	GD50	HS3	*R.nitidus*	-0.018927	0.067496
GD29	CS14	HS3	*R.nitidus*	0.149225	0.067089
SC35	GD50	HS3	*R.nitidus*	0.004934	0.066096
JW4	GD50	HS3	*R.nitidus*	0.139554	0.06964
JO7	CS14	HS3	*R.nitidus*	-0.004776	0.073068
JW6	GD50	HS3	*R.nitidus*	-0.061651	0.073596
SC32	GD50	HS3	*R.nitidus*	0.002402	0.070041
SC34	GD50	HS3	*R.nitidus*	0.020783	0.065533
CS12	CS14	HS3	*R.nitidus*	0.021598	0.070948
GD21	CS14	HS3	*R.nitidus*	0.150495	0.065908
GD26	CS14	HS3	*R.nitidus*	0.080327	0.067561
JW7	GD50	HS3	*R.nitidus*	-0.029528	0.07432
SC44	GD50	HS3	*R.nitidus*	-0.028037	0.069305
GD32	CS14	HS3	*R.nitidus*	0.050394	0.069357
CS11	CS14	HS3	*R.nitidus*	0.011507	0.069367
JO3	GD50	HS3	*R.nitidus*	-0.040073	0.072232
JO5	GD50	HS3	*R.nitidus*	-0.001657	0.067405
GD41	CS14	HS3	*R.nitidus*	0.016194	0.066454
GD30	CS14	HS3	*R.nitidus*	0.126749	0.070747
CS18	CS14	HS3	*R.nitidus*	-0.043605	0.068075
GD33	CS14	HS3	*R.nitidus*	0.035135	0.067148
CS13	CS14	HS3	*R.nitidus*	-0.043902	0.071678
GD45	CS14	HS3	*R.nitidus*	0.067977	0.073345
GD40	CS14	HS3	*R.nitidus*	0.060425	0.06652
GD31	CS14	HS3	*R.nitidus*	0.072622	0.069275
GD25	CS14	HS3	*R.nitidus*	0.077568	0.067607
GD42	CS14	HS3	*R.nitidus*	0.050859	0.067667
GD44	CS14	HS3	*R.nitidus*	0.039548	0.06718
GD35	CS14	HS3	*R.nitidus*	0.049451	0.067808
CS20	CS14	HS3	*R.nitidus*	-0.024948	0.067026
GD34	CS14	HS3	*R.nitidus*	0.020395	0.065847
GD48	CS14	HS3	*R.nitidus*	0.052863	0.068793
GD22	CS14	HS3	*R.nitidus*	0.090967	0.065829
GD46	CS14	HS3	*R.nitidus*	0.000713	0.06839
SC10	CS14	HS3	*R.nitidus*	0.065799	0.066608
CS21	CS14	HS3	*R.nitidus*	-0.037464	0.065391
GD50	CS14	HS3	*R.nitidus*	0.019802	0.072226
CS17	CS14	HS3	*R.nitidus*	0.036993	0.073699
SC6	CS14	HS3	*R.nitidus*	0.017781	0.067938
CS15	CS14	HS3	*R.nitidus*	0.068694	0.069547
GD49	CS14	HS3	*R.nitidus*	0.087363	0.071495
CS30	CS14	HS3	*R.nitidus*	-0.050185	0.068038
CS3	CS14	HS3	*R.nitidus*	-0.024	0.065771
SC12	CS14	HS3	*R.nitidus*	-0.007703	0.067696
CS16	CS14	HS3	*R.nitidus*	0.024678	0.068266
CS31	CS14	HS3	*R.nitidus*	-0.015186	0.069168
SC18	CS14	HS3	*R.nitidus*	0.022175	0.065751
SC14	CS14	HS3	*R.nitidus*	0.01158	0.067359
CS19	CS14	HS3	*R.nitidus*	-0.022137	0.069626
CS25	CS14	HS3	*R.nitidus*	-0.004386	0.0678
SC24	CS14	HS3	*R.nitidus*	-0.00561	0.066881
CS24	CS14	HS3	*R.nitidus*	-0.044719	0.067862
CS23	CS14	HS3	*R.nitidus*	-0.054814	0.066521
SC45	CS14	HS3	*R.nitidus*	0.032775	0.068845
CS28	CS14	HS3	*R.nitidus*	-0.026923	0.068399
SC28	CS14	HS3	*R.nitidus*	-0.026391	0.065514

CS22	CS14	HS3	*R.nitidus*	-0.008047	0.067529
SC5	CS14	HS3	*R.nitidus*	0.014716	0.070006
SC15	CS14	HS3	*R.nitidus*	0.019146	0.068543
SC32	CS14	HS3	*R.nitidus*	0.056769	0.06991
JW7	CS14	HS3	*R.nitidus*	0.037194	0.070995
CS11	CS15	HS3	*R.nitidus*	-0.052795	0.067925
SC31	CS14	HS3	*R.nitidus*	0.011604	0.065529
SC34	CS14	HS3	*R.nitidus*	0.040084	0.066235
CS4	CS14	HS3	*R.nitidus*	-0.020322	0.066394
SC27	CS14	HS3	*R.nitidus*	-0.009279	0.065548
JO8	CS15	HS3	*R.nitidus*	0.006536	0.072111
GD21	CS15	HS3	*R.nitidus*	0.110533	0.065161
GD26	CS15	HS3	*R.nitidus*	0.044206	0.067201
JO3	CS14	HS3	*R.nitidus*	0.042332	0.072143
JW6	CS14	HS3	*R.nitidus*	0.006446	0.073935
MY4	CS14	HS3	*R.nitidus*	-0.028592	0.067536
SC44	CS14	HS3	*R.nitidus*	0.00773	0.067048
SC26	CS14	HS3	*R.nitidus*	-0.034653	0.066104
GD32	CS15	HS3	*R.nitidus*	0.01947	0.068274
GD31	CS15	HS3	*R.nitidus*	0.050751	0.067553
JW4	CS14	HS3	*R.nitidus*	0.215046	0.067418
GD25	CS15	HS3	*R.nitidus*	0.04682	0.065951
SC42	CS14	HS3	*R.nitidus*	0.231153	0.062597
SC35	CS14	HS3	*R.nitidus*	0.048309	0.065875
GD42	CS15	HS3	*R.nitidus*	0.013032	0.068208
MY6	CS14	HS3	*R.nitidus*	0.000693	0.067365
GD30	CS15	HS3	*R.nitidus*	0.105478	0.069226
GD29	CS15	HS3	*R.nitidus*	0.100128	0.066317
GD34	CS15	HS3	*R.nitidus*	-0.021583	0.066682
CS6	CS14	HS3	*R.nitidus*	-0.053333	0.066303
JO7	CS15	HS3	*R.nitidus*	-0.045624	0.070965
CS12	CS15	HS3	*R.nitidus*	-0.029567	0.066131
GD22	CS15	HS3	*R.nitidus*	0.059412	0.066072
GD45	CS15	HS3	*R.nitidus*	0.049313	0.070812
GD33	CS15	HS3	*R.nitidus*	0.005337	0.066585
JO1	CS14	HS3	*R.nitidus*	-0.002144	0.067254
JO5	CS14	HS3	*R.nitidus*	0.039753	0.065831
GD35	CS15	HS3	*R.nitidus*	0.016892	0.066809
CS13	CS15	HS3	*R.nitidus*	-0.094283	0.070361
CS28	CS15	HS3	*R.nitidus*	-0.052469	0.067022
CS17	CS15	HS3	*R.nitidus*	-0.04386	0.068899
GD50	CS15	HS3	*R.nitidus*	0.00974	0.07128
GD44	CS15	HS3	*R.nitidus*	0.005559	0.066264
GD48	CS15	HS3	*R.nitidus*	0.024673	0.068347
CS14	CS15	HS3	*R.nitidus*	-0.068694	0.069547
CS19	CS15	HS3	*R.nitidus*	-0.059341	0.068418
SC10	CS15	HS3	*R.nitidus*	0.034359	0.066481
CS31	CS15	HS3	*R.nitidus*	-0.04403	0.067409
SC5	CS15	HS3	*R.nitidus*	-0.017968	0.069946
GD41	CS15	HS3	*R.nitidus*	-0.016097	0.065272
CS24	CS15	HS3	*R.nitidus*	-0.080838	0.066594
GD40	CS15	HS3	*R.nitidus*	0.032472	0.065432
CS16	CS15	HS3	*R.nitidus*	-0.045778	0.065007
GD49	CS15	HS3	*R.nitidus*	0.067247	0.068398
SC28	CS15	HS3	*R.nitidus*	-0.067606	0.064025
CS22	CS15	HS3	*R.nitidus*	-0.029391	0.065306

CS4	CS15	HS3	*R.nitidus*	-0.045205	0.064432
GD46	CS15	HS3	*R.nitidus*	-0.033126	0.067431
CS3	CS15	HS3	*R.nitidus*	-0.051613	0.066569
CS25	CS15	HS3	*R.nitidus*	-0.035636	0.069622
CS23	CS15	HS3	*R.nitidus*	-0.091743	0.066322
CS18	CS15	HS3	*R.nitidus*	-0.076867	0.067456
SC45	CS15	HS3	*R.nitidus*	0	0.068046
CS21	CS15	HS3	*R.nitidus*	-0.067416	0.065656
SC32	CS15	HS3	*R.nitidus*	0.020125	0.067868
SC6	CS15	HS3	*R.nitidus*	-0.025881	0.066644
CS20	CS15	HS3	*R.nitidus*	-0.058985	0.066951
SC24	CS15	HS3	*R.nitidus*	-0.038889	0.065587
JO1	CS15	HS3	*R.nitidus*	-0.036533	0.064482
SC12	CS15	HS3	*R.nitidus*	-0.043872	0.067769
JO3	CS15	HS3	*R.nitidus*	0.010023	0.069267
CS6	CS15	HS3	*R.nitidus*	-0.077372	0.066544
SC42	CS15	HS3	*R.nitidus*	0.207283	0.061983
CS30	CS15	HS3	*R.nitidus*	-0.091709	0.066944
SC18	CS15	HS3	*R.nitidus*	0.009366	0.065091
JW7	CS15	HS3	*R.nitidus*	-0.010779	0.069033
GD30	CS16	HS3	*R.nitidus*	0.12205	0.068704
JO8	CS16	HS3	*R.nitidus*	0.03164	0.072235
SC26	CS15	HS3	*R.nitidus*	-0.065341	0.065446
SC27	CS15	HS3	*R.nitidus*	-0.046132	0.065422
JW6	CS15	HS3	*R.nitidus*	-0.042727	0.070784
SC14	CS15	HS3	*R.nitidus*	-0.024931	0.067998
SC15	CS15	HS3	*R.nitidus*	-0.014535	0.068158
SC44	CS15	HS3	*R.nitidus*	-0.026038	0.066617
JW4	CS15	HS3	*R.nitidus*	0.175222	0.067007
SC31	CS15	HS3	*R.nitidus*	-0.02421	0.065256
SC34	CS15	HS3	*R.nitidus*	0.006878	0.065603
GD31	CS16	HS3	*R.nitidus*	0.064655	0.068205
GD29	CS16	HS3	*R.nitidus*	0.134566	0.064709
JO7	CS16	HS3	*R.nitidus*	-0.028409	0.072879
SC35	CS15	HS3	*R.nitidus*	0.014122	0.065611
GD22	CS16	HS3	*R.nitidus*	0.082383	0.063816
GD35	CS16	HS3	*R.nitidus*	0.046685	0.065291
MY4	CS15	HS3	*R.nitidus*	-0.064607	0.067237
MY6	CS15	HS3	*R.nitidus*	-0.026099	0.067243
GD25	CS16	HS3	*R.nitidus*	0.062588	0.066147
GD32	CS16	HS3	*R.nitidus*	0.053883	0.068332
CS11	CS16	HS3	*R.nitidus*	-0.007239	0.068236
JO5	CS15	HS3	*R.nitidus*	0.018493	0.066367
GD21	CS16	HS3	*R.nitidus*	0.124837	0.063958
GD42	CS16	HS3	*R.nitidus*	0.037293	0.065679
GD34	CS16	HS3	*R.nitidus*	0.011711	0.064195
GD40	CS16	HS3	*R.nitidus*	0.065529	0.064343
GD26	CS16	HS3	*R.nitidus*	0.066	0.065368
GD41	CS16	HS3	*R.nitidus*	0.005446	0.063028
GD45	CS16	HS3	*R.nitidus*	0.083534	0.071531
GD44	CS16	HS3	*R.nitidus*	0.024338	0.066156
CS25	CS16	HS3	*R.nitidus*	-0.011372	0.067864
CS3	CS16	HS3	*R.nitidus*	-0.027596	0.064288
CS14	CS16	HS3	*R.nitidus*	-0.024678	0.068266
CS15	CS16	HS3	*R.nitidus*	0.045778	0.065007
SC5	CS16	HS3	*R.nitidus*	0.002801	0.069406

CS28	CS16	HS3	*R.nitidus*	-0.031882	0.066419
CS12	CS16	HS3	*R.nitidus*	0.014908	0.065368
GD46	CS16	HS3	*R.nitidus*	-0.016231	0.067368
GD33	CS16	HS3	*R.nitidus*	0.023057	0.065425
CS18	CS16	HS3	*R.nitidus*	-0.035587	0.065657
SC14	CS16	HS3	*R.nitidus*	0.002068	0.066728
SC10	CS16	HS3	*R.nitidus*	0.071031	0.066288
GD50	CS16	HS3	*R.nitidus*	0.029827	0.069325
SC32	CS16	HS3	*R.nitidus*	0.040426	0.066686
CS24	CS16	HS3	*R.nitidus*	-0.055342	0.064988
CS19	CS16	HS3	*R.nitidus*	-0.021626	0.066235
CS20	CS16	HS3	*R.nitidus*	-0.027491	0.065456
GD49	CS16	HS3	*R.nitidus*	0.088441	0.068748
CS22	CS16	HS3	*R.nitidus*	-0.008059	0.066354
CS13	CS16	HS3	*R.nitidus*	-0.054634	0.068312
GD48	CS16	HS3	*R.nitidus*	0.051412	0.065876
SC6	CS16	HS3	*R.nitidus*	-0.006442	0.066249
CS21	CS16	HS3	*R.nitidus*	-0.045872	0.065089
SC26	CS16	HS3	*R.nitidus*	-0.039079	0.063672
CS23	CS16	HS3	*R.nitidus*	-0.051847	0.064512
CS17	CS16	HS3	*R.nitidus*	0.01105	0.070166
SC18	CS16	HS3	*R.nitidus*	0.02551	0.064871
SC24	CS16	HS3	*R.nitidus*	-0.012793	0.065028
SC28	CS16	HS3	*R.nitidus*	-0.046999	0.064272
CS31	CS16	HS3	*R.nitidus*	-0.028374	0.067312
SC34	CS16	HS3	*R.nitidus*	0.019485	0.064886
SC35	CS16	HS3	*R.nitidus*	0.041497	0.063884
JO3	CS16	HS3	*R.nitidus*	0.034161	0.070277
SC31	CS16	HS3	*R.nitidus*	-0.012363	0.06512
JO8	CS17	HS3	*R.nitidus*	0.001034	0.075211
CS30	CS16	HS3	*R.nitidus*	-0.068433	0.06502
SC15	CS16	HS3	*R.nitidus*	0.007418	0.067312
SC45	CS16	HS3	*R.nitidus*	0.02582	0.066674
CS4	CS16	HS3	*R.nitidus*	-0.025281	0.06471
SC12	CS16	HS3	*R.nitidus*	-0.02048	0.066297
SC27	CS16	HS3	*R.nitidus*	-0.007857	0.064264
JO1	CS16	HS3	*R.nitidus*	-0.008602	0.065387
GD29	CS17	HS3	*R.nitidus*	0.109267	0.067135
GD25	CS17	HS3	*R.nitidus*	0.043379	0.067806
MY6	CS16	HS3	*R.nitidus*	-0.006207	0.065114
CS11	CS17	HS3	*R.nitidus*	-0.026846	0.069294
MY4	CS16	HS3	*R.nitidus*	-0.037474	0.065455
CS6	CS16	HS3	*R.nitidus*	-0.053064	0.063746
GD22	CS17	HS3	*R.nitidus*	0.060298	0.065861
JW4	CS16	HS3	*R.nitidus*	0.216561	0.065663
JW6	CS16	HS3	*R.nitidus*	-0.00273	0.072134
SC42	CS16	HS3	*R.nitidus*	0.239413	0.061168
JW7	CS16	HS3	*R.nitidus*	0.033854	0.071541
GD31	CS17	HS3	*R.nitidus*	0.055342	0.071396
SC44	CS16	HS3	*R.nitidus*	-0.00143	0.064857
GD41	CS17	HS3	*R.nitidus*	-0.004338	0.068646
JO5	CS16	HS3	*R.nitidus*	0.036237	0.064518
CS12	CS17	HS3	*R.nitidus*	-0.005513	0.070649
GD45	CS17	HS3	*R.nitidus*	0.074576	0.071846
GD33	CS17	HS3	*R.nitidus*	0.007174	0.068306
GD49	CS17	HS3	*R.nitidus*	0.069959	0.072017

GD50	CS17	HS3	*R.nitidus*	-0.006908	0.072999
JO7	CS17	HS3	*R.nitidus*	-0.02229	0.075886
GD35	CS17	HS3	*R.nitidus*	0.035216	0.068039
CS18	CS17	HS3	*R.nitidus*	-0.064286	0.067157
GD30	CS17	HS3	*R.nitidus*	0.114382	0.07003
GD21	CS17	HS3	*R.nitidus*	0.109015	0.067386
CS14	CS17	HS3	*R.nitidus*	-0.036993	0.073699
GD44	CS17	HS3	*R.nitidus*	0.007651	0.071431
CS16	CS17	HS3	*R.nitidus*	-0.01105	0.070166
GD34	CS17	HS3	*R.nitidus*	-0.007785	0.068107
CS24	CS17	HS3	*R.nitidus*	-0.080565	0.068556
GD32	CS17	HS3	*R.nitidus*	0.022857	0.07146
GD48	CS17	HS3	*R.nitidus*	0.032437	0.070382
GD26	CS17	HS3	*R.nitidus*	0.043262	0.069184
CS13	CS17	HS3	*R.nitidus*	-0.068966	0.072251
CS25	CS17	HS3	*R.nitidus*	-0.040128	0.071502
CS23	CS17	HS3	*R.nitidus*	-0.081992	0.067007
CS15	CS17	HS3	*R.nitidus*	0.04386	0.068899
CS19	CS17	HS3	*R.nitidus*	-0.046191	0.06927
SC12	CS17	HS3	*R.nitidus*	-0.036953	0.069238
CS21	CS17	HS3	*R.nitidus*	-0.064567	0.067521
CS30	CS17	HS3	*R.nitidus*	-0.089063	0.06911
SC14	CS17	HS3	*R.nitidus*	-0.010728	0.068939
GD42	CS17	HS3	*R.nitidus*	0.012427	0.068956
GD40	CS17	HS3	*R.nitidus*	0.048423	0.066506
SC5	CS17	HS3	*R.nitidus*	-0.020532	0.070679
CS22	CS17	HS3	*R.nitidus*	-0.038522	0.069893
GD46	CS17	HS3	*R.nitidus*	-0.039347	0.069225
CS31	CS17	HS3	*R.nitidus*	-0.03827	0.070057
CS20	CS17	HS3	*R.nitidus*	-0.052793	0.067926
SC15	CS17	HS3	*R.nitidus*	-0.012955	0.069423
CS28	CS17	HS3	*R.nitidus*	-0.053691	0.06899
SC26	CS17	HS3	*R.nitidus*	-0.049923	0.067019
SC10	CS17	HS3	*R.nitidus*	0.042308	0.069435
SC28	CS17	HS3	*R.nitidus*	-0.063028	0.066546
SC24	CS17	HS3	*R.nitidus*	-0.036126	0.068183
SC6	CS17	HS3	*R.nitidus*	-0.023467	0.06885
SC44	CS17	HS3	*R.nitidus*	-0.023059	0.06749
CS3	CS17	HS3	*R.nitidus*	-0.046714	0.067867
JW4	CS17	HS3	*R.nitidus*	0.189759	0.069202
MY4	CS17	HS3	*R.nitidus*	-0.052233	0.068219
SC31	CS17	HS3	*R.nitidus*	-0.025934	0.067638
SC42	CS17	HS3	*R.nitidus*	0.208483	0.064025
SC32	CS17	HS3	*R.nitidus*	0.030612	0.070774
JW7	CS17	HS3	*R.nitidus*	0.008372	0.07227
CS4	CS17	HS3	*R.nitidus*	-0.044309	0.066638
JW6	CS17	HS3	*R.nitidus*	-0.027864	0.074003
GD22	CS18	HS3	*R.nitidus*	0.130719	0.064648
GD26	CS18	HS3	*R.nitidus*	0.106412	0.066352
MY6	CS17	HS3	*R.nitidus*	-0.014695	0.067783
SC18	CS17	HS3	*R.nitidus*	0.005287	0.068216
SC35	CS17	HS3	*R.nitidus*	0.014728	0.06657
SC27	CS17	HS3	*R.nitidus*	-0.048069	0.067272
JO3	CS17	HS3	*R.nitidus*	0.010221	0.070775
SC34	CS17	HS3	*R.nitidus*	0.005271	0.067774
GD21	CS18	HS3	*R.nitidus*	0.171905	0.065091

GD33	CS18	HS3	*R.nitidus*	0.064156	0.065228
CS6	CS17	HS3	*R.nitidus*	-0.046952	0.067787
JO1	CS17	HS3	*R.nitidus*	-0.043377	0.067801
SC45	CS17	HS3	*R.nitidus*	-0.001476	0.069073
GD30	CS18	HS3	*R.nitidus*	0.159055	0.067388
JO7	CS18	HS3	*R.nitidus*	0.022451	0.073856
JO8	CS18	HS3	*R.nitidus*	0.054348	0.073363
GD32	CS18	HS3	*R.nitidus*	0.089078	0.067917
GD31	CS18	HS3	*R.nitidus*	0.122405	0.067336
CS16	CS18	HS3	*R.nitidus*	0.035587	0.065657
GD25	CS18	HS3	*R.nitidus*	0.098439	0.064613
CS11	CS18	HS3	*R.nitidus*	0.042723	0.066988
GD34	CS18	HS3	*R.nitidus*	0.035837	0.064883
GD35	CS18	HS3	*R.nitidus*	0.077949	0.066245
GD49	CS18	HS3	*R.nitidus*	0.13454	0.069332
GD41	CS18	HS3	*R.nitidus*	0.044548	0.063568
GD42	CS18	HS3	*R.nitidus*	0.069907	0.066373
JO5	CS17	HS3	*R.nitidus*	0	0.068044
CS15	CS18	HS3	*R.nitidus*	0.076867	0.067456
GD50	CS18	HS3	*R.nitidus*	0.060181	0.068686
GD29	CS18	HS3	*R.nitidus*	0.163065	0.064088
CS14	CS18	HS3	*R.nitidus*	0.043605	0.068075
CS17	CS18	HS3	*R.nitidus*	0.064286	0.067157
GD48	CS18	HS3	*R.nitidus*	0.074265	0.068153
CS12	CS18	HS3	*R.nitidus*	0.038404	0.068016
GD40	CS18	HS3	*R.nitidus*	0.109091	0.064188
GD46	CS18	HS3	*R.nitidus*	0.029133	0.065867
GD45	CS18	HS3	*R.nitidus*	0.103721	0.071379
CS28	CS18	HS3	*R.nitidus*	0.007154	0.066862
CS3	CS18	HS3	*R.nitidus*	0.007557	0.064857
CS21	CS18	HS3	*R.nitidus*	0.009452	0.063401
CS31	CS18	HS3	*R.nitidus*	0.01514	0.064845
CS13	CS18	HS3	*R.nitidus*	0.003788	0.068204
GD44	CS18	HS3	*R.nitidus*	0.080377	0.066014
CS25	CS18	HS3	*R.nitidus*	0.040457	0.068579
CS20	CS18	HS3	*R.nitidus*	0.007958	0.0647
CS22	CS18	HS3	*R.nitidus*	0.038399	0.066011
SC10	CS18	HS3	*R.nitidus*	0.107223	0.065634
CS30	CS18	HS3	*R.nitidus*	-0.023112	0.064869
SC5	CS18	HS3	*R.nitidus*	0.04418	0.068887
SC42	CS18	HS3	*R.nitidus*	0.263977	0.061871
CS19	CS18	HS3	*R.nitidus*	0.005731	0.067003
CS4	CS18	HS3	*R.nitidus*	0.012589	0.062674
SC34	CS18	HS3	*R.nitidus*	0.072843	0.06457
SC24	CS18	HS3	*R.nitidus*	0.020863	0.065439
SC18	CS18	HS3	*R.nitidus*	0.071066	0.065307
CS24	CS18	HS3	*R.nitidus*	-0.014011	0.064944
SC26	CS18	HS3	*R.nitidus*	0.004237	0.064222
SC6	CS18	HS3	*R.nitidus*	0.036284	0.066667
SC45	CS18	HS3	*R.nitidus*	0.06916	0.065107
SC27	CS18	HS3	*R.nitidus*	0.034934	0.064366
CS23	CS18	HS3	*R.nitidus*	-0.018667	0.065377
MY4	CS18	HS3	*R.nitidus*	0.002894	0.066986
JO3	CS18	HS3	*R.nitidus*	0.0664	0.068376
CS6	CS18	HS3	*R.nitidus*	-0.016202	0.064229
SC35	CS18	HS3	*R.nitidus*	0.077739	0.063009

JO8	CS19	HS3	*R.nitidus*	0.058116	0.072053
JW7	CS18	HS3	*R.nitidus*	0.059829	0.069124
SC12	CS18	HS3	*R.nitidus*	0.020685	0.065938
SC44	CS18	HS3	*R.nitidus*	0.036906	0.064182
SC31	CS18	HS3	*R.nitidus*	0.037011	0.06587
JW6	CS18	HS3	*R.nitidus*	0.026268	0.071886
SC14	CS18	HS3	*R.nitidus*	0.03696	0.065274
JW4	CS18	HS3	*R.nitidus*	0.241625	0.066126
SC15	CS18	HS3	*R.nitidus*	0.045038	0.066096
JO1	CS18	HS3	*R.nitidus*	0.02521	0.065455
MY6	CS18	HS3	*R.nitidus*	0.036068	0.066215
SC32	CS18	HS3	*R.nitidus*	0.084709	0.065866
GD21	CS19	HS3	*R.nitidus*	0.153386	0.065051
JO5	CS18	HS3	*R.nitidus*	0.092554	0.066169
GD25	CS19	HS3	*R.nitidus*	0.07703	0.065959
GD22	CS19	HS3	*R.nitidus*	0.113095	0.066794
GD35	CS19	HS3	*R.nitidus*	0.059328	0.067358
JO7	CS19	HS3	*R.nitidus*	0.022578	0.073456
GD49	CS19	HS3	*R.nitidus*	0.100164	0.069057
SC28	CS18	HS3	*R.nitidus*	0.005654	0.065062
GD31	CS19	HS3	*R.nitidus*	0.121916	0.068335
GD26	CS19	HS3	*R.nitidus*	0.085193	0.066939
CS14	CS19	HS3	*R.nitidus*	0.022137	0.069626
GD32	CS19	HS3	*R.nitidus*	0.065334	0.068086
GD44	CS19	HS3	*R.nitidus*	0.06622	0.067637
GD29	CS19	HS3	*R.nitidus*	0.158537	0.065666
CS13	CS19	HS3	*R.nitidus*	-0.014118	0.070397
GD30	CS19	HS3	*R.nitidus*	0.138298	0.069807
GD40	CS19	HS3	*R.nitidus*	0.093772	0.065545
GD45	CS19	HS3	*R.nitidus*	0.083333	0.071332
CS11	CS19	HS3	*R.nitidus*	0.030943	0.067282
GD34	CS19	HS3	*R.nitidus*	0.028209	0.066001
CS25	CS19	HS3	*R.nitidus*	0.033133	0.069883
GD50	CS19	HS3	*R.nitidus*	0.063175	0.071294
GD48	CS19	HS3	*R.nitidus*	0.066154	0.067748
GD41	CS19	HS3	*R.nitidus*	0.028533	0.06577
CS12	CS19	HS3	*R.nitidus*	0.024845	0.06901
GD33	CS19	HS3	*R.nitidus*	0.046709	0.067587
CS23	CS19	HS3	*R.nitidus*	-0.024786	0.065215
GD42	CS19	HS3	*R.nitidus*	0.04878	0.067378
GD46	CS19	HS3	*R.nitidus*	0.01584	0.068174
CS18	CS19	HS3	*R.nitidus*	-0.005731	0.067003
CS28	CS19	HS3	*R.nitidus*	-0.0168	0.068742
CS3	CS19	HS3	*R.nitidus*	0.001704	0.065733
CS21	CS19	HS3	*R.nitidus*	-0.010417	0.065577
CS22	CS19	HS3	*R.nitidus*	0.017378	0.066839
SC12	CS19	HS3	*R.nitidus*	0.017618	0.066124
CS24	CS19	HS3	*R.nitidus*	-0.0075	0.067188
SC45	CS19	HS3	*R.nitidus*	0.066384	0.066965
CS17	CS19	HS3	*R.nitidus*	0.046191	0.06927
SC31	CS19	HS3	*R.nitidus*	0.030683	0.065447
CS15	CS19	HS3	*R.nitidus*	0.059341	0.068418
CS16	CS19	HS3	*R.nitidus*	0.021626	0.066235
SC6	CS19	HS3	*R.nitidus*	0.030043	0.067128
SC5	CS19	HS3	*R.nitidus*	0.033137	0.069155
SC28	CS19	HS3	*R.nitidus*	0.000708	0.065596

SC26	CS19	HS3	*R.nitidus*	-0.002144	0.065487
SC24	CS19	HS3	*R.nitidus*	0.019389	0.066475
SC18	CS19	HS3	*R.nitidus*	0.069519	0.065237
CS4	CS19	HS3	*R.nitidus*	0.010046	0.066566
SC10	CS19	HS3	*R.nitidus*	0.078534	0.068204
CS31	CS19	HS3	*R.nitidus*	0.010972	0.06726
SC15	CS19	HS3	*R.nitidus*	0.045386	0.067317
CS20	CS19	HS3	*R.nitidus*	-0.00417	0.067863
SC35	CS19	HS3	*R.nitidus*	0.08	0.064518
JW4	CS19	HS3	*R.nitidus*	0.235209	0.067882
SC32	CS19	HS3	*R.nitidus*	0.077827	0.067736
MY4	CS19	HS3	*R.nitidus*	-0.011878	0.069258
CS30	CS19	HS3	*R.nitidus*	-0.022342	0.066898
JW6	CS19	HS3	*R.nitidus*	0.056818	0.072015
SC14	CS19	HS3	*R.nitidus*	0.03574	0.067851
SC42	CS19	HS3	*R.nitidus*	0.258242	0.063409
SC27	CS19	HS3	*R.nitidus*	0.028302	0.066649
SC34	CS19	HS3	*R.nitidus*	0.066431	0.065869
JO3	CS19	HS3	*R.nitidus*	0.072072	0.069548
CS11	CS20	HS3	*R.nitidus*	0.031403	0.066433
JO7	CS20	HS3	*R.nitidus*	0.004476	0.070321
MY6	CS19	HS3	*R.nitidus*	0.031228	0.067002
JO8	CS20	HS3	*R.nitidus*	0.039234	0.071973
JO5	CS19	HS3	*R.nitidus*	0.067686	0.065382
JO1	CS19	HS3	*R.nitidus*	0.014025	0.067619
GD25	CS20	HS3	*R.nitidus*	0.094744	0.066882
CS6	CS19	HS3	*R.nitidus*	-0.02381	0.066926
JW7	CS19	HS3	*R.nitidus*	0.065532	0.068594
SC44	CS19	HS3	*R.nitidus*	0.030059	0.066997
GD29	CS20	HS3	*R.nitidus*	0.163903	0.065344
GD35	CS20	HS3	*R.nitidus*	0.069911	0.06689
GD21	CS20	HS3	*R.nitidus*	0.165049	0.064833
GD31	CS20	HS3	*R.nitidus*	0.113781	0.067006
GD42	CS20	HS3	*R.nitidus*	0.053876	0.066154
CS15	CS20	HS3	*R.nitidus*	0.058985	0.066951
GD44	CS20	HS3	*R.nitidus*	0.068474	0.06621
GD49	CS20	HS3	*R.nitidus*	0.145286	0.068456
GD50	CS20	HS3	*R.nitidus*	0.063518	0.070794
GD45	CS20	HS3	*R.nitidus*	0.087302	0.071388
GD34	CS20	HS3	*R.nitidus*	0.037433	0.066333
CS21	CS20	HS3	*R.nitidus*	-0.011029	0.063679
GD22	CS20	HS3	*R.nitidus*	0.125402	0.065085
GD30	CS20	HS3	*R.nitidus*	0.16382	0.067781
GD26	CS20	HS3	*R.nitidus*	0.094164	0.066827
CS24	CS20	HS3	*R.nitidus*	-0.005331	0.065274
GD46	CS20	HS3	*R.nitidus*	0.019372	0.064913
SC18	CS20	HS3	*R.nitidus*	0.066421	0.064781
SC10	CS20	HS3	*R.nitidus*	0.082976	0.066332
GD32	CS20	HS3	*R.nitidus*	0.075851	0.067603
CS16	CS20	HS3	*R.nitidus*	0.027491	0.065456
CS23	CS20	HS3	*R.nitidus*	-0.037101	0.064289
CS12	CS20	HS3	*R.nitidus*	0.020558	0.06728
GD40	CS20	HS3	*R.nitidus*	0.094976	0.063705
GD33	CS20	HS3	*R.nitidus*	0.05526	0.06617
CS17	CS20	HS3	*R.nitidus*	0.052793	0.067926
GD48	CS20	HS3	*R.nitidus*	0.063142	0.067381

CS19	CS20	HS3	*R.nitidus*	0.00417	0.067863
CS22	CS20	HS3	*R.nitidus*	0.029734	0.065336
GD41	CS20	HS3	*R.nitidus*	0.046588	0.064638
CS25	CS20	HS3	*R.nitidus*	0.018977	0.068763
CS13	CS20	HS3	*R.nitidus*	-0.004222	0.067727
CS3	CS20	HS3	*R.nitidus*	0.000992	0.062764
CS31	CS20	HS3	*R.nitidus*	0.00289	0.065973
SC35	CS20	HS3	*R.nitidus*	0.071864	0.062887
SC15	CS20	HS3	*R.nitidus*	0.034307	0.067608
CS14	CS20	HS3	*R.nitidus*	0.024948	0.067026
CS28	CS20	HS3	*R.nitidus*	-0.018939	0.067348
SC27	CS20	HS3	*R.nitidus*	0.024255	0.064658
SC44	CS20	HS3	*R.nitidus*	0.033755	0.064969
CS18	CS20	HS3	*R.nitidus*	-0.007958	0.0647
CS4	CS20	HS3	*R.nitidus*	0.01466	0.063025
SC26	CS20	HS3	*R.nitidus*	-0.005029	0.064893
JW4	CS20	HS3	*R.nitidus*	0.221682	0.06535
SC28	CS20	HS3	*R.nitidus*	0.001379	0.063959
SC24	CS20	HS3	*R.nitidus*	0.020043	0.064649
CS30	CS20	HS3	*R.nitidus*	-0.033358	0.064698
SC31	CS20	HS3	*R.nitidus*	0.028229	0.063215
SC5	CS20	HS3	*R.nitidus*	0.038596	0.067754
SC6	CS20	HS3	*R.nitidus*	0.02566	0.064841
SC45	CS20	HS3	*R.nitidus*	0.061107	0.065686
SC42	CS20	HS3	*R.nitidus*	0.250983	0.062245
SC12	CS20	HS3	*R.nitidus*	0.019567	0.065674
JW6	CS20	HS3	*R.nitidus*	-0.00087	0.069665
SC14	CS20	HS3	*R.nitidus*	0.034908	0.065126
JW7	CS20	HS3	*R.nitidus*	0.043406	0.068228
JO7	CS21	HS3	*R.nitidus*	0.030769	0.072498
GD31	CS21	HS3	*R.nitidus*	0.11174	0.067427
GD25	CS21	HS3	*R.nitidus*	0.097527	0.065397
SC34	CS20	HS3	*R.nitidus*	0.062331	0.063326
JO3	CS20	HS3	*R.nitidus*	0.054183	0.067398
CS11	CS21	HS3	*R.nitidus*	0.0349	0.064645
GD35	CS21	HS3	*R.nitidus*	0.074123	0.06592
CS6	CS20	HS3	*R.nitidus*	-0.0317	0.063057
GD30	CS21	HS3	*R.nitidus*	0.174014	0.067337
MY4	CS20	HS3	*R.nitidus*	-0.005559	0.06587
MY6	CS20	HS3	*R.nitidus*	0.024557	0.065338
GD33	CS21	HS3	*R.nitidus*	0.060667	0.065521
JO1	CS20	HS3	*R.nitidus*	0.011471	0.066731
SC32	CS20	HS3	*R.nitidus*	0.078947	0.064992
GD29	CS21	HS3	*R.nitidus*	0.165184	0.064836
JO5	CS20	HS3	*R.nitidus*	0.080623	0.063645
GD44	CS21	HS3	*R.nitidus*	0.065263	0.065418
CS12	CS21	HS3	*R.nitidus*	0.036955	0.065049
GD22	CS21	HS3	*R.nitidus*	0.121783	0.063963
CS18	CS21	HS3	*R.nitidus*	-0.009452	0.063401
JO8	CS21	HS3	*R.nitidus*	0.067362	0.071459
GD45	CS21	HS3	*R.nitidus*	0.094531	0.070612
GD32	CS21	HS3	*R.nitidus*	0.072893	0.069931
GD21	CS21	HS3	*R.nitidus*	0.165305	0.063459
GD49	CS21	HS3	*R.nitidus*	0.141487	0.067324
GD46	CS21	HS3	*R.nitidus*	0.027796	0.065539
CS13	CS21	HS3	*R.nitidus*	-0.004367	0.067127

GD26	CS21	HS3	*R.nitidus*	0.097577	0.066574
CS25	CS21	HS3	*R.nitidus*	0.027027	0.06949
CS24	CS21	HS3	*R.nitidus*	-0.005435	0.064804
CS17	CS21	HS3	*R.nitidus*	0.064567	0.067521
CS31	CS21	HS3	*R.nitidus*	0.008065	0.064845
CS20	CS21	HS3	*R.nitidus*	0.011029	0.063679
GD34	CS21	HS3	*R.nitidus*	0.03469	0.066074
GD50	CS21	HS3	*R.nitidus*	0.065653	0.06958
GD42	CS21	HS3	*R.nitidus*	0.07072	0.065744
GD41	CS21	HS3	*R.nitidus*	0.045514	0.063442
CS14	CS21	HS3	*R.nitidus*	0.037464	0.065391
GD40	CS21	HS3	*R.nitidus*	0.102341	0.062764
GD48	CS21	HS3	*R.nitidus*	0.083393	0.067642
CS19	CS21	HS3	*R.nitidus*	0.010417	0.065577
SC18	CS21	HS3	*R.nitidus*	0.068132	0.064097
SC14	CS21	HS3	*R.nitidus*	0.04669	0.065726
CS3	CS21	HS3	*R.nitidus*	0.004425	0.063914
CS30	CS21	HS3	*R.nitidus*	-0.024701	0.063276
CS15	CS21	HS3	*R.nitidus*	0.067416	0.065656
CS16	CS21	HS3	*R.nitidus*	0.045872	0.065089
SC34	CS21	HS3	*R.nitidus*	0.061892	0.062866
SC12	CS21	HS3	*R.nitidus*	0.021277	0.065975
JW7	CS21	HS3	*R.nitidus*	0.078747	0.067217
CS22	CS21	HS3	*R.nitidus*	0.035357	0.063769
SC6	CS21	HS3	*R.nitidus*	0.034407	0.064731
SC15	CS21	HS3	*R.nitidus*	0.040059	0.066608
CS23	CS21	HS3	*R.nitidus*	-0.024231	0.063685
SC32	CS21	HS3	*R.nitidus*	0.085999	0.063978
SC42	CS21	HS3	*R.nitidus*	0.259217	0.060189
JW6	CS21	HS3	*R.nitidus*	0.027853	0.070961
SC10	CS21	HS3	*R.nitidus*	0.092579	0.064708
CS4	CS21	HS3	*R.nitidus*	0.000765	0.063305
CS28	CS21	HS3	*R.nitidus*	-0.011774	0.065286
JO1	CS21	HS3	*R.nitidus*	0.028413	0.066196
SC5	CS21	HS3	*R.nitidus*	0.039804	0.066155
CS6	CS21	HS3	*R.nitidus*	-0.022645	0.064497
SC35	CS21	HS3	*R.nitidus*	0.087596	0.063733
SC28	CS21	HS3	*R.nitidus*	0.008451	0.064345
JO7	CS22	HS3	*R.nitidus*	-0.011204	0.0709
SC27	CS21	HS3	*R.nitidus*	0.028031	0.064553
GD21	CS22	HS3	*R.nitidus*	0.134514	0.062982
SC31	CS21	HS3	*R.nitidus*	0.045324	0.06442
JO8	CS22	HS3	*R.nitidus*	0.04431	0.07124
SC24	CS21	HS3	*R.nitidus*	0.021629	0.06499
SC26	CS21	HS3	*R.nitidus*	-0.002138	0.063772
CS11	CS22	HS3	*R.nitidus*	0.00438	0.068286
SC44	CS21	HS3	*R.nitidus*	0.040397	0.063103
GD25	CS22	HS3	*R.nitidus*	0.049169	0.064063
JW4	CS21	HS3	*R.nitidus*	0.244898	0.066459
GD22	CS22	HS3	*R.nitidus*	0.083496	0.064329
SC45	CS21	HS3	*R.nitidus*	0.063158	0.065749
MY6	CS21	HS3	*R.nitidus*	0.036136	0.065367
GD32	CS22	HS3	*R.nitidus*	0.062047	0.066864
JO3	CS21	HS3	*R.nitidus*	0.081487	0.06919
GD29	CS22	HS3	*R.nitidus*	0.137615	0.064074
CS12	CS22	HS3	*R.nitidus*	0.014515	0.065697

MY4	CS21	HS3	*R.nitidus*	-0.00288	0.066351
JO5	CS21	HS3	*R.nitidus*	0.083626	0.06379
GD33	CS22	HS3	*R.nitidus*	0.025624	0.065425
GD46	CS22	HS3	*R.nitidus*	-0.006198	0.063817
CS17	CS22	HS3	*R.nitidus*	0.038522	0.069893
CS14	CS22	HS3	*R.nitidus*	0.008047	0.067529
GD31	CS22	HS3	*R.nitidus*	0.077691	0.06684
GD41	CS22	HS3	*R.nitidus*	0.023874	0.063703
CS30	CS22	HS3	*R.nitidus*	-0.058868	0.065595
GD42	CS22	HS3	*R.nitidus*	0.0413	0.064211
GD50	CS22	HS3	*R.nitidus*	0.031199	0.068313
GD44	CS22	HS3	*R.nitidus*	0.061848	0.066226
CS24	CS22	HS3	*R.nitidus*	-0.047932	0.063038
CS19	CS22	HS3	*R.nitidus*	-0.017378	0.066839
CS18	CS22	HS3	*R.nitidus*	-0.038399	0.066011
GD35	CS22	HS3	*R.nitidus*	0.051055	0.06577
GD48	CS22	HS3	*R.nitidus*	0.044394	0.067632
CS23	CS22	HS3	*R.nitidus*	-0.057858	0.062863
GD30	CS22	HS3	*R.nitidus*	0.128492	0.066793
GD34	CS22	HS3	*R.nitidus*	0.016903	0.06421
GD26	CS22	HS3	*R.nitidus*	0.072514	0.065197
SC5	CS22	HS3	*R.nitidus*	0.025418	0.0681
GD45	CS22	HS3	*R.nitidus*	0.070204	0.06991
CS16	CS22	HS3	*R.nitidus*	0.008059	0.066354
CS20	CS22	HS3	*R.nitidus*	-0.029734	0.065336
GD40	CS22	HS3	*R.nitidus*	0.07014	0.062806
GD49	CS22	HS3	*R.nitidus*	0.094118	0.067474
SC6	CS22	HS3	*R.nitidus*	0.002187	0.065503
CS31	CS22	HS3	*R.nitidus*	-0.024261	0.065316
CS28	CS22	HS3	*R.nitidus*	-0.03451	0.066989
CS3	CS22	HS3	*R.nitidus*	-0.02271	0.064644
CS15	CS22	HS3	*R.nitidus*	0.029391	0.065306
CS13	CS22	HS3	*R.nitidus*	-0.045018	0.066829
CS4	CS22	HS3	*R.nitidus*	-0.019755	0.061745
CS21	CS22	HS3	*R.nitidus*	-0.035357	0.063769
SC26	CS22	HS3	*R.nitidus*	-0.032258	0.064878
SC35	CS22	HS3	*R.nitidus*	0.052399	0.062158
CS25	CS22	HS3	*R.nitidus*	-0.010228	0.068678
SC28	CS22	HS3	*R.nitidus*	-0.019737	0.063741
SC12	CS22	HS3	*R.nitidus*	-0.007305	0.063955
JO1	CS22	HS3	*R.nitidus*	0.003582	0.066082
SC15	CS22	HS3	*R.nitidus*	0.024261	0.065994
SC14	CS22	HS3	*R.nitidus*	0.017229	0.065137
MY4	CS22	HS3	*R.nitidus*	-0.029828	0.065464
CS6	CS22	HS3	*R.nitidus*	-0.043001	0.061692
SC45	CS22	HS3	*R.nitidus*	0.030238	0.063373
JW4	CS22	HS3	*R.nitidus*	0.231263	0.065083
SC27	CS22	HS3	*R.nitidus*	0.006637	0.064065
SC10	CS22	HS3	*R.nitidus*	0.08027	0.065044
JO5	CS22	HS3	*R.nitidus*	0.048711	0.064606
GD22	CS23	HS3	*R.nitidus*	0.135083	0.063332
SC34	CS22	HS3	*R.nitidus*	0.038301	0.06198
SC24	CS22	HS3	*R.nitidus*	-0.012857	0.064308
SC31	CS22	HS3	*R.nitidus*	0.011315	0.0647
SC18	CS22	HS3	*R.nitidus*	0.037975	0.0659
GD32	CS23	HS3	*R.nitidus*	0.103422	0.065755

MY6	CS22	HS3	*R.nitidus*	0.004351	0.064108
CS11	CS23	HS3	*R.nitidus*	0.061658	0.065744
SC42	CS22	HS3	*R.nitidus*	0.250892	0.060772
GD21	CS23	HS3	*R.nitidus*	0.178928	0.064435
SC32	CS22	HS3	*R.nitidus*	0.060044	0.064739
JO8	CS23	HS3	*R.nitidus*	0.065714	0.071769
JW7	CS22	HS3	*R.nitidus*	0.051349	0.067881
JW6	CS22	HS3	*R.nitidus*	0.034234	0.069708
GD26	CS23	HS3	*R.nitidus*	0.112052	0.064322
SC44	CS22	HS3	*R.nitidus*	0.003587	0.065036
JO7	CS23	HS3	*R.nitidus*	0.037615	0.071056
GD25	CS23	HS3	*R.nitidus*	0.109469	0.063944
GD31	CS23	HS3	*R.nitidus*	0.131228	0.066326
GD33	CS23	HS3	*R.nitidus*	0.076115	0.065073
JO3	CS22	HS3	*R.nitidus*	0.048567	0.068929
GD30	CS23	HS3	*R.nitidus*	0.160714	0.066978
GD42	CS23	HS3	*R.nitidus*	0.083445	0.066185
CS12	CS23	HS3	*R.nitidus*	0.051395	0.066309
GD41	CS23	HS3	*R.nitidus*	0.061599	0.063828
CS13	CS23	HS3	*R.nitidus*	0.020364	0.066013
GD29	CS23	HS3	*R.nitidus*	0.175711	0.062553
GD48	CS23	HS3	*R.nitidus*	0.09157	0.066742
GD46	CS23	HS3	*R.nitidus*	0.031511	0.063349
GD35	CS23	HS3	*R.nitidus*	0.094822	0.065391
CS21	CS23	HS3	*R.nitidus*	0.024231	0.063685
CS24	CS23	HS3	*R.nitidus*	0.01502	0.064619
SC5	CS23	HS3	*R.nitidus*	0.054092	0.067644
CS20	CS23	HS3	*R.nitidus*	0.037101	0.064289
CS18	CS23	HS3	*R.nitidus*	0.018667	0.065377
CS14	CS23	HS3	*R.nitidus*	0.054814	0.066521
SC12	CS23	HS3	*R.nitidus*	0.030942	0.064436
GD50	CS23	HS3	*R.nitidus*	0.070796	0.069595
GD45	CS23	HS3	*R.nitidus*	0.097692	0.071495
GD34	CS23	HS3	*R.nitidus*	0.061745	0.064394
GD49	CS23	HS3	*R.nitidus*	0.13948	0.06697
GD44	CS23	HS3	*R.nitidus*	0.085512	0.064767
GD40	CS23	HS3	*R.nitidus*	0.113575	0.063597
CS25	CS23	HS3	*R.nitidus*	0.038715	0.068567
CS17	CS23	HS3	*R.nitidus*	0.081992	0.067007
SC14	CS23	HS3	*R.nitidus*	0.057613	0.066312
SC26	CS23	HS3	*R.nitidus*	0.016678	0.06494
CS30	CS23	HS3	*R.nitidus*	-0.004508	0.064098
CS28	CS23	HS3	*R.nitidus*	0.017871	0.066674
CS19	CS23	HS3	*R.nitidus*	0.024786	0.065215
CS15	CS23	HS3	*R.nitidus*	0.091743	0.066322
SC15	CS23	HS3	*R.nitidus*	0.071429	0.065068
CS3	CS23	HS3	*R.nitidus*	0.033363	0.064451
CS6	CS23	HS3	*R.nitidus*	0.004344	0.064367
CS16	CS23	HS3	*R.nitidus*	0.051847	0.064512
SC42	CS23	HS3	*R.nitidus*	0.285225	0.059811
CS4	CS23	HS3	*R.nitidus*	0.03698	0.062209
JW7	CS23	HS3	*R.nitidus*	0.074442	0.065971
CS22	CS23	HS3	*R.nitidus*	0.057858	0.062863
SC6	CS23	HS3	*R.nitidus*	0.061074	0.065578
SC18	CS23	HS3	*R.nitidus*	0.089896	0.064702
SC24	CS23	HS3	*R.nitidus*	0.04089	0.064302

SC28	CS23	HS3	*R.nitidus*	0.006272	0.064311
JO7	CS24	HS3	*R.nitidus*	0.041785	0.071918
CS31	CS23	HS3	*R.nitidus*	0.032799	0.065677
CS11	CS24	HS3	*R.nitidus*	0.05291	0.066759
SC10	CS23	HS3	*R.nitidus*	0.117137	0.065911
JW6	CS23	HS3	*R.nitidus*	0.025152	0.070106
JO8	CS24	HS3	*R.nitidus*	0.055951	0.072076
JO1	CS23	HS3	*R.nitidus*	0.042224	0.066347
GD22	CS24	HS3	*R.nitidus*	0.131048	0.063
SC45	CS23	HS3	*R.nitidus*	0.080452	0.065176
SC31	CS23	HS3	*R.nitidus*	0.058123	0.063545
MY6	CS23	HS3	*R.nitidus*	0.053041	0.06401
SC44	CS23	HS3	*R.nitidus*	0.045969	0.063985
GD21	CS24	HS3	*R.nitidus*	0.181275	0.063636
SC34	CS23	HS3	*R.nitidus*	0.081476	0.063062
JW4	CS23	HS3	*R.nitidus*	0.246037	0.064086
SC35	CS23	HS3	*R.nitidus*	0.098053	0.063833
JO3	CS23	HS3	*R.nitidus*	0.081761	0.067939
SC32	CS23	HS3	*R.nitidus*	0.099024	0.06577
SC27	CS23	HS3	*R.nitidus*	0.045007	0.064378
GD31	CS24	HS3	*R.nitidus*	0.119306	0.066025
GD29	CS24	HS3	*R.nitidus*	0.173531	0.063567
GD30	CS24	HS3	*R.nitidus*	0.156402	0.065127
MY4	CS23	HS3	*R.nitidus*	0.017155	0.065025
GD46	CS24	HS3	*R.nitidus*	0.026148	0.065058
GD49	CS24	HS3	*R.nitidus*	0.131558	0.067457
JO5	CS23	HS3	*R.nitidus*	0.091037	0.063697
GD33	CS24	HS3	*R.nitidus*	0.069182	0.066827
CS12	CS24	HS3	*R.nitidus*	0.059809	0.066698
GD25	CS24	HS3	*R.nitidus*	0.086835	0.065295
CS15	CS24	HS3	*R.nitidus*	0.080838	0.066594
GD26	CS24	HS3	*R.nitidus*	0.109091	0.064107
GD40	CS24	HS3	*R.nitidus*	0.114685	0.061431
GD34	CS24	HS3	*R.nitidus*	0.053446	0.064595
CS28	CS24	HS3	*R.nitidus*	0.017059	0.067638
CS14	CS24	HS3	*R.nitidus*	0.044719	0.067862
GD32	CS24	HS3	*R.nitidus*	0.097403	0.065655
GD35	CS24	HS3	*R.nitidus*	0.088256	0.065637
GD48	CS24	HS3	*R.nitidus*	0.077922	0.067215
CS22	CS24	HS3	*R.nitidus*	0.047932	0.063038
GD45	CS24	HS3	*R.nitidus*	0.098441	0.069419
GD50	CS24	HS3	*R.nitidus*	0.048927	0.068307
SC12	CS24	HS3	*R.nitidus*	0.032329	0.064098
CS30	CS24	HS3	*R.nitidus*	-0.020635	0.064729
CS13	CS24	HS3	*R.nitidus*	0.009412	0.066795
GD42	CS24	HS3	*R.nitidus*	0.073602	0.066382
CS21	CS24	HS3	*R.nitidus*	0.005435	0.064804
GD41	CS24	HS3	*R.nitidus*	0.059786	0.064561
CS20	CS24	HS3	*R.nitidus*	0.005331	0.065274
CS19	CS24	HS3	*R.nitidus*	0.0075	0.067188
GD44	CS24	HS3	*R.nitidus*	0.090566	0.066701
CS17	CS24	HS3	*R.nitidus*	0.080565	0.068556
CS23	CS24	HS3	*R.nitidus*	-0.01502	0.064619
CS18	CS24	HS3	*R.nitidus*	0.014011	0.064944
SC10	CS24	HS3	*R.nitidus*	0.129713	0.063016
SC5	CS24	HS3	*R.nitidus*	0.061086	0.066873

CS31	CS24	HS3	*R.nitidus*	0.024883	0.064953
CS3	CS24	HS3	*R.nitidus*	0.004699	0.064781
SC6	CS24	HS3	*R.nitidus*	0.04814	0.064672
SC14	CS24	HS3	*R.nitidus*	0.052161	0.064265
CS16	CS24	HS3	*R.nitidus*	0.055342	0.064988
SC35	CS24	HS3	*R.nitidus*	0.091445	0.060943
CS25	CS24	HS3	*R.nitidus*	0.042587	0.068317
SC44	CS24	HS3	*R.nitidus*	0.053771	0.06531
SC45	CS24	HS3	*R.nitidus*	0.072238	0.06672
SC28	CS24	HS3	*R.nitidus*	0.018248	0.064618
CS6	CS24	HS3	*R.nitidus*	-0.002527	0.063938
JW7	CS24	HS3	*R.nitidus*	0.084685	0.065138
CS4	CS24	HS3	*R.nitidus*	0.032035	0.059992
MY4	CS24	HS3	*R.nitidus*	0.008869	0.065776
SC27	CS24	HS3	*R.nitidus*	0.036838	0.062854
SC26	CS24	HS3	*R.nitidus*	0.010877	0.063399
JO5	CS24	HS3	*R.nitidus*	0.084733	0.063932
JO7	CS25	HS3	*R.nitidus*	0.01215	0.071823
JO3	CS24	HS3	*R.nitidus*	0.072405	0.067836
CS11	CS25	HS3	*R.nitidus*	0.011808	0.068906
JW6	CS24	HS3	*R.nitidus*	0.068545	0.070229
SC18	CS24	HS3	*R.nitidus*	0.070465	0.064863
SC15	CS24	HS3	*R.nitidus*	0.058411	0.064426
SC24	CS24	HS3	*R.nitidus*	0.026686	0.062624
SC34	CS24	HS3	*R.nitidus*	0.08651	0.061504
SC32	CS24	HS3	*R.nitidus*	0.101515	0.064336
GD25	CS25	HS3	*R.nitidus*	0.082562	0.066338
GD34	CS25	HS3	*R.nitidus*	0.01717	0.065614
SC31	CS24	HS3	*R.nitidus*	0.055197	0.06364
JW4	CS24	HS3	*R.nitidus*	0.267946	0.064372
GD32	CS25	HS3	*R.nitidus*	0.054306	0.069076
SC42	CS24	HS3	*R.nitidus*	0.292436	0.060951
JO8	CS25	HS3	*R.nitidus*	0.03001	0.073061
GD21	CS25	HS3	*R.nitidus*	0.151575	0.06498
CS12	CS25	HS3	*R.nitidus*	0.006844	0.068512
GD41	CS25	HS3	*R.nitidus*	0.015272	0.066218
GD29	CS25	HS3	*R.nitidus*	0.152815	0.065853
GD22	CS25	HS3	*R.nitidus*	0.112299	0.067042
MY6	CS24	HS3	*R.nitidus*	0.045785	0.064528
GD26	CS25	HS3	*R.nitidus*	0.077808	0.066115
GD42	CS25	HS3	*R.nitidus*	0.037631	0.06868
JO1	CS24	HS3	*R.nitidus*	0.037572	0.065613
GD46	CS25	HS3	*R.nitidus*	-0.00677	0.067858
CS14	CS25	HS3	*R.nitidus*	0.004386	0.0678
CS15	CS25	HS3	*R.nitidus*	0.035636	0.069622
GD30	CS25	HS3	*R.nitidus*	0.129676	0.070928
GD48	CS25	HS3	*R.nitidus*	0.03914	0.069671
GD45	CS25	HS3	*R.nitidus*	0.076613	0.072583
CS24	CS25	HS3	*R.nitidus*	-0.042587	0.068317
CS28	CS25	HS3	*R.nitidus*	-0.038705	0.070182
CS22	CS25	HS3	*R.nitidus*	0.010228	0.068678
CS13	CS25	HS3	*R.nitidus*	-0.032114	0.070674
GD49	CS25	HS3	*R.nitidus*	0.108766	0.071791
GD31	CS25	HS3	*R.nitidus*	0.096154	0.06734
GD50	CS25	HS3	*R.nitidus*	0.045302	0.070052
CS23	CS25	HS3	*R.nitidus*	-0.038715	0.068567

SC5	CS25	HS3	*R.nitidus*	0.014947	0.069538
CS18	CS25	HS3	*R.nitidus*	-0.040457	0.068579
GD35	CS25	HS3	*R.nitidus*	0.044352	0.066707
GD33	CS25	HS3	*R.nitidus*	0.038678	0.067695
GD44	CS25	HS3	*R.nitidus*	0.057607	0.067454
SC12	CS25	HS3	*R.nitidus*	-0.009332	0.068386
GD40	CS25	HS3	*R.nitidus*	0.074024	0.065256
CS3	CS25	HS3	*R.nitidus*	-0.027419	0.066692
CS20	CS25	HS3	*R.nitidus*	-0.018977	0.068763
CS17	CS25	HS3	*R.nitidus*	0.040128	0.071502
CS16	CS25	HS3	*R.nitidus*	0.011372	0.067864
CS30	CS25	HS3	*R.nitidus*	-0.047059	0.066447
CS21	CS25	HS3	*R.nitidus*	-0.027027	0.06949
CS19	CS25	HS3	*R.nitidus*	-0.033133	0.069883
SC35	CS25	HS3	*R.nitidus*	0.052999	0.064234
SC42	CS25	HS3	*R.nitidus*	0.249556	0.064024
CS31	CS25	HS3	*R.nitidus*	-0.016479	0.067258
JW4	CS25	HS3	*R.nitidus*	0.214487	0.068566
CS4	CS25	HS3	*R.nitidus*	-0.001503	0.066903
SC6	CS25	HS3	*R.nitidus*	0.016105	0.067619
SC26	CS25	HS3	*R.nitidus*	-0.017033	0.065842
SC27	CS25	HS3	*R.nitidus*	0.005032	0.066376
JW6	CS25	HS3	*R.nitidus*	0.022907	0.071232
SC10	CS25	HS3	*R.nitidus*	0.045319	0.068985
SC31	CS25	HS3	*R.nitidus*	0.020308	0.06501
JO3	CS25	HS3	*R.nitidus*	0.055468	0.069185
GD30	CS28	HS3	*R.nitidus*	0.154569	0.069242
CS11	CS28	HS3	*R.nitidus*	0.036078	0.066852
JW7	CS25	HS3	*R.nitidus*	0.046809	0.070968
JO8	CS28	HS3	*R.nitidus*	0.043254	0.074528
SC14	CS25	HS3	*R.nitidus*	0.014184	0.067504
SC18	CS25	HS3	*R.nitidus*	0.045319	0.066521
SC15	CS25	HS3	*R.nitidus*	0.023952	0.067852
SC34	CS25	HS3	*R.nitidus*	0.042509	0.066025
JO5	CS25	HS3	*R.nitidus*	0.060738	0.066805
MY6	CS25	HS3	*R.nitidus*	0.013542	0.066958
MY4	CS25	HS3	*R.nitidus*	-0.02393	0.067442
GD31	CS28	HS3	*R.nitidus*	0.089714	0.067673
GD26	CS28	HS3	*R.nitidus*	0.101091	0.066818
SC24	CS25	HS3	*R.nitidus*	0.00438	0.065908
CS6	CS25	HS3	*R.nitidus*	-0.049331	0.065989
GD29	CS28	HS3	*R.nitidus*	0.162618	0.065843
CS13	CS28	HS3	*R.nitidus*	-0.01684	0.06986
SC44	CS25	HS3	*R.nitidus*	0	0.066675
GD33	CS28	HS3	*R.nitidus*	0.066278	0.06678
SC28	CS25	HS3	*R.nitidus*	-0.026429	0.06604
SC32	CS25	HS3	*R.nitidus*	0.063937	0.068769
SC45	CS25	HS3	*R.nitidus*	0.05	0.067006
GD44	CS28	HS3	*R.nitidus*	0.070933	0.067608
GD21	CS28	HS3	*R.nitidus*	0.156821	0.064666
JO7	CS28	HS3	*R.nitidus*	0.017068	0.072903
GD25	CS28	HS3	*R.nitidus*	0.088146	0.065527
JO1	CS25	HS3	*R.nitidus*	-0.005513	0.067332
GD32	CS28	HS3	*R.nitidus*	0.078254	0.069833
CS12	CS28	HS3	*R.nitidus*	0.028759	0.067885
GD22	CS28	HS3	*R.nitidus*	0.119781	0.066362

GD35	CS28	HS3	*R.nitidus*	0.0837	0.067745
GD50	CS28	HS3	*R.nitidus*	0.095745	0.070783
CS25	CS28	HS3	*R.nitidus*	0.038705	0.070182
GD46	CS28	HS3	*R.nitidus*	0.016284	0.067267
GD45	CS28	HS3	*R.nitidus*	0.102415	0.069135
CS3	CS28	HS3	*R.nitidus*	0.007103	0.067119
CS30	CS28	HS3	*R.nitidus*	-0.03209	0.068222
GD48	CS28	HS3	*R.nitidus*	0.080128	0.0698
CS23	CS28	HS3	*R.nitidus*	-0.017871	0.066674
GD34	CS28	HS3	*R.nitidus*	0.051699	0.066755
GD41	CS28	HS3	*R.nitidus*	0.043155	0.065826
GD42	CS28	HS3	*R.nitidus*	0.068216	0.067175
GD49	CS28	HS3	*R.nitidus*	0.112572	0.070673
GD40	CS28	HS3	*R.nitidus*	0.092806	0.064853
CS24	CS28	HS3	*R.nitidus*	-0.017059	0.067638
CS16	CS28	HS3	*R.nitidus*	0.031882	0.066419
SC6	CS28	HS3	*R.nitidus*	0.042969	0.066955
CS15	CS28	HS3	*R.nitidus*	0.052469	0.067022
SC24	CS28	HS3	*R.nitidus*	0.028302	0.065266
CS21	CS28	HS3	*R.nitidus*	0.011774	0.065286
SC5	CS28	HS3	*R.nitidus*	0.046332	0.068036
CS17	CS28	HS3	*R.nitidus*	0.053691	0.06899
CS31	CS28	HS3	*R.nitidus*	0.000993	0.068604
SC14	CS28	HS3	*R.nitidus*	0.040217	0.066614
SC31	CS28	HS3	*R.nitidus*	0.041471	0.065893
SC27	CS28	HS3	*R.nitidus*	0.026688	0.064697
CS18	CS28	HS3	*R.nitidus*	-0.007154	0.066862
CS14	CS28	HS3	*R.nitidus*	0.026923	0.068399
CS22	CS28	HS3	*R.nitidus*	0.03451	0.066989
JO8	CS30	HS3	*R.nitidus*	0.098619	0.068889
SC34	CS28	HS3	*R.nitidus*	0.077677	0.065917
SC26	CS28	HS3	*R.nitidus*	-0.006912	0.067062
SC45	CS28	HS3	*R.nitidus*	0.055679	0.068068
CS20	CS28	HS3	*R.nitidus*	0.018939	0.067348
SC28	CS28	HS3	*R.nitidus*	0.007849	0.064033
CS4	CS28	HS3	*R.nitidus*	0.015244	0.064391
CS19	CS28	HS3	*R.nitidus*	0.0168	0.068742
JW6	CS28	HS3	*R.nitidus*	0.048924	0.072042
JW7	CS28	HS3	*R.nitidus*	0.07757	0.070901
JW4	CS28	HS3	*R.nitidus*	0.246242	0.069323
CS6	CS28	HS3	*R.nitidus*	-0.007347	0.0664
SC42	CS28	HS3	*R.nitidus*	0.269962	0.061477
SC10	CS28	HS3	*R.nitidus*	0.098335	0.067509
SC32	CS28	HS3	*R.nitidus*	0.095351	0.066547
JO5	CS28	HS3	*R.nitidus*	0.078806	0.065627
MY4	CS28	HS3	*R.nitidus*	0.007728	0.067918
SC12	CS28	HS3	*R.nitidus*	0.023438	0.066402
JO7	CS30	HS3	*R.nitidus*	0.046489	0.072787
SC18	CS28	HS3	*R.nitidus*	0.070203	0.068272
SC15	CS28	HS3	*R.nitidus*	0.038779	0.068439
MY6	CS28	HS3	*R.nitidus*	0.038748	0.067925
CS11	CS30	HS3	*R.nitidus*	0.056328	0.067878
GD21	CS30	HS3	*R.nitidus*	0.195696	0.064369
GD22	CS30	HS3	*R.nitidus*	0.151333	0.063531
GD25	CS30	HS3	*R.nitidus*	0.113781	0.0658
SC44	CS28	HS3	*R.nitidus*	0.044996	0.066369

JO3	CS28	HS3	*R.nitidus*	0.077879	0.072156
SC35	CS28	HS3	*R.nitidus*	0.057927	0.065556
GD31	CS30	HS3	*R.nitidus*	0.136126	0.067889
GD40	CS30	HS3	*R.nitidus*	0.117246	0.063843
GD26	CS30	HS3	*R.nitidus*	0.127899	0.065094
JO1	CS28	HS3	*R.nitidus*	0.026709	0.066306
CS12	CS30	HS3	*R.nitidus*	0.062791	0.067713
GD30	CS30	HS3	*R.nitidus*	0.171938	0.067226
GD34	CS30	HS3	*R.nitidus*	0.069965	0.064322
GD35	CS30	HS3	*R.nitidus*	0.095007	0.066871
GD29	CS30	HS3	*R.nitidus*	0.190412	0.062467
CS18	CS30	HS3	*R.nitidus*	0.023112	0.064869
GD41	CS30	HS3	*R.nitidus*	0.080542	0.06429
GD33	CS30	HS3	*R.nitidus*	0.089583	0.066188
CS13	CS30	HS3	*R.nitidus*	0.025354	0.068251
CS14	CS30	HS3	*R.nitidus*	0.050185	0.068038
CS3	CS30	HS3	*R.nitidus*	0.03265	0.065007
CS25	CS30	HS3	*R.nitidus*	0.047059	0.066447
CS19	CS30	HS3	*R.nitidus*	0.022342	0.066898
GD49	CS30	HS3	*R.nitidus*	0.145663	0.067793
SC12	CS30	HS3	*R.nitidus*	0.035088	0.065707
GD46	CS30	HS3	*R.nitidus*	0.037857	0.06632
GD50	CS30	HS3	*R.nitidus*	0.086337	0.069764
CS16	CS30	HS3	*R.nitidus*	0.068433	0.06502
CS20	CS30	HS3	*R.nitidus*	0.033358	0.064698
GD42	CS30	HS3	*R.nitidus*	0.095481	0.065272
CS17	CS30	HS3	*R.nitidus*	0.089063	0.06911
GD45	CS30	HS3	*R.nitidus*	0.115702	0.068492
GD32	CS30	HS3	*R.nitidus*	0.093042	0.069943
SC18	CS30	HS3	*R.nitidus*	0.097037	0.064648
GD44	CS30	HS3	*R.nitidus*	0.079942	0.066524
SC26	CS30	HS3	*R.nitidus*	0.021567	0.064567
CS24	CS30	HS3	*R.nitidus*	0.020635	0.064729
CS21	CS30	HS3	*R.nitidus*	0.024701	0.063276
SC28	CS30	HS3	*R.nitidus*	0.023055	0.062789
CS4	CS30	HS3	*R.nitidus*	0.039677	0.062434
CS31	CS30	HS3	*R.nitidus*	0.053422	0.063481
SC10	CS30	HS3	*R.nitidus*	0.125931	0.065278
SC45	CS30	HS3	*R.nitidus*	0.087372	0.066005
GD48	CS30	HS3	*R.nitidus*	0.094874	0.068517
CS15	CS30	HS3	*R.nitidus*	0.091709	0.066944
SC34	CS30	HS3	*R.nitidus*	0.093007	0.063054
SC31	CS30	HS3	*R.nitidus*	0.06514	0.06561
MY4	CS30	HS3	*R.nitidus*	0.020863	0.066225
SC24	CS30	HS3	*R.nitidus*	0.046965	0.06574
CS6	CS30	HS3	*R.nitidus*	0.013037	0.065214
SC27	CS30	HS3	*R.nitidus*	0.053424	0.063465
CS23	CS30	HS3	*R.nitidus*	0.004508	0.064098
CS22	CS30	HS3	*R.nitidus*	0.058868	0.065595
CS28	CS30	HS3	*R.nitidus*	0.03209	0.068222
SC42	CS30	HS3	*R.nitidus*	0.287383	0.059852
SC5	CS30	HS3	*R.nitidus*	0.057692	0.069052
SC6	CS30	HS3	*R.nitidus*	0.069553	0.068125
JW7	CS30	HS3	*R.nitidus*	0.090753	0.067687
JW4	CS30	HS3	*R.nitidus*	0.264336	0.066189
JO3	CS30	HS3	*R.nitidus*	0.102041	0.069364

SC14	CS30	HS3	*R.nitidus*	0.061372	0.065062
SC15	CS30	HS3	*R.nitidus*	0.070108	0.066705
JO7	CS31	HS3	*R.nitidus*	0.016252	0.074136
JO5	CS30	HS3	*R.nitidus*	0.102115	0.063139
SC35	CS30	HS3	*R.nitidus*	0.09824	0.063103
SC32	CS30	HS3	*R.nitidus*	0.108728	0.06624
JO8	CS31	HS3	*R.nitidus*	0.063872	0.073296
JW6	CS30	HS3	*R.nitidus*	0.063037	0.069765
GD34	CS31	HS3	*R.nitidus*	0.022939	0.065653
GD30	CS31	HS3	*R.nitidus*	0.133774	0.067989
GD32	CS31	HS3	*R.nitidus*	0.070074	0.069221
GD29	CS31	HS3	*R.nitidus*	0.169697	0.064854
SC44	CS30	HS3	*R.nitidus*	0.065721	0.062886
GD26	CS31	HS3	*R.nitidus*	0.093418	0.065355
CS12	CS31	HS3	*R.nitidus*	0.033228	0.068877
GD21	CS31	HS3	*R.nitidus*	0.153846	0.064519
MY6	CS30	HS3	*R.nitidus*	0.057775	0.066
GD42	CS31	HS3	*R.nitidus*	0.041176	0.06672
GD31	CS31	HS3	*R.nitidus*	0.085301	0.067209
CS20	CS31	HS3	*R.nitidus*	-0.00289	0.065973
CS13	CS31	HS3	*R.nitidus*	-0.027111	0.068942
CS18	CS31	HS3	*R.nitidus*	-0.01514	0.064845
GD22	CS31	HS3	*R.nitidus*	0.107287	0.065808
JO1	CS30	HS3	*R.nitidus*	0.040433	0.065258
GD49	CS31	HS3	*R.nitidus*	0.123052	0.069993
GD48	CS31	HS3	*R.nitidus*	0.057515	0.068197
GD44	CS31	HS3	*R.nitidus*	0.062453	0.066773
CS11	CS31	HS3	*R.nitidus*	0.019084	0.069627
GD40	CS31	HS3	*R.nitidus*	0.085992	0.064232
GD35	CS31	HS3	*R.nitidus*	0.067148	0.066614
GD25	CS31	HS3	*R.nitidus*	0.08642	0.065411
CS14	CS31	HS3	*R.nitidus*	0.015186	0.069168
GD41	CS31	HS3	*R.nitidus*	0.034532	0.065318
CS16	CS31	HS3	*R.nitidus*	0.028374	0.067312
GD45	CS31	HS3	*R.nitidus*	0.105705	0.070266
CS15	CS31	HS3	*R.nitidus*	0.04403	0.067409
CS19	CS31	HS3	*R.nitidus*	-0.010972	0.06726
CS17	CS31	HS3	*R.nitidus*	0.03827	0.070057
GD33	CS31	HS3	*R.nitidus*	0.043964	0.065989
CS3	CS31	HS3	*R.nitidus*	0.000755	0.064986
SC12	CS31	HS3	*R.nitidus*	0.007236	0.06453
CS25	CS31	HS3	*R.nitidus*	0.016479	0.067258
CS21	CS31	HS3	*R.nitidus*	-0.008065	0.064845
SC10	CS31	HS3	*R.nitidus*	0.097117	0.065716
SC26	CS31	HS3	*R.nitidus*	-0.011577	0.064738
GD50	CS31	HS3	*R.nitidus*	0.059233	0.068142
SC14	CS31	HS3	*R.nitidus*	0.02959	0.06627
SC6	CS31	HS3	*R.nitidus*	0.029862	0.066635
CS4	CS31	HS3	*R.nitidus*	0.002884	0.063435
CS28	CS31	HS3	*R.nitidus*	-0.000993	0.068604
GD46	CS31	HS3	*R.nitidus*	0.003698	0.064989
JW6	CS31	HS3	*R.nitidus*	0.052431	0.071733
SC32	CS31	HS3	*R.nitidus*	0.074866	0.067295
CS24	CS31	HS3	*R.nitidus*	-0.024883	0.064953
CS23	CS31	HS3	*R.nitidus*	-0.032799	0.065677
SC35	CS31	HS3	*R.nitidus*	0.061269	0.063344

CS22	CS31	HS3	*R.nitidus*	0.024261	0.065316
CS30	CS31	HS3	*R.nitidus*	-0.053422	0.063481
SC34	CS31	HS3	*R.nitidus*	0.056318	0.06417
SC42	CS31	HS3	*R.nitidus*	0.270173	0.061249
SC24	CS31	HS3	*R.nitidus*	0	0.063904
SC5	CS31	HS3	*R.nitidus*	0.022895	0.069454
MY4	CS31	HS3	*R.nitidus*	-0.017778	0.066818
JW7	CS31	HS3	*R.nitidus*	0.080836	0.068975
SC27	CS31	HS3	*R.nitidus*	0.012821	0.064302
JO3	CS31	HS3	*R.nitidus*	0.054918	0.07029
CS6	CS31	HS3	*R.nitidus*	-0.022256	0.065351
SC44	CS31	HS3	*R.nitidus*	0.027571	0.06486
GD25	CS3	HS3	*R.nitidus*	0.08908	0.065209
MY6	CS31	HS3	*R.nitidus*	0.01748	0.067012
SC18	CS31	HS3	*R.nitidus*	0.058389	0.064775
SC15	CS31	HS3	*R.nitidus*	0.029549	0.068411
GD26	CS3	HS3	*R.nitidus*	0.097035	0.065687
SC45	CS31	HS3	*R.nitidus*	0.050761	0.065863
SC31	CS31	HS3	*R.nitidus*	0.033984	0.065333
JW4	CS31	HS3	*R.nitidus*	0.255423	0.066039
CS11	CS3	HS3	*R.nitidus*	0.034533	0.065784
JO7	CS3	HS3	*R.nitidus*	0.00542	0.071611
SC28	CS31	HS3	*R.nitidus*	-0.010324	0.063338
GD48	CS3	HS3	*R.nitidus*	0.06512	0.06696
GD21	CS3	HS3	*R.nitidus*	0.151005	0.063209
GD31	CS3	HS3	*R.nitidus*	0.110065	0.066039
JO8	CS3	HS3	*R.nitidus*	0.031823	0.071918
GD50	CS3	HS3	*R.nitidus*	0.070319	0.06853
CS13	CS3	HS3	*R.nitidus*	-0.008076	0.065648
JO1	CS31	HS3	*R.nitidus*	0.00365	0.065856
GD35	CS3	HS3	*R.nitidus*	0.052342	0.064882
JO5	CS31	HS3	*R.nitidus*	0.073295	0.064619
GD30	CS3	HS3	*R.nitidus*	0.149278	0.066454
GD45	CS3	HS3	*R.nitidus*	0.074819	0.070707
CS14	CS3	HS3	*R.nitidus*	0.024	0.065771
GD42	CS3	HS3	*R.nitidus*	0.057666	0.065113
GD40	CS3	HS3	*R.nitidus*	0.086713	0.062278
GD44	CS3	HS3	*R.nitidus*	0.059669	0.064206
GD49	CS3	HS3	*R.nitidus*	0.12988	0.06823
CS17	CS3	HS3	*R.nitidus*	0.046714	0.067867
GD32	CS3	HS3	*R.nitidus*	0.082612	0.068039
CS19	CS3	HS3	*R.nitidus*	-0.001704	0.065733
GD22	CS3	HS3	*R.nitidus*	0.120395	0.064186
CS25	CS3	HS3	*R.nitidus*	0.027419	0.066692
GD29	CS3	HS3	*R.nitidus*	0.167539	0.064276
CS12	CS3	HS3	*R.nitidus*	0.019742	0.065639
CS18	CS3	HS3	*R.nitidus*	-0.007557	0.064857
GD33	CS3	HS3	*R.nitidus*	0.06383	0.065443
CS31	CS3	HS3	*R.nitidus*	-0.000755	0.064986
GD34	CS3	HS3	*R.nitidus*	0.036986	0.065995
GD41	CS3	HS3	*R.nitidus*	0.050734	0.063349
CS22	CS3	HS3	*R.nitidus*	0.02271	0.064644
SC14	CS3	HS3	*R.nitidus*	0.040512	0.065427
CS16	CS3	HS3	*R.nitidus*	0.027596	0.064288
GD46	CS3	HS3	*R.nitidus*	0.005435	0.064225
CS24	CS3	HS3	*R.nitidus*	-0.004699	0.064781

SC10	CS3	HS3	*R.nitidus*	0.074129	0.063449
SC18	CS3	HS3	*R.nitidus*	0.072531	0.064833
SC6	CS3	HS3	*R.nitidus*	0.030191	0.063898
SC42	CS3	HS3	*R.nitidus*	0.253749	0.060443
SC12	CS3	HS3	*R.nitidus*	0.006479	0.064532
SC27	CS3	HS3	*R.nitidus*	0.013514	0.063091
CS4	CS3	HS3	*R.nitidus*	0.020548	0.063355
SC15	CS3	HS3	*R.nitidus*	0.046131	0.065788
CS30	CS3	HS3	*R.nitidus*	-0.03265	0.065007
SC26	CS3	HS3	*R.nitidus*	-0.007231	0.063577
CS15	CS3	HS3	*R.nitidus*	0.051613	0.066569
SC5	CS3	HS3	*R.nitidus*	0.021337	0.067787
MY6	CS3	HS3	*R.nitidus*	0.0163	0.063993
CS20	CS3	HS3	*R.nitidus*	-0.000992	0.062764
CS28	CS3	HS3	*R.nitidus*	-0.007103	0.067119
CS6	CS3	HS3	*R.nitidus*	-0.02997	0.060522
CS23	CS3	HS3	*R.nitidus*	-0.033363	0.064451
JO5	CS3	HS3	*R.nitidus*	0.069138	0.063407
SC35	CS3	HS3	*R.nitidus*	0.062632	0.063193
SC45	CS3	HS3	*R.nitidus*	0.050787	0.064701
JW4	CS3	HS3	*R.nitidus*	0.223479	0.065551
JO8	SC5	HS3	*R.nitidus*	0	0.075212
SC32	CS3	HS3	*R.nitidus*	0.078723	0.064911
CS21	CS3	HS3	*R.nitidus*	-0.004425	0.063914
JO3	CS3	HS3	*R.nitidus*	0.037551	0.068582
JO7	SC5	HS3	*R.nitidus*	-0.043112	0.073234
SC24	CS3	HS3	*R.nitidus*	0.024096	0.065411
SC28	CS3	HS3	*R.nitidus*	-0.01231	0.063269
GD25	SC5	HS3	*R.nitidus*	0.056407	0.06718
SC31	CS3	HS3	*R.nitidus*	0.019355	0.063842
SC34	CS3	HS3	*R.nitidus*	0.060056	0.063544
SC44	CS3	HS3	*R.nitidus*	0.020194	0.063776
JW6	CS3	HS3	*R.nitidus*	-0.00362	0.069923
CS13	SC5	HS3	*R.nitidus*	-0.039589	0.068771
GD50	SC5	HS3	*R.nitidus*	0.048696	0.072923
CS11	SC5	HS3	*R.nitidus*	-0.004184	0.069492
MY4	CS3	HS3	*R.nitidus*	-0.007914	0.064797
JW7	CS3	HS3	*R.nitidus*	0.056604	0.066715
GD32	SC5	HS3	*R.nitidus*	0.017915	0.072105
JO1	CS3	HS3	*R.nitidus*	0.008639	0.066289
GD30	SC5	HS3	*R.nitidus*	0.128931	0.070367
GD45	SC5	HS3	*R.nitidus*	0.075428	0.072377
GD42	SC5	HS3	*R.nitidus*	0.023994	0.068942
GD21	SC5	HS3	*R.nitidus*	0.123432	0.066723
GD41	SC5	HS3	*R.nitidus*	0.004213	0.067249
GD22	SC5	HS3	*R.nitidus*	0.096386	0.067039
CS16	SC5	HS3	*R.nitidus*	-0.002801	0.069406
CS12	SC5	HS3	*R.nitidus*	-0.014556	0.068086
GD48	SC5	HS3	*R.nitidus*	0.003065	0.070349
GD33	SC5	HS3	*R.nitidus*	0.012784	0.069554
GD26	SC5	HS3	*R.nitidus*	0.068966	0.066707
CS31	SC5	HS3	*R.nitidus*	-0.022895	0.069454
GD29	SC5	HS3	*R.nitidus*	0.137977	0.067595
CS17	SC5	HS3	*R.nitidus*	0.020532	0.070679
GD35	SC5	HS3	*R.nitidus*	0.038434	0.070527
CS23	SC5	HS3	*R.nitidus*	-0.054092	0.067644

GD46	SC5	HS3	*R.nitidus*	-0.018724	0.067672
GD31	SC5	HS3	*R.nitidus*	0.0707	0.068255
CS14	SC5	HS3	*R.nitidus*	-0.014716	0.070006
GD34	SC5	HS3	*R.nitidus*	0	0.069215
CS25	SC5	HS3	*R.nitidus*	-0.014947	0.069538
GD40	SC5	HS3	*R.nitidus*	0.064164	0.064955
CS18	SC5	HS3	*R.nitidus*	-0.04418	0.068887
SC12	SC5	HS3	*R.nitidus*	-0.023237	0.069829
CS19	SC5	HS3	*R.nitidus*	-0.033137	0.069155
CS28	SC5	HS3	*R.nitidus*	-0.046332	0.068036
CS21	SC5	HS3	*R.nitidus*	-0.039804	0.066155
GD44	SC5	HS3	*R.nitidus*	0.028295	0.069326
GD49	SC5	HS3	*R.nitidus*	0.102172	0.070713
CS3	SC5	HS3	*R.nitidus*	-0.021337	0.067787
SC6	SC5	HS3	*R.nitidus*	0.00301	0.069456
CS15	SC5	HS3	*R.nitidus*	0.017968	0.069946
SC10	SC5	HS3	*R.nitidus*	0.070533	0.070185
SC32	SC5	HS3	*R.nitidus*	0.045942	0.067431
CS24	SC5	HS3	*R.nitidus*	-0.061086	0.066873
CS30	SC5	HS3	*R.nitidus*	-0.057692	0.069052
CS20	SC5	HS3	*R.nitidus*	-0.038596	0.067754
CS22	SC5	HS3	*R.nitidus*	-0.025418	0.0681
SC18	SC5	HS3	*R.nitidus*	0.038554	0.067101
SC14	SC5	HS3	*R.nitidus*	0.004762	0.069354
SC35	SC5	HS3	*R.nitidus*	0.03532	0.066304
SC44	SC5	HS3	*R.nitidus*	0.00766	0.06671
SC24	SC5	HS3	*R.nitidus*	-0.006314	0.068982
JO3	SC5	HS3	*R.nitidus*	0.014515	0.073688
SC45	SC5	HS3	*R.nitidus*	0.033557	0.066903
SC34	SC5	HS3	*R.nitidus*	0.027437	0.065899
SC26	SC5	HS3	*R.nitidus*	-0.048653	0.068096
SC15	SC5	HS3	*R.nitidus*	0.013138	0.0695
MY6	SC5	HS3	*R.nitidus*	0.001423	0.066786
GD30	SC6	HS3	*R.nitidus*	0.138315	0.066901
SC31	SC5	HS3	*R.nitidus*	0.000744	0.068024
JW6	SC5	HS3	*R.nitidus*	-0.010233	0.074868
CS4	SC5	HS3	*R.nitidus*	-0.024425	0.06505
JO7	SC6	HS3	*R.nitidus*	-0.027103	0.070488
SC28	SC5	HS3	*R.nitidus*	-0.030782	0.066163
MY4	SC5	HS3	*R.nitidus*	-0.036982	0.067072
JW7	SC5	HS3	*R.nitidus*	0.025023	0.074178
SC27	SC5	HS3	*R.nitidus*	-0.007698	0.068261
GD25	SC6	HS3	*R.nitidus*	0.057463	0.064976
SC42	SC5	HS3	*R.nitidus*	0.247666	0.064779
JO5	SC5	HS3	*R.nitidus*	0.058188	0.066721
JW4	SC5	HS3	*R.nitidus*	0.190029	0.069798
GD45	SC6	HS3	*R.nitidus*	0.068775	0.070877
GD29	SC6	HS3	*R.nitidus*	0.139323	0.063678
GD32	SC6	HS3	*R.nitidus*	0.041569	0.068003
CS6	SC5	HS3	*R.nitidus*	-0.056245	0.066756
GD34	SC6	HS3	*R.nitidus*	0.004717	0.065218
GD41	SC6	HS3	*R.nitidus*	0.010989	0.065444
GD33	SC6	HS3	*R.nitidus*	0.020436	0.066756
GD31	SC6	HS3	*R.nitidus*	0.0625	0.0654
JO8	SC6	HS3	*R.nitidus*	0.018815	0.073166
CS11	SC6	HS3	*R.nitidus*	-0.007719	0.067225

GD26	SC6	HS3	*R.nitidus*	0.072011	0.066039
GD49	SC6	HS3	*R.nitidus*	0.102424	0.07097
JO1	SC5	HS3	*R.nitidus*	-0.015475	0.069245
GD22	SC6	HS3	*R.nitidus*	0.076826	0.066831
CS12	SC6	HS3	*R.nitidus*	0.011202	0.068753
GD21	SC6	HS3	*R.nitidus*	0.129156	0.066717
GD40	SC6	HS3	*R.nitidus*	0.067669	0.062515
GD35	SC6	HS3	*R.nitidus*	0.044521	0.064178
CS22	SC6	HS3	*R.nitidus*	-0.002187	0.065503
CS21	SC6	HS3	*R.nitidus*	-0.034407	0.064731
GD46	SC6	HS3	*R.nitidus*	-0.01669	0.064856
GD50	SC6	HS3	*R.nitidus*	0.03682	0.068245
CS16	SC6	HS3	*R.nitidus*	0.006442	0.066249
GD44	SC6	HS3	*R.nitidus*	0.023324	0.066917
CS23	SC6	HS3	*R.nitidus*	-0.061074	0.065578
GD42	SC6	HS3	*R.nitidus*	0.033081	0.066975
CS18	SC6	HS3	*R.nitidus*	-0.036284	0.066667
CS31	SC6	HS3	*R.nitidus*	-0.029862	0.066635
CS25	SC6	HS3	*R.nitidus*	-0.016105	0.067619
CS30	SC6	HS3	*R.nitidus*	-0.069553	0.068125
CS13	SC6	HS3	*R.nitidus*	-0.041783	0.068465
CS19	SC6	HS3	*R.nitidus*	-0.030043	0.067128
SC12	SC6	HS3	*R.nitidus*	-0.024793	0.065888
GD48	SC6	HS3	*R.nitidus*	0.041075	0.067748
SC18	SC6	HS3	*R.nitidus*	0.02405	0.065556
CS15	SC6	HS3	*R.nitidus*	0.025881	0.066644
CS17	SC6	HS3	*R.nitidus*	0.023467	0.06885
SC14	SC6	HS3	*R.nitidus*	0.004545	0.06845
CS14	SC6	HS3	*R.nitidus*	-0.017781	0.067938
SC31	SC6	HS3	*R.nitidus*	0.013393	0.066732
CS3	SC6	HS3	*R.nitidus*	-0.030191	0.063898
SC44	SC6	HS3	*R.nitidus*	0.000745	0.065767
SC10	SC6	HS3	*R.nitidus*	0.07837	0.067677
CS20	SC6	HS3	*R.nitidus*	-0.02566	0.064841
SC24	SC6	HS3	*R.nitidus*	-0.012327	0.064645
SC35	SC6	HS3	*R.nitidus*	0.038404	0.065266
SC26	SC6	HS3	*R.nitidus*	-0.05019	0.065466
CS28	SC6	HS3	*R.nitidus*	-0.042969	0.066955
SC5	SC6	HS3	*R.nitidus*	-0.00301	0.069456
SC45	SC6	HS3	*R.nitidus*	0.024006	0.066664
SC34	SC6	HS3	*R.nitidus*	0.029499	0.065762
CS24	SC6	HS3	*R.nitidus*	-0.04814	0.064672
SC28	SC6	HS3	*R.nitidus*	-0.052632	0.065707
JO5	SC6	HS3	*R.nitidus*	0.044853	0.066328
JO3	SC6	HS3	*R.nitidus*	0.025417	0.068735
SC42	SC6	HS3	*R.nitidus*	0.263253	0.061868
CS4	SC6	HS3	*R.nitidus*	-0.012474	0.063831
CS11	SC10	HS3	*R.nitidus*	-0.066762	0.066782
JO8	SC10	HS3	*R.nitidus*	-0.045643	0.07287
SC15	SC6	HS3	*R.nitidus*	-0.00312	0.066782
JO7	SC10	HS3	*R.nitidus*	-0.102041	0.073012
JW4	SC6	HS3	*R.nitidus*	0.207045	0.065694
SC27	SC6	HS3	*R.nitidus*	-0.013564	0.065412
MY4	SC6	HS3	*R.nitidus*	-0.039941	0.066497
SC32	SC6	HS3	*R.nitidus*	0.045593	0.064771
JW7	SC6	HS3	*R.nitidus*	0.028414	0.070333

GD25	SC10	HS3	*R.nitidus*	0.003655	0.063317
CS6	SC6	HS3	*R.nitidus*	-0.052356	0.06402
JO1	SC6	HS3	*R.nitidus*	-0.021084	0.066942
GD21	SC10	HS3	*R.nitidus*	0.078187	0.064906
CS13	SC10	HS3	*R.nitidus*	-0.099025	0.067709
GD30	SC10	HS3	*R.nitidus*	0.074813	0.06908
GD34	SC10	HS3	*R.nitidus*	-0.039409	0.066134
GD22	SC10	HS3	*R.nitidus*	0.017796	0.065901
JW6	SC6	HS3	*R.nitidus*	-0.024568	0.0713
MY6	SC6	HS3	*R.nitidus*	0	0.066688
CS12	SC10	HS3	*R.nitidus*	-0.085736	0.067668
GD26	SC10	HS3	*R.nitidus*	0.005658	0.066124
GD32	SC10	HS3	*R.nitidus*	-0.021531	0.066856
CS18	SC10	HS3	*R.nitidus*	-0.107223	0.065634
GD40	SC10	HS3	*R.nitidus*	0.00622	0.064856
GD35	SC10	HS3	*R.nitidus*	-0.016949	0.066244
GD46	SC10	HS3	*R.nitidus*	-0.067447	0.063649
GD49	SC10	HS3	*R.nitidus*	0.039872	0.069255
GD29	SC10	HS3	*R.nitidus*	0.066031	0.066149
GD33	SC10	HS3	*R.nitidus*	-0.023109	0.068276
GD31	SC10	HS3	*R.nitidus*	0.010347	0.068255
CS17	SC10	HS3	*R.nitidus*	-0.042308	0.069435
GD41	SC10	HS3	*R.nitidus*	-0.043262	0.064287
CS19	SC10	HS3	*R.nitidus*	-0.078534	0.068204
GD50	SC10	HS3	*R.nitidus*	-0.012853	0.07024
CS14	SC10	HS3	*R.nitidus*	-0.065799	0.066608
GD45	SC10	HS3	*R.nitidus*	0.00161	0.070532
GD44	SC10	HS3	*R.nitidus*	-0.014894	0.0662
CS31	SC10	HS3	*R.nitidus*	-0.097117	0.065716
GD48	SC10	HS3	*R.nitidus*	-0.019331	0.068568
GD42	SC10	HS3	*R.nitidus*	-0.024496	0.066583
CS22	SC10	HS3	*R.nitidus*	-0.08027	0.065044
SC34	SC10	HS3	*R.nitidus*	-0.020553	0.064269
SC6	SC10	HS3	*R.nitidus*	-0.07837	0.067677
CS24	SC10	HS3	*R.nitidus*	-0.129713	0.063016
CS25	SC10	HS3	*R.nitidus*	-0.045319	0.068985
CS15	SC10	HS3	*R.nitidus*	-0.034359	0.066481
SC5	SC10	HS3	*R.nitidus*	-0.070533	0.070185
CS21	SC10	HS3	*R.nitidus*	-0.092579	0.064708
CS20	SC10	HS3	*R.nitidus*	-0.082976	0.066332
CS28	SC10	HS3	*R.nitidus*	-0.098335	0.067509
SC44	SC10	HS3	*R.nitidus*	-0.071207	0.063804
SC31	SC10	HS3	*R.nitidus*	-0.065485	0.065363
CS30	SC10	HS3	*R.nitidus*	-0.125931	0.065278
SC15	SC10	HS3	*R.nitidus*	-0.058524	0.066854
CS23	SC10	HS3	*R.nitidus*	-0.117137	0.065911
JW6	SC10	HS3	*R.nitidus*	-0.077947	0.073369
CS3	SC10	HS3	*R.nitidus*	-0.074129	0.063449
JW7	SC10	HS3	*R.nitidus*	-0.008621	0.069556
SC42	SC10	HS3	*R.nitidus*	0.203704	0.061579
CS6	SC10	HS3	*R.nitidus*	-0.106015	0.064994
CS4	SC10	HS3	*R.nitidus*	-0.083629	0.063727
JO8	CS4	HS3	*R.nitidus*	0.032407	0.070006
SC24	SC10	HS3	*R.nitidus*	-0.09283	0.0655
CS16	SC10	HS3	*R.nitidus*	-0.071031	0.066288
SC27	SC10	HS3	*R.nitidus*	-0.078125	0.063544

JW4	SC10	HS3	*R.nitidus*	0.17731	0.066953
SC45	SC10	HS3	*R.nitidus*	-0.043818	0.066647
JO1	SC10	HS3	*R.nitidus*	-0.09324	0.063647
SC18	SC10	HS3	*R.nitidus*	-0.041296	0.066015
SC26	SC10	HS3	*R.nitidus*	-0.112142	0.063263
JO3	SC10	HS3	*R.nitidus*	-0.035714	0.07041
JO5	SC10	HS3	*R.nitidus*	-0.028549	0.065595
SC12	SC10	HS3	*R.nitidus*	-0.098695	0.062686
SC28	SC10	HS3	*R.nitidus*	-0.123525	0.063745
SC14	SC10	HS3	*R.nitidus*	-0.050901	0.066909
SC35	SC10	HS3	*R.nitidus*	-0.024143	0.065212
GD32	CS4	HS3	*R.nitidus*	0.077105	0.065446
GD21	CS4	HS3	*R.nitidus*	0.148194	0.061146
GD22	CS4	HS3	*R.nitidus*	0.106717	0.062053
SC32	SC10	HS3	*R.nitidus*	-0.018639	0.064491
GD31	CS4	HS3	*R.nitidus*	0.091346	0.064354
GD29	CS4	HS3	*R.nitidus*	0.154293	0.061629
JO7	CS4	HS3	*R.nitidus*	0.020942	0.06971
MY4	SC10	HS3	*R.nitidus*	-0.115385	0.066463
MY6	SC10	HS3	*R.nitidus*	-0.083333	0.066078
GD40	CS4	HS3	*R.nitidus*	0.092359	0.061919
CS11	CS4	HS3	*R.nitidus*	0.020729	0.066885
GD35	CS4	HS3	*R.nitidus*	0.056478	0.064314
GD34	CS4	HS3	*R.nitidus*	0.026157	0.061599
GD44	CS4	HS3	*R.nitidus*	0.073487	0.065761
GD26	CS4	HS3	*R.nitidus*	0.092359	0.063108
GD42	CS4	HS3	*R.nitidus*	0.040667	0.062398
CS12	CS4	HS3	*R.nitidus*	0.034357	0.064474
GD30	CS4	HS3	*R.nitidus*	0.132873	0.064663
GD46	CS4	HS3	*R.nitidus*	0.007246	0.06283
GD45	CS4	HS3	*R.nitidus*	0.086889	0.067063
GD25	CS4	HS3	*R.nitidus*	0.064624	0.063145
CS14	CS4	HS3	*R.nitidus*	0.020322	0.066394
CS16	CS4	HS3	*R.nitidus*	0.025281	0.06471
CS19	CS4	HS3	*R.nitidus*	-0.010046	0.066566
GD41	CS4	HS3	*R.nitidus*	0.035762	0.062079
GD50	CS4	HS3	*R.nitidus*	0.058964	0.065854
CS13	CS4	HS3	*R.nitidus*	-0.018012	0.066024
CS21	CS4	HS3	*R.nitidus*	-0.000765	0.063305
CS18	CS4	HS3	*R.nitidus*	-0.012589	0.062674
GD33	CS4	HS3	*R.nitidus*	0.048649	0.064657
CS22	CS4	HS3	*R.nitidus*	0.019755	0.061745
CS24	CS4	HS3	*R.nitidus*	-0.032035	0.059992
CS25	CS4	HS3	*R.nitidus*	0.001503	0.066903
CS3	CS4	HS3	*R.nitidus*	-0.020548	0.063355
CS30	CS4	HS3	*R.nitidus*	-0.039677	0.062434
GD49	CS4	HS3	*R.nitidus*	0.103448	0.066323
GD48	CS4	HS3	*R.nitidus*	0.047342	0.064915
SC24	CS4	HS3	*R.nitidus*	0.001414	0.063393
CS15	CS4	HS3	*R.nitidus*	0.045205	0.064432
SC34	CS4	HS3	*R.nitidus*	0.051713	0.061036
CS17	CS4	HS3	*R.nitidus*	0.044309	0.066638
SC27	CS4	HS3	*R.nitidus*	0.008978	0.062702
SC6	CS4	HS3	*R.nitidus*	0.012474	0.063831
CS23	CS4	HS3	*R.nitidus*	-0.03698	0.062209
JW6	CS4	HS3	*R.nitidus*	0.013734	0.068102

SC26	CS4	HS3	*R.nitidus*	-0.016238	0.061762
JW4	CS4	HS3	*R.nitidus*	0.223227	0.063534
SC42	CS4	HS3	*R.nitidus*	0.260563	0.060842
SC15	CS4	HS3	*R.nitidus*	0.03543	0.063481
SC31	CS4	HS3	*R.nitidus*	0.020181	0.062518
SC35	CS4	HS3	*R.nitidus*	0.049374	0.063754
SC32	CS4	HS3	*R.nitidus*	0.061441	0.062775
CS20	CS4	HS3	*R.nitidus*	-0.01466	0.063025
CS28	CS4	HS3	*R.nitidus*	-0.015244	0.064391
CS31	CS4	HS3	*R.nitidus*	-0.002884	0.063435
SC12	CS4	HS3	*R.nitidus*	-0.000699	0.062932
SC5	CS4	HS3	*R.nitidus*	0.024425	0.06505
SC44	CS4	HS3	*R.nitidus*	0.018207	0.062897
SC14	CS4	HS3	*R.nitidus*	0.019972	0.063681
SC10	CS4	HS3	*R.nitidus*	0.083629	0.063727
JW7	CS4	HS3	*R.nitidus*	0.041563	0.065469
SC18	CS4	HS3	*R.nitidus*	0.045487	0.062656
SC28	CS4	HS3	*R.nitidus*	-0.011127	0.062842
CS6	CS4	HS3	*R.nitidus*	-0.025893	0.062617
MY4	CS4	HS3	*R.nitidus*	-0.017417	0.065124
MY6	CS4	HS3	*R.nitidus*	0.02028	0.064605
GD21	SC12	HS3	*R.nitidus*	0.13505	0.063985
JO7	SC12	HS3	*R.nitidus*	0.004798	0.069038
CS11	SC12	HS3	*R.nitidus*	0.002867	0.066597
JO3	CS4	HS3	*R.nitidus*	0.057339	0.066408
GD25	SC12	HS3	*R.nitidus*	0.069832	0.06495
CS12	SC12	HS3	*R.nitidus*	0.011128	0.067669
GD22	SC12	HS3	*R.nitidus*	0.096609	0.062541
GD29	SC12	HS3	*R.nitidus*	0.136626	0.064619
SC45	CS4	HS3	*R.nitidus*	0.040383	0.06438
JO5	CS4	HS3	*R.nitidus*	0.061397	0.062255
GD45	SC12	HS3	*R.nitidus*	0.069413	0.068556
JO8	SC12	HS3	*R.nitidus*	0.046859	0.07022
GD35	SC12	HS3	*R.nitidus*	0.063758	0.065915
GD42	SC12	HS3	*R.nitidus*	0.042582	0.064428
JO1	CS4	HS3	*R.nitidus*	0.008463	0.063319
GD26	SC12	HS3	*R.nitidus*	0.087636	0.062421
GD31	SC12	HS3	*R.nitidus*	0.072329	0.066509
GD33	SC12	HS3	*R.nitidus*	0.038384	0.066736
GD44	SC12	HS3	*R.nitidus*	0.055021	0.064412
CS14	SC12	HS3	*R.nitidus*	0.007703	0.067696
GD30	SC12	HS3	*R.nitidus*	0.130709	0.067574
GD49	SC12	HS3	*R.nitidus*	0.091193	0.066389
GD41	SC12	HS3	*R.nitidus*	0.01828	0.06293
CS31	SC12	HS3	*R.nitidus*	-0.007236	0.06453
CS18	SC12	HS3	*R.nitidus*	-0.020685	0.065938
CS23	SC12	HS3	*R.nitidus*	-0.030942	0.064436
GD32	SC12	HS3	*R.nitidus*	0.059006	0.067264
GD40	SC12	HS3	*R.nitidus*	0.084712	0.064001
CS17	SC12	HS3	*R.nitidus*	0.036953	0.069238
CS15	SC12	HS3	*R.nitidus*	0.043872	0.067769
GD48	SC12	HS3	*R.nitidus*	0.055124	0.068285
CS16	SC12	HS3	*R.nitidus*	0.02048	0.066297
GD50	SC12	HS3	*R.nitidus*	0.070502	0.070396
GD34	SC12	HS3	*R.nitidus*	0.025726	0.065635
SC6	SC12	HS3	*R.nitidus*	0.024793	0.065888

CS20	SC12	HS3	*R.nitidus*	-0.019567	0.065674
CS30	SC12	HS3	*R.nitidus*	-0.035088	0.065707
CS22	SC12	HS3	*R.nitidus*	0.007305	0.063955
GD46	SC12	HS3	*R.nitidus*	-0.003397	0.064349
SC18	SC12	HS3	*R.nitidus*	0.069531	0.065202
SC24	SC12	HS3	*R.nitidus*	0.005279	0.064955
CS13	SC12	HS3	*R.nitidus*	-0.034582	0.067207
SC27	SC12	HS3	*R.nitidus*	0.013535	0.064181
SC26	SC12	HS3	*R.nitidus*	-0.025738	0.063762
SC5	SC12	HS3	*R.nitidus*	0.023237	0.069829
CS19	SC12	HS3	*R.nitidus*	-0.017618	0.066124
CS28	SC12	HS3	*R.nitidus*	-0.023438	0.066402
CS24	SC12	HS3	*R.nitidus*	-0.032329	0.064098
SC28	SC12	HS3	*R.nitidus*	-0.00939	0.064408
SC15	SC12	HS3	*R.nitidus*	0.030928	0.065701
CS4	SC12	HS3	*R.nitidus*	0.000699	0.062932
CS25	SC12	HS3	*R.nitidus*	0.009332	0.068386
SC32	SC12	HS3	*R.nitidus*	0.074074	0.066324
SC34	SC12	HS3	*R.nitidus*	0.061178	0.064258
MY6	SC12	HS3	*R.nitidus*	0.013177	0.06596
CS6	SC12	HS3	*R.nitidus*	-0.043511	0.065147
CS3	SC12	HS3	*R.nitidus*	-0.006479	0.064532
SC31	SC12	HS3	*R.nitidus*	0.022367	0.067129
JW4	SC12	HS3	*R.nitidus*	0.236936	0.064549
SC10	SC12	HS3	*R.nitidus*	0.098695	0.062686
CS21	SC12	HS3	*R.nitidus*	-0.021277	0.065975
SC44	SC12	HS3	*R.nitidus*	0.015323	0.063694
SC14	SC12	HS3	*R.nitidus*	0.018692	0.066145
SC42	SC12	HS3	*R.nitidus*	0.27149	0.059566
MY4	SC12	HS3	*R.nitidus*	-0.023065	0.064947
JO3	SC12	HS3	*R.nitidus*	0.07081	0.067234
JW7	SC12	HS3	*R.nitidus*	0.07024	0.066815
GD32	SC14	HS3	*R.nitidus*	0.028324	0.067878
JW6	SC12	HS3	*R.nitidus*	0.035349	0.06693
SC35	SC12	HS3	*R.nitidus*	0.073263	0.06194
JO1	SC12	HS3	*R.nitidus*	0.000756	0.065679
SC45	SC12	HS3	*R.nitidus*	0.054869	0.064178
GD34	SC14	HS3	*R.nitidus*	-0.005333	0.06562
GD40	SC14	HS3	*R.nitidus*	0.056728	0.063739
GD29	SC14	HS3	*R.nitidus*	0.124283	0.062495
GD26	SC14	HS3	*R.nitidus*	0.059172	0.066355
JO8	SC14	HS3	*R.nitidus*	0.015888	0.070564
GD42	SC14	HS3	*R.nitidus*	0.008978	0.065338
GD21	SC14	HS3	*R.nitidus*	0.117836	0.064316
GD35	SC14	HS3	*R.nitidus*	0.027229	0.066208
GD25	SC14	HS3	*R.nitidus*	0.054841	0.064317
JO7	SC14	HS3	*R.nitidus*	-0.012856	0.071341
JO5	SC12	HS3	*R.nitidus*	0.069644	0.064828
GD22	SC14	HS3	*R.nitidus*	0.081646	0.063672
CS11	SC14	HS3	*R.nitidus*	-0.015246	0.068633
GD48	SC14	HS3	*R.nitidus*	0.034182	0.067125
GD41	SC14	HS3	*R.nitidus*	0.006285	0.062674
CS12	SC14	HS3	*R.nitidus*	-0.010007	0.068559
GD50	SC14	HS3	*R.nitidus*	0.024213	0.067751
GD45	SC14	HS3	*R.nitidus*	0.040062	0.070881
GD30	SC14	HS3	*R.nitidus*	0.105551	0.066947

GD49	SC14	HS3	*R.nitidus*	0.070444	0.067593
CS13	SC14	HS3	*R.nitidus*	-0.049088	0.067912
GD31	SC14	HS3	*R.nitidus*	0.060124	0.064539
CS22	SC14	HS3	*R.nitidus*	-0.017229	0.065137
GD46	SC14	HS3	*R.nitidus*	-0.033243	0.064349
CS21	SC14	HS3	*R.nitidus*	-0.04669	0.065726
GD44	SC14	HS3	*R.nitidus*	0.034757	0.067034
SC10	SC14	HS3	*R.nitidus*	0.050901	0.066909
GD33	SC14	HS3	*R.nitidus*	0.005937	0.065544
CS20	SC14	HS3	*R.nitidus*	-0.034908	0.065126
CS24	SC14	HS3	*R.nitidus*	-0.052161	0.064265
CS31	SC14	HS3	*R.nitidus*	-0.02959	0.06627
SC12	SC14	HS3	*R.nitidus*	-0.018692	0.066145
CS18	SC14	HS3	*R.nitidus*	-0.03696	0.065274
CS14	SC14	HS3	*R.nitidus*	-0.01158	0.067359
SC5	SC14	HS3	*R.nitidus*	-0.004762	0.069354
CS15	SC14	HS3	*R.nitidus*	0.024931	0.067998
SC15	SC14	HS3	*R.nitidus*	0.0064	0.065858
CS19	SC14	HS3	*R.nitidus*	-0.03574	0.067851
CS4	SC14	HS3	*R.nitidus*	-0.019972	0.063681
CS17	SC14	HS3	*R.nitidus*	0.010728	0.068939
SC27	SC14	HS3	*R.nitidus*	-0.020555	0.06142
CS30	SC14	HS3	*R.nitidus*	-0.061372	0.065062
CS16	SC14	HS3	*R.nitidus*	-0.002068	0.066728
SC6	SC14	HS3	*R.nitidus*	-0.004545	0.06845
CS25	SC14	HS3	*R.nitidus*	-0.014184	0.067504
SC32	SC14	HS3	*R.nitidus*	0.043028	0.066594
CS23	SC14	HS3	*R.nitidus*	-0.057613	0.066312
CS3	SC14	HS3	*R.nitidus*	-0.040512	0.065427
CS28	SC14	HS3	*R.nitidus*	-0.040217	0.066614
CS6	SC14	HS3	*R.nitidus*	-0.058692	0.063936
SC44	SC14	HS3	*R.nitidus*	-0.005405	0.063578
GD21	SC15	HS3	*R.nitidus*	0.121935	0.065806
SC18	SC14	HS3	*R.nitidus*	0.027069	0.065854
MY4	SC14	HS3	*R.nitidus*	-0.045058	0.067229
SC34	SC14	HS3	*R.nitidus*	0.033133	0.06505
JO1	SC14	HS3	*R.nitidus*	-0.006623	0.06484
SC35	SC14	HS3	*R.nitidus*	0.040323	0.063832
SC45	SC14	HS3	*R.nitidus*	0.027027	0.065626
SC42	SC14	HS3	*R.nitidus*	0.250153	0.061452
GD26	SC15	HS3	*R.nitidus*	0.053425	0.065068
JO3	SC14	HS3	*R.nitidus*	0.033924	0.069276
JO8	SC15	HS3	*R.nitidus*	0.012133	0.072801
GD22	SC15	HS3	*R.nitidus*	0.074967	0.065083
SC24	SC14	HS3	*R.nitidus*	-0.022438	0.066293
SC26	SC14	HS3	*R.nitidus*	-0.045317	0.066149
GD29	SC15	HS3	*R.nitidus*	0.125337	0.065005
GD30	SC15	HS3	*R.nitidus*	0.110395	0.070441
SC28	SC14	HS3	*R.nitidus*	-0.039574	0.064444
SC31	SC14	HS3	*R.nitidus*	-0.003023	0.066791
JW6	SC14	HS3	*R.nitidus*	0.008333	0.069348
JW4	SC14	HS3	*R.nitidus*	0.208276	0.064774
GD25	SC15	HS3	*R.nitidus*	0.04821	0.065949
MY6	SC14	HS3	*R.nitidus*	-0.004871	0.064382
JO7	SC15	HS3	*R.nitidus*	-0.016457	0.072013
JW7	SC14	HS3	*R.nitidus*	0.041267	0.068764

CS11	SC15	HS3	*R.nitidus*	-0.017151	0.068066
GD32	SC15	HS3	*R.nitidus*	0.012235	0.068316
GD31	SC15	HS3	*R.nitidus*	0.05942	0.069223
CS12	SC15	HS3	*R.nitidus*	-0.005439	0.066723
JO5	SC14	HS3	*R.nitidus*	0.046048	0.06467
GD46	SC15	HS3	*R.nitidus*	-0.030891	0.065785
GD34	SC15	HS3	*R.nitidus*	0.005521	0.066704
CS18	SC15	HS3	*R.nitidus*	-0.045038	0.066096
GD41	SC15	HS3	*R.nitidus*	-0.002905	0.06377
GD50	SC15	HS3	*R.nitidus*	0.007719	0.070233
CS24	SC15	HS3	*R.nitidus*	-0.058411	0.064426
GD33	SC15	HS3	*R.nitidus*	0.013928	0.066016
GD49	SC15	HS3	*R.nitidus*	0.08744	0.069325
CS28	SC15	HS3	*R.nitidus*	-0.038779	0.068439
CS13	SC15	HS3	*R.nitidus*	-0.051632	0.067901
GD45	SC15	HS3	*R.nitidus*	0.036058	0.069412
CS22	SC15	HS3	*R.nitidus*	-0.024261	0.065994
GD35	SC15	HS3	*R.nitidus*	0.036792	0.06896
CS30	SC15	HS3	*R.nitidus*	-0.070108	0.066705
CS16	SC15	HS3	*R.nitidus*	-0.007418	0.067312
GD42	SC15	HS3	*R.nitidus*	0.034508	0.065652
CS31	SC15	HS3	*R.nitidus*	-0.029549	0.068411
GD40	SC15	HS3	*R.nitidus*	0.064428	0.063412
GD44	SC15	HS3	*R.nitidus*	0.011111	0.067051
CS15	SC15	HS3	*R.nitidus*	0.014535	0.068158
GD48	SC15	HS3	*R.nitidus*	0.033282	0.068785
CS14	SC15	HS3	*R.nitidus*	-0.019146	0.068543
CS21	SC15	HS3	*R.nitidus*	-0.040059	0.066608
CS4	SC15	HS3	*R.nitidus*	-0.03543	0.063481
CS20	SC15	HS3	*R.nitidus*	-0.034307	0.067608
CS23	SC15	HS3	*R.nitidus*	-0.071429	0.065068
SC5	SC15	HS3	*R.nitidus*	-0.013138	0.0695
SC12	SC15	HS3	*R.nitidus*	-0.030928	0.065701
CS25	SC15	HS3	*R.nitidus*	-0.023952	0.067852
CS17	SC15	HS3	*R.nitidus*	0.012955	0.069423
CS19	SC15	HS3	*R.nitidus*	-0.045386	0.067317
SC10	SC15	HS3	*R.nitidus*	0.058524	0.066854
CS3	SC15	HS3	*R.nitidus*	-0.046131	0.065788
JO3	SC15	HS3	*R.nitidus*	0.030731	0.070063
JW7	SC15	HS3	*R.nitidus*	0.033866	0.069733
JO1	SC15	HS3	*R.nitidus*	-0.028616	0.066495
SC44	SC15	HS3	*R.nitidus*	-0.010085	0.065623
SC26	SC15	HS3	*R.nitidus*	-0.050951	0.063411
SC45	SC15	HS3	*R.nitidus*	0.021788	0.066248
SC35	SC15	HS3	*R.nitidus*	0.035276	0.065009
SC27	SC15	HS3	*R.nitidus*	-0.017101	0.063906
SC6	SC15	HS3	*R.nitidus*	0.00312	0.066782
GD31	SC18	HS3	*R.nitidus*	0.032967	0.067003
SC34	SC15	HS3	*R.nitidus*	0.028594	0.065167
SC42	SC15	HS3	*R.nitidus*	0.253565	0.063218
CS6	SC15	HS3	*R.nitidus*	-0.065134	0.065244
JO7	SC18	HS3	*R.nitidus*	-0.036609	0.069717
GD29	SC18	HS3	*R.nitidus*	0.09712	0.063527
SC18	SC15	HS3	*R.nitidus*	0.026421	0.065946
SC24	SC15	HS3	*R.nitidus*	-0.029529	0.065671
JW4	SC15	HS3	*R.nitidus*	0.210753	0.066803

JO5	SC15	HS3	*R.nitidus*	0.041379	0.065502
GD30	SC18	HS3	*R.nitidus*	0.092084	0.067505
GD22	SC18	HS3	*R.nitidus*	0.05668	0.063868
JO8	SC18	HS3	*R.nitidus*	-0.006322	0.07037
GD26	SC18	HS3	*R.nitidus*	0.037842	0.063619
MY4	SC15	HS3	*R.nitidus*	-0.051493	0.066635
GD25	SC18	HS3	*R.nitidus*	0.032601	0.065568
SC28	SC15	HS3	*R.nitidus*	-0.041602	0.065257
SC31	SC15	HS3	*R.nitidus*	0	0.065457
SC32	SC15	HS3	*R.nitidus*	0.042755	0.06617
SC14	SC15	HS3	*R.nitidus*	-0.0064	0.065858
CS11	SC18	HS3	*R.nitidus*	-0.037196	0.065554
GD34	SC18	HS3	*R.nitidus*	-0.014593	0.065577
MY6	SC15	HS3	*R.nitidus*	-0.014001	0.065389
JW6	SC15	HS3	*R.nitidus*	0.008721	0.072936
CS15	SC18	HS3	*R.nitidus*	-0.009366	0.065091
GD45	SC18	HS3	*R.nitidus*	0.011494	0.068531
GD49	SC18	HS3	*R.nitidus*	0.055927	0.068266
GD21	SC18	HS3	*R.nitidus*	0.117296	0.064129
CS13	SC18	HS3	*R.nitidus*	-0.066278	0.067148
CS17	SC18	HS3	*R.nitidus*	-0.005287	0.068216
GD41	SC18	HS3	*R.nitidus*	-0.011544	0.064119
GD42	SC18	HS3	*R.nitidus*	0.014399	0.066131
CS14	SC18	HS3	*R.nitidus*	-0.022175	0.065751
GD44	SC18	HS3	*R.nitidus*	0.024115	0.064691
GD32	SC18	HS3	*R.nitidus*	-0.015523	0.066102
GD46	SC18	HS3	*R.nitidus*	-0.058023	0.062508
CS18	SC18	HS3	*R.nitidus*	-0.071066	0.065307
CS21	SC18	HS3	*R.nitidus*	-0.068132	0.064097
CS12	SC18	HS3	*R.nitidus*	-0.045727	0.06754
GD33	SC18	HS3	*R.nitidus*	0	0.065764
GD40	SC18	HS3	*R.nitidus*	0.033803	0.062071
GD35	SC18	HS3	*R.nitidus*	0.024478	0.065943
CS25	SC18	HS3	*R.nitidus*	-0.045319	0.066521
SC5	SC18	HS3	*R.nitidus*	-0.038554	0.067101
CS19	SC18	HS3	*R.nitidus*	-0.069519	0.065237
CS24	SC18	HS3	*R.nitidus*	-0.070465	0.064863
GD50	SC18	HS3	*R.nitidus*	0.008547	0.069836
GD48	SC18	HS3	*R.nitidus*	-0.000762	0.067267
CS23	SC18	HS3	*R.nitidus*	-0.089896	0.064702
CS30	SC18	HS3	*R.nitidus*	-0.097037	0.064648
SC34	SC18	HS3	*R.nitidus*	-0.000755	0.063799
CS22	SC18	HS3	*R.nitidus*	-0.037975	0.0659
CS31	SC18	HS3	*R.nitidus*	-0.058389	0.064775
SC44	SC18	HS3	*R.nitidus*	-0.050602	0.06253
CS3	SC18	HS3	*R.nitidus*	-0.072531	0.064833
SC24	SC18	HS3	*R.nitidus*	-0.07332	0.062783
CS4	SC18	HS3	*R.nitidus*	-0.045487	0.062656
SC42	SC18	HS3	*R.nitidus*	0.221728	0.062345
SC26	SC18	HS3	*R.nitidus*	-0.084047	0.065224
SC32	SC18	HS3	*R.nitidus*	0.011164	0.062895
SC6	SC18	HS3	*R.nitidus*	-0.02405	0.065556
SC15	SC18	HS3	*R.nitidus*	-0.026421	0.065946
JW4	SC18	HS3	*R.nitidus*	0.182284	0.065843
CS16	SC18	HS3	*R.nitidus*	-0.02551	0.064871
MY6	SC18	HS3	*R.nitidus*	-0.045942	0.062379

SC10	SC18	HS3	*R.nitidus*	0.041296	0.066015
CS28	SC18	HS3	*R.nitidus*	-0.070203	0.068272
SC14	SC18	HS3	*R.nitidus*	-0.027069	0.065854
SC28	SC18	HS3	*R.nitidus*	-0.086957	0.060938
SC12	SC18	HS3	*R.nitidus*	-0.069531	0.065202
SC35	SC18	HS3	*R.nitidus*	0.003794	0.062996
SC27	SC18	HS3	*R.nitidus*	-0.038043	0.065225
JO3	SC18	HS3	*R.nitidus*	0.011561	0.068149
CS20	SC18	HS3	*R.nitidus*	-0.066421	0.064781
SC45	SC18	HS3	*R.nitidus*	-0.015432	0.063889
MY4	SC18	HS3	*R.nitidus*	-0.08549	0.062827
SC31	SC18	HS3	*R.nitidus*	-0.033411	0.064587
GD25	SC24	HS3	*R.nitidus*	0.067643	0.064897
JO5	SC18	HS3	*R.nitidus*	0.014925	0.062503
CS6	SC18	HS3	*R.nitidus*	-0.094384	0.062775
JW6	SC18	HS3	*R.nitidus*	-0.003868	0.068701
CS11	SC24	HS3	*R.nitidus*	0.010722	0.066686
JW7	SC18	HS3	*R.nitidus*	0.026877	0.067633
JO7	SC24	HS3	*R.nitidus*	-0.01165	0.07087
JO1	SC18	HS3	*R.nitidus*	-0.050119	0.065504
GD21	SC24	HS3	*R.nitidus*	0.147362	0.064212
GD22	SC24	HS3	*R.nitidus*	0.103106	0.064023
JO8	SC24	HS3	*R.nitidus*	0.023162	0.070544
GD44	SC24	HS3	*R.nitidus*	0.061947	0.065939
GD30	SC24	HS3	*R.nitidus*	0.144984	0.06676
CS13	SC24	HS3	*R.nitidus*	-0.030824	0.067488
GD40	SC24	HS3	*R.nitidus*	0.072222	0.063086
GD45	SC24	HS3	*R.nitidus*	0.060222	0.068027
GD46	SC24	HS3	*R.nitidus*	-0.008397	0.063685
GD32	SC24	HS3	*R.nitidus*	0.055468	0.066309
GD33	SC24	HS3	*R.nitidus*	0.04186	0.064731
GD35	SC24	HS3	*R.nitidus*	0.061841	0.064675
GD42	SC24	HS3	*R.nitidus*	0.041549	0.063755
CS12	SC24	HS3	*R.nitidus*	0.00593	0.066904
GD49	SC24	HS3	*R.nitidus*	0.121309	0.066348
GD26	SC24	HS3	*R.nitidus*	0.086301	0.064445
CS15	SC24	HS3	*R.nitidus*	0.038889	0.065587
GD29	SC24	HS3	*R.nitidus*	0.143132	0.062678
GD31	SC24	HS3	*R.nitidus*	0.082116	0.066134
GD34	SC24	HS3	*R.nitidus*	0.026712	0.064446
CS17	SC24	HS3	*R.nitidus*	0.036126	0.068183
CS18	SC24	HS3	*R.nitidus*	-0.020863	0.065439
GD41	SC24	HS3	*R.nitidus*	0.03244	0.063048
GD48	SC24	HS3	*R.nitidus*	0.037707	0.066554
CS21	SC24	HS3	*R.nitidus*	-0.021629	0.06499
CS19	SC24	HS3	*R.nitidus*	-0.019389	0.066475
CS16	SC24	HS3	*R.nitidus*	0.012793	0.065028
CS28	SC24	HS3	*R.nitidus*	-0.028302	0.065266
GD50	SC24	HS3	*R.nitidus*	0.074897	0.067848
CS23	SC24	HS3	*R.nitidus*	-0.04089	0.064302
CS22	SC24	HS3	*R.nitidus*	0.012857	0.064308
CS31	SC24	HS3	*R.nitidus*	0	0.063904
CS24	SC24	HS3	*R.nitidus*	-0.026686	0.062624
JW6	SC24	HS3	*R.nitidus*	0.015326	0.071013
JO3	SC24	HS3	*R.nitidus*	0.060976	0.068452
SC31	SC24	HS3	*R.nitidus*	0.017203	0.064897

SC6	SC24	HS3	*R.nitidus*	0.012327	0.064645
SC15	SC24	HS3	*R.nitidus*	0.029529	0.065671
CS20	SC24	HS3	*R.nitidus*	-0.020043	0.064649
CS14	SC24	HS3	*R.nitidus*	0.00561	0.066881
CS30	SC24	HS3	*R.nitidus*	-0.046965	0.06574
JO5	SC24	HS3	*R.nitidus*	0.063913	0.063309
JO1	SC24	HS3	*R.nitidus*	0.003026	0.066068
CS25	SC24	HS3	*R.nitidus*	-0.00438	0.065908
GD21	SC26	HS3	*R.nitidus*	0.167404	0.063105
SC45	SC24	HS3	*R.nitidus*	0.048084	0.063256
CS3	SC24	HS3	*R.nitidus*	-0.024096	0.065411
CS4	SC24	HS3	*R.nitidus*	-0.001414	0.063393
SC14	SC24	HS3	*R.nitidus*	0.022438	0.066293
SC12	SC24	HS3	*R.nitidus*	-0.005279	0.064955
SC5	SC24	HS3	*R.nitidus*	0.006314	0.068982
SC26	SC24	HS3	*R.nitidus*	-0.027376	0.064295
JW4	SC24	HS3	*R.nitidus*	0.222062	0.065949
SC10	SC24	HS3	*R.nitidus*	0.09283	0.0655
SC44	SC24	HS3	*R.nitidus*	0.011655	0.064043
JO7	SC26	HS3	*R.nitidus*	0.026364	0.071069
JW7	SC24	HS3	*R.nitidus*	0.064161	0.068354
SC34	SC24	HS3	*R.nitidus*	0.050671	0.062607
GD33	SC26	HS3	*R.nitidus*	0.062331	0.065097
SC27	SC24	HS3	*R.nitidus*	0.017216	0.064995
MY6	SC24	HS3	*R.nitidus*	0.013453	0.064226
SC18	SC24	HS3	*R.nitidus*	0.07332	0.062783
GD25	SC26	HS3	*R.nitidus*	0.093515	0.06499
GD22	SC26	HS3	*R.nitidus*	0.112658	0.06337
MY4	SC24	HS3	*R.nitidus*	-0.030781	0.063565
SC35	SC24	HS3	*R.nitidus*	0.053914	0.063923
JO8	SC26	HS3	*R.nitidus*	0.096805	0.07153
GD35	SC26	HS3	*R.nitidus*	0.068687	0.064298
SC42	SC24	HS3	*R.nitidus*	0.260316	0.060853
GD29	SC26	HS3	*R.nitidus*	0.167519	0.063625
SC32	SC24	HS3	*R.nitidus*	0.068939	0.063467
SC28	SC24	HS3	*R.nitidus*	-0.027523	0.063155
CS6	SC24	HS3	*R.nitidus*	-0.052147	0.063404
CS11	SC26	HS3	*R.nitidus*	0.032328	0.065441
GD41	SC26	HS3	*R.nitidus*	0.042424	0.065708
CS12	SC26	HS3	*R.nitidus*	0.030008	0.066799
GD31	SC26	HS3	*R.nitidus*	0.103641	0.064681
GD34	SC26	HS3	*R.nitidus*	0.038514	0.063745
GD48	SC26	HS3	*R.nitidus*	0.078871	0.067364
GD45	SC26	HS3	*R.nitidus*	0.084848	0.068526
GD44	SC26	HS3	*R.nitidus*	0.065597	0.06629
GD30	SC26	HS3	*R.nitidus*	0.158915	0.06815
GD26	SC26	HS3	*R.nitidus*	0.102931	0.066004
GD32	SC26	HS3	*R.nitidus*	0.062549	0.066964
CS19	SC26	HS3	*R.nitidus*	0.002144	0.065487
GD49	SC26	HS3	*R.nitidus*	0.130233	0.069573
GD42	SC26	HS3	*R.nitidus*	0.05988	0.066374
CS23	SC26	HS3	*R.nitidus*	-0.016678	0.06494
SC18	SC26	HS3	*R.nitidus*	0.084047	0.065224
CS28	SC26	HS3	*R.nitidus*	0.006912	0.067062
GD50	SC26	HS3	*R.nitidus*	0.063218	0.06884
CS13	SC26	HS3	*R.nitidus*	-0.00369	0.066705

GD40	SC26	HS3	*R.nitidus*	0.095111	0.06161
CS17	SC26	HS3	*R.nitidus*	0.049923	0.067019
GD46	SC26	HS3	*R.nitidus*	0.008742	0.06264
SC6	SC26	HS3	*R.nitidus*	0.05019	0.065466
CS25	SC26	HS3	*R.nitidus*	0.017033	0.065842
CS21	SC26	HS3	*R.nitidus*	0.002138	0.063772
SC24	SC26	HS3	*R.nitidus*	0.027376	0.064295
CS20	SC26	HS3	*R.nitidus*	0.005029	0.064893
CS31	SC26	HS3	*R.nitidus*	0.011577	0.064738
CS24	SC26	HS3	*R.nitidus*	-0.010877	0.063399
CS15	SC26	HS3	*R.nitidus*	0.065341	0.065446
CS16	SC26	HS3	*R.nitidus*	0.039079	0.063672
CS3	SC26	HS3	*R.nitidus*	0.007231	0.063577
SC5	SC26	HS3	*R.nitidus*	0.048653	0.068096
SC27	SC26	HS3	*R.nitidus*	0.031832	0.064022
SC14	SC26	HS3	*R.nitidus*	0.045317	0.066149
SC28	SC26	HS3	*R.nitidus*	0.014937	0.062233
SC10	SC26	HS3	*R.nitidus*	0.112142	0.063263
SC15	SC26	HS3	*R.nitidus*	0.050951	0.063411
CS6	SC26	HS3	*R.nitidus*	-0.016035	0.063692
CS14	SC26	HS3	*R.nitidus*	0.034653	0.066104
CS30	SC26	HS3	*R.nitidus*	-0.021567	0.064567
MY4	SC26	HS3	*R.nitidus*	0.005727	0.064111
CS18	SC26	HS3	*R.nitidus*	-0.004237	0.064222
SC45	SC26	HS3	*R.nitidus*	0.082222	0.064972
JW4	SC26	HS3	*R.nitidus*	0.248954	0.064346
CS22	SC26	HS3	*R.nitidus*	0.032258	0.064878
CS4	SC26	HS3	*R.nitidus*	0.016238	0.061762
JO1	SC26	HS3	*R.nitidus*	0.021505	0.064198
SC31	SC26	HS3	*R.nitidus*	0.05136	0.064293
JW6	SC26	HS3	*R.nitidus*	0.048598	0.070005
SC12	SC26	HS3	*R.nitidus*	0.025738	0.063762
SC34	SC26	HS3	*R.nitidus*	0.077913	0.061826
JW7	SC26	HS3	*R.nitidus*	0.099543	0.067833
SC42	SC26	HS3	*R.nitidus*	0.273364	0.061142
SC35	SC26	HS3	*R.nitidus*	0.090976	0.06216
SC32	SC26	HS3	*R.nitidus*	0.086596	0.063361
MY6	SC26	HS3	*R.nitidus*	0.033934	0.063675
JO3	SC26	HS3	*R.nitidus*	0.085627	0.067703
SC44	SC26	HS3	*R.nitidus*	0.03997	0.06512
CS12	SC27	HS3	*R.nitidus*	-0.000757	0.066341
GD33	SC27	HS3	*R.nitidus*	0.026473	0.064458
GD26	SC27	HS3	*R.nitidus*	0.072464	0.064556
GD35	SC27	HS3	*R.nitidus*	0.050104	0.064907
JO7	SC27	HS3	*R.nitidus*	-0.010223	0.071346
GD29	SC27	HS3	*R.nitidus*	0.137662	0.063051
GD42	SC27	HS3	*R.nitidus*	0.033403	0.063667
GD30	SC27	HS3	*R.nitidus*	0.133125	0.066408
GD22	SC27	HS3	*R.nitidus*	0.090102	0.06204
JO5	SC26	HS3	*R.nitidus*	0.089325	0.062443
JO8	SC27	HS3	*R.nitidus*	0.021926	0.070437
GD44	SC27	HS3	*R.nitidus*	0.04717	0.066361
GD25	SC27	HS3	*R.nitidus*	0.076139	0.063005
GD34	SC27	HS3	*R.nitidus*	0.011394	0.065055
GD40	SC27	HS3	*R.nitidus*	0.070199	0.061758
CS14	SC27	HS3	*R.nitidus*	0.009279	0.065548

GD21	SC27	HS3	*R.nitidus*	0.133721	0.063823
GD49	SC27	HS3	*R.nitidus*	0.090208	0.066645
CS16	SC27	HS3	*R.nitidus*	0.007857	0.064264
CS11	SC27	HS3	*R.nitidus*	0.011603	0.066746
GD41	SC27	HS3	*R.nitidus*	0.024458	0.061772
CS20	SC27	HS3	*R.nitidus*	-0.024255	0.064658
CS19	SC27	HS3	*R.nitidus*	-0.028302	0.066649
GD31	SC27	HS3	*R.nitidus*	0.061841	0.065257
GD32	SC27	HS3	*R.nitidus*	0.035101	0.067273
GD50	SC27	HS3	*R.nitidus*	0.05892	0.065127
CS28	SC27	HS3	*R.nitidus*	-0.026688	0.064697
CS23	SC27	HS3	*R.nitidus*	-0.045007	0.064378
GD45	SC27	HS3	*R.nitidus*	0.05919	0.068904
CS31	SC27	HS3	*R.nitidus*	-0.012821	0.064302
SC6	SC27	HS3	*R.nitidus*	0.013564	0.065412
CS21	SC27	HS3	*R.nitidus*	-0.028031	0.064553
CS4	SC27	HS3	*R.nitidus*	-0.008978	0.062702
CS30	SC27	HS3	*R.nitidus*	-0.053424	0.063465
GD48	SC27	HS3	*R.nitidus*	0.037472	0.065971
GD46	SC27	HS3	*R.nitidus*	-0.00755	0.063566
CS13	SC27	HS3	*R.nitidus*	-0.03693	0.066827
SC15	SC27	HS3	*R.nitidus*	0.017101	0.063906
CS24	SC27	HS3	*R.nitidus*	-0.036838	0.062854
CS15	SC27	HS3	*R.nitidus*	0.046132	0.065422
CS3	SC27	HS3	*R.nitidus*	-0.013514	0.063091
SC26	SC27	HS3	*R.nitidus*	-0.031832	0.064022
SC31	SC27	HS3	*R.nitidus*	0.012792	0.064371
CS18	SC27	HS3	*R.nitidus*	-0.034934	0.064366
CS17	SC27	HS3	*R.nitidus*	0.048069	0.067272
SC18	SC27	HS3	*R.nitidus*	0.038043	0.065225
MY6	SC27	HS3	*R.nitidus*	0.006298	0.063803
SC24	SC27	HS3	*R.nitidus*	-0.017216	0.064995
JW7	SC27	HS3	*R.nitidus*	0.05345	0.067525
SC34	SC27	HS3	*R.nitidus*	0.045107	0.062927
CS22	SC27	HS3	*R.nitidus*	-0.006637	0.064065
CS25	SC27	HS3	*R.nitidus*	-0.005032	0.066376
CS6	SC27	HS3	*R.nitidus*	-0.04974	0.062426
JW4	SC27	HS3	*R.nitidus*	0.203263	0.065125
SC12	SC27	HS3	*R.nitidus*	-0.013535	0.064181
MY4	SC27	HS3	*R.nitidus*	-0.032212	0.064839
SC35	SC27	HS3	*R.nitidus*	0.058647	0.062844
SC44	SC27	HS3	*R.nitidus*	0.006187	0.062609
GD21	SC28	HS3	*R.nitidus*	0.15856	0.063453
SC14	SC27	HS3	*R.nitidus*	0.020555	0.06142
SC10	SC27	HS3	*R.nitidus*	0.078125	0.063544
JO7	SC28	HS3	*R.nitidus*	-0.013133	0.070762
CS11	SC28	HS3	*R.nitidus*	0.031052	0.065285
JO1	SC27	HS3	*R.nitidus*	-0.00074	0.063957
SC42	SC27	HS3	*R.nitidus*	0.263777	0.062086
SC28	SC27	HS3	*R.nitidus*	-0.031931	0.06347
JO8	SC28	HS3	*R.nitidus*	0.04467	0.071158
SC45	SC27	HS3	*R.nitidus*	0.031137	0.064902
SC5	SC27	HS3	*R.nitidus*	0.007698	0.068261
JO3	SC27	HS3	*R.nitidus*	0.03622	0.068382
GD22	SC28	HS3	*R.nitidus*	0.123077	0.063095
GD32	SC28	HS3	*R.nitidus*	0.056	0.06587

GD25	SC28	HS3	*R.nitidus*	0.090226	0.063935
SC32	SC27	HS3	*R.nitidus*	0.05456	0.063534
JW6	SC27	HS3	*R.nitidus*	0.014045	0.069823
JO5	SC27	HS3	*R.nitidus*	0.053118	0.065088
GD26	SC28	HS3	*R.nitidus*	0.098572	0.063754
GD40	SC28	HS3	*R.nitidus*	0.09825	0.06306
GD44	SC28	HS3	*R.nitidus*	0.072674	0.065887
GD33	SC28	HS3	*R.nitidus*	0.05	0.064194
GD34	SC28	HS3	*R.nitidus*	0.04192	0.062802
GD42	SC28	HS3	*R.nitidus*	0.066482	0.065435
CS19	SC28	HS3	*R.nitidus*	-0.000708	0.065596
GD31	SC28	HS3	*R.nitidus*	0.101418	0.06526
CS21	SC28	HS3	*R.nitidus*	-0.008451	0.064345
GD49	SC28	HS3	*R.nitidus*	0.120556	0.065156
GD29	SC28	HS3	*R.nitidus*	0.162655	0.064262
GD30	SC28	HS3	*R.nitidus*	0.159285	0.065668
GD35	SC28	HS3	*R.nitidus*	0.077922	0.064927
CS14	SC28	HS3	*R.nitidus*	0.026391	0.065514
CS13	SC28	HS3	*R.nitidus*	0.000733	0.06568
GD45	SC28	HS3	*R.nitidus*	0.090548	0.067275
GD41	SC28	HS3	*R.nitidus*	0.045839	0.064354
CS18	SC28	HS3	*R.nitidus*	-0.005654	0.065062
CS16	SC28	HS3	*R.nitidus*	0.046999	0.064272
CS20	SC28	HS3	*R.nitidus*	-0.001379	0.063959
CS31	SC28	HS3	*R.nitidus*	0.010324	0.063338
CS3	SC28	HS3	*R.nitidus*	0.01231	0.063269
CS15	SC28	HS3	*R.nitidus*	0.067606	0.064025
CS12	SC28	HS3	*R.nitidus*	0.039245	0.065414
SC15	SC28	HS3	*R.nitidus*	0.041602	0.065257
SC10	SC28	HS3	*R.nitidus*	0.123525	0.063745
CS17	SC28	HS3	*R.nitidus*	0.063028	0.066546
GD50	SC28	HS3	*R.nitidus*	0.071661	0.066704
GD48	SC28	HS3	*R.nitidus*	0.062044	0.067031
CS23	SC28	HS3	*R.nitidus*	-0.006272	0.064311
GD46	SC28	HS3	*R.nitidus*	0.020988	0.062458
SC26	SC28	HS3	*R.nitidus*	-0.014937	0.062233
SC5	SC28	HS3	*R.nitidus*	0.030782	0.066163
SC24	SC28	HS3	*R.nitidus*	0.027523	0.063155
CS28	SC28	HS3	*R.nitidus*	-0.007849	0.064033
CS4	SC28	HS3	*R.nitidus*	0.011127	0.062842
CS25	SC28	HS3	*R.nitidus*	0.026429	0.06604
CS24	SC28	HS3	*R.nitidus*	-0.018248	0.064618
SC6	SC28	HS3	*R.nitidus*	0.052632	0.065707
JW4	SC28	HS3	*R.nitidus*	0.238128	0.065183
CS22	SC28	HS3	*R.nitidus*	0.019737	0.063741
SC32	SC28	HS3	*R.nitidus*	0.086989	0.062775
SC34	SC28	HS3	*R.nitidus*	0.069666	0.062495
SC31	SC28	HS3	*R.nitidus*	0.037175	0.063187
SC27	SC28	HS3	*R.nitidus*	0.031931	0.06347
SC18	SC28	HS3	*R.nitidus*	0.086957	0.060938
CS30	SC28	HS3	*R.nitidus*	-0.023055	0.062789
SC12	SC28	HS3	*R.nitidus*	0.00939	0.064408
MY4	SC28	HS3	*R.nitidus*	-0.00578	0.06386
MY6	SC28	HS3	*R.nitidus*	0.0266	0.0618
SC44	SC28	HS3	*R.nitidus*	0.048534	0.05883
SC14	SC28	HS3	*R.nitidus*	0.039574	0.064444

SC35	SC28	HS3	*R.nitidus*	0.080029	0.062076
JW6	SC28	HS3	*R.nitidus*	0.018727	0.071786
GD25	SC31	HS3	*R.nitidus*	0.057026	0.063923
SC45	SC28	HS3	*R.nitidus*	0.053184	0.06352
SC42	SC28	HS3	*R.nitidus*	0.28737	0.060368
CS6	SC28	HS3	*R.nitidus*	-0.011967	0.061592
GD40	SC31	HS3	*R.nitidus*	0.06369	0.06458
JO5	SC28	HS3	*R.nitidus*	0.098993	0.061594
JW7	SC28	HS3	*R.nitidus*	0.074586	0.06706
CS11	SC31	HS3	*R.nitidus*	-0.006246	0.065456
GD46	SC31	HS3	*R.nitidus*	-0.015464	0.06321
GD32	SC31	HS3	*R.nitidus*	0.018911	0.068192
GD22	SC31	HS3	*R.nitidus*	0.08516	0.06445
GD33	SC31	HS3	*R.nitidus*	0.027388	0.066623
JO1	SC28	HS3	*R.nitidus*	0.001542	0.06415
JO3	SC28	HS3	*R.nitidus*	0.054988	0.067292
JO8	SC31	HS3	*R.nitidus*	-0.001867	0.071999
GD26	SC31	HS3	*R.nitidus*	0.056063	0.065062
GD30	SC31	HS3	*R.nitidus*	0.119301	0.067743
JO7	SC31	HS3	*R.nitidus*	-0.00878	0.071507
GD31	SC31	HS3	*R.nitidus*	0.062587	0.066447
GD29	SC31	HS3	*R.nitidus*	0.117978	0.06373
GD34	SC31	HS3	*R.nitidus*	0.000668	0.064305
GD48	SC31	HS3	*R.nitidus*	0.033213	0.068152
CS16	SC31	HS3	*R.nitidus*	0.012363	0.06512
GD21	SC31	HS3	*R.nitidus*	0.124538	0.064281
CS14	SC31	HS3	*R.nitidus*	-0.011604	0.065529
GD49	SC31	HS3	*R.nitidus*	0.100154	0.06889
CS12	SC31	HS3	*R.nitidus*	-0.007267	0.068294
GD35	SC31	HS3	*R.nitidus*	0.037231	0.065041
GD44	SC31	HS3	*R.nitidus*	0.019661	0.06535
GD42	SC31	HS3	*R.nitidus*	0.03148	0.066201
CS19	SC31	HS3	*R.nitidus*	-0.030683	0.065447
CS30	SC31	HS3	*R.nitidus*	-0.06514	0.06561
CS13	SC31	HS3	*R.nitidus*	-0.044881	0.067508
GD45	SC31	HS3	*R.nitidus*	0.04006	0.069621
GD41	SC31	HS3	*R.nitidus*	0.002026	0.063691
CS18	SC31	HS3	*R.nitidus*	-0.037011	0.06587
SC10	SC31	HS3	*R.nitidus*	0.065485	0.065363
CS23	SC31	HS3	*R.nitidus*	-0.058123	0.063545
CS20	SC31	HS3	*R.nitidus*	-0.028229	0.063215
GD50	SC31	HS3	*R.nitidus*	0.008648	0.069072
CS17	SC31	HS3	*R.nitidus*	0.025934	0.067638
CS25	SC31	HS3	*R.nitidus*	-0.020308	0.06501
CS21	SC31	HS3	*R.nitidus*	-0.045324	0.06442
CS28	SC31	HS3	*R.nitidus*	-0.041471	0.065893
SC34	SC31	HS3	*R.nitidus*	0.034043	0.065483
CS31	SC31	HS3	*R.nitidus*	-0.033984	0.065333
CS3	SC31	HS3	*R.nitidus*	-0.019355	0.063842
SC12	SC31	HS3	*R.nitidus*	-0.022367	0.067129
SC27	SC31	HS3	*R.nitidus*	-0.012792	0.064371
CS15	SC31	HS3	*R.nitidus*	0.02421	0.065256
CS22	SC31	HS3	*R.nitidus*	-0.011315	0.0647
SC24	SC31	HS3	*R.nitidus*	-0.017203	0.064897
SC26	SC31	HS3	*R.nitidus*	-0.05136	0.064293
CS24	SC31	HS3	*R.nitidus*	-0.055197	0.06364

SC14	SC31	HS3	*R.nitidus*	0.003023	0.066791
SC18	SC31	HS3	*R.nitidus*	0.033411	0.064587
SC42	SC31	HS3	*R.nitidus*	0.231228	0.062186
SC32	SC31	HS3	*R.nitidus*	0.047302	0.06506
SC44	SC31	HS3	*R.nitidus*	-0.00074	0.064203
JO1	SC31	HS3	*R.nitidus*	-0.016899	0.063422
JW4	SC31	HS3	*R.nitidus*	0.222378	0.067353
SC5	SC31	HS3	*R.nitidus*	-0.000744	0.068024
JO8	SC32	HS3	*R.nitidus*	0.009597	0.071602
SC6	SC31	HS3	*R.nitidus*	-0.013393	0.066732
GD29	SC32	HS3	*R.nitidus*	0.108892	0.064187
JO3	SC31	HS3	*R.nitidus*	0.039413	0.068806
JW6	SC31	HS3	*R.nitidus*	0.018568	0.071632
CS4	SC31	HS3	*R.nitidus*	-0.020181	0.062518
MY6	SC31	HS3	*R.nitidus*	0.000704	0.065537
SC28	SC31	HS3	*R.nitidus*	-0.037175	0.063187
JW7	SC31	HS3	*R.nitidus*	0.066545	0.066268
MY4	SC31	HS3	*R.nitidus*	-0.037966	0.065349
SC15	SC31	HS3	*R.nitidus*	0	0.065457
SC35	SC31	HS3	*R.nitidus*	0.032329	0.063898
SC45	SC31	HS3	*R.nitidus*	0.021939	0.064661
CS6	SC31	HS3	*R.nitidus*	-0.04964	0.065439
GD22	SC32	HS3	*R.nitidus*	0.044739	0.063132
JO5	SC31	HS3	*R.nitidus*	0.040146	0.063683
CS11	SC32	HS3	*R.nitidus*	-0.054913	0.068564
GD21	SC32	HS3	*R.nitidus*	0.090495	0.064917
GD34	SC32	HS3	*R.nitidus*	-0.030687	0.064605
JO7	SC32	HS3	*R.nitidus*	-0.051011	0.070176
GD31	SC32	HS3	*R.nitidus*	0.021636	0.065623
GD33	SC32	HS3	*R.nitidus*	-0.022957	0.065721
CS17	SC32	HS3	*R.nitidus*	-0.030612	0.070774
GD26	SC32	HS3	*R.nitidus*	0.026864	0.064474
GD44	SC32	HS3	*R.nitidus*	-0.016878	0.06708
GD45	SC32	HS3	*R.nitidus*	0.003934	0.068447
GD46	SC32	HS3	*R.nitidus*	-0.058944	0.06438
GD25	SC32	HS3	*R.nitidus*	0.02008	0.064165
GD42	SC32	HS3	*R.nitidus*	-0.017906	0.063991
GD30	SC32	HS3	*R.nitidus*	0.086381	0.068131
GD40	SC32	HS3	*R.nitidus*	0.023825	0.063623
GD48	SC32	HS3	*R.nitidus*	0.005216	0.067817
CS13	SC32	HS3	*R.nitidus*	-0.086207	0.069521
CS16	SC32	HS3	*R.nitidus*	-0.040426	0.066686
GD32	SC32	HS3	*R.nitidus*	-0.005587	0.067517
GD50	SC32	HS3	*R.nitidus*	-0.002402	0.070041
CS15	SC32	HS3	*R.nitidus*	-0.020125	0.067868
CS12	SC32	HS3	*R.nitidus*	-0.049401	0.069324
CS18	SC32	HS3	*R.nitidus*	-0.084709	0.065866
GD41	SC32	HS3	*R.nitidus*	-0.030096	0.062484
GD35	SC32	HS3	*R.nitidus*	-0.008021	0.065171
CS24	SC32	HS3	*R.nitidus*	-0.101515	0.064336
CS19	SC32	HS3	*R.nitidus*	-0.077827	0.067736
SC6	SC32	HS3	*R.nitidus*	-0.045593	0.064771
CS23	SC32	HS3	*R.nitidus*	-0.099024	0.06577
CS14	SC32	HS3	*R.nitidus*	-0.056769	0.06991
SC5	SC32	HS3	*R.nitidus*	-0.045942	0.067431
SC15	SC32	HS3	*R.nitidus*	-0.042755	0.06617

CS20	SC32	HS3	*R.nitidus*	-0.078947	0.064992
CS21	SC32	HS3	*R.nitidus*	-0.085999	0.063978
CS31	SC32	HS3	*R.nitidus*	-0.074866	0.067295
CS30	SC32	HS3	*R.nitidus*	-0.108728	0.06624
CS25	SC32	HS3	*R.nitidus*	-0.063937	0.068769
GD49	SC32	HS3	*R.nitidus*	0.058366	0.068381
SC35	SC32	HS3	*R.nitidus*	-0.010463	0.062343
SC12	SC32	HS3	*R.nitidus*	-0.074074	0.066324
SC26	SC32	HS3	*R.nitidus*	-0.086596	0.063361
MY4	SC32	HS3	*R.nitidus*	-0.084328	0.064629
SC18	SC32	HS3	*R.nitidus*	-0.011164	0.062895
CS6	SC32	HS3	*R.nitidus*	-0.101996	0.063954
SC28	SC32	HS3	*R.nitidus*	-0.086989	0.062775
JW7	SC32	HS3	*R.nitidus*	0.021154	0.068806
SC10	SC32	HS3	*R.nitidus*	0.018639	0.064491
CS28	SC32	HS3	*R.nitidus*	-0.095351	0.066547
CS22	SC32	HS3	*R.nitidus*	-0.060044	0.064739
SC34	SC32	HS3	*R.nitidus*	-0.002217	0.063364
CS4	SC32	HS3	*R.nitidus*	-0.061441	0.062775
JW4	SC32	HS3	*R.nitidus*	0.185501	0.064808
JO1	SC32	HS3	*R.nitidus*	-0.060139	0.065423
MY6	SC32	HS3	*R.nitidus*	-0.040656	0.064046
CS3	SC32	HS3	*R.nitidus*	-0.078723	0.064911
GD22	SC34	HS3	*R.nitidus*	0.052566	0.063072
SC24	SC32	HS3	*R.nitidus*	-0.068939	0.063467
SC14	SC32	HS3	*R.nitidus*	-0.043028	0.066594
SC27	SC32	HS3	*R.nitidus*	-0.05456	0.063534
SC44	SC32	HS3	*R.nitidus*	-0.04465	0.063957
GD30	SC34	HS3	*R.nitidus*	0.075818	0.067339
SC42	SC32	HS3	*R.nitidus*	0.23689	0.062328
JO8	SC34	HS3	*R.nitidus*	0.023386	0.070326
GD34	SC34	HS3	*R.nitidus*	-0.025049	0.064825
SC31	SC32	HS3	*R.nitidus*	-0.047302	0.06506
GD40	SC34	HS3	*R.nitidus*	0.02988	0.062352
JO5	SC32	HS3	*R.nitidus*	0.006461	0.063502
SC45	SC32	HS3	*R.nitidus*	-0.019912	0.065642
GD33	SC34	HS3	*R.nitidus*	-0.000662	0.063729
CS12	SC34	HS3	*R.nitidus*	-0.026198	0.064644
JW6	SC32	HS3	*R.nitidus*	-0.026465	0.070039
GD31	SC34	HS3	*R.nitidus*	0.031989	0.066101
JO7	SC34	HS3	*R.nitidus*	-0.027753	0.068113
JO3	SC32	HS3	*R.nitidus*	-0.007893	0.068483
GD29	SC34	HS3	*R.nitidus*	0.079818	0.063481
GD45	SC34	HS3	*R.nitidus*	0.014331	0.067464
GD25	SC34	HS3	*R.nitidus*	0.025589	0.063295
CS13	SC34	HS3	*R.nitidus*	-0.06744	0.068152
CS11	SC34	HS3	*R.nitidus*	-0.03678	0.066556
GD42	SC34	HS3	*R.nitidus*	-0.004118	0.06537
GD48	SC34	HS3	*R.nitidus*	0.008708	0.066791
GD35	SC34	HS3	*R.nitidus*	0.020353	0.064418
CS23	SC34	HS3	*R.nitidus*	-0.081476	0.063062
GD21	SC34	HS3	*R.nitidus*	0.101313	0.06475
CS14	SC34	HS3	*R.nitidus*	-0.040084	0.066235
GD26	SC34	HS3	*R.nitidus*	0.033686	0.064098
GD41	SC34	HS3	*R.nitidus*	-0.024624	0.063935
CS20	SC34	HS3	*R.nitidus*	-0.062331	0.063326

GD32	SC34	HS3	*R.nitidus*	0.015361	0.067242
CS30	SC34	HS3	*R.nitidus*	-0.093007	0.063054
CS16	SC34	HS3	*R.nitidus*	-0.019485	0.064886
GD46	SC34	HS3	*R.nitidus*	-0.049698	0.063343
CS15	SC34	HS3	*R.nitidus*	-0.006878	0.065603
CS24	SC34	HS3	*R.nitidus*	-0.08651	0.061504
CS18	SC34	HS3	*R.nitidus*	-0.072843	0.06457
GD50	SC34	HS3	*R.nitidus*	-0.020783	0.065533
CS21	SC34	HS3	*R.nitidus*	-0.061892	0.062866
GD49	SC34	HS3	*R.nitidus*	0.044479	0.065415
SC5	SC34	HS3	*R.nitidus*	-0.027437	0.065899
CS28	SC34	HS3	*R.nitidus*	-0.077677	0.065917
GD44	SC34	HS3	*R.nitidus*	-0.006263	0.066294
SC24	SC34	HS3	*R.nitidus*	-0.050671	0.062607
CS17	SC34	HS3	*R.nitidus*	-0.005271	0.067774
SC14	SC34	HS3	*R.nitidus*	-0.033133	0.06505
CS4	SC34	HS3	*R.nitidus*	-0.051713	0.061036
CS19	SC34	HS3	*R.nitidus*	-0.066431	0.065869
CS6	SC34	HS3	*R.nitidus*	-0.07164	0.063185
MY4	SC34	HS3	*R.nitidus*	-0.082202	0.06418
CS22	SC34	HS3	*R.nitidus*	-0.038301	0.06198
SC26	SC34	HS3	*R.nitidus*	-0.077913	0.061826
SC28	SC34	HS3	*R.nitidus*	-0.069666	0.062495
CS31	SC34	HS3	*R.nitidus*	-0.056318	0.06417
CS3	SC34	HS3	*R.nitidus*	-0.060056	0.063544
JW4	SC34	HS3	*R.nitidus*	0.189268	0.063247
SC6	SC34	HS3	*R.nitidus*	-0.029499	0.065762
SC35	SC34	HS3	*R.nitidus*	0.011722	0.062508
SC10	SC34	HS3	*R.nitidus*	0.020553	0.064269
SC42	SC34	HS3	*R.nitidus*	0.201846	0.060632
SC15	SC34	HS3	*R.nitidus*	-0.028594	0.065167
SC12	SC34	HS3	*R.nitidus*	-0.061178	0.064258
SC18	SC34	HS3	*R.nitidus*	0.000755	0.063799
CS25	SC34	HS3	*R.nitidus*	-0.042509	0.066025
SC32	SC34	HS3	*R.nitidus*	0.002217	0.063364
JO7	SC35	HS3	*R.nitidus*	-0.020833	0.069936
JO5	SC34	HS3	*R.nitidus*	0.017544	0.064836
SC27	SC34	HS3	*R.nitidus*	-0.045107	0.062927
SC31	SC34	HS3	*R.nitidus*	-0.034043	0.065483
MY6	SC34	HS3	*R.nitidus*	-0.03662	0.06266
CS11	SC35	HS3	*R.nitidus*	-0.042936	0.064651
GD31	SC35	HS3	*R.nitidus*	0.035565	0.064653
GD32	SC35	HS3	*R.nitidus*	-0.022481	0.066062
GD21	SC35	HS3	*R.nitidus*	0.092308	0.064481
SC44	SC34	HS3	*R.nitidus*	-0.045353	0.061771
GD30	SC35	HS3	*R.nitidus*	0.072699	0.066726
JW6	SC34	HS3	*R.nitidus*	-0.01629	0.067602
GD26	SC35	HS3	*R.nitidus*	0.031735	0.063835
SC45	SC34	HS3	*R.nitidus*	-0.002094	0.065611
GD41	SC35	HS3	*R.nitidus*	-0.031123	0.063401
GD44	SC35	HS3	*R.nitidus*	-0.013268	0.065005
JW7	SC34	HS3	*R.nitidus*	0.001762	0.066787
JO8	SC35	HS3	*R.nitidus*	0.007859	0.071451
GD25	SC35	HS3	*R.nitidus*	0.026518	0.065148
GD35	SC35	HS3	*R.nitidus*	0.011757	0.065884
GD33	SC35	HS3	*R.nitidus*	-0.005913	0.065595

CS12	SC35	HS3	*R.nitidus*	-0.047109	0.06528
GD22	SC35	HS3	*R.nitidus*	0.042513	0.062535
GD40	SC35	HS3	*R.nitidus*	0.020039	0.062428
GD34	SC35	HS3	*R.nitidus*	-0.028689	0.06488
GD29	SC35	HS3	*R.nitidus*	0.08447	0.063507
JO1	SC34	HS3	*R.nitidus*	-0.042385	0.064013
CS14	SC35	HS3	*R.nitidus*	-0.048309	0.065875
JO3	SC34	HS3	*R.nitidus*	0.031153	0.06756
CS16	SC35	HS3	*R.nitidus*	-0.041497	0.063884
GD46	SC35	HS3	*R.nitidus*	-0.061486	0.062027
CS15	SC35	HS3	*R.nitidus*	-0.014122	0.065611
GD48	SC35	HS3	*R.nitidus*	-0.015407	0.066855
GD42	SC35	HS3	*R.nitidus*	-0.006028	0.06392
CS20	SC35	HS3	*R.nitidus*	-0.071864	0.062887
CS21	SC35	HS3	*R.nitidus*	-0.087596	0.063733
CS17	SC35	HS3	*R.nitidus*	-0.014728	0.06657
GD50	SC35	HS3	*R.nitidus*	-0.004934	0.066096
GD45	SC35	HS3	*R.nitidus*	0.010828	0.067837
GD49	SC35	HS3	*R.nitidus*	0.032357	0.067722
CS25	SC35	HS3	*R.nitidus*	-0.052999	0.064234
SC26	SC35	HS3	*R.nitidus*	-0.090976	0.06216
SC12	SC35	HS3	*R.nitidus*	-0.073263	0.06194
CS13	SC35	HS3	*R.nitidus*	-0.08042	0.066423
CS22	SC35	HS3	*R.nitidus*	-0.052399	0.062158
SC27	SC35	HS3	*R.nitidus*	-0.058647	0.062844
SC24	SC35	HS3	*R.nitidus*	-0.053914	0.063923
CS24	SC35	HS3	*R.nitidus*	-0.091445	0.060943
SC18	SC35	HS3	*R.nitidus*	-0.003794	0.062996
CS30	SC35	HS3	*R.nitidus*	-0.09824	0.063103
SC10	SC35	HS3	*R.nitidus*	0.024143	0.065212
SC5	SC35	HS3	*R.nitidus*	-0.03532	0.066304
CS4	SC35	HS3	*R.nitidus*	-0.049374	0.063754
SC44	SC35	HS3	*R.nitidus*	-0.052006	0.062498
CS23	SC35	HS3	*R.nitidus*	-0.098053	0.063833
CS31	SC35	HS3	*R.nitidus*	-0.061269	0.063344
CS28	SC35	HS3	*R.nitidus*	-0.057927	0.065556
CS19	SC35	HS3	*R.nitidus*	-0.08	0.064518
CS18	SC35	HS3	*R.nitidus*	-0.077739	0.063009
JW4	SC35	HS3	*R.nitidus*	0.201285	0.06459
CS3	SC35	HS3	*R.nitidus*	-0.062632	0.063193
CS11	CS6	HS3	*R.nitidus*	0.03976	0.066183
SC42	SC35	HS3	*R.nitidus*	0.216135	0.062333
SC15	SC35	HS3	*R.nitidus*	-0.035276	0.065009
MY6	SC35	HS3	*R.nitidus*	-0.045259	0.063045
JW6	SC35	HS3	*R.nitidus*	-0.01275	0.069914
CS6	SC35	HS3	*R.nitidus*	-0.093796	0.062705
SC6	SC35	HS3	*R.nitidus*	-0.038404	0.065266
JO7	CS6	HS3	*R.nitidus*	0.053309	0.069003
SC45	SC35	HS3	*R.nitidus*	-0.008869	0.063411
JW7	SC35	HS3	*R.nitidus*	0.017148	0.065858
GD26	CS6	HS3	*R.nitidus*	0.106134	0.064547
GD29	CS6	HS3	*R.nitidus*	0.164894	0.063296
SC14	SC35	HS3	*R.nitidus*	-0.040323	0.063832
SC31	SC35	HS3	*R.nitidus*	-0.032329	0.063898
SC32	SC35	HS3	*R.nitidus*	0.010463	0.062343
SC34	SC35	HS3	*R.nitidus*	-0.011722	0.062508

JO1	SC35	HS3	*R.nitidus*	-0.06706	0.064014
SC28	SC35	HS3	*R.nitidus*	-0.080029	0.062076
GD25	CS6	HS3	*R.nitidus*	0.100072	0.063991
GD22	CS6	HS3	*R.nitidus*	0.137135	0.06473
MY4	SC35	HS3	*R.nitidus*	-0.089036	0.063403
JO8	CS6	HS3	*R.nitidus*	0.079412	0.071715
JO5	SC35	HS3	*R.nitidus*	0.005011	0.062537
JO3	SC35	HS3	*R.nitidus*	0.014343	0.068453
GD40	CS6	HS3	*R.nitidus*	0.11354	0.062852
GD41	CS6	HS3	*R.nitidus*	0.058123	0.062694
GD32	CS6	HS3	*R.nitidus*	0.09736	0.067596
GD42	CS6	HS3	*R.nitidus*	0.082686	0.064424
GD30	CS6	HS3	*R.nitidus*	0.159514	0.066657
GD21	CS6	HS3	*R.nitidus*	0.182361	0.062959
GD49	CS6	HS3	*R.nitidus*	0.125828	0.067477
CS22	CS6	HS3	*R.nitidus*	0.043001	0.061692
GD44	CS6	HS3	*R.nitidus*	0.08659	0.065662
CS20	CS6	HS3	*R.nitidus*	0.0317	0.063057
GD31	CS6	HS3	*R.nitidus*	0.115187	0.067097
GD35	CS6	HS3	*R.nitidus*	0.099436	0.065833
CS17	CS6	HS3	*R.nitidus*	0.046952	0.067787
CS25	CS6	HS3	*R.nitidus*	0.049331	0.065989
GD48	CS6	HS3	*R.nitidus*	0.085714	0.066467
GD33	CS6	HS3	*R.nitidus*	0.077135	0.064702
GD50	CS6	HS3	*R.nitidus*	0.065657	0.0691
CS21	CS6	HS3	*R.nitidus*	0.022645	0.064497
CS23	CS6	HS3	*R.nitidus*	-0.004344	0.064367
CS12	CS6	HS3	*R.nitidus*	0.054033	0.065593
GD45	CS6	HS3	*R.nitidus*	0.074935	0.069642
CS14	CS6	HS3	*R.nitidus*	0.053333	0.066303
CS3	CS6	HS3	*R.nitidus*	0.02997	0.060522
CS28	CS6	HS3	*R.nitidus*	0.007347	0.0664
GD34	CS6	HS3	*R.nitidus*	0.052047	0.065053
SC26	CS6	HS3	*R.nitidus*	0.016035	0.063692
CS13	CS6	HS3	*R.nitidus*	0.009797	0.068003
SC12	CS6	HS3	*R.nitidus*	0.043511	0.065147
SC10	CS6	HS3	*R.nitidus*	0.106015	0.064994
CS30	CS6	HS3	*R.nitidus*	-0.013037	0.065214
CS4	CS6	HS3	*R.nitidus*	0.025893	0.062617
SC14	CS6	HS3	*R.nitidus*	0.058692	0.063936
CS24	CS6	HS3	*R.nitidus*	0.002527	0.063938
SC6	CS6	HS3	*R.nitidus*	0.052356	0.06402
SC15	CS6	HS3	*R.nitidus*	0.065134	0.065244
SC31	CS6	HS3	*R.nitidus*	0.04964	0.065439
CS15	CS6	HS3	*R.nitidus*	0.077372	0.066544
CS16	CS6	HS3	*R.nitidus*	0.053064	0.063746
CS19	CS6	HS3	*R.nitidus*	0.02381	0.066926
JW4	CS6	HS3	*R.nitidus*	0.238335	0.064341
GD46	CS6	HS3	*R.nitidus*	0.027933	0.064323
CS18	CS6	HS3	*R.nitidus*	0.016202	0.064229
JO3	CS6	HS3	*R.nitidus*	0.106883	0.067581
SC5	CS6	HS3	*R.nitidus*	0.056245	0.066756
CS31	CS6	HS3	*R.nitidus*	0.022256	0.065351
SC45	CS6	HS3	*R.nitidus*	0.077206	0.064746
SC42	CS6	HS3	*R.nitidus*	0.264799	0.060641
SC27	CS6	HS3	*R.nitidus*	0.04974	0.062426

JO1	CS6	HS3	*R.nitidus*	0.034307	0.066582
GD30	SC42	HS3	*R.nitidus*	-0.116917	0.06389
SC44	CS6	HS3	*R.nitidus*	0.056231	0.061862
JW7	CS6	HS3	*R.nitidus*	0.089578	0.066816
SC18	CS6	HS3	*R.nitidus*	0.094384	0.062775
MY4	CS6	HS3	*R.nitidus*	0.006011	0.065373
JW6	CS6	HS3	*R.nitidus*	0.05893	0.069773
SC24	CS6	HS3	*R.nitidus*	0.052147	0.063404
SC35	CS6	HS3	*R.nitidus*	0.093796	0.062705
SC28	CS6	HS3	*R.nitidus*	0.011967	0.061592
SC32	CS6	HS3	*R.nitidus*	0.101996	0.063954
GD33	SC42	HS3	*R.nitidus*	-0.199027	0.064585
JO7	SC42	HS3	*R.nitidus*	-0.192216	0.066075
GD35	SC42	HS3	*R.nitidus*	-0.201865	0.063899
GD40	SC42	HS3	*R.nitidus*	-0.170366	0.062278
MY6	CS6	HS3	*R.nitidus*	0.046579	0.065425
SC34	CS6	HS3	*R.nitidus*	0.07164	0.063185
CS11	SC42	HS3	*R.nitidus*	-0.231973	0.061135
JO8	SC42	HS3	*R.nitidus*	-0.146444	0.068894
GD34	SC42	HS3	*R.nitidus*	-0.228022	0.062787
GD21	SC42	HS3	*R.nitidus*	-0.134409	0.060997
GD26	SC42	HS3	*R.nitidus*	-0.164071	0.062596
GD22	SC42	HS3	*R.nitidus*	-0.161428	0.06274
GD31	SC42	HS3	*R.nitidus*	-0.159488	0.065265
JO5	CS6	HS3	*R.nitidus*	0.096822	0.063399
CS13	SC42	HS3	*R.nitidus*	-0.266124	0.062744
GD45	SC42	HS3	*R.nitidus*	-0.186725	0.064882
GD46	SC42	HS3	*R.nitidus*	-0.239083	0.061598
GD42	SC42	HS3	*R.nitidus*	-0.224758	0.062013
GD50	SC42	HS3	*R.nitidus*	-0.199729	0.065535
CS15	SC42	HS3	*R.nitidus*	-0.207283	0.061983
GD44	SC42	HS3	*R.nitidus*	-0.203671	0.064122
GD48	SC42	HS3	*R.nitidus*	-0.177518	0.065919
GD25	SC42	HS3	*R.nitidus*	-0.166765	0.061552
GD49	SC42	HS3	*R.nitidus*	-0.15019	0.065822
CS17	SC42	HS3	*R.nitidus*	-0.208483	0.064025
CS12	SC42	HS3	*R.nitidus*	-0.238181	0.063664
GD29	SC42	HS3	*R.nitidus*	-0.102376	0.062218
CS28	SC42	HS3	*R.nitidus*	-0.269962	0.061477
GD32	SC42	HS3	*R.nitidus*	-0.192455	0.063971
CS23	SC42	HS3	*R.nitidus*	-0.285225	0.059811
CS14	SC42	HS3	*R.nitidus*	-0.231153	0.062597
SC5	SC42	HS3	*R.nitidus*	-0.247666	0.064779
GD41	SC42	HS3	*R.nitidus*	-0.227021	0.061892
CS3	SC42	HS3	*R.nitidus*	-0.253749	0.060443
CS21	SC42	HS3	*R.nitidus*	-0.259217	0.060189
CS20	SC42	HS3	*R.nitidus*	-0.250983	0.062245
CS16	SC42	HS3	*R.nitidus*	-0.239413	0.061168
SC28	SC42	HS3	*R.nitidus*	-0.28737	0.060368
CS22	SC42	HS3	*R.nitidus*	-0.250892	0.060772
SC24	SC42	HS3	*R.nitidus*	-0.260316	0.060853
SC14	SC42	HS3	*R.nitidus*	-0.250153	0.061452
SC27	SC42	HS3	*R.nitidus*	-0.263777	0.062086
SC6	SC42	HS3	*R.nitidus*	-0.263253	0.061868
CS18	SC42	HS3	*R.nitidus*	-0.263977	0.061871
CS30	SC42	HS3	*R.nitidus*	-0.287383	0.059852

CS19	SC42	HS3	*R.nitidus*	-0.258242	0.063409
SC34	SC42	HS3	*R.nitidus*	-0.201846	0.060632
JW7	SC42	HS3	*R.nitidus*	-0.186606	0.066234
SC45	SC42	HS3	*R.nitidus*	-0.2263	0.06286
SC18	SC42	HS3	*R.nitidus*	-0.221728	0.062345
SC32	SC42	HS3	*R.nitidus*	-0.23689	0.062328
CS6	SC42	HS3	*R.nitidus*	-0.264799	0.060641
JO1	SC42	HS3	*R.nitidus*	-0.262407	0.062564
MY6	SC42	HS3	*R.nitidus*	-0.251926	0.06272
CS24	SC42	HS3	*R.nitidus*	-0.292436	0.060951
CS25	SC42	HS3	*R.nitidus*	-0.249556	0.064024
CS11	SC44	HS3	*R.nitidus*	-0.002094	0.065551
CS31	SC42	HS3	*R.nitidus*	-0.270173	0.061249
JO8	SC44	HS3	*R.nitidus*	0.019019	0.070221
SC10	SC42	HS3	*R.nitidus*	-0.203704	0.061579
JW4	SC42	HS3	*R.nitidus*	-0.004353	0.064222
JO3	SC42	HS3	*R.nitidus*	-0.157123	0.064791
SC15	SC42	HS3	*R.nitidus*	-0.253565	0.063218
JO5	SC42	HS3	*R.nitidus*	-0.204433	0.063094
SC35	SC42	HS3	*R.nitidus*	-0.216135	0.062333
SC26	SC42	HS3	*R.nitidus*	-0.273364	0.061142
GD25	SC44	HS3	*R.nitidus*	0.056348	0.063139
SC12	SC42	HS3	*R.nitidus*	-0.27149	0.059566
JW6	SC42	HS3	*R.nitidus*	-0.177305	0.06635
SC44	SC42	HS3	*R.nitidus*	-0.25153	0.060203
SC31	SC42	HS3	*R.nitidus*	-0.231228	0.062186
GD22	SC44	HS3	*R.nitidus*	0.095206	0.062667
GD32	SC44	HS3	*R.nitidus*	0.017227	0.066145
CS4	SC42	HS3	*R.nitidus*	-0.260563	0.060842
MY4	SC42	HS3	*R.nitidus*	-0.275801	0.063599
GD26	SC44	HS3	*R.nitidus*	0.064605	0.063464
GD42	SC44	HS3	*R.nitidus*	0.02984	0.066019
GD33	SC44	HS3	*R.nitidus*	0.028513	0.064709
GD30	SC44	HS3	*R.nitidus*	0.108749	0.064197
JO7	SC44	HS3	*R.nitidus*	-0.001896	0.071962
GD31	SC44	HS3	*R.nitidus*	0.064167	0.065278
GD40	SC44	HS3	*R.nitidus*	0.068772	0.062637
CS14	SC44	HS3	*R.nitidus*	-0.00773	0.067048
GD48	SC44	HS3	*R.nitidus*	0.034483	0.066176
GD29	SC44	HS3	*R.nitidus*	0.124005	0.064957
GD44	SC44	HS3	*R.nitidus*	0.029542	0.066074
GD21	SC44	HS3	*R.nitidus*	0.124839	0.063867
GD34	SC44	HS3	*R.nitidus*	0.004196	0.064101
GD49	SC44	HS3	*R.nitidus*	0.082998	0.06577
CS22	SC44	HS3	*R.nitidus*	-0.003587	0.065036
CS12	SC44	HS3	*R.nitidus*	-0.007424	0.066149
CS13	SC44	HS3	*R.nitidus*	-0.035062	0.067003
CS21	SC44	HS3	*R.nitidus*	-0.040397	0.063103
CS3	SC44	HS3	*R.nitidus*	-0.020194	0.063776
GD35	SC44	HS3	*R.nitidus*	0.047216	0.065822
SC10	SC44	HS3	*R.nitidus*	0.071207	0.063804
CS20	SC44	HS3	*R.nitidus*	-0.033755	0.064969
CS23	SC44	HS3	*R.nitidus*	-0.045969	0.063985
CS17	SC44	HS3	*R.nitidus*	0.023059	0.06749
GD41	SC44	HS3	*R.nitidus*	0.007829	0.063736
GD45	SC44	HS3	*R.nitidus*	0.043165	0.067153

CS30	SC44	HS3	*R.nitidus*	-0.065721	0.062886
CS19	SC44	HS3	*R.nitidus*	-0.030059	0.066997
CS24	SC44	HS3	*R.nitidus*	-0.053771	0.06531
SC5	SC44	HS3	*R.nitidus*	-0.00766	0.06671
GD50	SC44	HS3	*R.nitidus*	0.028037	0.069305
CS16	SC44	HS3	*R.nitidus*	0.00143	0.064857
CS18	SC44	HS3	*R.nitidus*	-0.036906	0.064182
GD46	SC44	HS3	*R.nitidus*	-0.018156	0.064331
JW6	SC44	HS3	*R.nitidus*	0.015094	0.071258
CS28	SC44	HS3	*R.nitidus*	-0.044996	0.066369
SC31	SC44	HS3	*R.nitidus*	0.00074	0.064203
SC35	SC44	HS3	*R.nitidus*	0.052006	0.062498
SC18	SC44	HS3	*R.nitidus*	0.050602	0.06253
SC15	SC44	HS3	*R.nitidus*	0.010085	0.065623
CS31	SC44	HS3	*R.nitidus*	-0.027571	0.06486
SC45	SC44	HS3	*R.nitidus*	0.023503	0.064308
CS4	SC44	HS3	*R.nitidus*	-0.018207	0.062897
CS15	SC44	HS3	*R.nitidus*	0.026038	0.066617
SC24	SC44	HS3	*R.nitidus*	-0.011655	0.064043
CS25	SC44	HS3	*R.nitidus*	0	0.066675
SC14	SC44	HS3	*R.nitidus*	0.005405	0.063578
SC12	SC44	HS3	*R.nitidus*	-0.015323	0.063694
SC28	SC44	HS3	*R.nitidus*	-0.048534	0.05883
SC6	SC44	HS3	*R.nitidus*	-0.000745	0.065767
SC27	SC44	HS3	*R.nitidus*	-0.006187	0.062609
SC34	SC44	HS3	*R.nitidus*	0.045353	0.061771
SC26	SC44	HS3	*R.nitidus*	-0.03997	0.06512
JW4	SC44	HS3	*R.nitidus*	0.211119	0.065674
SC32	SC44	HS3	*R.nitidus*	0.04465	0.063957
JO3	SC44	HS3	*R.nitidus*	0.030794	0.06687
JO1	SC44	HS3	*R.nitidus*	-0.024024	0.065765
MY6	SC44	HS3	*R.nitidus*	-0.008746	0.063192
JO5	SC44	HS3	*R.nitidus*	0.059896	0.063024
SC42	SC44	HS3	*R.nitidus*	0.25153	0.060203
MY4	SC44	HS3	*R.nitidus*	-0.043542	0.06408
GD30	SC45	HS3	*R.nitidus*	0.097467	0.068812
GD22	SC45	HS3	*R.nitidus*	0.04886	0.065739
CS6	SC44	HS3	*R.nitidus*	-0.056231	0.061862
GD26	SC45	HS3	*R.nitidus*	0.033175	0.065499
GD31	SC45	HS3	*R.nitidus*	0.053147	0.067405
JW7	SC44	HS3	*R.nitidus*	0.036331	0.069338
GD29	SC45	HS3	*R.nitidus*	0.104193	0.06576
GD50	SC45	HS3	*R.nitidus*	0.002453	0.07073
GD33	SC45	HS3	*R.nitidus*	-0.019359	0.06578
CS11	SC45	HS3	*R.nitidus*	-0.026518	0.068451
GD32	SC45	HS3	*R.nitidus*	0.010008	0.066862
JO7	SC45	HS3	*R.nitidus*	-0.026591	0.070635
JO8	SC45	HS3	*R.nitidus*	0.010536	0.071929
GD44	SC45	HS3	*R.nitidus*	0.001422	0.066663
GD34	SC45	HS3	*R.nitidus*	-0.023684	0.065327
GD42	SC45	HS3	*R.nitidus*	-0.005464	0.065732
GD48	SC45	HS3	*R.nitidus*	0.015173	0.067085
GD41	SC45	HS3	*R.nitidus*	-0.020932	0.065142
GD35	SC45	HS3	*R.nitidus*	0.009453	0.06528
GD25	SC45	HS3	*R.nitidus*	0.03662	0.066474
GD21	SC45	HS3	*R.nitidus*	0.092088	0.062324

CS14	SC45	HS3	*R.nitidus*	-0.032775	0.068845
GD40	SC45	HS3	*R.nitidus*	0.036569	0.063876
CS21	SC45	HS3	*R.nitidus*	-0.063158	0.065749
CS24	SC45	HS3	*R.nitidus*	-0.072238	0.06672
CS12	SC45	HS3	*R.nitidus*	-0.046289	0.06779
CS15	SC45	HS3	*R.nitidus*	0	0.068046
GD45	SC45	HS3	*R.nitidus*	0.011952	0.069216
GD49	SC45	HS3	*R.nitidus*	0.060461	0.068386
CS18	SC45	HS3	*R.nitidus*	-0.06916	0.065107
CS16	SC45	HS3	*R.nitidus*	-0.02582	0.066674
CS20	SC45	HS3	*R.nitidus*	-0.061107	0.065686
SC10	SC45	HS3	*R.nitidus*	0.043818	0.066647
CS17	SC45	HS3	*R.nitidus*	0.001476	0.069073
SC28	SC45	HS3	*R.nitidus*	-0.053184	0.06352
CS13	SC45	HS3	*R.nitidus*	-0.060452	0.069428
GD46	SC45	HS3	*R.nitidus*	-0.047011	0.064678
CS28	SC45	HS3	*R.nitidus*	-0.055679	0.068068
CS3	SC45	HS3	*R.nitidus*	-0.050787	0.064701
CS31	SC45	HS3	*R.nitidus*	-0.050761	0.065863
CS19	SC45	HS3	*R.nitidus*	-0.066384	0.066965
CS4	SC45	HS3	*R.nitidus*	-0.040383	0.06438
MY6	SC45	HS3	*R.nitidus*	-0.031519	0.065845
CS6	SC45	HS3	*R.nitidus*	-0.077206	0.064746
SC5	SC45	HS3	*R.nitidus*	-0.033557	0.066903
JW4	SC45	HS3	*R.nitidus*	0.212473	0.063873
CS25	SC45	HS3	*R.nitidus*	-0.05	0.067006
CS22	SC45	HS3	*R.nitidus*	-0.030238	0.063373
JW7	SC45	HS3	*R.nitidus*	0.023417	0.069971
CS30	SC45	HS3	*R.nitidus*	-0.087372	0.066005
CS23	SC45	HS3	*R.nitidus*	-0.080452	0.065176
JW6	SC45	HS3	*R.nitidus*	-0.015525	0.070233
SC34	SC45	HS3	*R.nitidus*	0.002094	0.065611
SC14	SC45	HS3	*R.nitidus*	-0.027027	0.065626
SC42	SC45	HS3	*R.nitidus*	0.2263	0.06286
SC35	SC45	HS3	*R.nitidus*	0.008869	0.063411
SC6	SC45	HS3	*R.nitidus*	-0.024006	0.066664
GD31	MY4	HS3	*R.nitidus*	0.105491	0.066766
SC15	SC45	HS3	*R.nitidus*	-0.021788	0.066248
SC18	SC45	HS3	*R.nitidus*	0.015432	0.063889
GD30	MY4	HS3	*R.nitidus*	0.15655	0.066459
SC12	SC45	HS3	*R.nitidus*	-0.054869	0.064178
SC24	SC45	HS3	*R.nitidus*	-0.048084	0.063256
JO1	SC45	HS3	*R.nitidus*	-0.04491	0.067241
SC27	SC45	HS3	*R.nitidus*	-0.031137	0.064902
JO8	MY4	HS3	*R.nitidus*	0.073473	0.06973
SC26	SC45	HS3	*R.nitidus*	-0.082222	0.064972
GD32	MY4	HS3	*R.nitidus*	0.080032	0.067157
JO7	MY4	HS3	*R.nitidus*	0.032381	0.071636
SC32	SC45	HS3	*R.nitidus*	0.019912	0.065642
JO5	SC45	HS3	*R.nitidus*	0.011887	0.064649
GD29	MY4	HS3	*R.nitidus*	0.163226	0.063831
CS12	MY4	HS3	*R.nitidus*	0.044919	0.067819
JO3	SC45	HS3	*R.nitidus*	0.029157	0.06904
SC44	SC45	HS3	*R.nitidus*	-0.023503	0.064308
SC31	SC45	HS3	*R.nitidus*	-0.021939	0.064661
MY4	SC45	HS3	*R.nitidus*	-0.063538	0.065436

GD21	MY4	HS3	*R.nitidus*	0.17304	0.063684
GD22	MY4	HS3	*R.nitidus*	0.126467	0.064852
GD35	MY4	HS3	*R.nitidus*	0.08728	0.065035
GD50	MY4	HS3	*R.nitidus*	0.066015	0.068488
GD49	MY4	HS3	*R.nitidus*	0.119403	0.069145
GD41	MY4	HS3	*R.nitidus*	0.042538	0.062416
GD26	MY4	HS3	*R.nitidus*	0.09263	0.064548
GD34	MY4	HS3	*R.nitidus*	0.043243	0.064542
GD45	MY4	HS3	*R.nitidus*	0.083137	0.067346
GD25	MY4	HS3	*R.nitidus*	0.09868	0.063679
GD48	MY4	HS3	*R.nitidus*	0.069559	0.067393
CS15	MY4	HS3	*R.nitidus*	0.064607	0.067237
CS11	MY4	HS3	*R.nitidus*	0.033006	0.066317
CS14	MY4	HS3	*R.nitidus*	0.028592	0.067536
GD40	MY4	HS3	*R.nitidus*	0.109838	0.06282
GD44	MY4	HS3	*R.nitidus*	0.072307	0.066109
GD46	MY4	HS3	*R.nitidus*	0.015884	0.063938
CS21	MY4	HS3	*R.nitidus*	0.00288	0.066351
CS16	MY4	HS3	*R.nitidus*	0.037474	0.065455
CS18	MY4	HS3	*R.nitidus*	-0.002894	0.066986
GD33	MY4	HS3	*R.nitidus*	0.057394	0.065094
CS24	MY4	HS3	*R.nitidus*	-0.008869	0.065776
CS20	MY4	HS3	*R.nitidus*	0.005559	0.06587
GD42	MY4	HS3	*R.nitidus*	0.061495	0.064882
CS4	MY4	HS3	*R.nitidus*	0.017417	0.065124
SC14	MY4	HS3	*R.nitidus*	0.045058	0.067229
CS23	MY4	HS3	*R.nitidus*	-0.017155	0.065025
CS13	MY4	HS3	*R.nitidus*	-0.005162	0.067617
CS22	MY4	HS3	*R.nitidus*	0.029828	0.065464
SC24	MY4	HS3	*R.nitidus*	0.030781	0.063565
JW4	MY4	HS3	*R.nitidus*	0.252193	0.065912
SC18	MY4	HS3	*R.nitidus*	0.08549	0.062827
SC10	MY4	HS3	*R.nitidus*	0.115385	0.066463
CS30	MY4	HS3	*R.nitidus*	-0.020863	0.066225
SC12	MY4	HS3	*R.nitidus*	0.023065	0.064947
SC5	MY4	HS3	*R.nitidus*	0.036982	0.067072
CS3	MY4	HS3	*R.nitidus*	0.007914	0.064797
CS19	MY4	HS3	*R.nitidus*	0.011878	0.069258
CS17	MY4	HS3	*R.nitidus*	0.052233	0.068219
SC15	MY4	HS3	*R.nitidus*	0.051493	0.066635
SC42	MY4	HS3	*R.nitidus*	0.275801	0.063599
JW6	MY4	HS3	*R.nitidus*	0.06203	0.06887
SC44	MY4	HS3	*R.nitidus*	0.043542	0.06408
JW7	MY4	HS3	*R.nitidus*	0.082456	0.068465
SC26	MY4	HS3	*R.nitidus*	-0.005727	0.064111
SC27	MY4	HS3	*R.nitidus*	0.032212	0.064839
MY6	MY4	HS3	*R.nitidus*	0.050375	0.057386
SC31	MY4	HS3	*R.nitidus*	0.037966	0.065349
CS25	MY4	HS3	*R.nitidus*	0.02393	0.067442
SC45	MY4	HS3	*R.nitidus*	0.063538	0.065436
CS31	MY4	HS3	*R.nitidus*	0.017778	0.066818
SC6	MY4	HS3	*R.nitidus*	0.039941	0.066497
CS28	MY4	HS3	*R.nitidus*	-0.007728	0.067918
JO8	MY6	HS3	*R.nitidus*	0.029326	0.070024
JO5	MY4	HS3	*R.nitidus*	0.084831	0.064865
JO7	MY6	HS3	*R.nitidus*	-0.010436	0.071736

SC32	MY4	HS3	*R.nitidus*	0.084328	0.064629
JO3	MY4	HS3	*R.nitidus*	0.078352	0.06906
SC28	MY4	HS3	*R.nitidus*	0.00578	0.06386
SC35	MY4	HS3	*R.nitidus*	0.089036	0.063403
JO1	MY4	HS3	*R.nitidus*	0.023704	0.065256
SC34	MY4	HS3	*R.nitidus*	0.082202	0.06418
CS11	MY6	HS3	*R.nitidus*	-0.001366	0.066822
GD30	MY6	HS3	*R.nitidus*	0.121932	0.066252
CS6	MY4	HS3	*R.nitidus*	-0.006011	0.065373
GD42	MY6	HS3	*R.nitidus*	0.025	0.064518
CS12	MY6	HS3	*R.nitidus*	0.007829	0.067495
GD35	MY6	HS3	*R.nitidus*	0.054091	0.065392
GD32	MY6	HS3	*R.nitidus*	0.050343	0.066979
GD33	MY6	HS3	*R.nitidus*	0.02549	0.064618
GD26	MY6	HS3	*R.nitidus*	0.06471	0.065112
GD50	MY6	HS3	*R.nitidus*	0.018927	0.067496
CS16	MY6	HS3	*R.nitidus*	0.006207	0.065114
GD48	MY6	HS3	*R.nitidus*	0.028149	0.066195
GD21	MY6	HS3	*R.nitidus*	0.136015	0.064548
GD25	MY6	HS3	*R.nitidus*	0.056211	0.06386
GD45	MY6	HS3	*R.nitidus*	0.046384	0.068757
GD31	MY6	HS3	*R.nitidus*	0.073205	0.066544
GD22	MY6	HS3	*R.nitidus*	0.089571	0.06431
GD41	MY6	HS3	*R.nitidus*	0.003318	0.061311
SC6	MY6	HS3	*R.nitidus*	0	0.066688
CS14	MY6	HS3	*R.nitidus*	-0.000693	0.067365
GD29	MY6	HS3	*R.nitidus*	0.129747	0.063487
CS21	MY6	HS3	*R.nitidus*	-0.036136	0.065367
GD46	MY6	HS3	*R.nitidus*	-0.020791	0.063432
CS13	MY6	HS3	*R.nitidus*	-0.046329	0.067212
CS3	MY6	HS3	*R.nitidus*	-0.0163	0.063993
CS20	MY6	HS3	*R.nitidus*	-0.024557	0.065338
GD34	MY6	HS3	*R.nitidus*	0.014657	0.06405
CS17	MY6	HS3	*R.nitidus*	0.014695	0.067783
CS18	MY6	HS3	*R.nitidus*	-0.036068	0.066215
GD40	MY6	HS3	*R.nitidus*	0.068602	0.062358
GD44	MY6	HS3	*R.nitidus*	0.034605	0.067081
GD49	MY6	HS3	*R.nitidus*	0.094427	0.068244
CS28	MY6	HS3	*R.nitidus*	-0.038748	0.067925
CS25	MY6	HS3	*R.nitidus*	-0.013542	0.066958
CS23	MY6	HS3	*R.nitidus*	-0.053041	0.06401
SC10	MY6	HS3	*R.nitidus*	0.083333	0.066078
CS24	MY6	HS3	*R.nitidus*	-0.045785	0.064528
SC12	MY6	HS3	*R.nitidus*	-0.013177	0.06596
CS31	MY6	HS3	*R.nitidus*	-0.01748	0.067012
CS15	MY6	HS3	*R.nitidus*	0.026099	0.067243
CS4	MY6	HS3	*R.nitidus*	-0.02028	0.064605
CS19	MY6	HS3	*R.nitidus*	-0.031228	0.067002
SC28	MY6	HS3	*R.nitidus*	-0.0266	0.0618
SC34	MY6	HS3	*R.nitidus*	0.03662	0.06266
CS22	MY6	HS3	*R.nitidus*	-0.004351	0.064108
SC26	MY6	HS3	*R.nitidus*	-0.033934	0.063675
SC18	MY6	HS3	*R.nitidus*	0.045942	0.062379
SC14	MY6	HS3	*R.nitidus*	0.004871	0.064382
GD25	JW4	HS3	*R.nitidus*	-0.15575	0.066897
SC5	MY6	HS3	*R.nitidus*	-0.001423	0.066786

CS30	MY6	HS3	*R.nitidus*	-0.057775	0.066
JW7	MY6	HS3	*R.nitidus*	0.045217	0.069252
GD26	JW4	HS3	*R.nitidus*	-0.1606	0.068012
SC35	MY6	HS3	*R.nitidus*	0.045259	0.063045
SC32	MY6	HS3	*R.nitidus*	0.040656	0.064046
SC44	MY6	HS3	*R.nitidus*	0.008746	0.063192
JW4	MY6	HS3	*R.nitidus*	0.223837	0.065175
GD29	JW4	HS3	*R.nitidus*	-0.079398	0.065804
SC15	MY6	HS3	*R.nitidus*	0.014001	0.065389
SC24	MY6	HS3	*R.nitidus*	-0.013453	0.064226
JO3	MY6	HS3	*R.nitidus*	0.045344	0.068165
SC27	MY6	HS3	*R.nitidus*	-0.006298	0.063803
JW6	MY6	HS3	*R.nitidus*	0.024413	0.067995
SC31	MY6	HS3	*R.nitidus*	-0.000704	0.065537
MY4	MY6	HS3	*R.nitidus*	-0.050375	0.057386
CS11	JW4	HS3	*R.nitidus*	-0.199313	0.065977
GD32	JW4	HS3	*R.nitidus*	-0.184652	0.068081
CS6	MY6	HS3	*R.nitidus*	-0.046579	0.065425
JO5	MY6	HS3	*R.nitidus*	0.050212	0.06426
GD22	JW4	HS3	*R.nitidus*	-0.131839	0.064967
JO8	JW4	HS3	*R.nitidus*	-0.164717	0.070783
SC45	MY6	HS3	*R.nitidus*	0.031519	0.065845
GD48	JW4	HS3	*R.nitidus*	-0.163688	0.067544
JO7	JW4	HS3	*R.nitidus*	-0.192053	0.069265
SC42	MY6	HS3	*R.nitidus*	0.251926	0.06272
GD41	JW4	HS3	*R.nitidus*	-0.2	0.063143
GD21	JW4	HS3	*R.nitidus*	-0.085271	0.067292
GD49	JW4	HS3	*R.nitidus*	-0.132029	0.070245
CS14	JW4	HS3	*R.nitidus*	-0.215046	0.067418
GD31	JW4	HS3	*R.nitidus*	-0.12988	0.067949
CS17	JW4	HS3	*R.nitidus*	-0.189759	0.069202
JO1	MY6	HS3	*R.nitidus*	-0.019957	0.065025
GD42	JW4	HS3	*R.nitidus*	-0.172295	0.06465
GD33	JW4	HS3	*R.nitidus*	-0.199861	0.069009
GD30	JW4	HS3	*R.nitidus*	-0.096801	0.070315
GD44	JW4	HS3	*R.nitidus*	-0.160986	0.066916
CS23	JW4	HS3	*R.nitidus*	-0.246037	0.064086
GD46	JW4	HS3	*R.nitidus*	-0.226833	0.066509
CS12	JW4	HS3	*R.nitidus*	-0.204978	0.067213
GD45	JW4	HS3	*R.nitidus*	-0.148329	0.070874
CS18	JW4	HS3	*R.nitidus*	-0.241625	0.066126
GD35	JW4	HS3	*R.nitidus*	-0.176634	0.066057
GD34	JW4	HS3	*R.nitidus*	-0.210774	0.065893
GD40	JW4	HS3	*R.nitidus*	-0.14698	0.065773
CS13	JW4	HS3	*R.nitidus*	-0.255443	0.066172
GD50	JW4	HS3	*R.nitidus*	-0.139554	0.06964
CS20	JW4	HS3	*R.nitidus*	-0.221682	0.06535
CS30	JW4	HS3	*R.nitidus*	-0.264336	0.066189
CS28	JW4	HS3	*R.nitidus*	-0.246242	0.069323
SC28	JW4	HS3	*R.nitidus*	-0.238128	0.065183
SC10	JW4	HS3	*R.nitidus*	-0.17731	0.066953
SC6	JW4	HS3	*R.nitidus*	-0.207045	0.065694
CS15	JW4	HS3	*R.nitidus*	-0.175222	0.067007
CS16	JW4	HS3	*R.nitidus*	-0.216561	0.065663
CS25	JW4	HS3	*R.nitidus*	-0.214487	0.068566
SC5	JW4	HS3	*R.nitidus*	-0.190029	0.069798

SC32	JW4	HS3	*R.nitidus*	-0.185501	0.064808
SC14	JW4	HS3	*R.nitidus*	-0.208276	0.064774
CS31	JW4	HS3	*R.nitidus*	-0.255423	0.066039
SC18	JW4	HS3	*R.nitidus*	-0.182284	0.065843
CS24	JW4	HS3	*R.nitidus*	-0.267946	0.064372
CS22	JW4	HS3	*R.nitidus*	-0.231263	0.065083
CS21	JW4	HS3	*R.nitidus*	-0.244898	0.066459
CS6	JW4	HS3	*R.nitidus*	-0.238335	0.064341
CS19	JW4	HS3	*R.nitidus*	-0.235209	0.067882
SC15	JW4	HS3	*R.nitidus*	-0.210753	0.066803
JO5	JW4	HS3	*R.nitidus*	-0.15931	0.066752
SC42	JW4	HS3	*R.nitidus*	0.004353	0.064222
SC31	JW4	HS3	*R.nitidus*	-0.222378	0.067353
CS4	JW4	HS3	*R.nitidus*	-0.223227	0.063534
SC24	JW4	HS3	*R.nitidus*	-0.222062	0.065949
SC27	JW4	HS3	*R.nitidus*	-0.203263	0.065125
CS3	JW4	HS3	*R.nitidus*	-0.223479	0.065551
SC12	JW4	HS3	*R.nitidus*	-0.236936	0.064549
JW6	JW4	HS3	*R.nitidus*	-0.168653	0.069115
SC45	JW4	HS3	*R.nitidus*	-0.212473	0.063873
JO3	JW4	HS3	*R.nitidus*	-0.146362	0.068894
GD26	JW6	HS3	*R.nitidus*	0.076512	0.070235
MY6	JW4	HS3	*R.nitidus*	-0.223837	0.065175
GD25	JW6	HS3	*R.nitidus*	0.039595	0.070183
JO1	JW4	HS3	*R.nitidus*	-0.214038	0.066364
SC44	JW4	HS3	*R.nitidus*	-0.211119	0.065674
JO7	JW6	HS3	*R.nitidus*	0.029457	0.067791
SC26	JW4	HS3	*R.nitidus*	-0.248954	0.064346
SC34	JW4	HS3	*R.nitidus*	-0.189268	0.063247
JW7	JW4	HS3	*R.nitidus*	-0.134317	0.0679
SC35	JW4	HS3	*R.nitidus*	-0.201285	0.06459
JO8	JW6	HS3	*R.nitidus*	0.012195	0.070485
GD33	JW6	HS3	*R.nitidus*	0.009009	0.071592
GD45	JW6	HS3	*R.nitidus*	0.115304	0.074228
GD21	JW6	HS3	*R.nitidus*	0.129085	0.068391
GD32	JW6	HS3	*R.nitidus*	0.01712	0.071389
MY4	JW4	HS3	*R.nitidus*	-0.252193	0.065912
GD34	JW6	HS3	*R.nitidus*	-0.012853	0.069432
GD35	JW6	HS3	*R.nitidus*	0.044524	0.071488
GD46	JW6	HS3	*R.nitidus*	-0.041209	0.070103
GD29	JW6	HS3	*R.nitidus*	0.142123	0.068136
GD30	JW6	HS3	*R.nitidus*	0.140904	0.07304
GD41	JW6	HS3	*R.nitidus*	0.008272	0.068515
CS11	JW6	HS3	*R.nitidus*	-0.010909	0.07248
CS14	JW6	HS3	*R.nitidus*	-0.006446	0.073935
GD44	JW6	HS3	*R.nitidus*	0.028362	0.070651
GD22	JW6	HS3	*R.nitidus*	0.075519	0.06924
CS16	JW6	HS3	*R.nitidus*	0.00273	0.072134
GD50	JW6	HS3	*R.nitidus*	0.061651	0.073596
GD40	JW6	HS3	*R.nitidus*	0.045253	0.06935
CS23	JW6	HS3	*R.nitidus*	-0.025152	0.070106
CS22	JW6	HS3	*R.nitidus*	-0.034234	0.069708
CS25	JW6	HS3	*R.nitidus*	-0.022907	0.071232
CS28	JW6	HS3	*R.nitidus*	-0.048924	0.072042
CS4	JW6	HS3	*R.nitidus*	-0.013734	0.068102
GD48	JW6	HS3	*R.nitidus*	0.029384	0.071193

GD42	JW6	HS3	*R.nitidus*	0.048872	0.070732
CS13	JW6	HS3	*R.nitidus*	-0.037002	0.074089
CS12	JW6	HS3	*R.nitidus*	0.002822	0.072627
CS20	JW6	HS3	*R.nitidus*	0.00087	0.069665
CS24	JW6	HS3	*R.nitidus*	-0.068545	0.070229
CS18	JW6	HS3	*R.nitidus*	-0.026268	0.071886
GD31	JW6	HS3	*R.nitidus*	0.089826	0.071735
SC12	JW6	HS3	*R.nitidus*	-0.035349	0.06693
GD49	JW6	HS3	*R.nitidus*	0.084034	0.072435
CS19	JW6	HS3	*R.nitidus*	-0.056818	0.072015
SC18	JW6	HS3	*R.nitidus*	0.003868	0.068701
SC35	JW6	HS3	*R.nitidus*	0.01275	0.069914
SC14	JW6	HS3	*R.nitidus*	-0.008333	0.069348
SC6	JW6	HS3	*R.nitidus*	0.024568	0.0713
CS17	JW6	HS3	*R.nitidus*	0.027864	0.074003
CS21	JW6	HS3	*R.nitidus*	-0.027853	0.070961
CS3	JW6	HS3	*R.nitidus*	0.00362	0.069923
SC24	JW6	HS3	*R.nitidus*	-0.015326	0.071013
CS30	JW6	HS3	*R.nitidus*	-0.063037	0.069765
SC10	JW6	HS3	*R.nitidus*	0.077947	0.073369
CS15	JW6	HS3	*R.nitidus*	0.042727	0.070784
SC26	JW6	HS3	*R.nitidus*	-0.048598	0.070005
SC34	JW6	HS3	*R.nitidus*	0.01629	0.067602
SC5	JW6	HS3	*R.nitidus*	0.010233	0.074868
JO1	JW6	HS3	*R.nitidus*	-0.023679	0.070556
CS31	JW6	HS3	*R.nitidus*	-0.052431	0.071733
SC42	JW6	HS3	*R.nitidus*	0.177305	0.06635
CS6	JW6	HS3	*R.nitidus*	-0.05893	0.069773
JO3	JW6	HS3	*R.nitidus*	0.030435	0.072503
SC27	JW6	HS3	*R.nitidus*	-0.014045	0.069823
SC15	JW6	HS3	*R.nitidus*	-0.008721	0.072936
GD21	JW7	HS3	*R.nitidus*	0.10728	0.067245
SC31	JW6	HS3	*R.nitidus*	-0.018568	0.071632
SC28	JW6	HS3	*R.nitidus*	-0.018727	0.071786
SC32	JW6	HS3	*R.nitidus*	0.026465	0.070039
JO7	JW7	HS3	*R.nitidus*	-0.062626	0.072197
GD42	JW7	HS3	*R.nitidus*	0.022575	0.067902
JO8	JW7	HS3	*R.nitidus*	-0.028513	0.074176
MY4	JW6	HS3	*R.nitidus*	-0.06203	0.06887
GD26	JW7	HS3	*R.nitidus*	0.027265	0.070323
GD34	JW7	HS3	*R.nitidus*	-0.049206	0.069846
SC44	JW6	HS3	*R.nitidus*	-0.015094	0.071258
MY6	JW6	HS3	*R.nitidus*	-0.024413	0.067995
GD46	JW7	HS3	*R.nitidus*	-0.054591	0.067846
SC45	JW6	HS3	*R.nitidus*	0.015525	0.070233
GD31	JW7	HS3	*R.nitidus*	0.064198	0.070979
JO5	JW6	HS3	*R.nitidus*	0.05581	0.069611
GD32	JW7	HS3	*R.nitidus*	-0.00722	0.069438
GD25	JW7	HS3	*R.nitidus*	0.030911	0.069331
JW4	JW6	HS3	*R.nitidus*	0.168653	0.069115
GD41	JW7	HS3	*R.nitidus*	-0.02963	0.068159
GD22	JW7	HS3	*R.nitidus*	0.050414	0.067148
CS13	JW7	HS3	*R.nitidus*	-0.084265	0.0706
CS14	JW7	HS3	*R.nitidus*	-0.037194	0.070995
GD30	JW7	HS3	*R.nitidus*	0.101711	0.071647
JW7	JW6	HS3	*R.nitidus*	0.035538	0.071324

CS12	JW7	HS3	*R.nitidus*	-0.026126	0.071182
GD29	JW7	HS3	*R.nitidus*	0.108696	0.066393
GD40	JW7	HS3	*R.nitidus*	0.031671	0.067384
CS11	JW7	HS3	*R.nitidus*	-0.033015	0.070415
GD44	JW7	HS3	*R.nitidus*	0.000851	0.069945
GD35	JW7	HS3	*R.nitidus*	0.006436	0.070756
GD50	JW7	HS3	*R.nitidus*	0.029528	0.07432
CS21	JW7	HS3	*R.nitidus*	-0.078747	0.067217
GD45	JW7	HS3	*R.nitidus*	0.049856	0.072517
GD33	JW7	HS3	*R.nitidus*	-0.015823	0.070332
CS20	JW7	HS3	*R.nitidus*	-0.043406	0.068228
SC5	JW7	HS3	*R.nitidus*	-0.025023	0.074178
CS23	JW7	HS3	*R.nitidus*	-0.074442	0.065971
CS25	JW7	HS3	*R.nitidus*	-0.046809	0.070968
CS18	JW7	HS3	*R.nitidus*	-0.059829	0.069124
GD49	JW7	HS3	*R.nitidus*	0.021842	0.072547
CS28	JW7	HS3	*R.nitidus*	-0.07757	0.070901
GD48	JW7	HS3	*R.nitidus*	-0.00678	0.069791
CS17	JW7	HS3	*R.nitidus*	-0.008372	0.07227
CS19	JW7	HS3	*R.nitidus*	-0.065532	0.068594
CS30	JW7	HS3	*R.nitidus*	-0.090753	0.067687
SC28	JW7	HS3	*R.nitidus*	-0.074586	0.06706
SC10	JW7	HS3	*R.nitidus*	0.008621	0.069556
CS15	JW7	HS3	*R.nitidus*	0.010779	0.069033
CS31	JW7	HS3	*R.nitidus*	-0.080836	0.068975
JO3	JW7	HS3	*R.nitidus*	-0.01292	0.070104
CS6	JW7	HS3	*R.nitidus*	-0.089578	0.066816
CS16	JW7	HS3	*R.nitidus*	-0.033854	0.071541
SC44	JW7	HS3	*R.nitidus*	-0.036331	0.069338
CS22	JW7	HS3	*R.nitidus*	-0.051349	0.067881
SC12	JW7	HS3	*R.nitidus*	-0.07024	0.066815
JW4	JW7	HS3	*R.nitidus*	0.134317	0.0679
SC24	JW7	HS3	*R.nitidus*	-0.064161	0.068354
SC34	JW7	HS3	*R.nitidus*	-0.001762	0.066787
SC42	JW7	HS3	*R.nitidus*	0.186606	0.066234
CS24	JW7	HS3	*R.nitidus*	-0.084685	0.065138
SC35	JW7	HS3	*R.nitidus*	-0.017148	0.065858
SC6	JW7	HS3	*R.nitidus*	-0.028414	0.070333
SC45	JW7	HS3	*R.nitidus*	-0.023417	0.069971
CS3	JW7	HS3	*R.nitidus*	-0.056604	0.066715
SC18	JW7	HS3	*R.nitidus*	-0.026877	0.067633
GD21	JO1	HS3	*R.nitidus*	0.153061	0.064124
JO8	JO1	HS3	*R.nitidus*	0.028571	0.070847
JO5	JW7	HS3	*R.nitidus*	0.023831	0.069052
SC15	JW7	HS3	*R.nitidus*	-0.033866	0.069733
JO1	JW7	HS3	*R.nitidus*	-0.050137	0.067915
SC26	JW7	HS3	*R.nitidus*	-0.099543	0.067833
SC14	JW7	HS3	*R.nitidus*	-0.041267	0.068764
CS4	JW7	HS3	*R.nitidus*	-0.041563	0.065469
JW6	JW7	HS3	*R.nitidus*	-0.035538	0.071324
SC27	JW7	HS3	*R.nitidus*	-0.05345	0.067525
GD26	JO1	HS3	*R.nitidus*	0.089436	0.06497
SC31	JW7	HS3	*R.nitidus*	-0.066545	0.066268
GD33	JO1	HS3	*R.nitidus*	0.041444	0.065589
JO7	JO1	HS3	*R.nitidus*	-0.022341	0.069064
GD22	JO1	HS3	*R.nitidus*	0.099629	0.063676

GD25	JO1	HS3	*R.nitidus*	0.093575	0.06443
SC32	JW7	HS3	*R.nitidus*	-0.021154	0.068806
MY6	JW7	HS3	*R.nitidus*	-0.045217	0.069252
MY4	JW7	HS3	*R.nitidus*	-0.082456	0.068465
GD50	JO1	HS3	*R.nitidus*	0.06129	0.06928
CS12	JO1	HS3	*R.nitidus*	0.011512	0.066454
GD31	JO1	HS3	*R.nitidus*	0.094011	0.067177
CS18	JO1	HS3	*R.nitidus*	-0.02521	0.065455
GD41	JO1	HS3	*R.nitidus*	0.025624	0.063604
CS11	JO1	HS3	*R.nitidus*	0.002849	0.066751
GD29	JO1	HS3	*R.nitidus*	0.144955	0.063931
GD49	JO1	HS3	*R.nitidus*	0.09589	0.068524
GD34	JO1	HS3	*R.nitidus*	0.03455	0.063778
GD32	JO1	HS3	*R.nitidus*	0.054157	0.067013
CS19	JO1	HS3	*R.nitidus*	-0.014025	0.067619
GD30	JO1	HS3	*R.nitidus*	0.159684	0.065226
GD35	JO1	HS3	*R.nitidus*	0.059492	0.064388
GD44	JO1	HS3	*R.nitidus*	0.044554	0.066041
CS21	JO1	HS3	*R.nitidus*	-0.028413	0.066196
CS15	JO1	HS3	*R.nitidus*	0.036533	0.064482
GD45	JO1	HS3	*R.nitidus*	0.072141	0.068026
GD42	JO1	HS3	*R.nitidus*	0.046748	0.065771
CS16	JO1	HS3	*R.nitidus*	0.008602	0.065387
CS31	JO1	HS3	*R.nitidus*	-0.00365	0.065856
GD40	JO1	HS3	*R.nitidus*	0.084106	0.065255
CS14	JO1	HS3	*R.nitidus*	0.002144	0.067254
SC18	JO1	HS3	*R.nitidus*	0.050119	0.065504
GD46	JO1	HS3	*R.nitidus*	0.004057	0.063772
CS30	JO1	HS3	*R.nitidus*	-0.040433	0.065258
CS20	JO1	HS3	*R.nitidus*	-0.011471	0.066731
CS28	JO1	HS3	*R.nitidus*	-0.026709	0.066306
CS4	JO1	HS3	*R.nitidus*	-0.008463	0.063319
CS17	JO1	HS3	*R.nitidus*	0.043377	0.067801
GD48	JO1	HS3	*R.nitidus*	0.049311	0.06831
SC5	JO1	HS3	*R.nitidus*	0.015475	0.069245
SC24	JO1	HS3	*R.nitidus*	-0.003026	0.066068
SC6	JO1	HS3	*R.nitidus*	0.021084	0.066942
CS13	JO1	HS3	*R.nitidus*	-0.034107	0.066853
SC34	JO1	HS3	*R.nitidus*	0.042385	0.064013
SC27	JO1	HS3	*R.nitidus*	0.00074	0.063957
CS22	JO1	HS3	*R.nitidus*	-0.003582	0.066082
SC12	JO1	HS3	*R.nitidus*	-0.000756	0.065679
CS3	JO1	HS3	*R.nitidus*	-0.008639	0.066289
CS25	JO1	HS3	*R.nitidus*	0.005513	0.067332
SC45	JO1	HS3	*R.nitidus*	0.04491	0.067241
SC26	JO1	HS3	*R.nitidus*	-0.021505	0.064198
SC42	JO1	HS3	*R.nitidus*	0.262407	0.062564
CS6	JO1	HS3	*R.nitidus*	-0.034307	0.066582
CS23	JO1	HS3	*R.nitidus*	-0.042224	0.066347
CS24	JO1	HS3	*R.nitidus*	-0.037572	0.065613
SC10	JO1	HS3	*R.nitidus*	0.09324	0.063647
JW6	JO1	HS3	*R.nitidus*	0.023679	0.070556
GD30	JO3	HS3	*R.nitidus*	0.122841	0.071476
SC44	JO1	HS3	*R.nitidus*	0.024024	0.065765
SC14	JO1	HS3	*R.nitidus*	0.006623	0.06484
SC15	JO1	HS3	*R.nitidus*	0.028616	0.066495

JO7	JO3	HS3	*R.nitidus*	-0.035242	0.073819
JO5	JO1	HS3	*R.nitidus*	0.064588	0.064419
SC35	JO1	HS3	*R.nitidus*	0.06706	0.064014
MY6	JO1	HS3	*R.nitidus*	0.019957	0.065025
GD21	JO3	HS3	*R.nitidus*	0.092038	0.068283
SC28	JO1	HS3	*R.nitidus*	-0.001542	0.06415
SC32	JO1	HS3	*R.nitidus*	0.060139	0.065423
SC31	JO1	HS3	*R.nitidus*	0.016899	0.063422
JW4	JO1	HS3	*R.nitidus*	0.214038	0.066364
CS12	JO3	HS3	*R.nitidus*	-0.021757	0.070188
JO8	JO3	HS3	*R.nitidus*	-0.010011	0.073615
GD45	JO3	HS3	*R.nitidus*	0.052288	0.073571
GD32	JO3	HS3	*R.nitidus*	0.010018	0.070577
CS13	JO3	HS3	*R.nitidus*	-0.078759	0.071555
GD33	JO3	HS3	*R.nitidus*	-0.011811	0.069135
JW7	JO1	HS3	*R.nitidus*	0.050137	0.067915
GD25	JO3	HS3	*R.nitidus*	0.014155	0.067687
JO3	JO1	HS3	*R.nitidus*	0.055735	0.068414
GD40	JO3	HS3	*R.nitidus*	0.003084	0.069004
CS19	JO3	HS3	*R.nitidus*	-0.072072	0.069548
GD29	JO3	HS3	*R.nitidus*	0.103631	0.065808
CS11	JO3	HS3	*R.nitidus*	-0.043478	0.070974
GD22	JO3	HS3	*R.nitidus*	0.049475	0.067779
GD26	JO3	HS3	*R.nitidus*	0.031587	0.070047
CS17	JO3	HS3	*R.nitidus*	-0.010221	0.070775
GD34	JO3	HS3	*R.nitidus*	-0.03289	0.068457
GD44	JO3	HS3	*R.nitidus*	-0.002421	0.071475
CS15	JO3	HS3	*R.nitidus*	-0.010023	0.069267
GD41	JO3	HS3	*R.nitidus*	-0.024682	0.068526
GD31	JO3	HS3	*R.nitidus*	0.037563	0.071592
GD46	JO3	HS3	*R.nitidus*	-0.064071	0.067937
CS16	JO3	HS3	*R.nitidus*	-0.034161	0.070277
GD49	JO3	HS3	*R.nitidus*	0.025617	0.073534
CS23	JO3	HS3	*R.nitidus*	-0.081761	0.067939
CS30	JO3	HS3	*R.nitidus*	-0.102041	0.069364
GD42	JO3	HS3	*R.nitidus*	-0.001641	0.070629
MY4	JO1	HS3	*R.nitidus*	-0.023704	0.065256
CS31	JO3	HS3	*R.nitidus*	-0.054918	0.07029
GD50	JO3	HS3	*R.nitidus*	0.040073	0.072232
CS25	JO3	HS3	*R.nitidus*	-0.055468	0.069185
CS28	JO3	HS3	*R.nitidus*	-0.077879	0.072156
SC12	JO3	HS3	*R.nitidus*	-0.07081	0.067234
CS18	JO3	HS3	*R.nitidus*	-0.0664	0.068376
GD48	JO3	HS3	*R.nitidus*	0.017677	0.069485
CS14	JO3	HS3	*R.nitidus*	-0.042332	0.072143
GD35	JO3	HS3	*R.nitidus*	0.013799	0.070532
SC10	JO3	HS3	*R.nitidus*	0.035714	0.07041
SC28	JO3	HS3	*R.nitidus*	-0.054988	0.067292
SC24	JO3	HS3	*R.nitidus*	-0.060976	0.068452
CS20	JO3	HS3	*R.nitidus*	-0.054183	0.067398
CS4	JO3	HS3	*R.nitidus*	-0.057339	0.066408
SC6	JO3	HS3	*R.nitidus*	-0.025417	0.068735
JW7	JO3	HS3	*R.nitidus*	0.01292	0.070104
SC26	JO3	HS3	*R.nitidus*	-0.085627	0.067703
CS22	JO3	HS3	*R.nitidus*	-0.048567	0.068929
CS21	JO3	HS3	*R.nitidus*	-0.081487	0.06919

MY4	JO3	HS3	*R.nitidus*	-0.078352	0.06906
CS24	JO3	HS3	*R.nitidus*	-0.072405	0.067836
SC18	JO3	HS3	*R.nitidus*	-0.011561	0.068149
SC44	JO3	HS3	*R.nitidus*	-0.030794	0.06687
JO1	JO3	HS3	*R.nitidus*	-0.055735	0.068414
SC14	JO3	HS3	*R.nitidus*	-0.033924	0.069276
SC45	JO3	HS3	*R.nitidus*	-0.029157	0.06904
SC5	JO3	HS3	*R.nitidus*	-0.014515	0.073688
SC31	JO3	HS3	*R.nitidus*	-0.039413	0.068806
CS3	JO3	HS3	*R.nitidus*	-0.037551	0.068582
JO8	JO5	HS3	*R.nitidus*	-0.050098	0.070285
SC15	JO3	HS3	*R.nitidus*	-0.030731	0.070063
SC42	JO3	HS3	*R.nitidus*	0.157123	0.064791
SC27	JO3	HS3	*R.nitidus*	-0.03622	0.068382
CS11	JO5	HS3	*R.nitidus*	-0.030034	0.066371
GD32	JO5	HS3	*R.nitidus*	-0.023585	0.068308
SC32	JO3	HS3	*R.nitidus*	0.007893	0.068483
SC35	JO3	HS3	*R.nitidus*	-0.014343	0.068453
JO7	JO5	HS3	*R.nitidus*	-0.076383	0.068556
JW6	JO3	HS3	*R.nitidus*	-0.030435	0.072503
SC34	JO3	HS3	*R.nitidus*	-0.031153	0.06756
GD46	JO5	HS3	*R.nitidus*	-0.047941	0.063745
GD30	JO5	HS3	*R.nitidus*	0.083072	0.066567
GD40	JO5	HS3	*R.nitidus*	0.027027	0.063779
CS6	JO3	HS3	*R.nitidus*	-0.106883	0.067581
GD42	JO5	HS3	*R.nitidus*	-0.011173	0.065099
GD34	JO5	HS3	*R.nitidus*	-0.034137	0.064411
JW4	JO3	HS3	*R.nitidus*	0.146362	0.068894
GD26	JO5	HS3	*R.nitidus*	0.017426	0.064154
GD22	JO5	HS3	*R.nitidus*	0.039533	0.064195
GD50	JO5	HS3	*R.nitidus*	0.001657	0.067405
MY6	JO3	HS3	*R.nitidus*	-0.045344	0.068165
GD49	JO5	HS3	*R.nitidus*	0.071994	0.065983
JO5	JO3	HS3	*R.nitidus*	0.020946	0.069103
GD25	JO5	HS3	*R.nitidus*	0.029228	0.065429
GD35	JO5	HS3	*R.nitidus*	0.021262	0.066015
CS14	JO5	HS3	*R.nitidus*	-0.039753	0.065831
CS13	JO5	HS3	*R.nitidus*	-0.080946	0.066023
GD41	JO5	HS3	*R.nitidus*	-0.035762	0.063381
GD31	JO5	HS3	*R.nitidus*	0.045007	0.066123
GD21	JO5	HS3	*R.nitidus*	0.08375	0.063353
CS12	JO5	HS3	*R.nitidus*	-0.037834	0.065681
GD44	JO5	HS3	*R.nitidus*	-0.008493	0.065603
GD48	JO5	HS3	*R.nitidus*	-0.013304	0.067431
GD29	JO5	HS3	*R.nitidus*	0.098168	0.063876
CS19	JO5	HS3	*R.nitidus*	-0.067686	0.065382
GD33	JO5	HS3	*R.nitidus*	-0.02122	0.065749
GD45	JO5	HS3	*R.nitidus*	0.02809	0.068972
CS18	JO5	HS3	*R.nitidus*	-0.092554	0.066169
CS15	JO5	HS3	*R.nitidus*	-0.018493	0.066367
CS20	JO5	HS3	*R.nitidus*	-0.080623	0.063645
CS30	JO5	HS3	*R.nitidus*	-0.102115	0.063139
CS24	JO5	HS3	*R.nitidus*	-0.084733	0.063932
CS21	JO5	HS3	*R.nitidus*	-0.083626	0.06379
SC34	JO5	HS3	*R.nitidus*	-0.017544	0.064836
SC10	JO5	HS3	*R.nitidus*	0.028549	0.065595

JW6	JO5	HS3	*R.nitidus*	-0.05581	0.069611
CS4	JO5	HS3	*R.nitidus*	-0.061397	0.062255
SC14	JO5	HS3	*R.nitidus*	-0.046048	0.06467
CS28	JO5	HS3	*R.nitidus*	-0.078806	0.065627
SC31	JO5	HS3	*R.nitidus*	-0.040146	0.063683
CS23	JO5	HS3	*R.nitidus*	-0.091037	0.063697
SC6	JO5	HS3	*R.nitidus*	-0.044853	0.066328
SC45	JO5	HS3	*R.nitidus*	-0.011887	0.064649
CS16	JO5	HS3	*R.nitidus*	-0.036237	0.064518
JO3	JO5	HS3	*R.nitidus*	-0.020946	0.069103
SC26	JO5	HS3	*R.nitidus*	-0.089325	0.062443
SC24	JO5	HS3	*R.nitidus*	-0.063913	0.063309
CS17	JO5	HS3	*R.nitidus*	0	0.068044
SC5	JO5	HS3	*R.nitidus*	-0.058188	0.066721
CS22	JO5	HS3	*R.nitidus*	-0.048711	0.064606
GD25	JO7	HS5	*R.nitidus*	0.053611	0.077263
GD22	JO7	HS5	*R.nitidus*	0.061657	0.071681
CS3	JO5	HS3	*R.nitidus*	-0.069138	0.063407
CS31	JO5	HS3	*R.nitidus*	-0.073295	0.064619
CS25	JO5	HS3	*R.nitidus*	-0.060738	0.066805
MY4	JO5	HS3	*R.nitidus*	-0.084831	0.064865
SC32	JO5	HS3	*R.nitidus*	-0.006461	0.063502
JO1	JO5	HS3	*R.nitidus*	-0.064588	0.064419
GD30	JO7	HS5	*R.nitidus*	0.14375	0.077634
SC18	JO5	HS3	*R.nitidus*	-0.014925	0.062503
SC28	JO5	HS3	*R.nitidus*	-0.098993	0.061594
JO8	JO7	HS5	*R.nitidus*	-0.013453	0.075998
SC27	JO5	HS3	*R.nitidus*	-0.053118	0.065088
SC12	JO5	HS3	*R.nitidus*	-0.069644	0.064828
SC44	JO5	HS3	*R.nitidus*	-0.059896	0.063024
SC15	JO5	HS3	*R.nitidus*	-0.041379	0.065502
JW4	JO5	HS3	*R.nitidus*	0.15931	0.066752
MY6	JO5	HS3	*R.nitidus*	-0.050212	0.06426
GD32	JO7	HS5	*R.nitidus*	0.025463	0.079046
SC42	JO5	HS3	*R.nitidus*	0.204433	0.063094
GD34	JO7	HS5	*R.nitidus*	-0.011893	0.073623
GD50	JO7	HS5	*R.nitidus*	0.034965	0.078509
GD29	JO7	HS5	*R.nitidus*	0.149098	0.071708
CS6	JO5	HS3	*R.nitidus*	-0.096822	0.063399
CS12	JO7	HS5	*R.nitidus*	-0.011074	0.07706
SC35	JO5	HS3	*R.nitidus*	-0.005011	0.062537
GD21	JO7	HS5	*R.nitidus*	0.095149	0.073567
CS11	JO7	HS5	*R.nitidus*	-0.02335	0.076745
GD40	JO7	HS5	*R.nitidus*	0.052632	0.074687
GD45	JO7	HS5	*R.nitidus*	0.091743	0.079269
GD35	JO7	HS5	*R.nitidus*	0.024059	0.073783
GD26	JO7	HS5	*R.nitidus*	0.071358	0.076424
GD33	JO7	HS5	*R.nitidus*	0.003093	0.076441
GD31	JO7	HS5	*R.nitidus*	0.041341	0.076797
CS23	JO7	HS5	*R.nitidus*	-0.059244	0.075726
GD42	JO7	HS5	*R.nitidus*	0.04267	0.076138
CS14	JO7	HS5	*R.nitidus*	-0.012889	0.077781
CS13	JO7	HS5	*R.nitidus*	-0.04902	0.077046
GD48	JO7	HS5	*R.nitidus*	0.0348	0.075139
JW7	JO5	HS3	*R.nitidus*	-0.023831	0.069052
CS24	JO7	HS5	*R.nitidus*	-0.067308	0.074359

CS16	JO7	HS5	*R.nitidus*	-0.015201	0.076629
CS28	JO7	HS5	*R.nitidus*	-0.059226	0.07266
GD49	JO7	HS5	*R.nitidus*	0.060411	0.078631
CS3	JO7	HS5	*R.nitidus*	-0.030151	0.074671
CS21	JO7	HS5	*R.nitidus*	-0.044865	0.075177
CS17	JO7	HS5	*R.nitidus*	-0.00339	0.05064
GD46	JO7	HS5	*R.nitidus*	-0.031949	0.074256
CS18	JO7	HS5	*R.nitidus*	-0.058462	0.07615
SC10	JO7	HS5	*R.nitidus*	0.071116	0.076364
SC6	JO7	HS5	*R.nitidus*	-0.003181	0.074077
SC14	JO7	HS5	*R.nitidus*	-0.055046	0.072991
GD41	JO7	HS5	*R.nitidus*	-0.029167	0.07378
CS19	JO7	HS5	*R.nitidus*	-0.049112	0.077032
CS15	JO7	HS5	*R.nitidus*	-0.031712	0.075087
GD44	JO7	HS5	*R.nitidus*	-0.008264	0.074801
CS22	JO7	HS5	*R.nitidus*	-0.015021	0.074035
CS25	JO7	HS5	*R.nitidus*	-0.028956	0.076281
SC12	JO7	HS5	*R.nitidus*	-0.064894	0.071448
SC42	JO7	HS5	*R.nitidus*	0.131555	0.072751
SC5	JO7	HS5	*R.nitidus*	0.020534	0.075086
CS4	JO7	HS5	*R.nitidus*	-0.04501	0.073557
CS20	JO7	HS5	*R.nitidus*	-0.024851	0.073136
CS31	JO7	HS5	*R.nitidus*	-0.06531	0.07476
CS30	JO7	HS5	*R.nitidus*	-0.083149	0.074772
JO3	JO7	HS5	*R.nitidus*	0.015476	0.077806
JW6	JO7	HS5	*R.nitidus*	-0.042517	0.070832
SC35	JO7	HS5	*R.nitidus*	-0.006173	0.071812
MY4	JO7	HS5	*R.nitidus*	-0.066738	0.074512
CS6	JO7	HS5	*R.nitidus*	-0.067901	0.073166
SC24	JO7	HS5	*R.nitidus*	-0.005562	0.076418
GD29	JO8	HS5	*R.nitidus*	0.134945	0.071029
SC32	JO7	HS5	*R.nitidus*	0.017621	0.073102
SC44	JO7	HS5	*R.nitidus*	-0.055734	0.074091
JO7	JO8	HS5	*R.nitidus*	0.013453	0.075998
SC28	JO7	HS5	*R.nitidus*	-0.059385	0.071773
SC45	JO7	HS5	*R.nitidus*	0.006431	0.073595
SC15	JO7	HS5	*R.nitidus*	-0.047097	0.075464
SC18	JO7	HS5	*R.nitidus*	0.020225	0.074561
SC26	JO7	HS5	*R.nitidus*	-0.072464	0.072899
SC27	JO7	HS5	*R.nitidus*	-0.059252	0.074314
JW4	JO7	HS5	*R.nitidus*	0.142105	0.074582
MY6	JO7	HS5	*R.nitidus*	-0.014223	0.073515
SC34	JO7	HS5	*R.nitidus*	-0.007921	0.072866
JO5	JO7	HS5	*R.nitidus*	0.030333	0.071678
SC31	JO7	HS5	*R.nitidus*	-0.037661	0.07244
GD33	JO8	HS5	*R.nitidus*	-0.030917	0.077694
GD31	JO8	HS5	*R.nitidus*	0.003398	0.074233
CS11	JO8	HS5	*R.nitidus*	-0.009146	0.075089
GD32	JO8	HS5	*R.nitidus*	-0.011292	0.079232
JO1	JO7	HS5	*R.nitidus*	-0.023023	0.073376
GD30	JO8	HS5	*R.nitidus*	0.118439	0.078564
GD25	JO8	HS5	*R.nitidus*	0.041445	0.073443
GD45	JO8	HS5	*R.nitidus*	0.069987	0.050008
GD46	JO8	HS5	*R.nitidus*	-0.079153	0.071409
CS13	JO8	HS5	*R.nitidus*	-0.036026	0.079543
JW7	JO7	HS5	*R.nitidus*	0.032186	0.075032

GD35	JO8	HS5	*R.nitidus*	0.026824	0.074564
CS15	JO8	HS5	*R.nitidus*	-0.022941	0.075384
GD21	JO8	HS5	*R.nitidus*	0.085149	0.072511
GD34	JO8	HS5	*R.nitidus*	-0.040966	0.074851
GD26	JO8	HS5	*R.nitidus*	0.039871	0.074244
GD49	JO8	HS5	*R.nitidus*	0.033156	0.0503
CS28	JO8	HS5	*R.nitidus*	-0.034522	0.077737
CS20	JO8	HS5	*R.nitidus*	-0.034079	0.075083
GD22	JO8	HS5	*R.nitidus*	0.057809	0.075018
CS12	JO8	HS5	*R.nitidus*	-0.019016	0.07564
GD41	JO8	HS5	*R.nitidus*	-0.023861	0.074502
GD40	JO8	HS5	*R.nitidus*	0.055441	0.072136
GD44	JO8	HS5	*R.nitidus*	-0.003185	0.074986
GD42	JO8	HS5	*R.nitidus*	0.028857	0.076399
CS21	JO8	HS5	*R.nitidus*	-0.050936	0.073211
CS19	JO8	HS5	*R.nitidus*	-0.049217	0.078316
CS18	JO8	HS5	*R.nitidus*	-0.046892	0.074677
CS23	JO8	HS5	*R.nitidus*	-0.062048	0.075563
CS24	JO8	HS5	*R.nitidus*	-0.037671	0.07462
GD48	JO8	HS5	*R.nitidus*	0.032258	0.073747
SC26	JO8	HS5	*R.nitidus*	-0.091611	0.075167
CS17	JO8	HS5	*R.nitidus*	0.019676	0.079439
GD50	JO8	HS5	*R.nitidus*	0.022528	0.078878
SC10	JO8	HS5	*R.nitidus*	0.048643	0.074669
CS14	JO8	HS5	*R.nitidus*	-0.007392	0.077637
SC6	JO8	HS5	*R.nitidus*	-0.010661	0.076629
CS31	JO8	HS5	*R.nitidus*	-0.050167	0.075507
SC24	JO8	HS5	*R.nitidus*	-0.004603	0.075013
SC18	JO8	HS5	*R.nitidus*	0.009662	0.074711
CS4	JO8	HS5	*R.nitidus*	-0.022704	0.073964
CS6	JO8	HS5	*R.nitidus*	-0.051502	0.073804
CS16	JO8	HS5	*R.nitidus*	-0.027174	0.074548
SC5	JO8	HS5	*R.nitidus*	0.014508	0.079896
CS3	JO8	HS5	*R.nitidus*	-0.036458	0.076238
CS22	JO8	HS5	*R.nitidus*	-0.006772	0.072791
CS25	JO8	HS5	*R.nitidus*	-0.026461	0.075999
SC28	JO8	HS5	*R.nitidus*	-0.058036	0.073463
SC15	JO8	HS5	*R.nitidus*	-0.036077	0.075092
CS30	JO8	HS5	*R.nitidus*	-0.092511	0.073206
SC35	JO8	HS5	*R.nitidus*	0.016358	0.073372
SC42	JO8	HS5	*R.nitidus*	0.115135	0.073648
SC12	JO8	HS5	*R.nitidus*	-0.076582	0.070851
SC14	JO8	HS5	*R.nitidus*	-0.038622	0.073421
MY4	JO8	HS5	*R.nitidus*	-0.070175	0.07378
SC44	JO8	HS5	*R.nitidus*	-0.036344	0.073204
SC45	JO8	HS5	*R.nitidus*	0.009688	0.074979
SC31	JO8	HS5	*R.nitidus*	-0.025586	0.074054
JW7	JO8	HS5	*R.nitidus*	0.026846	0.075827
SC27	JO8	HS5	*R.nitidus*	-0.053079	0.07241
MY6	JO8	HS5	*R.nitidus*	-0.009019	0.073183
SC34	JO8	HS5	*R.nitidus*	-0.018538	0.071927
SC32	JO8	HS5	*R.nitidus*	-0.004396	0.074491
JO1	JO8	HS5	*R.nitidus*	-0.030655	0.074028
JW6	JO8	HS5	*R.nitidus*	0.010463	0.072137
JO5	JO8	HS5	*R.nitidus*	0.05074	0.071445
JO3	JO8	HS5	*R.nitidus*	0.023143	0.077574

JW4	JO8	HS5	*R.nitidus*	0.137527	0.071714
JO7	CS11	HS5	*R.nitidus*	0.02335	0.076745
GD21	CS11	HS5	*R.nitidus*	0.103592	0.070651
GD33	CS11	HS5	*R.nitidus*	0.006261	0.07433
CS12	CS11	HS5	*R.nitidus*	0.012853	0.073623
GD34	CS11	HS5	*R.nitidus*	0.007563	0.071493
GD40	CS11	HS5	*R.nitidus*	0.074106	0.069041
GD30	CS11	HS5	*R.nitidus*	0.138561	0.077624
GD25	CS11	HS5	*R.nitidus*	0.076147	0.07285
GD42	CS11	HS5	*R.nitidus*	0.052726	0.074231
GD35	CS11	HS5	*R.nitidus*	0.035587	0.070865
CS15	CS11	HS5	*R.nitidus*	0.006658	0.073591
CS14	CS11	HS5	*R.nitidus*	0.018732	0.077589
JO8	CS11	HS5	*R.nitidus*	0.009146	0.075089
GD45	CS11	HS5	*R.nitidus*	0.0931	0.050773
GD49	CS11	HS5	*R.nitidus*	0.055789	0.077952
GD22	CS11	HS5	*R.nitidus*	0.066946	0.073028
GD44	CS11	HS5	*R.nitidus*	0.022482	0.073056
CS22	CS11	HS5	*R.nitidus*	0.012093	0.072196
GD29	CS11	HS5	*R.nitidus*	0.147718	0.073325
GD26	CS11	HS5	*R.nitidus*	0.052038	0.073184
GD50	CS11	HS5	*R.nitidus*	0.038855	0.076229
SC5	CS11	HS5	*R.nitidus*	0.034265	0.076326
CS30	CS11	HS5	*R.nitidus*	-0.064667	0.072796
GD46	CS11	HS5	*R.nitidus*	-0.0045	0.074358
GD31	CS11	HS5	*R.nitidus*	0.069549	0.075099
GD32	CS11	HS5	*R.nitidus*	0.045409	0.075301
CS21	CS11	HS5	*R.nitidus*	-0.021858	0.073112
CS17	CS11	HS5	*R.nitidus*	0.062069	0.074642
CS24	CS11	HS5	*R.nitidus*	-0.044335	0.073914
CS20	CS11	HS5	*R.nitidus*	-0.019301	0.072127
SC6	CS11	HS5	*R.nitidus*	0.016053	0.073932
CS16	CS11	HS5	*R.nitidus*	-0.003942	0.074514
CS19	CS11	HS5	*R.nitidus*	-0.032738	0.072462
CS25	CS11	HS5	*R.nitidus*	-0.006686	0.074203
SC18	CS11	HS5	*R.nitidus*	0.035138	0.072506
GD48	CS11	HS5	*R.nitidus*	0.061773	0.074832
CS13	CS11	HS5	*R.nitidus*	-0.029948	0.077621
SC12	CS11	HS5	*R.nitidus*	-0.050832	0.07174
CS18	CS11	HS5	*R.nitidus*	-0.035138	0.072228
CS31	CS11	HS5	*R.nitidus*	-0.019861	0.074833
CS28	CS11	HS5	*R.nitidus*	-0.027439	0.072939
CS23	CS11	HS5	*R.nitidus*	-0.049639	0.072658
GD41	CS11	HS5	*R.nitidus*	0.012489	0.073085
SC10	CS11	HS5	*R.nitidus*	0.076336	0.074535
SC26	CS11	HS5	*R.nitidus*	-0.0519	0.072849
MY4	CS11	HS5	*R.nitidus*	-0.027372	0.073112
SC45	CS11	HS5	*R.nitidus*	0.021429	0.073179
SC27	CS11	HS5	*R.nitidus*	-0.031452	0.072443
CS4	CS11	HS5	*R.nitidus*	-0.000924	0.073351
JO8	GD21	HS5	*R.nitidus*	-0.085149	0.072511
CS3	CS11	HS5	*R.nitidus*	-0.03263	0.071728
SC15	CS11	HS5	*R.nitidus*	0.011881	0.074103
CS6	CS11	HS5	*R.nitidus*	-0.031761	0.071566
SC32	CS11	HS5	*R.nitidus*	0.042776	0.072549
CS11	GD21	HS5	*R.nitidus*	-0.103592	0.070651

SC31	CS11	HS5	*R.nitidus*	-0.021062	0.073112
SC14	CS11	HS5	*R.nitidus*	-0.0181	0.071997
SC34	CS11	HS5	*R.nitidus*	0.0053	0.073467
GD22	GD21	HS5	*R.nitidus*	-0.05597	0.068559
SC24	CS11	HS5	*R.nitidus*	-0.000924	0.073677
CS12	GD21	HS5	*R.nitidus*	-0.130713	0.06936
GD25	GD21	HS5	*R.nitidus*	-0.037736	0.069604
JO1	CS11	HS5	*R.nitidus*	-0.008435	0.072375
JW7	CS11	HS5	*R.nitidus*	0.045267	0.07564
GD32	GD21	HS5	*R.nitidus*	-0.0783	0.072024
JO3	CS11	HS5	*R.nitidus*	0.057522	0.073532
JW4	CS11	HS5	*R.nitidus*	0.148413	0.071113
SC28	CS11	HS5	*R.nitidus*	-0.047187	0.071464
JO7	GD21	HS5	*R.nitidus*	-0.095149	0.073567
SC35	CS11	HS5	*R.nitidus*	0.030946	0.072141
MY6	CS11	HS5	*R.nitidus*	0.009937	0.072362
SC42	CS11	HS5	*R.nitidus*	0.191108	0.06782
SC44	CS11	HS5	*R.nitidus*	-0.012658	0.073804
GD31	GD21	HS5	*R.nitidus*	-0.083077	0.069595
GD41	GD21	HS5	*R.nitidus*	-0.109005	0.065477
GD33	GD21	HS5	*R.nitidus*	-0.135394	0.069642
JW6	CS11	HS5	*R.nitidus*	0.024341	0.077854
GD35	GD21	HS5	*R.nitidus*	-0.079263	0.067978
GD34	GD21	HS5	*R.nitidus*	-0.127907	0.069218
GD26	GD21	HS5	*R.nitidus*	-0.065714	0.069337
CS13	GD21	HS5	*R.nitidus*	-0.120805	0.069688
GD30	GD21	HS5	*R.nitidus*	0.052903	0.07366
CS20	GD21	HS5	*R.nitidus*	-0.132826	0.069295
GD29	GD21	HS5	*R.nitidus*	0.01751	0.069535
GD44	GD21	HS5	*R.nitidus*	-0.120641	0.069679
JO5	CS11	HS5	*R.nitidus*	0.044003	0.071725
GD50	GD21	HS5	*R.nitidus*	-0.09434	0.072991
GD46	GD21	HS5	*R.nitidus*	-0.152242	0.066413
GD49	GD21	HS5	*R.nitidus*	-0.04662	0.075485
GD48	GD21	HS5	*R.nitidus*	-0.07449	0.069911
CS22	GD21	HS5	*R.nitidus*	-0.10052	0.068377
GD40	GD21	HS5	*R.nitidus*	-0.041815	0.069144
SC12	GD21	HS5	*R.nitidus*	-0.1508	0.067727
GD42	GD21	HS5	*R.nitidus*	-0.077699	0.067997
CS17	GD21	HS5	*R.nitidus*	-0.080645	0.073044
GD45	GD21	HS5	*R.nitidus*	-0.015276	0.072862
CS21	GD21	HS5	*R.nitidus*	-0.137386	0.068922
SC14	GD21	HS5	*R.nitidus*	-0.1185	0.068154
CS18	GD21	HS5	*R.nitidus*	-0.150602	0.06911
CS24	GD21	HS5	*R.nitidus*	-0.148116	0.068041
CS25	GD21	HS5	*R.nitidus*	-0.124017	0.071888
CS30	GD21	HS5	*R.nitidus*	-0.184466	0.067643
CS28	GD21	HS5	*R.nitidus*	-0.13506	0.07115
CS19	GD21	HS5	*R.nitidus*	-0.14412	0.069886
CS6	GD21	HS5	*R.nitidus*	-0.155306	0.066914
CS14	GD21	HS5	*R.nitidus*	-0.113891	0.071332
CS16	GD21	HS5	*R.nitidus*	-0.115083	0.067482
SC15	GD21	HS5	*R.nitidus*	-0.111603	0.070532
CS15	GD21	HS5	*R.nitidus*	-0.120205	0.068314
SC35	GD21	HS5	*R.nitidus*	-0.077711	0.068242
CS23	GD21	HS5	*R.nitidus*	-0.155684	0.069611

CS4	GD21	HS5	*R.nitidus*	-0.111111	0.06738
SC26	GD21	HS5	*R.nitidus*	-0.160034	0.06804
SC45	GD21	HS5	*R.nitidus*	-0.068936	0.067919
CS31	GD21	HS5	*R.nitidus*	-0.151268	0.069954
SC5	GD21	HS5	*R.nitidus*	-0.062112	0.072029
SC42	GD21	HS5	*R.nitidus*	0.108498	0.06858
SC6	GD21	HS5	*R.nitidus*	-0.099145	0.07021
SC18	GD21	HS5	*R.nitidus*	-0.092046	0.069731
SC31	GD21	HS5	*R.nitidus*	-0.136134	0.068636
MY4	GD21	HS5	*R.nitidus*	-0.154966	0.068994
JW7	GD21	HS5	*R.nitidus*	-0.060465	0.072075
SC32	GD21	HS5	*R.nitidus*	-0.073721	0.069602
SC10	GD21	HS5	*R.nitidus*	-0.048	0.069753
CS3	GD21	HS5	*R.nitidus*	-0.134697	0.068926
SC27	GD21	HS5	*R.nitidus*	-0.132689	0.067565
SC24	GD21	HS5	*R.nitidus*	-0.107732	0.069877
GD21	GD22	HS5	*R.nitidus*	0.05597	0.068559
CS11	GD22	HS5	*R.nitidus*	-0.066946	0.073028
JO8	GD22	HS5	*R.nitidus*	-0.057809	0.075018
JO5	GD21	HS5	*R.nitidus*	-0.045492	0.06683
JO3	GD21	HS5	*R.nitidus*	-0.063776	0.071668
MY6	GD21	HS5	*R.nitidus*	-0.114187	0.070098
SC28	GD21	HS5	*R.nitidus*	-0.147059	0.066762
SC34	GD21	HS5	*R.nitidus*	-0.104575	0.066637
GD25	GD22	HS5	*R.nitidus*	-0.005742	0.071251
JW6	GD21	HS5	*R.nitidus*	-0.083333	0.072082
JW4	GD21	HS5	*R.nitidus*	0.059969	0.070376
JO7	GD22	HS5	*R.nitidus*	-0.061657	0.071681
GD33	GD22	HS5	*R.nitidus*	-0.078261	0.068123
CS12	GD22	HS5	*R.nitidus*	-0.068203	0.071136
GD30	GD22	HS5	*R.nitidus*	0.10253	0.071998
SC44	GD21	HS5	*R.nitidus*	-0.115385	0.067863
GD29	GD22	HS5	*R.nitidus*	0.101493	0.066912
GD34	GD22	HS5	*R.nitidus*	-0.078242	0.067016
JO1	GD21	HS5	*R.nitidus*	-0.136667	0.069402
GD35	GD22	HS5	*R.nitidus*	-0.010309	0.070693
GD26	GD22	HS5	*R.nitidus*	0.003058	0.068601
GD42	GD22	HS5	*R.nitidus*	-0.030896	0.067735
GD49	GD22	HS5	*R.nitidus*	0.028807	0.074227
GD45	GD22	HS5	*R.nitidus*	0.017281	0.076522
CS16	GD22	HS5	*R.nitidus*	-0.071307	0.068888
GD32	GD22	HS5	*R.nitidus*	-0.048499	0.072562
CS14	GD22	HS5	*R.nitidus*	-0.057412	0.072034
CS30	GD22	HS5	*R.nitidus*	-0.132867	0.068129
GD31	GD22	HS5	*R.nitidus*	-0.010549	0.071713
GD44	GD22	HS5	*R.nitidus*	-0.063462	0.069089
CS22	GD22	HS5	*R.nitidus*	-0.051439	0.06726
CS19	GD22	HS5	*R.nitidus*	-0.096396	0.071411
SC6	GD22	HS5	*R.nitidus*	-0.04953	0.071117
GD50	GD22	HS5	*R.nitidus*	-0.030842	0.072876
CS15	GD22	HS5	*R.nitidus*	-0.07364	0.070797
GD40	GD22	HS5	*R.nitidus*	0.021183	0.068514
GD41	GD22	HS5	*R.nitidus*	-0.064155	0.068865
GD46	GD22	HS5	*R.nitidus*	-0.097319	0.068132
CS13	GD22	HS5	*R.nitidus*	-0.084996	0.072651
CS25	GD22	HS5	*R.nitidus*	-0.083408	0.071468

SC18	GD22	HS5	*R.nitidus*	-0.045208	0.070149
CS3	GD22	HS5	*R.nitidus*	-0.107021	0.069448
CS31	GD22	HS5	*R.nitidus*	-0.096916	0.071971
GD48	GD22	HS5	*R.nitidus*	-0.01206	0.070384
CS23	GD22	HS5	*R.nitidus*	-0.109787	0.06921
CS24	GD22	HS5	*R.nitidus*	-0.099027	0.068508
SC35	GD22	HS5	*R.nitidus*	-0.043215	0.06924
CS17	GD22	HS5	*R.nitidus*	-0.024862	0.073747
CS18	GD22	HS5	*R.nitidus*	-0.09869	0.067856
CS21	GD22	HS5	*R.nitidus*	-0.097338	0.066767
CS28	GD22	HS5	*R.nitidus*	-0.097806	0.074865
SC14	GD22	HS5	*R.nitidus*	-0.078283	0.070043
CS20	GD22	HS5	*R.nitidus*	-0.092359	0.068843
SC28	GD22	HS5	*R.nitidus*	-0.108894	0.068528
SC42	GD22	HS5	*R.nitidus*	0.129561	0.069699
SC45	GD22	HS5	*R.nitidus*	-0.031034	0.069523
SC10	GD22	HS5	*R.nitidus*	0.00463	0.071872
SC5	GD22	HS5	*R.nitidus*	-0.035398	0.075327
SC15	GD22	HS5	*R.nitidus*	-0.061837	0.071135
JO7	GD25	HS5	*R.nitidus*	-0.053611	0.077263
SC31	GD22	HS5	*R.nitidus*	-0.09182	0.072317
SC26	GD22	HS5	*R.nitidus*	-0.11234	0.069178
CS6	GD22	HS5	*R.nitidus*	-0.09282	0.070163
JO3	GD22	HS5	*R.nitidus*	-0.013937	0.069786
SC27	GD22	HS5	*R.nitidus*	-0.087704	0.069673
SC12	GD22	HS5	*R.nitidus*	-0.116379	0.069757
JW4	GD22	HS5	*R.nitidus*	0.10828	0.068103
SC44	GD22	HS5	*R.nitidus*	-0.08775	0.069965
CS4	GD22	HS5	*R.nitidus*	-0.071545	0.067039
MY6	GD22	HS5	*R.nitidus*	-0.063086	0.069638
GD22	GD25	HS5	*R.nitidus*	0.005742	0.071251
SC34	GD22	HS5	*R.nitidus*	-0.04878	0.069404
JO1	GD22	HS5	*R.nitidus*	-0.079968	0.072809
SC32	GD22	HS5	*R.nitidus*	-0.029889	0.071054
GD33	GD25	HS5	*R.nitidus*	-0.073469	0.073877
GD45	GD25	HS5	*R.nitidus*	0.06988	0.079575
CS11	GD25	HS5	*R.nitidus*	-0.076147	0.07285
GD21	GD25	HS5	*R.nitidus*	0.037736	0.069604
GD31	GD25	HS5	*R.nitidus*	-0.007519	0.073386
MY4	GD22	HS5	*R.nitidus*	-0.100433	0.069472
JW7	GD22	HS5	*R.nitidus*	-0.023445	0.07215
JO5	GD22	HS5	*R.nitidus*	0.006745	0.069345
JW6	GD22	HS5	*R.nitidus*	-0.045631	0.072092
GD40	GD25	HS5	*R.nitidus*	-0.004753	0.069874
GD44	GD25	HS5	*R.nitidus*	-0.062874	0.07235
JO8	GD25	HS5	*R.nitidus*	-0.041445	0.073443
SC24	GD22	HS5	*R.nitidus*	-0.069973	0.071021
CS23	GD25	HS5	*R.nitidus*	-0.113879	0.071716
GD48	GD25	HS5	*R.nitidus*	0.010549	0.070828
CS19	GD25	HS5	*R.nitidus*	-0.085126	0.072918
CS12	GD25	HS5	*R.nitidus*	-0.096057	0.070153
CS17	GD25	HS5	*R.nitidus*	-0.035751	0.074561
GD29	GD25	HS5	*R.nitidus*	0.084404	0.070072
CS30	GD25	HS5	*R.nitidus*	-0.130394	0.073299
GD41	GD25	HS5	*R.nitidus*	-0.033138	0.071775
GD50	GD25	HS5	*R.nitidus*	0.008111	0.072301

GD32	GD25	HS5	*R.nitidus*	-0.020202	0.074656
CS14	GD25	HS5	*R.nitidus*	-0.068224	0.075041
CS16	GD25	HS5	*R.nitidus*	-0.086093	0.07188
GD35	GD25	HS5	*R.nitidus*	-0.01749	0.067311
CS25	GD25	HS5	*R.nitidus*	-0.08642	0.072278
GD34	GD25	HS5	*R.nitidus*	-0.061836	0.071935
GD49	GD25	HS5	*R.nitidus*	0.020457	0.076087
GD26	GD25	HS5	*R.nitidus*	0.026812	0.071157
CS21	GD25	HS5	*R.nitidus*	-0.100539	0.07276
GD46	GD25	HS5	*R.nitidus*	-0.077228	0.071237
CS22	GD25	HS5	*R.nitidus*	-0.045497	0.070424
CS15	GD25	HS5	*R.nitidus*	-0.089649	0.071145
SC10	GD25	HS5	*R.nitidus*	0.001013	0.071892
CS18	GD25	HS5	*R.nitidus*	-0.090253	0.07228
SC26	GD25	HS5	*R.nitidus*	-0.114554	0.072403
CS13	GD25	HS5	*R.nitidus*	-0.085047	0.072805
SC12	GD25	HS5	*R.nitidus*	-0.106942	0.069136
GD42	GD25	HS5	*R.nitidus*	-0.009202	0.071466
SC6	GD25	HS5	*R.nitidus*	-0.053122	0.071506
SC24	GD25	HS5	*R.nitidus*	-0.055713	0.07166
CS31	GD25	HS5	*R.nitidus*	-0.105211	0.072627
SC15	GD25	HS5	*R.nitidus*	-0.072217	0.072882
CS28	GD25	HS5	*R.nitidus*	-0.090628	0.074052
CS4	GD25	HS5	*R.nitidus*	-0.055895	0.071578
CS3	GD25	HS5	*R.nitidus*	-0.09031	0.073334
SC5	GD25	HS5	*R.nitidus*	-0.027333	0.074376
CS20	GD25	HS5	*R.nitidus*	-0.086564	0.073365
CS24	GD25	HS5	*R.nitidus*	-0.079143	0.072672
SC42	GD25	HS5	*R.nitidus*	0.115894	0.068218
SC28	GD25	HS5	*R.nitidus*	-0.114391	0.069591
JW6	GD25	HS5	*R.nitidus*	-0.041801	0.074471
SC31	GD25	HS5	*R.nitidus*	-0.097606	0.070636
JO3	GD25	HS5	*R.nitidus*	-0.009497	0.072002
SC27	GD25	HS5	*R.nitidus*	-0.095595	0.069144
SC44	GD25	HS5	*R.nitidus*	-0.082464	0.069474
JW7	GD25	HS5	*R.nitidus*	-0.02381	0.074047
MY6	GD25	HS5	*R.nitidus*	-0.062619	0.071749
JO1	GD25	HS5	*R.nitidus*	-0.102658	0.072103
SC32	GD25	HS5	*R.nitidus*	-0.033395	0.068872
SC34	GD25	HS5	*R.nitidus*	-0.051075	0.071105
SC18	GD25	HS5	*R.nitidus*	-0.040385	0.07235
SC14	GD25	HS5	*R.nitidus*	-0.084259	0.069985
JW4	GD25	HS5	*R.nitidus*	0.099053	0.070779
GD40	GD26	HS5	*R.nitidus*	0.015903	0.069313
CS6	GD25	HS5	*R.nitidus*	-0.092507	0.071619
SC35	GD25	HS5	*R.nitidus*	-0.045923	0.070061
GD21	GD26	HS5	*R.nitidus*	0.065714	0.069337
GD32	GD26	HS5	*R.nitidus*	-0.023022	0.067293
GD30	GD26	HS5	*R.nitidus*	0.098465	0.073226
SC45	GD25	HS5	*R.nitidus*	-0.048991	0.070393
GD45	GD26	HS5	*R.nitidus*	0.050617	0.078311
GD44	GD26	HS5	*R.nitidus*	-0.049595	0.071333
GD49	GD26	HS5	*R.nitidus*	-0.011278	0.074592
GD29	GD26	HS5	*R.nitidus*	0.072874	0.06692
MY4	GD25	HS5	*R.nitidus*	-0.102097	0.071929
CS12	GD26	HS5	*R.nitidus*	-0.048644	0.071913

GD31	GD26	HS5	*R.nitidus*	0.005308	0.074201
JO7	GD26	HS5	*R.nitidus*	-0.071358	0.076424
CS18	GD26	HS5	*R.nitidus*	-0.099815	0.06902
JO8	GD26	HS5	*R.nitidus*	-0.039871	0.074244
JO5	GD25	HS5	*R.nitidus*	-0.01659	0.069502
CS15	GD26	HS5	*R.nitidus*	-0.066437	0.071103
CS11	GD26	HS5	*R.nitidus*	-0.052038	0.073184
CS21	GD26	HS5	*R.nitidus*	-0.082121	0.068133
GD33	GD26	HS5	*R.nitidus*	-0.081019	0.069094
GD22	GD26	HS5	*R.nitidus*	-0.003058	0.068601
GD34	GD26	HS5	*R.nitidus*	-0.069547	0.067828
CS13	GD26	HS5	*R.nitidus*	-0.073849	0.073292
GD35	GD26	HS5	*R.nitidus*	-0.021505	0.071808
CS23	GD26	HS5	*R.nitidus*	-0.106074	0.07002
CS31	GD26	HS5	*R.nitidus*	-0.094972	0.071432
GD48	GD26	HS5	*R.nitidus*	0	0.069779
CS30	GD26	HS5	*R.nitidus*	-0.126043	0.06935
CS20	GD26	HS5	*R.nitidus*	-0.085863	0.069256
GD50	GD26	HS5	*R.nitidus*	-0.019231	0.071032
CS24	GD26	HS5	*R.nitidus*	-0.09058	0.069554
GD41	GD26	HS5	*R.nitidus*	-0.062438	0.068772
SC6	GD26	HS5	*R.nitidus*	-0.057273	0.069465
GD42	GD26	HS5	*R.nitidus*	-0.00627	0.071341
CS3	GD26	HS5	*R.nitidus*	-0.097198	0.070206
GD46	GD26	HS5	*R.nitidus*	-0.092292	0.067449
CS17	GD26	HS5	*R.nitidus*	-0.014911	0.074092
SC5	GD26	HS5	*R.nitidus*	-0.024052	0.072581
CS28	GD26	HS5	*R.nitidus*	-0.091429	0.072087
CS14	GD26	HS5	*R.nitidus*	-0.052083	0.073976
SC26	GD26	HS5	*R.nitidus*	-0.110215	0.069864
CS22	GD26	HS5	*R.nitidus*	-0.061299	0.06971
CS19	GD26	HS5	*R.nitidus*	-0.092069	0.072495
SC14	GD26	HS5	*R.nitidus*	-0.083119	0.069593
CS16	GD26	HS5	*R.nitidus*	-0.07013	0.070079
CS25	GD26	HS5	*R.nitidus*	-0.071171	0.070504
CS6	GD26	HS5	*R.nitidus*	-0.085765	0.069044
GD25	GD26	HS5	*R.nitidus*	-0.026812	0.071157
SC42	GD26	HS5	*R.nitidus*	0.126357	0.068432
SC10	GD26	HS5	*R.nitidus*	0.004708	0.072611
SC34	GD26	HS5	*R.nitidus*	-0.04188	0.068678
CS4	GD26	HS5	*R.nitidus*	-0.069444	0.06835
SC27	GD26	HS5	*R.nitidus*	-0.091543	0.06908
SC15	GD26	HS5	*R.nitidus*	-0.061187	0.072034
JW6	GD26	HS5	*R.nitidus*	-0.06338	0.074205
GD21	GD29	HS5	*R.nitidus*	-0.01751	0.069535
SC12	GD26	HS5	*R.nitidus*	-0.114518	0.068039
GD31	GD29	HS5	*R.nitidus*	-0.067358	0.071336
GD45	GD29	HS5	*R.nitidus*	-0.021864	0.075275
SC18	GD26	HS5	*R.nitidus*	-0.030986	0.070166
SC28	GD26	HS5	*R.nitidus*	-0.109745	0.068522
SC24	GD26	HS5	*R.nitidus*	-0.060185	0.072193
JO5	GD26	HS5	*R.nitidus*	0.002568	0.069829
SC44	GD26	HS5	*R.nitidus*	-0.073386	0.069391
SC32	GD26	HS5	*R.nitidus*	-0.026738	0.071367
GD32	GD29	HS5	*R.nitidus*	-0.105505	0.070931
GD46	GD29	HS5	*R.nitidus*	-0.171488	0.066258

SC31	GD26	HS5	*R.nitidus*	-0.076857	0.070351
MY4	GD26	HS5	*R.nitidus*	-0.091644	0.070811
GD33	GD29	HS5	*R.nitidus*	-0.165422	0.070453
JO8	GD29	HS5	*R.nitidus*	-0.134945	0.071029
GD30	GD29	HS5	*R.nitidus*	0.033333	0.071682
CS11	GD29	HS5	*R.nitidus*	-0.147718	0.073325
SC35	GD26	HS5	*R.nitidus*	-0.047059	0.067845
MY6	GD26	HS5	*R.nitidus*	-0.059807	0.070641
SC45	GD26	HS5	*R.nitidus*	-0.029075	0.069889
JW7	GD26	HS5	*R.nitidus*	-0.021905	0.074054
GD25	GD29	HS5	*R.nitidus*	-0.084404	0.070072
JW4	GD26	HS5	*R.nitidus*	0.115869	0.069869
GD26	GD29	HS5	*R.nitidus*	-0.072874	0.06692
JO3	GD26	HS5	*R.nitidus*	-0.011324	0.07213
JO1	GD26	HS5	*R.nitidus*	-0.088926	0.071293
GD41	GD29	HS5	*R.nitidus*	-0.135214	0.065741
CS15	GD29	HS5	*R.nitidus*	-0.140408	0.070433
JO7	GD29	HS5	*R.nitidus*	-0.149098	0.071708
GD35	GD29	HS5	*R.nitidus*	-0.090411	0.071903
GD48	GD29	HS5	*R.nitidus*	-0.076297	0.067564
GD22	GD29	HS5	*R.nitidus*	-0.101493	0.066912
CS13	GD29	HS5	*R.nitidus*	-0.157155	0.071395
GD44	GD29	HS5	*R.nitidus*	-0.143514	0.068677
CS14	GD29	HS5	*R.nitidus*	-0.140833	0.073015
CS12	GD29	HS5	*R.nitidus*	-0.135993	0.070028
CS19	GD29	HS5	*R.nitidus*	-0.170344	0.069842
GD50	GD29	HS5	*R.nitidus*	-0.102417	0.069581
GD49	GD29	HS5	*R.nitidus*	-0.078031	0.072272
GD34	GD29	HS5	*R.nitidus*	-0.179825	0.066703
GD40	GD29	HS5	*R.nitidus*	-0.07971	0.068973
GD42	GD29	HS5	*R.nitidus*	-0.101578	0.06689
CS22	GD29	HS5	*R.nitidus*	-0.135856	0.068661
CS20	GD29	HS5	*R.nitidus*	-0.164167	0.068231
CS23	GD29	HS5	*R.nitidus*	-0.180976	0.067972
CS18	GD29	HS5	*R.nitidus*	-0.15942	0.067573
CS4	GD29	HS5	*R.nitidus*	-0.145575	0.067262
CS21	GD29	HS5	*R.nitidus*	-0.16145	0.06787
SC15	GD29	HS5	*R.nitidus*	-0.148248	0.071834
CS28	GD29	HS5	*R.nitidus*	-0.163588	0.072391
SC10	GD29	HS5	*R.nitidus*	-0.066487	0.071591
SC31	GD29	HS5	*R.nitidus*	-0.156536	0.069872
CS16	GD29	HS5	*R.nitidus*	-0.157107	0.068009
SC24	GD29	HS5	*R.nitidus*	-0.140638	0.069128
CS31	GD29	HS5	*R.nitidus*	-0.185764	0.068768
SC18	GD29	HS5	*R.nitidus*	-0.122776	0.068844
SC35	GD29	HS5	*R.nitidus*	-0.113793	0.066238
SC34	GD29	HS5	*R.nitidus*	-0.114698	0.067073
SC45	GD29	HS5	*R.nitidus*	-0.115798	0.069525
SC27	GD29	HS5	*R.nitidus*	-0.166955	0.067486
CS17	GD29	HS5	*R.nitidus*	-0.099001	0.073407
CS30	GD29	HS5	*R.nitidus*	-0.210113	0.065623
SC6	GD29	HS5	*R.nitidus*	-0.148918	0.069816
JO8	GD30	HS5	*R.nitidus*	-0.118439	0.078564
CS11	GD30	HS5	*R.nitidus*	-0.138561	0.077624
SC5	GD29	HS5	*R.nitidus*	-0.114883	0.072454
CS6	GD29	HS5	*R.nitidus*	-0.167808	0.067692

JW7	GD29	HS5	*R.nitidus*	-0.093107	0.070851
MY6	GD29	HS5	*R.nitidus*	-0.143564	0.06929
CS25	GD29	HS5	*R.nitidus*	-0.157391	0.070004
CS24	GD29	HS5	*R.nitidus*	-0.169002	0.067968
JO5	GD29	HS5	*R.nitidus*	-0.086739	0.067731
GD31	GD30	HS5	*R.nitidus*	-0.130268	0.075963
SC26	GD29	HS5	*R.nitidus*	-0.19848	0.069687
JO3	GD29	HS5	*R.nitidus*	-0.101462	0.068318
MY4	GD29	HS5	*R.nitidus*	-0.175042	0.068871
CS3	GD29	HS5	*R.nitidus*	-0.178002	0.068151
SC32	GD29	HS5	*R.nitidus*	-0.125	0.0689
SC12	GD29	HS5	*R.nitidus*	-0.187874	0.066881
SC14	GD29	HS5	*R.nitidus*	-0.159734	0.068294
SC28	GD29	HS5	*R.nitidus*	-0.184034	0.066411
GD41	GD30	HS5	*R.nitidus*	-0.113939	0.069661
JO7	GD30	HS5	*R.nitidus*	-0.14375	0.077634
SC42	GD29	HS5	*R.nitidus*	0.034911	0.068108
GD35	GD30	HS5	*R.nitidus*	-0.111498	0.072009
GD40	GD30	HS5	*R.nitidus*	-0.069988	0.072411
GD44	GD30	HS5	*R.nitidus*	-0.132353	0.073757
GD29	GD30	HS5	*R.nitidus*	-0.033333	0.071682
JO1	GD29	HS5	*R.nitidus*	-0.154791	0.069355
SC44	GD29	HS5	*R.nitidus*	-0.151695	0.069787
JW4	GD29	HS5	*R.nitidus*	0.040355	0.070107
GD32	GD30	HS5	*R.nitidus*	-0.143631	0.074352
JW6	GD29	HS5	*R.nitidus*	-0.131655	0.071651
GD22	GD30	HS5	*R.nitidus*	-0.10253	0.071998
GD26	GD30	HS5	*R.nitidus*	-0.098465	0.073226
GD34	GD30	HS5	*R.nitidus*	-0.171303	0.071519
CS14	GD30	HS5	*R.nitidus*	-0.117318	0.078341
CS22	GD30	HS5	*R.nitidus*	-0.110245	0.071643
GD25	GD30	HS5	*R.nitidus*	-0.114495	0.075778
GD21	GD30	HS5	*R.nitidus*	-0.052903	0.07366
CS18	GD30	HS5	*R.nitidus*	-0.150219	0.073418
CS12	GD30	HS5	*R.nitidus*	-0.136527	0.075262
GD46	GD30	HS5	*R.nitidus*	-0.168263	0.068208
GD49	GD30	HS5	*R.nitidus*	-0.125894	0.075727
CS13	GD30	HS5	*R.nitidus*	-0.135074	0.075801
GD33	GD30	HS5	*R.nitidus*	-0.126039	0.075703
GD42	GD30	HS5	*R.nitidus*	-0.102662	0.071695
SC5	GD30	HS5	*R.nitidus*	-0.097268	0.075393
CS15	GD30	HS5	*R.nitidus*	-0.129609	0.073258
CS19	GD30	HS5	*R.nitidus*	-0.147689	0.07464
CS25	GD30	HS5	*R.nitidus*	-0.147541	0.077208
CS3	GD30	HS5	*R.nitidus*	-0.14774	0.073494
CS17	GD30	HS5	*R.nitidus*	-0.107015	0.074964
CS20	GD30	HS5	*R.nitidus*	-0.145161	0.072741
GD45	GD30	HS5	*R.nitidus*	-0.03381	0.075443
CS24	GD30	HS5	*R.nitidus*	-0.158963	0.072429
SC34	GD30	HS5	*R.nitidus*	-0.099889	0.07321
CS30	GD30	HS5	*R.nitidus*	-0.18552	0.072073
CS16	GD30	HS5	*R.nitidus*	-0.144944	0.073805
CS4	GD30	HS5	*R.nitidus*	-0.114345	0.069965
GD50	GD30	HS5	*R.nitidus*	-0.110959	0.075725
GD48	GD30	HS5	*R.nitidus*	-0.046113	0.07581
SC14	GD30	HS5	*R.nitidus*	-0.140969	0.072576

SC15	GD30	HS5	*R.nitidus*	-0.125993	0.076003
SC6	GD30	HS5	*R.nitidus*	-0.133939	0.074265
CS21	GD30	HS5	*R.nitidus*	-0.181722	0.073658
CS23	GD30	HS5	*R.nitidus*	-0.153182	0.073701
CS6	GD30	HS5	*R.nitidus*	-0.12963	0.072495
SC18	GD30	HS5	*R.nitidus*	-0.098174	0.072822
SC26	GD30	HS5	*R.nitidus*	-0.175303	0.073423
JO1	GD30	HS5	*R.nitidus*	-0.170467	0.072971
CS28	GD30	HS5	*R.nitidus*	-0.166667	0.076265
SC10	GD30	HS5	*R.nitidus*	-0.07601	0.075682
SC45	GD30	HS5	*R.nitidus*	-0.112886	0.072391
MY4	GD30	HS5	*R.nitidus*	-0.17214	0.073842
SC28	GD30	HS5	*R.nitidus*	-0.15625	0.06957
JO3	GD30	HS5	*R.nitidus*	-0.108077	0.073873
CS31	GD30	HS5	*R.nitidus*	-0.156177	0.072776
SC44	GD30	HS5	*R.nitidus*	-0.130726	0.07093
JW7	GD30	HS5	*R.nitidus*	-0.089535	0.077801
SC35	GD30	HS5	*R.nitidus*	-0.088816	0.074155
SC27	GD30	HS5	*R.nitidus*	-0.169643	0.072799
JW4	GD30	HS5	*R.nitidus*	0.038895	0.076082
JO8	GD31	HS5	*R.nitidus*	-0.003398	0.074233
SC24	GD30	HS5	*R.nitidus*	-0.133333	0.071868
GD29	GD31	HS5	*R.nitidus*	0.067358	0.071336
SC31	GD30	HS5	*R.nitidus*	-0.15027	0.072211
GD22	GD31	HS5	*R.nitidus*	0.010549	0.071713
JW6	GD30	HS5	*R.nitidus*	-0.142678	0.075971
MY6	GD30	HS5	*R.nitidus*	-0.122172	0.073717
SC42	GD30	HS5	*R.nitidus*	0.052682	0.072646
CS11	GD31	HS5	*R.nitidus*	-0.069549	0.075099
SC32	GD30	HS5	*R.nitidus*	-0.102086	0.075683
SC12	GD30	HS5	*R.nitidus*	-0.165914	0.071495
JO7	GD31	HS5	*R.nitidus*	-0.041341	0.076797
GD25	GD31	HS5	*R.nitidus*	0.007519	0.073386
GD41	GD31	HS5	*R.nitidus*	-0.045891	0.073103
GD21	GD31	HS5	*R.nitidus*	0.083077	0.069595
GD44	GD31	HS5	*R.nitidus*	-0.026911	0.071224
CS12	GD31	HS5	*R.nitidus*	-0.054672	0.07256
GD49	GD31	HS5	*R.nitidus*	0.024548	0.07808
GD32	GD31	HS5	*R.nitidus*	-0.024038	0.075073
GD34	GD31	HS5	*R.nitidus*	-0.056564	0.07305
GD35	GD31	HS5	*R.nitidus*	-0.009336	0.072619
GD42	GD31	HS5	*R.nitidus*	0.011136	0.074215
JO5	GD30	HS5	*R.nitidus*	-0.057112	0.071544
CS17	GD31	HS5	*R.nitidus*	-0.033508	0.07807
GD33	GD31	HS5	*R.nitidus*	-0.059197	0.074738
CS19	GD31	HS5	*R.nitidus*	-0.11122	0.074164
GD26	GD31	HS5	*R.nitidus*	-0.005308	0.074201
GD30	GD31	HS5	*R.nitidus*	0.130268	0.075963
GD45	GD31	HS5	*R.nitidus*	0.032338	0.078076
CS23	GD31	HS5	*R.nitidus*	-0.10775	0.071122
CS28	GD31	HS5	*R.nitidus*	-0.073805	0.073301
CS22	GD31	HS5	*R.nitidus*	-0.051257	0.072105
CS16	GD31	HS5	*R.nitidus*	-0.067748	0.073686
SC5	GD31	HS5	*R.nitidus*	-0.021538	0.076987
CS13	GD31	HS5	*R.nitidus*	-0.085878	0.073622
SC18	GD31	HS5	*R.nitidus*	-0.024316	0.07249

CS18	GD31	HS5	*R.nitidus*	-0.096466	0.073766
CS30	GD31	HS5	*R.nitidus*	-0.128	0.073272
GD48	GD31	HS5	*R.nitidus*	0.025316	0.074872
SC6	GD31	HS5	*R.nitidus*	-0.048077	0.072294
GD50	GD31	HS5	*R.nitidus*	-0.003645	0.0751
GD46	GD31	HS5	*R.nitidus*	-0.051907	0.070927
GD40	GD31	HS5	*R.nitidus*	0.047331	0.071051
CS3	GD31	HS5	*R.nitidus*	-0.096929	0.072728
CS24	GD31	HS5	*R.nitidus*	-0.097087	0.071937
CS14	GD31	HS5	*R.nitidus*	-0.050332	0.07744
SC14	GD31	HS5	*R.nitidus*	-0.082613	0.072938
CS15	GD31	HS5	*R.nitidus*	-0.078008	0.073877
SC15	GD31	HS5	*R.nitidus*	-0.053946	0.074695
CS31	GD31	HS5	*R.nitidus*	-0.087539	0.073941
CS20	GD31	HS5	*R.nitidus*	-0.08034	0.074014
CS21	GD31	HS5	*R.nitidus*	-0.093661	0.072304
CS25	GD31	HS5	*R.nitidus*	-0.088632	0.072826
SC27	GD31	HS5	*R.nitidus*	-0.080661	0.071572
JO8	GD32	HS5	*R.nitidus*	0.011292	0.079232
JW4	GD31	HS5	*R.nitidus*	0.090992	0.072917
CS4	GD31	HS5	*R.nitidus*	-0.060998	0.070886
SC34	GD31	HS5	*R.nitidus*	-0.037665	0.0704
SC28	GD31	HS5	*R.nitidus*	-0.090737	0.071795
JW7	GD31	HS5	*R.nitidus*	-0.017051	0.075889
SC44	GD31	HS5	*R.nitidus*	-0.068571	0.072744
SC32	GD31	HS5	*R.nitidus*	-0.009276	0.073688
SC10	GD31	HS5	*R.nitidus*	0.016244	0.074677
SC42	GD31	HS5	*R.nitidus*	0.111853	0.070494
CS6	GD31	HS5	*R.nitidus*	-0.077897	0.073137
CS11	GD32	HS5	*R.nitidus*	-0.045409	0.075301
SC45	GD31	HS5	*R.nitidus*	-0.054924	0.072433
SC26	GD31	HS5	*R.nitidus*	-0.111753	0.073526
MY6	GD31	HS5	*R.nitidus*	-0.058594	0.073636
SC12	GD31	HS5	*R.nitidus*	-0.103896	0.070661
JW6	GD31	HS5	*R.nitidus*	-0.044728	0.074854
JO1	GD31	HS5	*R.nitidus*	-0.084326	0.071578
SC24	GD31	HS5	*R.nitidus*	-0.052478	0.074388
JO5	GD31	HS5	*R.nitidus*	-0.012116	0.072658
MY4	GD31	HS5	*R.nitidus*	-0.095098	0.07311
GD29	GD32	HS5	*R.nitidus*	0.105505	0.070931
SC31	GD31	HS5	*R.nitidus*	-0.075188	0.07341
GD25	GD32	HS5	*R.nitidus*	0.020202	0.074656
SC35	GD31	HS5	*R.nitidus*	-0.042333	0.071965
JO7	GD32	HS5	*R.nitidus*	-0.025463	0.079046
CS12	GD32	HS5	*R.nitidus*	-0.026824	0.073621
GD30	GD32	HS5	*R.nitidus*	0.143631	0.074352
GD26	GD32	HS5	*R.nitidus*	0.023022	0.067293
JO3	GD31	HS5	*R.nitidus*	0.017459	0.075559
GD40	GD32	HS5	*R.nitidus*	0.045016	0.070195
GD41	GD32	HS5	*R.nitidus*	-0.021542	0.072331
CS13	GD32	HS5	*R.nitidus*	-0.047666	0.073492
GD42	GD32	HS5	*R.nitidus*	0.010539	0.071913
GD33	GD32	HS5	*R.nitidus*	-0.045822	0.074014
GD21	GD32	HS5	*R.nitidus*	0.0783	0.072024
GD22	GD32	HS5	*R.nitidus*	0.048499	0.072562
CS23	GD32	HS5	*R.nitidus*	-0.086259	0.074215

GD31	GD32	HS5	*R.nitidus*	0.024038	0.075073
CS15	GD32	HS5	*R.nitidus*	-0.045778	0.075117
GD49	GD32	HS5	*R.nitidus*	0.061947	0.076966
CS17	GD32	HS5	*R.nitidus*	-0.002167	0.077899
GD35	GD32	HS5	*R.nitidus*	-0.003264	0.072602
GD50	GD32	HS5	*R.nitidus*	0.021305	0.073793
CS28	GD32	HS5	*R.nitidus*	-0.064586	0.075213
CS3	GD32	HS5	*R.nitidus*	-0.067554	0.073488
GD45	GD32	HS5	*R.nitidus*	0.080056	0.076924
CS19	GD32	HS5	*R.nitidus*	-0.056911	0.074679
CS20	GD32	HS5	*R.nitidus*	-0.049696	0.072314
SC5	GD32	HS5	*R.nitidus*	0.025184	0.076084
CS14	GD32	HS5	*R.nitidus*	-0.023279	0.076597
GD34	GD32	HS5	*R.nitidus*	-0.012077	0.070467
GD44	GD32	HS5	*R.nitidus*	-0.005787	0.072868
CS22	GD32	HS5	*R.nitidus*	-0.034339	0.071634
CS30	GD32	HS5	*R.nitidus*	-0.083595	0.07341
SC14	GD32	HS5	*R.nitidus*	-0.045596	0.071369
SC15	GD32	HS5	*R.nitidus*	-0.015974	0.075594
CS25	GD32	HS5	*R.nitidus*	-0.042531	0.075071
CS24	GD32	HS5	*R.nitidus*	-0.0842	0.071189
GD46	GD32	HS5	*R.nitidus*	-0.059908	0.071524
GD48	GD32	HS5	*R.nitidus*	0.028708	0.070328
CS18	GD32	HS5	*R.nitidus*	-0.068394	0.073102
SC24	GD32	HS5	*R.nitidus*	-0.026288	0.073169
SC18	GD32	HS5	*R.nitidus*	0.009804	0.073652
SC31	GD32	HS5	*R.nitidus*	-0.044625	0.072009
CS16	GD32	HS5	*R.nitidus*	-0.051653	0.073025
SC35	GD32	HS5	*R.nitidus*	0.0222	0.072198
CS21	GD32	HS5	*R.nitidus*	-0.058014	0.07291
SC12	GD32	HS5	*R.nitidus*	-0.088595	0.071188
SC27	GD32	HS5	*R.nitidus*	-0.059686	0.070122
MY4	GD32	HS5	*R.nitidus*	-0.067692	0.073659
SC10	GD32	HS5	*R.nitidus*	0.028785	0.071457
SC26	GD32	HS5	*R.nitidus*	-0.063918	0.071891
SC34	GD32	HS5	*R.nitidus*	-0.024072	0.072211
SC45	GD32	HS5	*R.nitidus*	0.002022	0.070733
SC6	GD32	HS5	*R.nitidus*	-0.032359	0.07335
JW4	GD32	HS5	*R.nitidus*	0.128372	0.071455
JO3	GD32	HS5	*R.nitidus*	0.008264	0.072835
CS6	GD32	HS5	*R.nitidus*	-0.070452	0.072903
SC42	GD32	HS5	*R.nitidus*	0.129204	0.071422
SC28	GD32	HS5	*R.nitidus*	-0.046201	0.070455
CS4	GD32	HS5	*R.nitidus*	-0.053427	0.068986
JW6	GD32	HS5	*R.nitidus*	-0.01479	0.075961
CS31	GD32	HS5	*R.nitidus*	-0.055495	0.074623
SC32	GD32	HS5	*R.nitidus*	0.002107	0.073302
SC44	GD32	HS5	*R.nitidus*	-0.011616	0.07181
GD40	CS12	HS5	*R.nitidus*	0.071845	0.070464
GD33	CS12	HS5	*R.nitidus*	-0.007767	0.072365
CS11	CS12	HS5	*R.nitidus*	-0.012853	0.073623
GD42	CS12	HS5	*R.nitidus*	0.049213	0.073136
JW7	GD32	HS5	*R.nitidus*	0.034664	0.071441
GD25	CS12	HS5	*R.nitidus*	0.096057	0.070153
GD21	CS12	HS5	*R.nitidus*	0.130713	0.06936
CS13	CS12	HS5	*R.nitidus*	-0.032432	0.076572

MY6	GD32	HS5	*R.nitidus*	-0.036036	0.074439
JO8	CS12	HS5	*R.nitidus*	0.019016	0.07564
GD45	CS12	HS5	*R.nitidus*	0.093182	0.076324
JO1	GD32	HS5	*R.nitidus*	-0.0583	0.072536
GD44	CS12	HS5	*R.nitidus*	0.030602	0.072681
GD34	CS12	HS5	*R.nitidus*	-0.001845	0.07149
GD29	CS12	HS5	*R.nitidus*	0.135993	0.070028
JO7	CS12	HS5	*R.nitidus*	0.011074	0.07706
GD26	CS12	HS5	*R.nitidus*	0.048644	0.071913
GD50	CS12	HS5	*R.nitidus*	0.046892	0.073831
JO5	GD32	HS5	*R.nitidus*	0.055276	0.069567
GD48	CS12	HS5	*R.nitidus*	0.061753	0.073513
CS25	CS12	HS5	*R.nitidus*	0.00804	0.074178
CS28	CS12	HS5	*R.nitidus*	0	0.073963
CS14	CS12	HS5	*R.nitidus*	0.013643	0.077386
GD41	CS12	HS5	*R.nitidus*	0.013308	0.071029
GD32	CS12	HS5	*R.nitidus*	0.026824	0.073621
GD30	CS12	HS5	*R.nitidus*	0.136527	0.075262
GD35	CS12	HS5	*R.nitidus*	0.050047	0.069407
GD22	CS12	HS5	*R.nitidus*	0.068203	0.071136
GD46	CS12	HS5	*R.nitidus*	-0.003972	0.071954
CS30	CS12	HS5	*R.nitidus*	-0.061013	0.073152
GD31	CS12	HS5	*R.nitidus*	0.054672	0.07256
CS19	CS12	HS5	*R.nitidus*	-0.017598	0.073455
SC12	CS12	HS5	*R.nitidus*	-0.04142	0.067744
CS17	CS12	HS5	*R.nitidus*	0.046809	0.075024
CS23	CS12	HS5	*R.nitidus*	-0.025194	0.072328
CS31	CS12	HS5	*R.nitidus*	-0.030851	0.073271
CS22	CS12	HS5	*R.nitidus*	0.008016	0.071458
GD49	CS12	HS5	*R.nitidus*	0.080275	0.07421
CS3	CS12	HS5	*R.nitidus*	-0.005946	0.071084
CS21	CS12	HS5	*R.nitidus*	-0.015504	0.071843
CS18	CS12	HS5	*R.nitidus*	-0.015091	0.072062
CS16	CS12	HS5	*R.nitidus*	-0.010703	0.069741
SC5	CS12	HS5	*R.nitidus*	0.052372	0.073418
SC18	CS12	HS5	*R.nitidus*	0.058409	0.071052
SC24	CS12	HS5	*R.nitidus*	0.019531	0.07145
CS15	CS12	HS5	*R.nitidus*	-0.001391	0.070419
SC10	CS12	HS5	*R.nitidus*	0.110302	0.071978
CS20	CS12	HS5	*R.nitidus*	0.003929	0.071925
CS24	CS12	HS5	*R.nitidus*	-0.044776	0.071336
SC28	CS12	HS5	*R.nitidus*	-0.045363	0.069718
SC42	CS12	HS5	*R.nitidus*	0.199497	0.070093
SC14	CS12	HS5	*R.nitidus*	-0.012322	0.071196
SC32	CS12	HS5	*R.nitidus*	0.045501	0.071901
JO5	CS12	HS5	*R.nitidus*	0.067764	0.070315
SC26	CS12	HS5	*R.nitidus*	-0.043825	0.070061
SC6	CS12	HS5	*R.nitidus*	-0.003064	0.0737
SC31	CS12	HS5	*R.nitidus*	-0.02266	0.074597
JW6	CS12	HS5	*R.nitidus*	0.009825	0.074343
CS6	CS12	HS5	*R.nitidus*	-0.028459	0.069074
SC34	CS12	HS5	*R.nitidus*	0.007866	0.069411
MY4	CS12	HS5	*R.nitidus*	-0.040959	0.073123
SC15	CS12	HS5	*R.nitidus*	0.005325	0.071661
JW4	CS12	HS5	*R.nitidus*	0.152528	0.072657
GD26	GD33	HS5	*R.nitidus*	0.081019	0.069094

SC45	CS12	HS5	*R.nitidus*	0.038348	0.069575
SC44	CS12	HS5	*R.nitidus*	0	0.069982
JO7	GD33	HS5	*R.nitidus*	-0.003093	0.076441
CS4	CS12	HS5	*R.nitidus*	-0.005792	0.069455
JO1	CS12	HS5	*R.nitidus*	-0.012183	0.071317
JO8	GD33	HS5	*R.nitidus*	0.030917	0.077694
JO3	CS12	HS5	*R.nitidus*	0.05278	0.072612
SC35	CS12	HS5	*R.nitidus*	0.04381	0.071144
GD31	GD33	HS5	*R.nitidus*	0.059197	0.074738
CS13	GD33	HS5	*R.nitidus*	-0.021486	0.074774
GD40	GD33	HS5	*R.nitidus*	0.076471	0.071342
GD21	GD33	HS5	*R.nitidus*	0.135394	0.069642
SC27	CS12	HS5	*R.nitidus*	-0.008016	0.06971
JW7	CS12	HS5	*R.nitidus*	0.051447	0.073586
GD35	GD33	HS5	*R.nitidus*	0.04878	0.07327
MY6	CS12	HS5	*R.nitidus*	0.005865	0.072235
GD45	GD33	HS5	*R.nitidus*	0.092883	0.077391
GD30	GD33	HS5	*R.nitidus*	0.126039	0.075703
GD49	GD33	HS5	*R.nitidus*	0.050505	0.078038
CS11	GD33	HS5	*R.nitidus*	-0.006261	0.07433
CS12	GD33	HS5	*R.nitidus*	0.007767	0.072365
GD46	GD33	HS5	*R.nitidus*	-0.017241	0.07211
GD25	GD33	HS5	*R.nitidus*	0.073469	0.073877
CS17	GD33	HS5	*R.nitidus*	0.039634	0.077113
GD22	GD33	HS5	*R.nitidus*	0.078261	0.068123
GD41	GD33	HS5	*R.nitidus*	0.008205	0.0691
GD29	GD33	HS5	*R.nitidus*	0.165422	0.070453
CS14	GD33	HS5	*R.nitidus*	0.006329	0.075877
CS28	GD33	HS5	*R.nitidus*	-0.036893	0.074364
GD48	GD33	HS5	*R.nitidus*	0.062147	0.074089
GD32	GD33	HS5	*R.nitidus*	0.045822	0.074014
CS19	GD33	HS5	*R.nitidus*	-0.024225	0.074106
GD42	GD33	HS5	*R.nitidus*	0.044276	0.0721
SC6	GD33	HS5	*R.nitidus*	0.008303	0.072129
CS21	GD33	HS5	*R.nitidus*	-0.024281	0.072734
GD34	GD33	HS5	*R.nitidus*	-0.015094	0.070662
GD44	GD33	HS5	*R.nitidus*	0.012109	0.072803
SC18	GD33	HS5	*R.nitidus*	0.024062	0.071389
SC12	GD33	HS5	*R.nitidus*	-0.053803	0.072199
CS18	GD33	HS5	*R.nitidus*	-0.028763	0.072091
GD50	GD33	HS5	*R.nitidus*	0.044064	0.073658
CS20	GD33	HS5	*R.nitidus*	-0.015219	0.072786
CS22	GD33	HS5	*R.nitidus*	0.011797	0.071731
CS4	GD33	HS5	*R.nitidus*	-0.008094	0.070837
CS23	GD33	HS5	*R.nitidus*	-0.049778	0.072727
CS16	GD33	HS5	*R.nitidus*	-0.00544	0.071331
CS6	GD33	HS5	*R.nitidus*	-0.038251	0.071755
SC32	GD33	HS5	*R.nitidus*	0.045581	0.073925
SC31	GD33	HS5	*R.nitidus*	-0.034608	0.072659
CS24	GD33	HS5	*R.nitidus*	-0.035055	0.072732
SC24	GD33	HS5	*R.nitidus*	-0.002765	0.072637
SC26	GD33	HS5	*R.nitidus*	-0.050233	0.070647
CS3	GD33	HS5	*R.nitidus*	-0.043318	0.072277
SC10	GD33	HS5	*R.nitidus*	0.062741	0.073175
SC14	GD33	HS5	*R.nitidus*	-0.012489	0.07356
SC42	GD33	HS5	*R.nitidus*	0.173713	0.070751

CS25	GD33	HS5	*R.nitidus*	-0.002904	0.074212
SC27	GD33	HS5	*R.nitidus*	-0.018116	0.071697
CS30	GD33	HS5	*R.nitidus*	-0.067219	0.071442
SC15	GD33	HS5	*R.nitidus*	-0.0065	0.074197
JW7	GD33	HS5	*R.nitidus*	0.057307	0.075346
CS31	GD33	HS5	*R.nitidus*	-0.0254	0.071777
SC5	GD33	HS5	*R.nitidus*	0.051973	0.07497
MY4	GD33	HS5	*R.nitidus*	-0.030055	0.073194
JW4	GD33	HS5	*R.nitidus*	0.186066	0.071948
SC45	GD33	HS5	*R.nitidus*	0.03664	0.073535
CS15	GD33	HS5	*R.nitidus*	-0.009892	0.070719
JO5	GD33	HS5	*R.nitidus*	0.06042	0.070016
SC28	GD33	HS5	*R.nitidus*	-0.033243	0.070827
GD30	GD34	HS5	*R.nitidus*	0.171303	0.071519
GD25	GD34	HS5	*R.nitidus*	0.061836	0.071935
GD22	GD34	HS5	*R.nitidus*	0.078242	0.067016
MY6	GD33	HS5	*R.nitidus*	0.003587	0.071988
CS12	GD34	HS5	*R.nitidus*	0.001845	0.07149
SC35	GD33	HS5	*R.nitidus*	0.015385	0.073275
GD21	GD34	HS5	*R.nitidus*	0.127907	0.069218
SC34	GD33	HS5	*R.nitidus*	-0.002664	0.070851
SC44	GD33	HS5	*R.nitidus*	-0.013649	0.071178
JO1	GD33	HS5	*R.nitidus*	-0.026738	0.073533
GD35	GD34	HS5	*R.nitidus*	0.037402	0.070487
JW6	GD33	HS5	*R.nitidus*	0.040456	0.075984
JO8	GD34	HS5	*R.nitidus*	0.040966	0.074851
GD26	GD34	HS5	*R.nitidus*	0.069547	0.067828
GD49	GD34	HS5	*R.nitidus*	0.05597	0.075273
CS11	GD34	HS5	*R.nitidus*	-0.007563	0.071493
GD42	GD34	HS5	*R.nitidus*	0.048649	0.06972
GD40	GD34	HS5	*R.nitidus*	0.088097	0.069476
GD41	GD34	HS5	*R.nitidus*	-0.00213	0.068705
JO3	GD33	HS5	*R.nitidus*	0.064428	0.073365
GD29	GD34	HS5	*R.nitidus*	0.179825	0.066703
GD31	GD34	HS5	*R.nitidus*	0.056564	0.07305
JO7	GD34	HS5	*R.nitidus*	0.011893	0.073623
GD46	GD34	HS5	*R.nitidus*	-0.024652	0.068925
GD48	GD34	HS5	*R.nitidus*	0.082579	0.068653
GD45	GD34	HS5	*R.nitidus*	0.106587	0.074703
GD50	GD34	HS5	*R.nitidus*	0.022274	0.074046
GD32	GD34	HS5	*R.nitidus*	0.012077	0.070467
CS15	GD34	HS5	*R.nitidus*	-0.005063	0.070765
CS24	GD34	HS5	*R.nitidus*	-0.041021	0.069284
CS18	GD34	HS5	*R.nitidus*	-0.029623	0.069089
CS16	GD34	HS5	*R.nitidus*	-0.009418	0.068836
CS13	GD34	HS5	*R.nitidus*	-0.016964	0.072111
GD33	GD34	HS5	*R.nitidus*	0.015094	0.070662
CS17	GD34	HS5	*R.nitidus*	0.024254	0.073437
CS20	GD34	HS5	*R.nitidus*	-0.023356	0.069962
GD44	GD34	HS5	*R.nitidus*	0.002962	0.071021
SC18	GD34	HS5	*R.nitidus*	0.013953	0.070836
CS31	GD34	HS5	*R.nitidus*	-0.038498	0.06824
CS19	GD34	HS5	*R.nitidus*	-0.039162	0.071076
CS21	GD34	HS5	*R.nitidus*	-0.023276	0.070606
CS22	GD34	HS5	*R.nitidus*	-0.001742	0.07136
SC10	GD34	HS5	*R.nitidus*	0.049571	0.072483

SC5	GD34	HS5	*R.nitidus*	0.040835	0.073043
SC27	GD34	HS5	*R.nitidus*	-0.035009	0.069752
CS14	GD34	HS5	*R.nitidus*	-0.008584	0.071731
SC45	GD34	HS5	*R.nitidus*	0.019845	0.070072
JW6	GD34	HS5	*R.nitidus*	0.031884	0.073915
SC14	GD34	HS5	*R.nitidus*	-0.020158	0.070138
CS3	GD34	HS5	*R.nitidus*	-0.039788	0.069333
SC12	GD34	HS5	*R.nitidus*	-0.068689	0.071928
CS28	GD34	HS5	*R.nitidus*	-0.046935	0.07407
JO5	GD34	HS5	*R.nitidus*	0.064879	0.068398
SC24	GD34	HS5	*R.nitidus*	-0.006536	0.072104
JO3	GD34	HS5	*R.nitidus*	0.050984	0.072125
SC34	GD34	HS5	*R.nitidus*	0.009624	0.070374
SC35	GD34	HS5	*R.nitidus*	0.016364	0.069477
CS4	GD34	HS5	*R.nitidus*	-0.009475	0.0691
CS25	GD34	HS5	*R.nitidus*	-0.016071	0.071594
CS23	GD34	HS5	*R.nitidus*	-0.045217	0.070836
SC26	GD34	HS5	*R.nitidus*	-0.053058	0.07112
CS30	GD34	HS5	*R.nitidus*	-0.082243	0.068356
JO8	GD35	HS5	*R.nitidus*	-0.026824	0.074564
MY4	GD34	HS5	*R.nitidus*	-0.04302	0.069905
SC6	GD34	HS5	*R.nitidus*	-0.002671	0.07081
JW4	GD34	HS5	*R.nitidus*	0.156225	0.069826
MY6	GD34	HS5	*R.nitidus*	-0.00613	0.070858
GD22	GD35	HS5	*R.nitidus*	0.010309	0.070693
JO1	GD34	HS5	*R.nitidus*	-0.037257	0.071169
SC15	GD34	HS5	*R.nitidus*	-0.010046	0.071387
SC44	GD34	HS5	*R.nitidus*	-0.01991	0.070111
SC28	GD34	HS5	*R.nitidus*	-0.046369	0.067579
SC31	GD34	HS5	*R.nitidus*	-0.029386	0.07032
SC32	GD34	HS5	*R.nitidus*	0.022361	0.071023
SC42	GD34	HS5	*R.nitidus*	0.184791	0.068798
GD25	GD35	HS5	*R.nitidus*	0.01749	0.067311
GD29	GD35	HS5	*R.nitidus*	0.090411	0.071903
GD21	GD35	HS5	*R.nitidus*	0.079263	0.067978
JO7	GD35	HS5	*R.nitidus*	-0.024059	0.073783
CS6	GD34	HS5	*R.nitidus*	-0.036092	0.069451
JW7	GD34	HS5	*R.nitidus*	0.060837	0.073912
CS11	GD35	HS5	*R.nitidus*	-0.035587	0.070865
GD40	GD35	HS5	*R.nitidus*	0.027938	0.066625
GD31	GD35	HS5	*R.nitidus*	0.009336	0.072619
GD32	GD35	HS5	*R.nitidus*	0.003264	0.072602
GD30	GD35	HS5	*R.nitidus*	0.111498	0.072009
CS16	GD35	HS5	*R.nitidus*	-0.046595	0.068122
GD44	GD35	HS5	*R.nitidus*	-0.035533	0.069894
GD34	GD35	HS5	*R.nitidus*	-0.037402	0.070487
CS18	GD35	HS5	*R.nitidus*	-0.063523	0.070444
GD33	GD35	HS5	*R.nitidus*	-0.04878	0.07327
GD26	GD35	HS5	*R.nitidus*	0.021505	0.071808
GD46	GD35	HS5	*R.nitidus*	-0.068136	0.069684
GD45	GD35	HS5	*R.nitidus*	0.067227	0.075395
CS15	GD35	HS5	*R.nitidus*	-0.039855	0.069542
GD41	GD35	HS5	*R.nitidus*	-0.037962	0.070127
GD42	GD35	HS5	*R.nitidus*	-0.00813	0.071646
CS28	GD35	HS5	*R.nitidus*	-0.067764	0.071195
CS20	GD35	HS5	*R.nitidus*	-0.055606	0.069612

GD49	GD35	HS5	*R.nitidus*	0.025404	0.074448
CS25	GD35	HS5	*R.nitidus*	-0.0377	0.071277
CS12	GD35	HS5	*R.nitidus*	-0.050047	0.069407
CS13	GD35	HS5	*R.nitidus*	-0.055198	0.071247
CS30	GD35	HS5	*R.nitidus*	-0.095281	0.070298
CS22	GD35	HS5	*R.nitidus*	-0.039252	0.069755
CS14	GD35	HS5	*R.nitidus*	-0.020966	0.069954
CS23	GD35	HS5	*R.nitidus*	-0.079494	0.069793
SC6	GD35	HS5	*R.nitidus*	-0.042457	0.068196
CS4	GD35	HS5	*R.nitidus*	-0.03286	0.068039
GD50	GD35	HS5	*R.nitidus*	-0.029904	0.071526
GD48	GD35	HS5	*R.nitidus*	0.01973	0.072835
SC12	GD35	HS5	*R.nitidus*	-0.095325	0.067819
CS31	GD35	HS5	*R.nitidus*	-0.072797	0.069851
SC10	GD35	HS5	*R.nitidus*	0.027436	0.06992
SC28	GD35	HS5	*R.nitidus*	-0.080543	0.070223
CS19	GD35	HS5	*R.nitidus*	-0.063107	0.071569
SC31	GD35	HS5	*R.nitidus*	-0.05821	0.068806
SC24	GD35	HS5	*R.nitidus*	-0.052478	0.069824
SC5	GD35	HS5	*R.nitidus*	0.007505	0.073287
CS24	GD35	HS5	*R.nitidus*	-0.073826	0.068944
SC34	GD35	HS5	*R.nitidus*	-0.030937	0.068363
CS17	GD35	HS5	*R.nitidus*	-0.006924	0.073752
CS21	GD35	HS5	*R.nitidus*	-0.060018	0.071086
CS3	GD35	HS5	*R.nitidus*	-0.047101	0.069142
SC32	GD35	HS5	*R.nitidus*	0.00366	0.070578
GD25	GD40	HS5	*R.nitidus*	0.004753	0.069874
JO3	GD35	HS5	*R.nitidus*	0	0.072248
JW4	GD35	HS5	*R.nitidus*	0.133559	0.06998
SC14	GD35	HS5	*R.nitidus*	-0.043916	0.069444
GD29	GD40	HS5	*R.nitidus*	0.07971	0.068973
SC15	GD35	HS5	*R.nitidus*	-0.040634	0.072714
SC18	GD35	HS5	*R.nitidus*	-0.021718	0.071148
JW7	GD35	HS5	*R.nitidus*	0.011583	0.071256
GD30	GD40	HS5	*R.nitidus*	0.069988	0.072411
JO8	GD40	HS5	*R.nitidus*	-0.055441	0.072136
JO7	GD40	HS5	*R.nitidus*	-0.052632	0.074687
SC26	GD35	HS5	*R.nitidus*	-0.079137	0.070387
GD26	GD40	HS5	*R.nitidus*	-0.015903	0.069313
CS6	GD35	HS5	*R.nitidus*	-0.07156	0.07007
GD21	GD40	HS5	*R.nitidus*	0.041815	0.069144
JO1	GD35	HS5	*R.nitidus*	-0.057743	0.06881
MY6	GD35	HS5	*R.nitidus*	-0.045956	0.071191
SC35	GD35	HS5	*R.nitidus*	-0.015962	0.07168
GD35	GD40	HS5	*R.nitidus*	-0.027938	0.066625
CS11	GD40	HS5	*R.nitidus*	-0.074106	0.069041
GD33	GD40	HS5	*R.nitidus*	-0.076471	0.071342
JW6	GD35	HS5	*R.nitidus*	-0.022941	0.073135
SC45	GD35	HS5	*R.nitidus*	-0.009033	0.068403
GD32	GD40	HS5	*R.nitidus*	-0.045016	0.070195
SC44	GD35	HS5	*R.nitidus*	-0.050326	0.070399
MY4	GD35	HS5	*R.nitidus*	-0.084914	0.071135
SC42	GD35	HS5	*R.nitidus*	0.162433	0.069001
CS12	GD40	HS5	*R.nitidus*	-0.071845	0.070464
GD41	GD40	HS5	*R.nitidus*	-0.079887	0.067899
GD45	GD40	HS5	*R.nitidus*	0.009091	0.074761

JO5	GD35	HS5	*R.nitidus*	0.013441	0.068842
CS13	GD40	HS5	*R.nitidus*	-0.093064	0.07079
GD49	GD40	HS5	*R.nitidus*	-0.002291	0.074377
GD22	GD40	HS5	*R.nitidus*	-0.021183	0.068514
GD42	GD40	HS5	*R.nitidus*	-0.045062	0.06917
GD31	GD40	HS5	*R.nitidus*	-0.047331	0.071051
GD44	GD40	HS5	*R.nitidus*	-0.078565	0.068115
CS14	GD40	HS5	*R.nitidus*	-0.05821	0.07165
GD46	GD40	HS5	*R.nitidus*	-0.092219	0.067872
CS24	GD40	HS5	*R.nitidus*	-0.12523	0.068302
GD34	GD40	HS5	*R.nitidus*	-0.088097	0.069476
CS22	GD40	HS5	*R.nitidus*	-0.073105	0.06828
CS18	GD40	HS5	*R.nitidus*	-0.115314	0.06751
SC10	GD40	HS5	*R.nitidus*	-0.010148	0.070581
CS25	GD40	HS5	*R.nitidus*	-0.083865	0.070205
CS3	GD40	HS5	*R.nitidus*	-0.105787	0.069539
GD50	GD40	HS5	*R.nitidus*	-0.040043	0.072497
GD48	GD40	HS5	*R.nitidus*	-0.02	0.070973
SC5	GD40	HS5	*R.nitidus*	-0.043358	0.070988
CS17	GD40	HS5	*R.nitidus*	-0.049951	0.072793
CS15	GD40	HS5	*R.nitidus*	-0.076787	0.069597
CS31	GD40	HS5	*R.nitidus*	-0.118812	0.068756
MY6	GD40	HS5	*R.nitidus*	-0.081882	0.067874
SC15	GD40	HS5	*R.nitidus*	-0.096774	0.069845
SC14	GD40	HS5	*R.nitidus*	-0.105727	0.070367
CS23	GD40	HS5	*R.nitidus*	-0.122667	0.068319
SC34	GD40	HS5	*R.nitidus*	-0.072695	0.06735
JW6	GD40	HS5	*R.nitidus*	-0.057561	0.072719
SC26	GD40	HS5	*R.nitidus*	-0.127773	0.069931
CS19	GD40	HS5	*R.nitidus*	-0.111628	0.069196
SC35	GD40	HS5	*R.nitidus*	-0.052768	0.068617
SC31	GD40	HS5	*R.nitidus*	-0.107081	0.067662
CS20	GD40	HS5	*R.nitidus*	-0.103448	0.069475
CS4	GD40	HS5	*R.nitidus*	-0.084848	0.068016
SC24	GD40	HS5	*R.nitidus*	-0.069159	0.067063
CS21	GD40	HS5	*R.nitidus*	-0.10614	0.069758
SC32	GD40	HS5	*R.nitidus*	-0.051601	0.067321
SC18	GD40	HS5	*R.nitidus*	-0.060976	0.067898
SC12	GD40	HS5	*R.nitidus*	-0.138298	0.068461
JO1	GD40	HS5	*R.nitidus*	-0.099405	0.069675
CS28	GD40	HS5	*R.nitidus*	-0.100659	0.069532
SC45	GD40	HS5	*R.nitidus*	-0.055846	0.068464
CS30	GD40	HS5	*R.nitidus*	-0.138596	0.067836
SC44	GD40	HS5	*R.nitidus*	-0.104736	0.068501
JO3	GD40	HS5	*R.nitidus*	-0.009666	0.070722
JW7	GD40	HS5	*R.nitidus*	-0.035917	0.072663
CS16	GD40	HS5	*R.nitidus*	-0.086796	0.069364
SC6	GD40	HS5	*R.nitidus*	-0.069509	0.06907
MY4	GD40	HS5	*R.nitidus*	-0.12816	0.068181
SC27	GD40	HS5	*R.nitidus*	-0.113879	0.06895
GD30	GD41	HS5	*R.nitidus*	0.113939	0.069661
GD25	GD41	HS5	*R.nitidus*	0.033138	0.071775
JW4	GD40	HS5	*R.nitidus*	0.081535	0.06947
SC42	GD40	HS5	*R.nitidus*	0.112941	0.067706
CS6	GD40	HS5	*R.nitidus*	-0.110911	0.068446
SC28	GD40	HS5	*R.nitidus*	-0.133568	0.068129

GD26	GD41	HS5	*R.nitidus*	0.062438	0.068772
JO7	GD41	HS5	*R.nitidus*	0.029167	0.07378
GD29	GD41	HS5	*R.nitidus*	0.135214	0.065741
JO5	GD40	HS5	*R.nitidus*	-0.013722	0.067938
GD31	GD41	HS5	*R.nitidus*	0.045891	0.073103
GD50	GD41	HS5	*R.nitidus*	0.032092	0.073449
CS12	GD41	HS5	*R.nitidus*	-0.013308	0.071029
GD33	GD41	HS5	*R.nitidus*	-0.008205	0.0691
GD32	GD41	HS5	*R.nitidus*	0.021542	0.072331
GD22	GD41	HS5	*R.nitidus*	0.064155	0.068865
GD44	GD41	HS5	*R.nitidus*	0.009464	0.071157
GD45	GD41	HS5	*R.nitidus*	0.066748	0.076862
GD48	GD41	HS5	*R.nitidus*	0.088428	0.069293
GD49	GD41	HS5	*R.nitidus*	0.078125	0.07547
GD35	GD41	HS5	*R.nitidus*	0.037962	0.070127
GD21	GD41	HS5	*R.nitidus*	0.109005	0.065477
CS11	GD41	HS5	*R.nitidus*	-0.012489	0.073085
GD42	GD41	HS5	*R.nitidus*	0.051491	0.066107
JO8	GD41	HS5	*R.nitidus*	0.023861	0.074502
CS19	GD41	HS5	*R.nitidus*	-0.032727	0.071948
CS14	GD41	HS5	*R.nitidus*	0.005333	0.073428
CS3	GD41	HS5	*R.nitidus*	-0.037294	0.069119
CS17	GD41	HS5	*R.nitidus*	0.024631	0.074135
CS4	GD41	HS5	*R.nitidus*	-0.014109	0.066796
CS13	GD41	HS5	*R.nitidus*	-0.023105	0.07143
SC10	GD41	HS5	*R.nitidus*	0.056475	0.070387
CS16	GD41	HS5	*R.nitidus*	-0.008182	0.069445
SC6	GD41	HS5	*R.nitidus*	-0.003666	0.069276
CS15	GD41	HS5	*R.nitidus*	-0.00713	0.070107
GD40	GD41	HS5	*R.nitidus*	0.079887	0.067899
SC18	GD41	HS5	*R.nitidus*	0.023622	0.067796
CS30	GD41	HS5	*R.nitidus*	-0.087406	0.06931
GD34	GD41	HS5	*R.nitidus*	0.00213	0.068705
CS23	GD41	HS5	*R.nitidus*	-0.055895	0.068861
CS25	GD41	HS5	*R.nitidus*	-0.007806	0.071317
CS22	GD41	HS5	*R.nitidus*	-0.012411	0.070063
SC34	GD41	HS5	*R.nitidus*	0	0.069685
GD46	GD41	HS5	*R.nitidus*	-0.038769	0.068648
SC28	GD41	HS5	*R.nitidus*	-0.048848	0.068261
SC12	GD41	HS5	*R.nitidus*	-0.059524	0.068554
CS21	GD41	HS5	*R.nitidus*	-0.026518	0.06846
SC24	GD41	HS5	*R.nitidus*	-0.018199	0.070319
CS31	GD41	HS5	*R.nitidus*	-0.040196	0.069702
CS18	GD41	HS5	*R.nitidus*	-0.032432	0.069022
CS24	GD41	HS5	*R.nitidus*	-0.041431	0.069562
SC32	GD41	HS5	*R.nitidus*	0.023963	0.070553
CS28	GD41	HS5	*R.nitidus*	-0.025692	0.072301
JO7	GD42	HS5	*R.nitidus*	-0.04267	0.076138
SC5	GD41	HS5	*R.nitidus*	0.030527	0.071599
MY4	GD41	HS5	*R.nitidus*	-0.040478	0.070166
CS6	GD41	HS5	*R.nitidus*	-0.033036	0.070439
MY6	GD41	HS5	*R.nitidus*	0	0.068938
SC26	GD41	HS5	*R.nitidus*	-0.055912	0.071802
CS20	GD41	HS5	*R.nitidus*	-0.026361	0.06955
SC15	GD41	HS5	*R.nitidus*	-0.004902	0.072783
GD25	GD42	HS5	*R.nitidus*	0.009202	0.071466

SC42	GD41	HS5	*R.nitidus*	0.180742	0.068098
SC35	GD41	HS5	*R.nitidus*	0.023853	0.068265
SC14	GD41	HS5	*R.nitidus*	-0.032498	0.070781
GD31	GD42	HS5	*R.nitidus*	-0.011136	0.074215
GD30	GD42	HS5	*R.nitidus*	0.102662	0.071695
GD21	GD42	HS5	*R.nitidus*	0.077699	0.067997
SC45	GD41	HS5	*R.nitidus*	0.017025	0.069937
JW7	GD41	HS5	*R.nitidus*	0.039487	0.072037
SC31	GD41	HS5	*R.nitidus*	-0.025594	0.06957
JW6	GD41	HS5	*R.nitidus*	0.002068	0.073025
GD41	GD42	HS5	*R.nitidus*	-0.051491	0.066107
GD26	GD42	HS5	*R.nitidus*	0.00627	0.071341
JW4	GD41	HS5	*R.nitidus*	0.157635	0.067098
SC27	GD41	HS5	*R.nitidus*	-0.042017	0.069141
JO5	GD41	HS5	*R.nitidus*	0.058459	0.067032
GD29	GD42	HS5	*R.nitidus*	0.101578	0.06689
JO1	GD41	HS5	*R.nitidus*	-0.029359	0.070463
SC44	GD41	HS5	*R.nitidus*	-0.022663	0.067667
CS15	GD42	HS5	*R.nitidus*	-0.062387	0.071532
CS11	GD42	HS5	*R.nitidus*	-0.052726	0.074231
JO3	GD41	HS5	*R.nitidus*	0.039234	0.069444
GD22	GD42	HS5	*R.nitidus*	0.030896	0.067735
GD35	GD42	HS5	*R.nitidus*	0.00813	0.071646
CS12	GD42	HS5	*R.nitidus*	-0.049213	0.073136
JO8	GD42	HS5	*R.nitidus*	-0.028857	0.076399
GD48	GD42	HS5	*R.nitidus*	0.031496	0.070249
GD32	GD42	HS5	*R.nitidus*	-0.010539	0.071913
GD50	GD42	HS5	*R.nitidus*	-0.01938	0.073613
CS14	GD42	HS5	*R.nitidus*	-0.052775	0.07291
GD49	GD42	HS5	*R.nitidus*	0.02843	0.074409
CS17	GD42	HS5	*R.nitidus*	-0.004955	0.075701
GD40	GD42	HS5	*R.nitidus*	0.045062	0.06917
CS23	GD42	HS5	*R.nitidus*	-0.090829	0.072272
CS25	GD42	HS5	*R.nitidus*	-0.058065	0.071569
GD33	GD42	HS5	*R.nitidus*	-0.044276	0.0721
CS21	GD42	HS5	*R.nitidus*	-0.073019	0.07199
GD34	GD42	HS5	*R.nitidus*	-0.048649	0.06972
CS20	GD42	HS5	*R.nitidus*	-0.053913	0.071524
CS30	GD42	HS5	*R.nitidus*	-0.122568	0.07047
GD44	GD42	HS5	*R.nitidus*	-0.035219	0.071111
CS13	GD42	HS5	*R.nitidus*	-0.068306	0.072726
CS31	GD42	HS5	*R.nitidus*	-0.063317	0.071911
GD46	GD42	HS5	*R.nitidus*	-0.079818	0.068301
CS4	GD42	HS5	*R.nitidus*	-0.039439	0.069307
SC42	GD42	HS5	*R.nitidus*	0.168149	0.068509
CS18	GD42	HS5	*R.nitidus*	-0.078467	0.069919
SC5	GD42	HS5	*R.nitidus*	-0.002778	0.072777
CS16	GD42	HS5	*R.nitidus*	-0.065317	0.070205
CS22	GD42	HS5	*R.nitidus*	-0.048128	0.070867
SC18	GD42	HS5	*R.nitidus*	-0.038	0.070998
SC26	GD42	HS5	*R.nitidus*	-0.096364	0.072933
JW6	GD42	HS5	*R.nitidus*	-0.04679	0.074817
SC31	GD42	HS5	*R.nitidus*	-0.081651	0.072264
SC14	GD42	HS5	*R.nitidus*	-0.064067	0.071454
SC24	GD42	HS5	*R.nitidus*	-0.044118	0.071097
CS19	GD42	HS5	*R.nitidus*	-0.066667	0.073594

CS24	GD42	HS5	*R.nitidus*	-0.081517	0.072415
SC35	GD42	HS5	*R.nitidus*	-0.015683	0.069587
SC34	GD42	HS5	*R.nitidus*	-0.028259	0.070707
GD45	GD42	HS5	*R.nitidus*	0.046482	0.076672
JO5	GD42	HS5	*R.nitidus*	0.015525	0.06801
CS6	GD42	HS5	*R.nitidus*	-0.073703	0.07099
SC10	GD42	HS5	*R.nitidus*	0.018719	0.0712
SC6	GD42	HS5	*R.nitidus*	-0.045539	0.07218
SC28	GD42	HS5	*R.nitidus*	-0.092322	0.070864
CS28	GD42	HS5	*R.nitidus*	-0.071142	0.071876
JW7	GD42	HS5	*R.nitidus*	-0.008081	0.072248
SC32	GD42	HS5	*R.nitidus*	-0.010436	0.071943
CS3	GD42	HS5	*R.nitidus*	-0.066308	0.071849
JW4	GD42	HS5	*R.nitidus*	0.12179	0.069375
JO8	GD44	HS5	*R.nitidus*	0.003185	0.074986
JO1	GD42	HS5	*R.nitidus*	-0.068841	0.072365
SC27	GD42	HS5	*R.nitidus*	-0.072642	0.070038
SC44	GD42	HS5	*R.nitidus*	-0.063953	0.070476
SC15	GD42	HS5	*R.nitidus*	-0.063353	0.074171
SC12	GD42	HS5	*R.nitidus*	-0.108513	0.068923
SC45	GD42	HS5	*R.nitidus*	-0.017658	0.072487
JO7	GD44	HS5	*R.nitidus*	0.008264	0.074801
GD25	GD44	HS5	*R.nitidus*	0.062874	0.07235
GD31	GD44	HS5	*R.nitidus*	0.026911	0.071224
MY6	GD42	HS5	*R.nitidus*	-0.04461	0.070807
GD42	GD44	HS5	*R.nitidus*	0.035219	0.071111
CS12	GD44	HS5	*R.nitidus*	-0.030602	0.072681
GD26	GD44	HS5	*R.nitidus*	0.049595	0.071333
MY4	GD42	HS5	*R.nitidus*	-0.079514	0.070844
JO3	GD42	HS5	*R.nitidus*	0.01912	0.072347
GD41	GD44	HS5	*R.nitidus*	-0.009464	0.071157
GD48	GD44	HS5	*R.nitidus*	0.062693	0.071619
GD22	GD44	HS5	*R.nitidus*	0.063462	0.069089
CS23	GD44	HS5	*R.nitidus*	-0.053162	0.070432
CS15	GD44	HS5	*R.nitidus*	-0.023235	0.071125
CS21	GD44	HS5	*R.nitidus*	-0.029864	0.070493
CS11	GD44	HS5	*R.nitidus*	-0.022482	0.073056
GD49	GD44	HS5	*R.nitidus*	0.080626	0.077013
GD21	GD44	HS5	*R.nitidus*	0.120641	0.069679
GD33	GD44	HS5	*R.nitidus*	-0.012109	0.072803
GD30	GD44	HS5	*R.nitidus*	0.132353	0.073757
GD34	GD44	HS5	*R.nitidus*	-0.002962	0.071021
GD32	GD44	HS5	*R.nitidus*	0.005787	0.072868
GD46	GD44	HS5	*R.nitidus*	-0.029502	0.071245
GD29	GD44	HS5	*R.nitidus*	0.143514	0.068677
CS13	GD44	HS5	*R.nitidus*	-0.045956	0.073933
CS31	GD44	HS5	*R.nitidus*	-0.049467	0.071243
CS19	GD44	HS5	*R.nitidus*	-0.040541	0.07315
GD40	GD44	HS5	*R.nitidus*	0.078565	0.068115
CS17	GD44	HS5	*R.nitidus*	0.026549	0.075191
GD35	GD44	HS5	*R.nitidus*	0.035533	0.069894
SC6	GD44	HS5	*R.nitidus*	0.008435	0.07085
CS16	GD44	HS5	*R.nitidus*	-0.022999	0.07096
GD45	GD44	HS5	*R.nitidus*	0.085025	0.076812
SC14	GD44	HS5	*R.nitidus*	-0.032505	0.073767
CS25	GD44	HS5	*R.nitidus*	-0.01223	0.074562

SC27	GD44	HS5	*R.nitidus*	-0.047619	0.072888
CS22	GD44	HS5	*R.nitidus*	-0.026341	0.071283
SC10	GD44	HS5	*R.nitidus*	0.050142	0.072962
GD50	GD44	HS5	*R.nitidus*	0.047619	0.075019
CS30	GD44	HS5	*R.nitidus*	-0.065619	0.071246
CS18	GD44	HS5	*R.nitidus*	-0.050279	0.07007
CS14	GD44	HS5	*R.nitidus*	-0.010811	0.073804
CS4	GD44	HS5	*R.nitidus*	-0.033852	0.067791
SC18	GD44	HS5	*R.nitidus*	0.004012	0.071194
SC5	GD44	HS5	*R.nitidus*	0.02737	0.075528
SC15	GD44	HS5	*R.nitidus*	0	0.073814
CS28	GD44	HS5	*R.nitidus*	-0.047337	0.073862
CS24	GD44	HS5	*R.nitidus*	-0.055133	0.069897
CS20	GD44	HS5	*R.nitidus*	-0.025294	0.070555
SC24	GD44	HS5	*R.nitidus*	-0.011858	0.072706
SC26	GD44	HS5	*R.nitidus*	-0.062044	0.072959
MY6	GD44	HS5	*R.nitidus*	-0.005535	0.07096
CS3	GD44	HS5	*R.nitidus*	-0.031789	0.070466
JO3	GD44	HS5	*R.nitidus*	0.024434	0.072131
JW4	GD44	HS5	*R.nitidus*	0.134012	0.072977
SC45	GD44	HS5	*R.nitidus*	0.014719	0.071685
SC12	GD44	HS5	*R.nitidus*	-0.072595	0.071582
SC28	GD44	HS5	*R.nitidus*	-0.067034	0.07113
JO7	GD45	HS5	*R.nitidus*	-0.091743	0.079269
JW6	GD44	HS5	*R.nitidus*	-0.015075	0.075101
MY4	GD44	HS5	*R.nitidus*	-0.045328	0.069567
GD26	GD45	HS5	*R.nitidus*	-0.050617	0.078311
GD30	GD45	HS5	*R.nitidus*	0.03381	0.075443
SC42	GD44	HS5	*R.nitidus*	0.172442	0.069668
JW7	GD44	HS5	*R.nitidus*	0.039275	0.073849
GD32	GD45	HS5	*R.nitidus*	-0.080056	0.076924
JO8	GD45	HS5	*R.nitidus*	-0.069987	0.050008
CS6	GD44	HS5	*R.nitidus*	-0.03966	0.070111
SC31	GD44	HS5	*R.nitidus*	-0.027507	0.071254
SC32	GD44	HS5	*R.nitidus*	0.030415	0.071738
SC34	GD44	HS5	*R.nitidus*	-0.003584	0.067765
GD25	GD45	HS5	*R.nitidus*	-0.06988	0.079575
GD22	GD45	HS5	*R.nitidus*	-0.017281	0.076522
CS12	GD45	HS5	*R.nitidus*	-0.093182	0.076324
JO5	GD44	HS5	*R.nitidus*	0.063964	0.070546
GD35	GD45	HS5	*R.nitidus*	-0.067227	0.075395
GD42	GD45	HS5	*R.nitidus*	-0.046482	0.076672
GD34	GD45	HS5	*R.nitidus*	-0.106587	0.074703
GD41	GD45	HS5	*R.nitidus*	-0.066748	0.076862
GD44	GD45	HS5	*R.nitidus*	-0.085025	0.076812
GD33	GD45	HS5	*R.nitidus*	-0.092883	0.077391
CS11	GD45	HS5	*R.nitidus*	-0.0931	0.050773
SC35	GD44	HS5	*R.nitidus*	0.024545	0.069353
GD40	GD45	HS5	*R.nitidus*	-0.009091	0.074761
CS15	GD45	HS5	*R.nitidus*	-0.081633	0.076918
JO1	GD44	HS5	*R.nitidus*	-0.03402	0.071766
GD21	GD45	HS5	*R.nitidus*	0.015276	0.072862
SC44	GD44	HS5	*R.nitidus*	-0.030217	0.073603
CS14	GD45	HS5	*R.nitidus*	-0.049438	0.051173
CS18	GD45	HS5	*R.nitidus*	-0.109649	0.076348
CS24	GD45	HS5	*R.nitidus*	-0.106383	0.076073

GD29	GD45	HS5	*R.nitidus*	0.021864	0.075275
GD31	GD45	HS5	*R.nitidus*	-0.032338	0.078076
CS17	GD45	HS5	*R.nitidus*	-0.06506	0.078857
CS20	GD45	HS5	*R.nitidus*	-0.077754	0.07844
CS28	GD45	HS5	*R.nitidus*	-0.093132	0.077724
GD46	GD45	HS5	*R.nitidus*	-0.114358	0.074261
GD49	GD45	HS5	*R.nitidus*	-0.017931	0.051615
SC24	GD45	HS5	*R.nitidus*	-0.054176	0.076951
CS13	GD45	HS5	*R.nitidus*	-0.082314	0.078184
CS30	GD45	HS5	*R.nitidus*	-0.128492	0.076249
CS25	GD45	HS5	*R.nitidus*	-0.095672	0.079781
GD48	GD45	HS5	*R.nitidus*	0.003861	0.07965
GD50	GD45	HS5	*R.nitidus*	-0.035135	0.051224
SC15	GD45	HS5	*R.nitidus*	-0.058231	0.075901
SC12	GD45	HS5	*R.nitidus*	-0.115756	0.07126
SC6	GD45	HS5	*R.nitidus*	-0.064302	0.076092
CS21	GD45	HS5	*R.nitidus*	-0.097849	0.075574
CS3	GD45	HS5	*R.nitidus*	-0.078861	0.077528
CS16	GD45	HS5	*R.nitidus*	-0.107744	0.078386
SC45	GD45	HS5	*R.nitidus*	-0.044809	0.072869
CS19	GD45	HS5	*R.nitidus*	-0.095238	0.077238
SC5	GD45	HS5	*R.nitidus*	-0.046719	0.07809
SC10	GD45	HS5	*R.nitidus*	-0.013348	0.07523
SC42	GD45	HS5	*R.nitidus*	0.123989	0.07256
CS23	GD45	HS5	*R.nitidus*	-0.092652	0.078049
CS4	GD45	HS5	*R.nitidus*	-0.075372	0.073834
CS22	GD45	HS5	*R.nitidus*	-0.070375	0.075119
JW6	GD45	HS5	*R.nitidus*	-0.103106	0.077318
SC35	GD45	HS5	*R.nitidus*	-0.046358	0.073732
SC31	GD45	HS5	*R.nitidus*	-0.090909	0.075392
SC18	GD45	HS5	*R.nitidus*	-0.016588	0.076896
CS31	GD45	HS5	*R.nitidus*	-0.129147	0.076516
SC44	GD45	HS5	*R.nitidus*	-0.068233	0.072364
SC14	GD45	HS5	*R.nitidus*	-0.09772	0.077084
SC34	GD45	HS5	*R.nitidus*	-0.052164	0.074139
GD25	CS13	HS5	*R.nitidus*	0.085047	0.072805
GD29	CS13	HS5	*R.nitidus*	0.157155	0.071395
SC32	GD45	HS5	*R.nitidus*	-0.036872	0.074407
MY6	GD45	HS5	*R.nitidus*	-0.054324	0.075573
JW7	GD45	HS5	*R.nitidus*	-0.043478	0.076292
SC26	GD45	HS5	*R.nitidus*	-0.113025	0.075359
SC27	GD45	HS5	*R.nitidus*	-0.099558	0.076912
JO1	GD45	HS5	*R.nitidus*	-0.092534	0.07708
MY4	GD45	HS5	*R.nitidus*	-0.08952	0.075191
GD26	CS13	HS5	*R.nitidus*	0.073849	0.073292
CS6	GD45	HS5	*R.nitidus*	-0.064516	0.076929
GD21	CS13	HS5	*R.nitidus*	0.120805	0.069688
JO8	CS13	HS5	*R.nitidus*	0.036026	0.079543
GD30	CS13	HS5	*R.nitidus*	0.135074	0.075801
JW4	GD45	HS5	*R.nitidus*	0.106275	0.074274
SC28	GD45	HS5	*R.nitidus*	-0.121246	0.074317
JO3	GD45	HS5	*R.nitidus*	-0.031868	0.077466
GD32	CS13	HS5	*R.nitidus*	0.047666	0.073492
JO7	CS13	HS5	*R.nitidus*	0.04902	0.077046
JO5	GD45	HS5	*R.nitidus*	-0.015436	0.07351
CS12	CS13	HS5	*R.nitidus*	0.032432	0.076572

GD34	CS13	HS5	*R.nitidus*	0.016964	0.072111
GD42	CS13	HS5	*R.nitidus*	0.068306	0.072726
GD33	CS13	HS5	*R.nitidus*	0.021486	0.074774
GD44	CS13	HS5	*R.nitidus*	0.045956	0.073933
CS16	CS13	HS5	*R.nitidus*	0.034264	0.075904
CS11	CS13	HS5	*R.nitidus*	0.029948	0.077621
GD22	CS13	HS5	*R.nitidus*	0.084996	0.072651
CS18	CS13	HS5	*R.nitidus*	-0.02008	0.075481
GD35	CS13	HS5	*R.nitidus*	0.055198	0.071247
GD48	CS13	HS5	*R.nitidus*	0.086916	0.076
GD49	CS13	HS5	*R.nitidus*	0.074972	0.078049
GD45	CS13	HS5	*R.nitidus*	0.082314	0.078184
CS23	CS13	HS5	*R.nitidus*	-0.029273	0.07494
CS31	CS13	HS5	*R.nitidus*	0.002035	0.076979
CS15	CS13	HS5	*R.nitidus*	0.030383	0.07583
SC14	CS13	HS5	*R.nitidus*	-0.011894	0.071993
CS14	CS13	HS5	*R.nitidus*	0.050473	0.051218
SC10	CS13	HS5	*R.nitidus*	0.088734	0.075393
GD46	CS13	HS5	*R.nitidus*	0.013812	0.074228
GD50	CS13	HS5	*R.nitidus*	0.051064	0.07651
GD31	CS13	HS5	*R.nitidus*	0.085878	0.073622
GD40	CS13	HS5	*R.nitidus*	0.093064	0.07079
CS24	CS13	HS5	*R.nitidus*	-0.032587	0.074586
CS28	CS13	HS5	*R.nitidus*	0	0.076333
GD41	CS13	HS5	*R.nitidus*	0.023105	0.07143
CS25	CS13	HS5	*R.nitidus*	0.019436	0.074667
CS17	CS13	HS5	*R.nitidus*	0.076487	0.078436
CS21	CS13	HS5	*R.nitidus*	-0.002841	0.075194
SC15	CS13	HS5	*R.nitidus*	0.020325	0.075417
SC28	CS13	HS5	*R.nitidus*	-0.033771	0.071226
SC24	CS13	HS5	*R.nitidus*	0.03022	0.07212
SC35	CS13	HS5	*R.nitidus*	0.048869	0.072734
CS19	CS13	HS5	*R.nitidus*	-0.012208	0.075245
SC45	CS13	HS5	*R.nitidus*	0.030217	0.072147
CS20	CS13	HS5	*R.nitidus*	-0.003756	0.074718
SC26	CS13	HS5	*R.nitidus*	-0.038536	0.073553
SC5	CS13	HS5	*R.nitidus*	0.057385	0.075181
CS22	CS13	HS5	*R.nitidus*	0.031609	0.073244
SC18	CS13	HS5	*R.nitidus*	0.056797	0.072297
CS30	CS13	HS5	*R.nitidus*	-0.049323	0.072699
SC34	CS13	HS5	*R.nitidus*	0.016667	0.072023
MY4	CS13	HS5	*R.nitidus*	-0.015549	0.075006
SC44	CS13	HS5	*R.nitidus*	-0.011257	0.07259
SC32	CS13	HS5	*R.nitidus*	0.058824	0.073309
JW4	CS13	HS5	*R.nitidus*	0.181056	0.072472
CS3	CS13	HS5	*R.nitidus*	-0.002863	0.072895
SC6	CS13	HS5	*R.nitidus*	0.029685	0.07367
CS4	CS13	HS5	*R.nitidus*	0.024074	0.071872
SC31	CS13	HS5	*R.nitidus*	-0.002783	0.073551
SC12	CS13	HS5	*R.nitidus*	-0.027831	0.071294
MY6	CS13	HS5	*R.nitidus*	0.034026	0.074364
JO7	GD46	HS5	*R.nitidus*	0.031949	0.074256
JO3	CS13	HS5	*R.nitidus*	0.08066	0.074569
GD42	GD46	HS5	*R.nitidus*	0.079818	0.068301
SC27	CS13	HS5	*R.nitidus*	-0.014124	0.070961
GD21	GD46	HS5	*R.nitidus*	0.152242	0.066413

GD29	GD46	HS5	*R.nitidus*	0.171488	0.066258
GD22	GD46	HS5	*R.nitidus*	0.097319	0.068132
JO8	GD46	HS5	*R.nitidus*	0.079153	0.071409
JW6	CS13	HS5	*R.nitidus*	0.025641	0.078502
SC42	CS13	HS5	*R.nitidus*	0.196329	0.06806
GD25	GD46	HS5	*R.nitidus*	0.077228	0.071237
JO5	CS13	HS5	*R.nitidus*	0.073411	0.07155
GD50	GD46	HS5	*R.nitidus*	0.052375	0.071719
JO1	CS13	HS5	*R.nitidus*	0.008515	0.072534
GD31	GD46	HS5	*R.nitidus*	0.051907	0.070927
CS11	GD46	HS5	*R.nitidus*	0.0045	0.074358
GD35	GD46	HS5	*R.nitidus*	0.068136	0.069684
GD45	GD46	HS5	*R.nitidus*	0.114358	0.074261
CS6	CS13	HS5	*R.nitidus*	-0.018664	0.072536
GD44	GD46	HS5	*R.nitidus*	0.029502	0.071245
CS14	GD46	HS5	*R.nitidus*	0.00565	0.075654
GD30	GD46	HS5	*R.nitidus*	0.168263	0.068208
JW7	CS13	HS5	*R.nitidus*	0.069416	0.074909
GD26	GD46	HS5	*R.nitidus*	0.092292	0.067449
GD34	GD46	HS5	*R.nitidus*	0.024652	0.068925
CS13	GD46	HS5	*R.nitidus*	-0.013812	0.074228
GD41	GD46	HS5	*R.nitidus*	0.038769	0.068648
CS12	GD46	HS5	*R.nitidus*	0.003972	0.071954
GD32	GD46	HS5	*R.nitidus*	0.059908	0.071524
CS17	GD46	HS5	*R.nitidus*	0.051515	0.074691
GD33	GD46	HS5	*R.nitidus*	0.017241	0.07211
CS18	GD46	HS5	*R.nitidus*	-0.031308	0.071034
CS20	GD46	HS5	*R.nitidus*	-0.016904	0.070194
GD40	GD46	HS5	*R.nitidus*	0.092219	0.067872
GD48	GD46	HS5	*R.nitidus*	0.111494	0.070303
CS22	GD46	HS5	*R.nitidus*	0.008287	0.070597
GD49	GD46	HS5	*R.nitidus*	0.074442	0.07446
CS24	GD46	HS5	*R.nitidus*	-0.021739	0.070308
SC10	GD46	HS5	*R.nitidus*	0.076851	0.07048
SC26	GD46	HS5	*R.nitidus*	-0.033759	0.070282
CS21	GD46	HS5	*R.nitidus*	-0.031167	0.071262
CS15	GD46	HS5	*R.nitidus*	-0.00466	0.072169
SC24	GD46	HS5	*R.nitidus*	0.018287	0.070159
SC31	GD46	HS5	*R.nitidus*	-0.016637	0.069971
SC6	GD46	HS5	*R.nitidus*	0.013158	0.070693
CS23	GD46	HS5	*R.nitidus*	-0.036082	0.070605
SC12	GD46	HS5	*R.nitidus*	-0.041894	0.068922
SC28	GD46	HS5	*R.nitidus*	-0.038113	0.06804
CS16	GD46	HS5	*R.nitidus*	-0.002849	0.071479
CS19	GD46	HS5	*R.nitidus*	-0.017528	0.072148
CS31	GD46	HS5	*R.nitidus*	-0.027861	0.069945
CS3	GD46	HS5	*R.nitidus*	-0.00976	0.070953
SC5	GD46	HS5	*R.nitidus*	0.048327	0.071539
SC27	GD46	HS5	*R.nitidus*	-0.016713	0.067983
CS25	GD46	HS5	*R.nitidus*	0.00363	0.072313
CS6	GD46	HS5	*R.nitidus*	-0.012809	0.069571
SC14	GD46	HS5	*R.nitidus*	-0.004558	0.068634
JO3	GD46	HS5	*R.nitidus*	0.06848	0.070398
CS28	GD46	HS5	*R.nitidus*	-0.021696	0.074741
MY6	GD46	HS5	*R.nitidus*	0.021062	0.070955
CS30	GD46	HS5	*R.nitidus*	-0.056497	0.071463

SC42	GD46	HS5	*R.nitidus*	0.195535	0.069009
SC15	GD46	HS5	*R.nitidus*	0.014521	0.073418
SC45	GD46	HS5	*R.nitidus*	0.032947	0.069229
MY4	GD46	HS5	*R.nitidus*	-0.027829	0.072814
CS4	GD46	HS5	*R.nitidus*	-0.003439	0.069943
GD30	GD48	HS5	*R.nitidus*	0.046113	0.07581
SC34	GD46	HS5	*R.nitidus*	0.014134	0.067991
SC44	GD46	HS5	*R.nitidus*	-0.009311	0.069828
GD26	GD48	HS5	*R.nitidus*	0	0.069779
SC18	GD46	HS5	*R.nitidus*	0.043435	0.066851
JO8	GD48	HS5	*R.nitidus*	-0.032258	0.073747
JO7	GD48	HS5	*R.nitidus*	-0.0348	0.075139
SC32	GD46	HS5	*R.nitidus*	0.044199	0.070838
GD33	GD48	HS5	*R.nitidus*	-0.062147	0.074089
JW6	GD46	HS5	*R.nitidus*	0.036649	0.073301
SC35	GD46	HS5	*R.nitidus*	0.035199	0.068293
GD21	GD48	HS5	*R.nitidus*	0.07449	0.069911
GD29	GD48	HS5	*R.nitidus*	0.076297	0.067564
GD32	GD48	HS5	*R.nitidus*	-0.028708	0.070328
GD35	GD48	HS5	*R.nitidus*	-0.01973	0.072835
GD31	GD48	HS5	*R.nitidus*	-0.025316	0.074872
JW4	GD46	HS5	*R.nitidus*	0.18797	0.070429
CS12	GD48	HS5	*R.nitidus*	-0.061753	0.073513
GD45	GD48	HS5	*R.nitidus*	-0.003861	0.07965
GD46	GD48	HS5	*R.nitidus*	-0.111494	0.070303
GD25	GD48	HS5	*R.nitidus*	-0.010549	0.070828
JW7	GD46	HS5	*R.nitidus*	0.067913	0.071325
CS11	GD48	HS5	*R.nitidus*	-0.061773	0.074832
GD40	GD48	HS5	*R.nitidus*	0.02	0.070973
GD22	GD48	HS5	*R.nitidus*	0.01206	0.070384
JO5	GD46	HS5	*R.nitidus*	0.061837	0.067177
GD49	GD48	HS5	*R.nitidus*	0.007528	0.077939
CS13	GD48	HS5	*R.nitidus*	-0.086916	0.076
CS16	GD48	HS5	*R.nitidus*	-0.074977	0.072459
CS23	GD48	HS5	*R.nitidus*	-0.110092	0.071852
GD34	GD48	HS5	*R.nitidus*	-0.082579	0.068653
GD44	GD48	HS5	*R.nitidus*	-0.062693	0.071619
CS17	GD48	HS5	*R.nitidus*	-0.043788	0.075101
GD41	GD48	HS5	*R.nitidus*	-0.088428	0.069293
CS18	GD48	HS5	*R.nitidus*	-0.086002	0.073021
GD42	GD48	HS5	*R.nitidus*	-0.031496	0.070249
CS14	GD48	HS5	*R.nitidus*	-0.041933	0.075597
CS19	GD48	HS5	*R.nitidus*	-0.084601	0.071626
SC18	GD48	HS5	*R.nitidus*	-0.022287	0.07145
CS24	GD48	HS5	*R.nitidus*	-0.092192	0.071001
SC10	GD48	HS5	*R.nitidus*	0.007576	0.071884
CS30	GD48	HS5	*R.nitidus*	-0.133396	0.072314
SC14	GD48	HS5	*R.nitidus*	-0.084809	0.070582
CS4	GD48	HS5	*R.nitidus*	-0.0563	0.068954
CS28	GD48	HS5	*R.nitidus*	-0.102081	0.072998
GD50	GD48	HS5	*R.nitidus*	-0.053593	0.075636
CS15	GD48	HS5	*R.nitidus*	-0.075592	0.071759
CS6	GD48	HS5	*R.nitidus*	-0.088725	0.069095
SC24	GD48	HS5	*R.nitidus*	-0.039961	0.07134
CS22	GD48	HS5	*R.nitidus*	-0.061922	0.07007
CS25	GD48	HS5	*R.nitidus*	-0.053622	0.073318

CS3	GD48	HS5	*R.nitidus*	-0.090909	0.071625
SC15	GD48	HS5	*R.nitidus*	-0.065686	0.073176
SC6	GD48	HS5	*R.nitidus*	-0.056039	0.072188
CS20	GD48	HS5	*R.nitidus*	-0.076077	0.070155
MY6	GD48	HS5	*R.nitidus*	-0.05536	0.072534
SC28	GD48	HS5	*R.nitidus*	-0.10221	0.069906
SC32	GD48	HS5	*R.nitidus*	-0.033739	0.070995
CS31	GD48	HS5	*R.nitidus*	-0.086622	0.071184
SC5	GD48	HS5	*R.nitidus*	0	0.075394
CS21	GD48	HS5	*R.nitidus*	-0.095281	0.070184
JW6	GD48	HS5	*R.nitidus*	-0.040881	0.073404
JO7	GD49	HS5	*R.nitidus*	-0.060411	0.078631
JO1	GD48	HS5	*R.nitidus*	-0.074306	0.071493
SC12	GD48	HS5	*R.nitidus*	-0.11883	0.070351
SC45	GD48	HS5	*R.nitidus*	-0.047402	0.070561
SC35	GD48	HS5	*R.nitidus*	-0.029163	0.068656
JO8	GD49	HS5	*R.nitidus*	-0.033156	0.0503
SC42	GD48	HS5	*R.nitidus*	0.10535	0.070878
SC34	GD48	HS5	*R.nitidus*	-0.060274	0.071062
GD29	GD49	HS5	*R.nitidus*	0.078031	0.072272
JO5	GD48	HS5	*R.nitidus*	0.008189	0.069542
CS11	GD49	HS5	*R.nitidus*	-0.055789	0.077952
MY4	GD48	HS5	*R.nitidus*	-0.097248	0.071554
SC27	GD48	HS5	*R.nitidus*	-0.078505	0.069198
GD26	GD49	HS5	*R.nitidus*	0.011278	0.074592
SC44	GD48	HS5	*R.nitidus*	-0.075889	0.070436
SC31	GD48	HS5	*R.nitidus*	-0.081596	0.071024
SC26	GD48	HS5	*R.nitidus*	-0.119376	0.071283
GD31	GD49	HS5	*R.nitidus*	-0.024548	0.07808
JW7	GD48	HS5	*R.nitidus*	-0.001959	0.07267
JO3	GD48	HS5	*R.nitidus*	-0.015009	0.072383
JW4	GD48	HS5	*R.nitidus*	0.114167	0.069244
GD30	GD49	HS5	*R.nitidus*	0.125894	0.075727
CS16	GD49	HS5	*R.nitidus*	-0.080874	0.076099
GD21	GD49	HS5	*R.nitidus*	0.04662	0.075485
GD35	GD49	HS5	*R.nitidus*	-0.025404	0.074448
GD40	GD49	HS5	*R.nitidus*	0.002291	0.074377
GD32	GD49	HS5	*R.nitidus*	-0.061947	0.076966
GD45	GD49	HS5	*R.nitidus*	0.017931	0.051615
GD46	GD49	HS5	*R.nitidus*	-0.074442	0.07446
CS13	GD49	HS5	*R.nitidus*	-0.074972	0.078049
CS17	GD49	HS5	*R.nitidus*	-0.015012	0.051098
CS14	GD49	HS5	*R.nitidus*	-0.03652	0.0787
GD22	GD49	HS5	*R.nitidus*	-0.028807	0.074227
GD41	GD49	HS5	*R.nitidus*	-0.078125	0.07547
CS25	GD49	HS5	*R.nitidus*	-0.055118	0.077364
GD25	GD49	HS5	*R.nitidus*	-0.020457	0.076087
GD42	GD49	HS5	*R.nitidus*	-0.02843	0.074409
GD34	GD49	HS5	*R.nitidus*	-0.05597	0.075273
CS12	GD49	HS5	*R.nitidus*	-0.080275	0.07421
CS31	GD49	HS5	*R.nitidus*	-0.100907	0.078046
GD44	GD49	HS5	*R.nitidus*	-0.080626	0.077013
CS18	GD49	HS5	*R.nitidus*	-0.084153	0.076886
SC6	GD49	HS5	*R.nitidus*	-0.048246	0.075747
SC12	GD49	HS5	*R.nitidus*	-0.099119	0.072188
CS15	GD49	HS5	*R.nitidus*	-0.067107	0.074254

CS23	GD49	HS5	*R.nitidus*	-0.09292	0.076346
GD50	GD49	HS5	*R.nitidus*	-0.034091	0.078927
CS21	GD49	HS5	*R.nitidus*	-0.110503	0.073343
GD48	GD49	HS5	*R.nitidus*	-0.007528	0.077939
CS24	GD49	HS5	*R.nitidus*	-0.087196	0.076772
GD33	GD49	HS5	*R.nitidus*	-0.050505	0.078038
CS28	GD49	HS5	*R.nitidus*	-0.068415	0.078175
SC18	GD49	HS5	*R.nitidus*	-0.00949	0.072869
SC27	GD49	HS5	*R.nitidus*	-0.088745	0.074895
CS3	GD49	HS5	*R.nitidus*	-0.108342	0.074411
CS22	GD49	HS5	*R.nitidus*	-0.040929	0.073848
CS30	GD49	HS5	*R.nitidus*	-0.120181	0.074903
SC5	GD49	HS5	*R.nitidus*	-0.029246	0.079523
CS4	GD49	HS5	*R.nitidus*	-0.049383	0.074075
CS19	GD49	HS5	*R.nitidus*	-0.065292	0.076711
SC15	GD49	HS5	*R.nitidus*	-0.067929	0.076582
CS20	GD49	HS5	*R.nitidus*	-0.091483	0.073912
JW6	GD49	HS5	*R.nitidus*	-0.066416	0.078618
JO1	GD49	HS5	*R.nitidus*	-0.069038	0.07618
JO5	GD49	HS5	*R.nitidus*	-0.013086	0.072197
SC45	GD49	HS5	*R.nitidus*	-0.027778	0.075841
MY4	GD49	HS5	*R.nitidus*	-0.067403	0.075353
SC26	GD49	HS5	*R.nitidus*	-0.106681	0.076703
JO7	GD50	HS5	*R.nitidus*	-0.034965	0.078509
GD22	GD50	HS5	*R.nitidus*	0.030842	0.072876
SC10	GD49	HS5	*R.nitidus*	0.005688	0.075701
JW7	GD49	HS5	*R.nitidus*	0.013905	0.078191
JW4	GD49	HS5	*R.nitidus*	0.106426	0.074818
MY6	GD49	HS5	*R.nitidus*	-0.055435	0.075726
SC14	GD49	HS5	*R.nitidus*	-0.067093	0.075314
SC24	GD49	HS5	*R.nitidus*	-0.062571	0.073863
SC31	GD49	HS5	*R.nitidus*	-0.112554	0.074333
SC28	GD49	HS5	*R.nitidus*	-0.094218	0.075299
SC44	GD49	HS5	*R.nitidus*	-0.063457	0.074038
JO3	GD49	HS5	*R.nitidus*	0.021077	0.076152
GD21	GD50	HS5	*R.nitidus*	0.09434	0.072991
SC32	GD49	HS5	*R.nitidus*	-0.028415	0.076712
CS6	GD49	HS5	*R.nitidus*	-0.062222	0.074328
JO8	GD50	HS5	*R.nitidus*	-0.022528	0.078878
CS11	GD50	HS5	*R.nitidus*	-0.038855	0.076229
GD32	GD50	HS5	*R.nitidus*	-0.021305	0.073793
GD34	GD50	HS5	*R.nitidus*	-0.022274	0.074046
SC42	GD49	HS5	*R.nitidus*	0.151346	0.072408
SC35	GD49	HS5	*R.nitidus*	-0.024973	0.074041
SC34	GD49	HS5	*R.nitidus*	-0.023429	0.072533
GD26	GD50	HS5	*R.nitidus*	0.019231	0.071032
CS17	GD50	HS5	*R.nitidus*	-0.006674	0.076564
GD29	GD50	HS5	*R.nitidus*	0.102417	0.069581
GD49	GD50	HS5	*R.nitidus*	0.034091	0.078927
CS22	GD50	HS5	*R.nitidus*	-0.030884	0.070484
CS13	GD50	HS5	*R.nitidus*	-0.051064	0.07651
GD42	GD50	HS5	*R.nitidus*	0.01938	0.073613
GD40	GD50	HS5	*R.nitidus*	0.040043	0.072497
CS14	GD50	HS5	*R.nitidus*	0	0.076116
GD25	GD50	HS5	*R.nitidus*	-0.008111	0.072301
GD45	GD50	HS5	*R.nitidus*	0.035135	0.051224

GD30	GD50	HS5	*R.nitidus*	0.110959	0.075725
SC5	GD50	HS5	*R.nitidus*	-0.024719	0.076932
GD31	GD50	HS5	*R.nitidus*	0.003645	0.0751
CS21	GD50	HS5	*R.nitidus*	-0.051205	0.074426
CS12	GD50	HS5	*R.nitidus*	-0.046892	0.073831
CS20	GD50	HS5	*R.nitidus*	-0.052472	0.074249
CS23	GD50	HS5	*R.nitidus*	-0.073737	0.0745
GD44	GD50	HS5	*R.nitidus*	-0.047619	0.075019
GD33	GD50	HS5	*R.nitidus*	-0.044064	0.073658
GD48	GD50	HS5	*R.nitidus*	0.053593	0.075636
GD35	GD50	HS5	*R.nitidus*	0.029904	0.071526
SC6	GD50	HS5	*R.nitidus*	-0.039404	0.073857
SC18	GD50	HS5	*R.nitidus*	-0.003344	0.074162
CS15	GD50	HS5	*R.nitidus*	-0.037525	0.073695
CS24	GD50	HS5	*R.nitidus*	-0.049127	0.072907
CS16	GD50	HS5	*R.nitidus*	-0.050403	0.073458
SC10	GD50	HS5	*R.nitidus*	0.023026	0.074509
GD46	GD50	HS5	*R.nitidus*	-0.052375	0.071719
CS25	GD50	HS5	*R.nitidus*	-0.046336	0.074754
SC31	GD50	HS5	*R.nitidus*	-0.054639	0.074896
SC34	GD50	HS5	*R.nitidus*	-0.025432	0.068786
CS4	GD50	HS5	*R.nitidus*	-0.047047	0.069718
GD41	GD50	HS5	*R.nitidus*	-0.032092	0.073449
CS19	GD50	HS5	*R.nitidus*	-0.065521	0.07685
CS30	GD50	HS5	*R.nitidus*	-0.110643	0.073793
SC12	GD50	HS5	*R.nitidus*	-0.11123	0.074158
CS18	GD50	HS5	*R.nitidus*	-0.056624	0.073621
SC28	GD50	HS5	*R.nitidus*	-0.077559	0.071946
SC35	GD50	HS5	*R.nitidus*	-0.023355	0.070488
JO1	GD50	HS5	*R.nitidus*	-0.064777	0.074489
JW6	GD50	HS5	*R.nitidus*	-0.032749	0.07744
CS3	GD50	HS5	*R.nitidus*	-0.070352	0.074119
CS6	GD50	HS5	*R.nitidus*	-0.044898	0.074212
JW4	GD50	HS5	*R.nitidus*	0.101449	0.074375
CS28	GD50	HS5	*R.nitidus*	-0.103837	0.077083
SC15	GD50	HS5	*R.nitidus*	-0.024096	0.076432
SC26	GD50	HS5	*R.nitidus*	-0.089151	0.074714
JW7	GD50	HS5	*R.nitidus*	0.00344	0.077152
SC14	GD50	HS5	*R.nitidus*	-0.067511	0.073013
SC24	GD50	HS5	*R.nitidus*	-0.054054	0.074389
SC27	GD50	HS5	*R.nitidus*	-0.087473	0.070514
MY6	GD50	HS5	*R.nitidus*	-0.024291	0.07355
CS31	GD50	HS5	*R.nitidus*	-0.072626	0.072809
MY4	GD50	HS5	*R.nitidus*	-0.070248	0.073417
SC42	GD50	HS5	*R.nitidus*	0.138965	0.07391
SC32	GD50	HS5	*R.nitidus*	-0.014523	0.074919
GD26	CS14	HS5	*R.nitidus*	0.052083	0.073976
GD32	CS14	HS5	*R.nitidus*	0.023279	0.076597
JO3	GD50	HS5	*R.nitidus*	-0.018443	0.07461
GD22	CS14	HS5	*R.nitidus*	0.057412	0.072034
CS11	CS14	HS5	*R.nitidus*	-0.018732	0.077589
SC44	GD50	HS5	*R.nitidus*	-0.048913	0.076426
GD25	CS14	HS5	*R.nitidus*	0.068224	0.075041
GD29	CS14	HS5	*R.nitidus*	0.140833	0.073015
GD49	CS14	HS5	*R.nitidus*	0.03652	0.0787
GD31	CS14	HS5	*R.nitidus*	0.050332	0.07744

JO7	CS14	HS5	*R.nitidus*	0.012889	0.077781
SC45	GD50	HS5	*R.nitidus*	-0.011399	0.070651
GD33	CS14	HS5	*R.nitidus*	-0.006329	0.075877
GD21	CS14	HS5	*R.nitidus*	0.113891	0.071332
GD30	CS14	HS5	*R.nitidus*	0.117318	0.078341
JO8	CS14	HS5	*R.nitidus*	0.007392	0.077637
CS16	CS14	HS5	*R.nitidus*	-0.012228	0.075253
JO5	GD50	HS5	*R.nitidus*	0.011411	0.071542
GD50	CS14	HS5	*R.nitidus*	0	0.076116
GD42	CS14	HS5	*R.nitidus*	0.052775	0.07291
GD35	CS14	HS5	*R.nitidus*	0.020966	0.069954
CS15	CS14	HS5	*R.nitidus*	-0.008708	0.079072
GD40	CS14	HS5	*R.nitidus*	0.05821	0.07165
GD48	CS14	HS5	*R.nitidus*	0.041933	0.075597
GD44	CS14	HS5	*R.nitidus*	0.010811	0.073804
CS17	CS14	HS5	*R.nitidus*	0.037594	0.079743
CS19	CS14	HS5	*R.nitidus*	-0.045862	0.07433
CS24	CS14	HS5	*R.nitidus*	-0.058163	0.074984
GD45	CS14	HS5	*R.nitidus*	0.049438	0.051173
CS23	CS14	HS5	*R.nitidus*	-0.061131	0.074244
CS22	CS14	HS5	*R.nitidus*	-0.013258	0.074696
CS20	CS14	HS5	*R.nitidus*	-0.025688	0.073806
CS18	CS14	HS5	*R.nitidus*	-0.047664	0.075086
CS25	CS14	HS5	*R.nitidus*	-0.010608	0.076395
GD34	CS14	HS5	*R.nitidus*	0.008584	0.071731
CS12	CS14	HS5	*R.nitidus*	-0.013643	0.077386
GD41	CS14	HS5	*R.nitidus*	-0.005333	0.073428
SC10	CS14	HS5	*R.nitidus*	0.059109	0.074396
CS13	CS14	HS5	*R.nitidus*	-0.050473	0.051218
GD46	CS14	HS5	*R.nitidus*	-0.00565	0.075654
SC34	CS14	HS5	*R.nitidus*	-0.008101	0.073102
SC12	CS14	HS5	*R.nitidus*	-0.065741	0.071423
CS28	CS14	HS5	*R.nitidus*	-0.043566	0.077331
SC18	CS14	HS5	*R.nitidus*	0.006673	0.072502
SC15	CS14	HS5	*R.nitidus*	-0.00782	0.075312
SC45	CS14	HS5	*R.nitidus*	0.00828	0.073525
SC5	CS14	HS5	*R.nitidus*	0.030387	0.07683
JW4	CS14	HS5	*R.nitidus*	0.149918	0.073063
CS21	CS14	HS5	*R.nitidus*	-0.038354	0.074107
SC14	CS14	HS5	*R.nitidus*	-0.031418	0.072914
SC42	CS14	HS5	*R.nitidus*	0.173415	0.069373
CS31	CS14	HS5	*R.nitidus*	-0.039565	0.076115
SC44	CS14	HS5	*R.nitidus*	-0.030415	0.073767
CS6	CS14	HS5	*R.nitidus*	-0.057561	0.072468
SC28	CS14	HS5	*R.nitidus*	-0.057038	0.072592
CS3	CS14	HS5	*R.nitidus*	-0.032926	0.072952
CS30	CS14	HS5	*R.nitidus*	-0.070463	0.072326
SC6	CS14	HS5	*R.nitidus*	0.013462	0.075742
CS4	CS14	HS5	*R.nitidus*	-0.010027	0.073433
SC27	CS14	HS5	*R.nitidus*	-0.043966	0.071597
JW6	CS14	HS5	*R.nitidus*	0.007292	0.078992
SC32	CS14	HS5	*R.nitidus*	0.035543	0.074034
GD22	CS15	HS5	*R.nitidus*	0.07364	0.070797
GD21	CS15	HS5	*R.nitidus*	0.120205	0.068314
JO1	CS14	HS5	*R.nitidus*	-0.020503	0.072478
GD30	CS15	HS5	*R.nitidus*	0.129609	0.073258

GD29	CS15	HS5	*R.nitidus*	0.140408	0.070433
SC31	CS14	HS5	*R.nitidus*	-0.026978	0.074717
GD25	CS15	HS5	*R.nitidus*	0.089649	0.071145
JW7	CS14	HS5	*R.nitidus*	0.031873	0.075893
MY6	CS14	HS5	*R.nitidus*	-0.002828	0.073994
SC26	CS14	HS5	*R.nitidus*	-0.068744	0.072734
GD41	CS15	HS5	*R.nitidus*	0.00713	0.070107
JO7	CS15	HS5	*R.nitidus*	0.031712	0.075087
SC35	CS14	HS5	*R.nitidus*	0.026221	0.073846
CS11	CS15	HS5	*R.nitidus*	-0.006658	0.073591
JO8	CS15	HS5	*R.nitidus*	0.022941	0.075384
GD33	CS15	HS5	*R.nitidus*	0.009892	0.070719
MY4	CS14	HS5	*R.nitidus*	-0.045062	0.076004
GD32	CS15	HS5	*R.nitidus*	0.045778	0.075117
SC24	CS14	HS5	*R.nitidus*	-0.006494	0.073639
CS12	CS15	HS5	*R.nitidus*	0.001391	0.070419
GD50	CS15	HS5	*R.nitidus*	0.037525	0.073695
JO5	CS14	HS5	*R.nitidus*	0.037522	0.070612
GD49	CS15	HS5	*R.nitidus*	0.067107	0.074254
GD42	CS15	HS5	*R.nitidus*	0.062387	0.071532
GD26	CS15	HS5	*R.nitidus*	0.066437	0.071103
GD34	CS15	HS5	*R.nitidus*	0.005063	0.070765
CS24	CS15	HS5	*R.nitidus*	-0.045954	0.071421
JO3	CS14	HS5	*R.nitidus*	0.057301	0.073595
CS21	CS15	HS5	*R.nitidus*	-0.024568	0.071959
GD44	CS15	HS5	*R.nitidus*	0.023235	0.071125
CS22	CS15	HS5	*R.nitidus*	0.017029	0.071027
CS20	CS15	HS5	*R.nitidus*	-0.016514	0.0696
GD40	CS15	HS5	*R.nitidus*	0.076787	0.069597
CS17	CS15	HS5	*R.nitidus*	0.027859	0.0764
GD31	CS15	HS5	*R.nitidus*	0.078008	0.073877
GD48	CS15	HS5	*R.nitidus*	0.075592	0.071759
GD35	CS15	HS5	*R.nitidus*	0.039855	0.069542
CS4	CS15	HS5	*R.nitidus*	0.007233	0.069888
GD45	CS15	HS5	*R.nitidus*	0.081633	0.076918
SC12	CS15	HS5	*R.nitidus*	-0.049091	0.07008
GD46	CS15	HS5	*R.nitidus*	0.00466	0.072169
CS16	CS15	HS5	*R.nitidus*	-0.009309	0.071685
CS3	CS15	HS5	*R.nitidus*	-0.018975	0.07038
CS28	CS15	HS5	*R.nitidus*	-0.00924	0.072875
SC14	CS15	HS5	*R.nitidus*	-0.025408	0.069919
SC10	CS15	HS5	*R.nitidus*	0.074784	0.072882
CS30	CS15	HS5	*R.nitidus*	-0.063107	0.071206
CS23	CS15	HS5	*R.nitidus*	-0.046875	0.071033
CS18	CS15	HS5	*R.nitidus*	-0.036027	0.071068
CS14	CS15	HS5	*R.nitidus*	0.008708	0.079072
CS19	CS15	HS5	*R.nitidus*	-0.035475	0.070734
SC26	CS15	HS5	*R.nitidus*	-0.053704	0.071065
SC24	CS15	HS5	*R.nitidus*	0.013612	0.070085
JW7	CS15	HS5	*R.nitidus*	0.035468	0.074153
SC27	CS15	HS5	*R.nitidus*	-0.026144	0.069229
SC18	CS15	HS5	*R.nitidus*	0.040797	0.069246
SC31	CS15	HS5	*R.nitidus*	-0.019749	0.071115
SC6	CS15	HS5	*R.nitidus*	0.009625	0.071464
JW4	CS15	HS5	*R.nitidus*	0.135458	0.071127
CS25	CS15	HS5	*R.nitidus*	0.002863	0.072572

CS13	CS15	HS5	*R.nitidus*	-0.030383	0.07583
CS6	CS15	HS5	*R.nitidus*	-0.032567	0.070202
SC44	CS15	HS5	*R.nitidus*	-0.012082	0.072007
SC35	CS15	HS5	*R.nitidus*	0.035808	0.0711
CS31	CS15	HS5	*R.nitidus*	-0.02	0.072819
JO3	CS15	HS5	*R.nitidus*	0.057399	0.071697
SC5	CS15	HS5	*R.nitidus*	0.049639	0.075346
SC45	CS15	HS5	*R.nitidus*	0.023339	0.071423
JW6	CS15	HS5	*R.nitidus*	0.01032	0.075445
SC34	CS15	HS5	*R.nitidus*	0.009709	0.071507
JO7	CS16	HS5	*R.nitidus*	0.015201	0.076629
SC32	CS15	HS5	*R.nitidus*	0.045746	0.070478
SC15	CS15	HS5	*R.nitidus*	0.004926	0.073893
SC42	CS15	HS5	*R.nitidus*	0.179231	0.067341
CS11	CS16	HS5	*R.nitidus*	0.003942	0.074514
GD32	CS16	HS5	*R.nitidus*	0.051653	0.073025
SC28	CS15	HS5	*R.nitidus*	-0.047926	0.069793
GD25	CS16	HS5	*R.nitidus*	0.086093	0.07188
GD21	CS16	HS5	*R.nitidus*	0.115083	0.067482
GD41	CS16	HS5	*R.nitidus*	0.008182	0.069445
MY4	CS15	HS5	*R.nitidus*	-0.035283	0.07374
GD33	CS16	HS5	*R.nitidus*	0.00544	0.071331
JO1	CS15	HS5	*R.nitidus*	-0.012195	0.06982
JO8	CS16	HS5	*R.nitidus*	0.027174	0.074548
MY6	CS15	HS5	*R.nitidus*	0.009276	0.071231
GD44	CS16	HS5	*R.nitidus*	0.022999	0.07096
GD31	CS16	HS5	*R.nitidus*	0.067748	0.073686
GD30	CS16	HS5	*R.nitidus*	0.144944	0.073805
JO5	CS15	HS5	*R.nitidus*	0.058312	0.069921
GD50	CS16	HS5	*R.nitidus*	0.050403	0.073458
GD26	CS16	HS5	*R.nitidus*	0.07013	0.070079
GD45	CS16	HS5	*R.nitidus*	0.107744	0.078386
GD46	CS16	HS5	*R.nitidus*	0.002849	0.071479
GD22	CS16	HS5	*R.nitidus*	0.071307	0.068888
GD42	CS16	HS5	*R.nitidus*	0.065317	0.070205
GD29	CS16	HS5	*R.nitidus*	0.157107	0.068009
CS20	CS16	HS5	*R.nitidus*	-0.008364	0.070966
CS12	CS16	HS5	*R.nitidus*	0.010703	0.069741
SC6	CS16	HS5	*R.nitidus*	0.001953	0.070575
CS21	CS16	HS5	*R.nitidus*	-0.036689	0.071613
GD35	CS16	HS5	*R.nitidus*	0.046595	0.068122
GD49	CS16	HS5	*R.nitidus*	0.080874	0.076099
GD40	CS16	HS5	*R.nitidus*	0.086796	0.069364
CS3	CS16	HS5	*R.nitidus*	-0.020115	0.07033
SC10	CS16	HS5	*R.nitidus*	0.096958	0.071503
CS18	CS16	HS5	*R.nitidus*	-0.020992	0.070835
GD34	CS16	HS5	*R.nitidus*	0.009418	0.068836
CS15	CS16	HS5	*R.nitidus*	0.009309	0.071685
CS30	CS16	HS5	*R.nitidus*	-0.067764	0.070487
CS24	CS16	HS5	*R.nitidus*	-0.042467	0.07124
CS31	CS16	HS5	*R.nitidus*	-0.025458	0.072924
SC26	CS16	HS5	*R.nitidus*	-0.047882	0.069002
CS19	CS16	HS5	*R.nitidus*	-0.01712	0.071317
CS13	CS16	HS5	*R.nitidus*	-0.034264	0.075904
CS22	CS16	HS5	*R.nitidus*	0.016083	0.071095
GD48	CS16	HS5	*R.nitidus*	0.074977	0.072459

SC5	CS16	HS5	*R.nitidus*	0.048644	0.074171
CS17	CS16	HS5	*R.nitidus*	0.058309	0.078843
CS14	CS16	HS5	*R.nitidus*	0.012228	0.075253
SC15	CS16	HS5	*R.nitidus*	0.001008	0.072317
SC18	CS16	HS5	*R.nitidus*	0.039761	0.069442
SC12	CS16	HS5	*R.nitidus*	-0.052288	0.069618
SC34	CS16	HS5	*R.nitidus*	0.002737	0.069501
CS6	CS16	HS5	*R.nitidus*	-0.037608	0.069332
CS25	CS16	HS5	*R.nitidus*	0.004721	0.074117
SC28	CS16	HS5	*R.nitidus*	-0.059434	0.068947
SC31	CS16	HS5	*R.nitidus*	-0.036551	0.073119
CS28	CS16	HS5	*R.nitidus*	-0.017189	0.072937
JW4	CS16	HS5	*R.nitidus*	0.147905	0.07057
SC42	CS16	HS5	*R.nitidus*	0.197974	0.067696
CS23	CS16	HS5	*R.nitidus*	-0.035714	0.069679
GD26	CS17	HS5	*R.nitidus*	0.014911	0.074092
GD30	CS17	HS5	*R.nitidus*	0.107015	0.074964
SC27	CS16	HS5	*R.nitidus*	-0.023787	0.068061
CS11	CS17	HS5	*R.nitidus*	-0.062069	0.074642
JW7	CS16	HS5	*R.nitidus*	0.034694	0.072716
JO8	CS17	HS5	*R.nitidus*	-0.019676	0.079439
SC14	CS16	HS5	*R.nitidus*	-0.028571	0.069075
SC35	CS16	HS5	*R.nitidus*	0.035746	0.068702
CS4	CS16	HS5	*R.nitidus*	0.002735	0.069037
SC24	CS16	HS5	*R.nitidus*	0.01434	0.071145
JO3	CS16	HS5	*R.nitidus*	0.059361	0.072054
JO1	CS16	HS5	*R.nitidus*	-0.000952	0.071086
SC32	CS16	HS5	*R.nitidus*	0.039962	0.072497
SC45	CS16	HS5	*R.nitidus*	0.026508	0.069864
MY4	CS16	HS5	*R.nitidus*	-0.033803	0.072341
JO5	CS16	HS5	*R.nitidus*	0.062387	0.069698
GD29	CS17	HS5	*R.nitidus*	0.099001	0.073407
SC44	CS16	HS5	*R.nitidus*	-0.012184	0.070369
GD32	CS17	HS5	*R.nitidus*	0.002167	0.077899
GD35	CS17	HS5	*R.nitidus*	0.006924	0.073752
GD25	CS17	HS5	*R.nitidus*	0.035751	0.074561
GD33	CS17	HS5	*R.nitidus*	-0.039634	0.077113
JW6	CS16	HS5	*R.nitidus*	0.006329	0.075885
MY6	CS16	HS5	*R.nitidus*	0.009461	0.070276
GD34	CS17	HS5	*R.nitidus*	-0.024254	0.073437
GD48	CS17	HS5	*R.nitidus*	0.043788	0.075101
JO7	CS17	HS5	*R.nitidus*	0.00339	0.05064
GD31	CS17	HS5	*R.nitidus*	0.033508	0.07807
GD40	CS17	HS5	*R.nitidus*	0.049951	0.072793
GD41	CS17	HS5	*R.nitidus*	-0.024631	0.074135
GD21	CS17	HS5	*R.nitidus*	0.080645	0.073044
GD22	CS17	HS5	*R.nitidus*	0.024862	0.073747
GD44	CS17	HS5	*R.nitidus*	-0.026549	0.075191
GD49	CS17	HS5	*R.nitidus*	0.015012	0.051098
GD46	CS17	HS5	*R.nitidus*	-0.051515	0.074691
CS16	CS17	HS5	*R.nitidus*	-0.058309	0.078843
CS19	CS17	HS5	*R.nitidus*	-0.061702	0.076676
CS12	CS17	HS5	*R.nitidus*	-0.046809	0.075024
CS31	CS17	HS5	*R.nitidus*	-0.066959	0.076112
GD50	CS17	HS5	*R.nitidus*	0.006674	0.076564
CS15	CS17	HS5	*R.nitidus*	-0.027859	0.0764

SC15	CS17	HS5	*R.nitidus*	-0.038168	0.077213
CS22	CS17	HS5	*R.nitidus*	-0.0409	0.075585
SC12	CS17	HS5	*R.nitidus*	-0.08483	0.074283
CS14	CS17	HS5	*R.nitidus*	-0.037594	0.079743
GD45	CS17	HS5	*R.nitidus*	0.06506	0.078857
SC6	CS17	HS5	*R.nitidus*	-0.029592	0.074853
SC18	CS17	HS5	*R.nitidus*	-0.006218	0.076464
CS24	CS17	HS5	*R.nitidus*	-0.096703	0.075139
CS25	CS17	HS5	*R.nitidus*	-0.059134	0.079153
GD42	CS17	HS5	*R.nitidus*	0.004955	0.075701
CS20	CS17	HS5	*R.nitidus*	-0.060204	0.075176
CS13	CS17	HS5	*R.nitidus*	-0.076487	0.078436
SC10	CS17	HS5	*R.nitidus*	0.044606	0.075746
CS28	CS17	HS5	*R.nitidus*	-0.062569	0.077677
SC31	CS17	HS5	*R.nitidus*	-0.070707	0.075145
SC28	CS17	HS5	*R.nitidus*	-0.094949	0.073624
SC5	CS17	HS5	*R.nitidus*	0.01	0.077491
CS18	CS17	HS5	*R.nitidus*	-0.072555	0.07473
CS21	CS17	HS5	*R.nitidus*	-0.053481	0.076045
SC27	CS17	HS5	*R.nitidus*	-0.080169	0.071029
CS23	CS17	HS5	*R.nitidus*	-0.082097	0.07453
JO8	CS18	HS5	*R.nitidus*	0.046892	0.074677
CS4	CS17	HS5	*R.nitidus*	-0.035433	0.073886
CS30	CS17	HS5	*R.nitidus*	-0.116615	0.075573
JO3	CS17	HS5	*R.nitidus*	0.01194	0.074772
CS3	CS17	HS5	*R.nitidus*	-0.067427	0.07573
SC14	CS17	HS5	*R.nitidus*	-0.05123	0.071006
SC44	CS17	HS5	*R.nitidus*	-0.052846	0.074166
MY6	CS17	HS5	*R.nitidus*	-0.02877	0.075942
GD22	CS18	HS5	*R.nitidus*	0.09869	0.067856
SC26	CS17	HS5	*R.nitidus*	-0.072854	0.073404
JW6	CS17	HS5	*R.nitidus*	-0.026498	0.079884
CS6	CS17	HS5	*R.nitidus*	-0.055854	0.074924
MY4	CS17	HS5	*R.nitidus*	-0.071501	0.076804
JO1	CS17	HS5	*R.nitidus*	-0.066047	0.074978
SC24	CS17	HS5	*R.nitidus*	-0.038027	0.073247
SC42	CS17	HS5	*R.nitidus*	0.153716	0.071065
SC34	CS17	HS5	*R.nitidus*	-0.035573	0.074904
GD29	CS18	HS5	*R.nitidus*	0.15942	0.067573
JO7	CS18	HS5	*R.nitidus*	0.058462	0.07615
GD31	CS18	HS5	*R.nitidus*	0.096466	0.073766
SC45	CS17	HS5	*R.nitidus*	-0.027001	0.07334
JW7	CS17	HS5	*R.nitidus*	0.008667	0.077415
JW4	CS17	HS5	*R.nitidus*	0.117545	0.072915
GD49	CS18	HS5	*R.nitidus*	0.084153	0.076886
GD40	CS18	HS5	*R.nitidus*	0.115314	0.06751
SC32	CS17	HS5	*R.nitidus*	0.016684	0.073988
GD42	CS18	HS5	*R.nitidus*	0.078467	0.069919
GD30	CS18	HS5	*R.nitidus*	0.150219	0.073418
GD35	CS18	HS5	*R.nitidus*	0.063523	0.070444
CS11	CS18	HS5	*R.nitidus*	0.035138	0.072228
JO5	CS17	HS5	*R.nitidus*	0.000962	0.07361
GD21	CS18	HS5	*R.nitidus*	0.150602	0.06911
CS17	CS18	HS5	*R.nitidus*	0.072555	0.07473
GD45	CS18	HS5	*R.nitidus*	0.109649	0.076348
CS15	CS18	HS5	*R.nitidus*	0.036027	0.071068

GD32	CS18	HS5	*R.nitidus*	0.068394	0.073102
CS16	CS18	HS5	*R.nitidus*	0.020992	0.070835
GD34	CS18	HS5	*R.nitidus*	0.029623	0.069089
GD25	CS18	HS5	*R.nitidus*	0.090253	0.07228
SC5	CS18	HS5	*R.nitidus*	0.070837	0.073951
CS12	CS18	HS5	*R.nitidus*	0.015091	0.072062
GD26	CS18	HS5	*R.nitidus*	0.099815	0.06902
CS21	CS18	HS5	*R.nitidus*	0.009501	0.068488
GD46	CS18	HS5	*R.nitidus*	0.031308	0.071034
GD41	CS18	HS5	*R.nitidus*	0.032432	0.069022
SC35	CS17	HS5	*R.nitidus*	0	0.07578
GD33	CS18	HS5	*R.nitidus*	0.028763	0.072091
CS14	CS18	HS5	*R.nitidus*	0.047664	0.075086
GD44	CS18	HS5	*R.nitidus*	0.050279	0.07007
CS28	CS18	HS5	*R.nitidus*	0.010267	0.075074
CS19	CS18	HS5	*R.nitidus*	-0.008642	0.069214
GD50	CS18	HS5	*R.nitidus*	0.056624	0.073621
CS25	CS18	HS5	*R.nitidus*	0.034871	0.073659
CS13	CS18	HS5	*R.nitidus*	0.02008	0.075481
CS22	CS18	HS5	*R.nitidus*	0.044681	0.070021
CS30	CS18	HS5	*R.nitidus*	-0.040394	0.069357
GD48	CS18	HS5	*R.nitidus*	0.086002	0.073021
CS3	CS18	HS5	*R.nitidus*	-0.004251	0.071103
SC18	CS18	HS5	*R.nitidus*	0.072478	0.068238
CS24	CS18	HS5	*R.nitidus*	-0.017301	0.069871
CS4	CS18	HS5	*R.nitidus*	0.023279	0.068924
CS6	CS18	HS5	*R.nitidus*	-0.006873	0.068105
SC27	CS18	HS5	*R.nitidus*	0.003872	0.070573
CS31	CS18	HS5	*R.nitidus*	-0.000982	0.070516
CS20	CS18	HS5	*R.nitidus*	0.008121	0.06878
SC6	CS18	HS5	*R.nitidus*	0.041829	0.071412
JW6	CS18	HS5	*R.nitidus*	0.029352	0.073515
SC31	CS18	HS5	*R.nitidus*	-0.001871	0.0708
CS23	CS18	HS5	*R.nitidus*	-0.022676	0.070481
SC10	CS18	HS5	*R.nitidus*	0.105723	0.072934
SC32	CS18	HS5	*R.nitidus*	0.072185	0.070148
SC26	CS18	HS5	*R.nitidus*	-0.028064	0.068961
SC28	CS18	HS5	*R.nitidus*	-0.023408	0.069548
JO1	CS18	HS5	*R.nitidus*	0.012785	0.073195
JW7	CS18	HS5	*R.nitidus*	0.060181	0.073476
SC12	CS18	HS5	*R.nitidus*	-0.029218	0.06941
SC15	CS18	HS5	*R.nitidus*	0.022088	0.071549
GD30	CS19	HS5	*R.nitidus*	0.147689	0.07464
SC14	CS18	HS5	*R.nitidus*	-0.006506	0.071539
SC35	CS18	HS5	*R.nitidus*	0.055347	0.068981
SC24	CS18	HS5	*R.nitidus*	0.028874	0.069214
MY6	CS18	HS5	*R.nitidus*	0.027675	0.071733
JO7	CS19	HS5	*R.nitidus*	0.049112	0.077032
GD25	CS19	HS5	*R.nitidus*	0.085126	0.072918
SC45	CS18	HS5	*R.nitidus*	0.050505	0.070306
GD31	CS19	HS5	*R.nitidus*	0.11122	0.074164
GD29	CS19	HS5	*R.nitidus*	0.170344	0.069842
GD32	CS19	HS5	*R.nitidus*	0.056911	0.074679
MY4	CS18	HS5	*R.nitidus*	-0.010456	0.07088
CS11	CS19	HS5	*R.nitidus*	0.032738	0.072462
SC42	CS18	HS5	*R.nitidus*	0.206022	0.069642

SC44	CS18	HS5	*R.nitidus*	0.003711	0.070376
JW4	CS18	HS5	*R.nitidus*	0.194183	0.067313
GD21	CS19	HS5	*R.nitidus*	0.14412	0.069886
JO8	CS19	HS5	*R.nitidus*	0.049217	0.078316
GD40	CS19	HS5	*R.nitidus*	0.111628	0.069196
GD26	CS19	HS5	*R.nitidus*	0.092069	0.072495
GD22	CS19	HS5	*R.nitidus*	0.096396	0.071411
GD42	CS19	HS5	*R.nitidus*	0.066667	0.073594
GD45	CS19	HS5	*R.nitidus*	0.095238	0.077238
GD34	CS19	HS5	*R.nitidus*	0.039162	0.071076
JO3	CS18	HS5	*R.nitidus*	0.072097	0.072327
JO5	CS18	HS5	*R.nitidus*	0.103884	0.069217
SC34	CS18	HS5	*R.nitidus*	0.028388	0.069331
CS20	CS19	HS5	*R.nitidus*	0.009978	0.073885
CS13	CS19	HS5	*R.nitidus*	0.012208	0.075245
CS16	CS19	HS5	*R.nitidus*	0.01712	0.071317
GD41	CS19	HS5	*R.nitidus*	0.032727	0.071948
GD48	CS19	HS5	*R.nitidus*	0.084601	0.071626
CS15	CS19	HS5	*R.nitidus*	0.035475	0.070734
CS12	CS19	HS5	*R.nitidus*	0.017598	0.073455
GD44	CS19	HS5	*R.nitidus*	0.040541	0.07315
GD35	CS19	HS5	*R.nitidus*	0.063107	0.071569
CS17	CS19	HS5	*R.nitidus*	0.061702	0.076676
CS24	CS19	HS5	*R.nitidus*	0.001119	0.070347
GD49	CS19	HS5	*R.nitidus*	0.065292	0.076711
CS28	CS19	HS5	*R.nitidus*	-0.021762	0.074006
GD50	CS19	HS5	*R.nitidus*	0.065521	0.07685
SC6	CS19	HS5	*R.nitidus*	0.039177	0.07223
SC35	CS19	HS5	*R.nitidus*	0.0621	0.070993
SC5	CS19	HS5	*R.nitidus*	0.068665	0.077083
SC31	CS19	HS5	*R.nitidus*	0.003633	0.072275
CS22	CS19	HS5	*R.nitidus*	0.035491	0.071785
SC12	CS19	HS5	*R.nitidus*	-0.014437	0.070918
CS18	CS19	HS5	*R.nitidus*	0.008642	0.069214
CS14	CS19	HS5	*R.nitidus*	0.045862	0.07433
GD46	CS19	HS5	*R.nitidus*	0.017528	0.072148
GD33	CS19	HS5	*R.nitidus*	0.024225	0.074106
SC10	CS19	HS5	*R.nitidus*	0.091716	0.074163
SC24	CS19	HS5	*R.nitidus*	0.039918	0.070985
CS30	CS19	HS5	*R.nitidus*	-0.037624	0.072149
CS21	CS19	HS5	*R.nitidus*	-0.004449	0.07189
MY6	CS19	HS5	*R.nitidus*	0.034417	0.072862
CS3	CS19	HS5	*R.nitidus*	0.008939	0.071633
SC34	CS19	HS5	*R.nitidus*	0.03255	0.070988
SC26	CS19	HS5	*R.nitidus*	-0.024621	0.072069
CS4	CS19	HS5	*R.nitidus*	0.020979	0.068971
SC18	CS19	HS5	*R.nitidus*	0.079381	0.071239
SC44	CS19	HS5	*R.nitidus*	0.007648	0.073671
CS31	CS19	HS5	*R.nitidus*	0.001015	0.071907
CS6	CS19	HS5	*R.nitidus*	-0.006912	0.069281
SC15	CS19	HS5	*R.nitidus*	0.033932	0.07399
CS23	CS19	HS5	*R.nitidus*	-0.014724	0.072003
GD21	CS20	HS5	*R.nitidus*	0.132826	0.069295
SC42	CS19	HS5	*R.nitidus*	0.214286	0.069211
SC32	CS19	HS5	*R.nitidus*	0.071921	0.072738
SC27	CS19	HS5	*R.nitidus*	0.002879	0.072483

MY4	CS19	HS5	*R.nitidus*	-0.004965	0.072952
SC45	CS19	HS5	*R.nitidus*	0.058824	0.073073
CS25	CS19	HS5	*R.nitidus*	0.036388	0.07717
JO7	CS20	HS5	*R.nitidus*	0.024851	0.073136
SC14	CS19	HS5	*R.nitidus*	0.003795	0.072458
GD30	CS20	HS5	*R.nitidus*	0.145161	0.072741
JW4	CS19	HS5	*R.nitidus*	0.19685	0.073668
SC28	CS19	HS5	*R.nitidus*	-0.016667	0.069863
GD22	CS20	HS5	*R.nitidus*	0.092359	0.068843
JO8	CS20	HS5	*R.nitidus*	0.034079	0.075083
CS12	CS20	HS5	*R.nitidus*	-0.003929	0.071925
JO5	CS19	HS5	*R.nitidus*	0.093371	0.071237
JW6	CS19	HS5	*R.nitidus*	0.050538	0.077173
CS11	CS20	HS5	*R.nitidus*	0.019301	0.072127
JO3	CS19	HS5	*R.nitidus*	0.092575	0.072257
JO1	CS19	HS5	*R.nitidus*	0.004558	0.071999
JW7	CS19	HS5	*R.nitidus*	0.077612	0.07251
GD31	CS20	HS5	*R.nitidus*	0.08034	0.074014
GD26	CS20	HS5	*R.nitidus*	0.085863	0.069256
GD35	CS20	HS5	*R.nitidus*	0.055606	0.069612
GD25	CS20	HS5	*R.nitidus*	0.086564	0.073365
GD32	CS20	HS5	*R.nitidus*	0.049696	0.072314
GD41	CS20	HS5	*R.nitidus*	0.026361	0.06955
GD40	CS20	HS5	*R.nitidus*	0.103448	0.069475
GD29	CS20	HS5	*R.nitidus*	0.164167	0.068231
GD33	CS20	HS5	*R.nitidus*	0.015219	0.072786
GD34	CS20	HS5	*R.nitidus*	0.023356	0.069962
CS15	CS20	HS5	*R.nitidus*	0.016514	0.0696
CS18	CS20	HS5	*R.nitidus*	-0.008121	0.06878
GD49	CS20	HS5	*R.nitidus*	0.091483	0.073912
GD45	CS20	HS5	*R.nitidus*	0.077754	0.07844
CS3	CS20	HS5	*R.nitidus*	-0.01933	0.067573
GD46	CS20	HS5	*R.nitidus*	0.016904	0.070194
CS21	CS20	HS5	*R.nitidus*	-0.015644	0.06823
GD44	CS20	HS5	*R.nitidus*	0.025294	0.070555
CS17	CS20	HS5	*R.nitidus*	0.060204	0.075176
SC6	CS20	HS5	*R.nitidus*	0.017875	0.06967
CS22	CS20	HS5	*R.nitidus*	0.023834	0.068996
CS16	CS20	HS5	*R.nitidus*	0.008364	0.070966
SC18	CS20	HS5	*R.nitidus*	0.058648	0.067167
SC12	CS20	HS5	*R.nitidus*	-0.034742	0.068826
GD48	CS20	HS5	*R.nitidus*	0.076077	0.070155
CS31	CS20	HS5	*R.nitidus*	-0.014327	0.071322
SC5	CS20	HS5	*R.nitidus*	0.06679	0.07299
GD42	CS20	HS5	*R.nitidus*	0.053913	0.071524
GD50	CS20	HS5	*R.nitidus*	0.052472	0.074249
SC15	CS20	HS5	*R.nitidus*	0.013699	0.071138
SC44	CS20	HS5	*R.nitidus*	0.002801	0.069265
JW4	CS20	HS5	*R.nitidus*	0.1825	0.069725
CS13	CS20	HS5	*R.nitidus*	0.003756	0.074718
SC10	CS20	HS5	*R.nitidus*	0.086124	0.072183
CS4	CS20	HS5	*R.nitidus*	0.021847	0.067536
SC28	CS20	HS5	*R.nitidus*	-0.029548	0.069912
SC26	CS20	HS5	*R.nitidus*	-0.042636	0.069903
CS14	CS20	HS5	*R.nitidus*	0.025688	0.073806
CS24	CS20	HS5	*R.nitidus*	-0.009128	0.069804

CS19	CS20	HS5	*R.nitidus*	-0.009978	0.073885
CS23	CS20	HS5	*R.nitidus*	-0.040909	0.069394
SC34	CS20	HS5	*R.nitidus*	0.021448	0.06909
CS28	CS20	HS5	*R.nitidus*	-0.019802	0.073634
CS25	CS20	HS5	*R.nitidus*	0.015368	0.074506
SC31	CS20	HS5	*R.nitidus*	-0.014939	0.06829
JO1	CS20	HS5	*R.nitidus*	-0.006195	0.07176
MY6	CS20	HS5	*R.nitidus*	0.011905	0.071858
SC14	CS20	HS5	*R.nitidus*	-0.009091	0.070274
JO8	CS21	HS5	*R.nitidus*	0.050936	0.073211
JW7	CS20	HS5	*R.nitidus*	0.059109	0.07229
SC24	CS20	HS5	*R.nitidus*	0.023256	0.069328
JO3	CS20	HS5	*R.nitidus*	0.068519	0.073221
CS30	CS20	HS5	*R.nitidus*	-0.052093	0.069612
SC32	CS20	HS5	*R.nitidus*	0.060038	0.071607
SC45	CS20	HS5	*R.nitidus*	0.038679	0.069801
SC27	CS20	HS5	*R.nitidus*	-0.011215	0.070141
GD25	CS21	HS5	*R.nitidus*	0.100539	0.07276
SC35	CS20	HS5	*R.nitidus*	0.046222	0.068643
SC42	CS20	HS5	*R.nitidus*	0.184748	0.069253
CS6	CS20	HS5	*R.nitidus*	-0.026731	0.065417
GD26	CS21	HS5	*R.nitidus*	0.082121	0.068133
GD21	CS21	HS5	*R.nitidus*	0.137386	0.068922
CS11	CS21	HS5	*R.nitidus*	0.021858	0.073112
GD45	CS21	HS5	*R.nitidus*	0.097849	0.075574
JW6	CS20	HS5	*R.nitidus*	0.024486	0.073366
GD29	CS21	HS5	*R.nitidus*	0.16145	0.06787
MY4	CS20	HS5	*R.nitidus*	-0.021043	0.071414
GD31	CS21	HS5	*R.nitidus*	0.093661	0.072304
JO7	CS21	HS5	*R.nitidus*	0.044865	0.075177
CS12	CS21	HS5	*R.nitidus*	0.015504	0.071843
GD46	CS21	HS5	*R.nitidus*	0.031167	0.071262
JO5	CS20	HS5	*R.nitidus*	0.097952	0.066652
GD40	CS21	HS5	*R.nitidus*	0.10614	0.069758
GD44	CS21	HS5	*R.nitidus*	0.029864	0.070493
CS18	CS21	HS5	*R.nitidus*	-0.009501	0.068488
GD42	CS21	HS5	*R.nitidus*	0.073019	0.07199
CS13	CS21	HS5	*R.nitidus*	0.002841	0.075194
CS14	CS21	HS5	*R.nitidus*	0.038354	0.074107
GD32	CS21	HS5	*R.nitidus*	0.058014	0.07291
GD30	CS21	HS5	*R.nitidus*	0.181722	0.073658
GD22	CS21	HS5	*R.nitidus*	0.097338	0.066767
CS25	CS21	HS5	*R.nitidus*	0.02043	0.075349
GD35	CS21	HS5	*R.nitidus*	0.060018	0.071086
GD33	CS21	HS5	*R.nitidus*	0.024281	0.072734
GD34	CS21	HS5	*R.nitidus*	0.023276	0.070606
CS17	CS21	HS5	*R.nitidus*	0.053481	0.076045
CS28	CS21	HS5	*R.nitidus*	-0.024218	0.07399
CS30	CS21	HS5	*R.nitidus*	-0.039735	0.068759
SC6	CS21	HS5	*R.nitidus*	0.026769	0.071759
CS15	CS21	HS5	*R.nitidus*	0.024568	0.071959
CS19	CS21	HS5	*R.nitidus*	0.004449	0.07189
GD41	CS21	HS5	*R.nitidus*	0.026518	0.06846
GD50	CS21	HS5	*R.nitidus*	0.051205	0.074426
GD48	CS21	HS5	*R.nitidus*	0.095281	0.070184
GD49	CS21	HS5	*R.nitidus*	0.110503	0.073343

CS23	CS21	HS5	*R.nitidus*	-0.027446	0.070108
CS24	CS21	HS5	*R.nitidus*	-0.005056	0.069428
SC15	CS21	HS5	*R.nitidus*	0.018465	0.071321
CS16	CS21	HS5	*R.nitidus*	0.036689	0.071613
CS31	CS21	HS5	*R.nitidus*	-0.011374	0.071578
SC5	CS21	HS5	*R.nitidus*	0.065589	0.072887
SC24	CS21	HS5	*R.nitidus*	0.037214	0.070741
CS20	CS21	HS5	*R.nitidus*	0.015644	0.06823
SC28	CS21	HS5	*R.nitidus*	-0.021978	0.070185
SC27	CS21	HS5	*R.nitidus*	-0.009234	0.069732
CS22	CS21	HS5	*R.nitidus*	0.038384	0.067762
SC42	CS21	HS5	*R.nitidus*	0.202381	0.068413
CS3	CS21	HS5	*R.nitidus*	-0.003367	0.070751
SC14	CS21	HS5	*R.nitidus*	0	0.071129
JW6	CS21	HS5	*R.nitidus*	0.029762	0.073
SC32	CS21	HS5	*R.nitidus*	0.073864	0.071947
JO1	CS21	HS5	*R.nitidus*	0.017086	0.073256
JW7	CS21	HS5	*R.nitidus*	0.079412	0.072719
SC35	CS21	HS5	*R.nitidus*	0.064428	0.068773
SC12	CS21	HS5	*R.nitidus*	-0.02639	0.069105
SC26	CS21	HS5	*R.nitidus*	-0.035481	0.070075
MY6	CS21	HS5	*R.nitidus*	0.032288	0.072094
CS4	CS21	HS5	*R.nitidus*	0.009718	0.067265
SC18	CS21	HS5	*R.nitidus*	0.067878	0.068993
SC45	CS21	HS5	*R.nitidus*	0.048913	0.070237
GD30	CS22	HS5	*R.nitidus*	0.110245	0.071643
MY4	CS21	HS5	*R.nitidus*	-0.01327	0.072776
SC44	CS21	HS5	*R.nitidus*	0.010979	0.070095
CS6	CS21	HS5	*R.nitidus*	-0.012415	0.07025
SC31	CS21	HS5	*R.nitidus*	0.005545	0.07027
JO8	CS22	HS5	*R.nitidus*	0.006772	0.072791
GD31	CS22	HS5	*R.nitidus*	0.051257	0.072105
CS12	CS22	HS5	*R.nitidus*	-0.008016	0.071458
SC10	CS21	HS5	*R.nitidus*	0.092131	0.071408
GD34	CS22	HS5	*R.nitidus*	0.001742	0.07136
GD32	CS22	HS5	*R.nitidus*	0.034339	0.071634
SC34	CS21	HS5	*R.nitidus*	0.021583	0.069951
GD21	CS22	HS5	*R.nitidus*	0.10052	0.068377
GD29	CS22	HS5	*R.nitidus*	0.135856	0.068661
JW4	CS21	HS5	*R.nitidus*	0.196355	0.068801
GD33	CS22	HS5	*R.nitidus*	-0.011797	0.071731
GD46	CS22	HS5	*R.nitidus*	-0.008287	0.070597
JO3	CS21	HS5	*R.nitidus*	0.085264	0.072052
GD45	CS22	HS5	*R.nitidus*	0.070375	0.075119
JO7	CS22	HS5	*R.nitidus*	0.015021	0.074035
GD48	CS22	HS5	*R.nitidus*	0.061922	0.07007
CS11	CS22	HS5	*R.nitidus*	-0.012093	0.072196
GD26	CS22	HS5	*R.nitidus*	0.061299	0.06971
GD25	CS22	HS5	*R.nitidus*	0.045497	0.070424
JO5	CS21	HS5	*R.nitidus*	0.097035	0.068452
GD22	CS22	HS5	*R.nitidus*	0.051439	0.06726
CS19	CS22	HS5	*R.nitidus*	-0.035491	0.071785
GD42	CS22	HS5	*R.nitidus*	0.048128	0.070867
GD44	CS22	HS5	*R.nitidus*	0.026341	0.071283
GD50	CS22	HS5	*R.nitidus*	0.030884	0.070484
GD41	CS22	HS5	*R.nitidus*	0.012411	0.070063

GD40	CS22	HS5	*R.nitidus*	0.073105	0.06828
CS25	CS22	HS5	*R.nitidus*	-0.011352	0.073474
GD35	CS22	HS5	*R.nitidus*	0.039252	0.069755
SC12	CS22	HS5	*R.nitidus*	-0.063054	0.068146
CS15	CS22	HS5	*R.nitidus*	-0.017029	0.071027
CS23	CS22	HS5	*R.nitidus*	-0.059426	0.069698
CS17	CS22	HS5	*R.nitidus*	0.0409	0.075585
CS14	CS22	HS5	*R.nitidus*	0.013258	0.074696
SC28	CS22	HS5	*R.nitidus*	-0.04707	0.071066
GD49	CS22	HS5	*R.nitidus*	0.040929	0.073848
CS18	CS22	HS5	*R.nitidus*	-0.044681	0.070021
CS13	CS22	HS5	*R.nitidus*	-0.031609	0.073244
CS24	CS22	HS5	*R.nitidus*	-0.053856	0.067426
CS28	CS22	HS5	*R.nitidus*	-0.029442	0.073544
SC31	CS22	HS5	*R.nitidus*	-0.028275	0.07141
CS21	CS22	HS5	*R.nitidus*	-0.038384	0.067762
SC18	CS22	HS5	*R.nitidus*	0.033033	0.070253
CS31	CS22	HS5	*R.nitidus*	-0.04458	0.070892
SC14	CS22	HS5	*R.nitidus*	-0.024178	0.071109
CS16	CS22	HS5	*R.nitidus*	-0.016083	0.071095
SC10	CS22	HS5	*R.nitidus*	0.088933	0.072896
SC24	CS22	HS5	*R.nitidus*	0.004826	0.070342
SC5	CS22	HS5	*R.nitidus*	0.047619	0.074187
SC34	CS22	HS5	*R.nitidus*	0.004579	0.069288
CS20	CS22	HS5	*R.nitidus*	-0.023834	0.068996
SC44	CS22	HS5	*R.nitidus*	-0.022857	0.072225
CS30	CS22	HS5	*R.nitidus*	-0.078973	0.06936
JW4	CS22	HS5	*R.nitidus*	0.16	0.06851
CS6	CS22	HS5	*R.nitidus*	-0.044341	0.068622
MY6	CS22	HS5	*R.nitidus*	0.000967	0.070885
SC27	CS22	HS5	*R.nitidus*	-0.026188	0.069769
SC6	CS22	HS5	*R.nitidus*	0.000975	0.071014
CS4	CS22	HS5	*R.nitidus*	-0.003774	0.066143
SC15	CS22	HS5	*R.nitidus*	0.006129	0.071667
GD21	CS23	HS5	*R.nitidus*	0.155684	0.069611
CS3	CS22	HS5	*R.nitidus*	-0.033138	0.071476
SC26	CS22	HS5	*R.nitidus*	-0.065777	0.068834
SC45	CS22	HS5	*R.nitidus*	0.017891	0.069411
SC32	CS22	HS5	*R.nitidus*	0.043265	0.071282
JW6	CS22	HS5	*R.nitidus*	0.023933	0.072368
JO1	CS22	HS5	*R.nitidus*	-0.008491	0.072745
MY4	CS22	HS5	*R.nitidus*	-0.039024	0.071315
SC35	CS22	HS5	*R.nitidus*	0.023553	0.069976
JO5	CS22	HS5	*R.nitidus*	0.066225	0.068857
JO7	CS23	HS5	*R.nitidus*	0.059244	0.075726
SC42	CS22	HS5	*R.nitidus*	0.18086	0.069275
GD31	CS23	HS5	*R.nitidus*	0.10775	0.071122
GD30	CS23	HS5	*R.nitidus*	0.153182	0.073701
GD26	CS23	HS5	*R.nitidus*	0.106074	0.07002
CS12	CS23	HS5	*R.nitidus*	0.025194	0.072328
GD41	CS23	HS5	*R.nitidus*	0.055895	0.068861
CS13	CS23	HS5	*R.nitidus*	0.029273	0.07494
GD33	CS23	HS5	*R.nitidus*	0.049778	0.072727
GD35	CS23	HS5	*R.nitidus*	0.079494	0.069793
GD25	CS23	HS5	*R.nitidus*	0.113879	0.071716
JW7	CS22	HS5	*R.nitidus*	0.043878	0.071757

JO3	CS22	HS5	*R.nitidus*	0.048579	0.072491
JO8	CS23	HS5	*R.nitidus*	0.062048	0.075563
GD22	CS23	HS5	*R.nitidus*	0.109787	0.06921
GD46	CS23	HS5	*R.nitidus*	0.036082	0.070605
CS11	CS23	HS5	*R.nitidus*	0.049639	0.072658
CS19	CS23	HS5	*R.nitidus*	0.014724	0.072003
CS18	CS23	HS5	*R.nitidus*	0.022676	0.070481
GD32	CS23	HS5	*R.nitidus*	0.086259	0.074215
GD48	CS23	HS5	*R.nitidus*	0.110092	0.071852
GD44	CS23	HS5	*R.nitidus*	0.053162	0.070432
GD45	CS23	HS5	*R.nitidus*	0.092652	0.078049
CS15	CS23	HS5	*R.nitidus*	0.046875	0.071033
GD42	CS23	HS5	*R.nitidus*	0.090829	0.072272
CS14	CS23	HS5	*R.nitidus*	0.061131	0.074244
CS20	CS23	HS5	*R.nitidus*	0.040909	0.069394
CS16	CS23	HS5	*R.nitidus*	0.035714	0.069679
GD29	CS23	HS5	*R.nitidus*	0.180976	0.067972
SC10	CS23	HS5	*R.nitidus*	0.11355	0.073382
GD40	CS23	HS5	*R.nitidus*	0.122667	0.068319
SC5	CS23	HS5	*R.nitidus*	0.078222	0.073778
CS25	CS23	HS5	*R.nitidus*	0.044276	0.075142
CS31	CS23	HS5	*R.nitidus*	0.016744	0.072634
GD49	CS23	HS5	*R.nitidus*	0.09292	0.076346
GD34	CS23	HS5	*R.nitidus*	0.045217	0.070836
GD50	CS23	HS5	*R.nitidus*	0.073737	0.0745
CS22	CS23	HS5	*R.nitidus*	0.059426	0.069698
CS21	CS23	HS5	*R.nitidus*	0.027446	0.070108
SC18	CS23	HS5	*R.nitidus*	0.090909	0.068913
SC27	CS23	HS5	*R.nitidus*	0.013979	0.069015
CS17	CS23	HS5	*R.nitidus*	0.082097	0.07453
CS28	CS23	HS5	*R.nitidus*	0.013807	0.074764
CS24	CS23	HS5	*R.nitidus*	0.009454	0.070624
CS4	CS23	HS5	*R.nitidus*	0.045726	0.067813
SC15	CS23	HS5	*R.nitidus*	0.056696	0.071274
SC24	CS23	HS5	*R.nitidus*	0.056622	0.070255
JO3	CS23	HS5	*R.nitidus*	0.097159	0.070537
MY4	CS23	HS5	*R.nitidus*	0.008303	0.07211
JW7	CS23	HS5	*R.nitidus*	0.091262	0.071543
CS30	CS23	HS5	*R.nitidus*	-0.020833	0.071019
CS6	CS23	HS5	*R.nitidus*	0.012101	0.070079
SC14	CS23	HS5	*R.nitidus*	0.017273	0.071012
SC31	CS23	HS5	*R.nitidus*	0.020683	0.070147
SC44	CS23	HS5	*R.nitidus*	0.018282	0.07016
CS3	CS23	HS5	*R.nitidus*	0.026437	0.068362
SC35	CS23	HS5	*R.nitidus*	0.077768	0.07064
SC6	CS23	HS5	*R.nitidus*	0.057597	0.070457
SC42	CS23	HS5	*R.nitidus*	0.233095	0.068906
SC45	CS23	HS5	*R.nitidus*	0.072222	0.070603
GD25	CS24	HS5	*R.nitidus*	0.079143	0.072672
GD31	CS24	HS5	*R.nitidus*	0.097087	0.071937
SC32	CS23	HS5	*R.nitidus*	0.08829	0.072909
SC12	CS23	HS5	*R.nitidus*	-0.015829	0.068604
SC34	CS23	HS5	*R.nitidus*	0.050137	0.069181
GD34	CS24	HS5	*R.nitidus*	0.041021	0.069284
GD35	CS24	HS5	*R.nitidus*	0.073826	0.068944
SC28	CS23	HS5	*R.nitidus*	-0.017352	0.06992

SC26	CS23	HS5	*R.nitidus*	-0.013953	0.071408
GD22	CS24	HS5	*R.nitidus*	0.099027	0.068508
CS11	CS24	HS5	*R.nitidus*	0.044335	0.073914
JW4	CS23	HS5	*R.nitidus*	0.205107	0.06917
MY6	CS23	HS5	*R.nitidus*	0.047882	0.071929
JO7	CS24	HS5	*R.nitidus*	0.067308	0.074359
JO8	CS24	HS5	*R.nitidus*	0.037671	0.07462
GD32	CS24	HS5	*R.nitidus*	0.0842	0.071189
GD26	CS24	HS5	*R.nitidus*	0.09058	0.069554
GD29	CS24	HS5	*R.nitidus*	0.169002	0.067968
GD30	CS24	HS5	*R.nitidus*	0.158963	0.072429
JO1	CS23	HS5	*R.nitidus*	0.031164	0.072232
JO5	CS23	HS5	*R.nitidus*	0.105168	0.067865
GD21	CS24	HS5	*R.nitidus*	0.148116	0.068041
JW6	CS23	HS5	*R.nitidus*	0.035054	0.07354
CS15	CS24	HS5	*R.nitidus*	0.045954	0.071421
GD33	CS24	HS5	*R.nitidus*	0.035055	0.072732
CS12	CS24	HS5	*R.nitidus*	0.044776	0.071336
GD41	CS24	HS5	*R.nitidus*	0.041431	0.069562
CS20	CS24	HS5	*R.nitidus*	0.009128	0.069804
CS13	CS24	HS5	*R.nitidus*	0.032587	0.074586
CS18	CS24	HS5	*R.nitidus*	0.017301	0.069871
CS17	CS24	HS5	*R.nitidus*	0.096703	0.075139
GD42	CS24	HS5	*R.nitidus*	0.081517	0.072415
GD40	CS24	HS5	*R.nitidus*	0.12523	0.068302
GD44	CS24	HS5	*R.nitidus*	0.055133	0.069897
GD45	CS24	HS5	*R.nitidus*	0.106383	0.076073
GD48	CS24	HS5	*R.nitidus*	0.092192	0.071001
SC10	CS24	HS5	*R.nitidus*	0.141717	0.07098
CS21	CS24	HS5	*R.nitidus*	0.005056	0.069428
SC26	CS24	HS5	*R.nitidus*	-0.019157	0.069298
CS22	CS24	HS5	*R.nitidus*	0.053856	0.067426
CS3	CS24	HS5	*R.nitidus*	-0.007209	0.070933
GD49	CS24	HS5	*R.nitidus*	0.087196	0.076772
CS31	CS24	HS5	*R.nitidus*	0.01147	0.071111
SC12	CS24	HS5	*R.nitidus*	-0.012859	0.069253
GD50	CS24	HS5	*R.nitidus*	0.049127	0.072907
CS23	CS24	HS5	*R.nitidus*	-0.009454	0.070624
SC35	CS24	HS5	*R.nitidus*	0.070678	0.068459
CS4	CS24	HS5	*R.nitidus*	0.045657	0.064584
SC34	CS24	HS5	*R.nitidus*	0.051625	0.067663
SC42	CS24	HS5	*R.nitidus*	0.236603	0.067271
CS30	CS24	HS5	*R.nitidus*	-0.042138	0.067459
SC5	CS24	HS5	*R.nitidus*	0.088294	0.073657
SC44	CS24	HS5	*R.nitidus*	0.031434	0.069472
CS19	CS24	HS5	*R.nitidus*	-0.001119	0.070347
SC32	CS24	HS5	*R.nitidus*	0.088845	0.07039
GD46	CS24	HS5	*R.nitidus*	0.021739	0.070308
CS14	CS24	HS5	*R.nitidus*	0.058163	0.074984
CS16	CS24	HS5	*R.nitidus*	0.042467	0.07124
CS28	CS24	HS5	*R.nitidus*	0.02234	0.07382
SC18	CS24	HS5	*R.nitidus*	0.073367	0.068808
SC31	CS24	HS5	*R.nitidus*	0.018904	0.072177
SC14	CS24	HS5	*R.nitidus*	0.007897	0.070582
JO7	CS25	HS5	*R.nitidus*	0.028956	0.076281
SC6	CS24	HS5	*R.nitidus*	0.054348	0.071086

SC28	CS24	HS5	*R.nitidus*	-0.00666	0.070037
CS6	CS24	HS5	*R.nitidus*	-0.002148	0.068622
CS25	CS24	HS5	*R.nitidus*	0.042531	0.073036
JW6	CS24	HS5	*R.nitidus*	0.057082	0.073894
MY6	CS24	HS5	*R.nitidus*	0.045499	0.07124
JW7	CS24	HS5	*R.nitidus*	0.082192	0.070902
SC15	CS24	HS5	*R.nitidus*	0.046536	0.071136
SC24	CS24	HS5	*R.nitidus*	0.04467	0.069386
SC27	CS24	HS5	*R.nitidus*	0.004028	0.068888
SC45	CS24	HS5	*R.nitidus*	0.051756	0.068027
JW4	CS24	HS5	*R.nitidus*	0.208403	0.068932
CS12	CS25	HS5	*R.nitidus*	-0.00804	0.074178
JO8	CS25	HS5	*R.nitidus*	0.026461	0.075999
JO1	CS24	HS5	*R.nitidus*	0.033645	0.072929
JO3	CS24	HS5	*R.nitidus*	0.087687	0.070731
GD45	CS25	HS5	*R.nitidus*	0.095672	0.079781
GD34	CS25	HS5	*R.nitidus*	0.016071	0.071594
CS14	CS25	HS5	*R.nitidus*	0.010608	0.076395
GD33	CS25	HS5	*R.nitidus*	0.002904	0.074212
GD46	CS25	HS5	*R.nitidus*	-0.00363	0.072313
MY4	CS24	HS5	*R.nitidus*	0.00779	0.071247
GD29	CS25	HS5	*R.nitidus*	0.157391	0.070004
GD32	CS25	HS5	*R.nitidus*	0.042531	0.075071
JO5	CS24	HS5	*R.nitidus*	0.108341	0.068301
GD41	CS25	HS5	*R.nitidus*	0.007806	0.071317
CS16	CS25	HS5	*R.nitidus*	-0.004721	0.074117
GD30	CS25	HS5	*R.nitidus*	0.147541	0.077208
CS11	CS25	HS5	*R.nitidus*	0.006686	0.074203
CS13	CS25	HS5	*R.nitidus*	-0.019436	0.074667
GD40	CS25	HS5	*R.nitidus*	0.083865	0.070205
GD44	CS25	HS5	*R.nitidus*	0.01223	0.074562
GD25	CS25	HS5	*R.nitidus*	0.08642	0.072278
GD48	CS25	HS5	*R.nitidus*	0.053622	0.073318
CS19	CS25	HS5	*R.nitidus*	-0.036388	0.07717
GD50	CS25	HS5	*R.nitidus*	0.046336	0.074754
GD22	CS25	HS5	*R.nitidus*	0.083408	0.071468
GD26	CS25	HS5	*R.nitidus*	0.071171	0.070504
GD49	CS25	HS5	*R.nitidus*	0.055118	0.077364
GD21	CS25	HS5	*R.nitidus*	0.124017	0.071888
CS17	CS25	HS5	*R.nitidus*	0.059134	0.079153
CS23	CS25	HS5	*R.nitidus*	-0.044276	0.075142
CS18	CS25	HS5	*R.nitidus*	-0.034871	0.073659
GD42	CS25	HS5	*R.nitidus*	0.058065	0.071569
CS20	CS25	HS5	*R.nitidus*	-0.015368	0.074506
GD31	CS25	HS5	*R.nitidus*	0.088632	0.072826
CS30	CS25	HS5	*R.nitidus*	-0.058654	0.073699
CS31	CS25	HS5	*R.nitidus*	-0.027972	0.073551
GD35	CS25	HS5	*R.nitidus*	0.0377	0.071277
SC6	CS25	HS5	*R.nitidus*	0.012072	0.074366
CS24	CS25	HS5	*R.nitidus*	-0.042531	0.073036
SC18	CS25	HS5	*R.nitidus*	0.047992	0.071605
CS15	CS25	HS5	*R.nitidus*	-0.002863	0.072572
SC15	CS25	HS5	*R.nitidus*	0.002901	0.074753
CS21	CS25	HS5	*R.nitidus*	-0.02043	0.075349
CS22	CS25	HS5	*R.nitidus*	0.011352	0.073474
SC14	CS25	HS5	*R.nitidus*	-0.025352	0.073055

JO1	CS25	HS5	*R.nitidus*	-0.021371	0.07285
SC44	CS25	HS5	*R.nitidus*	-0.032598	0.073736
JO7	CS28	HS5	*R.nitidus*	0.059226	0.07266
CS28	CS25	HS5	*R.nitidus*	-0.035454	0.073715
SC31	CS25	HS5	*R.nitidus*	-0.017986	0.073063
SC10	CS25	HS5	*R.nitidus*	0.052527	0.074746
GD22	CS28	HS5	*R.nitidus*	0.097806	0.074865
GD25	CS28	HS5	*R.nitidus*	0.090628	0.074052
CS3	CS25	HS5	*R.nitidus*	-0.037116	0.073498
SC5	CS25	HS5	*R.nitidus*	0.045923	0.077679
CS6	CS25	HS5	*R.nitidus*	-0.044824	0.071345
MY4	CS25	HS5	*R.nitidus*	-0.03445	0.072948
GD33	CS28	HS5	*R.nitidus*	0.036893	0.074364
CS12	CS28	HS5	*R.nitidus*	0	0.073963
CS4	CS25	HS5	*R.nitidus*	0.012758	0.072056
JW4	CS25	HS5	*R.nitidus*	0.162365	0.072553
JW6	CS25	HS5	*R.nitidus*	0.022267	0.076642
SC28	CS25	HS5	*R.nitidus*	-0.05174	0.072085
SC12	CS25	HS5	*R.nitidus*	-0.049904	0.070169
SC24	CS25	HS5	*R.nitidus*	0.022137	0.07182
MY6	CS25	HS5	*R.nitidus*	0.01131	0.071961
SC26	CS25	HS5	*R.nitidus*	-0.050285	0.072503
GD35	CS28	HS5	*R.nitidus*	0.067764	0.071195
JW7	CS25	HS5	*R.nitidus*	0.048563	0.075179
SC35	CS25	HS5	*R.nitidus*	0.034164	0.071688
JO5	CS25	HS5	*R.nitidus*	0.063889	0.07075
SC32	CS25	HS5	*R.nitidus*	0.049713	0.073406
GD40	CS28	HS5	*R.nitidus*	0.100659	0.069532
SC27	CS25	HS5	*R.nitidus*	-0.02644	0.071899
GD21	CS28	HS5	*R.nitidus*	0.13506	0.07115
CS11	CS28	HS5	*R.nitidus*	0.027439	0.072939
SC34	CS25	HS5	*R.nitidus*	0.002653	0.070395
GD26	CS28	HS5	*R.nitidus*	0.091429	0.072087
JO3	CS25	HS5	*R.nitidus*	0.073462	0.073382
SC42	CS25	HS5	*R.nitidus*	0.187092	0.070095
SC45	CS25	HS5	*R.nitidus*	0.026641	0.072172
GD32	CS28	HS5	*R.nitidus*	0.064586	0.075213
GD30	CS28	HS5	*R.nitidus*	0.166667	0.076265
JO8	CS28	HS5	*R.nitidus*	0.034522	0.077737
CS13	CS28	HS5	*R.nitidus*	0	0.076333
GD46	CS28	HS5	*R.nitidus*	0.021696	0.074741
GD29	CS28	HS5	*R.nitidus*	0.163588	0.072391
GD44	CS28	HS5	*R.nitidus*	0.047337	0.073862
GD31	CS28	HS5	*R.nitidus*	0.073805	0.073301
GD49	CS28	HS5	*R.nitidus*	0.068415	0.078175
GD50	CS28	HS5	*R.nitidus*	0.103837	0.077083
CS15	CS28	HS5	*R.nitidus*	0.00924	0.072875
GD34	CS28	HS5	*R.nitidus*	0.046935	0.07407
CS3	CS28	HS5	*R.nitidus*	0.002045	0.073627
CS17	CS28	HS5	*R.nitidus*	0.062569	0.077677
CS14	CS28	HS5	*R.nitidus*	0.043566	0.077331
GD41	CS28	HS5	*R.nitidus*	0.025692	0.072301
GD42	CS28	HS5	*R.nitidus*	0.071142	0.071876
GD45	CS28	HS5	*R.nitidus*	0.093132	0.077724
CS24	CS28	HS5	*R.nitidus*	-0.02234	0.07382
SC14	CS28	HS5	*R.nitidus*	-0.007202	0.074413

SC10	CS28	HS5	*R.nitidus*	0.104101	0.073353
GD48	CS28	HS5	*R.nitidus*	0.102081	0.072998
CS30	CS28	HS5	*R.nitidus*	-0.04811	0.071377
SC5	CS28	HS5	*R.nitidus*	0.075726	0.074524
SC31	CS28	HS5	*R.nitidus*	-0.007187	0.071857
CS19	CS28	HS5	*R.nitidus*	0.021762	0.074006
SC18	CS28	HS5	*R.nitidus*	0.071429	0.073758
SC24	CS28	HS5	*R.nitidus*	0.045696	0.072435
CS23	CS28	HS5	*R.nitidus*	-0.013807	0.074764
CS31	CS28	HS5	*R.nitidus*	-0.019711	0.074831
CS6	CS28	HS5	*R.nitidus*	-0.010204	0.071166
SC44	CS28	HS5	*R.nitidus*	0.016293	0.070217
SC12	CS28	HS5	*R.nitidus*	-0.026455	0.071519
SC15	CS28	HS5	*R.nitidus*	0.007584	0.074386
CS25	CS28	HS5	*R.nitidus*	0.035454	0.073715
CS16	CS28	HS5	*R.nitidus*	0.017189	0.072937
SC35	CS28	HS5	*R.nitidus*	0.04175	0.072368
SC42	CS28	HS5	*R.nitidus*	0.203704	0.069125
CS20	CS28	HS5	*R.nitidus*	0.019802	0.073634
CS18	CS28	HS5	*R.nitidus*	-0.010267	0.075074
CS21	CS28	HS5	*R.nitidus*	0.024218	0.07399
SC32	CS28	HS5	*R.nitidus*	0.072708	0.072118
MY6	CS28	HS5	*R.nitidus*	0.037717	0.073072
CS22	CS28	HS5	*R.nitidus*	0.029442	0.073544
SC27	CS28	HS5	*R.nitidus*	-0.001024	0.07252
JW6	CS28	HS5	*R.nitidus*	0.039216	0.074705
SC6	CS28	HS5	*R.nitidus*	0.043203	0.074922
JO5	CS28	HS5	*R.nitidus*	0.088381	0.070127
JW4	CS28	HS5	*R.nitidus*	0.189509	0.073733
CS4	CS28	HS5	*R.nitidus*	0.018756	0.072116
SC26	CS28	HS5	*R.nitidus*	-0.033161	0.073255
SC34	CS28	HS5	*R.nitidus*	0.039024	0.072334
SC28	CS28	HS5	*R.nitidus*	-0.009307	0.0698
MY4	CS28	HS5	*R.nitidus*	0.002049	0.073369
GD22	CS30	HS5	*R.nitidus*	0.132867	0.068129
JO7	CS30	HS5	*R.nitidus*	0.083149	0.074772
SC45	CS28	HS5	*R.nitidus*	0.032738	0.074231
JO8	CS30	HS5	*R.nitidus*	0.092511	0.073206
GD21	CS30	HS5	*R.nitidus*	0.184466	0.067643
GD25	CS30	HS5	*R.nitidus*	0.130394	0.073299
JO3	CS28	HS5	*R.nitidus*	0.097328	0.075913
GD26	CS30	HS5	*R.nitidus*	0.126043	0.06935
GD40	CS30	HS5	*R.nitidus*	0.138596	0.067836
CS12	CS30	HS5	*R.nitidus*	0.061013	0.073152
JO1	CS28	HS5	*R.nitidus*	0.00207	0.074212
GD29	CS30	HS5	*R.nitidus*	0.210113	0.065623
CS18	CS30	HS5	*R.nitidus*	0.040394	0.069357
GD49	CS30	HS5	*R.nitidus*	0.120181	0.074903
CS11	CS30	HS5	*R.nitidus*	0.064667	0.072796
GD33	CS30	HS5	*R.nitidus*	0.067219	0.071442
CS15	CS30	HS5	*R.nitidus*	0.063107	0.071206
GD42	CS30	HS5	*R.nitidus*	0.122568	0.07047
JW7	CS28	HS5	*R.nitidus*	0.075824	0.075971
GD45	CS30	HS5	*R.nitidus*	0.128492	0.076249
GD32	CS30	HS5	*R.nitidus*	0.083595	0.07341
GD30	CS30	HS5	*R.nitidus*	0.18552	0.072073

GD44	CS30	HS5	*R.nitidus*	0.065619	0.071246
CS22	CS30	HS5	*R.nitidus*	0.078973	0.06936
GD50	CS30	HS5	*R.nitidus*	0.110643	0.073793
GD31	CS30	HS5	*R.nitidus*	0.128	0.073272
CS14	CS30	HS5	*R.nitidus*	0.070463	0.072326
CS20	CS30	HS5	*R.nitidus*	0.052093	0.069612
CS21	CS30	HS5	*R.nitidus*	0.039735	0.068759
CS28	CS30	HS5	*R.nitidus*	0.04811	0.071377
GD35	CS30	HS5	*R.nitidus*	0.095281	0.070298
CS23	CS30	HS5	*R.nitidus*	0.020833	0.071019
GD48	CS30	HS5	*R.nitidus*	0.133396	0.072314
GD34	CS30	HS5	*R.nitidus*	0.082243	0.068356
CS17	CS30	HS5	*R.nitidus*	0.116615	0.075573
SC6	CS30	HS5	*R.nitidus*	0.088123	0.0692
GD41	CS30	HS5	*R.nitidus*	0.087406	0.06931
SC5	CS30	HS5	*R.nitidus*	0.104147	0.071603
CS13	CS30	HS5	*R.nitidus*	0.049323	0.072699
CS19	CS30	HS5	*R.nitidus*	0.037624	0.072149
CS25	CS30	HS5	*R.nitidus*	0.058654	0.073699
SC18	CS30	HS5	*R.nitidus*	0.099707	0.070171
GD46	CS30	HS5	*R.nitidus*	0.056497	0.071463
SC12	CS30	HS5	*R.nitidus*	0.008712	0.069037
CS16	CS30	HS5	*R.nitidus*	0.067764	0.070487
SC14	CS30	HS5	*R.nitidus*	0.040038	0.071098
CS3	CS30	HS5	*R.nitidus*	0.038462	0.069903
CS24	CS30	HS5	*R.nitidus*	0.042138	0.067459
SC31	CS30	HS5	*R.nitidus*	0.042751	0.071215
SC26	CS30	HS5	*R.nitidus*	0.007547	0.069564
CS31	CS30	HS5	*R.nitidus*	0.051592	0.06902
JO8	CS31	HS5	*R.nitidus*	0.050167	0.075507
SC15	CS30	HS5	*R.nitidus*	0.064615	0.0714
SC10	CS30	HS5	*R.nitidus*	0.14466	0.070791
SC27	CS30	HS5	*R.nitidus*	0.041543	0.068383
JO1	CS30	HS5	*R.nitidus*	0.043925	0.070485
SC45	CS30	HS5	*R.nitidus*	0.094321	0.070315
JW6	CS30	HS5	*R.nitidus*	0.069444	0.073374
SC35	CS30	HS5	*R.nitidus*	0.097303	0.069214
SC44	CS30	HS5	*R.nitidus*	0.054598	0.068118
CS4	CS30	HS5	*R.nitidus*	0.065903	0.066379
CS6	CS30	HS5	*R.nitidus*	0.035088	0.067918
SC34	CS30	HS5	*R.nitidus*	0.07326	0.067355
JW7	CS30	HS5	*R.nitidus*	0.122222	0.070527
SC24	CS30	HS5	*R.nitidus*	0.078278	0.071093
MY4	CS30	HS5	*R.nitidus*	0.031895	0.071773
CS12	CS31	HS5	*R.nitidus*	0.030851	0.073271
GD35	CS31	HS5	*R.nitidus*	0.072797	0.069851
SC32	CS30	HS5	*R.nitidus*	0.117135	0.070887
JO7	CS31	HS5	*R.nitidus*	0.06531	0.07476
GD25	CS31	HS5	*R.nitidus*	0.105211	0.072627
SC42	CS30	HS5	*R.nitidus*	0.251227	0.067498
GD22	CS31	HS5	*R.nitidus*	0.096916	0.071971
GD45	CS31	HS5	*R.nitidus*	0.129147	0.076516
CS11	CS31	HS5	*R.nitidus*	0.019861	0.074833
GD26	CS31	HS5	*R.nitidus*	0.094972	0.071432
MY6	CS30	HS5	*R.nitidus*	0.076851	0.070512
JW4	CS30	HS5	*R.nitidus*	0.227425	0.069295

GD48	CS31	HS5	*R.nitidus*	0.086622	0.071184
GD30	CS31	HS5	*R.nitidus*	0.156177	0.072776
GD33	CS31	HS5	*R.nitidus*	0.0254	0.071777
JO3	CS30	HS5	*R.nitidus*	0.130762	0.072725
GD49	CS31	HS5	*R.nitidus*	0.100907	0.078046
JO5	CS30	HS5	*R.nitidus*	0.122959	0.06651
GD40	CS31	HS5	*R.nitidus*	0.118812	0.068756
GD21	CS31	HS5	*R.nitidus*	0.151268	0.069954
GD34	CS31	HS5	*R.nitidus*	0.038498	0.06824
GD29	CS31	HS5	*R.nitidus*	0.185764	0.068768
GD50	CS31	HS5	*R.nitidus*	0.072626	0.072809
CS30	CS31	HS5	*R.nitidus*	-0.051592	0.06902
SC28	CS30	HS5	*R.nitidus*	0.015814	0.066762
GD46	CS31	HS5	*R.nitidus*	0.027861	0.069945
CS20	CS31	HS5	*R.nitidus*	0.014327	0.071322
GD31	CS31	HS5	*R.nitidus*	0.087539	0.073941
GD32	CS31	HS5	*R.nitidus*	0.055495	0.074623
CS4	CS31	HS5	*R.nitidus*	0.027698	0.068897
CS24	CS31	HS5	*R.nitidus*	-0.01147	0.071111
CS17	CS31	HS5	*R.nitidus*	0.066959	0.076112
GD42	CS31	HS5	*R.nitidus*	0.063317	0.071911
GD41	CS31	HS5	*R.nitidus*	0.040196	0.069702
GD44	CS31	HS5	*R.nitidus*	0.049467	0.071243
CS13	CS31	HS5	*R.nitidus*	-0.002035	0.076979
CS16	CS31	HS5	*R.nitidus*	0.025458	0.072924
CS18	CS31	HS5	*R.nitidus*	0.000982	0.070516
SC12	CS31	HS5	*R.nitidus*	-0.029894	0.067792
SC27	CS31	HS5	*R.nitidus*	0.002041	0.071702
SC28	CS31	HS5	*R.nitidus*	-0.012634	0.067636
CS19	CS31	HS5	*R.nitidus*	-0.001015	0.071907
CS3	CS31	HS5	*R.nitidus*	0.002938	0.071603
CS15	CS31	HS5	*R.nitidus*	0.02	0.072819
SC10	CS31	HS5	*R.nitidus*	0.119329	0.07151
SC6	CS31	HS5	*R.nitidus*	0.048515	0.0711
SC15	CS31	HS5	*R.nitidus*	0.03112	0.074438
SC18	CS31	HS5	*R.nitidus*	0.078411	0.071017
SC5	CS31	HS5	*R.nitidus*	0.069307	0.073133
CS22	CS31	HS5	*R.nitidus*	0.04458	0.070892
CS14	CS31	HS5	*R.nitidus*	0.039565	0.076115
JW4	CS31	HS5	*R.nitidus*	0.20332	0.069982
SC26	CS31	HS5	*R.nitidus*	-0.021463	0.072679
CS21	CS31	HS5	*R.nitidus*	0.011374	0.071578
SC14	CS31	HS5	*R.nitidus*	0.002028	0.071998
CS28	CS31	HS5	*R.nitidus*	0.019711	0.074831
SC24	CS31	HS5	*R.nitidus*	0.03009	0.069607
CS23	CS31	HS5	*R.nitidus*	-0.016744	0.072634
CS25	CS31	HS5	*R.nitidus*	0.027972	0.073551
SC32	CS31	HS5	*R.nitidus*	0.081407	0.072389
SC31	CS31	HS5	*R.nitidus*	0.010476	0.072563
SC42	CS31	HS5	*R.nitidus*	0.22807	0.068615
CS6	CS31	HS5	*R.nitidus*	-0.007007	0.071339
JO8	CS3	HS5	*R.nitidus*	0.036458	0.076238
JO7	CS3	HS5	*R.nitidus*	0.030151	0.074671
MY6	CS31	HS5	*R.nitidus*	0.03631	0.072051
GD31	CS3	HS5	*R.nitidus*	0.096929	0.072728
SC45	CS31	HS5	*R.nitidus*	0.048964	0.068973

JO5	CS31	HS5	*R.nitidus*	0.10596	0.067673
SC34	CS31	HS5	*R.nitidus*	0.032926	0.069889
JO1	CS31	HS5	*R.nitidus*	0.010397	0.072739
GD22	CS3	HS5	*R.nitidus*	0.107021	0.069448
GD29	CS3	HS5	*R.nitidus*	0.178002	0.068151
SC44	CS31	HS5	*R.nitidus*	0.017875	0.069229
GD21	CS3	HS5	*R.nitidus*	0.134697	0.068926
CS11	CS3	HS5	*R.nitidus*	0.03263	0.071728
MY4	CS31	HS5	*R.nitidus*	-0.005769	0.072534
GD44	CS3	HS5	*R.nitidus*	0.031789	0.070466
CS17	CS3	HS5	*R.nitidus*	0.067427	0.07573
SC35	CS31	HS5	*R.nitidus*	0.0625	0.070076
JO3	CS31	HS5	*R.nitidus*	0.07947	0.072031
JW6	CS31	HS5	*R.nitidus*	0.054025	0.07451
GD34	CS3	HS5	*R.nitidus*	0.039788	0.069333
GD35	CS3	HS5	*R.nitidus*	0.047101	0.069142
JW7	CS31	HS5	*R.nitidus*	0.095992	0.073882
GD42	CS3	HS5	*R.nitidus*	0.066308	0.071849
CS12	CS3	HS5	*R.nitidus*	0.005946	0.071084
CS15	CS3	HS5	*R.nitidus*	0.018975	0.07038
GD33	CS3	HS5	*R.nitidus*	0.043318	0.072277
CS13	CS3	HS5	*R.nitidus*	0.002863	0.072895
GD50	CS3	HS5	*R.nitidus*	0.070352	0.074119
GD25	CS3	HS5	*R.nitidus*	0.09031	0.073334
CS21	CS3	HS5	*R.nitidus*	0.003367	0.070751
GD26	CS3	HS5	*R.nitidus*	0.097198	0.070206
GD30	CS3	HS5	*R.nitidus*	0.14774	0.073494
GD41	CS3	HS5	*R.nitidus*	0.037294	0.069119
CS16	CS3	HS5	*R.nitidus*	0.020115	0.07033
GD49	CS3	HS5	*R.nitidus*	0.108342	0.074411
GD40	CS3	HS5	*R.nitidus*	0.105787	0.069539
GD45	CS3	HS5	*R.nitidus*	0.078861	0.077528
CS30	CS3	HS5	*R.nitidus*	-0.038462	0.069903
CS14	CS3	HS5	*R.nitidus*	0.032926	0.072952
GD32	CS3	HS5	*R.nitidus*	0.067554	0.073488
SC5	CS3	HS5	*R.nitidus*	0.060465	0.07335
CS22	CS3	HS5	*R.nitidus*	0.033138	0.071476
GD46	CS3	HS5	*R.nitidus*	0.00976	0.070953
CS25	CS3	HS5	*R.nitidus*	0.037116	0.073498
CS4	CS3	HS5	*R.nitidus*	0.044834	0.068877
CS31	CS3	HS5	*R.nitidus*	-0.002938	0.071603
CS23	CS3	HS5	*R.nitidus*	-0.026437	0.068362
CS28	CS3	HS5	*R.nitidus*	-0.002045	0.073627
CS19	CS3	HS5	*R.nitidus*	-0.008939	0.071633
CS18	CS3	HS5	*R.nitidus*	0.004251	0.071103
CS24	CS3	HS5	*R.nitidus*	0.007209	0.070933
SC42	CS3	HS5	*R.nitidus*	0.200666	0.067166
CS20	CS3	HS5	*R.nitidus*	0.01933	0.067573
SC14	CS3	HS5	*R.nitidus*	0.012241	0.069982
GD48	CS3	HS5	*R.nitidus*	0.090909	0.071625
JO5	CS3	HS5	*R.nitidus*	0.094409	0.066874
SC12	CS3	HS5	*R.nitidus*	-0.022158	0.069086
SC32	CS3	HS5	*R.nitidus*	0.075382	0.070428
SC26	CS3	HS5	*R.nitidus*	-0.022749	0.070943
SC27	CS3	HS5	*R.nitidus*	0.001898	0.069796
SC44	CS3	HS5	*R.nitidus*	0.00779	0.070444

MY6	CS3	HS5	*R.nitidus*	0.029026	0.07319
SC6	CS3	HS5	*R.nitidus*	0.034213	0.071468
JO3	CS3	HS5	*R.nitidus*	0.053421	0.072851
SC10	CS3	HS5	*R.nitidus*	0.100493	0.072166
SC24	CS3	HS5	*R.nitidus*	0.045726	0.071178
JO1	CS3	HS5	*R.nitidus*	0.001874	0.071781
SC15	CS3	HS5	*R.nitidus*	0.031465	0.073638
SC18	CS3	HS5	*R.nitidus*	0.07669	0.070875
JW7	CS3	HS5	*R.nitidus*	0.078095	0.070849
CS6	CS3	HS5	*R.nitidus*	0.007605	0.067174
JW4	CS3	HS5	*R.nitidus*	0.189482	0.069348
JW6	CS3	HS5	*R.nitidus*	0.02008	0.074424
GD34	SC5	HS5	*R.nitidus*	-0.040835	0.073043
SC34	CS3	HS5	*R.nitidus*	0.037615	0.070244
GD32	SC5	HS5	*R.nitidus*	-0.025184	0.076084
SC28	CS3	HS5	*R.nitidus*	-0.018027	0.068257
SC31	CS3	HS5	*R.nitidus*	-0.001873	0.070312
GD30	SC5	HS5	*R.nitidus*	0.097268	0.075393
GD29	SC5	HS5	*R.nitidus*	0.114883	0.072454
JO7	SC5	HS5	*R.nitidus*	-0.020534	0.075086
GD41	SC5	HS5	*R.nitidus*	-0.030527	0.071599
GD25	SC5	HS5	*R.nitidus*	0.027333	0.074376
SC35	CS3	HS5	*R.nitidus*	0.061638	0.070593
GD40	SC5	HS5	*R.nitidus*	0.043358	0.070988
JO8	SC5	HS5	*R.nitidus*	-0.014508	0.079896
MY4	CS3	HS5	*R.nitidus*	-0.00566	0.072064
SC45	CS3	HS5	*R.nitidus*	0.055243	0.070702
GD21	SC5	HS5	*R.nitidus*	0.062112	0.072029
GD26	SC5	HS5	*R.nitidus*	0.024052	0.072581
GD22	SC5	HS5	*R.nitidus*	0.035398	0.075327
GD33	SC5	HS5	*R.nitidus*	-0.051973	0.07497
CS11	SC5	HS5	*R.nitidus*	-0.034265	0.076326
CS13	SC5	HS5	*R.nitidus*	-0.057385	0.075181
GD35	SC5	HS5	*R.nitidus*	-0.007505	0.073287
GD42	SC5	HS5	*R.nitidus*	0.002778	0.072777
GD31	SC5	HS5	*R.nitidus*	0.021538	0.076987
CS20	SC5	HS5	*R.nitidus*	-0.06679	0.07299
GD50	SC5	HS5	*R.nitidus*	0.024719	0.076932
CS15	SC5	HS5	*R.nitidus*	-0.049639	0.075346
CS12	SC5	HS5	*R.nitidus*	-0.052372	0.073418
CS19	SC5	HS5	*R.nitidus*	-0.068665	0.077083
GD44	SC5	HS5	*R.nitidus*	-0.02737	0.075528
CS17	SC5	HS5	*R.nitidus*	-0.01	0.077491
CS30	SC5	HS5	*R.nitidus*	-0.104147	0.071603
CS3	SC5	HS5	*R.nitidus*	-0.060465	0.07335
SC6	SC5	HS5	*R.nitidus*	-0.024242	0.074269
CS21	SC5	HS5	*R.nitidus*	-0.065589	0.072887
SC15	SC5	HS5	*R.nitidus*	-0.050454	0.074964
CS22	SC5	HS5	*R.nitidus*	-0.047619	0.074187
CS31	SC5	HS5	*R.nitidus*	-0.069307	0.073133
GD48	SC5	HS5	*R.nitidus*	0	0.075394
SC14	SC5	HS5	*R.nitidus*	-0.06943	0.074715
SC27	SC5	HS5	*R.nitidus*	-0.072081	0.073391
CS25	SC5	HS5	*R.nitidus*	-0.045923	0.077679
GD49	SC5	HS5	*R.nitidus*	0.029246	0.079523
GD46	SC5	HS5	*R.nitidus*	-0.048327	0.071539

SC31	SC5	HS5	*R.nitidus*	-0.061584	0.073711
CS28	SC5	HS5	*R.nitidus*	-0.075726	0.074524
SC35	SC5	HS5	*R.nitidus*	-0.017561	0.072843
SC18	SC5	HS5	*R.nitidus*	-0.010582	0.073676
CS16	SC5	HS5	*R.nitidus*	-0.048644	0.074171
CS18	SC5	HS5	*R.nitidus*	-0.070837	0.073951
CS14	SC5	HS5	*R.nitidus*	-0.030387	0.07683
SC28	SC5	HS5	*R.nitidus*	-0.091603	0.072594
SC10	SC5	HS5	*R.nitidus*	0.052367	0.0765
SC26	SC5	HS5	*R.nitidus*	-0.107601	0.073362
CS23	SC5	HS5	*R.nitidus*	-0.078222	0.073778
MY4	SC5	HS5	*R.nitidus*	-0.075382	0.075429
CS24	SC5	HS5	*R.nitidus*	-0.088294	0.073657
JW4	SC5	HS5	*R.nitidus*	0.127148	0.074945
SC12	SC5	HS5	*R.nitidus*	-0.113898	0.072997
CS6	SC5	HS5	*R.nitidus*	-0.07635	0.072369
JW7	SC5	HS5	*R.nitidus*	0.014894	0.077021
CS4	SC5	HS5	*R.nitidus*	-0.048446	0.071763
SC24	SC5	HS5	*R.nitidus*	-0.025751	0.074477
SC34	SC5	HS5	*R.nitidus*	-0.032197	0.072769
JO5	SC5	HS5	*R.nitidus*	0.033084	0.072477
JW6	SC5	HS5	*R.nitidus*	-0.026558	0.07814
JO7	SC6	HS5	*R.nitidus*	0.003181	0.074077
JO8	SC6	HS5	*R.nitidus*	0.010661	0.076629
GD22	SC6	HS5	*R.nitidus*	0.04953	0.071117
CS11	SC6	HS5	*R.nitidus*	-0.016053	0.073932
SC32	SC5	HS5	*R.nitidus*	0.00304	0.075336
GD25	SC6	HS5	*R.nitidus*	0.053122	0.071506
GD32	SC6	HS5	*R.nitidus*	0.032359	0.07335
GD35	SC6	HS5	*R.nitidus*	0.042457	0.068196
SC44	SC5	HS5	*R.nitidus*	-0.052868	0.076019
SC42	SC5	HS5	*R.nitidus*	0.162371	0.072087
SC45	SC5	HS5	*R.nitidus*	-0.010516	0.07402
GD21	SC6	HS5	*R.nitidus*	0.099145	0.07021
CS12	SC6	HS5	*R.nitidus*	0.003064	0.0737
CS13	SC6	HS5	*R.nitidus*	-0.029685	0.07367
MY6	SC5	HS5	*R.nitidus*	-0.029907	0.07622
GD29	SC6	HS5	*R.nitidus*	0.148918	0.069816
JO3	SC5	HS5	*R.nitidus*	0.007005	0.077013
JO1	SC5	HS5	*R.nitidus*	-0.059558	0.074692
GD49	SC6	HS5	*R.nitidus*	0.048246	0.075747
GD33	SC6	HS5	*R.nitidus*	-0.008303	0.072129
GD40	SC6	HS5	*R.nitidus*	0.069509	0.06907
GD48	SC6	HS5	*R.nitidus*	0.056039	0.072188
GD46	SC6	HS5	*R.nitidus*	-0.013158	0.070693
CS25	SC6	HS5	*R.nitidus*	-0.012072	0.074366
GD30	SC6	HS5	*R.nitidus*	0.133939	0.074265
GD26	SC6	HS5	*R.nitidus*	0.057273	0.069465
GD41	SC6	HS5	*R.nitidus*	0.003666	0.069276
GD31	SC6	HS5	*R.nitidus*	0.048077	0.072294
CS18	SC6	HS5	*R.nitidus*	-0.041829	0.071412
GD42	SC6	HS5	*R.nitidus*	0.045539	0.07218
GD50	SC6	HS5	*R.nitidus*	0.039404	0.073857
CS19	SC6	HS5	*R.nitidus*	-0.039177	0.07223
GD45	SC6	HS5	*R.nitidus*	0.064302	0.076092
GD34	SC6	HS5	*R.nitidus*	0.002671	0.07081

GD44	SC6	HS5	*R.nitidus*	-0.008435	0.07085
CS23	SC6	HS5	*R.nitidus*	-0.057597	0.070457
CS21	SC6	HS5	*R.nitidus*	-0.026769	0.071759
CS17	SC6	HS5	*R.nitidus*	0.029592	0.074853
CS28	SC6	HS5	*R.nitidus*	-0.043203	0.074922
CS14	SC6	HS5	*R.nitidus*	-0.013462	0.075742
CS20	SC6	HS5	*R.nitidus*	-0.017875	0.06967
CS16	SC6	HS5	*R.nitidus*	-0.001953	0.070575
CS3	SC6	HS5	*R.nitidus*	-0.034213	0.071468
SC45	SC6	HS5	*R.nitidus*	0.00203	0.072114
SC42	SC6	HS5	*R.nitidus*	0.20489	0.067703
CS30	SC6	HS5	*R.nitidus*	-0.088123	0.0692
CS22	SC6	HS5	*R.nitidus*	-0.000975	0.071014
SC12	SC6	HS5	*R.nitidus*	-0.074566	0.068977
SC14	SC6	HS5	*R.nitidus*	-0.040796	0.07272
CS15	SC6	HS5	*R.nitidus*	-0.009625	0.071464
SC10	SC6	HS5	*R.nitidus*	0.08866	0.071728
CS4	SC6	HS5	*R.nitidus*	-0.003666	0.069028
SC27	SC6	HS5	*R.nitidus*	-0.044291	0.071296
SC26	SC6	HS5	*R.nitidus*	-0.082082	0.072344
SC5	SC6	HS5	*R.nitidus*	0.024242	0.074269
CS24	SC6	HS5	*R.nitidus*	-0.054348	0.071086
SC34	SC6	HS5	*R.nitidus*	0	0.07065
JO1	SC6	HS5	*R.nitidus*	-0.03631	0.073388
JW6	SC6	HS5	*R.nitidus*	-0.008197	0.074579
SC15	SC6	HS5	*R.nitidus*	-0.031992	0.071517
CS31	SC6	HS5	*R.nitidus*	-0.048515	0.0711
SC31	SC6	HS5	*R.nitidus*	-0.0251	0.071725
SC28	SC6	HS5	*R.nitidus*	-0.078093	0.070962
CS11	SC10	HS5	*R.nitidus*	-0.076336	0.074535
JO3	SC6	HS5	*R.nitidus*	0.041899	0.074016
SC35	SC6	HS5	*R.nitidus*	0.019115	0.069283
CS6	SC6	HS5	*R.nitidus*	-0.045187	0.068668
JO5	SC6	HS5	*R.nitidus*	0.055716	0.06995
SC24	SC6	HS5	*R.nitidus*	-0.003175	0.070818
JO7	SC10	HS5	*R.nitidus*	-0.071116	0.076364
MY4	SC6	HS5	*R.nitidus*	-0.040117	0.071408
JW4	SC6	HS5	*R.nitidus*	0.156034	0.070419
JO8	SC10	HS5	*R.nitidus*	-0.048643	0.074669
SC18	SC6	HS5	*R.nitidus*	0.01782	0.072015
SC32	SC6	HS5	*R.nitidus*	0.034197	0.073438
GD26	SC10	HS5	*R.nitidus*	-0.004708	0.072611
GD42	SC10	HS5	*R.nitidus*	-0.018719	0.0712
SC44	SC6	HS5	*R.nitidus*	-0.027136	0.070739
GD44	SC10	HS5	*R.nitidus*	-0.050142	0.072962
GD22	SC10	HS5	*R.nitidus*	-0.00463	0.071872
GD25	SC10	HS5	*R.nitidus*	-0.001013	0.071892
MY6	SC6	HS5	*R.nitidus*	-0.001955	0.072435
GD30	SC10	HS5	*R.nitidus*	0.07601	0.075682
JW7	SC6	HS5	*R.nitidus*	0.036638	0.074624
CS12	SC10	HS5	*R.nitidus*	-0.110302	0.071978
GD29	SC10	HS5	*R.nitidus*	0.066487	0.071591
GD34	SC10	HS5	*R.nitidus*	-0.049571	0.072483
GD49	SC10	HS5	*R.nitidus*	-0.005688	0.075701
GD45	SC10	HS5	*R.nitidus*	0.013348	0.07523
GD21	SC10	HS5	*R.nitidus*	0.048	0.069753

CS13	SC10	HS5	*R.nitidus*	-0.088734	0.075393
GD48	SC10	HS5	*R.nitidus*	-0.007576	0.071884
CS14	SC10	HS5	*R.nitidus*	-0.059109	0.074396
GD31	SC10	HS5	*R.nitidus*	-0.016244	0.074677
GD40	SC10	HS5	*R.nitidus*	0.010148	0.070581
CS30	SC10	HS5	*R.nitidus*	-0.14466	0.070791
SC14	SC10	HS5	*R.nitidus*	-0.090535	0.071279
GD50	SC10	HS5	*R.nitidus*	-0.023026	0.074509
GD32	SC10	HS5	*R.nitidus*	-0.028785	0.071457
CS28	SC10	HS5	*R.nitidus*	-0.104101	0.073353
GD33	SC10	HS5	*R.nitidus*	-0.062741	0.073175
SC6	SC10	HS5	*R.nitidus*	-0.08866	0.071728
GD35	SC10	HS5	*R.nitidus*	-0.027436	0.06992
SC31	SC10	HS5	*R.nitidus*	-0.108434	0.071377
CS25	SC10	HS5	*R.nitidus*	-0.052527	0.074746
CS22	SC10	HS5	*R.nitidus*	-0.088933	0.072896
CS24	SC10	HS5	*R.nitidus*	-0.141717	0.07098
GD46	SC10	HS5	*R.nitidus*	-0.076851	0.07048
CS3	SC10	HS5	*R.nitidus*	-0.100493	0.072166
CS6	SC10	HS5	*R.nitidus*	-0.097396	0.069657
CS16	SC10	HS5	*R.nitidus*	-0.096958	0.071503
SC5	SC10	HS5	*R.nitidus*	-0.052367	0.0765
SC24	SC10	HS5	*R.nitidus*	-0.084677	0.07108
CS17	SC10	HS5	*R.nitidus*	-0.044606	0.075746
CS18	SC10	HS5	*R.nitidus*	-0.105723	0.072934
CS21	SC10	HS5	*R.nitidus*	-0.092131	0.071408
CS15	SC10	HS5	*R.nitidus*	-0.074784	0.072882
GD41	SC10	HS5	*R.nitidus*	-0.056475	0.070387
CS20	SC10	HS5	*R.nitidus*	-0.086124	0.072183
SC12	SC10	HS5	*R.nitidus*	-0.148663	0.069327
CS19	SC10	HS5	*R.nitidus*	-0.091716	0.074163
SC34	SC10	HS5	*R.nitidus*	-0.062305	0.069481
SC28	SC10	HS5	*R.nitidus*	-0.154799	0.069289
SC15	SC10	HS5	*R.nitidus*	-0.083893	0.071872
CS23	SC10	HS5	*R.nitidus*	-0.11355	0.073382
SC35	SC10	HS5	*R.nitidus*	-0.040541	0.070389
SC45	SC10	HS5	*R.nitidus*	-0.050916	0.072151
SC44	SC10	HS5	*R.nitidus*	-0.103976	0.069778
SC27	SC10	HS5	*R.nitidus*	-0.120521	0.069696
CS31	SC10	HS5	*R.nitidus*	-0.119329	0.07151
JO5	SC10	HS5	*R.nitidus*	-0.026137	0.068385
CS4	SC10	HS5	*R.nitidus*	-0.089335	0.070827
SC32	SC10	HS5	*R.nitidus*	-0.042418	0.071771
JO8	CS4	HS5	*R.nitidus*	0.022704	0.073964
JW6	SC10	HS5	*R.nitidus*	-0.084926	0.076113
JW4	SC10	HS5	*R.nitidus*	0.099107	0.071811
SC18	SC10	HS5	*R.nitidus*	-0.055907	0.070615
GD22	CS4	HS5	*R.nitidus*	0.071545	0.067039
MY4	SC10	HS5	*R.nitidus*	-0.130172	0.071423
JW7	SC10	HS5	*R.nitidus*	-0.013187	0.070792
SC42	SC10	HS5	*R.nitidus*	0.125569	0.069364
GD30	CS4	HS5	*R.nitidus*	0.114345	0.069965
MY6	SC10	HS5	*R.nitidus*	-0.094433	0.071332
SC26	SC10	HS5	*R.nitidus*	-0.146114	0.070037
GD42	CS4	HS5	*R.nitidus*	0.039439	0.069307
GD29	CS4	HS5	*R.nitidus*	0.145575	0.067262

JO3	SC10	HS5	*R.nitidus*	-0.039063	0.071635
GD21	CS4	HS5	*R.nitidus*	0.111111	0.06738
CS11	CS4	HS5	*R.nitidus*	0.000924	0.073351
GD25	CS4	HS5	*R.nitidus*	0.055895	0.071578
GD31	CS4	HS5	*R.nitidus*	0.060998	0.070886
CS12	CS4	HS5	*R.nitidus*	0.005792	0.069455
GD50	CS4	HS5	*R.nitidus*	0.047047	0.069718
JO7	CS4	HS5	*R.nitidus*	0.04501	0.073557
GD44	CS4	HS5	*R.nitidus*	0.033852	0.067791
CS23	CS4	HS5	*R.nitidus*	-0.045726	0.067813
JO1	SC10	HS5	*R.nitidus*	-0.114257	0.071256
CS25	CS4	HS5	*R.nitidus*	-0.012758	0.072056
GD35	CS4	HS5	*R.nitidus*	0.03286	0.068039
GD33	CS4	HS5	*R.nitidus*	0.008094	0.070837
GD34	CS4	HS5	*R.nitidus*	0.009475	0.0691
GD45	CS4	HS5	*R.nitidus*	0.075372	0.073834
CS17	CS4	HS5	*R.nitidus*	0.035433	0.073886
CS30	CS4	HS5	*R.nitidus*	-0.065903	0.066379
SC5	CS4	HS5	*R.nitidus*	0.048446	0.071763
GD48	CS4	HS5	*R.nitidus*	0.0563	0.068954
CS24	CS4	HS5	*R.nitidus*	-0.045657	0.064584
GD32	CS4	HS5	*R.nitidus*	0.053427	0.068986
CS18	CS4	HS5	*R.nitidus*	-0.023279	0.068924
CS13	CS4	HS5	*R.nitidus*	-0.024074	0.071872
CS3	CS4	HS5	*R.nitidus*	-0.044834	0.068877
GD41	CS4	HS5	*R.nitidus*	0.014109	0.066796
CS15	CS4	HS5	*R.nitidus*	-0.007233	0.069888
CS31	CS4	HS5	*R.nitidus*	-0.027698	0.068897
CS19	CS4	HS5	*R.nitidus*	-0.020979	0.068971
CS22	CS4	HS5	*R.nitidus*	0.003774	0.066143
GD26	CS4	HS5	*R.nitidus*	0.069444	0.06835
GD40	CS4	HS5	*R.nitidus*	0.084848	0.068016
GD49	CS4	HS5	*R.nitidus*	0.049383	0.074075
GD46	CS4	HS5	*R.nitidus*	0.003439	0.069943
SC6	CS4	HS5	*R.nitidus*	0.003666	0.069028
SC12	CS4	HS5	*R.nitidus*	-0.055096	0.068218
SC24	CS4	HS5	*R.nitidus*	-0.000934	0.06882
CS14	CS4	HS5	*R.nitidus*	0.010027	0.073433
SC32	CS4	HS5	*R.nitidus*	0.042125	0.069954
SC26	CS4	HS5	*R.nitidus*	-0.051192	0.067544
CS16	CS4	HS5	*R.nitidus*	-0.002735	0.069037
SC27	CS4	HS5	*R.nitidus*	-0.025316	0.067966
CS21	CS4	HS5	*R.nitidus*	-0.009718	0.067265
CS20	CS4	HS5	*R.nitidus*	-0.021847	0.067536
CS28	CS4	HS5	*R.nitidus*	-0.018756	0.072116
SC15	CS4	HS5	*R.nitidus*	0.009294	0.068202
JW4	CS4	HS5	*R.nitidus*	0.156943	0.067154
SC10	CS4	HS5	*R.nitidus*	0.089335	0.070827
CS6	CS4	HS5	*R.nitidus*	-0.032694	0.065491
SC35	CS4	HS5	*R.nitidus*	0.023152	0.068831
SC28	CS4	HS5	*R.nitidus*	-0.043439	0.068697
SC18	CS4	HS5	*R.nitidus*	0.038789	0.067903
MY4	CS4	HS5	*R.nitidus*	-0.030726	0.070758
SC14	CS4	HS5	*R.nitidus*	-0.026056	0.069681
SC31	CS4	HS5	*R.nitidus*	-0.024107	0.069381
SC45	CS4	HS5	*R.nitidus*	0.019264	0.069653

GD22	SC12	HS5	*R.nitidus*	0.116379	0.069757
JO8	SC12	HS5	*R.nitidus*	0.076582	0.070851
SC44	CS4	HS5	*R.nitidus*	-0.015399	0.068348
GD34	SC12	HS5	*R.nitidus*	0.068689	0.071928
GD21	SC12	HS5	*R.nitidus*	0.1508	0.067727
GD25	SC12	HS5	*R.nitidus*	0.106942	0.069136
SC34	CS4	HS5	*R.nitidus*	0.012174	0.06696
GD26	SC12	HS5	*R.nitidus*	0.114518	0.068039
JW6	CS4	HS5	*R.nitidus*	0.014451	0.071127
SC42	CS4	HS5	*R.nitidus*	0.200479	0.067395
JO1	CS4	HS5	*R.nitidus*	-0.005405	0.069704
GD49	SC12	HS5	*R.nitidus*	0.099119	0.072188
JO7	SC12	HS5	*R.nitidus*	0.064894	0.071448
JO3	CS4	HS5	*R.nitidus*	0.059337	0.070019
CS11	SC12	HS5	*R.nitidus*	0.050832	0.07174
GD30	SC12	HS5	*R.nitidus*	0.165914	0.071495
GD31	SC12	HS5	*R.nitidus*	0.103896	0.070661
CS13	SC12	HS5	*R.nitidus*	0.027831	0.071294
GD32	SC12	HS5	*R.nitidus*	0.088595	0.071188
MY6	CS4	HS5	*R.nitidus*	0.004575	0.070103
JO5	CS4	HS5	*R.nitidus*	0.070716	0.067948
GD46	SC12	HS5	*R.nitidus*	0.041894	0.068922
JW7	CS4	HS5	*R.nitidus*	0.050766	0.068761
GD29	SC12	HS5	*R.nitidus*	0.187874	0.066881
CS14	SC12	HS5	*R.nitidus*	0.065741	0.071423
GD41	SC12	HS5	*R.nitidus*	0.059524	0.068554
GD33	SC12	HS5	*R.nitidus*	0.053803	0.072199
CS23	SC12	HS5	*R.nitidus*	0.015829	0.068604
CS12	SC12	HS5	*R.nitidus*	0.04142	0.067744
GD45	SC12	HS5	*R.nitidus*	0.115756	0.07126
GD35	SC12	HS5	*R.nitidus*	0.095325	0.067819
CS24	SC12	HS5	*R.nitidus*	0.012859	0.069253
GD40	SC12	HS5	*R.nitidus*	0.138298	0.068461
CS4	SC12	HS5	*R.nitidus*	0.055096	0.068218
GD42	SC12	HS5	*R.nitidus*	0.108513	0.068923
CS22	SC12	HS5	*R.nitidus*	0.063054	0.068146
CS31	SC12	HS5	*R.nitidus*	0.029894	0.067792
CS25	SC12	HS5	*R.nitidus*	0.049904	0.070169
CS19	SC12	HS5	*R.nitidus*	0.014437	0.070918
CS20	SC12	HS5	*R.nitidus*	0.034742	0.068826
GD50	SC12	HS5	*R.nitidus*	0.11123	0.074158
GD48	SC12	HS5	*R.nitidus*	0.11883	0.070351
CS28	SC12	HS5	*R.nitidus*	0.026455	0.071519
CS16	SC12	HS5	*R.nitidus*	0.052288	0.069618
CS18	SC12	HS5	*R.nitidus*	0.029218	0.06941
SC5	SC12	HS5	*R.nitidus*	0.113898	0.072997
SC6	SC12	HS5	*R.nitidus*	0.074566	0.068977
CS15	SC12	HS5	*R.nitidus*	0.049091	0.07008
CS30	SC12	HS5	*R.nitidus*	-0.008712	0.069037
CS21	SC12	HS5	*R.nitidus*	0.02639	0.069105
CS17	SC12	HS5	*R.nitidus*	0.08483	0.074283
GD44	SC12	HS5	*R.nitidus*	0.072595	0.071582
SC45	SC12	HS5	*R.nitidus*	0.090625	0.067191
SC31	SC12	HS5	*R.nitidus*	0.033302	0.072205
SC18	SC12	HS5	*R.nitidus*	0.102273	0.067255
SC15	SC12	HS5	*R.nitidus*	0.058152	0.071217

JO1	SC12	HS5	*R.nitidus*	0.036816	0.071405
SC14	SC12	HS5	*R.nitidus*	0.02795	0.070794
SC35	SC12	HS5	*R.nitidus*	0.103764	0.066267
SC10	SC12	HS5	*R.nitidus*	0.148663	0.069327
JW6	SC12	HS5	*R.nitidus*	0.058639	0.071284
SC42	SC12	HS5	*R.nitidus*	0.262111	0.065643
CS3	SC12	HS5	*R.nitidus*	0.022158	0.069086
SC34	SC12	HS5	*R.nitidus*	0.067677	0.066617
MY4	SC12	HS5	*R.nitidus*	0.015671	0.069276
SC24	SC12	HS5	*R.nitidus*	0.070922	0.069359
JO5	SC12	HS5	*R.nitidus*	0.125731	0.06686
SC26	SC12	HS5	*R.nitidus*	-0.009109	0.069031
SC27	SC12	HS5	*R.nitidus*	0.032258	0.067808
MY6	SC12	HS5	*R.nitidus*	0.053659	0.069802
SC28	SC12	HS5	*R.nitidus*	0.018614	0.067293
SC32	SC12	HS5	*R.nitidus*	0.109201	0.068781
SC44	SC12	HS5	*R.nitidus*	0.04385	0.068577
CS11	SC14	HS5	*R.nitidus*	0.0181	0.071997
JO7	SC14	HS5	*R.nitidus*	0.055046	0.072991
JW7	SC12	HS5	*R.nitidus*	0.12013	0.069735
GD26	SC14	HS5	*R.nitidus*	0.083119	0.069593
GD42	SC14	HS5	*R.nitidus*	0.064067	0.071454
CS6	SC12	HS5	*R.nitidus*	0.009891	0.069165
GD29	SC14	HS5	*R.nitidus*	0.159734	0.068294
GD34	SC14	HS5	*R.nitidus*	0.020158	0.070138
JO3	SC12	HS5	*R.nitidus*	0.111314	0.069323
JW4	SC12	HS5	*R.nitidus*	0.213668	0.066919
GD22	SC14	HS5	*R.nitidus*	0.078283	0.070043
CS12	SC14	HS5	*R.nitidus*	0.012322	0.071196
GD33	SC14	HS5	*R.nitidus*	0.012489	0.07356
GD21	SC14	HS5	*R.nitidus*	0.1185	0.068154
CS17	SC14	HS5	*R.nitidus*	0.05123	0.071006
GD45	SC14	HS5	*R.nitidus*	0.09772	0.077084
JO8	SC14	HS5	*R.nitidus*	0.038622	0.073421
GD30	SC14	HS5	*R.nitidus*	0.140969	0.072576
GD44	SC14	HS5	*R.nitidus*	0.032505	0.073767
GD25	SC14	HS5	*R.nitidus*	0.084259	0.069985
CS31	SC14	HS5	*R.nitidus*	-0.002028	0.071998
GD46	SC14	HS5	*R.nitidus*	0.004558	0.068634
GD35	SC14	HS5	*R.nitidus*	0.043916	0.069444
GD32	SC14	HS5	*R.nitidus*	0.045596	0.071369
GD49	SC14	HS5	*R.nitidus*	0.067093	0.075314
CS14	SC14	HS5	*R.nitidus*	0.031418	0.072914
CS18	SC14	HS5	*R.nitidus*	0.006506	0.071539
GD31	SC14	HS5	*R.nitidus*	0.082613	0.072938
GD40	SC14	HS5	*R.nitidus*	0.105727	0.070367
GD50	SC14	HS5	*R.nitidus*	0.067511	0.073013
GD41	SC14	HS5	*R.nitidus*	0.032498	0.070781
SC10	SC14	HS5	*R.nitidus*	0.090535	0.071279
SC18	SC14	HS5	*R.nitidus*	0.06237	0.070997
GD48	SC14	HS5	*R.nitidus*	0.084809	0.070582
CS24	SC14	HS5	*R.nitidus*	-0.007897	0.070582
CS23	SC14	HS5	*R.nitidus*	-0.017273	0.071012
CS13	SC14	HS5	*R.nitidus*	0.011894	0.071993
SC24	SC14	HS5	*R.nitidus*	0.0333	0.071571
CS19	SC14	HS5	*R.nitidus*	-0.003795	0.072458

CS22	SC14	HS5	*R.nitidus*	0.024178	0.071109
SC6	SC14	HS5	*R.nitidus*	0.040796	0.07272
CS21	SC14	HS5	*R.nitidus*	0	0.071129
CS30	SC14	HS5	*R.nitidus*	-0.040038	0.071098
CS15	SC14	HS5	*R.nitidus*	0.025408	0.069919
CS28	SC14	HS5	*R.nitidus*	0.007202	0.074413
CS16	SC14	HS5	*R.nitidus*	0.028571	0.069075
CS20	SC14	HS5	*R.nitidus*	0.009091	0.070274
SC5	SC14	HS5	*R.nitidus*	0.06943	0.074715
JW4	SC14	HS5	*R.nitidus*	0.176033	0.068918
SC31	SC14	HS5	*R.nitidus*	-0.004103	0.071442
CS25	SC14	HS5	*R.nitidus*	0.025352	0.073055
SC27	SC14	HS5	*R.nitidus*	-0.006849	0.06577
SC45	SC14	HS5	*R.nitidus*	0.050373	0.071487
SC15	SC14	HS5	*R.nitidus*	0.027513	0.072893
SC42	SC14	HS5	*R.nitidus*	0.217172	0.069918
CS3	SC14	HS5	*R.nitidus*	-0.012241	0.069982
CS6	SC14	HS5	*R.nitidus*	-0.003895	0.068511
SC12	SC14	HS5	*R.nitidus*	-0.02795	0.070794
CS4	SC14	HS5	*R.nitidus*	0.026056	0.069681
SC35	SC14	HS5	*R.nitidus*	0.061106	0.069806
JW6	SC14	HS5	*R.nitidus*	0.037832	0.073596
SC34	SC14	HS5	*R.nitidus*	0.03869	0.069952
SC28	SC14	HS5	*R.nitidus*	-0.016293	0.070953
JO5	SC14	HS5	*R.nitidus*	0.101079	0.068516
SC26	SC14	HS5	*R.nitidus*	-0.036706	0.070451
JW7	SC14	HS5	*R.nitidus*	0.079686	0.07072
GD21	SC15	HS5	*R.nitidus*	0.111603	0.070532
JO3	SC14	HS5	*R.nitidus*	0.071749	0.071737
GD41	SC15	HS5	*R.nitidus*	0.004902	0.072783
GD40	SC15	HS5	*R.nitidus*	0.096774	0.069845
JO1	SC14	HS5	*R.nitidus*	0.02176	0.073356
SC44	SC14	HS5	*R.nitidus*	0.013613	0.071265
GD22	SC15	HS5	*R.nitidus*	0.061837	0.071135
JO7	SC15	HS5	*R.nitidus*	0.047097	0.075464
MY4	SC14	HS5	*R.nitidus*	-0.017324	0.072636
SC32	SC14	HS5	*R.nitidus*	0.068822	0.069167
CS14	SC15	HS5	*R.nitidus*	0.00782	0.075312
MY6	SC14	HS5	*R.nitidus*	0.030841	0.072386
GD25	SC15	HS5	*R.nitidus*	0.072217	0.072882
GD44	SC15	HS5	*R.nitidus*	0	0.073814
CS17	SC15	HS5	*R.nitidus*	0.038168	0.077213
GD49	SC15	HS5	*R.nitidus*	0.067929	0.076582
GD31	SC15	HS5	*R.nitidus*	0.053946	0.074695
GD45	SC15	HS5	*R.nitidus*	0.058231	0.075901
GD33	SC15	HS5	*R.nitidus*	0.0065	0.074197
JO8	SC15	HS5	*R.nitidus*	0.036077	0.075092
GD46	SC15	HS5	*R.nitidus*	-0.014521	0.073418
GD30	SC15	HS5	*R.nitidus*	0.125993	0.076003
GD42	SC15	HS5	*R.nitidus*	0.063353	0.074171
GD26	SC15	HS5	*R.nitidus*	0.061187	0.072034
CS23	SC15	HS5	*R.nitidus*	-0.056696	0.071274
GD34	SC15	HS5	*R.nitidus*	0.010046	0.071387
GD32	SC15	HS5	*R.nitidus*	0.015974	0.075594
GD48	SC15	HS5	*R.nitidus*	0.065686	0.073176
GD29	SC15	HS5	*R.nitidus*	0.148248	0.071834

CS18	SC15	HS5	*R.nitidus*	-0.022088	0.071549
CS11	SC15	HS5	*R.nitidus*	-0.011881	0.074103
CS13	SC15	HS5	*R.nitidus*	-0.020325	0.075417
CS12	SC15	HS5	*R.nitidus*	-0.005325	0.071661
GD50	SC15	HS5	*R.nitidus*	0.024096	0.076432
GD35	SC15	HS5	*R.nitidus*	0.040634	0.072714
CS19	SC15	HS5	*R.nitidus*	-0.033932	0.07399
CS21	SC15	HS5	*R.nitidus*	-0.018465	0.071321
CS22	SC15	HS5	*R.nitidus*	-0.006129	0.071667
SC12	SC15	HS5	*R.nitidus*	-0.058152	0.071217
CS15	SC15	HS5	*R.nitidus*	-0.004926	0.073893
CS20	SC15	HS5	*R.nitidus*	-0.013699	0.071138
CS24	SC15	HS5	*R.nitidus*	-0.046536	0.071136
SC18	SC15	HS5	*R.nitidus*	0.047619	0.072934
CS3	SC15	HS5	*R.nitidus*	-0.031465	0.073638
SC28	SC15	HS5	*R.nitidus*	-0.05317	0.071476
SC14	SC15	HS5	*R.nitidus*	-0.027513	0.072893
SC26	SC15	HS5	*R.nitidus*	-0.058116	0.07039
SC10	SC15	HS5	*R.nitidus*	0.083893	0.071872
MY4	SC15	HS5	*R.nitidus*	-0.043011	0.073766
CS16	SC15	HS5	*R.nitidus*	-0.001008	0.072317
SC5	SC15	HS5	*R.nitidus*	0.050454	0.074964
CS28	SC15	HS5	*R.nitidus*	-0.007584	0.074386
CS31	SC15	HS5	*R.nitidus*	-0.03112	0.074438
CS30	SC15	HS5	*R.nitidus*	-0.064615	0.0714
SC24	SC15	HS5	*R.nitidus*	0.004255	0.071485
JO8	SC18	HS5	*R.nitidus*	-0.009662	0.074711
JO7	SC18	HS5	*R.nitidus*	-0.020225	0.074561
SC6	SC15	HS5	*R.nitidus*	0.031992	0.071517
SC34	SC15	HS5	*R.nitidus*	0.01758	0.072015
GD31	SC18	HS5	*R.nitidus*	0.024316	0.07249
GD22	SC18	HS5	*R.nitidus*	0.045208	0.070149
CS4	SC15	HS5	*R.nitidus*	-0.009294	0.068202
MY6	SC15	HS5	*R.nitidus*	-0.003941	0.073872
CS12	SC18	HS5	*R.nitidus*	-0.058409	0.071052
JW4	SC15	HS5	*R.nitidus*	0.169828	0.07292
SC27	SC15	HS5	*R.nitidus*	-0.029692	0.070386
SC44	SC15	HS5	*R.nitidus*	-0.013145	0.071712
CS25	SC15	HS5	*R.nitidus*	-0.002901	0.074753
SC31	SC15	HS5	*R.nitidus*	-0.021834	0.070391
JW7	SC15	HS5	*R.nitidus*	0.061798	0.075001
JO5	SC15	HS5	*R.nitidus*	0.071429	0.070243
GD30	SC18	HS5	*R.nitidus*	0.098174	0.072822
JO1	SC15	HS5	*R.nitidus*	-0.019427	0.074567
GD42	SC18	HS5	*R.nitidus*	0.038	0.070998
SC45	SC15	HS5	*R.nitidus*	0.032	0.074248
JO3	SC15	HS5	*R.nitidus*	0.079922	0.071449
GD25	SC18	HS5	*R.nitidus*	0.040385	0.07235
SC32	SC15	HS5	*R.nitidus*	0.052072	0.072331
CS11	SC18	HS5	*R.nitidus*	-0.035138	0.072506
SC35	SC15	HS5	*R.nitidus*	0.027778	0.071509
GD29	SC18	HS5	*R.nitidus*	0.122776	0.068844
GD26	SC18	HS5	*R.nitidus*	0.030986	0.070166
JW6	SC15	HS5	*R.nitidus*	0.022124	0.076904
CS13	SC18	HS5	*R.nitidus*	-0.056797	0.072297
GD46	SC18	HS5	*R.nitidus*	-0.043435	0.066851

GD49	SC18	HS5	*R.nitidus*	0.00949	0.072869
GD34	SC18	HS5	*R.nitidus*	-0.013953	0.070836
GD21	SC18	HS5	*R.nitidus*	0.092046	0.069731
SC42	SC15	HS5	*R.nitidus*	0.208191	0.070838
GD45	SC18	HS5	*R.nitidus*	0.016588	0.076896
CS6	SC15	HS5	*R.nitidus*	-0.029383	0.071209
GD44	SC18	HS5	*R.nitidus*	-0.004012	0.071194
GD41	SC18	HS5	*R.nitidus*	-0.023622	0.067796
GD40	SC18	HS5	*R.nitidus*	0.060976	0.067898
GD32	SC18	HS5	*R.nitidus*	-0.009804	0.073652
CS20	SC18	HS5	*R.nitidus*	-0.058648	0.067167
GD48	SC18	HS5	*R.nitidus*	0.022287	0.07145
GD33	SC18	HS5	*R.nitidus*	-0.024062	0.071389
GD50	SC18	HS5	*R.nitidus*	0.003344	0.074162
CS28	SC18	HS5	*R.nitidus*	-0.071429	0.073758
CS23	SC18	HS5	*R.nitidus*	-0.090909	0.068913
GD35	SC18	HS5	*R.nitidus*	0.021718	0.071148
CS14	SC18	HS5	*R.nitidus*	-0.006673	0.072502
CS17	SC18	HS5	*R.nitidus*	0.006218	0.076464
CS19	SC18	HS5	*R.nitidus*	-0.079381	0.071239
CS30	SC18	HS5	*R.nitidus*	-0.099707	0.070171
CS15	SC18	HS5	*R.nitidus*	-0.040797	0.069246
SC42	SC18	HS5	*R.nitidus*	0.164298	0.069144
SC14	SC18	HS5	*R.nitidus*	-0.06237	0.070997
SC5	SC18	HS5	*R.nitidus*	0.010582	0.073676
CS21	SC18	HS5	*R.nitidus*	-0.067878	0.068993
CS18	SC18	HS5	*R.nitidus*	-0.072478	0.068238
SC10	SC18	HS5	*R.nitidus*	0.055907	0.070615
SC26	SC18	HS5	*R.nitidus*	-0.105208	0.069452
CS16	SC18	HS5	*R.nitidus*	-0.039761	0.069442
CS3	SC18	HS5	*R.nitidus*	-0.07669	0.070875
CS31	SC18	HS5	*R.nitidus*	-0.078411	0.071017
SC15	SC18	HS5	*R.nitidus*	-0.047619	0.072934
CS22	SC18	HS5	*R.nitidus*	-0.033033	0.070253
SC32	SC18	HS5	*R.nitidus*	0.001068	0.070982
SC34	SC18	HS5	*R.nitidus*	-0.036072	0.069373
SC28	SC18	HS5	*R.nitidus*	-0.099471	0.067928
JO1	SC18	HS5	*R.nitidus*	-0.050725	0.072213
CS6	SC18	HS5	*R.nitidus*	-0.07996	0.067229
JW7	SC18	HS5	*R.nitidus*	0.016538	0.072047
SC31	SC18	HS5	*R.nitidus*	-0.070907	0.069933
CS4	SC18	HS5	*R.nitidus*	-0.038789	0.067903
SC27	SC18	HS5	*R.nitidus*	-0.06911	0.070167
JW6	SC18	HS5	*R.nitidus*	-0.011123	0.074214
CS25	SC18	HS5	*R.nitidus*	-0.047992	0.071605
JW4	SC18	HS5	*R.nitidus*	0.135159	0.06894
CS24	SC18	HS5	*R.nitidus*	-0.073367	0.068808
SC24	SC18	HS5	*R.nitidus*	-0.046639	0.067525
CS11	SC24	HS5	*R.nitidus*	0.000924	0.073677
SC6	SC18	HS5	*R.nitidus*	-0.01782	0.072015
JO3	SC18	HS5	*R.nitidus*	0.019305	0.071778
MY4	SC18	HS5	*R.nitidus*	-0.092228	0.069492
SC12	SC18	HS5	*R.nitidus*	-0.102273	0.067255
SC45	SC18	HS5	*R.nitidus*	-0.018274	0.072144
SC35	SC18	HS5	*R.nitidus*	-0.00402	0.068896
JO7	SC24	HS5	*R.nitidus*	0.005562	0.076418

SC44	SC18	HS5	*R.nitidus*	-0.072276	0.068748
JO5	SC18	HS5	*R.nitidus*	0.036364	0.067291
MY6	SC18	HS5	*R.nitidus*	-0.040123	0.070447
GD25	SC24	HS5	*R.nitidus*	0.055713	0.07166
GD45	SC24	HS5	*R.nitidus*	0.054176	0.076951
CS15	SC24	HS5	*R.nitidus*	-0.013612	0.070085
JO8	SC24	HS5	*R.nitidus*	0.004603	0.075013
GD33	SC24	HS5	*R.nitidus*	0.002765	0.072637
GD22	SC24	HS5	*R.nitidus*	0.069973	0.071021
GD35	SC24	HS5	*R.nitidus*	0.052478	0.069824
GD29	SC24	HS5	*R.nitidus*	0.140638	0.069128
GD48	SC24	HS5	*R.nitidus*	0.039961	0.07134
CS14	SC24	HS5	*R.nitidus*	0.006494	0.073639
GD30	SC24	HS5	*R.nitidus*	0.133333	0.071868
GD32	SC24	HS5	*R.nitidus*	0.026288	0.073169
GD49	SC24	HS5	*R.nitidus*	0.062571	0.073863
GD26	SC24	HS5	*R.nitidus*	0.060185	0.072193
GD46	SC24	HS5	*R.nitidus*	-0.018287	0.070159
CS20	SC24	HS5	*R.nitidus*	-0.023256	0.069328
CS21	SC24	HS5	*R.nitidus*	-0.037214	0.070741
GD31	SC24	HS5	*R.nitidus*	0.052478	0.074388
CS17	SC24	HS5	*R.nitidus*	0.038027	0.073247
GD34	SC24	HS5	*R.nitidus*	0.006536	0.072104
GD21	SC24	HS5	*R.nitidus*	0.107732	0.069877
CS12	SC24	HS5	*R.nitidus*	-0.019531	0.07145
CS25	SC24	HS5	*R.nitidus*	-0.022137	0.07182
GD44	SC24	HS5	*R.nitidus*	0.011858	0.072706
GD40	SC24	HS5	*R.nitidus*	0.069159	0.067063
GD42	SC24	HS5	*R.nitidus*	0.044118	0.071097
CS22	SC24	HS5	*R.nitidus*	-0.004826	0.070342
SC6	SC24	HS5	*R.nitidus*	0.003175	0.070818
CS16	SC24	HS5	*R.nitidus*	-0.01434	0.071145
CS28	SC24	HS5	*R.nitidus*	-0.045696	0.072435
GD50	SC24	HS5	*R.nitidus*	0.054054	0.074389
CS13	SC24	HS5	*R.nitidus*	-0.03022	0.07212
CS19	SC24	HS5	*R.nitidus*	-0.039918	0.070985
SC10	SC24	HS5	*R.nitidus*	0.084677	0.07108
GD41	SC24	HS5	*R.nitidus*	0.018199	0.070319
CS30	SC24	HS5	*R.nitidus*	-0.078278	0.071093
CS4	SC24	HS5	*R.nitidus*	0.000934	0.06882
SC14	SC24	HS5	*R.nitidus*	-0.0333	0.071571
CS24	SC24	HS5	*R.nitidus*	-0.04467	0.069386
SC5	SC24	HS5	*R.nitidus*	0.025751	0.074477
CS23	SC24	HS5	*R.nitidus*	-0.056622	0.070255
CS6	SC24	HS5	*R.nitidus*	-0.053678	0.068828
SC12	SC24	HS5	*R.nitidus*	-0.070922	0.069359
CS18	SC24	HS5	*R.nitidus*	-0.028874	0.069214
SC26	SC24	HS5	*R.nitidus*	-0.067005	0.071669
SC44	SC24	HS5	*R.nitidus*	-0.033058	0.067692
SC15	SC24	HS5	*R.nitidus*	-0.004255	0.071485
JW4	SC24	HS5	*R.nitidus*	0.155127	0.06973
CS3	SC24	HS5	*R.nitidus*	-0.045726	0.071178
CS31	SC24	HS5	*R.nitidus*	-0.03009	0.069607
JO7	SC26	HS5	*R.nitidus*	0.072464	0.072899
SC18	SC24	HS5	*R.nitidus*	0.046639	0.067525
JO3	SC24	HS5	*R.nitidus*	0.048571	0.071759

SC35	SC24	HS5	*R.nitidus*	0.016716	0.069998
SC31	SC24	HS5	*R.nitidus*	-0.038657	0.069713
JO8	SC26	HS5	*R.nitidus*	0.091611	0.075167
SC42	SC24	HS5	*R.nitidus*	0.196429	0.068033
SC45	SC24	HS5	*R.nitidus*	0.022245	0.072942
SC27	SC24	HS5	*R.nitidus*	-0.025381	0.069535
SC28	SC24	HS5	*R.nitidus*	-0.062749	0.069165
GD32	SC26	HS5	*R.nitidus*	0.063918	0.071891
SC32	SC24	HS5	*R.nitidus*	0.038934	0.070512
CS12	SC26	HS5	*R.nitidus*	0.043825	0.070061
JO1	SC24	HS5	*R.nitidus*	-0.016966	0.071598
GD29	SC26	HS5	*R.nitidus*	0.19848	0.069687
GD34	SC26	HS5	*R.nitidus*	0.053058	0.07112
SC34	SC24	HS5	*R.nitidus*	-0.003937	0.069083
GD41	SC26	HS5	*R.nitidus*	0.055912	0.071802
JW6	SC24	HS5	*R.nitidus*	-0.012088	0.075443
GD42	SC26	HS5	*R.nitidus*	0.096364	0.072933
GD48	SC26	HS5	*R.nitidus*	0.119376	0.071283
GD50	SC26	HS5	*R.nitidus*	0.089151	0.074714
MY4	SC24	HS5	*R.nitidus*	-0.054945	0.07114
CS13	SC26	HS5	*R.nitidus*	0.038536	0.073553
GD26	SC26	HS5	*R.nitidus*	0.110215	0.069864
GD45	SC26	HS5	*R.nitidus*	0.113025	0.075359
CS11	SC26	HS5	*R.nitidus*	0.0519	0.072849
JW7	SC24	HS5	*R.nitidus*	0.048458	0.072781
GD22	SC26	HS5	*R.nitidus*	0.11234	0.069178
GD25	SC26	HS5	*R.nitidus*	0.114554	0.072403
GD21	SC26	HS5	*R.nitidus*	0.160034	0.06804
MY6	SC24	HS5	*R.nitidus*	-0.008056	0.070589
GD30	SC26	HS5	*R.nitidus*	0.175303	0.073423
JO5	SC24	HS5	*R.nitidus*	0.056112	0.068347
GD35	SC26	HS5	*R.nitidus*	0.079137	0.070387
GD46	SC26	HS5	*R.nitidus*	0.033759	0.070282
GD40	SC26	HS5	*R.nitidus*	0.127773	0.069931
GD31	SC26	HS5	*R.nitidus*	0.111753	0.073526
CS14	SC26	HS5	*R.nitidus*	0.068744	0.072734
GD49	SC26	HS5	*R.nitidus*	0.106681	0.076703
GD33	SC26	HS5	*R.nitidus*	0.050233	0.070647
CS19	SC26	HS5	*R.nitidus*	0.024621	0.072069
CS18	SC26	HS5	*R.nitidus*	0.028064	0.068961
CS17	SC26	HS5	*R.nitidus*	0.072854	0.073404
GD44	SC26	HS5	*R.nitidus*	0.062044	0.072959
CS28	SC26	HS5	*R.nitidus*	0.033161	0.073255
SC6	SC26	HS5	*R.nitidus*	0.082082	0.072344
CS23	SC26	HS5	*R.nitidus*	0.013953	0.071408
CS25	SC26	HS5	*R.nitidus*	0.050285	0.072503
SC24	SC26	HS5	*R.nitidus*	0.067005	0.071669
CS20	SC26	HS5	*R.nitidus*	0.042636	0.069903
CS15	SC26	HS5	*R.nitidus*	0.053704	0.071065
CS24	SC26	HS5	*R.nitidus*	0.019157	0.069298
CS4	SC26	HS5	*R.nitidus*	0.051192	0.067544
SC5	SC26	HS5	*R.nitidus*	0.107601	0.073362
CS31	SC26	HS5	*R.nitidus*	0.021463	0.072679
SC12	SC26	HS5	*R.nitidus*	0.009109	0.069031
CS21	SC26	HS5	*R.nitidus*	0.035481	0.070075
CS22	SC26	HS5	*R.nitidus*	0.065777	0.068834

SC31	SC26	HS5	*R.nitidus*	0.045187	0.071021
SC14	SC26	HS5	*R.nitidus*	0.036706	0.070451
CS3	SC26	HS5	*R.nitidus*	0.022749	0.070943
CS6	SC26	HS5	*R.nitidus*	0.022663	0.068486
CS16	SC26	HS5	*R.nitidus*	0.047882	0.069002
SC45	SC26	HS5	*R.nitidus*	0.09372	0.070606
JO1	SC26	HS5	*R.nitidus*	0.036437	0.070928
SC27	SC26	HS5	*R.nitidus*	0.030644	0.07083
SC10	SC26	HS5	*R.nitidus*	0.146114	0.070037
CS30	SC26	HS5	*R.nitidus*	-0.007547	0.069564
JW4	SC26	HS5	*R.nitidus*	0.210133	0.069153
JO8	SC27	HS5	*R.nitidus*	0.053079	0.07241
JW7	SC26	HS5	*R.nitidus*	0.110993	0.072434
SC28	SC26	HS5	*R.nitidus*	0.020958	0.069569
SC35	SC26	HS5	*R.nitidus*	0.103582	0.070032
SC44	SC26	HS5	*R.nitidus*	0.035282	0.07175
JO7	SC27	HS5	*R.nitidus*	0.059252	0.074314
SC34	SC26	HS5	*R.nitidus*	0.07531	0.068071
GD21	SC27	HS5	*R.nitidus*	0.132689	0.067565
JW6	SC26	HS5	*R.nitidus*	0.062766	0.073757
MY6	SC26	HS5	*R.nitidus*	0.061033	0.07059
SC15	SC26	HS5	*R.nitidus*	0.058116	0.07039
SC18	SC26	HS5	*R.nitidus*	0.105208	0.069452
SC32	SC26	HS5	*R.nitidus*	0.096057	0.070616
GD29	SC27	HS5	*R.nitidus*	0.166955	0.067486
GD30	SC27	HS5	*R.nitidus*	0.169643	0.072799
CS11	SC27	HS5	*R.nitidus*	0.031452	0.072443
SC42	SC26	HS5	*R.nitidus*	0.239289	0.068477
GD25	SC27	HS5	*R.nitidus*	0.095595	0.069144
JO3	SC26	HS5	*R.nitidus*	0.107143	0.070872
GD26	SC27	HS5	*R.nitidus*	0.091543	0.06908
CS12	SC27	HS5	*R.nitidus*	0.008016	0.06971
JO5	SC26	HS5	*R.nitidus*	0.126437	0.066849
MY4	SC26	HS5	*R.nitidus*	0.028382	0.070127
GD33	SC27	HS5	*R.nitidus*	0.018116	0.071697
GD22	SC27	HS5	*R.nitidus*	0.087704	0.069673
GD48	SC27	HS5	*R.nitidus*	0.078505	0.069198
GD45	SC27	HS5	*R.nitidus*	0.099558	0.076912
GD50	SC27	HS5	*R.nitidus*	0.087473	0.070514
GD31	SC27	HS5	*R.nitidus*	0.080661	0.071572
CS17	SC27	HS5	*R.nitidus*	0.080169	0.071029
GD32	SC27	HS5	*R.nitidus*	0.059686	0.070122
GD42	SC27	HS5	*R.nitidus*	0.072642	0.070038
GD44	SC27	HS5	*R.nitidus*	0.047619	0.072888
GD46	SC27	HS5	*R.nitidus*	0.016713	0.067983
GD34	SC27	HS5	*R.nitidus*	0.035009	0.069752
GD35	SC27	HS5	*R.nitidus*	0.06174	0.068096
CS21	SC27	HS5	*R.nitidus*	0.009234	0.069732
CS16	SC27	HS5	*R.nitidus*	0.023787	0.068061
GD49	SC27	HS5	*R.nitidus*	0.088745	0.074895
CS24	SC27	HS5	*R.nitidus*	-0.004028	0.068888
GD40	SC27	HS5	*R.nitidus*	0.113879	0.06895
SC10	SC27	HS5	*R.nitidus*	0.120521	0.069696
CS13	SC27	HS5	*R.nitidus*	0.014124	0.070961
CS22	SC27	HS5	*R.nitidus*	0.026188	0.069769
CS14	SC27	HS5	*R.nitidus*	0.043966	0.071597

CS18	SC27	HS5	*R.nitidus*	-0.003872	0.070573
CS15	SC27	HS5	*R.nitidus*	0.026144	0.069229
SC5	SC27	HS5	*R.nitidus*	0.072081	0.073391
SC32	SC27	HS5	*R.nitidus*	0.067961	0.068061
GD41	SC27	HS5	*R.nitidus*	0.042017	0.069141
SC6	SC27	HS5	*R.nitidus*	0.044291	0.071296
CS3	SC27	HS5	*R.nitidus*	-0.001898	0.069796
SC14	SC27	HS5	*R.nitidus*	0.006849	0.06577
CS23	SC27	HS5	*R.nitidus*	-0.013979	0.069015
SC35	SC27	HS5	*R.nitidus*	0.068931	0.069933
CS30	SC27	HS5	*R.nitidus*	-0.041543	0.068383
SC24	SC27	HS5	*R.nitidus*	0.025381	0.069535
SC15	SC27	HS5	*R.nitidus*	0.029692	0.070386
CS19	SC27	HS5	*R.nitidus*	-0.002879	0.072483
JW4	SC27	HS5	*R.nitidus*	0.182348	0.067889
CS4	SC27	HS5	*R.nitidus*	0.025316	0.067966
CS28	SC27	HS5	*R.nitidus*	0.001024	0.07252
SC18	SC27	HS5	*R.nitidus*	0.06911	0.070167
CS25	SC27	HS5	*R.nitidus*	0.02644	0.071899
CS20	SC27	HS5	*R.nitidus*	0.011215	0.070141
SC42	SC27	HS5	*R.nitidus*	0.228188	0.068417
CS6	SC27	HS5	*R.nitidus*	-0.007797	0.068012
CS31	SC27	HS5	*R.nitidus*	-0.002041	0.071702
MY4	SC27	HS5	*R.nitidus*	-0.015947	0.07184
SC31	SC27	HS5	*R.nitidus*	0.004032	0.069409
JW6	SC27	HS5	*R.nitidus*	0.039791	0.073963
JO8	SC28	HS5	*R.nitidus*	0.058036	0.073463
GD26	SC28	HS5	*R.nitidus*	0.109745	0.068522
SC34	SC27	HS5	*R.nitidus*	0.047619	0.067714
SC26	SC27	HS5	*R.nitidus*	-0.030644	0.07083
SC28	SC27	HS5	*R.nitidus*	-0.021605	0.069211
SC12	SC27	HS5	*R.nitidus*	-0.032258	0.067808
JO1	SC27	HS5	*R.nitidus*	0.019212	0.070205
JO3	SC27	HS5	*R.nitidus*	0.073529	0.071271
JO5	SC27	HS5	*R.nitidus*	0.092864	0.067969
GD29	SC28	HS5	*R.nitidus*	0.184034	0.066411
CS12	SC28	HS5	*R.nitidus*	0.045363	0.069718
JW7	SC27	HS5	*R.nitidus*	0.075775	0.070447
CS11	SC28	HS5	*R.nitidus*	0.047187	0.071464
GD40	SC28	HS5	*R.nitidus*	0.133568	0.068129
SC44	SC27	HS5	*R.nitidus*	0.011317	0.069222
GD22	SC28	HS5	*R.nitidus*	0.108894	0.068528
MY6	SC27	HS5	*R.nitidus*	0.03318	0.07145
GD21	SC28	HS5	*R.nitidus*	0.147059	0.066762
GD32	SC28	HS5	*R.nitidus*	0.046201	0.070455
GD48	SC28	HS5	*R.nitidus*	0.10221	0.069906
SC45	SC27	HS5	*R.nitidus*	0.045802	0.070313
CS13	SC28	HS5	*R.nitidus*	0.033771	0.071226
GD33	SC28	HS5	*R.nitidus*	0.033243	0.070827
GD34	SC28	HS5	*R.nitidus*	0.046369	0.067579
JO7	SC28	HS5	*R.nitidus*	0.059385	0.071773
CS14	SC28	HS5	*R.nitidus*	0.057038	0.072592
GD45	SC28	HS5	*R.nitidus*	0.121246	0.074317
GD41	SC28	HS5	*R.nitidus*	0.048848	0.068261
GD25	SC28	HS5	*R.nitidus*	0.114391	0.069591
GD31	SC28	HS5	*R.nitidus*	0.090737	0.071795

GD30	SC28	HS5	*R.nitidus*	0.15625	0.06957
GD44	SC28	HS5	*R.nitidus*	0.067034	0.07113
CS21	SC28	HS5	*R.nitidus*	0.021978	0.070185
GD42	SC28	HS5	*R.nitidus*	0.092322	0.070864
CS25	SC28	HS5	*R.nitidus*	0.05174	0.072085
CS19	SC28	HS5	*R.nitidus*	0.016667	0.069863
CS18	SC28	HS5	*R.nitidus*	0.023408	0.069548
CS28	SC28	HS5	*R.nitidus*	0.009307	0.0698
CS17	SC28	HS5	*R.nitidus*	0.094949	0.073624
GD49	SC28	HS5	*R.nitidus*	0.094218	0.075299
CS24	SC28	HS5	*R.nitidus*	0.00666	0.070037
GD50	SC28	HS5	*R.nitidus*	0.077559	0.071946
GD35	SC28	HS5	*R.nitidus*	0.080543	0.070223
CS31	SC28	HS5	*R.nitidus*	0.012634	0.067636
GD46	SC28	HS5	*R.nitidus*	0.038113	0.06804
SC14	SC28	HS5	*R.nitidus*	0.016293	0.070953
CS15	SC28	HS5	*R.nitidus*	0.047926	0.069793
CS6	SC28	HS5	*R.nitidus*	0.028544	0.068376
SC18	SC28	HS5	*R.nitidus*	0.099471	0.067928
SC6	SC28	HS5	*R.nitidus*	0.078093	0.070962
CS20	SC28	HS5	*R.nitidus*	0.029548	0.069912
CS30	SC28	HS5	*R.nitidus*	-0.015814	0.066762
CS16	SC28	HS5	*R.nitidus*	0.059434	0.068947
MY4	SC28	HS5	*R.nitidus*	0.00932	0.07104
CS22	SC28	HS5	*R.nitidus*	0.04707	0.071066
CS23	SC28	HS5	*R.nitidus*	0.017352	0.06992
SC31	SC28	HS5	*R.nitidus*	0.031373	0.070034
SC34	SC28	HS5	*R.nitidus*	0.055877	0.067868
SC24	SC28	HS5	*R.nitidus*	0.062749	0.069165
SC26	SC28	HS5	*R.nitidus*	-0.020958	0.069569
SC45	SC28	HS5	*R.nitidus*	0.064769	0.06864
SC12	SC28	HS5	*R.nitidus*	-0.018614	0.067293
CS3	SC28	HS5	*R.nitidus*	0.018027	0.068257
JO7	SC31	HS5	*R.nitidus*	0.037661	0.07244
SC27	SC28	HS5	*R.nitidus*	0.021605	0.069211
JW4	SC28	HS5	*R.nitidus*	0.197148	0.068951
MY6	SC28	HS5	*R.nitidus*	0.046183	0.069279
SC10	SC28	HS5	*R.nitidus*	0.154799	0.069289
SC35	SC28	HS5	*R.nitidus*	0.079612	0.067193
CS4	SC28	HS5	*R.nitidus*	0.043439	0.068697
SC15	SC28	HS5	*R.nitidus*	0.05317	0.071476
JW7	SC28	HS5	*R.nitidus*	0.103037	0.070986
SC5	SC28	HS5	*R.nitidus*	0.091603	0.072594
SC44	SC28	HS5	*R.nitidus*	0.04183	0.064795
SC32	SC28	HS5	*R.nitidus*	0.101508	0.069124
SC42	SC28	HS5	*R.nitidus*	0.242347	0.067553
CS11	SC31	HS5	*R.nitidus*	0.021062	0.073112
JW6	SC28	HS5	*R.nitidus*	0.047569	0.073758
GD32	SC31	HS5	*R.nitidus*	0.044625	0.072009
GD26	SC31	HS5	*R.nitidus*	0.076857	0.070351
JO3	SC28	HS5	*R.nitidus*	0.079494	0.072432
GD21	SC31	HS5	*R.nitidus*	0.136134	0.068636
GD29	SC31	HS5	*R.nitidus*	0.156536	0.069872
GD31	SC31	HS5	*R.nitidus*	0.075188	0.07341
GD30	SC31	HS5	*R.nitidus*	0.15027	0.072211
GD22	SC31	HS5	*R.nitidus*	0.09182	0.072317

JO8	SC31	HS5	*R.nitidus*	0.025586	0.074054
JO1	SC28	HS5	*R.nitidus*	0.016064	0.068999
JO5	SC28	HS5	*R.nitidus*	0.137212	0.064809
GD34	SC31	HS5	*R.nitidus*	0.029386	0.07032
GD42	SC31	HS5	*R.nitidus*	0.081651	0.072264
GD40	SC31	HS5	*R.nitidus*	0.107081	0.067662
GD25	SC31	HS5	*R.nitidus*	0.097606	0.070636
CS31	SC31	HS5	*R.nitidus*	-0.010476	0.072563
GD44	SC31	HS5	*R.nitidus*	0.027507	0.071254
GD33	SC31	HS5	*R.nitidus*	0.034608	0.072659
CS16	SC31	HS5	*R.nitidus*	0.036551	0.073119
CS13	SC31	HS5	*R.nitidus*	0.002783	0.073551
CS18	SC31	HS5	*R.nitidus*	0.001871	0.0708
CS12	SC31	HS5	*R.nitidus*	0.02266	0.074597
CS23	SC31	HS5	*R.nitidus*	-0.020683	0.070147
GD48	SC31	HS5	*R.nitidus*	0.081596	0.071024
CS17	SC31	HS5	*R.nitidus*	0.070707	0.075145
GD41	SC31	HS5	*R.nitidus*	0.025594	0.06957
CS19	SC31	HS5	*R.nitidus*	-0.003633	0.072275
GD45	SC31	HS5	*R.nitidus*	0.090909	0.075392
GD35	SC31	HS5	*R.nitidus*	0.05821	0.068806
CS28	SC31	HS5	*R.nitidus*	0.007187	0.071857
SC26	SC31	HS5	*R.nitidus*	-0.045187	0.071021
SC14	SC31	HS5	*R.nitidus*	0.004103	0.071442
GD49	SC31	HS5	*R.nitidus*	0.112554	0.074333
GD46	SC31	HS5	*R.nitidus*	0.016637	0.069971
CS15	SC31	HS5	*R.nitidus*	0.019749	0.071115
CS3	SC31	HS5	*R.nitidus*	0.001873	0.070312
SC6	SC31	HS5	*R.nitidus*	0.0251	0.071725
CS20	SC31	HS5	*R.nitidus*	0.014939	0.06829
GD50	SC31	HS5	*R.nitidus*	0.054639	0.074896
CS4	SC31	HS5	*R.nitidus*	0.024107	0.069381
CS30	SC31	HS5	*R.nitidus*	-0.042751	0.071215
CS25	SC31	HS5	*R.nitidus*	0.017986	0.073063
SC24	SC31	HS5	*R.nitidus*	0.038657	0.069713
CS22	SC31	HS5	*R.nitidus*	0.028275	0.07141
CS14	SC31	HS5	*R.nitidus*	0.026978	0.074717
CS21	SC31	HS5	*R.nitidus*	-0.005545	0.07027
CS24	SC31	HS5	*R.nitidus*	-0.018904	0.072177
SC10	SC31	HS5	*R.nitidus*	0.108434	0.071377
SC32	SC31	HS5	*R.nitidus*	0.074479	0.071589
SC5	SC31	HS5	*R.nitidus*	0.061584	0.073711
SC12	SC31	HS5	*R.nitidus*	-0.033302	0.072205
SC27	SC31	HS5	*R.nitidus*	-0.004032	0.069409
SC35	SC31	HS5	*R.nitidus*	0.051675	0.069665
SC42	SC31	HS5	*R.nitidus*	0.201309	0.06865
SC45	SC31	HS5	*R.nitidus*	0.049486	0.072212
JW6	SC31	HS5	*R.nitidus*	0.045317	0.075362
SC34	SC31	HS5	*R.nitidus*	0.028945	0.0709
CS6	SC31	HS5	*R.nitidus*	-0.002768	0.070526
SC15	SC31	HS5	*R.nitidus*	0.021834	0.070391
SC28	SC31	HS5	*R.nitidus*	-0.031373	0.070034
GD31	SC32	HS5	*R.nitidus*	0.009276	0.073688
GD25	SC32	HS5	*R.nitidus*	0.033395	0.068872
CS11	SC32	HS5	*R.nitidus*	-0.042776	0.072549
JO8	SC32	HS5	*R.nitidus*	0.004396	0.074491

JO7	SC32	HS5	*R.nitidus*	-0.017621	0.073102
GD32	SC32	HS5	*R.nitidus*	-0.002107	0.073302
SC18	SC31	HS5	*R.nitidus*	0.070907	0.069933
MY4	SC31	HS5	*R.nitidus*	-0.010348	0.074103
JO3	SC31	HS5	*R.nitidus*	0.079452	0.072182
GD42	SC32	HS5	*R.nitidus*	0.010436	0.071943
JW4	SC31	HS5	*R.nitidus*	0.180247	0.070406
GD21	SC32	HS5	*R.nitidus*	0.073721	0.069602
SC44	SC31	HS5	*R.nitidus*	0.005859	0.072104
GD46	SC32	HS5	*R.nitidus*	-0.044199	0.070838
MY6	SC31	HS5	*R.nitidus*	0.034419	0.072378
JO1	SC31	HS5	*R.nitidus*	0.00291	0.073336
GD33	SC32	HS5	*R.nitidus*	-0.045581	0.073925
GD26	SC32	HS5	*R.nitidus*	0.026738	0.071367
JW7	SC31	HS5	*R.nitidus*	0.093448	0.069949
JO5	SC31	HS5	*R.nitidus*	0.091856	0.069295
GD45	SC32	HS5	*R.nitidus*	0.036872	0.074407
GD41	SC32	HS5	*R.nitidus*	-0.023963	0.070553
CS15	SC32	HS5	*R.nitidus*	-0.045746	0.070478
GD49	SC32	HS5	*R.nitidus*	0.028415	0.076712
GD22	SC32	HS5	*R.nitidus*	0.029889	0.071054
CS13	SC32	HS5	*R.nitidus*	-0.058824	0.073309
GD48	SC32	HS5	*R.nitidus*	0.033739	0.070995
GD40	SC32	HS5	*R.nitidus*	0.051601	0.067321
GD34	SC32	HS5	*R.nitidus*	-0.022361	0.071023
CS12	SC32	HS5	*R.nitidus*	-0.045501	0.071901
GD30	SC32	HS5	*R.nitidus*	0.102086	0.075683
CS21	SC32	HS5	*R.nitidus*	-0.073864	0.071947
GD35	SC32	HS5	*R.nitidus*	-0.00366	0.070578
CS22	SC32	HS5	*R.nitidus*	-0.043265	0.071282
CS28	SC32	HS5	*R.nitidus*	-0.072708	0.072118
GD29	SC32	HS5	*R.nitidus*	0.125	0.0689
CS20	SC32	HS5	*R.nitidus*	-0.060038	0.071607
CS19	SC32	HS5	*R.nitidus*	-0.071921	0.072738
CS18	SC32	HS5	*R.nitidus*	-0.072185	0.070148
GD50	SC32	HS5	*R.nitidus*	0.014523	0.074919
GD44	SC32	HS5	*R.nitidus*	-0.030415	0.071738
SC15	SC32	HS5	*R.nitidus*	-0.052072	0.072331
CS14	SC32	HS5	*R.nitidus*	-0.035543	0.074034
CS17	SC32	HS5	*R.nitidus*	-0.016684	0.073988
CS3	SC32	HS5	*R.nitidus*	-0.075382	0.070428
SC12	SC32	HS5	*R.nitidus*	-0.109201	0.068781
SC35	SC32	HS5	*R.nitidus*	-0.015167	0.068911
CS16	SC32	HS5	*R.nitidus*	-0.039962	0.072497
SC5	SC32	HS5	*R.nitidus*	-0.00304	0.075336
CS24	SC32	HS5	*R.nitidus*	-0.088845	0.07039
CS23	SC32	HS5	*R.nitidus*	-0.08829	0.072909
SC14	SC32	HS5	*R.nitidus*	-0.068822	0.069167
CS25	SC32	HS5	*R.nitidus*	-0.049713	0.073406
CS31	SC32	HS5	*R.nitidus*	-0.081407	0.072389
SC42	SC32	HS5	*R.nitidus*	0.190751	0.071126
SC31	SC32	HS5	*R.nitidus*	-0.074479	0.071589
CS30	SC32	HS5	*R.nitidus*	-0.117135	0.070887
SC6	SC32	HS5	*R.nitidus*	-0.034197	0.073438
SC34	SC32	HS5	*R.nitidus*	-0.025837	0.070043
SC10	SC32	HS5	*R.nitidus*	0.042418	0.071771

MY4	SC32	HS5	*R.nitidus*	-0.074111	0.068979
MY6	SC32	HS5	*R.nitidus*	-0.030505	0.069825
JW7	SC32	HS5	*R.nitidus*	0.023622	0.072594
SC27	SC32	HS5	*R.nitidus*	-0.067961	0.068061
JO8	SC34	HS5	*R.nitidus*	0.018538	0.071927
SC18	SC32	HS5	*R.nitidus*	-0.001068	0.070982
SC26	SC32	HS5	*R.nitidus*	-0.096057	0.070616
GD30	SC34	HS5	*R.nitidus*	0.099889	0.07321
JO7	SC34	HS5	*R.nitidus*	0.007921	0.072866
SC24	SC32	HS5	*R.nitidus*	-0.038934	0.070512
CS11	SC34	HS5	*R.nitidus*	-0.0053	0.073467
JW6	SC32	HS5	*R.nitidus*	-0.033514	0.072602
CS4	SC32	HS5	*R.nitidus*	-0.042125	0.069954
SC44	SC32	HS5	*R.nitidus*	-0.062887	0.069483
GD31	SC34	HS5	*R.nitidus*	0.037665	0.0704
JO1	SC32	HS5	*R.nitidus*	-0.053	0.070275
JO5	SC32	HS5	*R.nitidus*	0.031569	0.067983
SC28	SC32	HS5	*R.nitidus*	-0.101508	0.069124
SC45	SC32	HS5	*R.nitidus*	-0.02029	0.071992
JW4	SC32	HS5	*R.nitidus*	0.121867	0.069311
CS6	SC32	HS5	*R.nitidus*	-0.086106	0.069558
GD22	SC34	HS5	*R.nitidus*	0.04878	0.069404
GD35	SC34	HS5	*R.nitidus*	0.030937	0.068363
GD46	SC34	HS5	*R.nitidus*	-0.014134	0.067991
GD21	SC34	HS5	*R.nitidus*	0.104575	0.066637
GD25	SC34	HS5	*R.nitidus*	0.051075	0.071105
JO3	SC32	HS5	*R.nitidus*	0	0.071369
GD32	SC34	HS5	*R.nitidus*	0.024072	0.072211
CS19	SC34	HS5	*R.nitidus*	-0.03255	0.070988
GD26	SC34	HS5	*R.nitidus*	0.04188	0.068678
GD29	SC34	HS5	*R.nitidus*	0.114698	0.067073
GD44	SC34	HS5	*R.nitidus*	0.003584	0.067765
GD42	SC34	HS5	*R.nitidus*	0.028259	0.070707
GD40	SC34	HS5	*R.nitidus*	0.072695	0.06735
CS13	SC34	HS5	*R.nitidus*	-0.016667	0.072023
CS12	SC34	HS5	*R.nitidus*	-0.007866	0.069411
GD45	SC34	HS5	*R.nitidus*	0.052164	0.074139
GD34	SC34	HS5	*R.nitidus*	-0.009624	0.070374
CS30	SC34	HS5	*R.nitidus*	-0.07326	0.067355
CS31	SC34	HS5	*R.nitidus*	-0.032926	0.069889
CS15	SC34	HS5	*R.nitidus*	-0.009709	0.071507
GD33	SC34	HS5	*R.nitidus*	0.002664	0.070851
GD50	SC34	HS5	*R.nitidus*	0.025432	0.068786
CS17	SC34	HS5	*R.nitidus*	0.035573	0.074904
CS20	SC34	HS5	*R.nitidus*	-0.021448	0.06909
CS22	SC34	HS5	*R.nitidus*	-0.004579	0.069288
SC14	SC34	HS5	*R.nitidus*	-0.03869	0.069952
GD49	SC34	HS5	*R.nitidus*	0.023429	0.072533
CS23	SC34	HS5	*R.nitidus*	-0.050137	0.069181
CS25	SC34	HS5	*R.nitidus*	-0.002653	0.070395
CS18	SC34	HS5	*R.nitidus*	-0.028388	0.069331
CS4	SC34	HS5	*R.nitidus*	-0.012174	0.06696
CS14	SC34	HS5	*R.nitidus*	0.008101	0.073102
GD48	SC34	HS5	*R.nitidus*	0.060274	0.071062
SC12	SC34	HS5	*R.nitidus*	-0.067677	0.066617
SC24	SC34	HS5	*R.nitidus*	0.003937	0.069083

SC18	SC34	HS5	*R.nitidus*	0.036072	0.069373
SC42	SC34	HS5	*R.nitidus*	0.17893	0.068079
SC6	SC34	HS5	*R.nitidus*	0	0.07065
GD41	SC34	HS5	*R.nitidus*	0	0.069685
SC26	SC34	HS5	*R.nitidus*	-0.07531	0.068071
SC5	SC34	HS5	*R.nitidus*	0.032197	0.072769
CS16	SC34	HS5	*R.nitidus*	-0.002737	0.069501
SC35	SC34	HS5	*R.nitidus*	0.02737	0.067402
SC15	SC34	HS5	*R.nitidus*	-0.01758	0.072015
CS3	SC34	HS5	*R.nitidus*	-0.037615	0.070244
CS28	SC34	HS5	*R.nitidus*	-0.039024	0.072334
CS21	SC34	HS5	*R.nitidus*	-0.021583	0.069951
MY6	SC34	HS5	*R.nitidus*	0.001878	0.06861
JW6	SC34	HS5	*R.nitidus*	0	0.072039
SC10	SC34	HS5	*R.nitidus*	0.062305	0.069481
SC45	SC34	HS5	*R.nitidus*	0.020314	0.067929
JO5	SC34	HS5	*R.nitidus*	0.06391	0.066968
JO3	SC34	HS5	*R.nitidus*	0.071493	0.070583
JW4	SC34	HS5	*R.nitidus*	0.164395	0.068359
SC27	SC34	HS5	*R.nitidus*	-0.047619	0.067714
CS24	SC34	HS5	*R.nitidus*	-0.051625	0.067663
CS6	SC34	HS5	*R.nitidus*	-0.029665	0.068612
SC31	SC34	HS5	*R.nitidus*	-0.028945	0.0709
JO7	SC35	HS5	*R.nitidus*	0.006173	0.071812
MY4	SC34	HS5	*R.nitidus*	-0.050467	0.06933
SC32	SC34	HS5	*R.nitidus*	0.025837	0.070043
SC28	SC34	HS5	*R.nitidus*	-0.055877	0.067868
GD22	SC35	HS5	*R.nitidus*	0.043215	0.06924
GD21	SC35	HS5	*R.nitidus*	0.077711	0.068242
GD42	SC35	HS5	*R.nitidus*	0.015683	0.069587
GD33	SC35	HS5	*R.nitidus*	-0.015385	0.073275
GD30	SC35	HS5	*R.nitidus*	0.088816	0.074155
GD32	SC35	HS5	*R.nitidus*	-0.0222	0.072198
JW7	SC34	HS5	*R.nitidus*	0.045596	0.072418
GD41	SC35	HS5	*R.nitidus*	-0.023853	0.068265
SC44	SC34	HS5	*R.nitidus*	-0.032901	0.068793
CS14	SC35	HS5	*R.nitidus*	-0.026221	0.073846
GD31	SC35	HS5	*R.nitidus*	0.042333	0.071965
JO1	SC34	HS5	*R.nitidus*	-0.017807	0.070806
GD45	SC35	HS5	*R.nitidus*	0.046358	0.073732
CS18	SC35	HS5	*R.nitidus*	-0.055347	0.068981
JO8	SC35	HS5	*R.nitidus*	-0.016358	0.073372
CS12	SC35	HS5	*R.nitidus*	-0.04381	0.071144
GD46	SC35	HS5	*R.nitidus*	-0.035199	0.068293
GD50	SC35	HS5	*R.nitidus*	0.023355	0.070488
CS15	SC35	HS5	*R.nitidus*	-0.035808	0.0711
CS13	SC35	HS5	*R.nitidus*	-0.048869	0.072734
GD35	SC35	HS5	*R.nitidus*	0.015962	0.07168
GD44	SC35	HS5	*R.nitidus*	-0.024545	0.069353
GD25	SC35	HS5	*R.nitidus*	0.045923	0.070061
GD26	SC35	HS5	*R.nitidus*	0.047059	0.067845
CS25	SC35	HS5	*R.nitidus*	-0.034164	0.071688
CS11	SC35	HS5	*R.nitidus*	-0.030946	0.072141
GD49	SC35	HS5	*R.nitidus*	0.024973	0.074041
CS30	SC35	HS5	*R.nitidus*	-0.097303	0.069214
CS17	SC35	HS5	*R.nitidus*	0	0.07578

GD40	SC35	HS5	*R.nitidus*	0.052768	0.068617
CS20	SC35	HS5	*R.nitidus*	-0.046222	0.068643
CS21	SC35	HS5	*R.nitidus*	-0.064428	0.068773
CS31	SC35	HS5	*R.nitidus*	-0.0625	0.070076
SC10	SC35	HS5	*R.nitidus*	0.040541	0.070389
GD34	SC35	HS5	*R.nitidus*	-0.016364	0.069477
CS22	SC35	HS5	*R.nitidus*	-0.023553	0.069976
GD29	SC35	HS5	*R.nitidus*	0.113793	0.066238
GD48	SC35	HS5	*R.nitidus*	0.029163	0.068656
CS24	SC35	HS5	*R.nitidus*	-0.070678	0.068459
CS19	SC35	HS5	*R.nitidus*	-0.0621	0.070993
CS16	SC35	HS5	*R.nitidus*	-0.035746	0.068702
SC6	SC35	HS5	*R.nitidus*	-0.019115	0.069283
SC12	SC35	HS5	*R.nitidus*	-0.103764	0.066267
CS3	SC35	HS5	*R.nitidus*	-0.061638	0.070593
SC27	SC35	HS5	*R.nitidus*	-0.068931	0.069933
SC34	SC35	HS5	*R.nitidus*	-0.02737	0.067402
MY6	SC35	HS5	*R.nitidus*	-0.032258	0.070589
CS23	SC35	HS5	*R.nitidus*	-0.077768	0.07064
SC18	SC35	HS5	*R.nitidus*	0.00402	0.068896
JW4	SC35	HS5	*R.nitidus*	0.151104	0.068854
CS28	SC35	HS5	*R.nitidus*	-0.04175	0.072368
MY4	SC35	HS5	*R.nitidus*	-0.078846	0.071504
CS4	SC35	HS5	*R.nitidus*	-0.023152	0.068831
SC15	SC35	HS5	*R.nitidus*	-0.027778	0.071509
SC45	SC35	HS5	*R.nitidus*	-0.003902	0.068417
JO8	CS6	HS5	*R.nitidus*	0.051502	0.073804
SC26	SC35	HS5	*R.nitidus*	-0.103582	0.070032
SC5	SC35	HS5	*R.nitidus*	0.017561	0.072843
SC14	SC35	HS5	*R.nitidus*	-0.061106	0.069806
JO7	CS6	HS5	*R.nitidus*	0.067901	0.073166
SC32	SC35	HS5	*R.nitidus*	0.015167	0.068911
SC24	SC35	HS5	*R.nitidus*	-0.016716	0.069998
GD25	CS6	HS5	*R.nitidus*	0.092507	0.071619
CS6	SC35	HS5	*R.nitidus*	-0.070688	0.067708
GD32	CS6	HS5	*R.nitidus*	0.070452	0.072903
GD35	CS6	HS5	*R.nitidus*	0.07156	0.07007
SC44	SC35	HS5	*R.nitidus*	-0.058358	0.068512
SC28	SC35	HS5	*R.nitidus*	-0.079612	0.067193
GD26	CS6	HS5	*R.nitidus*	0.085765	0.069044
JW6	SC35	HS5	*R.nitidus*	-0.005176	0.072342
GD33	CS6	HS5	*R.nitidus*	0.038251	0.071755
JO5	SC35	HS5	*R.nitidus*	0.047025	0.068471
GD22	CS6	HS5	*R.nitidus*	0.09282	0.070163
SC42	SC35	HS5	*R.nitidus*	0.170732	0.069875
SC31	SC35	HS5	*R.nitidus*	-0.051675	0.069665
GD30	CS6	HS5	*R.nitidus*	0.12963	0.072495
JO3	SC35	HS5	*R.nitidus*	0.01959	0.071309
JO1	SC35	HS5	*R.nitidus*	-0.05103	0.071036
JW7	SC35	HS5	*R.nitidus*	0.027748	0.069484
GD29	CS6	HS5	*R.nitidus*	0.167808	0.067692
GD40	CS6	HS5	*R.nitidus*	0.110911	0.068446
GD34	CS6	HS5	*R.nitidus*	0.036092	0.069451
GD41	CS6	HS5	*R.nitidus*	0.033036	0.070439
GD49	CS6	HS5	*R.nitidus*	0.062222	0.074328
CS12	CS6	HS5	*R.nitidus*	0.028459	0.069074

GD50	CS6	HS5	*R.nitidus*	0.044898	0.074212
GD46	CS6	HS5	*R.nitidus*	0.012809	0.069571
GD31	CS6	HS5	*R.nitidus*	0.077897	0.073137
GD21	CS6	HS5	*R.nitidus*	0.155306	0.066914
CS16	CS6	HS5	*R.nitidus*	0.037608	0.069332
CS13	CS6	HS5	*R.nitidus*	0.018664	0.072536
GD45	CS6	HS5	*R.nitidus*	0.064516	0.076929
CS11	CS6	HS5	*R.nitidus*	0.031761	0.071566
CS21	CS6	HS5	*R.nitidus*	0.012415	0.07025
GD44	CS6	HS5	*R.nitidus*	0.03966	0.070111
CS17	CS6	HS5	*R.nitidus*	0.055854	0.074924
CS15	CS6	HS5	*R.nitidus*	0.032567	0.070202
SC6	CS6	HS5	*R.nitidus*	0.045187	0.068668
CS18	CS6	HS5	*R.nitidus*	0.006873	0.068105
GD42	CS6	HS5	*R.nitidus*	0.073703	0.07099
CS25	CS6	HS5	*R.nitidus*	0.044824	0.071345
SC24	CS6	HS5	*R.nitidus*	0.053678	0.068828
GD48	CS6	HS5	*R.nitidus*	0.088725	0.069095
CS22	CS6	HS5	*R.nitidus*	0.044341	0.068622
SC15	CS6	HS5	*R.nitidus*	0.029383	0.071209
SC10	CS6	HS5	*R.nitidus*	0.097396	0.069657
CS28	CS6	HS5	*R.nitidus*	0.010204	0.071166
SC26	CS6	HS5	*R.nitidus*	-0.022663	0.068486
CS20	CS6	HS5	*R.nitidus*	0.026731	0.065417
SC12	CS6	HS5	*R.nitidus*	-0.009891	0.069165
CS4	CS6	HS5	*R.nitidus*	0.032694	0.065491
SC5	CS6	HS5	*R.nitidus*	0.07635	0.072369
SC27	CS6	HS5	*R.nitidus*	0.007797	0.068012
CS23	CS6	HS5	*R.nitidus*	-0.012101	0.070079
CS19	CS6	HS5	*R.nitidus*	0.006912	0.069281
CS14	CS6	HS5	*R.nitidus*	0.057561	0.072468
JO1	CS6	HS5	*R.nitidus*	0.011204	0.072586
MY4	CS6	HS5	*R.nitidus*	-0.004812	0.06952
CS24	CS6	HS5	*R.nitidus*	0.002148	0.068622
CS30	CS6	HS5	*R.nitidus*	-0.035088	0.067918
JW4	CS6	HS5	*R.nitidus*	0.17632	0.067973
SC18	CS6	HS5	*R.nitidus*	0.07996	0.067229
SC42	CS6	HS5	*R.nitidus*	0.195894	0.067429
CS31	CS6	HS5	*R.nitidus*	0.007007	0.071339
JO8	SC42	HS5	*R.nitidus*	-0.115135	0.073648
SC34	CS6	HS5	*R.nitidus*	0.029665	0.068612
CS3	CS6	HS5	*R.nitidus*	-0.007605	0.067174
JW7	CS6	HS5	*R.nitidus*	0.083826	0.070783
GD31	SC42	HS5	*R.nitidus*	-0.111853	0.070494
SC14	CS6	HS5	*R.nitidus*	0.003895	0.068511
SC28	CS6	HS5	*R.nitidus*	-0.028544	0.068376
JO7	SC42	HS5	*R.nitidus*	-0.131555	0.072751
GD30	SC42	HS5	*R.nitidus*	-0.052682	0.072646
SC31	CS6	HS5	*R.nitidus*	0.002768	0.070526
GD29	SC42	HS5	*R.nitidus*	-0.034911	0.068108
GD32	SC42	HS5	*R.nitidus*	-0.129204	0.071422
CS11	SC42	HS5	*R.nitidus*	-0.191108	0.06782
JW6	CS6	HS5	*R.nitidus*	0.051724	0.072397
SC35	CS6	HS5	*R.nitidus*	0.070688	0.067708
SC44	CS6	HS5	*R.nitidus*	0.012609	0.068568
SC32	CS6	HS5	*R.nitidus*	0.086106	0.069558

MY6	CS6	HS5	*R.nitidus*	0.041905	0.070102
GD25	SC42	HS5	*R.nitidus*	-0.115894	0.068218
GD21	SC42	HS5	*R.nitidus*	-0.108498	0.06858
JO3	CS6	HS5	*R.nitidus*	0.106877	0.07183
SC45	CS6	HS5	*R.nitidus*	0.05949	0.070874
JO5	CS6	HS5	*R.nitidus*	0.103352	0.067537
GD26	SC42	HS5	*R.nitidus*	-0.126357	0.068432
GD41	SC42	HS5	*R.nitidus*	-0.180742	0.068098
GD49	SC42	HS5	*R.nitidus*	-0.151346	0.072408
CS12	SC42	HS5	*R.nitidus*	-0.199497	0.070093
GD40	SC42	HS5	*R.nitidus*	-0.112941	0.067706
GD35	SC42	HS5	*R.nitidus*	-0.162433	0.069001
CS13	SC42	HS5	*R.nitidus*	-0.196329	0.06806
GD22	SC42	HS5	*R.nitidus*	-0.129561	0.069699
GD33	SC42	HS5	*R.nitidus*	-0.173713	0.070751
CS21	SC42	HS5	*R.nitidus*	-0.202381	0.068413
SC6	SC42	HS5	*R.nitidus*	-0.20489	0.067703
CS19	SC42	HS5	*R.nitidus*	-0.214286	0.069211
CS28	SC42	HS5	*R.nitidus*	-0.203704	0.069125
GD50	SC42	HS5	*R.nitidus*	-0.138965	0.07391
GD45	SC42	HS5	*R.nitidus*	-0.123989	0.07256
CS23	SC42	HS5	*R.nitidus*	-0.233095	0.068906
GD44	SC42	HS5	*R.nitidus*	-0.172442	0.069668
GD48	SC42	HS5	*R.nitidus*	-0.10535	0.070878
GD42	SC42	HS5	*R.nitidus*	-0.168149	0.068509
CS22	SC42	HS5	*R.nitidus*	-0.18086	0.069275
GD34	SC42	HS5	*R.nitidus*	-0.184791	0.068798
SC31	SC42	HS5	*R.nitidus*	-0.201309	0.06865
CS14	SC42	HS5	*R.nitidus*	-0.173415	0.069373
CS18	SC42	HS5	*R.nitidus*	-0.206022	0.069642
GD46	SC42	HS5	*R.nitidus*	-0.195535	0.069009
CS25	SC42	HS5	*R.nitidus*	-0.187092	0.070095
CS20	SC42	HS5	*R.nitidus*	-0.184748	0.069253
CS15	SC42	HS5	*R.nitidus*	-0.179231	0.067341
SC15	SC42	HS5	*R.nitidus*	-0.208191	0.070838
CS30	SC42	HS5	*R.nitidus*	-0.251227	0.067498
CS24	SC42	HS5	*R.nitidus*	-0.236603	0.067271
CS16	SC42	HS5	*R.nitidus*	-0.197974	0.067696
CS31	SC42	HS5	*R.nitidus*	-0.22807	0.068615
SC5	SC42	HS5	*R.nitidus*	-0.162371	0.072087
CS4	SC42	HS5	*R.nitidus*	-0.200479	0.067395
SC45	SC42	HS5	*R.nitidus*	-0.17517	0.069024
JW7	SC42	HS5	*R.nitidus*	-0.120253	0.070987
SC18	SC42	HS5	*R.nitidus*	-0.164298	0.069144
CS3	SC42	HS5	*R.nitidus*	-0.200666	0.067166
CS6	SC42	HS5	*R.nitidus*	-0.195894	0.067429
SC10	SC42	HS5	*R.nitidus*	-0.125569	0.069364
SC34	SC42	HS5	*R.nitidus*	-0.17893	0.068079
SC12	SC42	HS5	*R.nitidus*	-0.262111	0.065643
SC24	SC42	HS5	*R.nitidus*	-0.196429	0.068033
JW6	SC42	HS5	*R.nitidus*	-0.127523	0.071707
SC26	SC42	HS5	*R.nitidus*	-0.239289	0.068477
SC14	SC42	HS5	*R.nitidus*	-0.217172	0.069918
SC32	SC42	HS5	*R.nitidus*	-0.190751	0.071126
JO1	SC42	HS5	*R.nitidus*	-0.220859	0.068397
SC35	SC42	HS5	*R.nitidus*	-0.170732	0.069875

JW4	SC42	HS5	*R.nitidus*	-0.019787	0.068492
CS17	SC42	HS5	*R.nitidus*	-0.153716	0.071065
SC27	SC42	HS5	*R.nitidus*	-0.228188	0.068417
MY4	SC42	HS5	*R.nitidus*	-0.221949	0.070146
JO5	SC42	HS5	*R.nitidus*	-0.123436	0.068921
MY6	SC42	HS5	*R.nitidus*	-0.192593	0.070223
CS11	SC44	HS5	*R.nitidus*	0.012658	0.073804
SC28	SC42	HS5	*R.nitidus*	-0.242347	0.067553
SC44	SC42	HS5	*R.nitidus*	-0.20541	0.066971
JO7	SC44	HS5	*R.nitidus*	0.055734	0.074091
CS12	SC44	HS5	*R.nitidus*	0	0.069982
GD22	SC44	HS5	*R.nitidus*	0.08775	0.069965
GD21	SC44	HS5	*R.nitidus*	0.115385	0.067863
GD45	SC44	HS5	*R.nitidus*	0.068233	0.072364
JO3	SC42	HS5	*R.nitidus*	-0.098251	0.069489
GD46	SC44	HS5	*R.nitidus*	0.009311	0.069828
GD34	SC44	HS5	*R.nitidus*	0.01991	0.070111
JO8	SC44	HS5	*R.nitidus*	0.036344	0.073204
GD30	SC44	HS5	*R.nitidus*	0.130726	0.07093
GD33	SC44	HS5	*R.nitidus*	0.013649	0.071178
GD25	SC44	HS5	*R.nitidus*	0.082464	0.069474
GD26	SC44	HS5	*R.nitidus*	0.073386	0.069391
GD31	SC44	HS5	*R.nitidus*	0.068571	0.072744
GD40	SC44	HS5	*R.nitidus*	0.104736	0.068501
GD32	SC44	HS5	*R.nitidus*	0.011616	0.07181
GD35	SC44	HS5	*R.nitidus*	0.050326	0.070399
CS16	SC44	HS5	*R.nitidus*	0.012184	0.070369
CS19	SC44	HS5	*R.nitidus*	-0.007648	0.073671
GD29	SC44	HS5	*R.nitidus*	0.151695	0.069787
CS22	SC44	HS5	*R.nitidus*	0.022857	0.072225
GD42	SC44	HS5	*R.nitidus*	0.063953	0.070476
CS20	SC44	HS5	*R.nitidus*	-0.002801	0.069265
CS21	SC44	HS5	*R.nitidus*	-0.010979	0.070095
CS31	SC44	HS5	*R.nitidus*	-0.017875	0.069229
GD44	SC44	HS5	*R.nitidus*	0.030217	0.073603
GD48	SC44	HS5	*R.nitidus*	0.075889	0.070436
SC6	SC44	HS5	*R.nitidus*	0.027136	0.070739
CS4	SC44	HS5	*R.nitidus*	0.015399	0.068348
GD49	SC44	HS5	*R.nitidus*	0.063457	0.074038
CS17	SC44	HS5	*R.nitidus*	0.052846	0.074166
SC31	SC44	HS5	*R.nitidus*	-0.005859	0.072104
GD50	SC44	HS5	*R.nitidus*	0.048913	0.076426
CS13	SC44	HS5	*R.nitidus*	0.011257	0.07259
SC15	SC44	HS5	*R.nitidus*	0.013145	0.071712
SC5	SC44	HS5	*R.nitidus*	0.052868	0.076019
SC12	SC44	HS5	*R.nitidus*	-0.04385	0.068577
CS18	SC44	HS5	*R.nitidus*	-0.003711	0.070376
CS15	SC44	HS5	*R.nitidus*	0.012082	0.072007
CS25	SC44	HS5	*R.nitidus*	0.032598	0.073736
CS14	SC44	HS5	*R.nitidus*	0.030415	0.073767
CS30	SC44	HS5	*R.nitidus*	-0.054598	0.068118
SC10	SC44	HS5	*R.nitidus*	0.103976	0.069778
JW4	SC44	HS5	*R.nitidus*	0.171453	0.070273
SC34	SC44	HS5	*R.nitidus*	0.032901	0.068793
SC32	SC44	HS5	*R.nitidus*	0.062887	0.069483
CS24	SC44	HS5	*R.nitidus*	-0.031434	0.069472

CS23	SC44	HS5	*R.nitidus*	-0.018282	0.07016
CS28	SC44	HS5	*R.nitidus*	-0.016293	0.070217
CS3	SC44	HS5	*R.nitidus*	-0.00779	0.070444
SC28	SC44	HS5	*R.nitidus*	-0.04183	0.064795
SC35	SC44	HS5	*R.nitidus*	0.058358	0.068512
SC14	SC44	HS5	*R.nitidus*	-0.013613	0.071265
SC18	SC44	HS5	*R.nitidus*	0.072276	0.068748
SC24	SC44	HS5	*R.nitidus*	0.033058	0.067692
CS6	SC44	HS5	*R.nitidus*	-0.012609	0.068568
MY4	SC44	HS5	*R.nitidus*	-0.030769	0.071856
GD30	SC45	HS5	*R.nitidus*	0.112886	0.072391
GD29	SC45	HS5	*R.nitidus*	0.115798	0.069525
JO3	SC44	HS5	*R.nitidus*	0.064186	0.071242
JO7	SC45	HS5	*R.nitidus*	-0.006431	0.073595
SC26	SC44	HS5	*R.nitidus*	-0.035282	0.07175
SC27	SC44	HS5	*R.nitidus*	-0.011317	0.069222
MY6	SC44	HS5	*R.nitidus*	0.015238	0.069871
JW6	SC44	HS5	*R.nitidus*	0.024287	0.073623
JO8	SC45	HS5	*R.nitidus*	-0.009688	0.074979
JO5	SC44	HS5	*R.nitidus*	0.102157	0.067033
JO1	SC44	HS5	*R.nitidus*	-0.012634	0.070324
GD21	SC45	HS5	*R.nitidus*	0.068936	0.067919
SC42	SC44	HS5	*R.nitidus*	0.20541	0.066971
GD35	SC45	HS5	*R.nitidus*	0.009033	0.068403
SC45	SC44	HS5	*R.nitidus*	0.03248	0.070453
JW7	SC44	HS5	*R.nitidus*	0.057264	0.071996
GD34	SC45	HS5	*R.nitidus*	-0.019845	0.070072
GD25	SC45	HS5	*R.nitidus*	0.048991	0.070393
GD50	SC45	HS5	*R.nitidus*	0.011399	0.070651
GD42	SC45	HS5	*R.nitidus*	0.017658	0.072487
CS11	SC45	HS5	*R.nitidus*	-0.021429	0.073179
CS17	SC45	HS5	*R.nitidus*	0.027001	0.07334
GD32	SC45	HS5	*R.nitidus*	-0.002022	0.070733
CS25	SC45	HS5	*R.nitidus*	-0.026641	0.072172
CS24	SC45	HS5	*R.nitidus*	-0.051756	0.068027
GD26	SC45	HS5	*R.nitidus*	0.029075	0.069889
GD22	SC45	HS5	*R.nitidus*	0.031034	0.069523
GD31	SC45	HS5	*R.nitidus*	0.054924	0.072433
GD45	SC45	HS5	*R.nitidus*	0.044809	0.072869
GD49	SC45	HS5	*R.nitidus*	0.027778	0.075841
CS13	SC45	HS5	*R.nitidus*	-0.030217	0.072147
GD44	SC45	HS5	*R.nitidus*	-0.014719	0.071685
CS12	SC45	HS5	*R.nitidus*	-0.038348	0.069575
GD40	SC45	HS5	*R.nitidus*	0.055846	0.068464
GD33	SC45	HS5	*R.nitidus*	-0.03664	0.073535
CS18	SC45	HS5	*R.nitidus*	-0.050505	0.070306
GD41	SC45	HS5	*R.nitidus*	-0.017025	0.069937
CS30	SC45	HS5	*R.nitidus*	-0.094321	0.070315
CS15	SC45	HS5	*R.nitidus*	-0.023339	0.071423
SC6	SC45	HS5	*R.nitidus*	-0.00203	0.072114
CS21	SC45	HS5	*R.nitidus*	-0.048913	0.070237
CS4	SC45	HS5	*R.nitidus*	-0.019264	0.069653
GD46	SC45	HS5	*R.nitidus*	-0.032947	0.069229
GD48	SC45	HS5	*R.nitidus*	0.047402	0.070561
CS3	SC45	HS5	*R.nitidus*	-0.055243	0.070702
SC27	SC45	HS5	*R.nitidus*	-0.045802	0.070313

CS20	SC45	HS5	*R.nitidus*	-0.038679	0.069801
CS22	SC45	HS5	*R.nitidus*	-0.017891	0.069411
SC28	SC45	HS5	*R.nitidus*	-0.064769	0.06864
SC18	SC45	HS5	*R.nitidus*	0.018274	0.072144
SC24	SC45	HS5	*R.nitidus*	-0.022245	0.072942
SC5	SC45	HS5	*R.nitidus*	0.010516	0.07402
CS14	SC45	HS5	*R.nitidus*	-0.00828	0.073525
SC10	SC45	HS5	*R.nitidus*	0.050916	0.072151
CS28	SC45	HS5	*R.nitidus*	-0.032738	0.074231
SC15	SC45	HS5	*R.nitidus*	-0.032	0.074248
JW4	SC45	HS5	*R.nitidus*	0.152861	0.067634
CS16	SC45	HS5	*R.nitidus*	-0.026508	0.069864
CS19	SC45	HS5	*R.nitidus*	-0.058824	0.073073
MY6	SC45	HS5	*R.nitidus*	-0.016822	0.07066
SC31	SC45	HS5	*R.nitidus*	-0.049486	0.072212
SC26	SC45	HS5	*R.nitidus*	-0.09372	0.070606
SC42	SC45	HS5	*R.nitidus*	0.17517	0.069024
CS31	SC45	HS5	*R.nitidus*	-0.048964	0.068973
CS23	SC45	HS5	*R.nitidus*	-0.072222	0.070603
JO7	MY4	HS5	*R.nitidus*	0.066738	0.074512
SC14	SC45	HS5	*R.nitidus*	-0.050373	0.071487
SC12	SC45	HS5	*R.nitidus*	-0.090625	0.067191
JW6	SC45	HS5	*R.nitidus*	-0.027112	0.073213
JW7	SC45	HS5	*R.nitidus*	0.011213	0.074387
SC44	SC45	HS5	*R.nitidus*	-0.03248	0.070453
GD30	MY4	HS5	*R.nitidus*	0.17214	0.073842
SC34	SC45	HS5	*R.nitidus*	-0.020314	0.067929
JO5	SC45	HS5	*R.nitidus*	0.036862	0.069256
SC32	SC45	HS5	*R.nitidus*	0.02029	0.071992
CS12	MY4	HS5	*R.nitidus*	0.040959	0.073123
SC35	SC45	HS5	*R.nitidus*	0.003902	0.068417
GD29	MY4	HS5	*R.nitidus*	0.175042	0.068871
GD21	MY4	HS5	*R.nitidus*	0.154966	0.068994
CS6	SC45	HS5	*R.nitidus*	-0.05949	0.070874
GD34	MY4	HS5	*R.nitidus*	0.04302	0.069905
GD40	MY4	HS5	*R.nitidus*	0.12816	0.068181
MY4	SC45	HS5	*R.nitidus*	-0.053016	0.07051
GD48	MY4	HS5	*R.nitidus*	0.097248	0.071554
GD26	MY4	HS5	*R.nitidus*	0.091644	0.070811
GD33	MY4	HS5	*R.nitidus*	0.030055	0.073194
GD22	MY4	HS5	*R.nitidus*	0.100433	0.069472
CS16	MY4	HS5	*R.nitidus*	0.033803	0.072341
JO8	MY4	HS5	*R.nitidus*	0.070175	0.07378
JO3	SC45	HS5	*R.nitidus*	0.033058	0.072012
CS20	MY4	HS5	*R.nitidus*	0.021043	0.071414
GD44	MY4	HS5	*R.nitidus*	0.045328	0.069567
JO1	SC45	HS5	*R.nitidus*	-0.037392	0.072436
GD50	MY4	HS5	*R.nitidus*	0.070248	0.073417
CS11	MY4	HS5	*R.nitidus*	0.027372	0.073112
GD42	MY4	HS5	*R.nitidus*	0.079514	0.070844
GD25	MY4	HS5	*R.nitidus*	0.102097	0.071929
GD32	MY4	HS5	*R.nitidus*	0.067692	0.073659
CS21	MY4	HS5	*R.nitidus*	0.01327	0.072776
GD31	MY4	HS5	*R.nitidus*	0.095098	0.07311
GD41	MY4	HS5	*R.nitidus*	0.040478	0.070166
CS30	MY4	HS5	*R.nitidus*	-0.031895	0.071773

CS22	MY4	HS5	*R.nitidus*	0.039024	0.071315
CS14	MY4	HS5	*R.nitidus*	0.045062	0.076004
GD45	MY4	HS5	*R.nitidus*	0.08952	0.075191
CS15	MY4	HS5	*R.nitidus*	0.035283	0.07374
GD35	MY4	HS5	*R.nitidus*	0.084914	0.071135
GD49	MY4	HS5	*R.nitidus*	0.067403	0.075353
CS31	MY4	HS5	*R.nitidus*	0.005769	0.072534
CS24	MY4	HS5	*R.nitidus*	-0.00779	0.071247
SC6	MY4	HS5	*R.nitidus*	0.040117	0.071408
GD46	MY4	HS5	*R.nitidus*	0.027829	0.072814
SC5	MY4	HS5	*R.nitidus*	0.075382	0.075429
CS13	MY4	HS5	*R.nitidus*	0.015549	0.075006
SC10	MY4	HS5	*R.nitidus*	0.130172	0.071423
SC15	MY4	HS5	*R.nitidus*	0.043011	0.073766
CS17	MY4	HS5	*R.nitidus*	0.071501	0.076804
CS18	MY4	HS5	*R.nitidus*	0.010456	0.07088
CS4	MY4	HS5	*R.nitidus*	0.030726	0.070758
SC32	MY4	HS5	*R.nitidus*	0.074111	0.068979
SC14	MY4	HS5	*R.nitidus*	0.017324	0.072636
CS25	MY4	HS5	*R.nitidus*	0.03445	0.072948
SC27	MY4	HS5	*R.nitidus*	0.015947	0.07184
SC12	MY4	HS5	*R.nitidus*	-0.015671	0.069276
MY6	MY4	HS5	*R.nitidus*	0.053221	0.063522
CS23	MY4	HS5	*R.nitidus*	-0.008303	0.07211
SC34	MY4	HS5	*R.nitidus*	0.050467	0.06933
CS19	MY4	HS5	*R.nitidus*	0.004965	0.072952
CS28	MY4	HS5	*R.nitidus*	-0.002049	0.073369
CS3	MY4	HS5	*R.nitidus*	0.00566	0.072064
JO7	MY6	HS5	*R.nitidus*	0.014223	0.073515
JW7	MY4	HS5	*R.nitidus*	0.1	0.072524
SC26	MY4	HS5	*R.nitidus*	-0.028382	0.070127
SC28	MY4	HS5	*R.nitidus*	-0.00932	0.07104
SC45	MY4	HS5	*R.nitidus*	0.053016	0.07051
GD30	MY6	HS5	*R.nitidus*	0.122172	0.073717
JO8	MY6	HS5	*R.nitidus*	0.009019	0.073183
SC24	MY4	HS5	*R.nitidus*	0.054945	0.07114
SC31	MY4	HS5	*R.nitidus*	0.010348	0.074103
CS12	MY6	HS5	*R.nitidus*	-0.005865	0.072235
SC18	MY4	HS5	*R.nitidus*	0.092228	0.069492
JO3	MY4	HS5	*R.nitidus*	0.087039	0.072277
CS11	MY6	HS5	*R.nitidus*	-0.009937	0.072362
SC44	MY4	HS5	*R.nitidus*	0.030769	0.071856
JW6	MY4	HS5	*R.nitidus*	0.071121	0.072497
CS6	MY4	HS5	*R.nitidus*	0.004812	0.06952
SC42	MY4	HS5	*R.nitidus*	0.221949	0.070146
GD29	MY6	HS5	*R.nitidus*	0.143564	0.06929
JW4	MY4	HS5	*R.nitidus*	0.201571	0.070077
GD33	MY6	HS5	*R.nitidus*	-0.003587	0.071988
GD42	MY6	HS5	*R.nitidus*	0.04461	0.070807
SC35	MY4	HS5	*R.nitidus*	0.078846	0.071504
JO5	MY4	HS5	*R.nitidus*	0.108133	0.069872
JO1	MY4	HS5	*R.nitidus*	0.018975	0.069895
GD31	MY6	HS5	*R.nitidus*	0.058594	0.073636
GD40	MY6	HS5	*R.nitidus*	0.081882	0.067874
CS17	MY6	HS5	*R.nitidus*	0.02877	0.075942
GD44	MY6	HS5	*R.nitidus*	0.005535	0.07096

GD21	MY6	HS5	*R.nitidus*	0.114187	0.070098
GD34	MY6	HS5	*R.nitidus*	0.00613	0.070858
GD25	MY6	HS5	*R.nitidus*	0.062619	0.071749
GD35	MY6	HS5	*R.nitidus*	0.045956	0.071191
GD49	MY6	HS5	*R.nitidus*	0.055435	0.075726
GD48	MY6	HS5	*R.nitidus*	0.05536	0.072534
CS16	MY6	HS5	*R.nitidus*	-0.009461	0.070276
GD26	MY6	HS5	*R.nitidus*	0.059807	0.070641
CS22	MY6	HS5	*R.nitidus*	-0.000967	0.070885
GD22	MY6	HS5	*R.nitidus*	0.063086	0.069638
CS21	MY6	HS5	*R.nitidus*	-0.032288	0.072094
GD32	MY6	HS5	*R.nitidus*	0.036036	0.074439
CS28	MY6	HS5	*R.nitidus*	-0.037717	0.073072
CS25	MY6	HS5	*R.nitidus*	-0.01131	0.071961
CS24	MY6	HS5	*R.nitidus*	-0.045499	0.07124
GD41	MY6	HS5	*R.nitidus*	0	0.068938
CS14	MY6	HS5	*R.nitidus*	0.002828	0.073994
GD45	MY6	HS5	*R.nitidus*	0.054324	0.075573
GD50	MY6	HS5	*R.nitidus*	0.024291	0.07355
CS31	MY6	HS5	*R.nitidus*	-0.03631	0.072051
CS30	MY6	HS5	*R.nitidus*	-0.076851	0.070512
CS23	MY6	HS5	*R.nitidus*	-0.047882	0.071929
CS13	MY6	HS5	*R.nitidus*	-0.034026	0.074364
GD46	MY6	HS5	*R.nitidus*	-0.021062	0.070955
CS15	MY6	HS5	*R.nitidus*	-0.009276	0.071231
CS20	MY6	HS5	*R.nitidus*	-0.011905	0.071858
SC5	MY6	HS5	*R.nitidus*	0.029907	0.07622
CS3	MY6	HS5	*R.nitidus*	-0.029026	0.07319
CS4	MY6	HS5	*R.nitidus*	-0.004575	0.070103
CS18	MY6	HS5	*R.nitidus*	-0.027675	0.071733
JW7	MY6	HS5	*R.nitidus*	0.061394	0.072854
CS19	MY6	HS5	*R.nitidus*	-0.034417	0.072862
SC15	MY6	HS5	*R.nitidus*	0.003941	0.073872
SC32	MY6	HS5	*R.nitidus*	0.030505	0.069825
SC27	MY6	HS5	*R.nitidus*	-0.03318	0.07145
SC12	MY6	HS5	*R.nitidus*	-0.053659	0.069802
SC14	MY6	HS5	*R.nitidus*	-0.030841	0.072386
CS6	MY6	HS5	*R.nitidus*	-0.041905	0.070102
SC10	MY6	HS5	*R.nitidus*	0.094433	0.071332
SC45	MY6	HS5	*R.nitidus*	0.016822	0.07066
SC24	MY6	HS5	*R.nitidus*	0.008056	0.070589
JO3	MY6	HS5	*R.nitidus*	0.051478	0.072821
SC34	MY6	HS5	*R.nitidus*	-0.001878	0.06861
JW6	MY6	HS5	*R.nitidus*	0.02603	0.072942
GD25	JW4	HS5	*R.nitidus*	-0.099053	0.070779
SC44	MY6	HS5	*R.nitidus*	-0.015238	0.069871
CS11	JW4	HS5	*R.nitidus*	-0.148413	0.071113
JO8	JW4	HS5	*R.nitidus*	-0.137527	0.071714
MY4	MY6	HS5	*R.nitidus*	-0.053221	0.063522
JO7	JW4	HS5	*R.nitidus*	-0.142105	0.074582
JW4	MY6	HS5	*R.nitidus*	0.15101	0.070477
SC6	MY6	HS5	*R.nitidus*	0.001955	0.072435
GD29	JW4	HS5	*R.nitidus*	-0.040355	0.070107
SC26	MY6	HS5	*R.nitidus*	-0.061033	0.07059
SC18	MY6	HS5	*R.nitidus*	0.040123	0.070447
GD30	JW4	HS5	*R.nitidus*	-0.038895	0.076082

SC35	MY6	HS5	*R.nitidus*	0.032258	0.070589
JO1	MY6	HS5	*R.nitidus*	-0.026266	0.069682
SC42	MY6	HS5	*R.nitidus*	0.192593	0.070223
GD22	JW4	HS5	*R.nitidus*	-0.10828	0.068103
SC28	MY6	HS5	*R.nitidus*	-0.046183	0.069279
SC31	MY6	HS5	*R.nitidus*	-0.034419	0.072378
GD31	JW4	HS5	*R.nitidus*	-0.090992	0.072917
GD32	JW4	HS5	*R.nitidus*	-0.128372	0.071455
GD49	JW4	HS5	*R.nitidus*	-0.106426	0.074818
GD40	JW4	HS5	*R.nitidus*	-0.081535	0.06947
GD21	JW4	HS5	*R.nitidus*	-0.059969	0.070376
CS18	JW4	HS5	*R.nitidus*	-0.194183	0.067313
JO5	MY6	HS5	*R.nitidus*	0.064995	0.068811
GD33	JW4	HS5	*R.nitidus*	-0.186066	0.071948
GD50	JW4	HS5	*R.nitidus*	-0.101449	0.074375
GD41	JW4	HS5	*R.nitidus*	-0.157635	0.067098
GD46	JW4	HS5	*R.nitidus*	-0.18797	0.070429
GD26	JW4	HS5	*R.nitidus*	-0.115869	0.069869
GD48	JW4	HS5	*R.nitidus*	-0.114167	0.069244
CS15	JW4	HS5	*R.nitidus*	-0.135458	0.071127
CS12	JW4	HS5	*R.nitidus*	-0.152528	0.072657
GD42	JW4	HS5	*R.nitidus*	-0.12179	0.069375
CS20	JW4	HS5	*R.nitidus*	-0.1825	0.069725
GD44	JW4	HS5	*R.nitidus*	-0.134012	0.072977
CS19	JW4	HS5	*R.nitidus*	-0.19685	0.073668
GD34	JW4	HS5	*R.nitidus*	-0.156225	0.069826
GD35	JW4	HS5	*R.nitidus*	-0.133559	0.06998
GD45	JW4	HS5	*R.nitidus*	-0.106275	0.074274
CS22	JW4	HS5	*R.nitidus*	-0.16	0.06851
CS21	JW4	HS5	*R.nitidus*	-0.196355	0.068801
CS28	JW4	HS5	*R.nitidus*	-0.189509	0.073733
CS25	JW4	HS5	*R.nitidus*	-0.162365	0.072553
CS13	JW4	HS5	*R.nitidus*	-0.181056	0.072472
CS30	JW4	HS5	*R.nitidus*	-0.227425	0.069295
CS3	JW4	HS5	*R.nitidus*	-0.189482	0.069348
CS17	JW4	HS5	*R.nitidus*	-0.117545	0.072915
CS14	JW4	HS5	*R.nitidus*	-0.149918	0.073063
CS16	JW4	HS5	*R.nitidus*	-0.147905	0.07057
CS4	JW4	HS5	*R.nitidus*	-0.156943	0.067154
SC26	JW4	HS5	*R.nitidus*	-0.210133	0.069153
SC12	JW4	HS5	*R.nitidus*	-0.213668	0.066919
CS31	JW4	HS5	*R.nitidus*	-0.20332	0.069982
SC18	JW4	HS5	*R.nitidus*	-0.135159	0.06894
SC6	JW4	HS5	*R.nitidus*	-0.156034	0.070419
SC24	JW4	HS5	*R.nitidus*	-0.155127	0.06973
JW6	JW4	HS5	*R.nitidus*	-0.141383	0.075247
SC10	JW4	HS5	*R.nitidus*	-0.099107	0.071811
SC14	JW4	HS5	*R.nitidus*	-0.176033	0.068918
JO5	JW4	HS5	*R.nitidus*	-0.09551	0.070166
CS24	JW4	HS5	*R.nitidus*	-0.208403	0.068932
CS23	JW4	HS5	*R.nitidus*	-0.205107	0.06917
SC27	JW4	HS5	*R.nitidus*	-0.182348	0.067889
JO1	JW4	HS5	*R.nitidus*	-0.155466	0.069483
SC32	JW4	HS5	*R.nitidus*	-0.121867	0.069311
SC34	JW4	HS5	*R.nitidus*	-0.164395	0.068359
SC5	JW4	HS5	*R.nitidus*	-0.127148	0.074945

SC28	JW4	HS5	*R.nitidus*	-0.197148	0.068951
JO3	JW4	HS5	*R.nitidus*	-0.096651	0.070119
SC44	JW4	HS5	*R.nitidus*	-0.171453	0.070273
CS6	JW4	HS5	*R.nitidus*	-0.17632	0.067973
SC15	JW4	HS5	*R.nitidus*	-0.169828	0.07292
JW7	JW4	HS5	*R.nitidus*	-0.086222	0.071656
MY6	JW4	HS5	*R.nitidus*	-0.15101	0.070477
MY4	JW4	HS5	*R.nitidus*	-0.201571	0.070077
JO7	JW6	HS5	*R.nitidus*	0.042517	0.070832
GD29	JW6	HS5	*R.nitidus*	0.131655	0.071651
SC42	JW4	HS5	*R.nitidus*	0.019787	0.068492
GD22	JW6	HS5	*R.nitidus*	0.045631	0.072092
SC31	JW4	HS5	*R.nitidus*	-0.180247	0.070406
JO8	JW6	HS5	*R.nitidus*	-0.010463	0.072137
SC45	JW4	HS5	*R.nitidus*	-0.152861	0.067634
GD41	JW6	HS5	*R.nitidus*	-0.002068	0.073025
GD26	JW6	HS5	*R.nitidus*	0.06338	0.074205
SC35	JW4	HS5	*R.nitidus*	-0.151104	0.068854
GD49	JW6	HS5	*R.nitidus*	0.066416	0.078618
CS15	JW6	HS5	*R.nitidus*	-0.01032	0.075445
GD44	JW6	HS5	*R.nitidus*	0.015075	0.075101
CS12	JW6	HS5	*R.nitidus*	-0.009825	0.074343
GD45	JW6	HS5	*R.nitidus*	0.103106	0.077318
GD33	JW6	HS5	*R.nitidus*	-0.040456	0.075984
CS16	JW6	HS5	*R.nitidus*	-0.006329	0.075885
GD30	JW6	HS5	*R.nitidus*	0.142678	0.075971
GD35	JW6	HS5	*R.nitidus*	0.022941	0.073135
GD31	JW6	HS5	*R.nitidus*	0.044728	0.074854
GD25	JW6	HS5	*R.nitidus*	0.041801	0.074471
CS11	JW6	HS5	*R.nitidus*	-0.024341	0.077854
GD21	JW6	HS5	*R.nitidus*	0.083333	0.072082
GD40	JW6	HS5	*R.nitidus*	0.057561	0.072719
GD32	JW6	HS5	*R.nitidus*	0.01479	0.075961
GD50	JW6	HS5	*R.nitidus*	0.032749	0.07744
CS21	JW6	HS5	*R.nitidus*	-0.029762	0.073
CS14	JW6	HS5	*R.nitidus*	-0.007292	0.078992
GD42	JW6	HS5	*R.nitidus*	0.04679	0.074817
CS28	JW6	HS5	*R.nitidus*	-0.039216	0.074705
GD34	JW6	HS5	*R.nitidus*	-0.031884	0.073915
GD48	JW6	HS5	*R.nitidus*	0.040881	0.073404
CS17	JW6	HS5	*R.nitidus*	0.026498	0.079884
GD46	JW6	HS5	*R.nitidus*	-0.036649	0.073301
CS22	JW6	HS5	*R.nitidus*	-0.023933	0.072368
CS13	JW6	HS5	*R.nitidus*	-0.025641	0.078502
CS24	JW6	HS5	*R.nitidus*	-0.057082	0.073894
CS23	JW6	HS5	*R.nitidus*	-0.035054	0.07354
SC15	JW6	HS5	*R.nitidus*	-0.022124	0.076904
CS31	JW6	HS5	*R.nitidus*	-0.054025	0.07451
SC27	JW6	HS5	*R.nitidus*	-0.039791	0.073963
CS30	JW6	HS5	*R.nitidus*	-0.069444	0.073374
CS18	JW6	HS5	*R.nitidus*	-0.029352	0.073515
CS3	JW6	HS5	*R.nitidus*	-0.02008	0.074424
SC12	JW6	HS5	*R.nitidus*	-0.058639	0.071284
SC18	JW6	HS5	*R.nitidus*	0.011123	0.074214
CS20	JW6	HS5	*R.nitidus*	-0.024486	0.073366
CS19	JW6	HS5	*R.nitidus*	-0.050538	0.077173

SC6	JW6	HS5	*R.nitidus*	0.008197	0.074579
SC32	JW6	HS5	*R.nitidus*	0.033514	0.072602
CS4	JW6	HS5	*R.nitidus*	-0.014451	0.071127
SC14	JW6	HS5	*R.nitidus*	-0.037832	0.073596
SC5	JW6	HS5	*R.nitidus*	0.026558	0.07814
CS25	JW6	HS5	*R.nitidus*	-0.022267	0.076642
SC42	JW6	HS5	*R.nitidus*	0.127523	0.071707
JW4	JW6	HS5	*R.nitidus*	0.141383	0.075247
SC34	JW6	HS5	*R.nitidus*	0	0.072039
CS6	JW6	HS5	*R.nitidus*	-0.051724	0.072397
JO5	JW6	HS5	*R.nitidus*	0.064975	0.070954
SC10	JW6	HS5	*R.nitidus*	0.084926	0.076113
SC35	JW6	HS5	*R.nitidus*	0.005176	0.072342
SC24	JW6	HS5	*R.nitidus*	0.012088	0.075443
MY4	JW6	HS5	*R.nitidus*	-0.071121	0.072497
SC44	JW6	HS5	*R.nitidus*	-0.024287	0.073623
SC45	JW6	HS5	*R.nitidus*	0.027112	0.073213
GD22	JW7	HS5	*R.nitidus*	0.023445	0.07215
GD25	JW7	HS5	*R.nitidus*	0.02381	0.074047
SC26	JW6	HS5	*R.nitidus*	-0.062766	0.073757
JO1	JW6	HS5	*R.nitidus*	-0.035461	0.074257
SC31	JW6	HS5	*R.nitidus*	-0.045317	0.075362
MY6	JW6	HS5	*R.nitidus*	-0.02603	0.072942
GD33	JW7	HS5	*R.nitidus*	-0.057307	0.075346
GD26	JW7	HS5	*R.nitidus*	0.021905	0.074054
SC28	JW6	HS5	*R.nitidus*	-0.047569	0.073758
JO3	JW6	HS5	*R.nitidus*	0.048349	0.074343
GD40	JW7	HS5	*R.nitidus*	0.035917	0.072663
GD29	JW7	HS5	*R.nitidus*	0.093107	0.070851
JO8	JW7	HS5	*R.nitidus*	-0.026846	0.075827
CS16	JW7	HS5	*R.nitidus*	-0.034694	0.072716
JO7	JW7	HS5	*R.nitidus*	-0.032186	0.075032
CS11	JW7	HS5	*R.nitidus*	-0.045267	0.07564
GD34	JW7	HS5	*R.nitidus*	-0.060837	0.073912
CS13	JW7	HS5	*R.nitidus*	-0.069416	0.074909
GD44	JW7	HS5	*R.nitidus*	-0.039275	0.073849
CS14	JW7	HS5	*R.nitidus*	-0.031873	0.075893
GD42	JW7	HS5	*R.nitidus*	0.008081	0.072248
GD48	JW7	HS5	*R.nitidus*	0.001959	0.07267
JW7	JW6	HS5	*R.nitidus*	0.040704	0.075979
GD31	JW7	HS5	*R.nitidus*	0.017051	0.075889
GD30	JW7	HS5	*R.nitidus*	0.089535	0.077801
GD35	JW7	HS5	*R.nitidus*	-0.011583	0.071256
GD49	JW7	HS5	*R.nitidus*	-0.013905	0.078191
GD21	JW7	HS5	*R.nitidus*	0.060465	0.072075
CS12	JW7	HS5	*R.nitidus*	-0.051447	0.073586
GD32	JW7	HS5	*R.nitidus*	-0.034664	0.071441
GD46	JW7	HS5	*R.nitidus*	-0.067913	0.071325
SC10	JW7	HS5	*R.nitidus*	0.013187	0.070792
CS22	JW7	HS5	*R.nitidus*	-0.043878	0.071757
CS30	JW7	HS5	*R.nitidus*	-0.122222	0.070527
GD45	JW7	HS5	*R.nitidus*	0.043478	0.076292
CS18	JW7	HS5	*R.nitidus*	-0.060181	0.073476
CS17	JW7	HS5	*R.nitidus*	-0.008667	0.077415
CS19	JW7	HS5	*R.nitidus*	-0.077612	0.07251
CS28	JW7	HS5	*R.nitidus*	-0.075824	0.075971

GD41	JW7	HS5	*R.nitidus*	-0.039487	0.072037
CS20	JW7	HS5	*R.nitidus*	-0.059109	0.07229
CS23	JW7	HS5	*R.nitidus*	-0.091262	0.071543
GD50	JW7	HS5	*R.nitidus*	-0.00344	0.077152
CS3	JW7	HS5	*R.nitidus*	-0.078095	0.070849
SC6	JW7	HS5	*R.nitidus*	-0.036638	0.074624
SC5	JW7	HS5	*R.nitidus*	-0.014894	0.077021
SC14	JW7	HS5	*R.nitidus*	-0.079686	0.07072
CS15	JW7	HS5	*R.nitidus*	-0.035468	0.074153
CS25	JW7	HS5	*R.nitidus*	-0.048563	0.075179
SC26	JW7	HS5	*R.nitidus*	-0.110993	0.072434
CS24	JW7	HS5	*R.nitidus*	-0.082192	0.070902
CS21	JW7	HS5	*R.nitidus*	-0.079412	0.072719
JO1	JW7	HS5	*R.nitidus*	-0.059829	0.07141
CS4	JW7	HS5	*R.nitidus*	-0.050766	0.068761
JW6	JW7	HS5	*R.nitidus*	-0.040704	0.075979
CS31	JW7	HS5	*R.nitidus*	-0.095992	0.073882
SC18	JW7	HS5	*R.nitidus*	-0.016538	0.072047
SC24	JW7	HS5	*R.nitidus*	-0.048458	0.072781
SC34	JW7	HS5	*R.nitidus*	-0.045596	0.072418
CS6	JW7	HS5	*R.nitidus*	-0.083826	0.070783
JO8	JO1	HS5	*R.nitidus*	0.030655	0.074028
JO7	JO1	HS5	*R.nitidus*	0.023023	0.073376
SC42	JW7	HS5	*R.nitidus*	0.120253	0.070987
SC12	JW7	HS5	*R.nitidus*	-0.12013	0.069735
GD26	JO1	HS5	*R.nitidus*	0.088926	0.071293
GD25	JO1	HS5	*R.nitidus*	0.102658	0.072103
JW4	JW7	HS5	*R.nitidus*	0.086222	0.071656
SC15	JW7	HS5	*R.nitidus*	-0.061798	0.075001
JO3	JW7	HS5	*R.nitidus*	-0.013514	0.072419
SC31	JW7	HS5	*R.nitidus*	-0.093448	0.069949
MY6	JW7	HS5	*R.nitidus*	-0.061394	0.072854
SC44	JW7	HS5	*R.nitidus*	-0.057264	0.071996
SC28	JW7	HS5	*R.nitidus*	-0.103037	0.070986
GD21	JO1	HS5	*R.nitidus*	0.136667	0.069402
SC32	JW7	HS5	*R.nitidus*	-0.023622	0.072594
GD30	JO1	HS5	*R.nitidus*	0.170467	0.072971
MY4	JW7	HS5	*R.nitidus*	-0.1	0.072524
SC35	JW7	HS5	*R.nitidus*	-0.027748	0.069484
GD44	JO1	HS5	*R.nitidus*	0.03402	0.071766
CS12	JO1	HS5	*R.nitidus*	0.012183	0.071317
GD31	JO1	HS5	*R.nitidus*	0.084326	0.071578
JO5	JW7	HS5	*R.nitidus*	0.020222	0.069547
CS14	JO1	HS5	*R.nitidus*	0.020503	0.072478
GD32	JO1	HS5	*R.nitidus*	0.0583	0.072536
CS11	JO1	HS5	*R.nitidus*	0.008435	0.072375
GD50	JO1	HS5	*R.nitidus*	0.064777	0.074489
GD35	JO1	HS5	*R.nitidus*	0.057743	0.06881
GD33	JO1	HS5	*R.nitidus*	0.026738	0.073533
SC45	JW7	HS5	*R.nitidus*	-0.011213	0.074387
GD49	JO1	HS5	*R.nitidus*	0.069038	0.07618
GD22	JO1	HS5	*R.nitidus*	0.079968	0.072809
GD40	JO1	HS5	*R.nitidus*	0.099405	0.069675
CS19	JO1	HS5	*R.nitidus*	-0.004558	0.071999
GD48	JO1	HS5	*R.nitidus*	0.074306	0.071493
SC5	JO1	HS5	*R.nitidus*	0.059558	0.074692

GD34	JO1	HS5	*R.nitidus*	0.037257	0.071169
GD46	JO1	HS5	*R.nitidus*	0.015915	0.07075
CS17	JO1	HS5	*R.nitidus*	0.066047	0.074978
CS13	JO1	HS5	*R.nitidus*	-0.008515	0.072534
GD41	JO1	HS5	*R.nitidus*	0.029359	0.070463
GD45	JO1	HS5	*R.nitidus*	0.092534	0.07708
CS21	JO1	HS5	*R.nitidus*	-0.017086	0.073256
GD42	JO1	HS5	*R.nitidus*	0.068841	0.072365
CS16	JO1	HS5	*R.nitidus*	0.000952	0.071086
CS15	JO1	HS5	*R.nitidus*	0.012195	0.06982
CS18	JO1	HS5	*R.nitidus*	-0.012785	0.073195
CS28	JO1	HS5	*R.nitidus*	-0.00207	0.074212
CS4	JO1	HS5	*R.nitidus*	0.005405	0.069704
CS22	JO1	HS5	*R.nitidus*	0.008491	0.072745
CS20	JO1	HS5	*R.nitidus*	0.006195	0.07176
CS30	JO1	HS5	*R.nitidus*	-0.043925	0.070485
CS25	JO1	HS5	*R.nitidus*	0.021371	0.07285
CS6	JO1	HS5	*R.nitidus*	-0.011204	0.072586
SC27	JO1	HS5	*R.nitidus*	-0.019212	0.070205
SC12	JO1	HS5	*R.nitidus*	-0.036816	0.071405
CS31	JO1	HS5	*R.nitidus*	-0.010397	0.072739
SC15	JO1	HS5	*R.nitidus*	0.019427	0.074567
SC34	JO1	HS5	*R.nitidus*	0.017807	0.070806
SC28	JO1	HS5	*R.nitidus*	-0.016064	0.068999
SC24	JO1	HS5	*R.nitidus*	0.016966	0.071598
CS24	JO1	HS5	*R.nitidus*	-0.033645	0.072929
CS3	JO1	HS5	*R.nitidus*	-0.001874	0.071781
CS23	JO1	HS5	*R.nitidus*	-0.031164	0.072232
JO3	JO1	HS5	*R.nitidus*	0.070698	0.071996
SC18	JO1	HS5	*R.nitidus*	0.050725	0.072213
MY6	JO1	HS5	*R.nitidus*	0.026266	0.069682
JW6	JO1	HS5	*R.nitidus*	0.035461	0.074257
SC6	JO1	HS5	*R.nitidus*	0.03631	0.073388
SC10	JO1	HS5	*R.nitidus*	0.114257	0.071256
SC31	JO1	HS5	*R.nitidus*	-0.00291	0.073336
GD32	JO3	HS5	*R.nitidus*	-0.008264	0.072835
MY4	JO1	HS5	*R.nitidus*	-0.018975	0.069895
SC44	JO1	HS5	*R.nitidus*	0.012634	0.070324
JO8	JO3	HS5	*R.nitidus*	-0.023143	0.077574
JW7	JO1	HS5	*R.nitidus*	0.059829	0.07141
GD30	JO3	HS5	*R.nitidus*	0.108077	0.073873
SC26	JO1	HS5	*R.nitidus*	-0.036437	0.070928
SC14	JO1	HS5	*R.nitidus*	-0.02176	0.073356
SC32	JO1	HS5	*R.nitidus*	0.053	0.070275
SC45	JO1	HS5	*R.nitidus*	0.037392	0.072436
SC35	JO1	HS5	*R.nitidus*	0.05103	0.071036
JO5	JO1	HS5	*R.nitidus*	0.088512	0.068518
SC42	JO1	HS5	*R.nitidus*	0.220859	0.068397
JW4	JO1	HS5	*R.nitidus*	0.155466	0.069483
GD44	JO3	HS5	*R.nitidus*	-0.024434	0.072131
GD21	JO3	HS5	*R.nitidus*	0.063776	0.071668
GD26	JO3	HS5	*R.nitidus*	0.011324	0.07213
CS11	JO3	HS5	*R.nitidus*	-0.057522	0.073532
CS14	JO3	HS5	*R.nitidus*	-0.057301	0.073595
GD22	JO3	HS5	*R.nitidus*	0.013937	0.069786
GD34	JO3	HS5	*R.nitidus*	-0.050984	0.072125

GD45	JO3	HS5	*R.nitidus*	0.031868	0.077466
JO7	JO3	HS5	*R.nitidus*	-0.015476	0.077806
GD41	JO3	HS5	*R.nitidus*	-0.039234	0.069444
CS17	JO3	HS5	*R.nitidus*	-0.01194	0.074772
GD50	JO3	HS5	*R.nitidus*	0.018443	0.07461
GD31	JO3	HS5	*R.nitidus*	-0.017459	0.075559
CS22	JO3	HS5	*R.nitidus*	-0.048579	0.072491
GD29	JO3	HS5	*R.nitidus*	0.101462	0.068318
GD46	JO3	HS5	*R.nitidus*	-0.06848	0.070398
GD40	JO3	HS5	*R.nitidus*	0.009666	0.070722
GD42	JO3	HS5	*R.nitidus*	-0.01912	0.072347
CS16	JO3	HS5	*R.nitidus*	-0.059361	0.072054
GD35	JO3	HS5	*R.nitidus*	0	0.072248
GD33	JO3	HS5	*R.nitidus*	-0.064428	0.073365
CS12	JO3	HS5	*R.nitidus*	-0.05278	0.072612
GD25	JO3	HS5	*R.nitidus*	0.009497	0.072002
CS13	JO3	HS5	*R.nitidus*	-0.08066	0.074569
GD48	JO3	HS5	*R.nitidus*	0.015009	0.072383
CS3	JO3	HS5	*R.nitidus*	-0.053421	0.072851
SC6	JO3	HS5	*R.nitidus*	-0.041899	0.074016
CS25	JO3	HS5	*R.nitidus*	-0.073462	0.073382
CS21	JO3	HS5	*R.nitidus*	-0.085264	0.072052
GD49	JO3	HS5	*R.nitidus*	-0.021077	0.076152
CS15	JO3	HS5	*R.nitidus*	-0.057399	0.071697
CS31	JO3	HS5	*R.nitidus*	-0.07947	0.072031
CS4	JO3	HS5	*R.nitidus*	-0.059337	0.070019
CS23	JO3	HS5	*R.nitidus*	-0.097159	0.070537
SC34	JO3	HS5	*R.nitidus*	-0.071493	0.070583
CS18	JO3	HS5	*R.nitidus*	-0.072097	0.072327
SC27	JO3	HS5	*R.nitidus*	-0.073529	0.071271
CS19	JO3	HS5	*R.nitidus*	-0.092575	0.072257
SC31	JO3	HS5	*R.nitidus*	-0.079452	0.072182
SC15	JO3	HS5	*R.nitidus*	-0.079922	0.071449
CS24	JO3	HS5	*R.nitidus*	-0.087687	0.070731
CS20	JO3	HS5	*R.nitidus*	-0.068519	0.073221
MY6	JO3	HS5	*R.nitidus*	-0.051478	0.072821
CS28	JO3	HS5	*R.nitidus*	-0.097328	0.075913
JW7	JO3	HS5	*R.nitidus*	0.013514	0.072419
JO7	JO5	HS5	*R.nitidus*	-0.030333	0.071678
SC10	JO3	HS5	*R.nitidus*	0.039063	0.071635
SC5	JO3	HS5	*R.nitidus*	-0.007005	0.077013
CS30	JO3	HS5	*R.nitidus*	-0.130762	0.072725
SC26	JO3	HS5	*R.nitidus*	-0.107143	0.070872
SC45	JO3	HS5	*R.nitidus*	-0.033058	0.072012
JW4	JO3	HS5	*R.nitidus*	0.096651	0.070119
SC32	JO3	HS5	*R.nitidus*	0	0.071369
JW6	JO3	HS5	*R.nitidus*	-0.048349	0.074343
SC42	JO3	HS5	*R.nitidus*	0.098251	0.069489
SC12	JO3	HS5	*R.nitidus*	-0.111314	0.069323
GD22	JO5	HS5	*R.nitidus*	-0.006745	0.069345
SC14	JO3	HS5	*R.nitidus*	-0.071749	0.071737
CS11	JO5	HS5	*R.nitidus*	-0.044003	0.071725
JO1	JO3	HS5	*R.nitidus*	-0.070698	0.071996
SC35	JO3	HS5	*R.nitidus*	-0.01959	0.071309
GD26	JO5	HS5	*R.nitidus*	-0.002568	0.069829
MY4	JO3	HS5	*R.nitidus*	-0.087039	0.072277

SC18	JO3	HS5	*R.nitidus*	-0.019305	0.071778
SC24	JO3	HS5	*R.nitidus*	-0.048571	0.071759
CS6	JO3	HS5	*R.nitidus*	-0.106877	0.07183
SC28	JO3	HS5	*R.nitidus*	-0.079494	0.072432
SC44	JO3	HS5	*R.nitidus*	-0.064186	0.071242
GD33	JO5	HS5	*R.nitidus*	-0.06042	0.070016
GD44	JO5	HS5	*R.nitidus*	-0.063964	0.070546
CS12	JO5	HS5	*R.nitidus*	-0.067764	0.070315
GD31	JO5	HS5	*R.nitidus*	0.012116	0.072658
GD21	JO5	HS5	*R.nitidus*	0.045492	0.06683
GD25	JO5	HS5	*R.nitidus*	0.01659	0.069502
JO8	JO5	HS5	*R.nitidus*	-0.05074	0.071445
GD42	JO5	HS5	*R.nitidus*	-0.015525	0.06801
GD34	JO5	HS5	*R.nitidus*	-0.064879	0.068398
JO5	JO3	HS5	*R.nitidus*	0.009709	0.070686
GD45	JO5	HS5	*R.nitidus*	0.015436	0.07351
GD30	JO5	HS5	*R.nitidus*	0.057112	0.071544
CS16	JO5	HS5	*R.nitidus*	-0.062387	0.069698
CS19	JO5	HS5	*R.nitidus*	-0.093371	0.071237
GD40	JO5	HS5	*R.nitidus*	0.013722	0.067938
GD35	JO5	HS5	*R.nitidus*	-0.013441	0.068842
CS20	JO5	HS5	*R.nitidus*	-0.097952	0.066652
GD29	JO5	HS5	*R.nitidus*	0.086739	0.067731
GD32	JO5	HS5	*R.nitidus*	-0.055276	0.069567
GD49	JO5	HS5	*R.nitidus*	0.013086	0.072197
CS22	JO5	HS5	*R.nitidus*	-0.066225	0.068857
GD41	JO5	HS5	*R.nitidus*	-0.058459	0.067032
GD46	JO5	HS5	*R.nitidus*	-0.061837	0.067177
CS13	JO5	HS5	*R.nitidus*	-0.073411	0.07155
CS4	JO5	HS5	*R.nitidus*	-0.070716	0.067948
CS3	JO5	HS5	*R.nitidus*	-0.094409	0.066874
CS23	JO5	HS5	*R.nitidus*	-0.105168	0.067865
CS17	JO5	HS5	*R.nitidus*	-0.000962	0.07361
CS18	JO5	HS5	*R.nitidus*	-0.103884	0.069217
GD50	JO5	HS5	*R.nitidus*	-0.011411	0.071542
GD48	JO5	HS5	*R.nitidus*	-0.008189	0.069542
SC6	JO5	HS5	*R.nitidus*	-0.055716	0.06995
CS15	JO5	HS5	*R.nitidus*	-0.058312	0.069921
SC18	JO5	HS5	*R.nitidus*	-0.036364	0.067291
CS14	JO5	HS5	*R.nitidus*	-0.037522	0.070612
SC28	JO5	HS5	*R.nitidus*	-0.137212	0.064809
SC5	JO5	HS5	*R.nitidus*	-0.033084	0.072477
SC24	JO5	HS5	*R.nitidus*	-0.056112	0.068347
MY6	JO5	HS5	*R.nitidus*	-0.064995	0.068811
SC42	JO5	HS5	*R.nitidus*	0.123436	0.068921
CS21	JO5	HS5	*R.nitidus*	-0.097035	0.068452
SC12	JO5	HS5	*R.nitidus*	-0.125731	0.06686
SC10	JO5	HS5	*R.nitidus*	0.026137	0.068385
JW4	JO5	HS5	*R.nitidus*	0.09551	0.070166
CS24	JO5	HS5	*R.nitidus*	-0.108341	0.068301
CS31	JO5	HS5	*R.nitidus*	-0.10596	0.067673
GD21	JO7	CS2	*R.nitidus*	0.1274	0.069897
JO3	JO5	HS5	*R.nitidus*	-0.009709	0.070686
CS28	JO5	HS5	*R.nitidus*	-0.088381	0.070127
JO8	JO7	CS2	*R.nitidus*	0.018841	0.073999
SC44	JO5	HS5	*R.nitidus*	-0.102157	0.067033

CS25	JO5	HS5	*R.nitidus*	-0.063889	0.07075
SC35	JO5	HS5	*R.nitidus*	-0.047025	0.068471
CS30	JO5	HS5	*R.nitidus*	-0.122959	0.06651
JW6	JO5	HS5	*R.nitidus*	-0.064975	0.070954
GD26	JO7	CS2	*R.nitidus*	0.094796	0.074087
SC26	JO5	HS5	*R.nitidus*	-0.126437	0.066849
SC31	JO5	HS5	*R.nitidus*	-0.091856	0.069295
SC15	JO5	HS5	*R.nitidus*	-0.071429	0.070243
GD22	JO7	CS2	*R.nitidus*	0.130199	0.070526
SC27	JO5	HS5	*R.nitidus*	-0.092864	0.067969
SC14	JO5	HS5	*R.nitidus*	-0.101079	0.068516
JO1	JO5	HS5	*R.nitidus*	-0.088512	0.068518
SC45	JO5	HS5	*R.nitidus*	-0.036862	0.069256
JW7	JO5	HS5	*R.nitidus*	-0.020222	0.069547
CS12	JO7	CS2	*R.nitidus*	0.036597	0.072897
CS6	JO5	HS5	*R.nitidus*	-0.103352	0.067537
SC32	JO5	HS5	*R.nitidus*	-0.031569	0.067983
SC34	JO5	HS5	*R.nitidus*	-0.06391	0.066968
CS11	JO7	CS2	*R.nitidus*	0.025448	0.074042
GD25	JO7	CS2	*R.nitidus*	0.090998	0.073916
MY4	JO5	HS5	*R.nitidus*	-0.108133	0.069872
GD29	JO7	CS2	*R.nitidus*	0.169794	0.069253
GD40	JO7	CS2	*R.nitidus*	0.0831	0.071899
GD46	JO7	CS2	*R.nitidus*	-0.021359	0.07144
GD41	JO7	CS2	*R.nitidus*	0.005808	0.073201
GD30	JO7	CS2	*R.nitidus*	0.174497	0.07324
CS17	JO7	CS2	*R.nitidus*	0.021538	0.077487
GD44	JO7	CS2	*R.nitidus*	0.035475	0.073045
GD31	JO7	CS2	*R.nitidus*	0.08619	0.074054
GD48	JO7	CS2	*R.nitidus*	0.04918	0.073274
GD35	JO7	CS2	*R.nitidus*	0.056818	0.073415
CS15	JO7	CS2	*R.nitidus*	0.038241	0.072885
GD32	JO7	CS2	*R.nitidus*	0.051227	0.075712
SC14	JO7	CS2	*R.nitidus*	0.014451	0.07286
GD33	JO7	CS2	*R.nitidus*	0.045412	0.073658
SC15	JO7	CS2	*R.nitidus*	-0.018386	0.073501
CS25	JO7	CS2	*R.nitidus*	-0.009921	0.074303
GD34	JO7	CS2	*R.nitidus*	0.026022	0.070487
CS18	JO7	CS2	*R.nitidus*	-0.011788	0.073407
CS21	JO7	CS2	*R.nitidus*	-0.021884	0.072394
CS28	JO7	CS2	*R.nitidus*	-0.005171	0.073009
CS4	JO7	CS2	*R.nitidus*	0.002817	0.071269
GD45	JO7	CS2	*R.nitidus*	0.137255	0.075329
SC10	JO7	CS2	*R.nitidus*	0.109645	0.073409
GD42	JO7	CS2	*R.nitidus*	0.078629	0.074051
GD49	JO7	CS2	*R.nitidus*	0.117376	0.074432
CS24	JO7	CS2	*R.nitidus*	-0.022267	0.073961
CS14	JO7	CS2	*R.nitidus*	0.029557	0.075482
GD50	JO7	CS2	*R.nitidus*	0.066376	0.07645
CS6	JO7	CS2	*R.nitidus*	-0.048031	0.071374
CS23	JO7	CS2	*R.nitidus*	-0.035853	0.073009
SC32	JO7	CS2	*R.nitidus*	0.048436	0.071995
CS22	JO7	CS2	*R.nitidus*	0.035538	0.07216
CS13	JO7	CS2	*R.nitidus*	-0.026946	0.073248
SC34	JO7	CS2	*R.nitidus*	0.034483	0.069441
CS19	JO7	CS2	*R.nitidus*	-0.003956	0.076164

SC26	JO7	CS2	*R.nitidus*	-0.024738	0.073125
CS16	JO7	CS2	*R.nitidus*	0.026627	0.074417
SC28	JO7	CS2	*R.nitidus*	0.01068	0.072032
CS20	JO7	CS2	*R.nitidus*	-0.007656	0.072725
SC35	JO7	CS2	*R.nitidus*	0.02243	0.069025
SC5	JO7	CS2	*R.nitidus*	0.044574	0.075421
CS31	JO7	CS2	*R.nitidus*	0.000983	0.073719
SC6	JO7	CS2	*R.nitidus*	0.01665	0.071097
SC31	JO7	CS2	*R.nitidus*	0.011215	0.072648
CS3	JO7	CS2	*R.nitidus*	0.009569	0.071836
SC12	JO7	CS2	*R.nitidus*	-0.008008	0.070561
GD22	JO8	CS2	*R.nitidus*	0.074488	0.071802
SC18	JO7	CS2	*R.nitidus*	0.044534	0.072823
MY4	JO7	CS2	*R.nitidus*	-0.024925	0.073902
GD26	JO8	CS2	*R.nitidus*	0.038652	0.072775
MY6	JO7	CS2	*R.nitidus*	0.027668	0.072602
SC27	JO7	CS2	*R.nitidus*	0.023166	0.072856
SC45	JO7	CS2	*R.nitidus*	0.045045	0.071576
SC24	JO7	CS2	*R.nitidus*	0.029263	0.073422
JW6	JO7	CS2	*R.nitidus*	-0.024038	0.070482
GD32	JO8	CS2	*R.nitidus*	-0.004454	0.077074
CS11	JO8	CS2	*R.nitidus*	-0.021905	0.073656
GD45	JO8	CS2	*R.nitidus*	0.076647	0.078242
GD44	JO8	CS2	*R.nitidus*	0.004921	0.073744
GD30	JO8	CS2	*R.nitidus*	0.11639	0.075864
JO1	JO7	CS2	*R.nitidus*	0.017824	0.071565
GD21	JO8	CS2	*R.nitidus*	0.086051	0.070711
SC42	JO7	CS2	*R.nitidus*	0.188442	0.068815
CS12	JO8	CS2	*R.nitidus*	0.003099	0.07515
JW4	JO7	CS2	*R.nitidus*	0.226824	0.071033
GD25	JO8	CS2	*R.nitidus*	0.056919	0.071042
SC44	JO7	CS2	*R.nitidus*	-0.001992	0.074304
JW7	JO7	CS2	*R.nitidus*	0.053088	0.07472
GD50	JO8	CS2	*R.nitidus*	0.026876	0.075053
GD40	JO8	CS2	*R.nitidus*	0.059109	0.072067
JO5	JO7	CS2	*R.nitidus*	0.089483	0.06972
JO7	JO8	CS2	*R.nitidus*	-0.018841	0.073999
GD41	JO8	CS2	*R.nitidus*	-0.005877	0.072613
JO3	JO7	CS2	*R.nitidus*	0.02593	0.075154
GD31	JO8	CS2	*R.nitidus*	0.02459	0.073119
CS25	JO8	CS2	*R.nitidus*	-0.022518	0.075485
GD29	JO8	CS2	*R.nitidus*	0.125232	0.069201
GD42	JO8	CS2	*R.nitidus*	0.039235	0.073482
CS20	JO8	CS2	*R.nitidus*	-0.037075	0.073541
GD46	JO8	CS2	*R.nitidus*	-0.082164	0.069477
CS24	JO8	CS2	*R.nitidus*	-0.034409	0.073052
CS17	JO8	CS2	*R.nitidus*	-0.003191	0.076054
GD48	JO8	CS2	*R.nitidus*	0.031968	0.070254
SC6	JO8	CS2	*R.nitidus*	-0.027695	0.073308
GD34	JO8	CS2	*R.nitidus*	-0.037428	0.070897
GD35	JO8	CS2	*R.nitidus*	0.036398	0.07296
GD49	JO8	CS2	*R.nitidus*	0.062574	0.07518
SC15	JO8	CS2	*R.nitidus*	-0.039236	0.07404
CS16	JO8	CS2	*R.nitidus*	-0.034908	0.074333
GD33	JO8	CS2	*R.nitidus*	0	0.07293
CS13	JO8	CS2	*R.nitidus*	-0.049949	0.076709

CS18	JO8	CS2	*R.nitidus*	-0.034126	0.073053
SC10	JO8	CS2	*R.nitidus*	0.068013	0.074626
SC5	JO8	CS2	*R.nitidus*	0.005842	0.077272
CS22	JO8	CS2	*R.nitidus*	-0.015658	0.073586
CS3	JO8	CS2	*R.nitidus*	-0.012384	0.073393
SC18	JO8	CS2	*R.nitidus*	0.016575	0.072663
CS15	JO8	CS2	*R.nitidus*	-0.010732	0.073308
SC24	JO8	CS2	*R.nitidus*	0.006309	0.071515
CS21	JO8	CS2	*R.nitidus*	-0.045045	0.071262
SC28	JO8	CS2	*R.nitidus*	-0.03538	0.072205
SC31	JO8	CS2	*R.nitidus*	0.004946	0.073871
CS31	JO8	CS2	*R.nitidus*	-0.044652	0.073073
CS19	JO8	CS2	*R.nitidus*	-0.039404	0.07263
CS30	JO8	CS2	*R.nitidus*	-0.088358	0.071008
CS23	JO8	CS2	*R.nitidus*	-0.063008	0.07213
SC14	JO8	CS2	*R.nitidus*	-0.008824	0.072307
CS6	JO8	CS2	*R.nitidus*	-0.069744	0.072221
JW6	JO8	CS2	*R.nitidus*	-0.021552	0.071975
SC35	JO8	CS2	*R.nitidus*	0	0.072686
CS28	JO8	CS2	*R.nitidus*	-0.030884	0.07605
JO5	JO8	CS2	*R.nitidus*	0.066327	0.07015
SC45	JO8	CS2	*R.nitidus*	0.015984	0.073343
SC44	JO8	CS2	*R.nitidus*	-0.009278	0.072275
SC32	JO8	CS2	*R.nitidus*	-0.008032	0.07337
CS14	JO8	CS2	*R.nitidus*	-0.003	0.075491
SC27	JO8	CS2	*R.nitidus*	-0.015968	0.071908
SC12	JO8	CS2	*R.nitidus*	-0.044792	0.072909
CS4	JO8	CS2	*R.nitidus*	-0.006849	0.070995
MY4	JO8	CS2	*R.nitidus*	-0.06841	0.072472
SC26	JO8	CS2	*R.nitidus*	-0.092199	0.071761
MY6	JO8	CS2	*R.nitidus*	-0.009298	0.073286
JW4	JO8	CS2	*R.nitidus*	0.199799	0.070877
SC34	JO8	CS2	*R.nitidus*	-0.008721	0.071394
SC42	JO8	CS2	*R.nitidus*	0.138381	0.070977
JO1	JO8	CS2	*R.nitidus*	-0.032258	0.07165
JW7	JO8	CS2	*R.nitidus*	0.02246	0.075088
JO3	JO8	CS2	*R.nitidus*	0.009346	0.073588
JO7	CS11	CS2	*R.nitidus*	-0.025448	0.074042
JO8	CS11	CS2	*R.nitidus*	0.021905	0.073656
GD34	CS11	CS2	*R.nitidus*	0.008632	0.066476
GD33	CS11	CS2	*R.nitidus*	0.019568	0.06791
GD44	CS11	CS2	*R.nitidus*	0.02439	0.069487
CS15	CS11	CS2	*R.nitidus*	0.031694	0.070186
GD46	CS11	CS2	*R.nitidus*	-0.01264	0.067153
GD26	CS11	CS2	*R.nitidus*	0.050103	0.067872
CS12	CS11	CS2	*R.nitidus*	0.017708	0.07097
CS14	CS11	CS2	*R.nitidus*	0.002398	0.070887
GD42	CS11	CS2	*R.nitidus*	0.039548	0.06824
GD40	CS11	CS2	*R.nitidus*	0.061695	0.065455
GD25	CS11	CS2	*R.nitidus*	0.073082	0.068173
GD29	CS11	CS2	*R.nitidus*	0.122691	0.065939
GD35	CS11	CS2	*R.nitidus*	0.041172	0.066522
GD45	CS11	CS2	*R.nitidus*	0.085597	0.070485
GD48	CS11	CS2	*R.nitidus*	0.049491	0.067967
CS25	CS11	CS2	*R.nitidus*	-0.012898	0.069716
GD31	CS11	CS2	*R.nitidus*	0.057513	0.068026

CS23	CS11	CS2	*R.nitidus*	-0.062225	0.065597
CS20	CS11	CS2	*R.nitidus*	-0.035456	0.068105
GD21	CS11	CS2	*R.nitidus*	0.116866	0.065664
GD32	CS11	CS2	*R.nitidus*	0.038192	0.070478
GD30	CS11	CS2	*R.nitidus*	0.125	0.070775
GD49	CS11	CS2	*R.nitidus*	0.079114	0.07189
CS3	CS11	CS2	*R.nitidus*	-0.032576	0.068224
GD22	CS11	CS2	*R.nitidus*	0.068943	0.068067
CS22	CS11	CS2	*R.nitidus*	0.004521	0.068519
GD41	CS11	CS2	*R.nitidus*	0.017265	0.065656
GD50	CS11	CS2	*R.nitidus*	0.012542	0.070865
CS13	CS11	CS2	*R.nitidus*	-0.055675	0.073145
CS16	CS11	CS2	*R.nitidus*	-0.001058	0.070151
CS31	CS11	CS2	*R.nitidus*	-0.018824	0.069478
SC18	CS11	CS2	*R.nitidus*	0.047689	0.066762
CS24	CS11	CS2	*R.nitidus*	-0.044601	0.067864
SC5	CS11	CS2	*R.nitidus*	-0.002146	0.071044
CS17	CS11	CS2	*R.nitidus*	0.029006	0.070148
SC14	CS11	CS2	*R.nitidus*	-0.003595	0.069058
SC31	CS11	CS2	*R.nitidus*	-0.007897	0.067973
SC26	CS11	CS2	*R.nitidus*	-0.037445	0.065387
CS18	CS11	CS2	*R.nitidus*	-0.029257	0.068014
CS19	CS11	CS2	*R.nitidus*	-0.021705	0.069261
CS28	CS11	CS2	*R.nitidus*	-0.023904	0.066429
SC12	CS11	CS2	*R.nitidus*	-0.013373	0.067905
SC15	CS11	CS2	*R.nitidus*	-0.021689	0.069104
CS21	CS11	CS2	*R.nitidus*	-0.024354	0.066578
CS6	CS11	CS2	*R.nitidus*	-0.042373	0.068014
SC10	CS11	CS2	*R.nitidus*	0.053571	0.069006
JO1	CS11	CS2	*R.nitidus*	-0.017634	0.06641
SC32	CS11	CS2	*R.nitidus*	0.028464	0.068831
CS30	CS11	CS2	*R.nitidus*	-0.061848	0.06803
JO8	GD21	CS2	*R.nitidus*	-0.086051	0.070711
SC35	CS11	CS2	*R.nitidus*	0.040283	0.06428
SC34	CS11	CS2	*R.nitidus*	0.023775	0.068109
SC24	CS11	CS2	*R.nitidus*	0	0.068851
JO5	CS11	CS2	*R.nitidus*	0.030112	0.067923
SC6	CS11	CS2	*R.nitidus*	-0.016825	0.067728
SC45	CS11	CS2	*R.nitidus*	0.017329	0.068332
MY4	CS11	CS2	*R.nitidus*	-0.036903	0.068409
JO3	CS11	CS2	*R.nitidus*	0.039548	0.071732
JW7	CS11	CS2	*R.nitidus*	0.01084	0.069725
SC27	CS11	CS2	*R.nitidus*	-0.014126	0.0669
JO7	GD21	CS2	*R.nitidus*	-0.1274	0.069897
SC44	CS11	CS2	*R.nitidus*	-0.003602	0.067004
CS4	CS11	CS2	*R.nitidus*	-0.008929	0.068108
JW4	CS11	CS2	*R.nitidus*	0.208598	0.067245
MY6	CS11	CS2	*R.nitidus*	0.001414	0.068006
SC28	CS11	CS2	*R.nitidus*	-0.047653	0.066976
CS11	GD21	CS2	*R.nitidus*	-0.116866	0.065664
GD45	GD21	CS2	*R.nitidus*	-0.028959	0.069147
GD42	GD21	CS2	*R.nitidus*	-0.094192	0.0636
GD26	GD21	CS2	*R.nitidus*	-0.078313	0.063643
JW6	CS11	CS2	*R.nitidus*	-0.003756	0.073151
GD25	GD21	CS2	*R.nitidus*	-0.024444	0.065018
SC42	CS11	CS2	*R.nitidus*	0.227142	0.061837

CS14	GD21	CS2	*R.nitidus*	-0.129771	0.066691
GD48	GD21	CS2	*R.nitidus*	-0.097293	0.066505
GD31	GD21	CS2	*R.nitidus*	-0.085123	0.065114
GD33	GD21	CS2	*R.nitidus*	-0.128487	0.063456
GD35	GD21	CS2	*R.nitidus*	-0.075182	0.06376
GD44	GD21	CS2	*R.nitidus*	-0.129129	0.064367
GD46	GD21	CS2	*R.nitidus*	-0.175207	0.061807
GD41	GD21	CS2	*R.nitidus*	-0.111862	0.063885
GD30	GD21	CS2	*R.nitidus*	0.03001	0.066862
GD32	GD21	CS2	*R.nitidus*	-0.078622	0.065608
CS16	GD21	CS2	*R.nitidus*	-0.115464	0.064353
CS15	GD21	CS2	*R.nitidus*	-0.106485	0.065902
GD22	GD21	CS2	*R.nitidus*	-0.054572	0.061136
CS12	GD21	CS2	*R.nitidus*	-0.121727	0.067331
GD29	GD21	CS2	*R.nitidus*	0.00858	0.064453
GD34	GD21	CS2	*R.nitidus*	-0.137386	0.062036
CS21	GD21	CS2	*R.nitidus*	-0.135593	0.06467
SC10	GD21	CS2	*R.nitidus*	-0.067431	0.06504
GD40	GD21	CS2	*R.nitidus*	-0.066996	0.064569
CS30	GD21	CS2	*R.nitidus*	-0.178822	0.064236
CS19	GD21	CS2	*R.nitidus*	-0.122734	0.066712
CS31	GD21	CS2	*R.nitidus*	-0.142452	0.064581
CS20	GD21	CS2	*R.nitidus*	-0.152965	0.065688
CS24	GD21	CS2	*R.nitidus*	-0.156727	0.064056
GD49	GD21	CS2	*R.nitidus*	-0.024955	0.067797
CS18	GD21	CS2	*R.nitidus*	-0.144	0.066405
GD50	GD21	CS2	*R.nitidus*	-0.124195	0.068252
CS13	GD21	CS2	*R.nitidus*	-0.150171	0.066045
SC28	GD21	CS2	*R.nitidus*	-0.152031	0.06437
SC5	GD21	CS2	*R.nitidus*	-0.106849	0.067868
CS23	GD21	CS2	*R.nitidus*	-0.162866	0.065355
CS22	GD21	CS2	*R.nitidus*	-0.107703	0.063593
SC14	GD21	CS2	*R.nitidus*	-0.11261	0.065917
CS25	GD21	CS2	*R.nitidus*	-0.136799	0.066452
CS6	GD21	CS2	*R.nitidus*	-0.161357	0.065038
CS17	GD21	CS2	*R.nitidus*	-0.100072	0.066855
CS4	GD21	CS2	*R.nitidus*	-0.122382	0.062875
CS28	GD21	CS2	*R.nitidus*	-0.134864	0.064739
SC18	GD21	CS2	*R.nitidus*	-0.099509	0.064563
SC45	GD21	CS2	*R.nitidus*	-0.076716	0.062686
SC6	GD21	CS2	*R.nitidus*	-0.129973	0.06471
SC32	GD21	CS2	*R.nitidus*	-0.099324	0.064145
SC24	GD21	CS2	*R.nitidus*	-0.121769	0.06563
CS3	GD21	CS2	*R.nitidus*	-0.129141	0.063621
MY6	GD21	CS2	*R.nitidus*	-0.118194	0.065051
MY4	GD21	CS2	*R.nitidus*	-0.162034	0.064385
JW4	GD21	CS2	*R.nitidus*	0.108345	0.06753
SC12	GD21	CS2	*R.nitidus*	-0.132013	0.064006
JW6	GD21	CS2	*R.nitidus*	-0.120451	0.07032
JW7	GD21	CS2	*R.nitidus*	-0.10425	0.06824
JO8	GD22	CS2	*R.nitidus*	-0.074488	0.071802
SC27	GD21	CS2	*R.nitidus*	-0.118805	0.06543
SC15	GD21	CS2	*R.nitidus*	-0.137621	0.066434
SC42	GD21	CS2	*R.nitidus*	0.149972	0.062218
SC26	GD21	CS2	*R.nitidus*	-0.157133	0.063361
SC31	GD21	CS2	*R.nitidus*	-0.122967	0.064837

SC34	GD21	CS2	*R.nitidus*	-0.084912	0.065118
JO5	GD21	CS2	*R.nitidus*	-0.060705	0.063859
GD31	GD22	CS2	*R.nitidus*	-0.03125	0.068339
SC35	GD21	CS2	*R.nitidus*	-0.079228	0.064785
GD25	GD22	CS2	*R.nitidus*	-0.002952	0.065513
JO7	GD22	CS2	*R.nitidus*	-0.130199	0.070526
SC44	GD21	CS2	*R.nitidus*	-0.115074	0.064562
GD26	GD22	CS2	*R.nitidus*	-0.026005	0.065347
JO1	GD21	CS2	*R.nitidus*	-0.14134	0.064659
GD21	GD22	CS2	*R.nitidus*	0.054572	0.061136
GD33	GD22	CS2	*R.nitidus*	-0.058577	0.064579
JO3	GD21	CS2	*R.nitidus*	-0.084027	0.069393
CS12	GD22	CS2	*R.nitidus*	-0.057281	0.067429
CS17	GD22	CS2	*R.nitidus*	-0.04577	0.06746
GD49	GD22	CS2	*R.nitidus*	0.035751	0.071
GD35	GD22	CS2	*R.nitidus*	-0.017366	0.065348
GD41	GD22	CS2	*R.nitidus*	-0.072713	0.06427
GD34	GD22	CS2	*R.nitidus*	-0.086844	0.061682
GD48	GD22	CS2	*R.nitidus*	-0.03974	0.065383
CS15	GD22	CS2	*R.nitidus*	-0.051813	0.066376
GD29	GD22	CS2	*R.nitidus*	0.065613	0.063833
GD45	GD22	CS2	*R.nitidus*	-0.020478	0.06868
GD30	GD22	CS2	*R.nitidus*	0.082852	0.065355
GD32	GD22	CS2	*R.nitidus*	-0.04741	0.066457
CS11	GD22	CS2	*R.nitidus*	-0.068943	0.068067
GD40	GD22	CS2	*R.nitidus*	-0.000699	0.064229
GD46	GD22	CS2	*R.nitidus*	-0.112121	0.061316
CS13	GD22	CS2	*R.nitidus*	-0.111489	0.065844
GD50	GD22	CS2	*R.nitidus*	-0.030132	0.069443
CS23	GD22	CS2	*R.nitidus*	-0.115669	0.064802
CS14	GD22	CS2	*R.nitidus*	-0.067541	0.06711
GD44	GD22	CS2	*R.nitidus*	-0.050835	0.066289
CS31	GD22	CS2	*R.nitidus*	-0.087744	0.066809
CS16	GD22	CS2	*R.nitidus*	-0.070059	0.064014
GD42	GD22	CS2	*R.nitidus*	-0.060995	0.064546
CS30	GD22	CS2	*R.nitidus*	-0.133241	0.064035
CS22	GD22	CS2	*R.nitidus*	-0.054054	0.065051
CS24	GD22	CS2	*R.nitidus*	-0.104635	0.064911
CS18	GD22	CS2	*R.nitidus*	-0.102389	0.065711
SC18	GD22	CS2	*R.nitidus*	-0.027046	0.064567
CS21	GD22	CS2	*R.nitidus*	-0.091984	0.06473
CS25	GD22	CS2	*R.nitidus*	-0.096301	0.067304
CS6	GD22	CS2	*R.nitidus*	-0.11955	0.065731
SC10	GD22	CS2	*R.nitidus*	-0.011204	0.067059
SC6	GD22	CS2	*R.nitidus*	-0.07487	0.066387
CS3	GD22	CS2	*R.nitidus*	-0.096327	0.065293
SC27	GD22	CS2	*R.nitidus*	-0.068783	0.0637
SC24	GD22	CS2	*R.nitidus*	-0.070383	0.066631
CS28	GD22	CS2	*R.nitidus*	-0.091672	0.067807
MY6	GD22	CS2	*R.nitidus*	-0.068302	0.065079
CS4	GD22	CS2	*R.nitidus*	-0.079523	0.062256
CS20	GD22	CS2	*R.nitidus*	-0.111036	0.066412
JW4	GD22	CS2	*R.nitidus*	0.157787	0.066308
CS19	GD22	CS2	*R.nitidus*	-0.082069	0.068065
SC28	GD22	CS2	*R.nitidus*	-0.11811	0.064393
JO5	GD22	CS2	*R.nitidus*	-0.012743	0.063775

JW7	GD22	CS2	*R.nitidus*	-0.059524	0.069098
JO1	GD22	CS2	*R.nitidus*	-0.093204	0.065238
JO8	GD25	CS2	*R.nitidus*	-0.056919	0.071042
SC15	GD22	CS2	*R.nitidus*	-0.084438	0.064926
GD21	GD25	CS2	*R.nitidus*	0.024444	0.065018
SC45	GD22	CS2	*R.nitidus*	-0.029332	0.065298
SC5	GD22	CS2	*R.nitidus*	-0.071972	0.068647
SC12	GD22	CS2	*R.nitidus*	-0.085733	0.063476
GD31	GD25	CS2	*R.nitidus*	-0.019417	0.065945
SC14	GD22	CS2	*R.nitidus*	-0.071334	0.064374
JW6	GD22	CS2	*R.nitidus*	-0.074496	0.070315
SC26	GD22	CS2	*R.nitidus*	-0.100396	0.064059
CS12	GD25	CS2	*R.nitidus*	-0.081853	0.06786
SC42	GD22	CS2	*R.nitidus*	0.167871	0.062516
SC31	GD22	CS2	*R.nitidus*	-0.081081	0.06571
MY4	GD22	CS2	*R.nitidus*	-0.110734	0.065751
SC35	GD22	CS2	*R.nitidus*	-0.022936	0.063695
SC34	GD22	CS2	*R.nitidus*	-0.03781	0.063174
GD49	GD25	CS2	*R.nitidus*	0.012422	0.06844
SC44	GD22	CS2	*R.nitidus*	-0.083682	0.063907
JO7	GD25	CS2	*R.nitidus*	-0.090998	0.073916
CS11	GD25	CS2	*R.nitidus*	-0.073082	0.068173
GD29	GD25	CS2	*R.nitidus*	0.044248	0.064594
SC32	GD22	CS2	*R.nitidus*	-0.049479	0.063183
CS13	GD25	CS2	*R.nitidus*	-0.115782	0.067299
JO3	GD22	CS2	*R.nitidus*	-0.033965	0.068337
GD30	GD25	CS2	*R.nitidus*	0.07852	0.068747
GD42	GD25	CS2	*R.nitidus*	-0.044728	0.065635
GD44	GD25	CS2	*R.nitidus*	-0.062303	0.067826
CS16	GD25	CS2	*R.nitidus*	-0.088041	0.067002
GD22	GD25	CS2	*R.nitidus*	0.002952	0.065513
GD35	GD25	CS2	*R.nitidus*	-0.030757	0.063867
GD32	GD25	CS2	*R.nitidus*	-0.03339	0.068556
GD40	GD25	CS2	*R.nitidus*	-0.026966	0.063849
GD26	GD25	CS2	*R.nitidus*	-0.021143	0.067199
CS19	GD25	CS2	*R.nitidus*	-0.086169	0.065465
GD33	GD25	CS2	*R.nitidus*	-0.067692	0.067978
GD46	GD25	CS2	*R.nitidus*	-0.106677	0.065558
GD41	GD25	CS2	*R.nitidus*	-0.05492	0.066438
GD45	GD25	CS2	*R.nitidus*	0.04344	0.070262
CS17	GD25	CS2	*R.nitidus*	-0.06089	0.068844
CS14	GD25	CS2	*R.nitidus*	-0.086672	0.068927
CS21	GD25	CS2	*R.nitidus*	-0.105866	0.067233
GD34	GD25	CS2	*R.nitidus*	-0.073004	0.066479
CS3	GD25	CS2	*R.nitidus*	-0.091244	0.066702
CS30	GD25	CS2	*R.nitidus*	-0.128205	0.065252
GD48	GD25	CS2	*R.nitidus*	-0.026473	0.06624
CS20	GD25	CS2	*R.nitidus*	-0.113571	0.06814
CS15	GD25	CS2	*R.nitidus*	-0.065562	0.066483
CS24	GD25	CS2	*R.nitidus*	-0.093411	0.065688
CS25	GD25	CS2	*R.nitidus*	-0.103935	0.066121
GD50	GD25	CS2	*R.nitidus*	-0.017874	0.069629
CS18	GD25	CS2	*R.nitidus*	-0.102528	0.065147
SC5	GD25	CS2	*R.nitidus*	-0.075036	0.069359
SC6	GD25	CS2	*R.nitidus*	-0.09299	0.067038
CS31	GD25	CS2	*R.nitidus*	-0.101427	0.066394

CS4	GD25	CS2	*R.nitidus*	-0.071034	0.063964
SC32	GD25	CS2	*R.nitidus*	-0.056826	0.064087
SC10	GD25	CS2	*R.nitidus*	-0.029784	0.066367
CS23	GD25	CS2	*R.nitidus*	-0.122991	0.06469
JW4	GD25	CS2	*R.nitidus*	0.146177	0.067437
SC26	GD25	CS2	*R.nitidus*	-0.111585	0.066198
CS22	GD25	CS2	*R.nitidus*	-0.052255	0.064903
CS28	GD25	CS2	*R.nitidus*	-0.090132	0.065737
SC15	GD25	CS2	*R.nitidus*	-0.096992	0.068991
JO1	GD25	CS2	*R.nitidus*	-0.120603	0.066035
SC24	GD25	CS2	*R.nitidus*	-0.068768	0.067438
JW6	GD25	CS2	*R.nitidus*	-0.080229	0.072634
SC12	GD25	CS2	*R.nitidus*	-0.093343	0.066439
SC35	GD25	CS2	*R.nitidus*	-0.042053	0.066114
MY4	GD25	CS2	*R.nitidus*	-0.117982	0.066273
SC28	GD25	CS2	*R.nitidus*	-0.124026	0.065046
SC18	GD25	CS2	*R.nitidus*	-0.040668	0.067129
SC42	GD25	CS2	*R.nitidus*	0.157704	0.063399
JO8	GD26	CS2	*R.nitidus*	-0.038652	0.072775
SC14	GD25	CS2	*R.nitidus*	-0.076064	0.065603
GD44	GD26	CS2	*R.nitidus*	-0.023256	0.068829
JO3	GD25	CS2	*R.nitidus*	-0.035928	0.070695
JW7	GD25	CS2	*R.nitidus*	-0.067603	0.069483
GD31	GD26	CS2	*R.nitidus*	0.021497	0.067402
SC27	GD25	CS2	*R.nitidus*	-0.091808	0.065036
MY6	GD25	CS2	*R.nitidus*	-0.073888	0.065839
GD42	GD26	CS2	*R.nitidus*	-0.004823	0.067643
SC31	GD25	CS2	*R.nitidus*	-0.082394	0.065075
CS11	GD26	CS2	*R.nitidus*	-0.050103	0.067872
SC34	GD25	CS2	*R.nitidus*	-0.039945	0.064913
CS6	GD25	CS2	*R.nitidus*	-0.10972	0.066181
SC45	GD25	CS2	*R.nitidus*	-0.063004	0.066769
JO7	GD26	CS2	*R.nitidus*	-0.094796	0.074087
GD41	GD26	CS2	*R.nitidus*	-0.046995	0.06595
GD25	GD26	CS2	*R.nitidus*	0.021143	0.067199
GD32	GD26	CS2	*R.nitidus*	-0.004484	0.064814
SC44	GD25	CS2	*R.nitidus*	-0.076696	0.065423
GD49	GD26	CS2	*R.nitidus*	0.036969	0.067392
GD48	GD26	CS2	*R.nitidus*	-0.02	0.067205
GD35	GD26	CS2	*R.nitidus*	-0.006163	0.066954
GD22	GD26	CS2	*R.nitidus*	0.026005	0.065347
JO5	GD25	CS2	*R.nitidus*	-0.038489	0.064767
GD34	GD26	CS2	*R.nitidus*	-0.057003	0.064438
GD29	GD26	CS2	*R.nitidus*	0.081017	0.063779
GD33	GD26	CS2	*R.nitidus*	-0.045815	0.066475
GD45	GD26	CS2	*R.nitidus*	0.053309	0.070271
GD30	GD26	CS2	*R.nitidus*	0.117647	0.067839
CS15	GD26	CS2	*R.nitidus*	-0.039917	0.067717
GD21	GD26	CS2	*R.nitidus*	0.078313	0.063643
GD40	GD26	CS2	*R.nitidus*	0.017686	0.062963
CS13	GD26	CS2	*R.nitidus*	-0.083747	0.067863
SC6	GD26	CS2	*R.nitidus*	-0.074386	0.06609
GD46	GD26	CS2	*R.nitidus*	-0.082118	0.062094
CS17	GD26	CS2	*R.nitidus*	-0.022448	0.069435
CS18	GD26	CS2	*R.nitidus*	-0.082985	0.068061
CS16	GD26	CS2	*R.nitidus*	-0.054899	0.066068

CS24	GD26	CS2	*R.nitidus*	-0.086831	0.065547
CS19	GD26	CS2	*R.nitidus*	-0.058659	0.069103
CS12	GD26	CS2	*R.nitidus*	-0.039445	0.067725
GD50	GD26	CS2	*R.nitidus*	-0.00947	0.0683
CS28	GD26	CS2	*R.nitidus*	-0.071271	0.06697
CS20	GD26	CS2	*R.nitidus*	-0.086088	0.06841
SC18	GD26	CS2	*R.nitidus*	-0.019679	0.064524
SC12	GD26	CS2	*R.nitidus*	-0.07839	0.063235
SC15	GD26	CS2	*R.nitidus*	-0.063559	0.066646
CS23	GD26	CS2	*R.nitidus*	-0.098372	0.065735
CS3	GD26	CS2	*R.nitidus*	-0.074669	0.067364
CS22	GD26	CS2	*R.nitidus*	-0.045198	0.066057
CS14	GD26	CS2	*R.nitidus*	-0.05784	0.067987
SC10	GD26	CS2	*R.nitidus*	-0.002855	0.067029
SC28	GD26	CS2	*R.nitidus*	-0.095938	0.066206
CS30	GD26	CS2	*R.nitidus*	-0.110473	0.066411
SC45	GD26	CS2	*R.nitidus*	-0.025352	0.066386
SC5	GD26	CS2	*R.nitidus*	-0.052899	0.069154
CS6	GD26	CS2	*R.nitidus*	-0.08662	0.066953
JW6	GD26	CS2	*R.nitidus*	-0.07032	0.071399
JO7	GD29	CS2	*R.nitidus*	-0.169794	0.069253
JO3	GD26	CS2	*R.nitidus*	-0.010367	0.070511
SC35	GD26	CS2	*R.nitidus*	-0.022603	0.064464
SC26	GD26	CS2	*R.nitidus*	-0.094274	0.066373
MY4	GD26	CS2	*R.nitidus*	-0.079521	0.066702
CS21	GD26	CS2	*R.nitidus*	-0.067568	0.06756
SC42	GD26	CS2	*R.nitidus*	0.180507	0.062683
CS25	GD26	CS2	*R.nitidus*	-0.069832	0.068106
JO8	GD29	CS2	*R.nitidus*	-0.125232	0.069201
SC32	GD26	CS2	*R.nitidus*	-0.030158	0.06572
JO5	GD26	CS2	*R.nitidus*	0.000698	0.065672
CS31	GD26	CS2	*R.nitidus*	-0.077259	0.067411
JW7	GD26	CS2	*R.nitidus*	-0.032205	0.071384
SC31	GD26	CS2	*R.nitidus*	-0.057162	0.066581
SC44	GD26	CS2	*R.nitidus*	-0.06082	0.065323
CS4	GD26	CS2	*R.nitidus*	-0.06323	0.064301
GD30	GD29	CS2	*R.nitidus*	0.034188	0.066437
SC24	GD26	CS2	*R.nitidus*	-0.055914	0.065415
SC14	GD26	CS2	*R.nitidus*	-0.06212	0.066792
JW4	GD26	CS2	*R.nitidus*	0.185893	0.068424
SC27	GD26	CS2	*R.nitidus*	-0.061486	0.06576
GD26	GD29	CS2	*R.nitidus*	-0.081017	0.063779
SC34	GD26	CS2	*R.nitidus*	-0.026027	0.065601
GD35	GD29	CS2	*R.nitidus*	-0.068149	0.064953
GD42	GD29	CS2	*R.nitidus*	-0.103529	0.064844
MY6	GD26	CS2	*R.nitidus*	-0.04726	0.067264
CS11	GD29	CS2	*R.nitidus*	-0.122691	0.065939
GD40	GD29	CS2	*R.nitidus*	-0.064748	0.061765
JO1	GD26	CS2	*R.nitidus*	-0.085178	0.064488
GD32	GD29	CS2	*R.nitidus*	-0.105171	0.066313
GD21	GD29	CS2	*R.nitidus*	-0.00858	0.064453
GD48	GD29	CS2	*R.nitidus*	-0.088797	0.064727
GD49	GD29	CS2	*R.nitidus*	-0.039126	0.068422
GD31	GD29	CS2	*R.nitidus*	-0.064593	0.066362
GD45	GD29	CS2	*R.nitidus*	-0.03273	0.067855
GD25	GD29	CS2	*R.nitidus*	-0.044248	0.064594

CS13	GD29	CS2	*R.nitidus*	-0.157191	0.066589
CS25	GD29	CS2	*R.nitidus*	-0.135794	0.065075
GD46	GD29	CS2	*R.nitidus*	-0.172999	0.06442
GD41	GD29	CS2	*R.nitidus*	-0.116705	0.063987
CS21	GD29	CS2	*R.nitidus*	-0.137633	0.063434
CS15	GD29	CS2	*R.nitidus*	-0.102681	0.065809
GD50	GD29	CS2	*R.nitidus*	-0.089981	0.067024
GD22	GD29	CS2	*R.nitidus*	-0.065613	0.063833
CS19	GD29	CS2	*R.nitidus*	-0.122348	0.066002
SC15	GD29	CS2	*R.nitidus*	-0.139567	0.065523
GD44	GD29	CS2	*R.nitidus*	-0.117045	0.065908
SC10	GD29	CS2	*R.nitidus*	-0.065007	0.066057
GD34	GD29	CS2	*R.nitidus*	-0.15471	0.063324
CS12	GD29	CS2	*R.nitidus*	-0.109944	0.065196
GD33	GD29	CS2	*R.nitidus*	-0.12741	0.064005
CS31	GD29	CS2	*R.nitidus*	-0.148892	0.065182
CS3	GD29	CS2	*R.nitidus*	-0.146375	0.063832
CS18	GD29	CS2	*R.nitidus*	-0.133378	0.064631
CS17	GD29	CS2	*R.nitidus*	-0.099156	0.066678
SC6	GD29	CS2	*R.nitidus*	-0.141988	0.063734
CS24	GD29	CS2	*R.nitidus*	-0.146461	0.064276
CS30	GD29	CS2	*R.nitidus*	-0.17561	0.063499
CS14	GD29	CS2	*R.nitidus*	-0.126406	0.067302
CS16	GD29	CS2	*R.nitidus*	-0.129887	0.065623
SC32	GD29	CS2	*R.nitidus*	-0.113531	0.065904
SC34	GD29	CS2	*R.nitidus*	-0.072839	0.063781
SC27	GD29	CS2	*R.nitidus*	-0.116857	0.063412
CS20	GD29	CS2	*R.nitidus*	-0.151031	0.064621
CS28	GD29	CS2	*R.nitidus*	-0.13627	0.066375
CS23	GD29	CS2	*R.nitidus*	-0.157104	0.062753
CS22	GD29	CS2	*R.nitidus*	-0.110215	0.064106
SC18	GD29	CS2	*R.nitidus*	-0.078059	0.065642
SC44	GD29	CS2	*R.nitidus*	-0.120055	0.06557
SC5	GD29	CS2	*R.nitidus*	-0.114246	0.06865
CS4	GD29	CS2	*R.nitidus*	-0.128378	0.062336
MY6	GD29	CS2	*R.nitidus*	-0.114883	0.064185
SC12	GD29	CS2	*R.nitidus*	-0.131333	0.065364
SC31	GD29	CS2	*R.nitidus*	-0.119248	0.063735
JO8	GD30	CS2	*R.nitidus*	-0.11639	0.075864
SC14	GD29	CS2	*R.nitidus*	-0.112352	0.063577
SC42	GD29	CS2	*R.nitidus*	0.11007	0.062795
JO3	GD29	CS2	*R.nitidus*	-0.086166	0.066576
MY4	GD29	CS2	*R.nitidus*	-0.153795	0.065158
SC35	GD29	CS2	*R.nitidus*	-0.073056	0.064438
GD22	GD30	CS2	*R.nitidus*	-0.082852	0.065355
SC26	GD29	CS2	*R.nitidus*	-0.158488	0.063347
SC24	GD29	CS2	*R.nitidus*	-0.117925	0.065104
JO7	GD30	CS2	*R.nitidus*	-0.174497	0.07324
CS11	GD30	CS2	*R.nitidus*	-0.125	0.070775
CS12	GD30	CS2	*R.nitidus*	-0.126065	0.071773
JW6	GD29	CS2	*R.nitidus*	-0.139091	0.068283
SC28	GD29	CS2	*R.nitidus*	-0.163102	0.064539
GD32	GD30	CS2	*R.nitidus*	-0.14	0.071238
JW4	GD29	CS2	*R.nitidus*	0.101132	0.066491
GD21	GD30	CS2	*R.nitidus*	-0.03001	0.066862
JO1	GD29	CS2	*R.nitidus*	-0.14106	0.063539

GD25	GD30	CS2	*R.nitidus*	-0.07852	0.068747
JW7	GD29	CS2	*R.nitidus*	-0.110592	0.067216
CS6	GD29	CS2	*R.nitidus*	-0.147484	0.06516
GD26	GD30	CS2	*R.nitidus*	-0.117647	0.067839
SC45	GD29	CS2	*R.nitidus*	-0.09377	0.064866
GD41	GD30	CS2	*R.nitidus*	-0.111508	0.06537
GD42	GD30	CS2	*R.nitidus*	-0.119205	0.066317
GD34	GD30	CS2	*R.nitidus*	-0.157128	0.063908
JO5	GD29	CS2	*R.nitidus*	-0.080897	0.064351
GD49	GD30	CS2	*R.nitidus*	-0.079836	0.07247
GD33	GD30	CS2	*R.nitidus*	-0.118881	0.067447
GD35	GD30	CS2	*R.nitidus*	-0.103066	0.068263
CS16	GD30	CS2	*R.nitidus*	-0.120393	0.068418
GD50	GD30	CS2	*R.nitidus*	-0.103665	0.071096
GD40	GD30	CS2	*R.nitidus*	-0.076323	0.065476
GD31	GD30	CS2	*R.nitidus*	-0.119854	0.068944
SC24	GD30	CS2	*R.nitidus*	-0.128981	0.067966
CS21	GD30	CS2	*R.nitidus*	-0.16575	0.067197
GD44	GD30	CS2	*R.nitidus*	-0.113309	0.069287
CS18	GD30	CS2	*R.nitidus*	-0.146965	0.068607
CS13	GD30	CS2	*R.nitidus*	-0.149666	0.069695
CS31	GD30	CS2	*R.nitidus*	-0.126572	0.06895
GD45	GD30	CS2	*R.nitidus*	-0.048403	0.0694
GD29	GD30	CS2	*R.nitidus*	-0.034188	0.066437
CS20	GD30	CS2	*R.nitidus*	-0.15873	0.067778
SC28	GD30	CS2	*R.nitidus*	-0.162141	0.065992
CS23	GD30	CS2	*R.nitidus*	-0.153175	0.067145
CS14	GD30	CS2	*R.nitidus*	-0.124481	0.072634
CS19	GD30	CS2	*R.nitidus*	-0.130471	0.070622
SC32	GD30	CS2	*R.nitidus*	-0.101426	0.068133
GD48	GD30	CS2	*R.nitidus*	-0.060576	0.068622
GD46	GD30	CS2	*R.nitidus*	-0.184815	0.065607
CS6	GD30	CS2	*R.nitidus*	-0.159278	0.067025
CS3	GD30	CS2	*R.nitidus*	-0.144833	0.067409
CS24	GD30	CS2	*R.nitidus*	-0.148971	0.065356
CS17	GD30	CS2	*R.nitidus*	-0.10408	0.070918
CS30	GD30	CS2	*R.nitidus*	-0.176568	0.067594
SC27	GD30	CS2	*R.nitidus*	-0.135308	0.066992
CS22	GD30	CS2	*R.nitidus*	-0.115756	0.067188
CS15	GD30	CS2	*R.nitidus*	-0.105565	0.070312
SC31	GD30	CS2	*R.nitidus*	-0.12989	0.067219
SC5	GD30	CS2	*R.nitidus*	-0.126511	0.070741
SC10	GD30	CS2	*R.nitidus*	-0.099746	0.070044
SC35	GD30	CS2	*R.nitidus*	-0.074451	0.067345
SC15	GD30	CS2	*R.nitidus*	-0.135607	0.069227
SC18	GD30	CS2	*R.nitidus*	-0.082158	0.067422
CS28	GD30	CS2	*R.nitidus*	-0.147668	0.068921
SC14	GD30	CS2	*R.nitidus*	-0.118357	0.067781
SC45	GD30	CS2	*R.nitidus*	-0.102282	0.066521
SC6	GD30	CS2	*R.nitidus*	-0.146461	0.066794
JW7	GD30	CS2	*R.nitidus*	-0.11379	0.070467
CS4	GD30	CS2	*R.nitidus*	-0.132549	0.065572
SC12	GD30	CS2	*R.nitidus*	-0.127642	0.06706
SC26	GD30	CS2	*R.nitidus*	-0.159524	0.066853
SC34	GD30	CS2	*R.nitidus*	-0.08646	0.067661
MY6	GD30	CS2	*R.nitidus*	-0.123177	0.066929

JW6	GD30	CS2	*R.nitidus*	-0.148148	0.071628
CS25	GD30	CS2	*R.nitidus*	-0.13255	0.071621
JO3	GD30	CS2	*R.nitidus*	-0.124756	0.071347
GD33	GD31	CS2	*R.nitidus*	-0.031128	0.068688
JW4	GD30	CS2	*R.nitidus*	0.095238	0.070587
JO1	GD30	CS2	*R.nitidus*	-0.159839	0.065423
MY4	GD30	CS2	*R.nitidus*	-0.166259	0.068364
GD30	GD31	CS2	*R.nitidus*	0.119854	0.068944
GD26	GD31	CS2	*R.nitidus*	-0.021497	0.067402
SC42	GD30	CS2	*R.nitidus*	0.117647	0.064204
SC44	GD30	CS2	*R.nitidus*	-0.11157	0.065882
JO7	GD31	CS2	*R.nitidus*	-0.08619	0.074054
GD25	GD31	CS2	*R.nitidus*	0.019417	0.065945
CS11	GD31	CS2	*R.nitidus*	-0.057513	0.068026
JO8	GD31	CS2	*R.nitidus*	-0.02459	0.073119
GD42	GD31	CS2	*R.nitidus*	-0.007494	0.067453
GD48	GD31	CS2	*R.nitidus*	0.003581	0.069418
CS17	GD31	CS2	*R.nitidus*	-0.042636	0.070274
JO5	GD30	CS2	*R.nitidus*	-0.080064	0.066656
GD45	GD31	CS2	*R.nitidus*	0.02952	0.069372
CS12	GD31	CS2	*R.nitidus*	-0.044343	0.068288
GD46	GD31	CS2	*R.nitidus*	-0.069712	0.064727
GD50	GD31	CS2	*R.nitidus*	-0.002913	0.070469
CS19	GD31	CS2	*R.nitidus*	-0.090977	0.069413
GD35	GD31	CS2	*R.nitidus*	-0.00077	0.066559
CS20	GD31	CS2	*R.nitidus*	-0.093499	0.06749
CS25	GD31	CS2	*R.nitidus*	-0.084683	0.068606
GD32	GD31	CS2	*R.nitidus*	-0.01	0.070513
GD29	GD31	CS2	*R.nitidus*	0.064593	0.066362
CS15	GD31	CS2	*R.nitidus*	-0.042121	0.067349
GD49	GD31	CS2	*R.nitidus*	0.051048	0.072282
CS16	GD31	CS2	*R.nitidus*	-0.050074	0.068087
GD22	GD31	CS2	*R.nitidus*	0.03125	0.068339
CS23	GD31	CS2	*R.nitidus*	-0.107065	0.065878
GD34	GD31	CS2	*R.nitidus*	-0.045234	0.068341
CS18	GD31	CS2	*R.nitidus*	-0.084821	0.06881
GD41	GD31	CS2	*R.nitidus*	-0.035427	0.067127
CS13	GD31	CS2	*R.nitidus*	-0.095808	0.068316
CS22	GD31	CS2	*R.nitidus*	-0.041481	0.067374
GD40	GD31	CS2	*R.nitidus*	0.018561	0.066347
GD44	GD31	CS2	*R.nitidus*	-0.038494	0.068278
SC15	GD31	CS2	*R.nitidus*	-0.068657	0.066819
GD21	GD31	CS2	*R.nitidus*	0.085123	0.065114
CS30	GD31	CS2	*R.nitidus*	-0.118865	0.067627
SC10	GD31	CS2	*R.nitidus*	0.003014	0.068043
CS3	GD31	CS2	*R.nitidus*	-0.079084	0.066895
CS21	GD31	CS2	*R.nitidus*	-0.076867	0.067177
CS4	GD31	CS2	*R.nitidus*	-0.060694	0.064937
CS14	GD31	CS2	*R.nitidus*	-0.054114	0.069716
CS28	GD31	CS2	*R.nitidus*	-0.057588	0.067203
CS24	GD31	CS2	*R.nitidus*	-0.088235	0.067702
SC26	GD31	CS2	*R.nitidus*	-0.087302	0.064774
SC45	GD31	CS2	*R.nitidus*	-0.042888	0.066468
SC32	GD31	CS2	*R.nitidus*	-0.027273	0.065988
CS31	GD31	CS2	*R.nitidus*	-0.071373	0.068632
SC27	GD31	CS2	*R.nitidus*	-0.045985	0.065875

SC31	GD31	CS2	*R.nitidus*	-0.059035	0.066796
JW6	GD31	CS2	*R.nitidus*	-0.074627	0.072466
SC34	GD31	CS2	*R.nitidus*	-0.016405	0.06654
SC6	GD31	CS2	*R.nitidus*	-0.062635	0.065291
SC18	GD31	CS2	*R.nitidus*	-0.007587	0.065792
SC5	GD31	CS2	*R.nitidus*	-0.052273	0.069508
JO3	GD31	CS2	*R.nitidus*	-0.013889	0.070795
SC28	GD31	CS2	*R.nitidus*	-0.099853	0.066094
SC12	GD31	CS2	*R.nitidus*	-0.063179	0.065989
SC35	GD31	CS2	*R.nitidus*	-0.020714	0.064952
SC44	GD31	CS2	*R.nitidus*	-0.051699	0.066165
SC14	GD31	CS2	*R.nitidus*	-0.055197	0.06607
JO1	GD31	CS2	*R.nitidus*	-0.090318	0.066285
SC24	GD31	CS2	*R.nitidus*	-0.051524	0.067428
MY6	GD31	CS2	*R.nitidus*	-0.053598	0.066875
JO7	GD32	CS2	*R.nitidus*	-0.051227	0.075712
JW7	GD31	CS2	*R.nitidus*	-0.06765	0.072355
CS11	GD32	CS2	*R.nitidus*	-0.038192	0.070478
JO8	GD32	CS2	*R.nitidus*	0.004454	0.077074
GD22	GD32	CS2	*R.nitidus*	0.04741	0.066457
MY4	GD31	CS2	*R.nitidus*	-0.091317	0.068412
CS12	GD32	CS2	*R.nitidus*	-0.019737	0.07026
JO5	GD31	CS2	*R.nitidus*	-0.022496	0.067549
GD21	GD32	CS2	*R.nitidus*	0.078622	0.065608
SC42	GD31	CS2	*R.nitidus*	0.184019	0.064447
GD41	GD32	CS2	*R.nitidus*	-0.033718	0.069381
GD26	GD32	CS2	*R.nitidus*	0.004484	0.064814
CS6	GD31	CS2	*R.nitidus*	-0.091867	0.068547
JW4	GD31	CS2	*R.nitidus*	0.154799	0.0694
GD30	GD32	CS2	*R.nitidus*	0.14	0.071238
GD29	GD32	CS2	*R.nitidus*	0.105171	0.066313
GD25	GD32	CS2	*R.nitidus*	0.03339	0.068556
GD45	GD32	CS2	*R.nitidus*	0.05307	0.07248
GD44	GD32	CS2	*R.nitidus*	0.00361	0.069807
GD48	GD32	CS2	*R.nitidus*	0.014451	0.066733
CS16	GD32	CS2	*R.nitidus*	-0.045082	0.069669
GD31	GD32	CS2	*R.nitidus*	0.01	0.070513
GD34	GD32	CS2	*R.nitidus*	-0.025328	0.065972
GD42	GD32	CS2	*R.nitidus*	-0.016173	0.069685
GD46	GD32	CS2	*R.nitidus*	-0.076991	0.067346
CS14	GD32	CS2	*R.nitidus*	-0.03745	0.071414
CS19	GD32	CS2	*R.nitidus*	-0.034865	0.068801
GD33	GD32	CS2	*R.nitidus*	-0.023279	0.0689
CS17	GD32	CS2	*R.nitidus*	-0.006656	0.072756
CS15	GD32	CS2	*R.nitidus*	-0.019033	0.069682
SC5	GD32	CS2	*R.nitidus*	0.000824	0.073149
GD40	GD32	CS2	*R.nitidus*	0.025126	0.066272
GD35	GD32	CS2	*R.nitidus*	0.008658	0.067883
CS3	GD32	CS2	*R.nitidus*	-0.06449	0.069006
SC6	GD32	CS2	*R.nitidus*	-0.037766	0.069409
GD50	GD32	CS2	*R.nitidus*	0.012766	0.072236
GD49	GD32	CS2	*R.nitidus*	0.076591	0.072052
CS13	GD32	CS2	*R.nitidus*	-0.064956	0.069672
CS18	GD32	CS2	*R.nitidus*	-0.063442	0.069586
CS23	GD32	CS2	*R.nitidus*	-0.085938	0.068974
CS20	GD32	CS2	*R.nitidus*	-0.063391	0.068501

SC31	GD32	CS2	*R.nitidus*	-0.025581	0.069551
SC24	GD32	CS2	*R.nitidus*	-0.023064	0.067973
SC14	GD32	CS2	*R.nitidus*	-0.014423	0.067934
SC18	GD32	CS2	*R.nitidus*	0.023529	0.068452
CS24	GD32	CS2	*R.nitidus*	-0.07055	0.067396
SC34	GD32	CS2	*R.nitidus*	-0.01014	0.06697
SC28	GD32	CS2	*R.nitidus*	-0.052075	0.067226
CS30	GD32	CS2	*R.nitidus*	-0.084967	0.070639
SC32	GD32	CS2	*R.nitidus*	0.006478	0.068619
MY6	GD32	CS2	*R.nitidus*	-0.025903	0.069458
JW7	GD32	CS2	*R.nitidus*	0.00455	0.07218
CS21	GD32	CS2	*R.nitidus*	-0.03867	0.070242
SC15	GD32	CS2	*R.nitidus*	-0.030885	0.069062
JW6	GD32	CS2	*R.nitidus*	-0.010246	0.073934
CS22	GD32	CS2	*R.nitidus*	-0.025472	0.066563
CS4	GD32	CS2	*R.nitidus*	-0.053055	0.066828
CS28	GD32	CS2	*R.nitidus*	-0.048495	0.069275
CS25	GD32	CS2	*R.nitidus*	-0.039872	0.071038
SC26	GD32	CS2	*R.nitidus*	-0.048348	0.067922
CS31	GD32	CS2	*R.nitidus*	-0.048822	0.070736
SC10	GD32	CS2	*R.nitidus*	0.02818	0.068929
SC42	GD32	CS2	*R.nitidus*	0.196347	0.065496
SC45	GD32	CS2	*R.nitidus*	0.00471	0.067448
JO7	CS12	CS2	*R.nitidus*	-0.036597	0.072897
SC12	GD32	CS2	*R.nitidus*	-0.046032	0.068274
JO1	GD32	CS2	*R.nitidus*	-0.053241	0.069284
SC27	GD32	CS2	*R.nitidus*	-0.013503	0.068529
GD31	CS12	CS2	*R.nitidus*	0.044343	0.068288
CS6	GD32	CS2	*R.nitidus*	-0.083056	0.068771
MY4	GD32	CS2	*R.nitidus*	-0.071545	0.071343
SC44	GD32	CS2	*R.nitidus*	-0.015139	0.067607
JO3	GD32	CS2	*R.nitidus*	0.01106	0.070776
SC35	GD32	CS2	*R.nitidus*	0.030806	0.06811
JW4	GD32	CS2	*R.nitidus*	0.199679	0.068907
GD22	CS12	CS2	*R.nitidus*	0.057281	0.067429
JO5	GD32	CS2	*R.nitidus*	0.041129	0.067868
GD26	CS12	CS2	*R.nitidus*	0.039445	0.067725
GD42	CS12	CS2	*R.nitidus*	0.018574	0.067816
GD33	CS12	CS2	*R.nitidus*	0	0.067959
GD30	CS12	CS2	*R.nitidus*	0.126065	0.071773
JO8	CS12	CS2	*R.nitidus*	-0.003099	0.07515
GD29	CS12	CS2	*R.nitidus*	0.109944	0.065196
GD45	CS12	CS2	*R.nitidus*	0.075758	0.070331
GD21	CS12	CS2	*R.nitidus*	0.121727	0.067331
GD46	CS12	CS2	*R.nitidus*	-0.02568	0.067527
GD35	CS12	CS2	*R.nitidus*	0.045487	0.067392
GD49	CS12	CS2	*R.nitidus*	0.088333	0.070739
GD50	CS12	CS2	*R.nitidus*	0.025619	0.071235
CS11	CS12	CS2	*R.nitidus*	-0.017708	0.07097
CS18	CS12	CS2	*R.nitidus*	-0.028861	0.06642
GD25	CS12	CS2	*R.nitidus*	0.081853	0.06786
GD40	CS12	CS2	*R.nitidus*	0.053468	0.066837
GD41	CS12	CS2	*R.nitidus*	0.007885	0.06561
CS15	CS12	CS2	*R.nitidus*	0.011025	0.065329
CS13	CS12	CS2	*R.nitidus*	-0.077754	0.071603
GD32	CS12	CS2	*R.nitidus*	0.019737	0.07026

GD44	CS12	CS2	*R.nitidus*	0.025424	0.069469
GD34	CS12	CS2	*R.nitidus*	-0.005	0.066397
CS3	CS12	CS2	*R.nitidus*	-0.021094	0.066378
CS14	CS12	CS2	*R.nitidus*	-0.017007	0.072288
CS31	CS12	CS2	*R.nitidus*	-0.038336	0.067898
CS19	CS12	CS2	*R.nitidus*	-0.011227	0.06978
GD48	CS12	CS2	*R.nitidus*	0.043887	0.068924
SC12	CS12	CS2	*R.nitidus*	-0.021358	0.068281
CS28	CS12	CS2	*R.nitidus*	-0.026667	0.068269
CS22	CS12	CS2	*R.nitidus*	-0.003962	0.066906
CS21	CS12	CS2	*R.nitidus*	-0.028396	0.065757
SC32	CS12	CS2	*R.nitidus*	0.020031	0.068976
SC5	CS12	CS2	*R.nitidus*	0.009804	0.067907
SC15	CS12	CS2	*R.nitidus*	-0.028962	0.066802
SC18	CS12	CS2	*R.nitidus*	0.052632	0.068415
CS30	CS12	CS2	*R.nitidus*	-0.075889	0.065588
SC14	CS12	CS2	*R.nitidus*	-0.007463	0.06784
CS17	CS12	CS2	*R.nitidus*	0.020431	0.070683
SC10	CS12	CS2	*R.nitidus*	0.06993	0.068683
CS16	CS12	CS2	*R.nitidus*	-0.020482	0.065331
CS20	CS12	CS2	*R.nitidus*	-0.029839	0.067388
CS25	CS12	CS2	*R.nitidus*	-0.007081	0.067938
SC44	CS12	CS2	*R.nitidus*	-0.003808	0.066656
JW6	CS12	CS2	*R.nitidus*	-0.025415	0.073469
CS23	CS12	CS2	*R.nitidus*	-0.04943	0.066133
CS24	CS12	CS2	*R.nitidus*	-0.05	0.066241
SC31	CS12	CS2	*R.nitidus*	-0.009782	0.068158
SC27	CS12	CS2	*R.nitidus*	-0.004677	0.065848
CS4	CS12	CS2	*R.nitidus*	-0.029096	0.066025
MY6	CS12	CS2	*R.nitidus*	-0.009608	0.067158
SC42	CS12	CS2	*R.nitidus*	0.237299	0.064474
JW4	CS12	CS2	*R.nitidus*	0.203148	0.06848
CS6	CS12	CS2	*R.nitidus*	-0.055336	0.065549
SC6	CS12	CS2	*R.nitidus*	-0.031056	0.069944
SC35	CS12	CS2	*R.nitidus*	0.039648	0.065209
MY4	CS12	CS2	*R.nitidus*	-0.054753	0.069213
SC28	CS12	CS2	*R.nitidus*	-0.059052	0.065932
SC24	CS12	CS2	*R.nitidus*	0.007616	0.067693
SC26	CS12	CS2	*R.nitidus*	-0.037606	0.066767
SC34	CS12	CS2	*R.nitidus*	0.01749	0.066721
CS11	GD33	CS2	*R.nitidus*	-0.019568	0.06791
GD32	GD33	CS2	*R.nitidus*	0.023279	0.0689
SC45	CS12	CS2	*R.nitidus*	0.031155	0.067142
JO1	CS12	CS2	*R.nitidus*	-0.02902	0.066408
GD40	GD33	CS2	*R.nitidus*	0.043578	0.063534
GD34	GD33	CS2	*R.nitidus*	-0.028818	0.064081
JO5	CS12	CS2	*R.nitidus*	0.037838	0.065963
GD22	GD33	CS2	*R.nitidus*	0.058577	0.064579
JW7	CS12	CS2	*R.nitidus*	-0.001867	0.072104
JO7	GD33	CS2	*R.nitidus*	-0.045412	0.073658
GD30	GD33	CS2	*R.nitidus*	0.118881	0.067447
JO8	GD33	CS2	*R.nitidus*	0	0.07293
GD35	GD33	CS2	*R.nitidus*	0.036925	0.064212
JO3	CS12	CS2	*R.nitidus*	0.014238	0.070351
GD31	GD33	CS2	*R.nitidus*	0.031128	0.068688
GD21	GD33	CS2	*R.nitidus*	0.128487	0.063456

GD26	GD33	CS2	*R.nitidus*	0.045815	0.066475
GD29	GD33	CS2	*R.nitidus*	0.12741	0.064005
CS19	GD33	CS2	*R.nitidus*	-0.021245	0.068802
GD45	GD33	CS2	*R.nitidus*	0.074208	0.06951
GD46	GD33	CS2	*R.nitidus*	-0.049759	0.06413
GD48	GD33	CS2	*R.nitidus*	0.023744	0.068485
CS23	GD33	CS2	*R.nitidus*	-0.063351	0.065633
CS30	GD33	CS2	*R.nitidus*	-0.079799	0.065631
GD49	GD33	CS2	*R.nitidus*	0.064457	0.070505
GD25	GD33	CS2	*R.nitidus*	0.067692	0.067978
GD44	GD33	CS2	*R.nitidus*	-0.004658	0.066781
SC5	GD33	CS2	*R.nitidus*	0	0.070956
CS28	GD33	CS2	*R.nitidus*	-0.040089	0.065166
GD42	GD33	CS2	*R.nitidus*	0.013136	0.067178
CS20	GD33	CS2	*R.nitidus*	-0.050659	0.067486
CS17	GD33	CS2	*R.nitidus*	0.003647	0.0689
CS24	GD33	CS2	*R.nitidus*	-0.046276	0.06765
CS12	GD33	CS2	*R.nitidus*	0	0.067959
CS31	GD33	CS2	*R.nitidus*	-0.031838	0.066921
GD41	GD33	CS2	*R.nitidus*	-0.001584	0.066919
GD50	GD33	CS2	*R.nitidus*	0.004794	0.07203
CS21	GD33	CS2	*R.nitidus*	-0.032258	0.066883
SC6	GD33	CS2	*R.nitidus*	-0.030921	0.066992
CS18	GD33	CS2	*R.nitidus*	-0.043103	0.065774
CS13	GD33	CS2	*R.nitidus*	-0.056747	0.067181
CS6	GD33	CS2	*R.nitidus*	-0.064011	0.066522
CS3	GD33	CS2	*R.nitidus*	-0.042857	0.066987
SC18	GD33	CS2	*R.nitidus*	0.014717	0.066262
CS15	GD33	CS2	*R.nitidus*	-0.00684	0.067941
CS16	GD33	CS2	*R.nitidus*	-0.01782	0.065916
CS14	GD33	CS2	*R.nitidus*	-0.017993	0.068002
SC27	GD33	CS2	*R.nitidus*	-0.015753	0.06604
SC28	GD33	CS2	*R.nitidus*	-0.051712	0.065631
SC14	GD33	CS2	*R.nitidus*	-0.010176	0.066502
CS4	GD33	CS2	*R.nitidus*	-0.026893	0.06577
CS25	GD33	CS2	*R.nitidus*	-0.029775	0.068129
JW6	GD33	CS2	*R.nitidus*	-0.002793	0.072503
CS22	GD33	CS2	*R.nitidus*	-0.00417	0.066861
SC10	GD33	CS2	*R.nitidus*	0.035966	0.068852
JW7	GD33	CS2	*R.nitidus*	0.00823	0.070101
SC26	GD33	CS2	*R.nitidus*	-0.050526	0.065915
SC15	GD33	CS2	*R.nitidus*	-0.028694	0.06518
SC42	GD33	CS2	*R.nitidus*	0.209994	0.06492
SC45	GD33	CS2	*R.nitidus*	0.024879	0.067293
SC31	GD33	CS2	*R.nitidus*	-0.029248	0.06842
JO3	GD33	CS2	*R.nitidus*	0.02188	0.069708
MY4	GD33	CS2	*R.nitidus*	-0.050385	0.066217
JO7	GD34	CS2	*R.nitidus*	-0.026022	0.070487
SC12	GD33	CS2	*R.nitidus*	-0.033779	0.066525
MY6	GD33	CS2	*R.nitidus*	-0.009466	0.06604
JO8	GD34	CS2	*R.nitidus*	0.037428	0.070897
SC44	GD33	CS2	*R.nitidus*	-0.026167	0.065613
SC24	GD33	CS2	*R.nitidus*	-0.018195	0.066132
SC34	GD33	CS2	*R.nitidus*	0.00823	0.063982
SC32	GD33	CS2	*R.nitidus*	0.006935	0.065989
GD25	GD34	CS2	*R.nitidus*	0.073004	0.066479

GD30	GD34	CS2	*R.nitidus*	0.157128	0.063908
SC35	GD33	CS2	*R.nitidus*	0.013477	0.065866
GD31	GD34	CS2	*R.nitidus*	0.045234	0.068341
GD44	GD34	CS2	*R.nitidus*	0.012461	0.068067
GD29	GD34	CS2	*R.nitidus*	0.15471	0.063324
GD22	GD34	CS2	*R.nitidus*	0.086844	0.061682
JO5	GD33	CS2	*R.nitidus*	0.035714	0.065773
JW4	GD33	CS2	*R.nitidus*	0.218571	0.068153
CS11	GD34	CS2	*R.nitidus*	-0.008632	0.066476
CS13	GD34	CS2	*R.nitidus*	-0.050169	0.067346
GD42	GD34	CS2	*R.nitidus*	0.018993	0.063901
JO1	GD33	CS2	*R.nitidus*	-0.042877	0.066481
GD21	GD34	CS2	*R.nitidus*	0.137386	0.062036
CS14	GD34	CS2	*R.nitidus*	-0.012129	0.066147
CS17	GD34	CS2	*R.nitidus*	0.008602	0.067716
GD26	GD34	CS2	*R.nitidus*	0.057003	0.064438
GD32	GD34	CS2	*R.nitidus*	0.025328	0.065972
GD33	GD34	CS2	*R.nitidus*	0.028818	0.064081
GD49	GD34	CS2	*R.nitidus*	0.086304	0.068479
CS21	GD34	CS2	*R.nitidus*	-0.021394	0.066208
CS24	GD34	CS2	*R.nitidus*	-0.042878	0.066665
CS15	GD34	CS2	*R.nitidus*	0.008707	0.066632
GD50	GD34	CS2	*R.nitidus*	0.019774	0.069997
CS30	GD34	CS2	*R.nitidus*	-0.071791	0.064919
GD48	GD34	CS2	*R.nitidus*	0.051724	0.065795
CS28	GD34	CS2	*R.nitidus*	-0.034328	0.066258
CS12	GD34	CS2	*R.nitidus*	0.005	0.066397
GD40	GD34	CS2	*R.nitidus*	0.065838	0.063662
GD45	GD34	CS2	*R.nitidus*	0.081923	0.067772
CS18	GD34	CS2	*R.nitidus*	-0.026931	0.066122
CS4	GD34	CS2	*R.nitidus*	-0.01385	0.06438
CS25	GD34	CS2	*R.nitidus*	-0.020423	0.06636
SC5	GD34	CS2	*R.nitidus*	0.002152	0.069247
GD35	GD34	CS2	*R.nitidus*	0.047473	0.065872
SC12	GD34	CS2	*R.nitidus*	-0.028926	0.066227
GD41	GD34	CS2	*R.nitidus*	0.007276	0.064982
CS31	GD34	CS2	*R.nitidus*	-0.01997	0.067092
CS16	GD34	CS2	*R.nitidus*	-0.016173	0.064939
CS22	GD34	CS2	*R.nitidus*	-0.004149	0.065657
GD46	GD34	CS2	*R.nitidus*	-0.030794	0.062695
SC34	GD34	CS2	*R.nitidus*	0.020548	0.065067
SC15	GD34	CS2	*R.nitidus*	-0.03196	0.066197
CS19	GD34	CS2	*R.nitidus*	-0.010885	0.067051
CS3	GD34	CS2	*R.nitidus*	-0.02964	0.067707
SC10	GD34	CS2	*R.nitidus*	0.034188	0.066964
SC42	GD34	CS2	*R.nitidus*	0.230505	0.063559
SC14	GD34	CS2	*R.nitidus*	-0.005495	0.065829
SC18	GD34	CS2	*R.nitidus*	0.023827	0.06554
CS20	GD34	CS2	*R.nitidus*	-0.044108	0.066684
SC26	GD34	CS2	*R.nitidus*	-0.042509	0.063655
SC28	GD34	CS2	*R.nitidus*	-0.053509	0.0638
CS23	GD34	CS2	*R.nitidus*	-0.059233	0.064174
SC44	GD34	CS2	*R.nitidus*	-0.013669	0.065492
SC31	GD34	CS2	*R.nitidus*	-0.013231	0.066009
CS6	GD34	CS2	*R.nitidus*	-0.05057	0.066158
SC45	GD34	CS2	*R.nitidus*	0.021247	0.065573

SC6	GD34	CS2	*R.nitidus*	-0.025105	0.066343
SC24	GD34	CS2	*R.nitidus*	-0.007179	0.065931
JO3	GD34	CS2	*R.nitidus*	0.032336	0.06921
JO8	GD35	CS2	*R.nitidus*	-0.036398	0.07296
JO5	GD34	CS2	*R.nitidus*	0.036755	0.064265
JW7	GD34	CS2	*R.nitidus*	0.032046	0.070747
MY4	GD34	CS2	*R.nitidus*	-0.046316	0.065663
SC35	GD34	CS2	*R.nitidus*	0.029944	0.064832
JO7	GD35	CS2	*R.nitidus*	-0.056818	0.073415
SC27	GD34	CS2	*R.nitidus*	-0.013167	0.066168
GD25	GD35	CS2	*R.nitidus*	0.030757	0.063867
SC32	GD34	CS2	*R.nitidus*	0.011708	0.064796
JW4	GD34	CS2	*R.nitidus*	0.21512	0.066259
JW6	GD34	CS2	*R.nitidus*	0.012613	0.069832
MY6	GD34	CS2	*R.nitidus*	-0.00885	0.065527
GD21	GD35	CS2	*R.nitidus*	0.075182	0.06376
CS12	GD35	CS2	*R.nitidus*	-0.045487	0.067392
GD33	GD35	CS2	*R.nitidus*	-0.036925	0.064212
JO1	GD34	CS2	*R.nitidus*	-0.043331	0.065107
CS13	GD35	CS2	*R.nitidus*	-0.077857	0.066318
GD26	GD35	CS2	*R.nitidus*	0.006163	0.066954
GD50	GD35	CS2	*R.nitidus*	-0.031946	0.068835
GD34	GD35	CS2	*R.nitidus*	-0.047473	0.065872
GD29	GD35	CS2	*R.nitidus*	0.068149	0.064953
GD45	GD35	CS2	*R.nitidus*	0.050442	0.067169
CS19	GD35	CS2	*R.nitidus*	-0.046392	0.067713
GD30	GD35	CS2	*R.nitidus*	0.103066	0.068263
CS11	GD35	CS2	*R.nitidus*	-0.041172	0.066522
GD22	GD35	CS2	*R.nitidus*	0.017366	0.065348
CS17	GD35	CS2	*R.nitidus*	-0.026846	0.068656
CS16	GD35	CS2	*R.nitidus*	-0.04795	0.065474
GD44	GD35	CS2	*R.nitidus*	-0.02492	0.067002
GD32	GD35	CS2	*R.nitidus*	-0.008658	0.067883
GD49	GD35	CS2	*R.nitidus*	0.050719	0.069683
CS14	GD35	CS2	*R.nitidus*	-0.044492	0.068802
CS20	GD35	CS2	*R.nitidus*	-0.076759	0.067499
GD46	GD35	CS2	*R.nitidus*	-0.083141	0.063613
GD42	GD35	CS2	*R.nitidus*	-0.021514	0.066096
GD48	GD35	CS2	*R.nitidus*	-0.011735	0.06757
CS24	GD35	CS2	*R.nitidus*	-0.078113	0.065805
GD40	GD35	CS2	*R.nitidus*	0.015994	0.063555
GD41	GD35	CS2	*R.nitidus*	-0.036462	0.065211
CS22	GD35	CS2	*R.nitidus*	-0.031513	0.067182
CS15	GD35	CS2	*R.nitidus*	-0.024238	0.067341
GD31	GD35	CS2	*R.nitidus*	0.00077	0.066559
SC12	GD35	CS2	*R.nitidus*	-0.067321	0.066064
CS4	GD35	CS2	*R.nitidus*	-0.04965	0.065519
SC32	GD35	CS2	*R.nitidus*	-0.012579	0.064442
SC5	GD35	CS2	*R.nitidus*	-0.033577	0.070909
CS18	GD35	CS2	*R.nitidus*	-0.064982	0.066509
CS30	GD35	CS2	*R.nitidus*	-0.096522	0.06748
CS28	GD35	CS2	*R.nitidus*	-0.070423	0.06755
SC14	GD35	CS2	*R.nitidus*	-0.033381	0.066736
SC31	GD35	CS2	*R.nitidus*	-0.049391	0.064733
CS21	GD35	CS2	*R.nitidus*	-0.061813	0.066311
CS25	GD35	CS2	*R.nitidus*	-0.050396	0.066317

SC34	GD35	CS2	*R.nitidus*	-0.028691	0.065302
CS6	GD35	CS2	*R.nitidus*	-0.097031	0.066201
SC27	GD35	CS2	*R.nitidus*	-0.052971	0.064522
SC28	GD35	CS2	*R.nitidus*	-0.080758	0.065705
CS31	GD35	CS2	*R.nitidus*	-0.068503	0.068466
CS23	GD35	CS2	*R.nitidus*	-0.094101	0.065002
SC15	GD35	CS2	*R.nitidus*	-0.057604	0.068384
SC24	GD35	CS2	*R.nitidus*	-0.049229	0.065266
JW4	GD35	CS2	*R.nitidus*	0.181429	0.06615
SC10	GD35	CS2	*R.nitidus*	0.012204	0.066775
SC35	GD35	CS2	*R.nitidus*	-0.018692	0.065689
CS3	GD35	CS2	*R.nitidus*	-0.053495	0.064533
JO8	GD40	CS2	*R.nitidus*	-0.059109	0.072067
JW6	GD35	CS2	*R.nitidus*	-0.051756	0.072699
SC6	GD35	CS2	*R.nitidus*	-0.059733	0.064102
SC18	GD35	CS2	*R.nitidus*	-0.019505	0.066931
MY4	GD35	CS2	*R.nitidus*	-0.093772	0.067396
SC26	GD35	CS2	*R.nitidus*	-0.073691	0.064086
SC42	GD35	CS2	*R.nitidus*	0.20194	0.064836
JO5	GD35	CS2	*R.nitidus*	-0.004947	0.06585
JO1	GD35	CS2	*R.nitidus*	-0.065835	0.06449
GD30	GD40	CS2	*R.nitidus*	0.076323	0.065476
CS11	GD40	CS2	*R.nitidus*	-0.061695	0.065455
SC44	GD35	CS2	*R.nitidus*	-0.052822	0.066223
JW7	GD35	CS2	*R.nitidus*	-0.031356	0.070611
GD41	GD40	CS2	*R.nitidus*	-0.049401	0.06418
GD22	GD40	CS2	*R.nitidus*	0.000699	0.064229
SC45	GD35	CS2	*R.nitidus*	-0.008984	0.065503
GD32	GD40	CS2	*R.nitidus*	-0.025126	0.066272
GD31	GD40	CS2	*R.nitidus*	-0.018561	0.066347
GD26	GD40	CS2	*R.nitidus*	-0.017686	0.062963
GD50	GD40	CS2	*R.nitidus*	-0.031001	0.068854
JO7	GD40	CS2	*R.nitidus*	-0.0831	0.071899
GD21	GD40	CS2	*R.nitidus*	0.066996	0.064569
MY6	GD35	CS2	*R.nitidus*	-0.049512	0.066652
GD34	GD40	CS2	*R.nitidus*	-0.065838	0.063662
GD44	GD40	CS2	*R.nitidus*	-0.052221	0.065042
GD42	GD40	CS2	*R.nitidus*	-0.035633	0.064757
GD29	GD40	CS2	*R.nitidus*	0.064748	0.061765
GD35	GD40	CS2	*R.nitidus*	-0.015994	0.063555
CS17	GD40	CS2	*R.nitidus*	-0.048708	0.067142
GD49	GD40	CS2	*R.nitidus*	0.026205	0.068071
CS25	GD40	CS2	*R.nitidus*	-0.076277	0.066549
CS12	GD40	CS2	*R.nitidus*	-0.053468	0.066837
CS16	GD40	CS2	*R.nitidus*	-0.073529	0.065716
GD46	GD40	CS2	*R.nitidus*	-0.087613	0.0623
GD33	GD40	CS2	*R.nitidus*	-0.043578	0.063534
GD45	GD40	CS2	*R.nitidus*	0.016835	0.067161
SC12	GD40	CS2	*R.nitidus*	-0.093243	0.064545
SC15	GD40	CS2	*R.nitidus*	-0.094767	0.064362
CS21	GD40	CS2	*R.nitidus*	-0.091794	0.064524
SC28	GD40	CS2	*R.nitidus*	-0.108815	0.063928
CS30	GD40	CS2	*R.nitidus*	-0.121574	0.062722
CS20	GD40	CS2	*R.nitidus*	-0.097787	0.066429
GD48	GD40	CS2	*R.nitidus*	-0.022599	0.066651
CS19	GD40	CS2	*R.nitidus*	-0.082073	0.066163

SC6	GD40	CS2	*R.nitidus*	-0.085915	0.06265
CS31	GD40	CS2	*R.nitidus*	-0.090077	0.066777
CS4	GD40	CS2	*R.nitidus*	-0.083916	0.06333
CS28	GD40	CS2	*R.nitidus*	-0.075347	0.064537
CS14	GD40	CS2	*R.nitidus*	-0.05514	0.067989
CS15	GD40	CS2	*R.nitidus*	-0.044098	0.066269
SC32	GD40	CS2	*R.nitidus*	-0.046322	0.063695
CS13	GD40	CS2	*R.nitidus*	-0.103833	0.066583
SC18	GD40	CS2	*R.nitidus*	-0.031295	0.063106
SC10	GD40	CS2	*R.nitidus*	-0.008386	0.066524
CS18	GD40	CS2	*R.nitidus*	-0.099783	0.065079
SC26	GD40	CS2	*R.nitidus*	-0.098428	0.063208
SC34	GD40	CS2	*R.nitidus*	-0.040328	0.064702
SC24	GD40	CS2	*R.nitidus*	-0.061107	0.064775
JW6	GD40	CS2	*R.nitidus*	-0.071429	0.070567
SC5	GD40	CS2	*R.nitidus*	-0.063326	0.067267
SC31	GD40	CS2	*R.nitidus*	-0.075625	0.063619
CS22	GD40	CS2	*R.nitidus*	-0.060942	0.063763
CS24	GD40	CS2	*R.nitidus*	-0.108206	0.06399
CS6	GD40	CS2	*R.nitidus*	-0.114576	0.065779
CS3	GD40	CS2	*R.nitidus*	-0.083333	0.064404
MY4	GD40	CS2	*R.nitidus*	-0.111567	0.064869
JW4	GD40	CS2	*R.nitidus*	0.150412	0.06589
JO3	GD40	CS2	*R.nitidus*	-0.018239	0.069746
JO1	GD40	CS2	*R.nitidus*	-0.093878	0.065426
MY6	GD40	CS2	*R.nitidus*	-0.063003	0.064425
CS23	GD40	CS2	*R.nitidus*	-0.110879	0.064724
SC14	GD40	CS2	*R.nitidus*	-0.065807	0.064824
SC27	GD40	CS2	*R.nitidus*	-0.07206	0.063513
GD22	GD41	CS2	*R.nitidus*	0.072713	0.06427
JO8	GD41	CS2	*R.nitidus*	0.005877	0.072613
GD25	GD41	CS2	*R.nitidus*	0.05492	0.066438
SC42	GD40	CS2	*R.nitidus*	0.172055	0.062956
JO7	GD41	CS2	*R.nitidus*	-0.005808	0.073201
JW7	GD40	CS2	*R.nitidus*	-0.055601	0.069851
SC45	GD40	CS2	*R.nitidus*	-0.038488	0.063525
SC35	GD40	CS2	*R.nitidus*	-0.024782	0.062196
GD21	GD41	CS2	*R.nitidus*	0.111862	0.063885
SC44	GD40	CS2	*R.nitidus*	-0.068327	0.063767
GD49	GD41	CS2	*R.nitidus*	0.093382	0.068812
GD33	GD41	CS2	*R.nitidus*	0.001584	0.066919
CS11	GD41	CS2	*R.nitidus*	-0.017265	0.065656
GD42	GD41	CS2	*R.nitidus*	0.029106	0.063287
GD31	GD41	CS2	*R.nitidus*	0.035427	0.067127
CS12	GD41	CS2	*R.nitidus*	-0.007885	0.06561
GD26	GD41	CS2	*R.nitidus*	0.046995	0.06595
GD44	GD41	CS2	*R.nitidus*	0.010806	0.067894
CS16	GD41	CS2	*R.nitidus*	-0.014851	0.063557
GD29	GD41	CS2	*R.nitidus*	0.116705	0.063987
GD45	GD41	CS2	*R.nitidus*	0.059299	0.068608
CS15	GD41	CS2	*R.nitidus*	0.008984	0.06621
GD46	GD41	CS2	*R.nitidus*	-0.053773	0.0628
JO5	GD40	CS2	*R.nitidus*	-0.019755	0.063923
CS21	GD41	CS2	*R.nitidus*	-0.033063	0.063586
GD50	GD41	CS2	*R.nitidus*	0.02229	0.069674
CS14	GD41	CS2	*R.nitidus*	-0.011822	0.066855

GD30	GD41	CS2	*R.nitidus*	0.111508	0.06537
CS25	GD41	CS2	*R.nitidus*	-0.020776	0.066966
CS17	GD41	CS2	*R.nitidus*	-0.000732	0.068593
CS18	GD41	CS2	*R.nitidus*	-0.031812	0.065206
GD32	GD41	CS2	*R.nitidus*	0.033718	0.069381
CS19	GD41	CS2	*R.nitidus*	-0.017569	0.066852
GD35	GD41	CS2	*R.nitidus*	0.036462	0.065211
CS13	GD41	CS2	*R.nitidus*	-0.054417	0.066401
GD40	GD41	CS2	*R.nitidus*	0.049401	0.06418
GD34	GD41	CS2	*R.nitidus*	-0.007276	0.064982
GD48	GD41	CS2	*R.nitidus*	0.05662	0.067143
SC18	GD41	CS2	*R.nitidus*	0.012696	0.066303
SC15	GD41	CS2	*R.nitidus*	-0.027347	0.066196
CS4	GD41	CS2	*R.nitidus*	-0.022663	0.063699
CS20	GD41	CS2	*R.nitidus*	-0.050069	0.066696
CS30	GD41	CS2	*R.nitidus*	-0.085312	0.064975
CS31	GD41	CS2	*R.nitidus*	-0.035928	0.065462
SC10	GD41	CS2	*R.nitidus*	0.035533	0.06623
JW7	GD41	CS2	*R.nitidus*	0.007772	0.0702
CS24	GD41	CS2	*R.nitidus*	-0.0522	0.064643
SC14	GD41	CS2	*R.nitidus*	-0.019466	0.064059
MY6	GD41	CS2	*R.nitidus*	-0.002786	0.063623
CS3	GD41	CS2	*R.nitidus*	-0.042391	0.065221
CS23	GD41	CS2	*R.nitidus*	-0.061084	0.06456
SC26	GD41	CS2	*R.nitidus*	-0.047751	0.067574
SC27	GD41	CS2	*R.nitidus*	-0.025751	0.064641
CS22	GD41	CS2	*R.nitidus*	-0.011963	0.064412
CS28	GD41	CS2	*R.nitidus*	-0.031061	0.067096
SC42	GD41	CS2	*R.nitidus*	0.226307	0.062498
JO7	GD42	CS2	*R.nitidus*	-0.078629	0.074051
SC35	GD41	CS2	*R.nitidus*	0.023448	0.064482
SC5	GD41	CS2	*R.nitidus*	-0.005789	0.068868
SC6	GD41	CS2	*R.nitidus*	-0.030346	0.066529
JW4	GD41	CS2	*R.nitidus*	0.209104	0.064633
JO3	GD41	CS2	*R.nitidus*	0.029678	0.069089
SC45	GD41	CS2	*R.nitidus*	0.014006	0.065354
SC32	GD41	CS2	*R.nitidus*	0.004916	0.063759
SC12	GD41	CS2	*R.nitidus*	-0.026112	0.065072
CS6	GD41	CS2	*R.nitidus*	-0.060825	0.065948
SC24	GD41	CS2	*R.nitidus*	-0.021183	0.065299
JO1	GD41	CS2	*R.nitidus*	-0.035714	0.065083
SC31	GD41	CS2	*R.nitidus*	-0.016807	0.064847
MY4	GD41	CS2	*R.nitidus*	-0.048621	0.06419
SC28	GD41	CS2	*R.nitidus*	-0.061908	0.065389
SC34	GD41	CS2	*R.nitidus*	0.019149	0.064914
GD29	GD42	CS2	*R.nitidus*	0.103529	0.064844
GD26	GD42	CS2	*R.nitidus*	0.004823	0.067643
JW6	GD41	CS2	*R.nitidus*	-0.014409	0.071297
JO5	GD41	CS2	*R.nitidus*	0.039392	0.063798
CS11	GD42	CS2	*R.nitidus*	-0.039548	0.06824
JO8	GD42	CS2	*R.nitidus*	-0.039235	0.073482
GD31	GD42	CS2	*R.nitidus*	0.007494	0.067453
SC44	GD41	CS2	*R.nitidus*	-0.019882	0.064759
GD25	GD42	CS2	*R.nitidus*	0.044728	0.065635
GD22	GD42	CS2	*R.nitidus*	0.060995	0.064546
GD33	GD42	CS2	*R.nitidus*	-0.013136	0.067178

GD35	GD42	CS2	*R.nitidus*	0.021514	0.066096
GD40	GD42	CS2	*R.nitidus*	0.035633	0.064757
GD41	GD42	CS2	*R.nitidus*	-0.029106	0.063287
GD34	GD42	CS2	*R.nitidus*	-0.018993	0.063901
GD45	GD42	CS2	*R.nitidus*	0.060886	0.067766
GD44	GD42	CS2	*R.nitidus*	-0.010842	0.067793
GD21	GD42	CS2	*R.nitidus*	0.094192	0.0636
CS19	GD42	CS2	*R.nitidus*	-0.040433	0.067052
CS12	GD42	CS2	*R.nitidus*	-0.018574	0.067816
CS14	GD42	CS2	*R.nitidus*	-0.045	0.068496
GD30	GD42	CS2	*R.nitidus*	0.119205	0.066317
CS16	GD42	CS2	*R.nitidus*	-0.041968	0.065783
CS25	GD42	CS2	*R.nitidus*	-0.043134	0.068178
GD50	GD42	CS2	*R.nitidus*	-0.015658	0.068392
CS17	GD42	CS2	*R.nitidus*	-0.01285	0.06958
GD32	GD42	CS2	*R.nitidus*	0.016173	0.069685
CS28	GD42	CS2	*R.nitidus*	-0.055982	0.068739
CS22	GD42	CS2	*R.nitidus*	-0.02805	0.065446
CS13	GD42	CS2	*R.nitidus*	-0.070815	0.067624
CS31	GD42	CS2	*R.nitidus*	-0.047104	0.065958
SC15	GD42	CS2	*R.nitidus*	-0.06577	0.067154
CS18	GD42	CS2	*R.nitidus*	-0.059151	0.067072
GD48	GD42	CS2	*R.nitidus*	0.014506	0.066502
CS4	GD42	CS2	*R.nitidus*	-0.037141	0.063965
CS24	GD42	CS2	*R.nitidus*	-0.06927	0.06609
CS23	GD42	CS2	*R.nitidus*	-0.084028	0.066662
SC10	GD42	CS2	*R.nitidus*	0.025148	0.06874
SC14	GD42	CS2	*R.nitidus*	-0.020878	0.065301
GD46	GD42	CS2	*R.nitidus*	-0.07016	0.062999
CS15	GD42	CS2	*R.nitidus*	-0.025568	0.067404
SC5	GD42	CS2	*R.nitidus*	-0.025566	0.068379
SC12	GD42	CS2	*R.nitidus*	-0.053725	0.066033
SC26	GD42	CS2	*R.nitidus*	-0.063961	0.067879
CS6	GD42	CS2	*R.nitidus*	-0.080233	0.065756
SC24	GD42	CS2	*R.nitidus*	-0.031111	0.066205
CS3	GD42	CS2	*R.nitidus*	-0.056298	0.065373
CS20	GD42	CS2	*R.nitidus*	-0.063361	0.066354
CS21	GD42	CS2	*R.nitidus*	-0.058661	0.065158
SC27	GD42	CS2	*R.nitidus*	-0.033141	0.065637
SC28	GD42	CS2	*R.nitidus*	-0.078024	0.065461
JW4	GD42	CS2	*R.nitidus*	0.186246	0.065657
CS30	GD42	CS2	*R.nitidus*	-0.097965	0.065
SC18	GD42	CS2	*R.nitidus*	-0.011346	0.066641
SC45	GD42	CS2	*R.nitidus*	0.005658	0.065972
SC6	GD42	CS2	*R.nitidus*	-0.052669	0.067591
SC34	GD42	CS2	*R.nitidus*	0.003556	0.066121
GD22	GD44	CS2	*R.nitidus*	0.050835	0.066289
SC35	GD42	CS2	*R.nitidus*	0.003453	0.064731
JW7	GD42	CS2	*R.nitidus*	-0.043248	0.069395
GD31	GD44	CS2	*R.nitidus*	0.038494	0.068278
JO8	GD44	CS2	*R.nitidus*	-0.004921	0.073744
SC32	GD42	CS2	*R.nitidus*	-0.012839	0.064922
CS11	GD44	CS2	*R.nitidus*	-0.02439	0.069487
SC44	GD42	CS2	*R.nitidus*	-0.035982	0.066299
JO7	GD44	CS2	*R.nitidus*	-0.035475	0.073045
JW6	GD42	CS2	*R.nitidus*	-0.062562	0.07112

GD34	GD44	CS2	*R.nitidus*	-0.012461	0.068067
JO3	GD42	CS2	*R.nitidus*	0	0.069921
JO1	GD42	CS2	*R.nitidus*	-0.057103	0.065668
GD25	GD44	CS2	*R.nitidus*	0.062303	0.067826
SC42	GD42	CS2	*R.nitidus*	0.229044	0.062934
SC31	GD42	CS2	*R.nitidus*	-0.049408	0.0665
GD26	GD44	CS2	*R.nitidus*	0.023256	0.068829
MY6	GD42	CS2	*R.nitidus*	-0.023977	0.064888
MY4	GD42	CS2	*R.nitidus*	-0.069242	0.065547
GD30	GD44	CS2	*R.nitidus*	0.113309	0.069287
JO5	GD42	CS2	*R.nitidus*	0.015011	0.06552
CS14	GD44	CS2	*R.nitidus*	-0.022399	0.068793
GD42	GD44	CS2	*R.nitidus*	0.010842	0.067793
CS19	GD44	CS2	*R.nitidus*	-0.03092	0.067814
CS12	GD44	CS2	*R.nitidus*	-0.025424	0.069469
GD29	GD44	CS2	*R.nitidus*	0.117045	0.065908
GD45	GD44	CS2	*R.nitidus*	0.054183	0.070891
GD32	GD44	CS2	*R.nitidus*	-0.00361	0.069807
GD21	GD44	CS2	*R.nitidus*	0.129129	0.064367
CS15	GD44	CS2	*R.nitidus*	0.000719	0.067965
GD41	GD44	CS2	*R.nitidus*	-0.010806	0.067894
GD35	GD44	CS2	*R.nitidus*	0.02492	0.067002
GD50	GD44	CS2	*R.nitidus*	0.01508	0.07058
GD33	GD44	CS2	*R.nitidus*	0.004658	0.066781
CS3	GD44	CS2	*R.nitidus*	-0.036284	0.06548
GD46	GD44	CS2	*R.nitidus*	-0.046512	0.066363
GD49	GD44	CS2	*R.nitidus*	0.074336	0.069207
CS17	GD44	CS2	*R.nitidus*	0.004605	0.071001
SC18	GD44	CS2	*R.nitidus*	-0.000777	0.067574
CS25	GD44	CS2	*R.nitidus*	-0.040121	0.068397
CS20	GD44	CS2	*R.nitidus*	-0.056934	0.067685
GD40	GD44	CS2	*R.nitidus*	0.052221	0.065042
CS22	GD44	CS2	*R.nitidus*	-0.031179	0.06678
CS18	GD44	CS2	*R.nitidus*	-0.051073	0.067661
CS13	GD44	CS2	*R.nitidus*	-0.077844	0.069634
CS24	GD44	CS2	*R.nitidus*	-0.060372	0.068888
CS16	GD44	CS2	*R.nitidus*	-0.017634	0.066653
SC31	GD44	CS2	*R.nitidus*	-0.023628	0.067492
SC6	GD44	CS2	*R.nitidus*	-0.030008	0.066516
MY4	GD44	CS2	*R.nitidus*	-0.06213	0.068033
CS21	GD44	CS2	*R.nitidus*	-0.032904	0.066588
GD48	GD44	CS2	*R.nitidus*	0.038593	0.067103
SC14	GD44	CS2	*R.nitidus*	-0.026987	0.067724
SC28	GD44	CS2	*R.nitidus*	-0.076006	0.067627
CS31	GD44	CS2	*R.nitidus*	-0.044275	0.067192
CS28	GD44	CS2	*R.nitidus*	-0.037896	0.067837
CS23	GD44	CS2	*R.nitidus*	-0.066812	0.065915
JW7	GD44	CS2	*R.nitidus*	-0.008913	0.070685
SC10	GD44	CS2	*R.nitidus*	0.024425	0.066701
SC5	GD44	CS2	*R.nitidus*	-0.013793	0.071805
SC12	GD44	CS2	*R.nitidus*	-0.050883	0.066003
CS4	GD44	CS2	*R.nitidus*	-0.043834	0.065396
CS6	GD44	CS2	*R.nitidus*	-0.06682	0.067202
SC27	GD44	CS2	*R.nitidus*	-0.032738	0.067475
SC32	GD44	CS2	*R.nitidus*	0.011536	0.068271
SC24	GD44	CS2	*R.nitidus*	-0.030211	0.068811

SC26	GD44	CS2	*R.nitidus*	-0.055994	0.06669
SC42	GD44	CS2	*R.nitidus*	0.208103	0.063531
CS30	GD44	CS2	*R.nitidus*	-0.071588	0.067636
GD22	GD45	CS2	*R.nitidus*	0.020478	0.06868
SC35	GD44	CS2	*R.nitidus*	0.020789	0.066317
JW4	GD44	CS2	*R.nitidus*	0.178862	0.068474
CS11	GD45	CS2	*R.nitidus*	-0.085597	0.070485
SC15	GD44	CS2	*R.nitidus*	-0.029163	0.068322
SC45	GD44	CS2	*R.nitidus*	0.007246	0.066278
JO7	GD45	CS2	*R.nitidus*	-0.137255	0.075329
JW6	GD44	CS2	*R.nitidus*	-0.027077	0.07195
GD30	GD45	CS2	*R.nitidus*	0.048403	0.0694
MY6	GD44	CS2	*R.nitidus*	-0.018745	0.067232
JO1	GD44	CS2	*R.nitidus*	-0.041485	0.066439
JO5	GD44	CS2	*R.nitidus*	0.034783	0.065975
SC34	GD44	CS2	*R.nitidus*	0.017279	0.066848
CS12	GD45	CS2	*R.nitidus*	-0.075758	0.070331
GD21	GD45	CS2	*R.nitidus*	0.028959	0.069147
GD26	GD45	CS2	*R.nitidus*	-0.053309	0.070271
GD40	GD45	CS2	*R.nitidus*	-0.016835	0.067161
JO8	GD45	CS2	*R.nitidus*	-0.076647	0.078242
GD29	GD45	CS2	*R.nitidus*	0.03273	0.067855
SC44	GD44	CS2	*R.nitidus*	-0.025641	0.066981
JO3	GD44	CS2	*R.nitidus*	0.004129	0.071703
GD25	GD45	CS2	*R.nitidus*	-0.04344	0.070262
GD35	GD45	CS2	*R.nitidus*	-0.050442	0.067169
GD32	GD45	CS2	*R.nitidus*	-0.05307	0.07248
CS18	GD45	CS2	*R.nitidus*	-0.102375	0.071612
CS23	GD45	CS2	*R.nitidus*	-0.098875	0.072883
CS19	GD45	CS2	*R.nitidus*	-0.085088	0.071931
GD42	GD45	CS2	*R.nitidus*	-0.060886	0.067766
GD50	GD45	CS2	*R.nitidus*	-0.052083	0.073178
GD49	GD45	CS2	*R.nitidus*	0.012257	0.073389
GD33	GD45	CS2	*R.nitidus*	-0.074208	0.06951
CS21	GD45	CS2	*R.nitidus*	-0.088997	0.069743
CS17	GD45	CS2	*R.nitidus*	-0.076115	0.070949
GD41	GD45	CS2	*R.nitidus*	-0.059299	0.068608
CS22	GD45	CS2	*R.nitidus*	-0.065436	0.06962
GD31	GD45	CS2	*R.nitidus*	-0.02952	0.069372
GD48	GD45	CS2	*R.nitidus*	-0.01494	0.071418
CS13	GD45	CS2	*R.nitidus*	-0.102349	0.070283
CS3	GD45	CS2	*R.nitidus*	-0.076355	0.070861
CS14	GD45	CS2	*R.nitidus*	-0.06929	0.073148
GD34	GD45	CS2	*R.nitidus*	-0.081923	0.067772
CS25	GD45	CS2	*R.nitidus*	-0.090448	0.073123
CS15	GD45	CS2	*R.nitidus*	-0.056114	0.07112
CS31	GD45	CS2	*R.nitidus*	-0.112931	0.071915
CS30	GD45	CS2	*R.nitidus*	-0.124155	0.069454
CS16	GD45	CS2	*R.nitidus*	-0.086192	0.071776
CS20	GD45	CS2	*R.nitidus*	-0.091058	0.071906
SC26	GD45	CS2	*R.nitidus*	-0.089342	0.068895
GD46	GD45	CS2	*R.nitidus*	-0.124411	0.06954
CS4	GD45	CS2	*R.nitidus*	-0.081566	0.068617
CS28	GD45	CS2	*R.nitidus*	-0.090372	0.069683
SC18	GD45	CS2	*R.nitidus*	-0.013525	0.070935
MY6	GD45	CS2	*R.nitidus*	-0.04898	0.069056

SC24	GD45	CS2	*R.nitidus*	-0.043265	0.069909
SC12	GD45	CS2	*R.nitidus*	-0.085241	0.069467
SC27	GD45	CS2	*R.nitidus*	-0.057799	0.069767
SC6	GD45	CS2	*R.nitidus*	-0.086608	0.071336
GD44	GD45	CS2	*R.nitidus*	-0.054183	0.070891
SC14	GD45	CS2	*R.nitidus*	-0.057891	0.070356
CS24	GD45	CS2	*R.nitidus*	-0.096965	0.069274
SC28	GD45	CS2	*R.nitidus*	-0.114727	0.068218
JO1	GD45	CS2	*R.nitidus*	-0.079653	0.069854
SC5	GD45	CS2	*R.nitidus*	-0.070683	0.072612
SC35	GD45	CS2	*R.nitidus*	-0.022293	0.069494
SC10	GD45	CS2	*R.nitidus*	-0.019769	0.070714
SC42	GD45	CS2	*R.nitidus*	0.186581	0.066369
JO3	GD45	CS2	*R.nitidus*	-0.045283	0.072868
SC15	GD45	CS2	*R.nitidus*	-0.070952	0.070012
SC32	GD45	CS2	*R.nitidus*	-0.024233	0.069815
JW7	GD45	CS2	*R.nitidus*	-0.082097	0.071741
SC34	GD45	CS2	*R.nitidus*	-0.034931	0.069231
JO7	CS13	CS2	*R.nitidus*	0.026946	0.073248
SC31	GD45	CS2	*R.nitidus*	-0.060748	0.069869
SC45	GD45	CS2	*R.nitidus*	-0.028759	0.068833
GD22	CS13	CS2	*R.nitidus*	0.111489	0.065844
SC44	GD45	CS2	*R.nitidus*	-0.061075	0.066894
GD25	CS13	CS2	*R.nitidus*	0.115782	0.067299
CS6	GD45	CS2	*R.nitidus*	-0.082529	0.070137
JO5	GD45	CS2	*R.nitidus*	-0.033031	0.069051
JW4	GD45	CS2	*R.nitidus*	0.135565	0.070197
GD45	CS13	CS2	*R.nitidus*	0.102349	0.070283
MY4	GD45	CS2	*R.nitidus*	-0.097087	0.069802
JW6	GD45	CS2	*R.nitidus*	-0.122318	0.073864
GD42	CS13	CS2	*R.nitidus*	0.070815	0.067624
GD26	CS13	CS2	*R.nitidus*	0.083747	0.067863
GD44	CS13	CS2	*R.nitidus*	0.077844	0.069634
GD41	CS13	CS2	*R.nitidus*	0.054417	0.066401
GD30	CS13	CS2	*R.nitidus*	0.149666	0.069695
CS11	CS13	CS2	*R.nitidus*	0.055675	0.073145
GD21	CS13	CS2	*R.nitidus*	0.150171	0.066045
GD31	CS13	CS2	*R.nitidus*	0.095808	0.068316
CS15	CS13	CS2	*R.nitidus*	0.080645	0.071695
JO8	CS13	CS2	*R.nitidus*	0.049949	0.076709
GD46	CS13	CS2	*R.nitidus*	0.033213	0.068253
CS16	CS13	CS2	*R.nitidus*	0.047619	0.069952
GD50	CS13	CS2	*R.nitidus*	0.064767	0.072329
GD35	CS13	CS2	*R.nitidus*	0.077857	0.066318
GD49	CS13	CS2	*R.nitidus*	0.101751	0.071239
GD29	CS13	CS2	*R.nitidus*	0.157191	0.066589
CS24	CS13	CS2	*R.nitidus*	0	0.067272
GD34	CS13	CS2	*R.nitidus*	0.050169	0.067346
GD32	CS13	CS2	*R.nitidus*	0.064956	0.069672
CS23	CS13	CS2	*R.nitidus*	-0.015957	0.067293
CS19	CS13	CS2	*R.nitidus*	0.027397	0.071598
CS12	CS13	CS2	*R.nitidus*	0.077754	0.071603
GD40	CS13	CS2	*R.nitidus*	0.103833	0.066583
CS21	CS13	CS2	*R.nitidus*	0.019505	0.068608
CS18	CS13	CS2	*R.nitidus*	0.014821	0.069447
CS28	CS13	CS2	*R.nitidus*	0.026961	0.069878

CS22	CS13	CS2	*R.nitidus*	0.059633	0.066961
GD33	CS13	CS2	*R.nitidus*	0.056747	0.067181
GD48	CS13	CS2	*R.nitidus*	0.085714	0.069562
CS30	CS13	CS2	*R.nitidus*	-0.028749	0.068259
CS3	CS13	CS2	*R.nitidus*	0.018141	0.068093
SC35	CS13	CS2	*R.nitidus*	0.08226	0.066668
JW6	CS13	CS2	*R.nitidus*	0.021526	0.076735
CS31	CS13	CS2	*R.nitidus*	0.01937	0.069566
SC42	CS13	CS2	*R.nitidus*	0.268765	0.062833
CS14	CS13	CS2	*R.nitidus*	0.057895	0.074495
CS17	CS13	CS2	*R.nitidus*	0.074576	0.07351
JW4	CS13	CS2	*R.nitidus*	0.257922	0.06689
CS20	CS13	CS2	*R.nitidus*	0.003674	0.069077
SC18	CS13	CS2	*R.nitidus*	0.078888	0.068596
JO3	CS13	CS2	*R.nitidus*	0.073432	0.072106
SC12	CS13	CS2	*R.nitidus*	0.028422	0.067862
SC26	CS13	CS2	*R.nitidus*	0.002256	0.066807
SC5	CS13	CS2	*R.nitidus*	0.034738	0.068345
CS25	CS13	CS2	*R.nitidus*	0.025542	0.071437
SC15	CS13	CS2	*R.nitidus*	0.023659	0.067763
SC10	CS13	CS2	*R.nitidus*	0.089231	0.068294
CS11	GD46	CS2	*R.nitidus*	0.01264	0.067153
CS4	CS13	CS2	*R.nitidus*	0.031963	0.066925
SC24	CS13	CS2	*R.nitidus*	0.048184	0.069303
SC32	CS13	CS2	*R.nitidus*	0.064391	0.070051
GD31	GD46	CS2	*R.nitidus*	0.069712	0.064727
SC34	CS13	CS2	*R.nitidus*	0.05878	0.067907
SC28	CS13	CS2	*R.nitidus*	-0.014925	0.067594
MY4	CS13	CS2	*R.nitidus*	-0.001535	0.068903
SC44	CS13	CS2	*R.nitidus*	0.026355	0.068402
JO7	GD46	CS2	*R.nitidus*	0.021359	0.07144
GD22	GD46	CS2	*R.nitidus*	0.112121	0.061316
SC14	CS13	CS2	*R.nitidus*	0.03663	0.067355
JO5	CS13	CS2	*R.nitidus*	0.087823	0.066856
SC31	CS13	CS2	*R.nitidus*	0.032023	0.067701
SC27	CS13	CS2	*R.nitidus*	0.040632	0.067714
GD35	GD46	CS2	*R.nitidus*	0.083141	0.063613
JW7	CS13	CS2	*R.nitidus*	0.059299	0.071512
JO8	GD46	CS2	*R.nitidus*	0.082164	0.069477
CS6	CS13	CS2	*R.nitidus*	-0.01098	0.069075
GD21	GD46	CS2	*R.nitidus*	0.175207	0.061807
MY6	CS13	CS2	*R.nitidus*	0.050633	0.068567
SC6	CS13	CS2	*R.nitidus*	0.02603	0.06911
SC45	CS13	CS2	*R.nitidus*	0.052513	0.068786
JO1	CS13	CS2	*R.nitidus*	0.02099	0.067814
GD29	GD46	CS2	*R.nitidus*	0.172999	0.06442
GD30	GD46	CS2	*R.nitidus*	0.184815	0.065607
GD34	GD46	CS2	*R.nitidus*	0.030794	0.062695
CS12	GD46	CS2	*R.nitidus*	0.02568	0.067527
GD25	GD46	CS2	*R.nitidus*	0.106677	0.065558
GD32	GD46	CS2	*R.nitidus*	0.076991	0.067346
GD48	GD46	CS2	*R.nitidus*	0.103664	0.065046
CS20	GD46	CS2	*R.nitidus*	-0.020964	0.066426
CS19	GD46	CS2	*R.nitidus*	0	0.06835
GD44	GD46	CS2	*R.nitidus*	0.046512	0.066363
GD50	GD46	CS2	*R.nitidus*	0.05888	0.068595

CS23	GD46	CS2	*R.nitidus*	-0.029054	0.064047
GD26	GD46	CS2	*R.nitidus*	0.082118	0.062094
CS14	GD46	CS2	*R.nitidus*	0.009545	0.069294
CS18	GD46	CS2	*R.nitidus*	-0.014778	0.066462
CS13	GD46	CS2	*R.nitidus*	-0.033213	0.068253
CS22	GD46	CS2	*R.nitidus*	0.021262	0.065615
GD33	GD46	CS2	*R.nitidus*	0.049759	0.06413
GD40	GD46	CS2	*R.nitidus*	0.087613	0.0623
SC6	GD46	CS2	*R.nitidus*	0.004332	0.065196
CS3	GD46	CS2	*R.nitidus*	0.007082	0.065318
CS17	GD46	CS2	*R.nitidus*	0.035633	0.069747
SC10	GD46	CS2	*R.nitidus*	0.061495	0.065756
GD41	GD46	CS2	*R.nitidus*	0.053773	0.0628
GD49	GD46	CS2	*R.nitidus*	0.114075	0.069011
GD42	GD46	CS2	*R.nitidus*	0.07016	0.062999
CS25	GD46	CS2	*R.nitidus*	0.005646	0.069473
GD45	GD46	CS2	*R.nitidus*	0.124411	0.06954
CS21	GD46	CS2	*R.nitidus*	-0.007612	0.065538
CS24	GD46	CS2	*R.nitidus*	-0.015419	0.065409
CS16	GD46	CS2	*R.nitidus*	0.010256	0.067319
SC14	GD46	CS2	*R.nitidus*	0.022551	0.065567
SC26	GD46	CS2	*R.nitidus*	-0.013755	0.064707
SC35	GD46	CS2	*R.nitidus*	0.060627	0.064294
CS30	GD46	CS2	*R.nitidus*	-0.040953	0.066092
SC31	GD46	CS2	*R.nitidus*	0.007333	0.064344
SC12	GD46	CS2	*R.nitidus*	-0.007757	0.065613
CS15	GD46	CS2	*R.nitidus*	0.031519	0.068006
SC28	GD46	CS2	*R.nitidus*	-0.03352	0.064564
CS31	GD46	CS2	*R.nitidus*	-0.00307	0.066777
CS28	GD46	CS2	*R.nitidus*	-0.003765	0.068018
JW4	GD46	CS2	*R.nitidus*	0.244986	0.066668
CS6	GD46	CS2	*R.nitidus*	-0.022513	0.065919
SC18	GD46	CS2	*R.nitidus*	0.068433	0.064822
JW6	GD46	CS2	*R.nitidus*	0.03445	0.070435
SC24	GD46	CS2	*R.nitidus*	0.025881	0.064936
SC45	GD46	CS2	*R.nitidus*	0.046961	0.066019
SC34	GD46	CS2	*R.nitidus*	0.047189	0.06404
SC5	GD46	CS2	*R.nitidus*	0.021186	0.067198
SC44	GD46	CS2	*R.nitidus*	0.006475	0.065395
JO1	GD46	CS2	*R.nitidus*	-0.01385	0.063949
CS4	GD46	CS2	*R.nitidus*	0.007623	0.064161
GD32	GD48	CS2	*R.nitidus*	-0.014451	0.066733
SC32	GD46	CS2	*R.nitidus*	0.036364	0.065861
JO7	GD48	CS2	*R.nitidus*	-0.04918	0.073274
SC15	GD46	CS2	*R.nitidus*	0.002963	0.066632
SC27	GD46	CS2	*R.nitidus*	0.011429	0.065806
MY4	GD46	CS2	*R.nitidus*	-0.017895	0.066279
JO3	GD46	CS2	*R.nitidus*	0.064435	0.067849
CS12	GD48	CS2	*R.nitidus*	-0.043887	0.068924
JO5	GD46	CS2	*R.nitidus*	0.061806	0.064632
JO8	GD48	CS2	*R.nitidus*	-0.031968	0.070254
GD34	GD48	CS2	*R.nitidus*	-0.051724	0.065795
MY6	GD46	CS2	*R.nitidus*	0.026001	0.065337
GD42	GD48	CS2	*R.nitidus*	-0.014506	0.066502
SC42	GD46	CS2	*R.nitidus*	0.238095	0.061992
GD50	GD48	CS2	*R.nitidus*	-0.042616	0.073755

GD33	GD48	CS2	*R.nitidus*	-0.023744	0.068485
GD21	GD48	CS2	*R.nitidus*	0.097293	0.066505
JW7	GD46	CS2	*R.nitidus*	0.034335	0.068455
CS11	GD48	CS2	*R.nitidus*	-0.049491	0.067967
CS17	GD48	CS2	*R.nitidus*	-0.041467	0.070812
GD49	GD48	CS2	*R.nitidus*	0.039961	0.071954
GD30	GD48	CS2	*R.nitidus*	0.060576	0.068622
GD45	GD48	CS2	*R.nitidus*	0.01494	0.071418
GD22	GD48	CS2	*R.nitidus*	0.03974	0.065383
GD29	GD48	CS2	*R.nitidus*	0.088797	0.064727
GD31	GD48	CS2	*R.nitidus*	-0.003581	0.069418
GD46	GD48	CS2	*R.nitidus*	-0.103664	0.065046
GD25	GD48	CS2	*R.nitidus*	0.026473	0.06624
GD35	GD48	CS2	*R.nitidus*	0.011735	0.06757
GD26	GD48	CS2	*R.nitidus*	0.02	0.067205
CS19	GD48	CS2	*R.nitidus*	-0.054816	0.068281
CS23	GD48	CS2	*R.nitidus*	-0.098496	0.068348
GD41	GD48	CS2	*R.nitidus*	-0.05662	0.067143
CS4	GD48	CS2	*R.nitidus*	-0.037206	0.065812
GD44	GD48	CS2	*R.nitidus*	-0.038593	0.067103
CS15	GD48	CS2	*R.nitidus*	-0.034226	0.069007
GD40	GD48	CS2	*R.nitidus*	0.022599	0.066651
SC24	GD48	CS2	*R.nitidus*	-0.025078	0.067858
CS31	GD48	CS2	*R.nitidus*	-0.061912	0.06763
CS24	GD48	CS2	*R.nitidus*	-0.068666	0.067144
CS22	GD48	CS2	*R.nitidus*	-0.036098	0.067964
CS21	GD48	CS2	*R.nitidus*	-0.069802	0.067012
SC31	GD48	CS2	*R.nitidus*	-0.052006	0.068846
SC15	GD48	CS2	*R.nitidus*	-0.066879	0.068002
CS13	GD48	CS2	*R.nitidus*	-0.085714	0.069562
CS20	GD48	CS2	*R.nitidus*	-0.071158	0.067011
CS30	GD48	CS2	*R.nitidus*	-0.094637	0.068639
SC10	GD48	CS2	*R.nitidus*	0.012772	0.068122
SC12	GD48	CS2	*R.nitidus*	-0.062823	0.068149
CS14	GD48	CS2	*R.nitidus*	-0.04858	0.069471
CS16	GD48	CS2	*R.nitidus*	-0.066517	0.066596
CS18	GD48	CS2	*R.nitidus*	-0.066066	0.068246
CS28	GD48	CS2	*R.nitidus*	-0.070388	0.068159
SC14	GD48	CS2	*R.nitidus*	-0.048872	0.067791
CS25	GD48	CS2	*R.nitidus*	-0.047843	0.070056
SC6	GD48	CS2	*R.nitidus*	-0.06141	0.067752
CS3	GD48	CS2	*R.nitidus*	-0.059322	0.06791
JW6	GD48	CS2	*R.nitidus*	-0.036027	0.071014
SC5	GD48	CS2	*R.nitidus*	-0.007075	0.071501
MY4	GD48	CS2	*R.nitidus*	-0.078387	0.069203
JO7	GD49	CS2	*R.nitidus*	-0.117376	0.074432
SC34	GD48	CS2	*R.nitidus*	-0.020974	0.065837
SC45	GD48	CS2	*R.nitidus*	-0.023686	0.066193
SC27	GD48	CS2	*R.nitidus*	-0.042392	0.066174
JO8	GD49	CS2	*R.nitidus*	-0.062574	0.07518
SC18	GD48	CS2	*R.nitidus*	0.00556	0.067918
SC44	GD48	CS2	*R.nitidus*	-0.045669	0.066301
JW4	GD48	CS2	*R.nitidus*	0.175258	0.068499
JO5	GD48	CS2	*R.nitidus*	0.018127	0.066625
GD32	GD49	CS2	*R.nitidus*	-0.076591	0.072052
SC26	GD48	CS2	*R.nitidus*	-0.081101	0.066814

SC28	GD48	CS2	*R.nitidus*	-0.079554	0.066506
JW7	GD48	CS2	*R.nitidus*	-0.017778	0.071148
GD29	GD49	CS2	*R.nitidus*	0.039126	0.068422
SC32	GD48	CS2	*R.nitidus*	-0.025738	0.067584
CS6	GD48	CS2	*R.nitidus*	-0.089506	0.067185
GD21	GD49	CS2	*R.nitidus*	0.024955	0.067797
GD25	GD49	CS2	*R.nitidus*	-0.012422	0.06844
SC42	GD48	CS2	*R.nitidus*	0.168035	0.064769
SC35	GD48	CS2	*R.nitidus*	0.005975	0.065778
GD45	GD49	CS2	*R.nitidus*	-0.012257	0.073389
GD34	GD49	CS2	*R.nitidus*	-0.086304	0.068479
GD22	GD49	CS2	*R.nitidus*	-0.035751	0.071
GD50	GD49	CS2	*R.nitidus*	-0.065076	0.07456
JO1	GD48	CS2	*R.nitidus*	-0.062266	0.068254
CS11	GD49	CS2	*R.nitidus*	-0.079114	0.07189
MY6	GD48	CS2	*R.nitidus*	-0.031655	0.067378
JO3	GD48	CS2	*R.nitidus*	-0.020942	0.070237
GD42	GD49	CS2	*R.nitidus*	-0.070501	0.068595
GD35	GD49	CS2	*R.nitidus*	-0.050719	0.069683
CS12	GD49	CS2	*R.nitidus*	-0.088333	0.070739
GD33	GD49	CS2	*R.nitidus*	-0.064457	0.070505
GD44	GD49	CS2	*R.nitidus*	-0.074336	0.069207
CS13	GD49	CS2	*R.nitidus*	-0.101751	0.071239
GD26	GD49	CS2	*R.nitidus*	-0.036969	0.067392
GD30	GD49	CS2	*R.nitidus*	0.079836	0.07247
CS19	GD49	CS2	*R.nitidus*	-0.072208	0.070226
CS15	GD49	CS2	*R.nitidus*	-0.048662	0.070434
GD31	GD49	CS2	*R.nitidus*	-0.051048	0.072282
CS14	GD49	CS2	*R.nitidus*	-0.064	0.072761
GD40	GD49	CS2	*R.nitidus*	-0.026205	0.068071
CS25	GD49	CS2	*R.nitidus*	-0.095477	0.073287
CS18	GD49	CS2	*R.nitidus*	-0.102917	0.069886
CS31	GD49	CS2	*R.nitidus*	-0.103302	0.071984
CS23	GD49	CS2	*R.nitidus*	-0.113655	0.068311
CS4	GD49	CS2	*R.nitidus*	-0.083201	0.066553
SC12	GD49	CS2	*R.nitidus*	-0.088997	0.066965
SC15	GD49	CS2	*R.nitidus*	-0.098856	0.068286
GD41	GD49	CS2	*R.nitidus*	-0.093382	0.068812
GD46	GD49	CS2	*R.nitidus*	-0.114075	0.069011
GD48	GD49	CS2	*R.nitidus*	-0.039961	0.071954
CS17	GD49	CS2	*R.nitidus*	-0.049372	0.07261
CS16	GD49	CS2	*R.nitidus*	-0.076061	0.070038
SC44	GD49	CS2	*R.nitidus*	-0.083542	0.068636
SC6	GD49	CS2	*R.nitidus*	-0.096878	0.069141
CS20	GD49	CS2	*R.nitidus*	-0.125997	0.069592
SC34	GD49	CS2	*R.nitidus*	-0.034073	0.066859
CS28	GD49	CS2	*R.nitidus*	-0.08969	0.071029
SC10	GD49	CS2	*R.nitidus*	-0.04095	0.07081
CS24	GD49	CS2	*R.nitidus*	-0.102819	0.068969
CS21	GD49	CS2	*R.nitidus*	-0.107553	0.067742
SC27	GD49	CS2	*R.nitidus*	-0.075277	0.068921
SC5	GD49	CS2	*R.nitidus*	-0.083748	0.072546
CS22	GD49	CS2	*R.nitidus*	-0.07074	0.0682
SC24	GD49	CS2	*R.nitidus*	-0.091362	0.068581
CS3	GD49	CS2	*R.nitidus*	-0.110927	0.068627
JO7	GD50	CS2	*R.nitidus*	-0.066376	0.07645

MY4	GD49	CS2	*R.nitidus*	-0.097502	0.070264
JO3	GD49	CS2	*R.nitidus*	-0.013793	0.073064
CS30	GD49	CS2	*R.nitidus*	-0.130544	0.069144
CS6	GD49	CS2	*R.nitidus*	-0.115905	0.068918
SC28	GD49	CS2	*R.nitidus*	-0.121332	0.067142
JO5	GD49	CS2	*R.nitidus*	-0.051345	0.067717
SC18	GD49	CS2	*R.nitidus*	-0.038698	0.069676
SC42	GD49	CS2	*R.nitidus*	0.172029	0.065996
JW6	GD49	CS2	*R.nitidus*	-0.096386	0.071521
JW7	GD49	CS2	*R.nitidus*	-0.032227	0.072223
GD26	GD50	CS2	*R.nitidus*	0.00947	0.0683
MY6	GD49	CS2	*R.nitidus*	-0.085714	0.069363
SC14	GD49	CS2	*R.nitidus*	-0.066298	0.069051
JO1	GD49	CS2	*R.nitidus*	-0.087981	0.068185
SC35	GD49	CS2	*R.nitidus*	-0.027712	0.067821
SC26	GD49	CS2	*R.nitidus*	-0.11901	0.069929
SC32	GD49	CS2	*R.nitidus*	-0.060365	0.067839
JO8	GD50	CS2	*R.nitidus*	-0.026876	0.075053
JW4	GD49	CS2	*R.nitidus*	0.155537	0.069989
SC31	GD49	CS2	*R.nitidus*	-0.103118	0.06874
GD29	GD50	CS2	*R.nitidus*	0.089981	0.067024
GD33	GD50	CS2	*R.nitidus*	-0.004794	0.07203
SC45	GD49	CS2	*R.nitidus*	-0.054276	0.068434
GD32	GD50	CS2	*R.nitidus*	-0.012766	0.072236
CS12	GD50	CS2	*R.nitidus*	-0.025619	0.071235
CS11	GD50	CS2	*R.nitidus*	-0.012542	0.070865
GD45	GD50	CS2	*R.nitidus*	0.052083	0.073178
GD22	GD50	CS2	*R.nitidus*	0.030132	0.069443
GD48	GD50	CS2	*R.nitidus*	0.042616	0.073755
GD44	GD50	CS2	*R.nitidus*	-0.01508	0.07058
GD25	GD50	CS2	*R.nitidus*	0.017874	0.069629
GD40	GD50	CS2	*R.nitidus*	0.031001	0.068854
CS15	GD50	CS2	*R.nitidus*	-0.008197	0.071347
GD21	GD50	CS2	*R.nitidus*	0.124195	0.068252
GD30	GD50	CS2	*R.nitidus*	0.103665	0.071096
GD42	GD50	CS2	*R.nitidus*	0.015658	0.068392
CS31	GD50	CS2	*R.nitidus*	-0.049002	0.069402
CS23	GD50	CS2	*R.nitidus*	-0.062765	0.069062
GD31	GD50	CS2	*R.nitidus*	0.002913	0.070469
GD49	GD50	CS2	*R.nitidus*	0.065076	0.07456
CS18	GD50	CS2	*R.nitidus*	-0.045068	0.069761
GD46	GD50	CS2	*R.nitidus*	-0.05888	0.068595
SC5	GD50	CS2	*R.nitidus*	-0.036972	0.074523
CS13	GD50	CS2	*R.nitidus*	-0.064767	0.072329
CS16	GD50	CS2	*R.nitidus*	-0.032389	0.069246
GD34	GD50	CS2	*R.nitidus*	-0.019774	0.069997
CS25	GD50	CS2	*R.nitidus*	-0.045572	0.070024
SC24	GD50	CS2	*R.nitidus*	-0.055085	0.068643
GD35	GD50	CS2	*R.nitidus*	0.031946	0.068835
CS24	GD50	CS2	*R.nitidus*	-0.031362	0.068465
GD41	GD50	CS2	*R.nitidus*	-0.02229	0.069674
CS4	GD50	CS2	*R.nitidus*	-0.039004	0.066408
CS22	GD50	CS2	*R.nitidus*	-0.016935	0.068749
SC26	GD50	CS2	*R.nitidus*	-0.065109	0.068939
CS28	GD50	CS2	*R.nitidus*	-0.075386	0.069805
SC31	GD50	CS2	*R.nitidus*	-0.02762	0.069182

SC15	GD50	CS2	*R.nitidus*	-0.038462	0.069836
CS30	GD50	CS2	*R.nitidus*	-0.086881	0.069174
CS17	GD50	CS2	*R.nitidus*	0.008696	0.071748
CS14	GD50	CS2	*R.nitidus*	-0.007595	0.071907
SC10	GD50	CS2	*R.nitidus*	0.007833	0.071758
SC32	GD50	CS2	*R.nitidus*	-0.01249	0.070307
CS21	GD50	CS2	*R.nitidus*	-0.048173	0.068808
CS19	GD50	CS2	*R.nitidus*	-0.040956	0.070669
SC18	GD50	CS2	*R.nitidus*	0	0.070043
CS20	GD50	CS2	*R.nitidus*	-0.05753	0.069799
JW4	GD50	CS2	*R.nitidus*	0.150473	0.070328
SC45	GD50	CS2	*R.nitidus*	-0.008489	0.069875
SC35	GD50	CS2	*R.nitidus*	-0.001679	0.067791
SC6	GD50	CS2	*R.nitidus*	-0.048593	0.067391
JO5	GD50	CS2	*R.nitidus*	0.001686	0.067852
SC42	GD50	CS2	*R.nitidus*	0.204451	0.066121
SC14	GD50	CS2	*R.nitidus*	-0.022425	0.067843
SC12	GD50	CS2	*R.nitidus*	-0.079125	0.068773
MY6	GD50	CS2	*R.nitidus*	-0.01638	0.067658
CS3	GD50	CS2	*R.nitidus*	-0.052676	0.06855
SC34	GD50	CS2	*R.nitidus*	0.015498	0.065987
SC28	GD50	CS2	*R.nitidus*	-0.089167	0.06754
JO3	GD50	CS2	*R.nitidus*	-0.026465	0.072575
CS6	GD50	CS2	*R.nitidus*	-0.068339	0.068747
MY4	GD50	CS2	*R.nitidus*	-0.074979	0.070046
SC27	GD50	CS2	*R.nitidus*	-0.049236	0.066654
SC44	GD50	CS2	*R.nitidus*	-0.043554	0.069147
JW6	GD50	CS2	*R.nitidus*	-0.063319	0.073915
JW7	GD50	CS2	*R.nitidus*	-0.037678	0.074507
JO7	CS14	CS2	*R.nitidus*	-0.029557	0.075482
CS12	CS14	CS2	*R.nitidus*	0.017007	0.072288
CS11	CS14	CS2	*R.nitidus*	-0.002398	0.070887
GD42	CS14	CS2	*R.nitidus*	0.045	0.068496
CS13	CS14	CS2	*R.nitidus*	-0.057895	0.074495
GD21	CS14	CS2	*R.nitidus*	0.129771	0.066691
GD44	CS14	CS2	*R.nitidus*	0.022399	0.068793
GD41	CS14	CS2	*R.nitidus*	0.011822	0.066855
GD30	CS14	CS2	*R.nitidus*	0.124481	0.072634
CS15	CS14	CS2	*R.nitidus*	0.03796	0.073013
JO8	CS14	CS2	*R.nitidus*	0.003	0.075491
GD26	CS14	CS2	*R.nitidus*	0.05784	0.067987
JO1	GD50	CS2	*R.nitidus*	-0.072925	0.06929
GD33	CS14	CS2	*R.nitidus*	0.017993	0.068002
GD50	CS14	CS2	*R.nitidus*	0.007595	0.071907
GD29	CS14	CS2	*R.nitidus*	0.126406	0.067302
GD32	CS14	CS2	*R.nitidus*	0.03745	0.071414
GD40	CS14	CS2	*R.nitidus*	0.05514	0.067989
GD46	CS14	CS2	*R.nitidus*	-0.009545	0.069294
GD35	CS14	CS2	*R.nitidus*	0.044492	0.068802
GD34	CS14	CS2	*R.nitidus*	0.012129	0.066147
GD45	CS14	CS2	*R.nitidus*	0.06929	0.073148
GD25	CS14	CS2	*R.nitidus*	0.086672	0.068927
GD22	CS14	CS2	*R.nitidus*	0.067541	0.06711
GD49	CS14	CS2	*R.nitidus*	0.064	0.072761
CS21	CS14	CS2	*R.nitidus*	-0.033133	0.067774
GD31	CS14	CS2	*R.nitidus*	0.054114	0.069716

CS20	CS14	CS2	*R.nitidus*	-0.03639	0.068841
GD48	CS14	CS2	*R.nitidus*	0.04858	0.069471
SC6	CS14	CS2	*R.nitidus*	-0.01487	0.069177
CS24	CS14	CS2	*R.nitidus*	-0.043165	0.068007
CS22	CS14	CS2	*R.nitidus*	-0.001522	0.06761
SC10	CS14	CS2	*R.nitidus*	0.048048	0.067904
SC42	CS14	CS2	*R.nitidus*	0.224525	0.063732
CS31	CS14	CS2	*R.nitidus*	-0.027473	0.069729
CS16	CS14	CS2	*R.nitidus*	0.002265	0.070642
CS17	CS14	CS2	*R.nitidus*	0.033292	0.075955
CS25	CS14	CS2	*R.nitidus*	-0.017504	0.069659
CS6	CS14	CS2	*R.nitidus*	-0.065536	0.067374
CS18	CS14	CS2	*R.nitidus*	-0.037965	0.068725
CS19	CS14	CS2	*R.nitidus*	-0.016548	0.07175
MY4	CS14	CS2	*R.nitidus*	-0.043997	0.07061
SC35	CS14	CS2	*R.nitidus*	0.038924	0.066179
CS30	CS14	CS2	*R.nitidus*	-0.063359	0.068107
SC27	CS14	CS2	*R.nitidus*	-0.01766	0.06717
SC31	CS14	CS2	*R.nitidus*	-0.007801	0.067071
CS28	CS14	CS2	*R.nitidus*	-0.029157	0.06914
CS4	CS14	CS2	*R.nitidus*	-0.013971	0.067389
SC26	CS14	CS2	*R.nitidus*	-0.04496	0.067517
JO3	CS14	CS2	*R.nitidus*	0.027569	0.07164
CS3	CS14	CS2	*R.nitidus*	-0.028593	0.068176
JW4	CS14	CS2	*R.nitidus*	0.208879	0.068741
SC44	CS14	CS2	*R.nitidus*	-0.009407	0.068944
SC18	CS14	CS2	*R.nitidus*	0.025604	0.068605
JO8	CS15	CS2	*R.nitidus*	0.010732	0.073308
CS23	CS14	CS2	*R.nitidus*	-0.065185	0.067289
GD21	CS15	CS2	*R.nitidus*	0.106485	0.065902
SC12	CS14	CS2	*R.nitidus*	-0.021245	0.069805
SC5	CS14	CS2	*R.nitidus*	0.007153	0.069688
JW6	CS14	CS2	*R.nitidus*	-0.02381	0.075454
GD30	CS15	CS2	*R.nitidus*	0.105565	0.070312
SC34	CS14	CS2	*R.nitidus*	0.02393	0.068056
JO7	CS15	CS2	*R.nitidus*	-0.038241	0.072885
JW7	CS14	CS2	*R.nitidus*	0.000891	0.072968
SC14	CS14	CS2	*R.nitidus*	-0.009213	0.067781
MY6	CS14	CS2	*R.nitidus*	-0.006541	0.069099
SC15	CS14	CS2	*R.nitidus*	-0.025443	0.069524
CS11	CS15	CS2	*R.nitidus*	-0.031694	0.070186
SC24	CS14	CS2	*R.nitidus*	0.005839	0.0702
JO5	CS14	CS2	*R.nitidus*	0.038732	0.068508
JO1	CS14	CS2	*R.nitidus*	-0.023512	0.068551
SC32	CS14	CS2	*R.nitidus*	0.023379	0.070959
GD29	CS15	CS2	*R.nitidus*	0.102681	0.065809
SC28	CS14	CS2	*R.nitidus*	-0.047101	0.067742
GD49	CS15	CS2	*R.nitidus*	0.048662	0.070434
SC45	CS14	CS2	*R.nitidus*	0.020803	0.069562
GD25	CS15	CS2	*R.nitidus*	0.065562	0.066483
GD31	CS15	CS2	*R.nitidus*	0.042121	0.067349
GD22	CS15	CS2	*R.nitidus*	0.051813	0.066376
GD45	CS15	CS2	*R.nitidus*	0.056114	0.07112
GD33	CS15	CS2	*R.nitidus*	0.00684	0.067941
GD48	CS15	CS2	*R.nitidus*	0.034226	0.069007
GD34	CS15	CS2	*R.nitidus*	-0.008707	0.066632

GD26	CS15	CS2	*R.nitidus*	0.039917	0.067717
GD32	CS15	CS2	*R.nitidus*	0.019033	0.069682
SC5	CS15	CS2	*R.nitidus*	-0.008445	0.070951
CS12	CS15	CS2	*R.nitidus*	-0.011025	0.065329
SC12	CS15	CS2	*R.nitidus*	-0.039046	0.068591
GD40	CS15	CS2	*R.nitidus*	0.044098	0.066269
CS20	CS15	CS2	*R.nitidus*	-0.052106	0.067777
CS18	CS15	CS2	*R.nitidus*	-0.052671	0.068185
GD35	CS15	CS2	*R.nitidus*	0.024238	0.067341
GD50	CS15	CS2	*R.nitidus*	0.008197	0.071347
CS4	CS15	CS2	*R.nitidus*	-0.024982	0.066318
CS14	CS15	CS2	*R.nitidus*	-0.03796	0.073013
CS21	CS15	CS2	*R.nitidus*	-0.044669	0.066546
SC31	CS15	CS2	*R.nitidus*	-0.027836	0.066215
CS17	CS15	CS2	*R.nitidus*	-0.018307	0.069565
CS13	CS15	CS2	*R.nitidus*	-0.080645	0.071695
GD42	CS15	CS2	*R.nitidus*	0.025568	0.067404
CS16	CS15	CS2	*R.nitidus*	-0.036286	0.066561
CS30	CS15	CS2	*R.nitidus*	-0.079699	0.067202
CS19	CS15	CS2	*R.nitidus*	-0.037206	0.069428
CS22	CS15	CS2	*R.nitidus*	-0.013423	0.066655
GD41	CS15	CS2	*R.nitidus*	-0.008984	0.06621
GD46	CS15	CS2	*R.nitidus*	-0.031519	0.068006
CS28	CS15	CS2	*R.nitidus*	-0.032437	0.068392
CS31	CS15	CS2	*R.nitidus*	-0.03577	0.06768
SC27	CS15	CS2	*R.nitidus*	-0.031387	0.06596
SC24	CS15	CS2	*R.nitidus*	-0.015173	0.066734
GD44	CS15	CS2	*R.nitidus*	-0.000719	0.067965
SC10	CS15	CS2	*R.nitidus*	0.02939	0.06753
SC15	CS15	CS2	*R.nitidus*	-0.035472	0.067867
CS23	CS15	CS2	*R.nitidus*	-0.081618	0.066775
MY4	CS15	CS2	*R.nitidus*	-0.062184	0.06813
CS25	CS15	CS2	*R.nitidus*	-0.03238	0.070182
SC32	CS15	CS2	*R.nitidus*	0.005743	0.068032
JW6	CS15	CS2	*R.nitidus*	-0.044007	0.073288
CS6	CS15	CS2	*R.nitidus*	-0.071158	0.067466
CS3	CS15	CS2	*R.nitidus*	-0.037834	0.068467
JW7	CS15	CS2	*R.nitidus*	-0.019828	0.071118
SC28	CS15	CS2	*R.nitidus*	-0.070087	0.066247
SC26	CS15	CS2	*R.nitidus*	-0.054506	0.065166
SC6	CS15	CS2	*R.nitidus*	-0.035101	0.066989
JW4	CS15	CS2	*R.nitidus*	0.186488	0.068464
MY6	CS15	CS2	*R.nitidus*	-0.017204	0.067599
SC14	CS15	CS2	*R.nitidus*	-0.028633	0.068172
SC34	CS15	CS2	*R.nitidus*	0.011404	0.06644
JO1	CS15	CS2	*R.nitidus*	-0.033898	0.065492
SC18	CS15	CS2	*R.nitidus*	0.030483	0.06667
CS24	CS15	CS2	*R.nitidus*	-0.060139	0.066505
JO7	CS16	CS2	*R.nitidus*	-0.026627	0.074417
CS11	CS16	CS2	*R.nitidus*	0.001058	0.070151
SC35	CS15	CS2	*R.nitidus*	0.024221	0.065536
JO5	CS15	CS2	*R.nitidus*	0.034752	0.067652
GD22	CS16	CS2	*R.nitidus*	0.070059	0.064014
SC44	CS15	CS2	*R.nitidus*	-0.020438	0.067509
SC42	CS15	CS2	*R.nitidus*	0.215777	0.062656
GD30	CS16	CS2	*R.nitidus*	0.120393	0.068418

SC45	CS15	CS2	*R.nitidus*	0.003546	0.068556
JO3	CS15	CS2	*R.nitidus*	0.013622	0.069073
GD25	CS16	CS2	*R.nitidus*	0.088041	0.067002
GD31	CS16	CS2	*R.nitidus*	0.050074	0.068087
JO8	CS16	CS2	*R.nitidus*	0.034908	0.074333
GD29	CS16	CS2	*R.nitidus*	0.129887	0.065623
GD40	CS16	CS2	*R.nitidus*	0.073529	0.065716
GD33	CS16	CS2	*R.nitidus*	0.01782	0.065916
GD32	CS16	CS2	*R.nitidus*	0.045082	0.069669
GD26	CS16	CS2	*R.nitidus*	0.054899	0.066068
CS17	CS16	CS2	*R.nitidus*	0.024887	0.07121
CS15	CS16	CS2	*R.nitidus*	0.036286	0.066561
GD45	CS16	CS2	*R.nitidus*	0.086192	0.071776
GD46	CS16	CS2	*R.nitidus*	-0.010256	0.067319
GD42	CS16	CS2	*R.nitidus*	0.041968	0.065783
GD50	CS16	CS2	*R.nitidus*	0.032389	0.069246
GD35	CS16	CS2	*R.nitidus*	0.04795	0.065474
CS19	CS16	CS2	*R.nitidus*	-0.001547	0.067986
GD21	CS16	CS2	*R.nitidus*	0.115464	0.064353
CS23	CS16	CS2	*R.nitidus*	-0.043059	0.065164
CS12	CS16	CS2	*R.nitidus*	0.020482	0.065331
CS20	CS16	CS2	*R.nitidus*	-0.024035	0.067399
GD34	CS16	CS2	*R.nitidus*	0.016173	0.064939
CS30	CS16	CS2	*R.nitidus*	-0.067732	0.064858
CS14	CS16	CS2	*R.nitidus*	-0.002265	0.070642
CS21	CS16	CS2	*R.nitidus*	-0.025055	0.066634
GD49	CS16	CS2	*R.nitidus*	0.076061	0.070038
CS22	CS16	CS2	*R.nitidus*	0.009119	0.067974
GD41	CS16	CS2	*R.nitidus*	0.014851	0.063557
GD48	CS16	CS2	*R.nitidus*	0.066517	0.066596
GD44	CS16	CS2	*R.nitidus*	0.017634	0.066653
CS13	CS16	CS2	*R.nitidus*	-0.047619	0.069952
SC12	CS16	CS2	*R.nitidus*	-0.021497	0.06656
SC10	CS16	CS2	*R.nitidus*	0.062682	0.067901
CS25	CS16	CS2	*R.nitidus*	-0.008863	0.068018
SC26	CS16	CS2	*R.nitidus*	-0.037091	0.064224
SC42	CS16	CS2	*R.nitidus*	0.242229	0.060678
CS18	CS16	CS2	*R.nitidus*	-0.014903	0.067153
CS3	CS16	CS2	*R.nitidus*	-0.015909	0.065764
CS24	CS16	CS2	*R.nitidus*	-0.039777	0.06609
SC35	CS16	CS2	*R.nitidus*	0.046165	0.06486
SC24	CS16	CS2	*R.nitidus*	0.009517	0.06596
SC45	CS16	CS2	*R.nitidus*	0.026354	0.066468
JO5	CS16	CS2	*R.nitidus*	0.053052	0.064121
SC6	CS16	CS2	*R.nitidus*	-0.017991	0.066708
SC5	CS16	CS2	*R.nitidus*	0.010174	0.07005
JO1	CS16	CS2	*R.nitidus*	-0.013443	0.065761
CS28	CS16	CS2	*R.nitidus*	-0.015176	0.066389
SC18	CS16	CS2	*R.nitidus*	0.041825	0.066682
SC32	CS16	CS2	*R.nitidus*	0.027612	0.067242
CS31	CS16	CS2	*R.nitidus*	-0.023923	0.067608
JW4	CS16	CS2	*R.nitidus*	0.227802	0.067558
JW7	CS16	CS2	*R.nitidus*	0.013761	0.071613
CS6	CS16	CS2	*R.nitidus*	-0.045348	0.066017
MY4	CS16	CS2	*R.nitidus*	-0.036664	0.068586
CS4	CS16	CS2	*R.nitidus*	-0.004435	0.066566

SC14	CS16	CS2	*R.nitidus*	-0.003644	0.066361
SC15	CS16	CS2	*R.nitidus*	-0.018913	0.067417
CS11	CS17	CS2	*R.nitidus*	-0.029006	0.070148
JW6	CS16	CS2	*R.nitidus*	-0.00289	0.07362
JO3	CS16	CS2	*R.nitidus*	0.037277	0.070413
SC27	CS16	CS2	*R.nitidus*	-0.002961	0.064922
SC28	CS16	CS2	*R.nitidus*	-0.056972	0.06554
MY6	CS16	CS2	*R.nitidus*	0.002878	0.066437
GD34	CS17	CS2	*R.nitidus*	-0.008602	0.067716
CS12	CS17	CS2	*R.nitidus*	-0.020431	0.070683
SC34	CS16	CS2	*R.nitidus*	0.02185	0.067316
SC31	CS16	CS2	*R.nitidus*	-0.019537	0.068198
JO7	CS17	CS2	*R.nitidus*	-0.021538	0.077487
JO8	CS17	CS2	*R.nitidus*	0.003191	0.076054
GD26	CS17	CS2	*R.nitidus*	0.022448	0.069435
SC44	CS16	CS2	*R.nitidus*	0	0.06589
GD21	CS17	CS2	*R.nitidus*	0.100072	0.066855
GD41	CS17	CS2	*R.nitidus*	0.000732	0.068593
CS14	CS17	CS2	*R.nitidus*	-0.033292	0.075955
GD50	CS17	CS2	*R.nitidus*	-0.008696	0.071748
GD31	CS17	CS2	*R.nitidus*	0.042636	0.070274
CS22	CS17	CS2	*R.nitidus*	-0.02	0.070056
CS13	CS17	CS2	*R.nitidus*	-0.074576	0.07351
GD35	CS17	CS2	*R.nitidus*	0.026846	0.068656
GD33	CS17	CS2	*R.nitidus*	-0.003647	0.0689
GD29	CS17	CS2	*R.nitidus*	0.099156	0.066678
GD22	CS17	CS2	*R.nitidus*	0.04577	0.06746
GD45	CS17	CS2	*R.nitidus*	0.076115	0.070949
GD30	CS17	CS2	*R.nitidus*	0.10408	0.070918
GD40	CS17	CS2	*R.nitidus*	0.048708	0.067142
GD25	CS17	CS2	*R.nitidus*	0.06089	0.068844
GD32	CS17	CS2	*R.nitidus*	0.006656	0.072756
CS15	CS17	CS2	*R.nitidus*	0.018307	0.069565
CS28	CS17	CS2	*R.nitidus*	-0.042642	0.069338
CS18	CS17	CS2	*R.nitidus*	-0.045008	0.069182
CS21	CS17	CS2	*R.nitidus*	-0.042037	0.069374
GD48	CS17	CS2	*R.nitidus*	0.041467	0.070812
GD46	CS17	CS2	*R.nitidus*	-0.035633	0.069747
SC6	CS17	CS2	*R.nitidus*	-0.033515	0.070127
GD44	CS17	CS2	*R.nitidus*	-0.004605	0.071001
CS4	CS17	CS2	*R.nitidus*	-0.030854	0.068562
CS23	CS17	CS2	*R.nitidus*	-0.070699	0.067625
GD42	CS17	CS2	*R.nitidus*	0.01285	0.06958
CS3	CS17	CS2	*R.nitidus*	-0.033898	0.0692
CS25	CS17	CS2	*R.nitidus*	-0.036304	0.072638
SC27	CS17	CS2	*R.nitidus*	-0.03974	0.067586
GD49	CS17	CS2	*R.nitidus*	0.049372	0.07261
SC12	CS17	CS2	*R.nitidus*	-0.03867	0.069788
SC10	CS17	CS2	*R.nitidus*	0.036135	0.069338
CS16	CS17	CS2	*R.nitidus*	-0.024887	0.07121
CS19	CS17	CS2	*R.nitidus*	-0.028854	0.070233
SC28	CS17	CS2	*R.nitidus*	-0.075851	0.066685
CS20	CS17	CS2	*R.nitidus*	-0.048069	0.069741
SC5	CS17	CS2	*R.nitidus*	-0.01926	0.070089
CS31	CS17	CS2	*R.nitidus*	-0.040782	0.06953
SC32	CS17	CS2	*R.nitidus*	0.019952	0.070229

CS24	CS17	CS2	*R.nitidus*	-0.070946	0.068717
SC15	CS17	CS2	*R.nitidus*	-0.042359	0.070855
SC18	CS17	CS2	*R.nitidus*	0.016241	0.069667
CS30	CS17	CS2	*R.nitidus*	-0.090476	0.068418
JW6	CS17	CS2	*R.nitidus*	-0.035106	0.075899
SC24	CS17	CS2	*R.nitidus*	-0.018853	0.069353
JW7	CS17	CS2	*R.nitidus*	-0.011472	0.072299
JW4	CS17	CS2	*R.nitidus*	0.197108	0.069529
SC14	CS17	CS2	*R.nitidus*	-0.016667	0.069917
SC45	CS17	CS2	*R.nitidus*	-0.003019	0.067411
SC42	CS17	CS2	*R.nitidus*	0.206642	0.064237
CS6	CS17	CS2	*R.nitidus*	-0.048061	0.069912
SC35	CS17	CS2	*R.nitidus*	0.018643	0.066684
SC34	CS17	CS2	*R.nitidus*	0.009962	0.069563
JO1	CS17	CS2	*R.nitidus*	-0.052632	0.068885
JO5	CS17	CS2	*R.nitidus*	0.004605	0.068196
JO8	CS18	CS2	*R.nitidus*	0.034126	0.073053
MY6	CS17	CS2	*R.nitidus*	-0.012763	0.069061
SC26	CS17	CS2	*R.nitidus*	-0.049728	0.068139
GD25	CS18	CS2	*R.nitidus*	0.102528	0.065147
GD41	CS18	CS2	*R.nitidus*	0.031812	0.065206
JO7	CS18	CS2	*R.nitidus*	0.011788	0.073407
GD22	CS18	CS2	*R.nitidus*	0.102389	0.065711
SC44	CS17	CS2	*R.nitidus*	-0.025842	0.067517
GD30	CS18	CS2	*R.nitidus*	0.146965	0.068607
MY4	CS17	CS2	*R.nitidus*	-0.054406	0.069199
GD32	CS18	CS2	*R.nitidus*	0.063442	0.069586
GD44	CS18	CS2	*R.nitidus*	0.051073	0.067661
SC31	CS17	CS2	*R.nitidus*	-0.036979	0.069998
CS11	CS18	CS2	*R.nitidus*	0.029257	0.068014
CS12	CS18	CS2	*R.nitidus*	0.028861	0.06642
GD21	CS18	CS2	*R.nitidus*	0.144	0.066405
JO3	CS17	CS2	*R.nitidus*	0.018325	0.072473
GD35	CS18	CS2	*R.nitidus*	0.064982	0.066509
GD34	CS18	CS2	*R.nitidus*	0.026931	0.066122
GD31	CS18	CS2	*R.nitidus*	0.084821	0.06881
GD29	CS18	CS2	*R.nitidus*	0.133378	0.064631
CS13	CS18	CS2	*R.nitidus*	-0.014821	0.069447
GD26	CS18	CS2	*R.nitidus*	0.082985	0.068061
GD50	CS18	CS2	*R.nitidus*	0.045068	0.069761
GD45	CS18	CS2	*R.nitidus*	0.102375	0.071612
GD40	CS18	CS2	*R.nitidus*	0.099783	0.065079
GD49	CS18	CS2	*R.nitidus*	0.102917	0.069886
GD33	CS18	CS2	*R.nitidus*	0.043103	0.065774
CS3	CS18	CS2	*R.nitidus*	0.003506	0.066938
CS15	CS18	CS2	*R.nitidus*	0.052671	0.068185
SC14	CS18	CS2	*R.nitidus*	0.015965	0.066304
GD42	CS18	CS2	*R.nitidus*	0.059151	0.067072
GD48	CS18	CS2	*R.nitidus*	0.066066	0.068246
SC6	CS18	CS2	*R.nitidus*	0.016442	0.067324
CS31	CS18	CS2	*R.nitidus*	0.003137	0.06636
CS21	CS18	CS2	*R.nitidus*	0.016569	0.063777
CS22	CS18	CS2	*R.nitidus*	0.038786	0.066478
CS24	CS18	CS2	*R.nitidus*	-0.019982	0.065609
CS28	CS18	CS2	*R.nitidus*	0.007171	0.067418
CS30	CS18	CS2	*R.nitidus*	-0.039106	0.064857

CS17	CS18	CS2	*R.nitidus*	0.045008	0.069182
CS14	CS18	CS2	*R.nitidus*	0.037965	0.068725
CS16	CS18	CS2	*R.nitidus*	0.014903	0.067153
SC24	CS18	CS2	*R.nitidus*	0.026198	0.065763
SC5	CS18	CS2	*R.nitidus*	0.03397	0.070362
CS19	CS18	CS2	*R.nitidus*	0.007874	0.06695
CS25	CS18	CS2	*R.nitidus*	0.020833	0.069463
SC15	CS18	CS2	*R.nitidus*	0.000793	0.06729
CS6	CS18	CS2	*R.nitidus*	-0.023452	0.064719
SC28	CS18	CS2	*R.nitidus*	-0.016105	0.066491
CS4	CS18	CS2	*R.nitidus*	0.015755	0.064269
SC26	CS18	CS2	*R.nitidus*	-0.01312	0.063666
CS23	CS18	CS2	*R.nitidus*	-0.02583	0.066304
SC34	CS18	CS2	*R.nitidus*	0.056976	0.064114
GD46	CS18	CS2	*R.nitidus*	0.014778	0.066462
SC44	CS18	CS2	*R.nitidus*	0.016899	0.065073
SC35	CS18	CS2	*R.nitidus*	0.067399	0.06256
CS20	CS18	CS2	*R.nitidus*	-0.007421	0.065994
SC12	CS18	CS2	*R.nitidus*	0.00224	0.065027
SC32	CS18	CS2	*R.nitidus*	0.053933	0.065107
SC10	CS18	CS2	*R.nitidus*	0.089871	0.066893
JW4	CS18	CS2	*R.nitidus*	0.247407	0.066662
JW7	CS18	CS2	*R.nitidus*	0.028169	0.070591
SC18	CS18	CS2	*R.nitidus*	0.071807	0.066788
JO3	CS18	CS2	*R.nitidus*	0.060017	0.068754
JW6	CS18	CS2	*R.nitidus*	0.010387	0.071395
GD26	CS19	CS2	*R.nitidus*	0.058659	0.069103
SC27	CS18	CS2	*R.nitidus*	0.024133	0.065722
JO1	CS18	CS2	*R.nitidus*	0.00366	0.066563
JO8	CS19	CS2	*R.nitidus*	0.039404	0.07263
JO7	CS19	CS2	*R.nitidus*	0.003956	0.076164
MY4	CS18	CS2	*R.nitidus*	-0.010526	0.068012
SC31	CS18	CS2	*R.nitidus*	0.014771	0.065824
GD22	CS19	CS2	*R.nitidus*	0.082069	0.068065
GD21	CS19	CS2	*R.nitidus*	0.122734	0.066712
SC42	CS18	CS2	*R.nitidus*	0.253722	0.063281
CS11	CS19	CS2	*R.nitidus*	0.021705	0.069261
GD25	CS19	CS2	*R.nitidus*	0.086169	0.065465
GD40	CS19	CS2	*R.nitidus*	0.082073	0.066163
SC45	CS18	CS2	*R.nitidus*	0.055514	0.065634
GD32	CS19	CS2	*R.nitidus*	0.034865	0.068801
GD45	CS19	CS2	*R.nitidus*	0.085088	0.071931
CS13	CS19	CS2	*R.nitidus*	-0.027397	0.071598
MY6	CS18	CS2	*R.nitidus*	0.029905	0.066638
GD31	CS19	CS2	*R.nitidus*	0.090977	0.069413
GD44	CS19	CS2	*R.nitidus*	0.03092	0.067814
GD30	CS19	CS2	*R.nitidus*	0.130471	0.070622
GD33	CS19	CS2	*R.nitidus*	0.021245	0.068802
CS12	CS19	CS2	*R.nitidus*	0.011227	0.06978
GD29	CS19	CS2	*R.nitidus*	0.122348	0.066002
GD42	CS19	CS2	*R.nitidus*	0.040433	0.067052
GD48	CS19	CS2	*R.nitidus*	0.054816	0.068281
GD50	CS19	CS2	*R.nitidus*	0.040956	0.070669
JO5	CS18	CS2	*R.nitidus*	0.089928	0.066234
GD35	CS19	CS2	*R.nitidus*	0.046392	0.067713
CS24	CS19	CS2	*R.nitidus*	-0.012059	0.069936

CS18	CS19	CS2	*R.nitidus*	-0.007874	0.06695
SC6	CS19	CS2	*R.nitidus*	0.005197	0.06879
CS25	CS19	CS2	*R.nitidus*	0.012422	0.071216
CS28	CS19	CS2	*R.nitidus*	-0.009717	0.069024
CS20	CS19	CS2	*R.nitidus*	-0.025907	0.069114
GD46	CS19	CS2	*R.nitidus*	0	0.06835
CS15	CS19	CS2	*R.nitidus*	0.037206	0.069428
CS14	CS19	CS2	*R.nitidus*	0.016548	0.07175
CS23	CS19	CS2	*R.nitidus*	-0.040434	0.066966
GD34	CS19	CS2	*R.nitidus*	0.010885	0.067051
CS21	CS19	CS2	*R.nitidus*	-0.006211	0.066242
GD49	CS19	CS2	*R.nitidus*	0.072208	0.070226
SC42	CS19	CS2	*R.nitidus*	0.252201	0.06394
SC28	CS19	CS2	*R.nitidus*	-0.020498	0.067062
SC24	CS19	CS2	*R.nitidus*	0.026174	0.068335
SC18	CS19	CS2	*R.nitidus*	0.06869	0.067534
CS16	CS19	CS2	*R.nitidus*	0.001547	0.067986
CS31	CS19	CS2	*R.nitidus*	-0.003252	0.069155
CS4	CS19	CS2	*R.nitidus*	0.008907	0.068553
SC14	CS19	CS2	*R.nitidus*	0.010463	0.0682
CS3	CS19	CS2	*R.nitidus*	-0.01049	0.066454
CS17	CS19	CS2	*R.nitidus*	0.028854	0.070233
SC27	CS19	CS2	*R.nitidus*	0.01435	0.068376
GD41	CS19	CS2	*R.nitidus*	0.017569	0.066852
SC31	CS19	CS2	*R.nitidus*	0.005128	0.067022
CS22	CS19	CS2	*R.nitidus*	0.021104	0.068522
JW4	CS19	CS2	*R.nitidus*	0.236842	0.068297
CS30	CS19	CS2	*R.nitidus*	-0.044071	0.067937
JO5	CS19	CS2	*R.nitidus*	0.066365	0.066191
SC32	CS19	CS2	*R.nitidus*	0.043177	0.068901
SC5	CS19	CS2	*R.nitidus*	0.019374	0.07158
SC45	CS19	CS2	*R.nitidus*	0.051433	0.06886
SC15	CS19	CS2	*R.nitidus*	0.004706	0.069316
MY4	CS19	CS2	*R.nitidus*	-0.025365	0.071179
CS6	CS19	CS2	*R.nitidus*	-0.037443	0.06671
SC12	CS19	CS2	*R.nitidus*	-0.000741	0.067002
SC44	CS19	CS2	*R.nitidus*	0.010558	0.06729
SC10	CS19	CS2	*R.nitidus*	0.063504	0.066256
JW6	CS19	CS2	*R.nitidus*	0.024096	0.074094
SC26	CS19	CS2	*R.nitidus*	-0.022861	0.066126
GD25	CS20	CS2	*R.nitidus*	0.113571	0.06814
JW7	CS19	CS2	*R.nitidus*	0.023111	0.070658
JO3	CS19	CS2	*R.nitidus*	0.048527	0.069781
SC34	CS19	CS2	*R.nitidus*	0.048727	0.067277
MY6	CS19	CS2	*R.nitidus*	0.022661	0.068955
JO1	CS19	CS2	*R.nitidus*	-0.010241	0.068199
GD45	CS20	CS2	*R.nitidus*	0.091058	0.071906
SC35	CS19	CS2	*R.nitidus*	0.065741	0.066395
GD32	CS20	CS2	*R.nitidus*	0.063391	0.068501
GD30	CS20	CS2	*R.nitidus*	0.15873	0.067778
GD34	CS20	CS2	*R.nitidus*	0.044108	0.066684
GD22	CS20	CS2	*R.nitidus*	0.111036	0.066412
GD35	CS20	CS2	*R.nitidus*	0.076759	0.067499
JO7	CS20	CS2	*R.nitidus*	0.007656	0.072725
JO8	CS20	CS2	*R.nitidus*	0.037075	0.073541
GD26	CS20	CS2	*R.nitidus*	0.086088	0.06841

GD41	CS20	CS2	*R.nitidus*	0.050069	0.066696
CS11	CS20	CS2	*R.nitidus*	0.035456	0.068105
GD40	CS20	CS2	*R.nitidus*	0.097787	0.066429
CS15	CS20	CS2	*R.nitidus*	0.052106	0.067777
GD29	CS20	CS2	*R.nitidus*	0.151031	0.064621
GD44	CS20	CS2	*R.nitidus*	0.056934	0.067685
CS16	CS20	CS2	*R.nitidus*	0.024035	0.067399
GD48	CS20	CS2	*R.nitidus*	0.071158	0.067011
GD46	CS20	CS2	*R.nitidus*	0.020964	0.066426
GD21	CS20	CS2	*R.nitidus*	0.152965	0.065688
GD42	CS20	CS2	*R.nitidus*	0.063361	0.066354
CS12	CS20	CS2	*R.nitidus*	0.029839	0.067388
CS25	CS20	CS2	*R.nitidus*	0.024957	0.068169
CS19	CS20	CS2	*R.nitidus*	0.025907	0.069114
CS21	CS20	CS2	*R.nitidus*	0.015489	0.064022
CS28	CS20	CS2	*R.nitidus*	-0.001554	0.068186
GD31	CS20	CS2	*R.nitidus*	0.093499	0.06749
GD49	CS20	CS2	*R.nitidus*	0.125997	0.069592
GD33	CS20	CS2	*R.nitidus*	0.050659	0.067486
CS24	CS20	CS2	*R.nitidus*	0.005573	0.067525
CS31	CS20	CS2	*R.nitidus*	0.004545	0.068063
GD50	CS20	CS2	*R.nitidus*	0.05753	0.069799
CS13	CS20	CS2	*R.nitidus*	-0.003674	0.069077
SC31	CS20	CS2	*R.nitidus*	0.022997	0.064813
CS18	CS20	CS2	*R.nitidus*	0.007421	0.065994
CS30	CS20	CS2	*R.nitidus*	-0.031037	0.066818
CS14	CS20	CS2	*R.nitidus*	0.03639	0.068841
CS3	CS20	CS2	*R.nitidus*	0.015658	0.061872
CS23	CS20	CS2	*R.nitidus*	-0.029676	0.066164
CS17	CS20	CS2	*R.nitidus*	0.048069	0.069741
SC6	CS20	CS2	*R.nitidus*	0.015849	0.064381
CS22	CS20	CS2	*R.nitidus*	0.0491	0.067553
JO3	CS20	CS2	*R.nitidus*	0.064298	0.067996
JW4	CS20	CS2	*R.nitidus*	0.24209	0.066869
JO8	CS21	CS2	*R.nitidus*	0.045045	0.071262
SC28	CS20	CS2	*R.nitidus*	-0.005785	0.06684
SC15	CS20	CS2	*R.nitidus*	0.006098	0.067874
JO7	CS21	CS2	*R.nitidus*	0.021884	0.072394
SC18	CS20	CS2	*R.nitidus*	0.078886	0.065793
SC35	CS20	CS2	*R.nitidus*	0.073239	0.063887
CS4	CS20	CS2	*R.nitidus*	0.031122	0.065474
SC5	CS20	CS2	*R.nitidus*	0.043822	0.068561
SC34	CS20	CS2	*R.nitidus*	0.0625	0.065688
SC27	CS20	CS2	*R.nitidus*	0.02886	0.065909
CS6	CS20	CS2	*R.nitidus*	-0.021472	0.063346
JW6	CS20	CS2	*R.nitidus*	-0.003683	0.073449
SC12	CS20	CS2	*R.nitidus*	0.013838	0.066693
SC10	CS20	CS2	*R.nitidus*	0.080119	0.066265
JO1	CS20	CS2	*R.nitidus*	0.004909	0.067026
SC24	CS20	CS2	*R.nitidus*	0.041199	0.065331
MY4	CS20	CS2	*R.nitidus*	-0.007267	0.068125
SC45	CS20	CS2	*R.nitidus*	0.062969	0.066105
SC14	CS20	CS2	*R.nitidus*	0.030714	0.065091
GD30	CS21	CS2	*R.nitidus*	0.16575	0.067197
SC26	CS20	CS2	*R.nitidus*	-0.009036	0.065567
SC44	CS20	CS2	*R.nitidus*	0.027516	0.066978

SC42	CS20	CS2	*R.nitidus*	0.257876	0.063148
SC32	CS20	CS2	*R.nitidus*	0.057971	0.065628
MY6	CS20	CS2	*R.nitidus*	0.030824	0.066781
GD32	CS21	CS2	*R.nitidus*	0.03867	0.070242
GD26	CS21	CS2	*R.nitidus*	0.067568	0.06756
JW7	CS20	CS2	*R.nitidus*	0.02535	0.069795
CS11	CS21	CS2	*R.nitidus*	0.024354	0.066578
JO5	CS20	CS2	*R.nitidus*	0.091549	0.065873
GD25	CS21	CS2	*R.nitidus*	0.105866	0.067233
GD31	CS21	CS2	*R.nitidus*	0.076867	0.067177
GD42	CS21	CS2	*R.nitidus*	0.058661	0.065158
GD22	CS21	CS2	*R.nitidus*	0.091984	0.06473
CS15	CS21	CS2	*R.nitidus*	0.044669	0.066546
GD46	CS21	CS2	*R.nitidus*	0.007612	0.065538
CS17	CS21	CS2	*R.nitidus*	0.042037	0.069374
GD48	CS21	CS2	*R.nitidus*	0.069802	0.067012
GD44	CS21	CS2	*R.nitidus*	0.032904	0.066588
CS12	CS21	CS2	*R.nitidus*	0.028396	0.065757
GD35	CS21	CS2	*R.nitidus*	0.061813	0.066311
CS14	CS21	CS2	*R.nitidus*	0.033133	0.067774
GD21	CS21	CS2	*R.nitidus*	0.135593	0.06467
GD29	CS21	CS2	*R.nitidus*	0.137633	0.063434
CS22	CS21	CS2	*R.nitidus*	0.032283	0.064936
CS3	CS21	CS2	*R.nitidus*	-0.002752	0.0657
GD45	CS21	CS2	*R.nitidus*	0.088997	0.069743
GD33	CS21	CS2	*R.nitidus*	0.032258	0.066883
CS19	CS21	CS2	*R.nitidus*	0.006211	0.066242
GD50	CS21	CS2	*R.nitidus*	0.048173	0.068808
CS13	CS21	CS2	*R.nitidus*	-0.019505	0.068608
CS16	CS21	CS2	*R.nitidus*	0.025055	0.066634
CS31	CS21	CS2	*R.nitidus*	-0.009878	0.066883
GD40	CS21	CS2	*R.nitidus*	0.091794	0.064524
CS25	CS21	CS2	*R.nitidus*	0.005226	0.068776
GD34	CS21	CS2	*R.nitidus*	0.021394	0.066208
CS30	CS21	CS2	*R.nitidus*	-0.047806	0.064183
CS4	CS21	CS2	*R.nitidus*	-0.005618	0.065445
GD41	CS21	CS2	*R.nitidus*	0.033063	0.063586
CS28	CS21	CS2	*R.nitidus*	-0.015079	0.065759
SC26	CS21	CS2	*R.nitidus*	-0.025926	0.063771
GD49	CS21	CS2	*R.nitidus*	0.107553	0.067742
CS18	CS21	CS2	*R.nitidus*	-0.016569	0.063777
CS20	CS21	CS2	*R.nitidus*	-0.015489	0.064022
CS24	CS21	CS2	*R.nitidus*	-0.014446	0.066097
SC34	CS21	CS2	*R.nitidus*	0.046143	0.064175
SC15	CS21	CS2	*R.nitidus*	-0.009195	0.066184
SC6	CS21	CS2	*R.nitidus*	0.00968	0.065317
SC10	CS21	CS2	*R.nitidus*	0.073263	0.065041
SC42	CS21	CS2	*R.nitidus*	0.249701	0.062137
CS23	CS21	CS2	*R.nitidus*	-0.042146	0.064009
JW7	CS21	CS2	*R.nitidus*	0.040245	0.068317
SC45	CS21	CS2	*R.nitidus*	0.048692	0.066072
SC5	CS21	CS2	*R.nitidus*	0.024322	0.067505
SC18	CS21	CS2	*R.nitidus*	0.064688	0.06645
MY6	CS21	CS2	*R.nitidus*	0.026657	0.066459
JO7	CS22	CS2	*R.nitidus*	-0.035538	0.07216
SC31	CS21	CS2	*R.nitidus*	0.019534	0.065928

SC32	CS21	CS2	*R.nitidus*	0.049556	0.06414
JO8	CS22	CS2	*R.nitidus*	0.015658	0.073586
JO1	CS21	CS2	*R.nitidus*	0.003579	0.066895
SC24	CS21	CS2	*R.nitidus*	0.023152	0.065122
JO3	CS21	CS2	*R.nitidus*	0.070833	0.06901
SC12	CS21	CS2	*R.nitidus*	-0.002195	0.065732
SC44	CS21	CS2	*R.nitidus*	0.014503	0.063561
SC14	CS21	CS2	*R.nitidus*	0.02603	0.065708
MY4	CS21	CS2	*R.nitidus*	-0.020958	0.06896
CS11	CS22	CS2	*R.nitidus*	-0.004521	0.068519
JW4	CS21	CS2	*R.nitidus*	0.247988	0.066818
SC27	CS21	CS2	*R.nitidus*	0.013091	0.064408
GD21	CS22	CS2	*R.nitidus*	0.107703	0.063593
GD42	CS22	CS2	*R.nitidus*	0.02805	0.065446
SC28	CS21	CS2	*R.nitidus*	-0.017404	0.065982
GD22	CS22	CS2	*R.nitidus*	0.054054	0.065051
CS6	CS21	CS2	*R.nitidus*	-0.037736	0.063901
GD29	CS22	CS2	*R.nitidus*	0.110215	0.064106
JW6	CS21	CS2	*R.nitidus*	0.00759	0.070346
GD25	CS22	CS2	*R.nitidus*	0.052255	0.064903
SC35	CS21	CS2	*R.nitidus*	0.070036	0.063388
GD35	CS22	CS2	*R.nitidus*	0.031513	0.067182
GD31	CS22	CS2	*R.nitidus*	0.041481	0.067374
GD34	CS22	CS2	*R.nitidus*	0.004149	0.065657
GD48	CS22	CS2	*R.nitidus*	0.036098	0.067964
CS14	CS22	CS2	*R.nitidus*	0.001522	0.06761
GD45	CS22	CS2	*R.nitidus*	0.065436	0.06962
GD26	CS22	CS2	*R.nitidus*	0.045198	0.066057
GD41	CS22	CS2	*R.nitidus*	0.011963	0.064412
GD30	CS22	CS2	*R.nitidus*	0.115756	0.067188
GD46	CS22	CS2	*R.nitidus*	-0.021262	0.065615
GD32	CS22	CS2	*R.nitidus*	0.025472	0.066563
GD44	CS22	CS2	*R.nitidus*	0.031179	0.06678
CS12	CS22	CS2	*R.nitidus*	0.003962	0.066906
GD33	CS22	CS2	*R.nitidus*	0.00417	0.066861
GD49	CS22	CS2	*R.nitidus*	0.07074	0.0682
GD50	CS22	CS2	*R.nitidus*	0.016935	0.068749
CS13	CS22	CS2	*R.nitidus*	-0.059633	0.066961
CS15	CS22	CS2	*R.nitidus*	0.013423	0.066655
JO5	CS21	CS2	*R.nitidus*	0.077922	0.064047
CS18	CS22	CS2	*R.nitidus*	-0.038786	0.066478
GD40	CS22	CS2	*R.nitidus*	0.060942	0.063763
CS3	CS22	CS2	*R.nitidus*	-0.02828	0.067056
SC6	CS22	CS2	*R.nitidus*	-0.02543	0.065565
CS19	CS22	CS2	*R.nitidus*	-0.021104	0.068522
CS24	CS22	CS2	*R.nitidus*	-0.058014	0.063752
SC5	CS22	CS2	*R.nitidus*	0.009694	0.069894
CS20	CS22	CS2	*R.nitidus*	-0.0491	0.067553
SC42	CS22	CS2	*R.nitidus*	0.243993	0.061597
SC12	CS22	CS2	*R.nitidus*	-0.031466	0.065786
CS30	CS22	CS2	*R.nitidus*	-0.074335	0.065928
SC18	CS22	CS2	*R.nitidus*	0.03125	0.066692
SC10	CS22	CS2	*R.nitidus*	0.055215	0.064922
SC27	CS22	CS2	*R.nitidus*	-0.008371	0.06481
CS28	CS22	CS2	*R.nitidus*	-0.038339	0.065674
CS31	CS22	CS2	*R.nitidus*	-0.039566	0.065223

CS6	CS22	CS2	*R.nitidus*	-0.057063	0.064858
CS17	CS22	CS2	*R.nitidus*	0.02	0.070056
SC15	CS22	CS2	*R.nitidus*	-0.025098	0.066894
CS23	CS22	CS2	*R.nitidus*	-0.069523	0.064712
SC35	CS22	CS2	*R.nitidus*	0.038783	0.063989
CS16	CS22	CS2	*R.nitidus*	-0.009119	0.067974
CS21	CS22	CS2	*R.nitidus*	-0.032283	0.064936
SC24	CS22	CS2	*R.nitidus*	-0.006696	0.065467
JW6	CS22	CS2	*R.nitidus*	0.012416	0.072669
SC14	CS22	CS2	*R.nitidus*	-0.006737	0.065462
SC45	CS22	CS2	*R.nitidus*	0.013363	0.065911
CS25	CS22	CS2	*R.nitidus*	-0.022654	0.068719
MY4	CS22	CS2	*R.nitidus*	-0.055215	0.06766
CS4	CS22	CS2	*R.nitidus*	-0.022527	0.063577
JW4	CS22	CS2	*R.nitidus*	0.225331	0.065921
JO1	CS22	CS2	*R.nitidus*	-0.022108	0.067099
SC26	CS22	CS2	*R.nitidus*	-0.053254	0.064783
SC28	CS22	CS2	*R.nitidus*	-0.046282	0.06549
SC32	CS22	CS2	*R.nitidus*	0.023059	0.064884
SC31	CS22	CS2	*R.nitidus*	-0.015173	0.065575
JO8	CS23	CS2	*R.nitidus*	0.063008	0.07213
GD25	CS23	CS2	*R.nitidus*	0.122991	0.06469
SC34	CS22	CS2	*R.nitidus*	0.018678	0.064538
GD35	CS23	CS2	*R.nitidus*	0.094101	0.065002
GD21	CS23	CS2	*R.nitidus*	0.162866	0.065355
GD26	CS23	CS2	*R.nitidus*	0.098372	0.065735
JO7	CS23	CS2	*R.nitidus*	0.035853	0.073009
SC44	CS22	CS2	*R.nitidus*	-0.016345	0.066096
CS12	CS23	CS2	*R.nitidus*	0.04943	0.066133
JO3	CS22	CS2	*R.nitidus*	0.037007	0.071729
GD31	CS23	CS2	*R.nitidus*	0.107065	0.065878
GD50	CS23	CS2	*R.nitidus*	0.062765	0.069062
MY6	CS22	CS2	*R.nitidus*	-0.007491	0.065855
JW7	CS22	CS2	*R.nitidus*	0.011701	0.068268
JO5	CS22	CS2	*R.nitidus*	0.039259	0.065701
GD44	CS23	CS2	*R.nitidus*	0.066812	0.065915
GD29	CS23	CS2	*R.nitidus*	0.157104	0.062753
CS17	CS23	CS2	*R.nitidus*	0.070699	0.067625
CS14	CS23	CS2	*R.nitidus*	0.065185	0.067289
CS25	CS23	CS2	*R.nitidus*	0.034217	0.068773
GD30	CS23	CS2	*R.nitidus*	0.153175	0.067145
GD42	CS23	CS2	*R.nitidus*	0.084028	0.066662
CS16	CS23	CS2	*R.nitidus*	0.043059	0.065164
CS11	CS23	CS2	*R.nitidus*	0.062225	0.065597
GD45	CS23	CS2	*R.nitidus*	0.098875	0.072883
GD22	CS23	CS2	*R.nitidus*	0.115669	0.064802
GD32	CS23	CS2	*R.nitidus*	0.085938	0.068974
GD34	CS23	CS2	*R.nitidus*	0.059233	0.064174
GD41	CS23	CS2	*R.nitidus*	0.061084	0.06456
GD46	CS23	CS2	*R.nitidus*	0.029054	0.064047
CS15	CS23	CS2	*R.nitidus*	0.081618	0.066775
CS13	CS23	CS2	*R.nitidus*	0.015957	0.067293
CS20	CS23	CS2	*R.nitidus*	0.029676	0.066164
CS3	CS23	CS2	*R.nitidus*	0.036792	0.064649
GD40	CS23	CS2	*R.nitidus*	0.110879	0.064724
GD33	CS23	CS2	*R.nitidus*	0.063351	0.065633

CS18	CS23	CS2	*R.nitidus*	0.02583	0.066304
CS21	CS23	CS2	*R.nitidus*	0.042146	0.064009
SC27	CS23	CS2	*R.nitidus*	0.044985	0.065103
SC18	CS23	CS2	*R.nitidus*	0.096824	0.065847
GD49	CS23	CS2	*R.nitidus*	0.113655	0.068311
CS24	CS23	CS2	*R.nitidus*	0.019818	0.065897
GD48	CS23	CS2	*R.nitidus*	0.098496	0.068348
CS31	CS23	CS2	*R.nitidus*	0.031202	0.066943
CS22	CS23	CS2	*R.nitidus*	0.069523	0.064712
SC10	CS23	CS2	*R.nitidus*	0.101417	0.067119
CS19	CS23	CS2	*R.nitidus*	0.040434	0.066966
SC24	CS23	CS2	*R.nitidus*	0.056138	0.064898
JO3	CS23	CS2	*R.nitidus*	0.085762	0.06723
SC26	CS23	CS2	*R.nitidus*	0.006526	0.065727
SC15	CS23	CS2	*R.nitidus*	0.038431	0.064679
MY4	CS23	CS2	*R.nitidus*	0.011967	0.06865
SC6	CS23	CS2	*R.nitidus*	0.045113	0.06606
CS28	CS23	CS2	*R.nitidus*	0.031175	0.066626
SC28	CS23	CS2	*R.nitidus*	-0.007908	0.065335
CS30	CS23	CS2	*R.nitidus*	-0.013407	0.065127
SC5	CS23	CS2	*R.nitidus*	0.053559	0.068906
SC31	CS23	CS2	*R.nitidus*	0.045622	0.065363
SC14	CS23	CS2	*R.nitidus*	0.047448	0.066048
SC32	CS23	CS2	*R.nitidus*	0.077988	0.064744
GD30	CS24	CS2	*R.nitidus*	0.148971	0.065356
CS6	CS23	CS2	*R.nitidus*	0.007273	0.065467
CS4	CS23	CS2	*R.nitidus*	0.048741	0.064175
SC12	CS23	CS2	*R.nitidus*	0.018328	0.064793
JW4	CS23	CS2	*R.nitidus*	0.25942	0.065733
GD26	CS24	CS2	*R.nitidus*	0.086831	0.065547
SC35	CS23	CS2	*R.nitidus*	0.096611	0.064958
GD25	CS24	CS2	*R.nitidus*	0.093411	0.065688
GD32	CS24	CS2	*R.nitidus*	0.07055	0.067396
GD21	CS24	CS2	*R.nitidus*	0.156727	0.064056
SC34	CS23	CS2	*R.nitidus*	0.076253	0.064362
JW7	CS23	CS2	*R.nitidus*	0.049957	0.068203
JO5	CS23	CS2	*R.nitidus*	0.102115	0.062801
GD40	CS24	CS2	*R.nitidus*	0.108206	0.06399
SC45	CS23	CS2	*R.nitidus*	0.077542	0.064719
JO7	CS24	CS2	*R.nitidus*	0.022267	0.073961
SC42	CS23	CS2	*R.nitidus*	0.287744	0.061763
GD35	CS24	CS2	*R.nitidus*	0.078113	0.065805
JW6	CS23	CS2	*R.nitidus*	0.017512	0.071741
SC44	CS23	CS2	*R.nitidus*	0.03639	0.065591
MY6	CS23	CS2	*R.nitidus*	0.052283	0.066124
CS11	CS24	CS2	*R.nitidus*	0.044601	0.067864
GD44	CS24	CS2	*R.nitidus*	0.060372	0.068888
JO8	CS24	CS2	*R.nitidus*	0.034409	0.073052
GD22	CS24	CS2	*R.nitidus*	0.104635	0.064911
GD31	CS24	CS2	*R.nitidus*	0.088235	0.067702
GD29	CS24	CS2	*R.nitidus*	0.146461	0.064276
CS16	CS24	CS2	*R.nitidus*	0.039777	0.06609
GD33	CS24	CS2	*R.nitidus*	0.046276	0.06765
GD45	CS24	CS2	*R.nitidus*	0.096965	0.069274
JO1	CS23	CS2	*R.nitidus*	0.031688	0.065833
GD41	CS24	CS2	*R.nitidus*	0.0522	0.064643

CS12	CS24	CS2	*R.nitidus*	0.05	0.066241
GD34	CS24	CS2	*R.nitidus*	0.042878	0.066665
GD50	CS24	CS2	*R.nitidus*	0.031362	0.068465
GD49	CS24	CS2	*R.nitidus*	0.102819	0.068969
GD46	CS24	CS2	*R.nitidus*	0.015419	0.065409
CS13	CS24	CS2	*R.nitidus*	0	0.067272
CS20	CS24	CS2	*R.nitidus*	-0.005573	0.067525
CS17	CS24	CS2	*R.nitidus*	0.070946	0.068717
CS28	CS24	CS2	*R.nitidus*	0.021613	0.066722
CS15	CS24	CS2	*R.nitidus*	0.060139	0.066505
SC18	CS24	CS2	*R.nitidus*	0.0726	0.066805
GD42	CS24	CS2	*R.nitidus*	0.06927	0.06609
CS14	CS24	CS2	*R.nitidus*	0.043165	0.068007
CS25	CS24	CS2	*R.nitidus*	0.032099	0.070209
CS31	CS24	CS2	*R.nitidus*	0.009631	0.067085
GD48	CS24	CS2	*R.nitidus*	0.068666	0.067144
SC42	CS24	CS2	*R.nitidus*	0.28801	0.060927
CS22	CS24	CS2	*R.nitidus*	0.058014	0.063752
CS30	CS24	CS2	*R.nitidus*	-0.031561	0.065257
CS19	CS24	CS2	*R.nitidus*	0.012059	0.069936
SC14	CS24	CS2	*R.nitidus*	0.028616	0.064477
CS4	CS24	CS2	*R.nitidus*	0.035584	0.061819
JO3	CS24	CS2	*R.nitidus*	0.063078	0.068573
CS18	CS24	CS2	*R.nitidus*	0.019982	0.065609
CS21	CS24	CS2	*R.nitidus*	0.014446	0.066097
SC26	CS24	CS2	*R.nitidus*	-0.006002	0.064497
CS3	CS24	CS2	*R.nitidus*	0.004036	0.066022
JO1	CS24	CS2	*R.nitidus*	0.020119	0.06729
CS23	CS24	CS2	*R.nitidus*	-0.019818	0.065897
SC12	CS24	CS2	*R.nitidus*	0.017843	0.065128
SC15	CS24	CS2	*R.nitidus*	0.019512	0.065108
SC34	CS24	CS2	*R.nitidus*	0.075	0.064683
SC31	CS24	CS2	*R.nitidus*	0.034892	0.065036
JO8	CS25	CS2	*R.nitidus*	0.022518	0.075485
SC6	CS24	CS2	*R.nitidus*	0.026987	0.06549
GD21	CS25	CS2	*R.nitidus*	0.136799	0.066452
SC5	CS24	CS2	*R.nitidus*	0.044857	0.068808
SC10	CS24	CS2	*R.nitidus*	0.111975	0.063606
MY4	CS24	CS2	*R.nitidus*	-0.004542	0.06765
JW4	CS24	CS2	*R.nitidus*	0.269174	0.065541
JO7	CS25	CS2	*R.nitidus*	0.009921	0.074303
SC45	CS24	CS2	*R.nitidus*	0.059471	0.066768
JW7	CS24	CS2	*R.nitidus*	0.053774	0.068045
SC44	CS24	CS2	*R.nitidus*	0.033515	0.064979
SC35	CS24	CS2	*R.nitidus*	0.082181	0.062971
GD22	CS25	CS2	*R.nitidus*	0.096301	0.067304
SC28	CS24	CS2	*R.nitidus*	-0.003776	0.064803
SC32	CS24	CS2	*R.nitidus*	0.068289	0.066061
JO5	CS24	CS2	*R.nitidus*	0.084091	0.065735
SC24	CS24	CS2	*R.nitidus*	0.035249	0.064721
SC27	CS24	CS2	*R.nitidus*	0.027624	0.063787
CS6	CS24	CS2	*R.nitidus*	-0.013123	0.066554
CS11	CS25	CS2	*R.nitidus*	0.012898	0.069716
JW6	CS24	CS2	*R.nitidus*	0.049	0.072324
MY6	CS24	CS2	*R.nitidus*	0.038031	0.066845
GD34	CS25	CS2	*R.nitidus*	0.020423	0.06636

GD26	CS25	CS2	*R.nitidus*	0.069832	0.068106
GD31	CS25	CS2	*R.nitidus*	0.084683	0.068606
GD46	CS25	CS2	*R.nitidus*	-0.005646	0.069473
GD25	CS25	CS2	*R.nitidus*	0.103935	0.066121
GD41	CS25	CS2	*R.nitidus*	0.020776	0.066966
GD35	CS25	CS2	*R.nitidus*	0.050396	0.066317
GD33	CS25	CS2	*R.nitidus*	0.029775	0.068129
CS12	CS25	CS2	*R.nitidus*	0.007081	0.067938
GD42	CS25	CS2	*R.nitidus*	0.043134	0.068178
CS13	CS25	CS2	*R.nitidus*	-0.025542	0.071437
GD30	CS25	CS2	*R.nitidus*	0.13255	0.071621
GD44	CS25	CS2	*R.nitidus*	0.040121	0.068397
CS19	CS25	CS2	*R.nitidus*	-0.012422	0.071216
SC5	CS25	CS2	*R.nitidus*	0.012968	0.07008
GD32	CS25	CS2	*R.nitidus*	0.039872	0.071038
SC10	CS25	CS2	*R.nitidus*	0.048229	0.069125
CS23	CS25	CS2	*R.nitidus*	-0.034217	0.068773
CS31	CS25	CS2	*R.nitidus*	-0.017188	0.069148
CS30	CS25	CS2	*R.nitidus*	-0.04751	0.067631
GD50	CS25	CS2	*R.nitidus*	0.045572	0.070024
CS28	CS25	CS2	*R.nitidus*	-0.025142	0.071698
CS22	CS25	CS2	*R.nitidus*	0.022654	0.068719
CS21	CS25	CS2	*R.nitidus*	-0.005226	0.068776
GD40	CS25	CS2	*R.nitidus*	0.076277	0.066549
GD29	CS25	CS2	*R.nitidus*	0.135794	0.065075
CS20	CS25	CS2	*R.nitidus*	-0.024957	0.068169
GD45	CS25	CS2	*R.nitidus*	0.090448	0.073123
CS16	CS25	CS2	*R.nitidus*	0.008863	0.068018
SC15	CS25	CS2	*R.nitidus*	-0.004651	0.069114
GD49	CS25	CS2	*R.nitidus*	0.095477	0.073287
GD48	CS25	CS2	*R.nitidus*	0.047843	0.070056
CS18	CS25	CS2	*R.nitidus*	-0.020833	0.069463
CS17	CS25	CS2	*R.nitidus*	0.036304	0.072638
CS24	CS25	CS2	*R.nitidus*	-0.032099	0.070209
CS14	CS25	CS2	*R.nitidus*	0.017504	0.069659
SC31	CS25	CS2	*R.nitidus*	0.011747	0.066512
SC24	CS25	CS2	*R.nitidus*	0.026576	0.068291
CS4	CS25	CS2	*R.nitidus*	0.014308	0.068699
SC32	CS25	CS2	*R.nitidus*	0.041541	0.0686
CS6	CS25	CS2	*R.nitidus*	-0.04087	0.066789
CS15	CS25	CS2	*R.nitidus*	0.03238	0.070182
SC34	CS25	CS2	*R.nitidus*	0.042857	0.066268
MY4	CS25	CS2	*R.nitidus*	-0.025602	0.067744
SC44	CS25	CS2	*R.nitidus*	-0.005291	0.066658
SC26	CS25	CS2	*R.nitidus*	-0.020573	0.066306
SC12	CS25	CS2	*R.nitidus*	-0.017293	0.067696
JW4	CS25	CS2	*R.nitidus*	0.229592	0.068822
CS3	CS25	CS2	*R.nitidus*	-0.018272	0.066409
SC6	CS25	CS2	*R.nitidus*	0.001519	0.06831
SC14	CS25	CS2	*R.nitidus*	0.006667	0.067042
JW6	CS25	CS2	*R.nitidus*	0.016807	0.074117
SC18	CS25	CS2	*R.nitidus*	0.05513	0.067808
SC27	CS25	CS2	*R.nitidus*	0.008234	0.066364
GD25	CS28	CS2	*R.nitidus*	0.090132	0.065737
SC45	CS25	CS2	*R.nitidus*	0.049144	0.066112
SC28	CS25	CS2	*R.nitidus*	-0.032258	0.067218

SC42	CS25	CS2	*R.nitidus*	0.248932	0.064731
JO7	CS28	CS2	*R.nitidus*	0.005171	0.073009
JO3	CS25	CS2	*R.nitidus*	0.05588	0.070508
JW7	CS25	CS2	*R.nitidus*	0.023256	0.071747
JO1	CS25	CS2	*R.nitidus*	-0.012839	0.067501
MY6	CS25	CS2	*R.nitidus*	0.017699	0.066994
CS13	CS28	CS2	*R.nitidus*	-0.026961	0.069878
GD26	CS28	CS2	*R.nitidus*	0.071271	0.06697
GD21	CS28	CS2	*R.nitidus*	0.134864	0.064739
CS11	CS28	CS2	*R.nitidus*	0.023904	0.066429
JO5	CS25	CS2	*R.nitidus*	0.07228	0.066035
GD48	CS28	CS2	*R.nitidus*	0.070388	0.068159
GD31	CS28	CS2	*R.nitidus*	0.057588	0.067203
JO8	CS28	CS2	*R.nitidus*	0.030884	0.07605
GD30	CS28	CS2	*R.nitidus*	0.147668	0.068921
SC35	CS25	CS2	*R.nitidus*	0.055556	0.065405
GD22	CS28	CS2	*R.nitidus*	0.091672	0.067807
GD49	CS28	CS2	*R.nitidus*	0.08969	0.071029
GD34	CS28	CS2	*R.nitidus*	0.034328	0.066258
GD45	CS28	CS2	*R.nitidus*	0.090372	0.069683
GD40	CS28	CS2	*R.nitidus*	0.075347	0.064537
GD44	CS28	CS2	*R.nitidus*	0.037896	0.067837
GD42	CS28	CS2	*R.nitidus*	0.055982	0.068739
CS17	CS28	CS2	*R.nitidus*	0.042642	0.069338
GD46	CS28	CS2	*R.nitidus*	0.003765	0.068018
GD29	CS28	CS2	*R.nitidus*	0.13627	0.066375
GD41	CS28	CS2	*R.nitidus*	0.031061	0.067096
CS12	CS28	CS2	*R.nitidus*	0.026667	0.068269
CS31	CS28	CS2	*R.nitidus*	-0.02008	0.068838
GD35	CS28	CS2	*R.nitidus*	0.070423	0.06755
CS18	CS28	CS2	*R.nitidus*	-0.007171	0.067418
GD33	CS28	CS2	*R.nitidus*	0.040089	0.065166
CS21	CS28	CS2	*R.nitidus*	0.015079	0.065759
GD32	CS28	CS2	*R.nitidus*	0.048495	0.069275
CS3	CS28	CS2	*R.nitidus*	0.002402	0.067706
GD50	CS28	CS2	*R.nitidus*	0.075386	0.069805
CS19	CS28	CS2	*R.nitidus*	0.009717	0.069024
SC31	CS28	CS2	*R.nitidus*	0.018415	0.06548
SC14	CS28	CS2	*R.nitidus*	0.018196	0.06714
CS30	CS28	CS2	*R.nitidus*	-0.051282	0.066793
SC15	CS28	CS2	*R.nitidus*	-0.008489	0.067771
CS15	CS28	CS2	*R.nitidus*	0.032437	0.068392
CS24	CS28	CS2	*R.nitidus*	-0.021613	0.066722
CS20	CS28	CS2	*R.nitidus*	0.001554	0.068186
CS14	CS28	CS2	*R.nitidus*	0.029157	0.06914
CS16	CS28	CS2	*R.nitidus*	0.015176	0.066389
SC28	CS28	CS2	*R.nitidus*	-0.016706	0.065682
SC26	CS28	CS2	*R.nitidus*	-0.022781	0.066397
SC10	CS28	CS2	*R.nitidus*	0.083401	0.067111
CS23	CS28	CS2	*R.nitidus*	-0.031175	0.066626
SC12	CS28	CS2	*R.nitidus*	-0.000792	0.066676
SC35	CS28	CS2	*R.nitidus*	0.046083	0.065666
SC6	CS28	CS2	*R.nitidus*	0.019952	0.067555
SC18	CS28	CS2	*R.nitidus*	0.0664	0.068276
CS22	CS28	CS2	*R.nitidus*	0.038339	0.065674
SC44	CS28	CS2	*R.nitidus*	0.021061	0.066102

SC42	CS28	CS2	*R.nitidus*	0.256377	0.063188
CS25	CS28	CS2	*R.nitidus*	0.025142	0.071698
CS4	CS28	CS2	*R.nitidus*	0.010244	0.06545
JO7	CS30	CS2	*R.nitidus*	0.032427	0.073809
JO5	CS28	CS2	*R.nitidus*	0.071651	0.065377
SC32	CS28	CS2	*R.nitidus*	0.061947	0.066756
SC5	CS28	CS2	*R.nitidus*	0.032157	0.068881
JW7	CS28	CS2	*R.nitidus*	0.043145	0.071646
JW4	CS28	CS2	*R.nitidus*	0.24	0.069967
MY4	CS28	CS2	*R.nitidus*	-0.016292	0.068641
JO3	CS28	CS2	*R.nitidus*	0.060965	0.071654
SC34	CS28	CS2	*R.nitidus*	0.065536	0.066523
JO1	CS28	CS2	*R.nitidus*	0.008013	0.066831
SC24	CS28	CS2	*R.nitidus*	0.024038	0.066049
JW6	CS28	CS2	*R.nitidus*	0.037074	0.072866
MY6	CS28	CS2	*R.nitidus*	0.025	0.068181
SC27	CS28	CS2	*R.nitidus*	0.015974	0.065141
GD29	CS30	CS2	*R.nitidus*	0.17561	0.063499
CS6	CS28	CS2	*R.nitidus*	-0.018197	0.067251
CS11	CS30	CS2	*R.nitidus*	0.061848	0.06803
GD26	CS30	CS2	*R.nitidus*	0.110473	0.066411
GD30	CS30	CS2	*R.nitidus*	0.176568	0.067594
GD25	CS30	CS2	*R.nitidus*	0.128205	0.065252
SC45	CS28	CS2	*R.nitidus*	0.039454	0.068604
GD50	CS30	CS2	*R.nitidus*	0.086881	0.069174
GD40	CS30	CS2	*R.nitidus*	0.121574	0.062722
GD45	CS30	CS2	*R.nitidus*	0.124155	0.069454
JO8	CS30	CS2	*R.nitidus*	0.088358	0.071008
CS12	CS30	CS2	*R.nitidus*	0.075889	0.065588
GD35	CS30	CS2	*R.nitidus*	0.096522	0.06748
GD31	CS30	CS2	*R.nitidus*	0.118865	0.067627
GD46	CS30	CS2	*R.nitidus*	0.040953	0.066092
GD48	CS30	CS2	*R.nitidus*	0.094637	0.068639
CS22	CS30	CS2	*R.nitidus*	0.074335	0.065928
CS13	CS30	CS2	*R.nitidus*	0.028749	0.068259
CS15	CS30	CS2	*R.nitidus*	0.079699	0.067202
GD21	CS30	CS2	*R.nitidus*	0.178822	0.064236
GD22	CS30	CS2	*R.nitidus*	0.133241	0.064035
CS20	CS30	CS2	*R.nitidus*	0.031037	0.066818
CS16	CS30	CS2	*R.nitidus*	0.067732	0.064858
GD41	CS30	CS2	*R.nitidus*	0.085312	0.064975
GD33	CS30	CS2	*R.nitidus*	0.079799	0.065631
CS14	CS30	CS2	*R.nitidus*	0.063359	0.068107
CS23	CS30	CS2	*R.nitidus*	0.013407	0.065127
SC10	CS30	CS2	*R.nitidus*	0.121746	0.066303
GD34	CS30	CS2	*R.nitidus*	0.071791	0.064919
GD44	CS30	CS2	*R.nitidus*	0.071588	0.067636
SC27	CS30	CS2	*R.nitidus*	0.054954	0.064444
GD42	CS30	CS2	*R.nitidus*	0.097965	0.065
SC12	CS30	CS2	*R.nitidus*	0.03265	0.065791
CS21	CS30	CS2	*R.nitidus*	0.047806	0.064183
SC18	CS30	CS2	*R.nitidus*	0.102167	0.066411
CS25	CS30	CS2	*R.nitidus*	0.04751	0.067631
CS28	CS30	CS2	*R.nitidus*	0.051282	0.066793
SC24	CS30	CS2	*R.nitidus*	0.069856	0.066106
GD32	CS30	CS2	*R.nitidus*	0.084967	0.070639

CS31	CS30	CS2	*R.nitidus*	0.052814	0.065065
GD49	CS30	CS2	*R.nitidus*	0.130544	0.069144
CS17	CS30	CS2	*R.nitidus*	0.090476	0.068418
CS18	CS30	CS2	*R.nitidus*	0.039106	0.064857
CS3	CS30	CS2	*R.nitidus*	0.044129	0.065519
SC5	CS30	CS2	*R.nitidus*	0.058466	0.070725
SC32	CS30	CS2	*R.nitidus*	0.084291	0.065272
SC42	CS30	CS2	*R.nitidus*	0.290049	0.060292
SC6	CS30	CS2	*R.nitidus*	0.049599	0.0673
CS19	CS30	CS2	*R.nitidus*	0.044071	0.067937
SC26	CS30	CS2	*R.nitidus*	0.02	0.064773
SC14	CS30	CS2	*R.nitidus*	0.051339	0.06533
SC34	CS30	CS2	*R.nitidus*	0.093023	0.06451
CS24	CS30	CS2	*R.nitidus*	0.031561	0.065257
JW7	CS30	CS2	*R.nitidus*	0.068627	0.067567
JO7	CS31	CS2	*R.nitidus*	-0.000983	0.073719
MY6	CS30	CS2	*R.nitidus*	0.067809	0.066635
SC35	CS30	CS2	*R.nitidus*	0.100752	0.06332
JW4	CS30	CS2	*R.nitidus*	0.265896	0.066339
CS6	CS30	CS2	*R.nitidus*	0.015873	0.066443
SC15	CS30	CS2	*R.nitidus*	0.041363	0.066166
CS4	CS30	CS2	*R.nitidus*	0.056765	0.063096
JO1	CS30	CS2	*R.nitidus*	0.031525	0.065831
JO3	CS30	CS2	*R.nitidus*	0.10477	0.067412
SC28	CS30	CS2	*R.nitidus*	0.016999	0.064961
GD22	CS31	CS2	*R.nitidus*	0.087744	0.066809
SC31	CS30	CS2	*R.nitidus*	0.058605	0.066483
JO5	CS30	CS2	*R.nitidus*	0.108108	0.06383
CS15	CS31	CS2	*R.nitidus*	0.03577	0.06768
GD46	CS31	CS2	*R.nitidus*	0.00307	0.066777
GD31	CS31	CS2	*R.nitidus*	0.071373	0.068632
GD25	CS31	CS2	*R.nitidus*	0.101427	0.066394
GD29	CS31	CS2	*R.nitidus*	0.148892	0.065182
SC44	CS30	CS2	*R.nitidus*	0.057737	0.062912
SC45	CS30	CS2	*R.nitidus*	0.087879	0.066625
JW6	CS30	CS2	*R.nitidus*	0.047716	0.070946
MY4	CS30	CS2	*R.nitidus*	0.019389	0.067351
JO8	CS31	CS2	*R.nitidus*	0.044652	0.073073
CS11	CS31	CS2	*R.nitidus*	0.018824	0.069478
GD21	CS31	CS2	*R.nitidus*	0.142452	0.064581
CS19	CS31	CS2	*R.nitidus*	0.003252	0.069155
GD40	CS31	CS2	*R.nitidus*	0.090077	0.066777
GD30	CS31	CS2	*R.nitidus*	0.126572	0.06895
CS21	CS31	CS2	*R.nitidus*	0.009878	0.066883
GD50	CS31	CS2	*R.nitidus*	0.049002	0.069402
CS14	CS31	CS2	*R.nitidus*	0.027473	0.069729
CS22	CS31	CS2	*R.nitidus*	0.039566	0.065223
CS13	CS31	CS2	*R.nitidus*	-0.01937	0.069566
GD26	CS31	CS2	*R.nitidus*	0.077259	0.067411
GD44	CS31	CS2	*R.nitidus*	0.044275	0.067192
GD48	CS31	CS2	*R.nitidus*	0.061912	0.06763
GD42	CS31	CS2	*R.nitidus*	0.047104	0.065958
CS16	CS31	CS2	*R.nitidus*	0.023923	0.067608
CS20	CS31	CS2	*R.nitidus*	-0.004545	0.068063
GD49	CS31	CS2	*R.nitidus*	0.103302	0.071984
CS18	CS31	CS2	*R.nitidus*	-0.003137	0.06636

CS12	CS31	CS2	*R.nitidus*	0.038336	0.067898
CS23	CS31	CS2	*R.nitidus*	-0.031202	0.066943
GD35	CS31	CS2	*R.nitidus*	0.068503	0.068466
GD45	CS31	CS2	*R.nitidus*	0.112931	0.071915
GD34	CS31	CS2	*R.nitidus*	0.01997	0.067092
GD41	CS31	CS2	*R.nitidus*	0.035928	0.065462
CS28	CS31	CS2	*R.nitidus*	0.02008	0.068838
SC14	CS31	CS2	*R.nitidus*	0.015748	0.067696
GD32	CS31	CS2	*R.nitidus*	0.048822	0.070736
CS3	CS31	CS2	*R.nitidus*	0.008661	0.066518
CS17	CS31	CS2	*R.nitidus*	0.040782	0.06953
SC12	CS31	CS2	*R.nitidus*	0.003026	0.065389
SC27	CS31	CS2	*R.nitidus*	0.015601	0.065093
SC6	CS31	CS2	*R.nitidus*	0.017437	0.067992
CS24	CS31	CS2	*R.nitidus*	-0.009631	0.067085
CS30	CS31	CS2	*R.nitidus*	-0.052814	0.065065
CS4	CS31	CS2	*R.nitidus*	0.015909	0.064238
JO7	CS3	CS2	*R.nitidus*	-0.009569	0.071836
SC15	CS31	CS2	*R.nitidus*	-0.005691	0.070001
CS6	CS31	CS2	*R.nitidus*	-0.01672	0.067153
CS25	CS31	CS2	*R.nitidus*	0.017188	0.069148
SC24	CS31	CS2	*R.nitidus*	0.02313	0.065789
JO3	CS31	CS2	*R.nitidus*	0.049914	0.070338
SC10	CS31	CS2	*R.nitidus*	0.088558	0.06625
SC5	CS31	CS2	*R.nitidus*	0.023449	0.071125
JW6	CS31	CS2	*R.nitidus*	0.024655	0.072443
SC28	CS31	CS2	*R.nitidus*	-0.019832	0.066099
JO8	CS3	CS2	*R.nitidus*	0.012384	0.073393
SC18	CS31	CS2	*R.nitidus*	0.068059	0.066289
SC42	CS31	CS2	*R.nitidus*	0.269089	0.061623
SC35	CS31	CS2	*R.nitidus*	0.062406	0.06438
SC45	CS31	CS2	*R.nitidus*	0.048653	0.066144
MY6	CS31	CS2	*R.nitidus*	0.024887	0.06763
SC26	CS31	CS2	*R.nitidus*	-0.015015	0.06595
GD21	CS3	CS2	*R.nitidus*	0.129141	0.063621
GD26	CS3	CS2	*R.nitidus*	0.074669	0.067364
GD40	CS3	CS2	*R.nitidus*	0.083333	0.064404
JW4	CS31	CS2	*R.nitidus*	0.255948	0.065823
GD30	CS3	CS2	*R.nitidus*	0.144833	0.067409
SC44	CS31	CS2	*R.nitidus*	0.02454	0.066951
GD22	CS3	CS2	*R.nitidus*	0.096327	0.065293
JO1	CS31	CS2	*R.nitidus*	-0.009819	0.067
SC34	CS31	CS2	*R.nitidus*	0.050263	0.06593
SC31	CS31	CS2	*R.nitidus*	0.025797	0.066385
JO5	CS31	CS2	*R.nitidus*	0.086466	0.064757
CS11	CS3	CS2	*R.nitidus*	0.032576	0.068224
MY4	CS31	CS2	*R.nitidus*	-0.018954	0.067547
SC32	CS31	CS2	*R.nitidus*	0.054075	0.06767
JW7	CS31	CS2	*R.nitidus*	0.057656	0.070937
GD25	CS3	CS2	*R.nitidus*	0.091244	0.066702
GD29	CS3	CS2	*R.nitidus*	0.146375	0.063832
CS15	CS3	CS2	*R.nitidus*	0.037834	0.068467
GD50	CS3	CS2	*R.nitidus*	0.052676	0.06855
GD32	CS3	CS2	*R.nitidus*	0.06449	0.069006
GD49	CS3	CS2	*R.nitidus*	0.110927	0.068627
GD45	CS3	CS2	*R.nitidus*	0.076355	0.070861

CS16	CS3	CS2	*R.nitidus*	0.015909	0.065764
CS17	CS3	CS2	*R.nitidus*	0.033898	0.0692
GD46	CS3	CS2	*R.nitidus*	-0.007082	0.065318
GD31	CS3	CS2	*R.nitidus*	0.079084	0.066895
GD44	CS3	CS2	*R.nitidus*	0.036284	0.06548
CS13	CS3	CS2	*R.nitidus*	-0.018141	0.068093
GD34	CS3	CS2	*R.nitidus*	0.02964	0.067707
CS12	CS3	CS2	*R.nitidus*	0.021094	0.066378
GD33	CS3	CS2	*R.nitidus*	0.042857	0.066987
GD42	CS3	CS2	*R.nitidus*	0.056298	0.065373
CS28	CS3	CS2	*R.nitidus*	-0.002402	0.067706
GD35	CS3	CS2	*R.nitidus*	0.053495	0.064533
CS31	CS3	CS2	*R.nitidus*	-0.008661	0.066518
GD41	CS3	CS2	*R.nitidus*	0.042391	0.065221
GD48	CS3	CS2	*R.nitidus*	0.059322	0.06791
SC10	CS3	CS2	*R.nitidus*	0.06459	0.064579
CS18	CS3	CS2	*R.nitidus*	-0.003506	0.066938
CS14	CS3	CS2	*R.nitidus*	0.028593	0.068176
CS30	CS3	CS2	*R.nitidus*	-0.044129	0.065519
CS23	CS3	CS2	*R.nitidus*	-0.036792	0.064649
CS19	CS3	CS2	*R.nitidus*	0.01049	0.066454
SC27	CS3	CS2	*R.nitidus*	0.009651	0.065193
CS21	CS3	CS2	*R.nitidus*	0.002752	0.0657
SC6	CS3	CS2	*R.nitidus*	0.009745	0.064527
CS4	CS3	CS2	*R.nitidus*	0.024721	0.065497
SC14	CS3	CS2	*R.nitidus*	0.023739	0.065991
CS20	CS3	CS2	*R.nitidus*	-0.015658	0.061872
SC12	CS3	CS2	*R.nitidus*	-0.005307	0.065452
SC28	CS3	CS2	*R.nitidus*	-0.026966	0.065038
SC26	CS3	CS2	*R.nitidus*	-0.018113	0.065026
SC18	CS3	CS2	*R.nitidus*	0.073132	0.065711
CS6	CS3	CS2	*R.nitidus*	-0.039749	0.062769
CS25	CS3	CS2	*R.nitidus*	0.018272	0.066409
SC24	CS3	CS2	*R.nitidus*	0.031882	0.066395
SC5	CS3	CS2	*R.nitidus*	0.011544	0.06853
CS22	CS3	CS2	*R.nitidus*	0.02828	0.067056
JW7	CS3	CS2	*R.nitidus*	0.028326	0.068178
SC15	CS3	CS2	*R.nitidus*	0	0.065994
SC31	CS3	CS2	*R.nitidus*	0.000748	0.064388
SC34	CS3	CS2	*R.nitidus*	0.052632	0.066326
JO5	CS3	CS2	*R.nitidus*	0.068463	0.064147
JW4	CS3	CS2	*R.nitidus*	0.22963	0.067384
JO1	CS3	CS2	*R.nitidus*	-0.011186	0.066045
JO8	SC5	CS2	*R.nitidus*	-0.005842	0.077272
CS24	CS3	CS2	*R.nitidus*	-0.004036	0.066022
SC35	CS3	CS2	*R.nitidus*	0.053936	0.063898
SC44	CS3	CS2	*R.nitidus*	0.008333	0.065317
JW6	CS3	CS2	*R.nitidus*	-0.021113	0.071182
SC32	CS3	CS2	*R.nitidus*	0.044477	0.065316
JO3	CS3	CS2	*R.nitidus*	0.030095	0.069483
GD21	SC5	CS2	*R.nitidus*	0.106849	0.067868
MY6	CS3	CS2	*R.nitidus*	0.017751	0.065685
MY4	CS3	CS2	*R.nitidus*	-0.018657	0.067405
SC42	CS3	CS2	*R.nitidus*	0.244336	0.060331
GD22	SC5	CS2	*R.nitidus*	0.071972	0.068647
SC45	CS3	CS2	*R.nitidus*	0.044709	0.065821

GD45	SC5	CS2	*R.nitidus*	0.070683	0.072612
GD50	SC5	CS2	*R.nitidus*	0.036972	0.074523
CS12	SC5	CS2	*R.nitidus*	-0.009804	0.067907
GD25	SC5	CS2	*R.nitidus*	0.075036	0.069359
JO7	SC5	CS2	*R.nitidus*	-0.044574	0.075421
GD29	SC5	CS2	*R.nitidus*	0.114246	0.06865
GD34	SC5	CS2	*R.nitidus*	-0.002152	0.069247
CS11	SC5	CS2	*R.nitidus*	0.002146	0.071044
GD40	SC5	CS2	*R.nitidus*	0.063326	0.067267
CS25	SC5	CS2	*R.nitidus*	-0.012968	0.07008
GD41	SC5	CS2	*R.nitidus*	0.005789	0.068868
GD30	SC5	CS2	*R.nitidus*	0.126511	0.070741
GD35	SC5	CS2	*R.nitidus*	0.033577	0.070909
GD26	SC5	CS2	*R.nitidus*	0.052899	0.069154
CS16	SC5	CS2	*R.nitidus*	-0.010174	0.07005
CS22	SC5	CS2	*R.nitidus*	-0.009694	0.069894
GD32	SC5	CS2	*R.nitidus*	-0.000824	0.073149
GD44	SC5	CS2	*R.nitidus*	0.013793	0.071805
GD31	SC5	CS2	*R.nitidus*	0.052273	0.069508
GD42	SC5	CS2	*R.nitidus*	0.025566	0.068379
CS20	SC5	CS2	*R.nitidus*	-0.043822	0.068561
CS4	SC5	CS2	*R.nitidus*	-0.013343	0.067652
CS15	SC5	CS2	*R.nitidus*	0.008445	0.070951
SC15	SC5	CS2	*R.nitidus*	-0.022275	0.070336
CS17	SC5	CS2	*R.nitidus*	0.01926	0.070089
GD33	SC5	CS2	*R.nitidus*	0	0.070956
CS23	SC5	CS2	*R.nitidus*	-0.053559	0.068906
CS13	SC5	CS2	*R.nitidus*	-0.034738	0.068345
GD49	SC5	CS2	*R.nitidus*	0.083748	0.072546
GD46	SC5	CS2	*R.nitidus*	-0.021186	0.067198
GD48	SC5	CS2	*R.nitidus*	0.007075	0.071501
CS18	SC5	CS2	*R.nitidus*	-0.03397	0.070362
SC6	SC5	CS2	*R.nitidus*	-0.014184	0.06966
SC10	SC5	CS2	*R.nitidus*	0.063556	0.070764
CS21	SC5	CS2	*R.nitidus*	-0.024322	0.067505
CS28	SC5	CS2	*R.nitidus*	-0.032157	0.068881
CS24	SC5	CS2	*R.nitidus*	-0.044857	0.068808
CS31	SC5	CS2	*R.nitidus*	-0.023449	0.071125
CS3	SC5	CS2	*R.nitidus*	-0.011544	0.06853
CS14	SC5	CS2	*R.nitidus*	-0.007153	0.069688
CS19	SC5	CS2	*R.nitidus*	-0.019374	0.07158
CS30	SC5	CS2	*R.nitidus*	-0.058466	0.070725
SC26	SC5	CS2	*R.nitidus*	-0.049574	0.068825
SC18	SC5	CS2	*R.nitidus*	0.045226	0.069793
SC31	SC5	CS2	*R.nitidus*	-0.008468	0.069576
SC24	SC5	CS2	*R.nitidus*	0.012235	0.070583
JW4	SC5	CS2	*R.nitidus*	0.204828	0.069787
SC35	SC5	CS2	*R.nitidus*	0.04006	0.066851
SC27	SC5	CS2	*R.nitidus*	-0.007962	0.068962
SC28	SC5	CS2	*R.nitidus*	-0.041773	0.068641
SC14	SC5	CS2	*R.nitidus*	-0.005719	0.070729
CS6	SC5	CS2	*R.nitidus*	-0.052437	0.067868
JW7	SC5	CS2	*R.nitidus*	0.007605	0.076613
GD29	SC6	CS2	*R.nitidus*	0.141988	0.063734
JO5	SC5	CS2	*R.nitidus*	0.070399	0.068749
SC12	SC5	CS2	*R.nitidus*	-0.028363	0.0696

JO8	SC6	CS2	*R.nitidus*	0.027695	0.073308
JO1	SC5	CS2	*R.nitidus*	-0.023592	0.069872
SC34	SC5	CS2	*R.nitidus*	0.024738	0.069055
CS11	SC6	CS2	*R.nitidus*	0.016825	0.067728
SC44	SC5	CS2	*R.nitidus*	-0.006146	0.068237
MY6	SC5	CS2	*R.nitidus*	0.007868	0.069655
SC45	SC5	CS2	*R.nitidus*	0.029141	0.06846
SC42	SC5	CS2	*R.nitidus*	0.253712	0.065361
SC32	SC5	CS2	*R.nitidus*	0.020619	0.068499
GD44	SC6	CS2	*R.nitidus*	0.030008	0.066516
MY4	SC5	CS2	*R.nitidus*	-0.037175	0.069606
GD42	SC6	CS2	*R.nitidus*	0.052669	0.067591
GD22	SC6	CS2	*R.nitidus*	0.07487	0.066387
GD25	SC6	CS2	*R.nitidus*	0.09299	0.067038
JW6	SC5	CS2	*R.nitidus*	-0.015195	0.076078
GD45	SC6	CS2	*R.nitidus*	0.086608	0.071336
GD40	SC6	CS2	*R.nitidus*	0.085915	0.06265
GD32	SC6	CS2	*R.nitidus*	0.037766	0.069409
GD31	SC6	CS2	*R.nitidus*	0.062635	0.065291
JO3	SC5	CS2	*R.nitidus*	0.007843	0.075363
JO7	SC6	CS2	*R.nitidus*	-0.01665	0.071097
GD21	SC6	CS2	*R.nitidus*	0.129973	0.06471
GD41	SC6	CS2	*R.nitidus*	0.030346	0.066529
GD46	SC6	CS2	*R.nitidus*	-0.004332	0.065196
GD34	SC6	CS2	*R.nitidus*	0.025105	0.066343
GD48	SC6	CS2	*R.nitidus*	0.06141	0.067752
CS14	SC6	CS2	*R.nitidus*	0.01487	0.069177
GD30	SC6	CS2	*R.nitidus*	0.146461	0.066794
CS23	SC6	CS2	*R.nitidus*	-0.045113	0.06606
GD26	SC6	CS2	*R.nitidus*	0.074386	0.06609
CS13	SC6	CS2	*R.nitidus*	-0.02603	0.06911
GD35	SC6	CS2	*R.nitidus*	0.059733	0.064102
CS12	SC6	CS2	*R.nitidus*	0.031056	0.069944
GD49	SC6	CS2	*R.nitidus*	0.096878	0.069141
GD33	SC6	CS2	*R.nitidus*	0.030921	0.066992
CS16	SC6	CS2	*R.nitidus*	0.017991	0.066708
SC14	SC6	CS2	*R.nitidus*	0.006364	0.066833
CS17	SC6	CS2	*R.nitidus*	0.033515	0.070127
GD50	SC6	CS2	*R.nitidus*	0.048593	0.067391
CS19	SC6	CS2	*R.nitidus*	-0.005197	0.06879
CS18	SC6	CS2	*R.nitidus*	-0.016442	0.067324
CS30	SC6	CS2	*R.nitidus*	-0.049599	0.0673
SC5	SC6	CS2	*R.nitidus*	0.014184	0.06966
CS15	SC6	CS2	*R.nitidus*	0.035101	0.066989
CS31	SC6	CS2	*R.nitidus*	-0.017437	0.067992
CS22	SC6	CS2	*R.nitidus*	0.02543	0.065565
SC24	SC6	CS2	*R.nitidus*	0.014575	0.06443
SC42	SC6	CS2	*R.nitidus*	0.275172	0.061403
CS25	SC6	CS2	*R.nitidus*	-0.001519	0.06831
CS24	SC6	CS2	*R.nitidus*	-0.026987	0.06549
SC12	SC6	CS2	*R.nitidus*	-0.011041	0.065864
CS28	SC6	CS2	*R.nitidus*	-0.019952	0.067555
CS21	SC6	CS2	*R.nitidus*	-0.00968	0.065317
CS3	SC6	CS2	*R.nitidus*	-0.009745	0.064527
JW4	SC6	CS2	*R.nitidus*	0.226766	0.066299
SC32	SC6	CS2	*R.nitidus*	0.040094	0.066599

CS4	SC6	CS2	*R.nitidus*	0.008689	0.064103
MY6	SC6	CS2	*R.nitidus*	0.019667	0.066772
JW6	SC6	CS2	*R.nitidus*	-0.015152	0.073024
SC45	SC6	CS2	*R.nitidus*	0.037618	0.065742
SC35	SC6	CS2	*R.nitidus*	0.056502	0.064341
JW7	SC6	CS2	*R.nitidus*	0.023033	0.070861
JO7	SC10	CS2	*R.nitidus*	-0.109645	0.073409
SC10	SC6	CS2	*R.nitidus*	0.090909	0.06771
JO1	SC6	CS2	*R.nitidus*	-0.019577	0.066996
CS6	SC6	CS2	*R.nitidus*	-0.03879	0.063761
MY4	SC6	CS2	*R.nitidus*	-0.026094	0.066251
JO3	SC6	CS2	*R.nitidus*	0.045833	0.069621
CS20	SC6	CS2	*R.nitidus*	-0.015849	0.064381
SC26	SC6	CS2	*R.nitidus*	-0.036892	0.065276
CS11	SC10	CS2	*R.nitidus*	-0.053571	0.069006
SC34	SC6	CS2	*R.nitidus*	0.044867	0.065663
SC44	SC6	CS2	*R.nitidus*	0.006168	0.065622
SC18	SC6	CS2	*R.nitidus*	0.052202	0.066125
SC15	SC6	CS2	*R.nitidus*	-0.011373	0.067139
SC28	SC6	CS2	*R.nitidus*	-0.044636	0.066227
SC27	SC6	CS2	*R.nitidus*	0.002335	0.064917
SC31	SC6	CS2	*R.nitidus*	0.021892	0.066635
JO8	SC10	CS2	*R.nitidus*	-0.068013	0.074626
GD31	SC10	CS2	*R.nitidus*	-0.003014	0.068043
JO5	SC6	CS2	*R.nitidus*	0.068027	0.06609
GD29	SC10	CS2	*R.nitidus*	0.065007	0.066057
GD30	SC10	CS2	*R.nitidus*	0.099746	0.070044
GD33	SC10	CS2	*R.nitidus*	-0.035966	0.068852
GD32	SC10	CS2	*R.nitidus*	-0.02818	0.068929
GD21	SC10	CS2	*R.nitidus*	0.067431	0.06504
GD26	SC10	CS2	*R.nitidus*	0.002855	0.067029
CS15	SC10	CS2	*R.nitidus*	-0.02939	0.06753
GD22	SC10	CS2	*R.nitidus*	0.011204	0.067059
GD34	SC10	CS2	*R.nitidus*	-0.034188	0.066964
GD35	SC10	CS2	*R.nitidus*	-0.012204	0.066775
CS19	SC10	CS2	*R.nitidus*	-0.063504	0.066256
GD48	SC10	CS2	*R.nitidus*	-0.012772	0.068122
CS13	SC10	CS2	*R.nitidus*	-0.089231	0.068294
GD44	SC10	CS2	*R.nitidus*	-0.024425	0.066701
GD25	SC10	CS2	*R.nitidus*	0.029784	0.066367
GD41	SC10	CS2	*R.nitidus*	-0.035533	0.06623
GD50	SC10	CS2	*R.nitidus*	-0.007833	0.071758
GD46	SC10	CS2	*R.nitidus*	-0.061495	0.065756
GD42	SC10	CS2	*R.nitidus*	-0.025148	0.06874
GD45	SC10	CS2	*R.nitidus*	0.019769	0.070714
CS20	SC10	CS2	*R.nitidus*	-0.080119	0.066265
CS17	SC10	CS2	*R.nitidus*	-0.036135	0.069338
CS12	SC10	CS2	*R.nitidus*	-0.06993	0.068683
GD49	SC10	CS2	*R.nitidus*	0.04095	0.07081
CS23	SC10	CS2	*R.nitidus*	-0.101417	0.067119
CS14	SC10	CS2	*R.nitidus*	-0.048048	0.067904
CS16	SC10	CS2	*R.nitidus*	-0.062682	0.067901
SC5	SC10	CS2	*R.nitidus*	-0.063556	0.070764
CS21	SC10	CS2	*R.nitidus*	-0.073263	0.065041
SC24	SC10	CS2	*R.nitidus*	-0.067083	0.066926
CS3	SC10	CS2	*R.nitidus*	-0.06459	0.064579

SC14	SC10	CS2	*R.nitidus*	-0.049674	0.068277
GD40	SC10	CS2	*R.nitidus*	0.008386	0.066524
SC42	SC10	CS2	*R.nitidus*	0.205601	0.063182
CS18	SC10	CS2	*R.nitidus*	-0.089871	0.066893
CS30	SC10	CS2	*R.nitidus*	-0.121746	0.066303
SC12	SC10	CS2	*R.nitidus*	-0.096774	0.063932
SC35	SC10	CS2	*R.nitidus*	-0.0192	0.065998
CS28	SC10	CS2	*R.nitidus*	-0.083401	0.067111
SC6	SC10	CS2	*R.nitidus*	-0.090909	0.06771
CS24	SC10	CS2	*R.nitidus*	-0.111975	0.063606
SC15	SC10	CS2	*R.nitidus*	-0.079086	0.067438
CS25	SC10	CS2	*R.nitidus*	-0.048229	0.069125
CS22	SC10	CS2	*R.nitidus*	-0.055215	0.064922
SC45	SC10	CS2	*R.nitidus*	-0.030008	0.066992
SC18	SC10	CS2	*R.nitidus*	-0.028381	0.06844
JO1	SC10	CS2	*R.nitidus*	-0.089968	0.065089
CS6	SC10	CS2	*R.nitidus*	-0.097918	0.066696
SC27	SC10	CS2	*R.nitidus*	-0.065124	0.064572
JO3	SC10	CS2	*R.nitidus*	-0.025551	0.070539
CS31	SC10	CS2	*R.nitidus*	-0.088558	0.06625
GD22	CS4	CS2	*R.nitidus*	0.079523	0.062256
CS4	SC10	CS2	*R.nitidus*	-0.064207	0.065234
MY6	SC10	CS2	*R.nitidus*	-0.069749	0.068051
SC32	SC10	CS2	*R.nitidus*	-0.02616	0.063893
MY4	SC10	CS2	*R.nitidus*	-0.108347	0.069496
SC26	SC10	CS2	*R.nitidus*	-0.1104	0.064113
SC31	SC10	CS2	*R.nitidus*	-0.070456	0.065171
SC44	SC10	CS2	*R.nitidus*	-0.074921	0.065058
SC28	SC10	CS2	*R.nitidus*	-0.120773	0.064485
JW7	SC10	CS2	*R.nitidus*	-0.026866	0.069528
SC34	SC10	CS2	*R.nitidus*	-0.01964	0.065502
GD21	CS4	CS2	*R.nitidus*	0.122382	0.062875
JW6	SC10	CS2	*R.nitidus*	-0.086053	0.074956
GD29	CS4	CS2	*R.nitidus*	0.128378	0.062336
GD32	CS4	CS2	*R.nitidus*	0.053055	0.066828
JO5	SC10	CS2	*R.nitidus*	-0.013323	0.065771
JO8	CS4	CS2	*R.nitidus*	0.006849	0.070995
JO7	CS4	CS2	*R.nitidus*	-0.002817	0.071269
GD40	CS4	CS2	*R.nitidus*	0.083916	0.06333
GD35	CS4	CS2	*R.nitidus*	0.04965	0.065519
GD30	CS4	CS2	*R.nitidus*	0.132549	0.065572
CS11	CS4	CS2	*R.nitidus*	0.008929	0.068108
GD33	CS4	CS2	*R.nitidus*	0.026893	0.06577
CS12	CS4	CS2	*R.nitidus*	0.029096	0.066025
CS13	CS4	CS2	*R.nitidus*	-0.031963	0.066925
GD26	CS4	CS2	*R.nitidus*	0.06323	0.064301
GD34	CS4	CS2	*R.nitidus*	0.01385	0.06438
CS14	CS4	CS2	*R.nitidus*	0.013971	0.067389
GD44	CS4	CS2	*R.nitidus*	0.043834	0.065396
GD31	CS4	CS2	*R.nitidus*	0.060694	0.064937
GD46	CS4	CS2	*R.nitidus*	-0.007623	0.064161
GD41	CS4	CS2	*R.nitidus*	0.022663	0.063699
CS16	CS4	CS2	*R.nitidus*	0.004435	0.066566
GD48	CS4	CS2	*R.nitidus*	0.037206	0.065812
CS20	CS4	CS2	*R.nitidus*	-0.031122	0.065474
GD25	CS4	CS2	*R.nitidus*	0.071034	0.063964

GD49	CS4	CS2	*R.nitidus*	0.083201	0.066553
SC15	CS4	CS2	*R.nitidus*	-0.009886	0.065079
SC6	CS4	CS2	*R.nitidus*	-0.008689	0.064103
CS24	CS4	CS2	*R.nitidus*	-0.035584	0.061819
CS15	CS4	CS2	*R.nitidus*	0.024982	0.066318
CS23	CS4	CS2	*R.nitidus*	-0.048741	0.064175
CS25	CS4	CS2	*R.nitidus*	-0.014308	0.068699
GD42	CS4	CS2	*R.nitidus*	0.037141	0.063965
SC26	CS4	CS2	*R.nitidus*	-0.029703	0.062578
CS28	CS4	CS2	*R.nitidus*	-0.010244	0.06545
GD50	CS4	CS2	*R.nitidus*	0.039004	0.066408
SC34	CS4	CS2	*R.nitidus*	0.037482	0.062714
CS17	CS4	CS2	*R.nitidus*	0.030854	0.068562
GD45	CS4	CS2	*R.nitidus*	0.081566	0.068617
CS21	CS4	CS2	*R.nitidus*	0.005618	0.065445
CS18	CS4	CS2	*R.nitidus*	-0.015755	0.064269
CS19	CS4	CS2	*R.nitidus*	-0.008907	0.068553
CS6	CS4	CS2	*R.nitidus*	-0.037488	0.064598
SC5	CS4	CS2	*R.nitidus*	0.013343	0.067652
SC42	CS4	CS2	*R.nitidus*	0.259327	0.060943
SC31	CS4	CS2	*R.nitidus*	-0.002896	0.064308
SC32	CS4	CS2	*R.nitidus*	0.030689	0.064969
CS22	CS4	CS2	*R.nitidus*	0.022527	0.063577
CS3	CS4	CS2	*R.nitidus*	-0.024721	0.065497
SC45	CS4	CS2	*R.nitidus*	0.030758	0.065394
JO3	CS4	CS2	*R.nitidus*	0.045714	0.068232
JW4	CS4	CS2	*R.nitidus*	0.224818	0.064884
SC24	CS4	CS2	*R.nitidus*	0.008197	0.065116
CS30	CS4	CS2	*R.nitidus*	-0.056765	0.063096
SC12	CS4	CS2	*R.nitidus*	-0.017673	0.064251
CS31	CS4	CS2	*R.nitidus*	-0.015909	0.064238
SC35	CS4	CS2	*R.nitidus*	0.039884	0.064693
SC10	CS4	CS2	*R.nitidus*	0.064207	0.065234
SC18	CS4	CS2	*R.nitidus*	0.044799	0.064658
SC14	CS4	CS2	*R.nitidus*	-0.000724	0.063615
GD30	SC12	CS2	*R.nitidus*	0.127642	0.06706
MY4	CS4	CS2	*R.nitidus*	-0.033962	0.066369
MY6	CS4	CS2	*R.nitidus*	0.011782	0.065343
SC28	CS4	CS2	*R.nitidus*	-0.037599	0.063633
GD25	SC12	CS2	*R.nitidus*	0.093343	0.066439
JW7	CS4	CS2	*R.nitidus*	0.006957	0.065308
JO5	CS4	CS2	*R.nitidus*	0.059854	0.062726
GD32	SC12	CS2	*R.nitidus*	0.046032	0.068274
SC27	CS4	CS2	*R.nitidus*	-0.000721	0.062715
JO1	CS4	CS2	*R.nitidus*	-0.017505	0.064916
JW6	CS4	CS2	*R.nitidus*	-0.012015	0.071607
GD26	SC12	CS2	*R.nitidus*	0.07839	0.063235
SC44	CS4	CS2	*R.nitidus*	-0.000729	0.062892
GD29	SC12	CS2	*R.nitidus*	0.131333	0.065364
GD31	SC12	CS2	*R.nitidus*	0.063179	0.065989
JO8	SC12	CS2	*R.nitidus*	0.044792	0.072909
JO7	SC12	CS2	*R.nitidus*	0.008008	0.070561
GD21	SC12	CS2	*R.nitidus*	0.132013	0.064006
CS12	SC12	CS2	*R.nitidus*	0.021358	0.068281
CS14	SC12	CS2	*R.nitidus*	0.021245	0.069805
GD45	SC12	CS2	*R.nitidus*	0.085241	0.069467

CS11	SC12	CS2	*R.nitidus*	0.013373	0.067905
GD22	SC12	CS2	*R.nitidus*	0.085733	0.063476
GD44	SC12	CS2	*R.nitidus*	0.050883	0.066003
GD46	SC12	CS2	*R.nitidus*	0.007757	0.065613
CS15	SC12	CS2	*R.nitidus*	0.039046	0.068591
CS21	SC12	CS2	*R.nitidus*	0.002195	0.065732
CS25	SC12	CS2	*R.nitidus*	0.017293	0.067696
GD33	SC12	CS2	*R.nitidus*	0.033779	0.066525
GD48	SC12	CS2	*R.nitidus*	0.062823	0.068149
GD50	SC12	CS2	*R.nitidus*	0.079125	0.068773
CS16	SC12	CS2	*R.nitidus*	0.021497	0.06656
GD49	SC12	CS2	*R.nitidus*	0.088997	0.066965
CS13	SC12	CS2	*R.nitidus*	-0.028422	0.067862
GD34	SC12	CS2	*R.nitidus*	0.028926	0.066227
GD40	SC12	CS2	*R.nitidus*	0.093243	0.064545
CS19	SC12	CS2	*R.nitidus*	0.000741	0.067002
CS3	SC12	CS2	*R.nitidus*	0.005307	0.065452
GD35	SC12	CS2	*R.nitidus*	0.067321	0.066064
GD41	SC12	CS2	*R.nitidus*	0.026112	0.065072
GD42	SC12	CS2	*R.nitidus*	0.053725	0.066033
CS28	SC12	CS2	*R.nitidus*	0.000792	0.066676
SC28	SC12	CS2	*R.nitidus*	-0.014481	0.06408
SC14	SC12	CS2	*R.nitidus*	0.013147	0.066002
CS30	SC12	CS2	*R.nitidus*	-0.03265	0.065791
CS23	SC12	CS2	*R.nitidus*	-0.018328	0.064793
SC24	SC12	CS2	*R.nitidus*	0.033281	0.064704
CS17	SC12	CS2	*R.nitidus*	0.03867	0.069788
SC18	SC12	CS2	*R.nitidus*	0.092834	0.065528
CS22	SC12	CS2	*R.nitidus*	0.031466	0.065786
CS18	SC12	CS2	*R.nitidus*	-0.00224	0.065027
CS4	SC12	CS2	*R.nitidus*	0.017673	0.064251
SC5	SC12	CS2	*R.nitidus*	0.028363	0.0696
SC15	SC12	CS2	*R.nitidus*	0.004898	0.066435
SC34	SC12	CS2	*R.nitidus*	0.060678	0.06578
CS24	SC12	CS2	*R.nitidus*	-0.017843	0.065128
CS6	SC12	CS2	*R.nitidus*	-0.030469	0.064918
SC27	SC12	CS2	*R.nitidus*	0.022708	0.064255
SC35	SC12	CS2	*R.nitidus*	0.081839	0.061663
CS20	SC12	CS2	*R.nitidus*	-0.013838	0.066693
SC32	SC12	CS2	*R.nitidus*	0.05873	0.06556
JO1	SC12	CS2	*R.nitidus*	-0.003928	0.065651
CS31	SC12	CS2	*R.nitidus*	-0.003026	0.065389
SC42	SC12	CS2	*R.nitidus*	0.281853	0.060294
JW7	SC12	CS2	*R.nitidus*	0.062317	0.068304
JO8	SC14	CS2	*R.nitidus*	0.008824	0.072307
JO7	SC14	CS2	*R.nitidus*	-0.014451	0.07286
SC26	SC12	CS2	*R.nitidus*	-0.022709	0.06377
SC45	SC12	CS2	*R.nitidus*	0.0544	0.065059
SC6	SC12	CS2	*R.nitidus*	0.011041	0.065864
SC10	SC12	CS2	*R.nitidus*	0.096774	0.063932
JW4	SC12	CS2	*R.nitidus*	0.261161	0.06528
GD30	SC14	CS2	*R.nitidus*	0.118357	0.067781
MY6	SC12	CS2	*R.nitidus*	0.025699	0.066334
SC31	SC12	CS2	*R.nitidus*	0.015957	0.066306
JW6	SC12	CS2	*R.nitidus*	0.036965	0.071438
GD25	SC14	CS2	*R.nitidus*	0.076064	0.065603

MY4	SC12	CS2	*R.nitidus*	-0.019069	0.064626
GD29	SC14	CS2	*R.nitidus*	0.112352	0.063577
GD26	SC14	CS2	*R.nitidus*	0.06212	0.066792
GD31	SC14	CS2	*R.nitidus*	0.055197	0.06607
JO3	SC12	CS2	*R.nitidus*	0.077815	0.068246
GD33	SC14	CS2	*R.nitidus*	0.010176	0.066502
JO5	SC12	CS2	*R.nitidus*	0.08716	0.063622
GD32	SC14	CS2	*R.nitidus*	0.014423	0.067934
CS11	SC14	CS2	*R.nitidus*	0.003595	0.069058
CS13	SC14	CS2	*R.nitidus*	-0.03663	0.067355
SC44	SC12	CS2	*R.nitidus*	0.013344	0.064434
GD21	SC14	CS2	*R.nitidus*	0.11261	0.065917
CS20	SC14	CS2	*R.nitidus*	-0.030714	0.065091
GD45	SC14	CS2	*R.nitidus*	0.057891	0.070356
GD41	SC14	CS2	*R.nitidus*	0.019466	0.064059
GD49	SC14	CS2	*R.nitidus*	0.066298	0.069051
CS15	SC14	CS2	*R.nitidus*	0.028633	0.068172
GD46	SC14	CS2	*R.nitidus*	-0.022551	0.065567
GD22	SC14	CS2	*R.nitidus*	0.071334	0.064374
CS17	SC14	CS2	*R.nitidus*	0.016667	0.069917
CS12	SC14	CS2	*R.nitidus*	0.007463	0.06784
GD44	SC14	CS2	*R.nitidus*	0.026987	0.067724
GD42	SC14	CS2	*R.nitidus*	0.020878	0.065301
GD34	SC14	CS2	*R.nitidus*	0.005495	0.065829
SC5	SC14	CS2	*R.nitidus*	0.005719	0.070729
GD35	SC14	CS2	*R.nitidus*	0.033381	0.066736
GD40	SC14	CS2	*R.nitidus*	0.065807	0.064824
GD48	SC14	CS2	*R.nitidus*	0.048872	0.067791
CS25	SC14	CS2	*R.nitidus*	-0.006667	0.067042
CS21	SC14	CS2	*R.nitidus*	-0.02603	0.065708
CS14	SC14	CS2	*R.nitidus*	0.009213	0.067781
CS23	SC14	CS2	*R.nitidus*	-0.047448	0.066048
GD50	SC14	CS2	*R.nitidus*	0.022425	0.067843
CS30	SC14	CS2	*R.nitidus*	-0.051339	0.06533
CS16	SC14	CS2	*R.nitidus*	0.003644	0.066361
CS19	SC14	CS2	*R.nitidus*	-0.010463	0.0682
SC15	SC14	CS2	*R.nitidus*	-0.018364	0.066341
CS22	SC14	CS2	*R.nitidus*	0.006737	0.065462
SC35	SC14	CS2	*R.nitidus*	0.05142	0.064436
CS31	SC14	CS2	*R.nitidus*	-0.015748	0.067696
CS18	SC14	CS2	*R.nitidus*	-0.015965	0.066304
CS28	SC14	CS2	*R.nitidus*	-0.018196	0.06714
SC18	SC14	CS2	*R.nitidus*	0.041667	0.067137
CS6	SC14	CS2	*R.nitidus*	-0.048913	0.064115
SC12	SC14	CS2	*R.nitidus*	-0.013147	0.066002
CS24	SC14	CS2	*R.nitidus*	-0.028616	0.064477
JW6	SC14	CS2	*R.nitidus*	0.004789	0.069692
SC28	SC14	CS2	*R.nitidus*	-0.038156	0.065263
SC32	SC14	CS2	*R.nitidus*	0.03104	0.06667
MY4	SC14	CS2	*R.nitidus*	-0.040449	0.067981
JW7	SC14	CS2	*R.nitidus*	0.03	0.069403
SC10	SC14	CS2	*R.nitidus*	0.049674	0.068277
CS3	SC14	CS2	*R.nitidus*	-0.023739	0.065991
JO3	SC14	CS2	*R.nitidus*	0.044498	0.068801
SC6	SC14	CS2	*R.nitidus*	-0.006364	0.066833
JO1	SC14	CS2	*R.nitidus*	-0.00768	0.065064

SC44	SC14	CS2	*R.nitidus*	-0.005632	0.06457
SC27	SC14	CS2	*R.nitidus*	-0.004315	0.060661
SC24	SC14	CS2	*R.nitidus*	0.00716	0.066624
SC34	SC14	CS2	*R.nitidus*	0.037618	0.066249
JW4	SC14	CS2	*R.nitidus*	0.223088	0.065272
SC26	SC14	CS2	*R.nitidus*	-0.037529	0.065238
CS4	SC14	CS2	*R.nitidus*	0.000724	0.063615
JO5	SC14	CS2	*R.nitidus*	0.064593	0.065428
GD32	SC15	CS2	*R.nitidus*	0.030885	0.069062
SC45	SC14	CS2	*R.nitidus*	0.033482	0.066992
SC31	SC14	CS2	*R.nitidus*	-0.007206	0.06678
GD34	SC15	CS2	*R.nitidus*	0.03196	0.066197
GD21	SC15	CS2	*R.nitidus*	0.137621	0.066434
SC42	SC14	CS2	*R.nitidus*	0.255198	0.0625
GD22	SC15	CS2	*R.nitidus*	0.084438	0.064926
MY6	SC14	CS2	*R.nitidus*	0.007236	0.066616
JO8	SC15	CS2	*R.nitidus*	0.039236	0.07404
GD33	SC15	CS2	*R.nitidus*	0.028694	0.06518
JO7	SC15	CS2	*R.nitidus*	0.018386	0.073501
GD26	SC15	CS2	*R.nitidus*	0.063559	0.066646
GD42	SC15	CS2	*R.nitidus*	0.06577	0.067154
GD30	SC15	CS2	*R.nitidus*	0.135607	0.069227
GD40	SC15	CS2	*R.nitidus*	0.094767	0.064362
CS11	SC15	CS2	*R.nitidus*	0.021689	0.069104
GD45	SC15	CS2	*R.nitidus*	0.070952	0.070012
CS13	SC15	CS2	*R.nitidus*	-0.023659	0.067763
GD49	SC15	CS2	*R.nitidus*	0.098856	0.068286
GD50	SC15	CS2	*R.nitidus*	0.038462	0.069836
GD44	SC15	CS2	*R.nitidus*	0.029163	0.068322
CS16	SC15	CS2	*R.nitidus*	0.018913	0.067417
GD25	SC15	CS2	*R.nitidus*	0.096992	0.068991
CS23	SC15	CS2	*R.nitidus*	-0.038431	0.064679
GD29	SC15	CS2	*R.nitidus*	0.139567	0.065523
CS20	SC15	CS2	*R.nitidus*	-0.006098	0.067874
CS12	SC15	CS2	*R.nitidus*	0.028962	0.066802
GD31	SC15	CS2	*R.nitidus*	0.068657	0.066819
GD48	SC15	CS2	*R.nitidus*	0.066879	0.068002
SC18	SC15	CS2	*R.nitidus*	0.063131	0.067036
GD46	SC15	CS2	*R.nitidus*	-0.002963	0.066632
CS18	SC15	CS2	*R.nitidus*	-0.000793	0.06729
CS19	SC15	CS2	*R.nitidus*	-0.004706	0.069316
CS24	SC15	CS2	*R.nitidus*	-0.019512	0.065108
CS21	SC15	CS2	*R.nitidus*	0.009195	0.066184
CS25	SC15	CS2	*R.nitidus*	0.004651	0.069114
GD41	SC15	CS2	*R.nitidus*	0.027347	0.066196
GD35	SC15	CS2	*R.nitidus*	0.057604	0.068384
CS30	SC15	CS2	*R.nitidus*	-0.041363	0.066166
CS15	SC15	CS2	*R.nitidus*	0.035472	0.067867
SC27	SC15	CS2	*R.nitidus*	0.017964	0.064098
CS14	SC15	CS2	*R.nitidus*	0.025443	0.069524
SC28	SC15	CS2	*R.nitidus*	-0.022152	0.067439
CS28	SC15	CS2	*R.nitidus*	0.008489	0.067771
SC5	SC15	CS2	*R.nitidus*	0.022275	0.070336
SC35	SC15	CS2	*R.nitidus*	0.067729	0.065687
SC31	SC15	CS2	*R.nitidus*	0.017197	0.066671
CS31	SC15	CS2	*R.nitidus*	0.005691	0.070001

CS17	SC15	CS2	*R.nitidus*	0.042359	0.070855
CS22	SC15	CS2	*R.nitidus*	0.025098	0.066894
SC6	SC15	CS2	*R.nitidus*	0.011373	0.067139
CS4	SC15	CS2	*R.nitidus*	0.009886	0.065079
SC44	SC15	CS2	*R.nitidus*	0.012749	0.065601
SC32	SC15	CS2	*R.nitidus*	0.056432	0.066584
SC12	SC15	CS2	*R.nitidus*	-0.004898	0.066435
CS3	SC15	CS2	*R.nitidus*	0	0.065994
SC24	SC15	CS2	*R.nitidus*	0.023529	0.067697
CS6	SC15	CS2	*R.nitidus*	-0.03236	0.06552
SC26	SC15	CS2	*R.nitidus*	-0.026835	0.062734
SC10	SC15	CS2	*R.nitidus*	0.079086	0.067438
SC45	SC15	CS2	*R.nitidus*	0.055075	0.06713
SC34	SC15	CS2	*R.nitidus*	0.057258	0.066616
GD21	SC18	CS2	*R.nitidus*	0.099509	0.064563
CS12	SC18	CS2	*R.nitidus*	-0.052632	0.068415
MY6	SC15	CS2	*R.nitidus*	0.022516	0.066605
JO8	SC18	CS2	*R.nitidus*	-0.016575	0.072663
JW6	SC15	CS2	*R.nitidus*	0.027495	0.07313
SC42	SC15	CS2	*R.nitidus*	0.284884	0.062179
JO7	SC18	CS2	*R.nitidus*	-0.044534	0.072823
GD33	SC18	CS2	*R.nitidus*	-0.014717	0.066262
JO1	SC15	CS2	*R.nitidus*	-0.002408	0.067173
GD35	SC18	CS2	*R.nitidus*	0.019505	0.066931
JO5	SC15	CS2	*R.nitidus*	0.085714	0.064896
GD34	SC18	CS2	*R.nitidus*	-0.023827	0.06554
JW4	SC15	CS2	*R.nitidus*	0.241354	0.067705
GD31	SC18	CS2	*R.nitidus*	0.007587	0.065792
GD30	SC18	CS2	*R.nitidus*	0.082158	0.067422
JO3	SC15	CS2	*R.nitidus*	0.06626	0.070067
JW7	SC15	CS2	*R.nitidus*	0.04451	0.070023
MY4	SC15	CS2	*R.nitidus*	-0.021722	0.067778
GD32	SC18	CS2	*R.nitidus*	-0.023529	0.068452
SC14	SC15	CS2	*R.nitidus*	0.018364	0.066341
GD29	SC18	CS2	*R.nitidus*	0.078059	0.065642
CS14	SC18	CS2	*R.nitidus*	-0.025604	0.068605
GD48	SC18	CS2	*R.nitidus*	-0.00556	0.067918
GD22	SC18	CS2	*R.nitidus*	0.027046	0.064567
GD45	SC18	CS2	*R.nitidus*	0.013525	0.070935
CS11	SC18	CS2	*R.nitidus*	-0.047689	0.066762
GD25	SC18	CS2	*R.nitidus*	0.040668	0.067129
GD42	SC18	CS2	*R.nitidus*	0.011346	0.066641
CS13	SC18	CS2	*R.nitidus*	-0.078888	0.068596
GD26	SC18	CS2	*R.nitidus*	0.019679	0.064524
GD50	SC18	CS2	*R.nitidus*	0	0.070043
GD40	SC18	CS2	*R.nitidus*	0.031295	0.063106
GD49	SC18	CS2	*R.nitidus*	0.038698	0.069676
CS20	SC18	CS2	*R.nitidus*	-0.078886	0.065793
GD46	SC18	CS2	*R.nitidus*	-0.068433	0.064822
GD41	SC18	CS2	*R.nitidus*	-0.012696	0.066303
GD44	SC18	CS2	*R.nitidus*	0.000777	0.067574
CS30	SC18	CS2	*R.nitidus*	-0.102167	0.066411
CS19	SC18	CS2	*R.nitidus*	-0.06869	0.067534
CS21	SC18	CS2	*R.nitidus*	-0.064688	0.06645
SC6	SC18	CS2	*R.nitidus*	-0.052202	0.066125
SC5	SC18	CS2	*R.nitidus*	-0.045226	0.069793

CS15	SC18	CS2	*R.nitidus*	-0.030483	0.06667
SC27	SC18	CS2	*R.nitidus*	-0.042869	0.065609
MY4	SC18	CS2	*R.nitidus*	-0.100895	0.063255
CS17	SC18	CS2	*R.nitidus*	-0.016241	0.069667
CS16	SC18	CS2	*R.nitidus*	-0.041825	0.066682
SC42	SC18	CS2	*R.nitidus*	0.218543	0.063592
CS6	SC18	CS2	*R.nitidus*	-0.101926	0.064546
SC10	SC18	CS2	*R.nitidus*	0.028381	0.06844
CS22	SC18	CS2	*R.nitidus*	-0.03125	0.066692
CS28	SC18	CS2	*R.nitidus*	-0.0664	0.068276
JW7	SC18	CS2	*R.nitidus*	0.004897	0.069453
CS24	SC18	CS2	*R.nitidus*	-0.0726	0.066805
SC35	SC18	CS2	*R.nitidus*	-0.005582	0.062906
SC12	SC18	CS2	*R.nitidus*	-0.092834	0.065528
CS4	SC18	CS2	*R.nitidus*	-0.044799	0.064658
JO3	SC18	CS2	*R.nitidus*	0.002575	0.070635
SC34	SC18	CS2	*R.nitidus*	-0.012668	0.065616
JO5	SC18	CS2	*R.nitidus*	0.01381	0.065339
SC26	SC18	CS2	*R.nitidus*	-0.104613	0.064888
SC24	SC18	CS2	*R.nitidus*	-0.068376	0.063364
CS25	SC18	CS2	*R.nitidus*	-0.05513	0.067808
JW6	SC18	CS2	*R.nitidus*	-0.020263	0.07235
CS23	SC18	CS2	*R.nitidus*	-0.096824	0.065847
SC15	SC18	CS2	*R.nitidus*	-0.063131	0.067036
JO8	SC24	CS2	*R.nitidus*	-0.006309	0.071515
CS31	SC18	CS2	*R.nitidus*	-0.068059	0.066289
SC14	SC18	CS2	*R.nitidus*	-0.041667	0.067137
SC31	SC18	CS2	*R.nitidus*	-0.053834	0.065349
CS3	SC18	CS2	*R.nitidus*	-0.073132	0.065711
JW4	SC18	CS2	*R.nitidus*	0.200917	0.067877
JO1	SC18	CS2	*R.nitidus*	-0.07084	0.066895
JO7	SC24	CS2	*R.nitidus*	-0.029263	0.073422
SC32	SC18	CS2	*R.nitidus*	-0.018441	0.062559
CS18	SC18	CS2	*R.nitidus*	-0.071807	0.066788
GD25	SC24	CS2	*R.nitidus*	0.068768	0.067438
GD32	SC24	CS2	*R.nitidus*	0.023064	0.067973
MY6	SC18	CS2	*R.nitidus*	-0.05427	0.064372
SC28	SC18	CS2	*R.nitidus*	-0.105	0.063427
SC44	SC18	CS2	*R.nitidus*	-0.071669	0.063402
SC45	SC18	CS2	*R.nitidus*	-0.025122	0.066515
CS11	SC24	CS2	*R.nitidus*	0	0.068851
GD26	SC24	CS2	*R.nitidus*	0.055914	0.065415
GD50	SC24	CS2	*R.nitidus*	0.055085	0.068643
GD44	SC24	CS2	*R.nitidus*	0.030211	0.068811
GD31	SC24	CS2	*R.nitidus*	0.051524	0.067428
CS20	SC24	CS2	*R.nitidus*	-0.041199	0.065331
GD21	SC24	CS2	*R.nitidus*	0.121769	0.06563
CS14	SC24	CS2	*R.nitidus*	-0.005839	0.0702
CS15	SC24	CS2	*R.nitidus*	0.015173	0.066734
GD46	SC24	CS2	*R.nitidus*	-0.025881	0.064936
GD30	SC24	CS2	*R.nitidus*	0.128981	0.067966
GD22	SC24	CS2	*R.nitidus*	0.070383	0.066631
CS18	SC24	CS2	*R.nitidus*	-0.026198	0.065763
GD34	SC24	CS2	*R.nitidus*	0.007179	0.065931
CS4	SC24	CS2	*R.nitidus*	-0.008197	0.065116
CS12	SC24	CS2	*R.nitidus*	-0.007616	0.067693

CS22	SC24	CS2	*R.nitidus*	0.006696	0.065467
SC12	SC24	CS2	*R.nitidus*	-0.033281	0.064704
GD45	SC24	CS2	*R.nitidus*	0.043265	0.069909
GD49	SC24	CS2	*R.nitidus*	0.091362	0.068581
GD29	SC24	CS2	*R.nitidus*	0.117925	0.065104
CS17	SC24	CS2	*R.nitidus*	0.018853	0.069353
GD40	SC24	CS2	*R.nitidus*	0.061107	0.064775
CS24	SC24	CS2	*R.nitidus*	-0.035249	0.064721
CS30	SC24	CS2	*R.nitidus*	-0.069856	0.066106
GD41	SC24	CS2	*R.nitidus*	0.021183	0.065299
GD48	SC24	CS2	*R.nitidus*	0.025078	0.067858
SC5	SC24	CS2	*R.nitidus*	-0.012235	0.070583
CS19	SC24	CS2	*R.nitidus*	-0.026174	0.068335
CS21	SC24	CS2	*R.nitidus*	-0.023152	0.065122
CS3	SC24	CS2	*R.nitidus*	-0.031882	0.066395
GD35	SC24	CS2	*R.nitidus*	0.049229	0.065266
SC14	SC24	CS2	*R.nitidus*	-0.00716	0.066624
GD42	SC24	CS2	*R.nitidus*	0.031111	0.066205
CS31	SC24	CS2	*R.nitidus*	-0.02313	0.065789
SC18	SC24	CS2	*R.nitidus*	0.068376	0.063364
GD33	SC24	CS2	*R.nitidus*	0.018195	0.066132
CS13	SC24	CS2	*R.nitidus*	-0.048184	0.069303
SC6	SC24	CS2	*R.nitidus*	-0.014575	0.06443
CS6	SC24	CS2	*R.nitidus*	-0.059477	0.063987
SC28	SC24	CS2	*R.nitidus*	-0.057052	0.063997
CS16	SC24	CS2	*R.nitidus*	-0.009517	0.06596
CS23	SC24	CS2	*R.nitidus*	-0.056138	0.064898
CS28	SC24	CS2	*R.nitidus*	-0.024038	0.066049
CS25	SC24	CS2	*R.nitidus*	-0.026576	0.068291
SC10	SC24	CS2	*R.nitidus*	0.067083	0.066926
SC42	SC24	CS2	*R.nitidus*	0.256297	0.061571
JW6	SC24	CS2	*R.nitidus*	-0.014014	0.073464
SC45	SC24	CS2	*R.nitidus*	0.03115	0.063424
JO7	SC26	CS2	*R.nitidus*	0.024738	0.073125
SC27	SC24	CS2	*R.nitidus*	-0.000797	0.064769
JO3	SC24	CS2	*R.nitidus*	0.044764	0.068973
JW4	SC24	CS2	*R.nitidus*	0.227886	0.067128
SC44	SC24	CS2	*R.nitidus*	-0.015335	0.064362
SC31	SC24	CS2	*R.nitidus*	-0.014241	0.065527
GD32	SC26	CS2	*R.nitidus*	0.048348	0.067922
SC35	SC24	CS2	*R.nitidus*	0.038192	0.063564
JW7	SC24	CS2	*R.nitidus*	0.026975	0.070524
SC15	SC24	CS2	*R.nitidus*	-0.023529	0.067697
SC32	SC24	CS2	*R.nitidus*	0.029295	0.064063
GD30	SC26	CS2	*R.nitidus*	0.159524	0.066853
MY6	SC24	CS2	*R.nitidus*	-0.003858	0.066168
GD29	SC26	CS2	*R.nitidus*	0.158488	0.063347
MY4	SC24	CS2	*R.nitidus*	-0.05251	0.066013
SC34	SC24	CS2	*R.nitidus*	0.026709	0.065465
JO8	SC26	CS2	*R.nitidus*	0.092199	0.071761
SC26	SC24	CS2	*R.nitidus*	-0.053643	0.064249
GD31	SC26	CS2	*R.nitidus*	0.087302	0.064774
GD35	SC26	CS2	*R.nitidus*	0.073691	0.064086
GD25	SC26	CS2	*R.nitidus*	0.111585	0.066198
CS12	SC26	CS2	*R.nitidus*	0.037606	0.066767
CS15	SC26	CS2	*R.nitidus*	0.054506	0.065166

GD42	SC26	CS2	*R.nitidus*	0.063961	0.067879
GD21	SC26	CS2	*R.nitidus*	0.157133	0.063361
GD46	SC26	CS2	*R.nitidus*	0.013755	0.064707
GD33	SC26	CS2	*R.nitidus*	0.050526	0.065915
GD44	SC26	CS2	*R.nitidus*	0.055994	0.06669
CS13	SC26	CS2	*R.nitidus*	-0.002256	0.066807
CS14	SC26	CS2	*R.nitidus*	0.04496	0.067517
JO1	SC24	CS2	*R.nitidus*	-0.027624	0.065462
GD22	SC26	CS2	*R.nitidus*	0.100396	0.064059
GD48	SC26	CS2	*R.nitidus*	0.081101	0.066814
GD34	SC26	CS2	*R.nitidus*	0.042509	0.063655
CS11	SC26	CS2	*R.nitidus*	0.037445	0.065387
GD50	SC26	CS2	*R.nitidus*	0.065109	0.068939
CS19	SC26	CS2	*R.nitidus*	0.022861	0.066126
GD26	SC26	CS2	*R.nitidus*	0.094274	0.066373
GD45	SC26	CS2	*R.nitidus*	0.089342	0.068895
GD41	SC26	CS2	*R.nitidus*	0.047751	0.067574
CS16	SC26	CS2	*R.nitidus*	0.037091	0.064224
CS20	SC26	CS2	*R.nitidus*	0.009036	0.065567
GD49	SC26	CS2	*R.nitidus*	0.11901	0.069929
GD40	SC26	CS2	*R.nitidus*	0.098428	0.063208
CS22	SC26	CS2	*R.nitidus*	0.053254	0.064783
JO5	SC24	CS2	*R.nitidus*	0.057003	0.063901
CS23	SC26	CS2	*R.nitidus*	-0.006526	0.065727
SC15	SC26	CS2	*R.nitidus*	0.026835	0.062734
SC44	SC26	CS2	*R.nitidus*	0.034564	0.065086
SC18	SC26	CS2	*R.nitidus*	0.104613	0.064888
SC14	SC26	CS2	*R.nitidus*	0.037529	0.065238
SC24	SC26	CS2	*R.nitidus*	0.053643	0.064249
CS31	SC26	CS2	*R.nitidus*	0.015015	0.06595
CS3	SC26	CS2	*R.nitidus*	0.018113	0.065026
SC6	SC26	CS2	*R.nitidus*	0.036892	0.065276
CS30	SC26	CS2	*R.nitidus*	-0.02	0.064773
CS21	SC26	CS2	*R.nitidus*	0.025926	0.063771
CS18	SC26	CS2	*R.nitidus*	0.01312	0.063666
CS17	SC26	CS2	*R.nitidus*	0.049728	0.068139
SC34	SC26	CS2	*R.nitidus*	0.081404	0.062864
SC42	SC26	CS2	*R.nitidus*	0.277946	0.06116
JW7	SC26	CS2	*R.nitidus*	0.083095	0.068926
JW4	SC26	CS2	*R.nitidus*	0.264642	0.065192
CS24	SC26	CS2	*R.nitidus*	0.006002	0.064497
MY6	SC26	CS2	*R.nitidus*	0.041908	0.064713
SC45	SC26	CS2	*R.nitidus*	0.083916	0.065961
CS28	SC26	CS2	*R.nitidus*	0.022781	0.066397
CS6	SC26	CS2	*R.nitidus*	-0.003805	0.065208
JO1	SC26	CS2	*R.nitidus*	0.015212	0.064682
CS25	SC26	CS2	*R.nitidus*	0.020573	0.066306
SC35	SC26	CS2	*R.nitidus*	0.097859	0.061034
SC5	SC26	CS2	*R.nitidus*	0.049574	0.068825
SC27	SC26	CS2	*R.nitidus*	0.033898	0.063483
SC10	SC26	CS2	*R.nitidus*	0.1104	0.064113
SC32	SC26	CS2	*R.nitidus*	0.068482	0.064425
JW6	SC26	CS2	*R.nitidus*	0.038573	0.071209
CS4	SC26	CS2	*R.nitidus*	0.029703	0.062578
GD21	SC27	CS2	*R.nitidus*	0.118805	0.06543
MY4	SC26	CS2	*R.nitidus*	0.005201	0.064479

SC12	SC26	CS2	*R.nitidus*	0.022709	0.06377
GD31	SC27	CS2	*R.nitidus*	0.045985	0.065875
SC28	SC26	CS2	*R.nitidus*	0.005405	0.064014
GD25	SC27	CS2	*R.nitidus*	0.091808	0.065036
SC31	SC26	CS2	*R.nitidus*	0.041335	0.064488
JO8	SC27	CS2	*R.nitidus*	0.015968	0.071908
CS12	SC27	CS2	*R.nitidus*	0.004677	0.065848
GD41	SC27	CS2	*R.nitidus*	0.025751	0.064641
JO3	SC26	CS2	*R.nitidus*	0.09178	0.067967
GD34	SC27	CS2	*R.nitidus*	0.013167	0.066168
JO7	SC27	CS2	*R.nitidus*	-0.023166	0.072856
CS11	SC27	CS2	*R.nitidus*	0.014126	0.0669
CS15	SC27	CS2	*R.nitidus*	0.031387	0.06596
GD30	SC27	CS2	*R.nitidus*	0.135308	0.066992
GD33	SC27	CS2	*R.nitidus*	0.015753	0.06604
CS14	SC27	CS2	*R.nitidus*	0.01766	0.06717
GD44	SC27	CS2	*R.nitidus*	0.032738	0.067475
GD42	SC27	CS2	*R.nitidus*	0.033141	0.065637
GD29	SC27	CS2	*R.nitidus*	0.116857	0.063412
GD35	SC27	CS2	*R.nitidus*	0.052971	0.064522
JO5	SC26	CS2	*R.nitidus*	0.095599	0.063143
GD49	SC27	CS2	*R.nitidus*	0.075277	0.068921
GD45	SC27	CS2	*R.nitidus*	0.057799	0.069767
CS17	SC27	CS2	*R.nitidus*	0.03974	0.067586
GD46	SC27	CS2	*R.nitidus*	-0.011429	0.065806
GD40	SC27	CS2	*R.nitidus*	0.07206	0.063513
GD26	SC27	CS2	*R.nitidus*	0.061486	0.06576
GD32	SC27	CS2	*R.nitidus*	0.013503	0.068529
GD50	SC27	CS2	*R.nitidus*	0.049236	0.066654
GD48	SC27	CS2	*R.nitidus*	0.042392	0.066174
CS18	SC27	CS2	*R.nitidus*	-0.024133	0.065722
CS24	SC27	CS2	*R.nitidus*	-0.027624	0.063787
CS25	SC27	CS2	*R.nitidus*	-0.008234	0.066364
CS20	SC27	CS2	*R.nitidus*	-0.02886	0.065909
CS22	SC27	CS2	*R.nitidus*	0.008371	0.06481
SC12	SC27	CS2	*R.nitidus*	-0.022708	0.064255
GD22	SC27	CS2	*R.nitidus*	0.068783	0.0637
CS13	SC27	CS2	*R.nitidus*	-0.040632	0.067714
CS21	SC27	CS2	*R.nitidus*	-0.013091	0.064408
SC6	SC27	CS2	*R.nitidus*	-0.002335	0.064917
CS30	SC27	CS2	*R.nitidus*	-0.054954	0.064444
SC18	SC27	CS2	*R.nitidus*	0.042869	0.065609
CS19	SC27	CS2	*R.nitidus*	-0.01435	0.068376
CS28	SC27	CS2	*R.nitidus*	-0.015974	0.065141
SC10	SC27	CS2	*R.nitidus*	0.065124	0.064572
CS4	SC27	CS2	*R.nitidus*	0.000721	0.062715
CS3	SC27	CS2	*R.nitidus*	-0.009651	0.065193
MY4	SC27	CS2	*R.nitidus*	-0.037834	0.066539
SC14	SC27	CS2	*R.nitidus*	0.004315	0.060661
SC32	SC27	CS2	*R.nitidus*	0.032451	0.064562
CS23	SC27	CS2	*R.nitidus*	-0.044985	0.065103
MY6	SC27	CS2	*R.nitidus*	0.009407	0.065336
SC15	SC27	CS2	*R.nitidus*	-0.017964	0.064098
CS16	SC27	CS2	*R.nitidus*	0.002961	0.064922
CS31	SC27	CS2	*R.nitidus*	-0.015601	0.065093
SC5	SC27	CS2	*R.nitidus*	0.007962	0.068962

JW7	SC27	CS2	*R.nitidus*	0.025329	0.068137
GD26	SC28	CS2	*R.nitidus*	0.095938	0.066206
JO5	SC27	CS2	*R.nitidus*	0.061502	0.064793
SC34	SC27	CS2	*R.nitidus*	0.039714	0.063239
SC31	SC27	CS2	*R.nitidus*	-0.00319	0.065888
JO7	SC28	CS2	*R.nitidus*	-0.01068	0.072032
GD25	SC28	CS2	*R.nitidus*	0.124026	0.065046
JO3	SC27	CS2	*R.nitidus*	0.032706	0.068483
JO8	SC28	CS2	*R.nitidus*	0.03538	0.072205
GD21	SC28	CS2	*R.nitidus*	0.152031	0.06437
SC26	SC27	CS2	*R.nitidus*	-0.033898	0.063483
SC45	SC27	CS2	*R.nitidus*	0.030098	0.066549
SC24	SC27	CS2	*R.nitidus*	0.000797	0.064769
GD33	SC28	CS2	*R.nitidus*	0.051712	0.065631
JO1	SC27	CS2	*R.nitidus*	-0.006178	0.06463
SC28	SC27	CS2	*R.nitidus*	-0.043796	0.065683
GD34	SC28	CS2	*R.nitidus*	0.053509	0.0638
GD30	SC28	CS2	*R.nitidus*	0.162141	0.065992
SC44	SC27	CS2	*R.nitidus*	-0.008026	0.06418
CS6	SC27	CS2	*R.nitidus*	-0.049307	0.063763
SC42	SC27	CS2	*R.nitidus*	0.261646	0.062919
SC35	SC27	CS2	*R.nitidus*	0.059795	0.063198
JW4	SC27	CS2	*R.nitidus*	0.214136	0.064817
JW6	SC27	CS2	*R.nitidus*	0.003902	0.0708
GD46	SC28	CS2	*R.nitidus*	0.03352	0.064564
GD32	SC28	CS2	*R.nitidus*	0.052075	0.067226
CS11	SC28	CS2	*R.nitidus*	0.047653	0.066976
CS19	SC28	CS2	*R.nitidus*	0.020498	0.067062
GD35	SC28	CS2	*R.nitidus*	0.080758	0.065705
GD22	SC28	CS2	*R.nitidus*	0.11811	0.064393
CS12	SC28	CS2	*R.nitidus*	0.059052	0.065932
GD42	SC28	CS2	*R.nitidus*	0.078024	0.065461
GD41	SC28	CS2	*R.nitidus*	0.061908	0.065389
GD29	SC28	CS2	*R.nitidus*	0.163102	0.064539
GD48	SC28	CS2	*R.nitidus*	0.079554	0.066506
CS17	SC28	CS2	*R.nitidus*	0.075851	0.066685
GD49	SC28	CS2	*R.nitidus*	0.121332	0.067142
GD45	SC28	CS2	*R.nitidus*	0.114727	0.068218
CS13	SC28	CS2	*R.nitidus*	0.014925	0.067594
GD31	SC28	CS2	*R.nitidus*	0.099853	0.066094
GD50	SC28	CS2	*R.nitidus*	0.089167	0.06754
CS16	SC28	CS2	*R.nitidus*	0.056972	0.06554
CS20	SC28	CS2	*R.nitidus*	0.005785	0.06684
CS15	SC28	CS2	*R.nitidus*	0.070087	0.066247
GD44	SC28	CS2	*R.nitidus*	0.076006	0.067627
CS21	SC28	CS2	*R.nitidus*	0.017404	0.065982
SC15	SC28	CS2	*R.nitidus*	0.022152	0.067439
CS14	SC28	CS2	*R.nitidus*	0.047101	0.067742
CS18	SC28	CS2	*R.nitidus*	0.016105	0.066491
GD40	SC28	CS2	*R.nitidus*	0.108815	0.063928
CS24	SC28	CS2	*R.nitidus*	0.003776	0.064803
SC32	SC28	CS2	*R.nitidus*	0.078599	0.063098
CS31	SC28	CS2	*R.nitidus*	0.019832	0.066099
SC31	SC28	CS2	*R.nitidus*	0.038402	0.064818
SC18	SC28	CS2	*R.nitidus*	0.105	0.063427
CS23	SC28	CS2	*R.nitidus*	0.007908	0.065335

CS25	SC28	CS2	*R.nitidus*	0.032258	0.067218
CS30	SC28	CS2	*R.nitidus*	-0.016999	0.064961
CS22	SC28	CS2	*R.nitidus*	0.046282	0.06549
SC35	SC28	CS2	*R.nitidus*	0.092551	0.063168
CS28	SC28	CS2	*R.nitidus*	0.016706	0.065682
SC6	SC28	CS2	*R.nitidus*	0.044636	0.066227
CS4	SC28	CS2	*R.nitidus*	0.037599	0.063633
JW6	SC28	CS2	*R.nitidus*	0.021463	0.073342
SC5	SC28	CS2	*R.nitidus*	0.041773	0.068641
SC27	SC28	CS2	*R.nitidus*	0.043796	0.065683
SC24	SC28	CS2	*R.nitidus*	0.057052	0.063997
SC12	SC28	CS2	*R.nitidus*	0.014481	0.06408
CS6	SC28	CS2	*R.nitidus*	-0.001526	0.064369
SC34	SC28	CS2	*R.nitidus*	0.078078	0.064654
SC10	SC28	CS2	*R.nitidus*	0.120773	0.064485
CS3	SC28	CS2	*R.nitidus*	0.026966	0.065038
MY4	SC28	CS2	*R.nitidus*	0.002972	0.06654
MY6	SC28	CS2	*R.nitidus*	0.04646	0.06434
SC26	SC28	CS2	*R.nitidus*	-0.005405	0.064014
JO5	SC28	CS2	*R.nitidus*	0.123371	0.061611
JO3	SC28	CS2	*R.nitidus*	0.05578	0.068887
SC14	SC28	CS2	*R.nitidus*	0.038156	0.065263
JW4	SC28	CS2	*R.nitidus*	0.251249	0.066506
GD32	SC31	CS2	*R.nitidus*	0.025581	0.069551
SC44	SC28	CS2	*R.nitidus*	0.053854	0.05973
JO8	SC31	CS2	*R.nitidus*	-0.004946	0.073871
CS11	SC31	CS2	*R.nitidus*	0.007897	0.067973
SC45	SC28	CS2	*R.nitidus*	0.065284	0.064242
JO1	SC28	CS2	*R.nitidus*	0.003187	0.066041
SC42	SC28	CS2	*R.nitidus*	0.29256	0.061595
GD26	SC31	CS2	*R.nitidus*	0.057162	0.066581
GD41	SC31	CS2	*R.nitidus*	0.016807	0.064847
GD48	SC31	CS2	*R.nitidus*	0.052006	0.068846
CS12	SC31	CS2	*R.nitidus*	0.009782	0.068158
JW7	SC28	CS2	*R.nitidus*	0.057115	0.068784
GD45	SC31	CS2	*R.nitidus*	0.060748	0.069869
JO7	SC31	CS2	*R.nitidus*	-0.011215	0.072648
GD25	SC31	CS2	*R.nitidus*	0.082394	0.065075
GD34	SC31	CS2	*R.nitidus*	0.013231	0.066009
GD44	SC31	CS2	*R.nitidus*	0.023628	0.067492
GD31	SC31	CS2	*R.nitidus*	0.059035	0.066796
CS14	SC31	CS2	*R.nitidus*	0.007801	0.067071
GD35	SC31	CS2	*R.nitidus*	0.049391	0.064733
GD21	SC31	CS2	*R.nitidus*	0.122967	0.064837
GD30	SC31	CS2	*R.nitidus*	0.12989	0.067219
GD29	SC31	CS2	*R.nitidus*	0.119248	0.063735
CS22	SC31	CS2	*R.nitidus*	0.015173	0.065575
GD22	SC31	CS2	*R.nitidus*	0.081081	0.06571
CS30	SC31	CS2	*R.nitidus*	-0.058605	0.066483
GD33	SC31	CS2	*R.nitidus*	0.029248	0.06842
CS18	SC31	CS2	*R.nitidus*	-0.014771	0.065824
GD49	SC31	CS2	*R.nitidus*	0.103118	0.06874
CS13	SC31	CS2	*R.nitidus*	-0.032023	0.067701
GD42	SC31	CS2	*R.nitidus*	0.049408	0.0665
GD46	SC31	CS2	*R.nitidus*	-0.007333	0.064344
CS16	SC31	CS2	*R.nitidus*	0.019537	0.068198

CS15	SC31	CS2	*R.nitidus*	0.027836	0.066215
GD40	SC31	CS2	*R.nitidus*	0.075625	0.063619
CS31	SC31	CS2	*R.nitidus*	-0.025797	0.066385
CS21	SC31	CS2	*R.nitidus*	-0.019534	0.065928
SC6	SC31	CS2	*R.nitidus*	-0.021892	0.066635
GD50	SC31	CS2	*R.nitidus*	0.02762	0.069182
SC12	SC31	CS2	*R.nitidus*	-0.015957	0.066306
CS17	SC31	CS2	*R.nitidus*	0.036979	0.069998
CS24	SC31	CS2	*R.nitidus*	-0.034892	0.065036
SC42	SC31	CS2	*R.nitidus*	0.241315	0.062491
SC32	SC31	CS2	*R.nitidus*	0.038491	0.064806
SC10	SC31	CS2	*R.nitidus*	0.070456	0.065171
CS25	SC31	CS2	*R.nitidus*	-0.011747	0.066512
CS20	SC31	CS2	*R.nitidus*	-0.022997	0.064813
CS23	SC31	CS2	*R.nitidus*	-0.045622	0.065363
SC28	SC31	CS2	*R.nitidus*	-0.038402	0.064818
SC18	SC31	CS2	*R.nitidus*	0.053834	0.065349
SC15	SC31	CS2	*R.nitidus*	-0.017197	0.066671
SC14	SC31	CS2	*R.nitidus*	0.007206	0.06678
CS28	SC31	CS2	*R.nitidus*	-0.018415	0.06548
CS3	SC31	CS2	*R.nitidus*	-0.000748	0.064388
CS19	SC31	CS2	*R.nitidus*	-0.005128	0.067022
SC27	SC31	CS2	*R.nitidus*	0.00319	0.065888
SC26	SC31	CS2	*R.nitidus*	-0.041335	0.064488
JO8	SC32	CS2	*R.nitidus*	0.008032	0.07337
SC44	SC31	CS2	*R.nitidus*	0.000774	0.06557
JO7	SC32	CS2	*R.nitidus*	-0.048436	0.071995
JW4	SC31	CS2	*R.nitidus*	0.242711	0.066377
SC5	SC31	CS2	*R.nitidus*	0.008468	0.069576
SC34	SC31	CS2	*R.nitidus*	0.038835	0.066855
CS4	SC31	CS2	*R.nitidus*	0.002896	0.064308
CS11	SC32	CS2	*R.nitidus*	-0.028464	0.068831
CS6	SC31	CS2	*R.nitidus*	-0.037538	0.06683
JW6	SC31	CS2	*R.nitidus*	0.014995	0.073954
GD29	SC32	CS2	*R.nitidus*	0.113531	0.065904
CS12	SC32	CS2	*R.nitidus*	-0.020031	0.068976
JO5	SC31	CS2	*R.nitidus*	0.056146	0.064869
GD40	SC32	CS2	*R.nitidus*	0.046322	0.063695
MY6	SC31	CS2	*R.nitidus*	0.015544	0.065993
JW7	SC31	CS2	*R.nitidus*	0.055238	0.068002
JO1	SC31	CS2	*R.nitidus*	-0.013783	0.06503
SC24	SC31	CS2	*R.nitidus*	0.014241	0.065527
GD21	SC32	CS2	*R.nitidus*	0.099324	0.064145
CS13	SC32	CS2	*R.nitidus*	-0.064391	0.070051
SC35	SC31	CS2	*R.nitidus*	0.051184	0.065841
GD35	SC32	CS2	*R.nitidus*	0.012579	0.064442
SC45	SC31	CS2	*R.nitidus*	0.037258	0.065347
MY4	SC31	CS2	*R.nitidus*	-0.029674	0.06528
JO3	SC31	CS2	*R.nitidus*	0.053279	0.069403
GD30	SC32	CS2	*R.nitidus*	0.101426	0.068133
GD34	SC32	CS2	*R.nitidus*	-0.011708	0.064796
GD33	SC32	CS2	*R.nitidus*	-0.006935	0.065989
CS17	SC32	CS2	*R.nitidus*	-0.019952	0.070229
GD22	SC32	CS2	*R.nitidus*	0.049479	0.063183
GD31	SC32	CS2	*R.nitidus*	0.027273	0.065988
CS19	SC32	CS2	*R.nitidus*	-0.043177	0.068901

GD41	SC32	CS2	*R.nitidus*	-0.004916	0.063759
GD26	SC32	CS2	*R.nitidus*	0.030158	0.06572
GD25	SC32	CS2	*R.nitidus*	0.056826	0.064087
CS25	SC32	CS2	*R.nitidus*	-0.041541	0.0686
GD32	SC32	CS2	*R.nitidus*	-0.006478	0.068619
CS21	SC32	CS2	*R.nitidus*	-0.049556	0.06414
GD50	SC32	CS2	*R.nitidus*	0.01249	0.070307
CS14	SC32	CS2	*R.nitidus*	-0.023379	0.070959
CS30	SC32	CS2	*R.nitidus*	-0.084291	0.065272
CS16	SC32	CS2	*R.nitidus*	-0.027612	0.067242
GD49	SC32	CS2	*R.nitidus*	0.060365	0.067839
CS31	SC32	CS2	*R.nitidus*	-0.054075	0.06767
GD42	SC32	CS2	*R.nitidus*	0.012839	0.064922
GD44	SC32	CS2	*R.nitidus*	-0.011536	0.068271
GD45	SC32	CS2	*R.nitidus*	0.024233	0.069815
GD48	SC32	CS2	*R.nitidus*	0.025738	0.067584
CS24	SC32	CS2	*R.nitidus*	-0.068289	0.066061
SC42	SC32	CS2	*R.nitidus*	0.250628	0.063002
SC12	SC32	CS2	*R.nitidus*	-0.05873	0.06556
CS18	SC32	CS2	*R.nitidus*	-0.053933	0.065107
CS15	SC32	CS2	*R.nitidus*	-0.005743	0.068032
CS28	SC32	CS2	*R.nitidus*	-0.061947	0.066756
SC18	SC32	CS2	*R.nitidus*	0.018441	0.062559
CS20	SC32	CS2	*R.nitidus*	-0.057971	0.065628
SC24	SC32	CS2	*R.nitidus*	-0.029295	0.064063
SC34	SC32	CS2	*R.nitidus*	0.012346	0.064818
CS4	SC32	CS2	*R.nitidus*	-0.030689	0.064969
SC14	SC32	CS2	*R.nitidus*	-0.03104	0.06667
SC15	SC32	CS2	*R.nitidus*	-0.056432	0.066584
JO3	SC32	CS2	*R.nitidus*	0.013169	0.068932
CS22	SC32	CS2	*R.nitidus*	-0.023059	0.064884
SC26	SC32	CS2	*R.nitidus*	-0.068482	0.064425
CS23	SC32	CS2	*R.nitidus*	-0.077988	0.064744
GD46	SC32	CS2	*R.nitidus*	-0.036364	0.065861
SC44	SC32	CS2	*R.nitidus*	-0.032905	0.063562
JW6	SC32	CS2	*R.nitidus*	-0.023645	0.072165
MY6	SC32	CS2	*R.nitidus*	-0.017817	0.063464
JO8	SC34	CS2	*R.nitidus*	0.008721	0.071394
JW4	SC32	CS2	*R.nitidus*	0.208517	0.064157
JW7	SC32	CS2	*R.nitidus*	0.017928	0.07031
SC5	SC32	CS2	*R.nitidus*	-0.020619	0.068499
SC6	SC32	CS2	*R.nitidus*	-0.040094	0.066599
SC10	SC32	CS2	*R.nitidus*	0.02616	0.063893
JO1	SC32	CS2	*R.nitidus*	-0.046326	0.065058
SC31	SC32	CS2	*R.nitidus*	-0.038491	0.064806
CS6	SC32	CS2	*R.nitidus*	-0.082371	0.063846
SC27	SC32	CS2	*R.nitidus*	-0.032451	0.064562
SC28	SC32	CS2	*R.nitidus*	-0.078599	0.063098
SC35	SC32	CS2	*R.nitidus*	0.010963	0.063114
CS3	SC32	CS2	*R.nitidus*	-0.044477	0.065316
MY4	SC32	CS2	*R.nitidus*	-0.066103	0.065542
GD26	SC34	CS2	*R.nitidus*	0.026027	0.065601
GD29	SC34	CS2	*R.nitidus*	0.072839	0.063781
GD22	SC34	CS2	*R.nitidus*	0.03781	0.063174
GD21	SC34	CS2	*R.nitidus*	0.084912	0.065118
JO5	SC32	CS2	*R.nitidus*	0.035153	0.063756

SC45	SC32	CS2	*R.nitidus*	0.001547	0.065305
GD25	SC34	CS2	*R.nitidus*	0.039945	0.064913
GD30	SC34	CS2	*R.nitidus*	0.08646	0.067661
JO7	SC34	CS2	*R.nitidus*	-0.034483	0.069441
GD33	SC34	CS2	*R.nitidus*	-0.00823	0.063982
GD45	SC34	CS2	*R.nitidus*	0.034931	0.069231
GD32	SC34	CS2	*R.nitidus*	0.01014	0.06697
GD42	SC34	CS2	*R.nitidus*	-0.003556	0.066121
GD50	SC34	CS2	*R.nitidus*	-0.015498	0.065987
GD31	SC34	CS2	*R.nitidus*	0.016405	0.06654
GD49	SC34	CS2	*R.nitidus*	0.034073	0.066859
GD40	SC34	CS2	*R.nitidus*	0.040328	0.064702
CS11	SC34	CS2	*R.nitidus*	-0.023775	0.068109
GD35	SC34	CS2	*R.nitidus*	0.028691	0.065302
CS14	SC34	CS2	*R.nitidus*	-0.02393	0.068056
GD48	SC34	CS2	*R.nitidus*	0.020974	0.065837
CS16	SC34	CS2	*R.nitidus*	-0.02185	0.067316
GD41	SC34	CS2	*R.nitidus*	-0.019149	0.064914
CS17	SC34	CS2	*R.nitidus*	-0.009962	0.069563
CS18	SC34	CS2	*R.nitidus*	-0.056976	0.064114
CS21	SC34	CS2	*R.nitidus*	-0.046143	0.064175
CS20	SC34	CS2	*R.nitidus*	-0.0625	0.065688
CS19	SC34	CS2	*R.nitidus*	-0.048727	0.067277
CS12	SC34	CS2	*R.nitidus*	-0.01749	0.066721
CS31	SC34	CS2	*R.nitidus*	-0.050263	0.06593
GD46	SC34	CS2	*R.nitidus*	-0.047189	0.06404
CS15	SC34	CS2	*R.nitidus*	-0.011404	0.06644
GD44	SC34	CS2	*R.nitidus*	-0.017279	0.066848
SC42	SC34	CS2	*R.nitidus*	0.200378	0.060491
CS13	SC34	CS2	*R.nitidus*	-0.05878	0.067907
GD34	SC34	CS2	*R.nitidus*	-0.020548	0.065067
SC10	SC34	CS2	*R.nitidus*	0.01964	0.065502
SC6	SC34	CS2	*R.nitidus*	-0.044867	0.065663
CS22	SC34	CS2	*R.nitidus*	-0.018678	0.064538
SC31	SC34	CS2	*R.nitidus*	-0.038835	0.066855
SC35	SC34	CS2	*R.nitidus*	0.013595	0.063548
CS28	SC34	CS2	*R.nitidus*	-0.065536	0.066523
CS30	SC34	CS2	*R.nitidus*	-0.093023	0.06451
SC12	SC34	CS2	*R.nitidus*	-0.060678	0.06578
CS4	SC34	CS2	*R.nitidus*	-0.037482	0.062714
SC32	SC34	CS2	*R.nitidus*	-0.012346	0.064818
SC27	SC34	CS2	*R.nitidus*	-0.039714	0.063239
CS23	SC34	CS2	*R.nitidus*	-0.076253	0.064362
CS3	SC34	CS2	*R.nitidus*	-0.052632	0.066326
CS25	SC34	CS2	*R.nitidus*	-0.042857	0.066268
SC15	SC34	CS2	*R.nitidus*	-0.057258	0.066616
CS24	SC34	CS2	*R.nitidus*	-0.075	0.064683
SC26	SC34	CS2	*R.nitidus*	-0.081404	0.062864
CS6	SC34	CS2	*R.nitidus*	-0.06535	0.064264
SC45	SC34	CS2	*R.nitidus*	-0.002177	0.06637
SC28	SC34	CS2	*R.nitidus*	-0.078078	0.064654
JO7	SC35	CS2	*R.nitidus*	-0.02243	0.069025
JW4	SC34	CS2	*R.nitidus*	0.204748	0.06431
GD21	SC35	CS2	*R.nitidus*	0.079228	0.064785
SC18	SC34	CS2	*R.nitidus*	0.012668	0.065616
MY6	SC34	CS2	*R.nitidus*	-0.028825	0.064633

JW6	SC34	CS2	*R.nitidus*	-0.017094	0.070205
SC24	SC34	CS2	*R.nitidus*	-0.026709	0.065465
SC44	SC34	CS2	*R.nitidus*	-0.046851	0.062968
SC5	SC34	CS2	*R.nitidus*	-0.024738	0.069055
SC14	SC34	CS2	*R.nitidus*	-0.037618	0.066249
GD31	SC35	CS2	*R.nitidus*	0.020714	0.064952
JO3	SC34	CS2	*R.nitidus*	0.034426	0.067584
JW7	SC34	CS2	*R.nitidus*	-0.019231	0.069074
JO1	SC34	CS2	*R.nitidus*	-0.051243	0.065521
JO8	SC35	CS2	*R.nitidus*	0	0.072686
CS11	SC35	CS2	*R.nitidus*	-0.040283	0.06428
GD22	SC35	CS2	*R.nitidus*	0.022936	0.063695
GD29	SC35	CS2	*R.nitidus*	0.073056	0.064438
MY4	SC34	CS2	*R.nitidus*	-0.07991	0.065456
GD41	SC35	CS2	*R.nitidus*	-0.023448	0.064482
GD35	SC35	CS2	*R.nitidus*	0.018692	0.065689
GD25	SC35	CS2	*R.nitidus*	0.042053	0.066114
JO5	SC34	CS2	*R.nitidus*	0.027715	0.066186
GD26	SC35	CS2	*R.nitidus*	0.022603	0.064464
GD44	SC35	CS2	*R.nitidus*	-0.020789	0.066317
GD34	SC35	CS2	*R.nitidus*	-0.029944	0.064832
GD30	SC35	CS2	*R.nitidus*	0.074451	0.067345
GD33	SC35	CS2	*R.nitidus*	-0.013477	0.065866
GD50	SC35	CS2	*R.nitidus*	0.001679	0.067791
GD45	SC35	CS2	*R.nitidus*	0.022293	0.069494
GD48	SC35	CS2	*R.nitidus*	-0.005975	0.065778
CS12	SC35	CS2	*R.nitidus*	-0.039648	0.065209
CS25	SC35	CS2	*R.nitidus*	-0.055556	0.065405
GD42	SC35	CS2	*R.nitidus*	-0.003453	0.064731
SC6	SC35	CS2	*R.nitidus*	-0.056502	0.064341
GD32	SC35	CS2	*R.nitidus*	-0.030806	0.06811
CS16	SC35	CS2	*R.nitidus*	-0.046165	0.06486
CS14	SC35	CS2	*R.nitidus*	-0.038924	0.066179
CS24	SC35	CS2	*R.nitidus*	-0.082181	0.062971
CS28	SC35	CS2	*R.nitidus*	-0.046083	0.065666
GD40	SC35	CS2	*R.nitidus*	0.024782	0.062196
CS30	SC35	CS2	*R.nitidus*	-0.100752	0.06332
CS22	SC35	CS2	*R.nitidus*	-0.038783	0.063989
CS4	SC35	CS2	*R.nitidus*	-0.039884	0.064693
GD49	SC35	CS2	*R.nitidus*	0.027712	0.067821
GD46	SC35	CS2	*R.nitidus*	-0.060627	0.064294
CS13	SC35	CS2	*R.nitidus*	-0.08226	0.066668
CS31	SC35	CS2	*R.nitidus*	-0.062406	0.06438
CS15	SC35	CS2	*R.nitidus*	-0.024221	0.065536
CS19	SC35	CS2	*R.nitidus*	-0.065741	0.066395
SC12	SC35	CS2	*R.nitidus*	-0.081839	0.061663
SC24	SC35	CS2	*R.nitidus*	-0.038192	0.063564
SC27	SC35	CS2	*R.nitidus*	-0.059795	0.063198
SC5	SC35	CS2	*R.nitidus*	-0.04006	0.066851
SC31	SC35	CS2	*R.nitidus*	-0.051184	0.065841
CS17	SC35	CS2	*R.nitidus*	-0.018643	0.066684
CS21	SC35	CS2	*R.nitidus*	-0.070036	0.063388
CS6	SC35	CS2	*R.nitidus*	-0.09633	0.0634
SC10	SC35	CS2	*R.nitidus*	0.0192	0.065998
MY4	SC35	CS2	*R.nitidus*	-0.094011	0.064502
CS20	SC35	CS2	*R.nitidus*	-0.073239	0.063887

SC34	SC35	CS2	*R.nitidus*	-0.013595	0.063548
CS23	SC35	CS2	*R.nitidus*	-0.096611	0.064958
SC14	SC35	CS2	*R.nitidus*	-0.05142	0.064436
SC28	SC35	CS2	*R.nitidus*	-0.092551	0.063168
SC26	SC35	CS2	*R.nitidus*	-0.097859	0.061034
CS18	SC35	CS2	*R.nitidus*	-0.067399	0.06256
CS3	SC35	CS2	*R.nitidus*	-0.053936	0.063898
MY6	SC35	CS2	*R.nitidus*	-0.042222	0.064636
SC18	SC35	CS2	*R.nitidus*	0.005582	0.062906
SC42	SC35	CS2	*R.nitidus*	0.219315	0.063456
SC44	SC35	CS2	*R.nitidus*	-0.058238	0.062129
JW6	SC35	CS2	*R.nitidus*	-0.01983	0.070101
JO7	CS6	CS2	*R.nitidus*	0.048031	0.071374
SC45	SC35	CS2	*R.nitidus*	-0.012413	0.064539
SC15	SC35	CS2	*R.nitidus*	-0.067729	0.065687
JO3	SC35	CS2	*R.nitidus*	0.019851	0.069373
GD21	CS6	CS2	*R.nitidus*	0.161357	0.065038
SC32	SC35	CS2	*R.nitidus*	-0.010963	0.063114
JW7	SC35	CS2	*R.nitidus*	-0.002814	0.065754
JO5	SC35	CS2	*R.nitidus*	0.012658	0.063963
JW4	SC35	CS2	*R.nitidus*	0.219369	0.065255
JO1	SC35	CS2	*R.nitidus*	-0.077863	0.064706
GD25	CS6	CS2	*R.nitidus*	0.10972	0.066181
GD30	CS6	CS2	*R.nitidus*	0.159278	0.067025
GD29	CS6	CS2	*R.nitidus*	0.147484	0.06516
CS14	CS6	CS2	*R.nitidus*	0.065536	0.067374
JO8	CS6	CS2	*R.nitidus*	0.069744	0.072221
GD48	CS6	CS2	*R.nitidus*	0.089506	0.067185
GD33	CS6	CS2	*R.nitidus*	0.064011	0.066522
CS12	CS6	CS2	*R.nitidus*	0.055336	0.065549
CS21	CS6	CS2	*R.nitidus*	0.037736	0.063901
GD32	CS6	CS2	*R.nitidus*	0.083056	0.068771
CS18	CS6	CS2	*R.nitidus*	0.023452	0.064719
GD22	CS6	CS2	*R.nitidus*	0.11955	0.065731
GD26	CS6	CS2	*R.nitidus*	0.08662	0.066953
GD31	CS6	CS2	*R.nitidus*	0.091867	0.068547
CS20	CS6	CS2	*R.nitidus*	0.021472	0.063346
CS16	CS6	CS2	*R.nitidus*	0.045348	0.066017
CS15	CS6	CS2	*R.nitidus*	0.071158	0.067466
CS31	CS6	CS2	*R.nitidus*	0.01672	0.067153
GD40	CS6	CS2	*R.nitidus*	0.114576	0.065779
GD41	CS6	CS2	*R.nitidus*	0.060825	0.065948
CS23	CS6	CS2	*R.nitidus*	-0.007273	0.065467
CS19	CS6	CS2	*R.nitidus*	0.037443	0.06671
CS13	CS6	CS2	*R.nitidus*	0.01098	0.069075
GD46	CS6	CS2	*R.nitidus*	0.022513	0.065919
CS24	CS6	CS2	*R.nitidus*	0.013123	0.066554
GD34	CS6	CS2	*R.nitidus*	0.05057	0.066158
GD35	CS6	CS2	*R.nitidus*	0.097031	0.066201
CS22	CS6	CS2	*R.nitidus*	0.057063	0.064858
GD42	CS6	CS2	*R.nitidus*	0.080233	0.065756
SC5	CS6	CS2	*R.nitidus*	0.052437	0.067868
CS11	CS6	CS2	*R.nitidus*	0.042373	0.068014
GD49	CS6	CS2	*R.nitidus*	0.115905	0.068918
CS3	CS6	CS2	*R.nitidus*	0.039749	0.062769
GD45	CS6	CS2	*R.nitidus*	0.082529	0.070137

GD44	CS6	CS2	*R.nitidus*	0.06682	0.067202
CS17	CS6	CS2	*R.nitidus*	0.048061	0.069912
SC18	CS6	CS2	*R.nitidus*	0.101926	0.064546
GD50	CS6	CS2	*R.nitidus*	0.068339	0.068747
CS25	CS6	CS2	*R.nitidus*	0.04087	0.066789
SC10	CS6	CS2	*R.nitidus*	0.097918	0.066696
SC12	CS6	CS2	*R.nitidus*	0.030469	0.064918
SC24	CS6	CS2	*R.nitidus*	0.059477	0.063987
CS30	CS6	CS2	*R.nitidus*	-0.015873	0.066443
SC6	CS6	CS2	*R.nitidus*	0.03879	0.063761
SC34	CS6	CS2	*R.nitidus*	0.06535	0.064264
CS4	CS6	CS2	*R.nitidus*	0.037488	0.064598
CS28	CS6	CS2	*R.nitidus*	0.018197	0.067251
SC15	CS6	CS2	*R.nitidus*	0.03236	0.06552
MY4	CS6	CS2	*R.nitidus*	0.000772	0.067396
SC32	CS6	CS2	*R.nitidus*	0.082371	0.063846
SC31	CS6	CS2	*R.nitidus*	0.037538	0.06683
SC45	CS6	CS2	*R.nitidus*	0.074159	0.065453
SC35	CS6	CS2	*R.nitidus*	0.09633	0.0634
CS11	SC42	CS2	*R.nitidus*	-0.227142	0.061837
SC14	CS6	CS2	*R.nitidus*	0.048913	0.064115
JO3	CS6	CS2	*R.nitidus*	0.105532	0.069085
GD21	SC42	CS2	*R.nitidus*	-0.149972	0.062218
GD31	SC42	CS2	*R.nitidus*	-0.184019	0.064447
MY6	CS6	CS2	*R.nitidus*	0.046757	0.066193
JW7	CS6	CS2	*R.nitidus*	0.067918	0.067942
JO5	CS6	CS2	*R.nitidus*	0.103085	0.064212
SC26	CS6	CS2	*R.nitidus*	0.003805	0.065208
SC28	CS6	CS2	*R.nitidus*	0.001526	0.064369
GD35	SC42	CS2	*R.nitidus*	-0.20194	0.064836
SC27	CS6	CS2	*R.nitidus*	0.049307	0.063763
JO1	CS6	CS2	*R.nitidus*	0.016541	0.067574
GD30	SC42	CS2	*R.nitidus*	-0.117647	0.064204
GD48	SC42	CS2	*R.nitidus*	-0.168035	0.064769
SC42	CS6	CS2	*R.nitidus*	0.262402	0.061749
GD26	SC42	CS2	*R.nitidus*	-0.180507	0.062683
JO7	SC42	CS2	*R.nitidus*	-0.188442	0.068815
SC44	CS6	CS2	*R.nitidus*	0.046995	0.063062
GD33	SC42	CS2	*R.nitidus*	-0.209994	0.06492
JW6	CS6	CS2	*R.nitidus*	0.043726	0.070615
CS14	SC42	CS2	*R.nitidus*	-0.224525	0.063732
GD22	SC42	CS2	*R.nitidus*	-0.167871	0.062516
GD50	SC42	CS2	*R.nitidus*	-0.204451	0.066121
CS17	SC42	CS2	*R.nitidus*	-0.206642	0.064237
GD41	SC42	CS2	*R.nitidus*	-0.226307	0.062498
GD40	SC42	CS2	*R.nitidus*	-0.172055	0.062956
GD29	SC42	CS2	*R.nitidus*	-0.11007	0.062795
CS16	SC42	CS2	*R.nitidus*	-0.242229	0.060678
GD32	SC42	CS2	*R.nitidus*	-0.196347	0.065496
GD25	SC42	CS2	*R.nitidus*	-0.157704	0.063399
GD34	SC42	CS2	*R.nitidus*	-0.230505	0.063559
GD42	SC42	CS2	*R.nitidus*	-0.229044	0.062934
GD45	SC42	CS2	*R.nitidus*	-0.186581	0.066369
CS19	SC42	CS2	*R.nitidus*	-0.252201	0.06394
CS12	SC42	CS2	*R.nitidus*	-0.237299	0.064474
GD49	SC42	CS2	*R.nitidus*	-0.172029	0.065996

JW4	CS6	CS2	*R.nitidus*	0.252434	0.06602
GD44	SC42	CS2	*R.nitidus*	-0.208103	0.063531
CS24	SC42	CS2	*R.nitidus*	-0.28801	0.060927
CS23	SC42	CS2	*R.nitidus*	-0.287744	0.061763
SC6	SC42	CS2	*R.nitidus*	-0.275172	0.061403
GD46	SC42	CS2	*R.nitidus*	-0.238095	0.061992
CS13	SC42	CS2	*R.nitidus*	-0.268765	0.062833
SC5	SC42	CS2	*R.nitidus*	-0.253712	0.065361
CS15	SC42	CS2	*R.nitidus*	-0.215777	0.062656
CS18	SC42	CS2	*R.nitidus*	-0.253722	0.063281
CS4	SC42	CS2	*R.nitidus*	-0.259327	0.060943
CS30	SC42	CS2	*R.nitidus*	-0.290049	0.060292
CS20	SC42	CS2	*R.nitidus*	-0.257876	0.063148
CS31	SC42	CS2	*R.nitidus*	-0.269089	0.061623
MY6	SC42	CS2	*R.nitidus*	-0.24665	0.062564
SC24	SC42	CS2	*R.nitidus*	-0.256297	0.061571
SC10	SC42	CS2	*R.nitidus*	-0.205601	0.063182
CS22	SC42	CS2	*R.nitidus*	-0.243993	0.061597
JO7	SC44	CS2	*R.nitidus*	0.001992	0.074304
SC18	SC42	CS2	*R.nitidus*	-0.218543	0.063592
CS21	SC42	CS2	*R.nitidus*	-0.249701	0.062137
CS28	SC42	CS2	*R.nitidus*	-0.256377	0.063188
SC34	SC42	CS2	*R.nitidus*	-0.200378	0.060491
SC28	SC42	CS2	*R.nitidus*	-0.29256	0.061595
CS3	SC42	CS2	*R.nitidus*	-0.244336	0.060331
SC27	SC42	CS2	*R.nitidus*	-0.261646	0.062919
SC15	SC42	CS2	*R.nitidus*	-0.284884	0.062179
SC31	SC42	CS2	*R.nitidus*	-0.241315	0.062491
JW7	SC42	CS2	*R.nitidus*	-0.191738	0.067252
SC26	SC42	CS2	*R.nitidus*	-0.277946	0.06116
SC44	SC42	CS2	*R.nitidus*	-0.265903	0.060858
JW6	SC42	CS2	*R.nitidus*	-0.176568	0.067926
JO1	SC42	CS2	*R.nitidus*	-0.267352	0.063254
JW4	SC42	CS2	*R.nitidus*	0.011598	0.065093
CS25	SC42	CS2	*R.nitidus*	-0.248932	0.064731
JO5	SC42	CS2	*R.nitidus*	-0.194904	0.06456
SC14	SC42	CS2	*R.nitidus*	-0.255198	0.0625
SC12	SC42	CS2	*R.nitidus*	-0.281853	0.060294
JO3	SC42	CS2	*R.nitidus*	-0.148827	0.065523
SC45	SC42	CS2	*R.nitidus*	-0.225765	0.063302
MY4	SC42	CS2	*R.nitidus*	-0.280122	0.063487
GD34	SC44	CS2	*R.nitidus*	0.013669	0.065492
GD33	SC44	CS2	*R.nitidus*	0.026167	0.065613
JO8	SC44	CS2	*R.nitidus*	0.009278	0.072275
SC32	SC42	CS2	*R.nitidus*	-0.250628	0.063002
SC35	SC42	CS2	*R.nitidus*	-0.219315	0.063456
CS11	SC44	CS2	*R.nitidus*	0.003602	0.067004
CS6	SC42	CS2	*R.nitidus*	-0.262402	0.061749
GD26	SC44	CS2	*R.nitidus*	0.06082	0.065323
GD40	SC44	CS2	*R.nitidus*	0.068327	0.063767
GD22	SC44	CS2	*R.nitidus*	0.083682	0.063907
GD35	SC44	CS2	*R.nitidus*	0.052822	0.066223
GD21	SC44	CS2	*R.nitidus*	0.115074	0.064562
GD29	SC44	CS2	*R.nitidus*	0.120055	0.06557
GD48	SC44	CS2	*R.nitidus*	0.045669	0.066301
GD50	SC44	CS2	*R.nitidus*	0.043554	0.069147

GD45	SC44	CS2	*R.nitidus*	0.061075	0.066894
GD30	SC44	CS2	*R.nitidus*	0.11157	0.065882
CS12	SC44	CS2	*R.nitidus*	0.003808	0.066656
GD25	SC44	CS2	*R.nitidus*	0.076696	0.065423
CS20	SC44	CS2	*R.nitidus*	-0.027516	0.066978
GD49	SC44	CS2	*R.nitidus*	0.083542	0.068636
CS24	SC44	CS2	*R.nitidus*	-0.033515	0.064979
CS15	SC44	CS2	*R.nitidus*	0.020438	0.067509
GD46	SC44	CS2	*R.nitidus*	-0.006475	0.065395
CS28	SC44	CS2	*R.nitidus*	-0.021061	0.066102
GD32	SC44	CS2	*R.nitidus*	0.015139	0.067607
CS18	SC44	CS2	*R.nitidus*	-0.016899	0.065073
CS13	SC44	CS2	*R.nitidus*	-0.026355	0.068402
GD31	SC44	CS2	*R.nitidus*	0.051699	0.066165
GD41	SC44	CS2	*R.nitidus*	0.019882	0.064759
CS14	SC44	CS2	*R.nitidus*	0.009407	0.068944
CS19	SC44	CS2	*R.nitidus*	-0.010558	0.06729
CS22	SC44	CS2	*R.nitidus*	0.016345	0.066096
GD42	SC44	CS2	*R.nitidus*	0.035982	0.066299
GD44	SC44	CS2	*R.nitidus*	0.025641	0.066981
SC26	SC44	CS2	*R.nitidus*	-0.034564	0.065086
SC15	SC44	CS2	*R.nitidus*	-0.012749	0.065601
SC5	SC44	CS2	*R.nitidus*	0.006146	0.068237
CS17	SC44	CS2	*R.nitidus*	0.025842	0.067517
SC42	SC44	CS2	*R.nitidus*	0.265903	0.060858
SC14	SC44	CS2	*R.nitidus*	0.005632	0.06457
SC18	SC44	CS2	*R.nitidus*	0.071669	0.063402
CS16	SC44	CS2	*R.nitidus*	0	0.06589
CS3	SC44	CS2	*R.nitidus*	-0.008333	0.065317
SC34	SC44	CS2	*R.nitidus*	0.046851	0.062968
SC10	SC44	CS2	*R.nitidus*	0.074921	0.065058
SC31	SC44	CS2	*R.nitidus*	-0.000774	0.06557
CS25	SC44	CS2	*R.nitidus*	0.005291	0.066658
CS21	SC44	CS2	*R.nitidus*	-0.014503	0.063561
JW7	SC44	CS2	*R.nitidus*	0.030447	0.070217
SC24	SC44	CS2	*R.nitidus*	0.015335	0.064362
SC32	SC44	CS2	*R.nitidus*	0.032905	0.063562
JO1	SC44	CS2	*R.nitidus*	-0.025443	0.06553
CS30	SC44	CS2	*R.nitidus*	-0.057737	0.062912
SC6	SC44	CS2	*R.nitidus*	-0.006168	0.065622
SC27	SC44	CS2	*R.nitidus*	0.008026	0.06418
CS6	SC44	CS2	*R.nitidus*	-0.046995	0.063062
CS31	SC44	CS2	*R.nitidus*	-0.02454	0.066951
CS23	SC44	CS2	*R.nitidus*	-0.03639	0.065591
CS4	SC44	CS2	*R.nitidus*	0.000729	0.062892
SC28	SC44	CS2	*R.nitidus*	-0.053854	0.05973
SC12	SC44	CS2	*R.nitidus*	-0.013344	0.064434
GD22	SC45	CS2	*R.nitidus*	0.029332	0.065298
JW6	SC44	CS2	*R.nitidus*	0.014677	0.073778
MY6	SC44	CS2	*R.nitidus*	0.005247	0.065789
GD26	SC45	CS2	*R.nitidus*	0.025352	0.066386
JO5	SC44	CS2	*R.nitidus*	0.069058	0.06363
SC35	SC44	CS2	*R.nitidus*	0.058238	0.062129
GD25	SC45	CS2	*R.nitidus*	0.063004	0.066769
GD21	SC45	CS2	*R.nitidus*	0.076716	0.062686
JO3	SC44	CS2	*R.nitidus*	0.034942	0.068414

GD42	SC45	CS2	*R.nitidus*	-0.005658	0.065972
SC45	SC44	CS2	*R.nitidus*	0.025761	0.065584
GD30	SC45	CS2	*R.nitidus*	0.102282	0.066521
MY4	SC44	CS2	*R.nitidus*	-0.036697	0.066951
JW4	SC44	CS2	*R.nitidus*	0.232389	0.065371
JO8	SC45	CS2	*R.nitidus*	-0.015984	0.073343
JO7	SC45	CS2	*R.nitidus*	-0.045045	0.071576
GD33	SC45	CS2	*R.nitidus*	-0.024879	0.067293
GD40	SC45	CS2	*R.nitidus*	0.038488	0.063525
GD45	SC45	CS2	*R.nitidus*	0.028759	0.068833
GD44	SC45	CS2	*R.nitidus*	-0.007246	0.066278
CS15	SC45	CS2	*R.nitidus*	-0.003546	0.068556
CS21	SC45	CS2	*R.nitidus*	-0.048692	0.066072
CS11	SC45	CS2	*R.nitidus*	-0.017329	0.068332
CS19	SC45	CS2	*R.nitidus*	-0.051433	0.06886
GD29	SC45	CS2	*R.nitidus*	0.09377	0.064866
GD31	SC45	CS2	*R.nitidus*	0.042888	0.066468
GD41	SC45	CS2	*R.nitidus*	-0.014006	0.065354
GD49	SC45	CS2	*R.nitidus*	0.054276	0.068434
CS12	SC45	CS2	*R.nitidus*	-0.031155	0.067142
CS24	SC45	CS2	*R.nitidus*	-0.059471	0.066768
CS22	SC45	CS2	*R.nitidus*	-0.013363	0.065911
CS25	SC45	CS2	*R.nitidus*	-0.049144	0.066112
CS18	SC45	CS2	*R.nitidus*	-0.055514	0.065634
CS31	SC45	CS2	*R.nitidus*	-0.048653	0.066144
GD35	SC45	CS2	*R.nitidus*	0.008984	0.065503
GD50	SC45	CS2	*R.nitidus*	0.008489	0.069875
GD34	SC45	CS2	*R.nitidus*	-0.021247	0.065573
CS13	SC45	CS2	*R.nitidus*	-0.052513	0.068786
SC5	SC45	CS2	*R.nitidus*	-0.029141	0.06846
GD48	SC45	CS2	*R.nitidus*	0.023686	0.066193
CS28	SC45	CS2	*R.nitidus*	-0.039454	0.068604
CS16	SC45	CS2	*R.nitidus*	-0.026354	0.066468
GD46	SC45	CS2	*R.nitidus*	-0.046961	0.066019
GD32	SC45	CS2	*R.nitidus*	-0.00471	0.067448
SC14	SC45	CS2	*R.nitidus*	-0.033482	0.066992
CS20	SC45	CS2	*R.nitidus*	-0.062969	0.066105
SC24	SC45	CS2	*R.nitidus*	-0.03115	0.063424
SC28	SC45	CS2	*R.nitidus*	-0.065284	0.064242
SC15	SC45	CS2	*R.nitidus*	-0.055075	0.06713
CS23	SC45	CS2	*R.nitidus*	-0.077542	0.064719
CS14	SC45	CS2	*R.nitidus*	-0.020803	0.069562
SC10	SC45	CS2	*R.nitidus*	0.030008	0.066992
SC31	SC45	CS2	*R.nitidus*	-0.037258	0.065347
CS17	SC45	CS2	*R.nitidus*	0.003019	0.067411
JW4	SC45	CS2	*R.nitidus*	0.219076	0.064731
SC18	SC45	CS2	*R.nitidus*	0.025122	0.066515
SC42	SC45	CS2	*R.nitidus*	0.225765	0.063302
CS30	SC45	CS2	*R.nitidus*	-0.087879	0.066625
CS3	SC45	CS2	*R.nitidus*	-0.044709	0.065821
CS6	SC45	CS2	*R.nitidus*	-0.074159	0.065453
JW7	SC45	CS2	*R.nitidus*	-0.003656	0.07116
SC12	SC45	CS2	*R.nitidus*	-0.0544	0.065059
SC6	SC45	CS2	*R.nitidus*	-0.037618	0.065742
SC44	SC45	CS2	*R.nitidus*	-0.025761	0.065584
JW6	SC45	CS2	*R.nitidus*	-0.027645	0.07226

JO1	SC45	CS2	*R.nitidus*	-0.053042	0.066639
SC26	SC45	CS2	*R.nitidus*	-0.083916	0.065961
JO3	SC45	CS2	*R.nitidus*	0.026316	0.069028
CS11	MY4	CS2	*R.nitidus*	0.036903	0.068409
CS4	SC45	CS2	*R.nitidus*	-0.030758	0.065394
SC27	SC45	CS2	*R.nitidus*	-0.030098	0.066549
GD26	MY4	CS2	*R.nitidus*	0.079521	0.066702
GD22	MY4	CS2	*R.nitidus*	0.110734	0.065751
SC34	SC45	CS2	*R.nitidus*	0.002177	0.06637
GD25	MY4	CS2	*R.nitidus*	0.117982	0.066273
JO8	MY4	CS2	*R.nitidus*	0.06841	0.072472
SC35	SC45	CS2	*R.nitidus*	0.012413	0.064539
GD32	MY4	CS2	*R.nitidus*	0.071545	0.071343
SC32	SC45	CS2	*R.nitidus*	-0.001547	0.065305
CS12	MY4	CS2	*R.nitidus*	0.054753	0.069213
MY4	SC45	CS2	*R.nitidus*	-0.069248	0.06615
GD30	MY4	CS2	*R.nitidus*	0.166259	0.068364
JO7	MY4	CS2	*R.nitidus*	0.024925	0.073902
MY6	SC45	CS2	*R.nitidus*	-0.028127	0.06658
GD45	MY4	CS2	*R.nitidus*	0.097087	0.069802
JO5	SC45	CS2	*R.nitidus*	0.024672	0.065418
GD29	MY4	CS2	*R.nitidus*	0.153795	0.065158
GD49	MY4	CS2	*R.nitidus*	0.097502	0.070264
GD33	MY4	CS2	*R.nitidus*	0.050385	0.066217
GD40	MY4	CS2	*R.nitidus*	0.111567	0.064869
GD31	MY4	CS2	*R.nitidus*	0.091317	0.068412
GD35	MY4	CS2	*R.nitidus*	0.093772	0.067396
CS19	MY4	CS2	*R.nitidus*	0.025365	0.071179
CS24	MY4	CS2	*R.nitidus*	0.004542	0.06765
GD21	MY4	CS2	*R.nitidus*	0.162034	0.064385
GD42	MY4	CS2	*R.nitidus*	0.069242	0.065547
CS22	MY4	CS2	*R.nitidus*	0.055215	0.06766
GD48	MY4	CS2	*R.nitidus*	0.078387	0.069203
GD34	MY4	CS2	*R.nitidus*	0.046316	0.065663
CS16	MY4	CS2	*R.nitidus*	0.036664	0.068586
CS14	MY4	CS2	*R.nitidus*	0.043997	0.07061
CS23	MY4	CS2	*R.nitidus*	-0.011967	0.06865
GD46	MY4	CS2	*R.nitidus*	0.017895	0.066279
CS15	MY4	CS2	*R.nitidus*	0.062184	0.06813
CS13	MY4	CS2	*R.nitidus*	0.001535	0.068903
CS17	MY4	CS2	*R.nitidus*	0.054406	0.069199
GD50	MY4	CS2	*R.nitidus*	0.074979	0.070046
SC14	MY4	CS2	*R.nitidus*	0.040449	0.067981
CS21	MY4	CS2	*R.nitidus*	0.020958	0.06896
CS18	MY4	CS2	*R.nitidus*	0.010526	0.068012
GD44	MY4	CS2	*R.nitidus*	0.06213	0.068033
GD41	MY4	CS2	*R.nitidus*	0.048621	0.06419
SC5	MY4	CS2	*R.nitidus*	0.037175	0.069606
SC6	MY4	CS2	*R.nitidus*	0.026094	0.066251
CS25	MY4	CS2	*R.nitidus*	0.025602	0.067744
CS3	MY4	CS2	*R.nitidus*	0.018657	0.067405
JW6	MY4	CS2	*R.nitidus*	0.06601	0.07107
CS4	MY4	CS2	*R.nitidus*	0.033962	0.066369
SC32	MY4	CS2	*R.nitidus*	0.066103	0.065542
JW4	MY4	CS2	*R.nitidus*	0.271483	0.066468
SC44	MY4	CS2	*R.nitidus*	0.036697	0.066951

SC34	MY4	CS2	*R.nitidus*	0.07991	0.065456
JO1	MY4	CS2	*R.nitidus*	0.016756	0.066276
SC26	MY4	CS2	*R.nitidus*	-0.005201	0.064479
CS20	MY4	CS2	*R.nitidus*	0.007267	0.068125
SC31	MY4	CS2	*R.nitidus*	0.029674	0.06528
SC27	MY4	CS2	*R.nitidus*	0.037834	0.066539
SC18	MY4	CS2	*R.nitidus*	0.100895	0.063255
JO5	MY4	CS2	*R.nitidus*	0.096421	0.066673
JW7	MY4	CS2	*R.nitidus*	0.068431	0.070646
CS30	MY4	CS2	*R.nitidus*	-0.019389	0.067351
SC35	MY4	CS2	*R.nitidus*	0.094011	0.064502
CS28	MY4	CS2	*R.nitidus*	0.016292	0.068641
SC12	MY4	CS2	*R.nitidus*	0.019069	0.064626
SC15	MY4	CS2	*R.nitidus*	0.021722	0.067778
CS6	MY4	CS2	*R.nitidus*	-0.000772	0.067396
CS31	MY4	CS2	*R.nitidus*	0.018954	0.067547
SC10	MY4	CS2	*R.nitidus*	0.108347	0.069496
JO8	MY6	CS2	*R.nitidus*	0.009298	0.073286
SC24	MY4	CS2	*R.nitidus*	0.05251	0.066013
JO7	MY6	CS2	*R.nitidus*	-0.027668	0.072602
SC28	MY4	CS2	*R.nitidus*	-0.002972	0.06654
SC42	MY4	CS2	*R.nitidus*	0.280122	0.063487
CS12	MY6	CS2	*R.nitidus*	0.009608	0.067158
JO3	MY4	CS2	*R.nitidus*	0.086844	0.071153
SC45	MY4	CS2	*R.nitidus*	0.069248	0.06615
GD26	MY6	CS2	*R.nitidus*	0.04726	0.067264
MY6	MY4	CS2	*R.nitidus*	0.060241	0.059584
GD45	MY6	CS2	*R.nitidus*	0.04898	0.069056
GD50	MY6	CS2	*R.nitidus*	0.01638	0.067658
GD41	MY6	CS2	*R.nitidus*	0.002786	0.063623
GD30	MY6	CS2	*R.nitidus*	0.123177	0.066929
GD42	MY6	CS2	*R.nitidus*	0.023977	0.064888
GD21	MY6	CS2	*R.nitidus*	0.118194	0.065051
GD32	MY6	CS2	*R.nitidus*	0.025903	0.069458
GD40	MY6	CS2	*R.nitidus*	0.063003	0.064425
CS18	MY6	CS2	*R.nitidus*	-0.029905	0.066638
CS11	MY6	CS2	*R.nitidus*	-0.001414	0.068006
CS24	MY6	CS2	*R.nitidus*	-0.038031	0.066845
GD22	MY6	CS2	*R.nitidus*	0.068302	0.065079
GD49	MY6	CS2	*R.nitidus*	0.085714	0.069363
GD48	MY6	CS2	*R.nitidus*	0.031655	0.067378
SC10	MY6	CS2	*R.nitidus*	0.069749	0.068051
GD33	MY6	CS2	*R.nitidus*	0.009466	0.06604
GD29	MY6	CS2	*R.nitidus*	0.114883	0.064185
GD35	MY6	CS2	*R.nitidus*	0.049512	0.066652
CS21	MY6	CS2	*R.nitidus*	-0.026657	0.066459
GD25	MY6	CS2	*R.nitidus*	0.073888	0.065839
GD31	MY6	CS2	*R.nitidus*	0.053598	0.066875
CS15	MY6	CS2	*R.nitidus*	0.017204	0.067599
CS13	MY6	CS2	*R.nitidus*	-0.050633	0.068567
CS17	MY6	CS2	*R.nitidus*	0.012763	0.069061
GD44	MY6	CS2	*R.nitidus*	0.018745	0.067232
GD34	MY6	CS2	*R.nitidus*	0.00885	0.065527
CS30	MY6	CS2	*R.nitidus*	-0.067809	0.066635
SC5	MY6	CS2	*R.nitidus*	-0.007868	0.069655
CS22	MY6	CS2	*R.nitidus*	0.007491	0.065855

GD46	MY6	CS2	*R.nitidus*	-0.026001	0.065337
CS23	MY6	CS2	*R.nitidus*	-0.052283	0.066124
SC27	MY6	CS2	*R.nitidus*	-0.009407	0.065336
CS19	MY6	CS2	*R.nitidus*	-0.022661	0.068955
CS20	MY6	CS2	*R.nitidus*	-0.030824	0.066781
SC12	MY6	CS2	*R.nitidus*	-0.025699	0.066334
CS3	MY6	CS2	*R.nitidus*	-0.017751	0.065685
SC28	MY6	CS2	*R.nitidus*	-0.04646	0.06434
SC34	MY6	CS2	*R.nitidus*	0.028825	0.064633
CS25	MY6	CS2	*R.nitidus*	-0.017699	0.066994
CS31	MY6	CS2	*R.nitidus*	-0.024887	0.06763
SC6	MY6	CS2	*R.nitidus*	-0.019667	0.066772
CS28	MY6	CS2	*R.nitidus*	-0.025	0.068181
CS14	MY6	CS2	*R.nitidus*	0.006541	0.069099
SC45	MY6	CS2	*R.nitidus*	0.028127	0.06658
CS16	MY6	CS2	*R.nitidus*	-0.002878	0.066437
GD22	JW4	CS2	*R.nitidus*	-0.157787	0.066308
SC32	MY6	CS2	*R.nitidus*	0.017817	0.063464
CS4	MY6	CS2	*R.nitidus*	-0.011782	0.065343
JO1	MY6	CS2	*R.nitidus*	-0.033358	0.065201
JO7	JW4	CS2	*R.nitidus*	-0.226824	0.071033
CS6	MY6	CS2	*R.nitidus*	-0.046757	0.066193
SC44	MY6	CS2	*R.nitidus*	-0.005247	0.065789
SC42	MY6	CS2	*R.nitidus*	0.24665	0.062564
JO5	MY6	CS2	*R.nitidus*	0.056277	0.066099
MY4	MY6	CS2	*R.nitidus*	-0.060241	0.059584
JW7	MY6	CS2	*R.nitidus*	0.012927	0.070217
SC15	MY6	CS2	*R.nitidus*	-0.022516	0.066605
GD21	JW4	CS2	*R.nitidus*	-0.108345	0.06753
GD30	JW4	CS2	*R.nitidus*	-0.095238	0.070587
SC18	MY6	CS2	*R.nitidus*	0.05427	0.064372
SC24	MY6	CS2	*R.nitidus*	0.003858	0.066168
CS11	JW4	CS2	*R.nitidus*	-0.208598	0.067245
JO8	JW4	CS2	*R.nitidus*	-0.199799	0.070877
SC14	MY6	CS2	*R.nitidus*	-0.007236	0.066616
JO3	MY6	CS2	*R.nitidus*	0.034775	0.067811
SC31	MY6	CS2	*R.nitidus*	-0.015544	0.065993
SC26	MY6	CS2	*R.nitidus*	-0.041908	0.064713
GD29	JW4	CS2	*R.nitidus*	-0.101132	0.066491
JW4	MY6	CS2	*R.nitidus*	0.226998	0.066046
SC35	MY6	CS2	*R.nitidus*	0.042222	0.064636
JW6	MY6	CS2	*R.nitidus*	0.01382	0.071508
GD33	JW4	CS2	*R.nitidus*	-0.218571	0.068153
GD41	JW4	CS2	*R.nitidus*	-0.209104	0.064633
CS12	JW4	CS2	*R.nitidus*	-0.203148	0.06848
GD35	JW4	CS2	*R.nitidus*	-0.181429	0.06615
GD25	JW4	CS2	*R.nitidus*	-0.146177	0.067437
GD40	JW4	CS2	*R.nitidus*	-0.150412	0.06589
GD45	JW4	CS2	*R.nitidus*	-0.135565	0.070197
GD32	JW4	CS2	*R.nitidus*	-0.199679	0.068907
GD50	JW4	CS2	*R.nitidus*	-0.150473	0.070328
GD26	JW4	CS2	*R.nitidus*	-0.185893	0.068424
GD44	JW4	CS2	*R.nitidus*	-0.178862	0.068474
GD49	JW4	CS2	*R.nitidus*	-0.155537	0.069989
GD31	JW4	CS2	*R.nitidus*	-0.154799	0.0694
GD46	JW4	CS2	*R.nitidus*	-0.244986	0.066668

CS13	JW4	CS2	*R.nitidus*	-0.257922	0.06689
CS24	JW4	CS2	*R.nitidus*	-0.269174	0.065541
GD48	JW4	CS2	*R.nitidus*	-0.175258	0.068499
CS14	JW4	CS2	*R.nitidus*	-0.208879	0.068741
GD34	JW4	CS2	*R.nitidus*	-0.21512	0.066259
CS16	JW4	CS2	*R.nitidus*	-0.227802	0.067558
GD42	JW4	CS2	*R.nitidus*	-0.186246	0.065657
SC27	JW4	CS2	*R.nitidus*	-0.214136	0.064817
CS15	JW4	CS2	*R.nitidus*	-0.186488	0.068464
SC6	JW4	CS2	*R.nitidus*	-0.226766	0.066299
CS3	JW4	CS2	*R.nitidus*	-0.22963	0.067384
CS23	JW4	CS2	*R.nitidus*	-0.25942	0.065733
CS28	JW4	CS2	*R.nitidus*	-0.24	0.069967
SC15	JW4	CS2	*R.nitidus*	-0.241354	0.067705
SC5	JW4	CS2	*R.nitidus*	-0.204828	0.069787
CS25	JW4	CS2	*R.nitidus*	-0.229592	0.068822
CS18	JW4	CS2	*R.nitidus*	-0.247407	0.066662
SC18	JW4	CS2	*R.nitidus*	-0.200917	0.067877
SC42	JW4	CS2	*R.nitidus*	-0.011598	0.065093
CS21	JW4	CS2	*R.nitidus*	-0.247988	0.066818
CS20	JW4	CS2	*R.nitidus*	-0.24209	0.066869
CS30	JW4	CS2	*R.nitidus*	-0.265896	0.066339
CS17	JW4	CS2	*R.nitidus*	-0.197108	0.069529
CS19	JW4	CS2	*R.nitidus*	-0.236842	0.068297
SC32	JW4	CS2	*R.nitidus*	-0.208517	0.064157
CS31	JW4	CS2	*R.nitidus*	-0.255948	0.065823
CS22	JW4	CS2	*R.nitidus*	-0.225331	0.065921
SC24	JW4	CS2	*R.nitidus*	-0.227886	0.067128
SC26	JW4	CS2	*R.nitidus*	-0.264642	0.065192
CS4	JW4	CS2	*R.nitidus*	-0.224818	0.064884
SC10	JW4	CS2	*R.nitidus*	-0.174312	0.06643
GD22	JW6	CS2	*R.nitidus*	0.074496	0.070315
MY4	JW4	CS2	*R.nitidus*	-0.271483	0.066468
SC12	JW4	CS2	*R.nitidus*	-0.261161	0.06528
SC14	JW4	CS2	*R.nitidus*	-0.223088	0.065272
MY6	JW4	CS2	*R.nitidus*	-0.226998	0.066046
SC28	JW4	CS2	*R.nitidus*	-0.251249	0.066506
CS6	JW4	CS2	*R.nitidus*	-0.252434	0.06602
GD26	JW6	CS2	*R.nitidus*	0.07032	0.071399
JO3	JW4	CS2	*R.nitidus*	-0.161905	0.069282
JO8	JW6	CS2	*R.nitidus*	0.021552	0.071975
GD25	JW6	CS2	*R.nitidus*	0.080229	0.072634
SC45	JW4	CS2	*R.nitidus*	-0.219076	0.064731
SC31	JW4	CS2	*R.nitidus*	-0.242711	0.066377
GD32	JW6	CS2	*R.nitidus*	0.010246	0.073934
SC35	JW4	CS2	*R.nitidus*	-0.219369	0.065255
SC44	JW4	CS2	*R.nitidus*	-0.232389	0.065371
GD40	JW6	CS2	*R.nitidus*	0.071429	0.070567
JO1	JW4	CS2	*R.nitidus*	-0.23099	0.065179
CS11	JW6	CS2	*R.nitidus*	0.003756	0.073151
JW7	JW4	CS2	*R.nitidus*	-0.153414	0.068749
JW6	JW4	CS2	*R.nitidus*	-0.200952	0.070292
GD34	JW6	CS2	*R.nitidus*	-0.012613	0.069832
JO5	JW4	CS2	*R.nitidus*	-0.169395	0.066283
GD33	JW6	CS2	*R.nitidus*	0.002793	0.072503
GD41	JW6	CS2	*R.nitidus*	0.014409	0.071297

GD29	JW6	CS2	*R.nitidus*	0.139091	0.068283
GD21	JW6	CS2	*R.nitidus*	0.120451	0.07032
GD30	JW6	CS2	*R.nitidus*	0.148148	0.071628
JO7	JW6	CS2	*R.nitidus*	0.024038	0.070482
CS14	JW6	CS2	*R.nitidus*	0.02381	0.075454
SC34	JW4	CS2	*R.nitidus*	-0.204748	0.06431
GD46	JW6	CS2	*R.nitidus*	-0.03445	0.070435
GD44	JW6	CS2	*R.nitidus*	0.027077	0.07195
GD42	JW6	CS2	*R.nitidus*	0.062562	0.07112
GD35	JW6	CS2	*R.nitidus*	0.051756	0.072699
GD50	JW6	CS2	*R.nitidus*	0.063319	0.073915
GD31	JW6	CS2	*R.nitidus*	0.074627	0.072466
CS21	JW6	CS2	*R.nitidus*	-0.00759	0.070346
CS12	JW6	CS2	*R.nitidus*	0.025415	0.073469
GD45	JW6	CS2	*R.nitidus*	0.122318	0.073864
CS16	JW6	CS2	*R.nitidus*	0.00289	0.07362
CS13	JW6	CS2	*R.nitidus*	-0.021526	0.076735
CS23	JW6	CS2	*R.nitidus*	-0.017512	0.071741
GD48	JW6	CS2	*R.nitidus*	0.036027	0.071014
CS22	JW6	CS2	*R.nitidus*	-0.012416	0.072669
CS17	JW6	CS2	*R.nitidus*	0.035106	0.075899
GD49	JW6	CS2	*R.nitidus*	0.096386	0.071521
CS3	JW6	CS2	*R.nitidus*	0.021113	0.071182
CS19	JW6	CS2	*R.nitidus*	-0.024096	0.074094
SC6	JW6	CS2	*R.nitidus*	0.015152	0.073024
CS20	JW6	CS2	*R.nitidus*	0.003683	0.073449
SC26	JW6	CS2	*R.nitidus*	-0.038573	0.071209
CS18	JW6	CS2	*R.nitidus*	-0.010387	0.071395
CS4	JW6	CS2	*R.nitidus*	0.012015	0.071607
SC18	JW6	CS2	*R.nitidus*	0.020263	0.07235
SC27	JW6	CS2	*R.nitidus*	-0.003902	0.0708
SC14	JW6	CS2	*R.nitidus*	-0.004789	0.069692
SC12	JW6	CS2	*R.nitidus*	-0.036965	0.071438
CS24	JW6	CS2	*R.nitidus*	-0.049	0.072324
SC28	JW6	CS2	*R.nitidus*	-0.021463	0.073342
SC45	JW6	CS2	*R.nitidus*	0.027645	0.07226
SC24	JW6	CS2	*R.nitidus*	0.014014	0.073464
JO1	JW6	CS2	*R.nitidus*	-0.018234	0.072554
SC34	JW6	CS2	*R.nitidus*	0.017094	0.070205
CS31	JW6	CS2	*R.nitidus*	-0.024655	0.072443
SC44	JW6	CS2	*R.nitidus*	-0.014677	0.073778
CS28	JW6	CS2	*R.nitidus*	-0.037074	0.072866
CS6	JW6	CS2	*R.nitidus*	-0.043726	0.070615
CS25	JW6	CS2	*R.nitidus*	-0.016807	0.074117
SC10	JW6	CS2	*R.nitidus*	0.086053	0.074956
SC5	JW6	CS2	*R.nitidus*	0.015195	0.076078
SC42	JW6	CS2	*R.nitidus*	0.176568	0.067926
JO3	JW6	CS2	*R.nitidus*	0.032621	0.073255
SC35	JW6	CS2	*R.nitidus*	0.01983	0.070101
GD25	JW7	CS2	*R.nitidus*	0.067603	0.069483
CS15	JW6	CS2	*R.nitidus*	0.044007	0.073288
CS30	JW6	CS2	*R.nitidus*	-0.047716	0.070946
SC15	JW6	CS2	*R.nitidus*	-0.027495	0.07313
JO8	JW7	CS2	*R.nitidus*	-0.02246	0.075088
SC31	JW6	CS2	*R.nitidus*	-0.014995	0.073954
JO7	JW7	CS2	*R.nitidus*	-0.053088	0.07472

JO5	JW6	CS2	*R.nitidus*	0.082778	0.070391
MY6	JW6	CS2	*R.nitidus*	-0.01382	0.071508
SC32	JW6	CS2	*R.nitidus*	0.023645	0.072165
GD30	JW7	CS2	*R.nitidus*	0.11379	0.070467
JW4	JW6	CS2	*R.nitidus*	0.200952	0.070292
JW7	JW6	CS2	*R.nitidus*	0.025316	0.074115
MY4	JW6	CS2	*R.nitidus*	-0.06601	0.07107
GD31	JW7	CS2	*R.nitidus*	0.06765	0.072355
CS12	JW7	CS2	*R.nitidus*	0.001867	0.072104
GD33	JW7	CS2	*R.nitidus*	-0.00823	0.070101
GD34	JW7	CS2	*R.nitidus*	-0.032046	0.070747
GD29	JW7	CS2	*R.nitidus*	0.110592	0.067216
GD49	JW7	CS2	*R.nitidus*	0.032227	0.072223
GD26	JW7	CS2	*R.nitidus*	0.032205	0.071384
GD35	JW7	CS2	*R.nitidus*	0.031356	0.070611
GD21	JW7	CS2	*R.nitidus*	0.10425	0.06824
CS11	JW7	CS2	*R.nitidus*	-0.01084	0.069725
CS22	JW7	CS2	*R.nitidus*	-0.011701	0.068268
GD22	JW7	CS2	*R.nitidus*	0.059524	0.069098
GD50	JW7	CS2	*R.nitidus*	0.037678	0.074507
GD48	JW7	CS2	*R.nitidus*	0.017778	0.071148
GD45	JW7	CS2	*R.nitidus*	0.082097	0.071741
GD44	JW7	CS2	*R.nitidus*	0.008913	0.070685
GD32	JW7	CS2	*R.nitidus*	-0.00455	0.07218
CS21	JW7	CS2	*R.nitidus*	-0.040245	0.068317
GD40	JW7	CS2	*R.nitidus*	0.055601	0.069851
CS16	JW7	CS2	*R.nitidus*	-0.013761	0.071613
GD46	JW7	CS2	*R.nitidus*	-0.034335	0.068455
CS20	JW7	CS2	*R.nitidus*	-0.02535	0.069795
GD42	JW7	CS2	*R.nitidus*	0.043248	0.069395
GD41	JW7	CS2	*R.nitidus*	-0.007772	0.0702
CS18	JW7	CS2	*R.nitidus*	-0.028169	0.070591
CS14	JW7	CS2	*R.nitidus*	-0.000891	0.072968
CS13	JW7	CS2	*R.nitidus*	-0.059299	0.071512
CS28	JW7	CS2	*R.nitidus*	-0.043145	0.071646
CS15	JW7	CS2	*R.nitidus*	0.019828	0.071118
CS23	JW7	CS2	*R.nitidus*	-0.049957	0.068203
SC12	JW7	CS2	*R.nitidus*	-0.062317	0.068304
CS3	JW7	CS2	*R.nitidus*	-0.028326	0.068178
CS31	JW7	CS2	*R.nitidus*	-0.057656	0.070937
SC5	JW7	CS2	*R.nitidus*	-0.007605	0.076613
SC6	JW7	CS2	*R.nitidus*	-0.023033	0.070861
CS17	JW7	CS2	*R.nitidus*	0.011472	0.072299
SC10	JW7	CS2	*R.nitidus*	0.026866	0.069528
SC15	JW7	CS2	*R.nitidus*	-0.04451	0.070023
CS19	JW7	CS2	*R.nitidus*	-0.023111	0.070658
CS24	JW7	CS2	*R.nitidus*	-0.053774	0.068045
SC18	JW7	CS2	*R.nitidus*	-0.004897	0.069453
CS4	JW7	CS2	*R.nitidus*	-0.006957	0.065308
CS25	JW7	CS2	*R.nitidus*	-0.023256	0.071747
SC14	JW7	CS2	*R.nitidus*	-0.03	0.069403
CS30	JW7	CS2	*R.nitidus*	-0.068627	0.067567
SC31	JW7	CS2	*R.nitidus*	-0.055238	0.068002
JW6	JW7	CS2	*R.nitidus*	-0.025316	0.074115
SC28	JW7	CS2	*R.nitidus*	-0.057115	0.068784
JO5	JW7	CS2	*R.nitidus*	0.038603	0.068739

SC34	JW7	CS2	*R.nitidus*	0.019231	0.069074
SC42	JW7	CS2	*R.nitidus*	0.191738	0.067252
JO8	JO1	CS2	*R.nitidus*	0.032258	0.07165
SC26	JW7	CS2	*R.nitidus*	-0.083095	0.068926
CS11	JO1	CS2	*R.nitidus*	0.017634	0.06641
SC44	JW7	CS2	*R.nitidus*	-0.030447	0.070217
SC32	JW7	CS2	*R.nitidus*	-0.017928	0.07031
SC27	JW7	CS2	*R.nitidus*	-0.025329	0.068137
JO1	JW7	CS2	*R.nitidus*	-0.041588	0.069669
SC45	JW7	CS2	*R.nitidus*	0.003656	0.07116
SC35	JW7	CS2	*R.nitidus*	0.002814	0.065754
JO7	JO1	CS2	*R.nitidus*	-0.017824	0.071565
JO3	JW7	CS2	*R.nitidus*	0.011797	0.069665
GD41	JO1	CS2	*R.nitidus*	0.035714	0.065083
CS6	JW7	CS2	*R.nitidus*	-0.067918	0.067942
GD21	JO1	CS2	*R.nitidus*	0.14134	0.064659
JW4	JW7	CS2	*R.nitidus*	0.153414	0.068749
CS12	JO1	CS2	*R.nitidus*	0.02902	0.066408
GD40	JO1	CS2	*R.nitidus*	0.093878	0.065426
SC24	JW7	CS2	*R.nitidus*	-0.026975	0.070524
GD30	JO1	CS2	*R.nitidus*	0.159839	0.065423
MY6	JW7	CS2	*R.nitidus*	-0.012927	0.070217
GD29	JO1	CS2	*R.nitidus*	0.14106	0.063539
GD22	JO1	CS2	*R.nitidus*	0.093204	0.065238
MY4	JW7	CS2	*R.nitidus*	-0.068431	0.070646
GD26	JO1	CS2	*R.nitidus*	0.085178	0.064488
GD32	JO1	CS2	*R.nitidus*	0.053241	0.069284
GD31	JO1	CS2	*R.nitidus*	0.090318	0.066285
GD46	JO1	CS2	*R.nitidus*	0.01385	0.063949
GD34	JO1	CS2	*R.nitidus*	0.043331	0.065107
GD35	JO1	CS2	*R.nitidus*	0.065835	0.06449
CS19	JO1	CS2	*R.nitidus*	0.010241	0.068199
CS21	JO1	CS2	*R.nitidus*	-0.003579	0.066895
GD45	JO1	CS2	*R.nitidus*	0.079653	0.069854
GD25	JO1	CS2	*R.nitidus*	0.120603	0.066035
CS18	JO1	CS2	*R.nitidus*	-0.00366	0.066563
CS16	JO1	CS2	*R.nitidus*	0.013443	0.065761
CS31	JO1	CS2	*R.nitidus*	0.009819	0.067
CS28	JO1	CS2	*R.nitidus*	-0.008013	0.066831
GD50	JO1	CS2	*R.nitidus*	0.072925	0.06929
GD33	JO1	CS2	*R.nitidus*	0.042877	0.066481
CS30	JO1	CS2	*R.nitidus*	-0.031525	0.065831
GD44	JO1	CS2	*R.nitidus*	0.041485	0.066439
GD48	JO1	CS2	*R.nitidus*	0.062266	0.068254
GD49	JO1	CS2	*R.nitidus*	0.087981	0.068185
GD42	JO1	CS2	*R.nitidus*	0.057103	0.065668
CS22	JO1	CS2	*R.nitidus*	0.022108	0.067099
CS13	JO1	CS2	*R.nitidus*	-0.02099	0.067814
CS17	JO1	CS2	*R.nitidus*	0.052632	0.068885
CS14	JO1	CS2	*R.nitidus*	0.023512	0.068551
CS15	JO1	CS2	*R.nitidus*	0.033898	0.065492
CS4	JO1	CS2	*R.nitidus*	0.017505	0.064916
SC18	JO1	CS2	*R.nitidus*	0.07084	0.066895
CS25	JO1	CS2	*R.nitidus*	0.012839	0.067501
SC10	JO1	CS2	*R.nitidus*	0.089968	0.065089
SC12	JO1	CS2	*R.nitidus*	0.003928	0.065651

CS20	JO1	CS2	*R.nitidus*	-0.004909	0.067026
SC5	JO1	CS2	*R.nitidus*	0.023592	0.069872
SC35	JO1	CS2	*R.nitidus*	0.077863	0.064706
SC14	JO1	CS2	*R.nitidus*	0.00768	0.065064
JW6	JO1	CS2	*R.nitidus*	0.018234	0.072554
MY4	JO1	CS2	*R.nitidus*	-0.016756	0.066276
SC27	JO1	CS2	*R.nitidus*	0.006178	0.06463
SC6	JO1	CS2	*R.nitidus*	0.019577	0.066996
CS23	JO1	CS2	*R.nitidus*	-0.031688	0.065833
CS24	JO1	CS2	*R.nitidus*	-0.020119	0.06729
JO8	JO3	CS2	*R.nitidus*	-0.009346	0.073588
JW4	JO1	CS2	*R.nitidus*	0.23099	0.065179
SC24	JO1	CS2	*R.nitidus*	0.027624	0.065462
SC32	JO1	CS2	*R.nitidus*	0.046326	0.065058
JW7	JO1	CS2	*R.nitidus*	0.041588	0.069669
MY6	JO1	CS2	*R.nitidus*	0.033358	0.065201
CS6	JO1	CS2	*R.nitidus*	-0.016541	0.067574
CS11	JO3	CS2	*R.nitidus*	-0.039548	0.071732
SC26	JO1	CS2	*R.nitidus*	-0.015212	0.064682
SC34	JO1	CS2	*R.nitidus*	0.051243	0.065521
JO3	JO1	CS2	*R.nitidus*	0.072925	0.069464
CS3	JO1	CS2	*R.nitidus*	0.011186	0.066045
SC31	JO1	CS2	*R.nitidus*	0.013783	0.06503
GD32	JO3	CS2	*R.nitidus*	-0.01106	0.070776
SC15	JO1	CS2	*R.nitidus*	0.002408	0.067173
SC45	JO1	CS2	*R.nitidus*	0.053042	0.066639
GD25	JO3	CS2	*R.nitidus*	0.035928	0.070695
SC44	JO1	CS2	*R.nitidus*	0.025443	0.06553
GD26	JO3	CS2	*R.nitidus*	0.010367	0.070511
GD31	JO3	CS2	*R.nitidus*	0.013889	0.070795
GD21	JO3	CS2	*R.nitidus*	0.084027	0.069393
SC28	JO1	CS2	*R.nitidus*	-0.003187	0.066041
CS12	JO3	CS2	*R.nitidus*	-0.014238	0.070351
GD46	JO3	CS2	*R.nitidus*	-0.064435	0.067849
SC42	JO1	CS2	*R.nitidus*	0.267352	0.063254
GD33	JO3	CS2	*R.nitidus*	-0.02188	0.069708
GD34	JO3	CS2	*R.nitidus*	-0.032336	0.06921
GD45	JO3	CS2	*R.nitidus*	0.045283	0.072868
GD50	JO3	CS2	*R.nitidus*	0.026465	0.072575
JO5	JO1	CS2	*R.nitidus*	0.084681	0.064706
GD30	JO3	CS2	*R.nitidus*	0.124756	0.071347
JO7	JO3	CS2	*R.nitidus*	-0.02593	0.075154
GD22	JO3	CS2	*R.nitidus*	0.033965	0.068337
CS13	JO3	CS2	*R.nitidus*	-0.073432	0.072106
CS17	JO3	CS2	*R.nitidus*	-0.018325	0.072473
CS16	JO3	CS2	*R.nitidus*	-0.037277	0.070413
GD29	JO3	CS2	*R.nitidus*	0.086166	0.066576
GD48	JO3	CS2	*R.nitidus*	0.020942	0.070237
CS31	JO3	CS2	*R.nitidus*	-0.049914	0.070338
CS28	JO3	CS2	*R.nitidus*	-0.060965	0.071654
GD35	JO3	CS2	*R.nitidus*	0.021794	0.070193
CS14	JO3	CS2	*R.nitidus*	-0.027569	0.07164
GD40	JO3	CS2	*R.nitidus*	0.018239	0.069746
CS18	JO3	CS2	*R.nitidus*	-0.060017	0.068754
CS15	JO3	CS2	*R.nitidus*	-0.013622	0.069073
CS30	JO3	CS2	*R.nitidus*	-0.10477	0.067412

CS24	JO3	CS2	*R.nitidus*	-0.063078	0.068573
GD49	JO3	CS2	*R.nitidus*	0.013793	0.073064
GD41	JO3	CS2	*R.nitidus*	-0.029678	0.069089
SC12	JO3	CS2	*R.nitidus*	-0.077815	0.068246
CS20	JO3	CS2	*R.nitidus*	-0.064298	0.067996
GD44	JO3	CS2	*R.nitidus*	-0.004129	0.071703
SC6	JO3	CS2	*R.nitidus*	-0.045833	0.069621
GD42	JO3	CS2	*R.nitidus*	0	0.069921
SC15	JO3	CS2	*R.nitidus*	-0.06626	0.070067
CS21	JO3	CS2	*R.nitidus*	-0.070833	0.06901
CS23	JO3	CS2	*R.nitidus*	-0.085762	0.06723
CS19	JO3	CS2	*R.nitidus*	-0.048527	0.069781
SC44	JO3	CS2	*R.nitidus*	-0.034942	0.068414
SC32	JO3	CS2	*R.nitidus*	-0.013169	0.068932
SC18	JO3	CS2	*R.nitidus*	-0.002575	0.070635
SC5	JO3	CS2	*R.nitidus*	-0.007843	0.075363
SC45	JO3	CS2	*R.nitidus*	-0.026316	0.069028
CS25	JO3	CS2	*R.nitidus*	-0.05588	0.070508
SC27	JO3	CS2	*R.nitidus*	-0.032706	0.068483
SC24	JO3	CS2	*R.nitidus*	-0.044764	0.068973
CS22	JO3	CS2	*R.nitidus*	-0.037007	0.071729
CS3	JO3	CS2	*R.nitidus*	-0.030095	0.069483
SC26	JO3	CS2	*R.nitidus*	-0.09178	0.067967
SC14	JO3	CS2	*R.nitidus*	-0.044498	0.068801
SC35	JO3	CS2	*R.nitidus*	-0.019851	0.069373
JO7	JO5	CS2	*R.nitidus*	-0.089483	0.06972
JW4	JO3	CS2	*R.nitidus*	0.161905	0.069282
SC28	JO3	CS2	*R.nitidus*	-0.05578	0.068887
JW7	JO3	CS2	*R.nitidus*	-0.011797	0.069665
CS4	JO3	CS2	*R.nitidus*	-0.045714	0.068232
JW6	JO3	CS2	*R.nitidus*	-0.032621	0.073255
SC31	JO3	CS2	*R.nitidus*	-0.053279	0.069403
SC10	JO3	CS2	*R.nitidus*	0.025551	0.070539
JO8	JO5	CS2	*R.nitidus*	-0.066327	0.07015
MY4	JO3	CS2	*R.nitidus*	-0.086844	0.071153
MY6	JO3	CS2	*R.nitidus*	-0.034775	0.067811
SC42	JO3	CS2	*R.nitidus*	0.148827	0.065523
CS6	JO3	CS2	*R.nitidus*	-0.105532	0.069085
GD31	JO5	CS2	*R.nitidus*	0.022496	0.067549
GD34	JO5	CS2	*R.nitidus*	-0.036755	0.064265
GD41	JO5	CS2	*R.nitidus*	-0.039392	0.063798
SC34	JO3	CS2	*R.nitidus*	-0.034426	0.067584
GD32	JO5	CS2	*R.nitidus*	-0.041129	0.067868
GD33	JO5	CS2	*R.nitidus*	-0.035714	0.065773
JO5	JO3	CS2	*R.nitidus*	0.037097	0.069025
JO1	JO3	CS2	*R.nitidus*	-0.072925	0.069464
CS11	JO5	CS2	*R.nitidus*	-0.030112	0.067923
CS12	JO5	CS2	*R.nitidus*	-0.037838	0.065963
CS15	JO5	CS2	*R.nitidus*	-0.034752	0.067652
GD22	JO5	CS2	*R.nitidus*	0.012743	0.063775
GD48	JO5	CS2	*R.nitidus*	-0.018127	0.066625
GD35	JO5	CS2	*R.nitidus*	0.004947	0.06585
GD50	JO5	CS2	*R.nitidus*	-0.001686	0.067852
GD21	JO5	CS2	*R.nitidus*	0.060705	0.063859
GD44	JO5	CS2	*R.nitidus*	-0.034783	0.065975
CS14	JO5	CS2	*R.nitidus*	-0.038732	0.068508

GD30	JO5	CS2	*R.nitidus*	0.080064	0.066656
GD45	JO5	CS2	*R.nitidus*	0.033031	0.069051
GD46	JO5	CS2	*R.nitidus*	-0.061806	0.064632
GD26	JO5	CS2	*R.nitidus*	-0.000698	0.065672
GD25	JO5	CS2	*R.nitidus*	0.038489	0.064767
GD29	JO5	CS2	*R.nitidus*	0.080897	0.064351
GD49	JO5	CS2	*R.nitidus*	0.051345	0.067717
CS13	JO5	CS2	*R.nitidus*	-0.087823	0.066856
GD42	JO5	CS2	*R.nitidus*	-0.015011	0.06552
CS24	JO5	CS2	*R.nitidus*	-0.084091	0.065735
CS18	JO5	CS2	*R.nitidus*	-0.089928	0.066234
CS17	JO5	CS2	*R.nitidus*	-0.004605	0.068196
CS23	JO5	CS2	*R.nitidus*	-0.102115	0.062801
CS21	JO5	CS2	*R.nitidus*	-0.077922	0.064047
GD40	JO5	CS2	*R.nitidus*	0.019755	0.063923
CS19	JO5	CS2	*R.nitidus*	-0.066365	0.066191
SC12	JO5	CS2	*R.nitidus*	-0.08716	0.063622
SC34	JO5	CS2	*R.nitidus*	-0.027715	0.066186
CS30	JO5	CS2	*R.nitidus*	-0.108108	0.06383
SC31	JO5	CS2	*R.nitidus*	-0.056146	0.064869
SC28	JO5	CS2	*R.nitidus*	-0.123371	0.061611
SC14	JO5	CS2	*R.nitidus*	-0.064593	0.065428
CS16	JO5	CS2	*R.nitidus*	-0.053052	0.064121
CS20	JO5	CS2	*R.nitidus*	-0.091549	0.065873
CS3	JO5	CS2	*R.nitidus*	-0.068463	0.064147
SC5	JO5	CS2	*R.nitidus*	-0.070399	0.068749
CS22	JO5	CS2	*R.nitidus*	-0.039259	0.065701
SC15	JO5	CS2	*R.nitidus*	-0.085714	0.064896
CS31	JO5	CS2	*R.nitidus*	-0.086466	0.064757
SC35	JO5	CS2	*R.nitidus*	-0.012658	0.063963
CS28	JO5	CS2	*R.nitidus*	-0.071651	0.065377
SC6	JO5	CS2	*R.nitidus*	-0.068027	0.06609
CS6	JO5	CS2	*R.nitidus*	-0.103085	0.064212
CS25	JO5	CS2	*R.nitidus*	-0.07228	0.066035
SC10	JO5	CS2	*R.nitidus*	0.013323	0.065771
SC45	JO5	CS2	*R.nitidus*	-0.024672	0.065418
MY4	JO5	CS2	*R.nitidus*	-0.096421	0.066673
MY6	JO5	CS2	*R.nitidus*	-0.056277	0.066099
JO1	JO5	CS2	*R.nitidus*	-0.084681	0.064706
SC26	JO5	CS2	*R.nitidus*	-0.095599	0.063143
SC42	JO5	CS2	*R.nitidus*	0.194904	0.06456
SC24	JO5	CS2	*R.nitidus*	-0.057003	0.063901
SC18	JO5	CS2	*R.nitidus*	-0.01381	0.065339
CS4	JO5	CS2	*R.nitidus*	-0.059854	0.062726
GD22	JO7	CS26	*R.nitidus*	0.109336	0.070401
JW6	JO5	CS2	*R.nitidus*	-0.082778	0.070391
GD21	JO7	CS26	*R.nitidus*	0.105949	0.069327
SC32	JO5	CS2	*R.nitidus*	-0.035153	0.063756
JO3	JO5	CS2	*R.nitidus*	-0.037097	0.069025
GD31	JO7	CS26	*R.nitidus*	0.080556	0.070615
GD40	JO7	CS26	*R.nitidus*	0.055938	0.069045
SC27	JO5	CS2	*R.nitidus*	-0.061502	0.064793
JW7	JO5	CS2	*R.nitidus*	-0.038603	0.068739
GD48	JO7	CS26	*R.nitidus*	0.018987	0.071755
CS11	JO7	CS26	*R.nitidus*	-0.00708	0.072288
GD44	JO7	CS26	*R.nitidus*	0.012624	0.071473

GD26	JO7	CS26	*R.nitidus*	0.073128	0.071868
GD30	JO7	CS26	*R.nitidus*	0.154261	0.072328
JO8	JO7	CS26	*R.nitidus*	0.008086	0.073928
CS12	JO7	CS26	*R.nitidus*	0.003791	0.0731
SC44	JO5	CS2	*R.nitidus*	-0.069058	0.06363
JW4	JO5	CS2	*R.nitidus*	0.169395	0.066283
GD35	JO7	CS26	*R.nitidus*	0.032143	0.072103
GD49	JO7	CS26	*R.nitidus*	0.108315	0.07333
GD33	JO7	CS26	*R.nitidus*	0.031306	0.07217
GD25	JO7	CS26	*R.nitidus*	0.069661	0.071572
CS18	JO7	CS26	*R.nitidus*	-0.055861	0.072651
GD41	JO7	CS26	*R.nitidus*	-0.022321	0.070677
GD29	JO7	CS26	*R.nitidus*	0.156977	0.067819
GD42	JO7	CS26	*R.nitidus*	0.050142	0.072278
GD45	JO7	CS26	*R.nitidus*	0.10596	0.074578
CS13	JO7	CS26	*R.nitidus*	-0.052484	0.073599
GD32	JO7	CS26	*R.nitidus*	0.024415	0.073146
SC10	JO7	CS26	*R.nitidus*	0.096466	0.072201
CS25	JO7	CS26	*R.nitidus*	-0.044527	0.072449
GD34	JO7	CS26	*R.nitidus*	0.007772	0.069524
GD50	JO7	CS26	*R.nitidus*	0.062308	0.075119
CS16	JO7	CS26	*R.nitidus*	0.004634	0.073205
GD46	JO7	CS26	*R.nitidus*	-0.023339	0.070161
CS19	JO7	CS26	*R.nitidus*	-0.04944	0.073533
CS28	JO7	CS26	*R.nitidus*	-0.036275	0.072401
CS30	JO7	CS26	*R.nitidus*	-0.05695	0.070198
CS15	JO7	CS26	*R.nitidus*	0.01086	0.07146
CS14	JO7	CS26	*R.nitidus*	-0.008451	0.073987
SC44	JO7	CS26	*R.nitidus*	-0.000921	0.072289
CS20	JO7	CS26	*R.nitidus*	-0.035305	0.070267
CS17	JO7	CS26	*R.nitidus*	-0.010784	0.076453
CS31	JO7	CS26	*R.nitidus*	-0.035218	0.072883
CS23	JO7	CS26	*R.nitidus*	-0.06036	0.071071
CS22	JO7	CS26	*R.nitidus*	0.000924	0.071447
SC15	JO7	CS26	*R.nitidus*	-0.024528	0.072756
SC5	JO7	CS26	*R.nitidus*	0.0217	0.07431
SC45	JO7	CS26	*R.nitidus*	0.029548	0.070147
SC12	JO7	CS26	*R.nitidus*	-0.027829	0.068935
SC27	JO7	CS26	*R.nitidus*	-0.016889	0.071487
CS21	JO7	CS26	*R.nitidus*	-0.049601	0.072054
JO1	JO7	CS26	*R.nitidus*	0.006969	0.068734
CS3	JO7	CS26	*R.nitidus*	-0.013274	0.069918
CS4	JO7	CS26	*R.nitidus*	-0.039965	0.069866
SC24	JO7	CS26	*R.nitidus*	0.001864	0.072051
JW4	JO7	CS26	*R.nitidus*	0.190299	0.070167
SC42	JO7	CS26	*R.nitidus*	0.177482	0.068231
SC26	JO7	CS26	*R.nitidus*	-0.036155	0.071226
SC34	JO7	CS26	*R.nitidus*	-0.002558	0.068545
CS24	JO7	CS26	*R.nitidus*	-0.054511	0.071824
SC35	JO7	CS26	*R.nitidus*	0.002641	0.06928
GD21	JO8	CS26	*R.nitidus*	0.062712	0.06944
SC14	JO7	CS26	*R.nitidus*	-0.014172	0.071459
SC6	JO7	CS26	*R.nitidus*	0.007299	0.069948
MY4	JO7	CS26	*R.nitidus*	-0.058394	0.073411
JO5	JO7	CS26	*R.nitidus*	0.063738	0.067632
JO3	JO7	CS26	*R.nitidus*	0.006376	0.073504

JW6	JO7	CS26	*R.nitidus*	-0.032787	0.067249
GD22	JO8	CS26	*R.nitidus*	0.060764	0.071431
CS11	JO8	CS26	*R.nitidus*	-0.040564	0.071622
SC31	JO7	CS26	*R.nitidus*	-0.014744	0.070995
SC28	JO7	CS26	*R.nitidus*	0.005415	0.070563
SC18	JO7	CS26	*R.nitidus*	0.026168	0.070088
SC32	JO7	CS26	*R.nitidus*	0.030812	0.070984
GD26	JO8	CS26	*R.nitidus*	0.01885	0.070472
CS6	JO7	CS26	*R.nitidus*	-0.06812	0.068941
GD29	JO8	CS26	*R.nitidus*	0.111401	0.067477
CS12	JO8	CS26	*R.nitidus*	-0.024248	0.073048
MY6	JO7	CS26	*R.nitidus*	0.00184	0.07137
JW7	JO7	CS26	*R.nitidus*	0.046443	0.071784
JO7	JO8	CS26	*R.nitidus*	-0.008086	0.073928
GD34	JO8	CS26	*R.nitidus*	-0.05814	0.069713
GD44	JO8	CS26	*R.nitidus*	-0.009217	0.071699
GD42	JO8	CS26	*R.nitidus*	0.006648	0.072411
GD32	JO8	CS26	*R.nitidus*	-0.040426	0.075447
GD25	JO8	CS26	*R.nitidus*	0.049405	0.067946
GD49	JO8	CS26	*R.nitidus*	0.050056	0.072678
CS13	JO8	CS26	*R.nitidus*	-0.06501	0.074797
GD31	JO8	CS26	*R.nitidus*	0.019157	0.070621
CS25	JO8	CS26	*R.nitidus*	-0.063688	0.072786
CS22	JO8	CS26	*R.nitidus*	-0.052889	0.069309
GD30	JO8	CS26	*R.nitidus*	0.09375	0.076091
CS14	JO8	CS26	*R.nitidus*	-0.023518	0.073841
GD45	JO8	CS26	*R.nitidus*	0.03588	0.076637
SC12	JO8	CS26	*R.nitidus*	-0.069431	0.070402
CS17	JO8	CS26	*R.nitidus*	-0.019211	0.074841
SC14	JO8	CS26	*R.nitidus*	-0.041856	0.070548
GD41	JO8	CS26	*R.nitidus*	-0.043278	0.069974
CS19	JO8	CS26	*R.nitidus*	-0.083416	0.072913
CS30	JO8	CS26	*R.nitidus*	-0.114754	0.069198
CS21	JO8	CS26	*R.nitidus*	-0.079851	0.070782
GD50	JO8	CS26	*R.nitidus*	0.021186	0.074653
GD33	JO8	CS26	*R.nitidus*	-0.022769	0.072306
CS24	JO8	CS26	*R.nitidus*	-0.071856	0.071434
GD35	JO8	CS26	*R.nitidus*	0.006318	0.072069
CS23	JO8	CS26	*R.nitidus*	-0.082166	0.071563
GD48	JO8	CS26	*R.nitidus*	0.002841	0.070513
GD46	JO8	CS26	*R.nitidus*	-0.088785	0.068844
CS3	JO8	CS26	*R.nitidus*	-0.040528	0.071683
SC15	JO8	CS26	*R.nitidus*	-0.045231	0.072324
GD40	JO8	CS26	*R.nitidus*	0.034203	0.069743
CS4	JO8	CS26	*R.nitidus*	-0.05215	0.069648
CS16	JO8	CS26	*R.nitidus*	-0.043396	0.072834
CS15	JO8	CS26	*R.nitidus*	-0.030965	0.071426
CS20	JO8	CS26	*R.nitidus*	-0.06391	0.072316
SC28	JO8	CS26	*R.nitidus*	-0.051406	0.070627
CS28	JO8	CS26	*R.nitidus*	-0.06163	0.073144
SC6	JO8	CS26	*R.nitidus*	-0.033058	0.072907
SC26	JO8	CS26	*R.nitidus*	-0.102804	0.07063
SC5	JO8	CS26	*R.nitidus*	-0.020128	0.074678
MY4	JO8	CS26	*R.nitidus*	-0.094183	0.070786
SC18	JO8	CS26	*R.nitidus*	-0.009128	0.070383
SC35	JO8	CS26	*R.nitidus*	-0.020696	0.069962

JW7	JO8	CS26	*R.nitidus*	0.019019	0.074423
CS31	JO8	CS26	*R.nitidus*	-0.081652	0.071605
CS18	JO8	CS26	*R.nitidus*	-0.074324	0.071405
SC10	JO8	CS26	*R.nitidus*	0.043173	0.072609
SC42	JO8	CS26	*R.nitidus*	0.126214	0.069536
SC44	JO8	CS26	*R.nitidus*	-0.016144	0.071684
CS6	JO8	CS26	*R.nitidus*	-0.083571	0.070697
MY6	JO8	CS26	*R.nitidus*	-0.028763	0.071222
SC32	JO8	CS26	*R.nitidus*	-0.023321	0.07109
SC31	JO8	CS26	*R.nitidus*	-0.015511	0.072321
JW4	JO8	CS26	*R.nitidus*	0.162417	0.069777
SC27	JO8	CS26	*R.nitidus*	-0.050738	0.070068
SC24	JO8	CS26	*R.nitidus*	-0.025987	0.069996
SC34	JO8	CS26	*R.nitidus*	-0.049911	0.069553
SC45	JO8	CS26	*R.nitidus*	-0.002775	0.071409
JW6	JO8	CS26	*R.nitidus*	-0.017219	0.070566
JO3	JO8	CS26	*R.nitidus*	-0.005429	0.073173
JO5	JO8	CS26	*R.nitidus*	0.041825	0.068803
JO1	JO8	CS26	*R.nitidus*	-0.04588	0.070042
JO8	CS11	CS26	*R.nitidus*	0.040564	0.071622
GD30	CS11	CS26	*R.nitidus*	0.132669	0.070132
GD50	CS11	CS26	*R.nitidus*	0.024642	0.071403
GD26	CS11	CS26	*R.nitidus*	0.047557	0.067098
GD42	CS11	CS26	*R.nitidus*	0.036618	0.066704
GD49	CS11	CS26	*R.nitidus*	0.089619	0.069871
GD21	CS11	CS26	*R.nitidus*	0.112984	0.06545
GD40	CS11	CS26	*R.nitidus*	0.067568	0.064437
CS22	CS11	CS26	*R.nitidus*	0.002134	0.066386
JO7	CS11	CS26	*R.nitidus*	0.00708	0.072288
GD25	CS11	CS26	*R.nitidus*	0.085324	0.067085
GD31	CS11	CS26	*R.nitidus*	0.069959	0.067882
GD32	CS11	CS26	*R.nitidus*	0.030551	0.068856
GD29	CS11	CS26	*R.nitidus*	0.133746	0.066248
GD35	CS11	CS26	*R.nitidus*	0.030901	0.06598
CS12	CS11	CS26	*R.nitidus*	0.013807	0.070472
CS23	CS11	CS26	*R.nitidus*	-0.065889	0.06554
GD33	CS11	CS26	*R.nitidus*	0.020343	0.067395
GD22	CS11	CS26	*R.nitidus*	0.075483	0.066891
GD34	CS11	CS26	*R.nitidus*	0.00682	0.065626
GD45	CS11	CS26	*R.nitidus*	0.085023	0.069255
GD41	CS11	CS26	*R.nitidus*	0.016276	0.064942
CS16	CS11	CS26	*R.nitidus*	0.011881	0.069001
CS18	CS11	CS26	*R.nitidus*	-0.049365	0.066687
CS17	CS11	CS26	*R.nitidus*	0.016993	0.068441
CS25	CS11	CS26	*R.nitidus*	-0.031746	0.068459
CS21	CS11	CS26	*R.nitidus*	-0.034746	0.064049
CS4	CS11	CS26	*R.nitidus*	-0.020294	0.066143
SC6	CS11	CS26	*R.nitidus*	0.001373	0.06746
GD44	CS11	CS26	*R.nitidus*	0.024933	0.068061
CS20	CS11	CS26	*R.nitidus*	-0.039563	0.067286
CS28	CS11	CS26	*R.nitidus*	-0.032012	0.065535
CS13	CS11	CS26	*R.nitidus*	-0.057884	0.070812
GD48	CS11	CS26	*R.nitidus*	0.043329	0.066928
CS15	CS11	CS26	*R.nitidus*	0.025329	0.067324
GD46	CS11	CS26	*R.nitidus*	0.001315	0.066047
CS14	CS11	CS26	*R.nitidus*	-0.010239	0.068539

CS3	CS11	CS26	*R.nitidus*	-0.033476	0.06681
SC24	CS11	CS26	*R.nitidus*	-0.005498	0.066152
SC27	CS11	CS26	*R.nitidus*	-0.01669	0.064883
CS30	CS11	CS26	*R.nitidus*	-0.058865	0.066873
SC5	CS11	CS26	*R.nitidus*	-0.002019	0.069158
SC10	CS11	CS26	*R.nitidus*	0.062674	0.066429
CS19	CS11	CS26	*R.nitidus*	-0.04482	0.066093
SC15	CS11	CS26	*R.nitidus*	-0.002155	0.067203
SC14	CS11	CS26	*R.nitidus*	0.003376	0.066627
CS24	CS11	CS26	*R.nitidus*	-0.05117	0.066159
CS31	CS11	CS26	*R.nitidus*	-0.028169	0.069526
SC26	CS11	CS26	*R.nitidus*	-0.028179	0.063541
SC18	CS11	CS26	*R.nitidus*	0.04242	0.065573
SC44	CS11	CS26	*R.nitidus*	0.008103	0.065971
SC12	CS11	CS26	*R.nitidus*	-0.003482	0.065607
MY4	CS11	CS26	*R.nitidus*	-0.033602	0.066392
JO7	GD21	CS26	*R.nitidus*	-0.105949	0.069327
GD26	GD21	CS26	*R.nitidus*	-0.079251	0.063384
SC45	CS11	CS26	*R.nitidus*	0.023777	0.066829
JW4	CS11	CS26	*R.nitidus*	0.206734	0.066086
GD30	GD21	CS26	*R.nitidus*	0.034906	0.06733
JO8	GD21	CS26	*R.nitidus*	-0.062712	0.06944
GD31	GD21	CS26	*R.nitidus*	-0.072932	0.063204
JW6	CS11	CS26	*R.nitidus*	0.022948	0.071238
GD25	GD21	CS26	*R.nitidus*	-0.026723	0.06468
SC32	CS11	CS26	*R.nitidus*	0.04507	0.065982
MY6	CS11	CS26	*R.nitidus*	0.006609	0.066618
GD32	GD21	CS26	*R.nitidus*	-0.087436	0.063607
SC28	CS11	CS26	*R.nitidus*	-0.033631	0.06455
JO3	CS11	CS26	*R.nitidus*	0.03976	0.069082
GD44	GD21	CS26	*R.nitidus*	-0.12491	0.063149
SC31	CS11	CS26	*R.nitidus*	-0.004695	0.06596
SC34	CS11	CS26	*R.nitidus*	0.017461	0.065652
CS6	CS11	CS26	*R.nitidus*	-0.04185	0.06602
SC35	CS11	CS26	*R.nitidus*	0.038281	0.063297
JO1	CS11	CS26	*R.nitidus*	-0.005521	0.065845
SC42	CS11	CS26	*R.nitidus*	0.236334	0.06137
GD48	GD21	CS26	*R.nitidus*	-0.1096	0.06716
JW7	CS11	CS26	*R.nitidus*	0.037194	0.06942
GD35	GD21	CS26	*R.nitidus*	-0.083333	0.063006
GD50	GD21	CS26	*R.nitidus*	-0.126205	0.068137
GD22	GD21	CS26	*R.nitidus*	-0.044568	0.0609
JO5	CS11	CS26	*R.nitidus*	0.032537	0.065587
GD41	GD21	CS26	*R.nitidus*	-0.114041	0.061962
GD49	GD21	CS26	*R.nitidus*	-0.010213	0.067645
CS13	GD21	CS26	*R.nitidus*	-0.149743	0.065483
GD29	GD21	CS26	*R.nitidus*	0.009601	0.063693
CS11	GD21	CS26	*R.nitidus*	-0.112984	0.06545
GD46	GD21	CS26	*R.nitidus*	-0.160187	0.061125
GD40	GD21	CS26	*R.nitidus*	-0.065276	0.06478
CS25	GD21	CS26	*R.nitidus*	-0.152687	0.066191
CS12	GD21	CS26	*R.nitidus*	-0.123732	0.066884
GD42	GD21	CS26	*R.nitidus*	-0.097451	0.06349
CS17	GD21	CS26	*R.nitidus*	-0.095238	0.067457
GD34	GD21	CS26	*R.nitidus*	-0.1369	0.060303
GD33	GD21	CS26	*R.nitidus*	-0.1247	0.063183

SC10	GD21	CS26	*R.nitidus*	-0.054974	0.064717
GD45	GD21	CS26	*R.nitidus*	-0.034217	0.06968
CS18	GD21	CS26	*R.nitidus*	-0.164856	0.064537
CS19	GD21	CS26	*R.nitidus*	-0.149035	0.065499
CS16	GD21	CS26	*R.nitidus*	-0.104381	0.063911
CS23	GD21	CS26	*R.nitidus*	-0.165115	0.064237
CS20	GD21	CS26	*R.nitidus*	-0.15483	0.063918
SC12	GD21	CS26	*R.nitidus*	-0.11677	0.064019
SC15	GD21	CS26	*R.nitidus*	-0.119079	0.065351
CS15	GD21	CS26	*R.nitidus*	-0.108039	0.06569
CS21	GD21	CS26	*R.nitidus*	-0.143742	0.063016
CS14	GD21	CS26	*R.nitidus*	-0.130804	0.066133
SC35	GD21	CS26	*R.nitidus*	-0.079696	0.063351
CS31	GD21	CS26	*R.nitidus*	-0.148223	0.063892
CS24	GD21	CS26	*R.nitidus*	-0.162958	0.063538
SC34	GD21	CS26	*R.nitidus*	-0.096237	0.064537
CS28	GD21	CS26	*R.nitidus*	-0.139904	0.064466
SC18	GD21	CS26	*R.nitidus*	-0.094577	0.064866
CS22	GD21	CS26	*R.nitidus*	-0.111688	0.062933
CS6	GD21	CS26	*R.nitidus*	-0.163373	0.063159
CS4	GD21	CS26	*R.nitidus*	-0.133045	0.061295
JO1	GD21	CS26	*R.nitidus*	-0.129537	0.063347
MY4	GD21	CS26	*R.nitidus*	-0.157827	0.063876
CS30	GD21	CS26	*R.nitidus*	-0.176744	0.063899
SC32	GD21	CS26	*R.nitidus*	-0.081946	0.063212
CS3	GD21	CS26	*R.nitidus*	-0.133376	0.063023
SC5	GD21	CS26	*R.nitidus*	-0.103761	0.066848
JO3	GD21	CS26	*R.nitidus*	-0.086586	0.068024
SC6	GD21	CS26	*R.nitidus*	-0.112374	0.0649
JO8	GD22	CS26	*R.nitidus*	-0.060764	0.071431
MY6	GD21	CS26	*R.nitidus*	-0.11041	0.065037
SC28	GD21	CS26	*R.nitidus*	-0.135152	0.063015
SC14	GD21	CS26	*R.nitidus*	-0.103647	0.064774
GD21	GD22	CS26	*R.nitidus*	0.044568	0.0609
SC42	GD21	CS26	*R.nitidus*	0.152524	0.061049
SC45	GD21	CS26	*R.nitidus*	-0.073955	0.062175
SC27	GD21	CS26	*R.nitidus*	-0.122265	0.064637
JO5	GD21	CS26	*R.nitidus*	-0.057585	0.063165
CS11	GD22	CS26	*R.nitidus*	-0.075483	0.066891
GD25	GD22	CS26	*R.nitidus*	0.001404	0.06345
SC26	GD21	CS26	*R.nitidus*	-0.148976	0.062562
SC24	GD21	CS26	*R.nitidus*	-0.126356	0.064114
GD32	GD22	CS26	*R.nitidus*	-0.064902	0.066002
SC44	GD21	CS26	*R.nitidus*	-0.100824	0.064354
JW4	GD21	CS26	*R.nitidus*	0.098922	0.065323
SC31	GD21	CS26	*R.nitidus*	-0.118654	0.064146
JW7	GD21	CS26	*R.nitidus*	-0.090703	0.067199
JO7	GD22	CS26	*R.nitidus*	-0.109336	0.070401
GD30	GD22	CS26	*R.nitidus*	0.085126	0.063674
JW6	GD21	CS26	*R.nitidus*	-0.10778	0.068381
GD44	GD22	CS26	*R.nitidus*	-0.058526	0.064363
GD42	GD22	CS26	*R.nitidus*	-0.070336	0.063943
GD40	GD22	CS26	*R.nitidus*	-0.008536	0.062523
GD34	GD22	CS26	*R.nitidus*	-0.093997	0.060749
GD41	GD22	CS26	*R.nitidus*	-0.080206	0.062725
GD49	GD22	CS26	*R.nitidus*	0.056585	0.069022

GD35	GD22	CS26	*R.nitidus*	-0.030199	0.063342
GD33	GD22	CS26	*R.nitidus*	-0.066771	0.063018
GD48	GD22	CS26	*R.nitidus*	-0.054795	0.064861
CS12	GD22	CS26	*R.nitidus*	-0.066099	0.066585
CS13	GD22	CS26	*R.nitidus*	-0.121212	0.064861
GD29	GD22	CS26	*R.nitidus*	0.067708	0.063241
CS16	GD22	CS26	*R.nitidus*	-0.070068	0.063817
GD31	GD22	CS26	*R.nitidus*	-0.024499	0.066371
GD26	GD22	CS26	*R.nitidus*	-0.03037	0.062879
GD50	GD22	CS26	*R.nitidus*	-0.032057	0.067857
CS17	GD22	CS26	*R.nitidus*	-0.052632	0.066632
CS18	GD22	CS26	*R.nitidus*	-0.126525	0.064629
CS15	GD22	CS26	*R.nitidus*	-0.061249	0.065508
GD45	GD22	CS26	*R.nitidus*	-0.021104	0.067785
GD46	GD22	CS26	*R.nitidus*	-0.104181	0.060835
CS24	GD22	CS26	*R.nitidus*	-0.118533	0.063723
CS19	GD22	CS26	*R.nitidus*	-0.112492	0.066261
CS14	GD22	CS26	*R.nitidus*	-0.081014	0.066196
CS31	GD22	CS26	*R.nitidus*	-0.101761	0.065575
CS28	GD22	CS26	*R.nitidus*	-0.105508	0.065943
CS20	GD22	CS26	*R.nitidus*	-0.119469	0.064943
CS4	GD22	CS26	*R.nitidus*	-0.096535	0.062469
SC24	GD22	CS26	*R.nitidus*	-0.084462	0.064895
SC5	GD22	CS26	*R.nitidus*	-0.082628	0.066818
SC14	GD22	CS26	*R.nitidus*	-0.071827	0.063898
SC12	GD22	CS26	*R.nitidus*	-0.083386	0.062884
SC32	GD22	CS26	*R.nitidus*	-0.039586	0.062979
CS25	GD22	CS26	*R.nitidus*	-0.121212	0.066405
SC28	GD22	CS26	*R.nitidus*	-0.108844	0.06335
SC26	GD22	CS26	*R.nitidus*	-0.097111	0.063439
CS22	GD22	CS26	*R.nitidus*	-0.067308	0.064579
CS30	GD22	CS26	*R.nitidus*	-0.135538	0.063407
SC18	GD22	CS26	*R.nitidus*	-0.04064	0.064418
SC45	GD22	CS26	*R.nitidus*	-0.035646	0.063881
CS23	GD22	CS26	*R.nitidus*	-0.128157	0.063814
MY6	GD22	CS26	*R.nitidus*	-0.070143	0.064232
CS21	GD22	CS26	*R.nitidus*	-0.107341	0.064273
SC6	GD22	CS26	*R.nitidus*	-0.067361	0.066292
CS3	GD22	CS26	*R.nitidus*	-0.106821	0.063979
JO5	GD22	CS26	*R.nitidus*	-0.016311	0.063808
SC35	GD22	CS26	*R.nitidus*	-0.034077	0.063
SC42	GD22	CS26	*R.nitidus*	0.166316	0.061665
JW6	GD22	CS26	*R.nitidus*	-0.065359	0.069413
SC10	GD22	CS26	*R.nitidus*	-0.006653	0.066121
JO8	GD25	CS26	*R.nitidus*	-0.049405	0.067946
GD32	GD25	CS26	*R.nitidus*	-0.054681	0.067848
JW7	GD22	CS26	*R.nitidus*	-0.046494	0.067847
SC15	GD22	CS26	*R.nitidus*	-0.076471	0.064554
SC31	GD22	CS26	*R.nitidus*	-0.086716	0.06537
SC27	GD22	CS26	*R.nitidus*	-0.080247	0.063509
SC34	GD22	CS26	*R.nitidus*	-0.052503	0.062721
SC44	GD22	CS26	*R.nitidus*	-0.076122	0.063236
GD41	GD25	CS26	*R.nitidus*	-0.064214	0.064457
JO7	GD25	CS26	*R.nitidus*	-0.069661	0.071572
JO1	GD22	CS26	*R.nitidus*	-0.085145	0.063913
CS6	GD22	CS26	*R.nitidus*	-0.127575	0.064536

GD30	GD25	CS26	*R.nitidus*	0.079464	0.068558
JW4	GD22	CS26	*R.nitidus*	0.140283	0.063361
MY4	GD22	CS26	*R.nitidus*	-0.112232	0.065749
GD31	GD25	CS26	*R.nitidus*	-0.01542	0.064301
GD34	GD25	CS26	*R.nitidus*	-0.074339	0.065128
JO3	GD22	CS26	*R.nitidus*	-0.048744	0.066647
GD26	GD25	CS26	*R.nitidus*	-0.026906	0.065377
CS13	GD25	CS26	*R.nitidus*	-0.119558	0.066858
CS12	GD25	CS26	*R.nitidus*	-0.091575	0.067343
GD40	GD25	CS26	*R.nitidus*	-0.033708	0.062561
GD21	GD25	CS26	*R.nitidus*	0.026723	0.06468
GD50	GD25	CS26	*R.nitidus*	-0.022282	0.069098
GD22	GD25	CS26	*R.nitidus*	-0.001404	0.06345
CS18	GD25	CS26	*R.nitidus*	-0.127646	0.064438
CS11	GD25	CS26	*R.nitidus*	-0.085324	0.067085
GD46	GD25	CS26	*R.nitidus*	-0.094653	0.063335
GD45	GD25	CS26	*R.nitidus*	0.029711	0.069609
GD29	GD25	CS26	*R.nitidus*	0.055014	0.064592
CS23	GD25	CS26	*R.nitidus*	-0.133245	0.063895
GD44	GD25	CS26	*R.nitidus*	-0.067315	0.066137
GD42	GD25	CS26	*R.nitidus*	-0.05424	0.065048
GD33	GD25	CS26	*R.nitidus*	-0.07402	0.066513
GD49	GD25	CS26	*R.nitidus*	0.023789	0.068691
GD35	GD25	CS26	*R.nitidus*	-0.045556	0.063385
CS20	GD25	CS26	*R.nitidus*	-0.123407	0.066423
CS28	GD25	CS26	*R.nitidus*	-0.108793	0.065153
CS15	GD25	CS26	*R.nitidus*	-0.078912	0.065756
CS30	GD25	CS26	*R.nitidus*	-0.130374	0.063824
CS21	GD25	CS26	*R.nitidus*	-0.116589	0.065507
CS25	GD25	CS26	*R.nitidus*	-0.12535	0.066351
SC31	GD25	CS26	*R.nitidus*	-0.089533	0.063844
SC34	GD25	CS26	*R.nitidus*	-0.059857	0.063292
SC28	GD25	CS26	*R.nitidus*	-0.112827	0.063792
SC5	GD25	CS26	*R.nitidus*	-0.084574	0.067978
GD48	GD25	CS26	*R.nitidus*	-0.036829	0.064603
CS16	GD25	CS26	*R.nitidus*	-0.081395	0.066483
CS3	GD25	CS26	*R.nitidus*	-0.107809	0.065719
SC14	GD25	CS26	*R.nitidus*	-0.081242	0.06455
SC10	GD25	CS26	*R.nitidus*	-0.022001	0.063584
CS17	GD25	CS26	*R.nitidus*	-0.069373	0.06805
SC12	GD25	CS26	*R.nitidus*	-0.088633	0.064796
CS14	GD25	CS26	*R.nitidus*	-0.093728	0.067332
CS19	GD25	CS26	*R.nitidus*	-0.109664	0.066309
CS6	GD25	CS26	*R.nitidus*	-0.124118	0.06406
SC44	GD25	CS26	*R.nitidus*	-0.074409	0.063397
CS22	GD25	CS26	*R.nitidus*	-0.062373	0.06454
MY6	GD25	CS26	*R.nitidus*	-0.077852	0.064142
JW4	GD25	CS26	*R.nitidus*	0.131597	0.065364
CS24	GD25	CS26	*R.nitidus*	-0.104833	0.06512
SC42	GD25	CS26	*R.nitidus*	0.155581	0.062635
SC26	GD25	CS26	*R.nitidus*	-0.108739	0.06562
SC18	GD25	CS26	*R.nitidus*	-0.056905	0.065985
JO5	GD25	CS26	*R.nitidus*	-0.046875	0.063815
SC6	GD25	CS26	*R.nitidus*	-0.081481	0.06546
JO3	GD25	CS26	*R.nitidus*	-0.038186	0.06807
CS31	GD25	CS26	*R.nitidus*	-0.114894	0.066235

CS4	GD25	CS26	*R.nitidus*	-0.082138	0.063428
SC15	GD25	CS26	*R.nitidus*	-0.085694	0.066561
SC35	GD25	CS26	*R.nitidus*	-0.047361	0.064029
JO1	GD25	CS26	*R.nitidus*	-0.113079	0.063997
SC45	GD25	CS26	*R.nitidus*	-0.061379	0.064513
SC32	GD25	CS26	*R.nitidus*	-0.048271	0.062716
SC24	GD25	CS26	*R.nitidus*	-0.084746	0.06558
SC27	GD25	CS26	*R.nitidus*	-0.097948	0.064252
JW6	GD25	CS26	*R.nitidus*	-0.064748	0.070707
MY4	GD25	CS26	*R.nitidus*	-0.122667	0.06419
GD25	GD26	CS26	*R.nitidus*	0.026906	0.065377
GD22	GD26	CS26	*R.nitidus*	0.03037	0.062879
GD21	GD26	CS26	*R.nitidus*	0.079251	0.063384
GD49	GD26	CS26	*R.nitidus*	0.04682	0.067298
CS11	GD26	CS26	*R.nitidus*	-0.047557	0.067098
JW7	GD25	CS26	*R.nitidus*	-0.056726	0.067669
JO8	GD26	CS26	*R.nitidus*	-0.01885	0.070472
GD33	GD26	CS26	*R.nitidus*	-0.04142	0.065146
GD45	GD26	CS26	*R.nitidus*	0.044683	0.068722
GD35	GD26	CS26	*R.nitidus*	-0.011103	0.064818
GD30	GD26	CS26	*R.nitidus*	0.115455	0.066853
GD48	GD26	CS26	*R.nitidus*	-0.021531	0.066289
CS17	GD26	CS26	*R.nitidus*	-0.024339	0.068638
GD32	GD26	CS26	*R.nitidus*	-0.010718	0.06284
JO7	GD26	CS26	*R.nitidus*	-0.073128	0.071868
GD40	GD26	CS26	*R.nitidus*	0.016807	0.062634
CS12	GD26	CS26	*R.nitidus*	-0.04123	0.065574
GD41	GD26	CS26	*R.nitidus*	-0.046563	0.063017
GD29	GD26	CS26	*R.nitidus*	0.077971	0.063369
CS20	GD26	CS26	*R.nitidus*	-0.086162	0.066929
GD50	GD26	CS26	*R.nitidus*	-0.010949	0.067562
SC14	GD26	CS26	*R.nitidus*	-0.051956	0.06543
SC5	GD26	CS26	*R.nitidus*	-0.048464	0.066983
CS19	GD26	CS26	*R.nitidus*	-0.074766	0.06723
GD31	GD26	CS26	*R.nitidus*	0.040243	0.065877
GD44	GD26	CS26	*R.nitidus*	-0.020849	0.066267
CS13	GD26	CS26	*R.nitidus*	-0.086512	0.067032
GD34	GD26	CS26	*R.nitidus*	-0.059414	0.062966
CS15	GD26	CS26	*R.nitidus*	-0.038663	0.06648
CS14	GD26	CS26	*R.nitidus*	-0.061238	0.067286
SC28	GD26	CS26	*R.nitidus*	-0.076821	0.06394
GD42	GD26	CS26	*R.nitidus*	-0.007728	0.064887
CS16	GD26	CS26	*R.nitidus*	-0.050393	0.064462
CS21	GD26	CS26	*R.nitidus*	-0.074407	0.066271
GD46	GD26	CS26	*R.nitidus*	-0.067202	0.060682
CS3	GD26	CS26	*R.nitidus*	-0.075397	0.065488
SC32	GD26	CS26	*R.nitidus*	-0.016383	0.064692
CS28	GD26	CS26	*R.nitidus*	-0.078556	0.066226
CS31	GD26	CS26	*R.nitidus*	-0.07931	0.06611
SC31	GD26	CS26	*R.nitidus*	-0.051151	0.065611
CS18	GD26	CS26	*R.nitidus*	-0.096192	0.066538
CS25	GD26	CS26	*R.nitidus*	-0.076203	0.067107
SC26	GD26	CS26	*R.nitidus*	-0.082392	0.065436
SC15	GD26	CS26	*R.nitidus*	-0.046527	0.064908
SC27	GD26	CS26	*R.nitidus*	-0.063267	0.065396
SC18	GD26	CS26	*R.nitidus*	-0.017361	0.064307

SC35	GD26	CS26	*R.nitidus*	-0.027541	0.063185
CS30	GD26	CS26	*R.nitidus*	-0.105915	0.064019
CS24	GD26	CS26	*R.nitidus*	-0.08898	0.065474
SC10	GD26	CS26	*R.nitidus*	0.010345	0.066272
CS22	GD26	CS26	*R.nitidus*	-0.051213	0.065568
SC34	GD26	CS26	*R.nitidus*	-0.030908	0.063717
CS23	GD26	CS26	*R.nitidus*	-0.099227	0.064435
SC6	GD26	CS26	*R.nitidus*	-0.060788	0.065738
SC12	GD26	CS26	*R.nitidus*	-0.06221	0.063508
JW4	GD26	CS26	*R.nitidus*	0.180168	0.066744
JW6	GD26	CS26	*R.nitidus*	-0.05464	0.070297
CS4	GD26	CS26	*R.nitidus*	-0.072454	0.062901
SC24	GD26	CS26	*R.nitidus*	-0.057627	0.064719
JO3	GD26	CS26	*R.nitidus*	-0.021196	0.069384
MY6	GD26	CS26	*R.nitidus*	-0.042913	0.065932
JO1	GD26	CS26	*R.nitidus*	-0.066709	0.063972
GD21	GD29	CS26	*R.nitidus*	-0.009601	0.063693
GD30	GD29	CS26	*R.nitidus*	0.034232	0.064621
GD25	GD29	CS26	*R.nitidus*	-0.055014	0.064592
SC44	GD26	CS26	*R.nitidus*	-0.041389	0.063638
CS11	GD29	CS26	*R.nitidus*	-0.133746	0.066248
JO7	GD29	CS26	*R.nitidus*	-0.156977	0.067819
GD40	GD29	CS26	*R.nitidus*	-0.07367	0.062903
SC42	GD26	CS26	*R.nitidus*	0.185841	0.061889
GD32	GD29	CS26	*R.nitidus*	-0.111959	0.065204
CS6	GD26	CS26	*R.nitidus*	-0.087517	0.065354
JW7	GD26	CS26	*R.nitidus*	-0.011839	0.070748
GD35	GD29	CS26	*R.nitidus*	-0.080897	0.064718
SC45	GD26	CS26	*R.nitidus*	-0.014608	0.064454
MY4	GD26	CS26	*R.nitidus*	-0.07672	0.065364
JO8	GD29	CS26	*R.nitidus*	-0.111401	0.067477
GD44	GD29	CS26	*R.nitidus*	-0.127424	0.064375
CS12	GD29	CS26	*R.nitidus*	-0.118	0.064917
CS15	GD29	CS26	*R.nitidus*	-0.111043	0.06569
GD22	GD29	CS26	*R.nitidus*	-0.067708	0.063241
GD48	GD29	CS26	*R.nitidus*	-0.099219	0.063386
JO5	GD26	CS26	*R.nitidus*	0.009192	0.063883
GD31	GD29	CS26	*R.nitidus*	-0.050414	0.065141
CS17	GD29	CS26	*R.nitidus*	-0.109103	0.067322
GD34	GD29	CS26	*R.nitidus*	-0.164251	0.061651
CS23	GD29	CS26	*R.nitidus*	-0.16986	0.062227
CS25	GD29	CS26	*R.nitidus*	-0.161669	0.064311
GD33	GD29	CS26	*R.nitidus*	-0.132492	0.063747
GD49	GD29	CS26	*R.nitidus*	-0.033563	0.068462
GD26	GD29	CS26	*R.nitidus*	-0.077971	0.063369
GD50	GD29	CS26	*R.nitidus*	-0.098057	0.06615
GD42	GD29	CS26	*R.nitidus*	-0.118732	0.064215
CS13	GD29	CS26	*R.nitidus*	-0.165721	0.065576
CS18	GD29	CS26	*R.nitidus*	-0.160383	0.063102
GD41	GD29	CS26	*R.nitidus*	-0.122845	0.06302
CS16	GD29	CS26	*R.nitidus*	-0.129492	0.064473
CS24	GD29	CS26	*R.nitidus*	-0.15974	0.063705
GD45	GD29	CS26	*R.nitidus*	-0.035333	0.067235
CS30	GD29	CS26	*R.nitidus*	-0.182895	0.06209
CS14	GD29	CS26	*R.nitidus*	-0.141782	0.066314
SC12	GD29	CS26	*R.nitidus*	-0.131004	0.064642

CS22	GD29	CS26	*R.nitidus*	-0.12081	0.06465
CS19	GD29	CS26	*R.nitidus*	-0.157964	0.06541
CS20	GD29	CS26	*R.nitidus*	-0.161351	0.064771
SC6	GD29	CS26	*R.nitidus*	-0.131096	0.063947
CS4	GD29	CS26	*R.nitidus*	-0.144828	0.061559
SC32	GD29	CS26	*R.nitidus*	-0.105525	0.063818
CS21	GD29	CS26	*R.nitidus*	-0.155569	0.063955
MY4	GD29	CS26	*R.nitidus*	-0.159502	0.063861
CS28	GD29	CS26	*R.nitidus*	-0.148148	0.065776
SC31	GD29	CS26	*R.nitidus*	-0.125304	0.063005
JW6	GD29	CS26	*R.nitidus*	-0.130143	0.067019
JW4	GD29	CS26	*R.nitidus*	0.090909	0.065857
CS3	GD29	CS26	*R.nitidus*	-0.158232	0.063167
SC15	GD29	CS26	*R.nitidus*	-0.133946	0.065002
SC10	GD29	CS26	*R.nitidus*	-0.061048	0.065565
JO8	GD30	CS26	*R.nitidus*	-0.09375	0.076091
CS31	GD29	CS26	*R.nitidus*	-0.16353	0.065029
SC28	GD29	CS26	*R.nitidus*	-0.157729	0.063864
SC42	GD29	CS26	*R.nitidus*	0.106145	0.062006
SC5	GD29	CS26	*R.nitidus*	-0.12637	0.067254
SC44	GD29	CS26	*R.nitidus*	-0.1139	0.065184
SC24	GD29	CS26	*R.nitidus*	-0.135508	0.063848
SC18	GD29	CS26	*R.nitidus*	-0.091089	0.064948
CS11	GD30	CS26	*R.nitidus*	-0.132669	0.070132
MY6	GD29	CS26	*R.nitidus*	-0.118478	0.063113
JO3	GD29	CS26	*R.nitidus*	-0.102715	0.065083
SC26	GD29	CS26	*R.nitidus*	-0.153321	0.063935
GD34	GD30	CS26	*R.nitidus*	-0.157	0.063415
SC14	GD29	CS26	*R.nitidus*	-0.118086	0.062153
GD22	GD30	CS26	*R.nitidus*	-0.085126	0.063674
JO5	GD29	CS26	*R.nitidus*	-0.086042	0.062856
SC35	GD29	CS26	*R.nitidus*	-0.084284	0.063611
SC27	GD29	CS26	*R.nitidus*	-0.131847	0.06326
SC34	GD29	CS26	*R.nitidus*	-0.090852	0.06333
JO7	GD30	CS26	*R.nitidus*	-0.154261	0.072328
GD33	GD30	CS26	*R.nitidus*	-0.11546	0.067743
GD35	GD30	CS26	*R.nitidus*	-0.107203	0.067364
CS6	GD29	CS26	*R.nitidus*	-0.156799	0.063817
GD26	GD30	CS26	*R.nitidus*	-0.115455	0.066853
GD25	GD30	CS26	*R.nitidus*	-0.079464	0.068558
JO1	GD29	CS26	*R.nitidus*	-0.132252	0.062848
SC45	GD29	CS26	*R.nitidus*	-0.098391	0.064379
GD21	GD30	CS26	*R.nitidus*	-0.034906	0.06733
JW7	GD29	CS26	*R.nitidus*	-0.102131	0.066908
GD32	GD30	CS26	*R.nitidus*	-0.151724	0.070203
GD31	GD30	CS26	*R.nitidus*	-0.098302	0.067911
GD40	GD30	CS26	*R.nitidus*	-0.079661	0.06488
GD50	GD30	CS26	*R.nitidus*	-0.098059	0.070904
CS24	GD30	CS26	*R.nitidus*	-0.159777	0.064683
GD42	GD30	CS26	*R.nitidus*	-0.115527	0.065893
CS12	GD30	CS26	*R.nitidus*	-0.124688	0.070952
GD29	GD30	CS26	*R.nitidus*	-0.034232	0.064621
CS14	GD30	CS26	*R.nitidus*	-0.127711	0.071182
GD49	GD30	CS26	*R.nitidus*	-0.064741	0.071327
CS17	GD30	CS26	*R.nitidus*	-0.107114	0.070036
GD46	GD30	CS26	*R.nitidus*	-0.164899	0.06435

GD48	GD30	CS26	*R.nitidus*	-0.076035	0.067147
CS16	GD30	CS26	*R.nitidus*	-0.119142	0.06746
CS13	GD30	CS26	*R.nitidus*	-0.15935	0.068285
CS23	GD30	CS26	*R.nitidus*	-0.153139	0.065667
CS28	GD30	CS26	*R.nitidus*	-0.153199	0.067943
CS15	GD30	CS26	*R.nitidus*	-0.114467	0.06966
CS19	GD30	CS26	*R.nitidus*	-0.141821	0.069054
CS3	GD30	CS26	*R.nitidus*	-0.140379	0.06677
SC26	GD30	CS26	*R.nitidus*	-0.149962	0.066772
GD41	GD30	CS26	*R.nitidus*	-0.11399	0.064162
SC5	GD30	CS26	*R.nitidus*	-0.126848	0.069708
GD44	GD30	CS26	*R.nitidus*	-0.118943	0.067559
SC34	GD30	CS26	*R.nitidus*	-0.095426	0.066365
CS25	GD30	CS26	*R.nitidus*	-0.152066	0.070716
SC44	GD30	CS26	*R.nitidus*	-0.103948	0.064786
CS20	GD30	CS26	*R.nitidus*	-0.157329	0.066676
GD45	GD30	CS26	*R.nitidus*	-0.047037	0.068343
SC18	GD30	CS26	*R.nitidus*	-0.088474	0.067582
JW6	GD30	CS26	*R.nitidus*	-0.129979	0.072098
CS4	GD30	CS26	*R.nitidus*	-0.140791	0.064729
CS18	GD30	CS26	*R.nitidus*	-0.154383	0.067106
SC35	GD30	CS26	*R.nitidus*	-0.080978	0.066987
CS21	GD30	CS26	*R.nitidus*	-0.16325	0.066567
CS31	GD30	CS26	*R.nitidus*	-0.140394	0.068448
SC6	GD30	CS26	*R.nitidus*	-0.142176	0.067114
CS6	GD30	CS26	*R.nitidus*	-0.159711	0.065756
SC32	GD30	CS26	*R.nitidus*	-0.093292	0.067927
SC10	GD30	CS26	*R.nitidus*	-0.081967	0.069124
CS22	GD30	CS26	*R.nitidus*	-0.125786	0.067408
JO8	GD31	CS26	*R.nitidus*	-0.019157	0.070621
CS30	GD30	CS26	*R.nitidus*	-0.170161	0.067261
SC14	GD30	CS26	*R.nitidus*	-0.1146	0.066791
JO3	GD30	CS26	*R.nitidus*	-0.125119	0.069992
JO5	GD30	CS26	*R.nitidus*	-0.07866	0.06637
SC12	GD30	CS26	*R.nitidus*	-0.122642	0.06683
SC42	GD30	CS26	*R.nitidus*	0.121666	0.064368
SC27	GD30	CS26	*R.nitidus*	-0.143646	0.066419
MY6	GD30	CS26	*R.nitidus*	-0.122161	0.065836
SC15	GD30	CS26	*R.nitidus*	-0.124595	0.069483
JW7	GD30	CS26	*R.nitidus*	-0.11477	0.070216
SC31	GD30	CS26	*R.nitidus*	-0.129323	0.066631
SC24	GD30	CS26	*R.nitidus*	-0.145074	0.067359
SC28	GD30	CS26	*R.nitidus*	-0.150579	0.065647
GD33	GD31	CS26	*R.nitidus*	-0.048601	0.065583
SC45	GD30	CS26	*R.nitidus*	-0.099388	0.066439
GD30	GD31	CS26	*R.nitidus*	0.098302	0.067911
CS11	GD31	CS26	*R.nitidus*	-0.069959	0.067882
MY4	GD30	CS26	*R.nitidus*	-0.1595	0.067235
JW4	GD30	CS26	*R.nitidus*	0.094684	0.069807
GD22	GD31	CS26	*R.nitidus*	0.024499	0.066371
CS12	GD31	CS26	*R.nitidus*	-0.058694	0.066885
JO7	GD31	CS26	*R.nitidus*	-0.080556	0.070615
JO1	GD30	CS26	*R.nitidus*	-0.15625	0.064745
GD32	GD31	CS26	*R.nitidus*	-0.042907	0.069024
GD25	GD31	CS26	*R.nitidus*	0.01542	0.064301
CS21	GD31	CS26	*R.nitidus*	-0.09751	0.066727

GD26	GD31	CS26	*R.nitidus*	-0.040243	0.065877
GD41	GD31	CS26	*R.nitidus*	-0.05295	0.065002
GD49	GD31	CS26	*R.nitidus*	0.04682	0.070516
GD35	GD31	CS26	*R.nitidus*	-0.025111	0.064699
GD40	GD31	CS26	*R.nitidus*	0.010249	0.065184
CS30	GD31	CS26	*R.nitidus*	-0.131924	0.066862
CS18	GD31	CS26	*R.nitidus*	-0.115331	0.066941
GD21	GD31	CS26	*R.nitidus*	0.072932	0.063204
CS13	GD31	CS26	*R.nitidus*	-0.112766	0.067337
GD29	GD31	CS26	*R.nitidus*	0.050414	0.065141
SC6	GD31	CS26	*R.nitidus*	-0.059823	0.064016
GD42	GD31	CS26	*R.nitidus*	-0.027978	0.065734
GD44	GD31	CS26	*R.nitidus*	-0.051917	0.067006
GD46	GD31	CS26	*R.nitidus*	-0.069351	0.063707
GD48	GD31	CS26	*R.nitidus*	-0.027825	0.067957
GD45	GD31	CS26	*R.nitidus*	0.006206	0.069876
GD34	GD31	CS26	*R.nitidus*	-0.056402	0.06502
GD50	GD31	CS26	*R.nitidus*	-0.023788	0.068968
CS28	GD31	CS26	*R.nitidus*	-0.088939	0.065999
CS14	GD31	CS26	*R.nitidus*	-0.068454	0.069199
CS25	GD31	CS26	*R.nitidus*	-0.110408	0.066766
CS23	GD31	CS26	*R.nitidus*	-0.122053	0.065818
CS22	GD31	CS26	*R.nitidus*	-0.06788	0.06625
CS3	GD31	CS26	*R.nitidus*	-0.096436	0.066546
CS24	GD31	CS26	*R.nitidus*	-0.109142	0.065944
CS16	GD31	CS26	*R.nitidus*	-0.057759	0.066824
CS15	GD31	CS26	*R.nitidus*	-0.058011	0.066392
CS17	GD31	CS26	*R.nitidus*	-0.054315	0.070301
SC28	GD31	CS26	*R.nitidus*	-0.095833	0.064527
CS19	GD31	CS26	*R.nitidus*	-0.126165	0.067751
CS4	GD31	CS26	*R.nitidus*	-0.080272	0.064436
SC24	GD31	CS26	*R.nitidus*	-0.071623	0.066965
SC10	GD31	CS26	*R.nitidus*	-0.000719	0.066657
SC5	GD31	CS26	*R.nitidus*	-0.073782	0.068149
SC27	GD31	CS26	*R.nitidus*	-0.063624	0.065538
SC45	GD31	CS26	*R.nitidus*	-0.054073	0.065671
SC12	GD31	CS26	*R.nitidus*	-0.063348	0.064774
CS6	GD31	CS26	*R.nitidus*	-0.108477	0.06795
CS20	GD31	CS26	*R.nitidus*	-0.110497	0.066753
SC32	GD31	CS26	*R.nitidus*	-0.021898	0.06476
CS31	GD31	CS26	*R.nitidus*	-0.094144	0.067429
SC18	GD31	CS26	*R.nitidus*	-0.027417	0.066193
SC26	GD31	CS26	*R.nitidus*	-0.088195	0.064008
SC35	GD31	CS26	*R.nitidus*	-0.036153	0.06349
SC31	GD31	CS26	*R.nitidus*	-0.069925	0.065501
SC42	GD31	CS26	*R.nitidus*	0.163963	0.063858
SC14	GD31	CS26	*R.nitidus*	-0.065041	0.065096
JW4	GD31	CS26	*R.nitidus*	0.135252	0.067413
SC15	GD31	CS26	*R.nitidus*	-0.065911	0.06667
GD30	GD32	CS26	*R.nitidus*	0.151724	0.070203
JO1	GD31	CS26	*R.nitidus*	-0.088175	0.065312
JO7	GD32	CS26	*R.nitidus*	-0.024415	0.073146
JO8	GD32	CS26	*R.nitidus*	0.040426	0.075447
JW7	GD31	CS26	*R.nitidus*	-0.064956	0.06944
GD21	GD32	CS26	*R.nitidus*	0.087436	0.063607
SC44	GD31	CS26	*R.nitidus*	-0.058574	0.064511

SC34	GD31	CS26	*R.nitidus*	-0.040163	0.065162
GD29	GD32	CS26	*R.nitidus*	0.111959	0.065204
MY4	GD31	CS26	*R.nitidus*	-0.101408	0.067122
GD42	GD32	CS26	*R.nitidus*	-0.006157	0.067389
JW6	GD31	CS26	*R.nitidus*	-0.066608	0.070782
JO5	GD31	CS26	*R.nitidus*	-0.035887	0.066322
JO3	GD31	CS26	*R.nitidus*	-0.029009	0.070339
MY6	GD31	CS26	*R.nitidus*	-0.06358	0.06624
GD44	GD32	CS26	*R.nitidus*	0.007874	0.067759
GD33	GD32	CS26	*R.nitidus*	-0.01564	0.067361
GD26	GD32	CS26	*R.nitidus*	0.010718	0.06284
GD35	GD32	CS26	*R.nitidus*	0.014975	0.067466
GD45	GD32	CS26	*R.nitidus*	0.050302	0.072011
GD40	GD32	CS26	*R.nitidus*	0.037995	0.065749
GD22	GD32	CS26	*R.nitidus*	0.064902	0.066002
GD50	GD32	CS26	*R.nitidus*	0.026804	0.070728
GD41	GD32	CS26	*R.nitidus*	-0.028278	0.067406
CS11	GD32	CS26	*R.nitidus*	-0.030551	0.068856
CS17	GD32	CS26	*R.nitidus*	0.001603	0.071793
CS14	GD32	CS26	*R.nitidus*	-0.030817	0.068771
GD25	GD32	CS26	*R.nitidus*	0.054681	0.067848
CS20	GD32	CS26	*R.nitidus*	-0.053394	0.066989
CS25	GD32	CS26	*R.nitidus*	-0.045385	0.069802
GD31	GD32	CS26	*R.nitidus*	0.042907	0.069024
CS15	GD32	CS26	*R.nitidus*	-0.012158	0.067171
GD48	GD32	CS26	*R.nitidus*	0.010223	0.065025
GD34	GD32	CS26	*R.nitidus*	-0.015357	0.06435
CS19	GD32	CS26	*R.nitidus*	-0.050305	0.067852
CS13	GD32	CS26	*R.nitidus*	-0.057055	0.068514
CS18	GD32	CS26	*R.nitidus*	-0.072893	0.06907
CS28	GD32	CS26	*R.nitidus*	-0.045491	0.069151
CS12	GD32	CS26	*R.nitidus*	-0.009486	0.069332
GD46	GD32	CS26	*R.nitidus*	-0.053254	0.064671
GD49	GD32	CS26	*R.nitidus*	0.097917	0.07077
SC5	GD32	CS26	*R.nitidus*	0.007109	0.070659
CS23	GD32	CS26	*R.nitidus*	-0.07958	0.06808
CS3	GD32	CS26	*R.nitidus*	-0.052469	0.068016
SC10	GD32	CS26	*R.nitidus*	0.052673	0.066898
SC14	GD32	CS26	*R.nitidus*	-0.00154	0.066868
CS30	GD32	CS26	*R.nitidus*	-0.065821	0.068124
SC24	GD32	CS26	*R.nitidus*	-0.023511	0.066219
CS21	GD32	CS26	*R.nitidus*	-0.040059	0.068759
CS16	GD32	CS26	*R.nitidus*	-0.031758	0.068635
CS4	GD32	CS26	*R.nitidus*	-0.048762	0.065112
SC15	GD32	CS26	*R.nitidus*	0	0.067515
SC6	GD32	CS26	*R.nitidus*	-0.018997	0.067711
CS24	GD32	CS26	*R.nitidus*	-0.064314	0.065973
CS22	GD32	CS26	*R.nitidus*	-0.022134	0.066574
SC18	GD32	CS26	*R.nitidus*	0.043124	0.067346
SC31	GD32	CS26	*R.nitidus*	-0.008902	0.068504
SC26	GD32	CS26	*R.nitidus*	-0.028331	0.066653
CS31	GD32	CS26	*R.nitidus*	-0.050243	0.068882
MY4	GD32	CS26	*R.nitidus*	-0.058915	0.069115
SC34	GD32	CS26	*R.nitidus*	-0.005979	0.065844
CS6	GD32	CS26	*R.nitidus*	-0.068604	0.068313
SC27	GD32	CS26	*R.nitidus*	-0.016166	0.067711

JW7	GD32	CS26	*R.nitidus*	0.020158	0.068665
JO7	CS12	CS26	*R.nitidus*	-0.003791	0.0731
SC12	GD32	CS26	*R.nitidus*	-0.031866	0.065819
SC35	GD32	CS26	*R.nitidus*	0.037262	0.06611
SC32	GD32	CS26	*R.nitidus*	0.02795	0.067204
SC45	GD32	CS26	*R.nitidus*	0.018868	0.066228
GD30	CS12	CS26	*R.nitidus*	0.124688	0.070952
GD29	CS12	CS26	*R.nitidus*	0.118	0.064917
MY6	GD32	CS26	*R.nitidus*	-0.018072	0.06835
CS11	CS12	CS26	*R.nitidus*	-0.013807	0.070472
GD21	CS12	CS26	*R.nitidus*	0.123732	0.066884
SC28	GD32	CS26	*R.nitidus*	-0.023456	0.065801
JO1	GD32	CS26	*R.nitidus*	-0.025074	0.067165
GD25	CS12	CS26	*R.nitidus*	0.091575	0.067343
SC42	GD32	CS26	*R.nitidus*	0.215909	0.063611
JW6	GD32	CS26	*R.nitidus*	0	0.071757
GD49	CS12	CS26	*R.nitidus*	0.098055	0.069639
JW4	GD32	CS26	*R.nitidus*	0.193972	0.066992
GD22	CS12	CS26	*R.nitidus*	0.066099	0.066585
GD45	CS12	CS26	*R.nitidus*	0.068966	0.06931
SC44	GD32	CS26	*R.nitidus*	0.016064	0.067033
GD35	CS12	CS26	*R.nitidus*	0.039392	0.067021
JO3	GD32	CS26	*R.nitidus*	-0.001765	0.069225
JO8	CS12	CS26	*R.nitidus*	0.024248	0.073048
GD40	CS12	CS26	*R.nitidus*	0.059151	0.064932
GD31	CS12	CS26	*R.nitidus*	0.058694	0.066885
GD33	CS12	CS26	*R.nitidus*	0.004118	0.066552
JO5	GD32	CS26	*R.nitidus*	0.060046	0.066507
GD41	CS12	CS26	*R.nitidus*	0.006106	0.064901
GD34	CS12	CS26	*R.nitidus*	-0.002694	0.064697
GD50	CS12	CS26	*R.nitidus*	0.027273	0.071424
GD42	CS12	CS26	*R.nitidus*	0.019704	0.066493
CS16	CS12	CS26	*R.nitidus*	-0.003375	0.064769
GD26	CS12	CS26	*R.nitidus*	0.04123	0.065574
CS22	CS12	CS26	*R.nitidus*	-0.005275	0.065801
CS18	CS12	CS26	*R.nitidus*	-0.04698	0.066576
GD32	CS12	CS26	*R.nitidus*	0.009486	0.069332
CS20	CS12	CS26	*R.nitidus*	-0.030679	0.067445
SC10	CS12	CS26	*R.nitidus*	0.084257	0.06732
CS15	CS12	CS26	*R.nitidus*	0.009434	0.06513
GD46	CS12	CS26	*R.nitidus*	-0.010691	0.065878
CS21	CS12	CS26	*R.nitidus*	-0.032282	0.065311
CS17	CS12	CS26	*R.nitidus*	0.008686	0.070345
GD44	CS12	CS26	*R.nitidus*	0.027778	0.066993
SC18	CS12	CS26	*R.nitidus*	0.055556	0.067138
CS28	CS12	CS26	*R.nitidus*	-0.026527	0.06637
GD48	CS12	CS26	*R.nitidus*	0.039552	0.067672
CS13	CS12	CS26	*R.nitidus*	-0.077079	0.07039
CS24	CS12	CS26	*R.nitidus*	-0.05439	0.065302
SC12	CS12	CS26	*R.nitidus*	-0.004329	0.06605
CS31	CS12	CS26	*R.nitidus*	-0.048249	0.066756
CS25	CS12	CS26	*R.nitidus*	-0.030257	0.066969
CS19	CS12	CS26	*R.nitidus*	-0.036952	0.06818
SC42	CS12	CS26	*R.nitidus*	0.245227	0.063323
SC6	CS12	CS26	*R.nitidus*	-0.013304	0.068681
CS4	CS12	CS26	*R.nitidus*	-0.034884	0.063694

SC14	CS12	CS26	*R.nitidus*	0.002126	0.067966
SC15	CS12	CS26	*R.nitidus*	-0.008359	0.067138
SC24	CS12	CS26	*R.nitidus*	0.001436	0.066062
CS14	CS12	CS26	*R.nitidus*	-0.023707	0.071053
SC28	CS12	CS26	*R.nitidus*	-0.037037	0.064431
JW6	CS12	CS26	*R.nitidus*	0.001854	0.071499
JO5	CS12	CS26	*R.nitidus*	0.050074	0.064768
CS23	CS12	CS26	*R.nitidus*	-0.050798	0.065712
MY4	CS12	CS26	*R.nitidus*	-0.047076	0.068369
CS30	CS12	CS26	*R.nitidus*	-0.063588	0.064876
CS3	CS12	CS26	*R.nitidus*	-0.017857	0.065385
SC45	CS12	CS26	*R.nitidus*	0.043478	0.066676
SC34	CS12	CS26	*R.nitidus*	0.012319	0.063232
SC32	CS12	CS26	*R.nitidus*	0.042868	0.067739
MY6	CS12	CS26	*R.nitidus*	-0.000697	0.066676
SC5	CS12	CS26	*R.nitidus*	0.008571	0.067543
SC27	CS12	CS26	*R.nitidus*	0	0.065724
JO3	CS12	CS26	*R.nitidus*	0.020602	0.070075
JO1	CS12	CS26	*R.nitidus*	-0.00824	0.065873
JO7	GD33	CS26	*R.nitidus*	-0.031306	0.07217
SC26	CS12	CS26	*R.nitidus*	-0.028343	0.066394
JW4	CS12	CS26	*R.nitidus*	0.205755	0.066748
JW7	CS12	CS26	*R.nitidus*	0.02039	0.071322
GD35	GD33	CS26	*R.nitidus*	0.026753	0.06382
GD25	GD33	CS26	*R.nitidus*	0.07402	0.066513
GD21	GD33	CS26	*R.nitidus*	0.1247	0.063183
SC35	CS12	CS26	*R.nitidus*	0.042747	0.064319
GD29	GD33	CS26	*R.nitidus*	0.132492	0.063747
CS6	CS12	CS26	*R.nitidus*	-0.05136	0.064721
JO8	GD33	CS26	*R.nitidus*	0.022769	0.072306
CS12	GD33	CS26	*R.nitidus*	-0.004118	0.066552
SC31	CS12	CS26	*R.nitidus*	-0.008529	0.067913
SC44	CS12	CS26	*R.nitidus*	0.011611	0.066007
GD44	GD33	CS26	*R.nitidus*	-0.001485	0.064653
CS11	GD33	CS26	*R.nitidus*	-0.020343	0.067395
GD41	GD33	CS26	*R.nitidus*	-0.004481	0.064535
GD30	GD33	CS26	*R.nitidus*	0.11546	0.067743
GD45	GD33	CS26	*R.nitidus*	0.074042	0.06949
GD32	GD33	CS26	*R.nitidus*	0.01564	0.067361
GD22	GD33	CS26	*R.nitidus*	0.066771	0.063018
GD34	GD33	CS26	*R.nitidus*	-0.03176	0.064308
CS13	GD33	CS26	*R.nitidus*	-0.057312	0.066928
GD31	GD33	CS26	*R.nitidus*	0.048601	0.065583
CS14	GD33	CS26	*R.nitidus*	-0.020395	0.06717
GD42	GD33	CS26	*R.nitidus*	0.007087	0.066454
GD26	GD33	CS26	*R.nitidus*	0.04142	0.065146
CS19	GD33	CS26	*R.nitidus*	-0.043326	0.067931
GD50	GD33	CS26	*R.nitidus*	0.009149	0.070947
GD40	GD33	CS26	*R.nitidus*	0.043165	0.064056
CS20	GD33	CS26	*R.nitidus*	-0.05036	0.066326
GD46	GD33	CS26	*R.nitidus*	-0.034901	0.062446
CS15	GD33	CS26	*R.nitidus*	-0.009038	0.066508
SC5	GD33	CS26	*R.nitidus*	-0.006901	0.068092
CS28	GD33	CS26	*R.nitidus*	-0.051209	0.065198
CS17	GD33	CS26	*R.nitidus*	0.007719	0.068021
CS30	GD33	CS26	*R.nitidus*	-0.075085	0.065474

CS31	GD33	CS26	*R.nitidus*	-0.039699	0.064815
GD48	GD33	CS26	*R.nitidus*	0.016253	0.067294
CS24	GD33	CS26	*R.nitidus*	-0.053668	0.066636
SC10	GD33	CS26	*R.nitidus*	0.041781	0.067535
GD49	GD33	CS26	*R.nitidus*	0.084806	0.070178
CS3	GD33	CS26	*R.nitidus*	-0.04304	0.065168
CS22	GD33	CS26	*R.nitidus*	-0.00728	0.065028
CS21	GD33	CS26	*R.nitidus*	-0.042691	0.065327
SC15	GD33	CS26	*R.nitidus*	-0.016349	0.065129
CS18	GD33	CS26	*R.nitidus*	-0.059701	0.065363
CS16	GD33	CS26	*R.nitidus*	-0.00898	0.063997
JW4	GD33	CS26	*R.nitidus*	0.200136	0.066646
SC27	GD33	CS26	*R.nitidus*	-0.01696	0.064766
JW6	GD33	CS26	*R.nitidus*	0.007036	0.070838
CS23	GD33	CS26	*R.nitidus*	-0.064036	0.065497
SC35	GD33	CS26	*R.nitidus*	0.014103	0.064963
CS25	GD33	CS26	*R.nitidus*	-0.046592	0.067099
SC12	GD33	CS26	*R.nitidus*	-0.021768	0.06582
SC28	GD33	CS26	*R.nitidus*	-0.035146	0.064237
CS4	GD33	CS26	*R.nitidus*	-0.036472	0.063958
JO1	GD33	CS26	*R.nitidus*	-0.029145	0.06507
MY4	GD33	CS26	*R.nitidus*	-0.046967	0.065308
SC34	GD33	CS26	*R.nitidus*	-0.003899	0.063556
SC6	GD33	CS26	*R.nitidus*	-0.014628	0.065751
SC42	GD33	CS26	*R.nitidus*	0.214632	0.063525
MY6	GD33	CS26	*R.nitidus*	-0.006365	0.064521
CS6	GD33	CS26	*R.nitidus*	-0.064626	0.064888
SC31	GD33	CS26	*R.nitidus*	-0.024215	0.0668
SC44	GD33	CS26	*R.nitidus*	-0.007324	0.065292
SC14	GD33	CS26	*R.nitidus*	0	0.064684
SC18	GD33	CS26	*R.nitidus*	0.011847	0.065459
JO3	GD33	CS26	*R.nitidus*	0.013007	0.068472
SC45	GD33	CS26	*R.nitidus*	0.031434	0.065482
GD25	GD34	CS26	*R.nitidus*	0.074339	0.065128
SC24	GD33	CS26	*R.nitidus*	-0.022295	0.064326
SC26	GD33	CS26	*R.nitidus*	-0.043593	0.064984
GD21	GD34	CS26	*R.nitidus*	0.1369	0.060303
SC32	GD33	CS26	*R.nitidus*	0.024869	0.064751
JO7	GD34	CS26	*R.nitidus*	-0.007772	0.069524
JO5	GD33	CS26	*R.nitidus*	0.044545	0.064197
CS12	GD34	CS26	*R.nitidus*	0.002694	0.064697
JO8	GD34	CS26	*R.nitidus*	0.05814	0.069713
JW7	GD33	CS26	*R.nitidus*	0.024106	0.068484
CS11	GD34	CS26	*R.nitidus*	-0.00682	0.065626
GD30	GD34	CS26	*R.nitidus*	0.157	0.063415
GD33	GD34	CS26	*R.nitidus*	0.03176	0.064308
GD41	GD34	CS26	*R.nitidus*	0.006813	0.062313
GD32	GD34	CS26	*R.nitidus*	0.015357	0.06435
GD45	GD34	CS26	*R.nitidus*	0.081995	0.067376
GD44	GD34	CS26	*R.nitidus*	0.015453	0.065715
CS18	GD34	CS26	*R.nitidus*	-0.041251	0.064549
GD46	GD34	CS26	*R.nitidus*	-0.015873	0.060821
GD40	GD34	CS26	*R.nitidus*	0.066436	0.063836
GD22	GD34	CS26	*R.nitidus*	0.093997	0.060749
GD35	GD34	CS26	*R.nitidus*	0.038322	0.065111
CS28	GD34	CS26	*R.nitidus*	-0.04311	0.065617

GD31	GD34	CS26	*R.nitidus*	0.056402	0.06502
GD29	GD34	CS26	*R.nitidus*	0.164251	0.061651
GD26	GD34	CS26	*R.nitidus*	0.059414	0.062966
GD49	GD34	CS26	*R.nitidus*	0.09823	0.066808
CS31	GD34	CS26	*R.nitidus*	-0.027624	0.065462
GD48	GD34	CS26	*R.nitidus*	0.042123	0.063773
GD50	GD34	CS26	*R.nitidus*	0.020628	0.067699
CS24	GD34	CS26	*R.nitidus*	-0.046309	0.065525
CS17	GD34	CS26	*R.nitidus*	0.008814	0.066735
CS15	GD34	CS26	*R.nitidus*	0.009416	0.064812
CS22	GD34	CS26	*R.nitidus*	-0.005829	0.064688
CS14	GD34	CS26	*R.nitidus*	-0.015903	0.065378
GD42	GD34	CS26	*R.nitidus*	0.020265	0.063001
CS25	GD34	CS26	*R.nitidus*	-0.035216	0.064929
CS16	GD34	CS26	*R.nitidus*	-0.005009	0.062306
SC15	GD34	CS26	*R.nitidus*	-0.016118	0.066102
CS13	GD34	CS26	*R.nitidus*	-0.045252	0.066386
SC18	GD34	CS26	*R.nitidus*	0.020394	0.065234
SC5	GD34	CS26	*R.nitidus*	0.001331	0.067569
CS3	GD34	CS26	*R.nitidus*	-0.031662	0.065978
CS21	GD34	CS26	*R.nitidus*	-0.029773	0.064889
SC28	GD34	CS26	*R.nitidus*	-0.037158	0.06283
SC35	GD34	CS26	*R.nitidus*	0.027833	0.064065
CS19	GD34	CS26	*R.nitidus*	-0.036685	0.065081
JW6	GD34	CS26	*R.nitidus*	0.027569	0.068215
SC31	GD34	CS26	*R.nitidus*	-0.007129	0.064143
CS20	GD34	CS26	*R.nitidus*	-0.042774	0.064891
SC44	GD34	CS26	*R.nitidus*	0.00538	0.064526
CS23	GD34	CS26	*R.nitidus*	-0.060784	0.062919
SC45	GD34	CS26	*R.nitidus*	0.027617	0.064225
CS4	GD34	CS26	*R.nitidus*	-0.021992	0.062586
SC27	GD34	CS26	*R.nitidus*	-0.014351	0.064846
MY4	GD34	CS26	*R.nitidus*	-0.042969	0.064311
CS30	GD34	CS26	*R.nitidus*	-0.065663	0.063454
JW4	GD34	CS26	*R.nitidus*	0.205997	0.064623
SC32	GD34	CS26	*R.nitidus*	0.02652	0.063066
SC14	GD34	CS26	*R.nitidus*	0.003224	0.064322
SC6	GD34	CS26	*R.nitidus*	-0.007134	0.064528
SC10	GD34	CS26	*R.nitidus*	0.044867	0.065833
CS6	GD34	CS26	*R.nitidus*	-0.049832	0.065
SC12	GD34	CS26	*R.nitidus*	-0.018566	0.065147
SC24	GD34	CS26	*R.nitidus*	-0.015344	0.06525
SC26	GD34	CS26	*R.nitidus*	-0.030679	0.063184
MY6	GD34	CS26	*R.nitidus*	-0.003189	0.063558
JW7	GD34	CS26	*R.nitidus*	0.049421	0.068646
CS11	GD35	CS26	*R.nitidus*	-0.030901	0.06598
JO8	GD35	CS26	*R.nitidus*	-0.006318	0.072069
JO1	GD34	CS26	*R.nitidus*	-0.026482	0.063664
SC34	GD34	CS26	*R.nitidus*	0.014022	0.064373
SC42	GD34	CS26	*R.nitidus*	0.237029	0.062882
GD29	GD35	CS26	*R.nitidus*	0.080897	0.064718
JO7	GD35	CS26	*R.nitidus*	-0.032143	0.072103
GD42	GD35	CS26	*R.nitidus*	-0.018939	0.064451
GD22	GD35	CS26	*R.nitidus*	0.030199	0.063342
GD50	GD35	CS26	*R.nitidus*	-0.02112	0.067252
GD25	GD35	CS26	*R.nitidus*	0.045556	0.063385

GD21	GD35	CS26	*R.nitidus*	0.083333	0.063006
GD30	GD35	CS26	*R.nitidus*	0.107203	0.067364
GD31	GD35	CS26	*R.nitidus*	0.025111	0.064699
CS12	GD35	CS26	*R.nitidus*	-0.039392	0.067021
GD45	GD35	CS26	*R.nitidus*	0.054468	0.067183
JO3	GD34	CS26	*R.nitidus*	0.026435	0.067019
GD32	GD35	CS26	*R.nitidus*	-0.014975	0.067466
GD26	GD35	CS26	*R.nitidus*	0.011103	0.064818
GD41	GD35	CS26	*R.nitidus*	-0.030657	0.062688
GD33	GD35	CS26	*R.nitidus*	-0.026753	0.06382
GD44	GD35	CS26	*R.nitidus*	-0.019757	0.065831
JO5	GD34	CS26	*R.nitidus*	0.047341	0.062302
CS17	GD35	CS26	*R.nitidus*	-0.024443	0.068294
GD40	GD35	CS26	*R.nitidus*	0.024602	0.063109
GD34	GD35	CS26	*R.nitidus*	-0.038322	0.065111
CS14	GD35	CS26	*R.nitidus*	-0.039031	0.067194
GD48	GD35	CS26	*R.nitidus*	-0.013481	0.066235
CS13	GD35	CS26	*R.nitidus*	-0.071477	0.066166
GD46	GD35	CS26	*R.nitidus*	-0.060694	0.062768
SC5	GD35	CS26	*R.nitidus*	-0.029006	0.069022
CS20	GD35	CS26	*R.nitidus*	-0.068521	0.066725
GD49	GD35	CS26	*R.nitidus*	0.068627	0.068977
CS16	GD35	CS26	*R.nitidus*	-0.032873	0.064046
CS21	GD35	CS26	*R.nitidus*	-0.060904	0.064996
CS30	GD35	CS26	*R.nitidus*	-0.087312	0.065635
CS25	GD35	CS26	*R.nitidus*	-0.060648	0.066519
CS24	GD35	CS26	*R.nitidus*	-0.077244	0.064985
CS3	GD35	CS26	*R.nitidus*	-0.043185	0.064037
CS19	GD35	CS26	*R.nitidus*	-0.065771	0.067009
CS15	GD35	CS26	*R.nitidus*	-0.017763	0.066604
CS4	GD35	CS26	*R.nitidus*	-0.047051	0.063641
CS23	GD35	CS26	*R.nitidus*	-0.089155	0.064271
CS18	GD35	CS26	*R.nitidus*	-0.076072	0.064981
SC14	GD35	CS26	*R.nitidus*	-0.019947	0.065545
SC12	GD35	CS26	*R.nitidus*	-0.048716	0.064963
SC45	GD35	CS26	*R.nitidus*	0.001317	0.063585
JO7	GD40	CS26	*R.nitidus*	-0.055938	0.069045
SC15	GD35	CS26	*R.nitidus*	-0.037417	0.067105
CS28	GD35	CS26	*R.nitidus*	-0.071891	0.066628
CS22	GD35	CS26	*R.nitidus*	-0.034899	0.066261
MY6	GD35	CS26	*R.nitidus*	-0.039125	0.065577
CS31	GD35	CS26	*R.nitidus*	-0.069356	0.066759
GD30	GD40	CS26	*R.nitidus*	0.079661	0.06488
SC6	GD35	CS26	*R.nitidus*	-0.04064	0.063703
JW6	GD35	CS26	*R.nitidus*	-0.030435	0.070762
JO8	GD40	CS26	*R.nitidus*	-0.034203	0.069743
SC32	GD35	CS26	*R.nitidus*	0.00857	0.063063
SC42	GD35	CS26	*R.nitidus*	0.213974	0.063111
JO3	GD35	CS26	*R.nitidus*	-0.015008	0.06904
JO1	GD35	CS26	*R.nitidus*	-0.043025	0.063567
JW4	GD35	CS26	*R.nitidus*	0.187797	0.063958
JO5	GD35	CS26	*R.nitidus*	0.004704	0.064519
SC26	GD35	CS26	*R.nitidus*	-0.055665	0.063638
SC10	GD35	CS26	*R.nitidus*	0.027121	0.064848
SC18	GD35	CS26	*R.nitidus*	-0.016393	0.065875
SC28	GD35	CS26	*R.nitidus*	-0.059294	0.062964

CS6	GD35	CS26	*R.nitidus*	-0.087622	0.065572
SC24	GD35	CS26	*R.nitidus*	-0.051282	0.064733
GD22	GD40	CS26	*R.nitidus*	0.008536	0.062523
GD25	GD40	CS26	*R.nitidus*	0.033708	0.062561
SC35	GD35	CS26	*R.nitidus*	-0.007453	0.064364
GD32	GD40	CS26	*R.nitidus*	-0.037995	0.065749
SC27	GD35	CS26	*R.nitidus*	-0.044278	0.06327
SC31	GD35	CS26	*R.nitidus*	-0.039693	0.064398
[truncated: 453,006 more chars]
